# Genetically predicted immune cells and metabolites mediate the causal relationship between inflammatory factors and rheumatoid arthritis

**DOI:** 10.1097/MD.0000000000044574

**Published:** 2025-09-12

**Authors:** Tianyang Li, Jinpeng Wei, Hua Wu, Chen Chen

**Affiliations:** a Department of Orthopedics, Third Hospital of Shanxi Medical University, Shanxi Bethune Hospital, Shanxi Academy of Medical Sciences, Tongji Shanxi Hospital, Taiyuan, China.

**Keywords:** immune cells, inflammatory factors, Mendelian randomization, metabolites, rheumatoid arthritis

## Abstract

This study investigates the causal relationship between inflammatory factors and rheumatoid arthritis (RA) and potential immune cells and metabolites mediator using Mendelian randomization. A bidirectional Mendelian randomization study was conducted using statistics from a genome-wide association study database to explore the causal relationship between inflammatory factors and RA, combined with mediation analysis, to discover potential immune cells and metabolites with mediating effects, and to estimate the proportion of total effects mediated by each immune cell and metabolite. The level of the inflammatory factor CD40L receptor was positively associated with an elevated risk of RA. Mediation analysis showed evidence of indirect effect of CD40L receptor levels on RA through CD14^+^CD16^−^ monocyte% monocyte (OR 1.022, 95% CI, 1.003–1.042; *P* = .021), X-24757 levels (OR 1.168, 95% CI, 1.035–1.317; *P* = .011) and IgD on IgD ^+^ CD38^−^ unswitched memory (OR 1.088, 95% CI, 1.010–1.173; *P* = .026), with a mediated proportion of 1.89%, 10.6%, and 11.6% of the total effect, respectively. Our study identified the causal relationship between inflammatory factor CD40L receptor levels and RA and the potential mediating role of 2 immune cells and 1 metabolite.

## 1. Introduction

Rheumatoid arthritis (RA) is a relatively common disease, occurring in about 1% of the population. It is conspicuously more frequent in females than in males, with a prevalence ratio of roughly 2.5:1.^[1]^ RA can occur at any age, but the prevalence of the disease tends to increase with age and is most prevalent among individuals aged between 40 and 70.^[2]^ RA has an insidious onset and its main clinical manifestations include stiffness, pain and swelling of the surrounding joints. The disease is characterized by synovitis, aggressive damage to articular cartilage and bone, which may ultimately result in irreversible deformity and loss of function of the joints. Bone erosion, as the main pathologic alteration in RA, is observed even in the early stages of the disease in over 45% of patients with RA.^[3]^

There is a significant relationship between inflammatory factors and RA. First, inflammatory factors are one of the key factors in the development of RA. In the joints of RA patients, inflammatory factors such as tumor necrosis factor and interleukin are released in substantial quantities, thereby activating the immune system and initiating inflammatory responses in the synovium of the joints. These inflammatory reactions can give rise to symptoms such as joint swelling, pain, and stiffness, which can significantly impact the patient’s quality of life.^[4]^ Second, inflammatory factors also exert an influence on the progression and severity of RA. In the course of the pathogenesis of RA, inflammatory factors promote the proliferation and differentiation of synovial cells, resulting in synovial thickening and the formation of vascular opacities. These pathological alterations will further exacerbate inflammation and destruction of joints, leading to the damage of articular cartilage and bone, and even causing joint deformity and dysfunction.^[5]^ In addition, inflammatory factors are implicated in the systemic inflammatory response to RA. RA is not only a localized joint disease, but also causes a systemic inflammatory response. Inflammatory factors can permeate organs and tissues throughout the body via blood circulation, triggering a systemic inflammatory response and immune disorders. This may lead to systemic symptoms such as fever, anemia, enlarged lymph nodes, etc, further exacerbating the severity of the disease. In summary, inflammatory factors exert an important role in the onset, progression and severity of RA.^[6]^ Consequently, therapeutic strategies focusing on inflammatory factors hold significant importance in the treatment of RA. By inhibiting the production and action of inflammatory factors, joint inflammation can be effectively mitigated, pain alleviated, joint function improved, and the quality of life of patients enhanced.

Both innate immune cells, such as macrophages, neutrophils and monocytes, as well as adaptive immune cells, such as B lymphocytes and T lymphocytes, exhibit heightened activity in the joint tissue of RA patients.^[7–9]^ The significant markers of RA involve the presence of autoantibodies, such as rheumatoid factor and anti-citrullinated protein antibodies. However, the specific mechanism of autoimmune response and autoantibody production in RA remains ambiguous and needs further study.

The occurrence of RA involves the dysfunction of diverse metabolic pathways. Previous studies have found that glucose metabolism, lipid metabolism and amino acid metabolism are intimately correlated with the degree of disease activity.^[10–12]^ Analyzing the associated intermediate metabolites can provide valuable predictive information. They may be employed as biomarkers to predict disease progression and treatment response in RA.

Mendelian randomization (MR) serves as a methodology that employs genetic variation as an instrumental variable to infer causal relationships between exposure and outcome.^[13]^ In the light of the important effects of inflammatory factors, immune cells, and metabolites in RA, research group conducted an MR study to predict the causal relationship between inflammatory factors and RA, as well as the potential mediation of immune cells and metabolites.

## 2. Materials and methods

### 2.1. Study design

**Figure 1.**
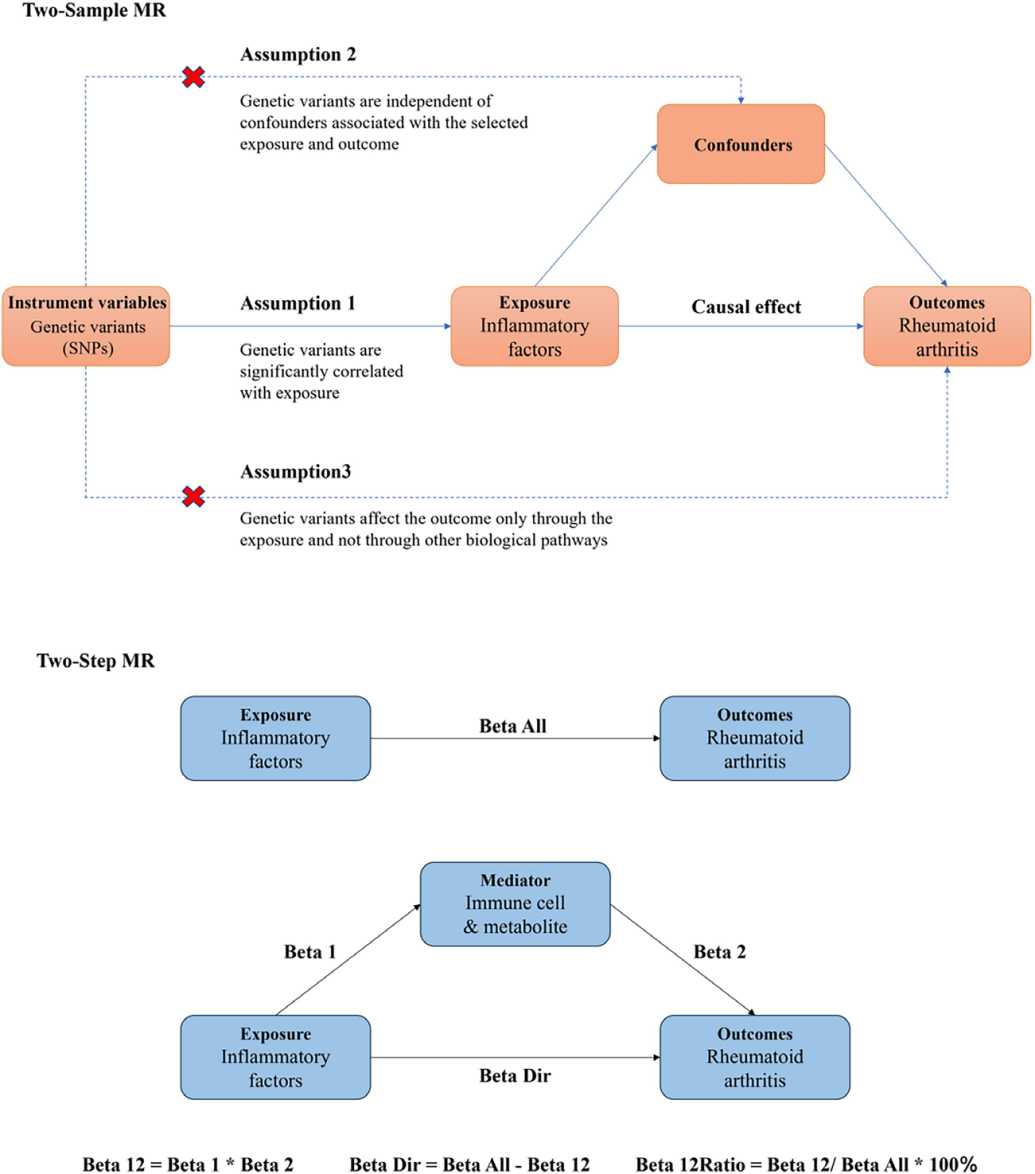
Flowchart of the analysis performed.

We first assessed the causal relationship between exposure (91 inflammatory factors) and outcome (RA) using a two-sample MR study, and then used a mediated (two-step approach) MR study to examine the role of immune cells and metabolites as mediators between exposure and outcome. MR represents risk factors through genetic variation, and 3 key assumptions must be met by instrumental variables (IVs) in causal inference: IVs must be strongly associated with exposure; IVs are independent of possible confounders between exposure and outcome; and Genetic variation does not affect outcome through pathways other than exposure (Fig. 1). We obtained summary data from publicly available GWAS databases, which were approved by the relevant institutional review boards for the data analyzed in the study, and ethical approval and informed consent were obtained.

### 2.2. Data sources

The summary data for the 91 inflammatory factors included in the GWAS catalog (https://ftp.ebi.ac.uk/pub/databases/gwas/summary_statistics/), covering association studies between a wide range of inflammatory factors and genetic variants, helped us gain insight into the role of inflammatory factors in the development and progression of diseases such as RA.^[14]^ Second, we obtained GWAS data for 731 immunophenotypes (https://ftp.ebi.ac.uk/pub/databases/gwas/ summary_statistics/) and 1400 plasma metabolites from the GWAS catalog, (https://ftp.ebi.ac.uk/pub/databases/gwas/summary_statistics/). These data cover association studies between immunophenotypes and plasma metabolites with genetic variants.^[15]^ Finally, the FinnGen Consortium R10 version (FinnGen Consortium/Institute for Molecular Medicine Finland [FIMM], University of Helsinki, Helsinki, Uusimaa, Finland) provides GWAS summary statistics for RA. This version contains 13,621 cases of RA and 262,844 control individuals.^[16]^

### 2.3. Instrument variables selection

The selection of IVs required rigorous quality control procedures to ensure that sufficient SNPs were available for MR analysis and data reliability. Therefore, based on previous studies, we set the genome-wide threshold (CD40L receptor level) for exposure-related SNPs to 1 × 10^−5^.^[17,18]^ Meanwhile, irrelevant SNPs were excluded by applying the linkage disequilibrium (*R*^2^ < 0.001 and clumping distance = 10,000 kb) strategy to ensure that the IVs were independent of each other.^[14]^ During the screening process, we focused on SNPs with minor allele frequency > 0.01 and excluded palindromic SNPs.^[19]^ Finally, by calculating and excluding *F*-statistics (*F* = R2(n − k − 1)/k(1 − R2); R2, exposure variance explained by selected instrumental variables, R2 value obtained in MR Steiger directionality test; n, sample size; *k*, number of IVs) <10 SNPs to minimize the bias of weak instrumental variables on the results.^[20]^

### 2.4. Statistical analysis

#### 2.4.1. MR statistical analysis

There are multiple tests in this study to determine whether there is a causal relationship between exposure and outcome, including inverse variance weighting (IVW), MR-Egger, weighted median, weighted modeling, and simple modeling methods.^[21]^ Among these, IVW is a method of combining the Wald estimates for each SNP through meta-analysis to obtain an overall estimate of the effect of exposure on outcome. And when horizontal pleiotropy is not present, unbiased causal estimates can be obtained by IVW linear regression. Thus, the IVW method is considered the core method for assessing the presence of causality, with the remaining 4 models serving as auxiliary analytical tools, *P* < .05 showed that there was a potential causal relationship between exposure and outcome.^[22]^ False discovery rate (FDR) correction is a statistical method used in multiple hypothesis testing to control the false discovery rate, that is, the proportion of hypothesis tests that are incorrectly judged to be significant.^[23]^ Bayesian model averaging (BMA) avoids the limitations of the traditional logistic regression method and further validates the reliability of the results.^[24]^ In this study we used both methods to test the results of the MR study.

#### 2.4.2. Mediation analysis

In traditional mediation analysis, the establishment of a mediator effect relies on several assumptions: the absence of confounding factors between variables; that the exposure does not introduce additional confounders; and no interaction between the exposure and the mediator. One key advantage of MR is its ability to eliminate confounding effects, thereby yielding a clean causal estimate. We used IVW as our primary method to assess the effect of inflammatory factors on immune cells and metabolites (β1) and multivariable MR to assess the effect of each immune cell and metabolite on RA risk to adjust for the genetic effect of inflammatory factors (β2). The indirect mediating effect of inflammatory factors on RA outcomes was calculated using the coefficient product method as the primary method, that is, the incidental effect of inflammatory factors on outcomes via immune cells and metabolites (β12 = β1 × β2). The direct effect was an estimate of inflammatory factors on the individually adjusted outcome for each mediator (β Dir), and the total effect was the sum of the direct and indirect effects (β all=β12+β Dir). Therefore, the proportion of the total effect mediated by each immune cell and metabolite was estimated by dividing the indirect effect by the total effect (β12 Ratio=β12/β all). All MR analyses were conducted in R (version 4.3.2; R Foundation for Statistical Computing, https://www.r-project.org/foundation/).^[25]^

#### 2.4.3. Sensitivity analysis

In this study, we examined the horizontal pleiotropy of the results by using the Mendelian Randomization Pleiotropy RESidual Sum and Outlier (MR-PRESSO) test and MR-Egger regression intercept analysis.^[26]^ Heterogeneity of SNP was tested using Cochran Q statistic, with *P*-values < .05 indicating significant heterogeneity.^[27]^ In addition, to avoid horizontal pleiotropy caused by a single SNP, we performed a “leave-one-out” sensitivity analysis to ensure the reliability of the results.^[28]^ Finally, we determined whether reverse causality existed through reverse MR and conducted subsequent mediated MR studies.

## 3. Results

Summary data on inflammatory factors, metabolites, and immune cells were screened for SNPs and then analyzed by MR using thresholds (*P* < 1 × 10^−5^), linkage disequilibrium standards and *F*-statistic values. Detailed IVs data are shown in Tables S1–S3, Supplemental Digital Content, https://links.lww.com/MD/Q8.

### 3.1. Exploration of the causal effect of inflammatory factors on RA risk

**Figure 2.**
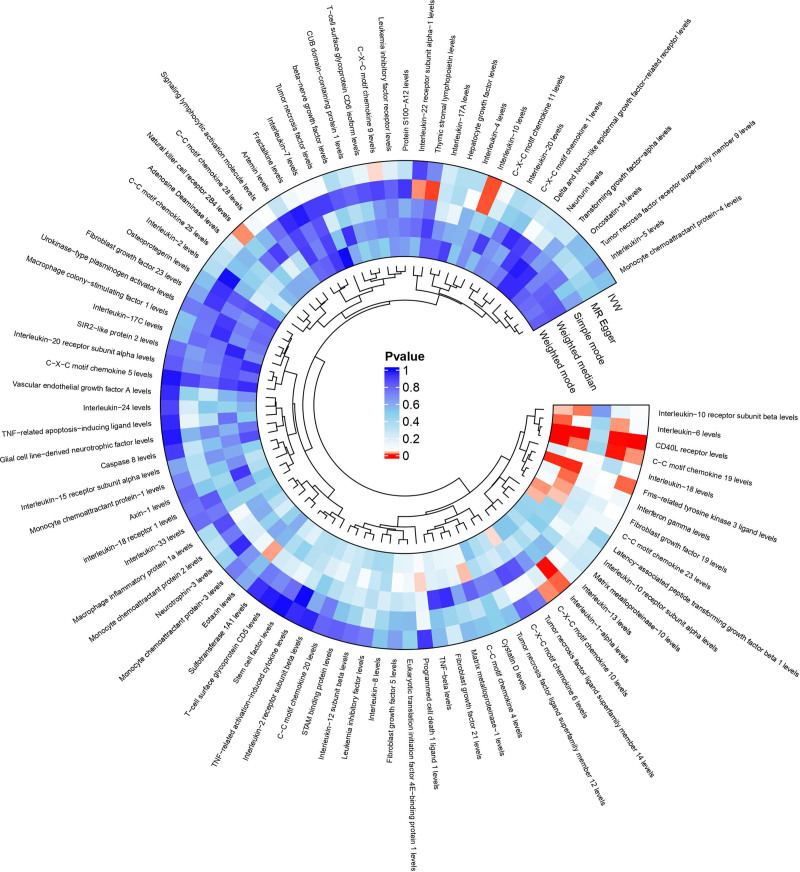
Visualization of the causal relationship between inflammatory factors and RA. RA = rheumatoid arthritis.

To understand the causal effect of inflammatory factors on RA, we conducted a two-way two-sample MR study (Fig. 2, Table S4, Supplemental Digital Content, https://links.lww.com/MD/Q8).

**Figure 3.**
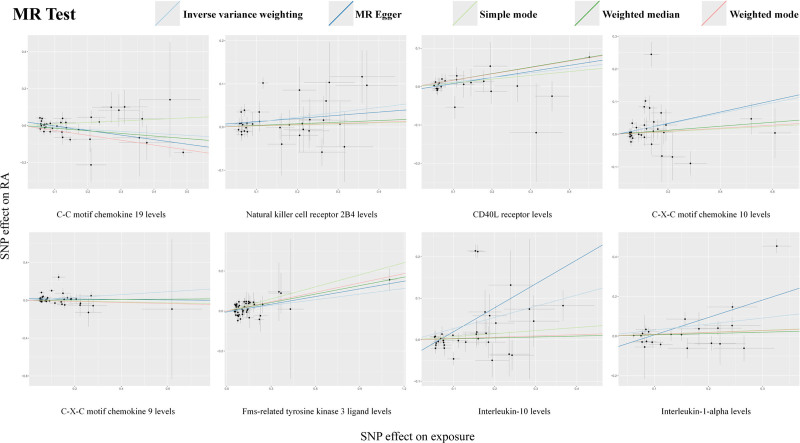
Scatterplot of causal effects on exposure and outcome.

**Figure 4.**
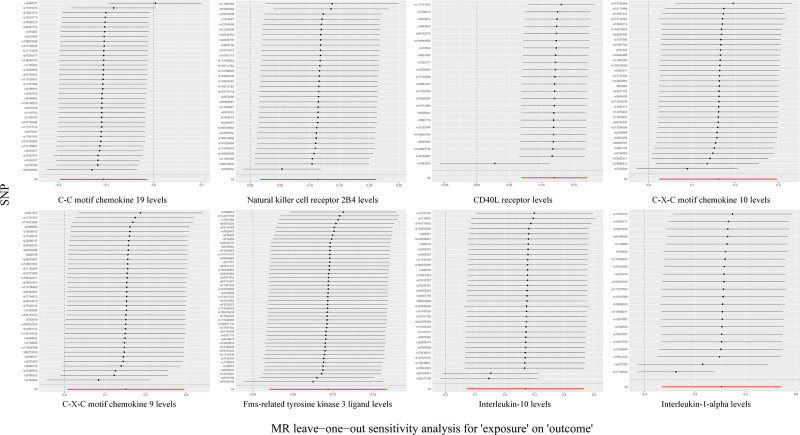
Leave-one-out plots of exposure and outcome causal effects.

By MR analysis, we identified a causal effect between 8 inflammatory factors and RA: C-C motif chemokine 19 levels (OR 0.900, 95% CI, 0.822–0.985; *P* = .022), Natural killer cell receptor 2B4 levels (OR 1.120, 95% CI, 1.017–1.234; *P* = .021), CD40L receptor levels (OR 1.126, 95% CI, 1.071–1.185; *P* = 3.734 × 10^-6^), C-X-C motif chemokine 10 levels (OR 1.174, 95% CI, 1.025–1.345; *P* = .021), C-X-C motif chemokine 9 levels (OR 1.163, 95% CI, 1.007–1.344; *P* = .040), Fms-related tyrosine kinase 3 ligand levels (OR 1.098, 95% CI, 1.021–1.182; *P* = .012), interleukin-10 levels (OR 1.308, 95% CI, 1.074–1.594; *P* = .008), interleukin-1-alpha levels (OR 1.355, 95% CI, 1.071–1.715; *P* = .011) (Table 1). Potential horizontal pleiotropy was excluded using the MR-egger intercept and MR-PRESSO test, and the IV-exposure-outcome triangulation visualized in scatterplots methodologically confirmed the robustness of linear dose–response relationships across instrumental variables (Fig. 3). Furthermore, leave-one-out sensitivity analyses systematically confirmed the absence of influential SNPs through iterative reestimation of causal effects, with all leave-one-out estimates remaining within 95% confidence intervals of the primary analysis (Fig. 4). In addition, the BMA verification of the results was performed to ensure the reliability of the results. The Bayesian results showed that there were 3 inflammatory factors with *P* > .05 after BMA verification, respectively: Natural killer cell receptor 2B4 levels, *P* = .062; C-X-C motif chemokine 10 levels, *P* = .092; Interleukin-10 levels, *P* = .792 (Table S5, Supplemental Digital Content, https://links.lww.com/MD/Q8). After correction for FDR (*P* < .05), we identified an inflammatory factor (CD40L receptor levels, OR 1.126, 95% CI, 1.071–1.185; *P* = 3.734 × 10^-6^) that was significantly causally associated to disease.

**Table 1:** Results of Mendelian randomization analysis of the association between 8 inflammatory factors and rheumatoid arthritis.

Exposure	Method	nSNP	OR	95% CI	*P*-value	FDR-P	Pleiotropy
C-C motif chemokine 19 levels	IVW	36	0.9	0.822–0.985	.022	2.002	0.011
Natural killer cell receptor 2B4 levels	IVW	33	1.12	1.017–1.234	.021	1.911	0.627
CD40L receptor levels	IVW	23	1.126	1.071–1.185	3.734 × 10^−6^	3.398 × 10^-4^	0.143
C-X-C motif chemokine 10 levels	IVW	33	1.174	1.025–1.345	.021	1.911	0.899
C-X-C motif chemokine 9 levels	IVW	37	1.163	1.007–1.344	.04	3.64	0.234
FMS-related tyrosine kinase 3 ligand levels	IVW	45	1.098	1.021–1.182	.012	1.092	0.512
Interleukin-10 levels	IVW	33	1.308	1.074–1.594	.008	0.728	0.096
Interleukin-1-alpha levels	IVW	22	1.355	1.071–1.715	.011	1.001	0.004

FDR = false discovery rate, IVW = inverse variance weighting.

### 3.2. Mediation analysis of immune cells and metabolites

**Figure 5.**
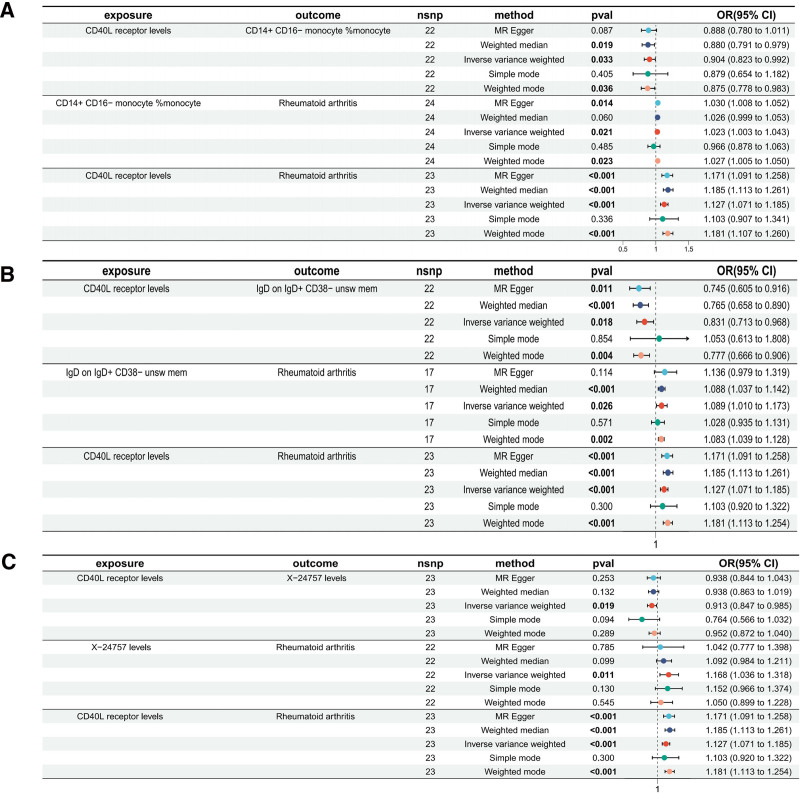
Forest plot of the causal relationship between CD40L receptor levels and RA mediated by 3 different mediators (inverse variance weighted, IVW, *P* < .01). (A) CD14+ CD16− monocyte% monocyte (OR 1.022, 95% CI, 1.003–1.042; *P* = .021). (B) IgD on IgD+ CD38− unsw mem (OR 1.088, 95% CI, 1.010–1.173; *P* = .026). (C) X-24757 levels (OR 1.168, 95% CI, 1.035–1.317; *P* = .011). IVW = inverse variance weighting, RA = rheumatoid arthritis, unsw mem = unswitched memory.

To explore the mediating role of immune cells and metabolites between the causal effects of inflammatory factors and RA, we performed subsequent mediating effect analyses of CD40L receptor levels. First, we performed MR analysis between inflammatory factors and immune cells and metabolites to identify immune cells and metabolites that were causally associated with CD40L receptor levels. The results showed that CD40L receptor levels were causally associated with 74 immune cells and 50 metabolites (Tables S6 and S7, Supplemental Digital Content, https://links.lww.com/MD/Q8). We then used the obtained immune cells and metabolites for further causal analysis with RA, and there existed 7 immune cells and 1 metabolite that were causally associated with RA. After pleiotropy testing of the two-step MR described above, the results showed the presence of 2 immune cells and a metabolite genetically predicted to be associated with an elevated risk of RA: the proportion of CD14⁺CD16⁻ classical monocytes within the total monocyte population (CD14^+^CD16⁻ monocyte% monocyte; OR 1.022, 95% CI, 1.003–1.042; *P* = .021), IgD expression levels on IgD⁺CD38⁻ unswitched memory B cells (IgD on IgD^+^ CD38⁻ unsw mem; OR 1.088, 95% CI, 1.010–1.173; *P* = .026), and an unnamed metabolite identified via plasma metabolomics profiling (X-24757 levels; OR 1.168, 95% CI, 1.035–1.317; *P* = .011) (Fig. 5).

Finally, we performed a mediation effect proportion assessment. The results showed that CD40L receptor levels mediated their effect on RA through CD14^+^ CD16⁻ monocyte% monocyte and IgD on IgD^+^CD38⁻ unsw mem in the proportions of 1.89% and 11.6%. CD40L receptor levels also mediated their effect on RA through X-24757 levels mediated its effect on RA in 10.6%.

### 3.3. Sensitivity analysis

We examined heterogeneity and horizontal pleiotropy for the entirety of this study. There was no evidence of heterogeneity or asymmetry in causality observed for these SNPs using Cochran *Q*-test and funnel plots (Tables S8–S12, Supplemental Digital Content, https://links.lww.com/MD/Q7; Figs. S1–S5, Supplemental Digital Content, https://links.lww.com/MD/Q8). The MR-PRESSO global test and MR egger intercept test did not detect potential horizontal pleiotropy (Tables S13–17, Supplemental Digital Content, https://links.lww.com/MD/Q8, Tables S18–S22, Supplemental Digital Content, https://links.lww.com/MD/Q8). In addition, we used leave-one-out analysis to validate the effect of each SNP on the overall causal estimation, and the results showed that the inclusion of all SNPs contributed significantly to the establishment of causality (Figs. S6–S10, Supplemental Digital Content, https://links.lww.com/MD/Q7). The results of all MR analyses were visualized using scatterplots (Figs. S11–S15, Supplemental Digital Content, https://links.lww.com/MD/Q7).

## 4. Discussion

In recent years, the exploration of the complex relationship between inflammatory factors, immune cells and metabolites, and their contribution to disease development has become a focus in scientific research. Immune cells and metabolites undertake the functions of immune defense and immune surveillance under the regulation of inflammatory factors. They cooperate to maintain the immune equilibrium within the human body, and the disruption of homeostasis will lead to the development of autoimmune diseases.^[29]^ This study used an MR approach based on summary data from the GWAS database to delve into the relationship between inflammatory factors and RA. The mediating role of immune cells and metabolites was also explored in this context, revealing the complex genetic relationships among inflammatory factors, immune cells, and RA. This study confirms that CD40L receptor levels can act on RA mediated by specific immune cells and metabolites. This is the first time that genetic prediction methods were used to investigate the potential causal and mediating roles between the above factors. The findings of this research could facilitate a better understanding of RA and provide novel insights for the prevention and management of the disease.

CD40L (also known as CD154, gp39, T-BAM, or TRAP) activates CD40, which is primarily expressed on activated CD4^+^ T cells. Its molecular weight ranges between 240 and 274 amino acids.^[30]^ CD40L, as a potential immune-enhancing molecule, can also be expressed on the cell surface of monocytes, B cells, natural killer cells, CD8^+^ cells, mast cells, and basophils.^[31,32]^ The role of the CD40/CD40L signaling pathway is crucial in the pathogenesis of RA. This signaling pathway has the ability to induce autoantibody production and class switching, as well as promoting the expression of co-stimulatory molecules such as inflammatory cytokines, chemokines, and adhesion molecules. In addition, the pathway is also directly involved in the pathological process of tissue injury by stimulating the expression of matrix metalloproteinase and receptor activator of nuclear factor kappa-B ligand.^[33]^ A further study analyzed the expression of multiple factors in the pathway by collecting synovial tissue and blood/serum samples from patients and applying disease activity score and nuclear magnetic resonance techniques. The results of the study indicated that patients with RA exhibited elevated levels of CD40L expression compared to healthy controls, and it was positively correlated with the degree of disease activity and the severity of the disease.^[34]^ Liu et al found that synovial fibroblasts enhanced the production of tumor necrosis factor-a through the CD40 signaling pathway. This may further influence and activate macrophages to produce persistent inflammation. And inhibition of synovial fibroblast activation by the CD40–CD40L interaction may be a potential therapeutic approach.^[35]^

This study employed MR studies to explore potential causal effects between inflammatory factors and RA. The study analyzed the relationship between 91 common inflammatory factors and RA and showed that 8 inflammatory factors bore a causal effect with RA. After correcting FDR, it showed that CD40L receptor level displayed the most significant causal relationship with RA (*P* = 3.734 × 10^−6^). And it was observed that an elevated CD40L receptor level was a risk factor for RA (OR 1.126, 95% CI, 1.071–1.185). Considering the crucial immunomodulatory role that CD40L receptor level exhibits in RA, it may be an important target for therapeutic strategies in RA.

Human blood monocytes can be classified into 3 functionally distinct subpopulations, CD14^+^CD16^−^ (classical), CD14^+^CD16^+^ (intermediate), and CD14^dim^CD16^−^ (atypical). Among circulating monocytes, CD14^+^CD16^−^ monocytes dominate, accounting for about 85% of the total, while the remaining 2 subpopulations each occupy about 5% to 8%. These subpopulations respond rapidly during the acute inflammatory phase, particularly during bacterial infection.^[36]^ A clear association between focal bone erosion in RA and overactivation of localized osteoclasts has been demonstrated according to available studies.^[37]^ Osteoclasts, as a type of multinucleated cells, are mainly derived from monocyte/macrophage lineage, especially CD14^+^CD16^−^ monocytes.^[38]^ Studies have indicated that CD16^+^ monocytes have a high rate of spontaneous apoptosis and a more significant expression of apoptosis-related genes. Apoptosis-related proteins, such as Bax, Bid, and cytochrome c, are expressed at higher levels in these cells. CD16^+^ monocytes are exposed to relatively high levels of oxidative stress, which leads to their spontaneous apoptosis. In contrast, CD16^−^ monocytes have higher expression of antioxidant enzymes and are able to resist oxidative stress and show greater survival. The investigation of the differential apoptotic mechanisms of CD16^+^ and CD16^−^ monocyte subpopulations in homeostatic and infected states has contributed to an in-depth comprehension of the regulatory role of these subpopulations under RA pathological conditions.^[39]^ One study analyzed the immunophenotypic and transcriptomic characteristics of CD14^+^ monocytes in the bone marrow of patients with multiple myeloma. The results showed that the quantity of CD14^+^CD16^+^ monocytes was higher in patients with multiple myeloma in contrast to the normal population, and this number was associated with increased bone destruction and bone resorption.^[40]^ In addition, some studies have explored CD16^+^ as a potential marker of bone resorption precursor cells in patients with psoriatic arthritis (PSA). It was found that the percentage of CD14^+^CD16^+^ cells in the peripheral blood of PSA patients was higher than that of healthy controls, and the expression level of CD16^+^ in PSA was positively correlated with the degree of bone erosion.^[41]^ In further studies by comparing the bone resorption capacity of CD14^+^CD16^+^ and CD14^+^CD16^−^ monocytes in RA patients, it was shown that CD14^+^CD16^−^ monocytes dominated the bone resorption process in RA patients. It was also revealed that the increased expression of Tyro3TK in CD14^+^CD16^−^ monocytes was closely associated with the clinical and immunologic characteristics of RA patients.^[42]^

Non-switched memory B cells are cells with antigen experience, mainly expressing the memory marker CD27 as well as IgM/IgD immunoglobulin, without conversion to other immunoglobulin subtypes. The heterogeneity of these cells has complicated the definition of their function in humoral immunity. In recent years, studies on the role of non-switched memory B cells in autoimmune diseases and viral infections have gradually increased, revealing their significance in healthy and disease conditions.^[43]^ Some studies have shown a significant deviation of VDJ gene use in plasma cells and non-switched memory B cells in patients with systemic lupus erythematosus, especially because the high use of IGHV 4 to 34 gene is closely associated with disease activity.^[44]^ A comparative study analyzing peripheral blood samples from healthy controls and RA patients demonstrated significantly reduced levels of total memory B cells, IgM memory B cells, and unswitched memory B cells in RA patients. These findings suggest that the depletion of these B cell subsets may promote excessive production of autoantibodies, such as anti-cyclic citrullinated peptide antibodies and rheumatoid factor, thereby amplifying autoimmune-mediated tissue damage and perpetuating disease pathogenesis.^[45]^ IgD expression is associated with B cell activation and proliferation, which may lead to dysregulated immune responses and exacerbate inflammation in autoimmune conditions patients. This suggests that some B cell subsets may not only be ineffective in anti-inflammatory aspects, but may even lead to dysregulated immune response that aggravate the condition.

In this study, we used MR mediation analysis to explore the mediating effect of immune cells between CD40L receptor levels and RA. First, we analyzed the causal relationship between CD40L receptor levels and 731 common immune cells, and then we explored another MR causal analysis between immune cells and RA. The result is that 2 types of immune cells have a potential mediating role, including the CD14^+^ CD16^−^ monocyte% monocyte (OR 1.022, 95% CI, 1.003–1.042; *P* = .021) and the IgD on IgD^+^CD38^−^ unsw mem (OR 1.088, 95% CI, 1.010–1.173; *P* = .026) (Fig. 5A and B). This result may further corroborate the conclusion that elevated CD14^+^CD16^−^ monocytes are more strongly associated with RA progression. However, after reviewing the relevant literature, we did not find any studies related to IgD on IgD^+^CD38^−^ unsw mem, which may become a research direction for subsequent basic studies.

In recent years, metabolomics has achieved remarkable results in the study of RA, and a large number of studies have found significant differences in metabolic profiles between RA patients and healthy controls.^[46,47]^ In one study, collagen-induced arthritis rats were used as a model, and their hydrated metabolites were analyzed by nuclear magnetic resonance spectroscopy. The results showed that the levels of lactate, lysine, branched-chain amino acids and creatinine were significantly altered in collagen-induced arthritis rats, suggesting that metabolic abnormalities may play an important role in the pathogenesis of RA.^[48]^ Another study used 1H nuclear magnetic resonance technique to perform metabolomic analysis of the joint fluid phases of osteoarthritis (OA) and RA and found significant metabolic differences between the 2 diseases. A total of 50 metabolites were detected in the study, 32 of which were significantly different between the 2 groups. These differences may be related to inflammation, cartilage wear and tear, and the pathologic process of OA. The study also found significant differences in urinary levels of metabolites such as bile acids, amino acids and glycosylation end products between OA and RA patients.^[10]^ RA patients are usually accompanied by multiple metabolic diseases, and one study comparing lipid metabolism differences in different diseases by multi-omics and clinical data analysis further suggests a crucial role of dysregulated lipid metabolism in RA, as well as an upregulation of the expression of the gut microbiota associated with lipid metabolism.^[49]^

In the present study we concluded from MR studies that X-24757 levels, a metabolite that plays a mediating role between CD40L receptor levels and RA (Fig. 5C), is an unnamed metabolite identified via plasma metabolomics profiling that has not been studied in relevant experiments, or the meaning of which will be further explored in subsequent scientific work. The inclusion of X-24757 testing in upfront screening may help identify populations at high risk of RA and actively carry out relevant prevention work in the future.

Overall, the strength of this study is that MR analysis using multiple statistical methods exempted the results from confounding factors or other bias, and it is concluded that a genetic causal relationship between inflammatory factors and RA, and identified 3 mediators that play important roles. However, there are some limitations in this study. First, the GWAS data were obtained from the European database and the main participants were Europeans, considering the possible genetic variation among different populations, there may be some limitations in the promotion of this study. Second, the lack of sex-stratified GWAS analyses may obscure sex-specific genetic associations in RA, potentially limiting biological insights into its pronounced female predominance and compromising clinical applicability across genders. This limitation underscores the need for future studies to systematically investigate sex-differentiated genetic architectures to address epidemiological disparities in RA.

## 5. Conclusion

In conclusion, the present study explored the causal relationship between inflammatory cytokines and RA, as well as the effects of immune cells and metabolites as mediators. The results showed that there was a significant causal relationship between CD40L receptor levels and RA. All 3 mediators, CD14^+^CD16^−^ monocyte% monocyte, X-24757 levels, and IgD on IgD^+^CD38^−^ unsw mem, increase the risk of developing RA. This provides new evidence for future studies in the field of RA at the genetic level, and provides new ideas for research on inflammatory markers and potential therapeutic targets in RA. In the future, the discovered inflammatory factors and mediators can be applied to the comprehensive evaluation and prediction of RA clinical diagnosis and treatment process. Moreover, it is necessary to further explore and find new strategies for the prevention and treatment of RA targeting potential therapeutic targets.

## Acknowledgments

We would like to thank the FinnGen Consortium, GWAS Catalog. We would like to thank the anonymous reviewers for their constructive comments.

## Author contributions

**Conceptualization:** Tianyang Li, Jinpeng Wei, Chen Chen.

**Data curation:** Tianyang Li, Jinpeng Wei, Chen Chen.

**Formal analysis:** Tianyang Li, Jinpeng Wei.

**Investigation:** Tianyang Li, Jinpeng Wei.

**Methodology:** Tianyang Li, Jinpeng Wei.

**Project administration:** Hua Wu, Chen Chen.

**Resources:** Jinpeng Wei.

**Software:** Tianyang Li, Jinpeng Wei.

**Supervision:** Hua Wu, Chen Chen.

**Validation:** Hua Wu, Chen Chen.

**Visualization:** Hua Wu, Chen Chen.

**Writing – original draft:** Tianyang Li, Jinpeng Wei.

**Writing – review & editing:** Hua Wu, Chen Chen.

## Supplementary Material


