# Supplementary material for: Genetically predicted immune cells and metabolites mediate the causal relationship between inflammatory factors and rheumatoid arthritis
Source: Medicine (Baltimore). 2025 Sep 12;104(37):e44574. doi: 10.1097/MD.0000000000044574 (PMC12440540; doi:10.1097/MD.0000000000044574)
Supplement: Supplementary file 2 [file medi-104-e44574-s002.pdf]

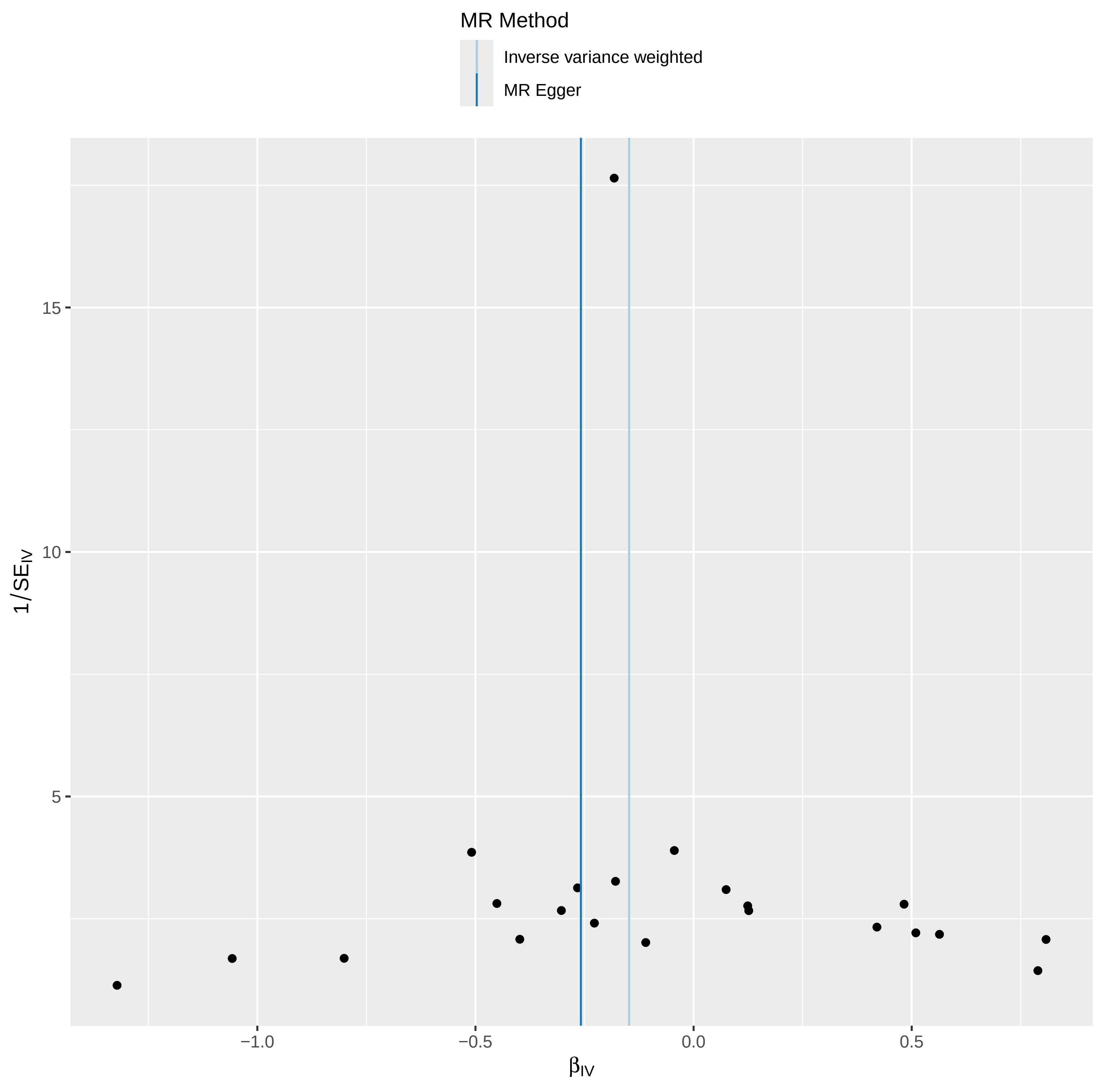
MR Method
Inverse variance weighted
MR Egger
15
10
5
1/SE_IV
−1.0
−0.5
0.0
0.5
β_IV

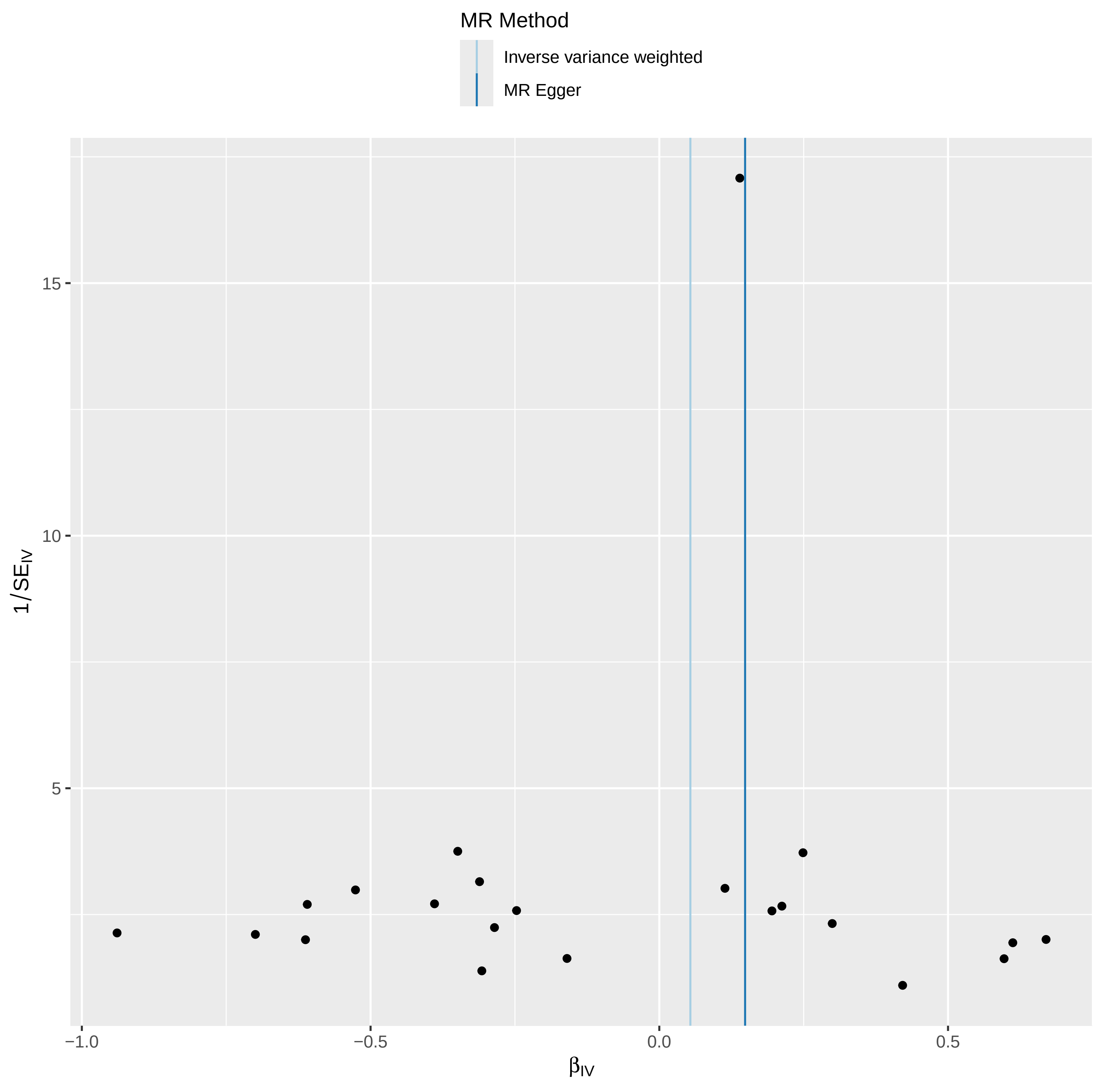
MR Method
Inverse variance weighted
MR Egger
15
10
5
1/SE_IV
−1.0
−0.5
0.0
0.5
β_IV

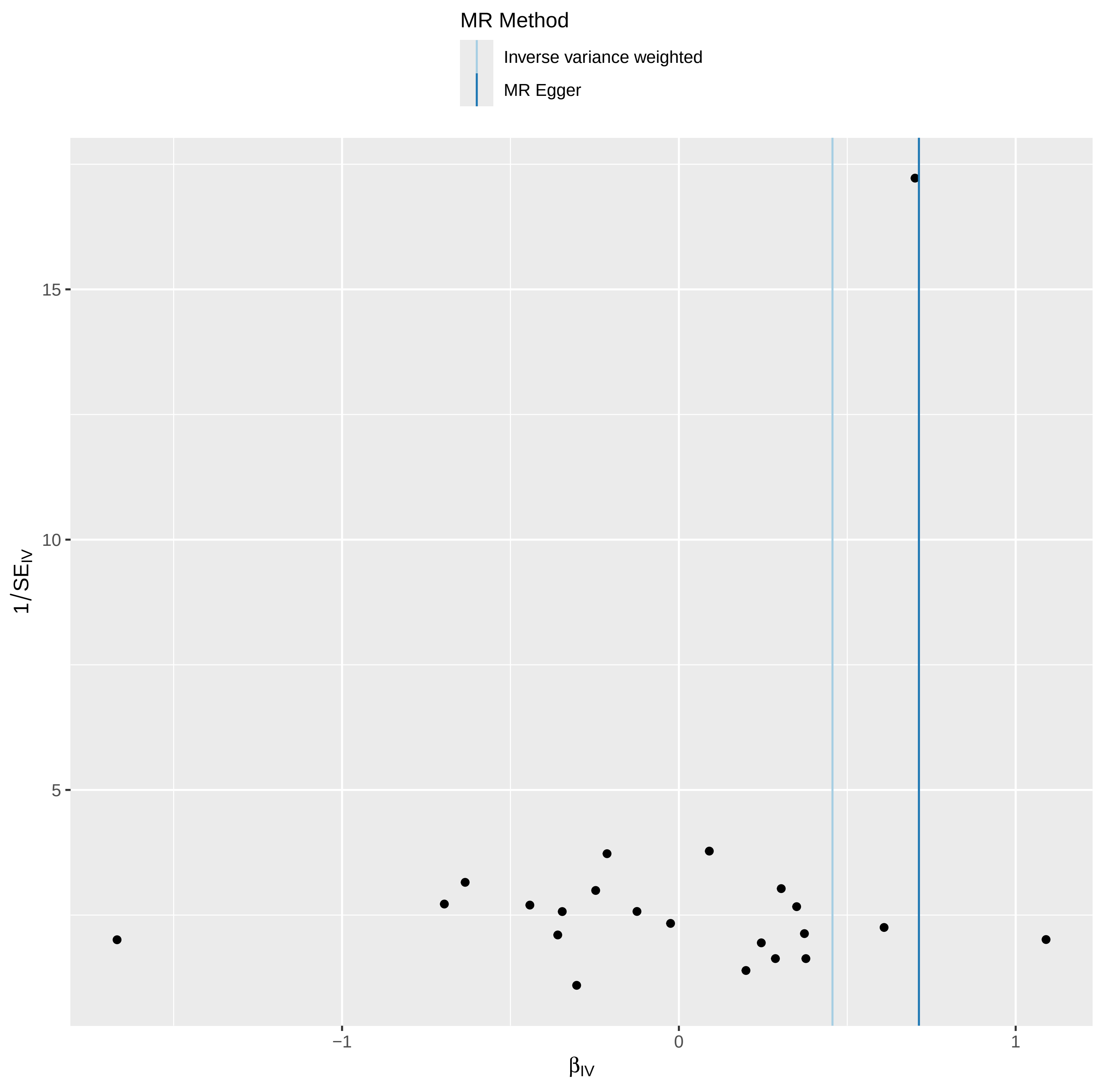
MR Method
Inverse variance weighted
MR Egger
15
10
5
1/SE_IV
−1
0
1
β_IV

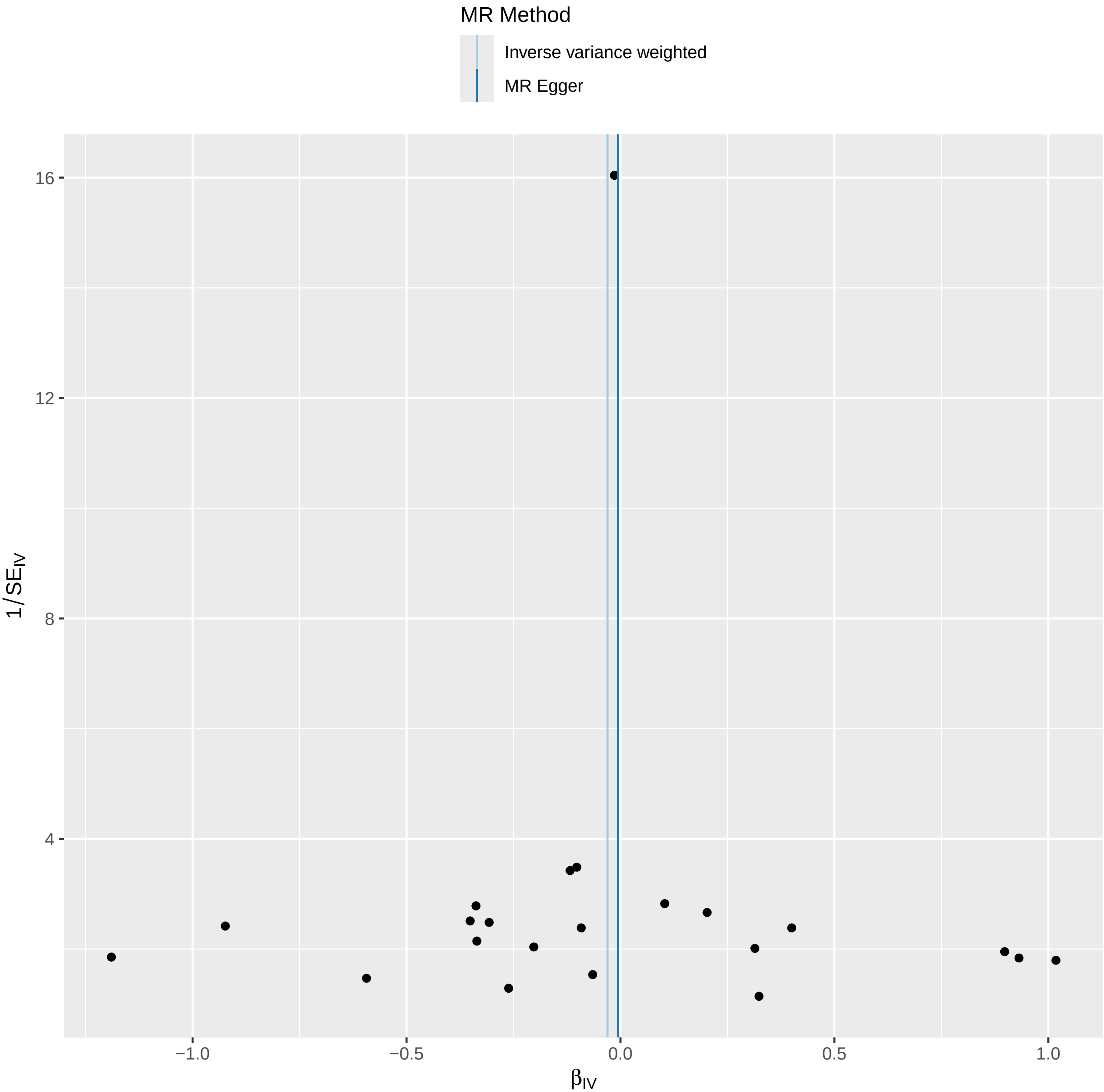
MR Method
Inverse variance weighted
MR Egger
16
12
8
4
1/SE_IV
−1.0
−0.5
0.0
0.5
1.0
β_IV

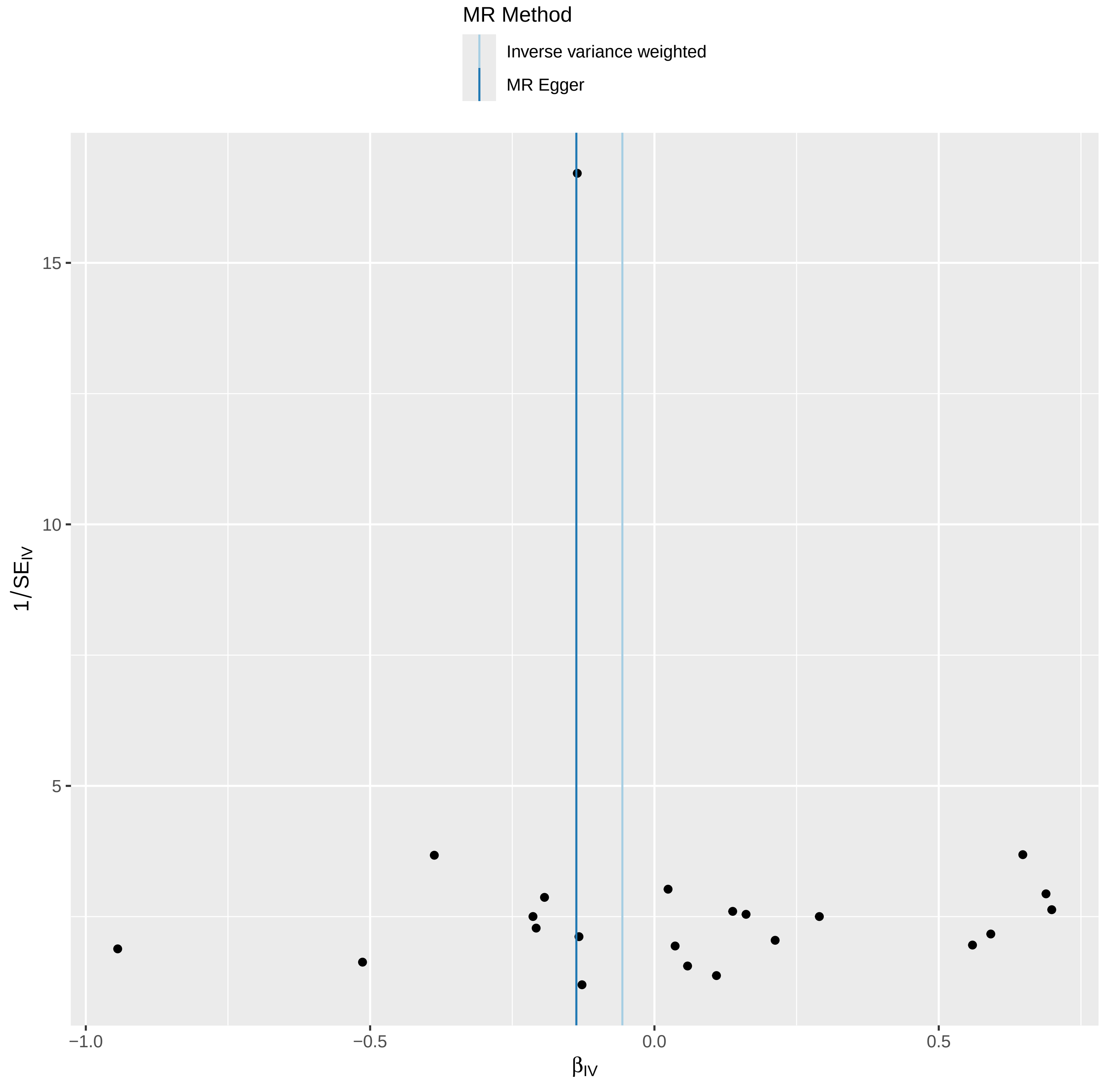

MR Method
Inverse variance weighted
MR Egger
15
10
5
1/SE_IV
−1.0
−0.5
0.0
0.5
β_IV

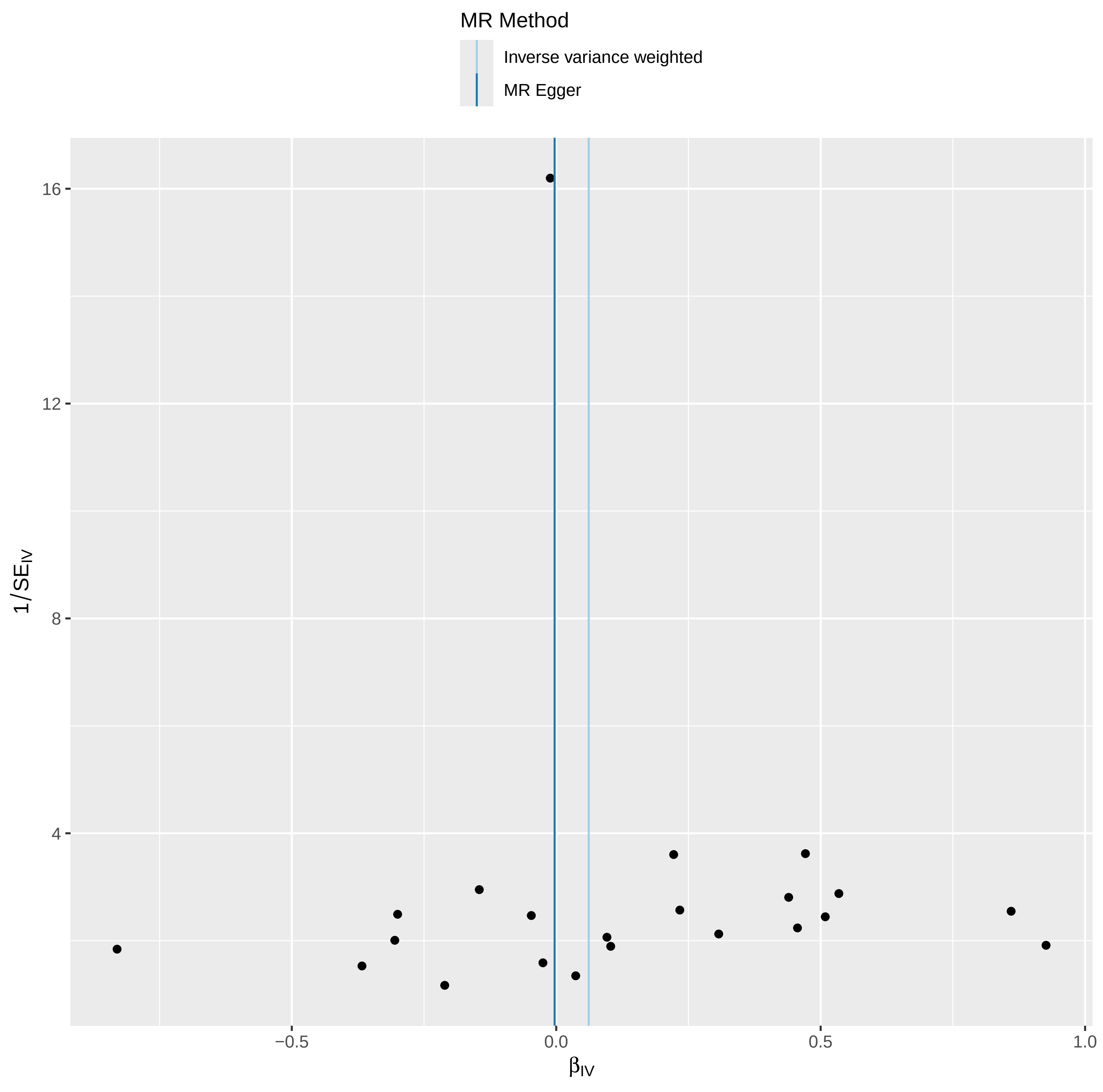
MR Method
Inverse variance weighted
MR Egger
16
12
8
4
$1/SE_{IV}$
−0.5
0.0
0.5
1.0
β_{IV}

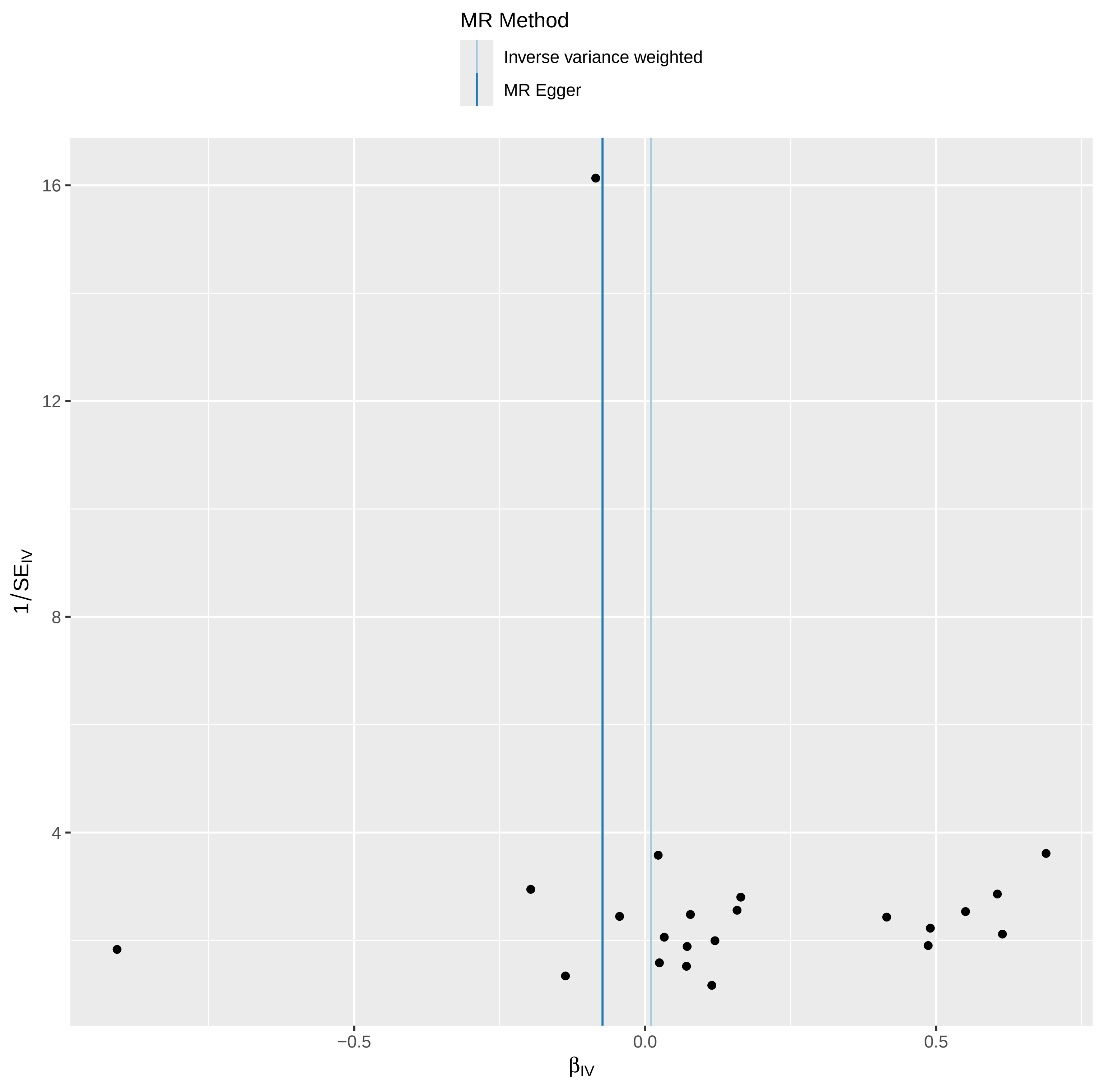

MR Method
Inverse variance weighted
MR Egger
16
12
8
4
1/SE$_{IV}$
−0.5
0.0
0.5
β$_{IV}$

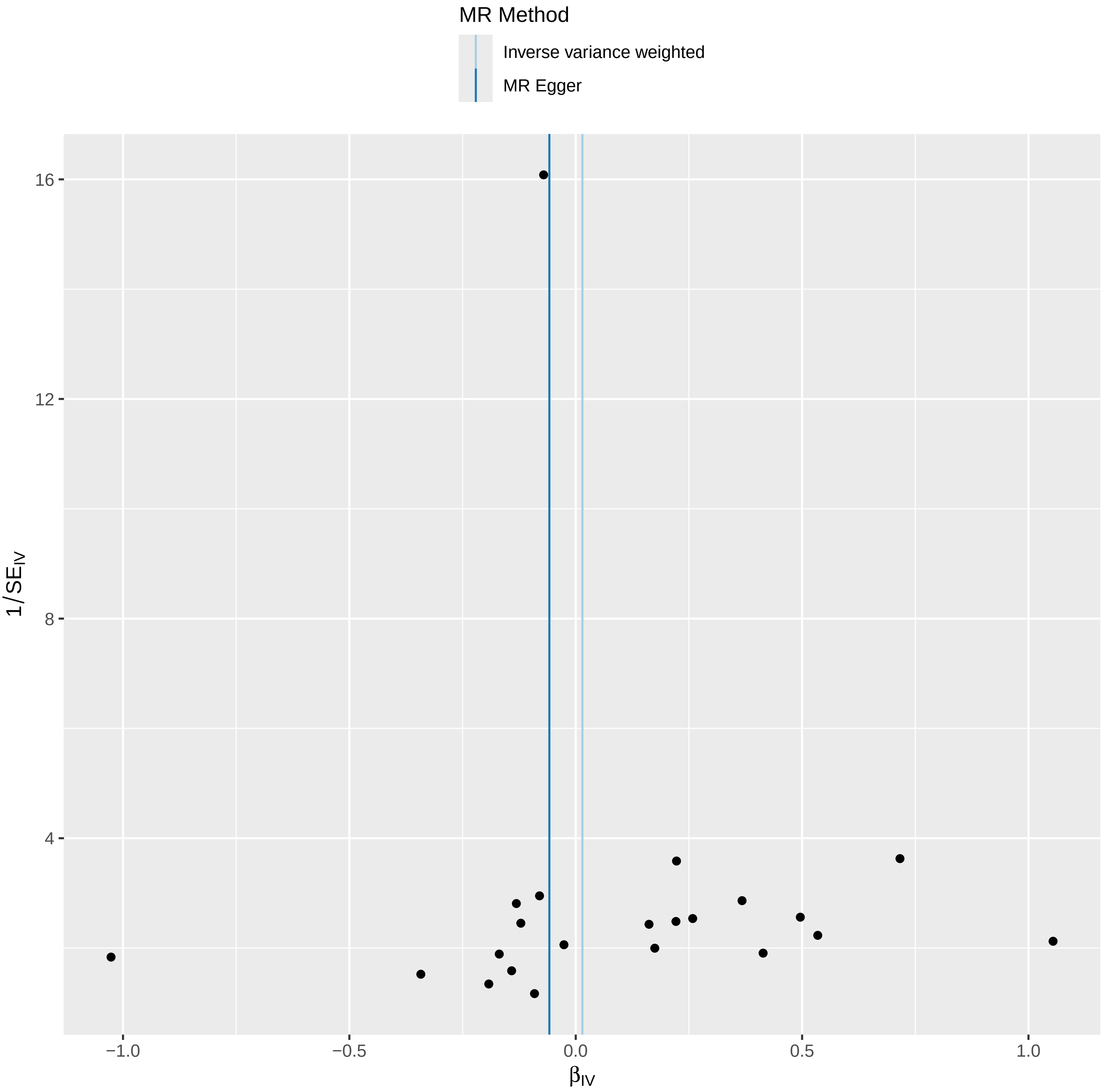

MR Method
Inverse variance weighted
MR Egger
16
12
8
4
1/SE$_{IV}$
−1.0
−0.5
0.0
0.5
1.0
β_{IV}

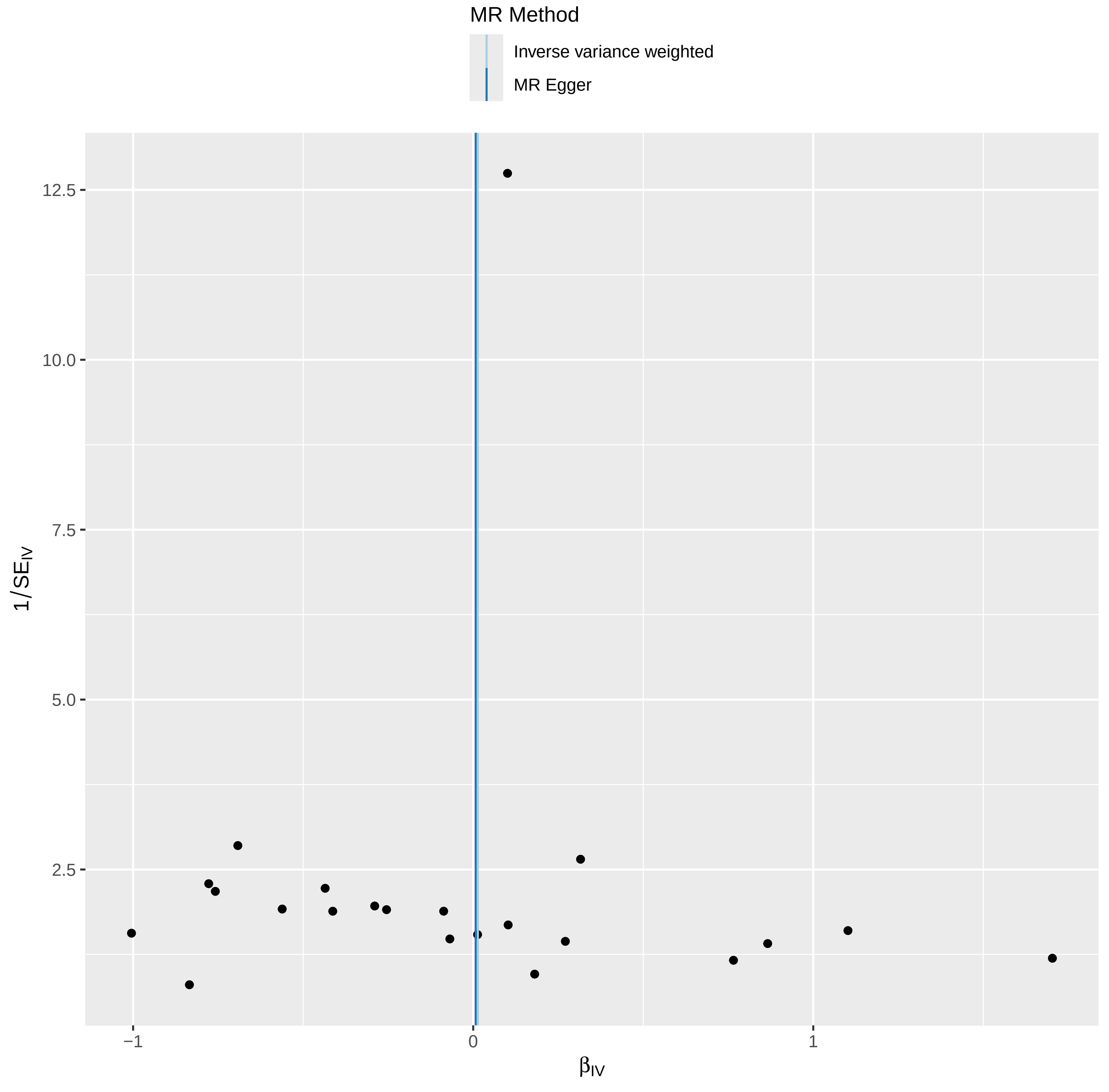
MR Method
Inverse variance weighted
MR Egger
12.5
10.0
7.5
5.0
2.5
$1/SE_{IV}$
−1
0
1
β_{IV}

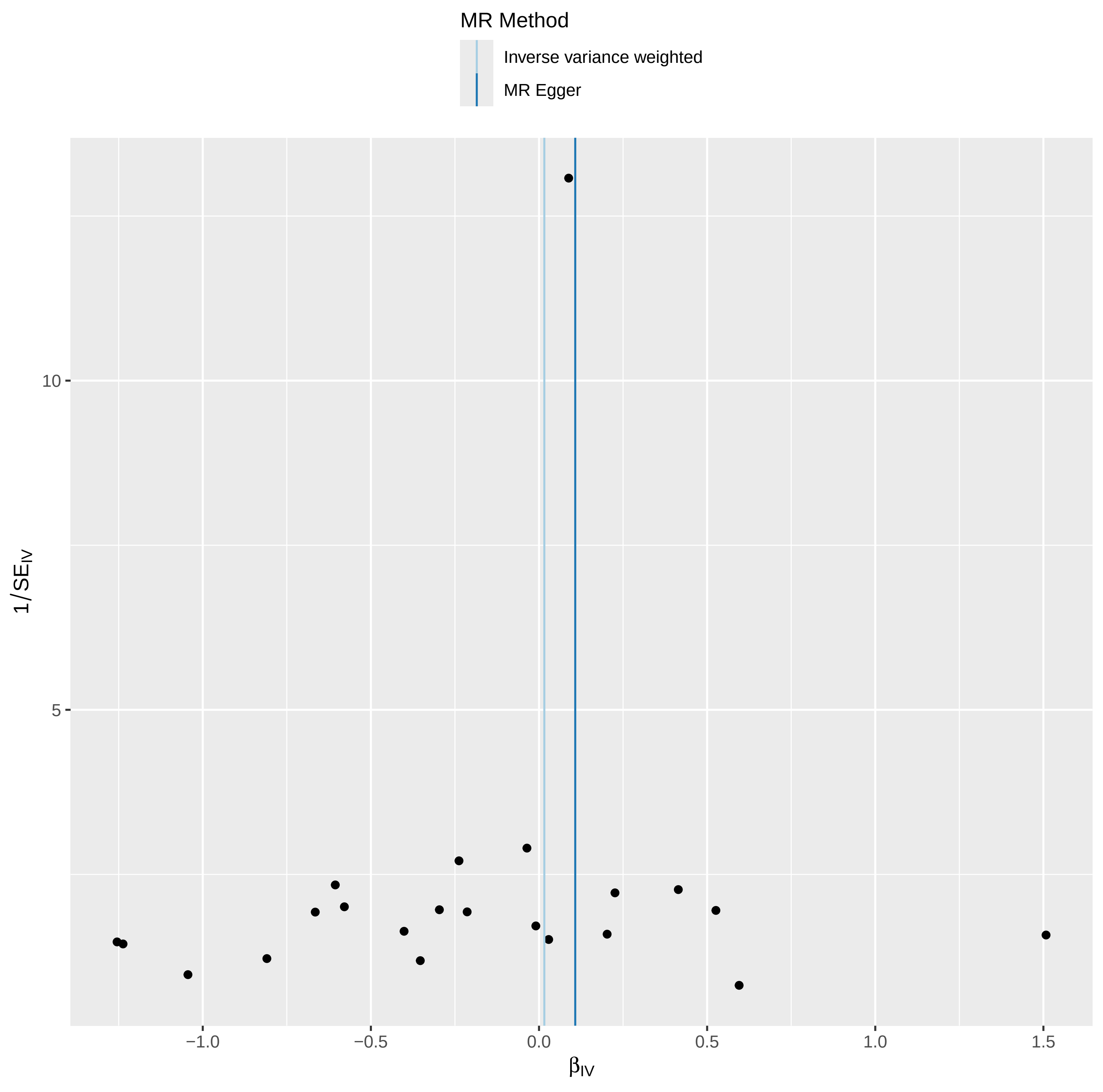

MR Method
Inverse variance weighted
MR Egger
10
5
$1/SE_{IV}$
−1.0
−0.5
0.0
0.5
1.0
1.5
β_{IV}

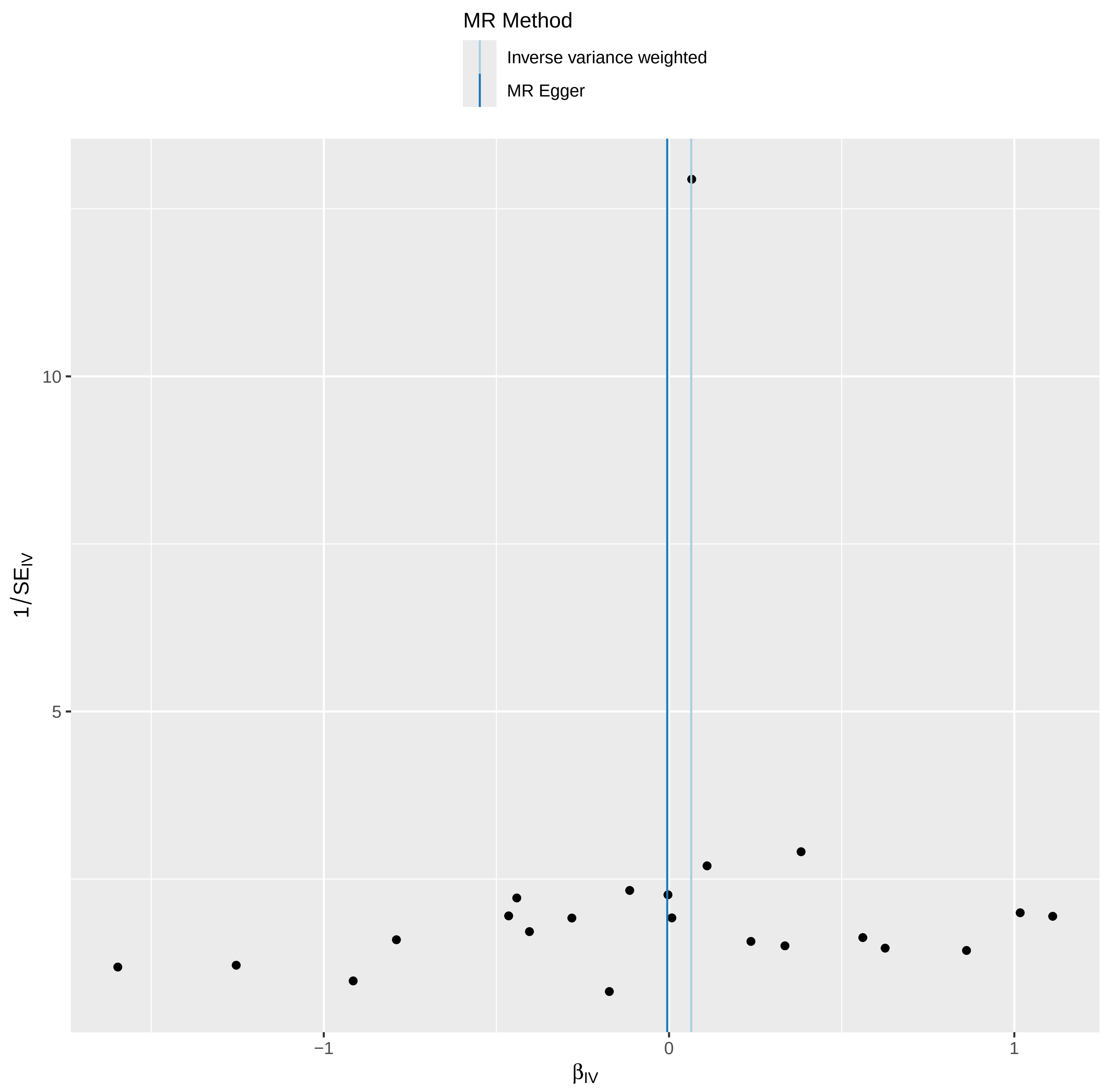
MR Method
Inverse variance weighted
MR Egger
10
5
1/SE$_{IV}$
−1
0
1
β_{IV}

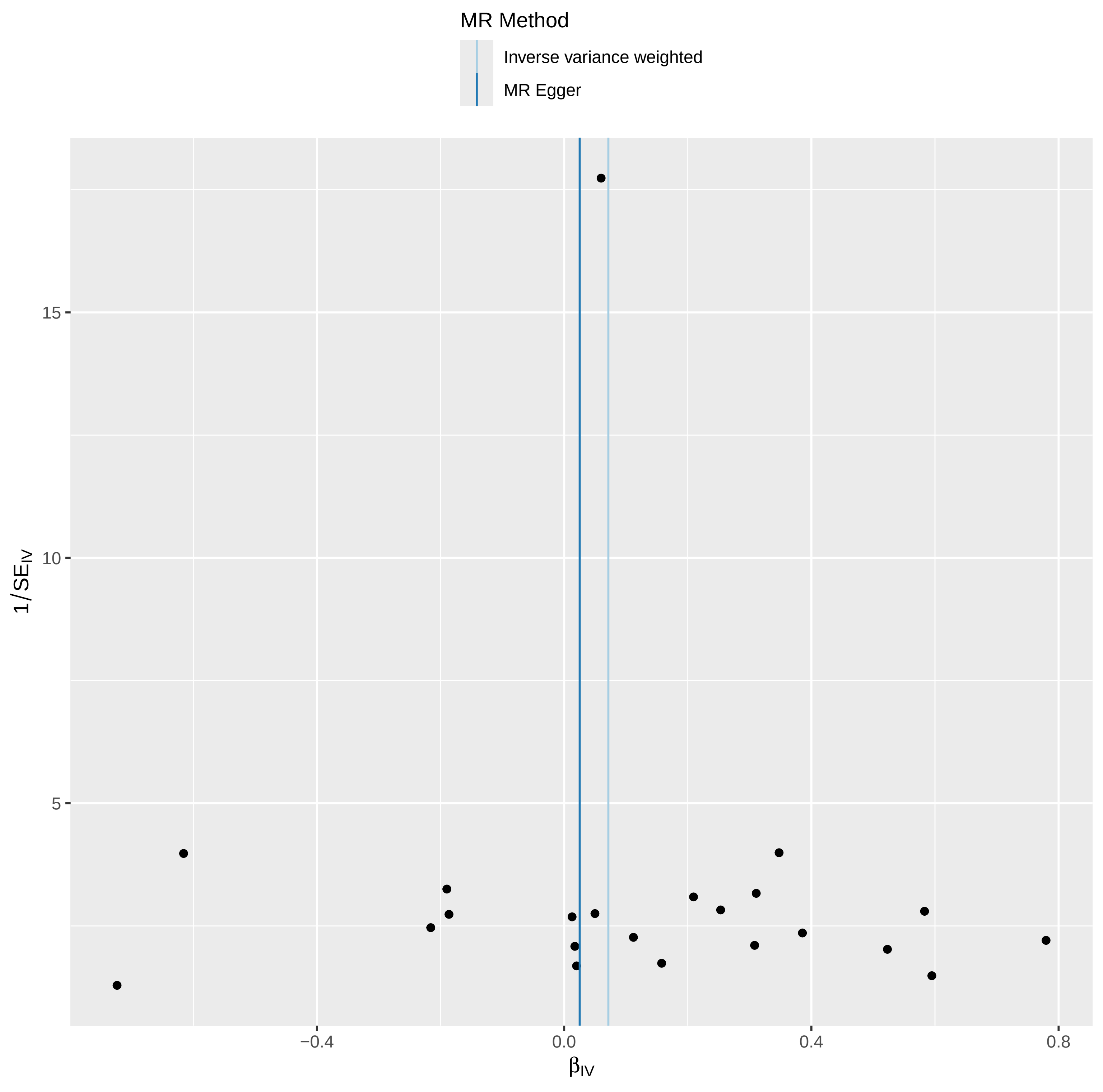
MR Method
Inverse variance weighted
MR Egger
15
10
5
1/SE$_{IV}$
−0.4
0.0
0.4
0.8
β_{IV}

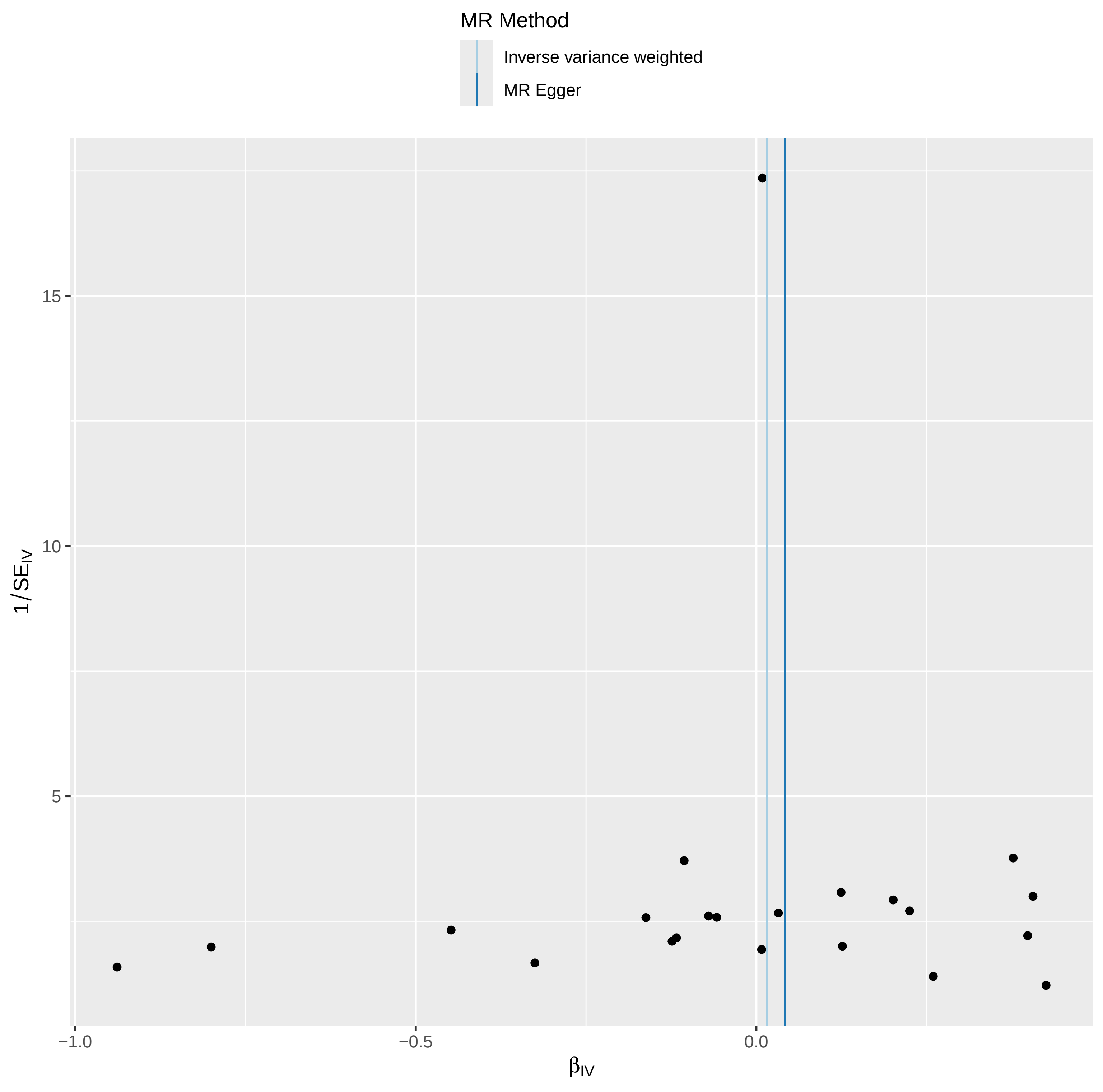
MR Method
Inverse variance weighted
MR Egger
15
10
5
$1/SE_{IV}$
−1.0
−0.5
0.0
β_{IV}

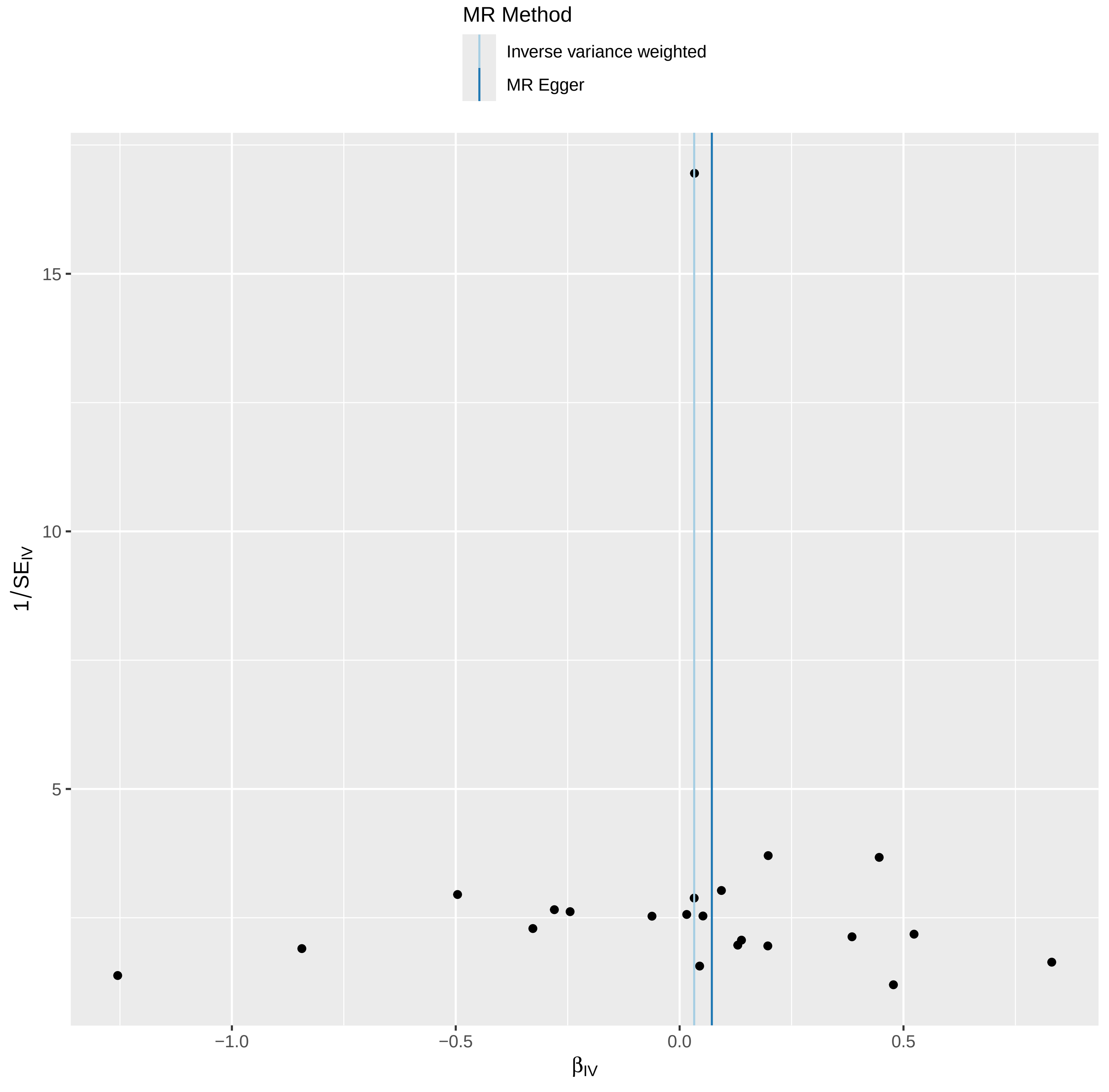
MR Method
Inverse variance weighted
MR Egger
15
10
5
1/SE$_{IV}$
−1.0
−0.5
0.0
0.5
β$_{IV}$

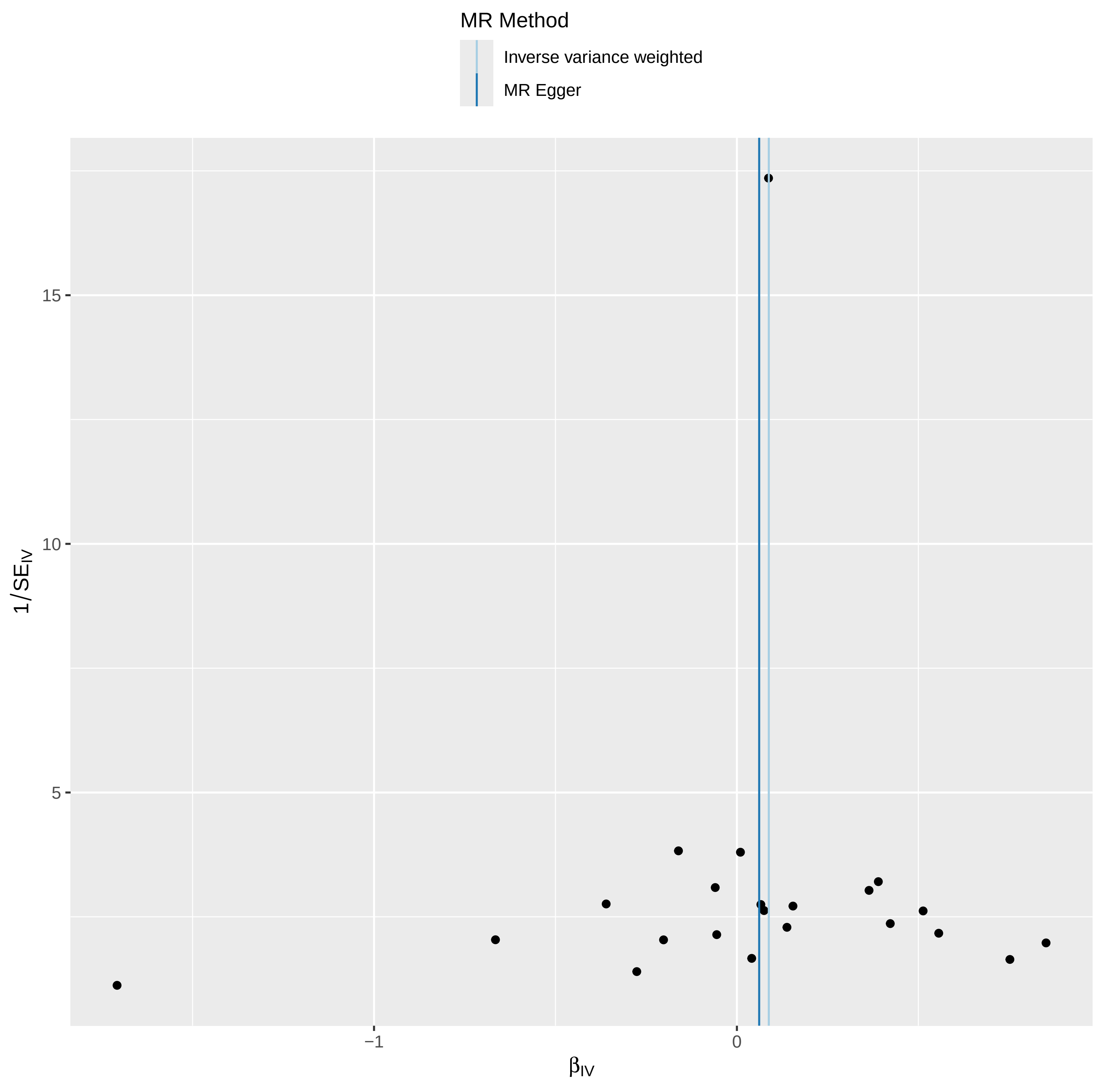
MR Method
Inverse variance weighted
MR Egger
15
10
5
1/SE_IV
−1
0
β_IV

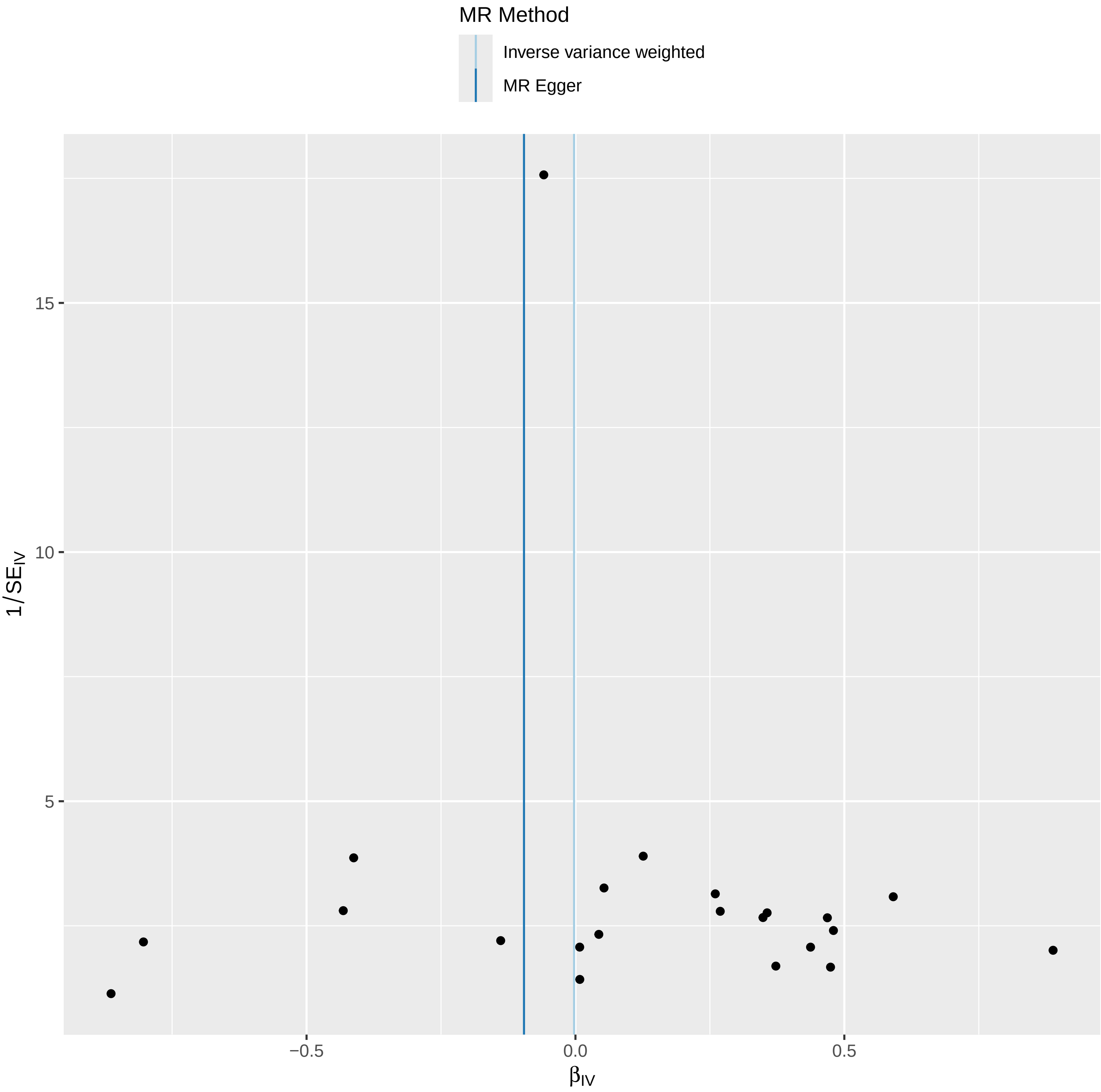

MR Method
Inverse variance weighted
MR Egger
15
10
5
−0.5
0.0
0.5
$1/SE_{IV}$
β_{IV}

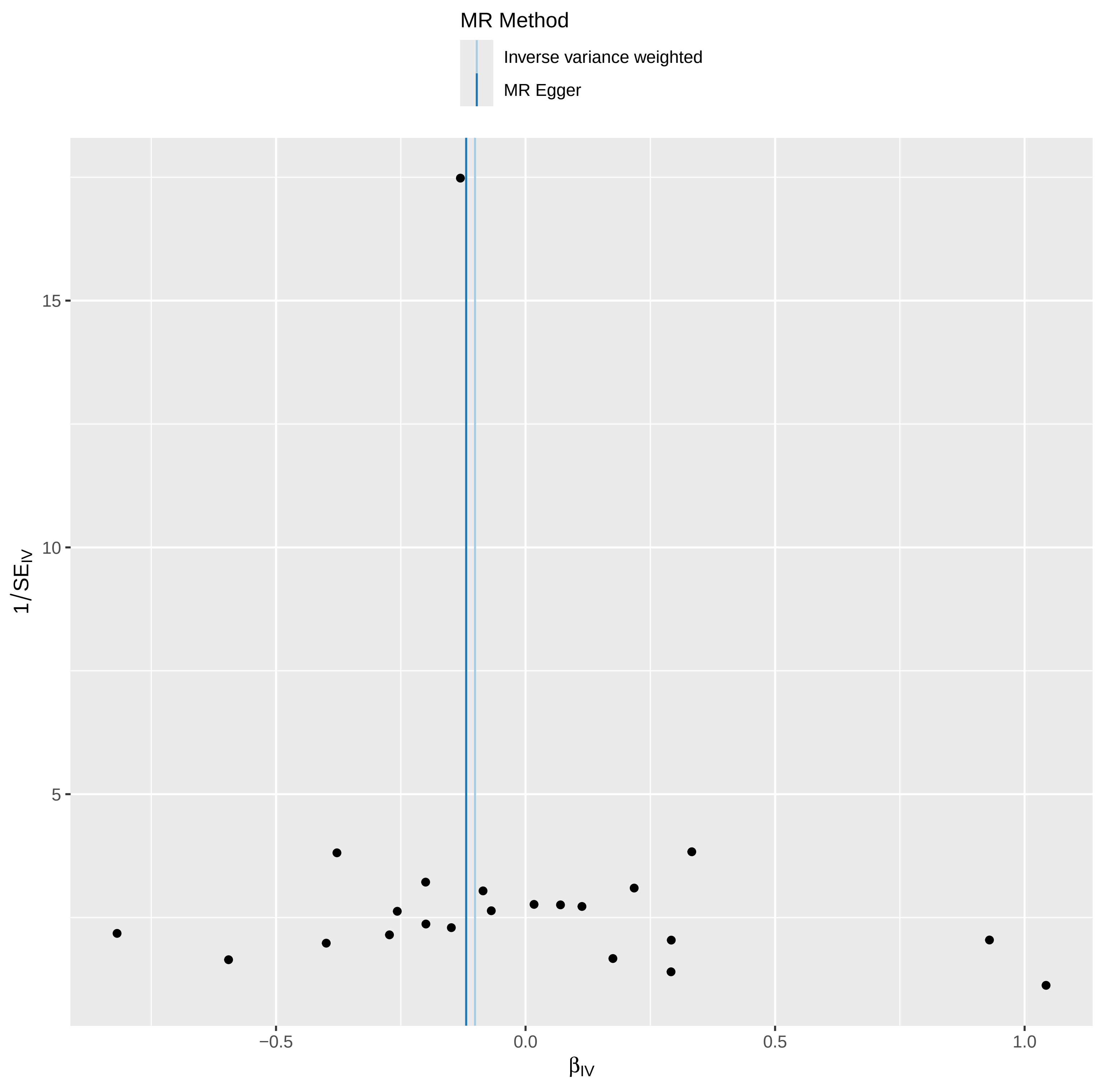
MR Method
Inverse variance weighted
MR Egger
15
10
5
1/SE_IV
−0.5
0.0
0.5
1.0
β_IV

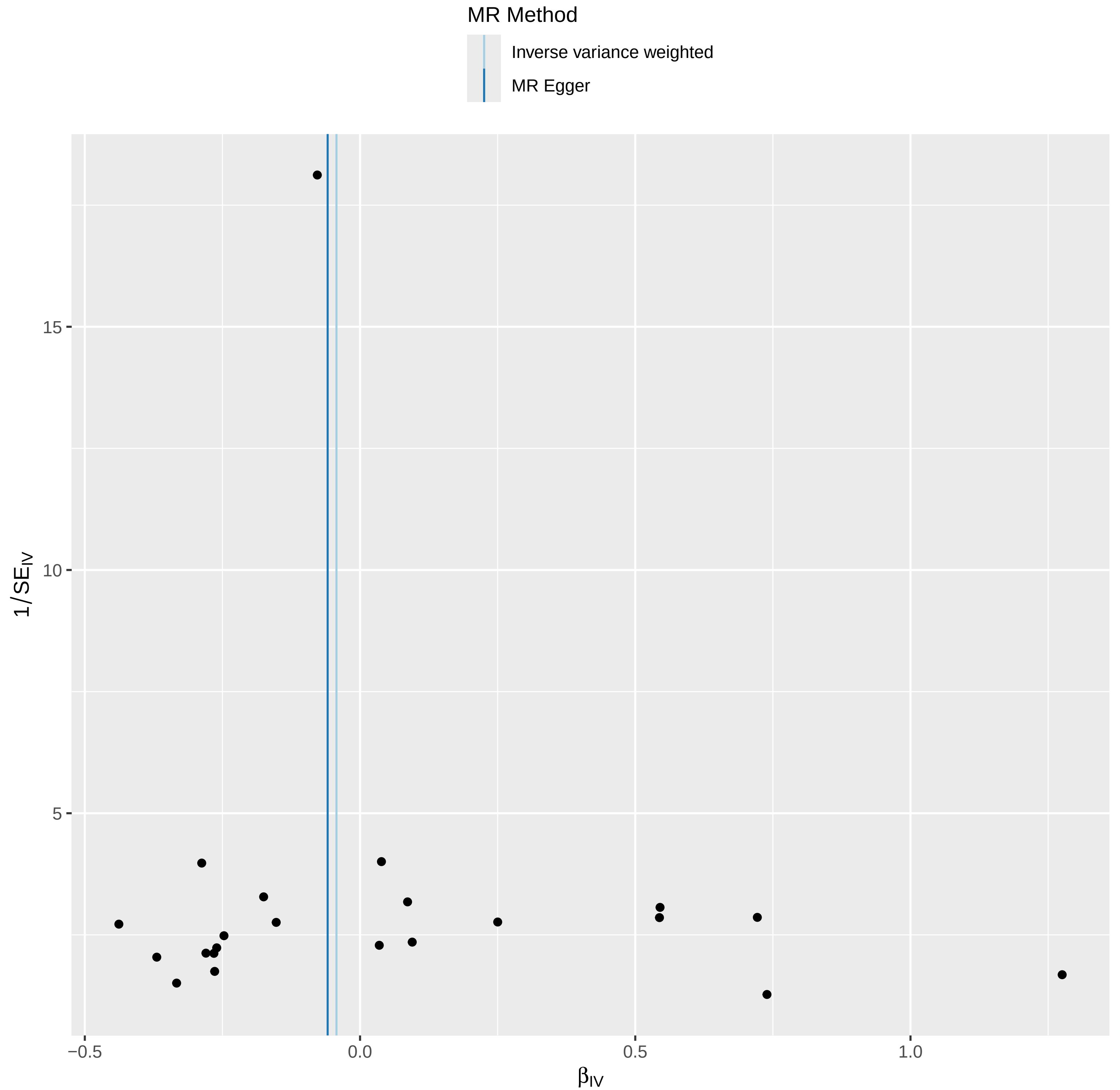

MR Method
Inverse variance weighted
MR Egger
15
10
5
1/SE_IV
−0.5
0.0
0.5
1.0
β_IV

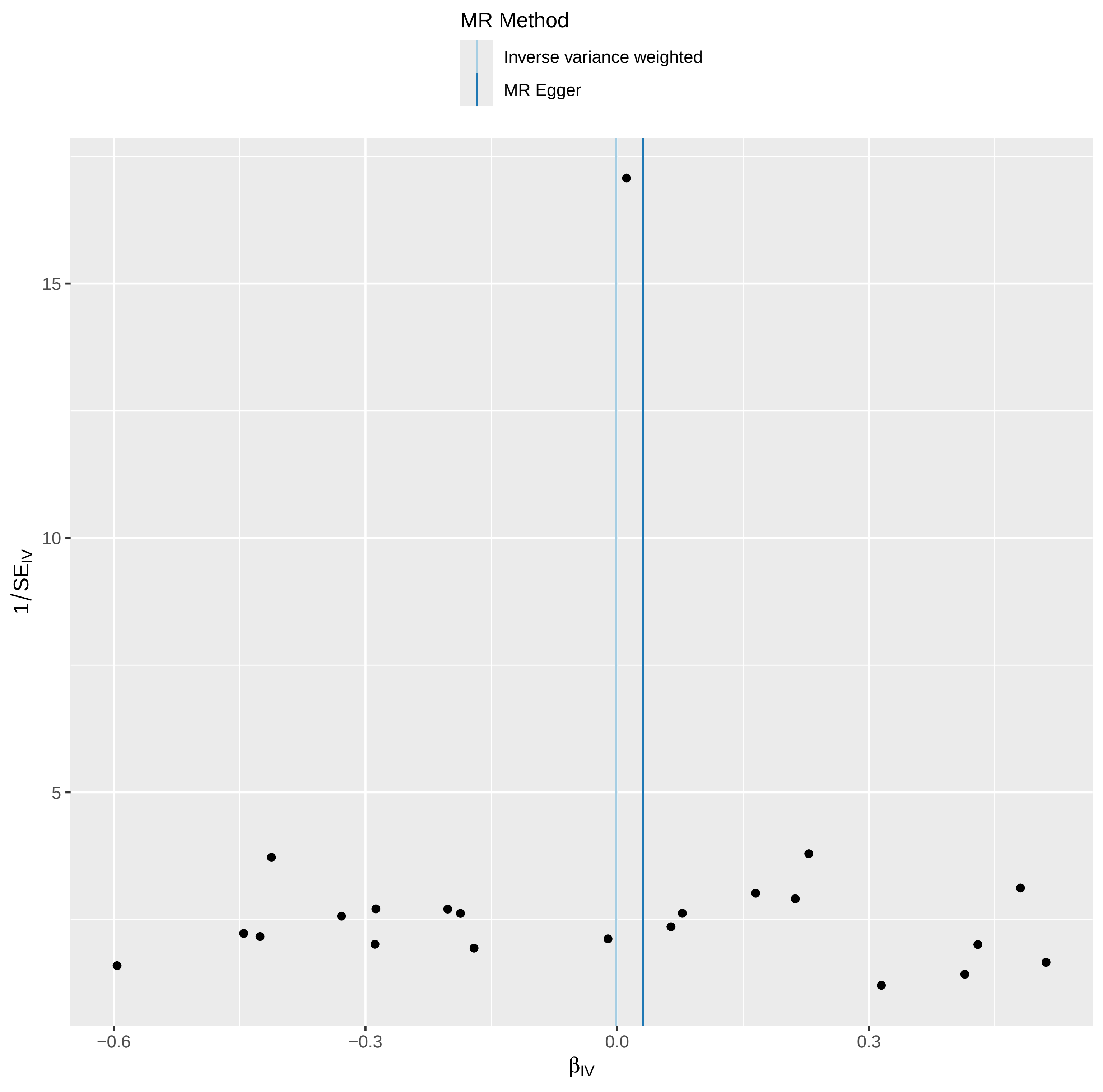
MR Method
Inverse variance weighted
MR Egger
15
10
5
1/SE_IV
−0.6
−0.3
0.0
0.3
β_IV

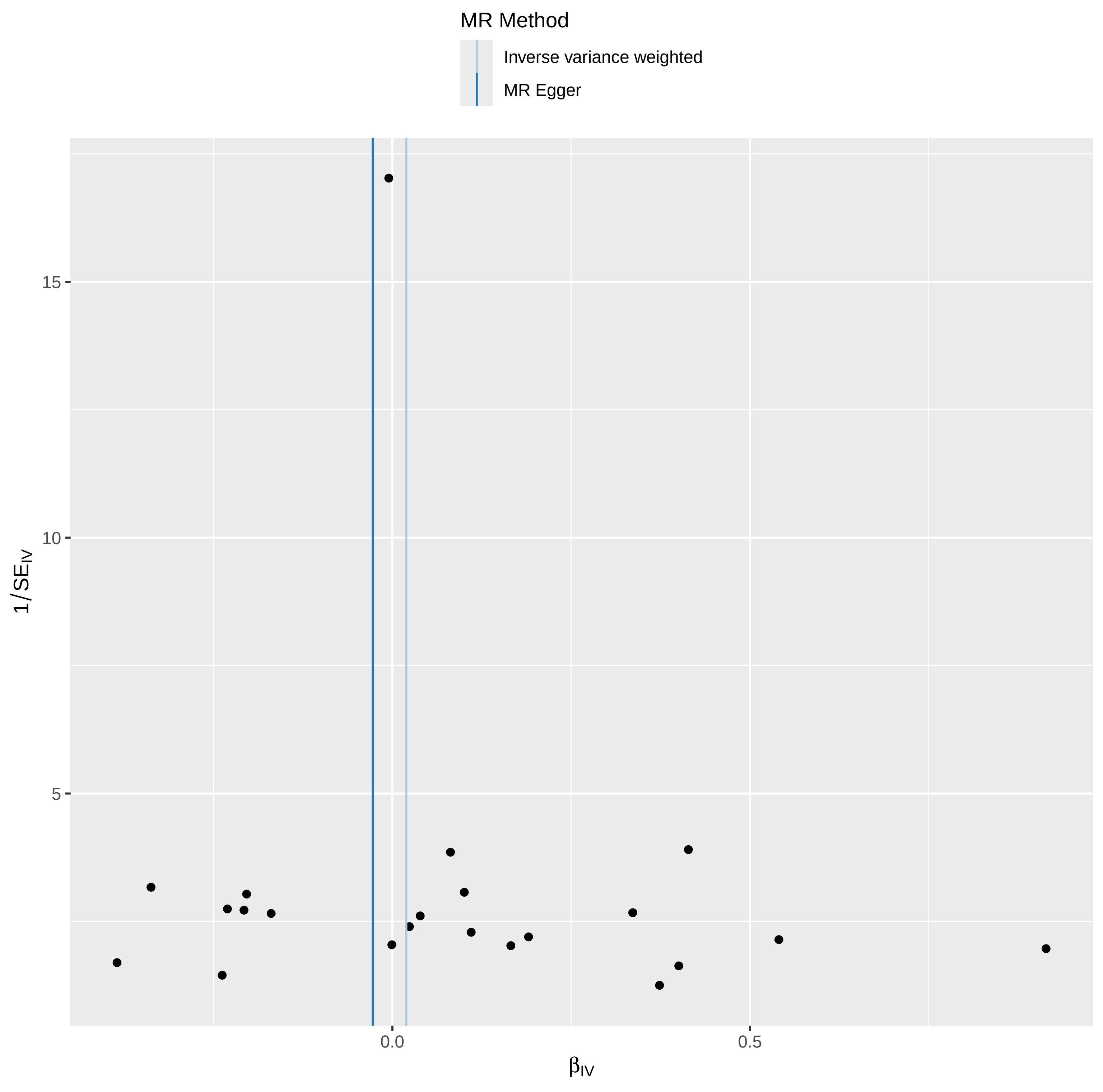
MR Method
Inverse variance weighted
MR Egger
15
10
5
1/SEIV
0.0
0.5
βIV

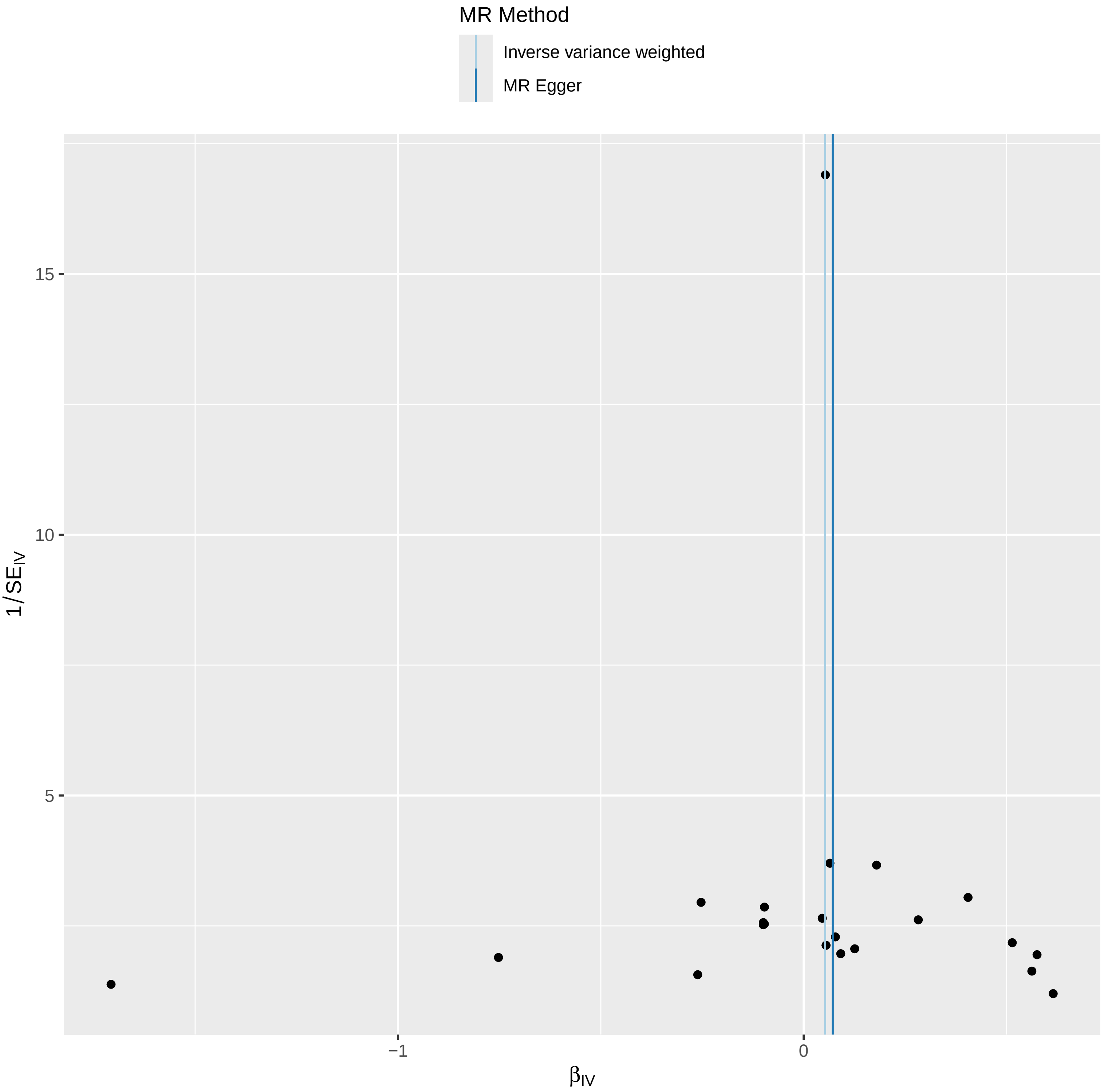

MR Method
Inverse variance weighted
MR Egger
15
10
5
1/SE_IV
−1
0
β_IV

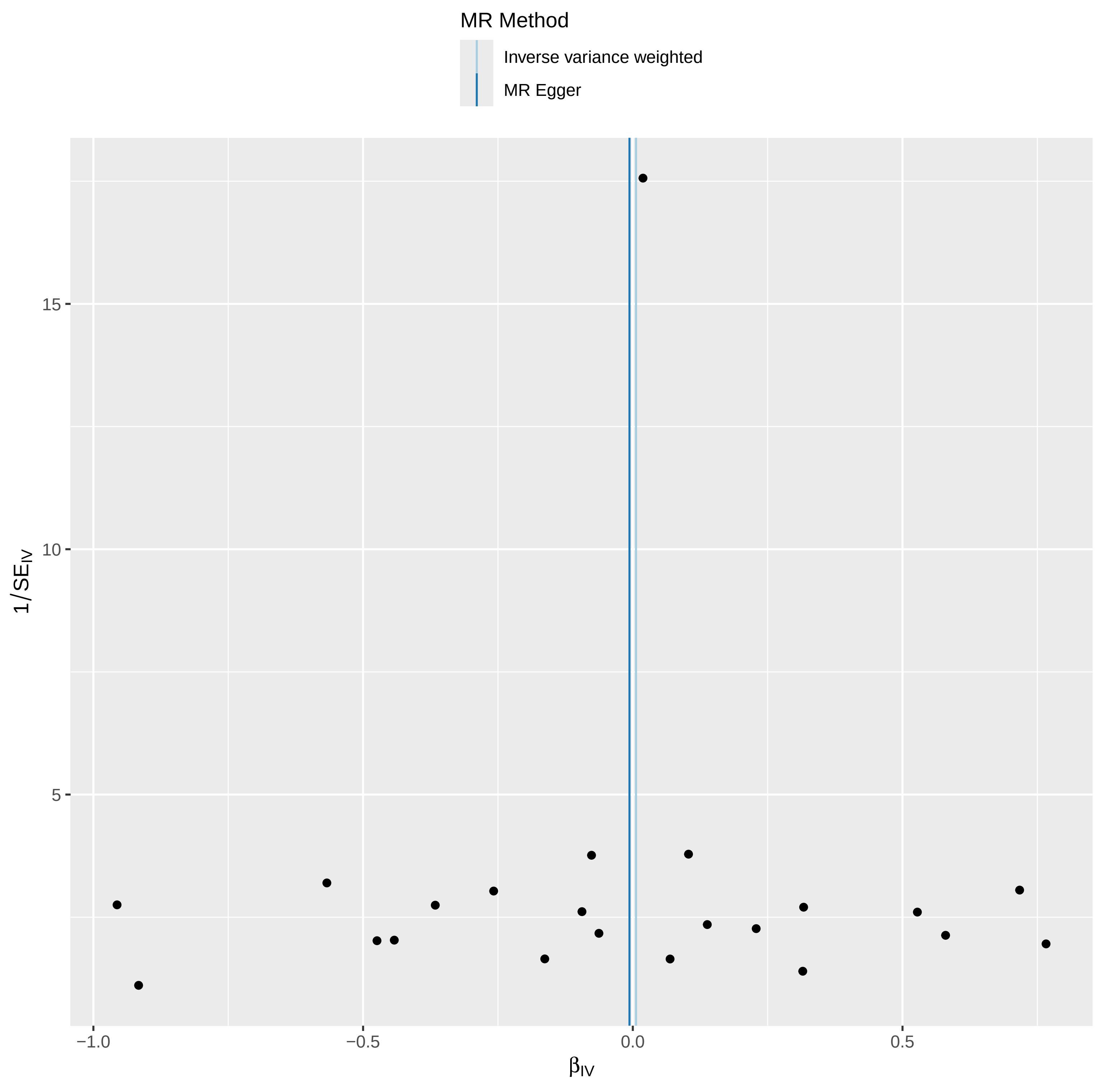
MR Method
Inverse variance weighted
MR Egger
15
10
5
1/SE$_{IV}$
−1.0
−0.5
0.0
0.5
β$_{IV}$

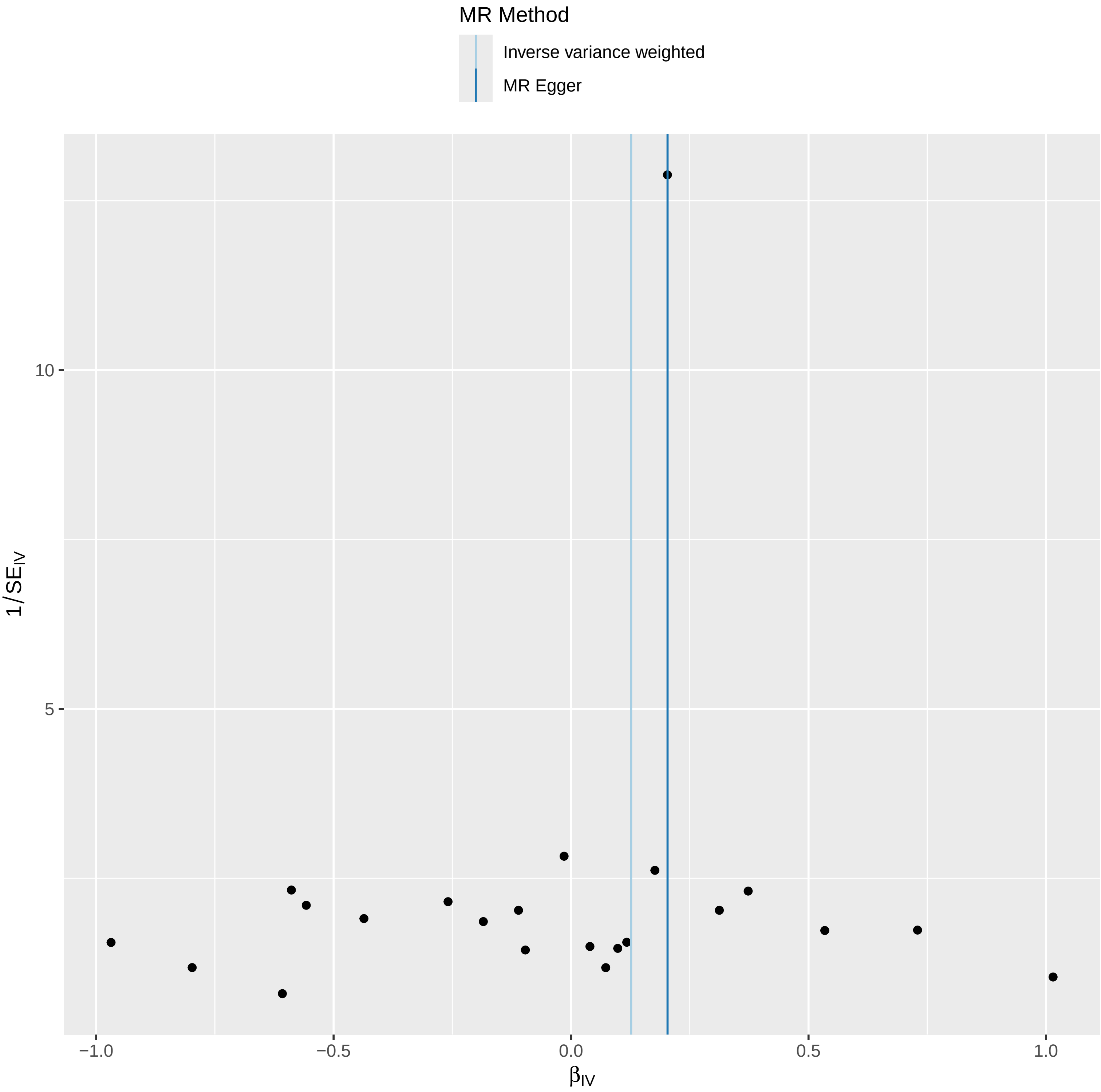
MR Method
Inverse variance weighted
MR Egger
10
5
1/SE_IV
−1.0
−0.5
0.0
0.5
1.0
β_IV

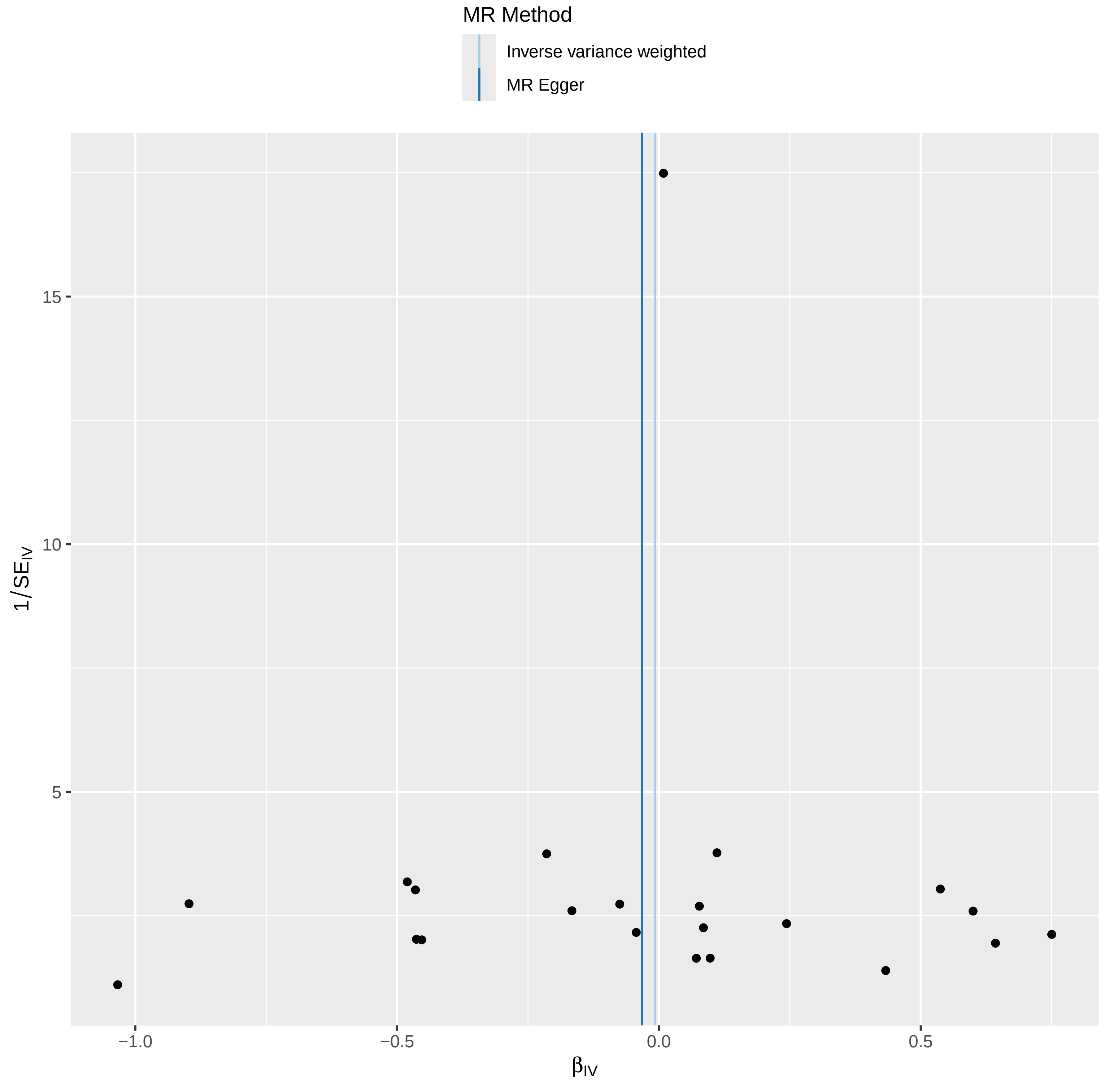
MR Method
Inverse variance weighted
MR Egger
15
10
5
$1/SE_{IV}$
−1.0
−0.5
0.0
0.5
β_{IV}

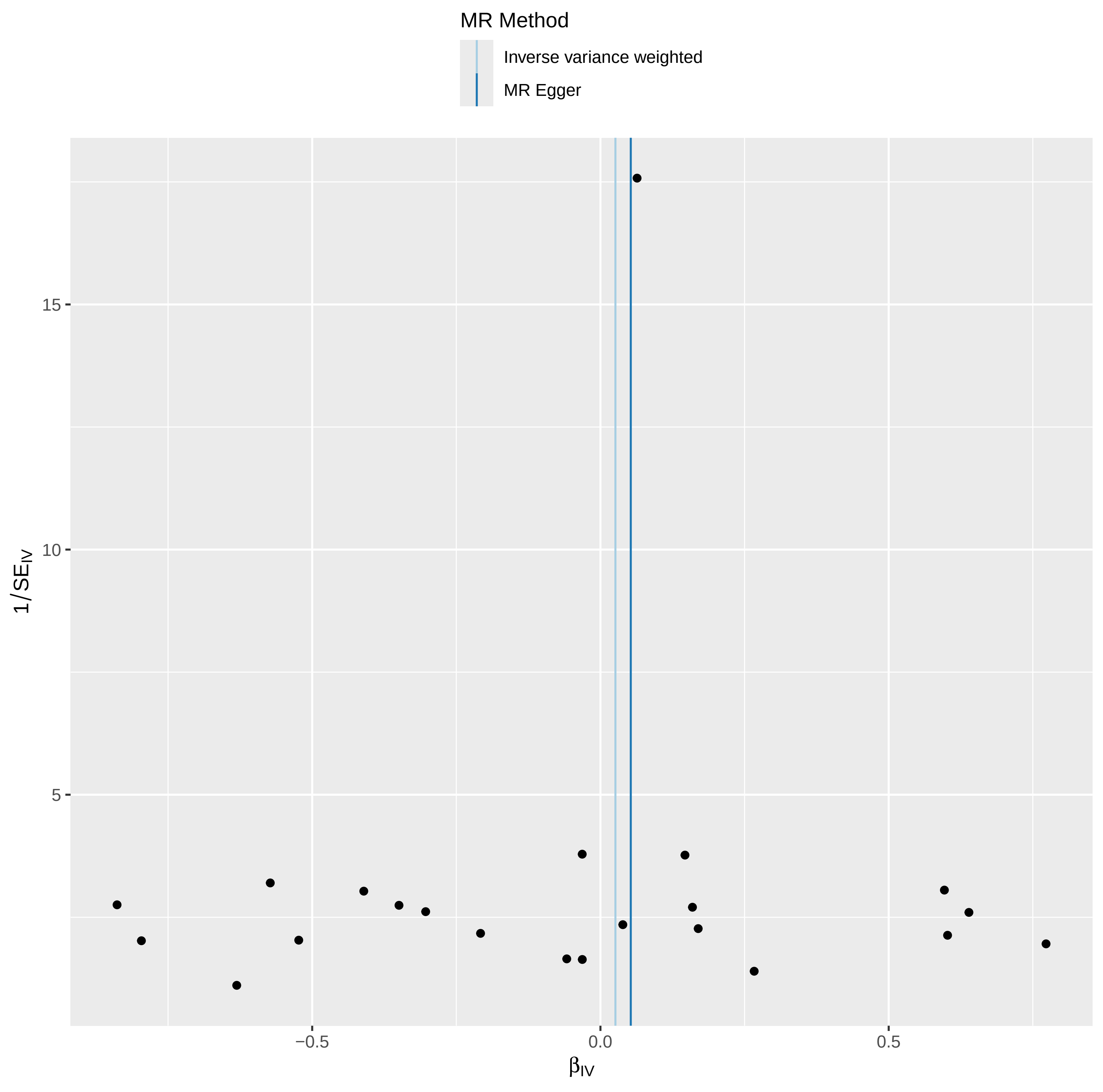
MR Method
Inverse variance weighted
MR Egger
15
10
5
−0.5
0.0
0.5
1/SE$_{IV}$
β$_{IV}$

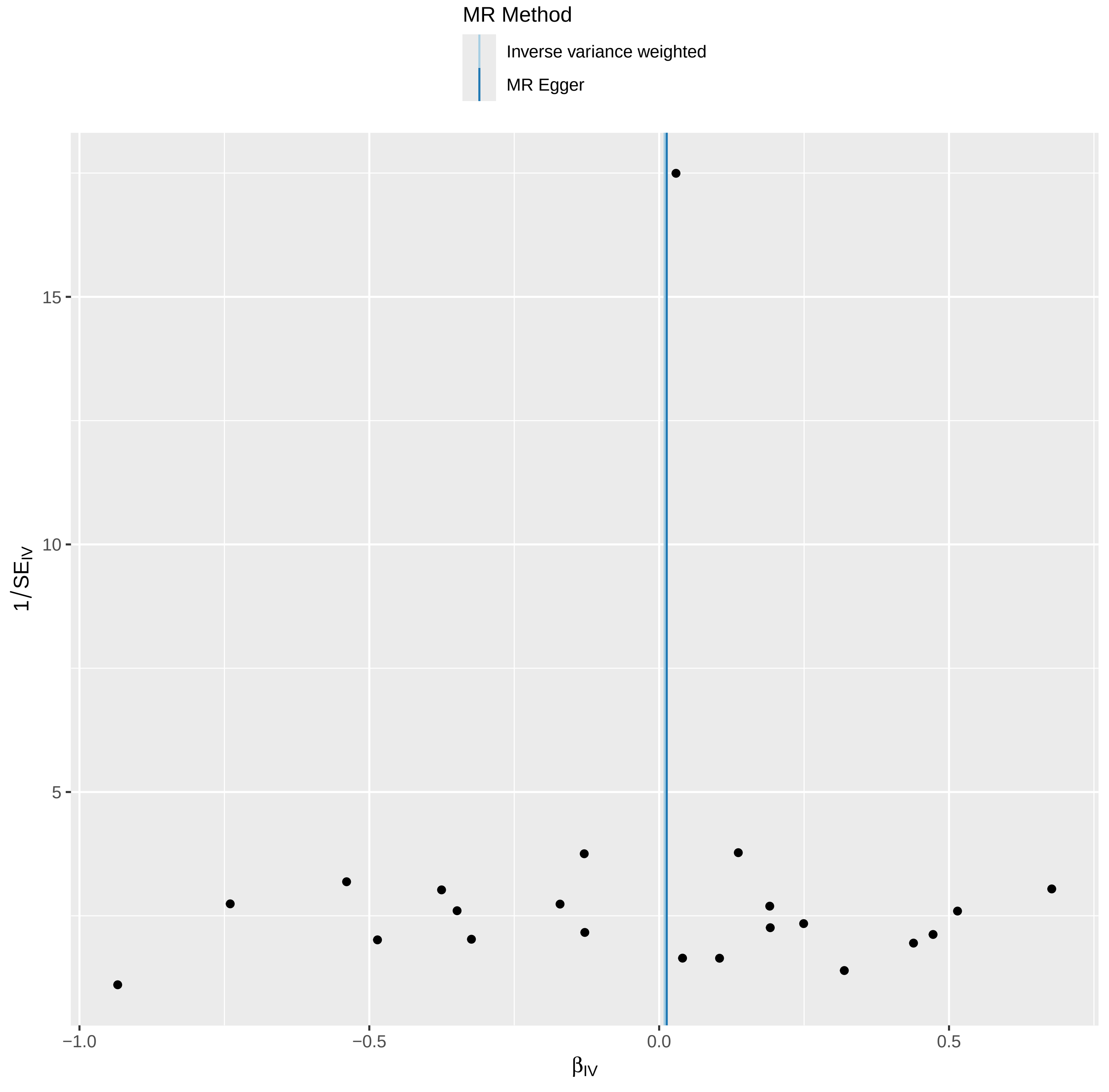
MR Method
Inverse variance weighted
MR Egger
15
10
5
1/SE$_{IV}$
−1.0
−0.5
0.0
0.5
β$_{IV}$

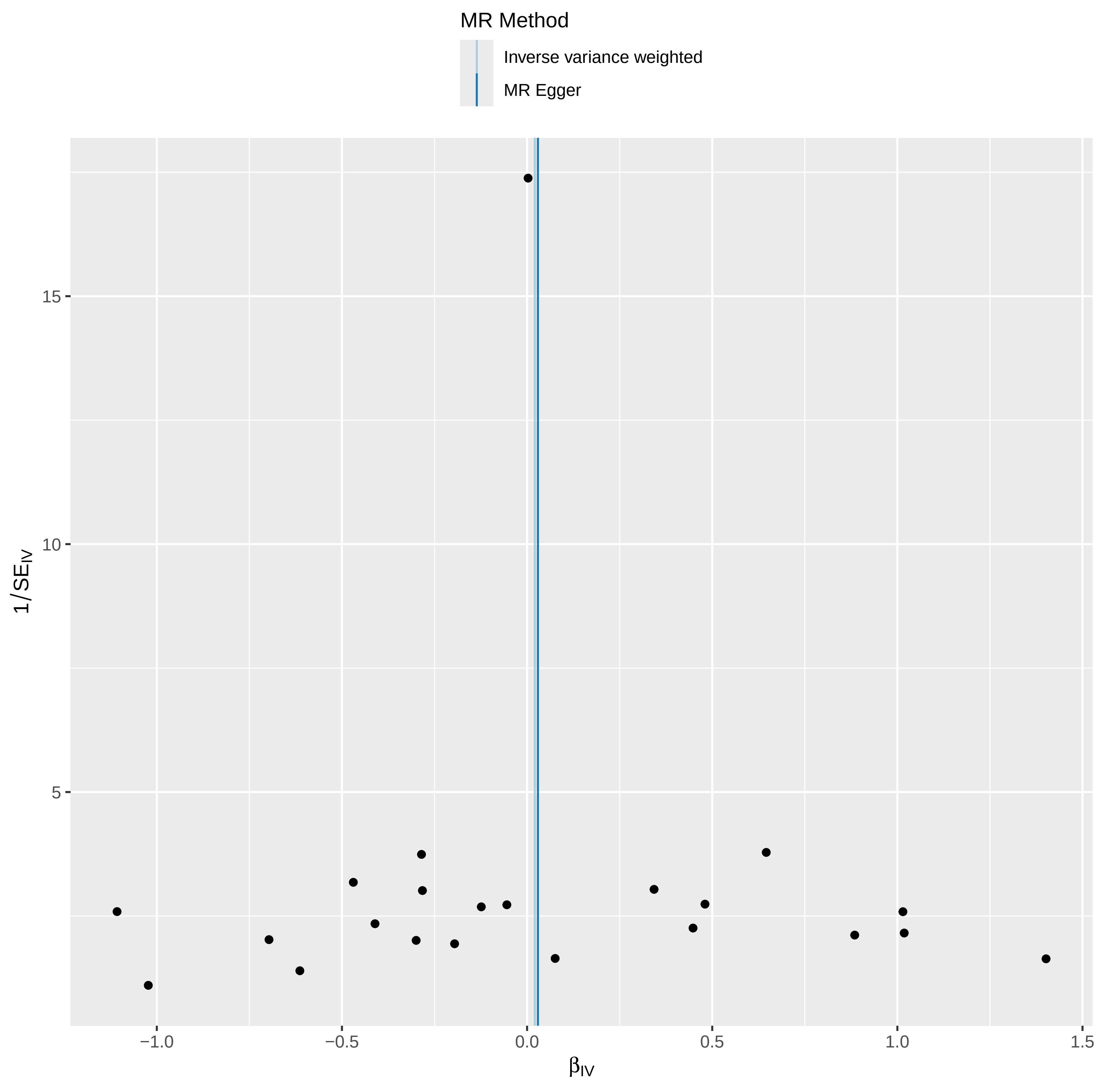

MR Method
Inverse variance weighted
MR Egger
15
10
5
1/SE$_{IV}$
−1.0
−0.5
0.0
0.5
1.0
1.5
β$_{IV}$

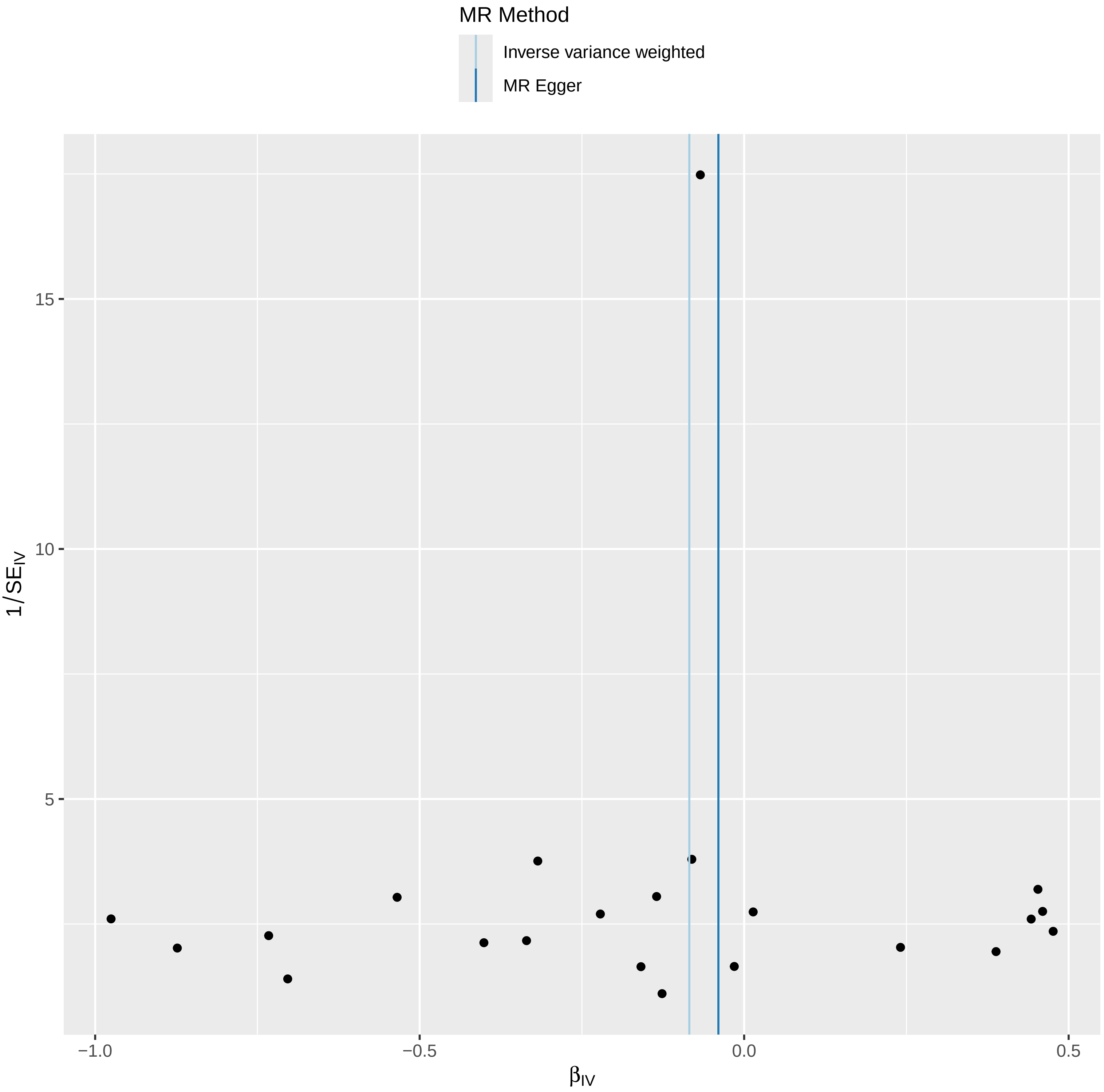
MR Method
Inverse variance weighted
MR Egger
1/SE$_{IV}$
β_{IV}
15
10
5
−1.0
−0.5
0.0
0.5

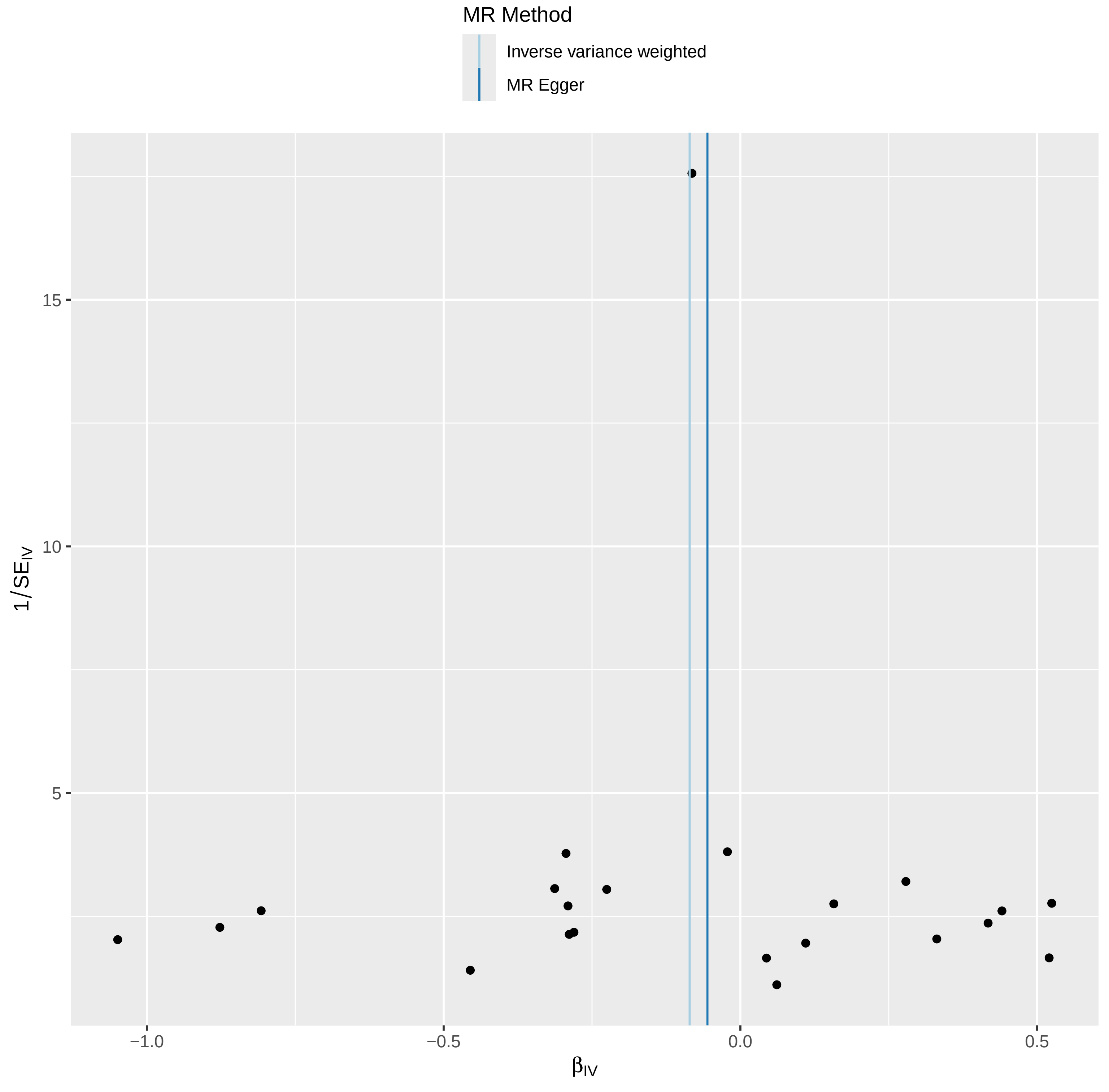

MR Method
Inverse variance weighted
MR Egger
15
10
5
$1/SE_{IV}$
−1.0
−0.5
0.0
0.5
β_{IV}

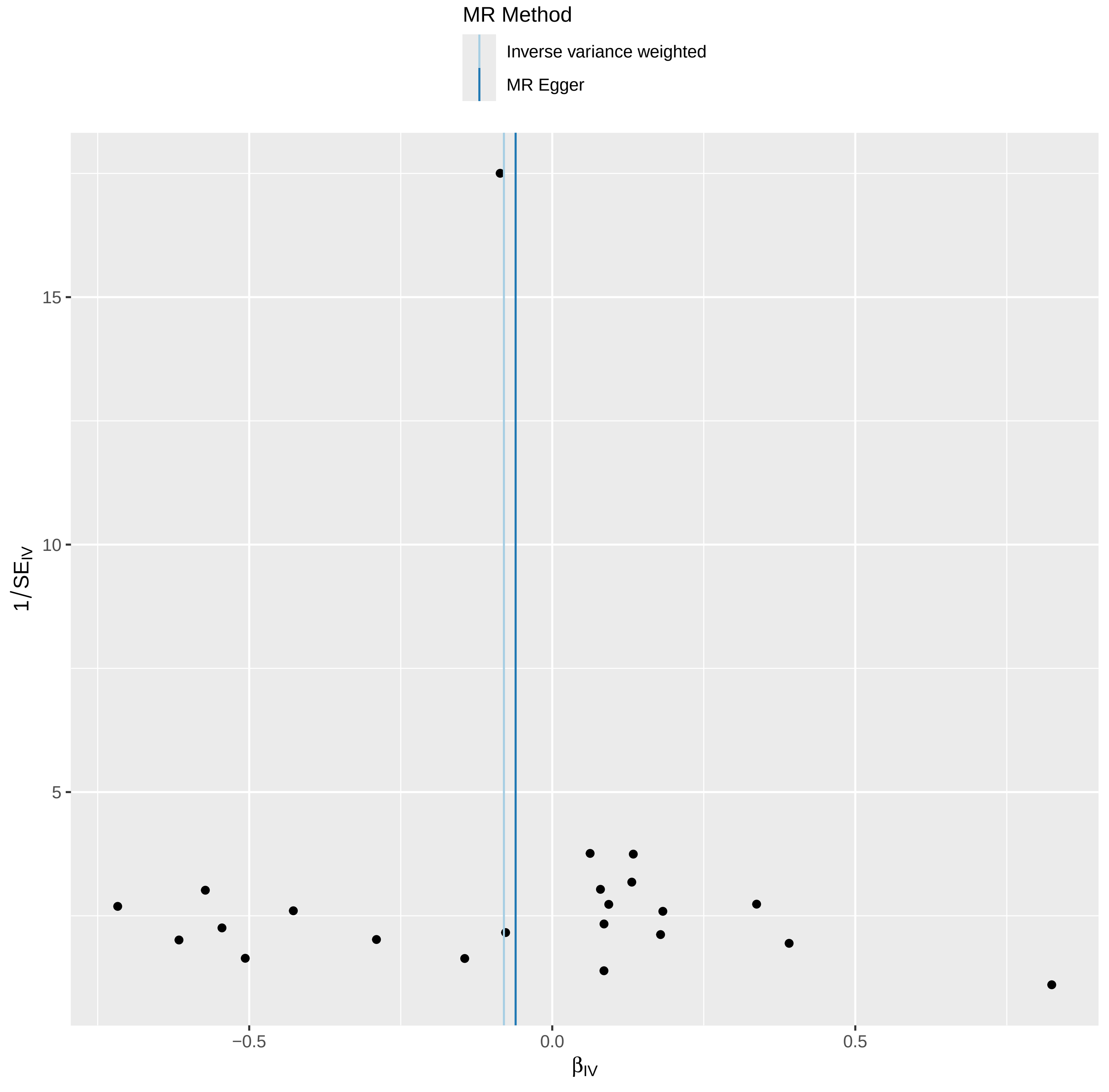

MR Method
Inverse variance weighted
MR Egger
15
10
5
1/SE_IV
−0.5
0.0
0.5
β_IV

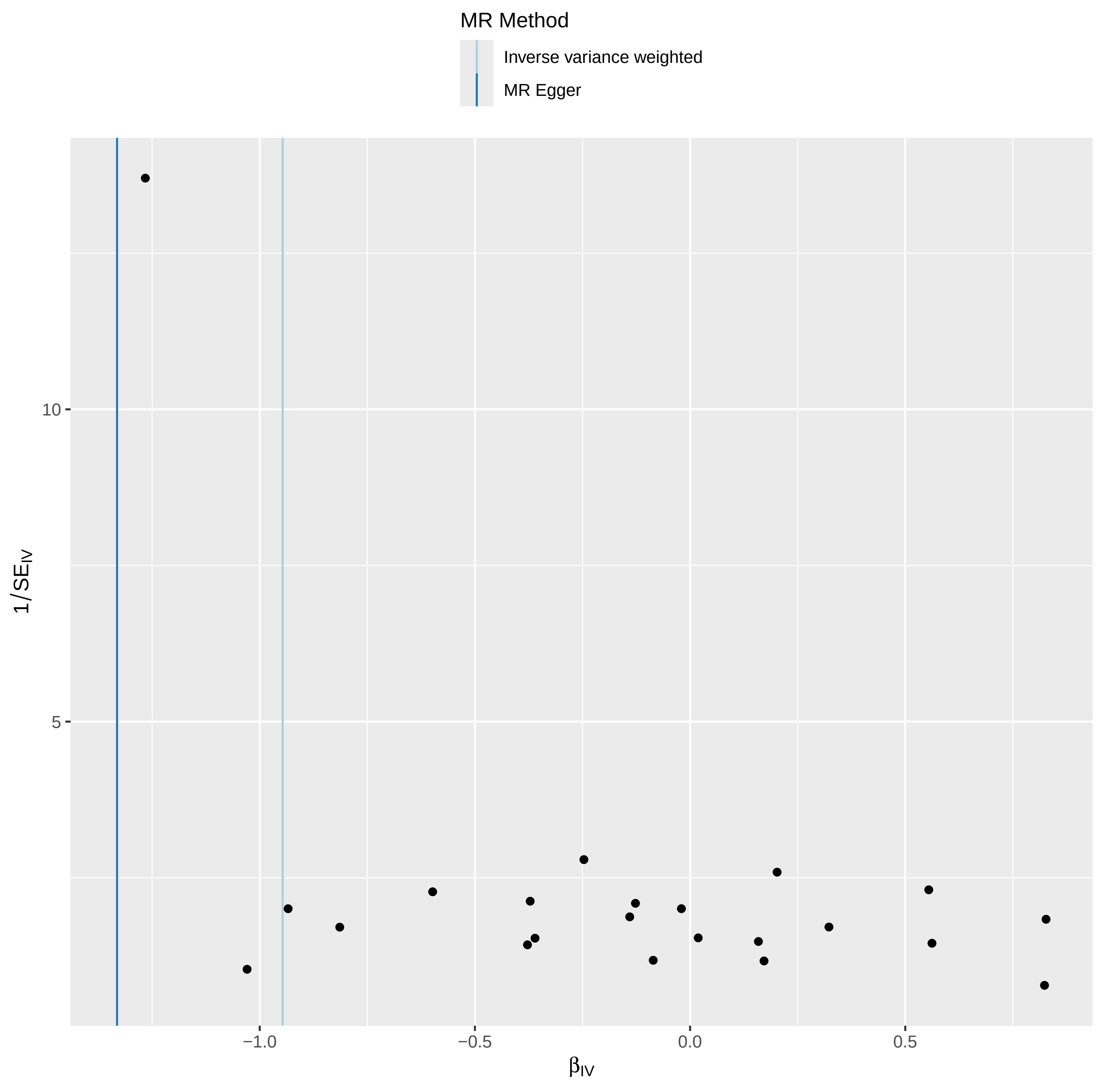
MR Method
Inverse variance weighted
MR Egger
1/SE$_{IV}$
10
5
β$_{IV}$
−1.0
−0.5
0.0
0.5

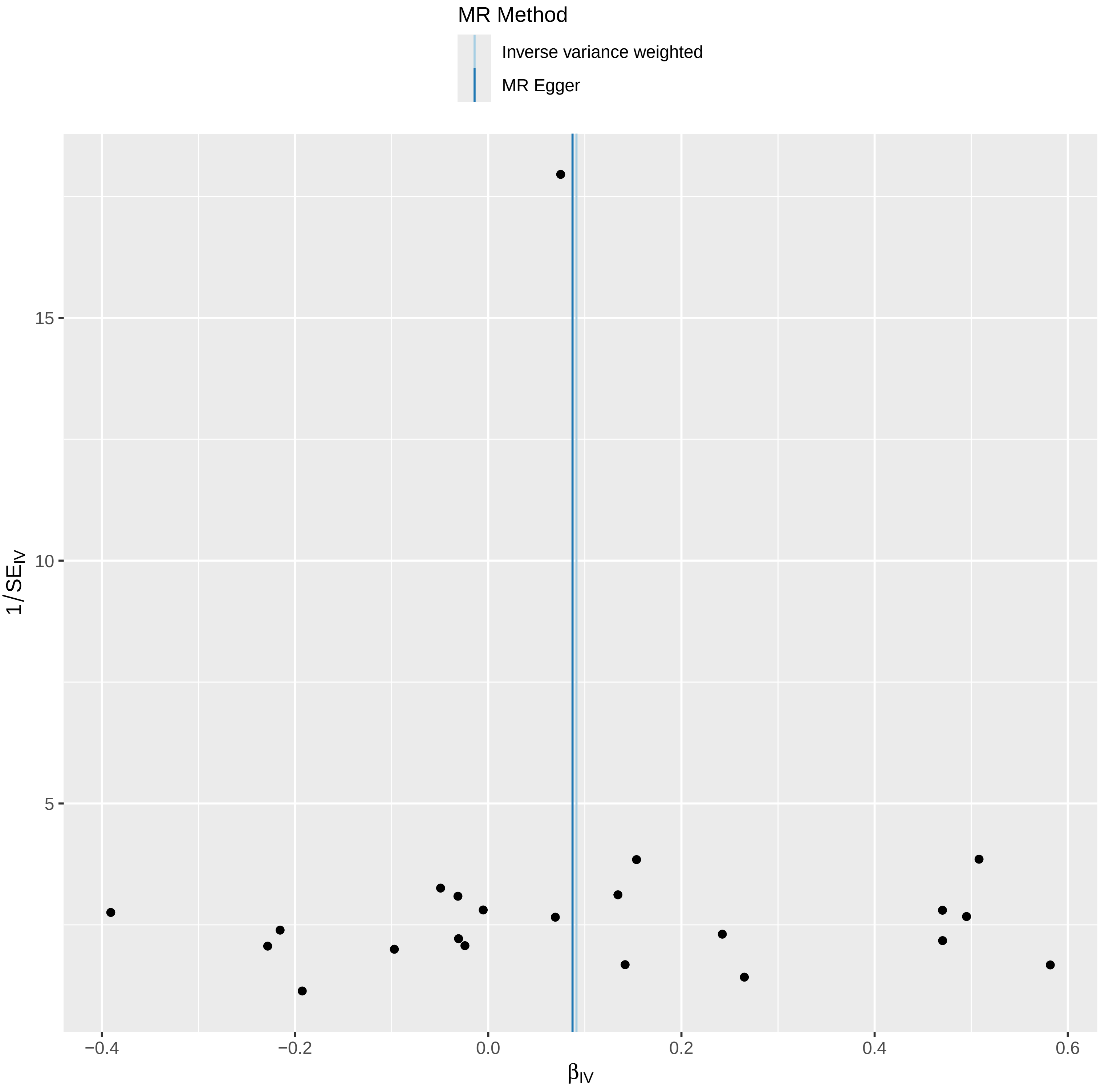
MR Method
Inverse variance weighted
MR Egger
15
10
5
1/SE$_{IV}$
−0.4
−0.2
0.0
0.2
0.4
0.6
β$_{IV}$

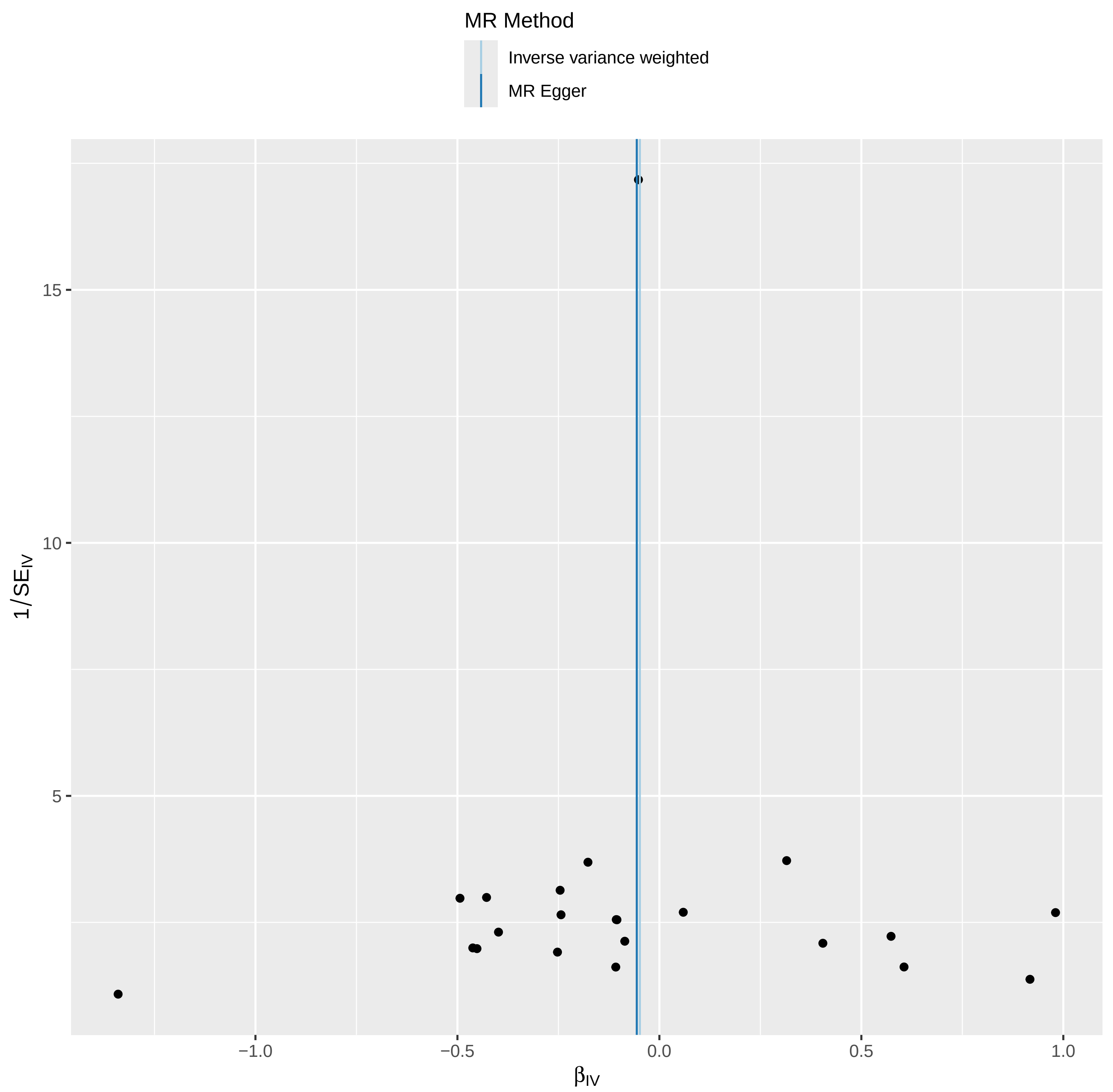

MR Method
Inverse variance weighted
MR Egger
15
10
5
1/SE_IV
−1.0
−0.5
0.0
0.5
1.0
β_IV

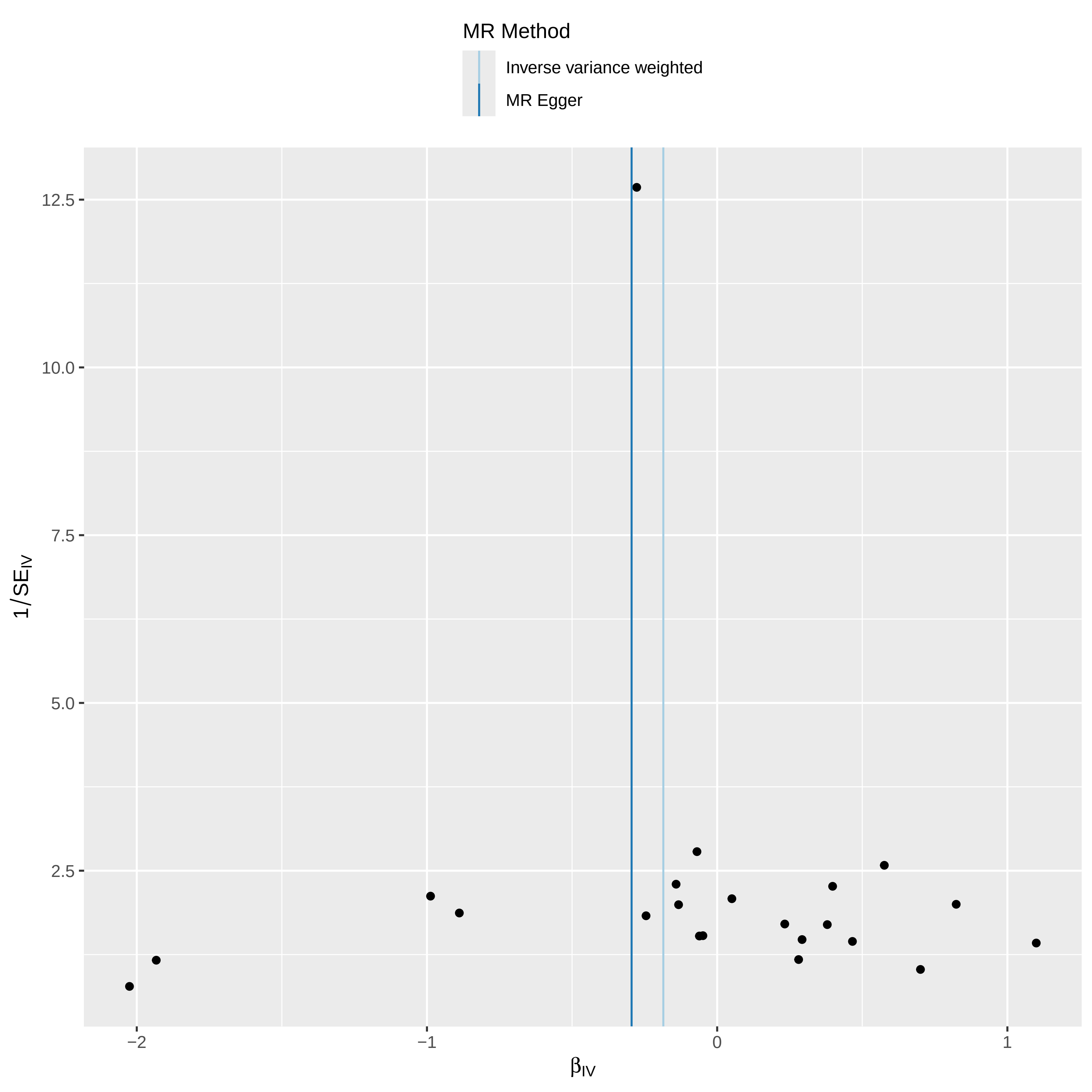
MR Method
Inverse variance weighted
MR Egger
12.5
10.0
7.5
5.0
2.5
$1/SE_{IV}$
−2
−1
0
1
β_{IV}

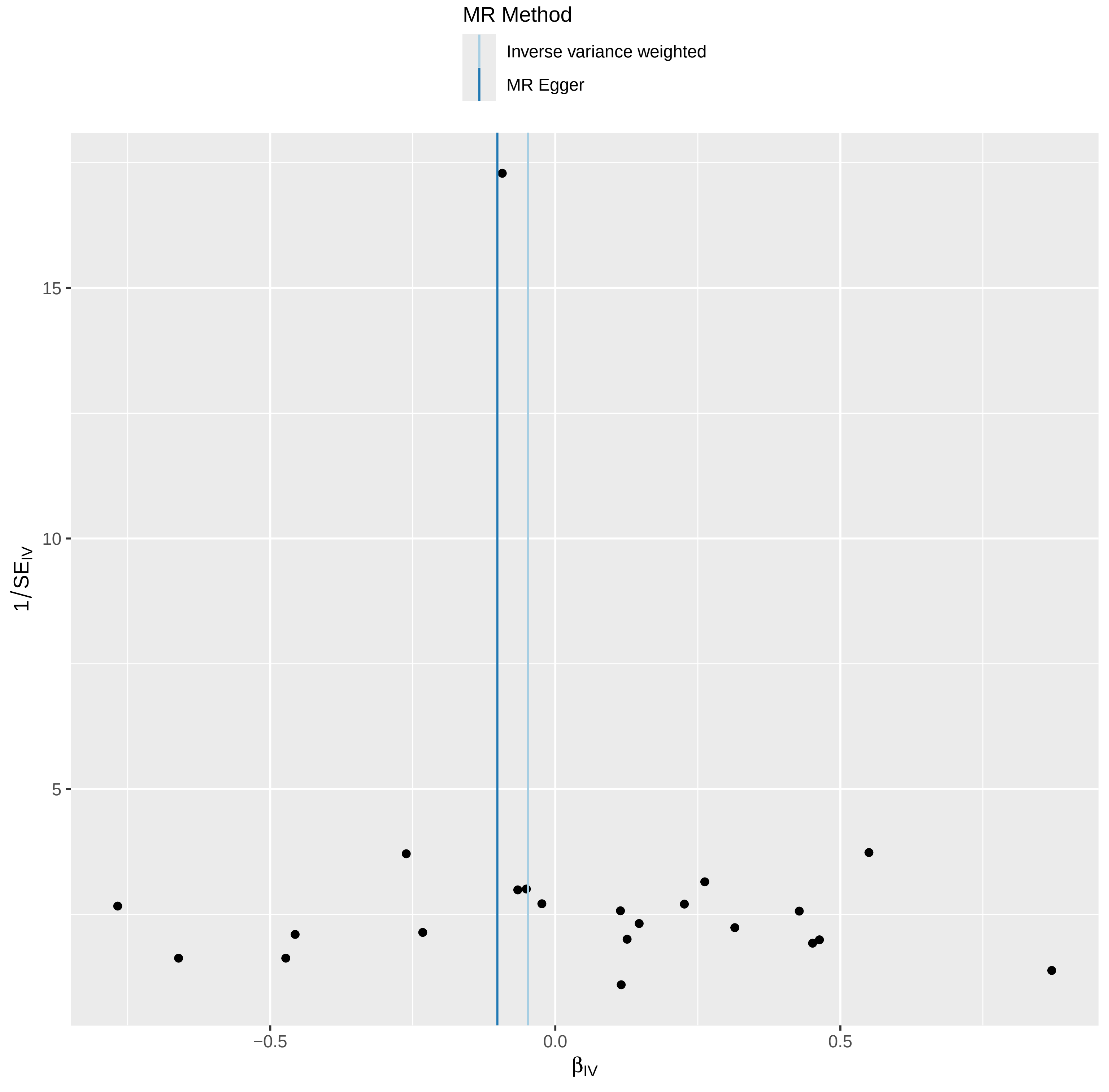
MR Method
Inverse variance weighted
MR Egger
15
10
5
$1/SE_{IV}$
−0.5
0.0
0.5
β_{IV}

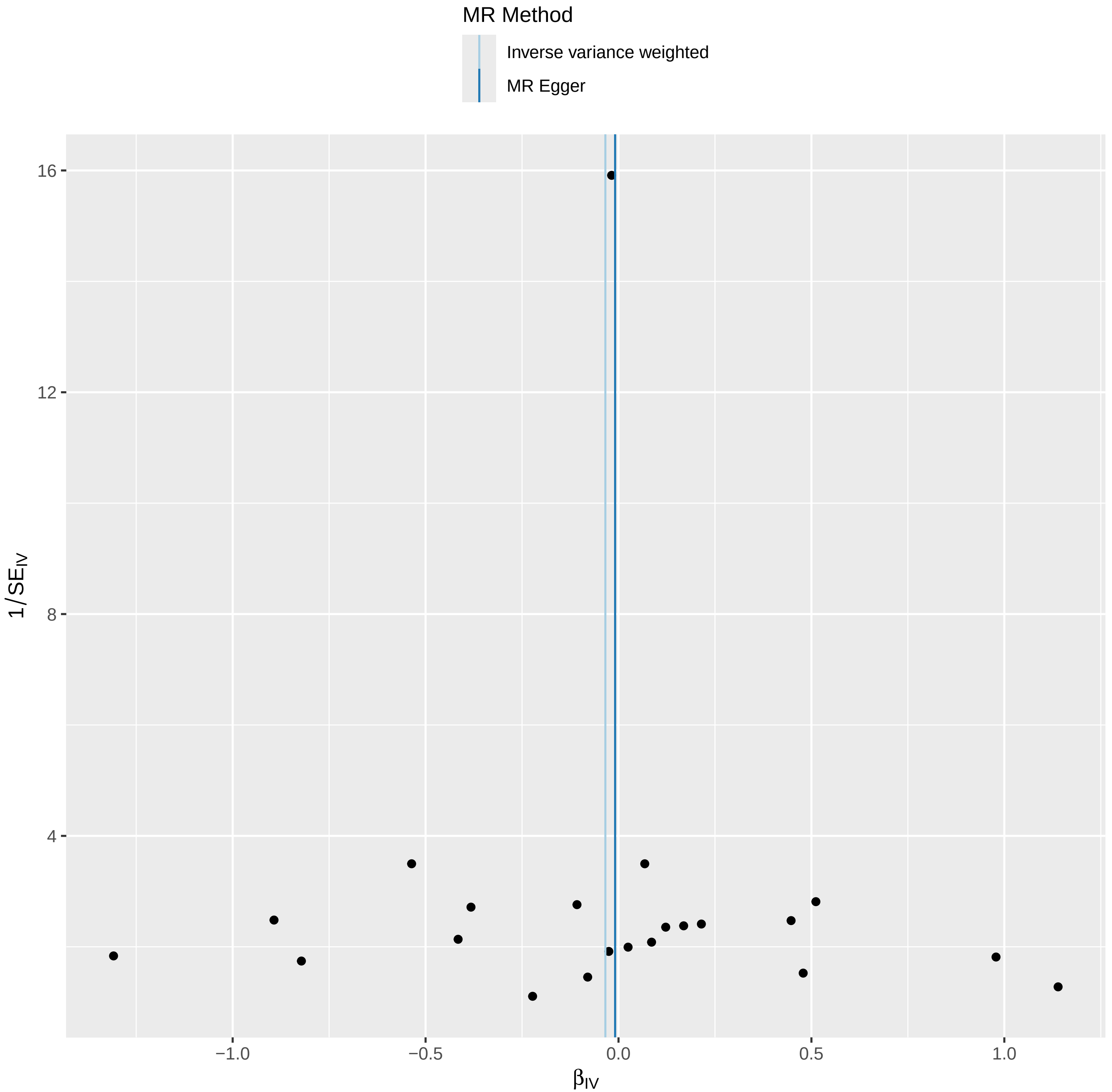
MR Method
Inverse variance weighted
MR Egger
16
12
8
4
1/SE_IV
−1.0
−0.5
0.0
0.5
1.0
β_IV

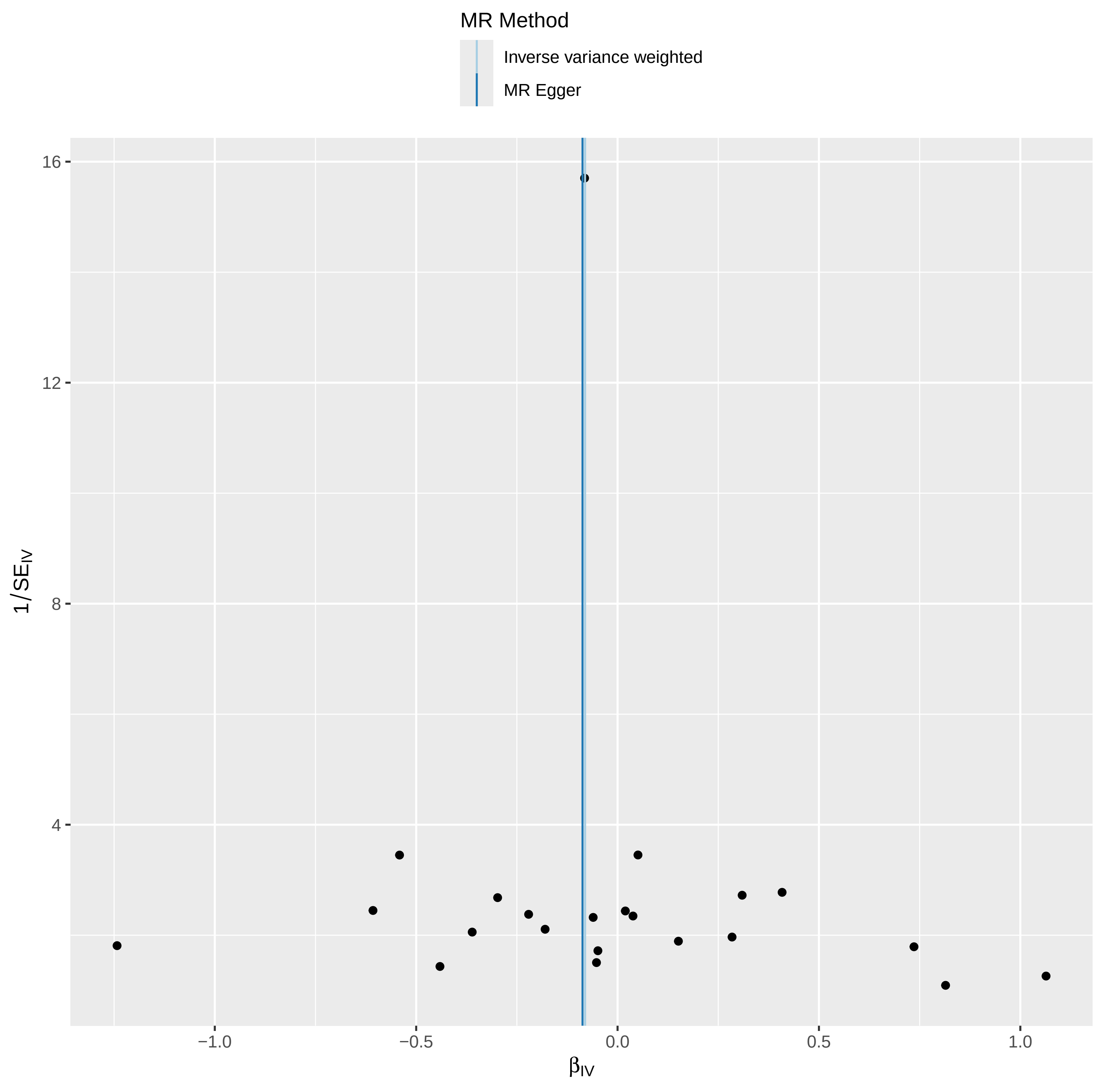

MR Method
Inverse variance weighted
MR Egger
16
12
8
4
1/SE_IV
−1.0
−0.5
0.0
0.5
1.0
β_IV

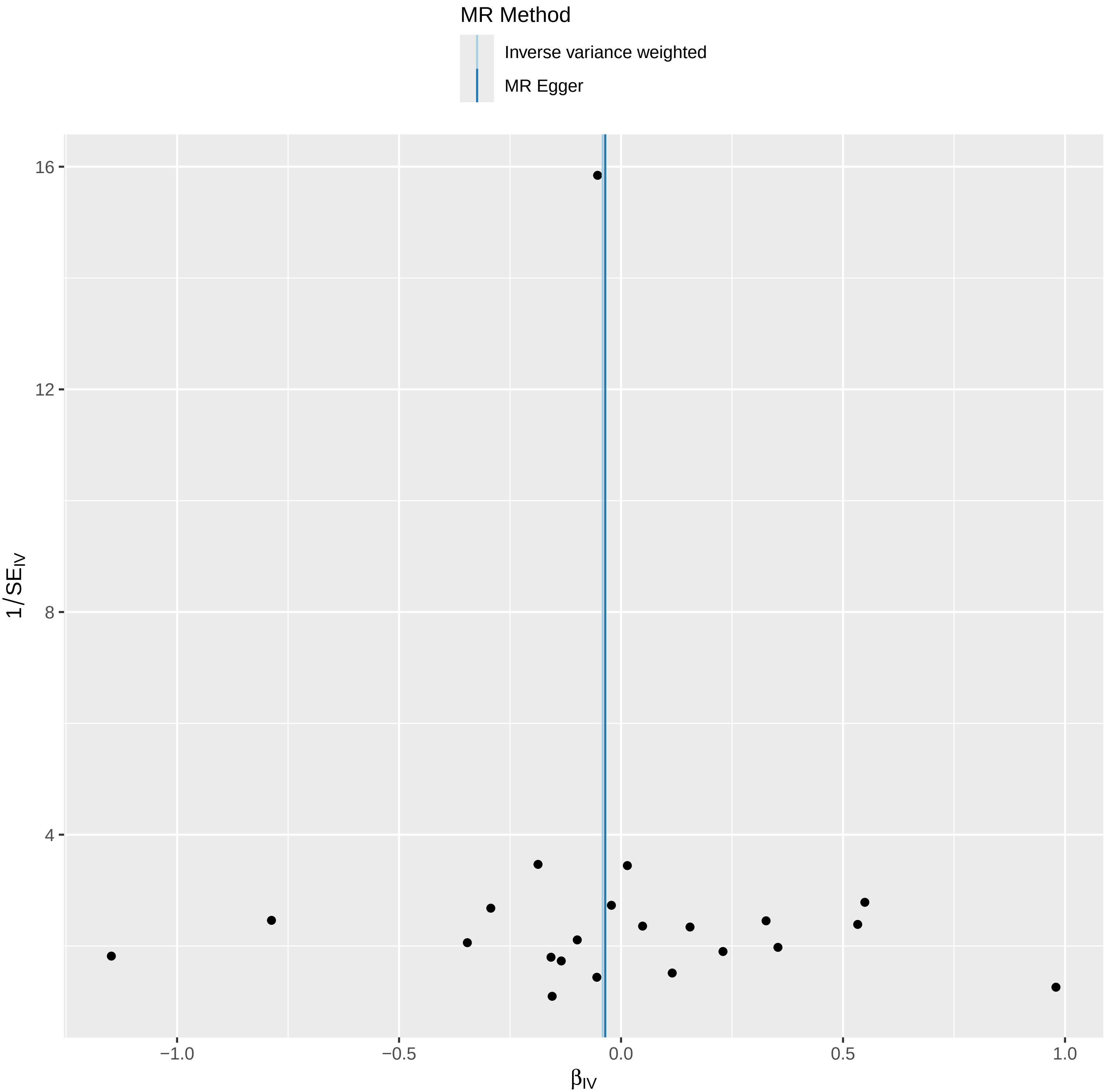

MR Method
Inverse variance weighted
MR Egger
16
12
8
4
1/SE_IV
−1.0
−0.5
0.0
0.5
1.0
β_IV

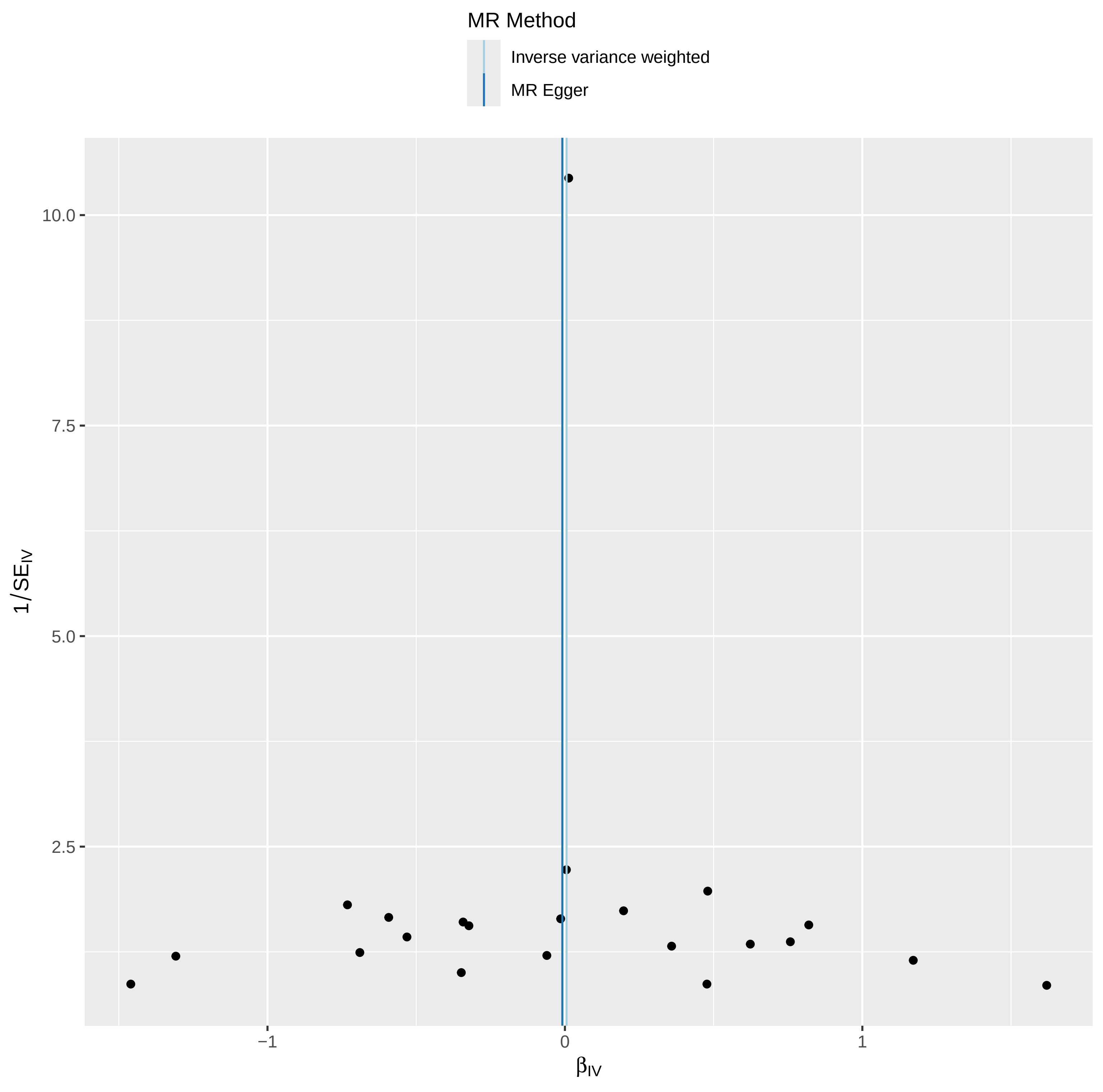
MR Method
Inverse variance weighted
MR Egger
10.0
7.5
5.0
2.5
1/SE_IV
−1
0
1
β_IV

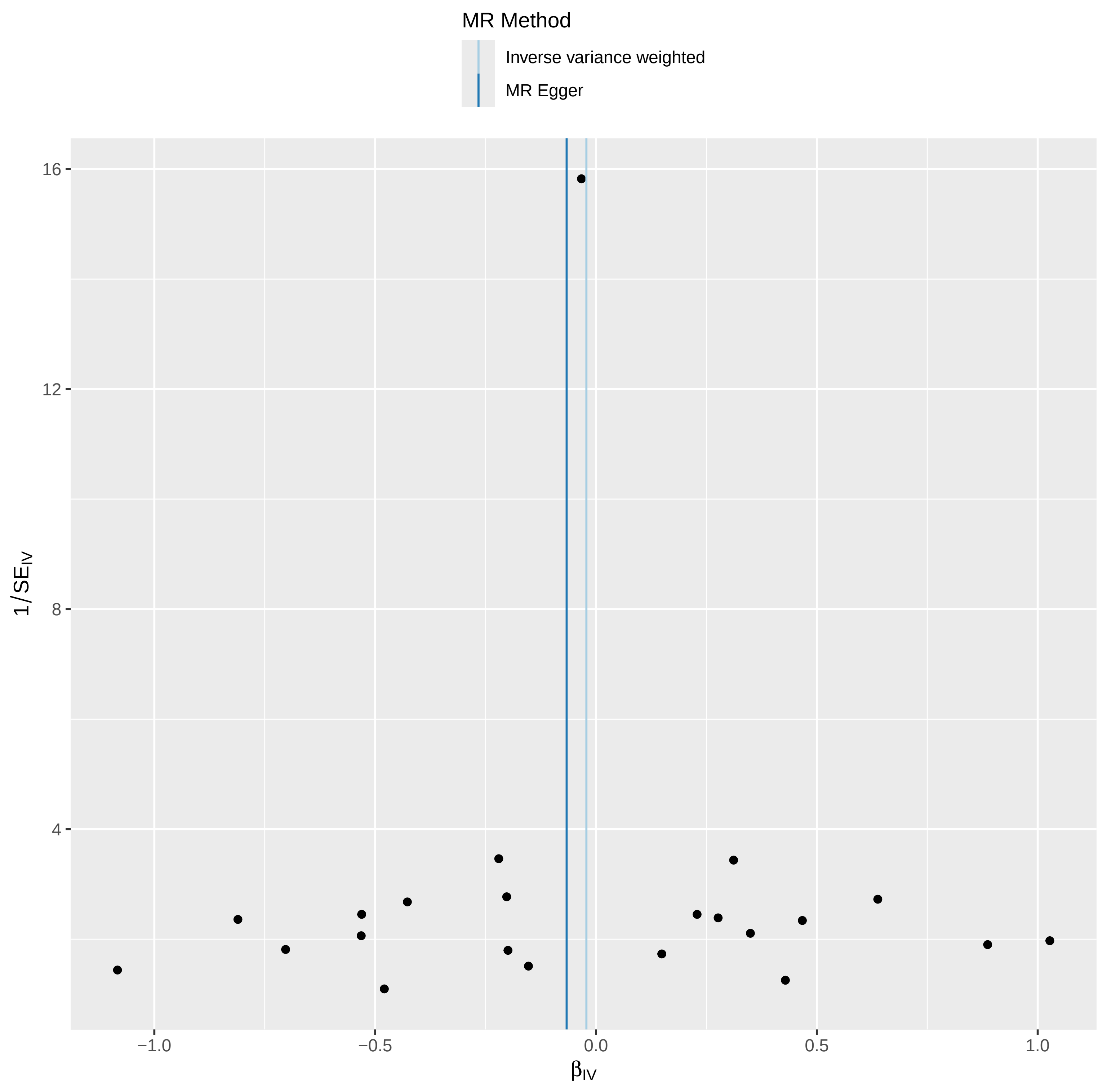
MR Method
Inverse variance weighted
MR Egger
16
12
8
4
$1/SE_{IV}$
−1.0
−0.5
0.0
0.5
1.0
β_{IV}

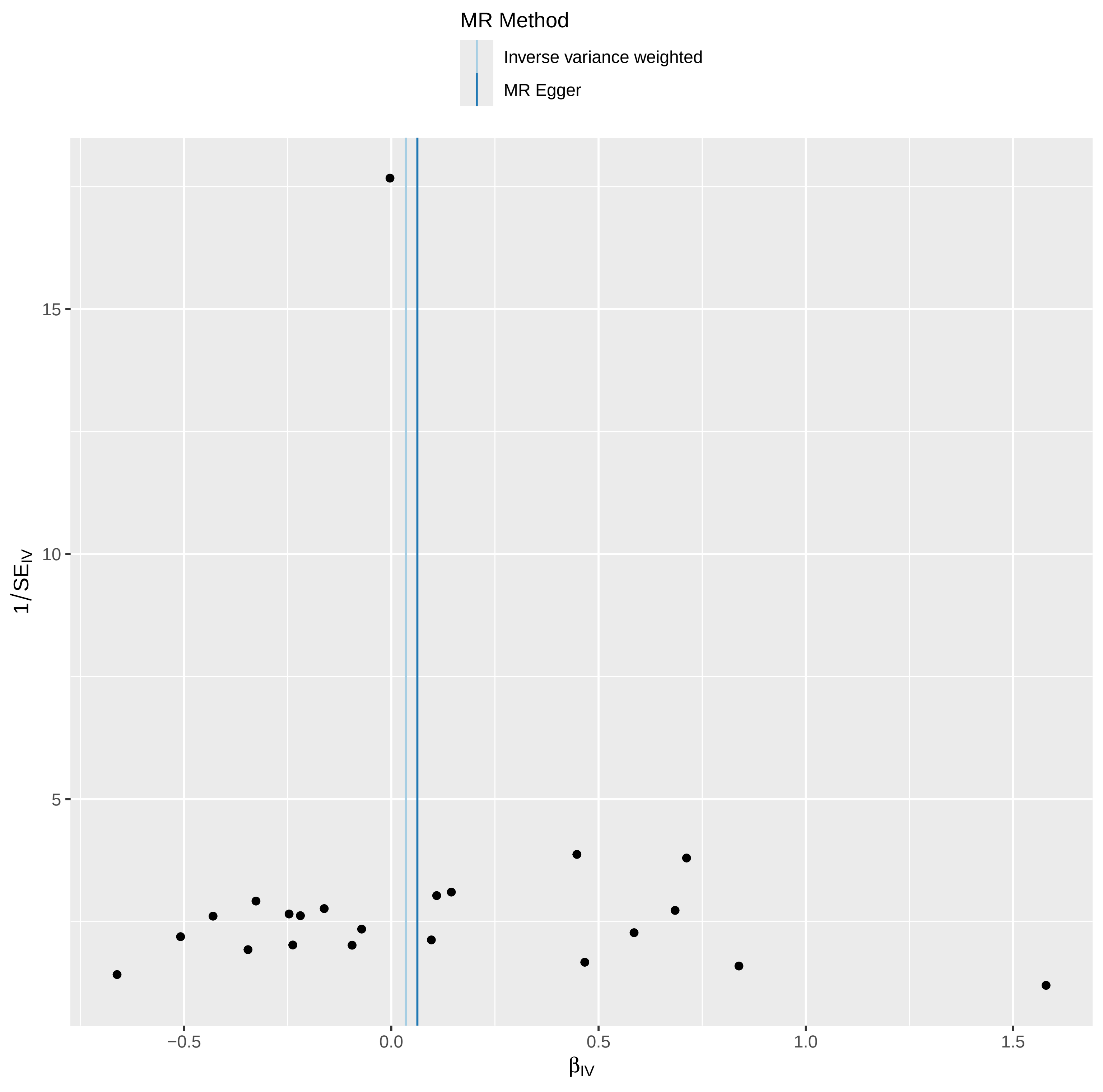

MR Method
Inverse variance weighted
MR Egger
15
10
5
1/SE$_{IV}$
−0.5
0.0
0.5
1.0
1.5
β$_{IV}$

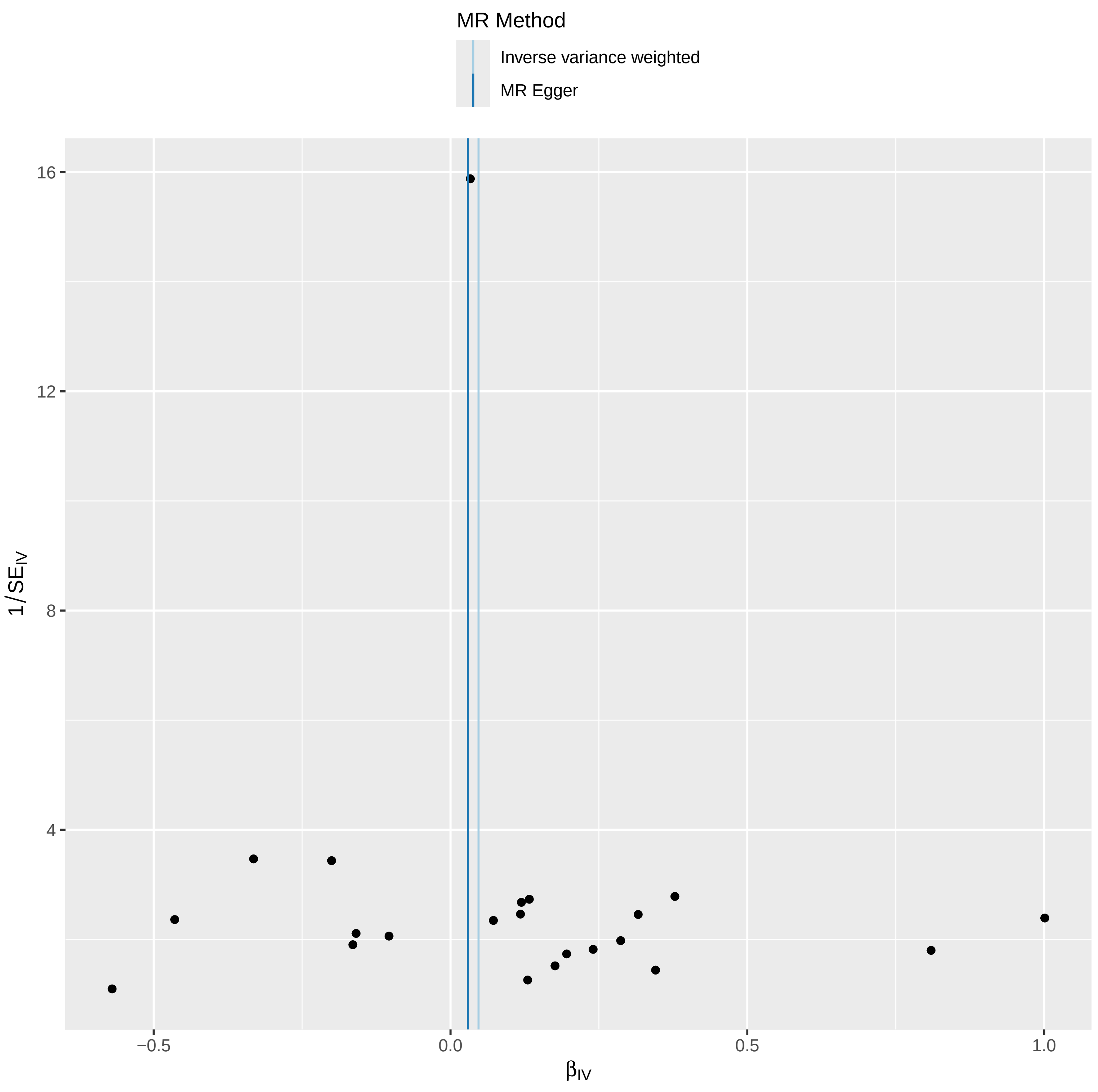

MR Method
Inverse variance weighted
MR Egger
16
12
8
4
1/SE_IV
−0.5
0.0
0.5
1.0
β_IV

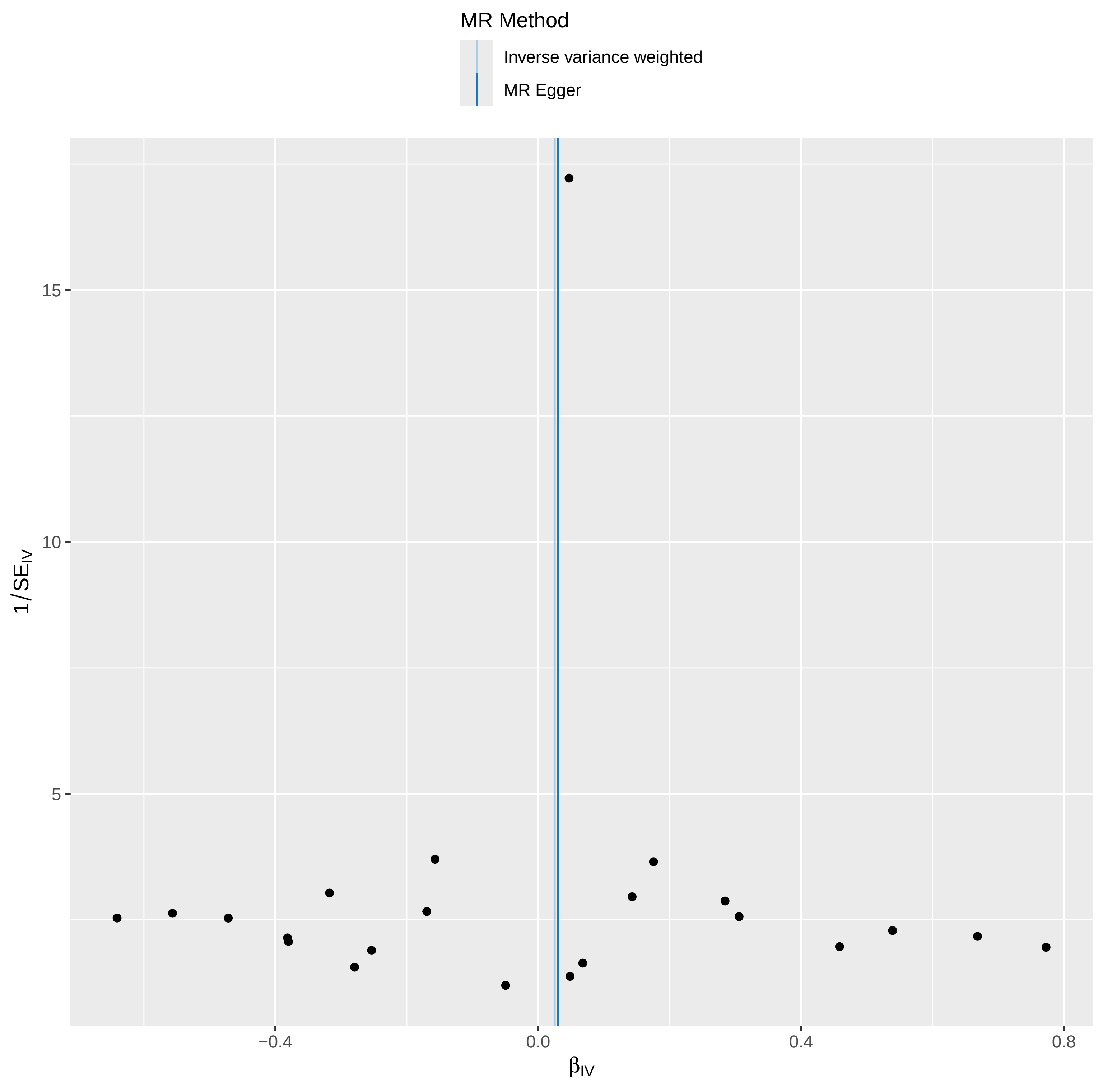

MR Method
Inverse variance weighted
MR Egger
15
10
5
$1/SE_{IV}$
−0.4
0.0
0.4
0.8
β_{IV}

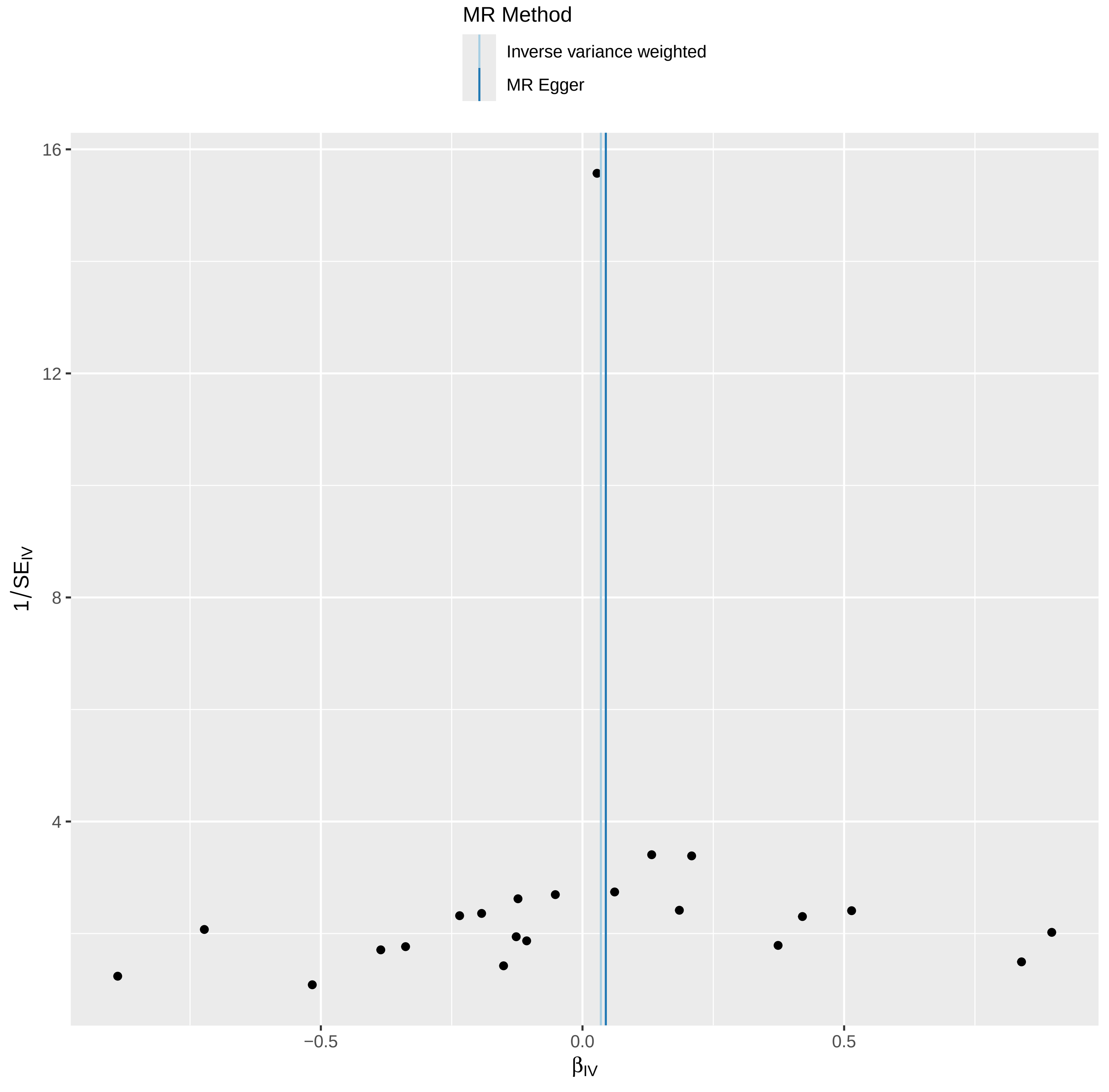
MR Method
Inverse variance weighted
MR Egger
16
12
8
4
1/SE_IV
−0.5
0.0
0.5
β_IV

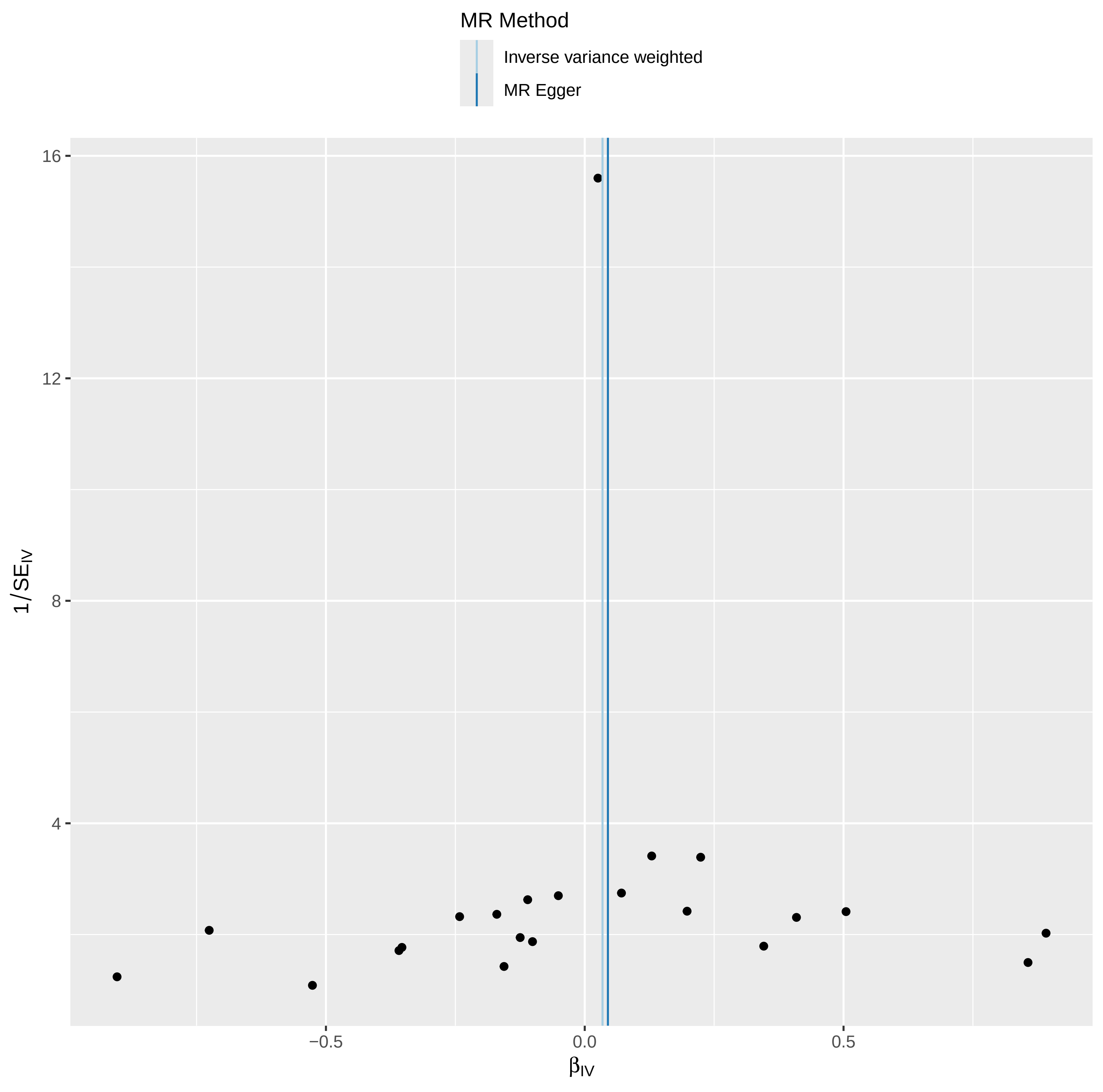
MR Method
Inverse variance weighted
MR Egger
16
12
8
4
$1/SE_{IV}$
−0.5
0.0
0.5
β_{IV}

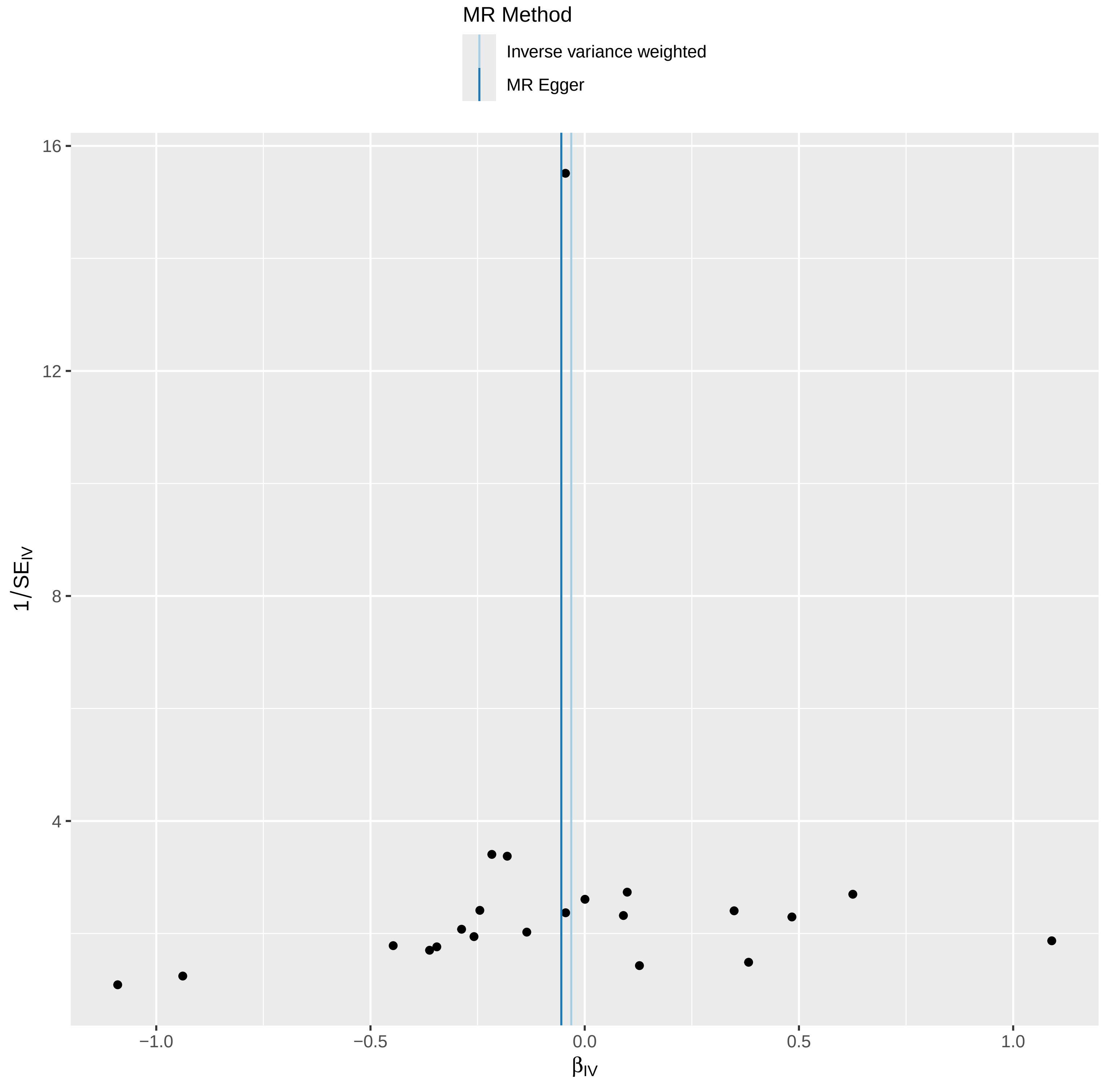

MR Method
Inverse variance weighted
MR Egger
16
12
8
4
$1/SE_{IV}$
−1.0
−0.5
0.0
0.5
1.0
β_{IV}

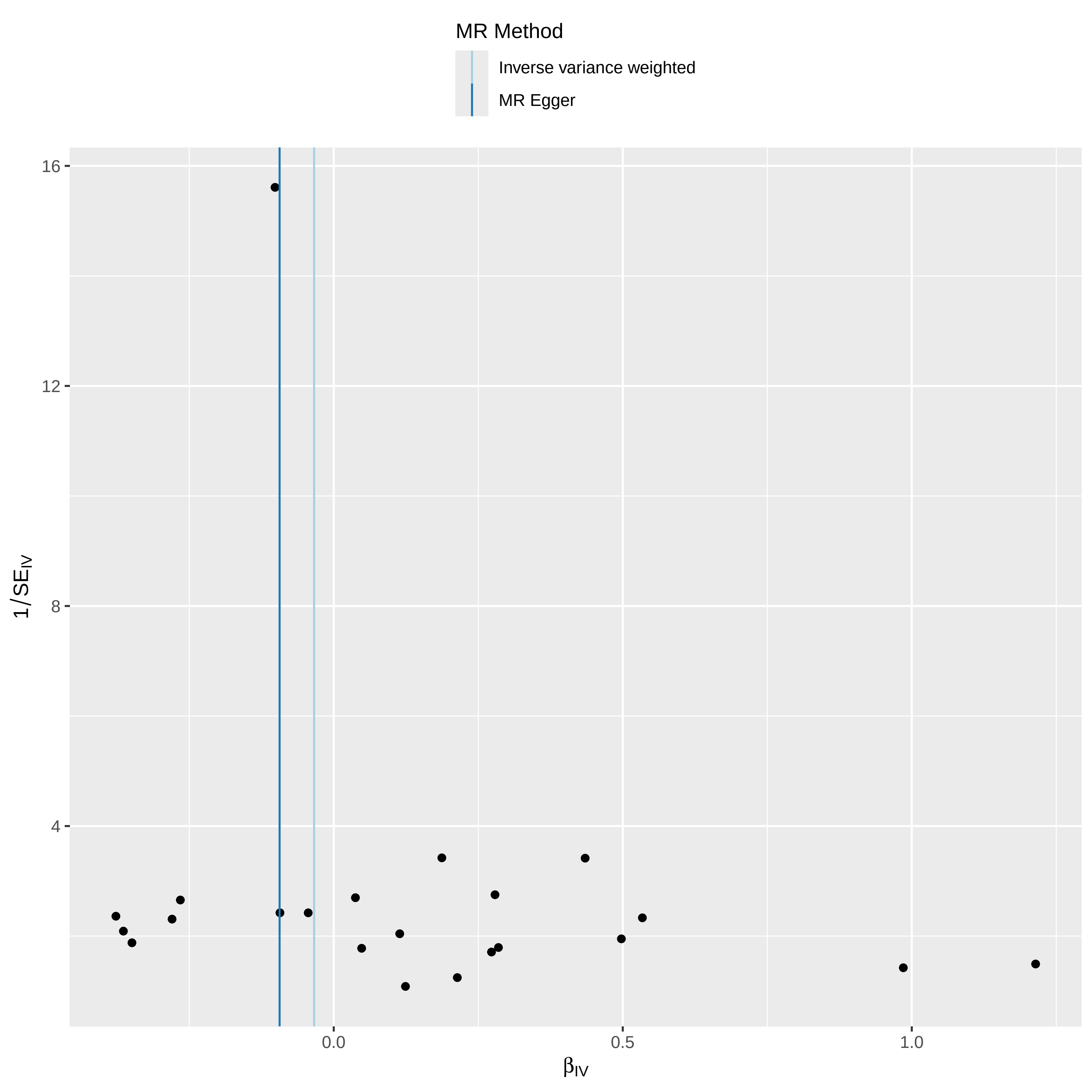

MR Method
Inverse variance weighted
MR Egger
16
12
8
4
$1/SE_{IV}$
0.0
0.5
1.0
β_{IV}

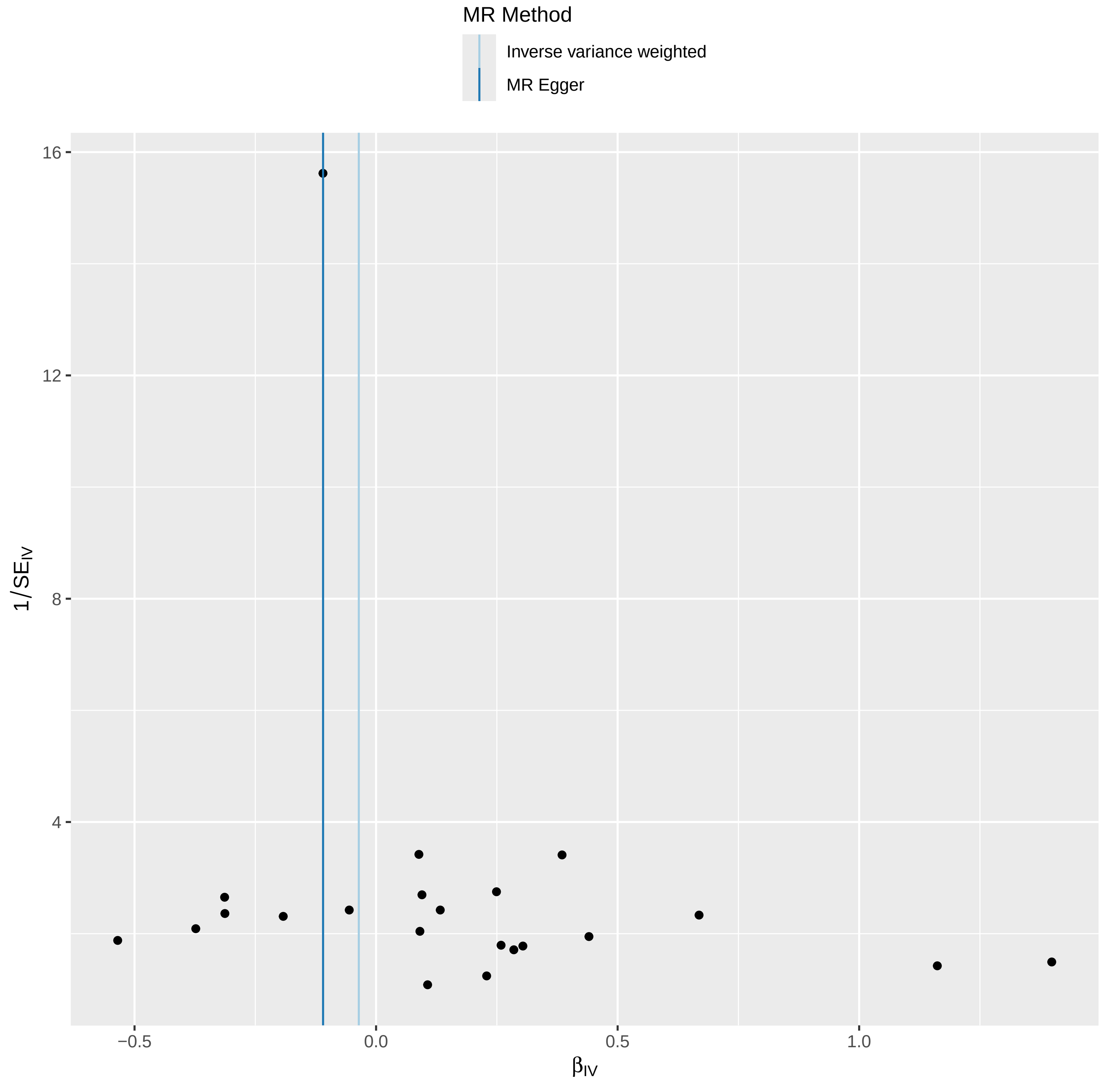

MR Method
Inverse variance weighted
MR Egger
16
12
8
4
1/SE_IV
−0.5
0.0
0.5
1.0
β_IV

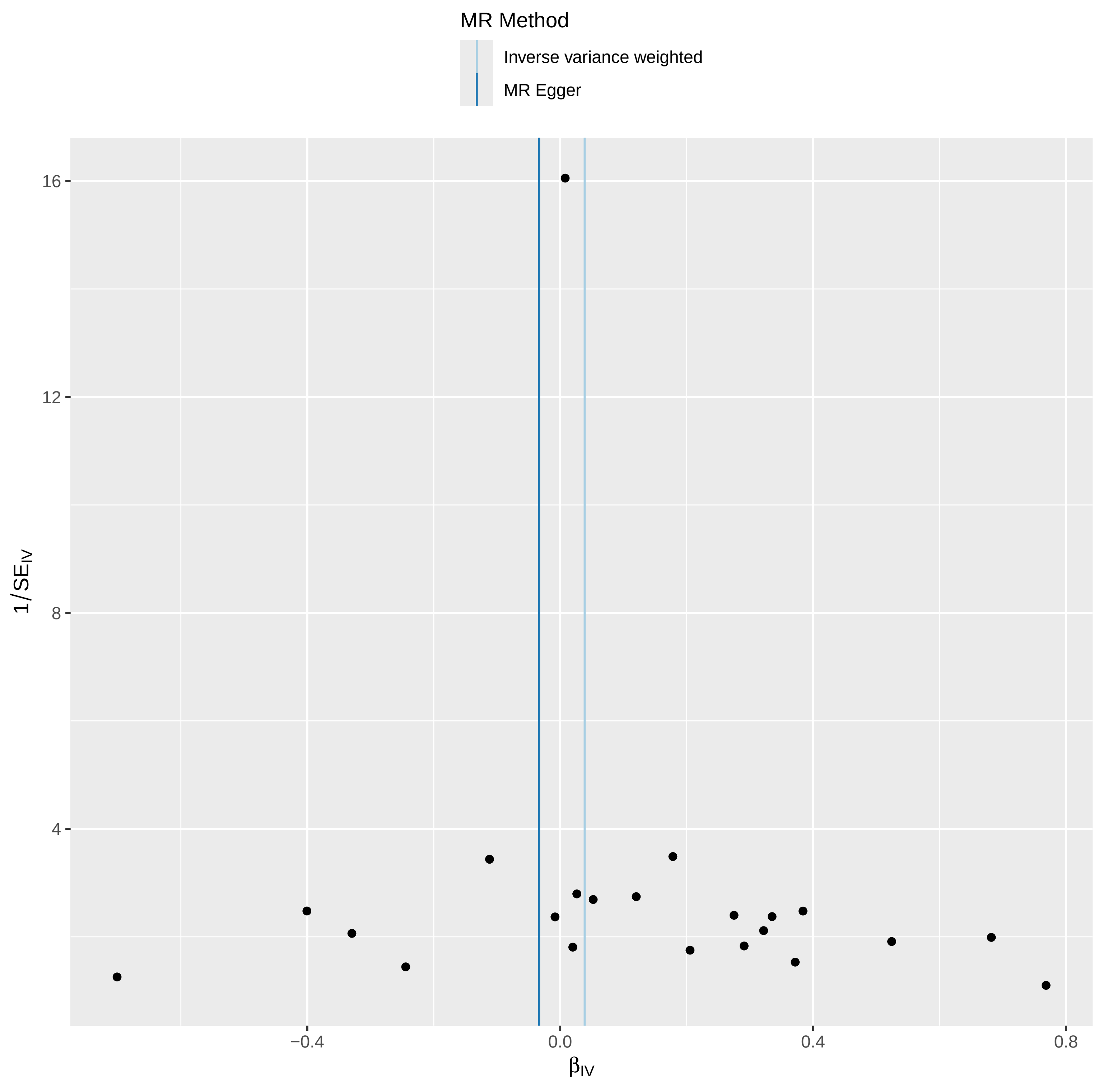
MR Method
Inverse variance weighted
MR Egger
1/SE_IV
16
12
8
4
−0.4
0.0
0.4
0.8
β_IV

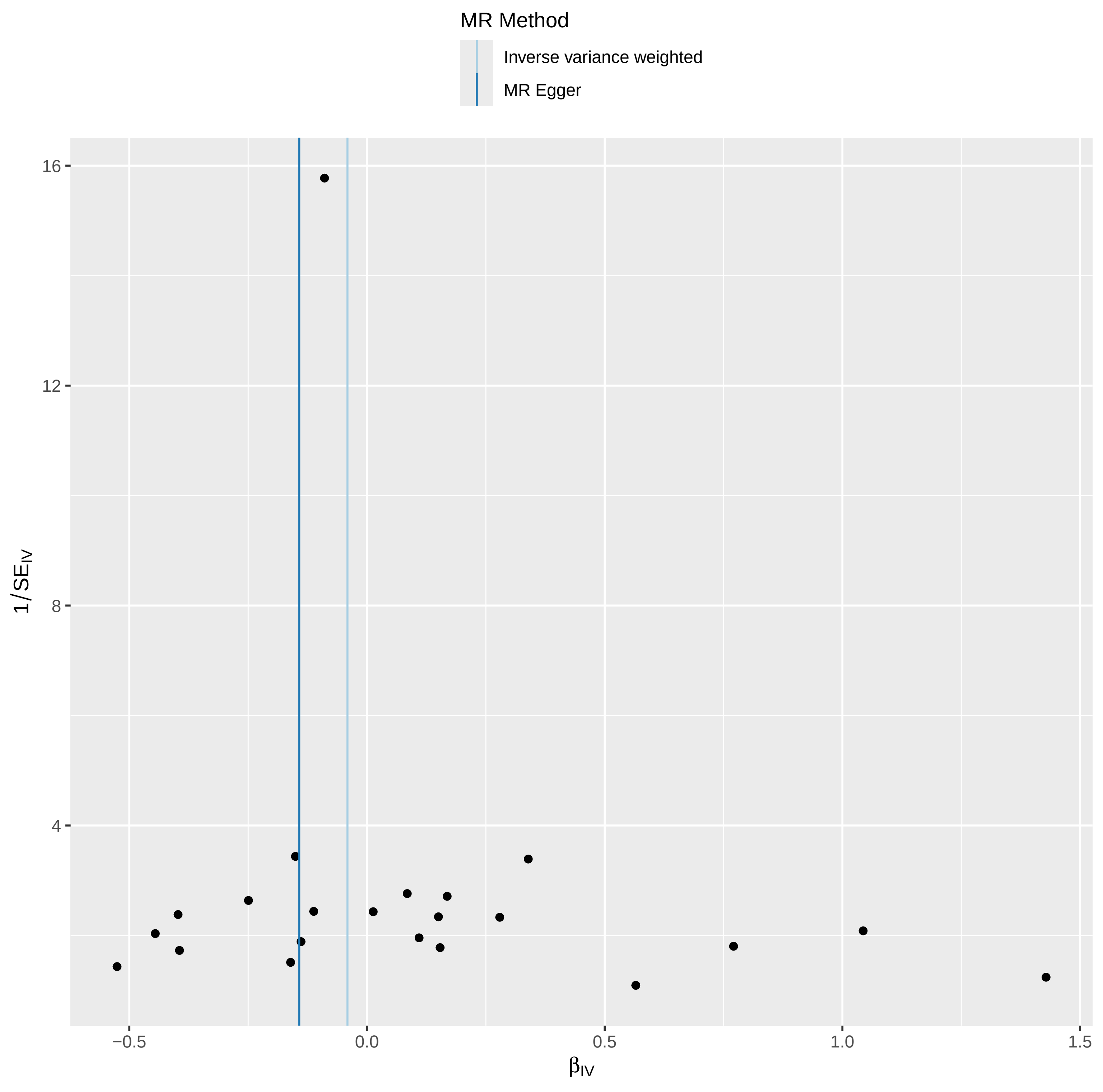
MR Method
Inverse variance weighted
MR Egger
16
12
8
4
1/SE_{IV}
−0.5
0.0
0.5
1.0
1.5
β_{IV}

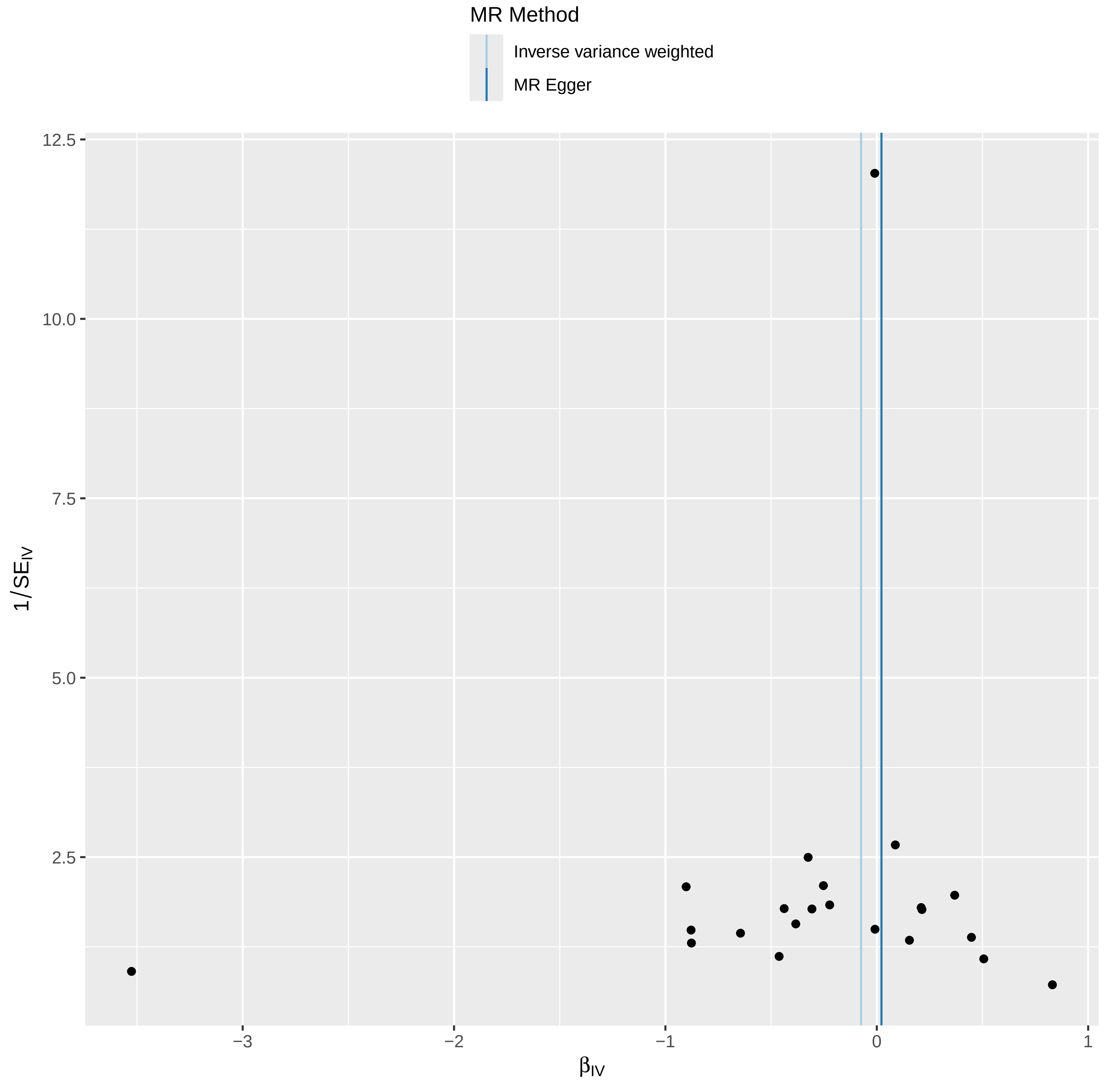

MR Method
Inverse variance weighted
MR Egger
12.5
10.0
7.5
5.0
2.5
1/SE$_{IV}$
−3
−2
−1
0
1
β$_{IV}$

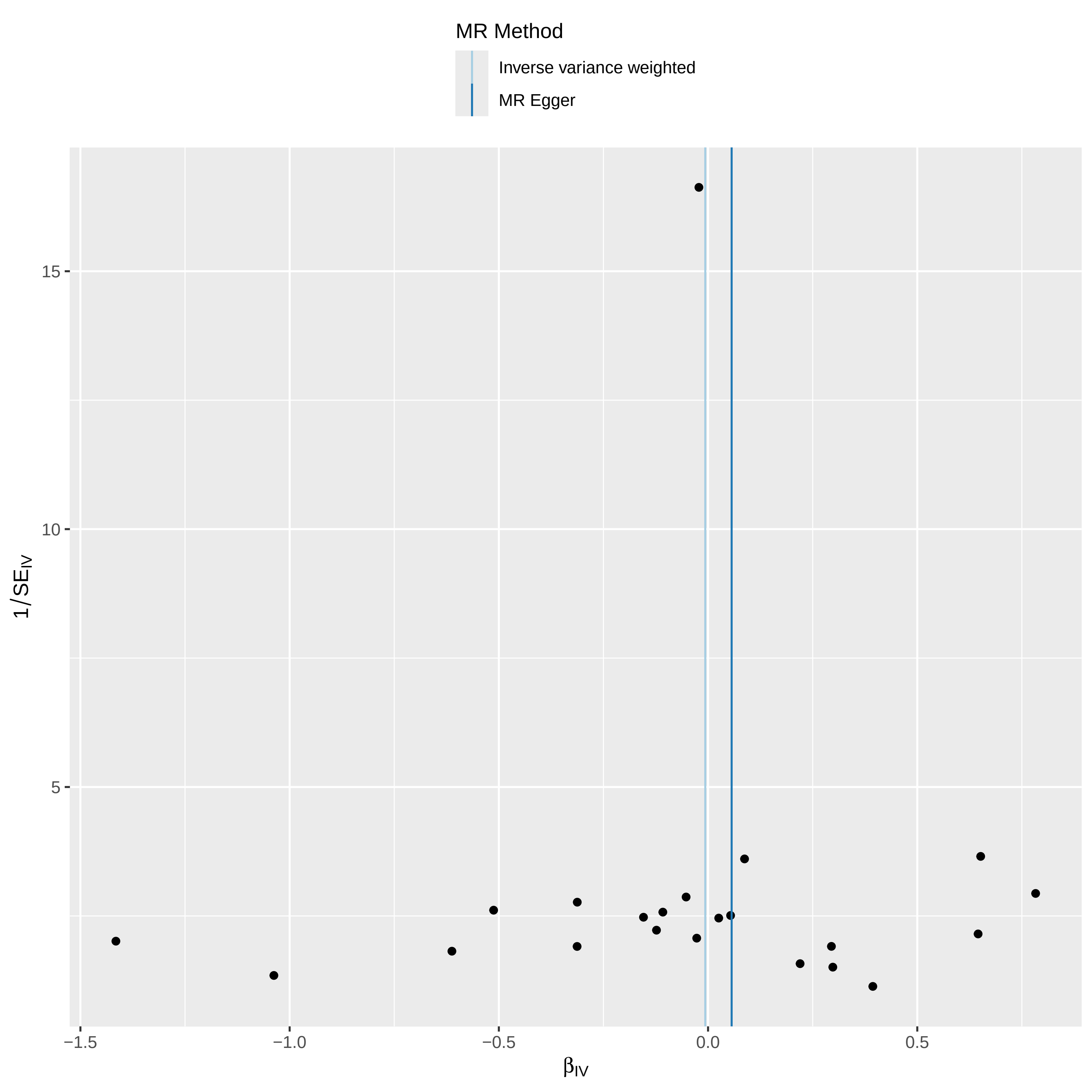
MR Method
Inverse variance weighted
MR Egger
1/SE_IV
β_IV
15
10
5
−1.5
−1.0
−0.5
0.0
0.5

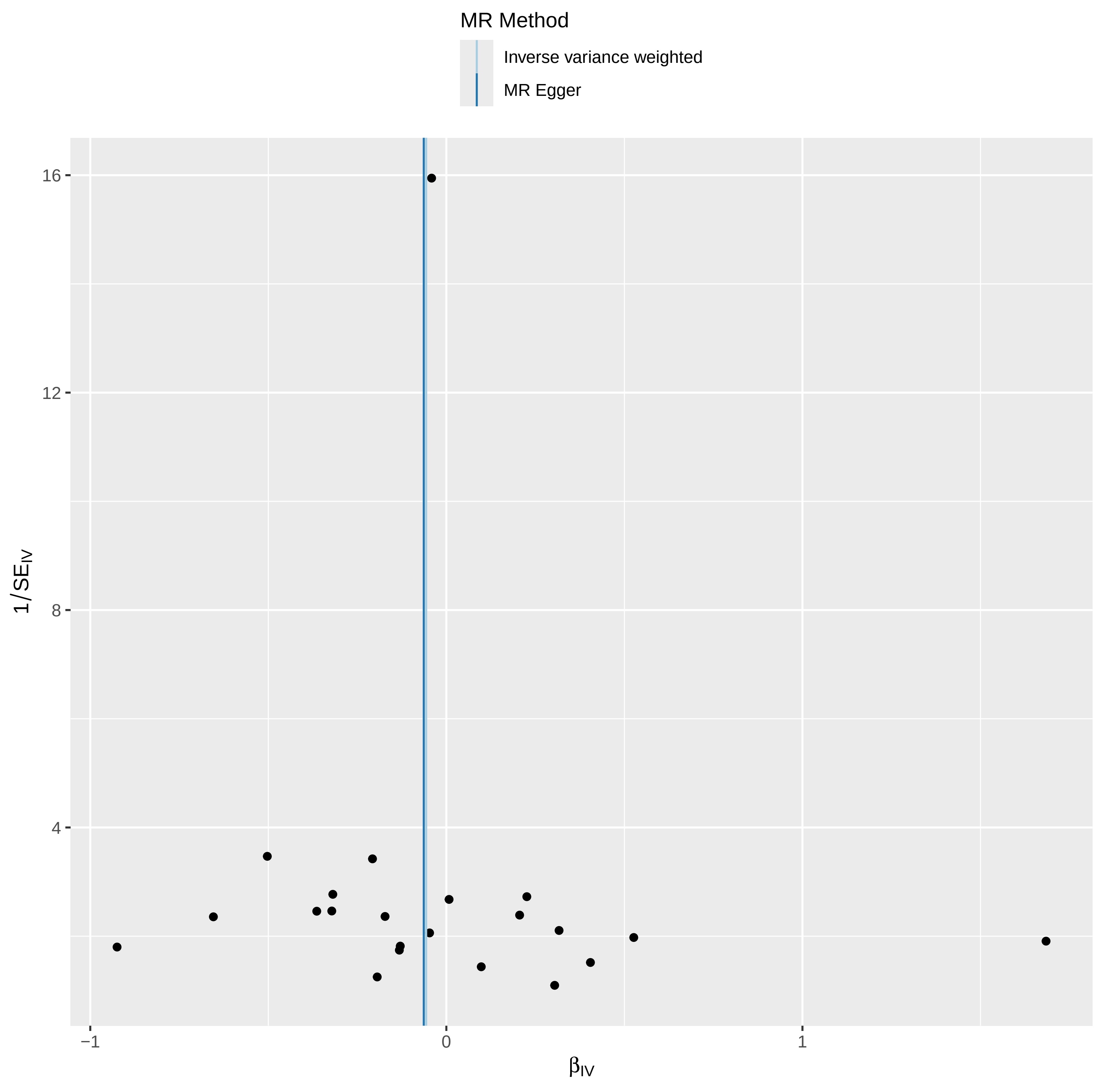

MR Method
Inverse variance weighted
MR Egger
16
12
8
4
1/SE_{IV}
−1
0
1
β_{IV}

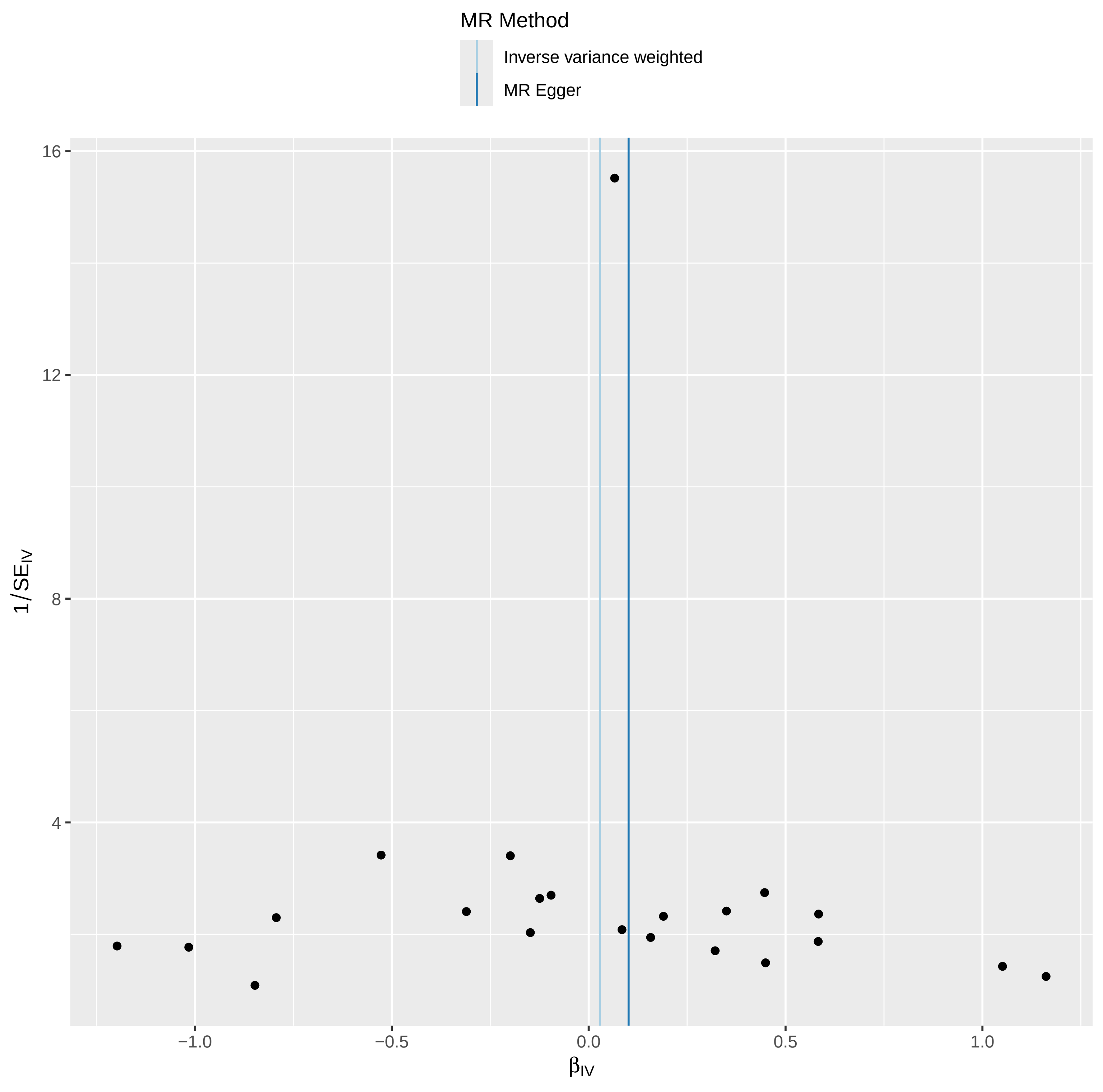

MR Method
Inverse variance weighted
MR Egger
16
12
8
4
1/SE_IV
−1.0
−0.5
0.0
0.5
1.0
β_IV

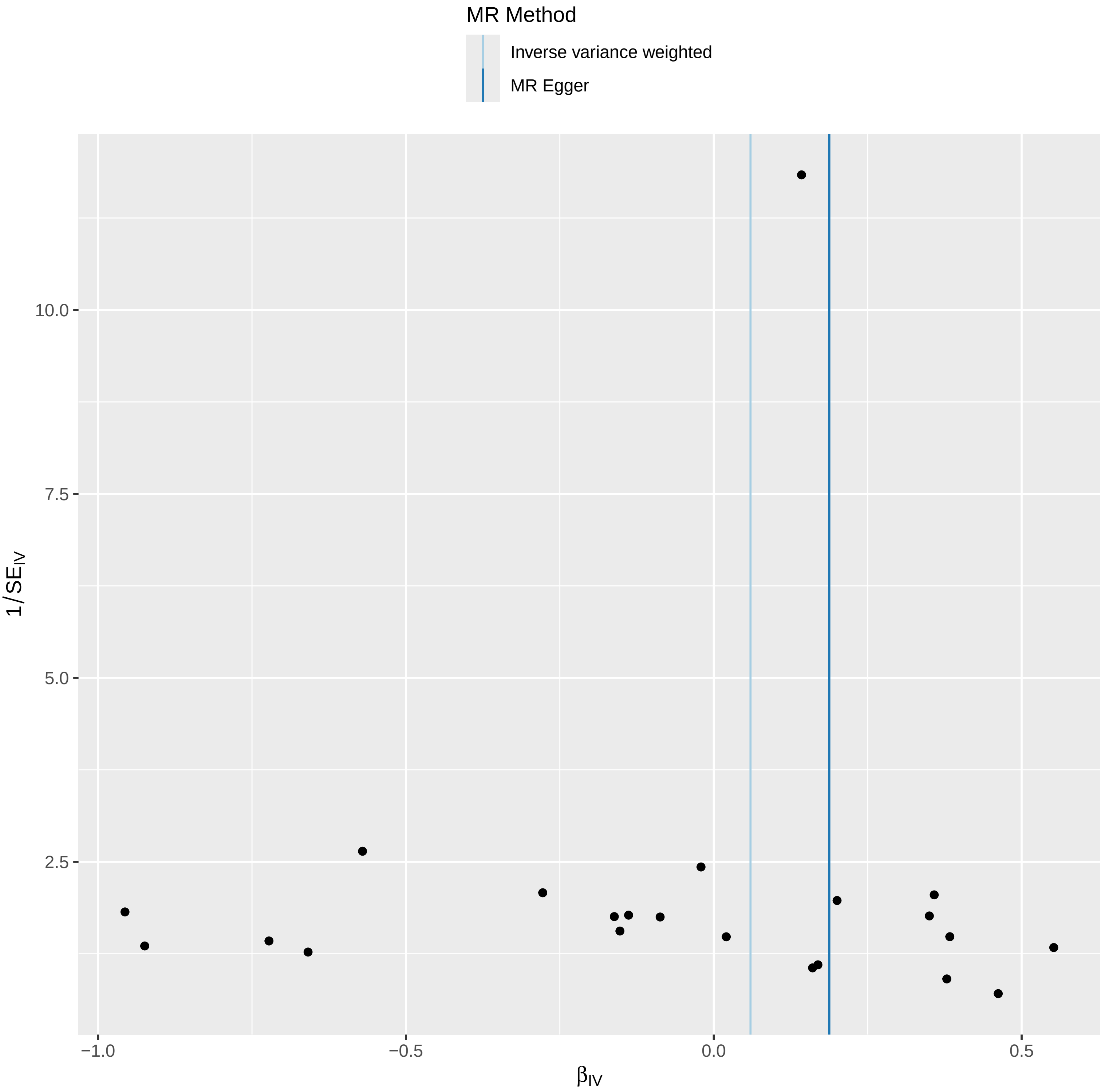

MR Method
Inverse variance weighted
MR Egger
1/SE_IV
10.0
7.5
5.0
2.5
−1.0
−0.5
0.0
0.5
β_IV

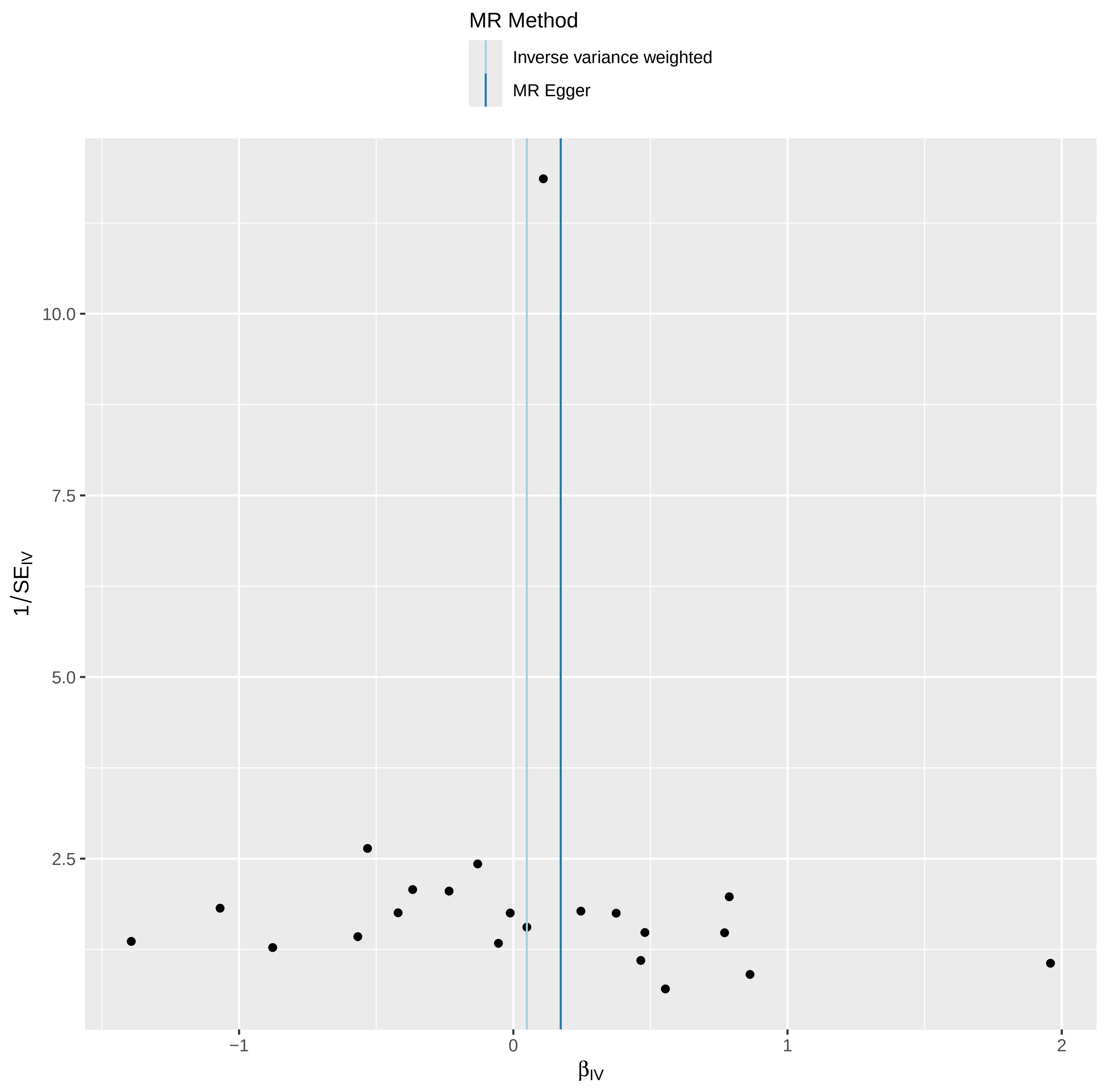

MR Method
Inverse variance weighted
MR Egger
10.0
7.5
5.0
2.5
1/SE$_{IV}$
−1
0
1
2
β$_{IV}$

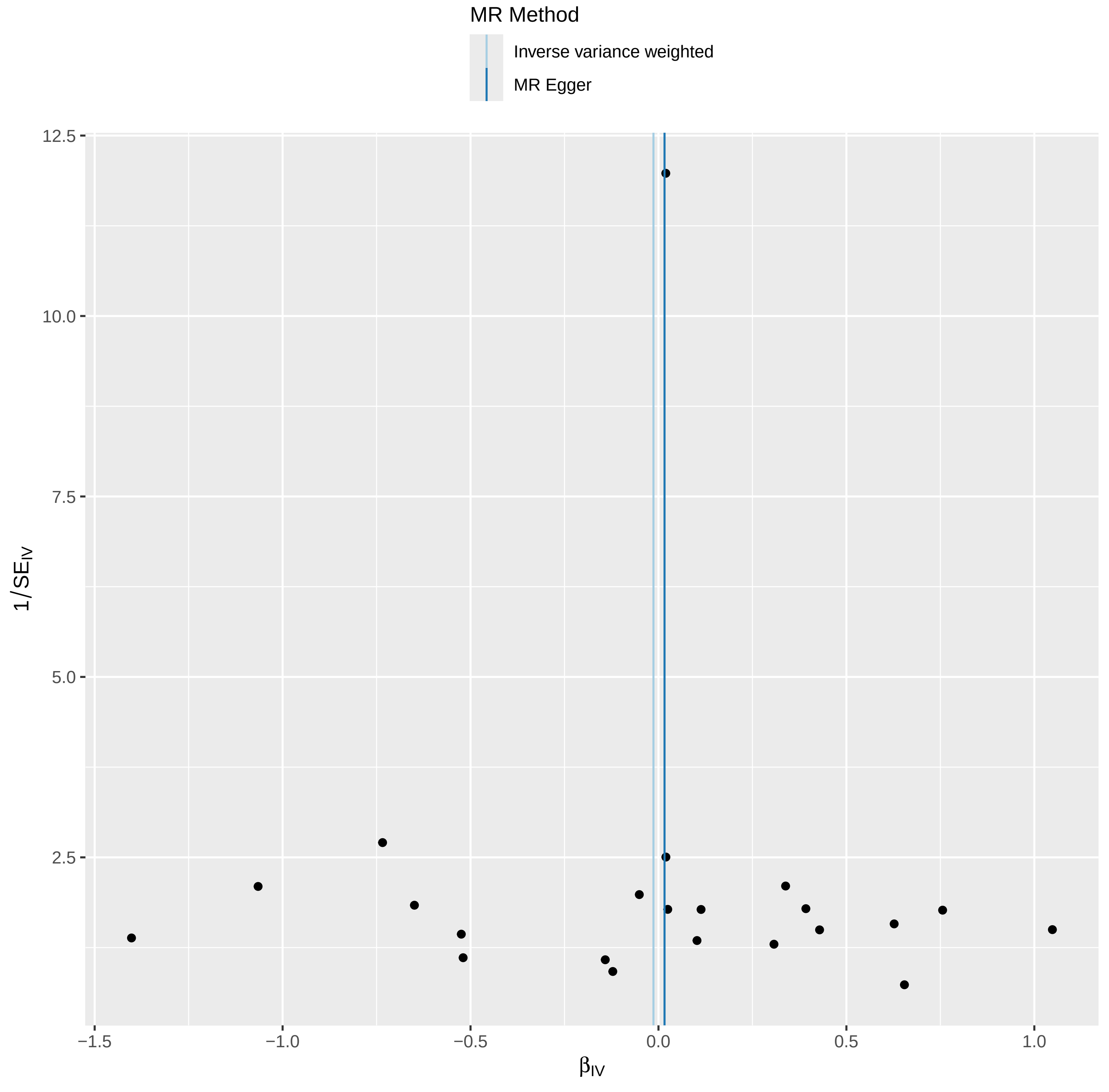
MR Method
Inverse variance weighted
MR Egger
12.5
10.0
7.5
5.0
2.5
1/SE$_{IV}$
−1.5
−1.0
−0.5
0.0
0.5
1.0
β$_{IV}$

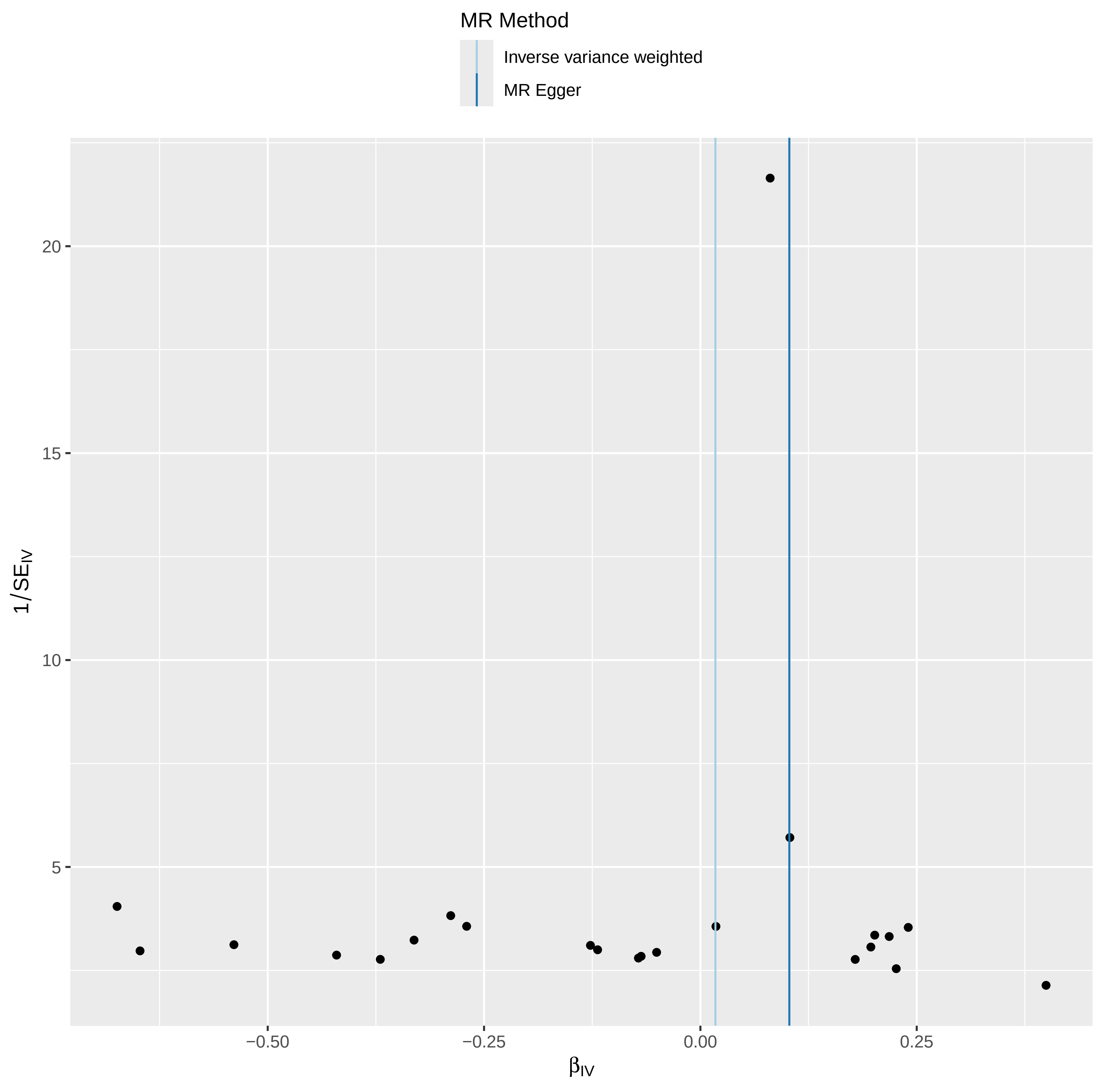

MR Method
Inverse variance weighted
MR Egger
20
15
10
5
1/SE_IV
−0.50
−0.25
0.00
0.25
β_IV

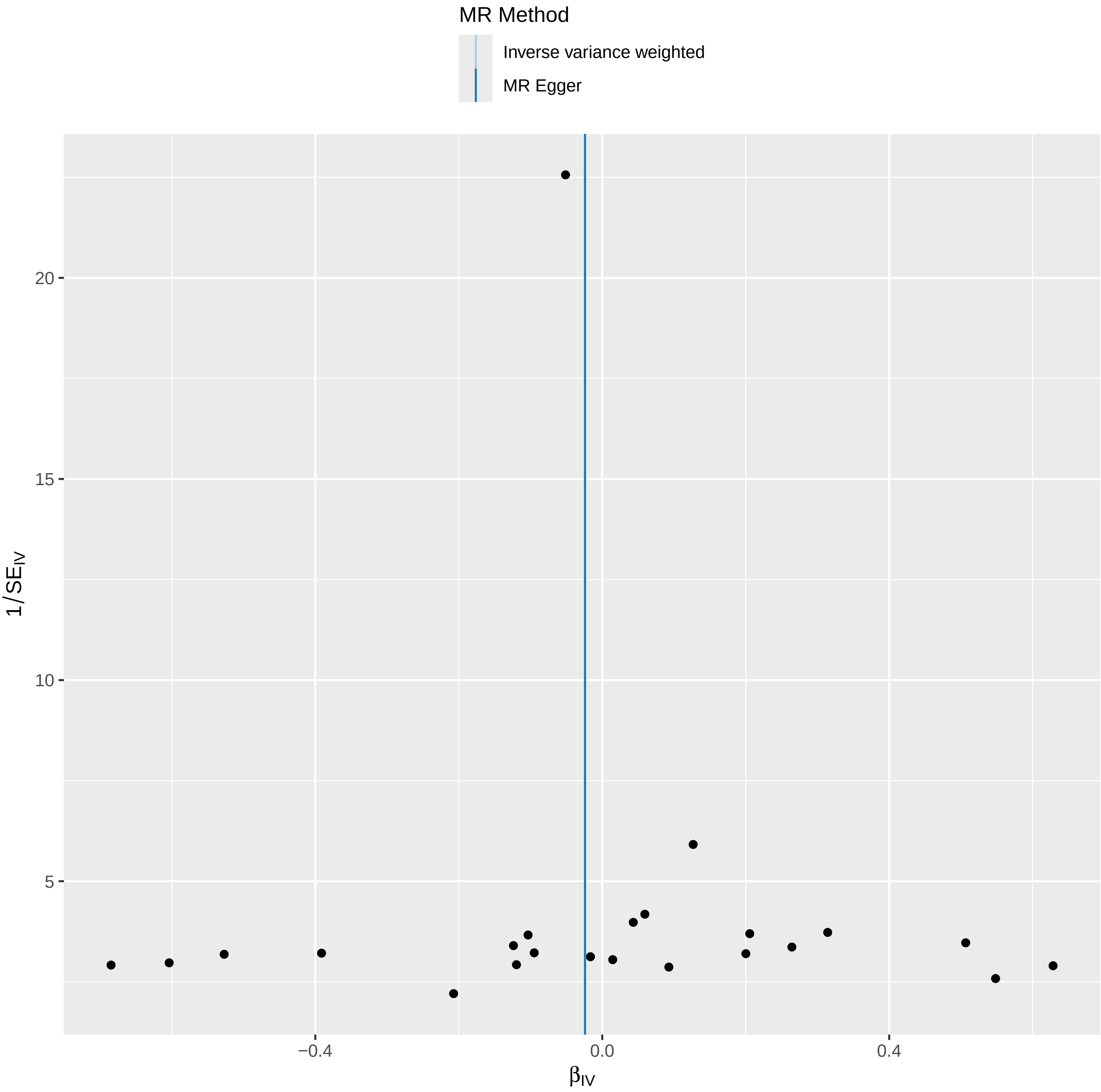
MR Method
Inverse variance weighted
MR Egger
20
15
10
5
1/SE_IV
−0.4
0.0
0.4
β_IV

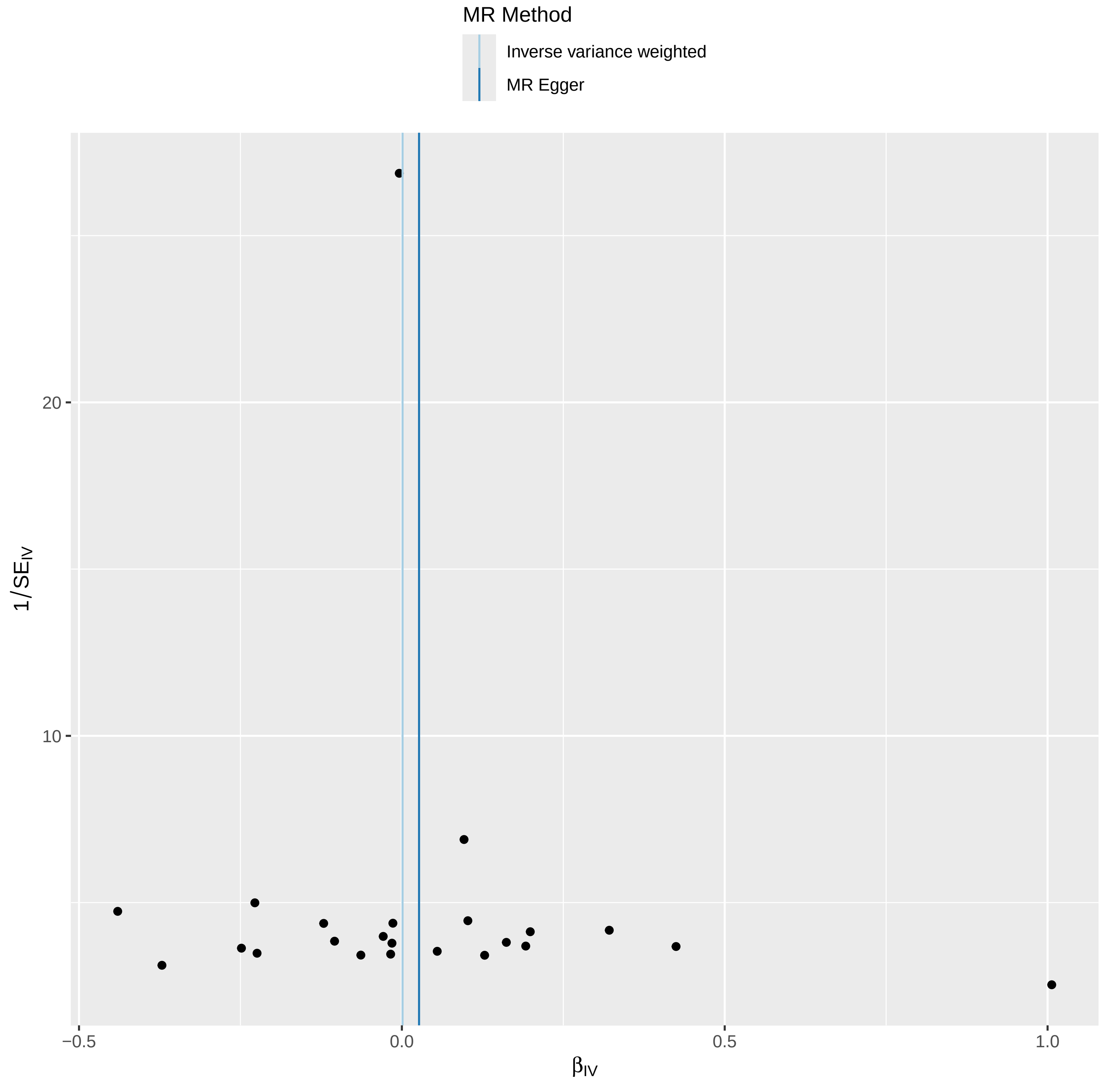
MR Method
Inverse variance weighted
MR Egger
20
10
1/SE_IV
−0.5
0.0
0.5
1.0
β_IV

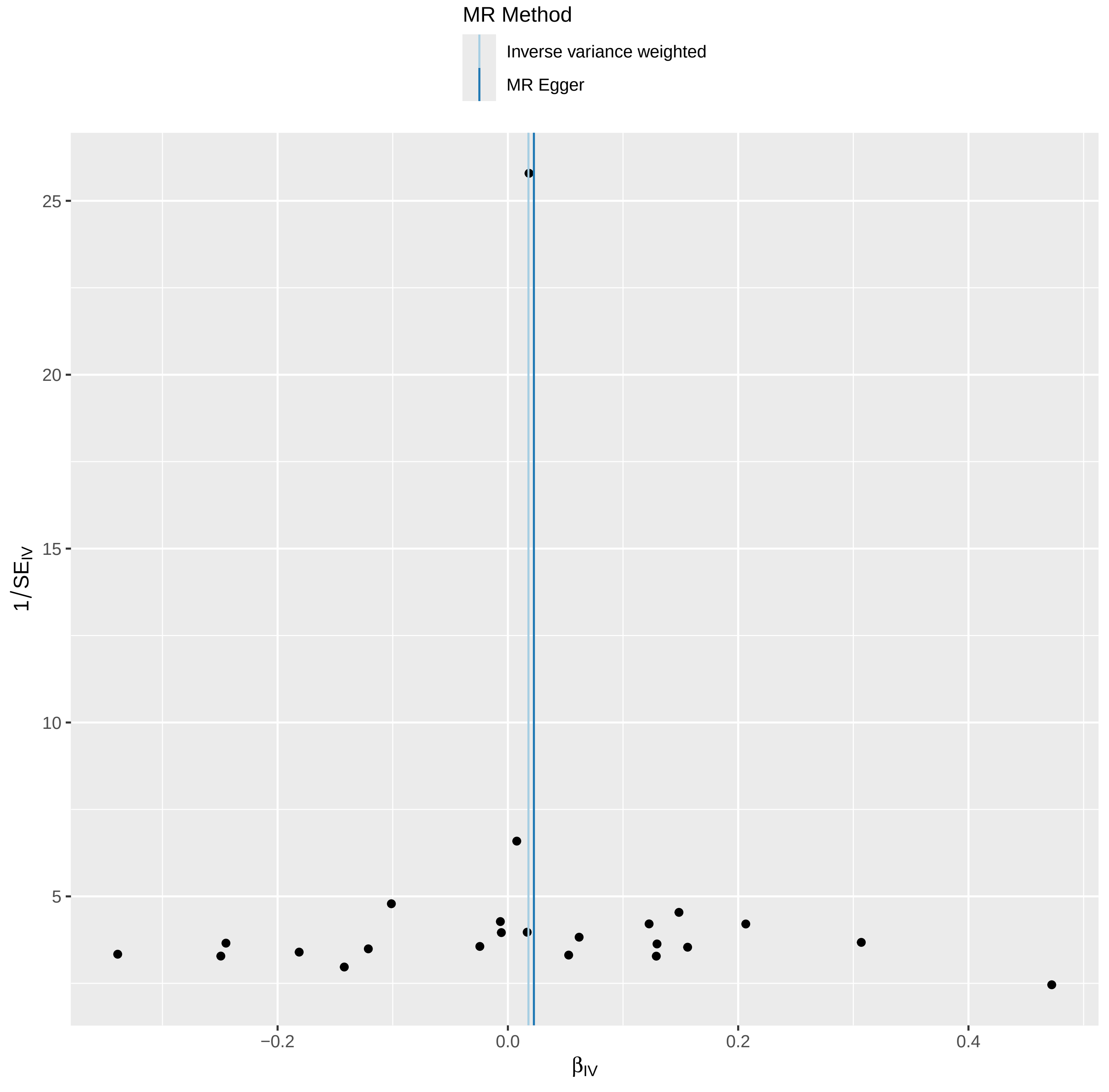
MR Method
Inverse variance weighted
MR Egger
1/SE_IV
25
20
15
10
5
−0.2
0.0
0.2
0.4
β_IV

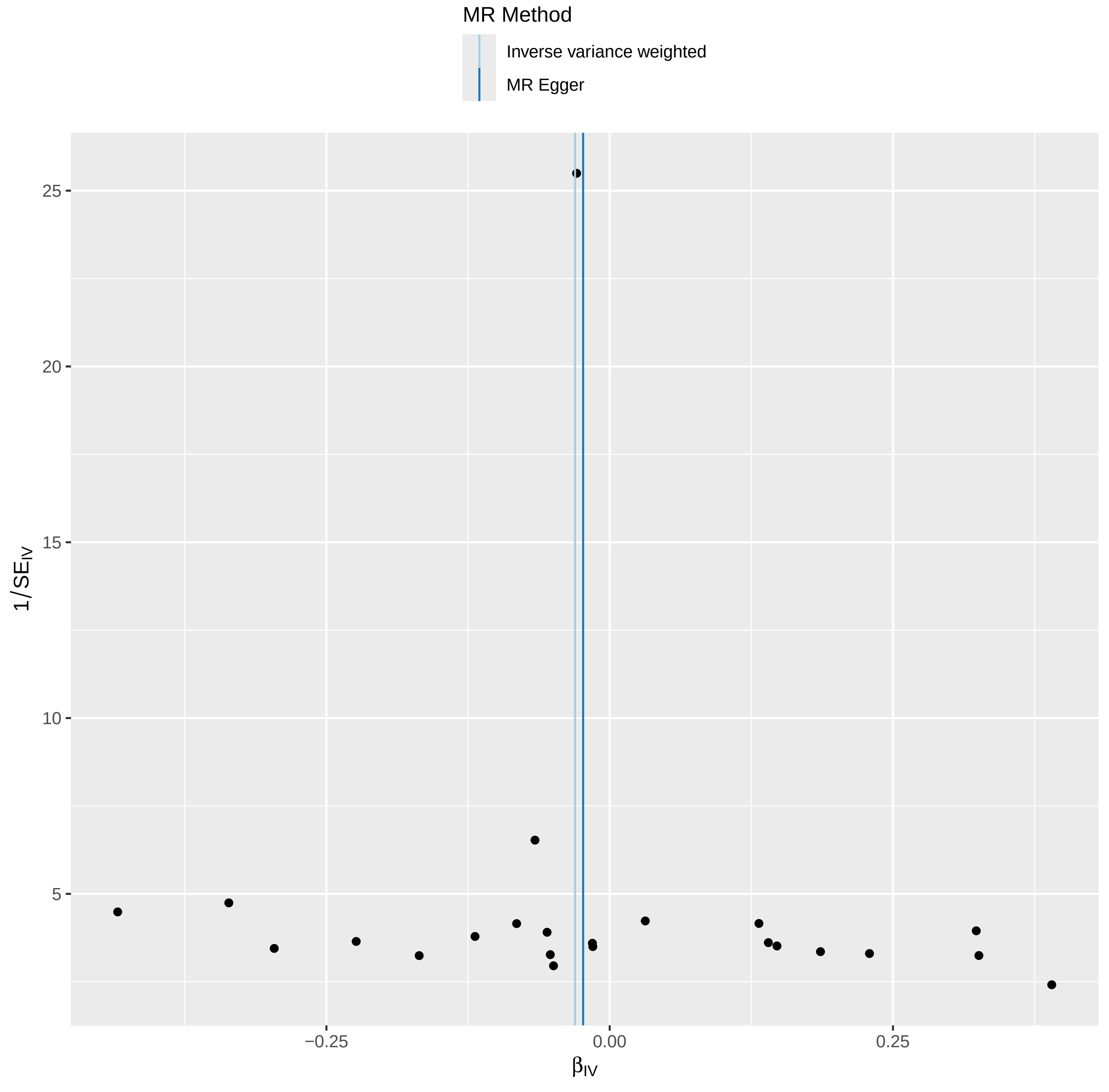

MR Method
Inverse variance weighted
MR Egger
25
20
15
10
5
1/SE_IV
−0.25
0.00
0.25
β_IV

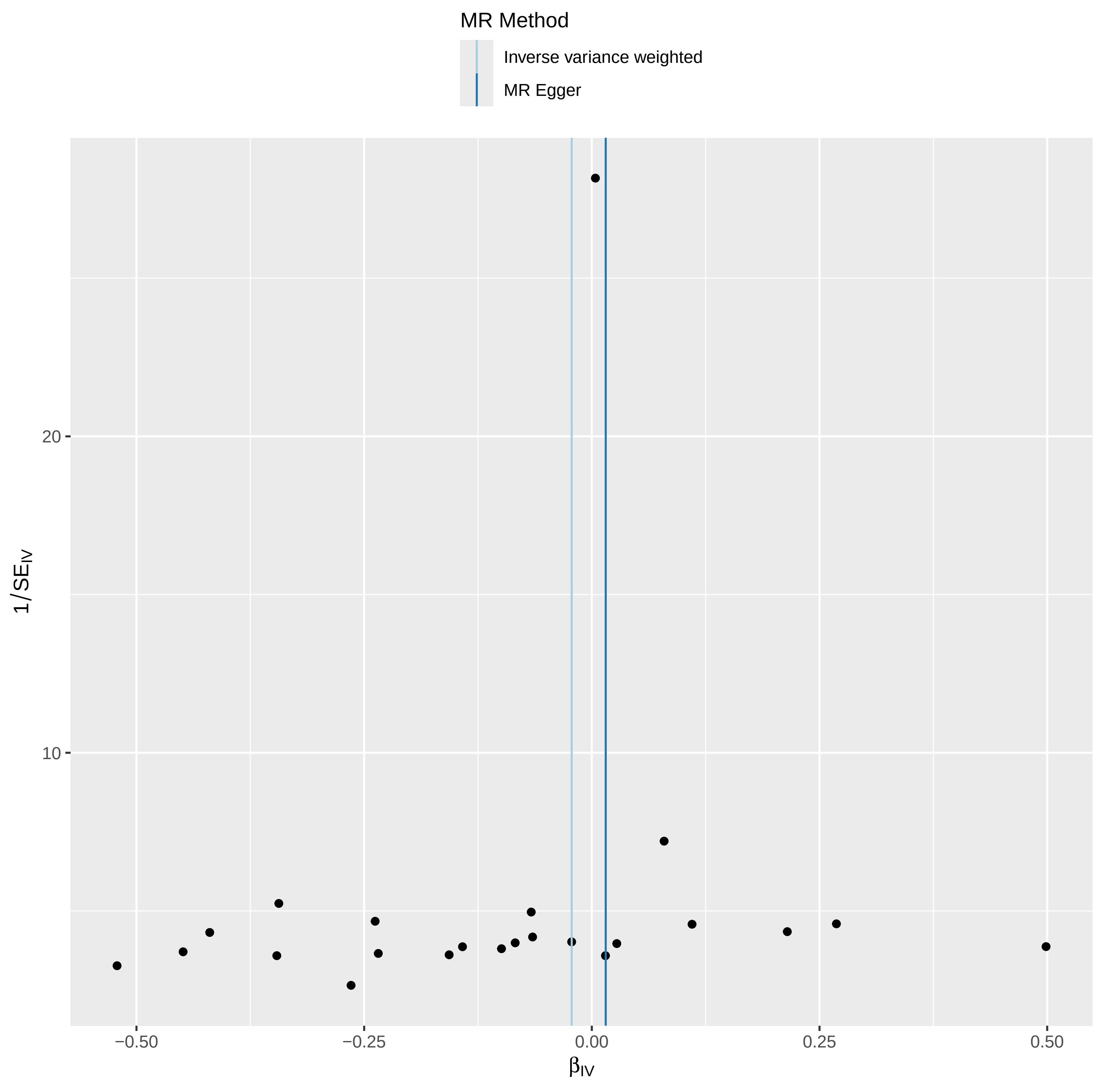
MR Method
Inverse variance weighted
MR Egger
20
10
$1/SE_{IV}$
−0.50
−0.25
0.00
0.25
0.50
β_{IV}

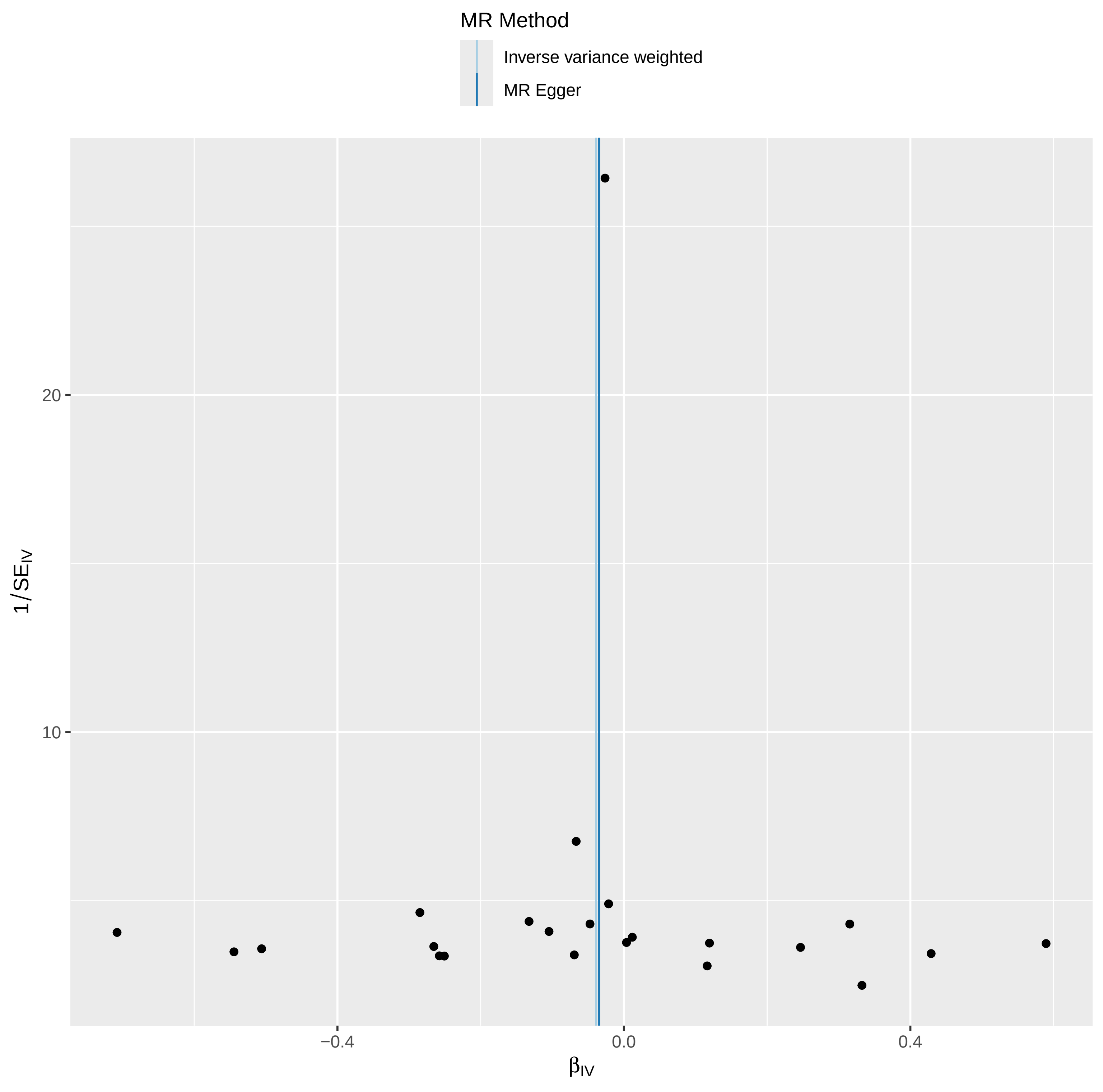

MR Method
Inverse variance weighted
MR Egger
20
10
1/SE$_{IV}$
−0.4
0.0
0.4
β$_{IV}$

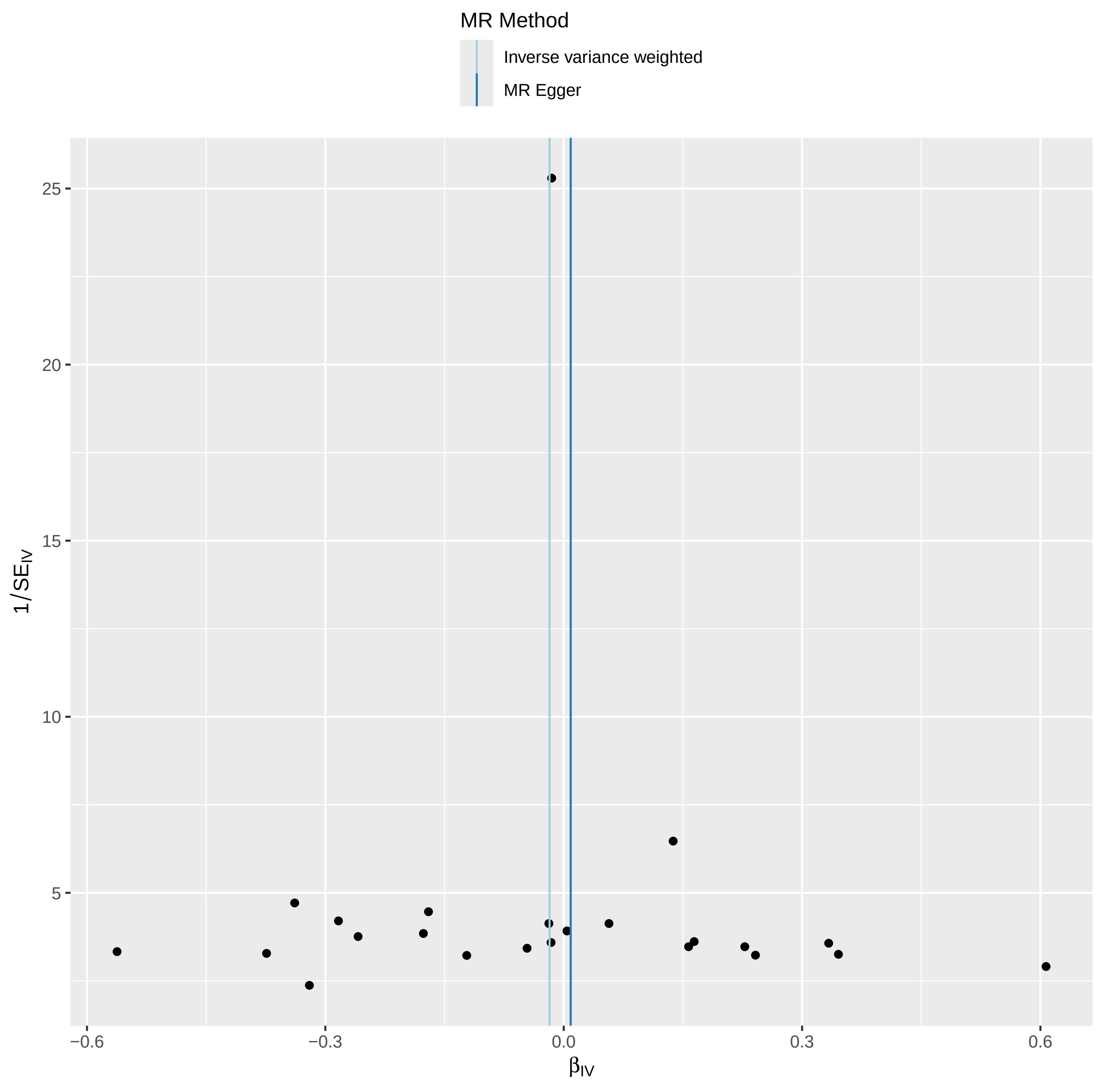

MR Method
Inverse variance weighted
MR Egger
25
20
15
10
5
$1/SE_{IV}$
−0.6
−0.3
0.0
0.3
0.6
β_{IV}

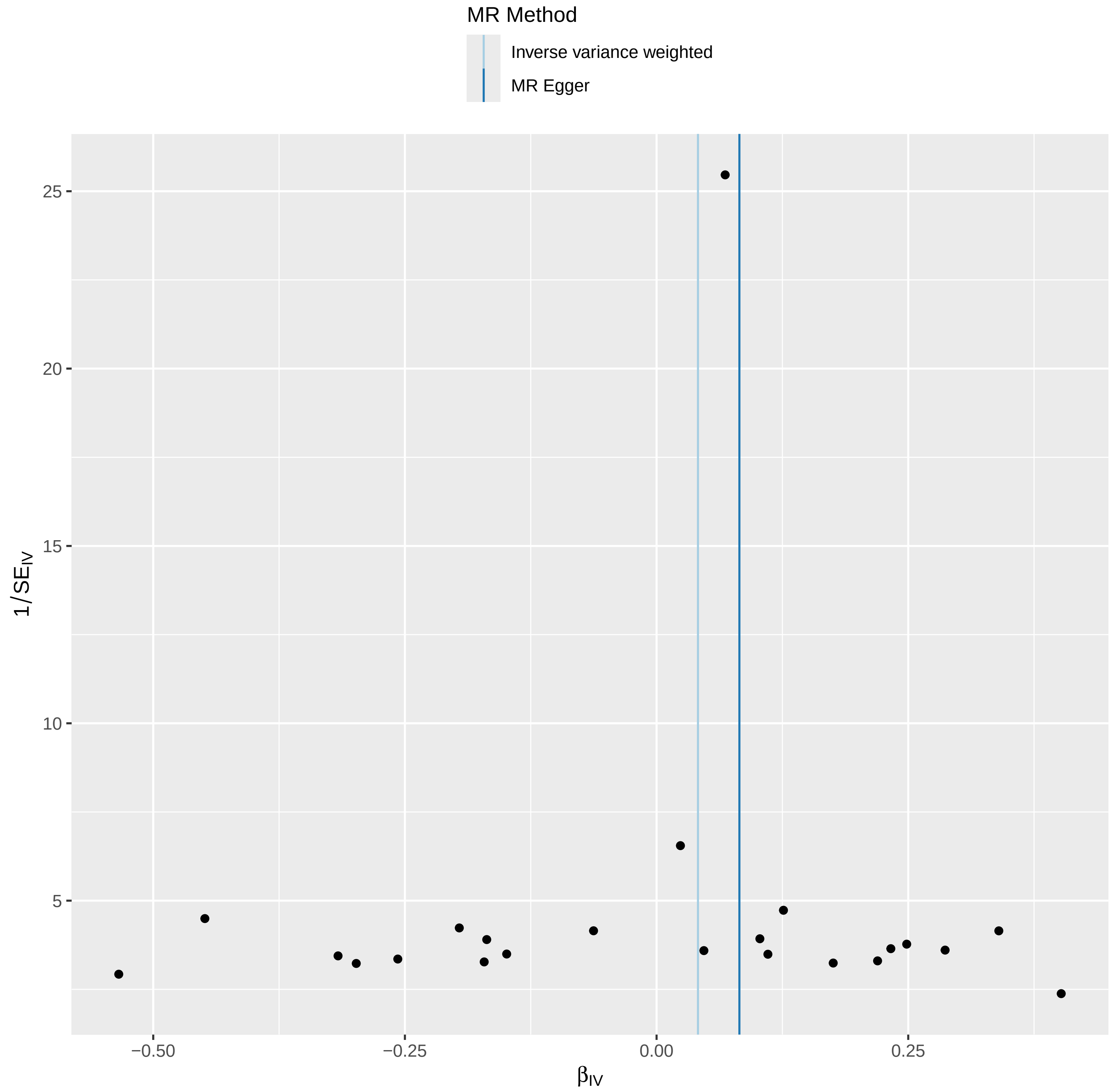

MR Method
Inverse variance weighted
MR Egger
25
20
15
10
5
1/SE$_{IV}$
−0.50
−0.25
0.00
0.25
β_{IV}

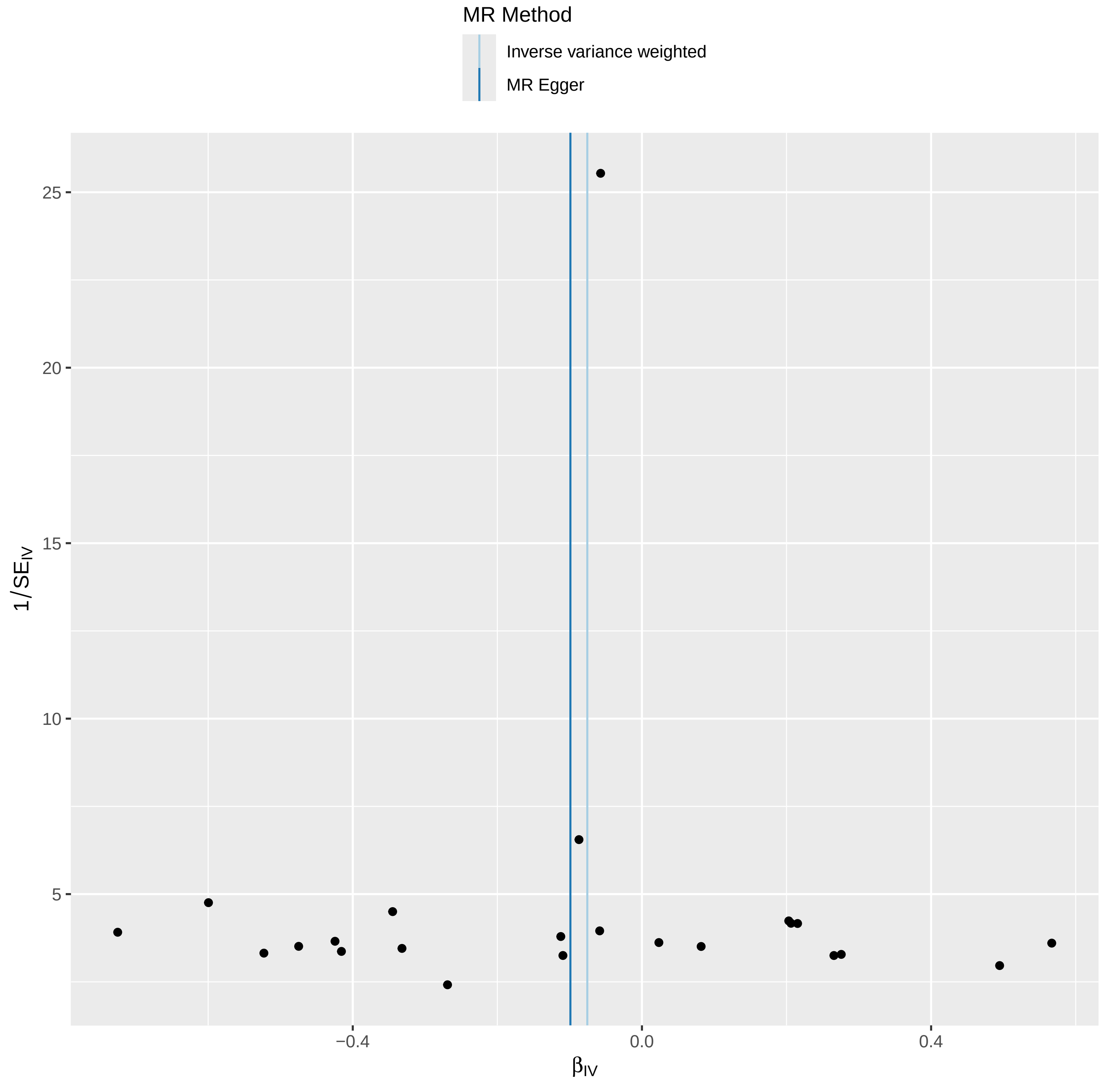
MR Method
Inverse variance weighted
MR Egger
25
20
15
10
5
1/SE_IV
−0.4
0.0
0.4
β_IV

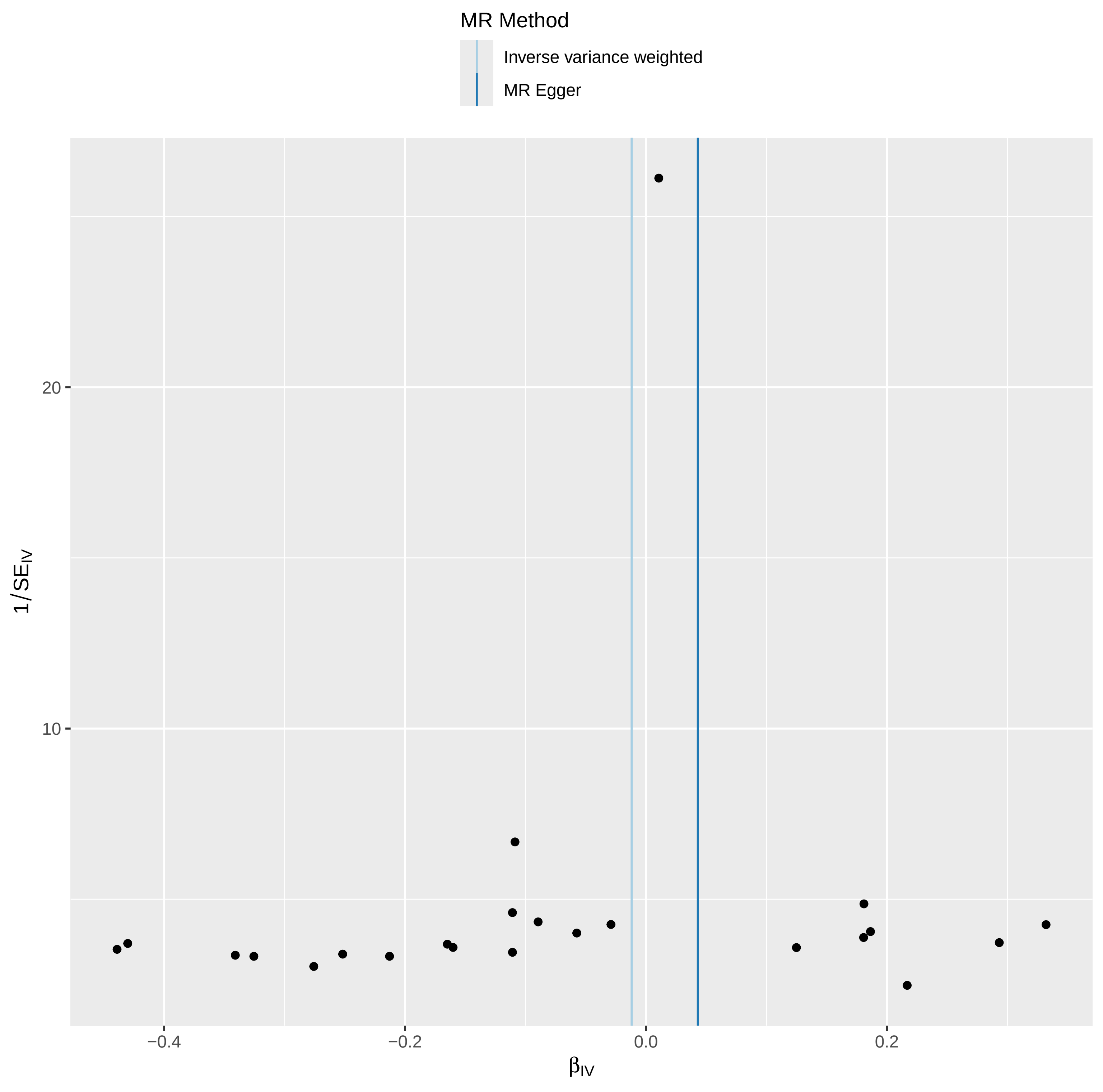
MR Method
Inverse variance weighted
MR Egger
20
10
1/SE_{IV}
−0.4
−0.2
0.0
0.2
β_{IV}

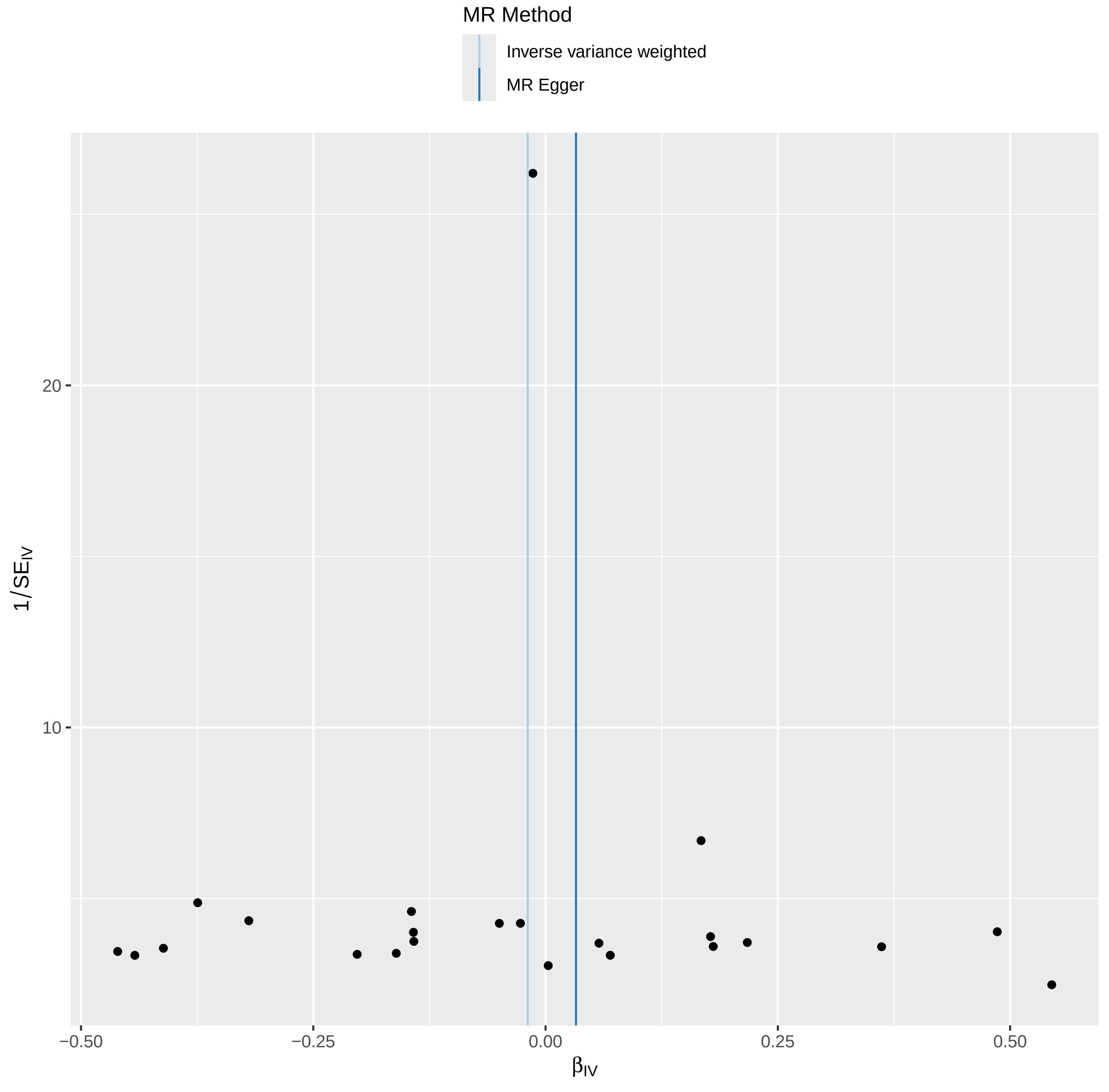

MR Method
Inverse variance weighted
MR Egger
20
10
1/SE_IV
−0.50
−0.25
0.00
0.25
0.50
β_IV

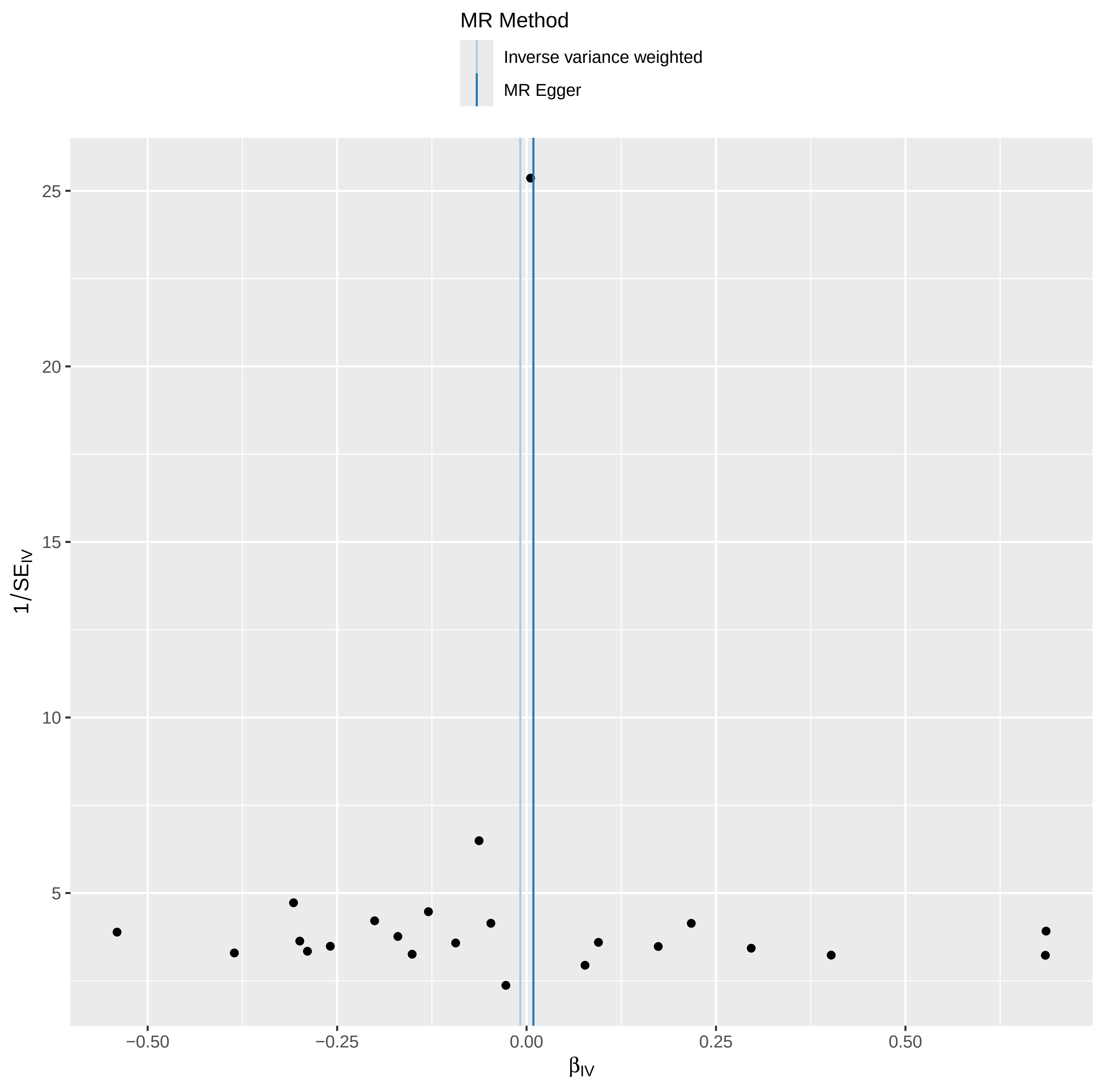

MR Method
Inverse variance weighted
MR Egger
1/SE_IV
β_IV
25
20
15
10
5
−0.50
−0.25
0.00
0.25
0.50

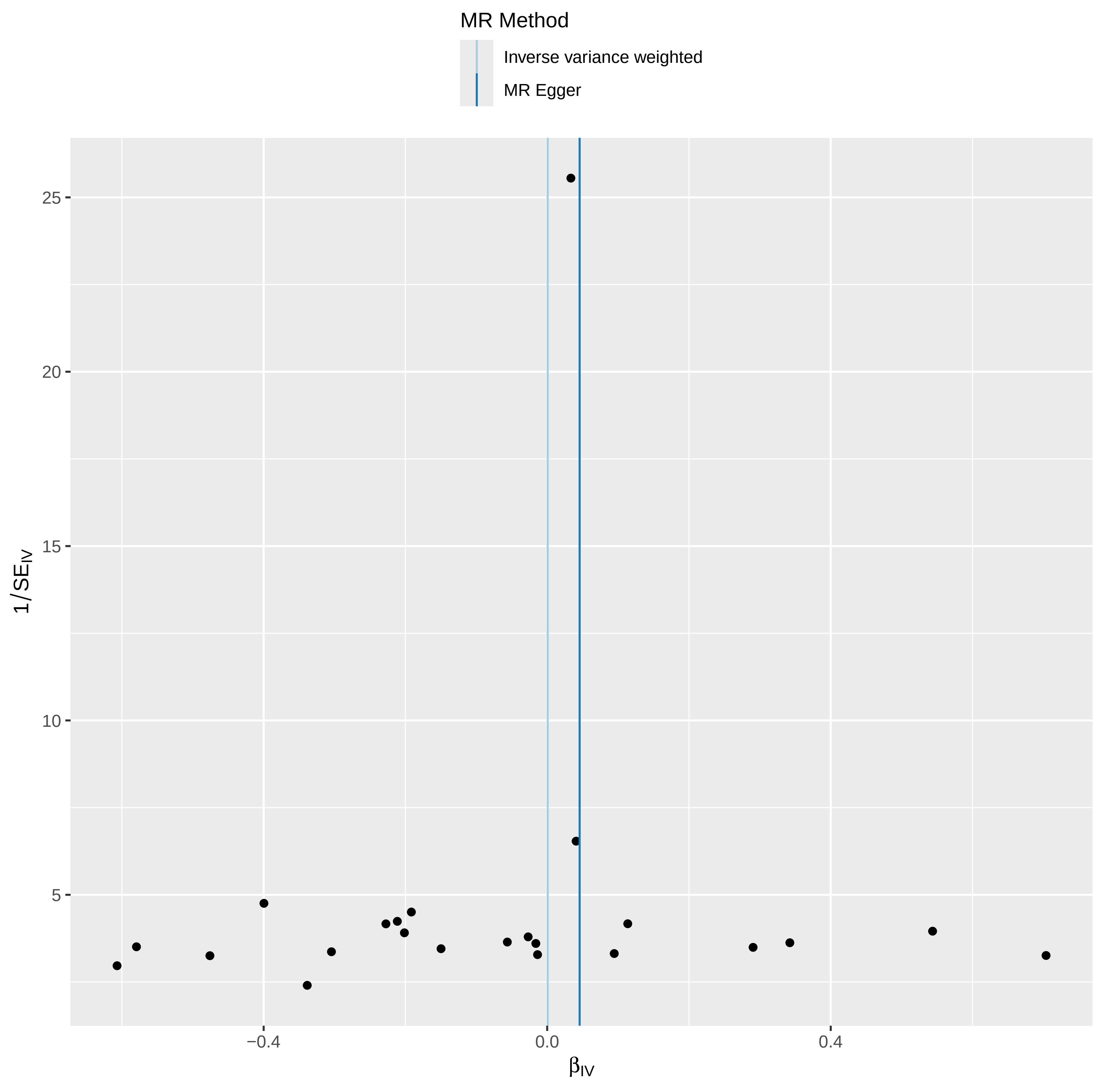
MR Method
Inverse variance weighted
MR Egger
25
20
15
10
5
1/SE$_{IV}$
−0.4
0.0
0.4
β$_{IV}$

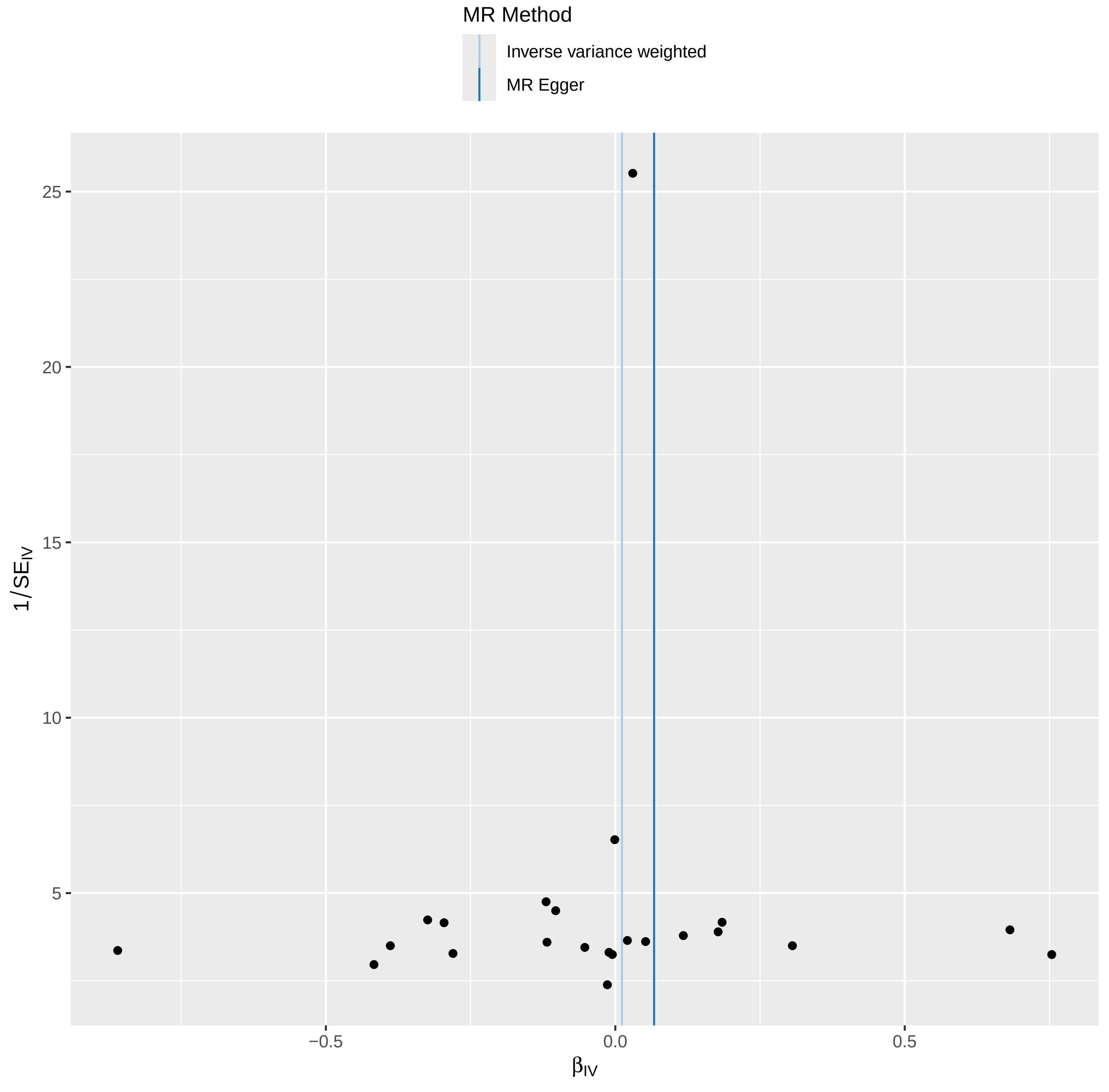

MR Method
Inverse variance weighted
MR Egger
25
20
15
10
5
1/SE$_{IV}$
−0.5
0.0
0.5
β$_{IV}$

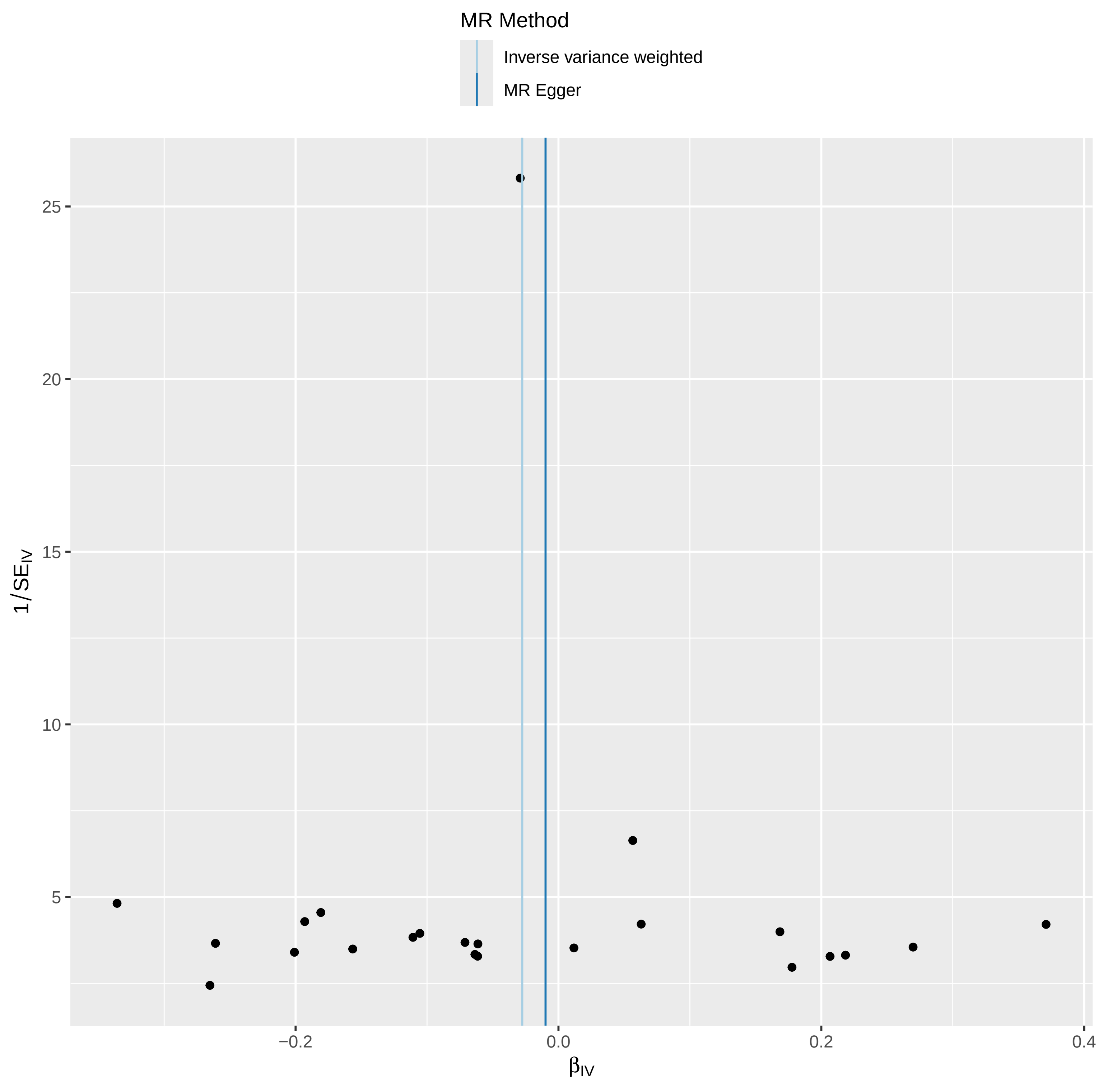

MR Method
Inverse variance weighted
MR Egger
25
20
15
10
5
1/SE$_{IV}$
−0.2
0.0
0.2
0.4
β$_{IV}$

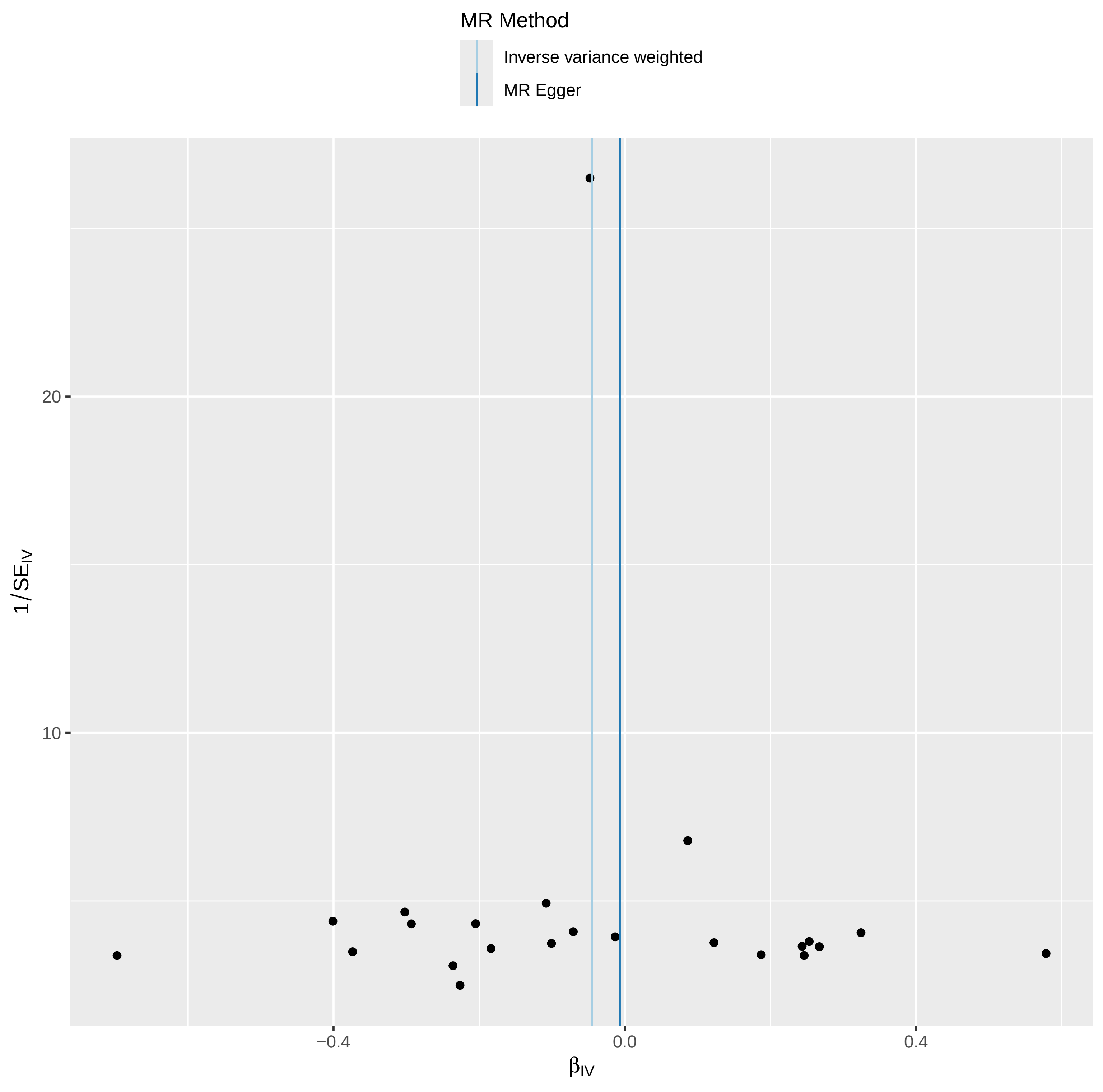
MR Method
Inverse variance weighted
MR Egger
20
10
1/SE$_{IV}$
−0.4
0.0
0.4
β$_{IV}$

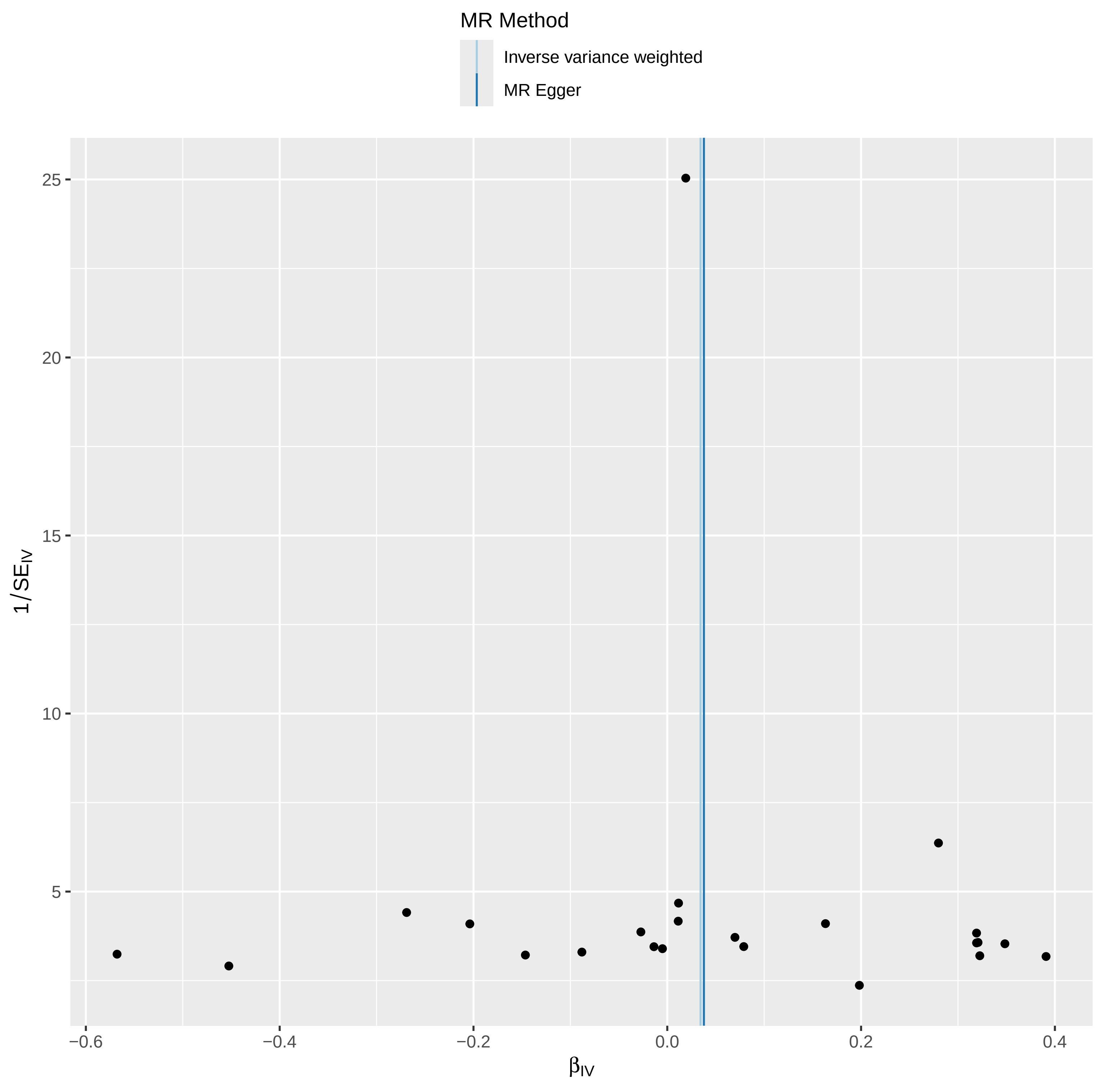
MR Method
Inverse variance weighted
MR Egger
25
20
15
10
5
1/SE$_{IV}$
−0.6
−0.4
−0.2
0.0
0.2
0.4
β$_{IV}$

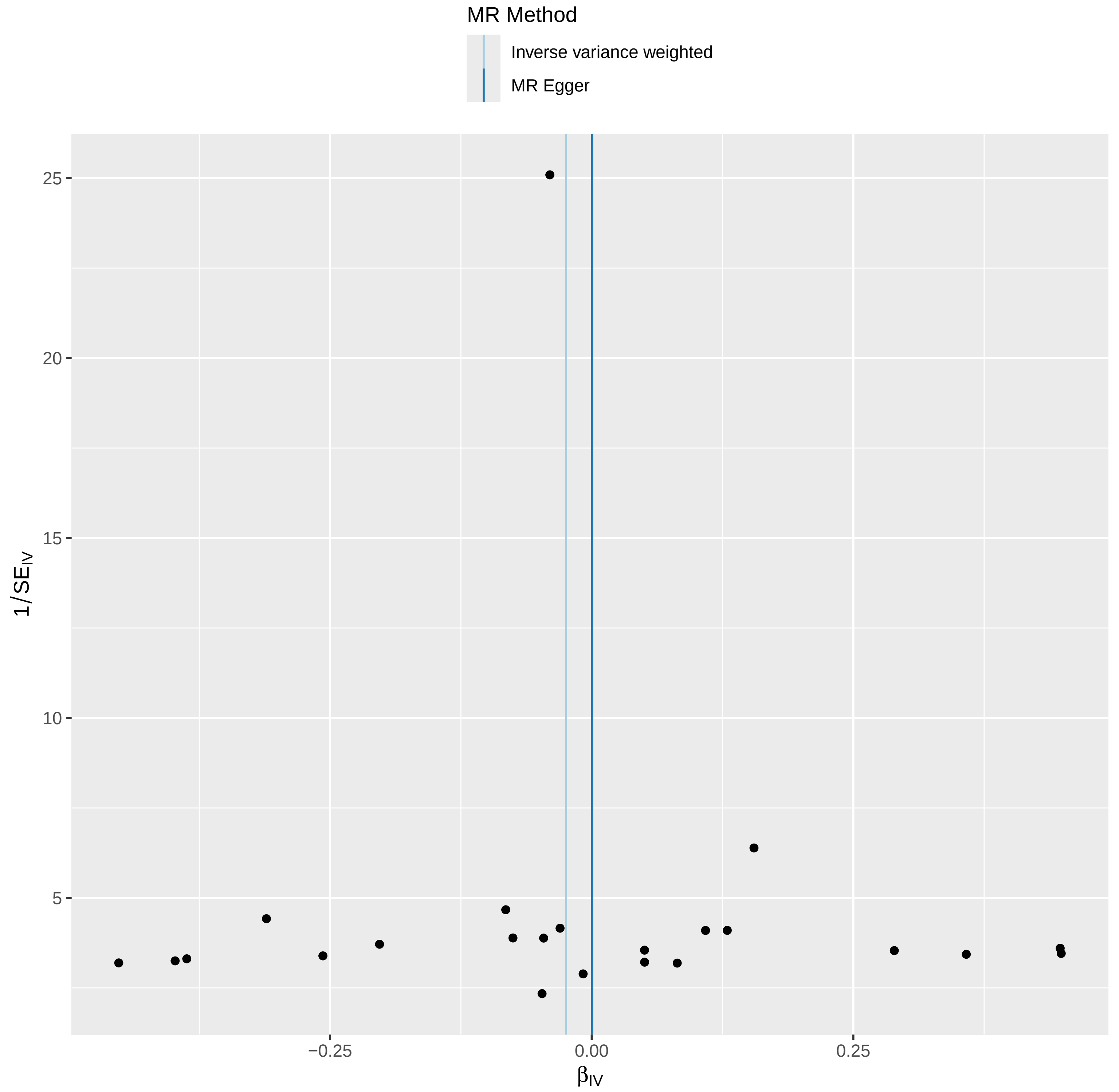

MR Method
Inverse variance weighted
MR Egger
25
20
15
10
5
1/SE$_{IV}$
−0.25
0.00
0.25
β$_{IV}$

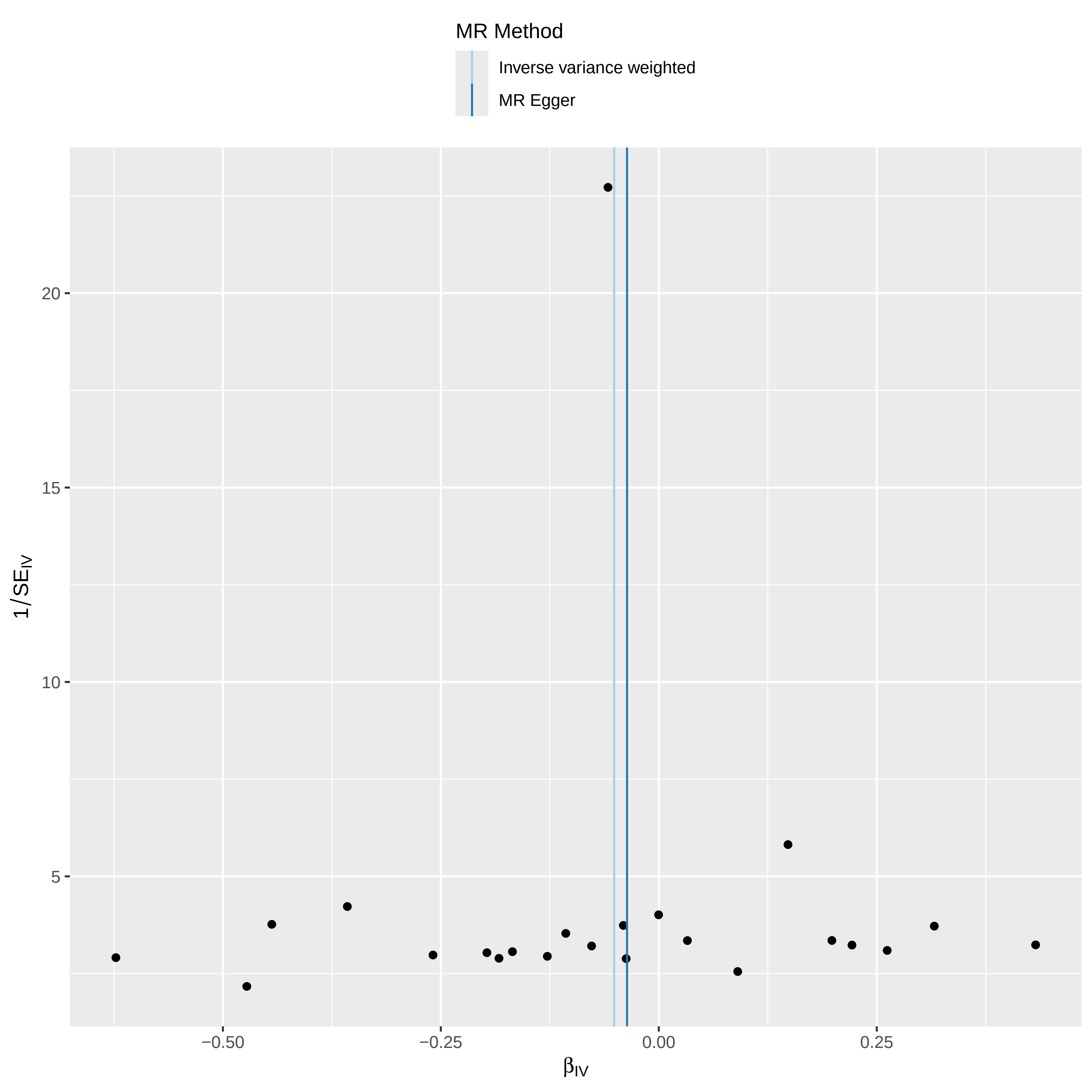

MR Method
Inverse variance weighted
MR Egger
20
15
10
5
1/SE_IV
−0.50
−0.25
0.00
0.25
β_IV

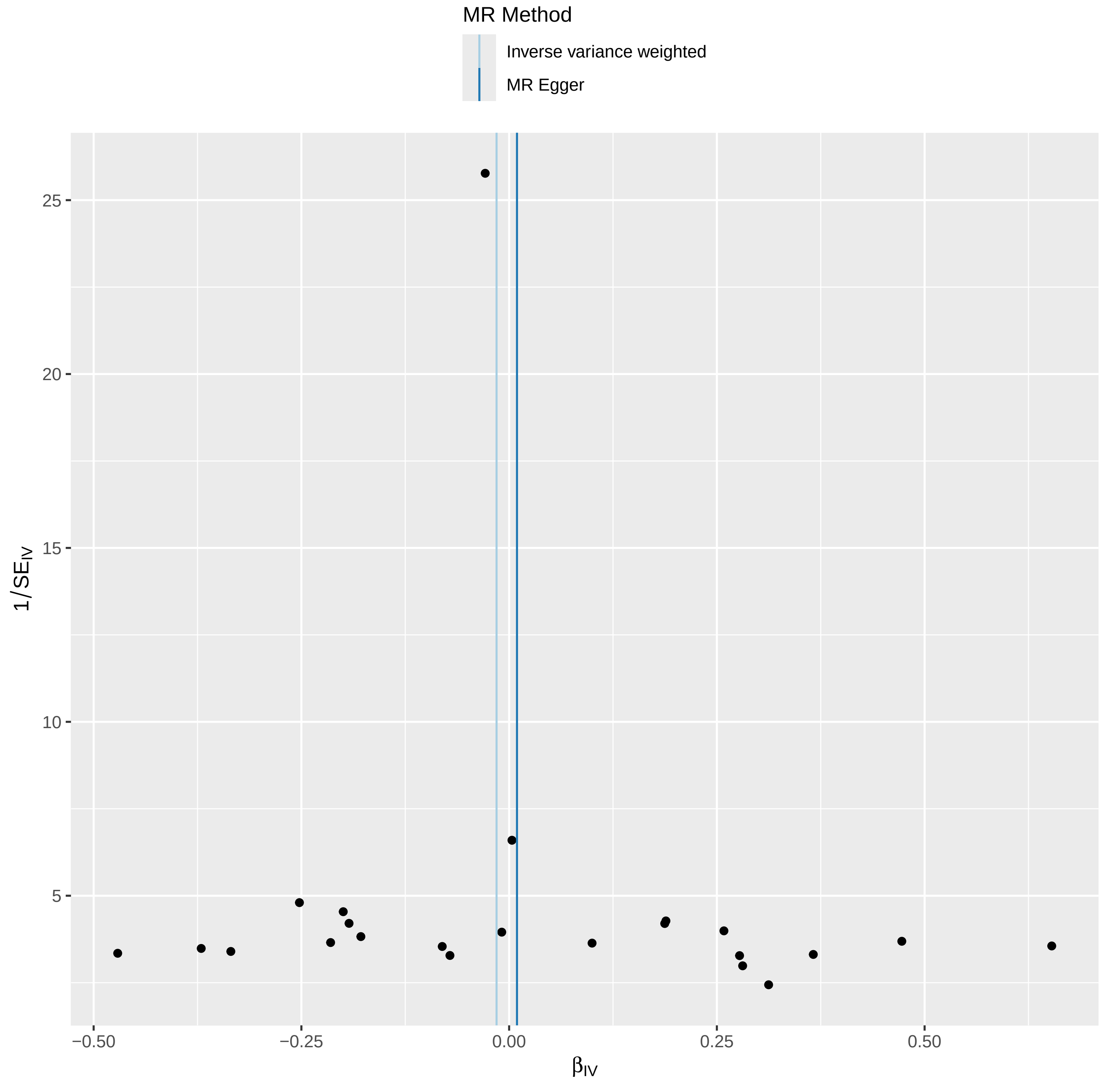

MR Method
Inverse variance weighted
MR Egger
25
20
15
10
5
1/SE_IV
−0.50
−0.25
0.00
0.25
0.50
β_IV

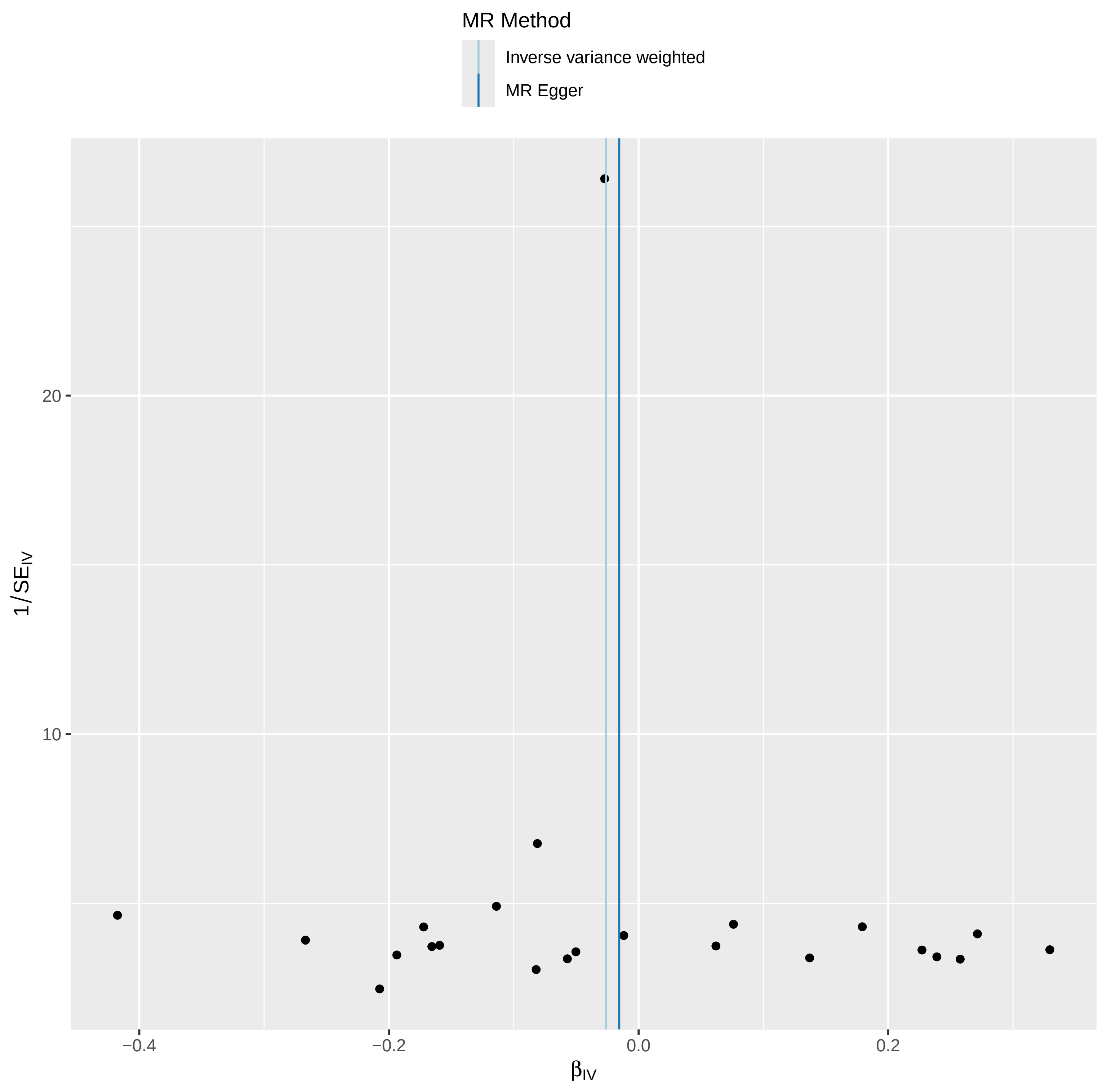

MR Method
Inverse variance weighted
MR Egger
20
10
1/SE_{IV}
−0.4
−0.2
0.0
0.2
β_{IV}

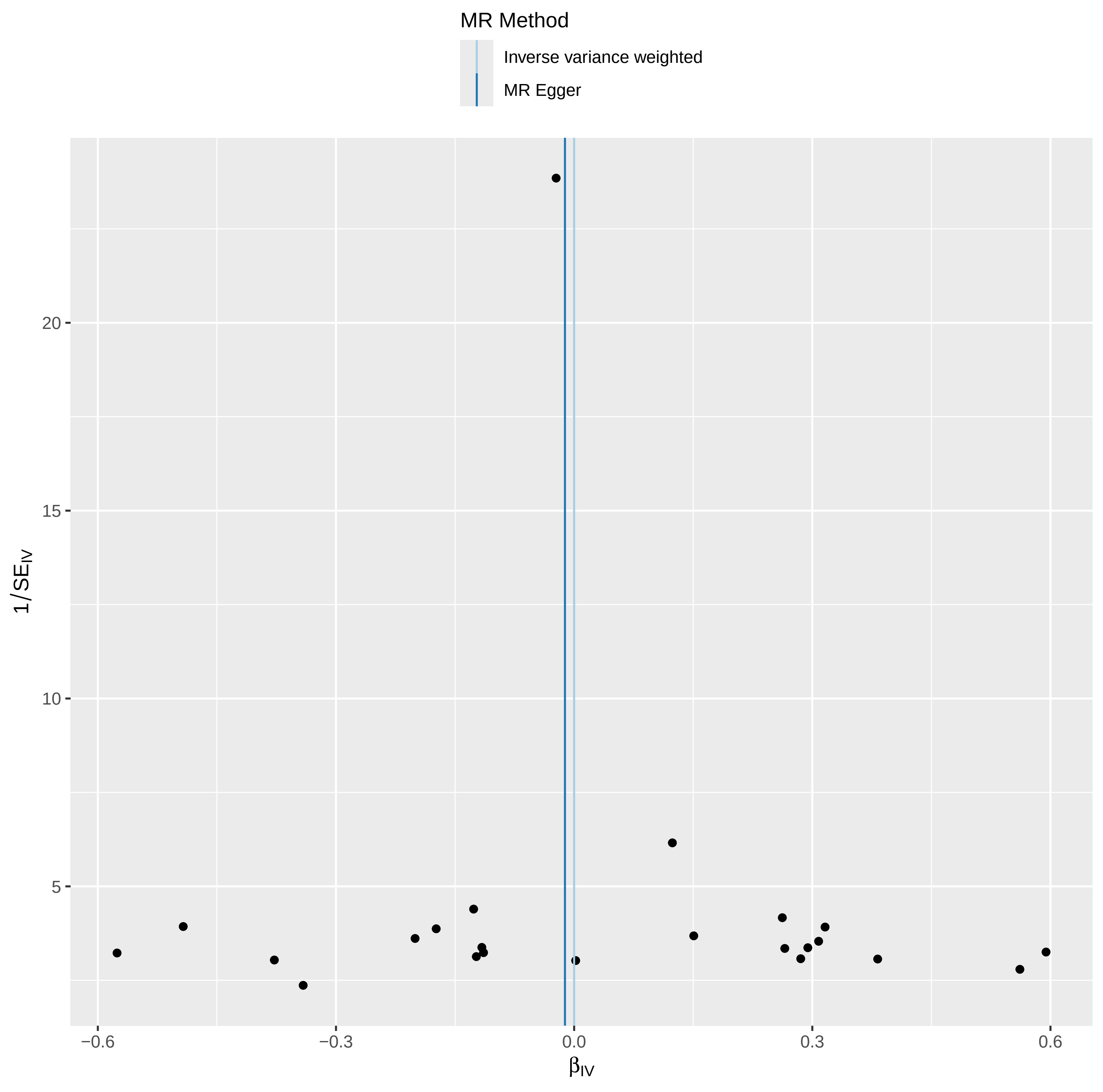

MR Method
Inverse variance weighted
MR Egger
20
15
10
5
1/SE_IV
−0.6
−0.3
0.0
0.3
0.6
β_IV

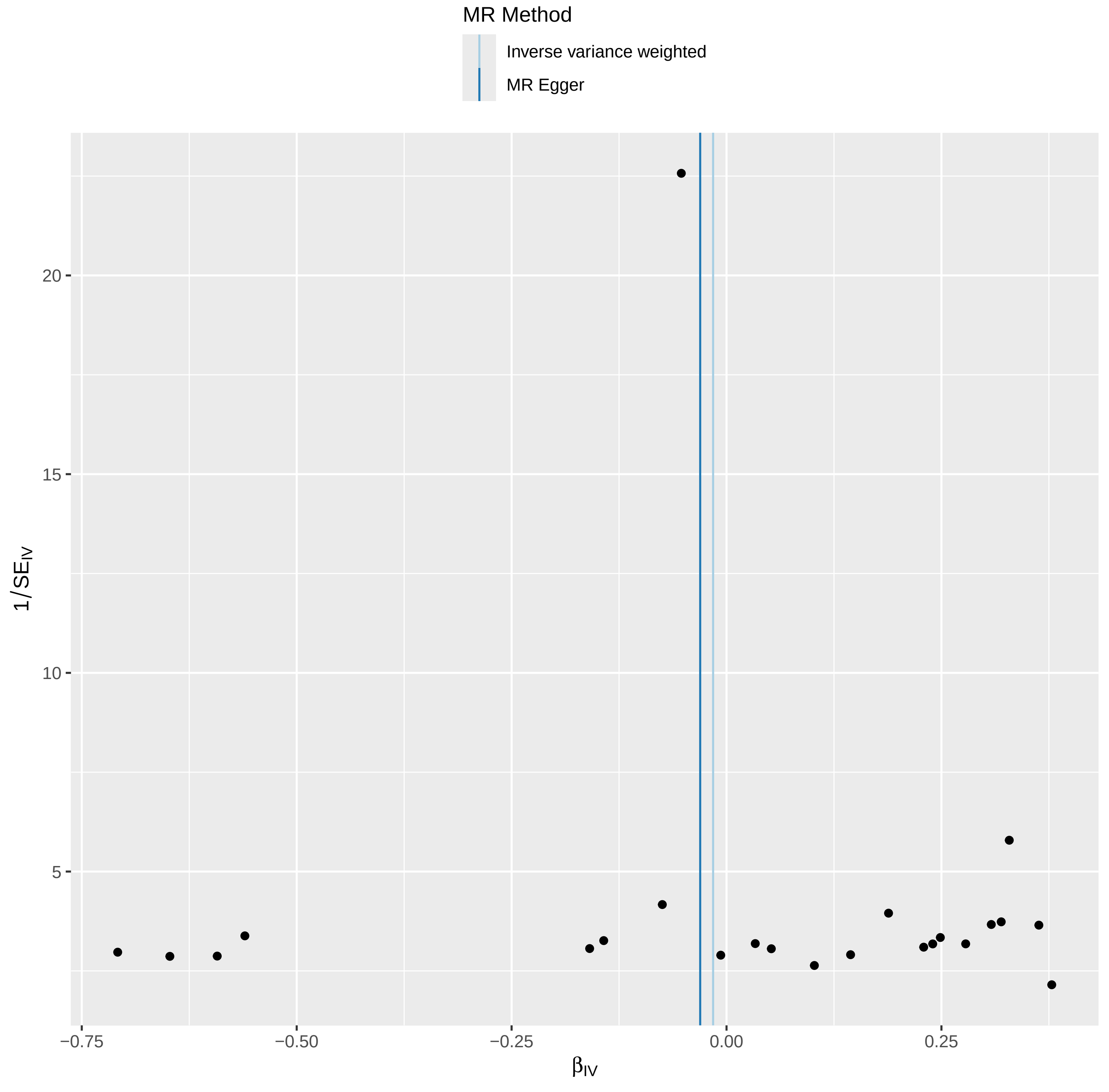

MR Method
Inverse variance weighted
MR Egger
20
15
10
5
1/SE_IV
−0.75
−0.50
−0.25
0.00
0.25
β_IV

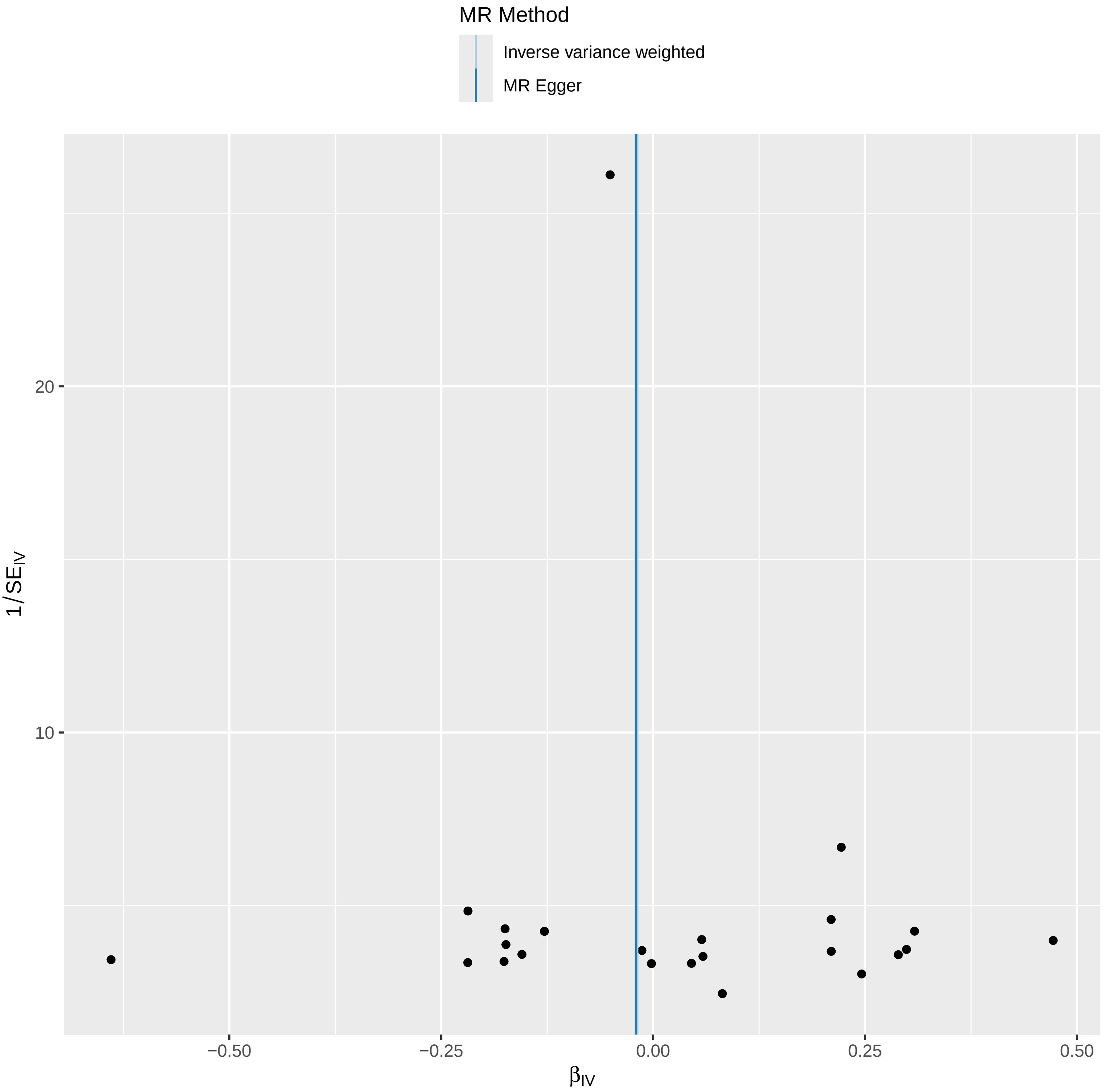
MR Method
Inverse variance weighted
MR Egger
20
10
1/SE_IV
−0.50
−0.25
0.00
0.25
0.50
β_IV

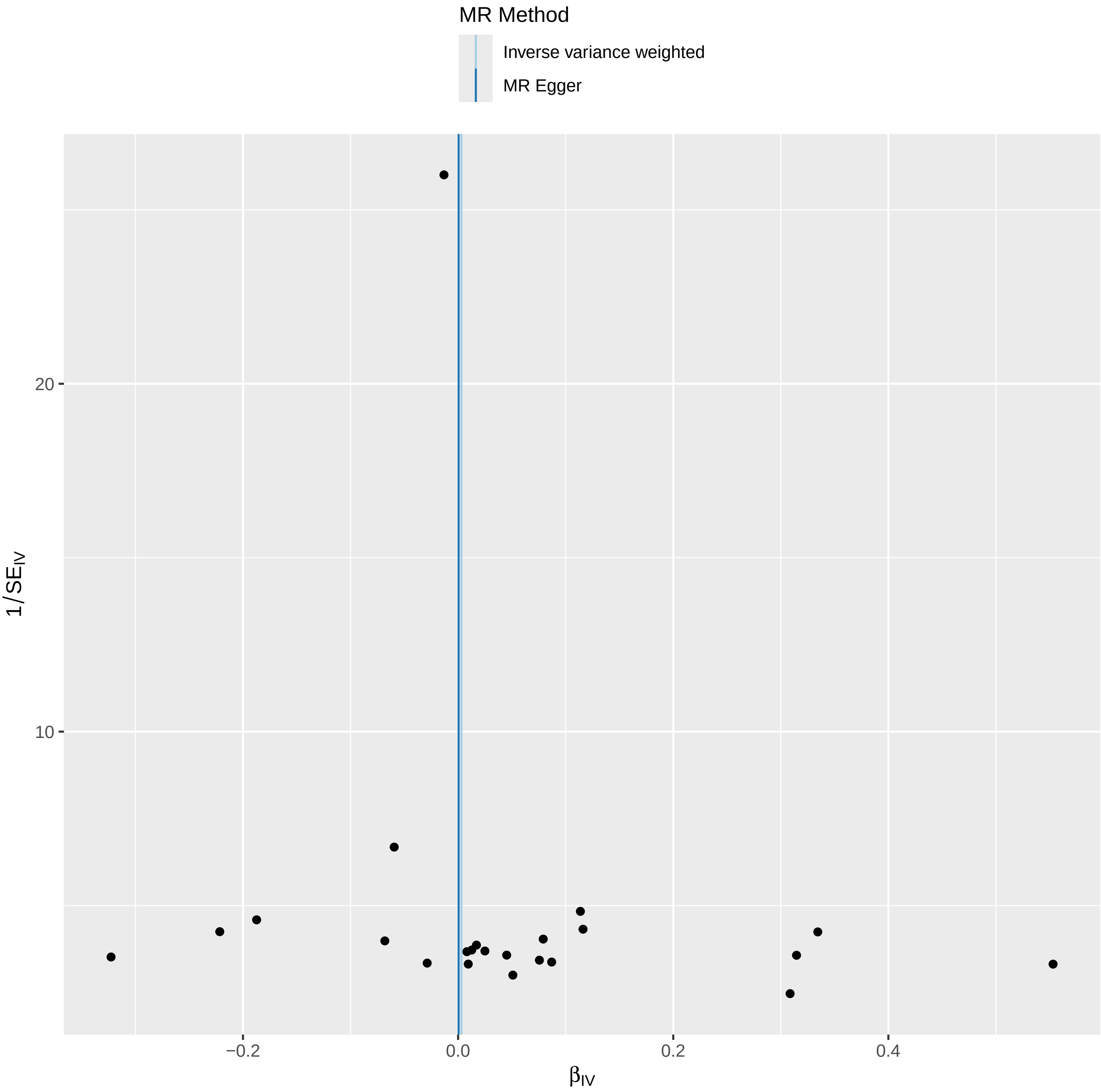

MR Method
Inverse variance weighted
MR Egger
20
10
1/SE$_{IV}$
−0.2
0.0
0.2
0.4
β$_{IV}$

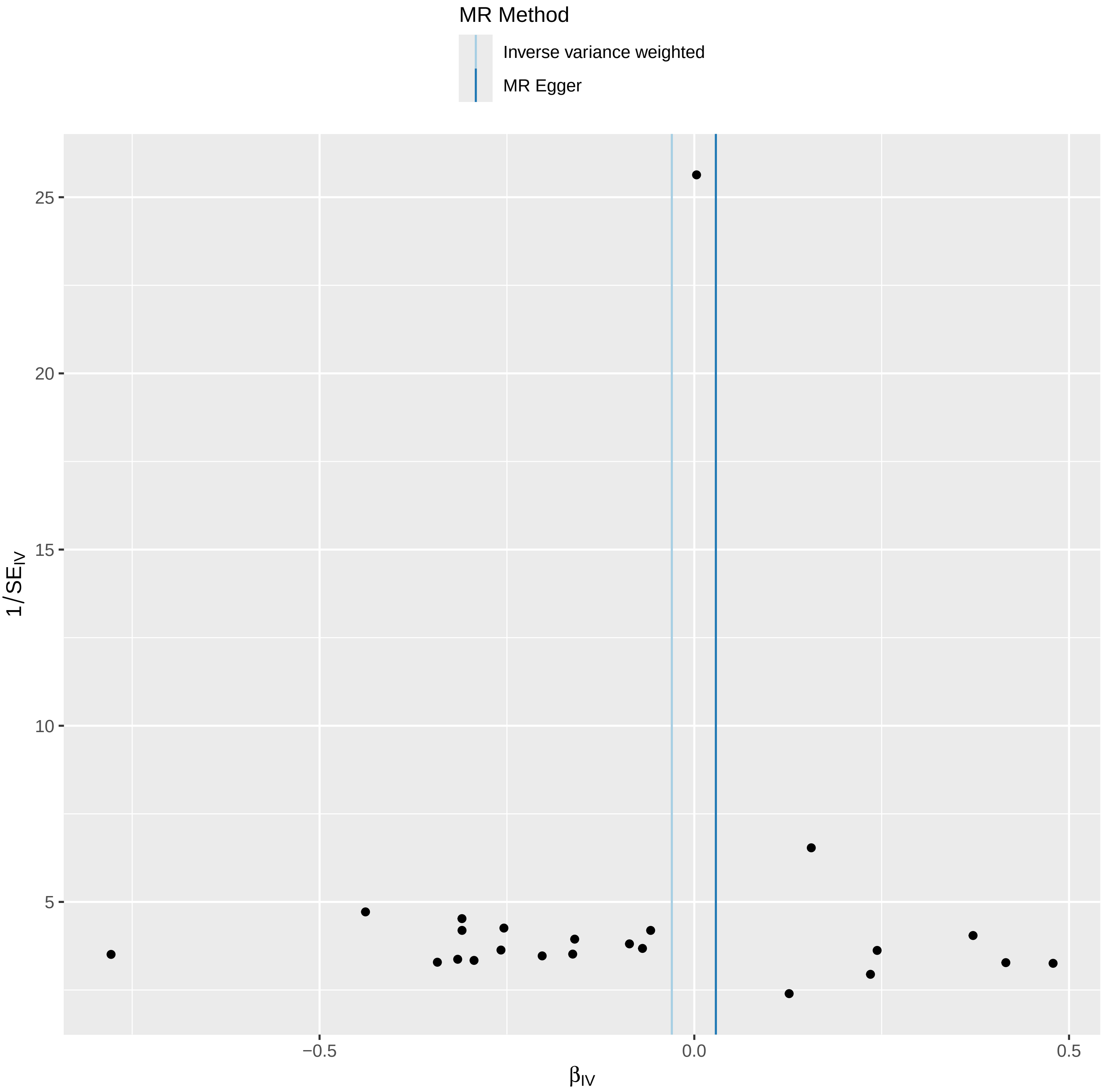

MR Method
Inverse variance weighted
MR Egger
25
20
15
10
5
1/SE_IV
−0.5
0.0
0.5
β_IV

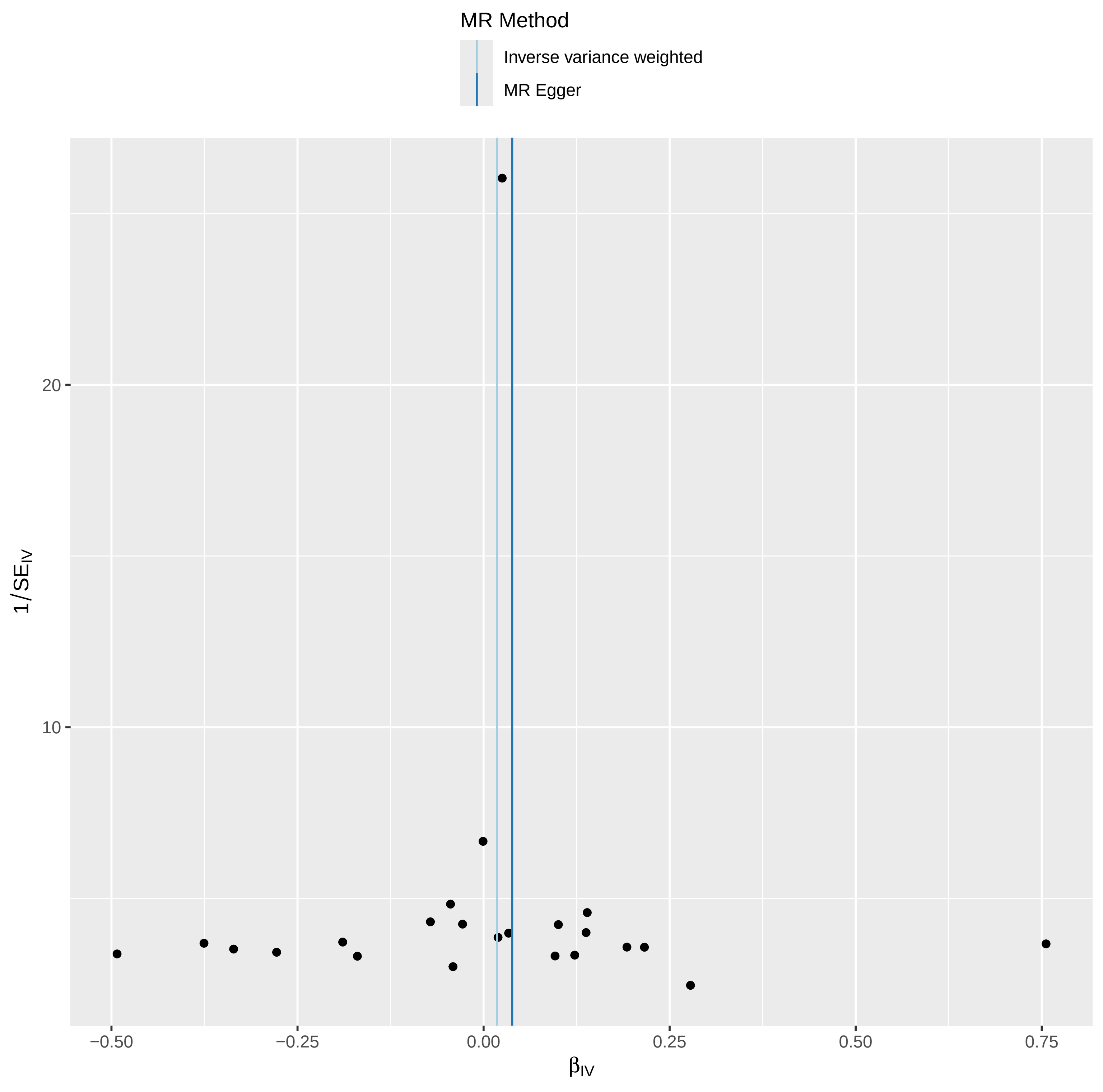
MR Method
Inverse variance weighted
MR Egger
20
10
1/SE_IV
−0.50
−0.25
0.00
0.25
0.50
0.75
β_IV

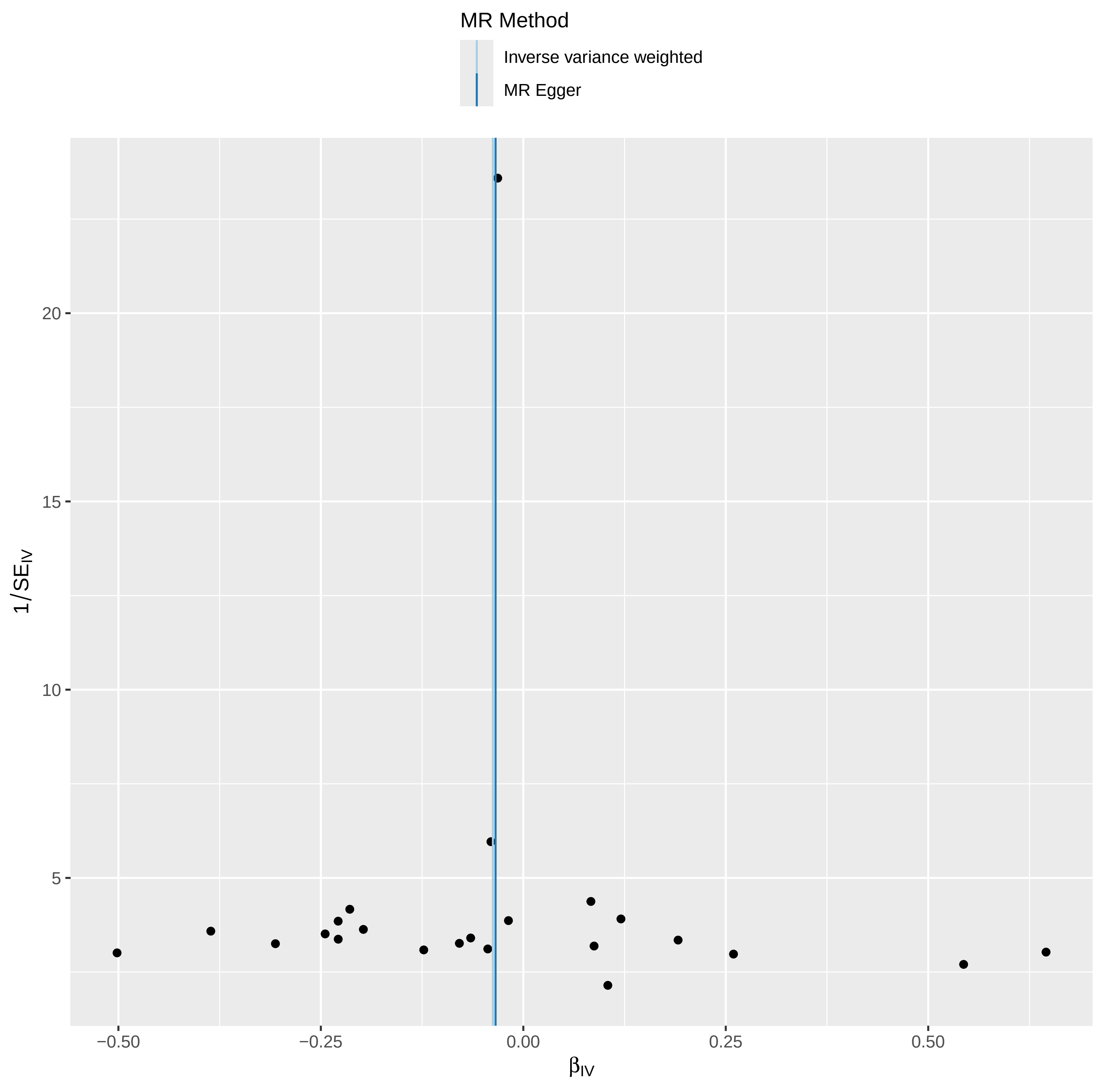

MR Method
Inverse variance weighted
MR Egger
20
15
10
5
1/SE$_{IV}$
−0.50
−0.25
0.00
0.25
0.50
β$_{IV}$

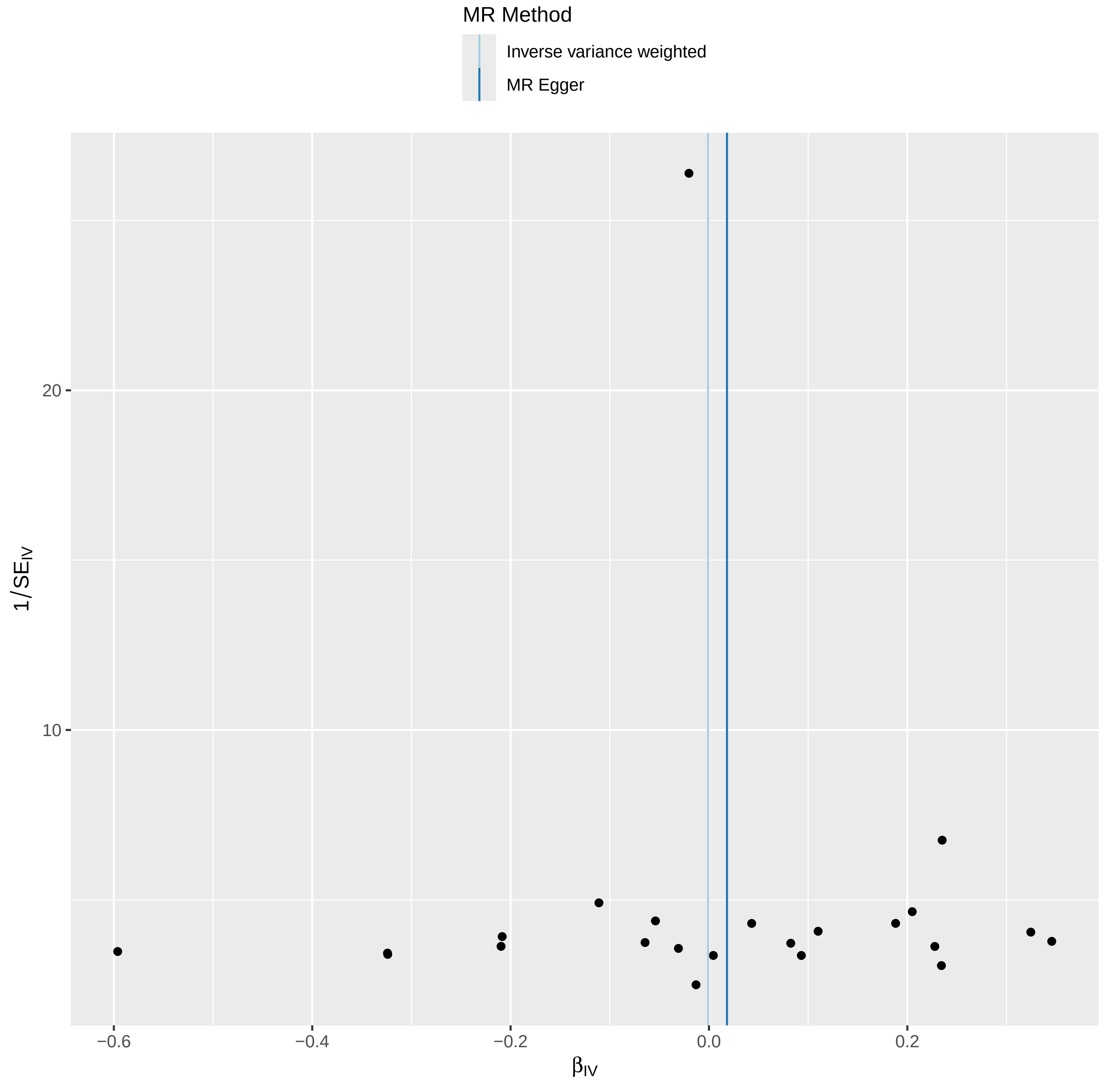
MR Method
Inverse variance weighted
MR Egger
20
10
1/SE$_{IV}$
−0.6
−0.4
−0.2
0.0
0.2
β$_{IV}$

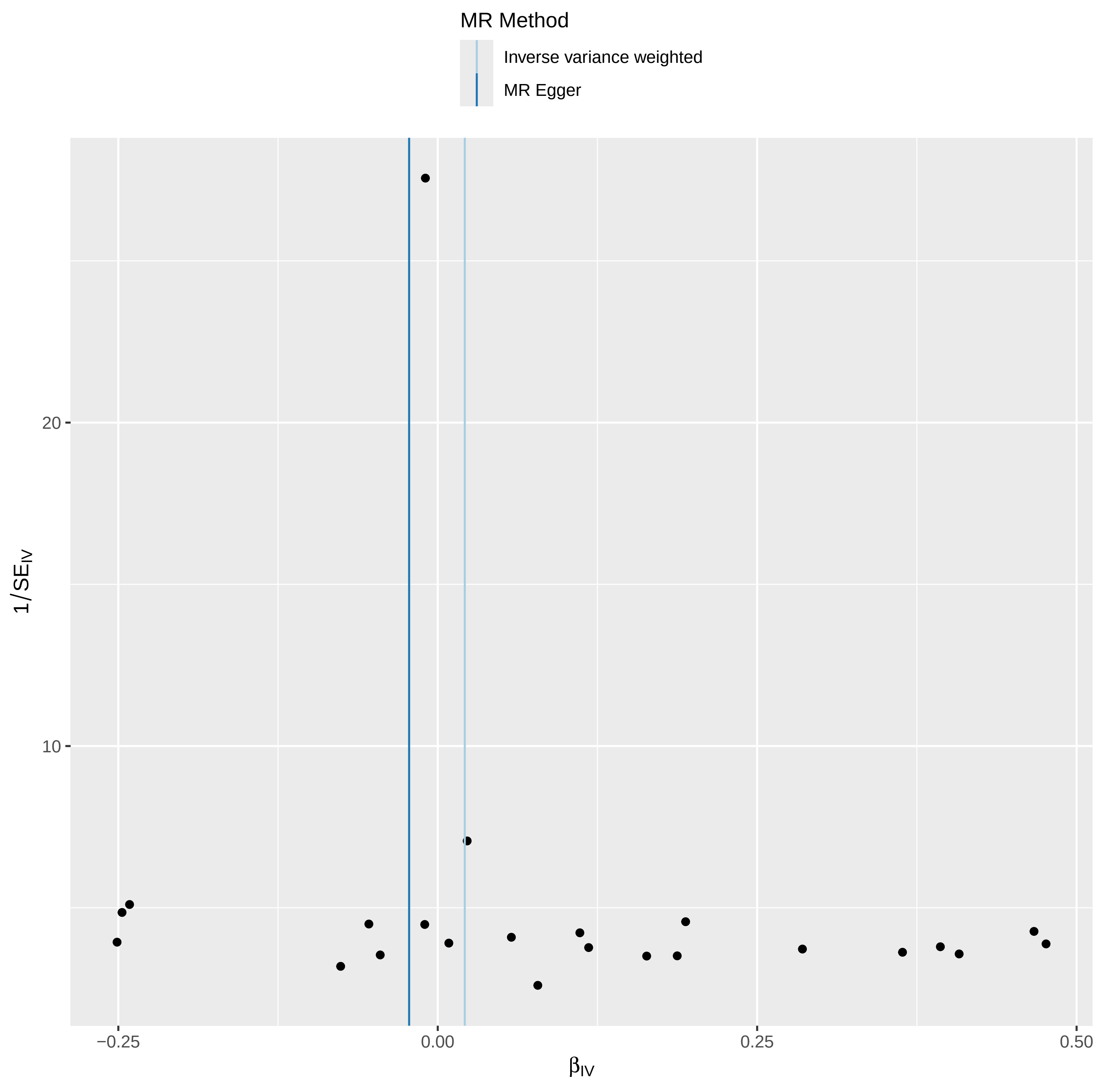
MR Method
Inverse variance weighted
MR Egger
20
10
1/SE_IV
−0.25
0.00
0.25
0.50
β_IV

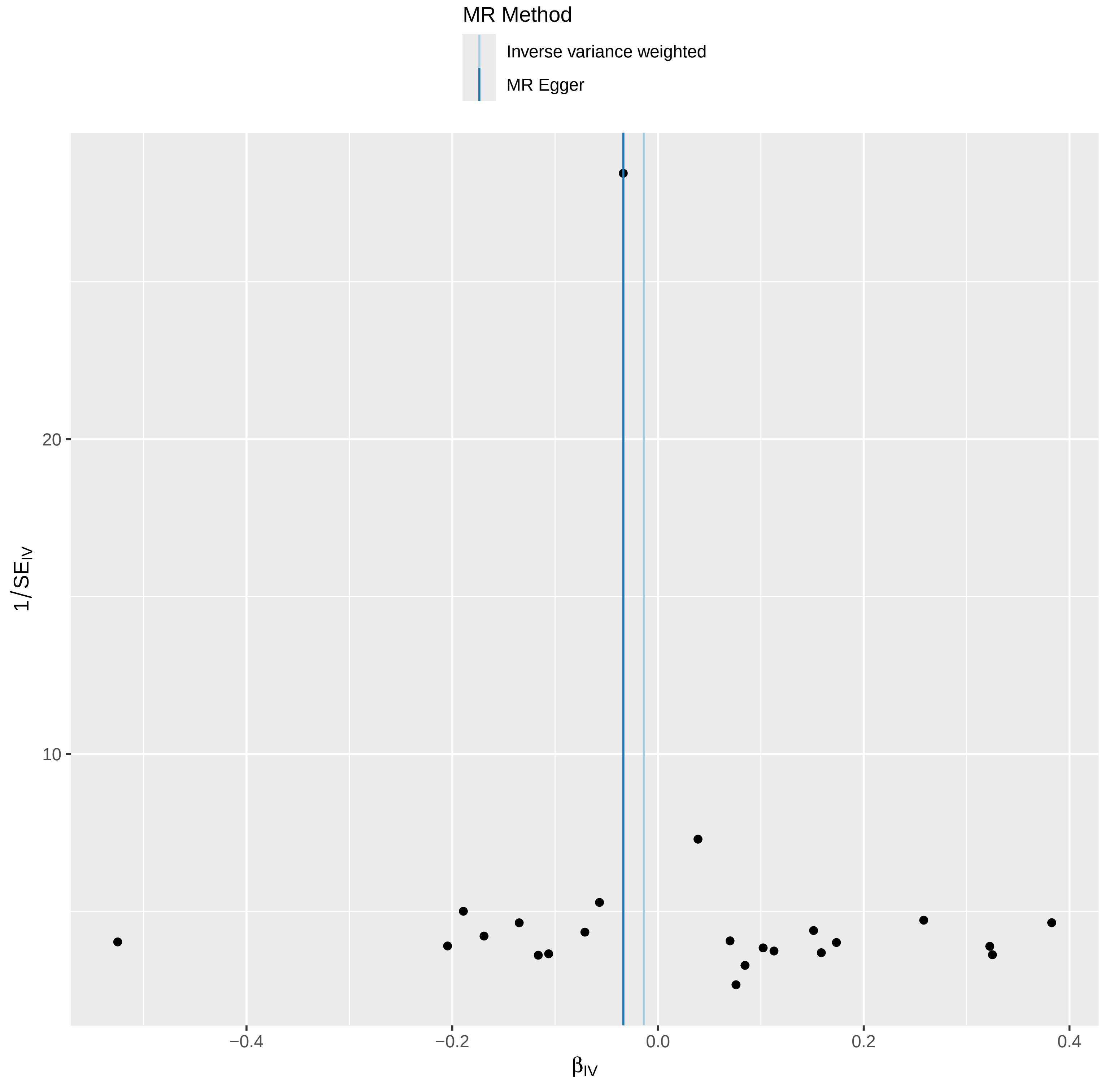

MR Method
Inverse variance weighted
MR Egger
20
10
1/SE$_{IV}$
−0.4
−0.2
0.0
0.2
0.4
β$_{IV}$

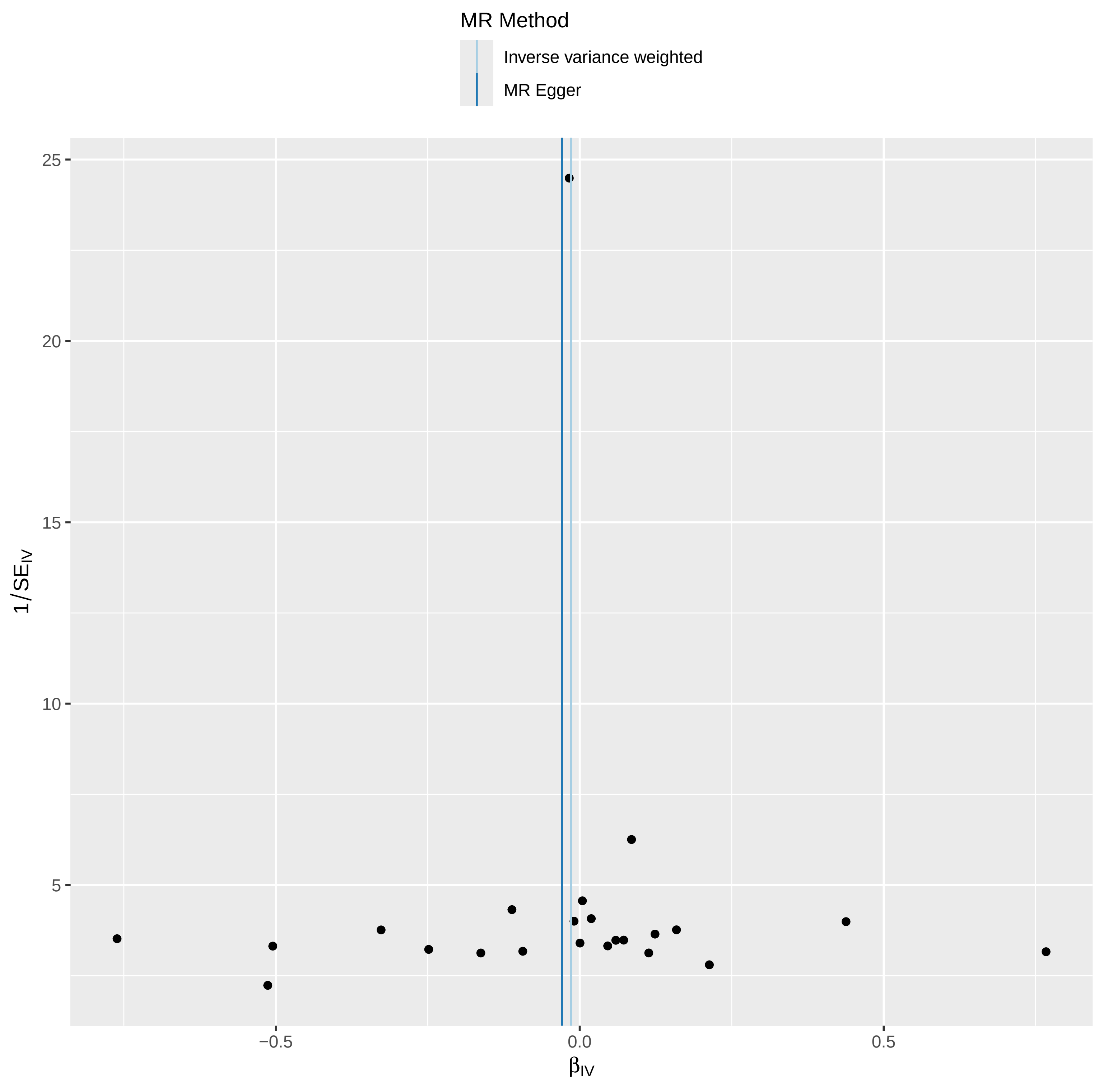

MR Method
Inverse variance weighted
MR Egger
25
20
15
10
5
1/SE_IV
−0.5
0.0
0.5
β_IV

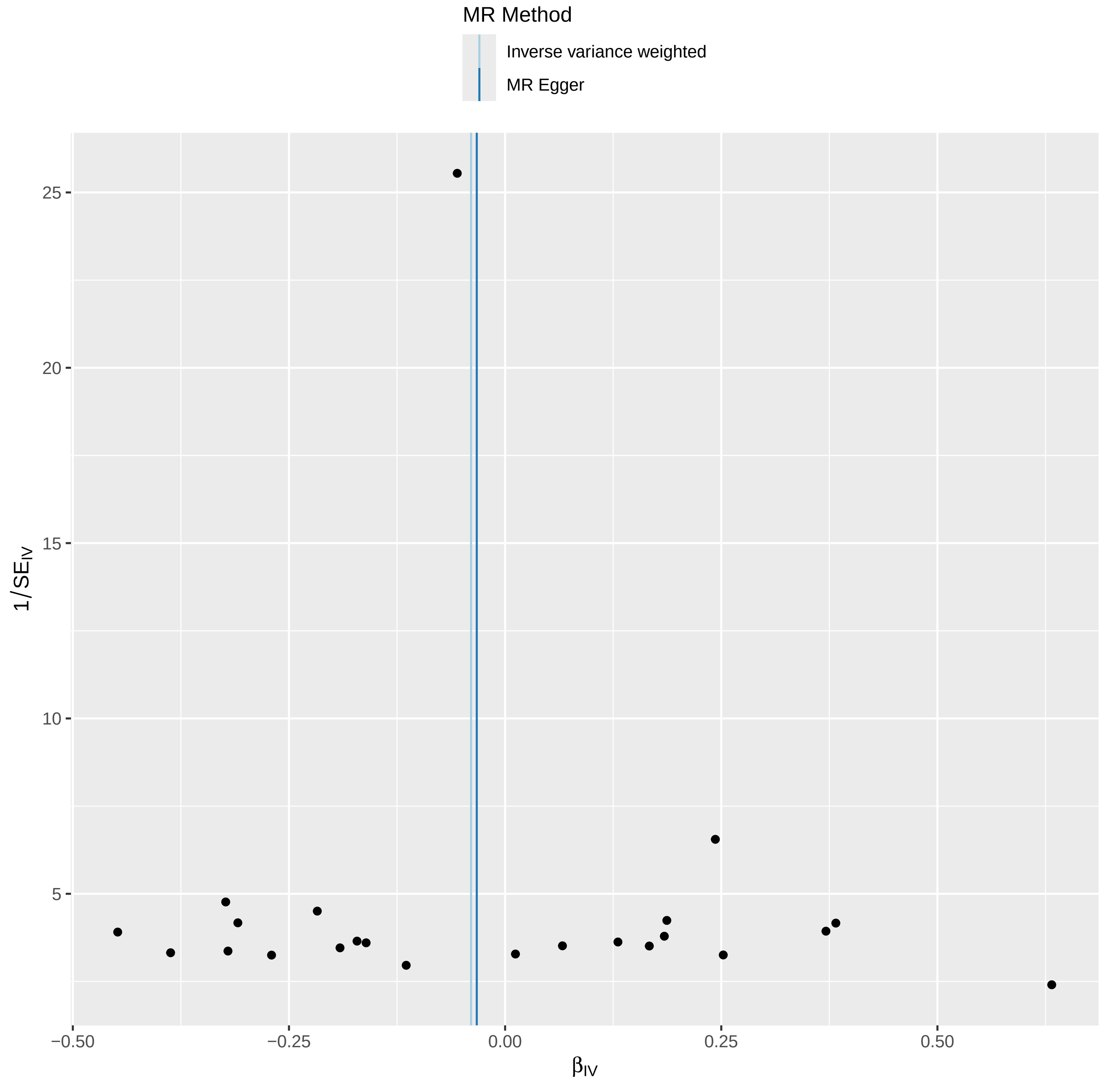
MR Method
Inverse variance weighted
MR Egger
25
20
15
10
5
1/SE$_{IV}$
−0.50
−0.25
0.00
0.25
0.50
β$_{IV}$

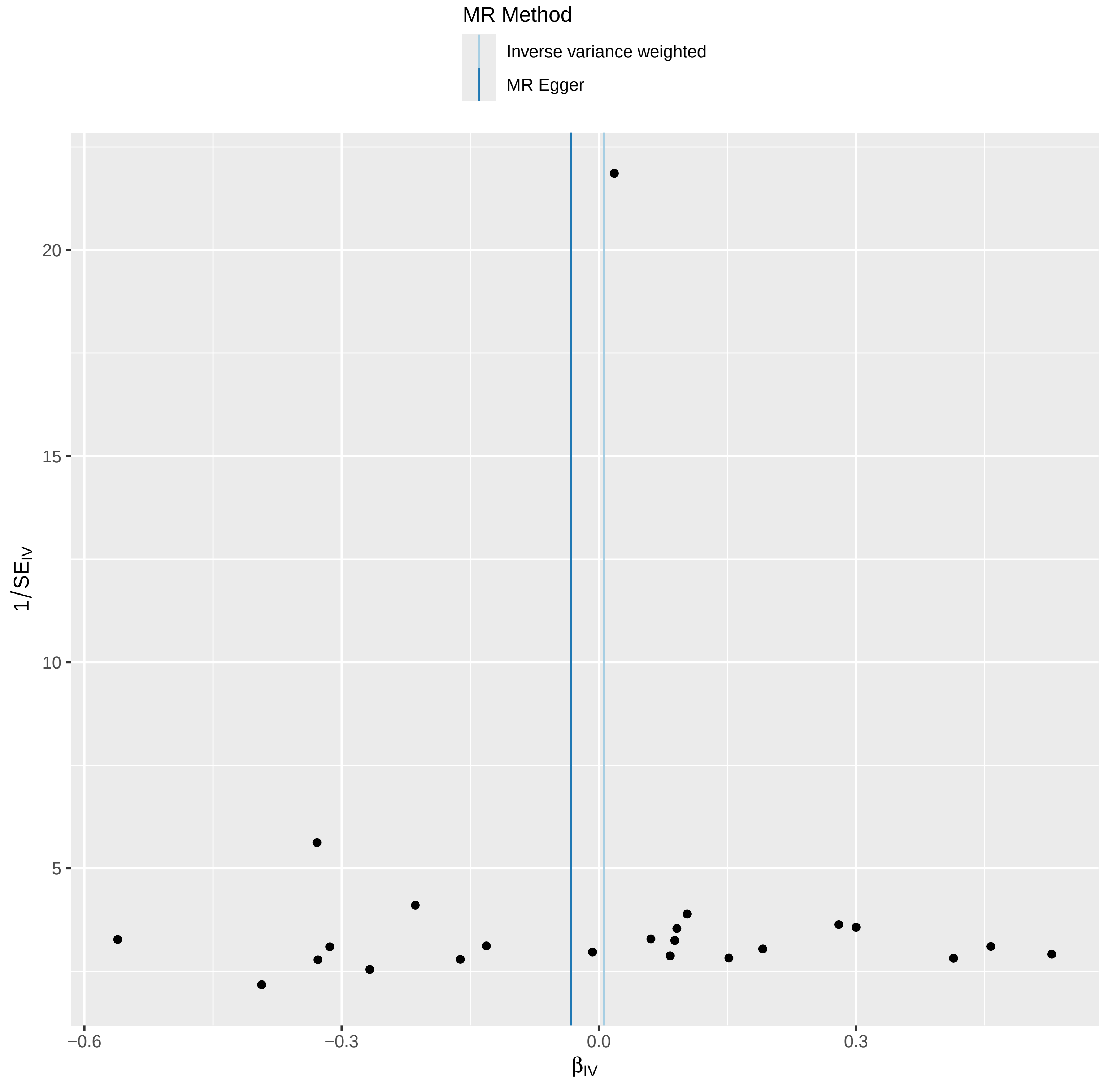

MR Method
Inverse variance weighted
MR Egger
20
15
10
5
1/SE$_{IV}$
−0.6
−0.3
0.0
0.3
β$_{IV}$

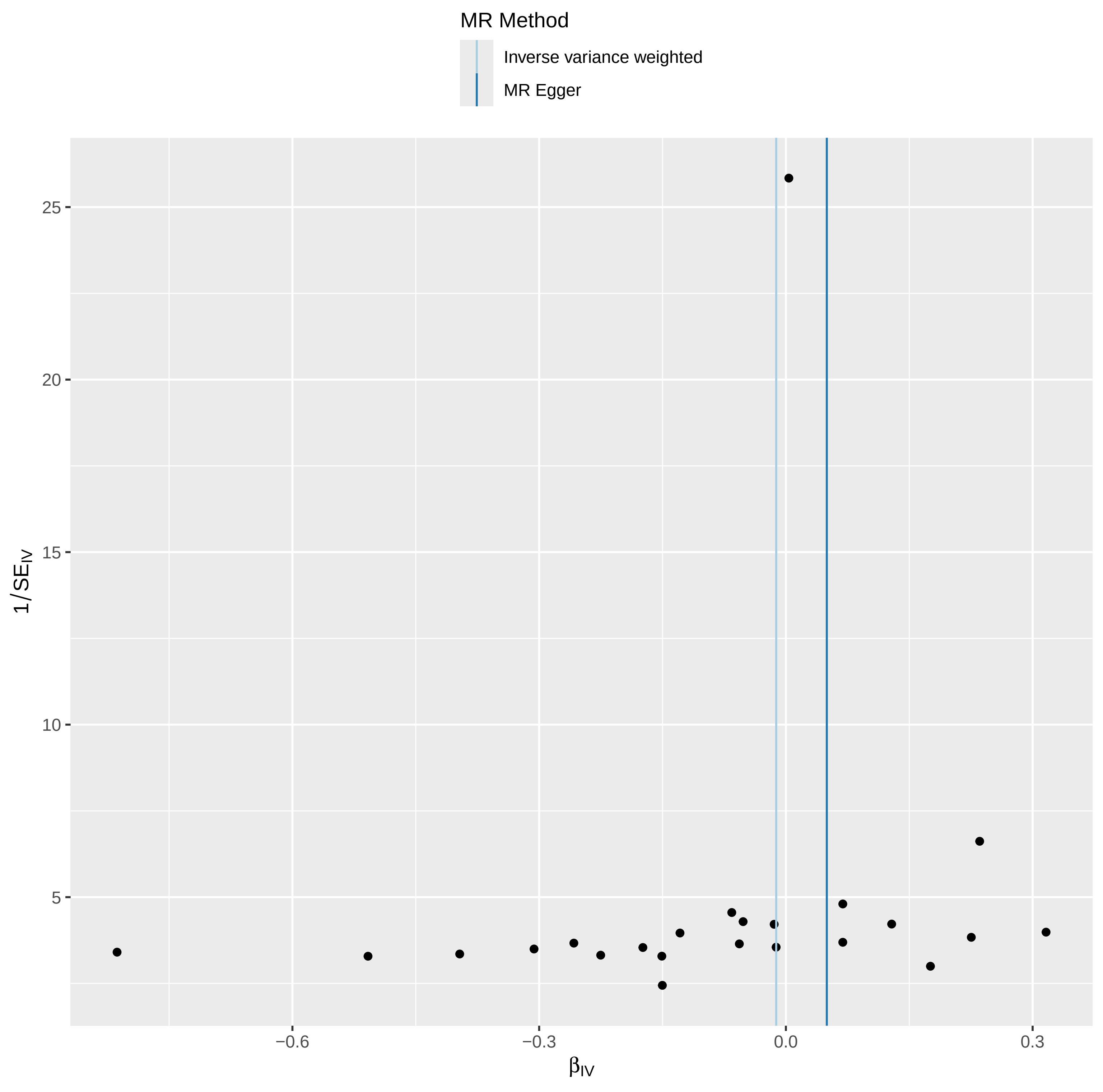
MR Method
Inverse variance weighted
MR Egger
25
20
15
10
5
1/SE_IV
−0.6
−0.3
0.0
0.3
β_IV

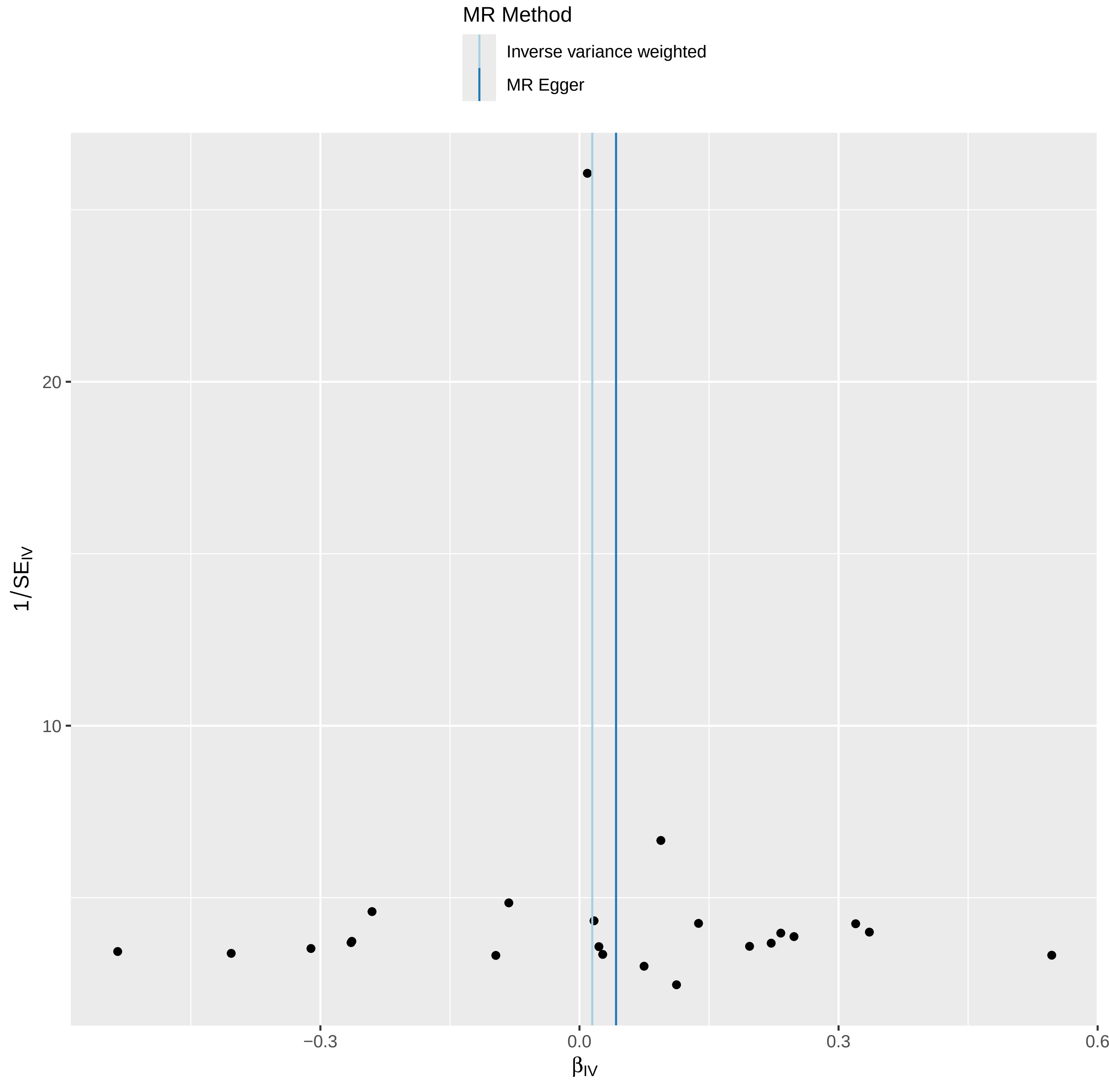
MR Method
Inverse variance weighted
MR Egger
20
10
1/SE$_{IV}$
−0.3
0.0
0.3
0.6
β$_{IV}$

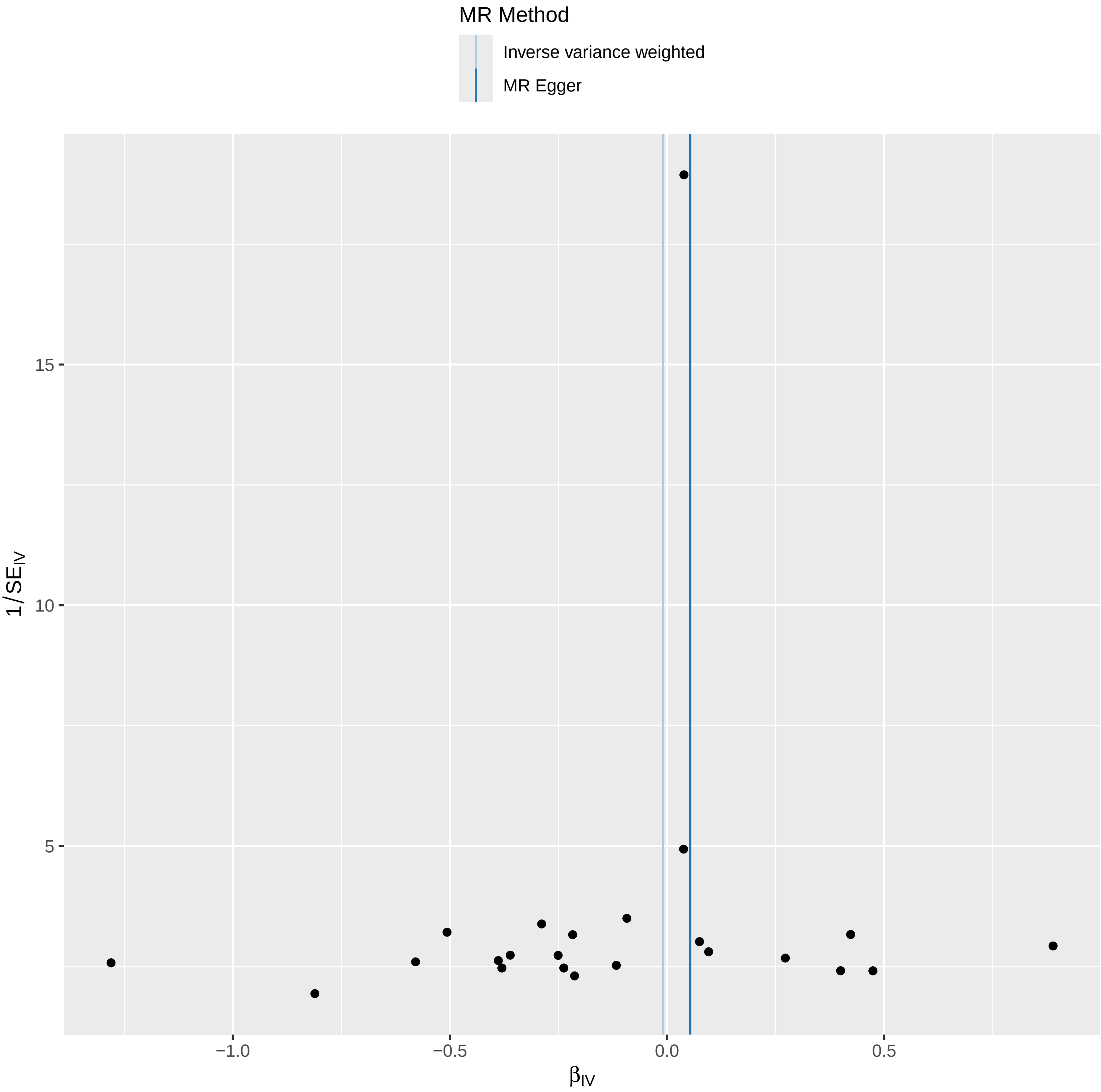
MR Method
Inverse variance weighted
MR Egger
1/SE$_{IV}$
15
10
5
−1.0
−0.5
0.0
0.5
β$_{IV}$

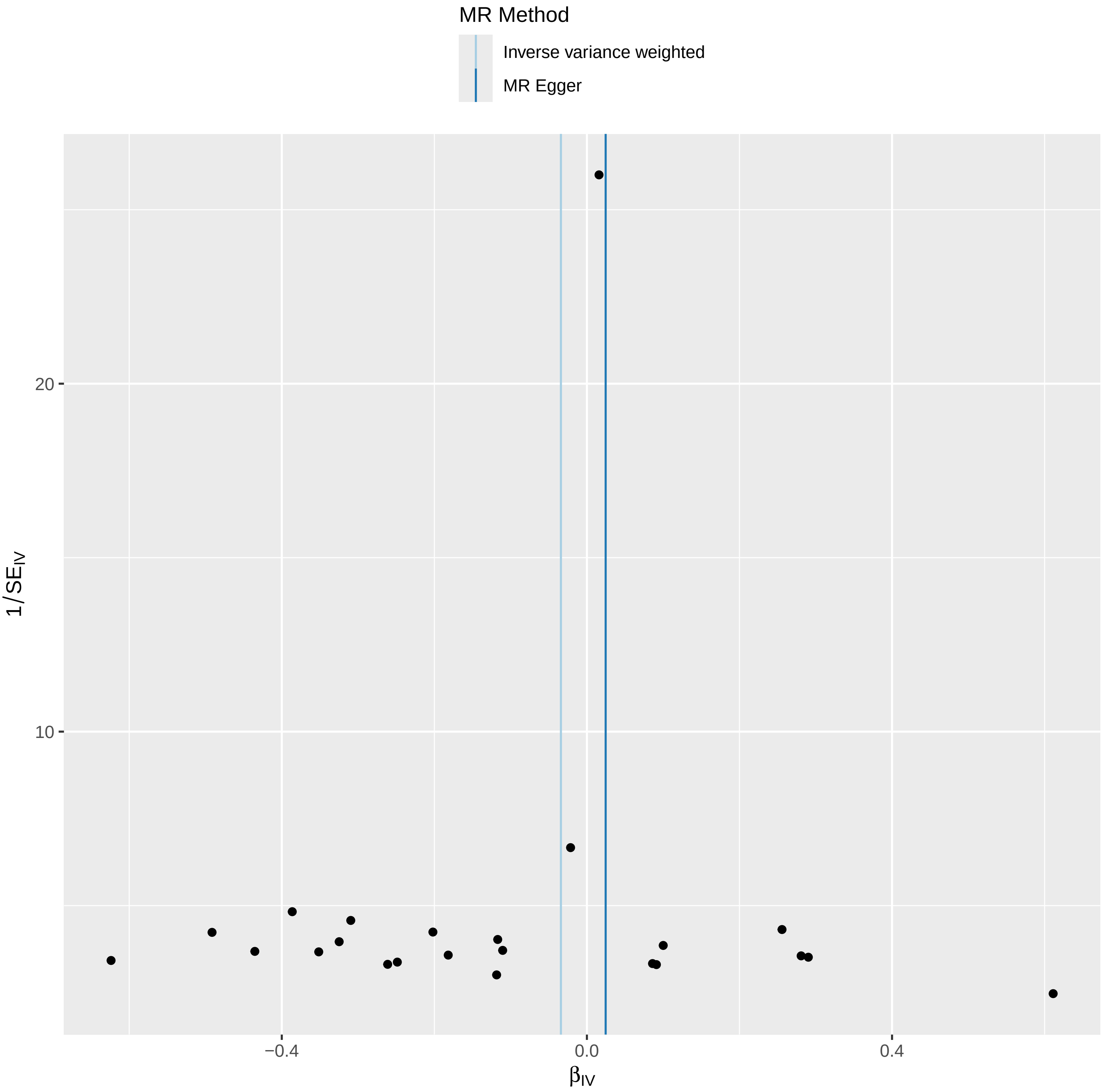
MR Method
Inverse variance weighted
MR Egger
20
10
1/SE$_{IV}$
−0.4
0.0
0.4
β_{IV}

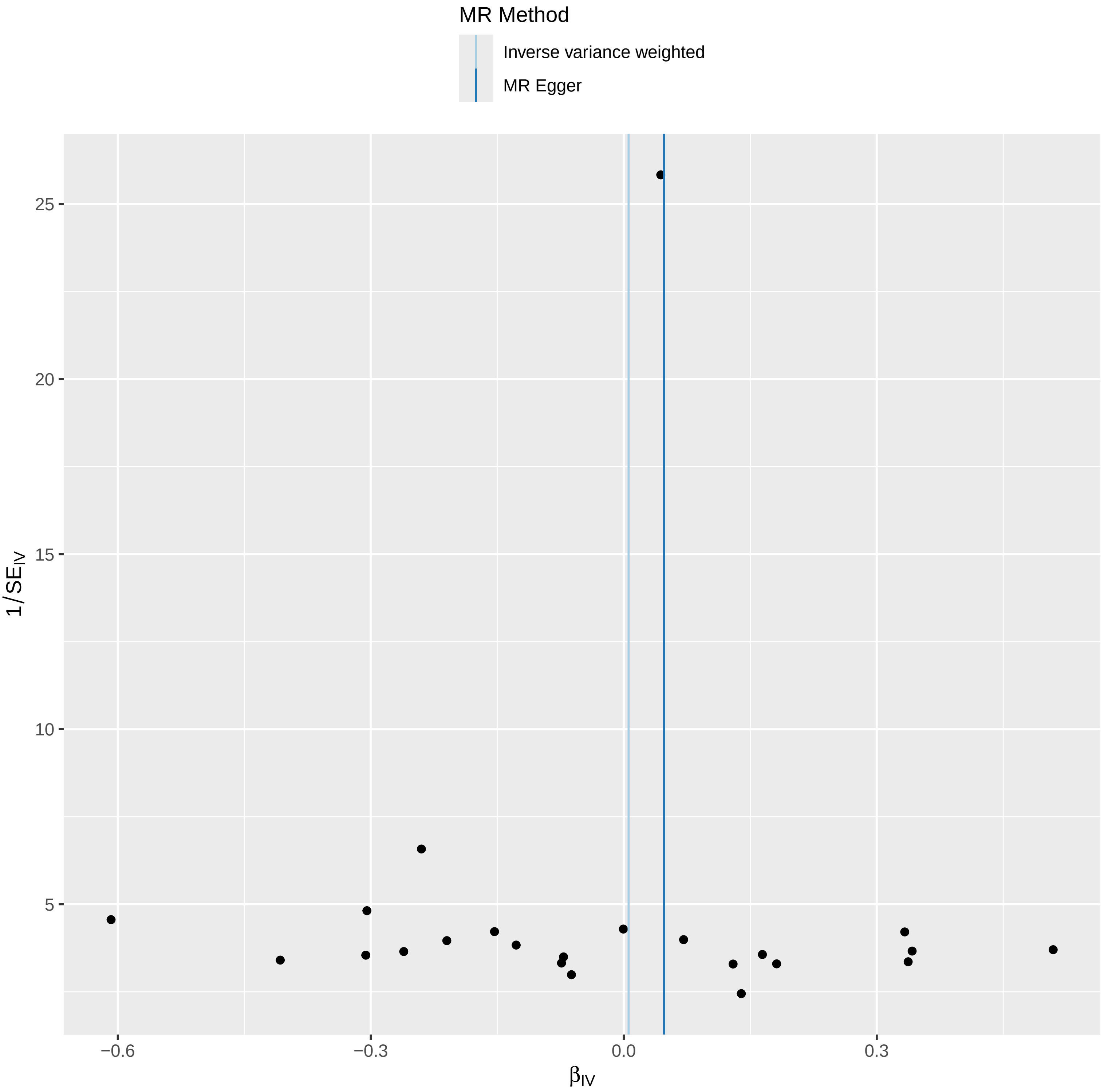

MR Method
Inverse variance weighted
MR Egger
25
20
15
10
5
1/SE$_{IV}$
−0.6
−0.3
0.0
0.3
β$_{IV}$

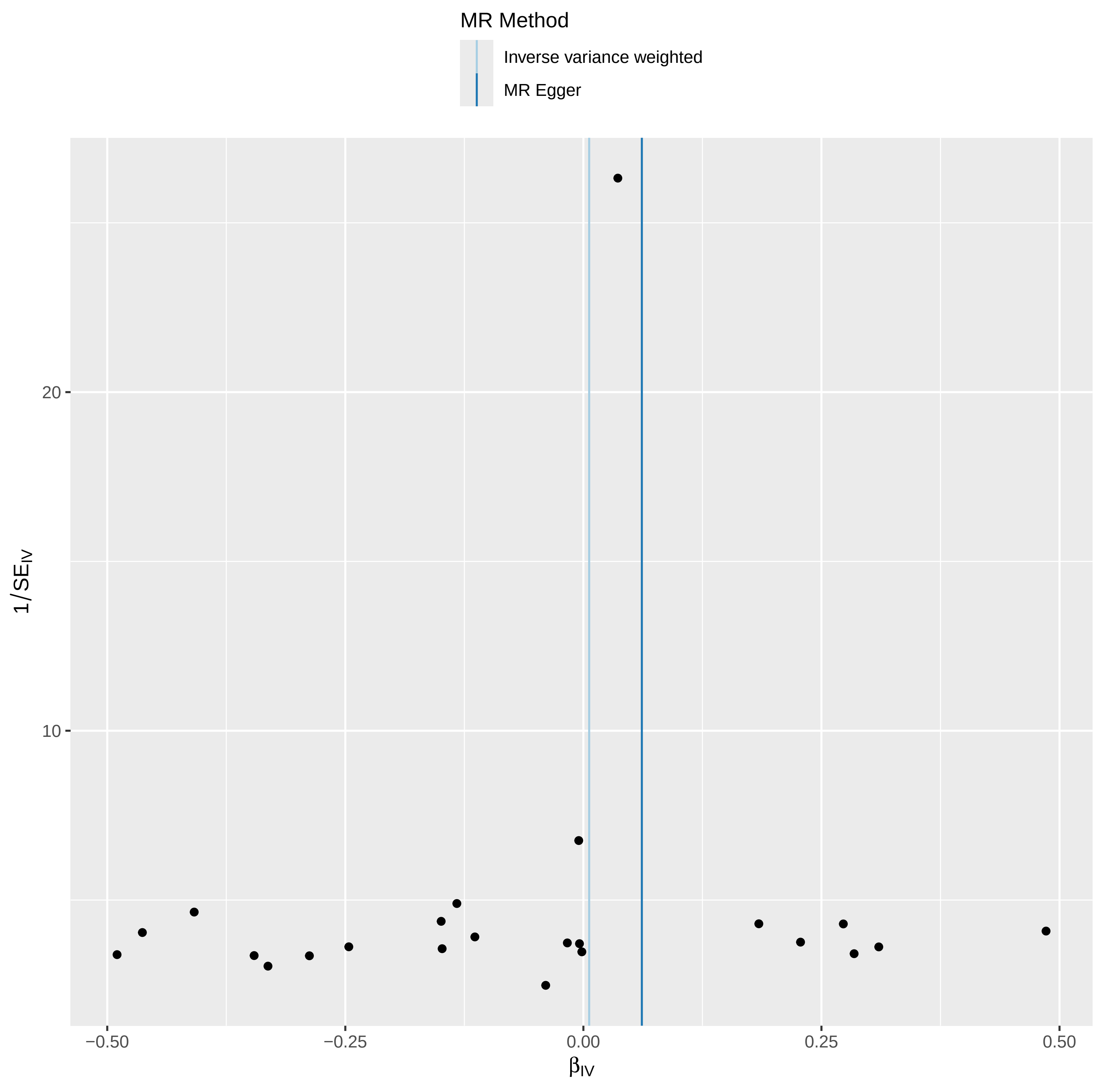
MR Method
Inverse variance weighted
MR Egger
20
10
1/SE_IV
−0.50
−0.25
0.00
0.25
0.50
β_IV

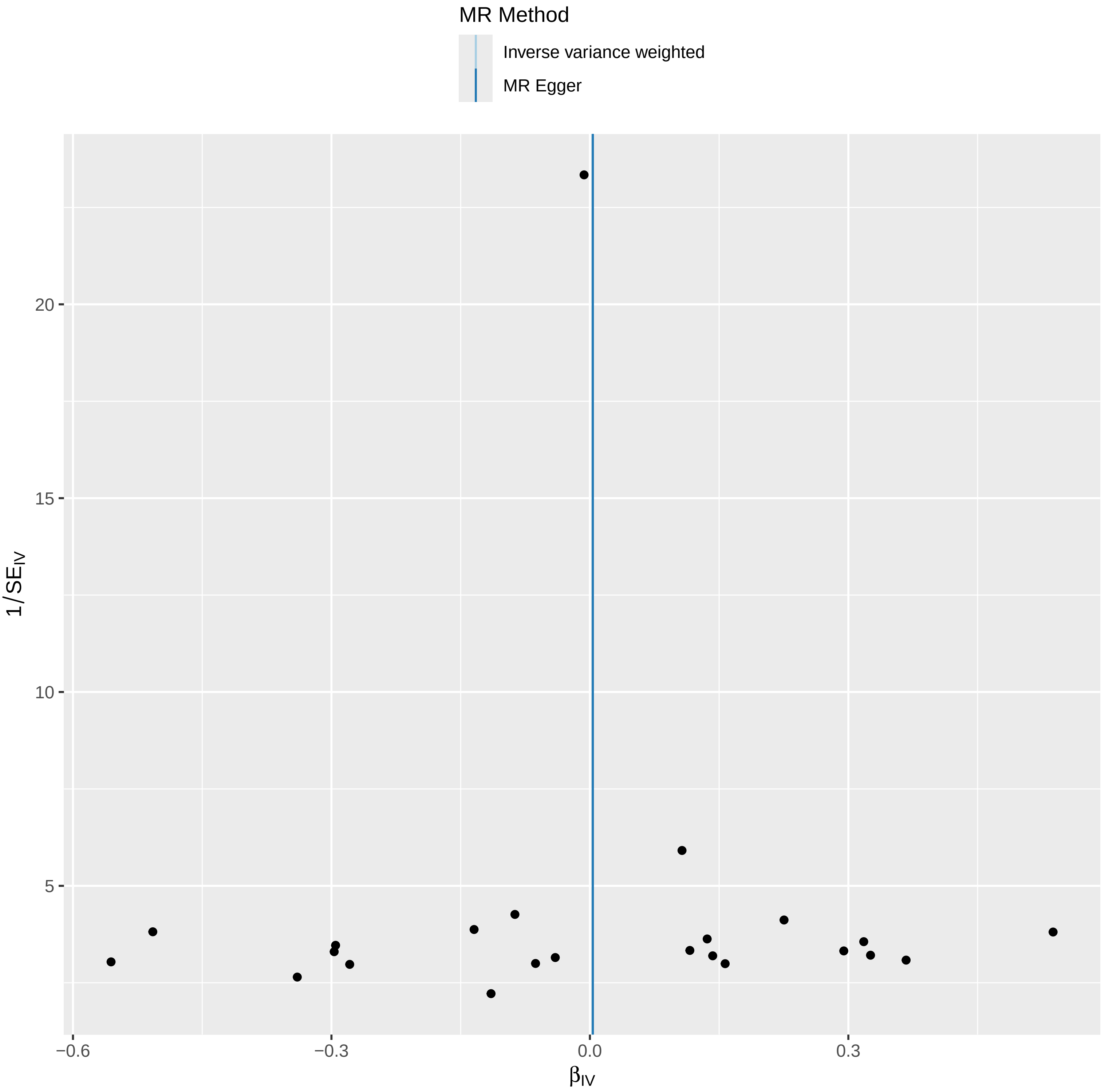
MR Method
Inverse variance weighted
MR Egger
20
15
10
5
1/SE_IV
−0.6
−0.3
0.0
0.3
β_IV

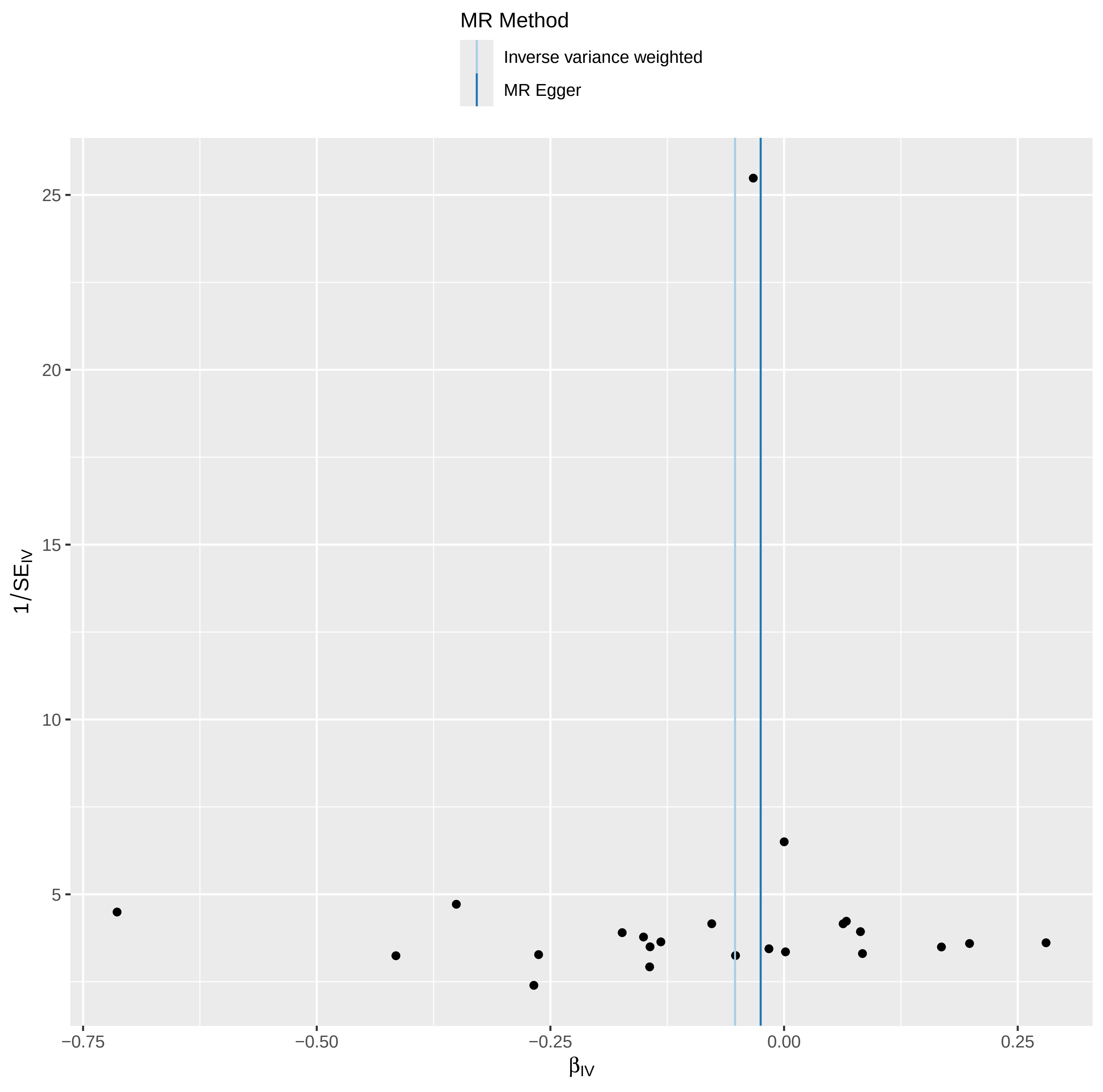

MR Method
Inverse variance weighted
MR Egger
25
20
15
10
5
1/SE$_{IV}$
−0.75
−0.50
−0.25
0.00
0.25
β$_{IV}$

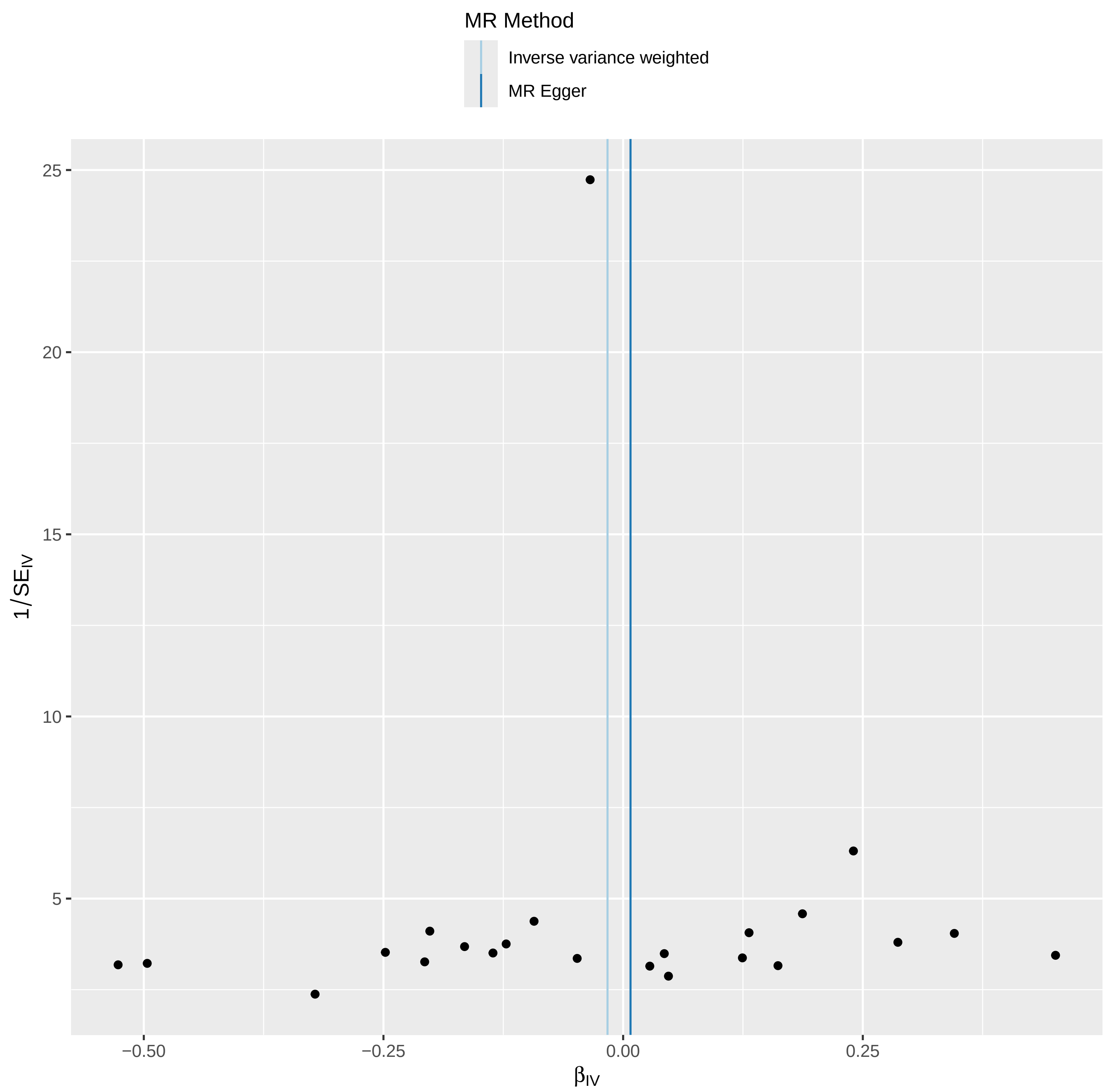
MR Method
Inverse variance weighted
MR Egger
25
20
15
10
5
1/SE$_{IV}$
−0.50
−0.25
0.00
0.25
β_{IV}

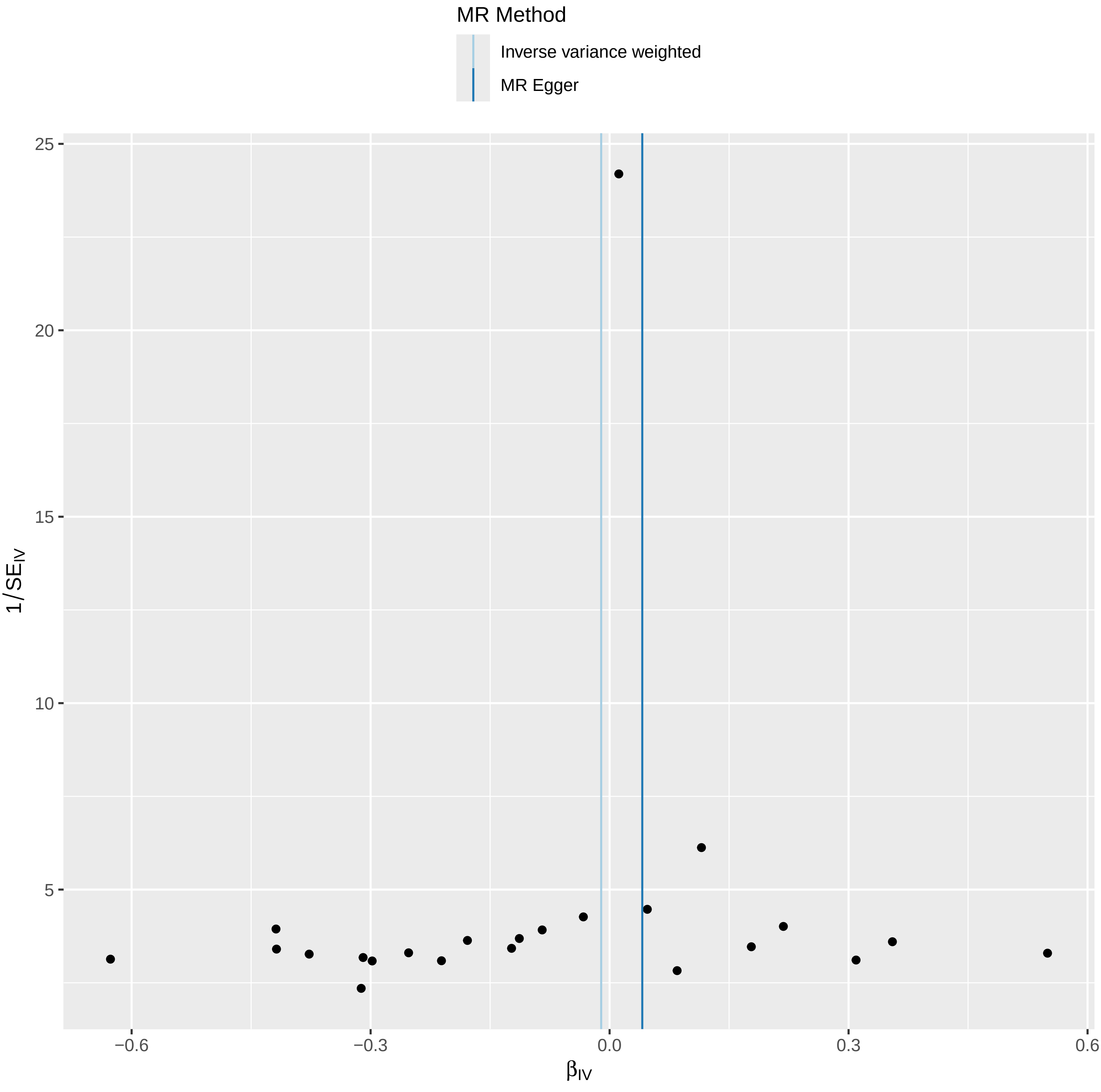
MR Method
Inverse variance weighted
MR Egger
25
20
15
10
5
$1/SE_{IV}$
−0.6
−0.3
0.0
0.3
0.6
β_{IV}

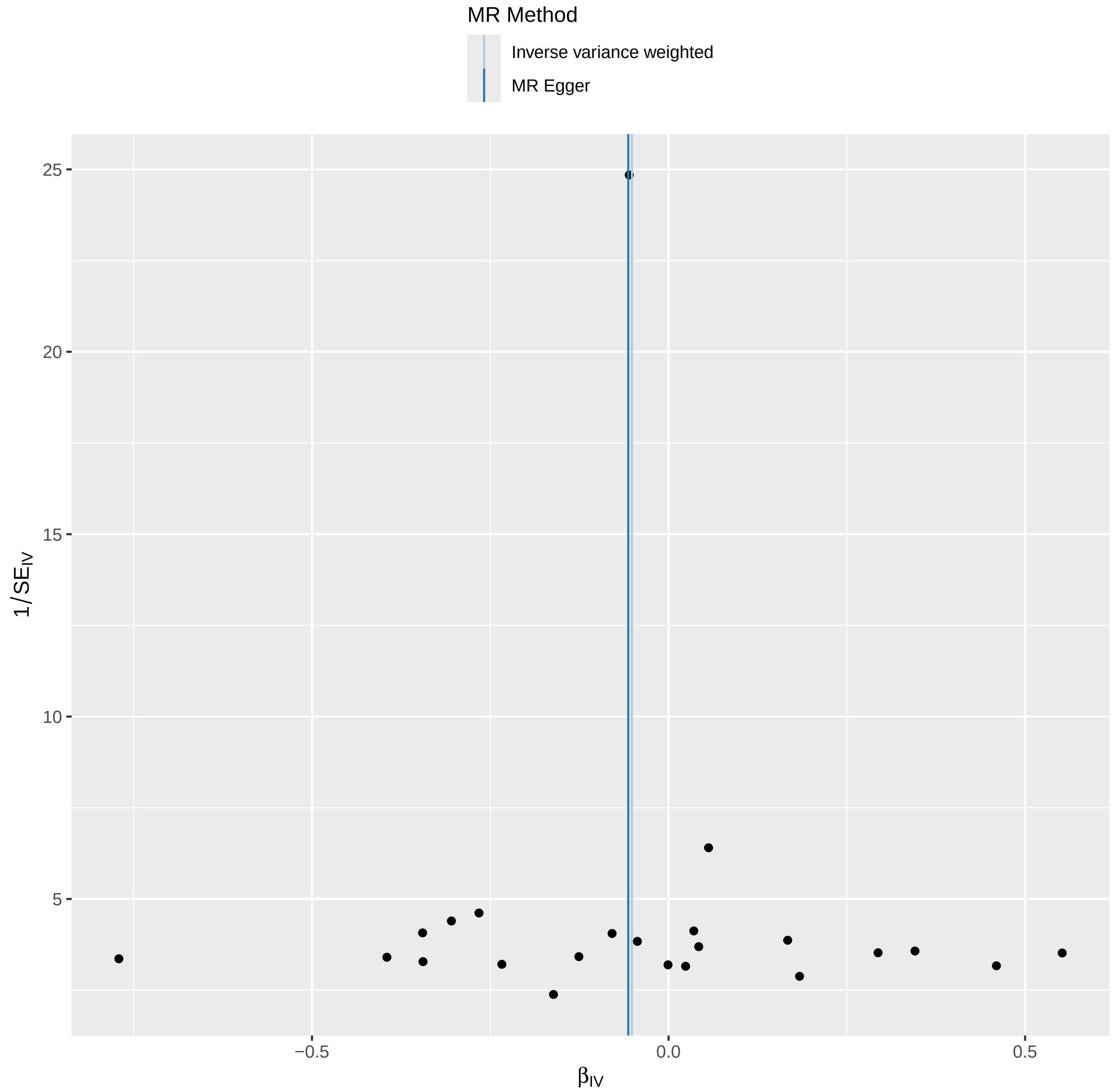
MR Method
Inverse variance weighted
MR Egger
25
20
15
10
5
1/SE$_{IV}$
−0.5
0.0
0.5
β$_{IV}$

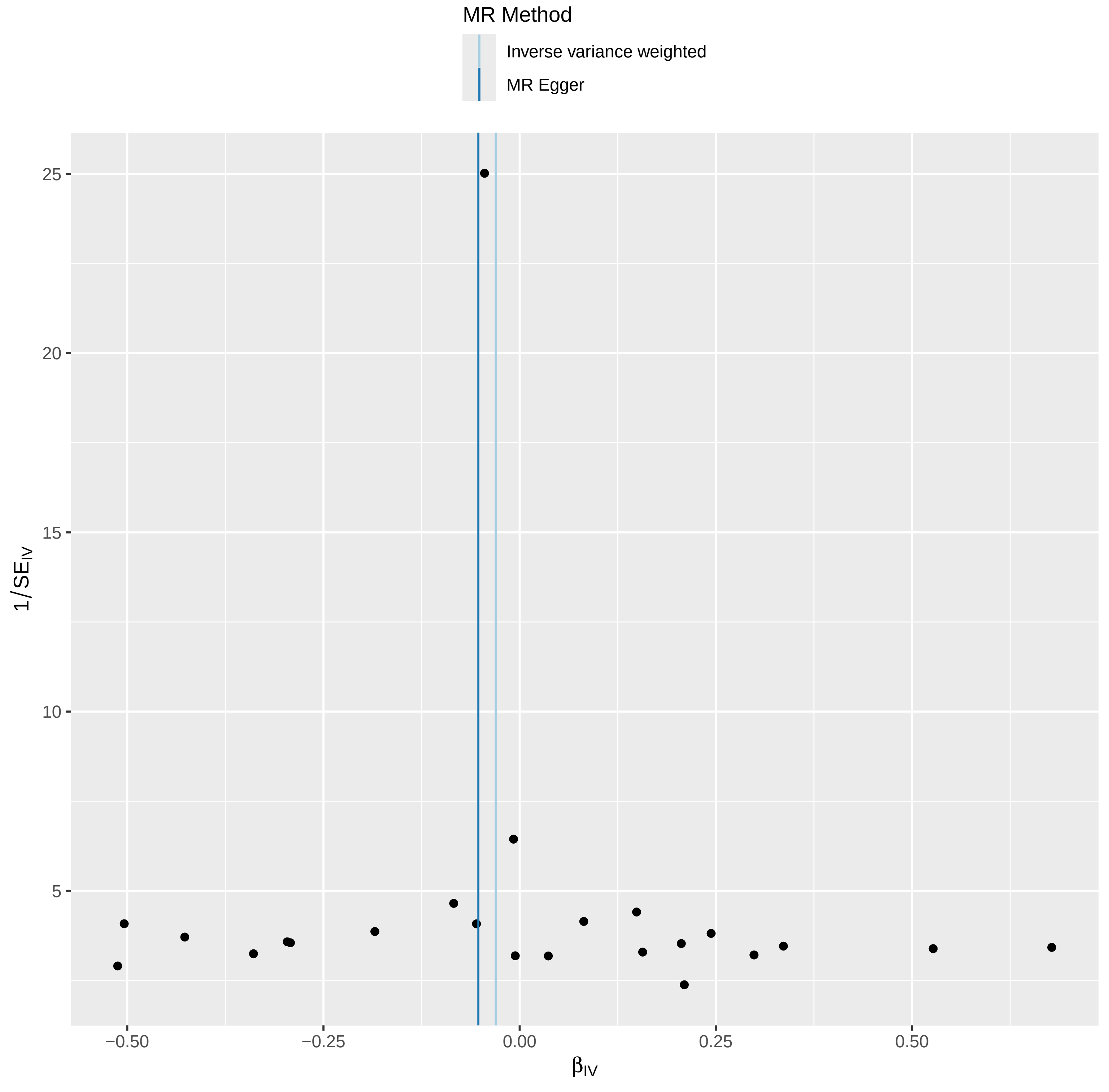

MR Method
Inverse variance weighted
MR Egger
25
20
15
10
5
1/SE_IV
−0.50
−0.25
0.00
0.25
0.50
β_IV

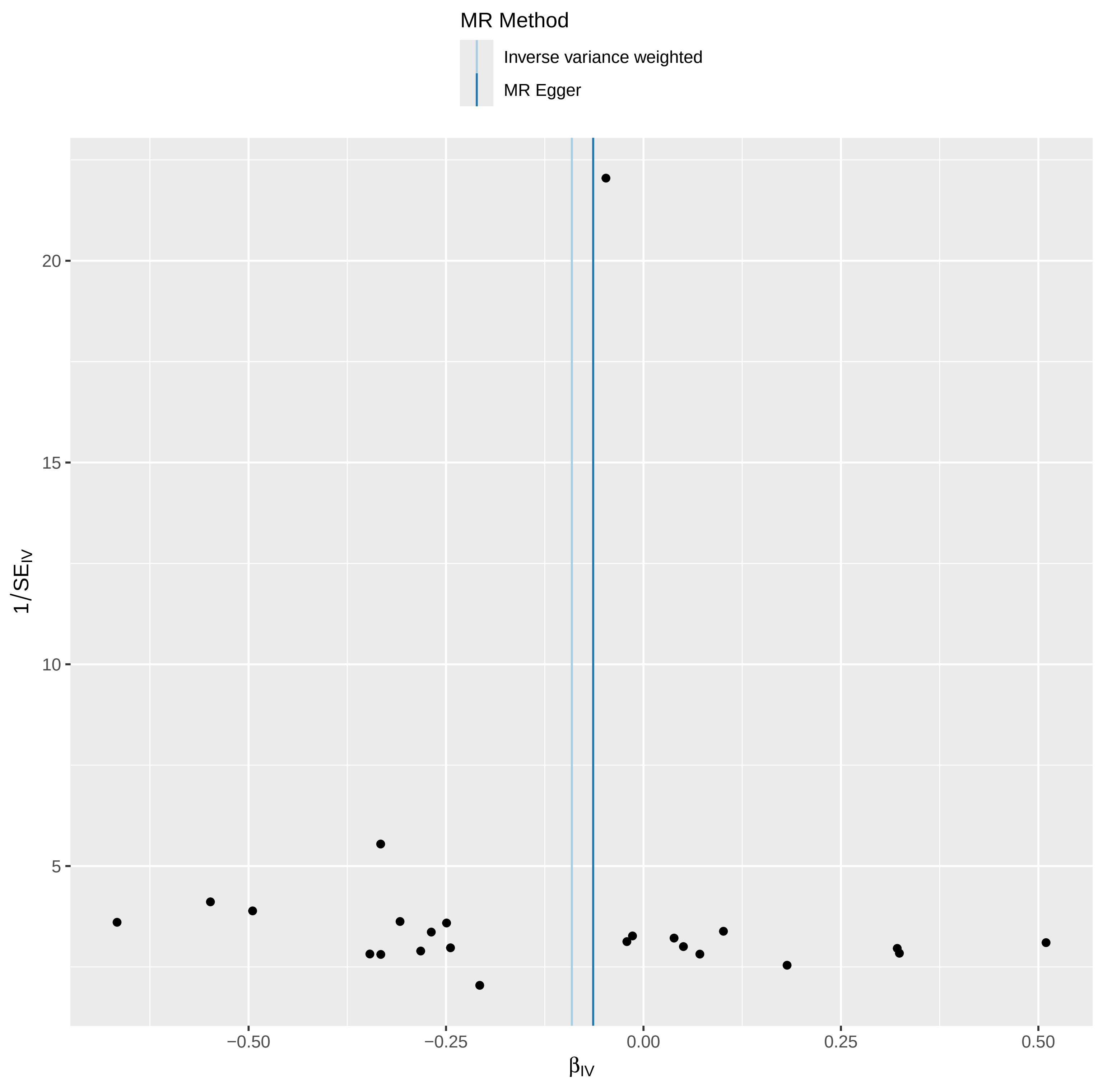

MR Method
Inverse variance weighted
MR Egger
20
15
10
5
1/SE_IV
−0.50
−0.25
0.00
0.25
0.50
β_IV

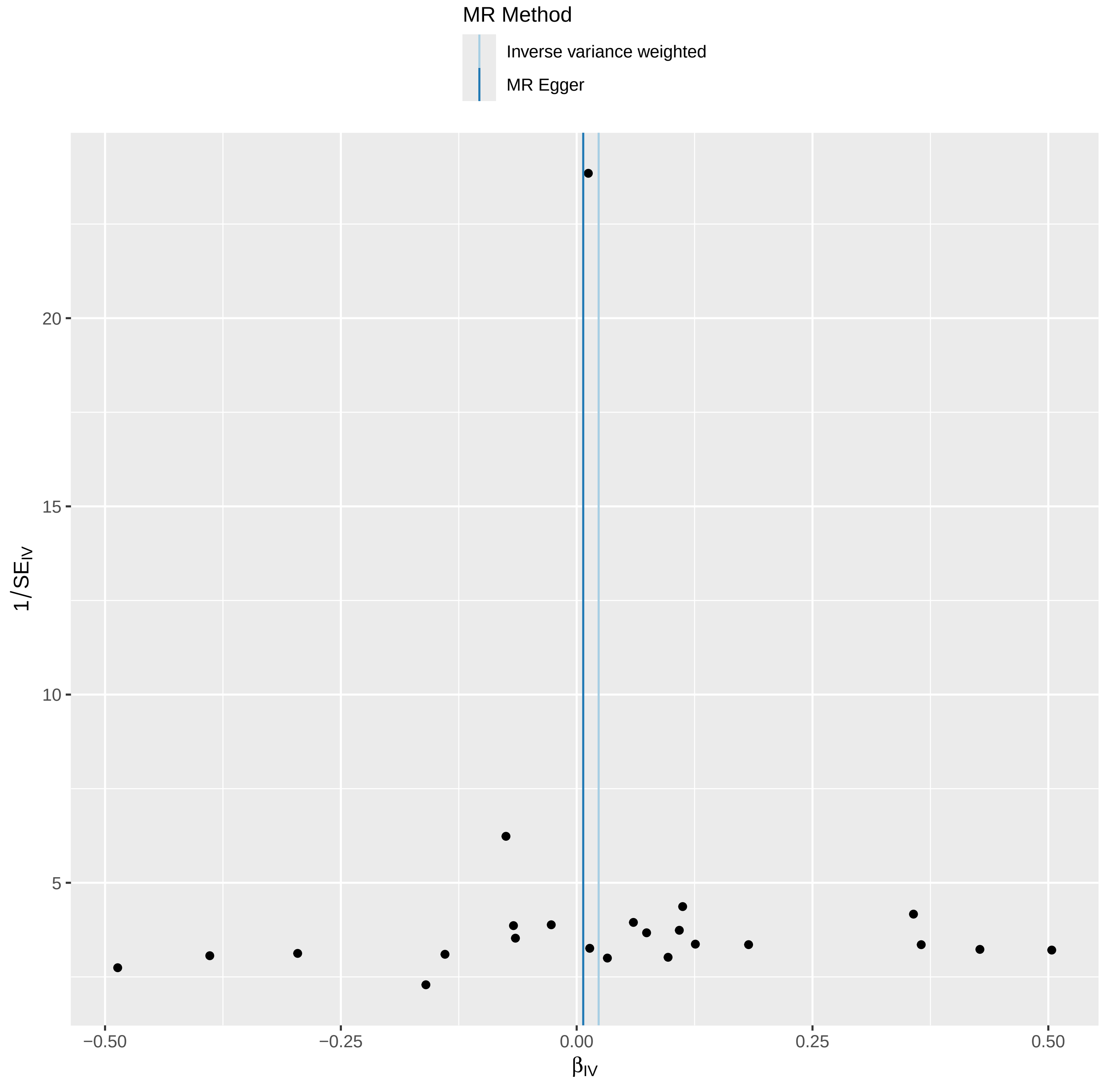

MR Method
Inverse variance weighted
MR Egger
20
15
10
5
1/SE$_{IV}$
−0.50
−0.25
0.00
0.25
0.50
β$_{IV}$

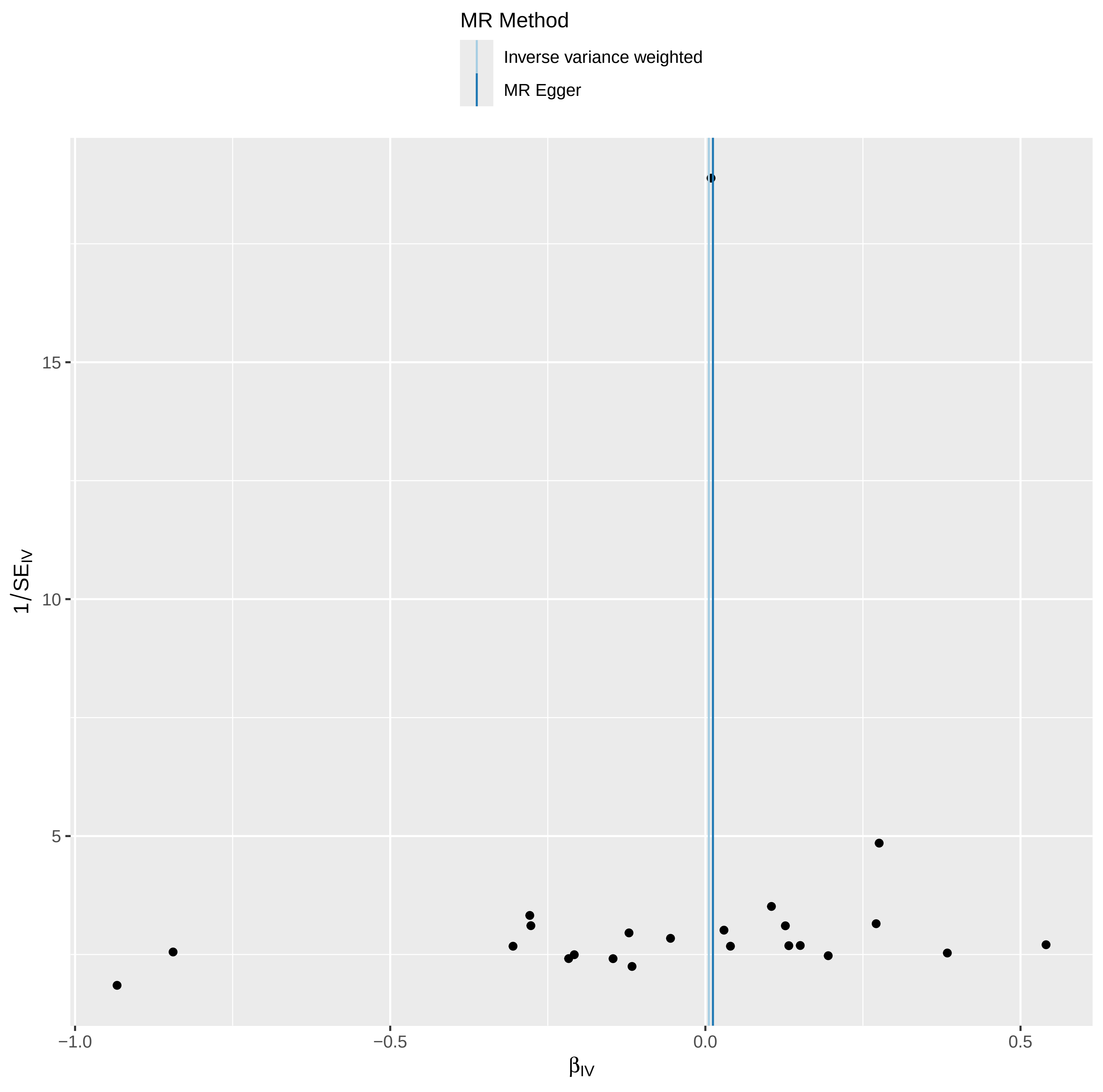

MR Method
Inverse variance weighted
MR Egger
15
10
5
$1/SE_{IV}$
−1.0
−0.5
0.0
0.5
β_{IV}

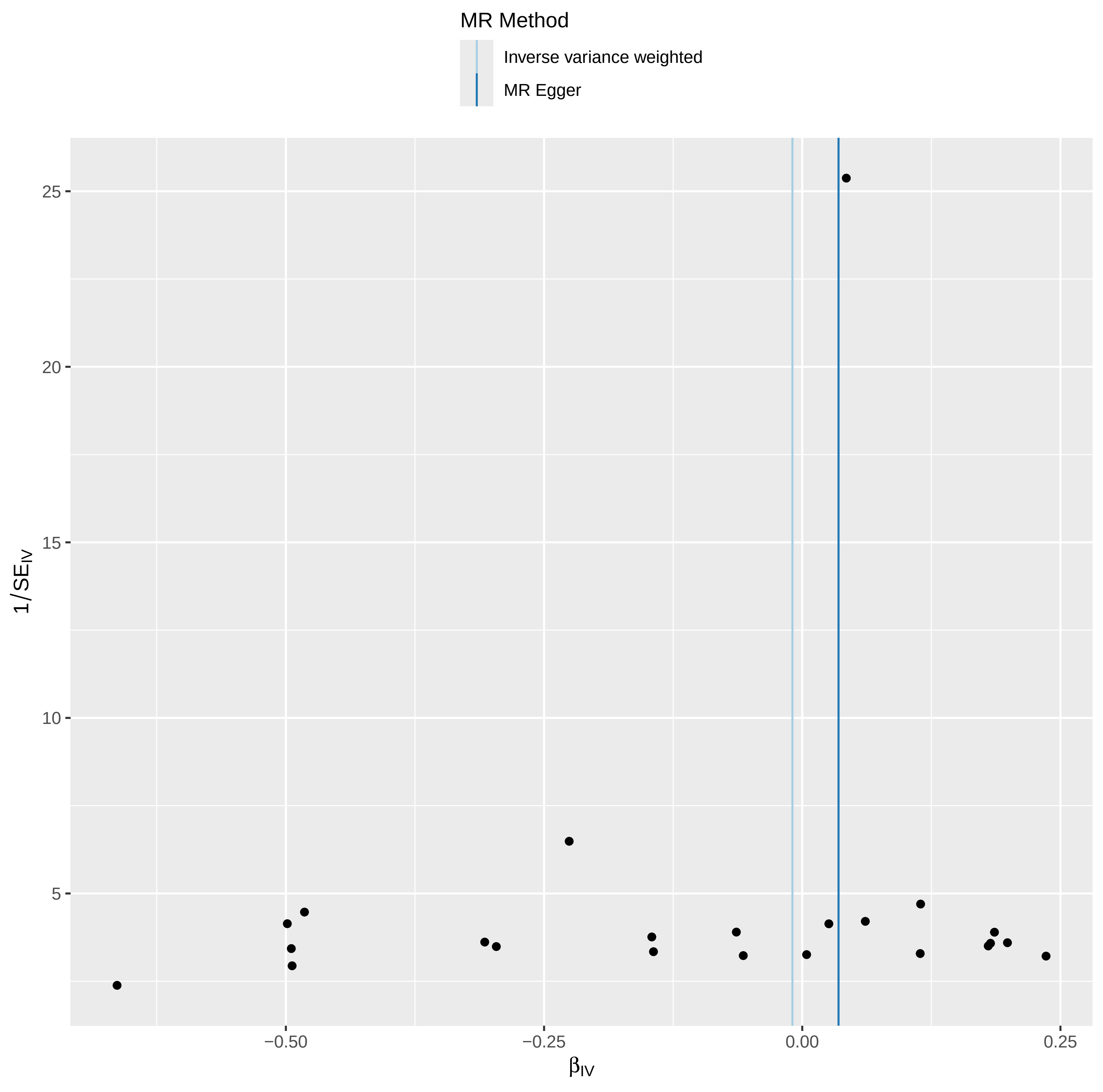

MR Method
Inverse variance weighted
MR Egger
25
20
15
10
5
1/SE$_{IV}$
−0.50
−0.25
0.00
0.25
β_{IV}

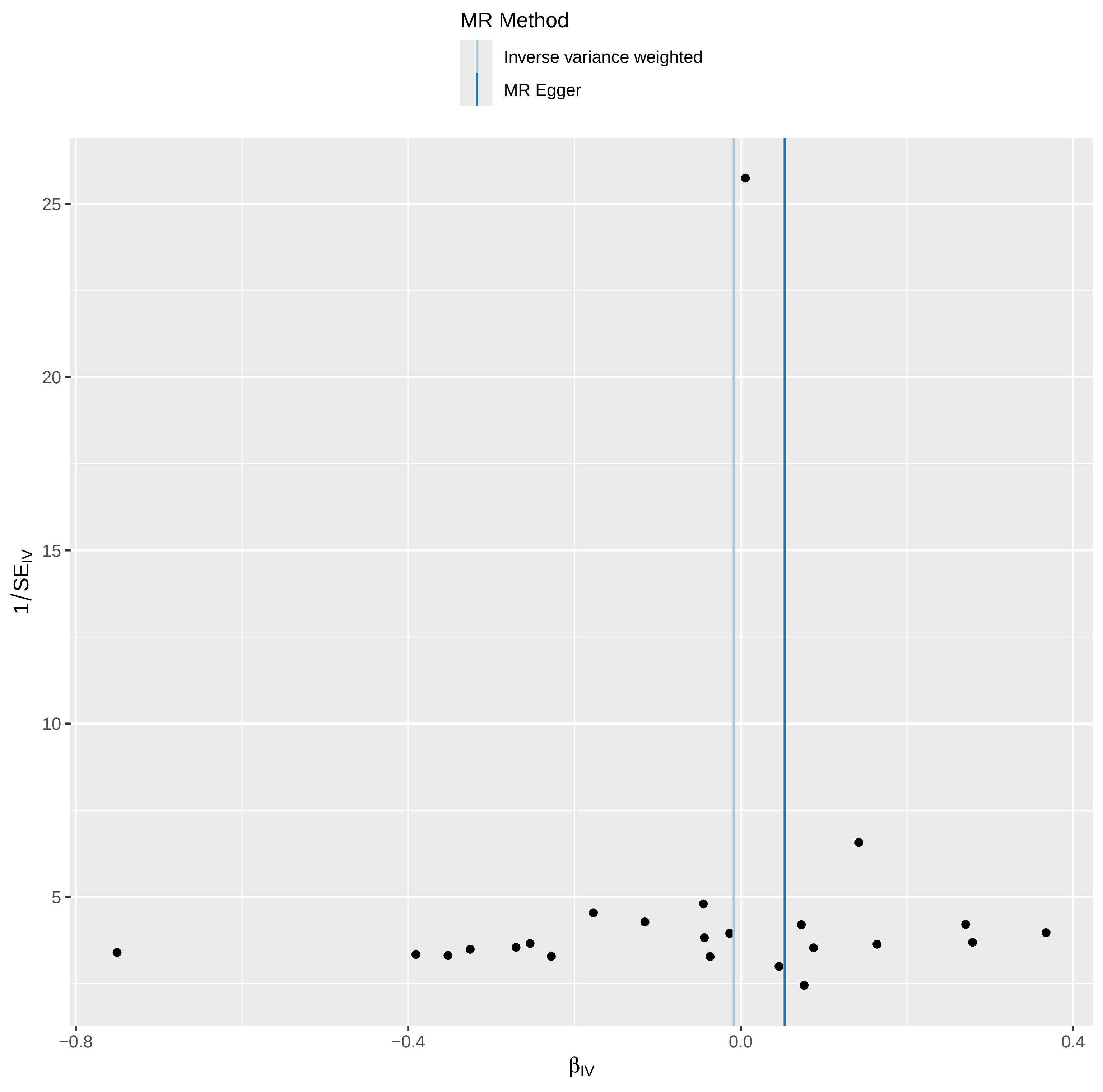
MR Method
Inverse variance weighted
MR Egger
25
20
15
10
5
1/SE$_{IV}$
−0.8
−0.4
0.0
0.4
β$_{IV}$

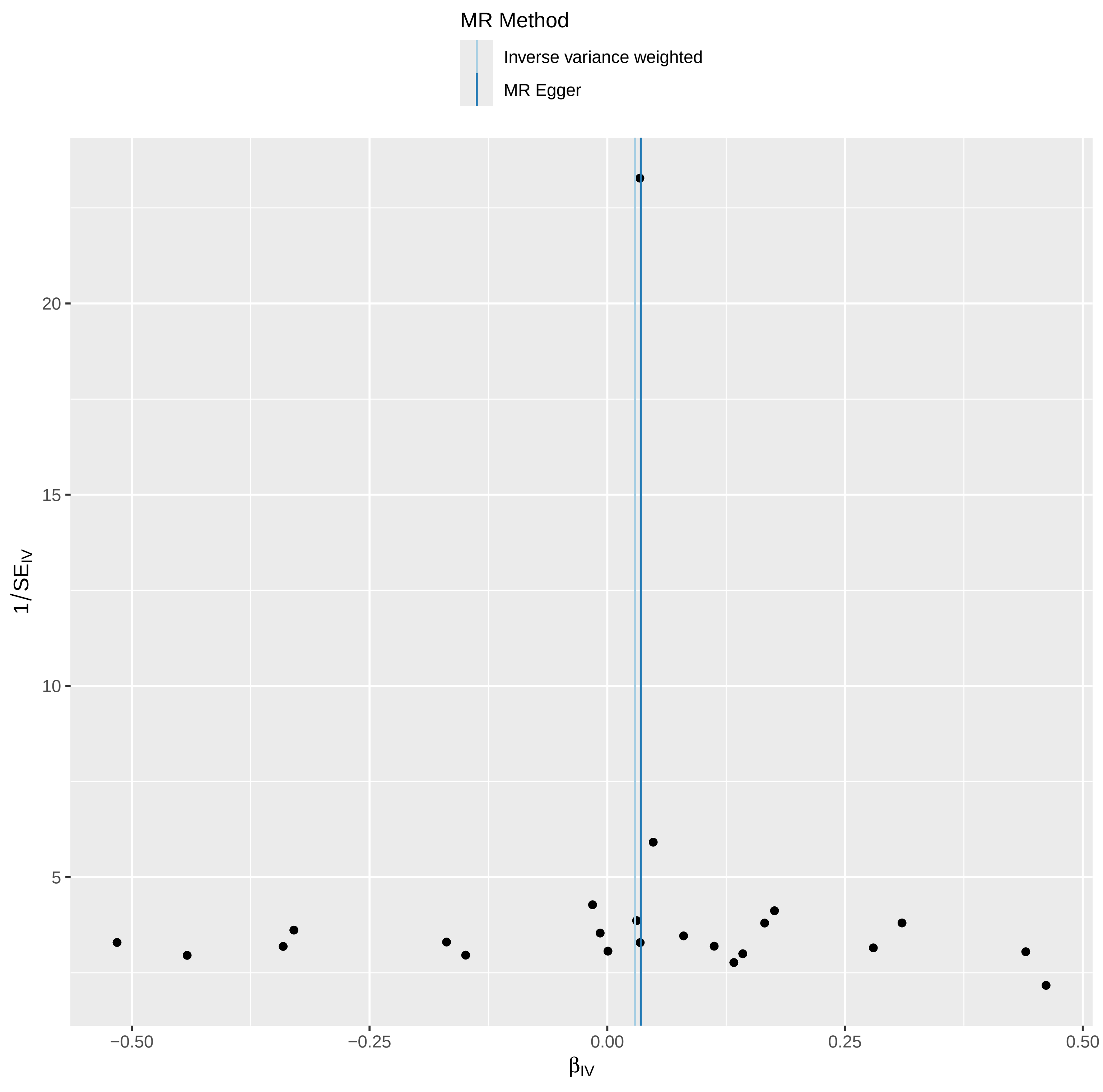
MR Method
Inverse variance weighted
MR Egger
20
15
10
5
−0.50
−0.25
0.00
0.25
0.50
$1/SE_{IV}$
β_{IV}

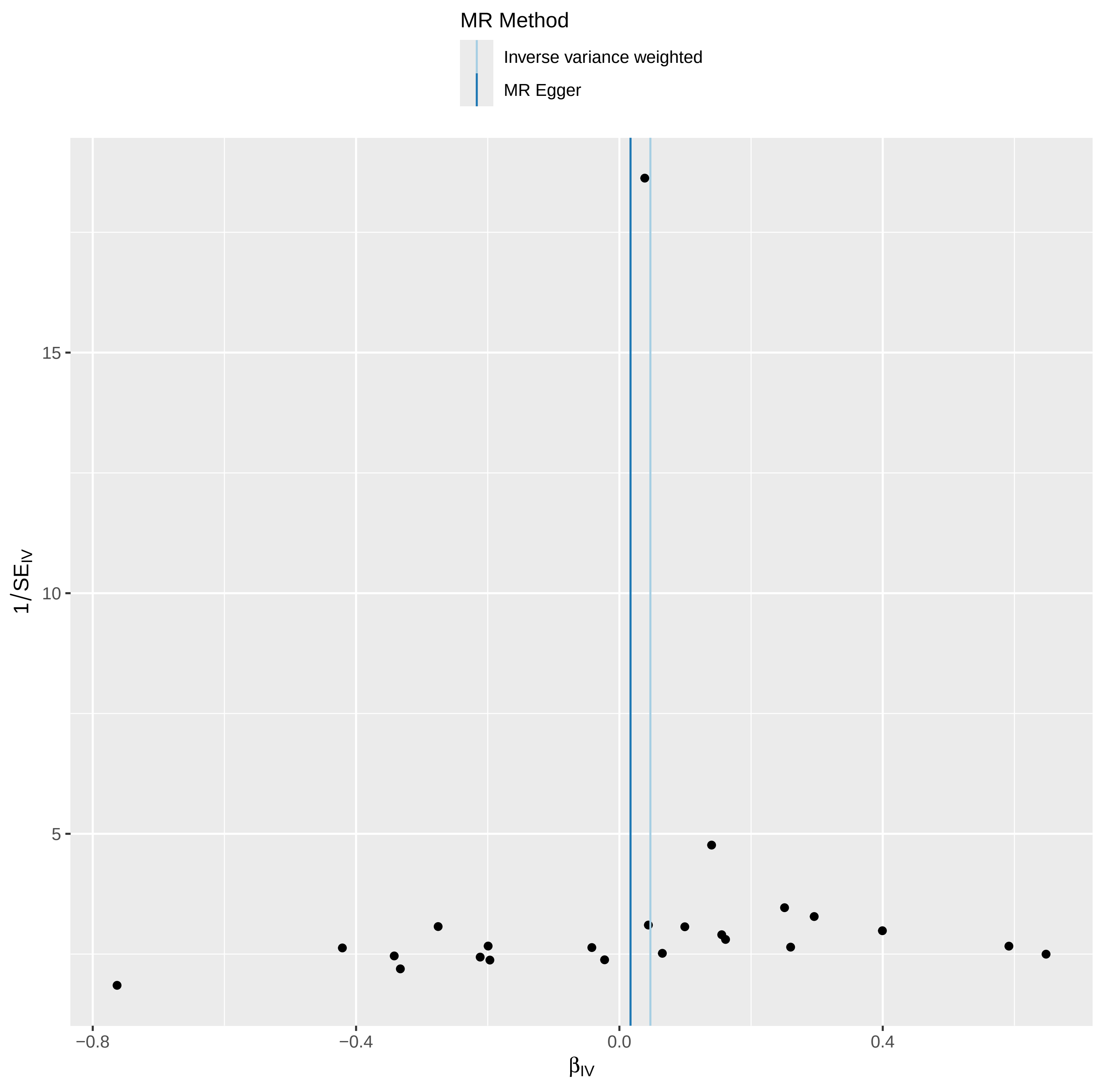
MR Method
Inverse variance weighted
MR Egger
1/SE_IV
15
10
5
−0.8
−0.4
0.0
0.4
β_IV

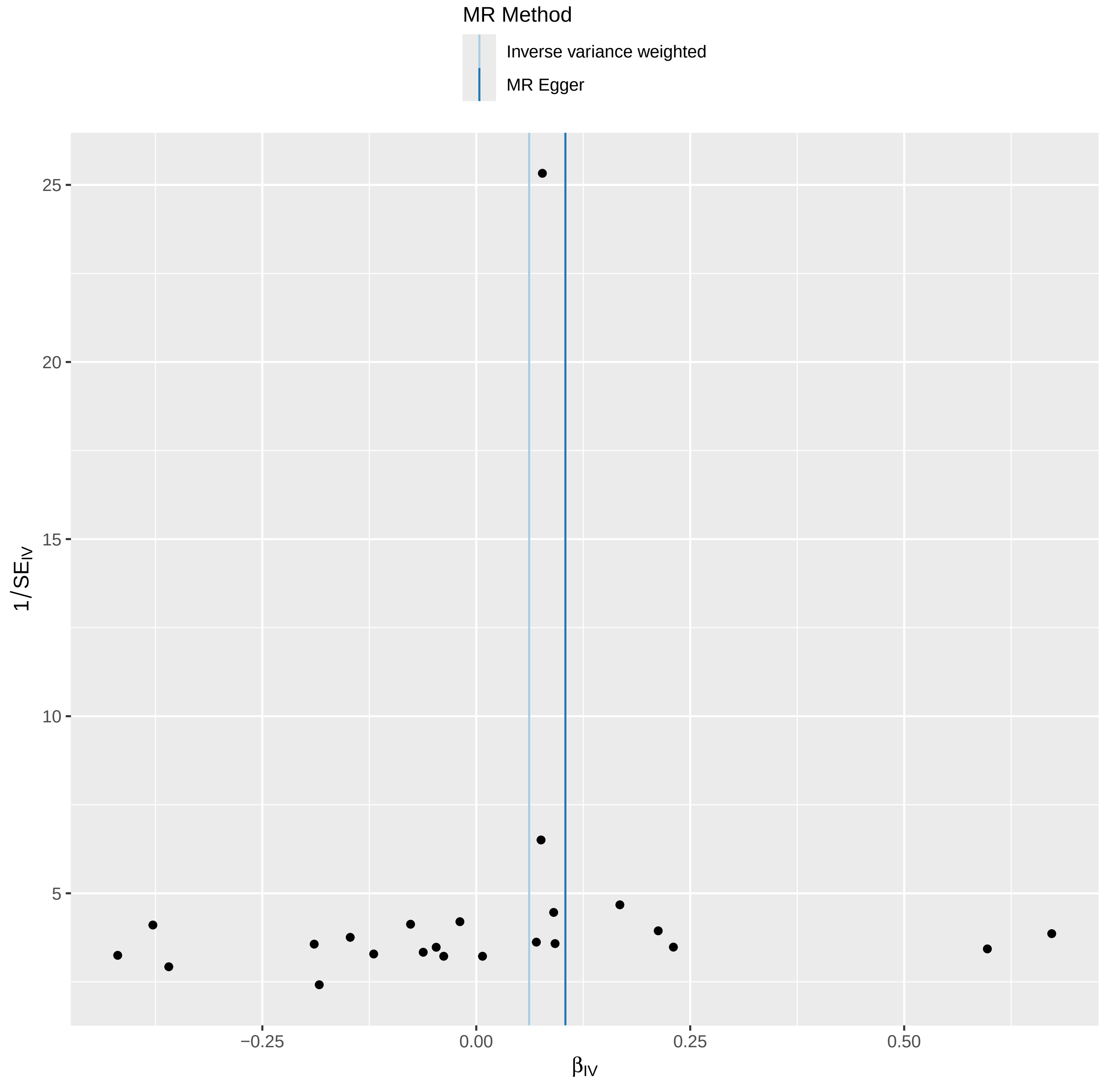
MR Method
Inverse variance weighted
MR Egger
25
20
15
10
5
1/SE_IV
−0.25
0.00
0.25
0.50
β_IV

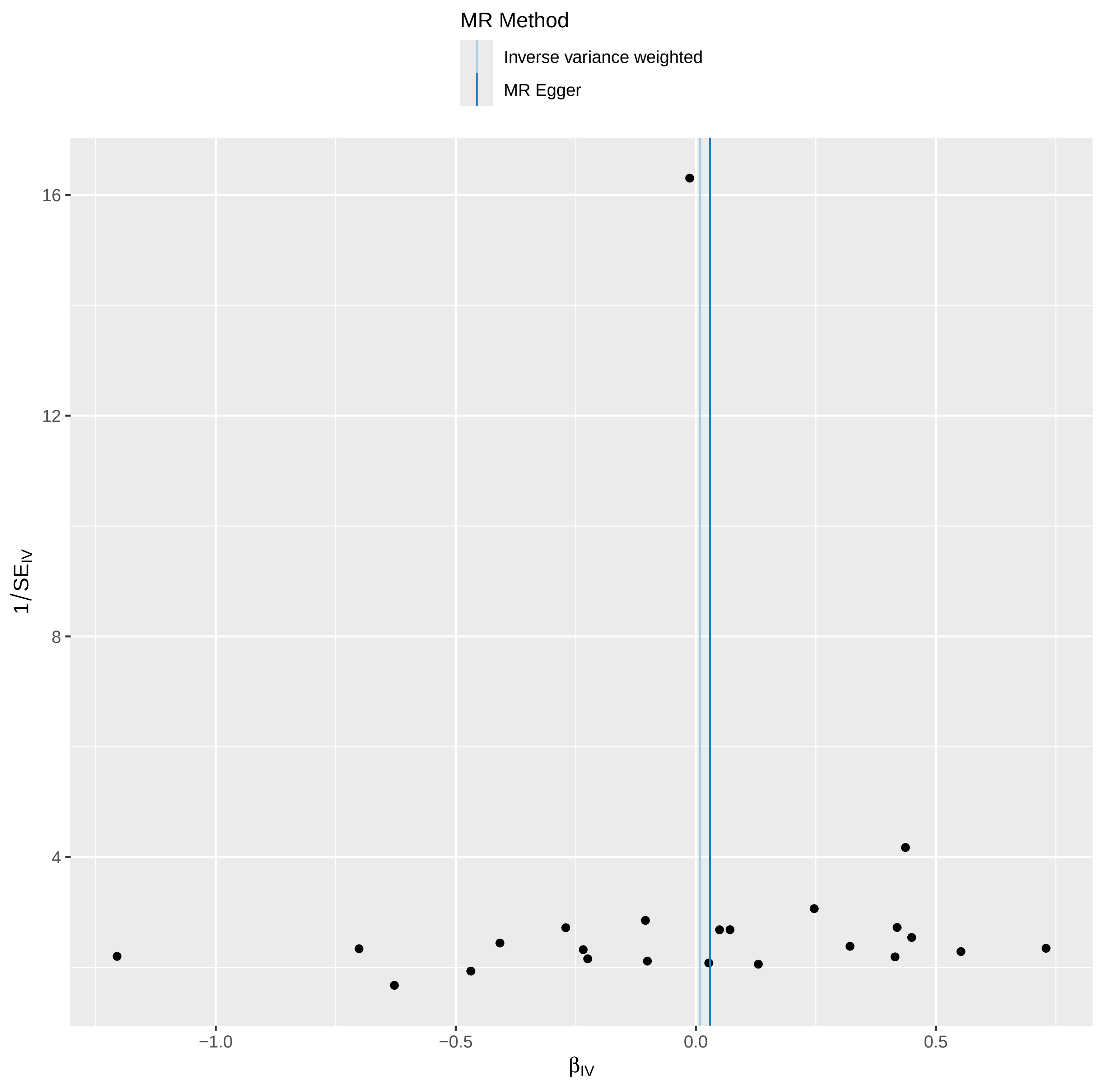

MR Method
Inverse variance weighted
MR Egger
16
12
8
4
1/SE$_{IV}$
−1.0
−0.5
0.0
0.5
β$_{IV}$

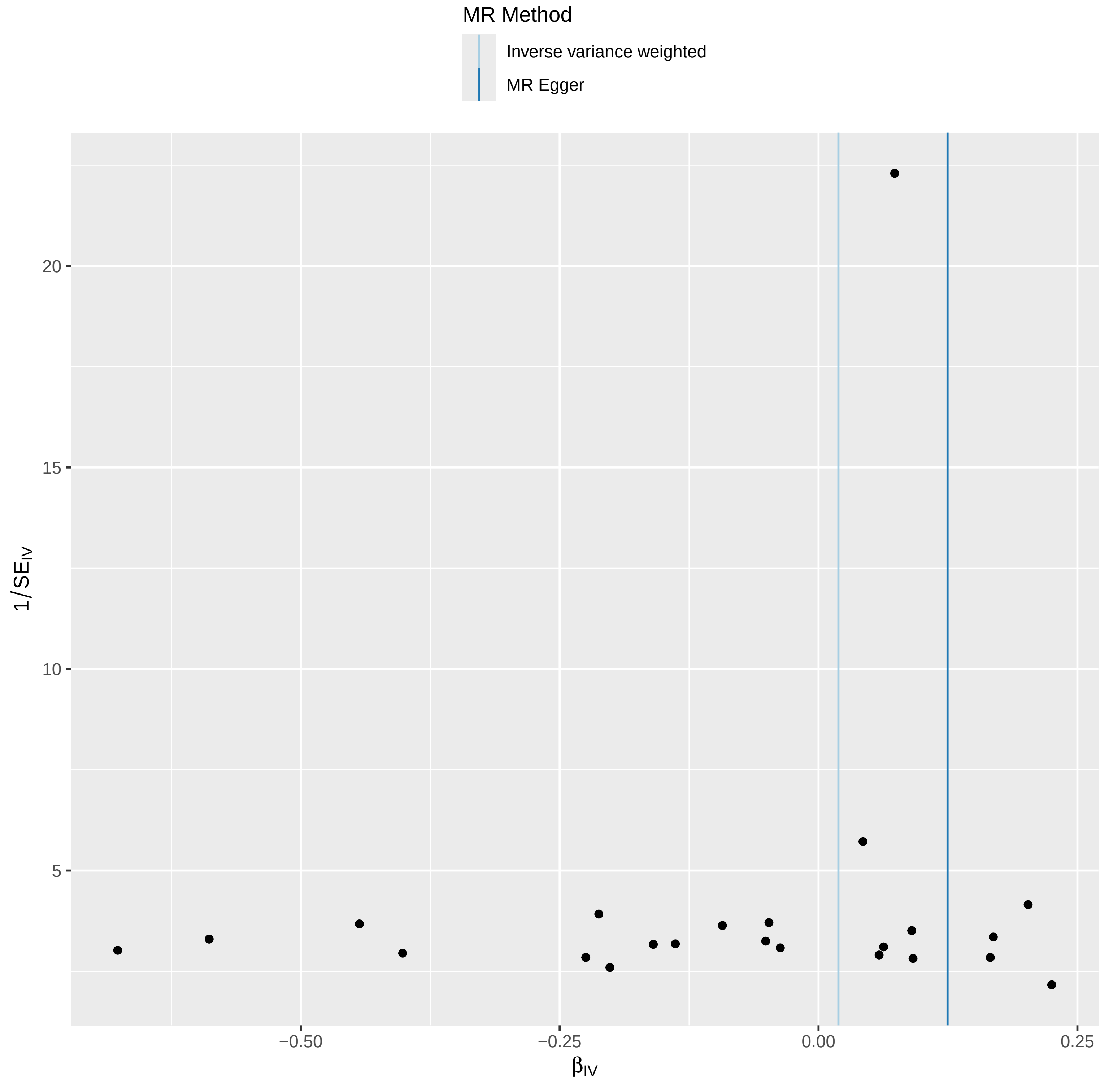

MR Method
Inverse variance weighted
MR Egger
20
15
10
5
−0.50
−0.25
0.00
0.25
β_{IV}
$1/SE_{IV}$

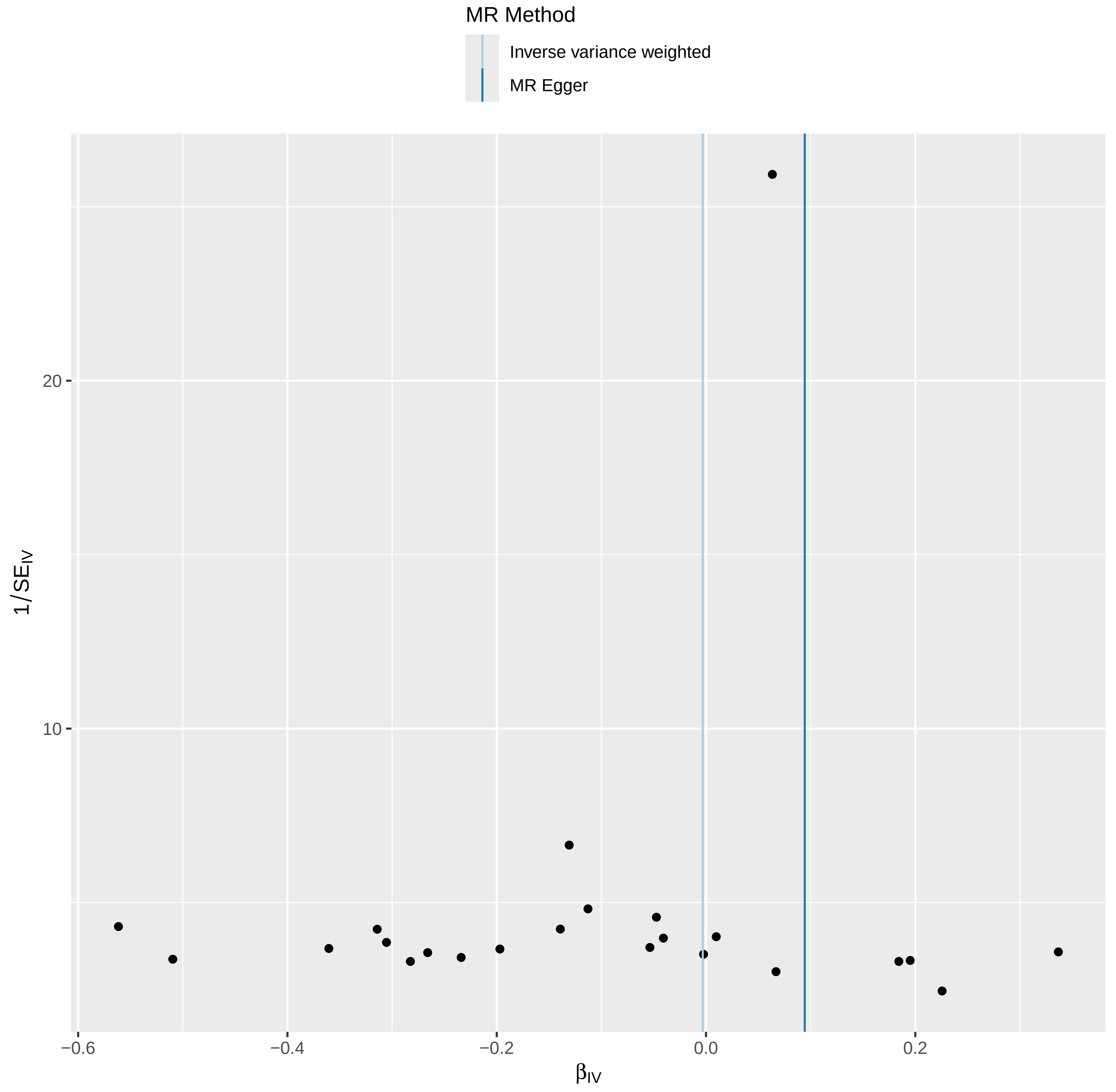

MR Method
Inverse variance weighted
MR Egger
20
10
1/SE$_{IV}$
−0.6
−0.4
−0.2
0.0
0.2
β_{IV}

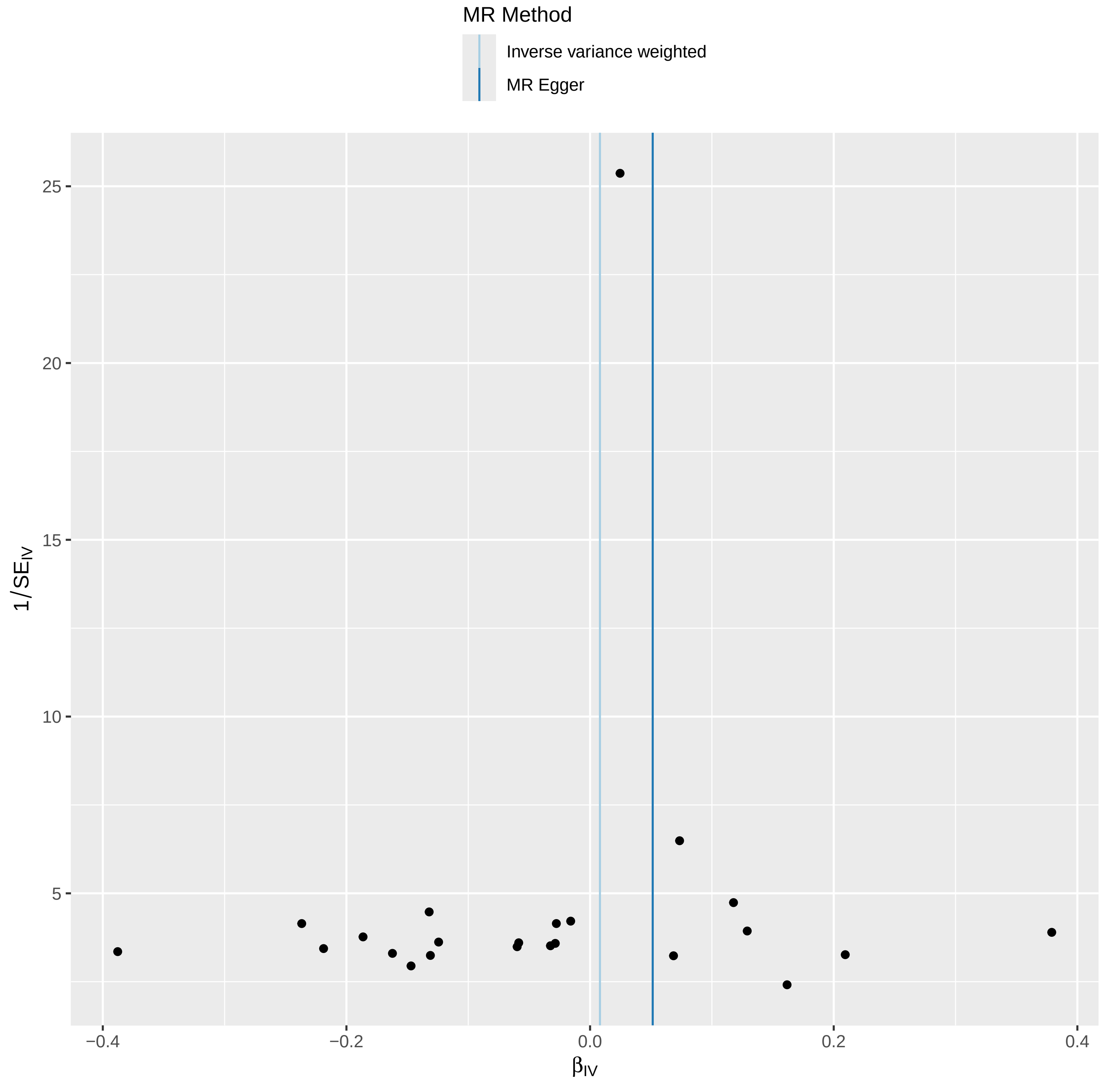
MR Method
Inverse variance weighted
MR Egger
25
20
15
10
5
1/SE_IV
−0.4
−0.2
0.0
0.2
0.4
β_IV

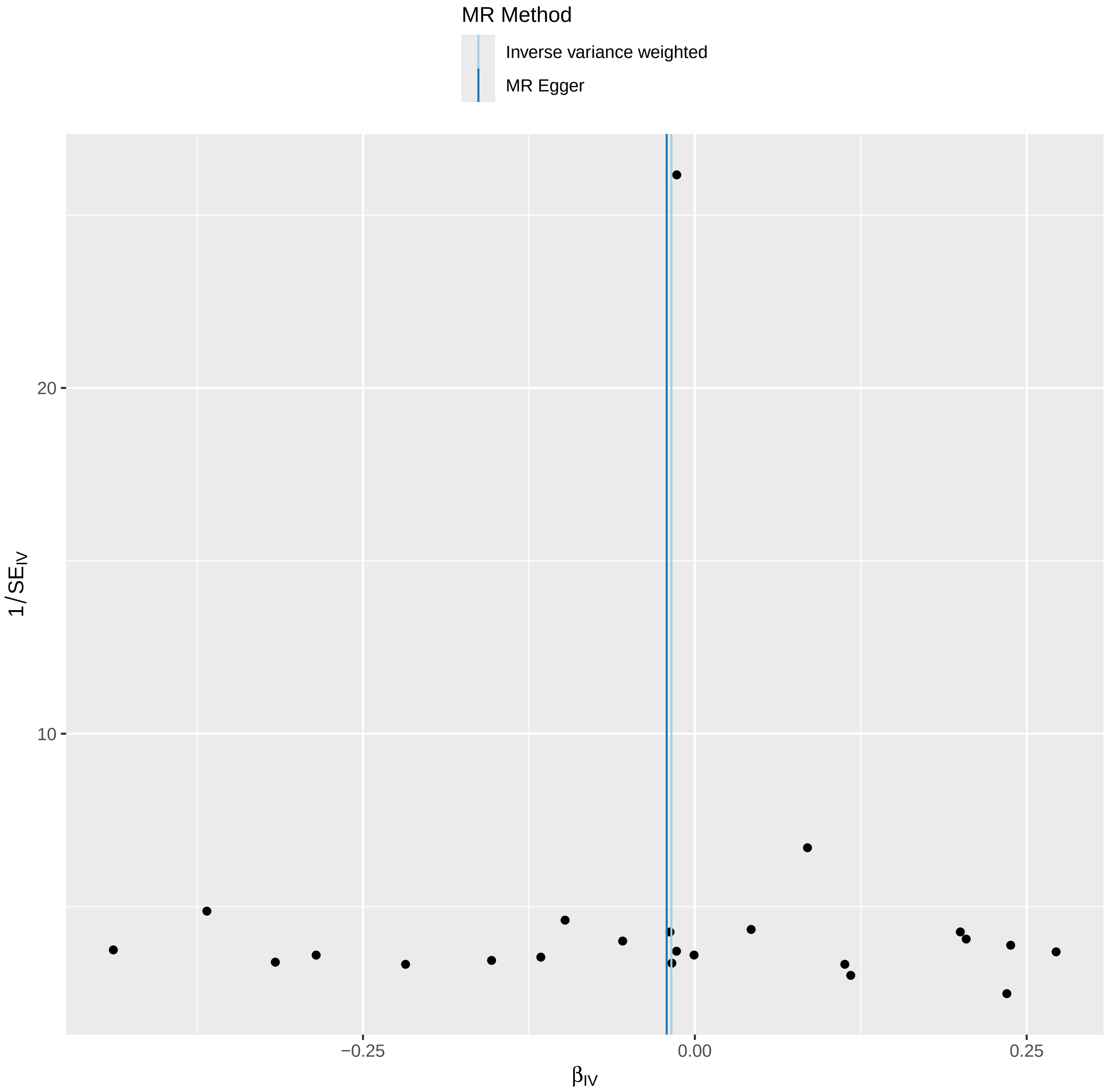

MR Method
Inverse variance weighted
MR Egger
20
10
1/SE$_{IV}$
−0.25
0.00
0.25
β$_{IV}$

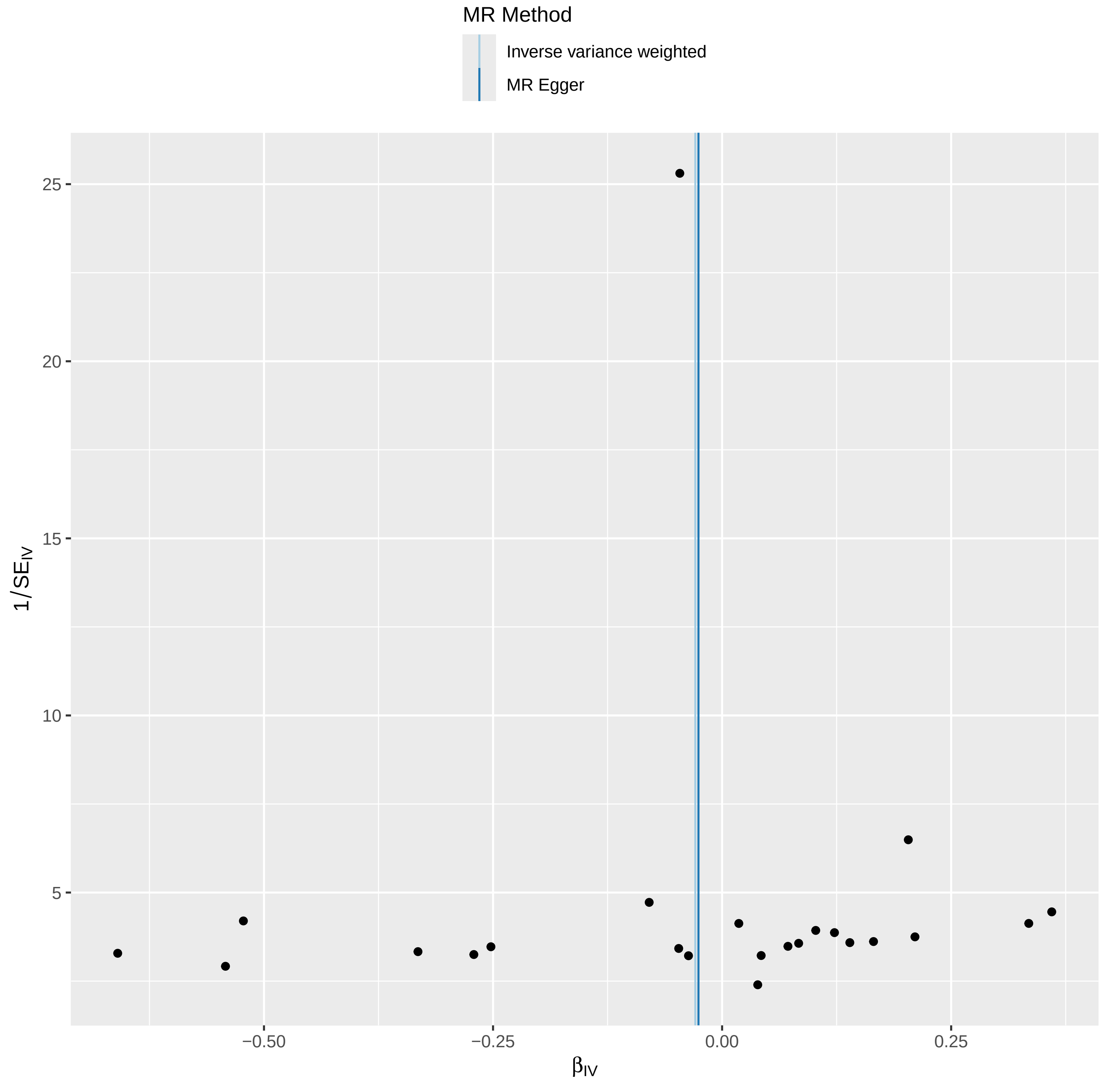

MR Method
Inverse variance weighted
MR Egger
25
20
15
10
5
1/SE_IV
−0.50
−0.25
0.00
0.25
β_IV

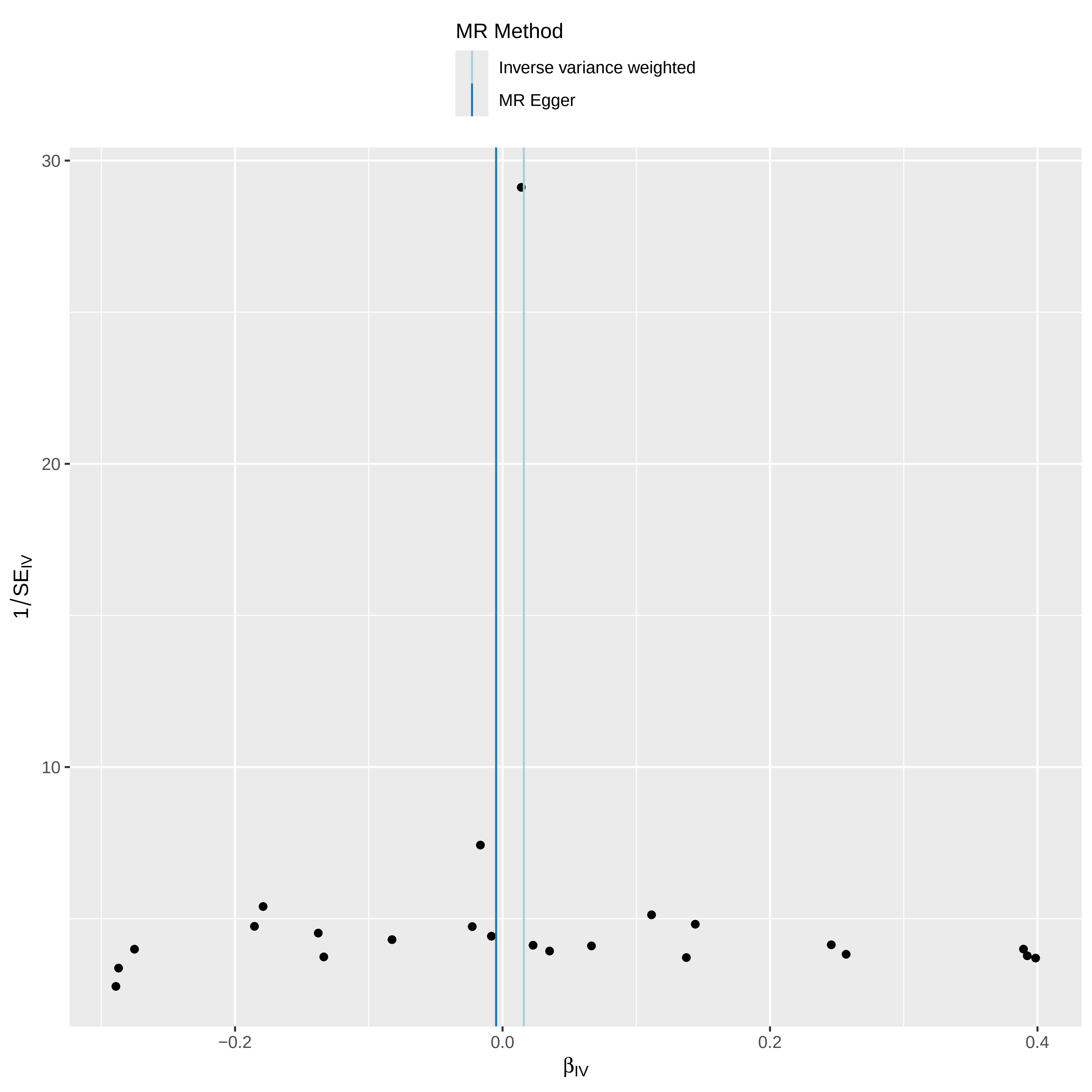

MR Method
Inverse variance weighted
MR Egger
30
20
10
1/SE$_{IV}$
−0.2
0.0
0.2
0.4
β$_{IV}$

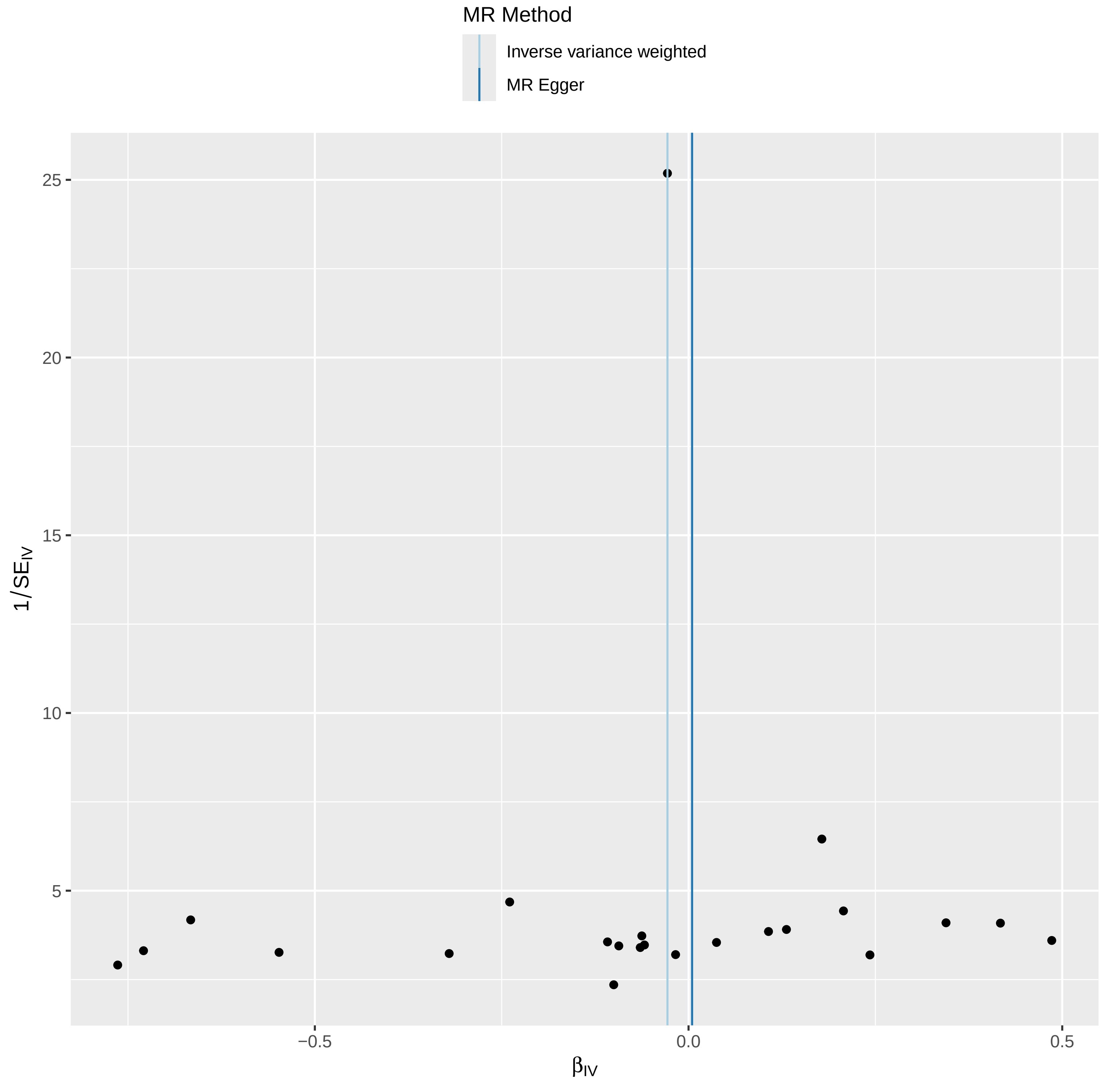

MR Method
Inverse variance weighted
MR Egger
25
20
15
10
5
1/SE_IV
−0.5
0.0
0.5
β_IV

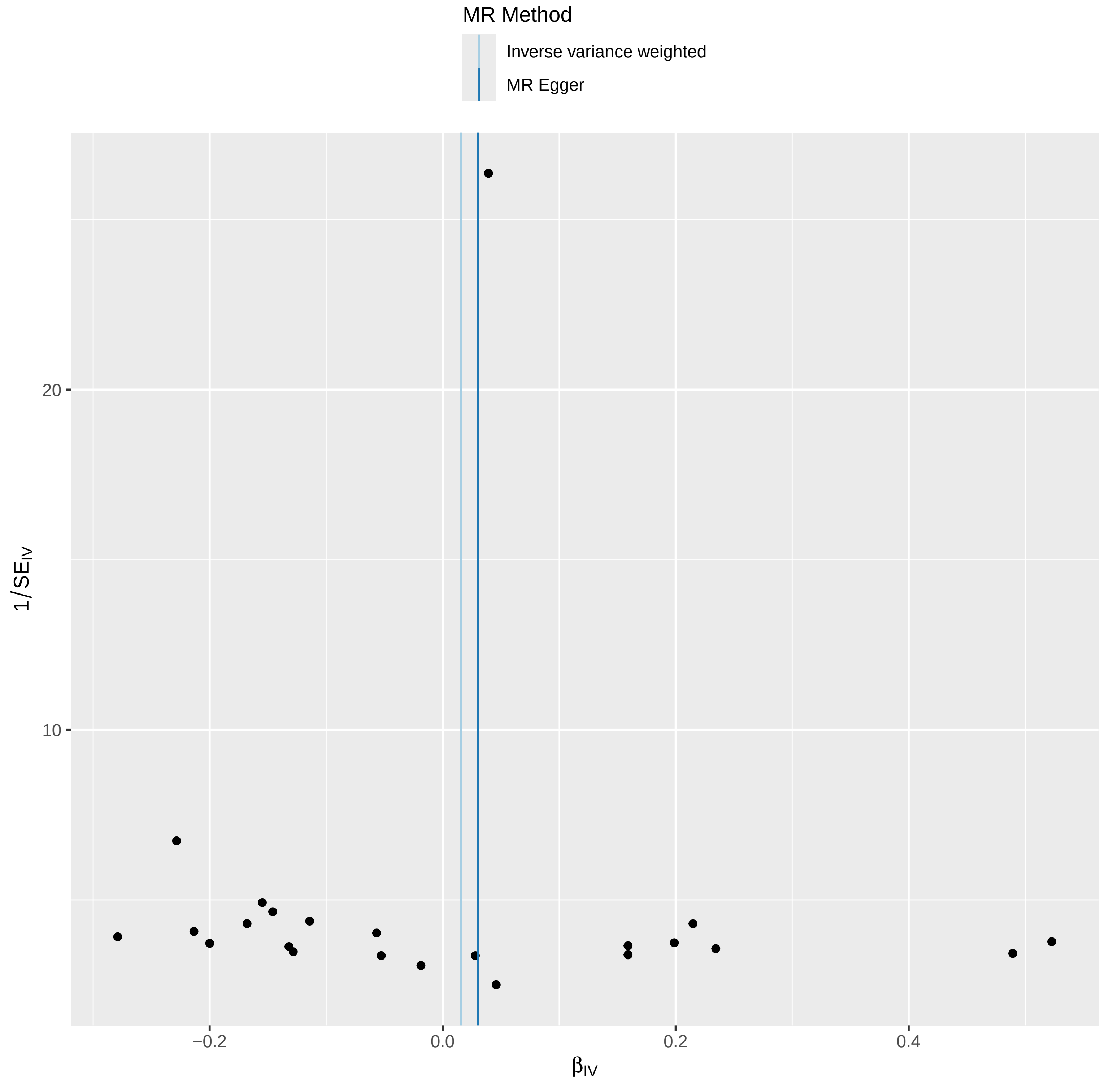

MR Method
Inverse variance weighted
MR Egger
20
10
1/SE_IV
−0.2
0.0
0.2
0.4
β_IV

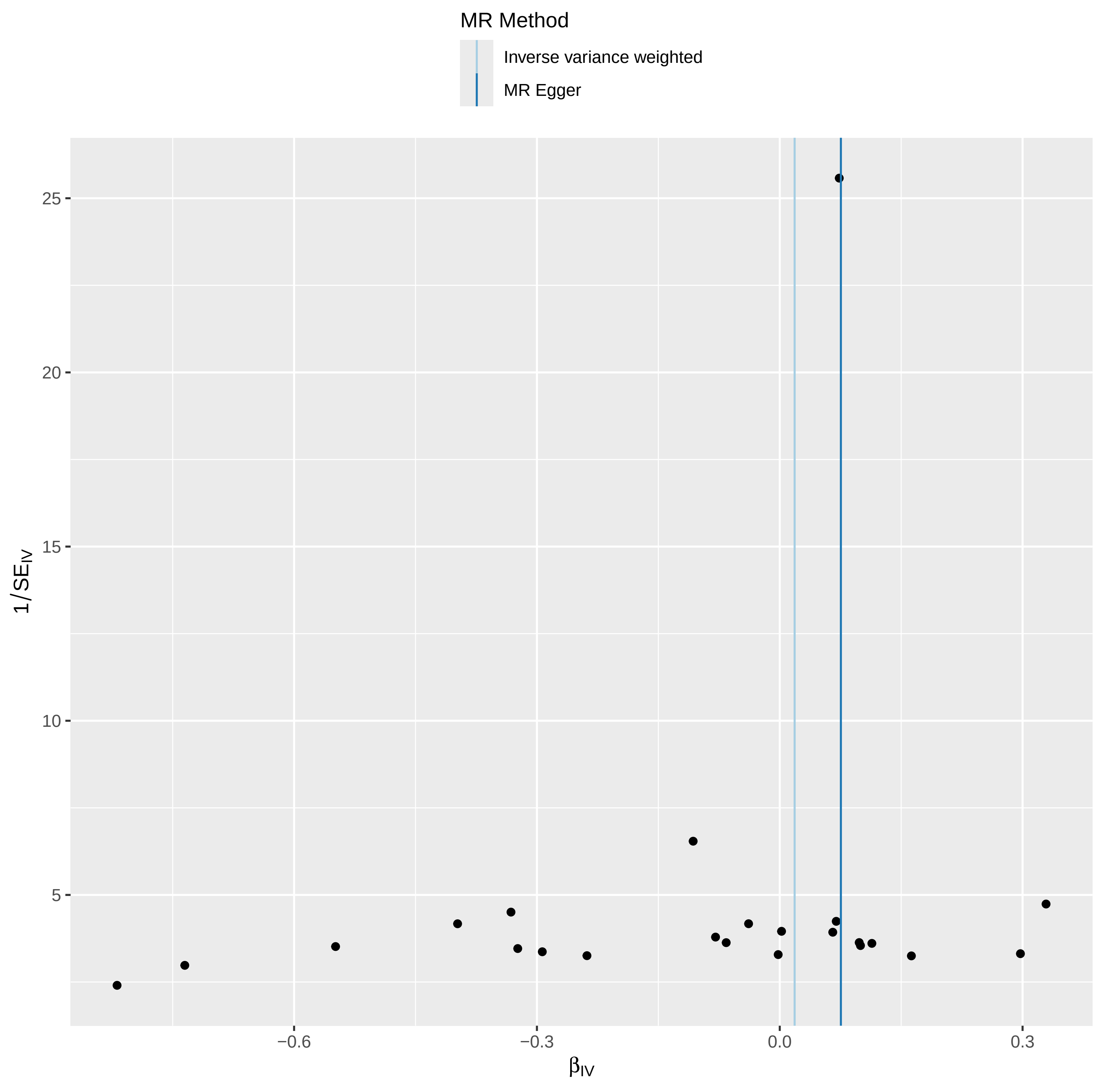
MR Method
Inverse variance weighted
MR Egger
25
20
15
10
5
1/SE$_{IV}$
−0.6
−0.3
0.0
0.3
β$_{IV}$

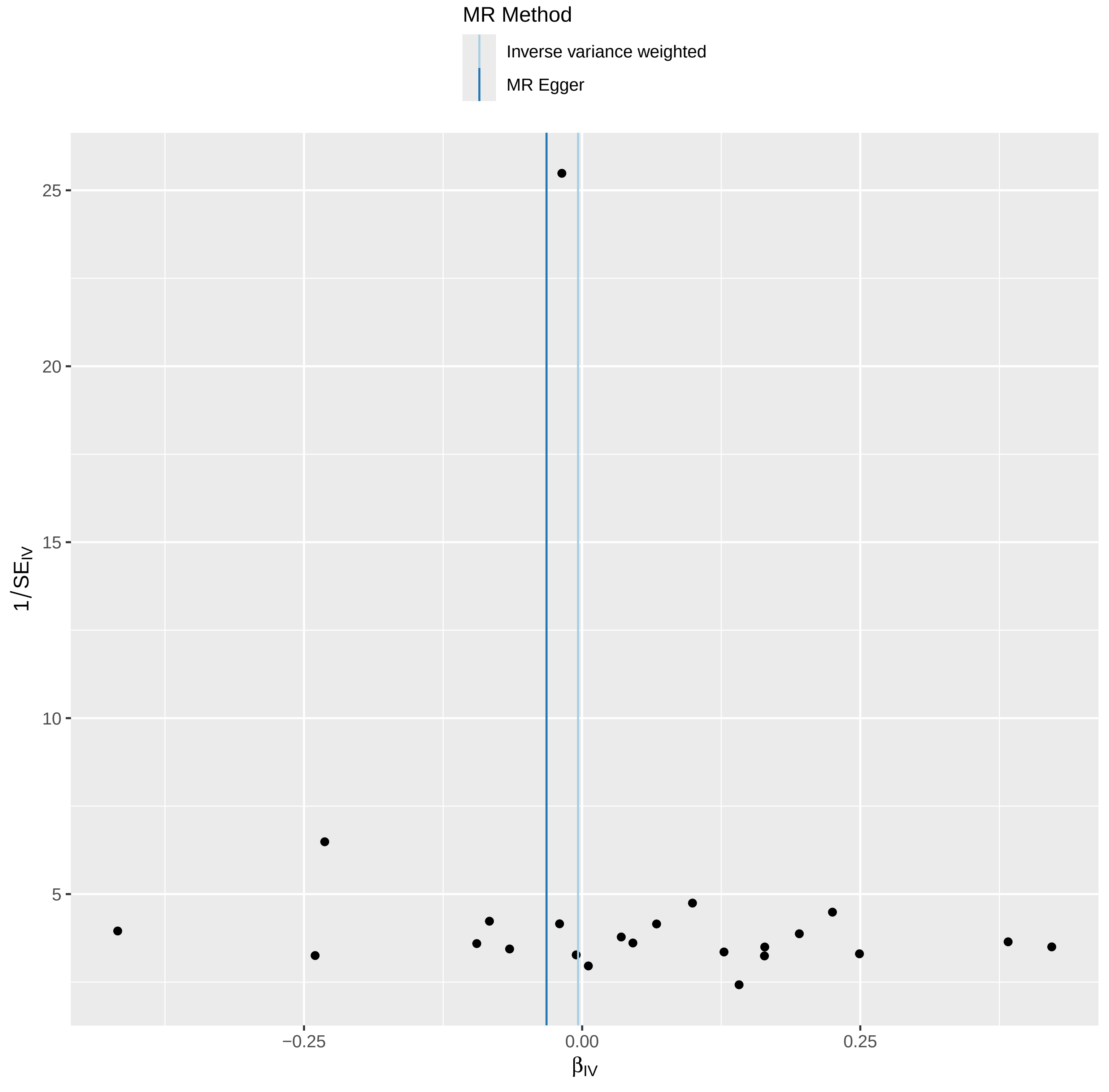
MR Method
Inverse variance weighted
MR Egger
25
20
15
10
5
$1/SE_{IV}$
−0.25
0.00
0.25
β_{IV}

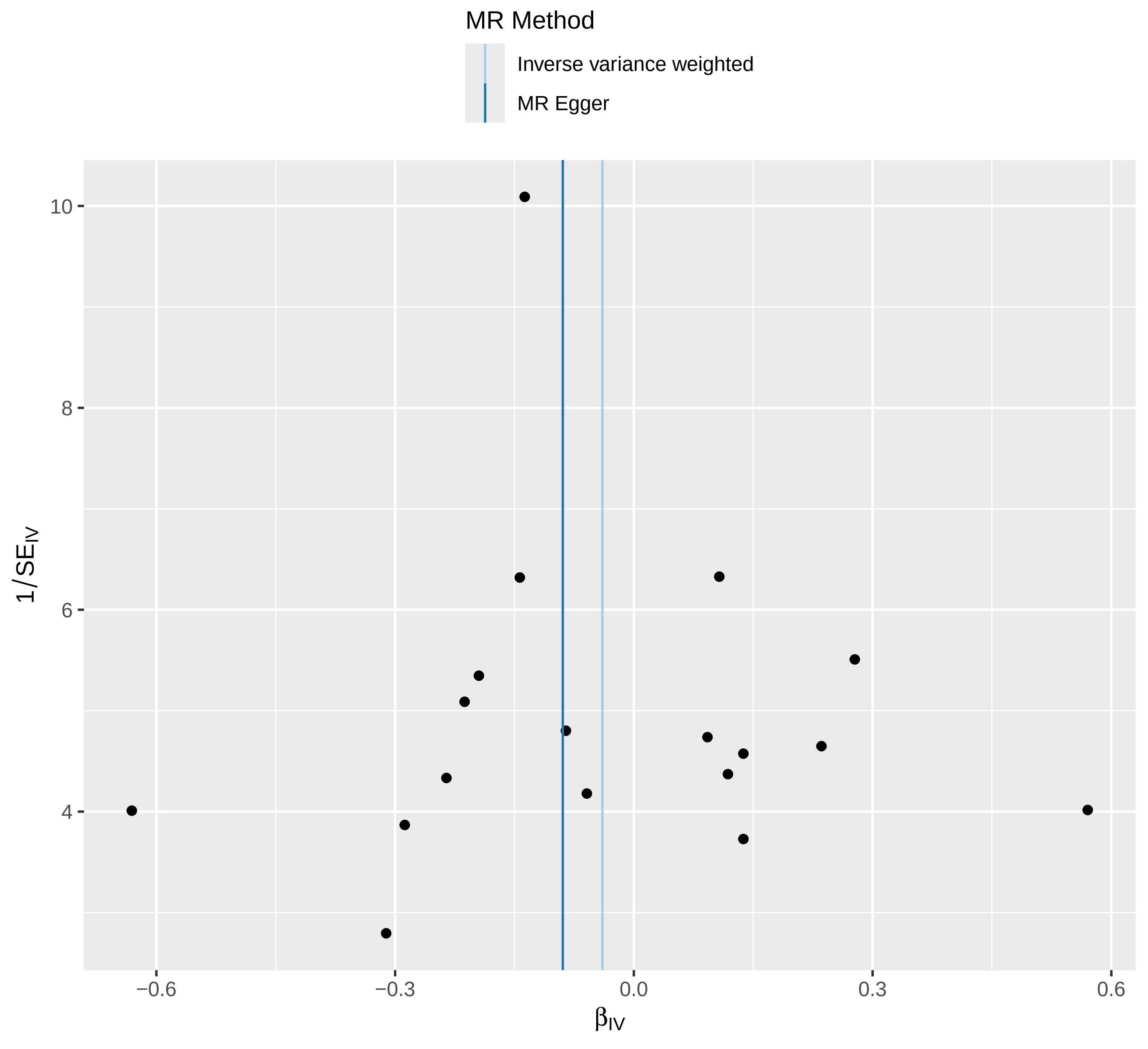

MR Method
Inverse variance weighted
MR Egger
10
8
6
4
1/SE$_{IV}$
−0.6
−0.3
0.0
0.3
0.6
β$_{IV}$

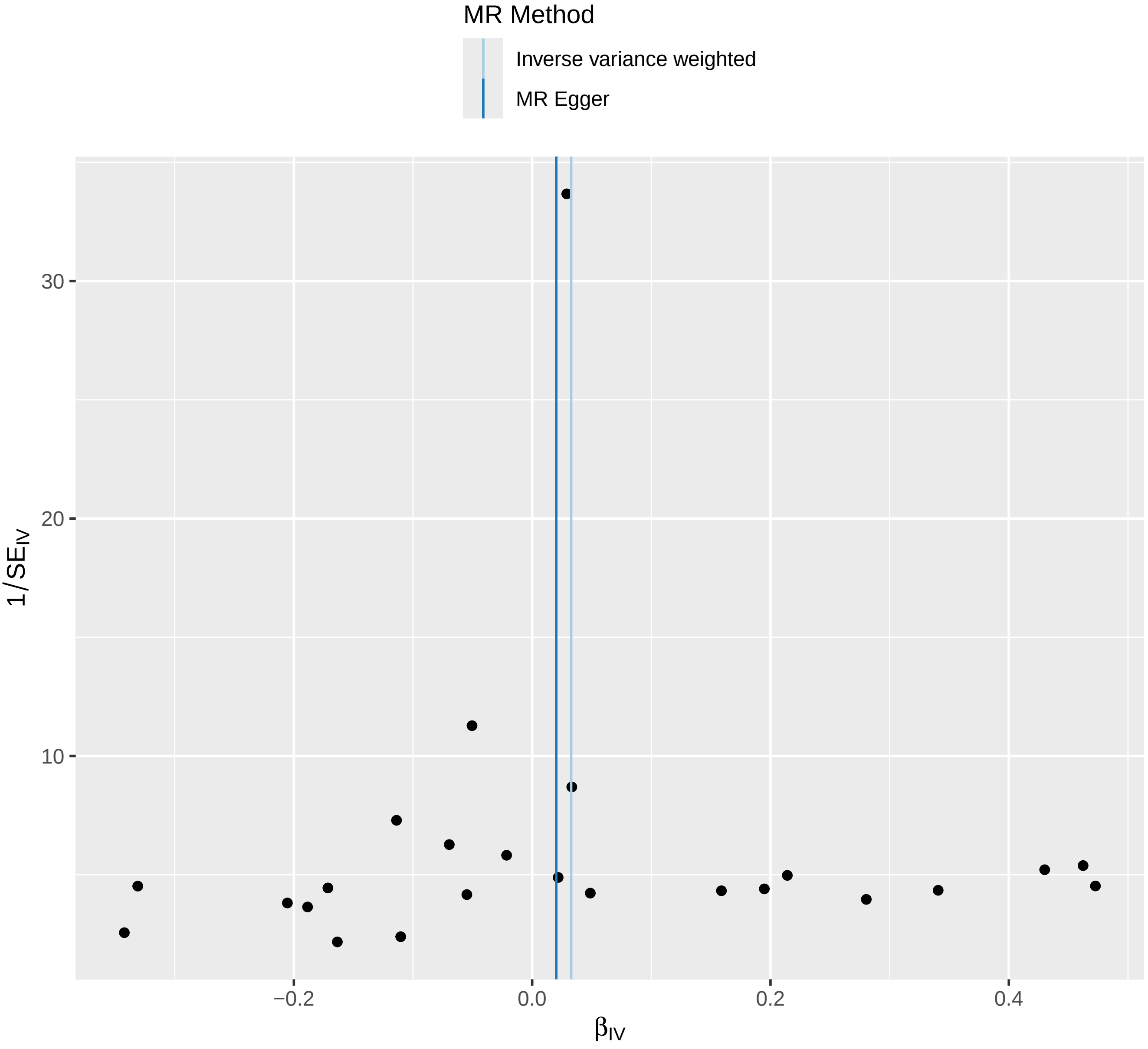
MR Method
Inverse variance weighted
MR Egger
30
20
10
1/SE_IV
−0.2
0.0
0.2
0.4
β_IV

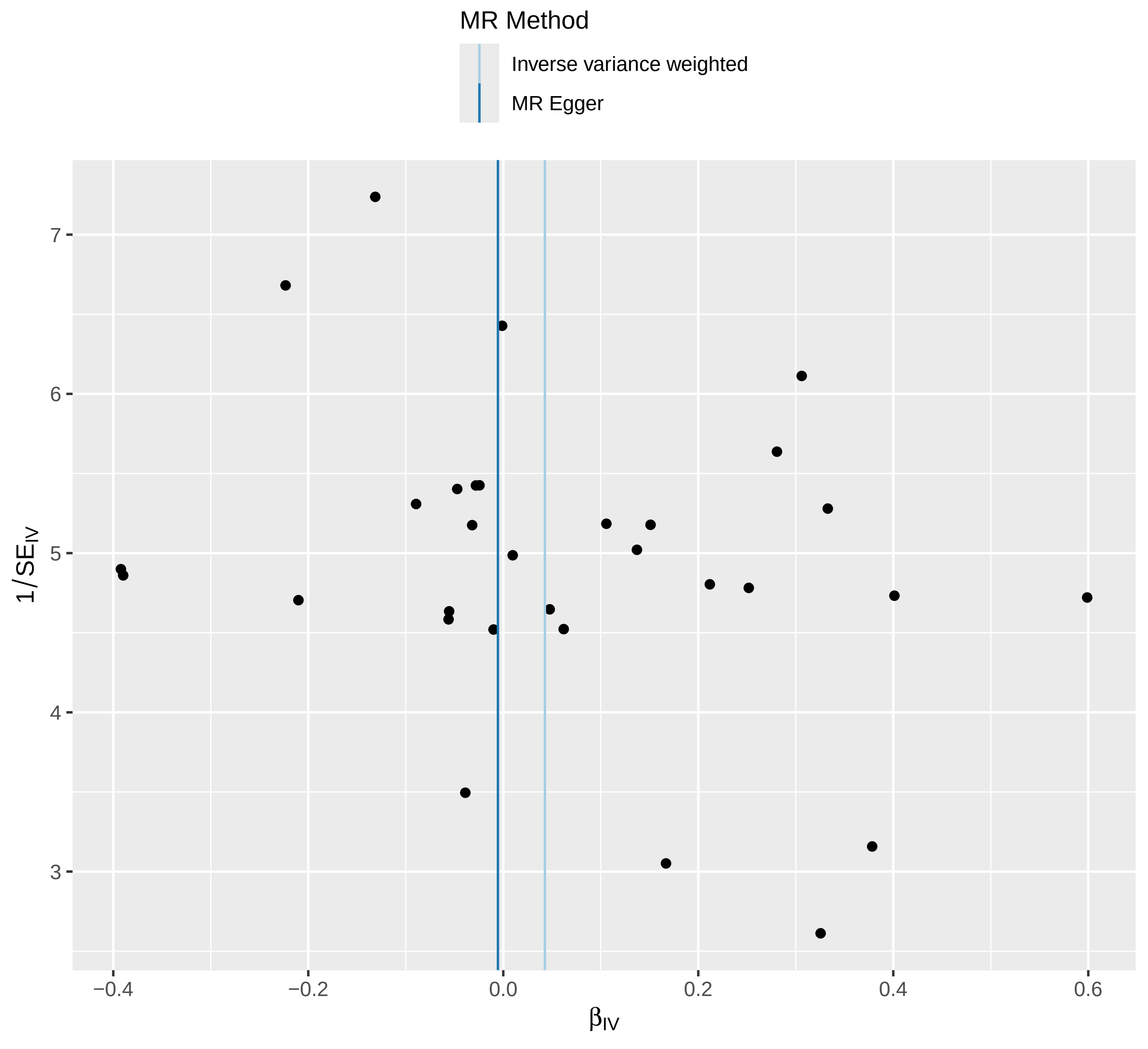
MR Method
Inverse variance weighted
MR Egger
1/SE_IV
β_IV
7
6
5
4
3
−0.4
−0.2
0.0
0.2
0.4
0.6

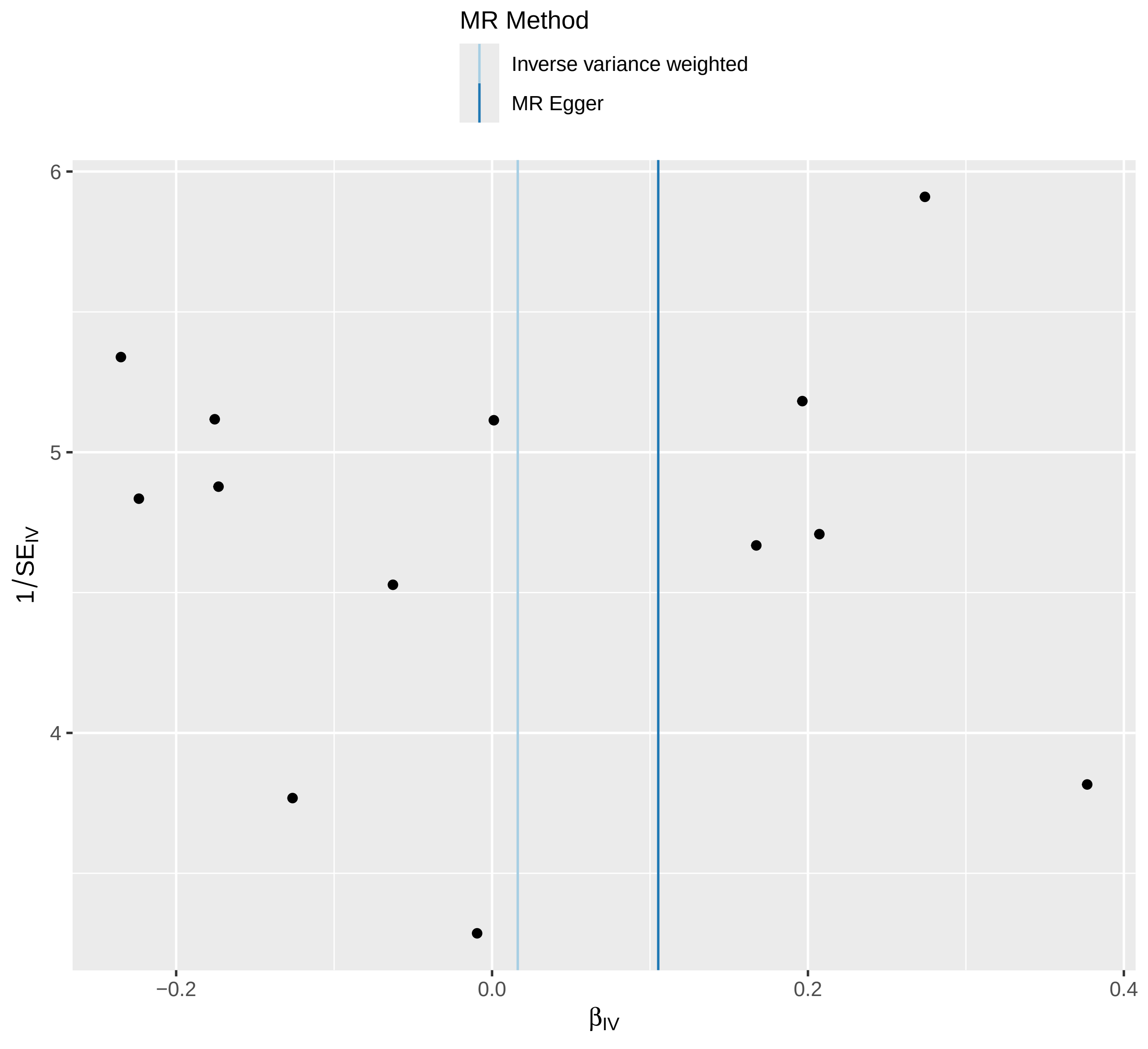
MR Method
Inverse variance weighted
MR Egger
6
5
4
$1/SE_{IV}$
−0.2
0.0
0.2
0.4
β_{IV}

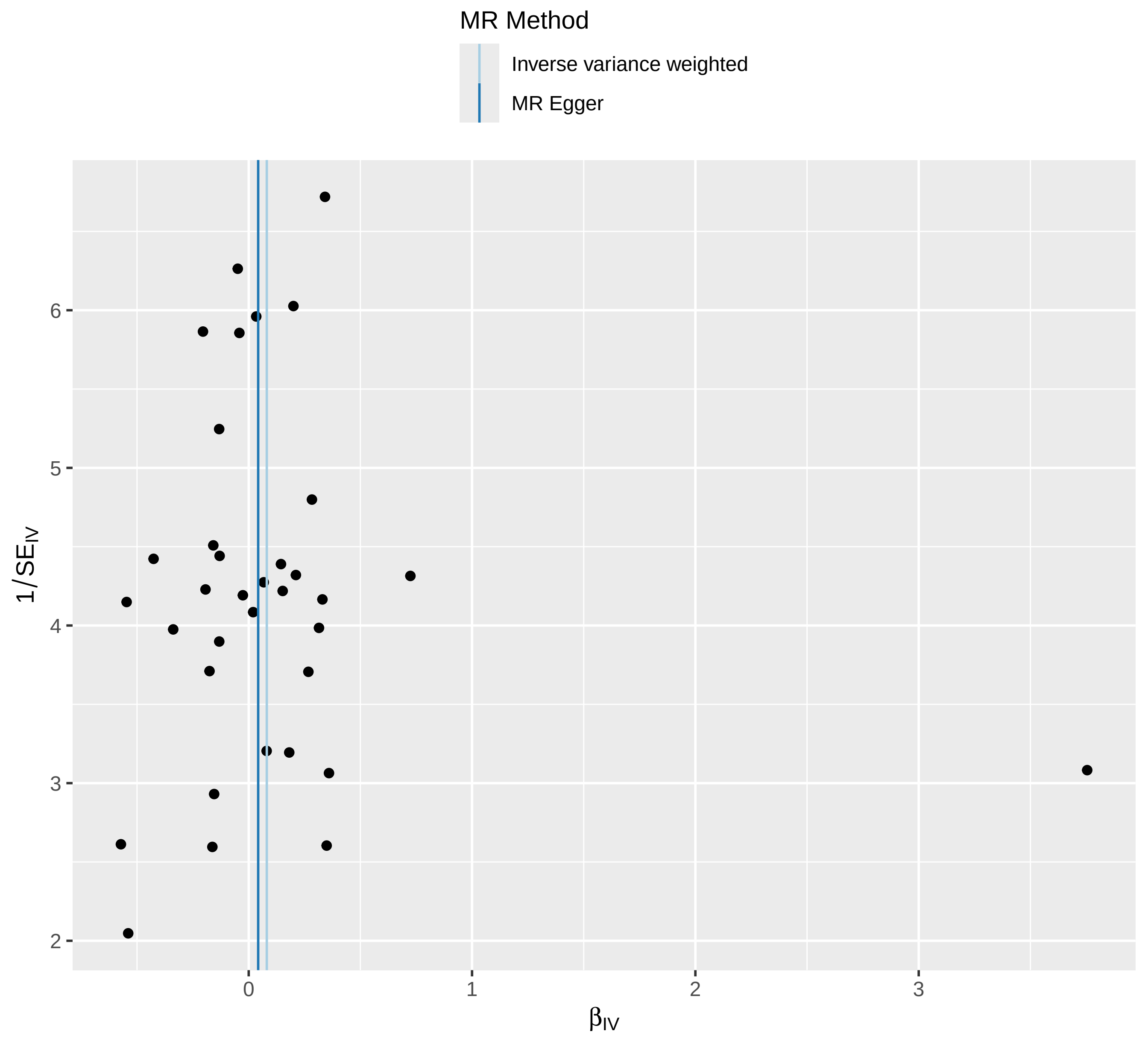

MR Method
Inverse variance weighted
MR Egger
6
5
4
3
2
0
1
2
3
1/SE_IV
β_IV

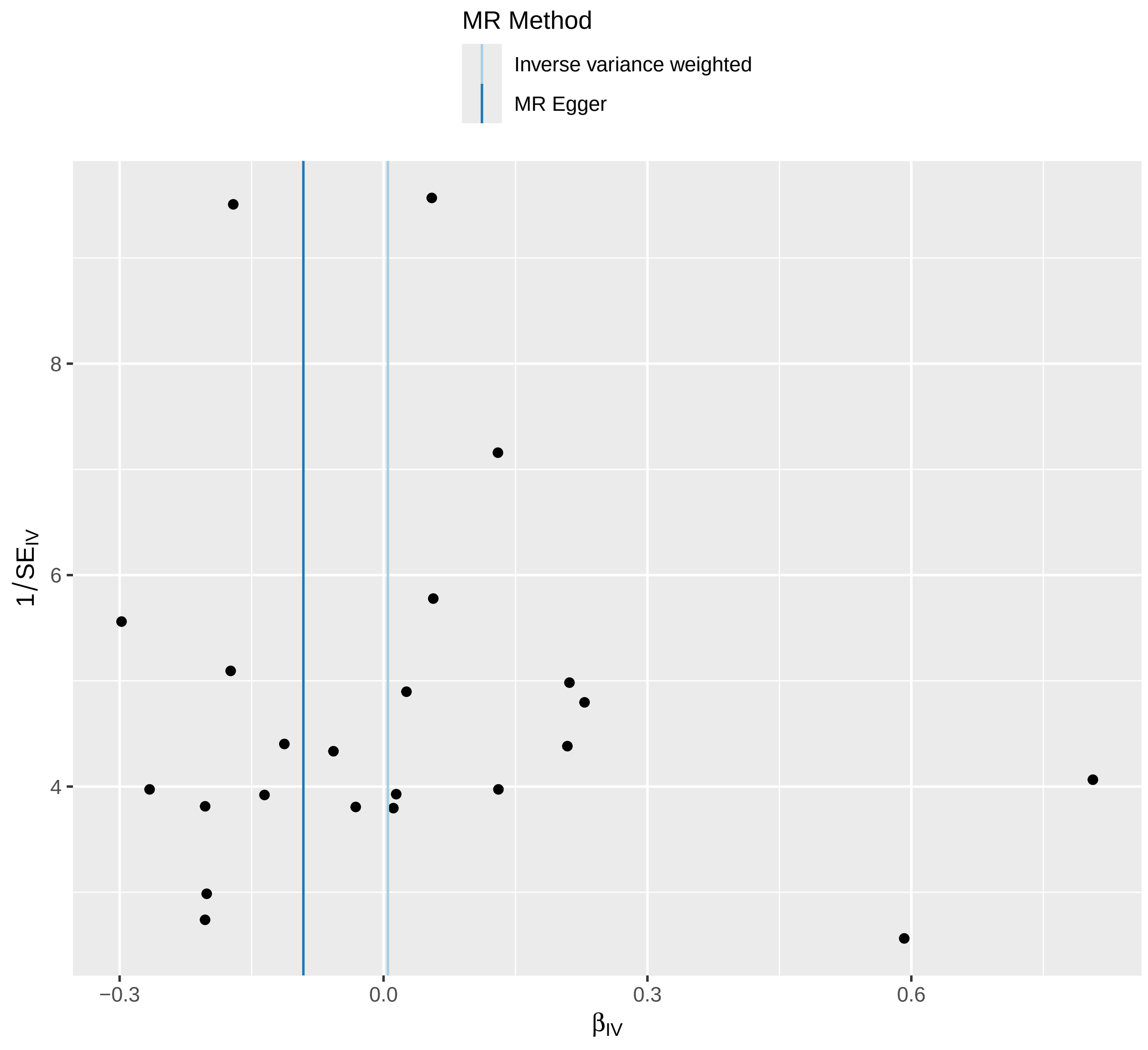
MR Method
Inverse variance weighted
MR Egger
1/SE_{IV}
8
6
4
−0.3
0.0
0.3
0.6
β_{IV}

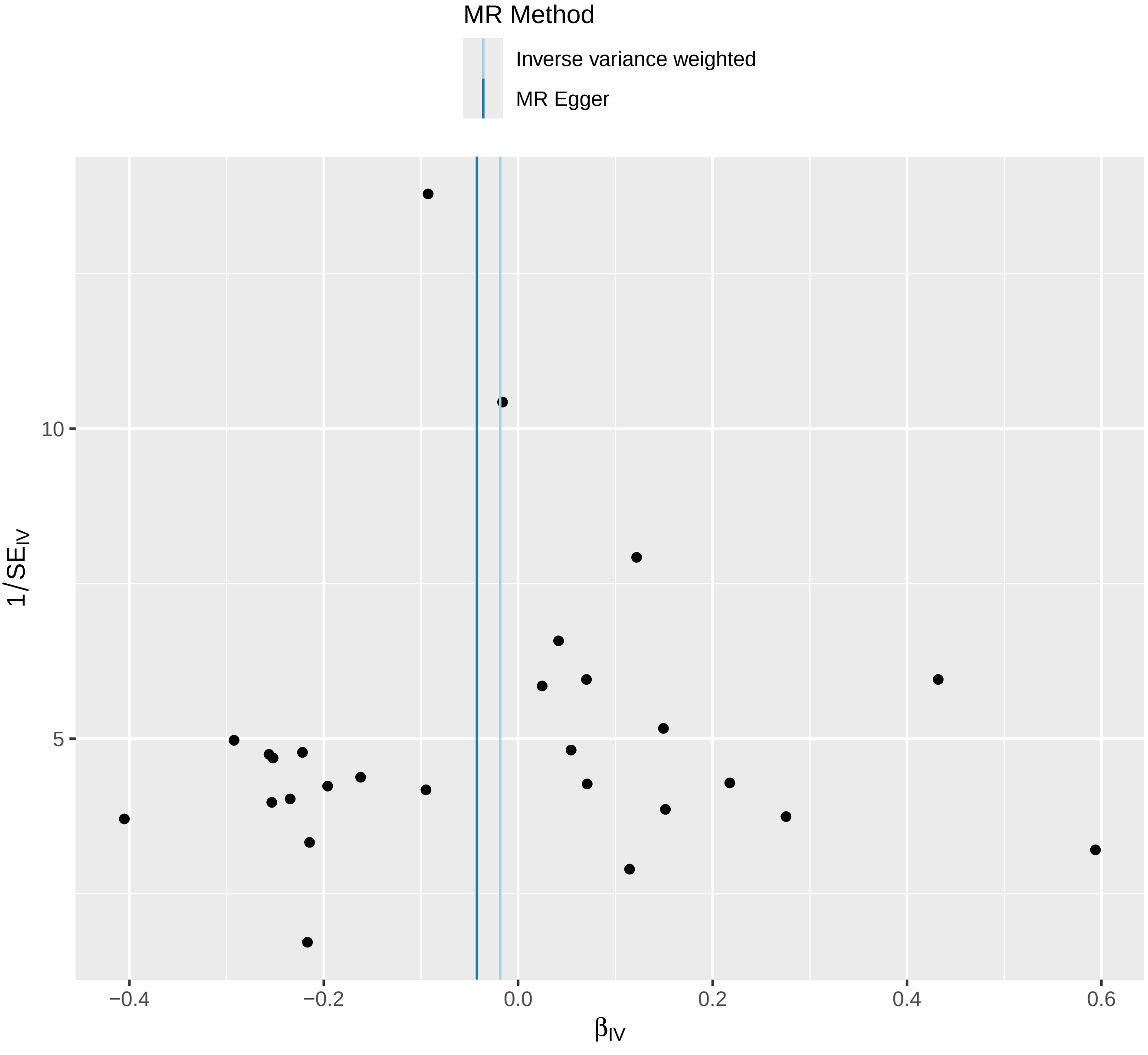
MR Method
Inverse variance weighted
MR Egger
10
5
1/SE_IV
−0.4
−0.2
0.0
0.2
0.4
0.6
β_IV

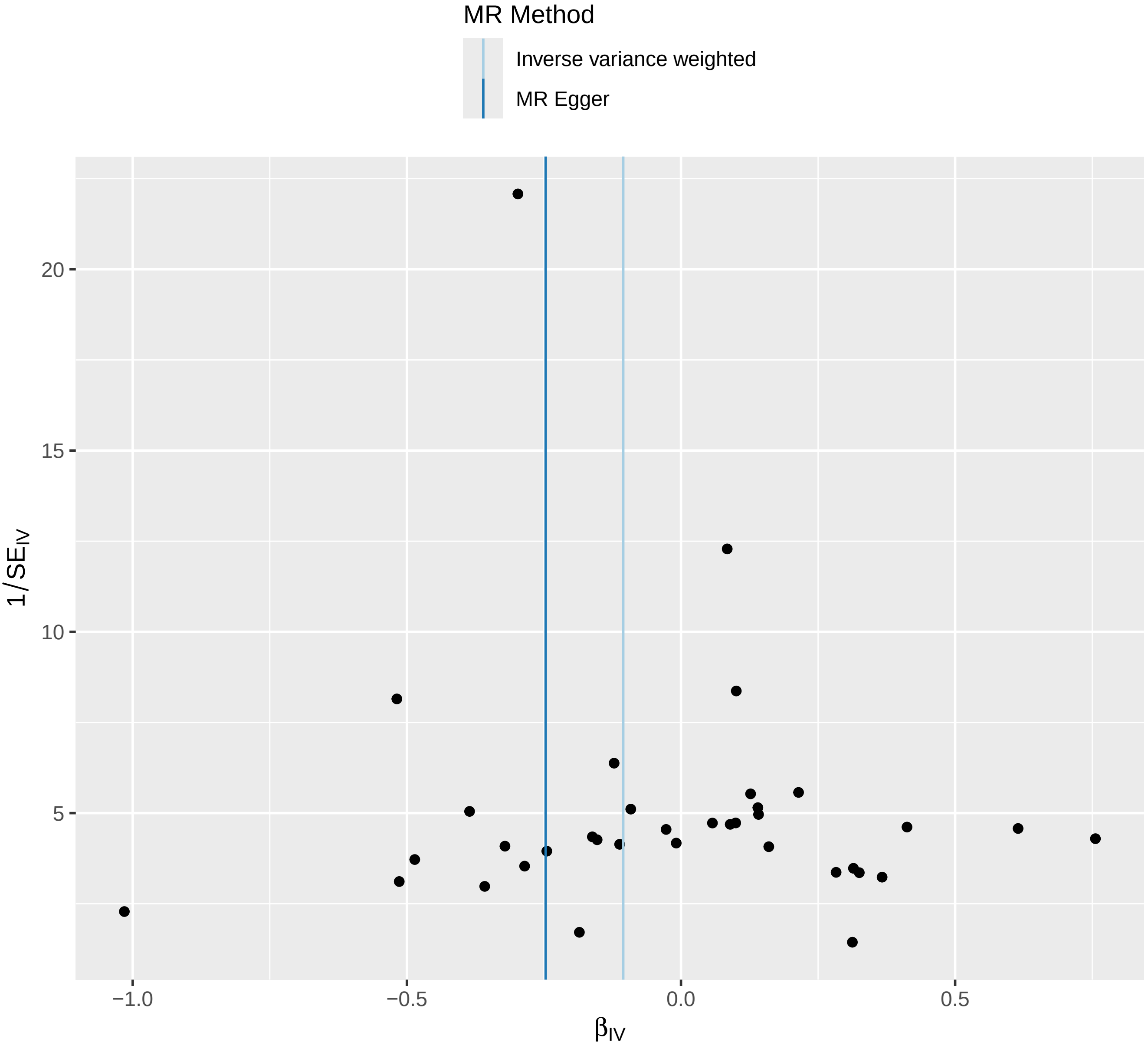

MR Method
Inverse variance weighted
MR Egger
20
15
10
5
1/SE$_{IV}$
−1.0
−0.5
0.0
0.5
β$_{IV}$

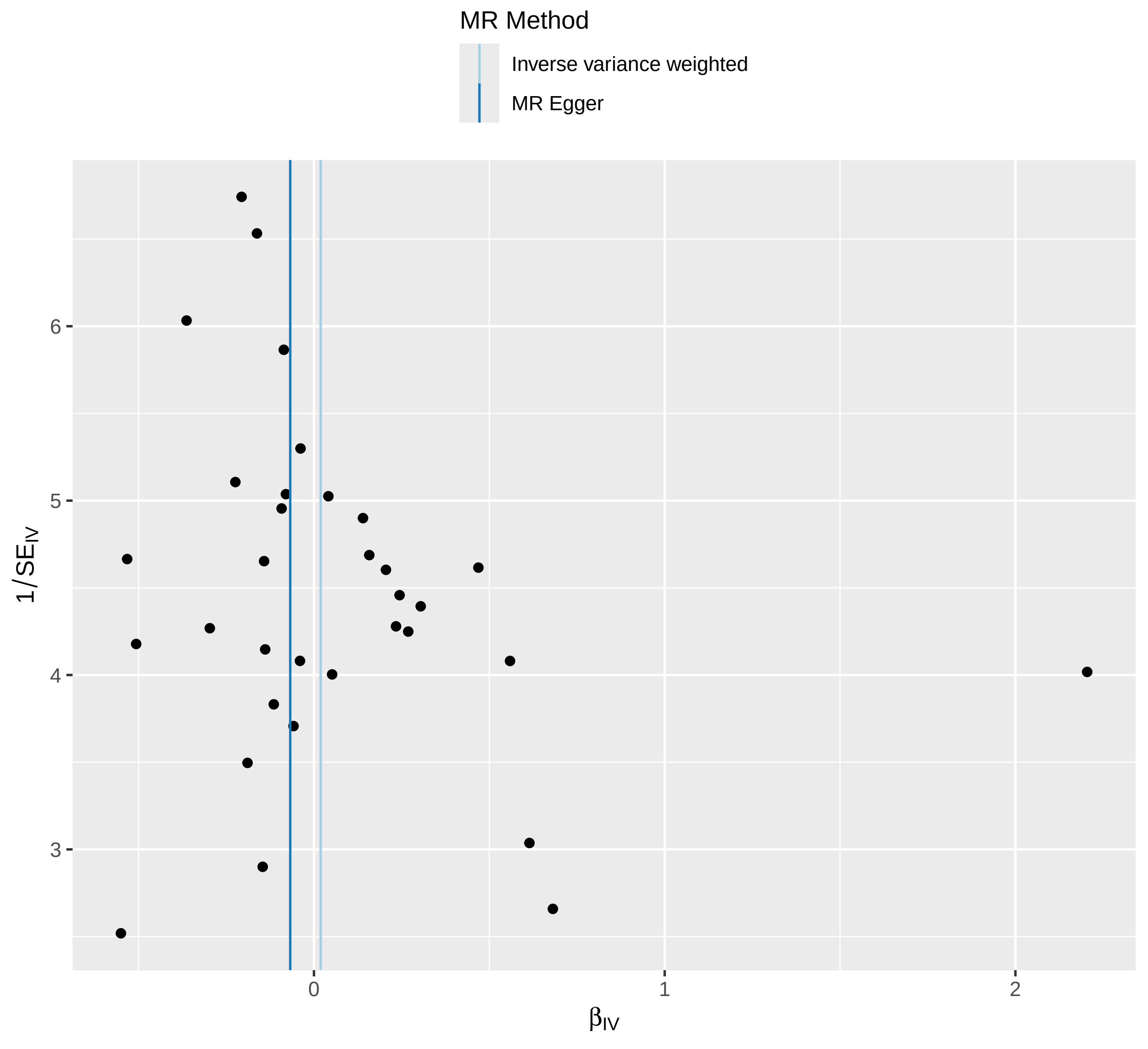

MR Method
Inverse variance weighted
MR Egger
6
5
4
3
1/SE_IV
0
1
2
β_IV

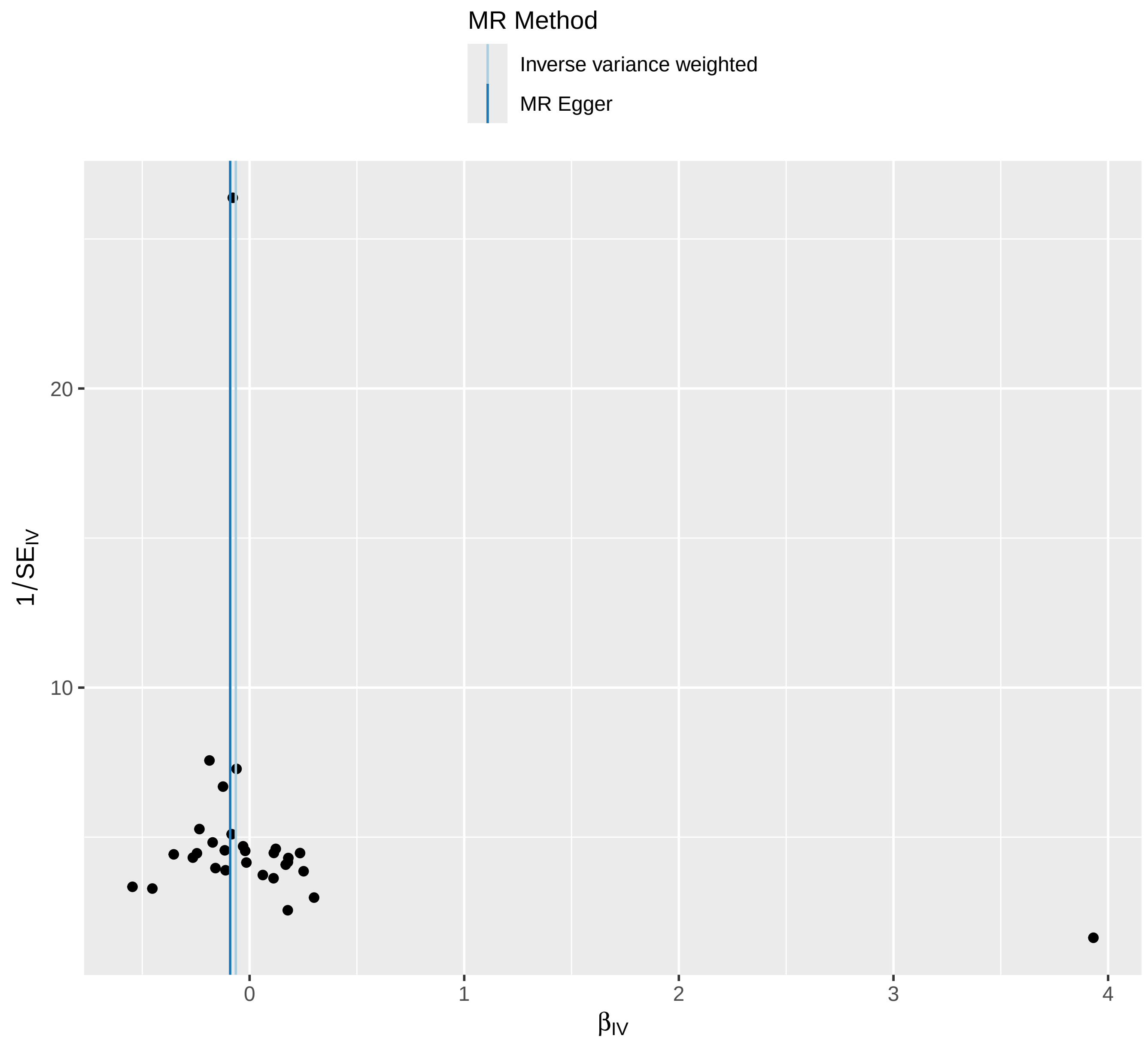
MR Method
Inverse variance weighted
MR Egger
20
10
0
1
2
3
4
1/SE_IV
β_IV

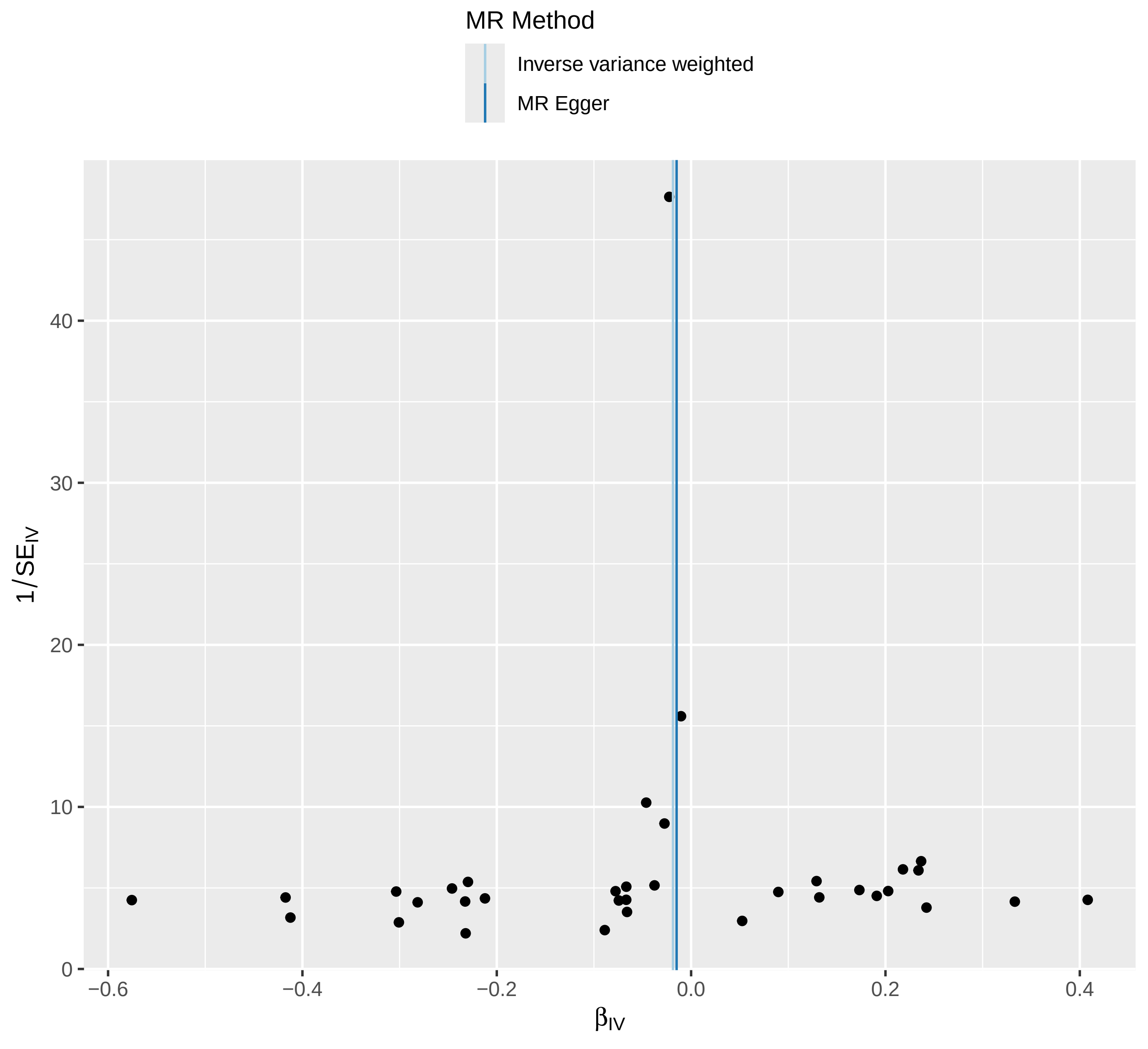

MR Method
Inverse variance weighted
MR Egger
1/SE_IV
β_IV
0
10
20
30
40
−0.6
−0.4
−0.2
0.0
0.2
0.4

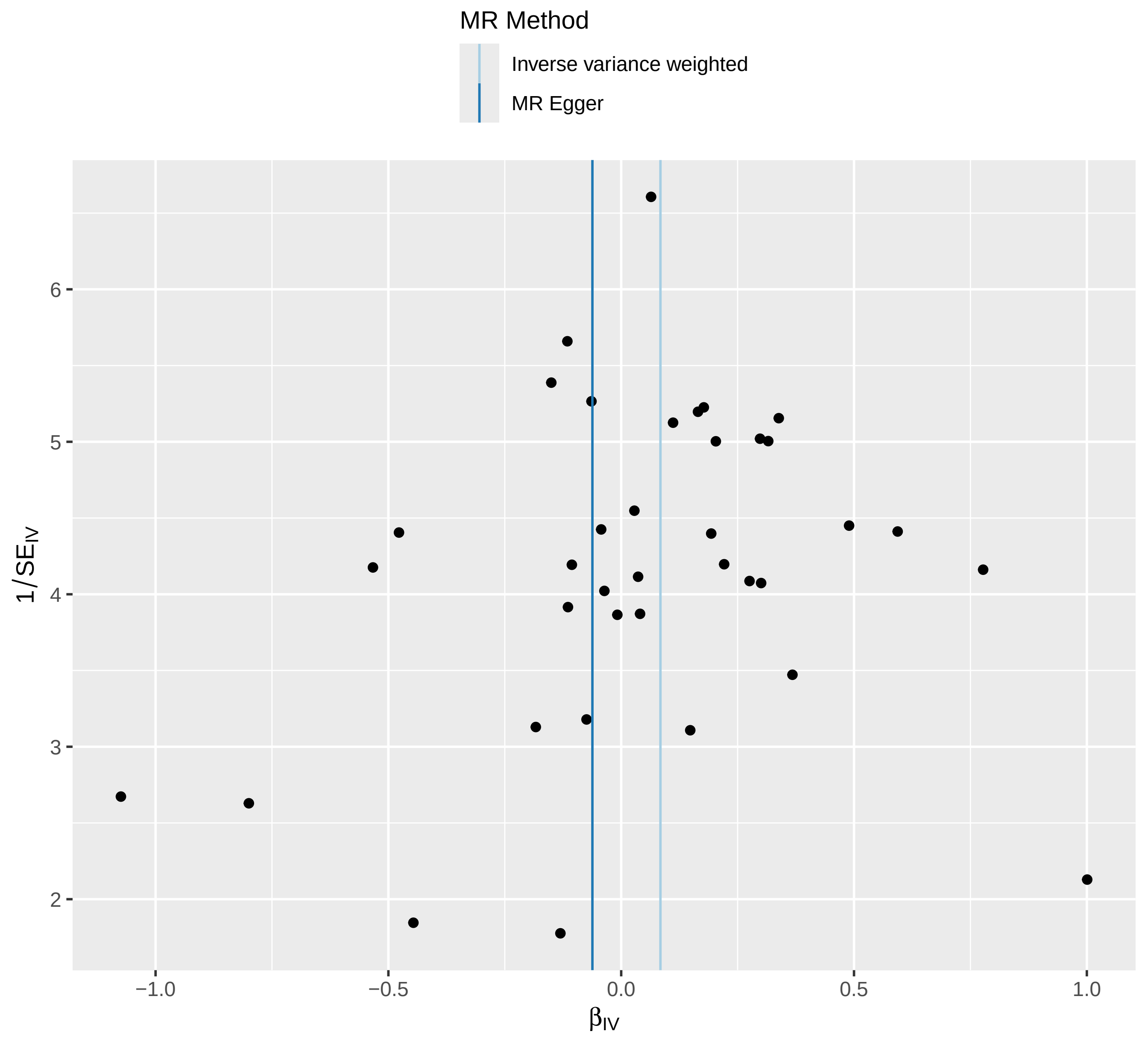
MR Method
Inverse variance weighted
MR Egger
6
5
4
3
2
1/SE_IV
−1.0
−0.5
0.0
0.5
1.0
β_IV

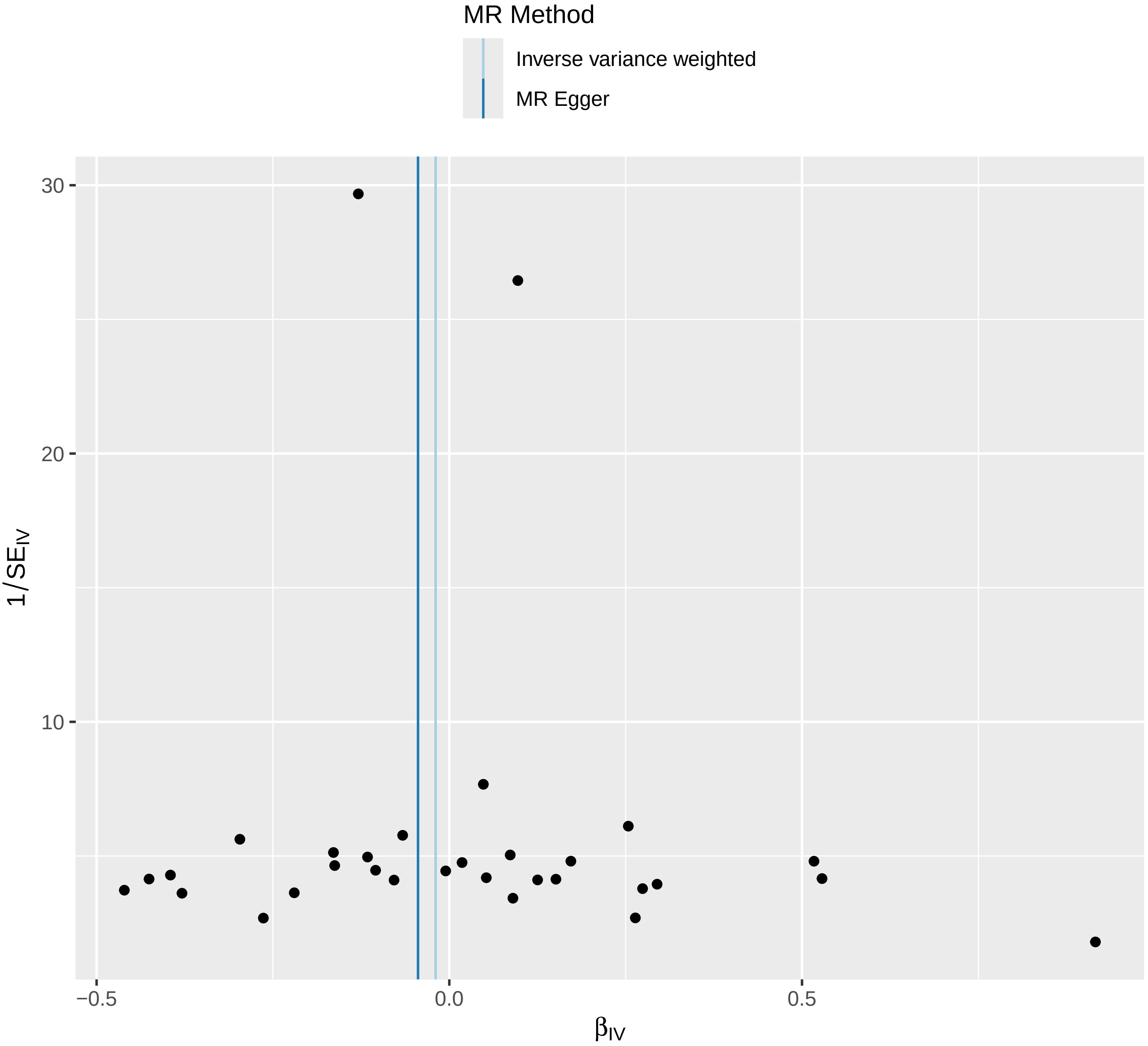
MR Method
Inverse variance weighted
MR Egger
30
20
10
1/SE_IV
−0.5
0.0
0.5
β_IV

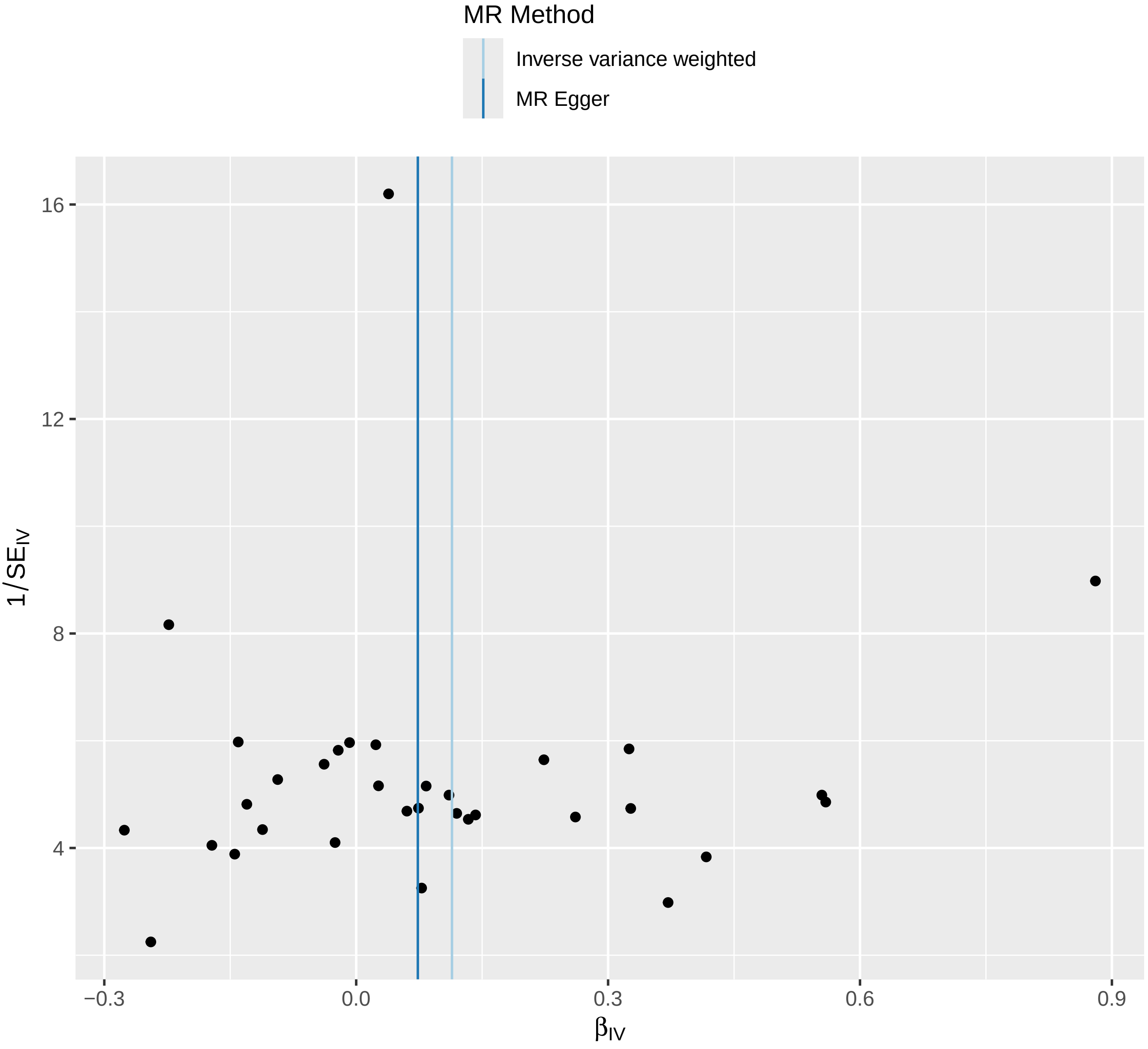

MR Method
Inverse variance weighted
MR Egger
16
12
8
4
1/SE_IV
−0.3
0.0
0.3
0.6
0.9
β_IV

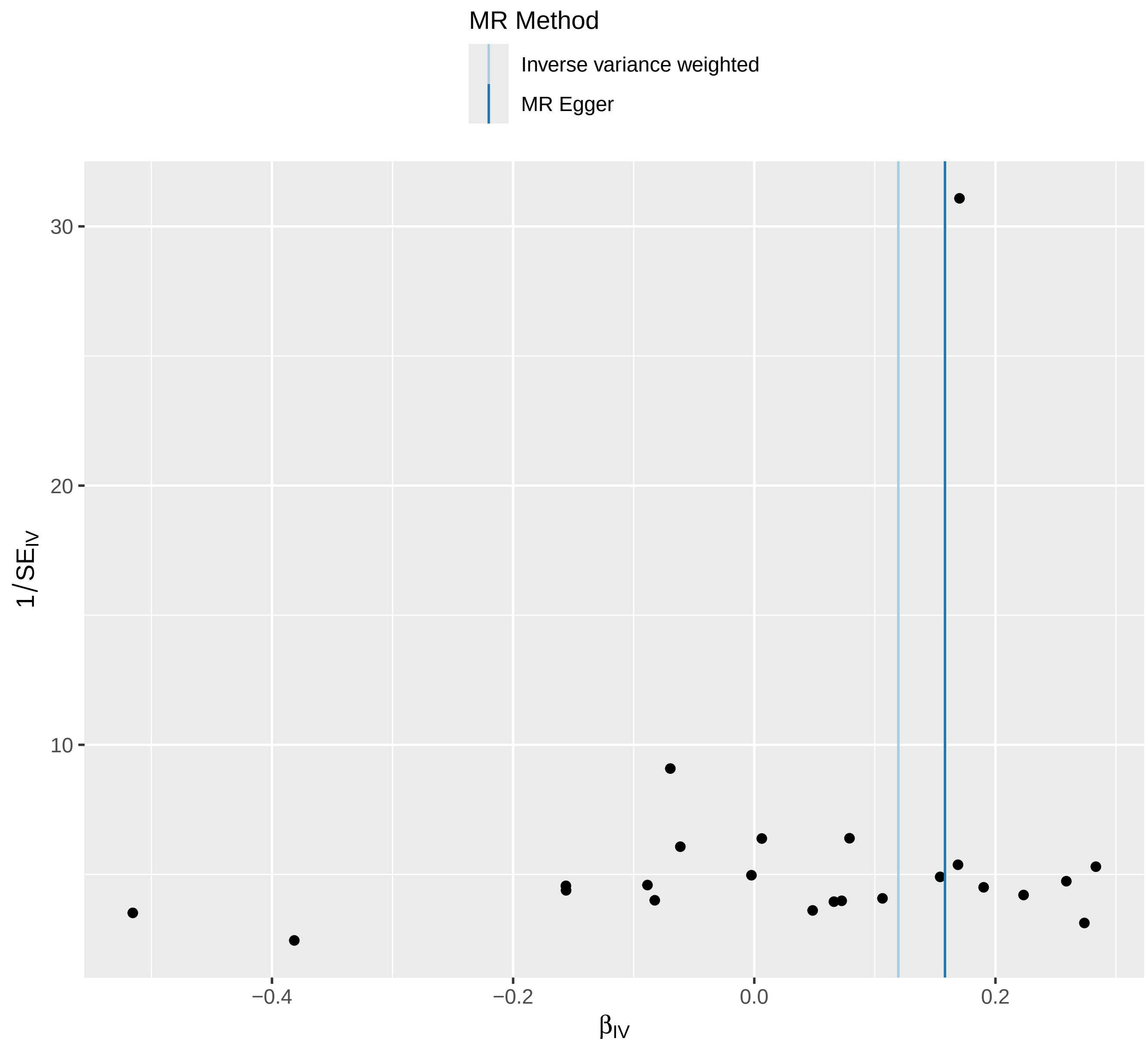
MR Method
Inverse variance weighted
MR Egger
30
20
10
−0.4
−0.2
0.0
0.2
$1/SE_{IV}$
β_{IV}

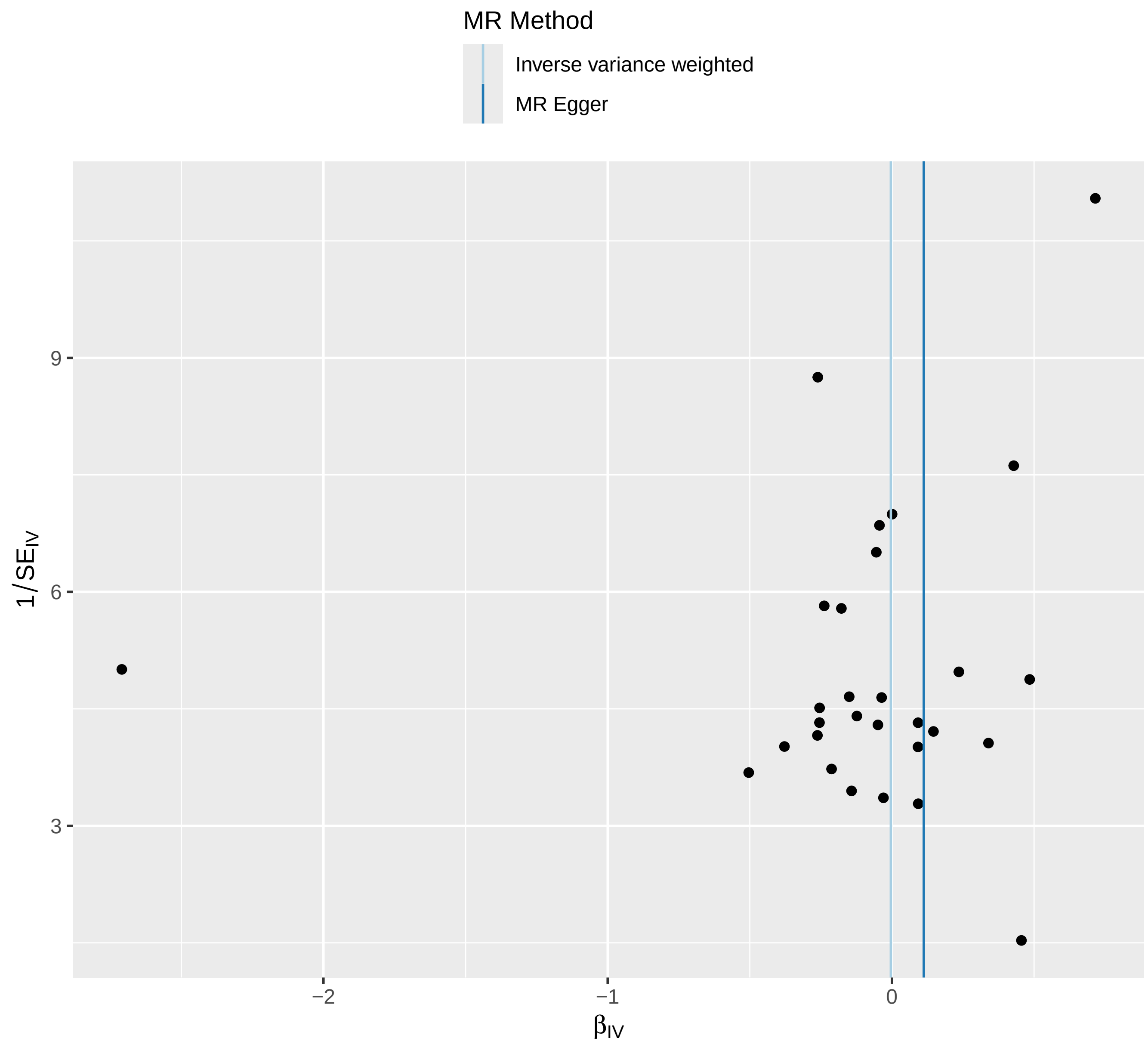
MR Method
Inverse variance weighted
MR Egger
1/SE_IV
9
6
3
−2
−1
0
β_IV

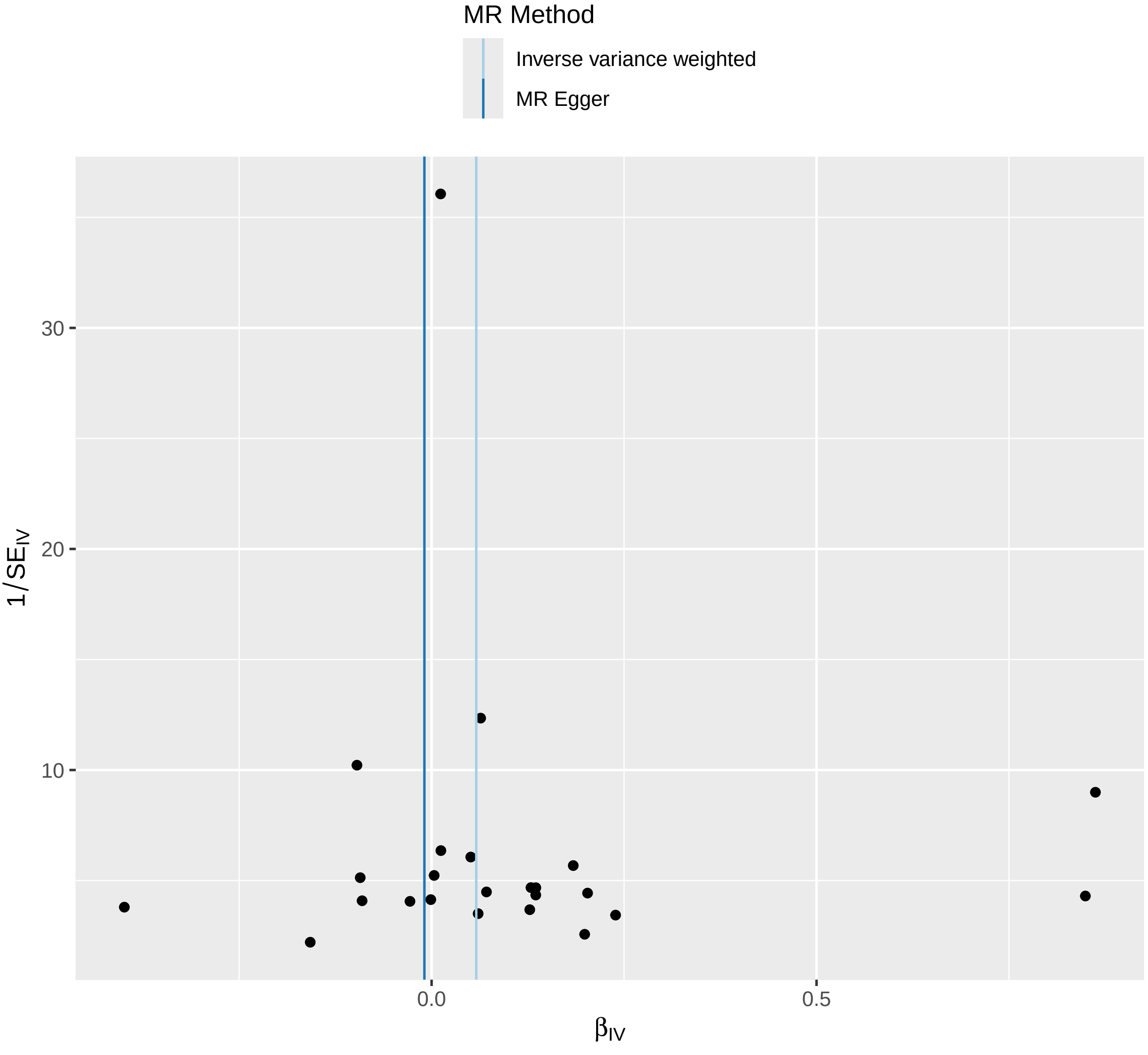
MR Method
Inverse variance weighted
MR Egger
30
20
10
0.0
0.5
1/SE$_{IV}$
β$_{IV}$

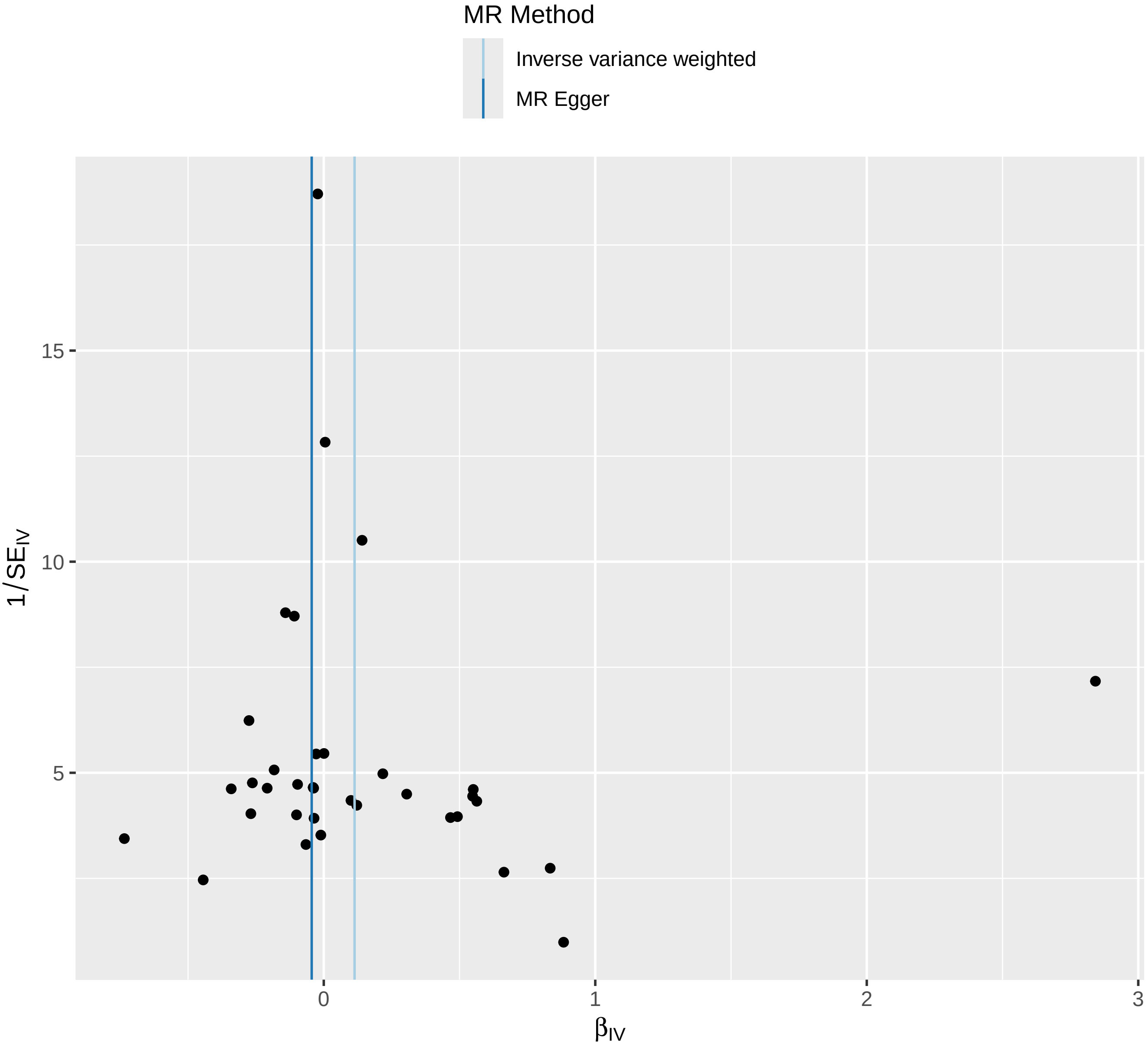
MR Method
Inverse variance weighted
MR Egger
15
10
5
1/SE$_{IV}$
0
1
2
3
β$_{IV}$

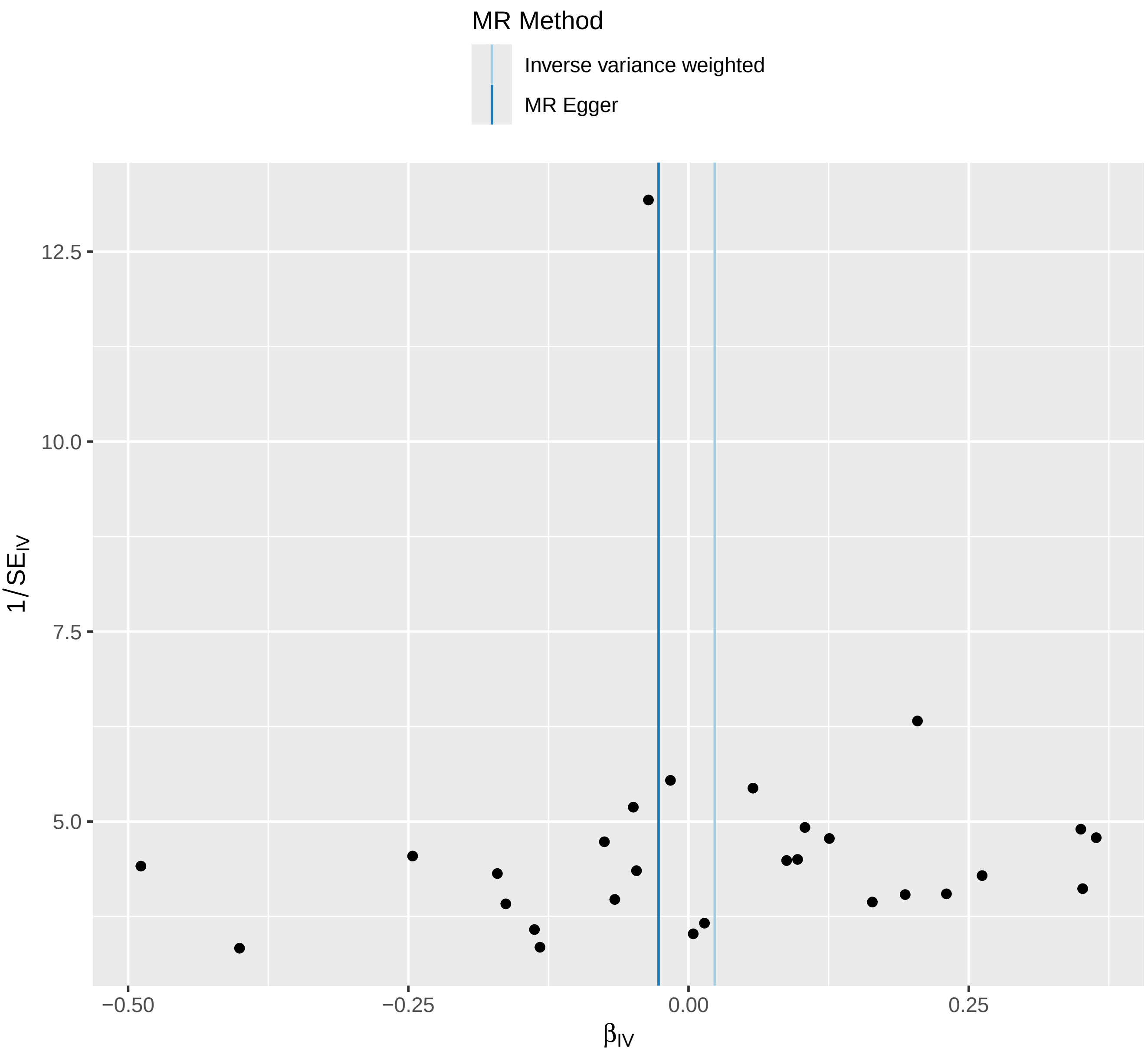
MR Method
Inverse variance weighted
MR Egger
12.5
10.0
7.5
5.0
1/SE$_{IV}$
−0.50
−0.25
0.00
0.25
β$_{IV}$

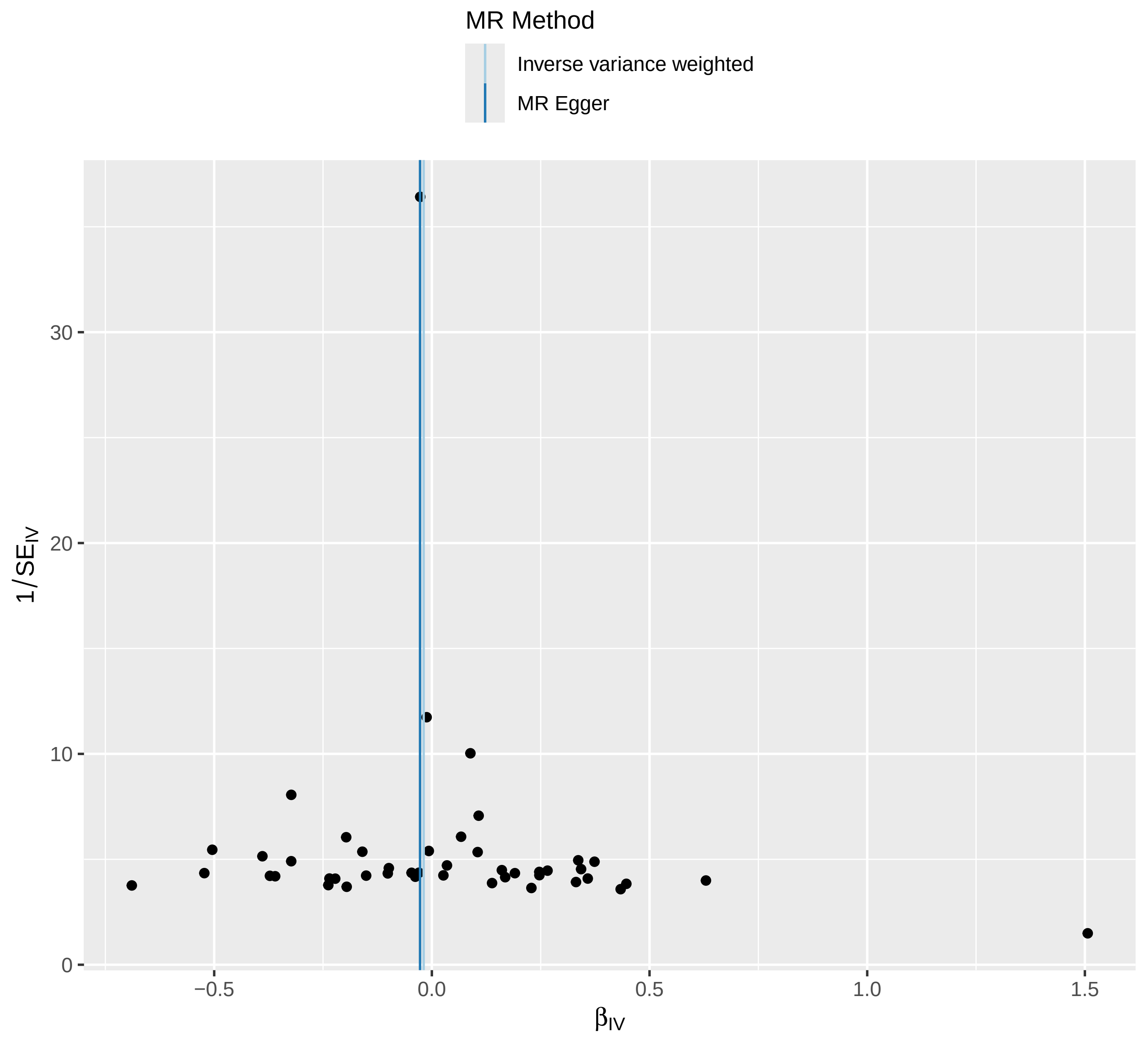
MR Method
Inverse variance weighted
MR Egger
30
20
10
0
1/SE_IV
−0.5
0.0
0.5
1.0
1.5
β_IV

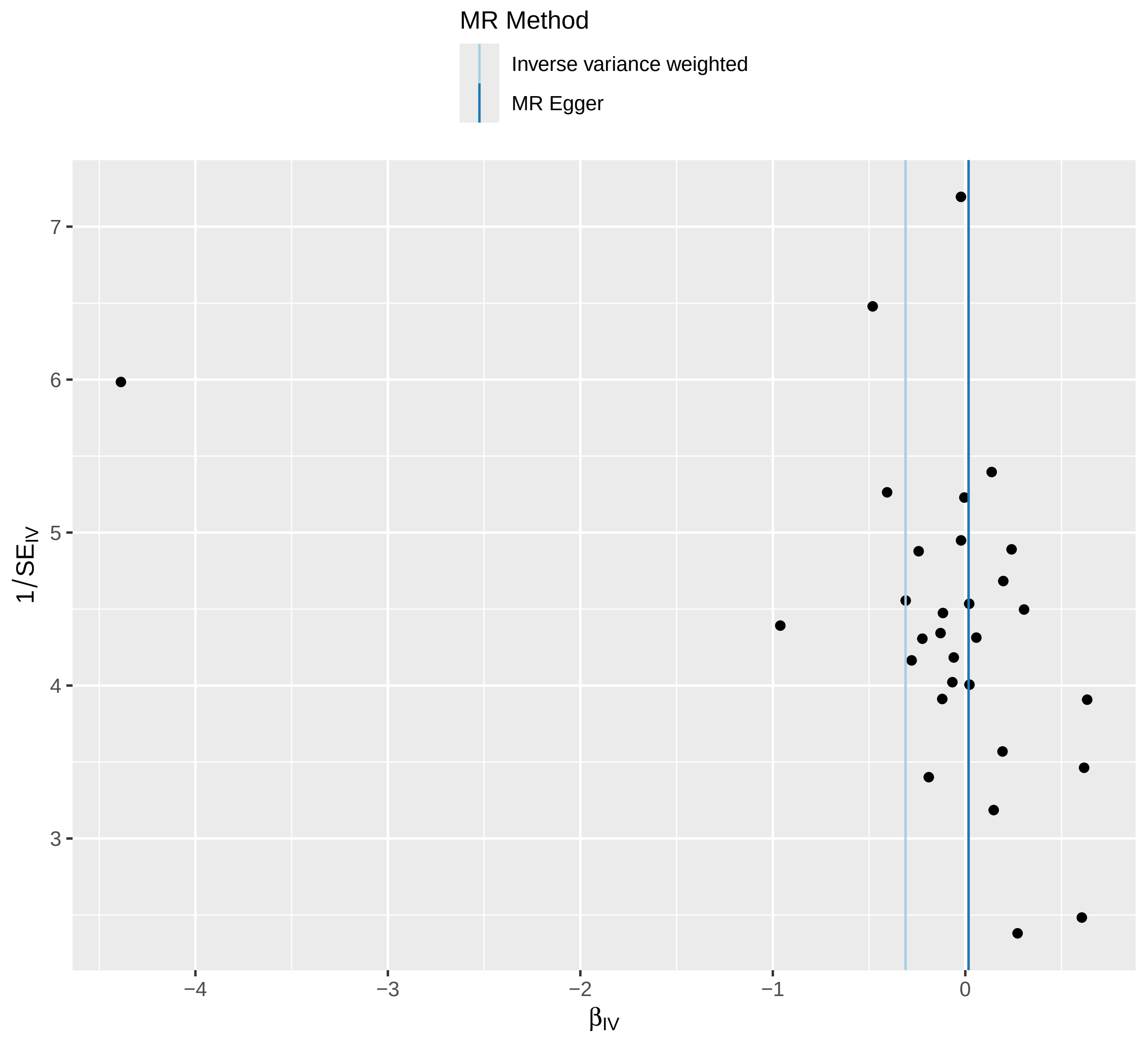
MR Method
Inverse variance weighted
MR Egger
7
6
5
4
3
1/SE$_{IV}$
−4
−3
−2
−1
0
β$_{IV}$

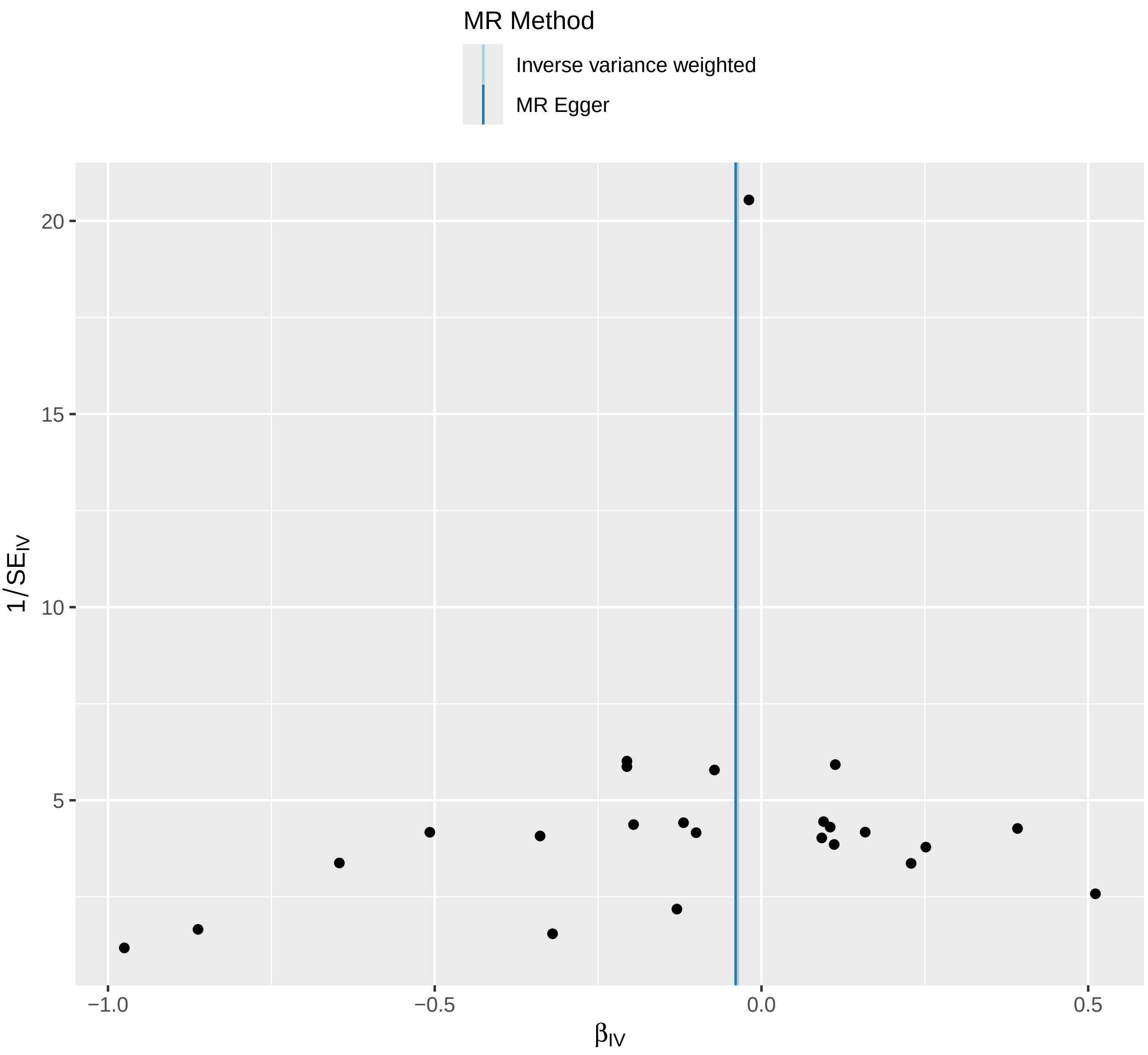
MR Method
Inverse variance weighted
MR Egger
20
15
10
5
1/SE_IV
−1.0
−0.5
0.0
0.5
β_IV

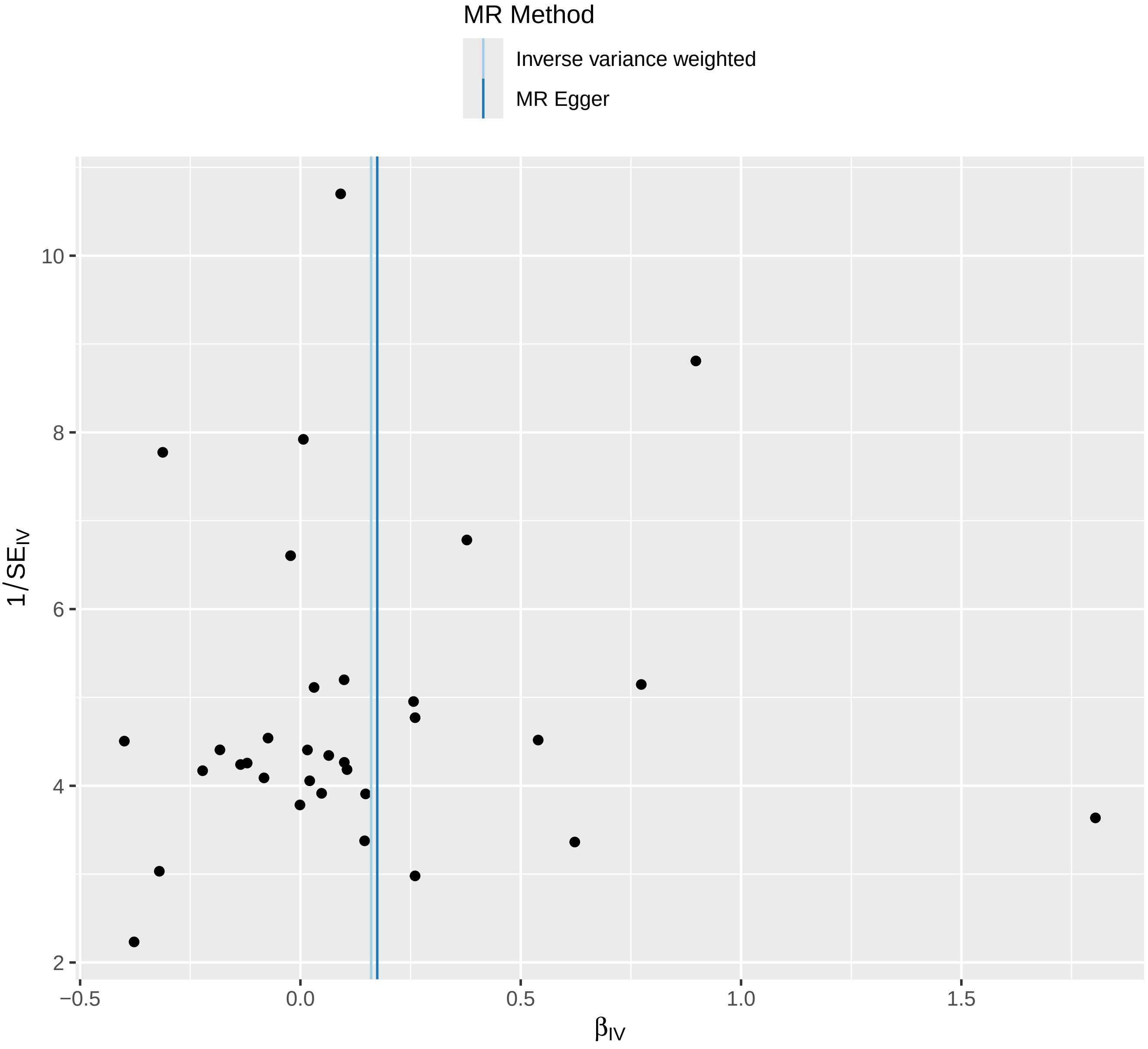
MR Method
Inverse variance weighted
MR Egger
1/SE_IV
β_IV
10
8
6
4
2
−0.5
0.0
0.5
1.0
1.5

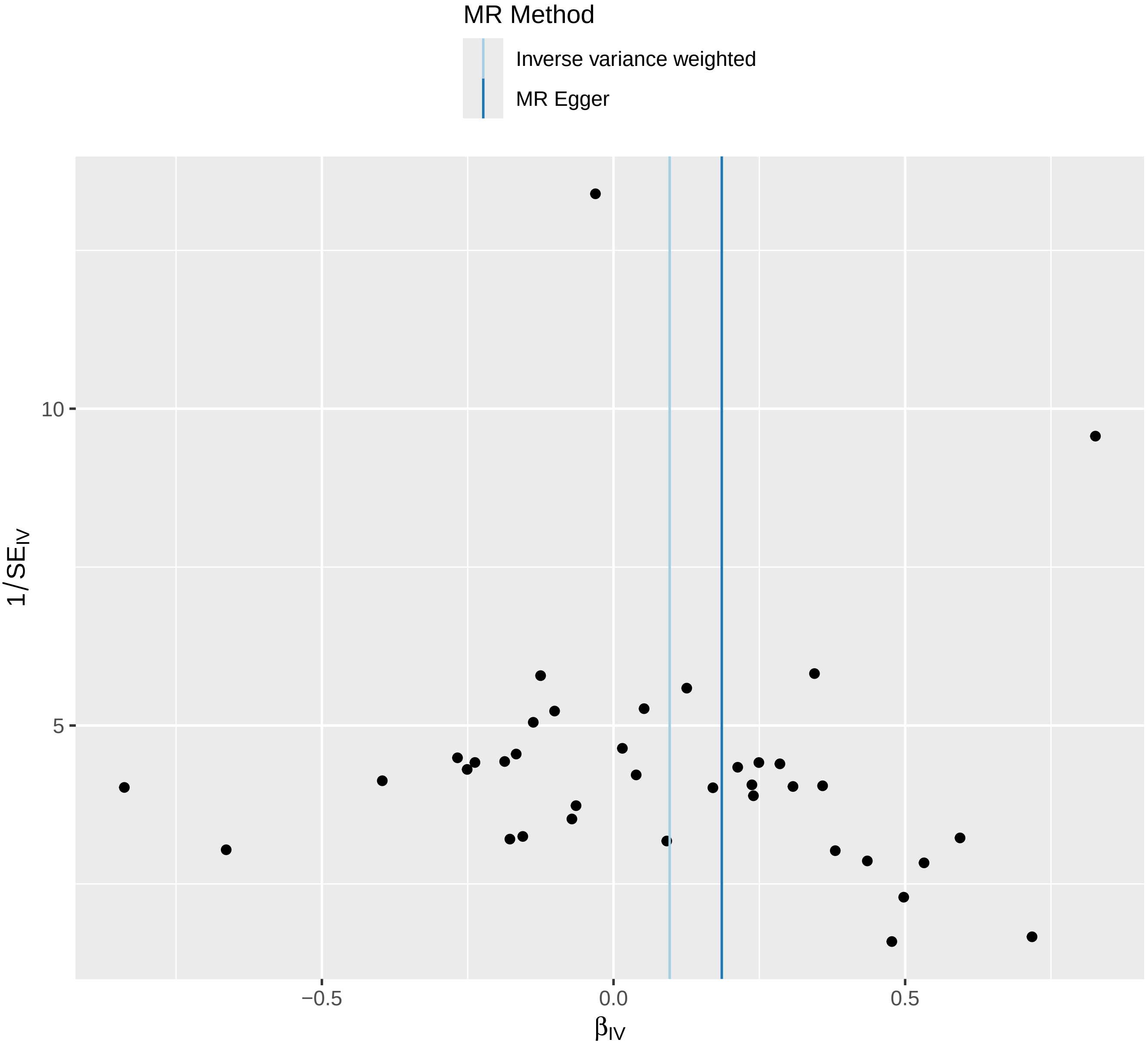
MR Method
Inverse variance weighted
MR Egger
10
5
1/SE_IV
−0.5
0.0
0.5
β_IV

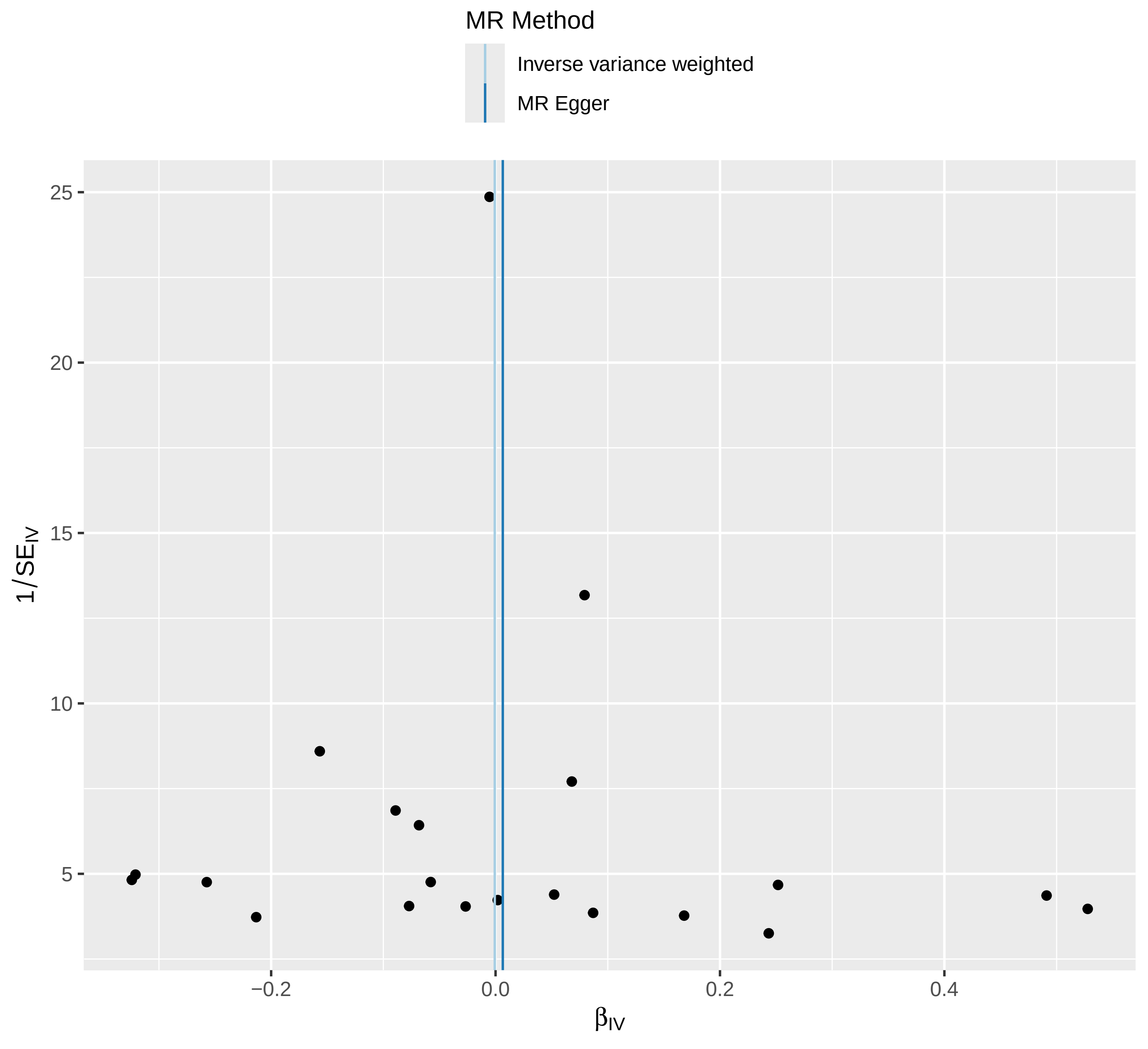
MR Method
Inverse variance weighted
MR Egger
25
20
15
10
5
$1/SE_{IV}$
−0.2
0.0
0.2
0.4
β_{IV}

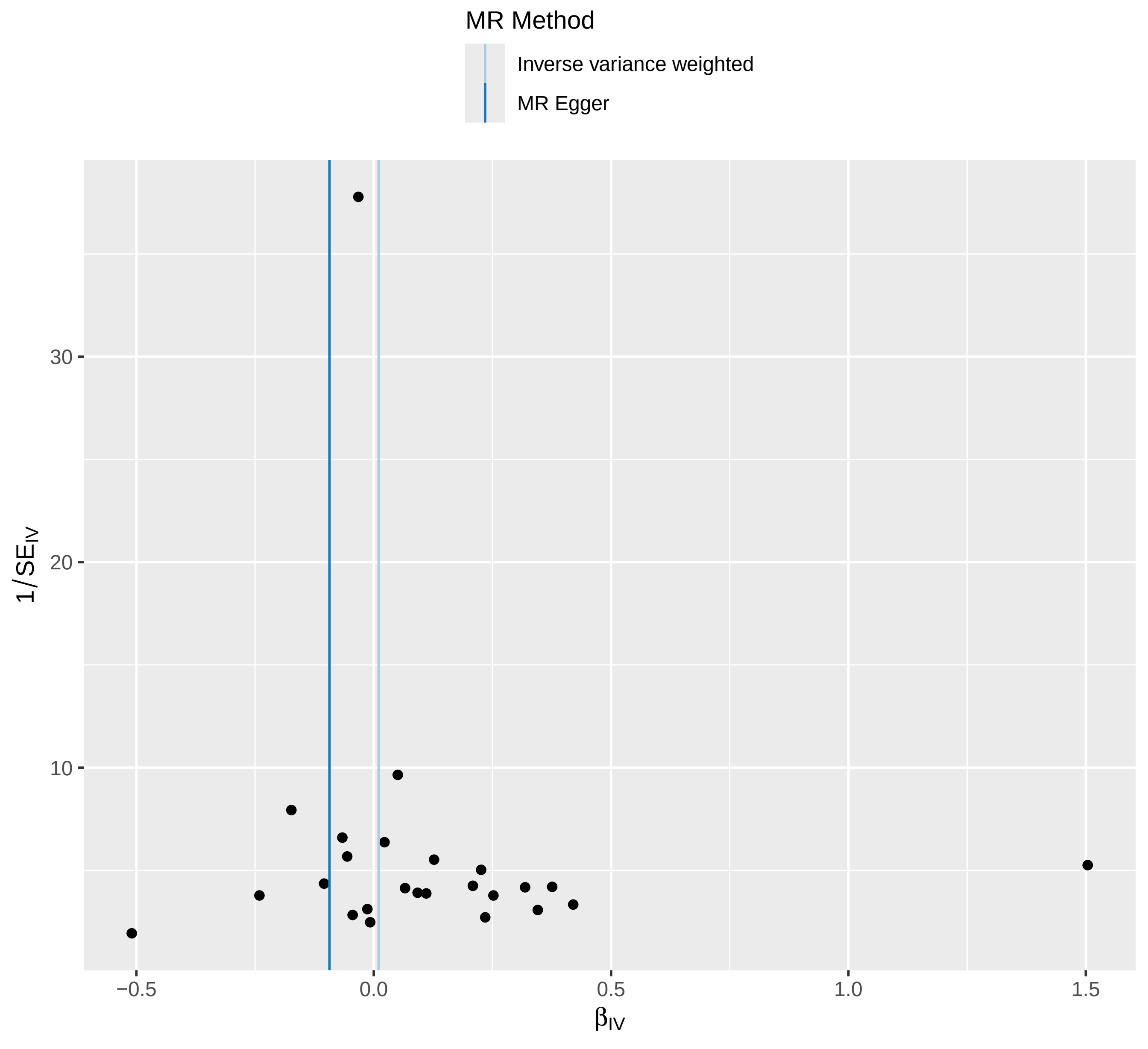
MR Method
Inverse variance weighted
MR Egger
1/SE_IV
30
20
10
−0.5
0.0
0.5
1.0
1.5
β_IV

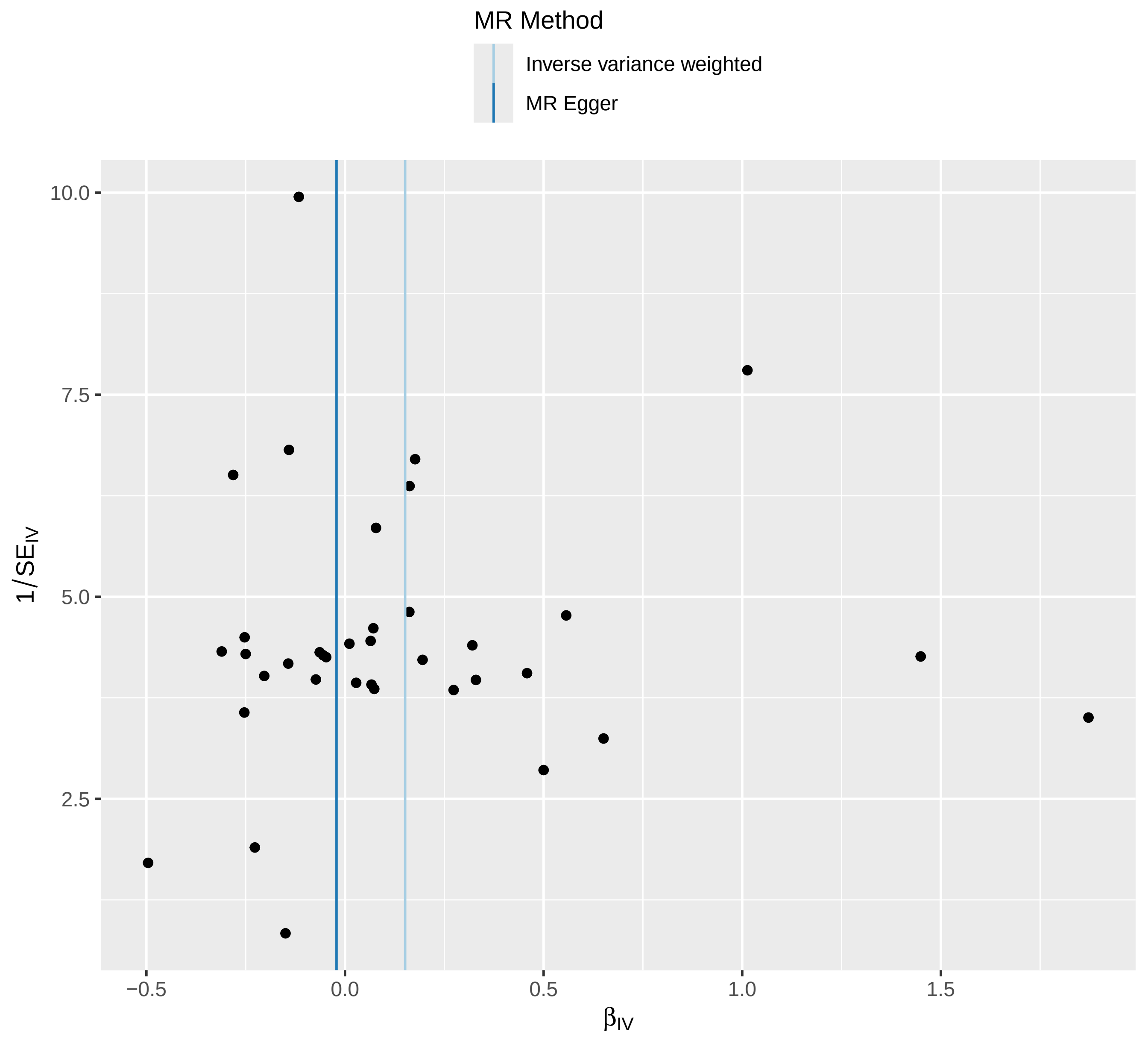

MR Method
Inverse variance weighted
MR Egger
10.0
7.5
5.0
2.5
1/SE_IV
−0.5
0.0
0.5
1.0
1.5
β_IV

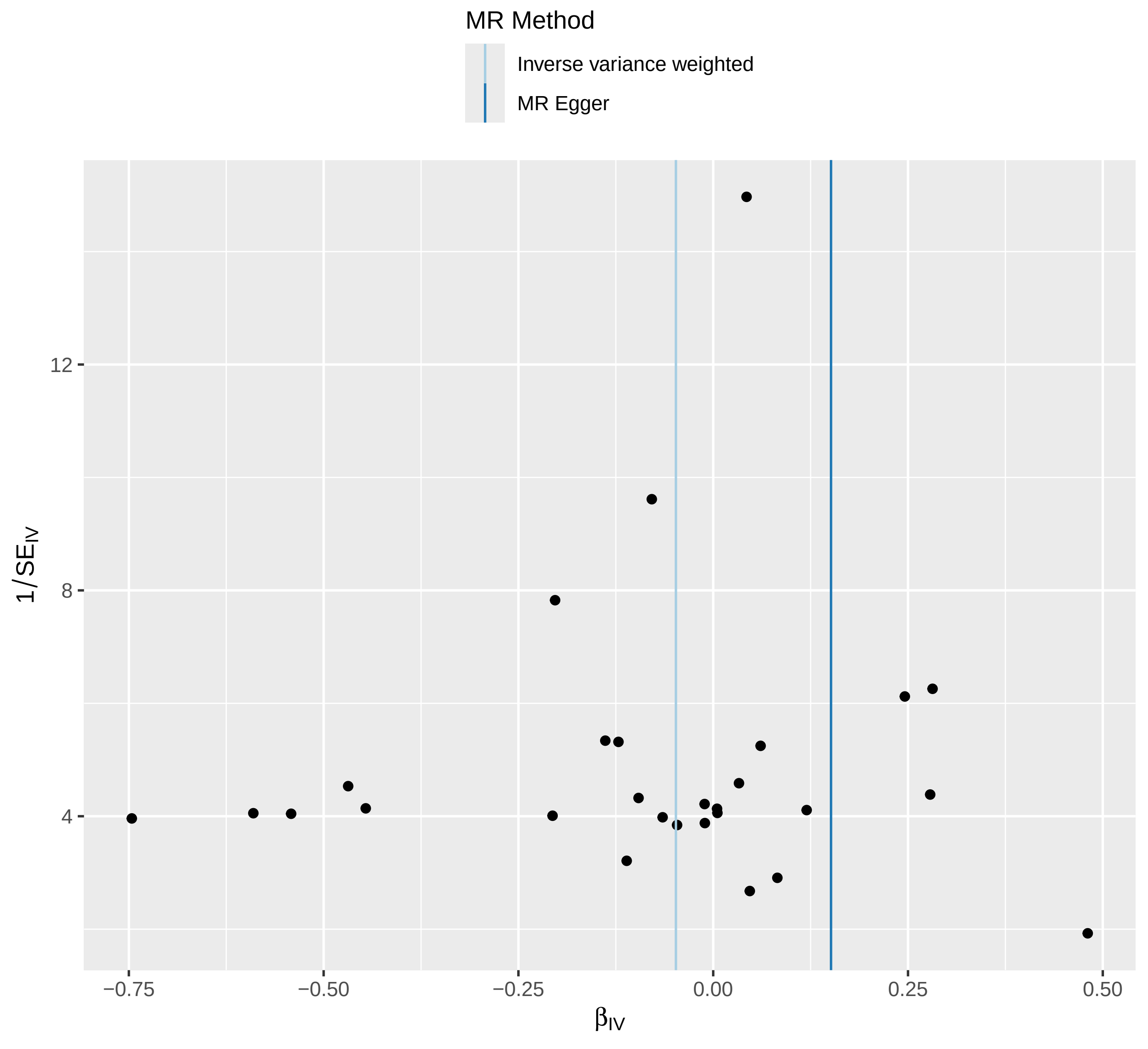

MR Method
Inverse variance weighted
MR Egger
12
8
4
1/SE_IV
−0.75
−0.50
−0.25
0.00
0.25
0.50
β_IV

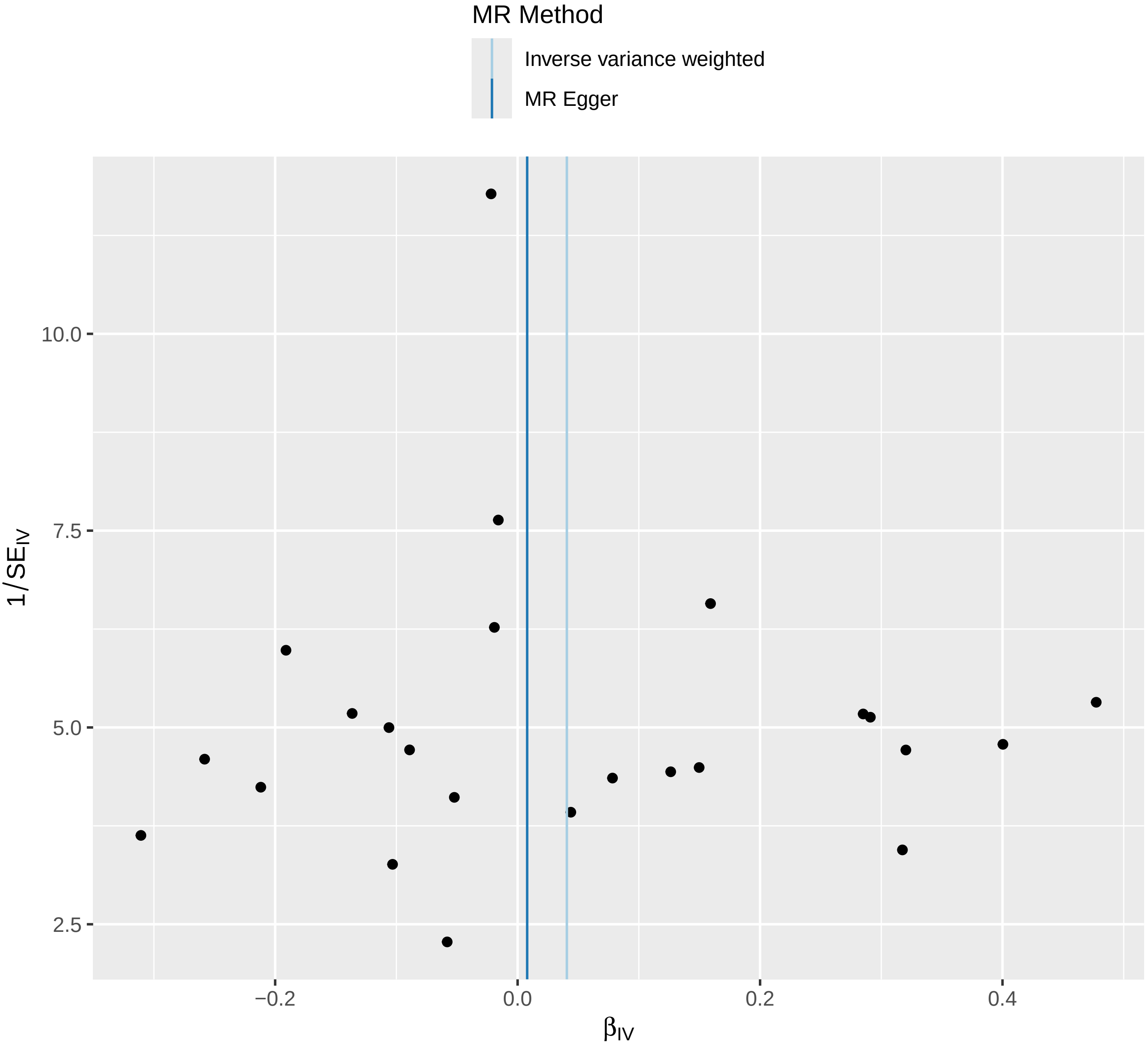
MR Method
Inverse variance weighted
MR Egger
10.0
7.5
5.0
2.5
1/SE$_{IV}$
−0.2
0.0
0.2
0.4
β$_{IV}$

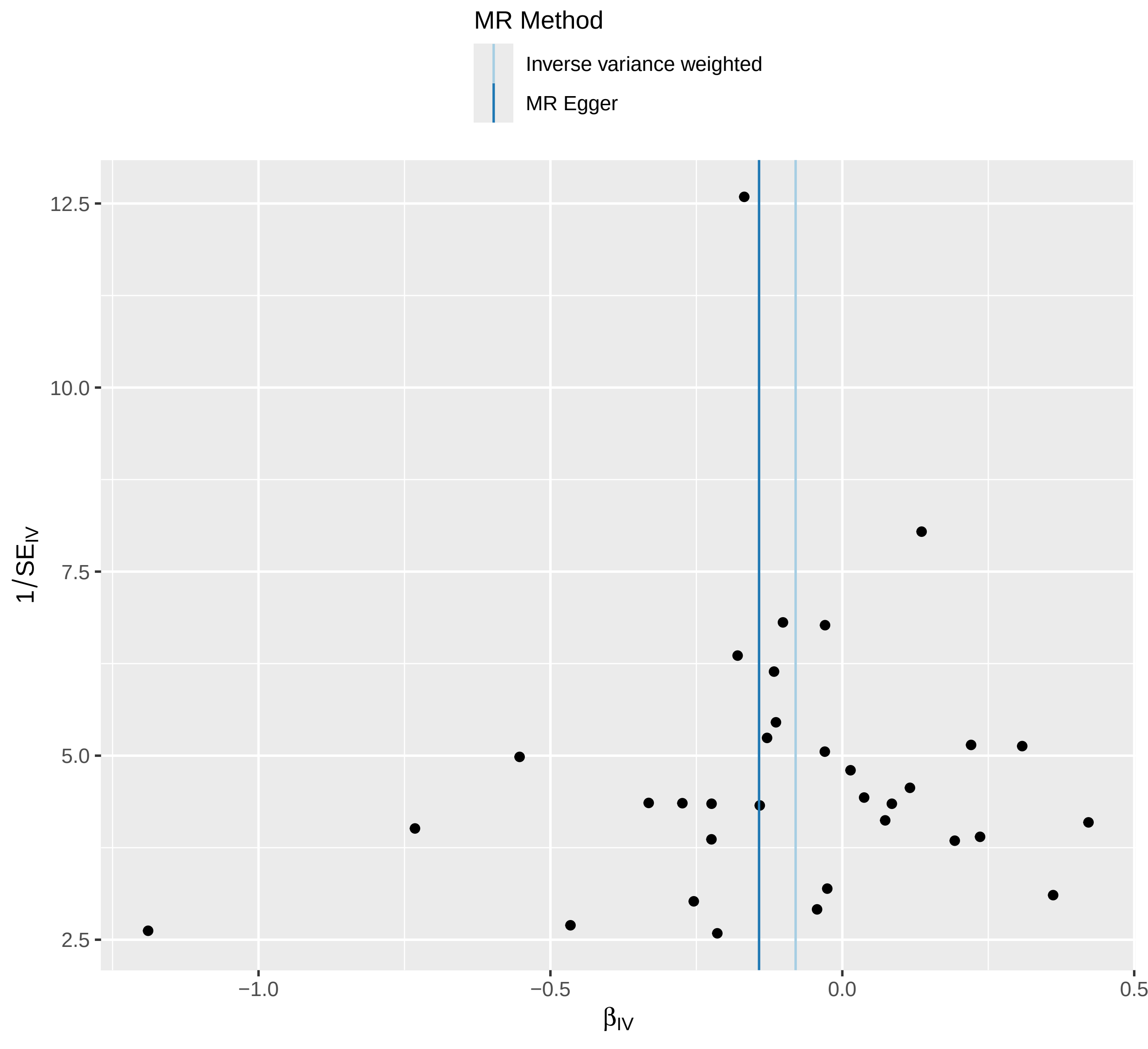
MR Method
Inverse variance weighted
MR Egger
12.5
10.0
7.5
5.0
2.5
$1/SE_{IV}$
−1.0
−0.5
0.0
0.5
β_{IV}

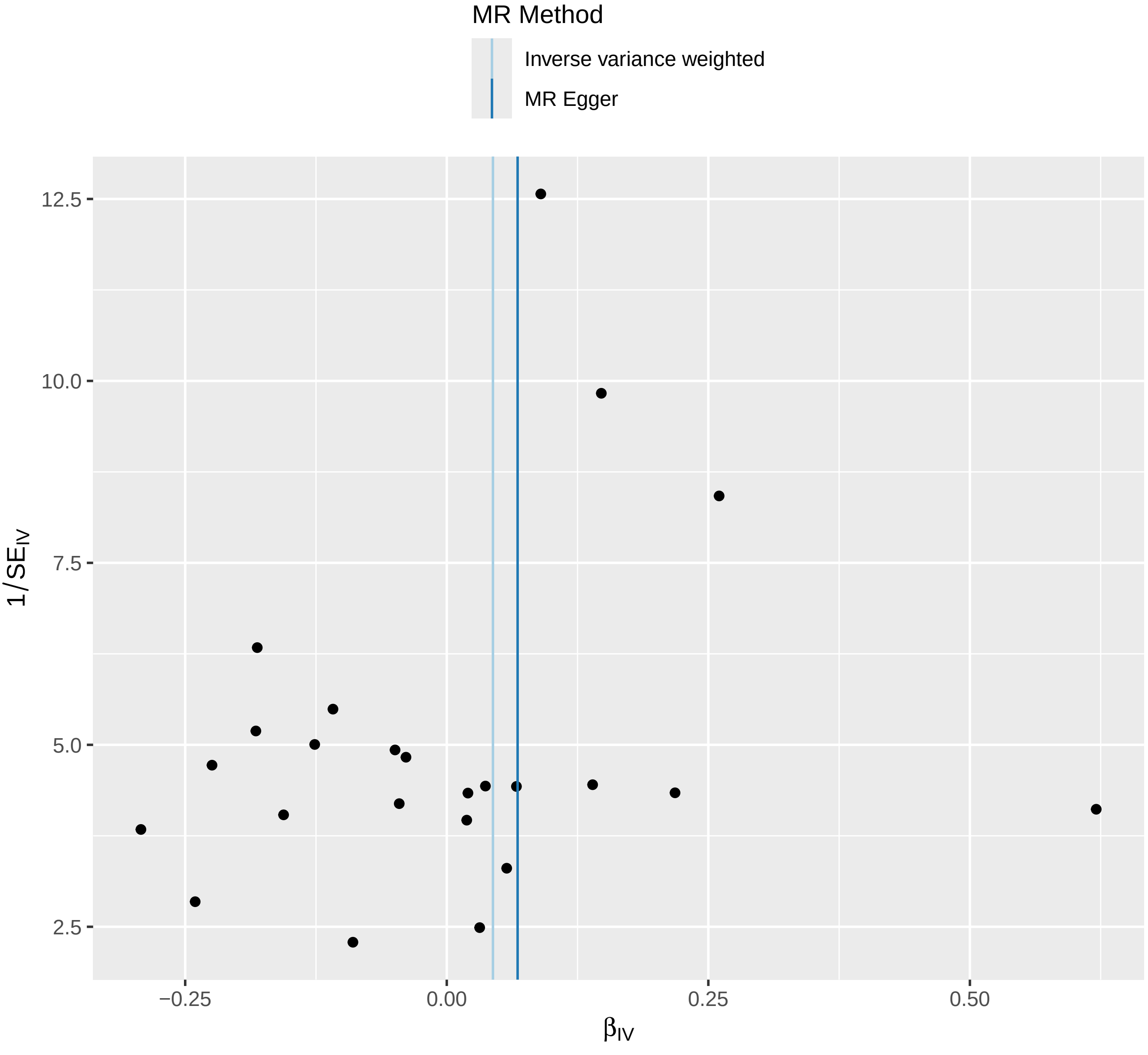
MR Method
Inverse variance weighted
MR Egger
12.5
10.0
7.5
5.0
2.5
1/SE_IV
−0.25
0.00
0.25
0.50
β_IV

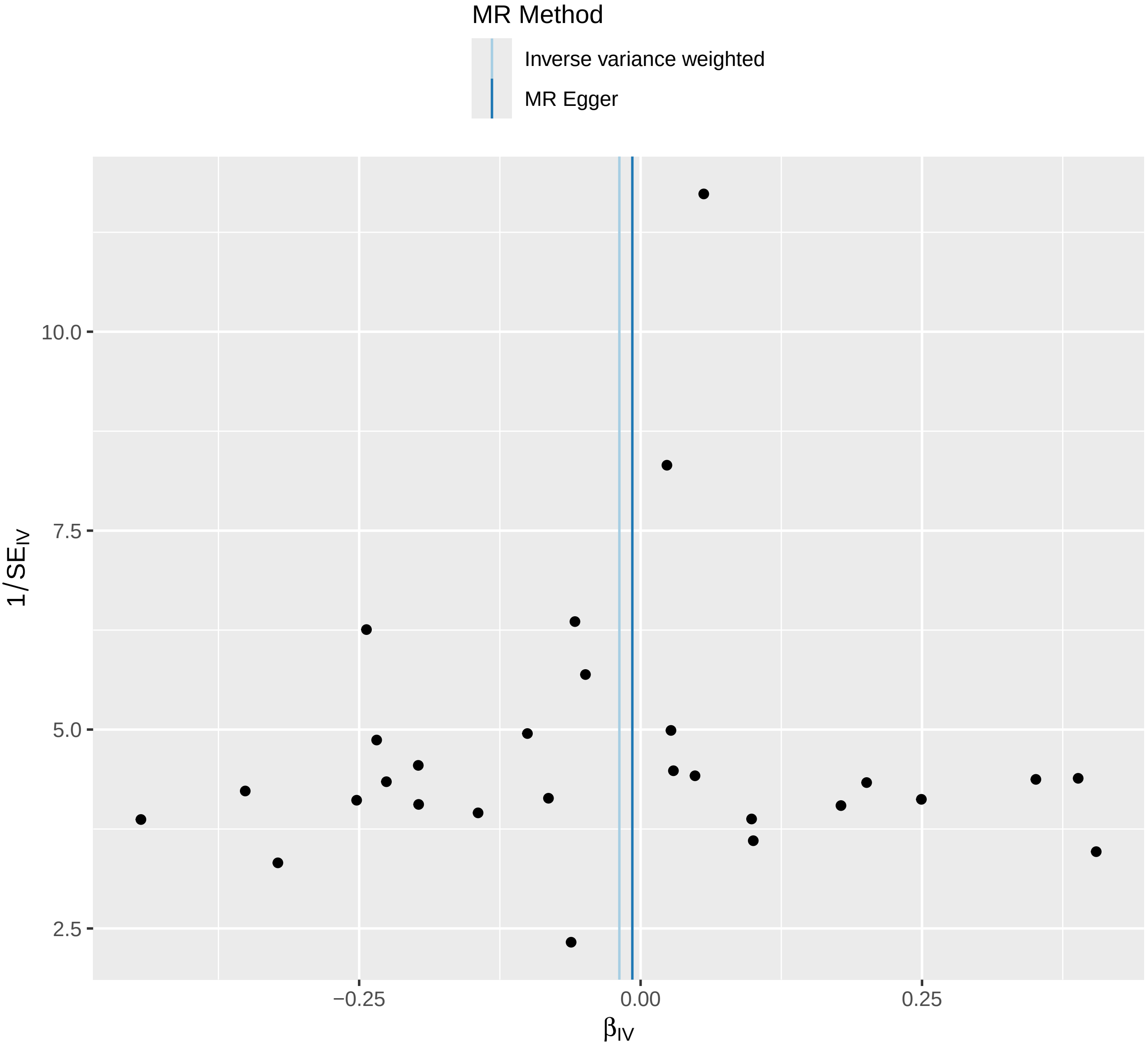
MR Method
Inverse variance weighted
MR Egger
10.0
7.5
5.0
2.5
$1/SE_{IV}$
−0.25
0.00
0.25
β_{IV}

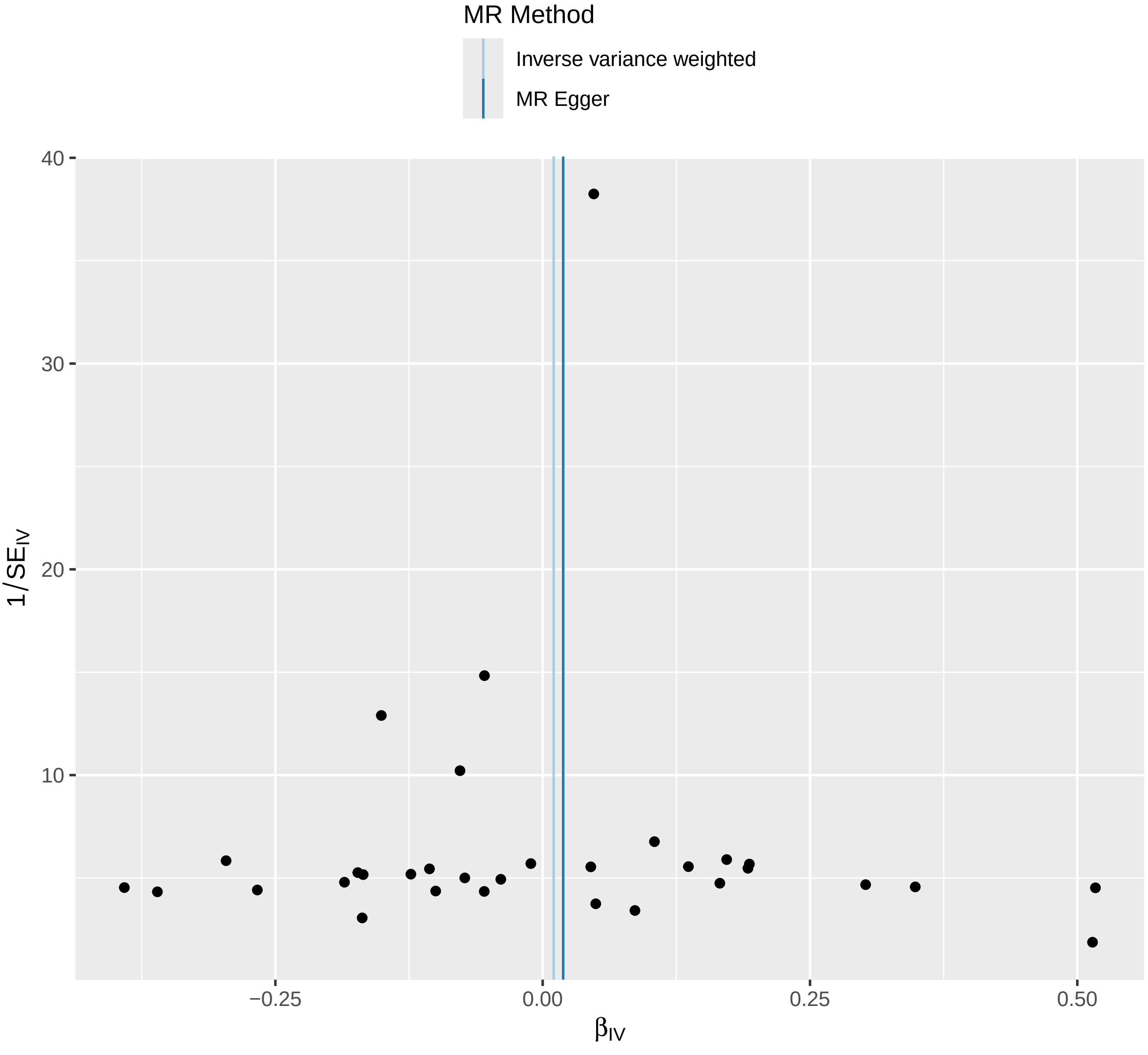
MR Method
Inverse variance weighted
MR Egger
40
30
20
10
1/SE$_{IV}$
−0.25
0.00
0.25
0.50
β$_{IV}$

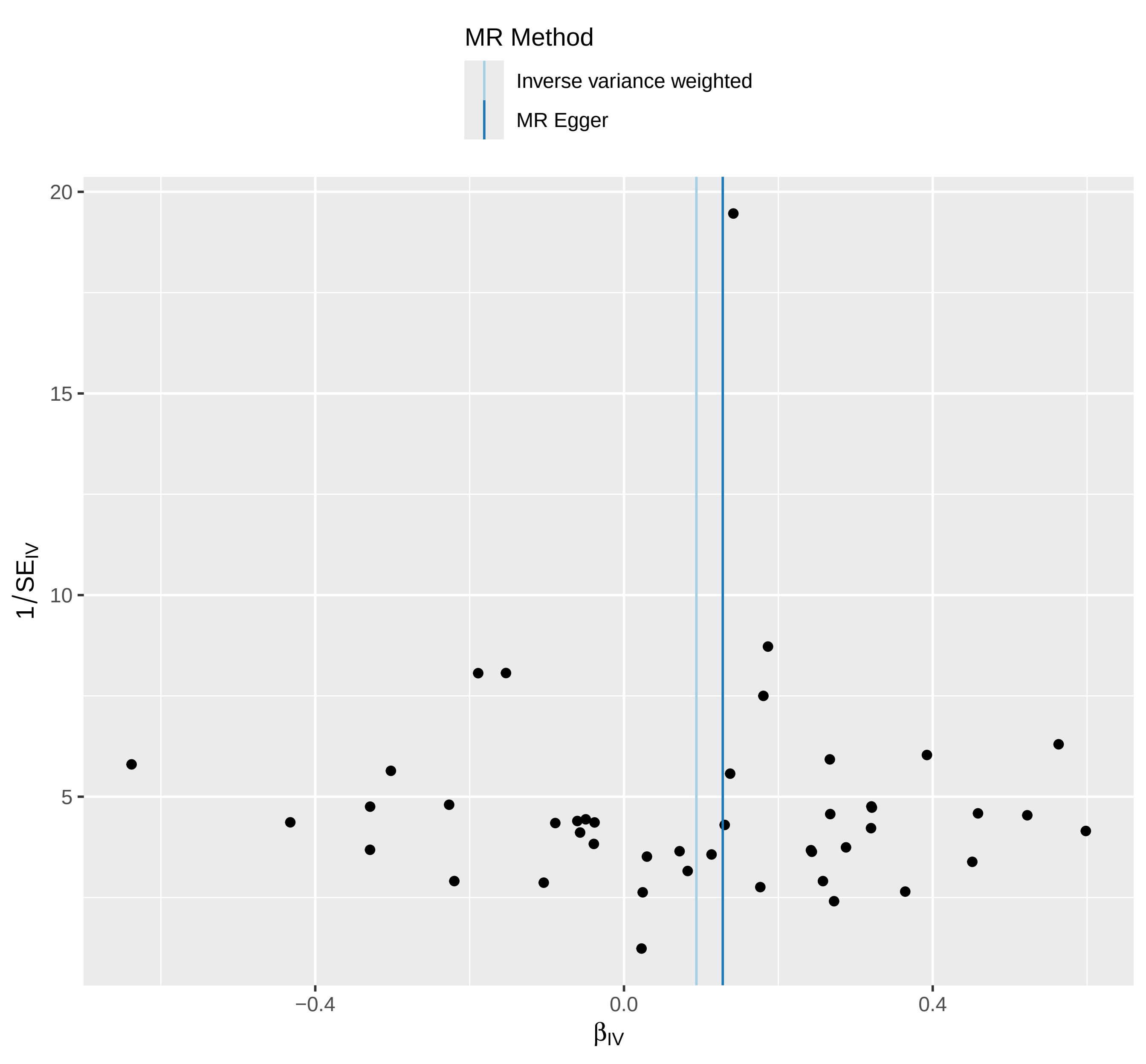

MR Method
Inverse variance weighted
MR Egger
20
15
10
5
1/SE_IV
−0.4
0.0
0.4
β_IV

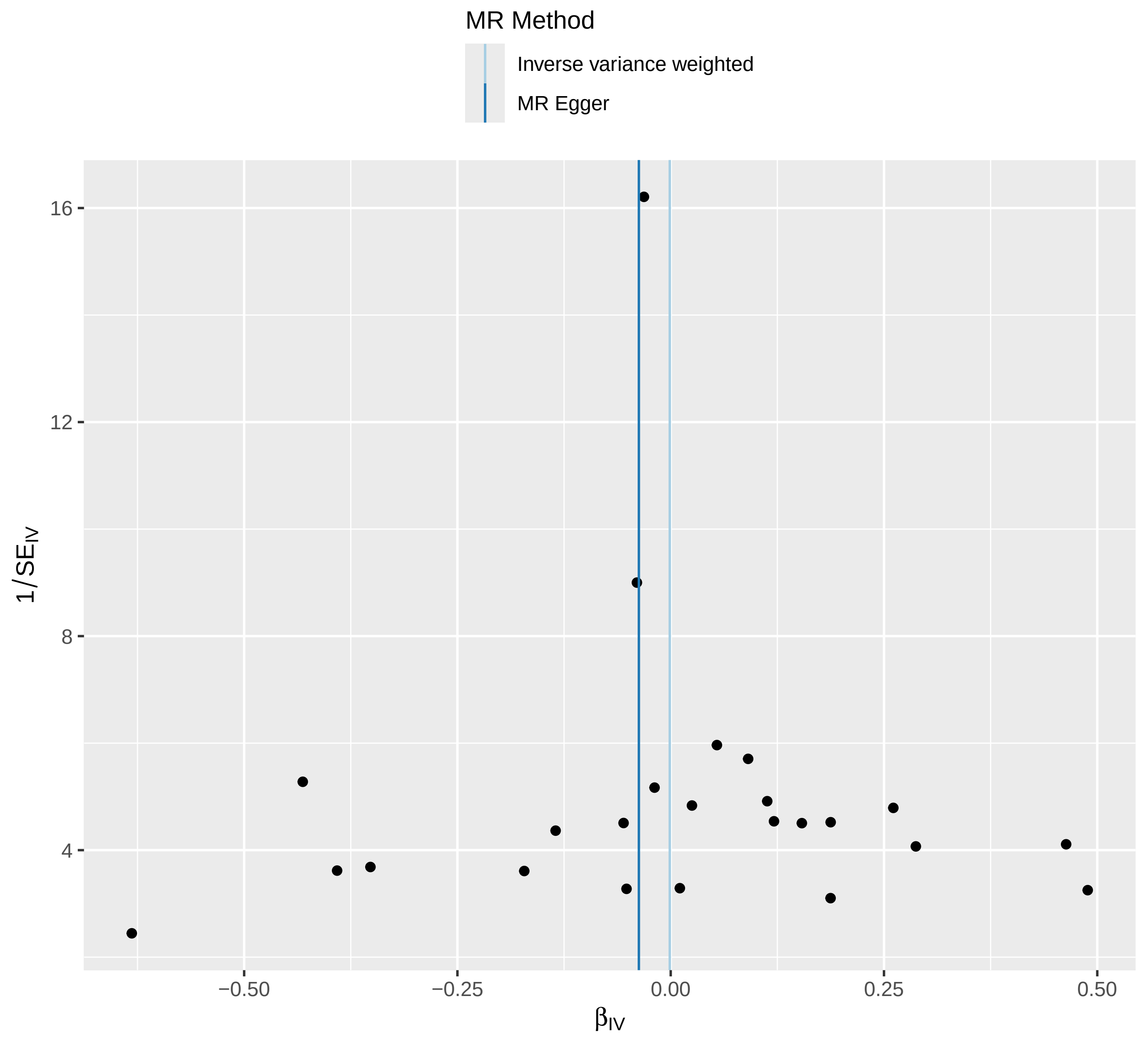
MR Method
Inverse variance weighted
MR Egger
16
12
8
4
1/SE$_{IV}$
−0.50
−0.25
0.00
0.25
0.50
β$_{IV}$

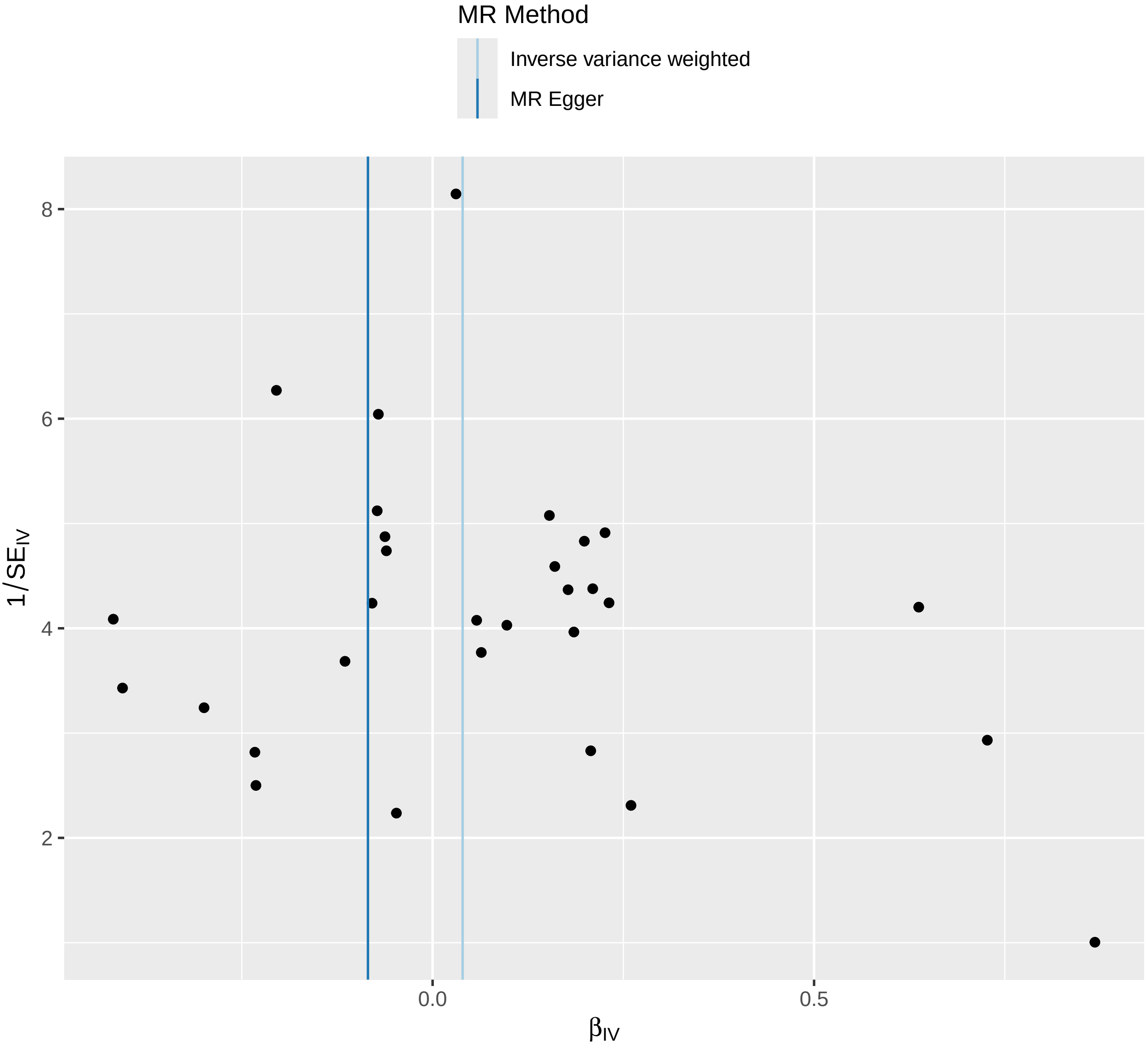
MR Method
Inverse variance weighted
MR Egger
8
6
4
2
1/SE_IV
0.0
0.5
β_IV

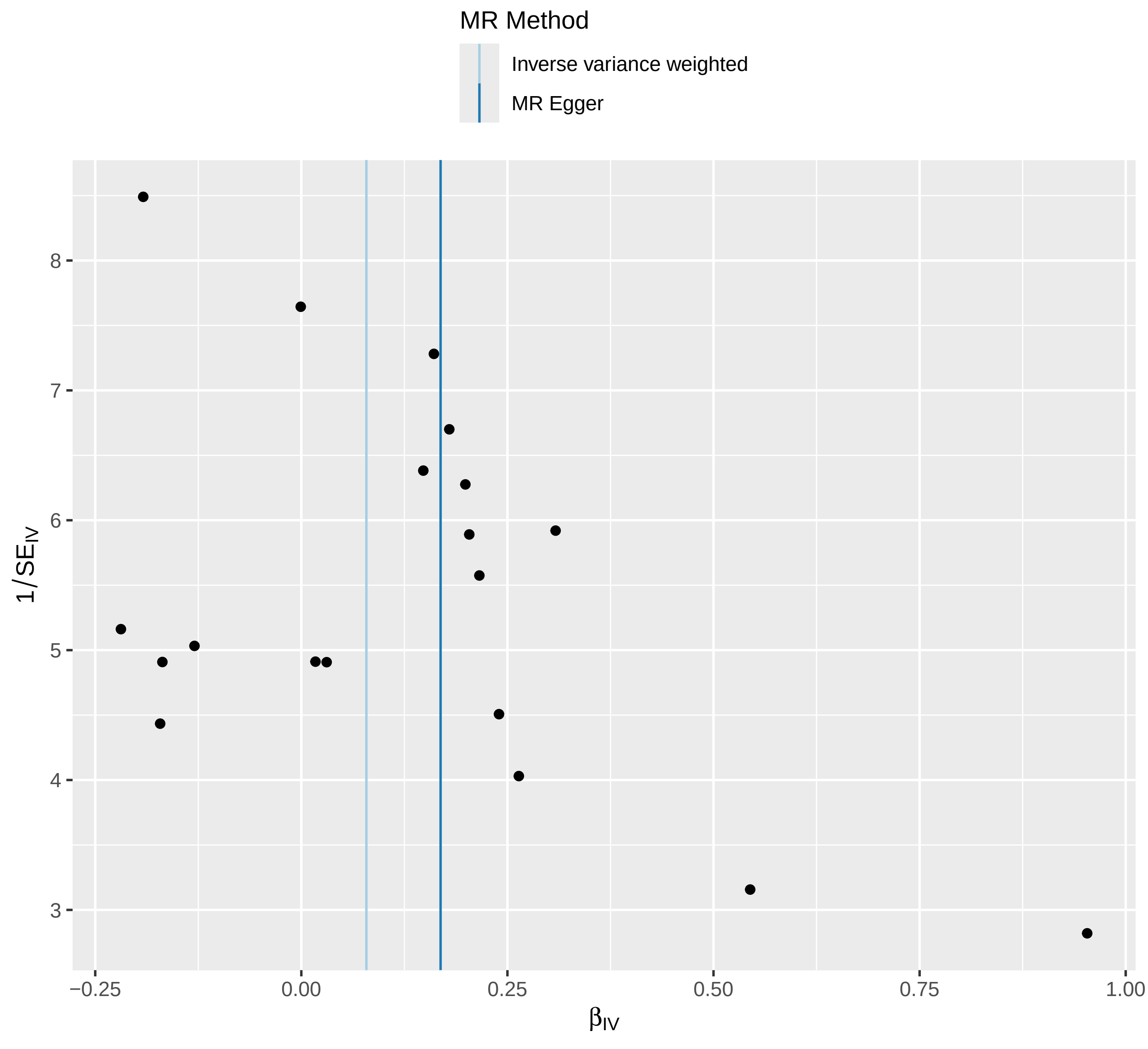
MR Method
Inverse variance weighted
MR Egger
$1/SE_{IV}$
8
7
6
5
4
3
−0.25
0.00
0.25
0.50
0.75
1.00
β_{IV}

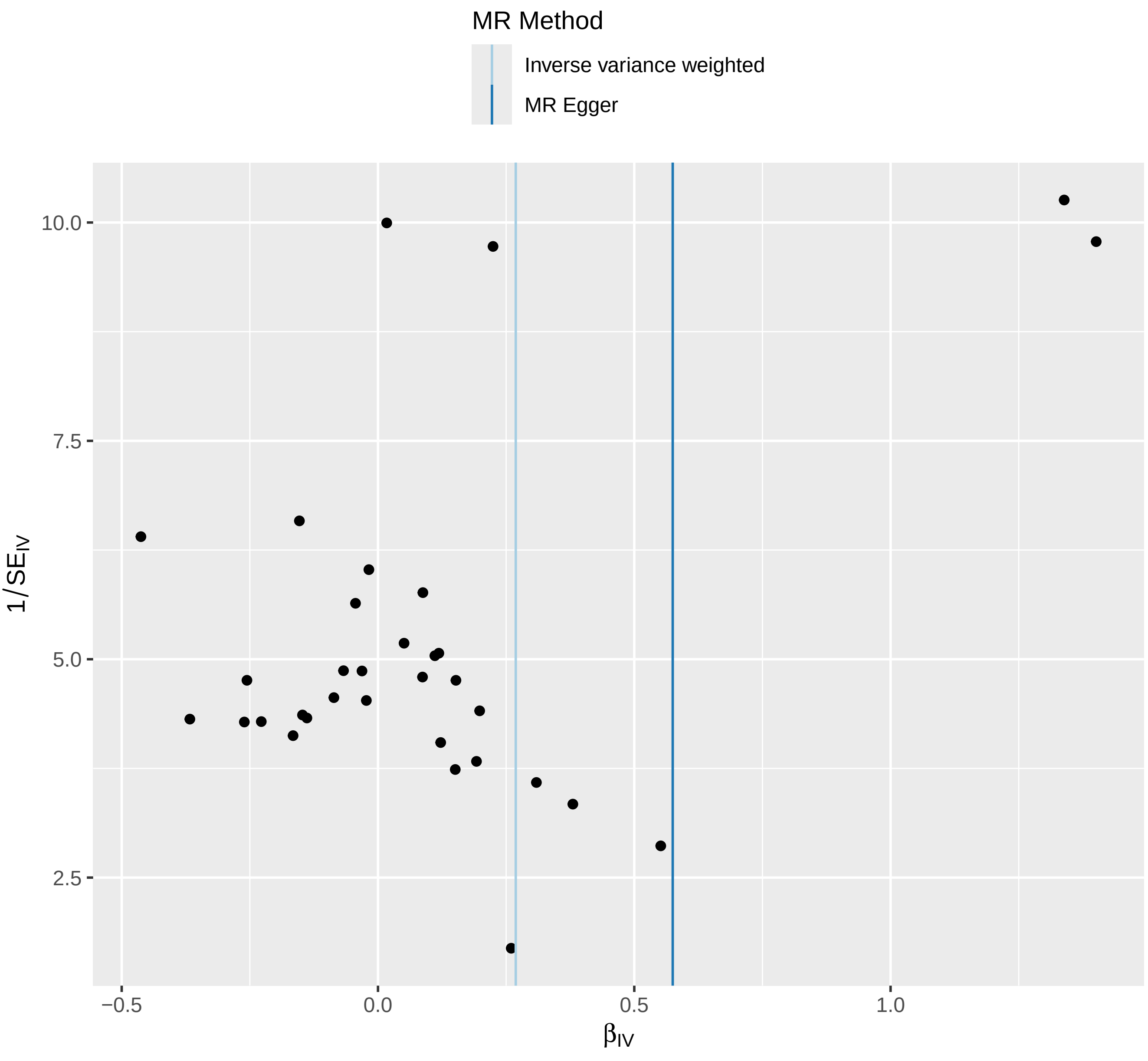

MR Method
Inverse variance weighted
MR Egger
10.0
7.5
5.0
2.5
1/SE_IV
−0.5
0.0
0.5
1.0
β_IV

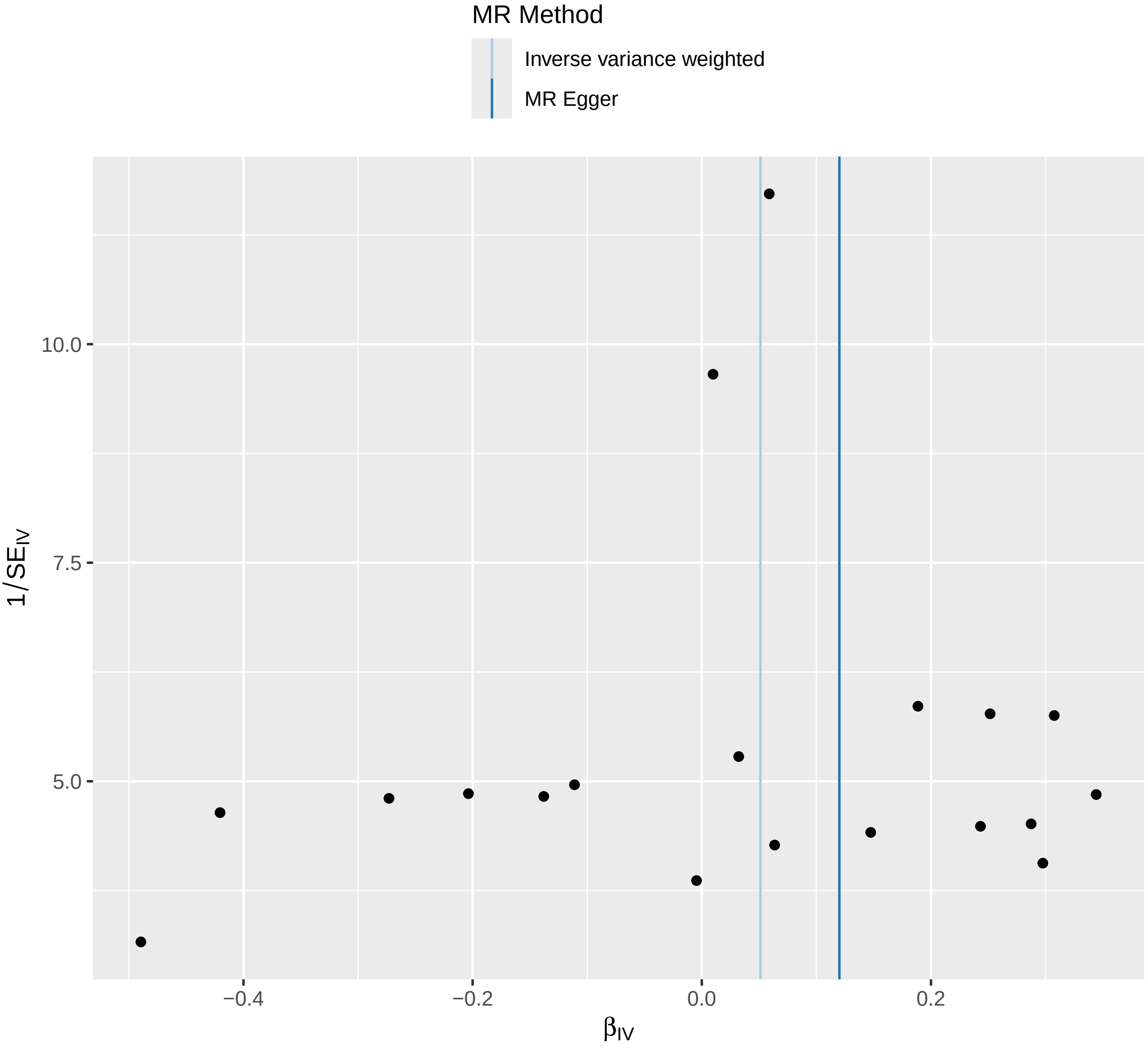
MR Method
Inverse variance weighted
MR Egger
10.0
7.5
5.0
1/SE_IV
−0.4
−0.2
0.0
0.2
β_IV

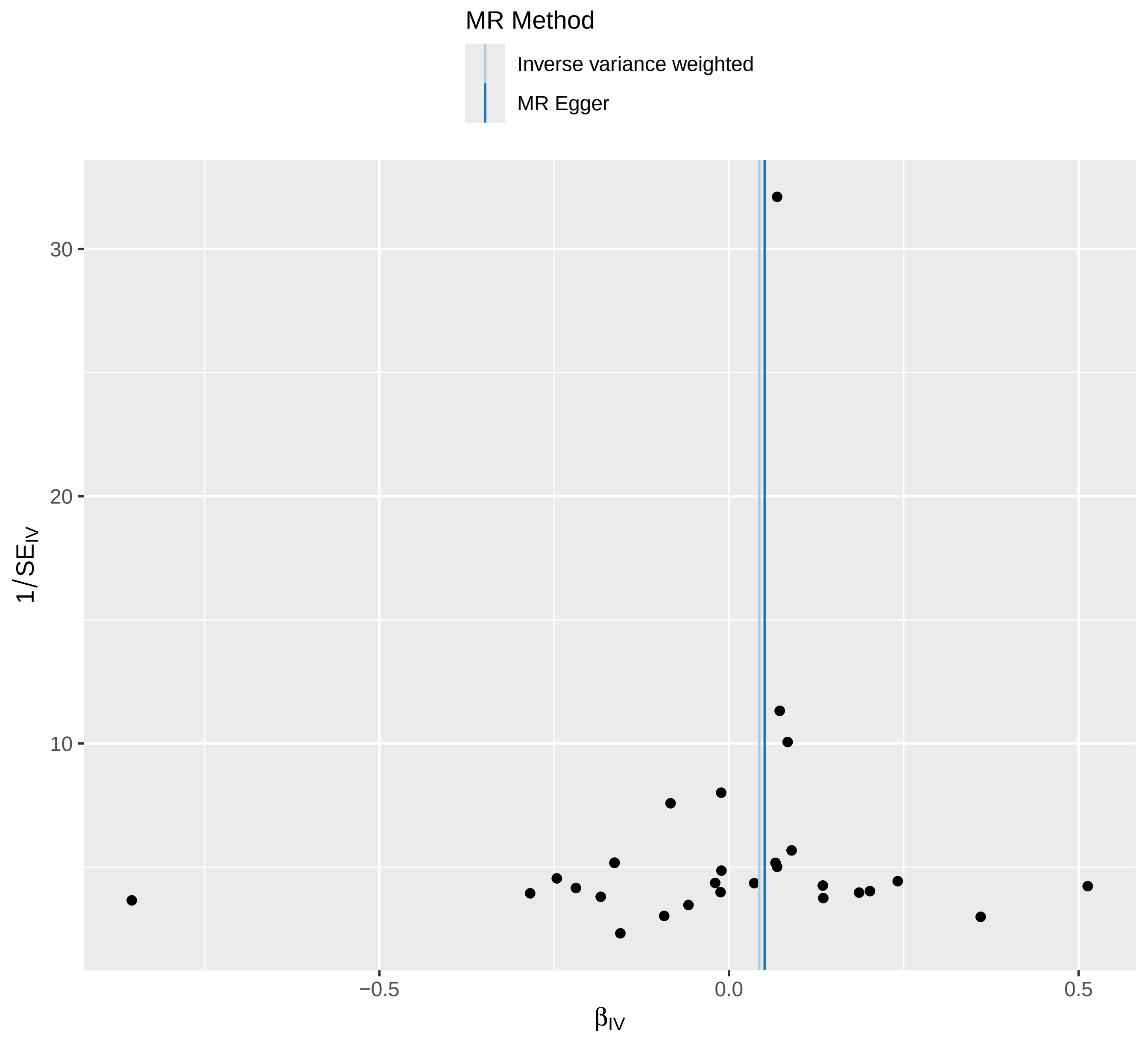

MR Method
Inverse variance weighted
MR Egger
30
20
10
1/SE_IV
−0.5
0.0
0.5
β_IV

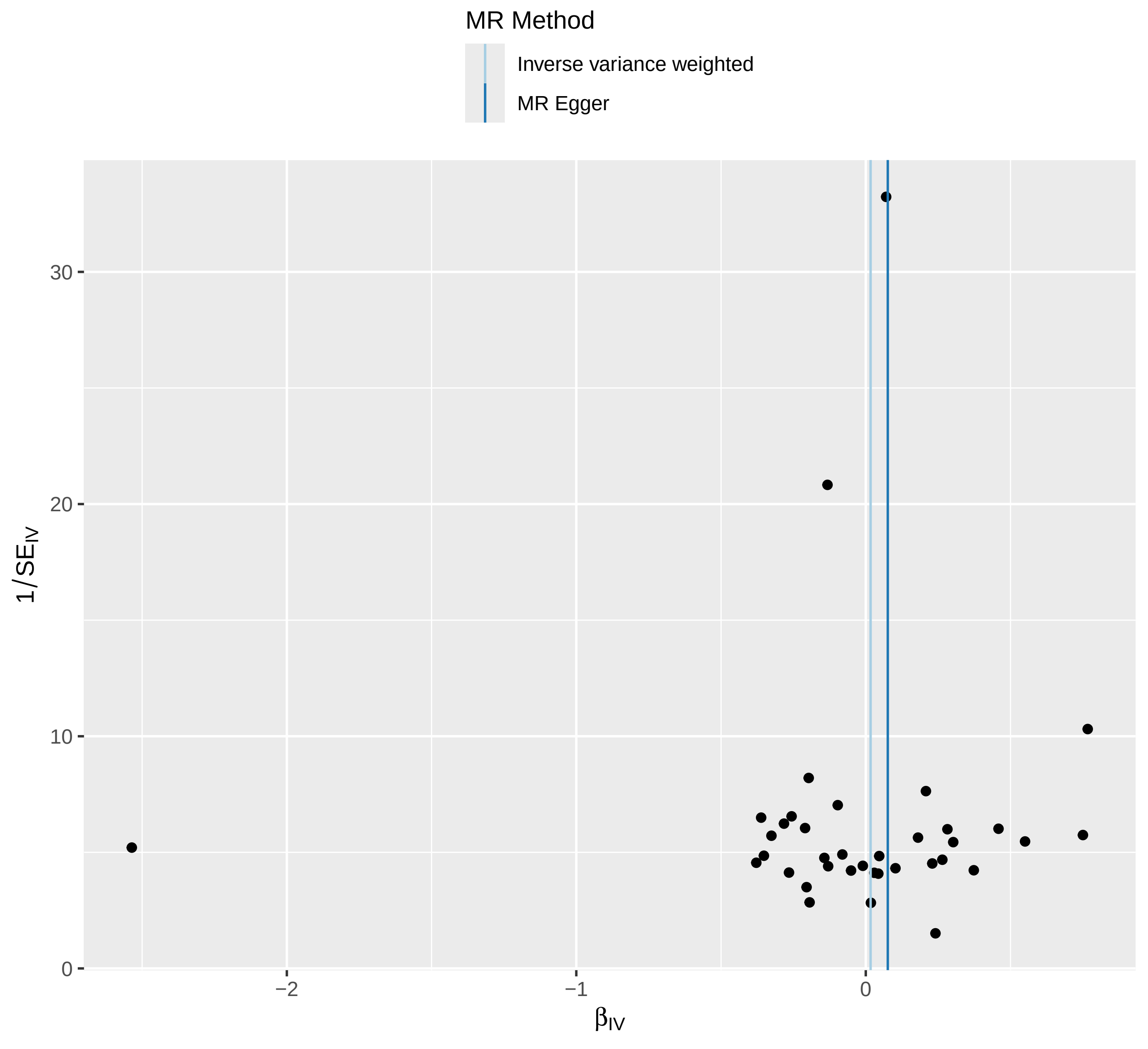
MR Method
Inverse variance weighted
MR Egger
30
20
10
0
−2
−1
0
1/SE_IV
β_IV

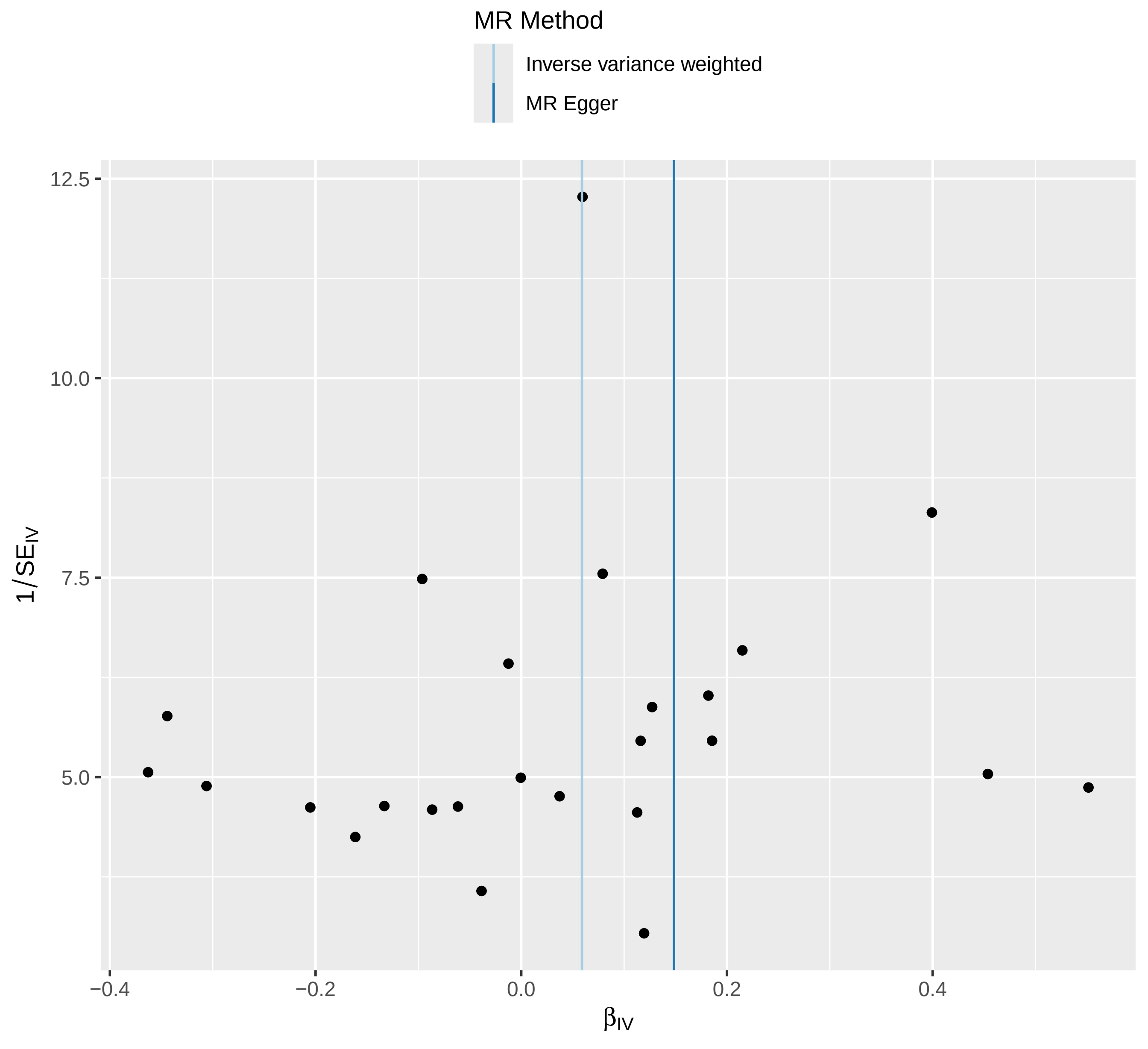
MR Method
Inverse variance weighted
MR Egger
12.5
10.0
7.5
5.0
1/SE_IV
−0.4
−0.2
0.0
0.2
0.4
β_IV

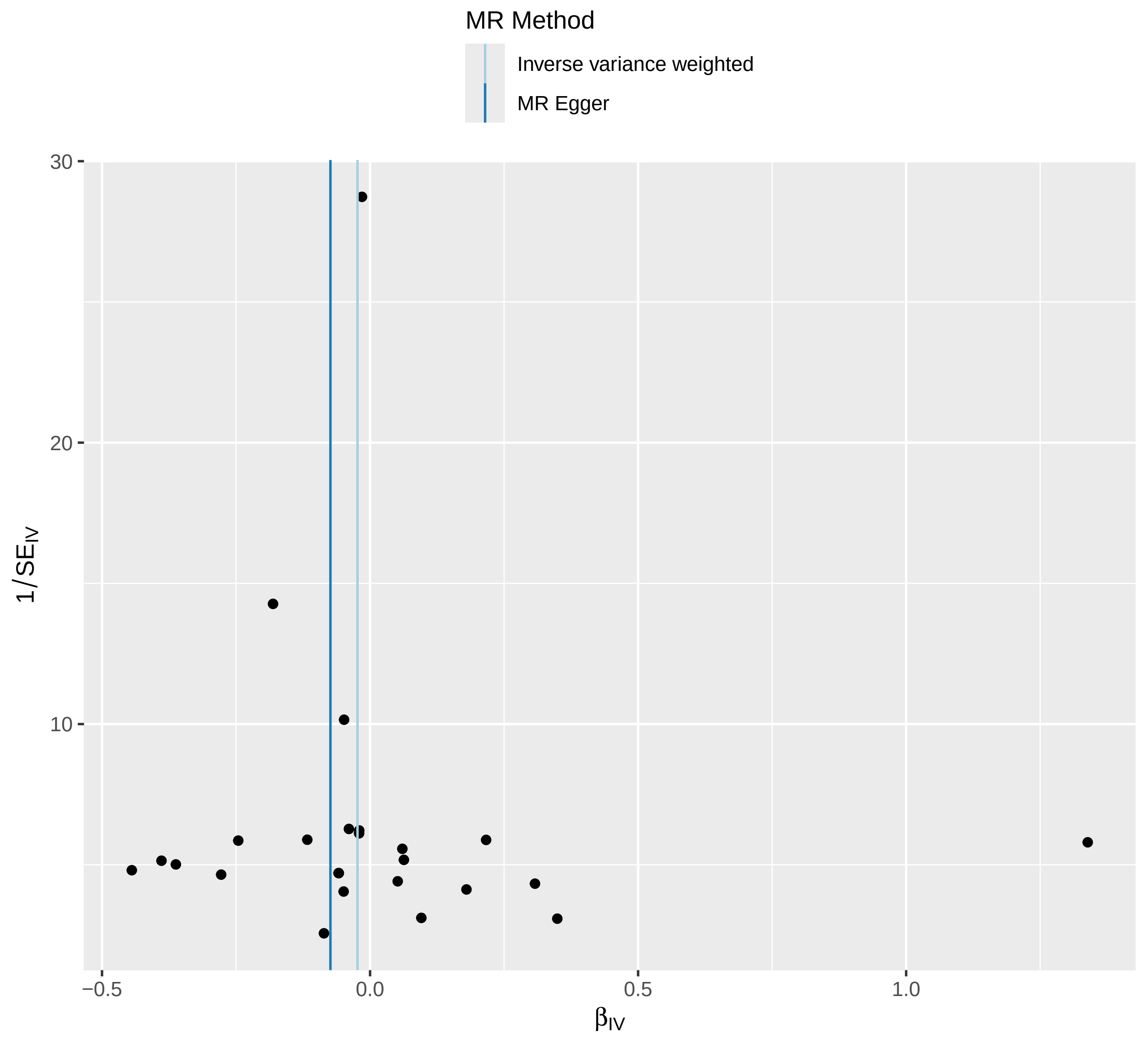

MR Method
Inverse variance weighted
MR Egger
30
20
10
1/SE_IV
−0.5
0.0
0.5
1.0
β_IV

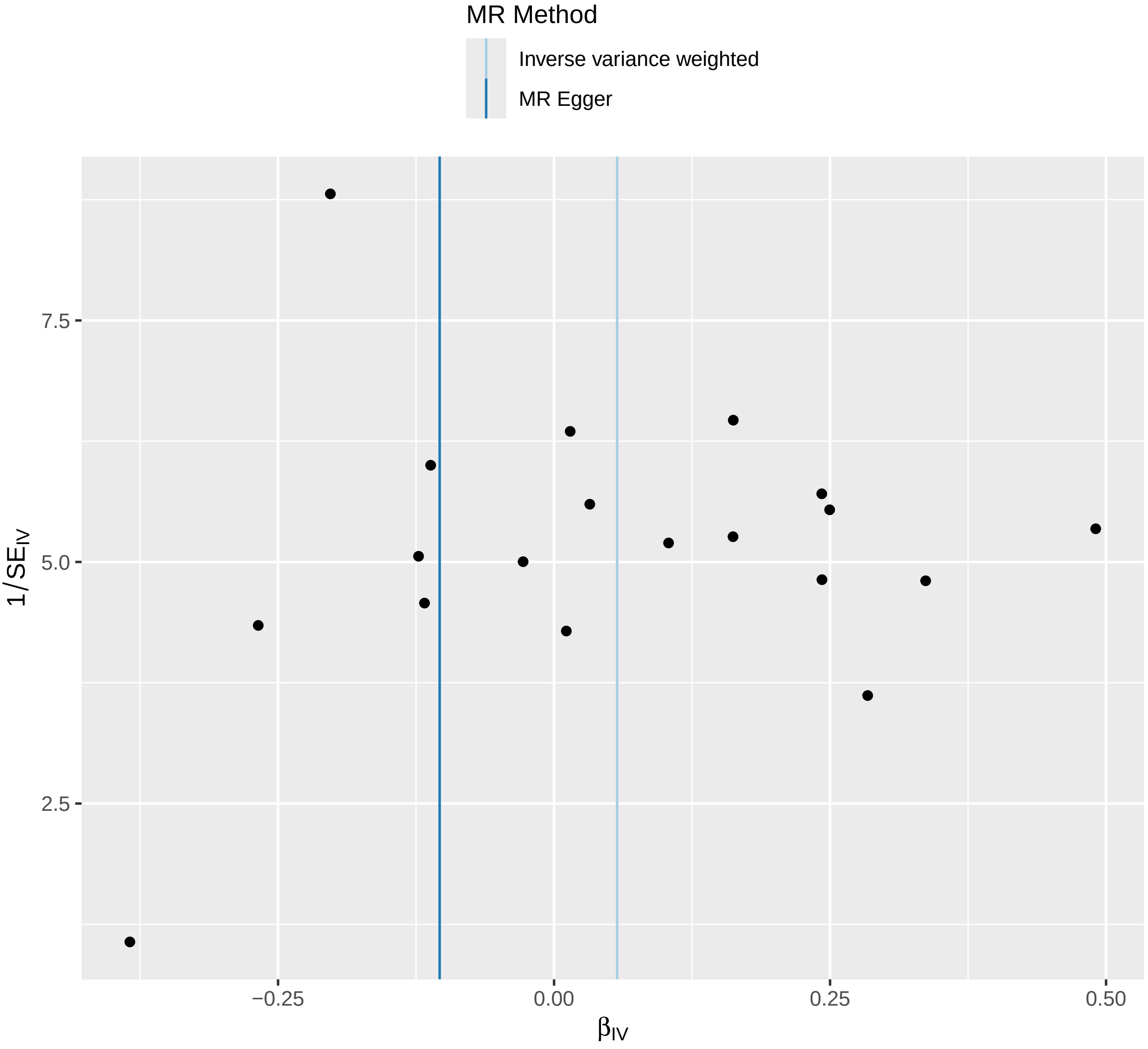

MR Method
Inverse variance weighted
MR Egger
7.5
5.0
2.5
1/SE_IV
−0.25
0.00
0.25
0.50
β_IV

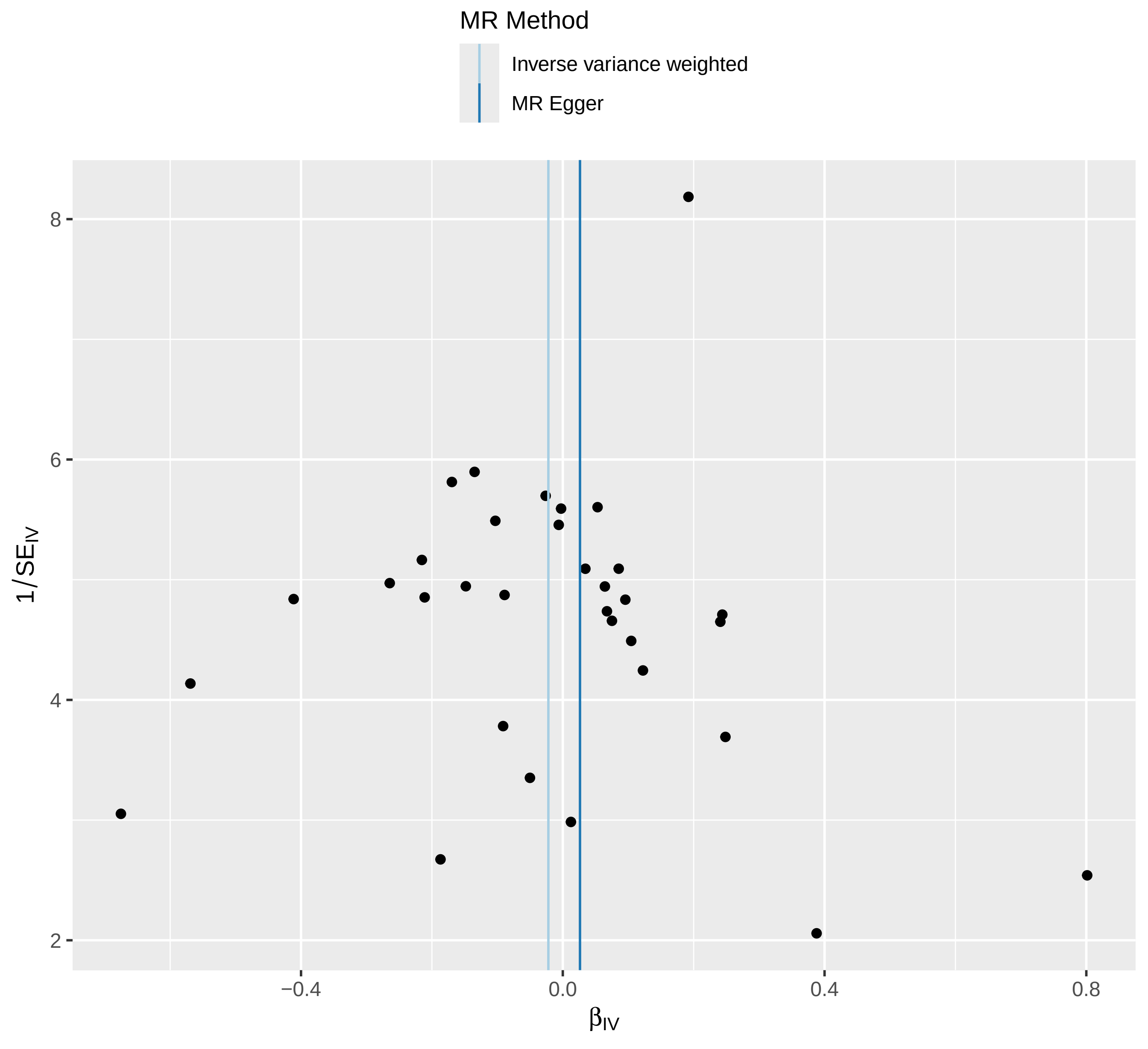
MR Method
Inverse variance weighted
MR Egger
1/SE$_{IV}$
8
6
4
2
−0.4
0.0
0.4
0.8
β$_{IV}$

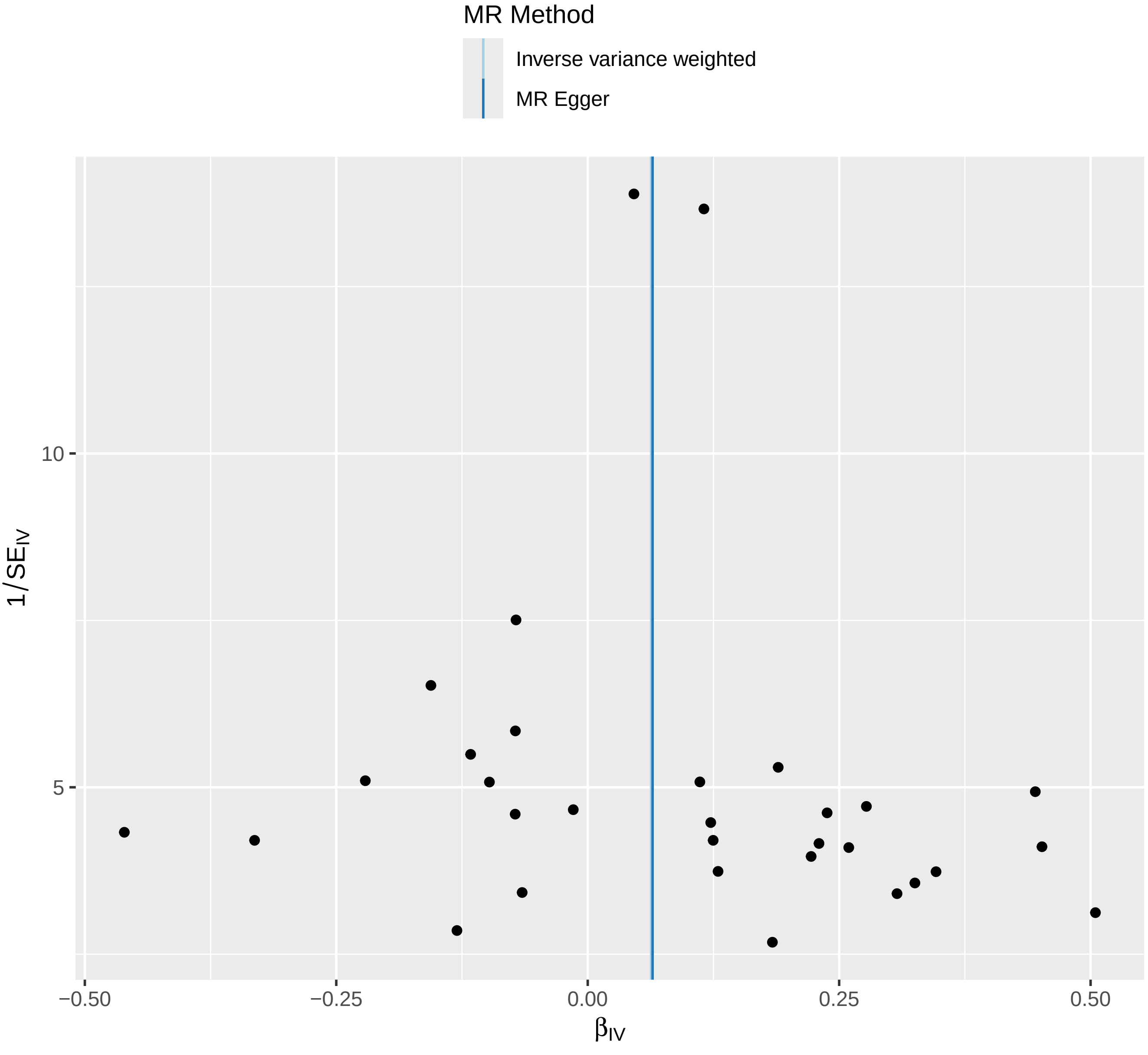
MR Method
Inverse variance weighted
MR Egger
1/SE_IV
10
5
−0.50
−0.25
0.00
0.25
0.50
β_IV

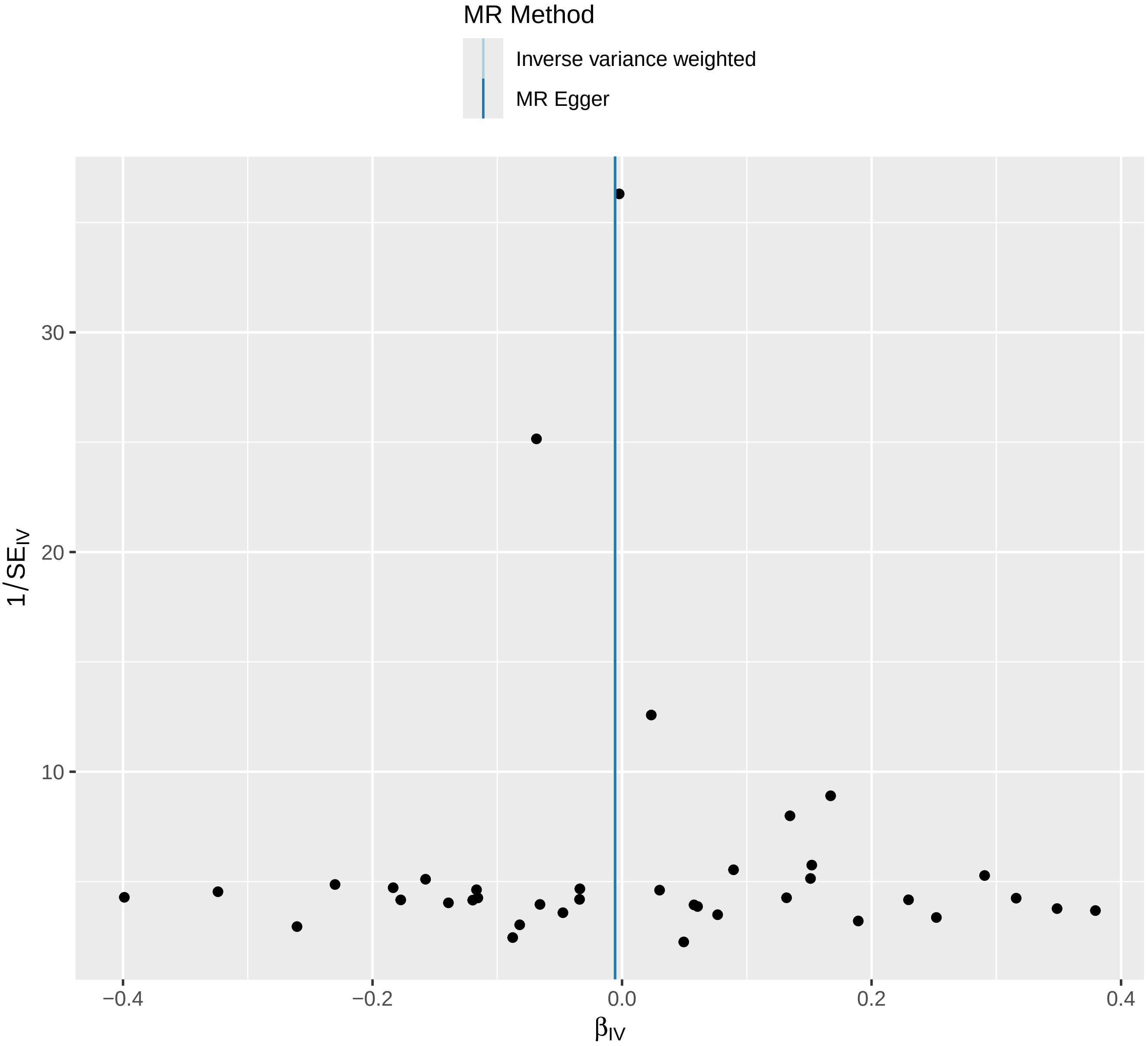
MR Method
Inverse variance weighted
MR Egger
1/SE$_{IV}$
β_{IV}
30
20
10
−0.4
−0.2
0.0
0.2
0.4

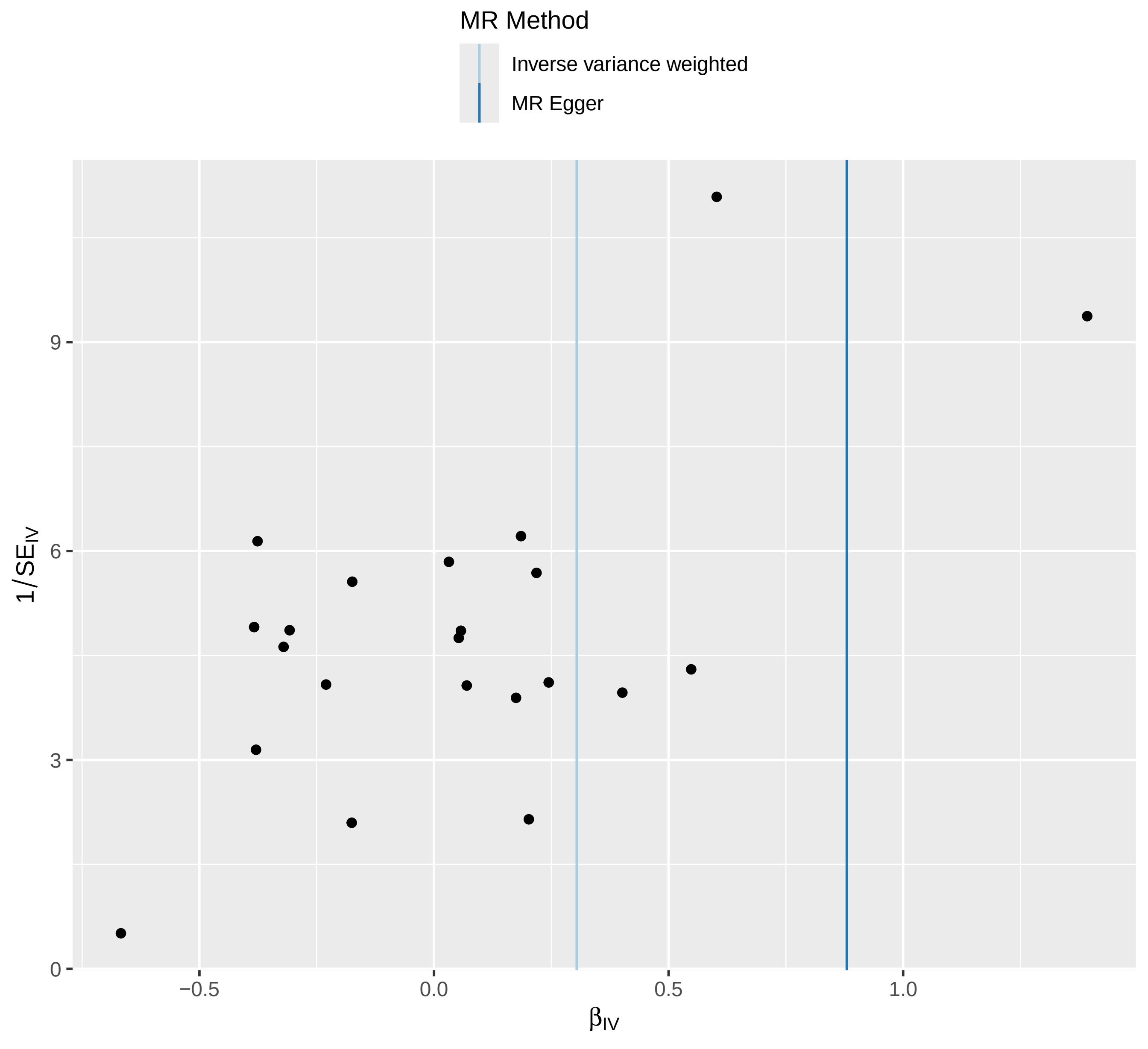
MR Method
Inverse variance weighted
MR Egger
1/SE_IV
9
6
3
0
−0.5
0.0
0.5
1.0
β_IV

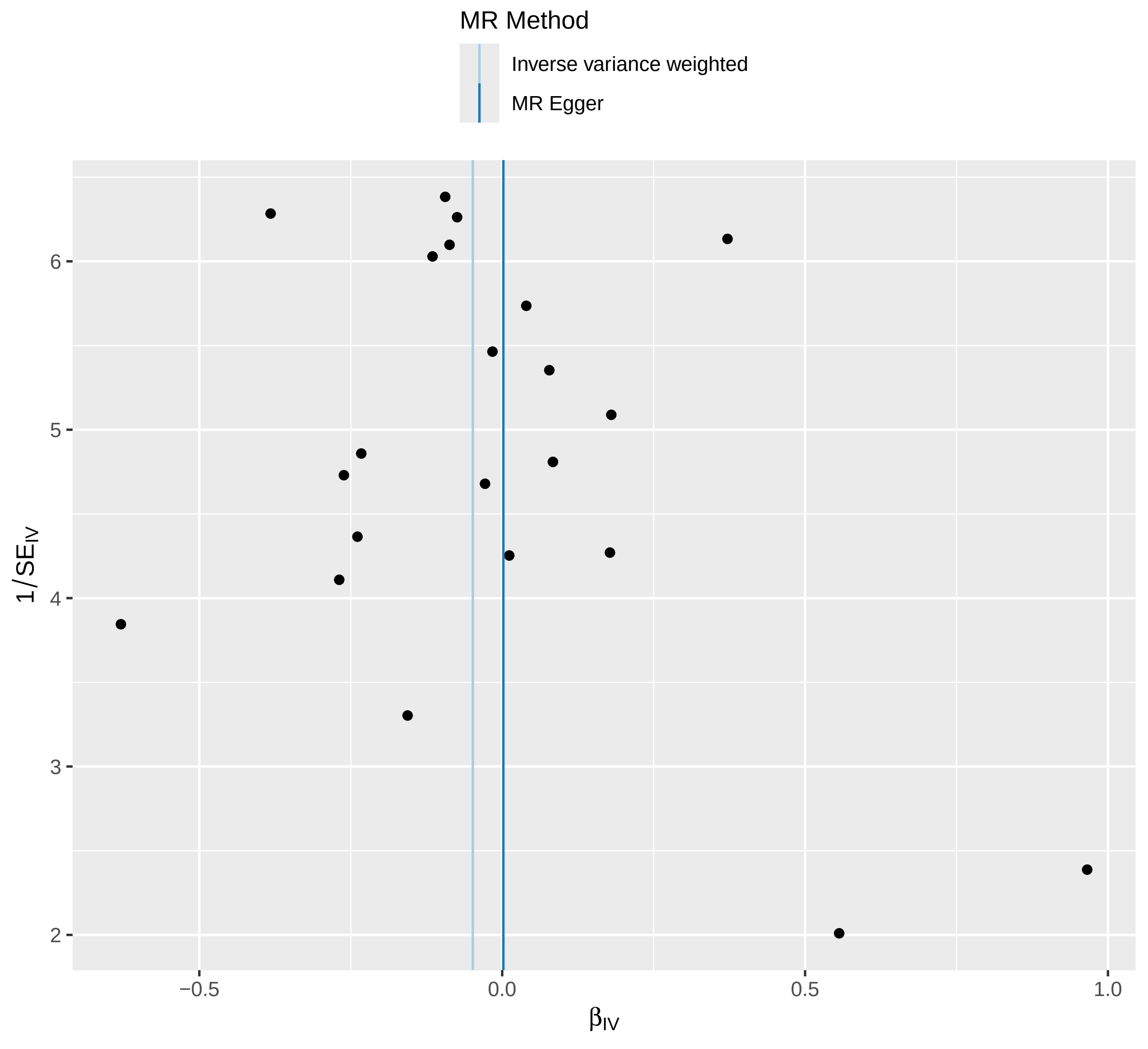
MR Method
Inverse variance weighted
MR Egger
1/SE_IV
6
5
4
3
2
−0.5
0.0
0.5
1.0
β_IV

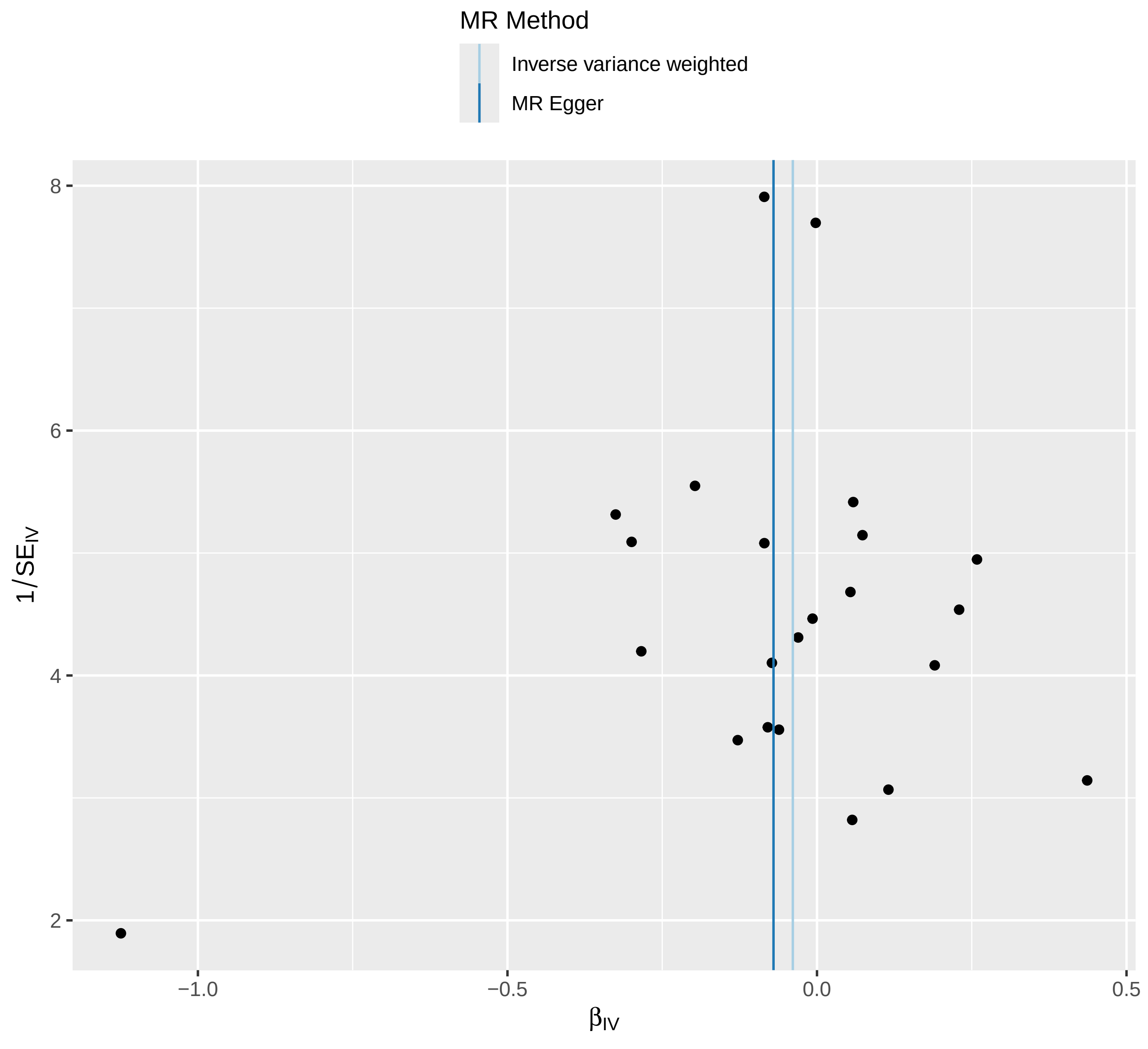

MR Method
Inverse variance weighted
MR Egger
8
6
4
2
1/SE$_{IV}$
−1.0
−0.5
0.0
0.5
β$_{IV}$

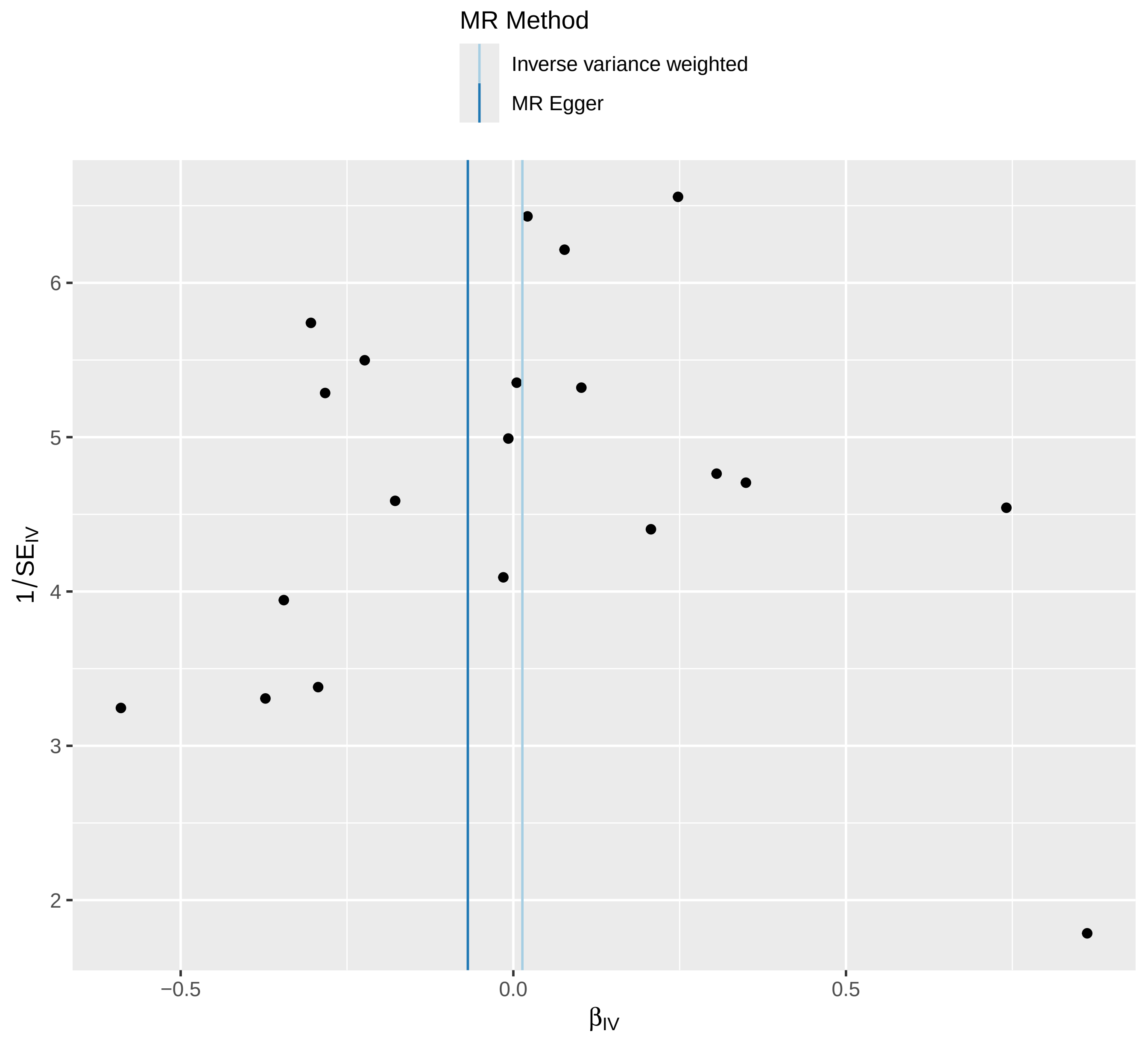
MR Method
Inverse variance weighted
MR Egger
1/SE$_{IV}$
6
5
4
3
2
−0.5
0.0
0.5
β_{IV}

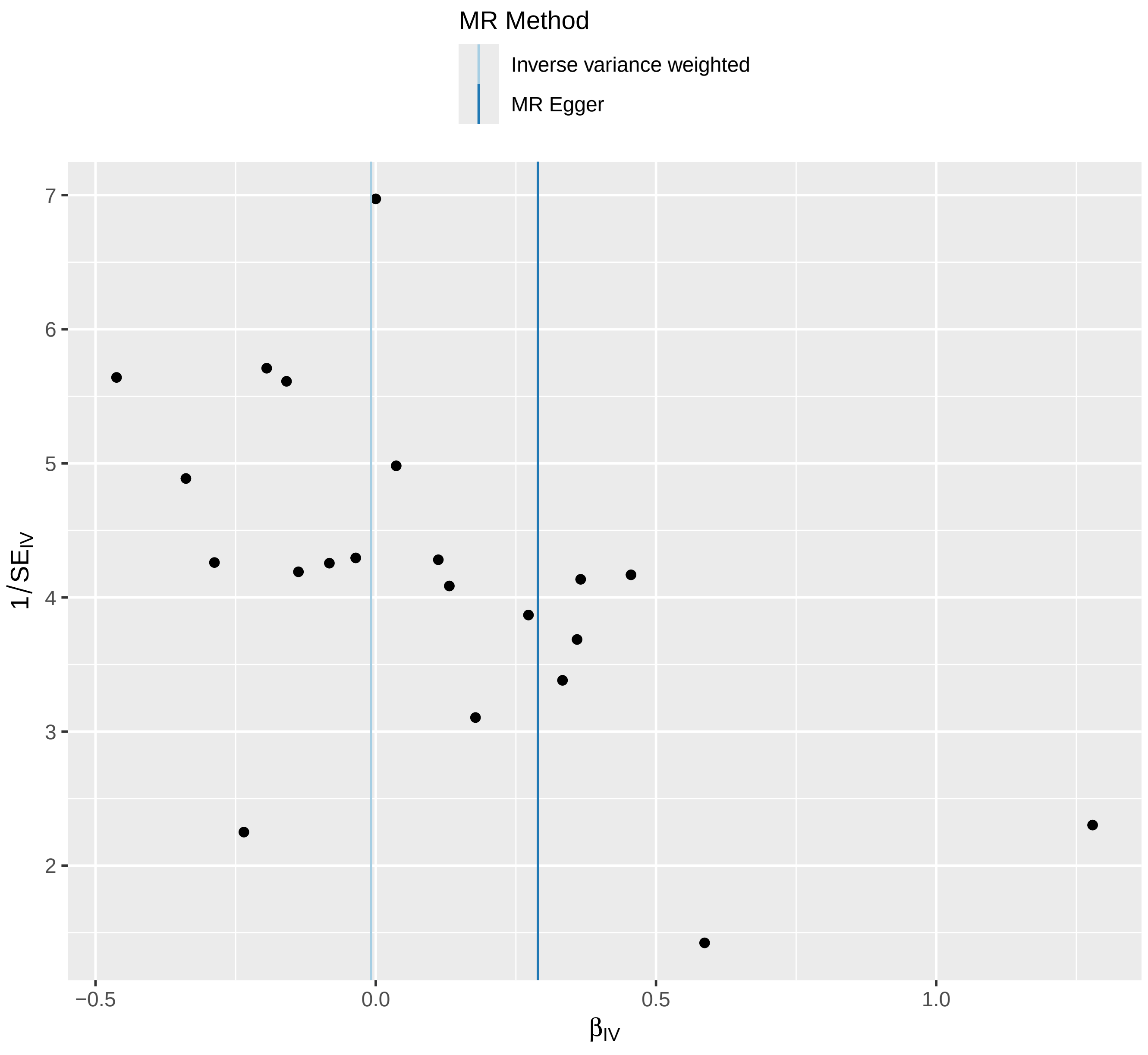
MR Method
Inverse variance weighted
MR Egger
1/SE_IV
β_IV
7
6
5
4
3
2
−0.5
0.0
0.5
1.0

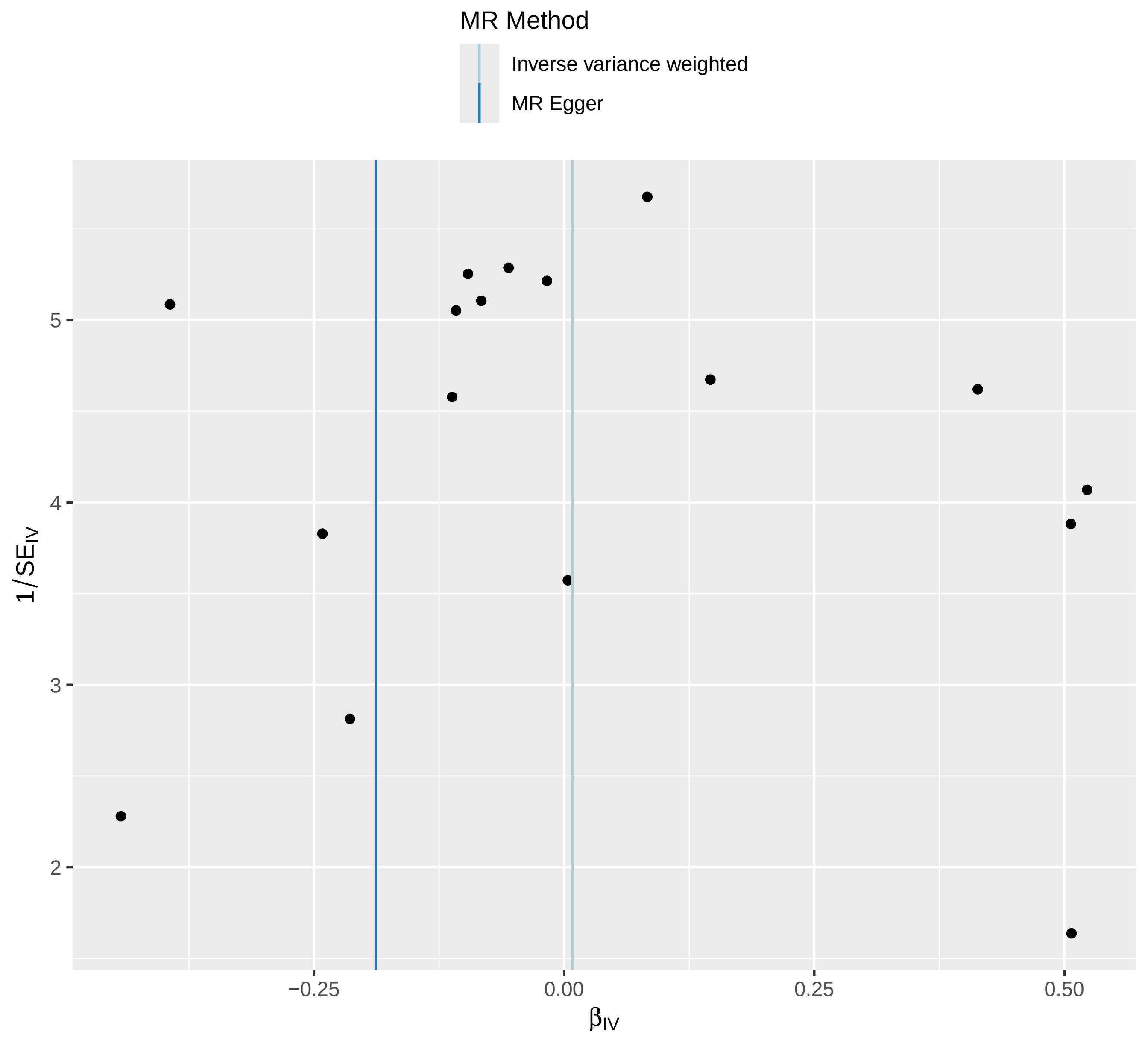

MR Method
Inverse variance weighted
MR Egger
1/SE_IV
β_IV
5
4
3
2
−0.25
0.00
0.25
0.50

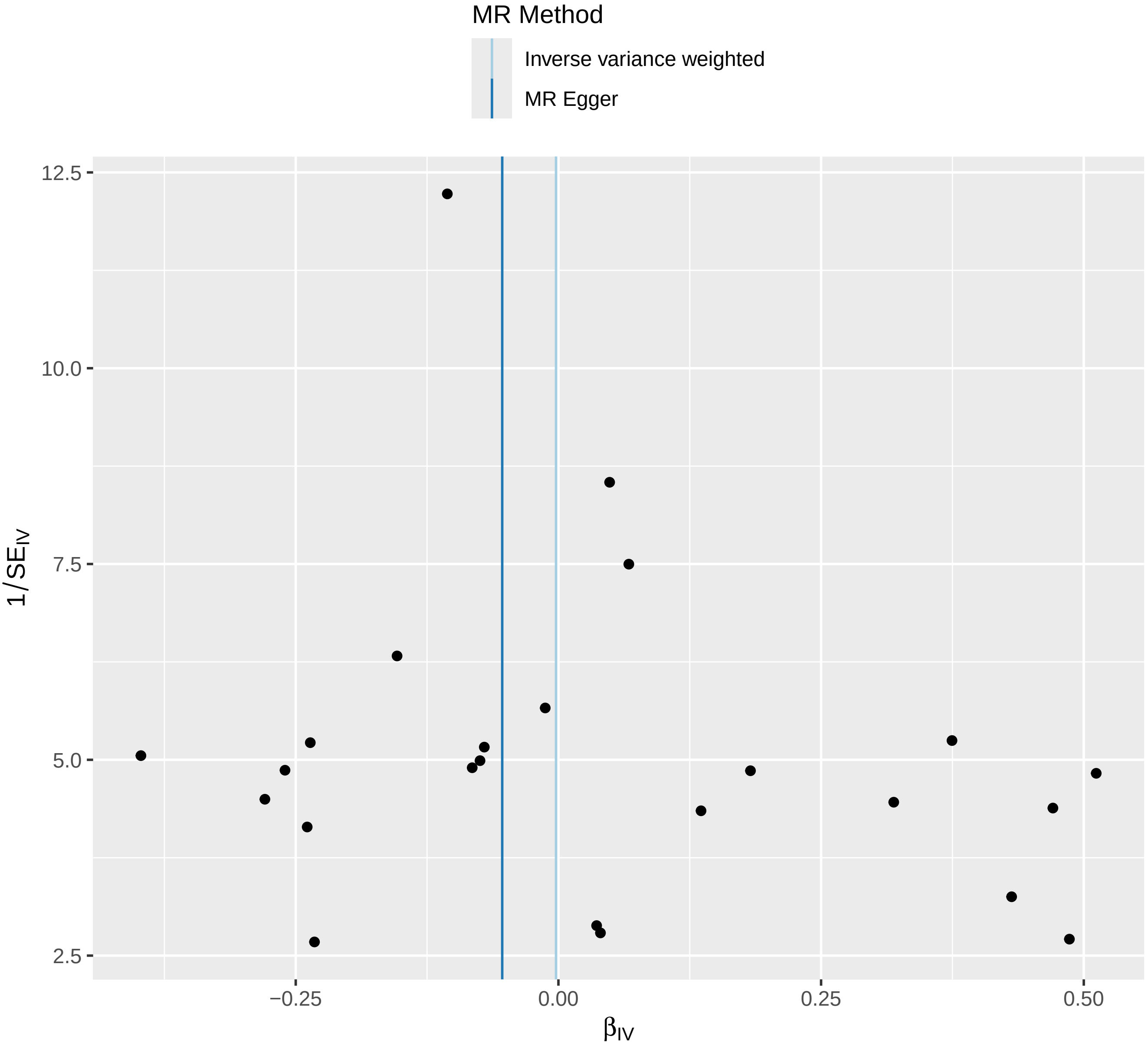
MR Method
Inverse variance weighted
MR Egger
12.5
10.0
7.5
5.0
2.5
1/SE_IV
−0.25
0.00
0.25
0.50
β_IV

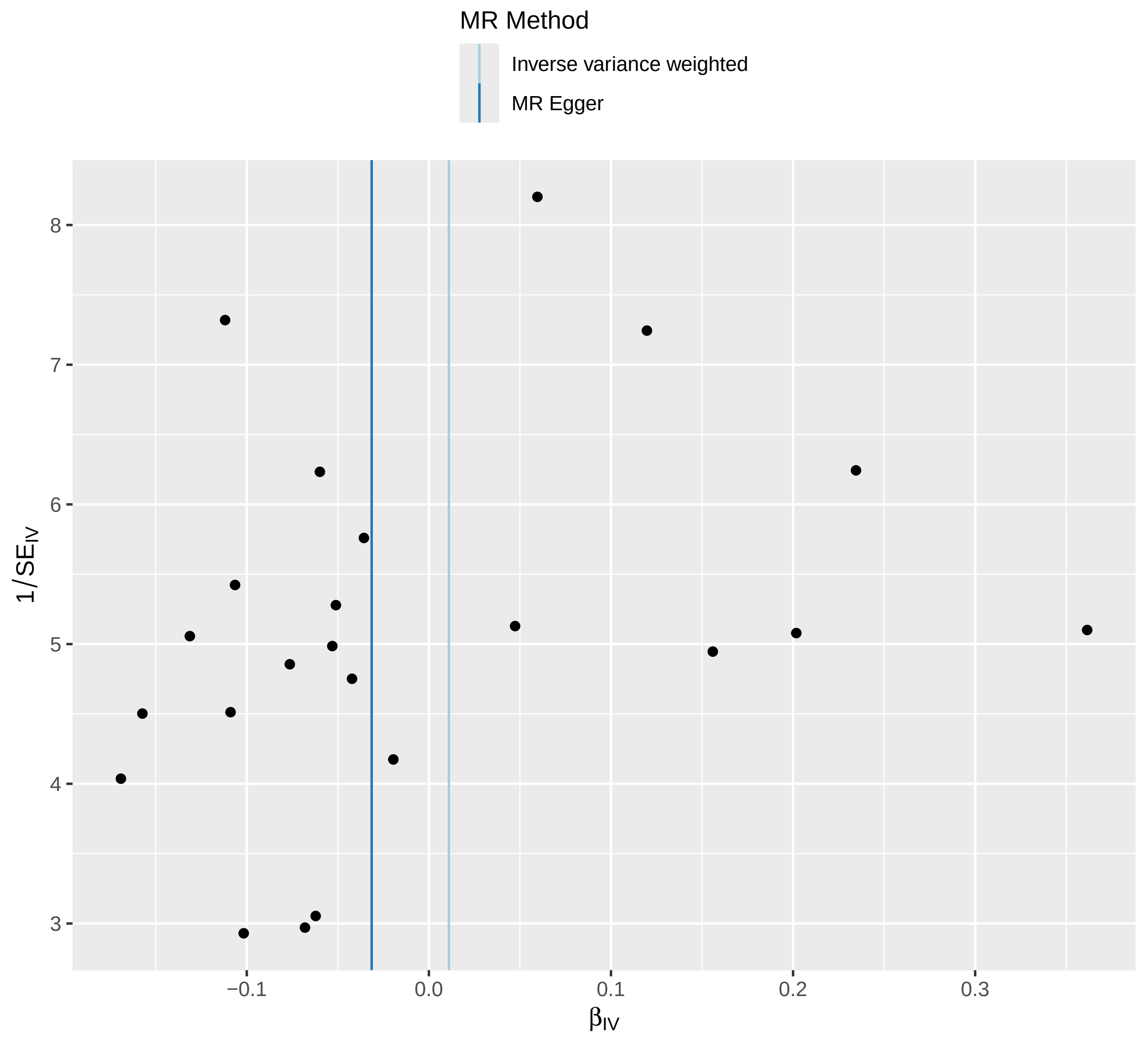

MR Method
Inverse variance weighted
MR Egger
8
7
6
5
4
3
1/SE$_{IV}$
−0.1
0.0
0.1
0.2
0.3
β$_{IV}$

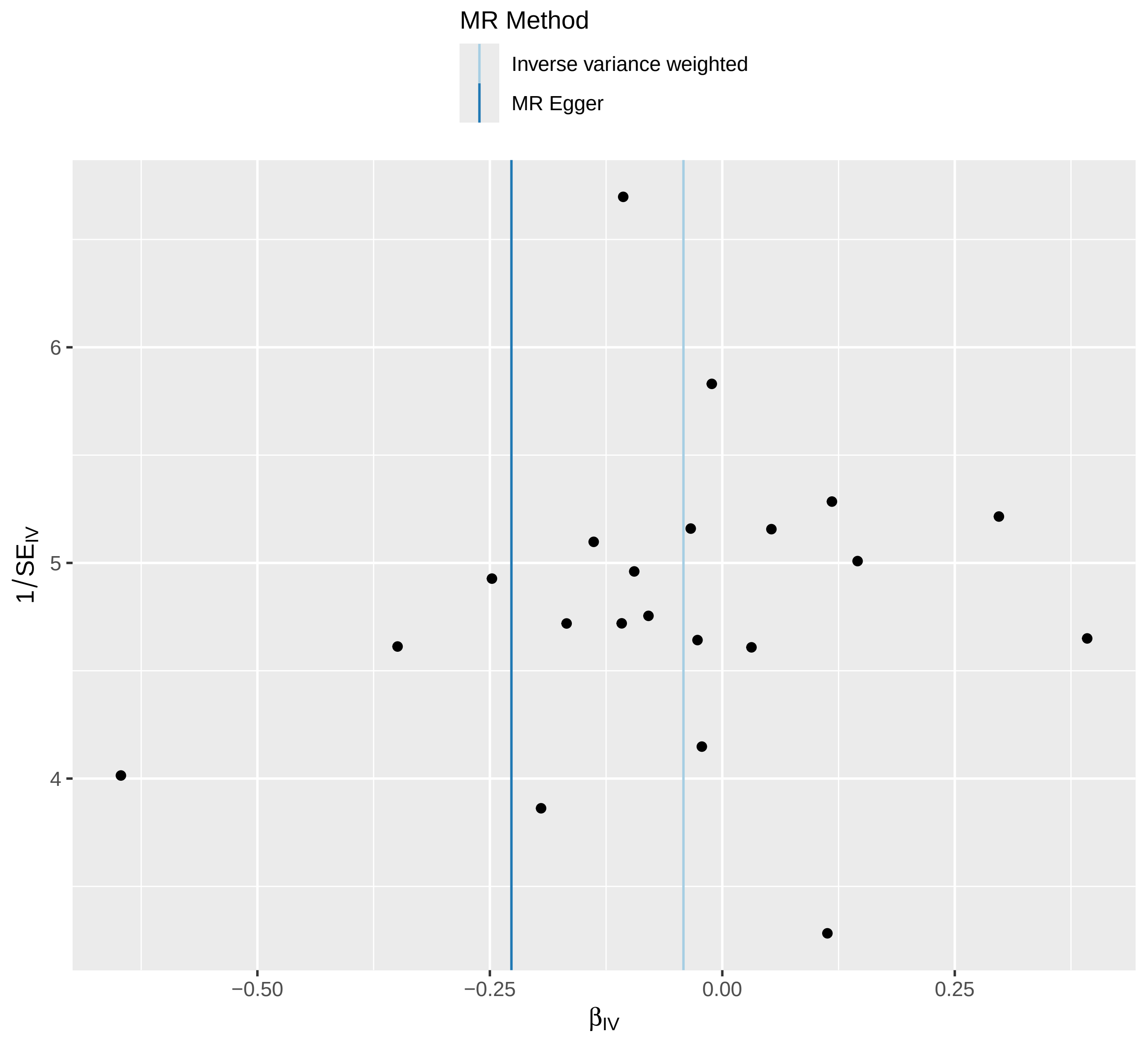
MR Method
Inverse variance weighted
MR Egger
1/SE_IV
6
5
4
−0.50
−0.25
0.00
0.25
β_IV

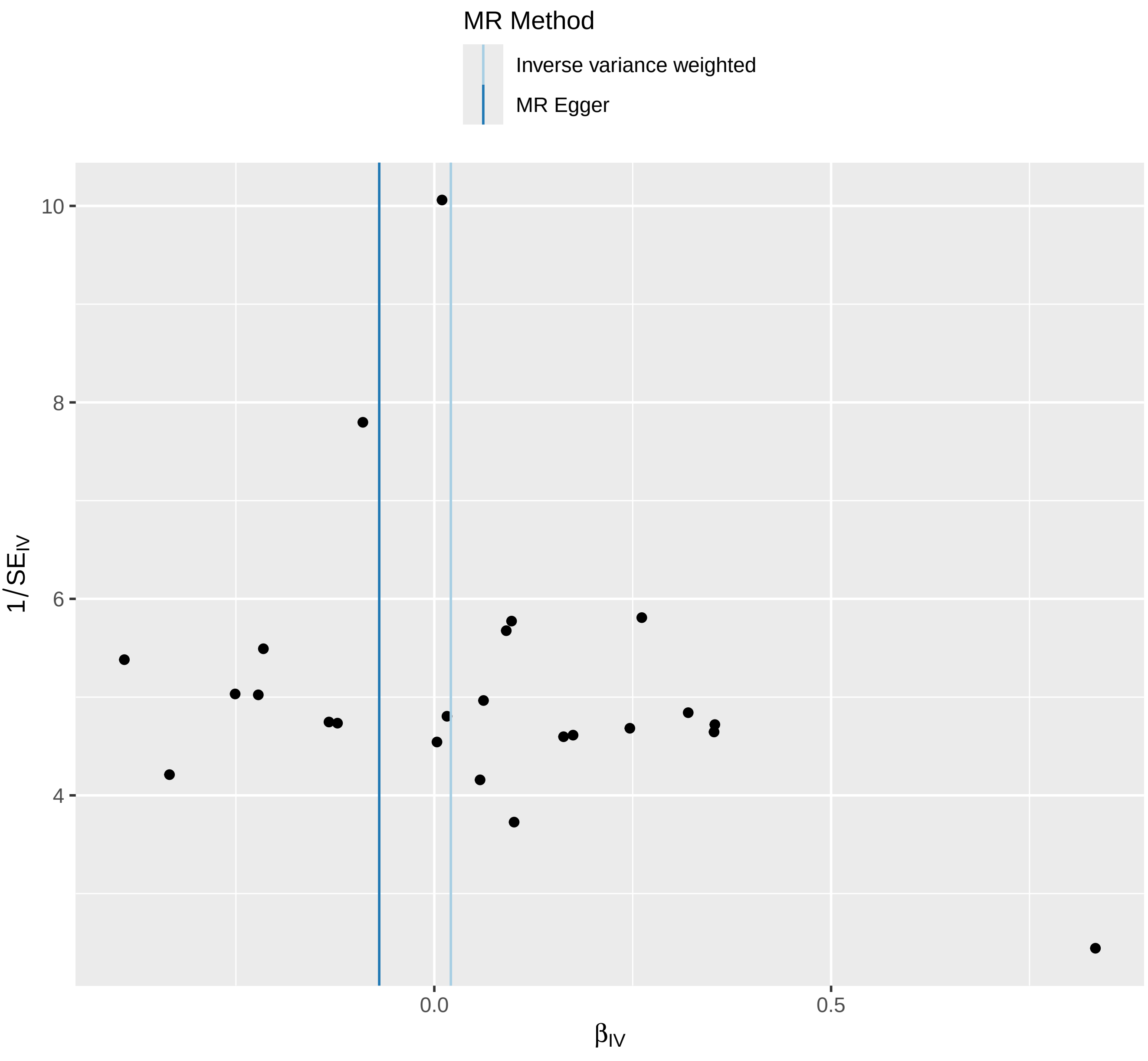

MR Method
Inverse variance weighted
MR Egger
10
8
6
4
1/SE$_{IV}$
0.0
0.5
β$_{IV}$

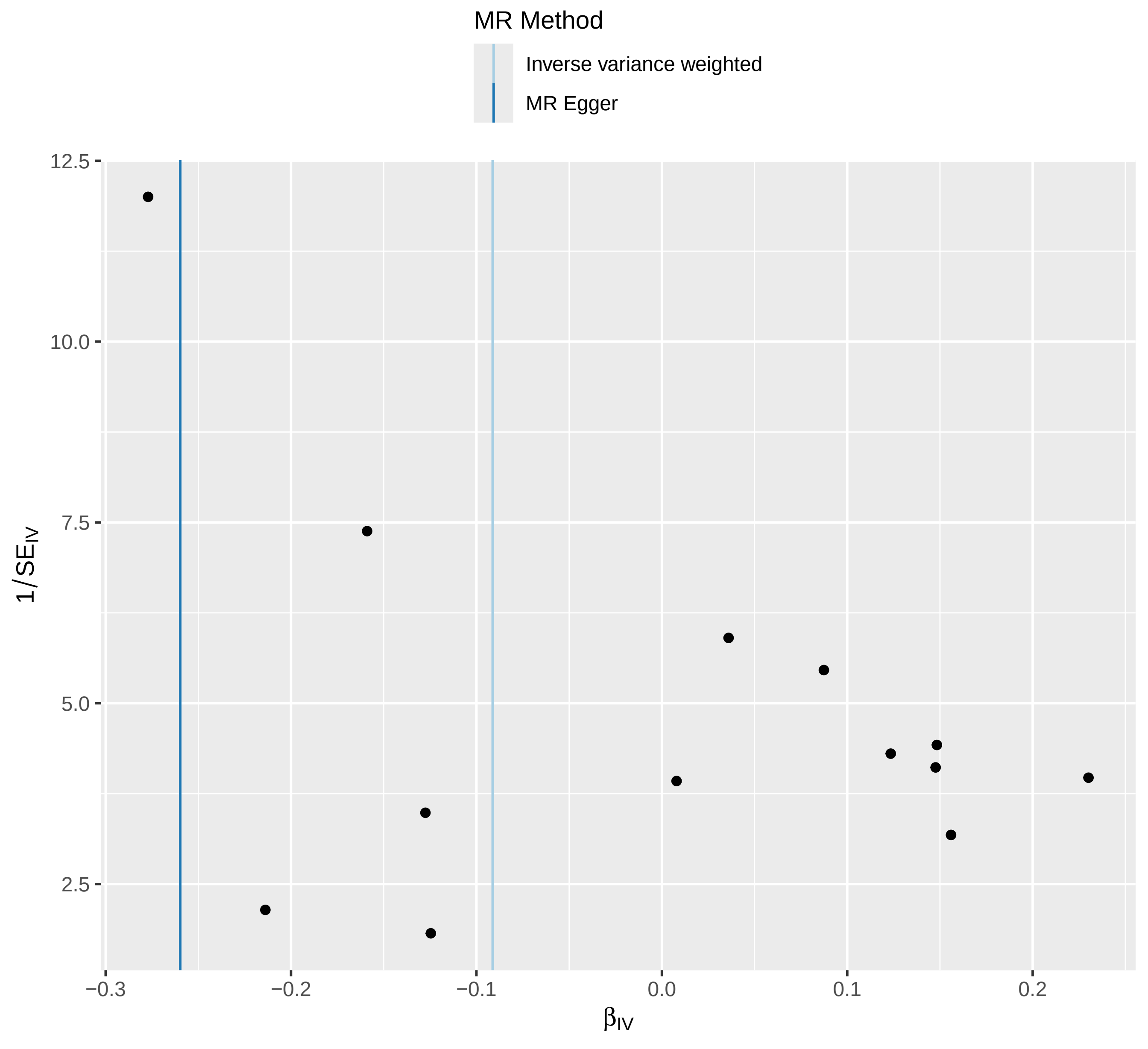

MR Method
Inverse variance weighted
MR Egger
12.5
10.0
7.5
5.0
2.5
1/SE_IV
−0.3
−0.2
−0.1
0.0
0.1
0.2
β_IV

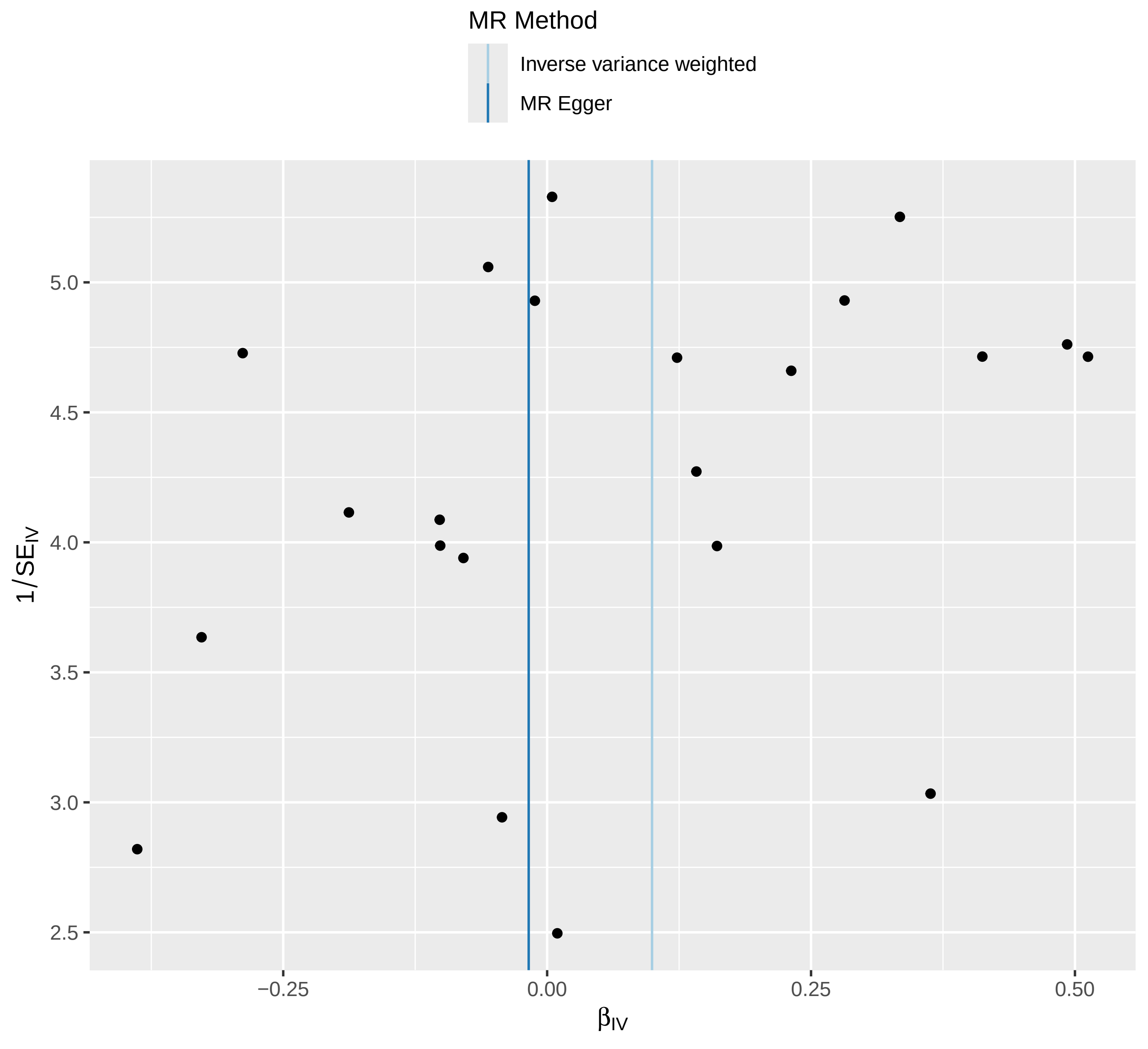
MR Method
Inverse variance weighted
MR Egger
1/SE_IV
5.0
4.5
4.0
3.5
3.0
2.5
−0.25
0.00
0.25
0.50
β_IV

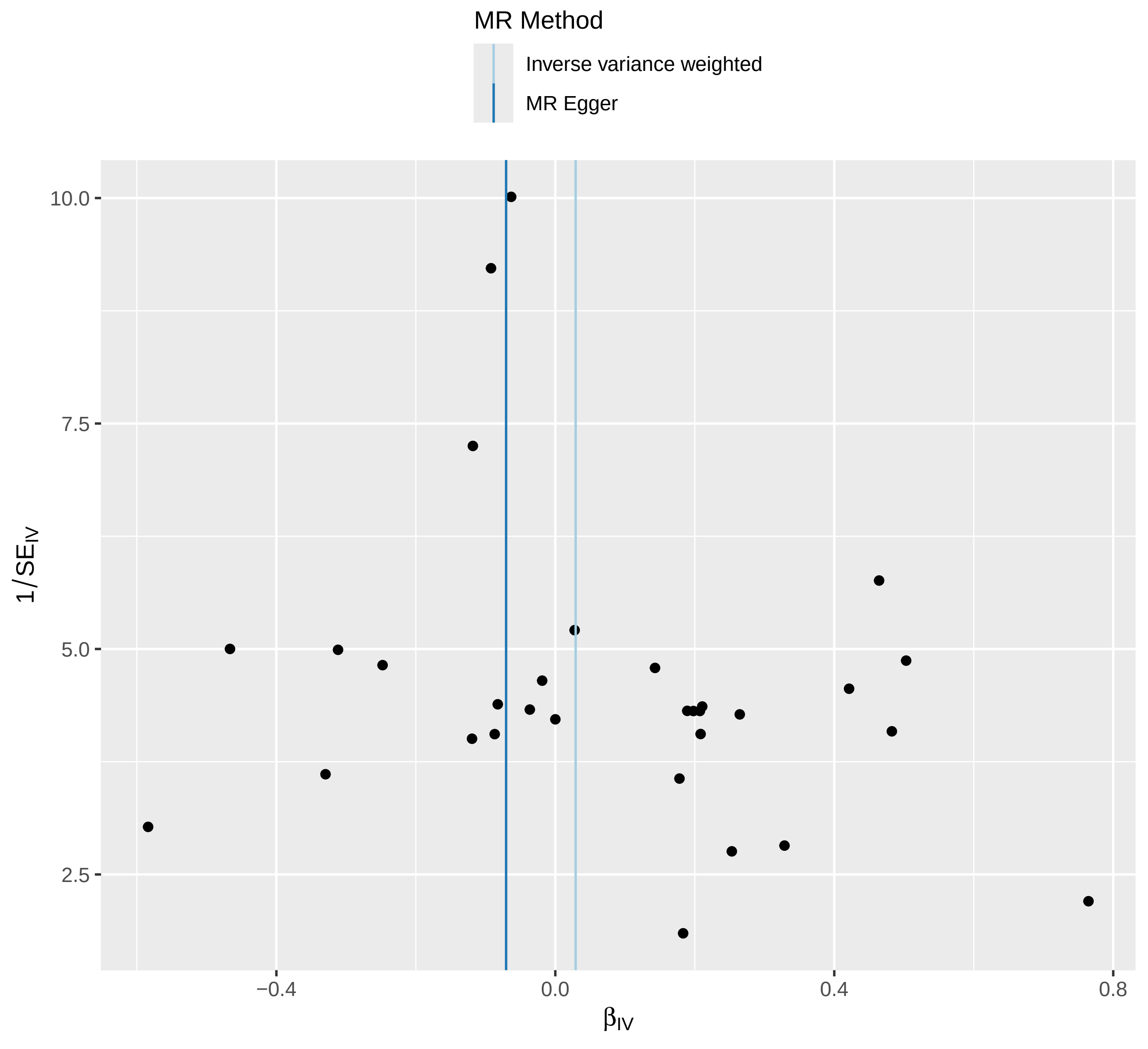

MR Method
Inverse variance weighted
MR Egger
10.0
7.5
5.0
2.5
1/SE_IV
−0.4
0.0
0.4
0.8
β_IV

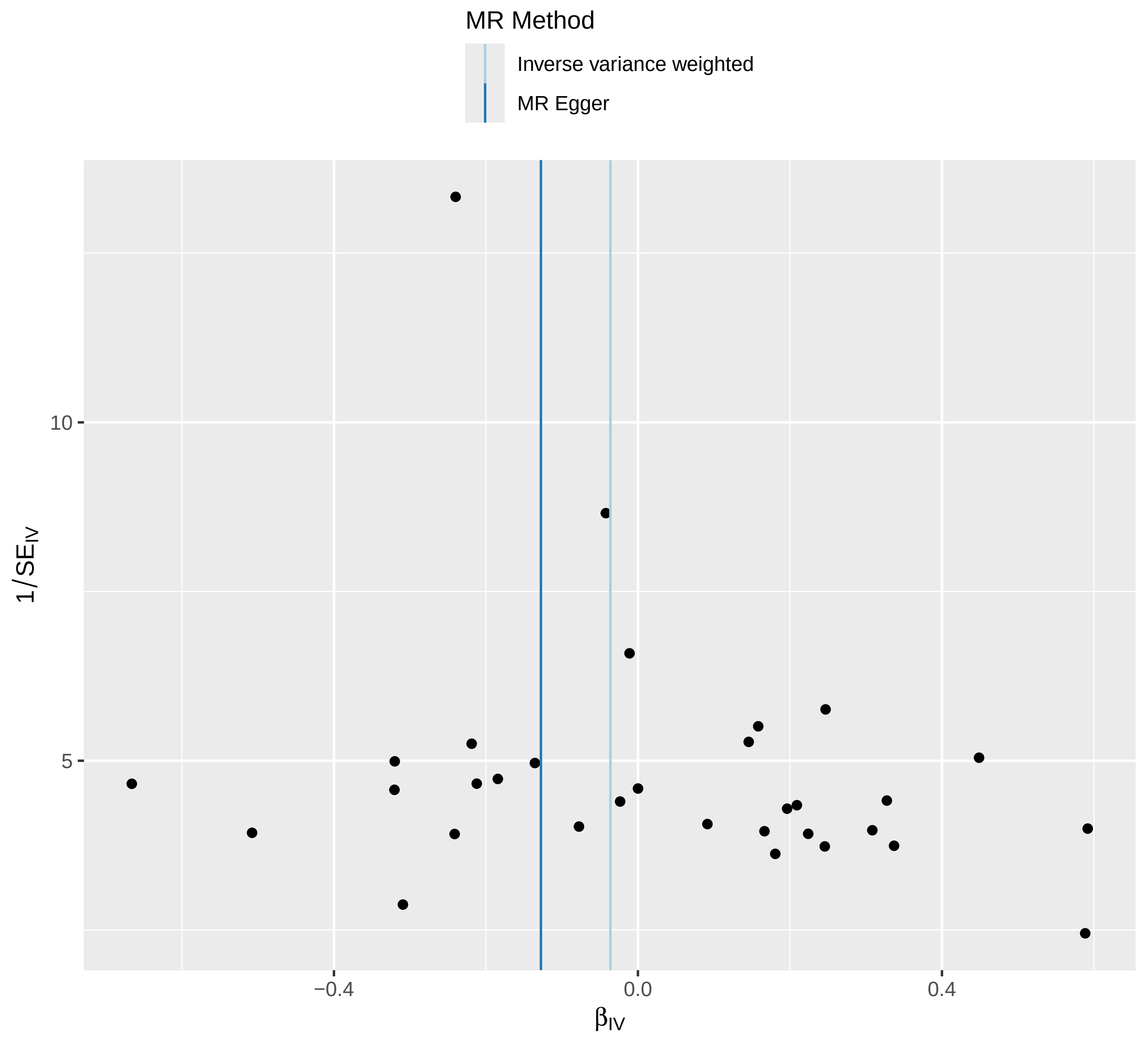
MR Method
Inverse variance weighted
MR Egger
10
5
1/SE$_{IV}$
−0.4
0.0
0.4
β$_{IV}$

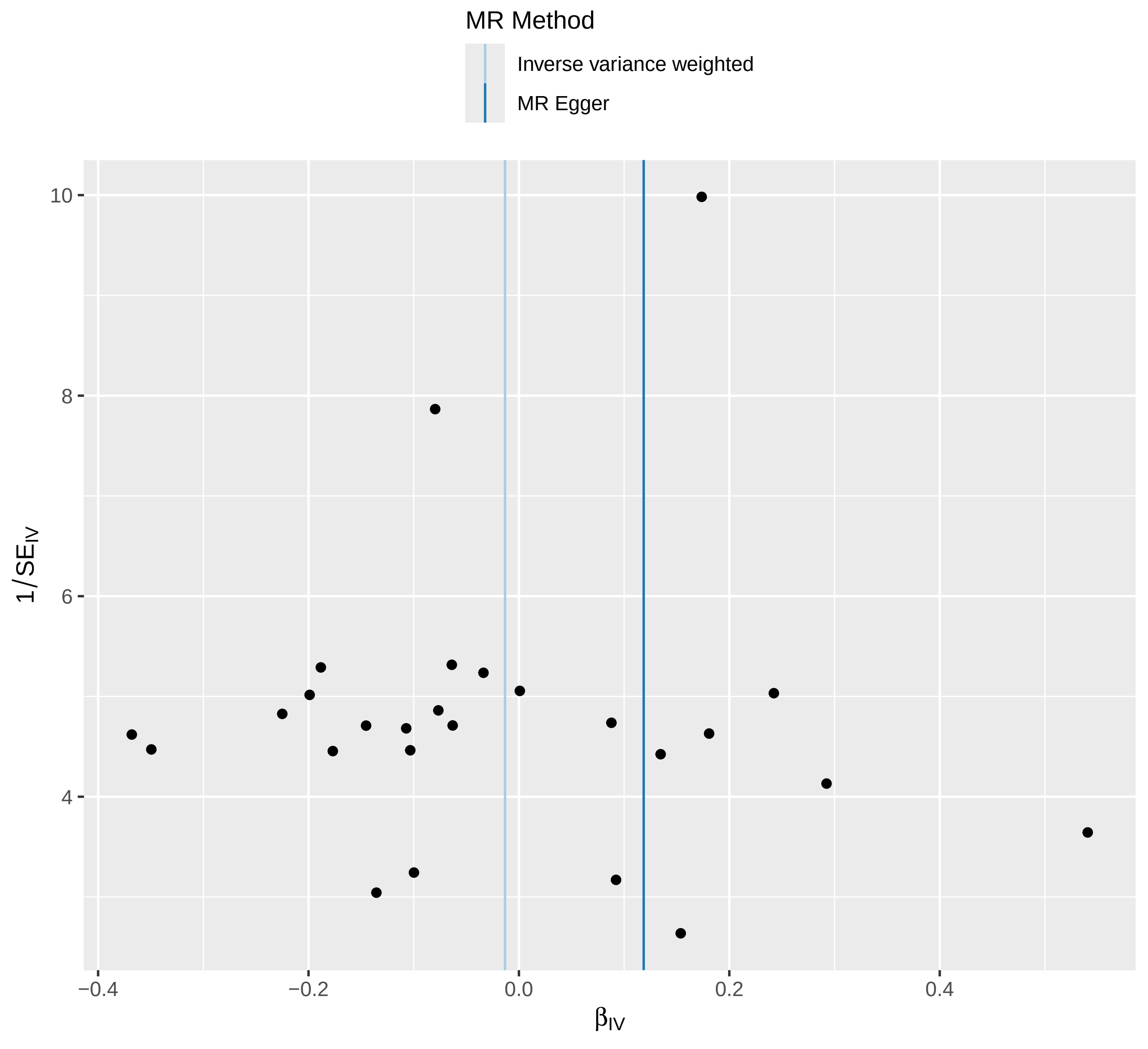
MR Method
Inverse variance weighted
MR Egger
10
8
6
4
1/SE_IV
−0.4
−0.2
0.0
0.2
0.4
β_IV

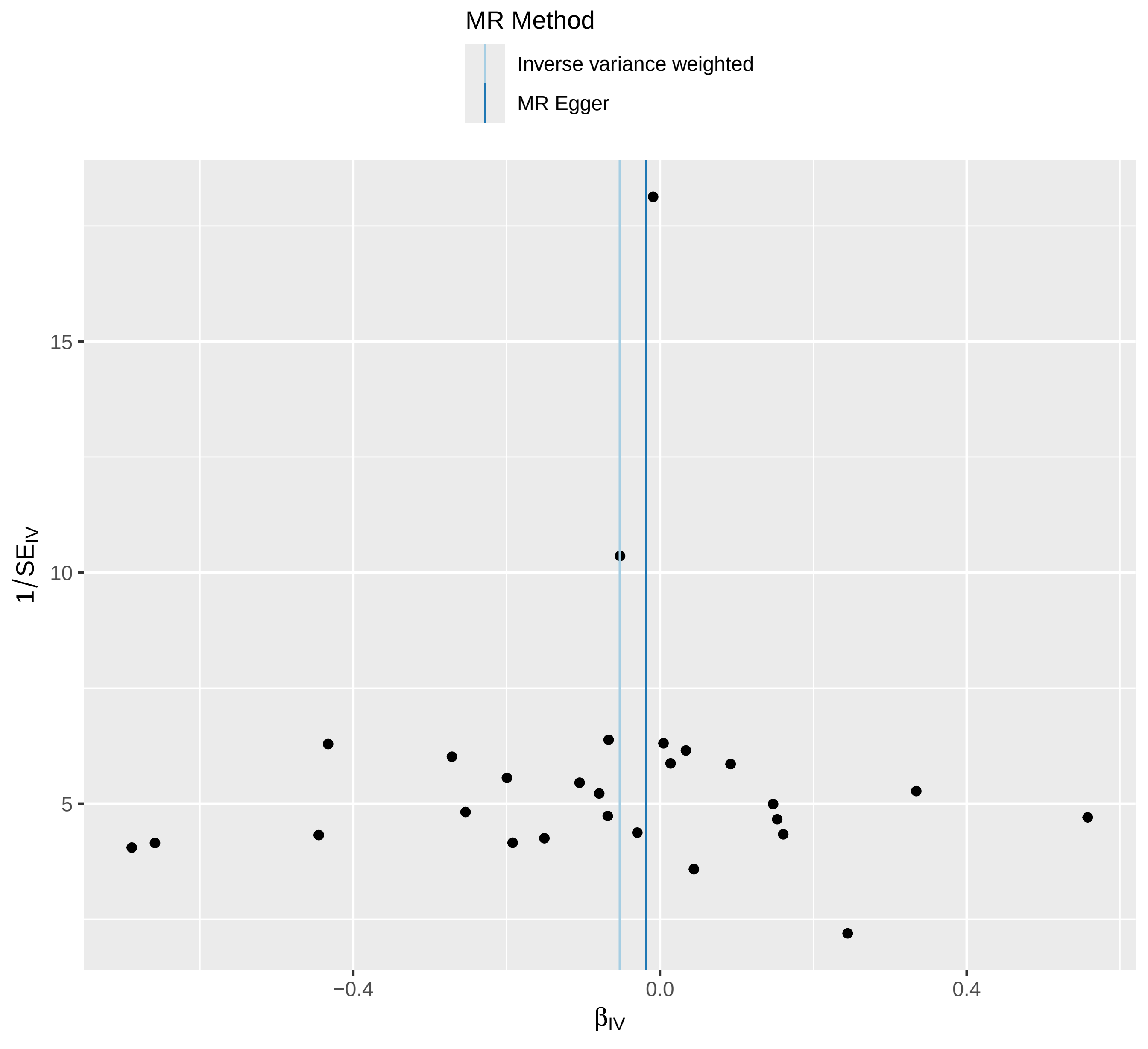
MR Method
Inverse variance weighted
MR Egger
15
10
5
−0.4
0.0
0.4
$1/SE_{IV}$
β_{IV}

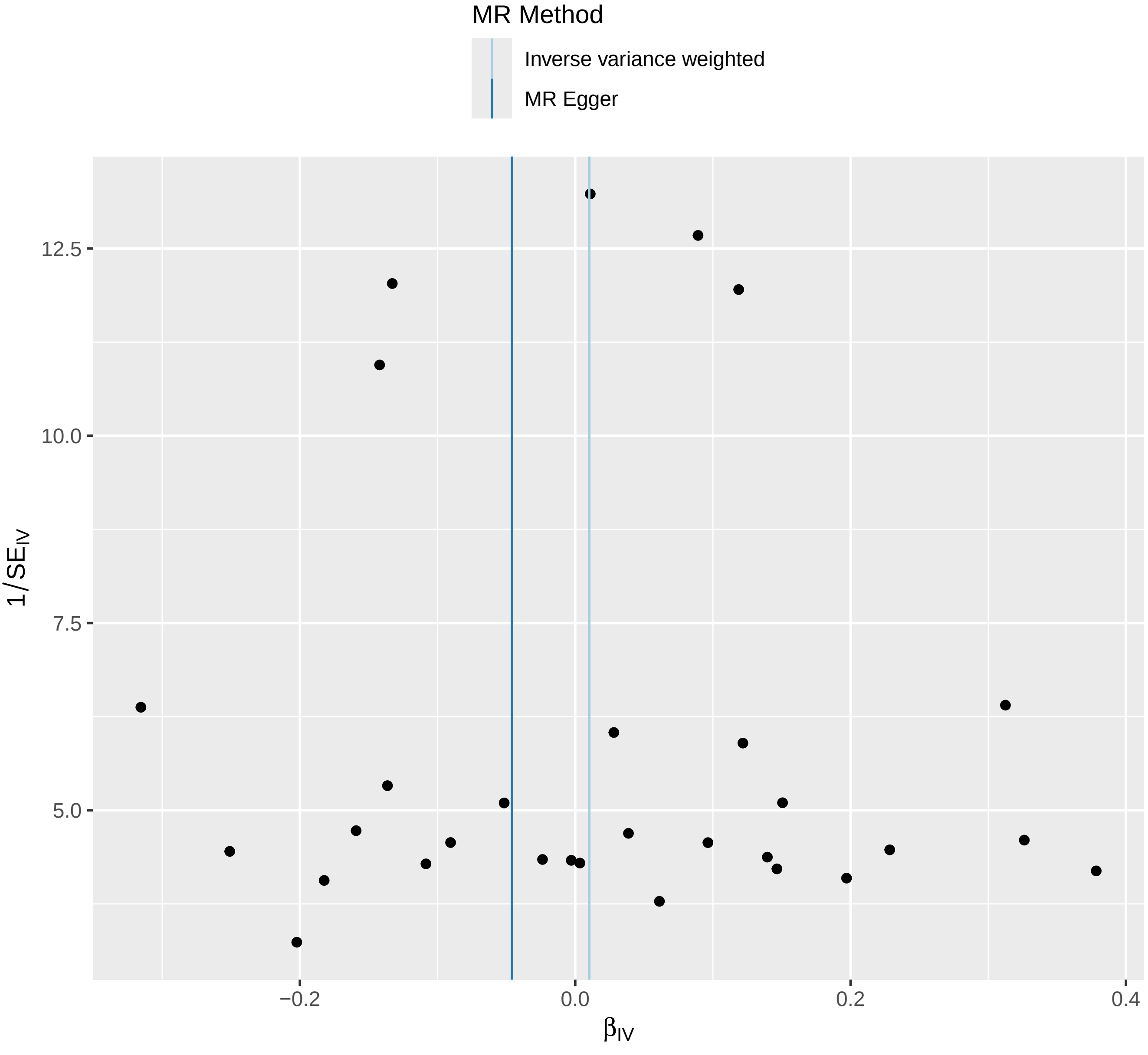
MR Method
Inverse variance weighted
MR Egger
12.5
10.0
7.5
5.0
1/SE_IV
−0.2
0.0
0.2
0.4
β_IV

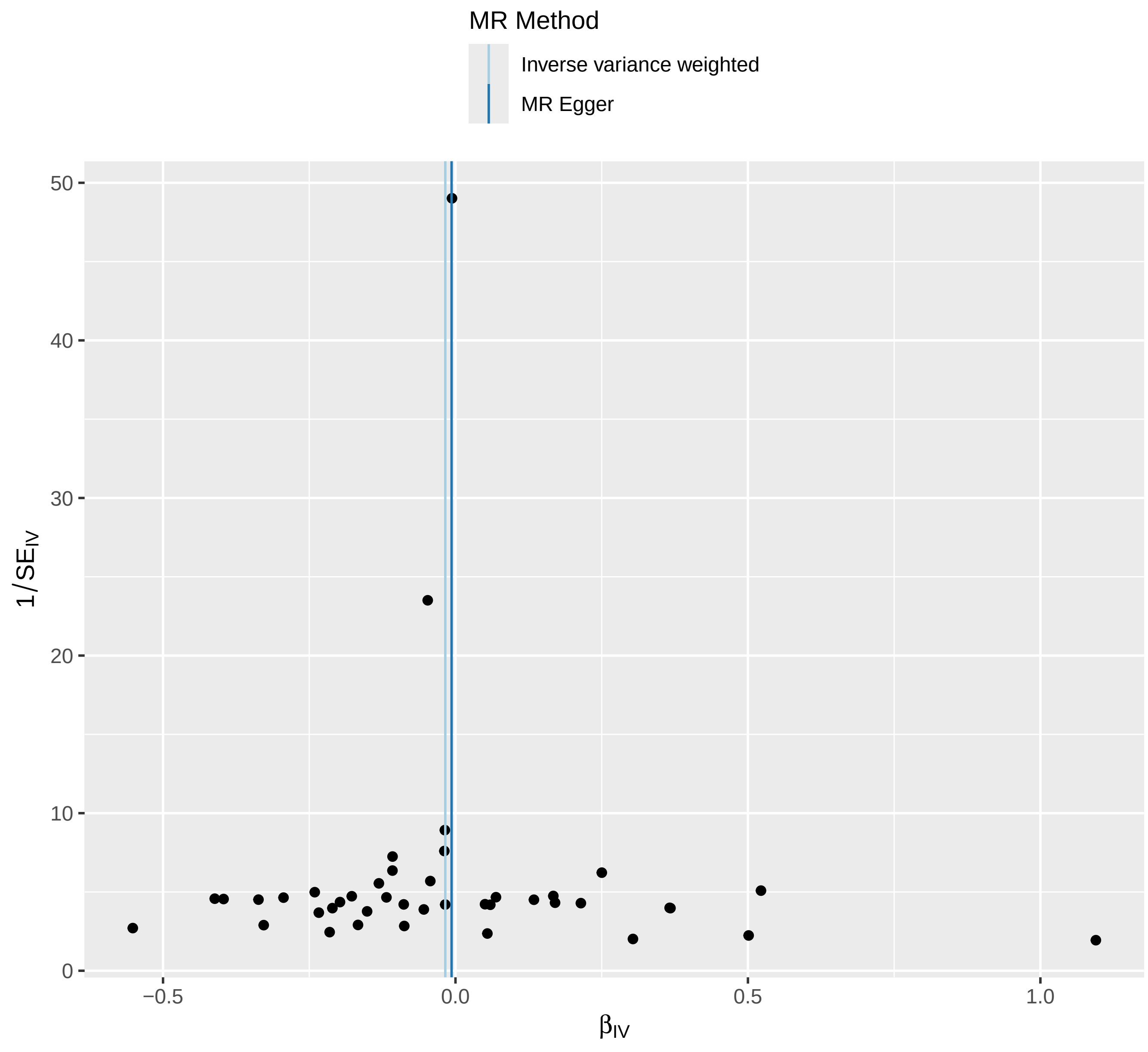
MR Method
Inverse variance weighted
MR Egger
50
40
30
20
10
0
1/SE_IV
−0.5
0.0
0.5
1.0
β_IV

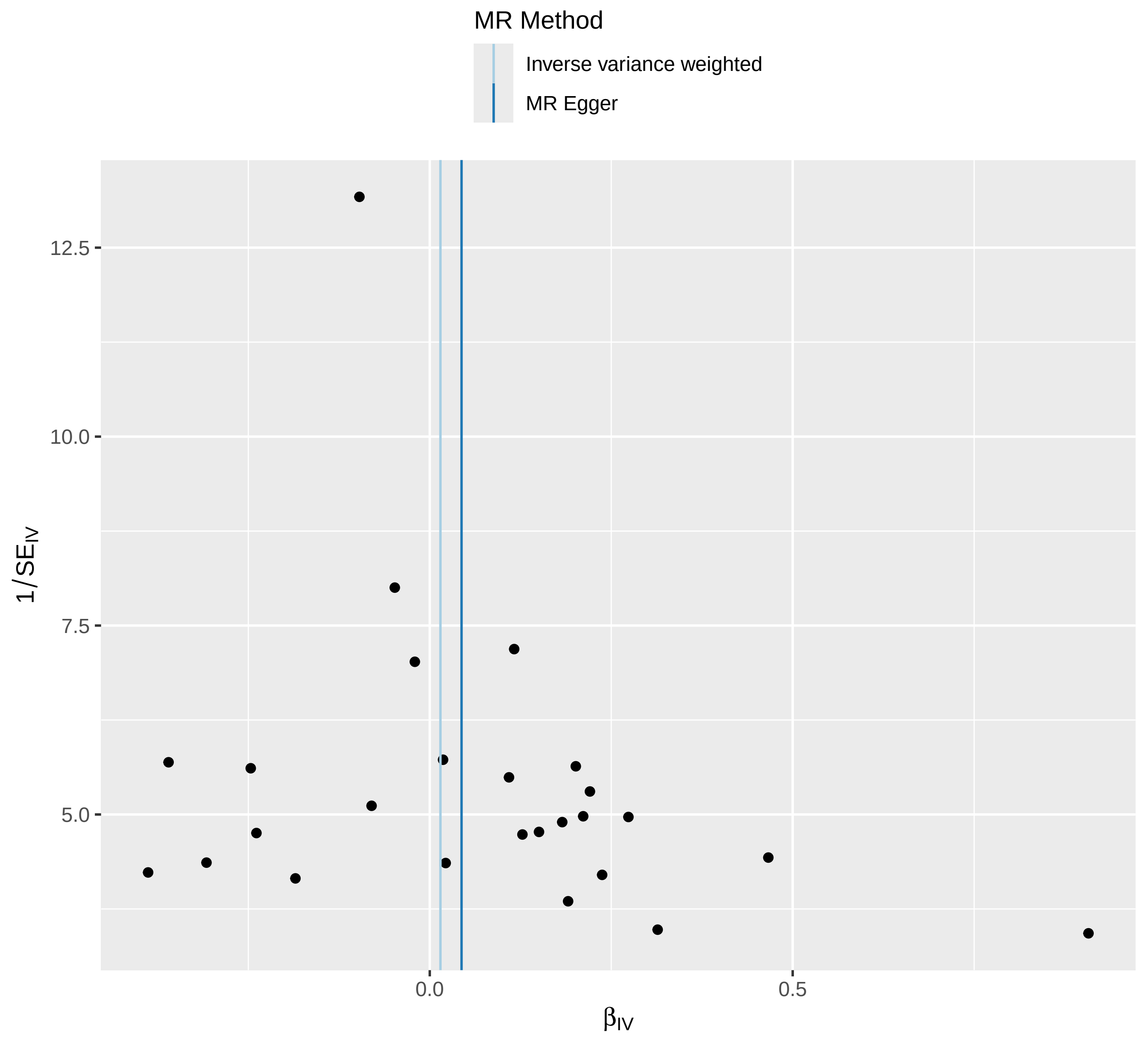
MR Method
Inverse variance weighted
MR Egger
12.5
10.0
7.5
5.0
1/SE_IV
0.0
0.5
β_IV

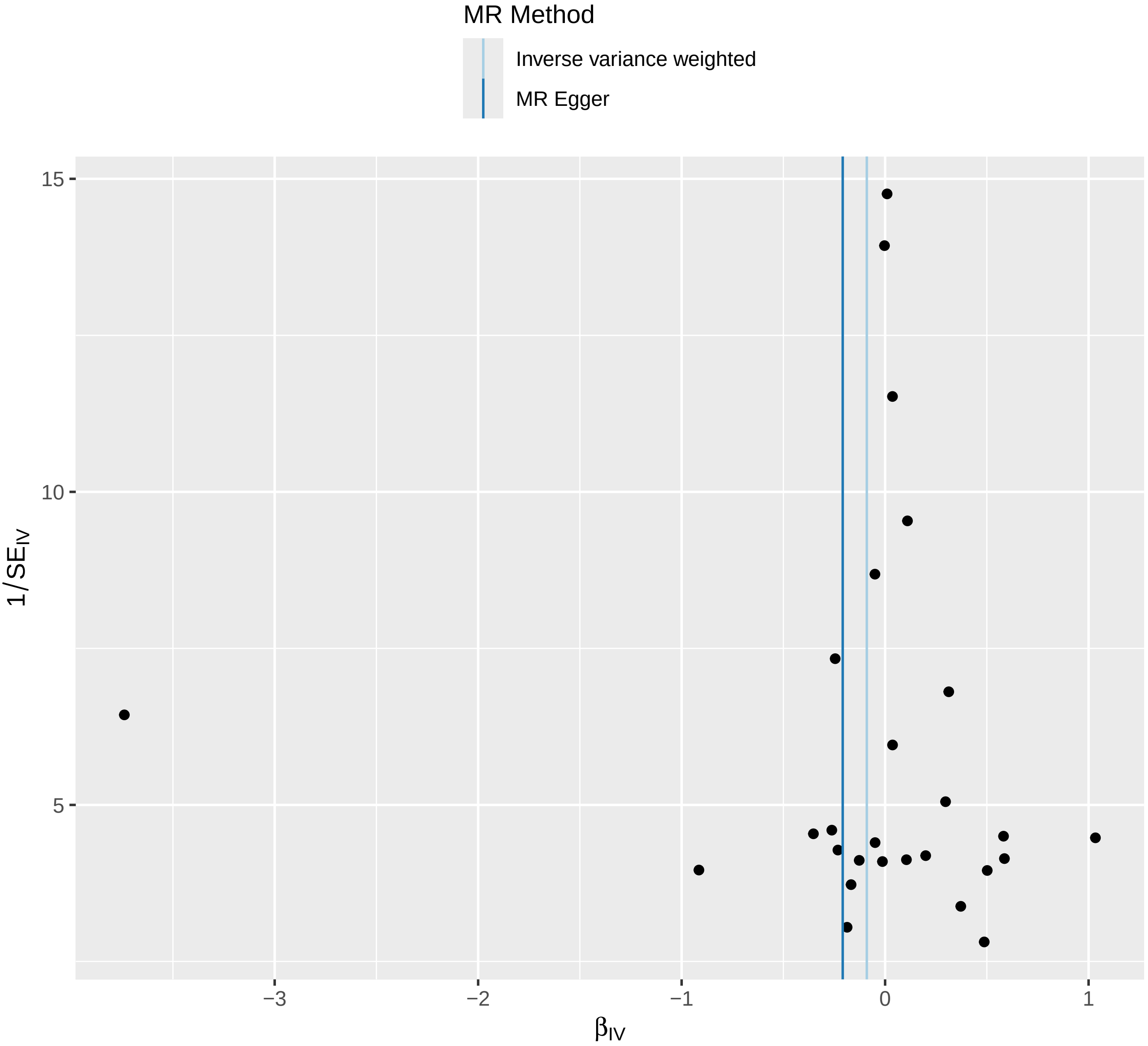
MR Method
Inverse variance weighted
MR Egger
15
10
5
1/SE$_{IV}$
−3
−2
−1
0
1
β$_{IV}$

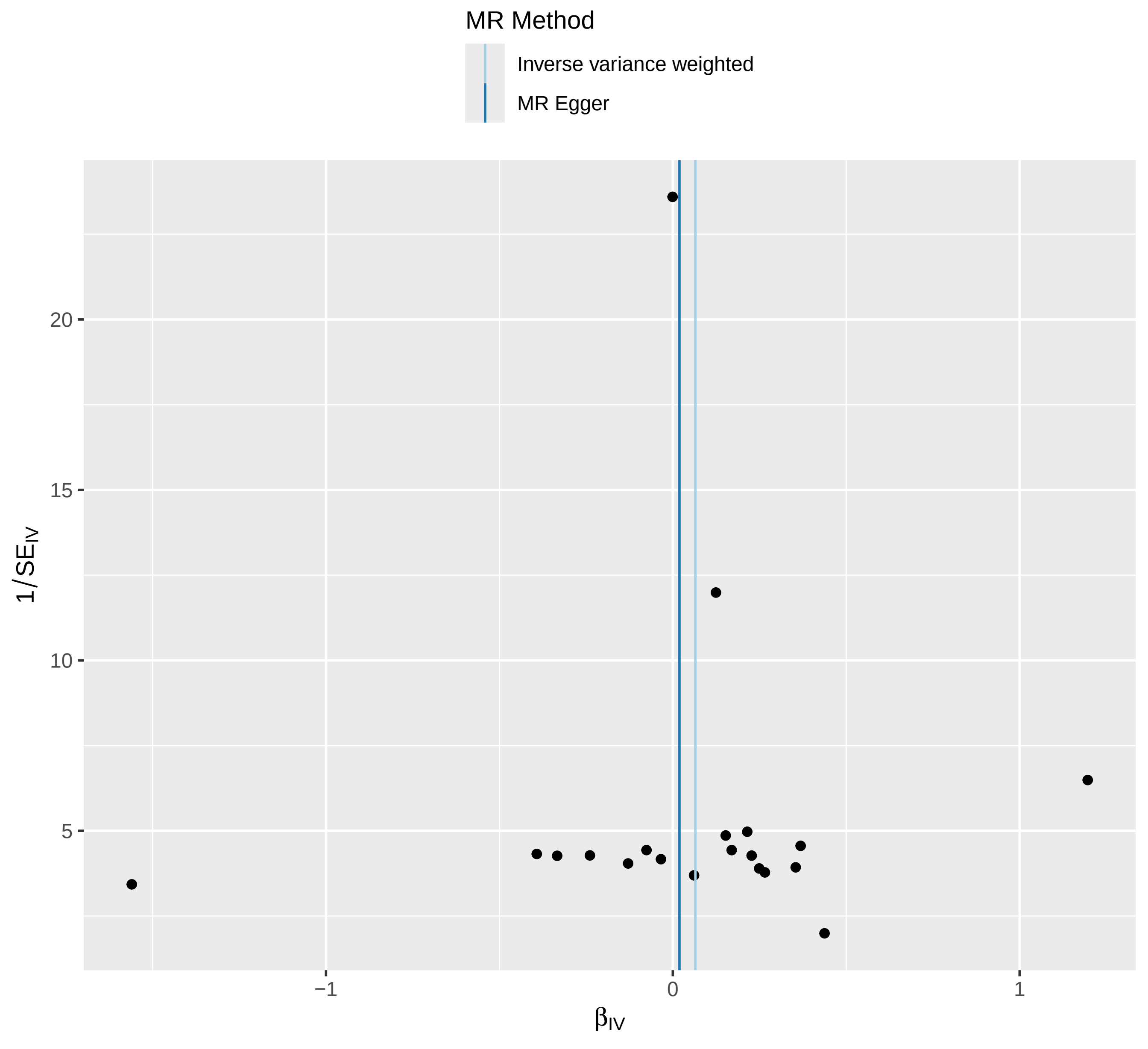
MR Method
Inverse variance weighted
MR Egger
20
15
10
5
1/SE_IV
−1
0
1
β_IV

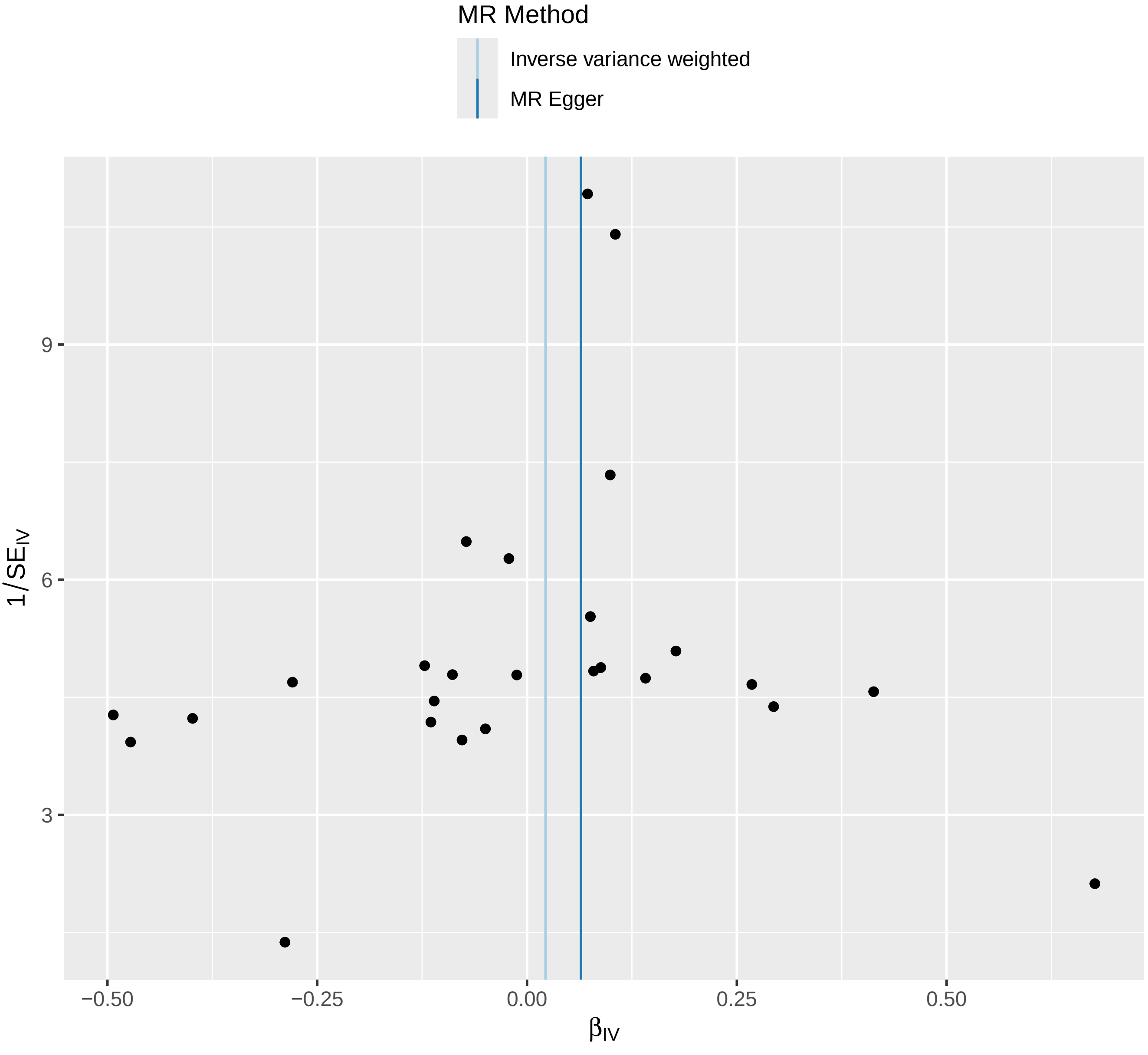
MR Method
Inverse variance weighted
MR Egger
9
6
3
1/SE$_{IV}$
−0.50
−0.25
0.00
0.25
0.50
β$_{IV}$

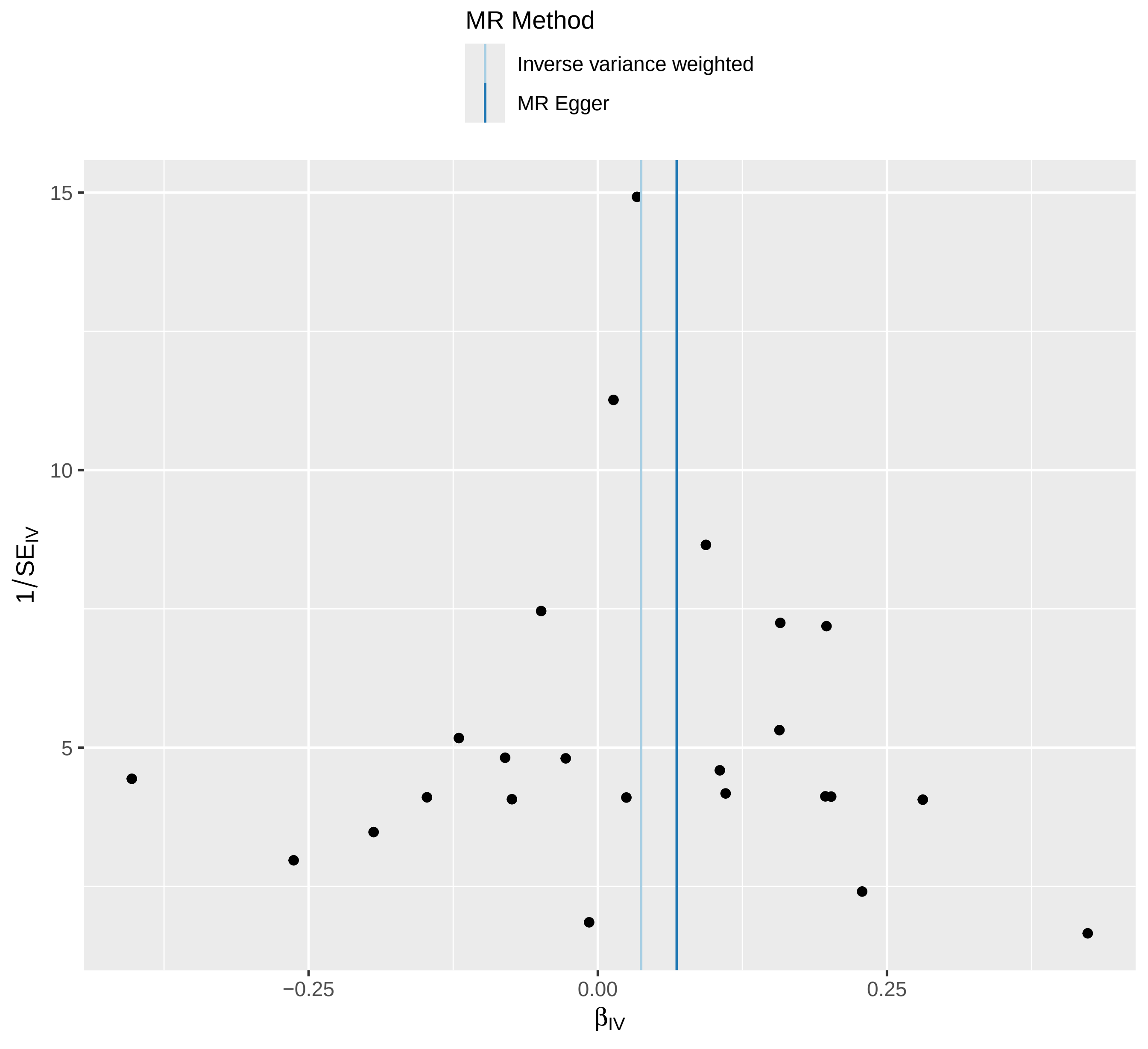
MR Method
Inverse variance weighted
MR Egger
15
10
5
1/SE$_{IV}$
−0.25
0.00
0.25
β$_{IV}$

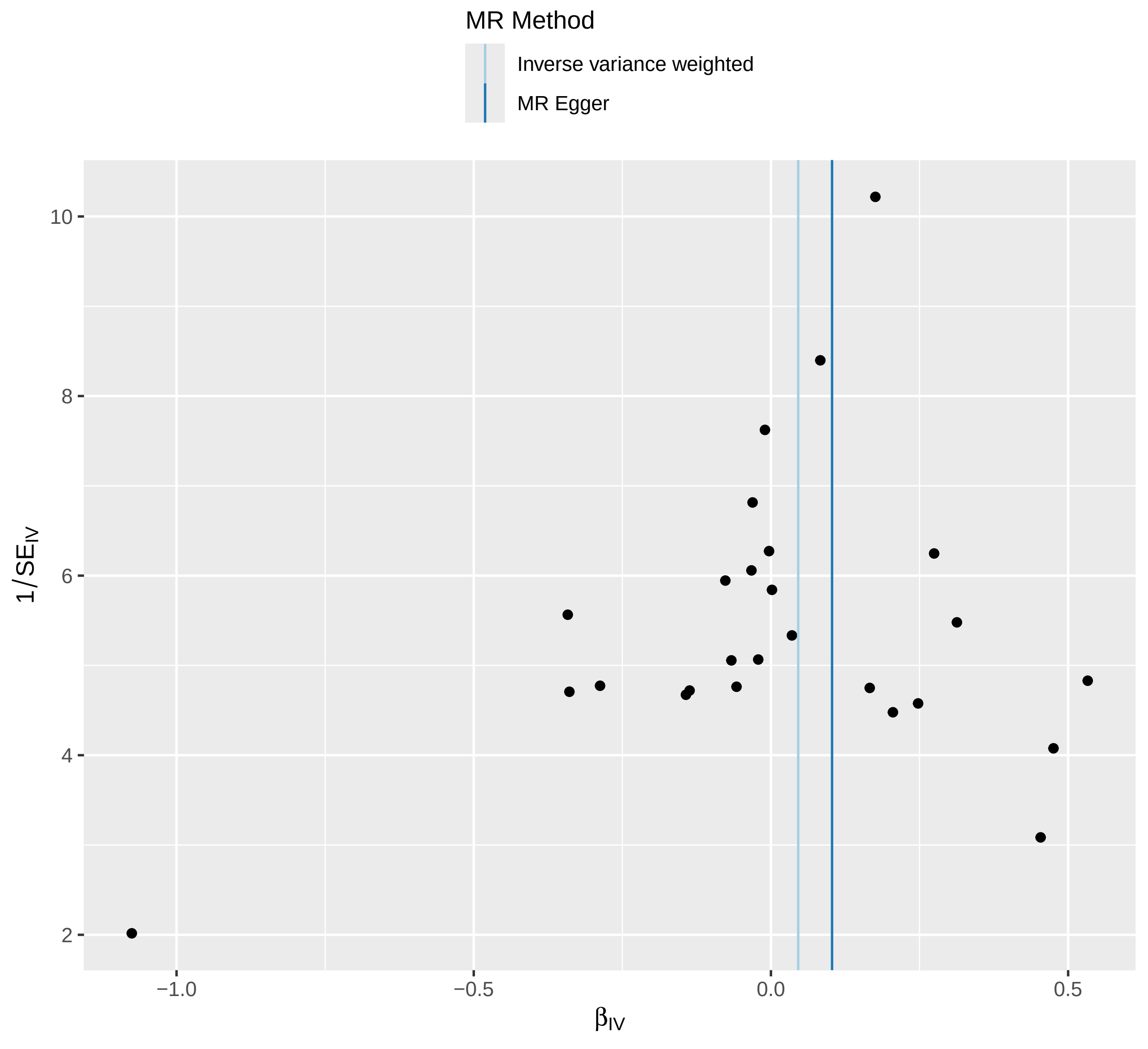

MR Method
Inverse variance weighted
MR Egger
10
8
6
4
2
1/SE_IV
−1.0
−0.5
0.0
0.5
β_IV

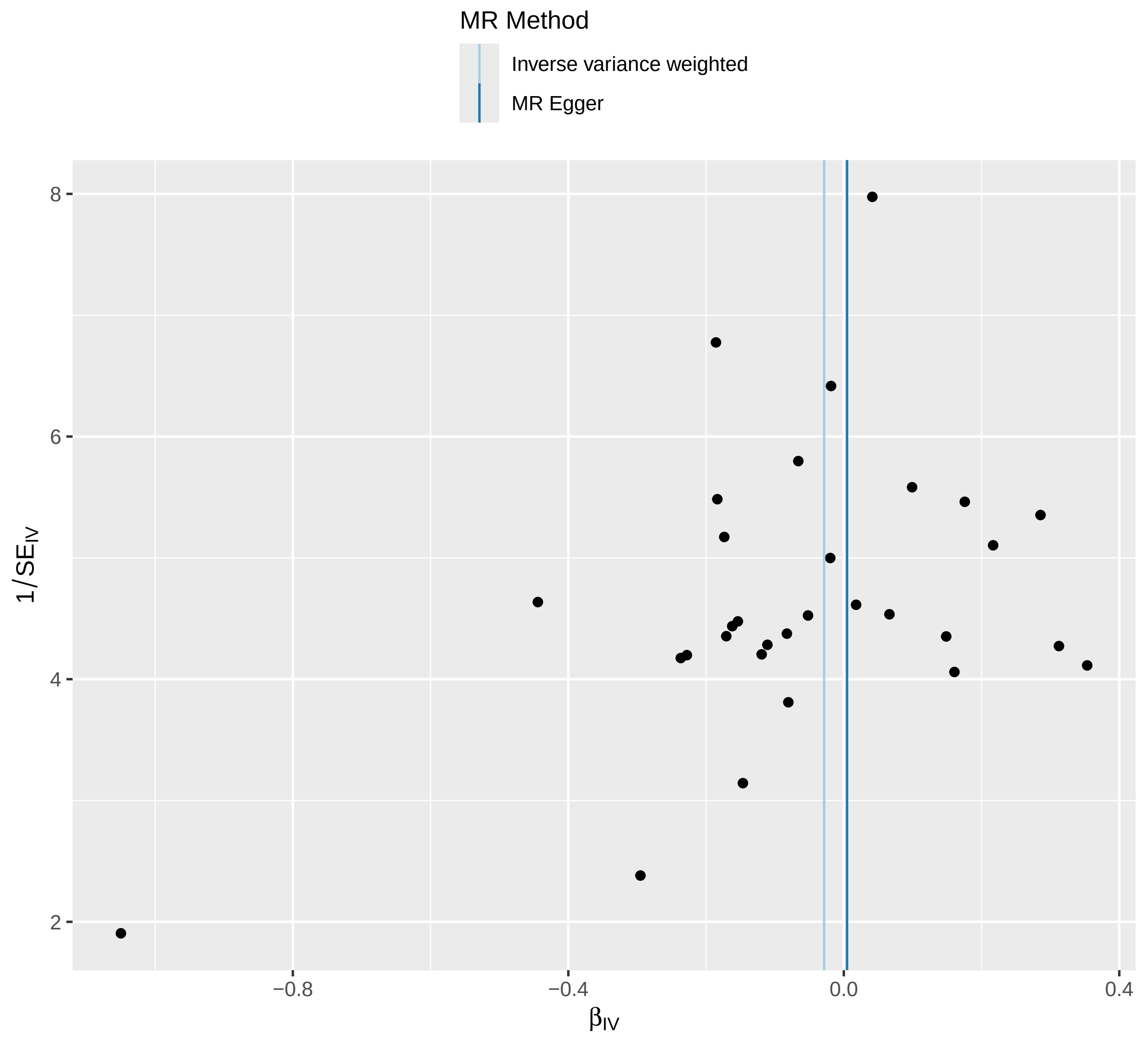
MR Method
Inverse variance weighted
MR Egger
8
6
4
2
$1/SE_{IV}$
−0.8
−0.4
0.0
0.4
β_{IV}

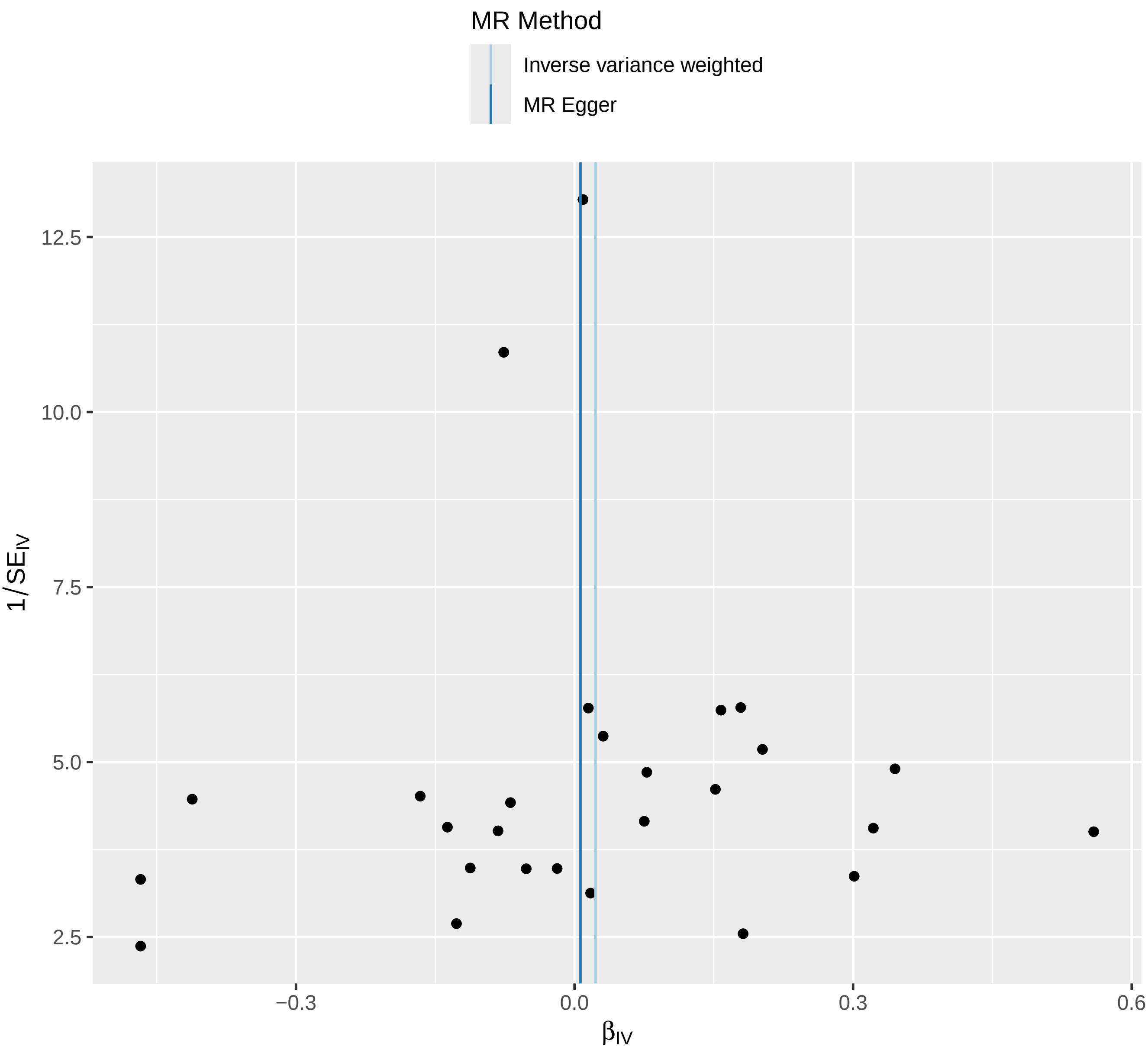
MR Method
Inverse variance weighted
MR Egger
12.5
10.0
7.5
5.0
2.5
1/SE_IV
−0.3
0.0
0.3
0.6
β_IV

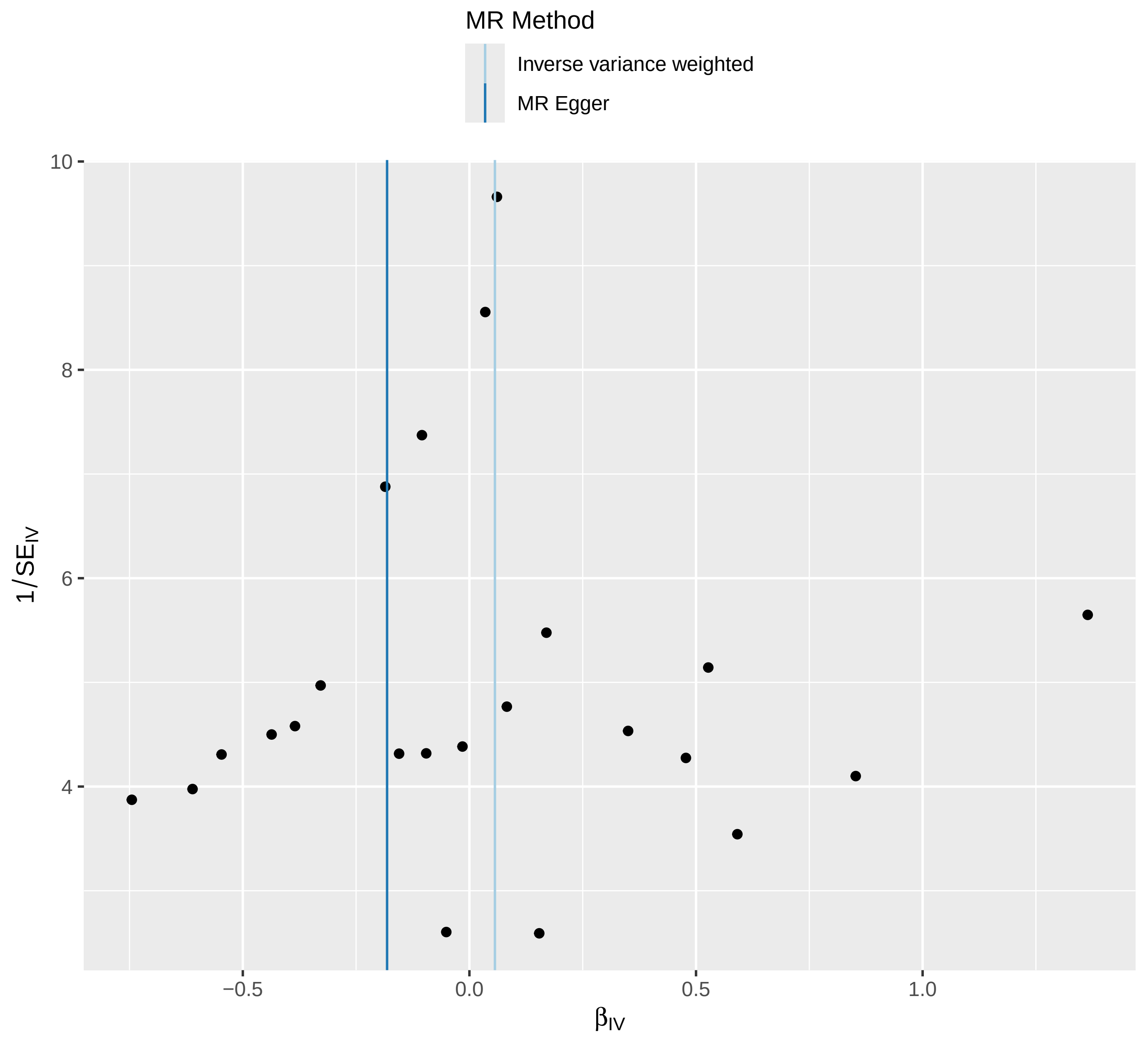
MR Method
Inverse variance weighted
MR Egger
10
8
6
4
−0.5
0.0
0.5
1.0
$1/SE_{IV}$
β_{IV}

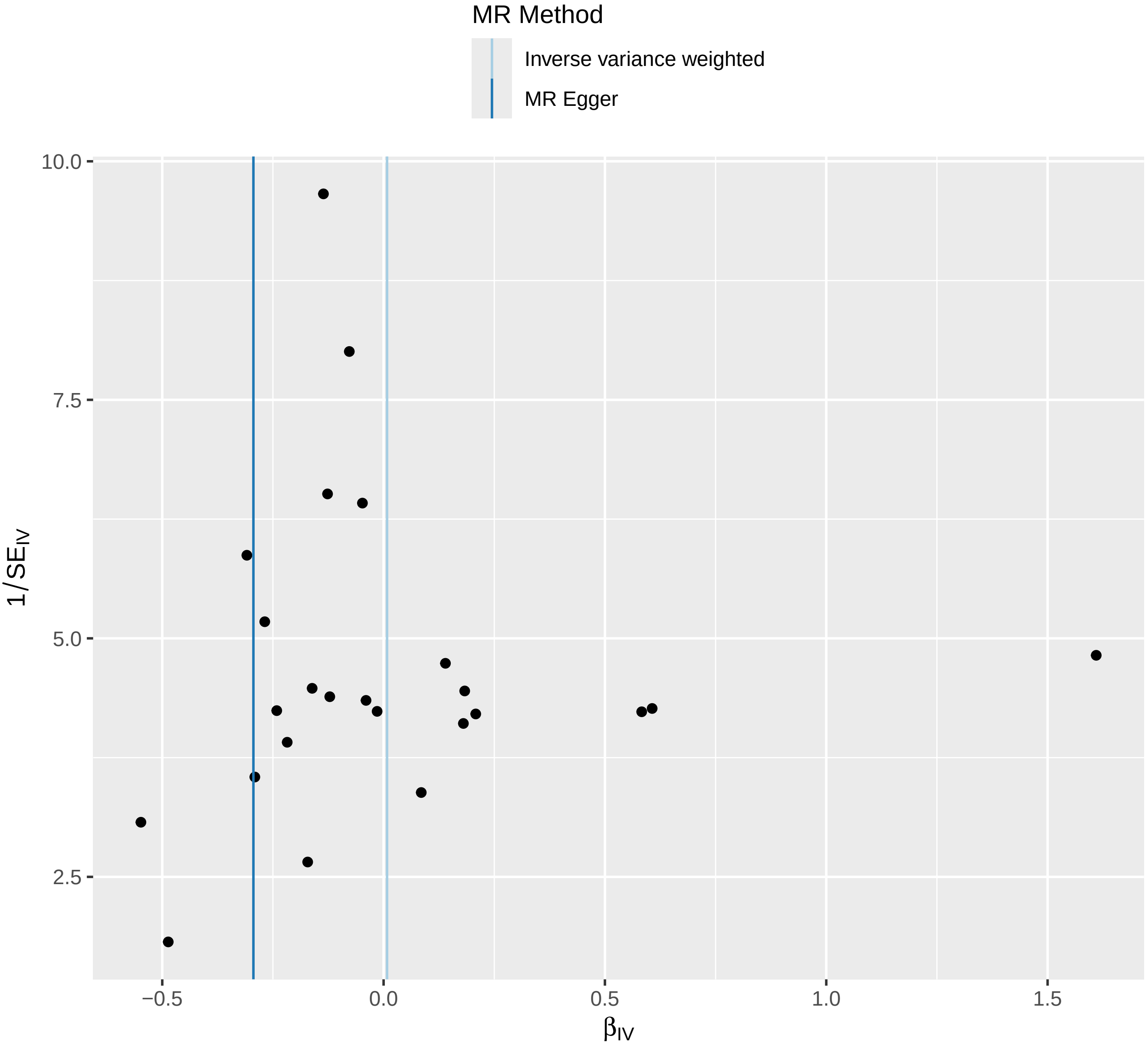
MR Method
Inverse variance weighted
MR Egger
10.0
7.5
5.0
2.5
1/SE_{IV}
−0.5
0.0
0.5
1.0
1.5
β_{IV}

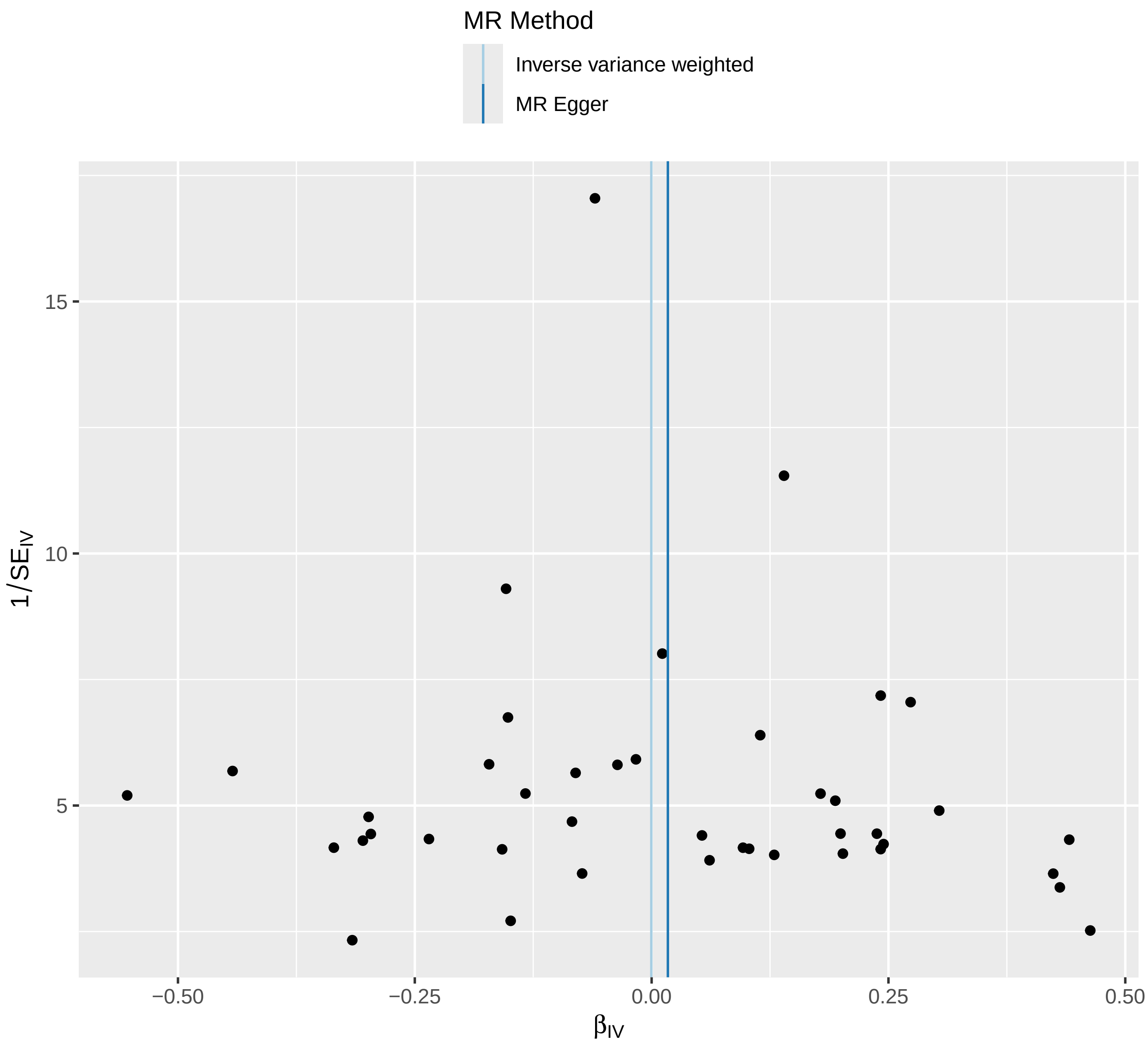
MR Method
Inverse variance weighted
MR Egger
15
10
5
1/SE$_{IV}$
−0.50
−0.25
0.00
0.25
0.50
β$_{IV}$

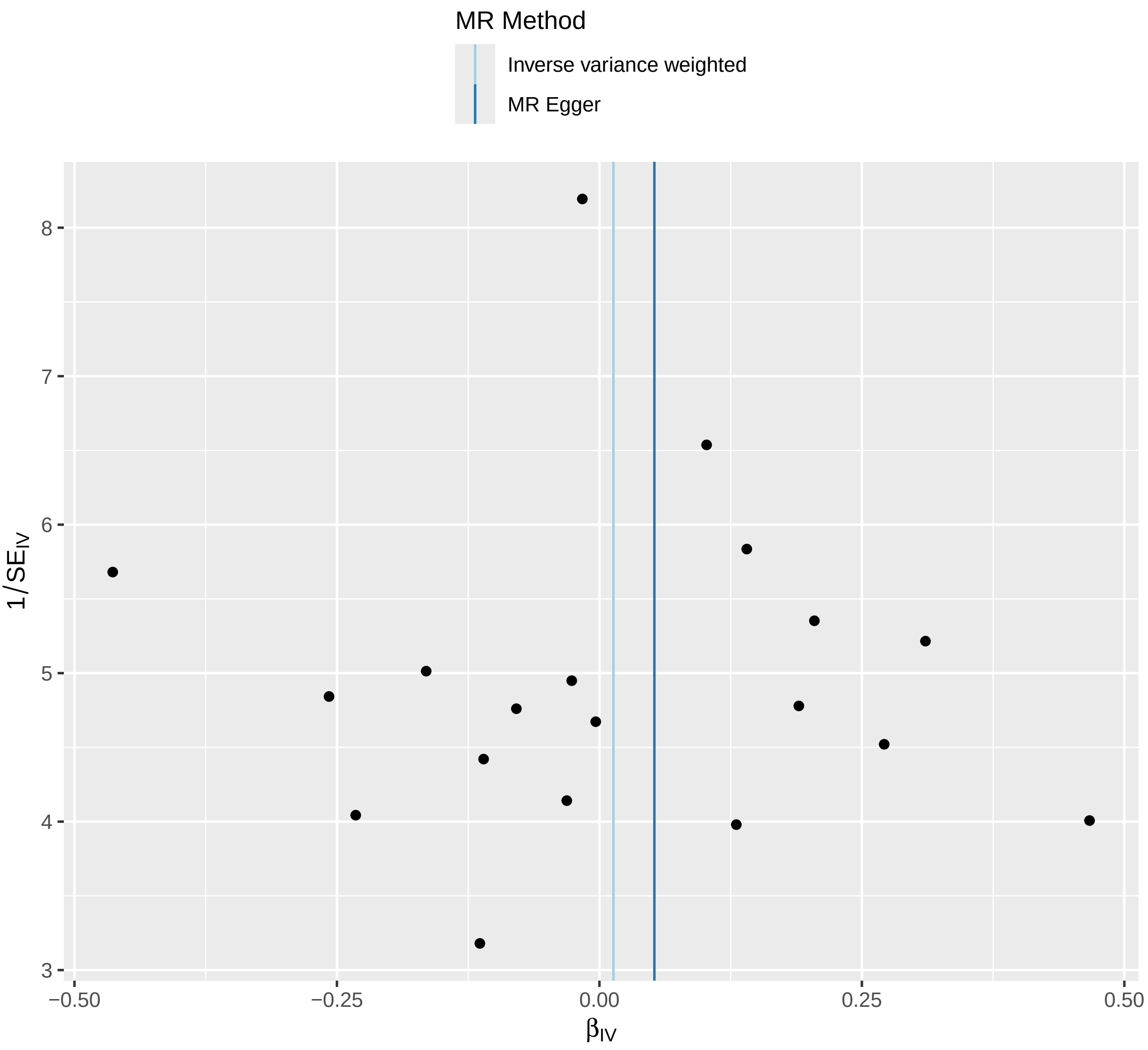

MR Method
Inverse variance weighted
MR Egger
8
7
6
5
4
3
1/SE_IV
−0.50
−0.25
0.00
0.25
0.50
β_IV

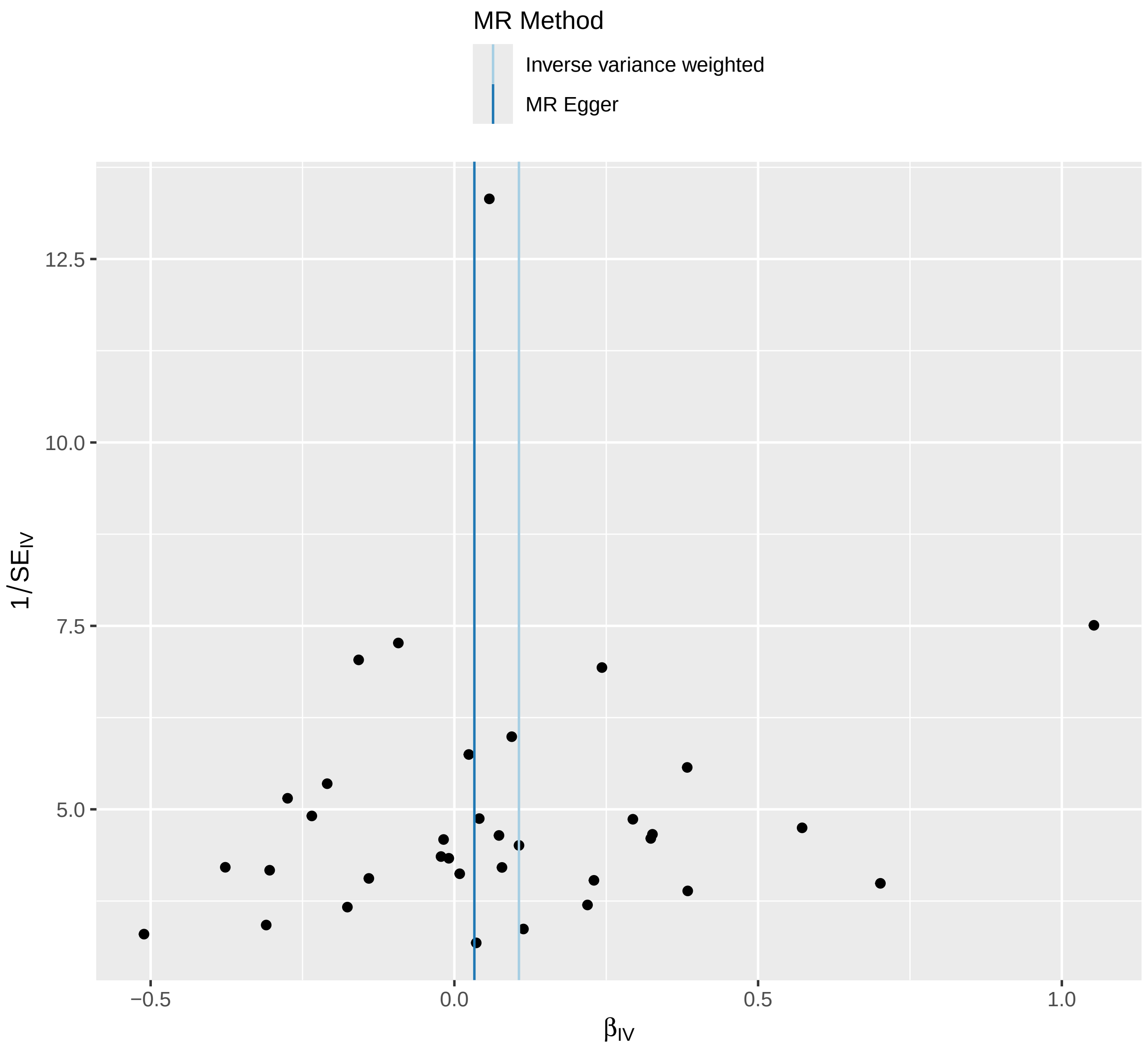

MR Method
Inverse variance weighted
MR Egger
12.5
10.0
7.5
5.0
1/SE$_{IV}$
−0.5
0.0
0.5
1.0
β$_{IV}$

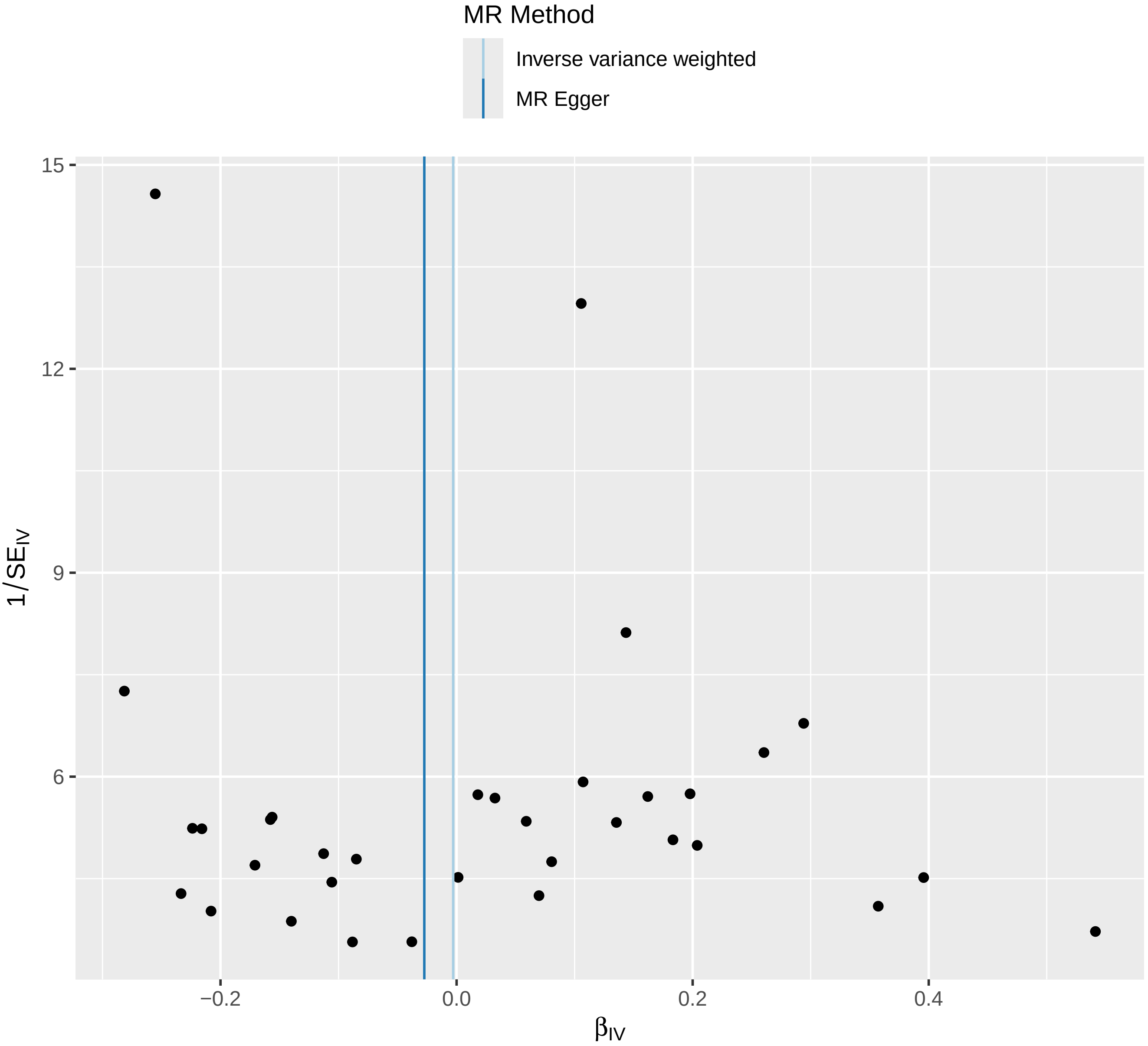
MR Method
Inverse variance weighted
MR Egger
15
12
9
6
$1/SE_{IV}$
−0.2
0.0
0.2
0.4
β_{IV}

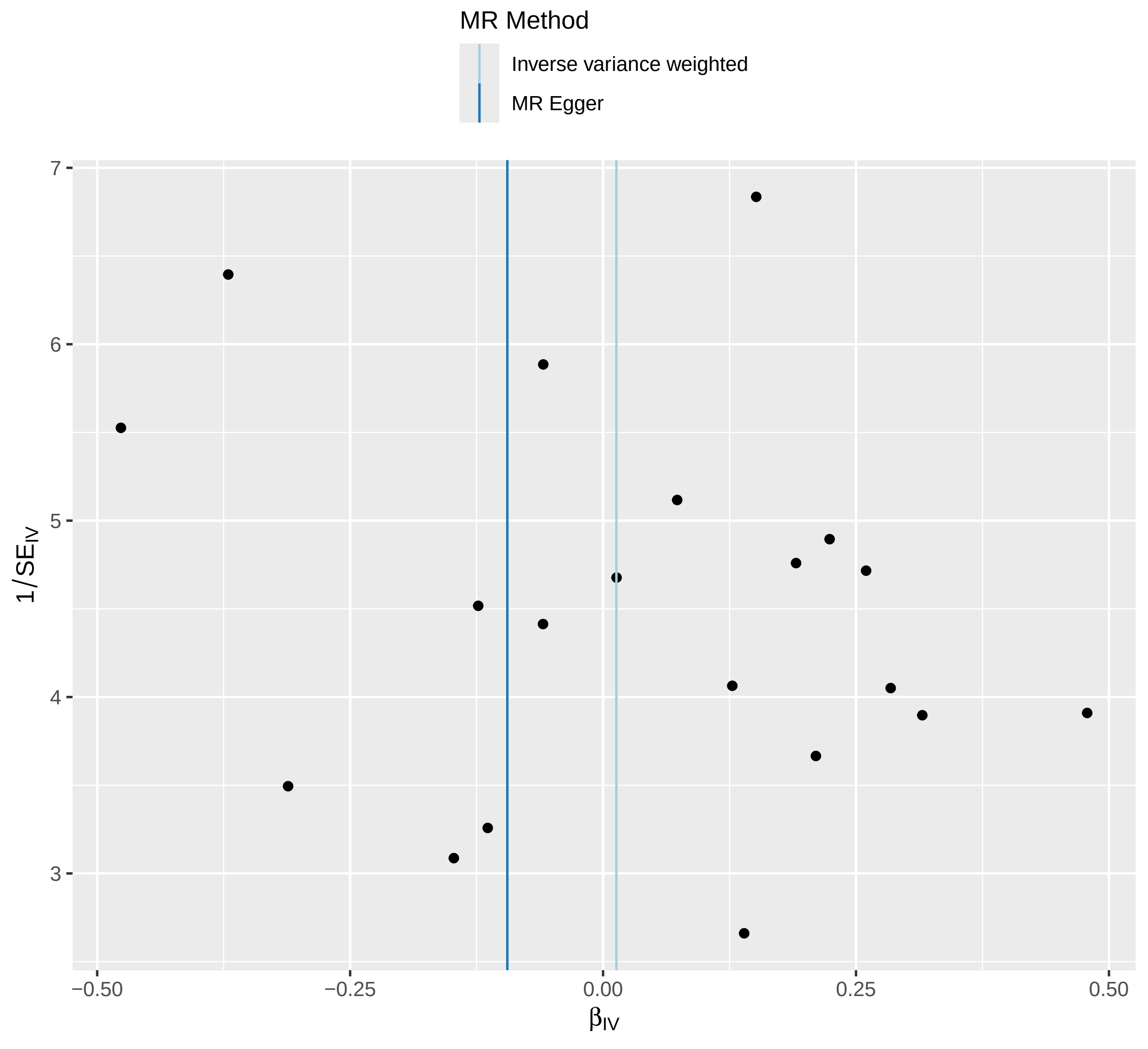

MR Method
Inverse variance weighted
MR Egger
7
6
5
4
3
1/SE_IV
−0.50
−0.25
0.00
0.25
0.50
β_IV

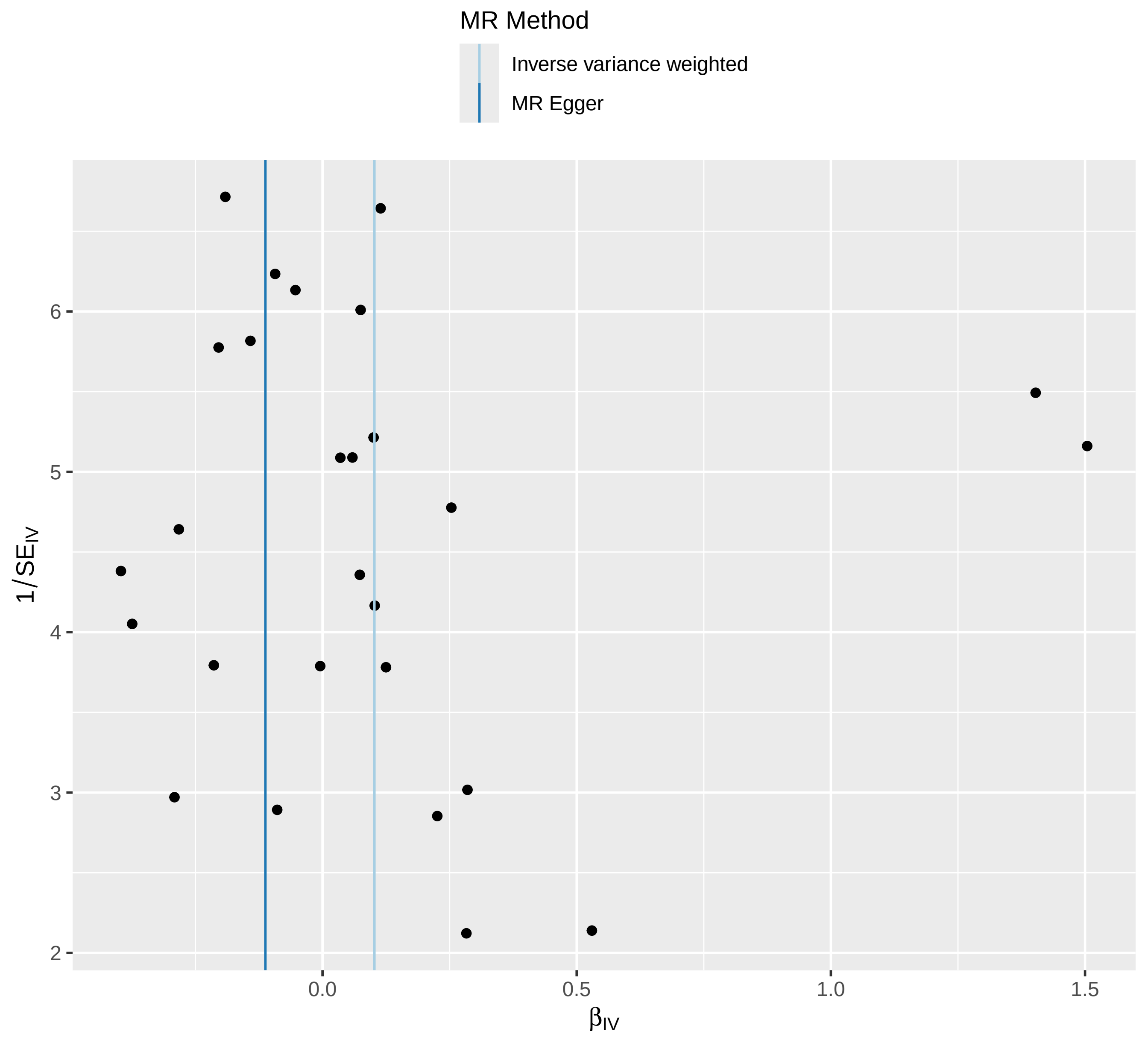
MR Method
Inverse variance weighted
MR Egger
1/SE$_{IV}$
6
5
4
3
2
0.0
0.5
1.0
1.5
β$_{IV}$

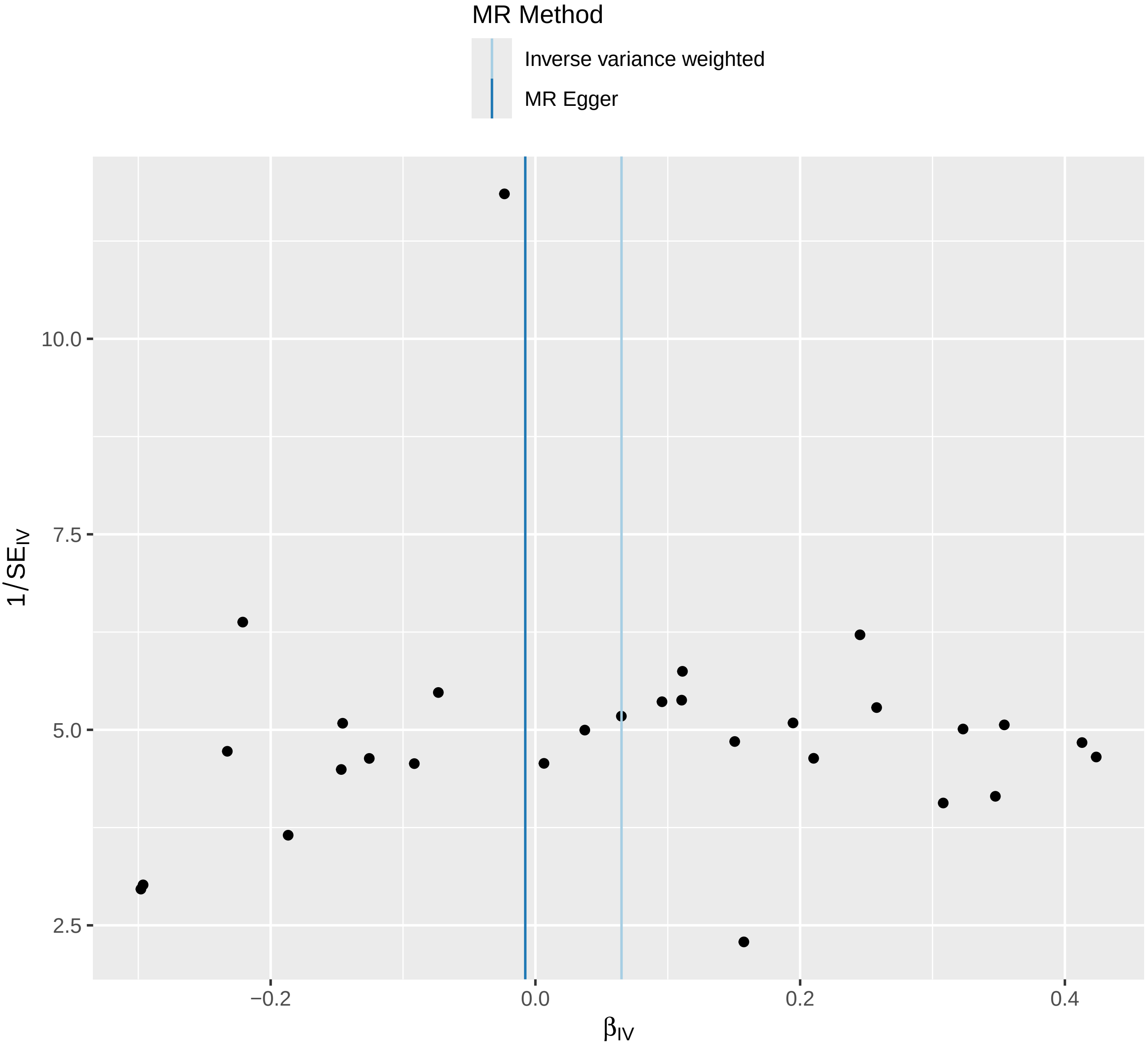
MR Method
Inverse variance weighted
MR Egger
10.0
7.5
5.0
2.5
$1/SE_{IV}$
−0.2
0.0
0.2
0.4
β_{IV}

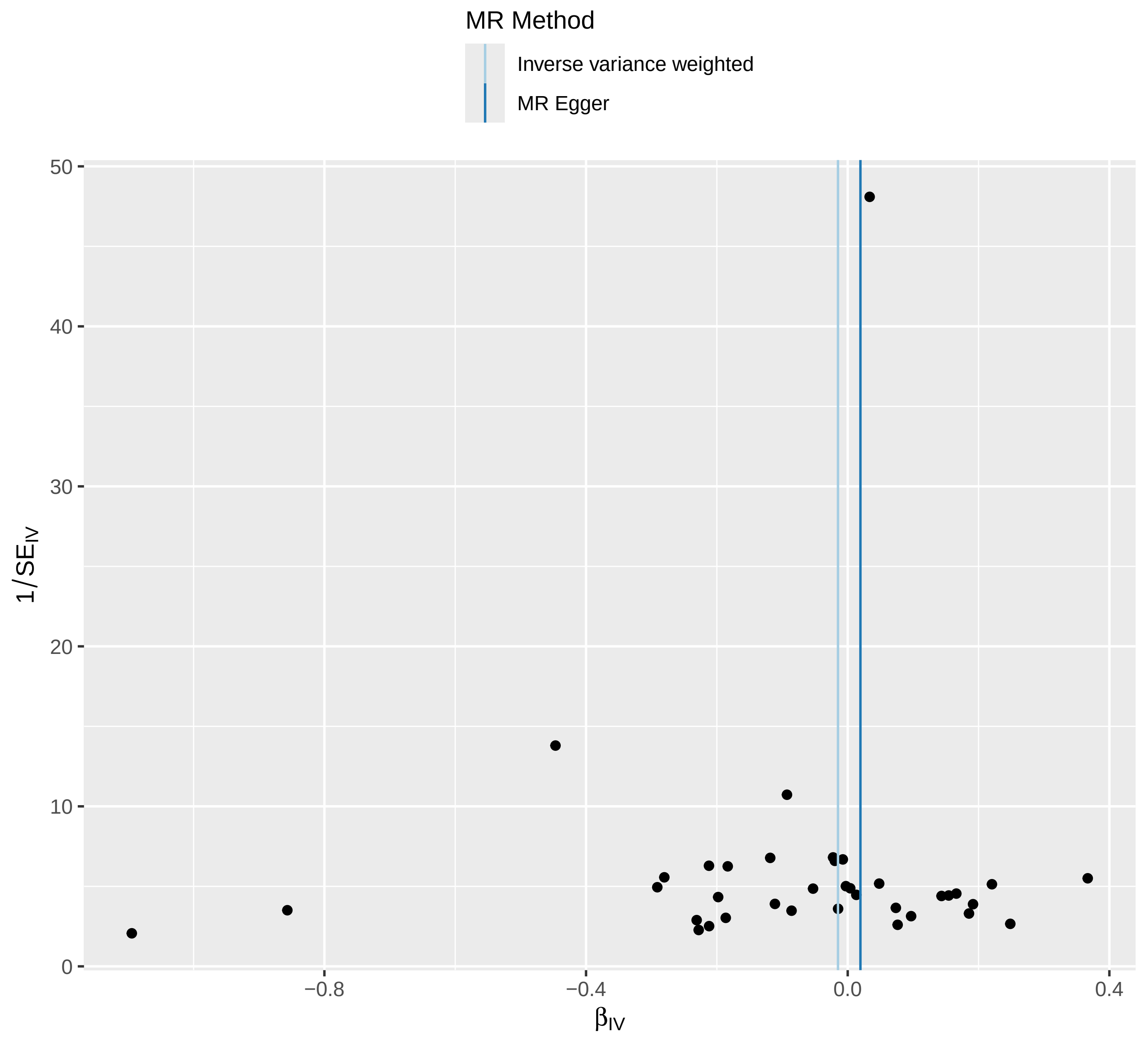

MR Method
Inverse variance weighted
MR Egger
50
40
30
20
10
0
1/SE_IV
−0.8
−0.4
0.0
0.4
β_IV

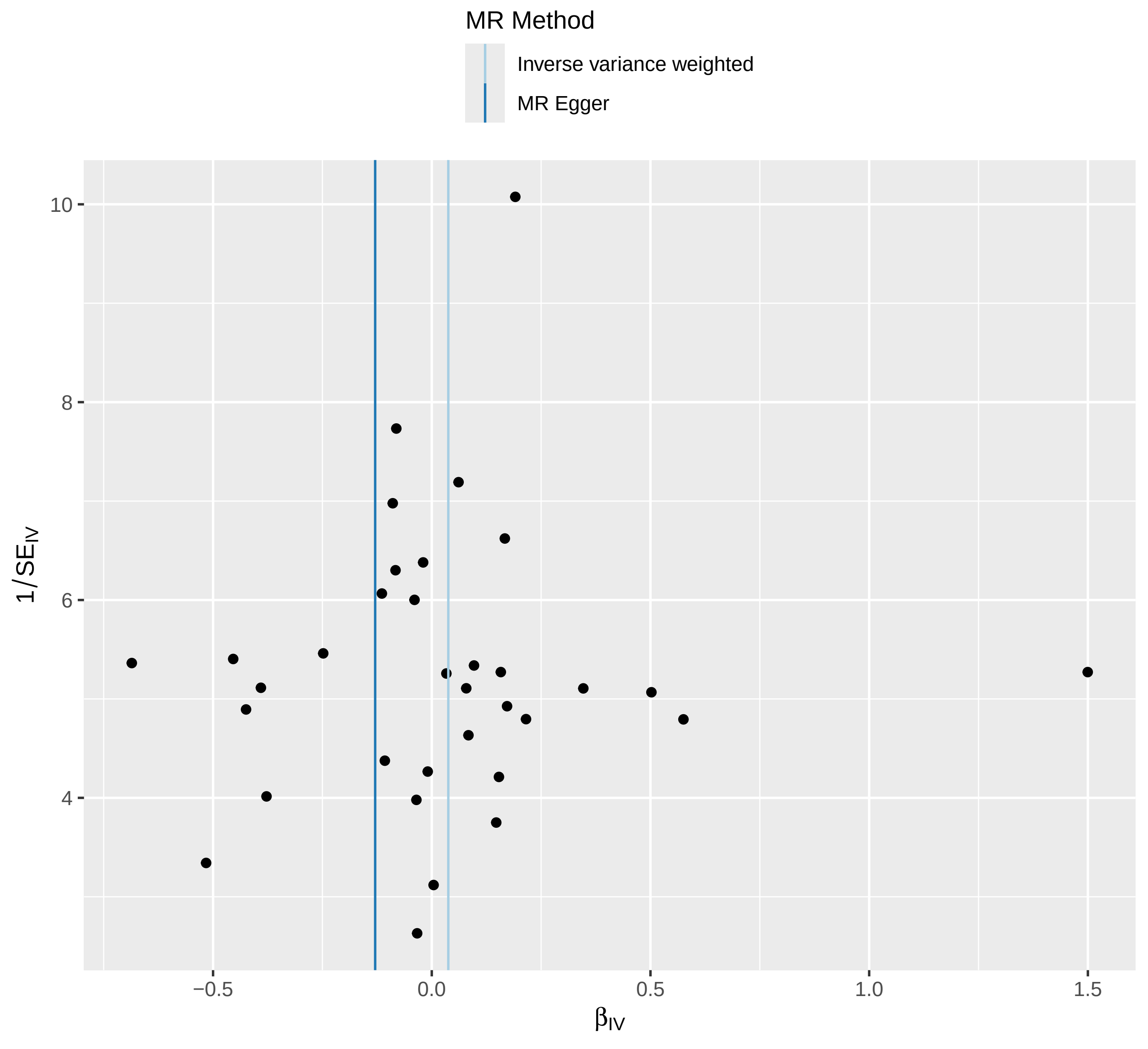

MR Method
Inverse variance weighted
MR Egger
10
8
6
4
1/SE$_{IV}$
−0.5
0.0
0.5
1.0
1.5
β$_{IV}$

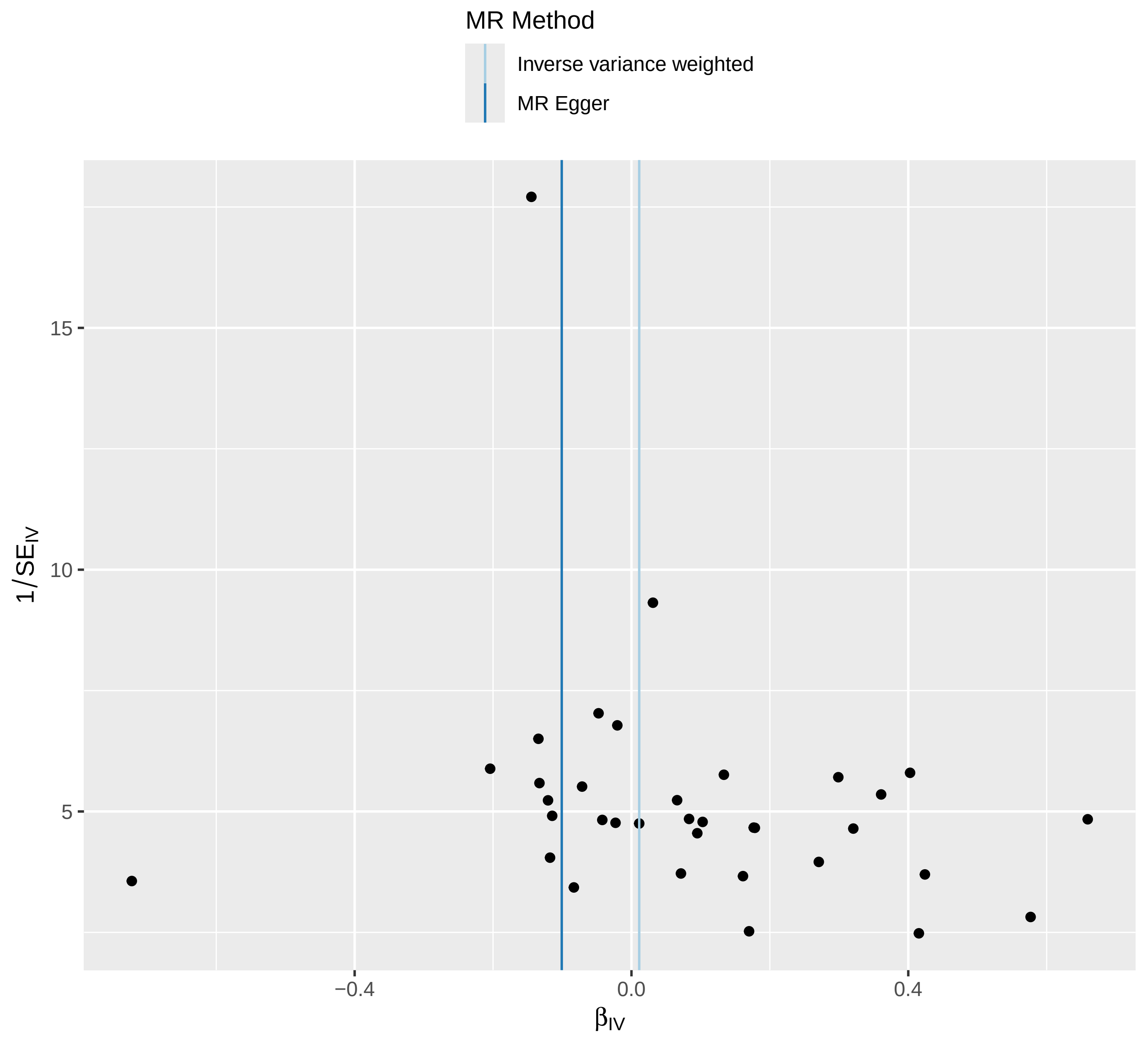

MR Method
Inverse variance weighted
MR Egger
15
10
5
1/SE_IV
−0.4
0.0
0.4
β_IV

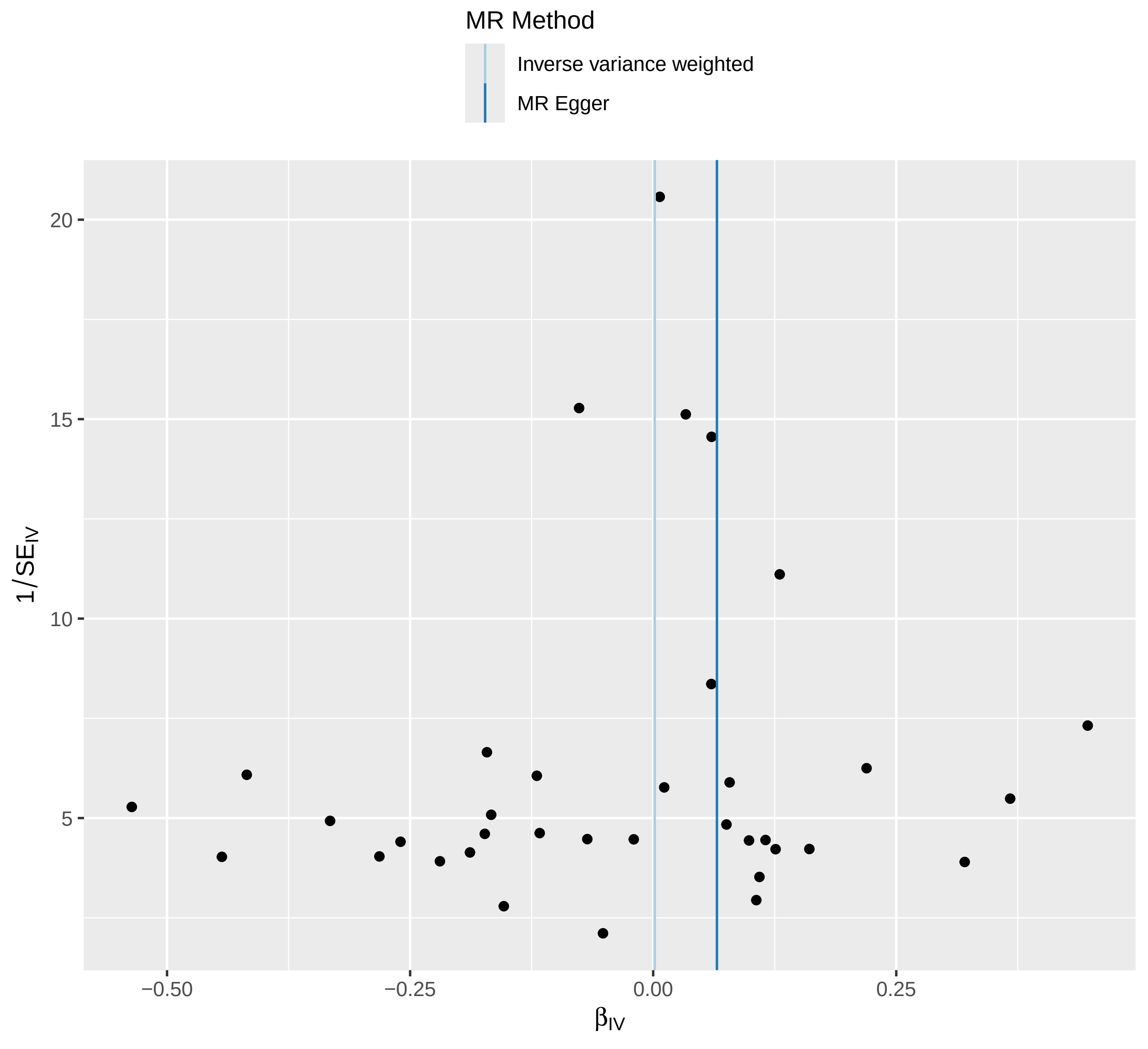

MR Method
Inverse variance weighted
MR Egger
1/SE$_{IV}$
20
15
10
5
−0.50
−0.25
0.00
0.25
β$_{IV}$

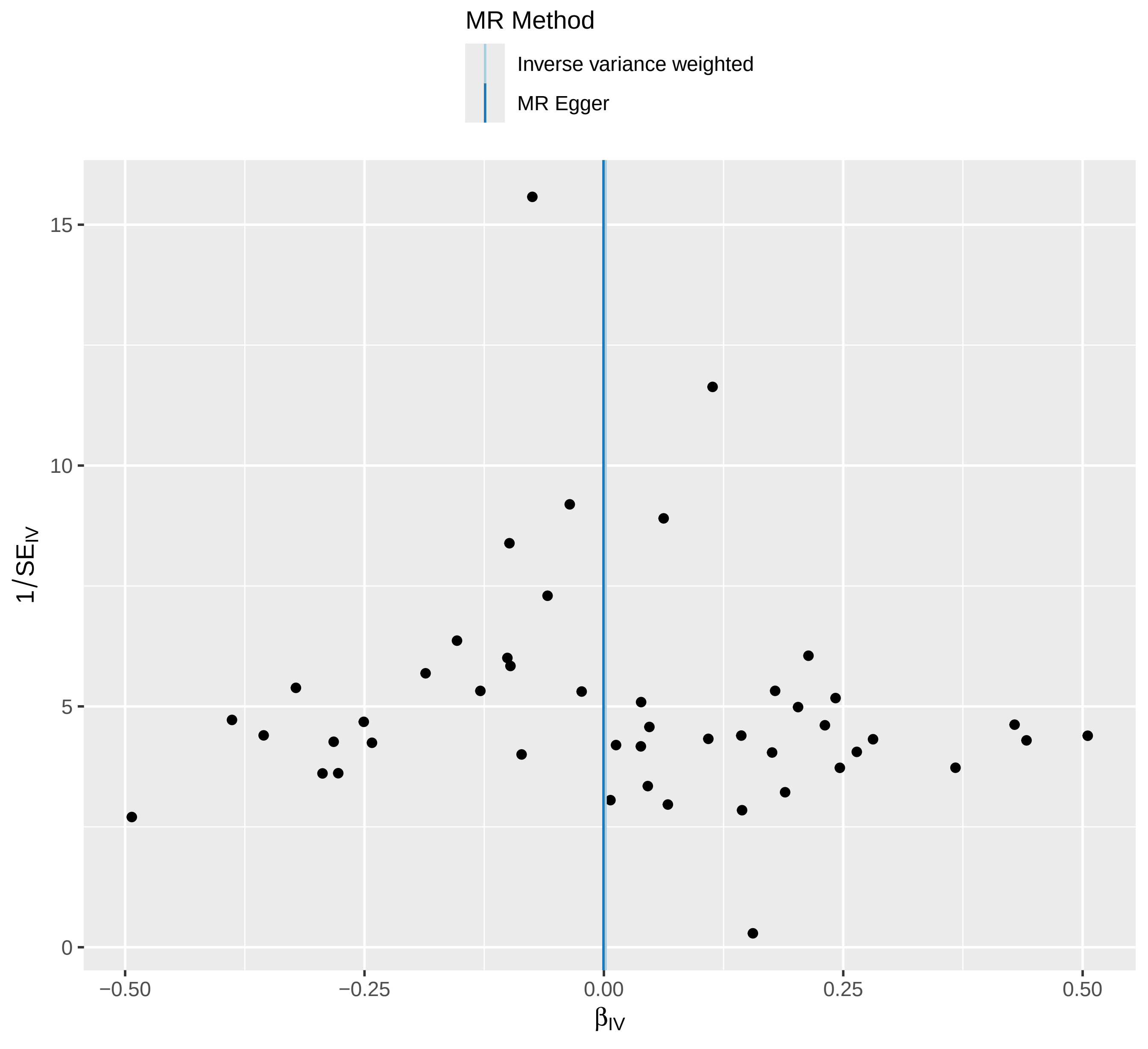

MR Method
Inverse variance weighted
MR Egger
15
10
5
0
1/SE_IV
−0.50
−0.25
0.00
0.25
0.50
β_IV

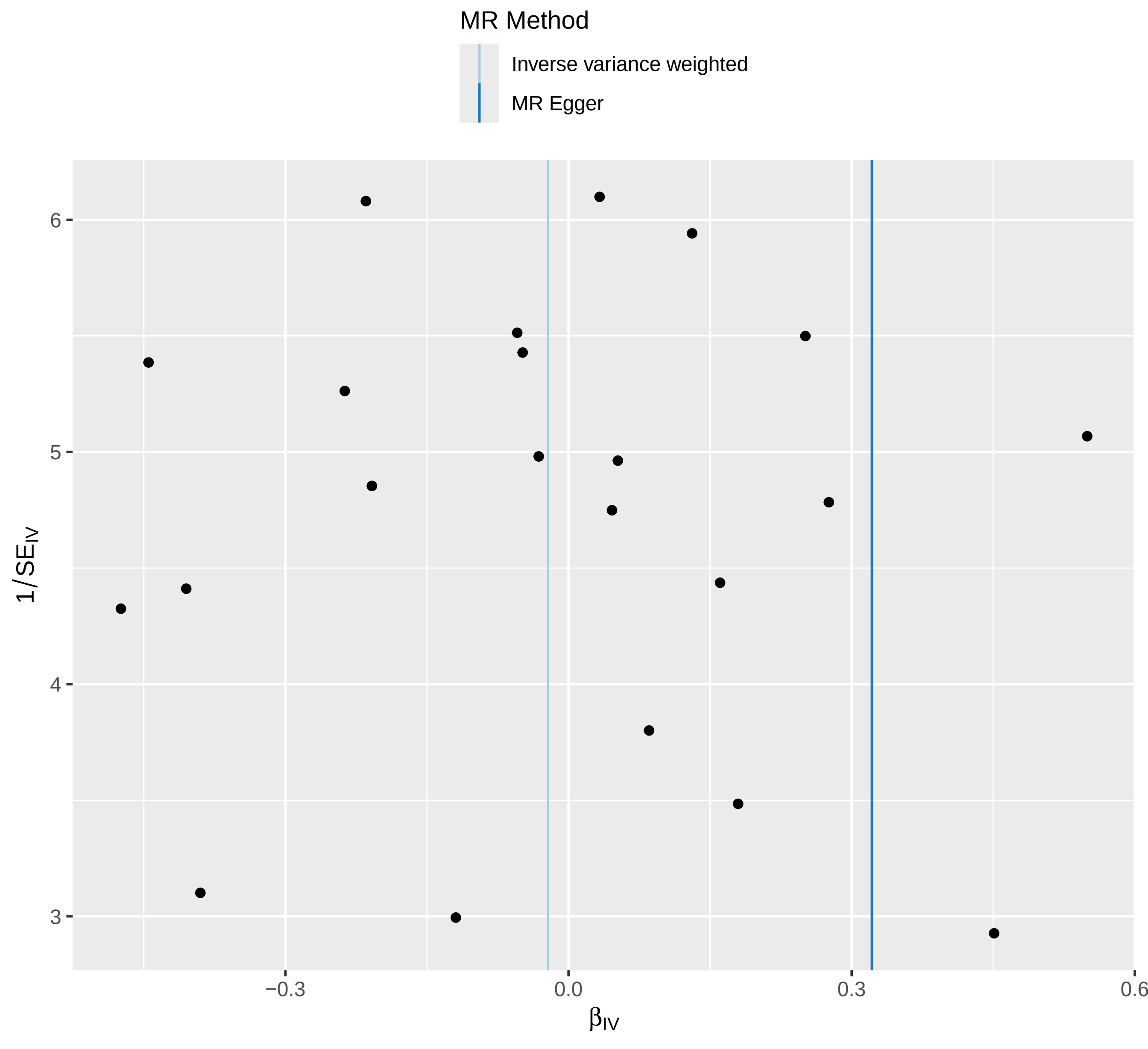

MR Method
Inverse variance weighted
MR Egger
6
5
4
3
1/SE_IV
−0.3
0.0
0.3
0.6
β_IV

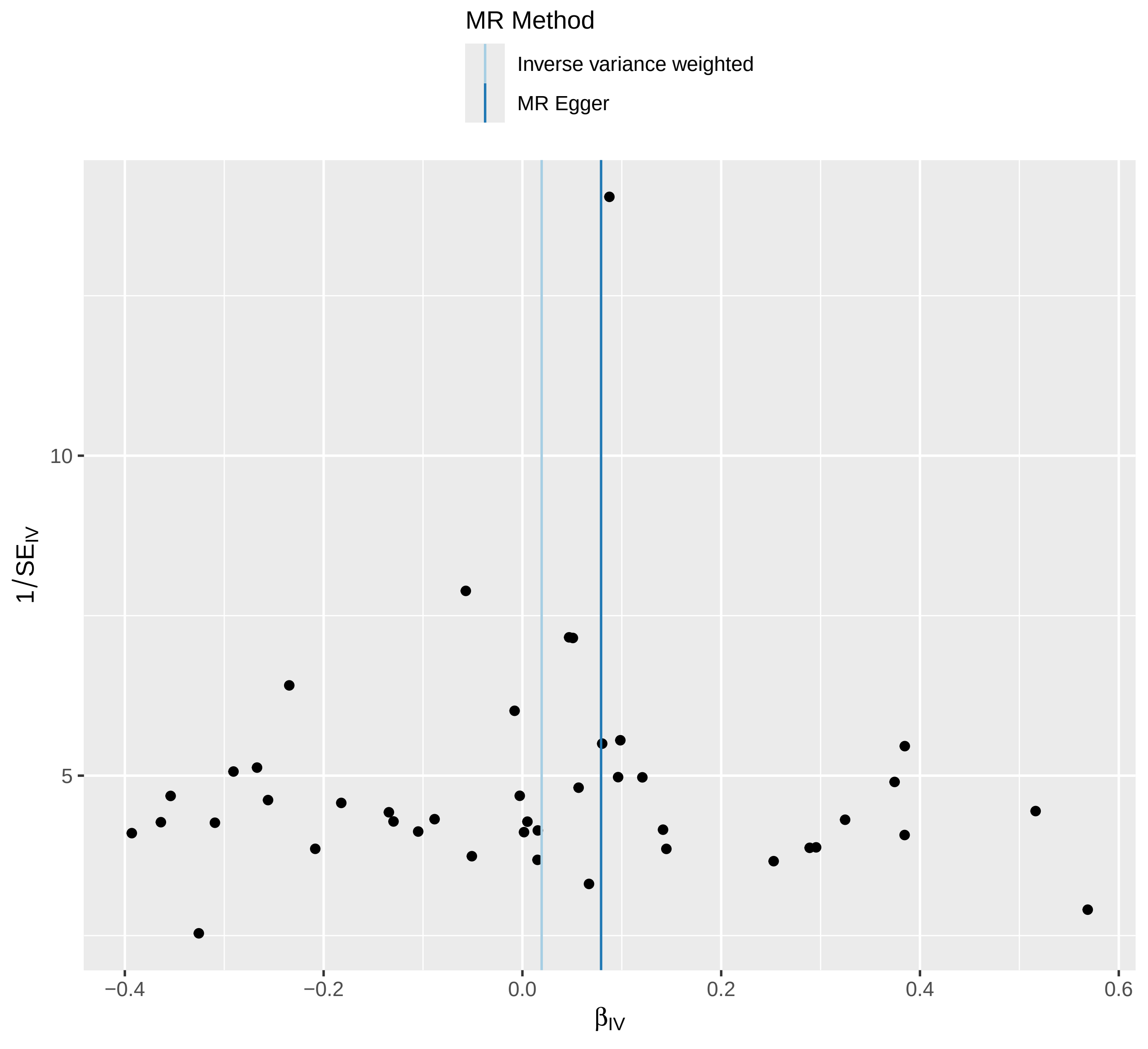
MR Method
Inverse variance weighted
MR Egger
1/SE_IV
10
5
−0.4
−0.2
0.0
0.2
0.4
0.6
β_IV

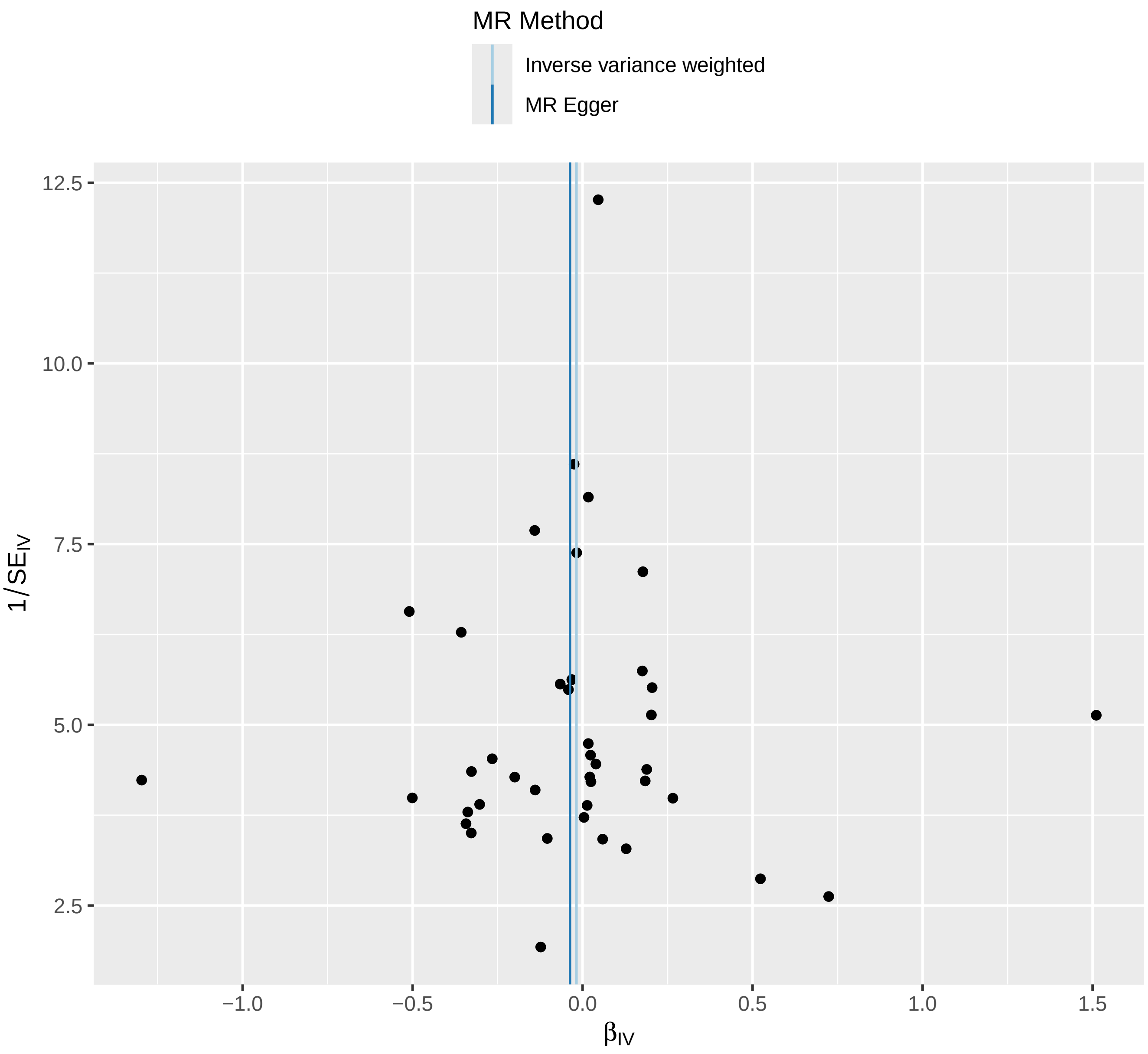
MR Method
Inverse variance weighted
MR Egger
12.5
10.0
7.5
5.0
2.5
1/SE_IV
−1.0
−0.5
0.0
0.5
1.0
1.5
β_IV

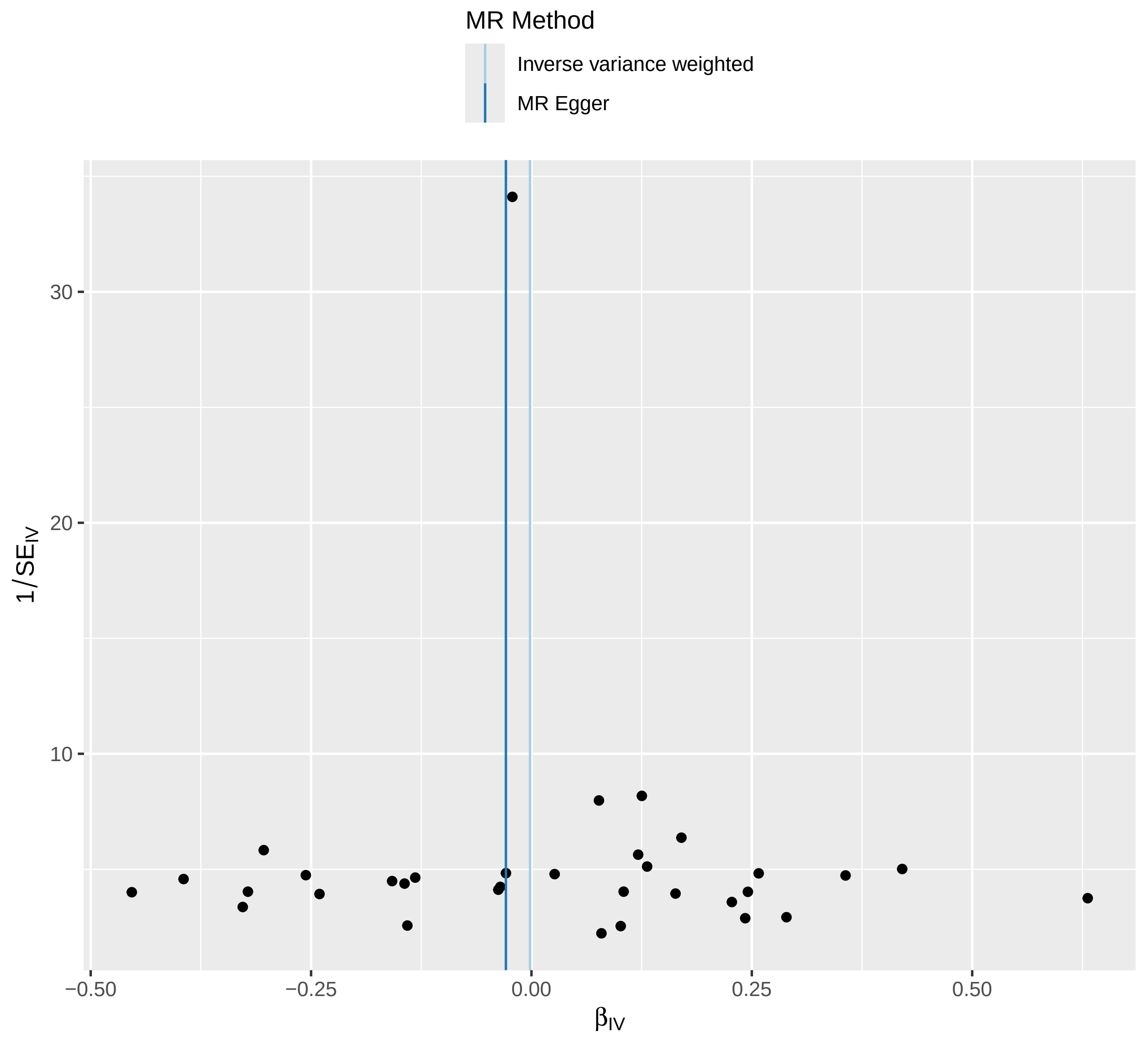
MR Method
Inverse variance weighted
MR Egger
1/SE_IV
30
20
10
−0.50
−0.25
0.00
0.25
0.50
β_IV

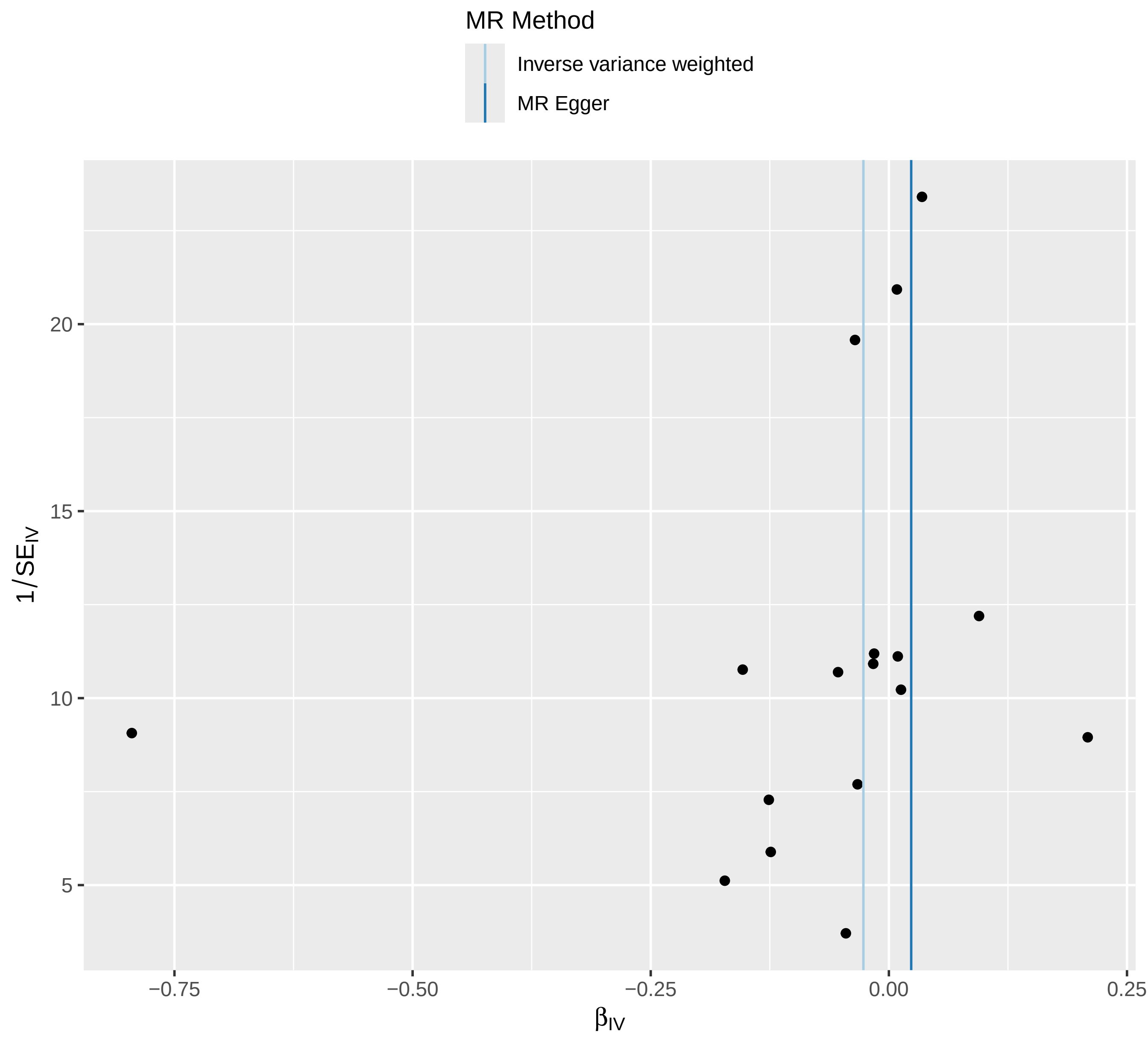
MR Method
Inverse variance weighted
MR Egger
20
15
10
5
1/SE_IV
−0.75
−0.50
−0.25
0.00
0.25
β_IV

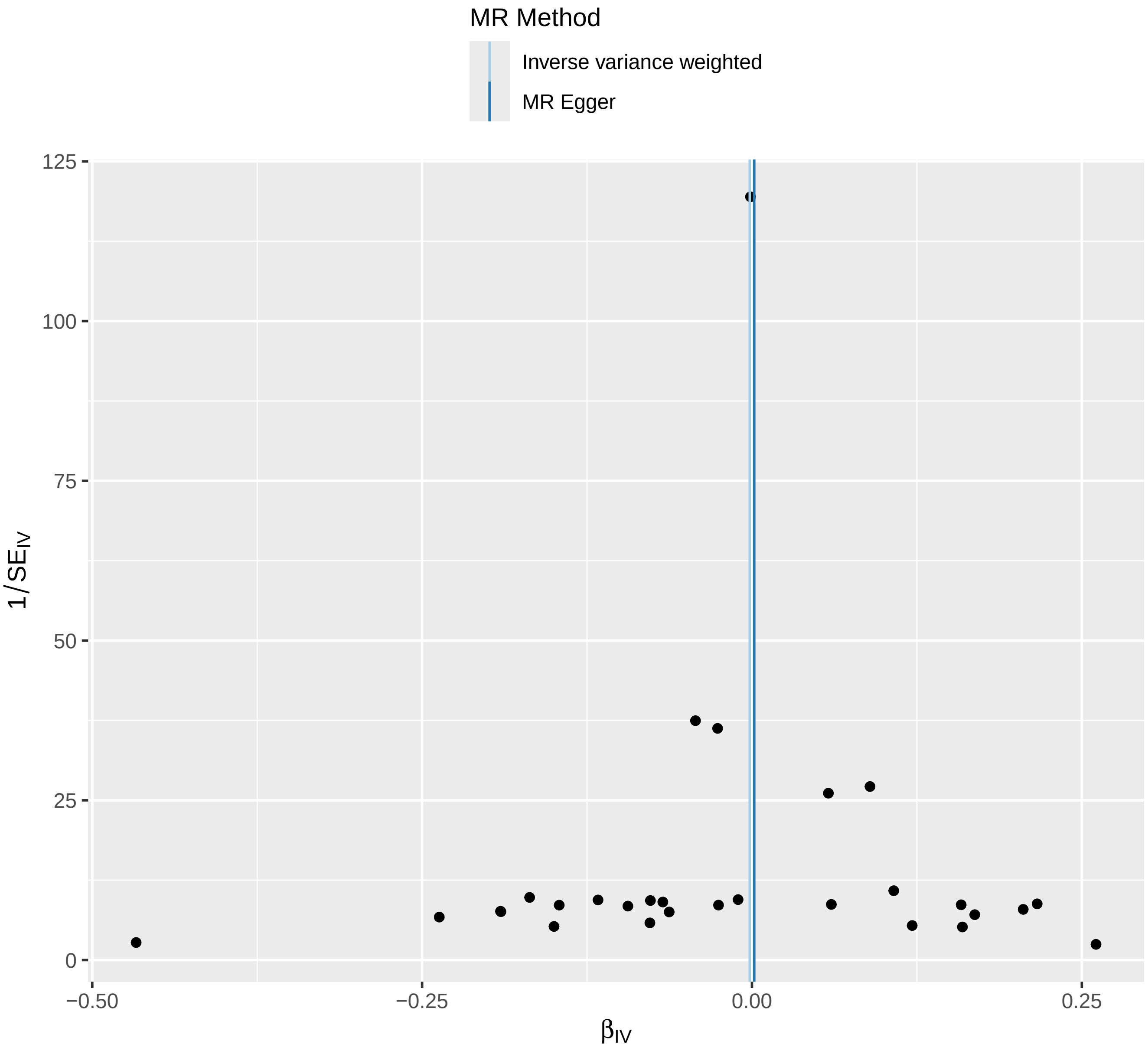
MR Method
Inverse variance weighted
MR Egger
125
100
75
50
25
0
1/SE_IV
−0.50
−0.25
0.00
0.25
β_IV

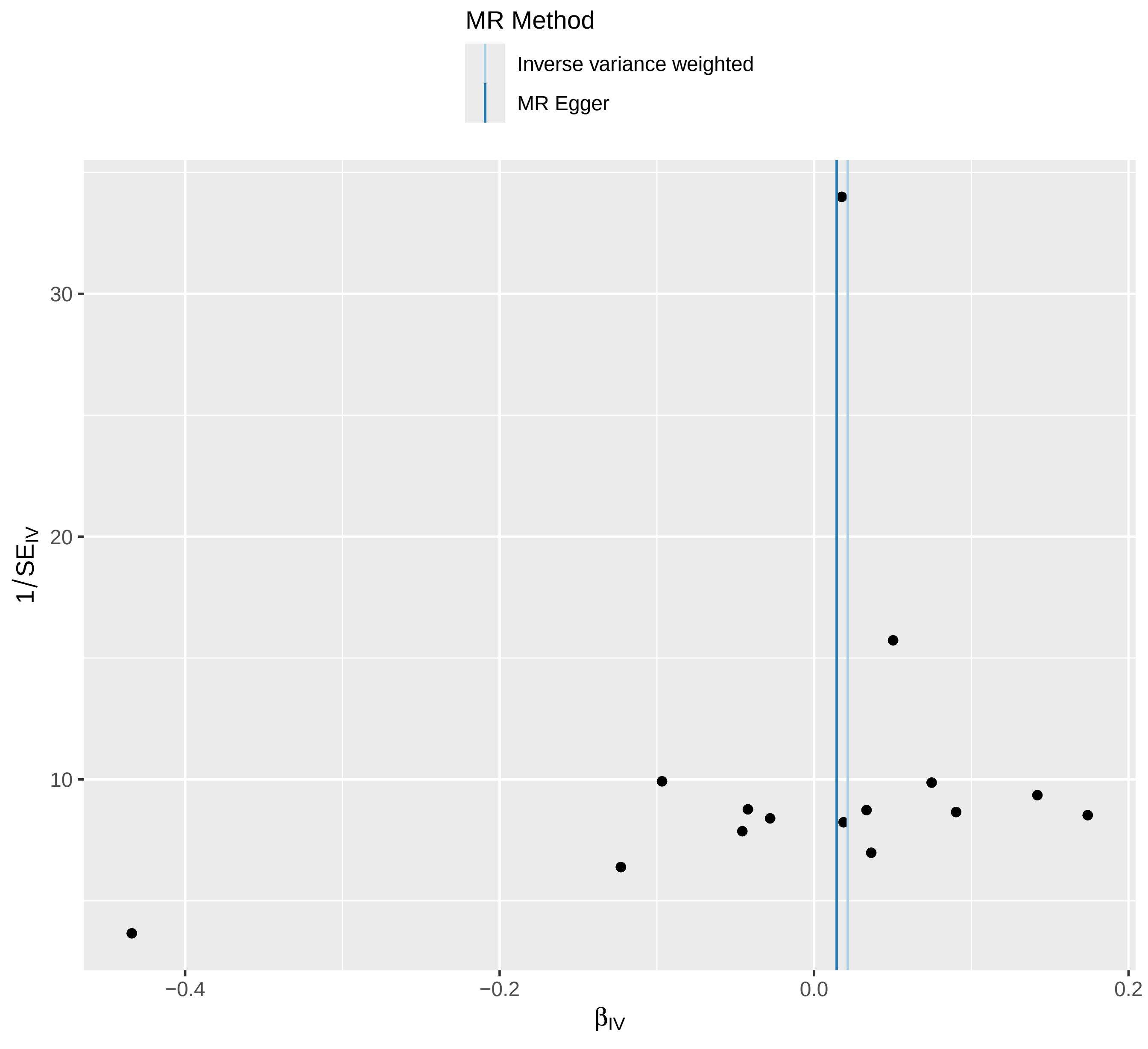
MR Method
Inverse variance weighted
MR Egger
30
20
10
$1/SE_{IV}$
−0.4
−0.2
0.0
0.2
β_{IV}

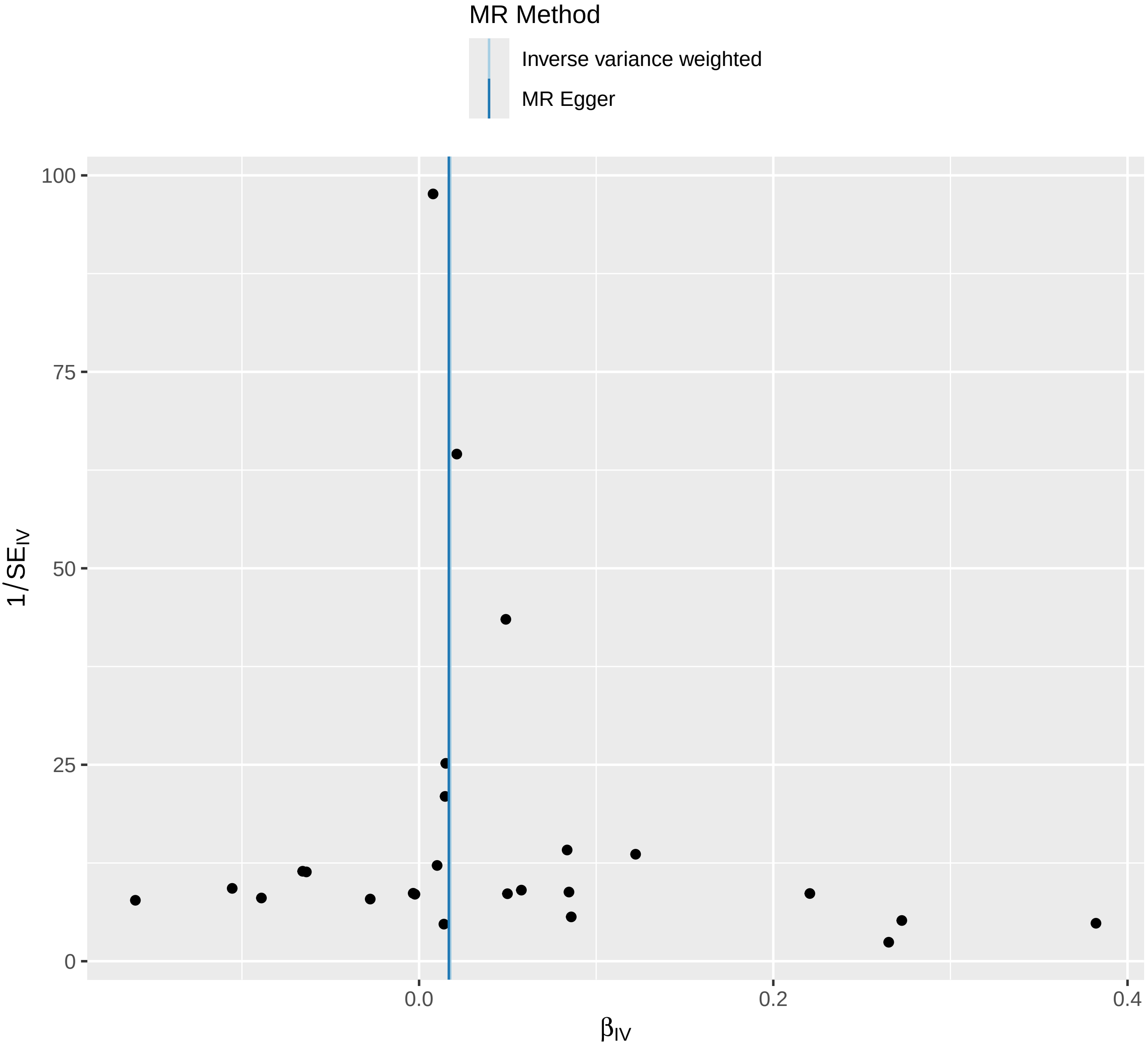
MR Method
Inverse variance weighted
MR Egger
100
75
50
25
0
1/SE_IV
0.0
0.2
0.4
β_IV

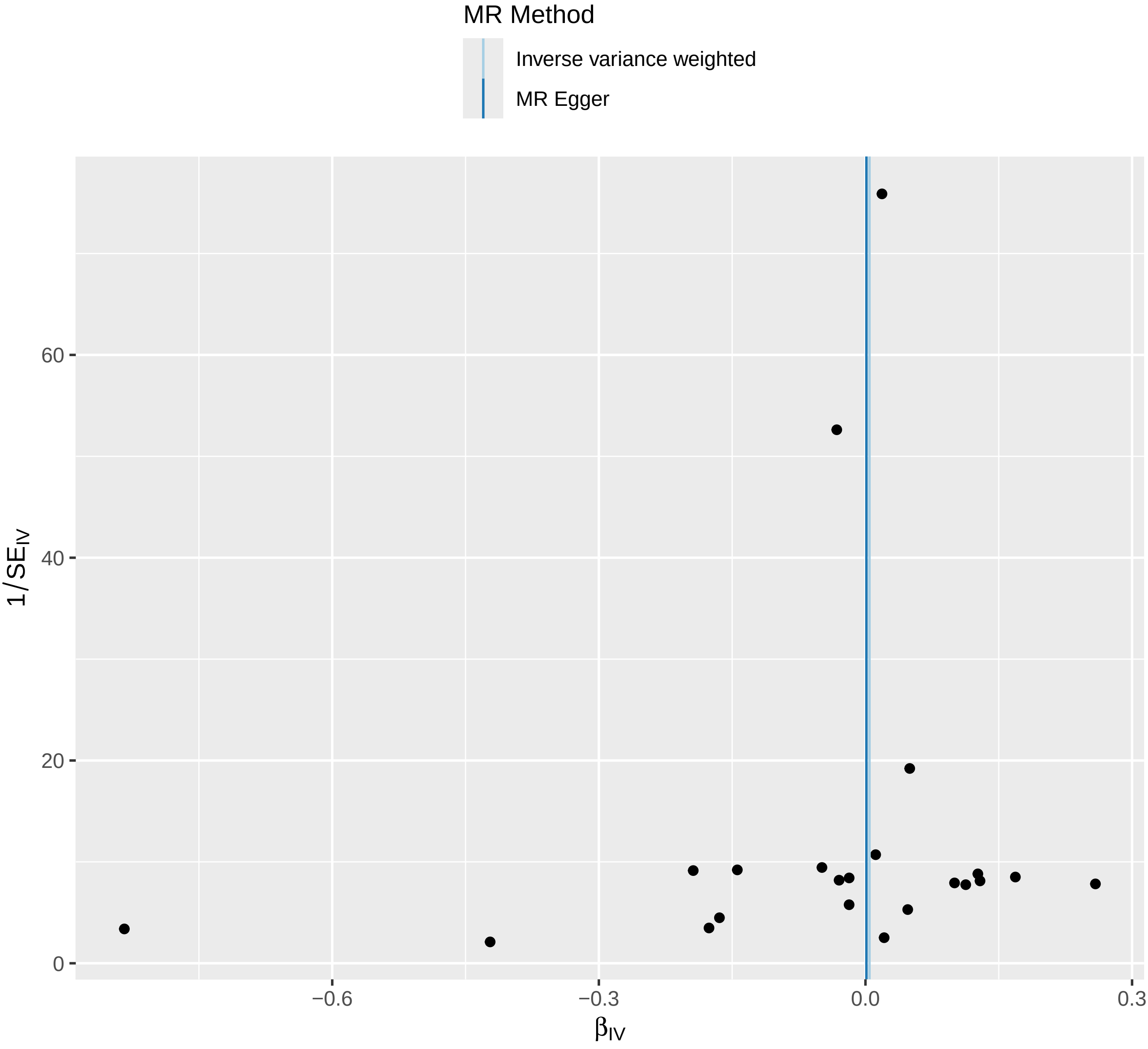

MR Method
Inverse variance weighted
MR Egger
60
40
20
0
1/SE$_{IV}$
−0.6
−0.3
0.0
0.3
β$_{IV}$

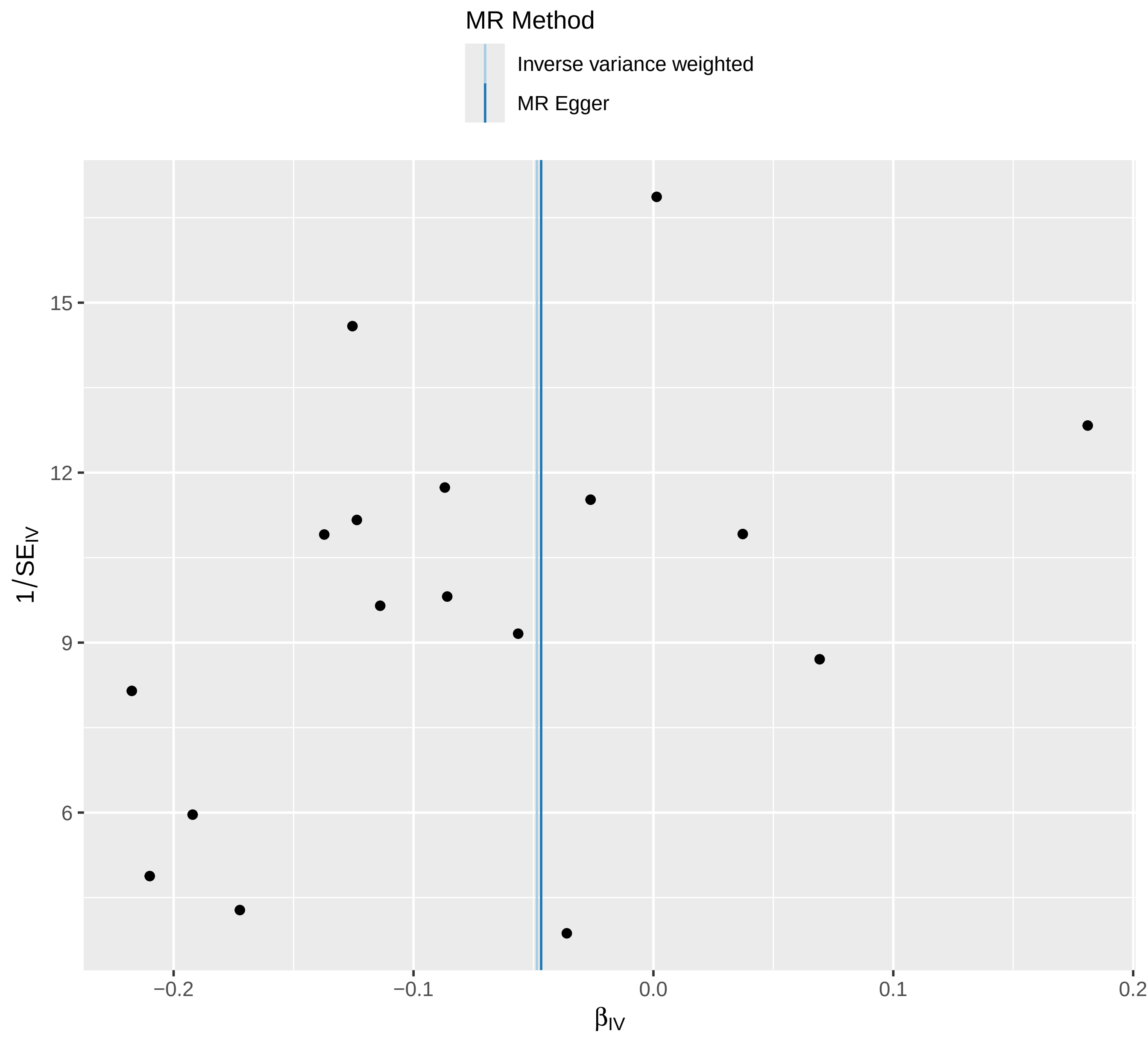
MR Method
Inverse variance weighted
MR Egger
15
12
9
6
1/SE_IV
−0.2
−0.1
0.0
0.1
0.2
β_IV

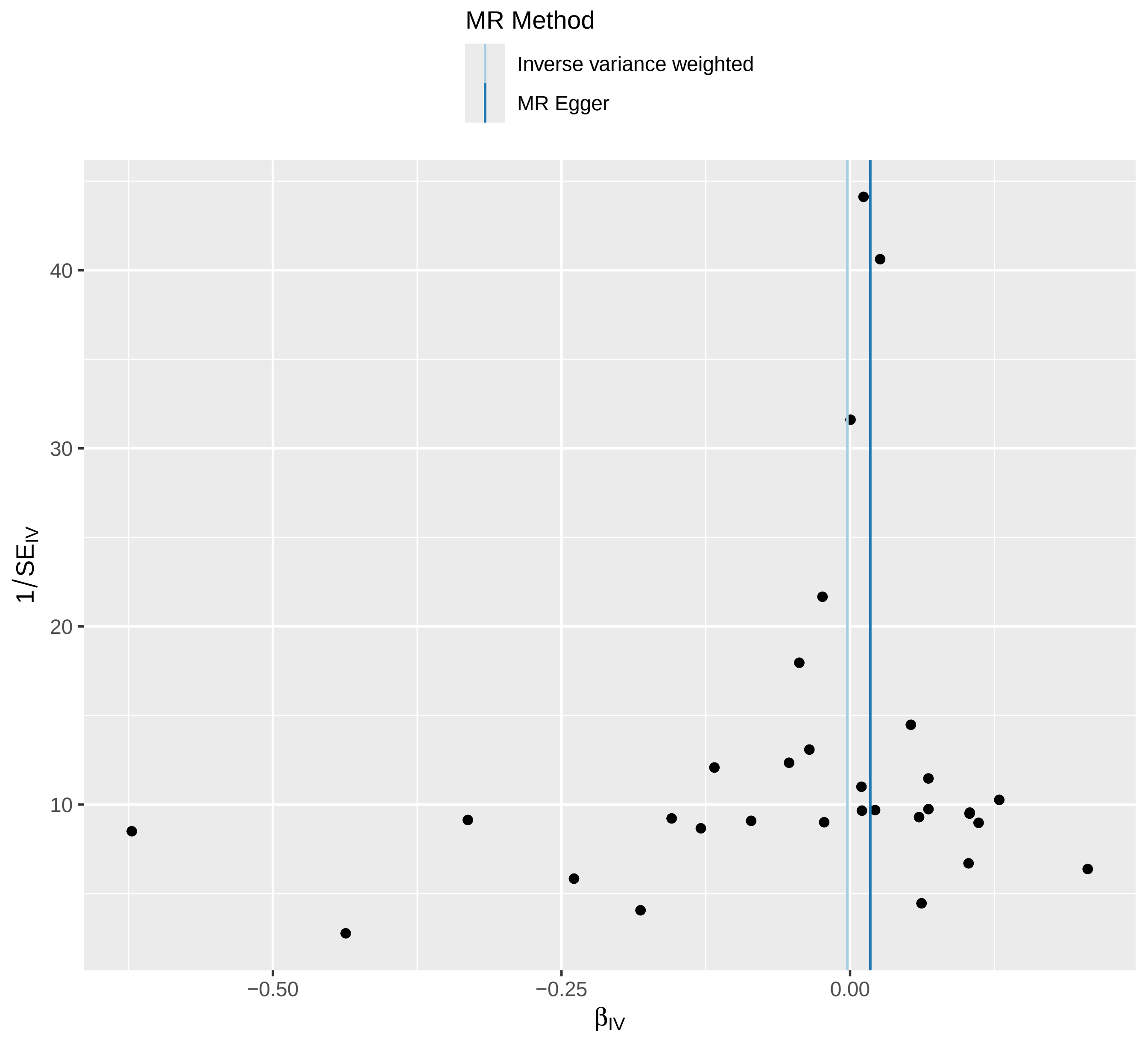

MR Method
Inverse variance weighted
MR Egger
1/SE_IV
40
30
20
10
−0.50
−0.25
0.00
β_IV

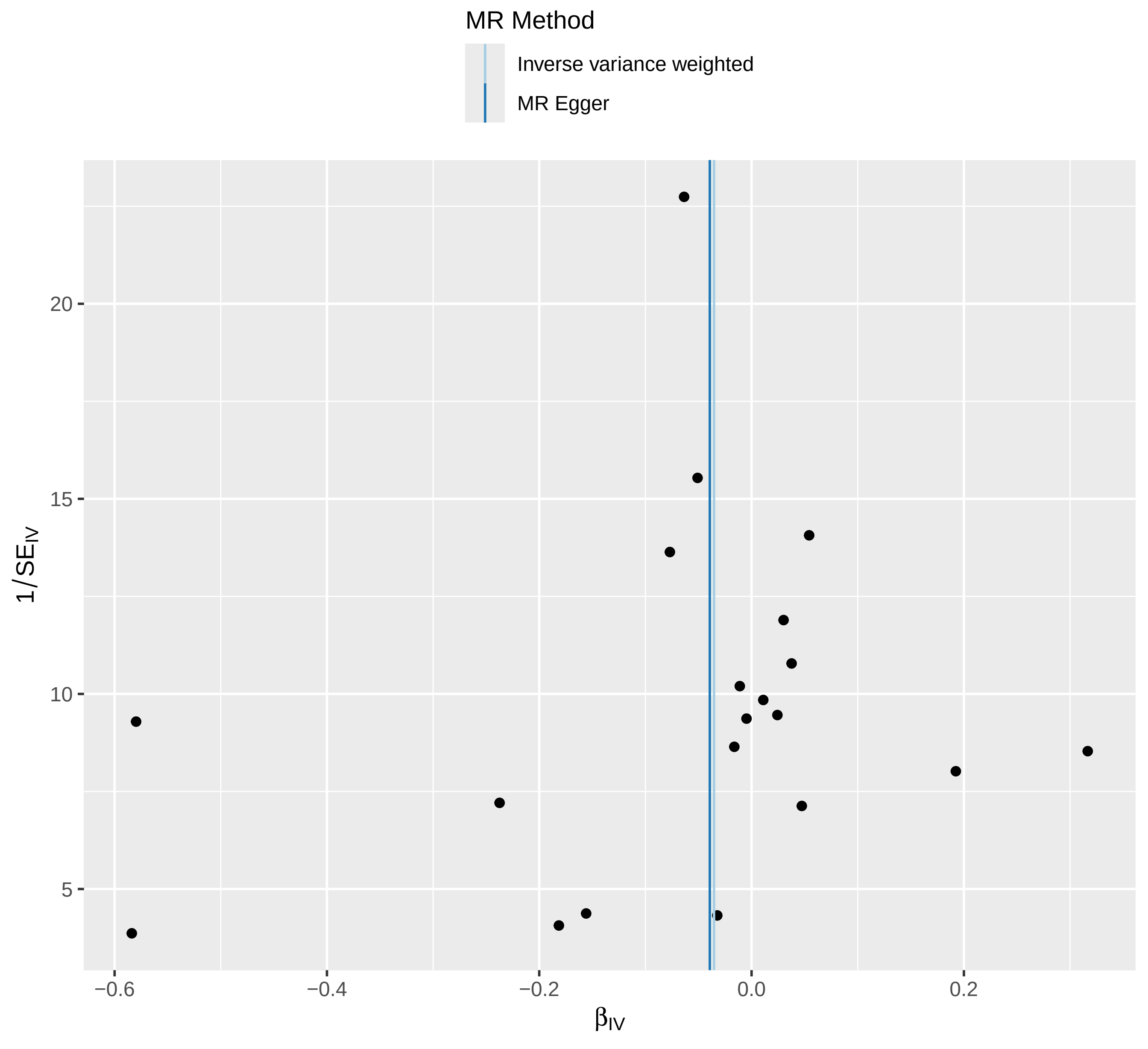
MR Method
Inverse variance weighted
MR Egger
1/SE_{IV}
20
15
10
5
−0.6
−0.4
−0.2
0.0
0.2
β_{IV}

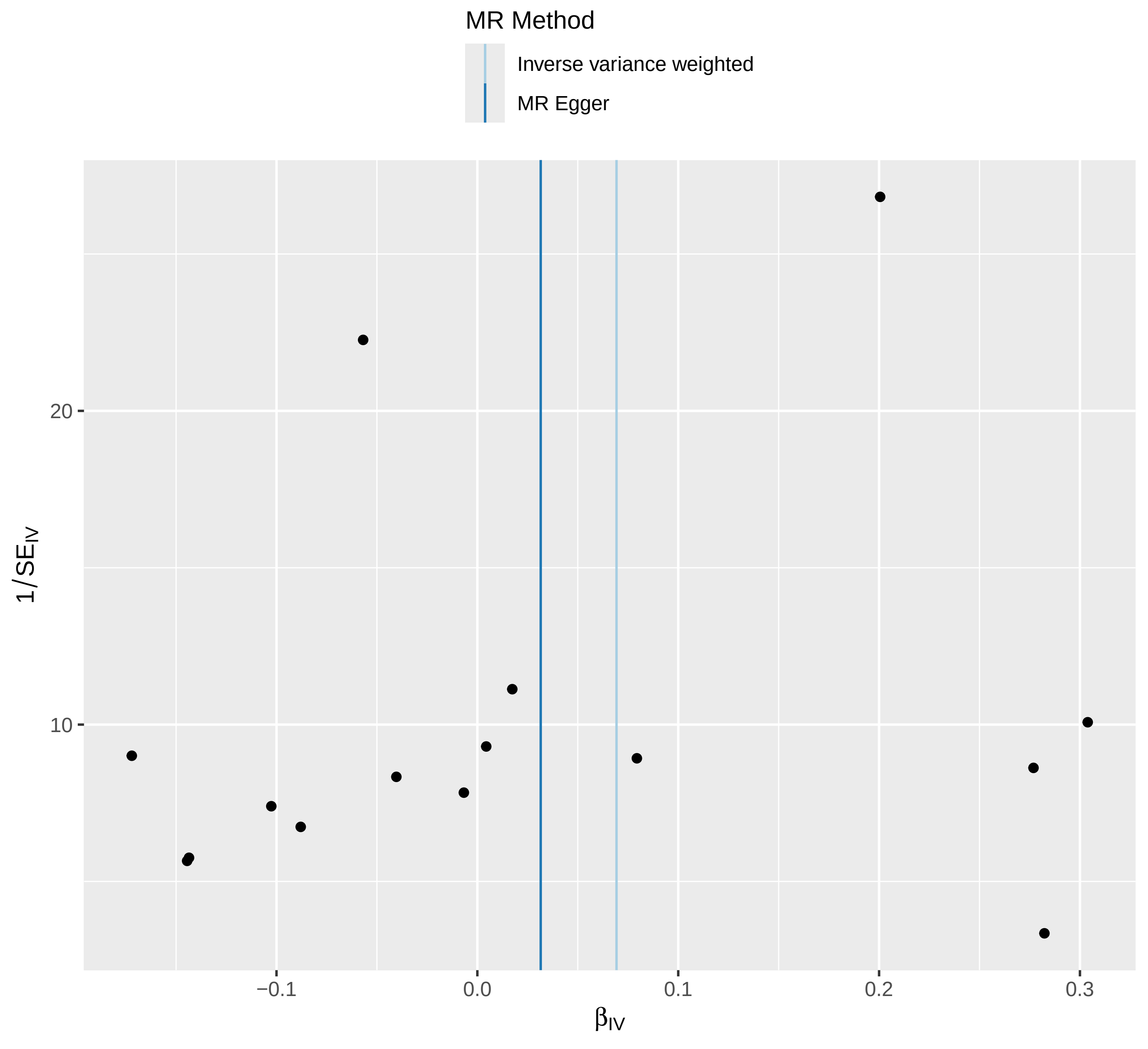
MR Method
Inverse variance weighted
MR Egger
20
10
1/SE_IV
−0.1
0.0
0.1
0.2
0.3
β_IV

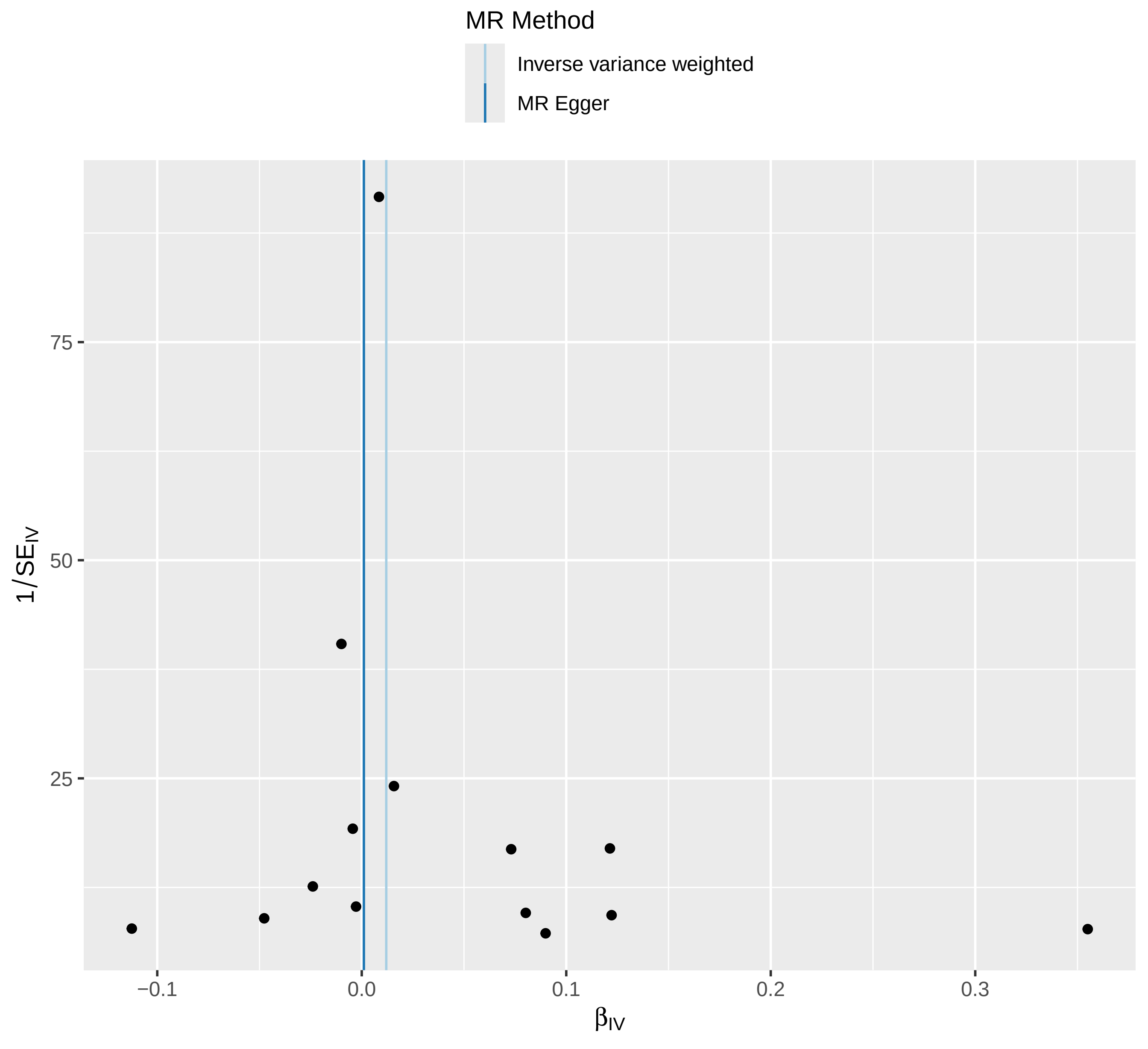
MR Method
Inverse variance weighted
MR Egger
75
50
25
1/SE_IV
−0.1
0.0
0.1
0.2
0.3
β_IV

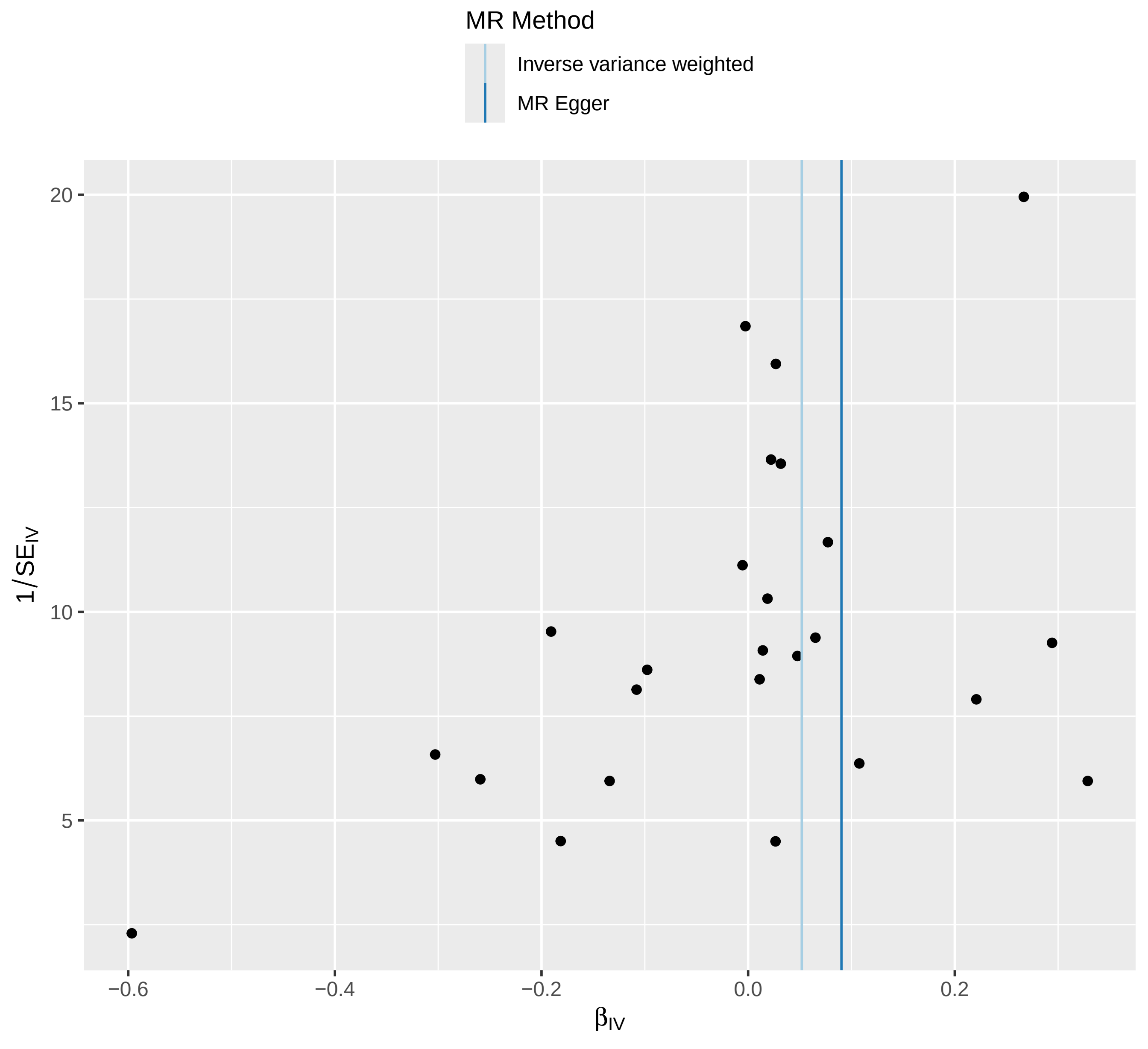
MR Method
Inverse variance weighted
MR Egger
20
15
10
5
1/SE$_{IV}$
−0.6
−0.4
−0.2
0.0
0.2
β$_{IV}$

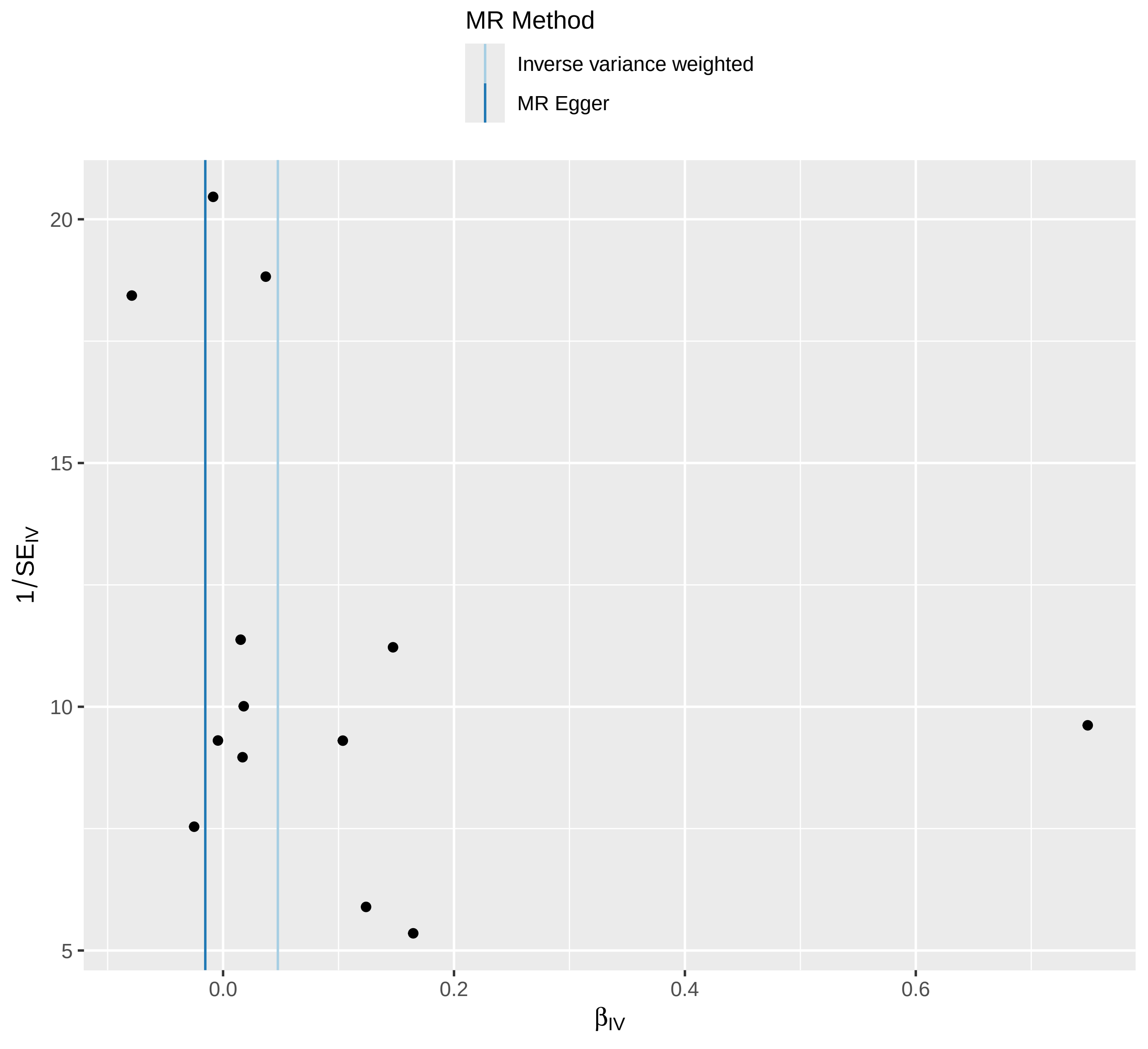

MR Method
Inverse variance weighted
MR Egger
20
15
10
5
$1/SE_{IV}$
0.0
0.2
0.4
0.6
β_{IV}

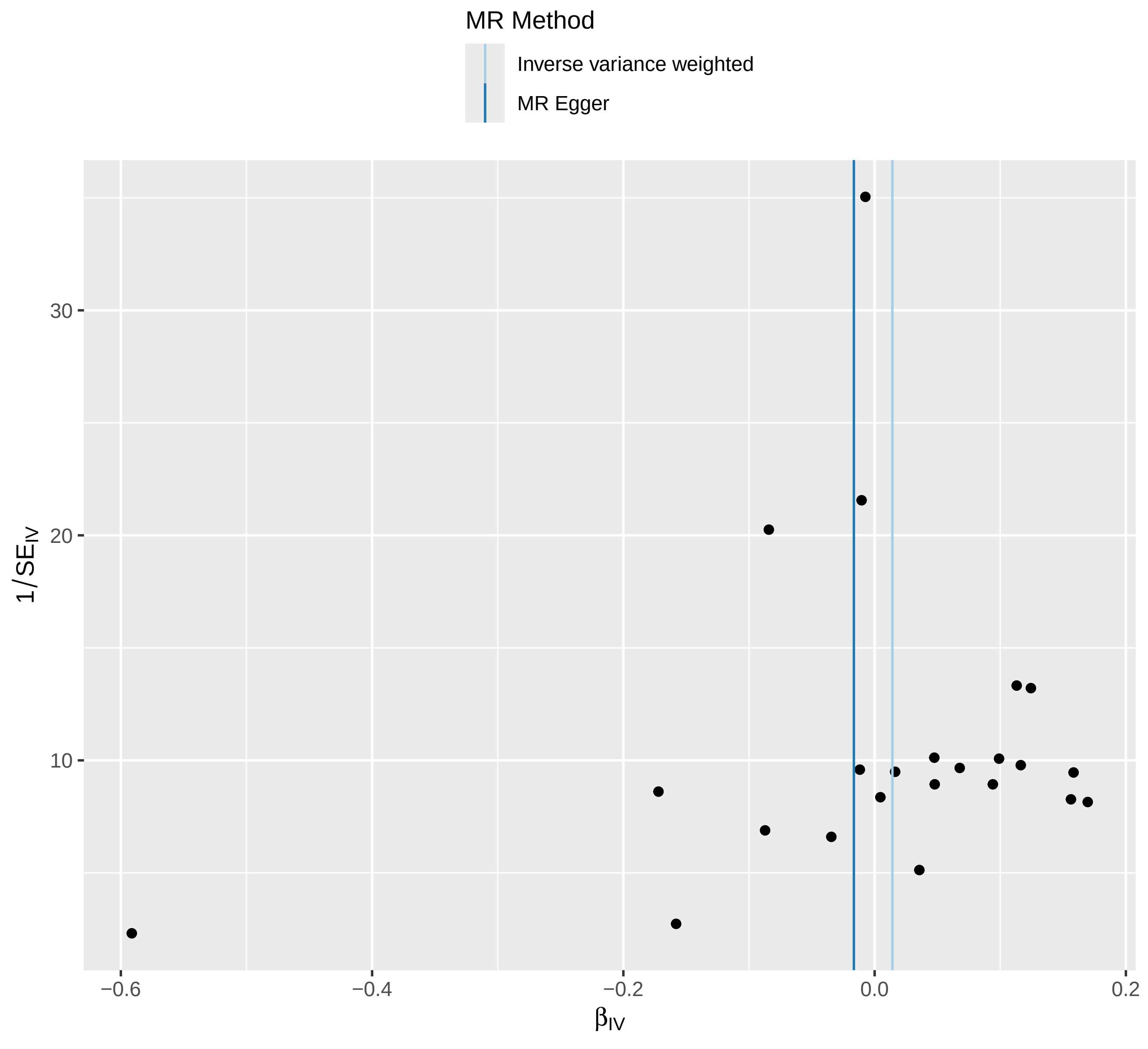
MR Method
Inverse variance weighted
MR Egger
30
20
10
1/SE_IV
−0.6
−0.4
−0.2
0.0
0.2
β_IV

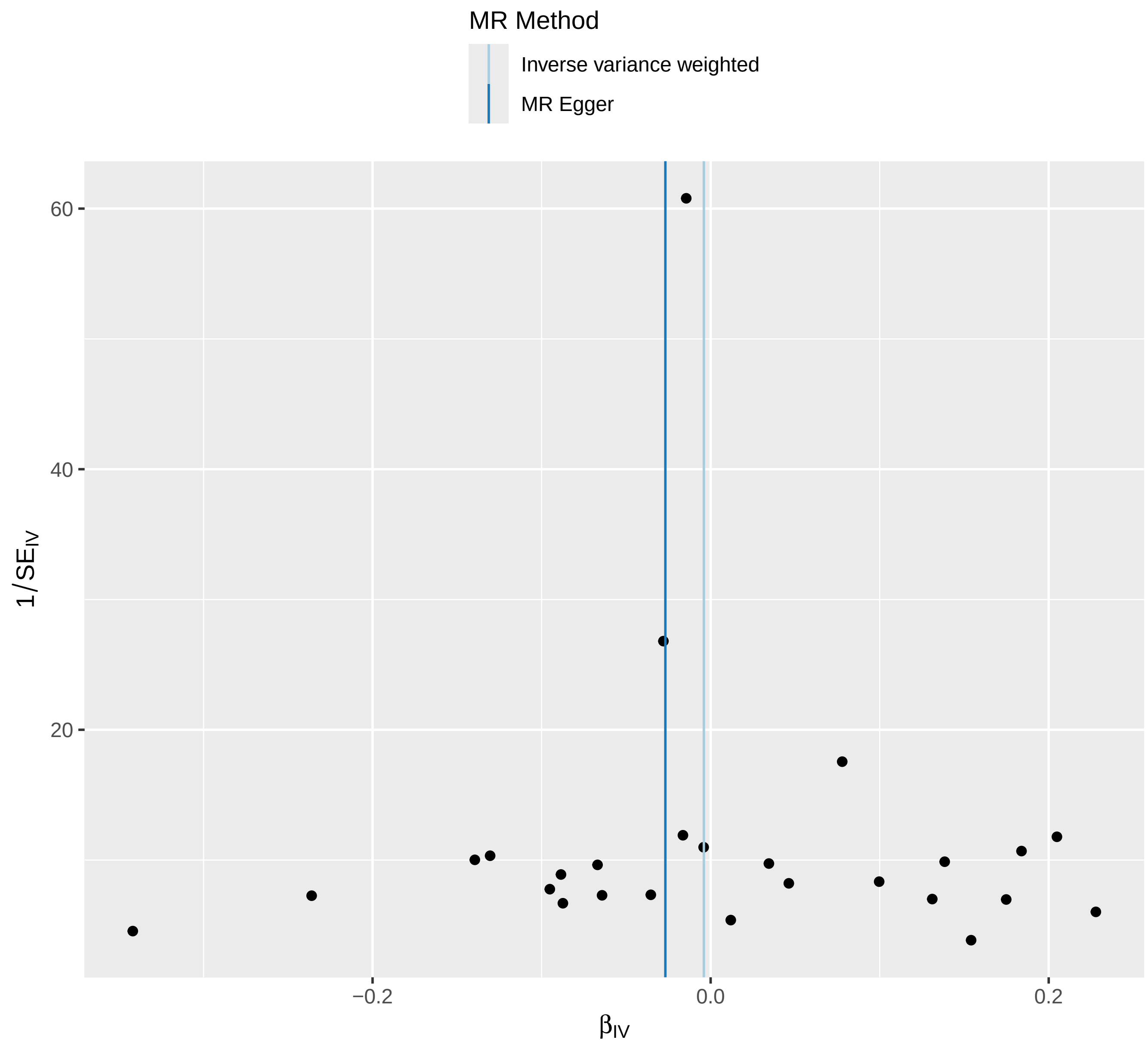
MR Method
Inverse variance weighted
MR Egger
60
40
20
1/SE$_{IV}$
−0.2
0.0
0.2
β$_{IV}$

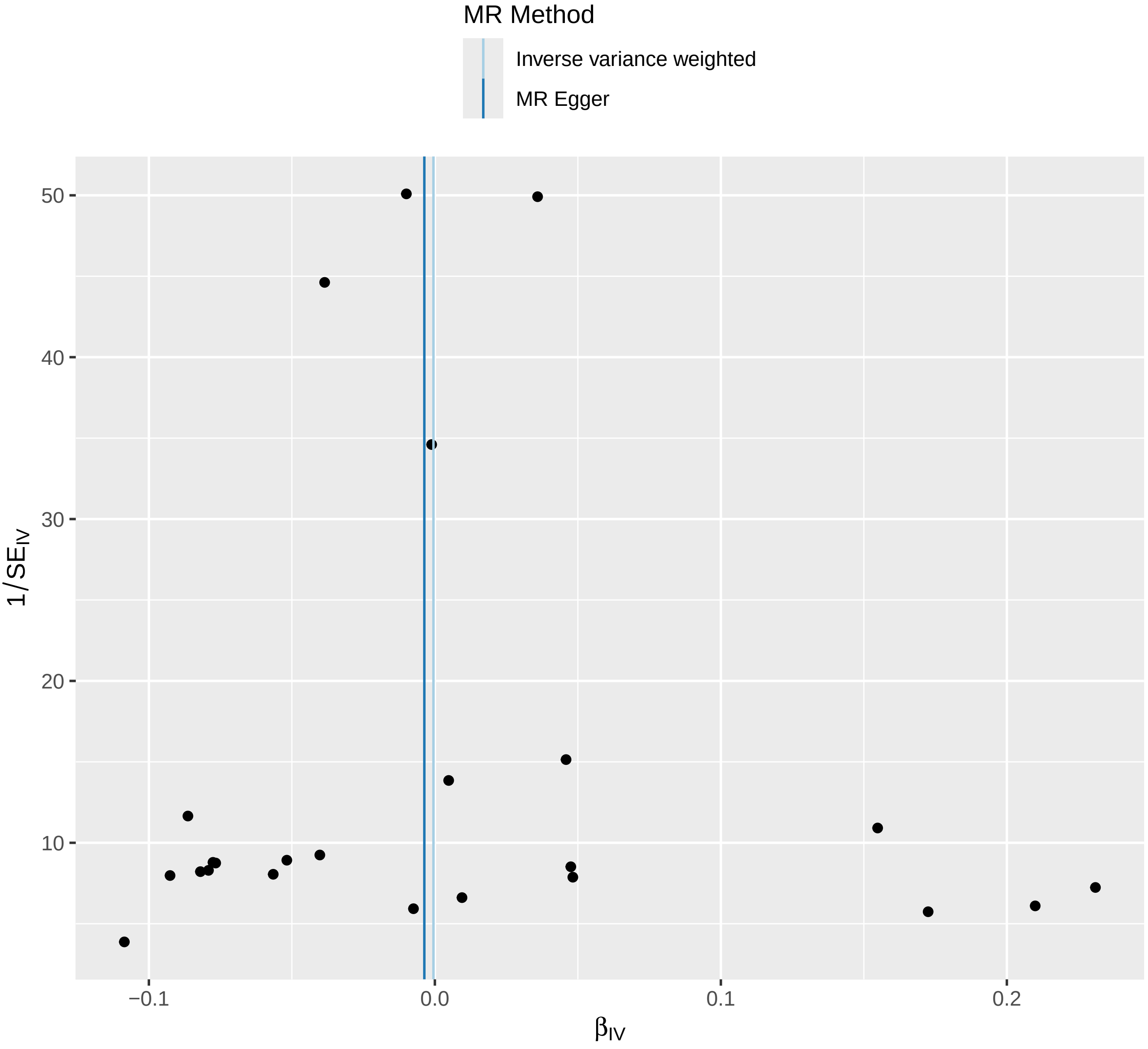
MR Method
Inverse variance weighted
MR Egger
50
40
30
20
10
1/SE_IV
−0.1
0.0
0.1
0.2
β_IV

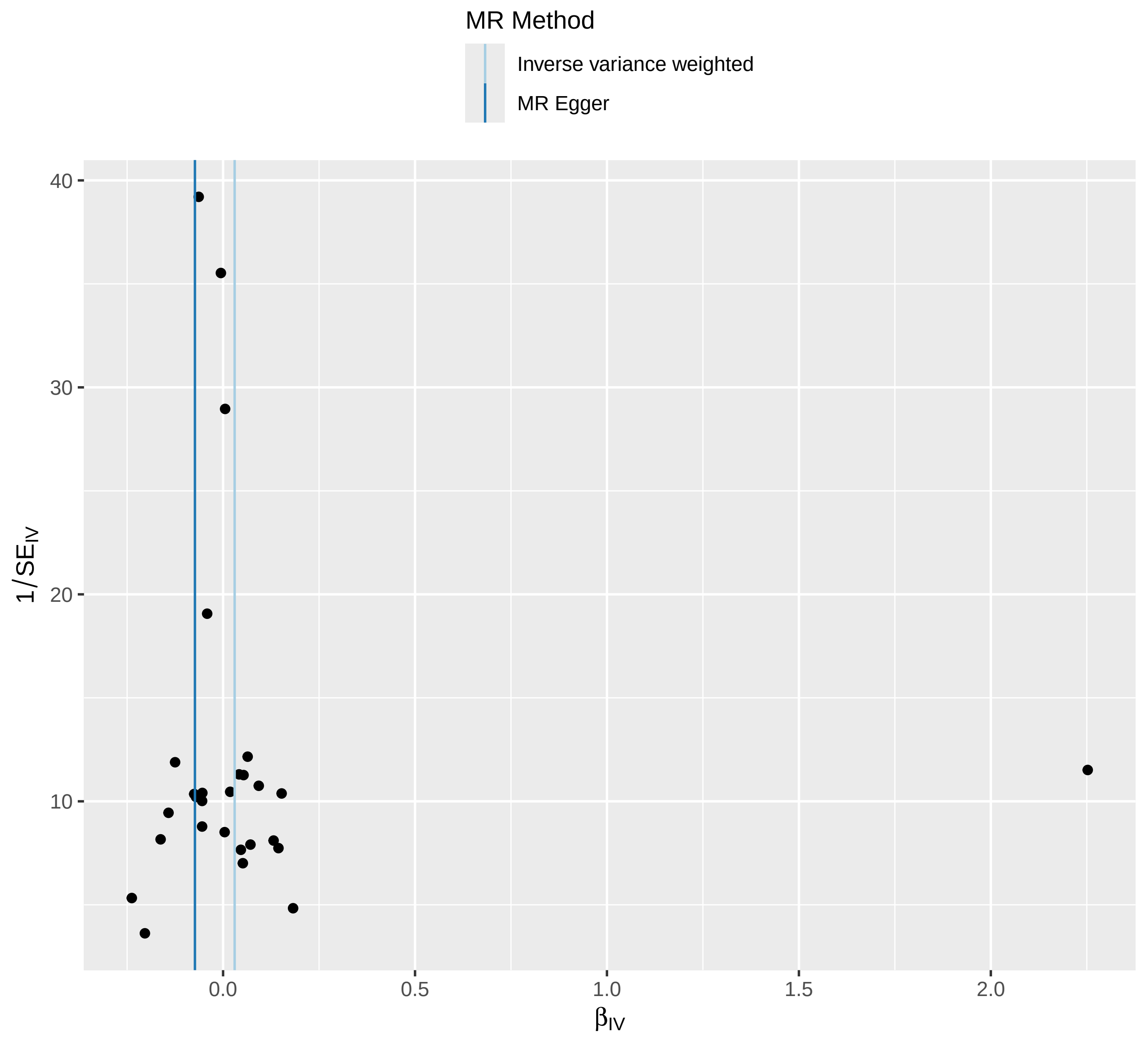
MR Method
Inverse variance weighted
MR Egger
40
30
20
10
1/SE$_{IV}$
0.0
0.5
1.0
1.5
2.0
β$_{IV}$

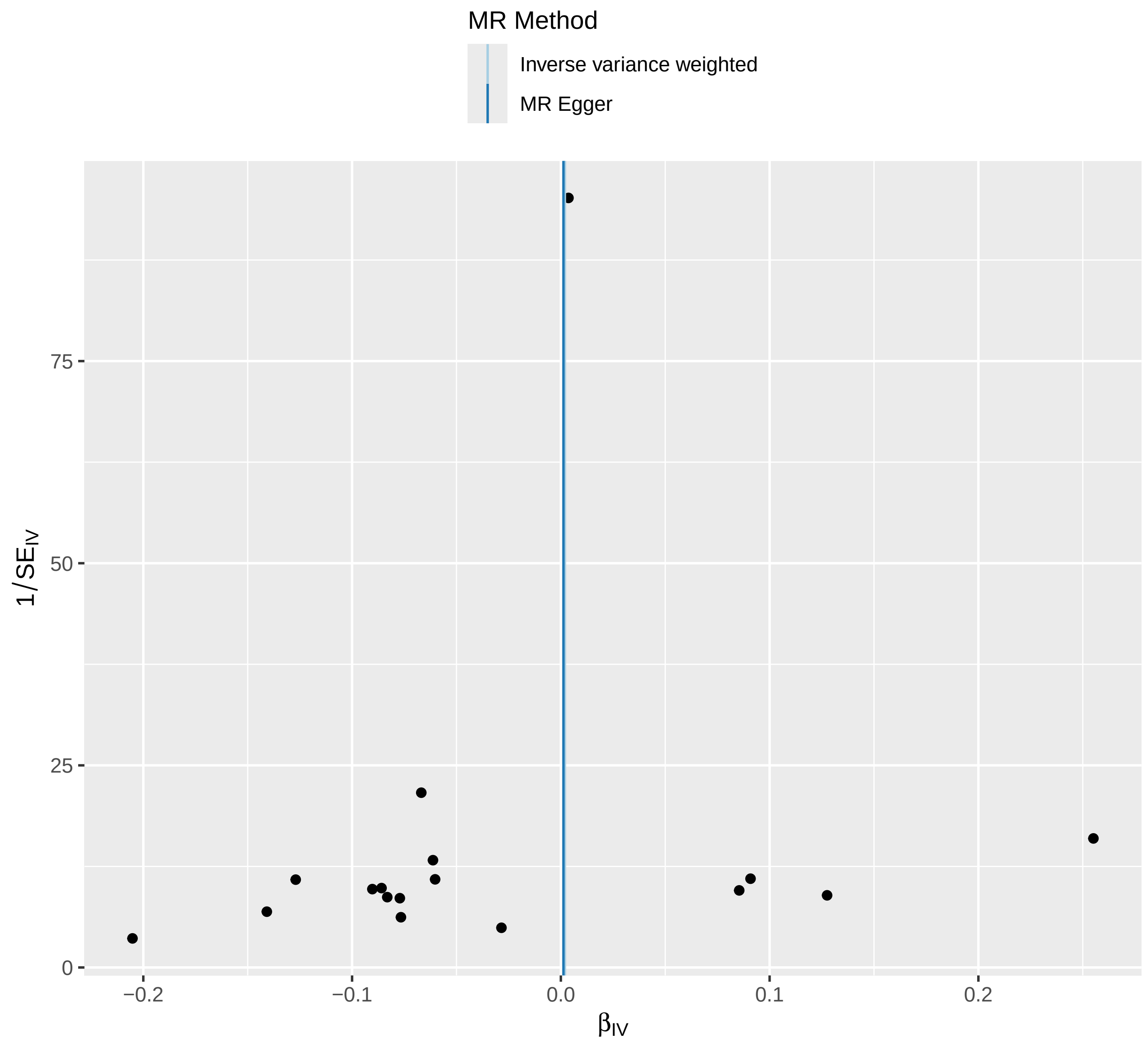

MR Method
Inverse variance weighted
MR Egger
75
50
25
0
1/SE$_{IV}$
−0.2
−0.1
0.0
0.1
0.2
β$_{IV}$

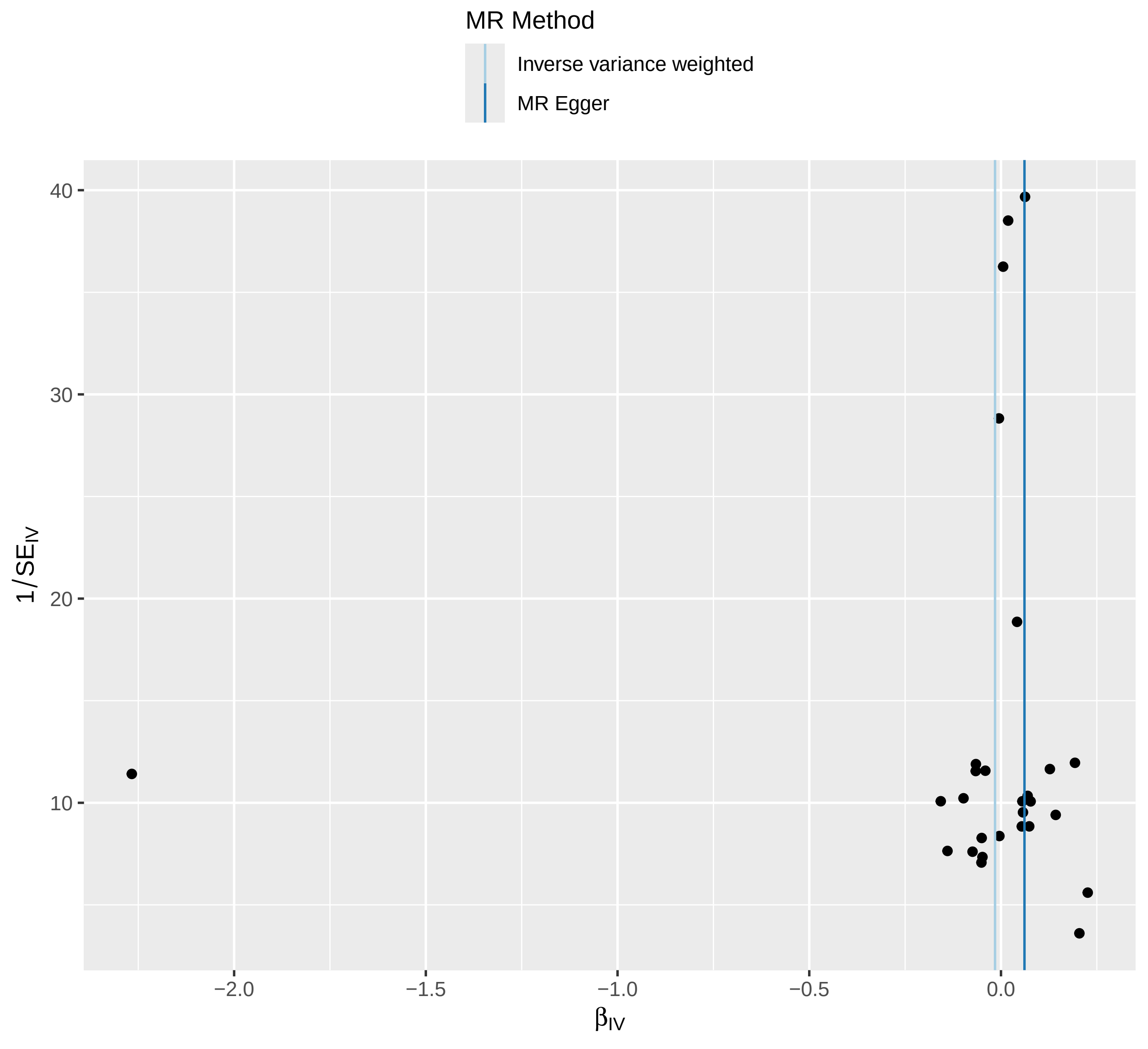
MR Method
Inverse variance weighted
MR Egger
40
30
20
10
−2.0
−1.5
−1.0
−0.5
0.0
1/SE_IV
β_IV

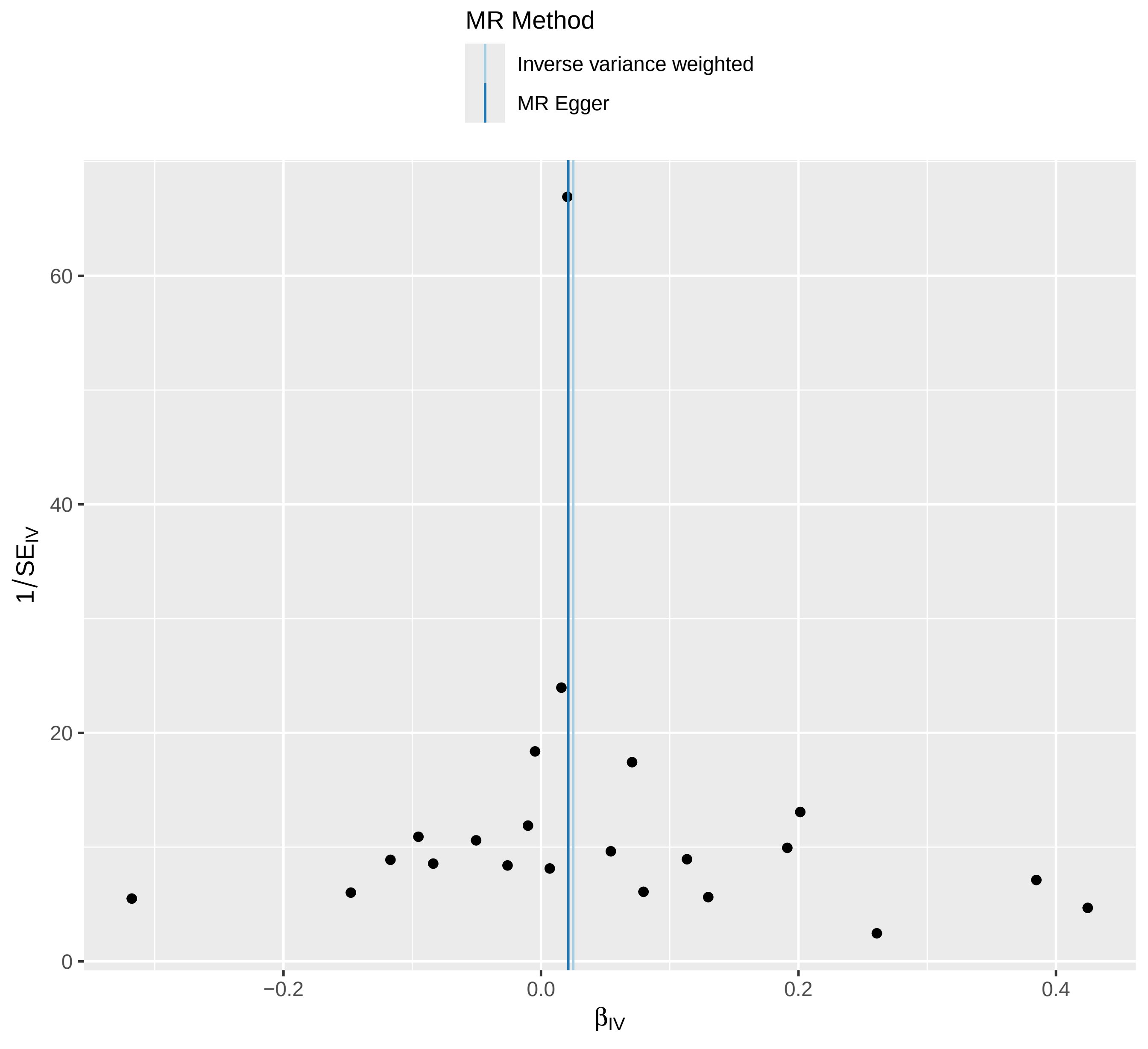

MR Method
Inverse variance weighted
MR Egger
60
40
20
0
$1/SE_{IV}$
−0.2
0.0
0.2
0.4
β_{IV}

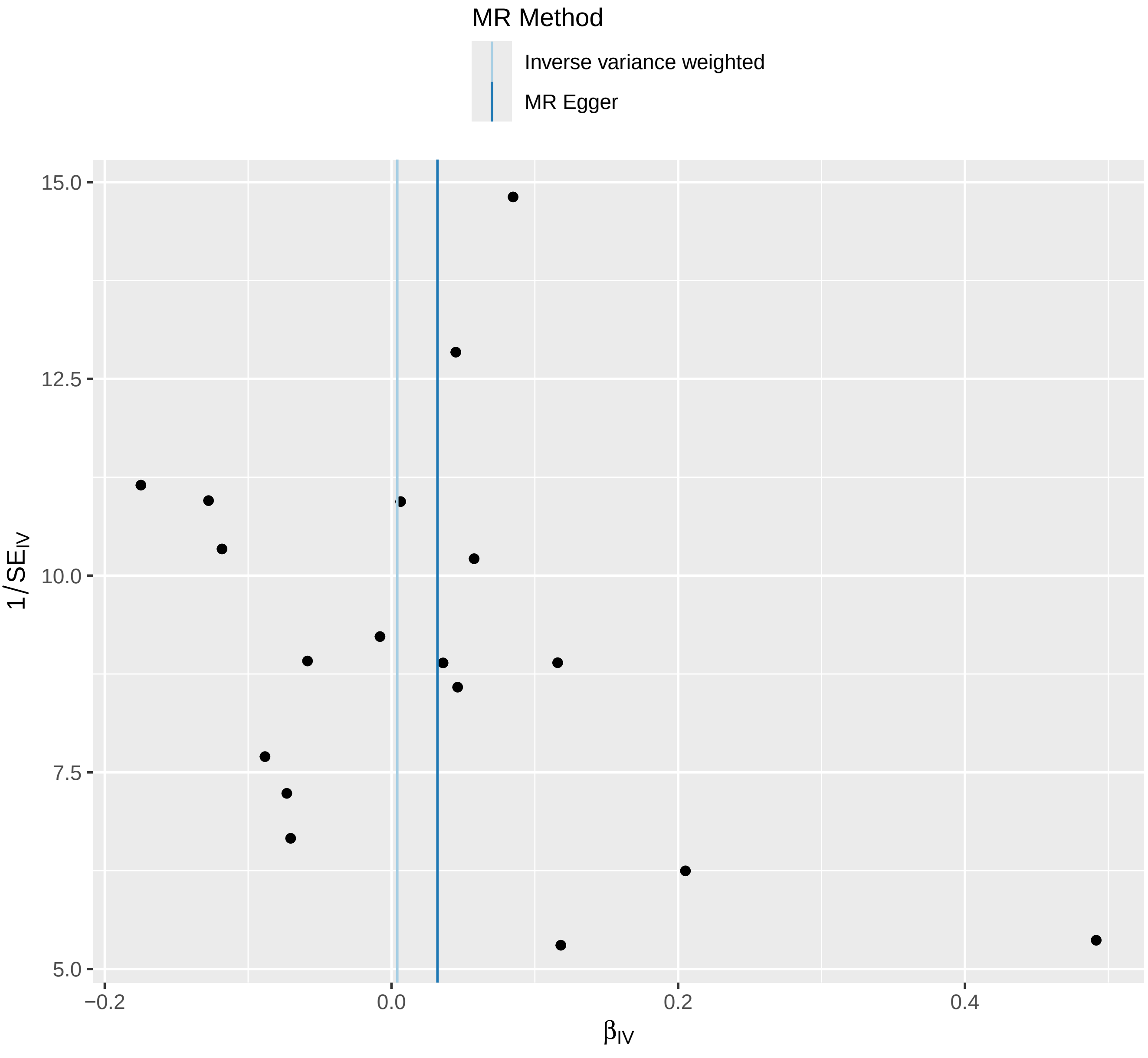
MR Method
Inverse variance weighted
MR Egger
15.0
12.5
10.0
7.5
5.0
1/SE_IV
−0.2
0.0
0.2
0.4
β_IV

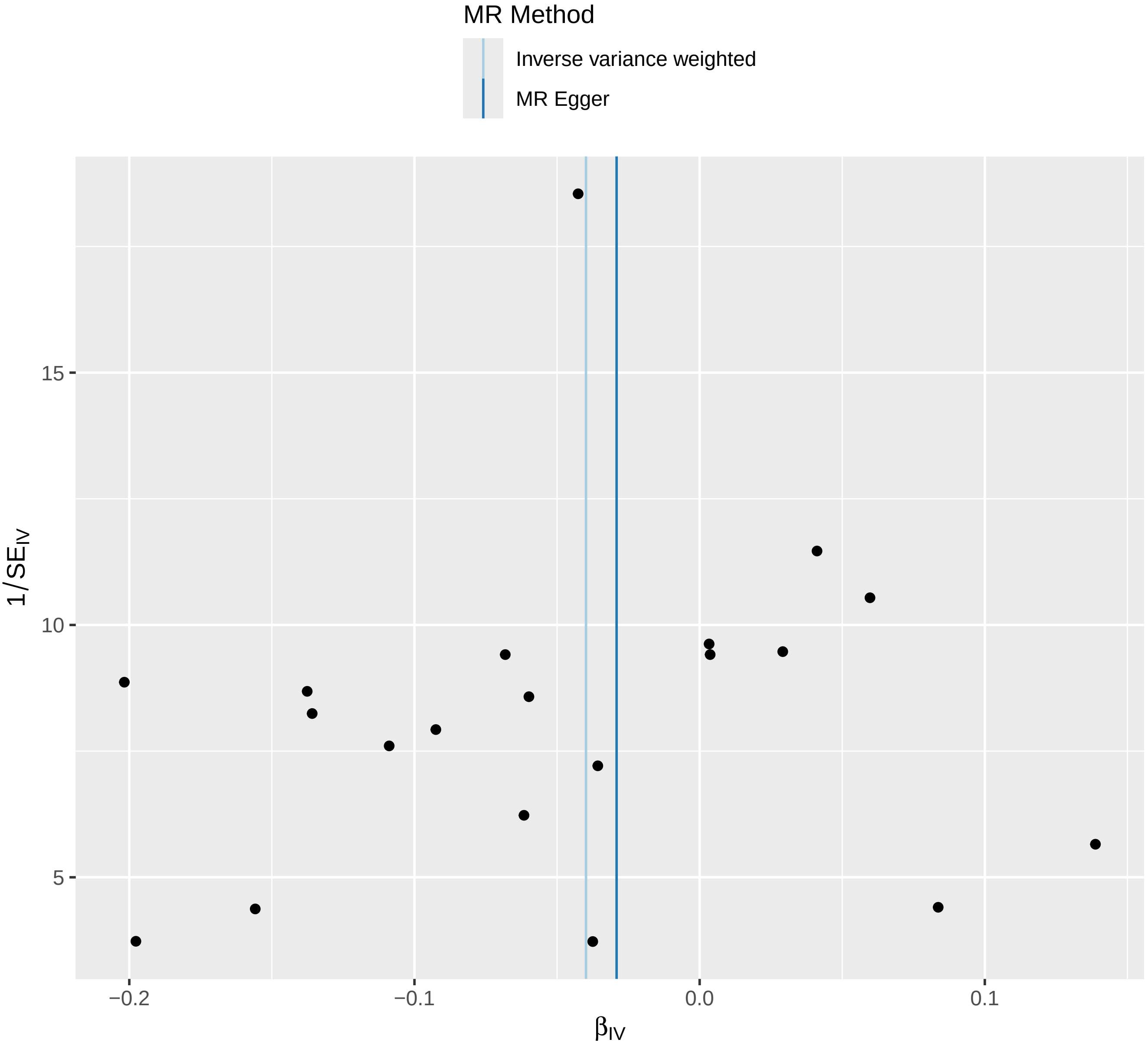

MR Method
Inverse variance weighted
MR Egger
1/SE_IV
15
10
5
−0.2
−0.1
0.0
0.1
β_IV

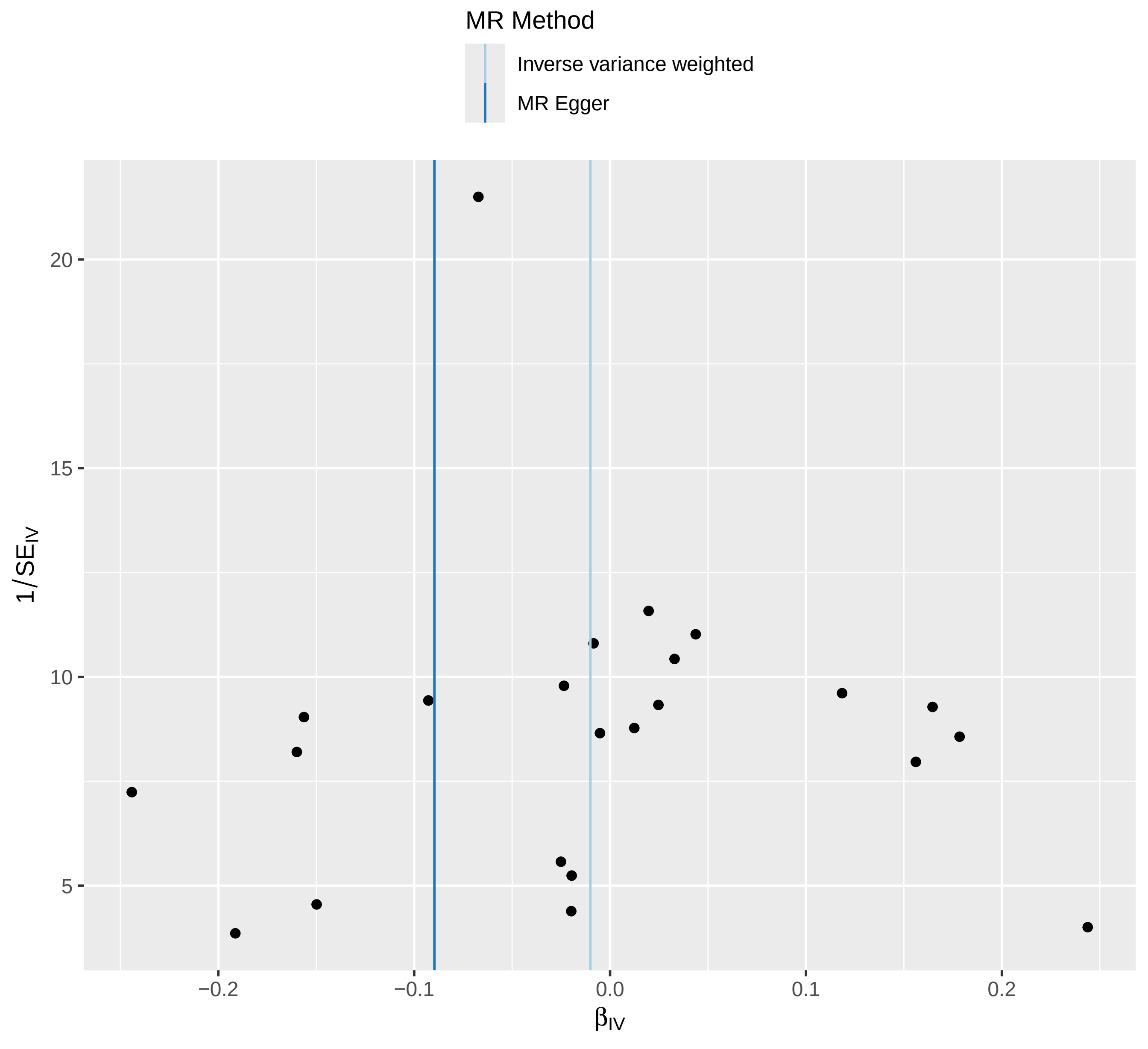
MR Method
Inverse variance weighted
MR Egger
20
15
10
5
1/SE_IV
−0.2
−0.1
0.0
0.1
0.2
β_IV

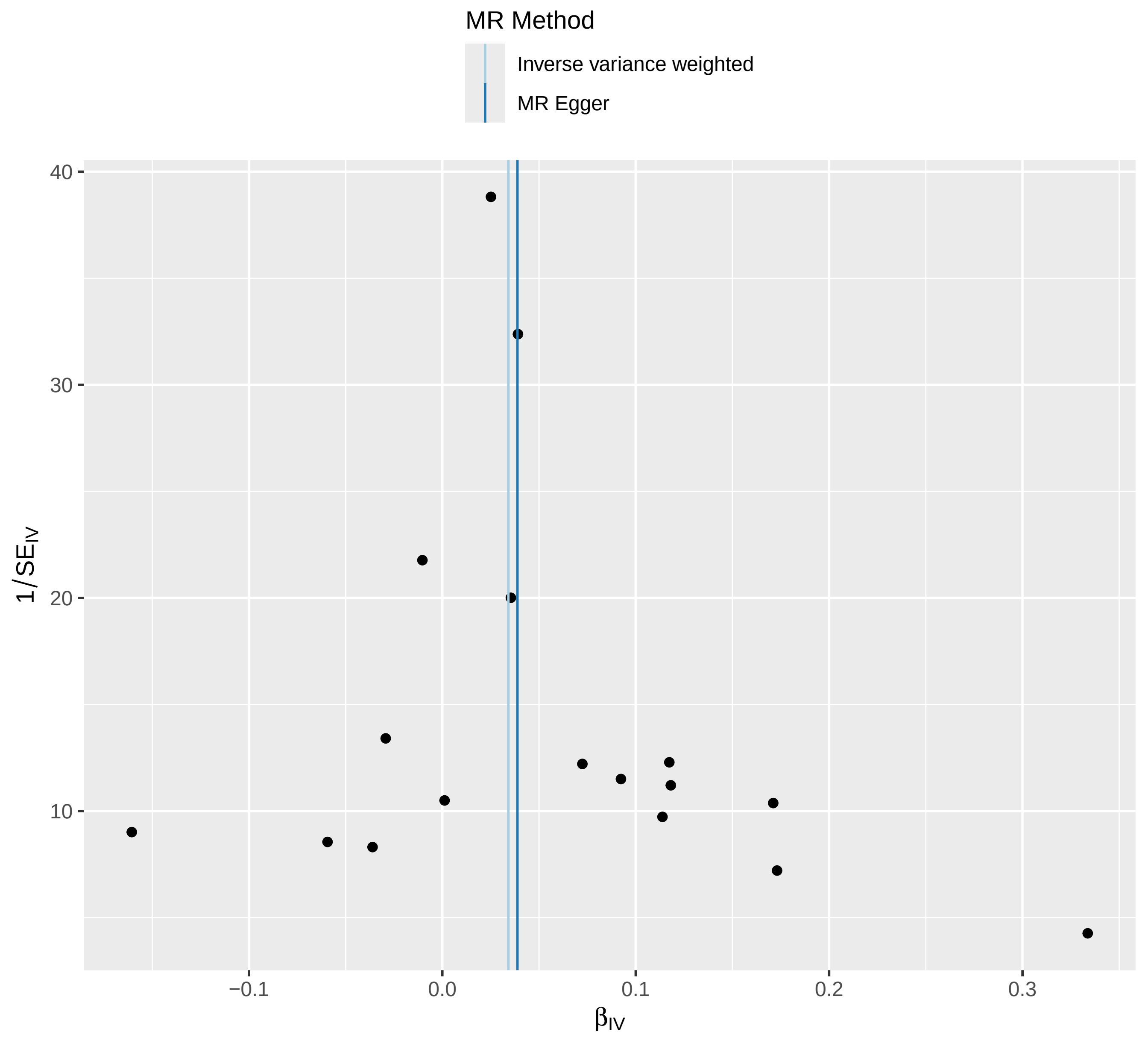
MR Method
Inverse variance weighted
MR Egger
40
30
20
10
1/SE$_{IV}$
−0.1
0.0
0.1
0.2
0.3
β$_{IV}$

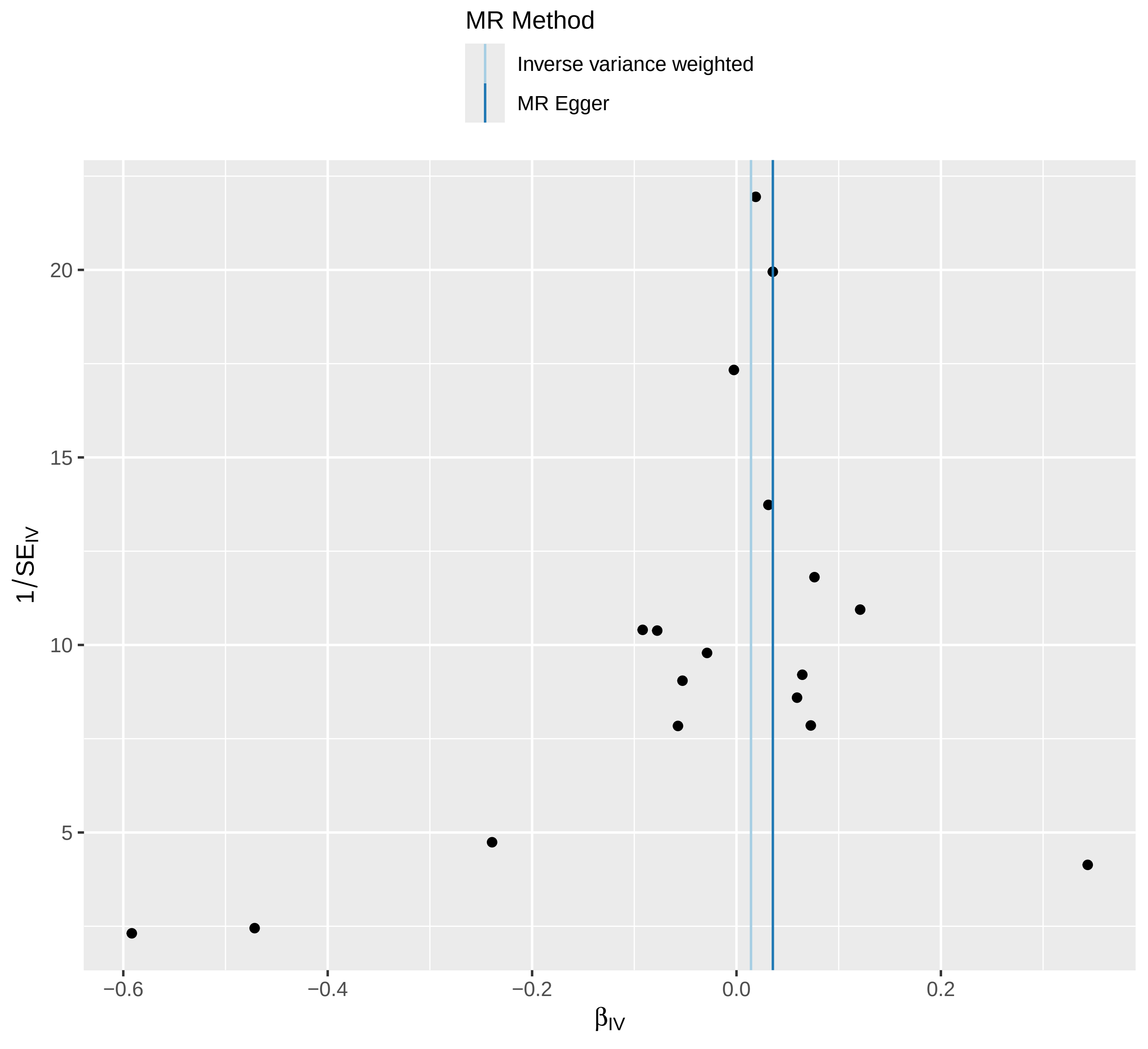
MR Method
Inverse variance weighted
MR Egger
20
15
10
5
1/SE_IV
−0.6
−0.4
−0.2
0.0
0.2
β_IV

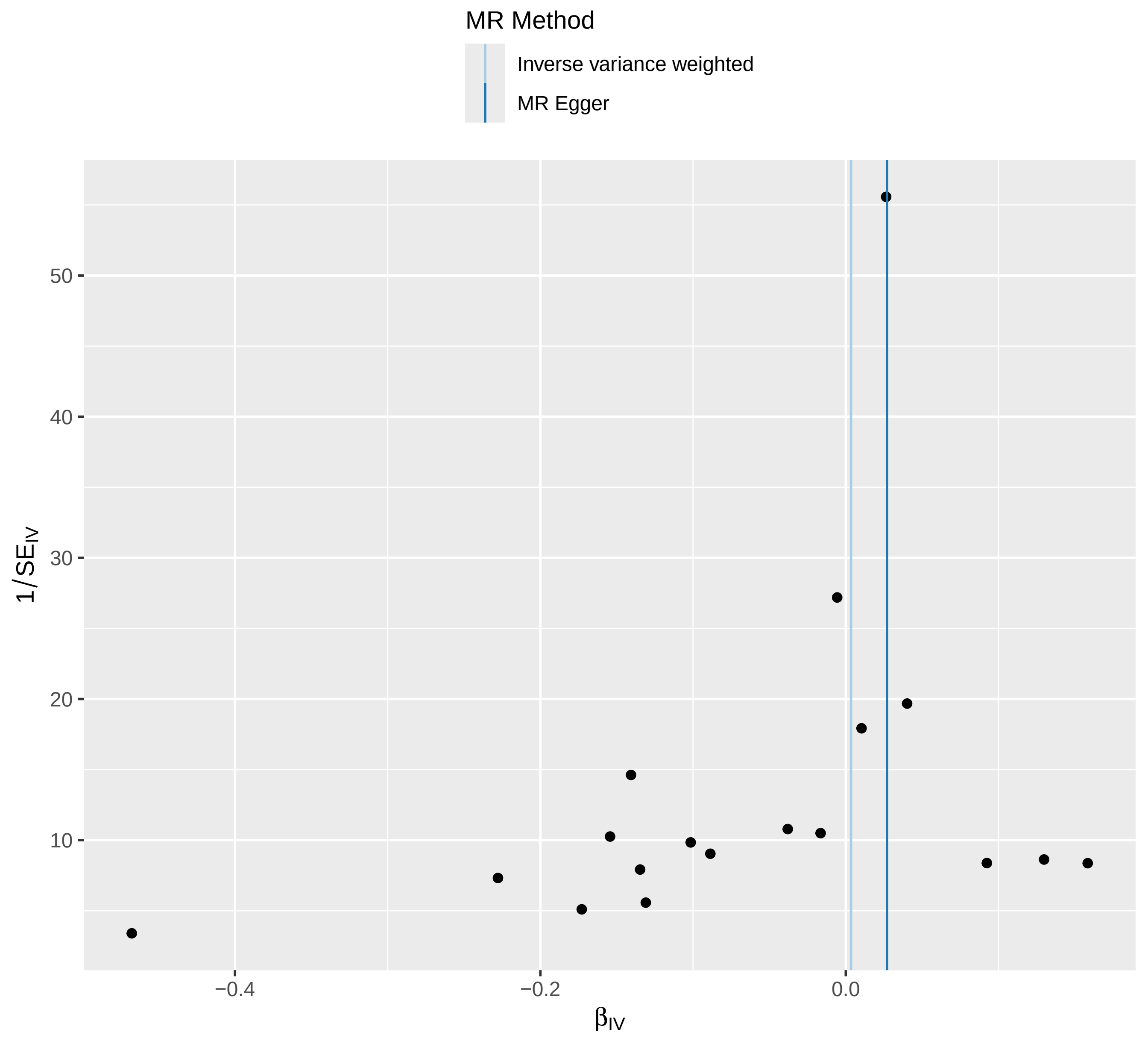
MR Method
Inverse variance weighted
MR Egger
50
40
30
20
10
$1/SE_{IV}$
−0.4
−0.2
0.0
β_{IV}

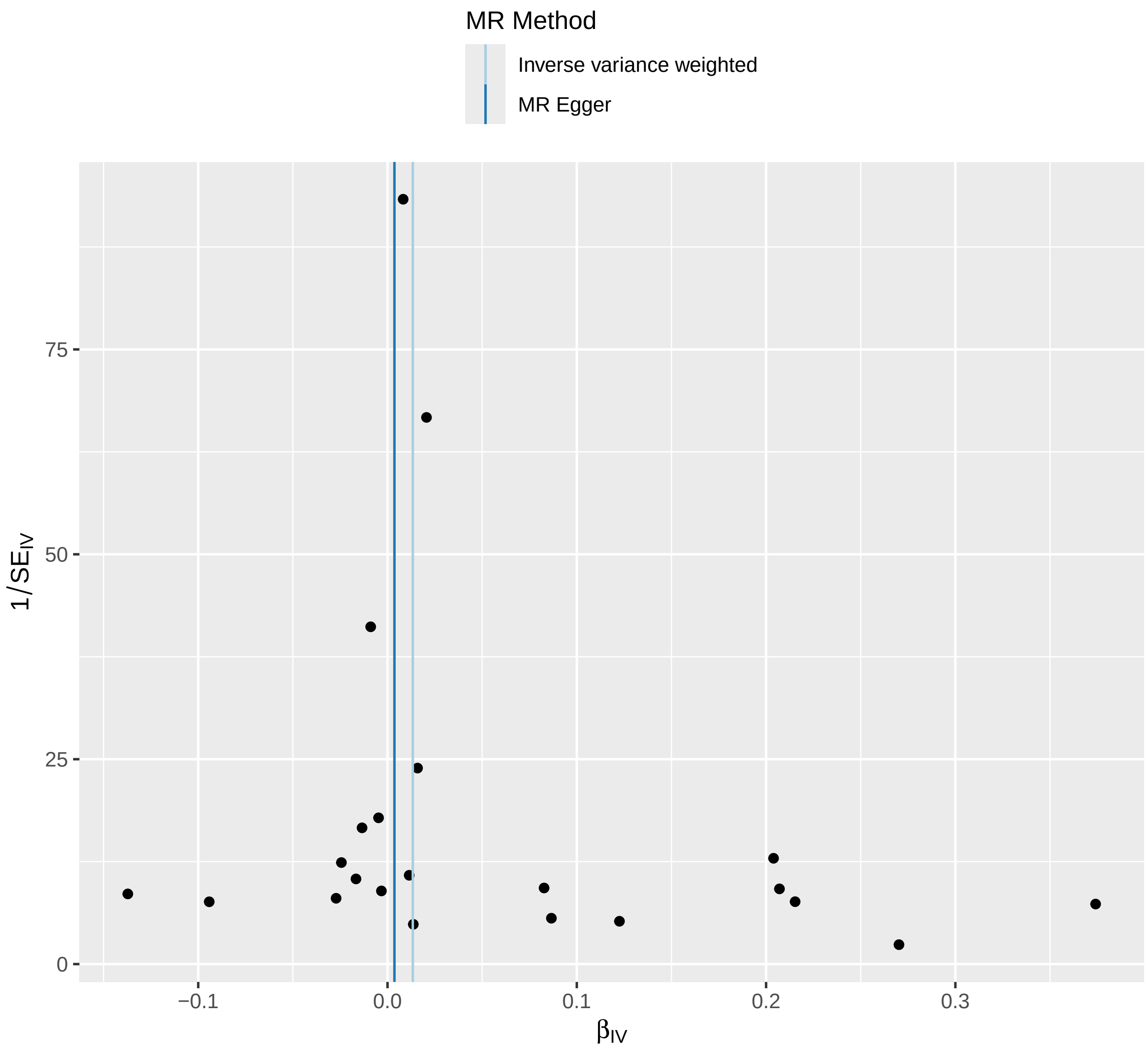

MR Method
Inverse variance weighted
MR Egger
1/SE$_{IV}$
0
25
50
75
−0.1
0.0
0.1
0.2
0.3
β$_{IV}$

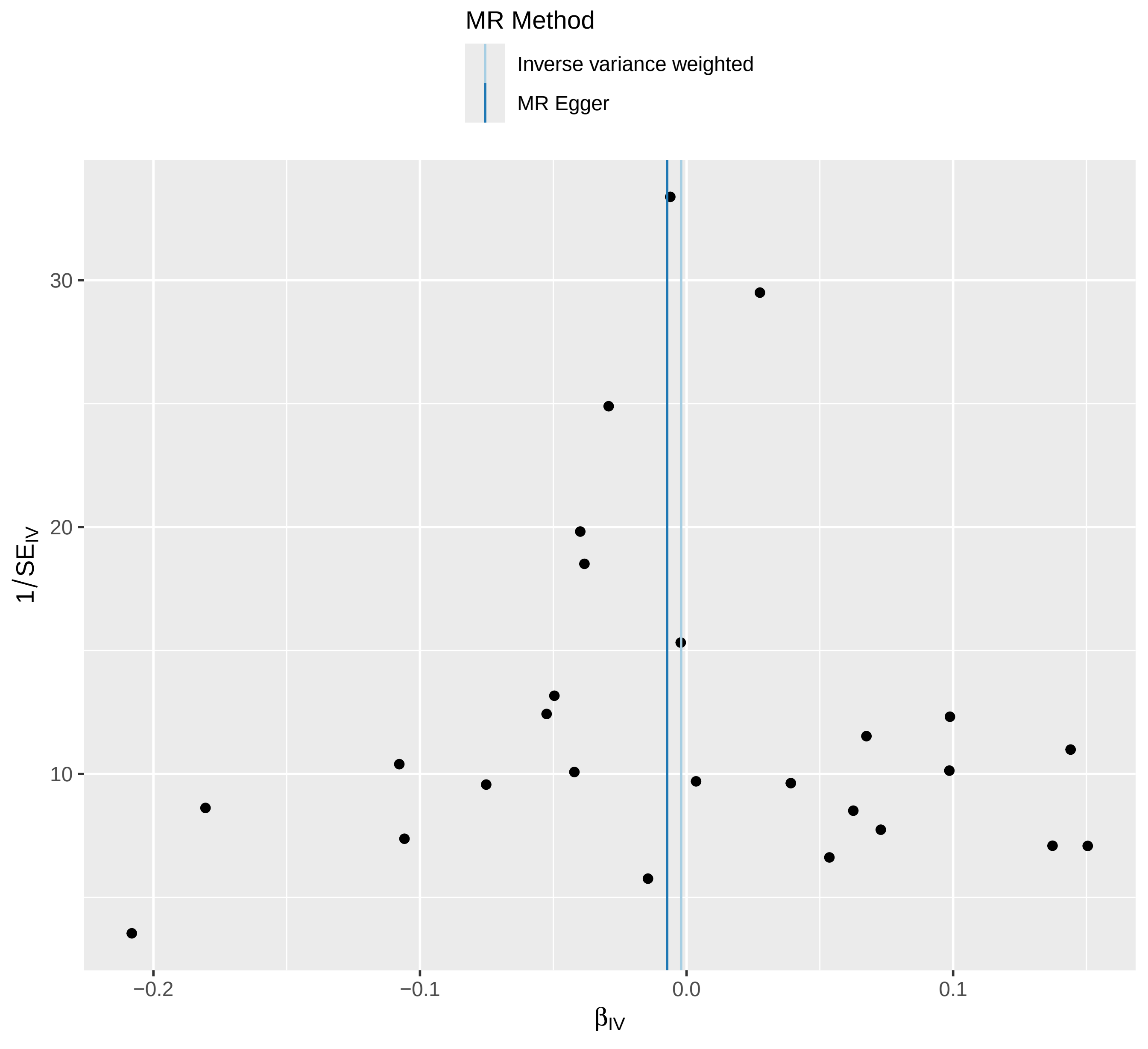
MR Method
Inverse variance weighted
MR Egger
30
20
10
$1/SE_{IV}$
−0.2
−0.1
0.0
0.1
β_{IV}

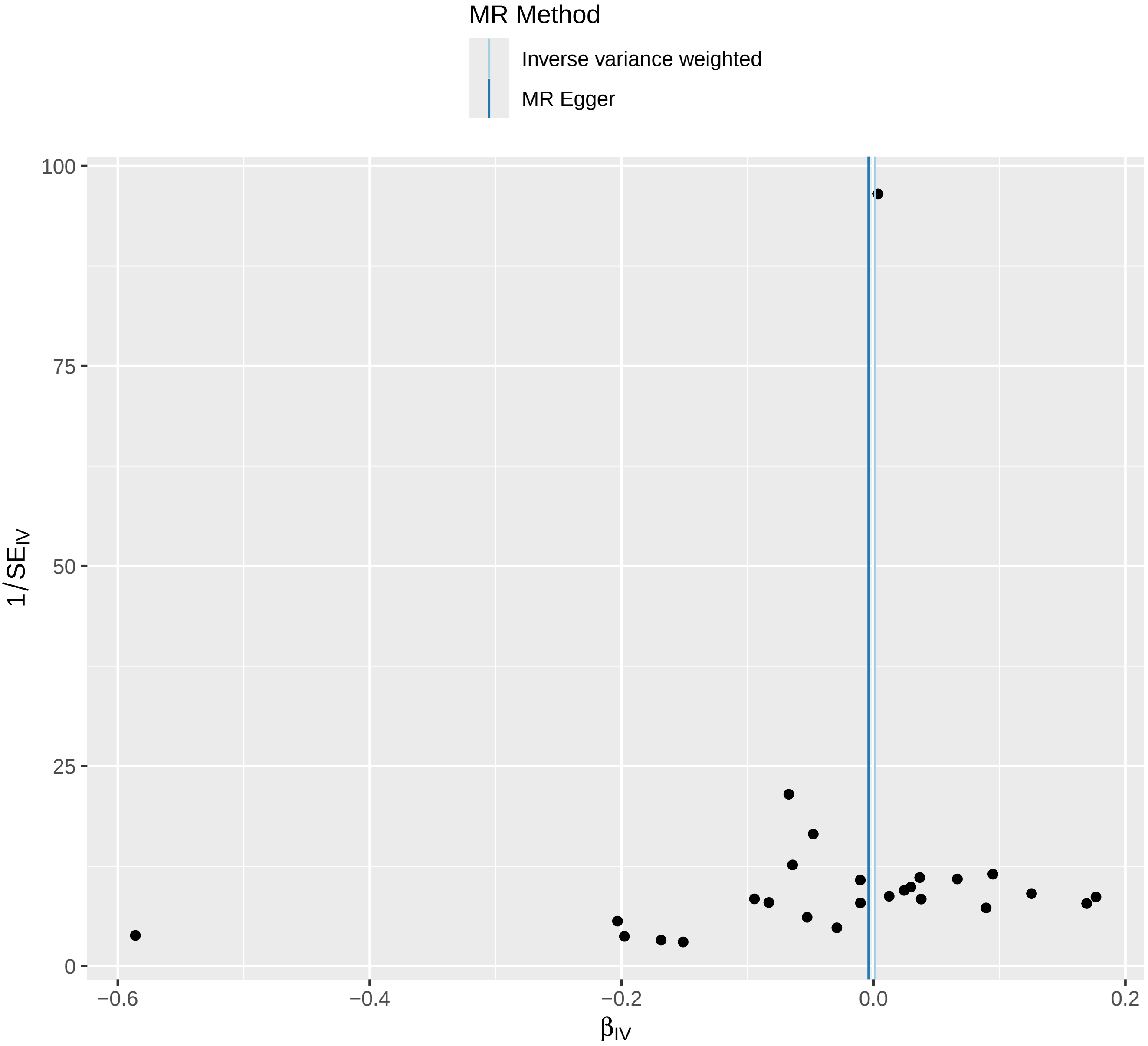
MR Method
Inverse variance weighted
MR Egger
100
75
50
25
0
1/SE_IV
−0.6
−0.4
−0.2
0.0
0.2
β_IV

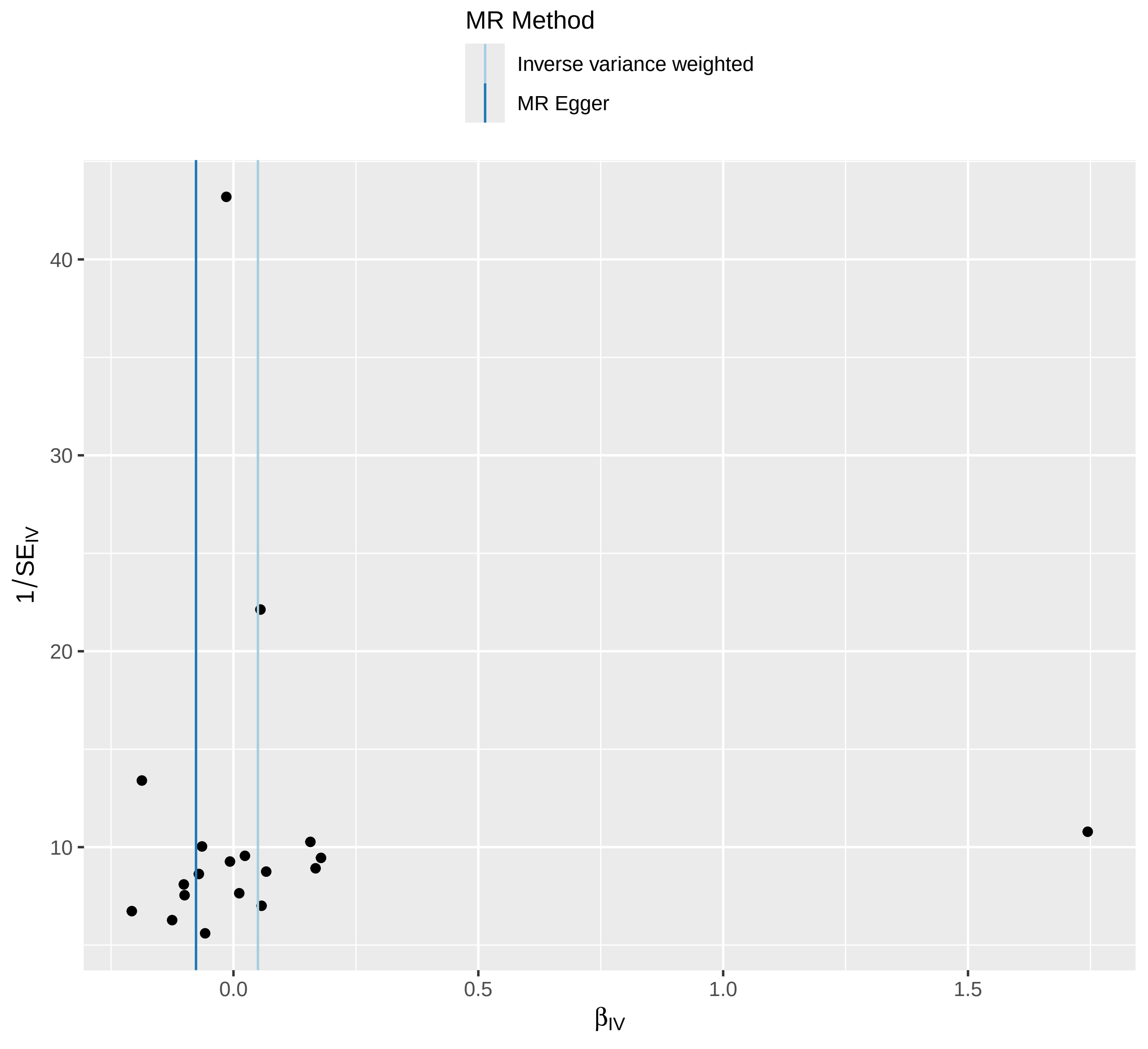
MR Method
Inverse variance weighted
MR Egger
40
30
20
10
1/SE_IV
0.0
0.5
1.0
1.5
β_IV

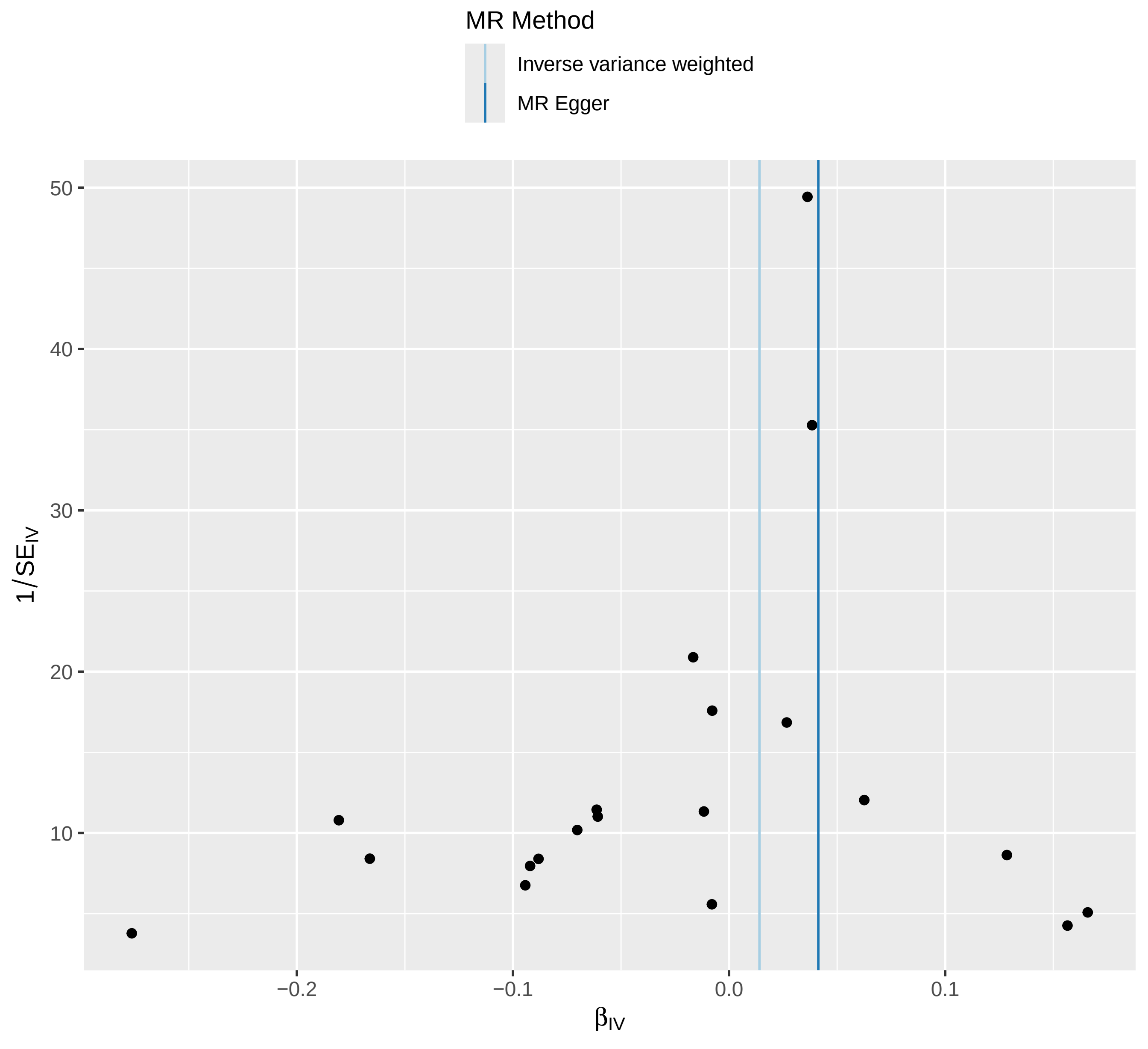
MR Method
Inverse variance weighted
MR Egger
50
40
30
20
10
1/SE_IV
−0.2
−0.1
0.0
0.1
β_IV

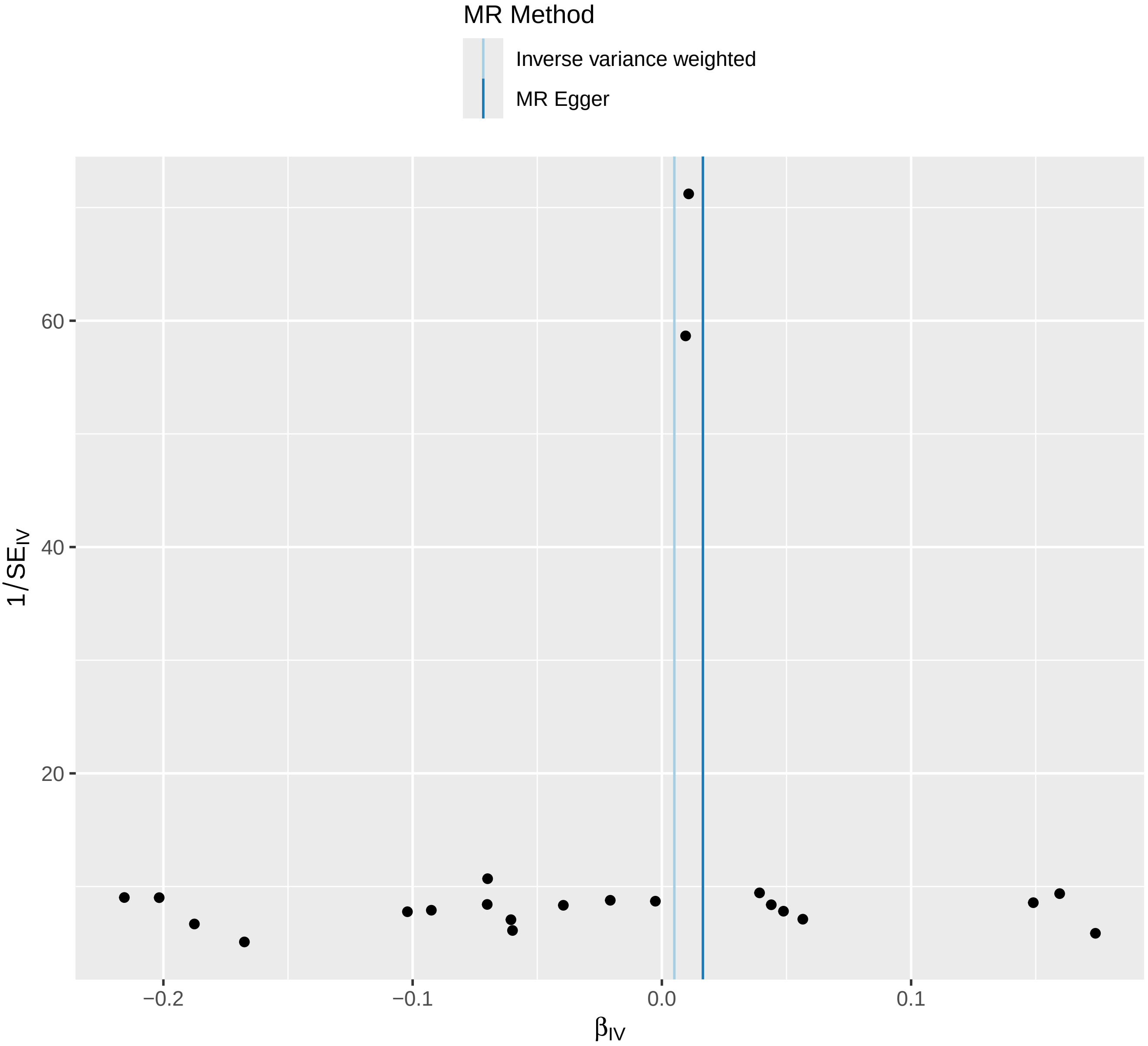
MR Method
Inverse variance weighted
MR Egger
60
40
20
1/SE$_{IV}$
−0.2
−0.1
0.0
0.1
β$_{IV}$

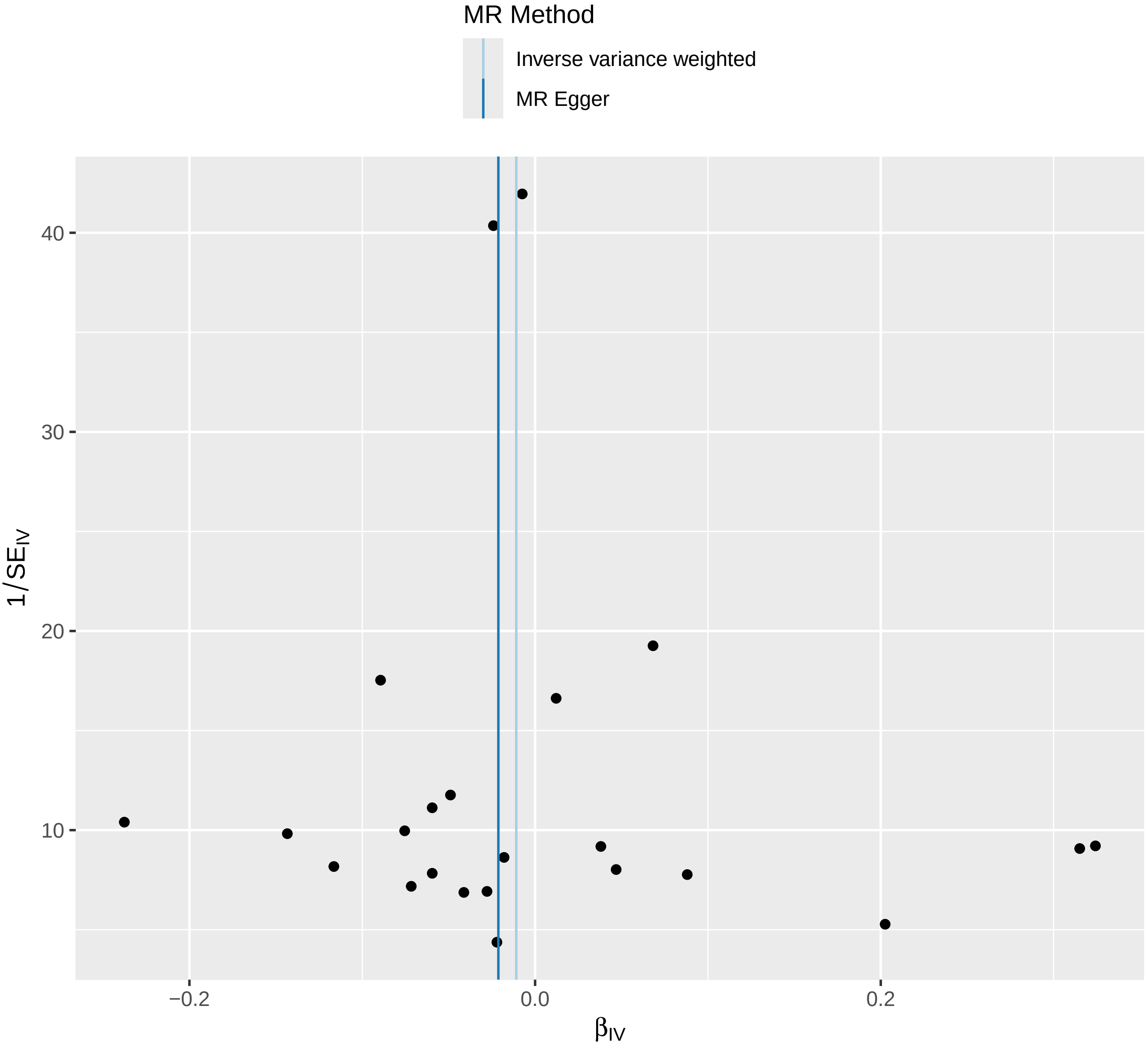
MR Method
Inverse variance weighted
MR Egger
40
30
20
10
1/SE_{IV}
−0.2
0.0
0.2
β_{IV}

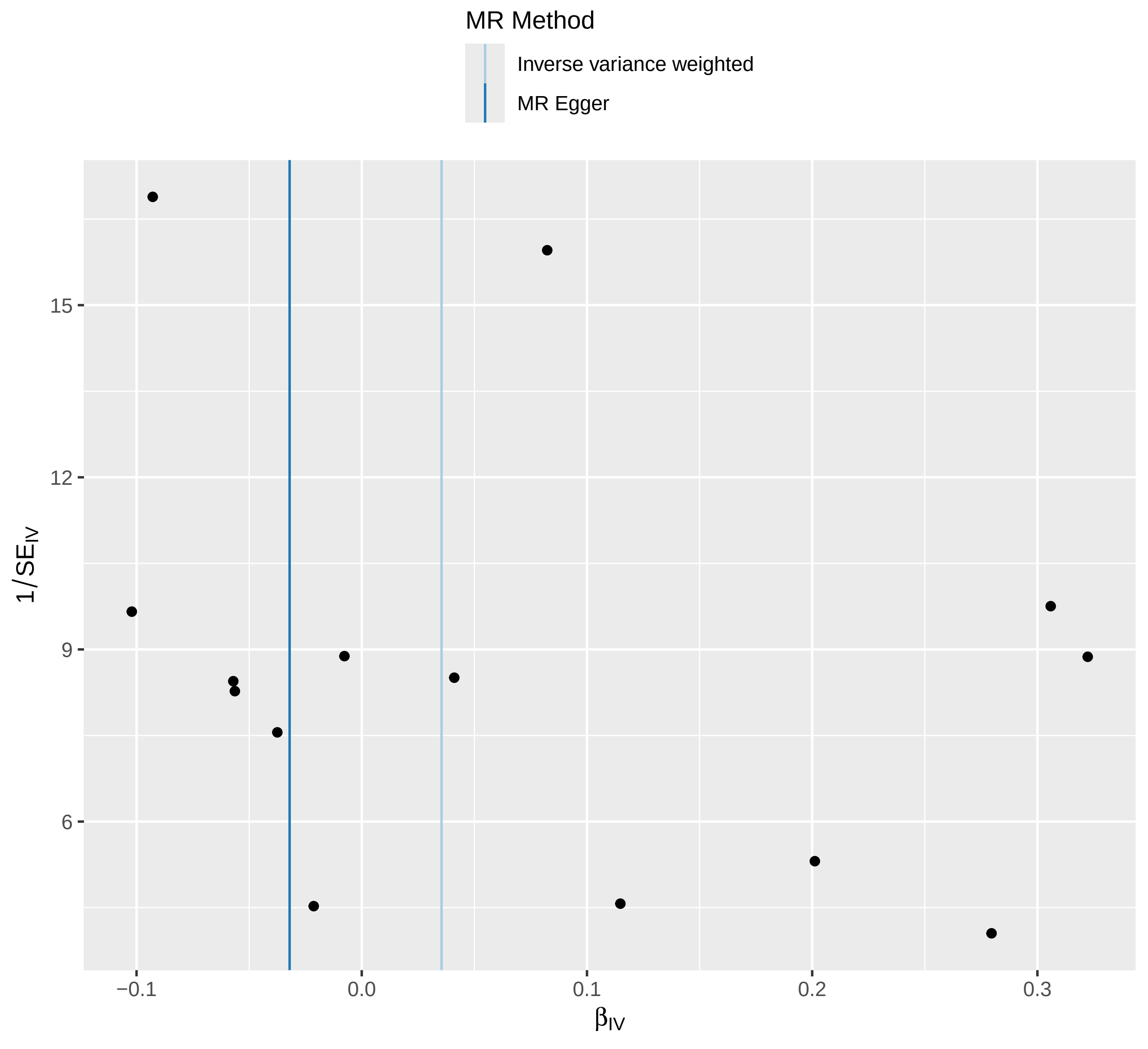
MR Method
Inverse variance weighted
MR Egger
15
12
9
6
1/SE_IV
−0.1
0.0
0.1
0.2
0.3
β_IV

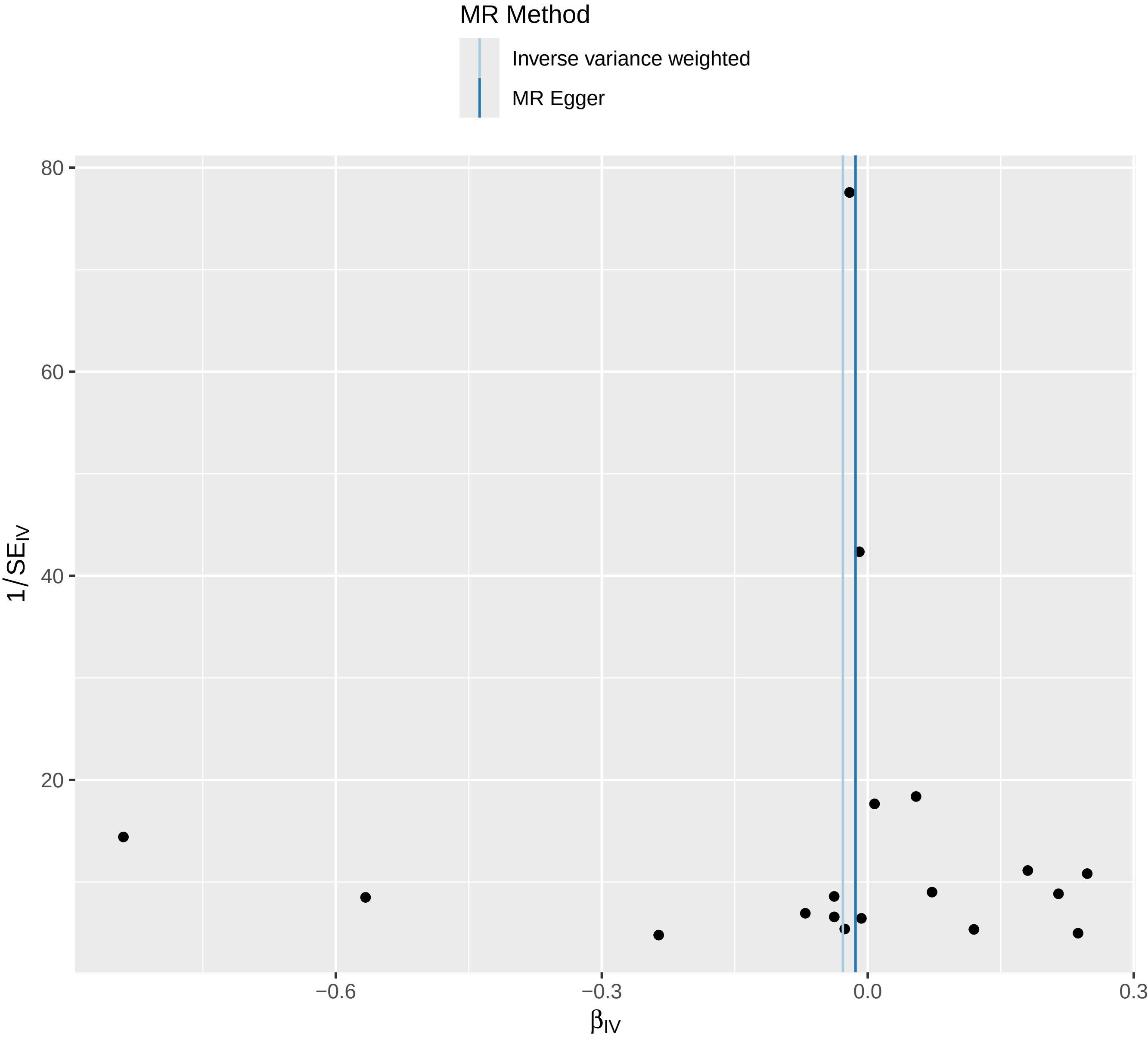

MR Method
Inverse variance weighted
MR Egger
80
60
40
20
1/SE_IV
−0.6
−0.3
0.0
0.3
β_IV

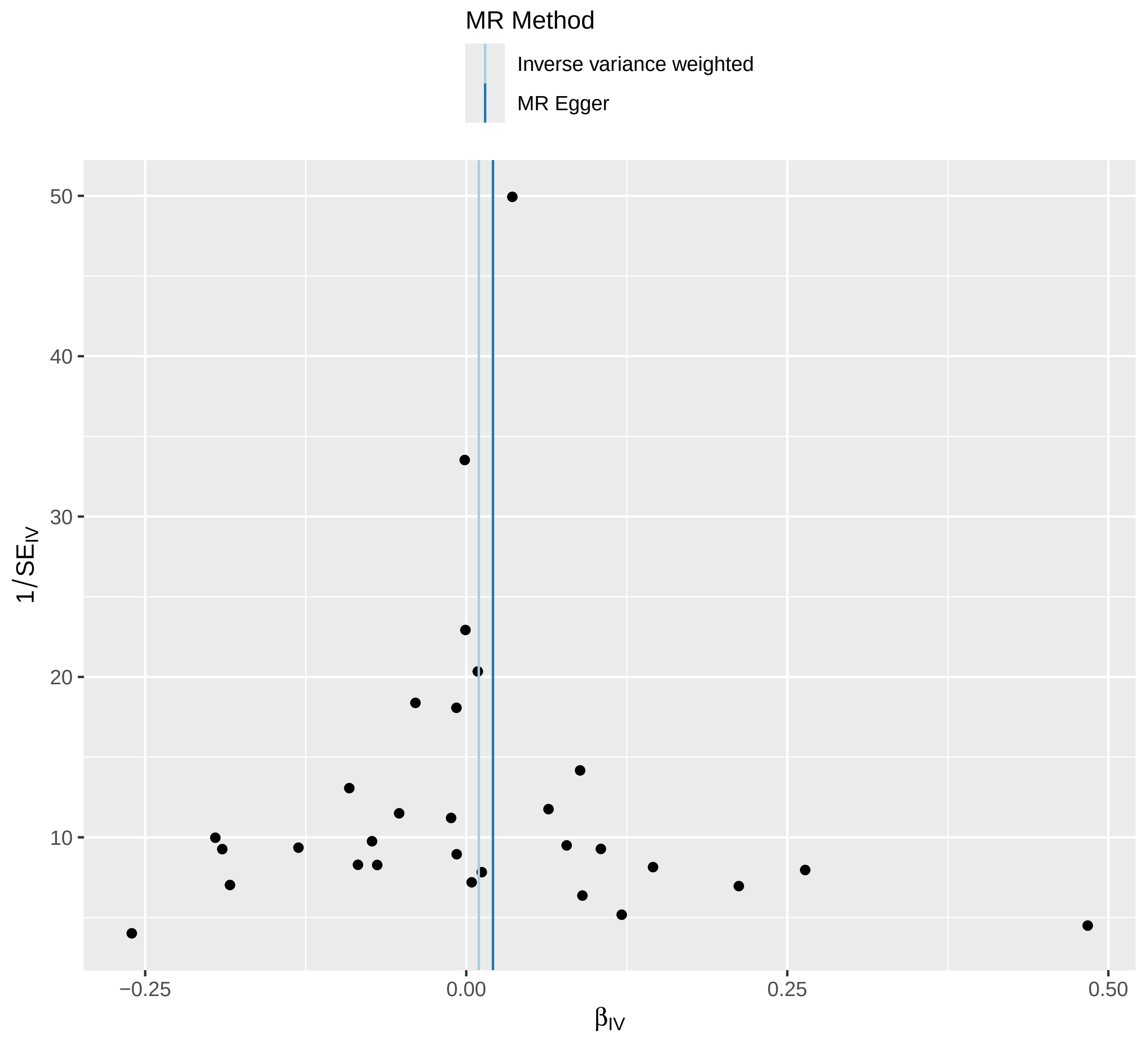

MR Method
Inverse variance weighted
MR Egger
50
40
30
20
10
1/SE_IV
−0.25
0.00
0.25
0.50
β_IV

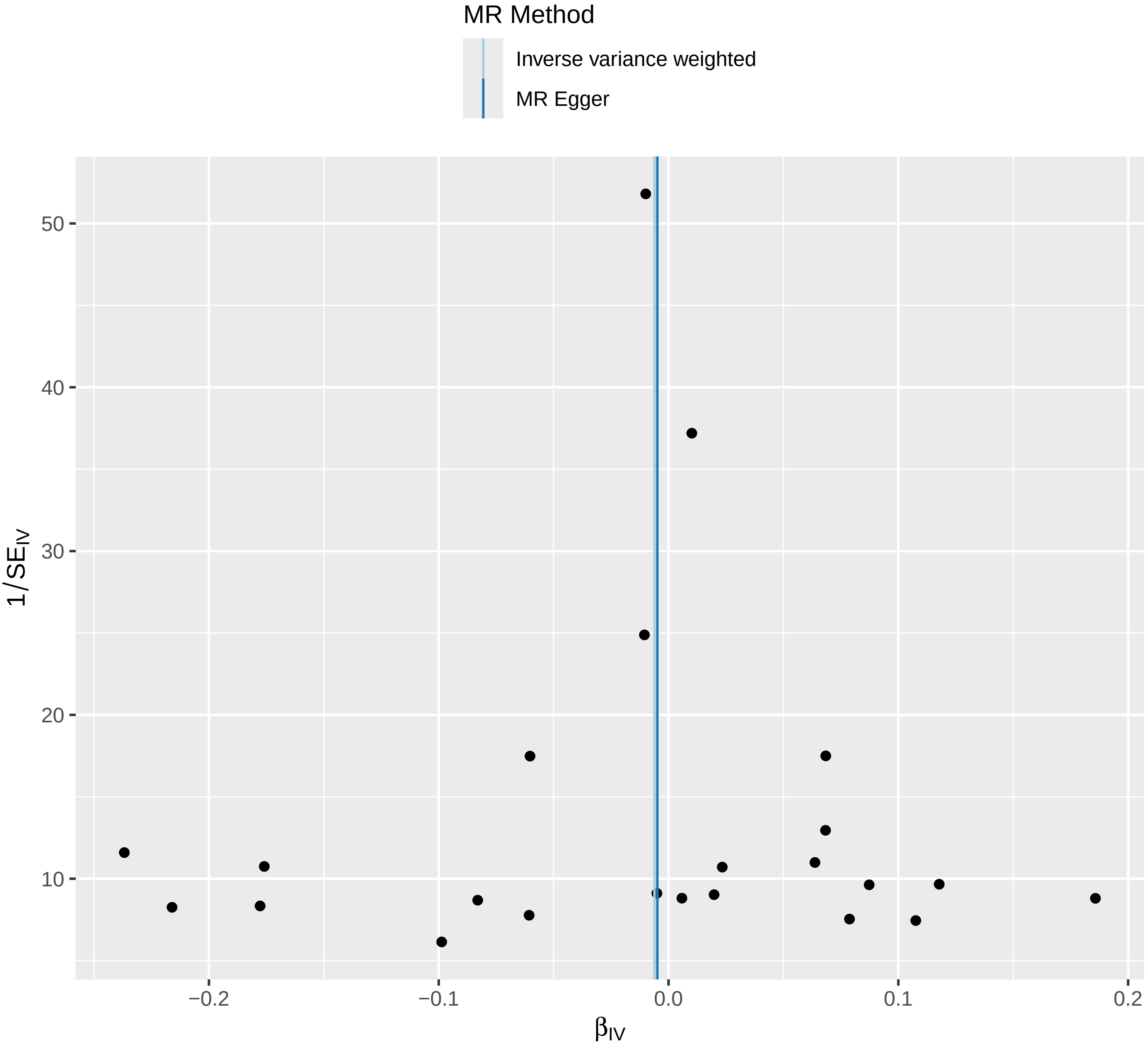
MR Method
Inverse variance weighted
MR Egger
1/SE_IV
50
40
30
20
10
β_IV
−0.2
−0.1
0.0
0.1
0.2

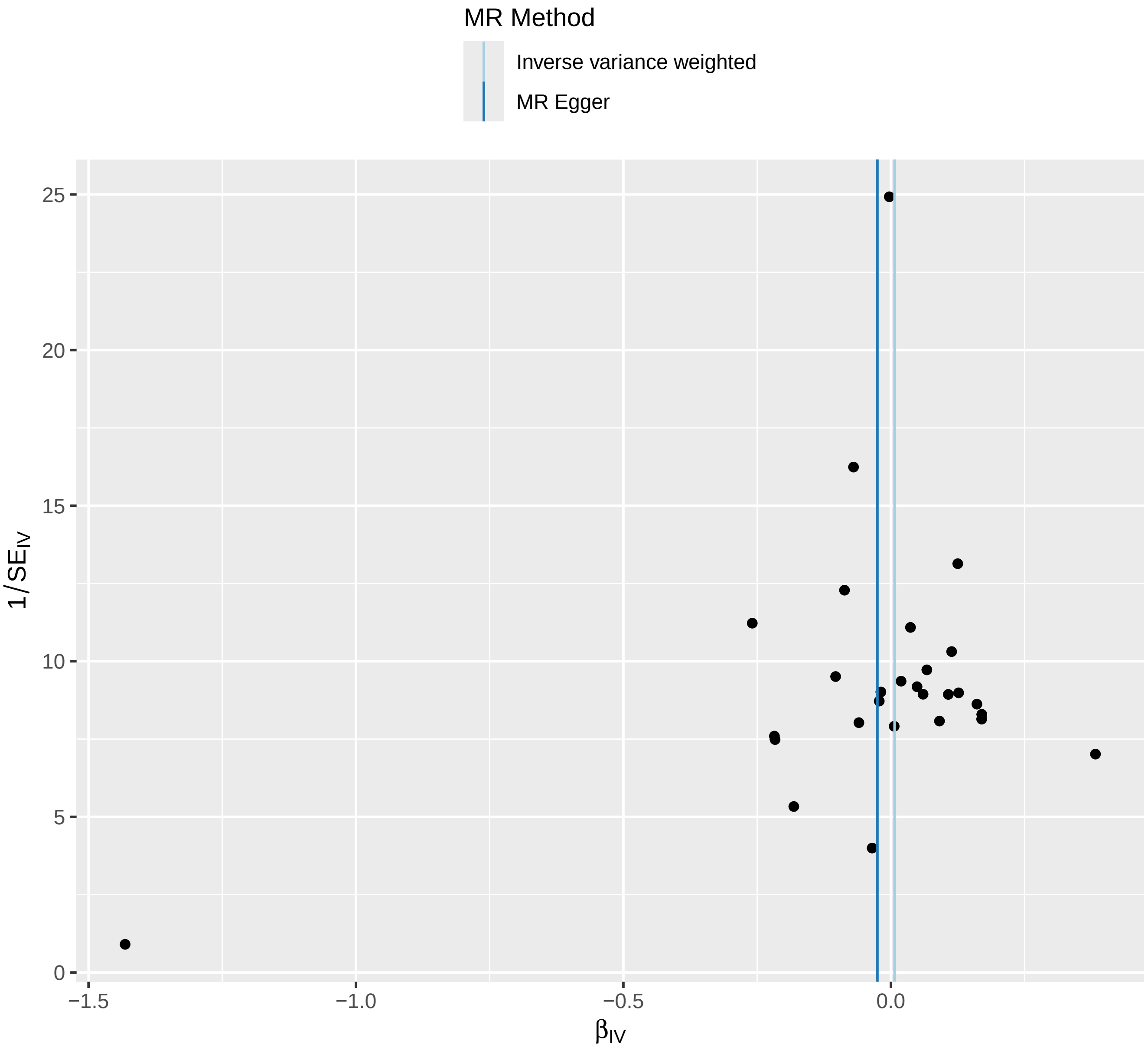

MR Method
Inverse variance weighted
MR Egger
25
20
15
10
5
0
1/SE_IV
−1.5
−1.0
−0.5
0.0
β_IV

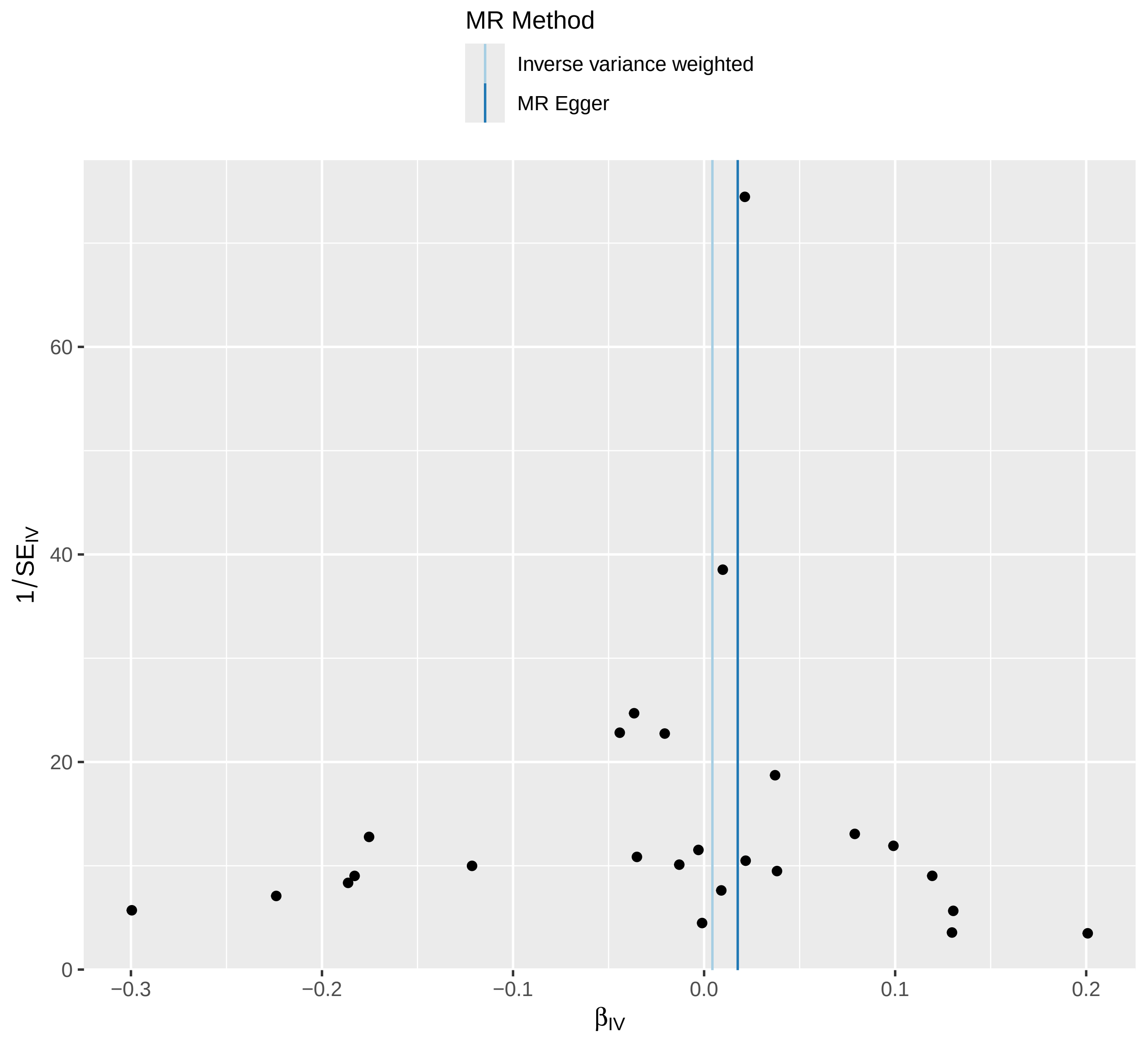

MR Method
Inverse variance weighted
MR Egger
1/SE_IV
60
40
20
0
−0.3
−0.2
−0.1
0.0
0.1
0.2
β_IV

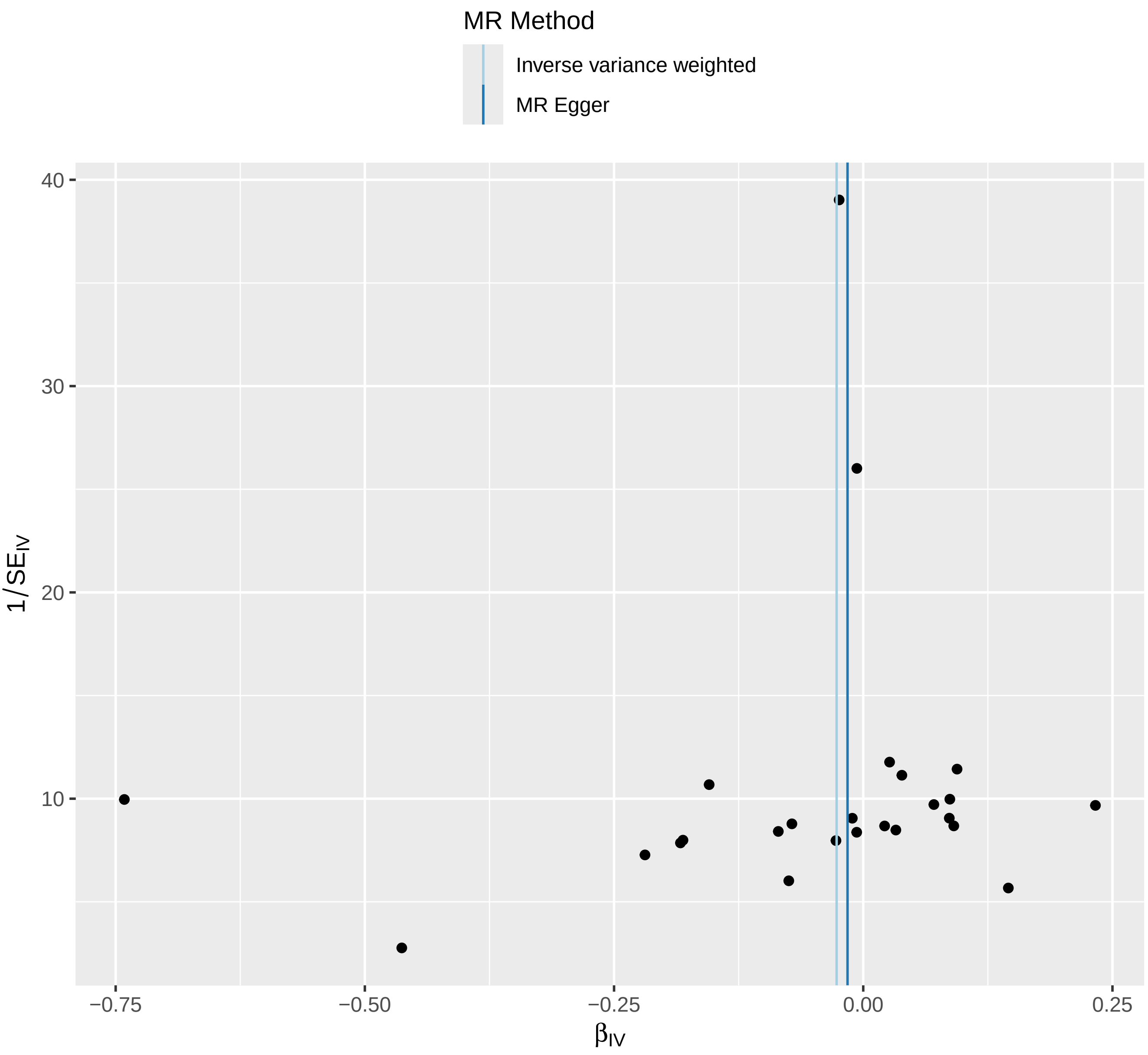

MR Method
Inverse variance weighted
MR Egger
40
30
20
10
1/SE_IV
−0.75
−0.50
−0.25
0.00
0.25
β_IV

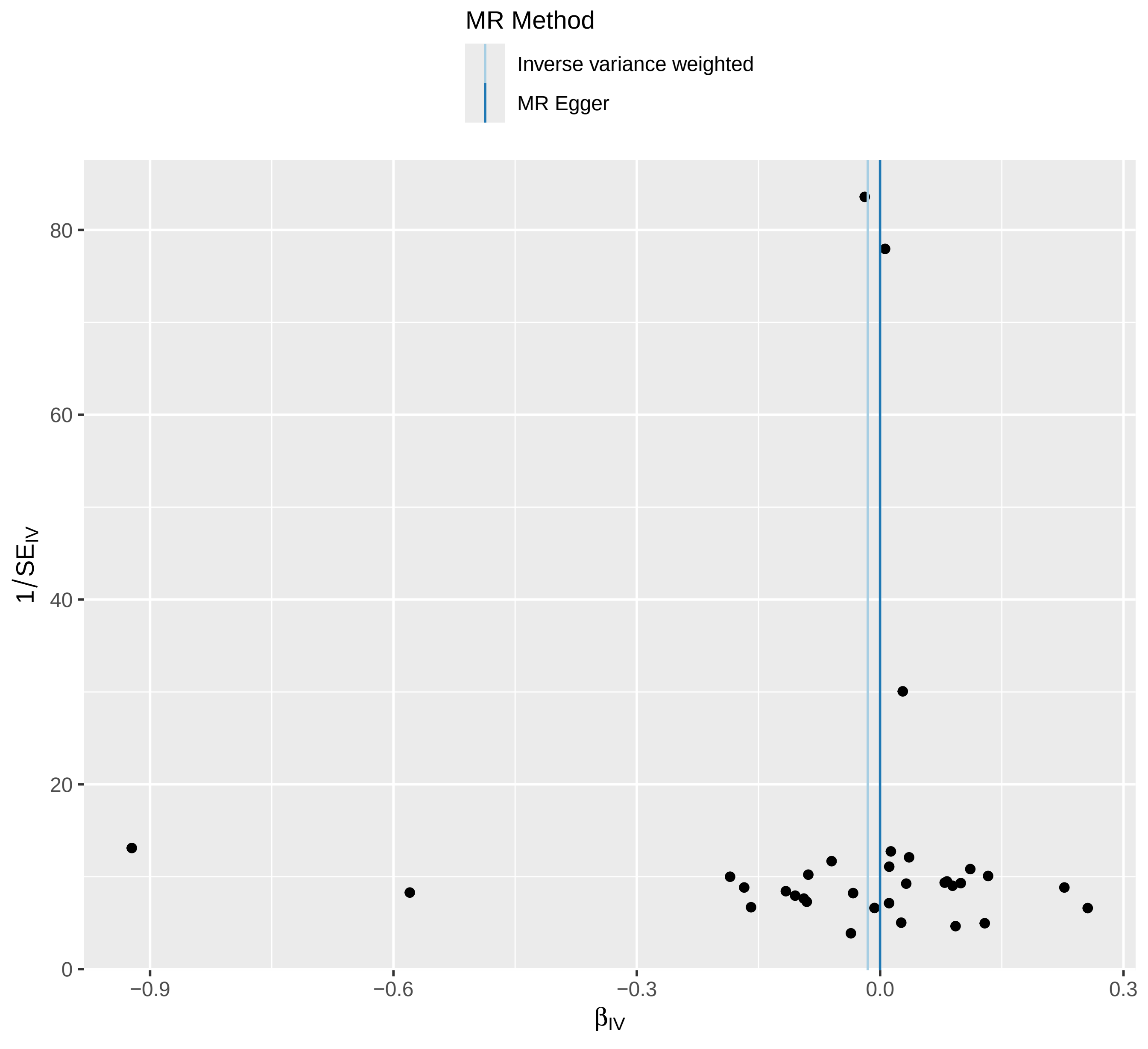
MR Method
Inverse variance weighted
MR Egger
80
60
40
20
0
1/SE_IV
−0.9
−0.6
−0.3
0.0
0.3
β_IV

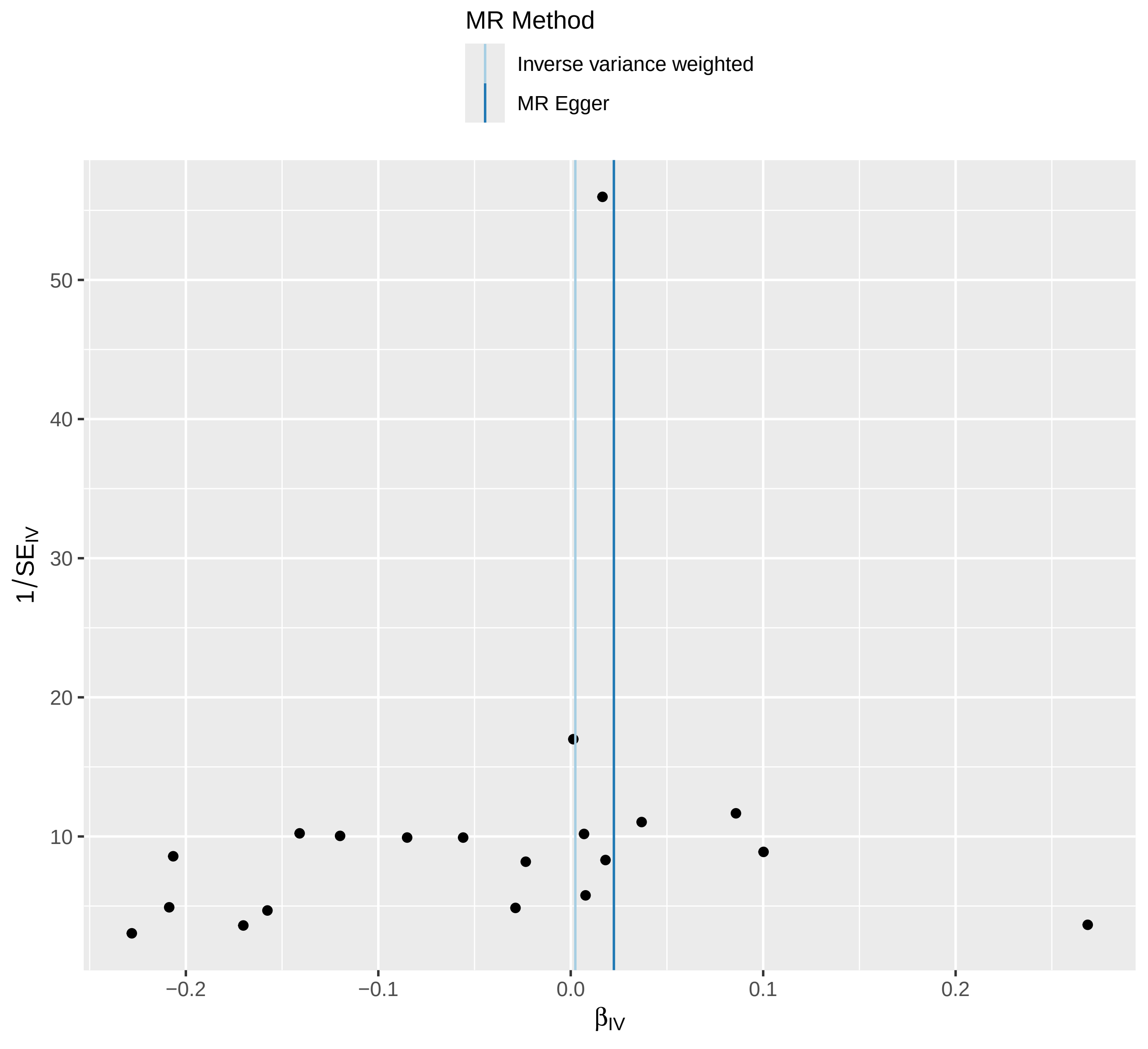

MR Method
Inverse variance weighted
MR Egger
50
40
30
20
10
1/SE_IV
−0.2
−0.1
0.0
0.1
0.2
β_IV

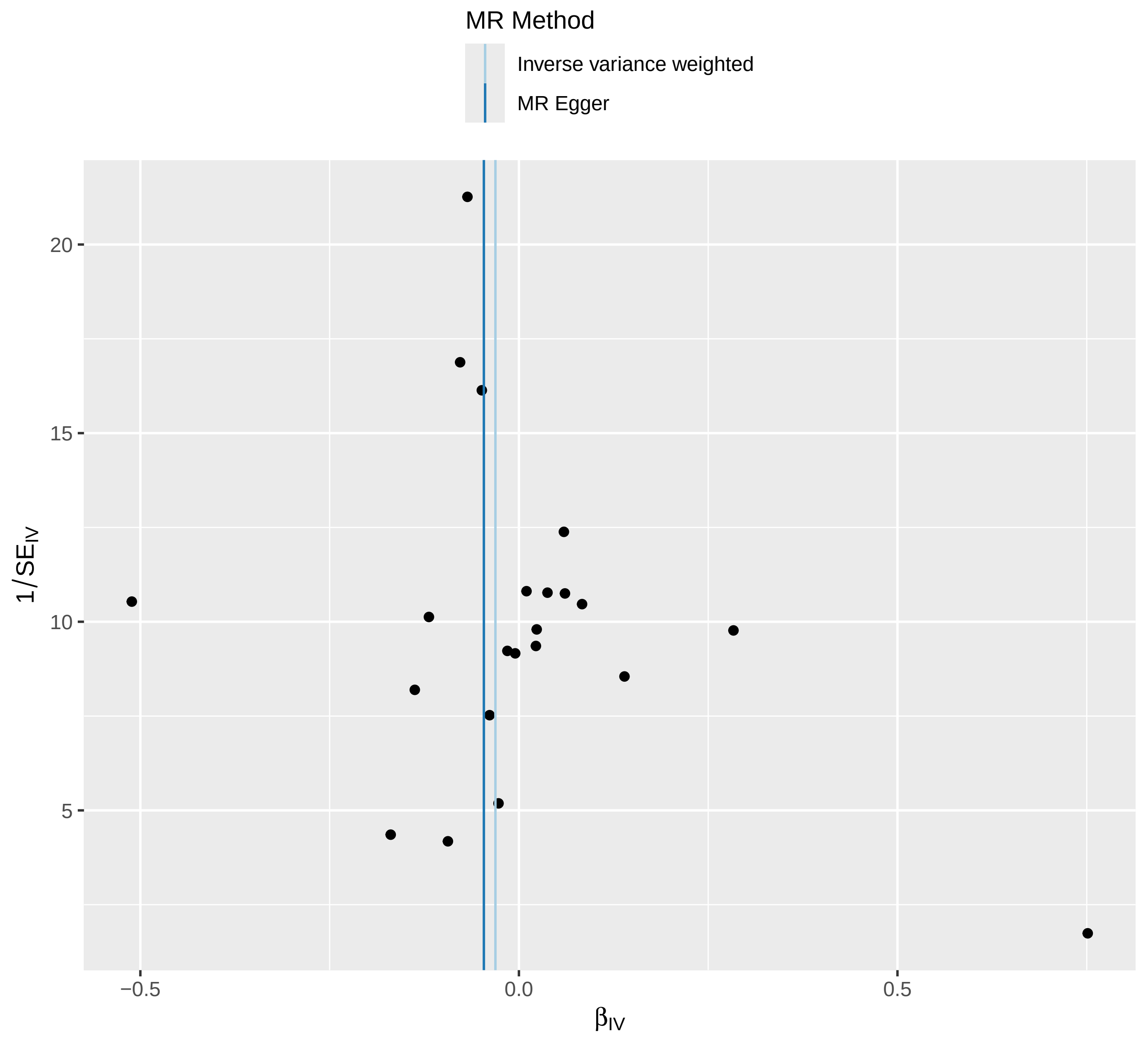
MR Method
Inverse variance weighted
MR Egger
20
15
10
5
1/SE_IV
−0.5
0.0
0.5
β_IV

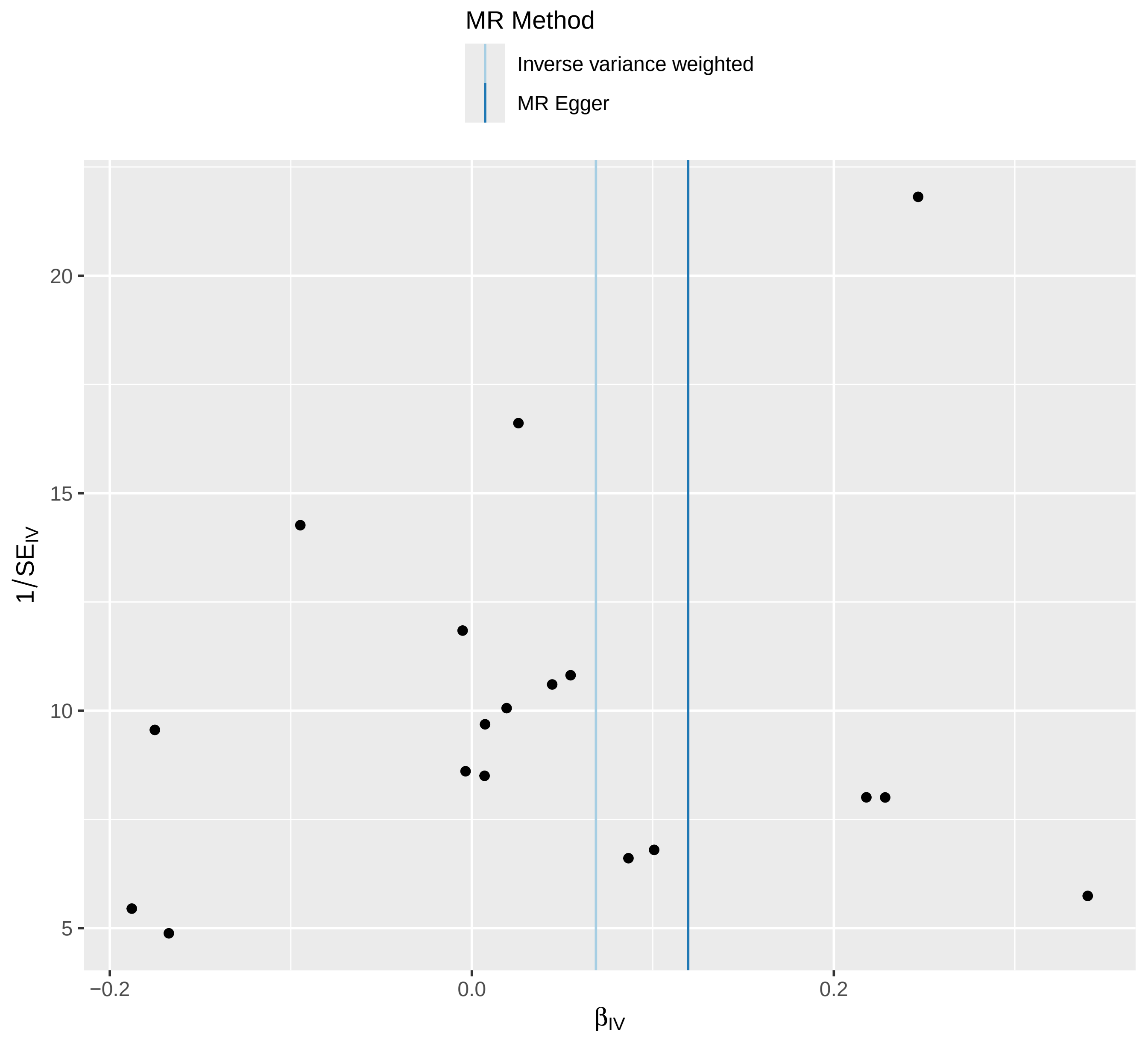
MR Method
Inverse variance weighted
MR Egger
20
15
10
5
−0.2
0.0
0.2
1/SE_IV
β_IV

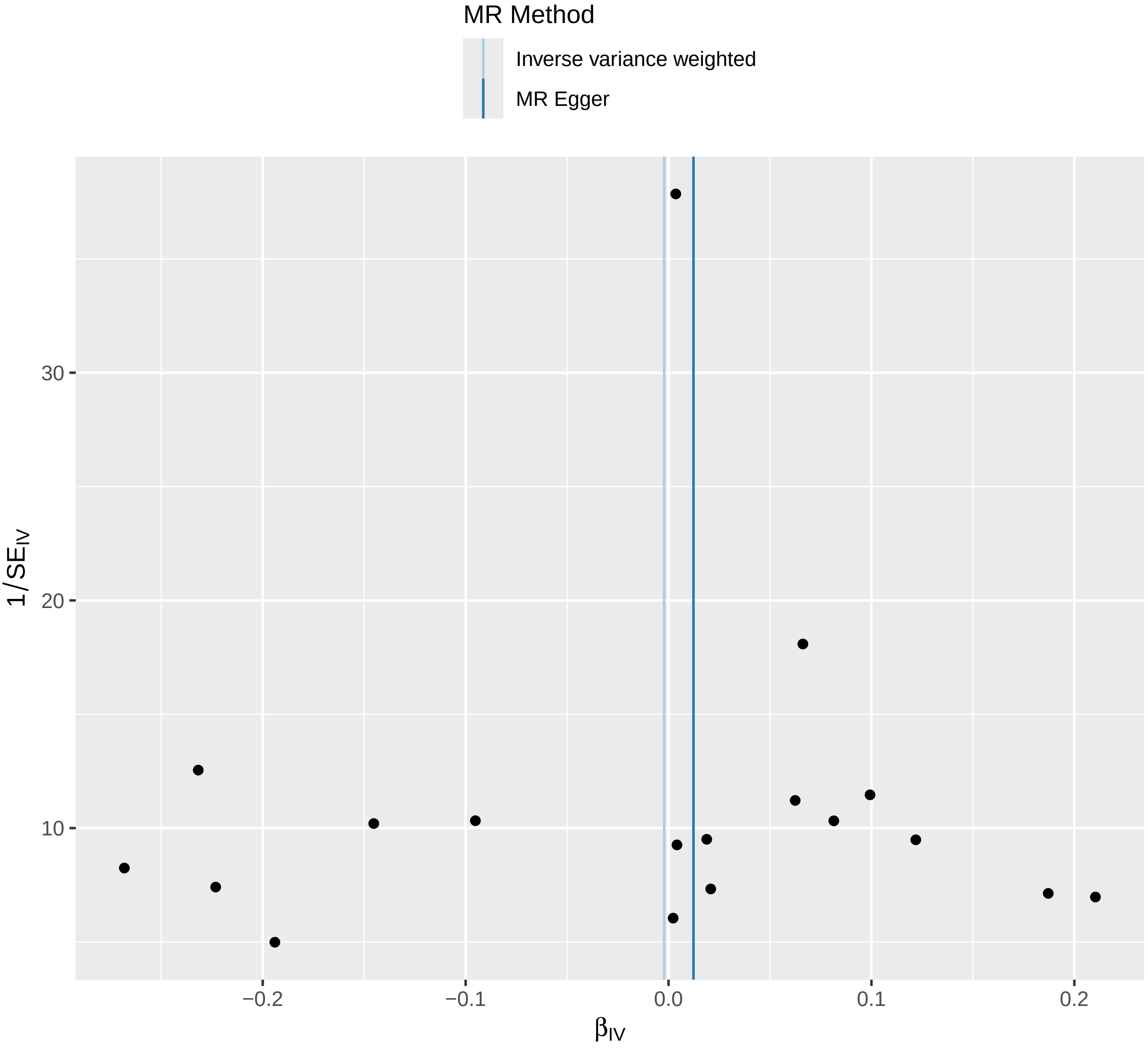
MR Method
Inverse variance weighted
MR Egger
30
20
10
1/SE$_{IV}$
−0.2
−0.1
0.0
0.1
0.2
β$_{IV}$

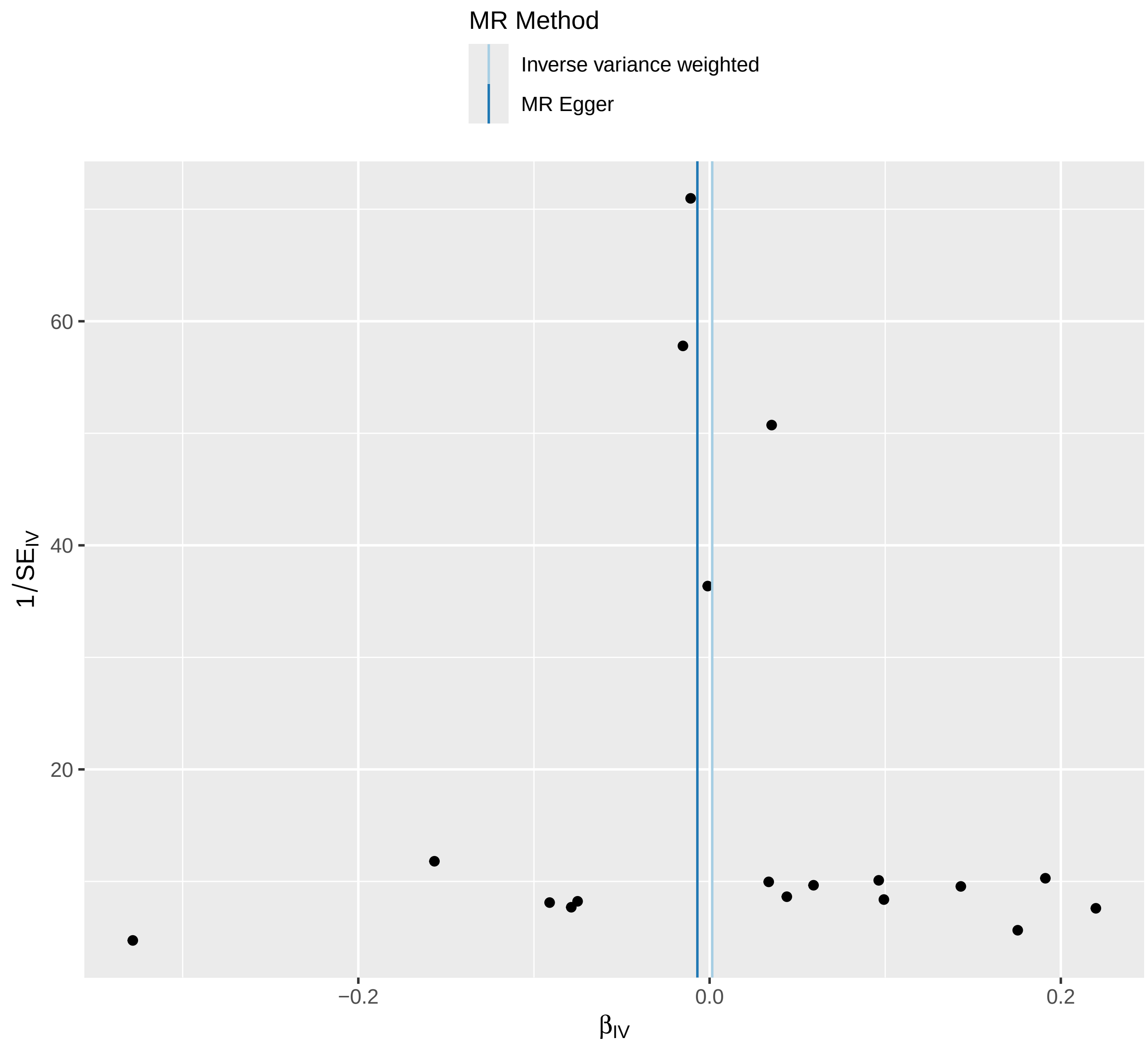
MR Method
Inverse variance weighted
MR Egger
60
40
20
1/SE$_{IV}$
−0.2
0.0
0.2
β_{IV}

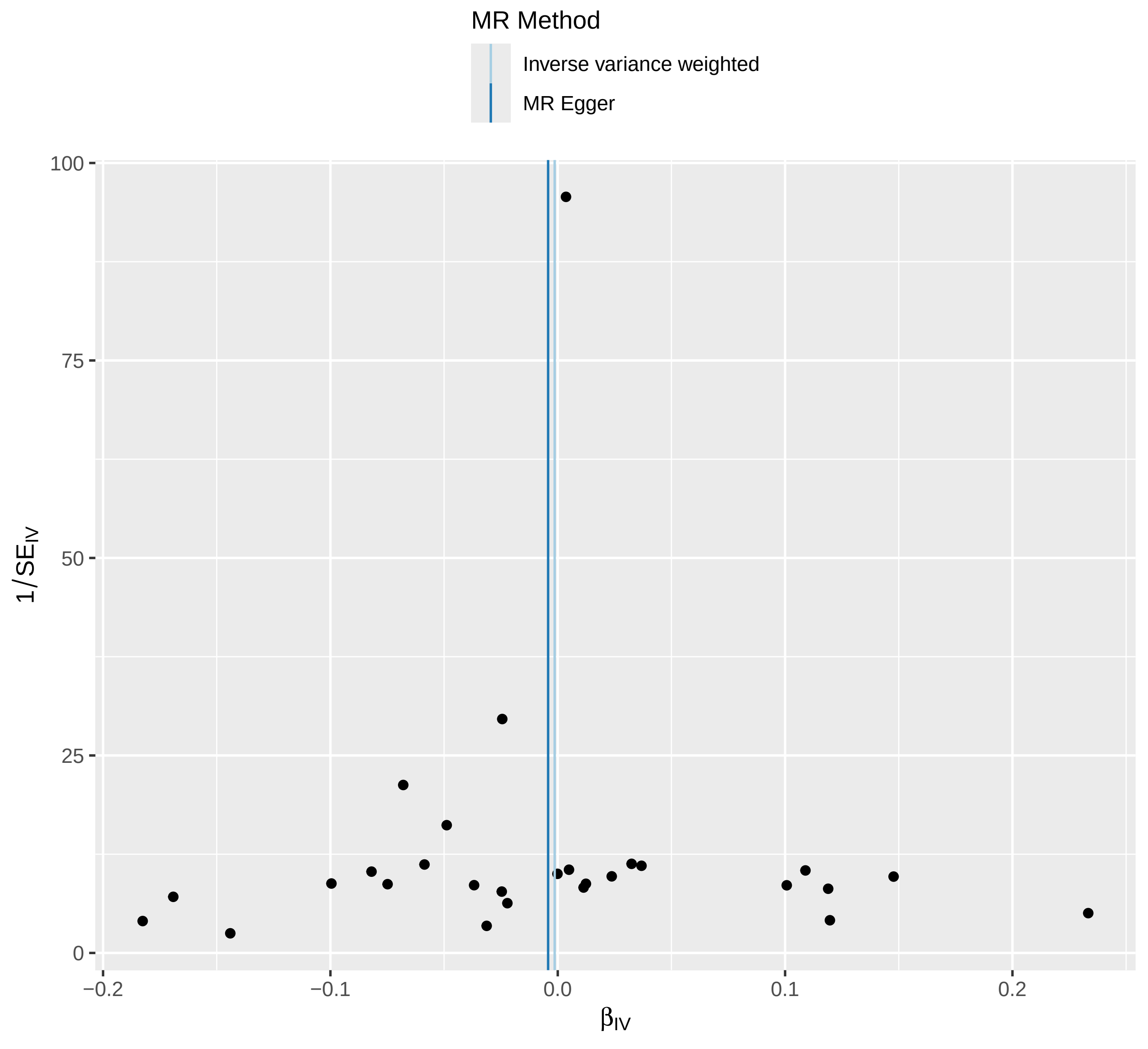
MR Method
Inverse variance weighted
MR Egger
100
75
50
25
0
1/SE$_{IV}$
−0.2
−0.1
0.0
0.1
0.2
β$_{IV}$

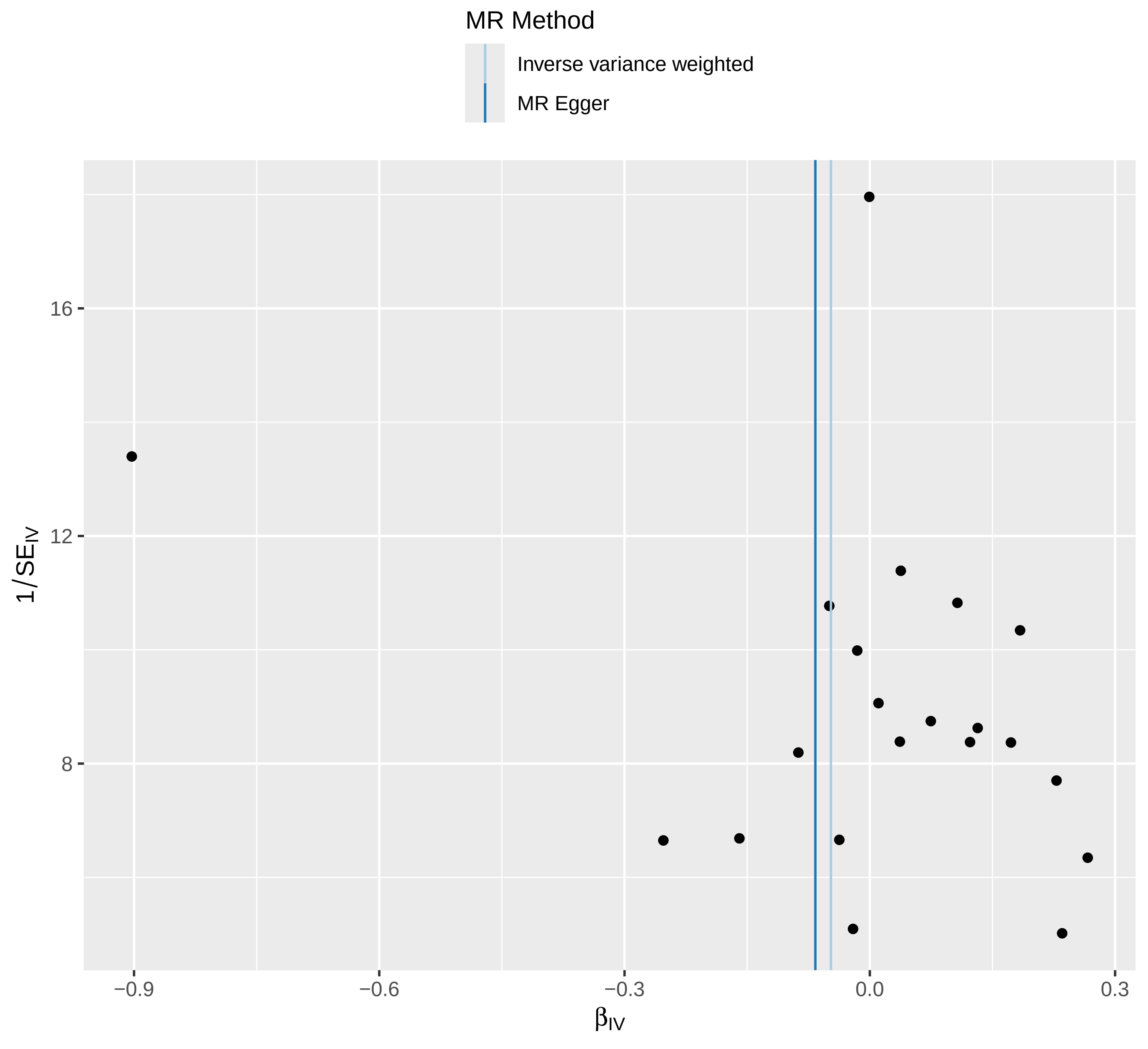
MR Method
Inverse variance weighted
MR Egger
16
12
8
1/SE$_{IV}$
−0.9
−0.6
−0.3
0.0
0.3
β$_{IV}$

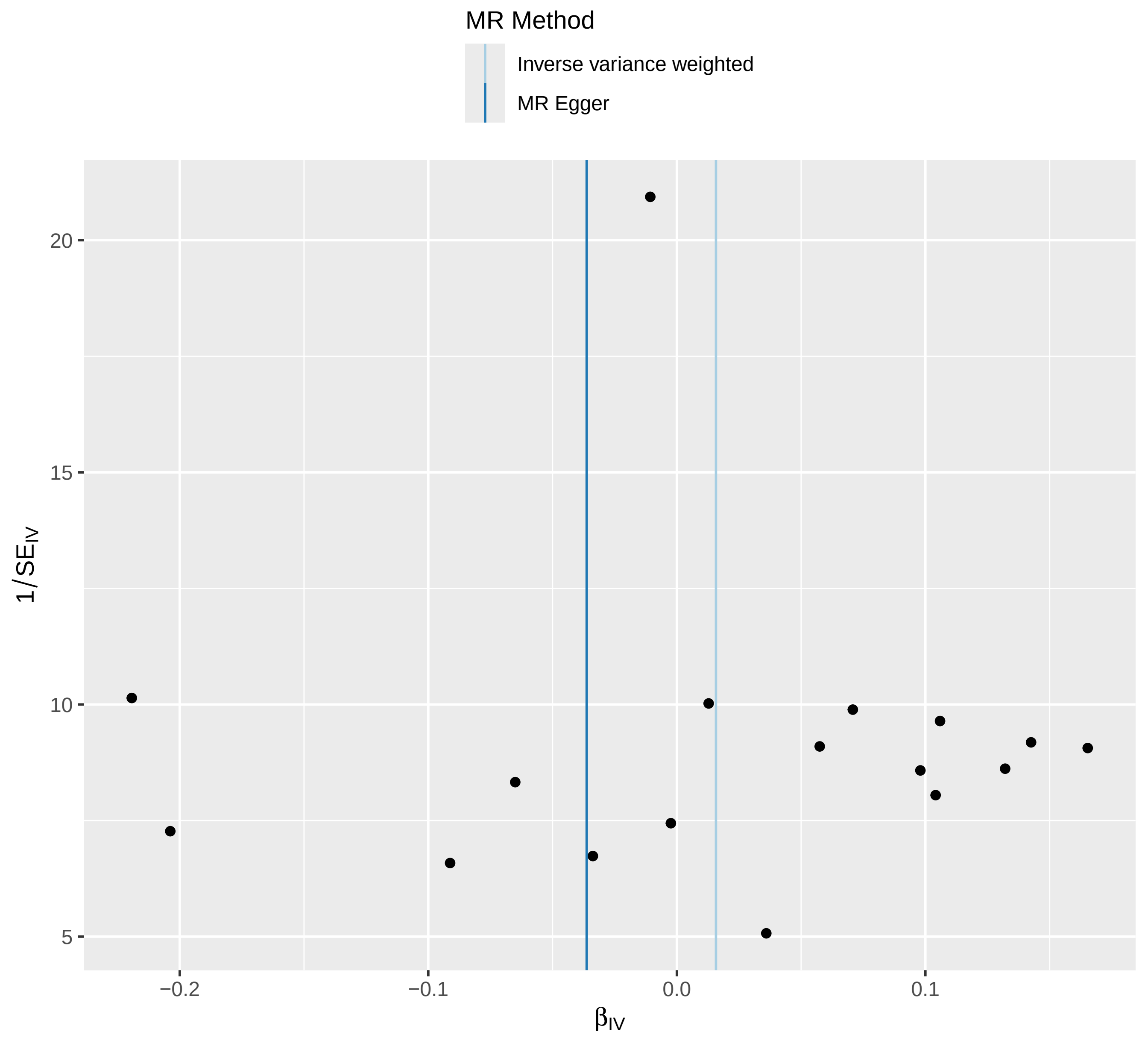
MR Method
Inverse variance weighted
MR Egger
20
15
10
5
1/SE_IV
−0.2
−0.1
0.0
0.1
β_IV

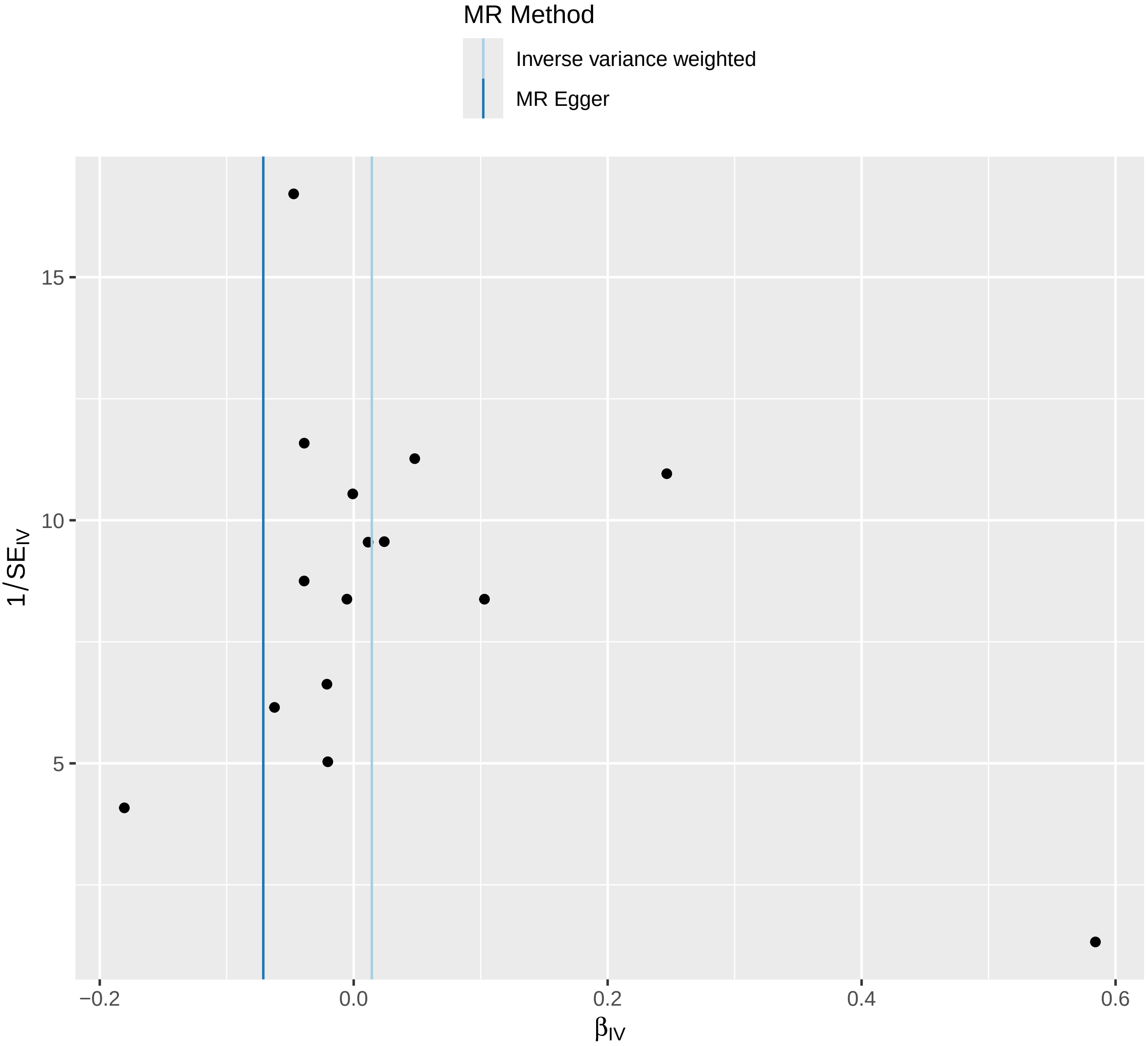
MR Method
Inverse variance weighted
MR Egger
15
10
5
$1/SE_{IV}$
−0.2
0.0
0.2
0.4
0.6
β_{IV}

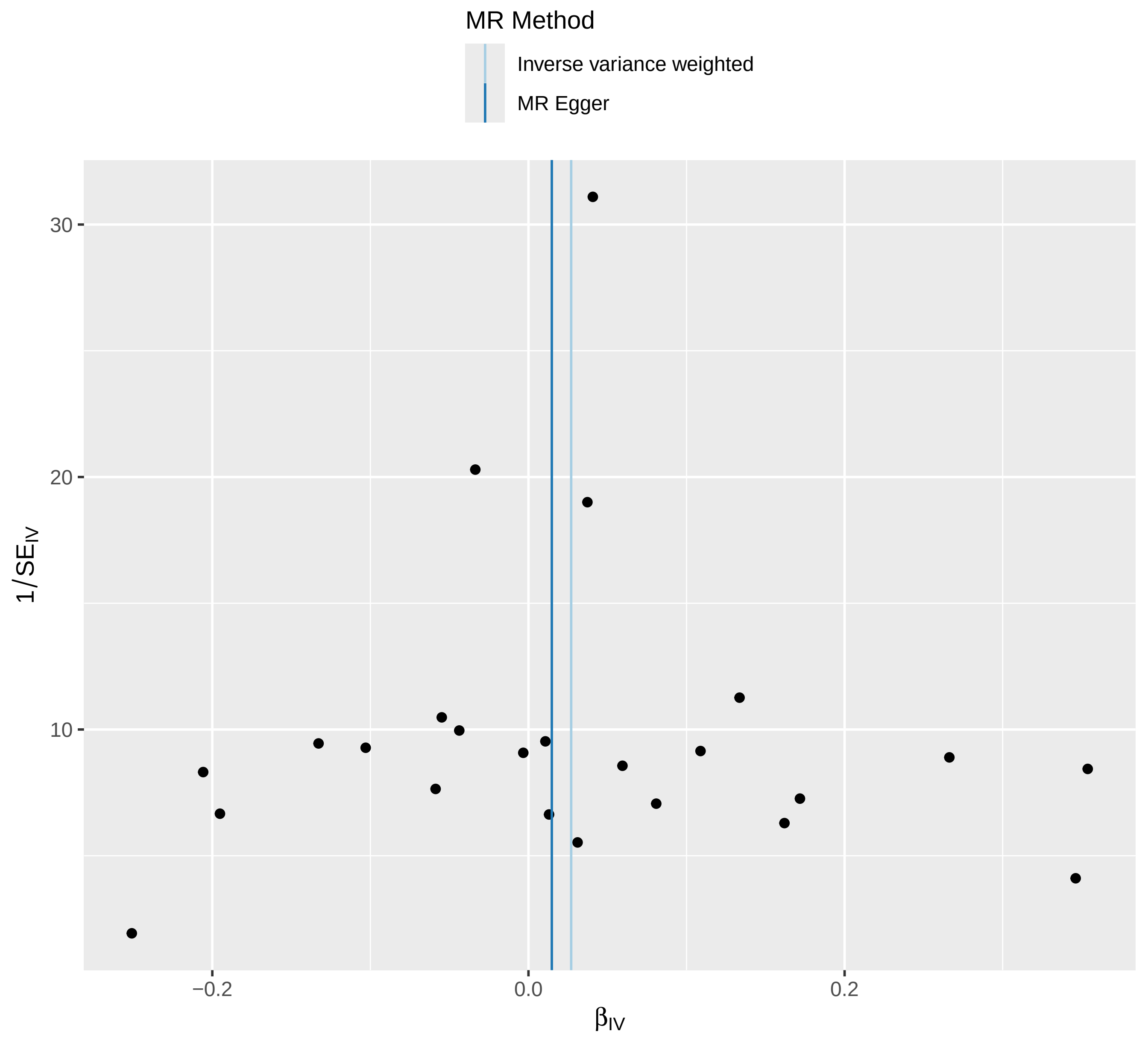
MR Method
Inverse variance weighted
MR Egger
30
20
10
1/SE$_{IV}$
−0.2
0.0
0.2
β$_{IV}$

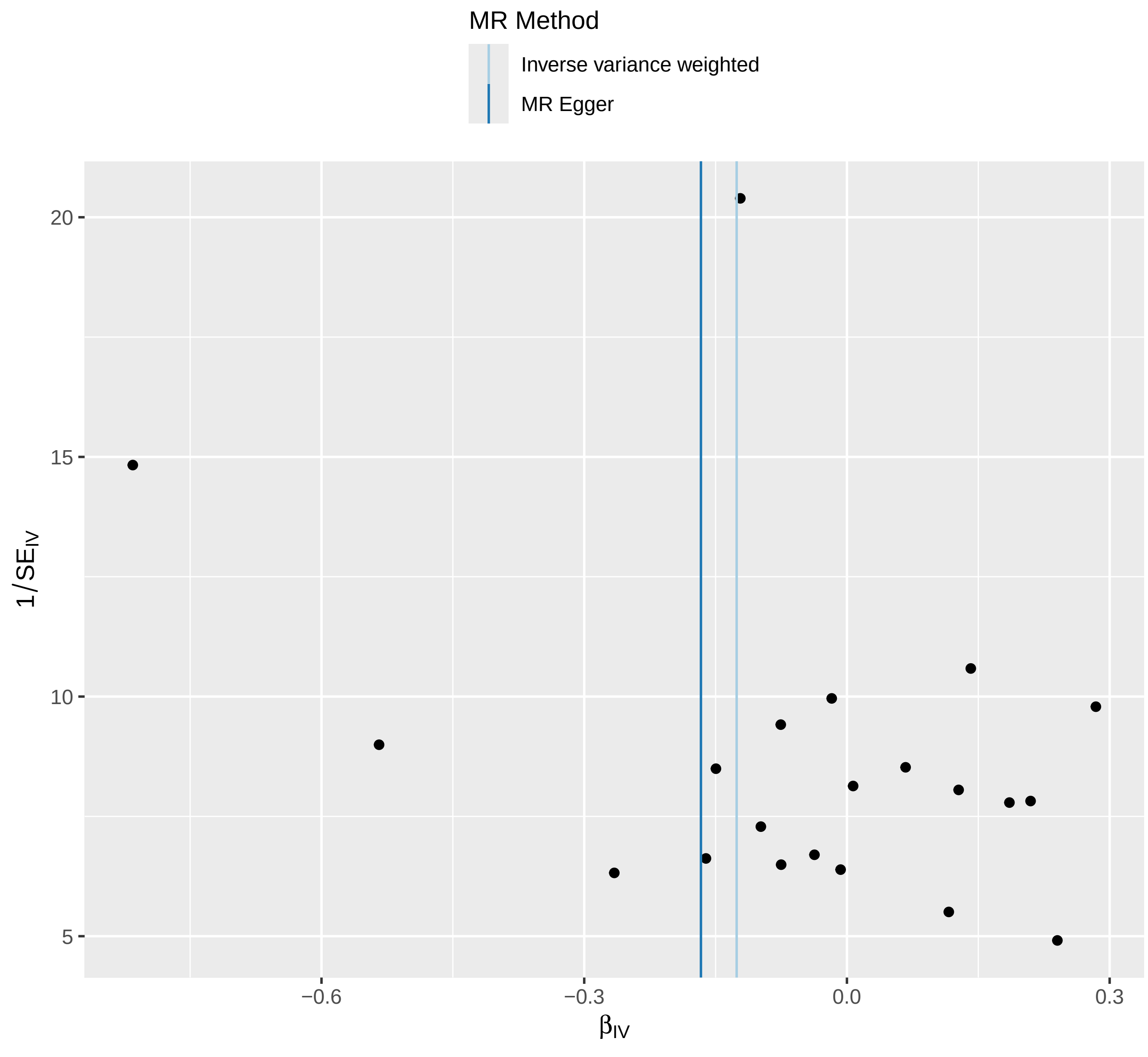
MR Method
Inverse variance weighted
MR Egger
20
15
10
5
1/SE$_{IV}$
−0.6
−0.3
0.0
0.3
β$_{IV}$

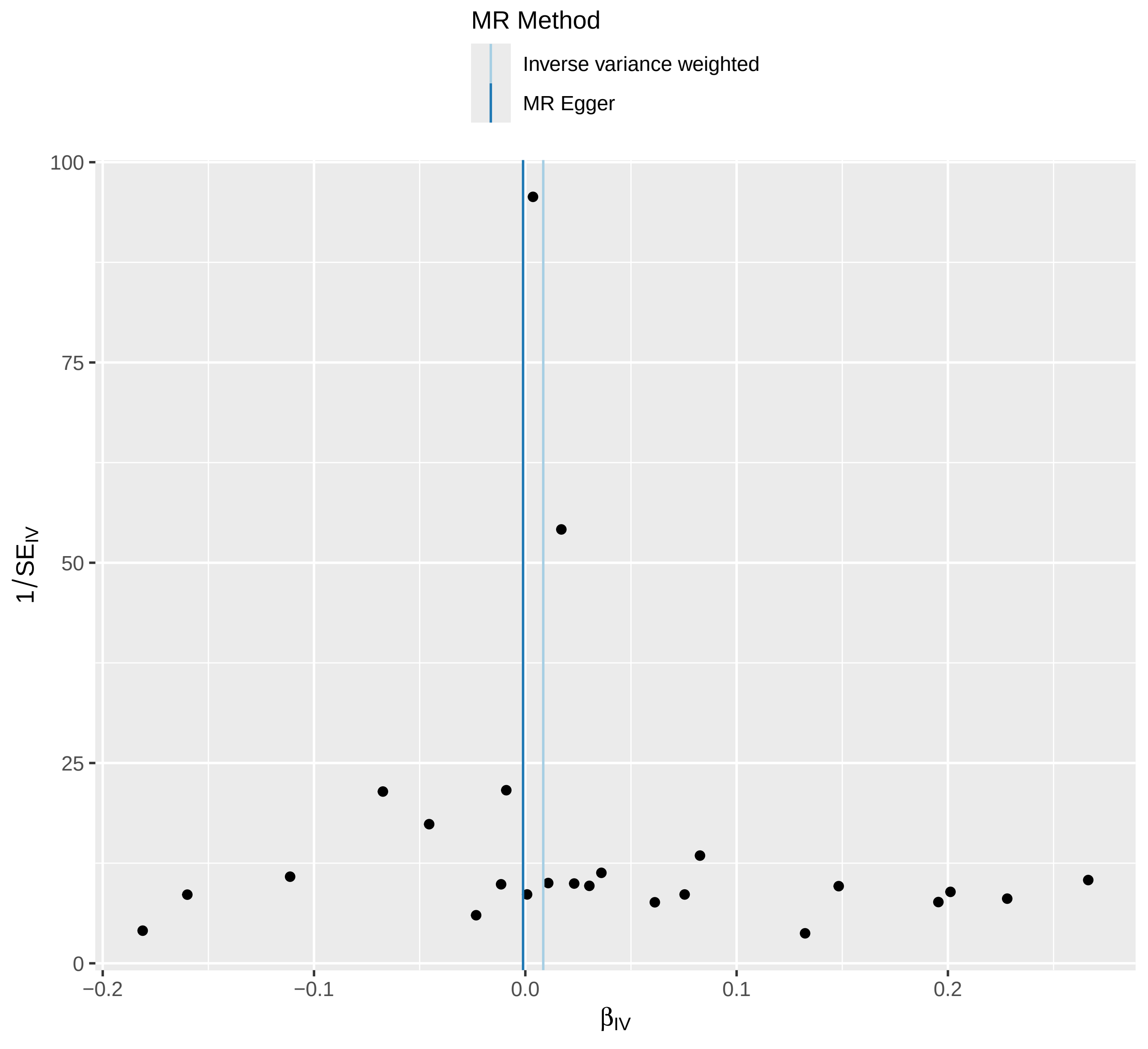

MR Method
Inverse variance weighted
MR Egger
100
75
50
25
0
1/SE$_{IV}$
−0.2
−0.1
0.0
0.1
0.2
β$_{IV}$

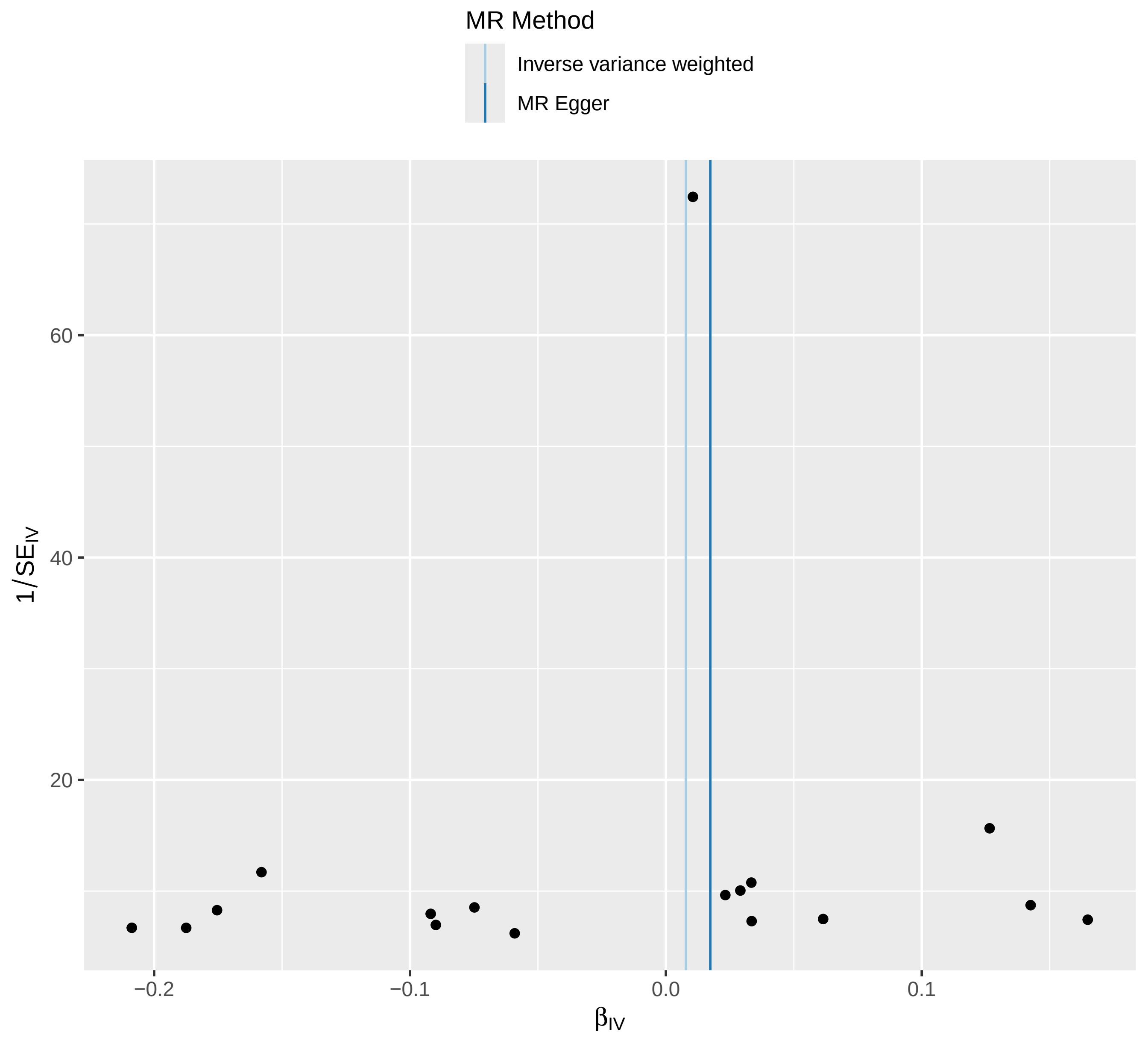
MR Method
Inverse variance weighted
MR Egger
60
40
20
1/SE_{IV}
−0.2
−0.1
0.0
0.1
β_{IV}

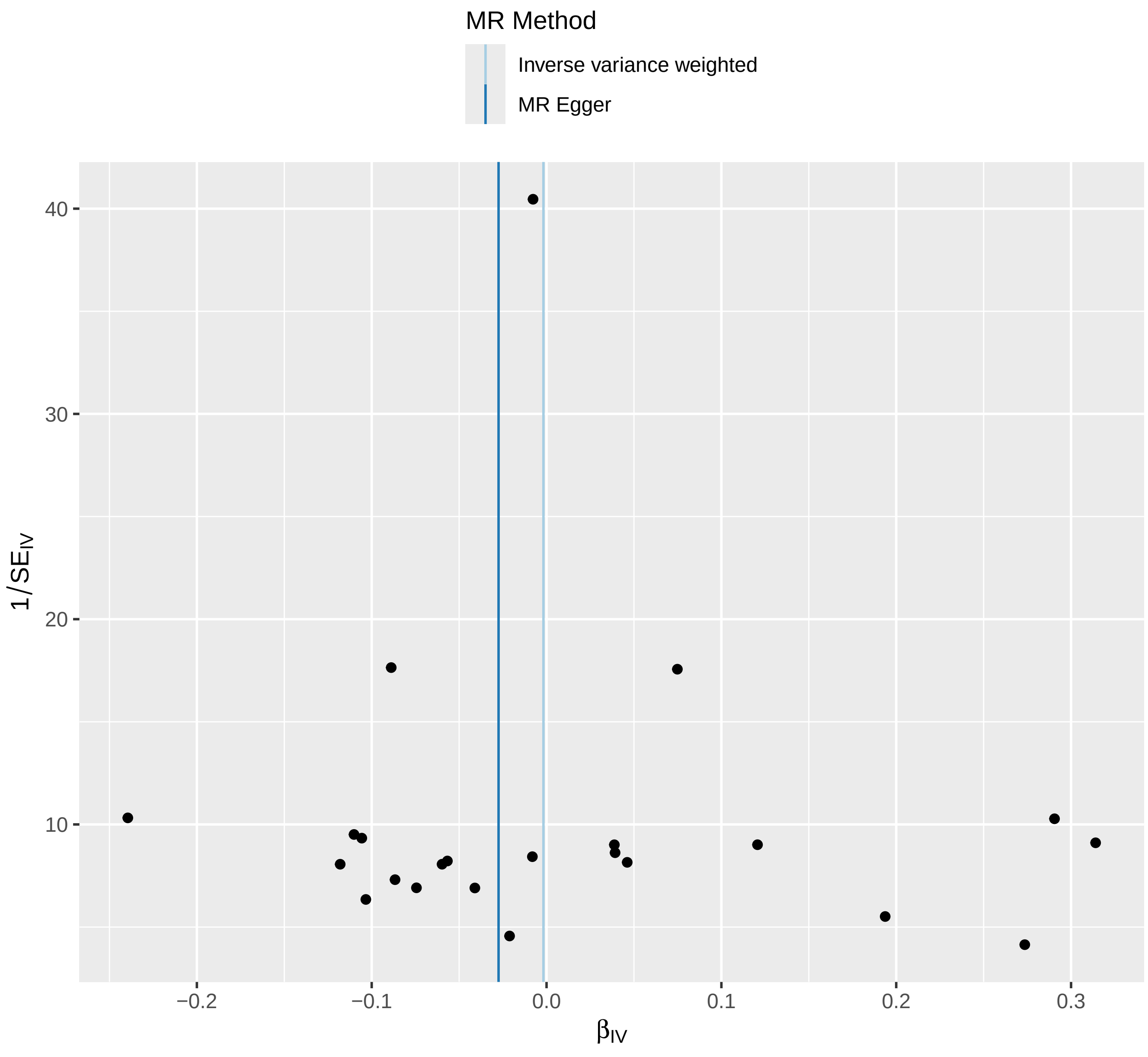

MR Method
Inverse variance weighted
MR Egger
40
30
20
10
1/SE_IV
−0.2
−0.1
0.0
0.1
0.2
0.3
β_IV

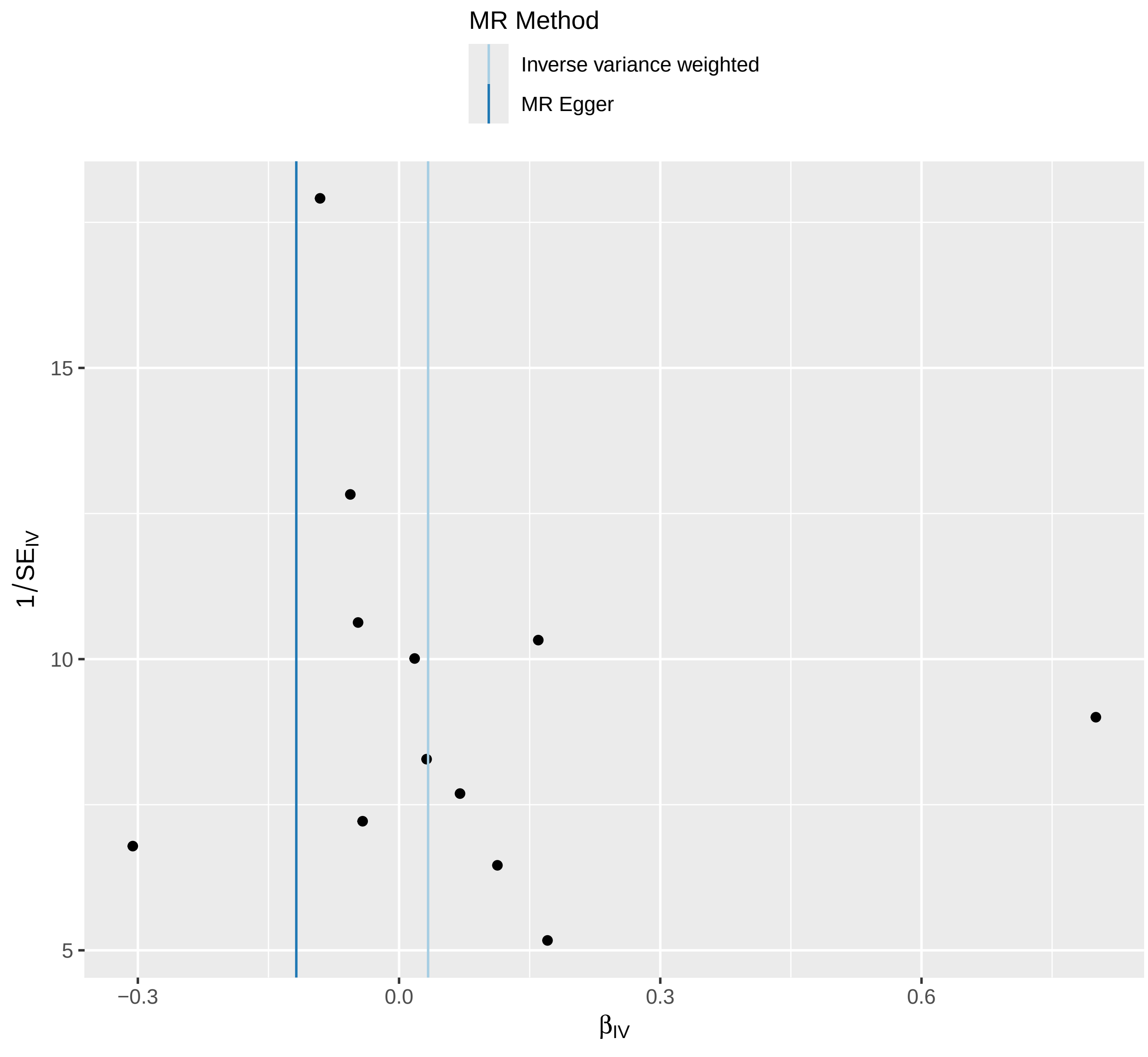
MR Method
Inverse variance weighted
MR Egger
1/SE_IV
15
10
5
−0.3
0.0
0.3
0.6
β_IV

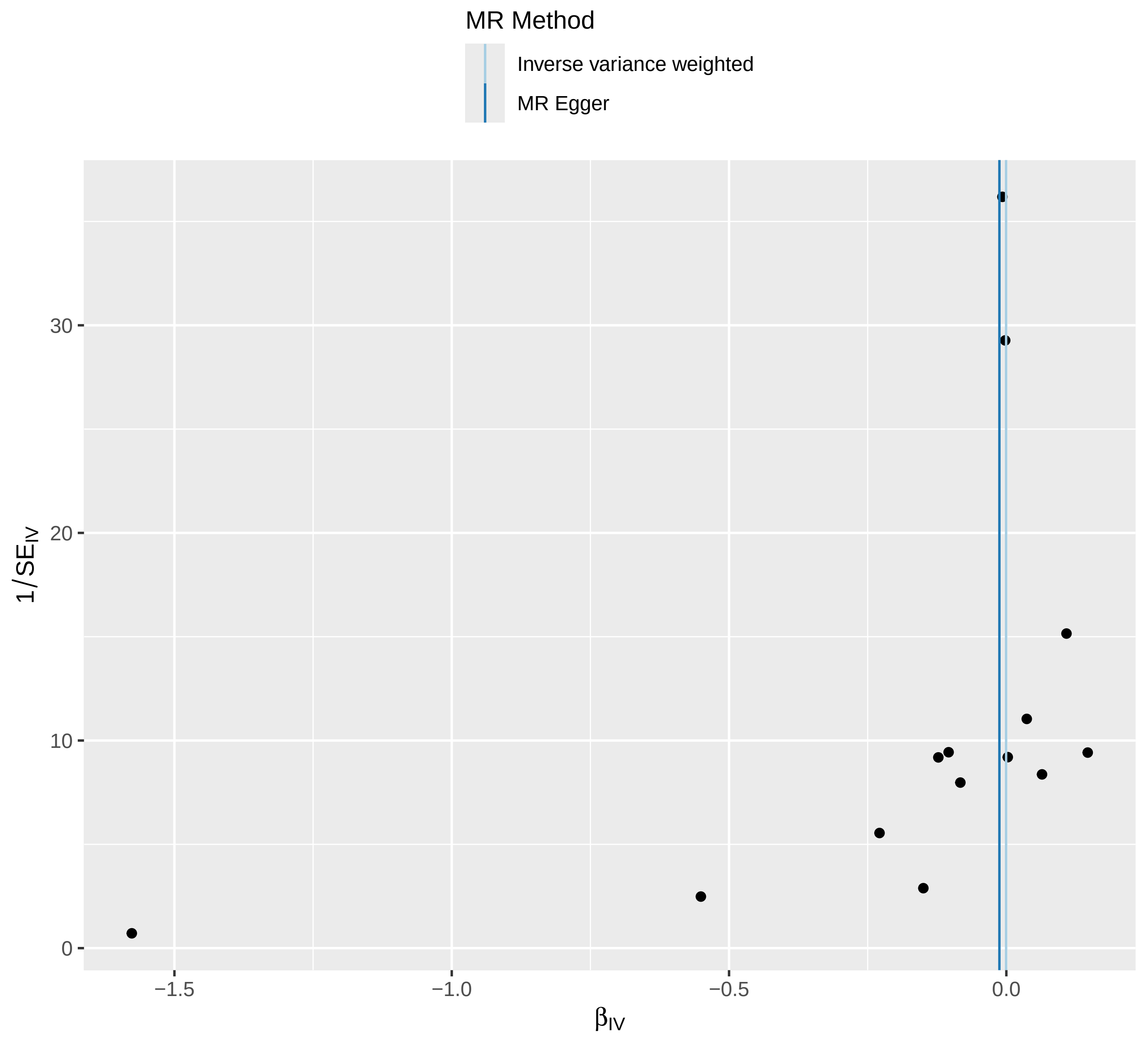
MR Method
Inverse variance weighted
MR Egger
$1/SE_{IV}$
30
20
10
0
−1.5
−1.0
−0.5
0.0
β_{IV}

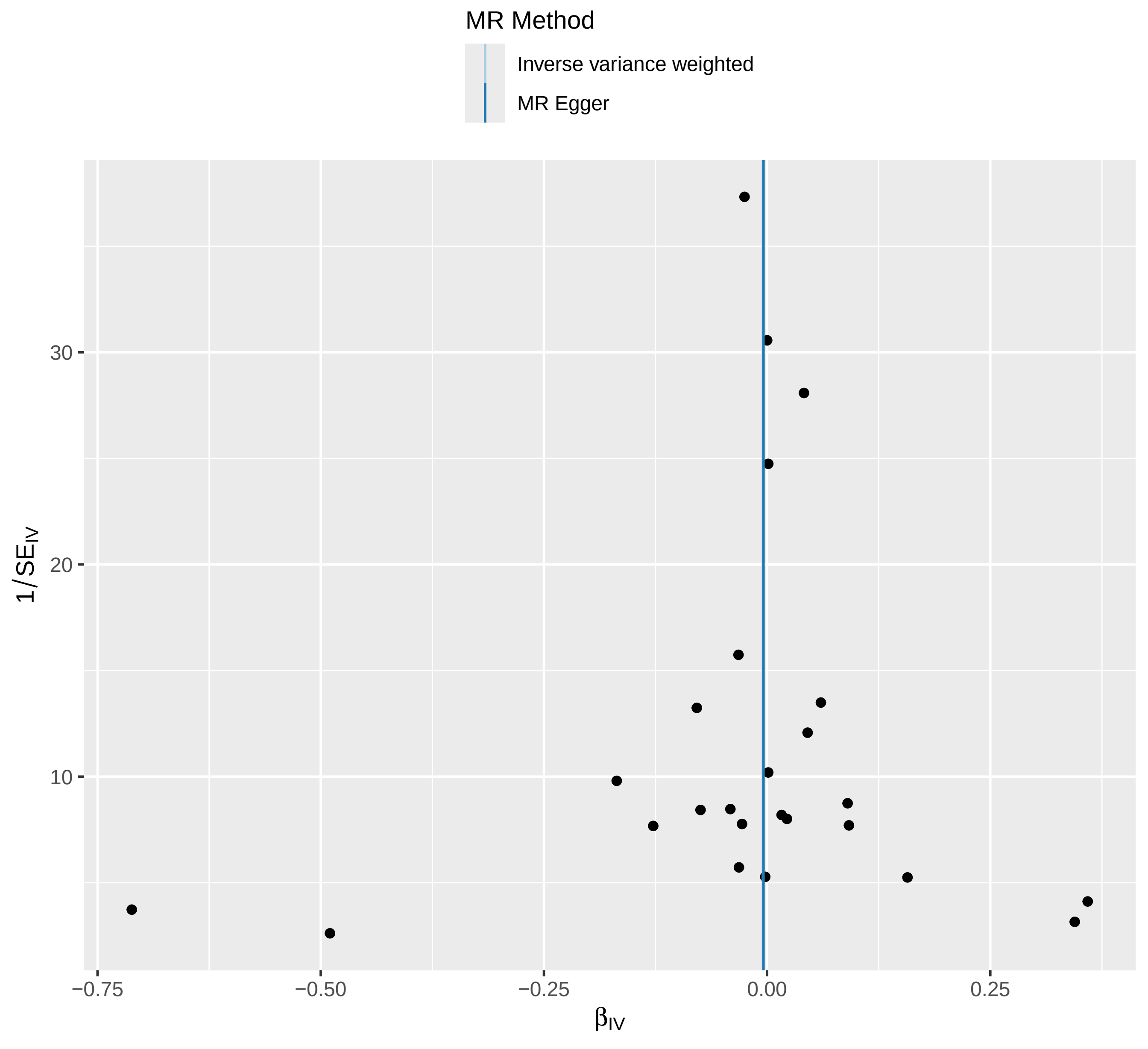

MR Method
Inverse variance weighted
MR Egger
30
20
10
1/SE$_{IV}$
−0.75
−0.50
−0.25
0.00
0.25
β$_{IV}$

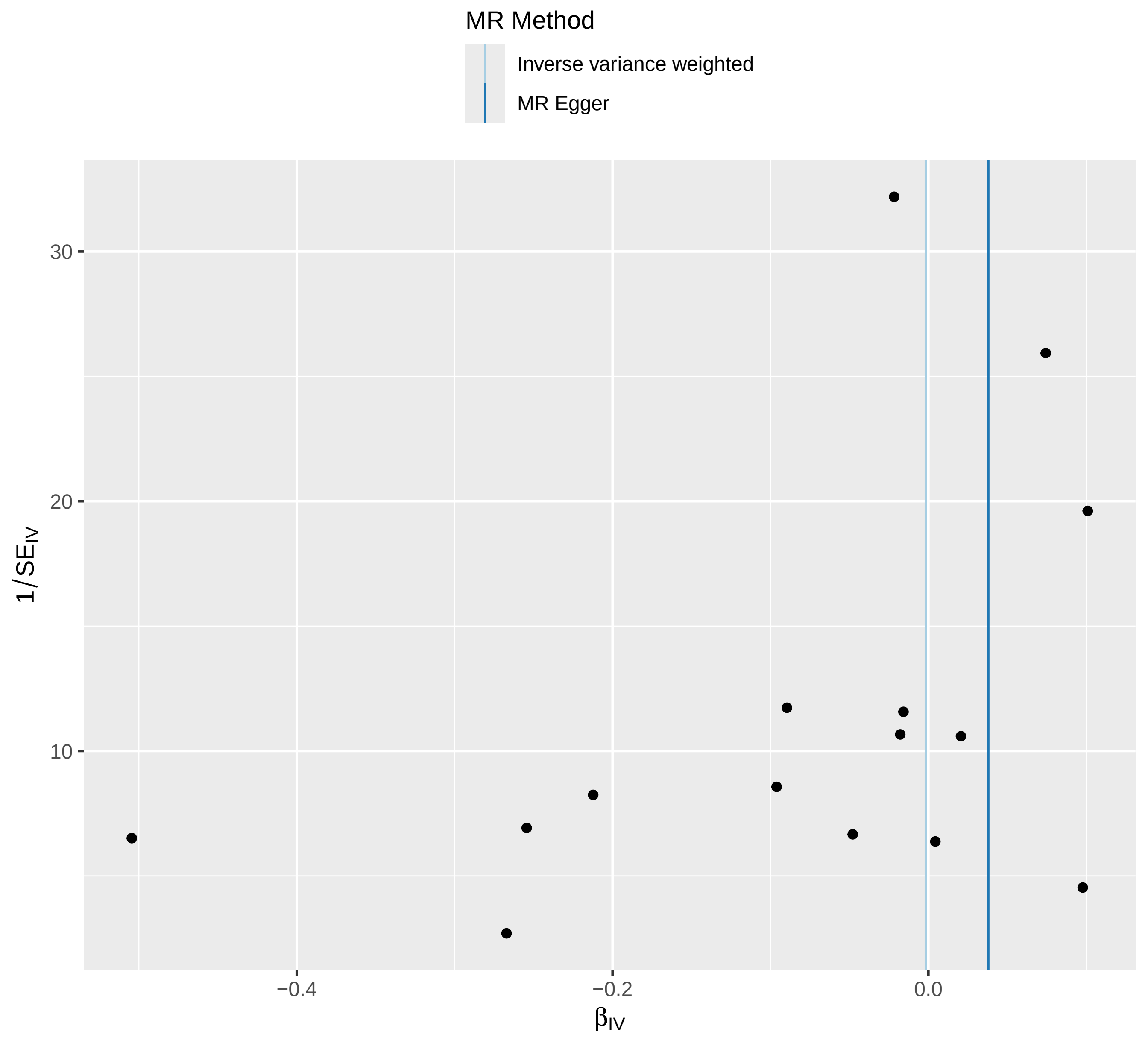
MR Method
Inverse variance weighted
MR Egger
30
20
10
1/SE$_{IV}$
−0.4
−0.2
0.0
β$_{IV}$

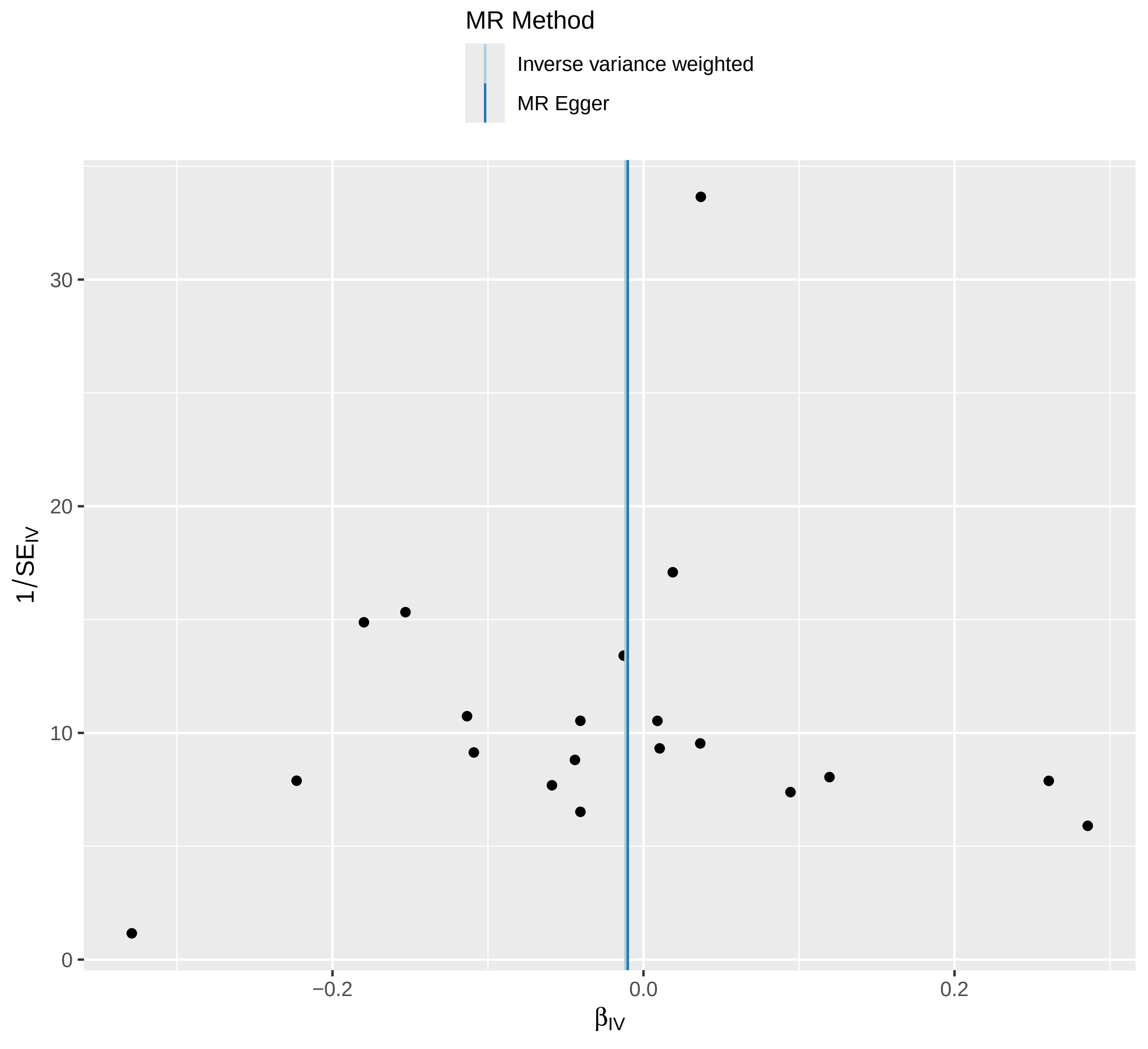
MR Method
Inverse variance weighted
MR Egger
30
20
10
0
1/SE_IV
−0.2
0.0
0.2
β_IV

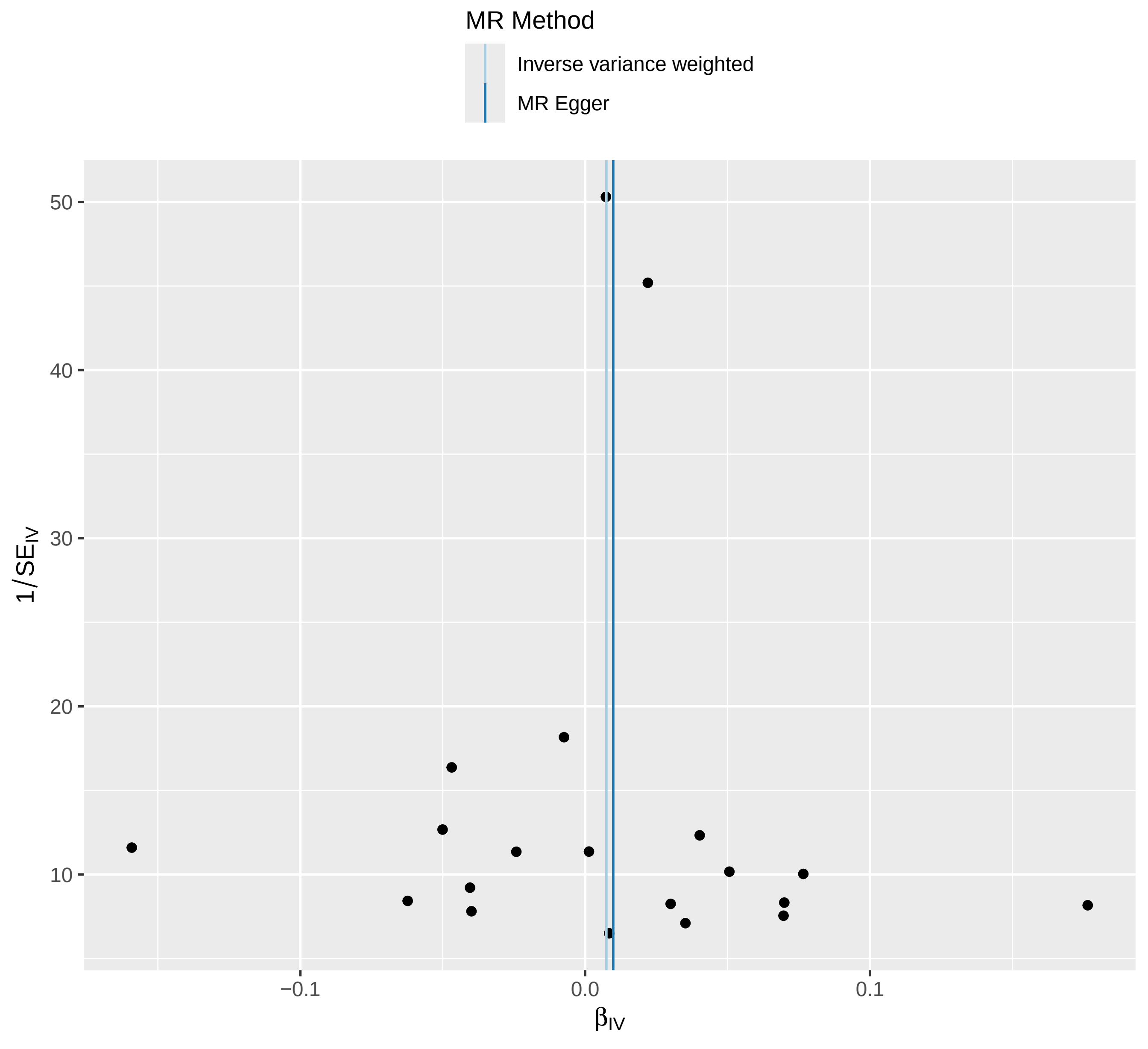
MR Method
Inverse variance weighted
MR Egger
50
40
30
20
10
$1/SE_{IV}$
−0.1
0.0
0.1
β_{IV}

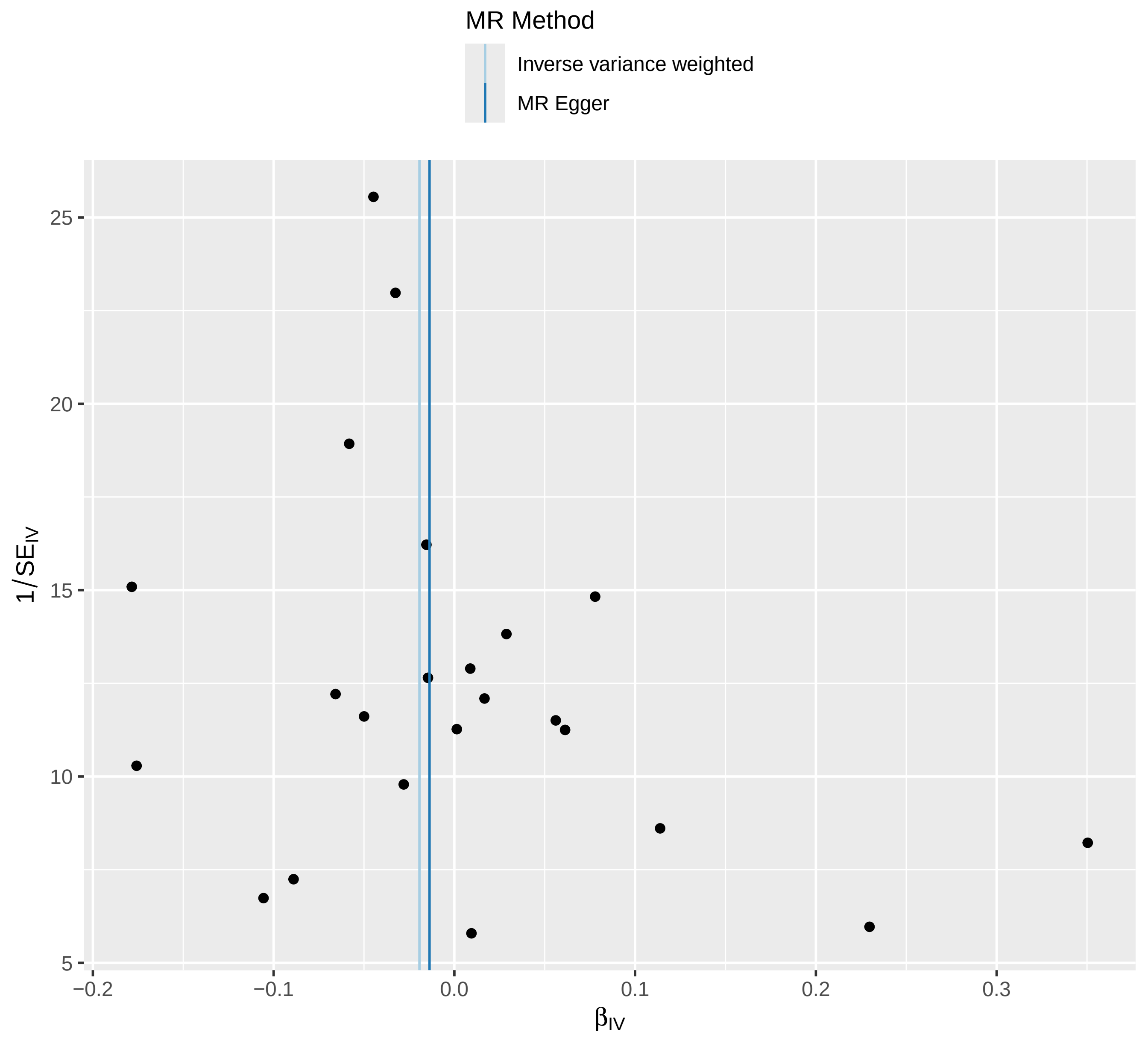
MR Method
Inverse variance weighted
MR Egger
25
20
15
10
5
−0.2
−0.1
0.0
0.1
0.2
0.3
1/SE_IV
β_IV

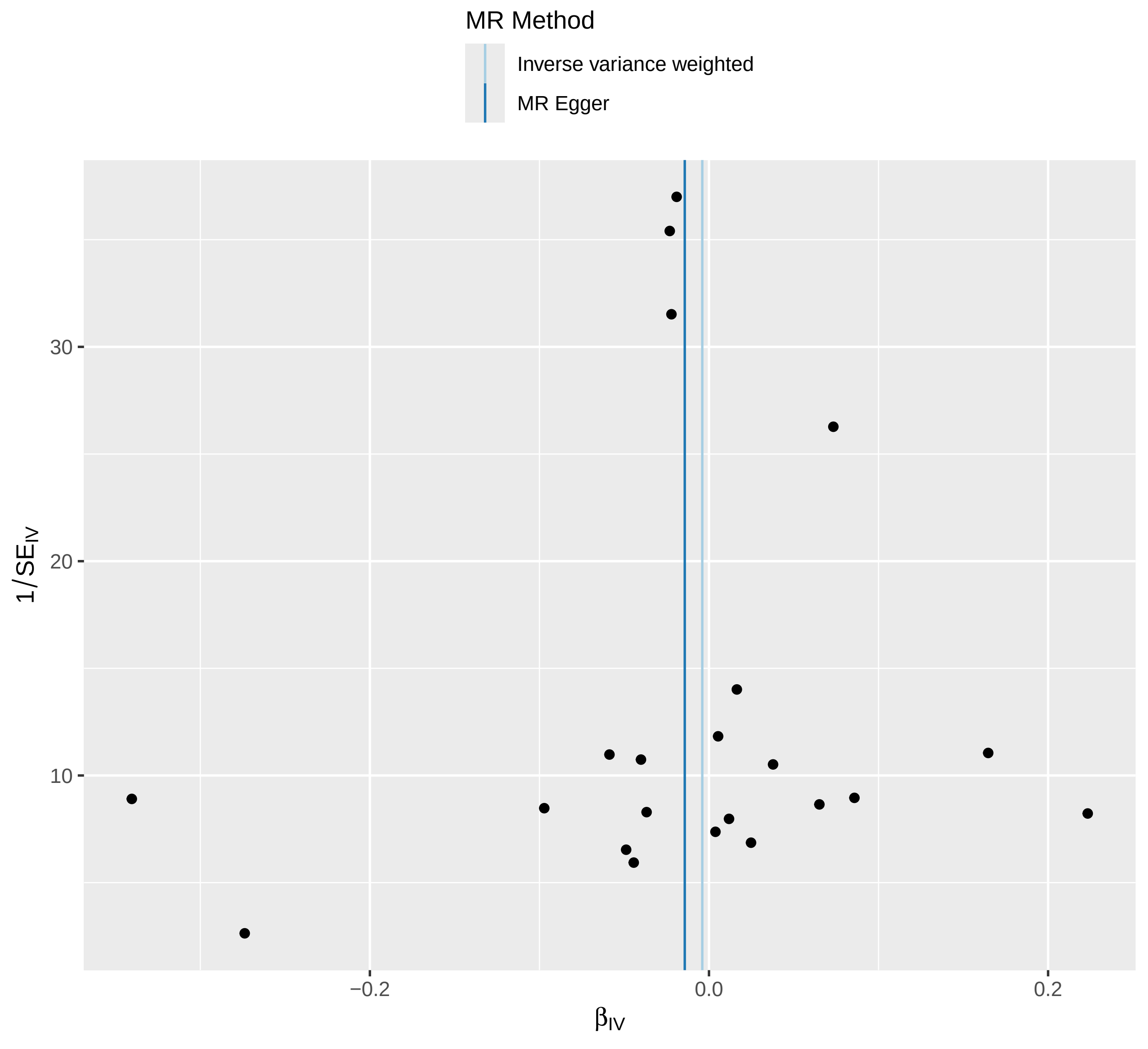
MR Method
Inverse variance weighted
MR Egger
$1/SE_{IV}$
30
20
10
−0.2
0.0
0.2
β_{IV}

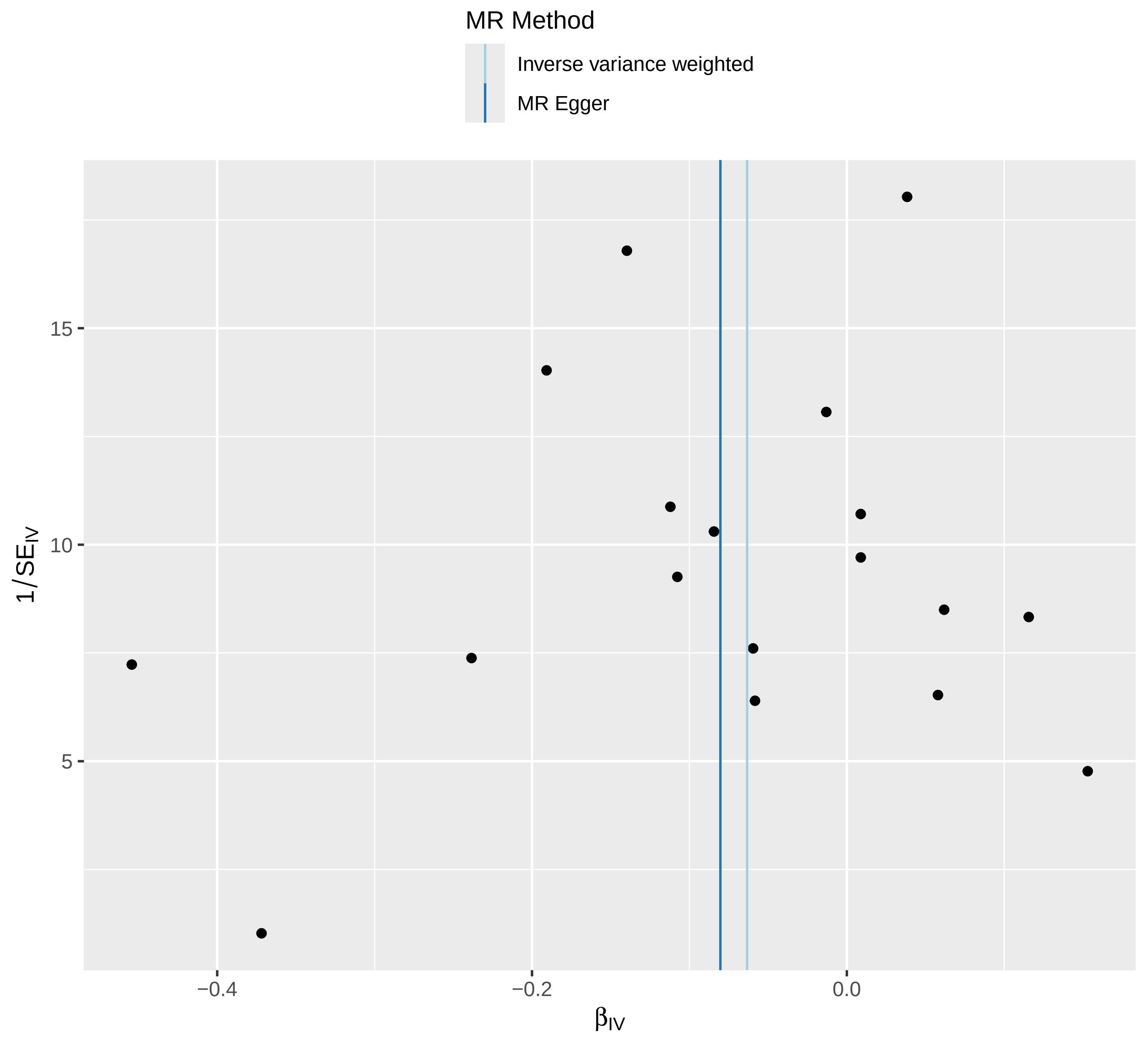

MR Method
Inverse variance weighted
MR Egger
15
10
5
1/SE_IV
−0.4
−0.2
0.0
β_IV

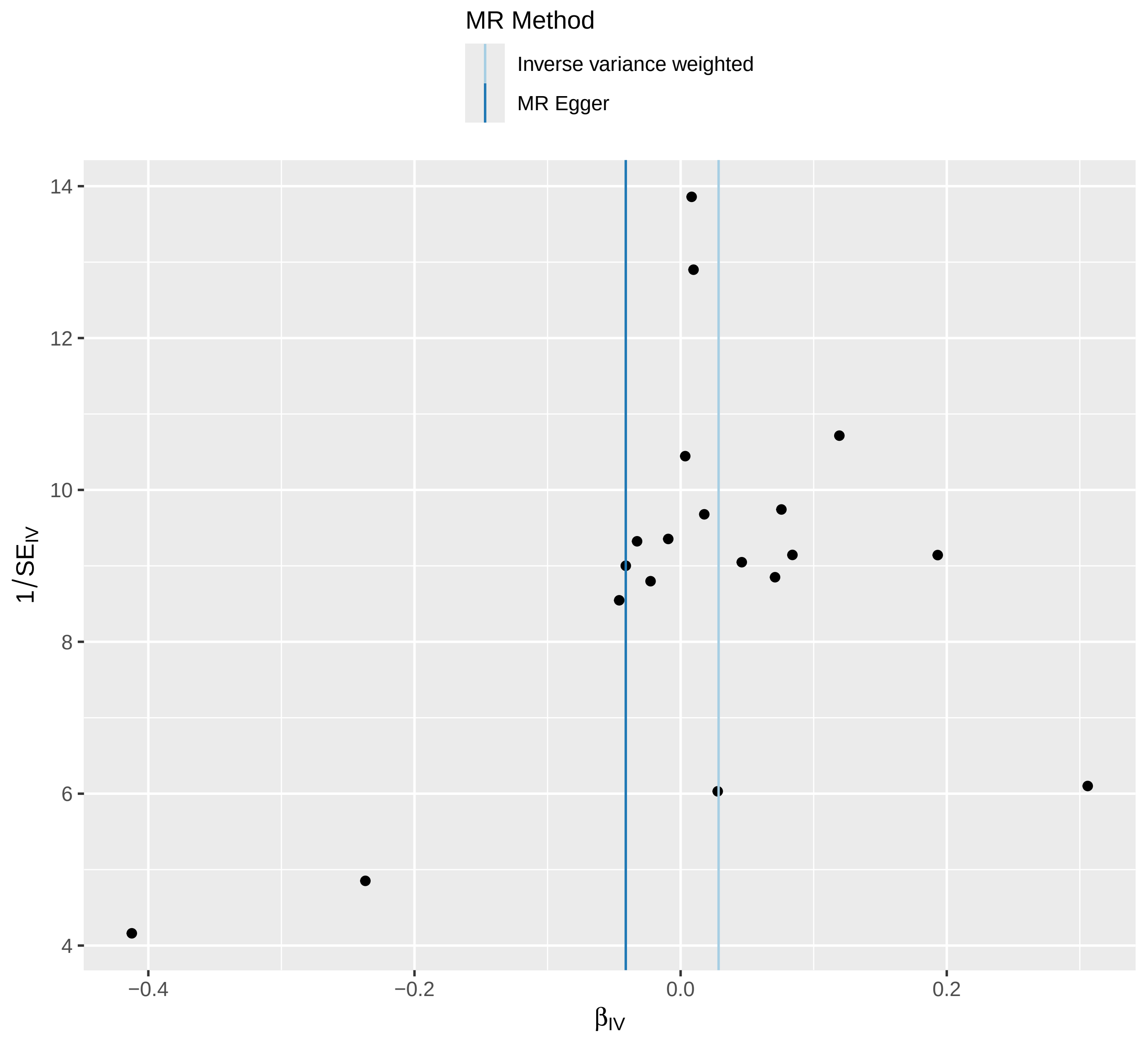
MR Method
Inverse variance weighted
MR Egger
14
12
10
8
6
4
1/SE_IV
−0.4
−0.2
0.0
0.2
β_IV

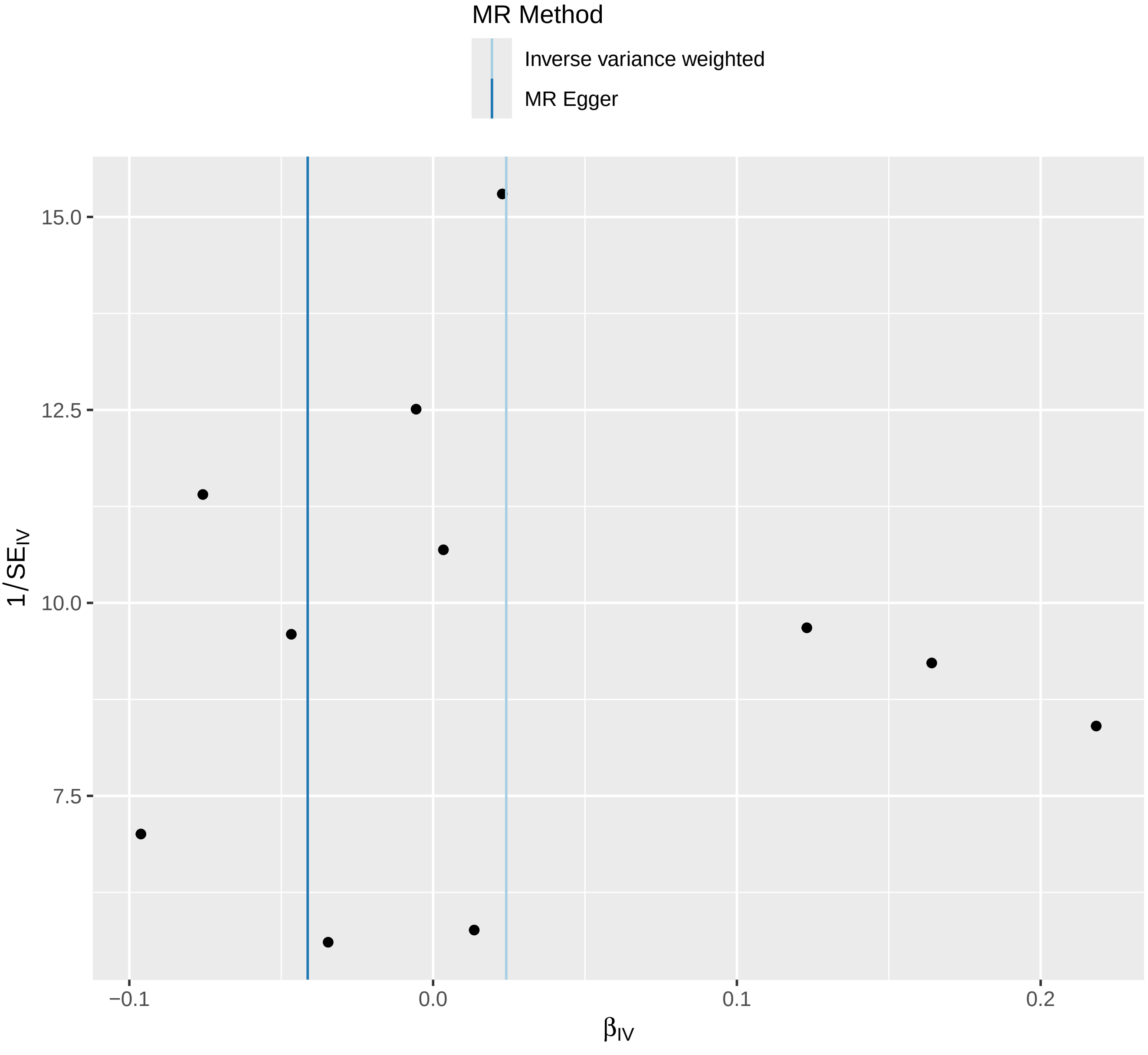
MR Method
Inverse variance weighted
MR Egger
15.0
12.5
10.0
7.5
1/SE_IV
−0.1
0.0
0.1
0.2
β_IV

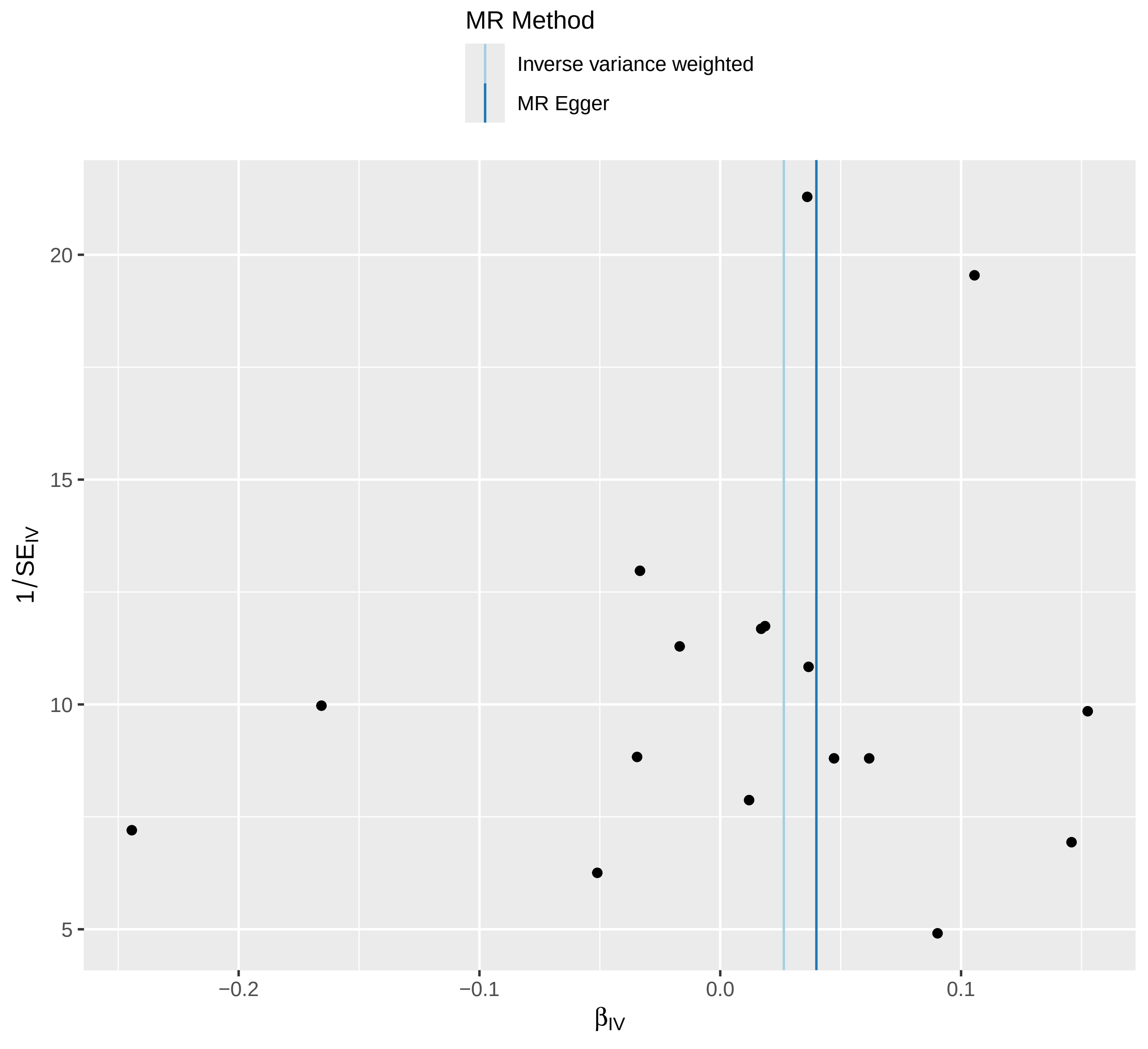

MR Method
Inverse variance weighted
MR Egger
20
15
10
5
1/SE$_{IV}$
−0.2
−0.1
0.0
0.1
β$_{IV}$

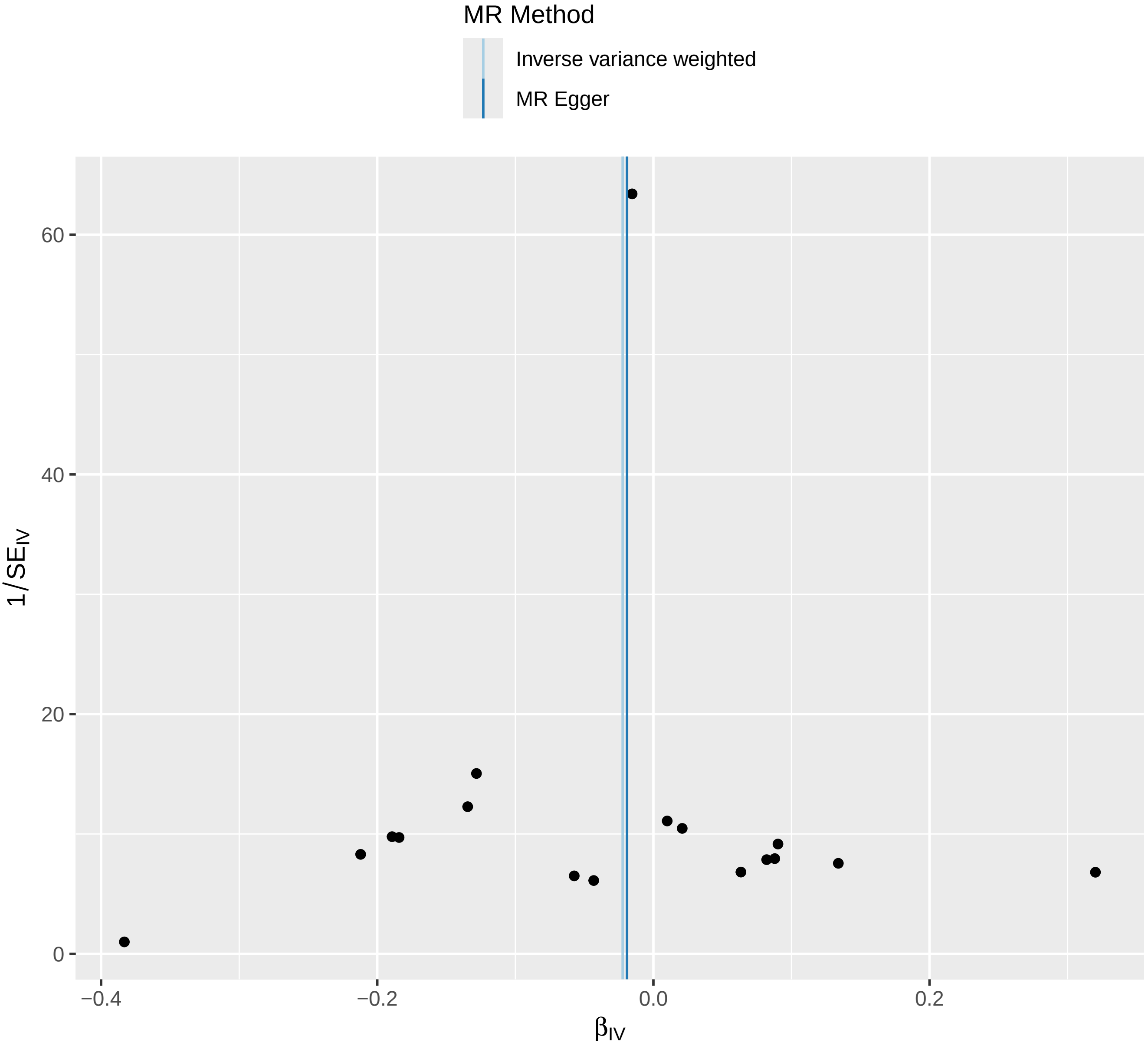
MR Method
Inverse variance weighted
MR Egger
60
40
20
0
1/SE_IV
−0.4
−0.2
0.0
0.2
β_IV

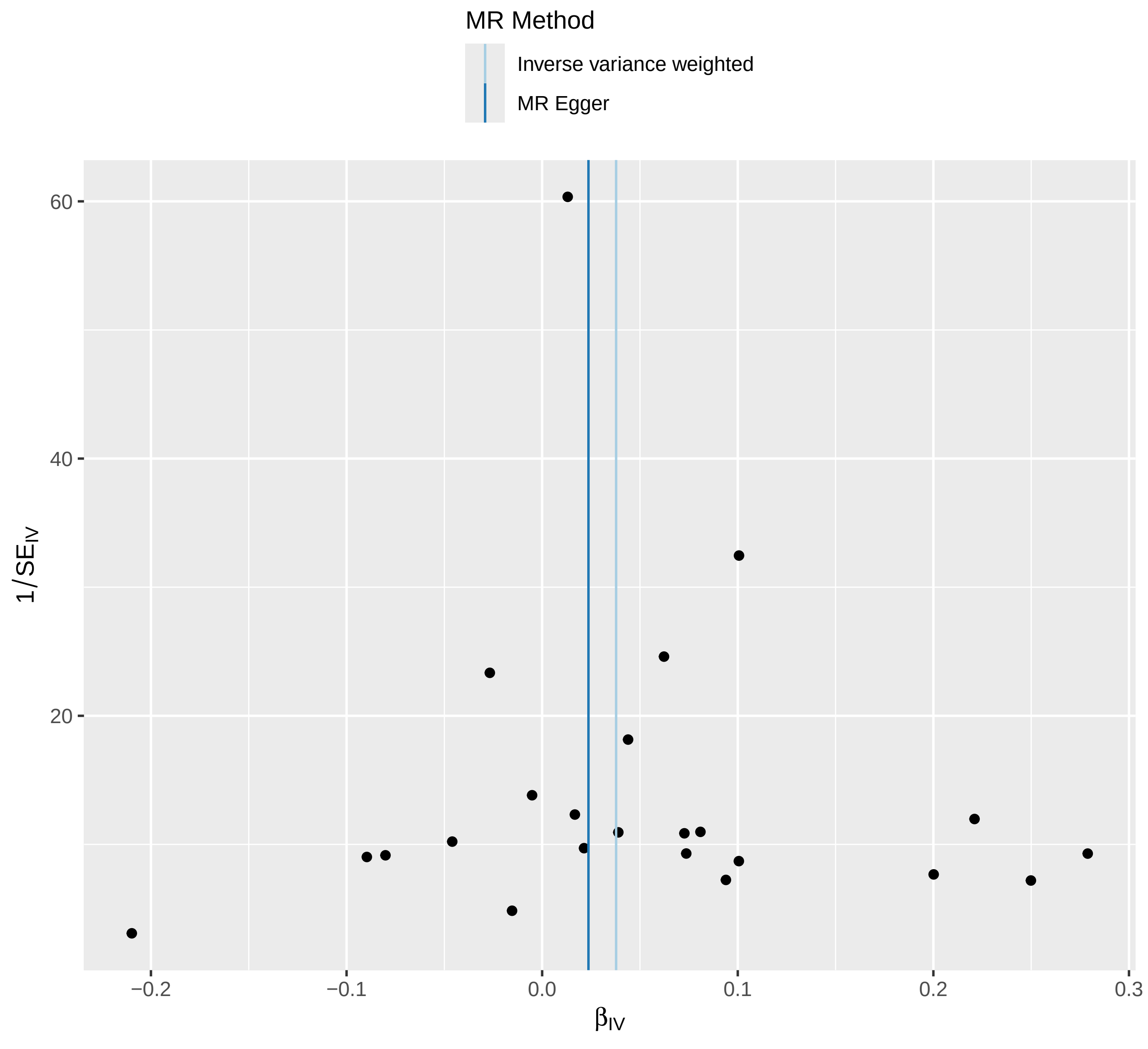
MR Method
Inverse variance weighted
MR Egger
60
40
20
1/SE$_{IV}$
−0.2
−0.1
0.0
0.1
0.2
0.3
β$_{IV}$

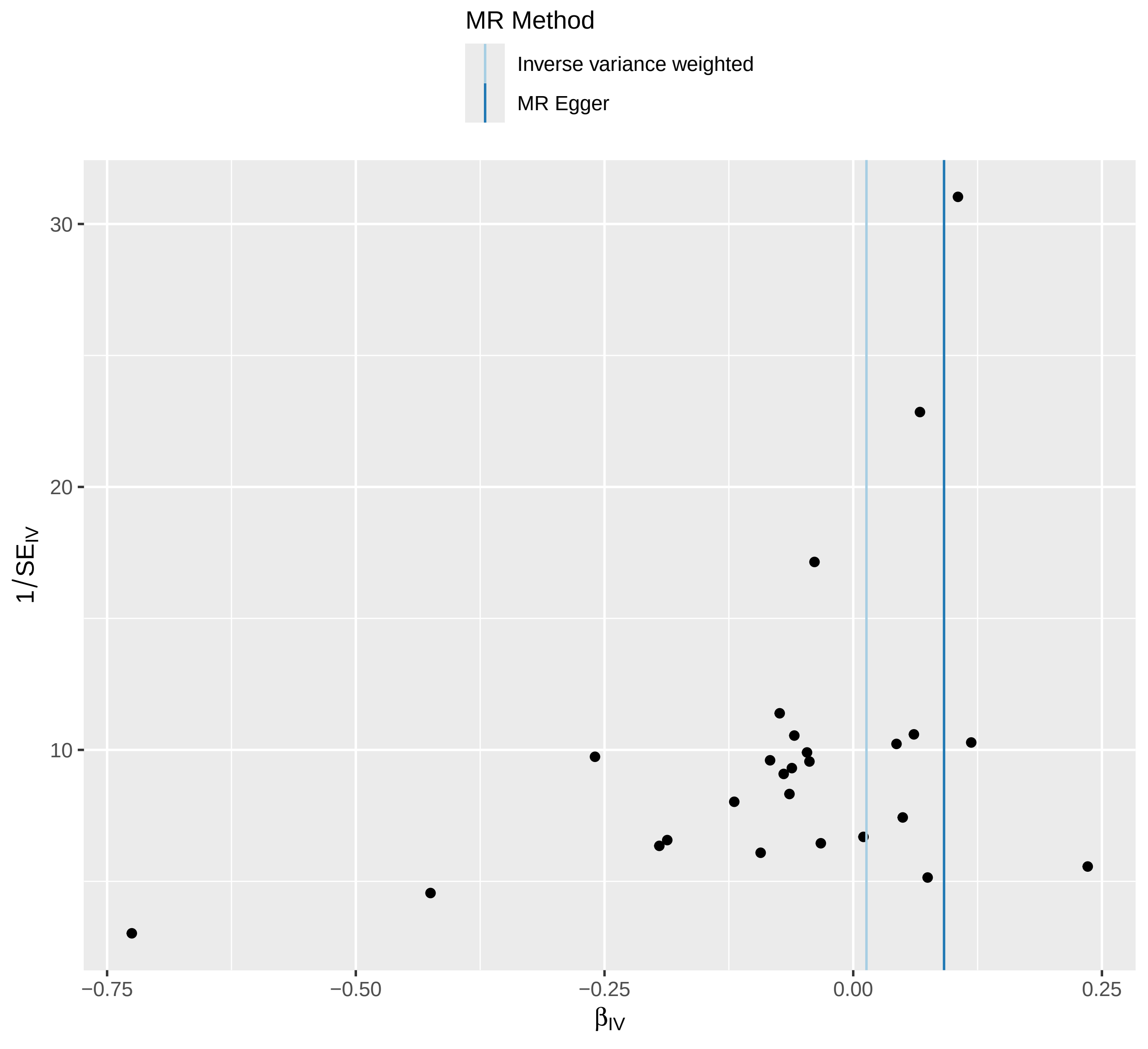
MR Method
Inverse variance weighted
MR Egger
30
20
10
1/SE$_{IV}$
−0.75
−0.50
−0.25
0.00
0.25
β$_{IV}$

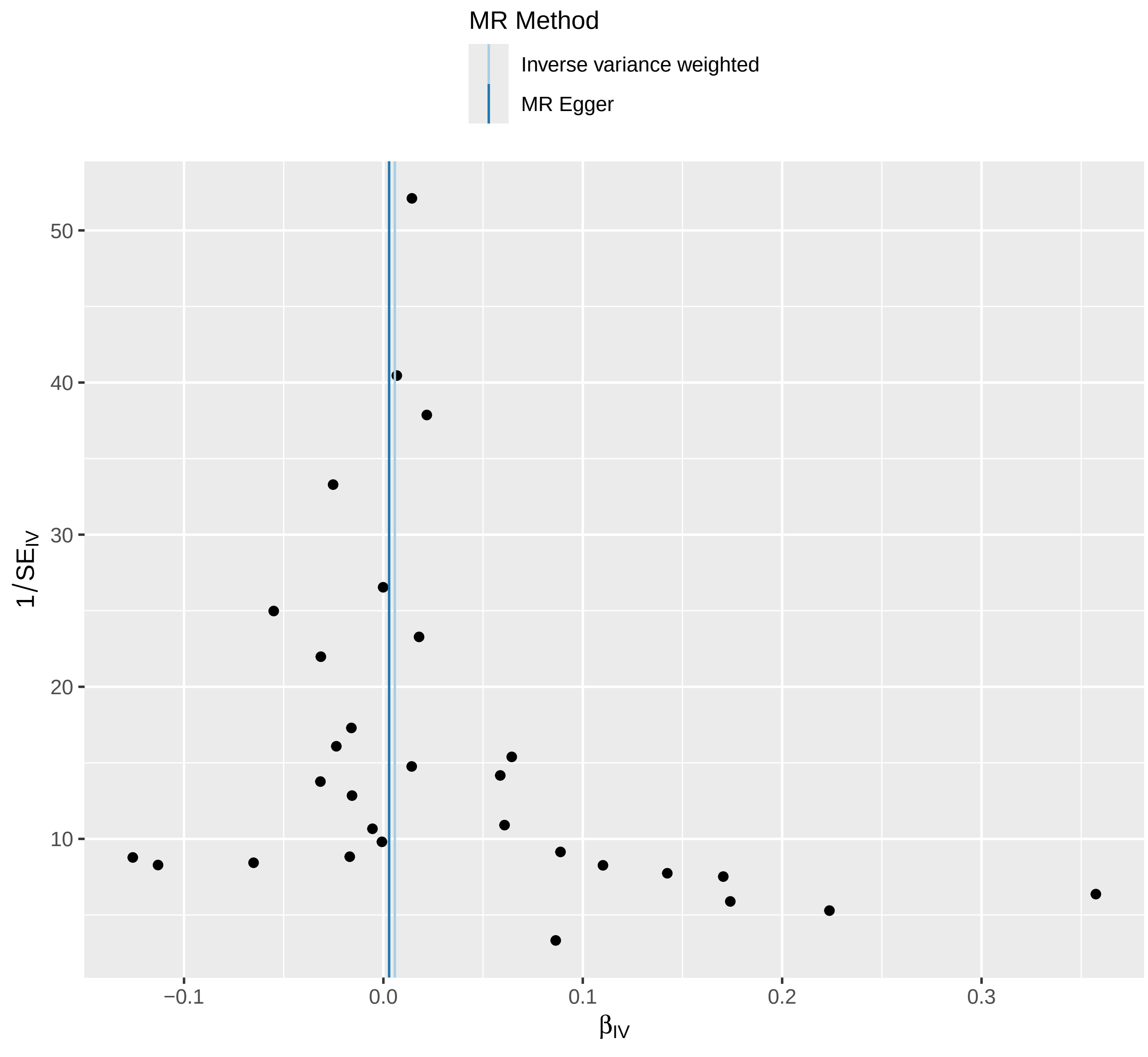
MR Method
Inverse variance weighted
MR Egger
50
40
30
20
10
$1/SE_{IV}$
−0.1
0.0
0.1
0.2
0.3
β_{IV}

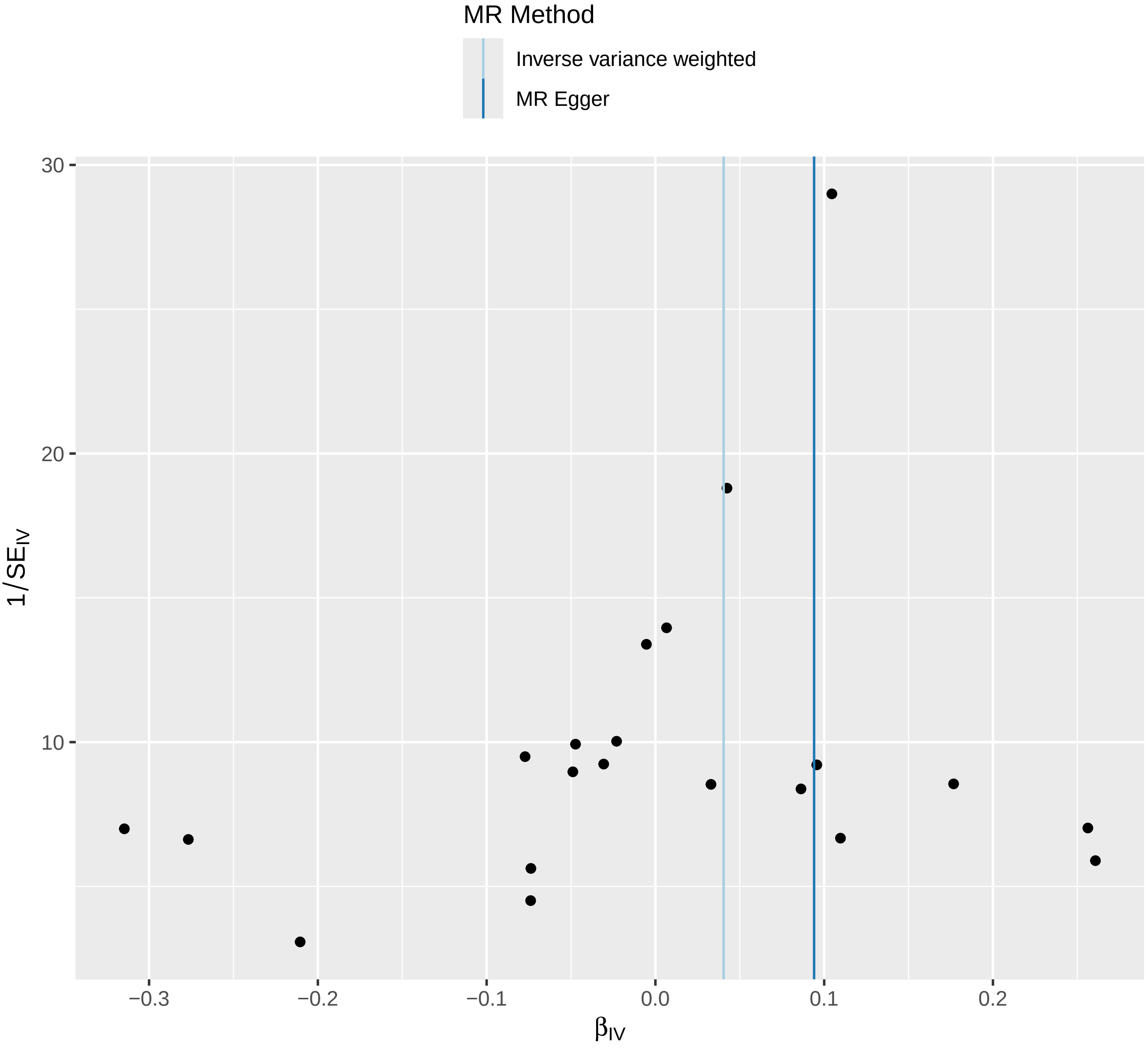
MR Method
Inverse variance weighted
MR Egger
30
20
10
1/SE$_{IV}$
−0.3
−0.2
−0.1
0.0
0.1
0.2
β$_{IV}$

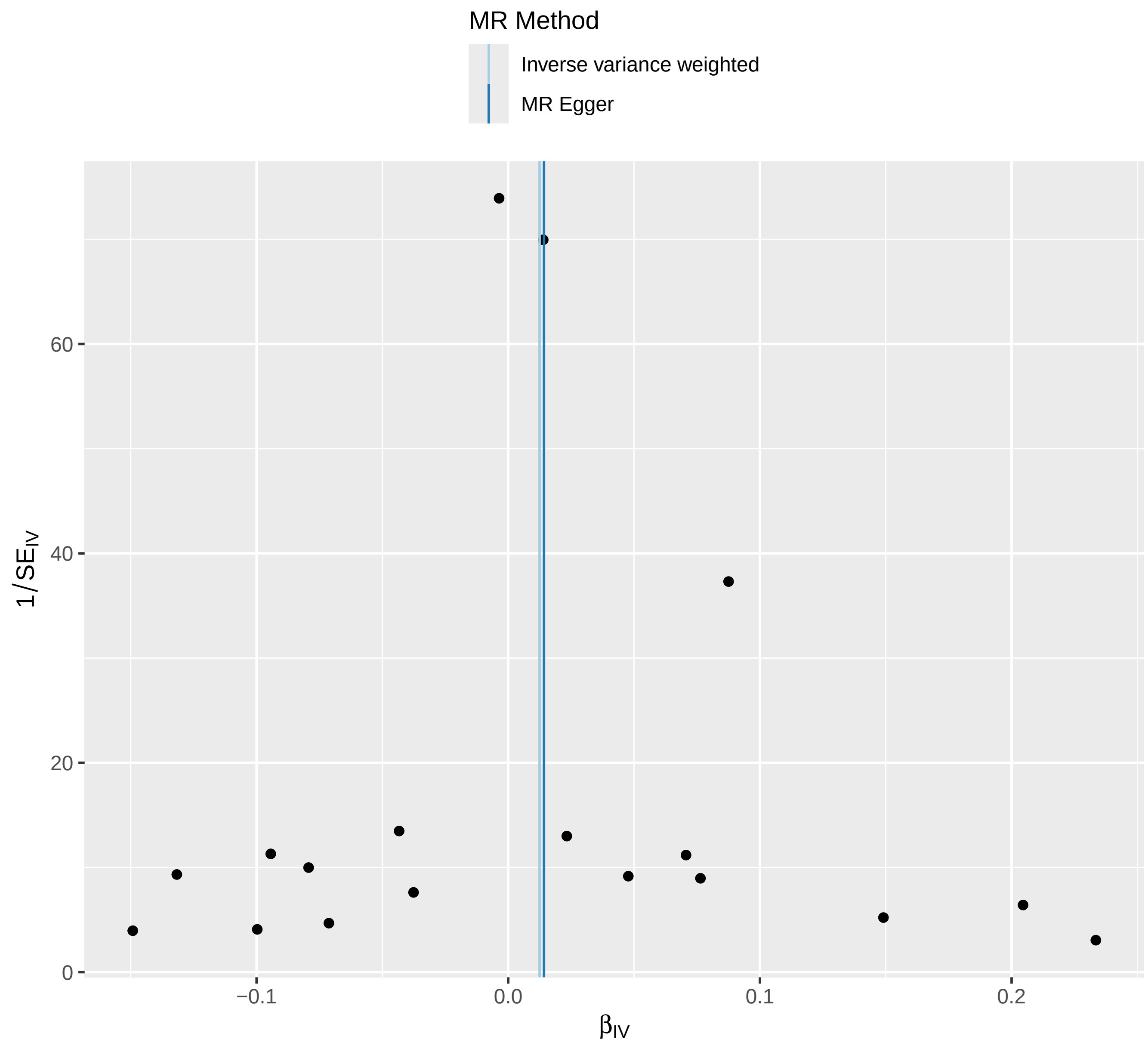
MR Method
Inverse variance weighted
MR Egger
60
40
20
0
−0.1
0.0
0.1
0.2
1/SE_IV
β_IV

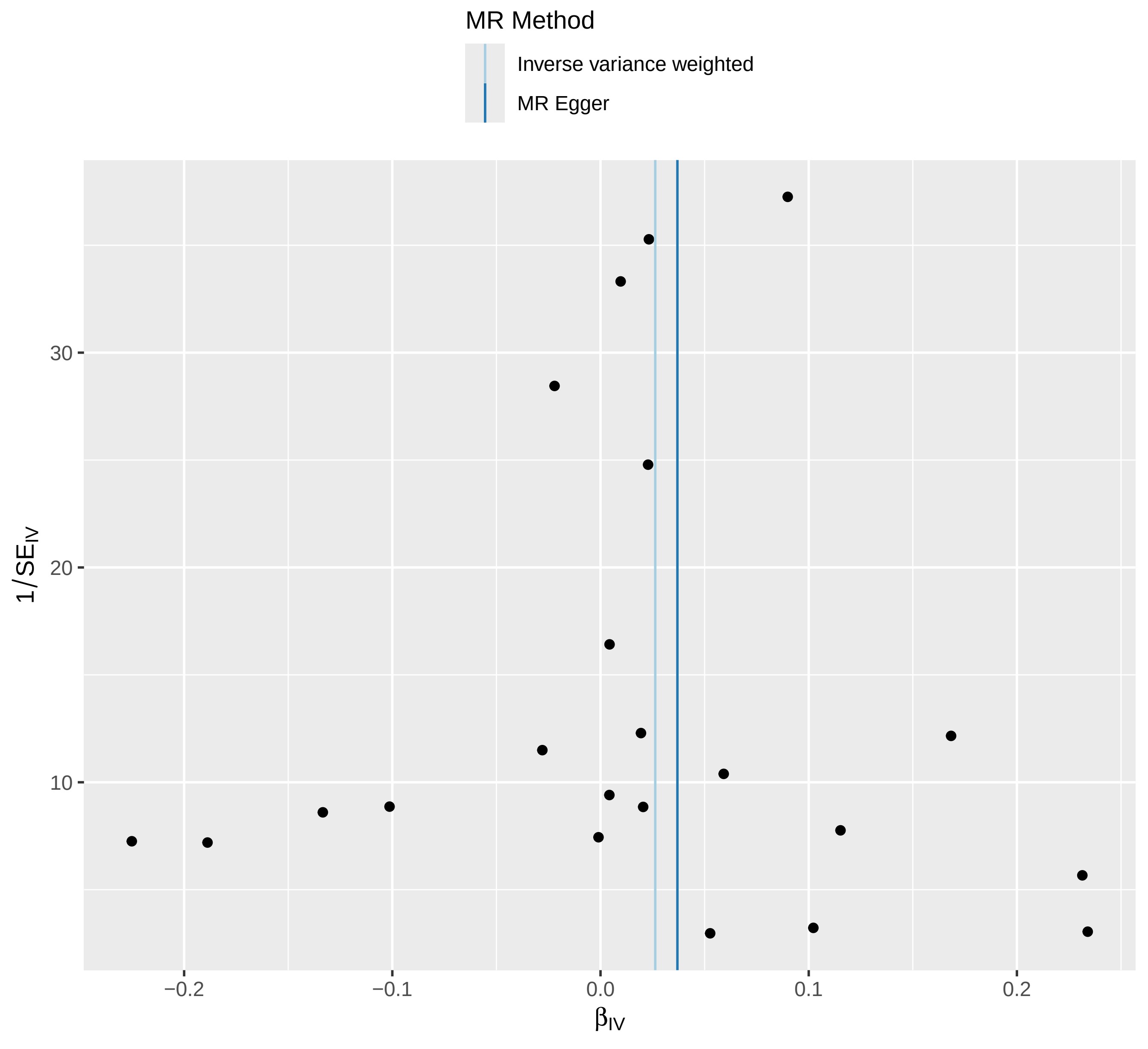
MR Method
Inverse variance weighted
MR Egger
30
20
10
1/SE$_{IV}$
−0.2
−0.1
0.0
0.1
0.2
β$_{IV}$

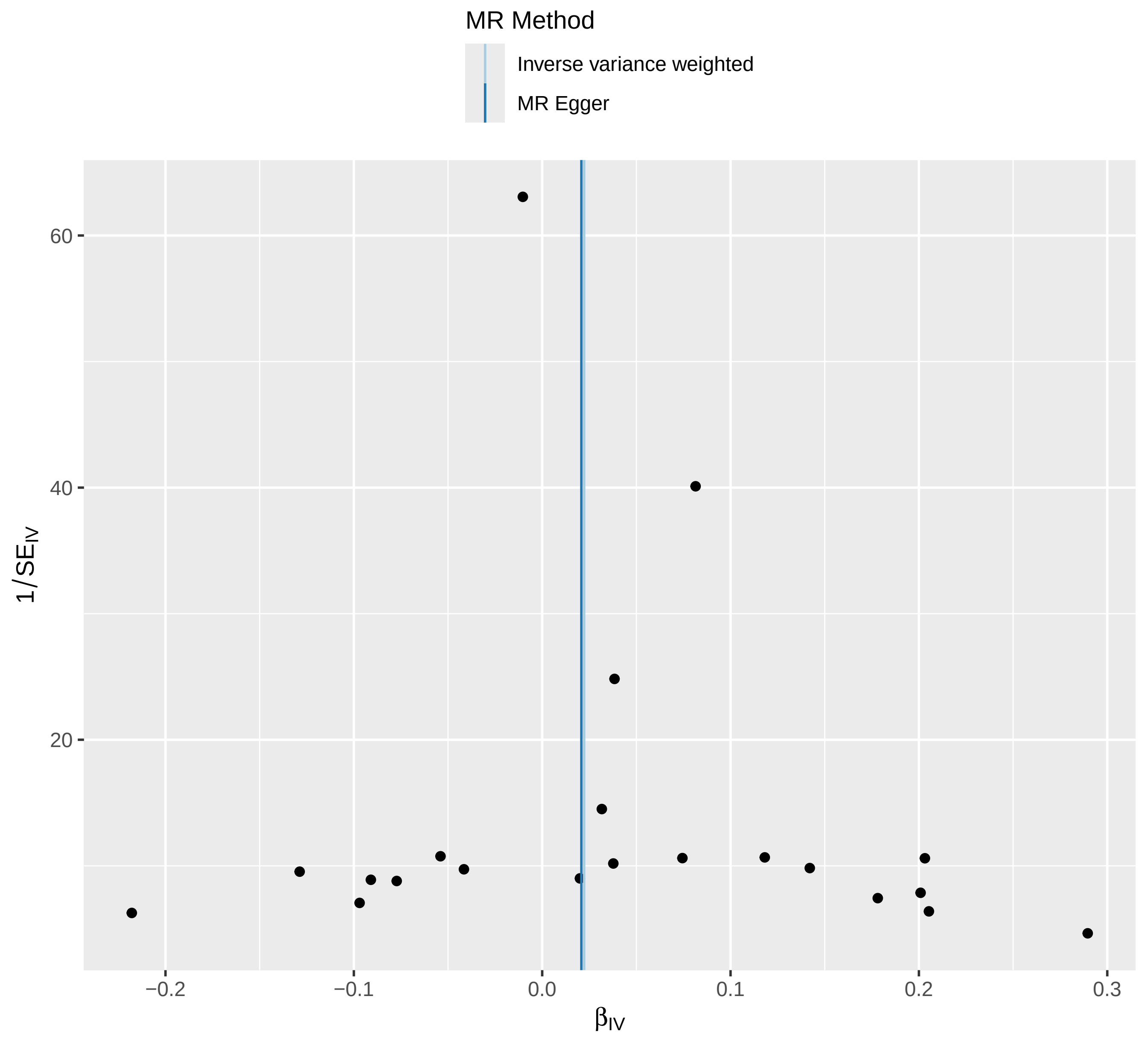
MR Method
Inverse variance weighted
MR Egger
60
40
20
1/SE$_{IV}$
−0.2
−0.1
0.0
0.1
0.2
0.3
β$_{IV}$

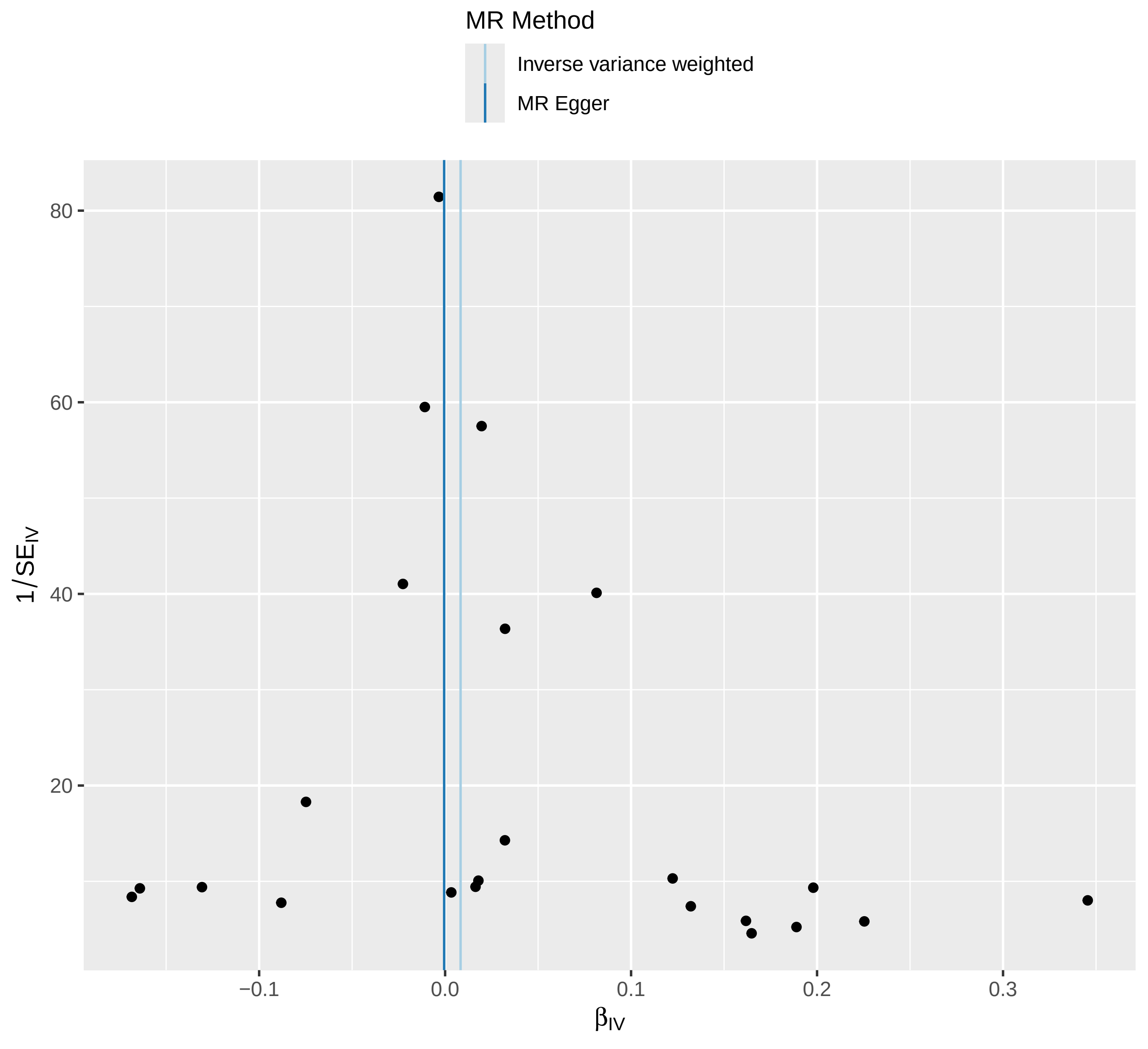
MR Method
Inverse variance weighted
MR Egger
80
60
40
20
1/SE_IV
−0.1
0.0
0.1
0.2
0.3
β_IV

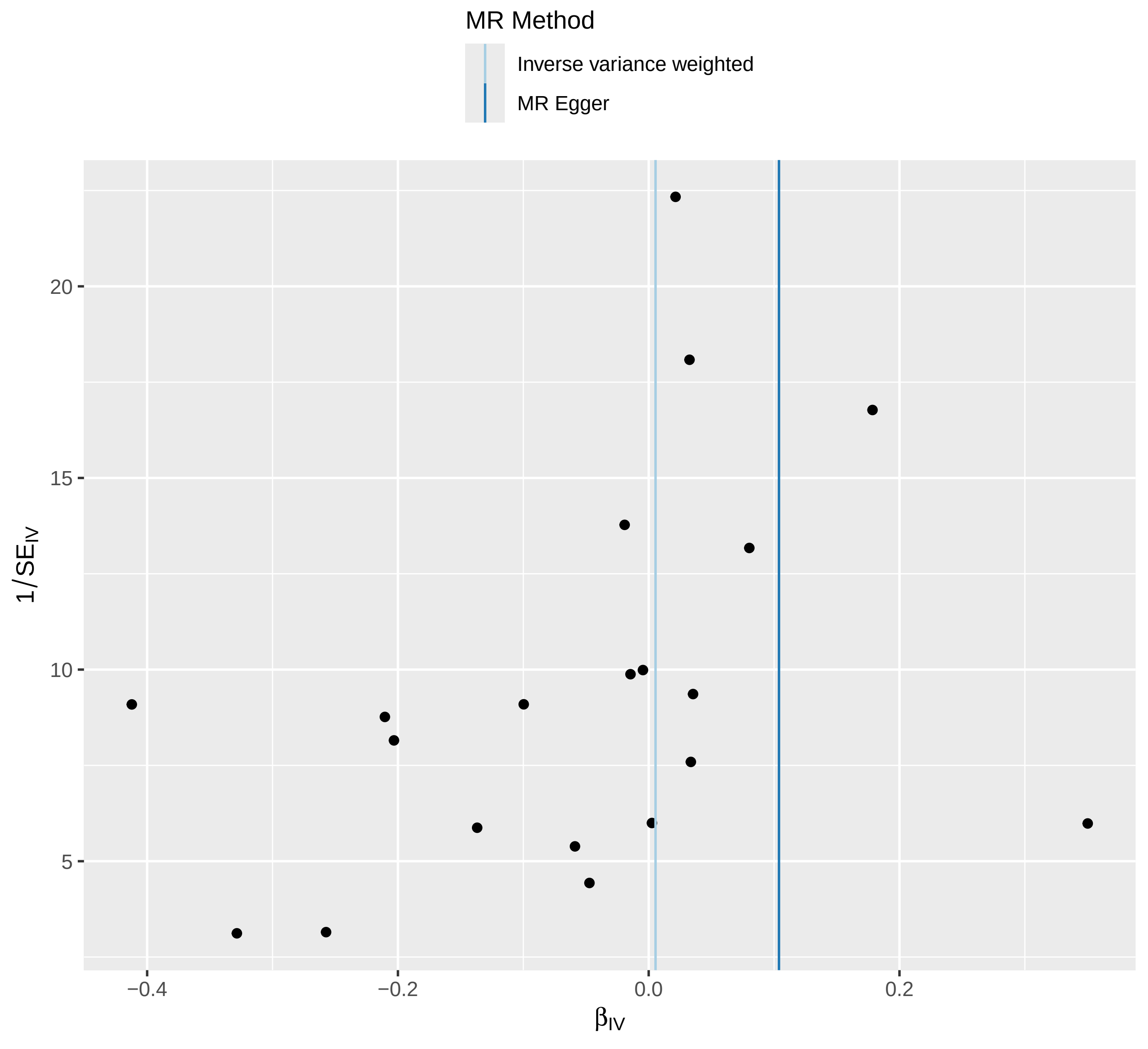
MR Method
Inverse variance weighted
MR Egger
20
15
10
5
1/SE_IV
−0.4
−0.2
0.0
0.2
β_IV

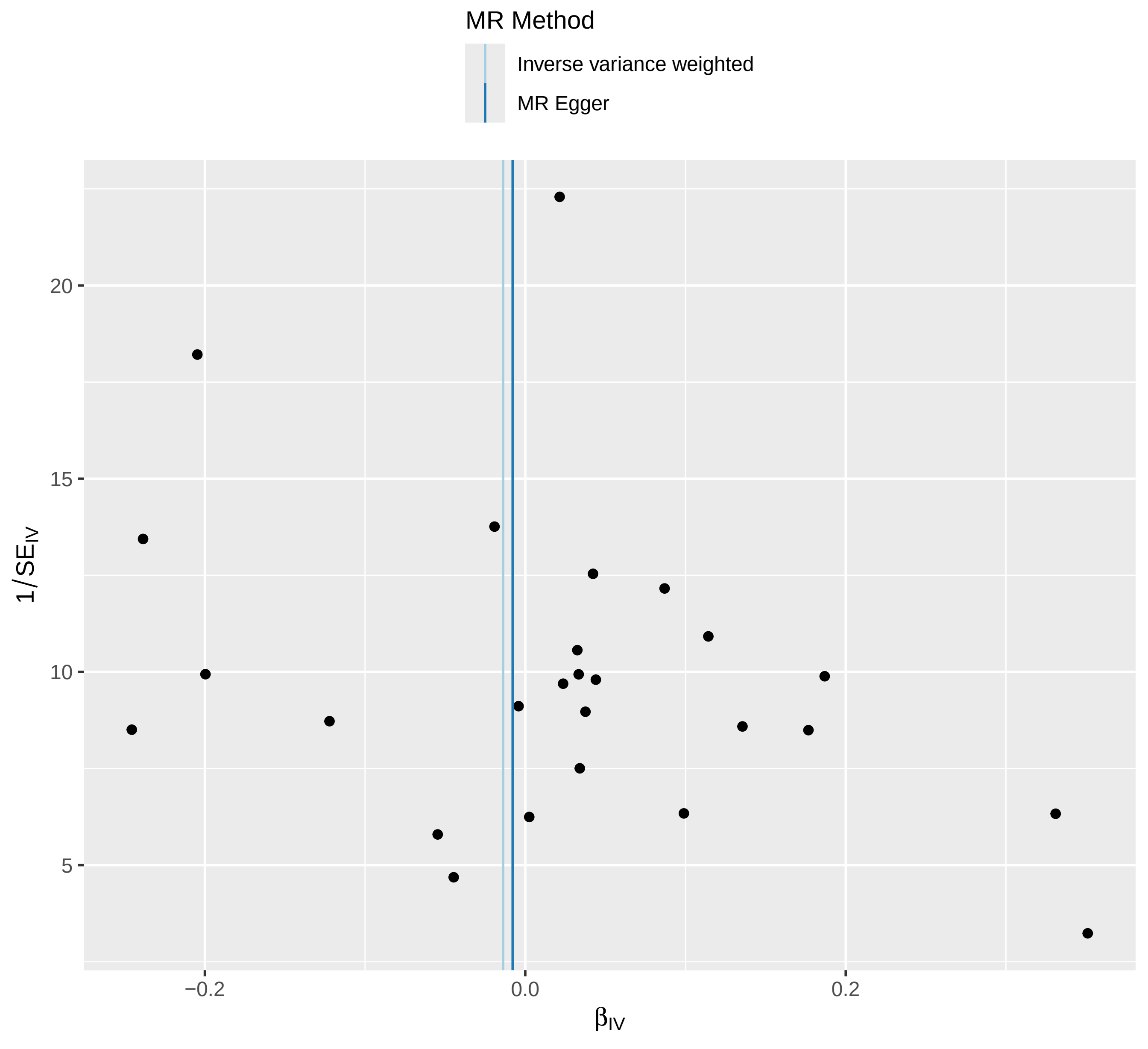
MR Method
Inverse variance weighted
MR Egger
20
15
10
5
−0.2
0.0
0.2
1/SE_IV
β_IV

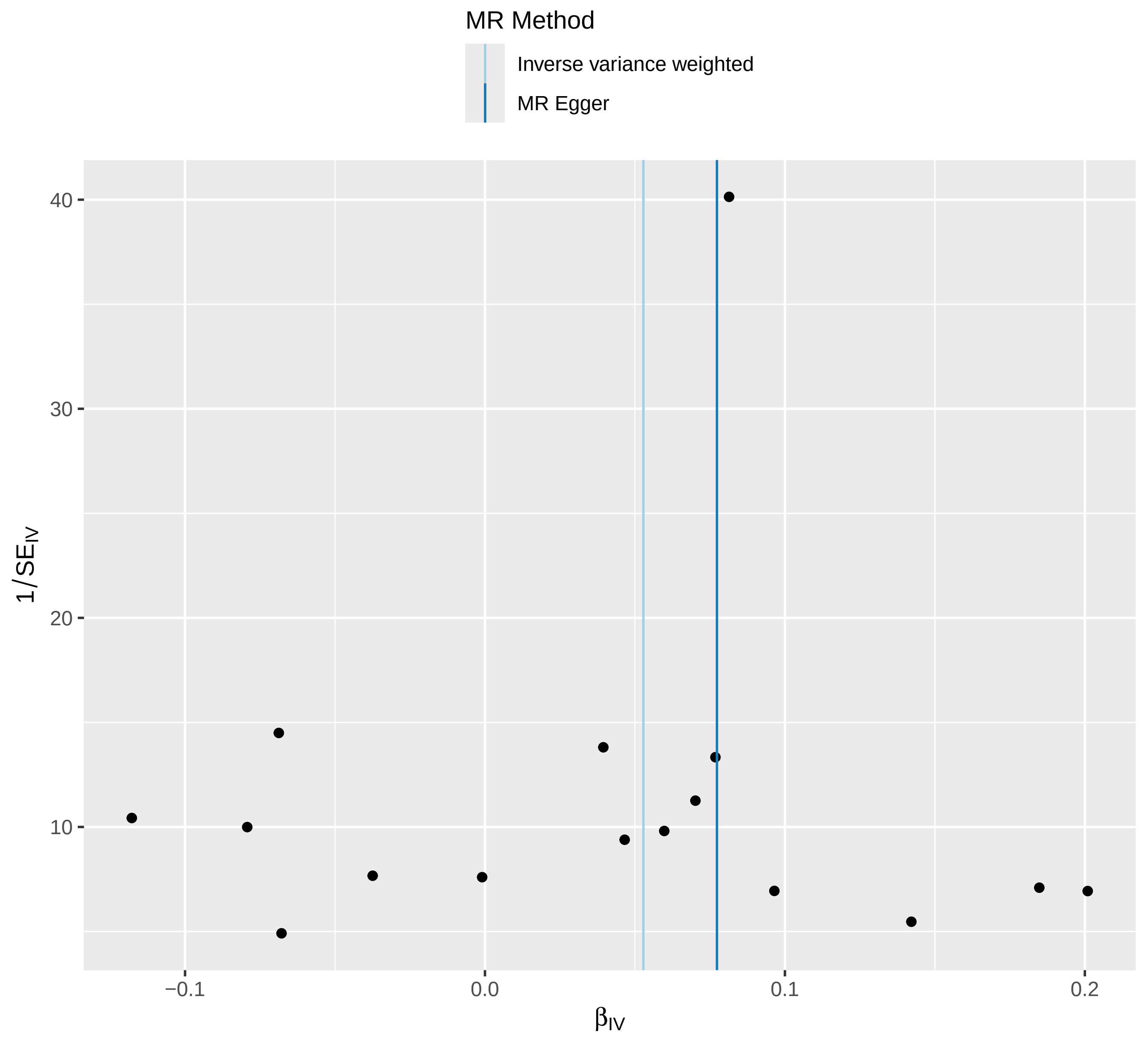
MR Method
Inverse variance weighted
MR Egger
40
30
20
10
−0.1
0.0
0.1
0.2
$1/SE_{IV}$
β_{IV}

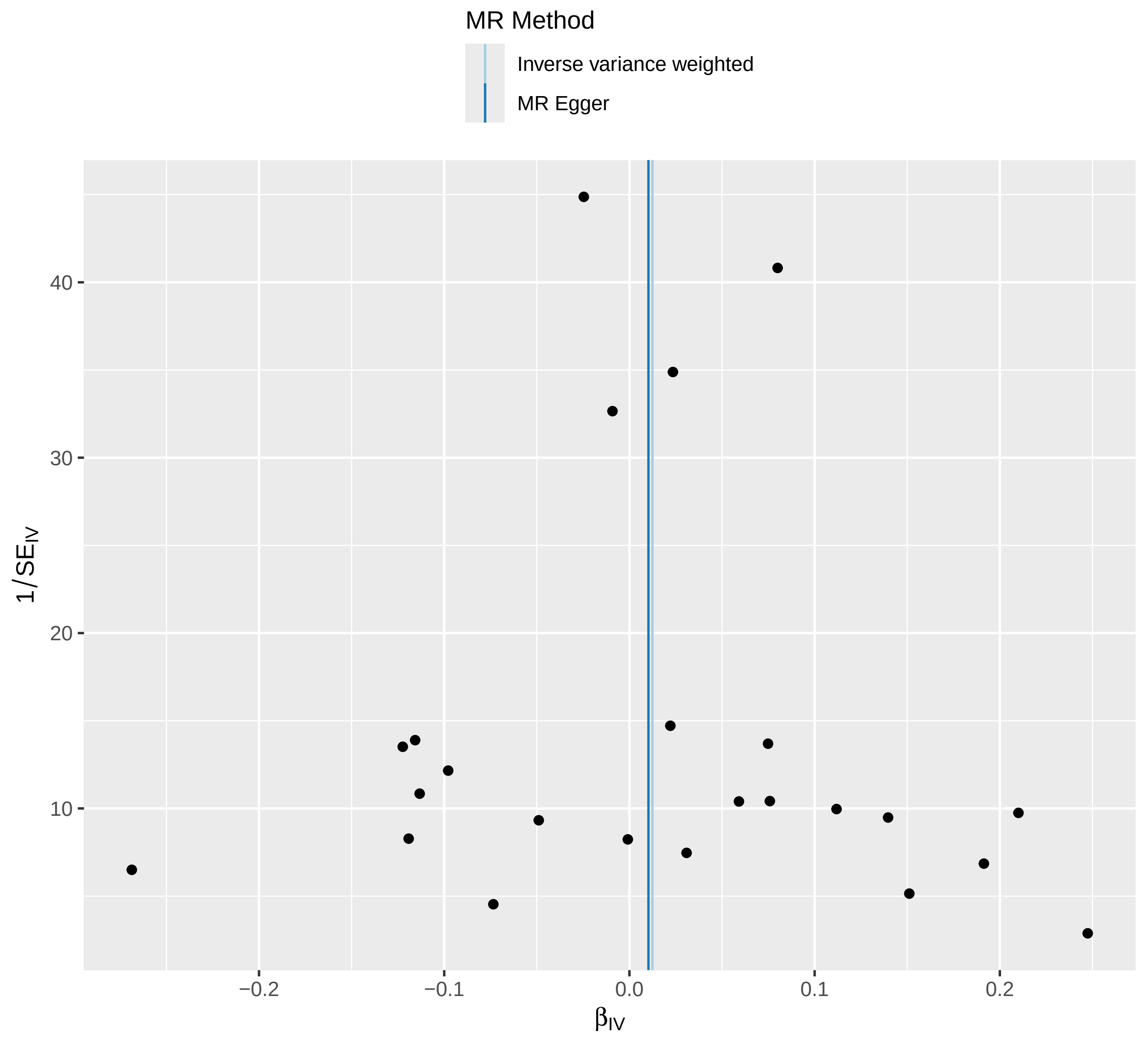
MR Method
Inverse variance weighted
MR Egger
40
30
20
10
1/SE_IV
−0.2
−0.1
0.0
0.1
0.2
β_IV

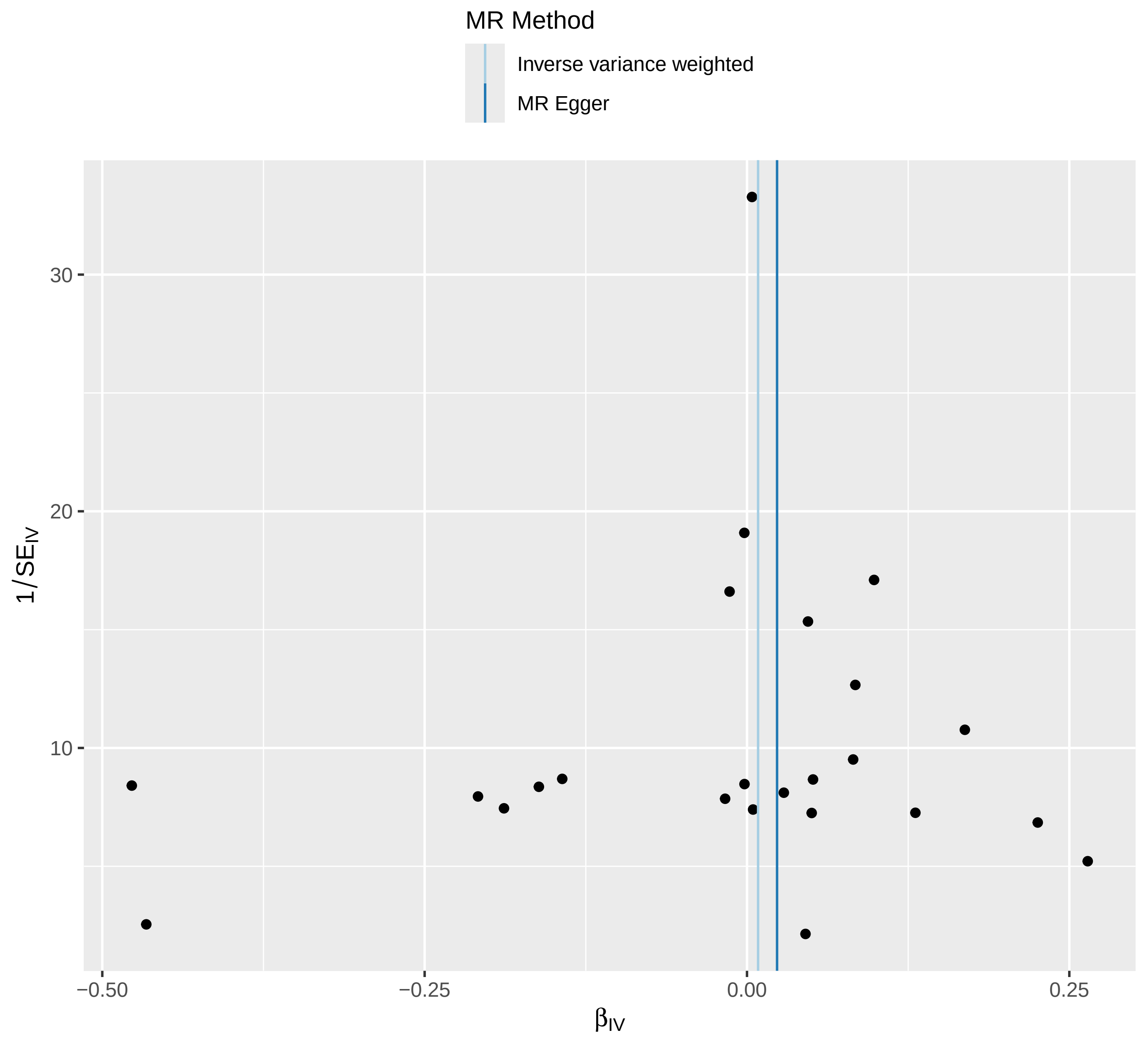

MR Method
Inverse variance weighted
MR Egger
30
20
10
1/SE_IV
−0.50
−0.25
0.00
0.25
β_IV

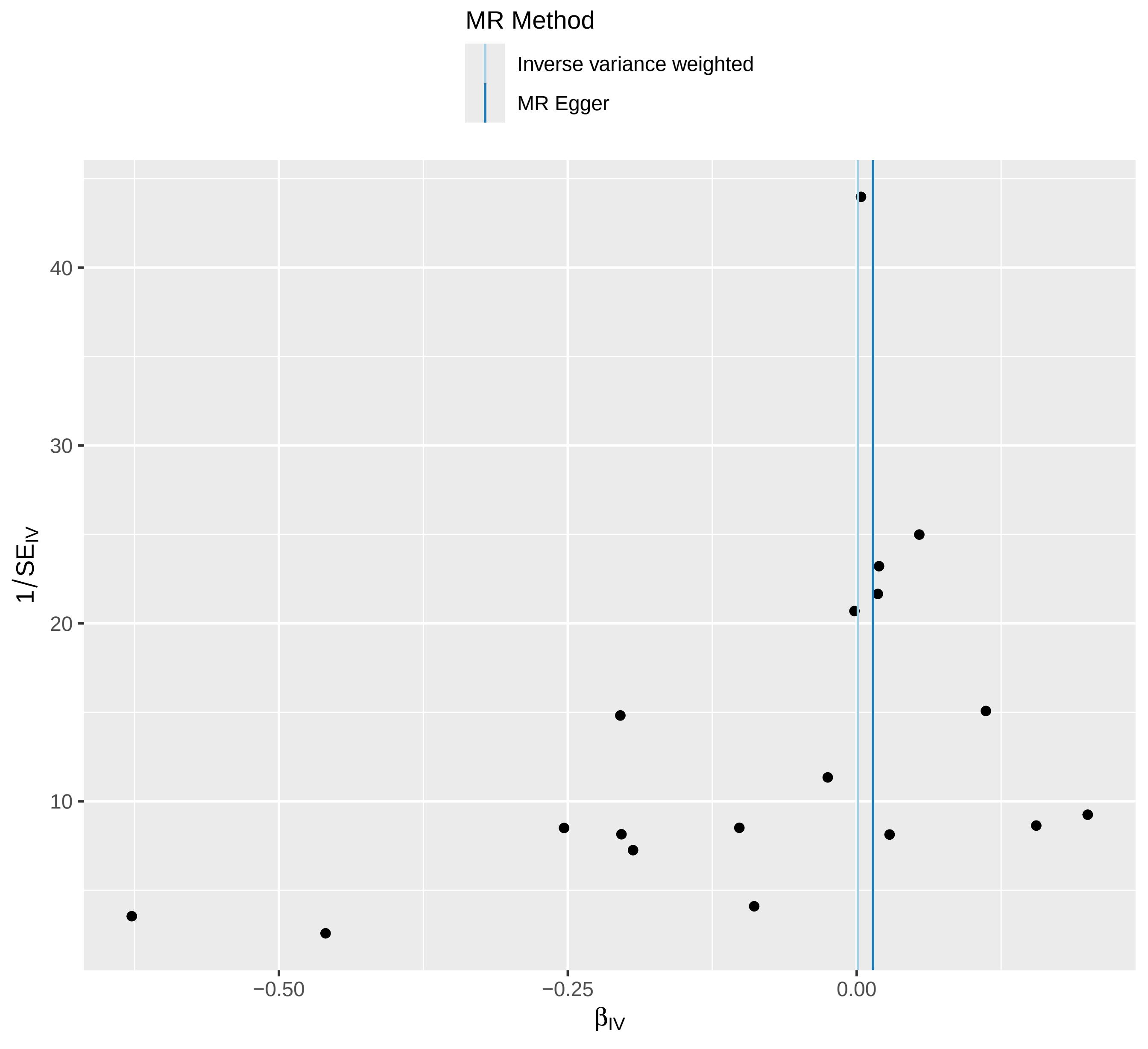
MR Method
Inverse variance weighted
MR Egger
40
30
20
10
−0.50
−0.25
0.00
1/SE_IV
β_IV

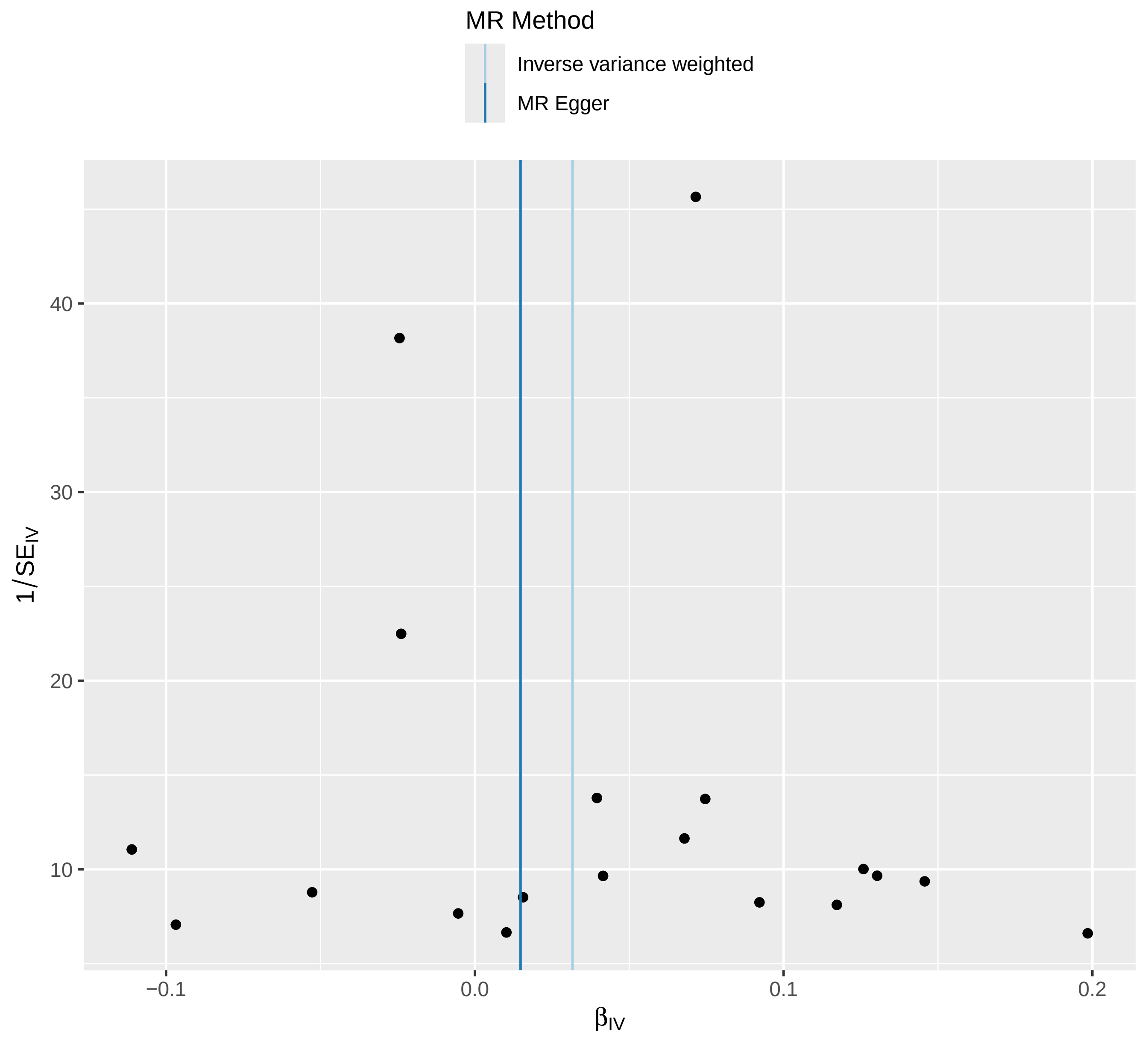
MR Method
Inverse variance weighted
MR Egger
40
30
20
10
1/SE$_{IV}$
−0.1
0.0
0.1
0.2
β$_{IV}$

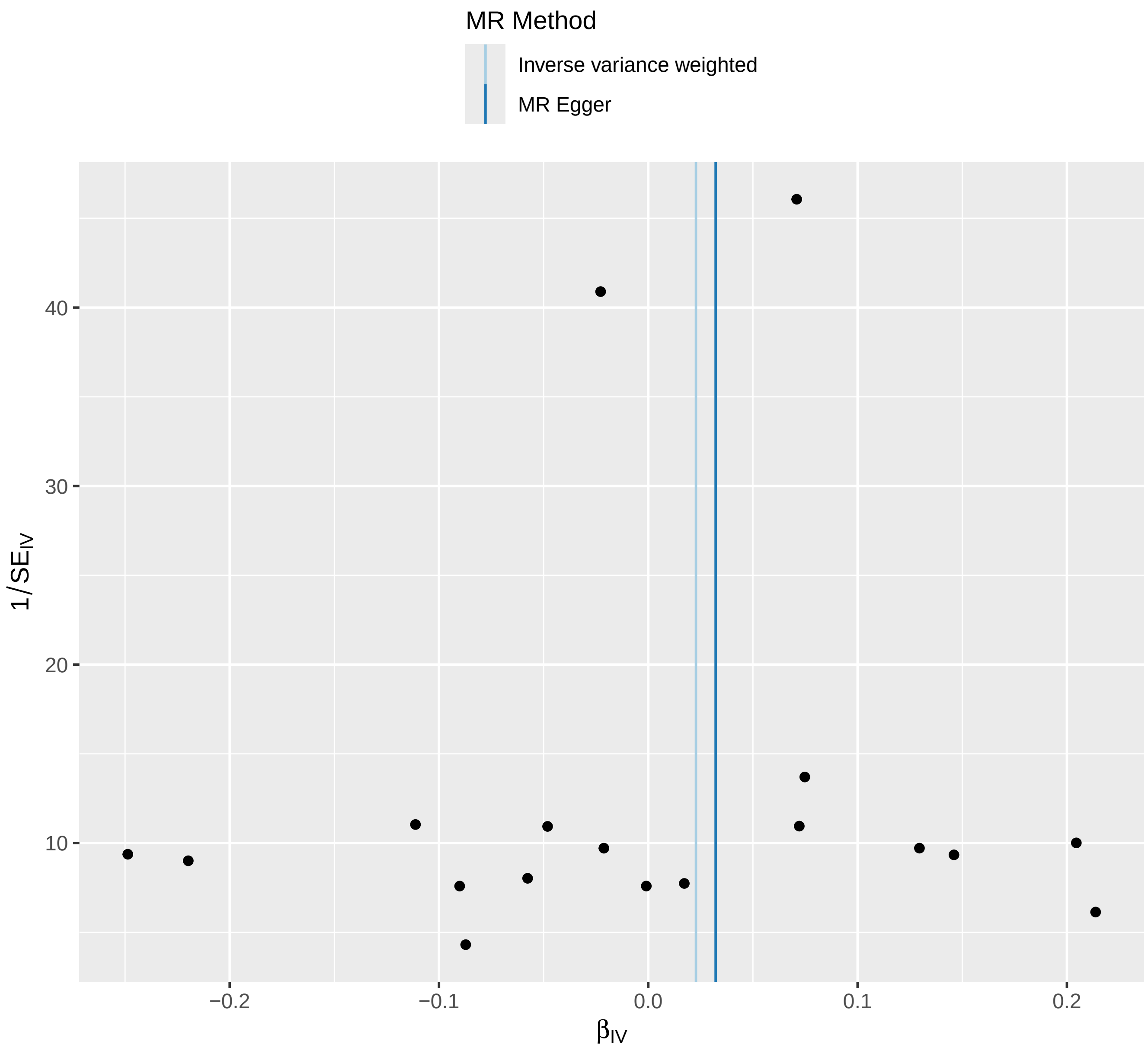
MR Method
Inverse variance weighted
MR Egger
1/SE_IV
40
30
20
10
−0.2
−0.1
0.0
0.1
0.2
β_IV

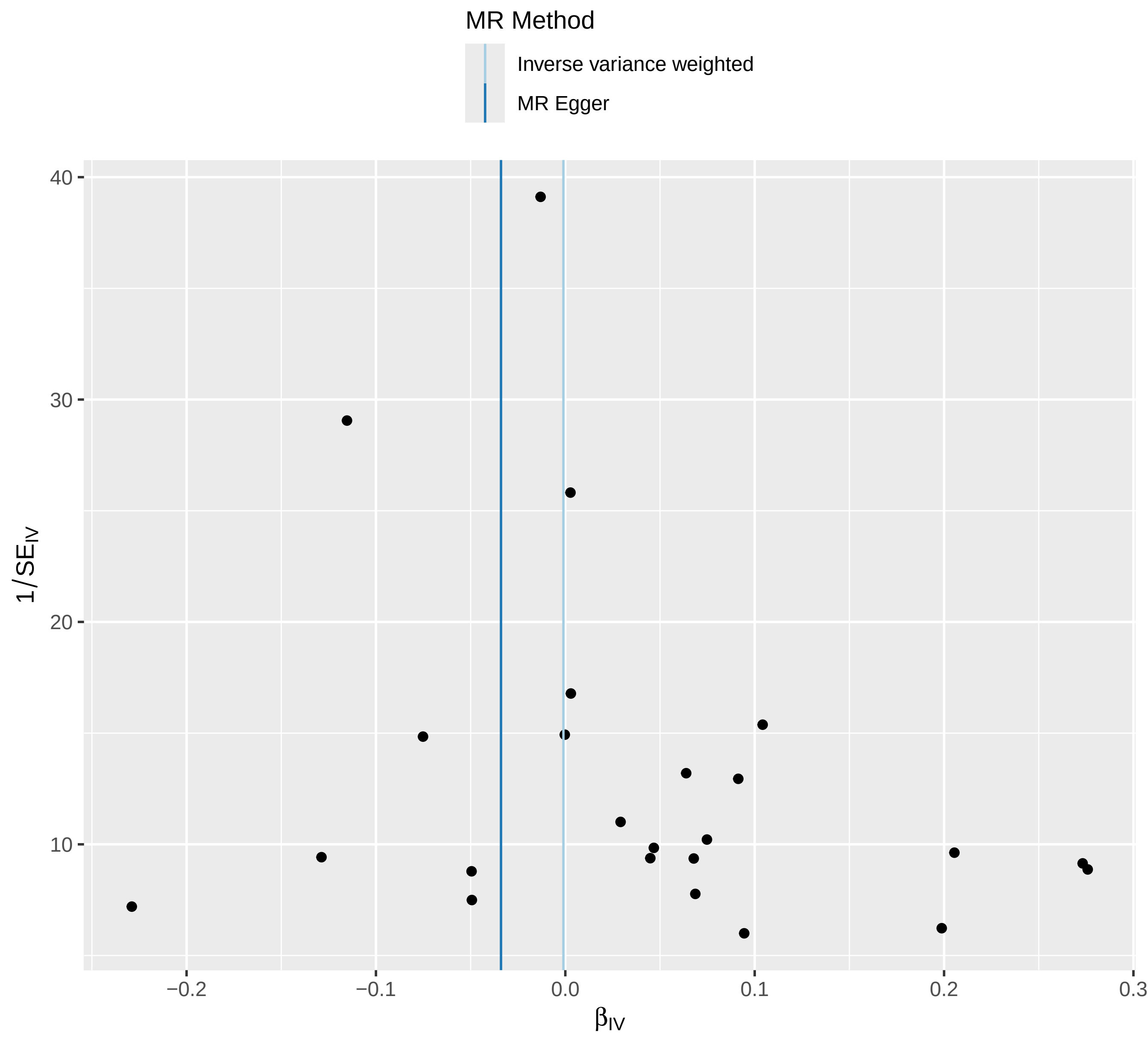
MR Method
Inverse variance weighted
MR Egger
40
30
20
10
1/SE_IV
−0.2
−0.1
0.0
0.1
0.2
0.3
β_IV

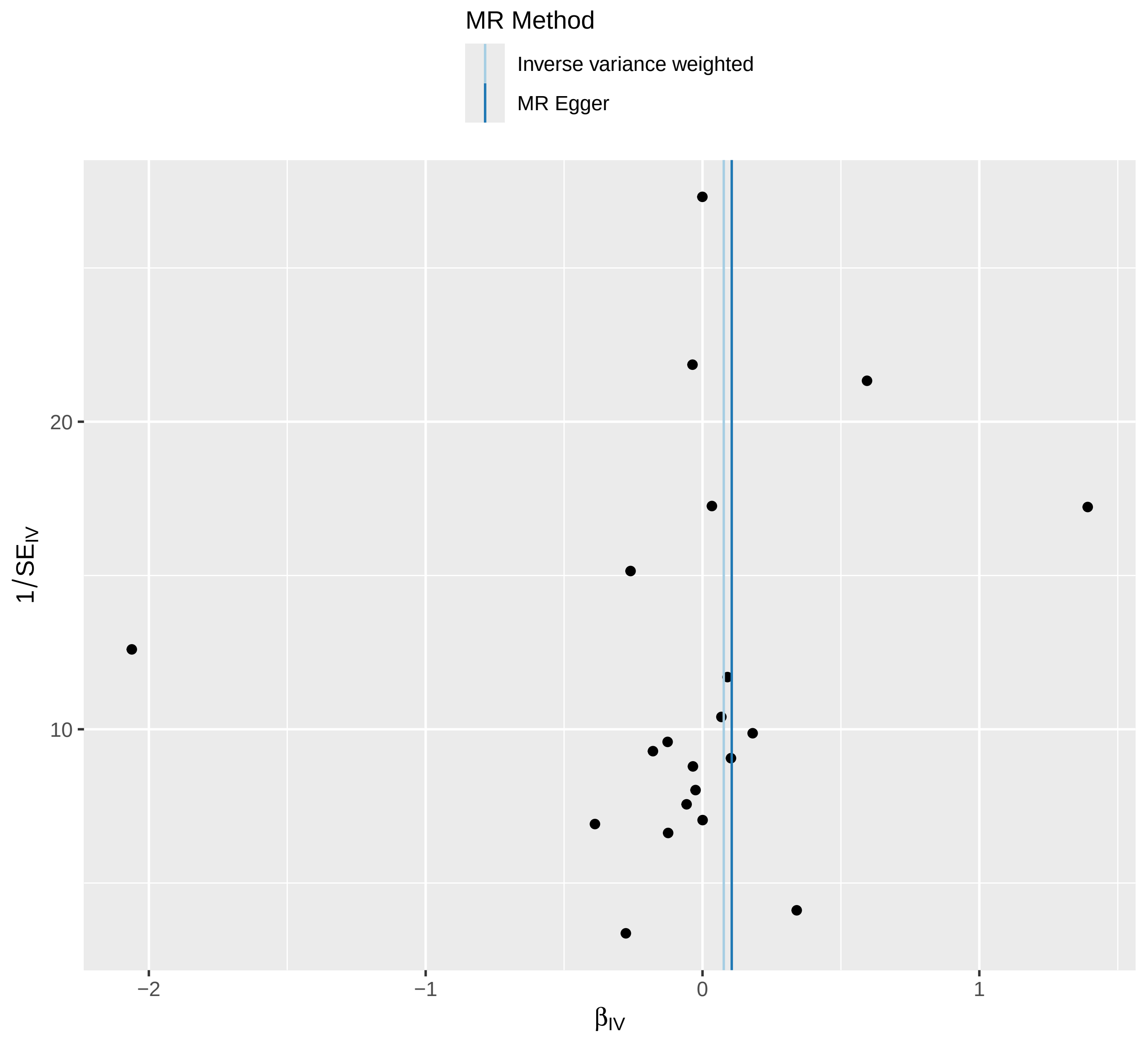
MR Method
Inverse variance weighted
MR Egger
20
10
$1/SE_{IV}$
−2
−1
0
1
β_{IV}

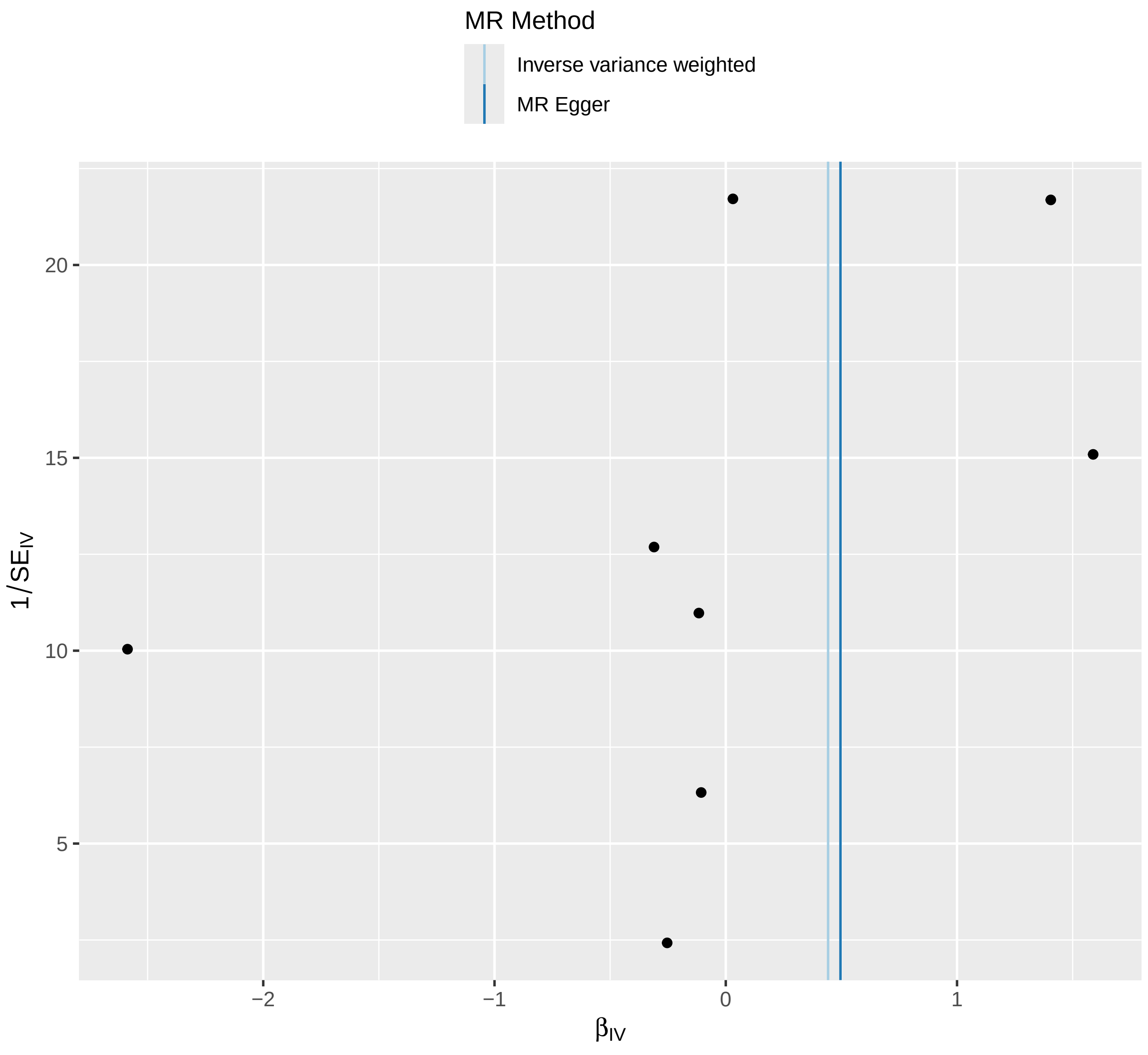

MR Method
Inverse variance weighted
MR Egger
20
15
10
5
$1/SE_{IV}$
−2
−1
0
1
β_{IV}

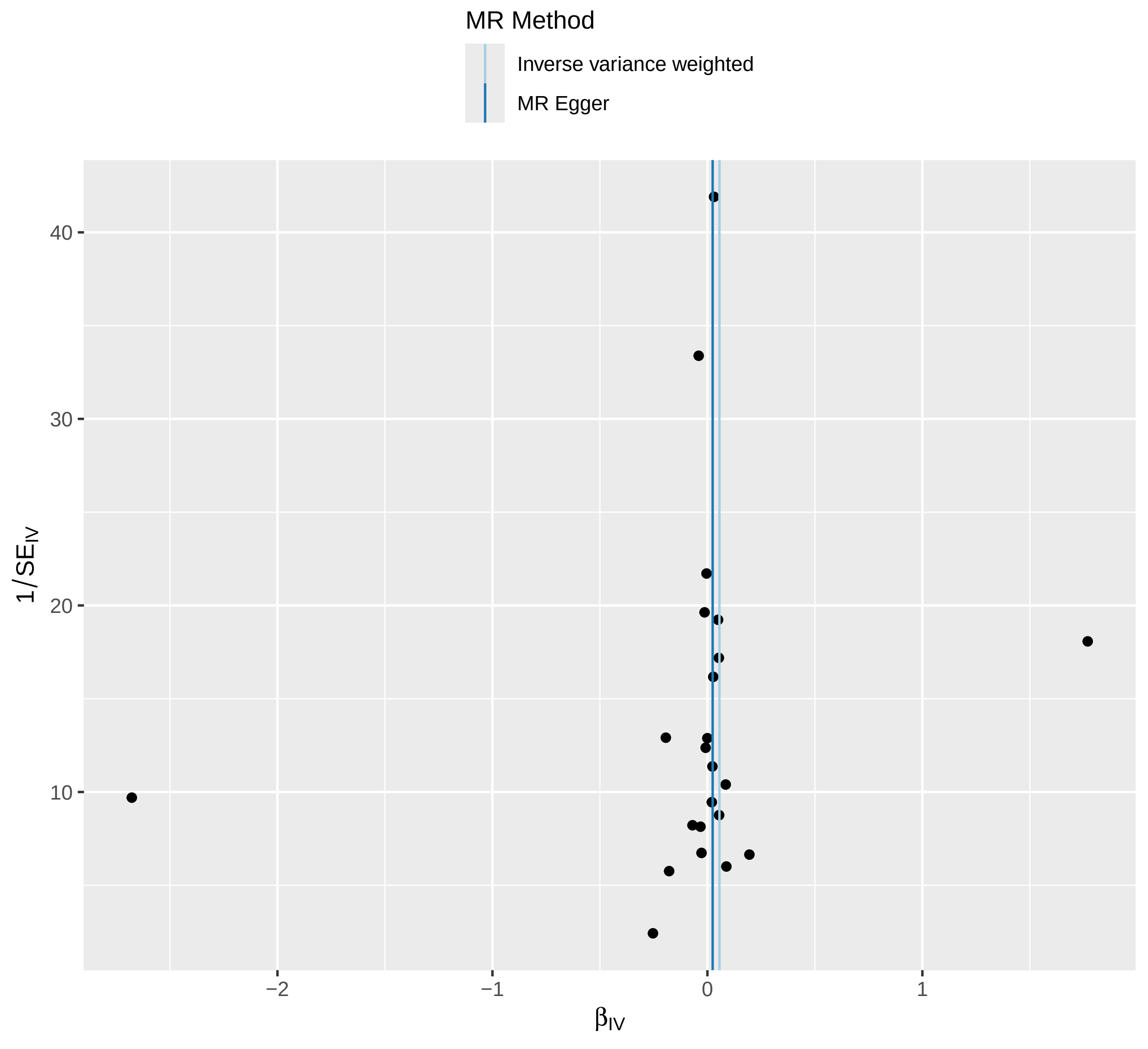
MR Method
Inverse variance weighted
MR Egger
40
30
20
10
1/SE$_{IV}$
−2
−1
0
1
β$_{IV}$

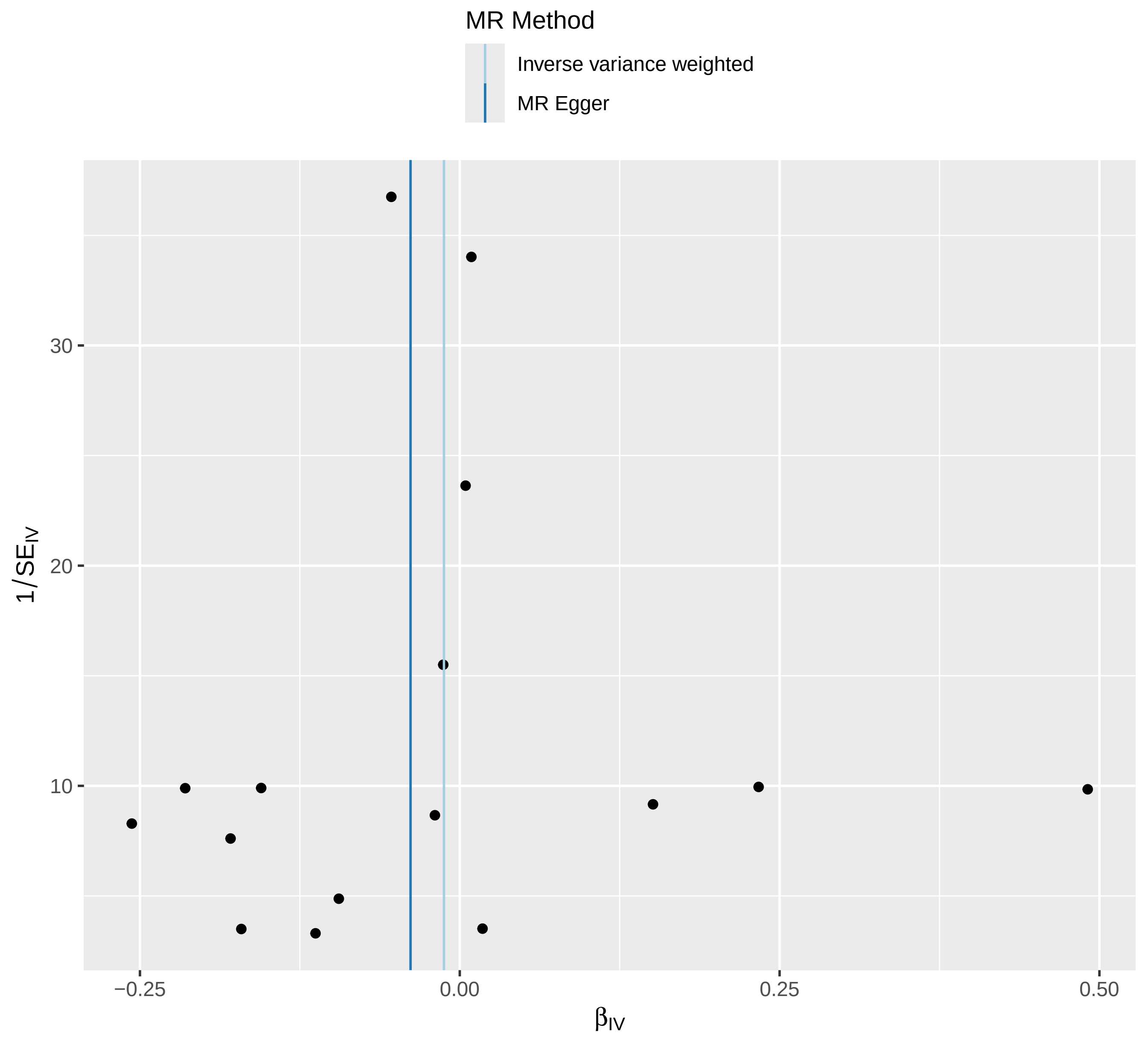
MR Method
Inverse variance weighted
MR Egger
30
20
10
1/SE_IV
−0.25
0.00
0.25
0.50
β_IV

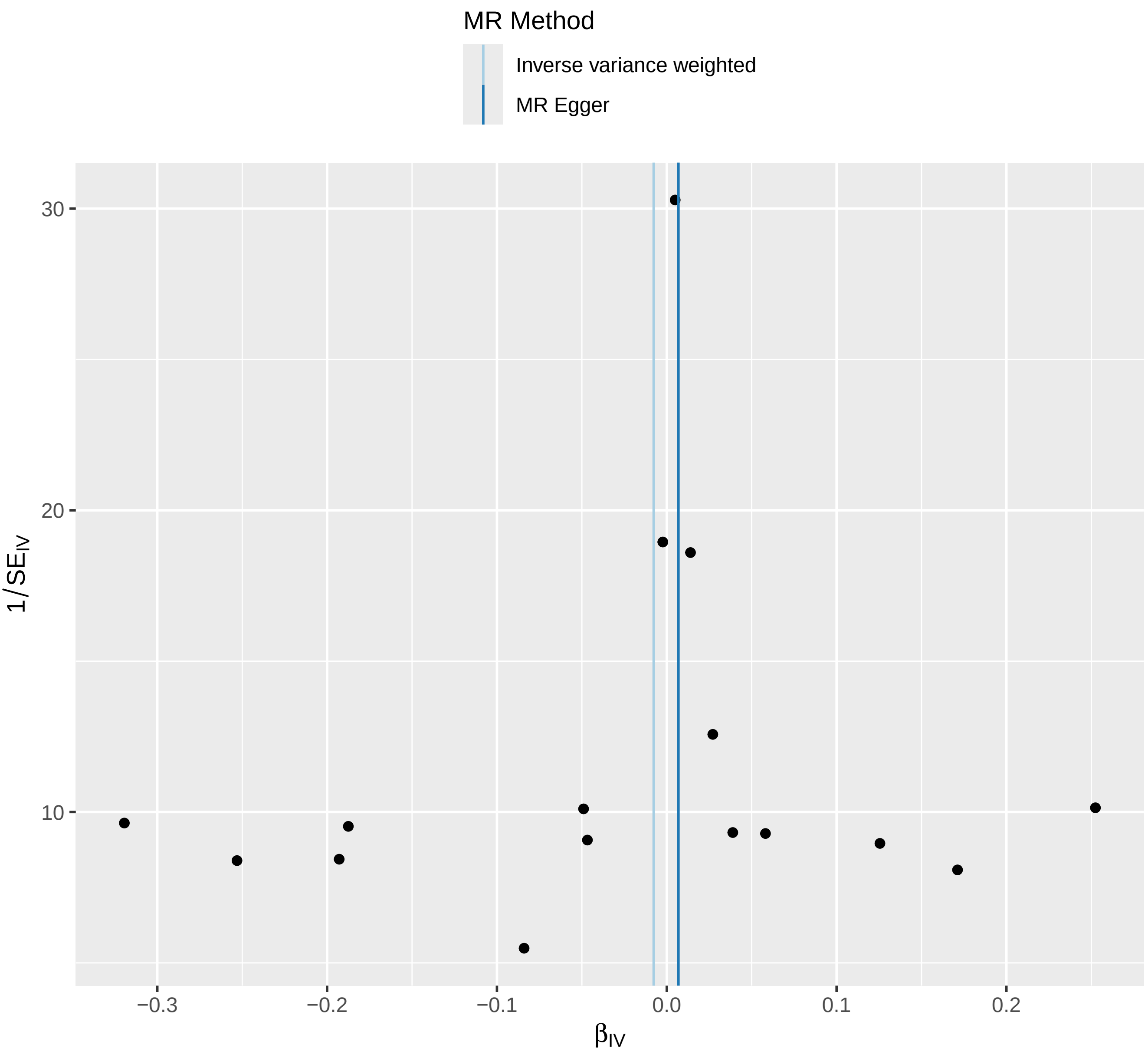

MR Method
Inverse variance weighted
MR Egger
30
20
10
1/SE_IV
−0.3
−0.2
−0.1
0.0
0.1
0.2
β_IV

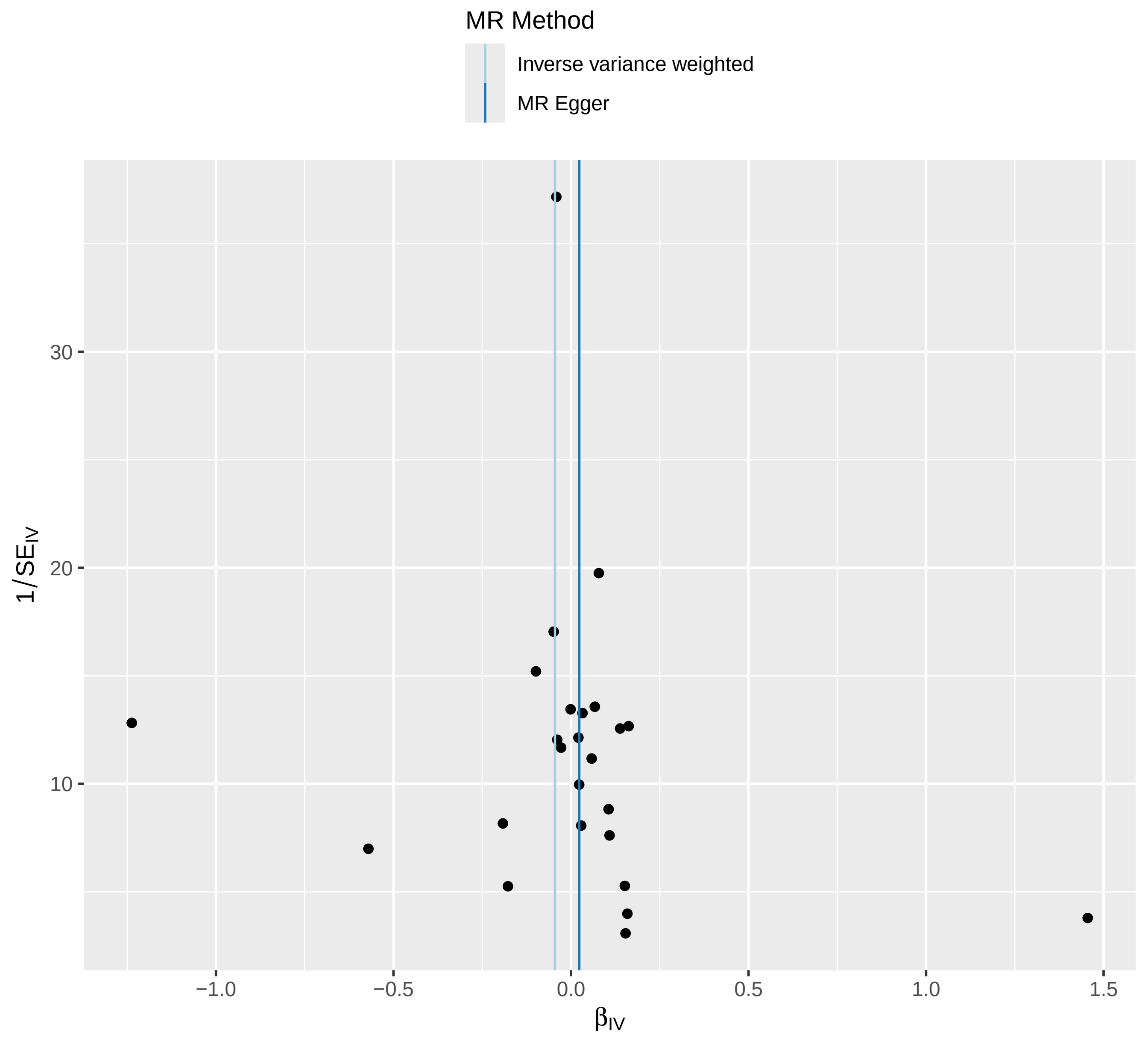
MR Method
Inverse variance weighted
MR Egger
30
20
10
1/SE_IV
−1.0
−0.5
0.0
0.5
1.0
1.5
β_IV

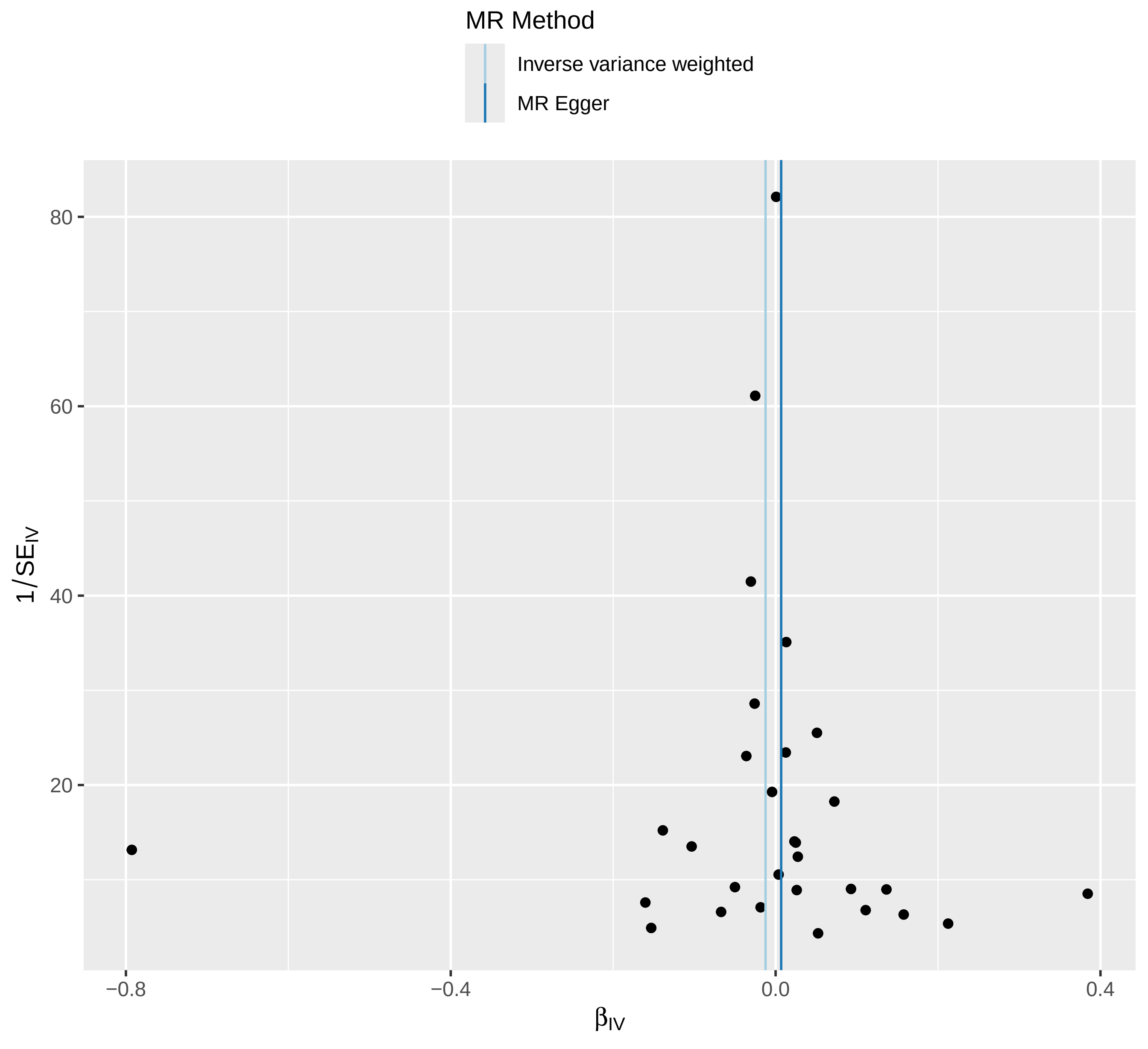
MR Method
Inverse variance weighted
MR Egger
80
60
40
20
$1/SE_{IV}$
−0.8
−0.4
0.0
0.4
β_{IV}

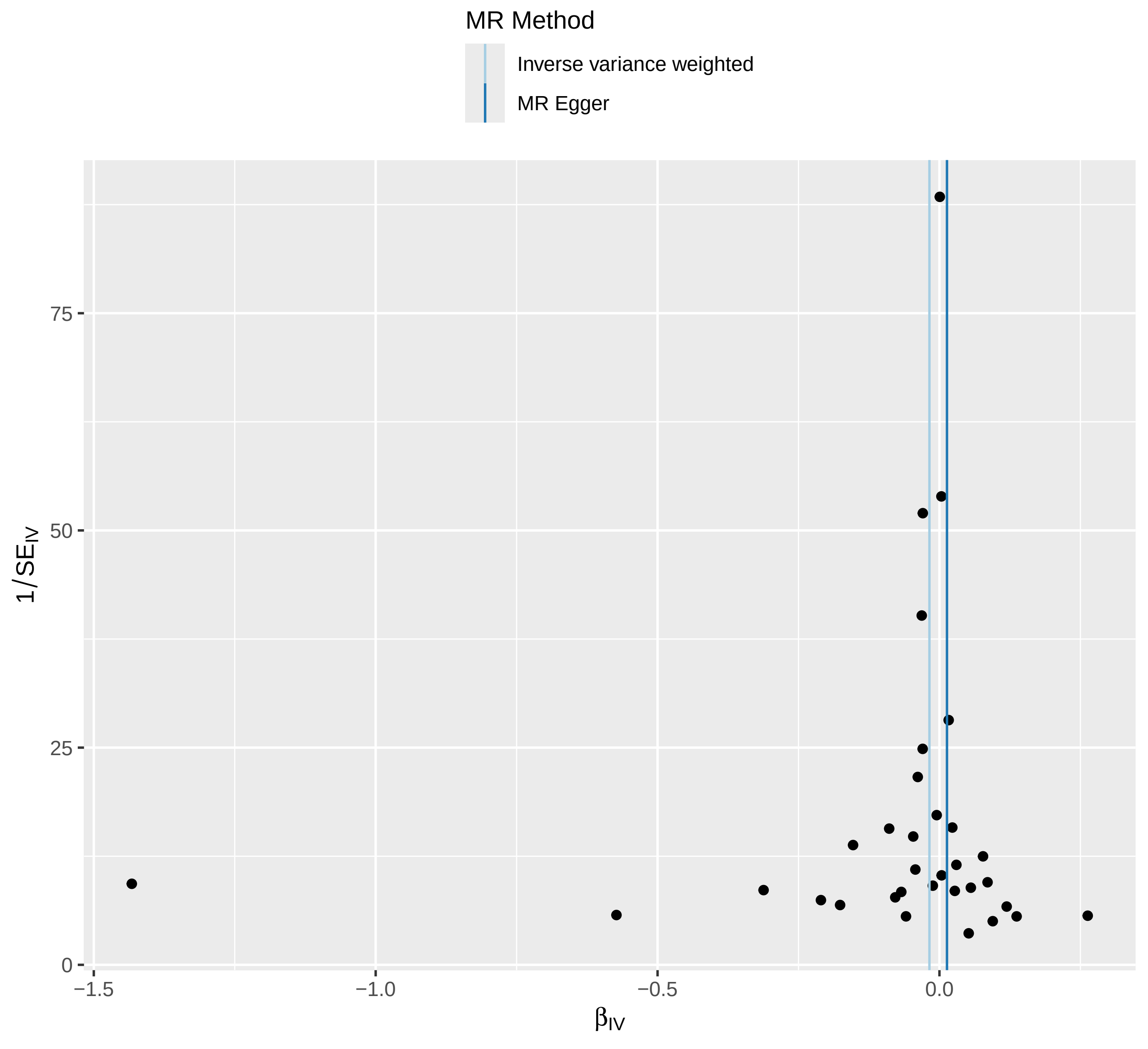
MR Method
Inverse variance weighted
MR Egger
1/SE_IV
0
25
50
75
β_IV
−1.5
−1.0
−0.5
0.0

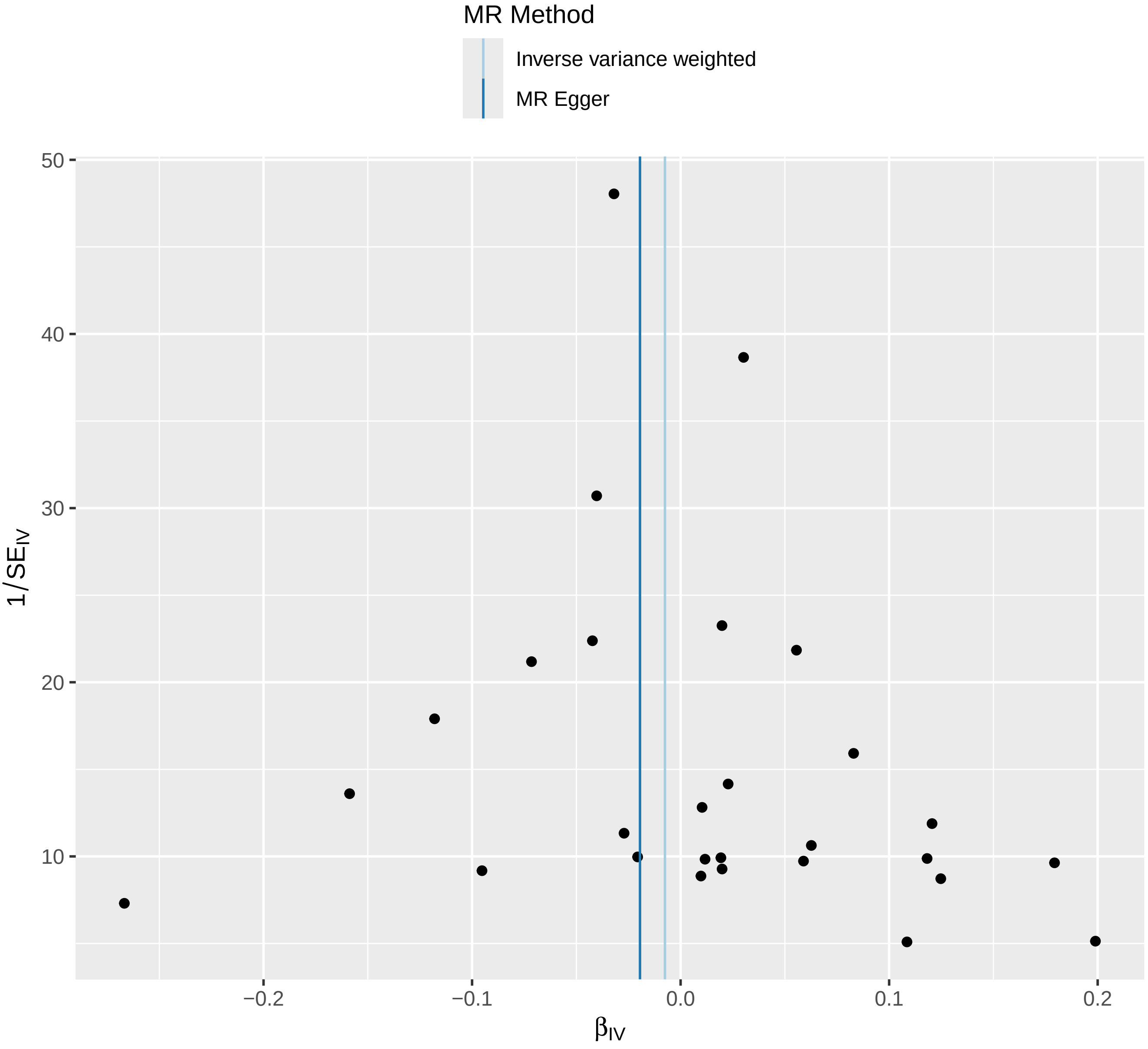
MR Method
Inverse variance weighted
MR Egger
50
40
30
20
10
1/SE$_{IV}$
−0.2
−0.1
0.0
0.1
0.2
β$_{IV}$

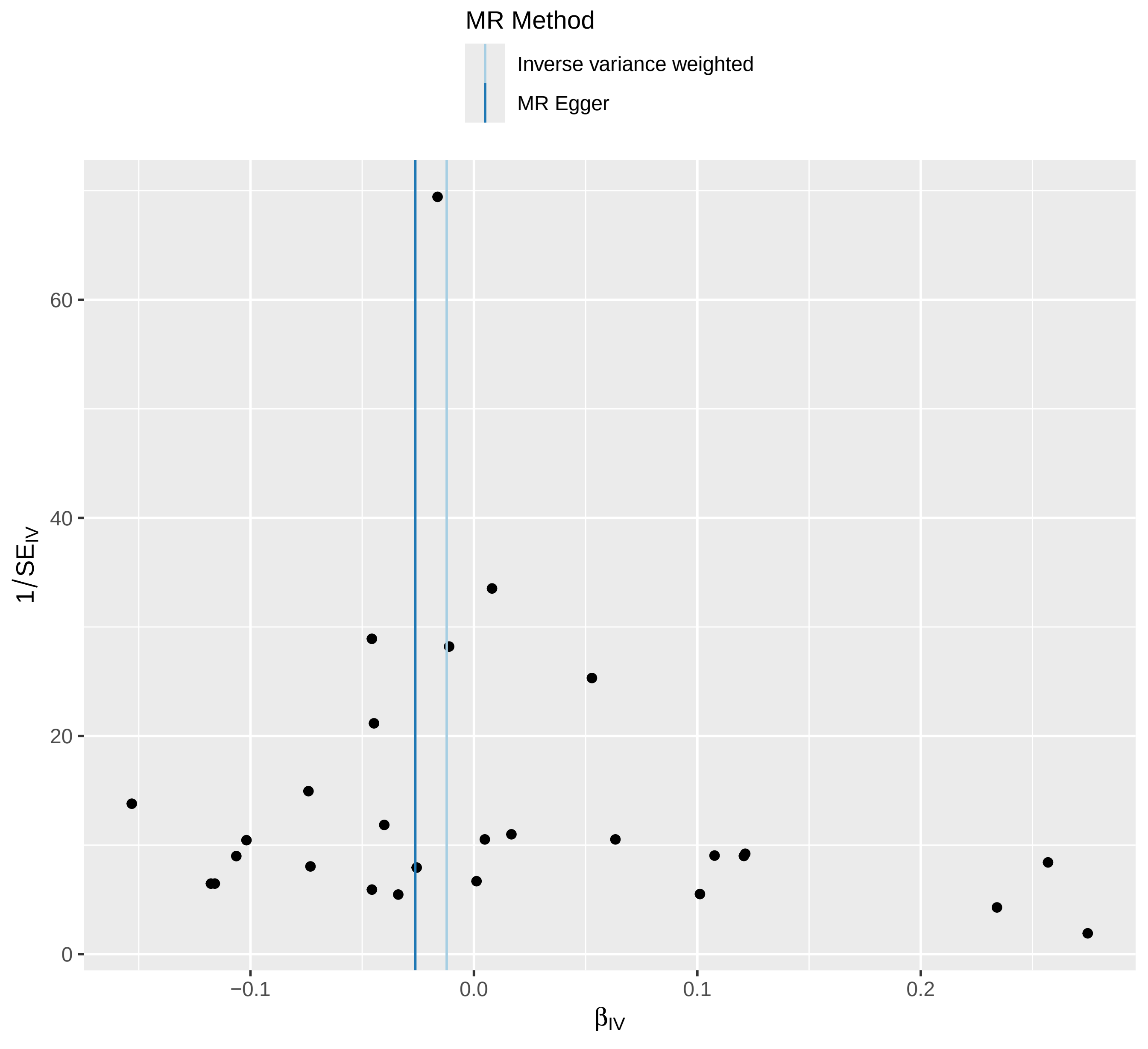

MR Method
Inverse variance weighted
MR Egger
60
40
20
0
1/SE_IV
−0.1
0.0
0.1
0.2
β_IV

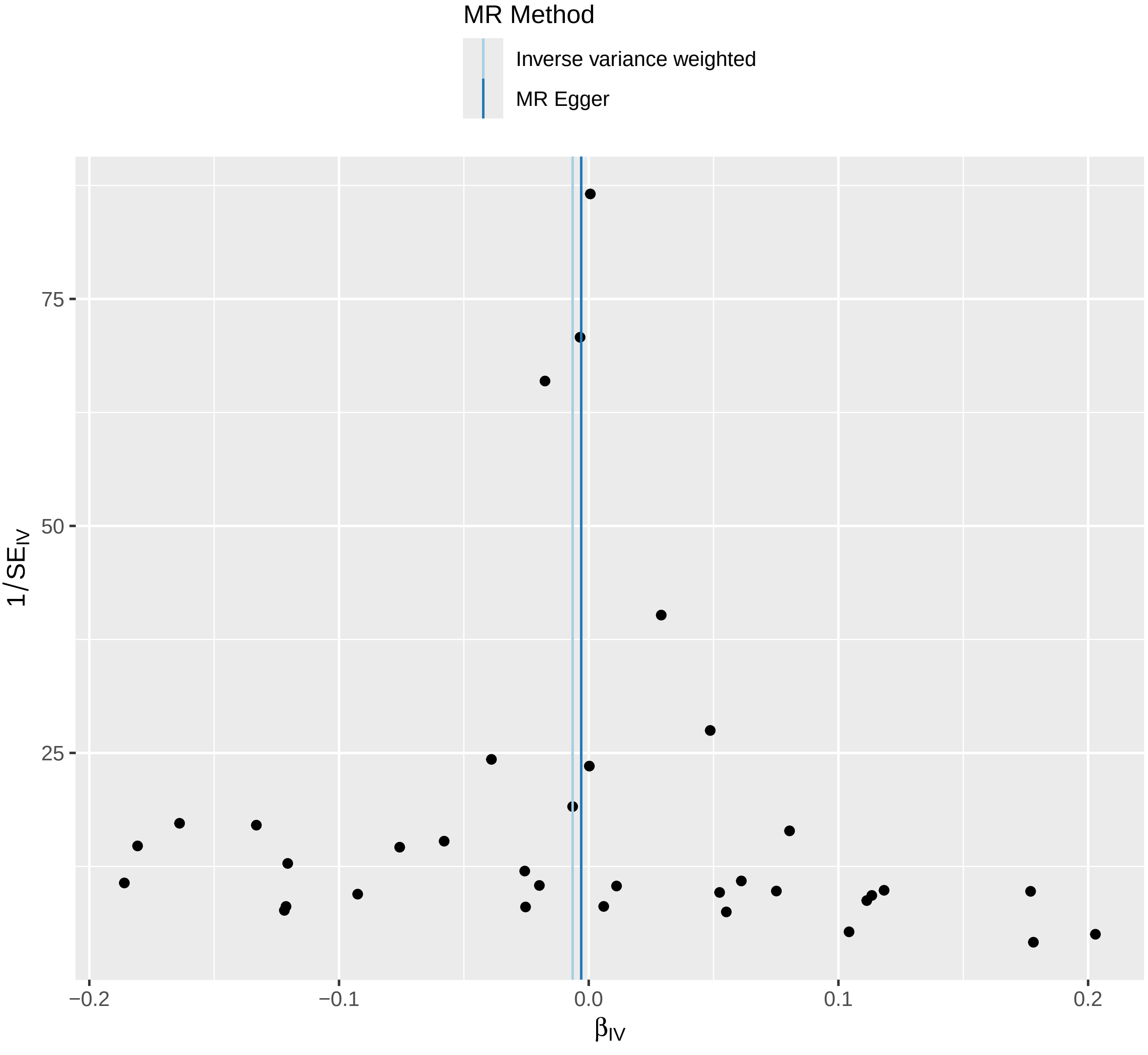

MR Method
Inverse variance weighted
MR Egger
75
50
25
$1/SE_{IV}$
−0.2
−0.1
0.0
0.1
0.2
β_{IV}

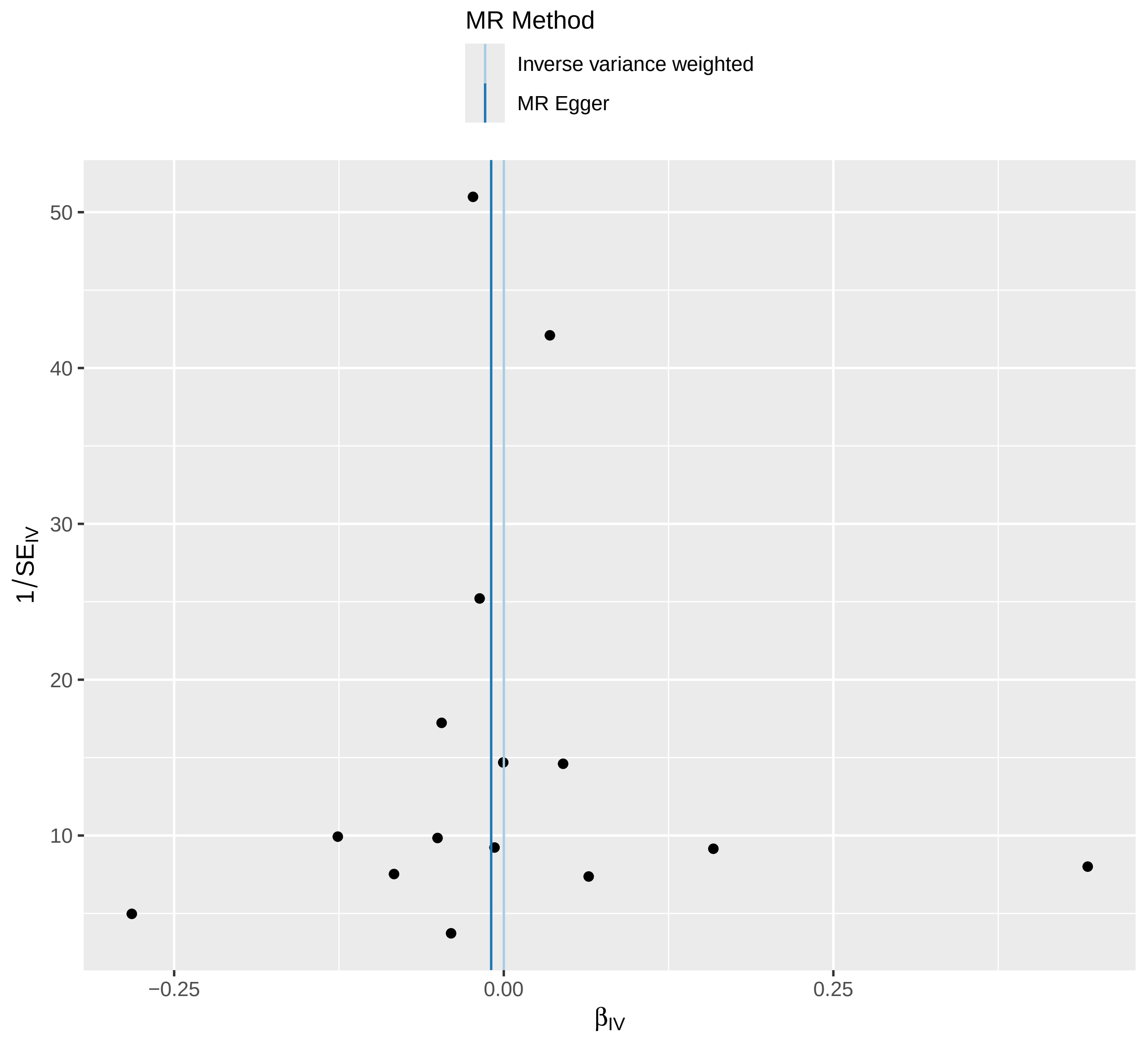
MR Method
Inverse variance weighted
MR Egger
50
40
30
20
10
1/SE_IV
−0.25
0.00
0.25
β_IV

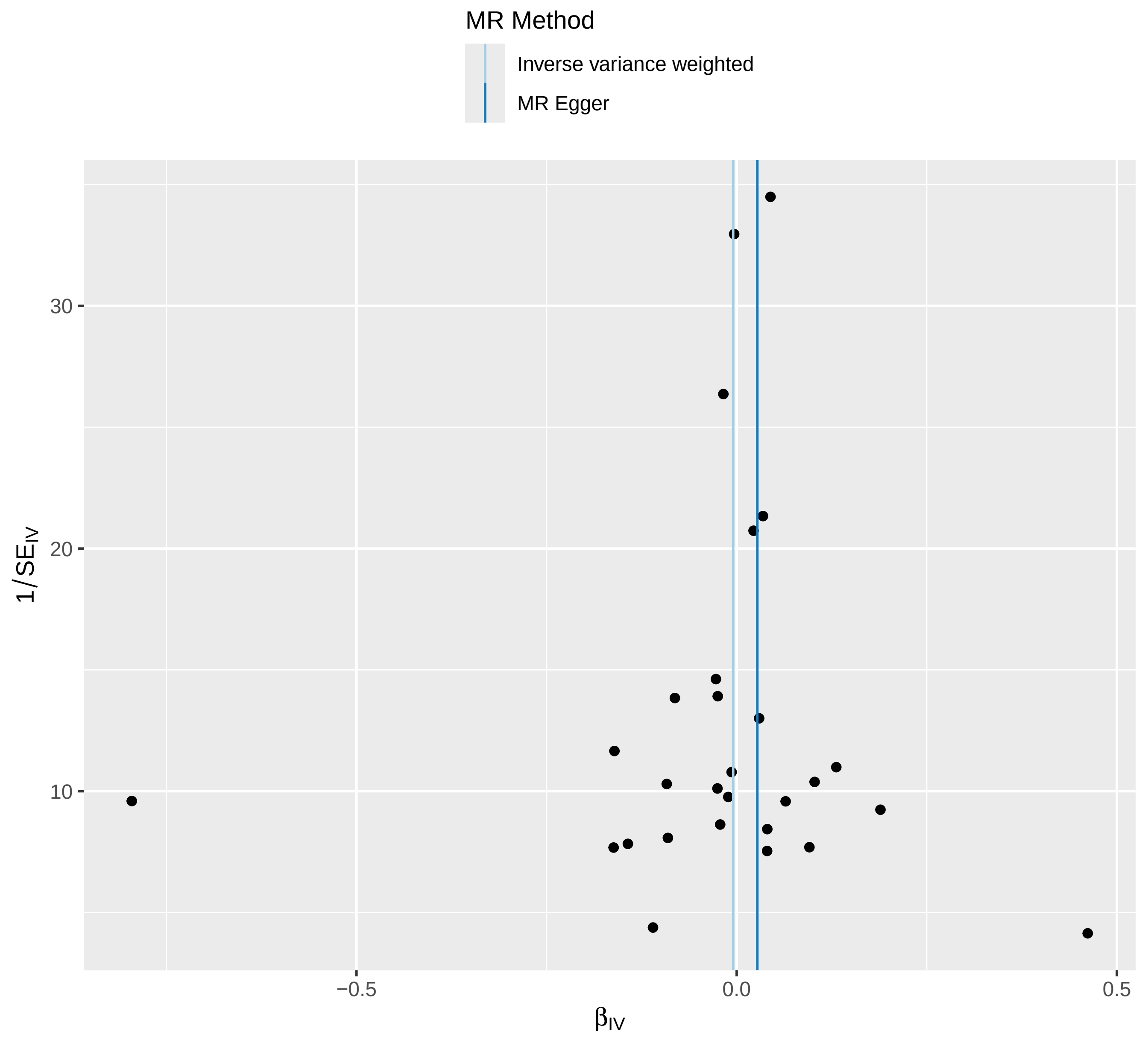
MR Method
Inverse variance weighted
MR Egger
30
20
10
1/SE_IV
−0.5
0.0
0.5
β_IV

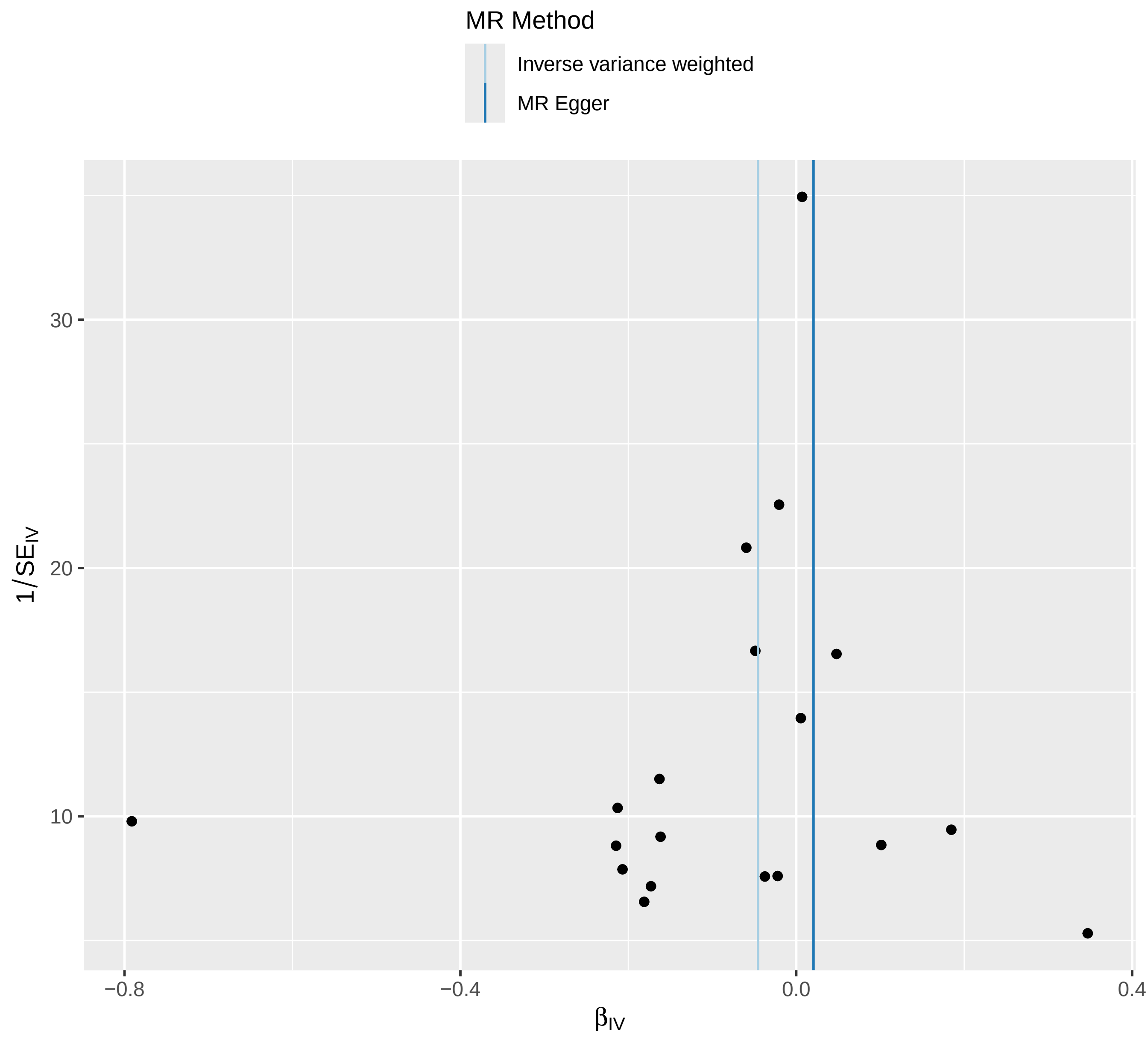
MR Method
Inverse variance weighted
MR Egger
30
20
10
1/SE_IV
−0.8
−0.4
0.0
0.4
β_IV

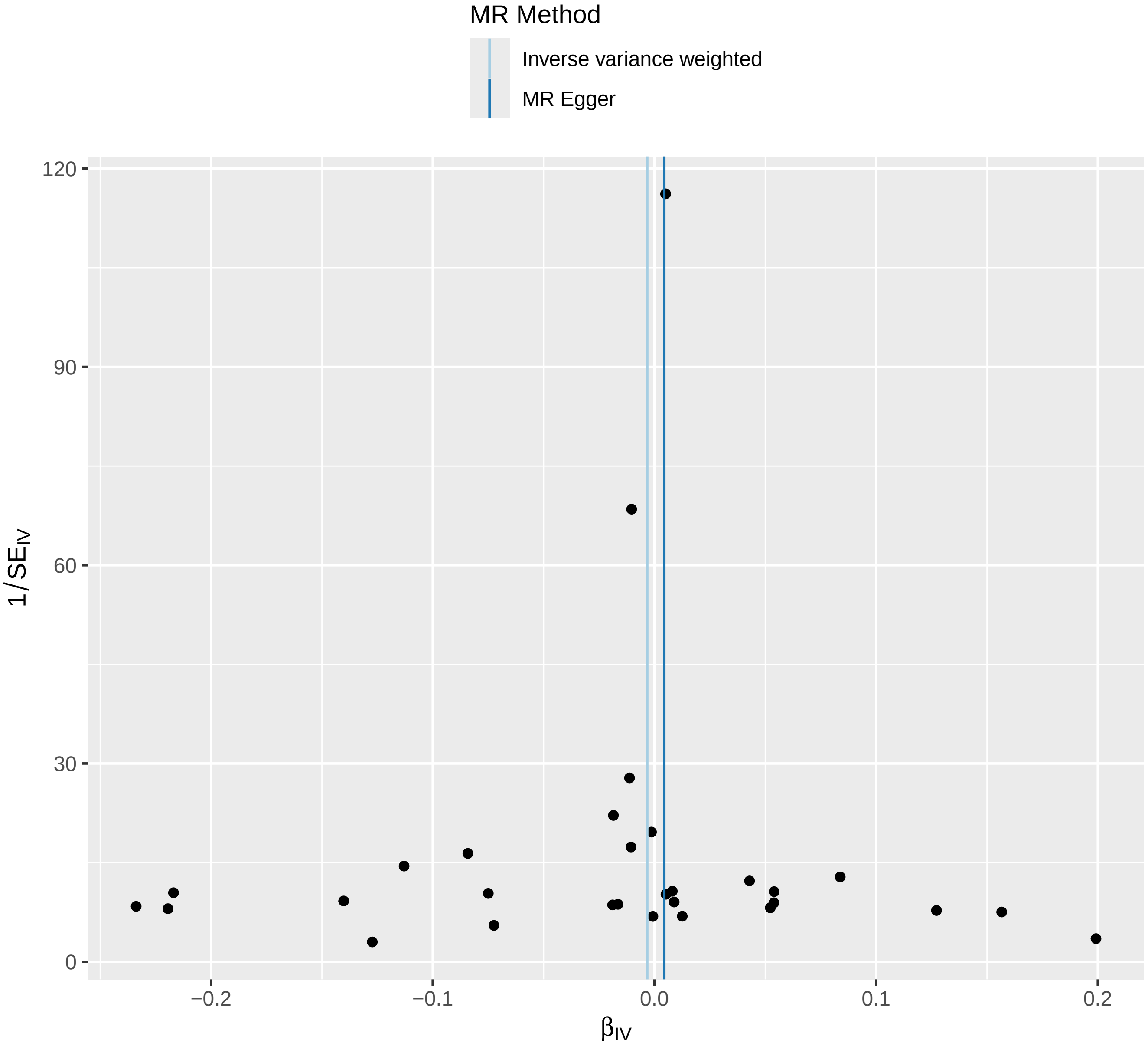

MR Method
Inverse variance weighted
MR Egger
120
90
60
30
0
1/SE$_{IV}$
−0.2
−0.1
0.0
0.1
0.2
β$_{IV}$

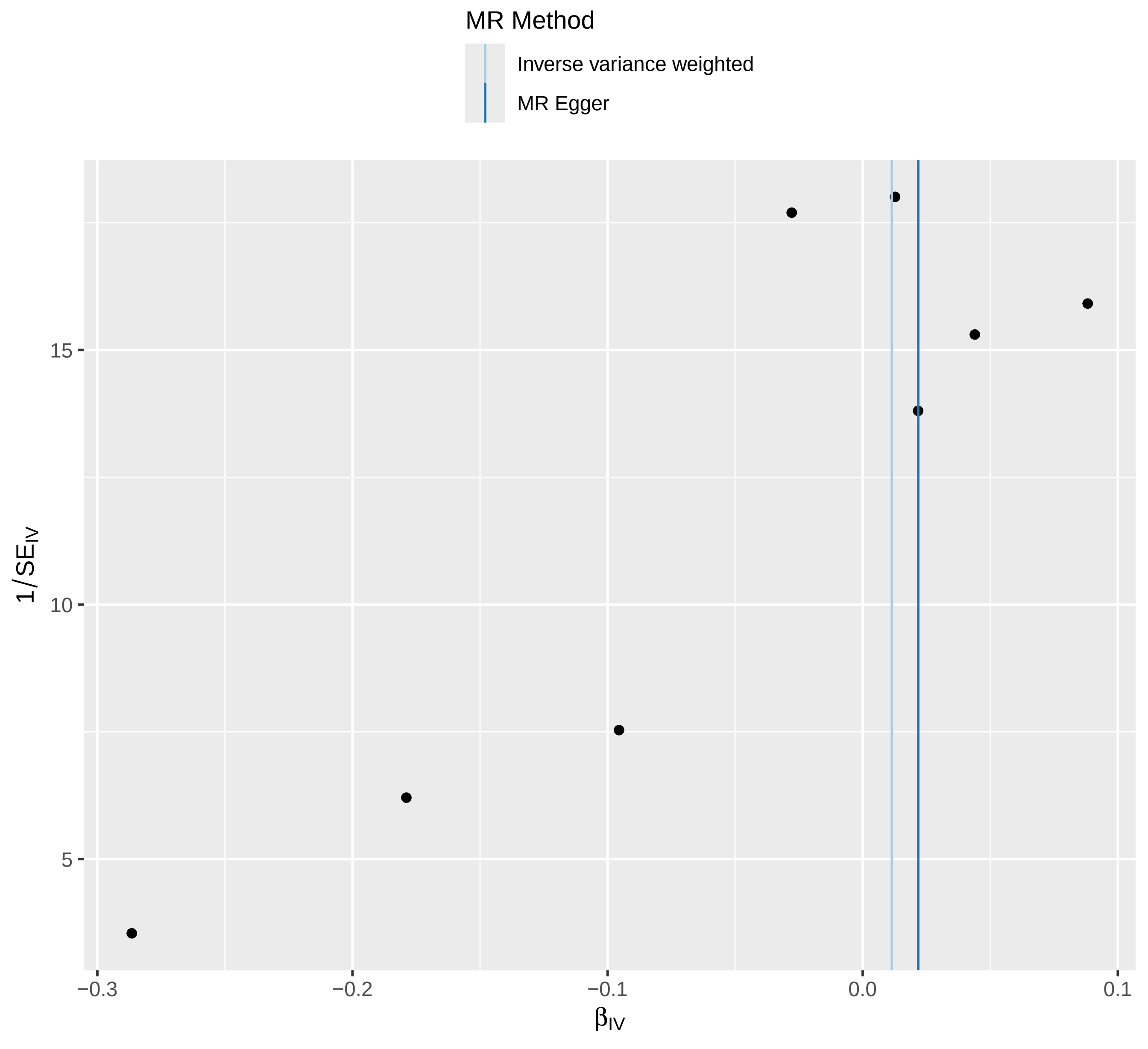
MR Method
Inverse variance weighted
MR Egger
15
10
5
1/SE_IV
−0.3
−0.2
−0.1
0.0
0.1
β_IV

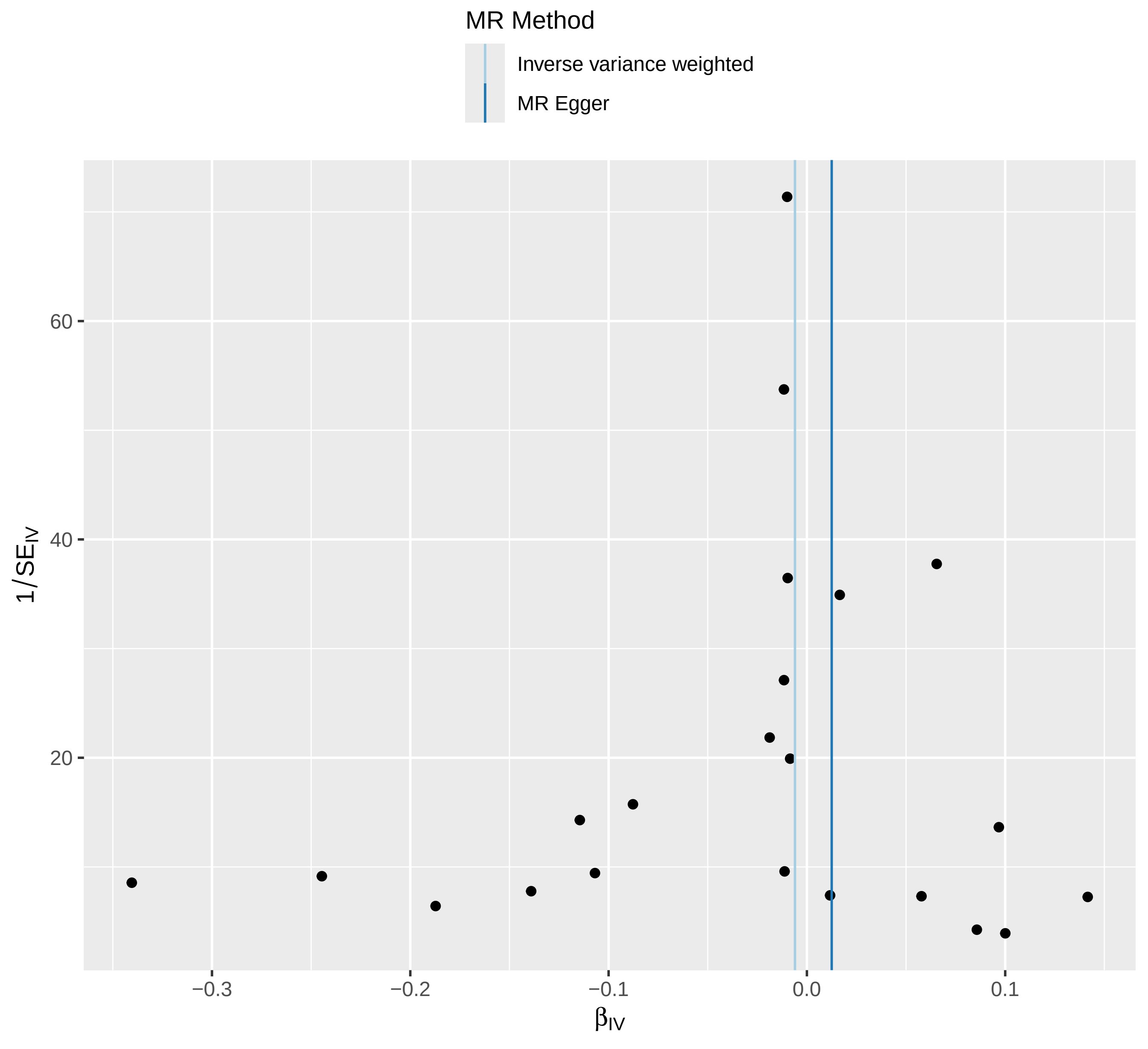
MR Method
Inverse variance weighted
MR Egger
60
40
20
1/SE_IV
−0.3
−0.2
−0.1
0.0
0.1
β_IV

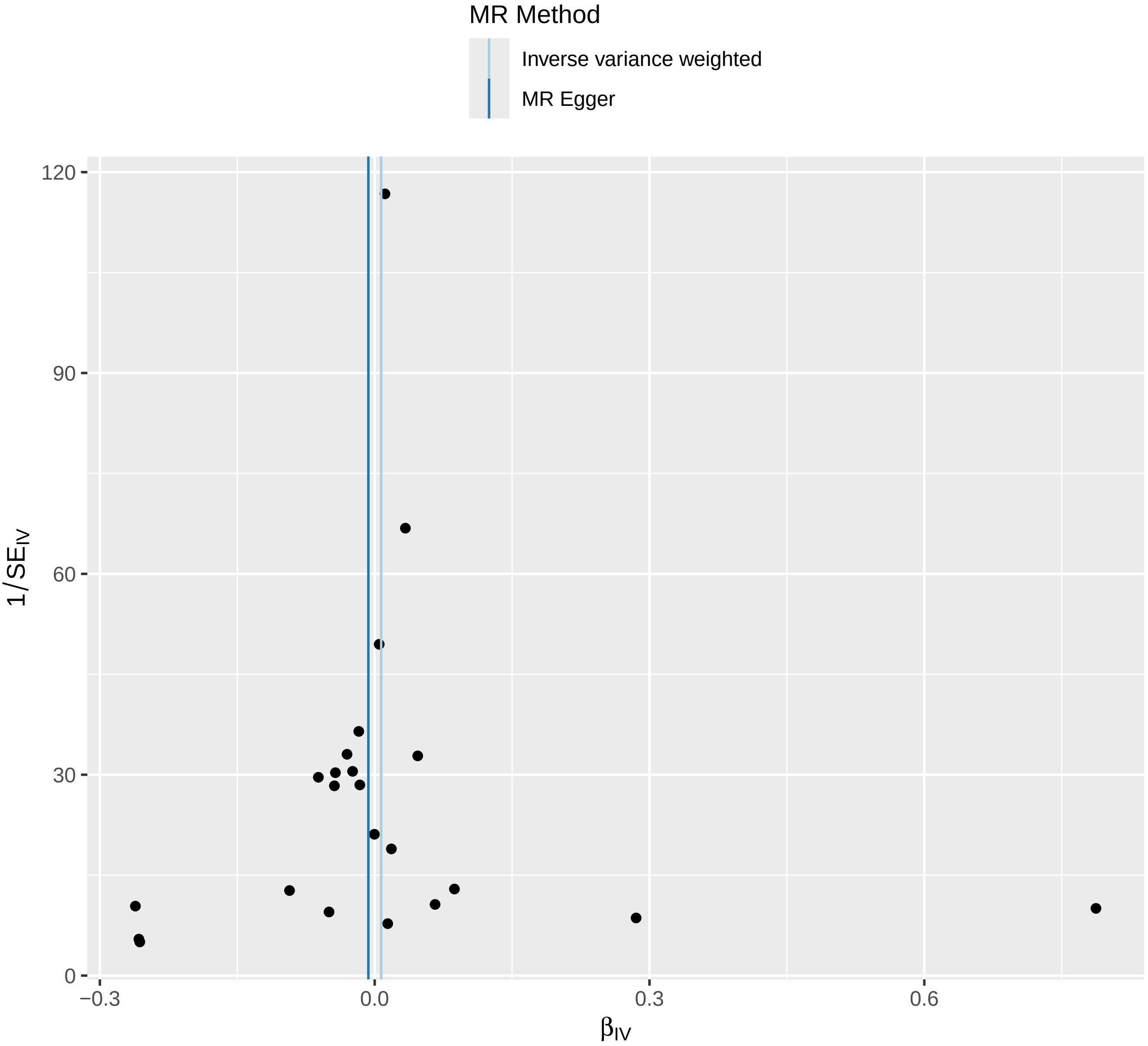
MR Method
Inverse variance weighted
MR Egger
120
90
60
30
0
1/SE_IV
−0.3
0.0
0.3
0.6
β_IV

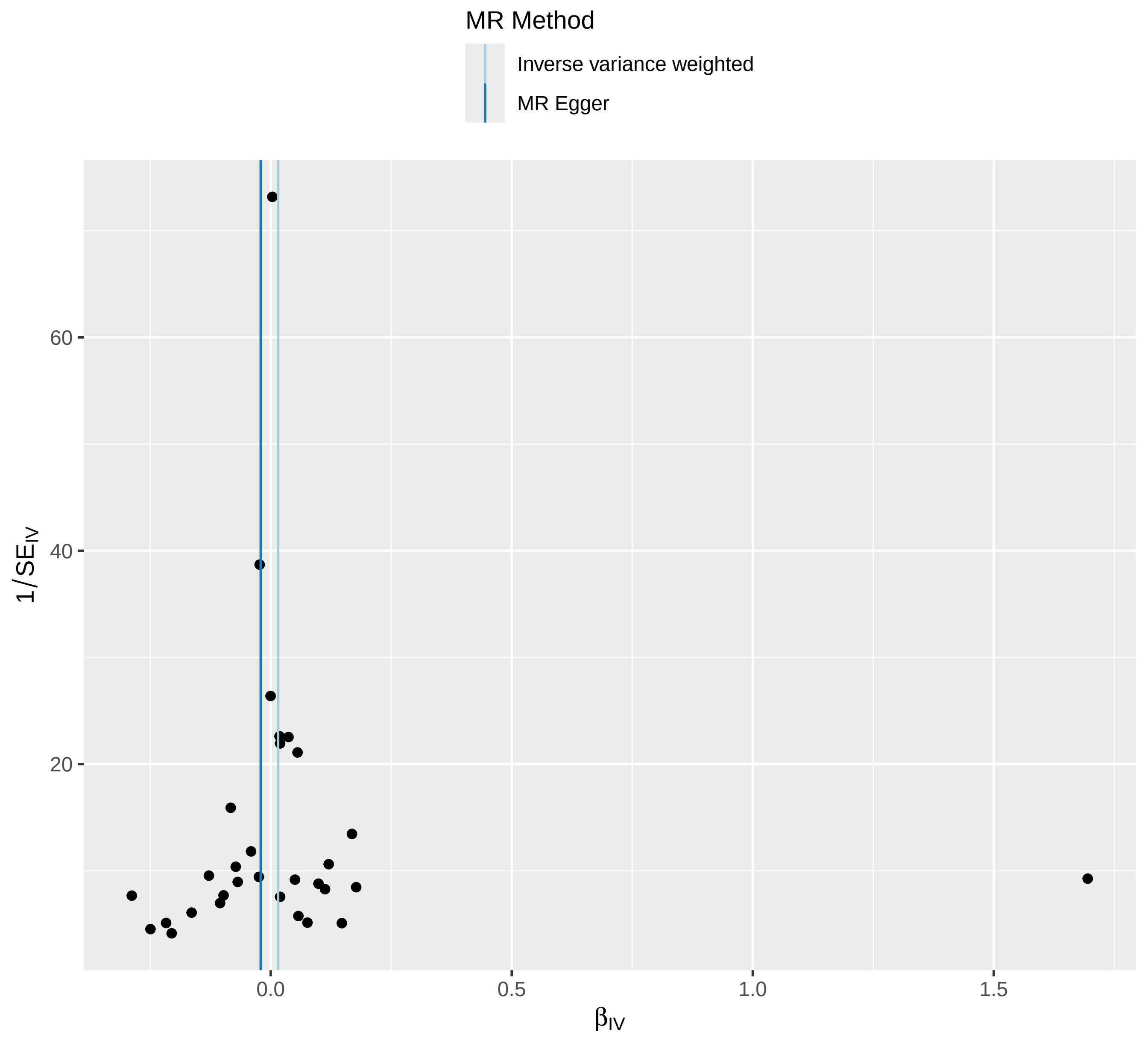
MR Method
Inverse variance weighted
MR Egger
60
40
20
1/SE_IV
0.0
0.5
1.0
1.5
β_IV

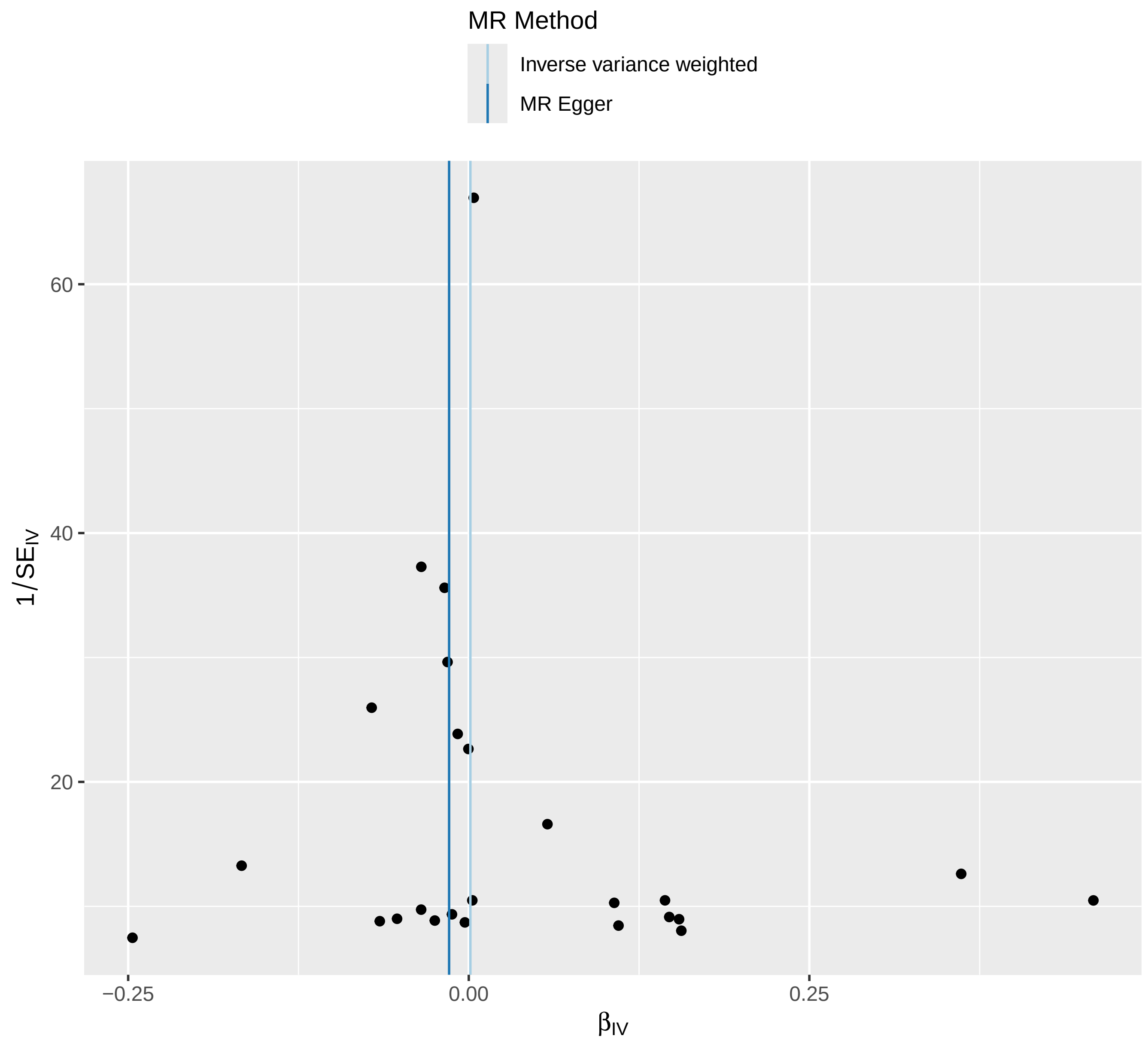
MR Method
Inverse variance weighted
MR Egger
60
40
20
1/SE$_{IV}$
−0.25
0.00
0.25
β$_{IV}$

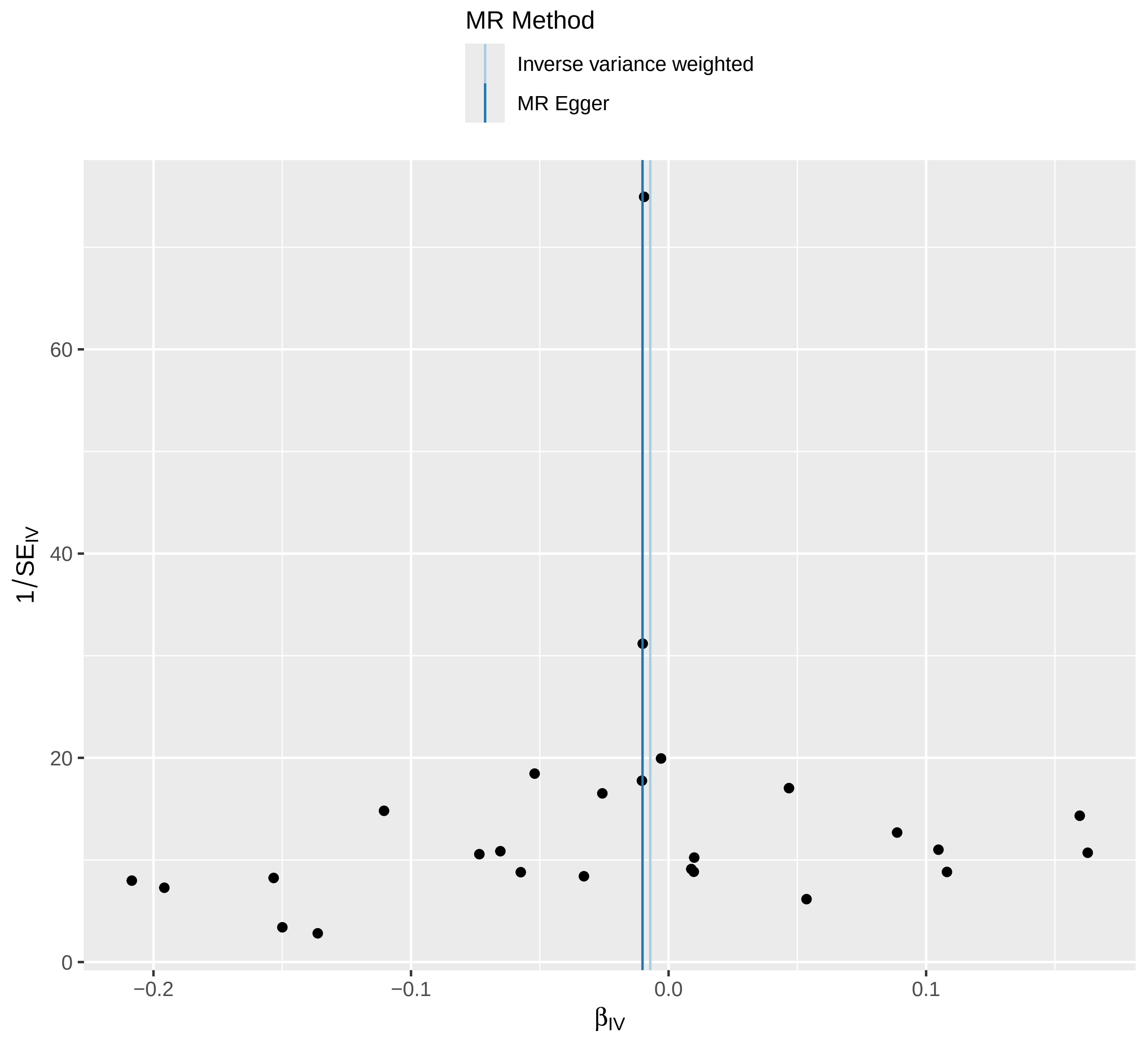

MR Method
Inverse variance weighted
MR Egger
60
40
20
0
1/SE_IV
−0.2
−0.1
0.0
0.1
β_IV

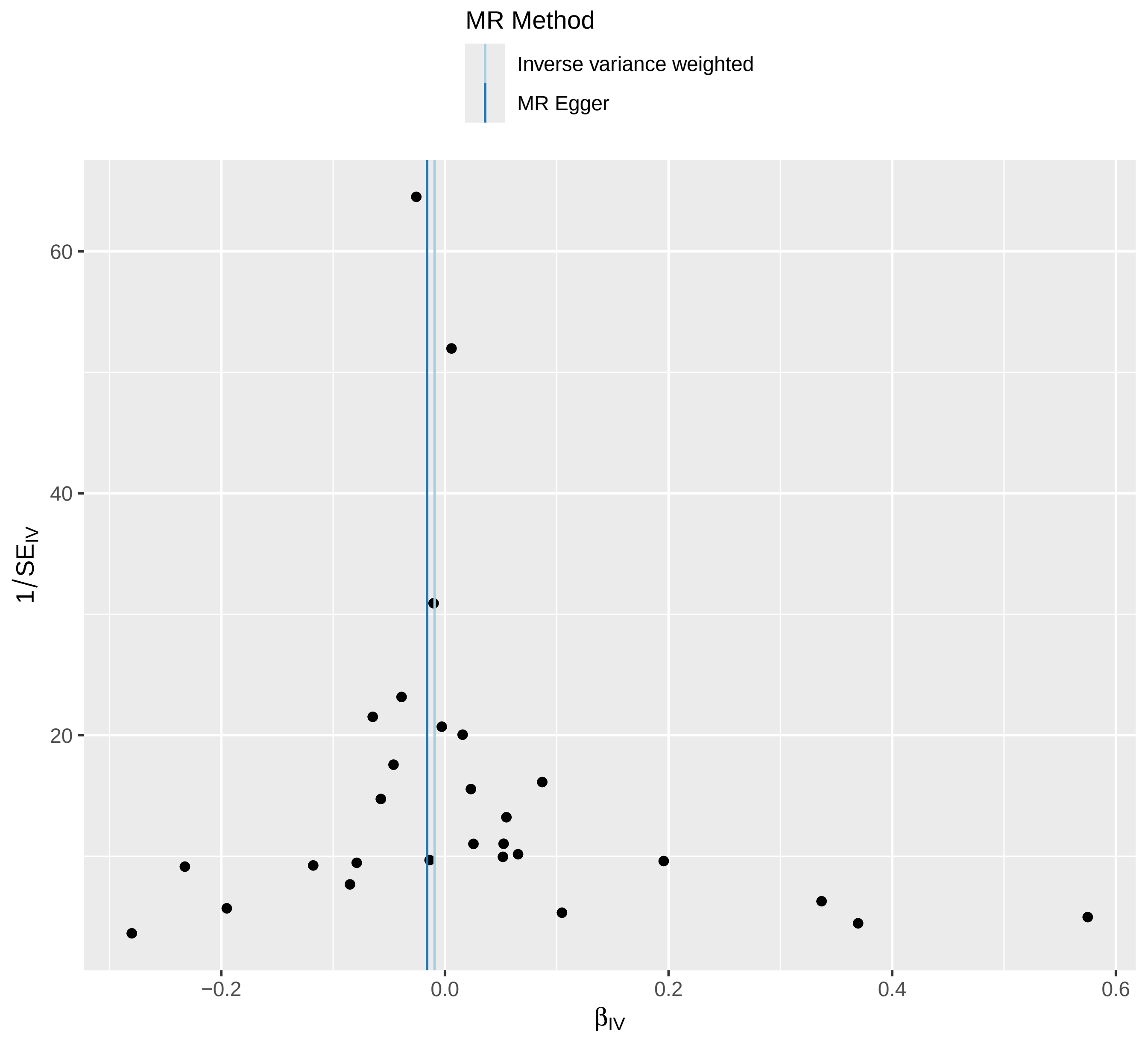
MR Method
Inverse variance weighted
MR Egger
60
40
20
1/SE_IV
−0.2
0.0
0.2
0.4
0.6
β_IV

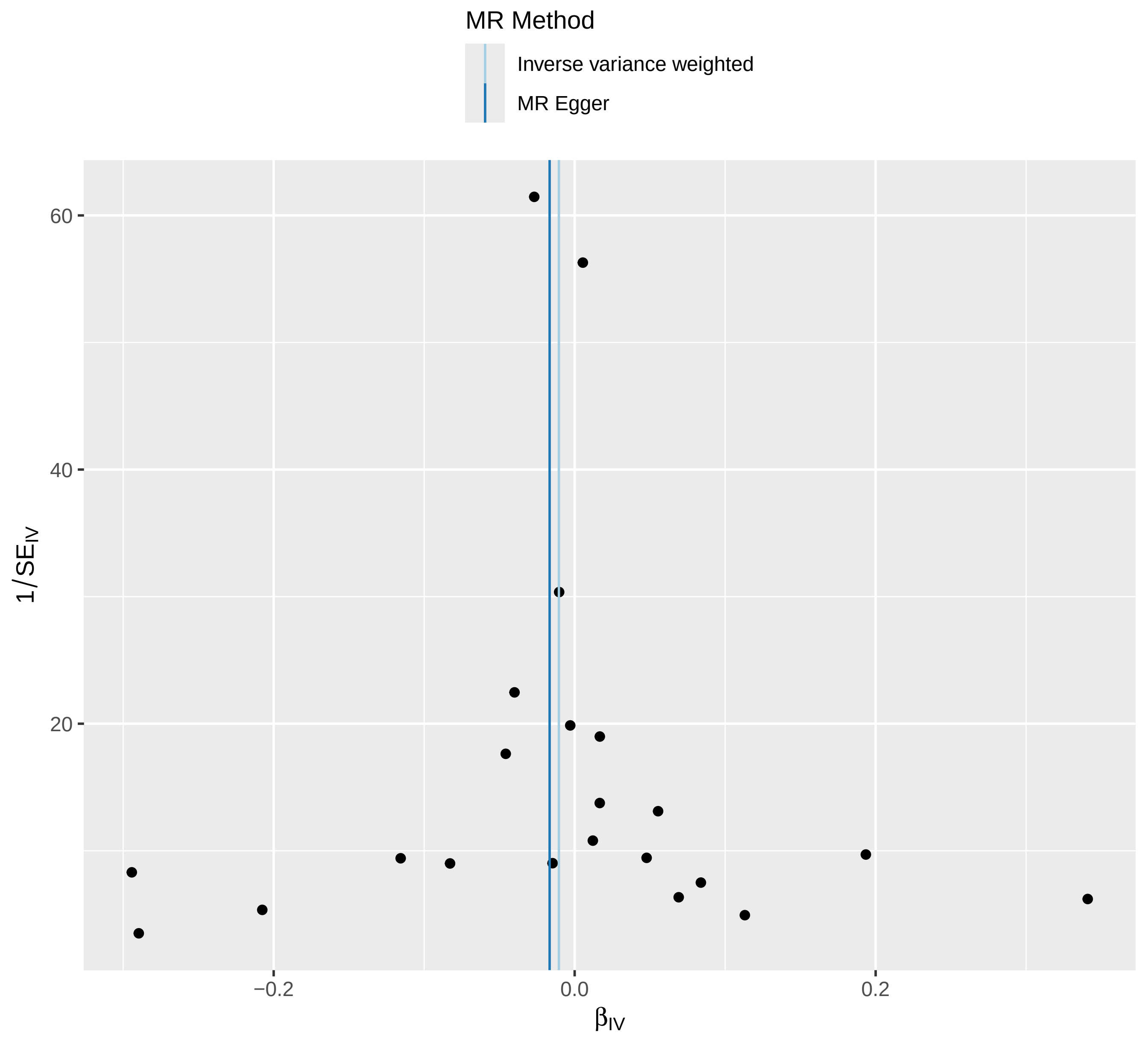
MR Method
Inverse variance weighted
MR Egger
60
40
20
1/SE_IV
−0.2
0.0
0.2
β_IV

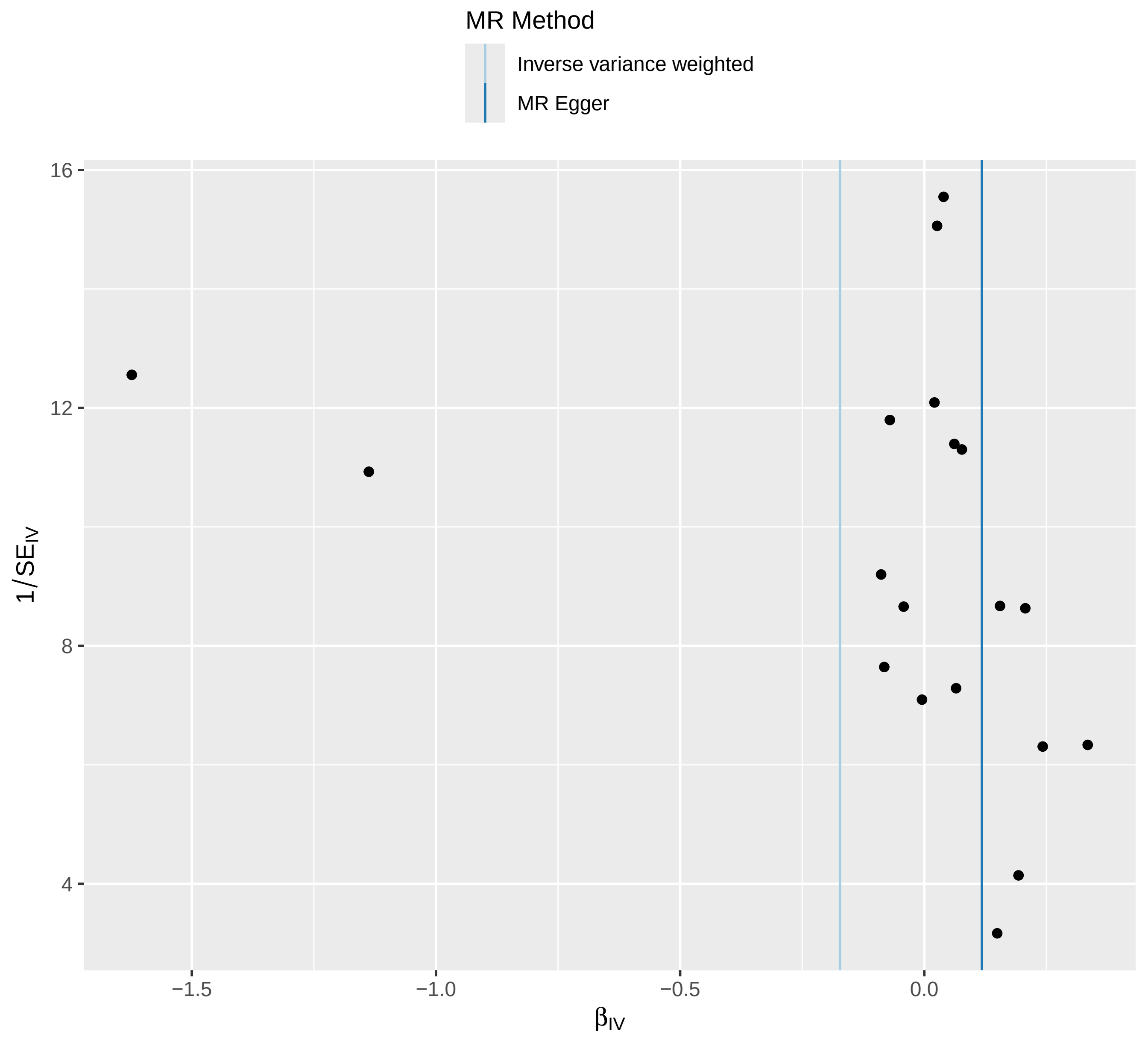
MR Method
Inverse variance weighted
MR Egger
16
12
8
4
1/SE_IV
−1.5
−1.0
−0.5
0.0
β_IV

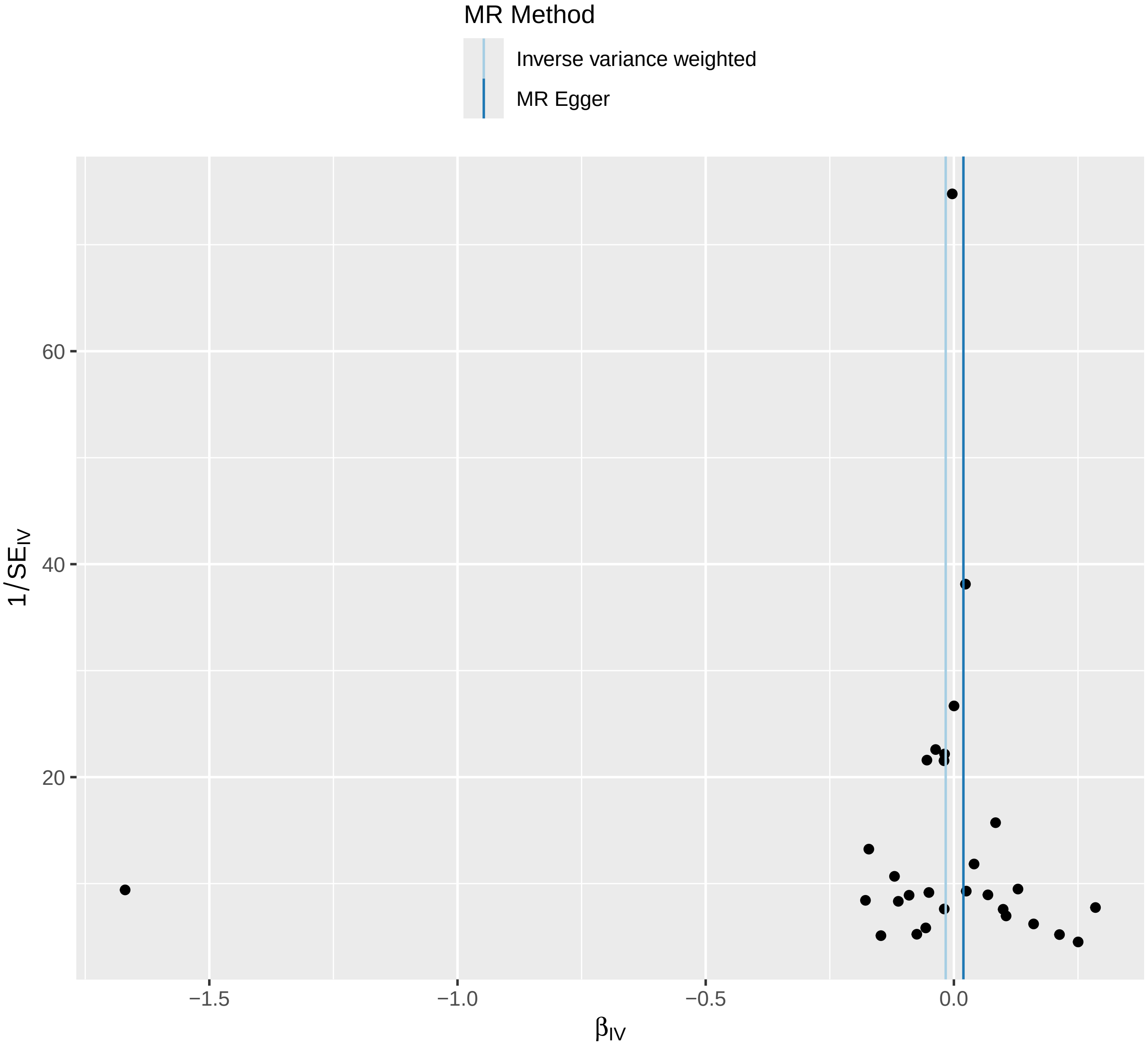
MR Method
Inverse variance weighted
MR Egger
60
40
20
1/SE$_{IV}$
−1.5
−1.0
−0.5
0.0
β$_{IV}$

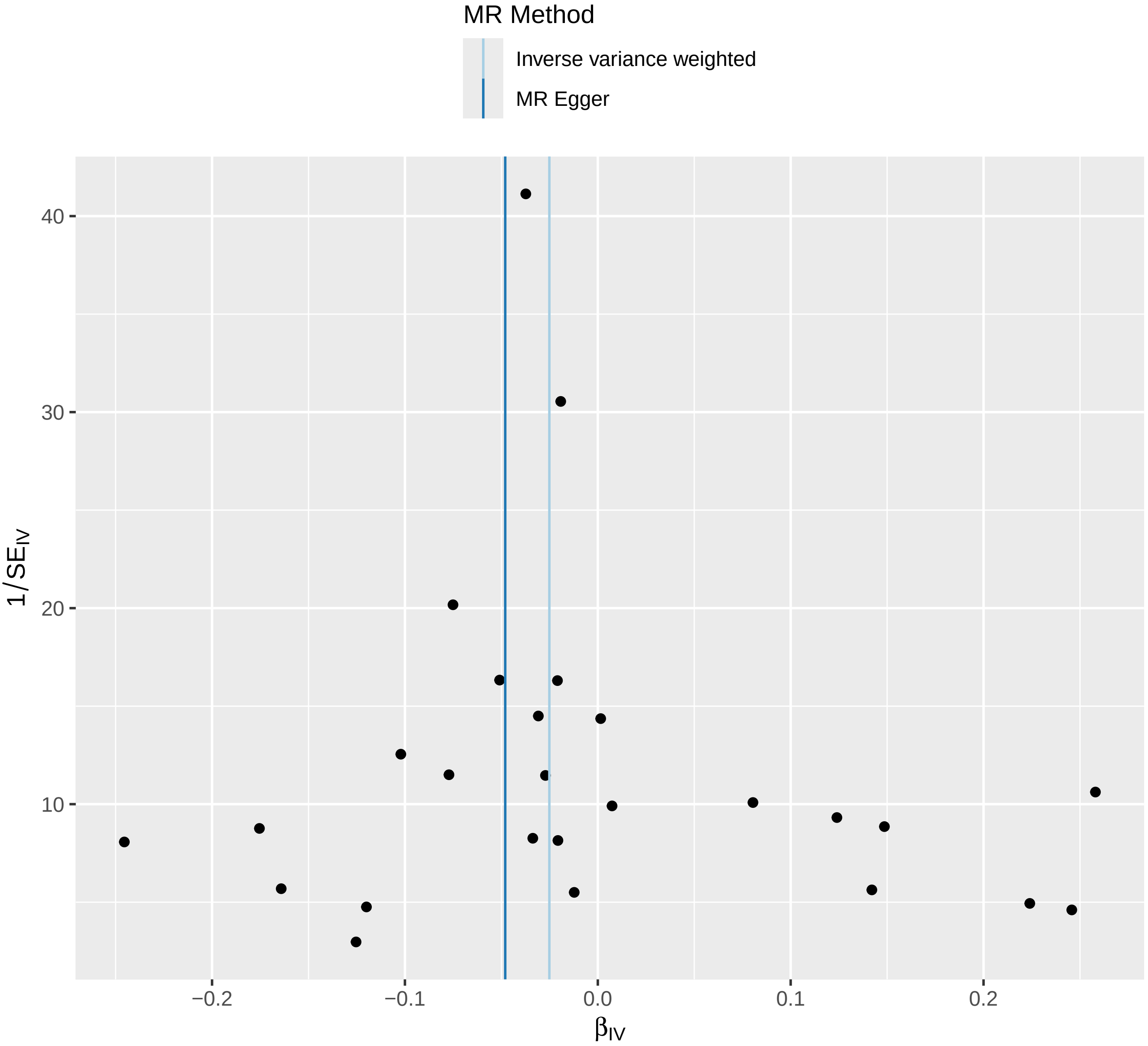
MR Method
Inverse variance weighted
MR Egger
40
30
20
10
1/SE_IV
−0.2
−0.1
0.0
0.1
0.2
β_IV

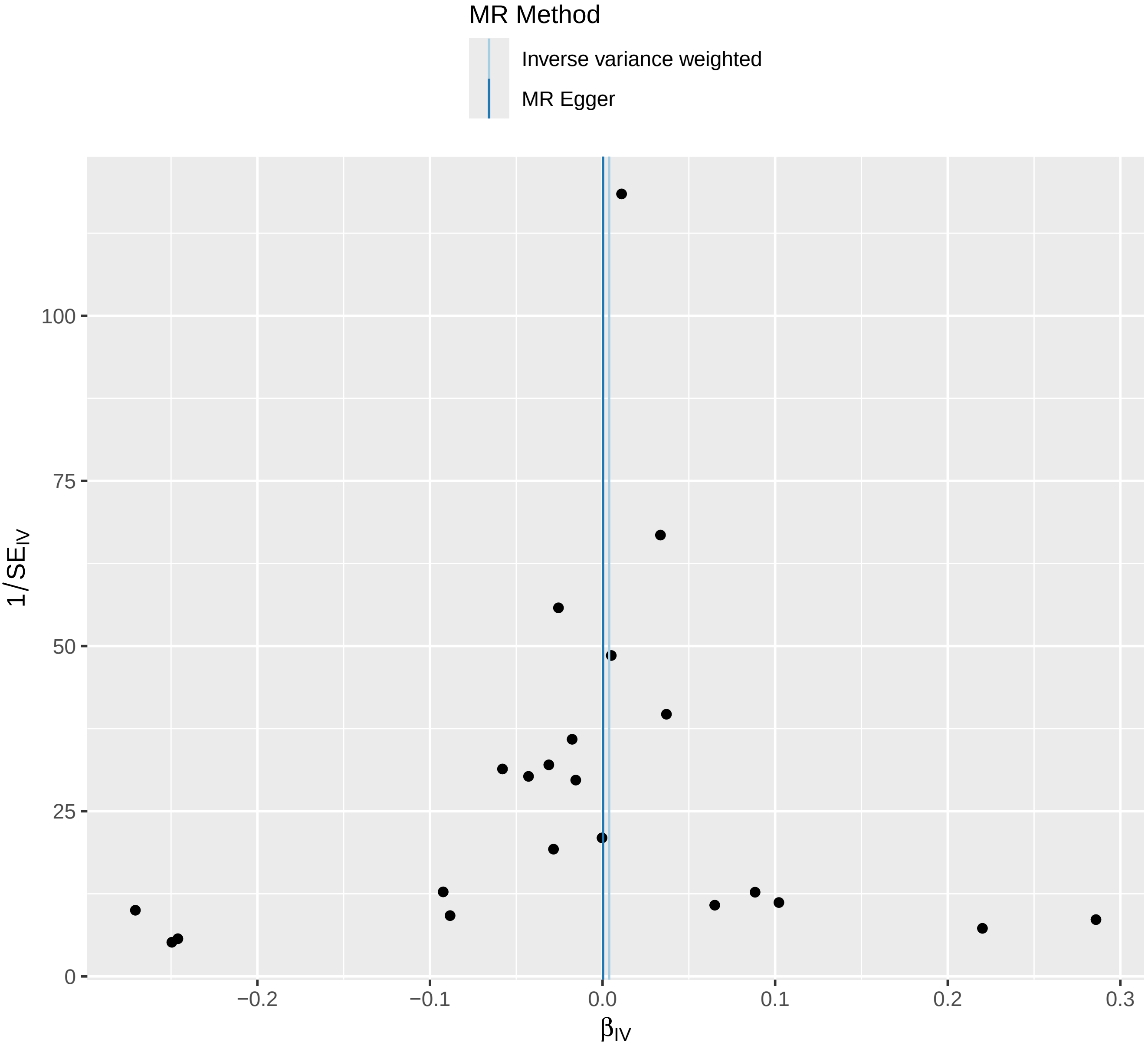
MR Method
Inverse variance weighted
MR Egger
1/SE$_{IV}$
0
25
50
75
100
−0.2
−0.1
0.0
0.1
0.2
0.3
β$_{IV}$

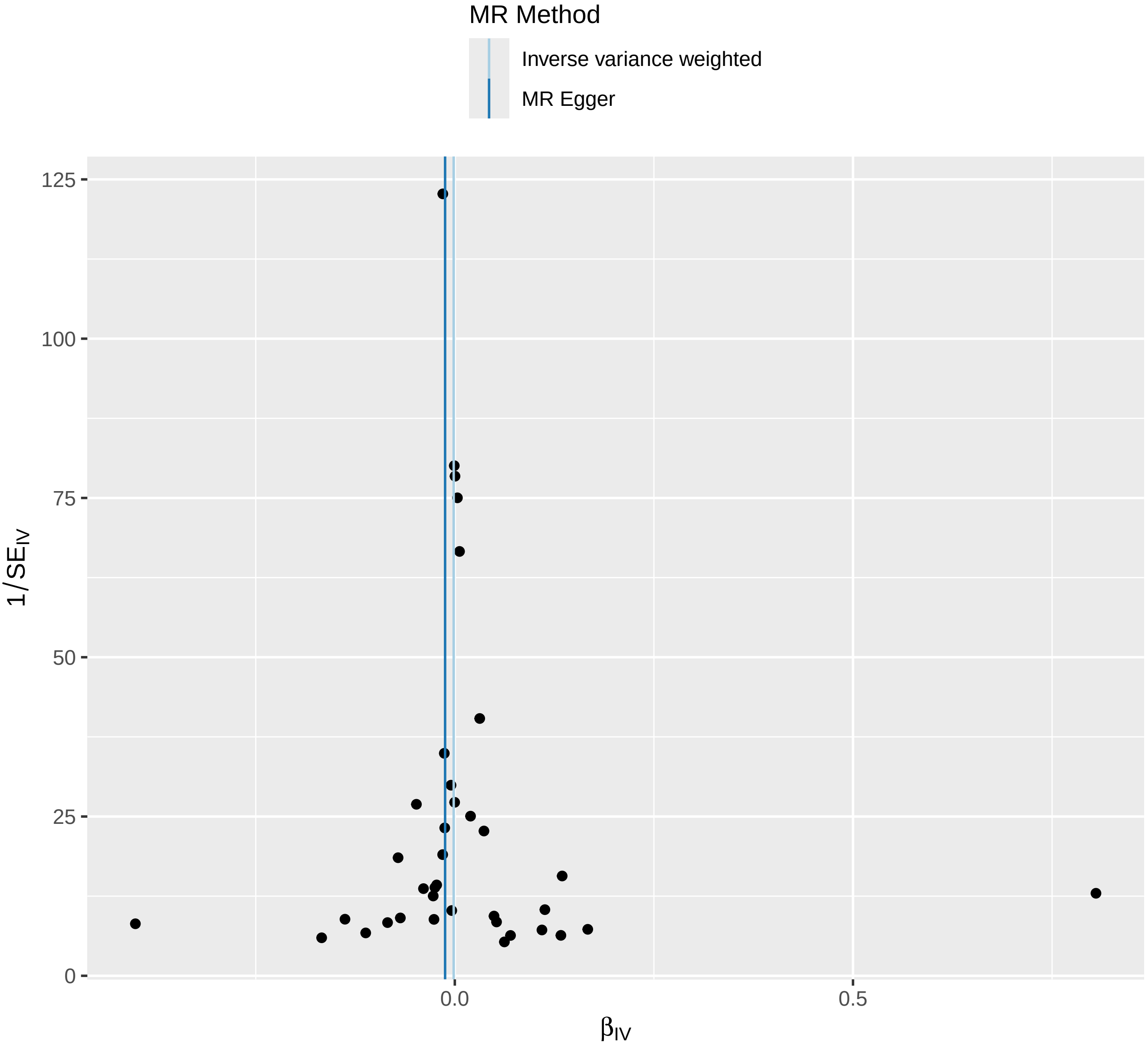
MR Method
Inverse variance weighted
MR Egger
125
100
75
50
25
0
1/SE$_{IV}$
0.0
0.5
β$_{IV}$

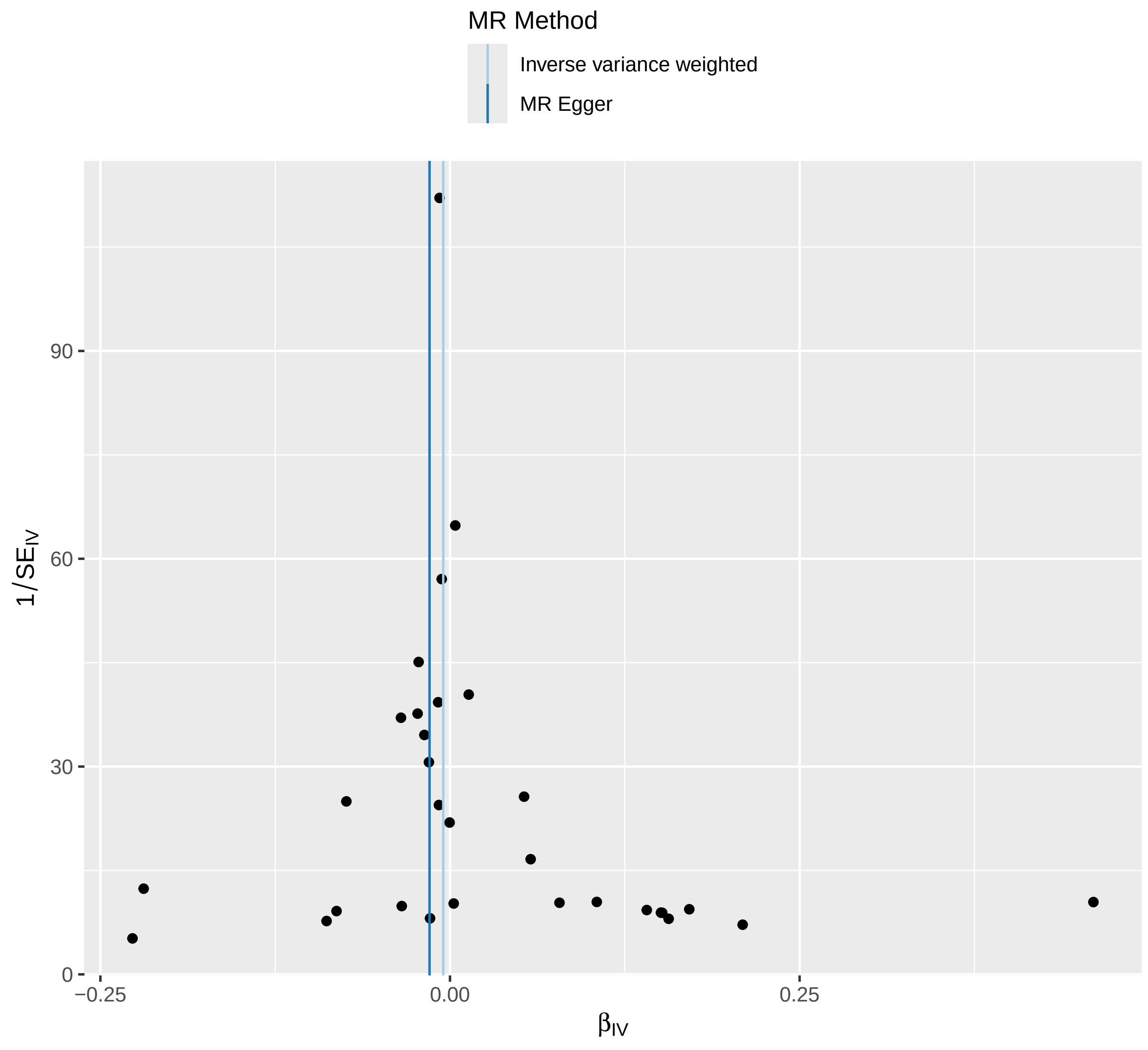

MR Method
Inverse variance weighted
MR Egger
1/SE_IV
90
60
30
0
−0.25
0.00
0.25
β_IV

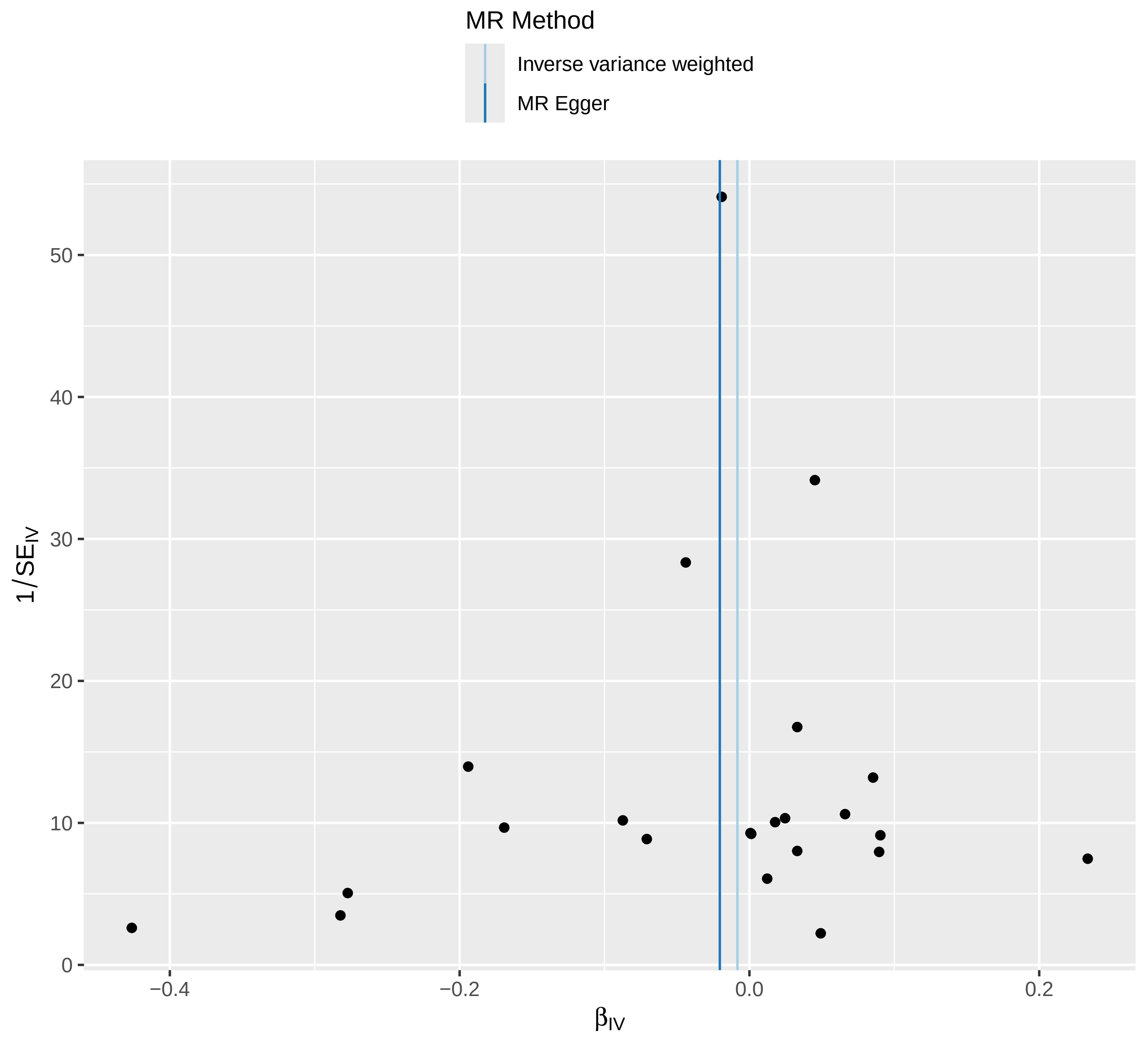

MR Method
Inverse variance weighted
MR Egger
50
40
30
20
10
0
1/SE_IV
−0.4
−0.2
0.0
0.2
β_IV

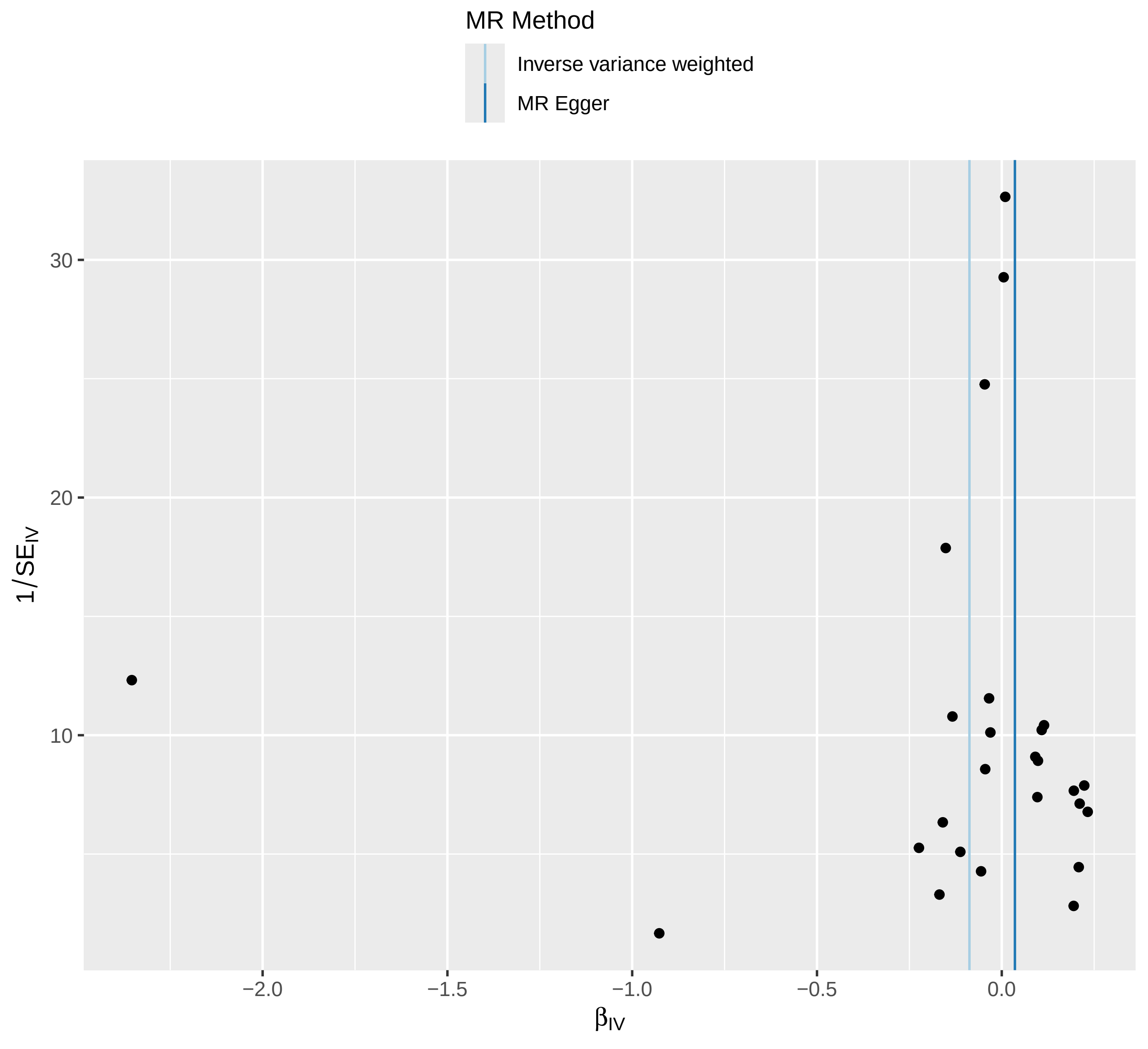
MR Method
Inverse variance weighted
MR Egger
30
20
10
1/SE_IV
−2.0
−1.5
−1.0
−0.5
0.0
β_IV

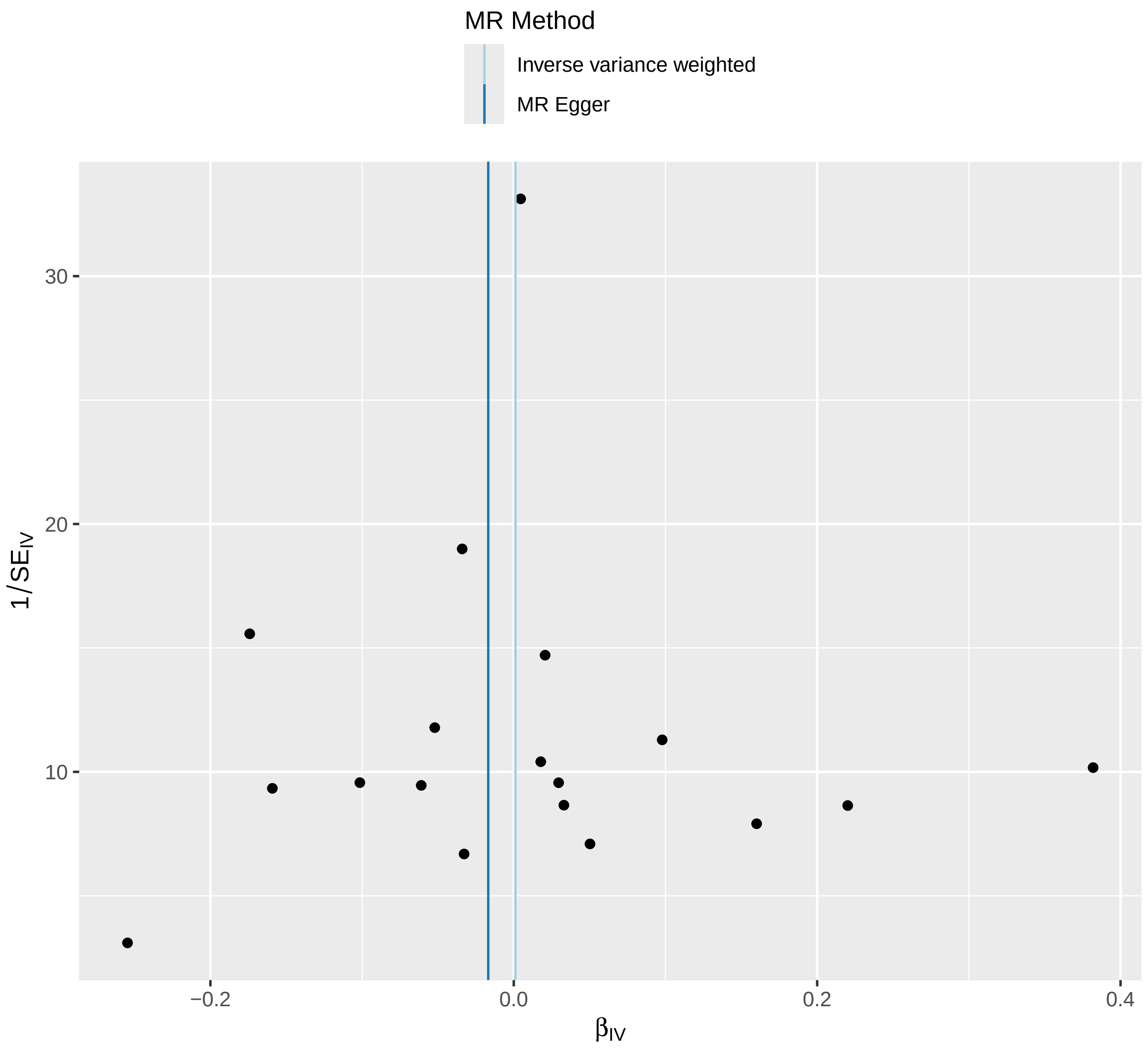
MR Method
Inverse variance weighted
MR Egger
30
20
10
1/SE_IV
−0.2
0.0
0.2
0.4
β_IV

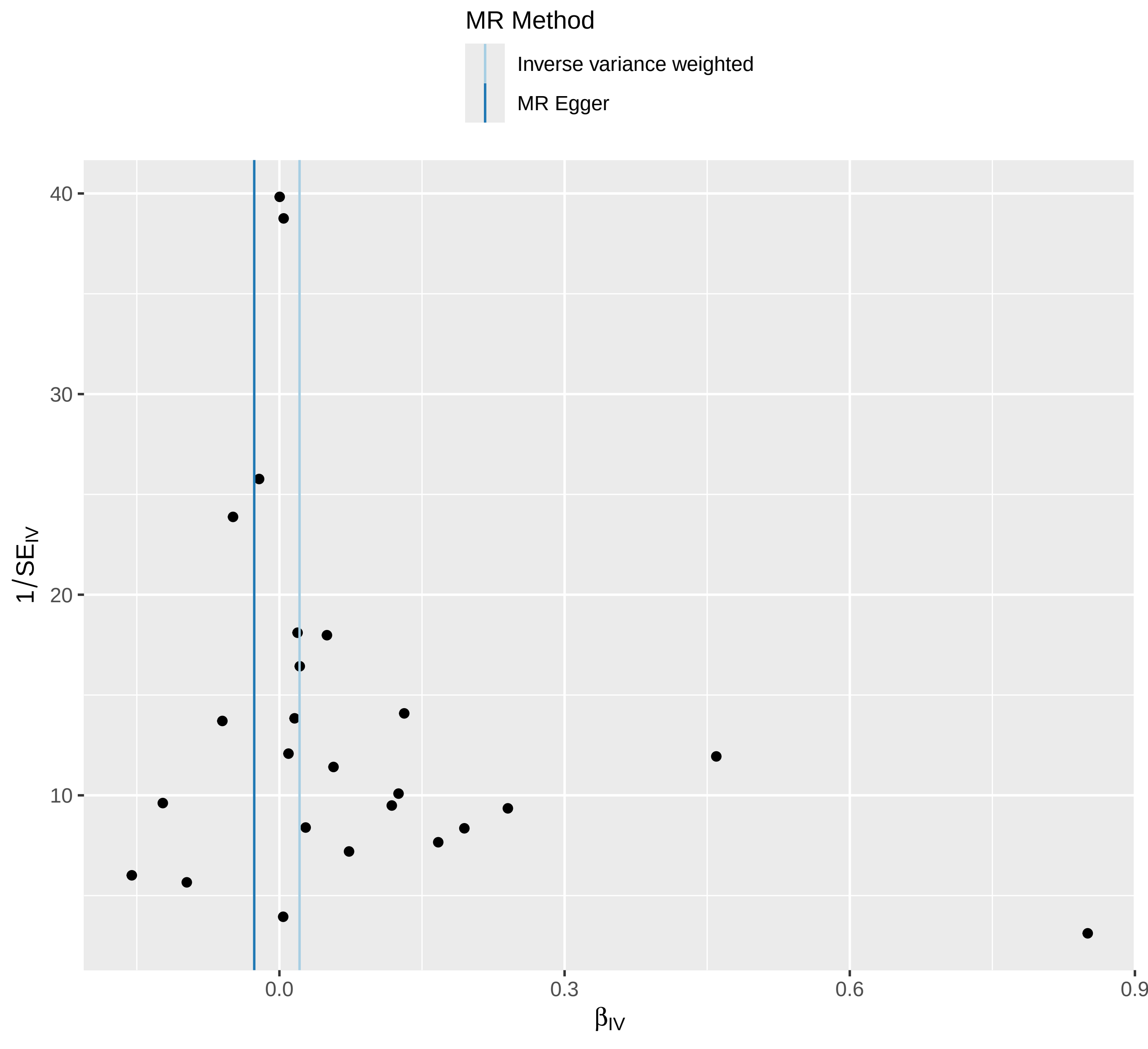
MR Method
Inverse variance weighted
MR Egger
40
30
20
10
0.0
0.3
0.6
0.9
$1/SE_{IV}$
β_{IV}

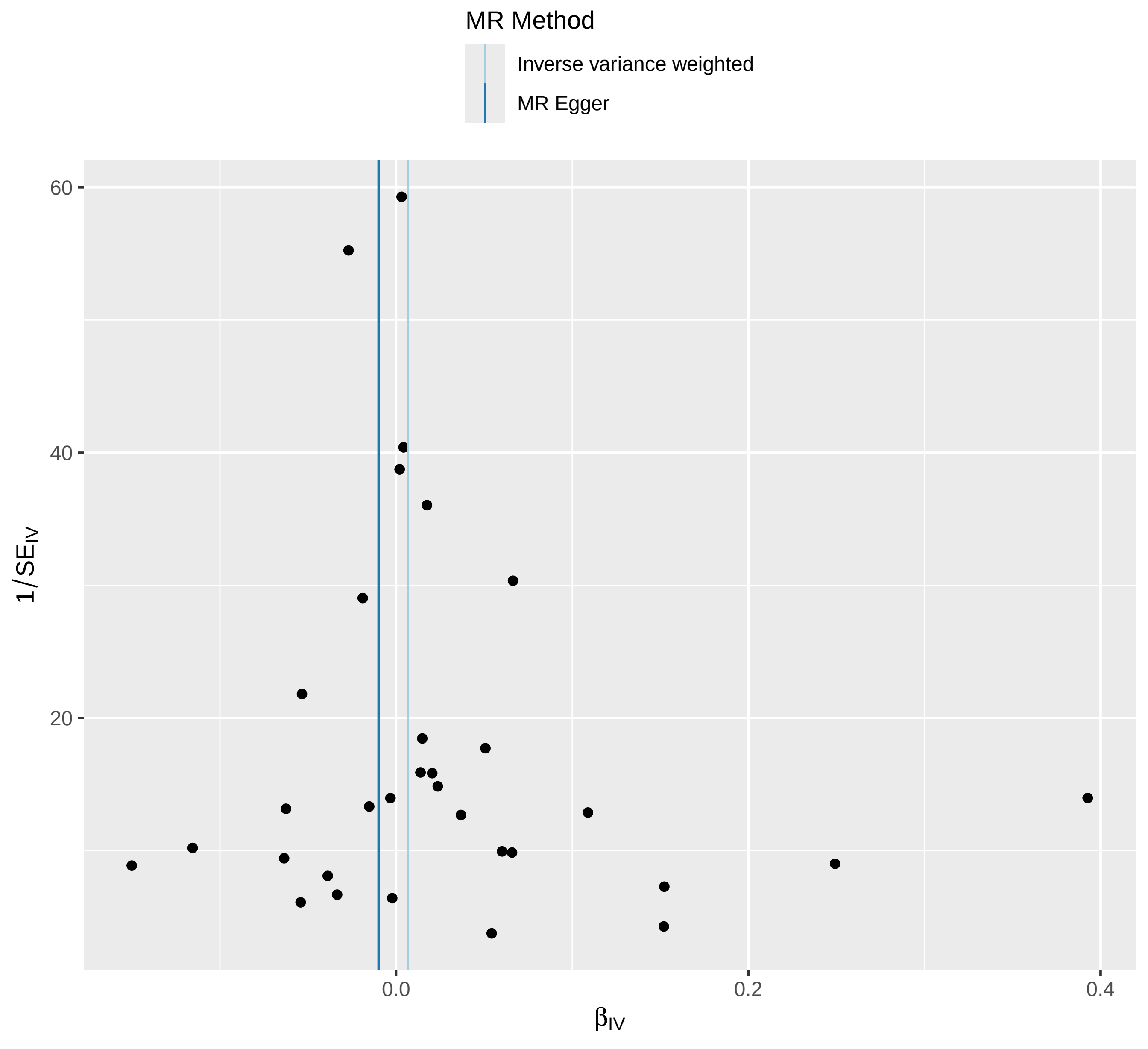

MR Method
Inverse variance weighted
MR Egger
60
40
20
0.0
0.2
0.4
1/SE_IV
β_IV

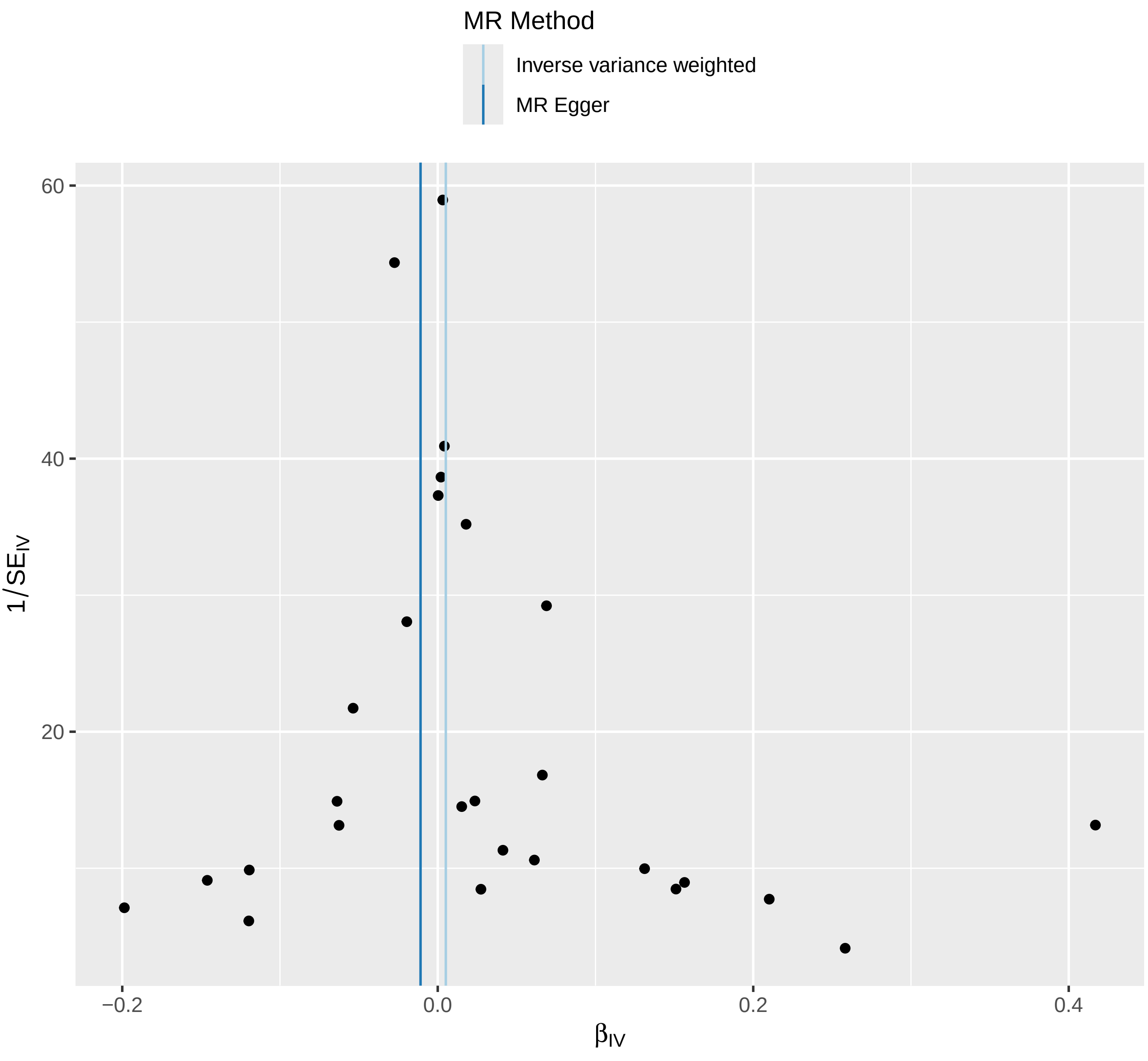
MR Method
Inverse variance weighted
MR Egger
60
40
20
1/SE$_{IV}$
−0.2
0.0
0.2
0.4
β$_{IV}$

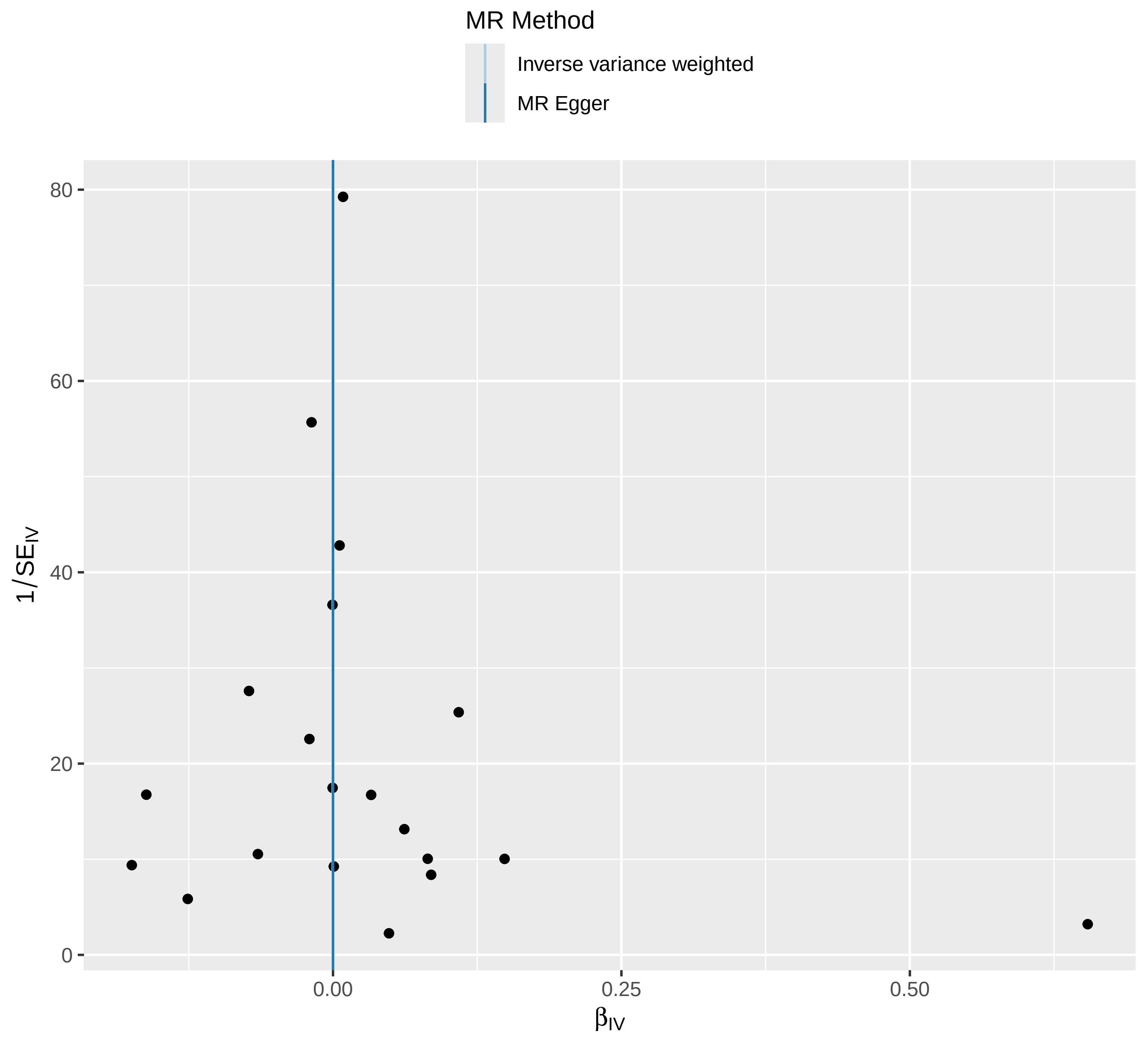
MR Method
Inverse variance weighted
MR Egger
80
60
40
20
0
0.00
0.25
0.50
1/SE_IV
β_IV

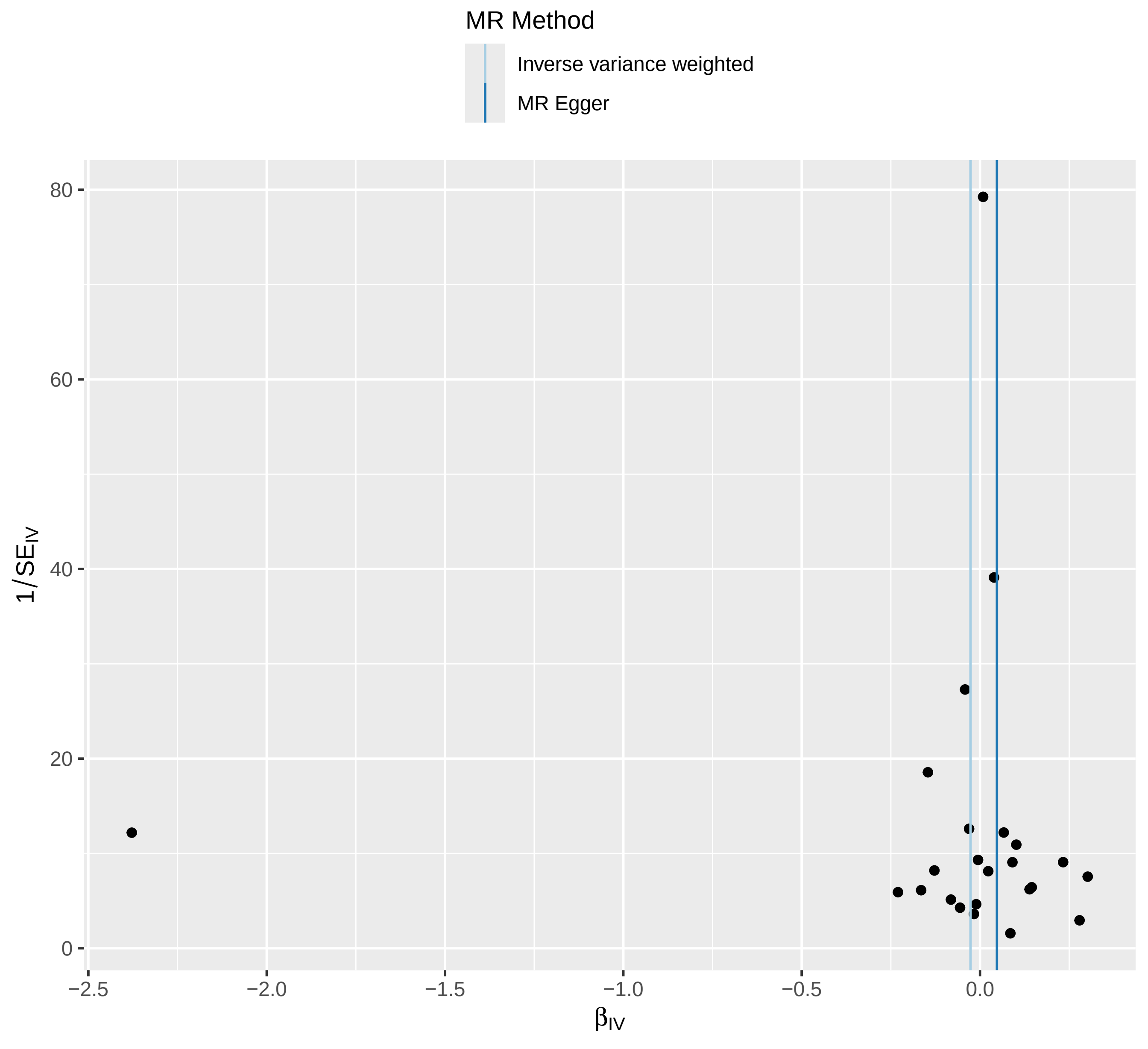
MR Method
Inverse variance weighted
MR Egger
80
60
40
20
0
1/SE_IV
−2.5
−2.0
−1.5
−1.0
−0.5
0.0
β_IV

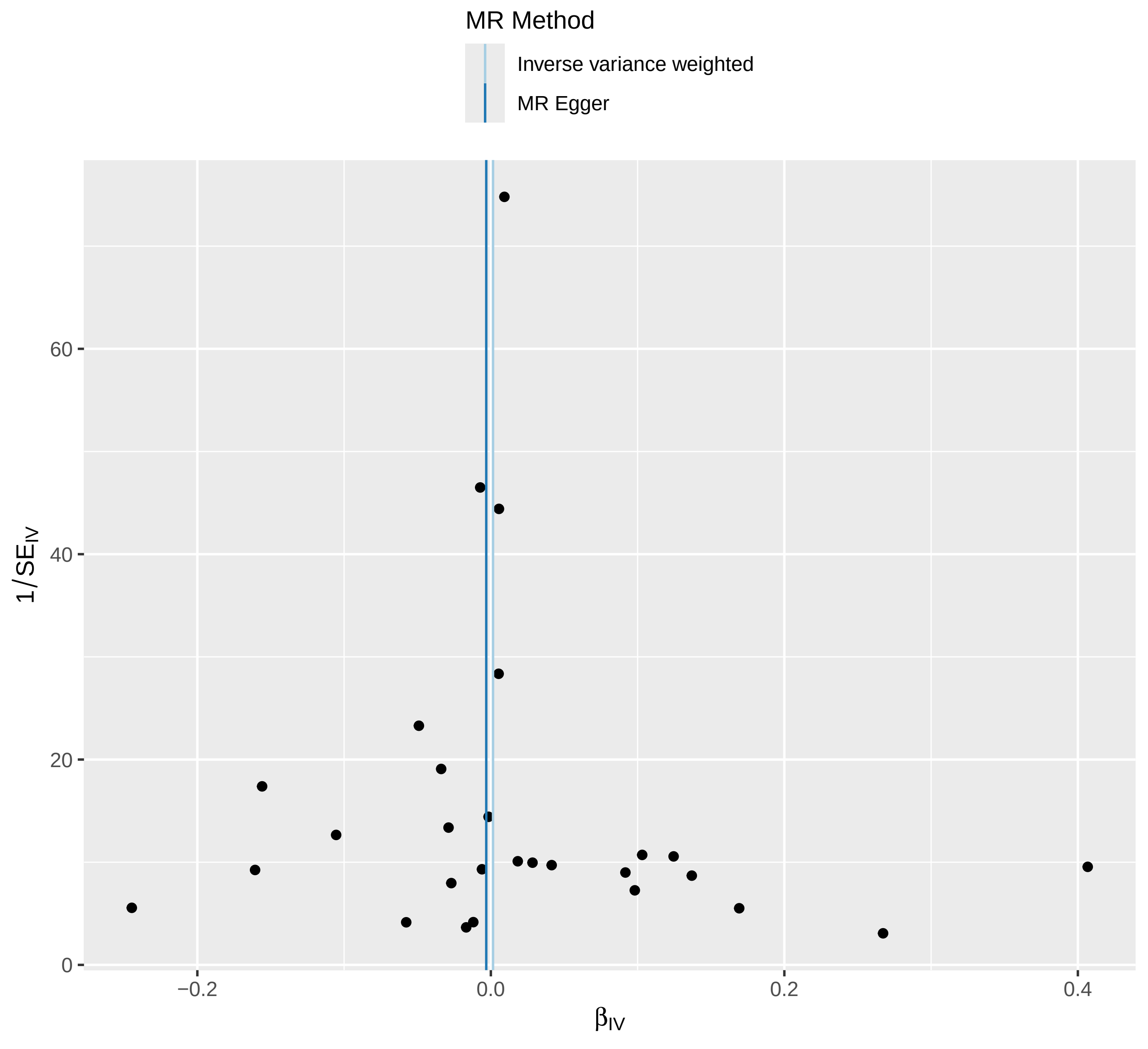
MR Method
Inverse variance weighted
MR Egger
1/SE$_{IV}$
60
40
20
0
−0.2
0.0
0.2
0.4
β$_{IV}$

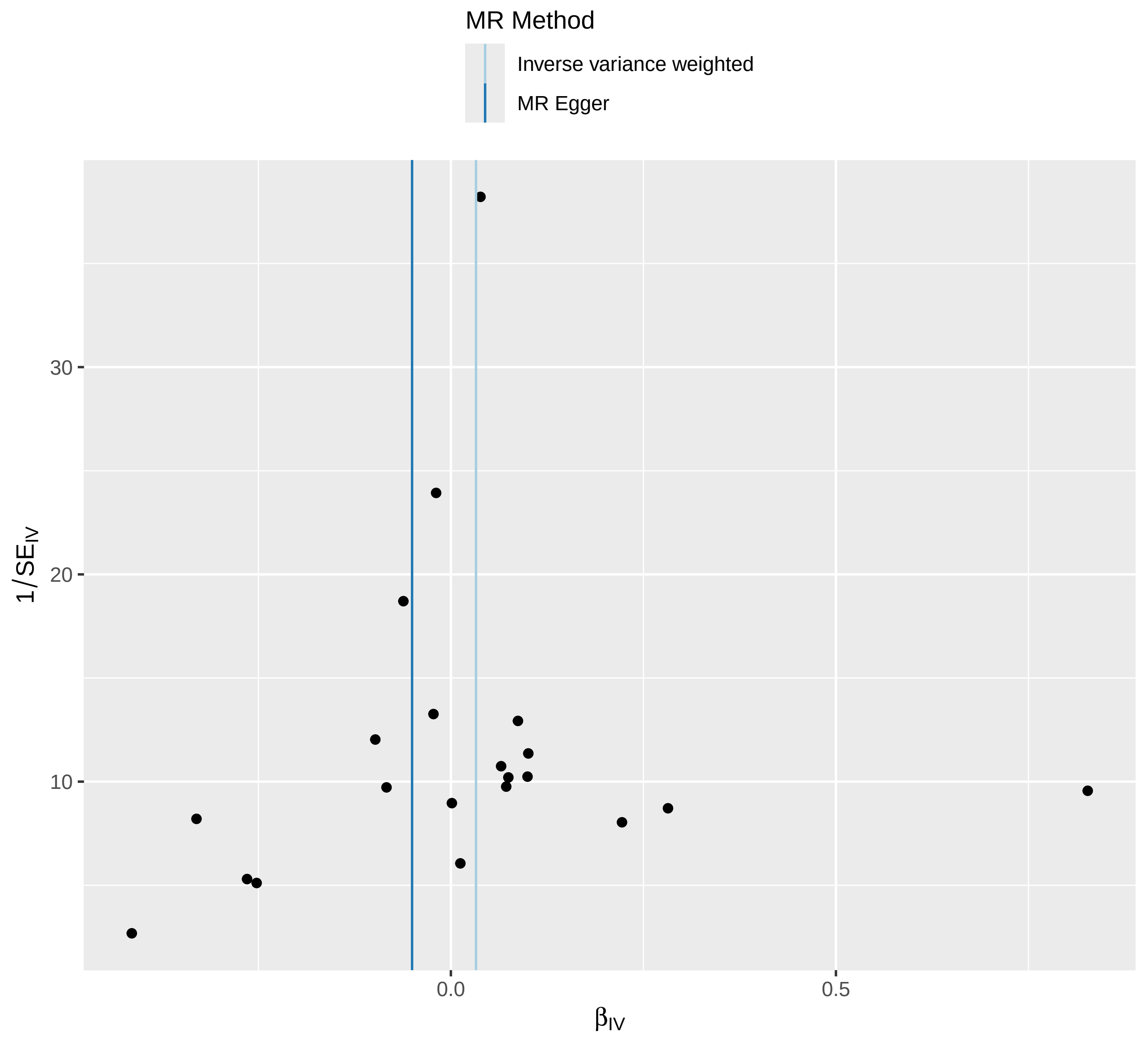
MR Method
Inverse variance weighted
MR Egger
30
20
10
0.0
0.5
1/SE_IV
β_IV

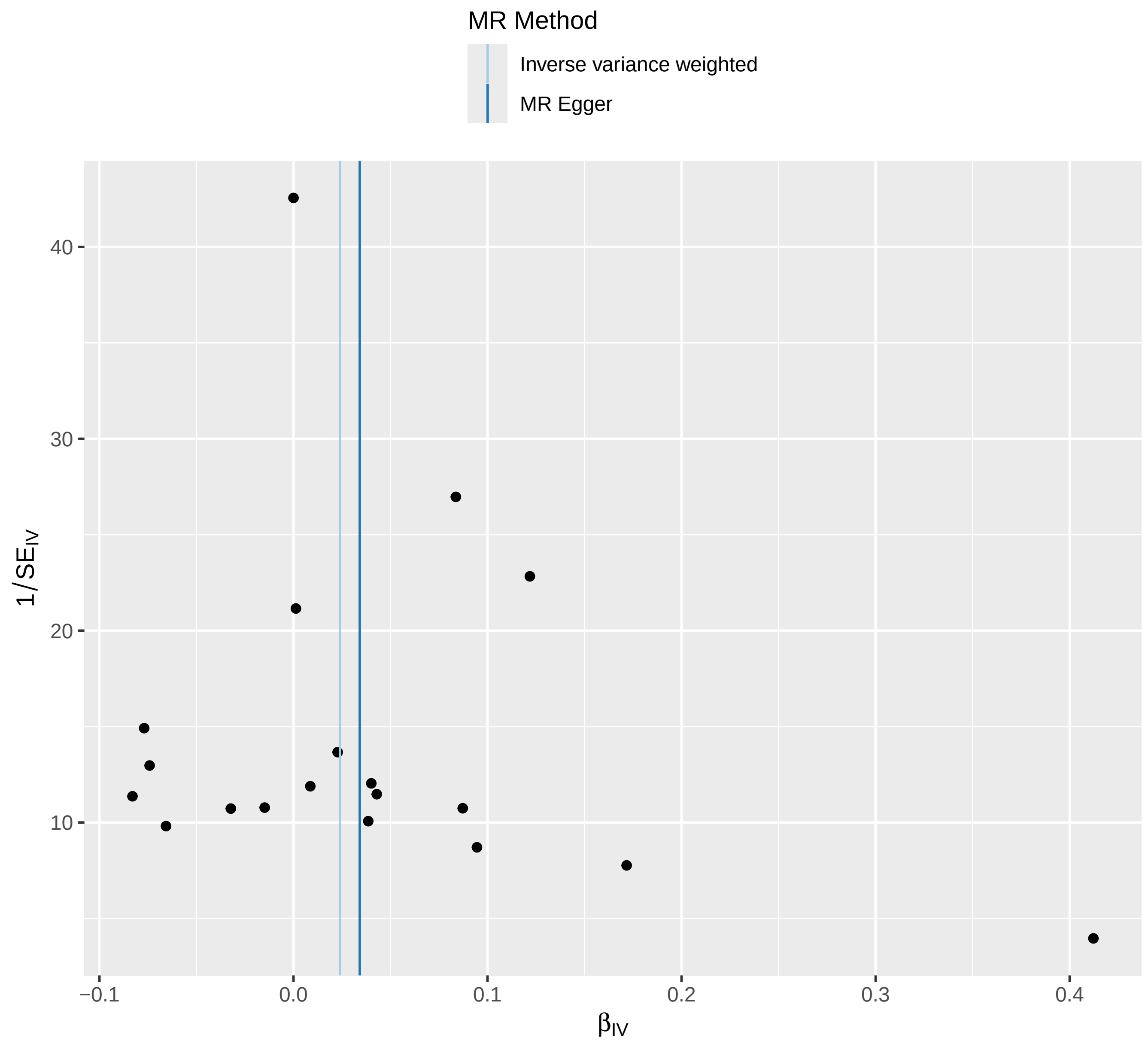
MR Method
Inverse variance weighted
MR Egger
1/SE_IV
40
30
20
10
−0.1
0.0
0.1
0.2
0.3
0.4
β_IV

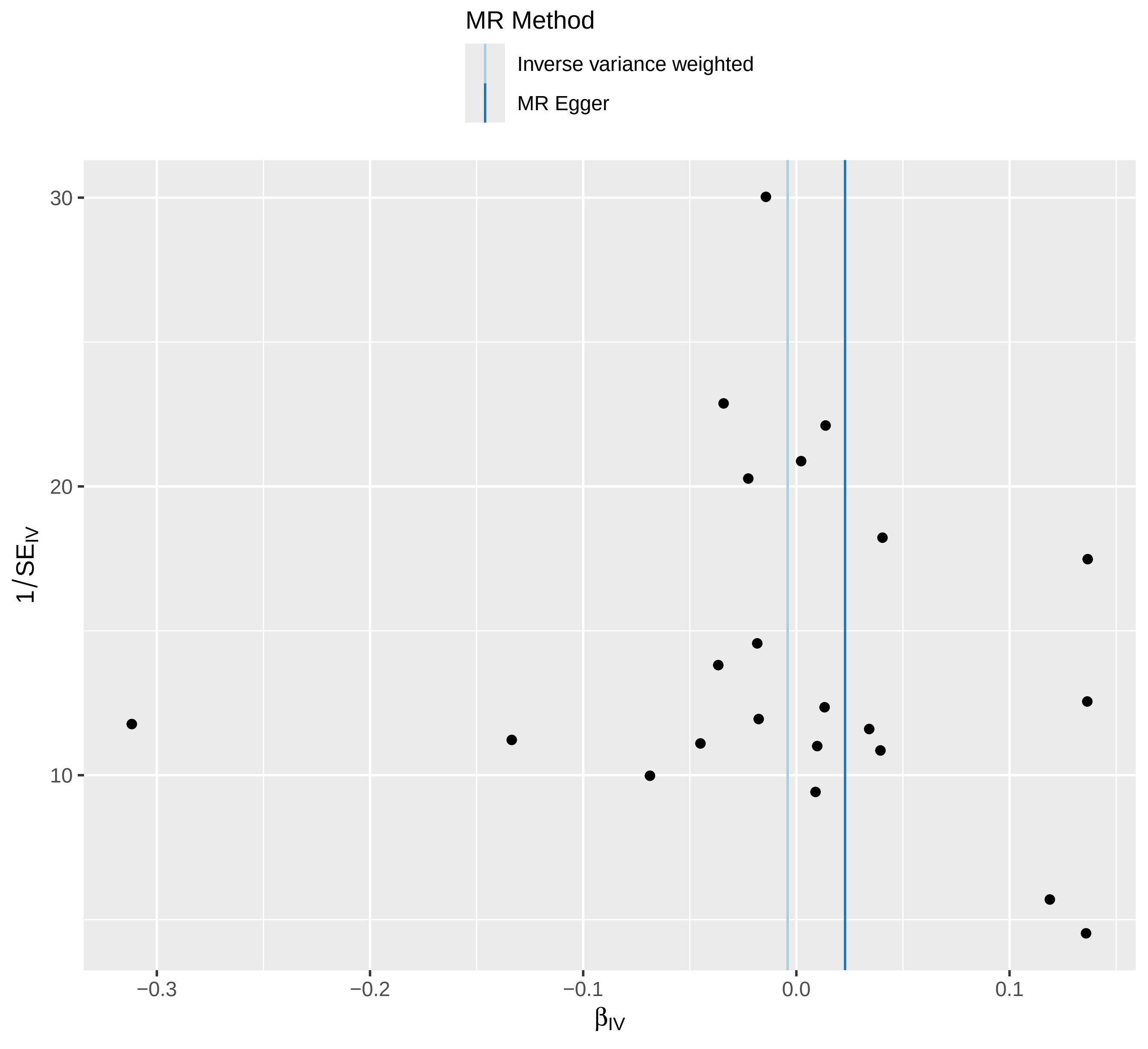

MR Method
Inverse variance weighted
MR Egger
30
20
10
1/SE$_{IV}$
−0.3
−0.2
−0.1
0.0
0.1
β$_{IV}$

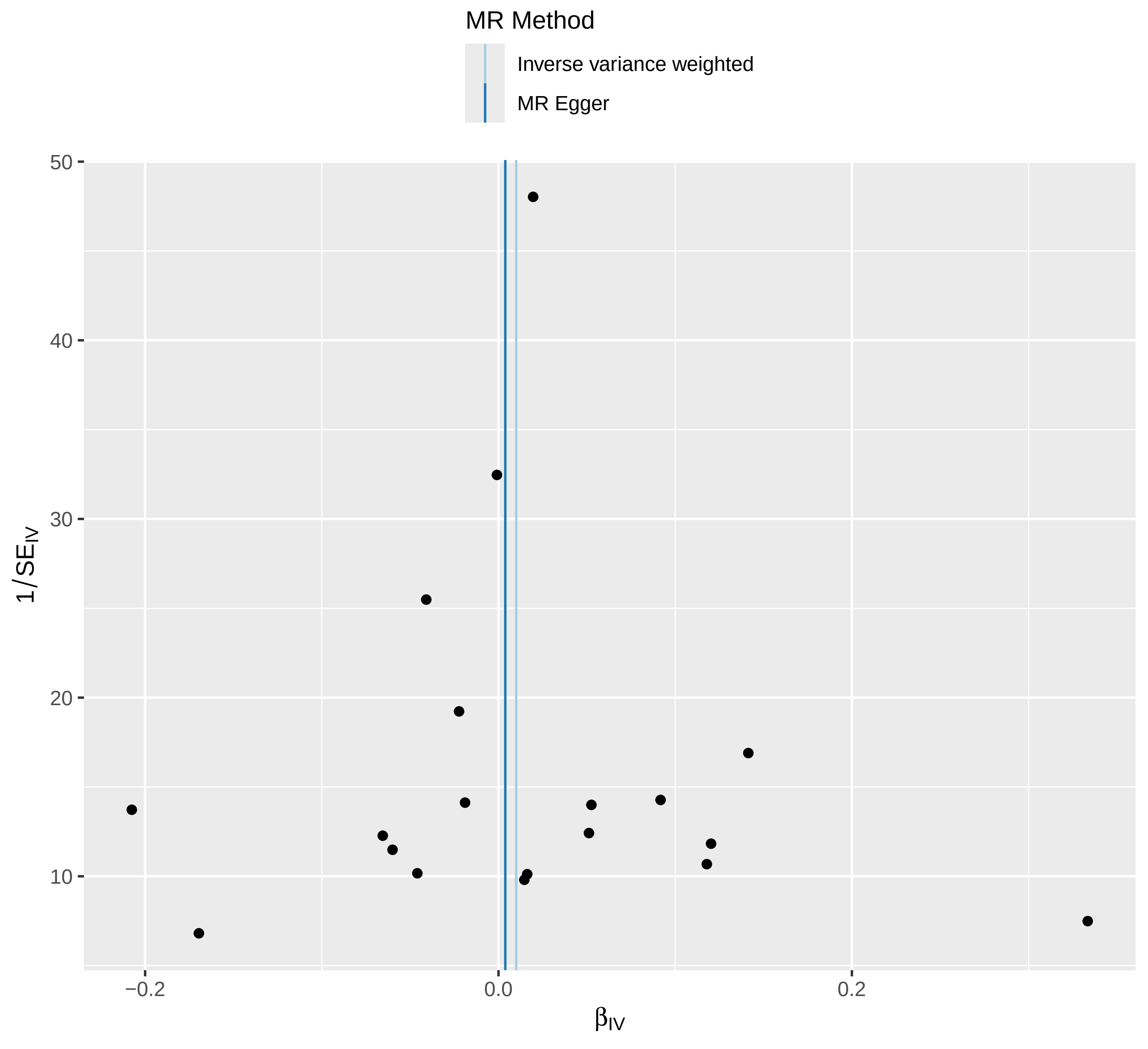
MR Method
Inverse variance weighted
MR Egger
50
40
30
20
10
$1/SE_{IV}$
−0.2
0.0
0.2
β_{IV}

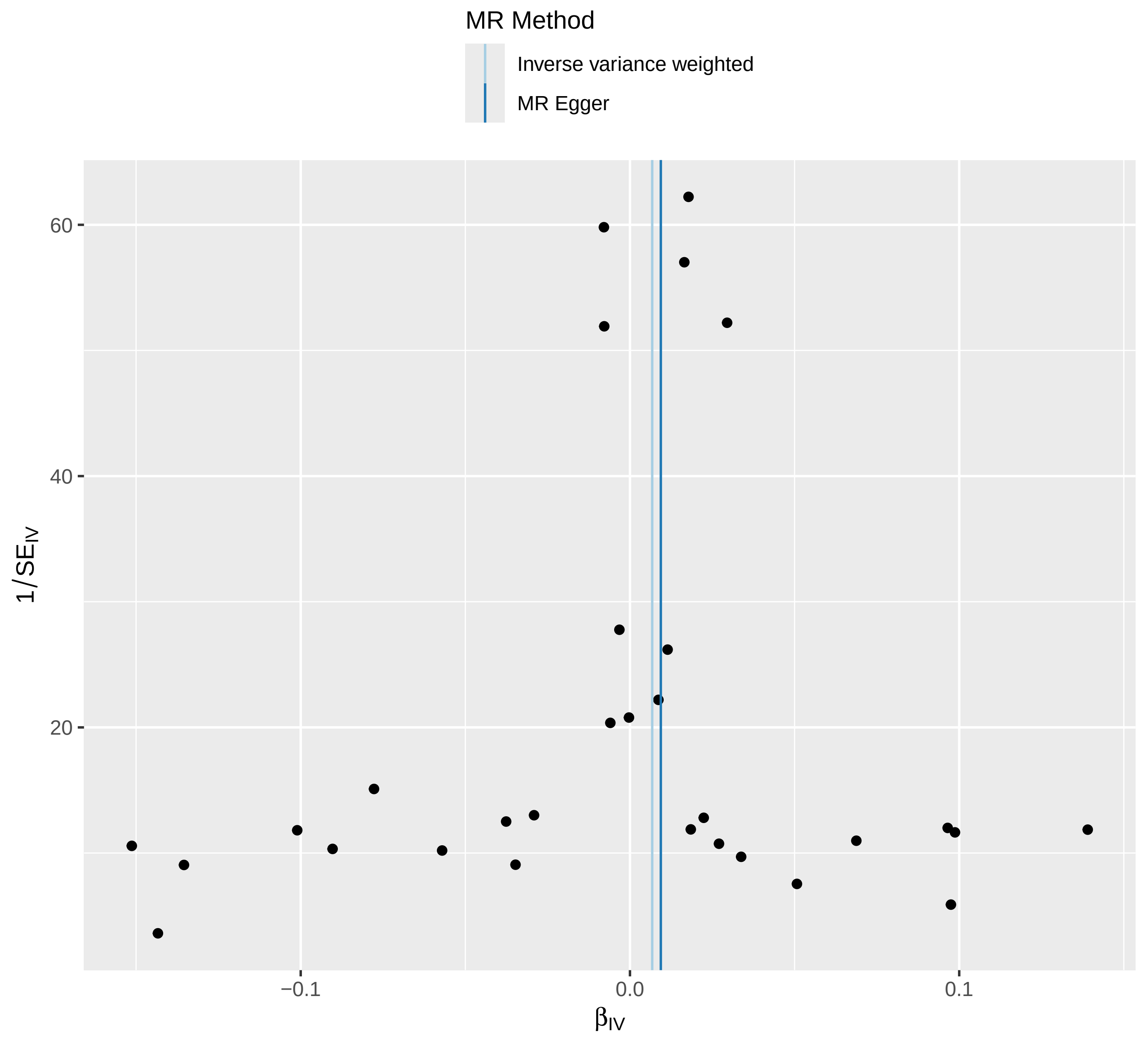

MR Method
Inverse variance weighted
MR Egger
60
40
20
1/SE_IV
−0.1
0.0
0.1
β_IV

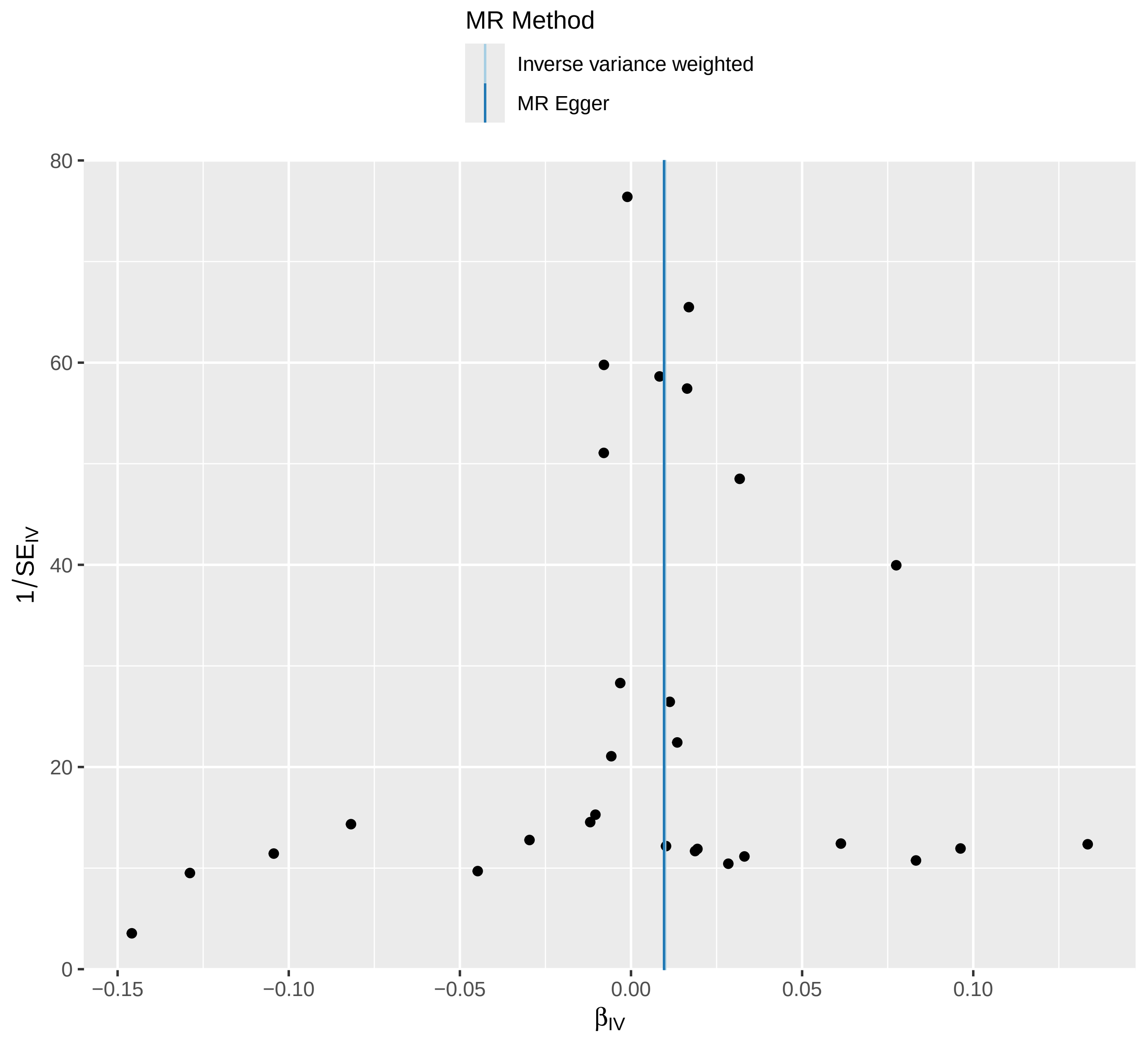
MR Method
Inverse variance weighted
MR Egger
80
60
40
20
0
$1/SE_{IV}$
−0.15
−0.10
−0.05
0.00
0.05
0.10
β_{IV}

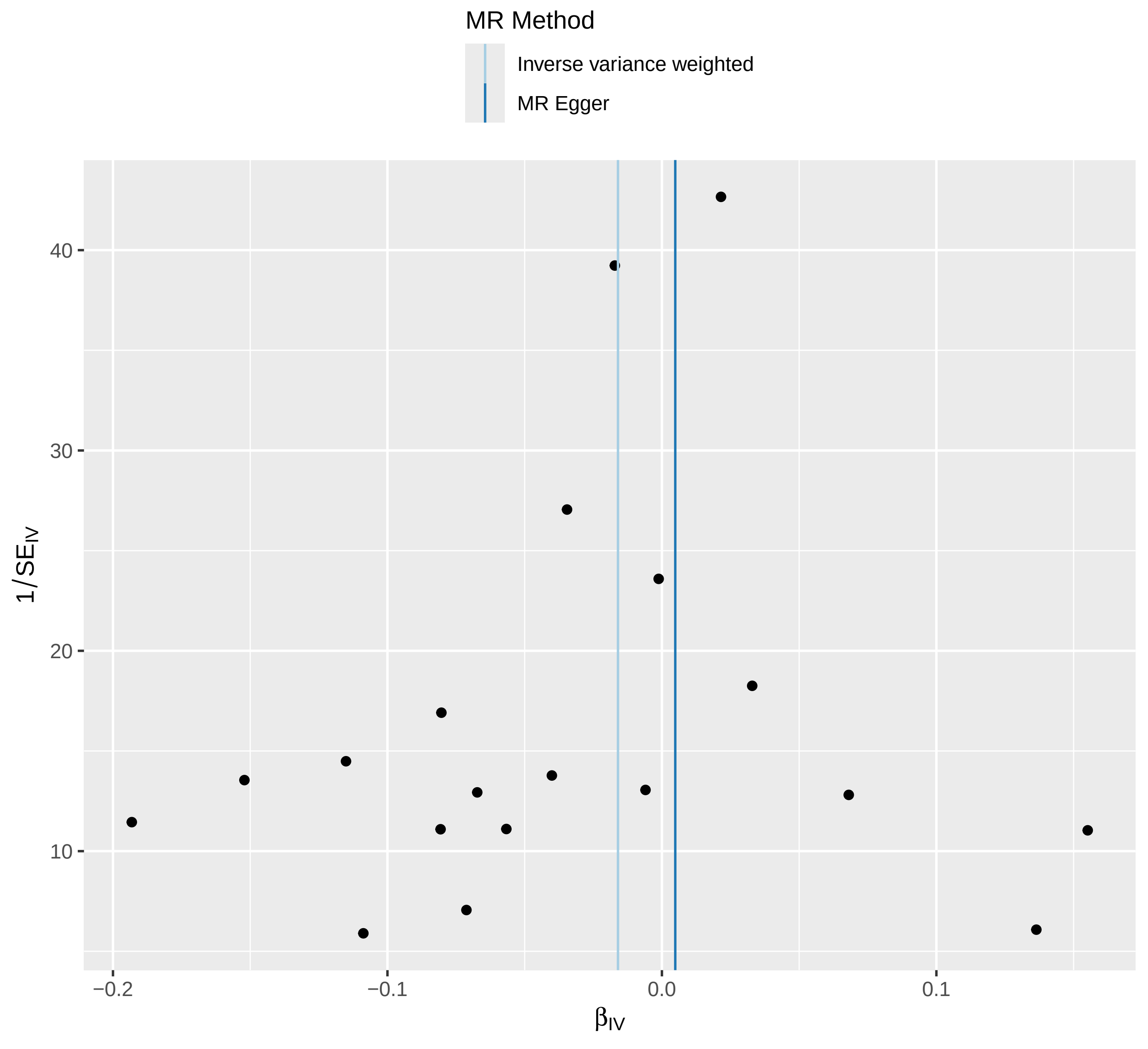
MR Method
Inverse variance weighted
MR Egger
40
30
20
10
1/SE_IV
−0.2
−0.1
0.0
0.1
β_IV

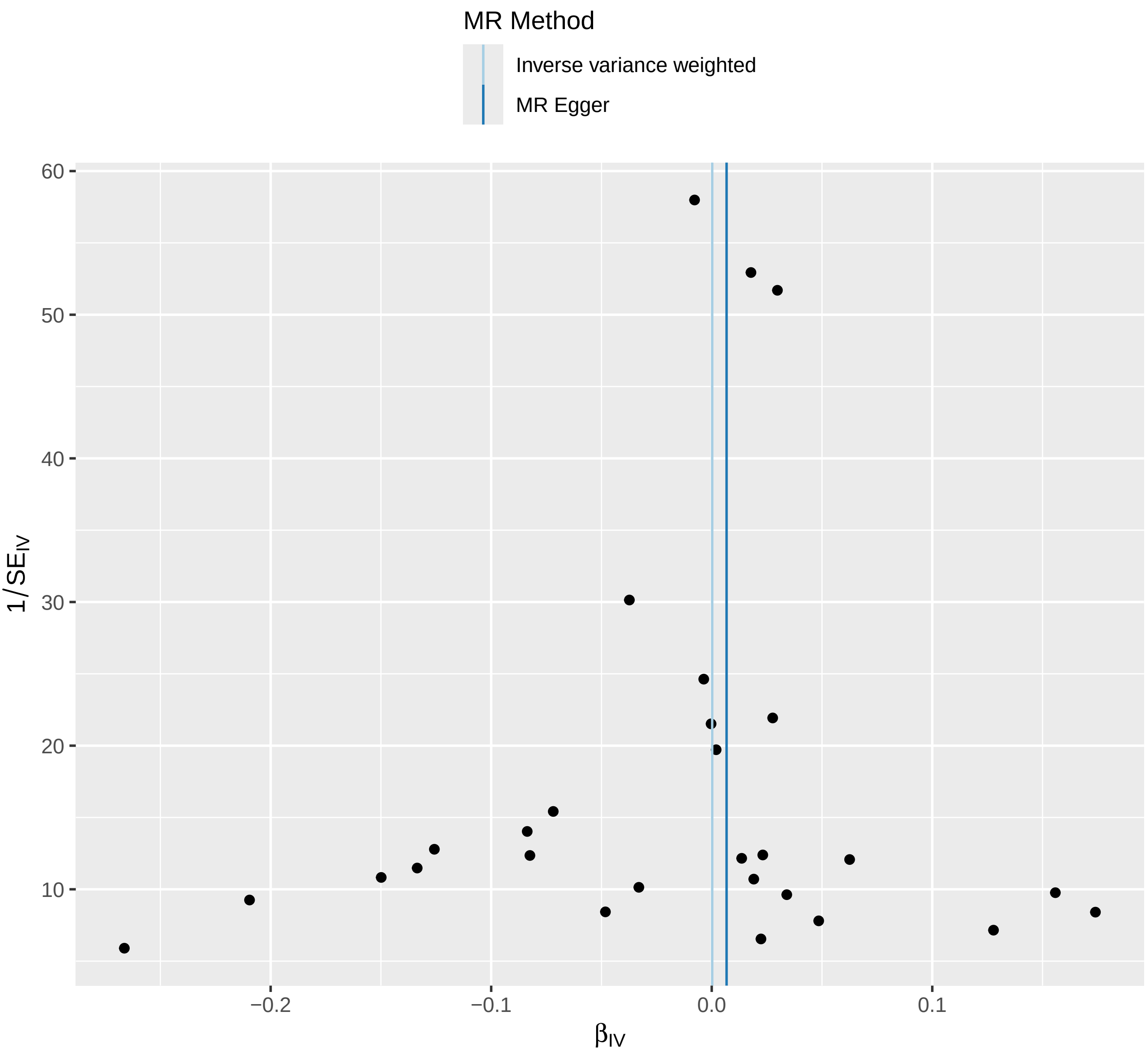

MR Method
Inverse variance weighted
MR Egger
60
50
40
30
20
10
1/SE_IV
−0.2
−0.1
0.0
0.1
β_IV

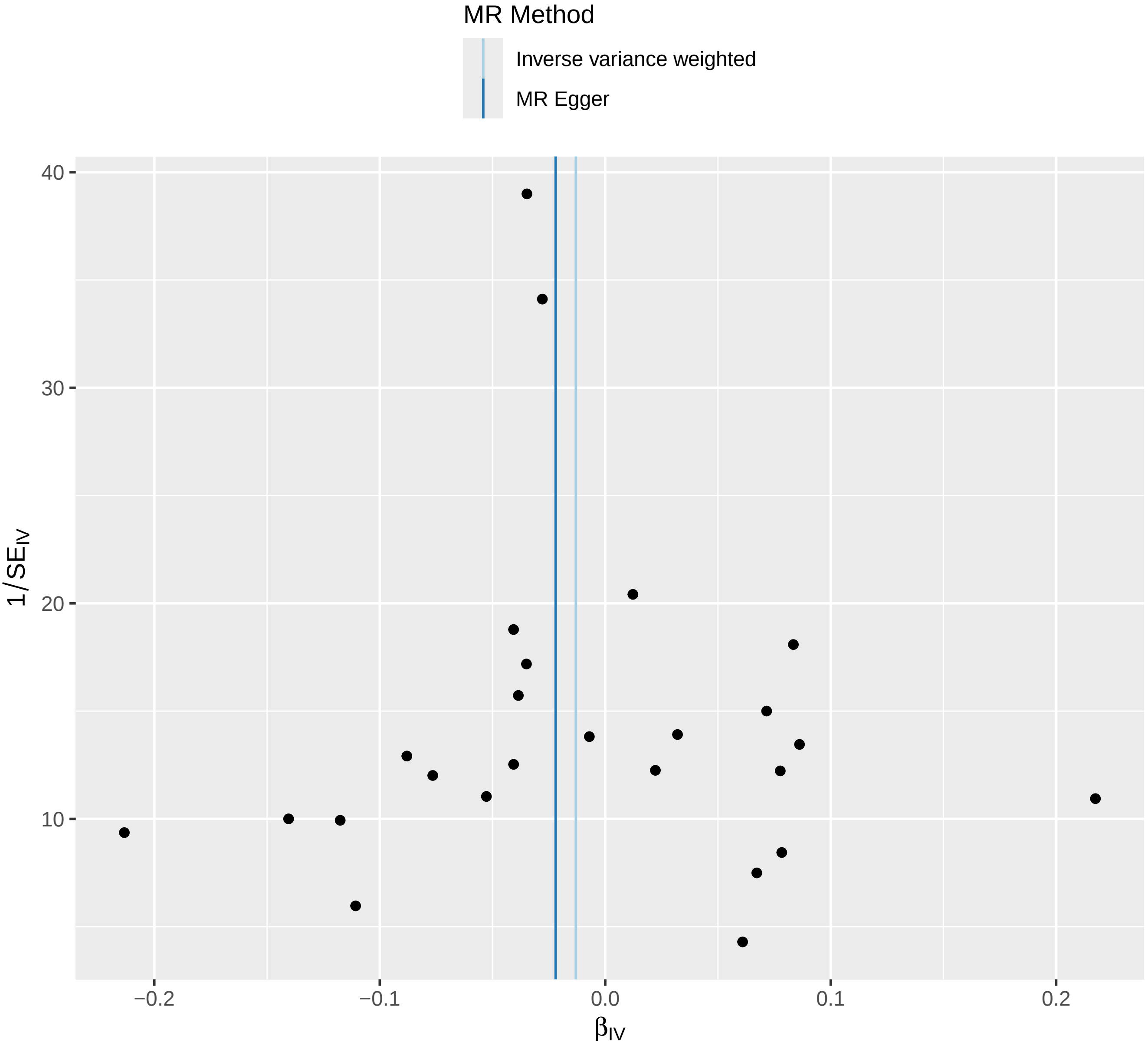
MR Method
Inverse variance weighted
MR Egger
40
30
20
10
1/SE_IV
−0.2
−0.1
0.0
0.1
0.2
β_IV

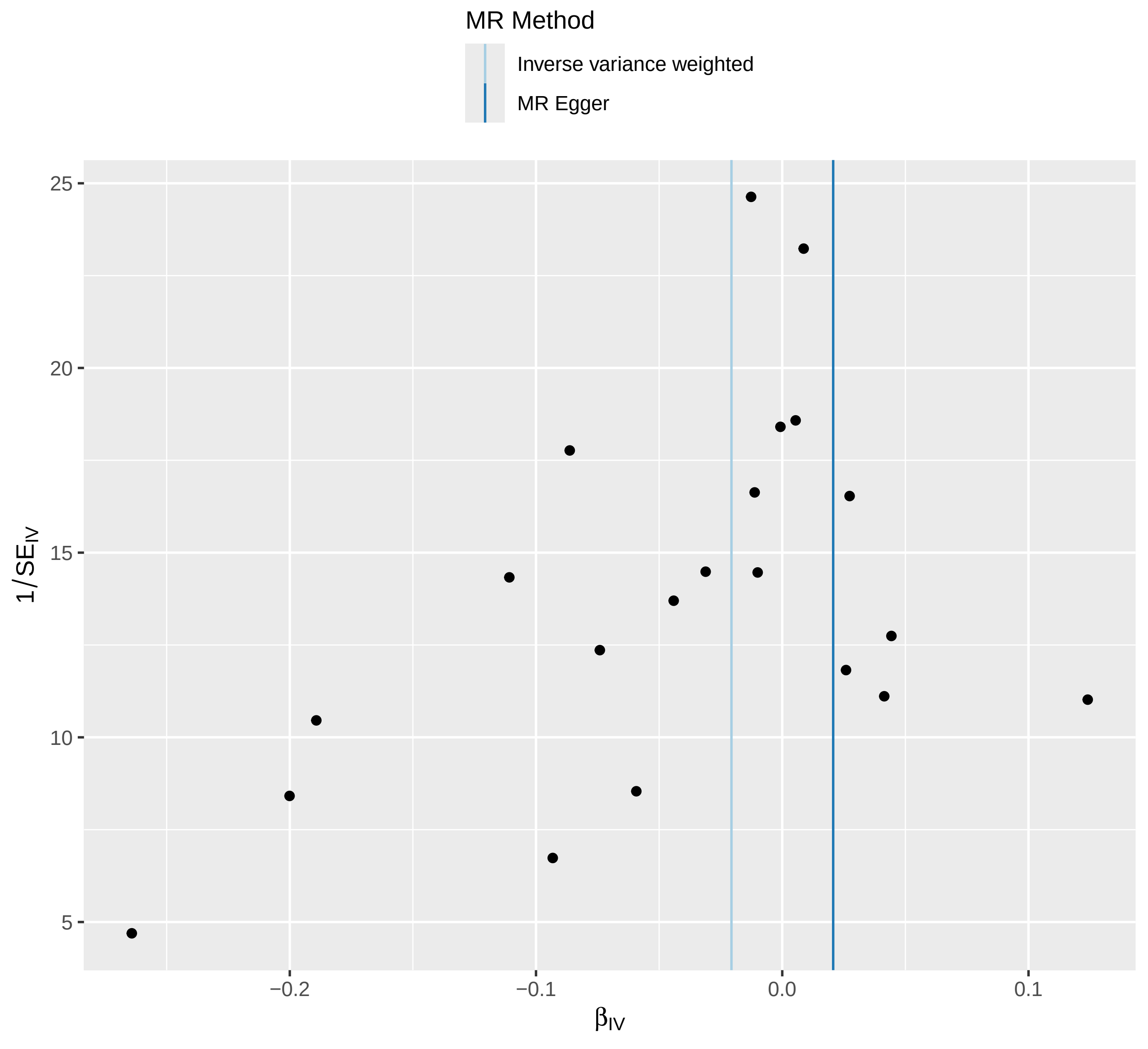
MR Method
Inverse variance weighted
MR Egger
25
20
15
10
5
1/SE_IV
−0.2
−0.1
0.0
0.1
β_IV

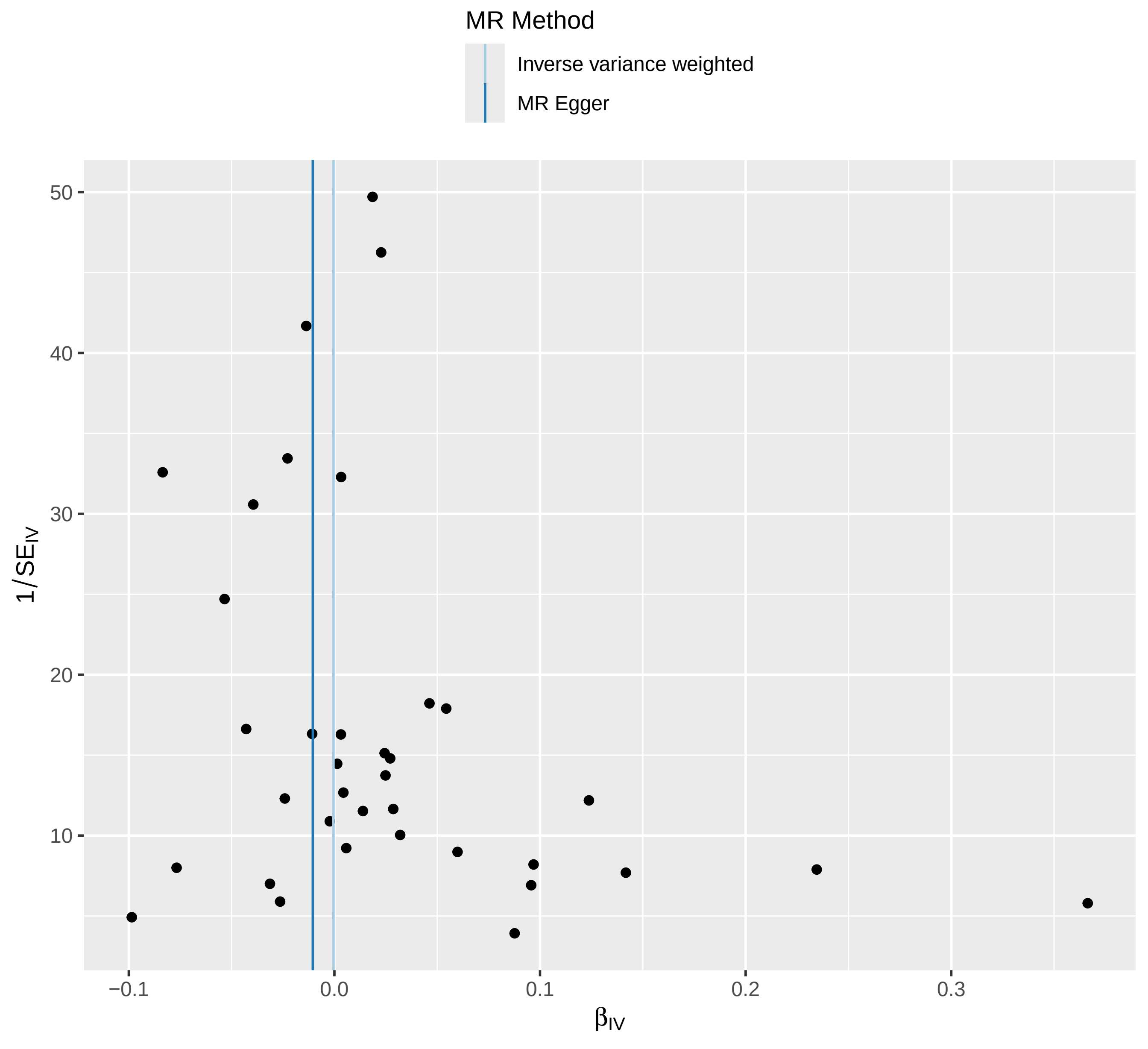
MR Method
Inverse variance weighted
MR Egger
50
40
30
20
10
1/SE$_{IV}$
−0.1
0.0
0.1
0.2
0.3
β$_{IV}$

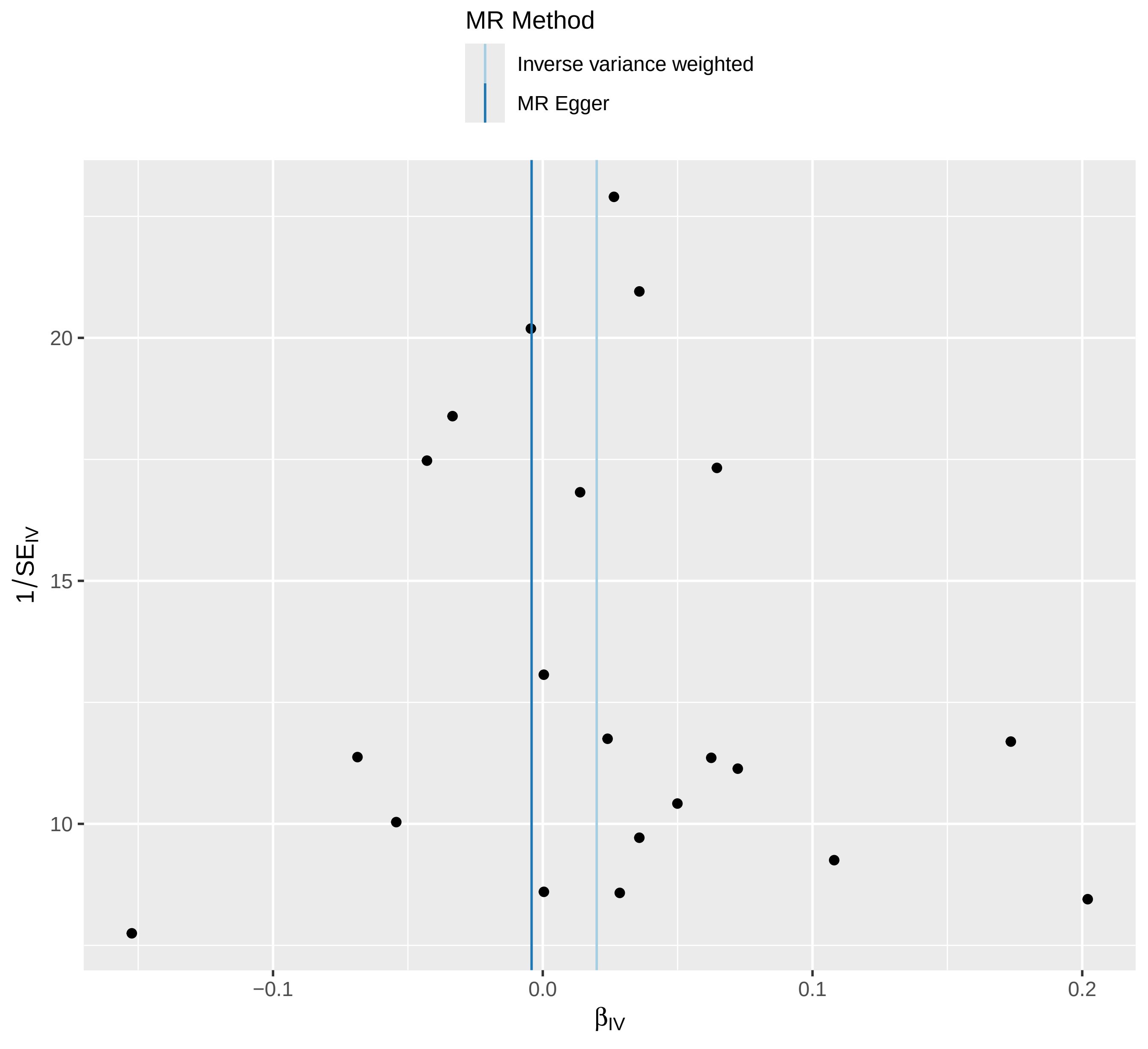
MR Method
Inverse variance weighted
MR Egger
1/SE_IV
20
15
10
−0.1
0.0
0.1
0.2
β_IV

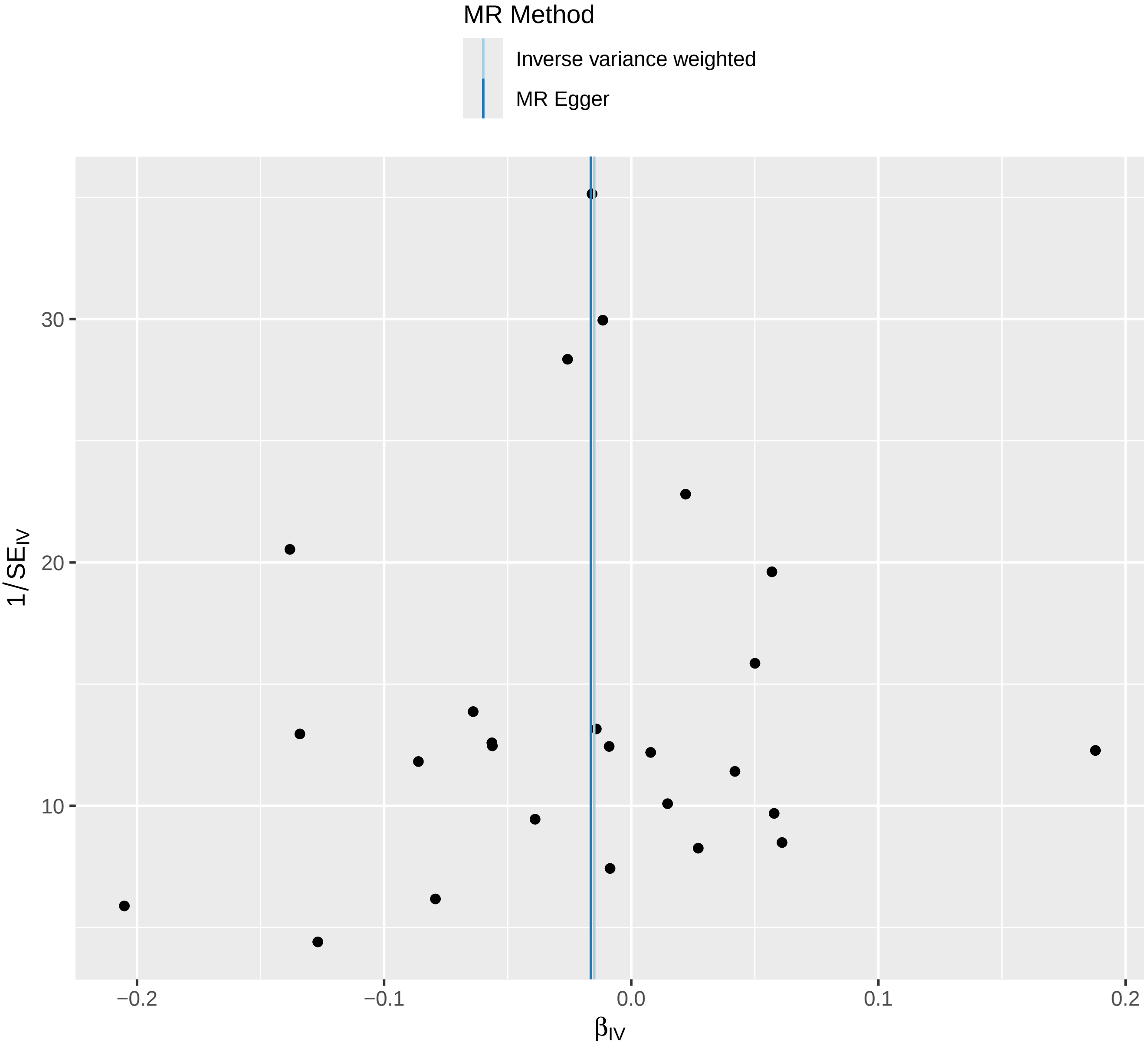

MR Method
Inverse variance weighted
MR Egger
30
20
10
1/SE_IV
−0.2
−0.1
0.0
0.1
0.2
β_IV

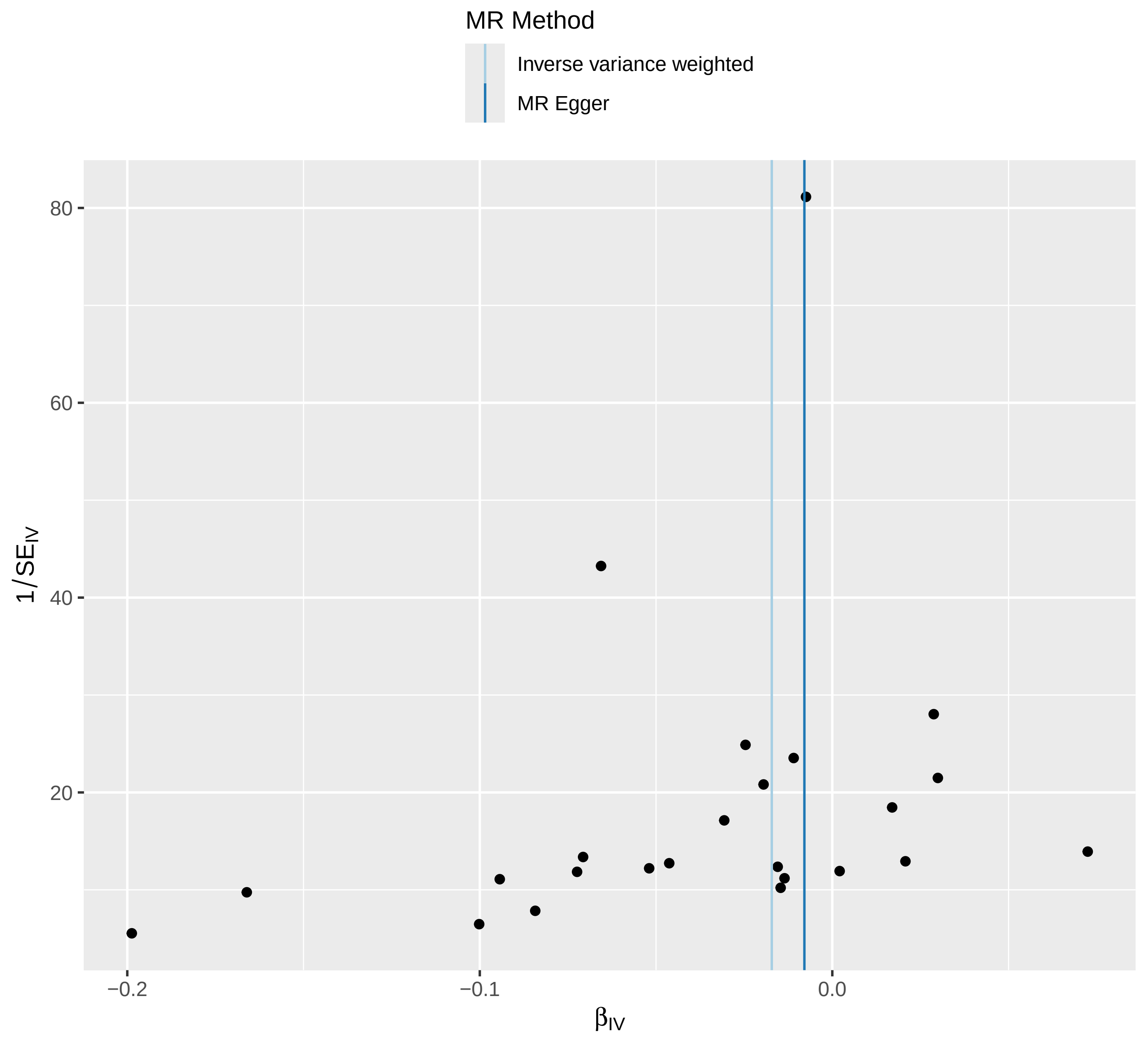

MR Method
Inverse variance weighted
MR Egger
80
60
40
20
1/SE_IV
−0.2
−0.1
0.0
β_IV

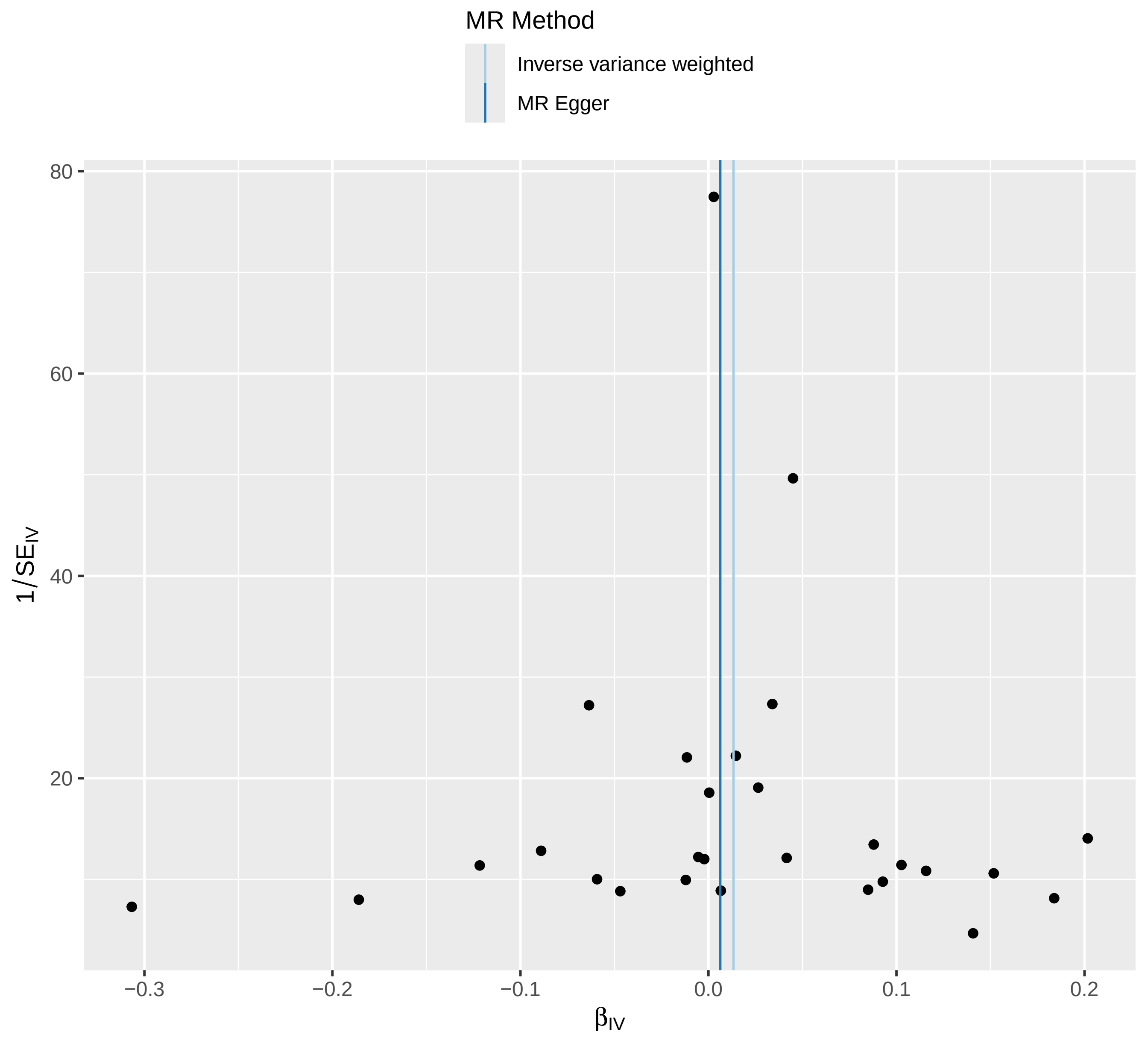
MR Method
Inverse variance weighted
MR Egger
80
60
40
20
1/SE_IV
−0.3
−0.2
−0.1
0.0
0.1
0.2
β_IV

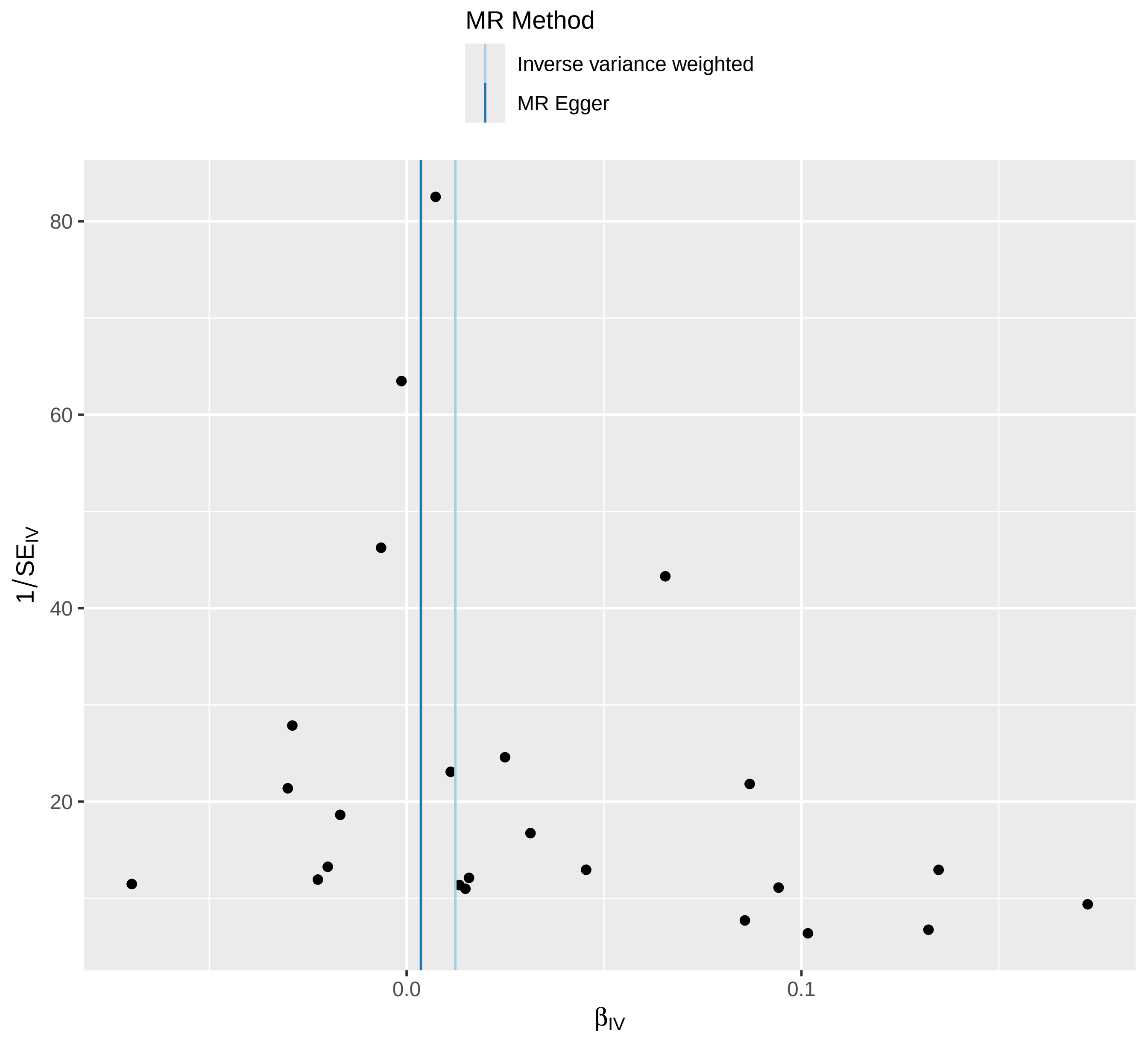
MR Method
Inverse variance weighted
MR Egger
80
60
40
20
1/SE$_{IV}$
0.0
0.1
β$_{IV}$

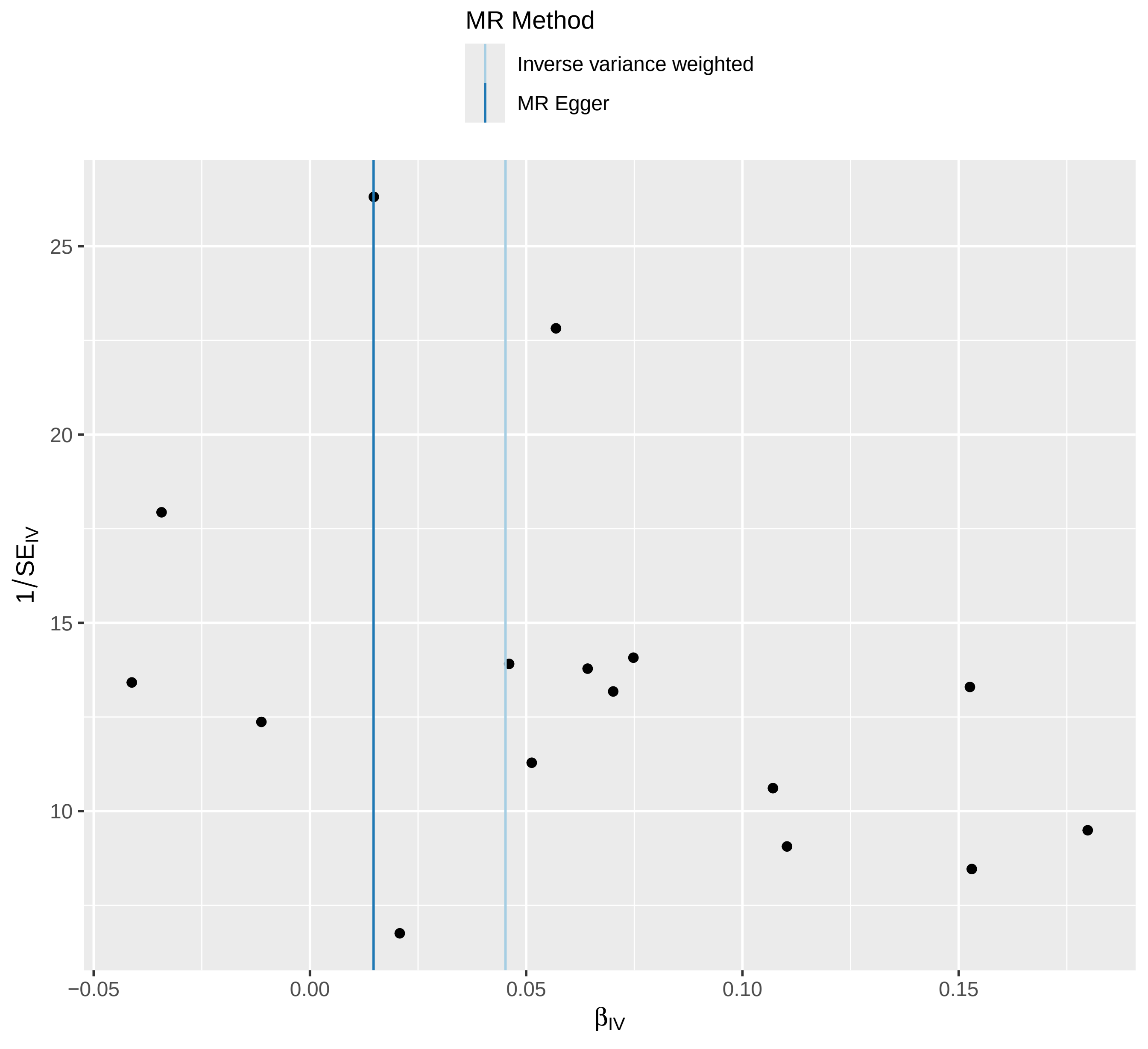
MR Method
Inverse variance weighted
MR Egger
25
20
15
10
−0.05
0.00
0.05
0.10
0.15
$1/SE_{IV}$
β_{IV}

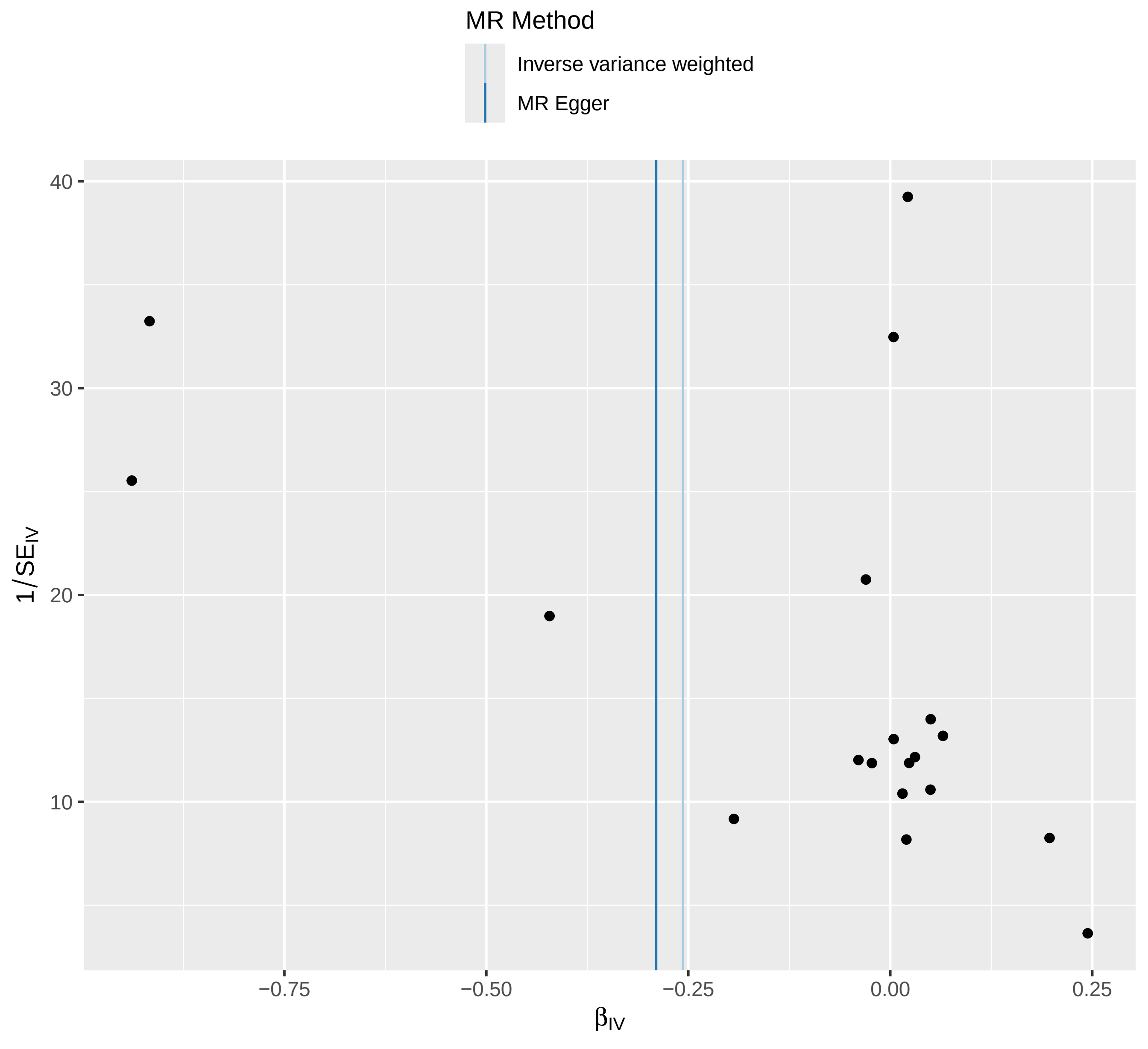
MR Method
Inverse variance weighted
MR Egger
1/SE_IV
40
30
20
10
−0.75
−0.50
−0.25
0.00
0.25
β_IV

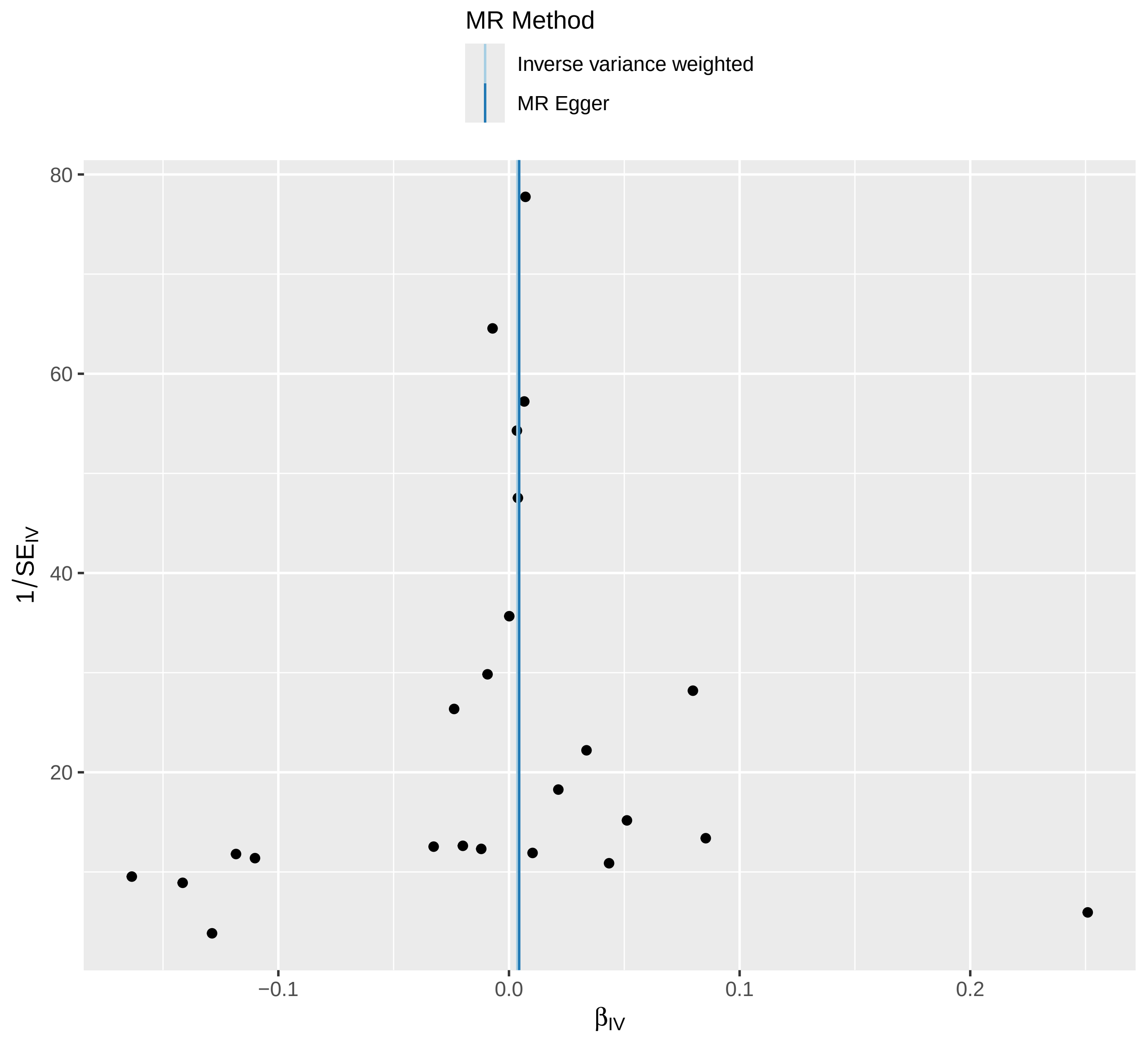

MR Method
Inverse variance weighted
MR Egger
80
60
40
20
1/SE_IV
−0.1
0.0
0.1
0.2
β_IV

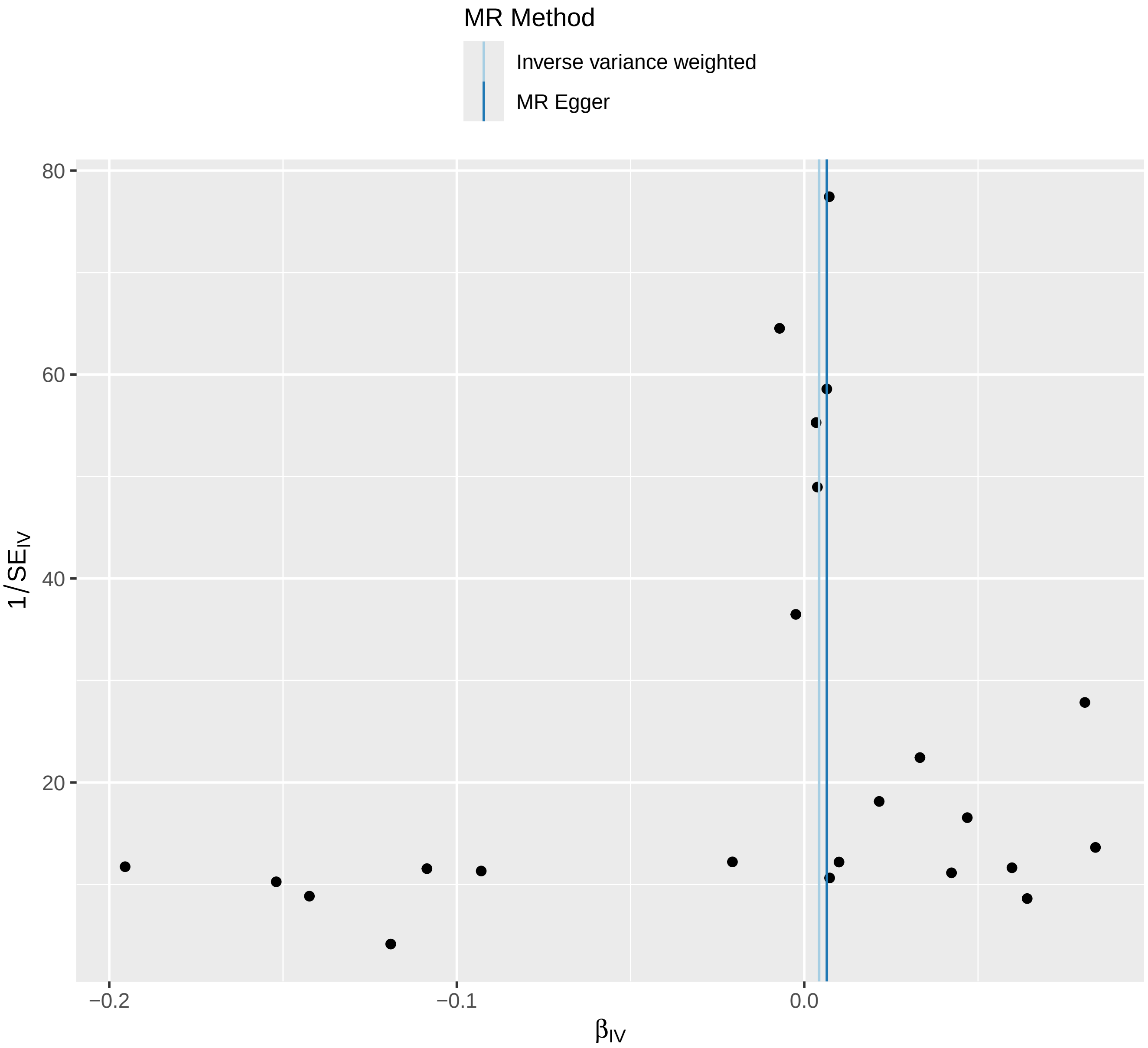
MR Method
Inverse variance weighted
MR Egger
80
60
40
20
$1/SE_{IV}$
−0.2
−0.1
0.0
β_{IV}

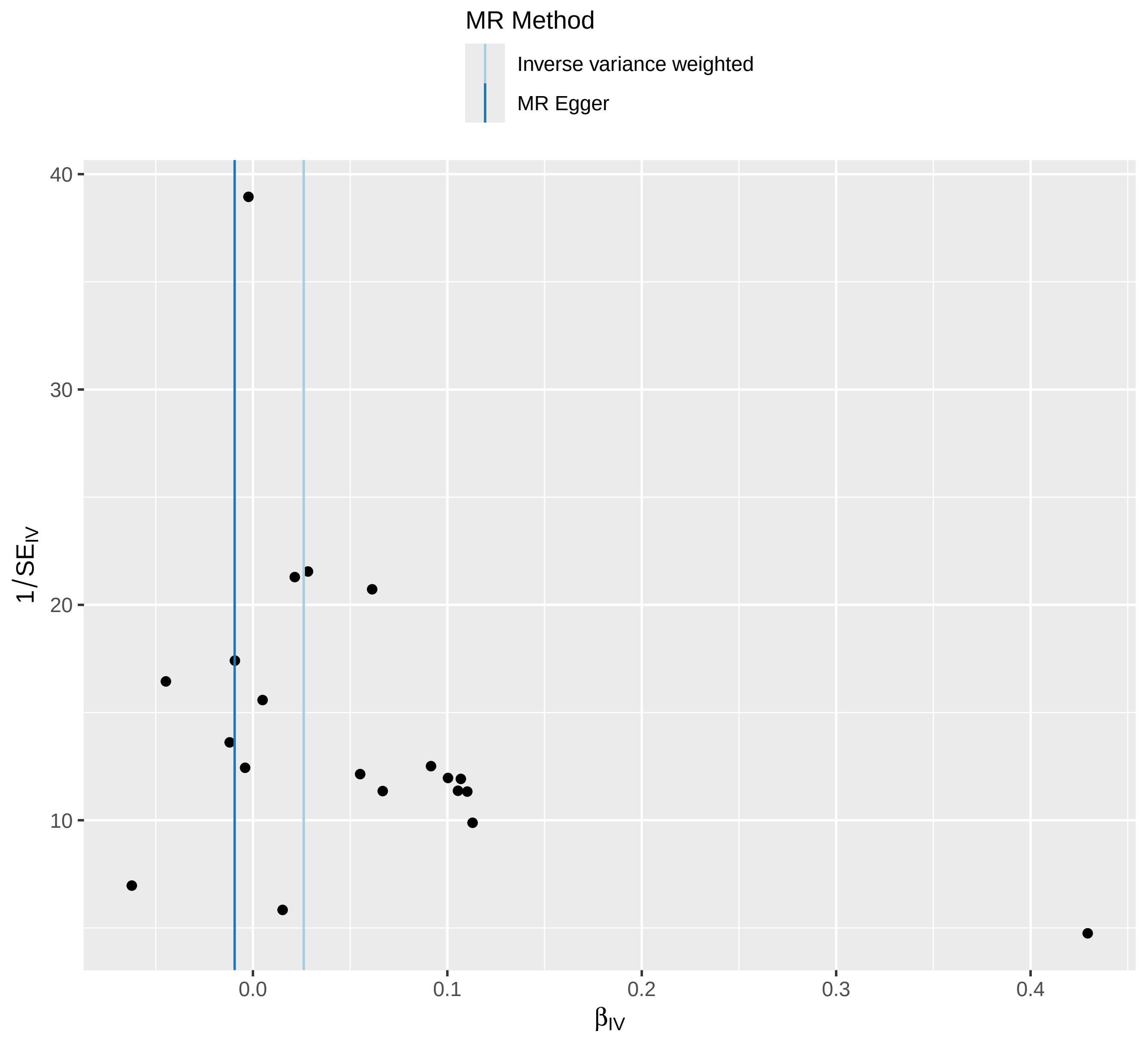
MR Method
Inverse variance weighted
MR Egger
40
30
20
10
1/SE_IV
0.0
0.1
0.2
0.3
0.4
β_IV

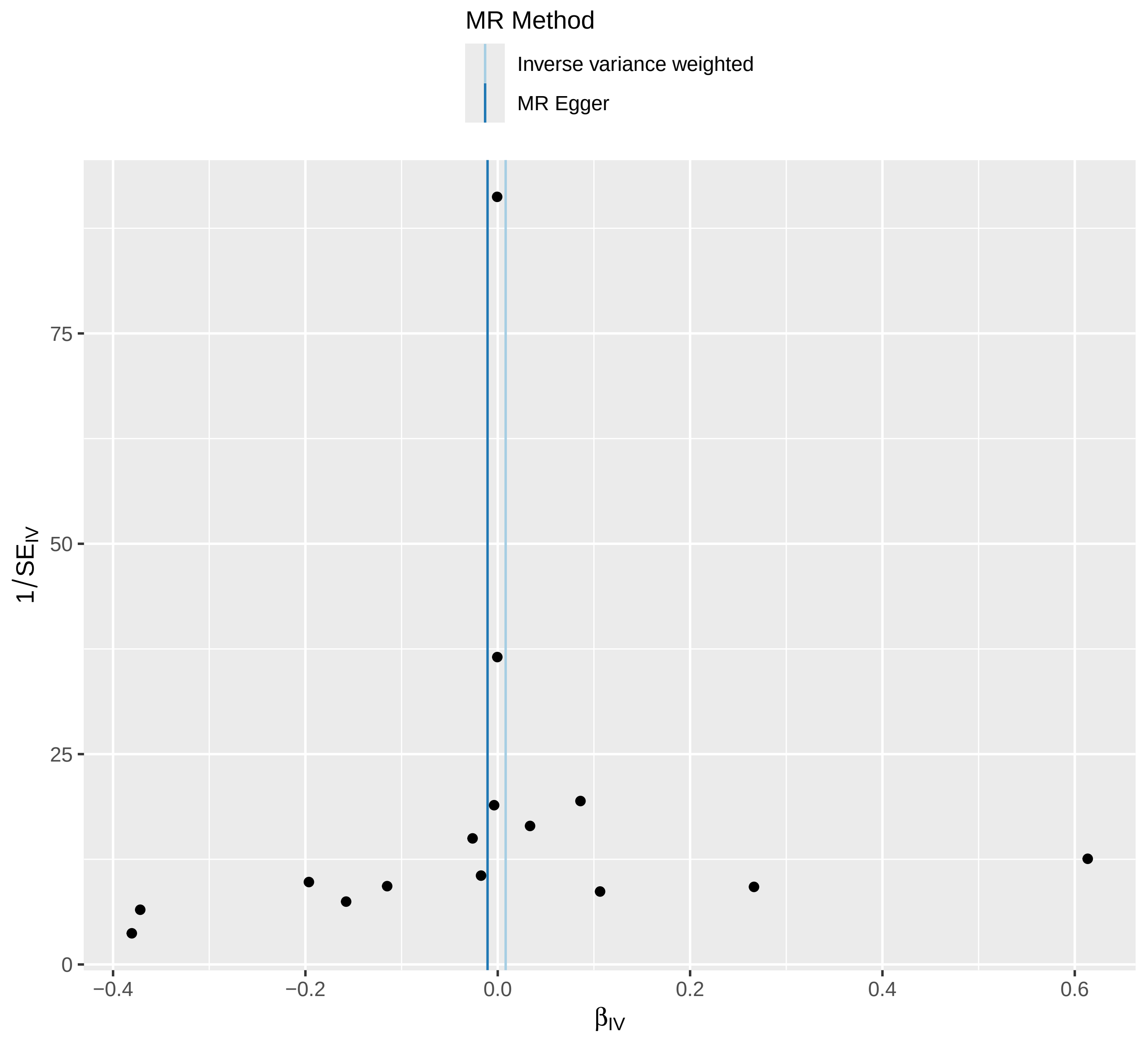
MR Method
Inverse variance weighted
MR Egger
1/SE_IV
75
50
25
0
−0.4
−0.2
0.0
0.2
0.4
0.6
β_IV

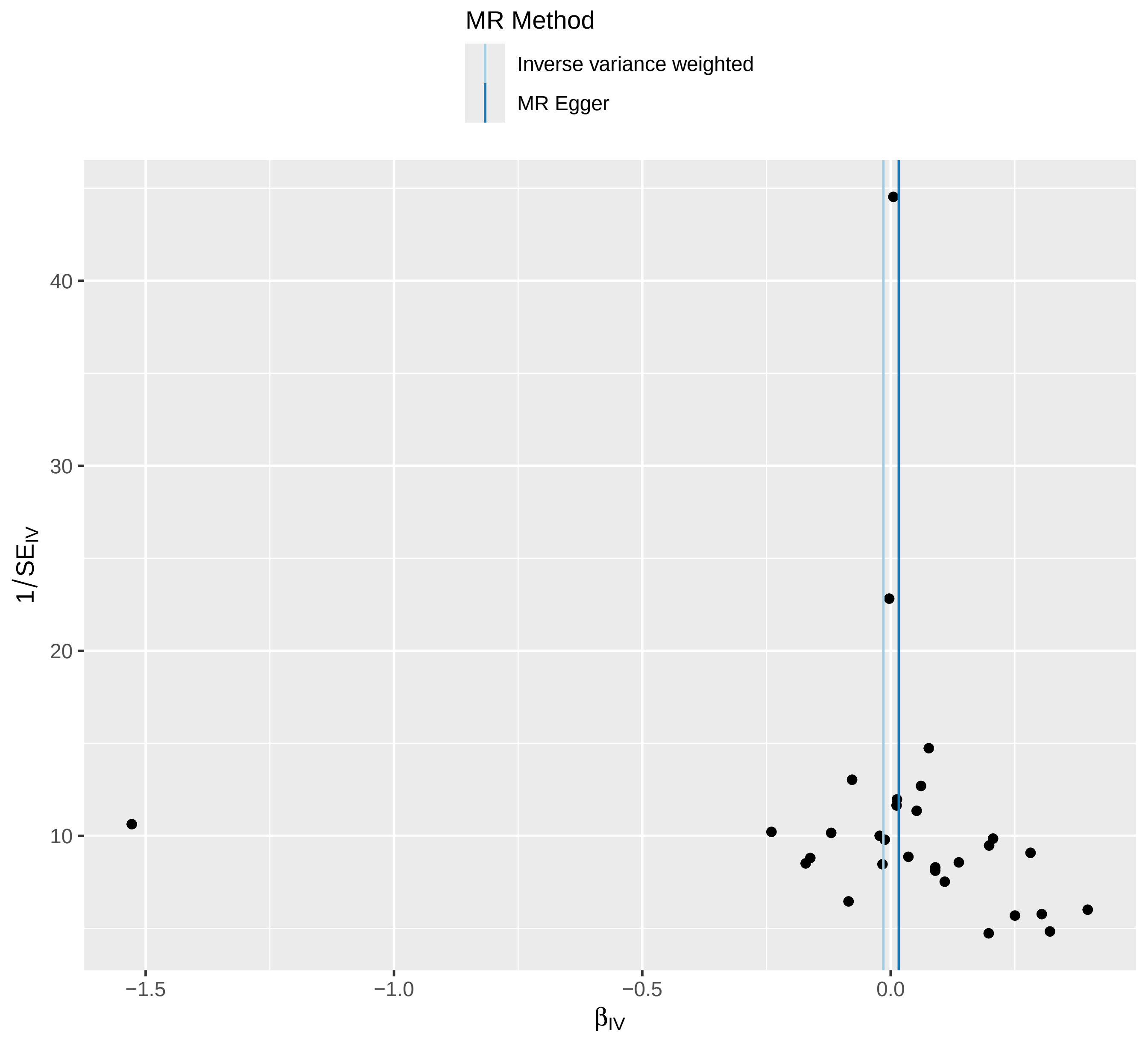
MR Method
Inverse variance weighted
MR Egger
40
30
20
10
1/SE_IV
−1.5
−1.0
−0.5
0.0
β_IV

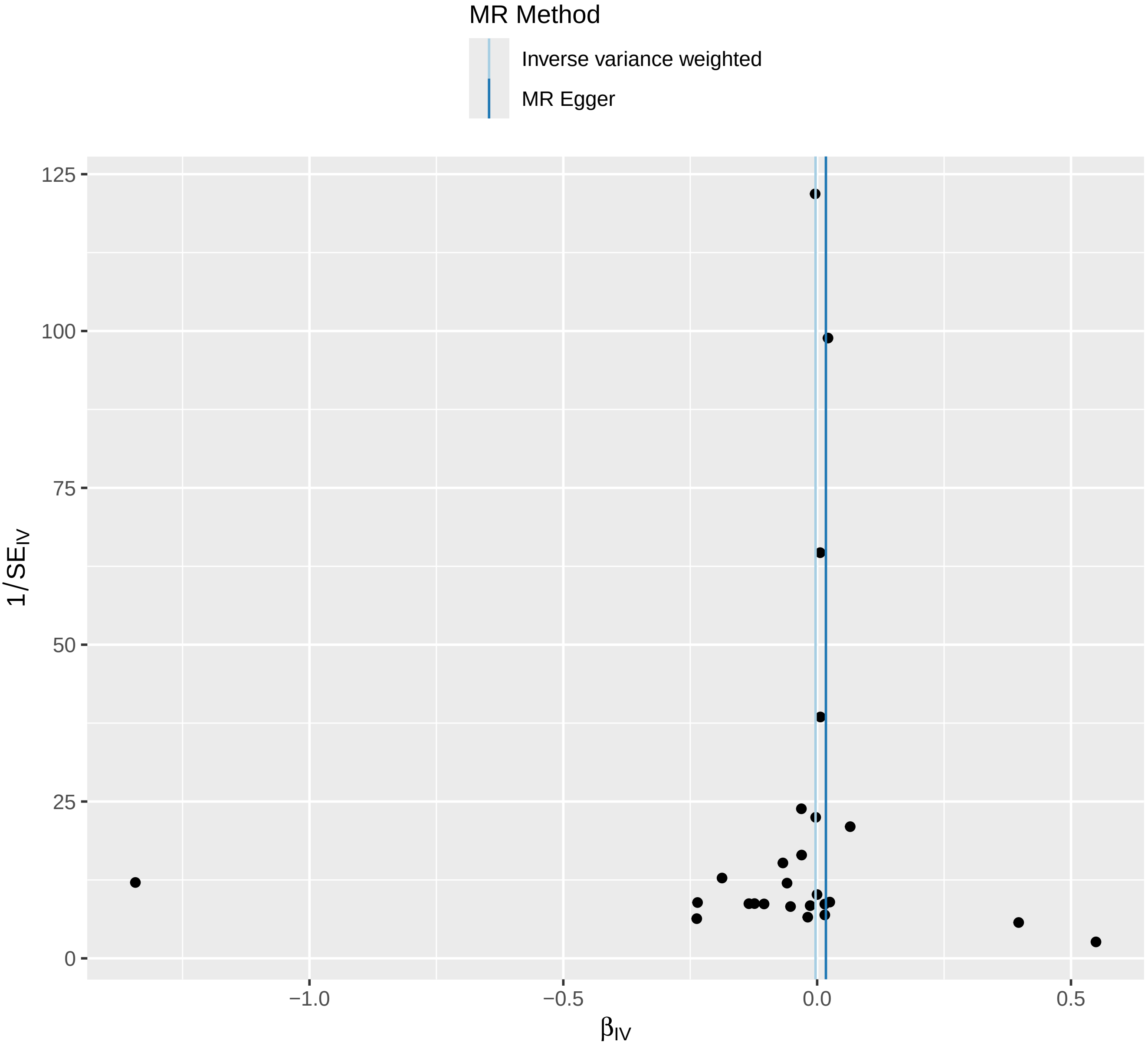
MR Method
Inverse variance weighted
MR Egger
125
100
75
50
25
0
1/SE_IV
−1.0
−0.5
0.0
0.5
β_IV

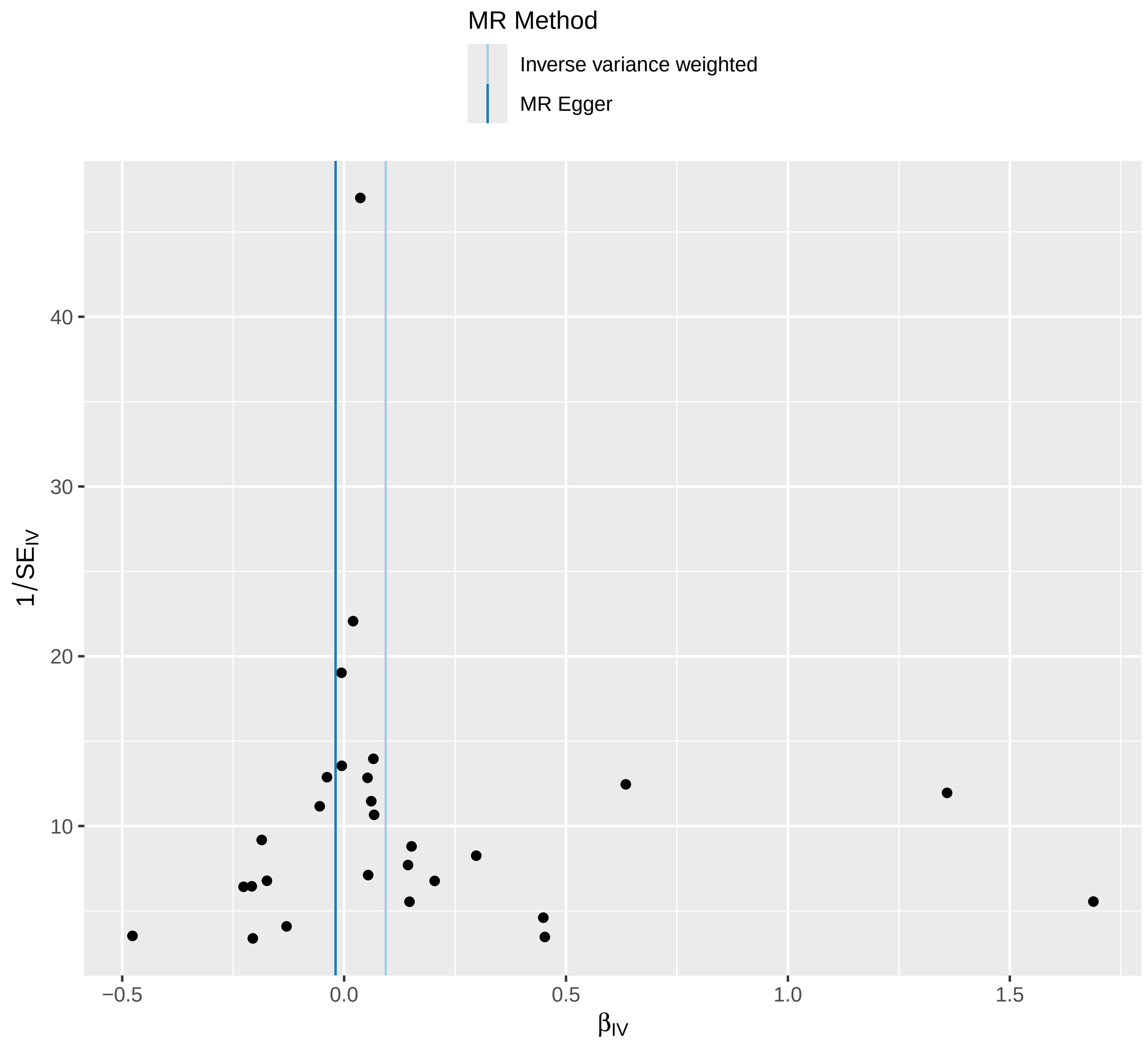
MR Method
Inverse variance weighted
MR Egger
1/SE_IV
40
30
20
10
−0.5
0.0
0.5
1.0
1.5
β_IV

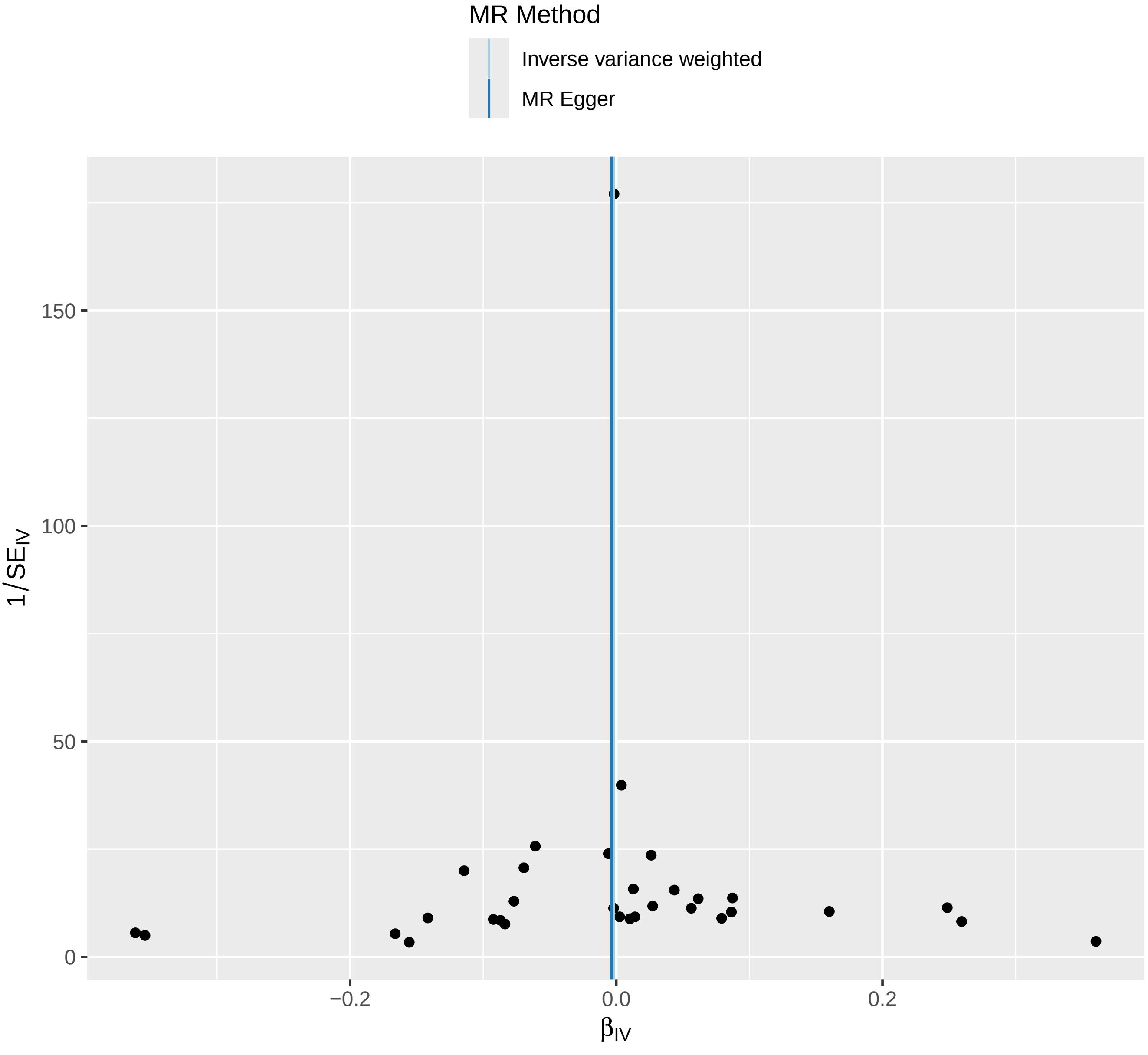

MR Method
Inverse variance weighted
MR Egger
150
100
50
0
1/SE_IV
−0.2
0.0
0.2
β_IV

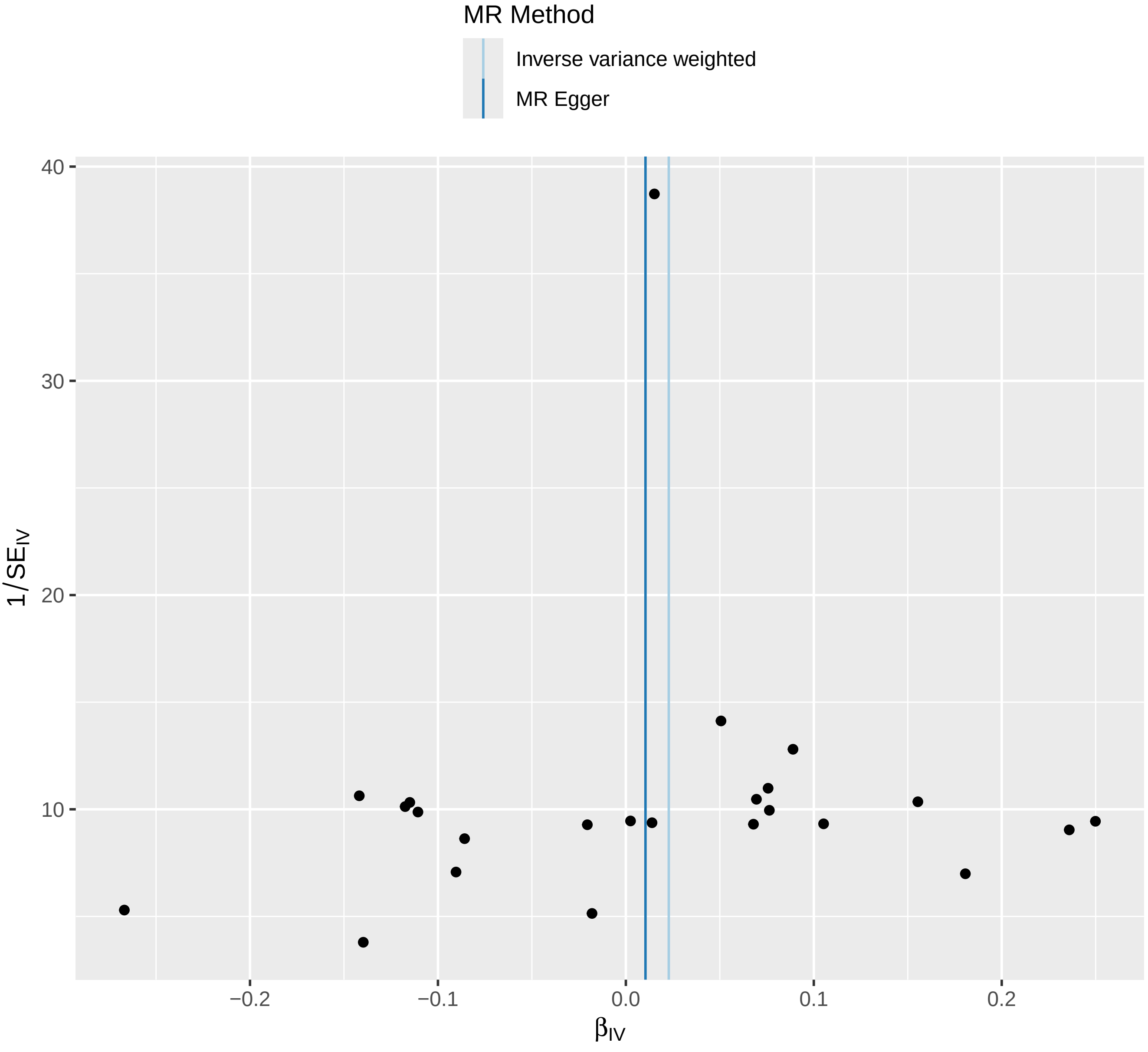
MR Method
Inverse variance weighted
MR Egger
40
30
20
10
1/SE_IV
−0.2
−0.1
0.0
0.1
0.2
β_IV

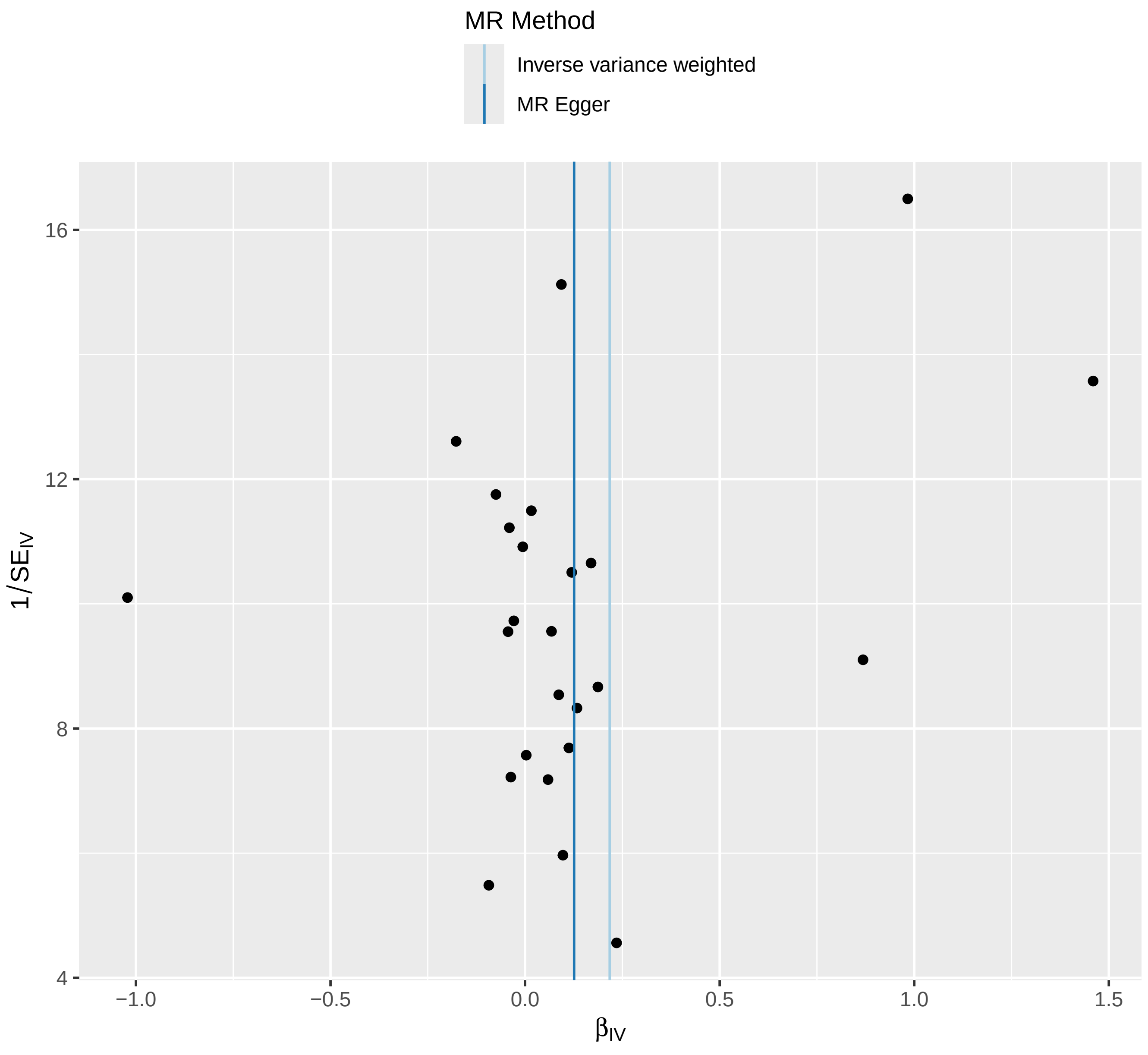
MR Method
Inverse variance weighted
MR Egger
16
12
8
4
1/SE$_{IV}$
−1.0
−0.5
0.0
0.5
1.0
1.5
β$_{IV}$

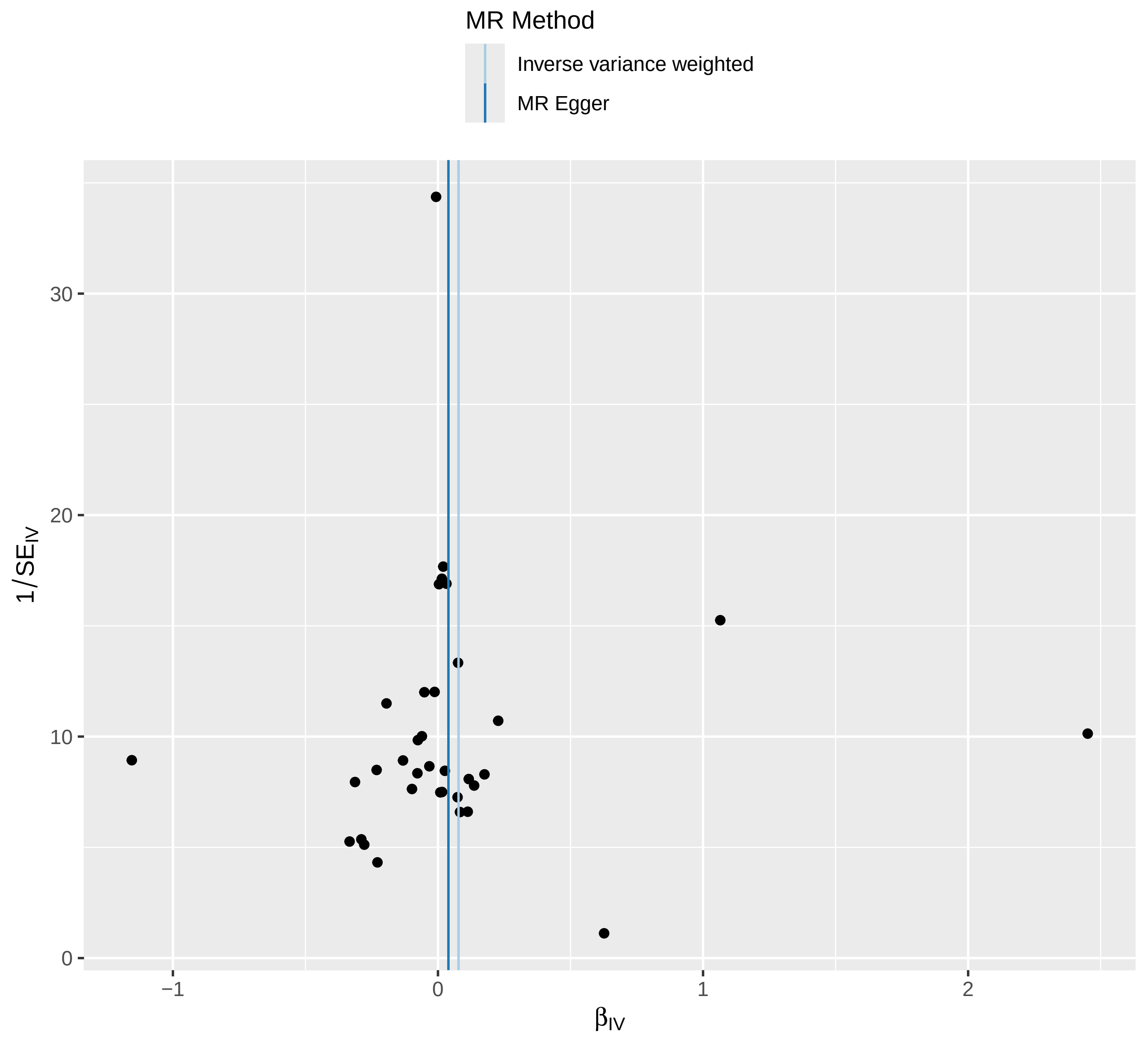
MR Method
Inverse variance weighted
MR Egger
30
20
10
0
1/SE_IV
−1
0
1
2
β_IV

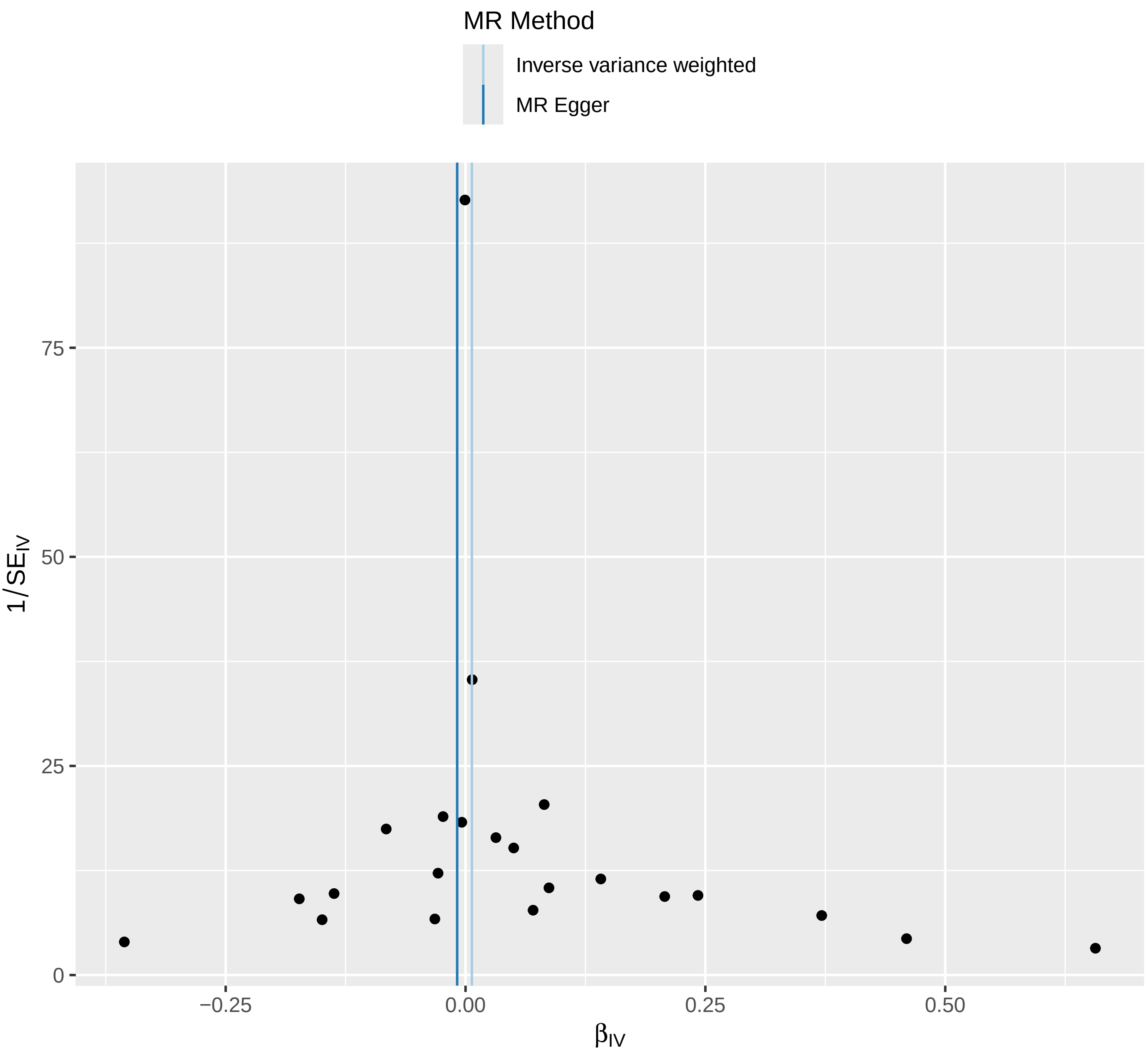
MR Method
Inverse variance weighted
MR Egger
75
50
25
0
1/SE_IV
−0.25
0.00
0.25
0.50
β_IV

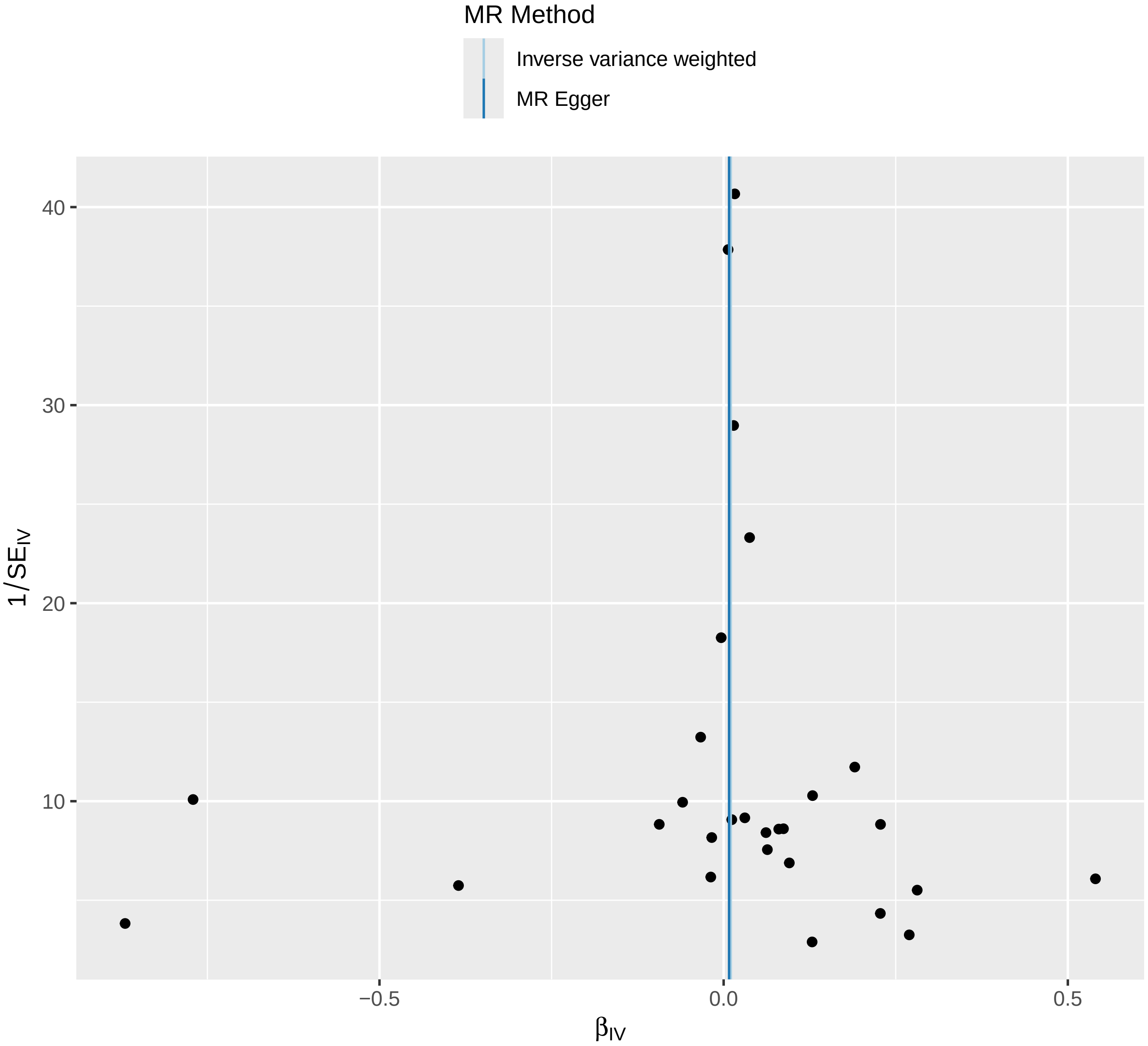

MR Method
Inverse variance weighted
MR Egger
40
30
20
10
1/SE_IV
−0.5
0.0
0.5
β_IV

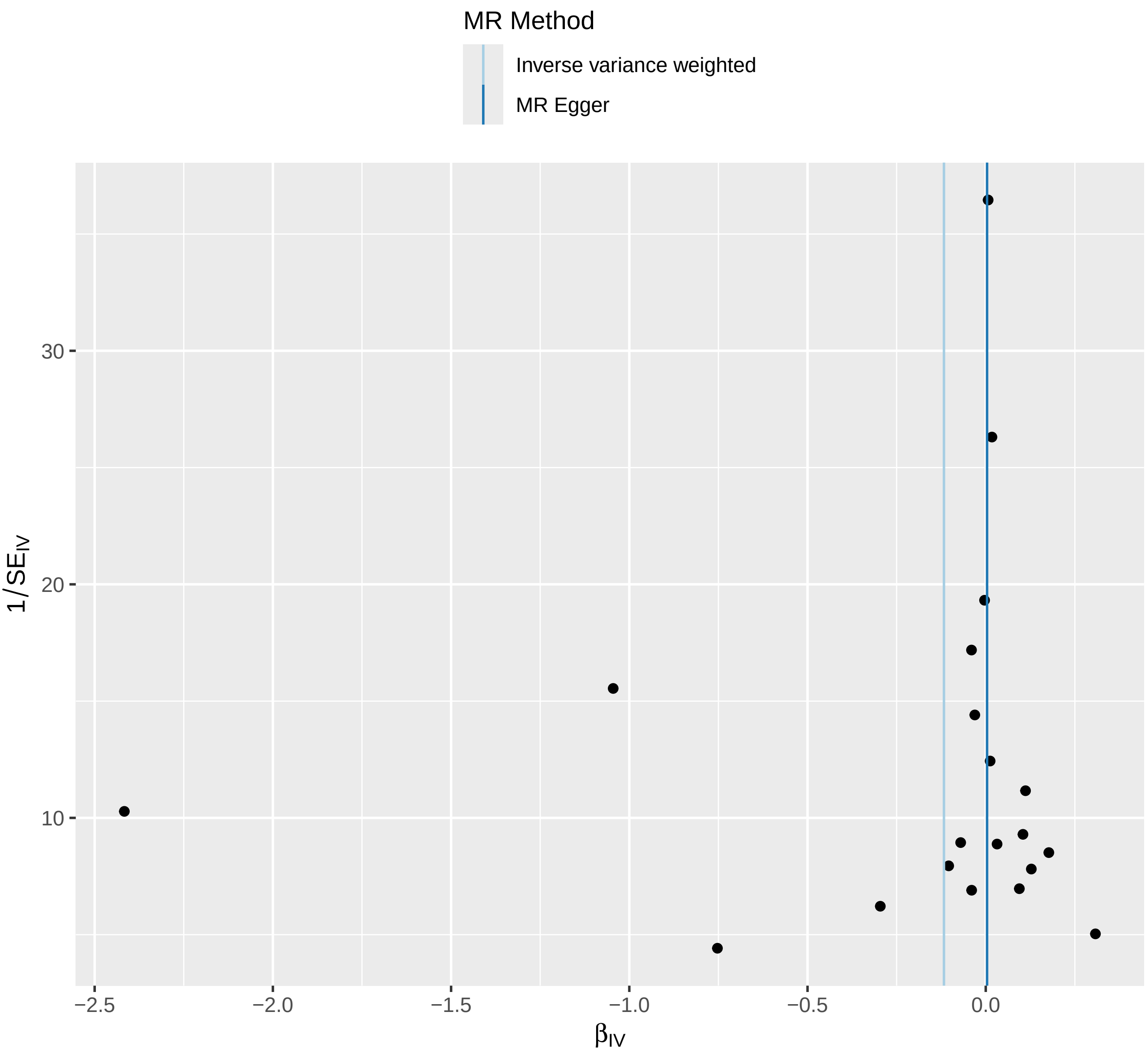
MR Method
Inverse variance weighted
MR Egger
30
20
10
1/SE_IV
−2.5
−2.0
−1.5
−1.0
−0.5
0.0
β_IV

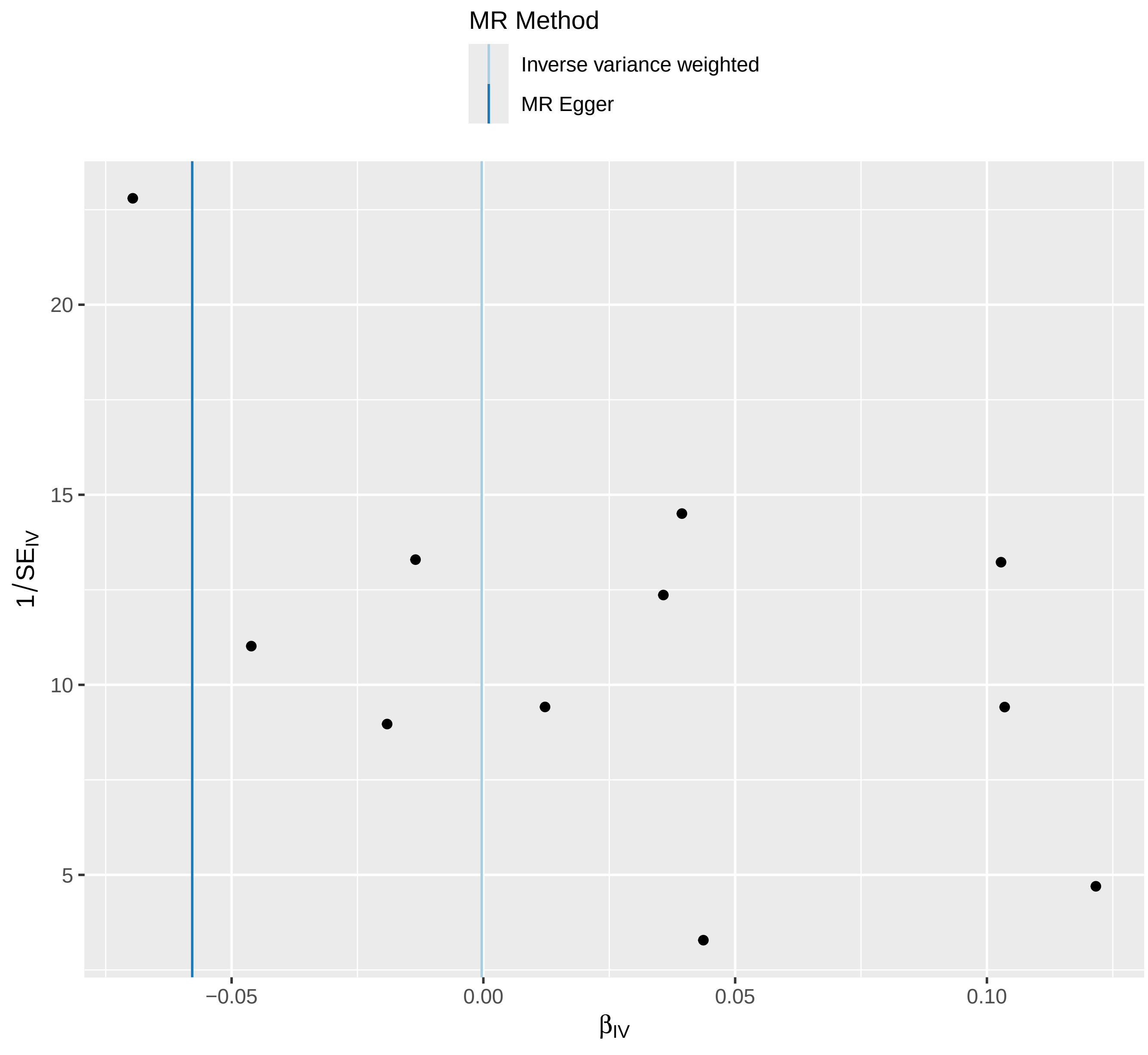

MR Method
Inverse variance weighted
MR Egger
1/SE$_{IV}$
20
15
10
5
−0.05
0.00
0.05
0.10
β$_{IV}$

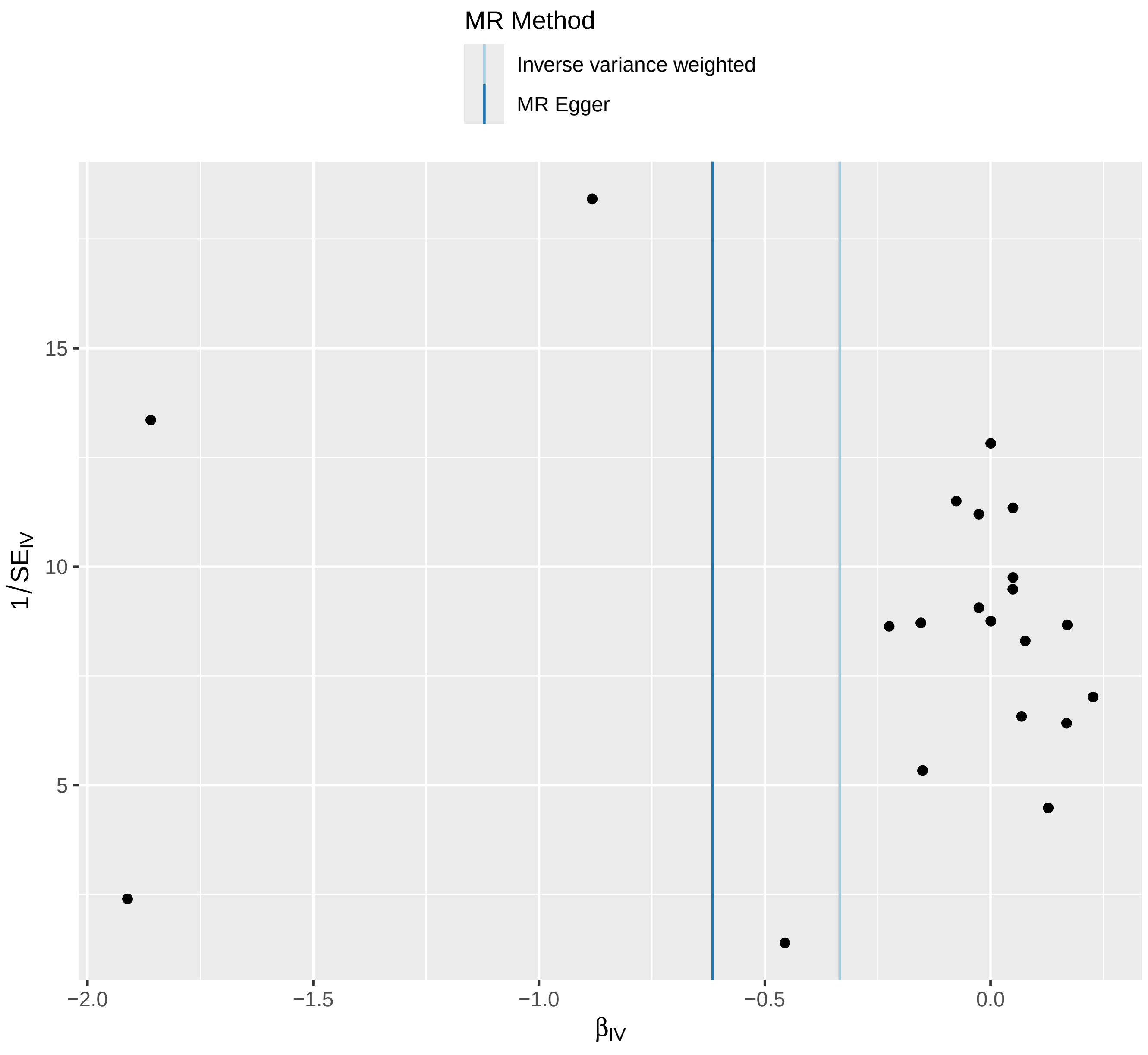

MR Method
Inverse variance weighted
MR Egger
15
10
5
1/SE$_{IV}$
−2.0
−1.5
−1.0
−0.5
0.0
β$_{IV}$

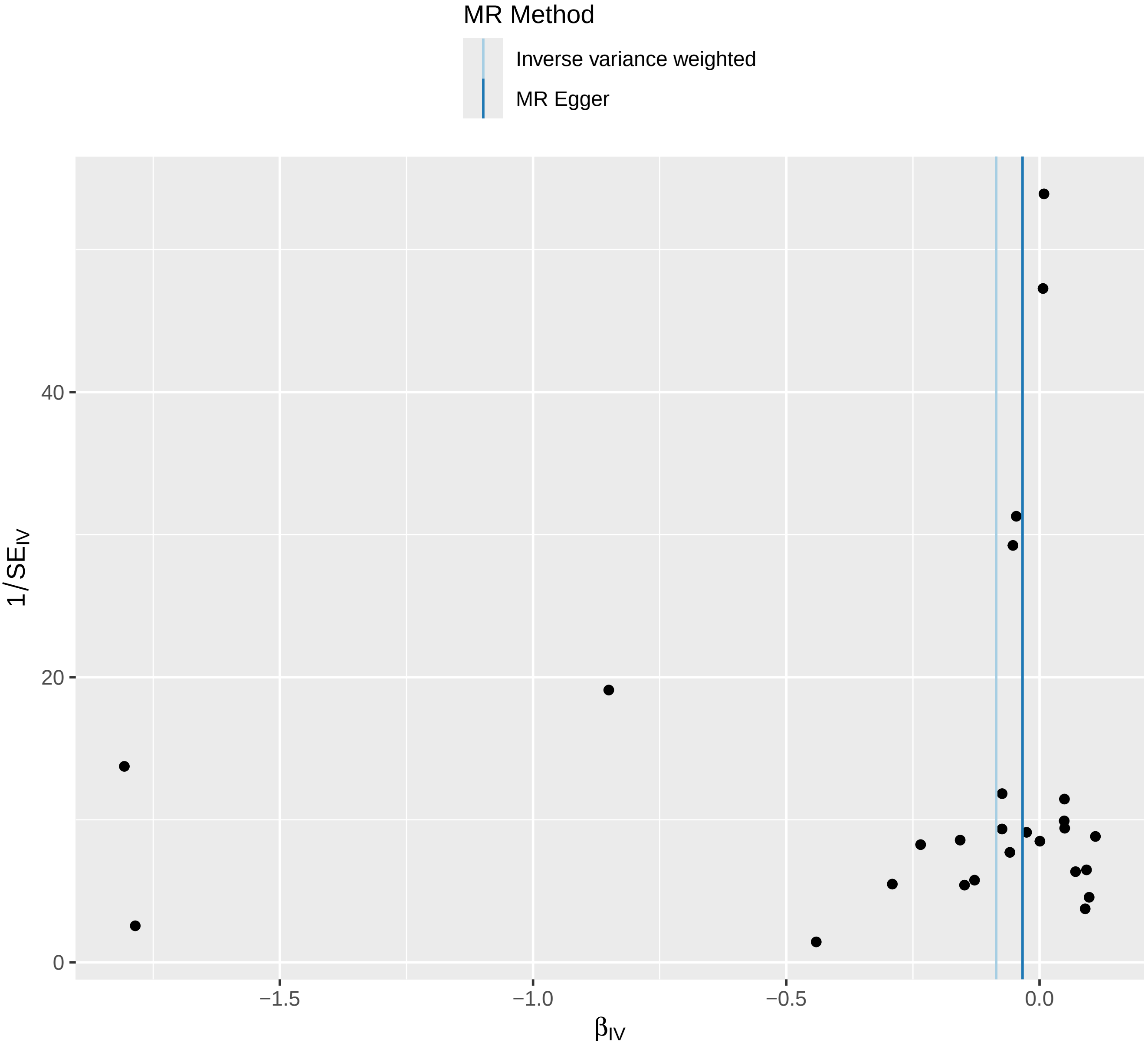

MR Method
Inverse variance weighted
MR Egger
1/SE$_{IV}$
β_{IV}
0
20
40
−1.5
−1.0
−0.5
0.0

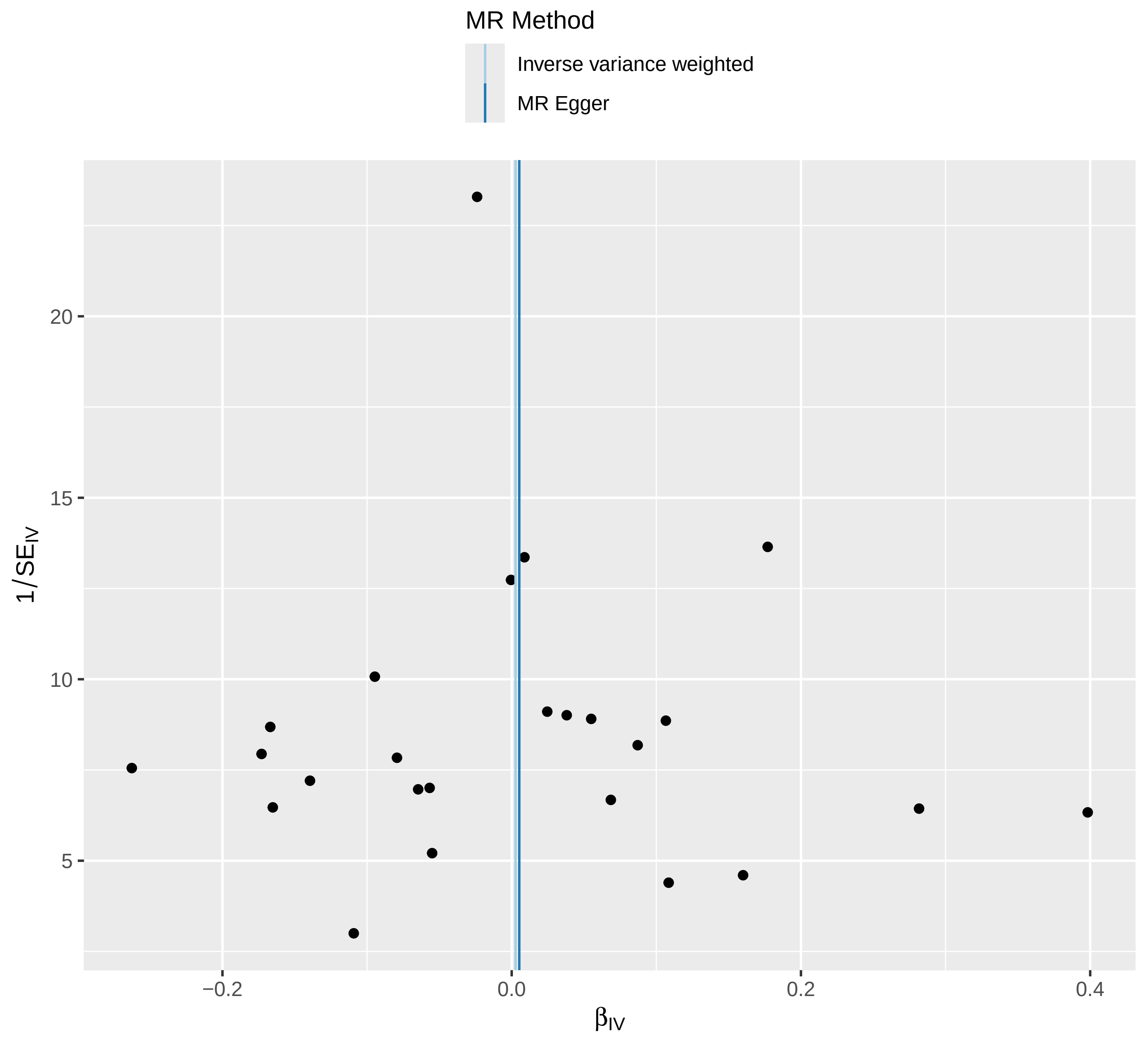
MR Method
Inverse variance weighted
MR Egger
1/SE$_{IV}$
20
15
10
5
−0.2
0.0
0.2
0.4
β$_{IV}$

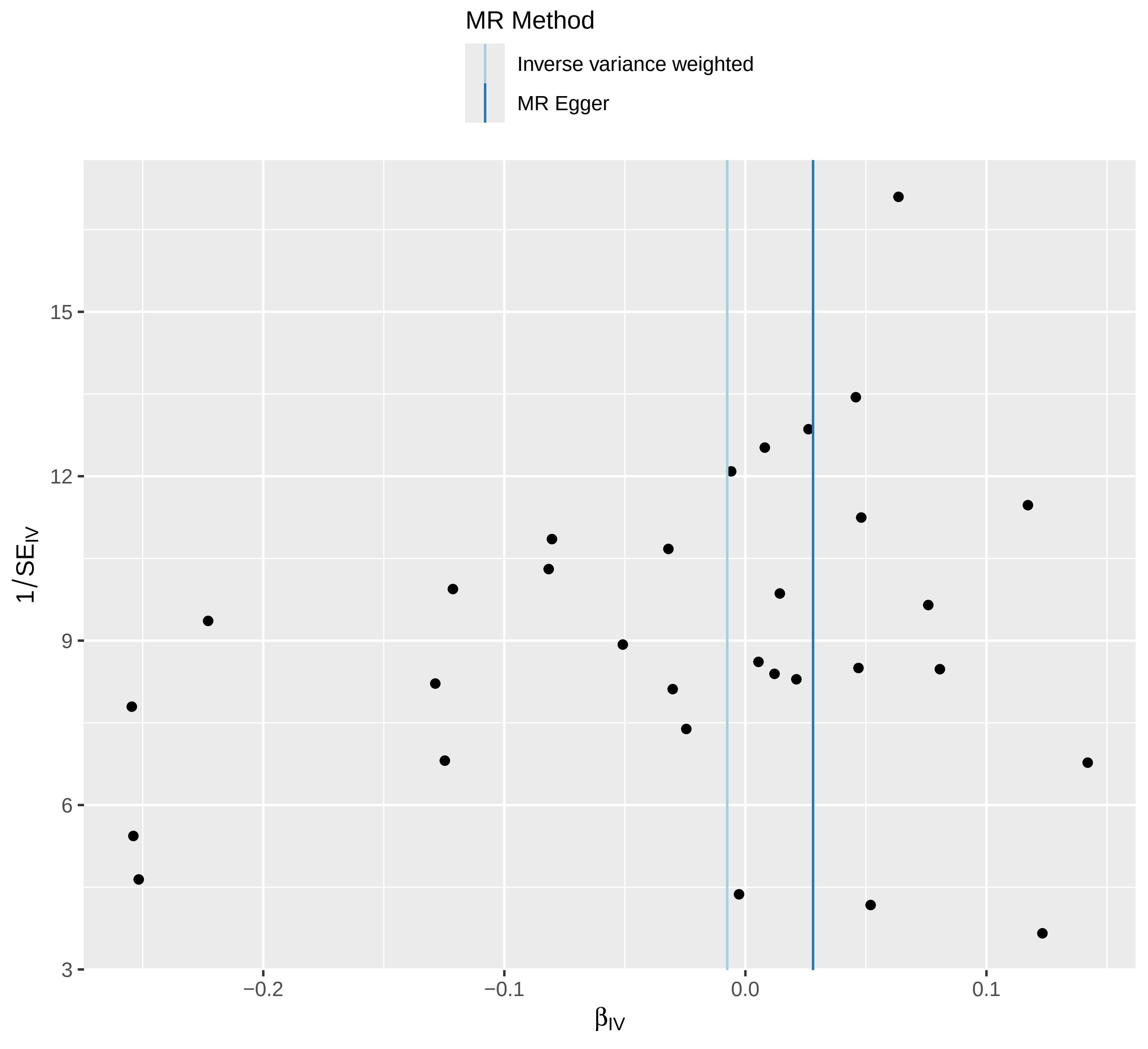
MR Method
Inverse variance weighted
MR Egger
1/SE_IV
15
12
9
6
3
−0.2
−0.1
0.0
0.1
β_IV

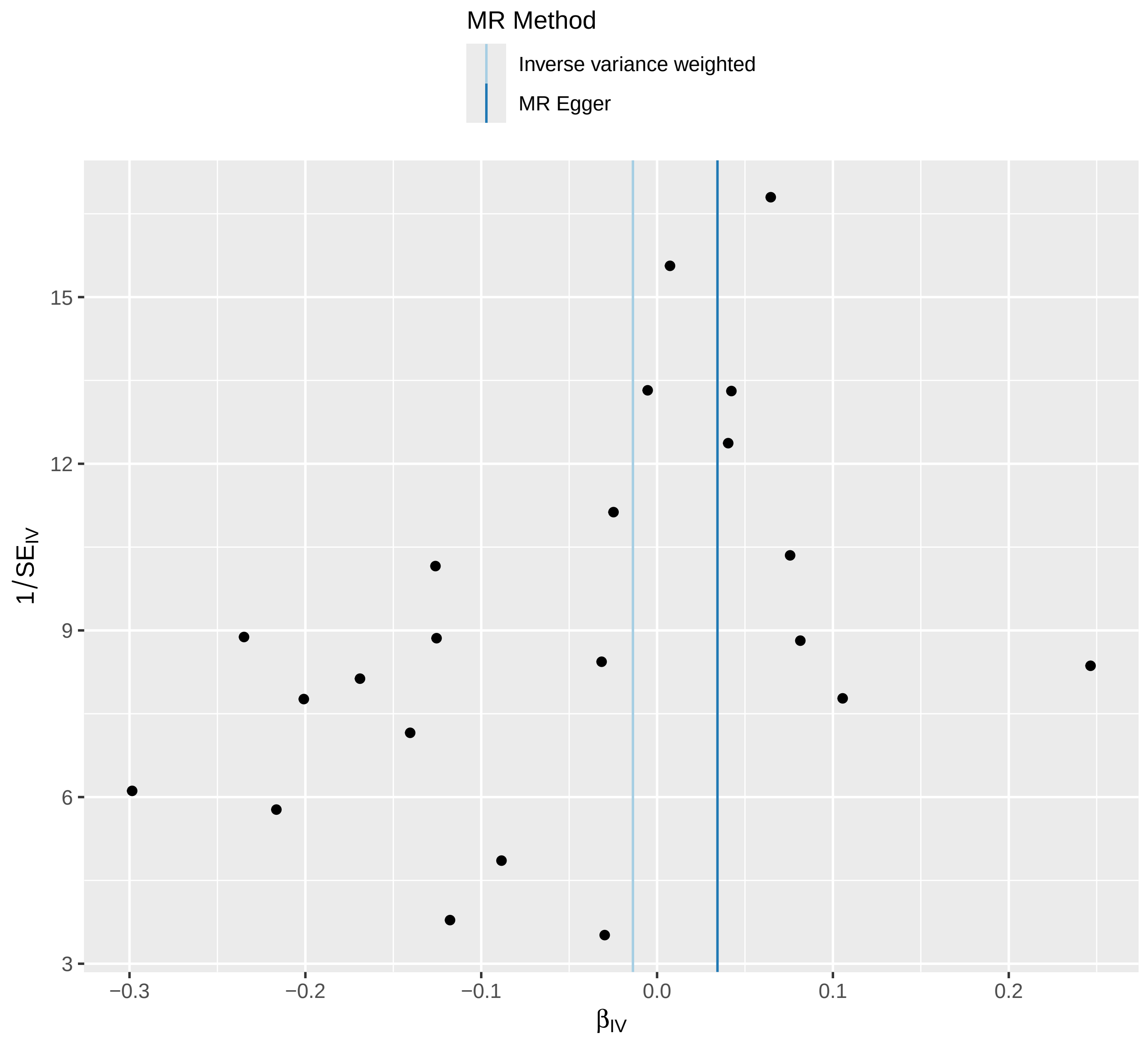

MR Method
Inverse variance weighted
MR Egger
15
12
9
6
3
1/SE$_{IV}$
−0.3
−0.2
−0.1
0.0
0.1
0.2
β$_{IV}$

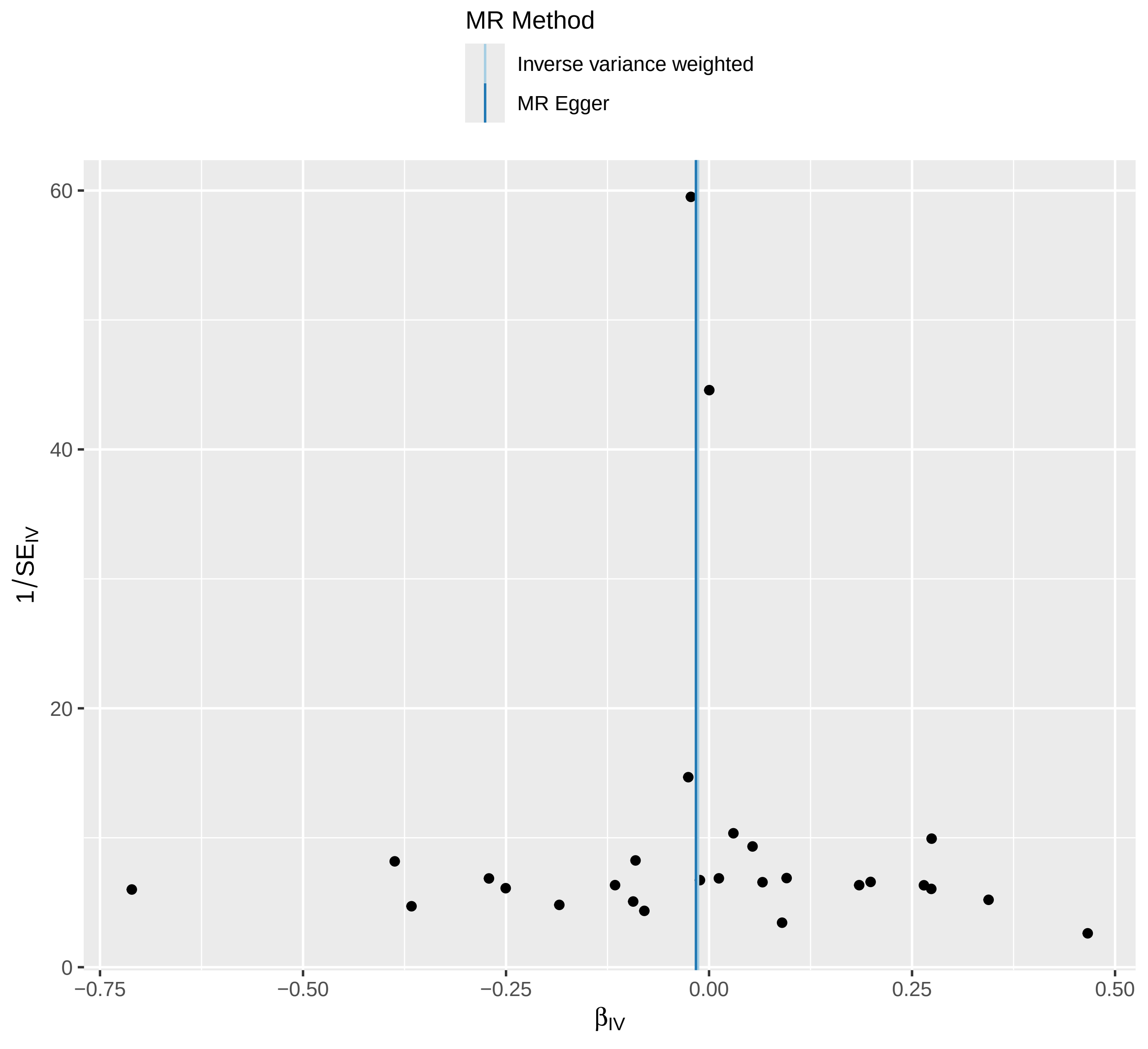

MR Method
Inverse variance weighted
MR Egger
60
40
20
0
1/SE$_{IV}$
−0.75
−0.50
−0.25
0.00
0.25
0.50
β$_{IV}$

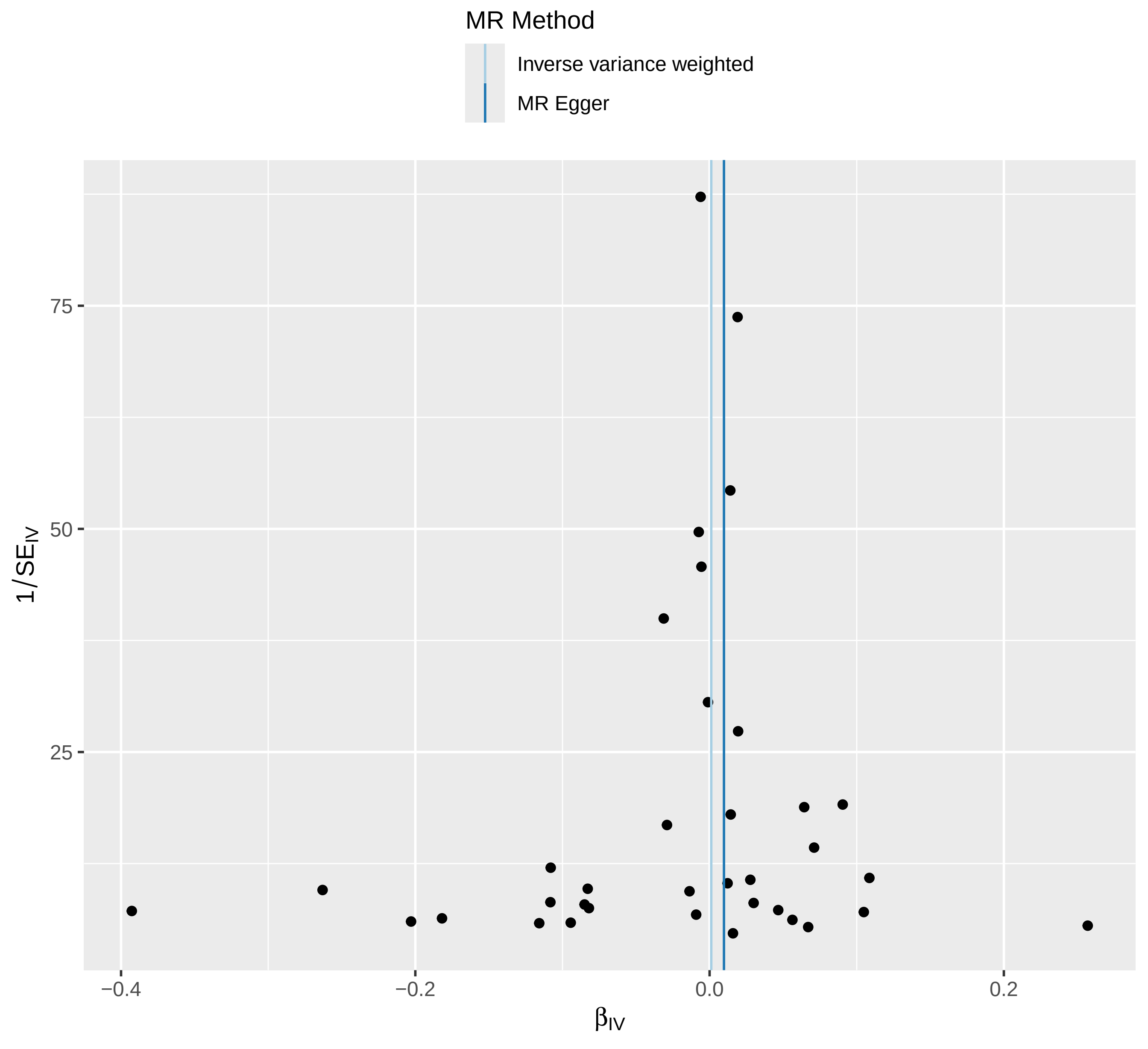

MR Method
Inverse variance weighted
MR Egger
75
50
25
1/SE_IV
−0.4
−0.2
0.0
0.2
β_IV

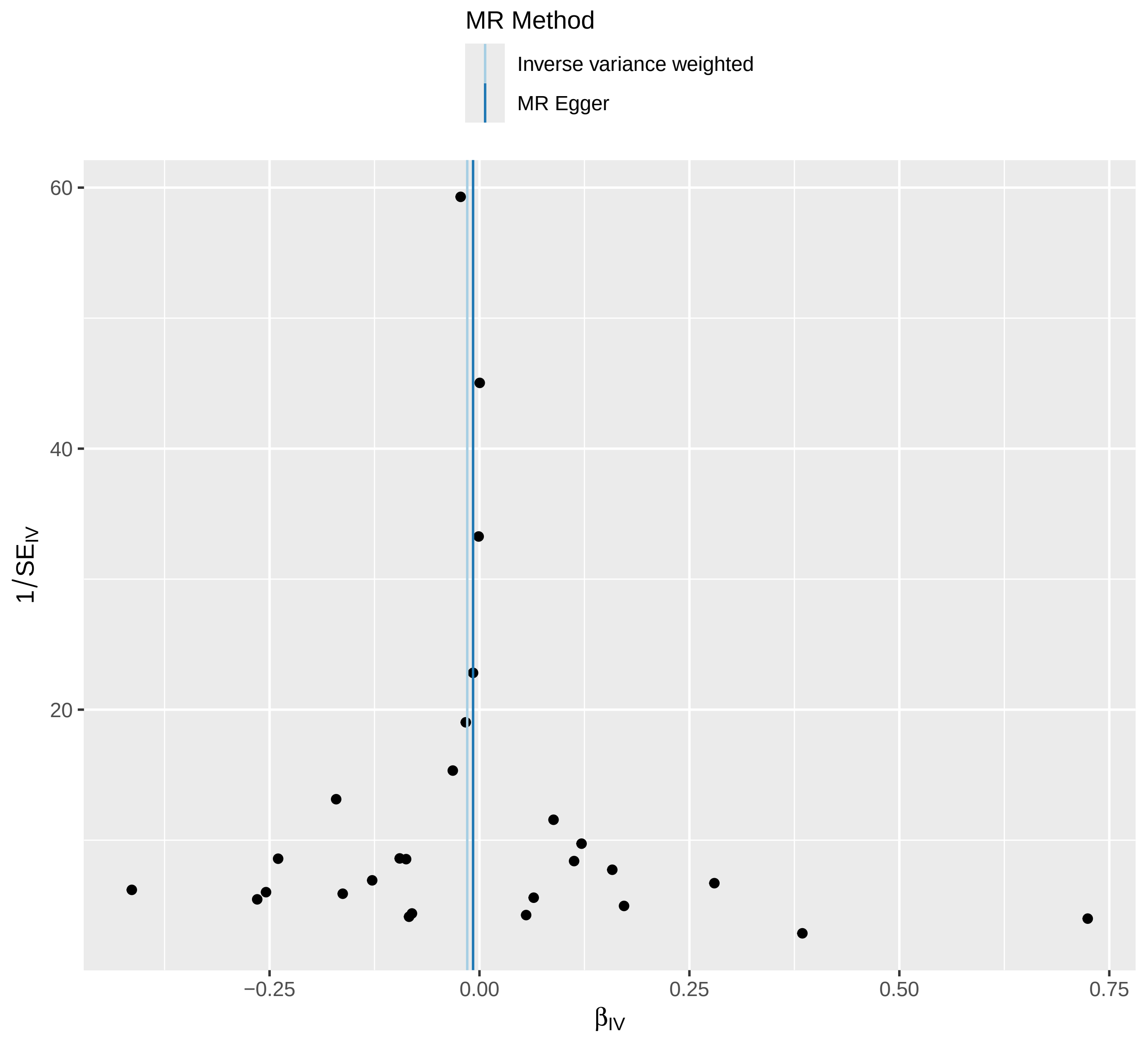
MR Method
Inverse variance weighted
MR Egger
60
40
20
1/SE$_{IV}$
−0.25
0.00
0.25
0.50
0.75
β$_{IV}$

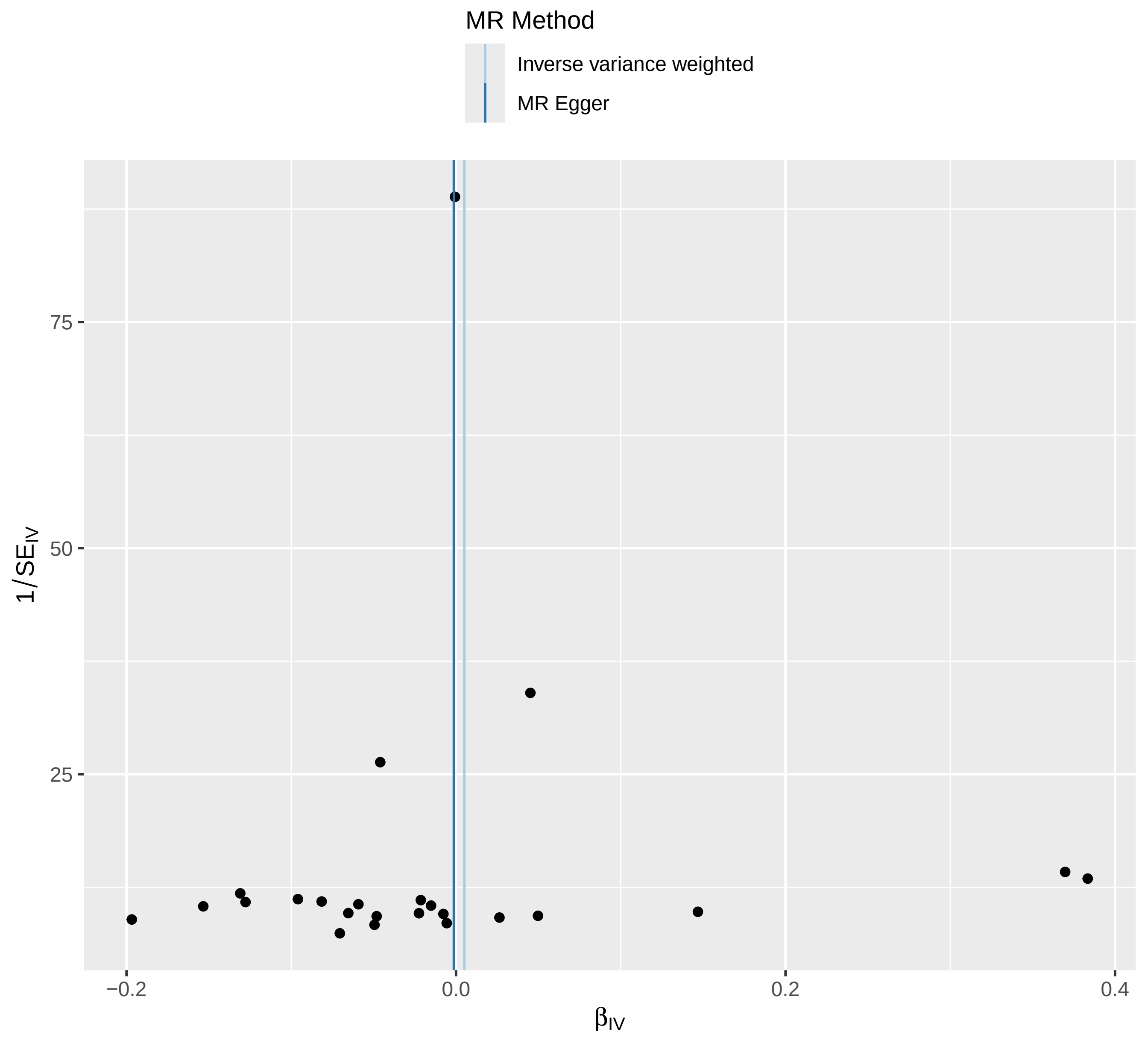

MR Method
Inverse variance weighted
MR Egger
75
50
25
1/SE$_{IV}$
−0.2
0.0
0.2
0.4
β$_{IV}$

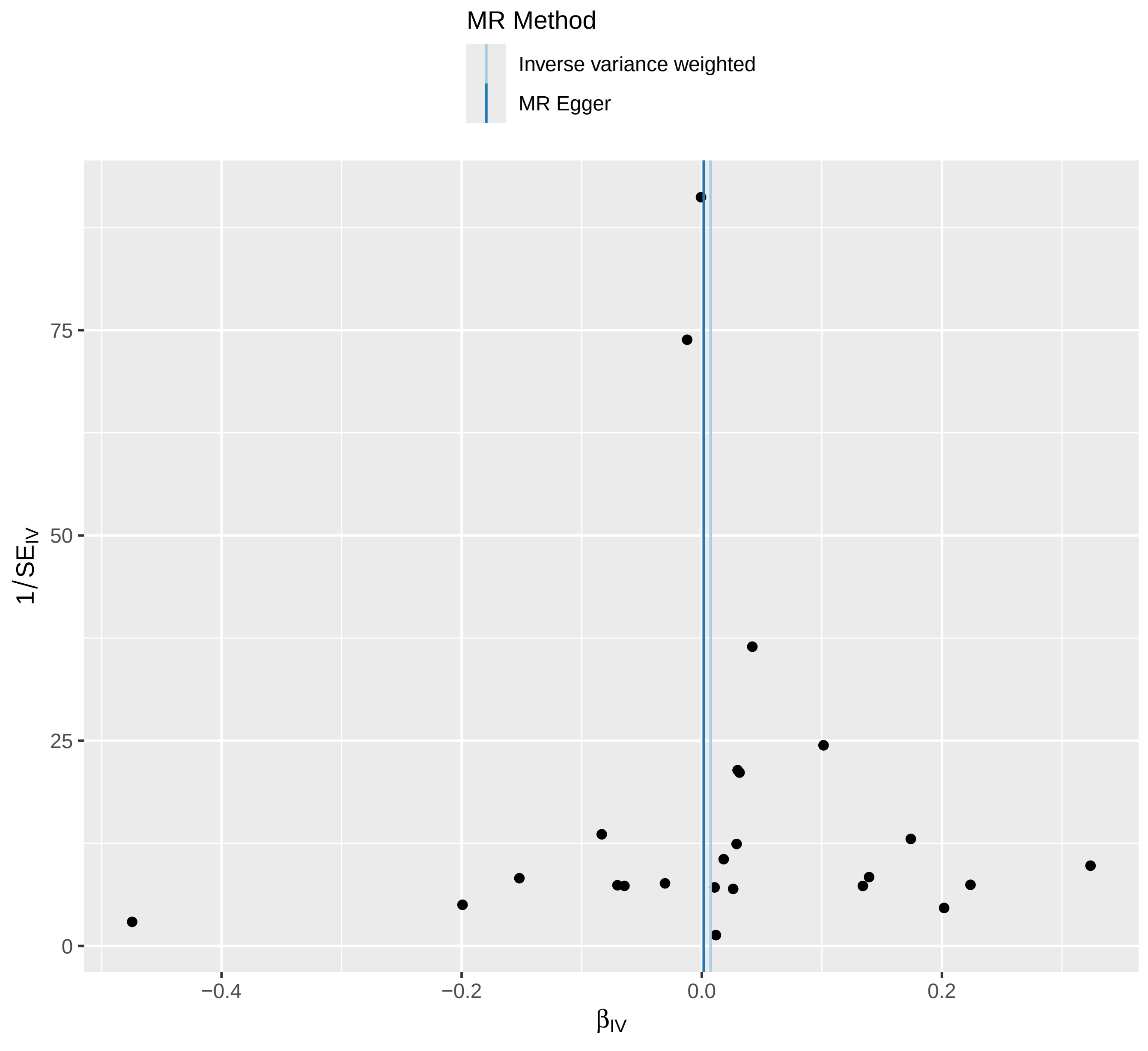
MR Method
Inverse variance weighted
MR Egger
75
50
25
0
1/SE$_{IV}$
−0.4
−0.2
0.0
0.2
β$_{IV}$

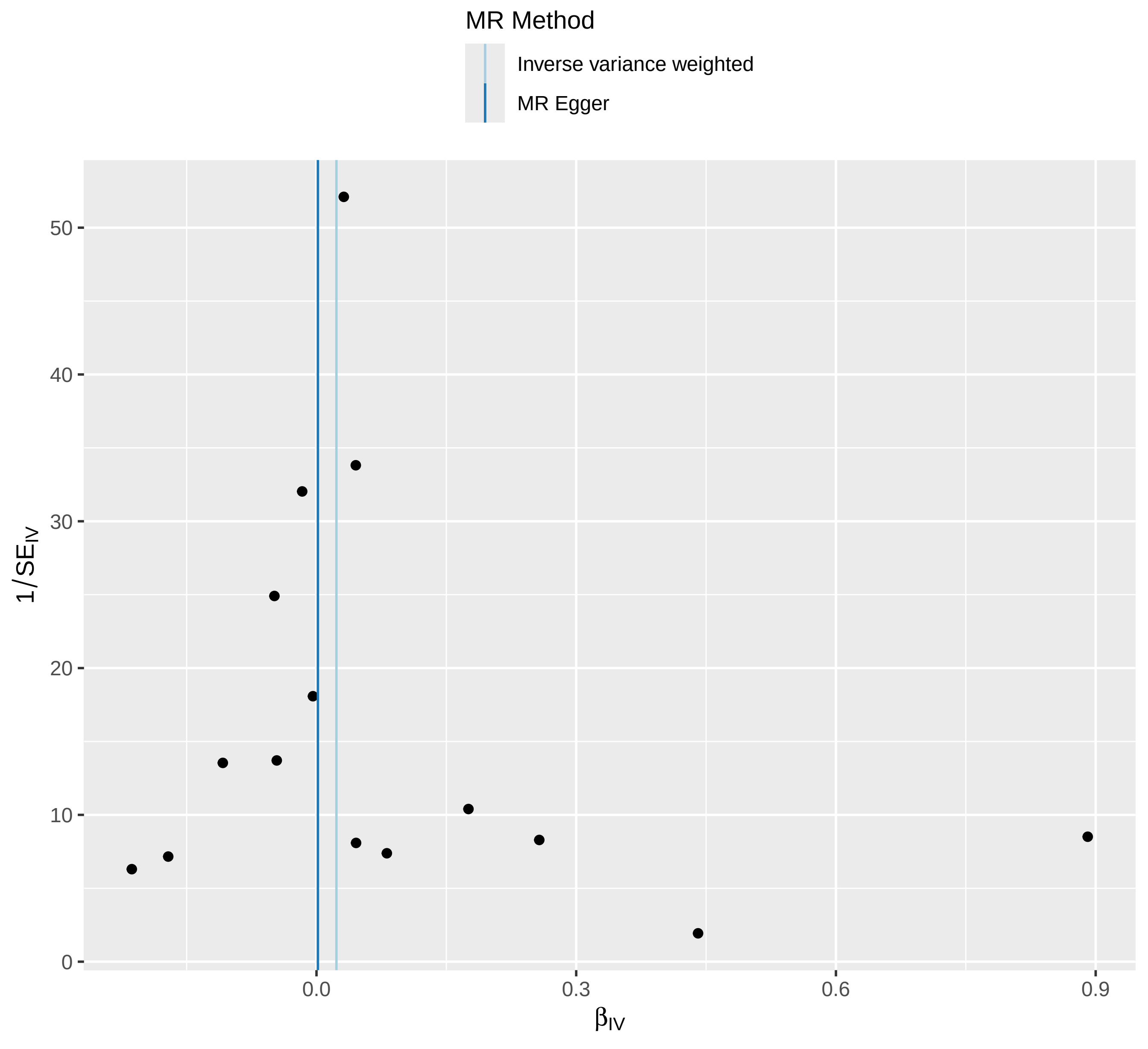

MR Method
Inverse variance weighted
MR Egger
50
40
30
20
10
0
0.0
0.3
0.6
0.9
1/SE_IV
β_IV

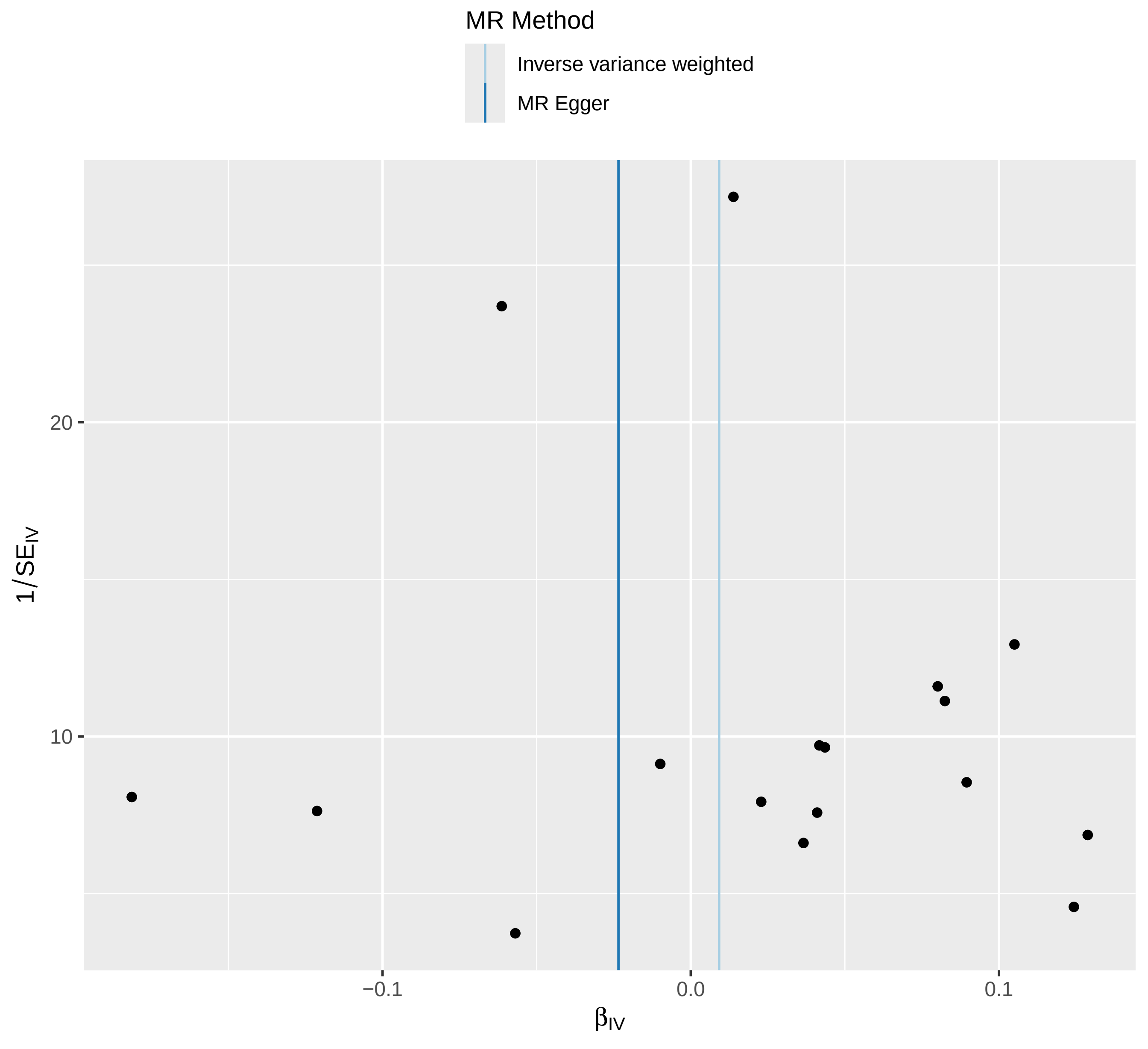

MR Method
Inverse variance weighted
MR Egger
20
10
1/SE_IV
−0.1
0.0
0.1
β_IV

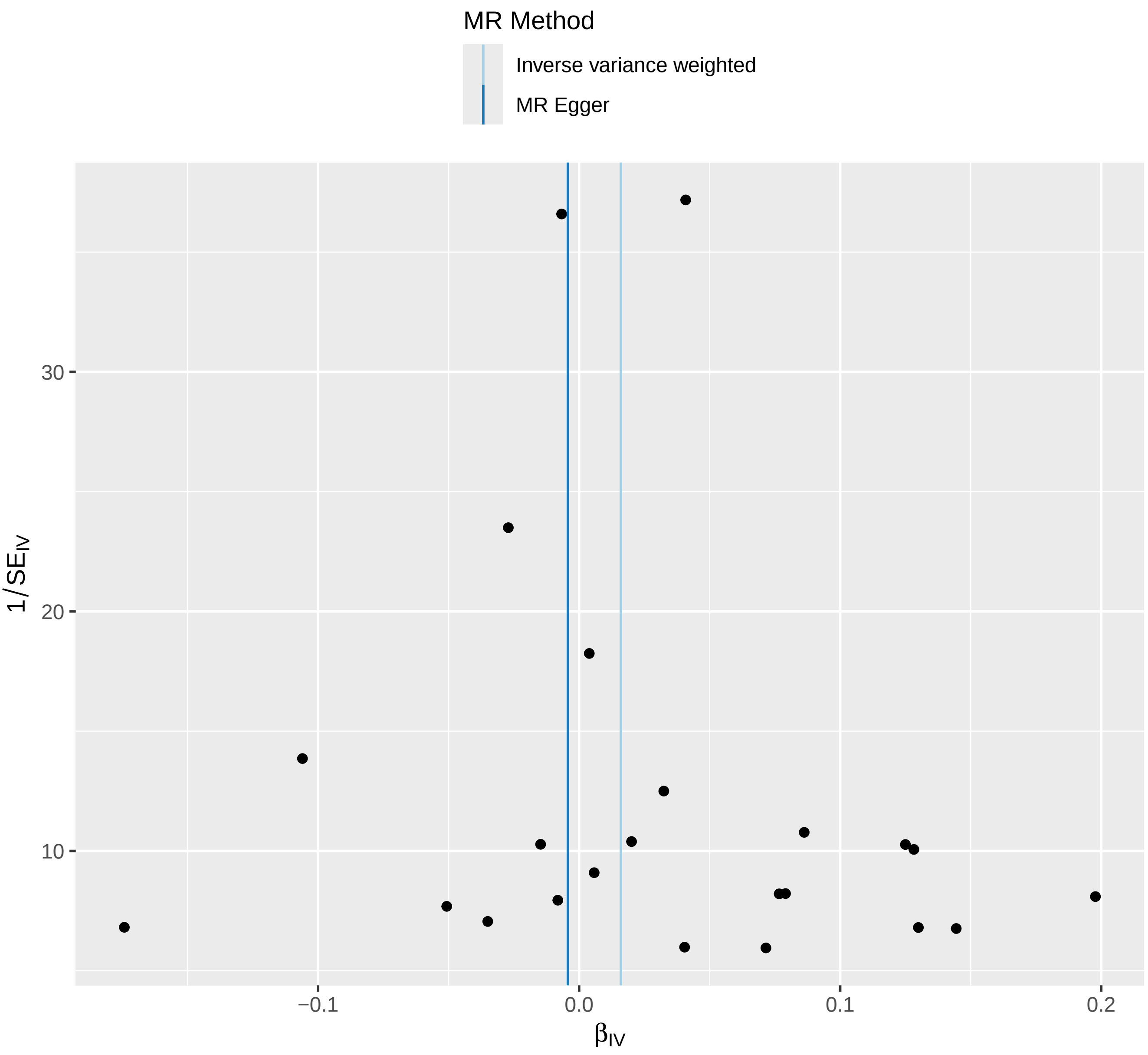
MR Method
Inverse variance weighted
MR Egger
1/SE$_{IV}$
30
20
10
−0.1
0.0
0.1
0.2
β$_{IV}$

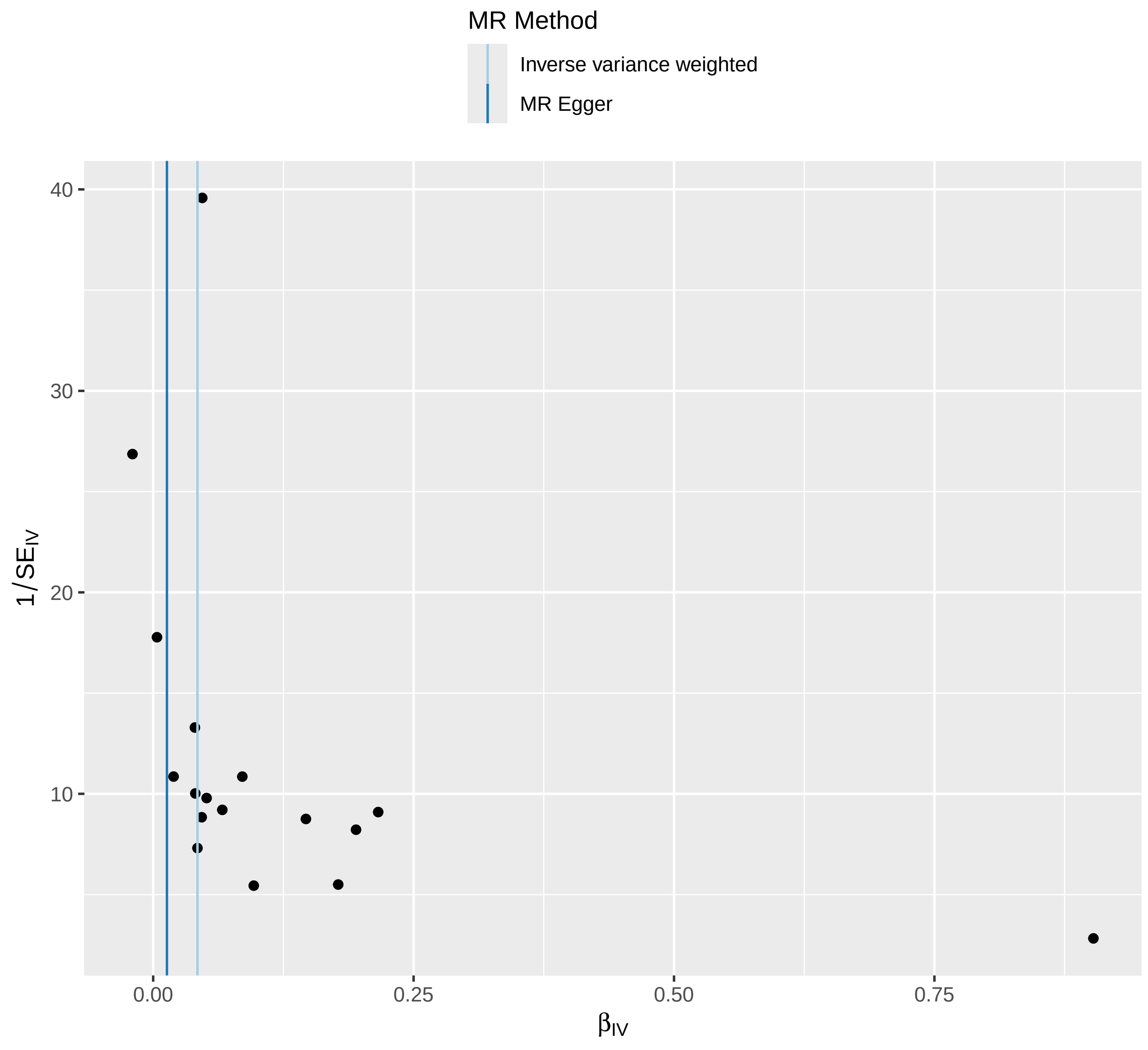
MR Method
Inverse variance weighted
MR Egger
40
30
20
10
1/SE_IV
0.00
0.25
0.50
0.75
β_IV

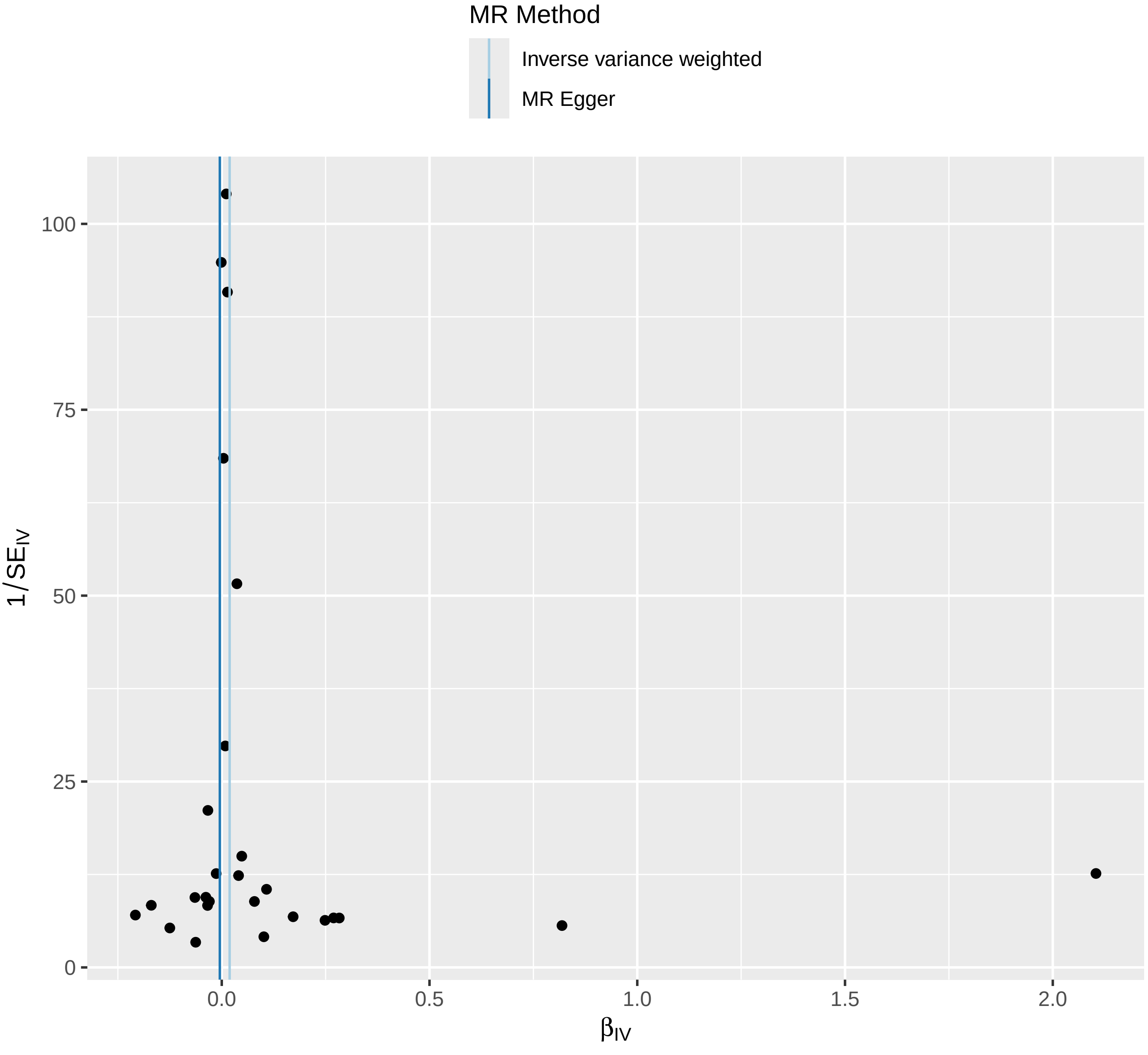
MR Method
Inverse variance weighted
MR Egger
100
75
50
25
0
0.0
0.5
1.0
1.5
2.0
1/SE_IV
β_IV

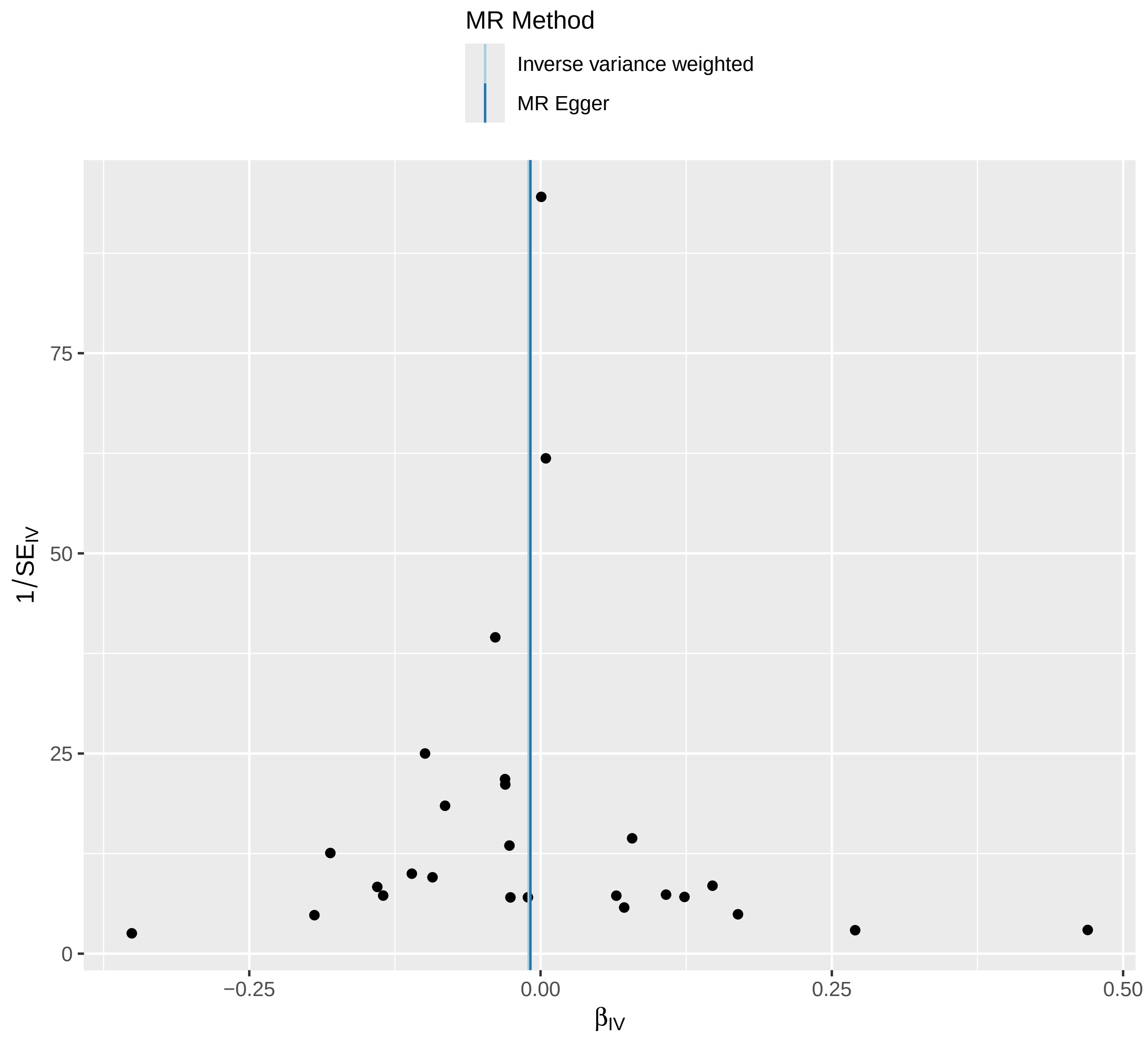
MR Method
Inverse variance weighted
MR Egger
1/SE$_{IV}$
0
25
50
75
−0.25
0.00
0.25
0.50
β$_{IV}$

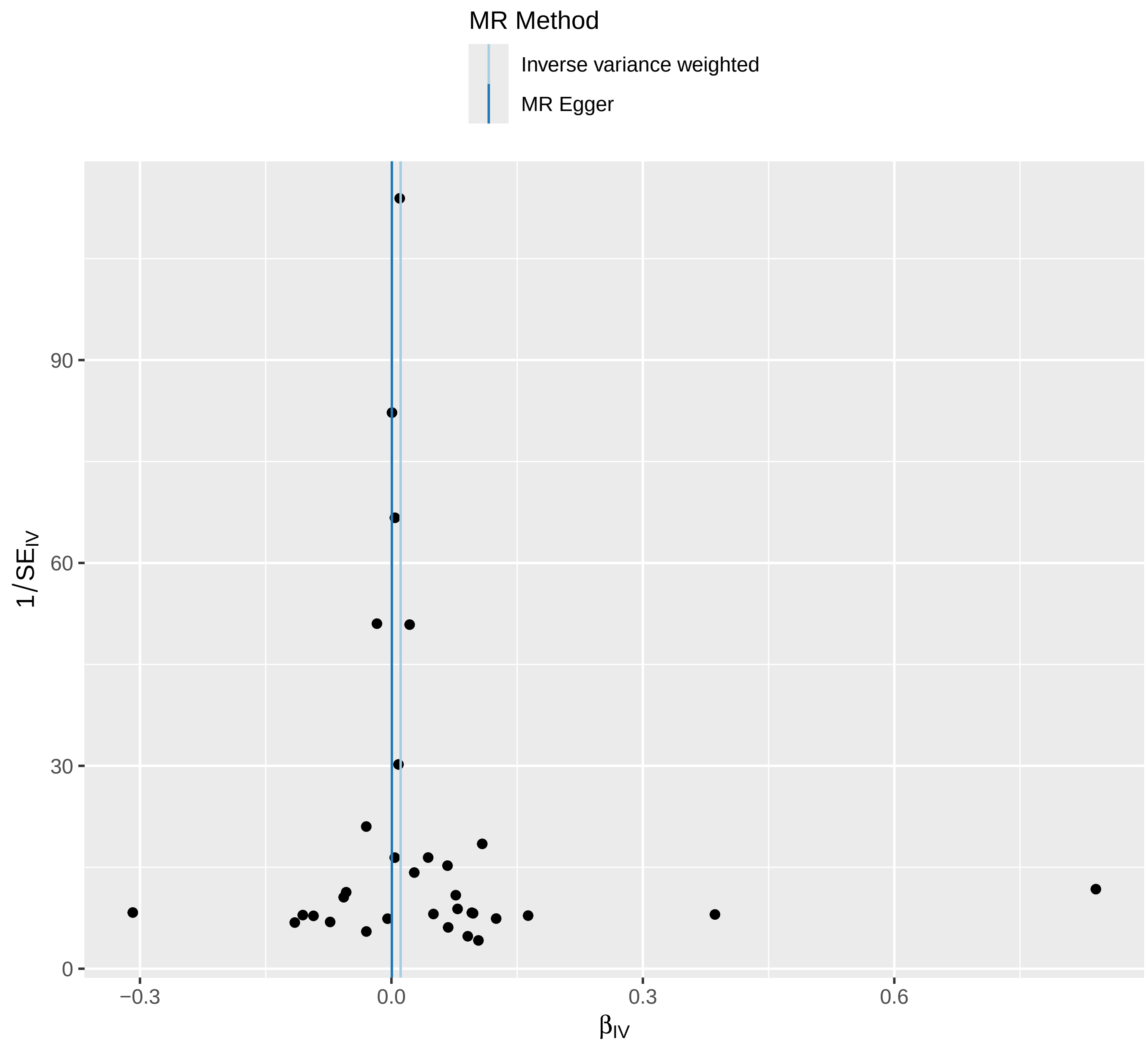
MR Method
Inverse variance weighted
MR Egger
90
60
30
0
1/SE$_{IV}$
−0.3
0.0
0.3
0.6
β$_{IV}$

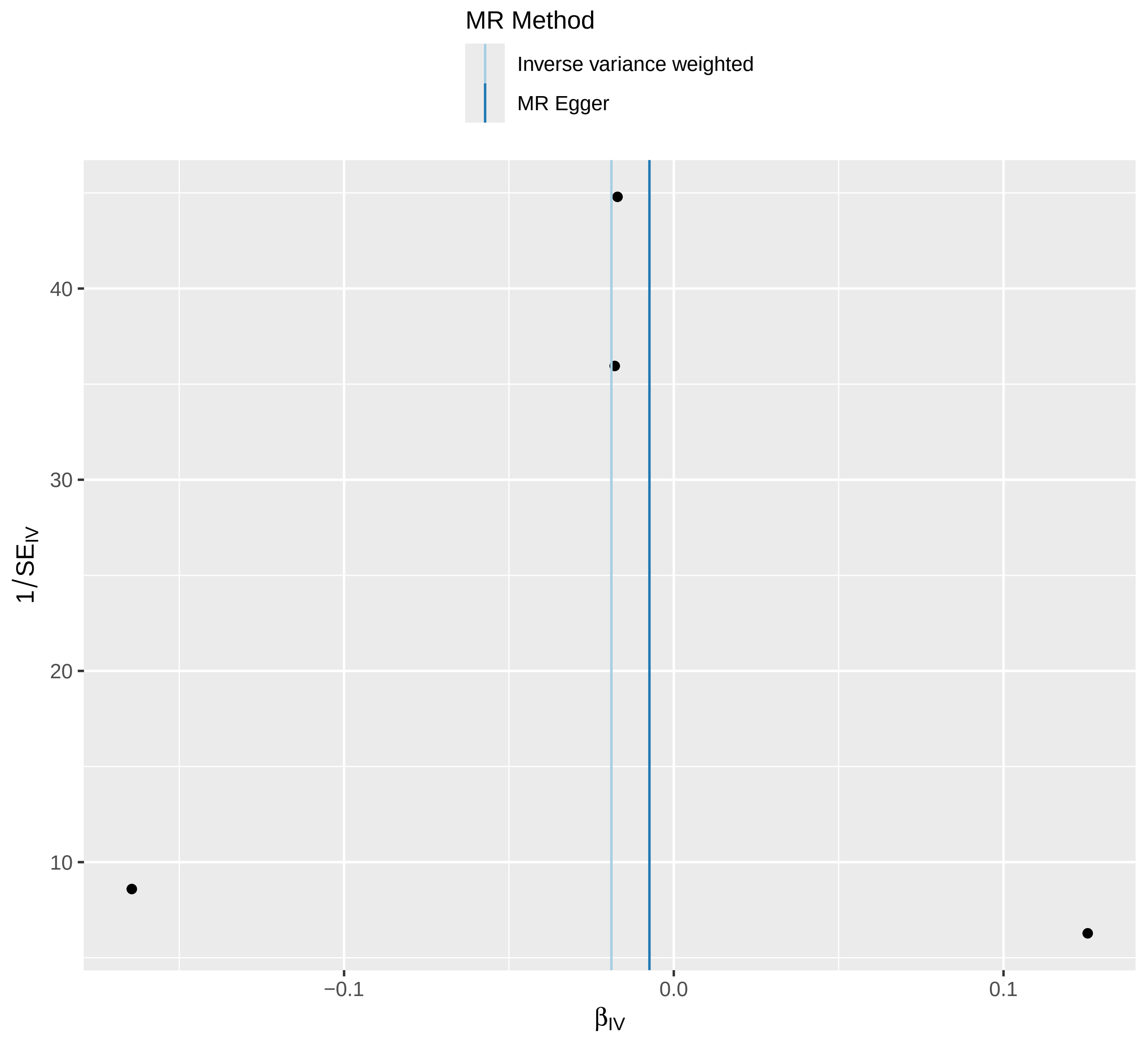
MR Method
Inverse variance weighted
MR Egger
40
30
20
10
1/SE_IV
−0.1
0.0
0.1
β_IV

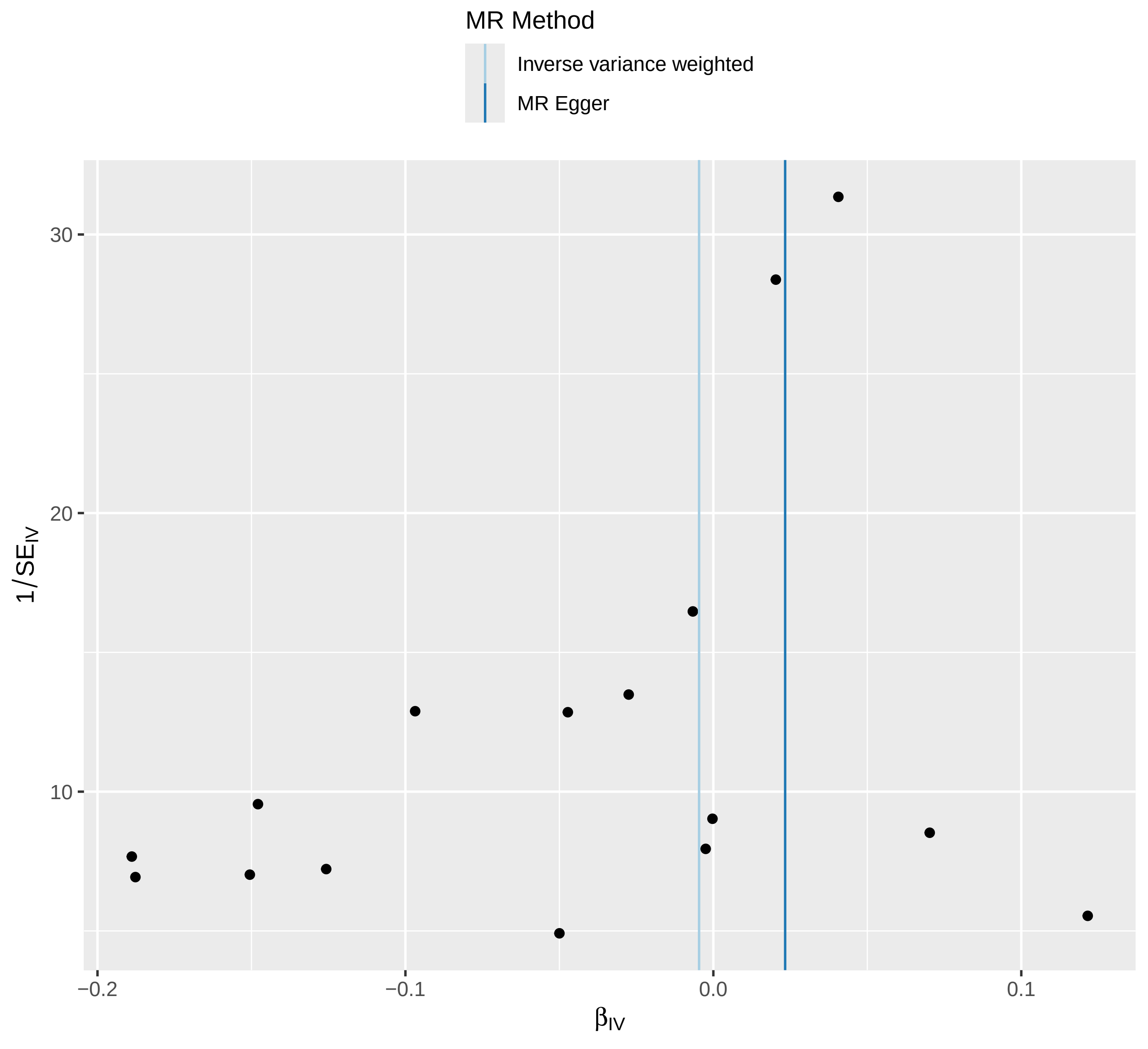

MR Method
Inverse variance weighted
MR Egger
30
20
10
1/SE$_{IV}$
−0.2
−0.1
0.0
0.1
β$_{IV}$

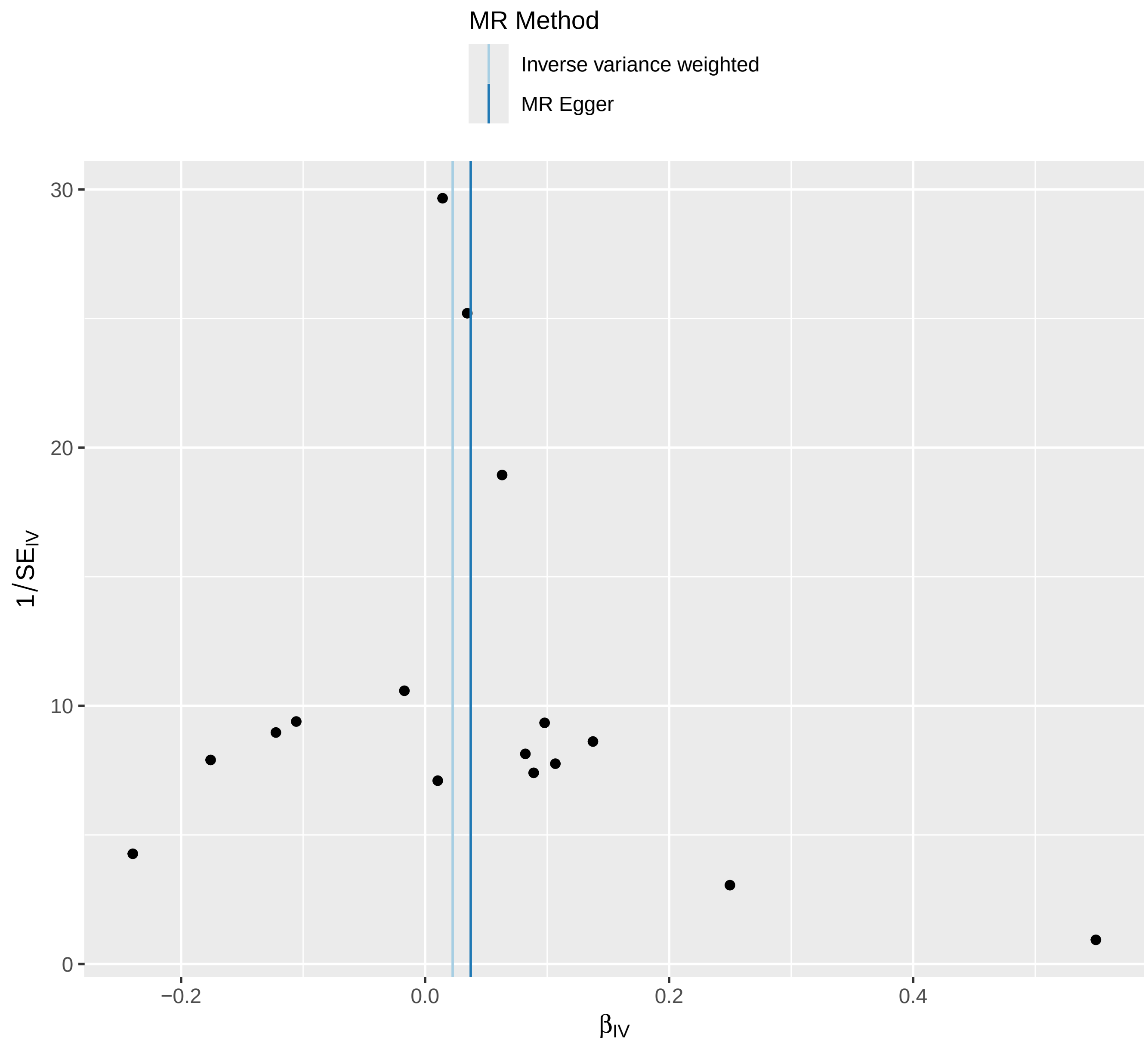
MR Method
Inverse variance weighted
MR Egger
30
20
10
0
1/SE_IV
−0.2
0.0
0.2
0.4
β_IV

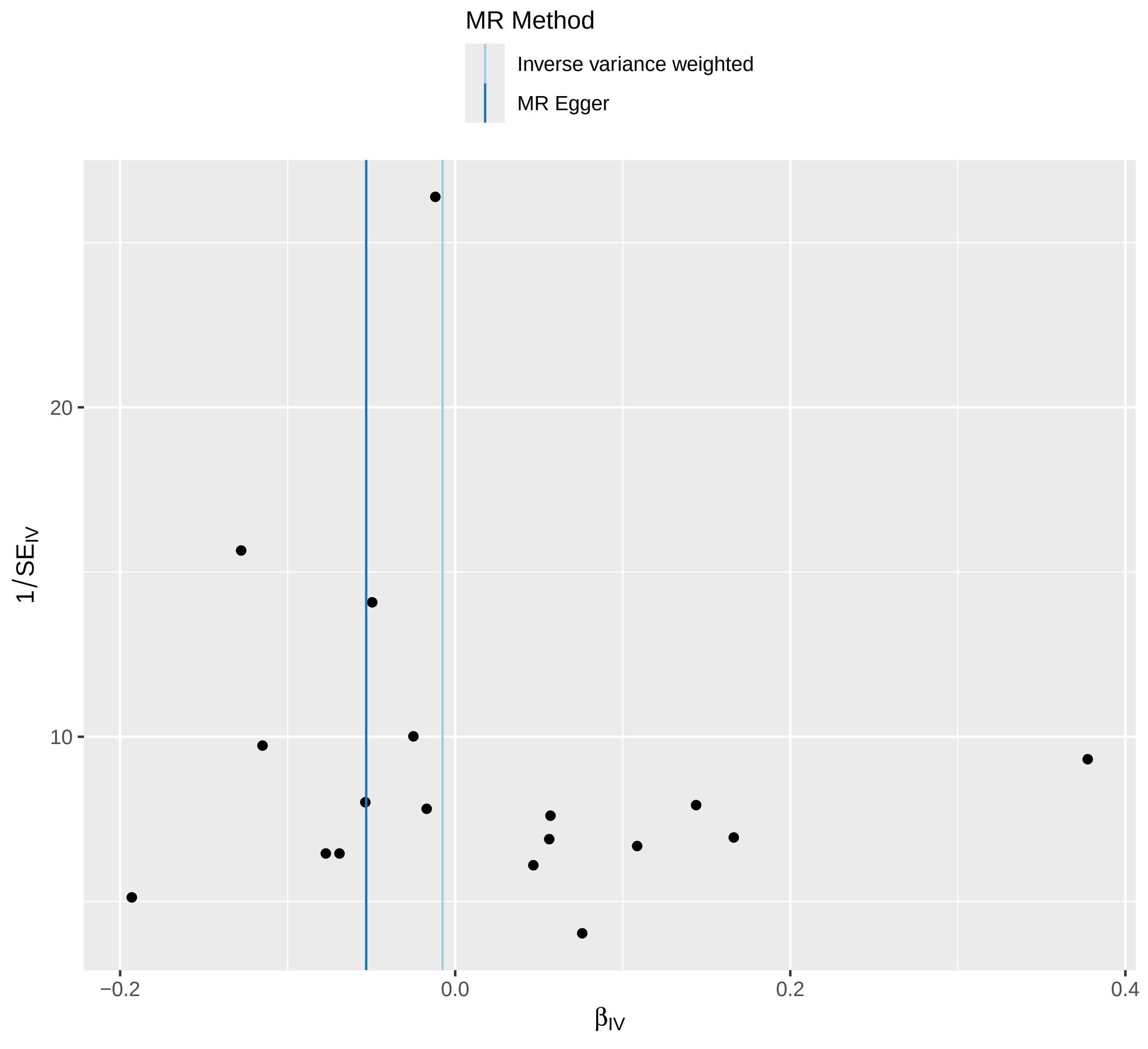
MR Method
Inverse variance weighted
MR Egger
20
10
1/SE_IV
−0.2
0.0
0.2
0.4
β_IV

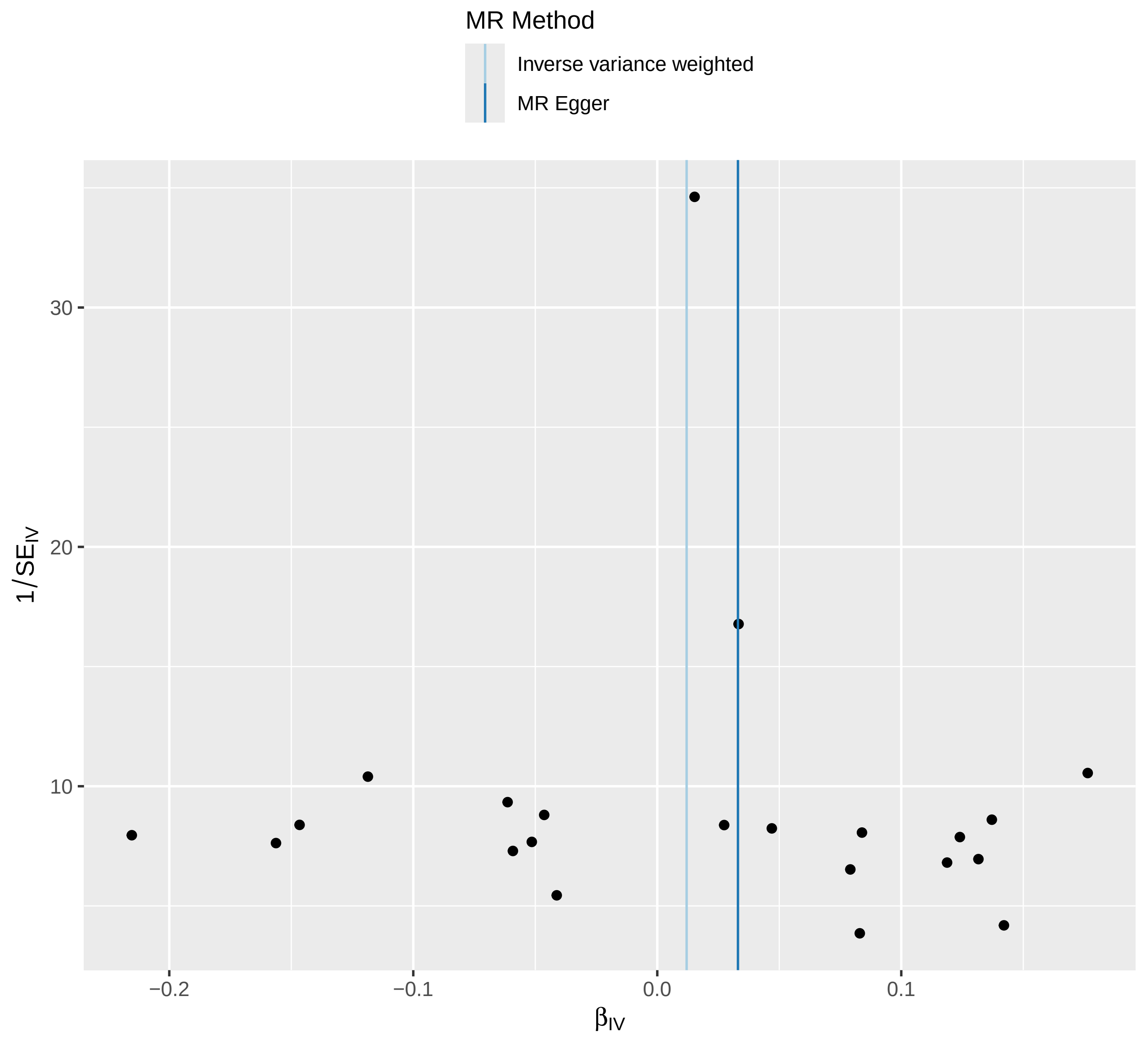

MR Method
Inverse variance weighted
MR Egger
30
20
10
1/SE_IV
−0.2
−0.1
0.0
0.1
β_IV

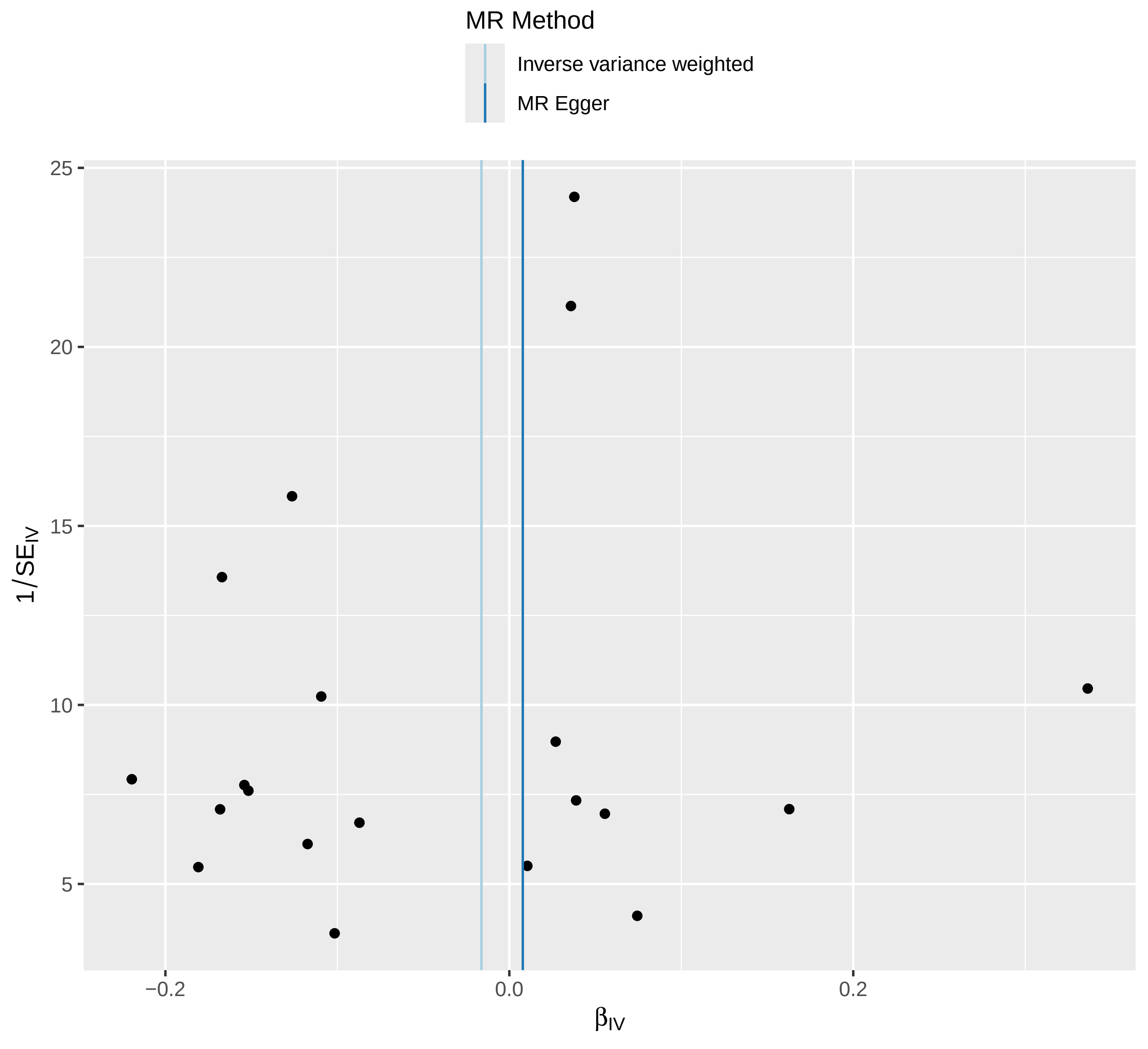
MR Method
Inverse variance weighted
MR Egger
25
20
15
10
5
1/SE_IV
−0.2
0.0
0.2
β_IV

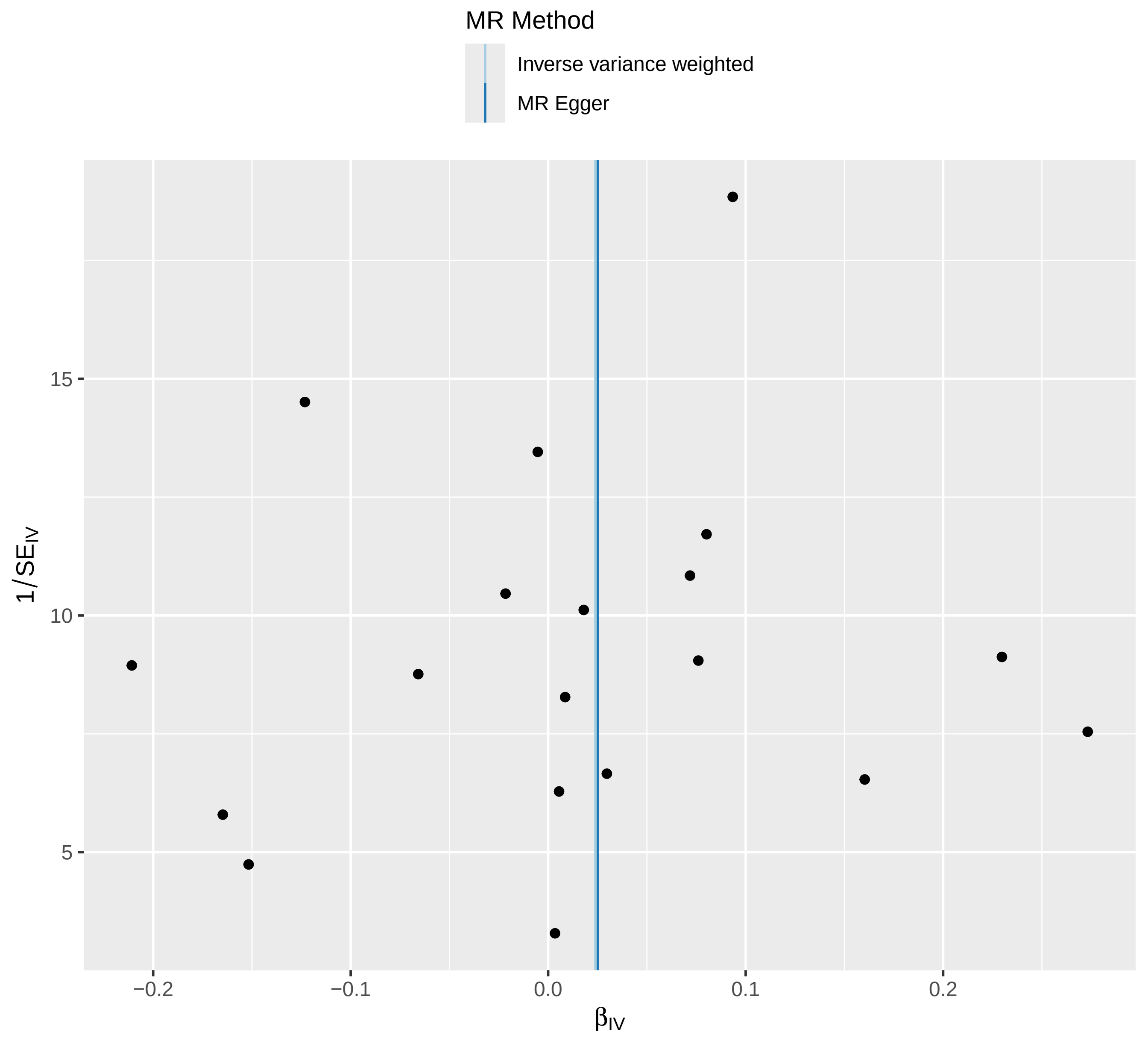

MR Method
Inverse variance weighted
MR Egger
15
10
5
1/SE$_{IV}$
−0.2
−0.1
0.0
0.1
0.2
β$_{IV}$

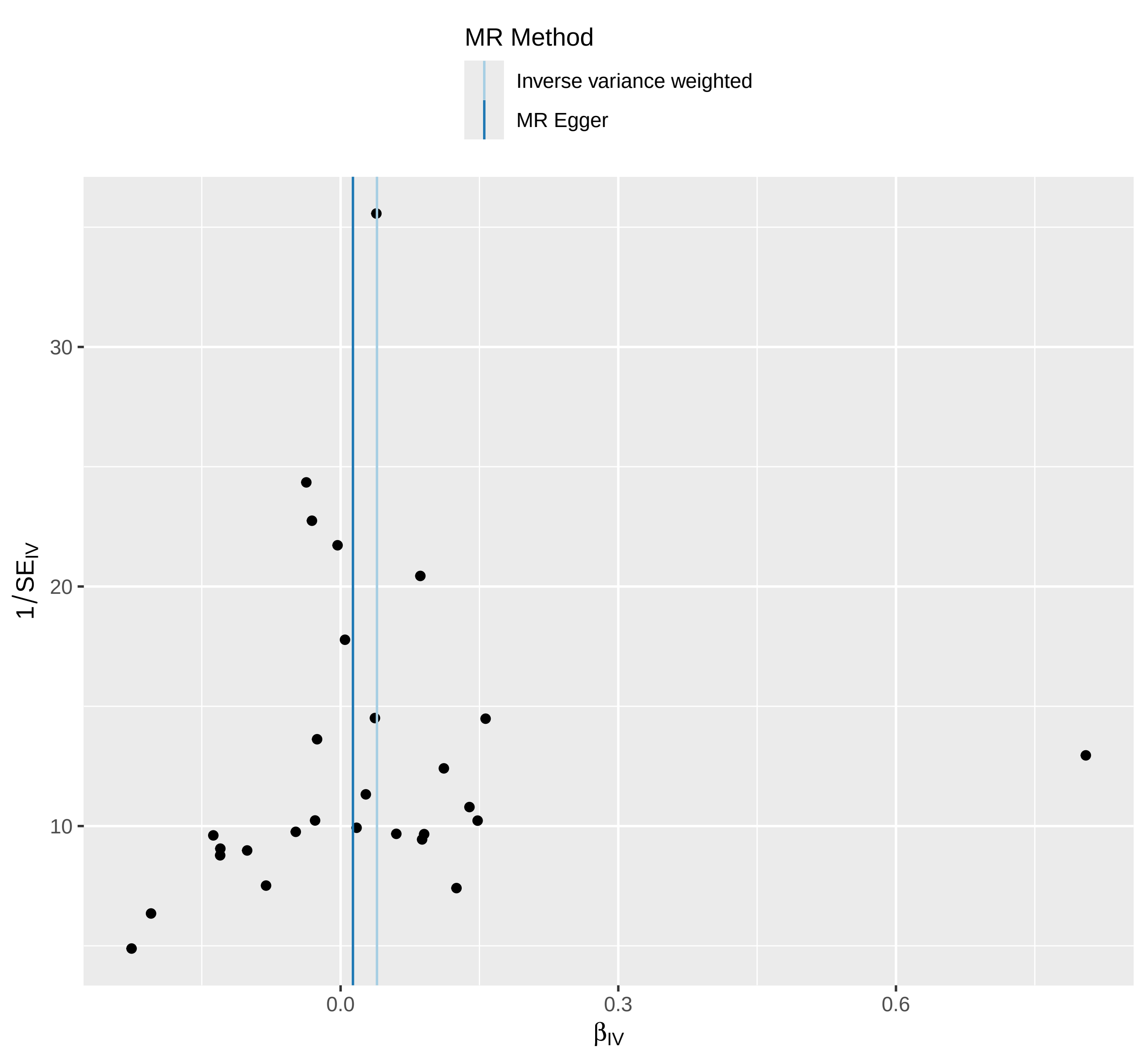
MR Method
Inverse variance weighted
MR Egger
30
20
10
1/SE$_{IV}$
0.0
0.3
0.6
β$_{IV}$

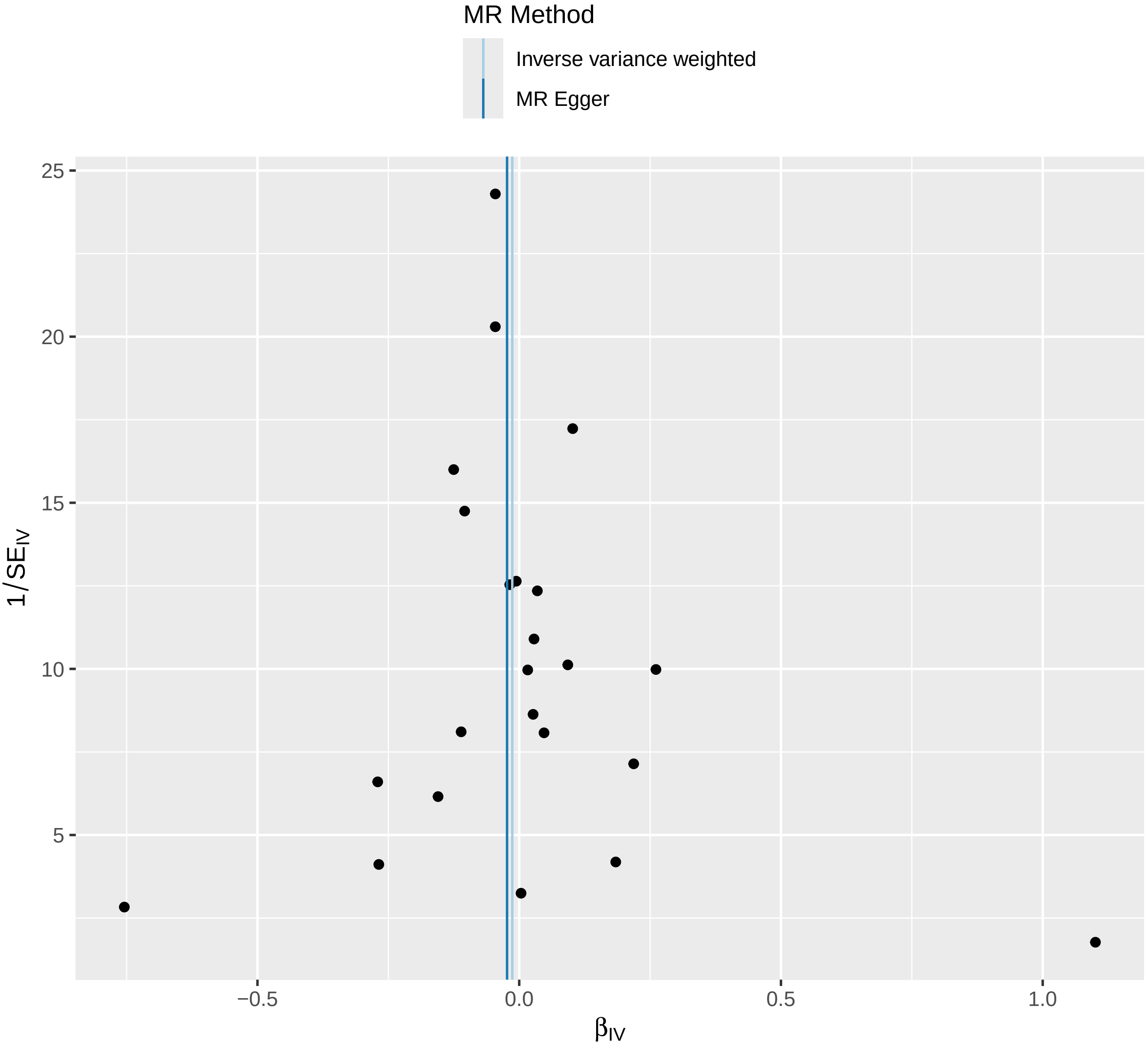
MR Method
Inverse variance weighted
MR Egger
25
20
15
10
5
1/SE$_{IV}$
−0.5
0.0
0.5
1.0
β$_{IV}$

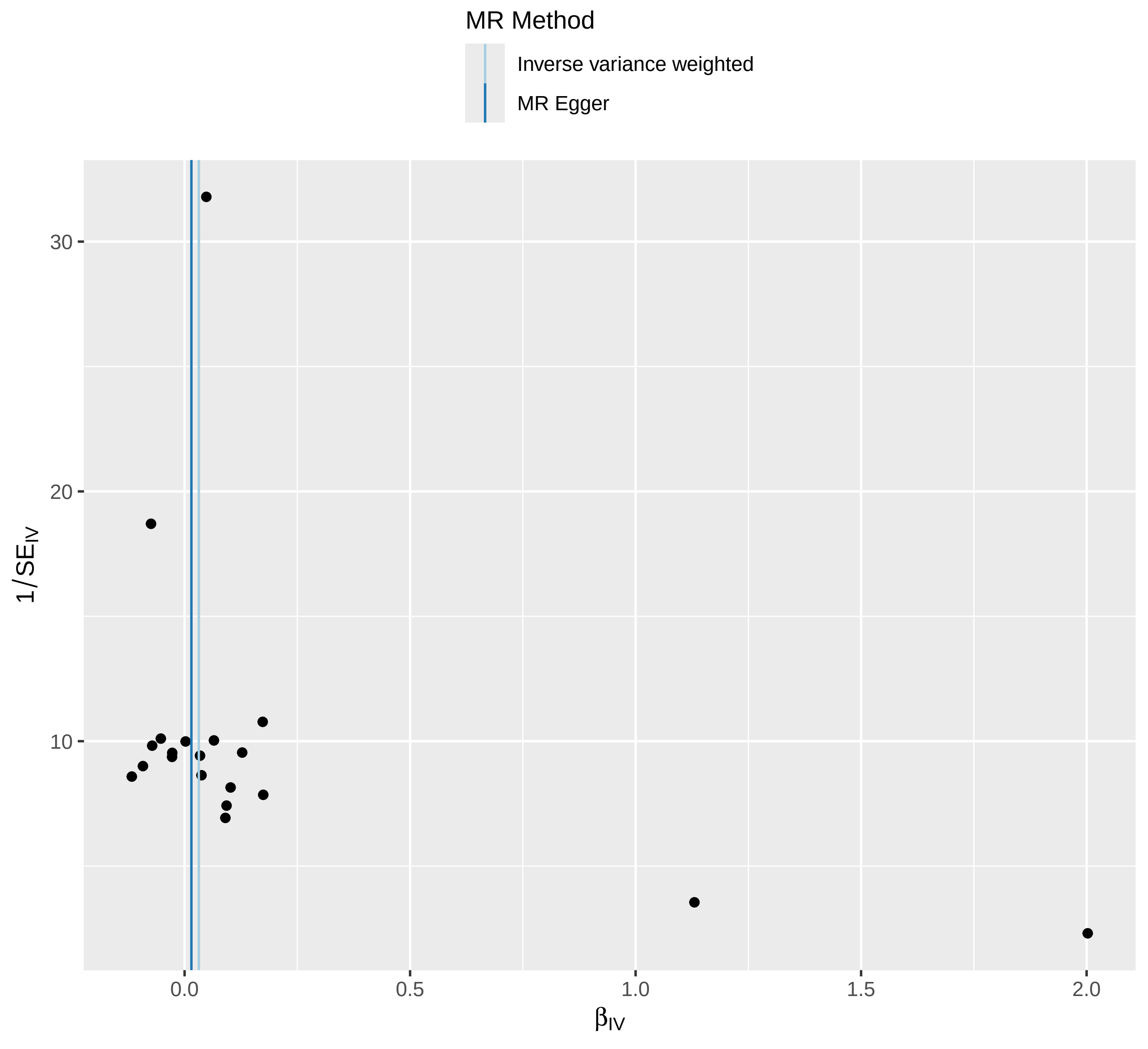
MR Method
Inverse variance weighted
MR Egger
30
20
10
1/SE_IV
0.0
0.5
1.0
1.5
2.0
β_IV

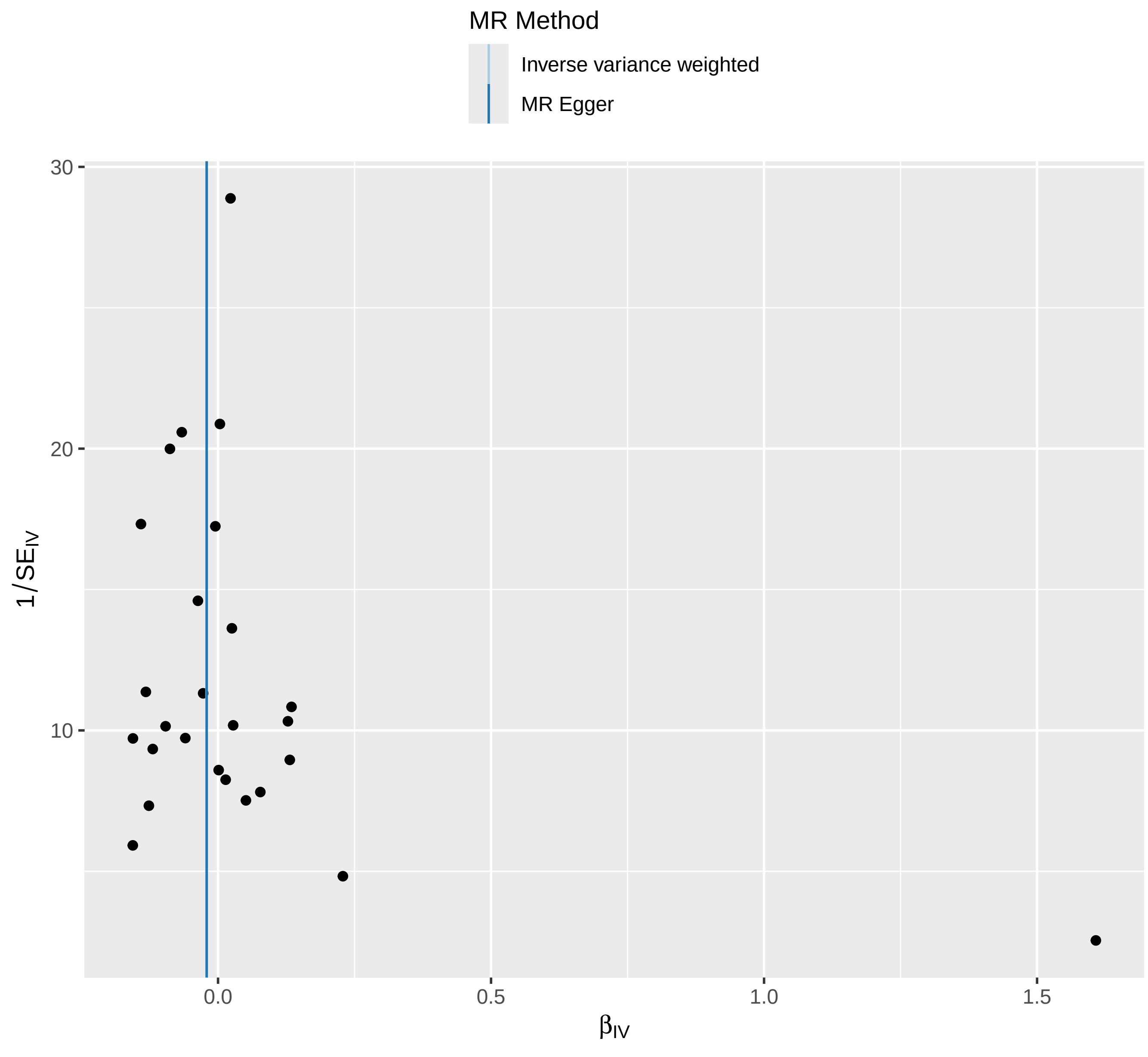
MR Method
Inverse variance weighted
MR Egger
30
20
10
0.0
0.5
1.0
1.5
1/SE_IV
β_IV

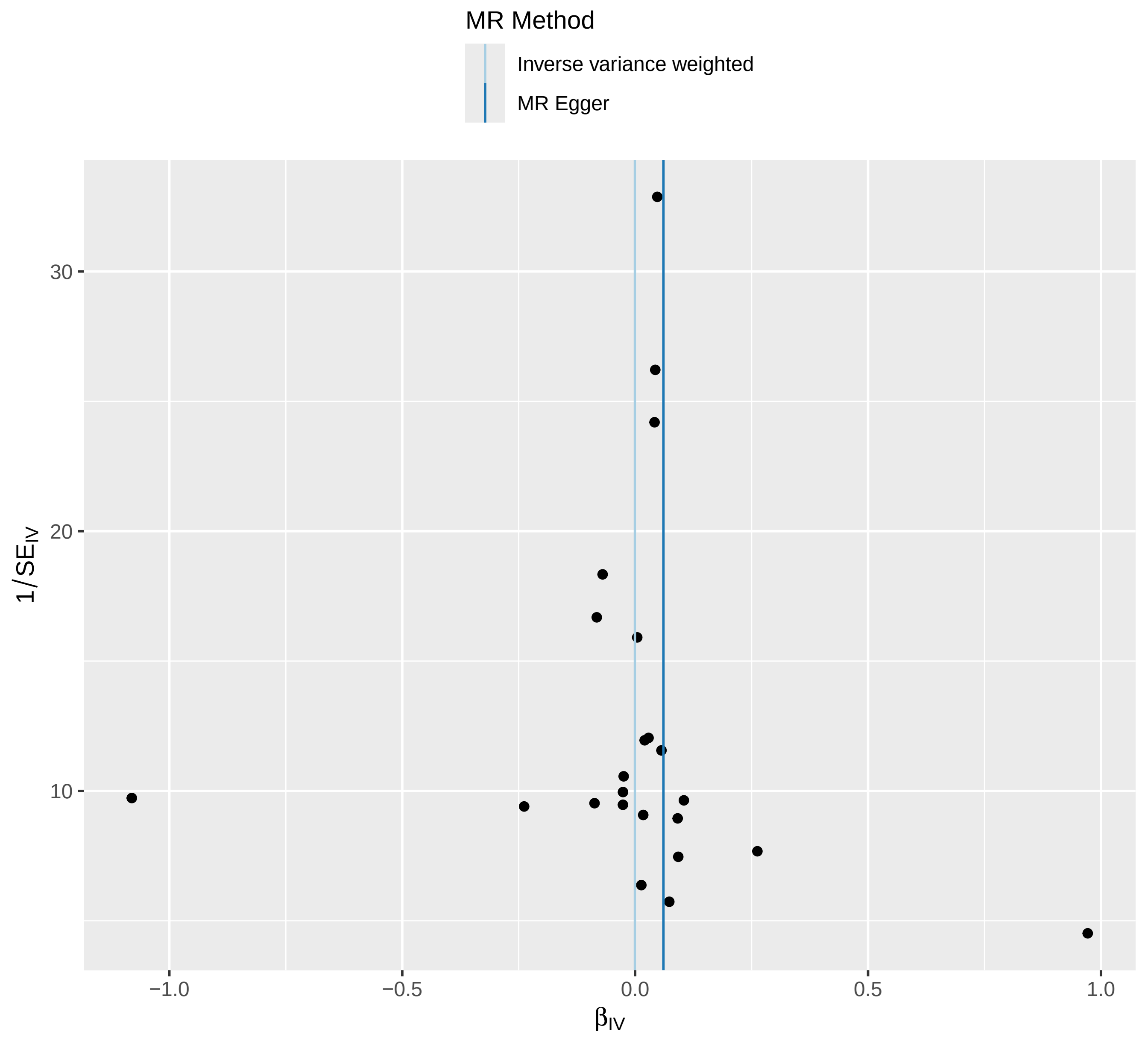
MR Method
Inverse variance weighted
MR Egger
30
20
10
1/SE$_{IV}$
−1.0
−0.5
0.0
0.5
1.0
β_{IV}

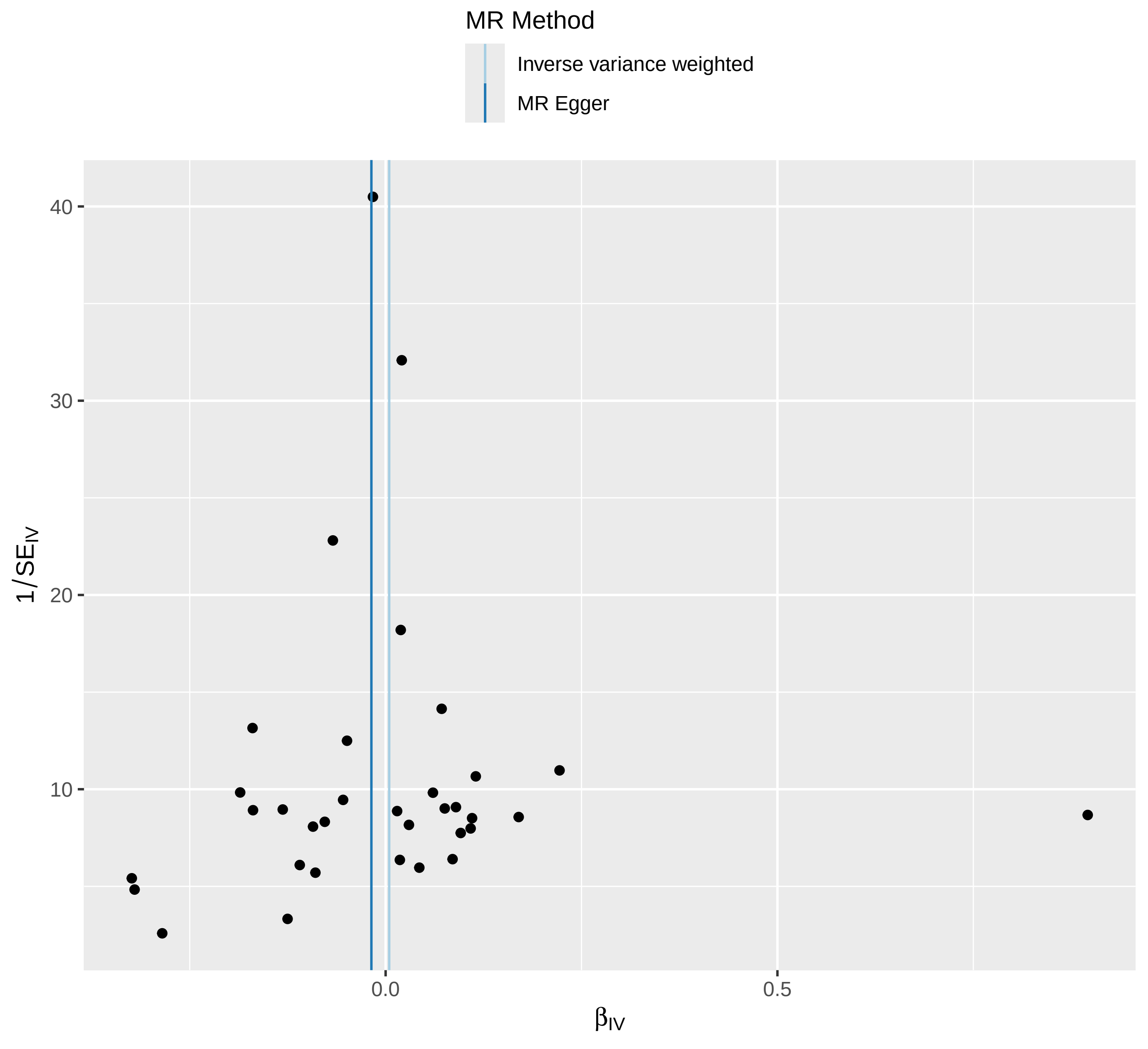
MR Method
Inverse variance weighted
MR Egger
40
30
20
10
0.0
0.5
1/SE_IV
β_IV

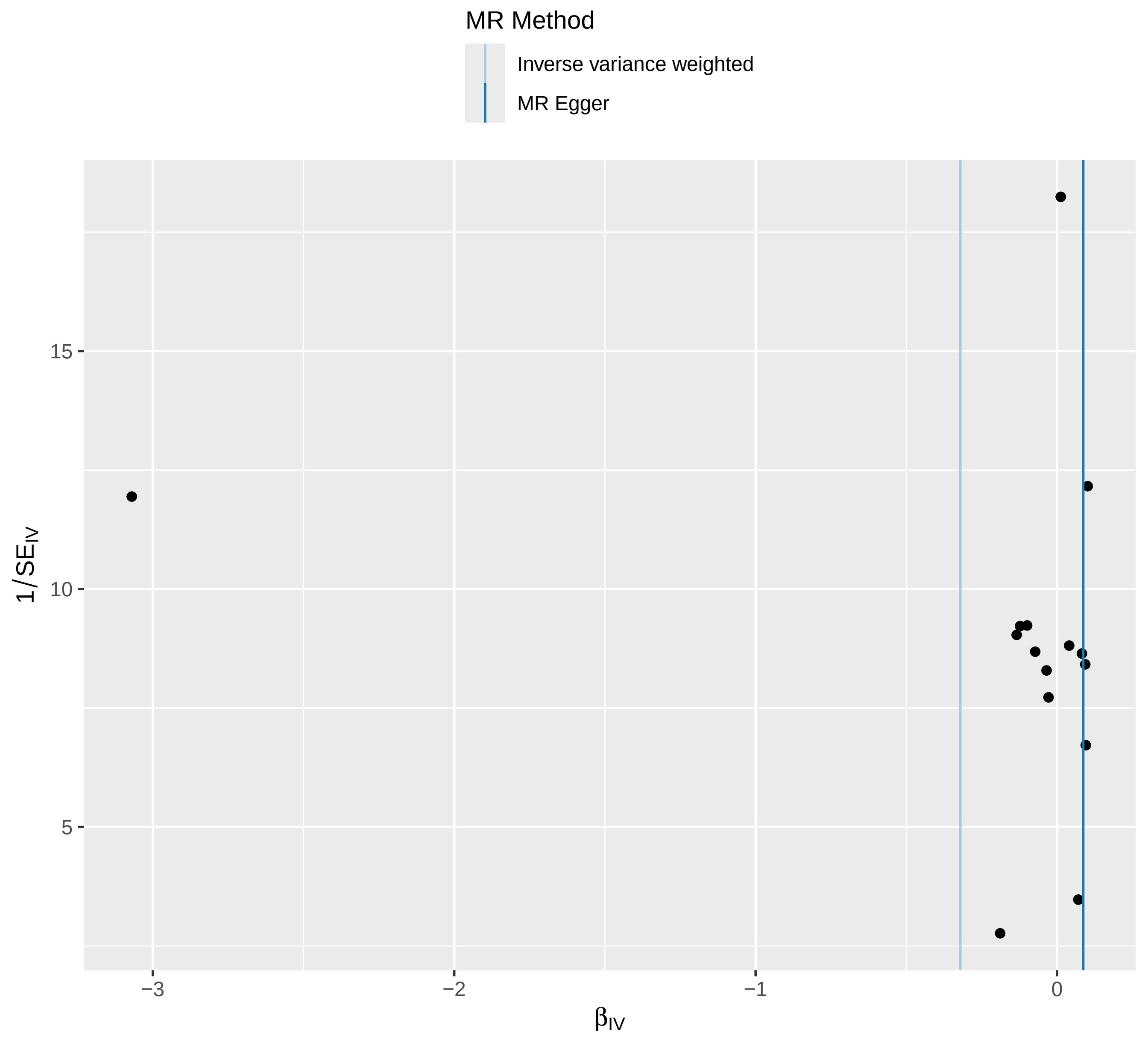
MR Method
Inverse variance weighted
MR Egger
15
10
5
−3
−2
−1
0
$1/SE_{IV}$
β_{IV}

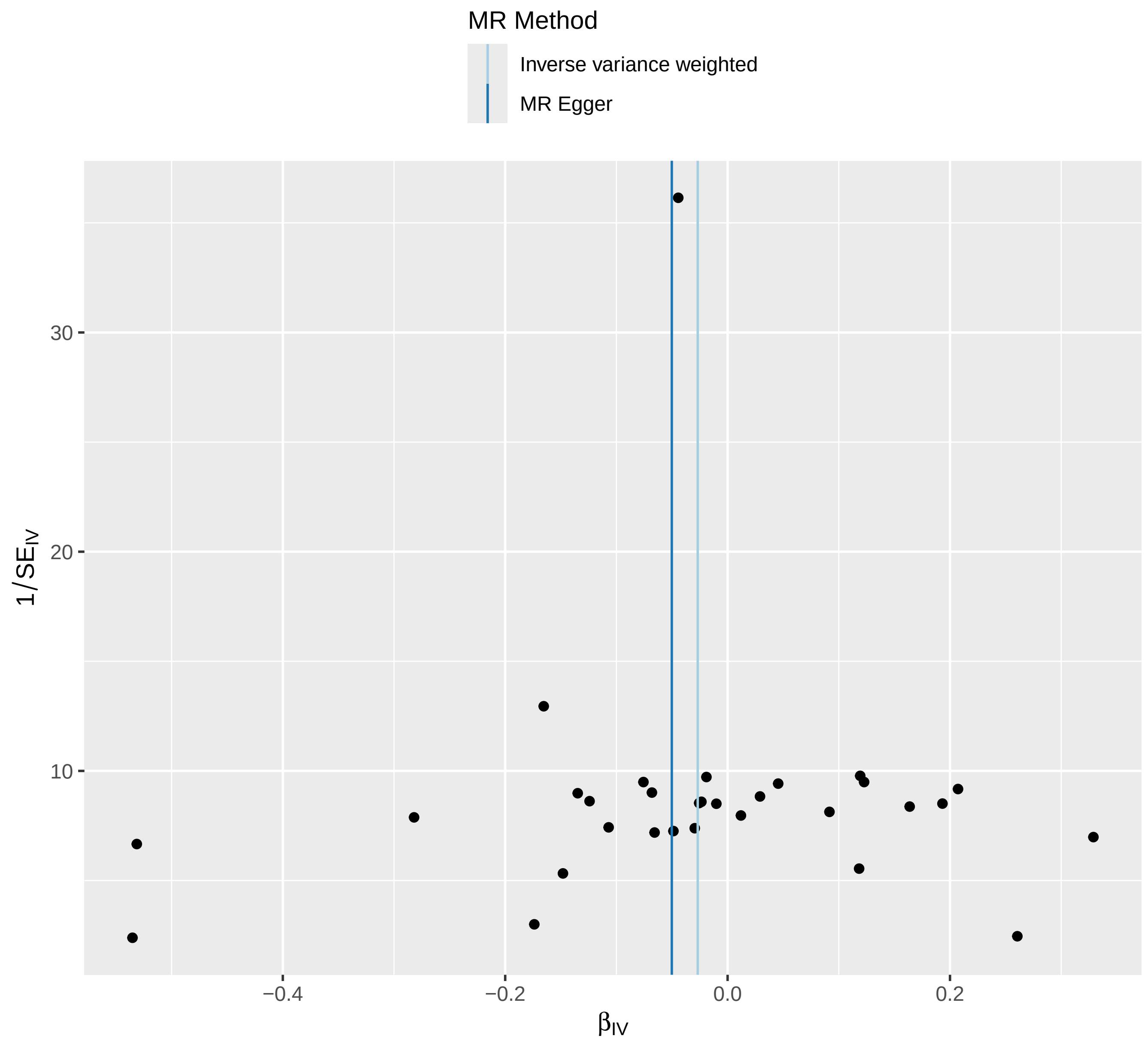

MR Method
Inverse variance weighted
MR Egger
30
20
10
1/SE_IV
−0.4
−0.2
0.0
0.2
β_IV

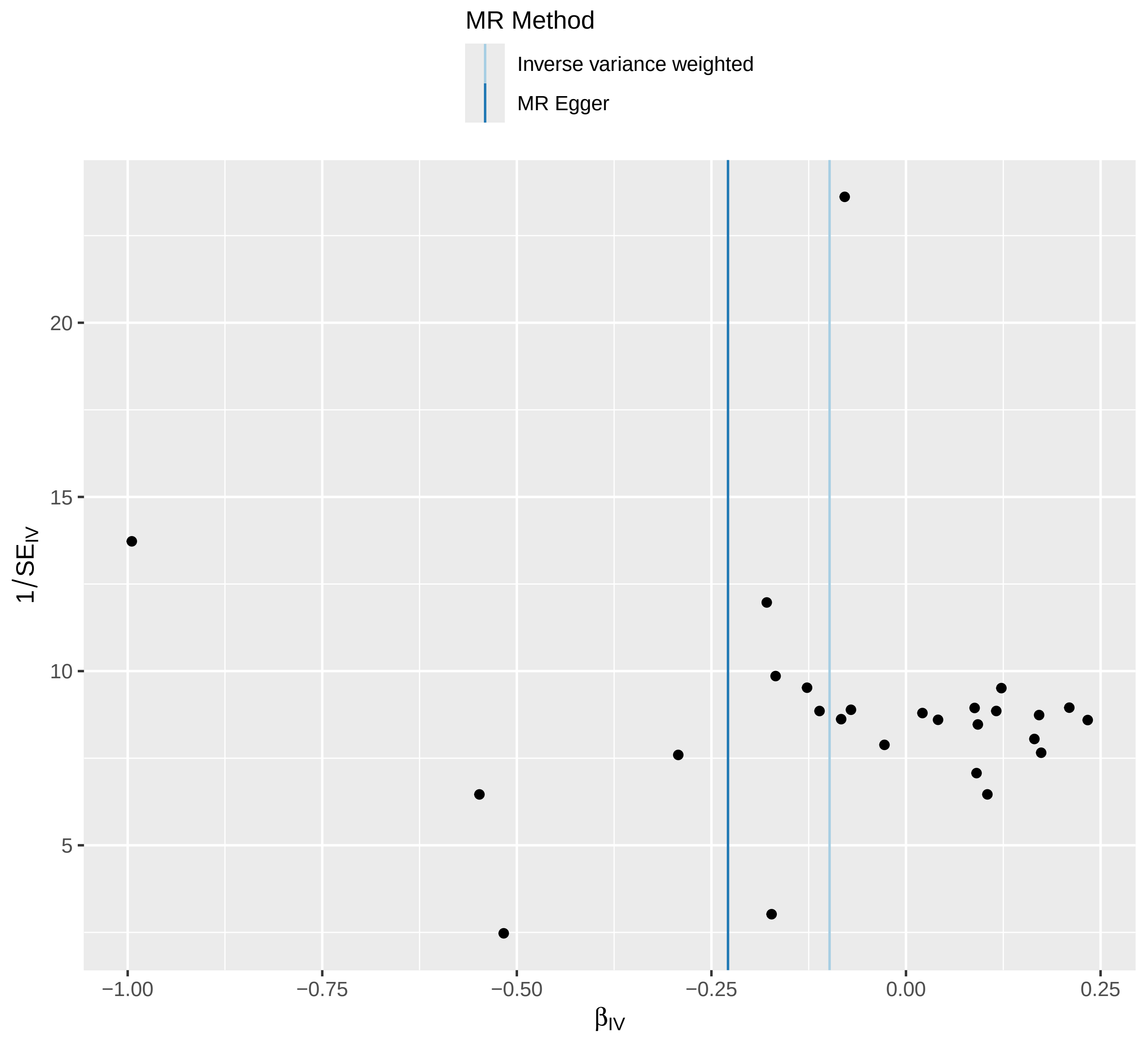

MR Method
Inverse variance weighted
MR Egger
20
15
10
5
1/SE$_{IV}$
−1.00
−0.75
−0.50
−0.25
0.00
0.25
β$_{IV}$

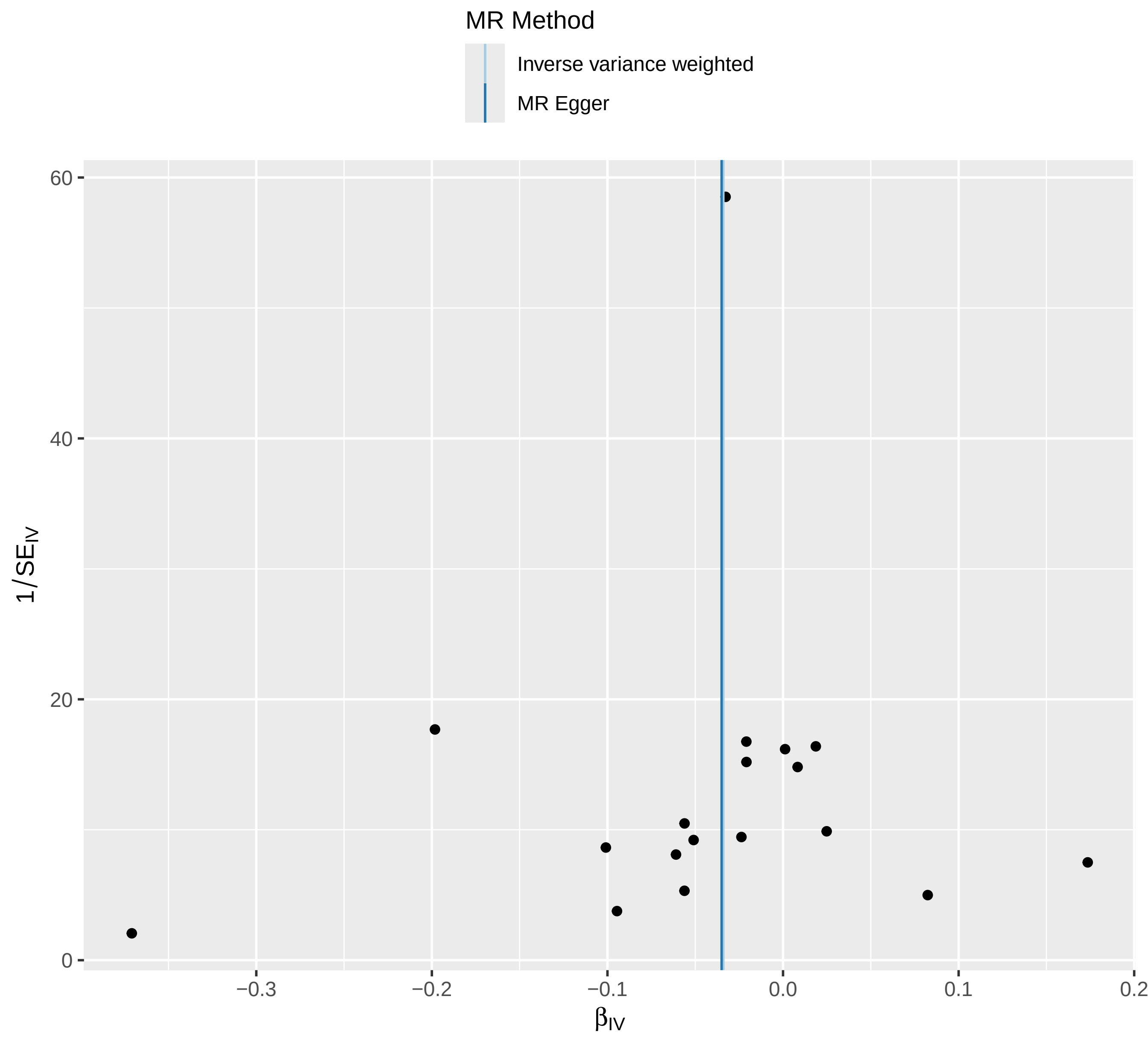

MR Method
Inverse variance weighted
MR Egger
60
40
20
0
$1/SE_{IV}$
−0.3
−0.2
−0.1
0.0
0.1
0.2
β_{IV}

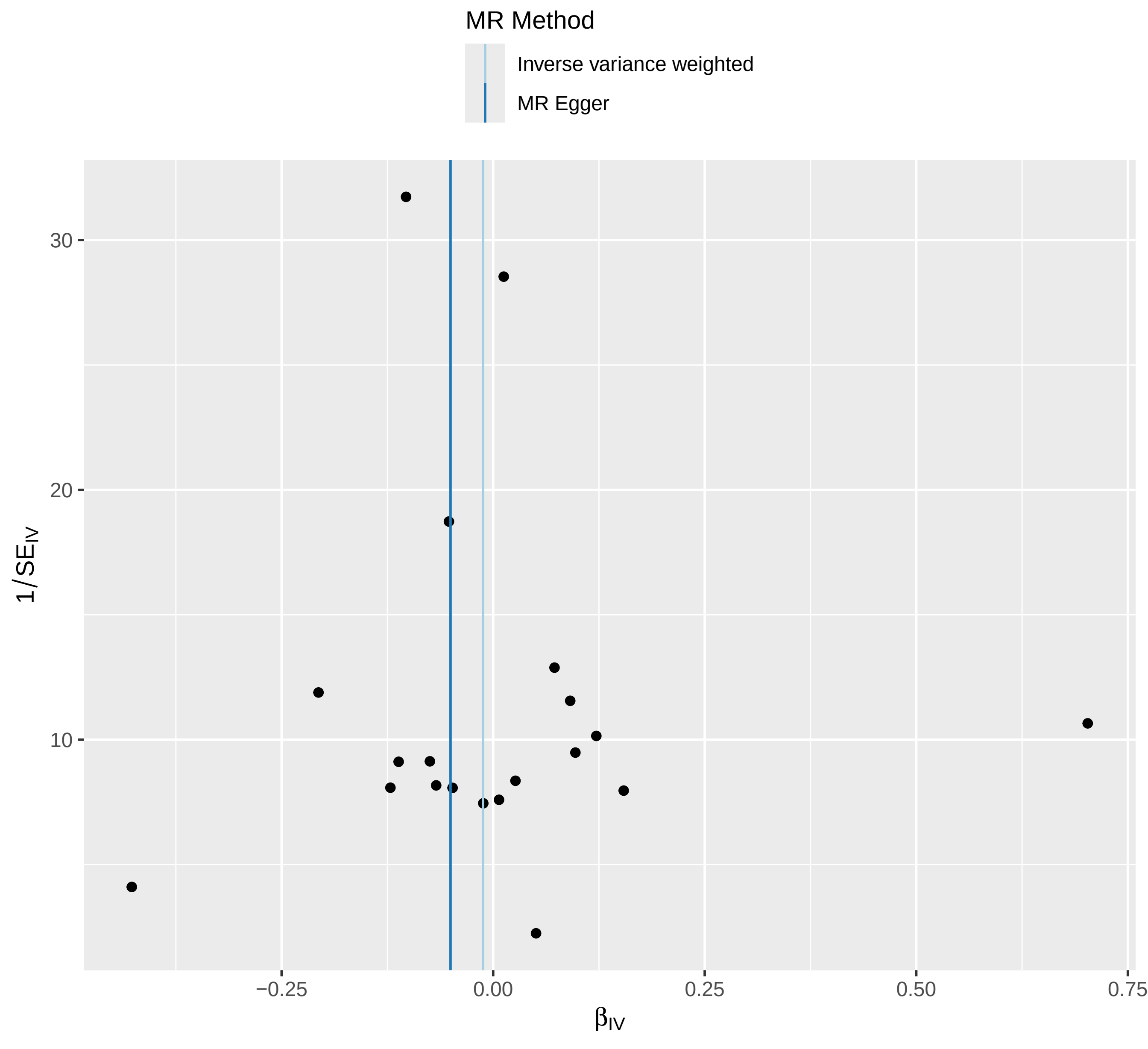

MR Method
Inverse variance weighted
MR Egger
30
20
10
1/SE$_{IV}$
−0.25
0.00
0.25
0.50
0.75
β$_{IV}$

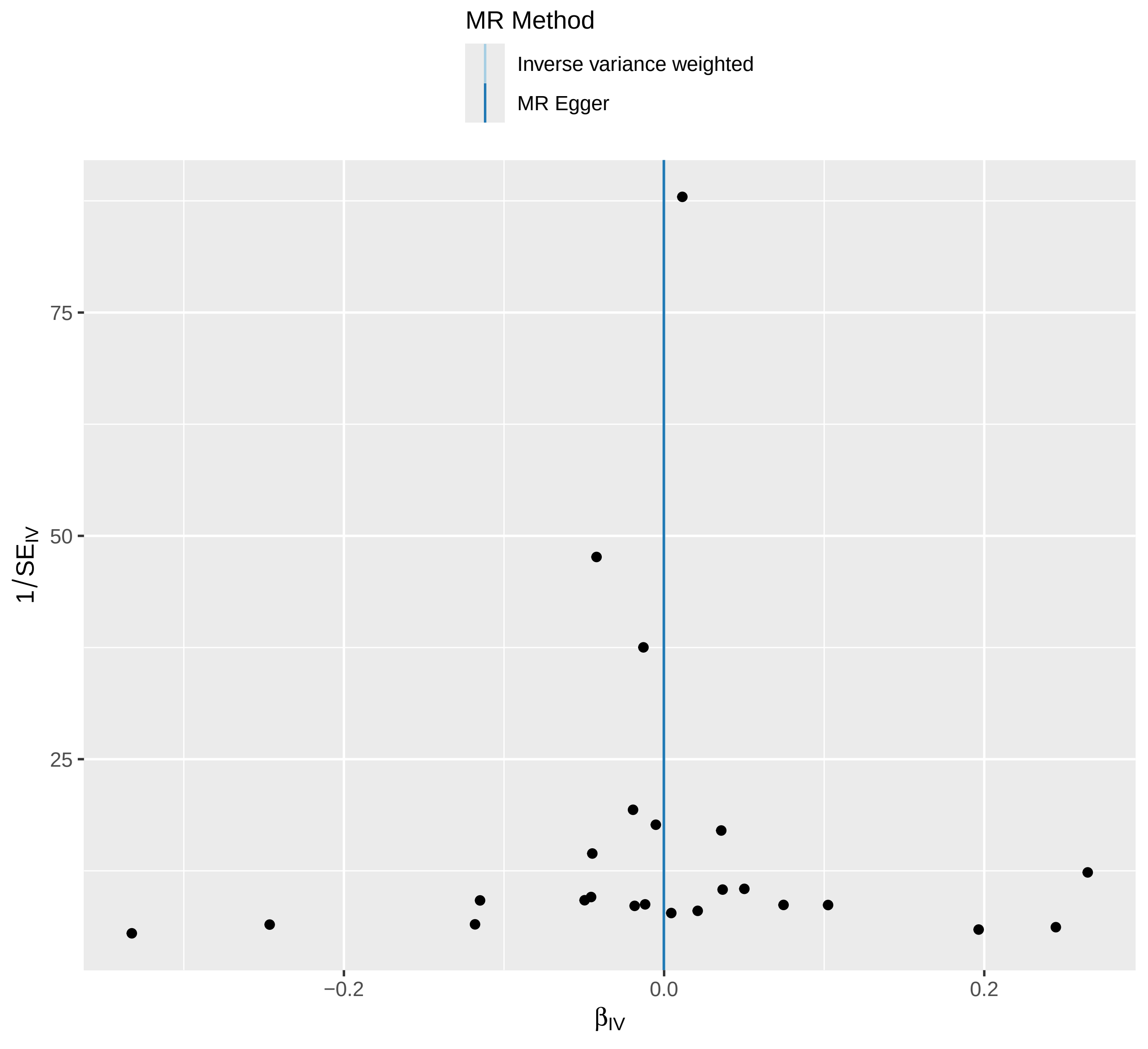
MR Method
Inverse variance weighted
MR Egger
75
50
25
1/SE$_{IV}$
−0.2
0.0
0.2
β$_{IV}$

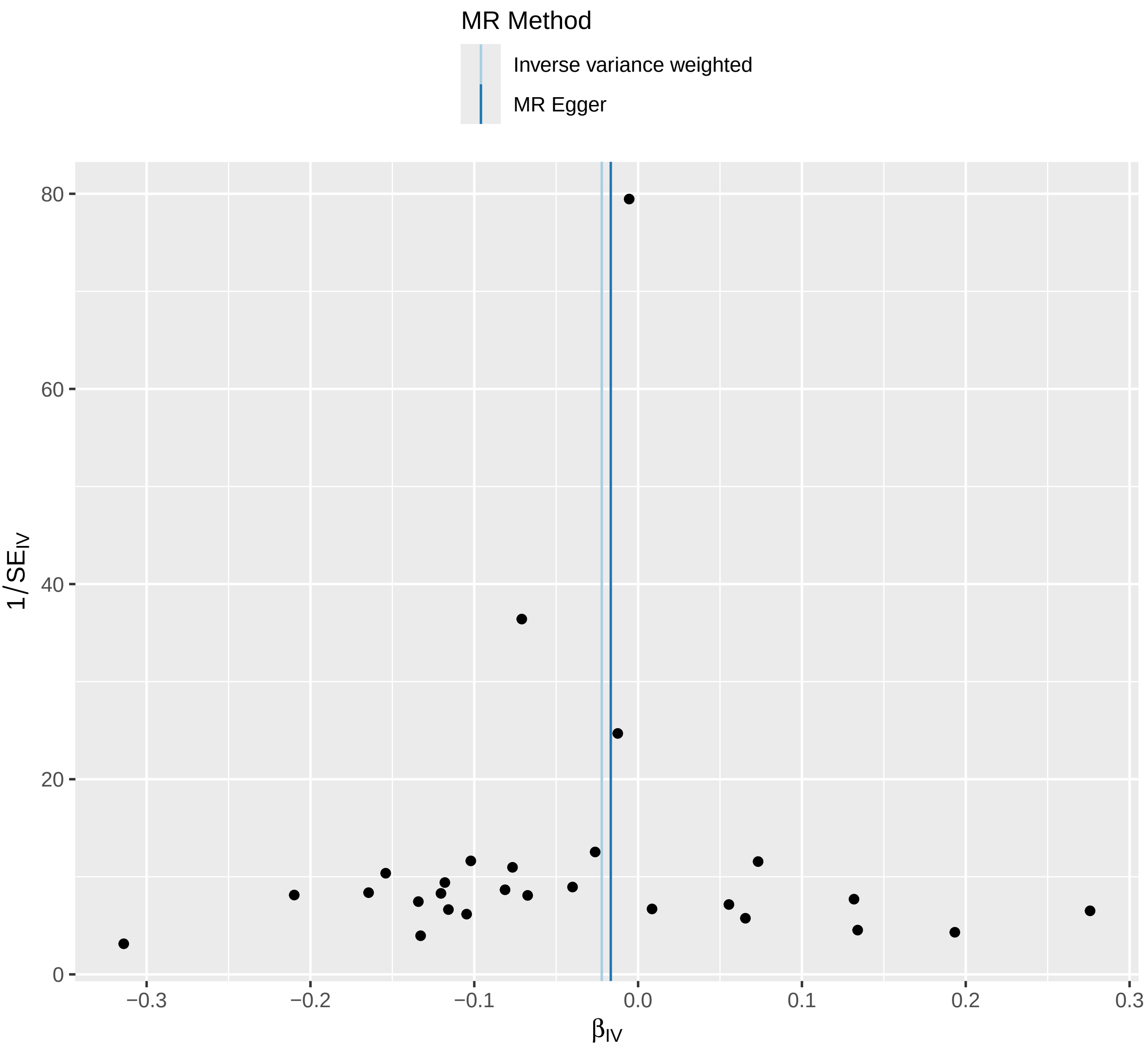
MR Method
Inverse variance weighted
MR Egger
1/SE_IV
80
60
40
20
0
−0.3
−0.2
−0.1
0.0
0.1
0.2
0.3
β_IV

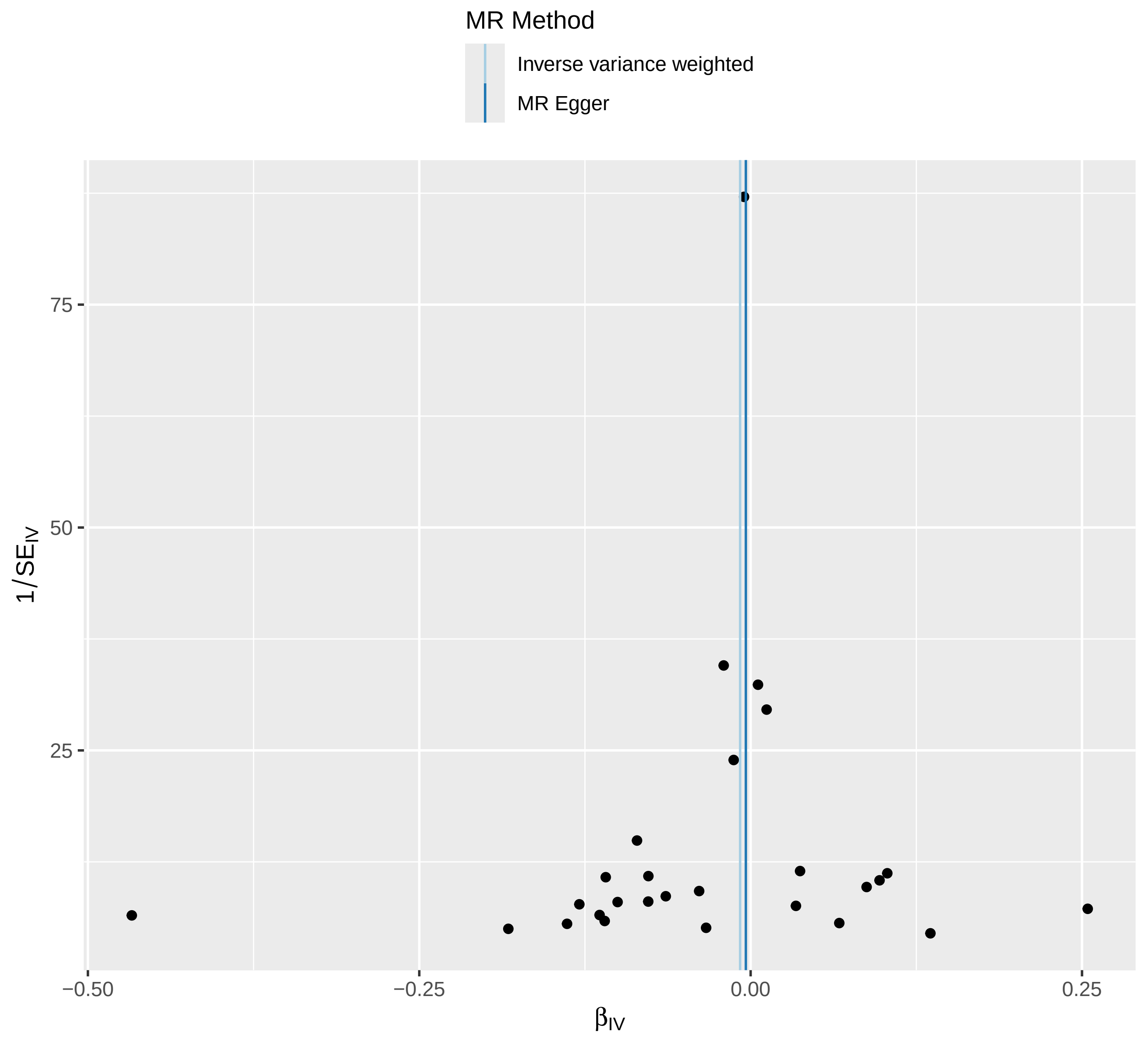
MR Method
Inverse variance weighted
MR Egger
75
50
25
$1/SE_{IV}$
−0.50
−0.25
0.00
0.25
β_{IV}

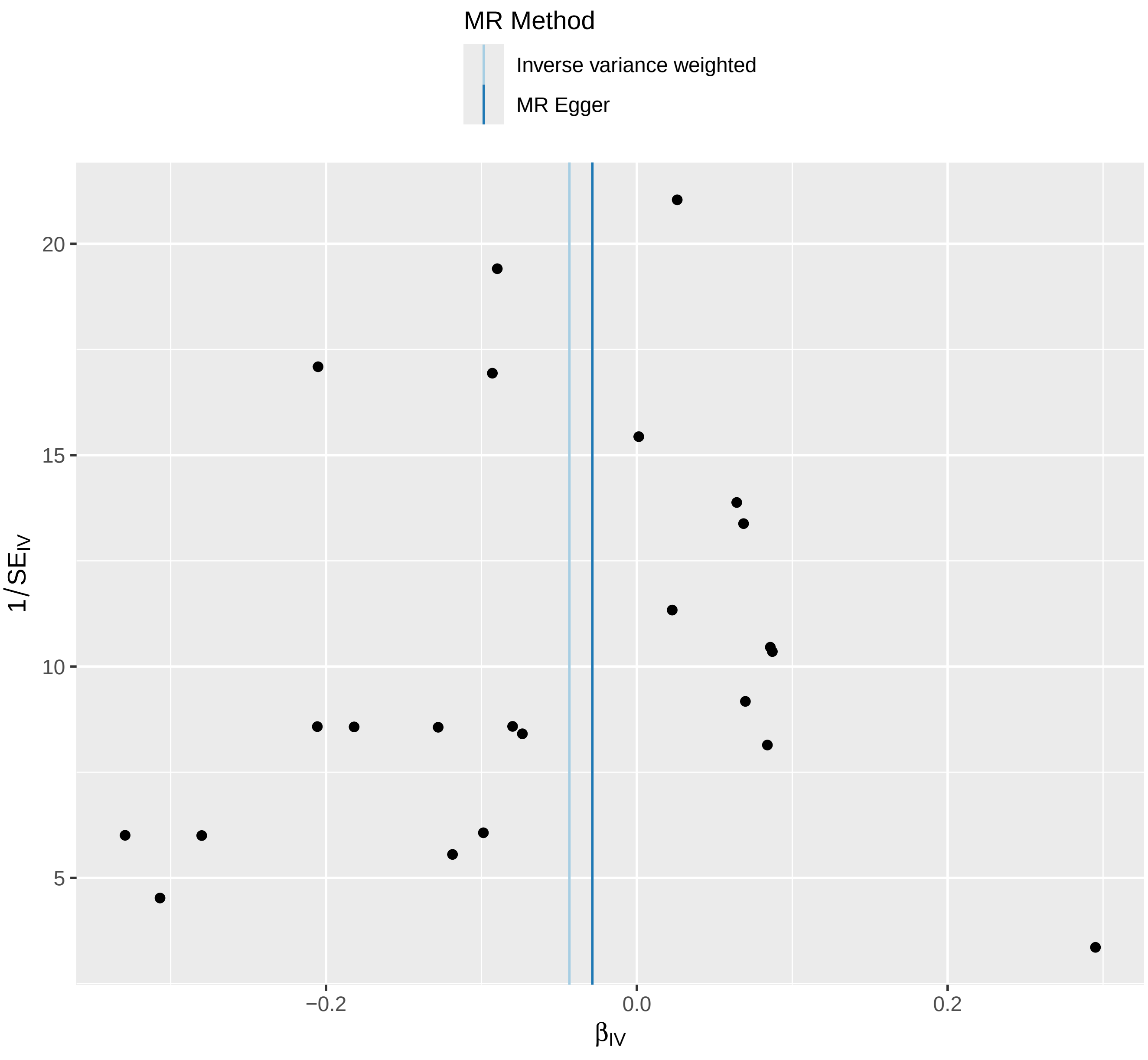
MR Method
Inverse variance weighted
MR Egger
20
15
10
5
1/SE$_{IV}$
−0.2
0.0
0.2
β$_{IV}$

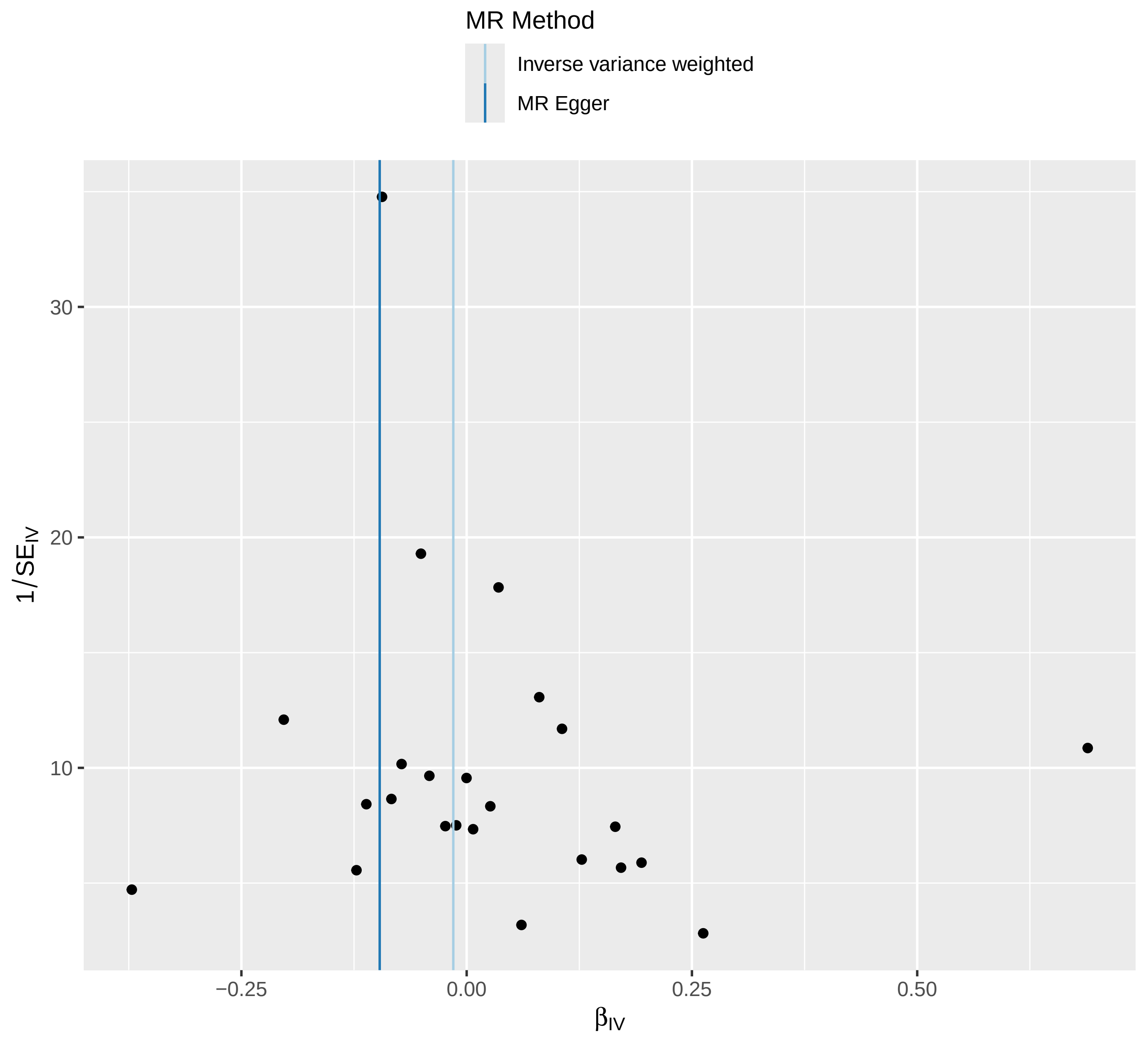
MR Method
Inverse variance weighted
MR Egger
1/SE_IV
30
20
10
−0.25
0.00
0.25
0.50
β_IV

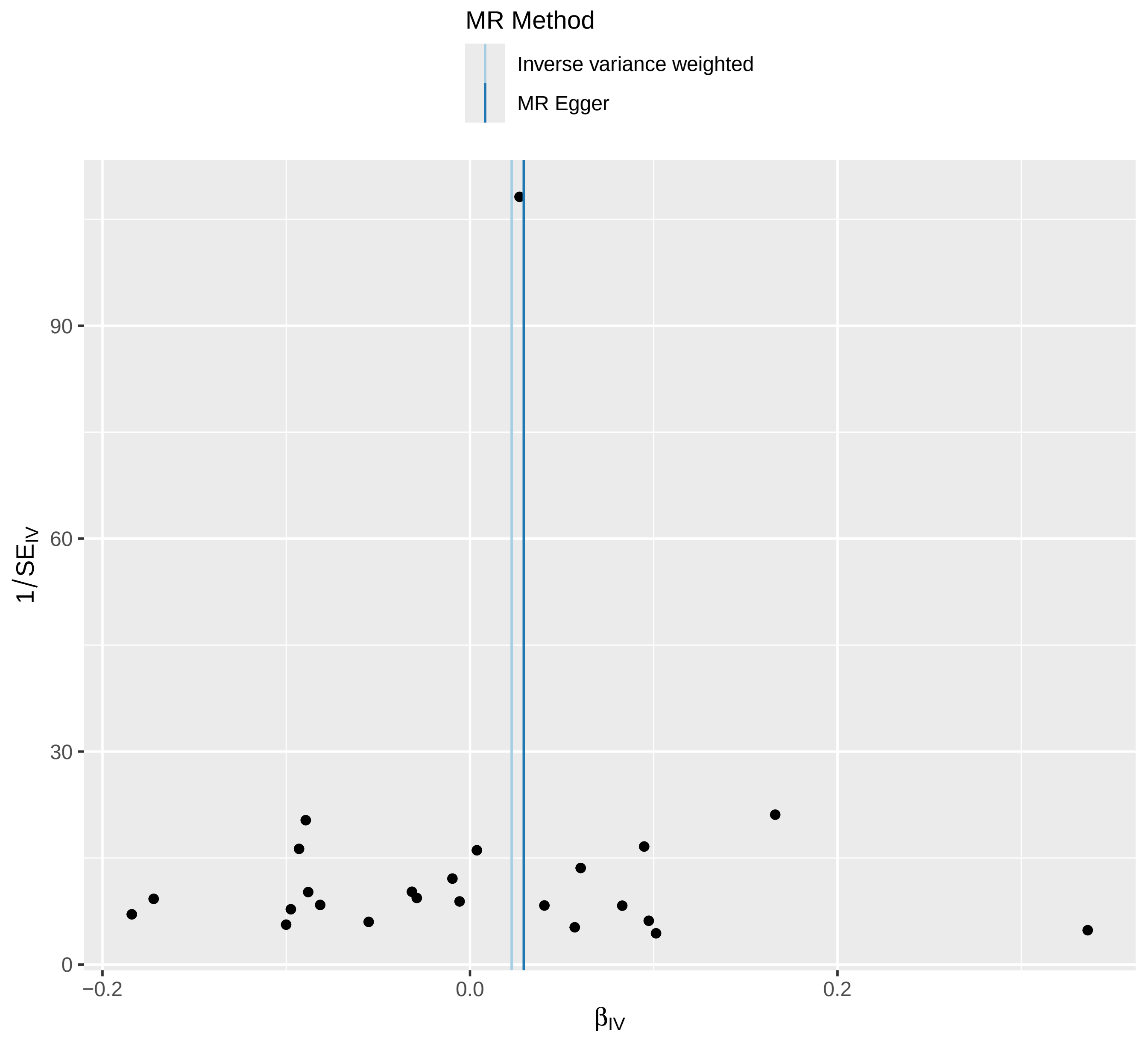

MR Method
Inverse variance weighted
MR Egger
90
60
30
0
1/SE$_{IV}$
−0.2
0.0
0.2
β$_{IV}$

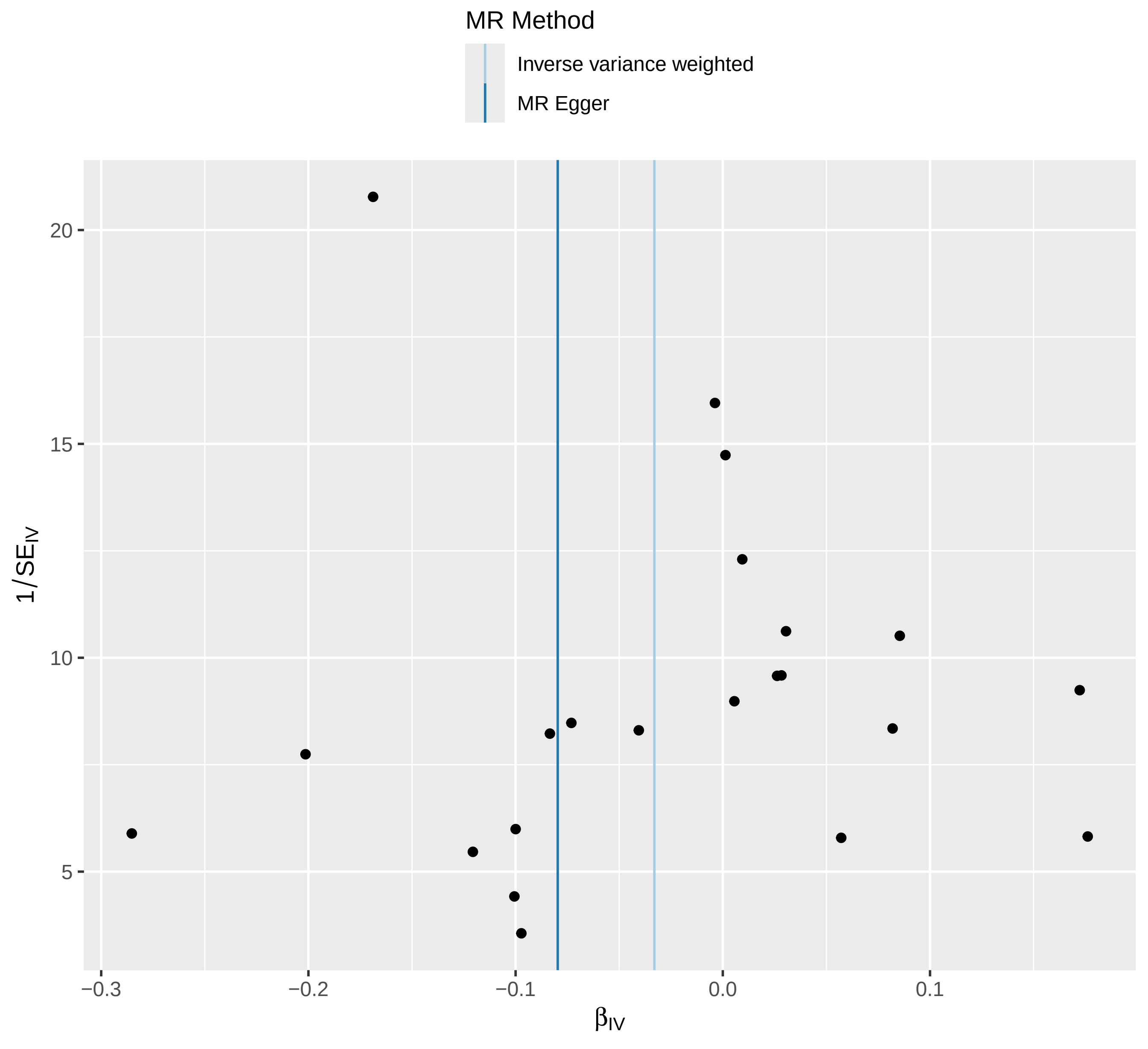

MR Method
Inverse variance weighted
MR Egger
20
15
10
5
1/SE$_{IV}$
−0.3
−0.2
−0.1
0.0
0.1
β$_{IV}$

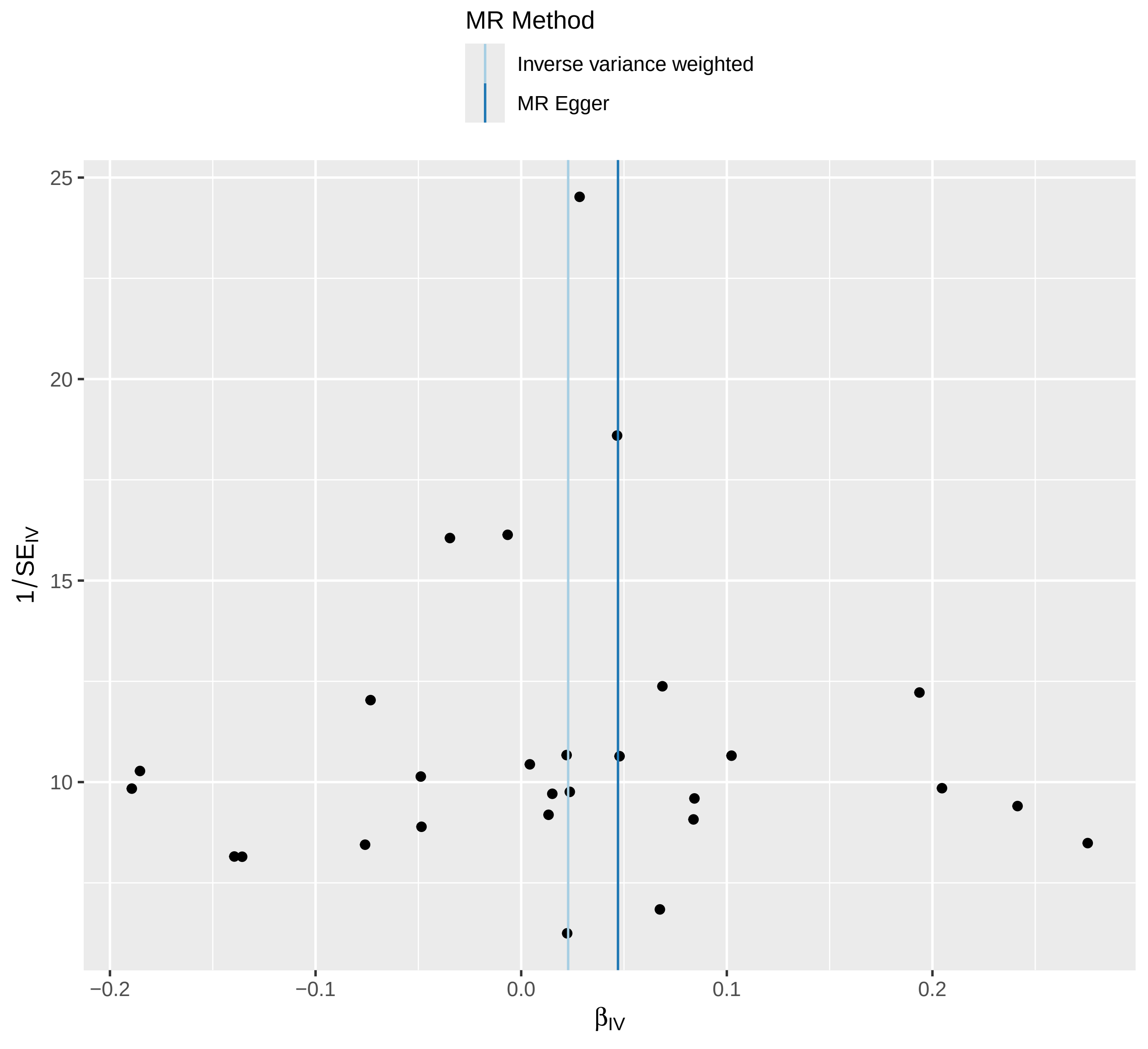
MR Method
Inverse variance weighted
MR Egger
25
20
15
10
1/SE_IV
−0.2
−0.1
0.0
0.1
0.2
β_IV

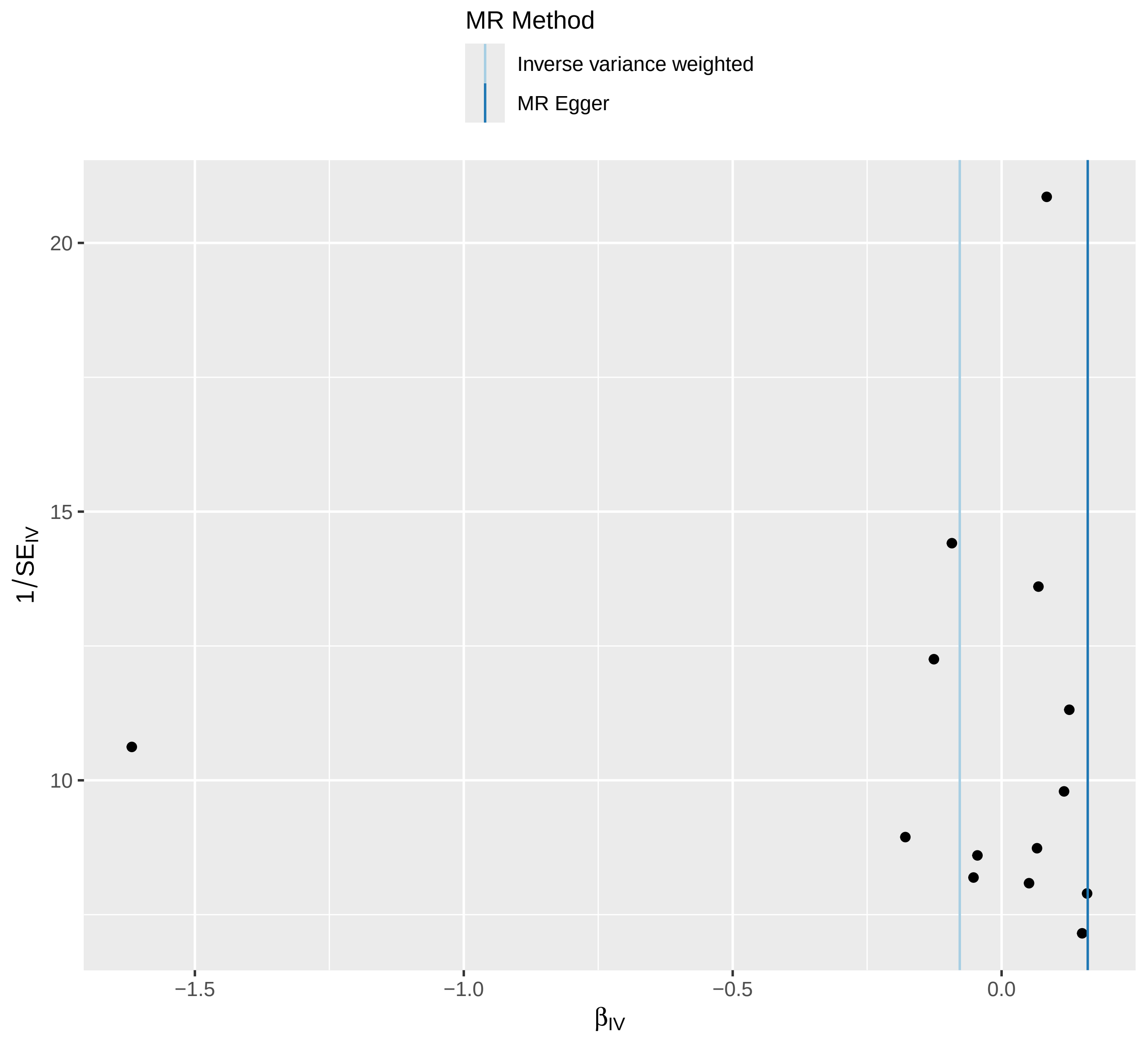
MR Method
Inverse variance weighted
MR Egger
20
15
10
−1.5
−1.0
−0.5
0.0
$1/SE_{IV}$
β_{IV}

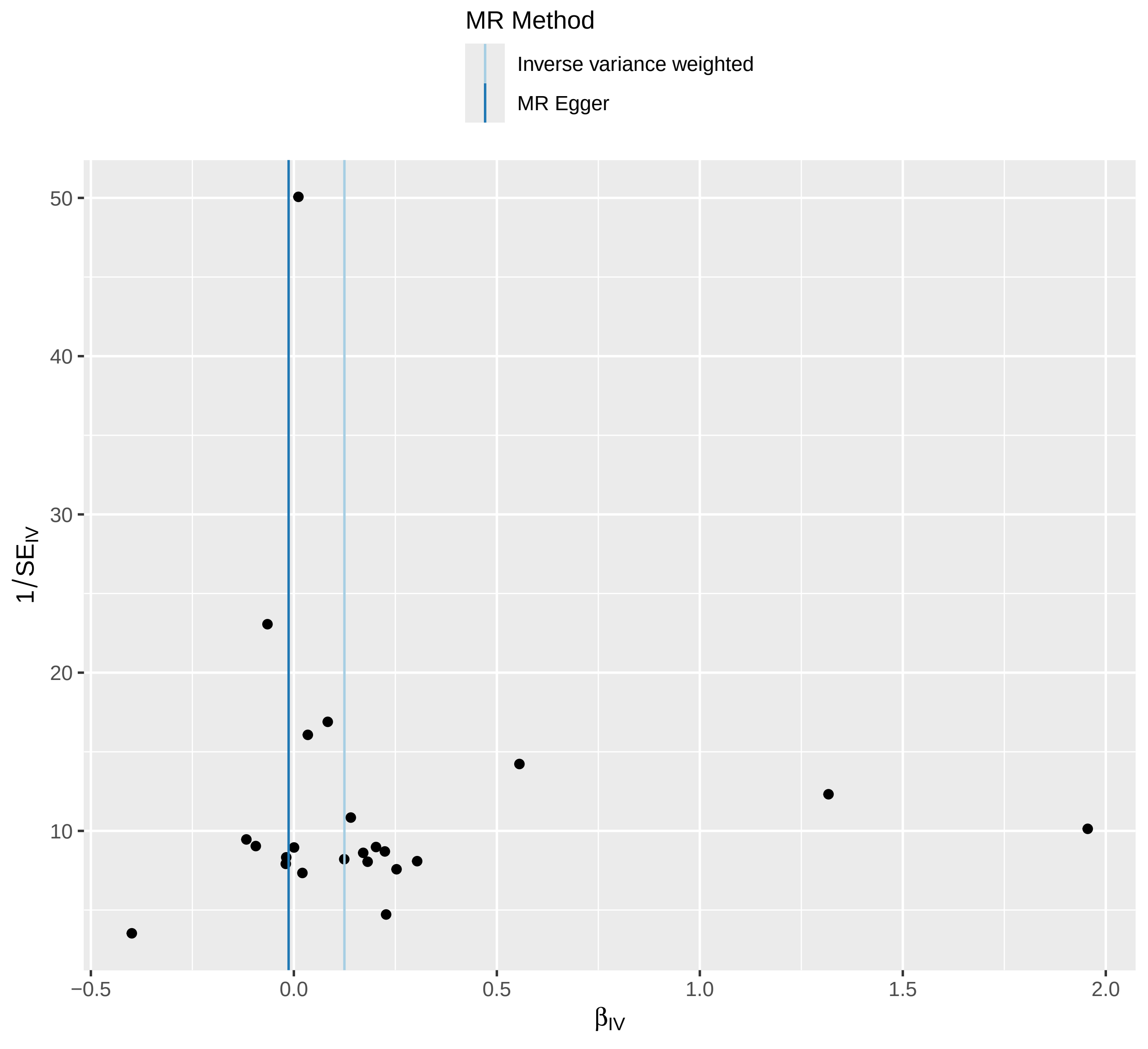
MR Method
Inverse variance weighted
MR Egger
1/SE_{IV}
50
40
30
20
10
−0.5
0.0
0.5
1.0
1.5
2.0
β_{IV}

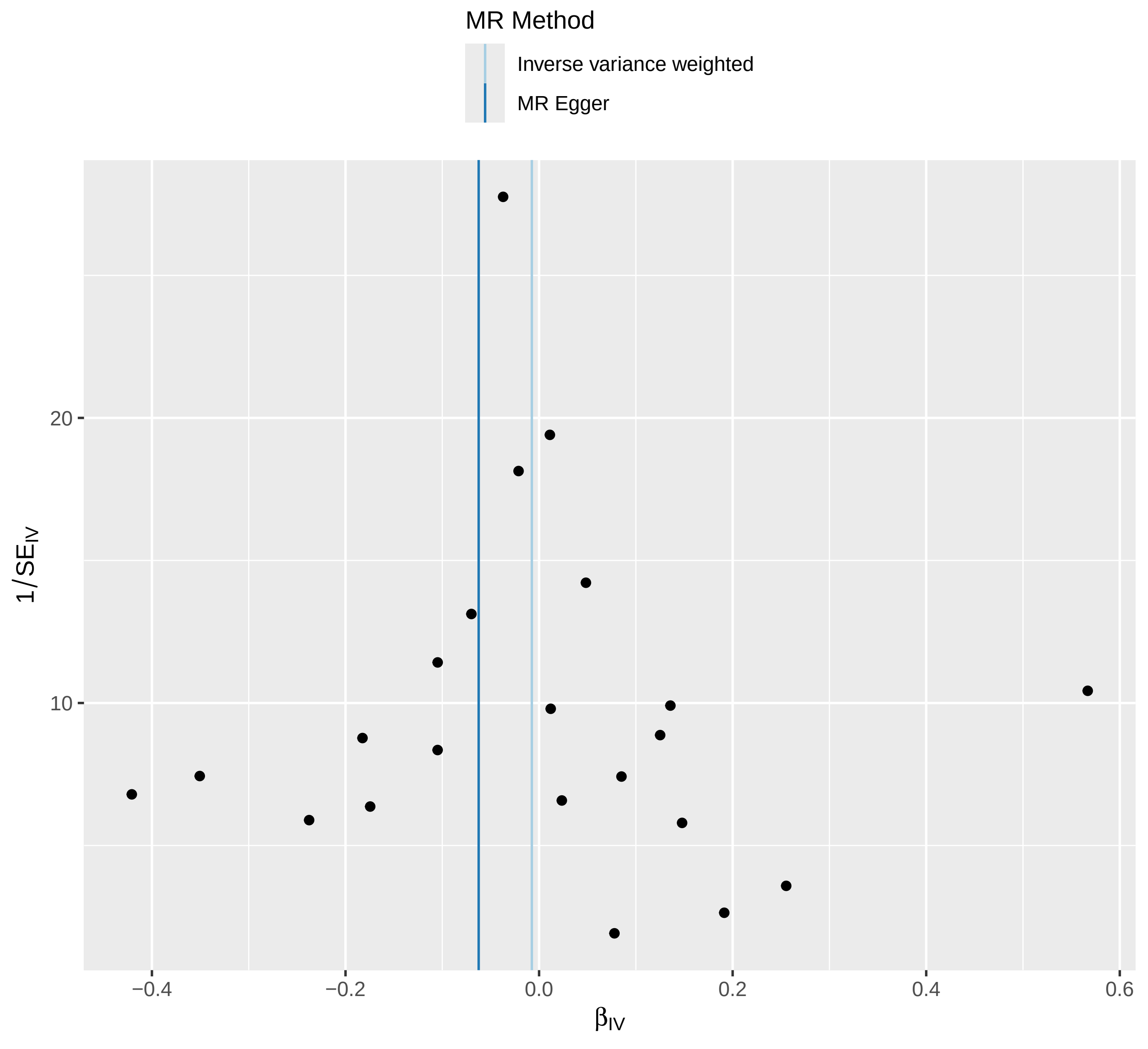
MR Method
Inverse variance weighted
MR Egger
$1/SE_{IV}$
20
10
−0.4
−0.2
0.0
0.2
0.4
0.6
β_{IV}

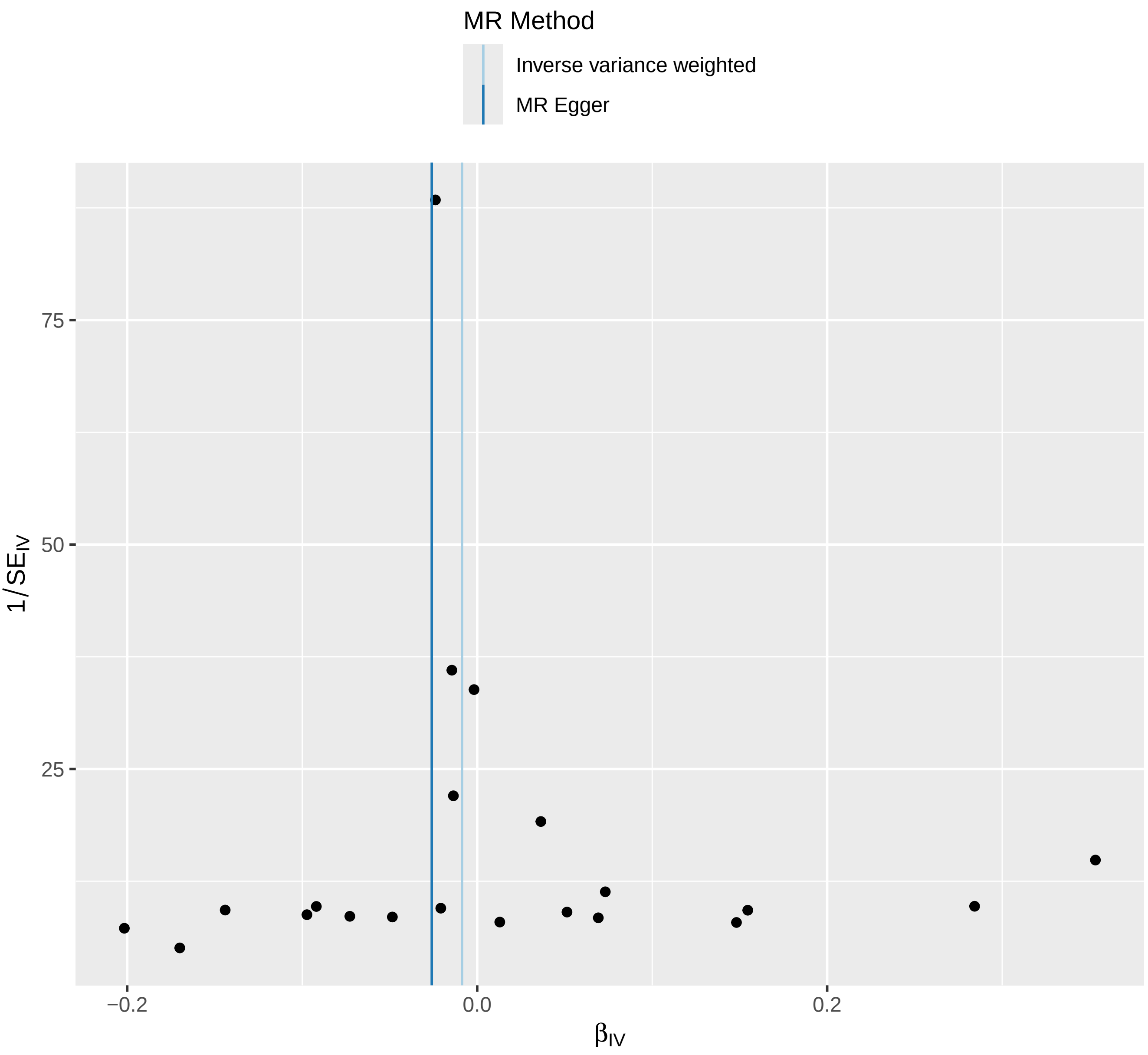
MR Method
Inverse variance weighted
MR Egger
75
50
25
1/SE_IV
−0.2
0.0
0.2
β_IV

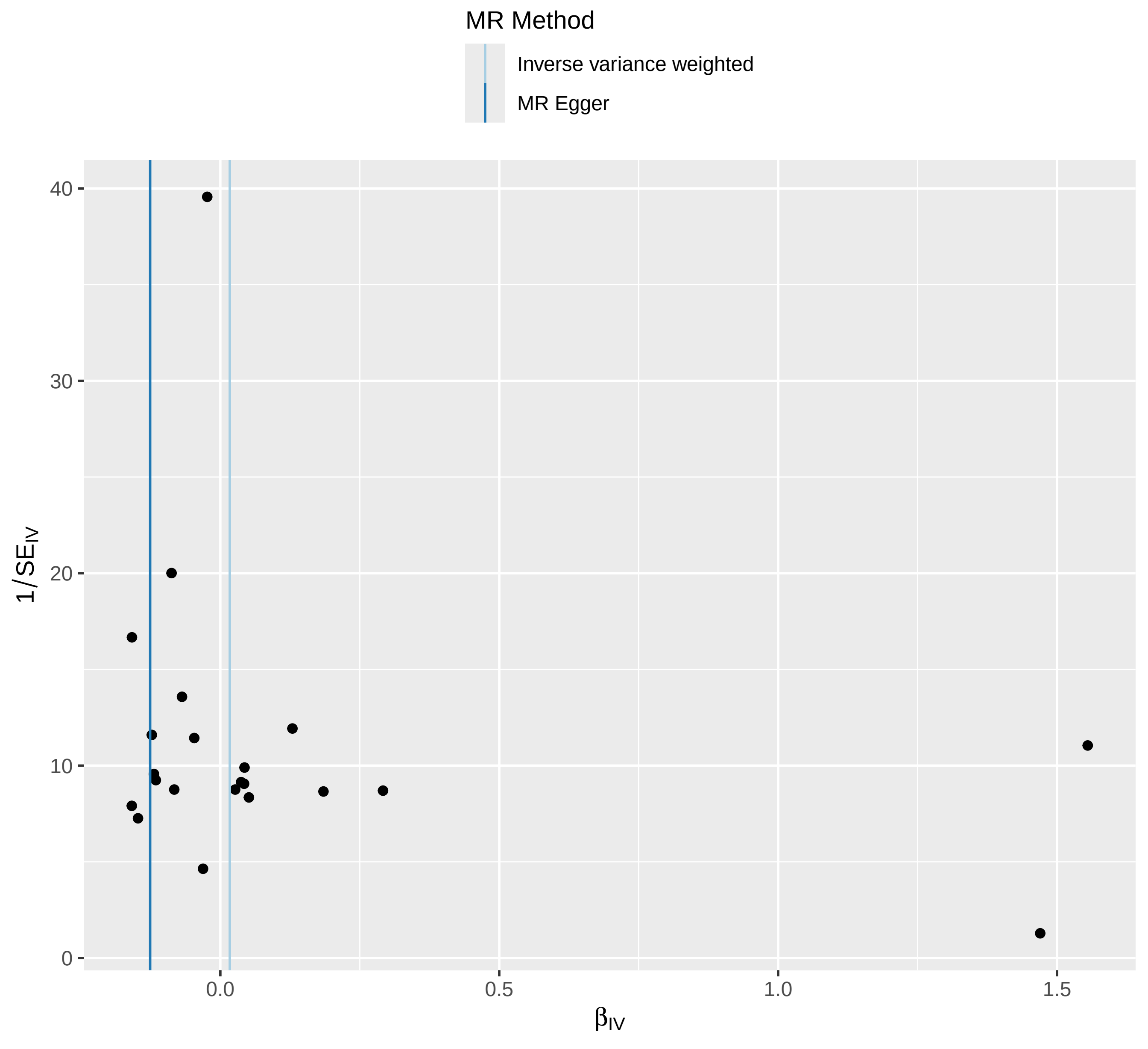
MR Method
Inverse variance weighted
MR Egger
40
30
20
10
0
$1/SE_{IV}$
0.0
0.5
1.0
1.5
β_{IV}

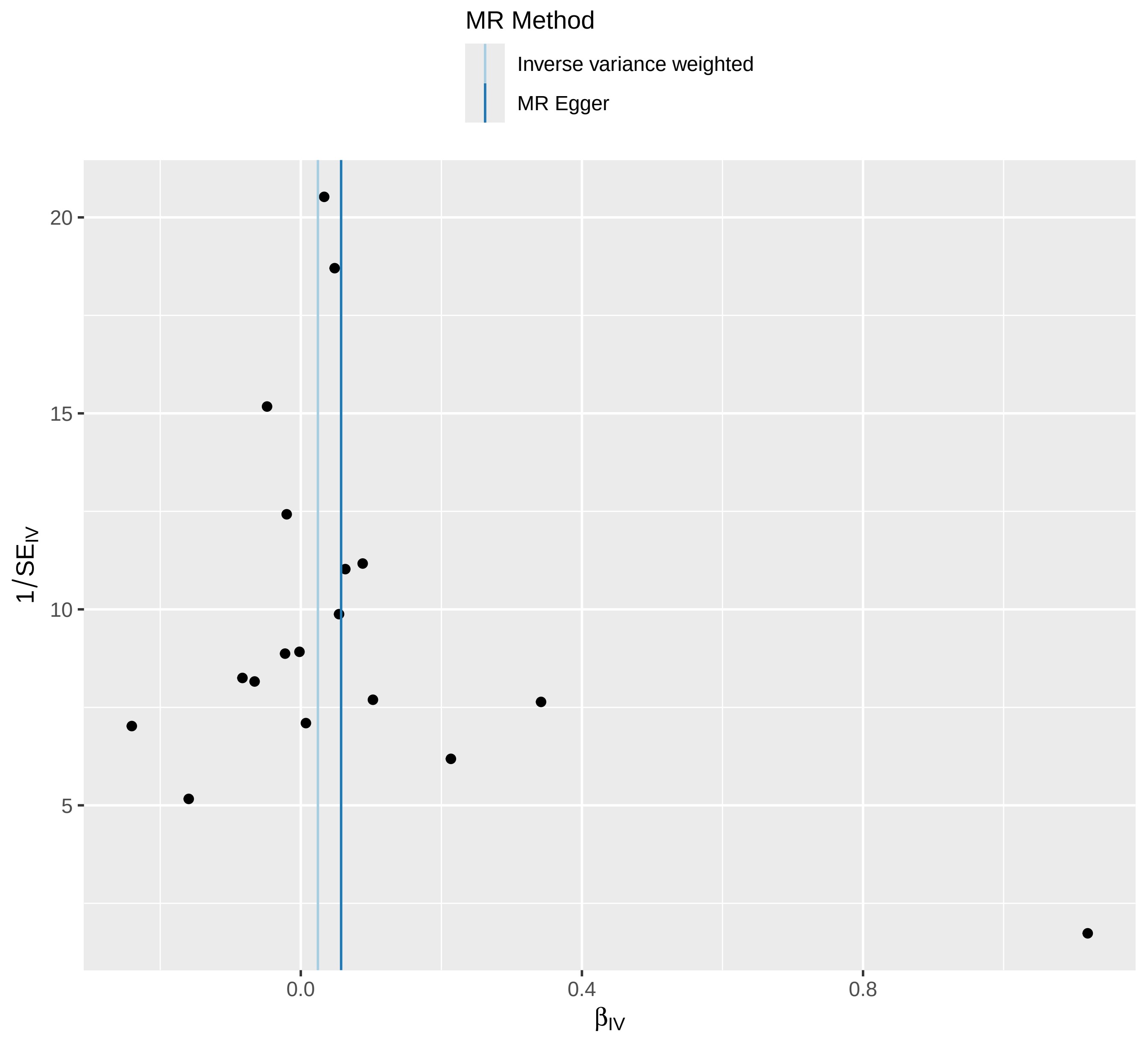
MR Method
Inverse variance weighted
MR Egger
20
15
10
5
1/SE_IV
0.0
0.4
0.8
β_IV

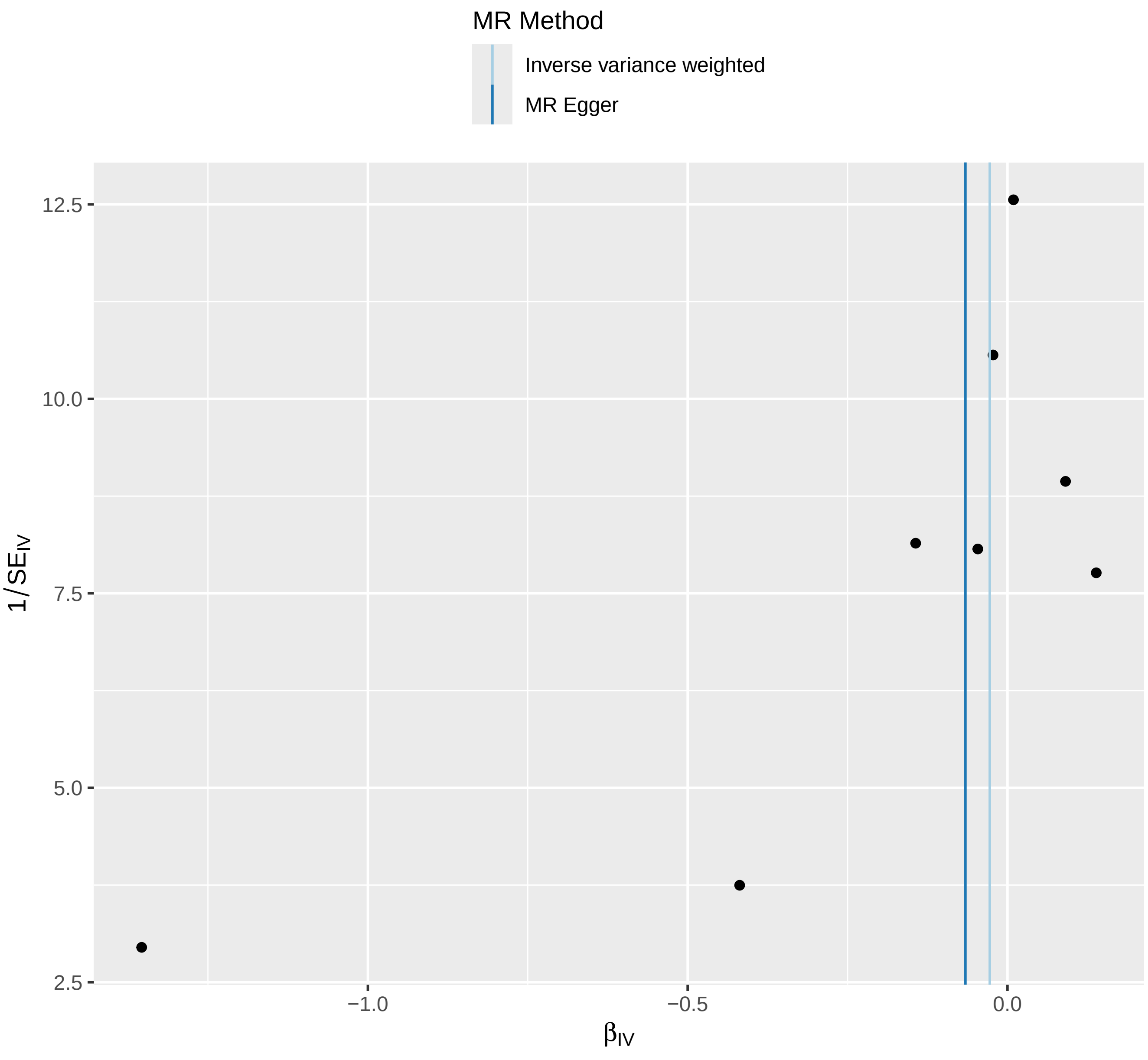

MR Method
Inverse variance weighted
MR Egger
12.5
10.0
7.5
5.0
2.5
1/SE_IV
−1.0
−0.5
0.0
β_IV

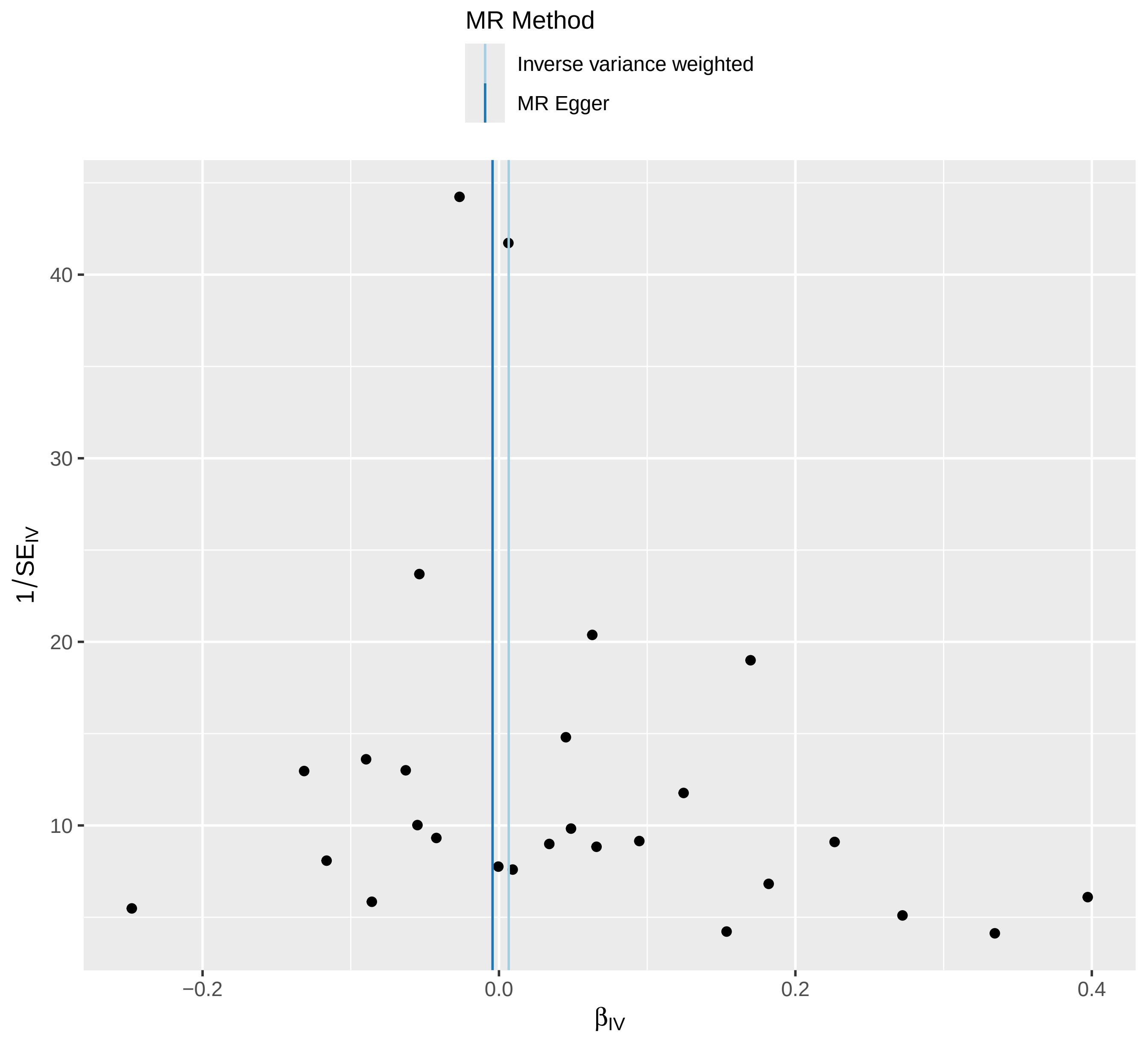
MR Method
Inverse variance weighted
MR Egger
40
30
20
10
1/SE$_{IV}$
−0.2
0.0
0.2
0.4
β_{IV}

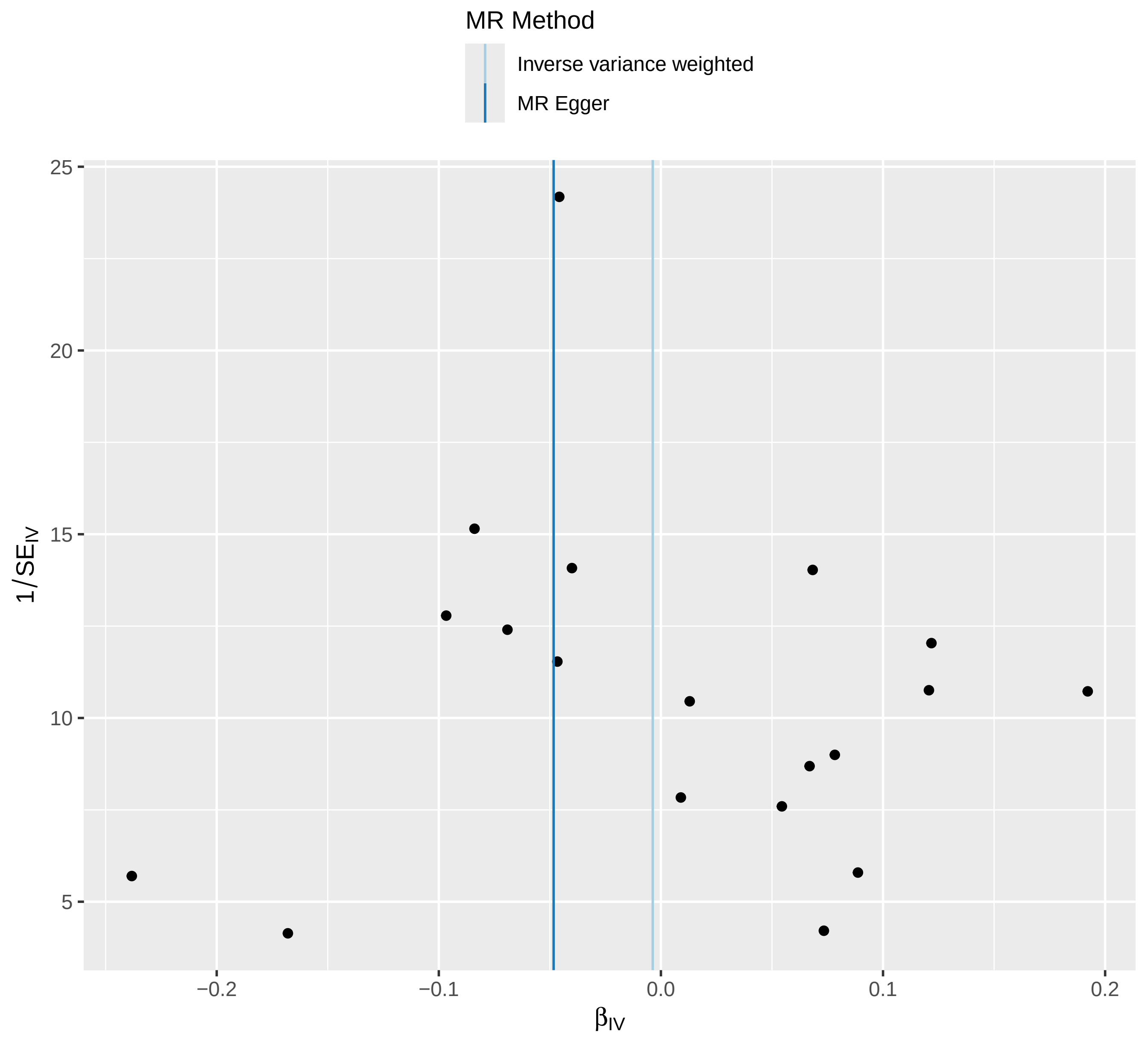
MR Method
Inverse variance weighted
MR Egger
25
20
15
10
5
−0.2
−0.1
0.0
0.1
0.2
β_{IV}
$1/SE_{IV}$

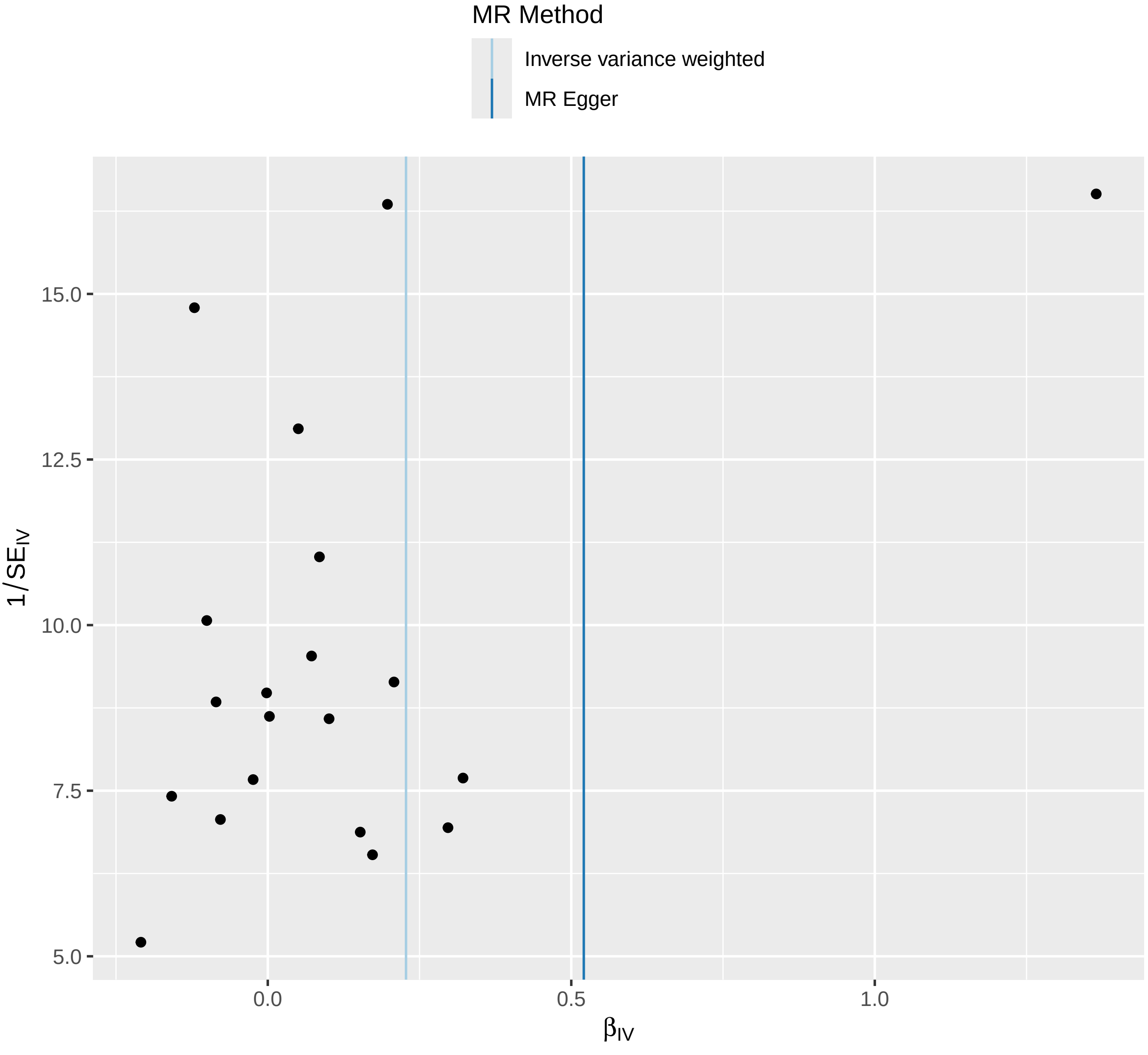
MR Method
Inverse variance weighted
MR Egger
15.0
12.5
10.0
7.5
5.0
$1/SE_{IV}$
0.0
0.5
1.0
β_{IV}

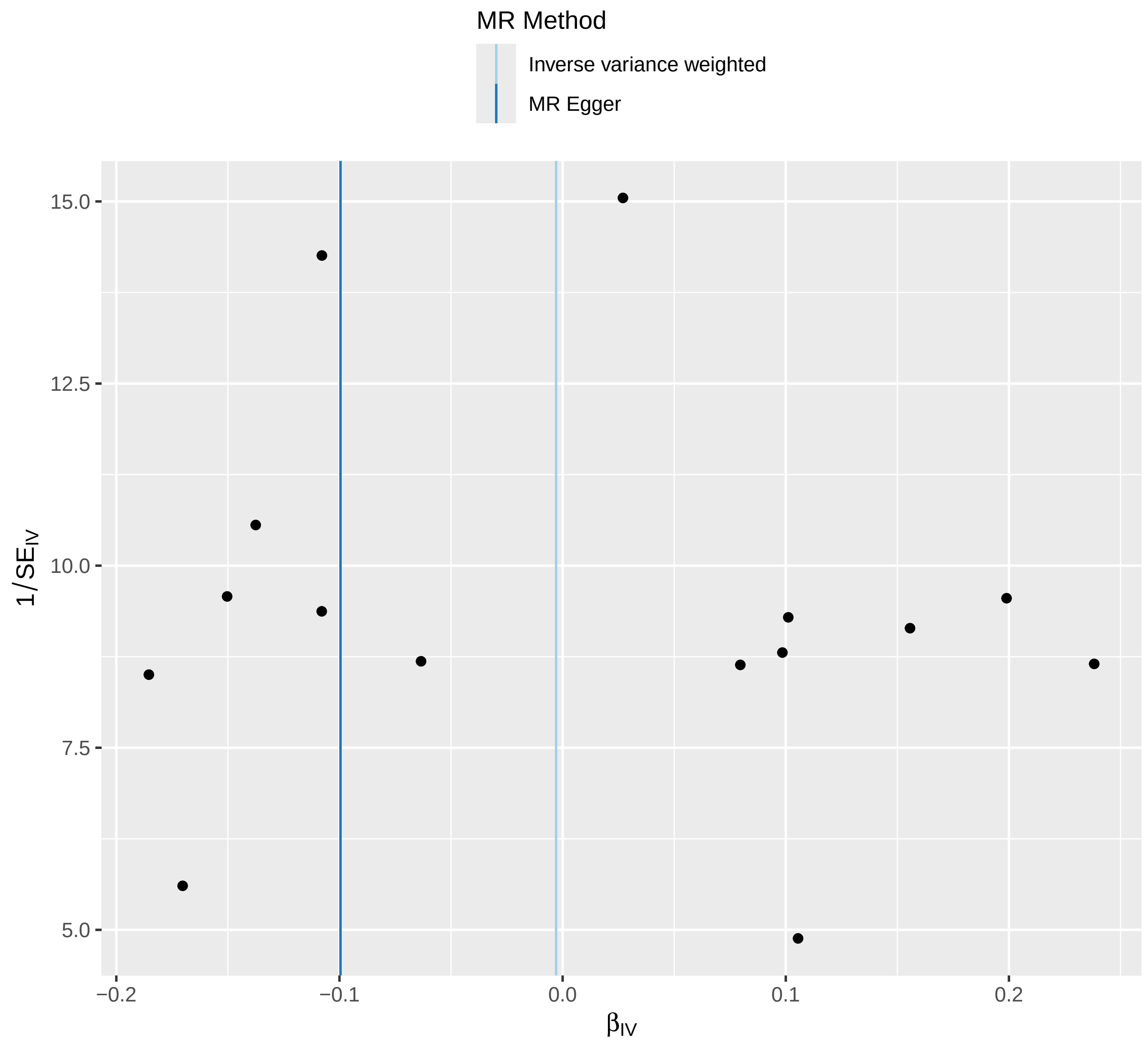
MR Method
Inverse variance weighted
MR Egger
15.0
12.5
10.0
7.5
5.0
$1/SE_{IV}$
−0.2
−0.1
0.0
0.1
0.2
β_{IV}

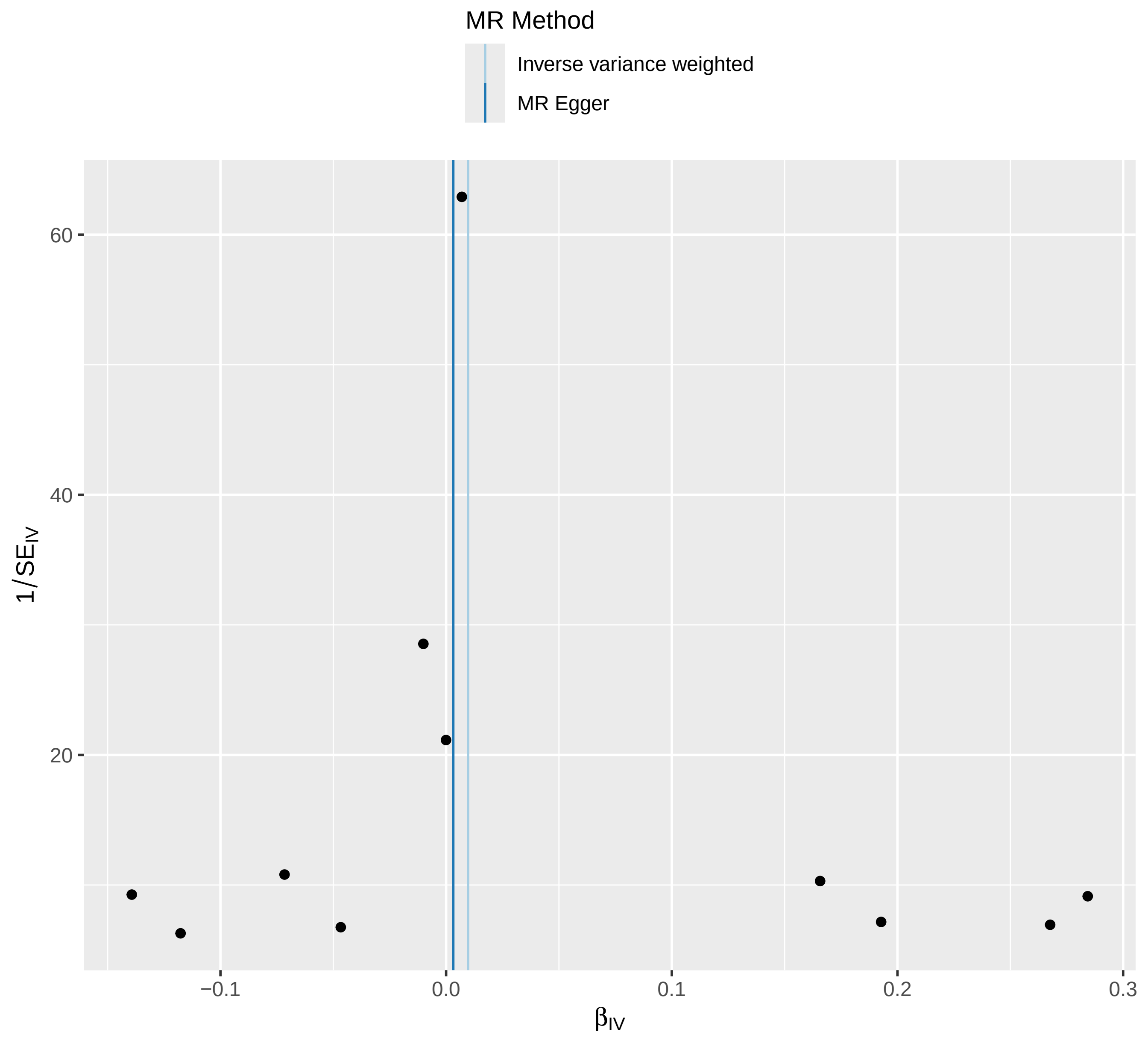
MR Method
Inverse variance weighted
MR Egger
60
40
20
$1/SE_{IV}$
−0.1
0.0
0.1
0.2
0.3
β_{IV}

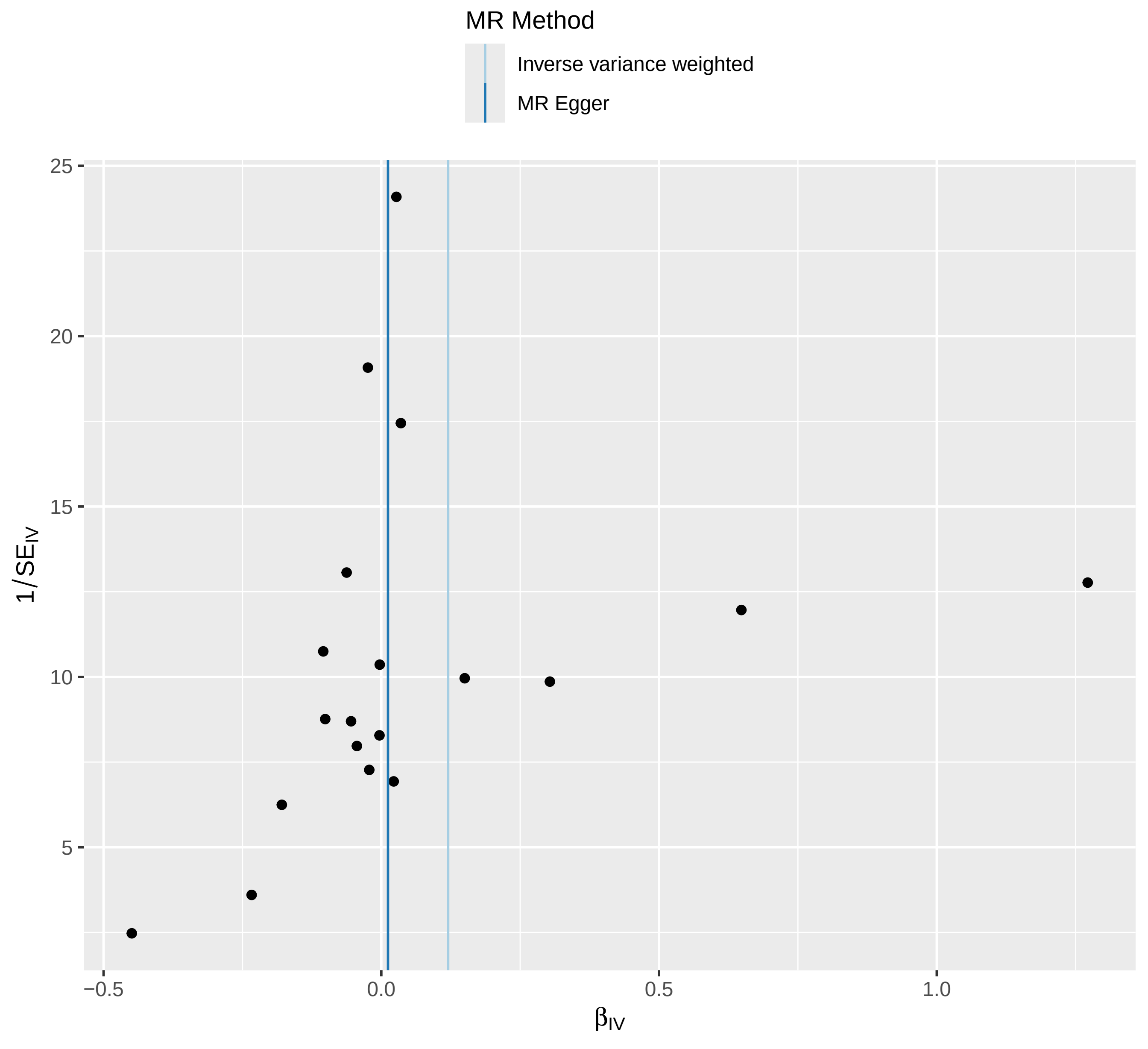
MR Method
Inverse variance weighted
MR Egger
25
20
15
10
5
1/SE$_{IV}$
−0.5
0.0
0.5
1.0
β$_{IV}$

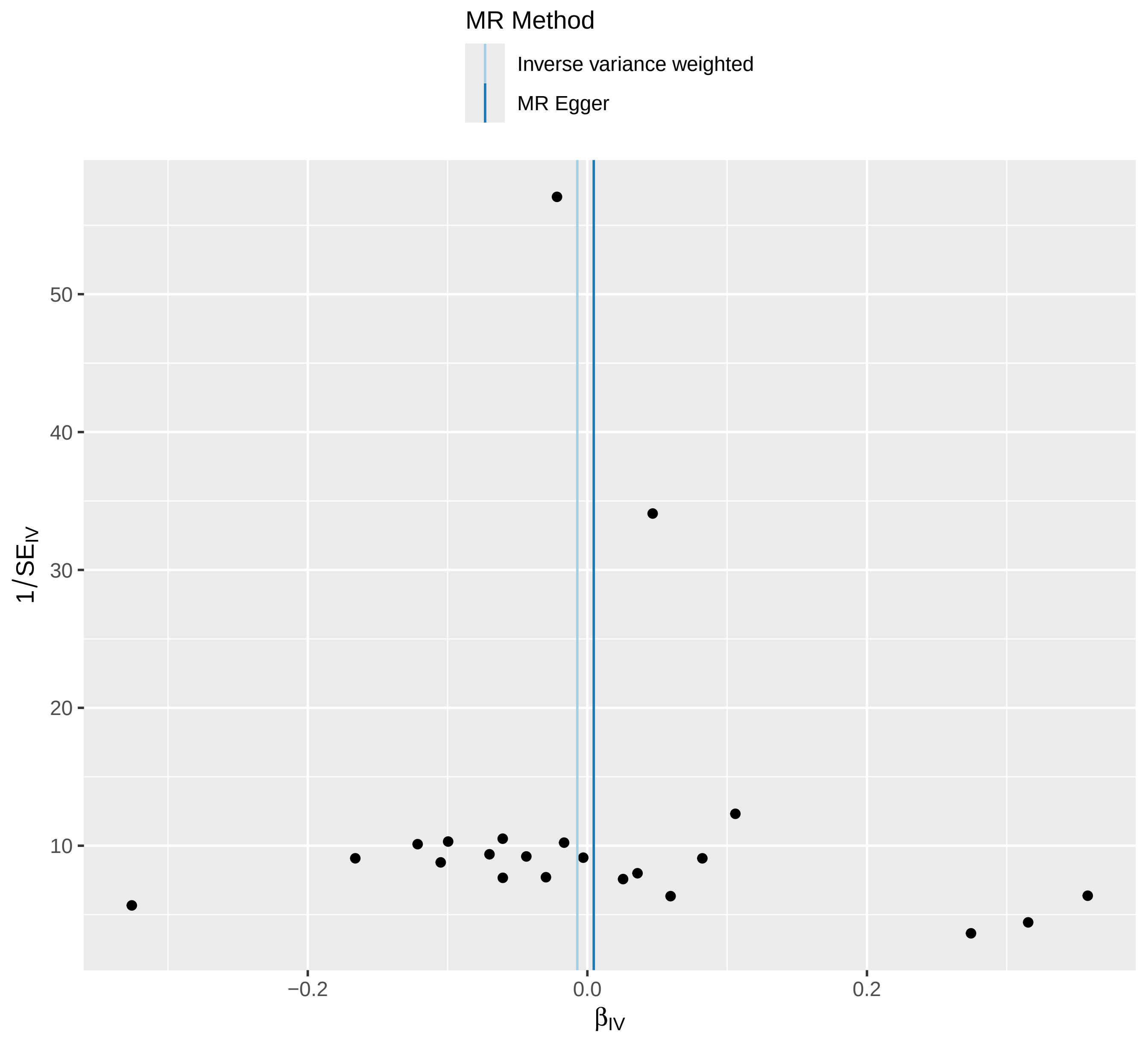
MR Method
Inverse variance weighted
MR Egger
50
40
30
20
10
1/SE_IV
−0.2
0.0
0.2
β_IV

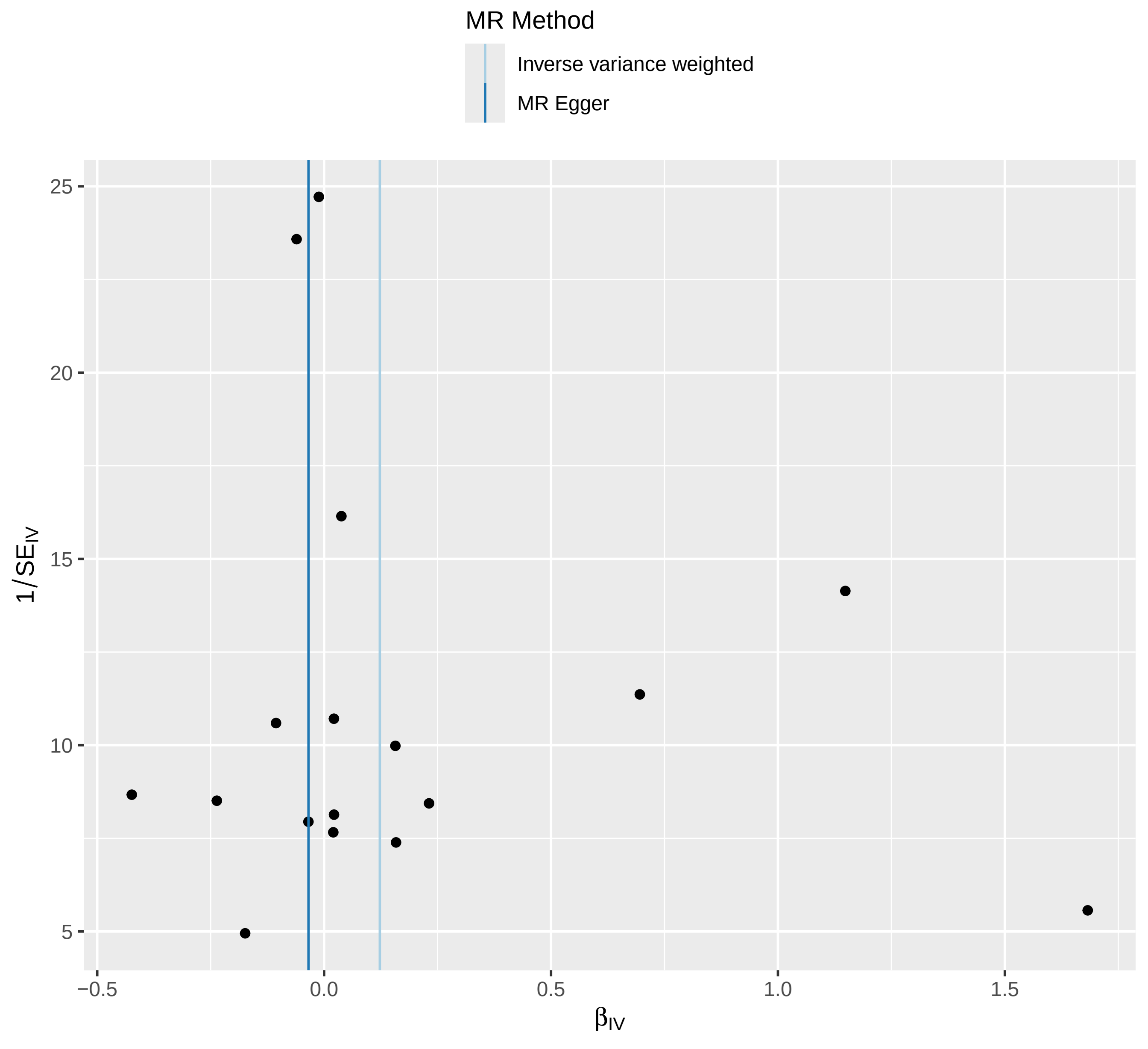
MR Method
Inverse variance weighted
MR Egger
25
20
15
10
5
1/SE$_{IV}$
−0.5
0.0
0.5
1.0
1.5
β$_{IV}$

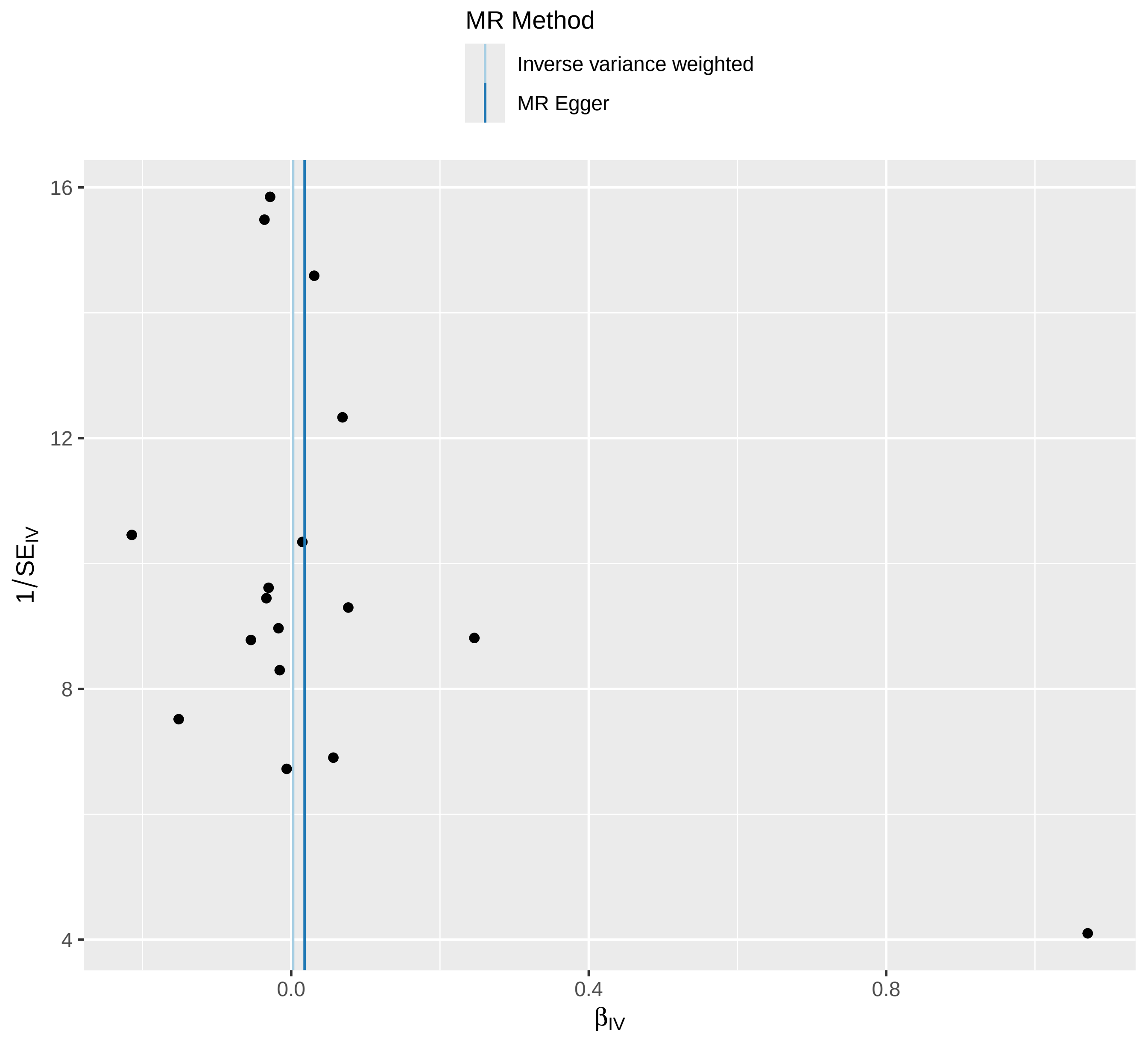
MR Method
Inverse variance weighted
MR Egger
1/SE_IV
16
12
8
4
0.0
0.4
0.8
β_IV

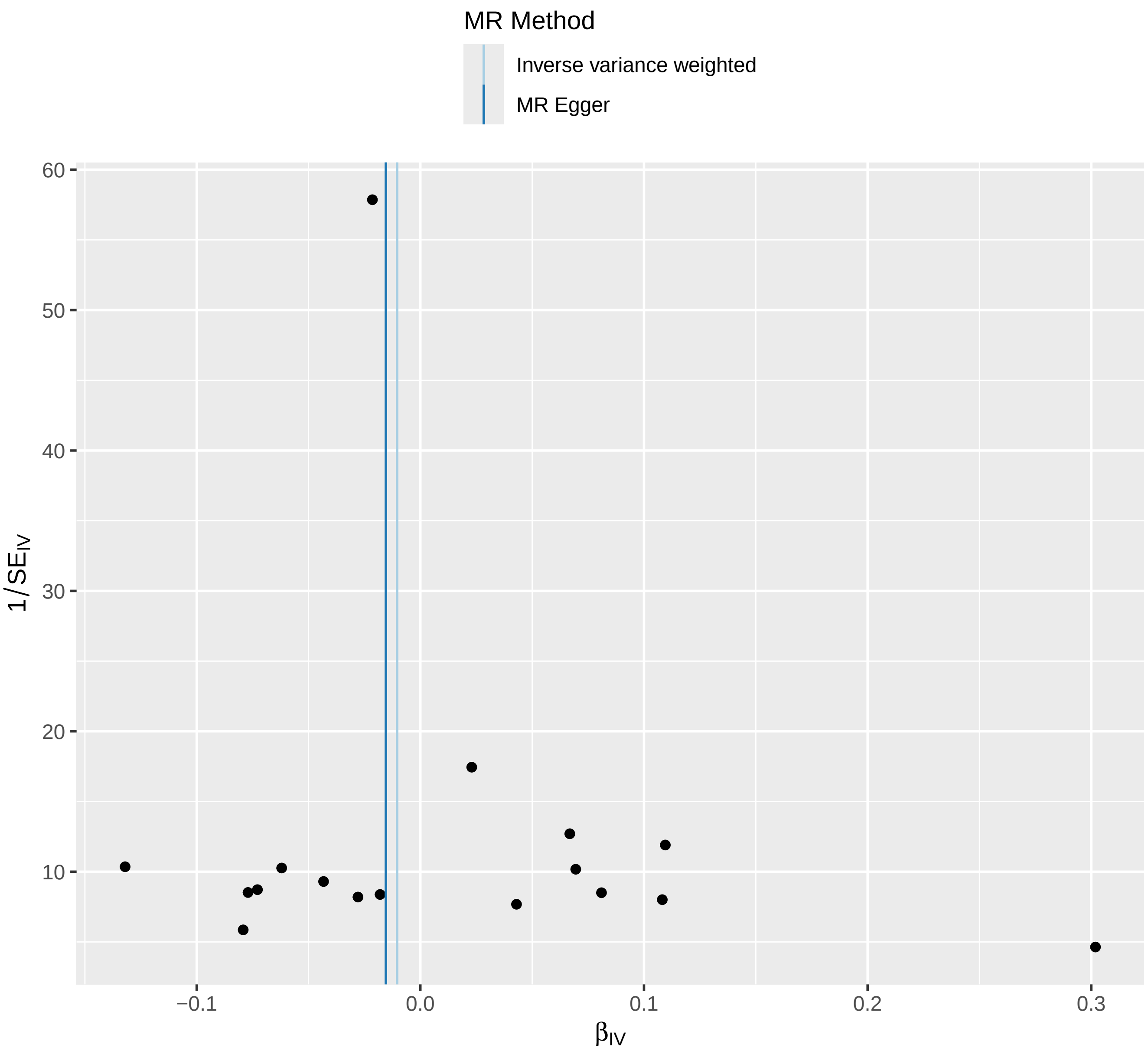

MR Method
Inverse variance weighted
MR Egger
60
50
40
30
20
10
1/SE_IV
−0.1
0.0
0.1
0.2
0.3
β_IV

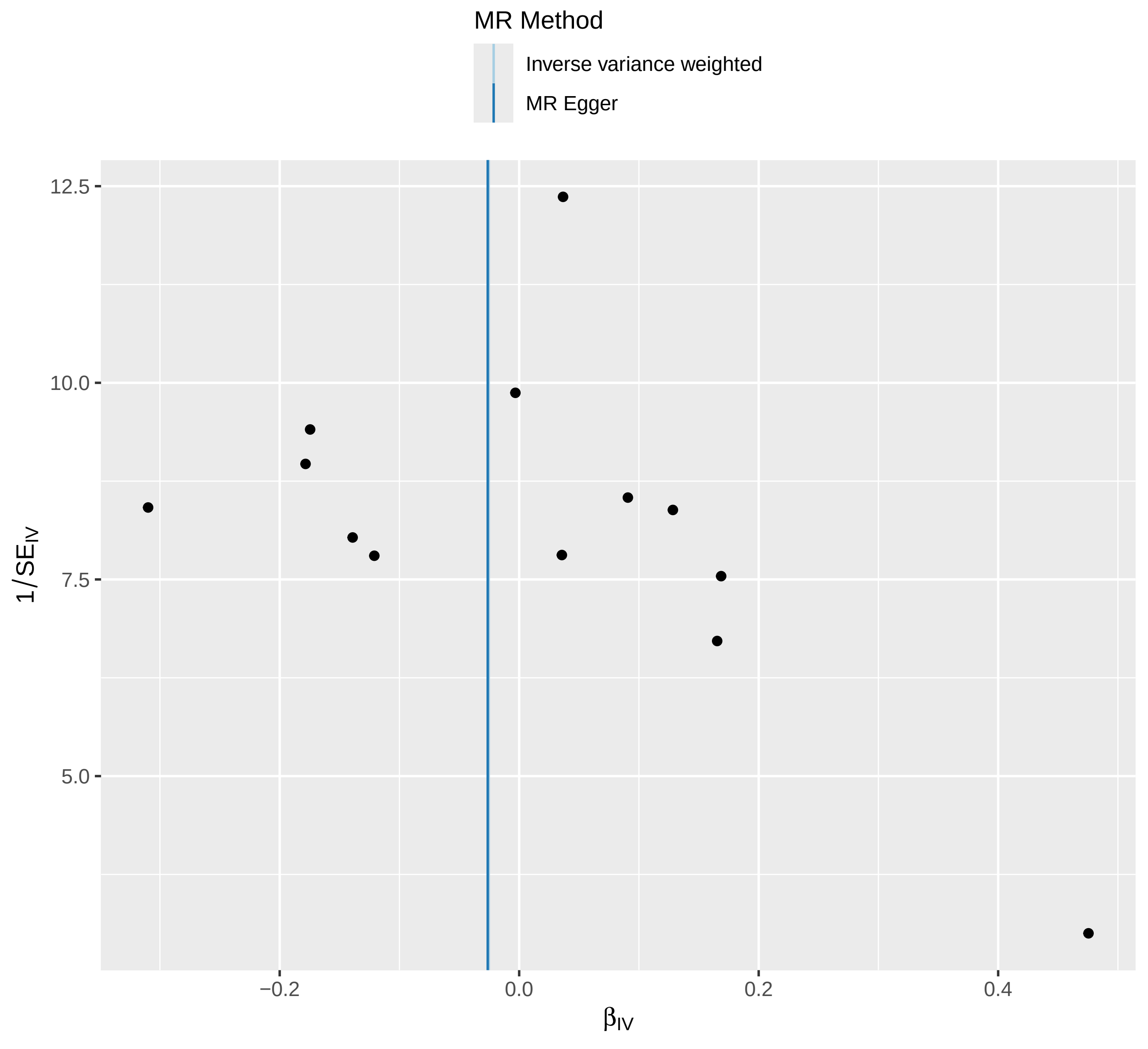
MR Method
Inverse variance weighted
MR Egger
12.5
10.0
7.5
5.0
$1/SE_{IV}$
−0.2
0.0
0.2
0.4
β_{IV}

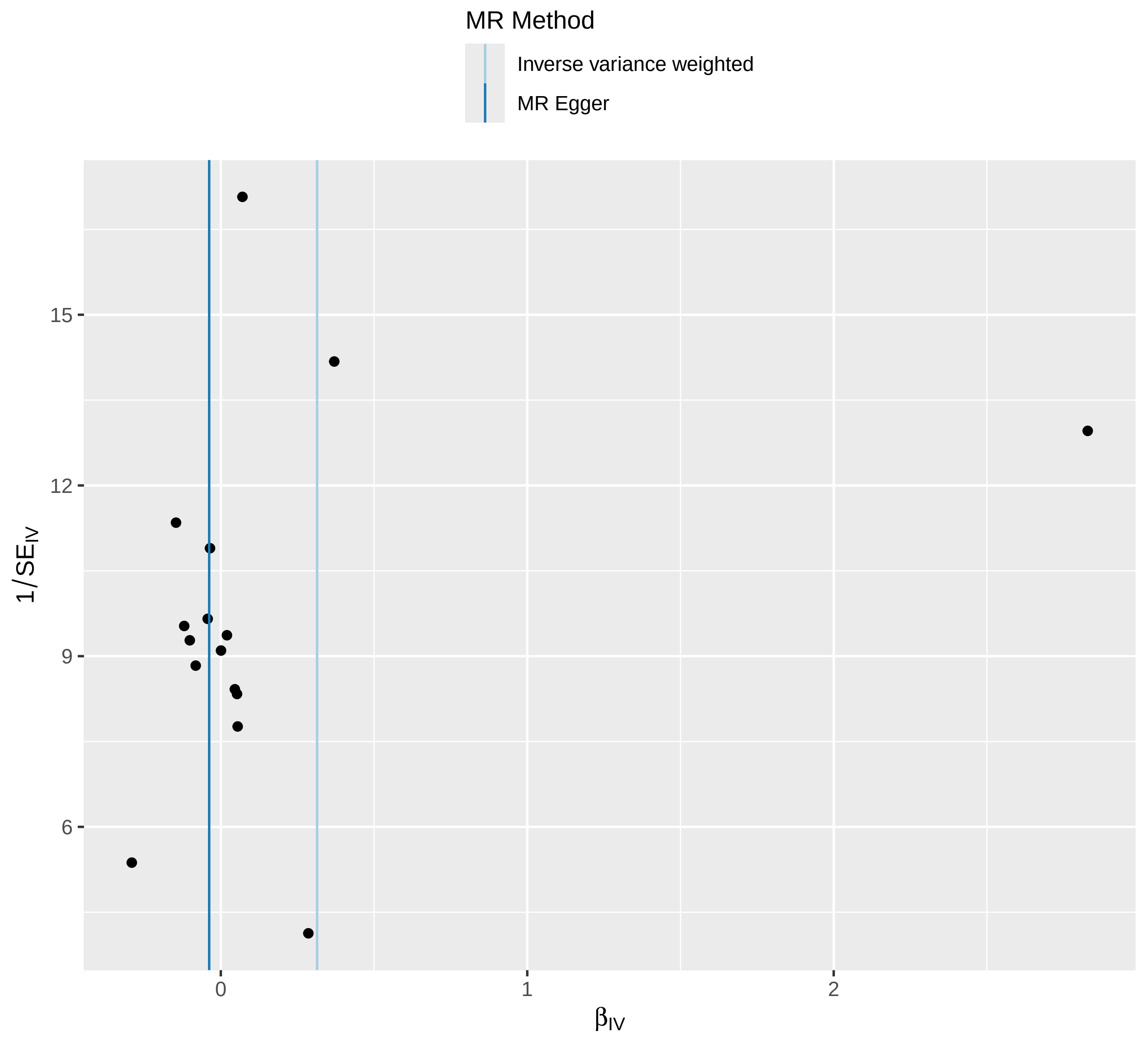

MR Method
Inverse variance weighted
MR Egger
15
12
9
6
0
1
2
1/SE_IV
β_IV

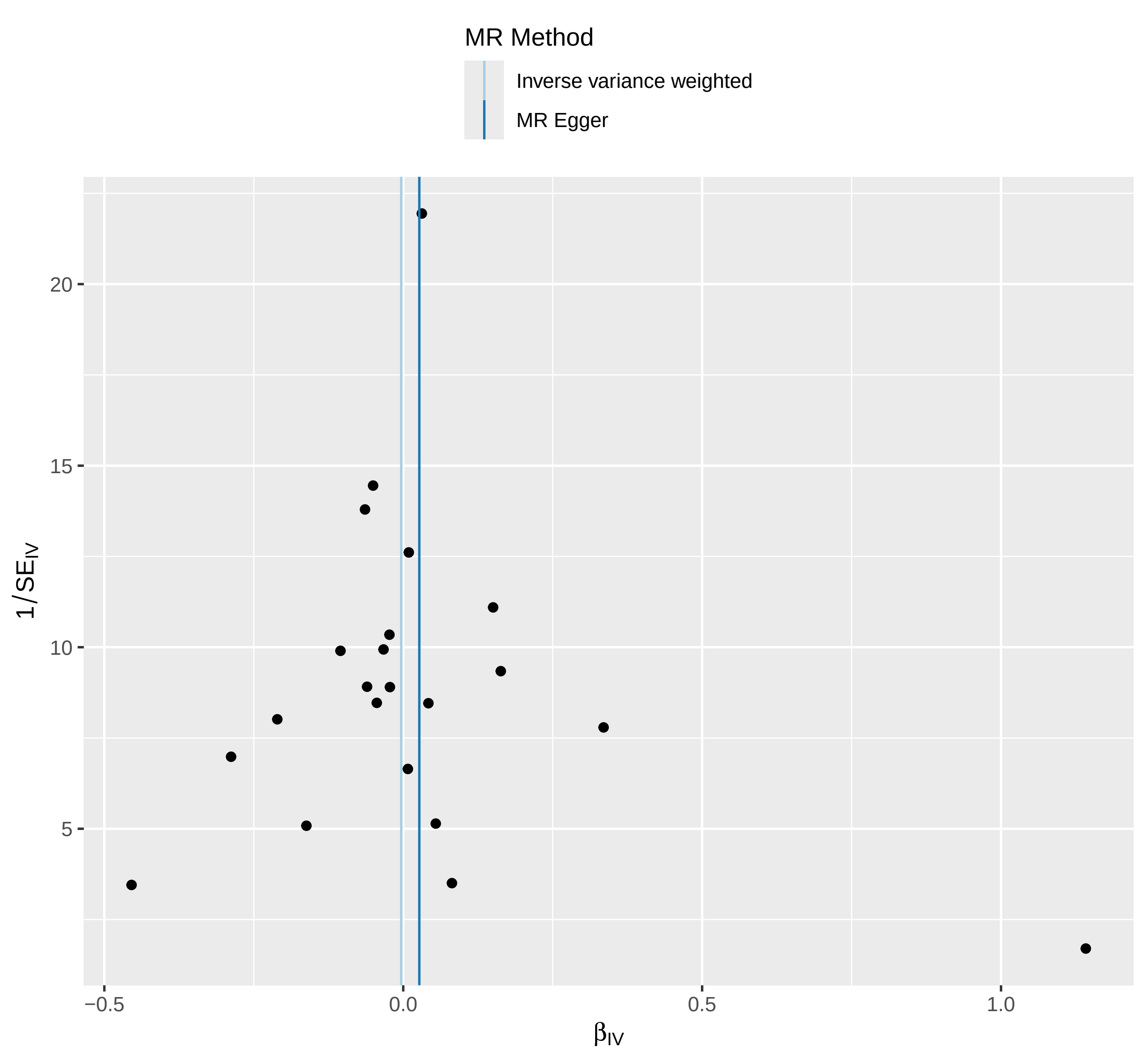
MR Method
Inverse variance weighted
MR Egger
20
15
10
5
1/SE_IV
−0.5
0.0
0.5
1.0
β_IV

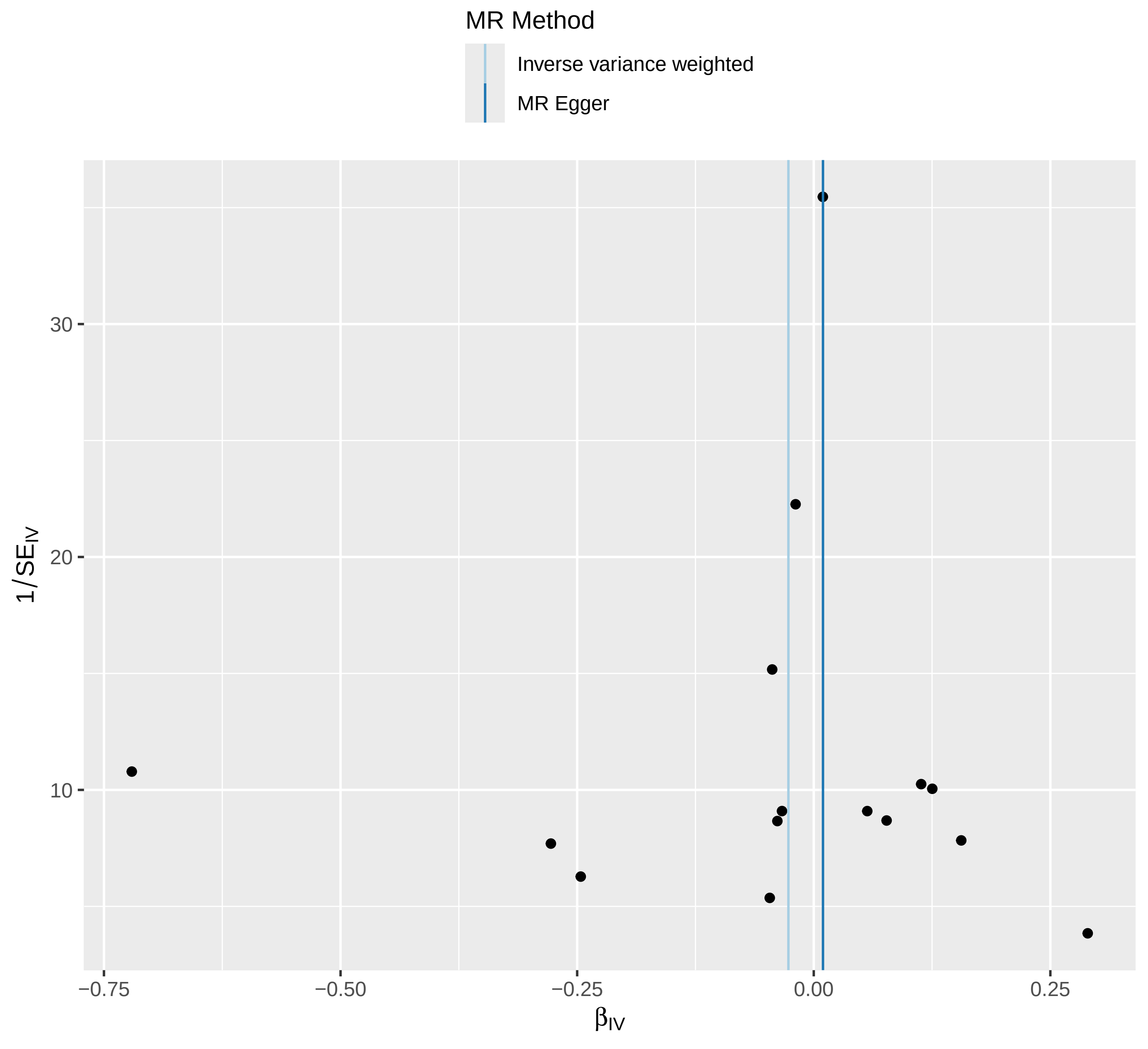

MR Method
Inverse variance weighted
MR Egger
30
20
10
1/SE$_{IV}$
−0.75
−0.50
−0.25
0.00
0.25
β$_{IV}$

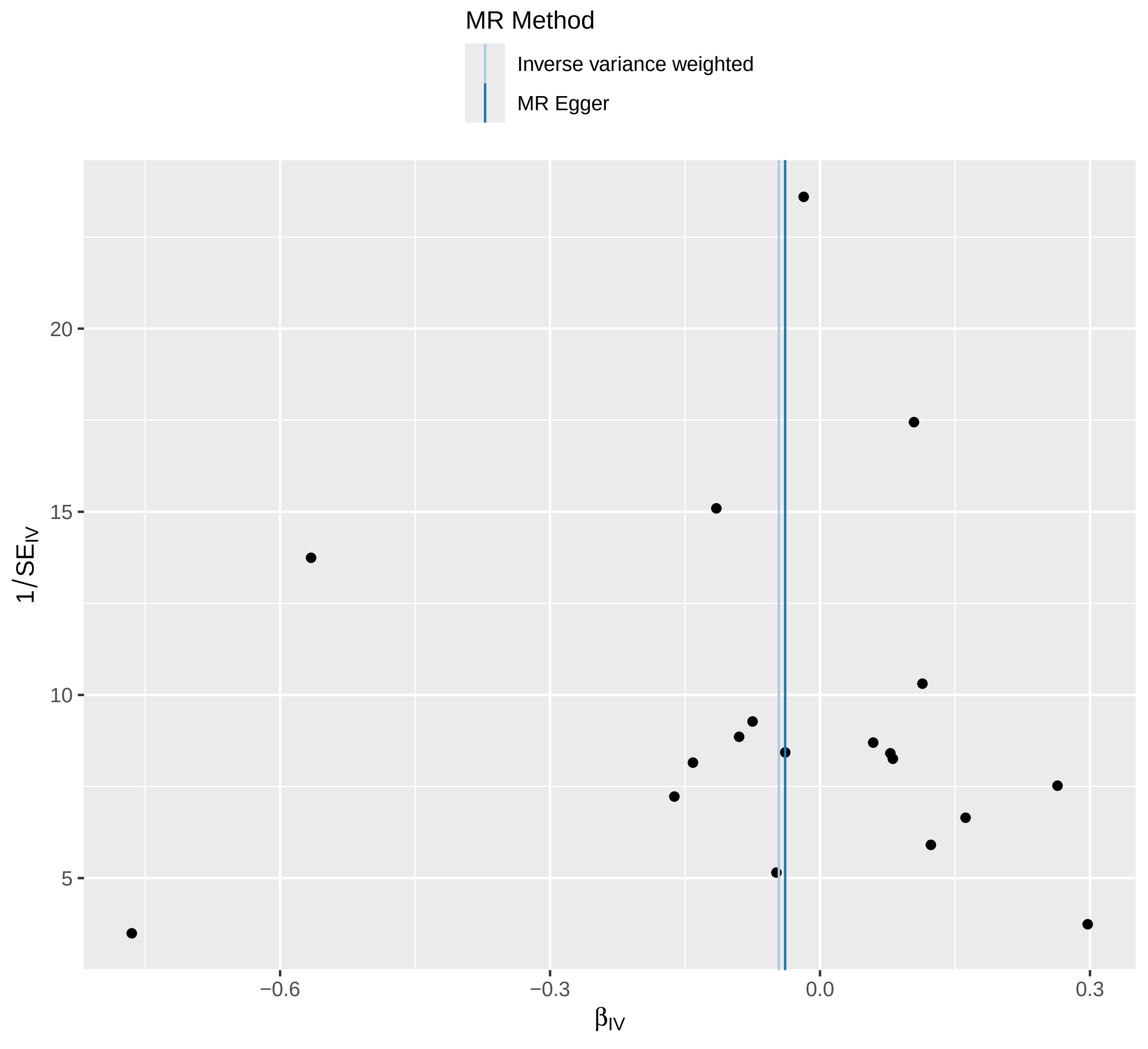
MR Method
Inverse variance weighted
MR Egger
20
15
10
5
1/SE$_{IV}$
−0.6
−0.3
0.0
0.3
β$_{IV}$

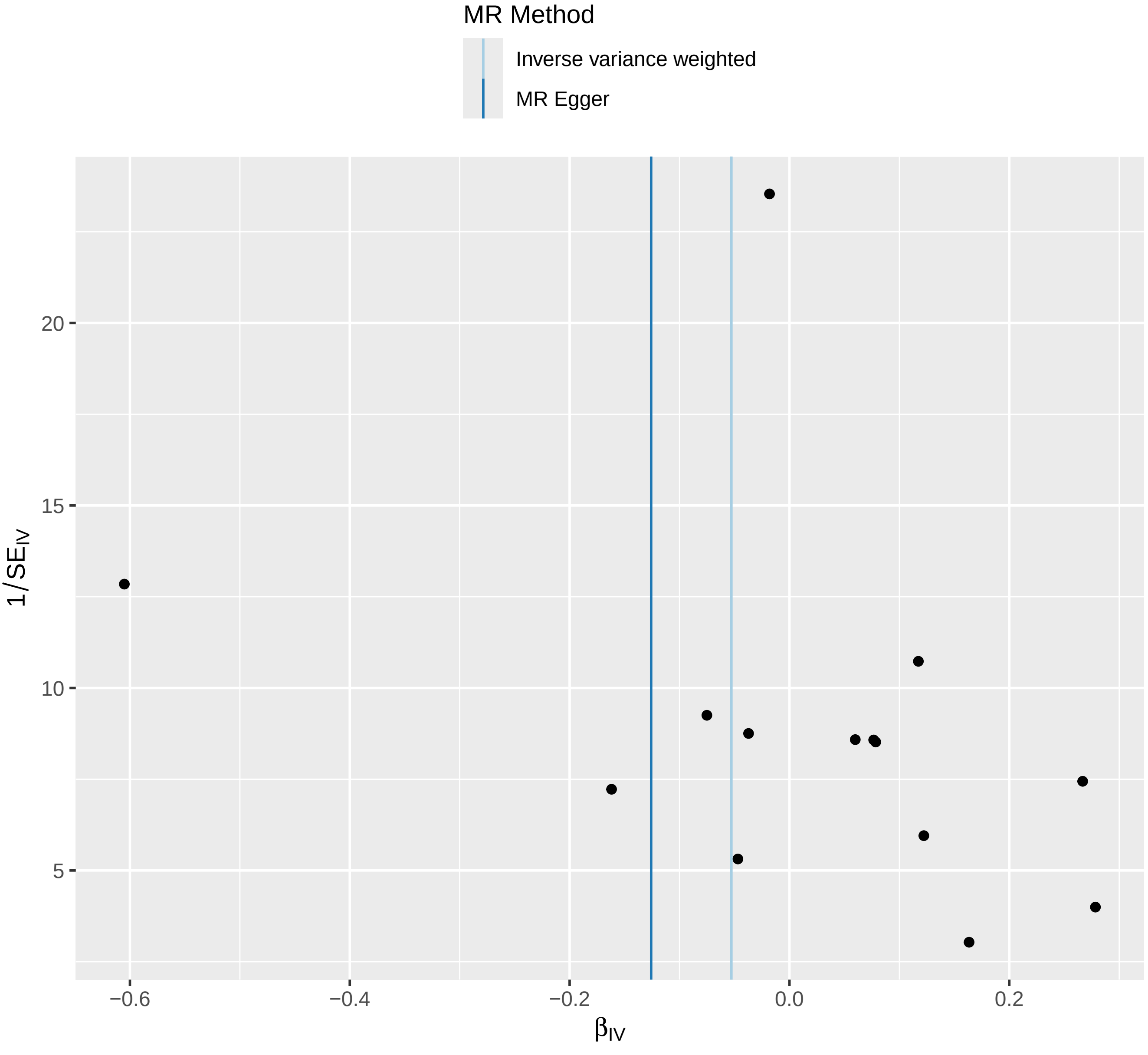

MR Method
Inverse variance weighted
MR Egger
20
15
10
5
1/SE$_{IV}$
−0.6
−0.4
−0.2
0.0
0.2
β$_{IV}$

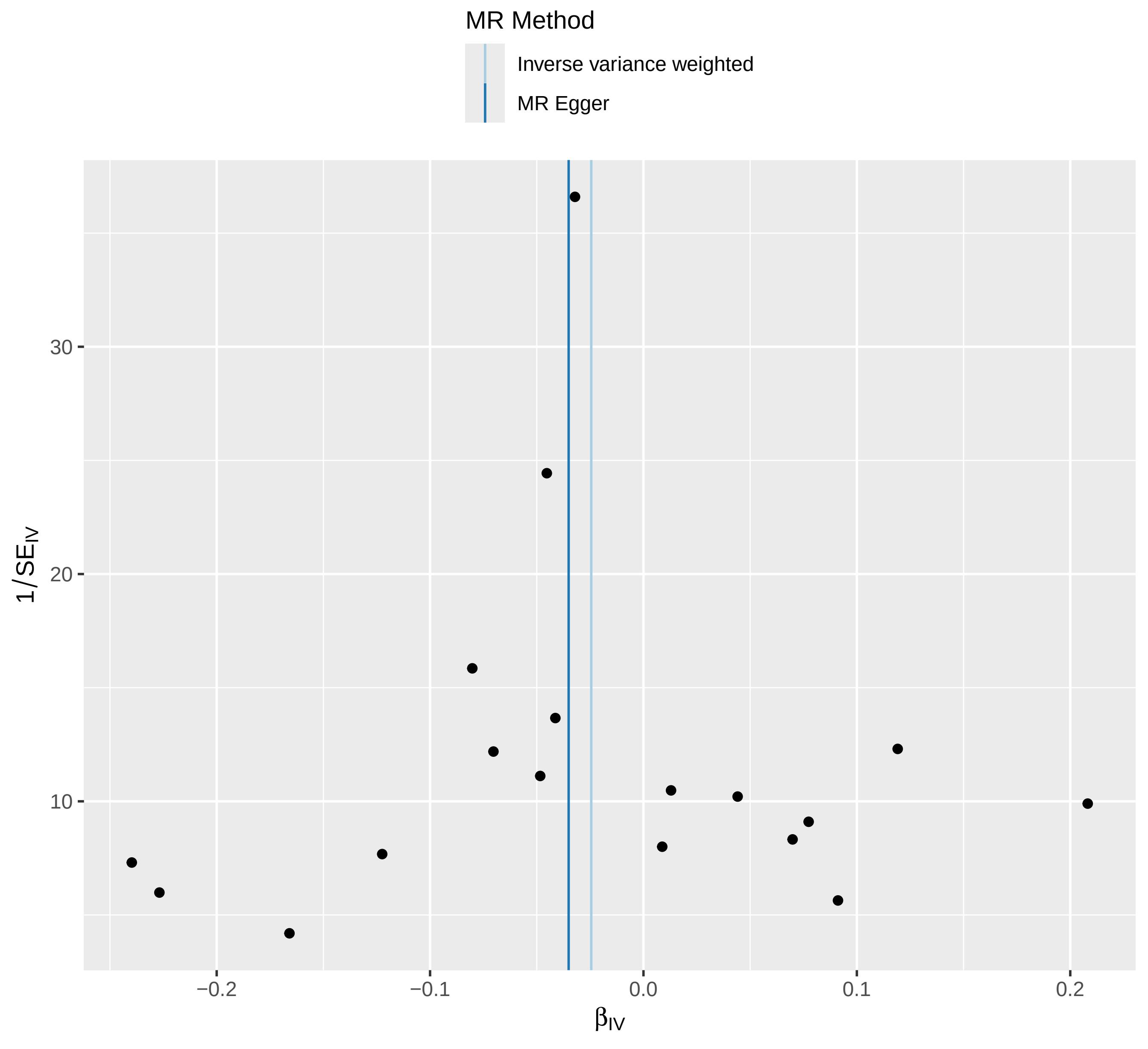
MR Method
Inverse variance weighted
MR Egger
1/SE_IV
30
20
10
−0.2
−0.1
0.0
0.1
0.2
β_IV

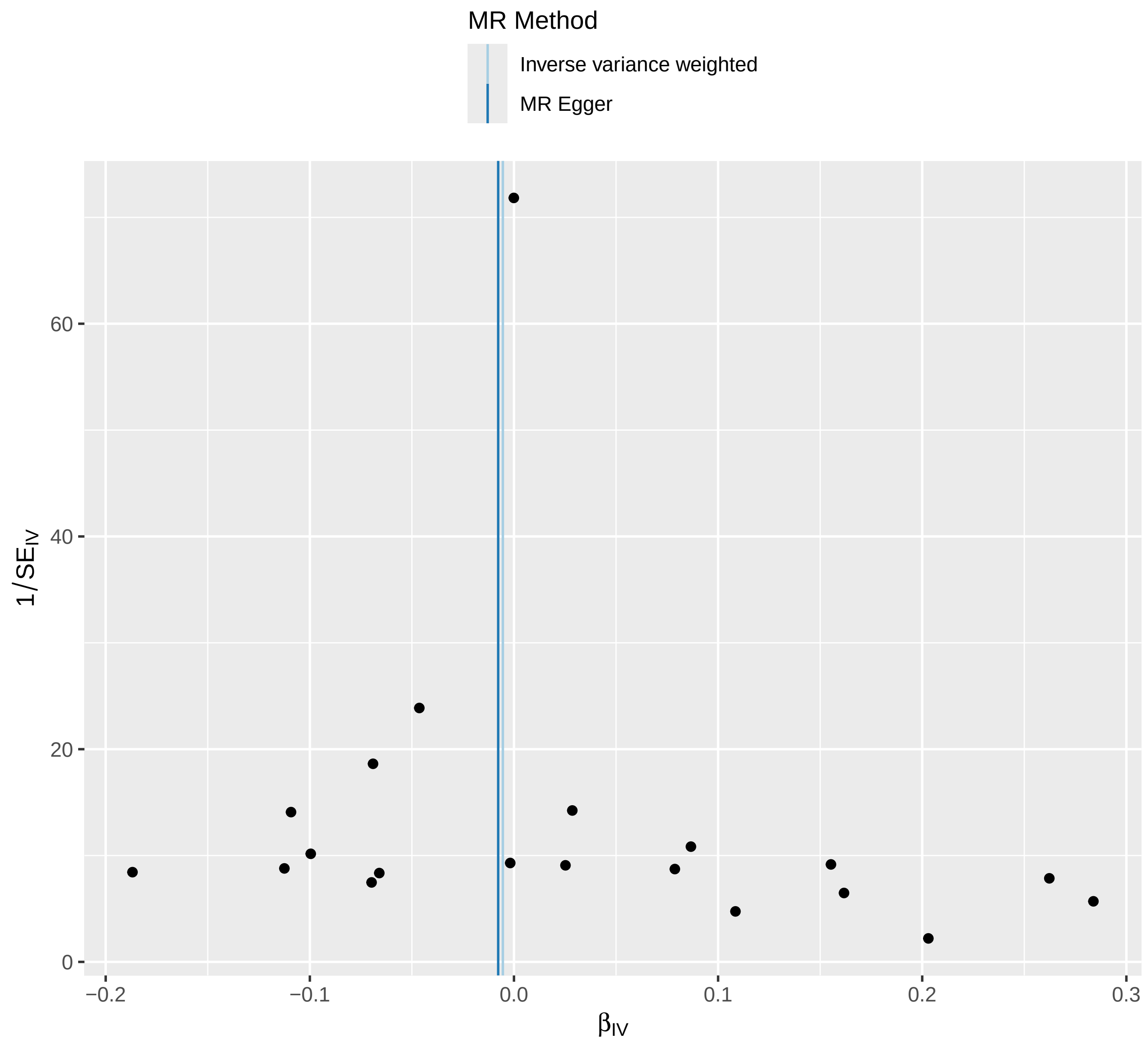
MR Method
Inverse variance weighted
MR Egger
60
40
20
0
$1/SE_{IV}$
−0.2
−0.1
0.0
0.1
0.2
0.3
β_{IV}

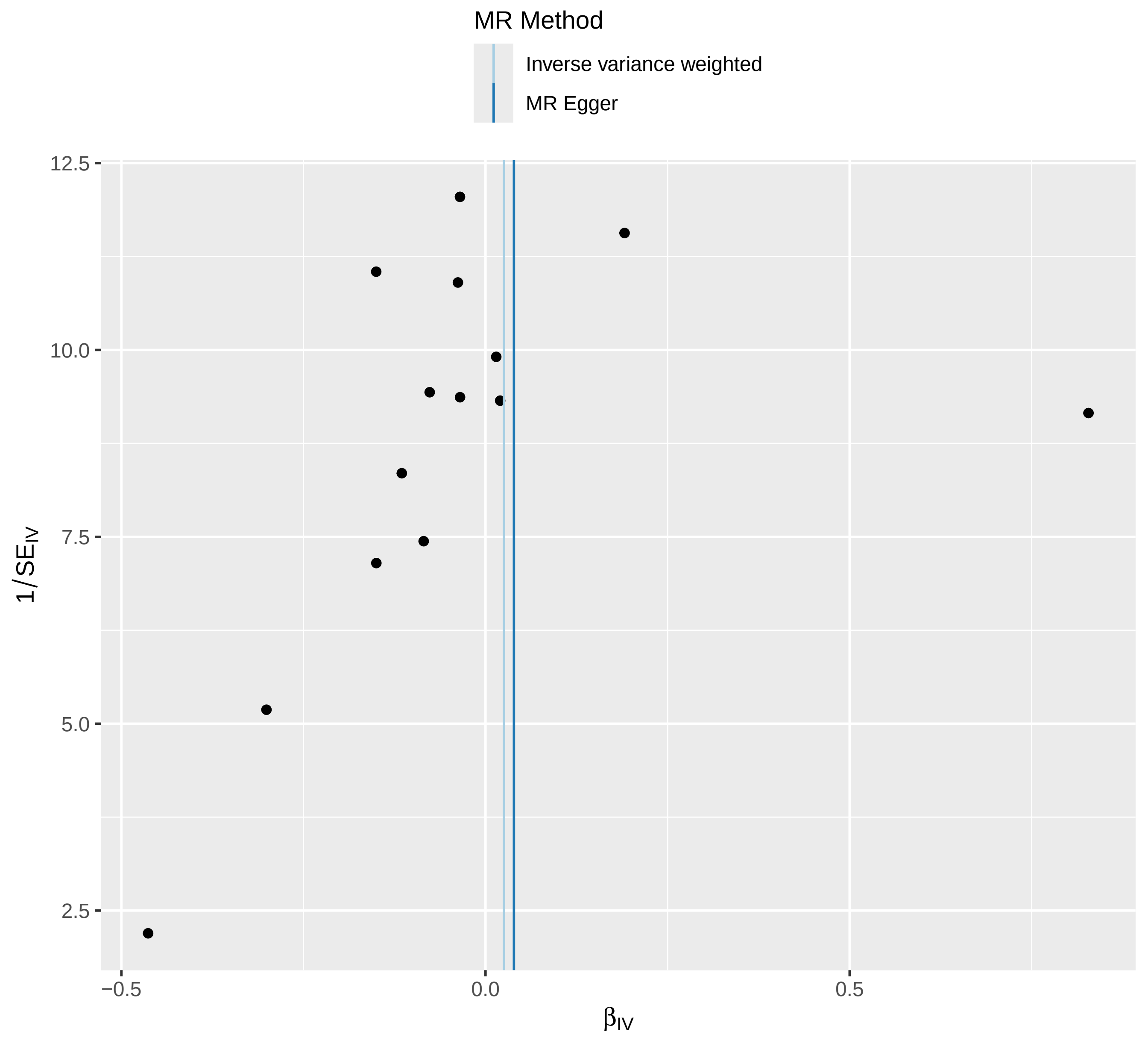
MR Method
Inverse variance weighted
MR Egger
12.5
10.0
7.5
5.0
2.5
1/SE$_{IV}$
−0.5
0.0
0.5
β$_{IV}$

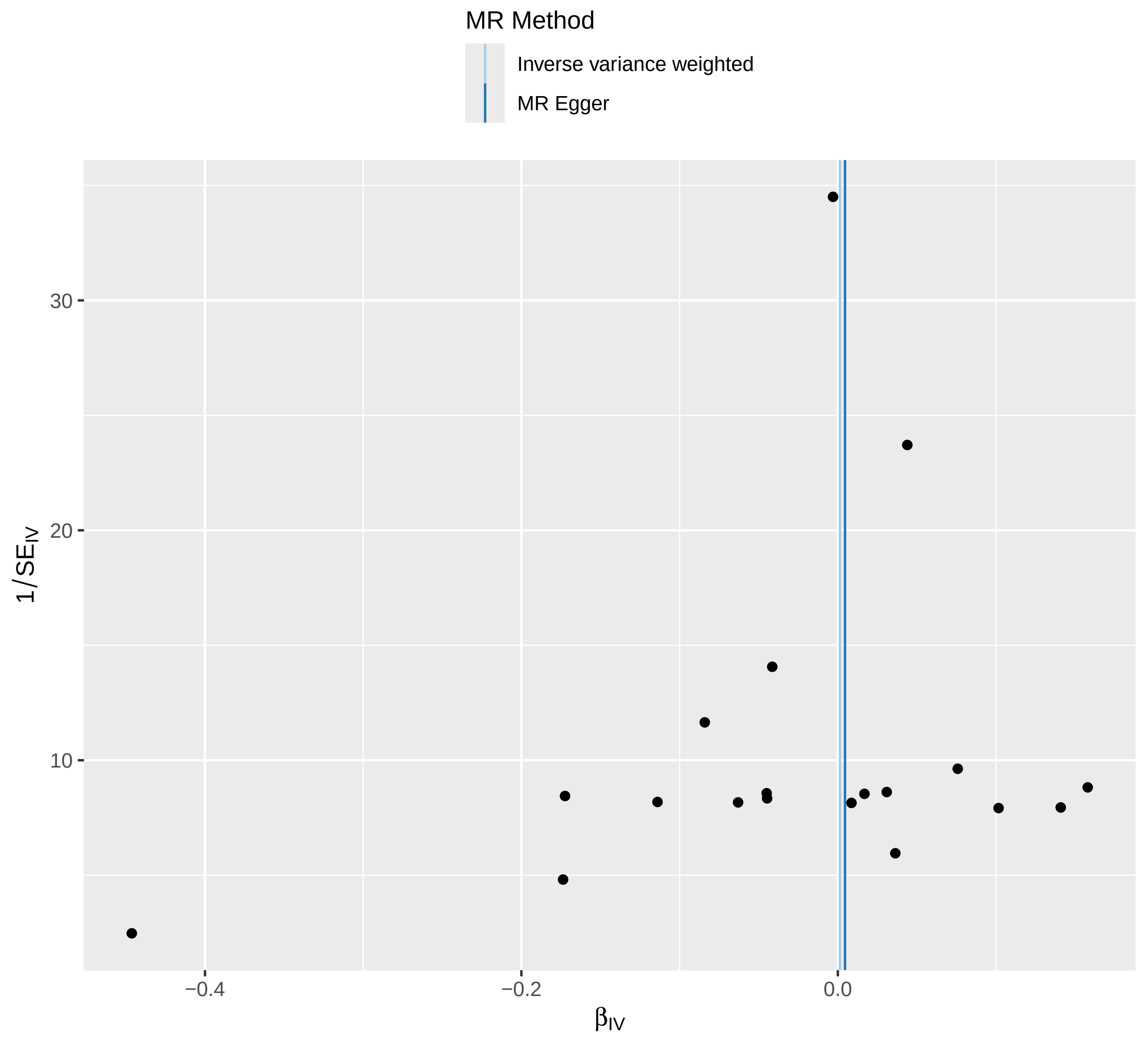
MR Method
Inverse variance weighted
MR Egger
30
20
10
1/SE_IV
−0.4
−0.2
0.0
β_IV

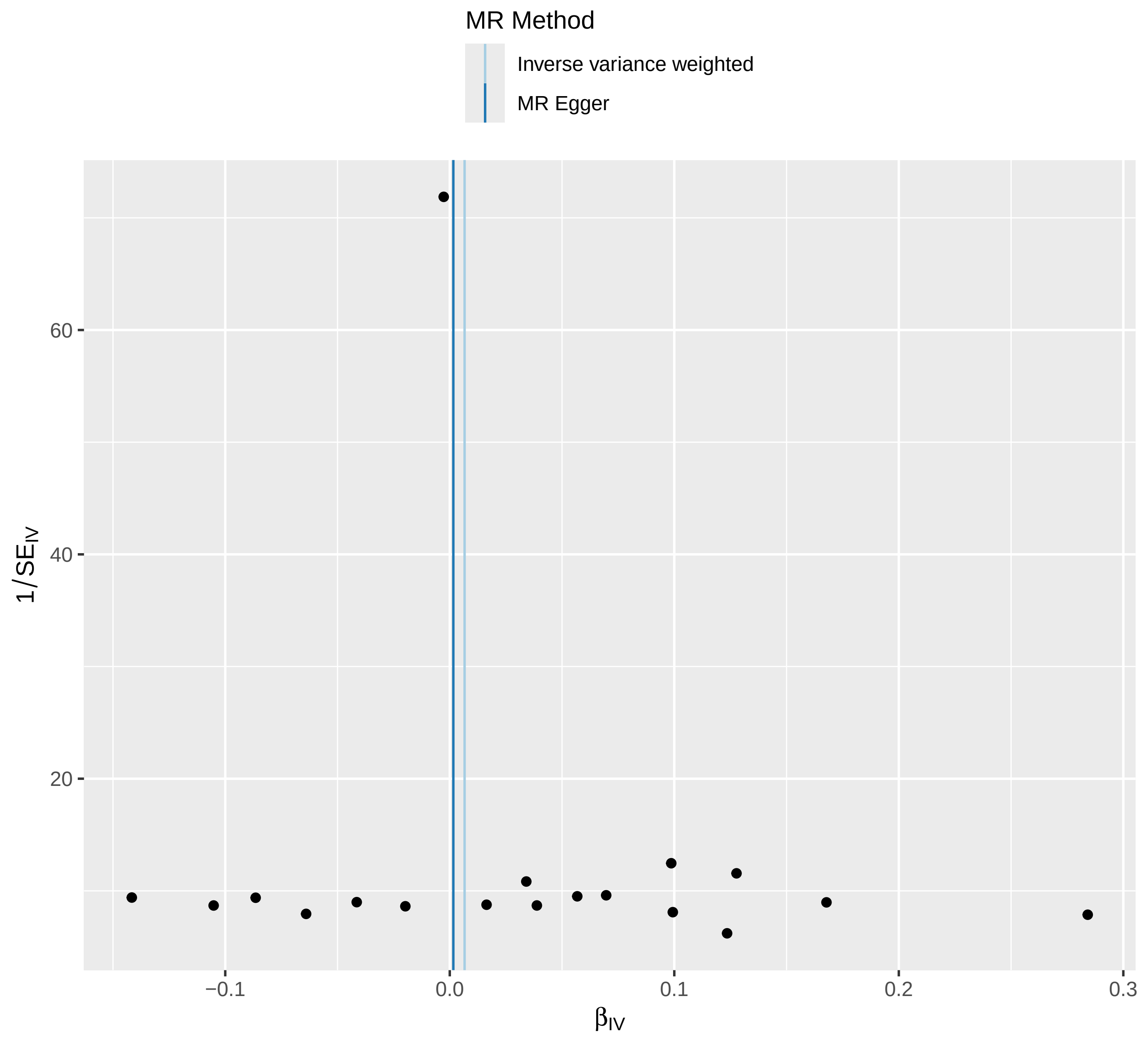

MR Method
Inverse variance weighted
MR Egger
60
40
20
1/SE$_{IV}$
−0.1
0.0
0.1
0.2
0.3
β$_{IV}$

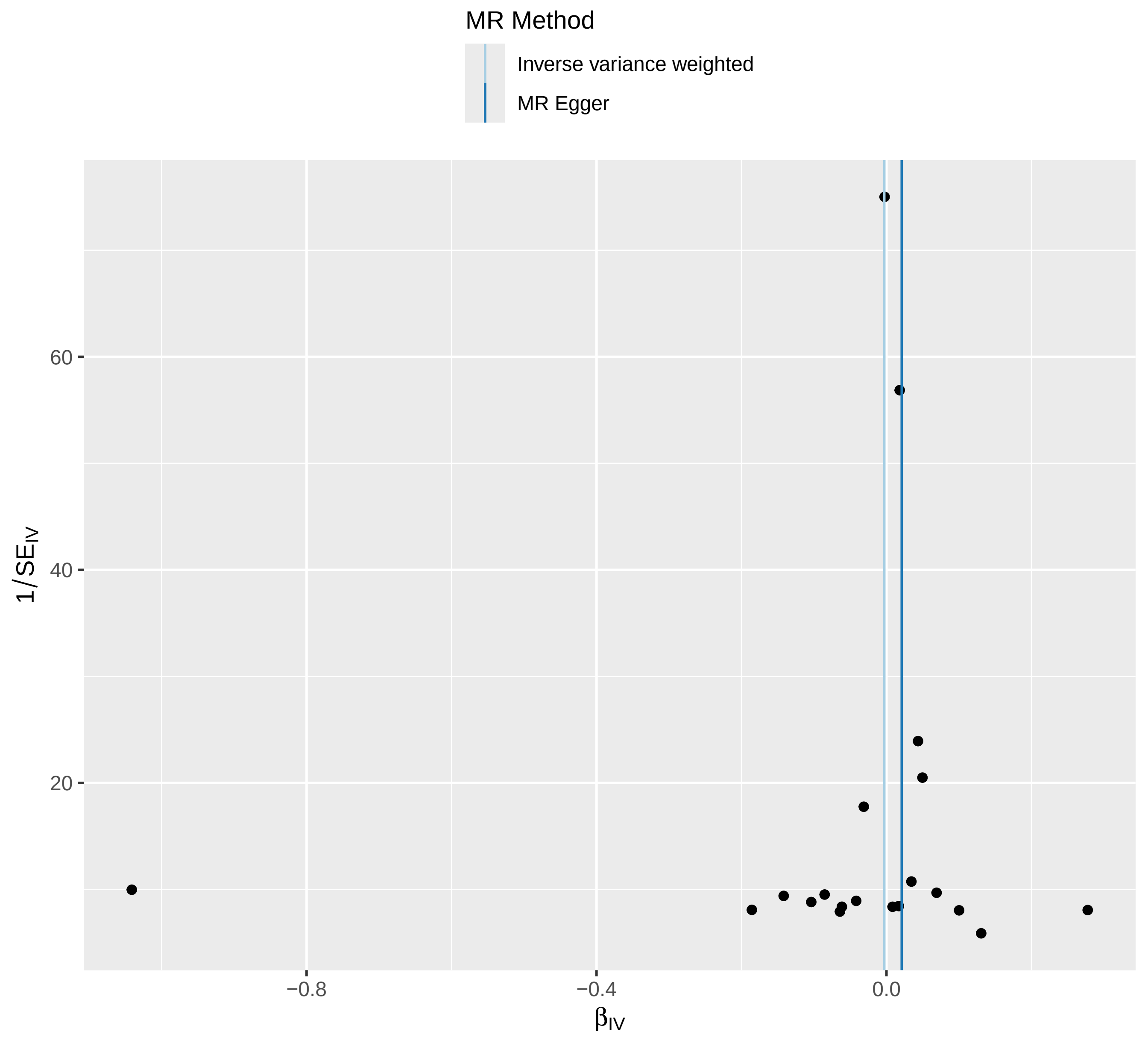
MR Method
Inverse variance weighted
MR Egger
60
40
20
1/SE$_{IV}$
−0.8
−0.4
0.0
β$_{IV}$

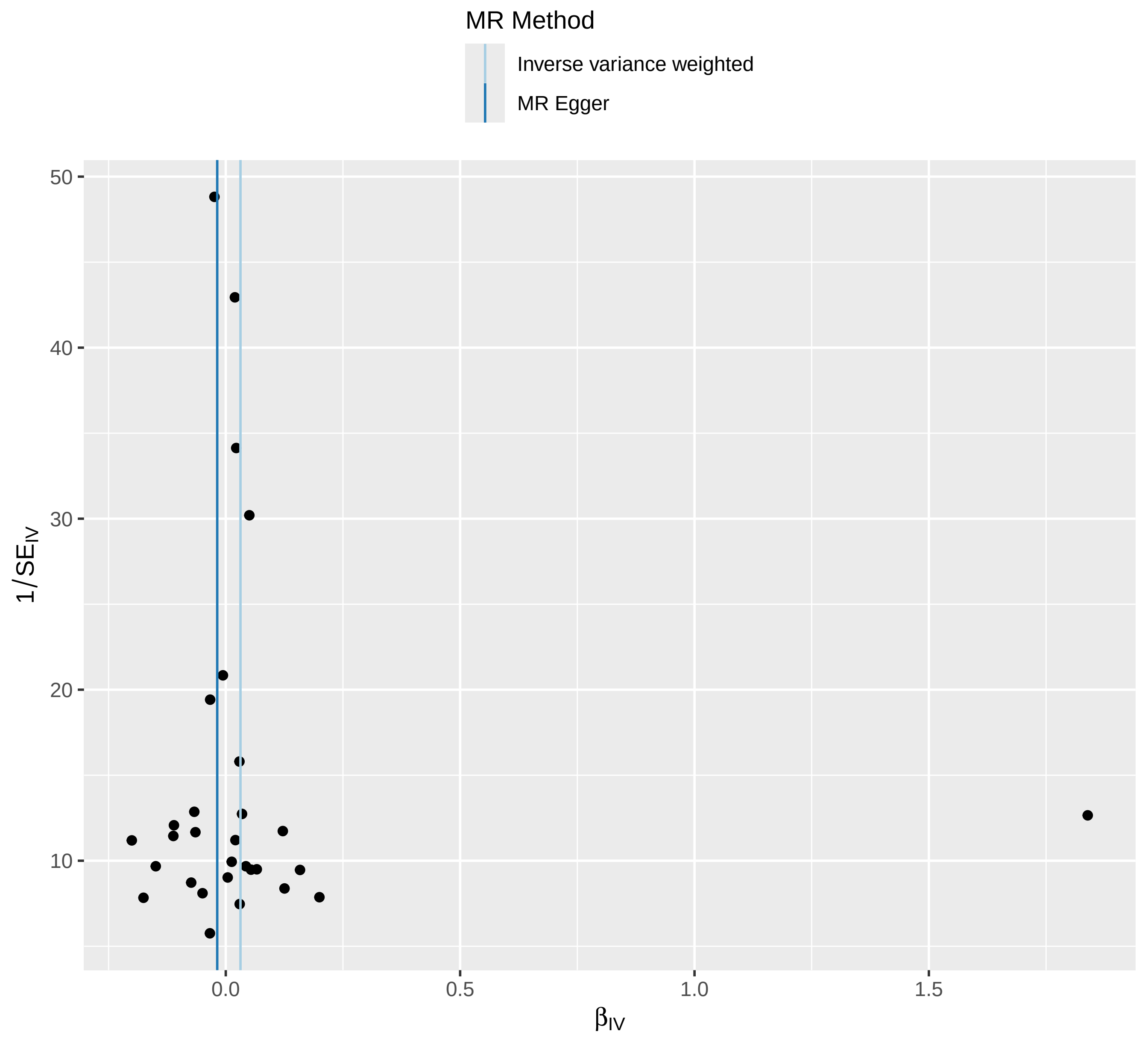

MR Method
Inverse variance weighted
MR Egger
50
40
30
20
10
1/SE_IV
0.0
0.5
1.0
1.5
β_IV

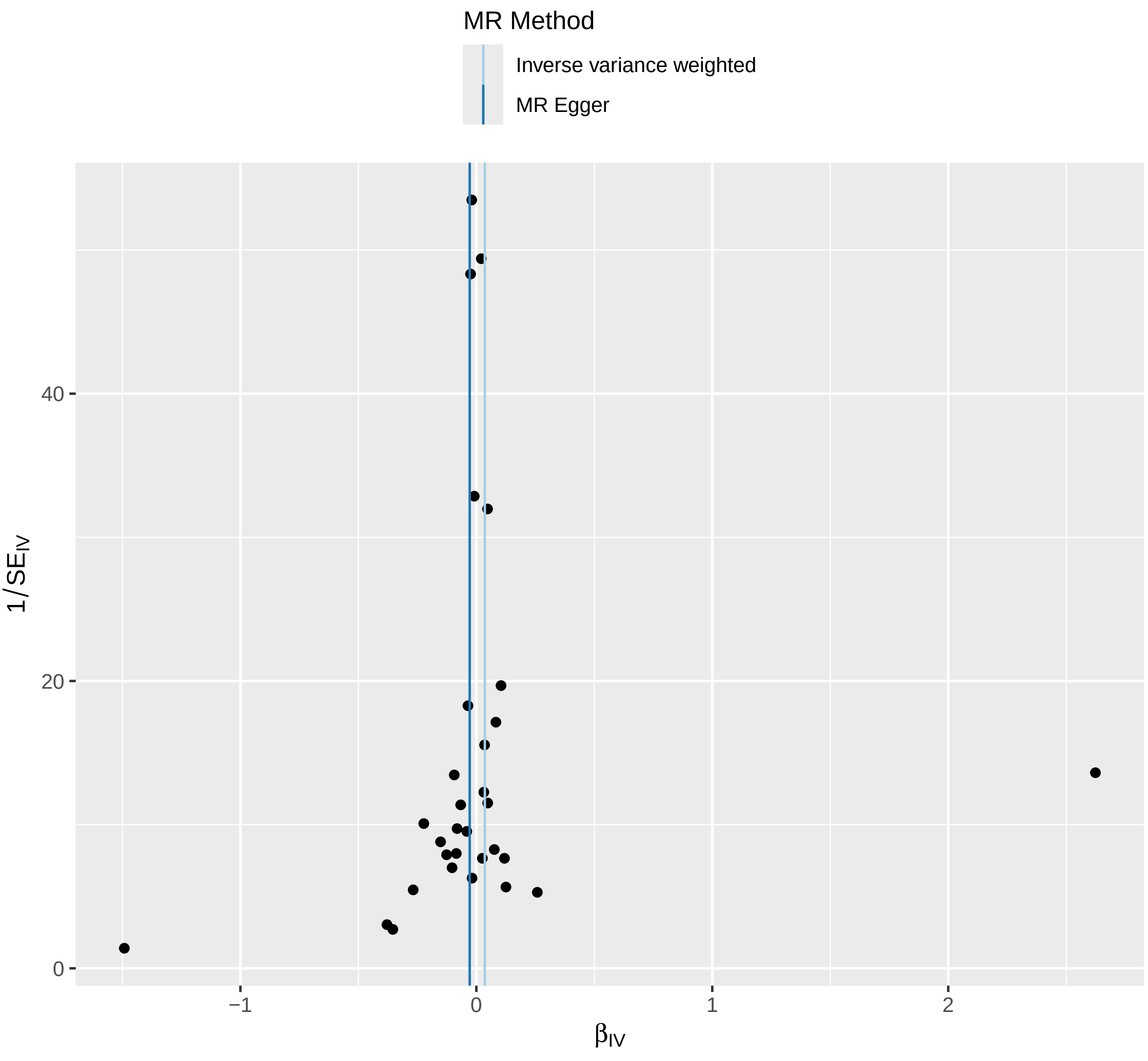
MR Method
Inverse variance weighted
MR Egger
1/SE_IV
β_IV
40
20
0
−1
0
1
2

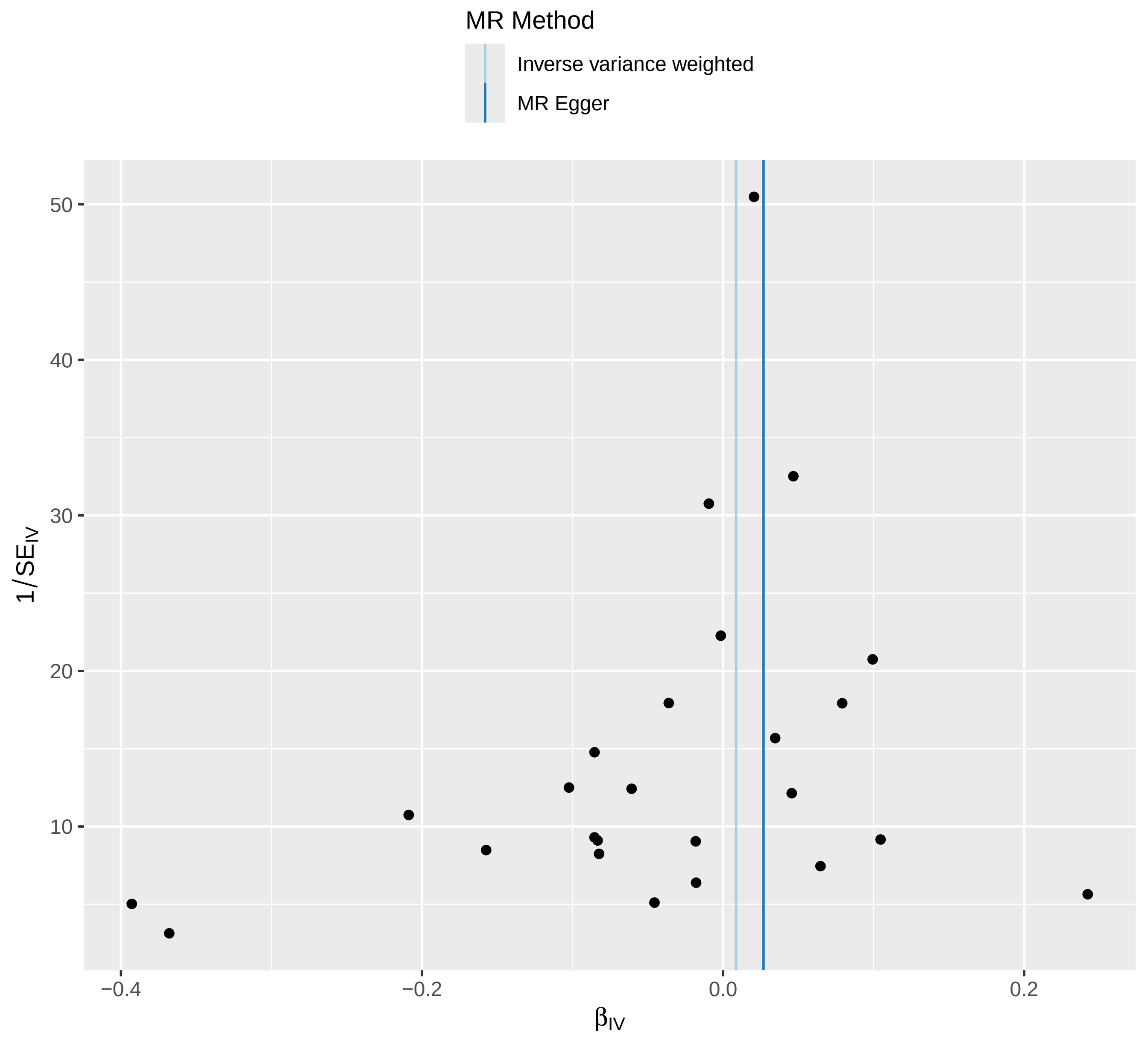

MR Method
Inverse variance weighted
MR Egger
50
40
30
20
10
1/SE_IV
−0.4
−0.2
0.0
0.2
β_IV

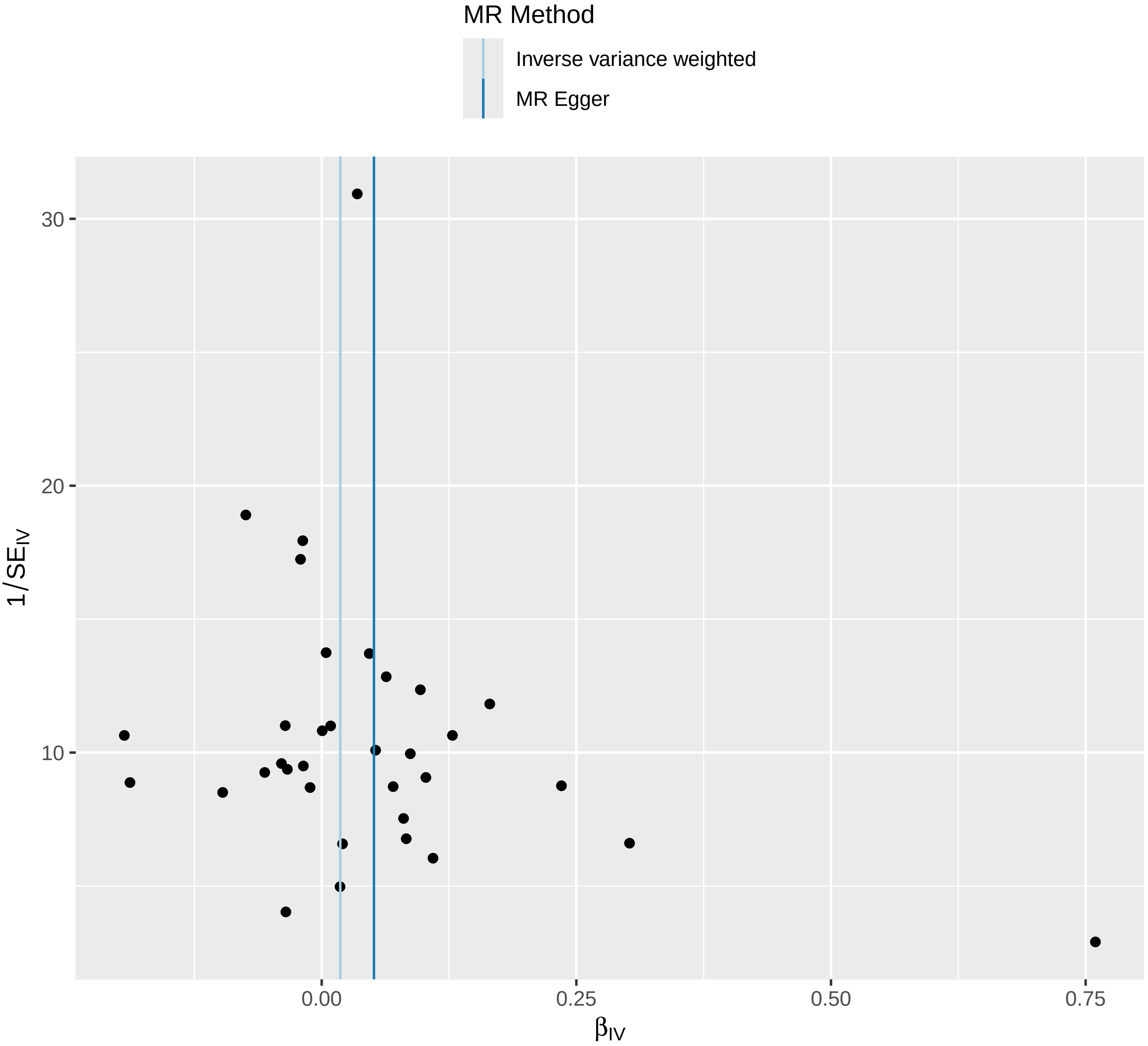
MR Method
Inverse variance weighted
MR Egger
30
20
10
0.00
0.25
0.50
0.75
1/SE_IV
β_IV

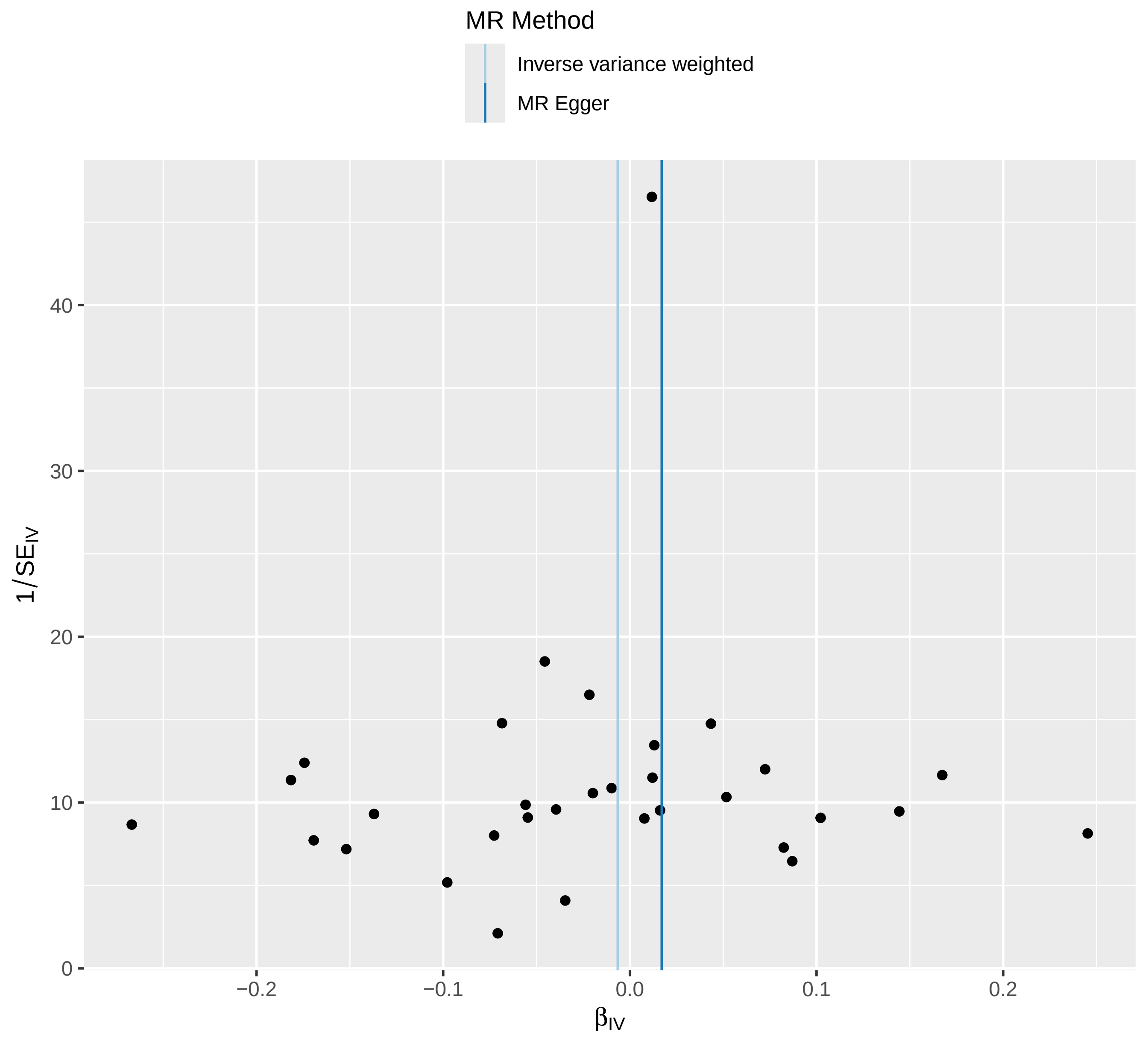
MR Method
Inverse variance weighted
MR Egger
40
30
20
10
0
1/SE$_{IV}$
−0.2
−0.1
0.0
0.1
0.2
β$_{IV}$

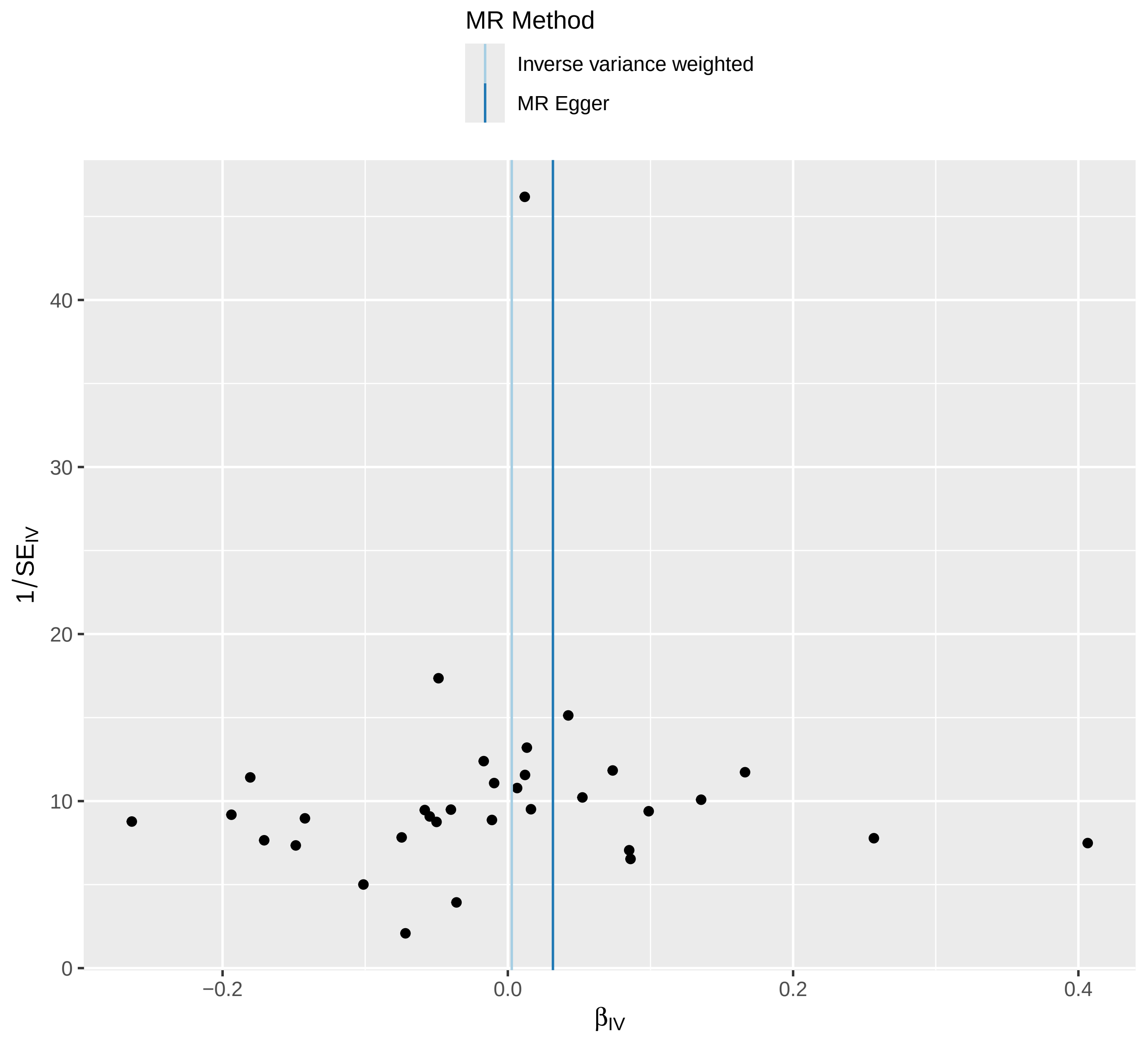
MR Method
Inverse variance weighted
MR Egger
40
30
20
10
0
1/SE_{IV}
−0.2
0.0
0.2
0.4
β_{IV}

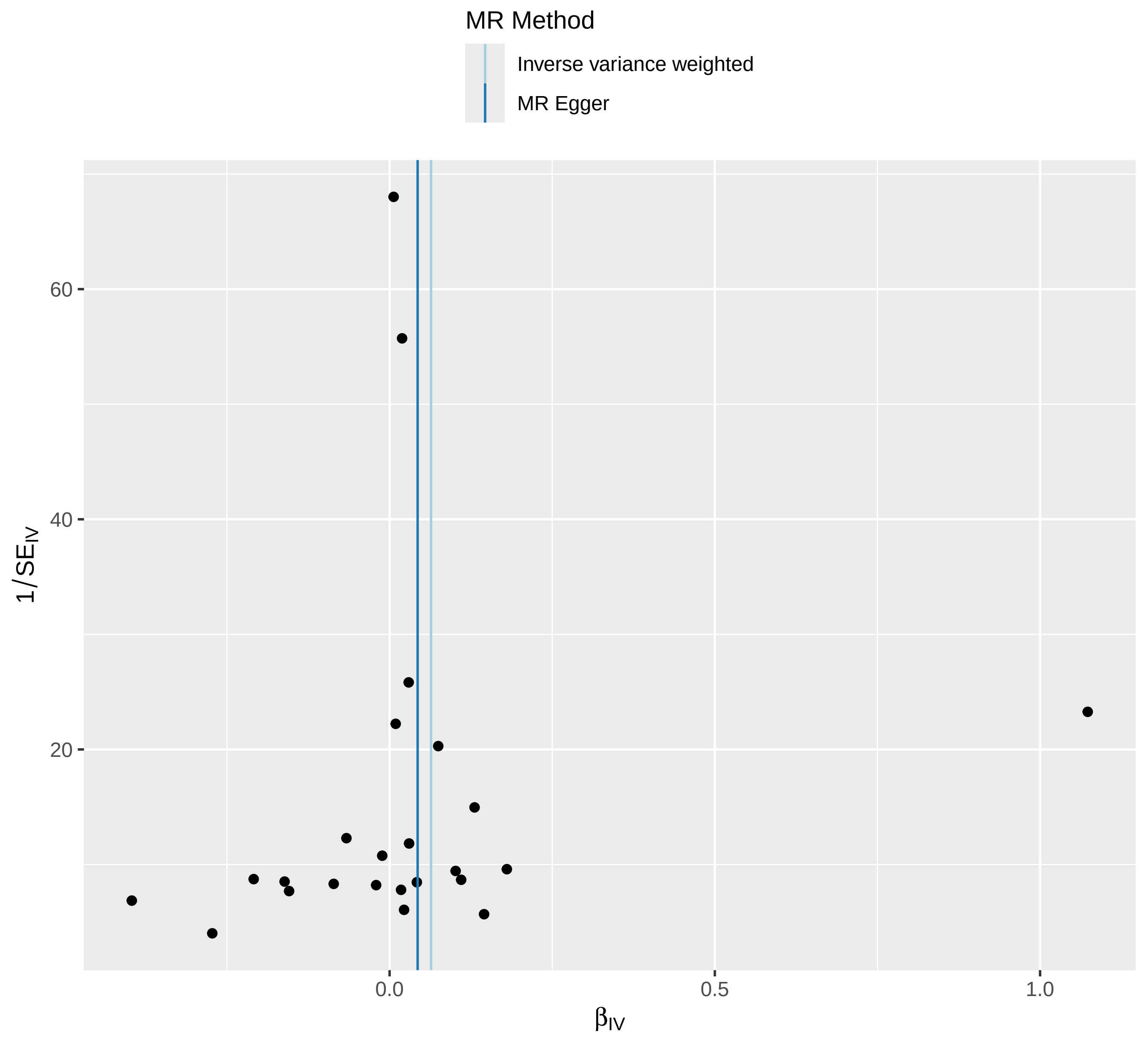
MR Method
Inverse variance weighted
MR Egger
60
40
20
1/SE$_{IV}$
0.0
0.5
1.0
β$_{IV}$

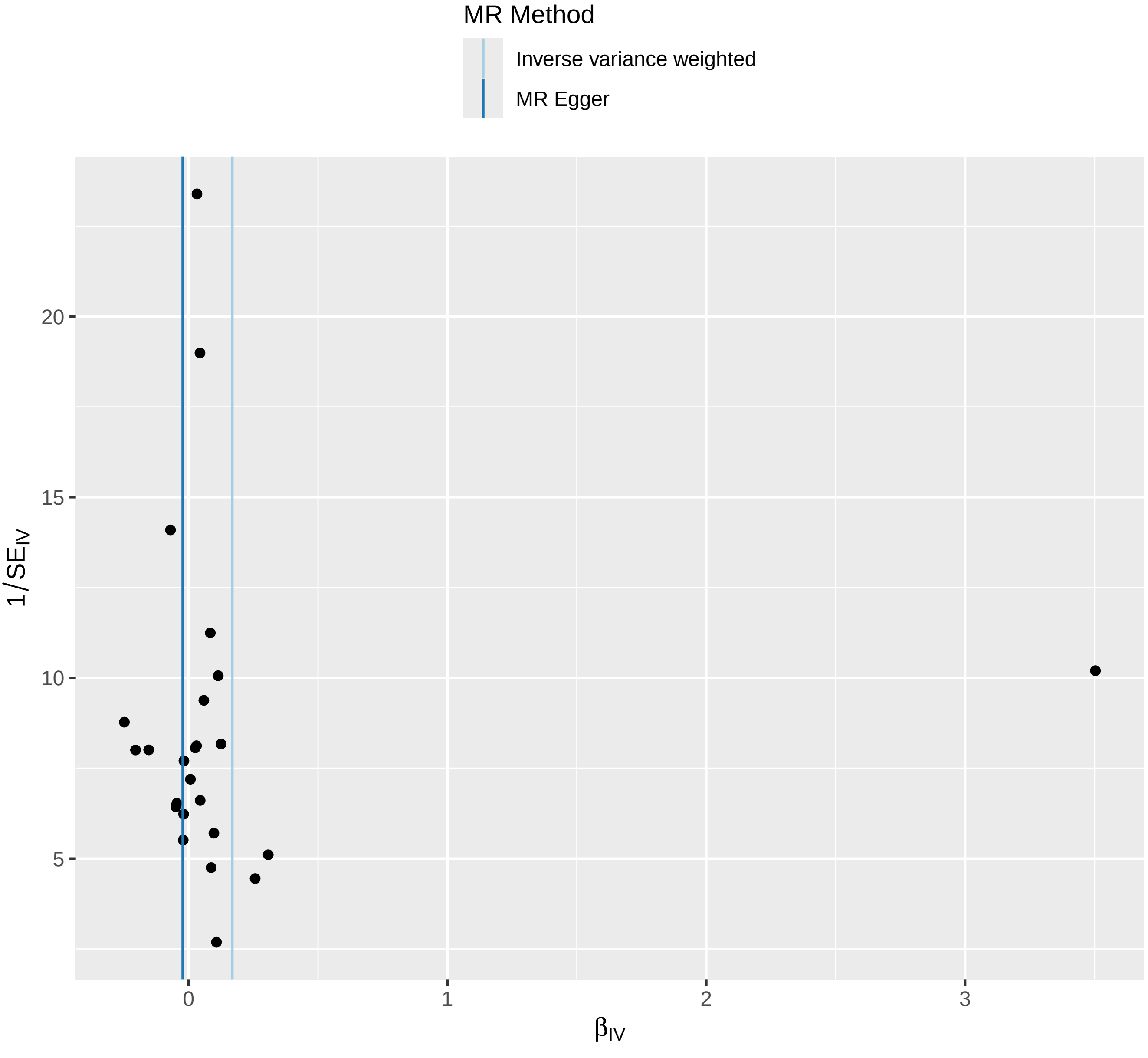
MR Method
Inverse variance weighted
MR Egger
1/SE_IV
20
15
10
5
0
1
2
3
β_IV

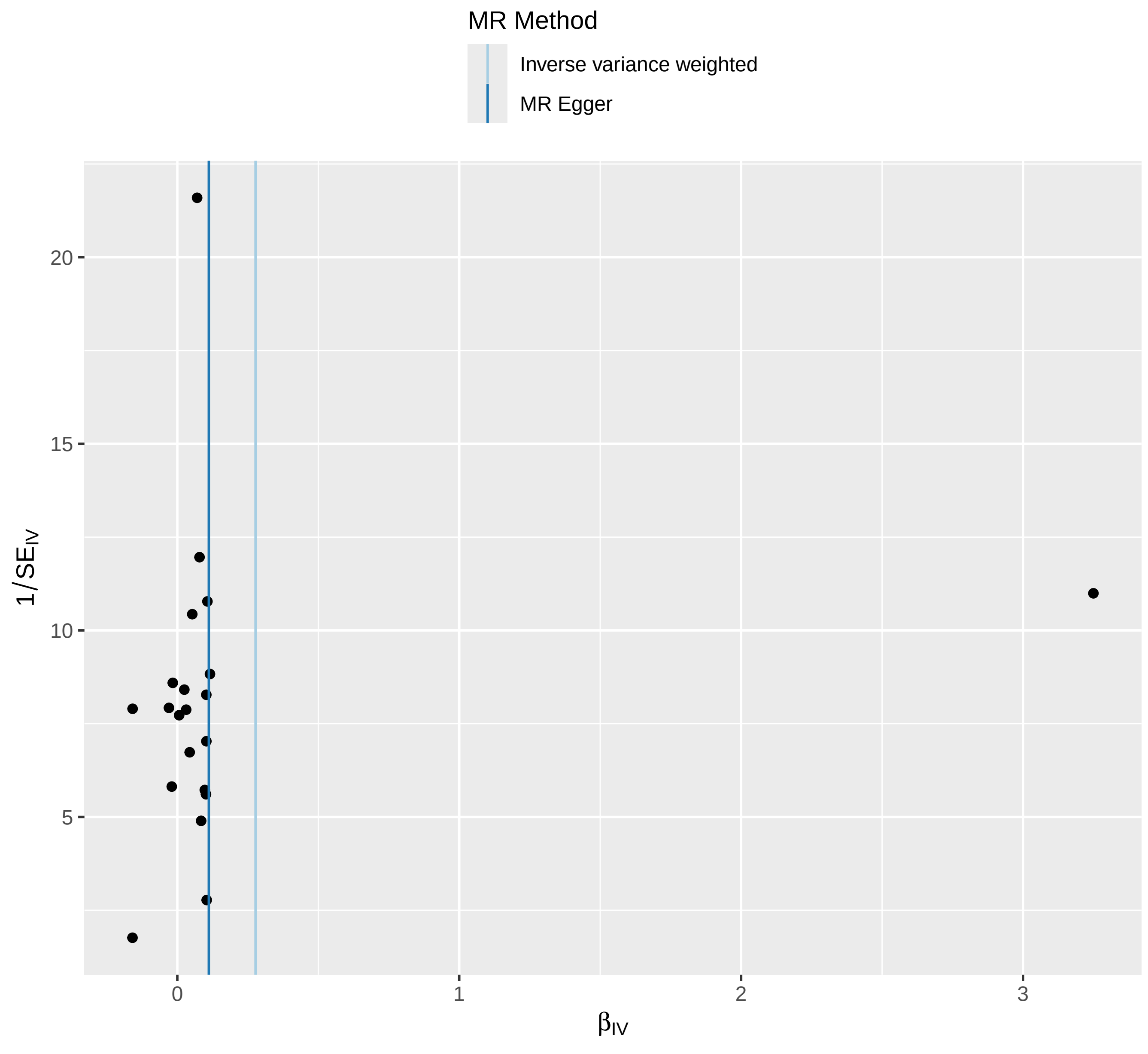

MR Method
Inverse variance weighted
MR Egger
20
15
10
5
1/SE_IV
0
1
2
3
β_IV

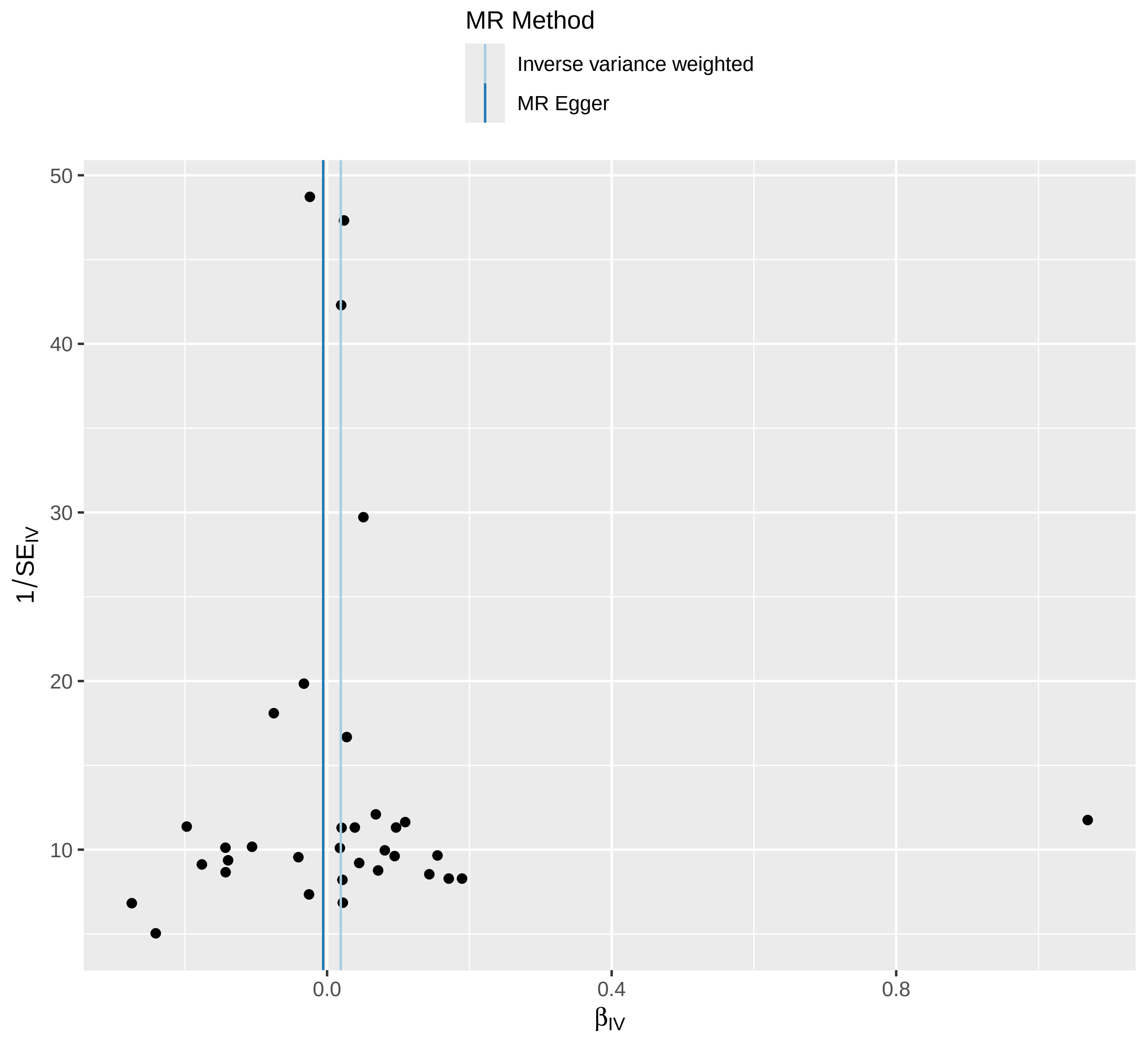
MR Method
Inverse variance weighted
MR Egger
50
40
30
20
10
1/SE$_{IV}$
0.0
0.4
0.8
β$_{IV}$

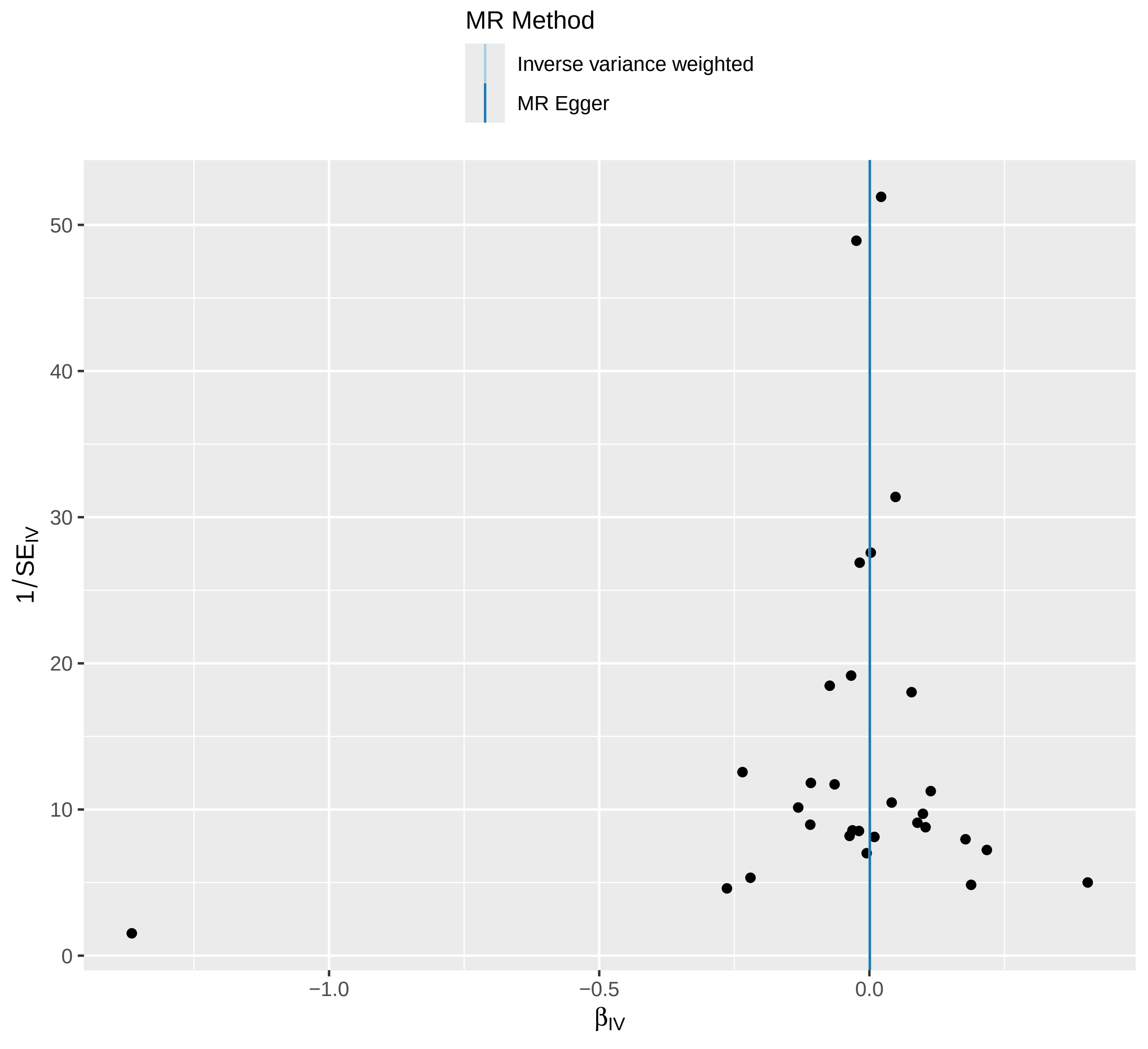
MR Method
Inverse variance weighted
MR Egger
1/SE_IV
50
40
30
20
10
0
−1.0
−0.5
0.0
β_IV

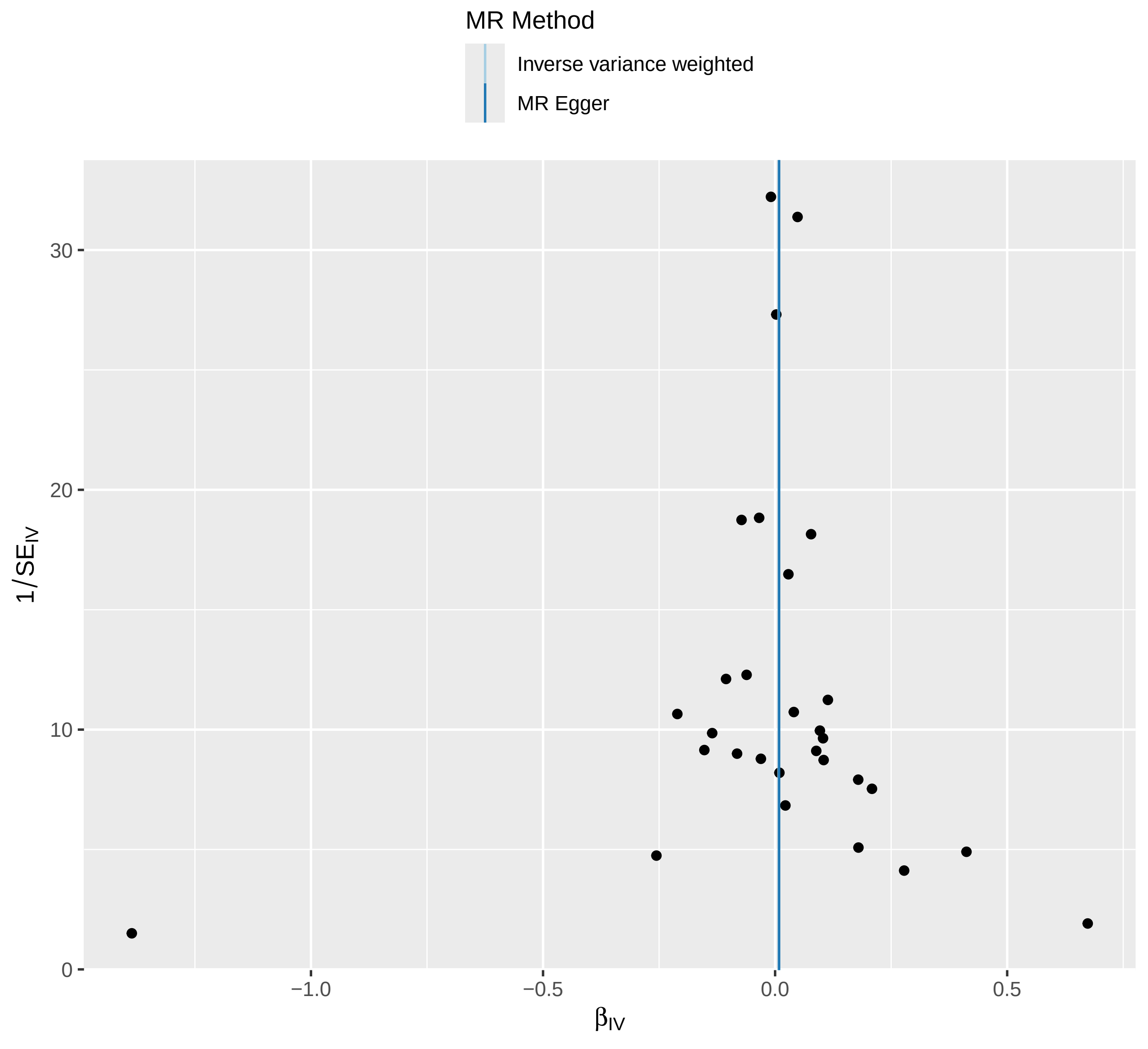

MR Method
Inverse variance weighted
MR Egger
30
20
10
0
1/SE_IV
−1.0
−0.5
0.0
0.5
β_IV

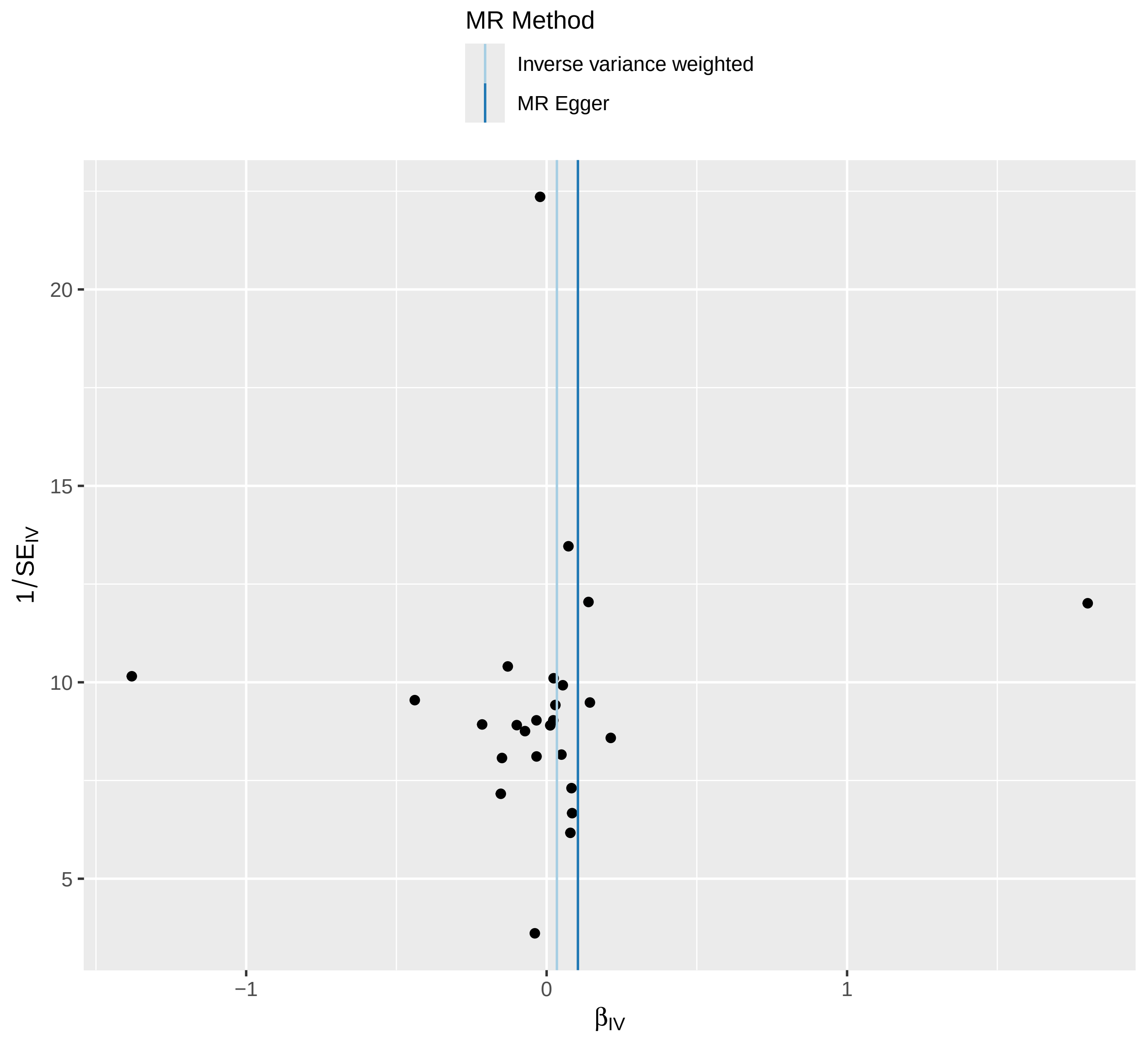
MR Method
Inverse variance weighted
MR Egger
20
15
10
5
−1
0
1
$1/SE_{IV}$
β_{IV}

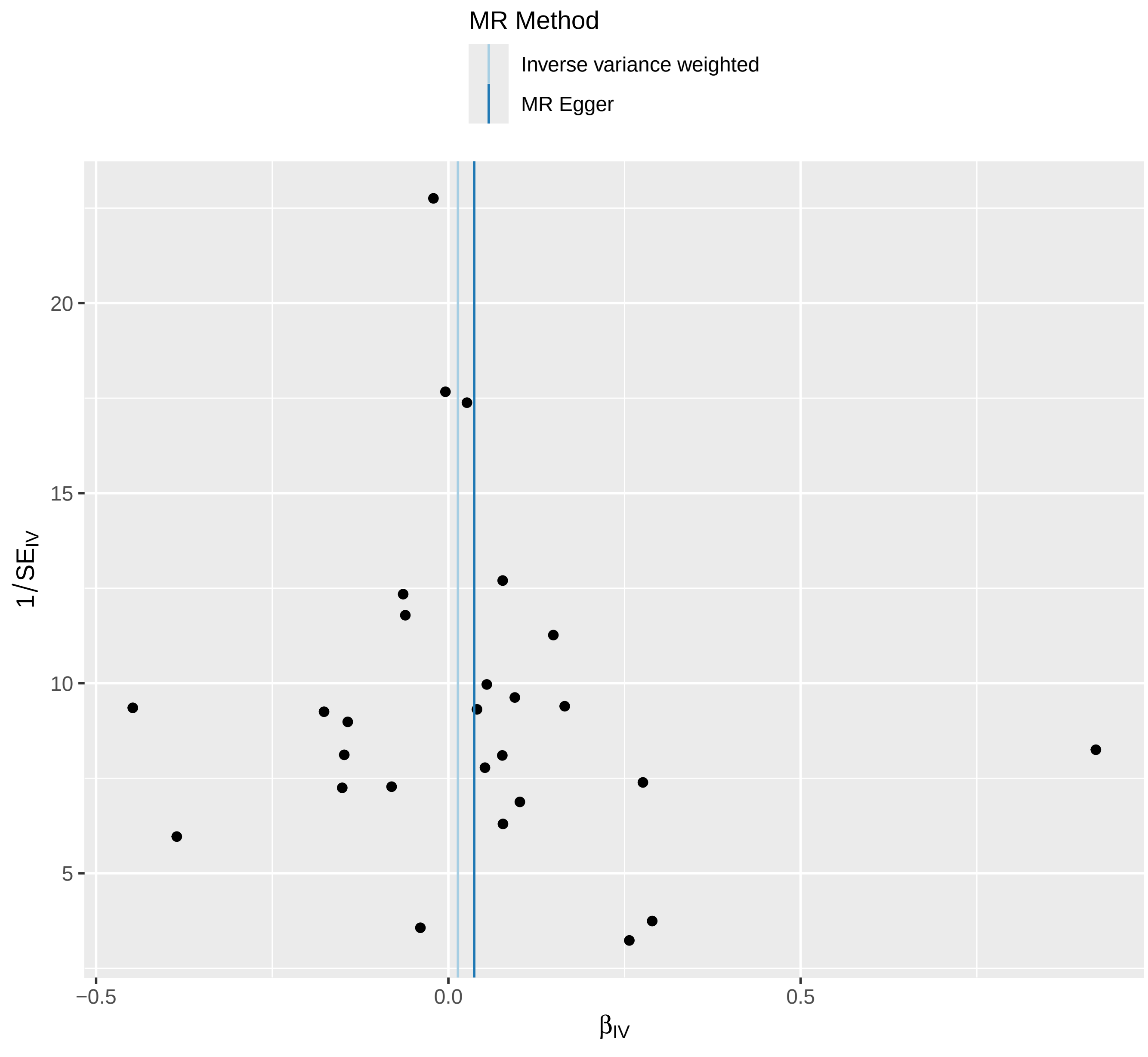
MR Method
Inverse variance weighted
MR Egger
20
15
10
5
1/SE$_{IV}$
−0.5
0.0
0.5
β$_{IV}$

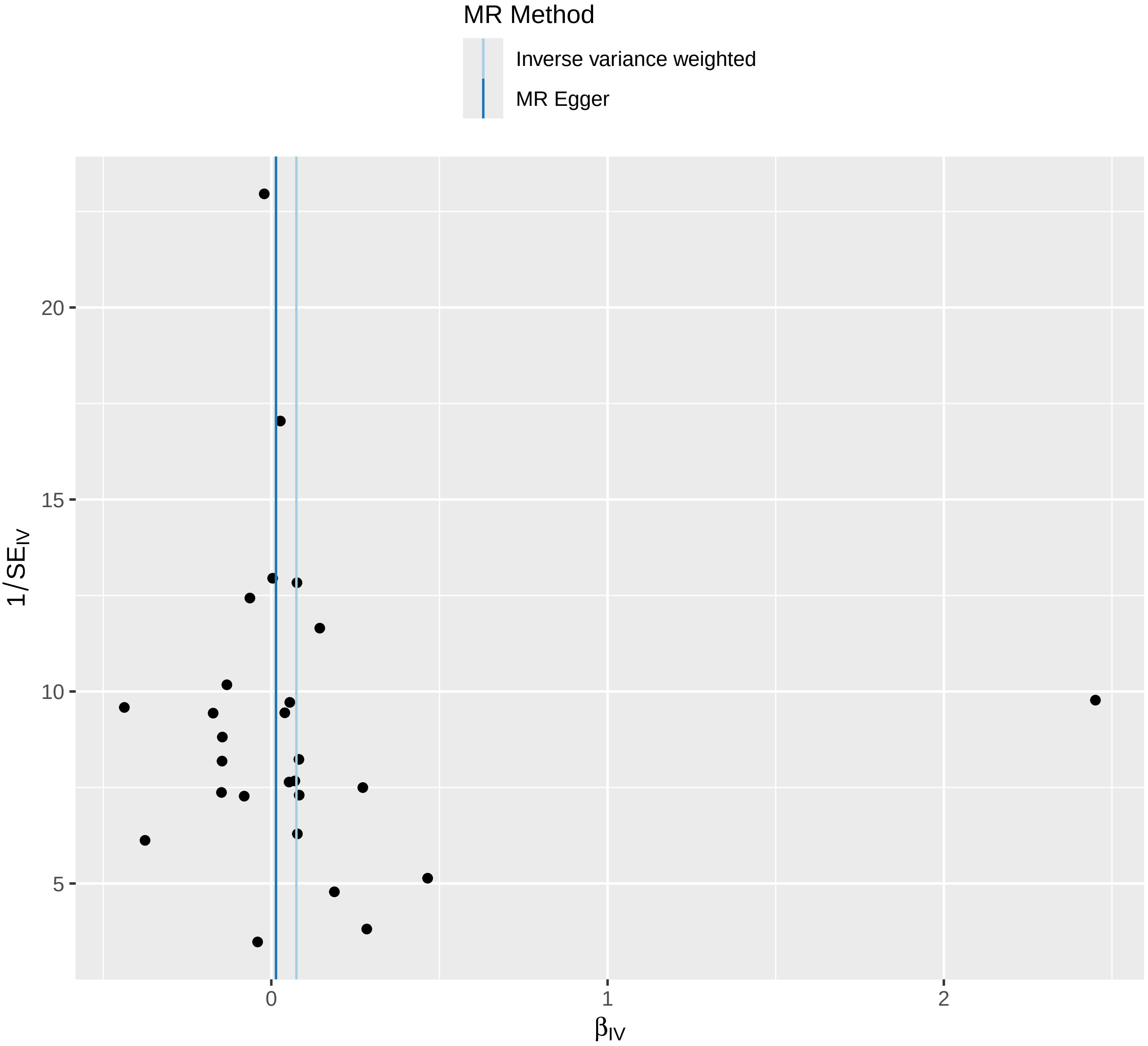

MR Method
Inverse variance weighted
MR Egger
20
15
10
5
$1/SE_{IV}$
0
1
2
β_{IV}

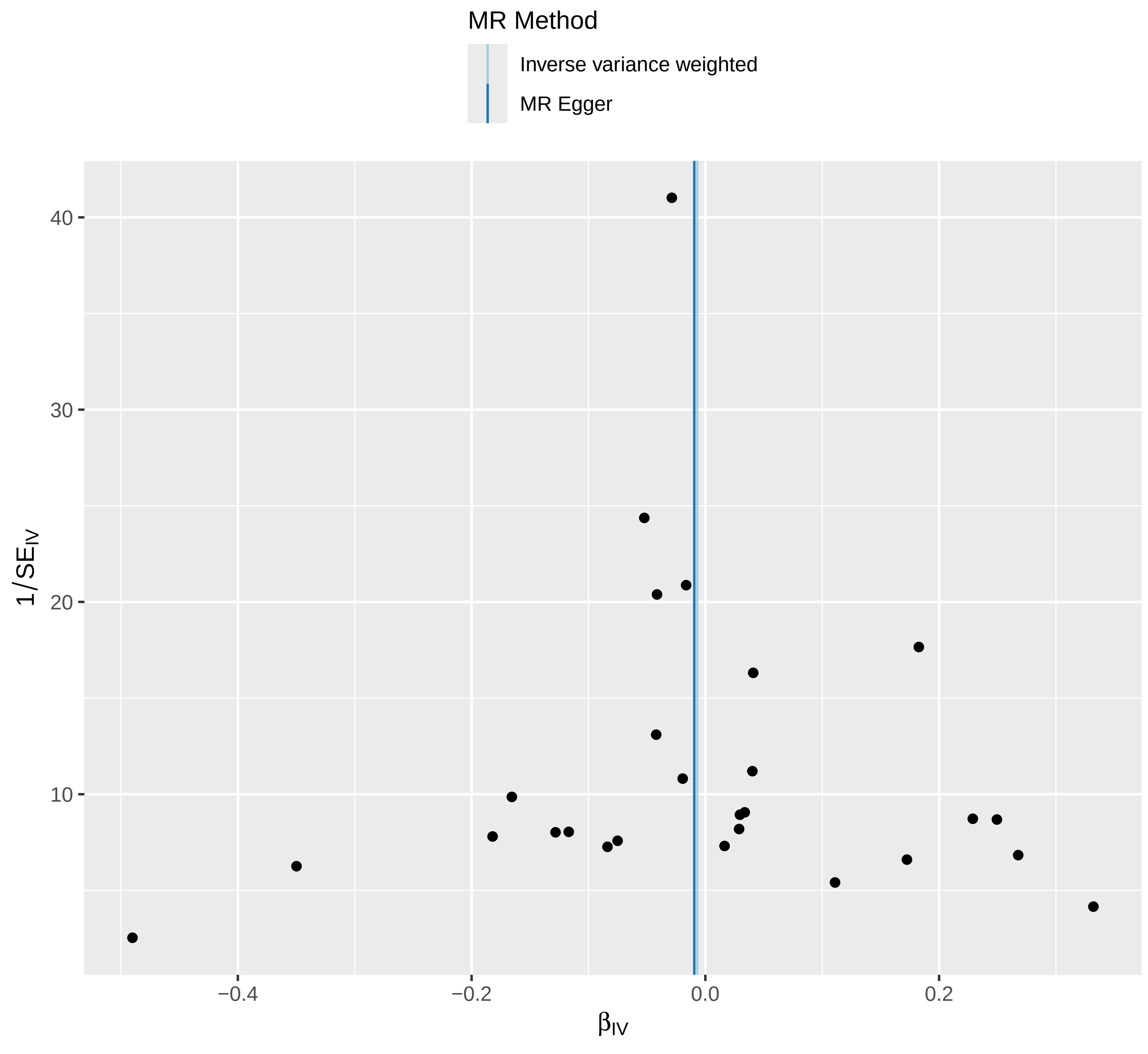

MR Method
Inverse variance weighted
MR Egger
40
30
20
10
1/SE_IV
−0.4
−0.2
0.0
0.2
β_IV

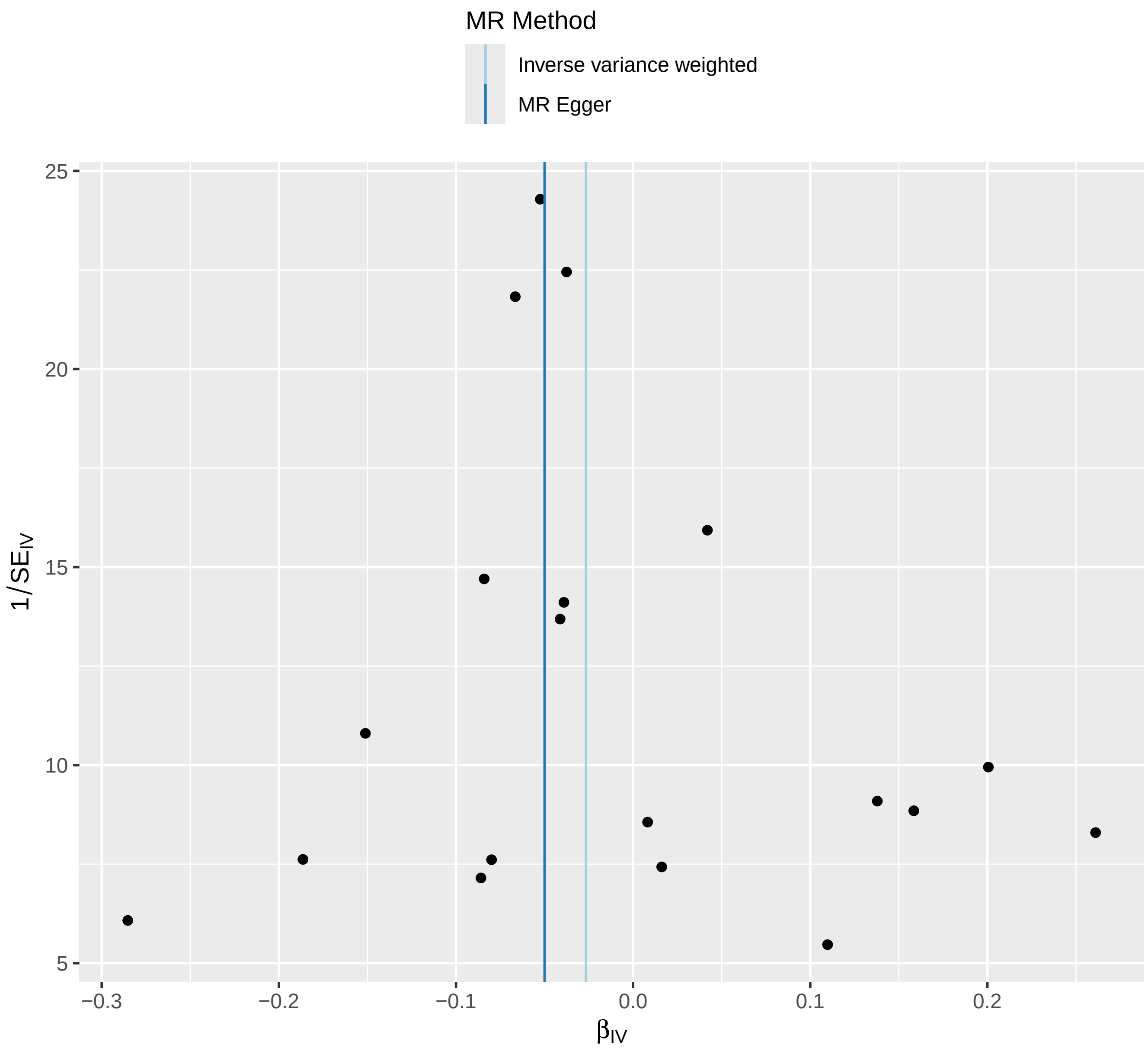
MR Method
Inverse variance weighted
MR Egger
25
20
15
10
5
$1/SE_{IV}$
−0.3
−0.2
−0.1
0.0
0.1
0.2
β_{IV}

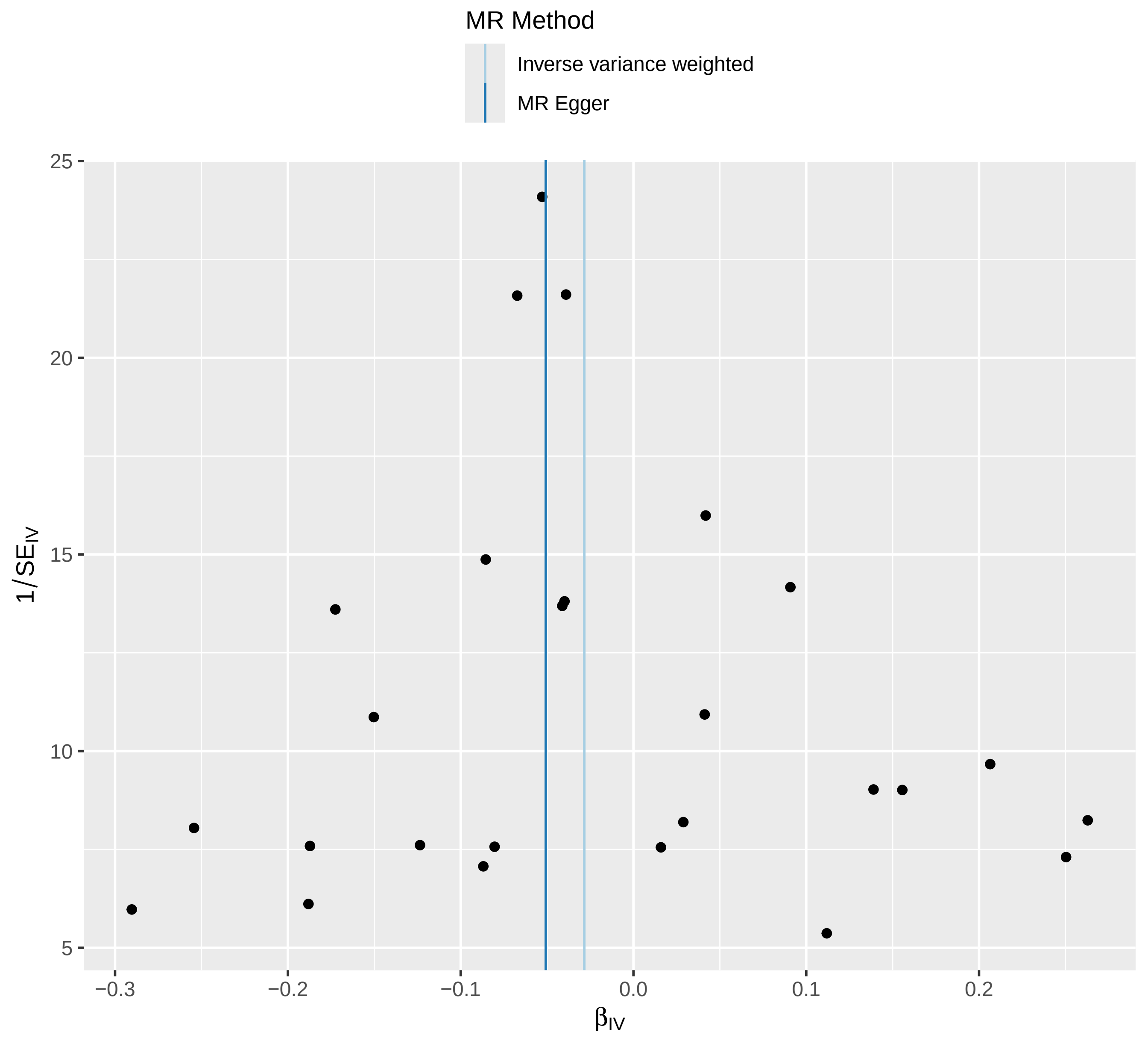
MR Method
Inverse variance weighted
MR Egger
25
20
15
10
5
1/SE$_{IV}$
−0.3
−0.2
−0.1
0.0
0.1
0.2
β$_{IV}$

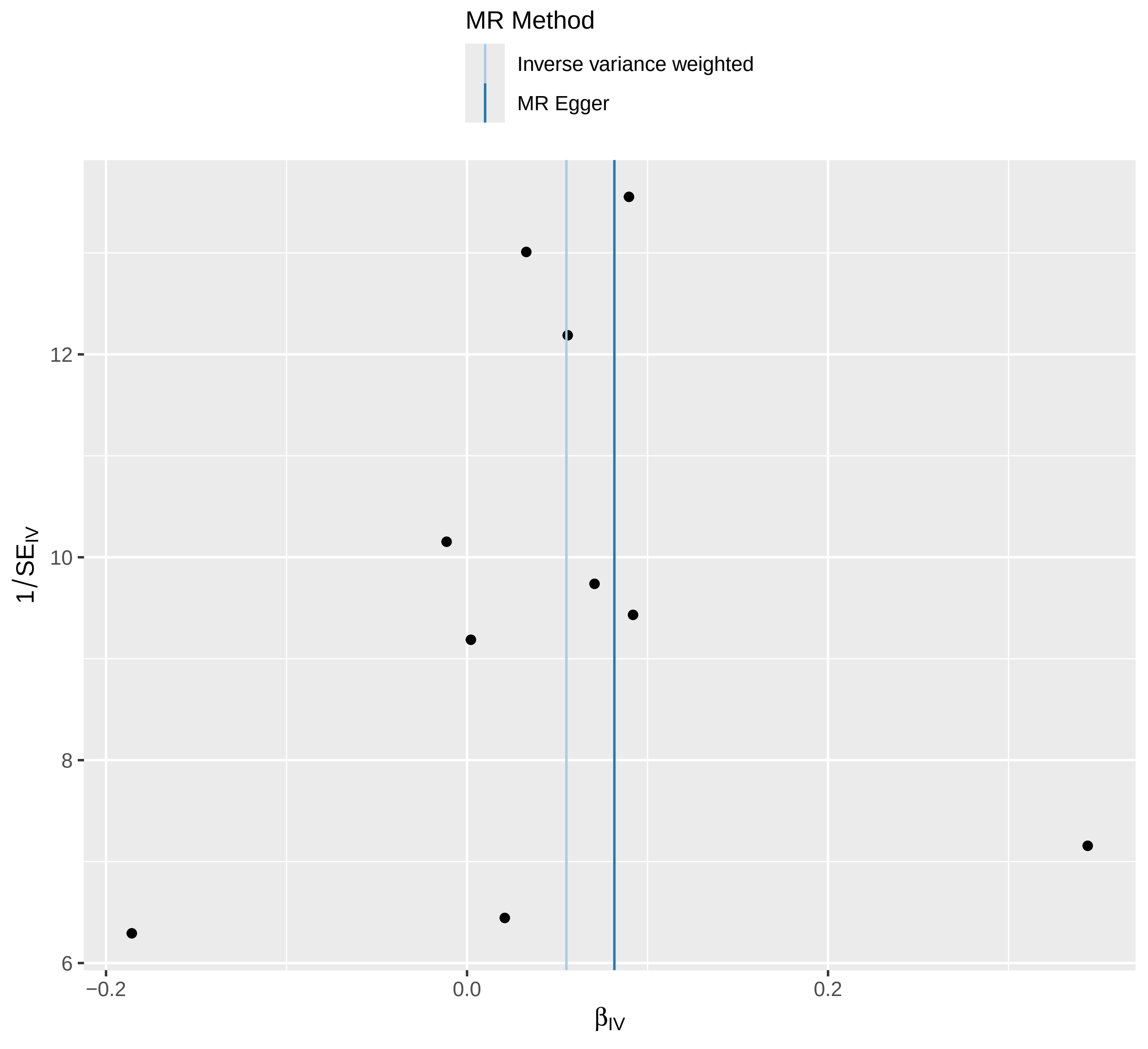
MR Method
Inverse variance weighted
MR Egger
12
10
8
6
1/SE_IV
−0.2
0.0
0.2
β_IV

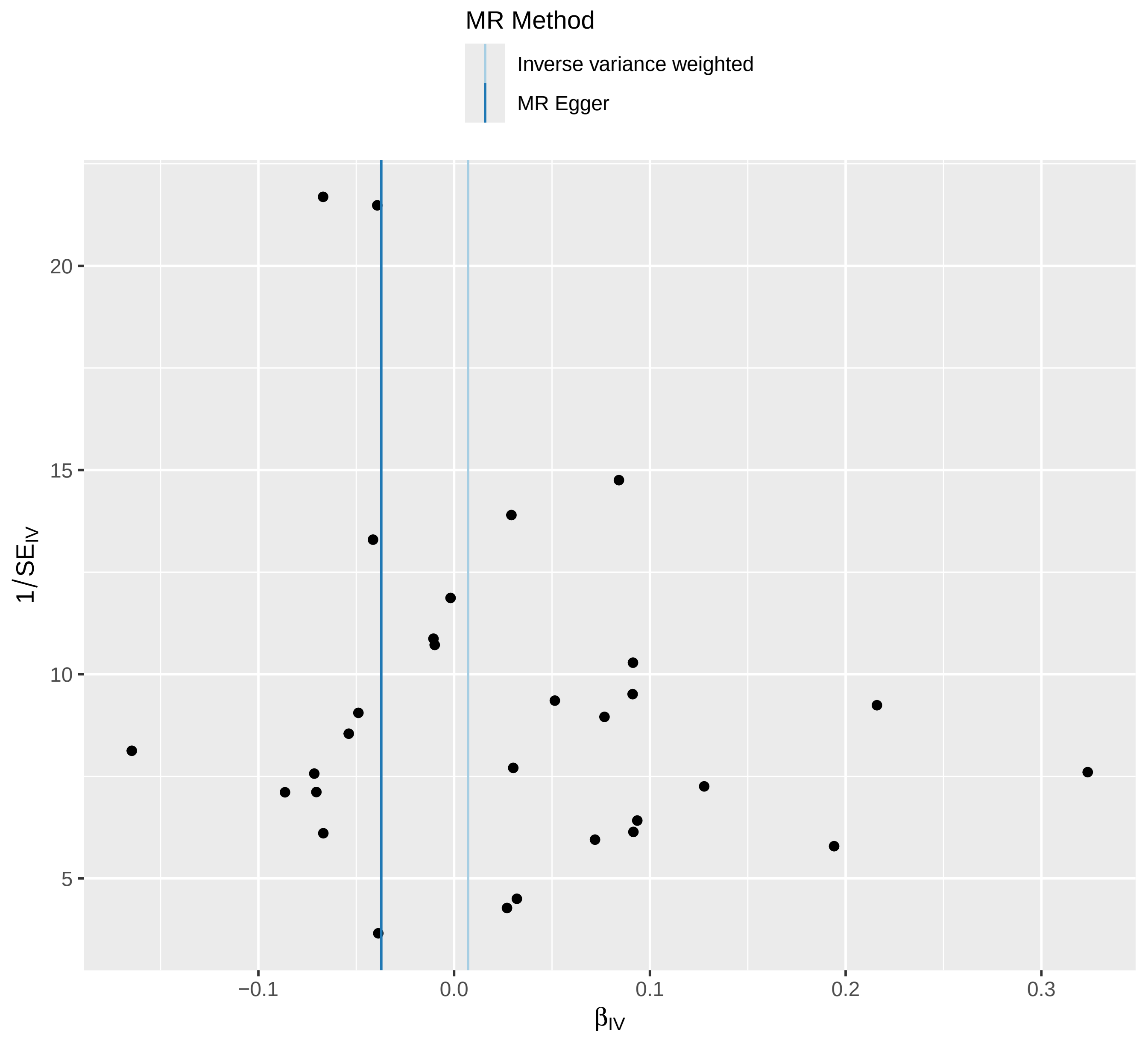
MR Method
Inverse variance weighted
MR Egger
20
15
10
5
1/SE_IV
−0.1
0.0
0.1
0.2
0.3
β_IV

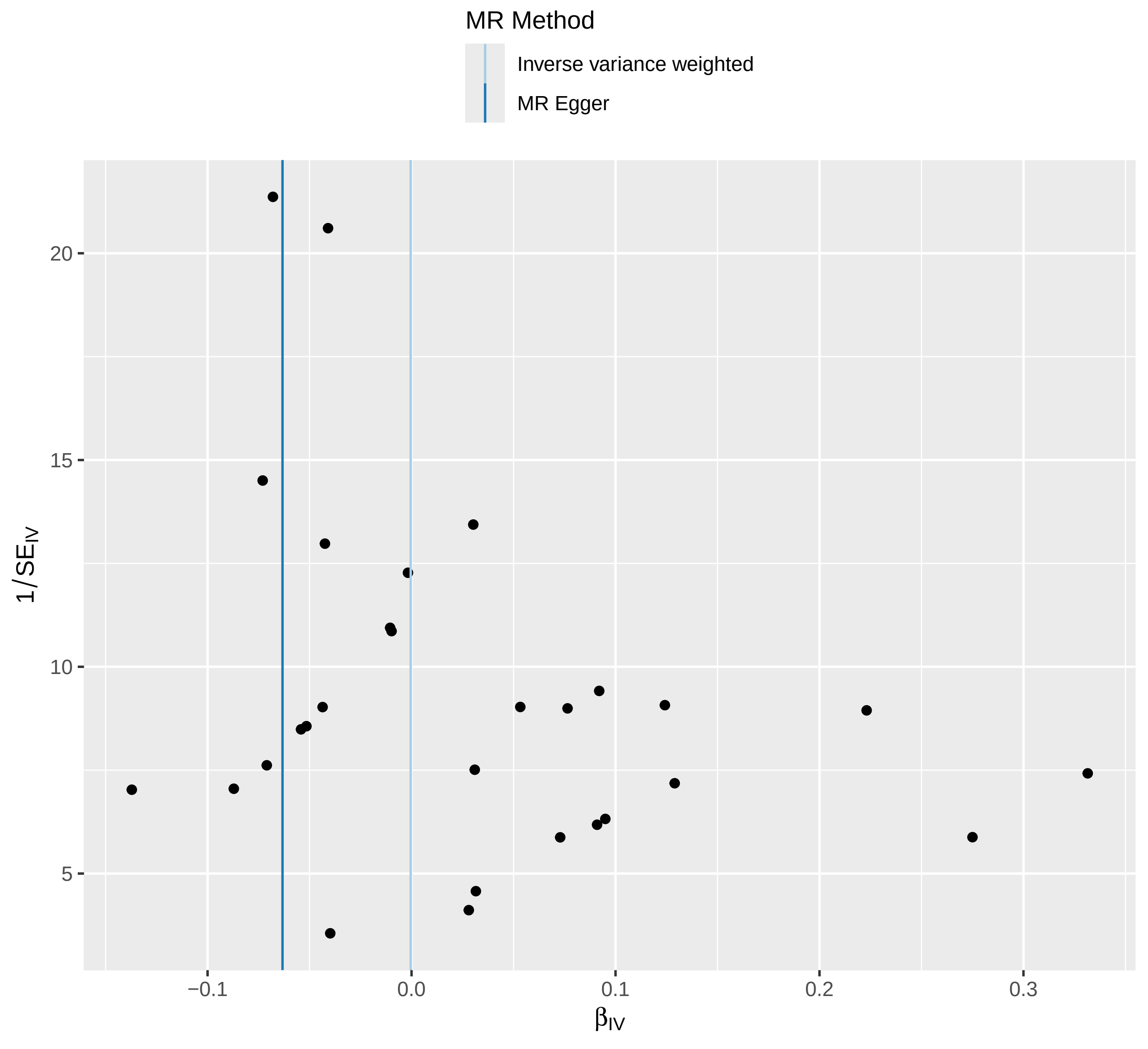
MR Method
Inverse variance weighted
MR Egger
1/SE_IV
β_IV
20
15
10
5
−0.1
0.0
0.1
0.2
0.3

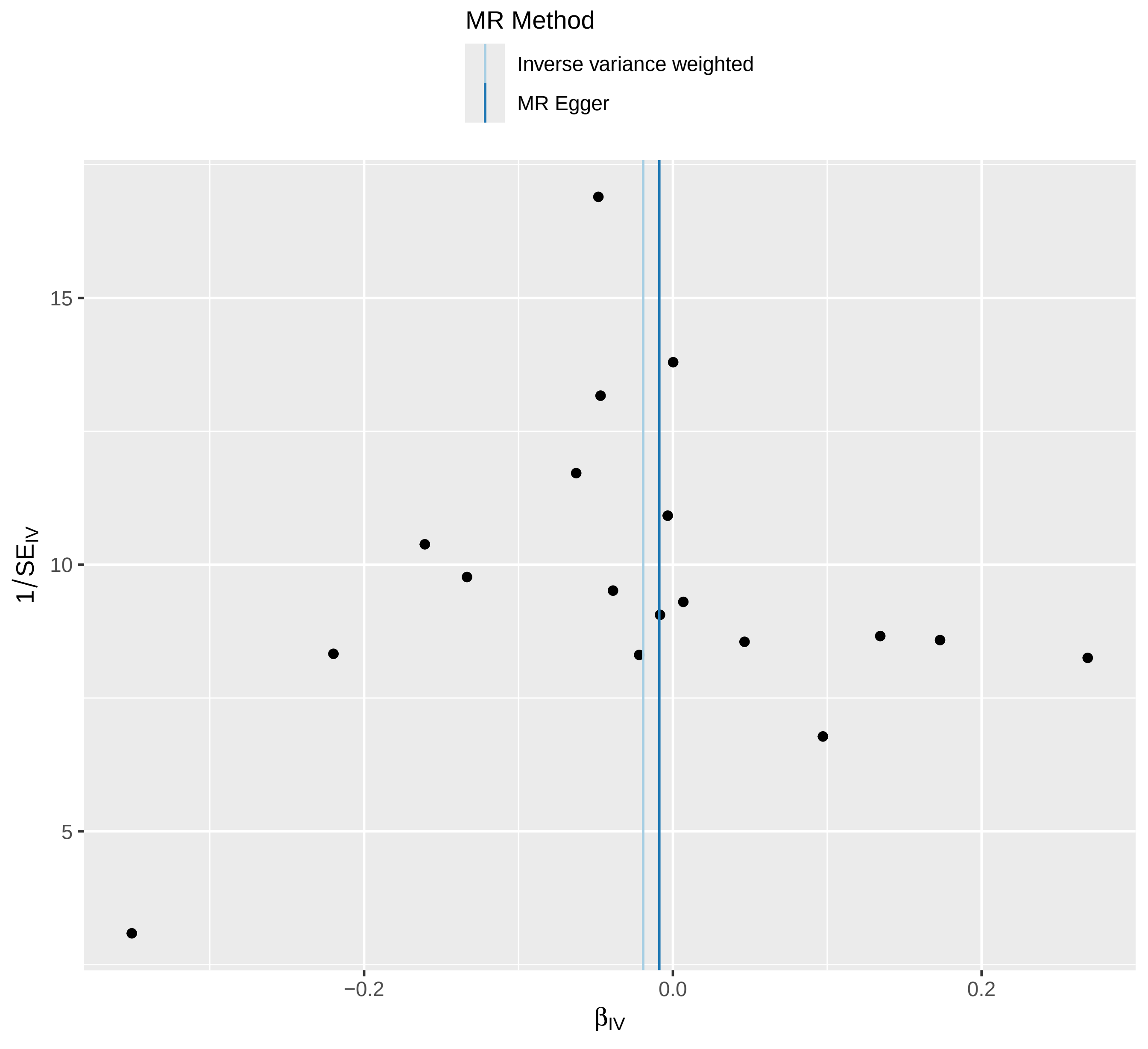
MR Method
Inverse variance weighted
MR Egger
15
10
5
1/SE_IV
−0.2
0.0
0.2
β_IV

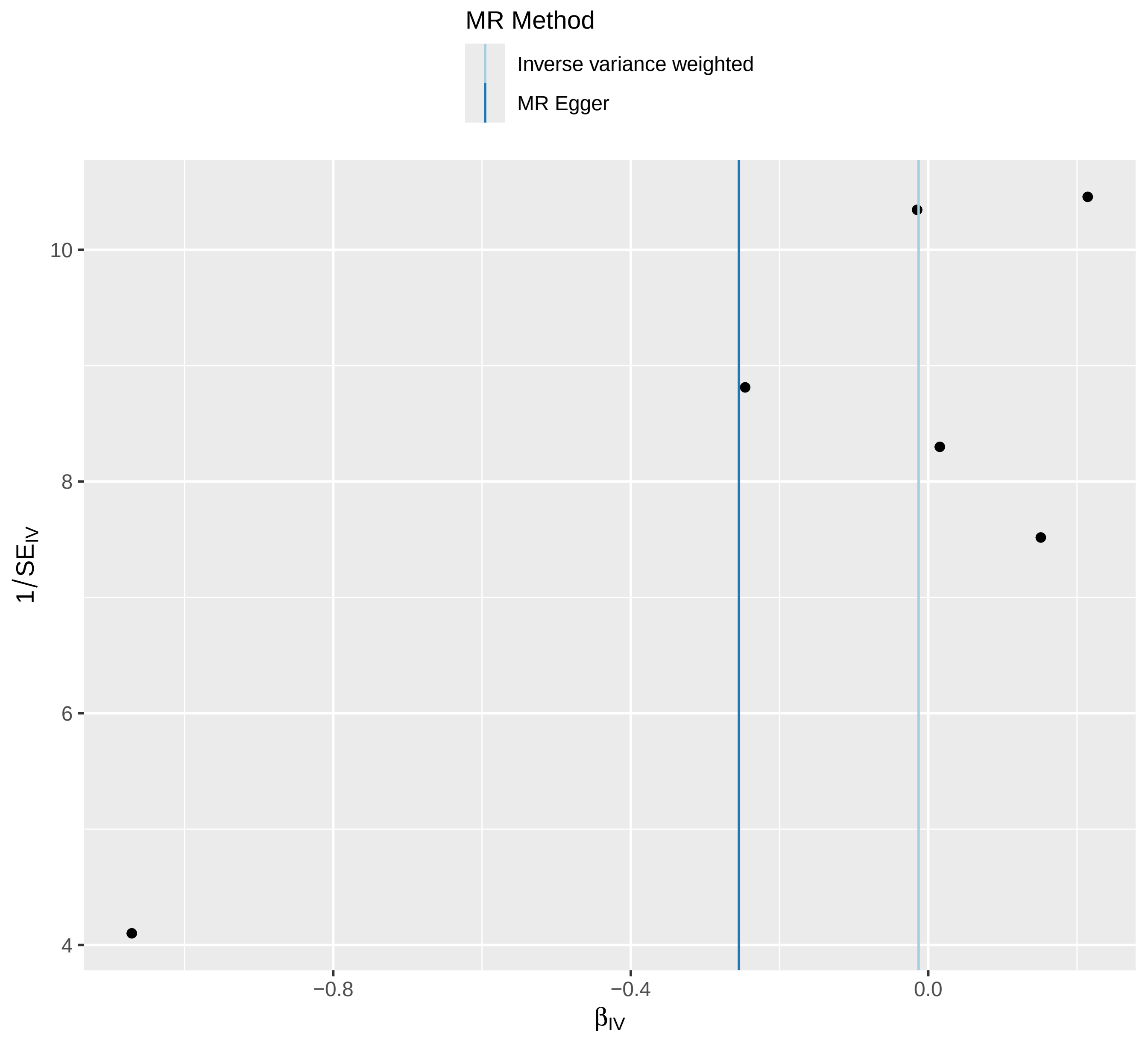
MR Method
Inverse variance weighted
MR Egger
10
8
6
4
1/SE_IV
−0.8
−0.4
0.0
β_IV

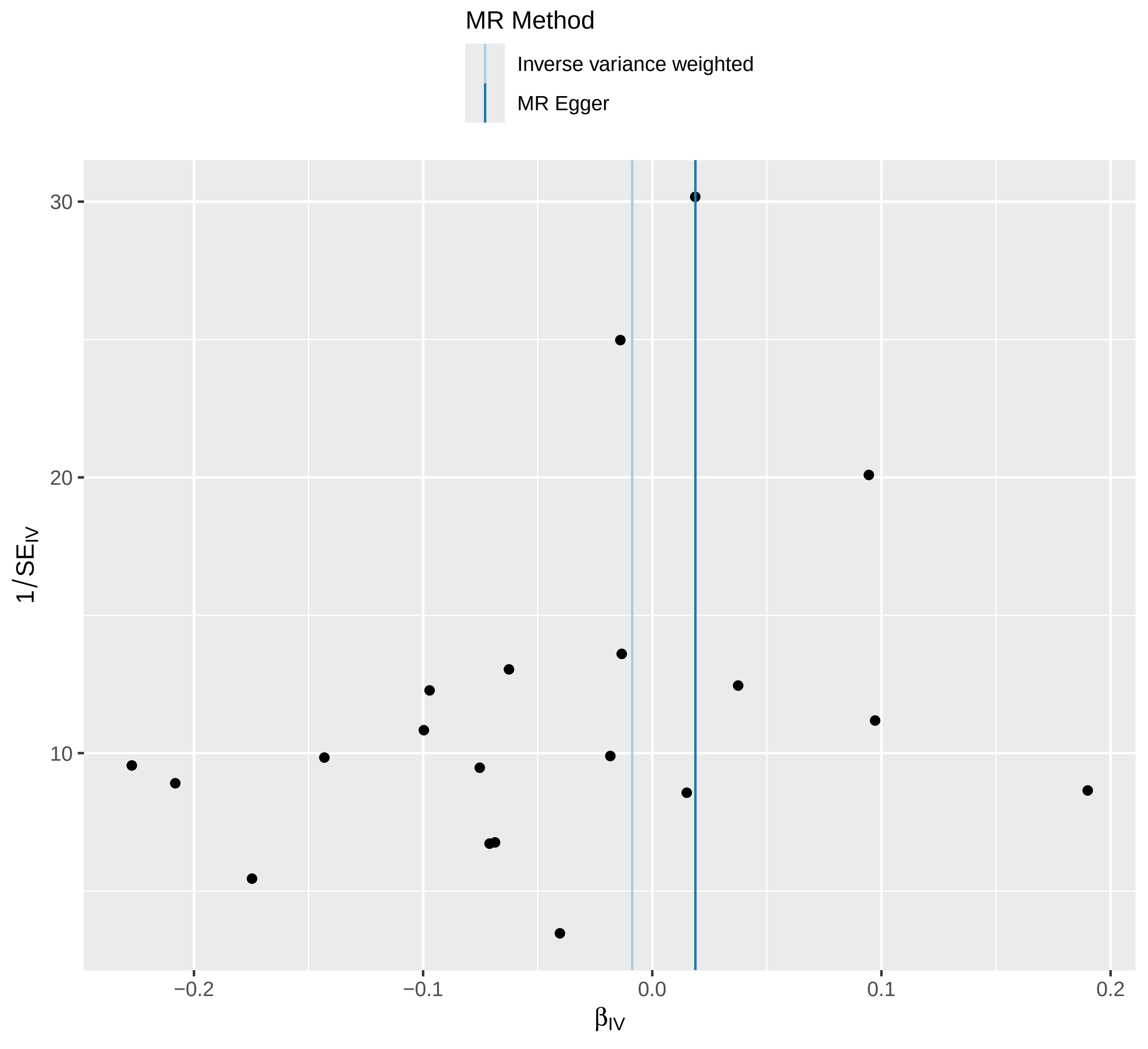

MR Method
Inverse variance weighted
MR Egger
30
20
10
1/SE_IV
−0.2
−0.1
0.0
0.1
0.2
β_IV

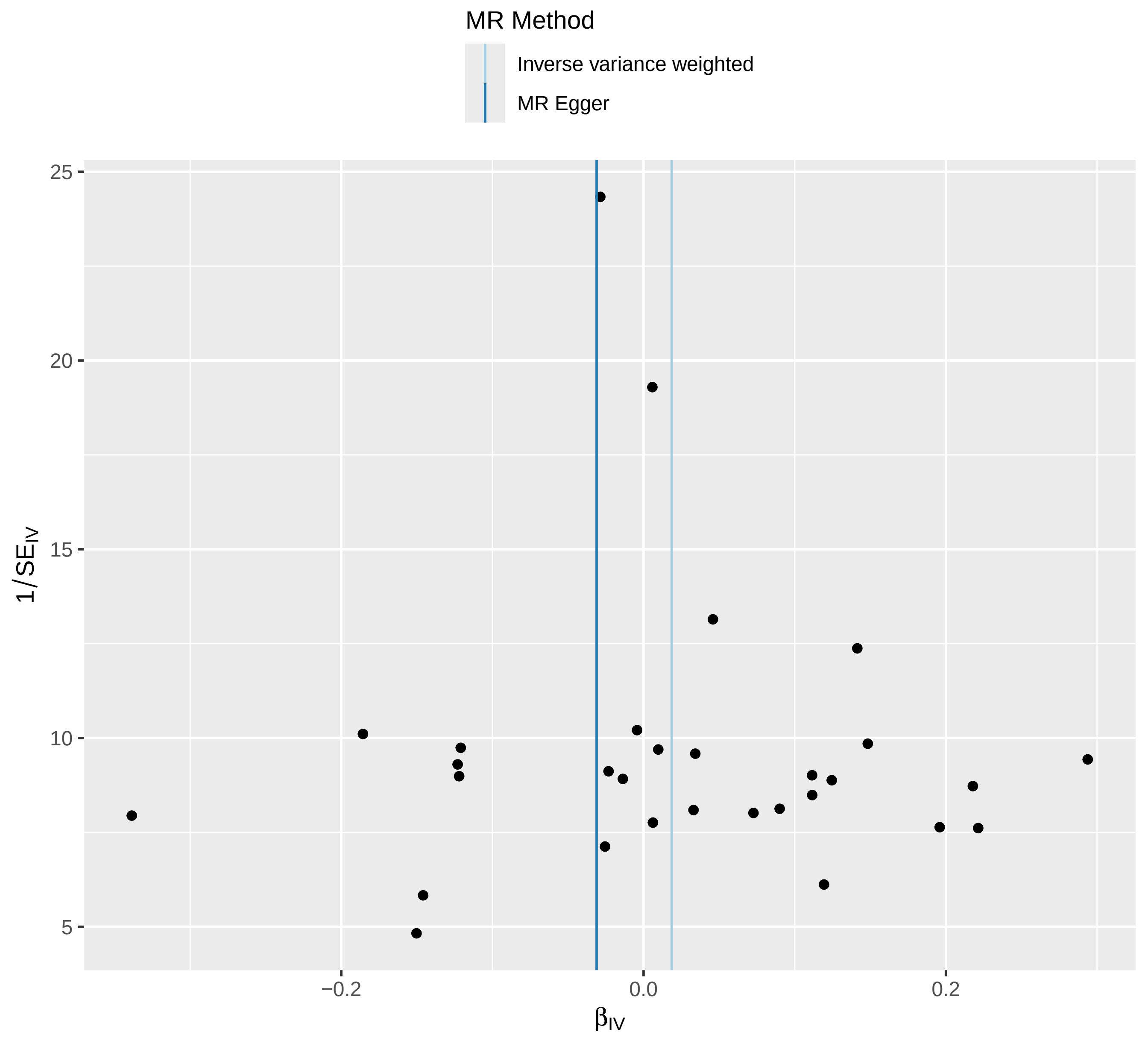

MR Method
Inverse variance weighted
MR Egger
25
20
15
10
5
$1/SE_{IV}$
−0.2
0.0
0.2
β_{IV}

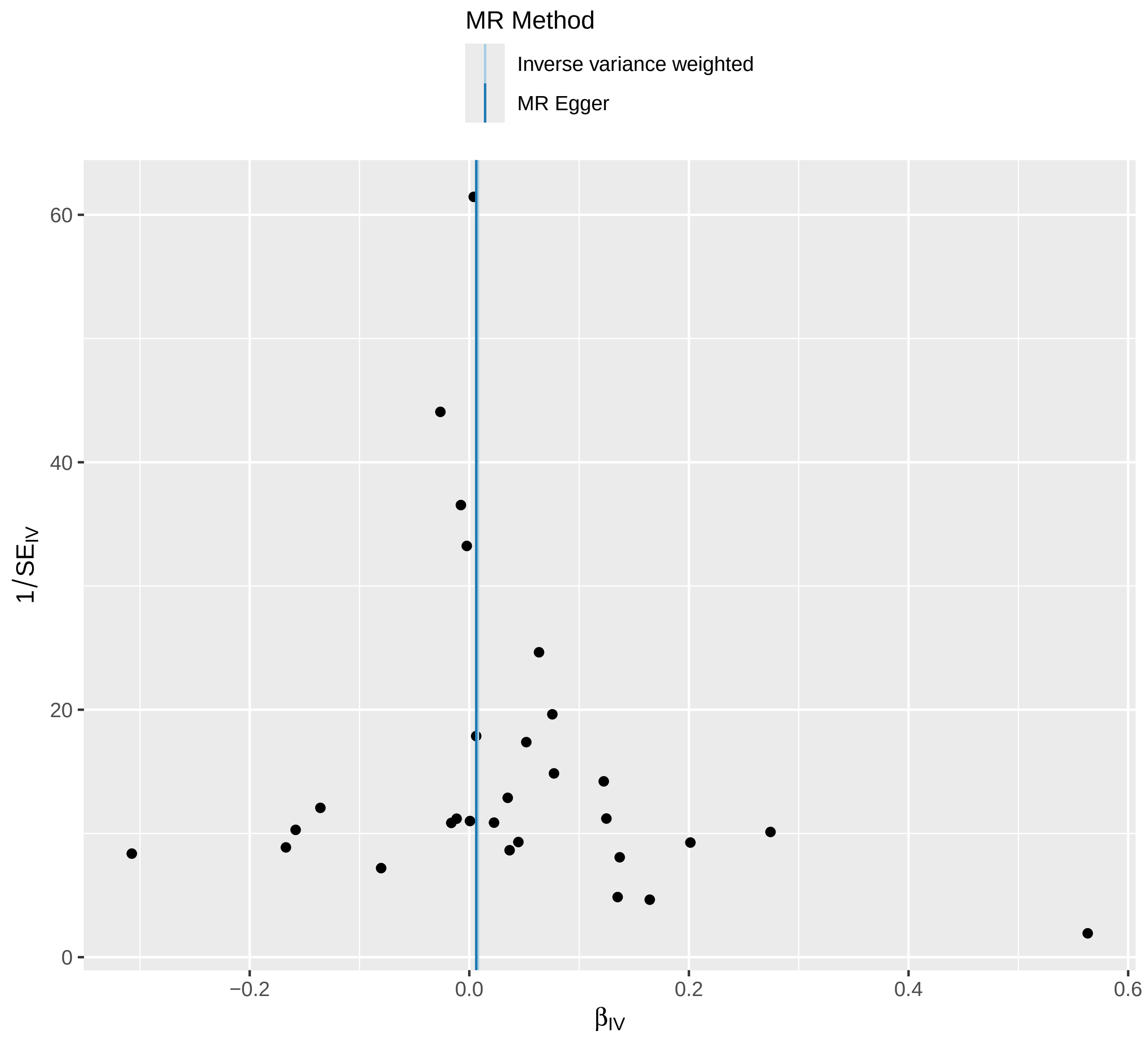
MR Method
Inverse variance weighted
MR Egger
60
40
20
0
−0.2
0.0
0.2
0.4
0.6
1/SE_IV
β_IV

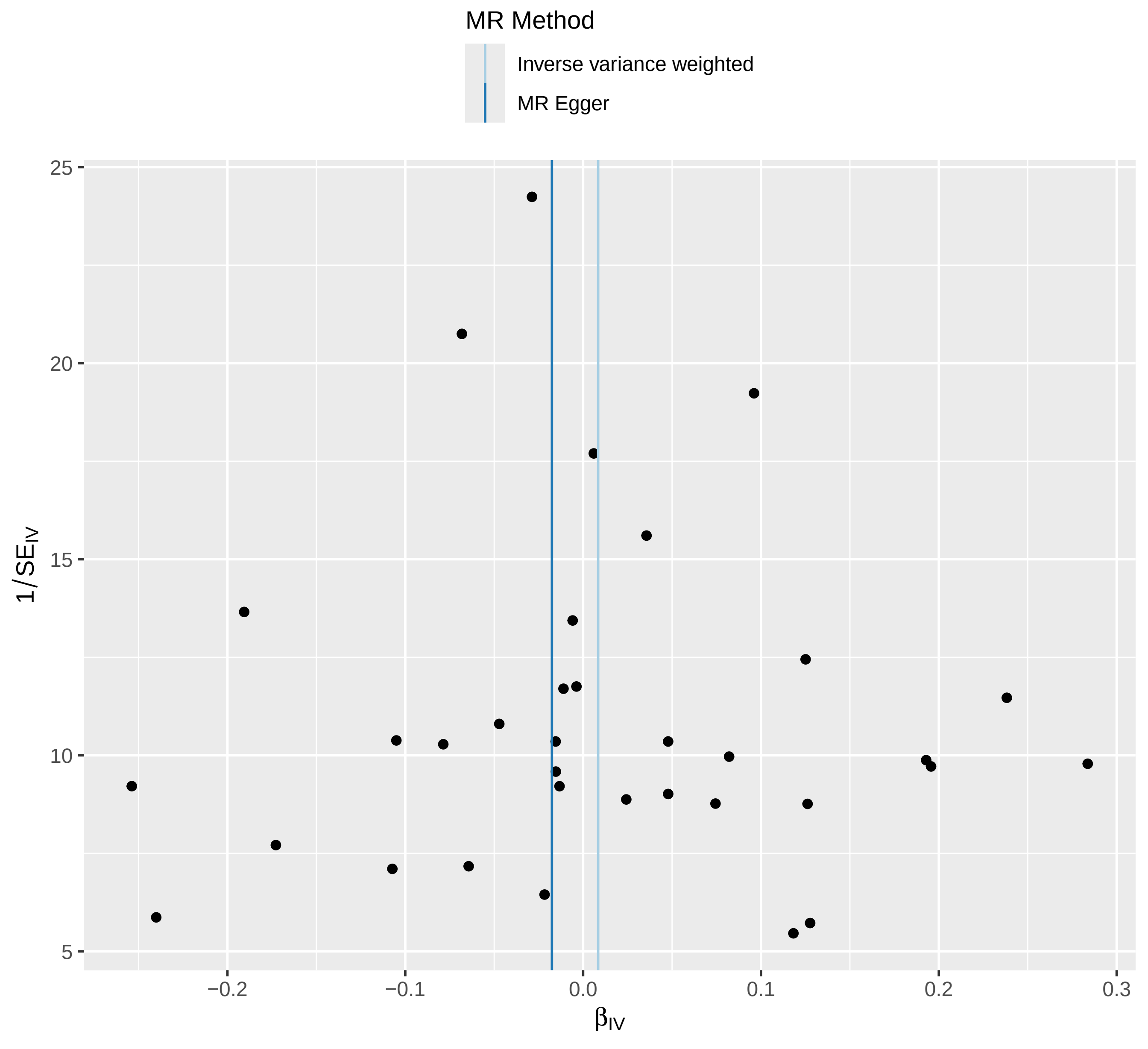
MR Method
Inverse variance weighted
MR Egger
25
20
15
10
5
1/SE$_{IV}$
−0.2
−0.1
0.0
0.1
0.2
0.3
β$_{IV}$

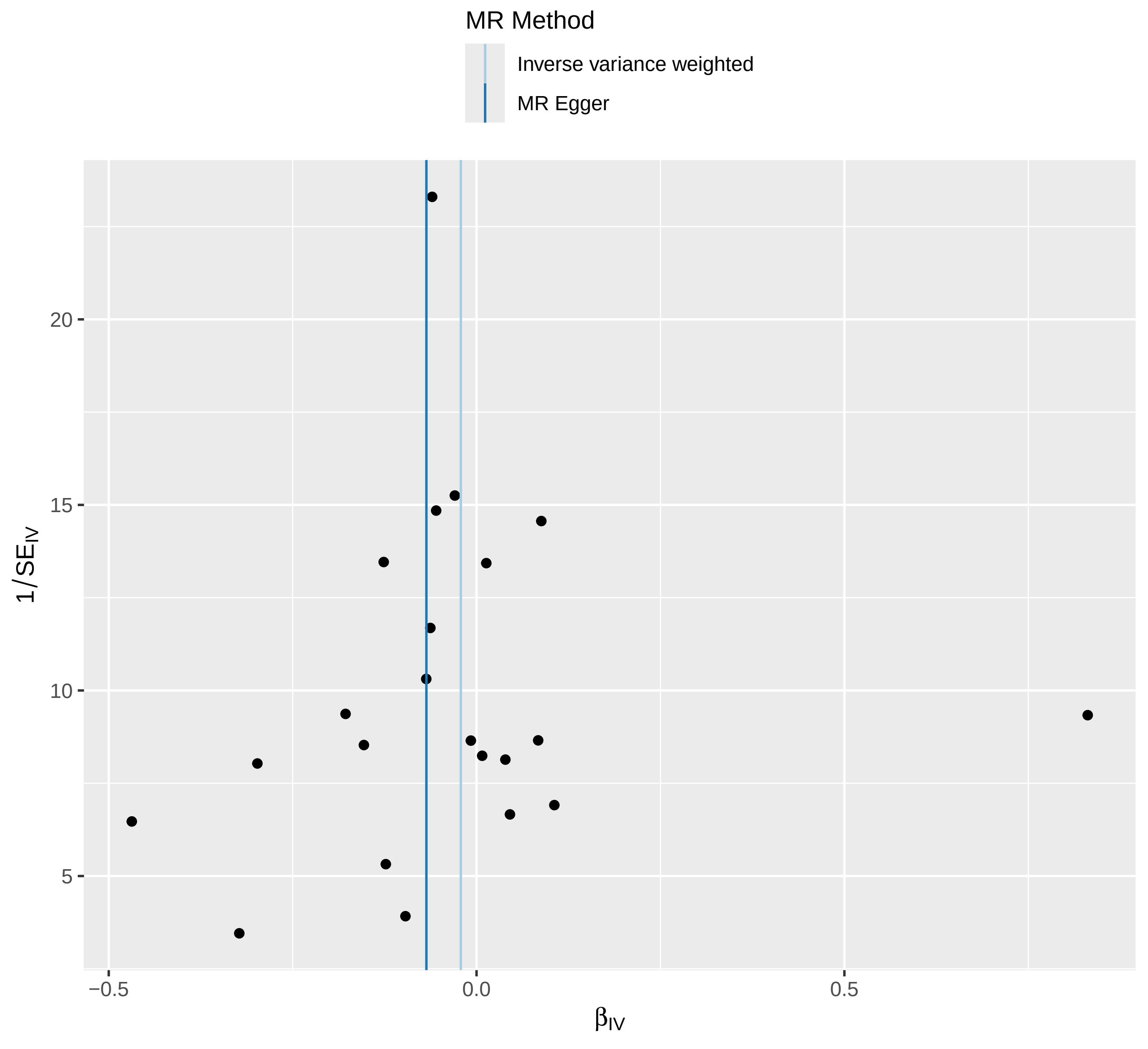
MR Method
Inverse variance weighted
MR Egger
20
15
10
5
1/SE_IV
−0.5
0.0
0.5
β_IV

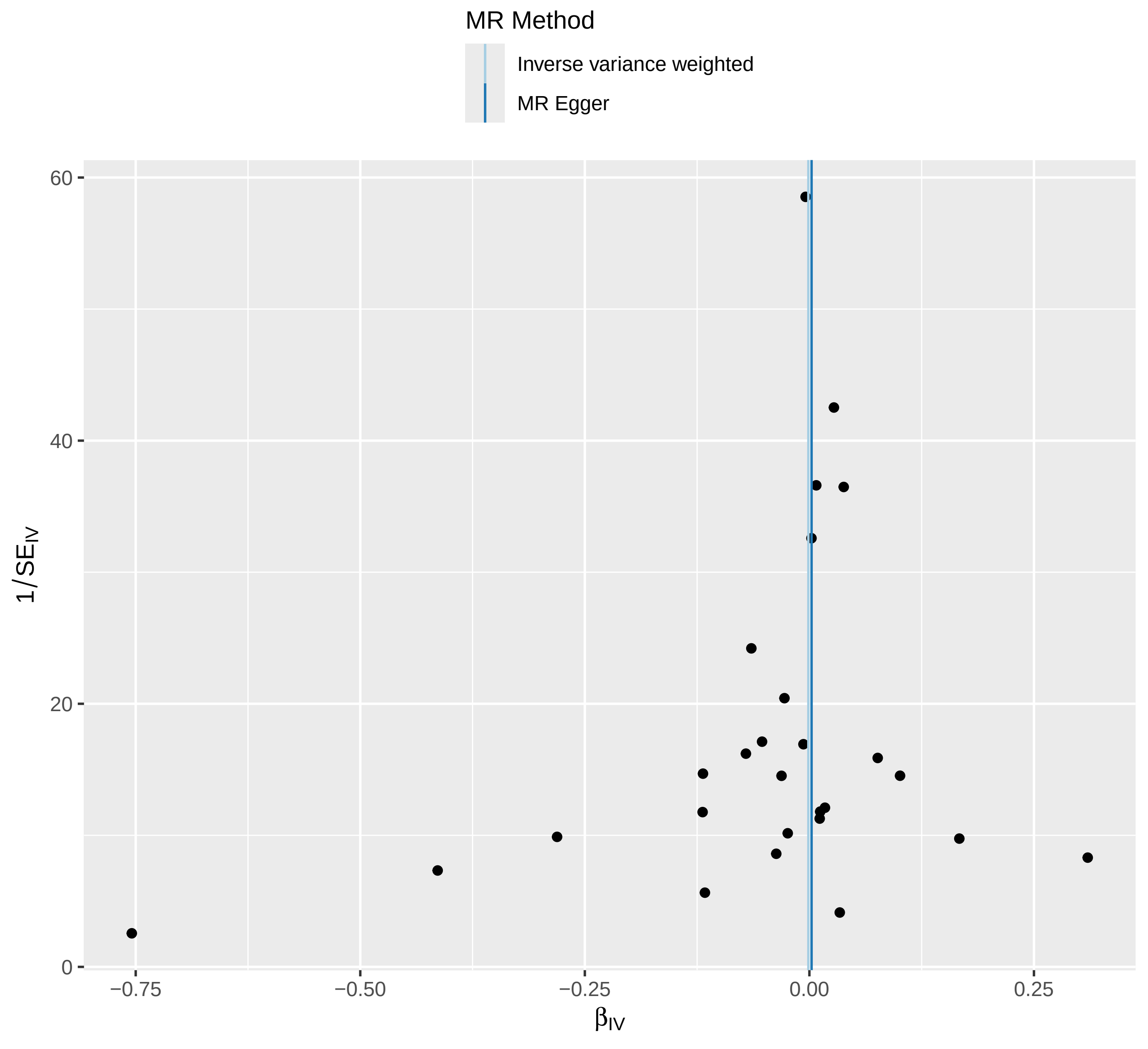
MR Method
Inverse variance weighted
MR Egger
60
40
20
0
1/SE$_{IV}$
−0.75
−0.50
−0.25
0.00
0.25
β$_{IV}$

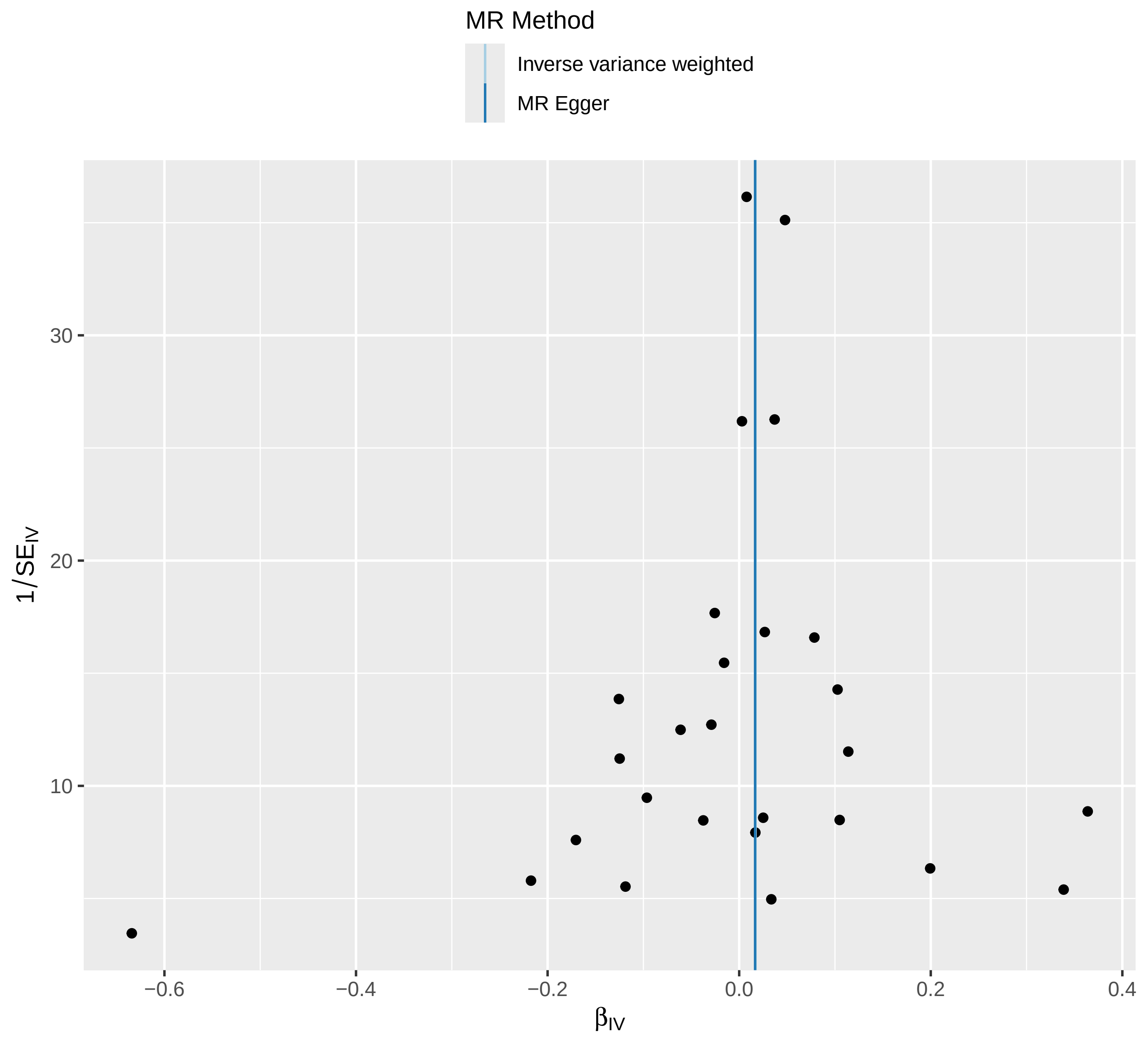
MR Method
Inverse variance weighted
MR Egger
30
20
10
1/SE_IV
−0.6
−0.4
−0.2
0.0
0.2
0.4
β_IV

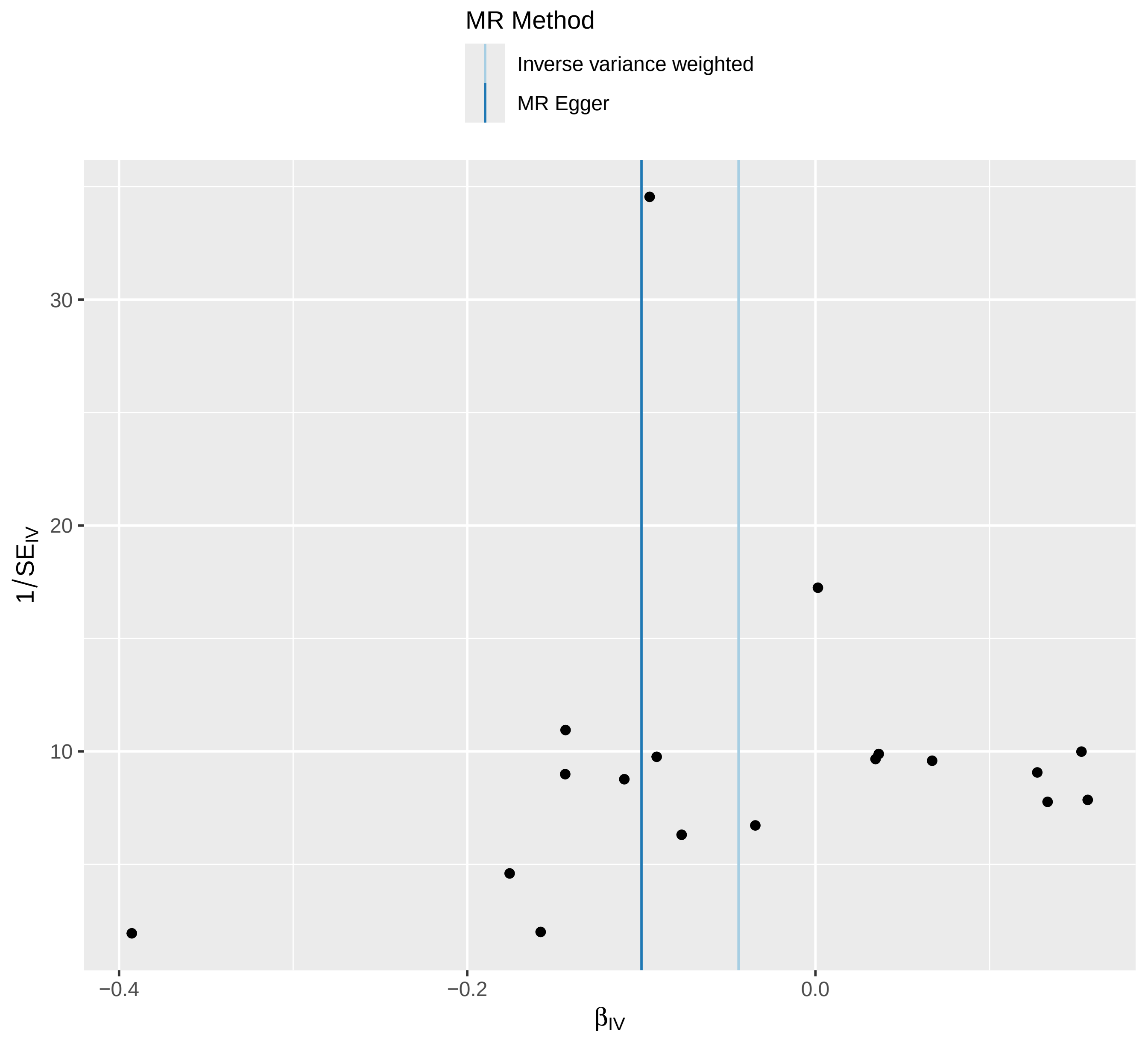
MR Method
Inverse variance weighted
MR Egger
30
20
10
1/SE_IV
−0.4
−0.2
0.0
β_IV

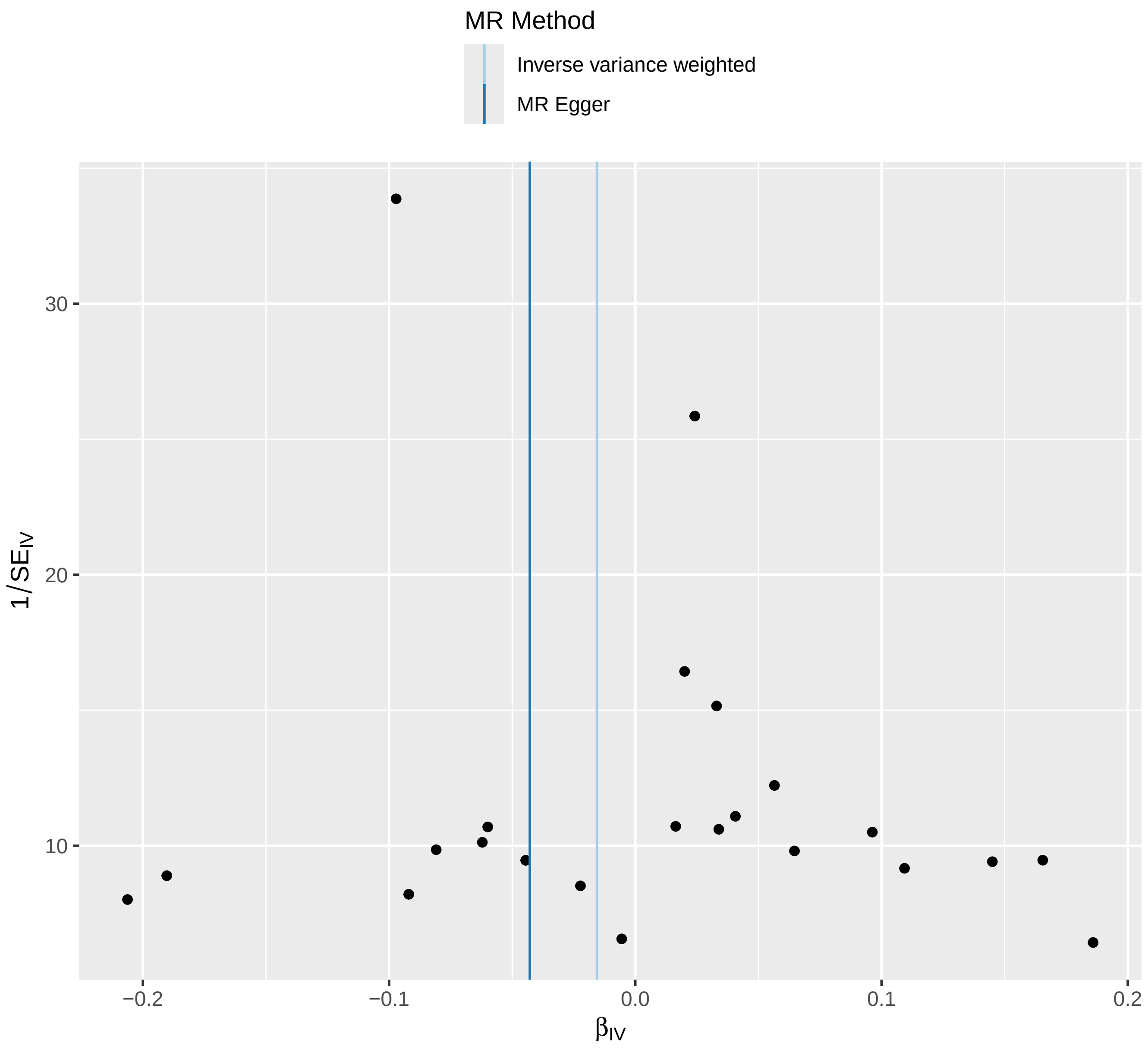
MR Method
Inverse variance weighted
MR Egger
1/SE_IV
30
20
10
−0.2
−0.1
0.0
0.1
0.2
β_IV

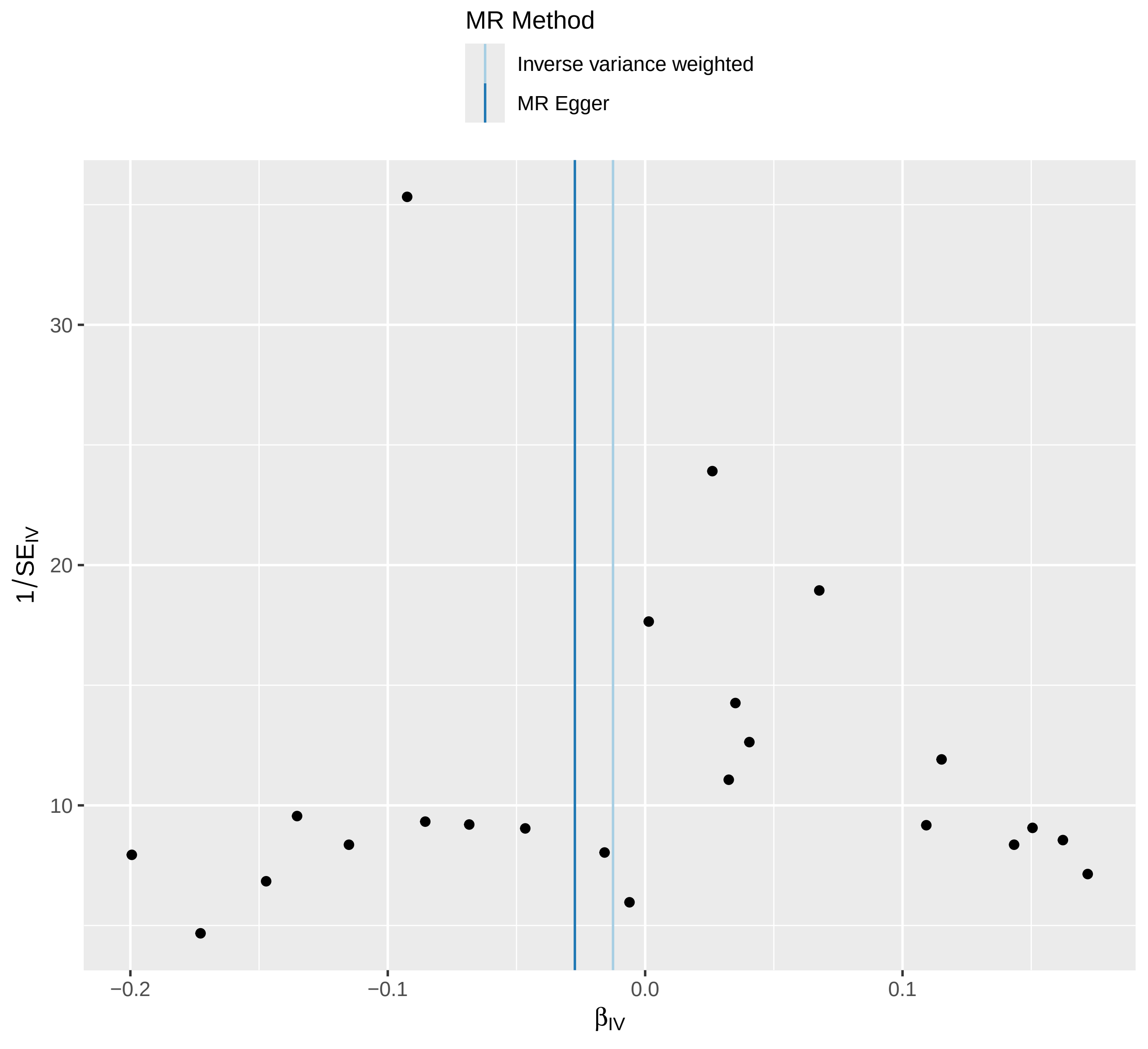
MR Method
Inverse variance weighted
MR Egger
30
20
10
1/SE_IV
−0.2
−0.1
0.0
0.1
β_IV

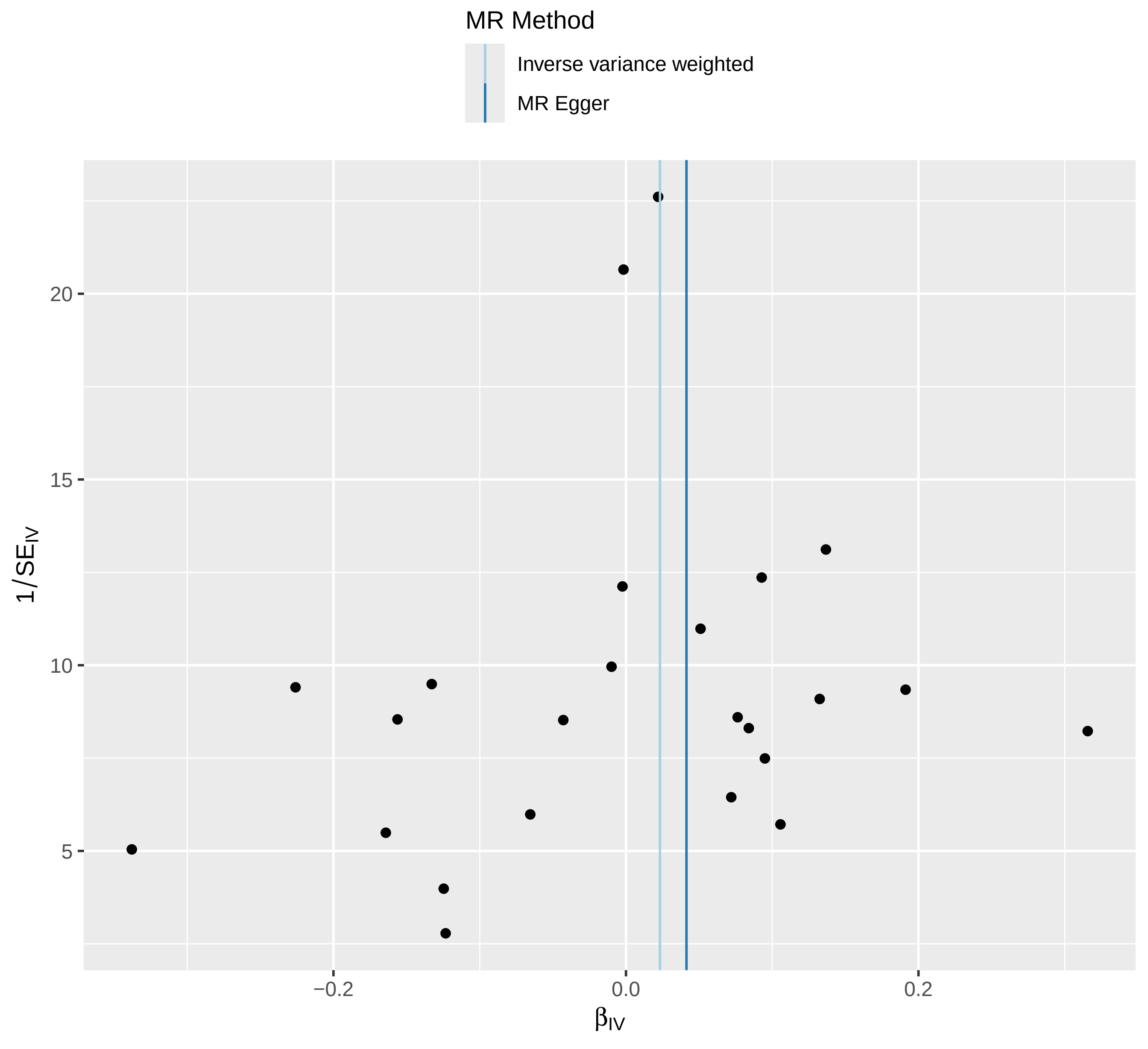

MR Method
Inverse variance weighted
MR Egger
20
15
10
5
1/SE$_{IV}$
−0.2
0.0
0.2
β$_{IV}$

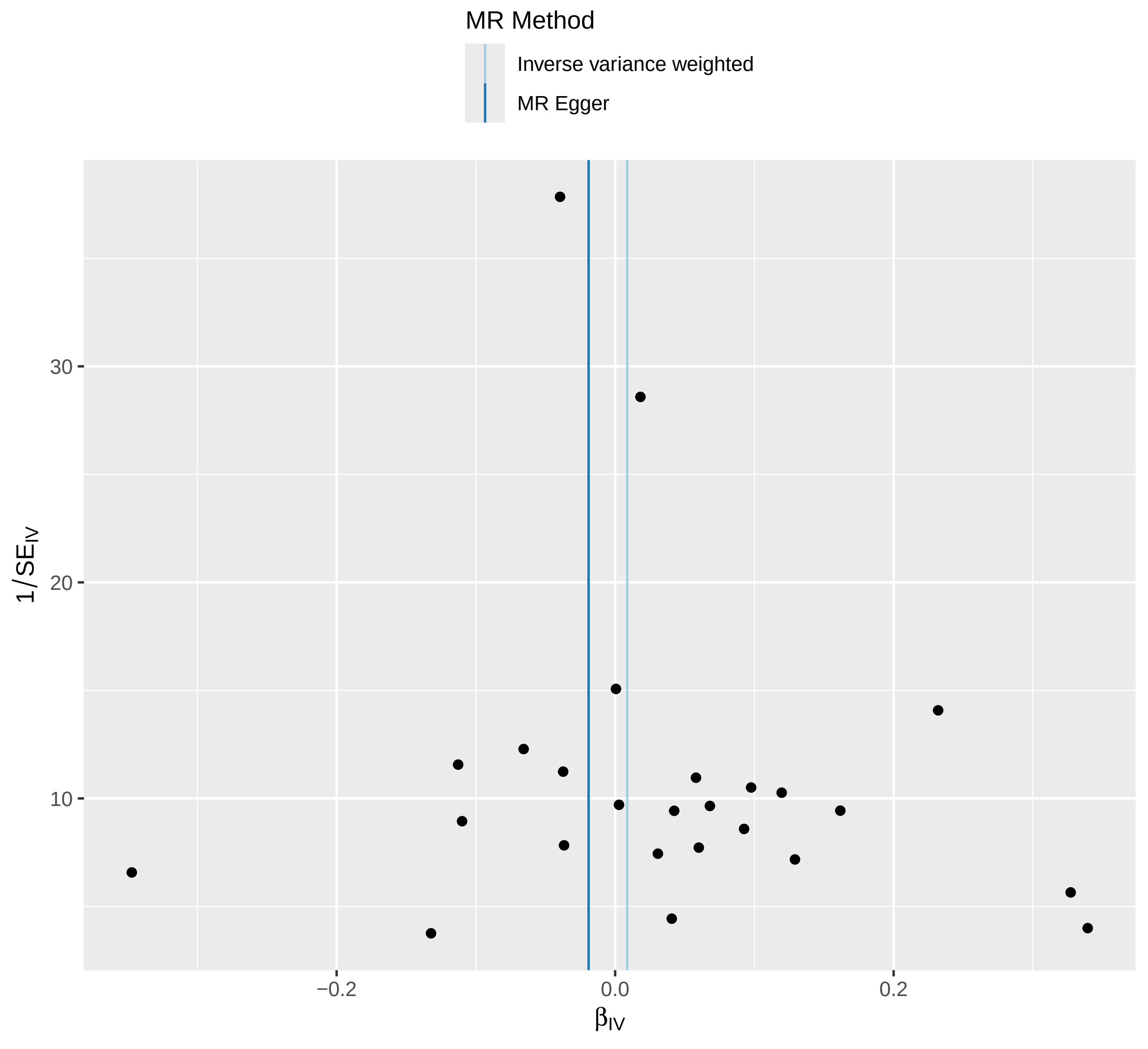
MR Method
Inverse variance weighted
MR Egger
30
20
10
$1/SE_{IV}$
−0.2
0.0
0.2
β_{IV}

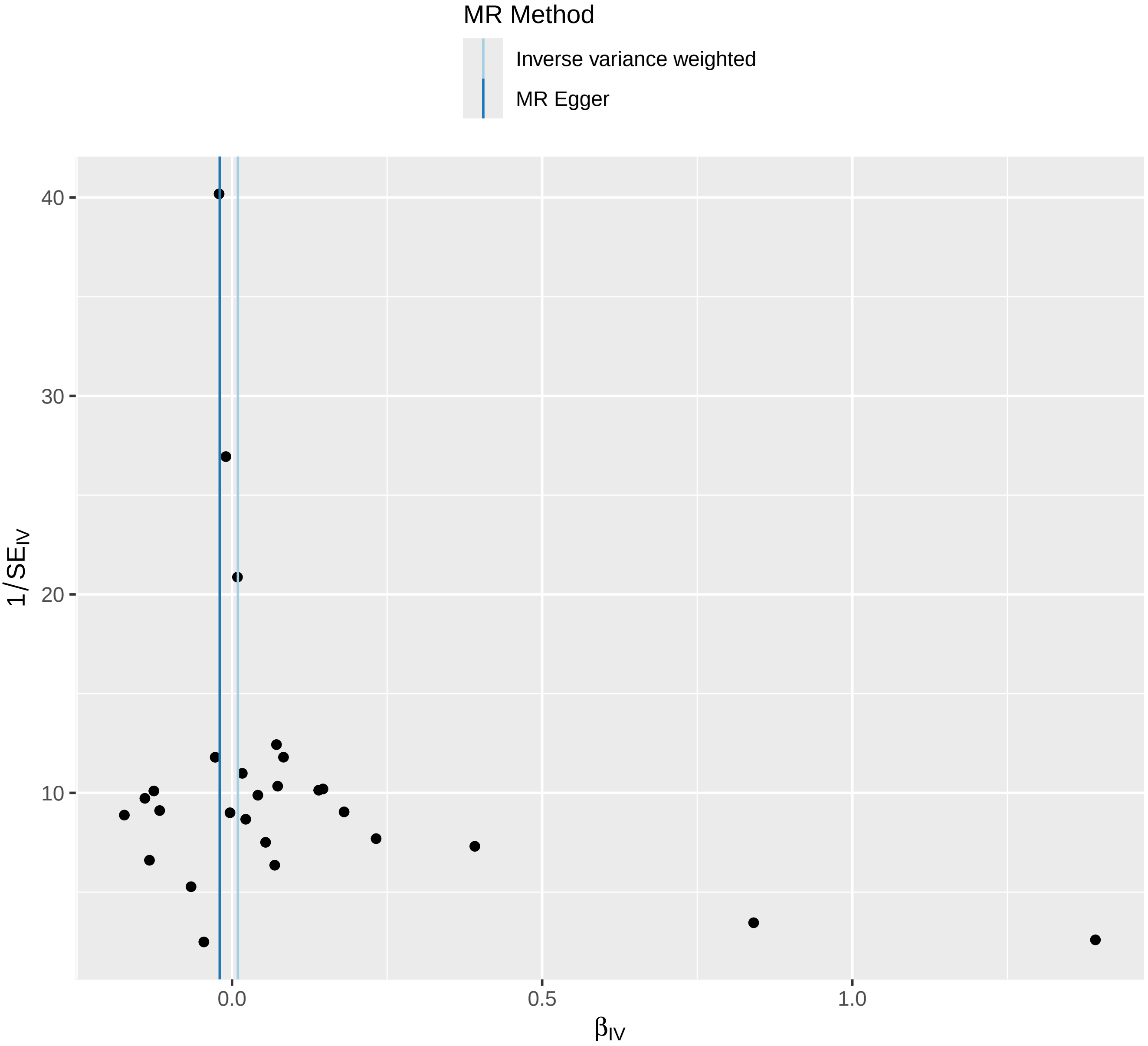
MR Method
Inverse variance weighted
MR Egger
40
30
20
10
1/SE$_{IV}$
0.0
0.5
1.0
β$_{IV}$

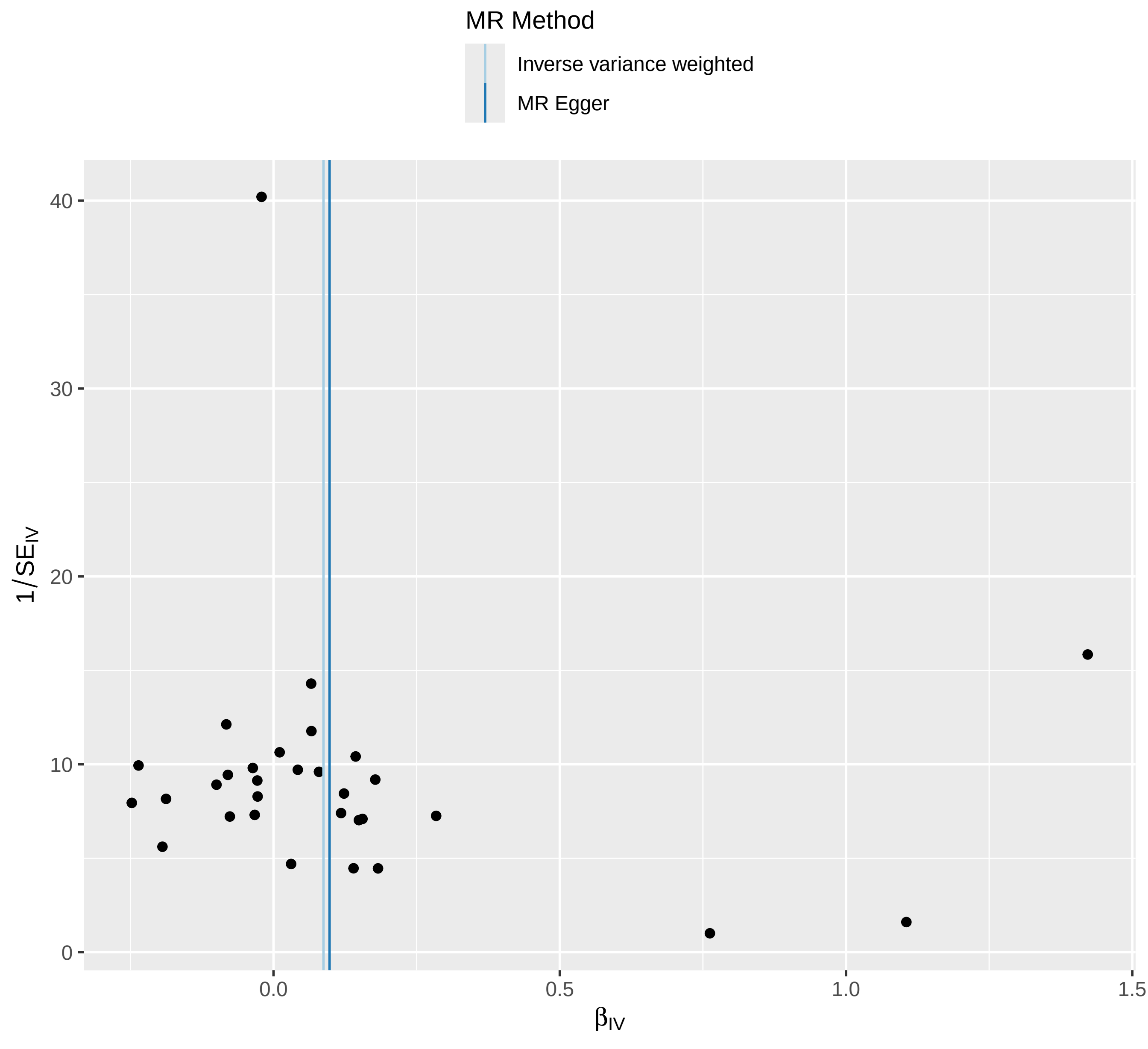

MR Method
Inverse variance weighted
MR Egger
40
30
20
10
0
1/SE$_{IV}$
0.0
0.5
1.0
1.5
β$_{IV}$

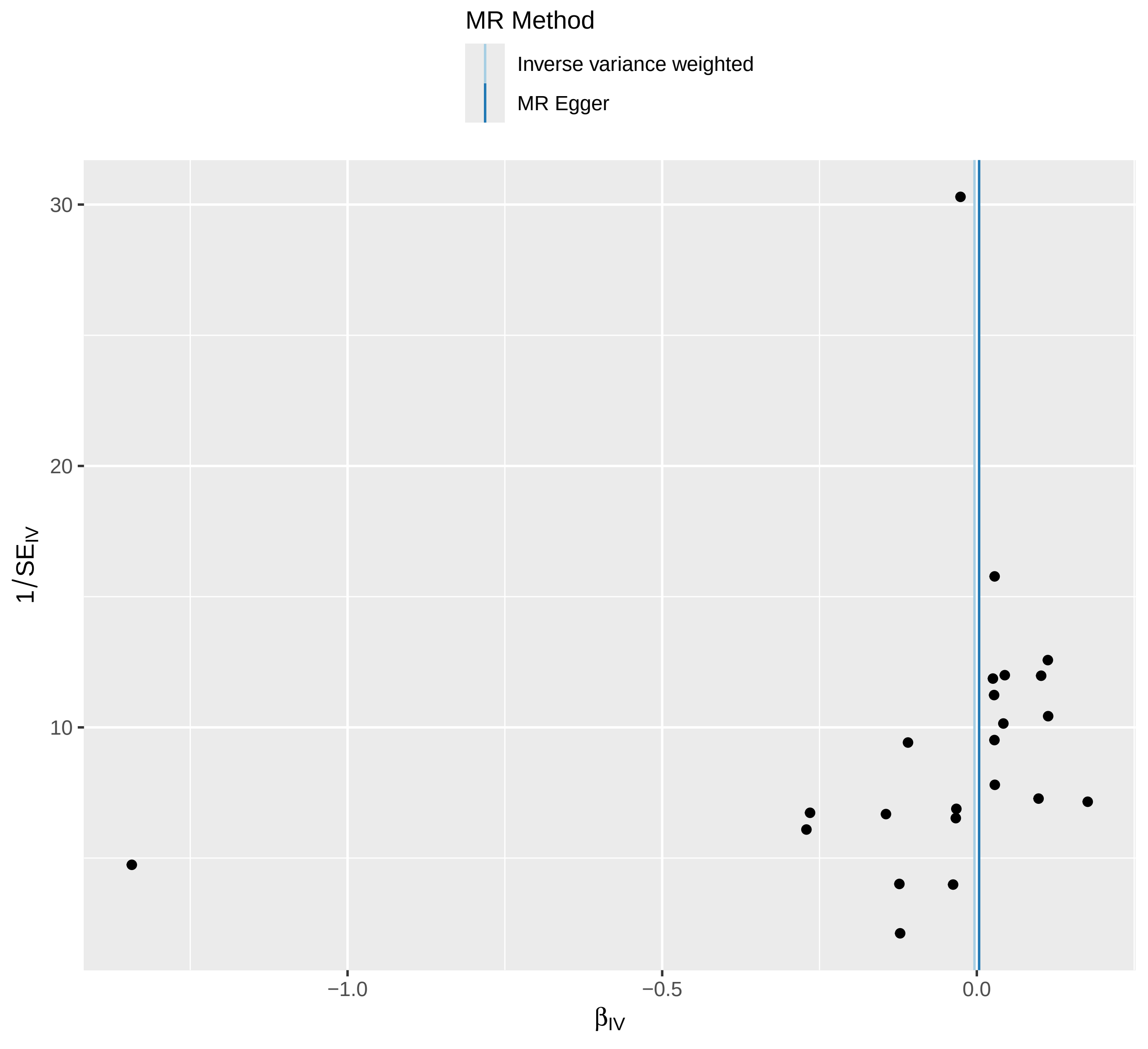

MR Method
Inverse variance weighted
MR Egger
30
20
10
1/SE$_{IV}$
−1.0
−0.5
0.0
β$_{IV}$

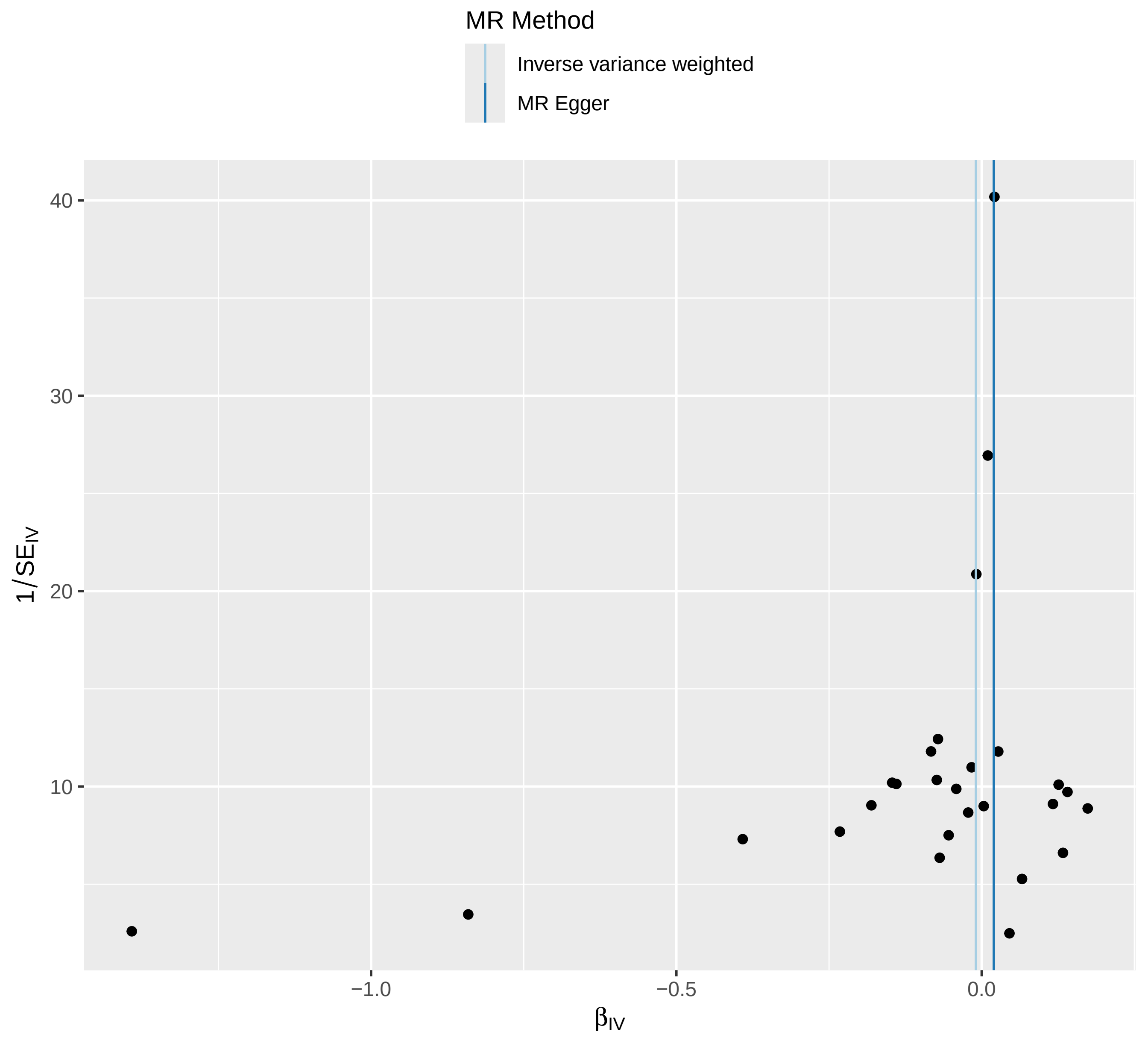
MR Method
Inverse variance weighted
MR Egger
40
30
20
10
1/SE_IV
−1.0
−0.5
0.0
β_IV

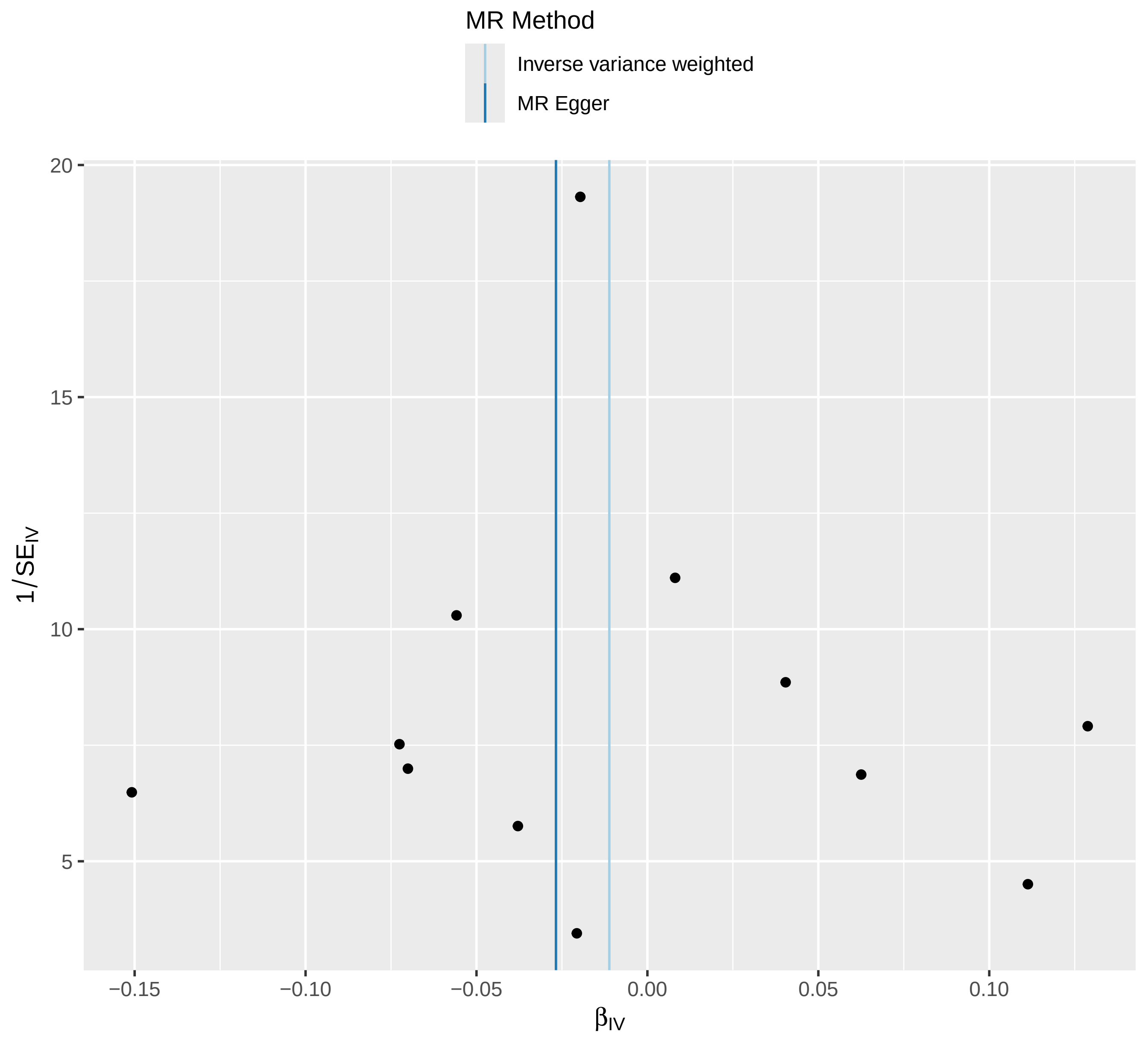

MR Method
Inverse variance weighted
MR Egger
20
15
10
5
1/SE_IV
−0.15
−0.10
−0.05
0.00
0.05
0.10
β_IV

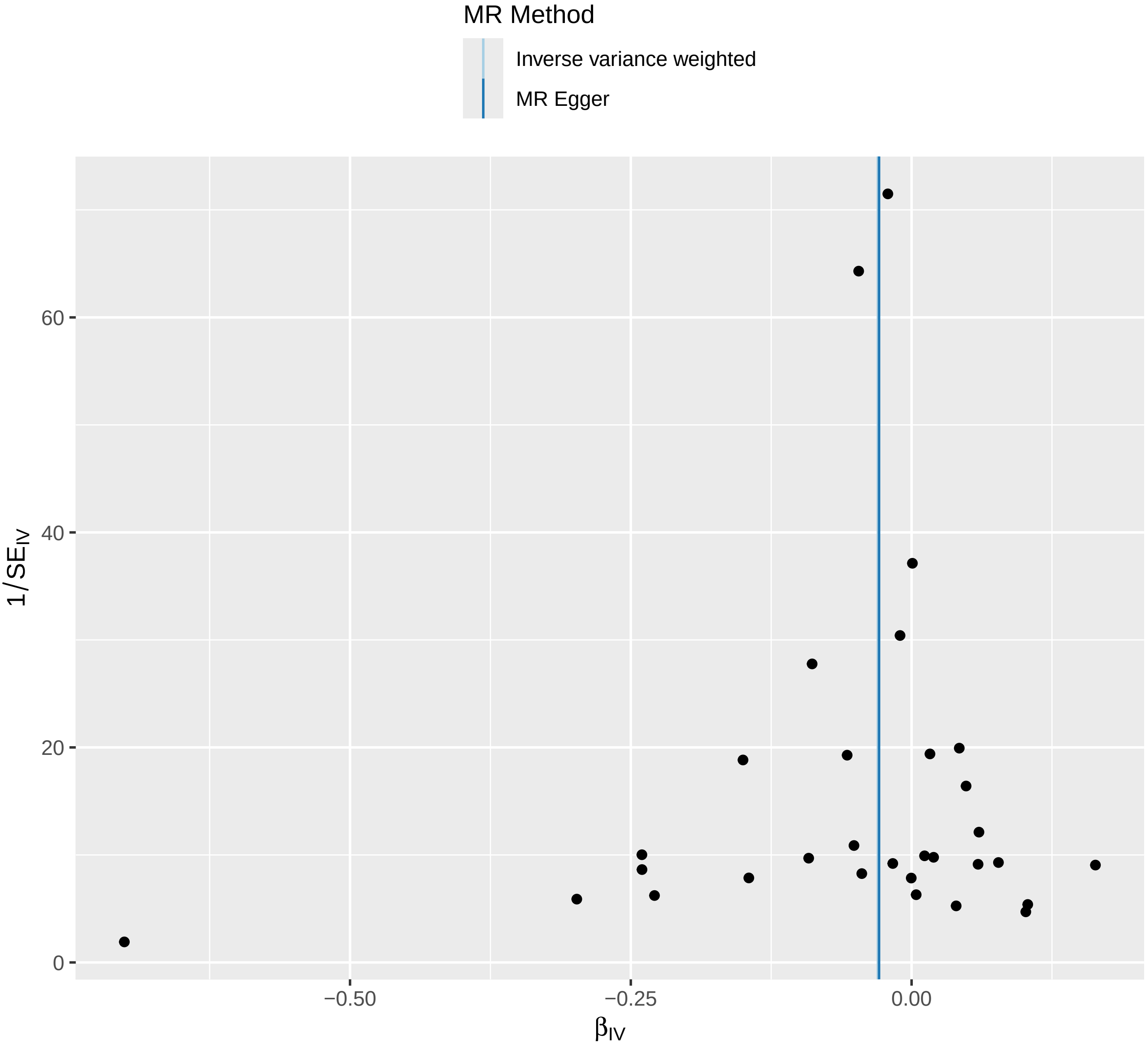
MR Method
Inverse variance weighted
MR Egger
60
40
20
0
1/SE_IV
−0.50
−0.25
0.00
β_IV

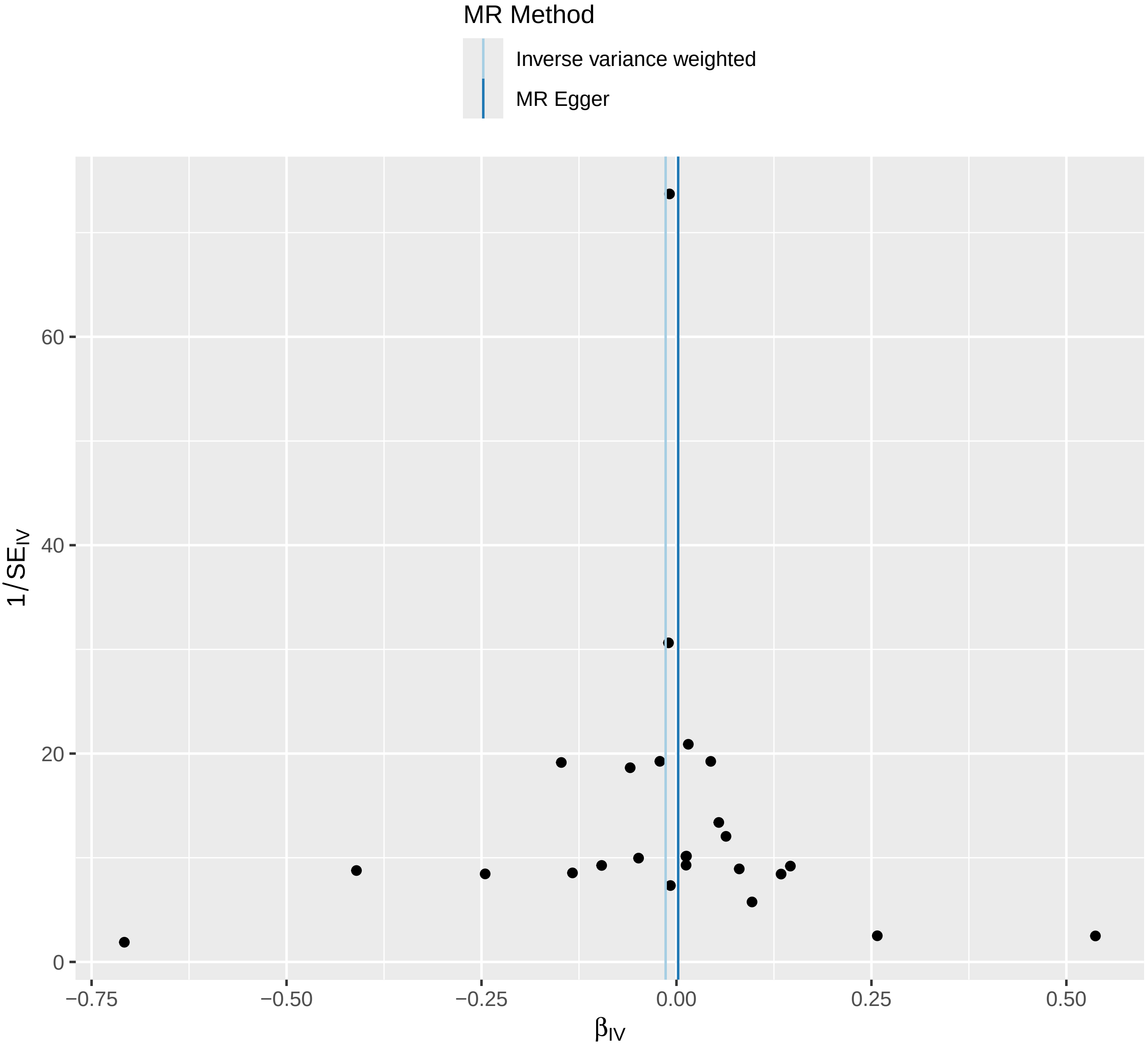
MR Method
Inverse variance weighted
MR Egger
60
40
20
0
1/SE_IV
−0.75
−0.50
−0.25
0.00
0.25
0.50
β_IV

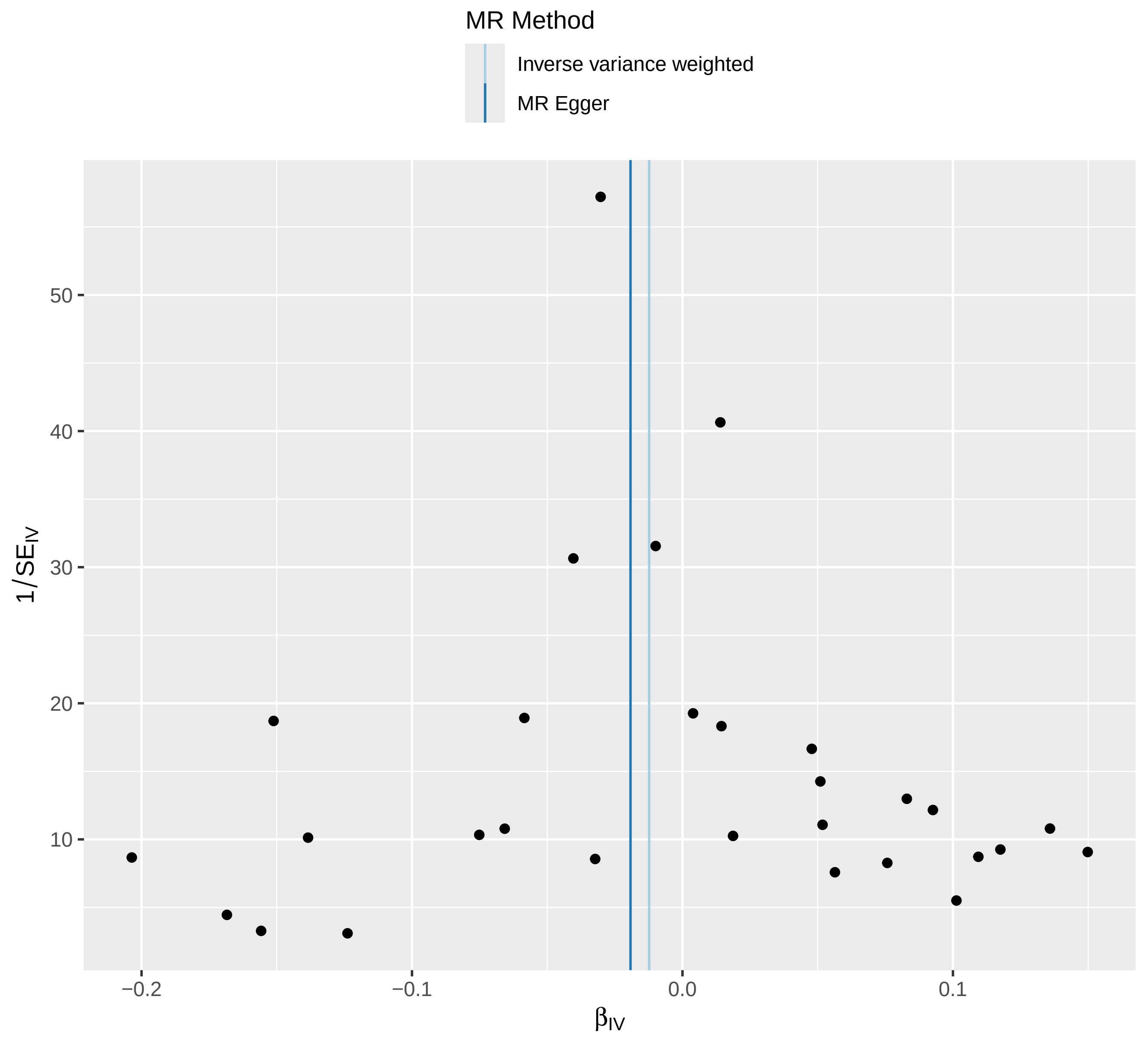

MR Method
Inverse variance weighted
MR Egger
50
40
30
20
10
1/SE_IV
−0.2
−0.1
0.0
0.1
β_IV

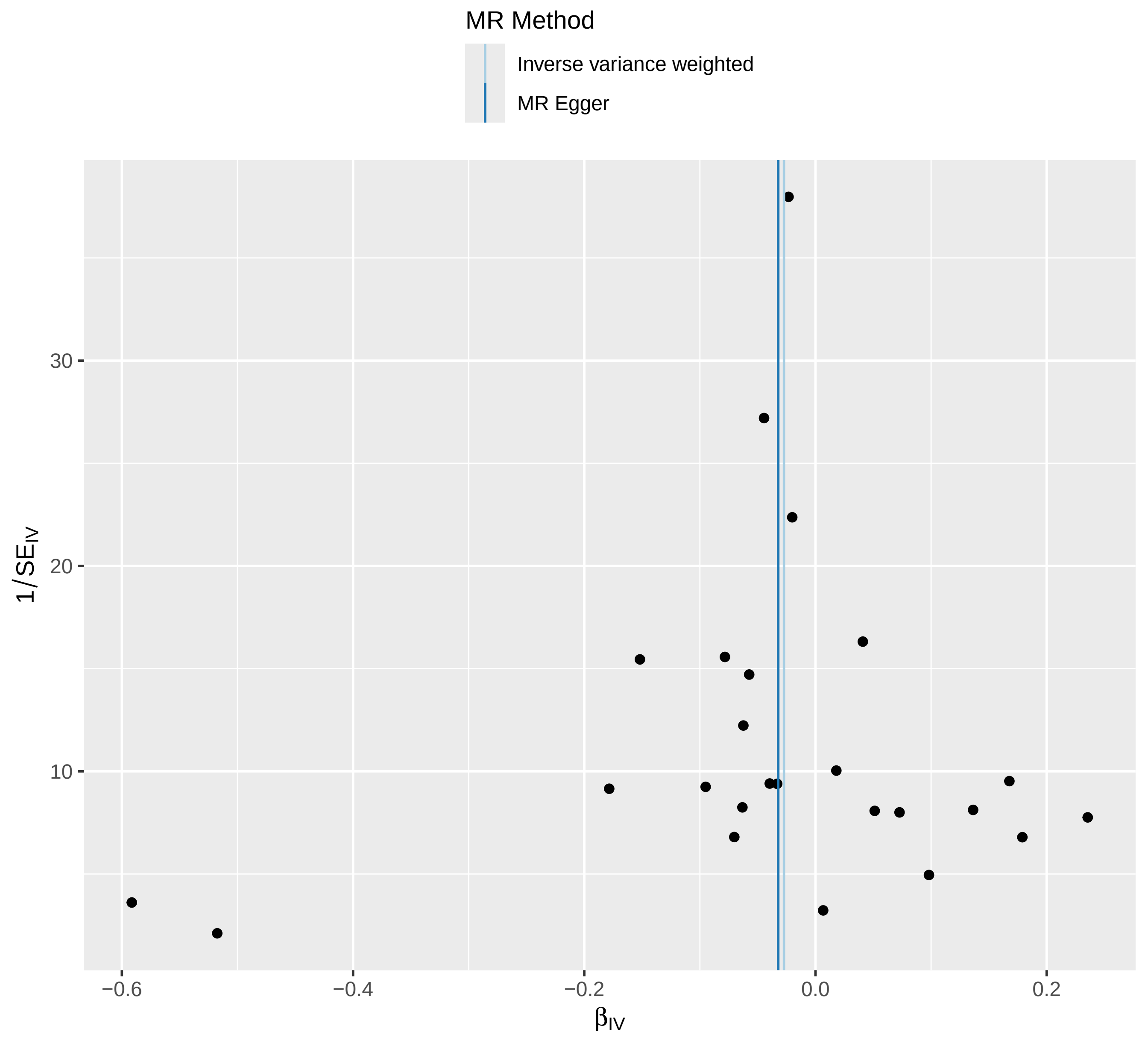

MR Method
Inverse variance weighted
MR Egger
30
20
10
1/SE_IV
−0.6
−0.4
−0.2
0.0
0.2
β_IV

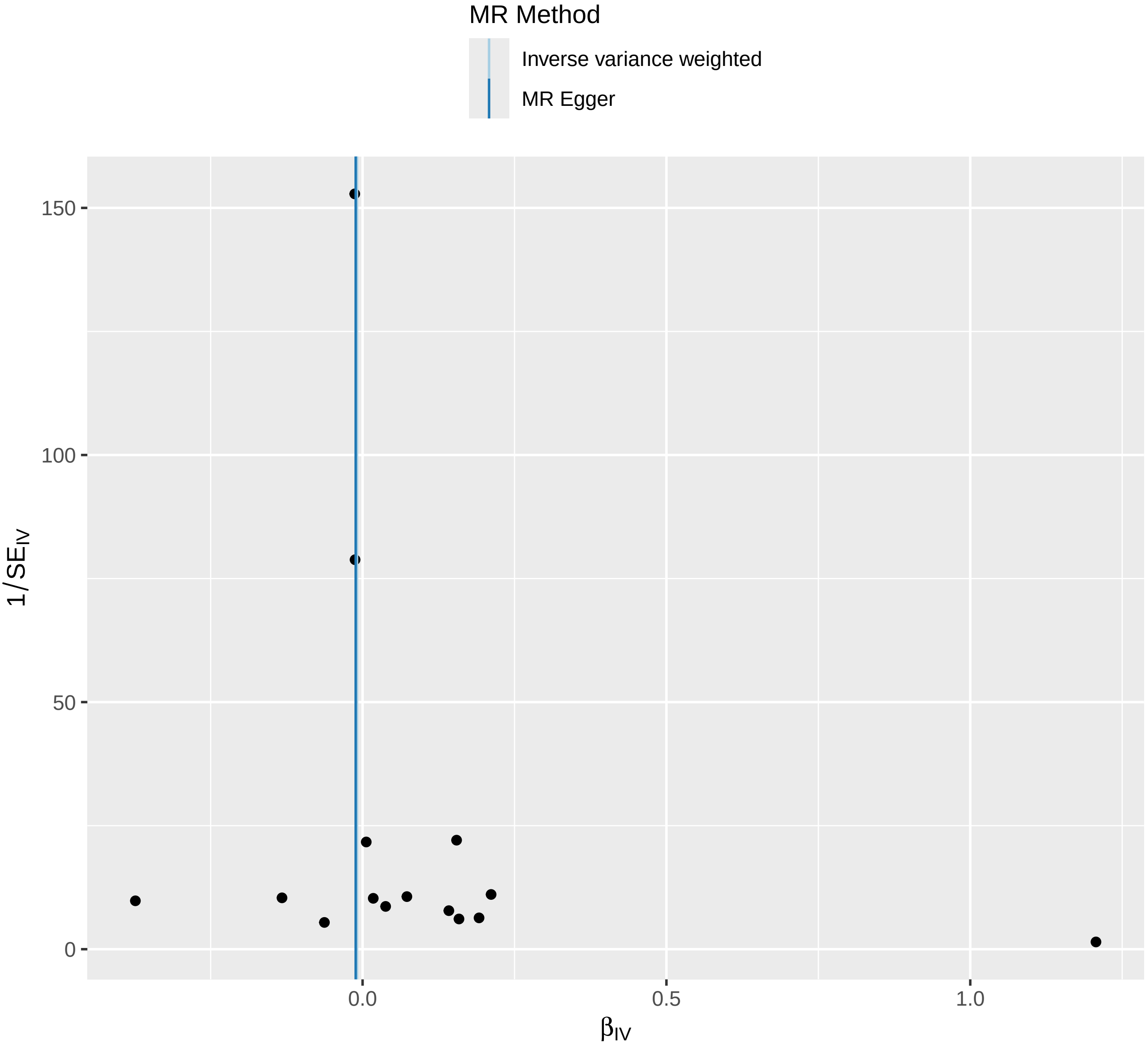
MR Method
Inverse variance weighted
MR Egger
150
100
50
0
$1/SE_{IV}$
0.0
0.5
1.0
β_{IV}

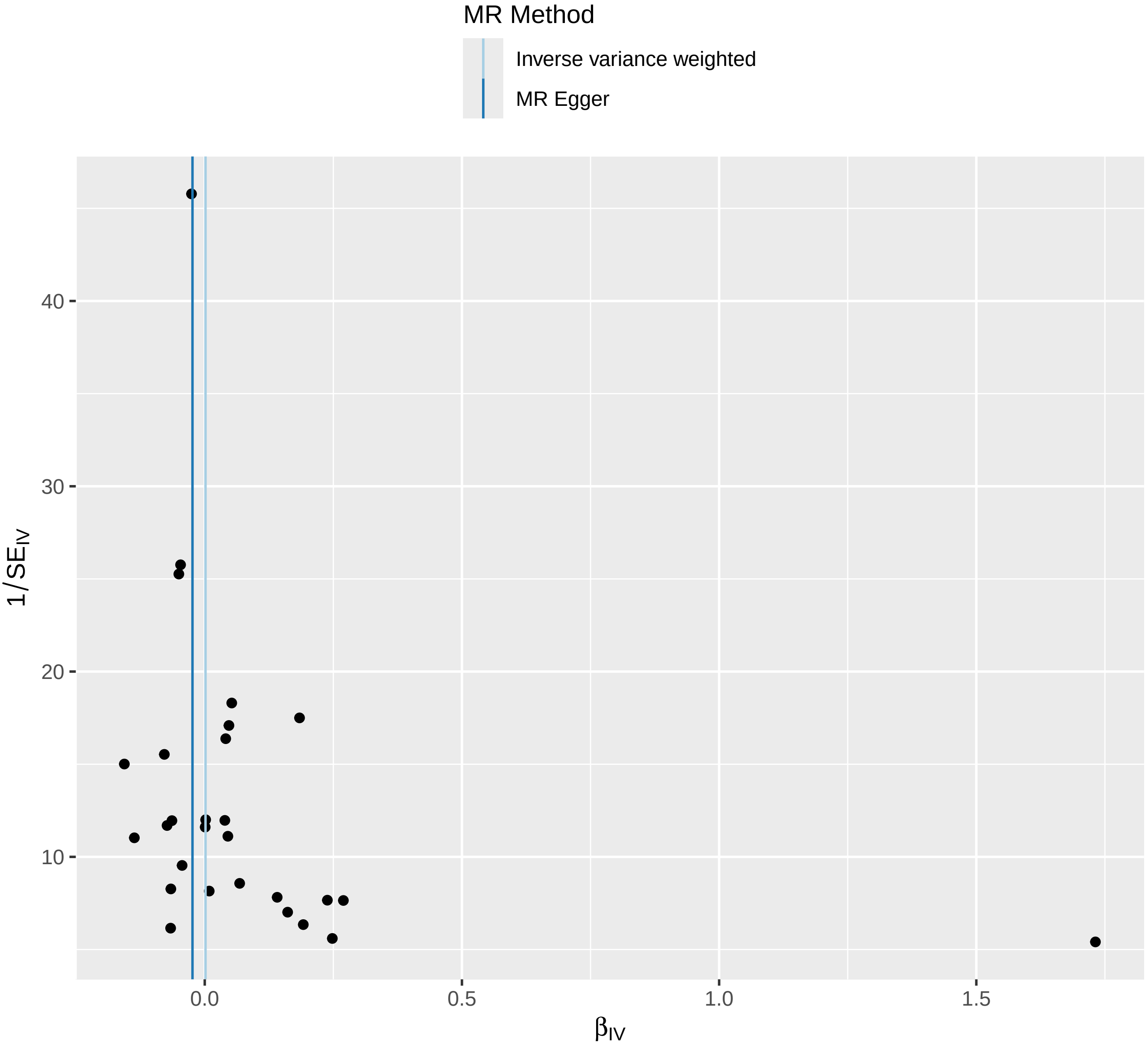
MR Method
Inverse variance weighted
MR Egger
1/SE_IV
40
30
20
10
0.0
0.5
1.0
1.5
β_IV

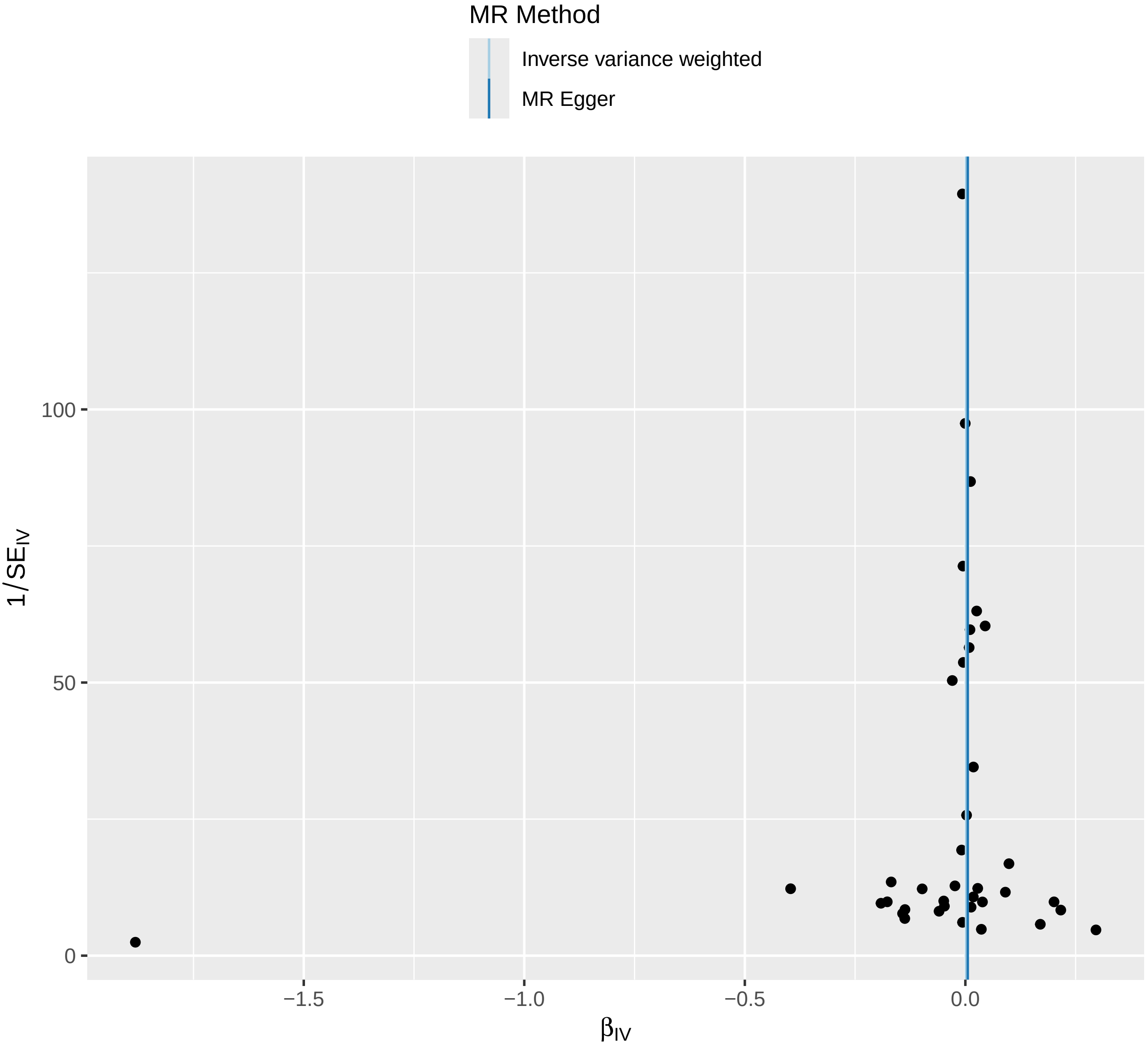
MR Method
Inverse variance weighted
MR Egger
1/SE_IV
100
50
0
−1.5
−1.0
−0.5
0.0
β_IV

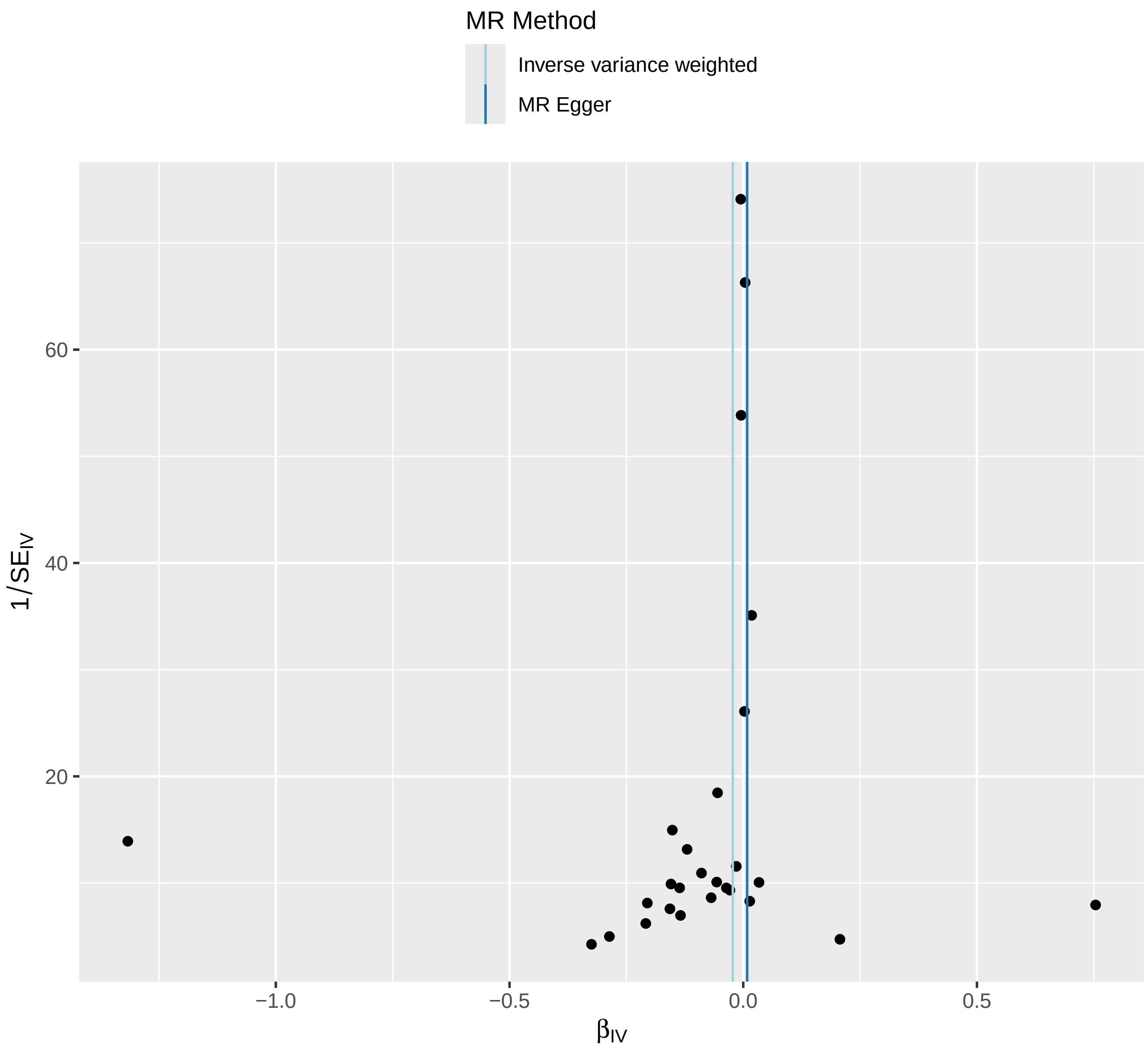
MR Method
Inverse variance weighted
MR Egger
60
40
20
1/SE_IV
−1.0
−0.5
0.0
0.5
β_IV

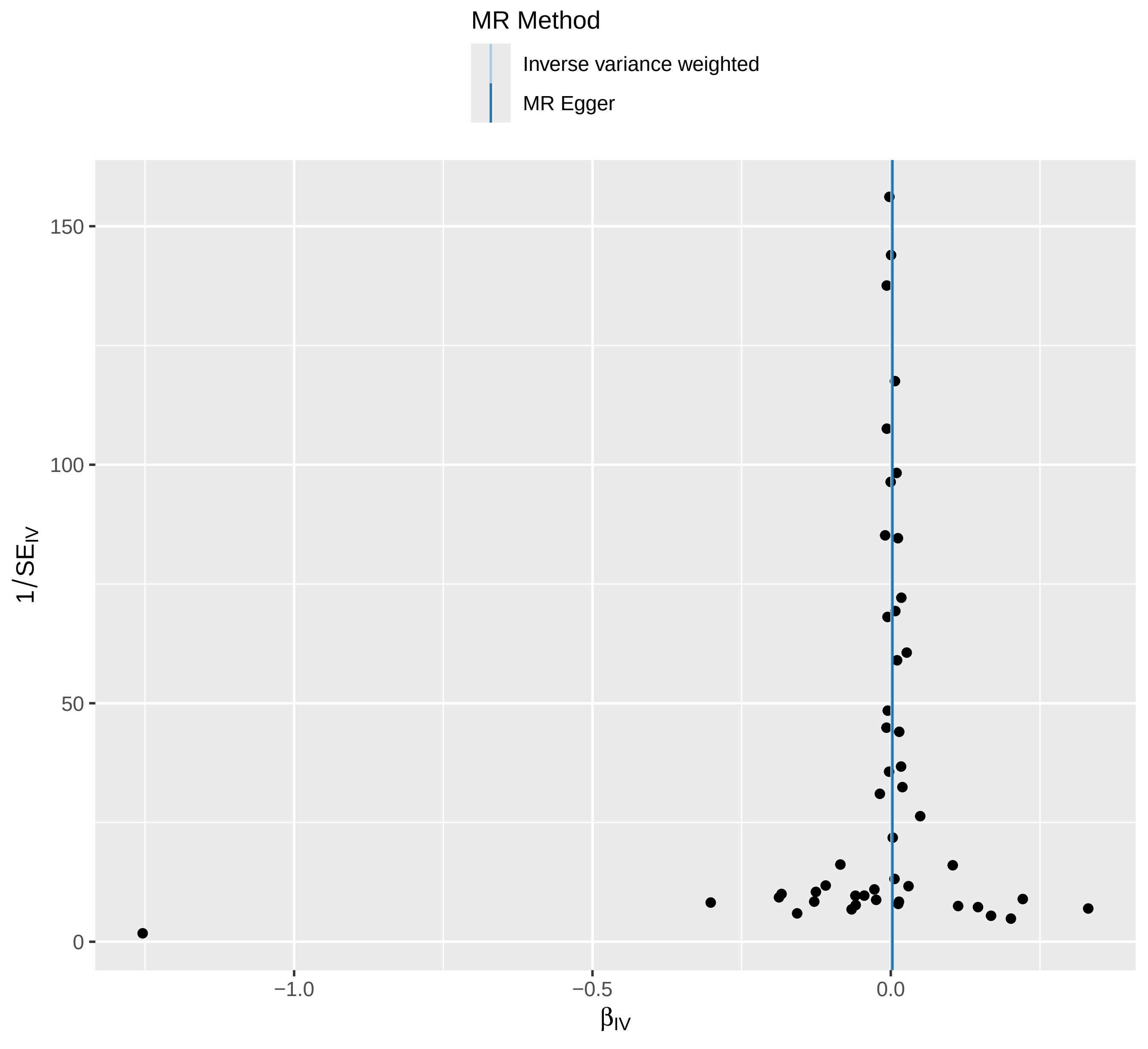

MR Method
Inverse variance weighted
MR Egger
150
100
50
0
1/SE_IV
−1.0
−0.5
0.0
β_IV

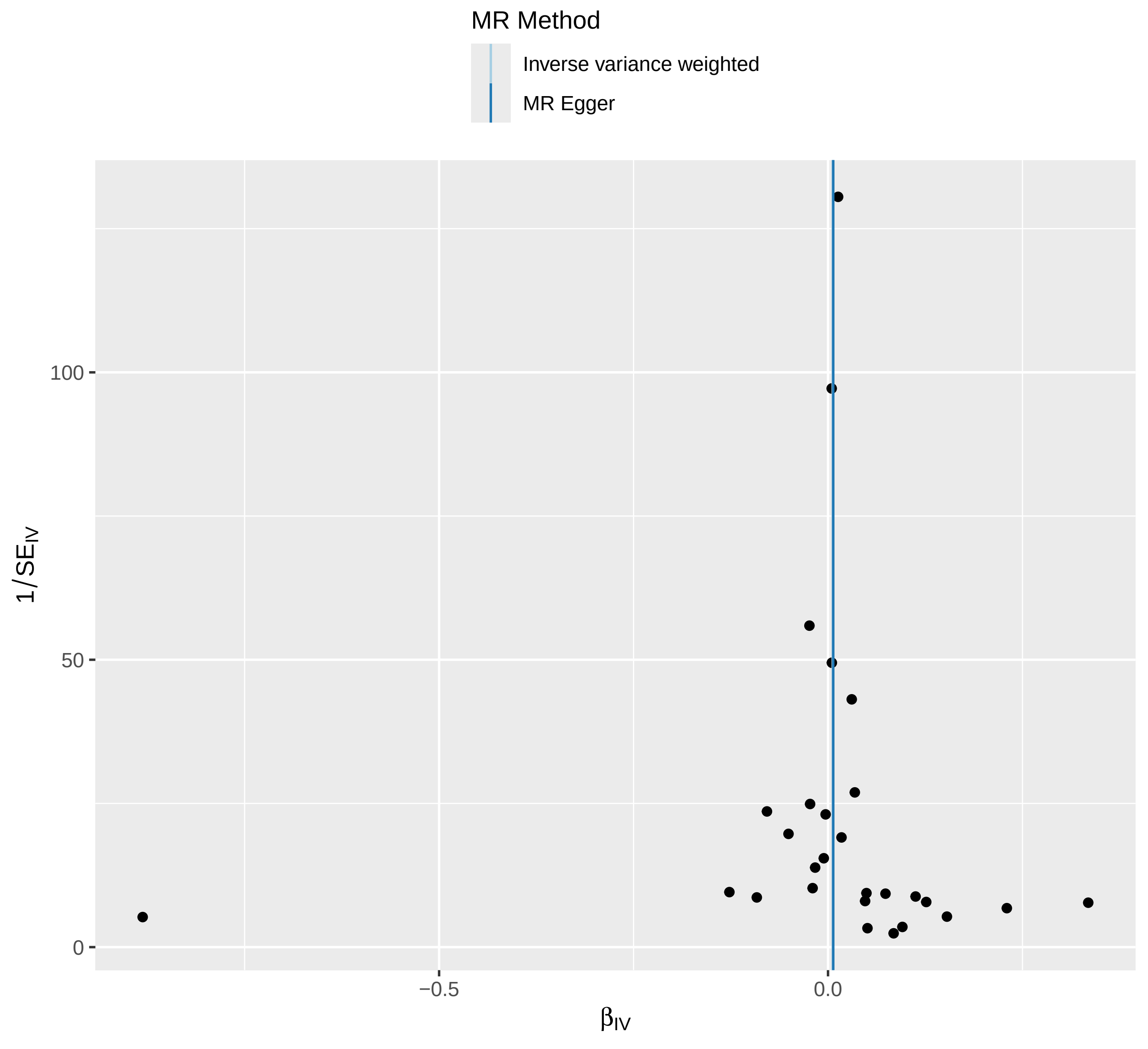
MR Method
Inverse variance weighted
MR Egger
1/SE$_{IV}$
100
50
0
−0.5
0.0
β$_{IV}$

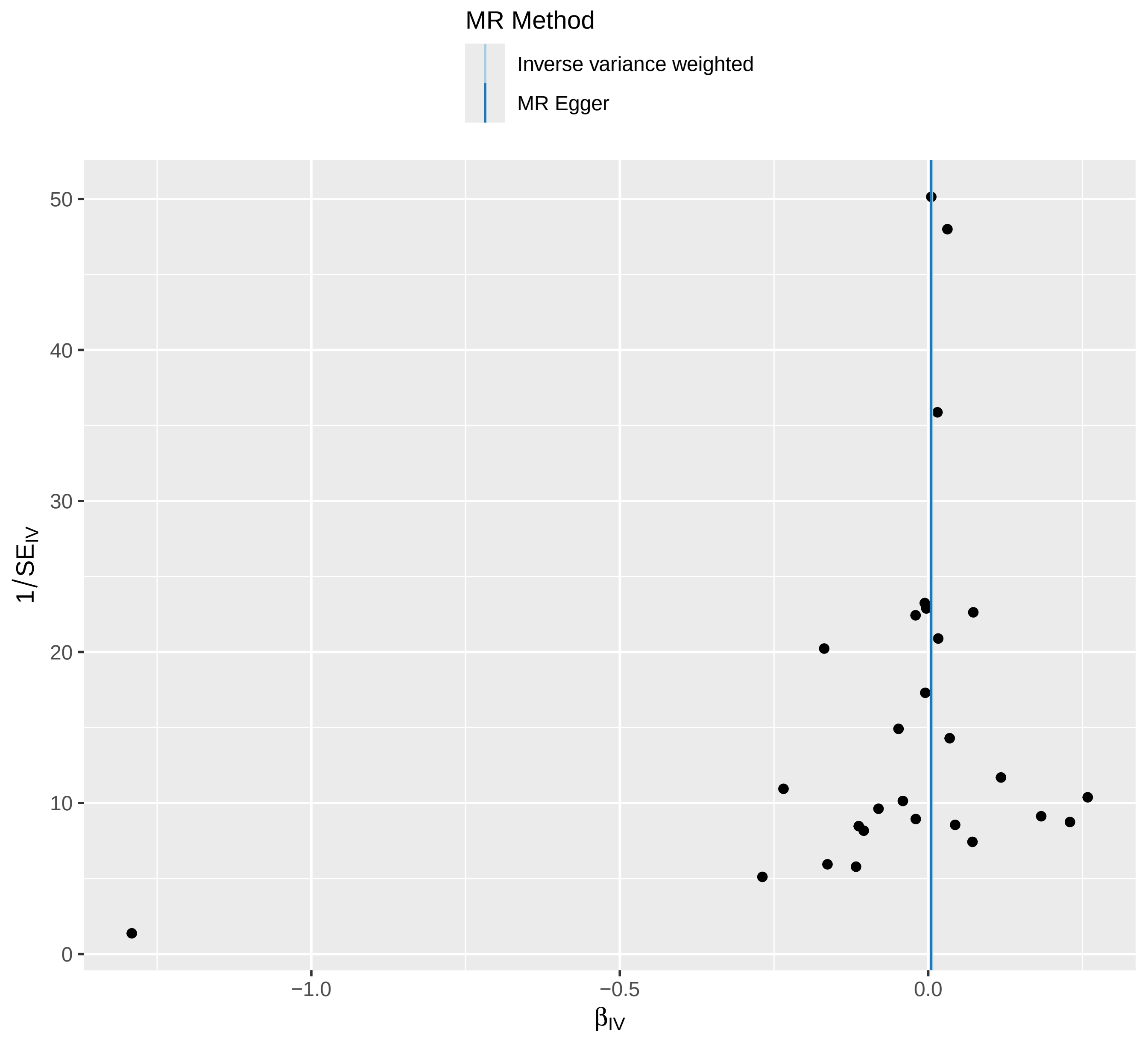
MR Method
Inverse variance weighted
MR Egger
50
40
30
20
10
0
$1/SE_{IV}$
−1.0
−0.5
0.0
β_{IV}

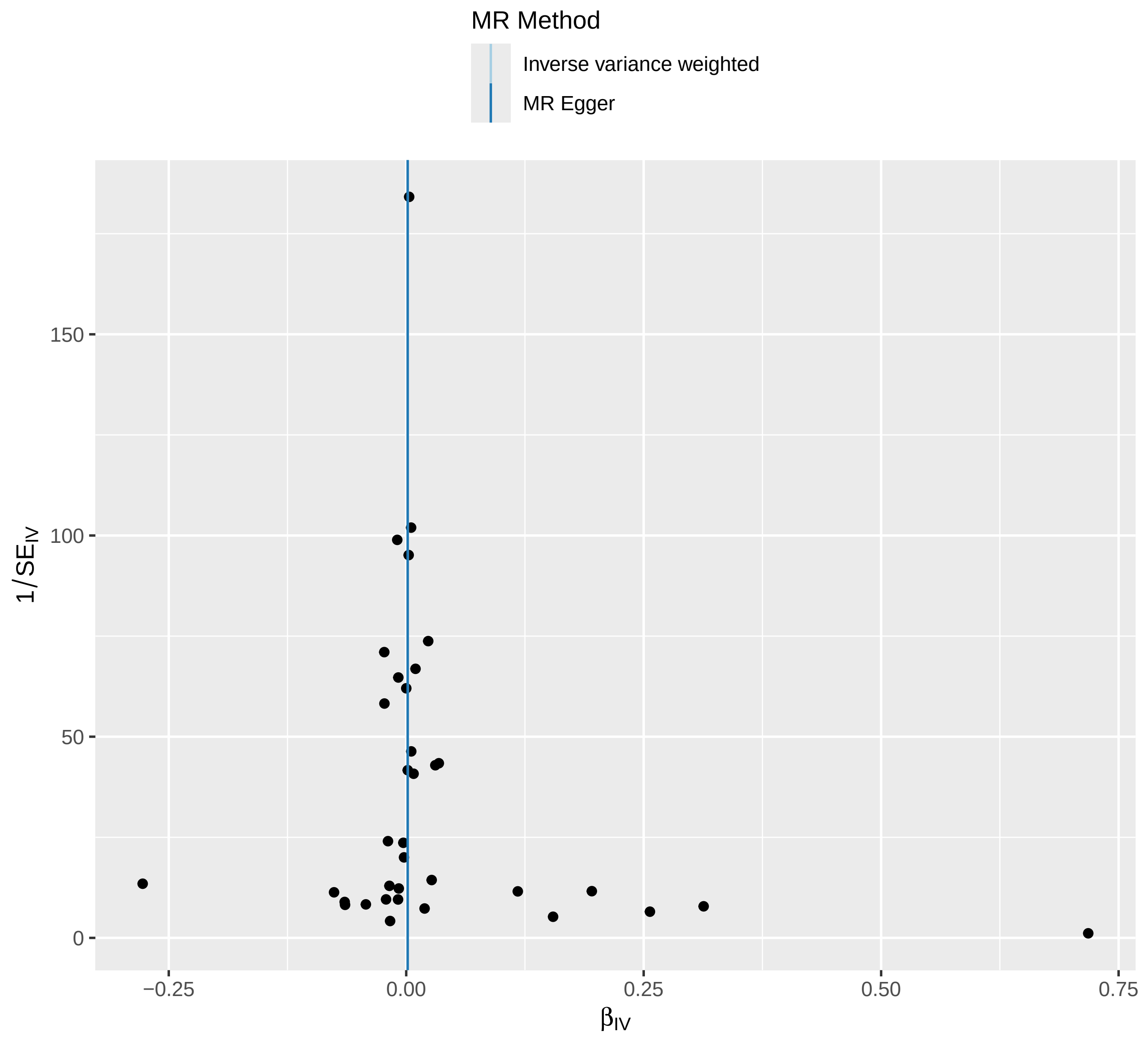
MR Method
Inverse variance weighted
MR Egger
150
100
50
0
1/SE_IV
−0.25
0.00
0.25
0.50
0.75
β_IV

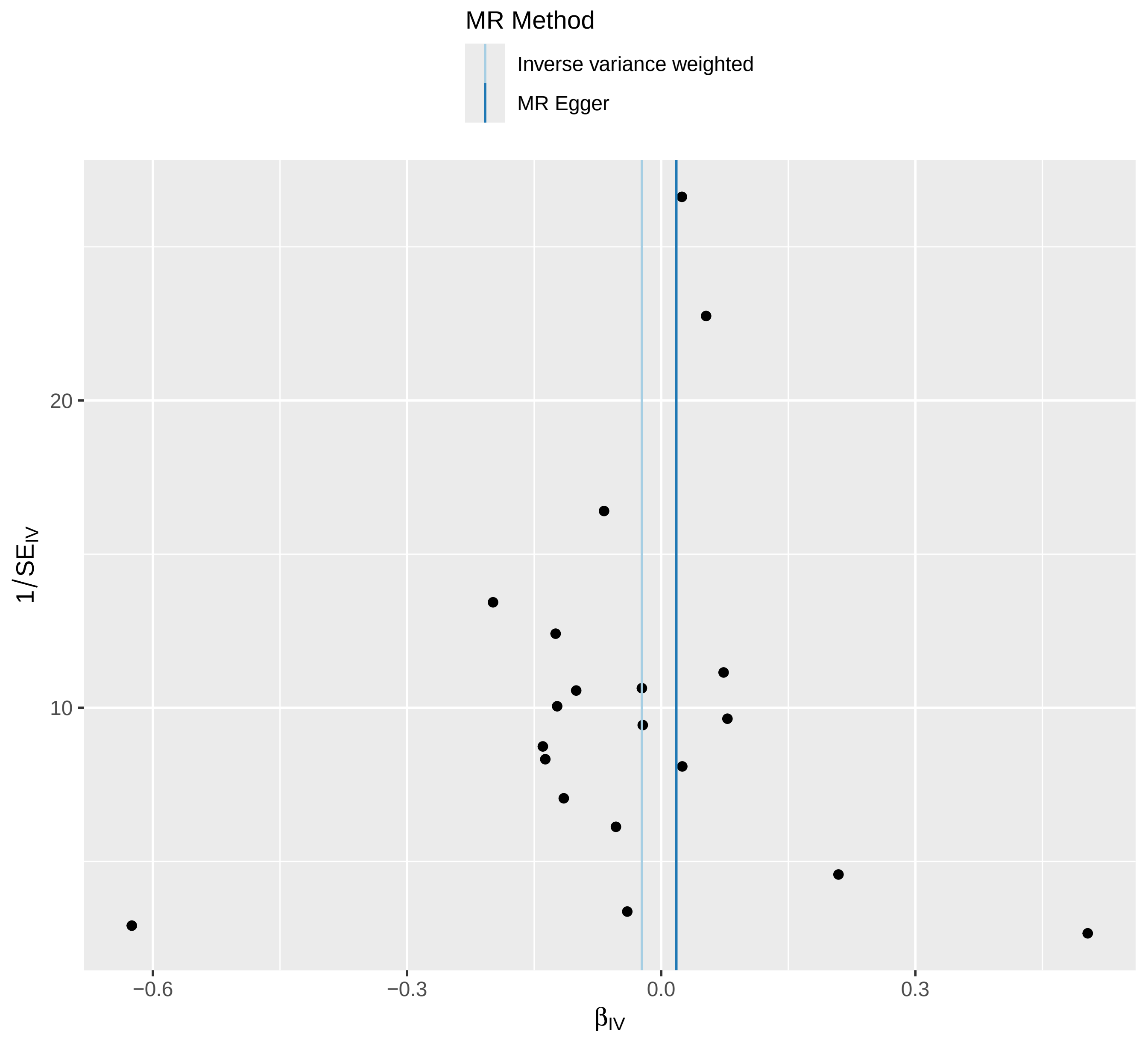
MR Method
Inverse variance weighted
MR Egger
20
10
1/SE_IV
−0.6
−0.3
0.0
0.3
β_IV

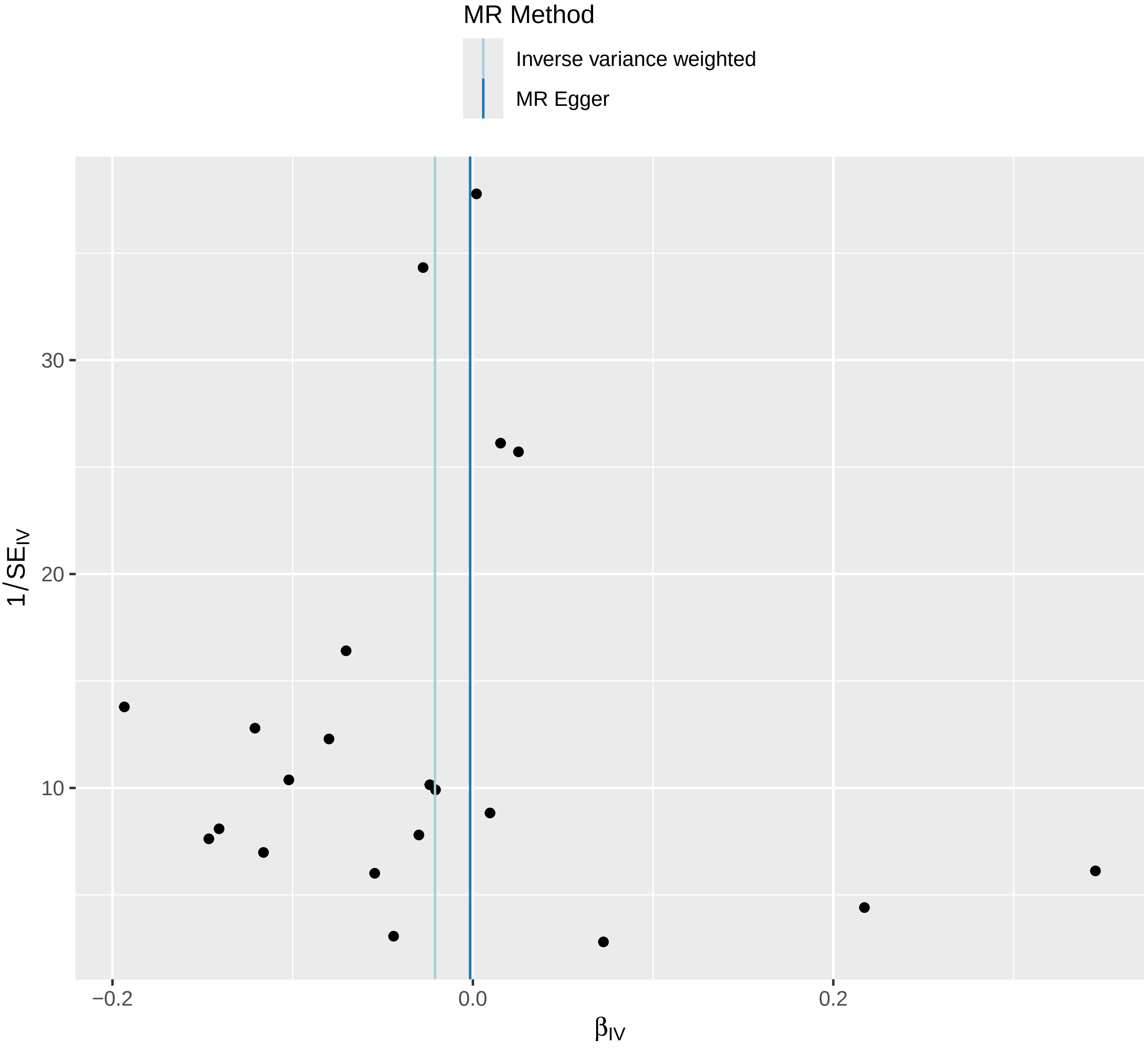
MR Method
Inverse variance weighted
MR Egger
30
20
10
1/SE_IV
−0.2
0.0
0.2
β_IV

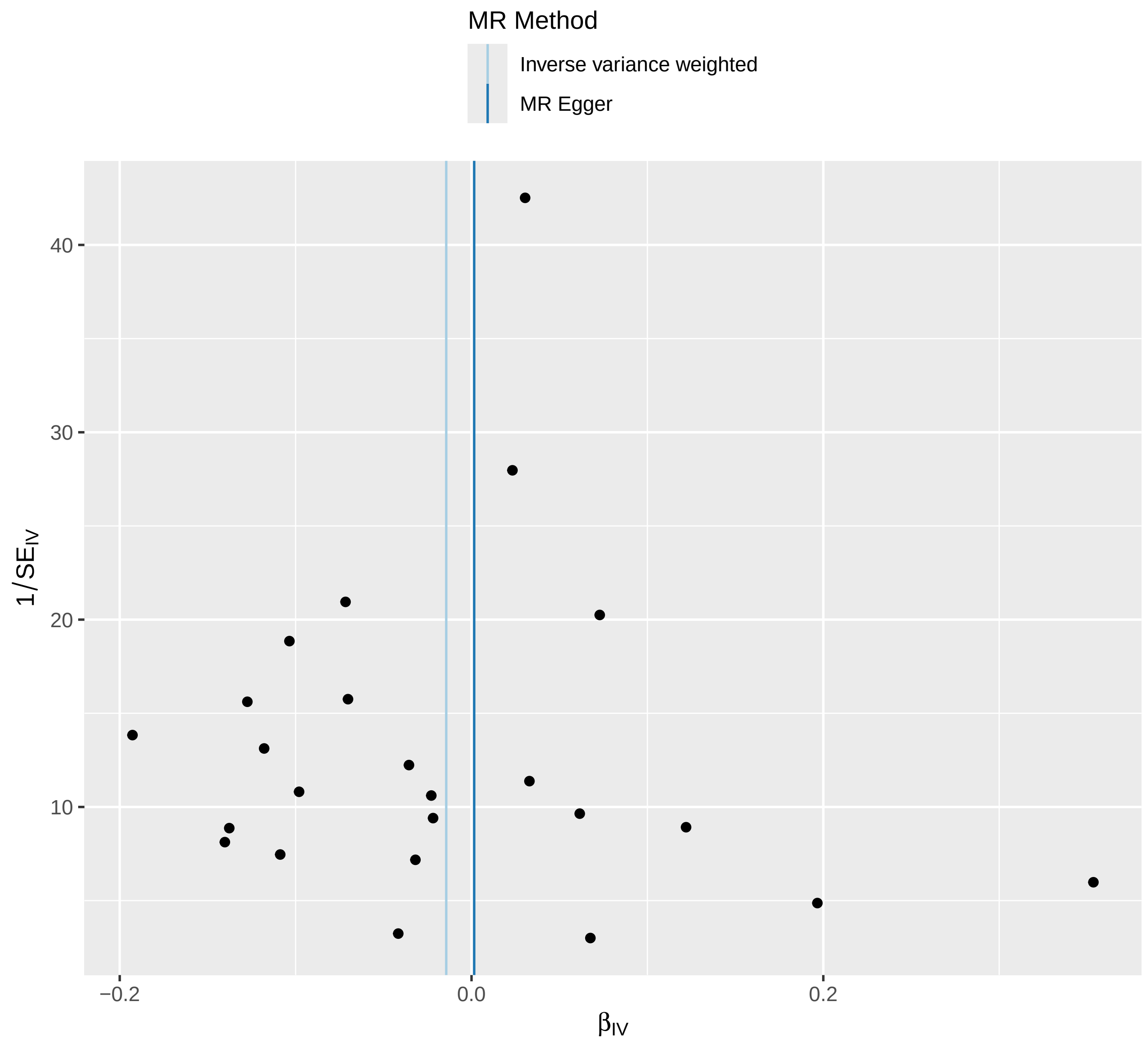
MR Method
Inverse variance weighted
MR Egger
40
30
20
10
1/SE$_{IV}$
−0.2
0.0
0.2
β$_{IV}$

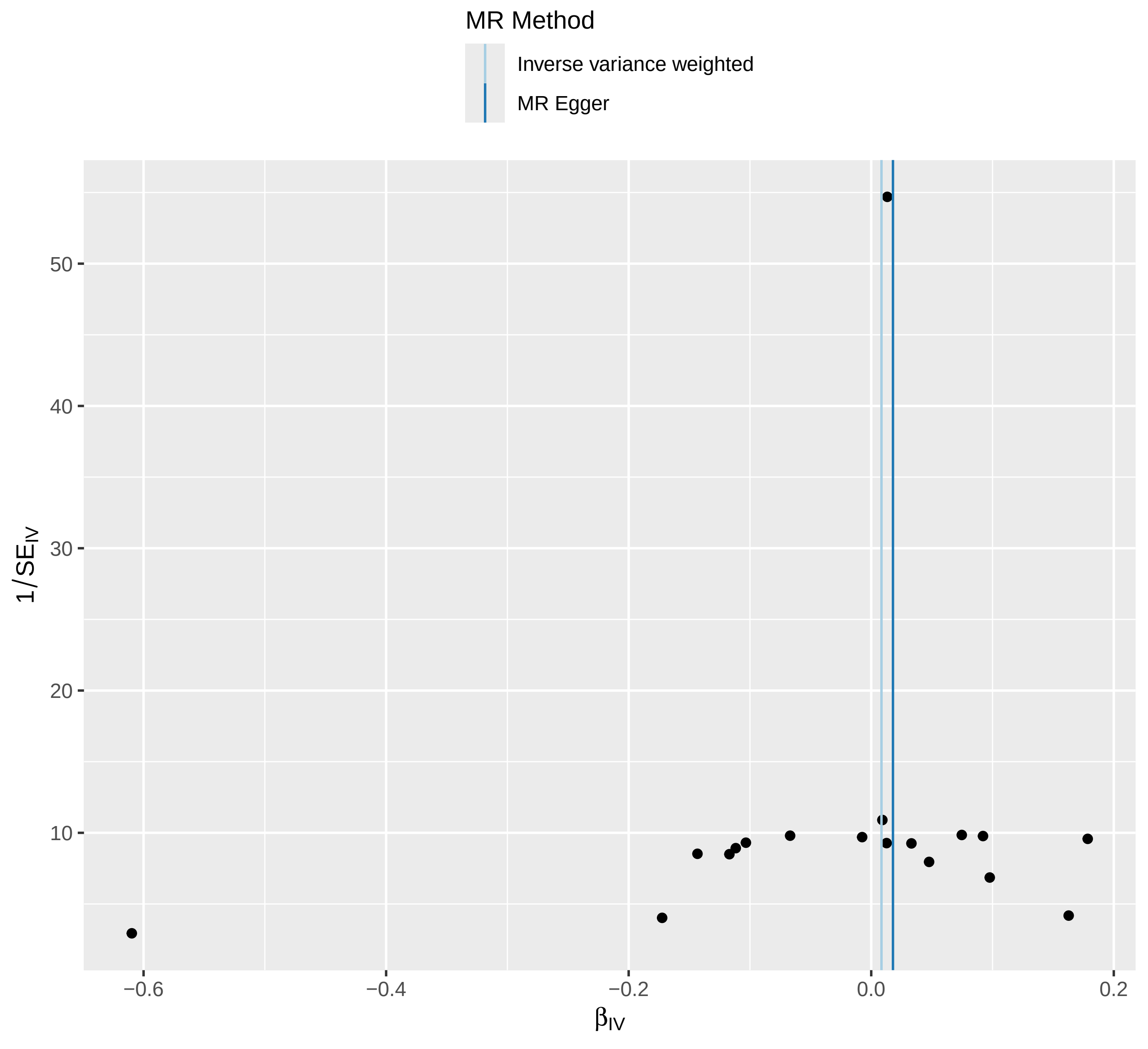
MR Method
Inverse variance weighted
MR Egger
50
40
30
20
10
1/SE_IV
−0.6
−0.4
−0.2
0.0
0.2
β_IV

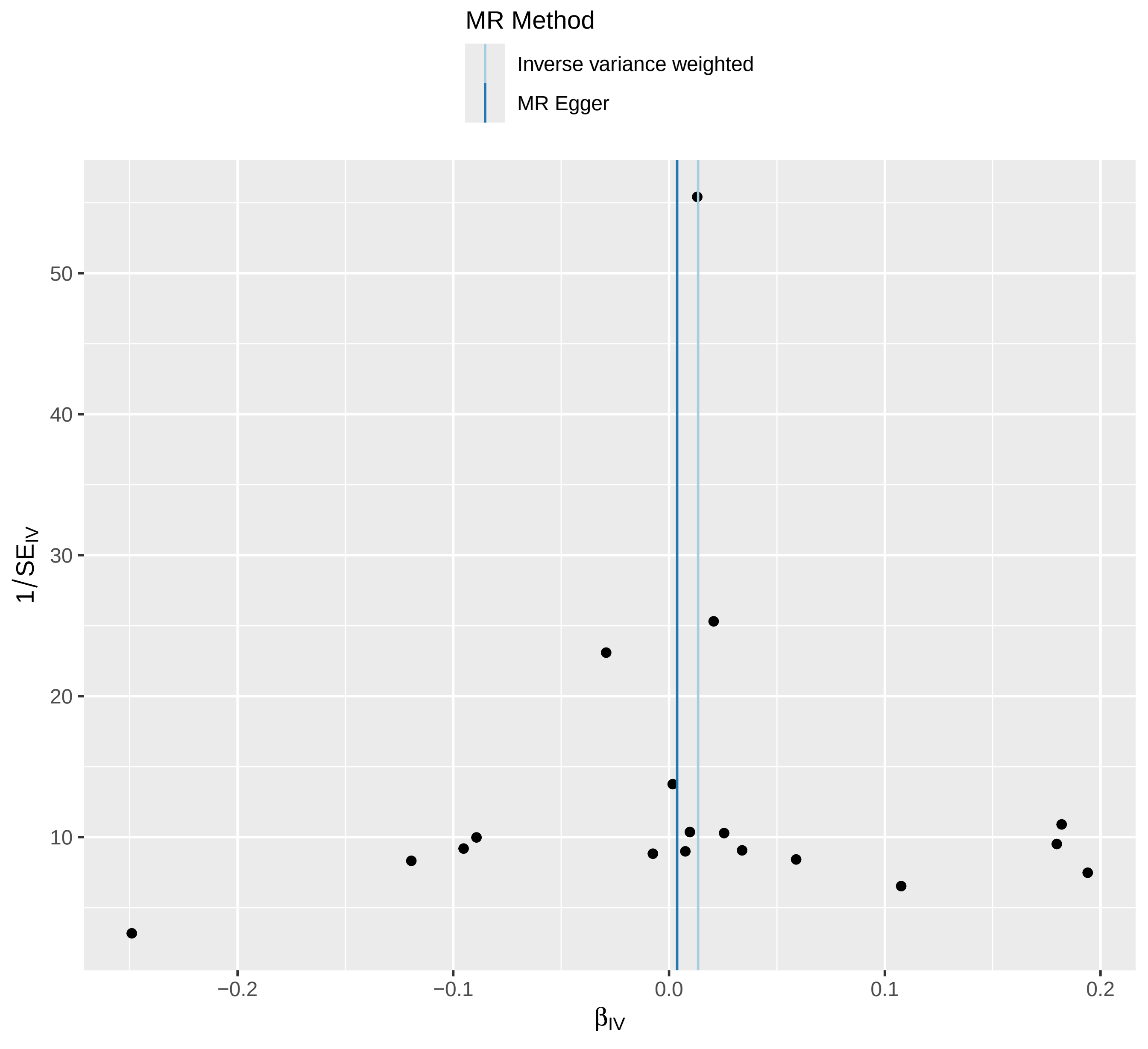
MR Method
Inverse variance weighted
MR Egger
50
40
30
20
10
1/SE_IV
−0.2
−0.1
0.0
0.1
0.2
β_IV

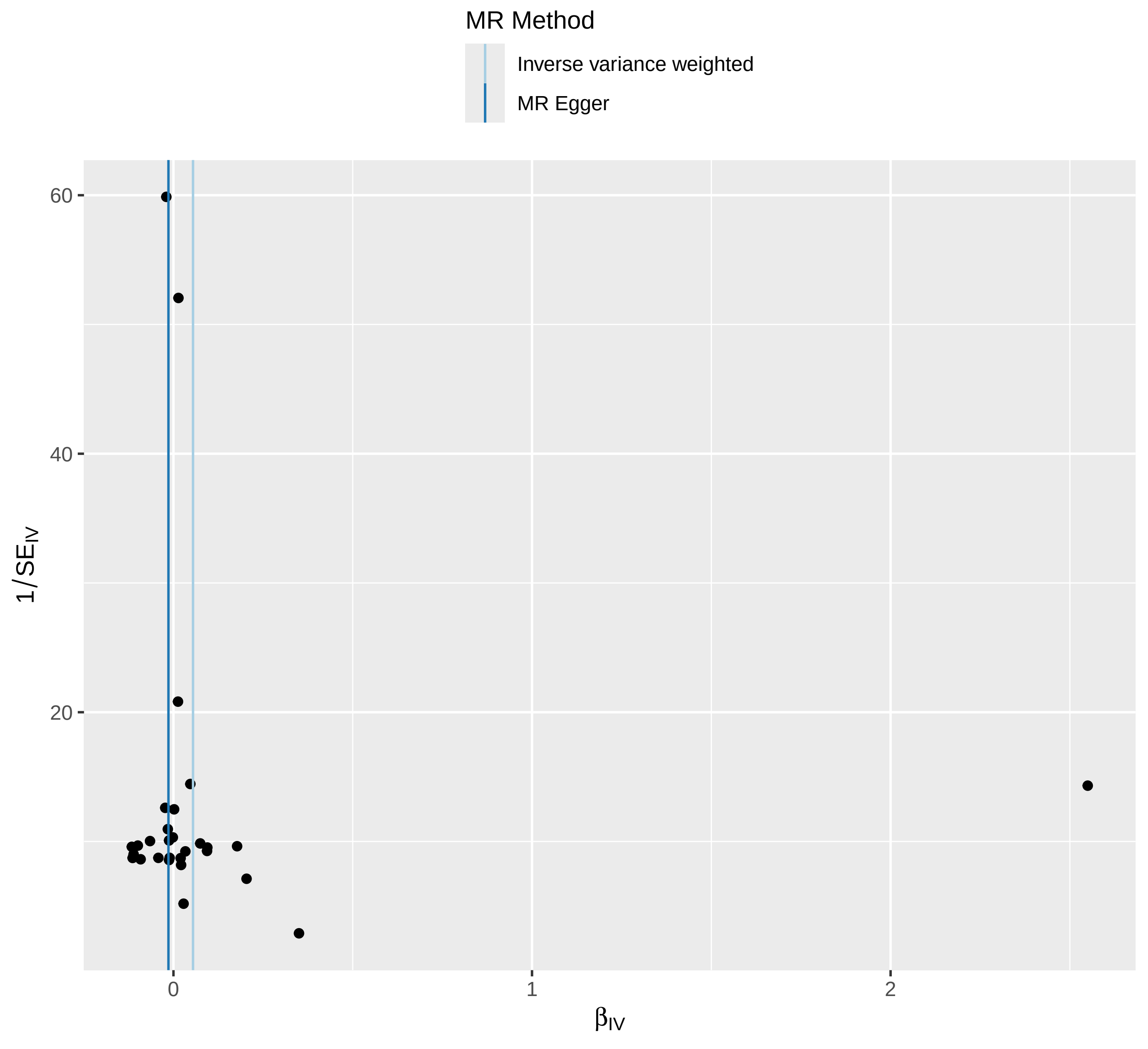
MR Method
Inverse variance weighted
MR Egger
60
40
20
0
1
2
1/SE_{IV}
β_{IV}

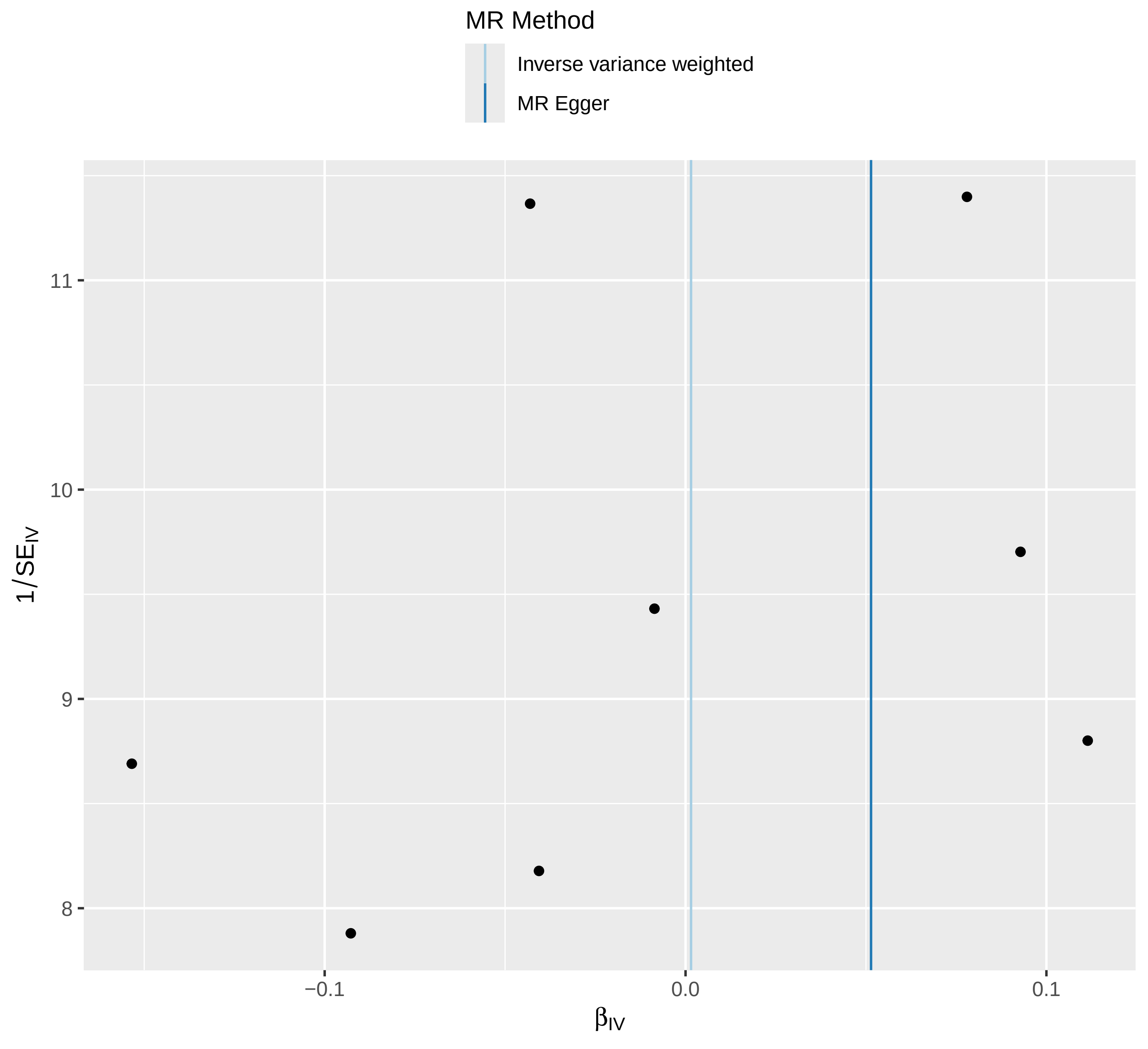
MR Method
Inverse variance weighted
MR Egger
11
10
9
8
1/SE$_{IV}$
−0.1
0.0
0.1
β$_{IV}$

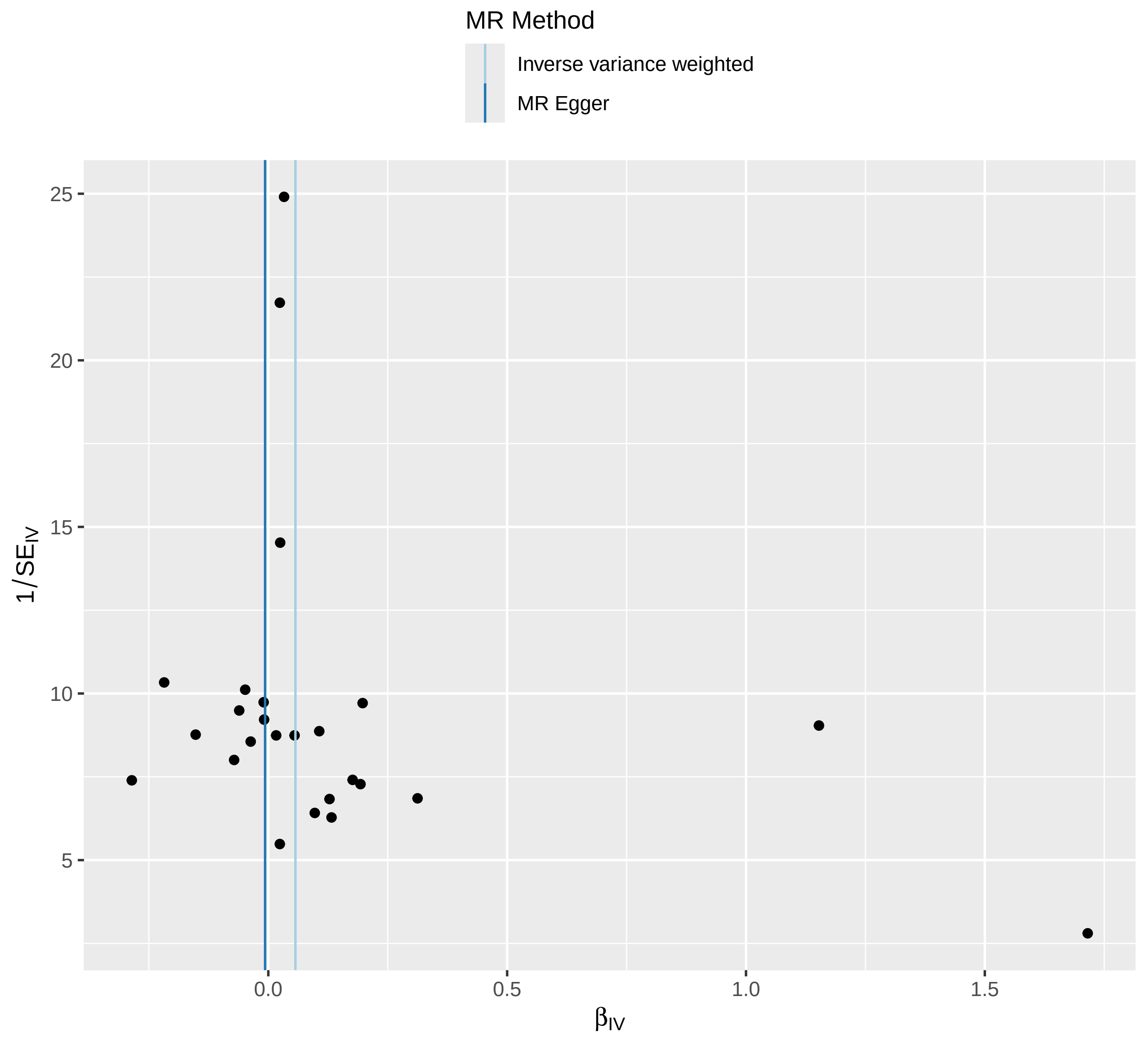
MR Method
Inverse variance weighted
MR Egger
1/SE$_{IV}$
25
20
15
10
5
0.0
0.5
1.0
1.5
β$_{IV}$

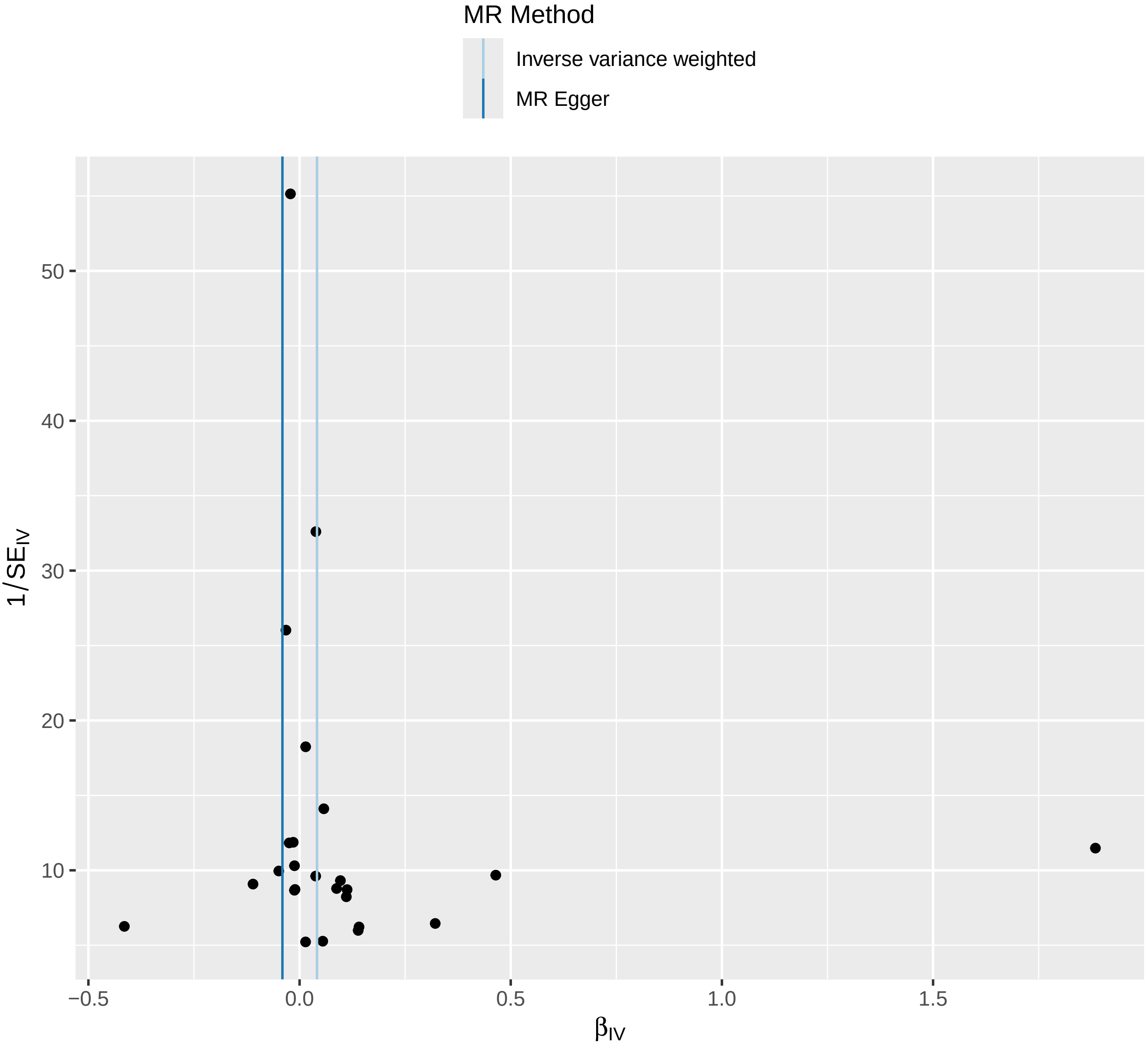

MR Method
Inverse variance weighted
MR Egger
50
40
30
20
10
$1/SE_{IV}$
−0.5
0.0
0.5
1.0
1.5
β_{IV}

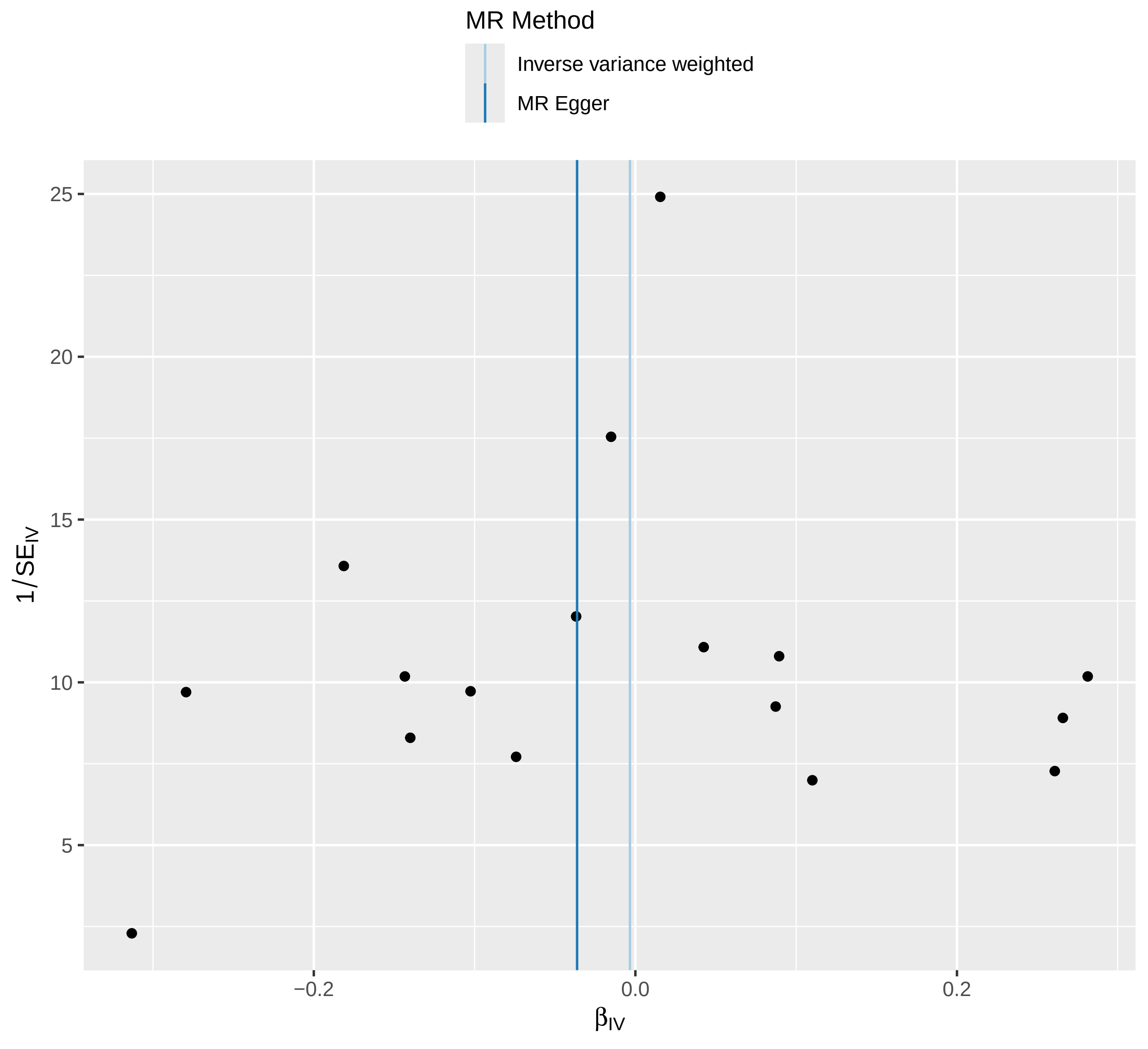
MR Method
Inverse variance weighted
MR Egger
25
20
15
10
5
1/SE$_{IV}$
−0.2
0.0
0.2
β$_{IV}$

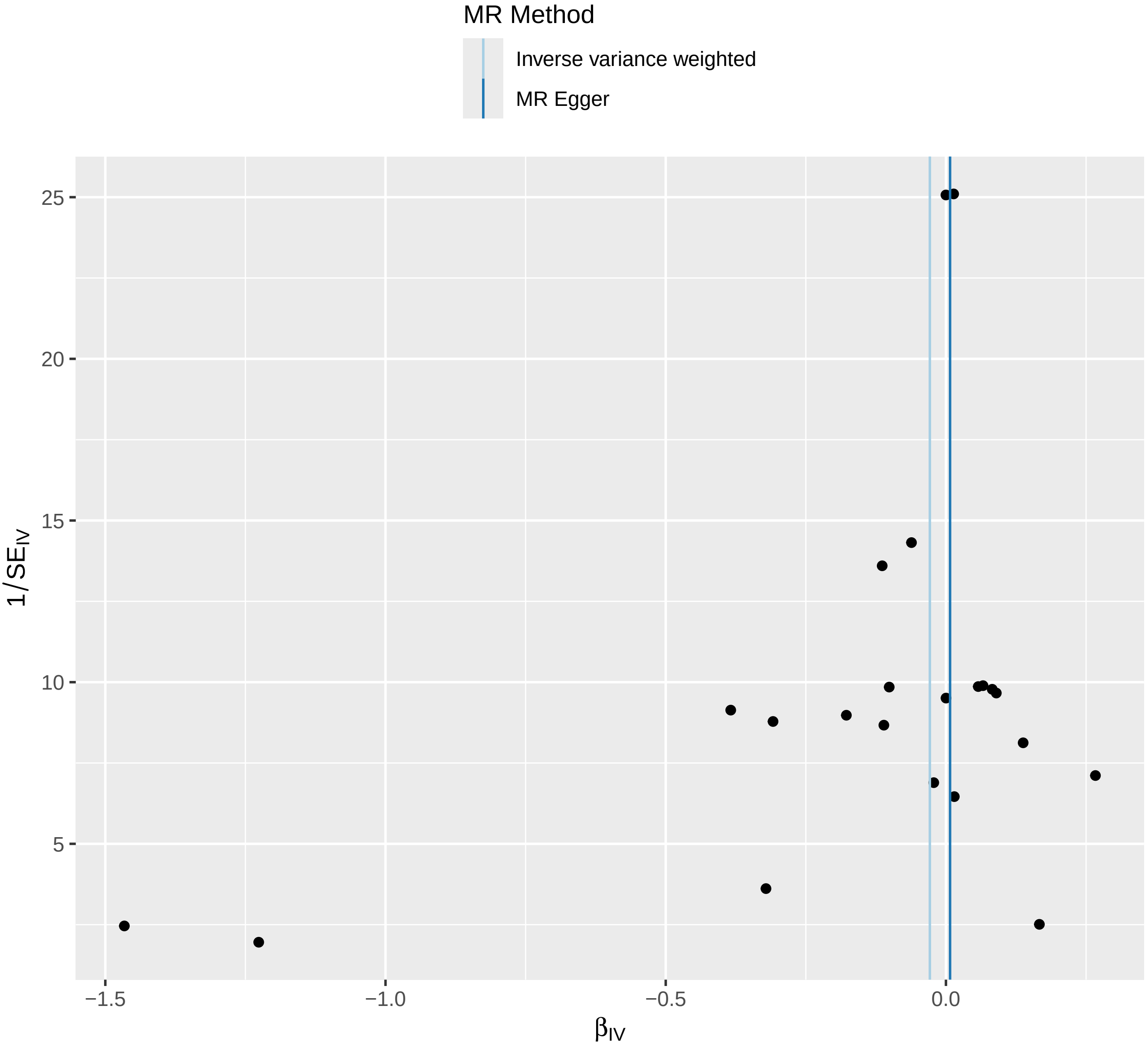
MR Method
Inverse variance weighted
MR Egger
25
20
15
10
5
$1/SE_{IV}$
−1.5
−1.0
−0.5
0.0
β_{IV}

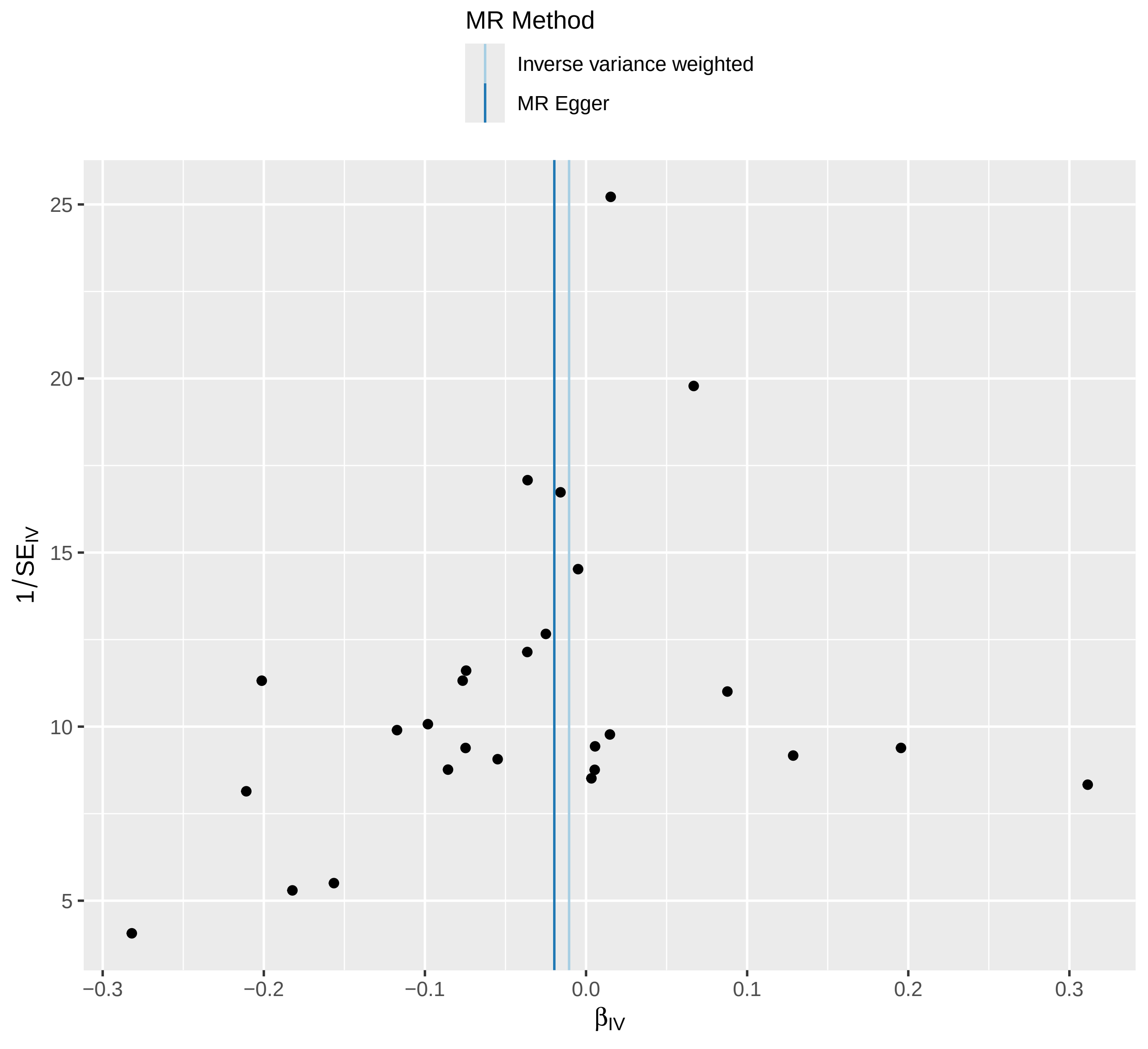

MR Method
Inverse variance weighted
MR Egger
25
20
15
10
5
$1/SE_{IV}$
−0.3
−0.2
−0.1
0.0
0.1
0.2
0.3
β_{IV}

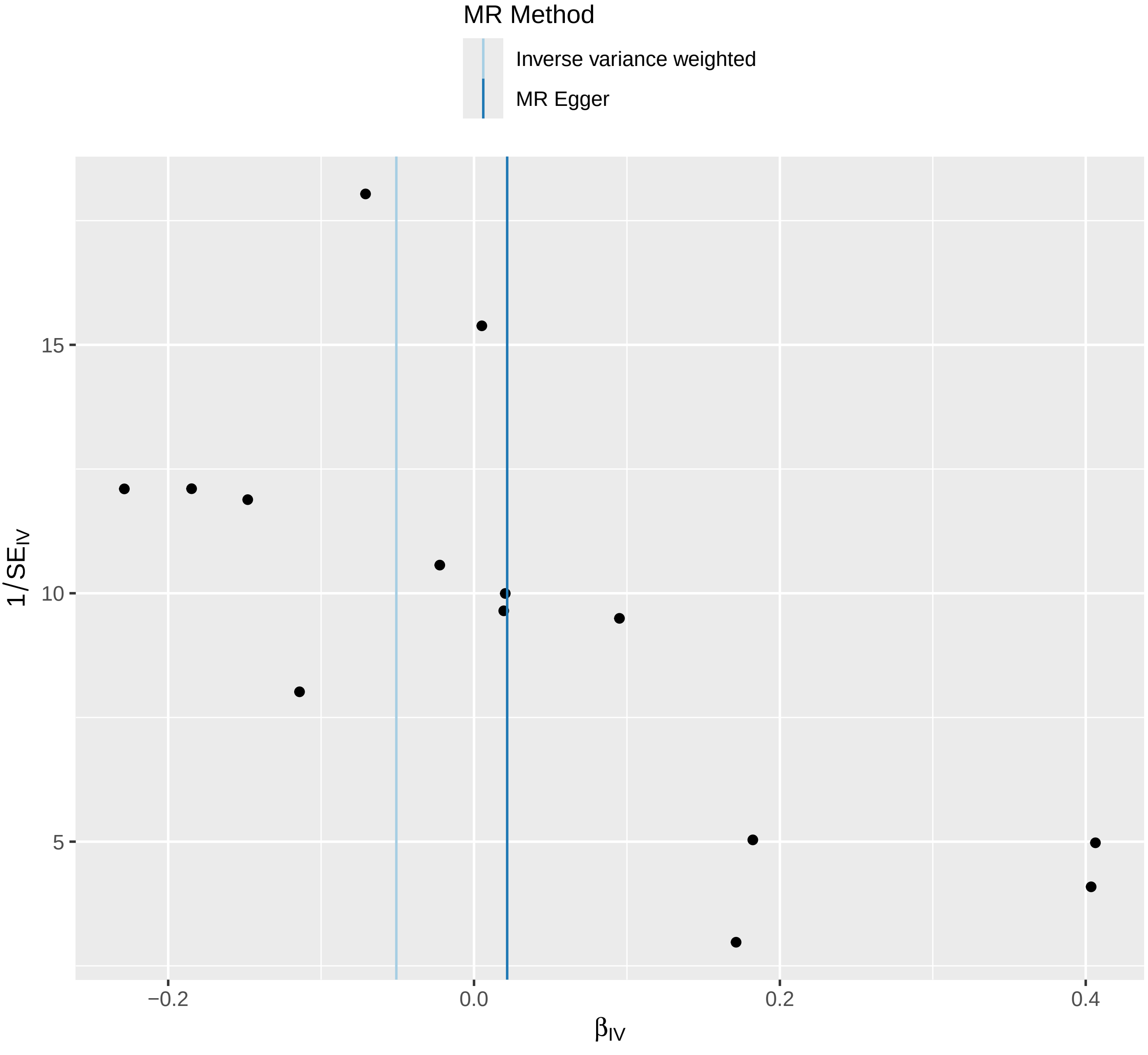

MR Method
Inverse variance weighted
MR Egger
1/SE_IV
15
10
5
−0.2
0.0
0.2
0.4
β_IV

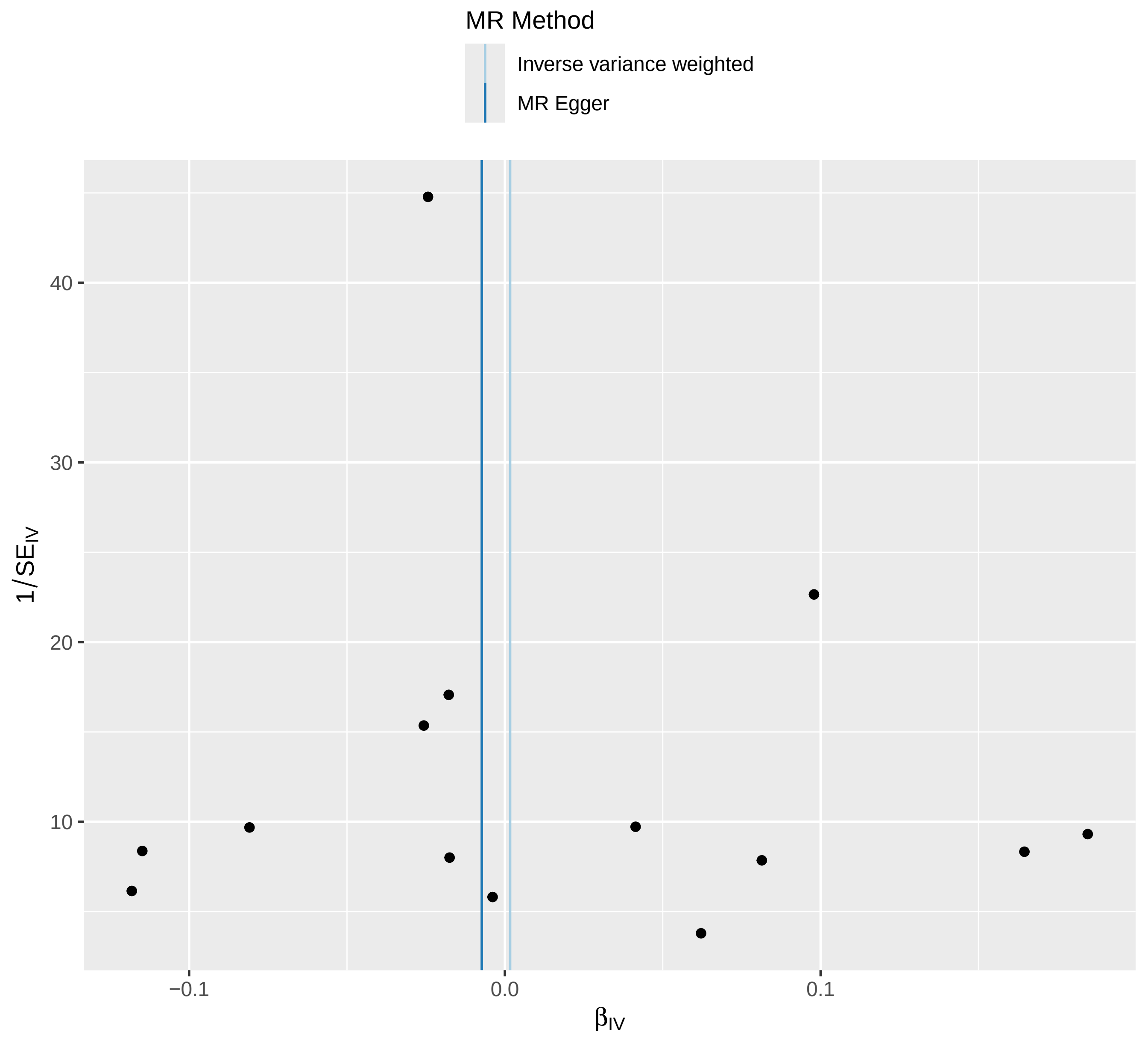
MR Method
Inverse variance weighted
MR Egger
1/SE$_{IV}$
40
30
20
10
−0.1
0.0
0.1
β$_{IV}$

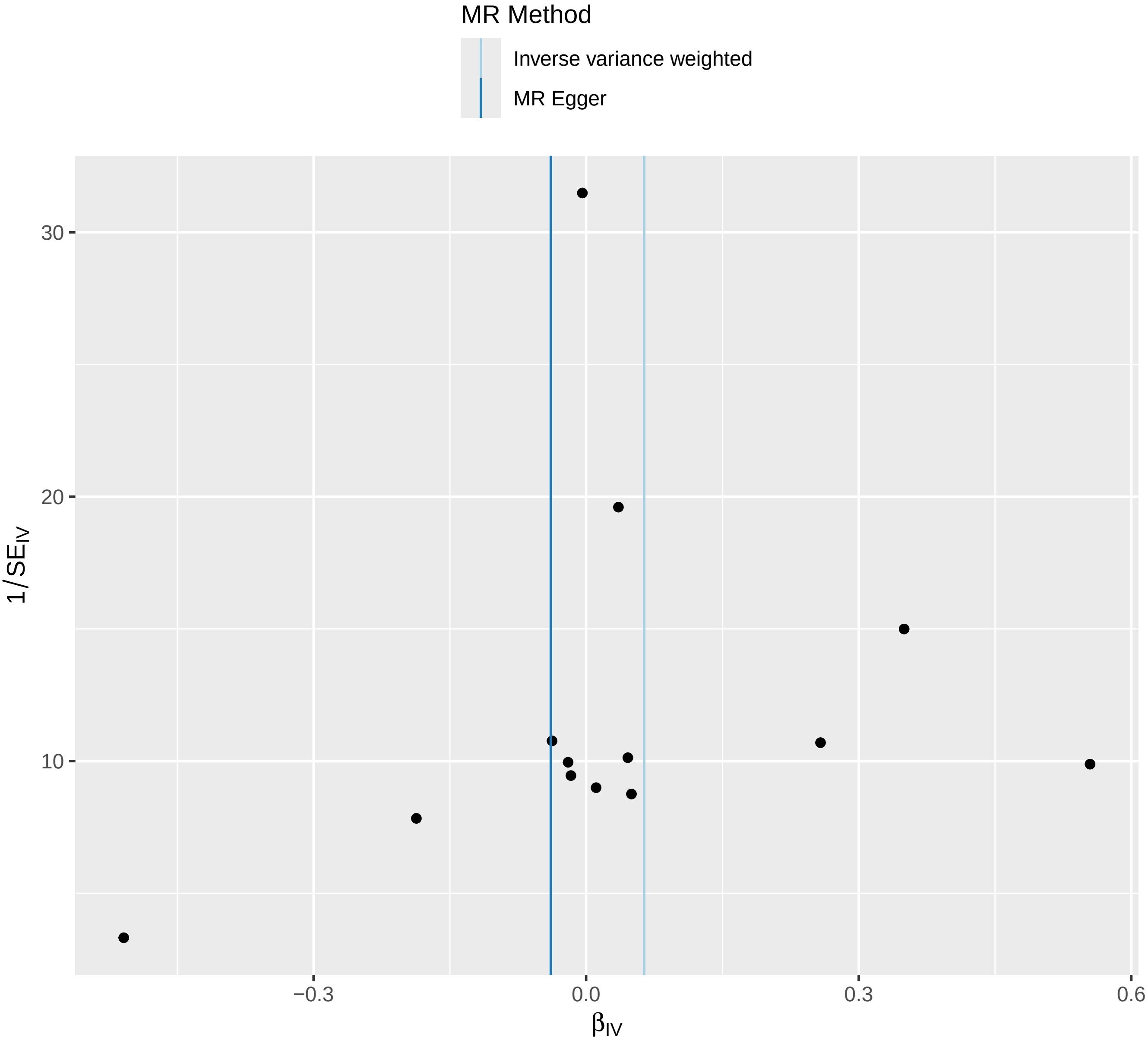
MR Method
Inverse variance weighted
MR Egger
30
20
10
1/SE_{IV}
−0.3
0.0
0.3
0.6
$β_{IV}$

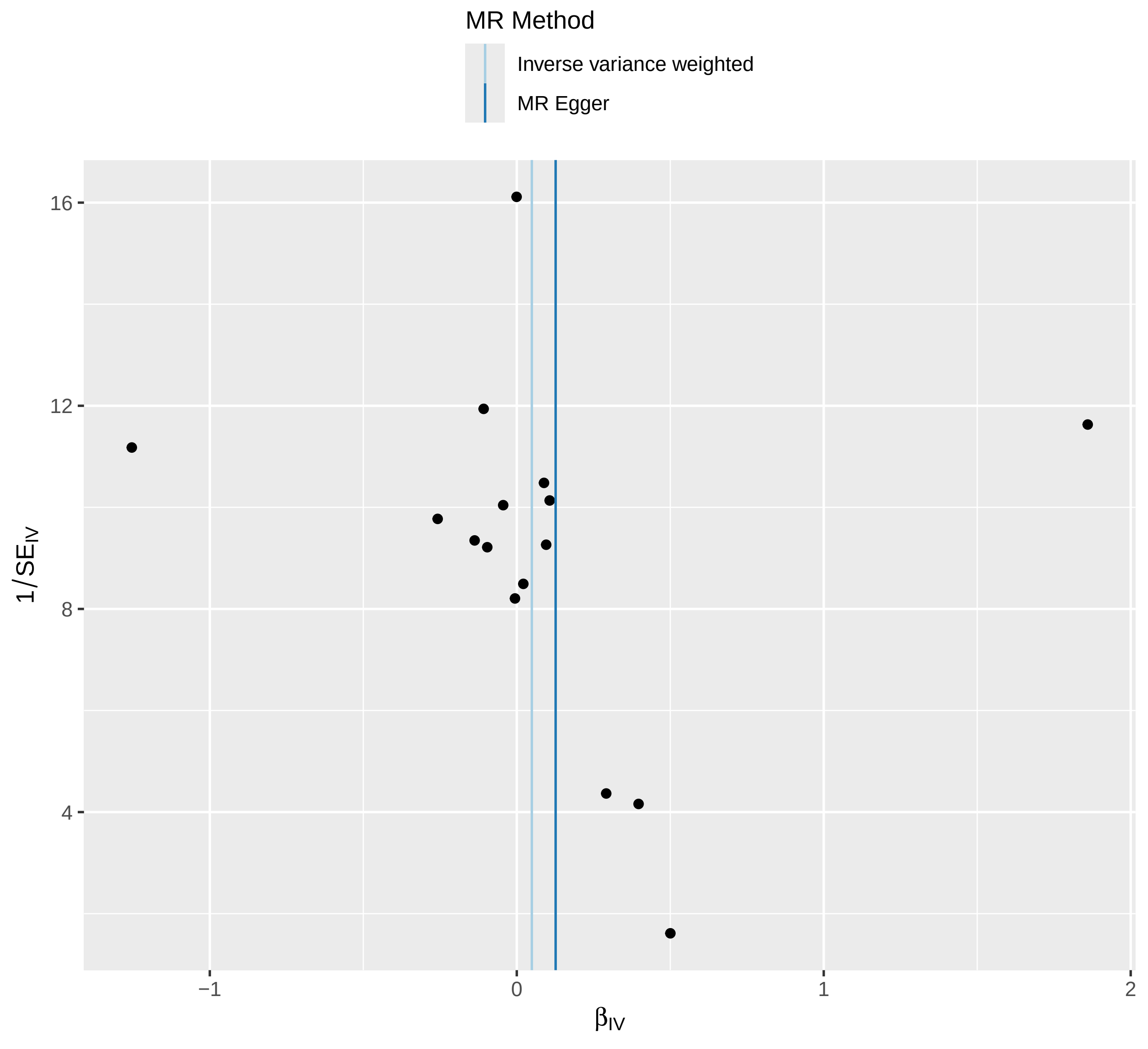
MR Method
Inverse variance weighted
MR Egger
16
12
8
4
−1
0
1
2
$1/SE_{IV}$
β_{IV}

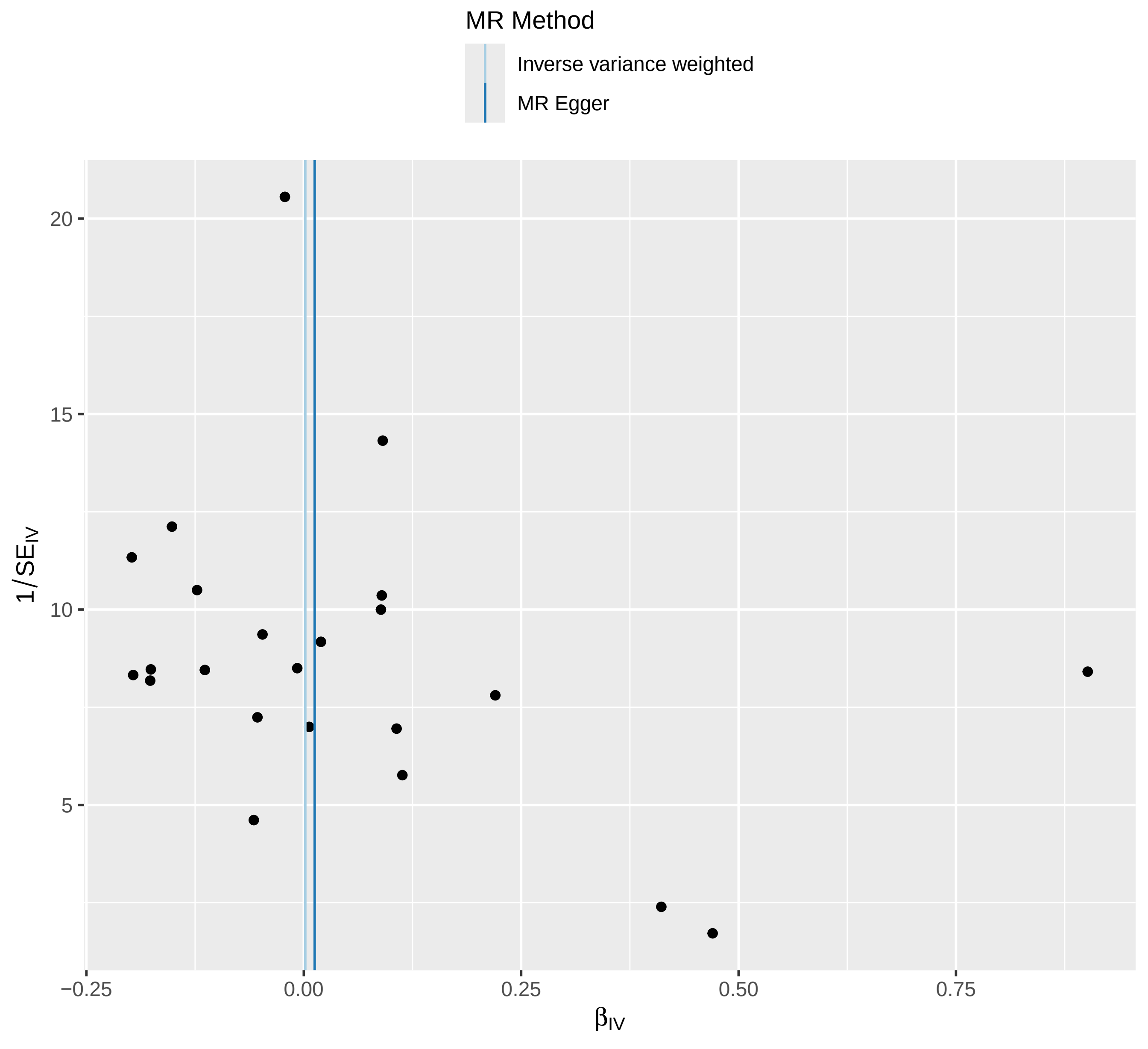

MR Method
Inverse variance weighted
MR Egger
20
15
10
5
1/SE_IV
−0.25
0.00
0.25
0.50
0.75
β_IV

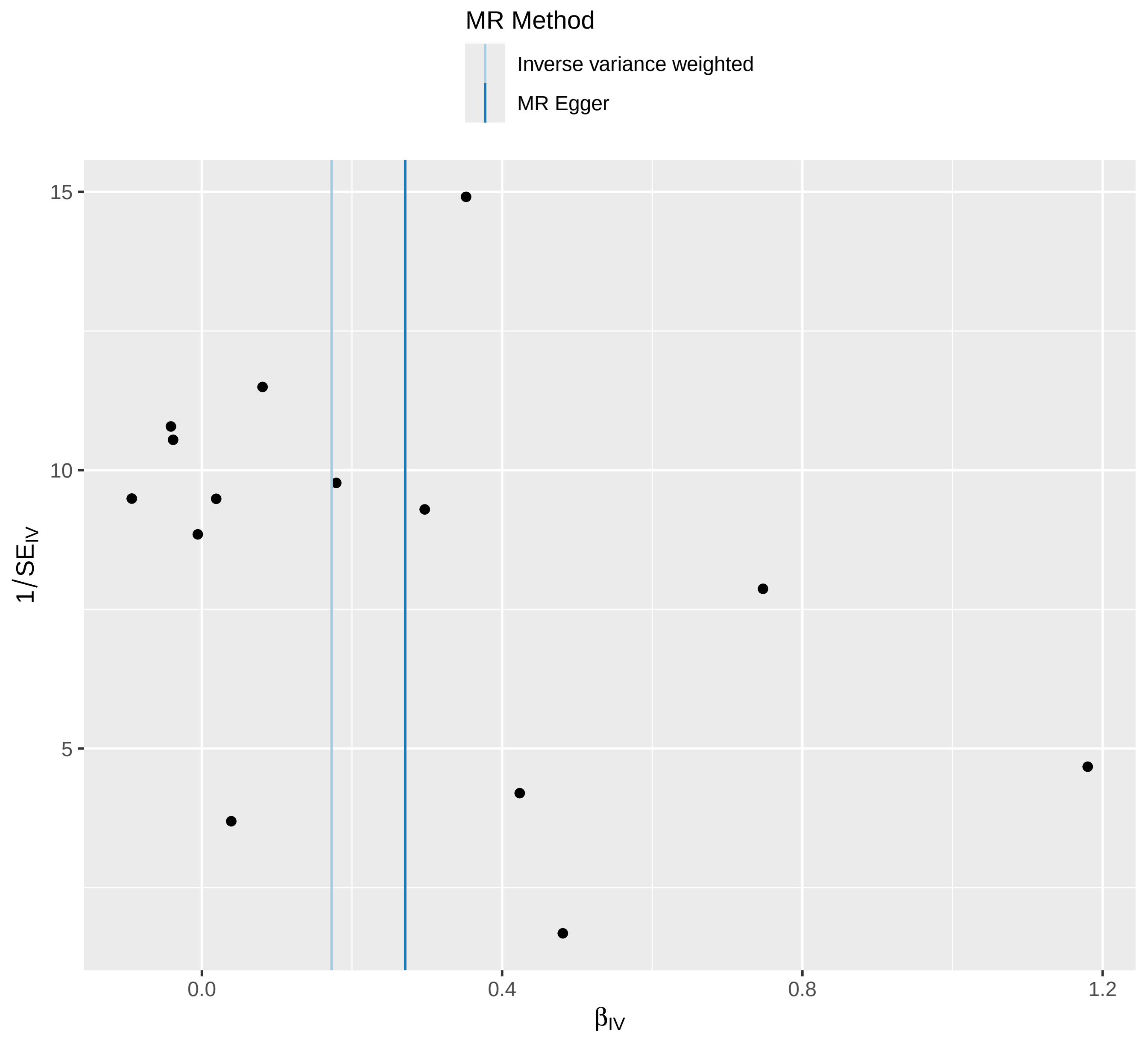
MR Method
Inverse variance weighted
MR Egger
15
10
5
1/SE_IV
0.0
0.4
0.8
1.2
β_IV

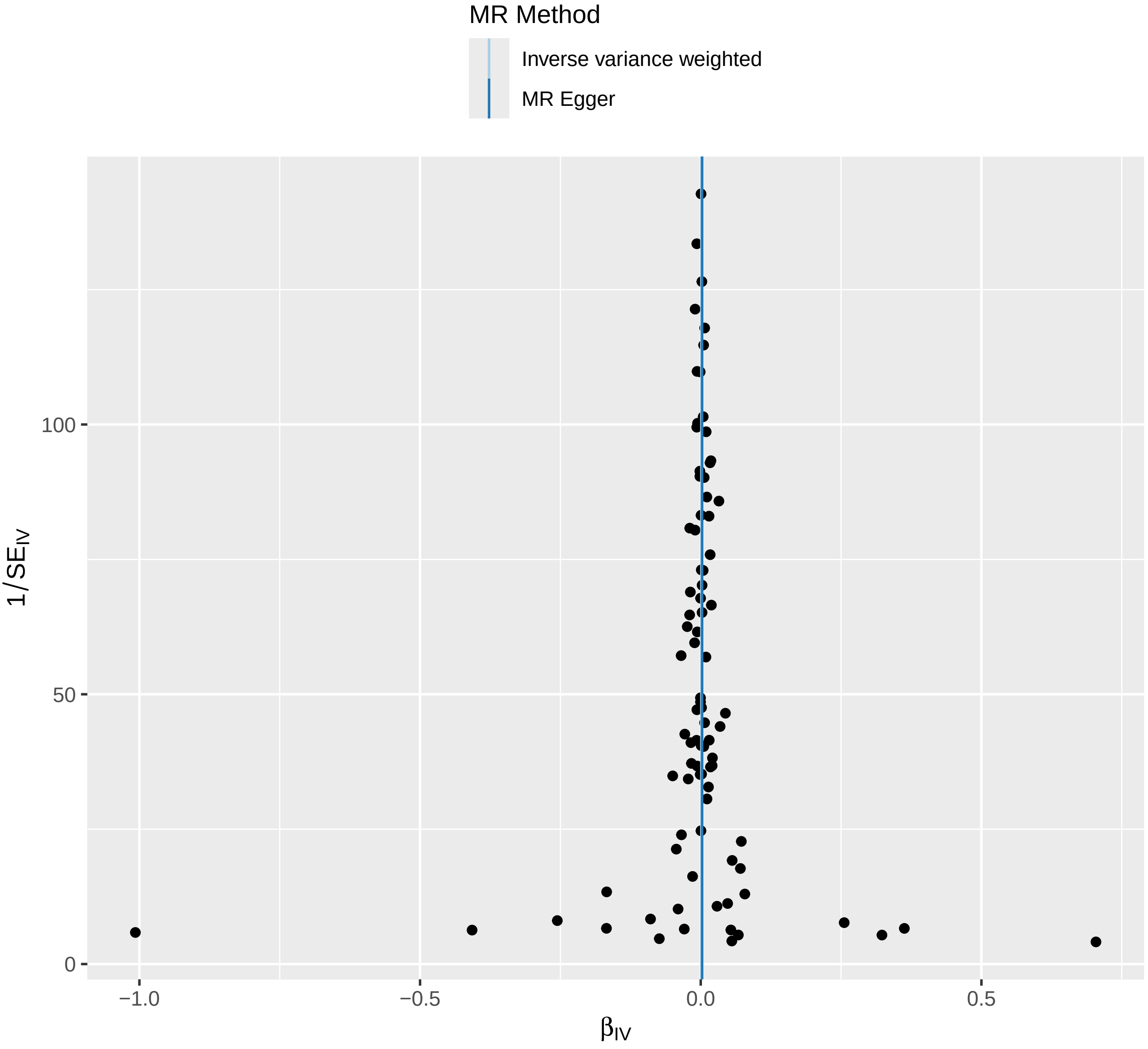

MR Method
Inverse variance weighted
MR Egger
1/SE_IV
100
50
0
−1.0
−0.5
0.0
0.5
β_IV

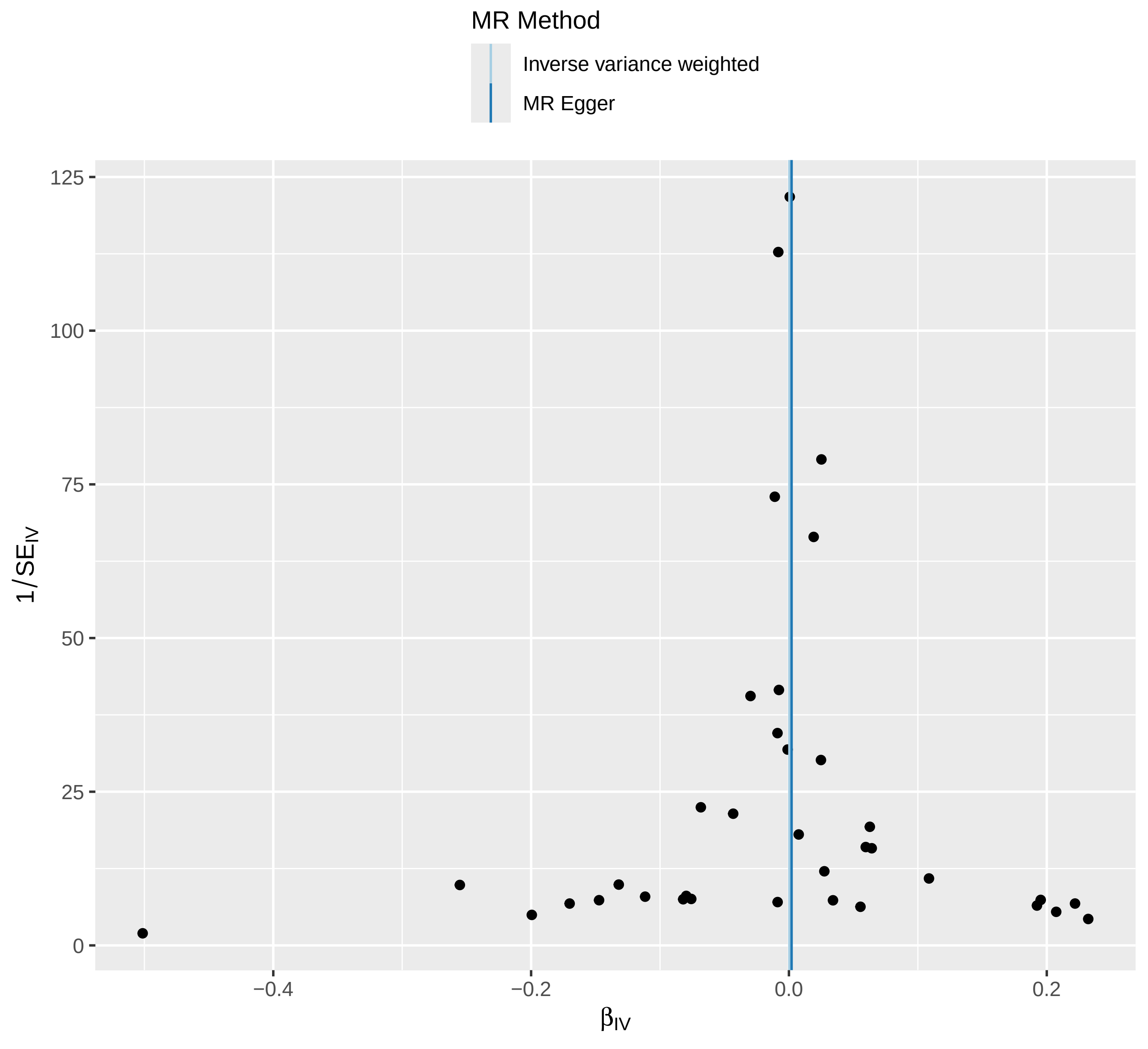
MR Method
Inverse variance weighted
MR Egger
125
100
75
50
25
0
−0.4
−0.2
0.0
0.2
$1/SE_{IV}$
β_{IV}

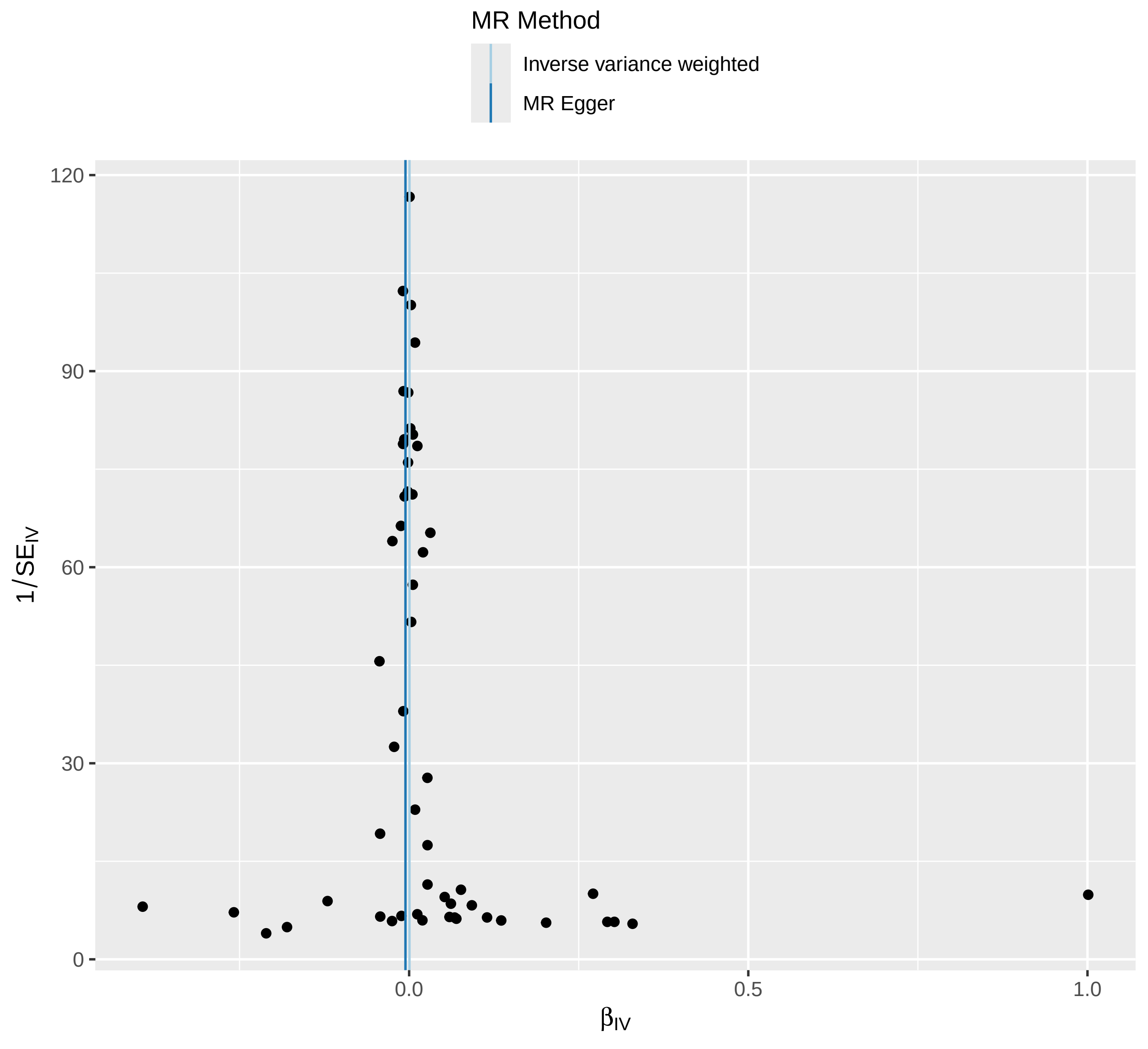
MR Method
Inverse variance weighted
MR Egger
120
90
60
30
0
1/SE_IV
0.0
0.5
1.0
β_IV

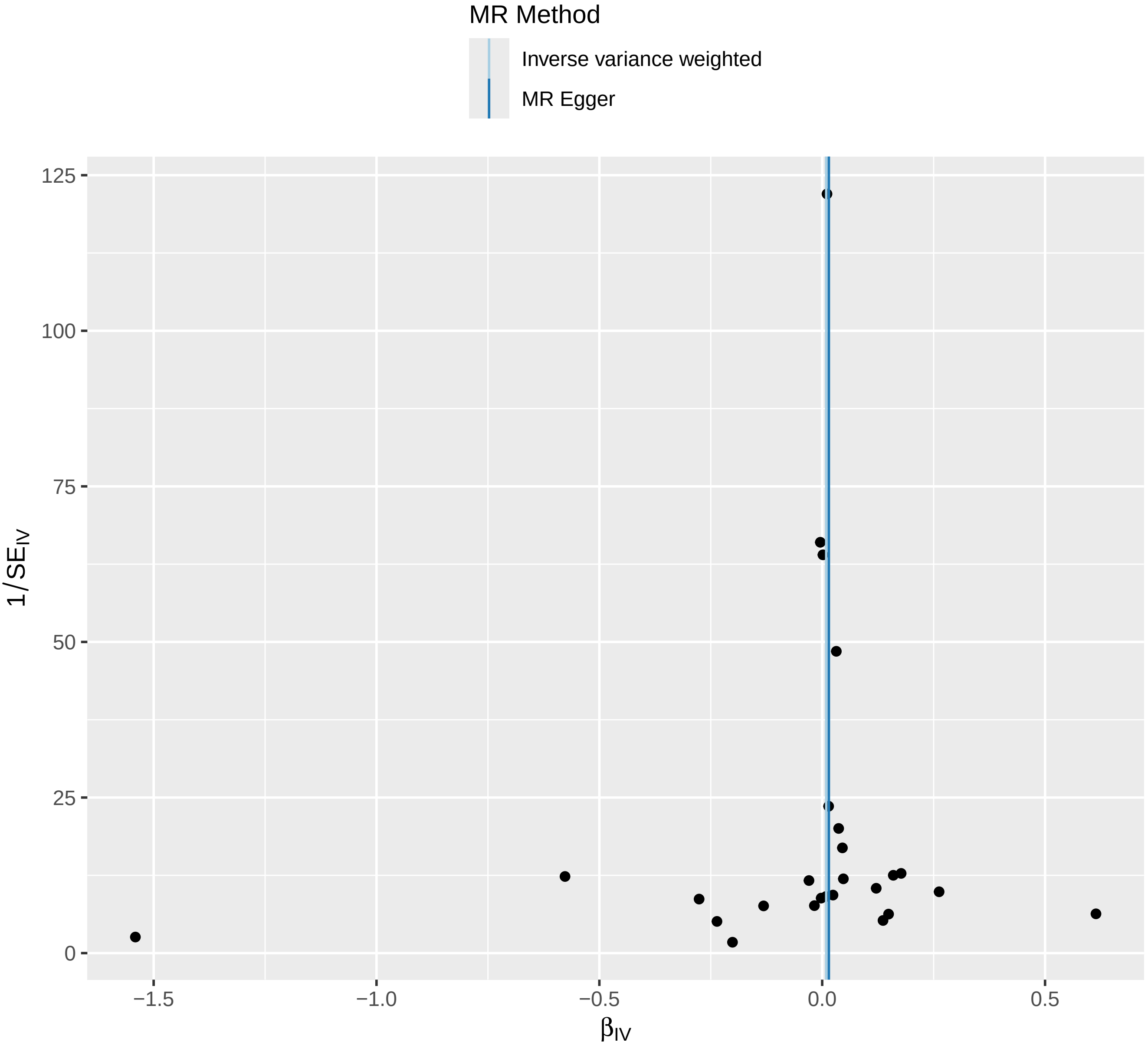

MR Method
Inverse variance weighted
MR Egger
125
100
75
50
25
0
1/SE$_{IV}$
−1.5
−1.0
−0.5
0.0
0.5
β$_{IV}$

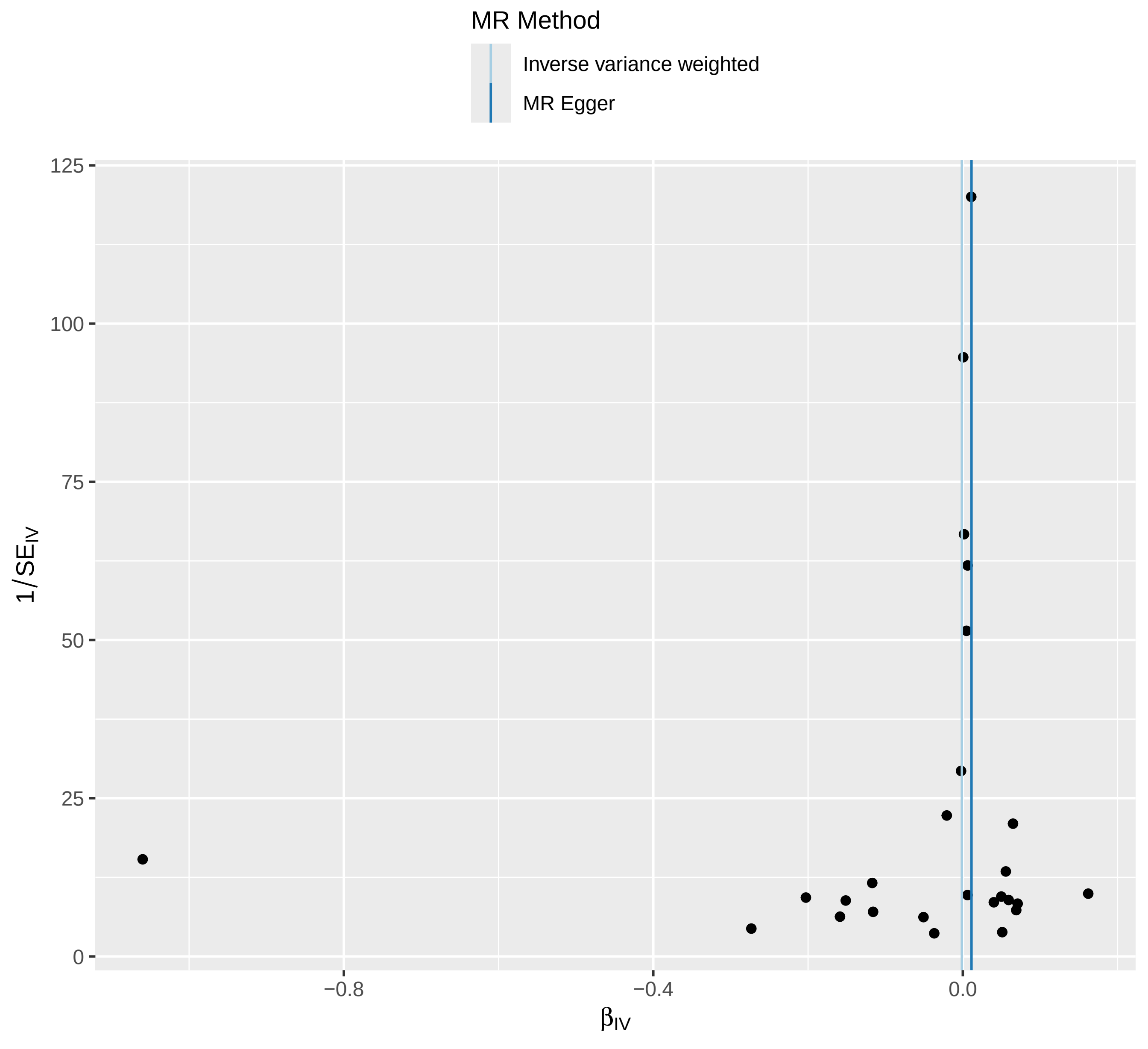
MR Method
Inverse variance weighted
MR Egger
125
100
75
50
25
0
1/SE$_{IV}$
−0.8
−0.4
0.0
β$_{IV}$

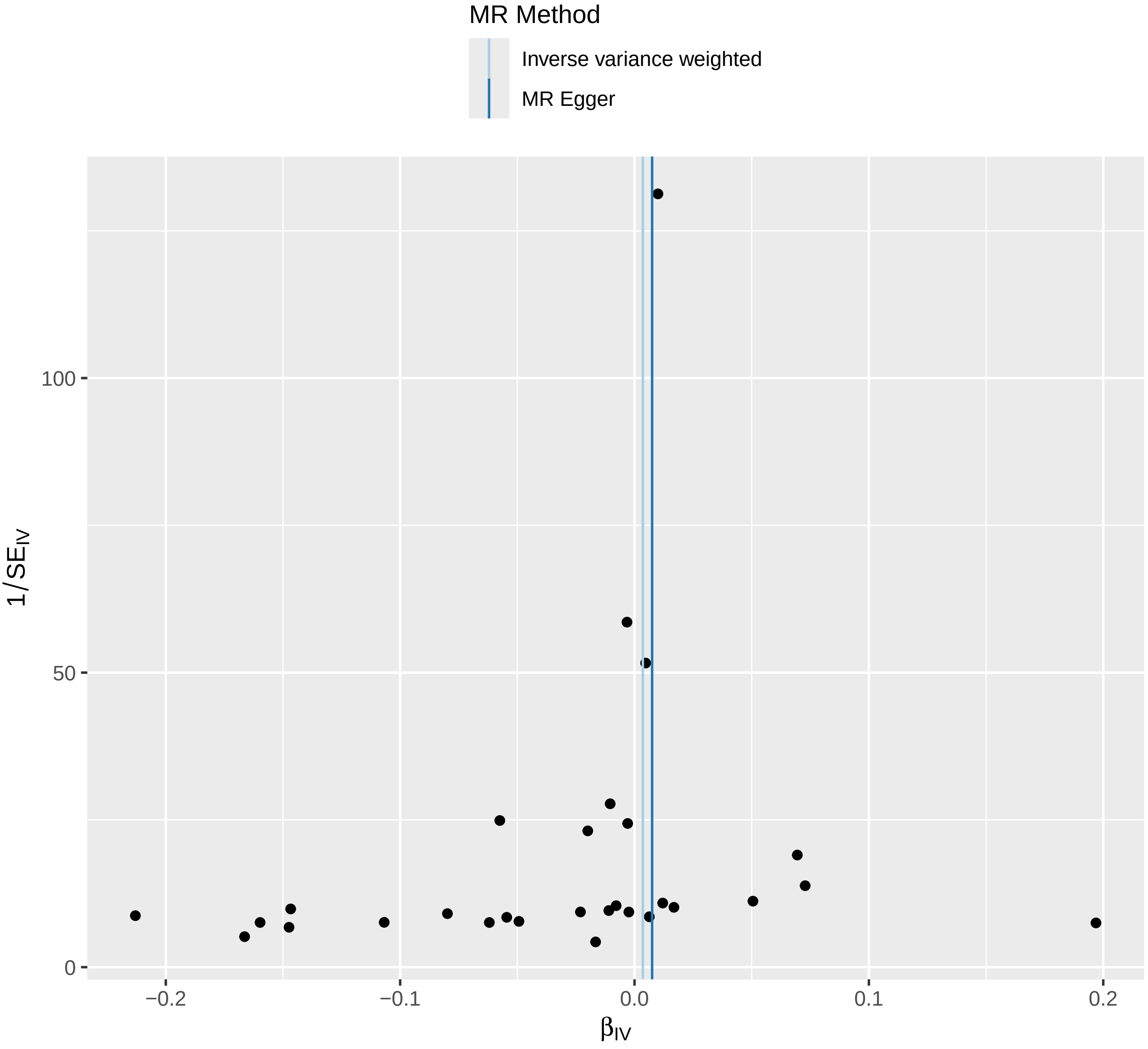
MR Method
Inverse variance weighted
MR Egger
100
50
0
1/SE$_{IV}$
−0.2
−0.1
0.0
0.1
0.2
β$_{IV}$

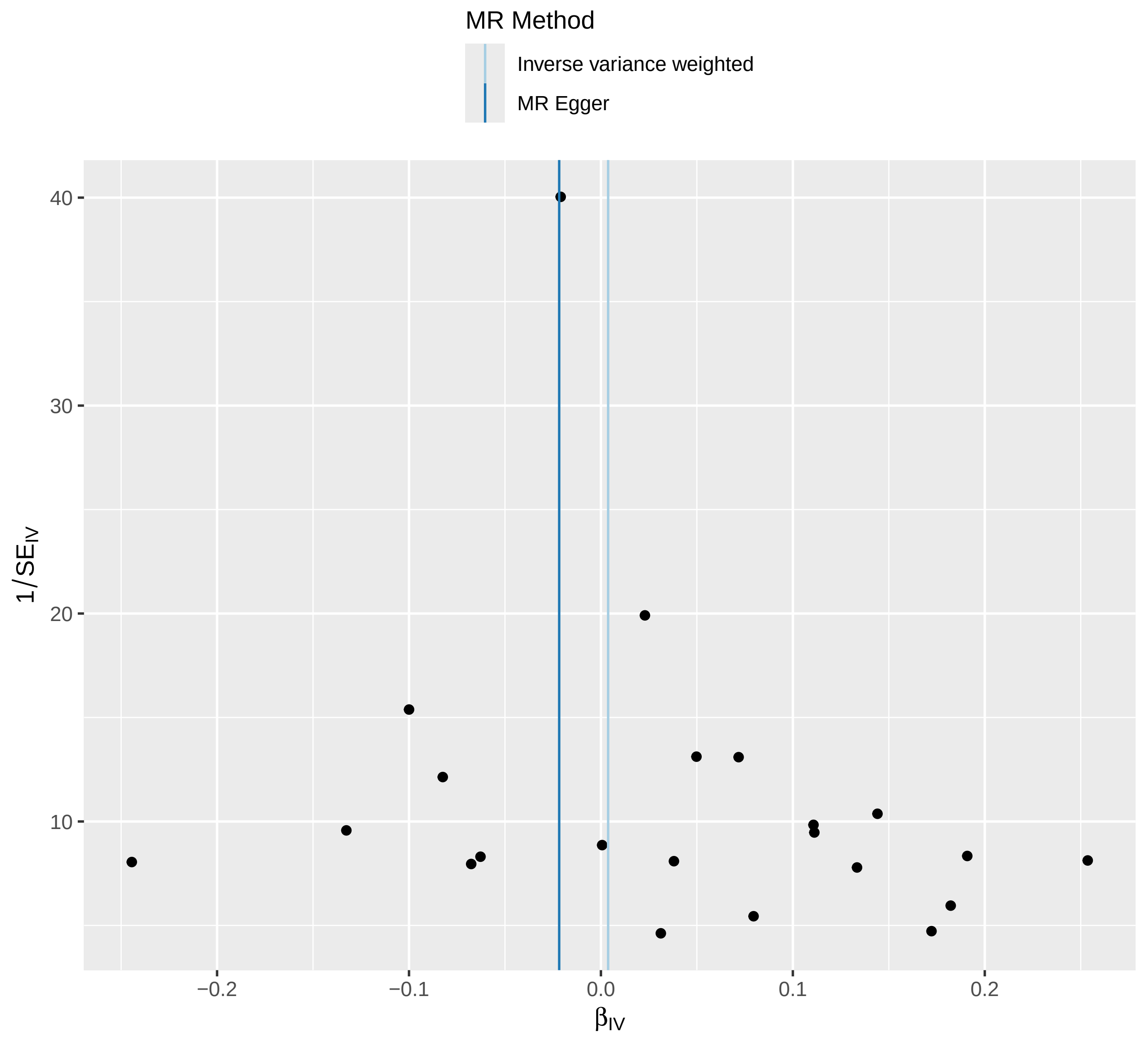
MR Method
Inverse variance weighted
MR Egger
40
30
20
10
1/SE$_{IV}$
−0.2
−0.1
0.0
0.1
0.2
β$_{IV}$

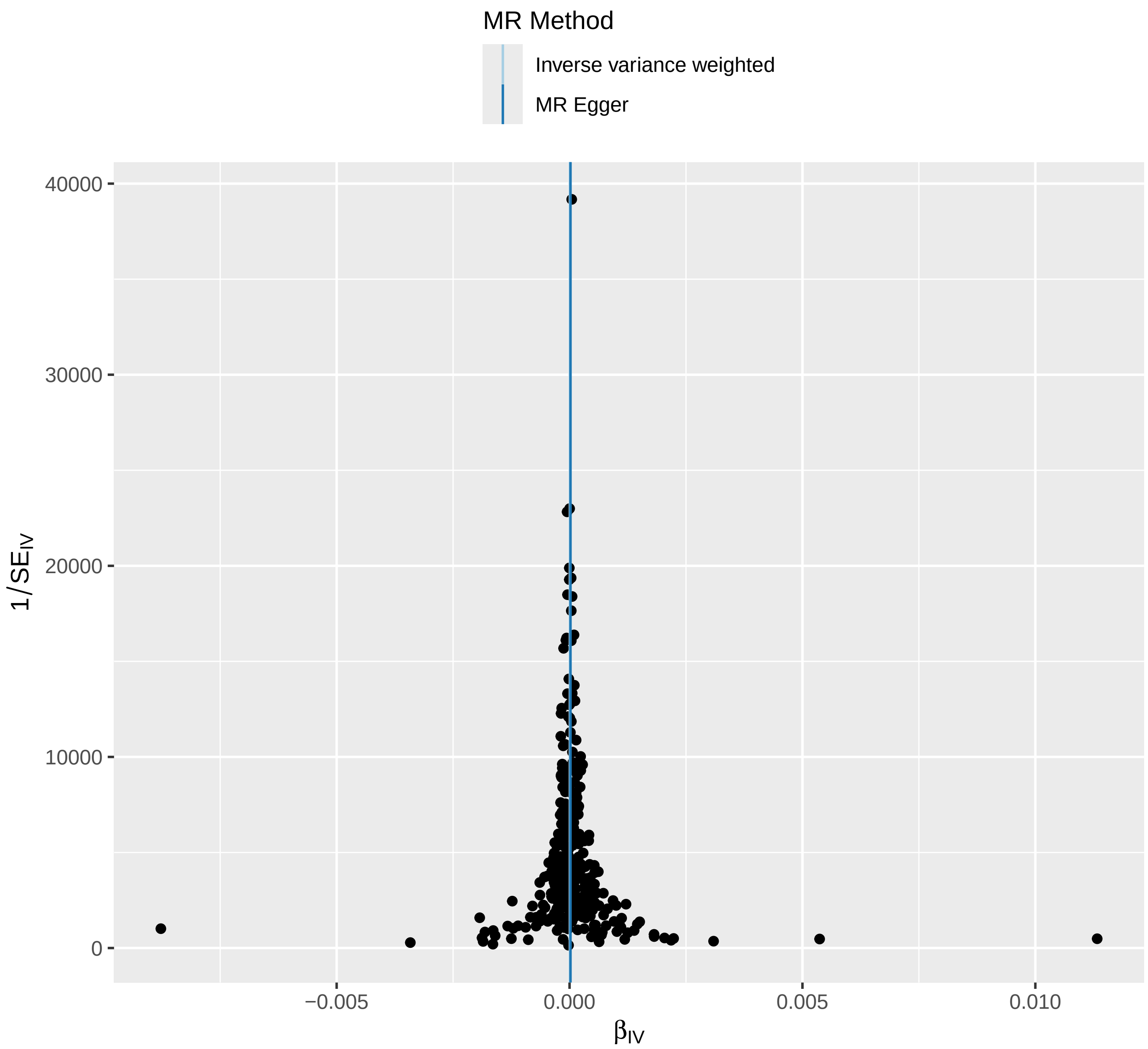
MR Method
Inverse variance weighted
MR Egger
40000
30000
20000
10000
0
$1/SE_{IV}$
−0.005
0.000
0.005
0.010
β_{IV}

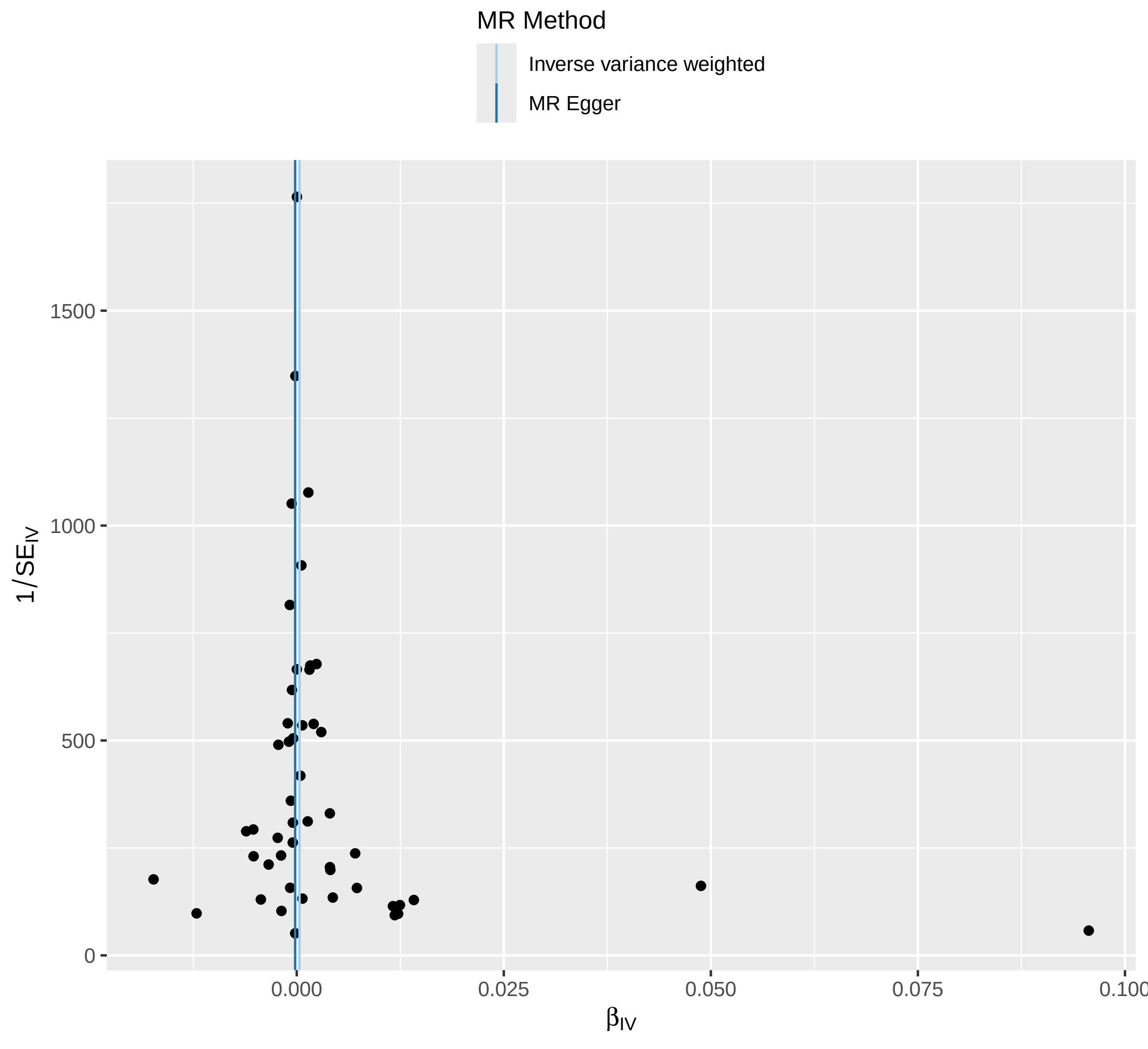
MR Method
Inverse variance weighted
MR Egger
1500
1000
500
0
0.000
0.025
0.050
0.075
0.100
$1/SE_{IV}$
β_{IV}

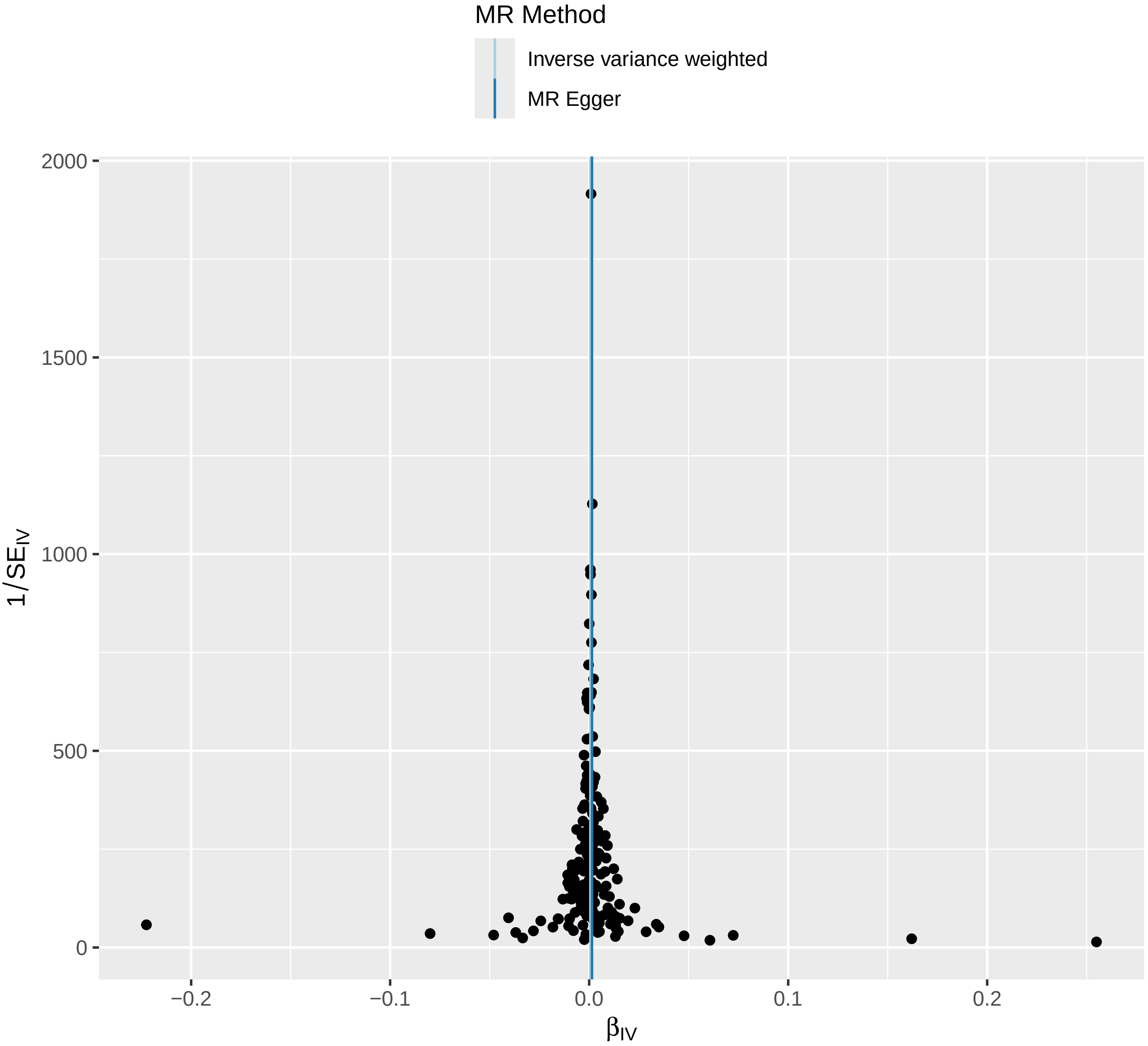
MR Method
Inverse variance weighted
MR Egger
1/SE$_{IV}$
2000
1500
1000
500
0
−0.2
−0.1
0.0
0.1
0.2
β$_{IV}$

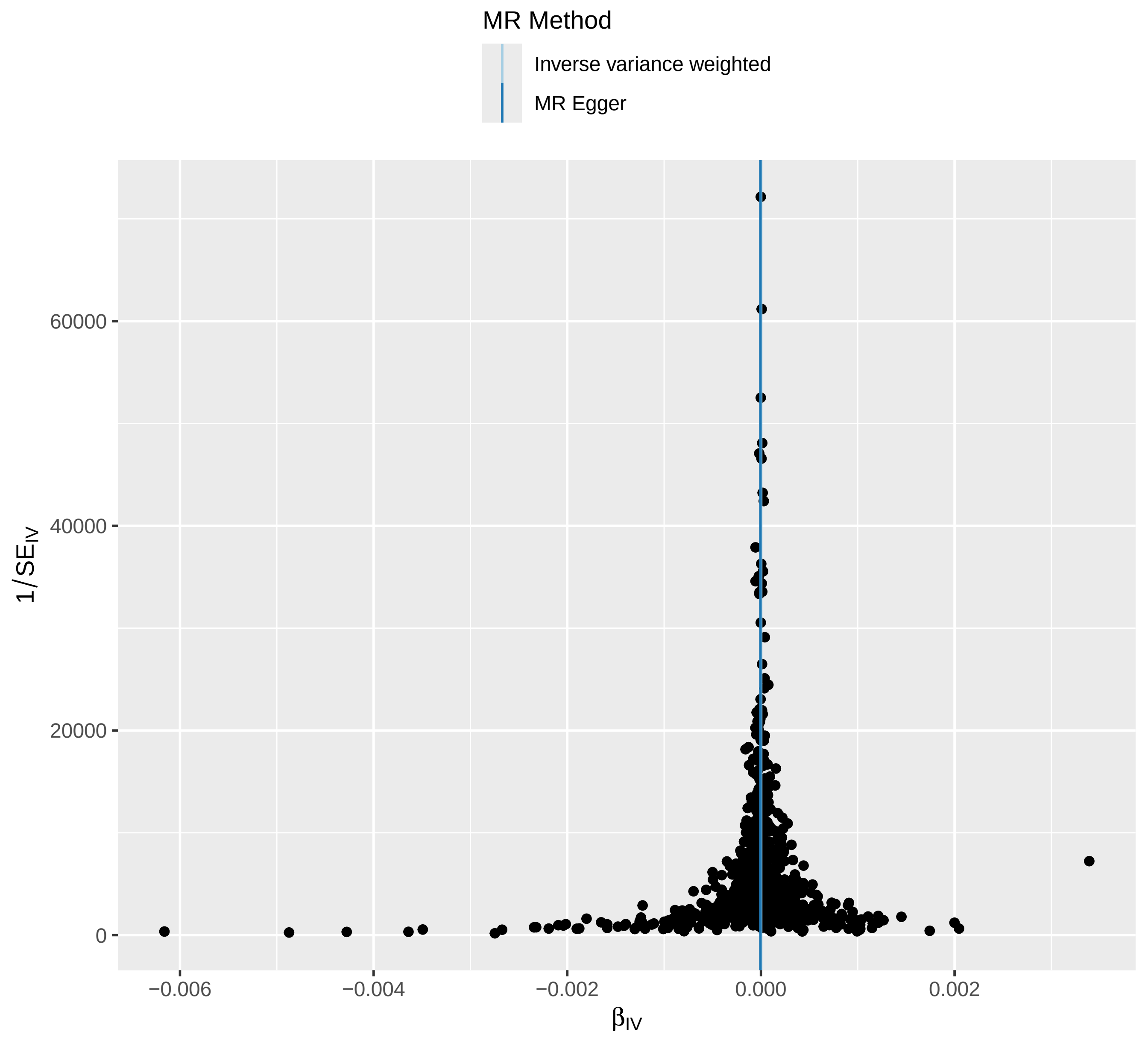
MR Method
Inverse variance weighted
MR Egger
1/SE_IV
60000
40000
20000
0
−0.006
−0.004
−0.002
0.000
0.002
β_IV

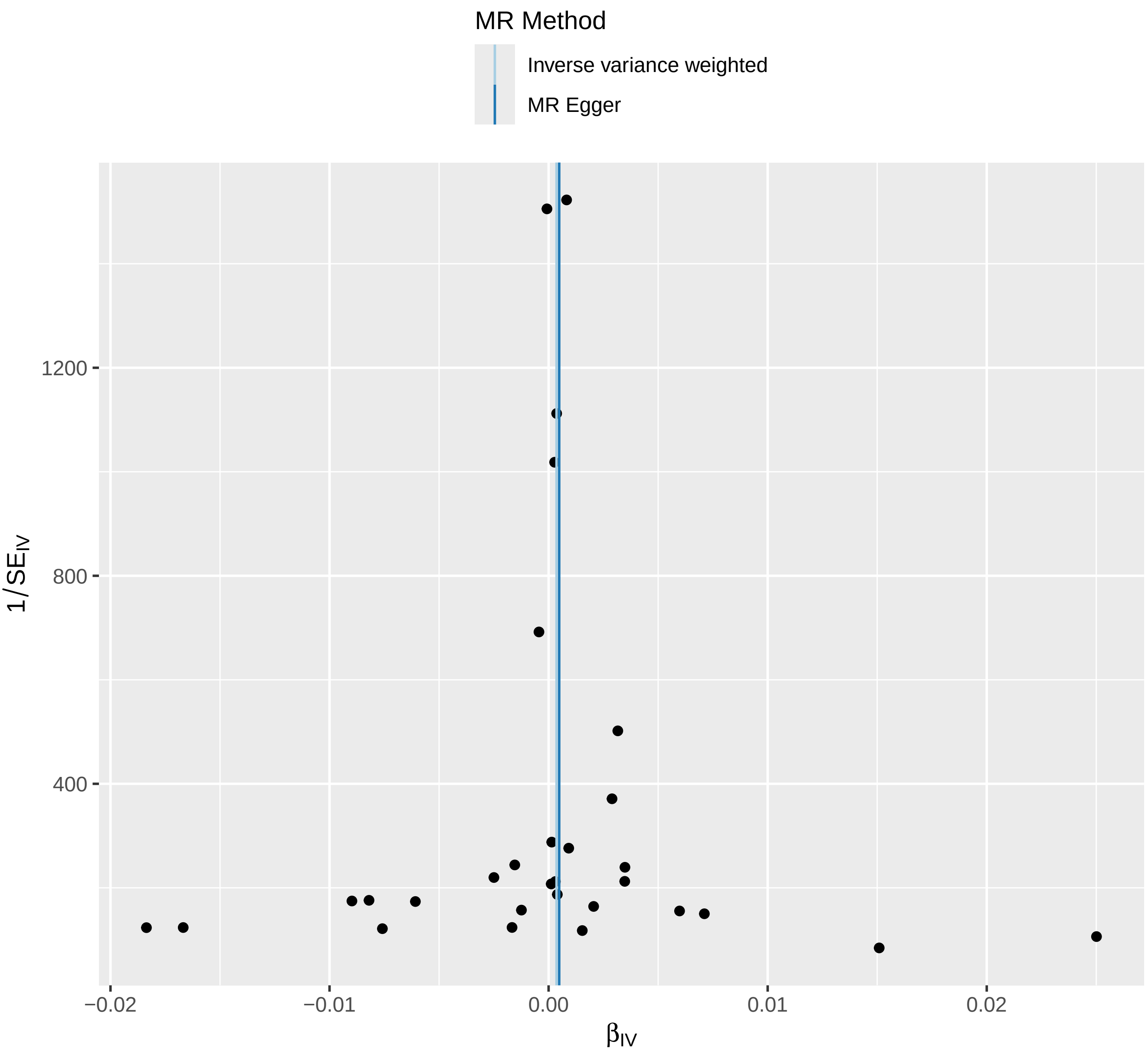

MR Method
Inverse variance weighted
MR Egger
1200
800
400
1/SE_IV
−0.02
−0.01
0.00
0.01
0.02
β_IV

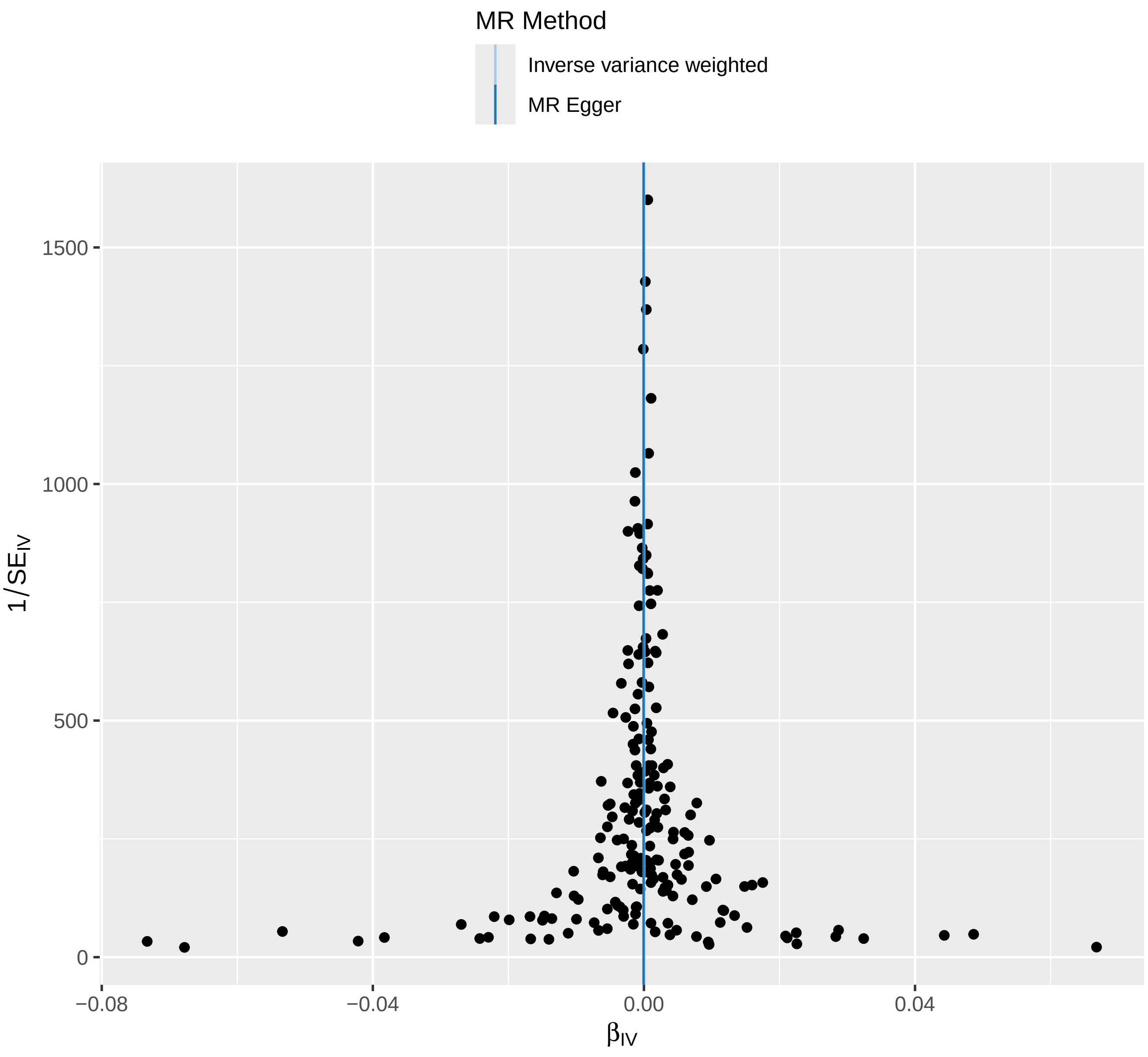
MR Method
Inverse variance weighted
MR Egger
1500
1000
500
0
1/SE$_{IV}$
−0.08
−0.04
0.00
0.04
β$_{IV}$

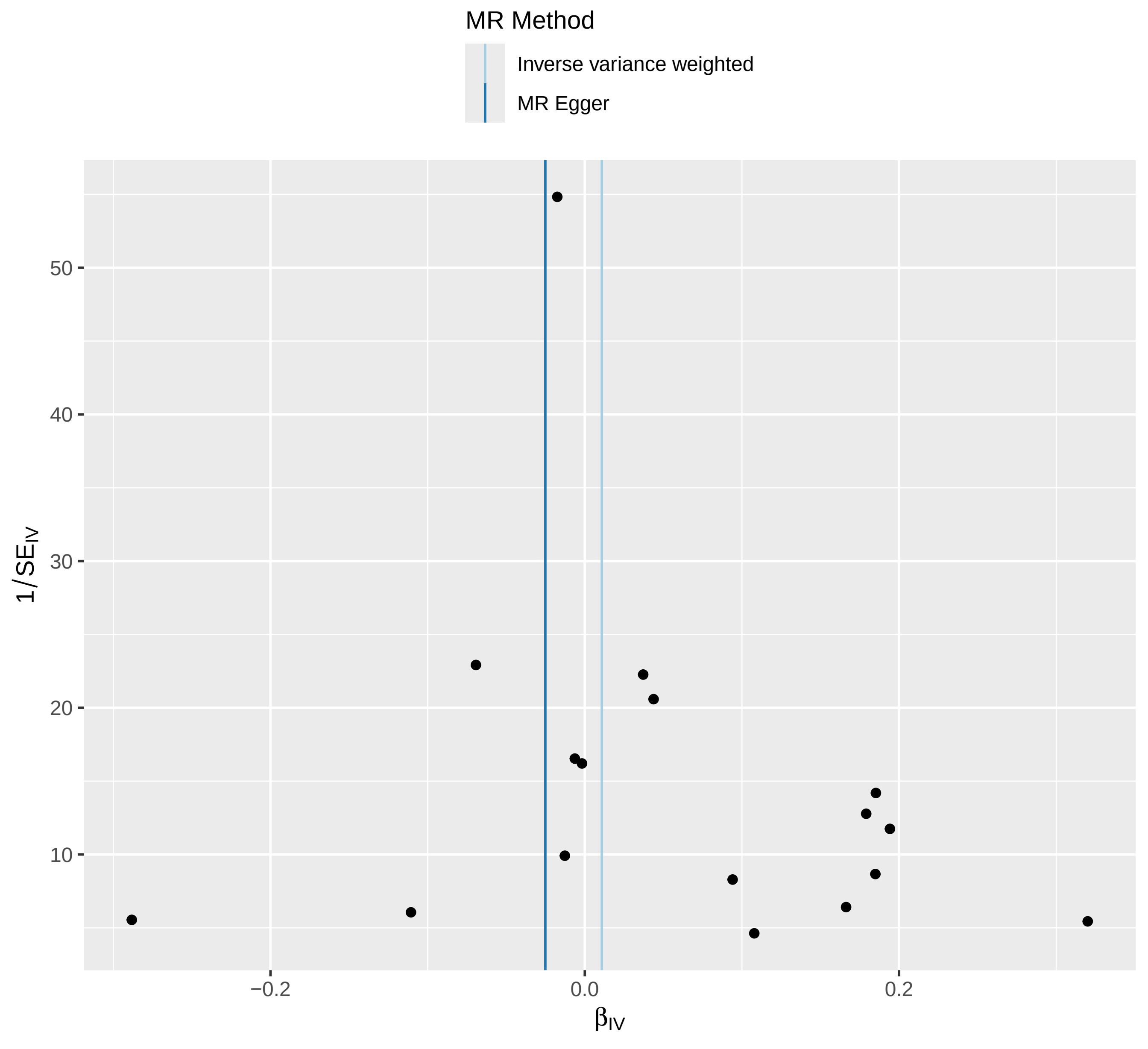
MR Method
Inverse variance weighted
MR Egger
50
40
30
20
10
1/SE_IV
−0.2
0.0
0.2
β_IV

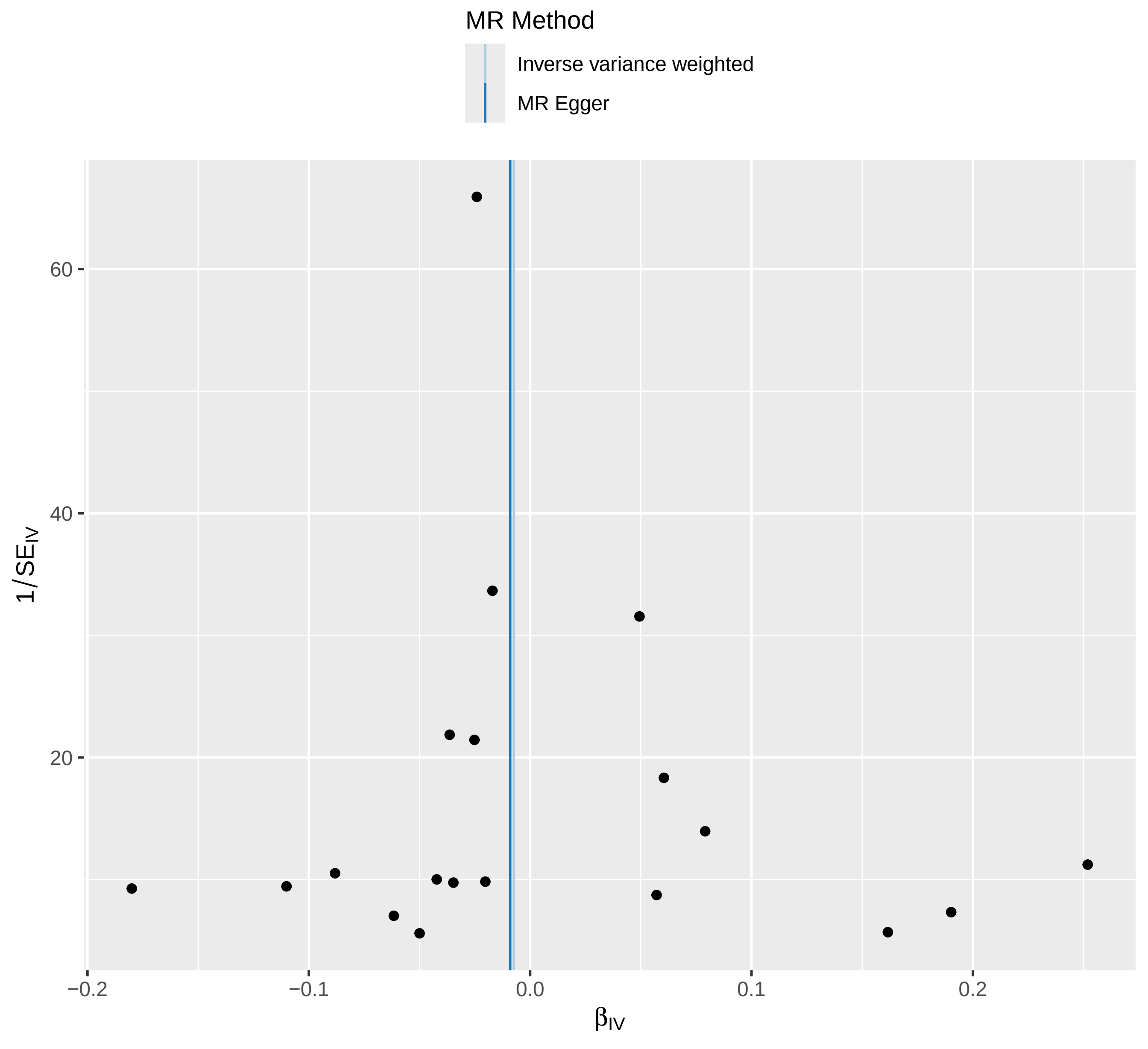

MR Method
Inverse variance weighted
MR Egger
60
40
20
1/SE$_{IV}$
−0.2
−0.1
0.0
0.1
0.2
β$_{IV}$

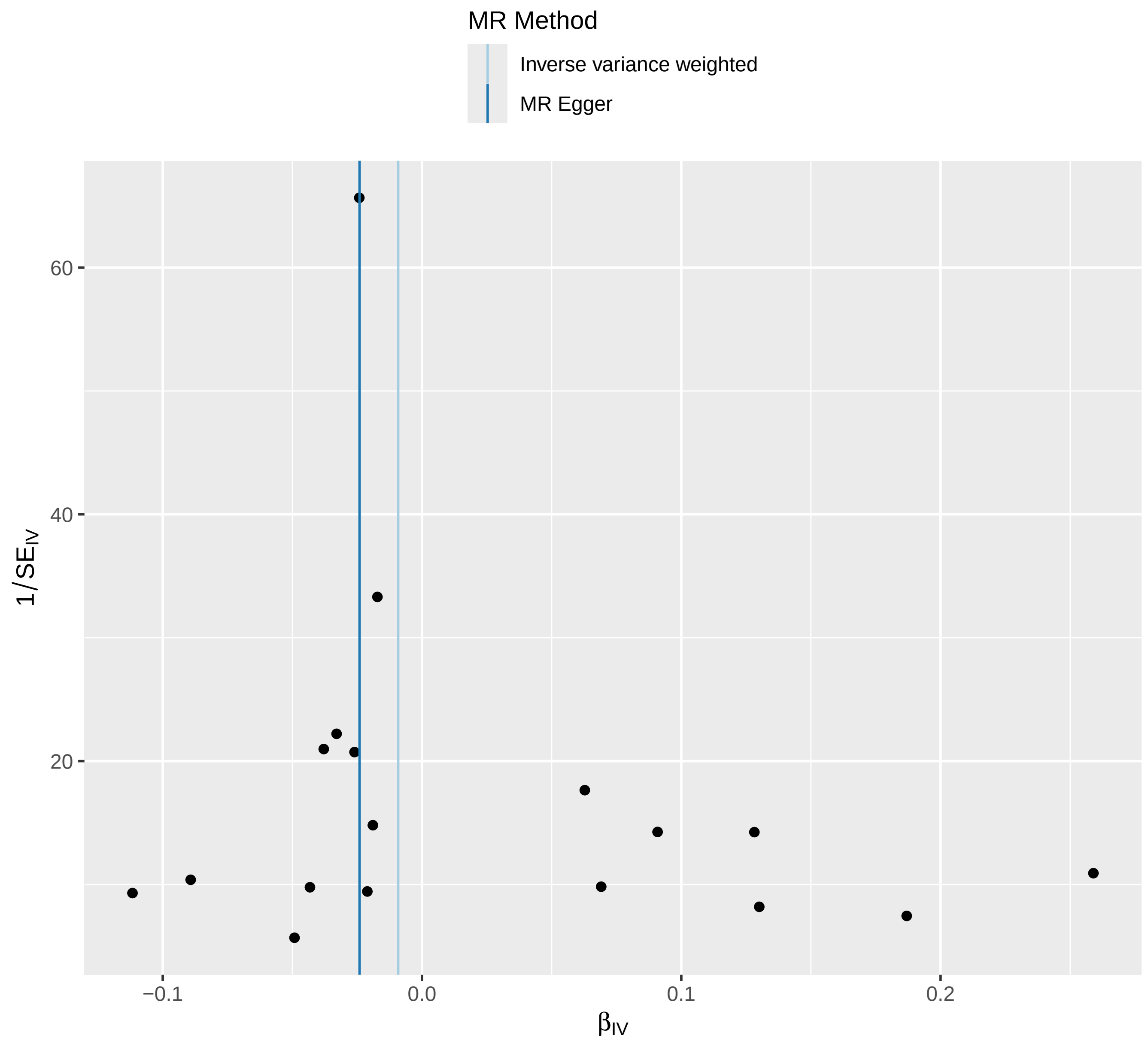

MR Method
Inverse variance weighted
MR Egger
60
40
20
1/SE_IV
−0.1
0.0
0.1
0.2
β_IV

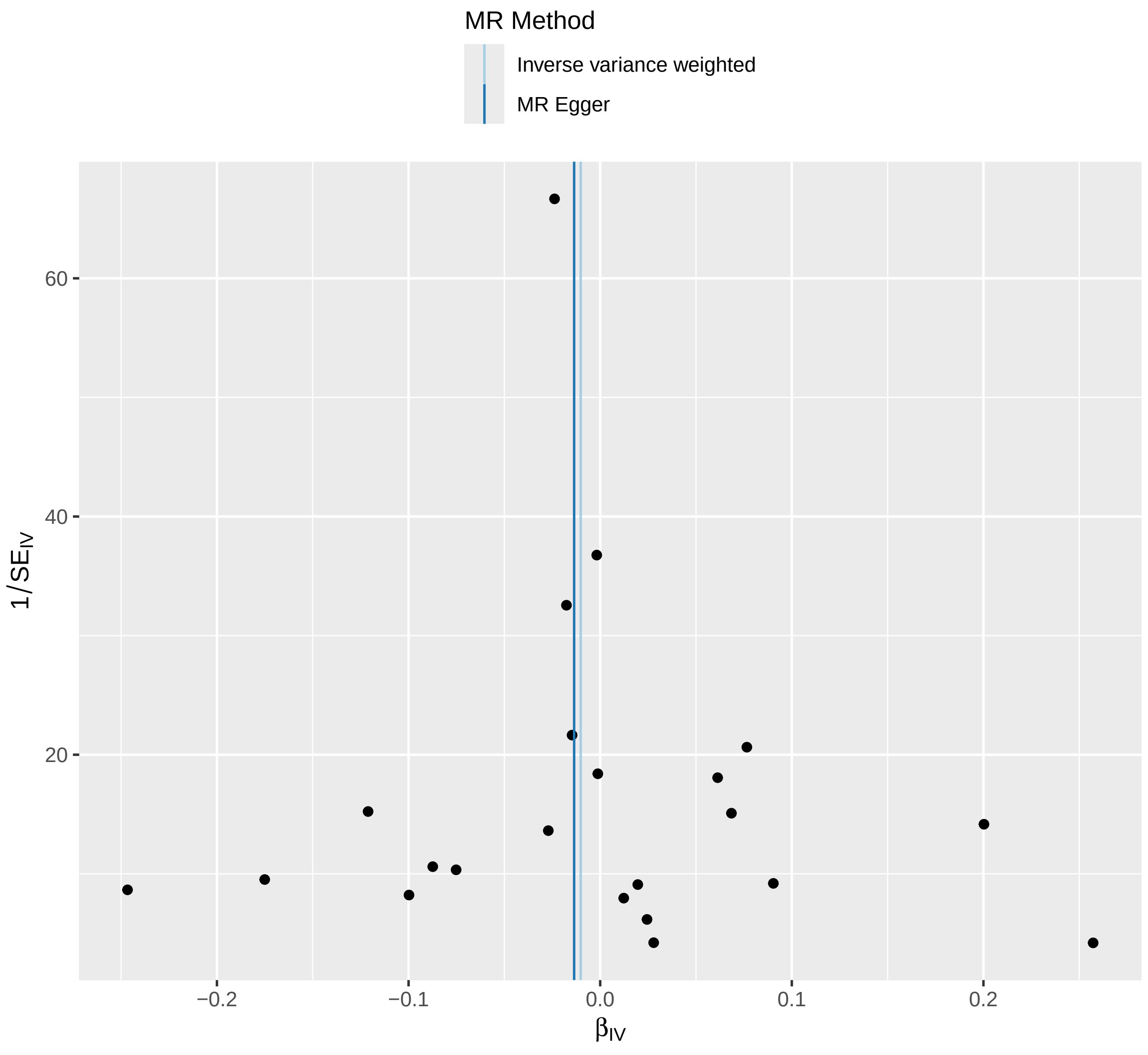
MR Method
Inverse variance weighted
MR Egger
60
40
20
1/SE_IV
−0.2
−0.1
0.0
0.1
0.2
β_IV

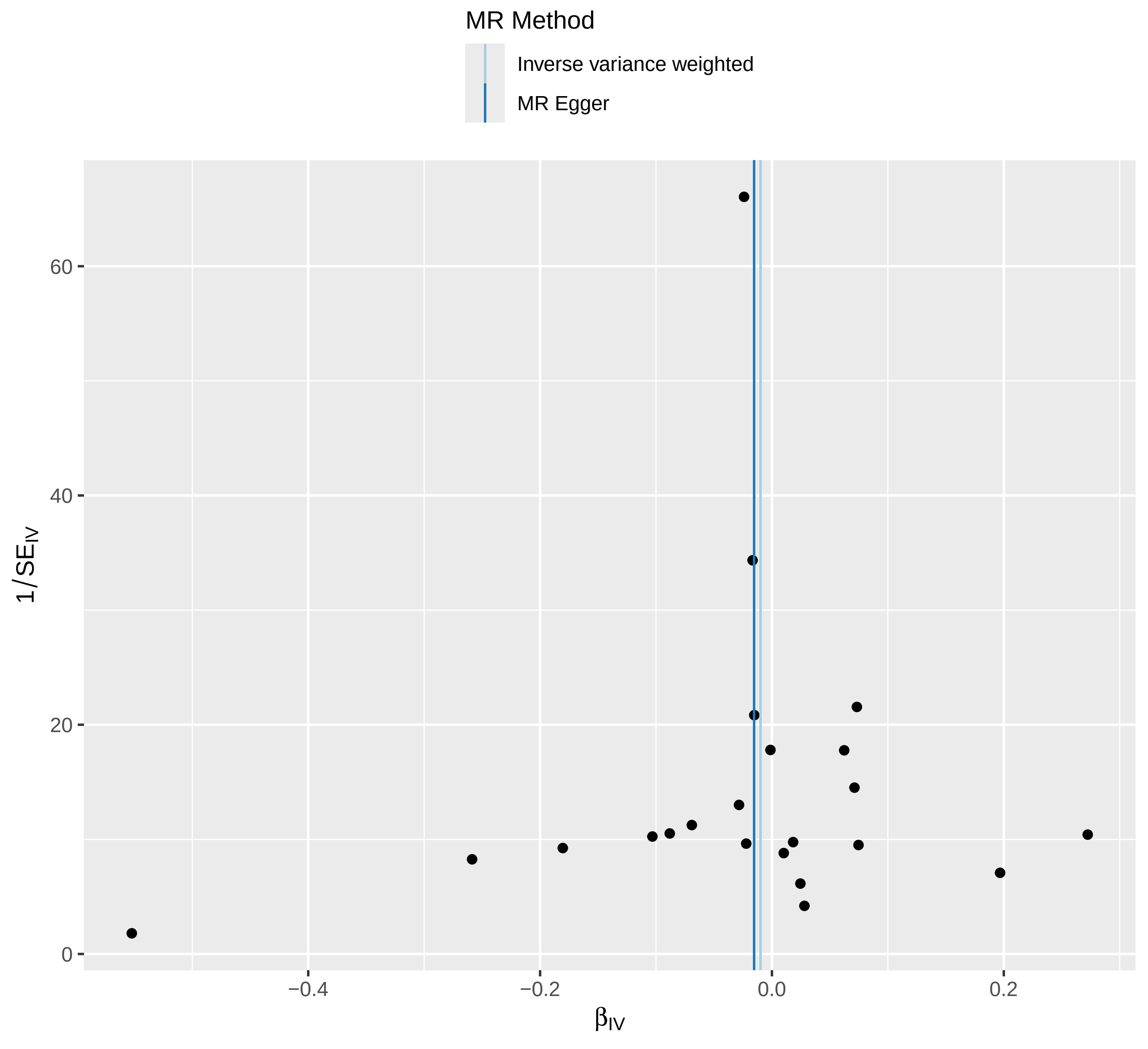
MR Method
Inverse variance weighted
MR Egger
60
40
20
0
1/SE$_{IV}$
−0.4
−0.2
0.0
0.2
β$_{IV}$

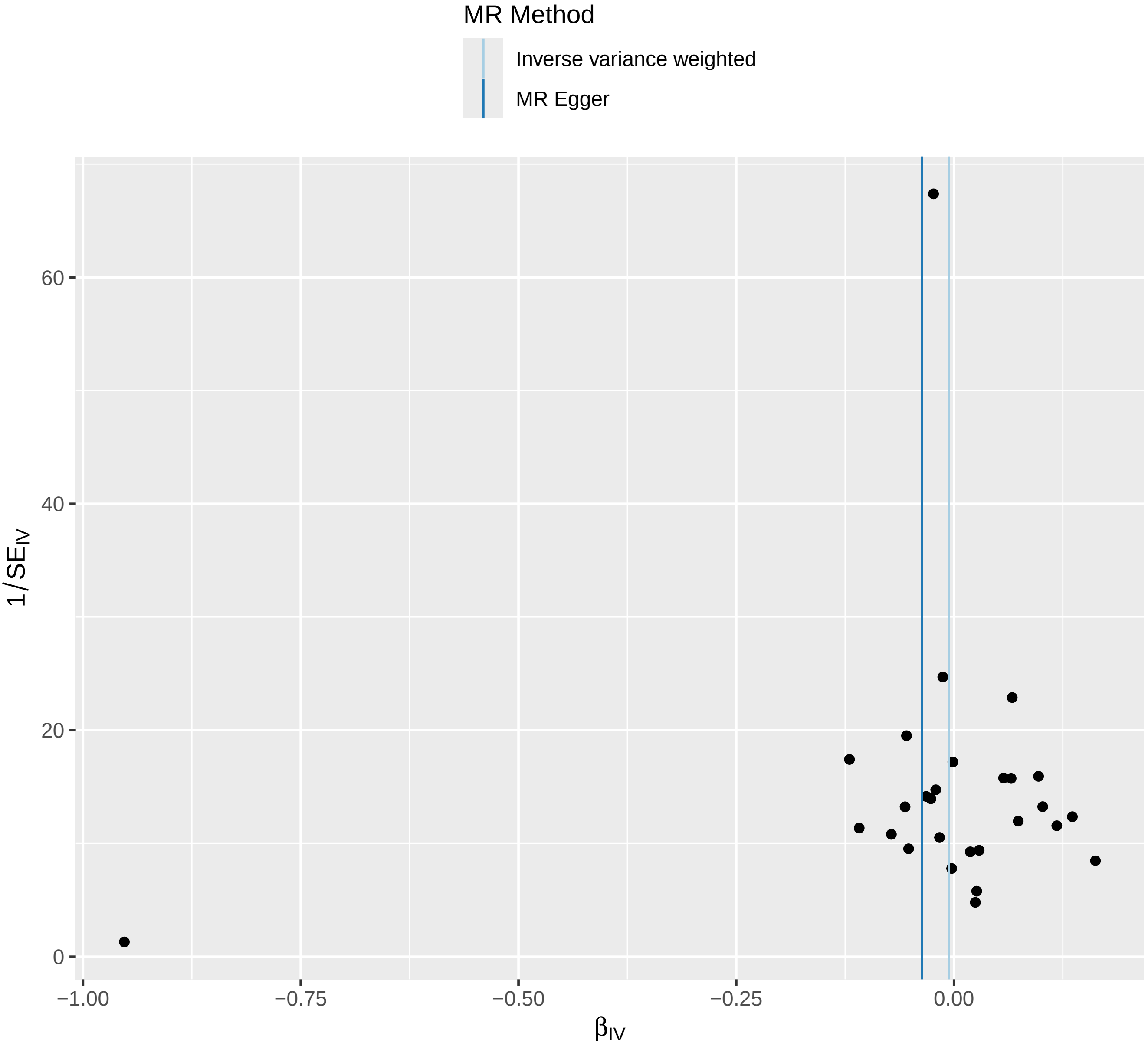
MR Method
Inverse variance weighted
MR Egger
60
40
20
0
1/SE_IV
−1.00
−0.75
−0.50
−0.25
0.00
β_IV

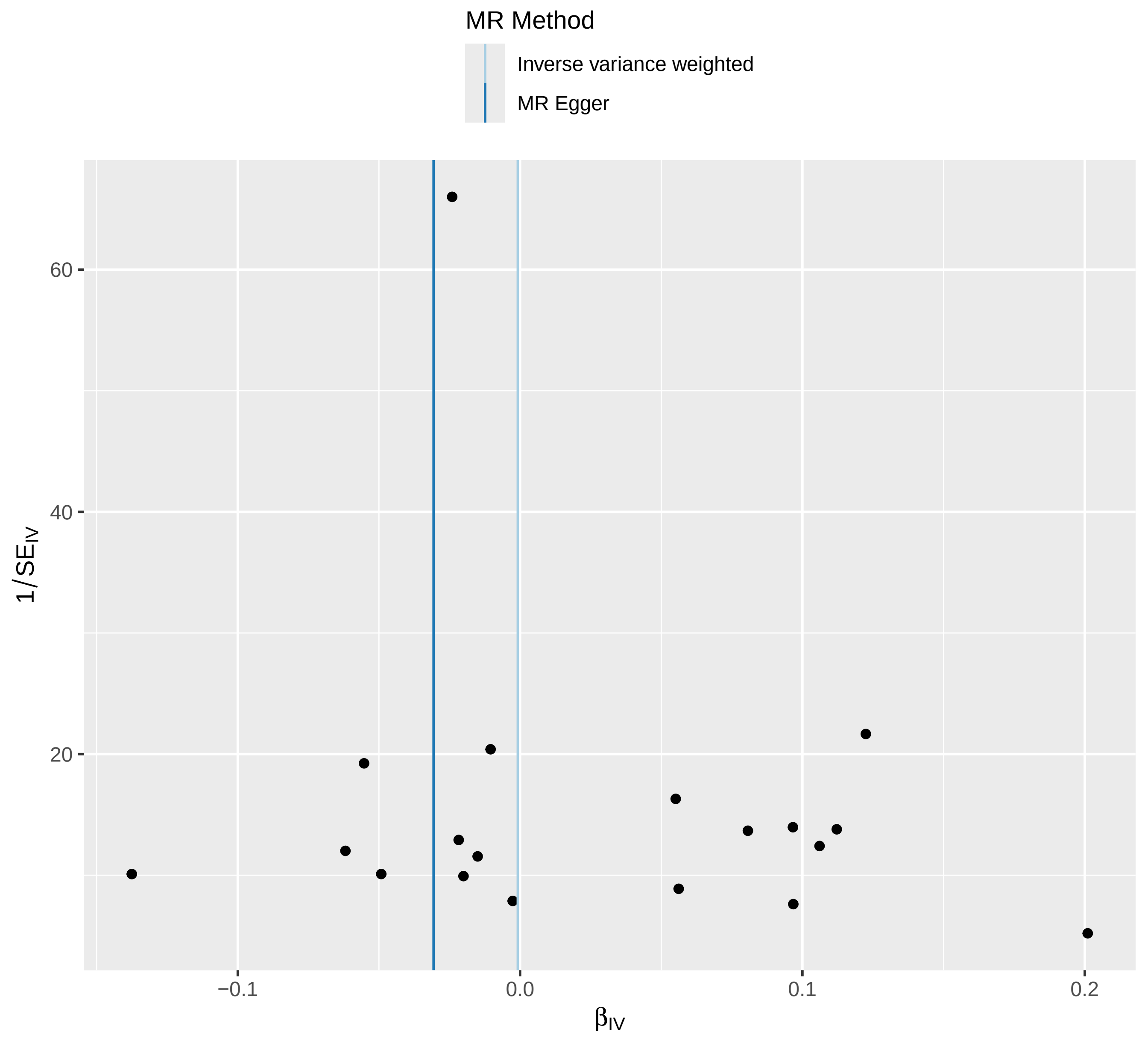
MR Method
Inverse variance weighted
MR Egger
60
40
20
1/SE$_{IV}$
−0.1
0.0
0.1
0.2
β$_{IV}$

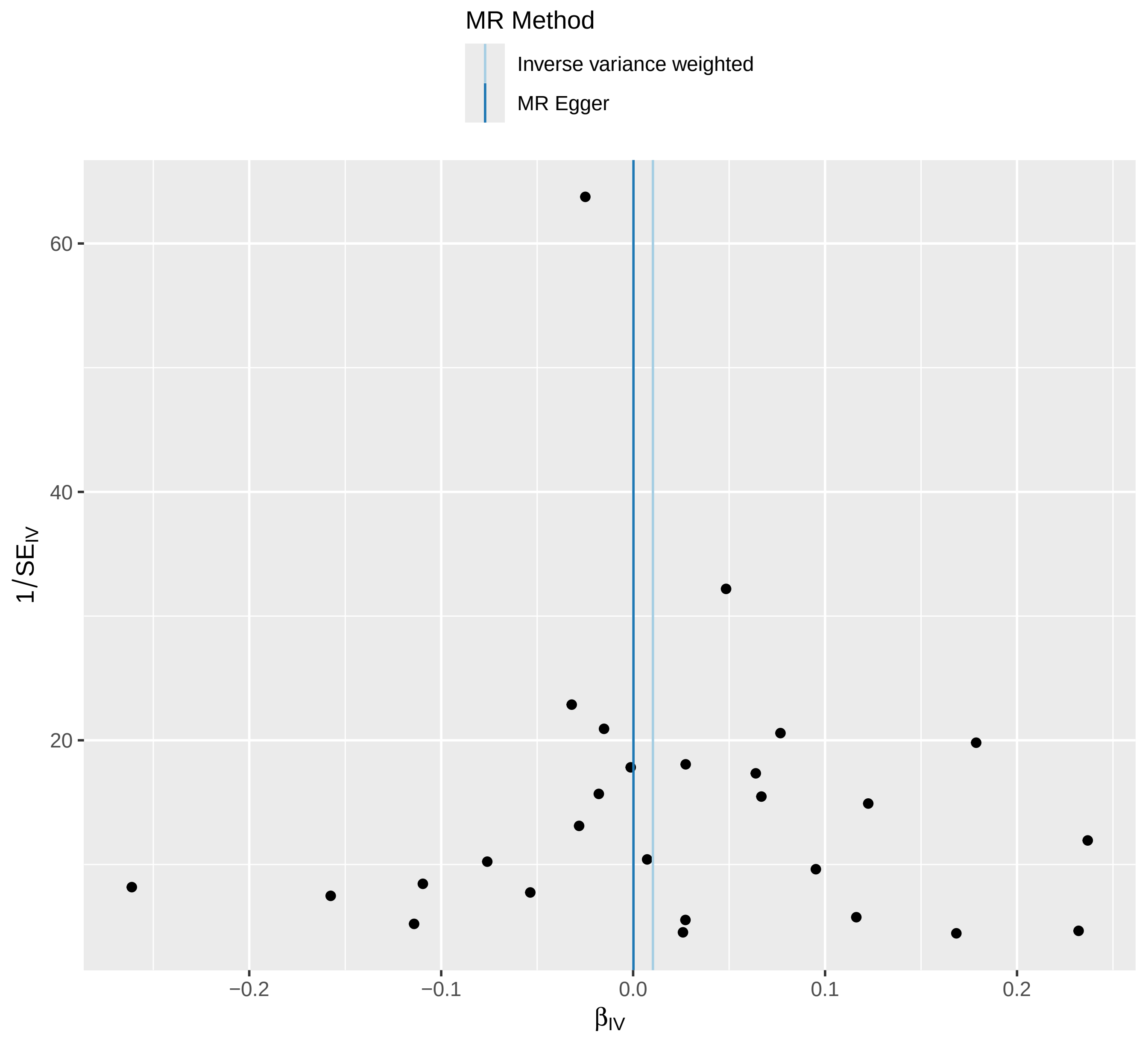
MR Method
Inverse variance weighted
MR Egger
60
40
20
1/SE$_{IV}$
−0.2
−0.1
0.0
0.1
0.2
β$_{IV}$

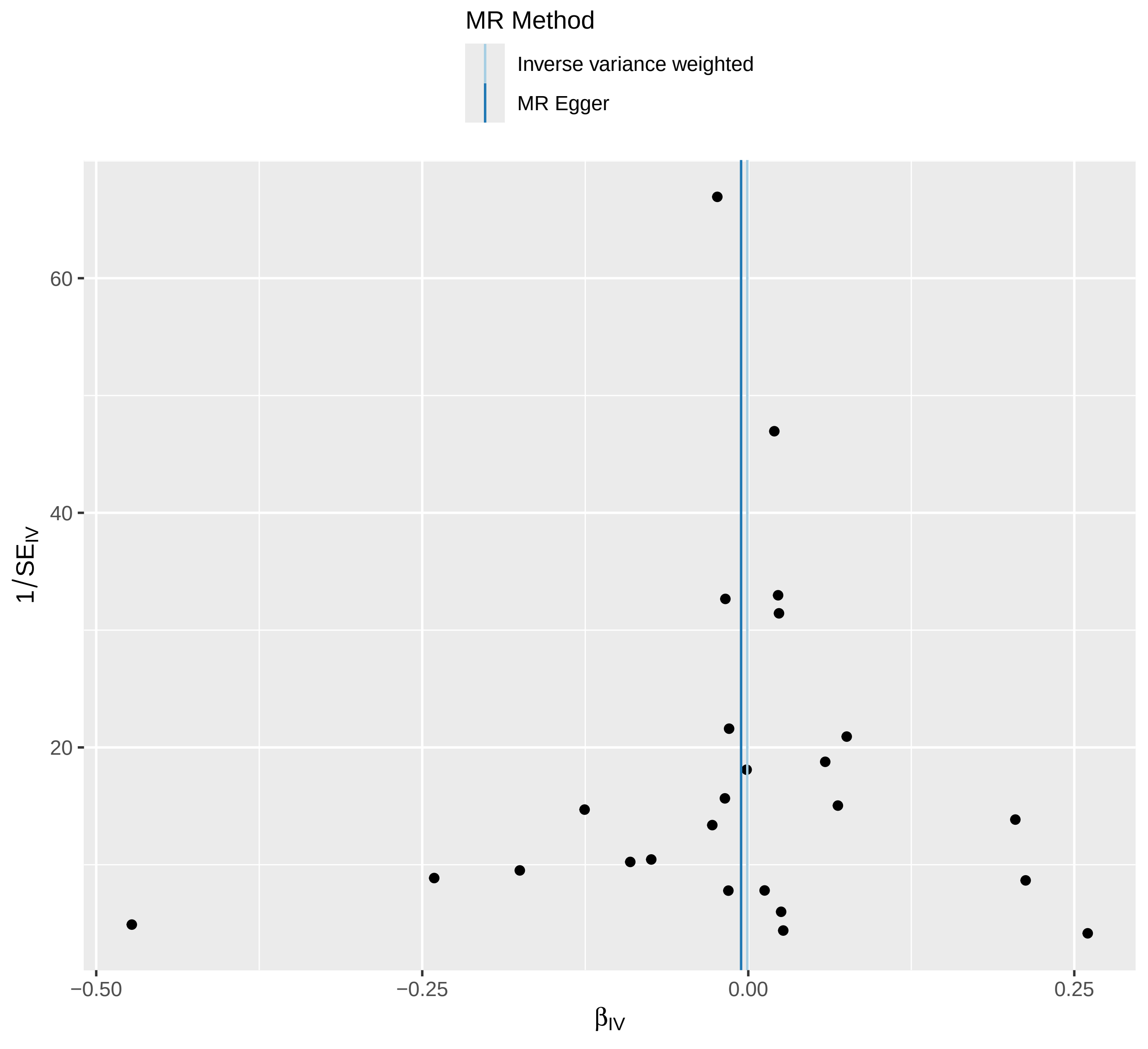

MR Method
Inverse variance weighted
MR Egger
60
40
20
1/SE_IV
−0.50
−0.25
0.00
0.25
β_IV

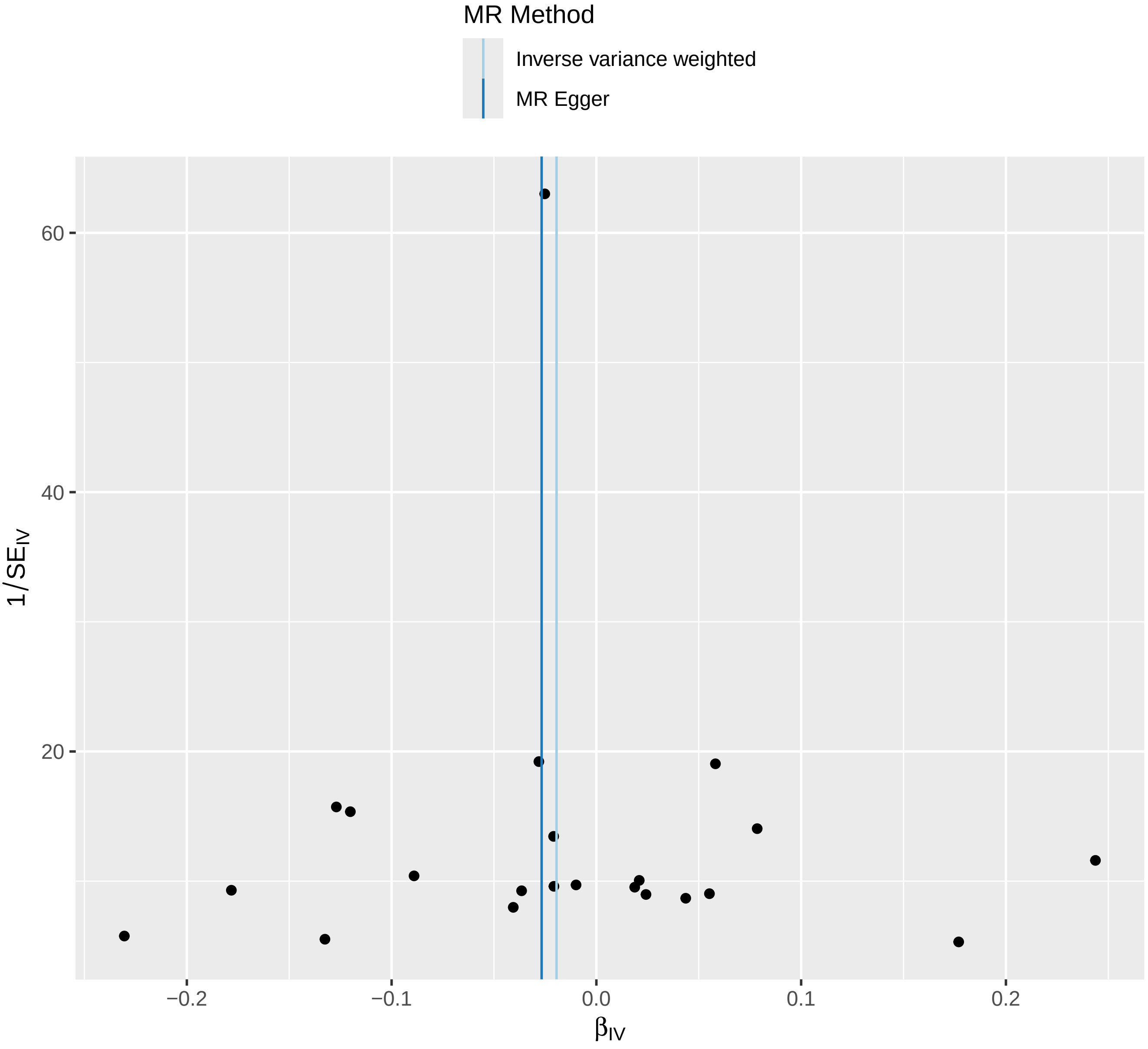
MR Method
Inverse variance weighted
MR Egger
60
40
20
1/SE$_{IV}$
−0.2
−0.1
0.0
0.1
0.2
β$_{IV}$

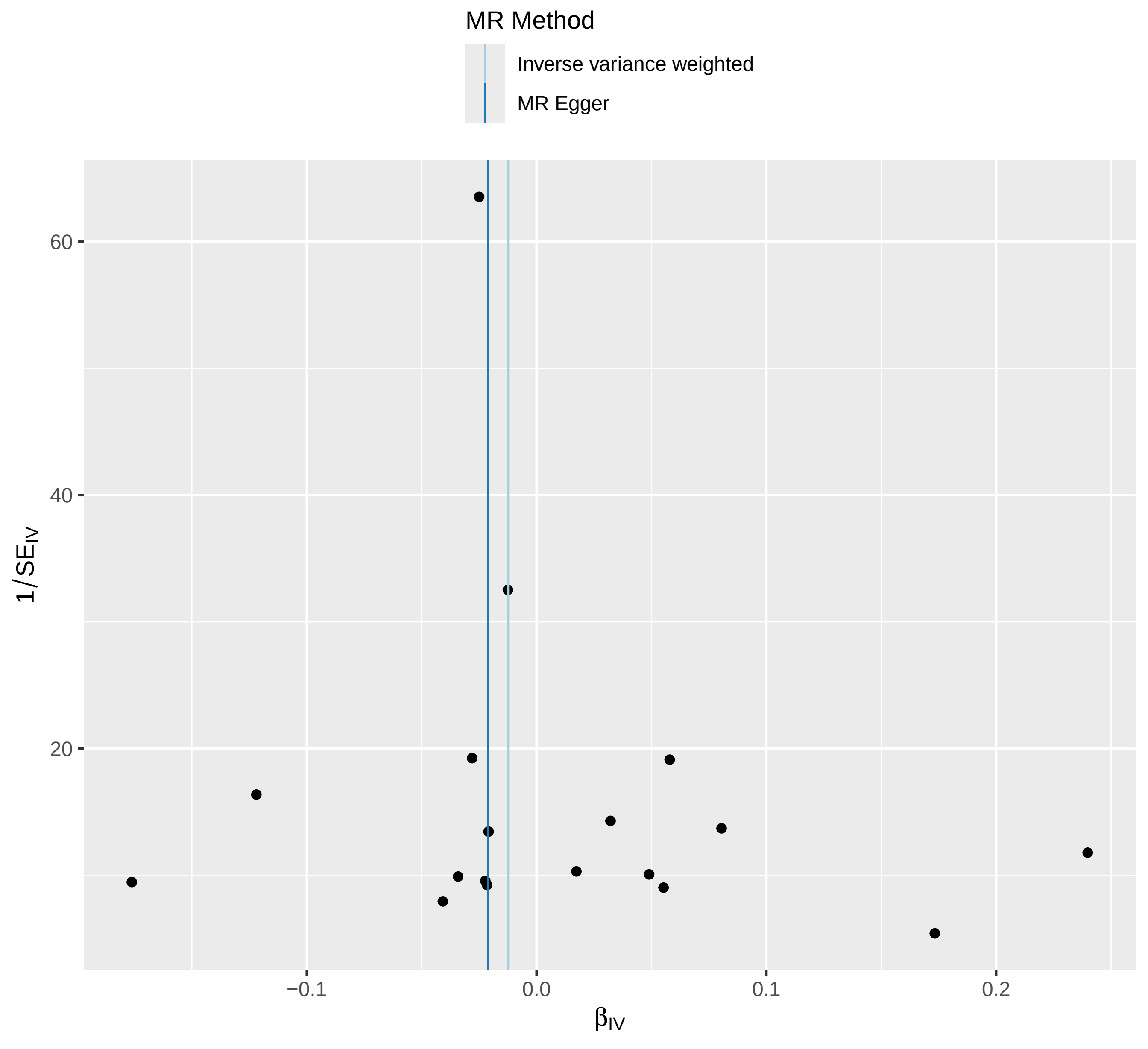
MR Method
Inverse variance weighted
MR Egger
60
40
20
1/SE$_{IV}$
−0.1
0.0
0.1
0.2
β$_{IV}$

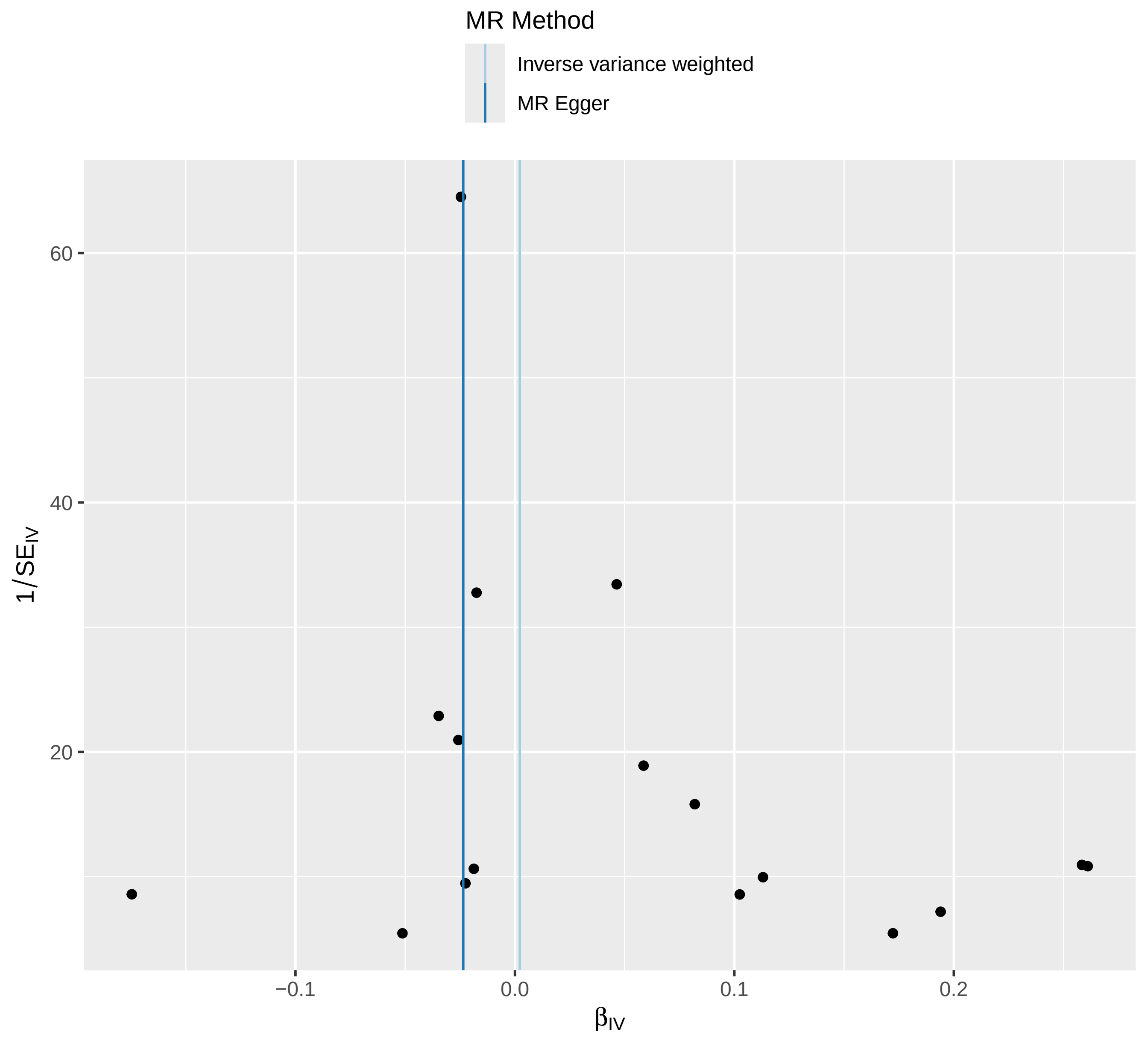
MR Method
Inverse variance weighted
MR Egger
60
40
20
1/SE$_{IV}$
−0.1
0.0
0.1
0.2
β$_{IV}$

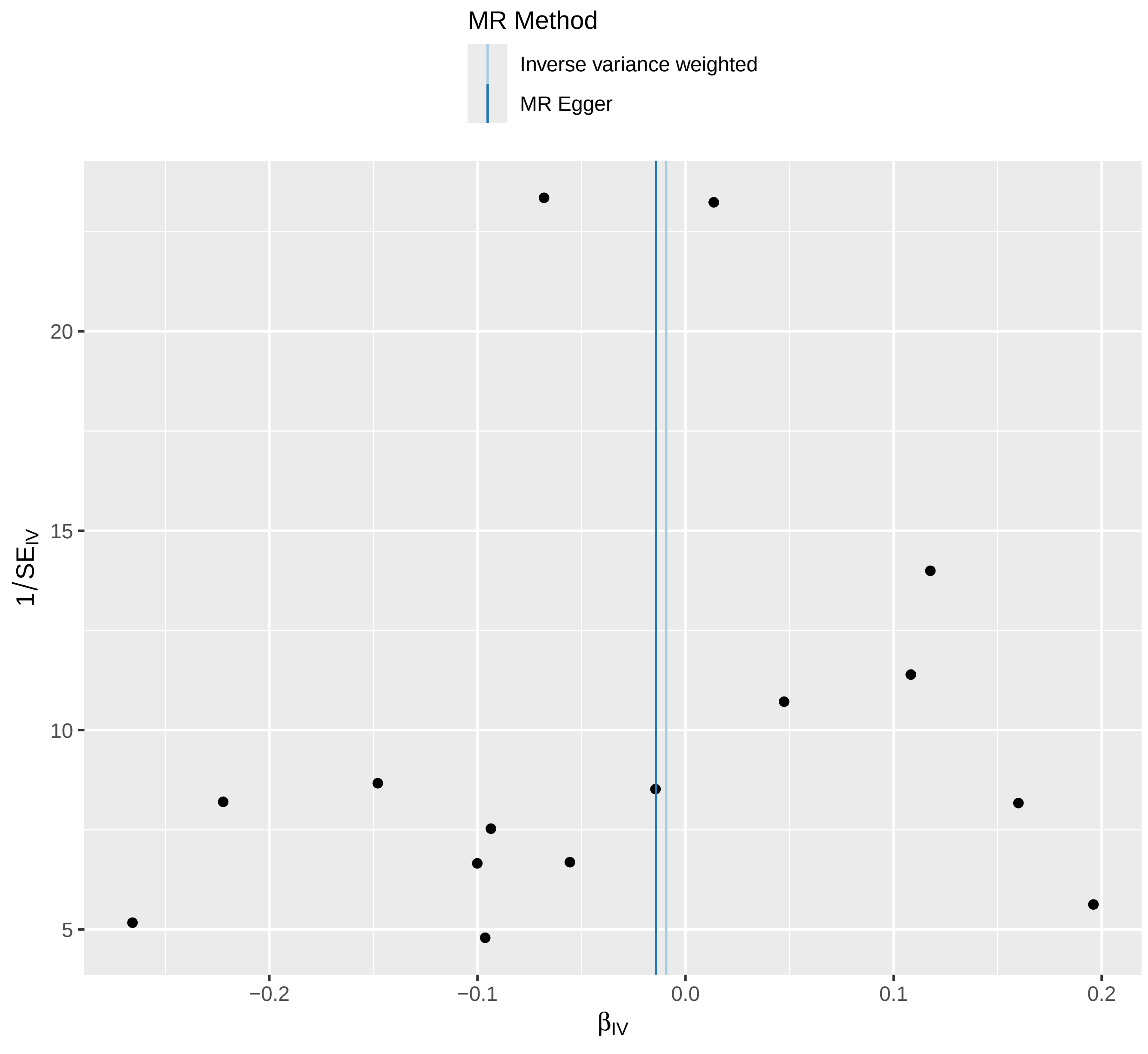
MR Method
Inverse variance weighted
MR Egger
20
15
10
5
1/SE_IV
−0.2
−0.1
0.0
0.1
0.2
β_IV

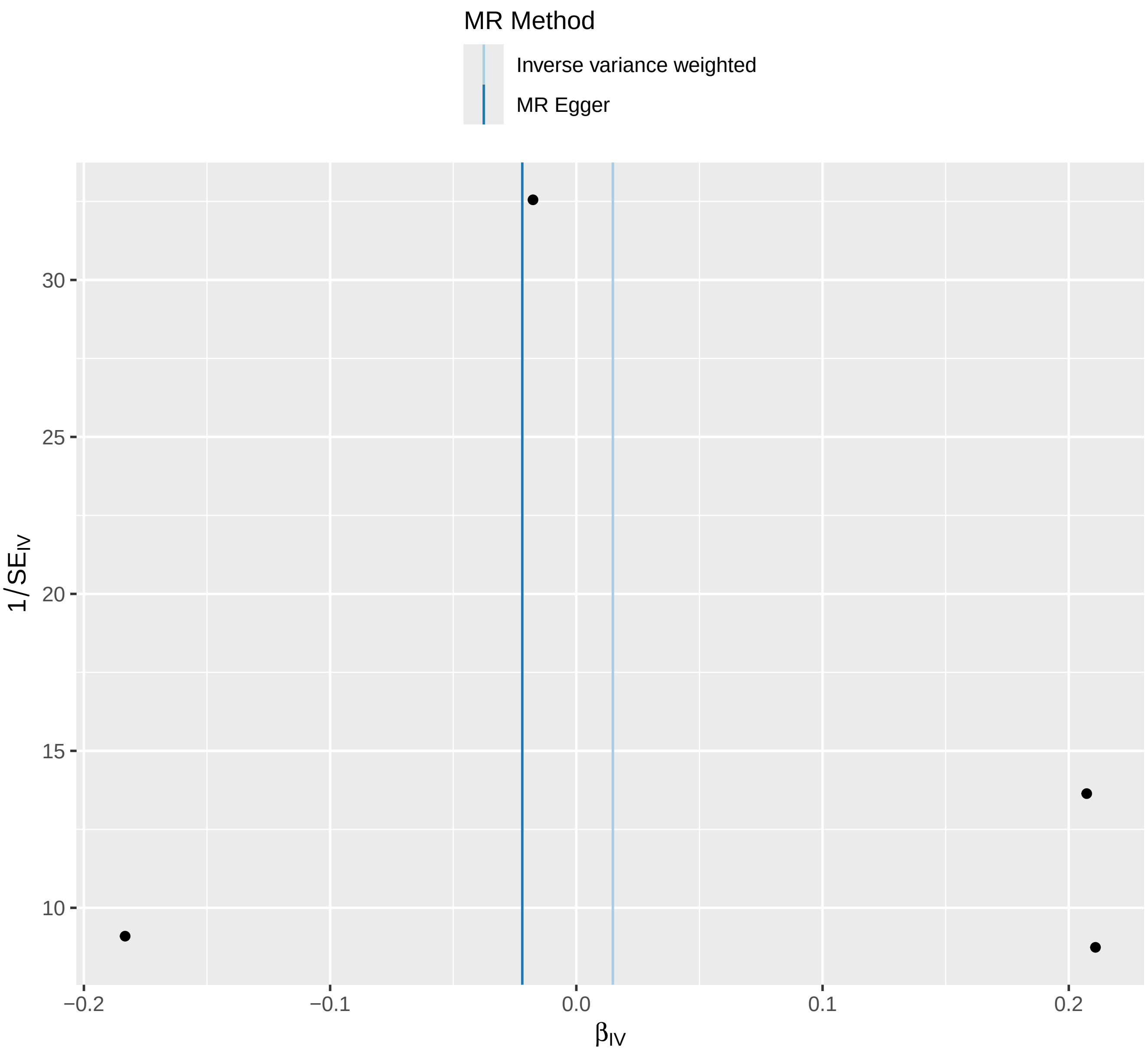
MR Method
Inverse variance weighted
MR Egger
30
25
20
15
10
1/SE_IV
−0.2
−0.1
0.0
0.1
0.2
β_IV

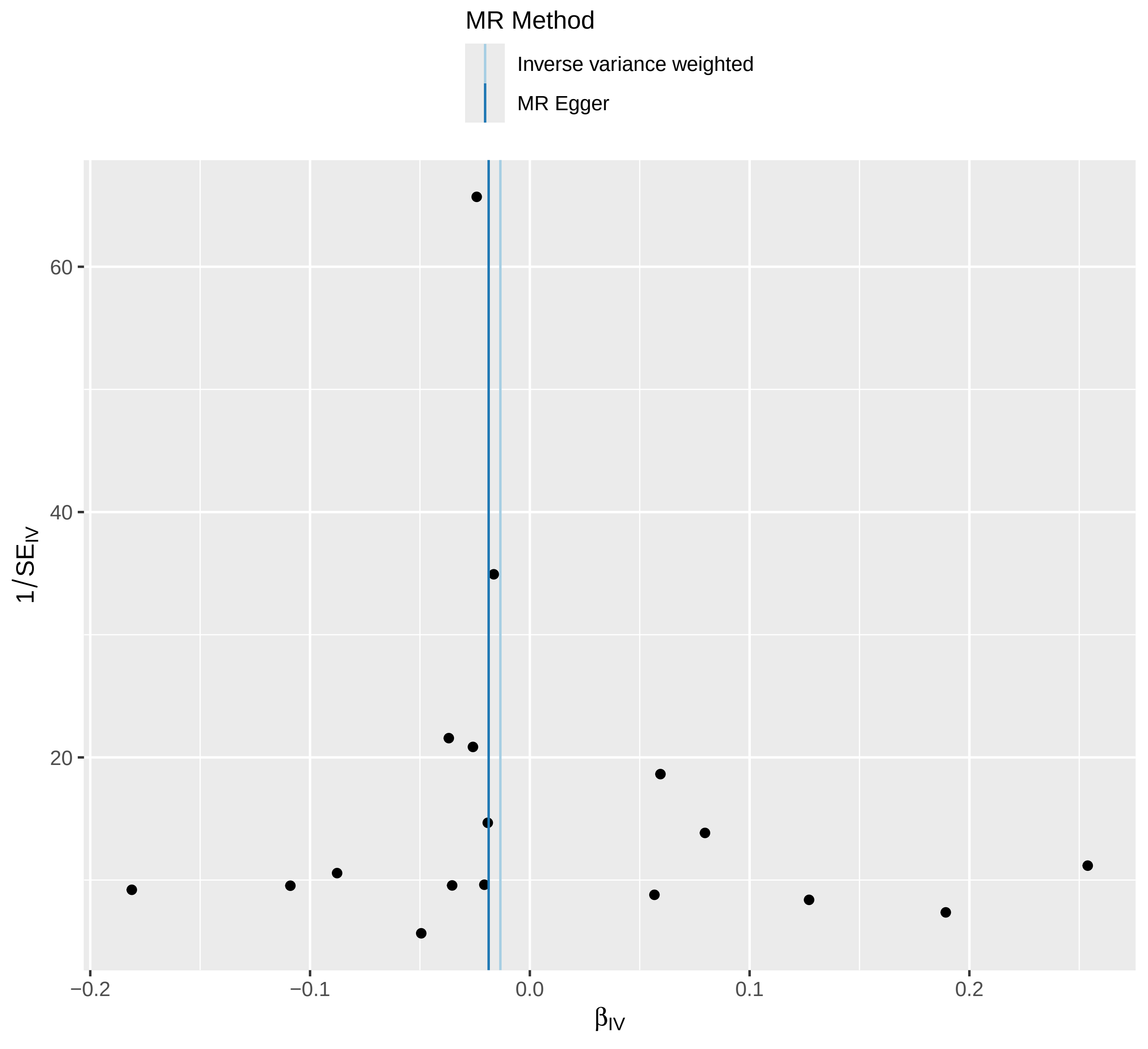
MR Method
Inverse variance weighted
MR Egger
60
40
20
1/SE$_{IV}$
−0.2
−0.1
0.0
0.1
0.2
β$_{IV}$

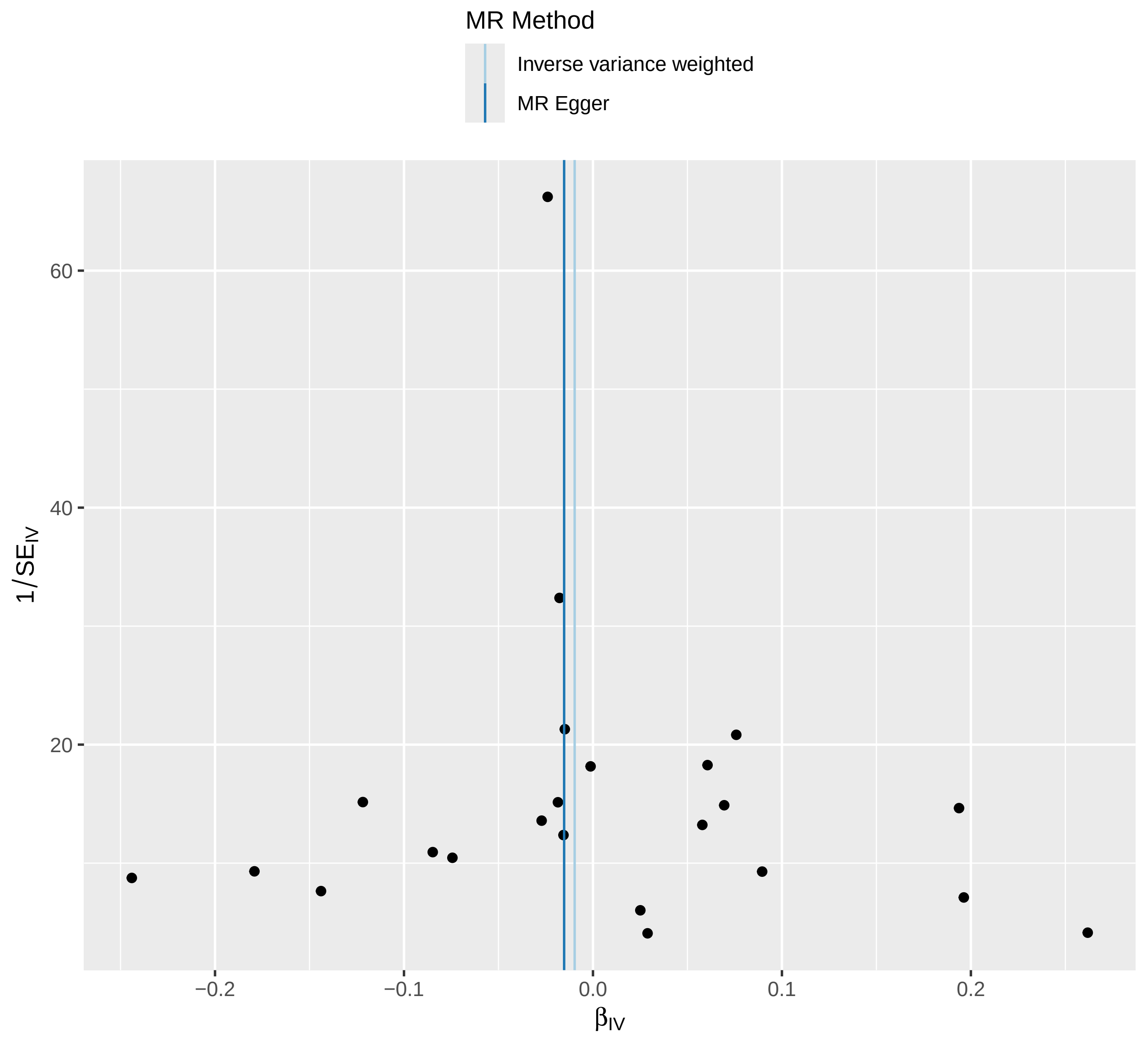

MR Method
Inverse variance weighted
MR Egger
60
40
20
1/SE_IV
−0.2
−0.1
0.0
0.1
0.2
β_IV

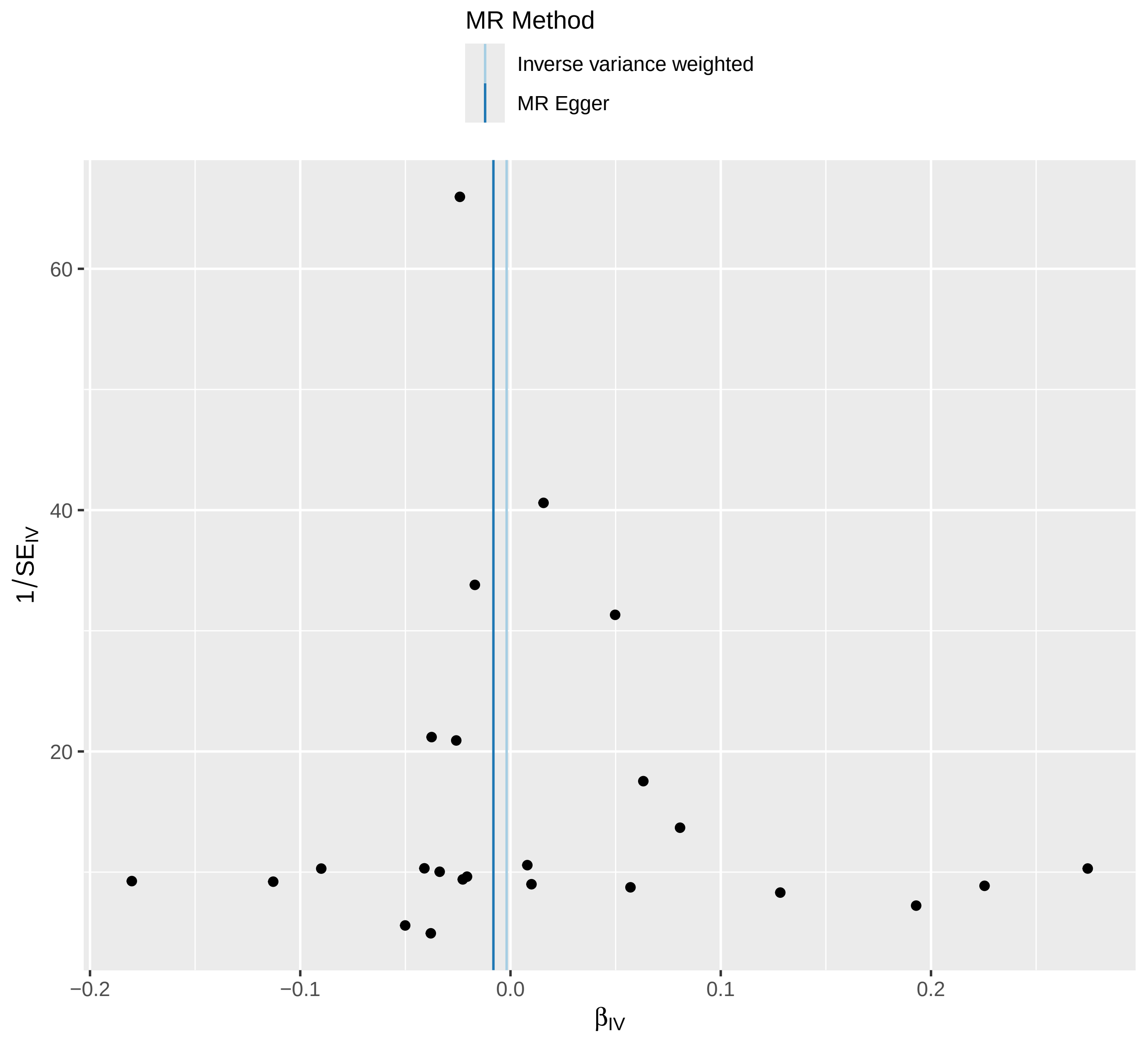
MR Method
Inverse variance weighted
MR Egger
60
40
20
1/SE$_{IV}$
−0.2
−0.1
0.0
0.1
0.2
β$_{IV}$

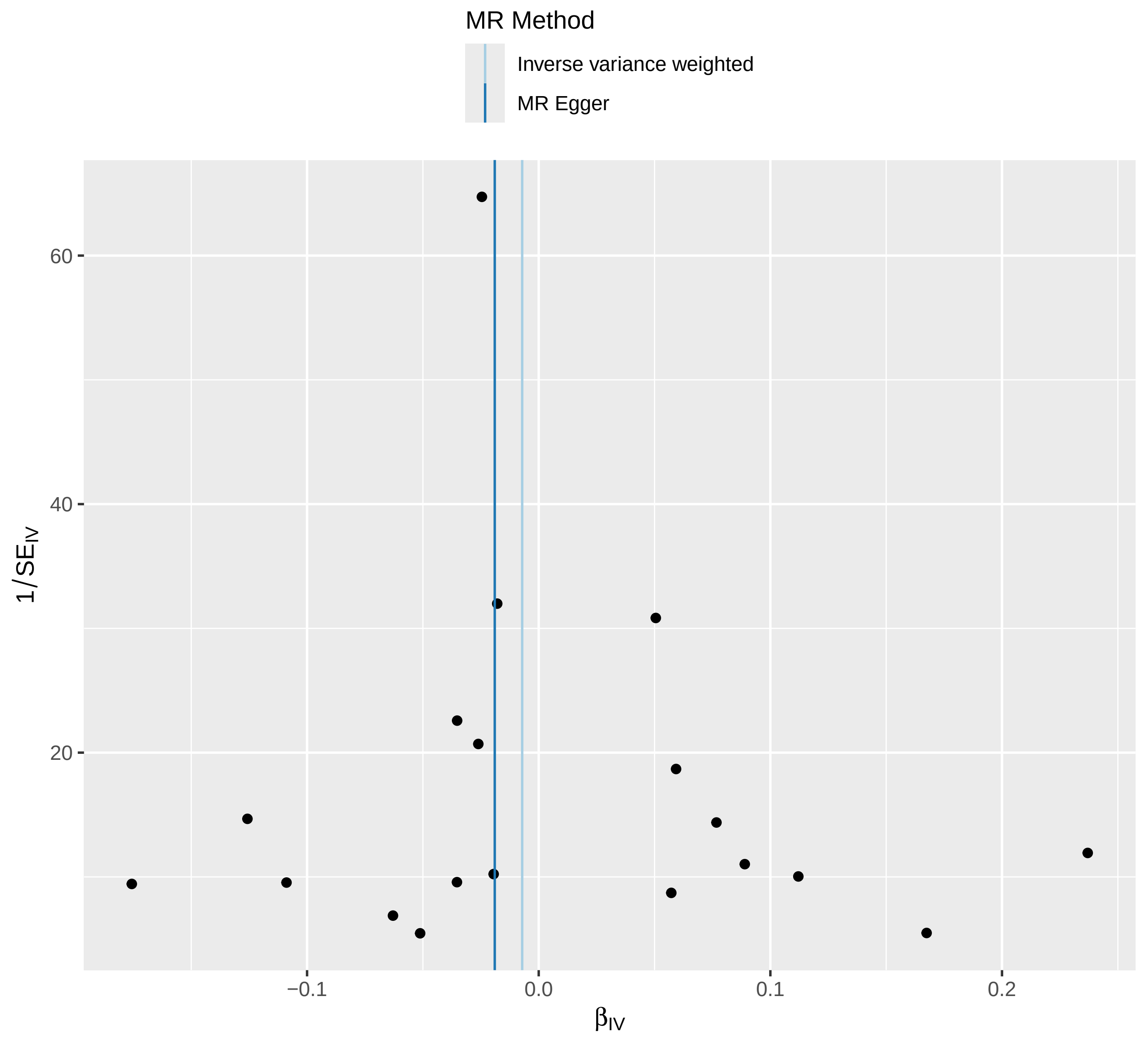
MR Method
Inverse variance weighted
MR Egger
60
40
20
$1/SE_{IV}$
−0.1
0.0
0.1
0.2
β_{IV}

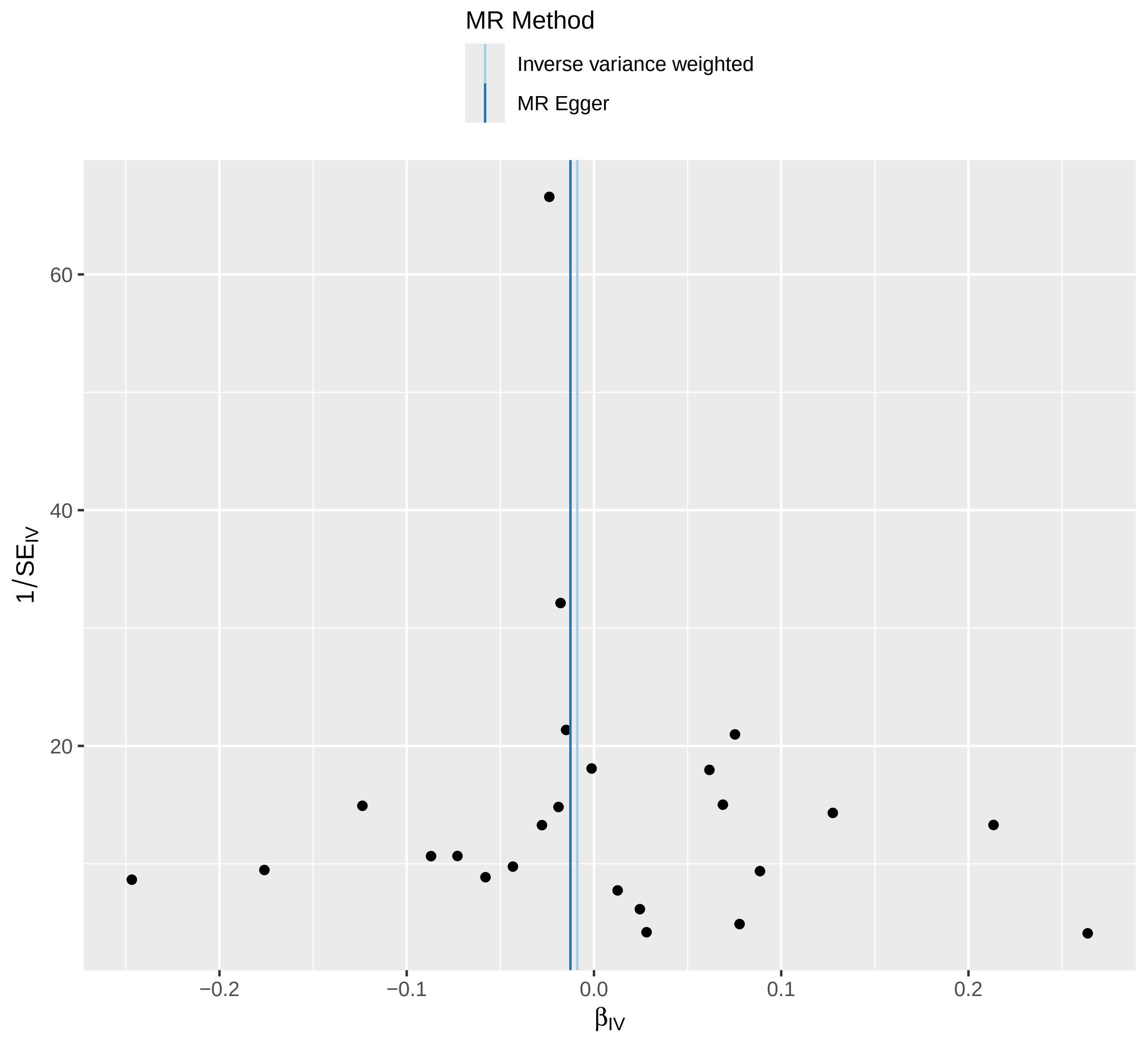

MR Method
Inverse variance weighted
MR Egger
60
40
20
1/SE_IV
−0.2
−0.1
0.0
0.1
0.2
β_IV

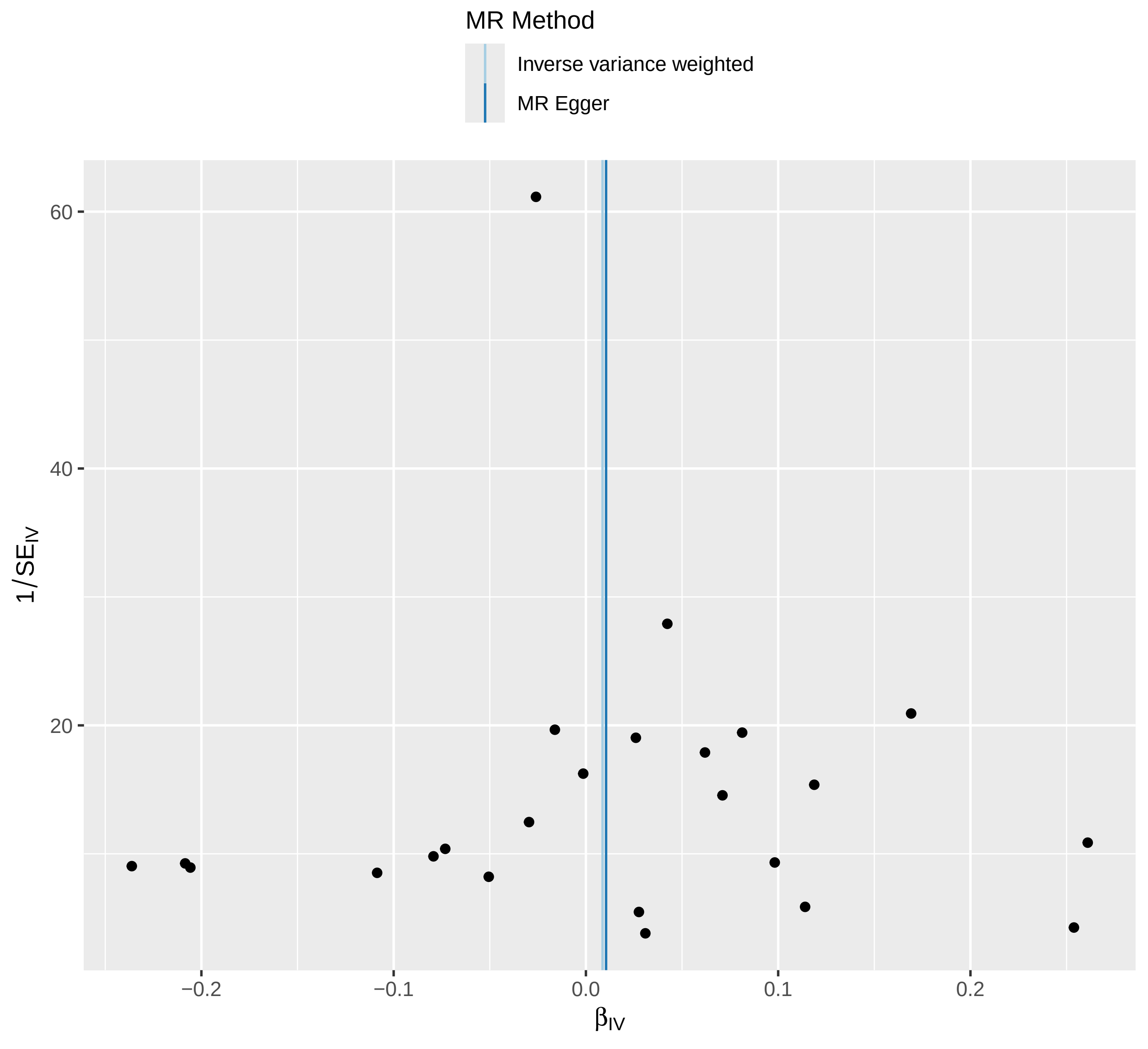
MR Method
Inverse variance weighted
MR Egger
60
40
20
1/SE_IV
−0.2
−0.1
0.0
0.1
0.2
β_IV

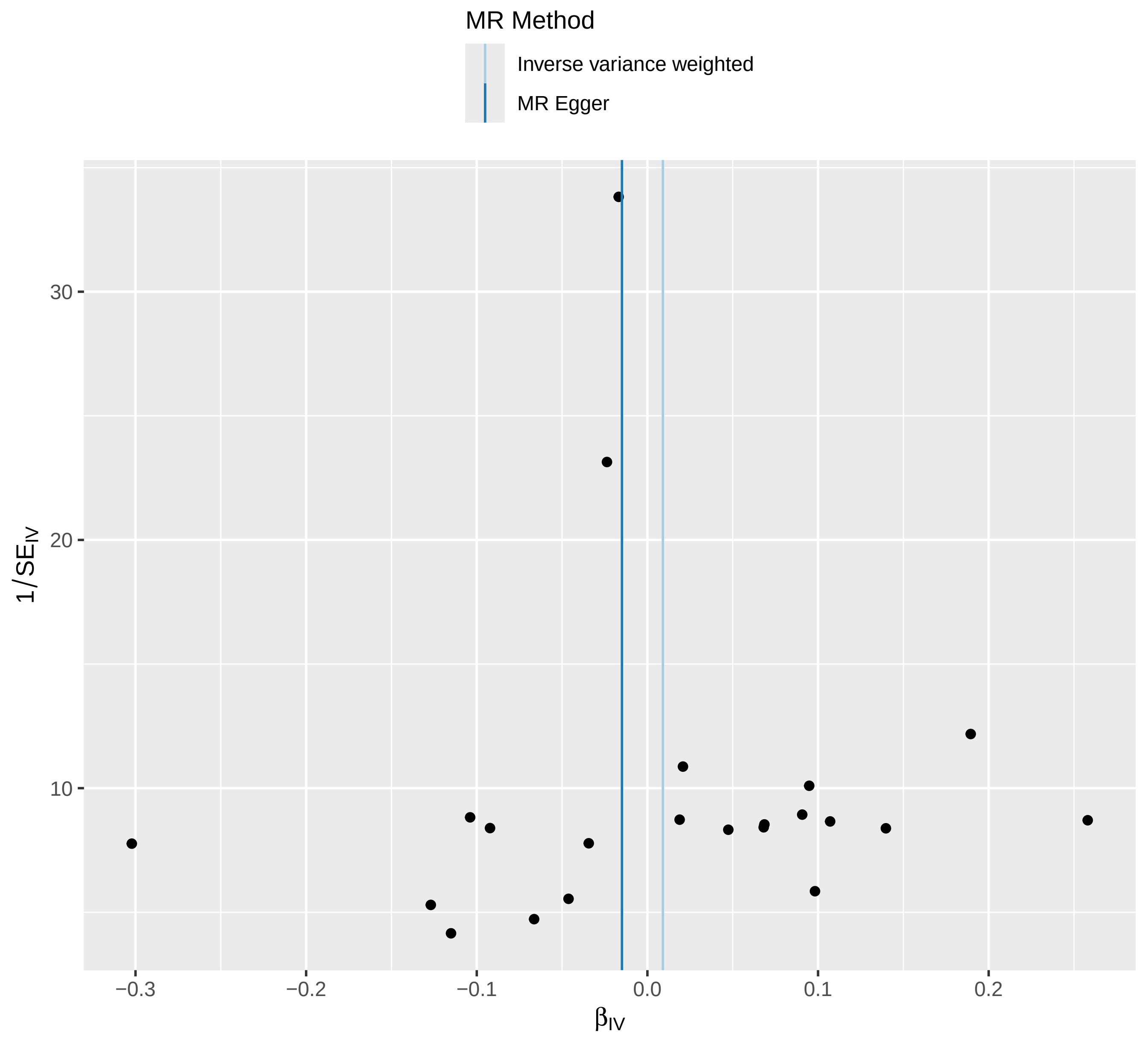

MR Method
Inverse variance weighted
MR Egger
30
20
10
$1/SE_{IV}$
−0.3
−0.2
−0.1
0.0
0.1
0.2
β_{IV}

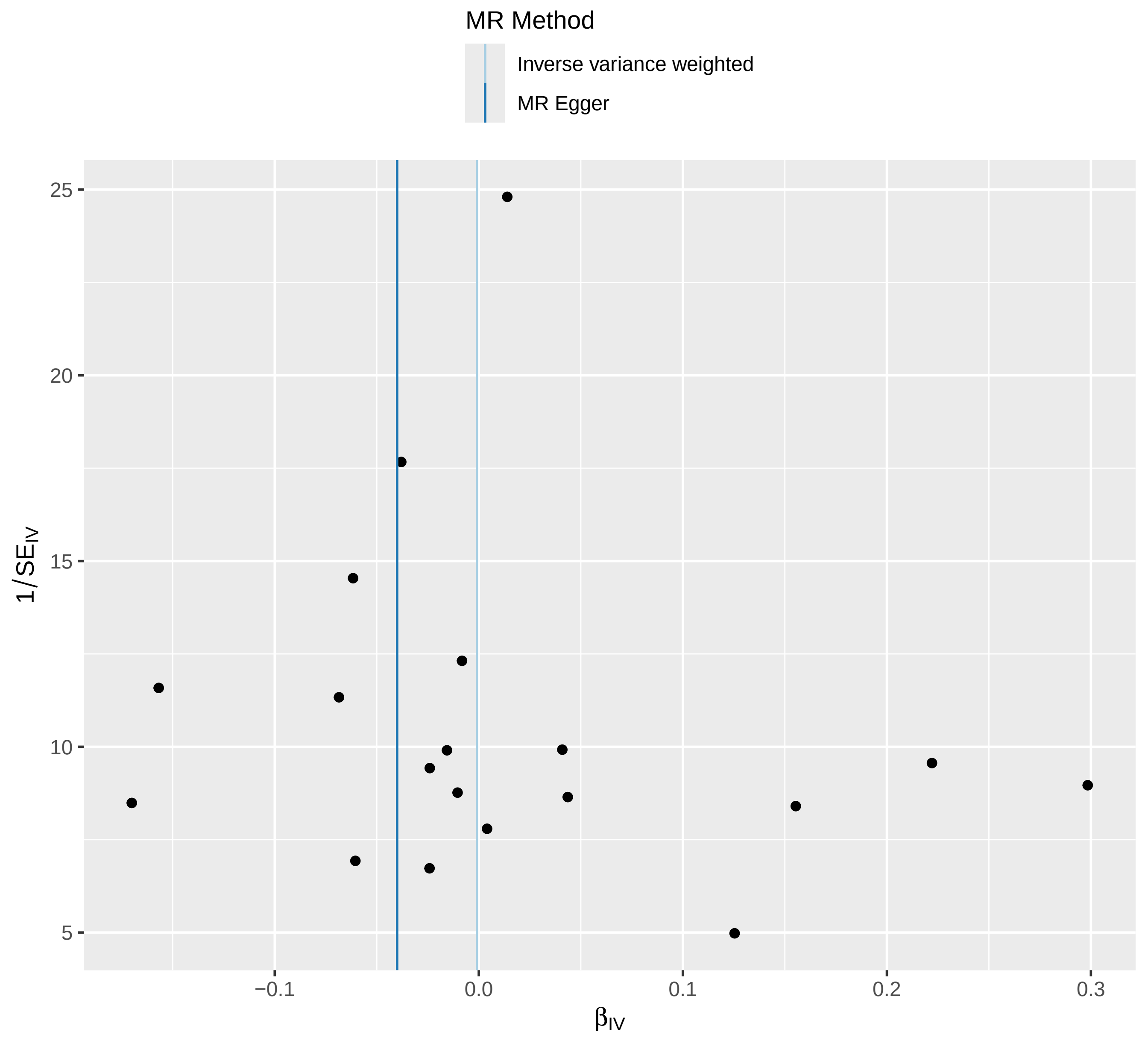
MR Method
Inverse variance weighted
MR Egger
25
20
15
10
5
1/SE$_{IV}$
−0.1
0.0
0.1
0.2
0.3
β$_{IV}$

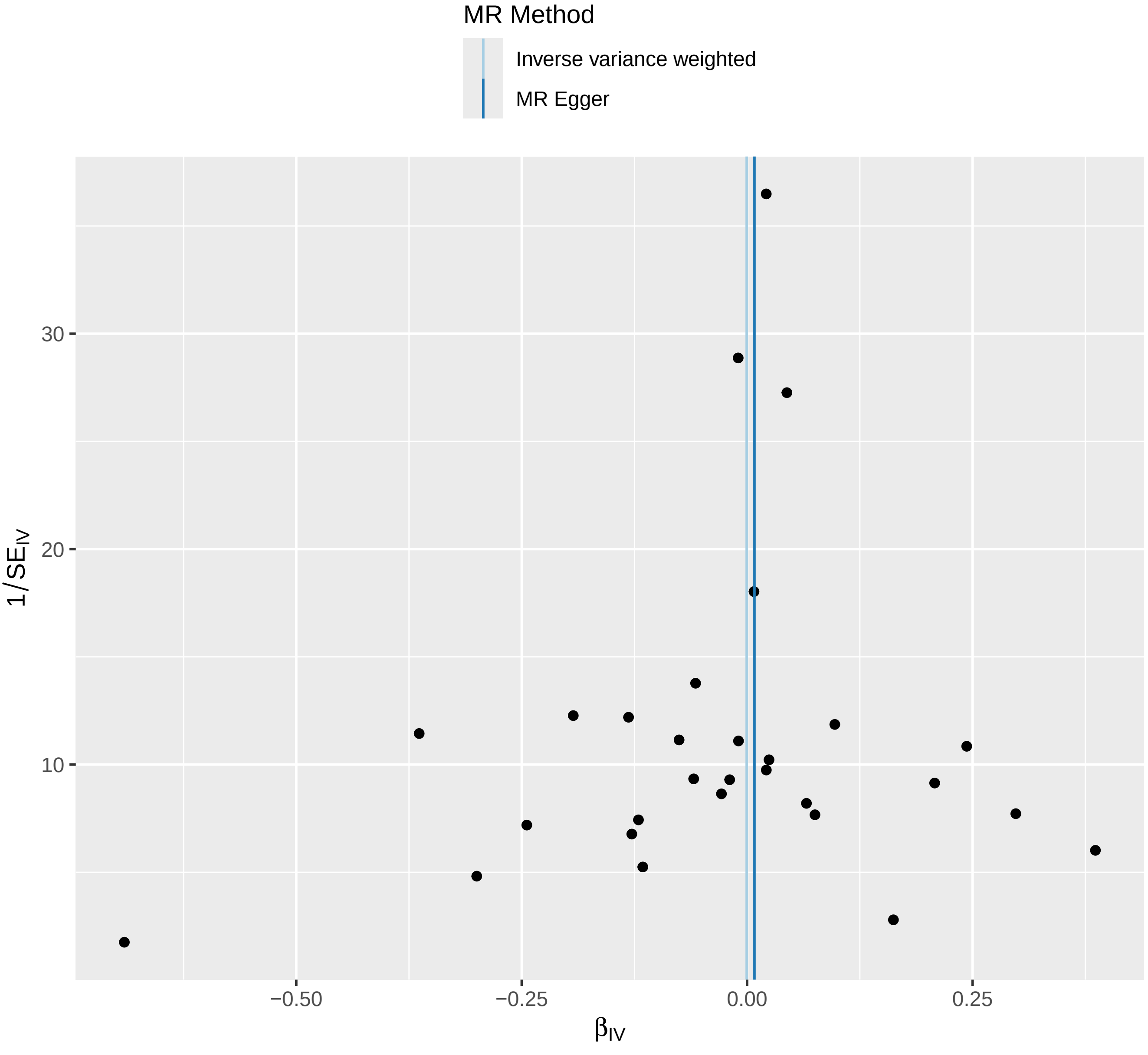
MR Method
Inverse variance weighted
MR Egger
1/SE_IV
30
20
10
−0.50
−0.25
0.00
0.25
β_IV

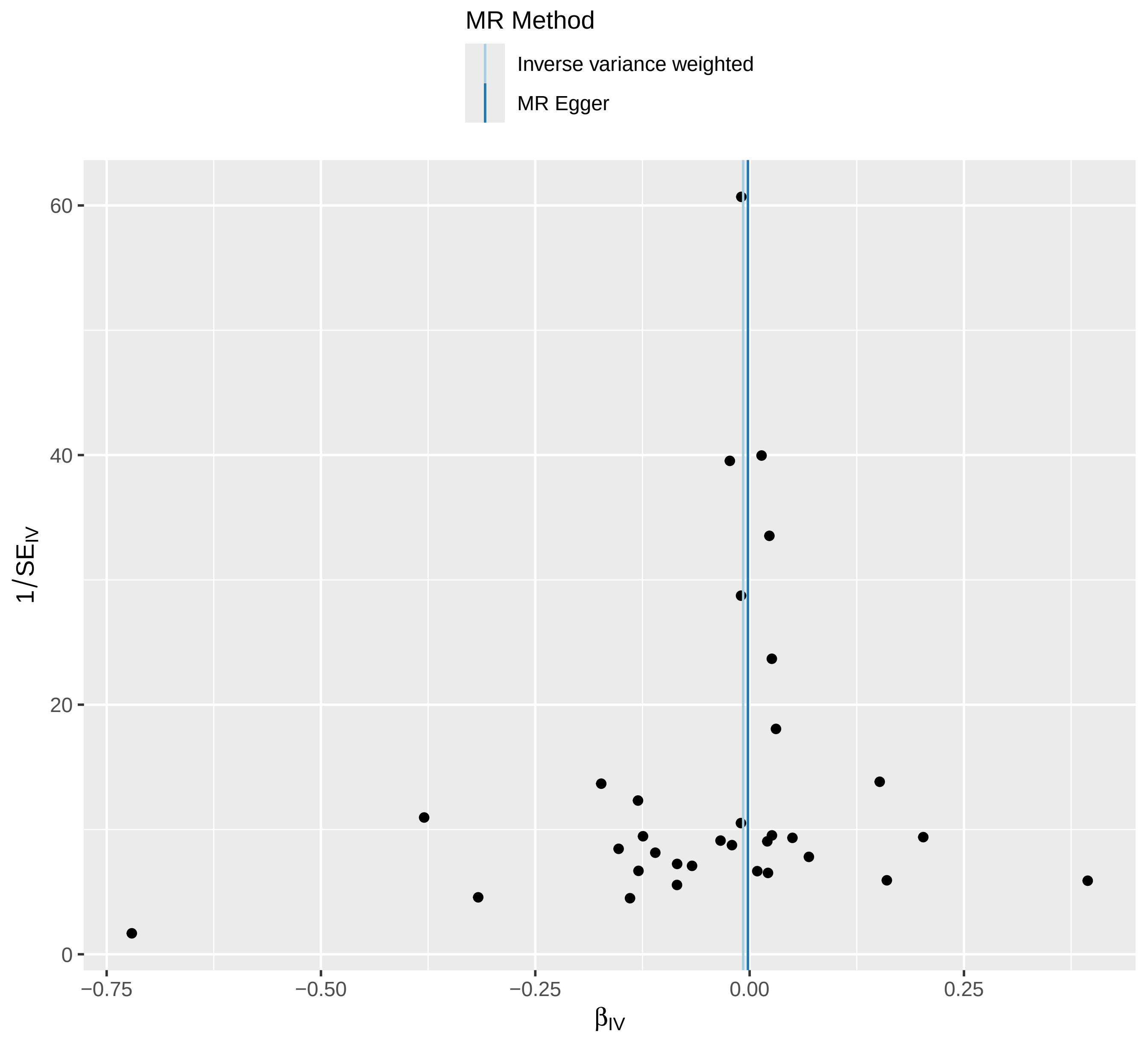
MR Method
Inverse variance weighted
MR Egger
1/SE_IV
β_IV
60
40
20
0
−0.75
−0.50
−0.25
0.00
0.25

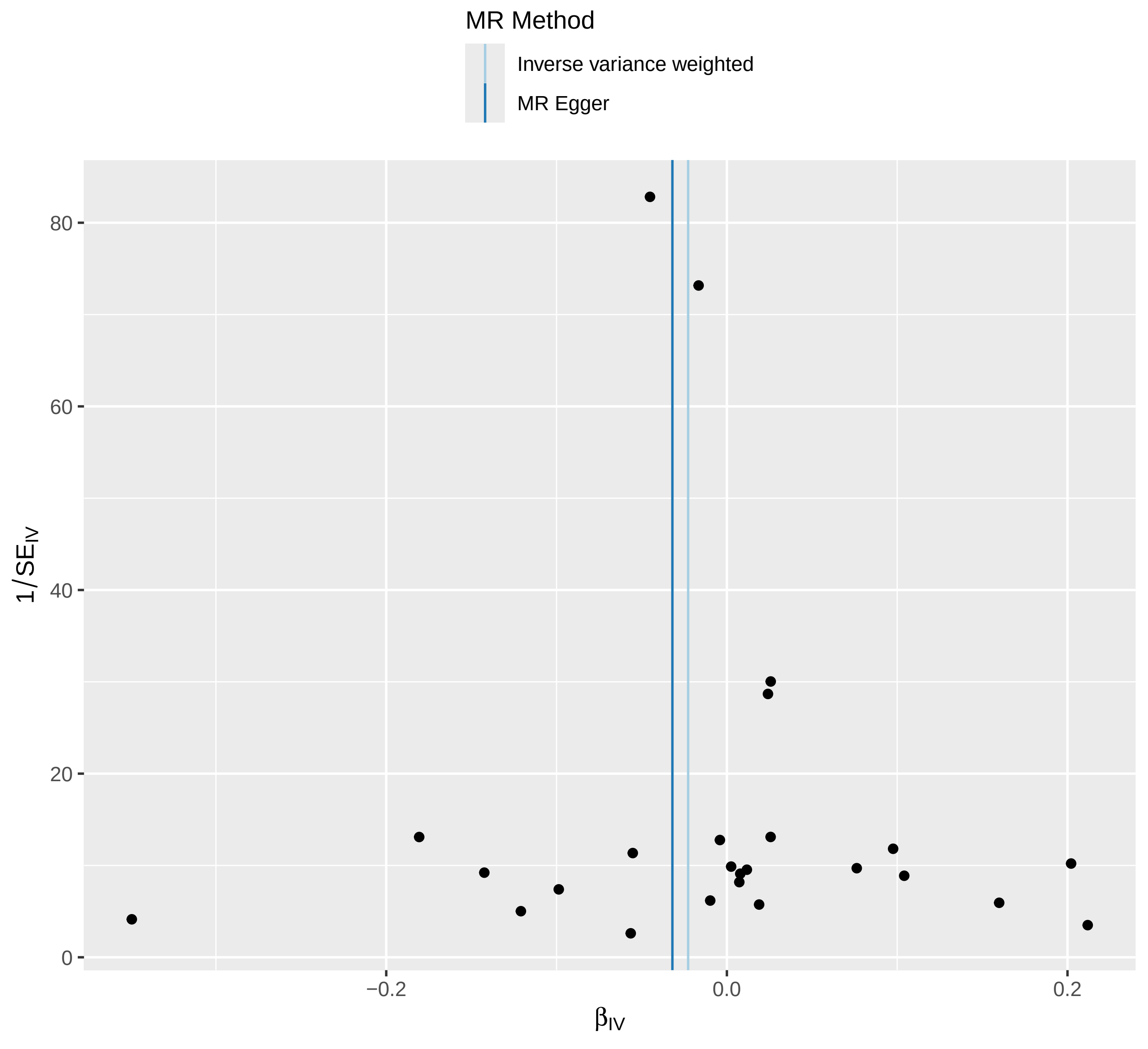
MR Method
Inverse variance weighted
MR Egger
1/SE_IV
80
60
40
20
0
−0.2
0.0
0.2
β_IV

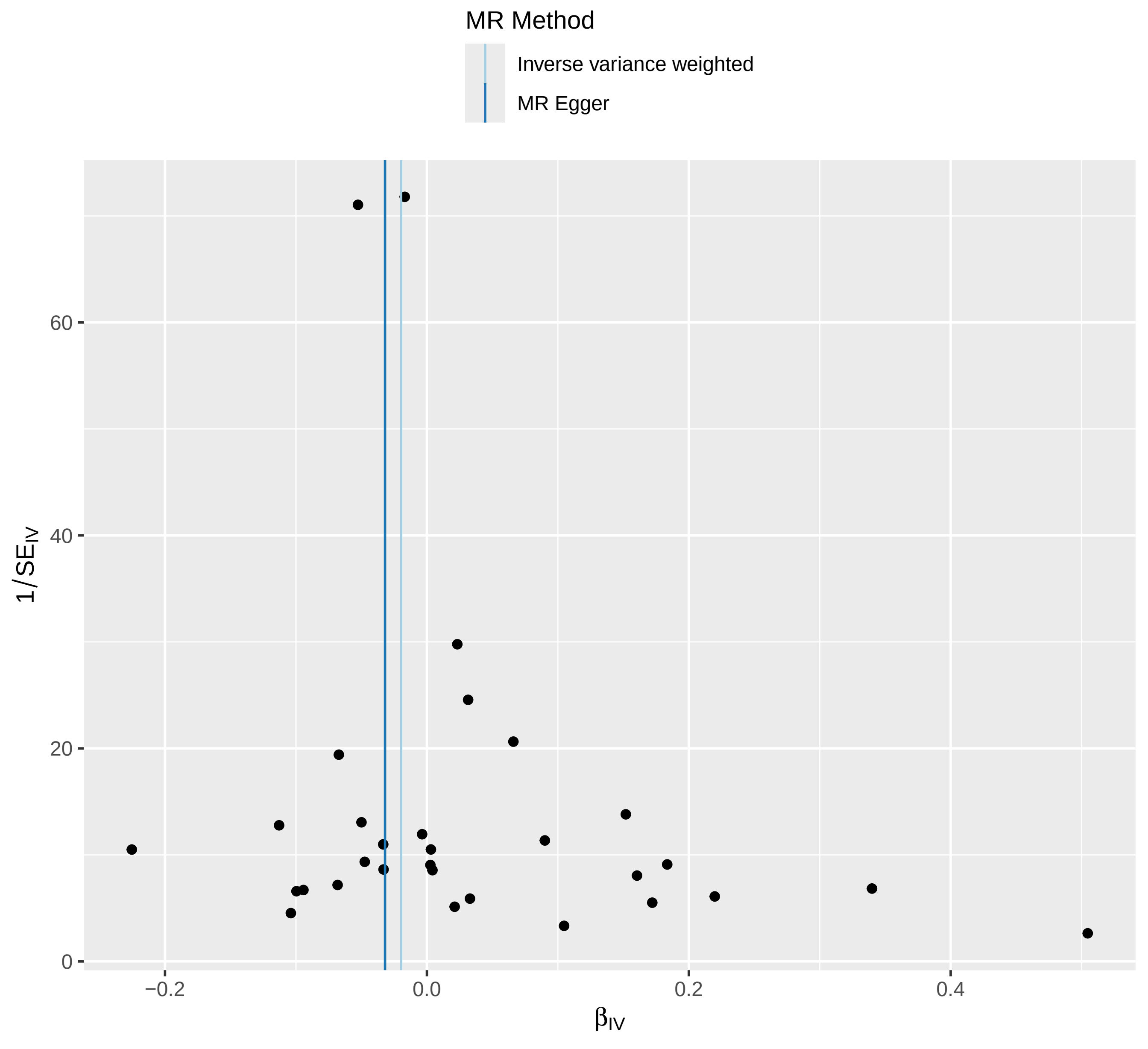
MR Method
Inverse variance weighted
MR Egger
60
40
20
0
1/SE_IV
−0.2
0.0
0.2
0.4
β_IV

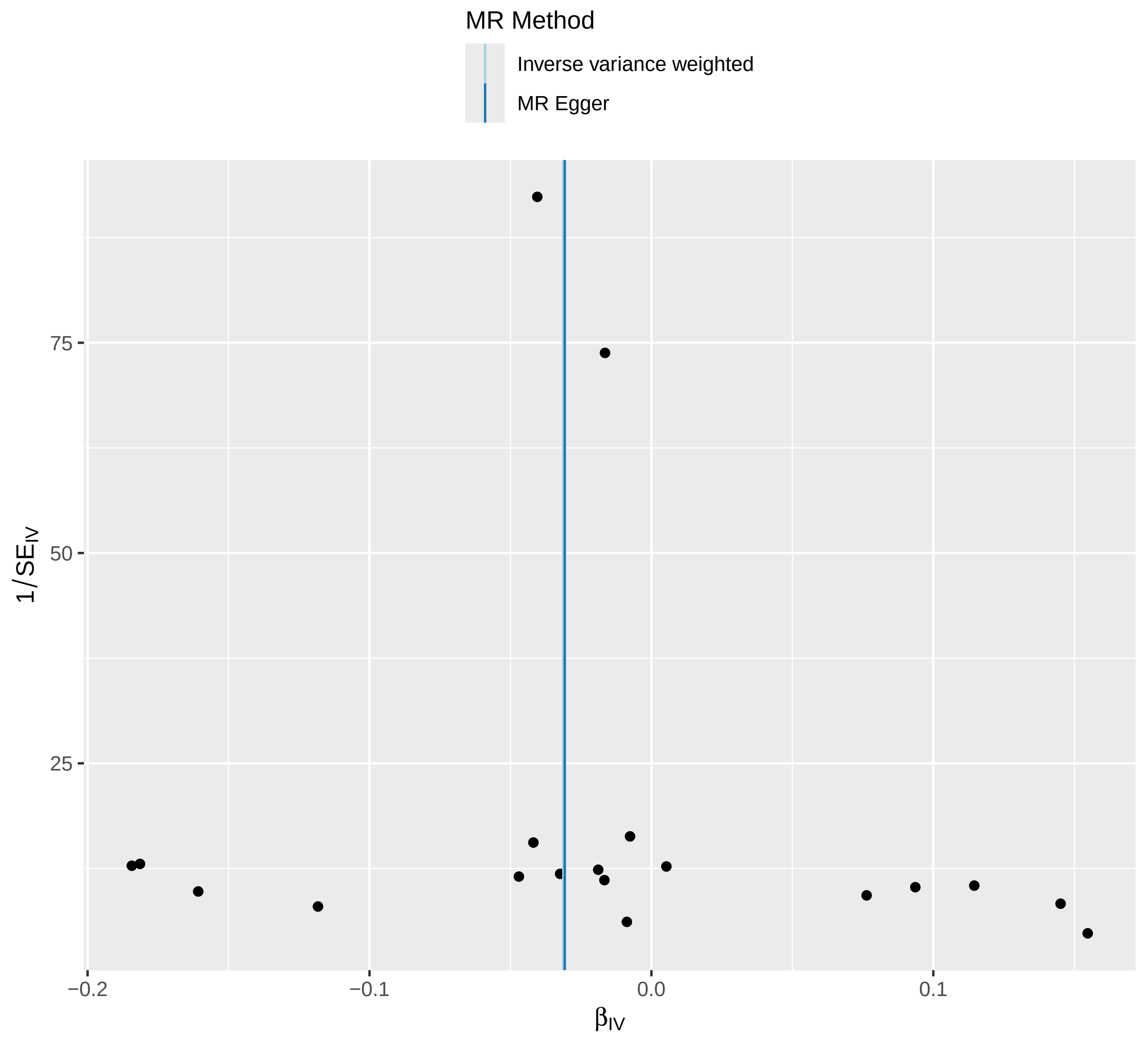

MR Method
Inverse variance weighted
MR Egger
1/SE_IV
75
50
25
−0.2
−0.1
0.0
0.1
β_IV

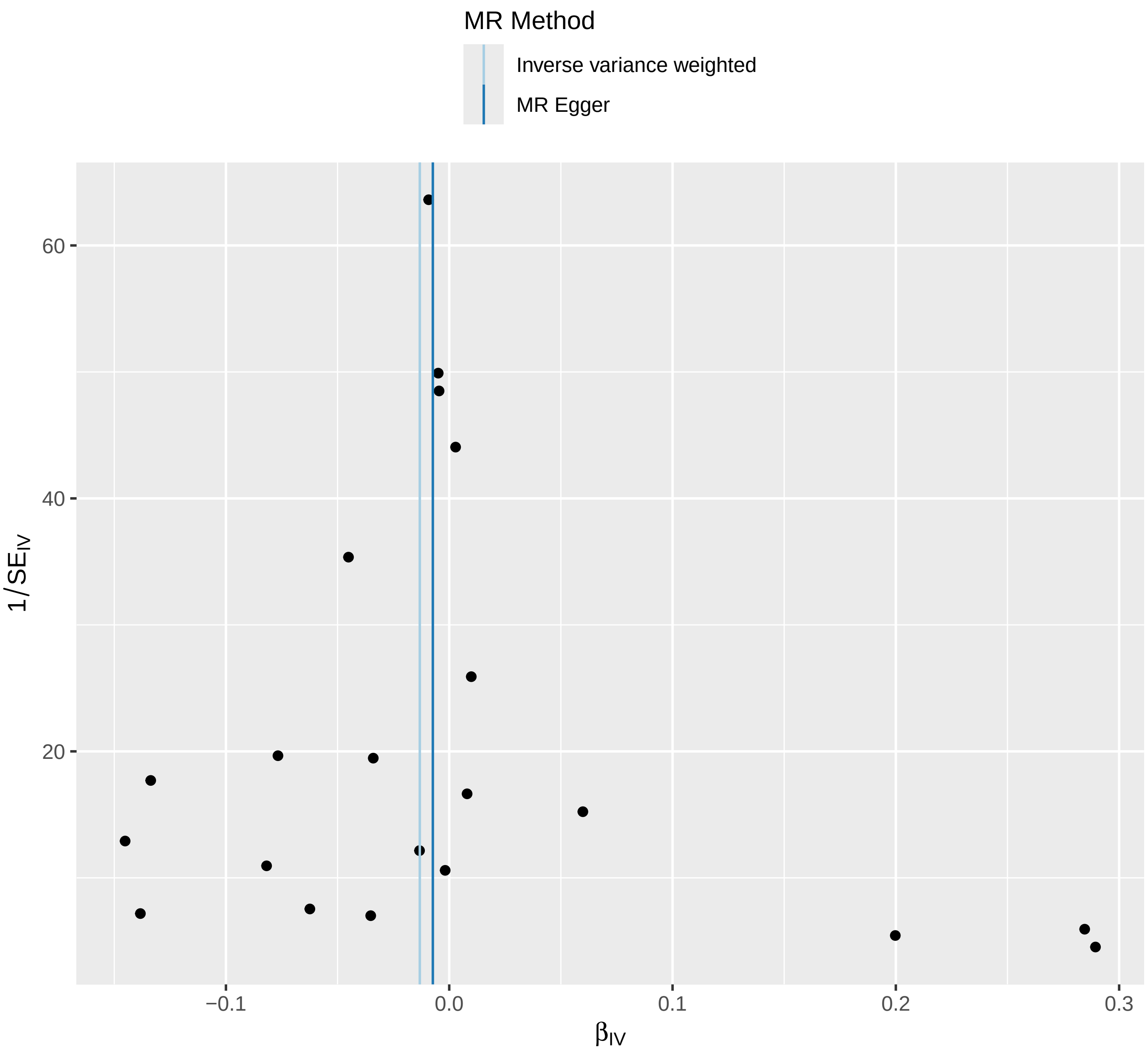
MR Method
Inverse variance weighted
MR Egger
60
40
20
1/SE_IV
−0.1
0.0
0.1
0.2
0.3
β_IV

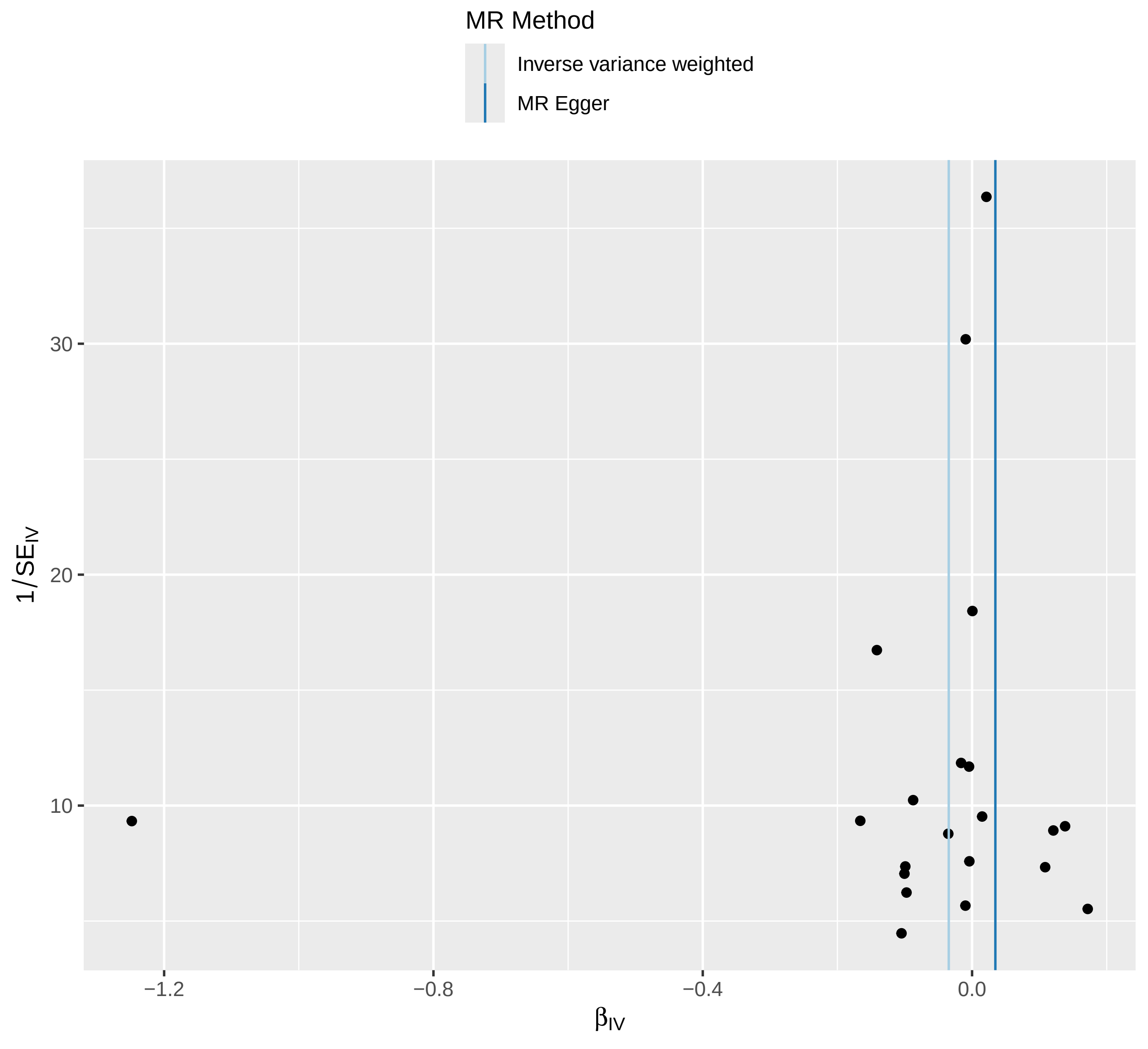
MR Method
Inverse variance weighted
MR Egger
30
20
10
1/SE_IV
−1.2
−0.8
−0.4
0.0
β_IV

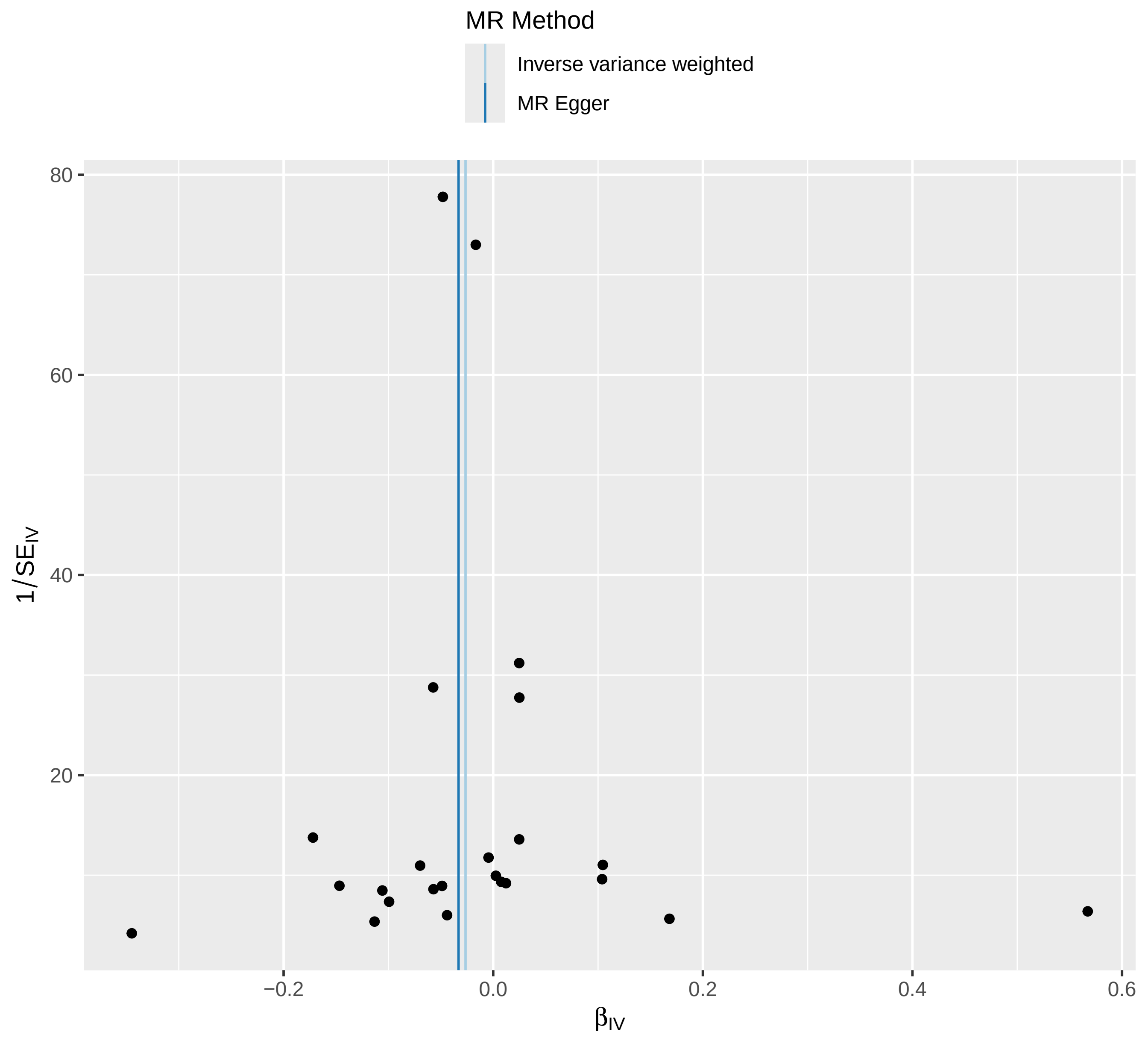

MR Method
Inverse variance weighted
MR Egger
80
60
40
20
$1/SE_{IV}$
−0.2
0.0
0.2
0.4
0.6
β_{IV}

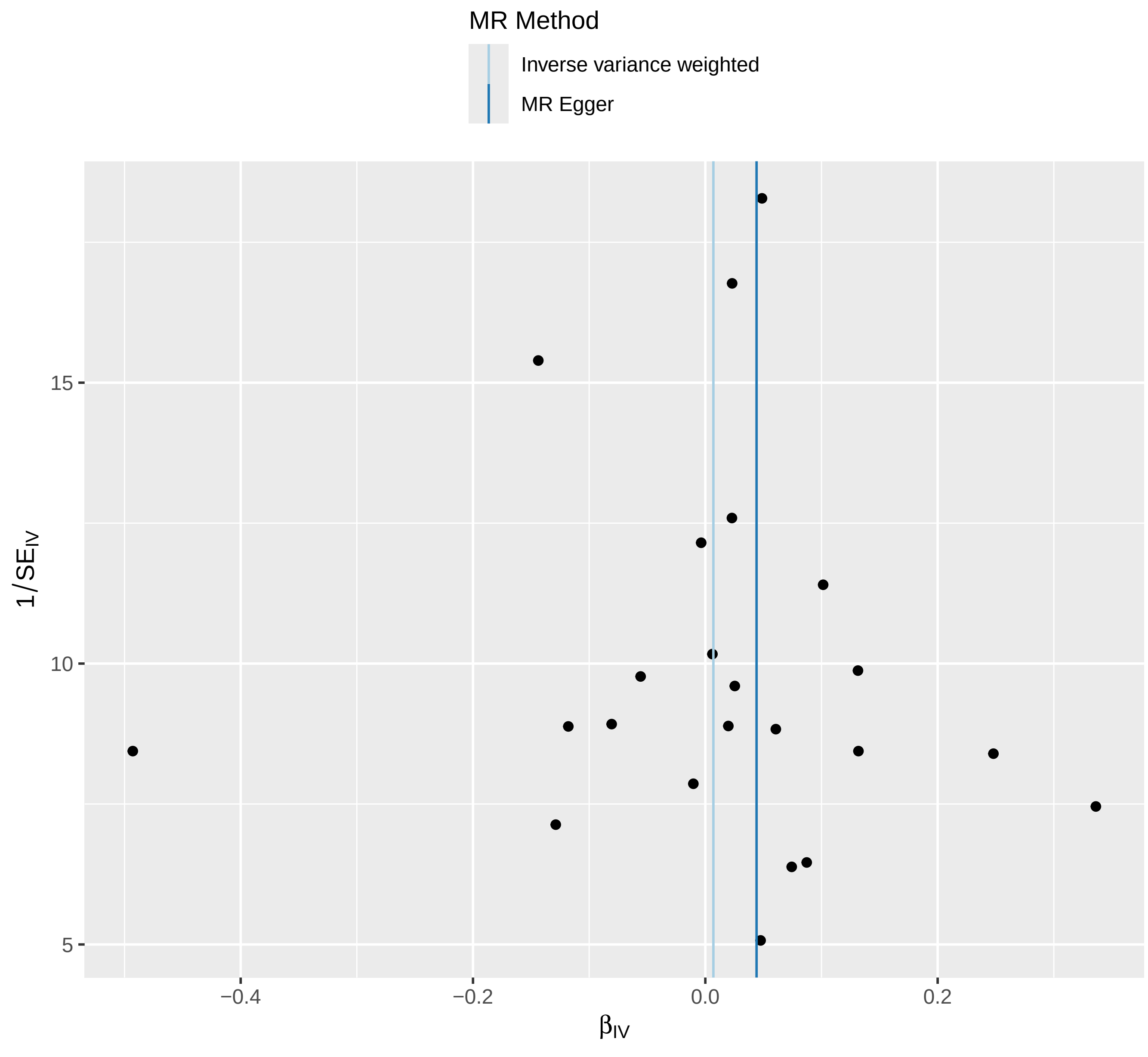

MR Method
Inverse variance weighted
MR Egger
15
10
5
1/SE_IV
−0.4
−0.2
0.0
0.2
β_IV

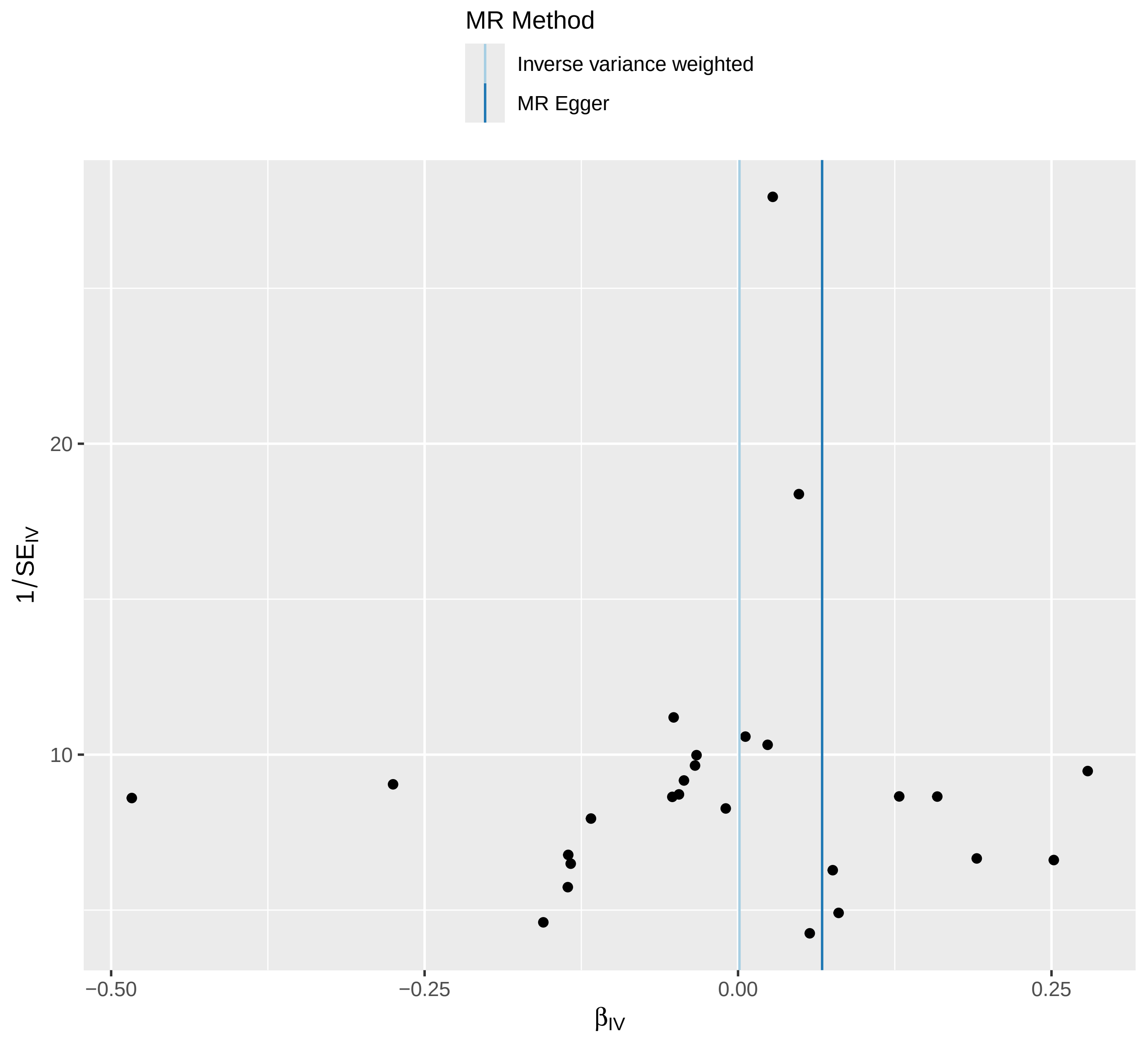
MR Method
Inverse variance weighted
MR Egger
20
10
$1/SE_{IV}$
−0.50
−0.25
0.00
0.25
β_{IV}

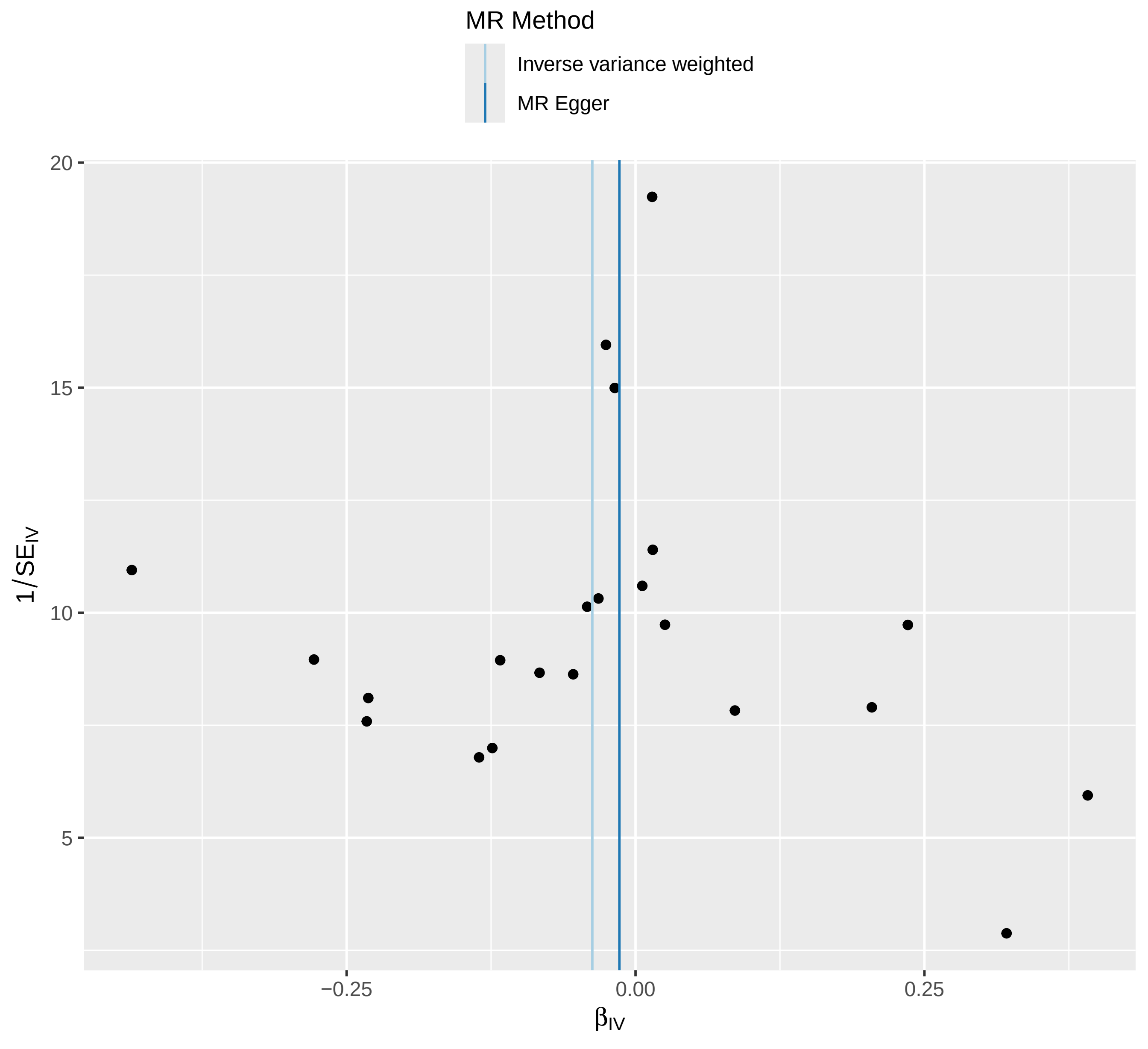
MR Method
Inverse variance weighted
MR Egger
20
15
10
5
$1/SE_{IV}$
−0.25
0.00
0.25
β_{IV}

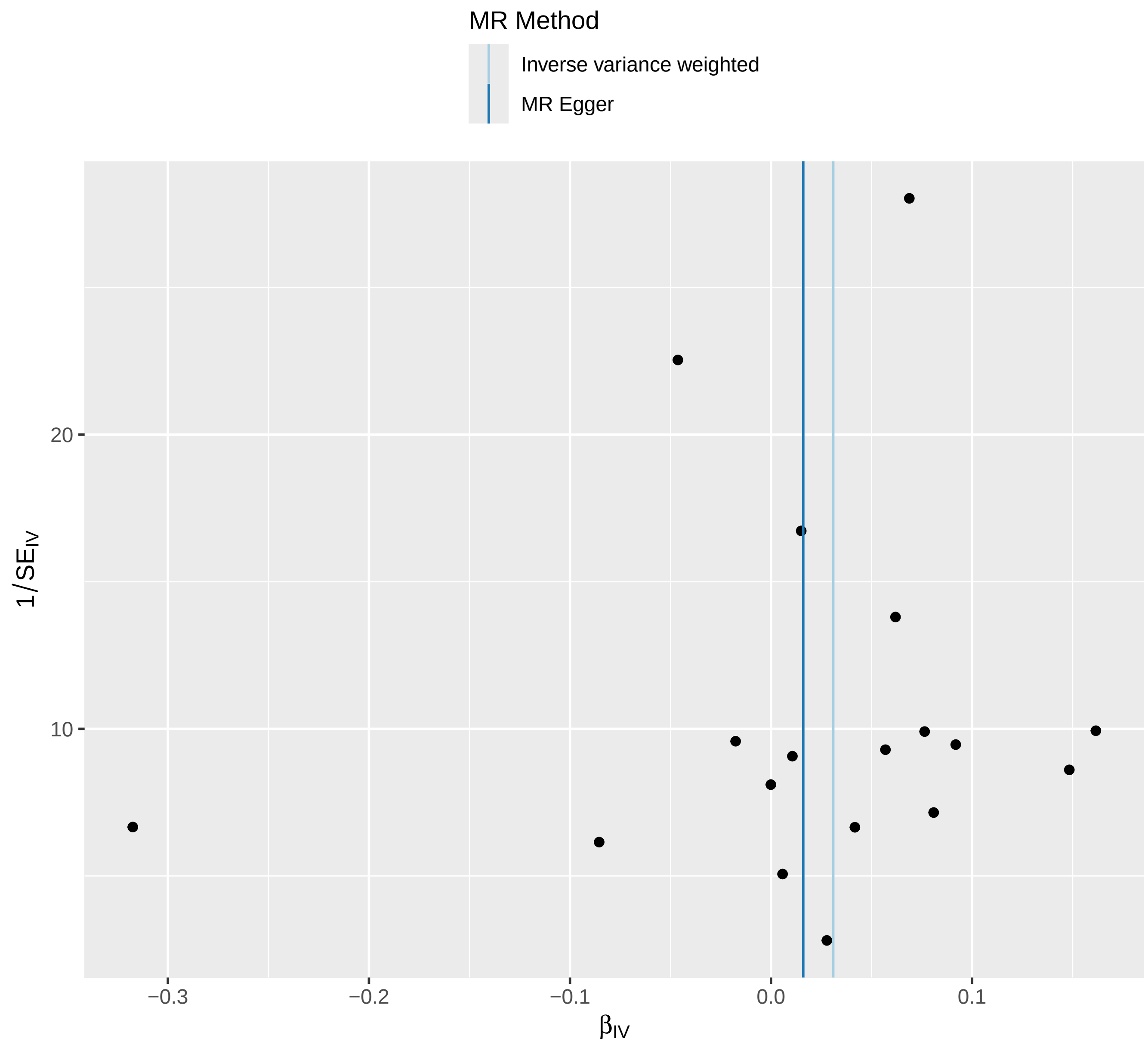

MR Method
Inverse variance weighted
MR Egger
20
10
$1/SE_{IV}$
−0.3
−0.2
−0.1
0.0
0.1
β_{IV}

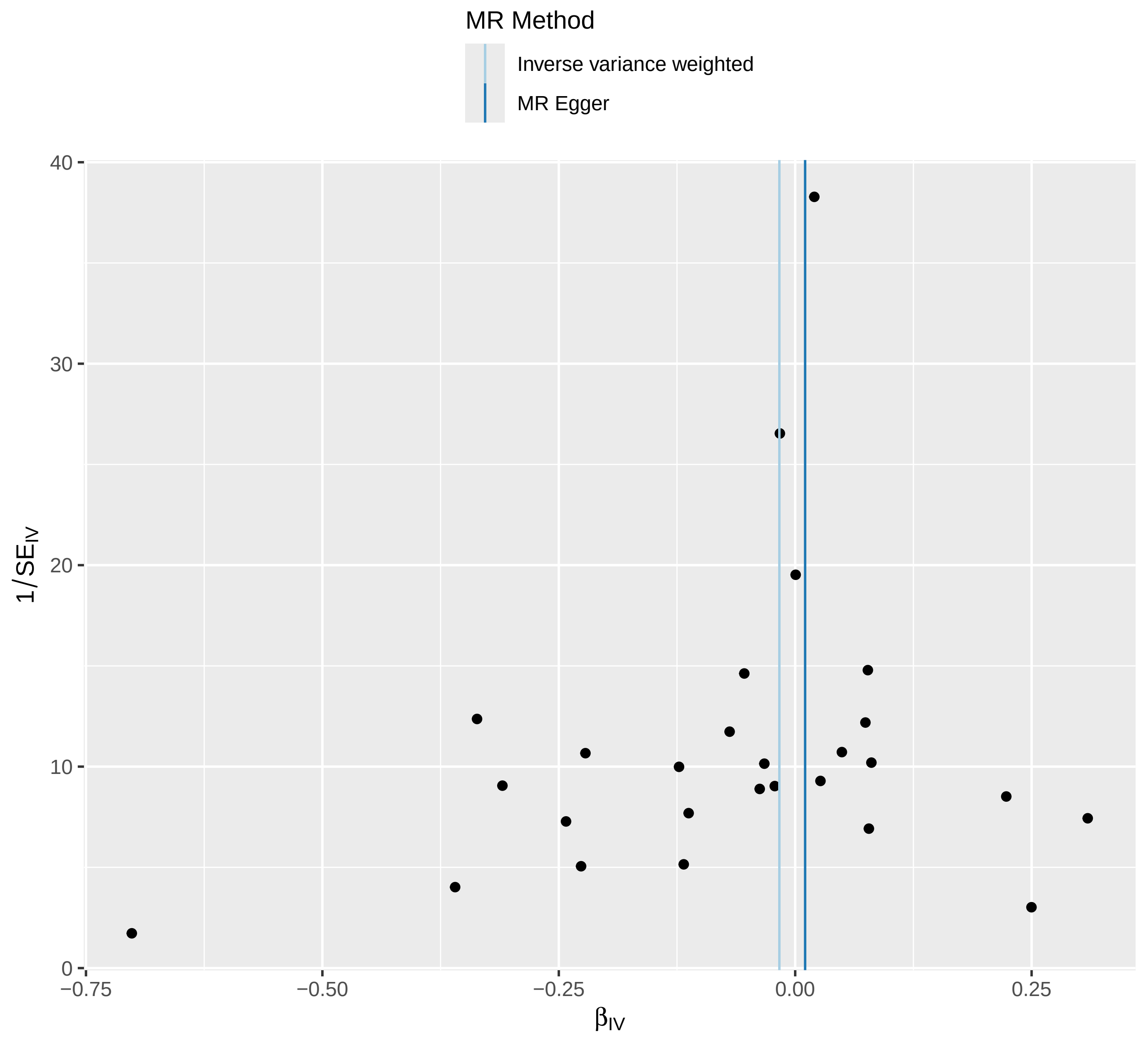
MR Method
Inverse variance weighted
MR Egger
40
30
20
10
0
1/SE_IV
−0.75
−0.50
−0.25
0.00
0.25
β_IV

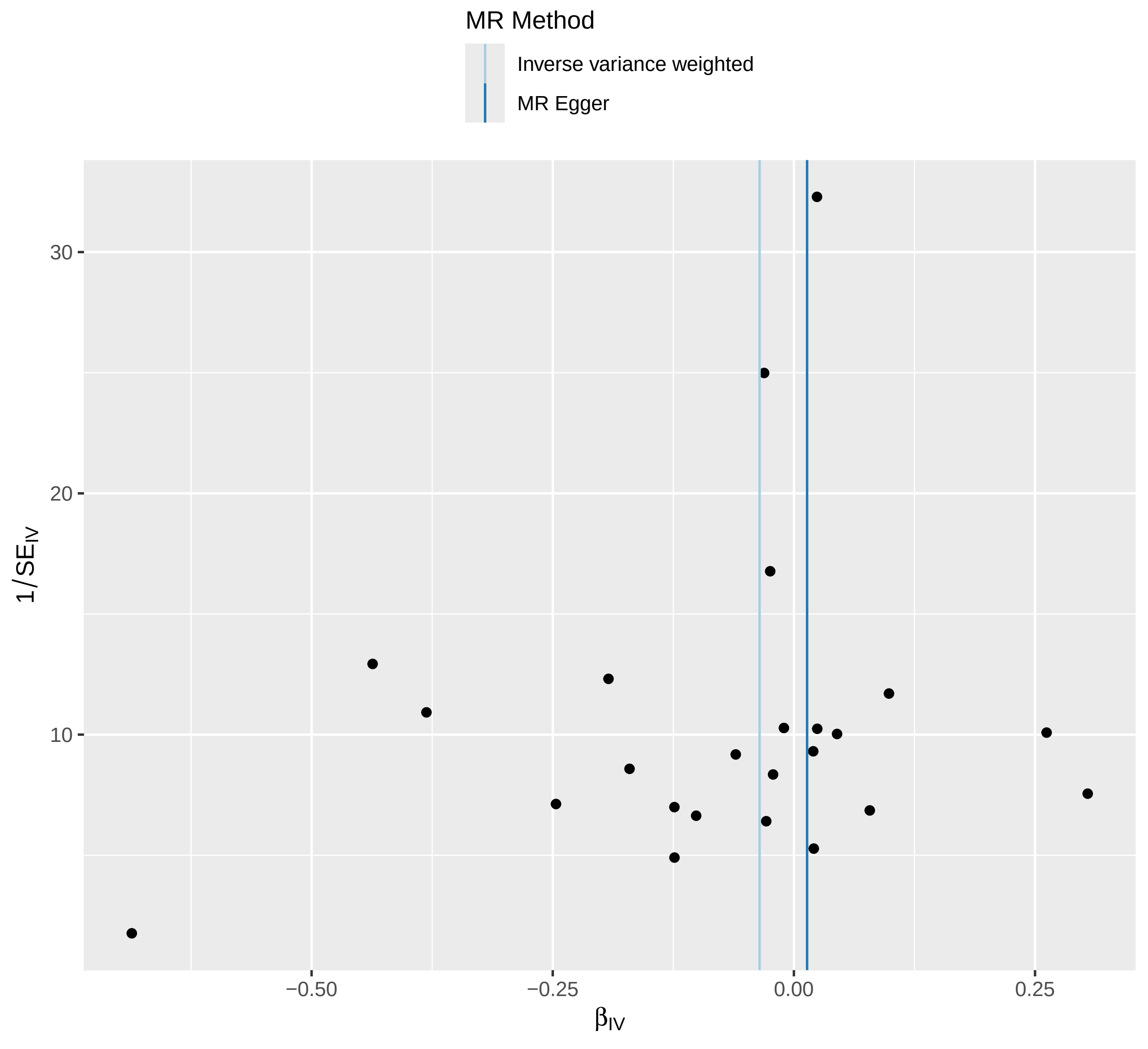

MR Method
Inverse variance weighted
MR Egger
30
20
10
1/SE$_{IV}$
−0.50
−0.25
0.00
0.25
β$_{IV}$

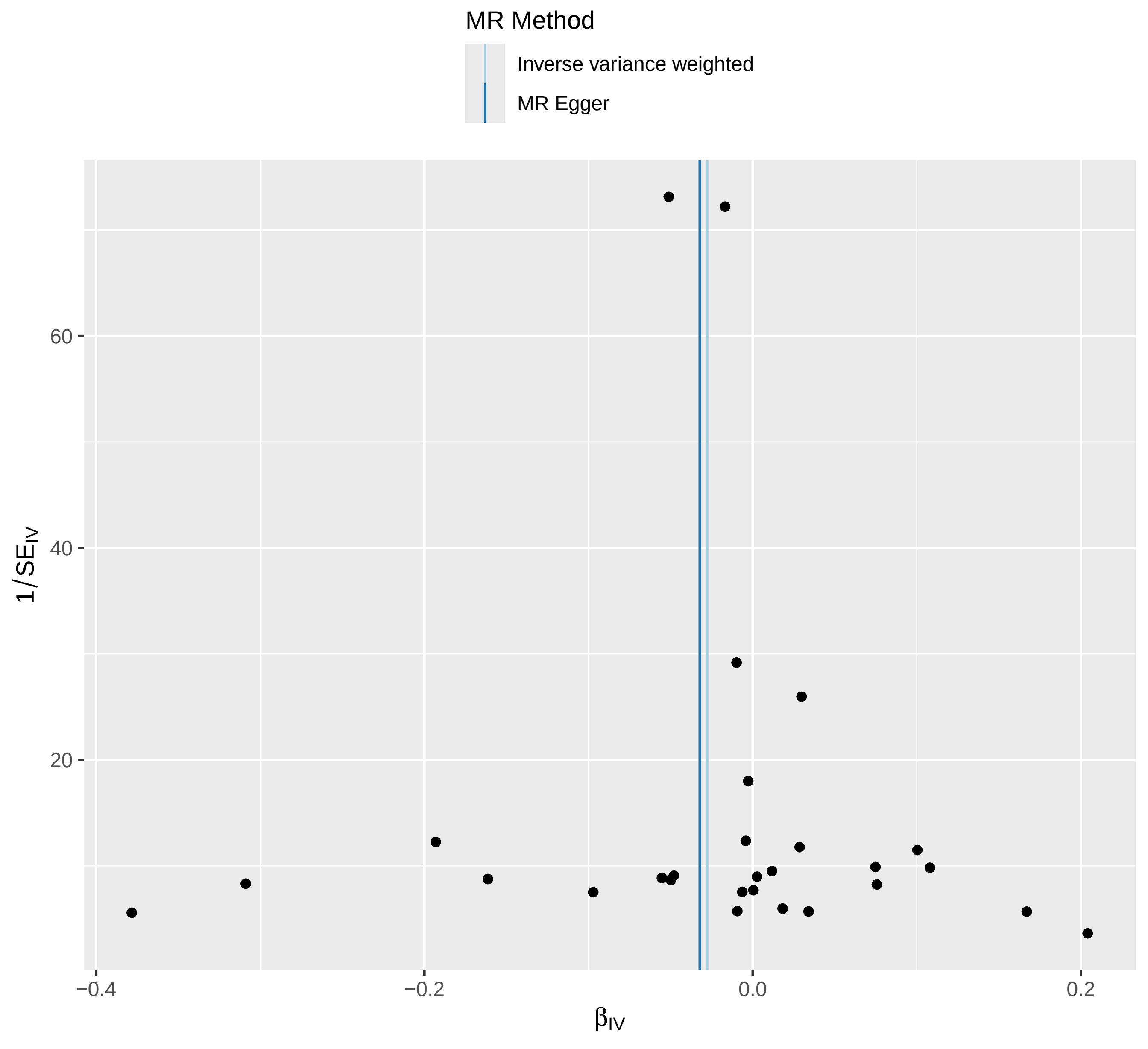
MR Method
Inverse variance weighted
MR Egger
60
40
20
1/SE$_{IV}$
−0.4
−0.2
0.0
0.2
β$_{IV}$

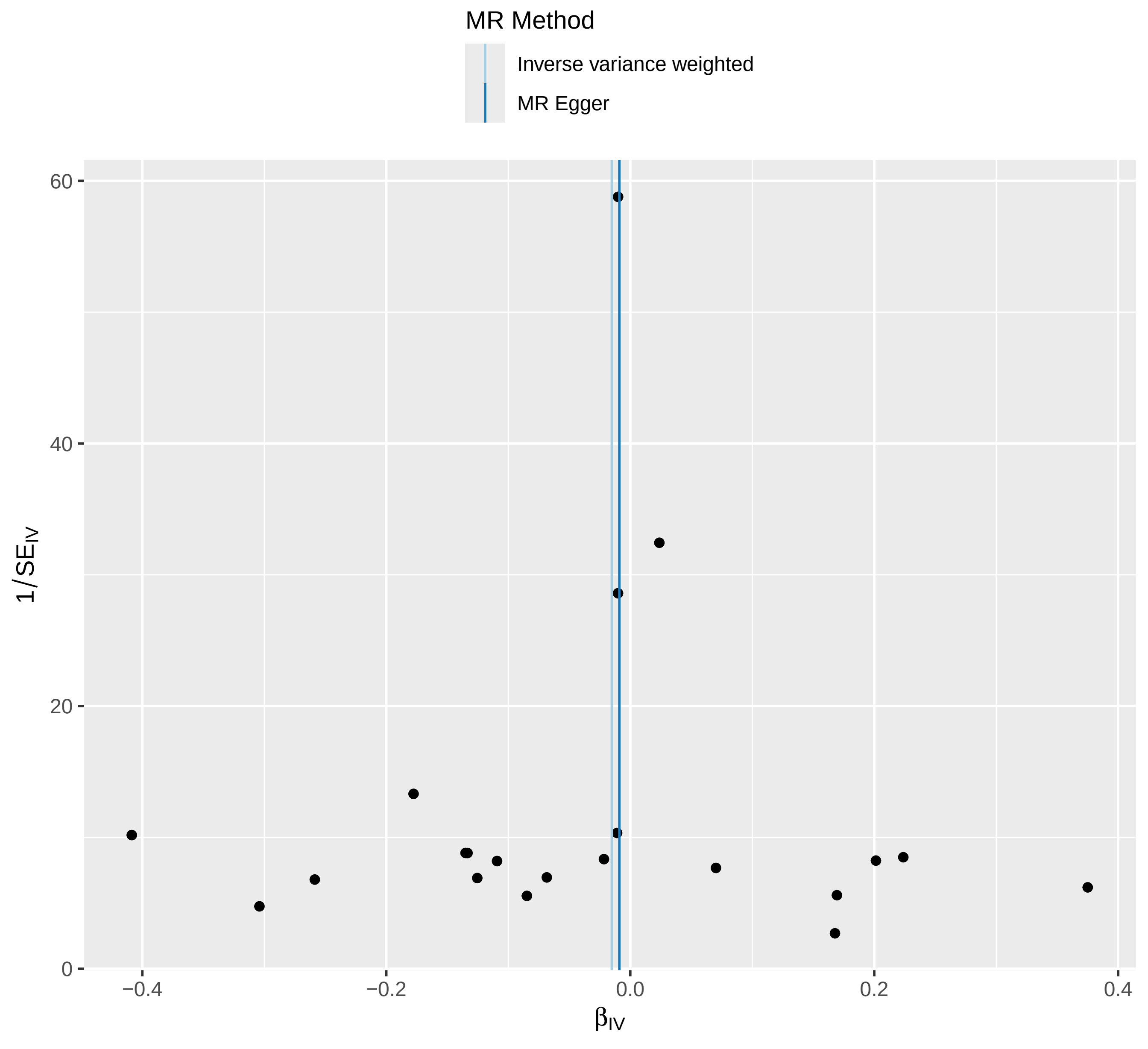
MR Method
Inverse variance weighted
MR Egger
60
40
20
0
1/SE_IV
−0.4
−0.2
0.0
0.2
0.4
β_IV

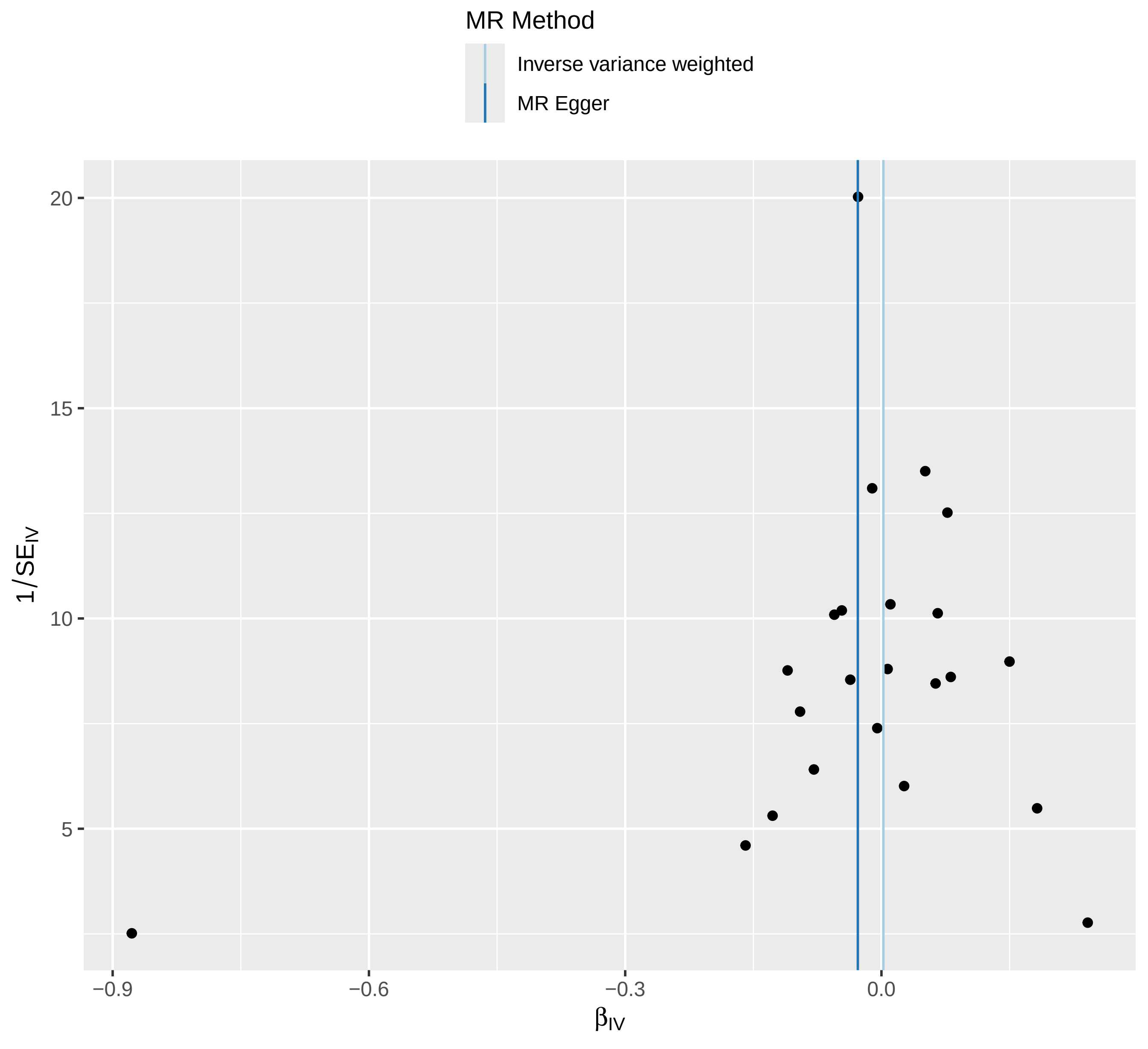
MR Method
Inverse variance weighted
MR Egger
20
15
10
5
1/SE_IV
−0.9
−0.6
−0.3
0.0
β_IV

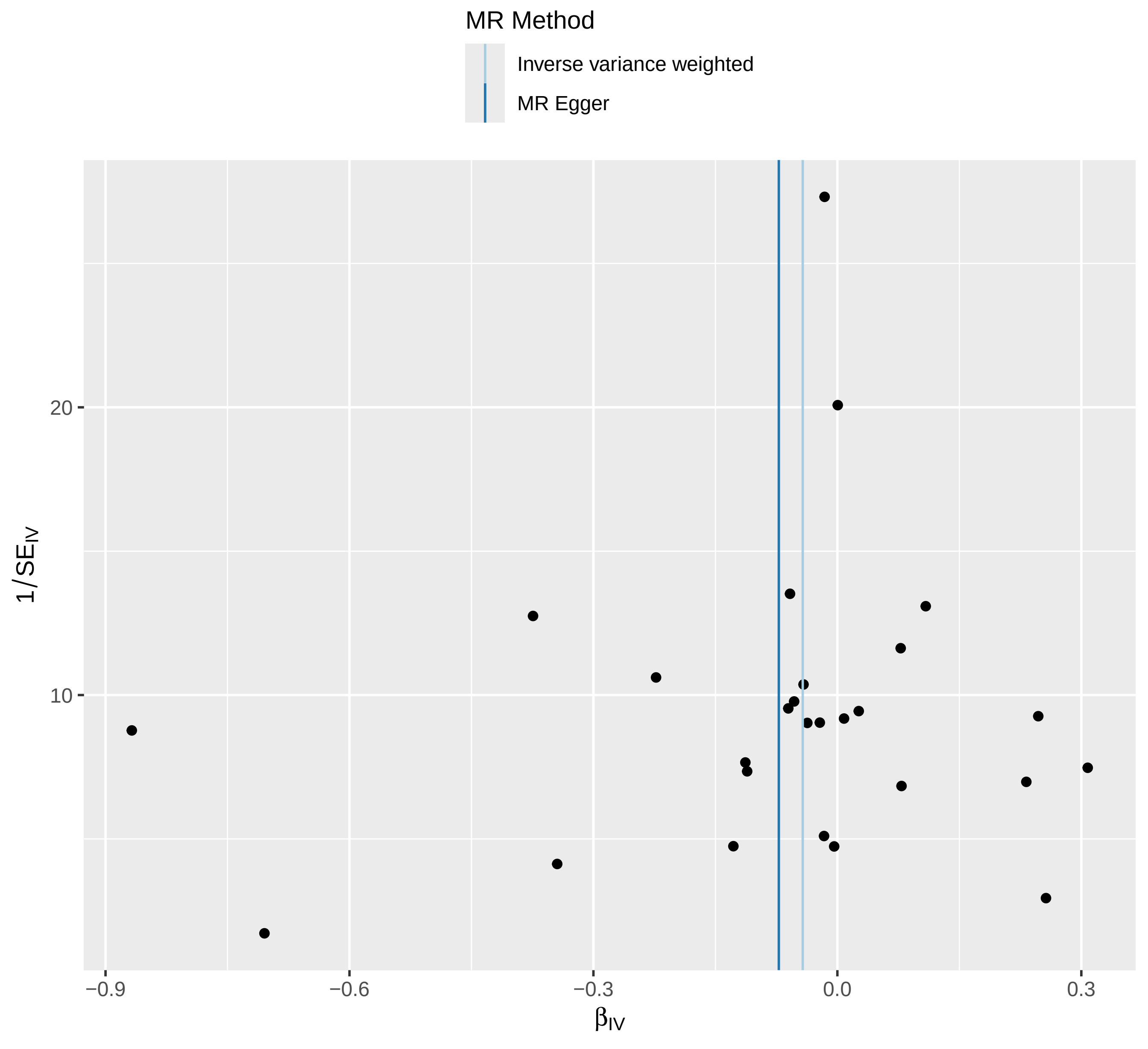

MR Method
Inverse variance weighted
MR Egger
20
10
1/SE_IV
−0.9
−0.6
−0.3
0.0
0.3
β_IV

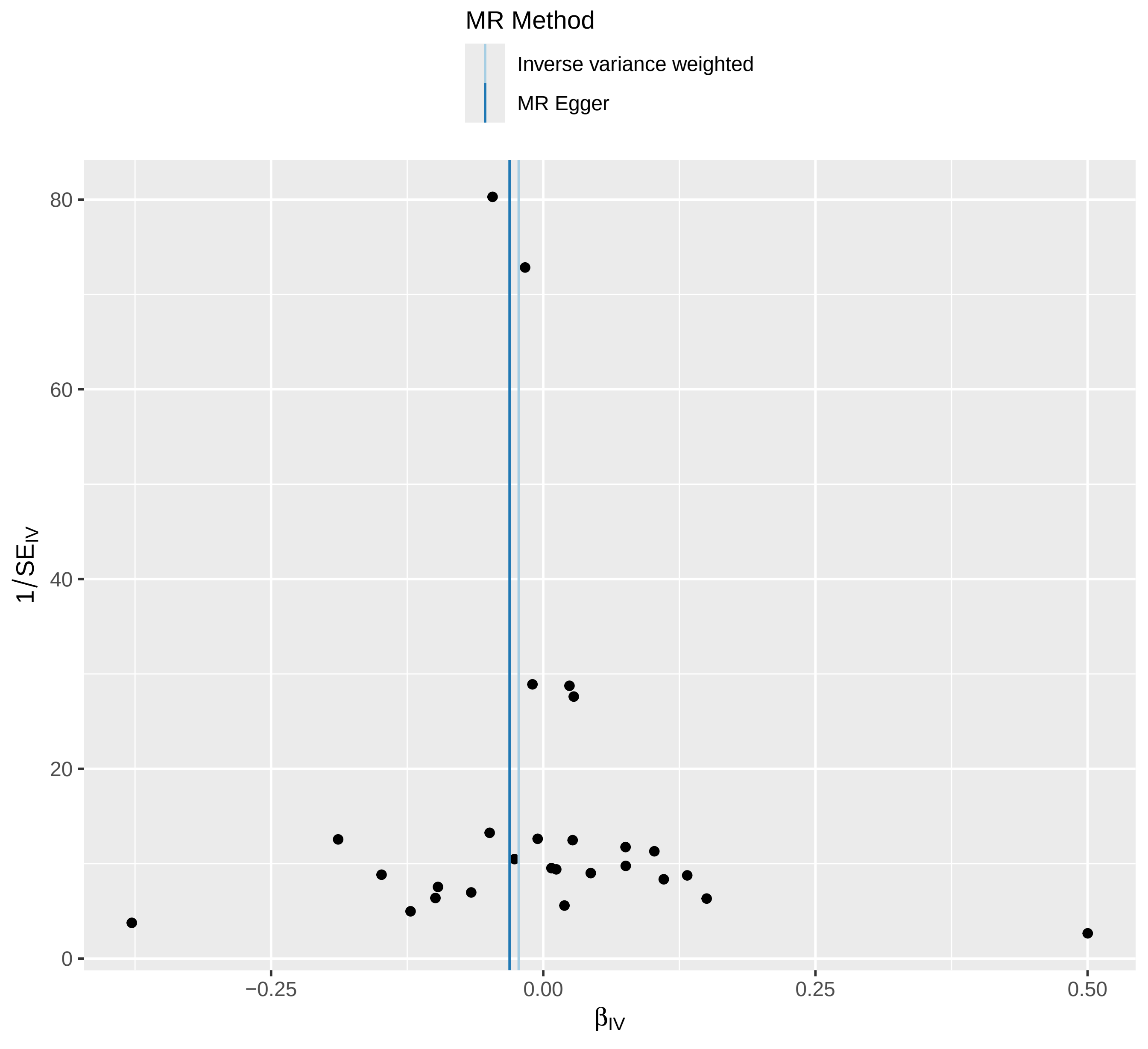

MR Method
Inverse variance weighted
MR Egger
80
60
40
20
0
$1/SE_{IV}$
−0.25
0.00
0.25
0.50
β_{IV}

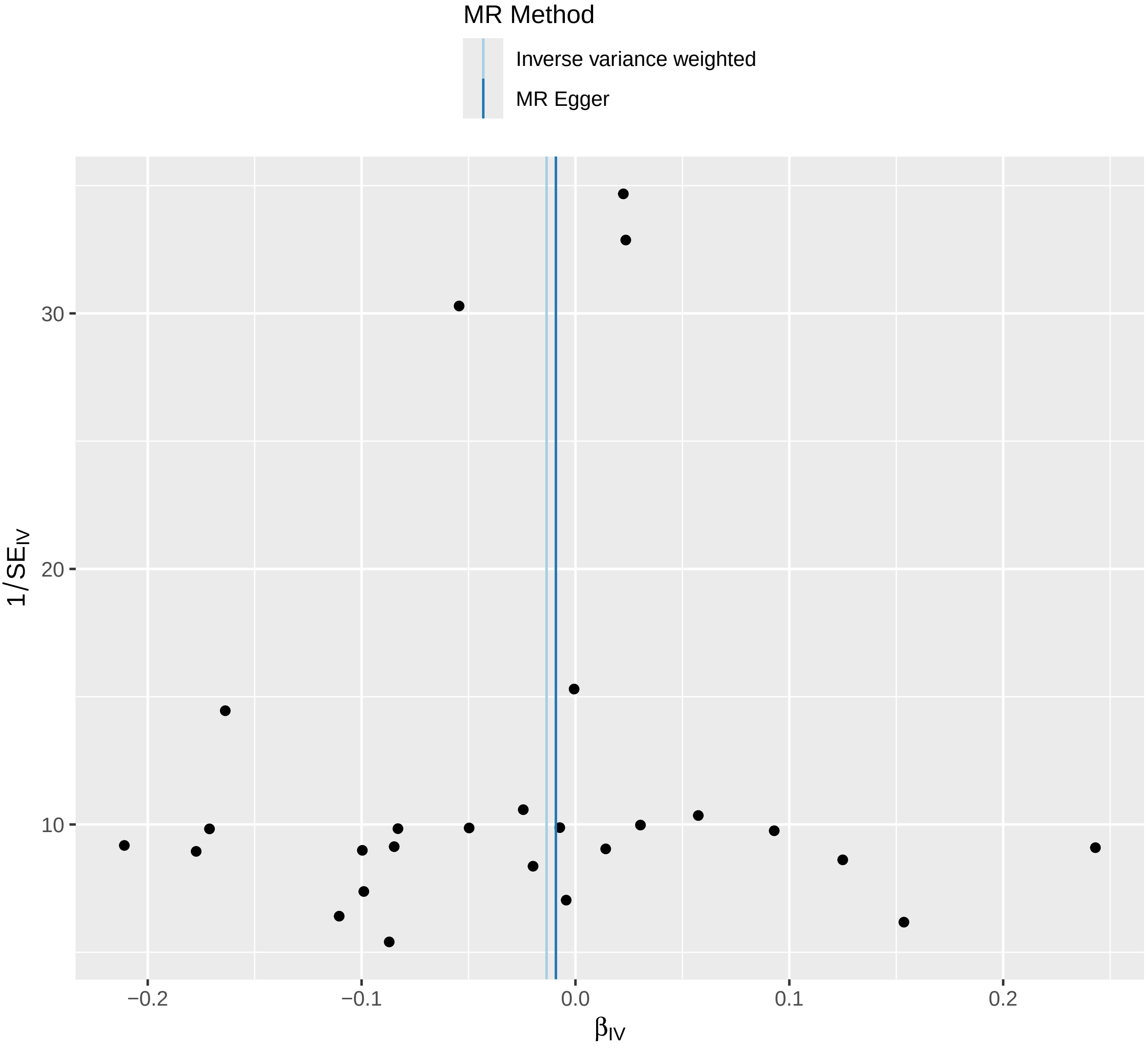
MR Method
Inverse variance weighted
MR Egger
30
20
10
1/SE$_{IV}$
−0.2
−0.1
0.0
0.1
0.2
β$_{IV}$

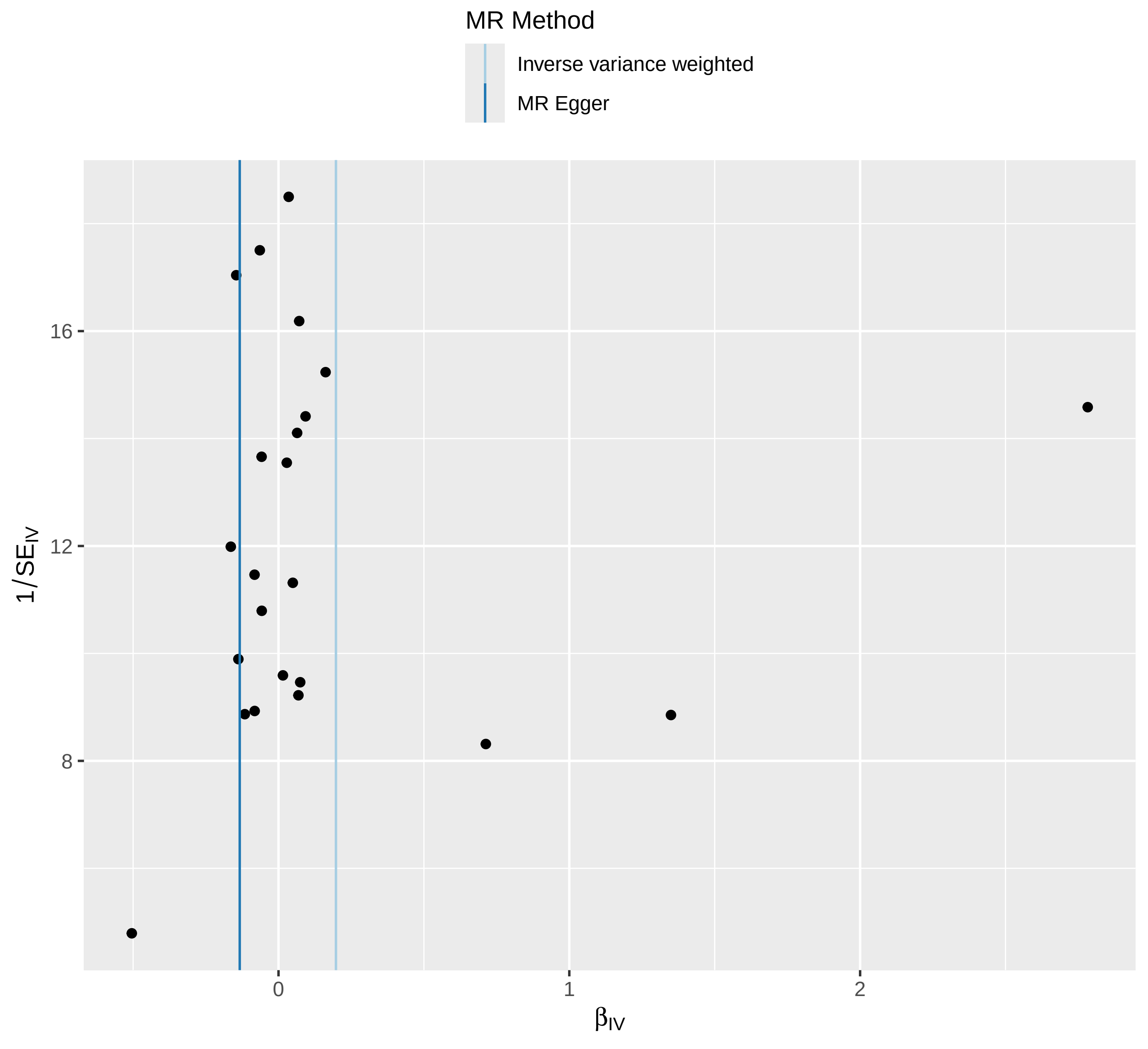
MR Method
Inverse variance weighted
MR Egger
16
12
8
1/SE$_{IV}$
0
1
2
β$_{IV}$

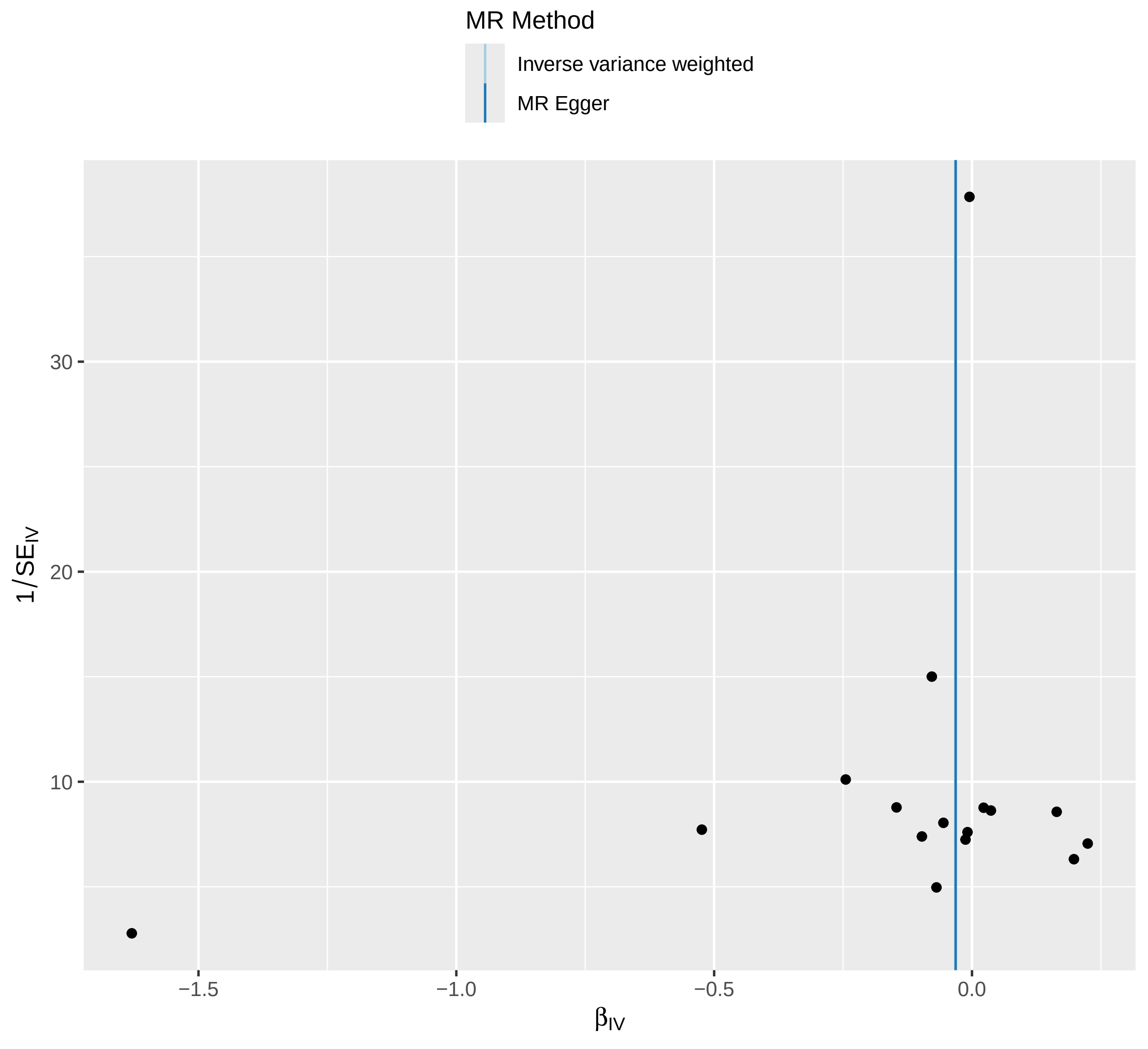
MR Method
Inverse variance weighted
MR Egger
1/SE_IV
30
20
10
−1.5
−1.0
−0.5
0.0
β_IV

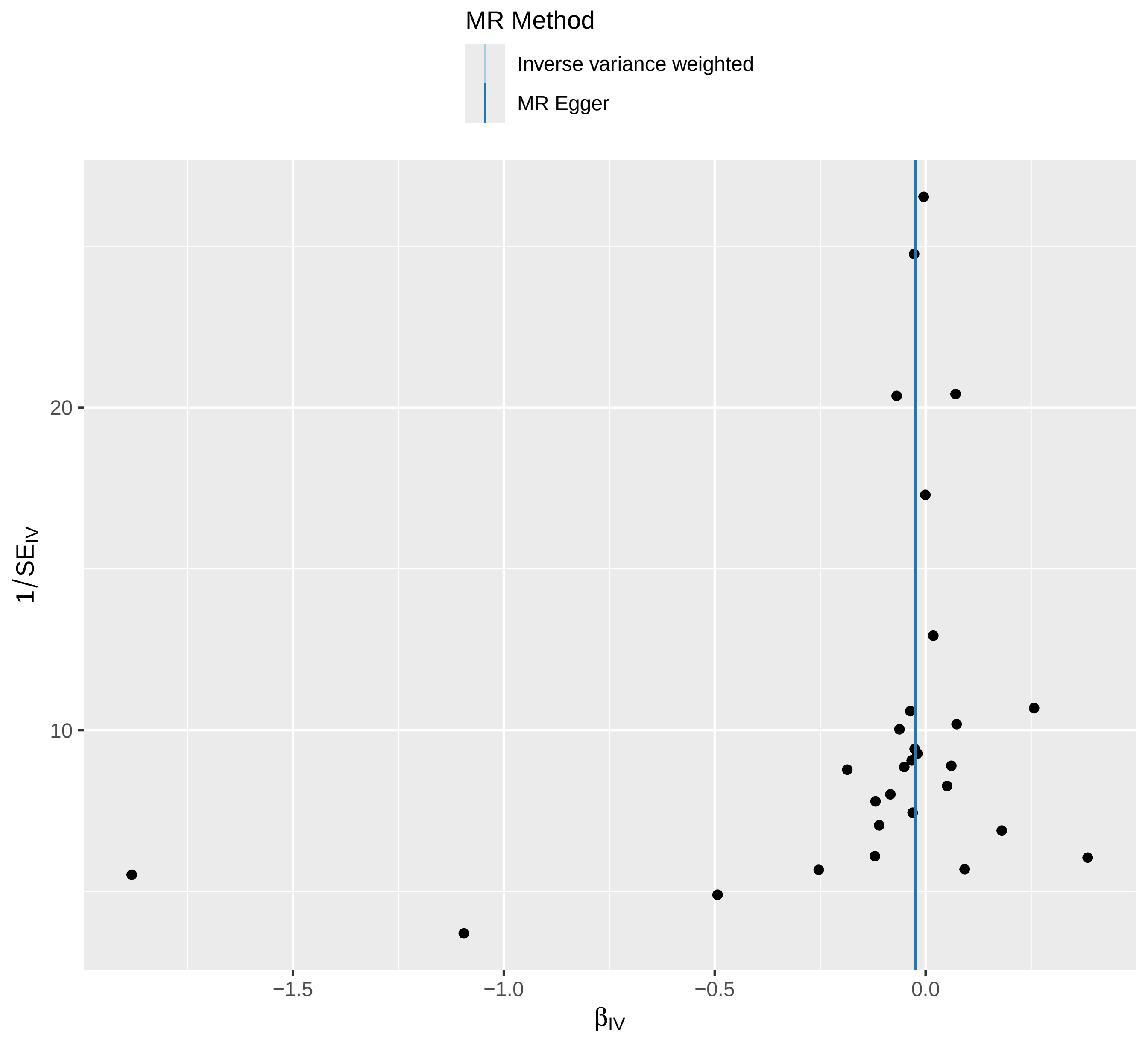

MR Method
Inverse variance weighted
MR Egger
20
10
$1/SE_{IV}$
−1.5
−1.0
−0.5
0.0
β_{IV}

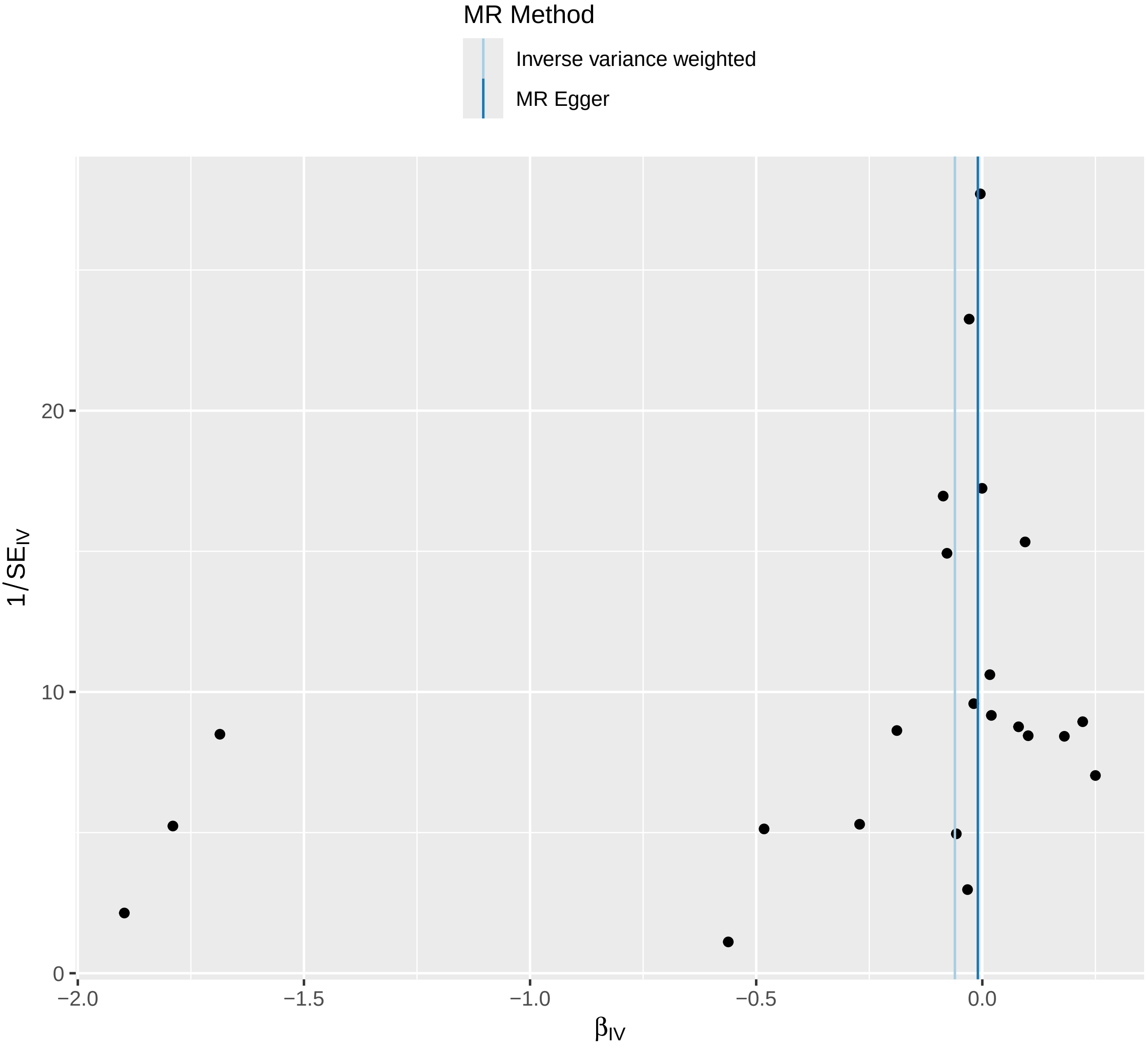
MR Method
Inverse variance weighted
MR Egger
1/SE_IV
20
10
0
−2.0
−1.5
−1.0
−0.5
0.0
β_IV

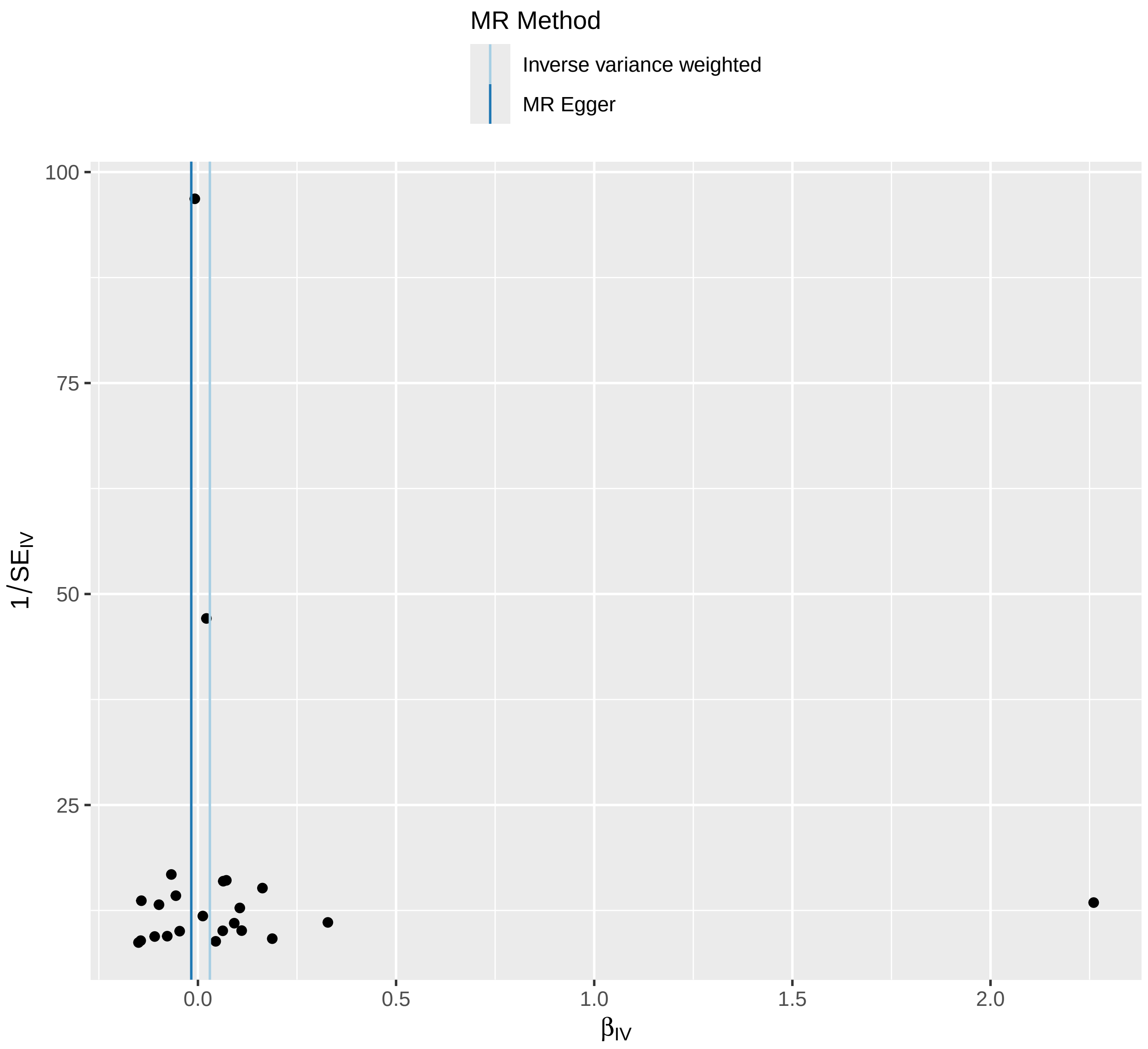
MR Method
Inverse variance weighted
MR Egger
100
75
50
25
1/SE_IV
0.0
0.5
1.0
1.5
2.0
β_IV

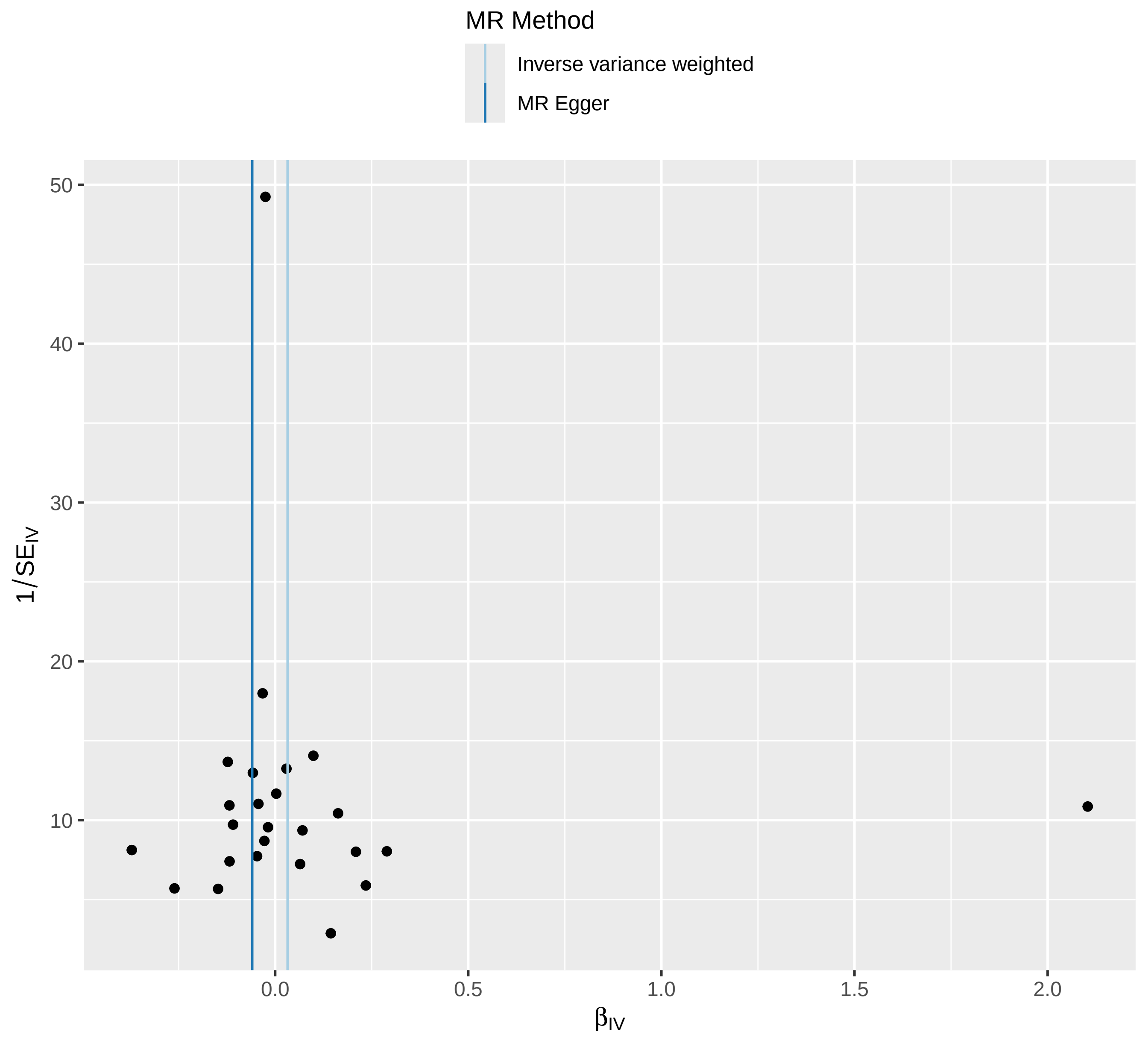

MR Method
Inverse variance weighted
MR Egger
50
40
30
20
10
1/SE_IV
0.0
0.5
1.0
1.5
2.0
β_IV

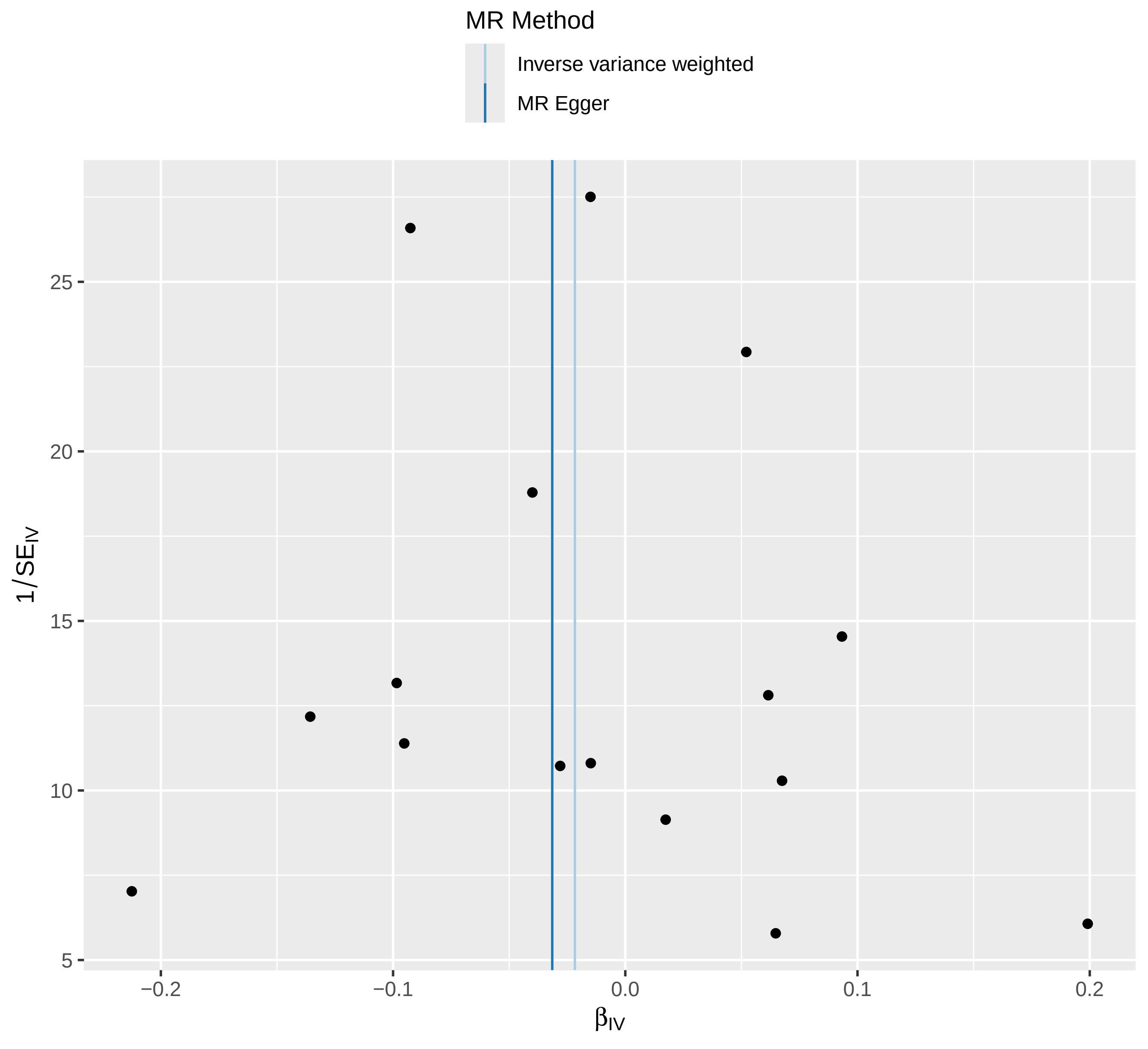
MR Method
Inverse variance weighted
MR Egger
1/SE_IV
25
20
15
10
5
−0.2
−0.1
0.0
0.1
0.2
β_IV

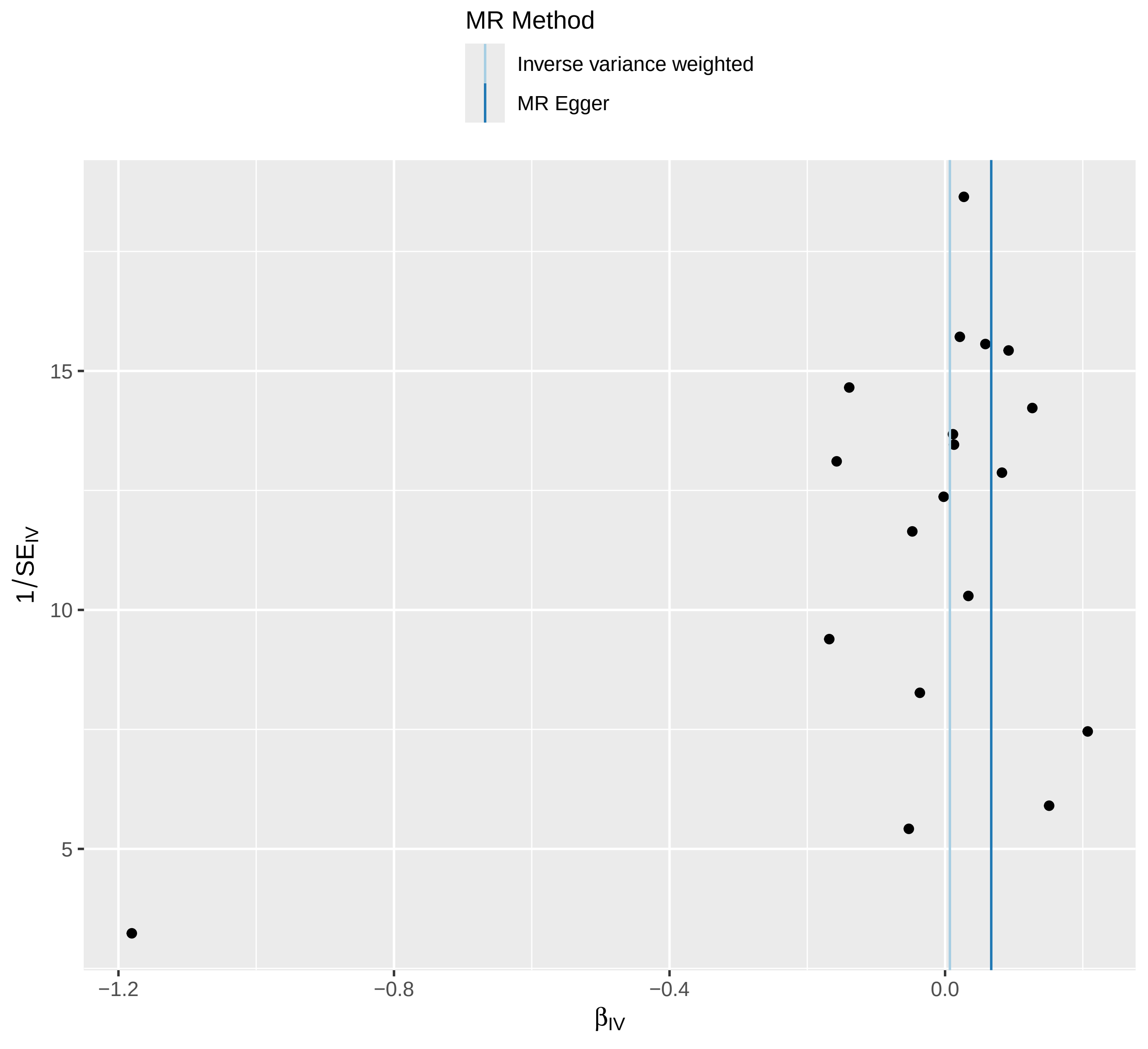
MR Method
Inverse variance weighted
MR Egger
15
10
5
1/SE_IV
−1.2
−0.8
−0.4
0.0
β_IV

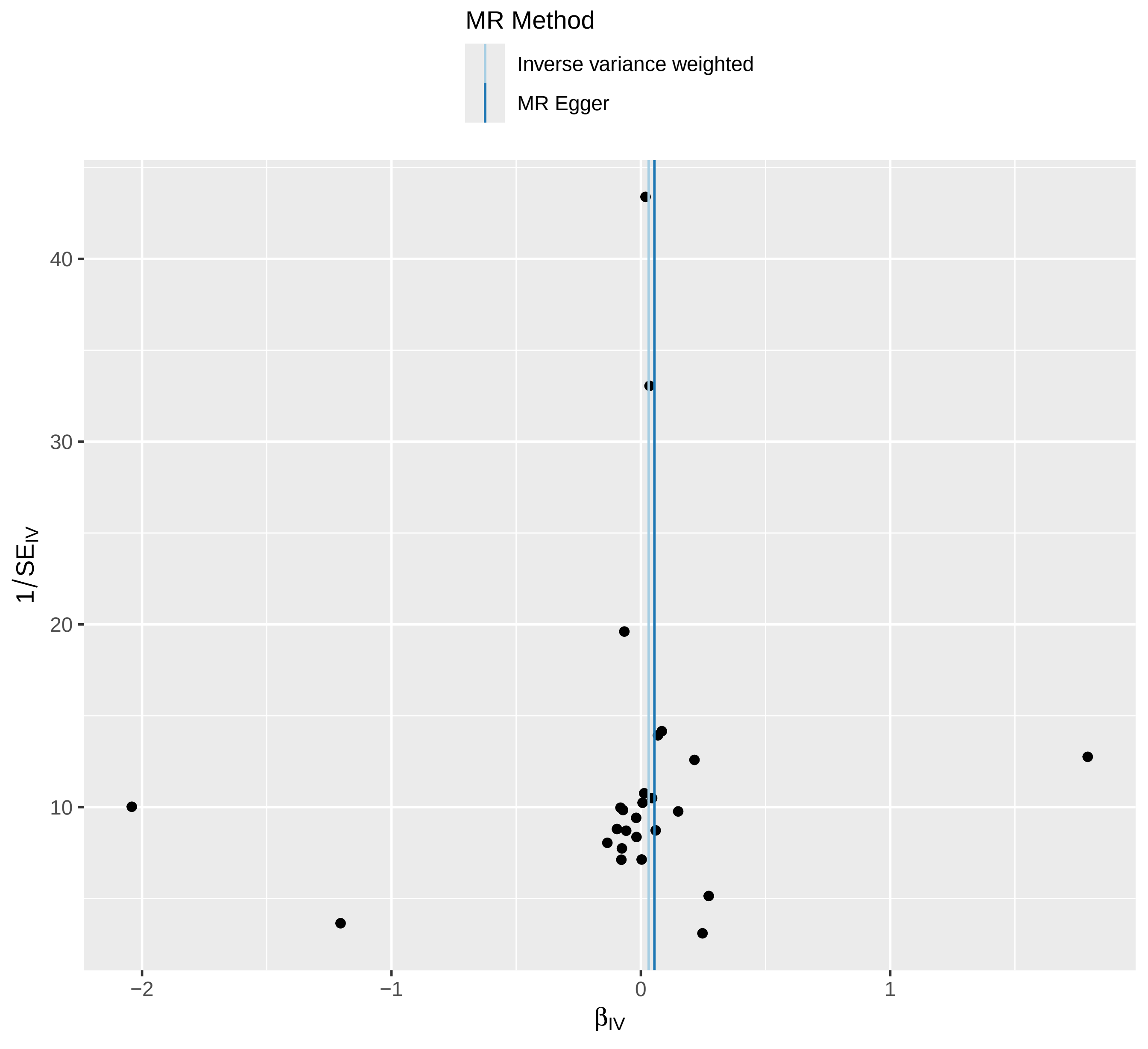
MR Method
Inverse variance weighted
MR Egger
1/SE_IV
40
30
20
10
β_IV
−2
−1
0
1

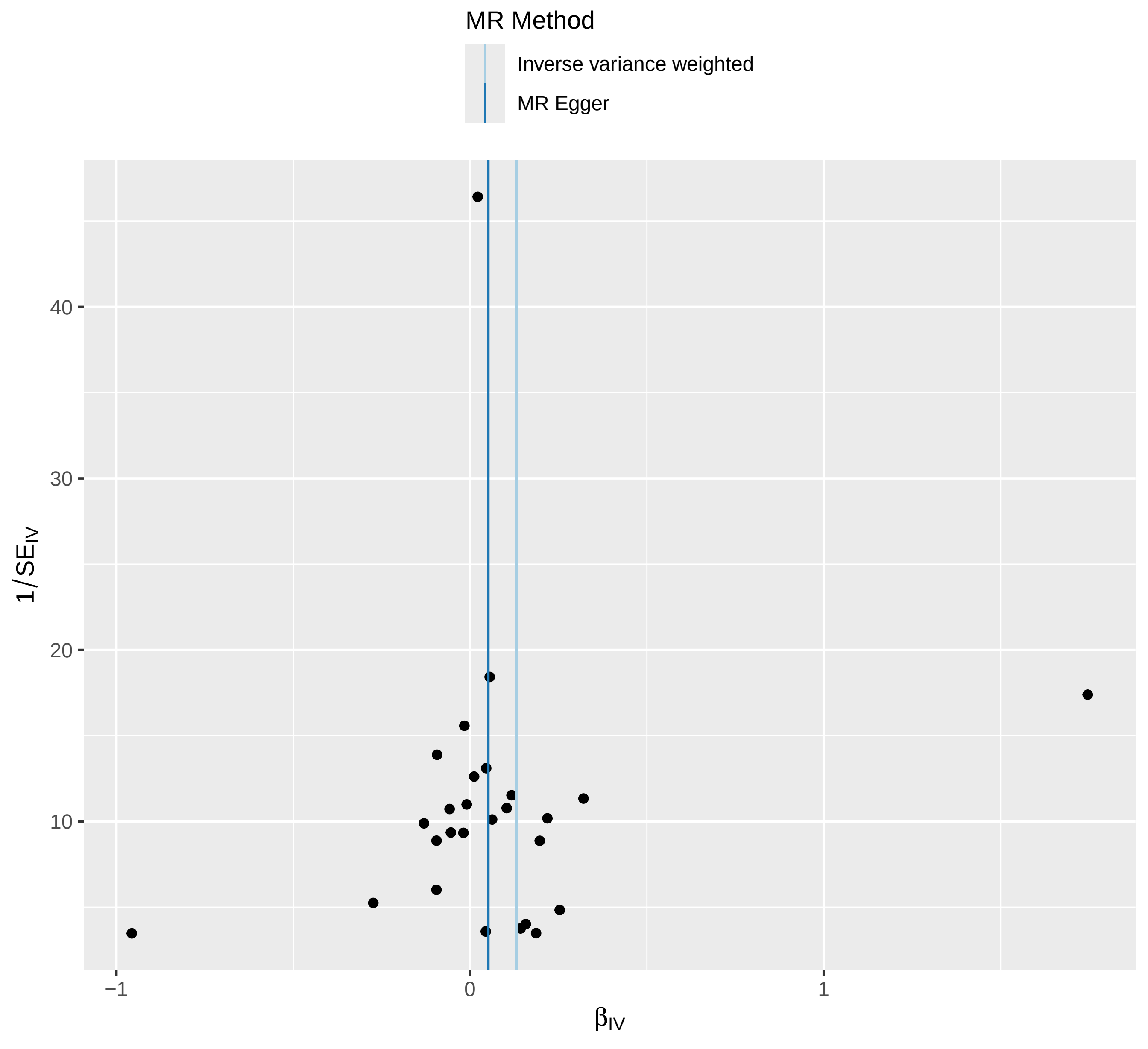

MR Method
Inverse variance weighted
MR Egger
40
30
20
10
1/SE$_{IV}$
−1
0
1
β$_{IV}$

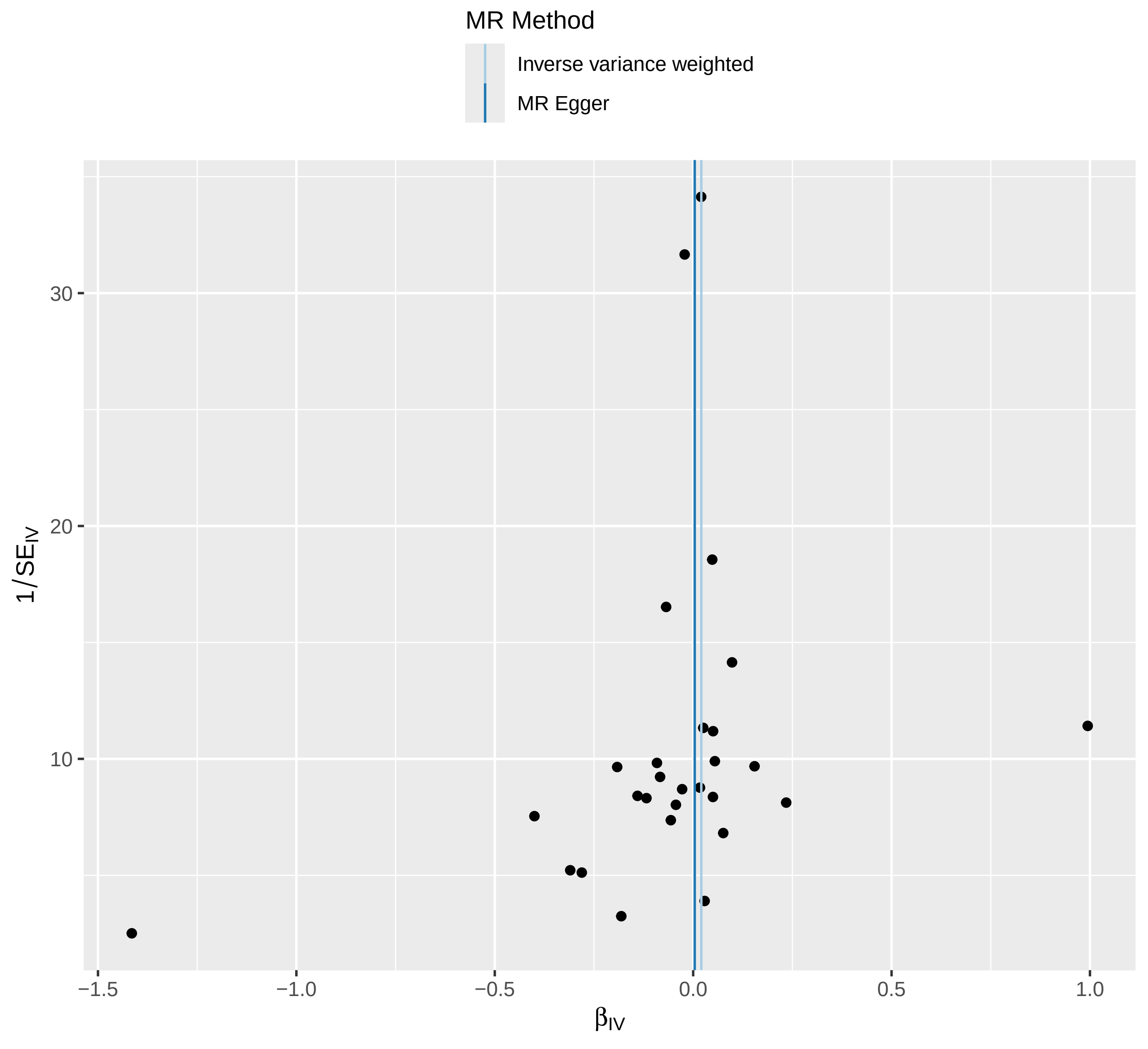

MR Method
Inverse variance weighted
MR Egger
30
20
10
1/SE_IV
−1.5
−1.0
−0.5
0.0
0.5
1.0
β_IV

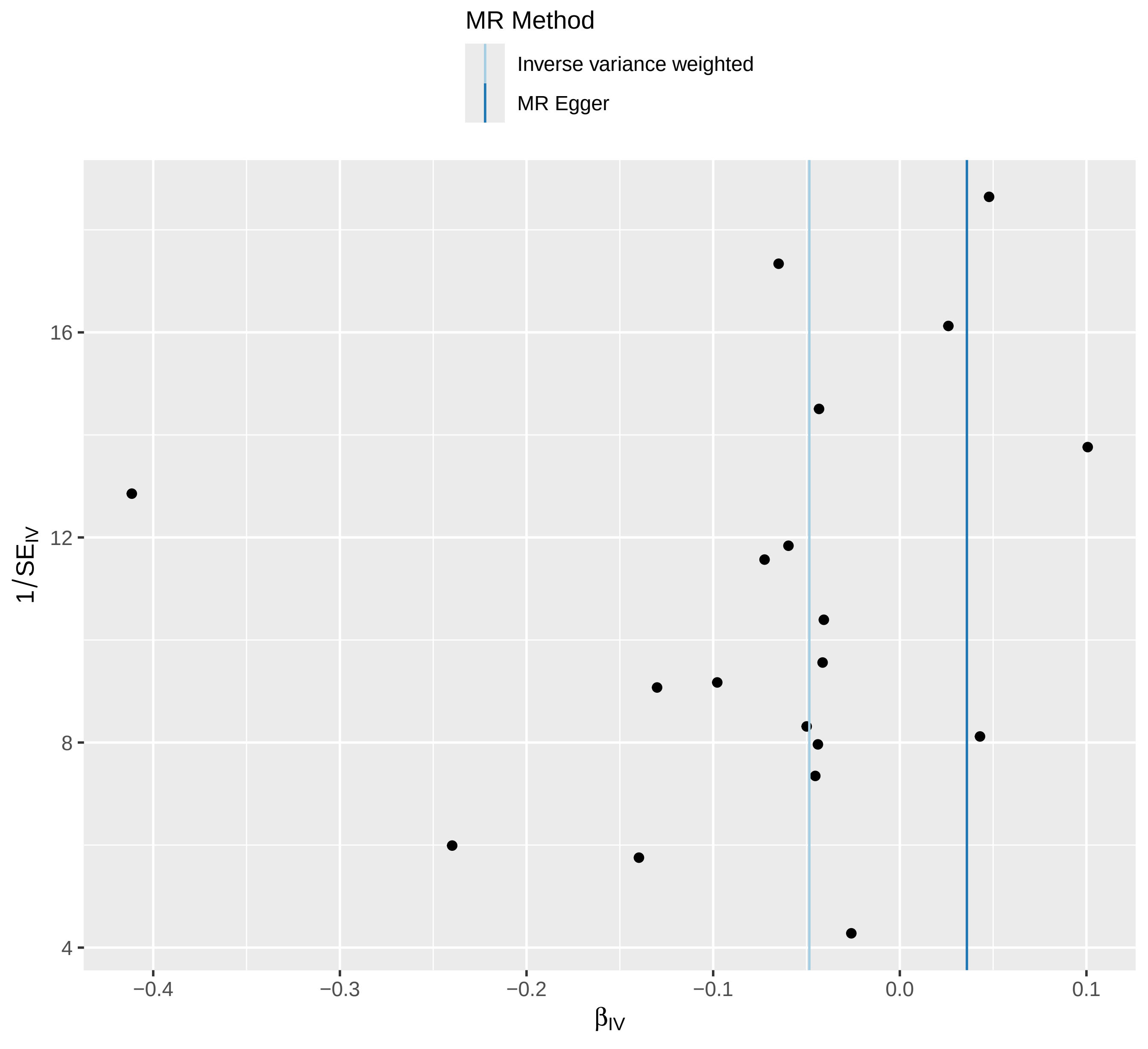
MR Method
Inverse variance weighted
MR Egger
1/SE$_{IV}$
16
12
8
4
−0.4
−0.3
−0.2
−0.1
0.0
0.1
β$_{IV}$

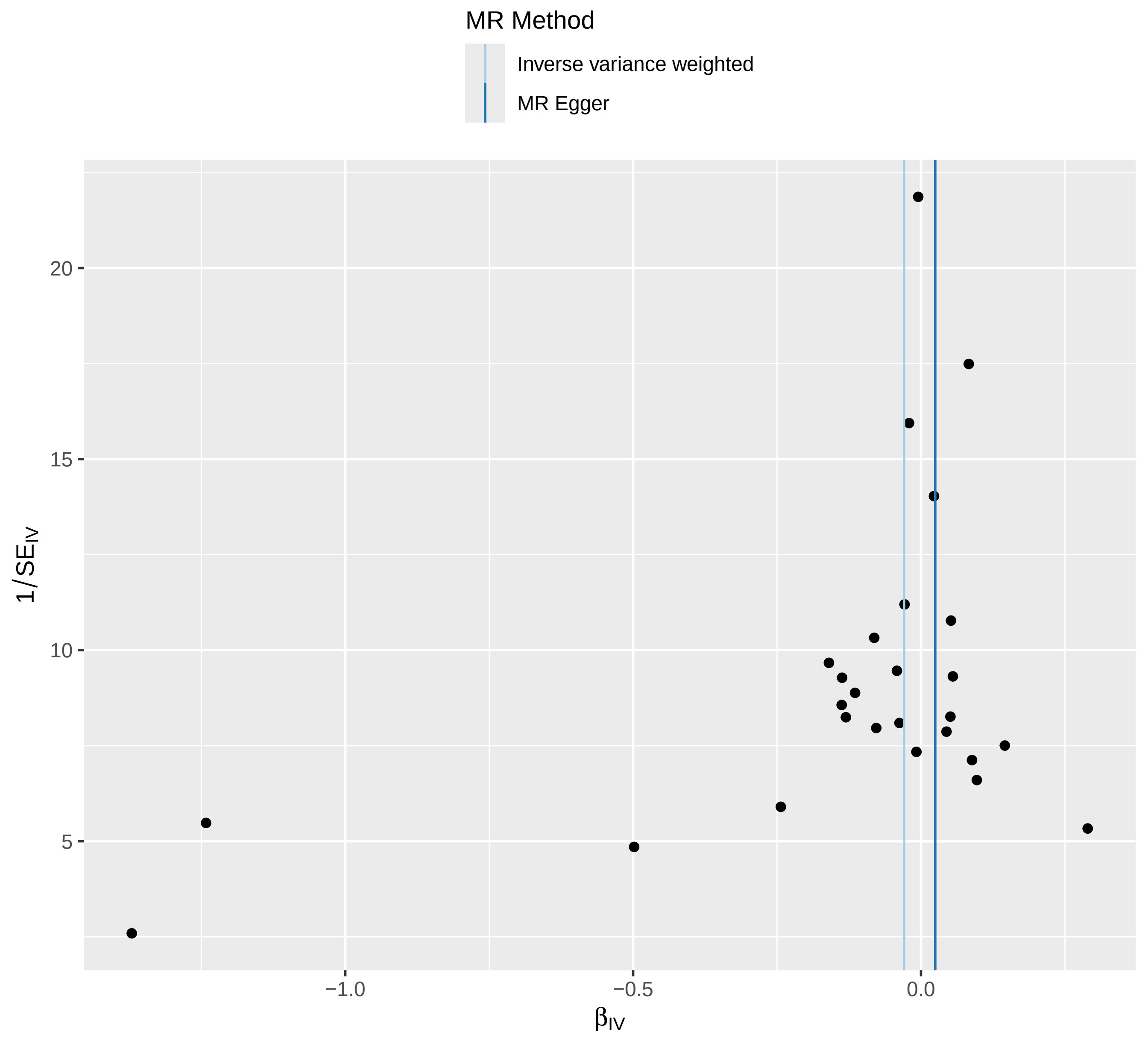
MR Method
Inverse variance weighted
MR Egger
20
15
10
5
1/SE_IV
−1.0
−0.5
0.0
β_IV

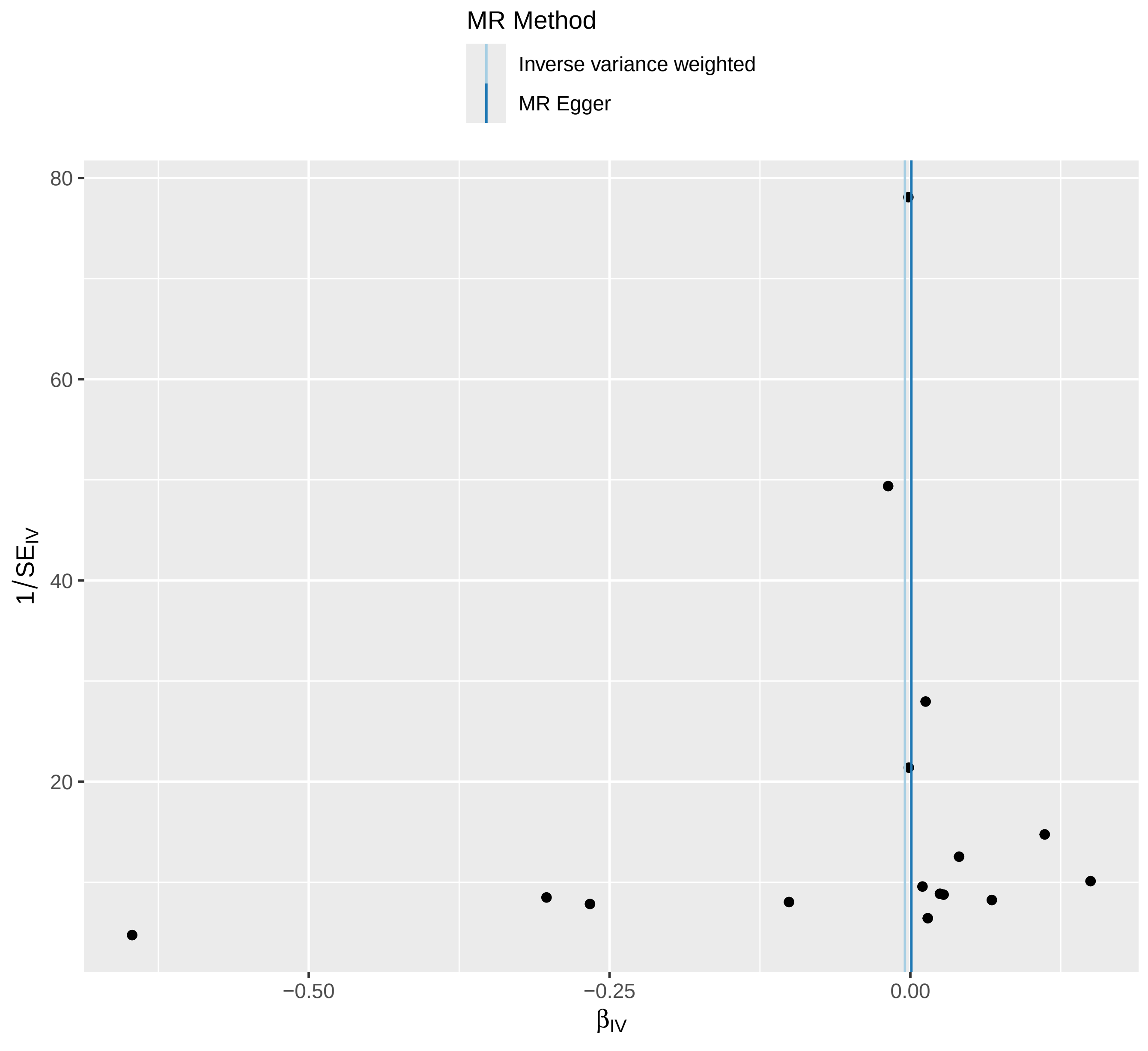
MR Method
Inverse variance weighted
MR Egger
80
60
40
20
1/SE_IV
−0.50
−0.25
0.00
β_IV

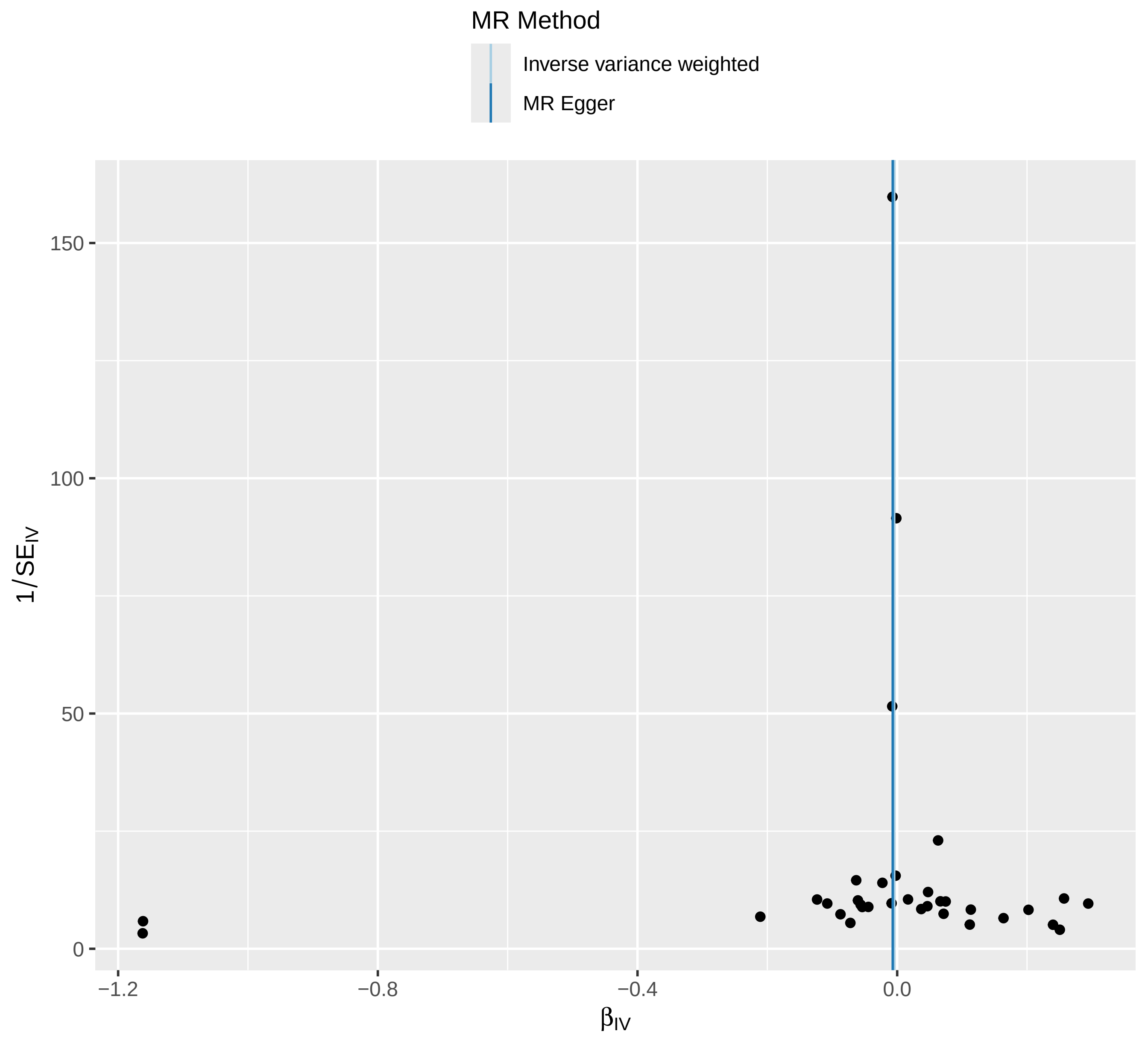

MR Method
Inverse variance weighted
MR Egger
150
100
50
0
−1.2
−0.8
−0.4
0.0
$1/SE_{IV}$
β_{IV}

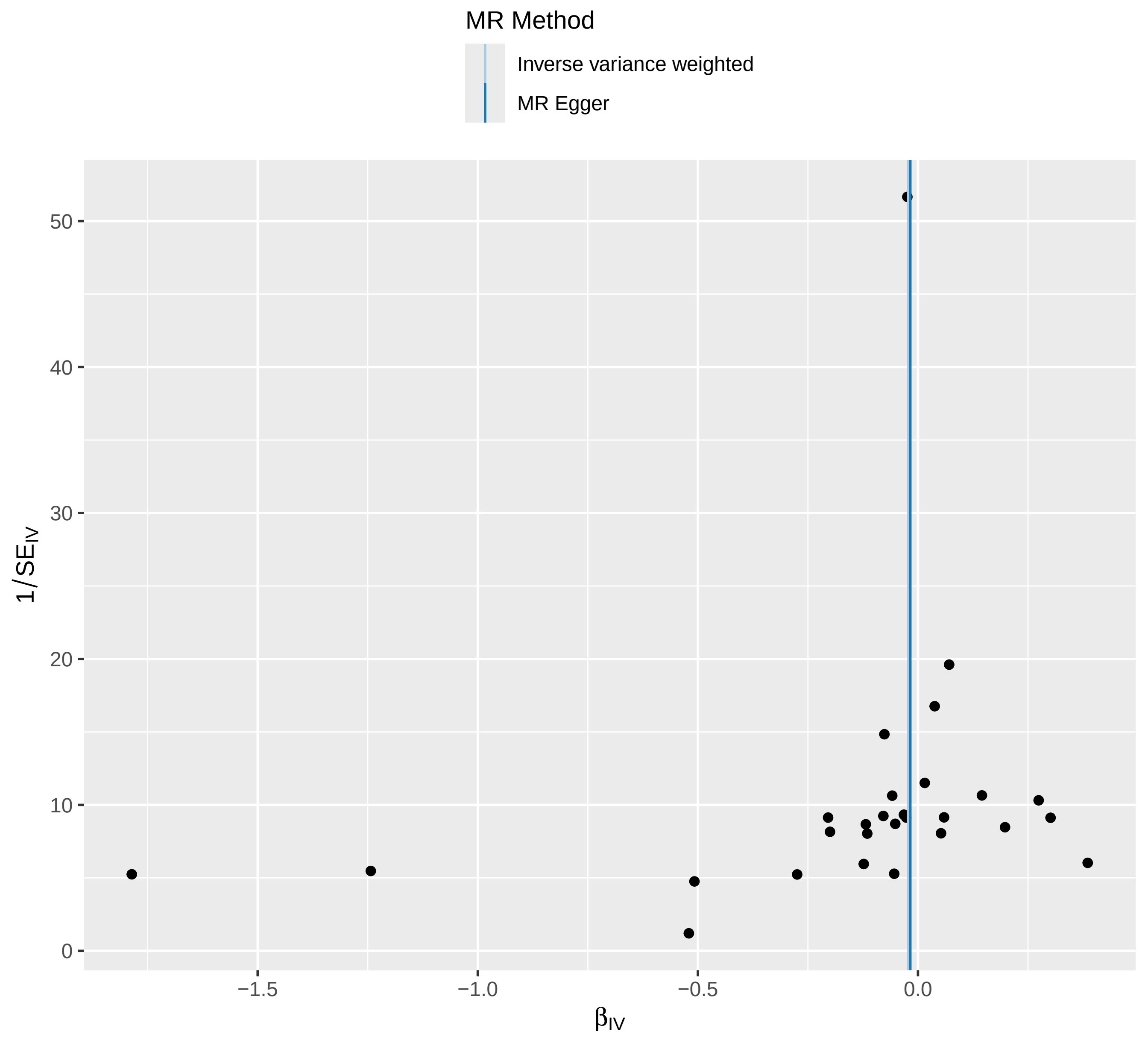
MR Method
Inverse variance weighted
MR Egger
1/SE_IV
β_IV
50
40
30
20
10
0
−1.5
−1.0
−0.5
0.0

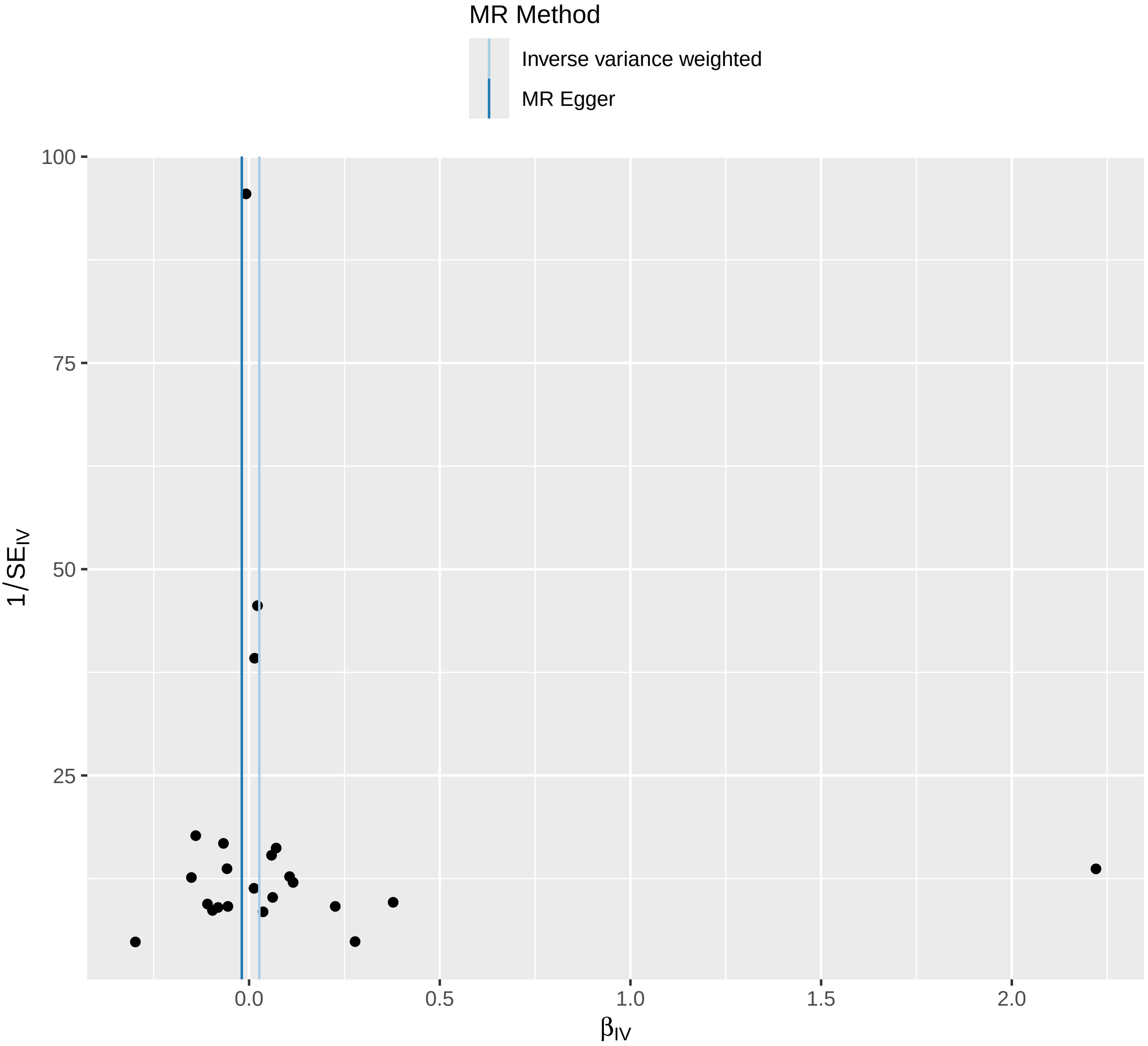

MR Method
Inverse variance weighted
MR Egger
100
75
50
25
1/SE_IV
0.0
0.5
1.0
1.5
2.0
β_IV

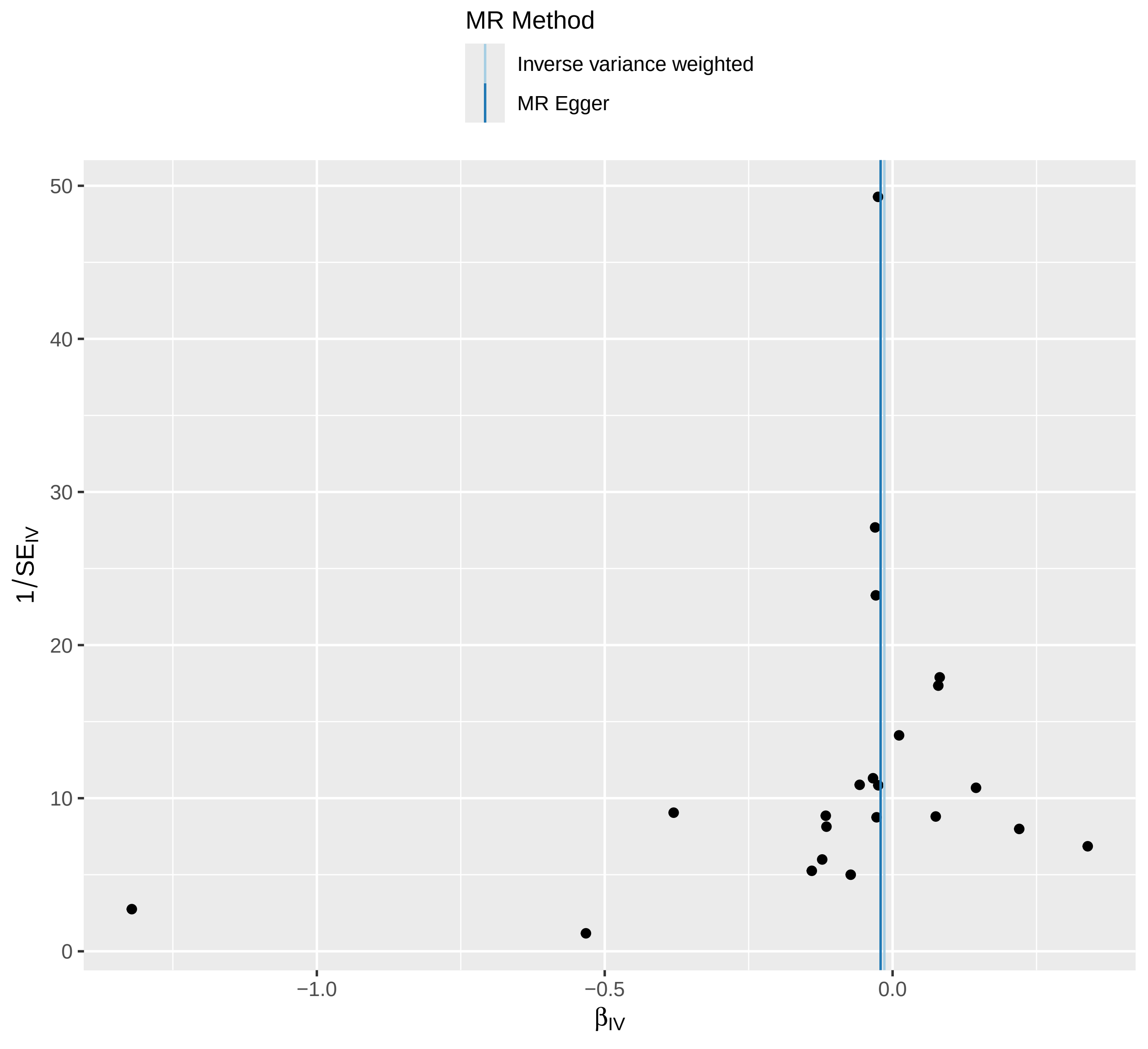
MR Method
Inverse variance weighted
MR Egger
1/SE_{IV}
50
40
30
20
10
0
−1.0
−0.5
0.0
β_{IV}

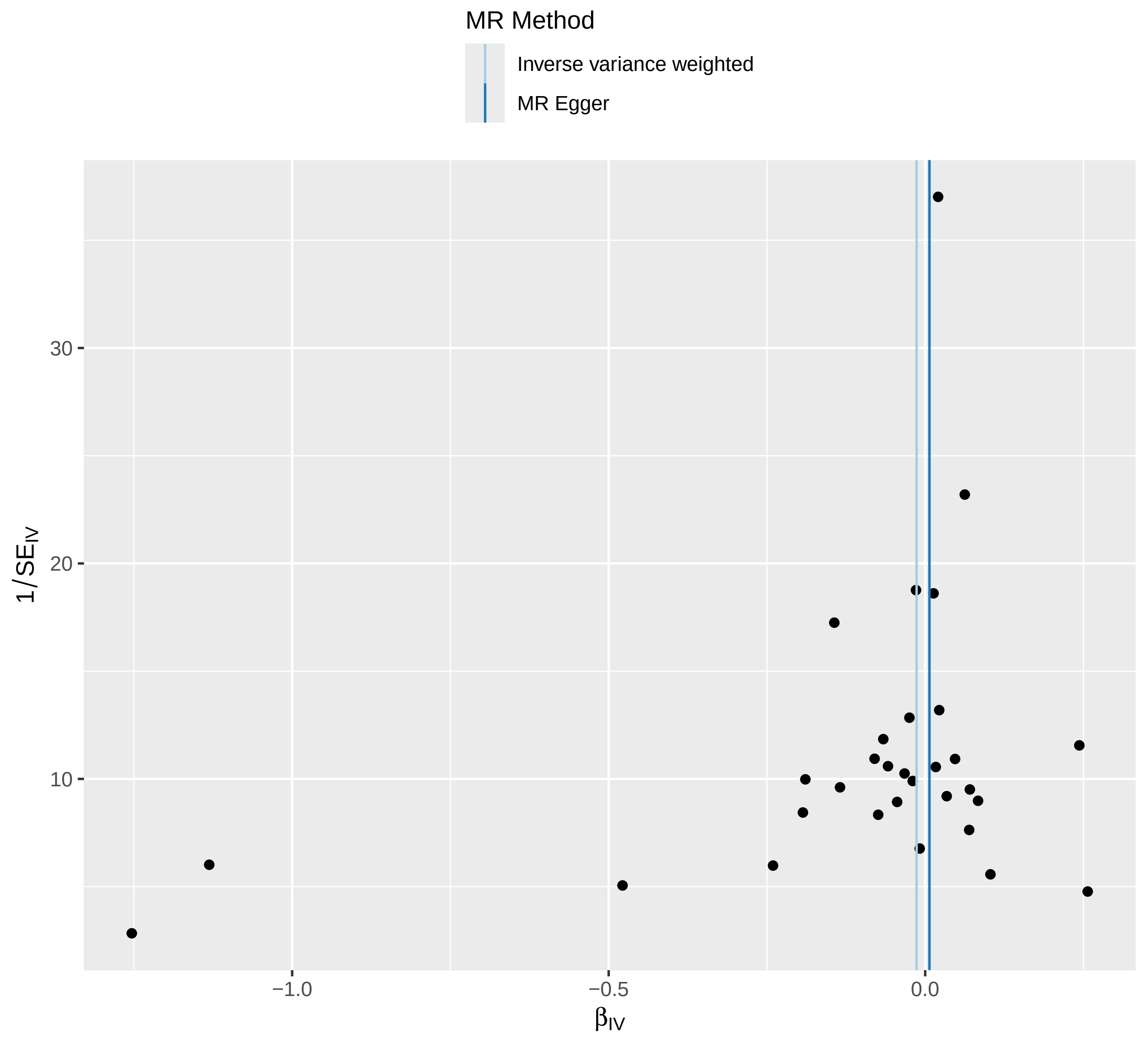

MR Method
Inverse variance weighted
MR Egger
1/SE$_{IV}$
30
20
10
−1.0
−0.5
0.0
β$_{IV}$

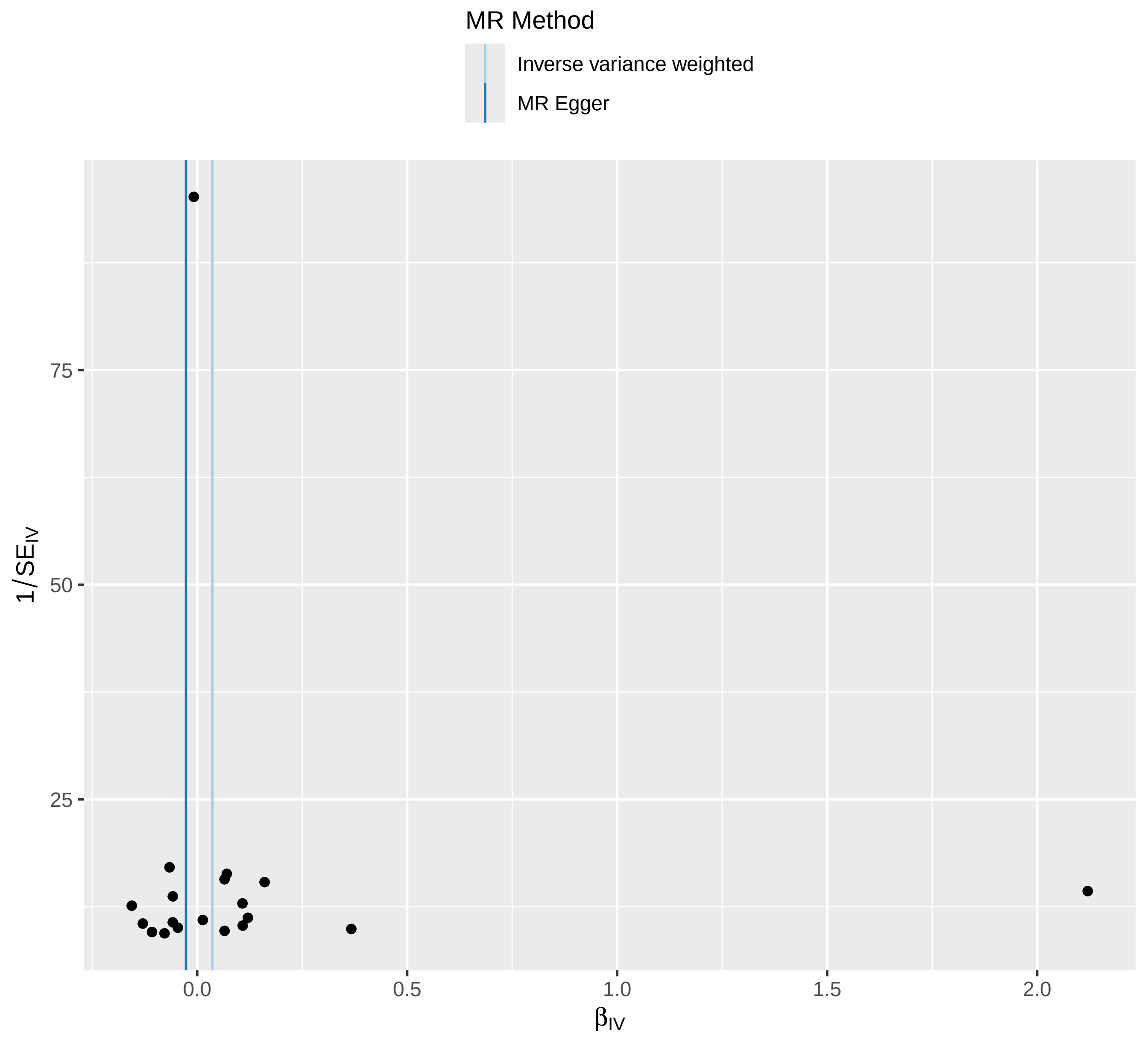

MR Method
Inverse variance weighted
MR Egger
1/SE_IV
75
50
25
0.0
0.5
1.0
1.5
2.0
β_IV

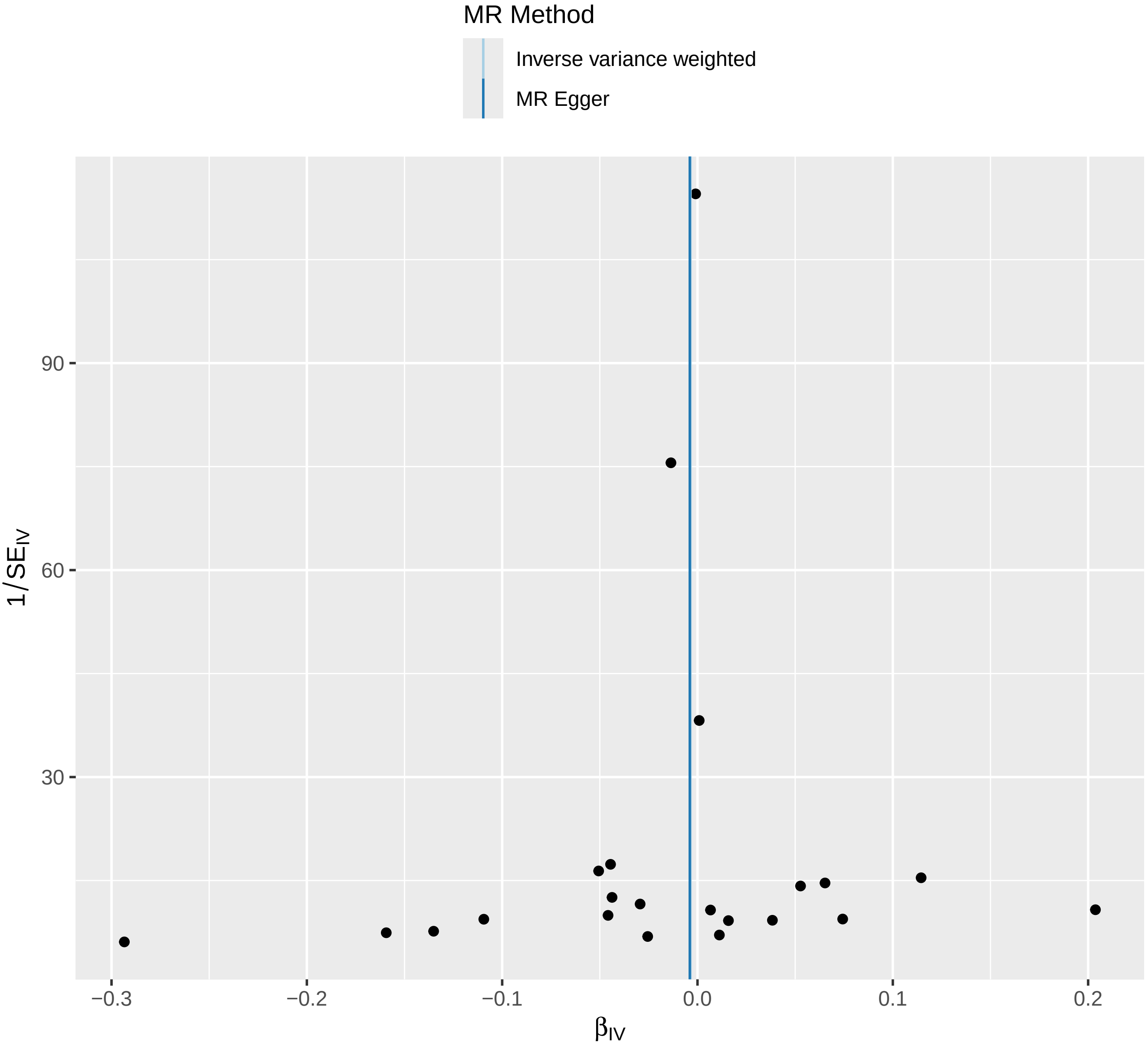
MR Method
Inverse variance weighted
MR Egger
90
60
30
1/SE_IV
−0.3
−0.2
−0.1
0.0
0.1
0.2
β_IV

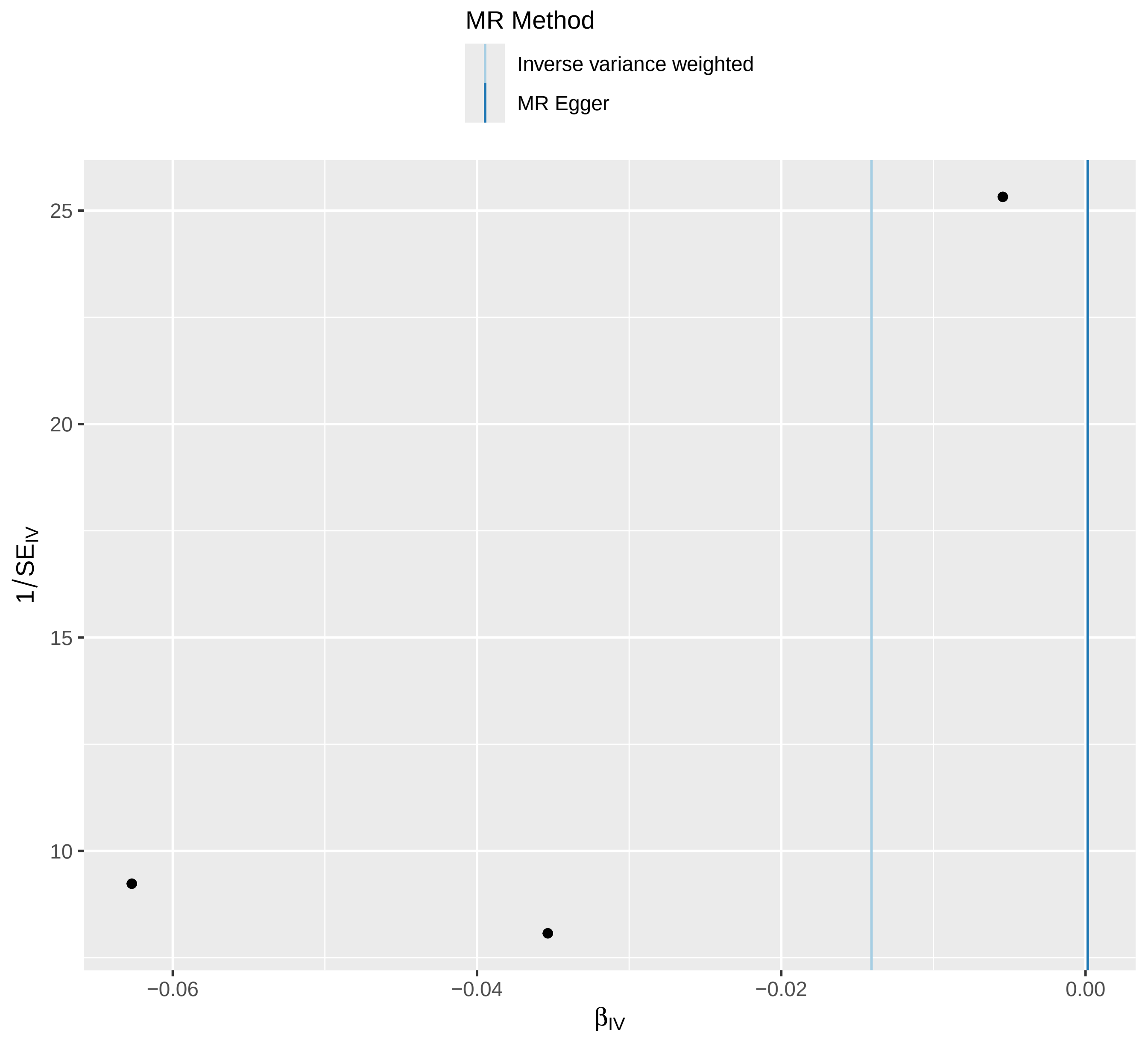
MR Method
Inverse variance weighted
MR Egger
25
20
15
10
1/SE_IV
−0.06
−0.04
−0.02
0.00
β_IV

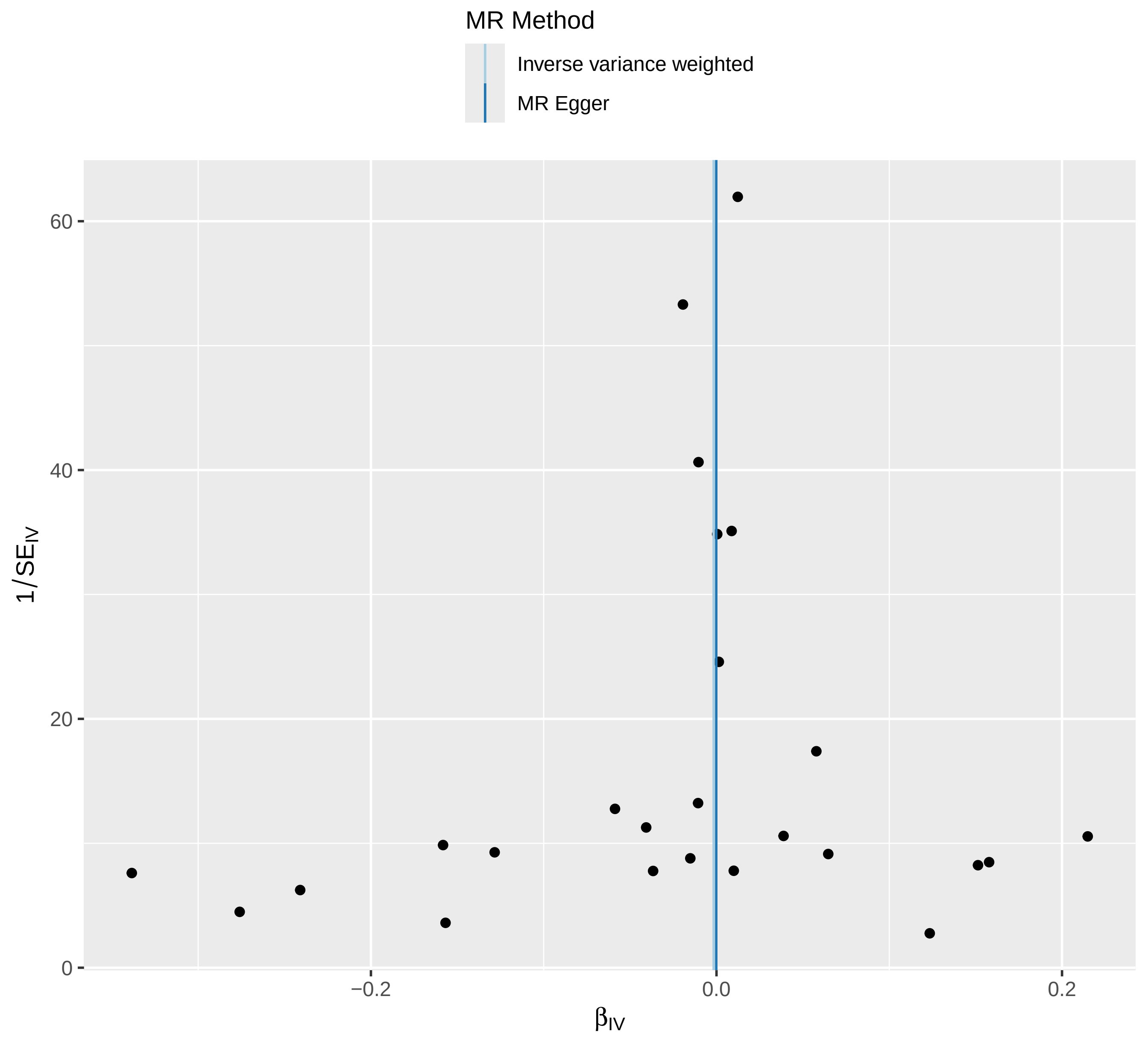
MR Method
Inverse variance weighted
MR Egger
60
40
20
0
−0.2
0.0
0.2
1/SE_IV
β_IV

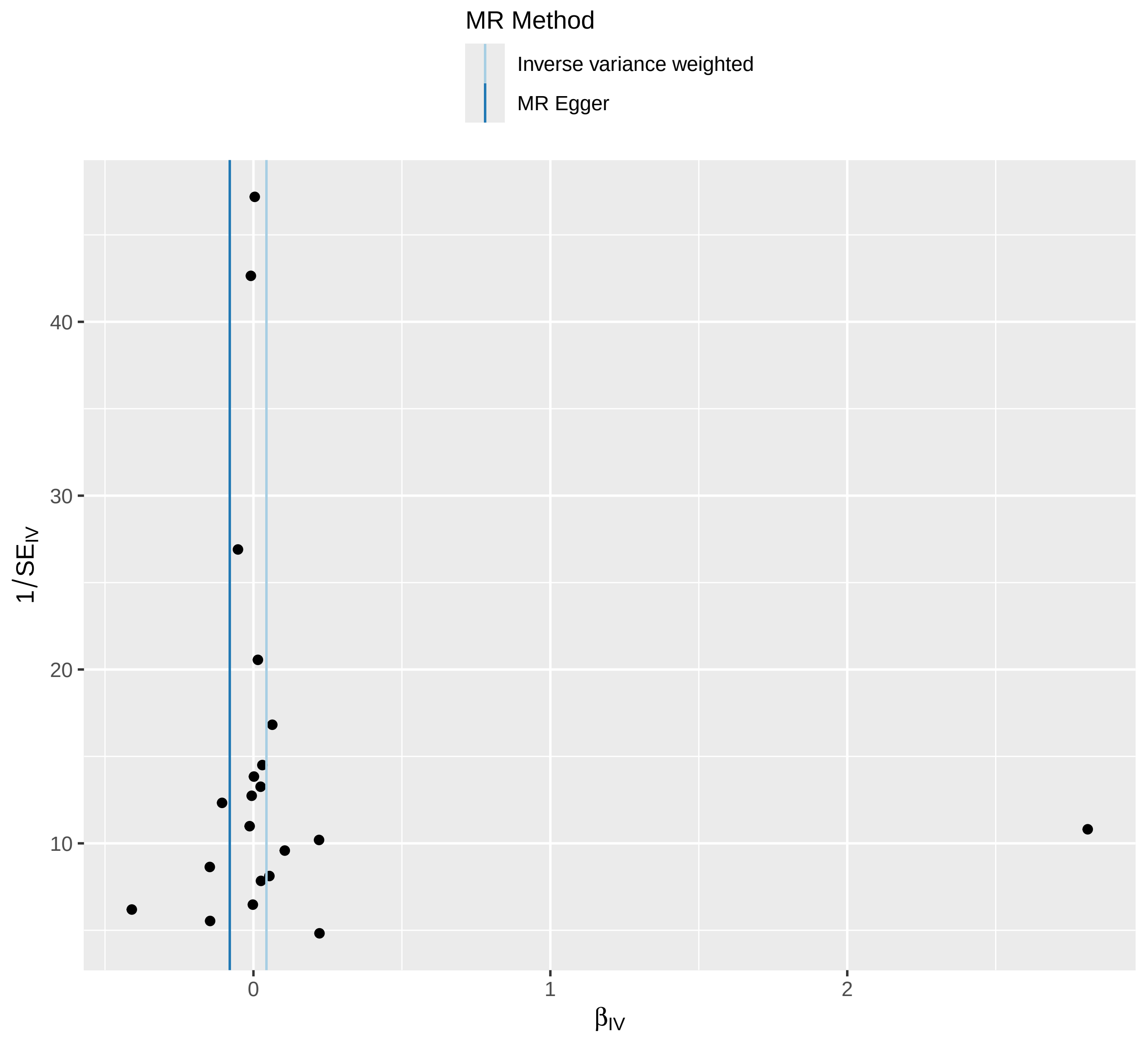
MR Method
Inverse variance weighted
MR Egger
40
30
20
10
1/SE$_{IV}$
0
1
2
β$_{IV}$

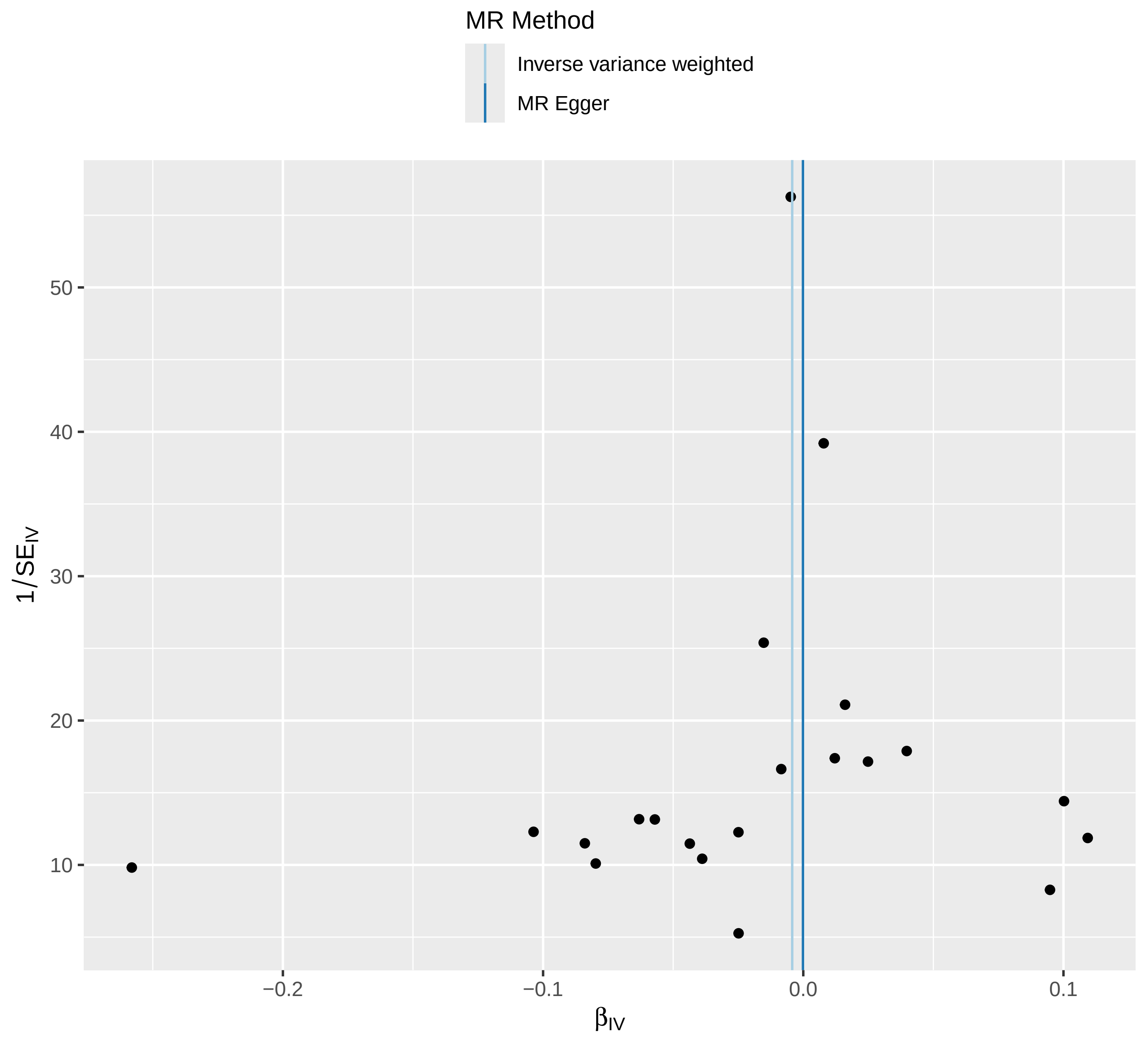

MR Method
Inverse variance weighted
MR Egger
50
40
30
20
10
1/SE_IV
−0.2
−0.1
0.0
0.1
β_IV

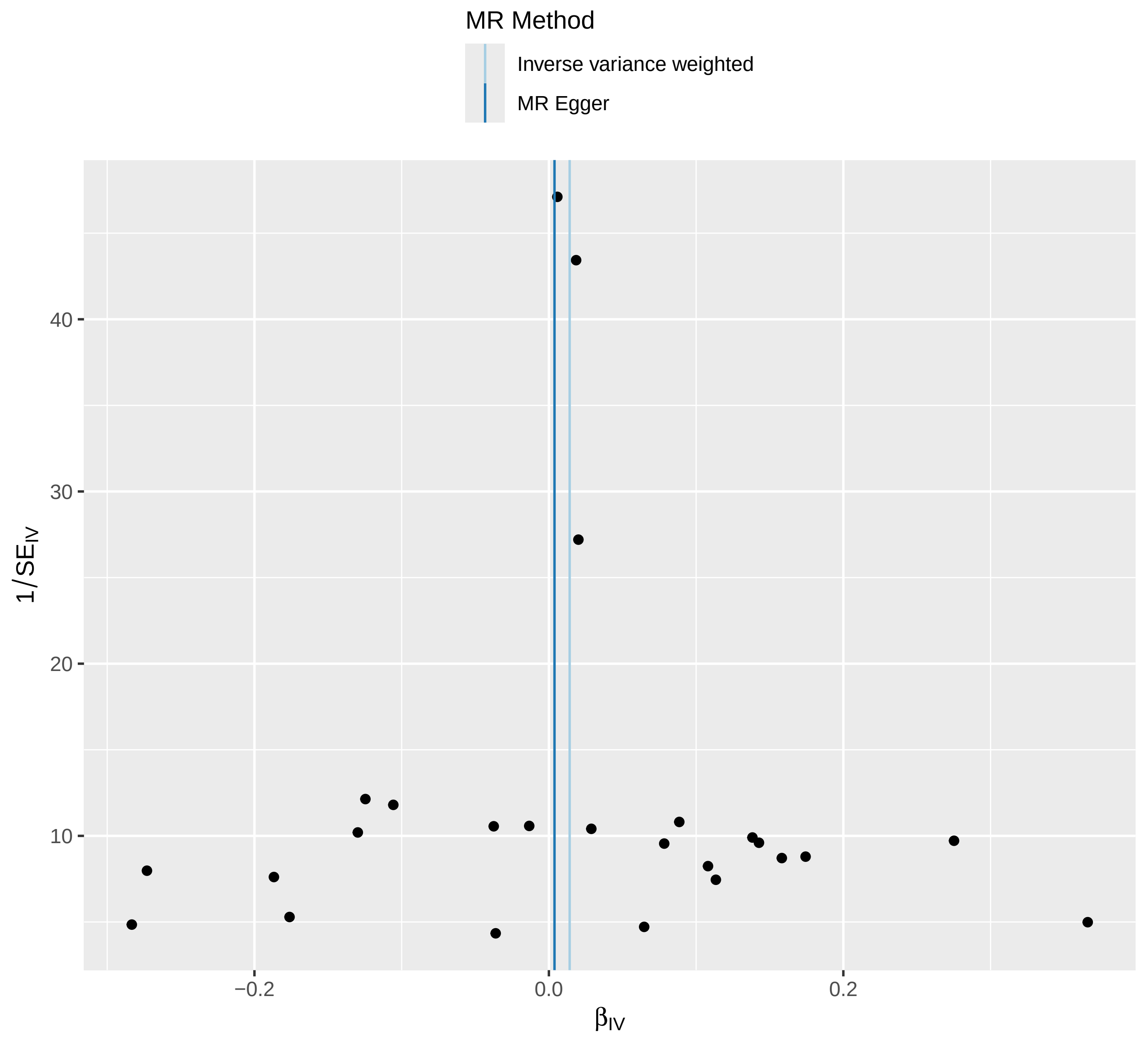
MR Method
Inverse variance weighted
MR Egger
1/SE_IV
40
30
20
10
−0.2
0.0
0.2
β_IV

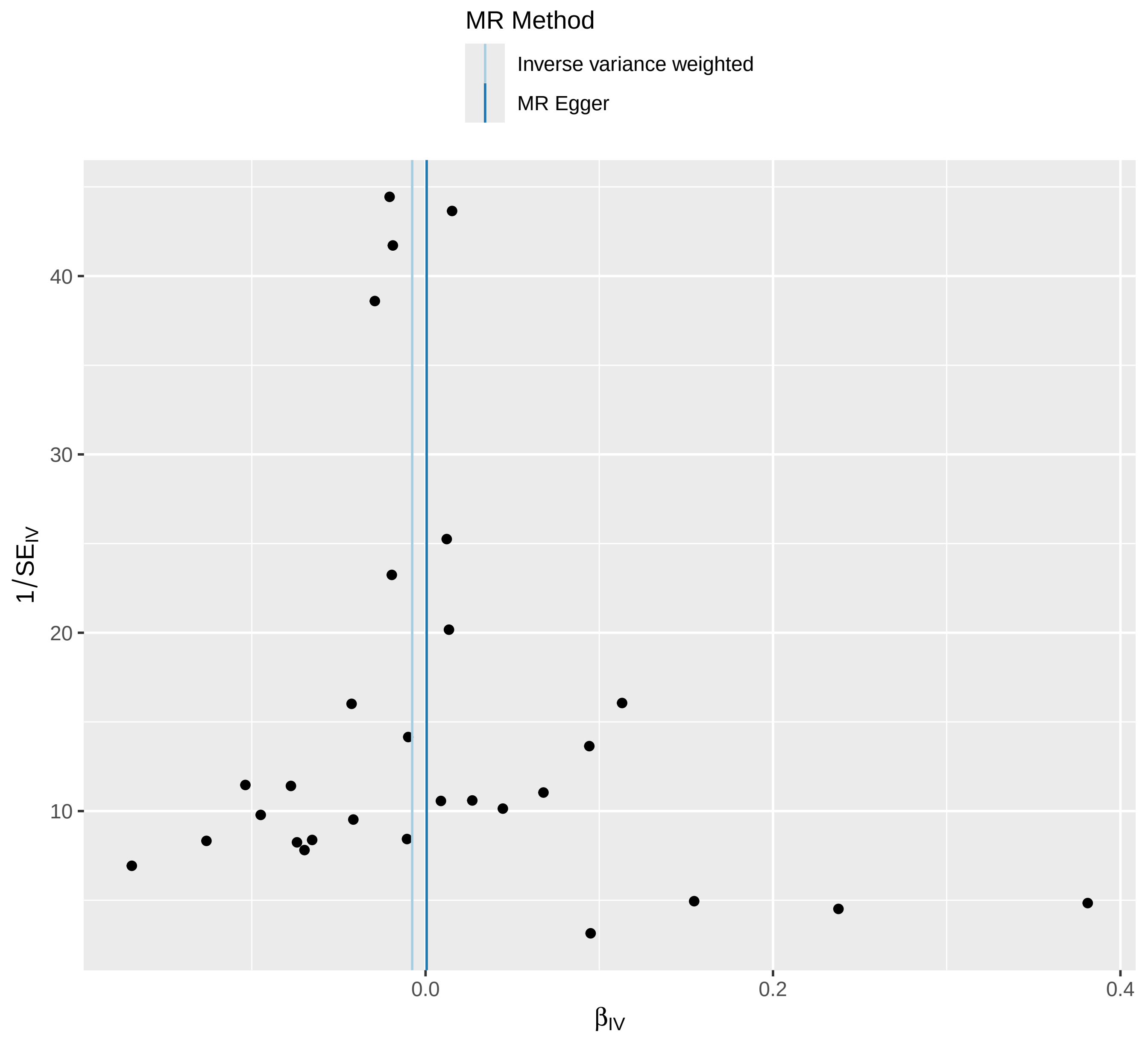
MR Method
Inverse variance weighted
MR Egger
40
30
20
10
0.0
0.2
0.4
$1/SE_{IV}$
β_{IV}

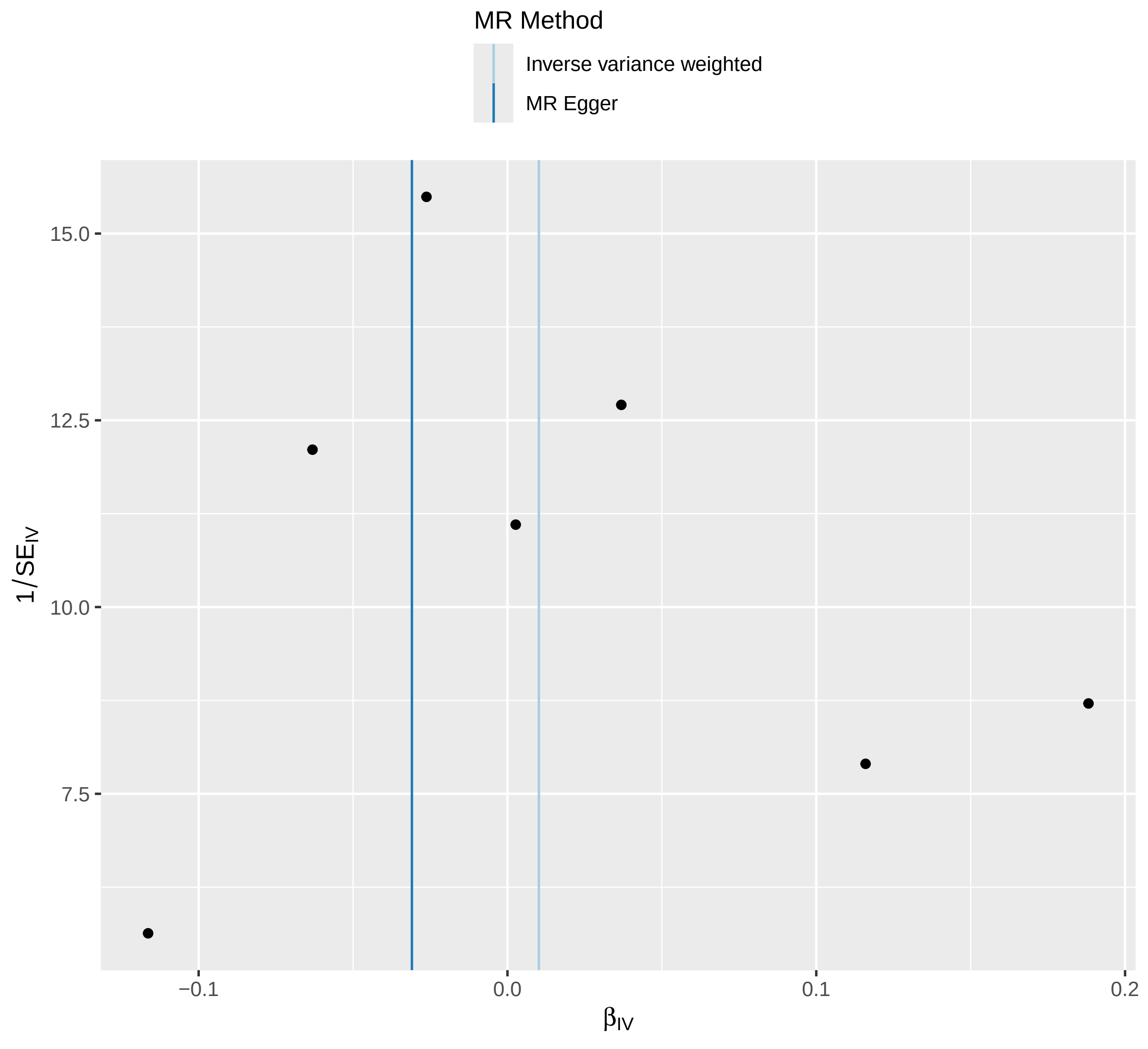
MR Method
Inverse variance weighted
MR Egger
15.0
12.5
10.0
7.5
1/SE_IV
−0.1
0.0
0.1
0.2
β_IV

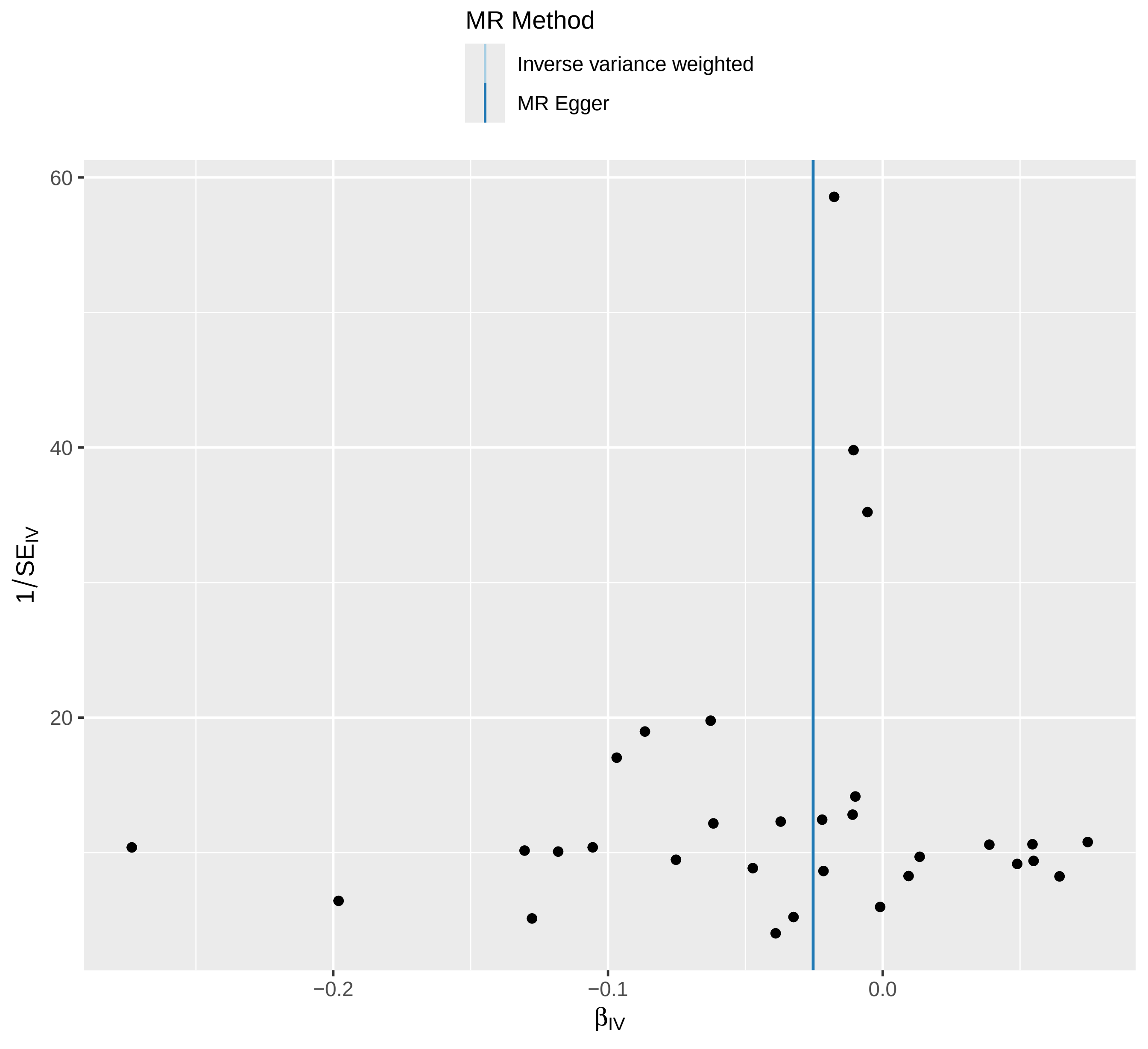
MR Method
Inverse variance weighted
MR Egger
60
40
20
1/SE_IV
−0.2
−0.1
0.0
β_IV

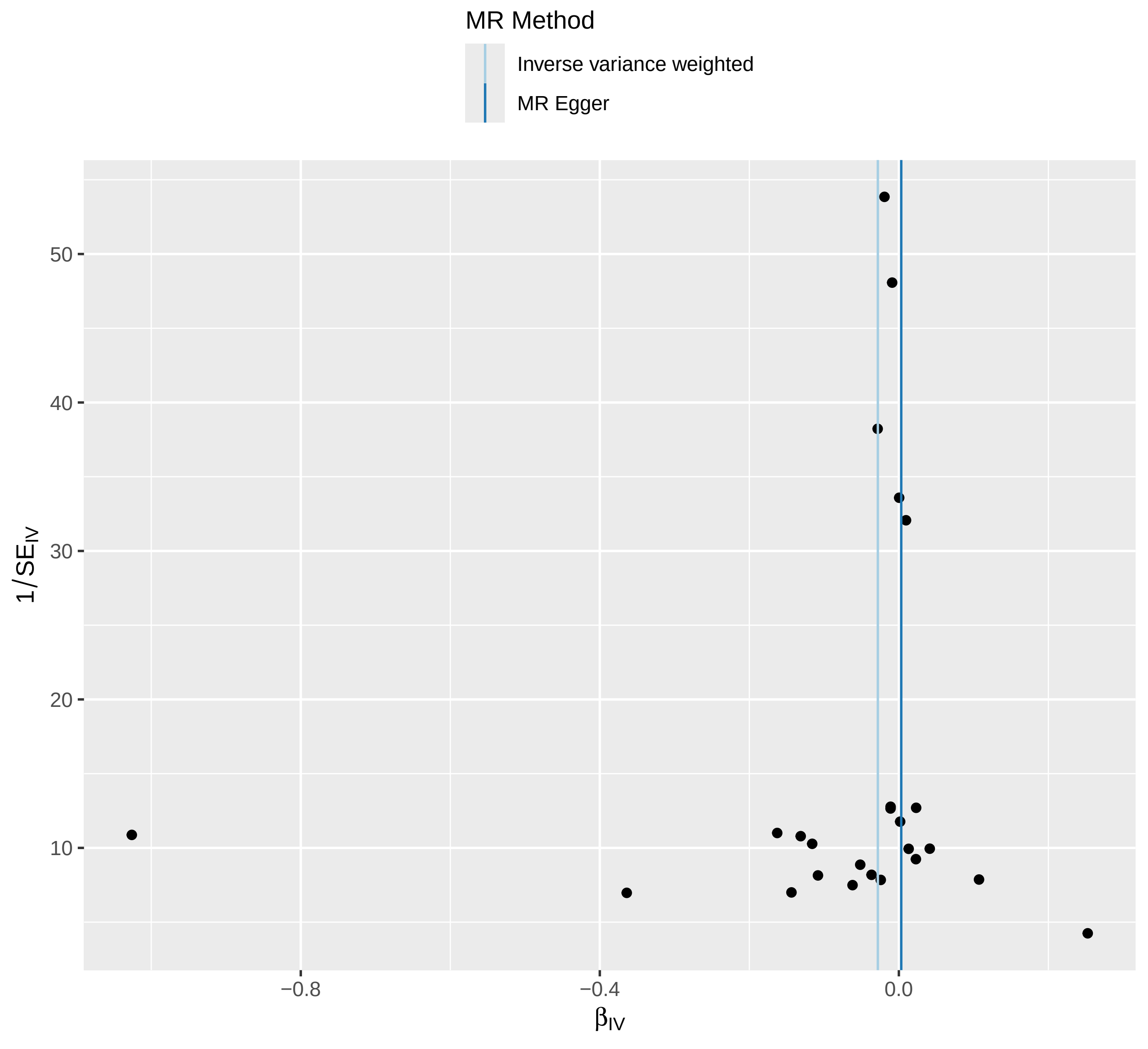
MR Method
Inverse variance weighted
MR Egger
1/SE_IV
β_IV
50
40
30
20
10
−0.8
−0.4
0.0

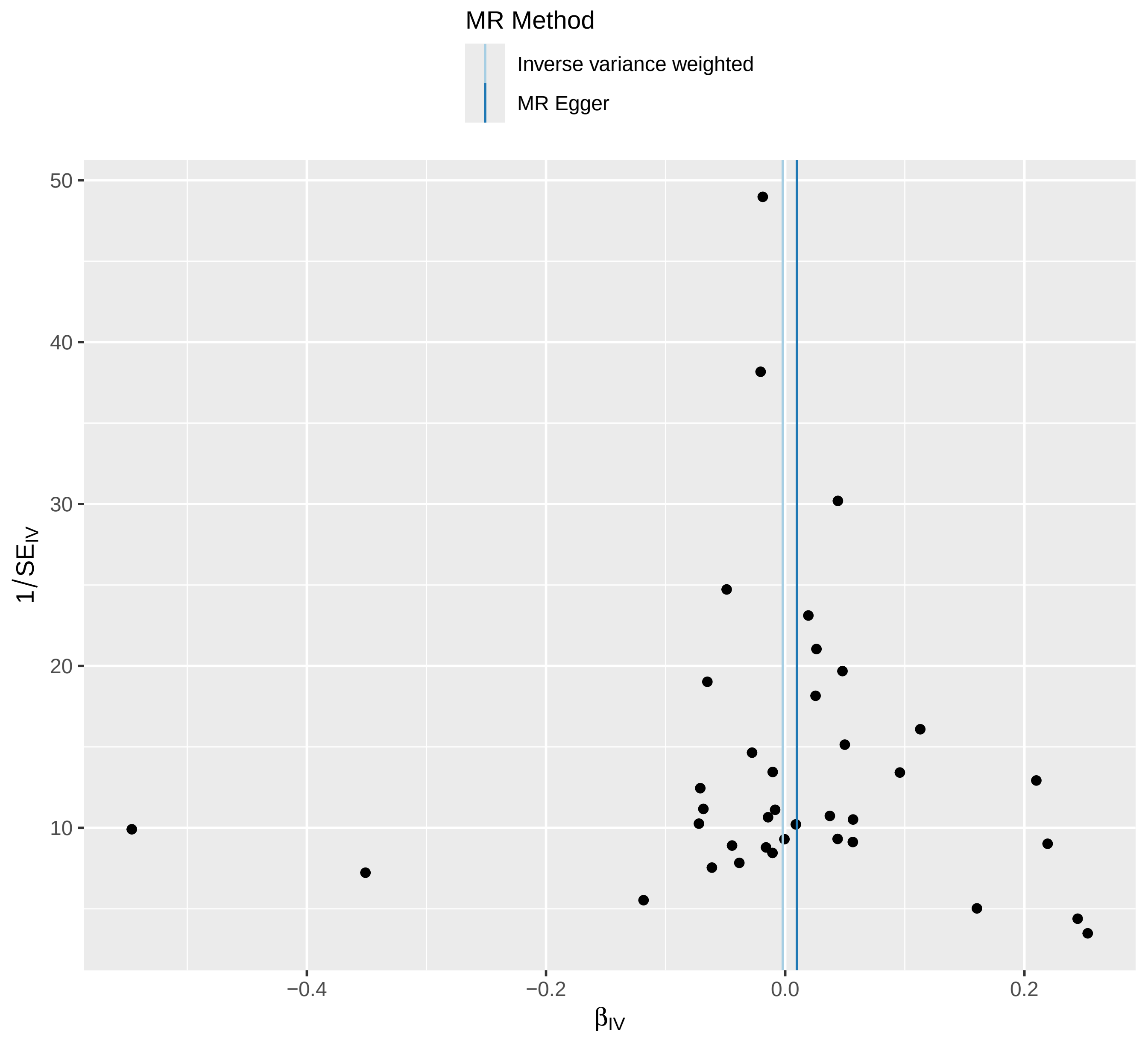
MR Method
Inverse variance weighted
MR Egger
50
40
30
20
10
1/SE_IV
−0.4
−0.2
0.0
0.2
β_IV

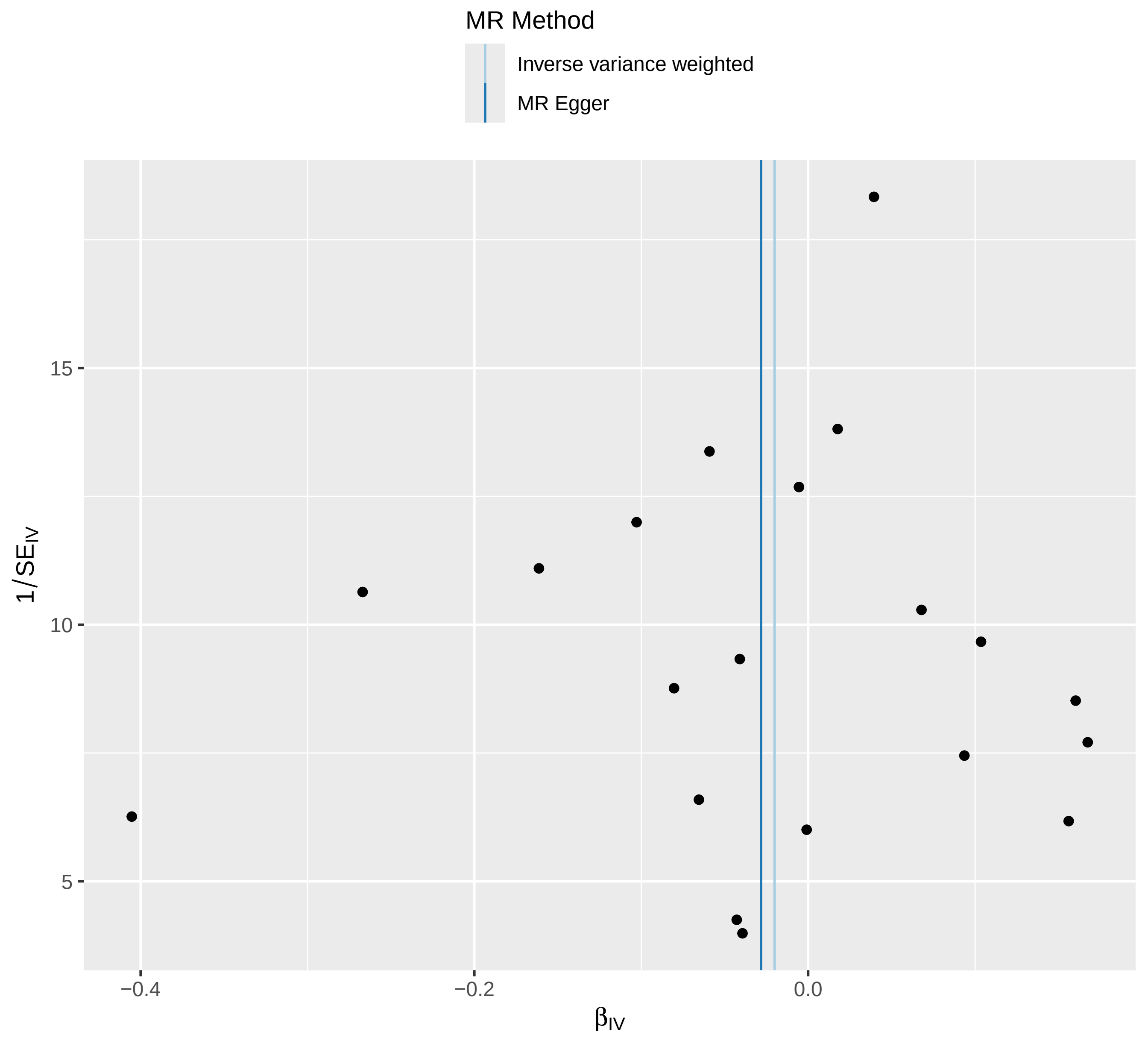

MR Method
Inverse variance weighted
MR Egger
15
10
5
1/SE_IV
−0.4
−0.2
0.0
β_IV

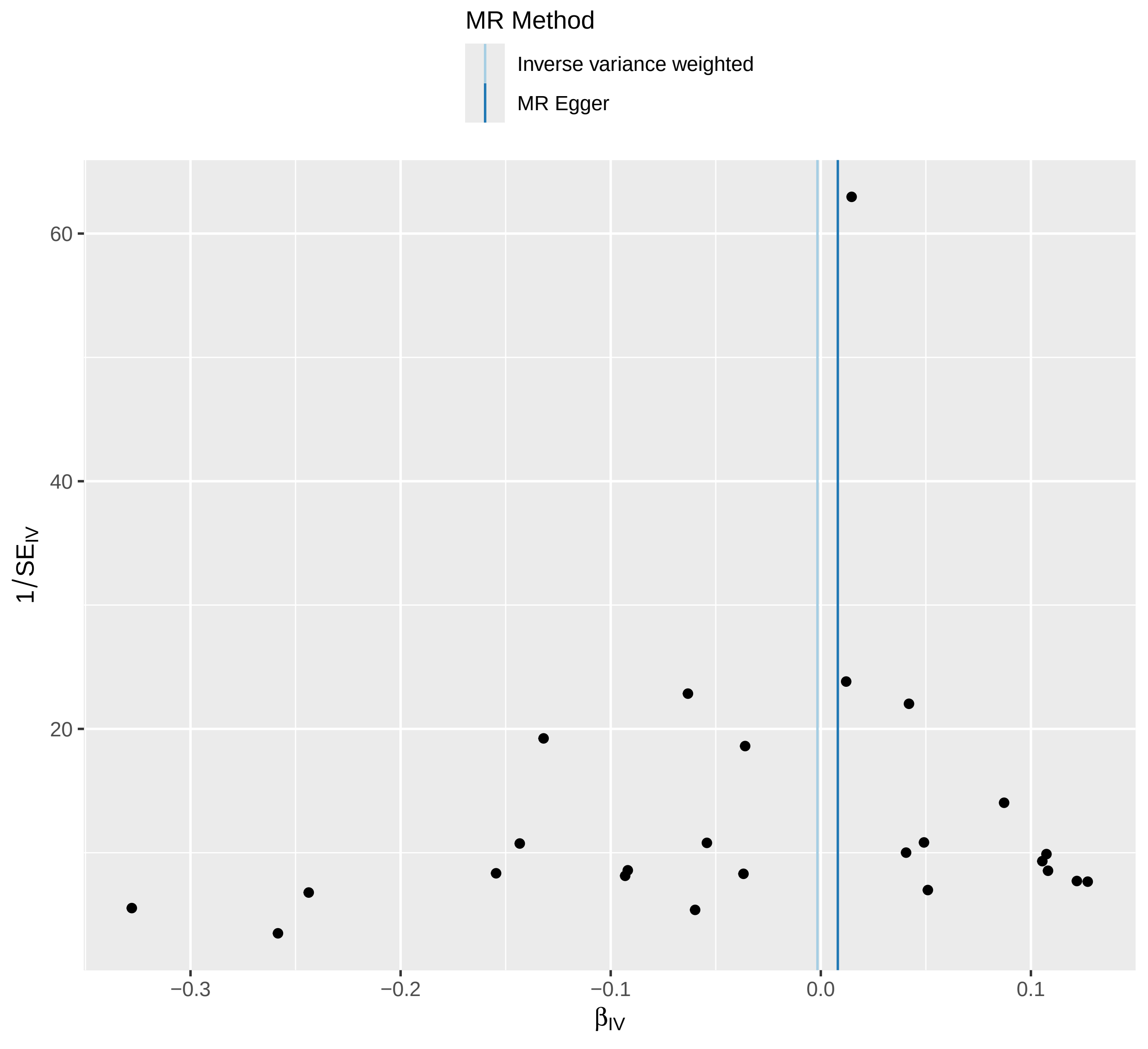

MR Method
Inverse variance weighted
MR Egger
60
40
20
1/SE$_{IV}$
−0.3
−0.2
−0.1
0.0
0.1
β$_{IV}$

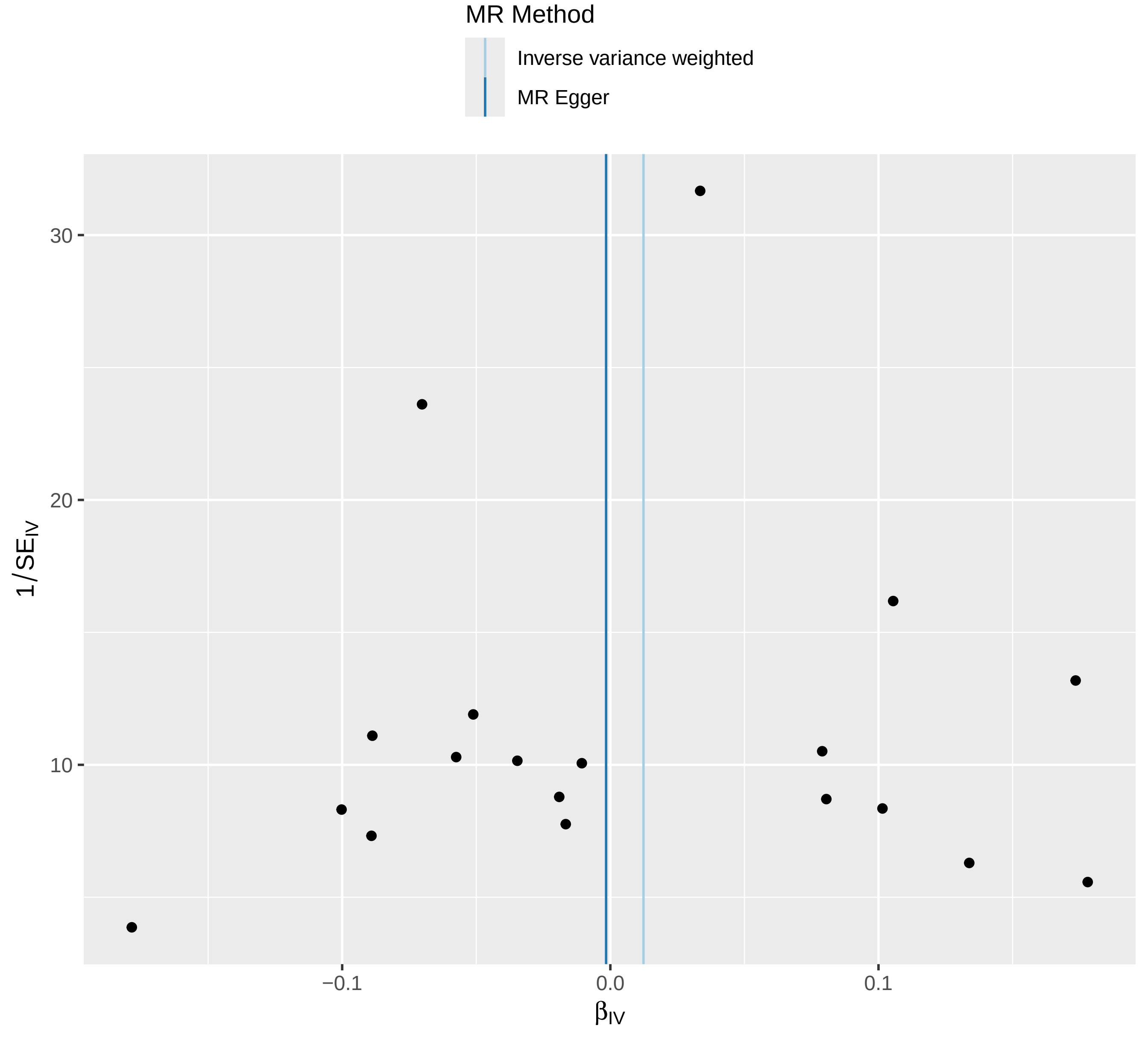

MR Method
Inverse variance weighted
MR Egger
30
20
10
1/SE$_{IV}$
−0.1
0.0
0.1
β$_{IV}$

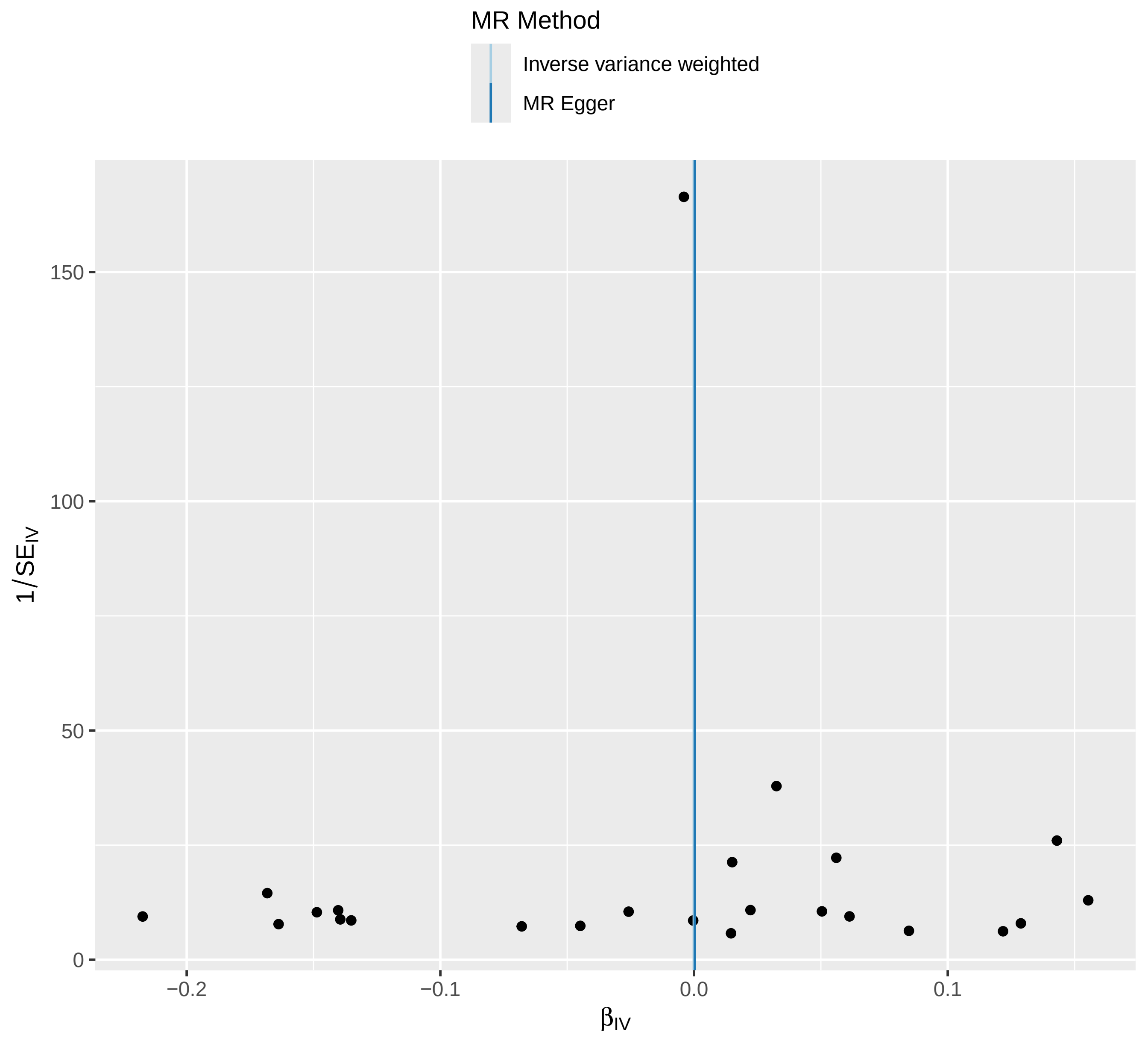
MR Method
Inverse variance weighted
MR Egger
150
100
50
0
1/SE_{IV}
−0.2
−0.1
0.0
0.1
β_{IV}

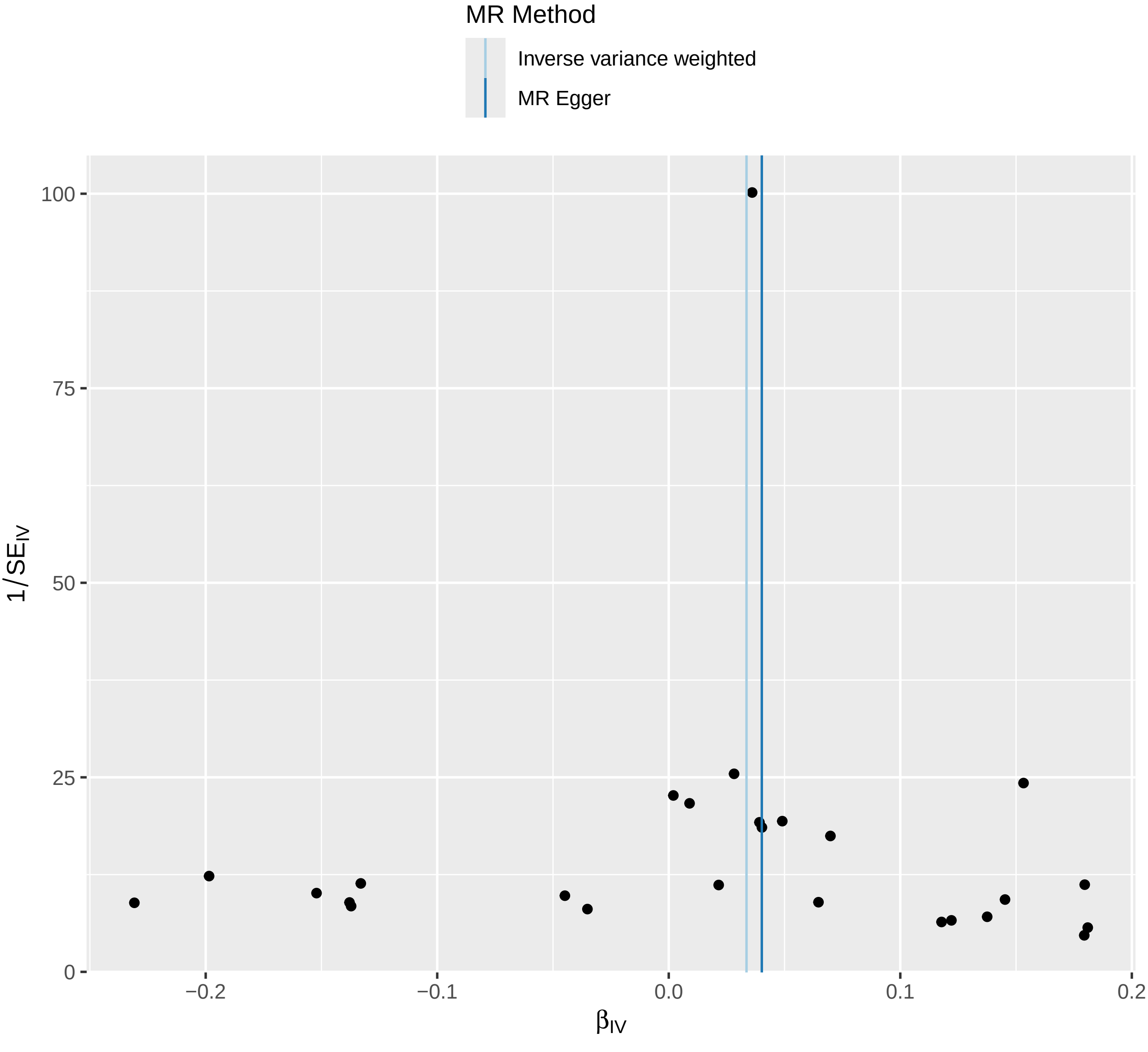
MR Method
Inverse variance weighted
MR Egger
100
75
50
25
0
1/SE$_{IV}$
−0.2
−0.1
0.0
0.1
0.2
β$_{IV}$

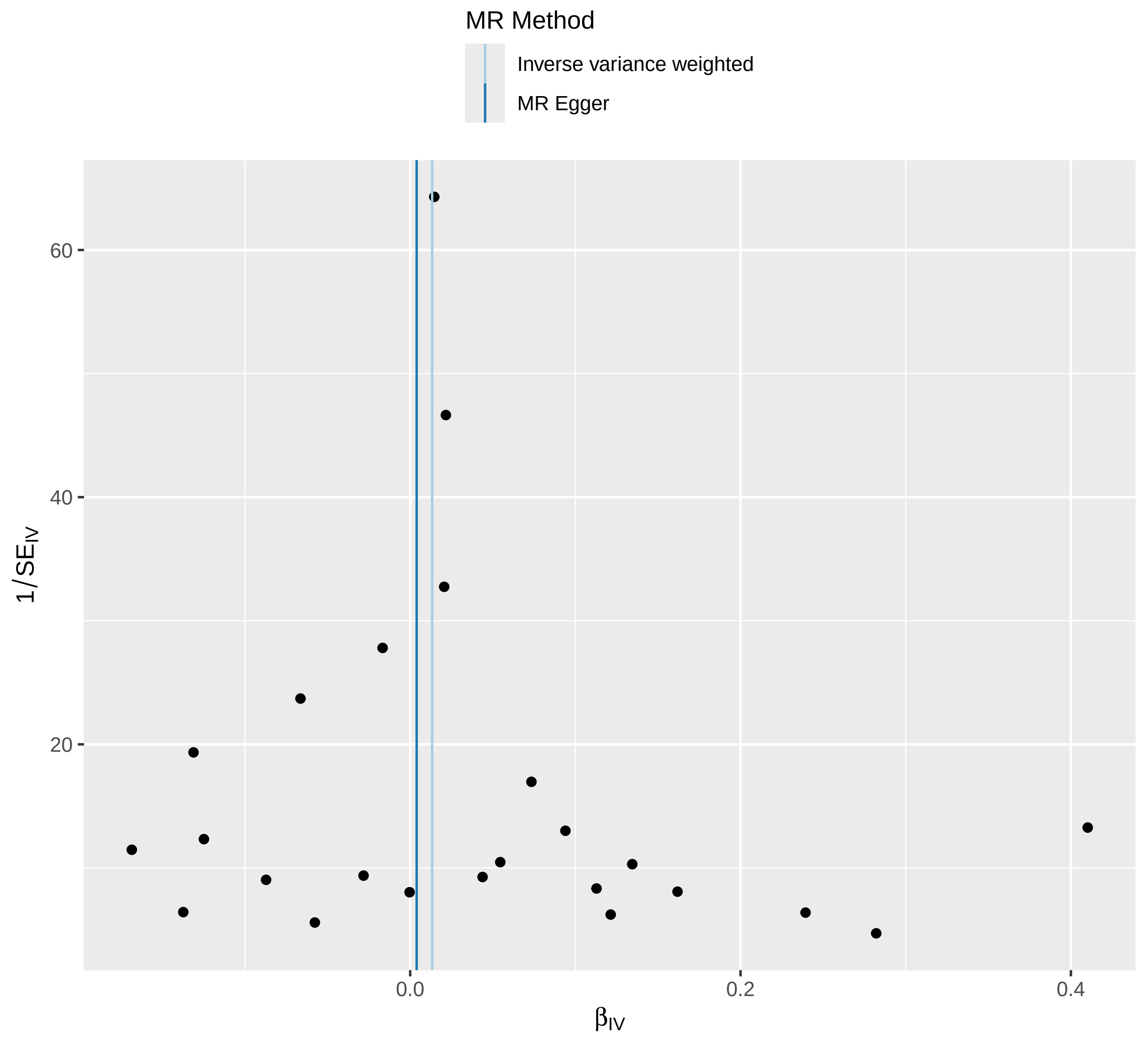
MR Method
Inverse variance weighted
MR Egger
60
40
20
1/SE$_{IV}$
0.0
0.2
0.4
β$_{IV}$

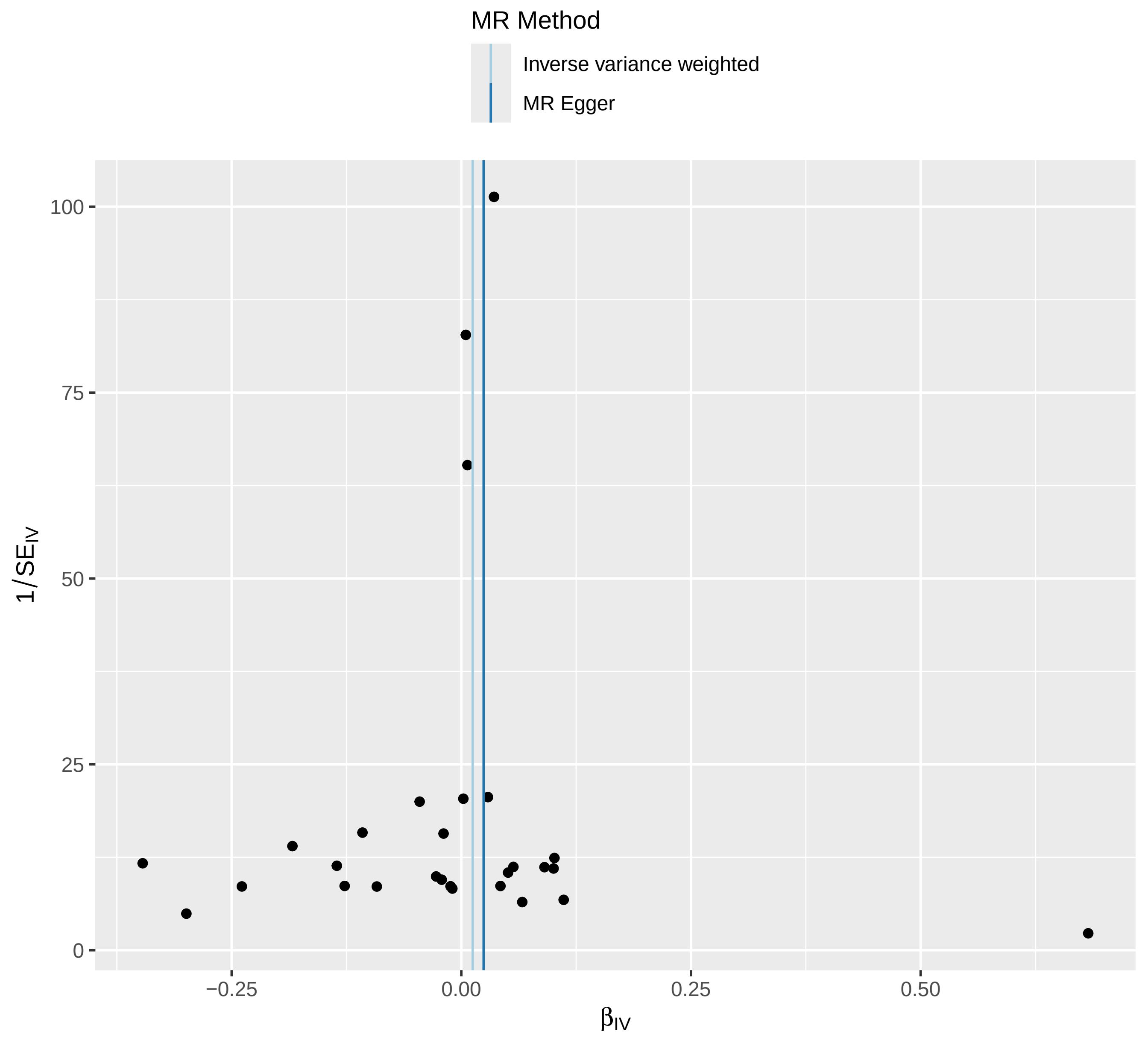
MR Method
Inverse variance weighted
MR Egger
100
75
50
25
0
1/SE_IV
−0.25
0.00
0.25
0.50
β_IV

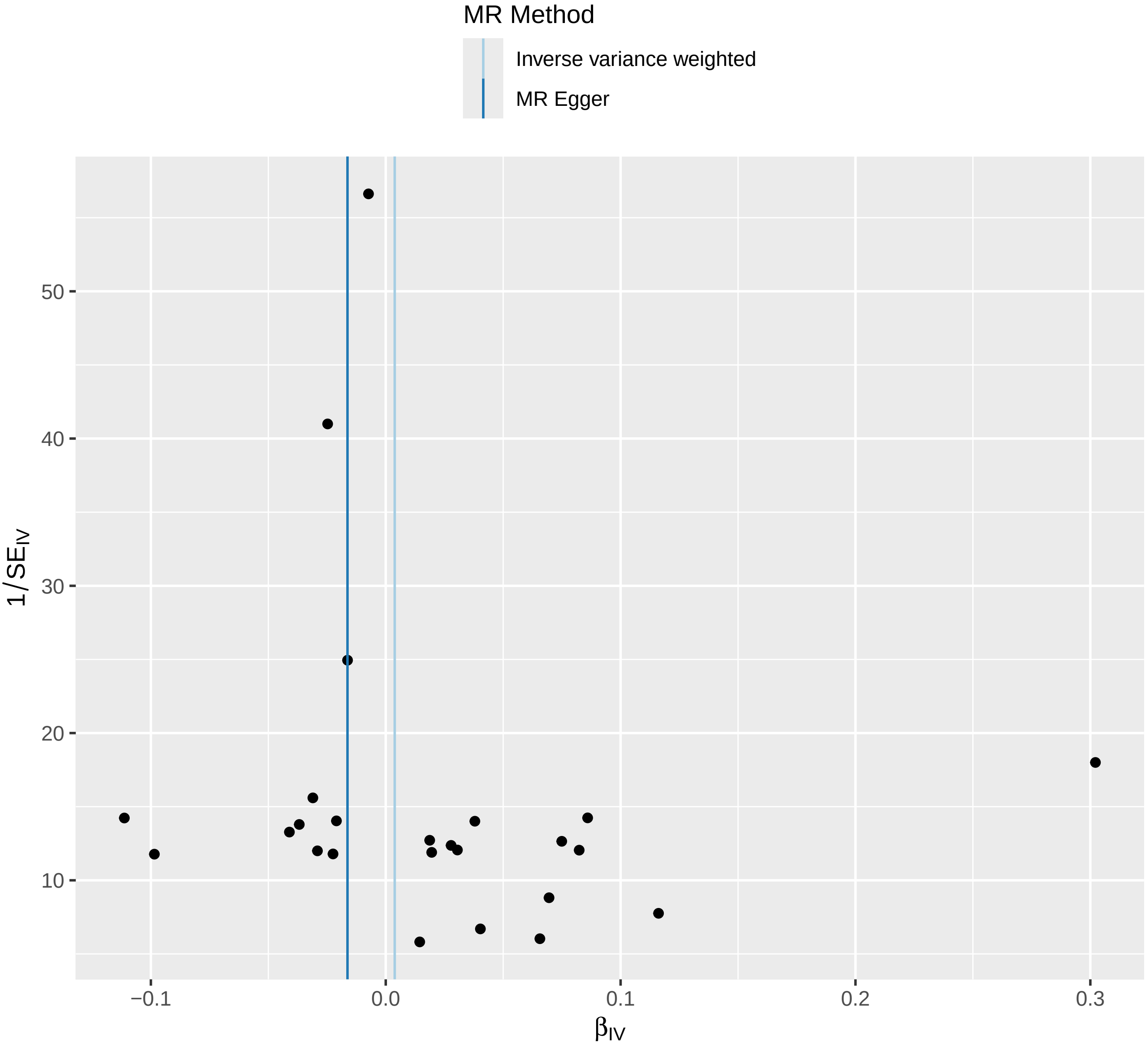
MR Method
Inverse variance weighted
MR Egger
50
40
30
20
10
1/SE$_{IV}$
−0.1
0.0
0.1
0.2
0.3
β$_{IV}$

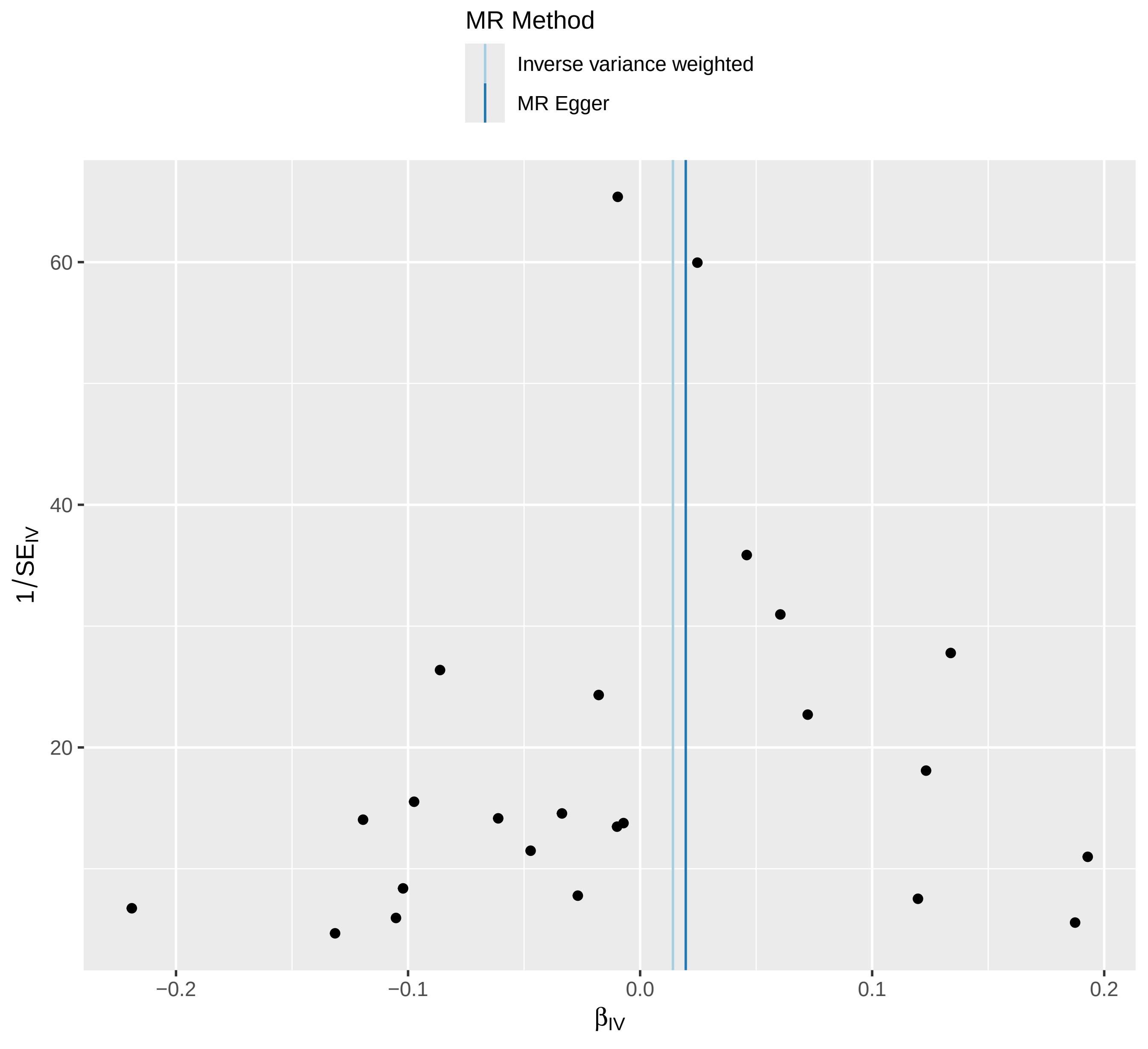

MR Method
Inverse variance weighted
MR Egger
60
40
20
1/SE$_{IV}$
−0.2
−0.1
0.0
0.1
0.2
β_{IV}

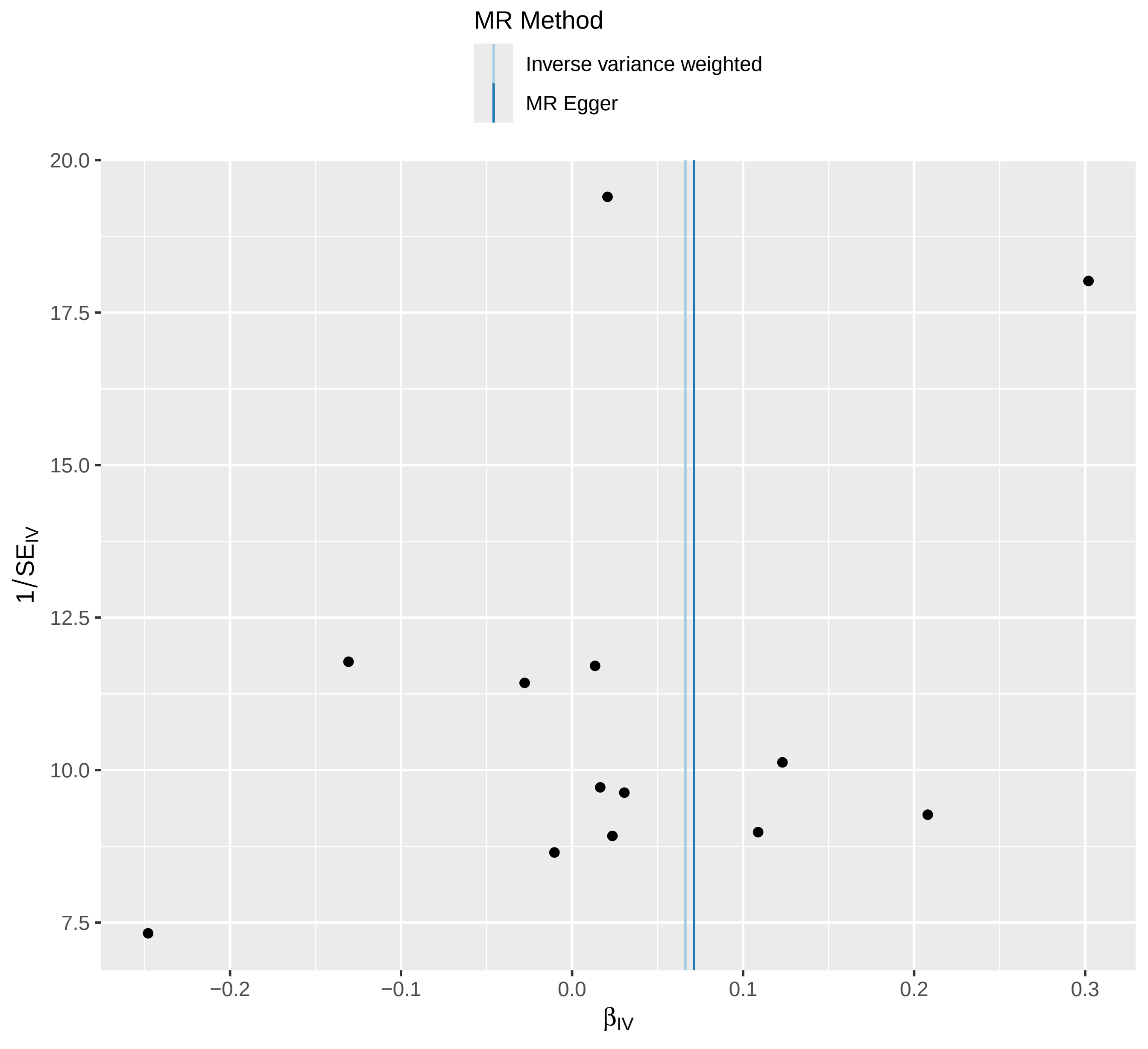
MR Method
Inverse variance weighted
MR Egger
20.0
17.5
15.0
12.5
10.0
7.5
1/SE_IV
−0.2
−0.1
0.0
0.1
0.2
0.3
β_IV

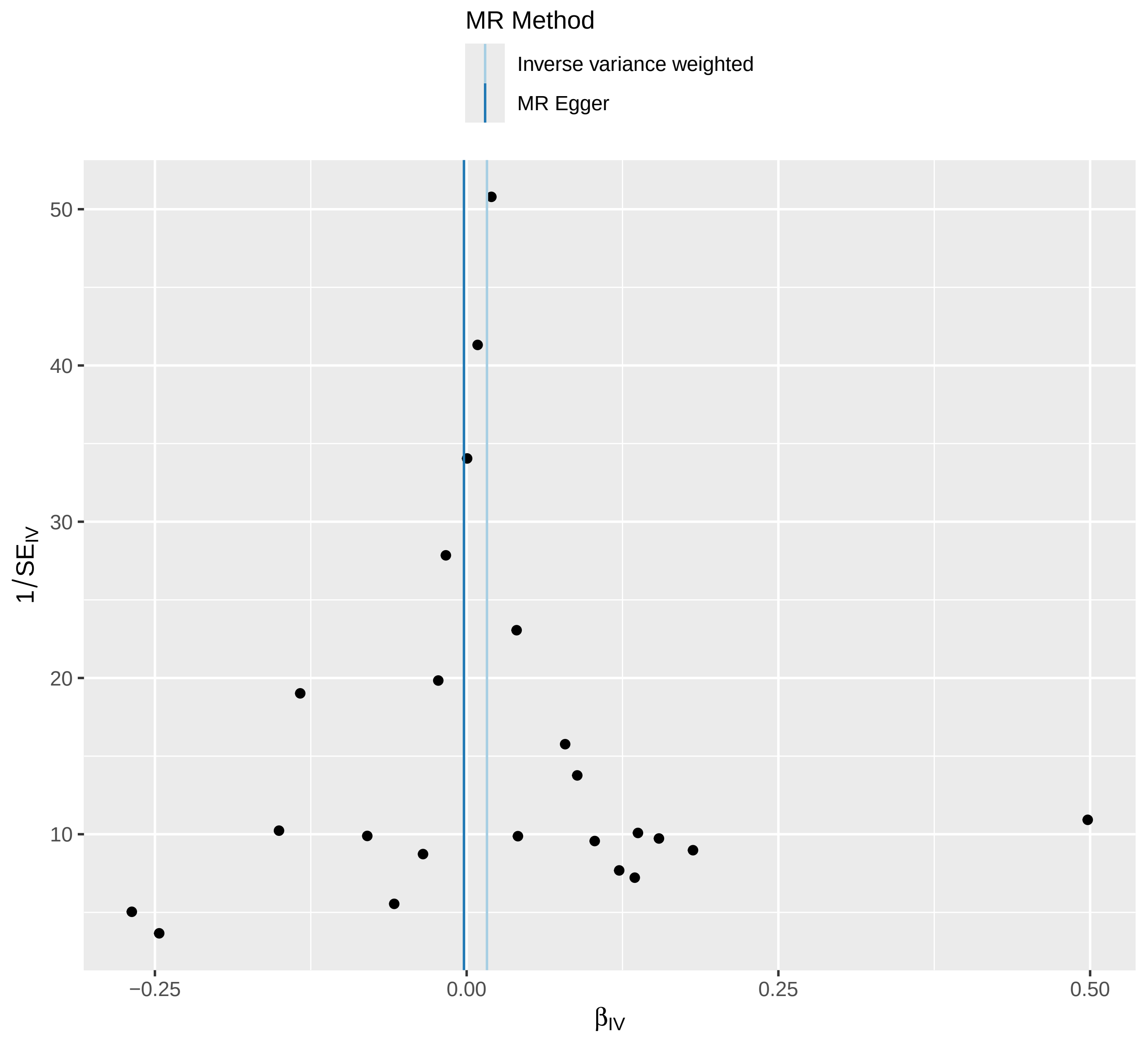

MR Method
Inverse variance weighted
MR Egger
50
40
30
20
10
$1/SE_{IV}$
−0.25
0.00
0.25
0.50
β_{IV}

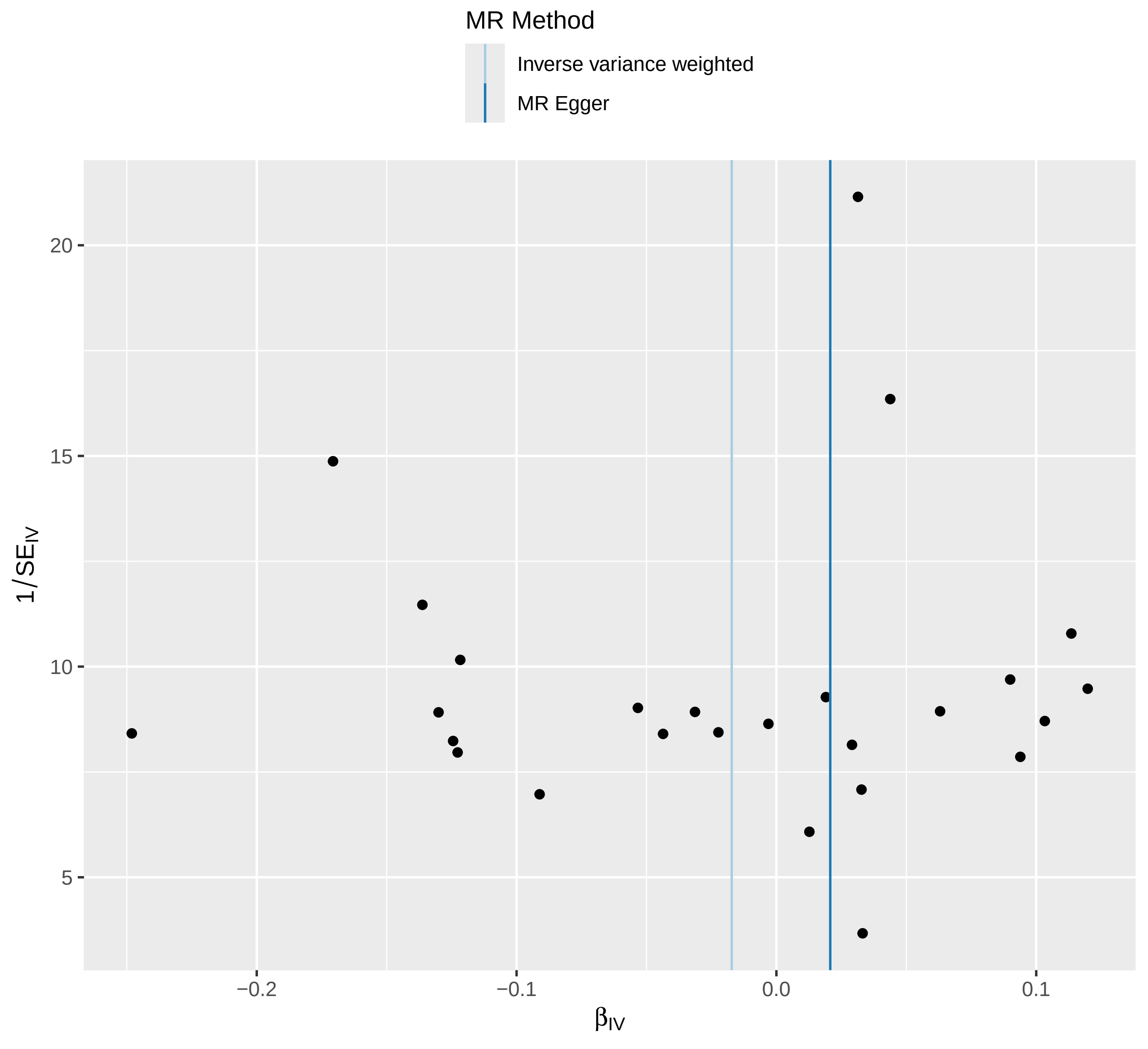

MR Method
Inverse variance weighted
MR Egger
20
15
10
5
1/SE_IV
−0.2
−0.1
0.0
0.1
β_IV

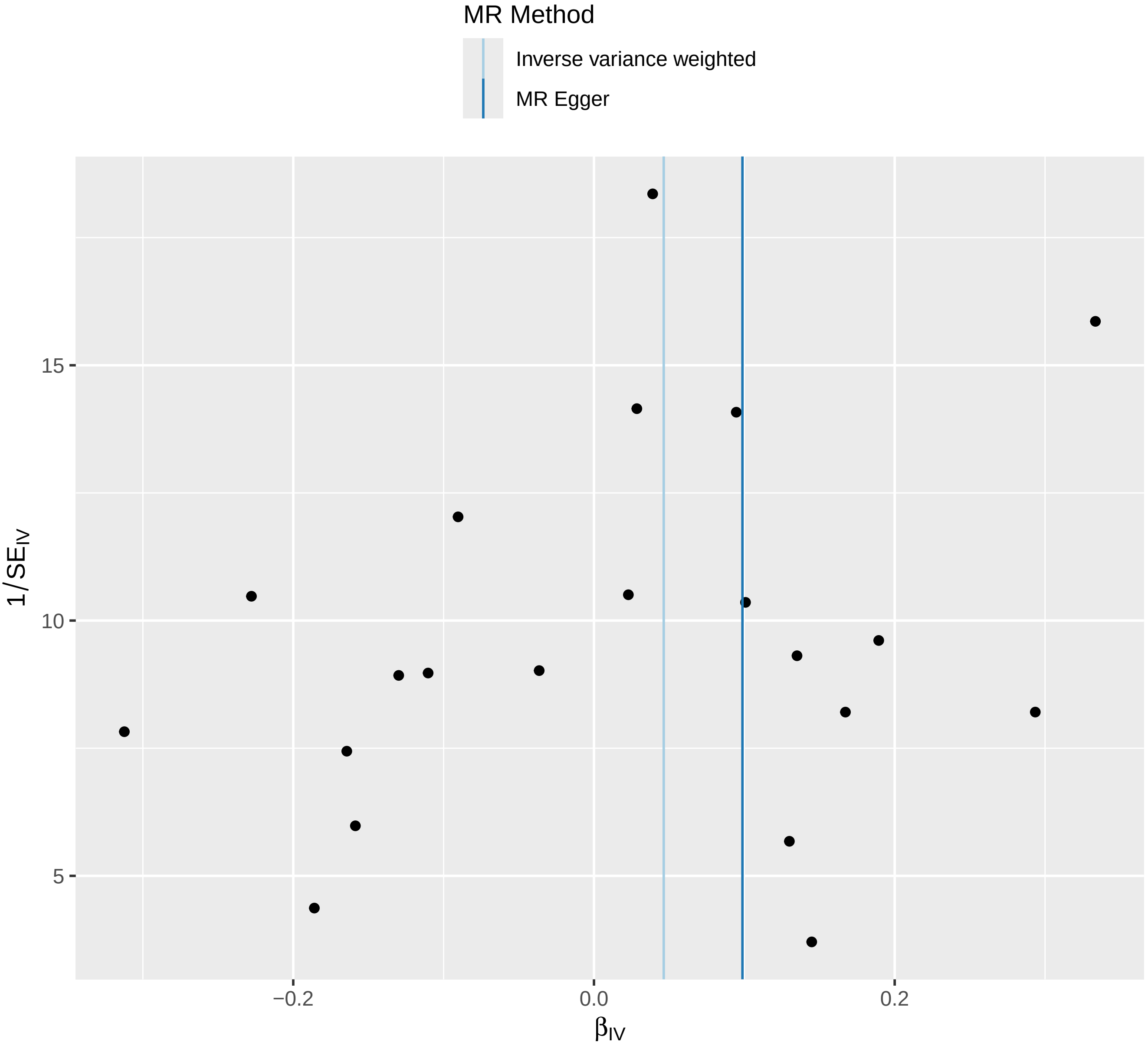
MR Method
Inverse variance weighted
MR Egger
1/SE$_{IV}$
15
10
5
−0.2
0.0
0.2
β$_{IV}$

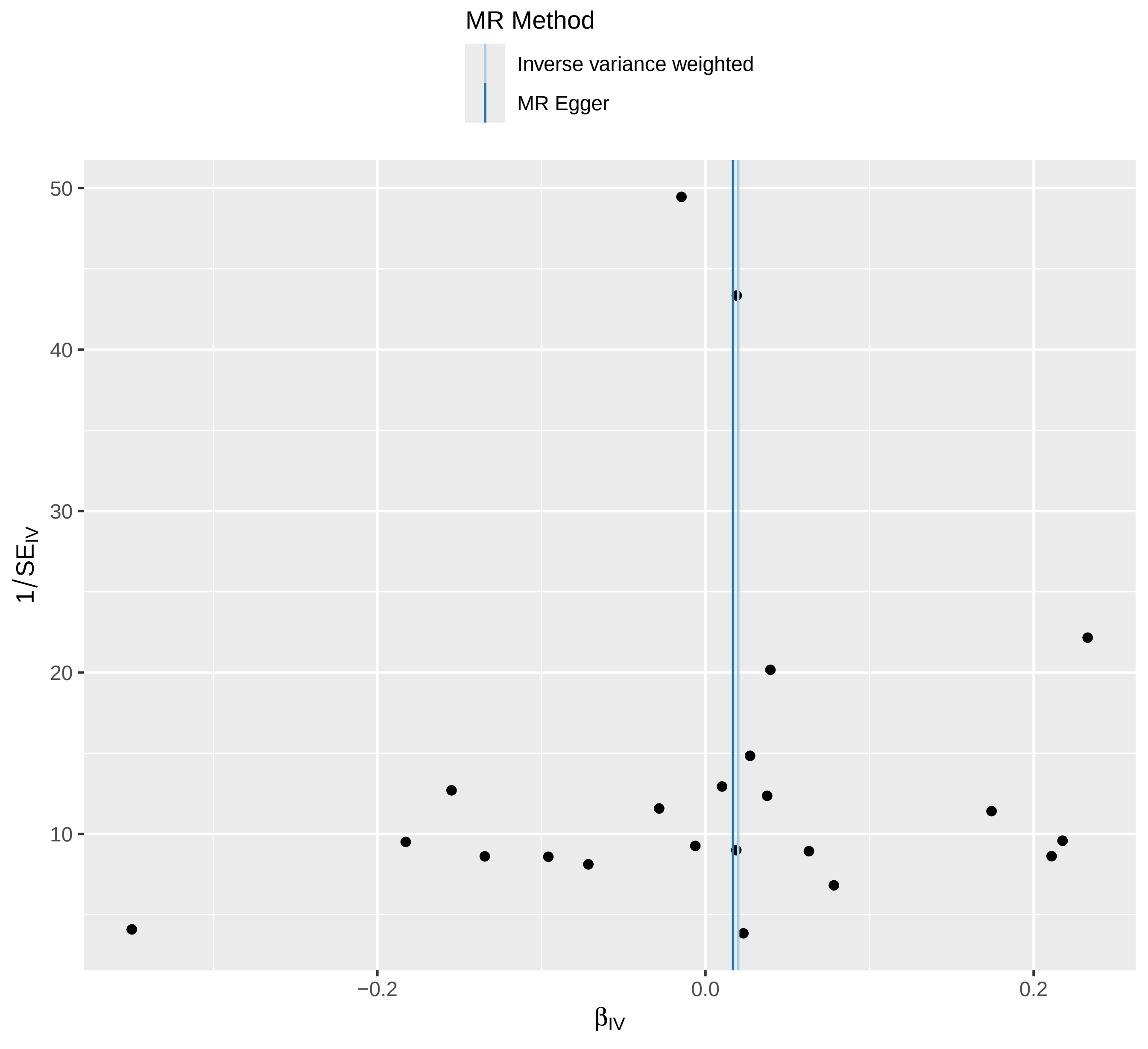
MR Method
Inverse variance weighted
MR Egger
50
40
30
20
10
1/SE$_{IV}$
−0.2
0.0
0.2
β$_{IV}$

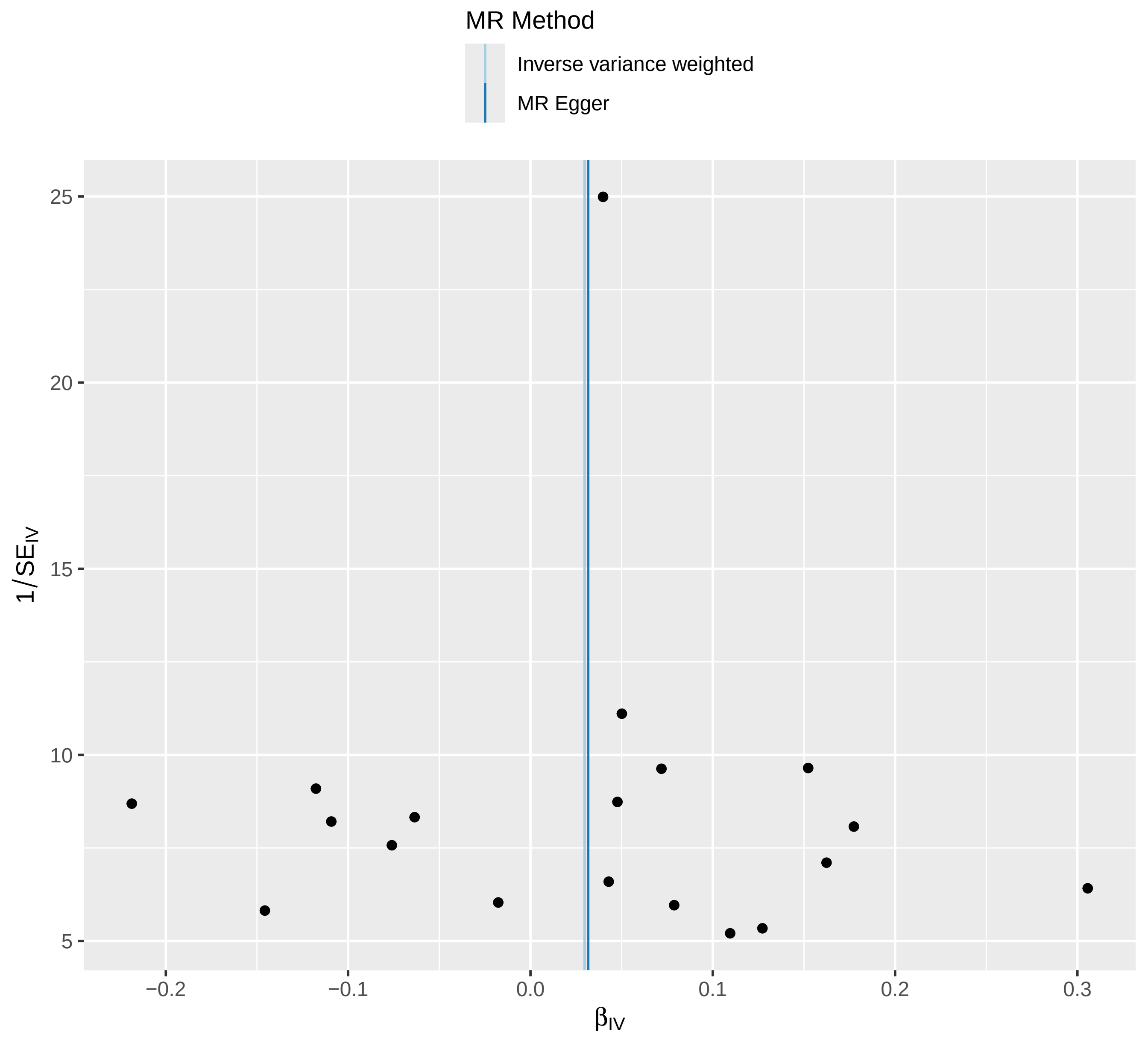

MR Method
Inverse variance weighted
MR Egger
25
20
15
10
5
1/SE_IV
−0.2
−0.1
0.0
0.1
0.2
0.3
β_IV

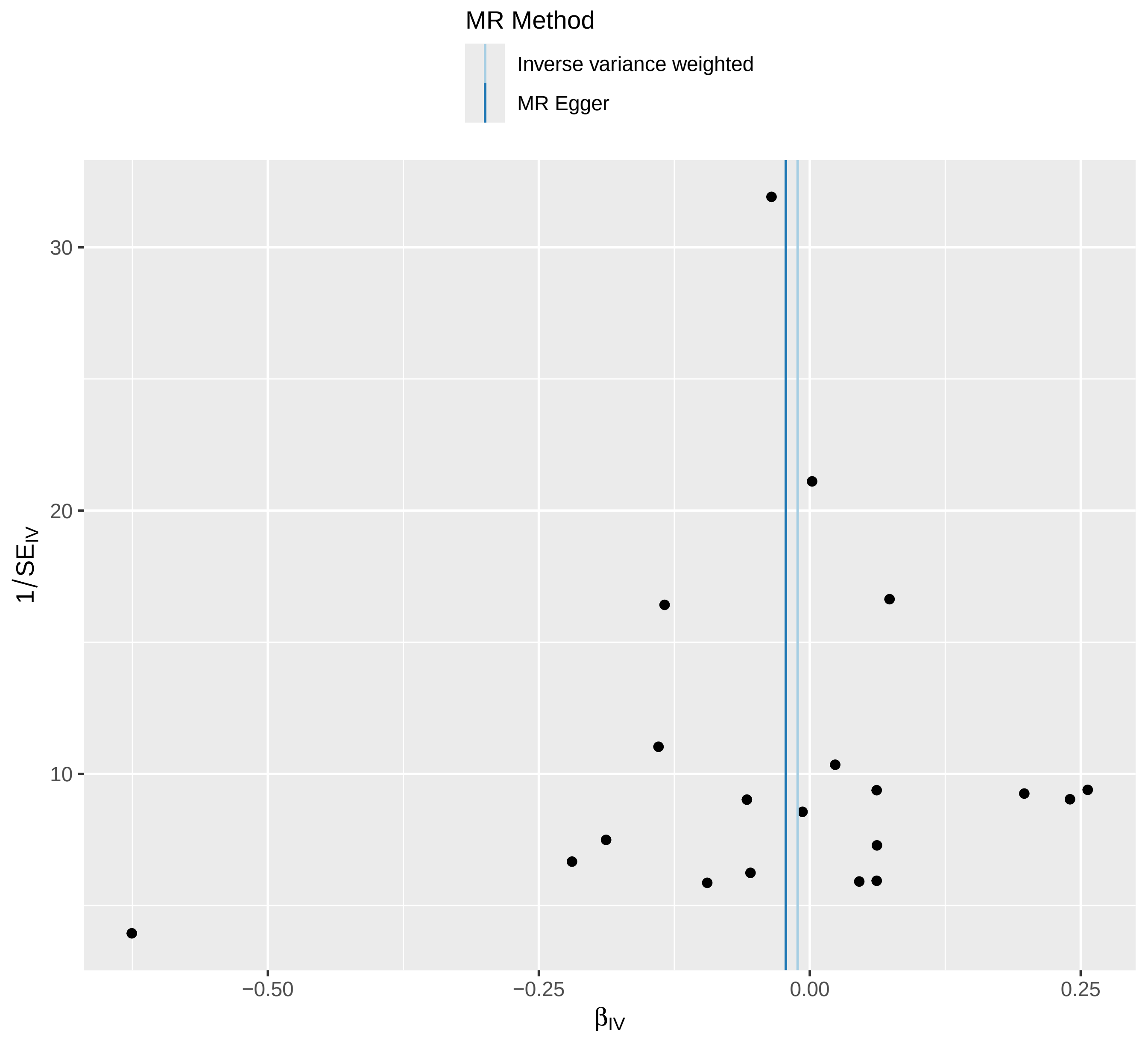
MR Method
Inverse variance weighted
MR Egger
30
20
10
1/SE$_{IV}$
−0.50
−0.25
0.00
0.25
β$_{IV}$

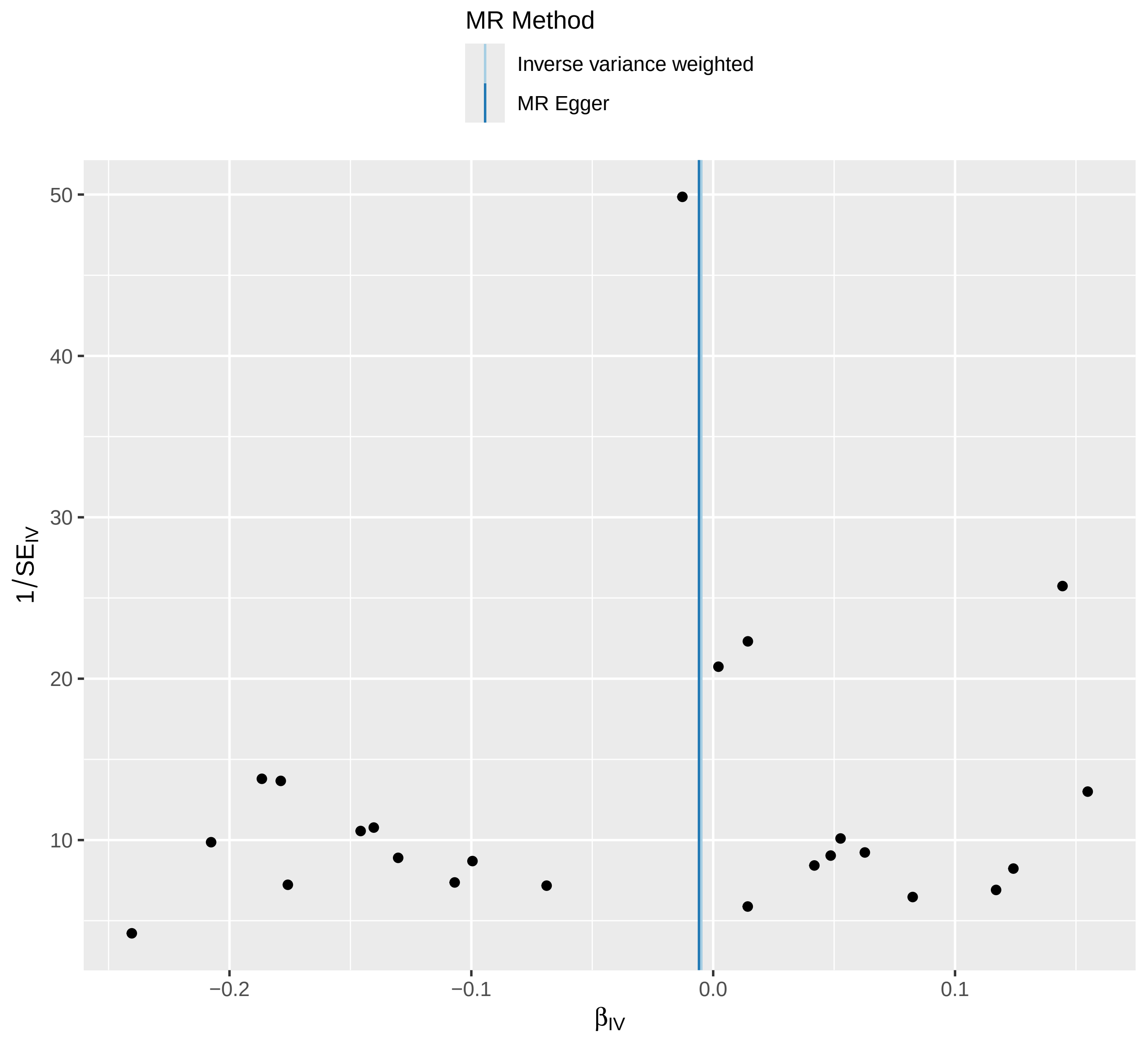
MR Method
Inverse variance weighted
MR Egger
50
40
30
20
10
1/SE$_{IV}$
−0.2
−0.1
0.0
0.1
β$_{IV}$

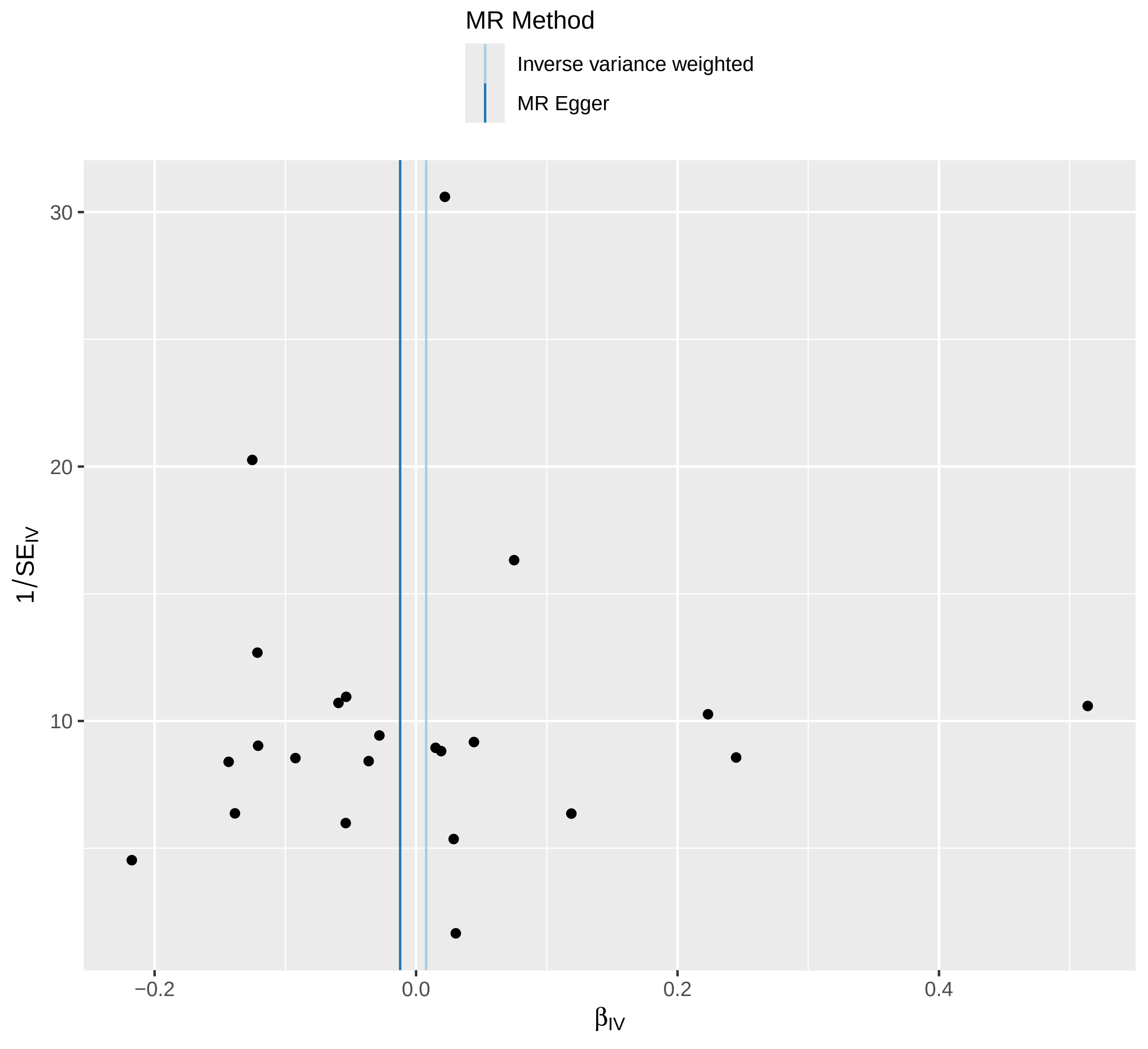
MR Method
Inverse variance weighted
MR Egger
30
20
10
1/SE$_{IV}$
−0.2
0.0
0.2
0.4
β$_{IV}$

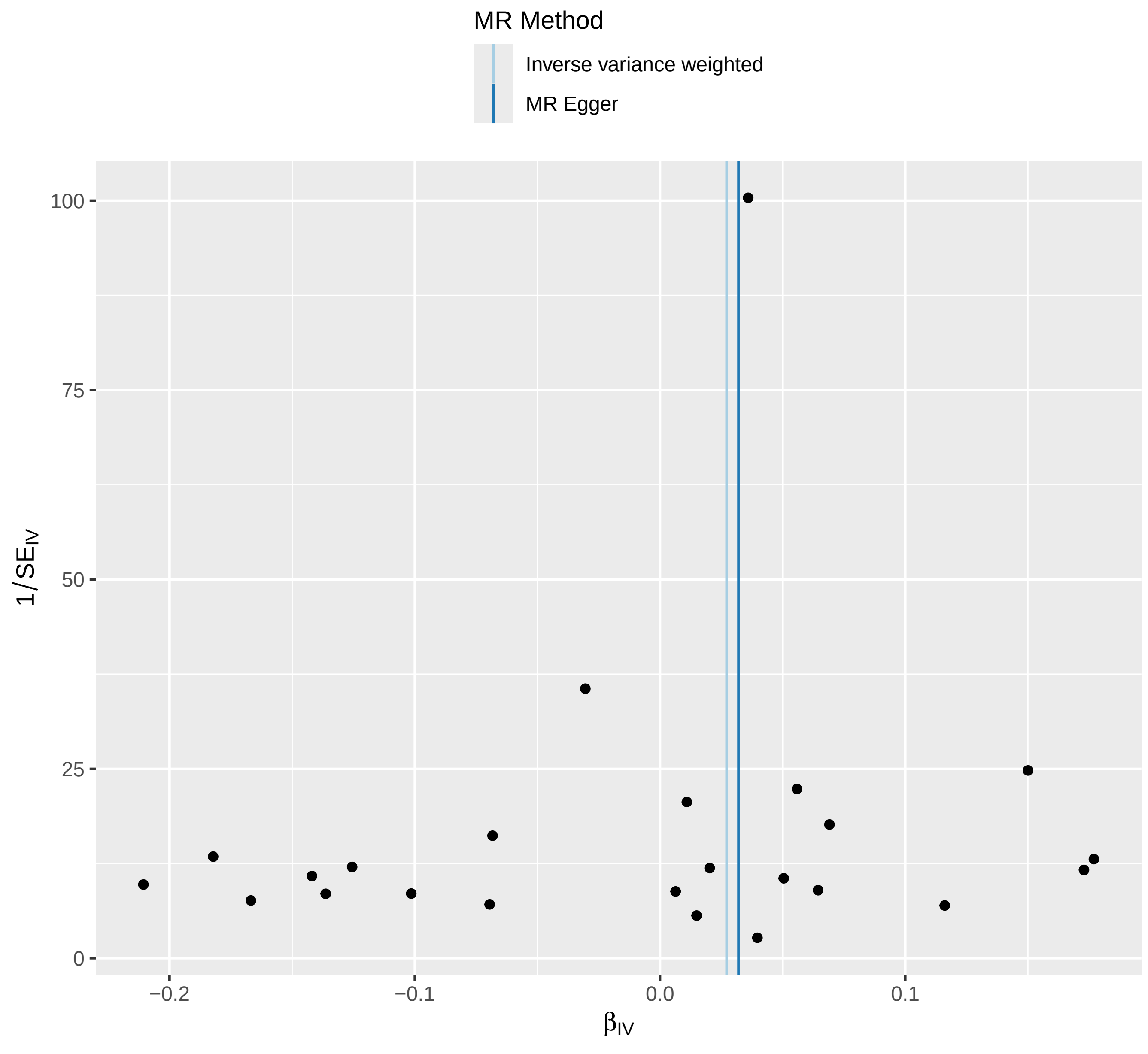
MR Method
Inverse variance weighted
MR Egger
100
75
50
25
0
1/SE$_{IV}$
−0.2
−0.1
0.0
0.1
β$_{IV}$

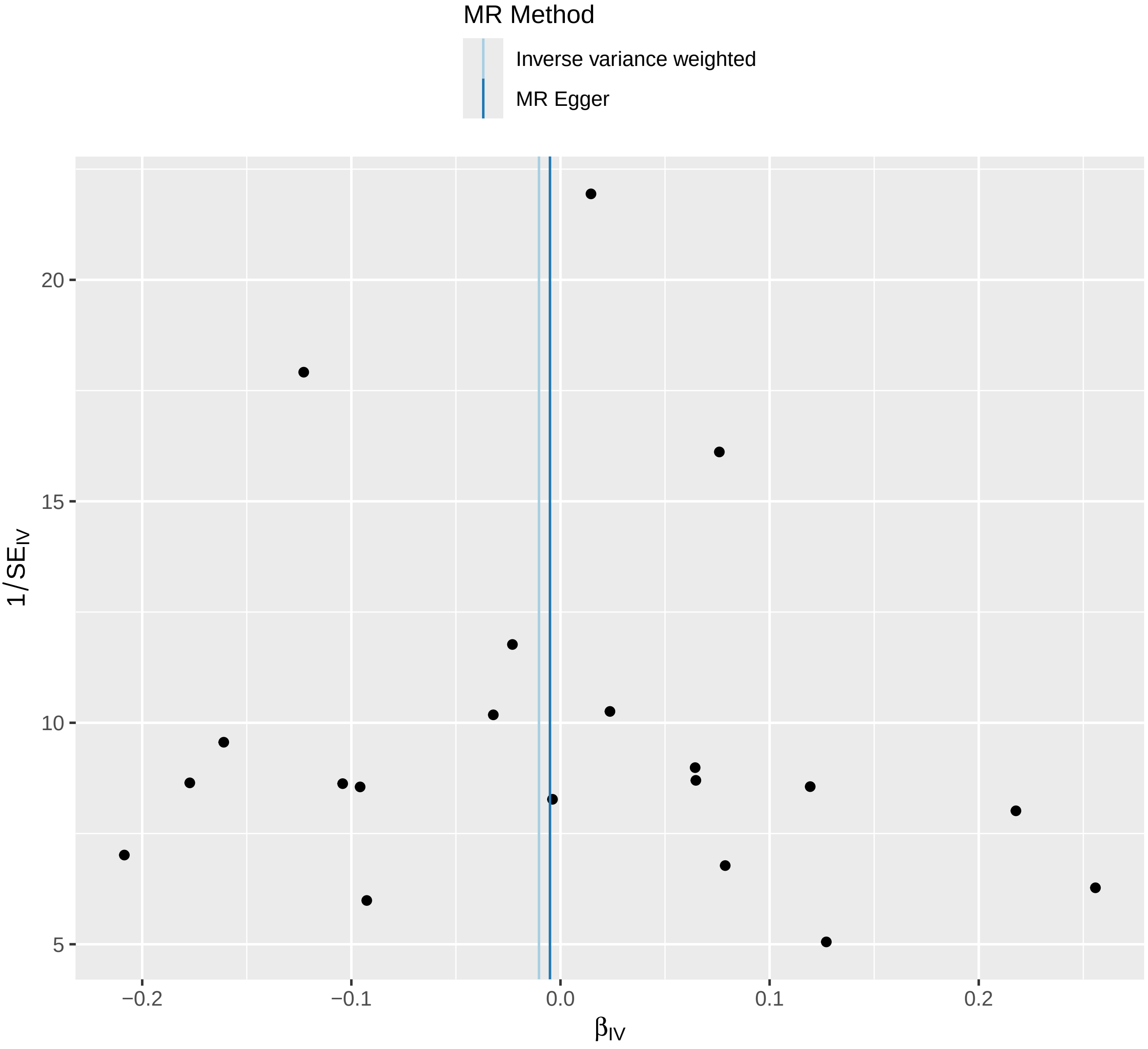
MR Method
Inverse variance weighted
MR Egger
1/SE$_{IV}$
20
15
10
5
−0.2
−0.1
0.0
0.1
0.2
β$_{IV}$

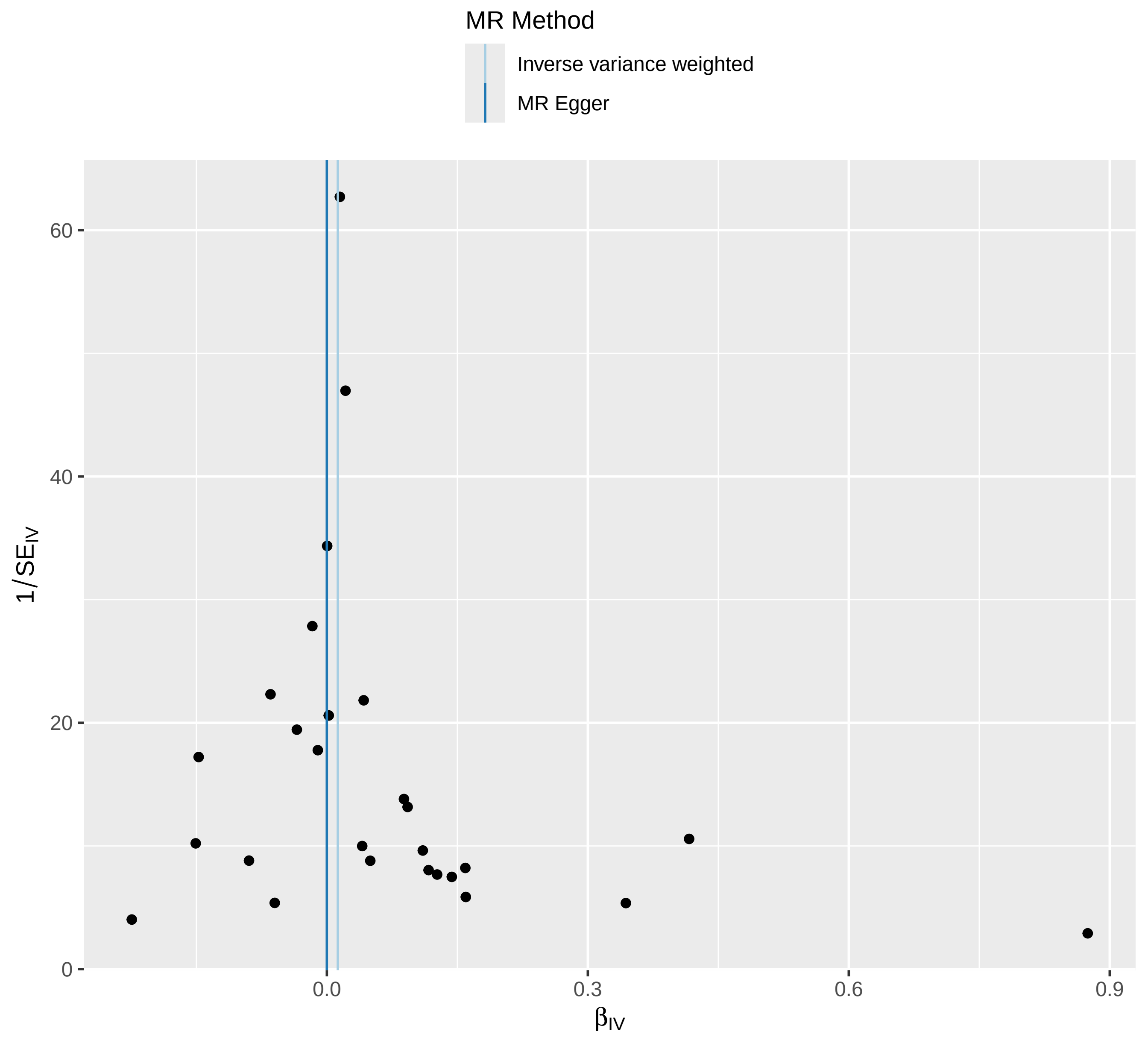

MR Method
Inverse variance weighted
MR Egger
60
40
20
0
1/SE_IV
0.0
0.3
0.6
0.9
β_IV

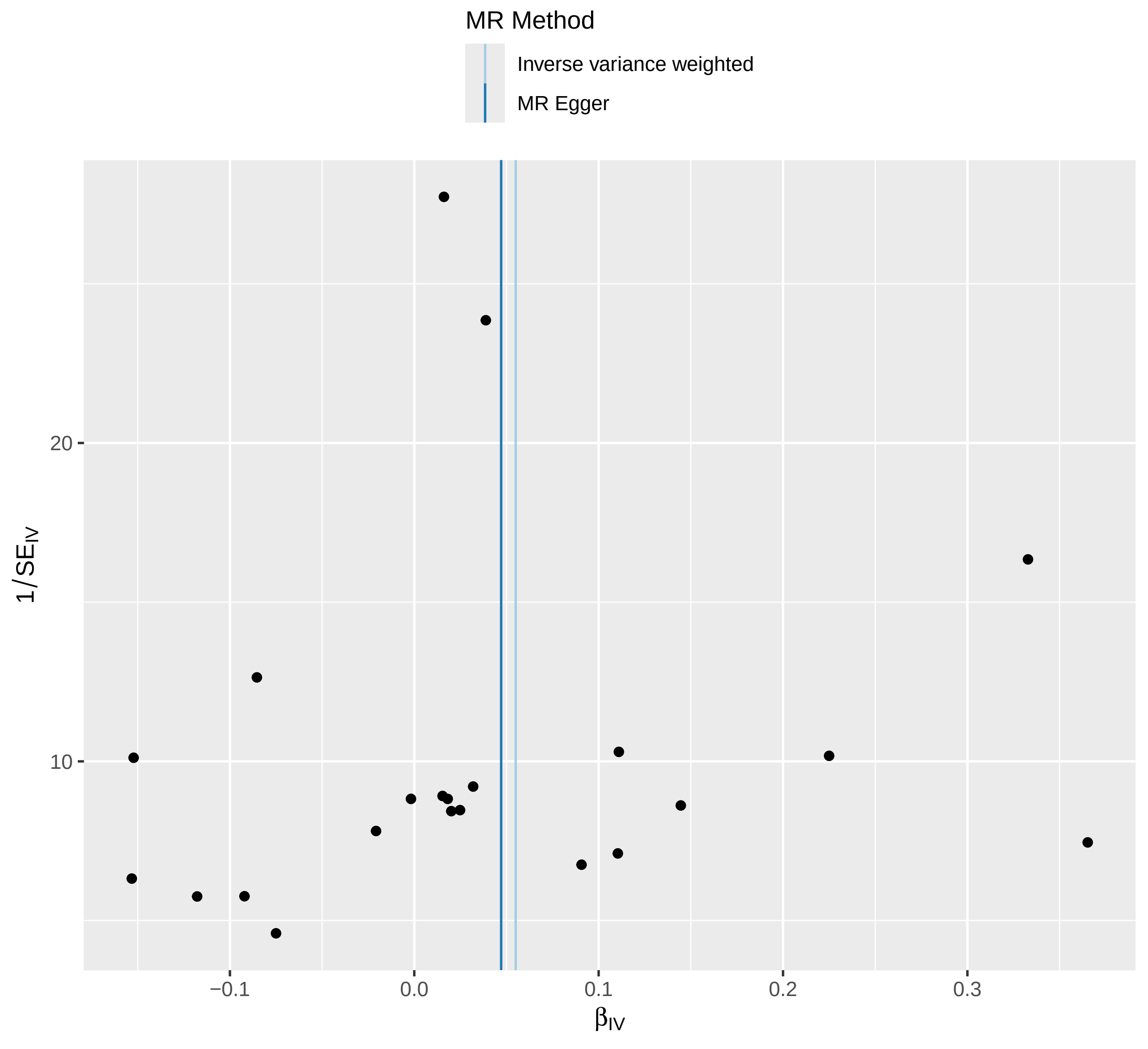
MR Method
Inverse variance weighted
MR Egger
20
10
1/SE_IV
−0.1
0.0
0.1
0.2
0.3
β_IV

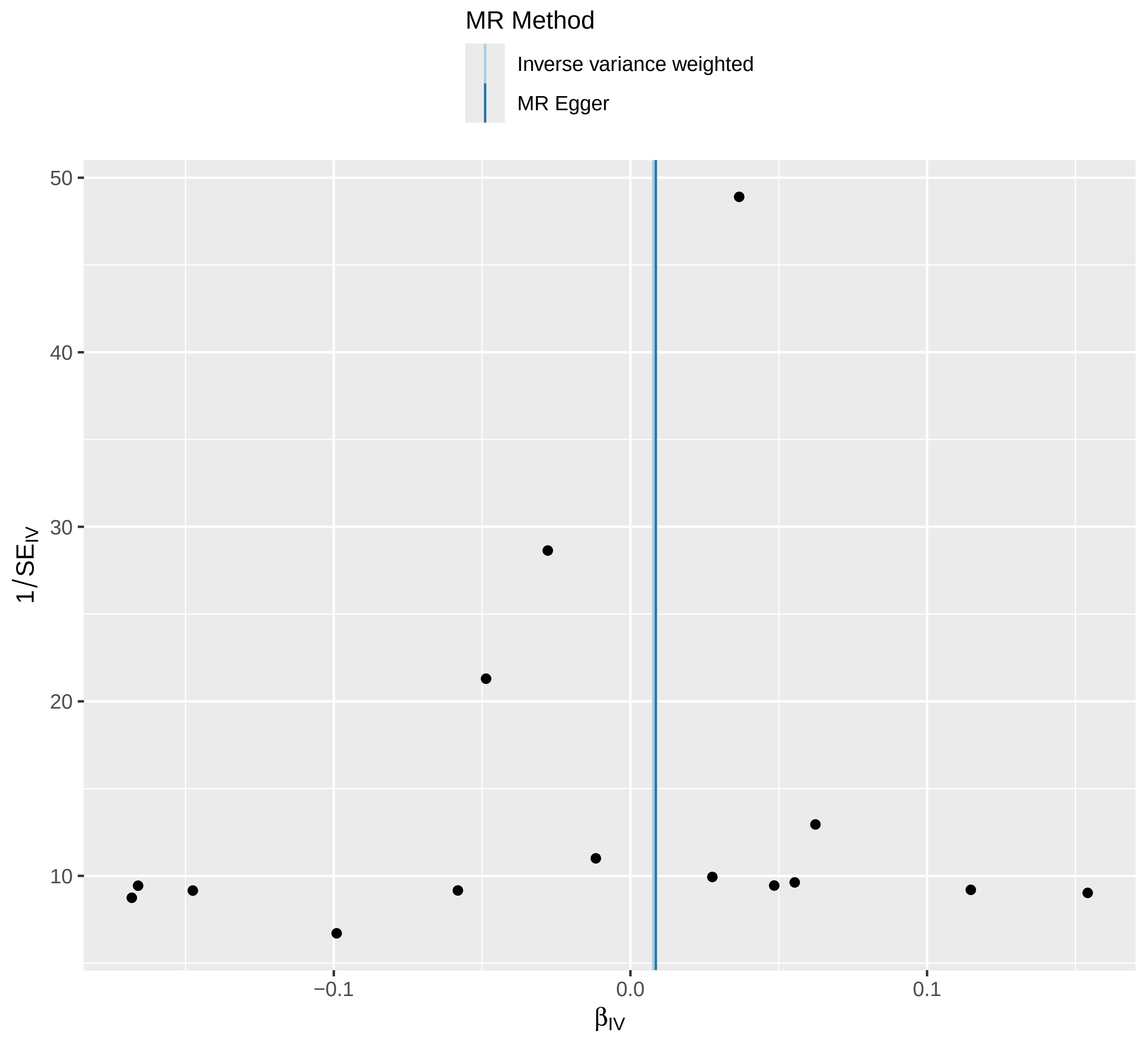
MR Method
Inverse variance weighted
MR Egger
50
40
30
20
10
1/SE$_{IV}$
−0.1
0.0
0.1
β$_{IV}$

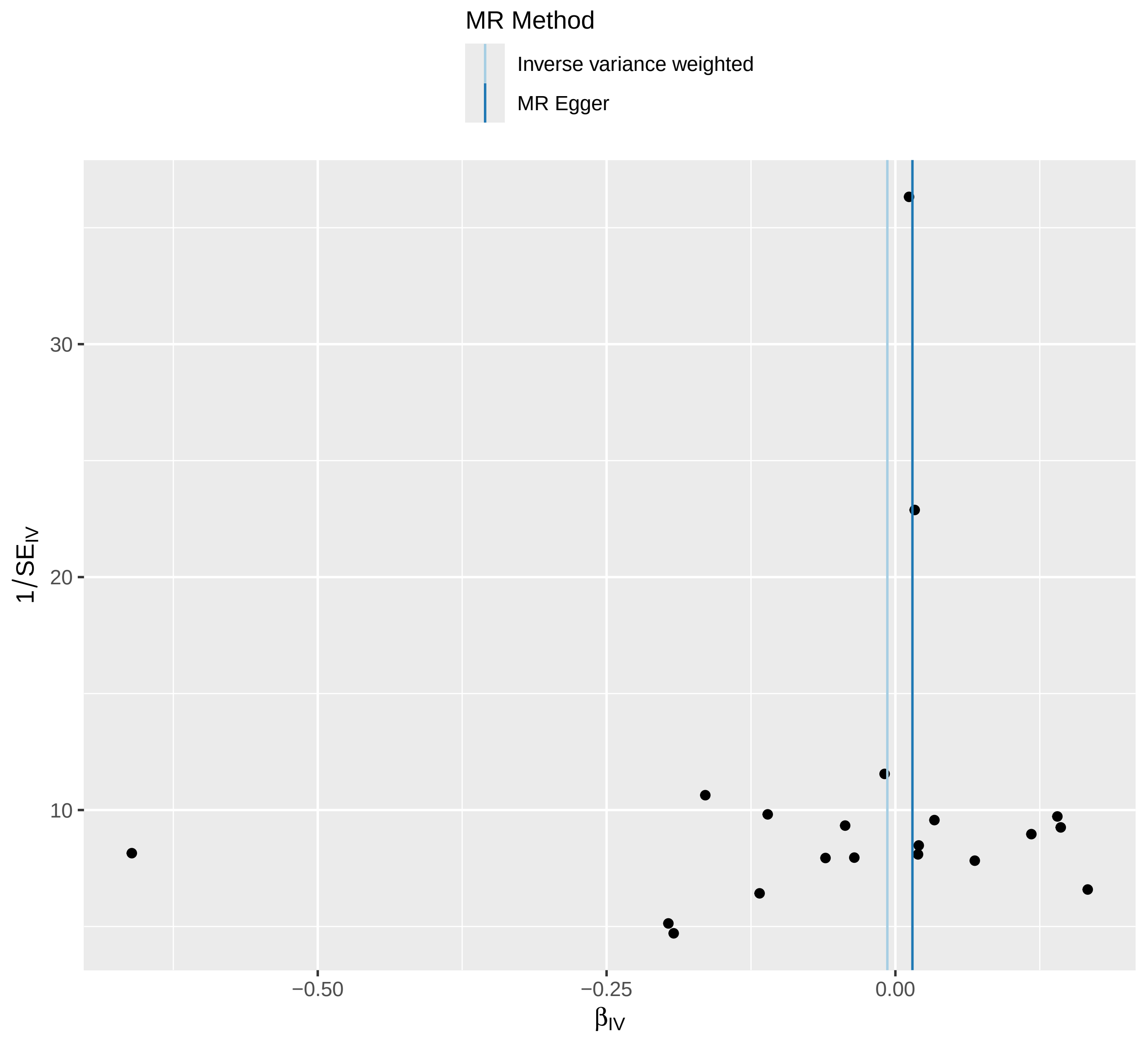

MR Method
Inverse variance weighted
MR Egger
30
20
10
1/SE_IV
−0.50
−0.25
0.00
β_IV

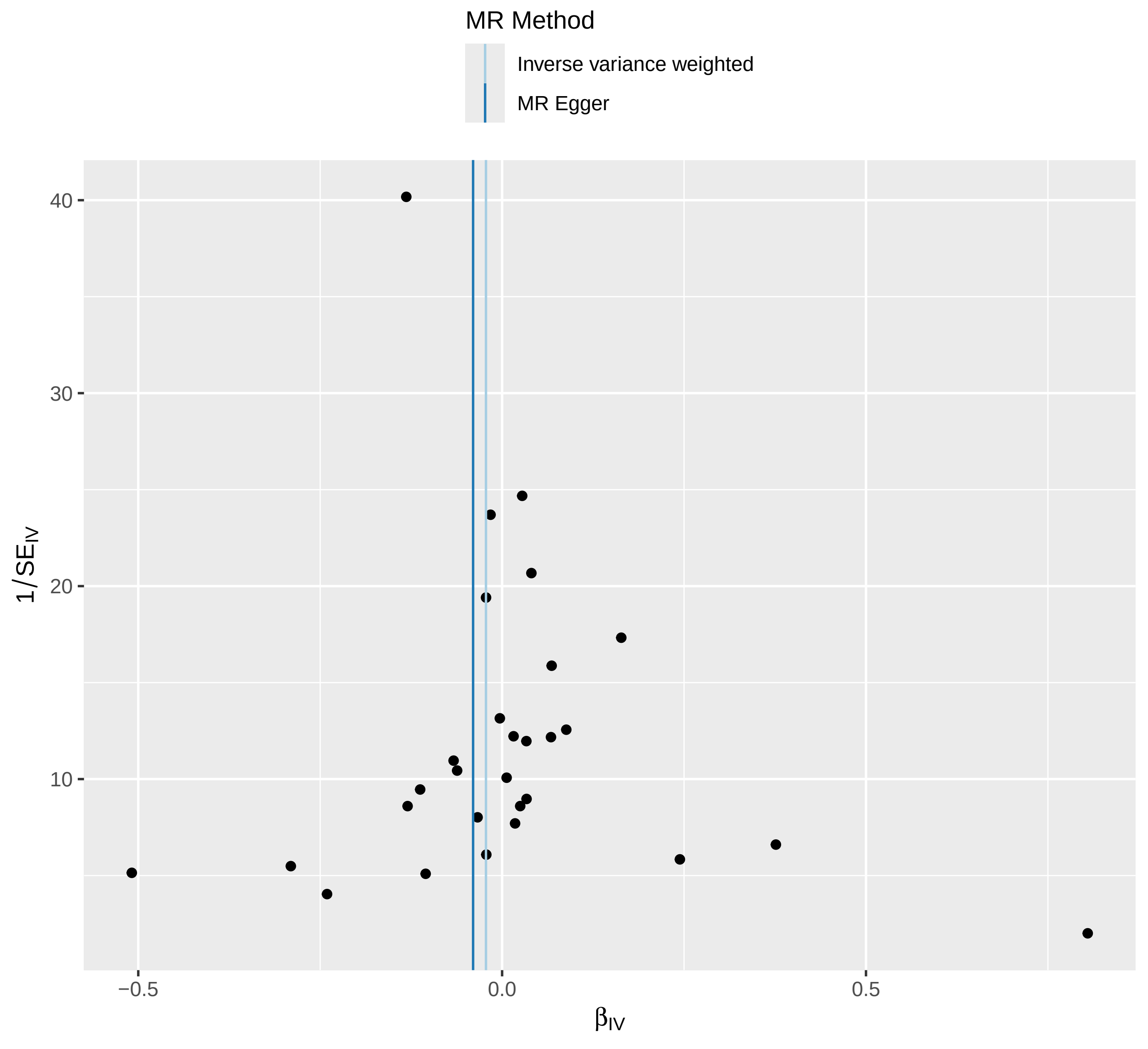
MR Method
Inverse variance weighted
MR Egger
40
30
20
10
1/SE_IV
−0.5
0.0
0.5
β_IV

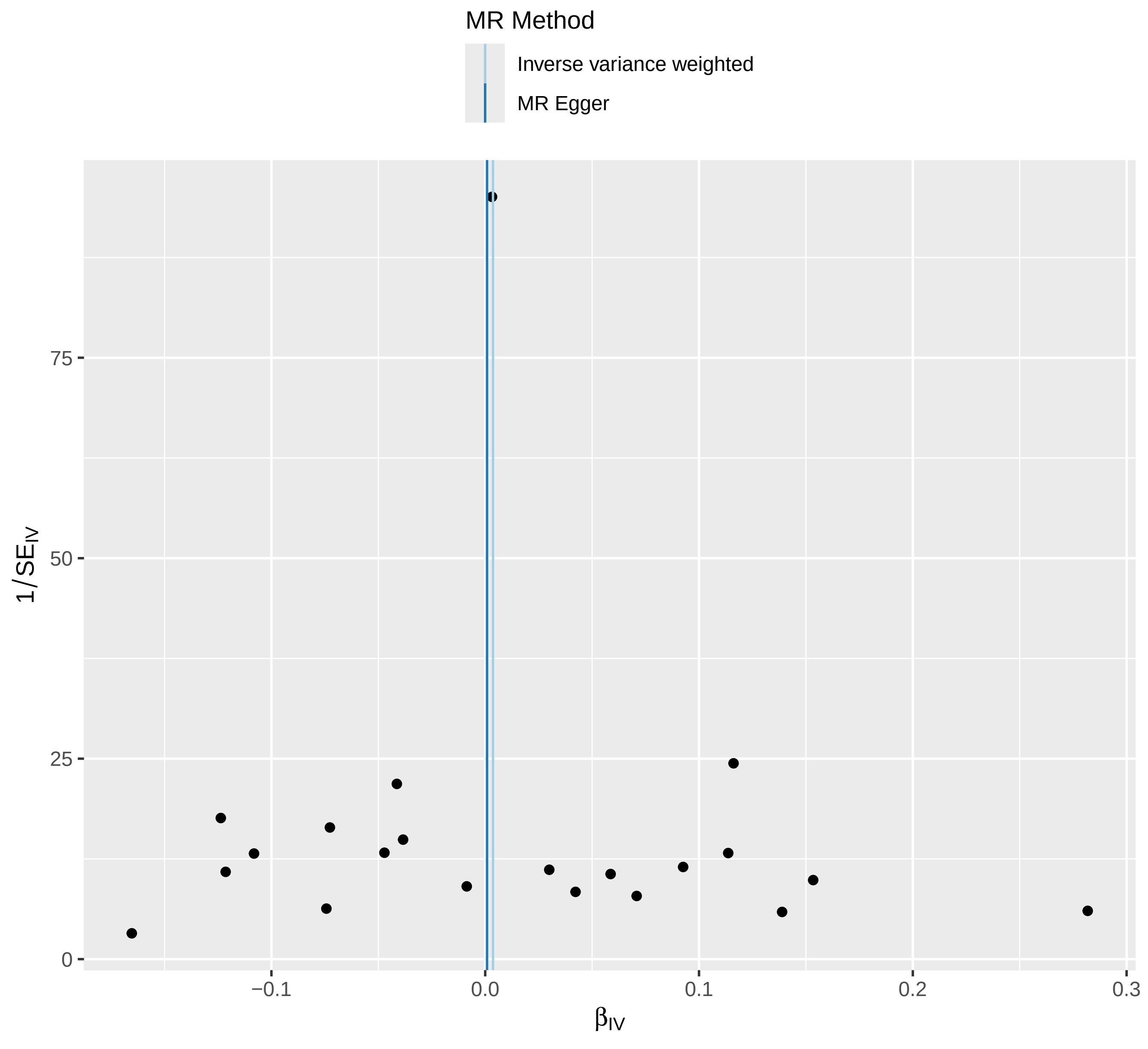
MR Method
Inverse variance weighted
MR Egger
1/SE_IV
75
50
25
0
−0.1
0.0
0.1
0.2
0.3
β_IV

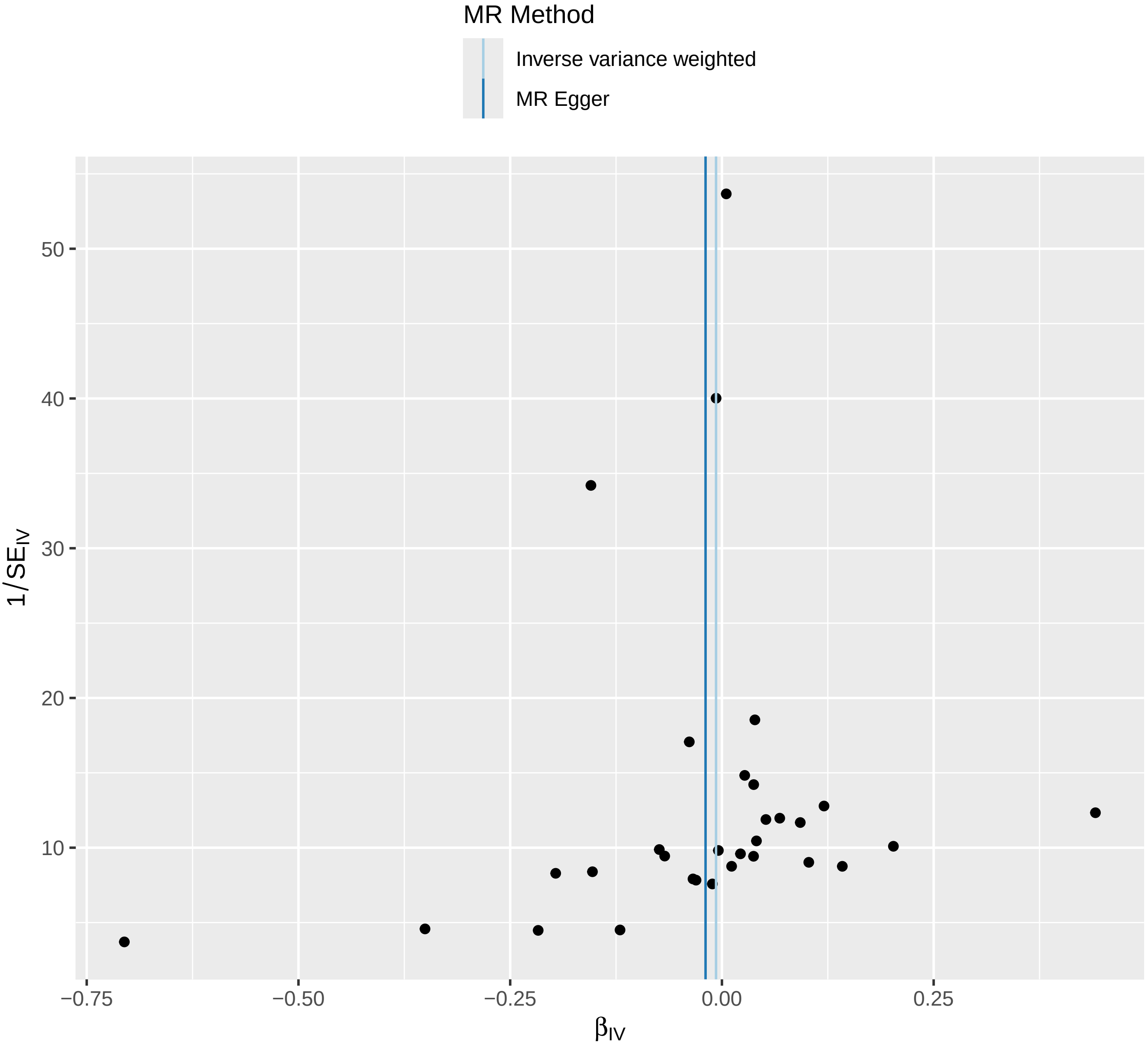

MR Method
Inverse variance weighted
MR Egger
1/SE_IV
50
40
30
20
10
−0.75
−0.50
−0.25
0.00
0.25
β_IV

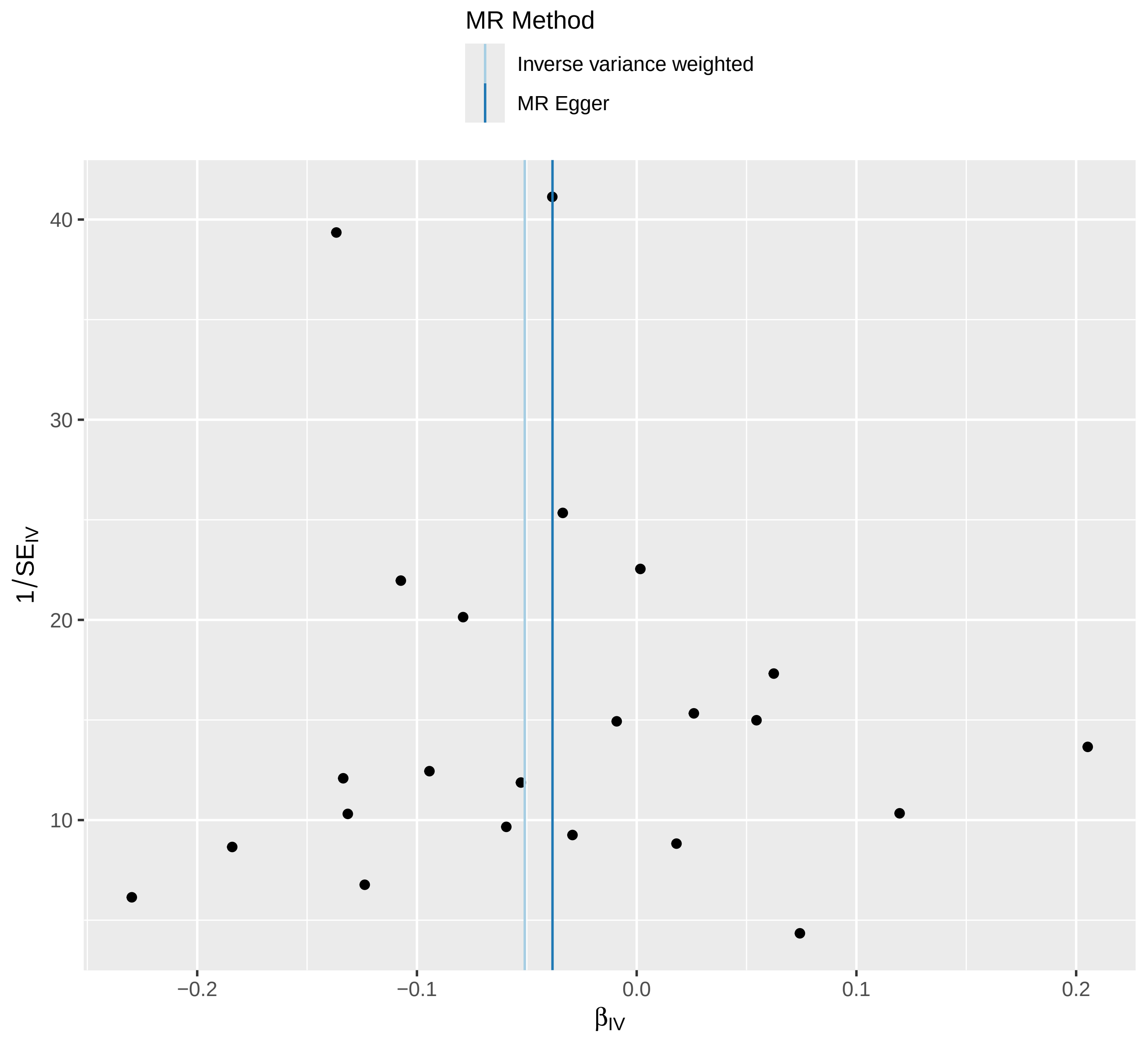

MR Method
Inverse variance weighted
MR Egger
40
30
20
10
1/SE_IV
−0.2
−0.1
0.0
0.1
0.2
β_IV

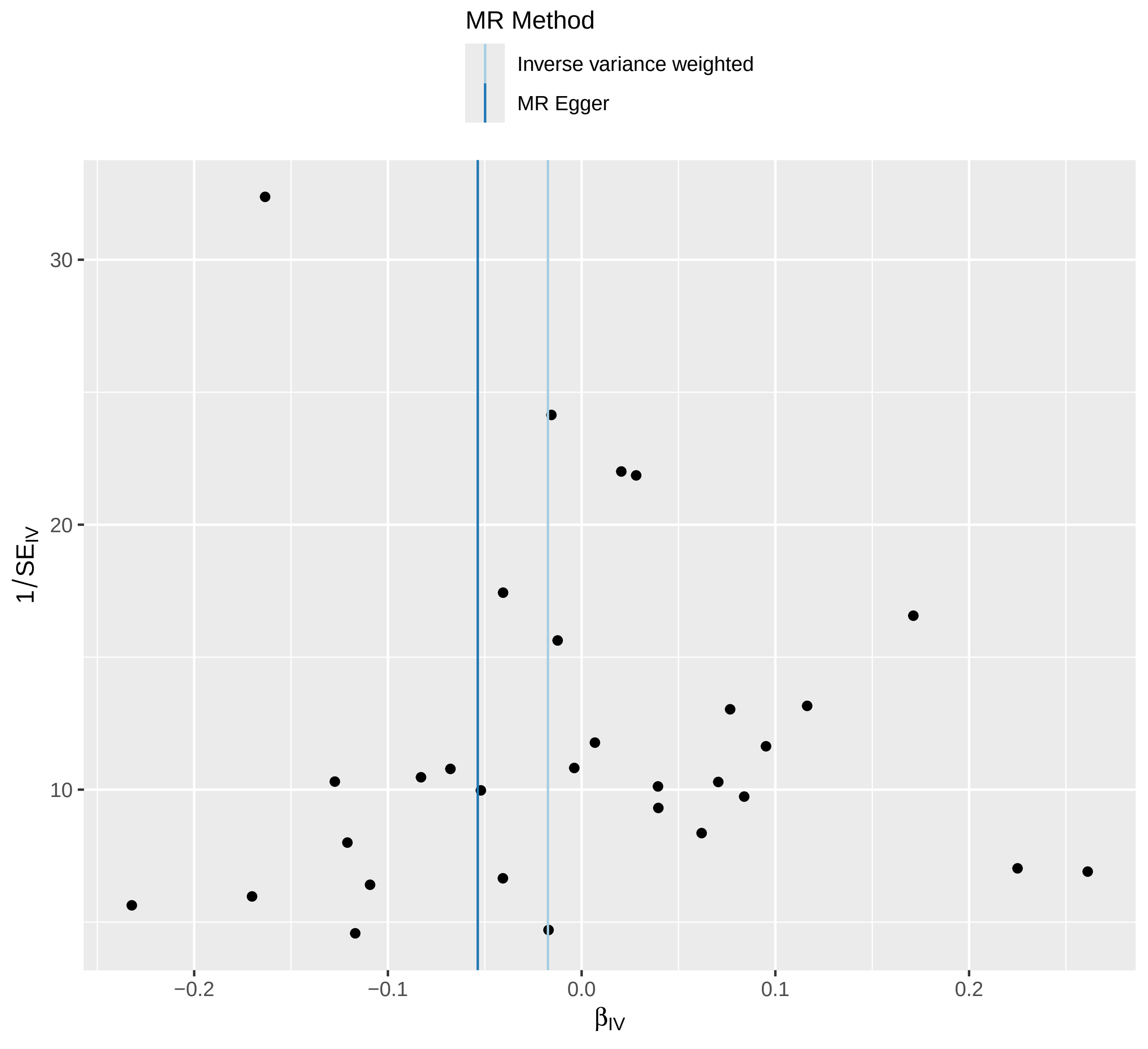
MR Method
Inverse variance weighted
MR Egger
30
20
10
1/SE_IV
−0.2
−0.1
0.0
0.1
0.2
β_IV

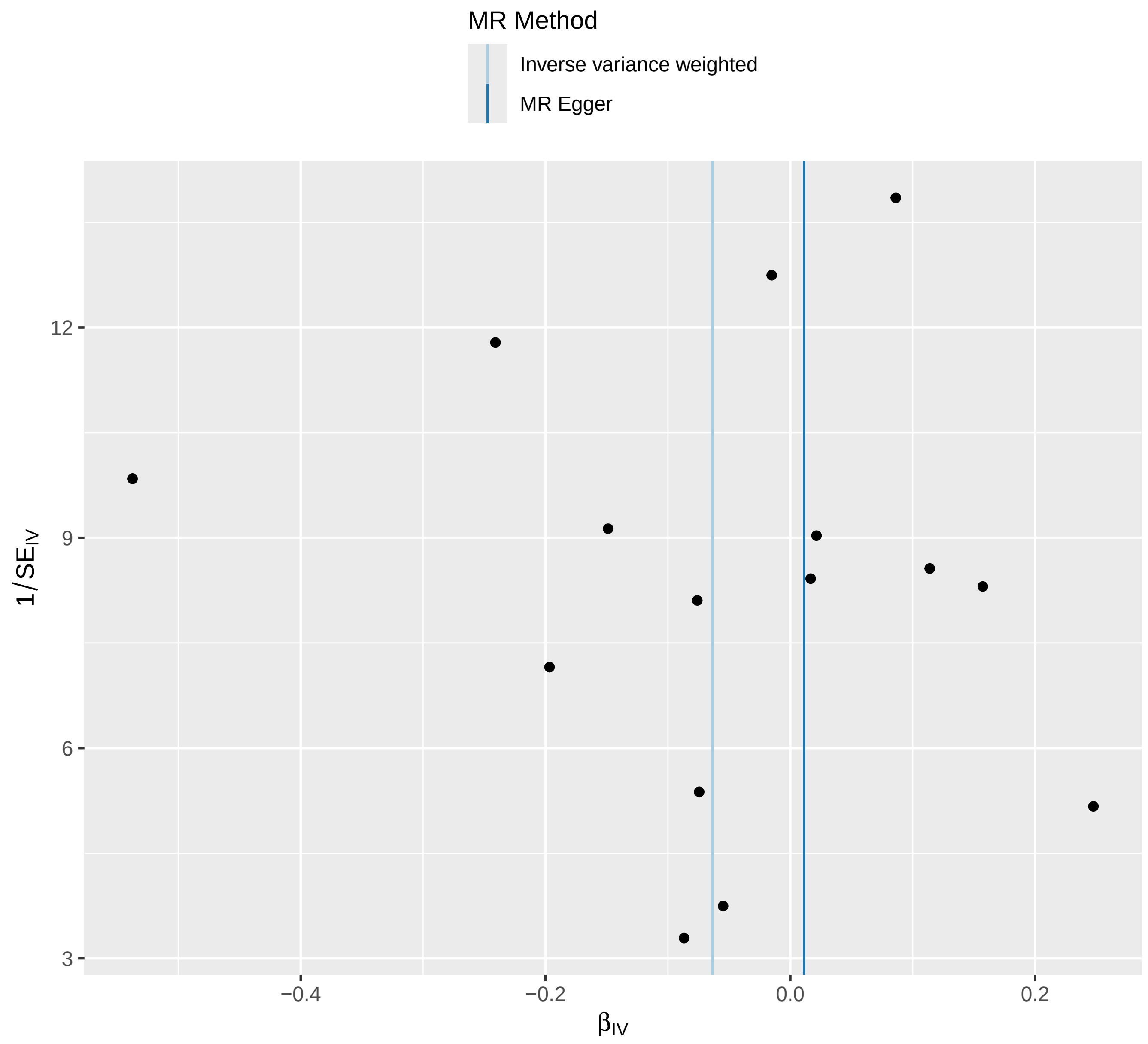
MR Method
Inverse variance weighted
MR Egger
12
9
6
3
$1/SE_{IV}$
−0.4
−0.2
0.0
0.2
β_{IV}

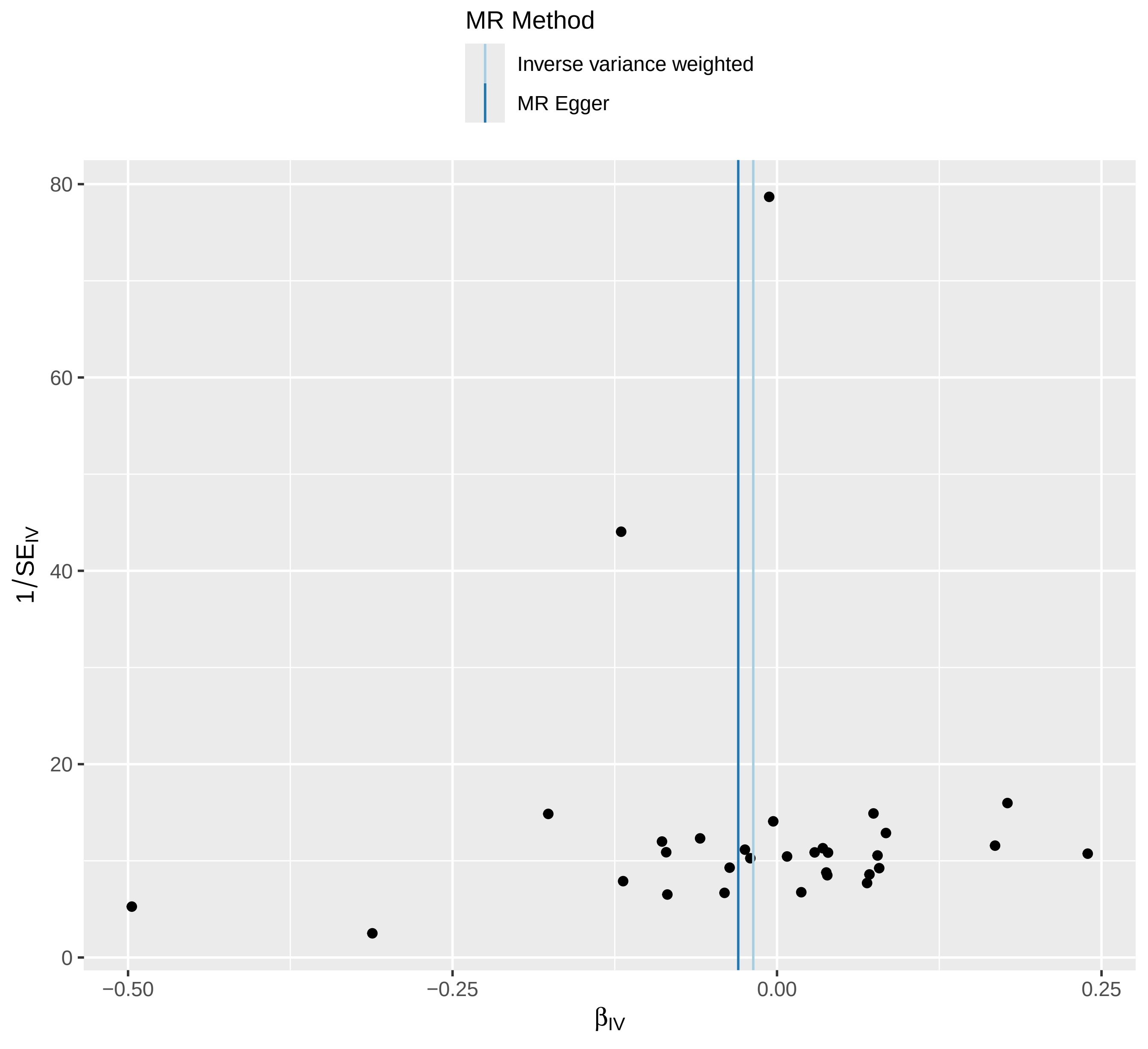
MR Method
Inverse variance weighted
MR Egger
1/SE_IV
80
60
40
20
0
−0.50
−0.25
0.00
0.25
β_IV

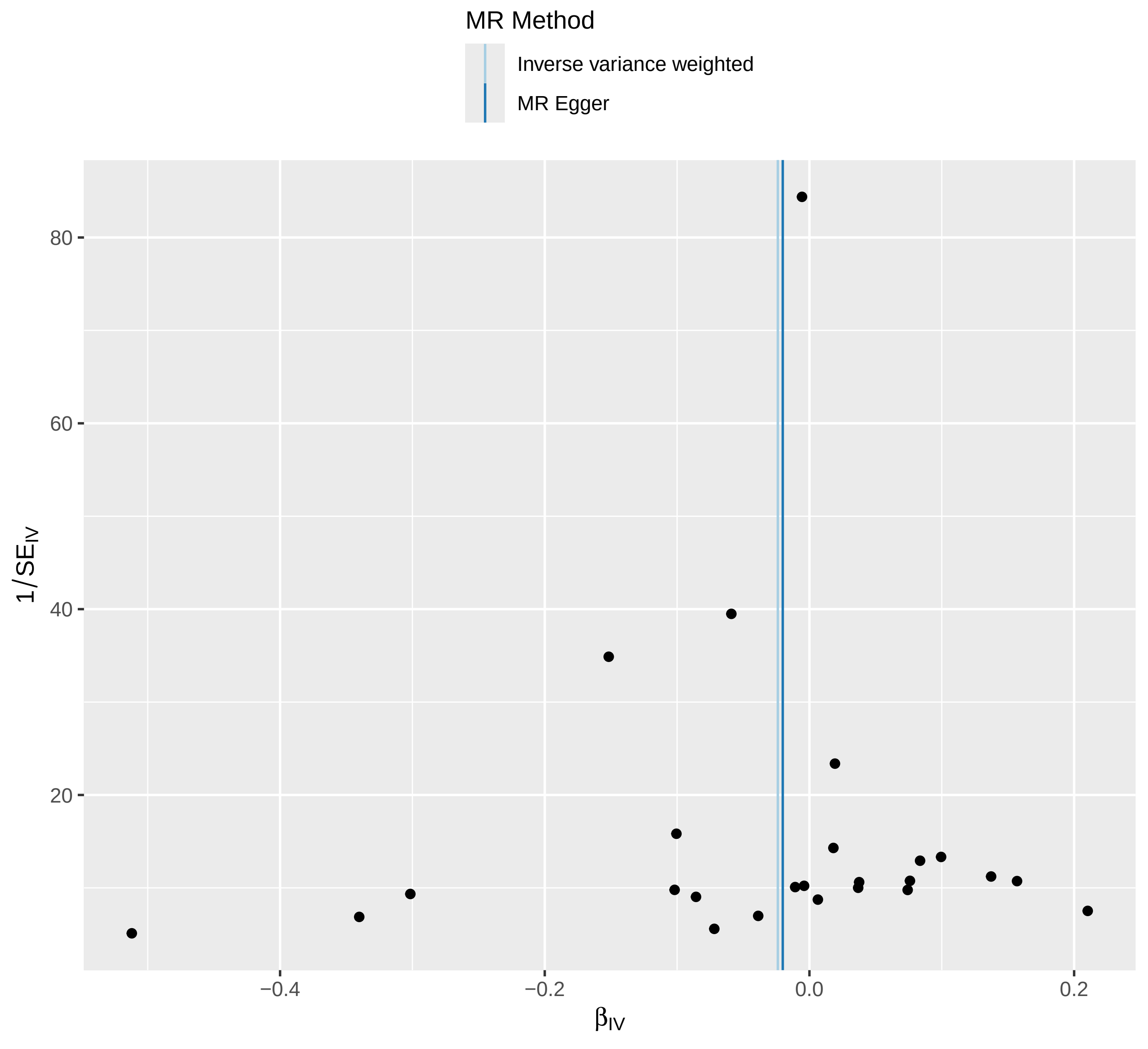
MR Method
Inverse variance weighted
MR Egger
80
60
40
20
1/SE_IV
−0.4
−0.2
0.0
0.2
β_IV

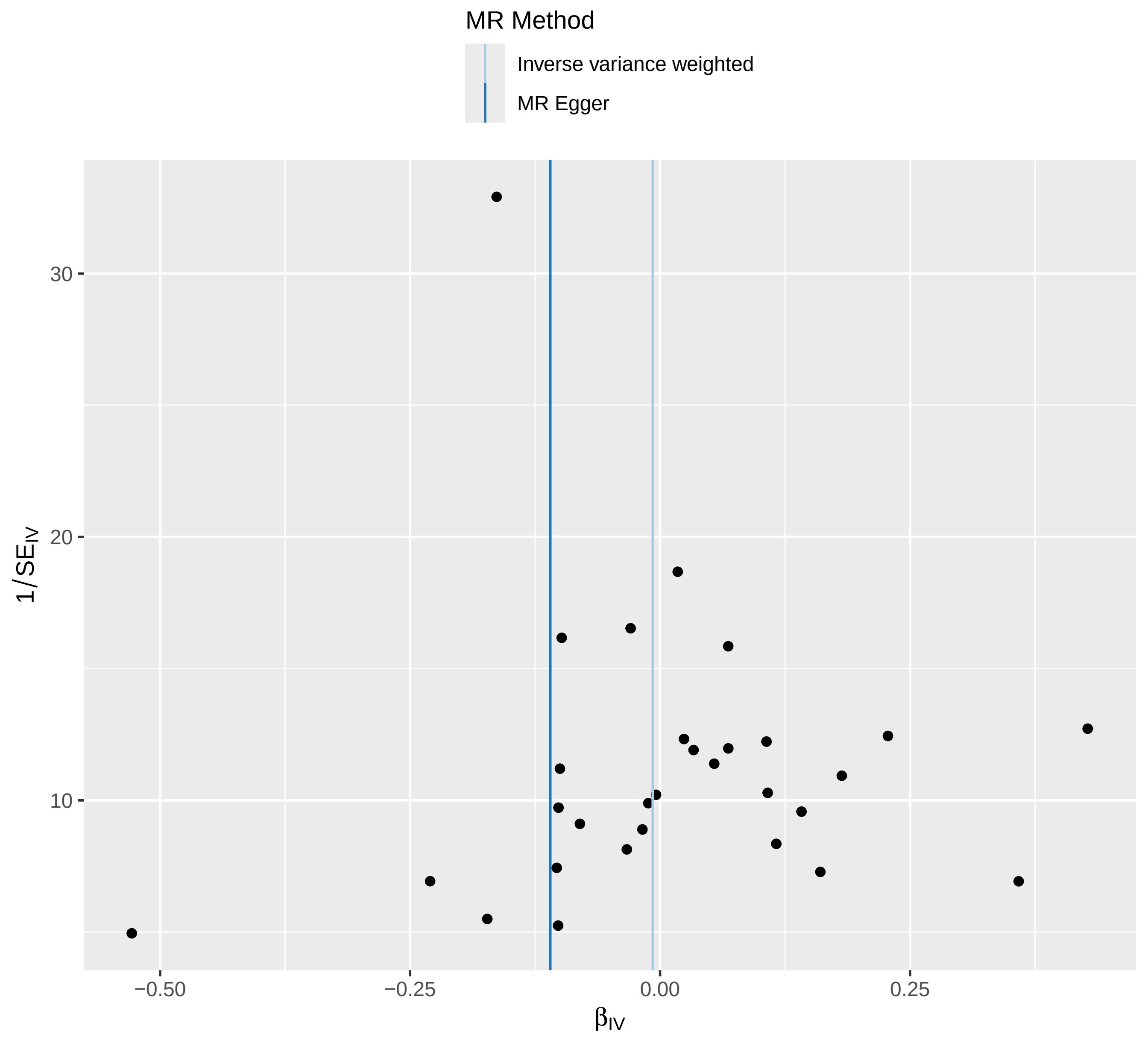
MR Method
Inverse variance weighted
MR Egger
30
20
10
$1/SE_{IV}$
−0.50
−0.25
0.00
0.25
β_{IV}

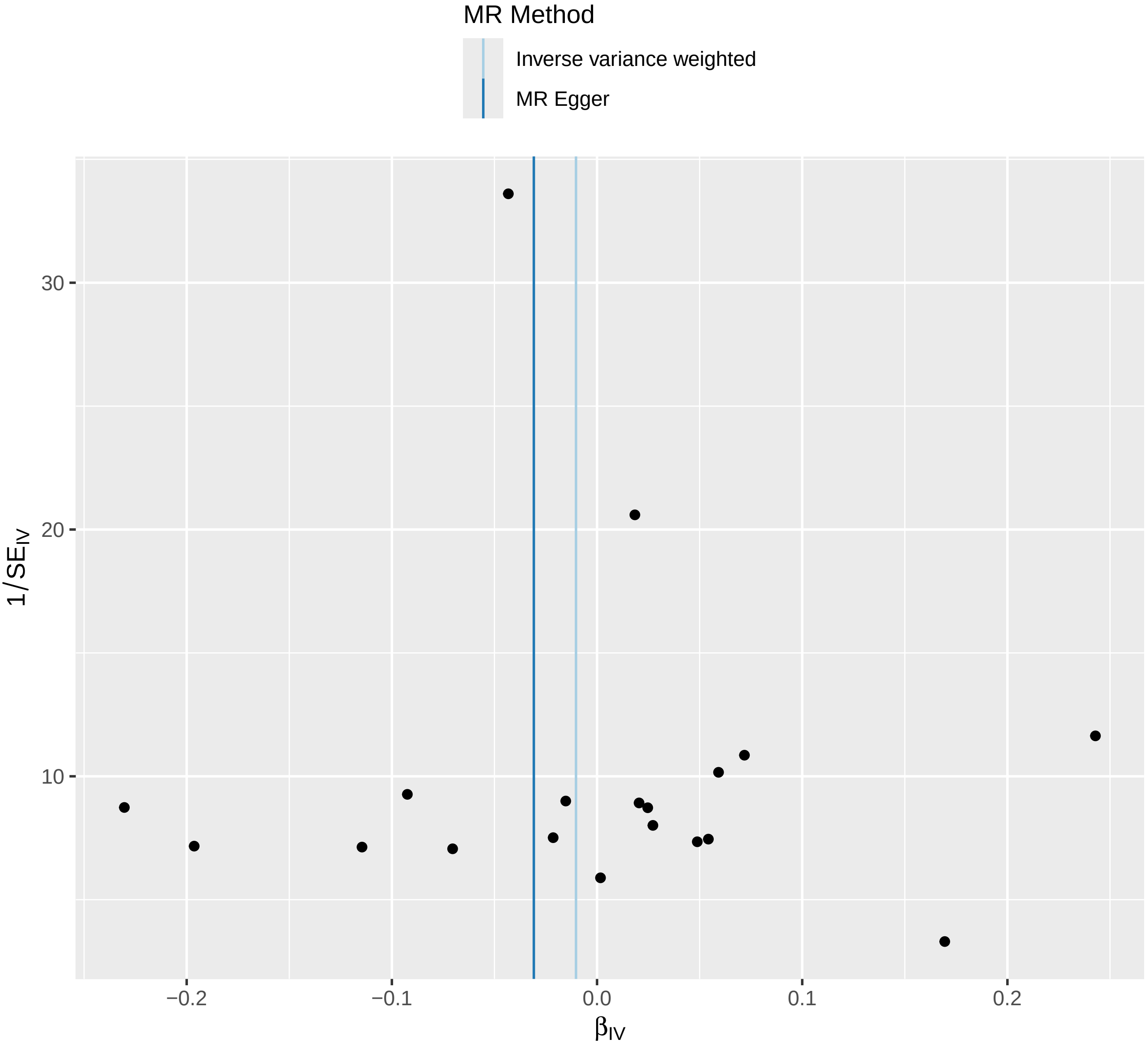

MR Method
Inverse variance weighted
MR Egger
30
20
10
1/SE$_{IV}$
−0.2
−0.1
0.0
0.1
0.2
β$_{IV}$

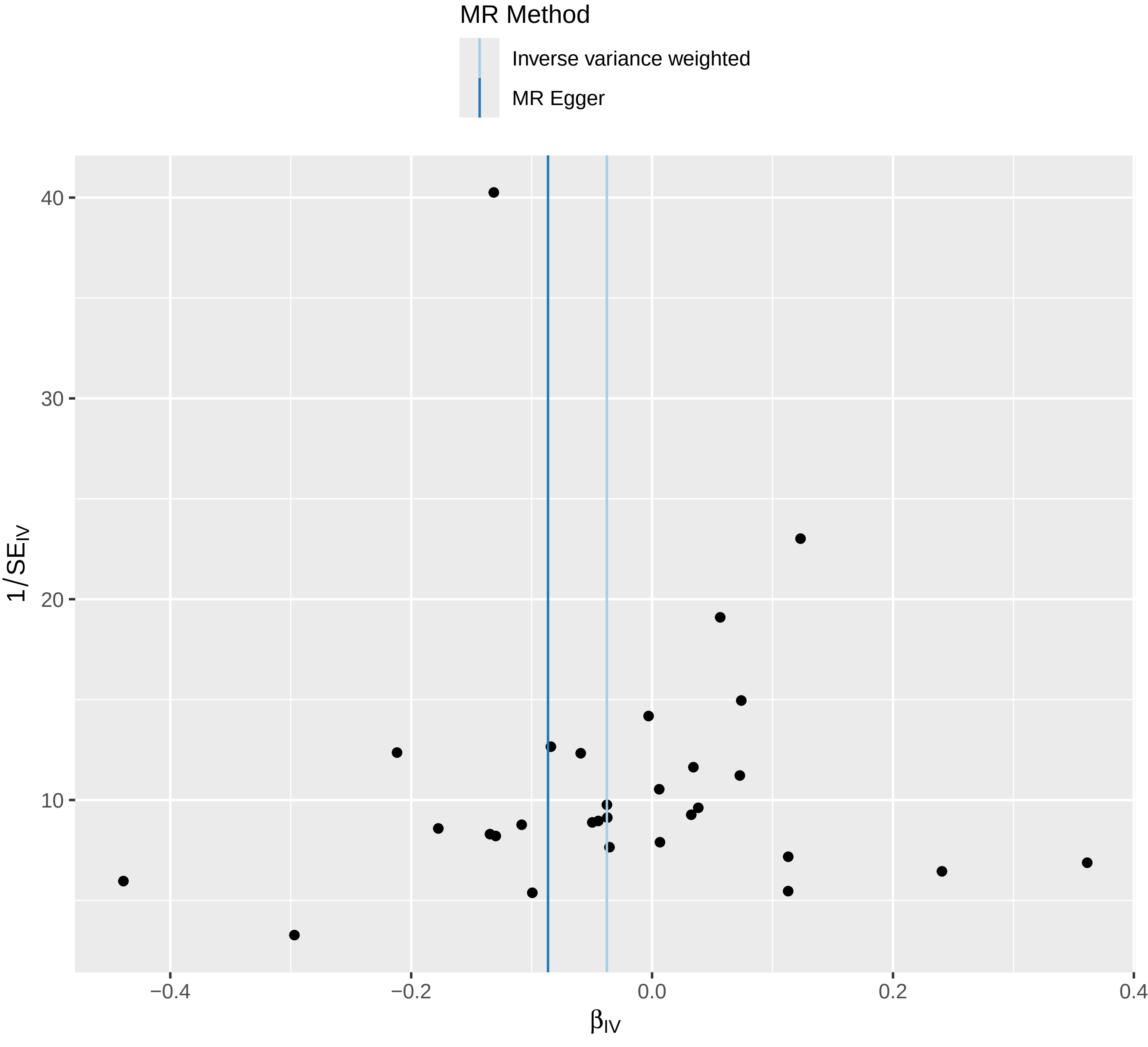
MR Method
Inverse variance weighted
MR Egger
40
30
20
10
$1/SE_{IV}$
−0.4
−0.2
0.0
0.2
0.4
β_{IV}

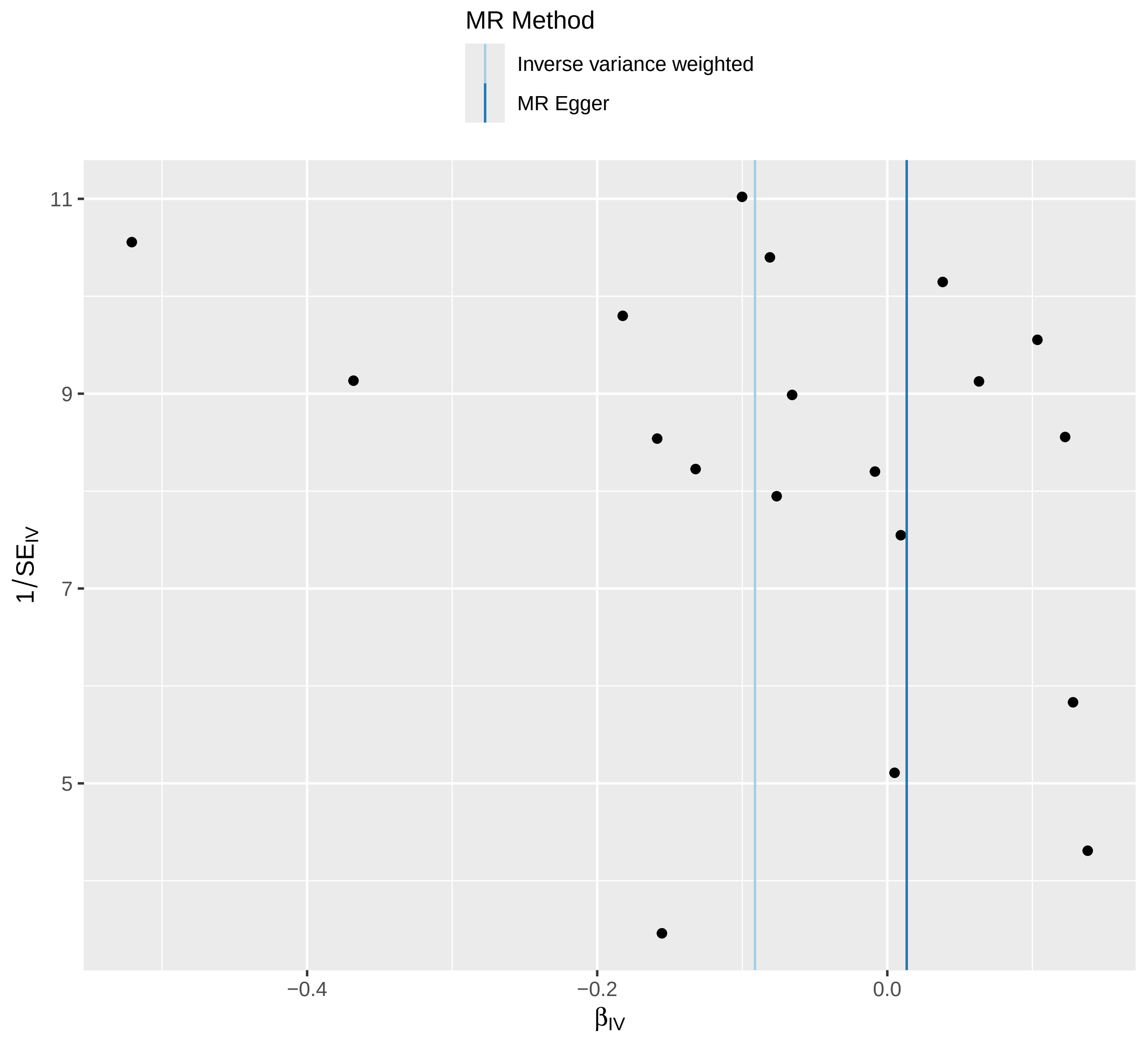
MR Method
Inverse variance weighted
MR Egger
11
9
7
5
1/SE_IV
−0.4
−0.2
0.0
β_IV

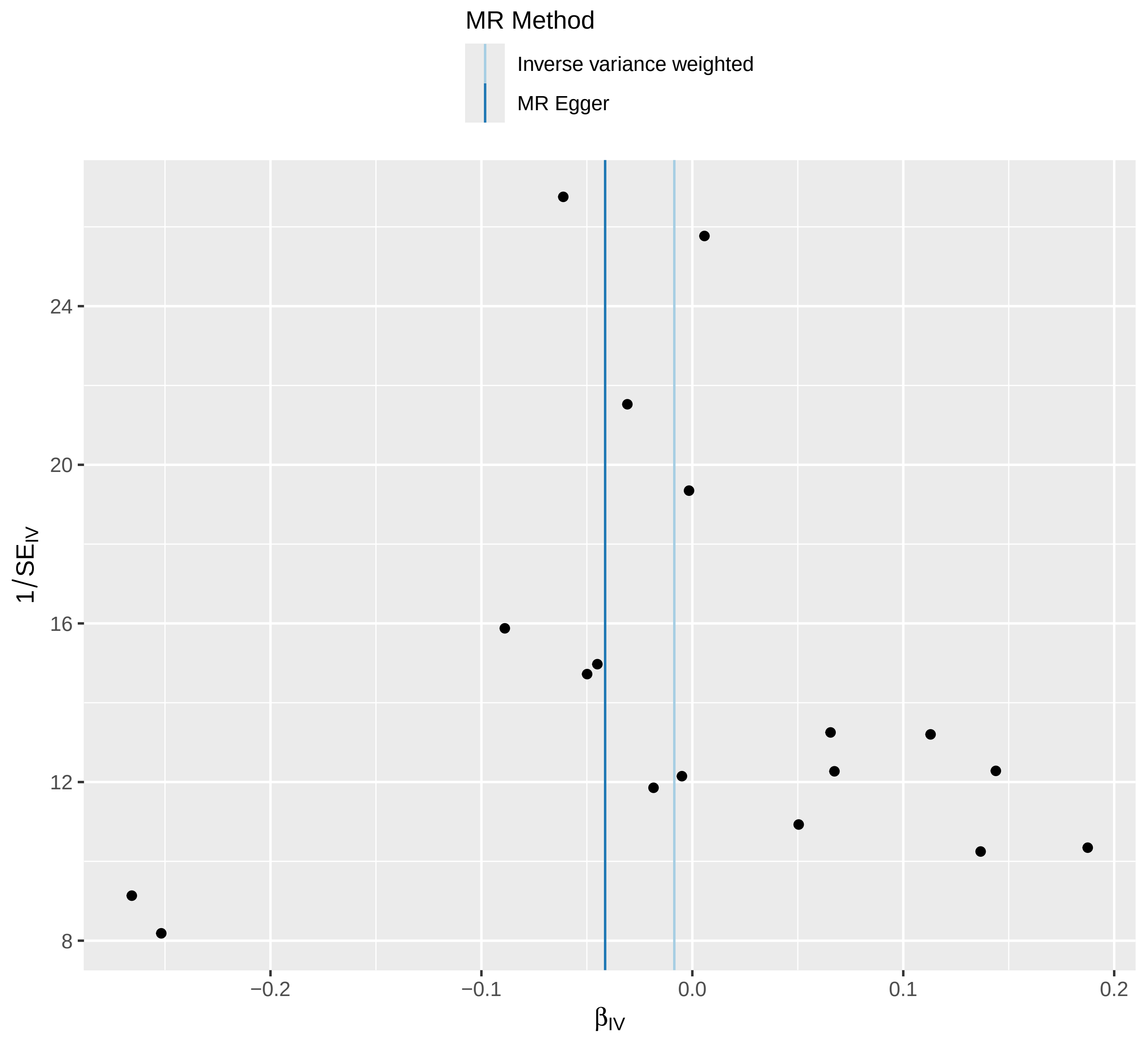
MR Method
Inverse variance weighted
MR Egger
24
20
16
12
8
1/SE_IV
−0.2
−0.1
0.0
0.1
0.2
β_IV

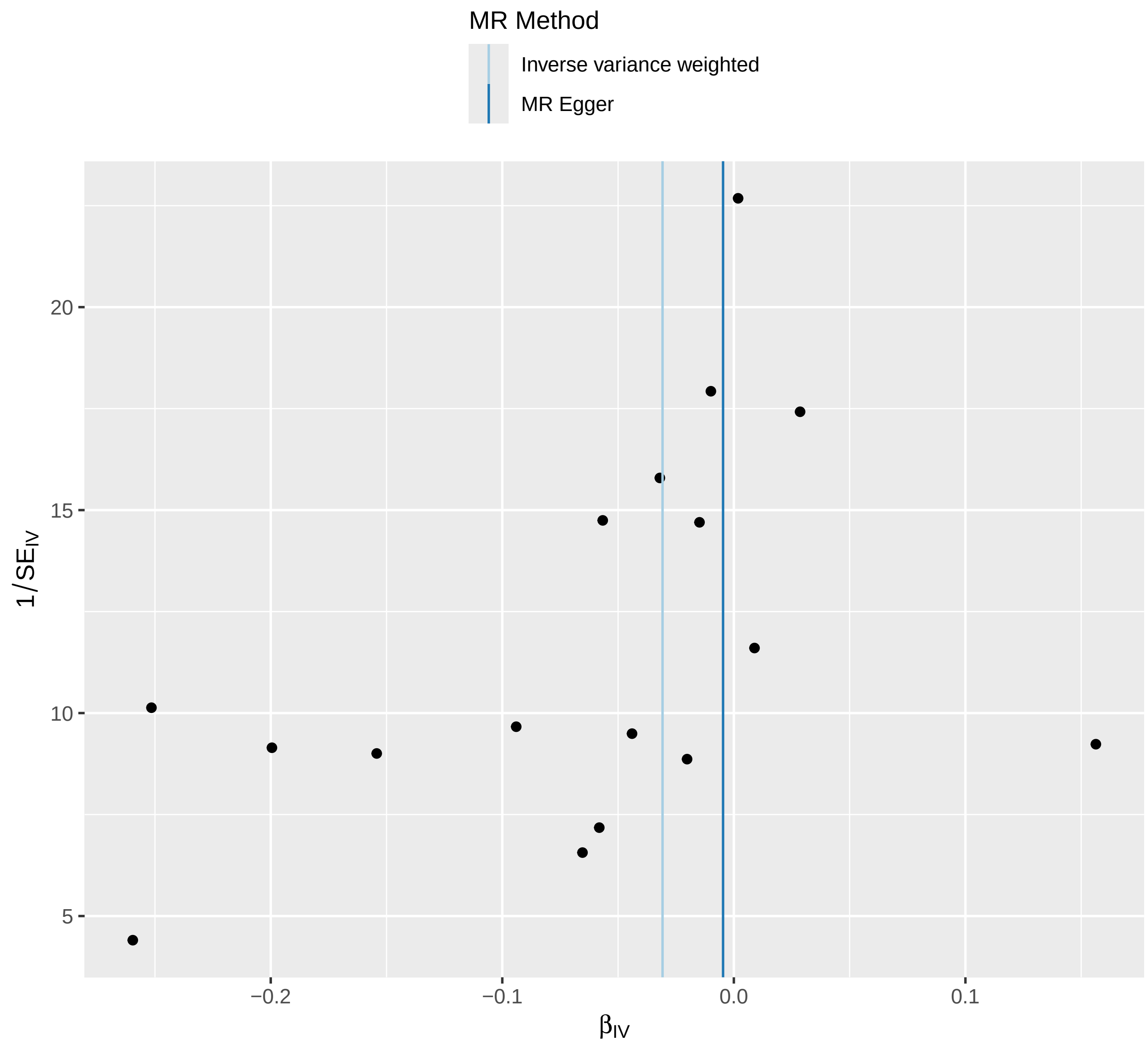
MR Method
Inverse variance weighted
MR Egger
20
15
10
5
1/SE_IV
−0.2
−0.1
0.0
0.1
β_IV

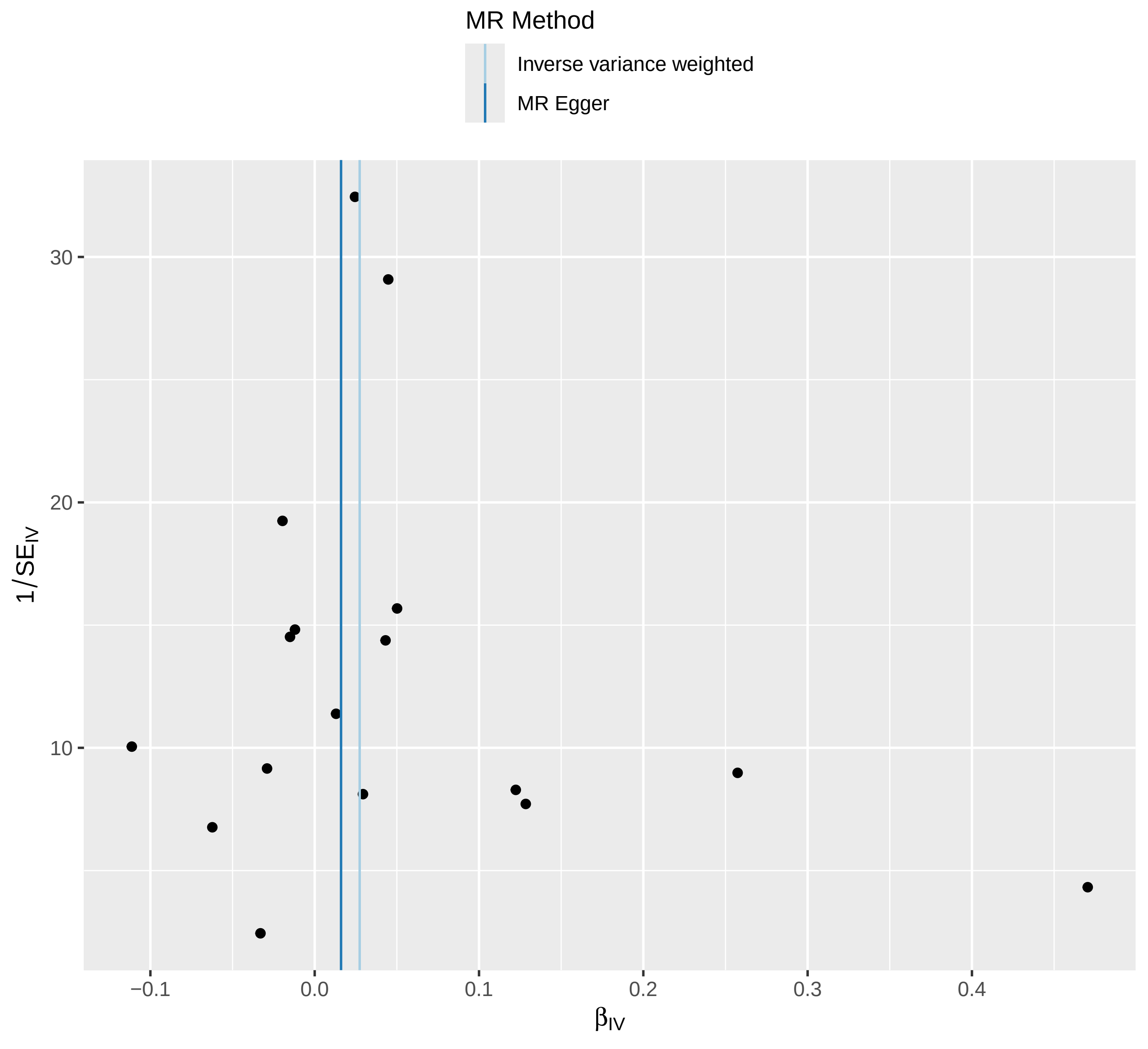
MR Method
Inverse variance weighted
MR Egger
30
20
10
1/SE_IV
−0.1
0.0
0.1
0.2
0.3
0.4
β_IV

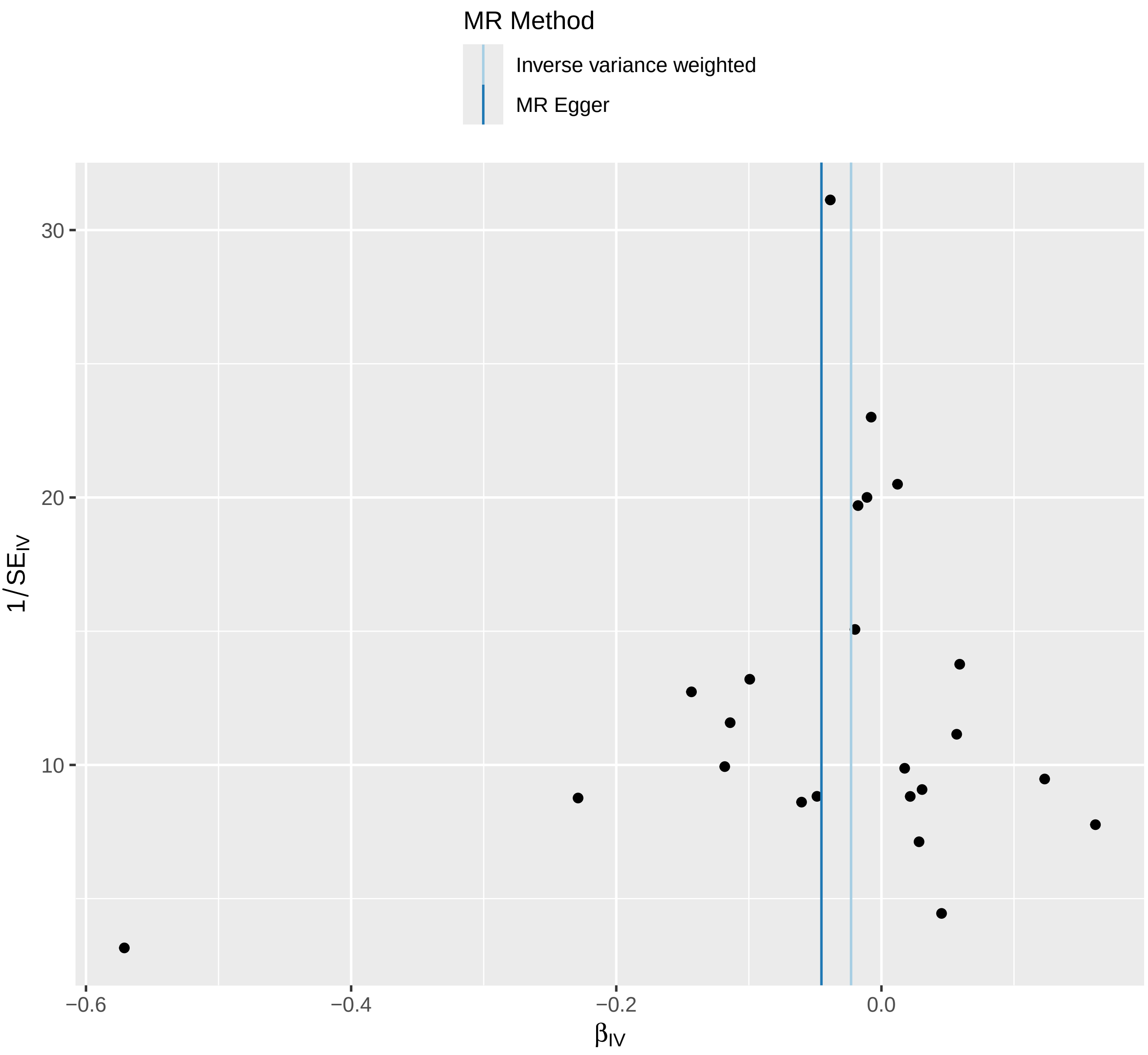
MR Method
Inverse variance weighted
MR Egger
30
20
10
1/SE_IV
−0.6
−0.4
−0.2
0.0
β_IV

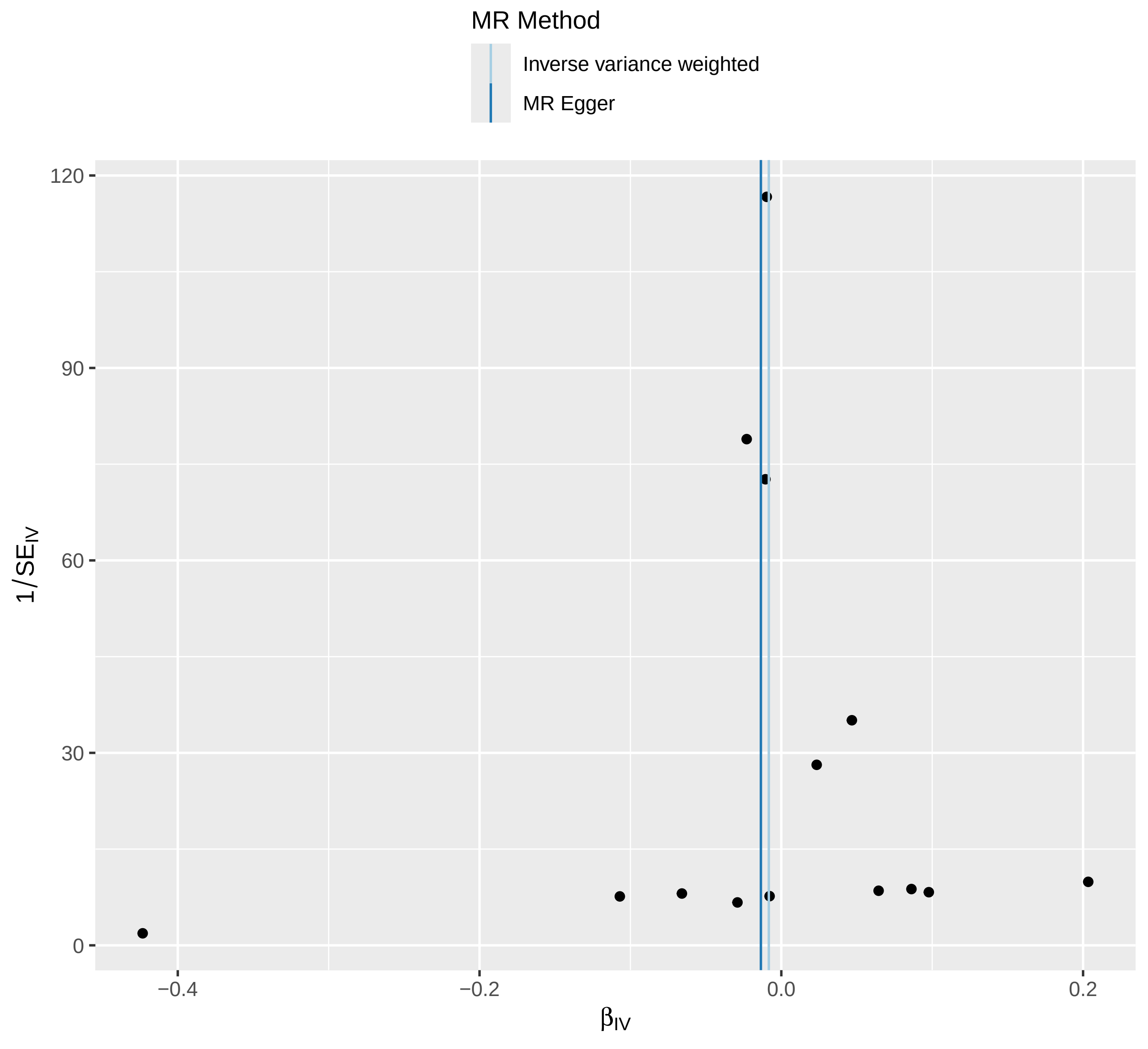
MR Method
Inverse variance weighted
MR Egger
120
90
60
30
0
1/SE$_{IV}$
−0.4
−0.2
0.0
0.2
β$_{IV}$

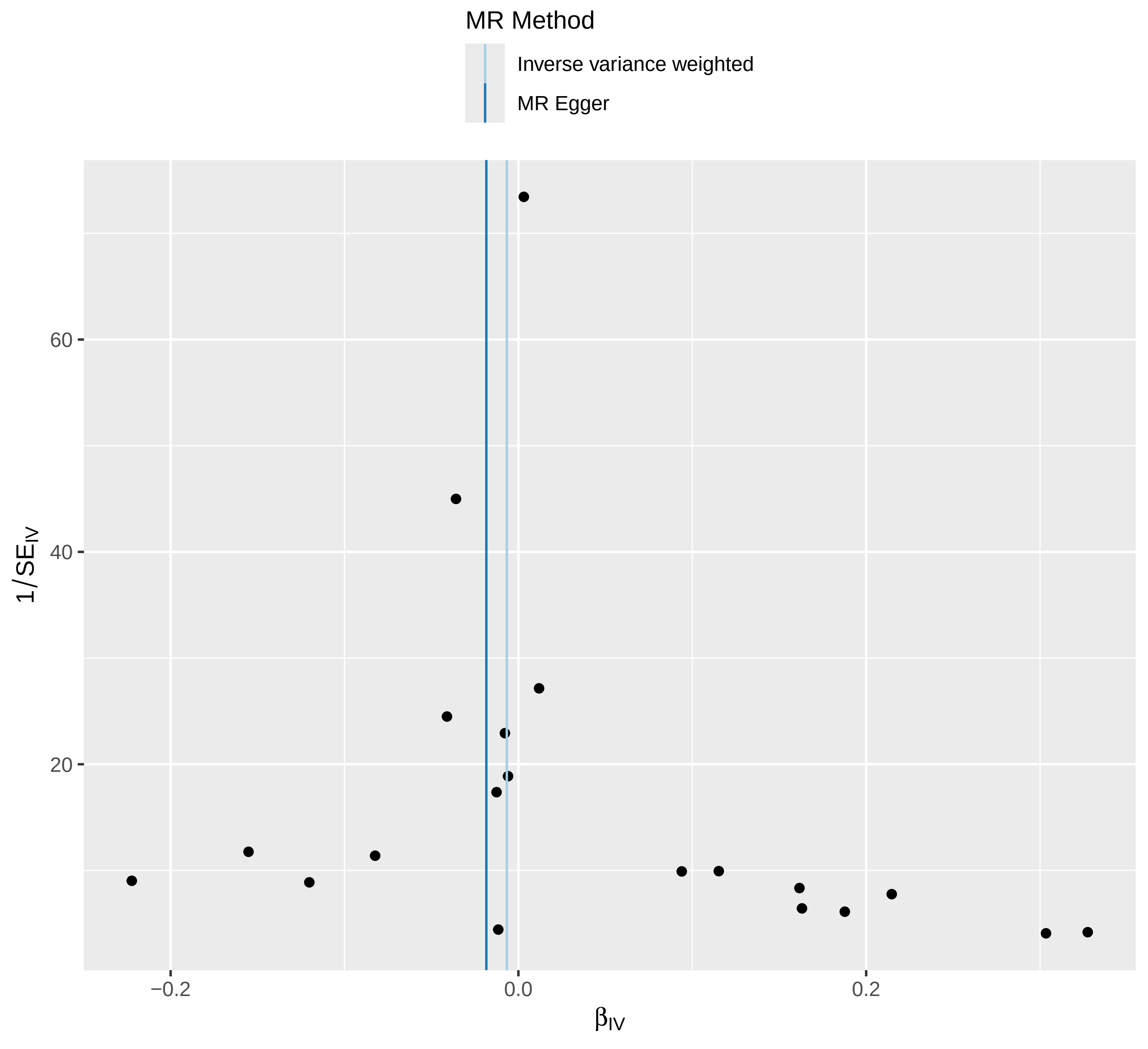
MR Method
Inverse variance weighted
MR Egger
60
40
20
1/SE_IV
−0.2
0.0
0.2
β_IV

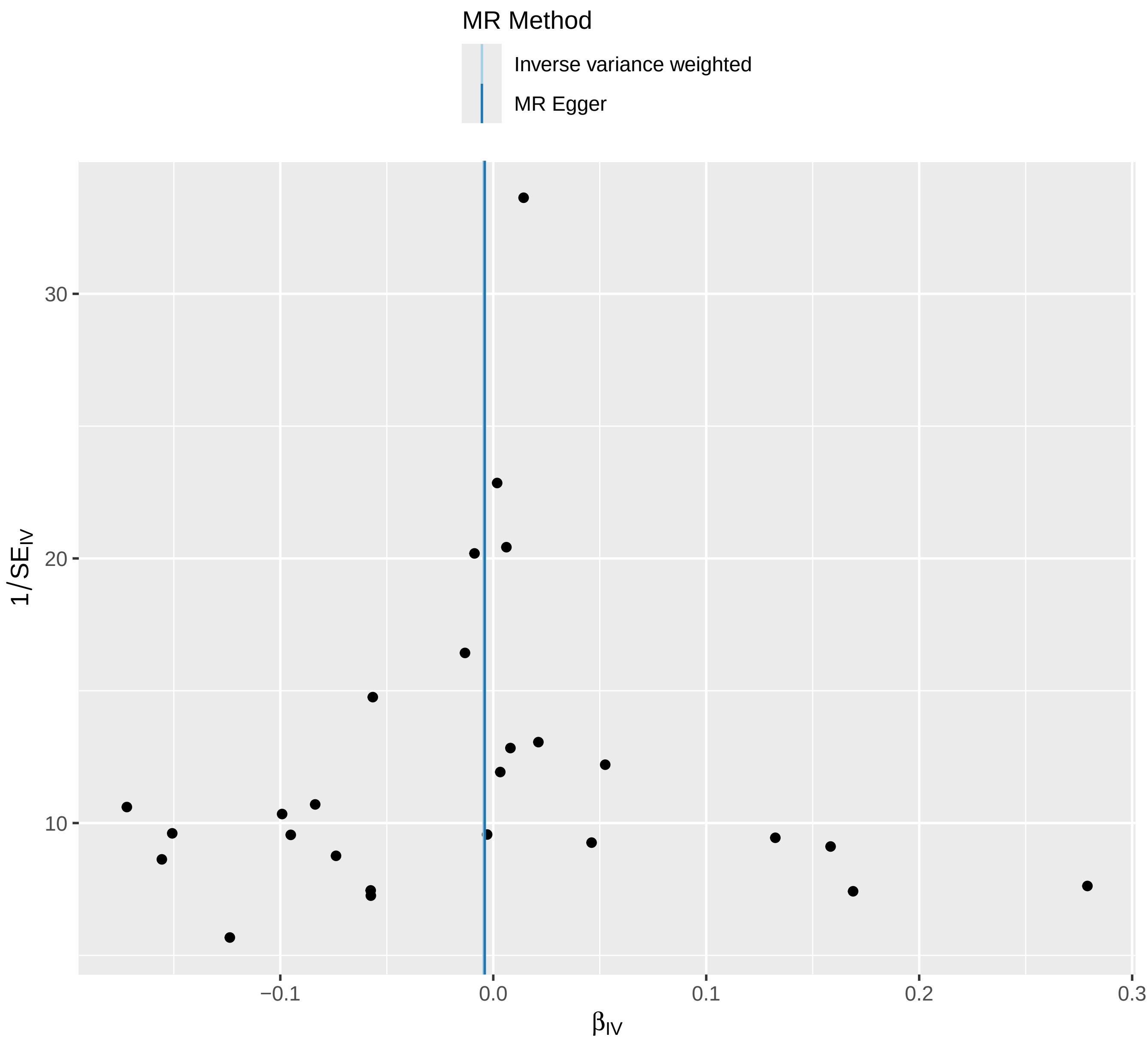
MR Method
Inverse variance weighted
MR Egger
1/SE$_{IV}$
30
20
10
β$_{IV}$
−0.1
0.0
0.1
0.2
0.3

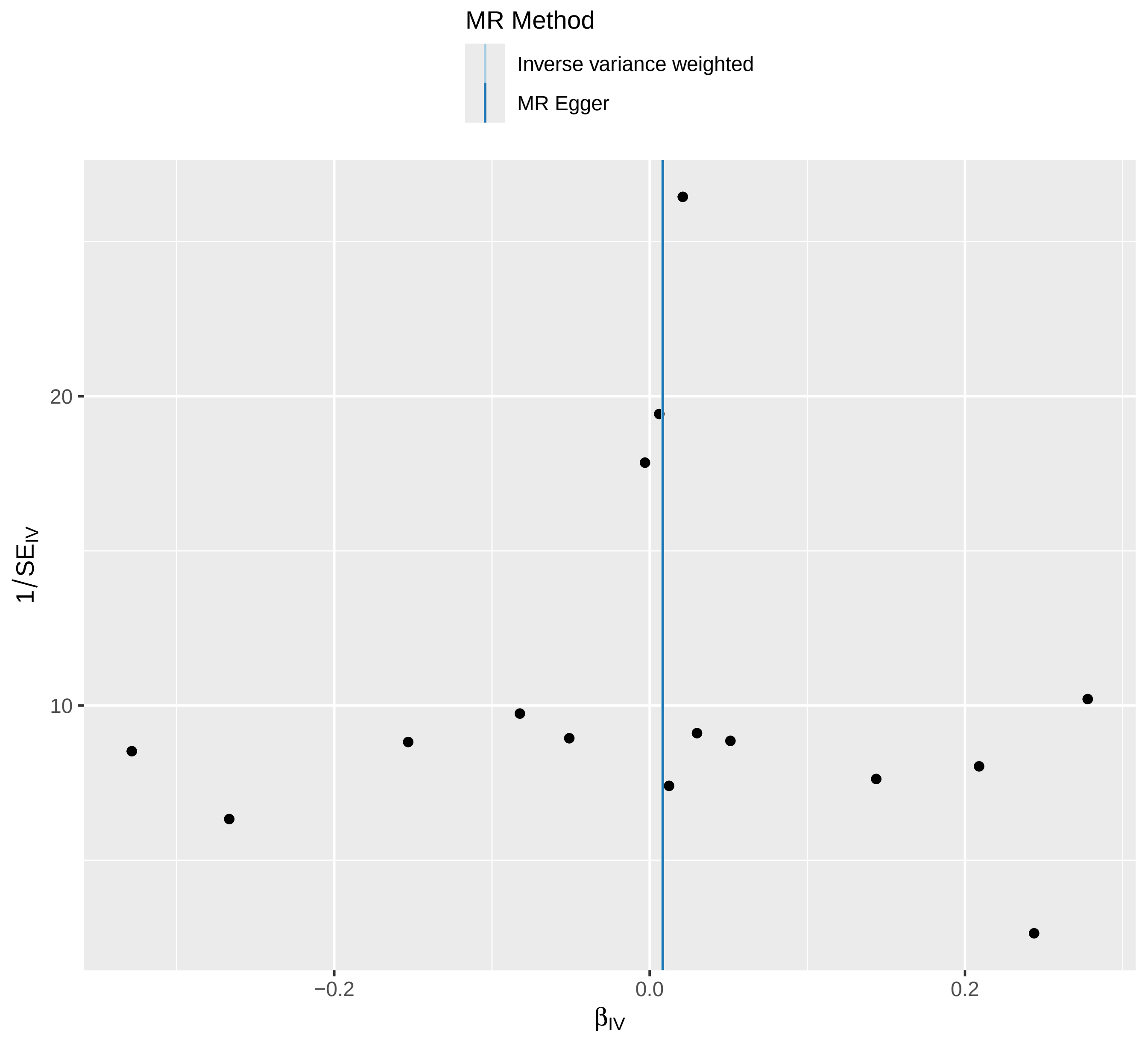

MR Method
Inverse variance weighted
MR Egger
20
10
1/SE$_{IV}$
−0.2
0.0
0.2
β$_{IV}$

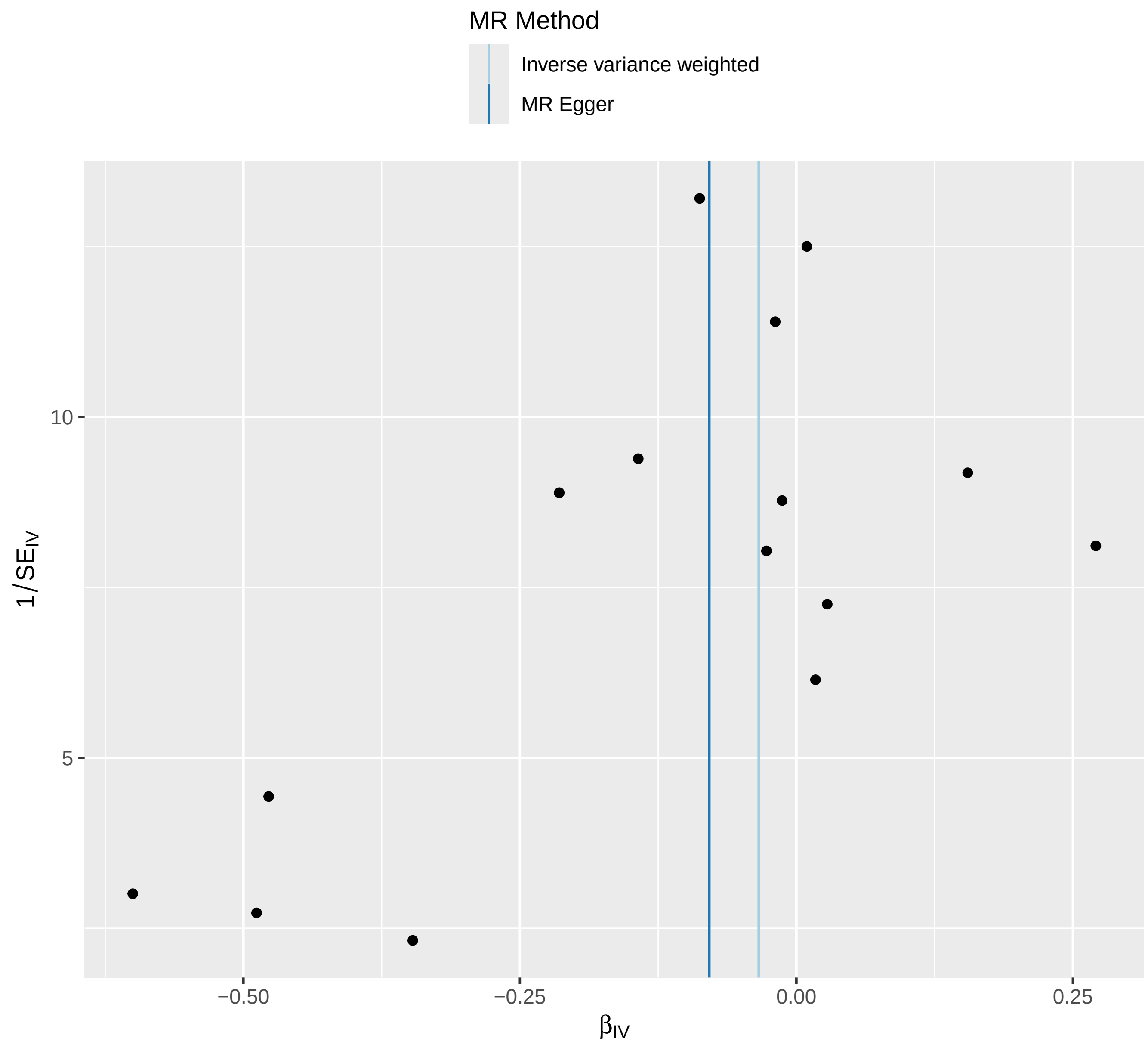
MR Method
Inverse variance weighted
MR Egger
10
5
1/SE_IV
−0.50
−0.25
0.00
0.25
β_IV

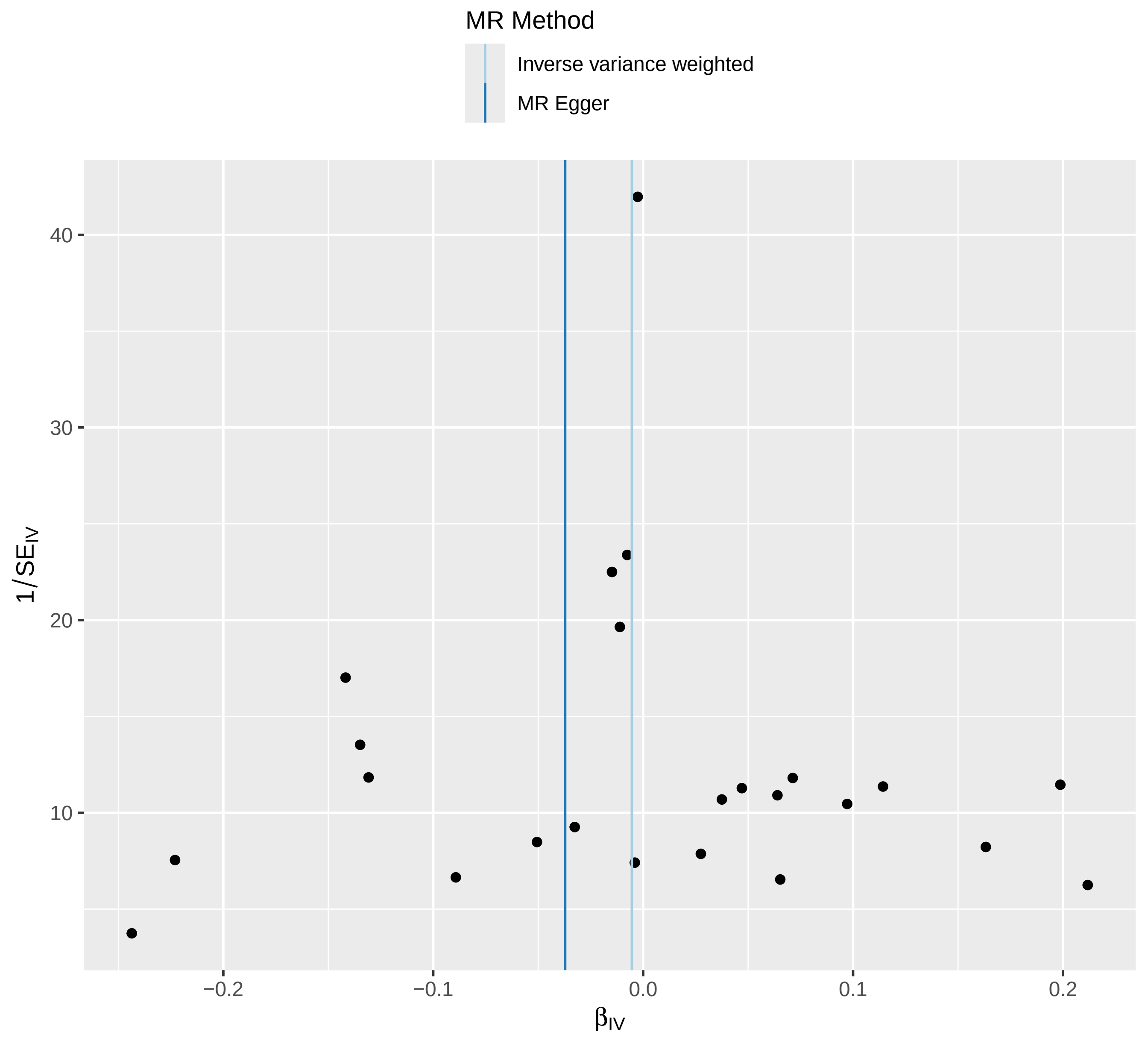
MR Method
Inverse variance weighted
MR Egger
1/SE$_{IV}$
40
30
20
10
−0.2
−0.1
0.0
0.1
0.2
β$_{IV}$

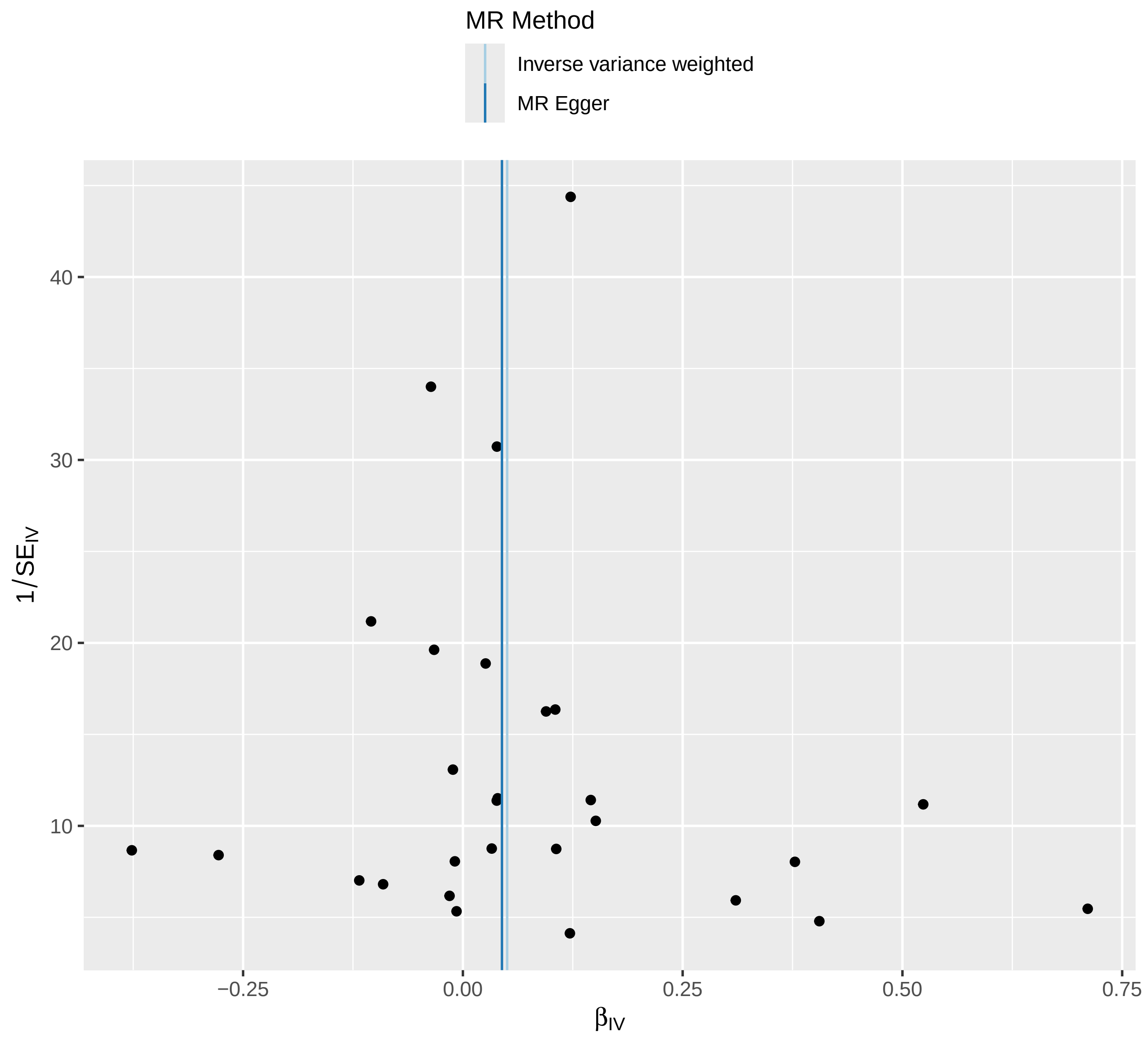
MR Method
Inverse variance weighted
MR Egger
1/SE$_{IV}$
40
30
20
10
β$_{IV}$
−0.25
0.00
0.25
0.50
0.75

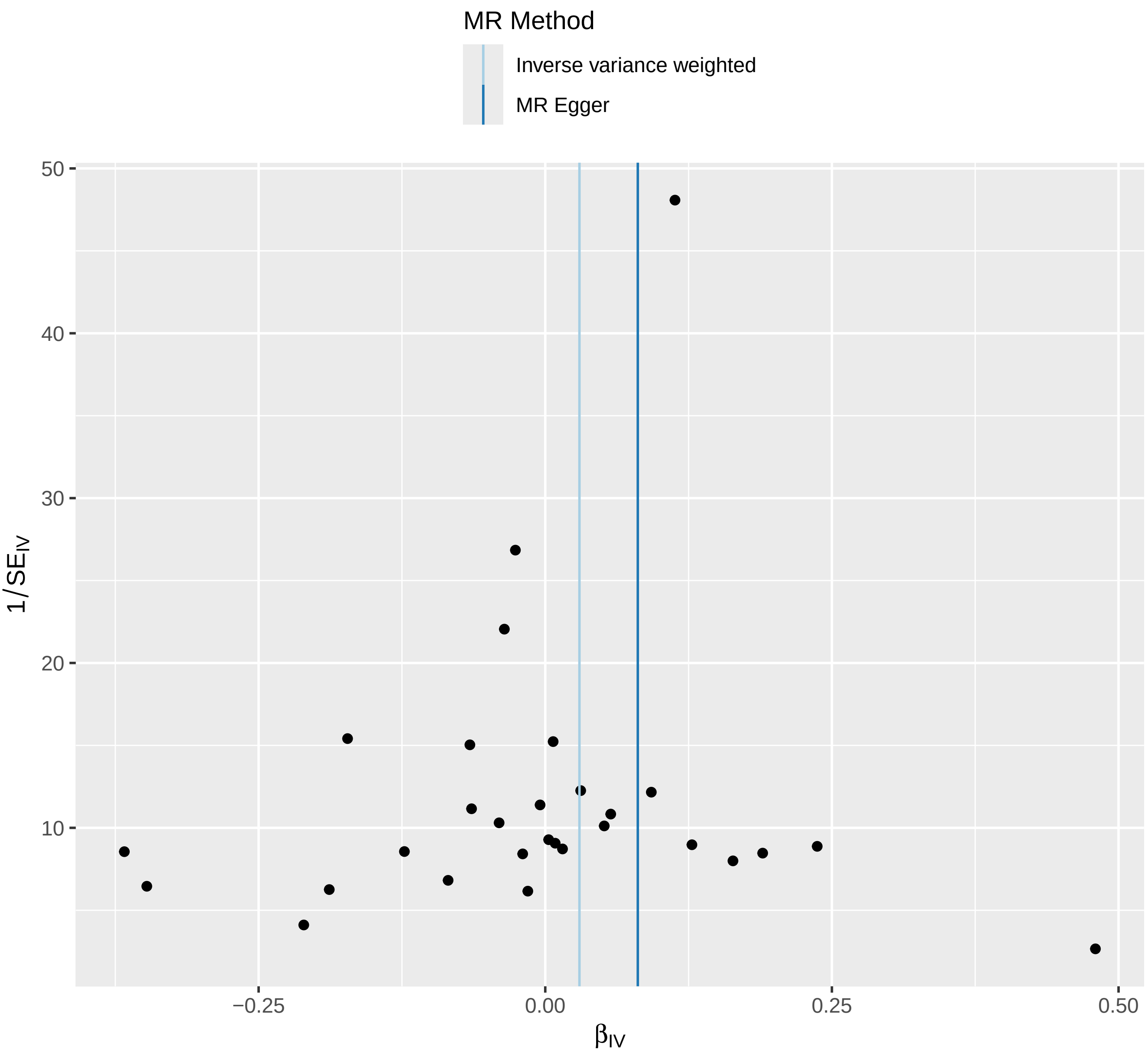
MR Method
Inverse variance weighted
MR Egger
50
40
30
20
10
1/SE_IV
−0.25
0.00
0.25
0.50
β_IV

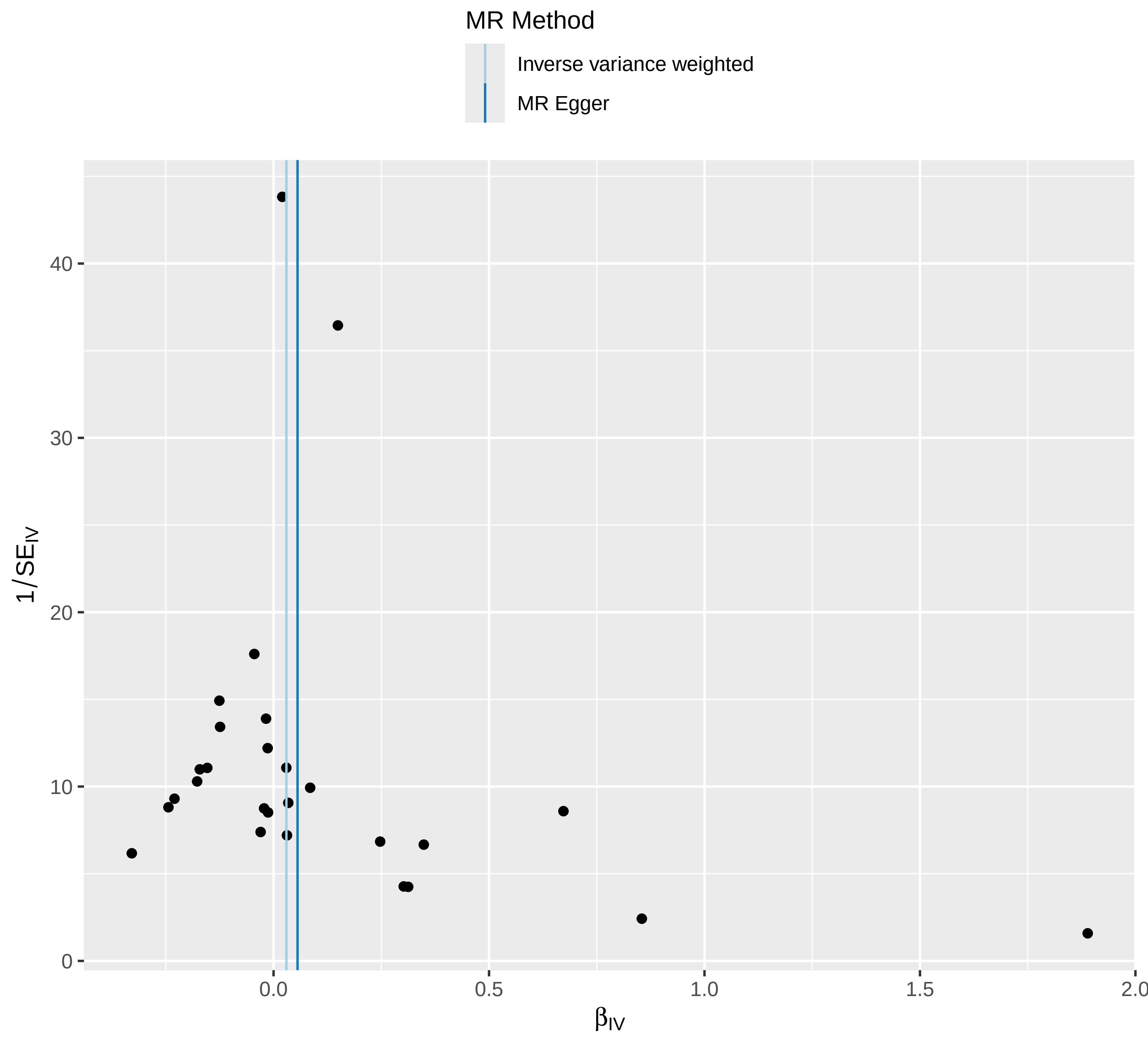
MR Method
Inverse variance weighted
MR Egger
40
30
20
10
0
$1/SE_{IV}$
0.0
0.5
1.0
1.5
2.0
β_{IV}

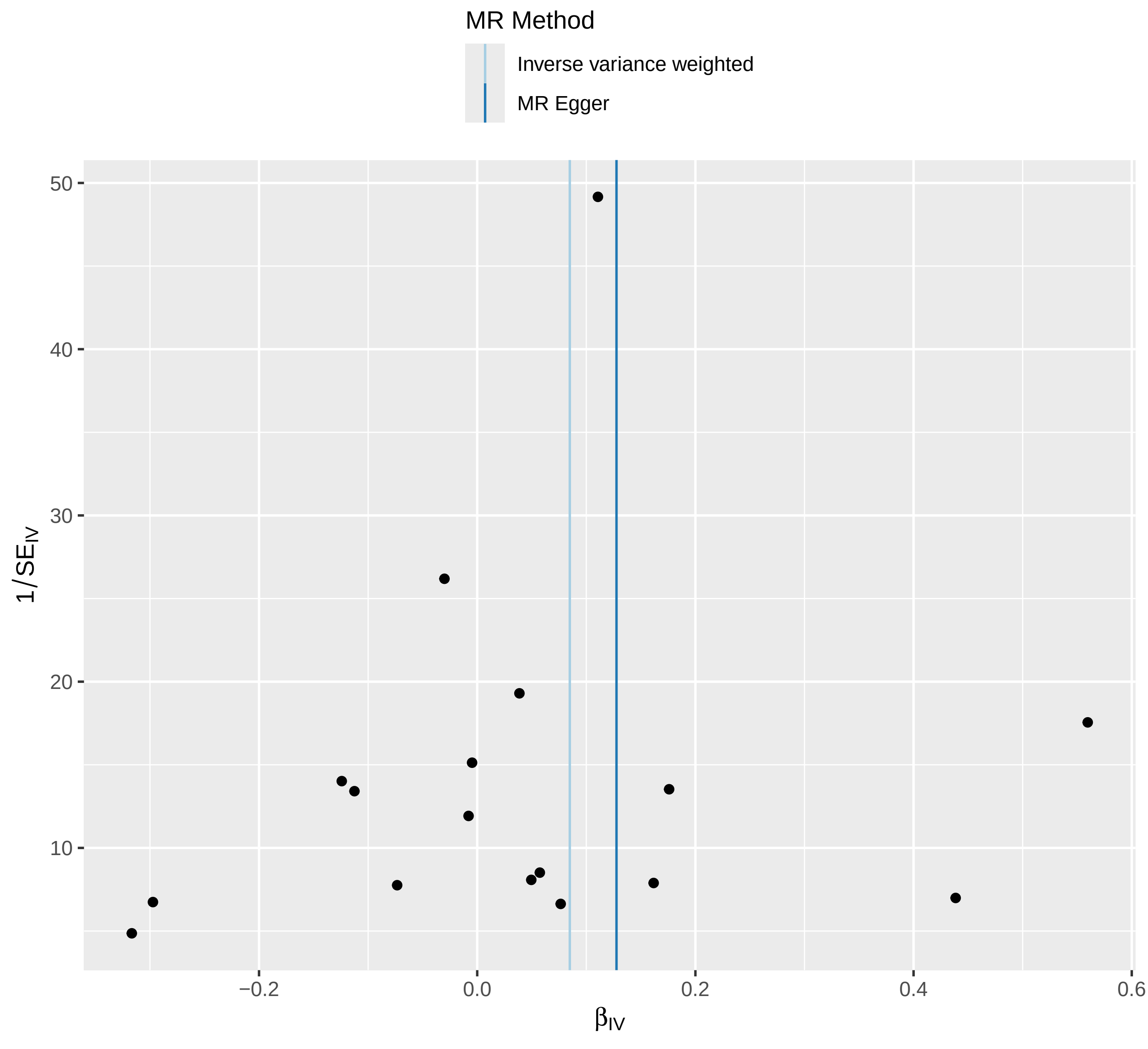

MR Method
Inverse variance weighted
MR Egger
1/SE_IV
β_IV
50
40
30
20
10
−0.2
0.0
0.2
0.4
0.6

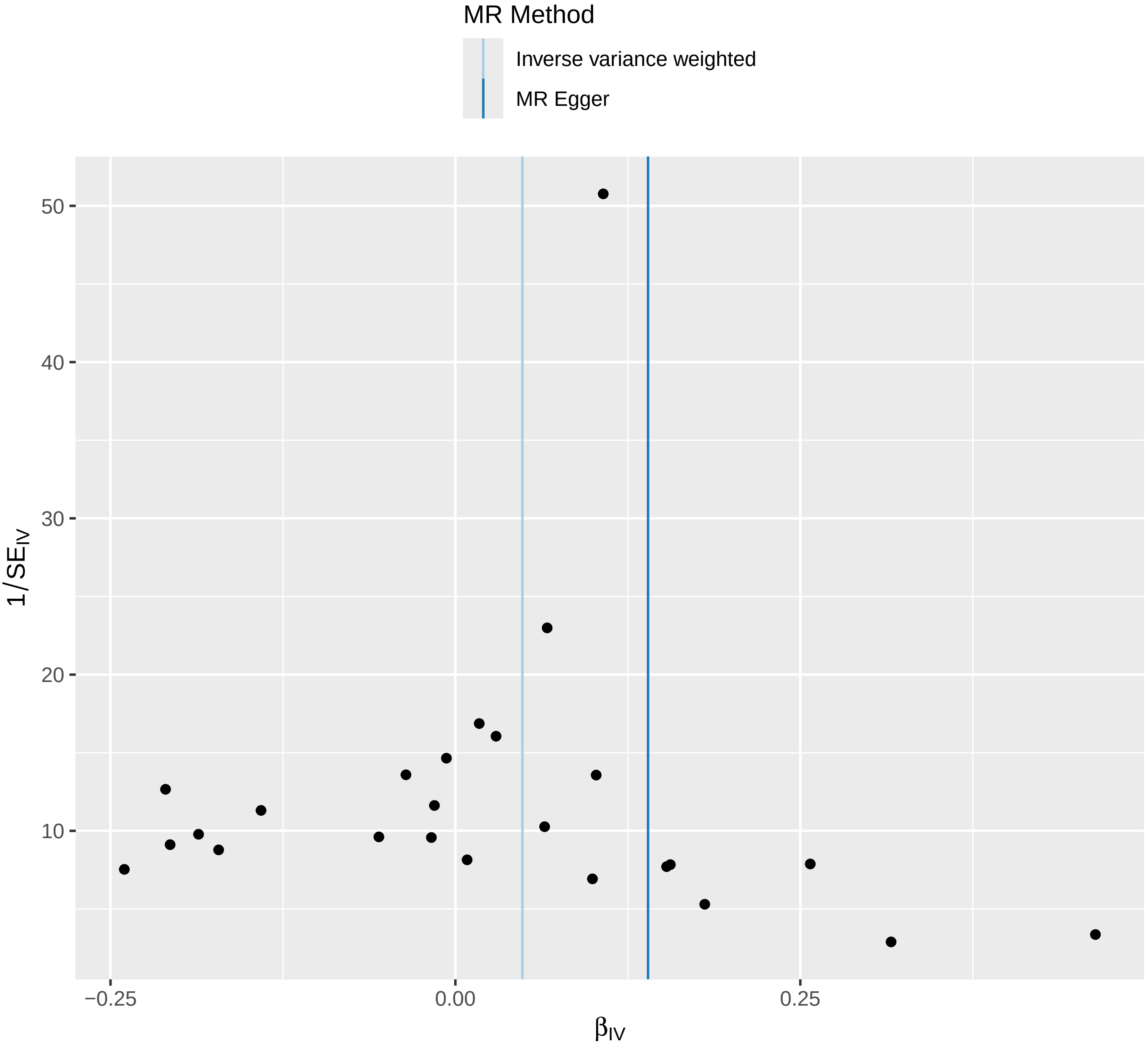
MR Method
Inverse variance weighted
MR Egger
50
40
30
20
10
1/SE$_{IV}$
−0.25
0.00
0.25
β$_{IV}$

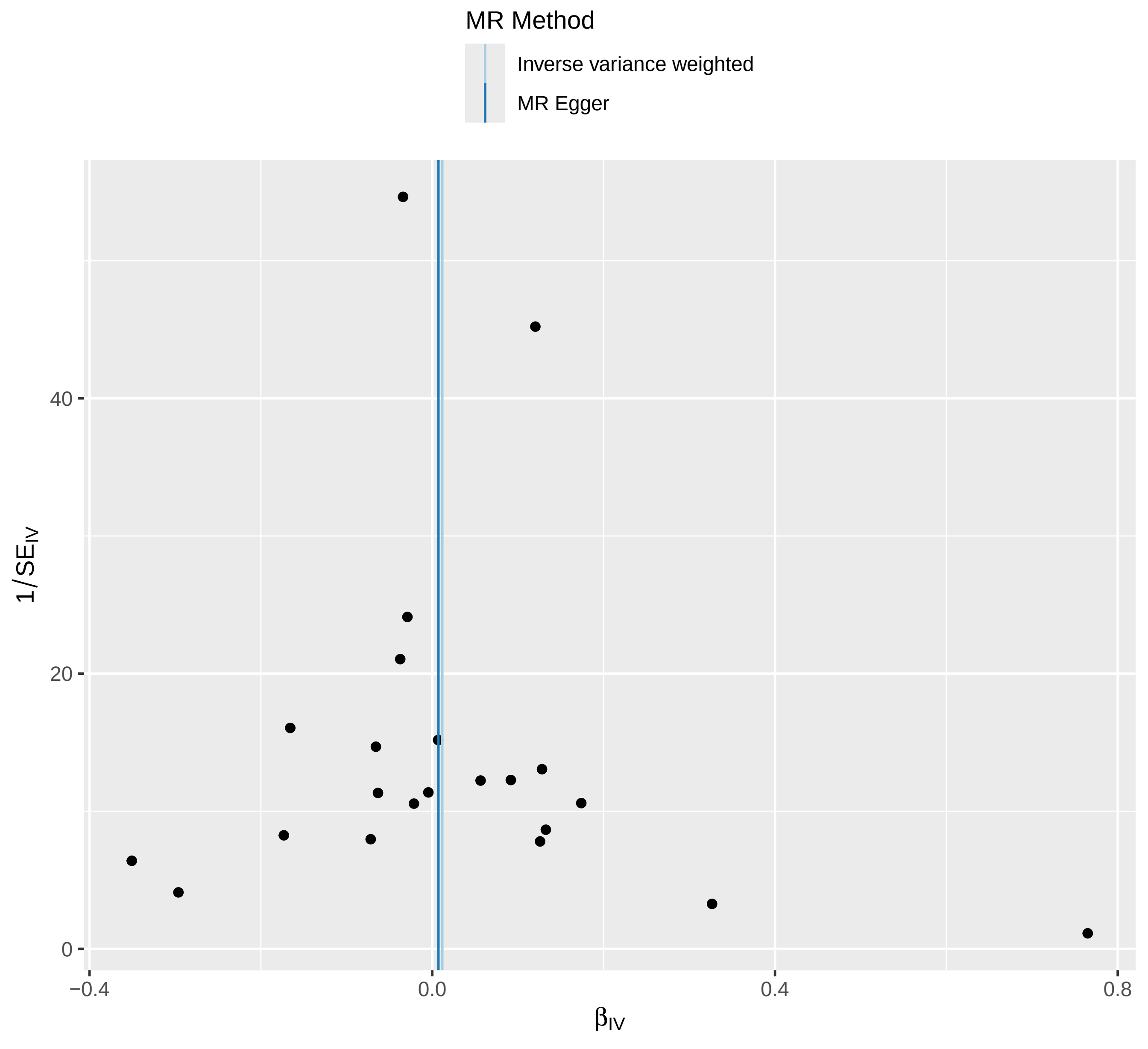

MR Method
Inverse variance weighted
MR Egger
40
20
0
1/SE$_{IV}$
−0.4
0.0
0.4
0.8
β$_{IV}$

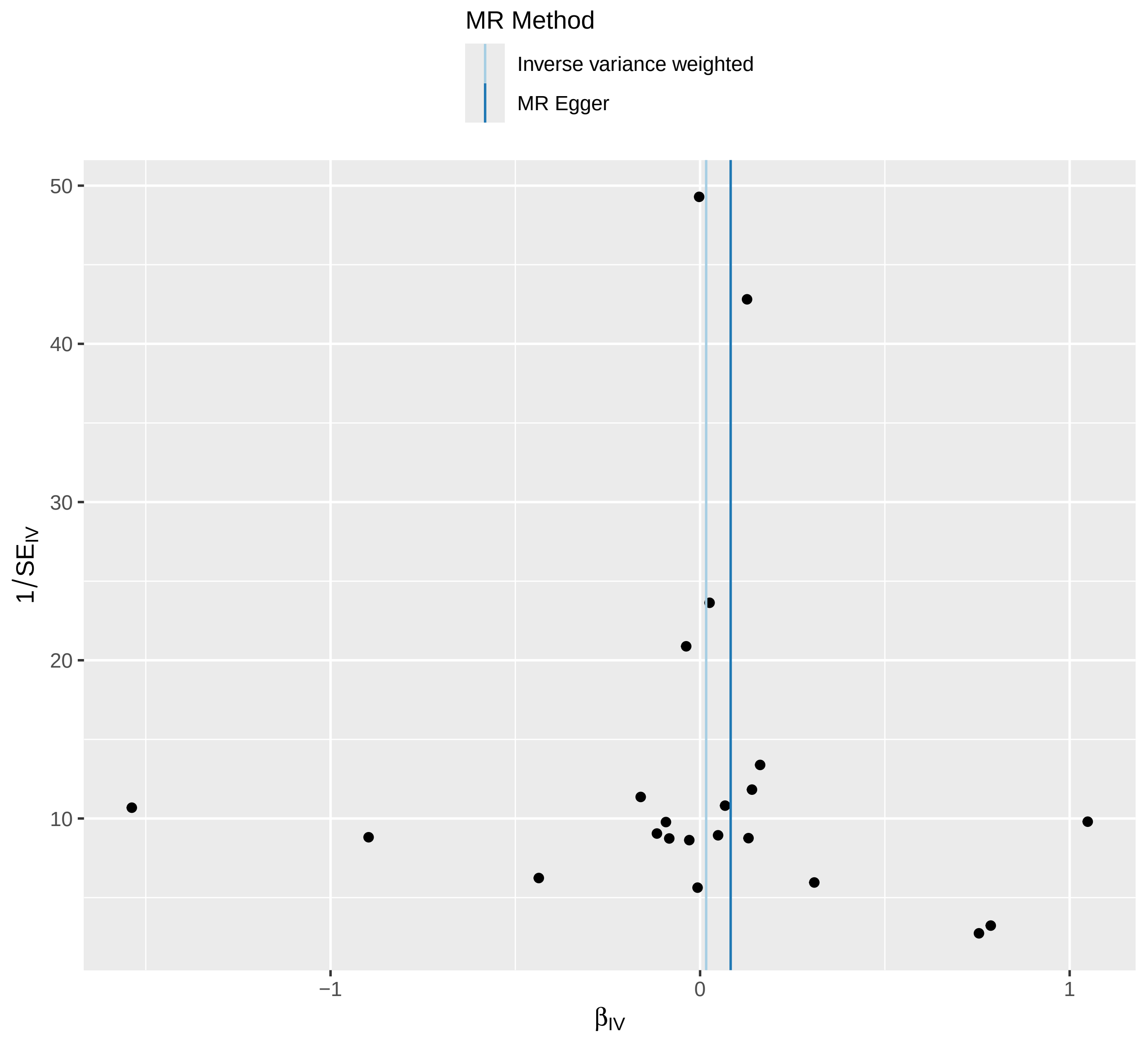
MR Method
Inverse variance weighted
MR Egger
1/SE_{IV}
50
40
30
20
10
−1
0
1
β_{IV}

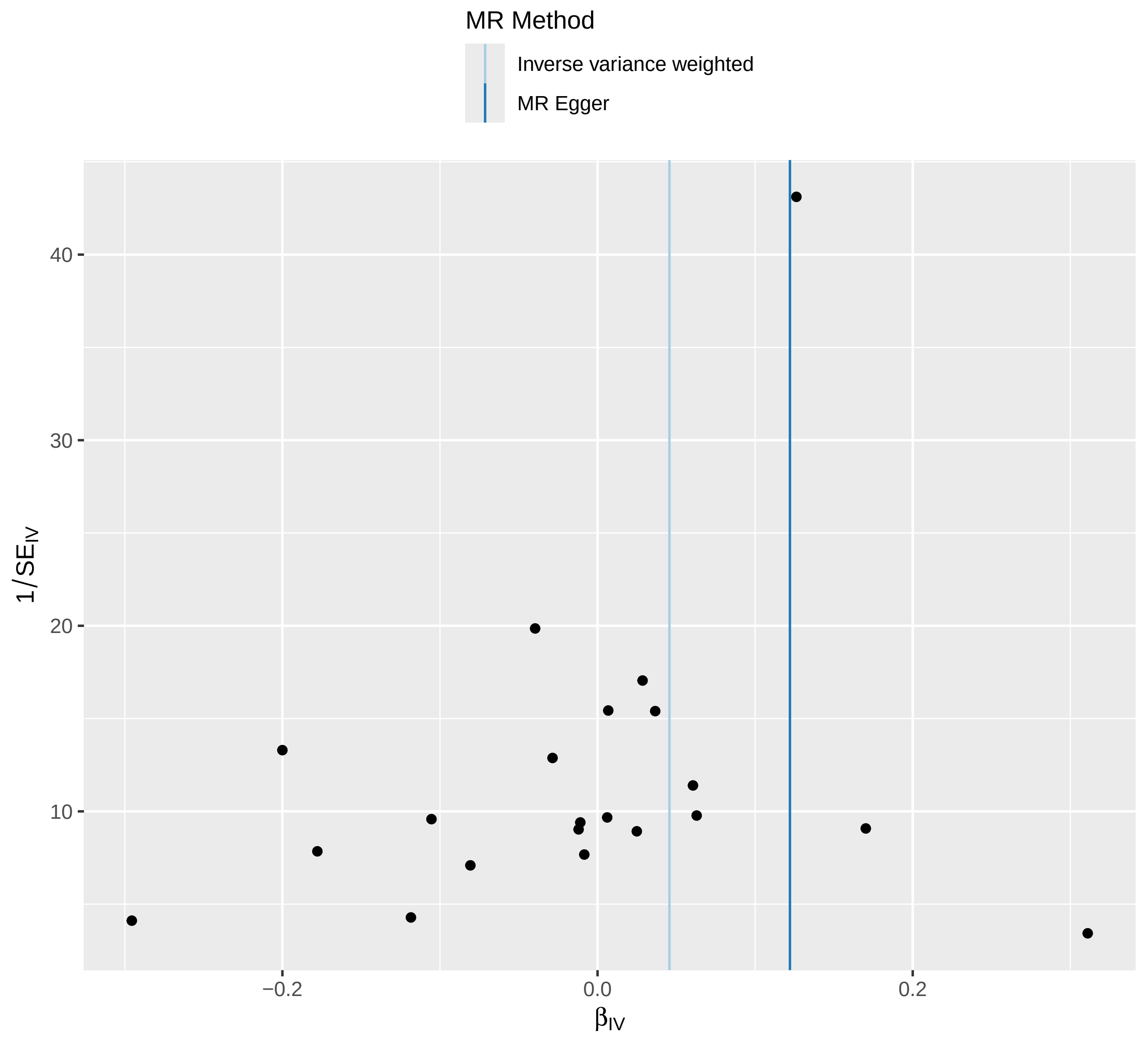
MR Method
Inverse variance weighted
MR Egger
40
30
20
10
1/SE_IV
−0.2
0.0
0.2
β_IV

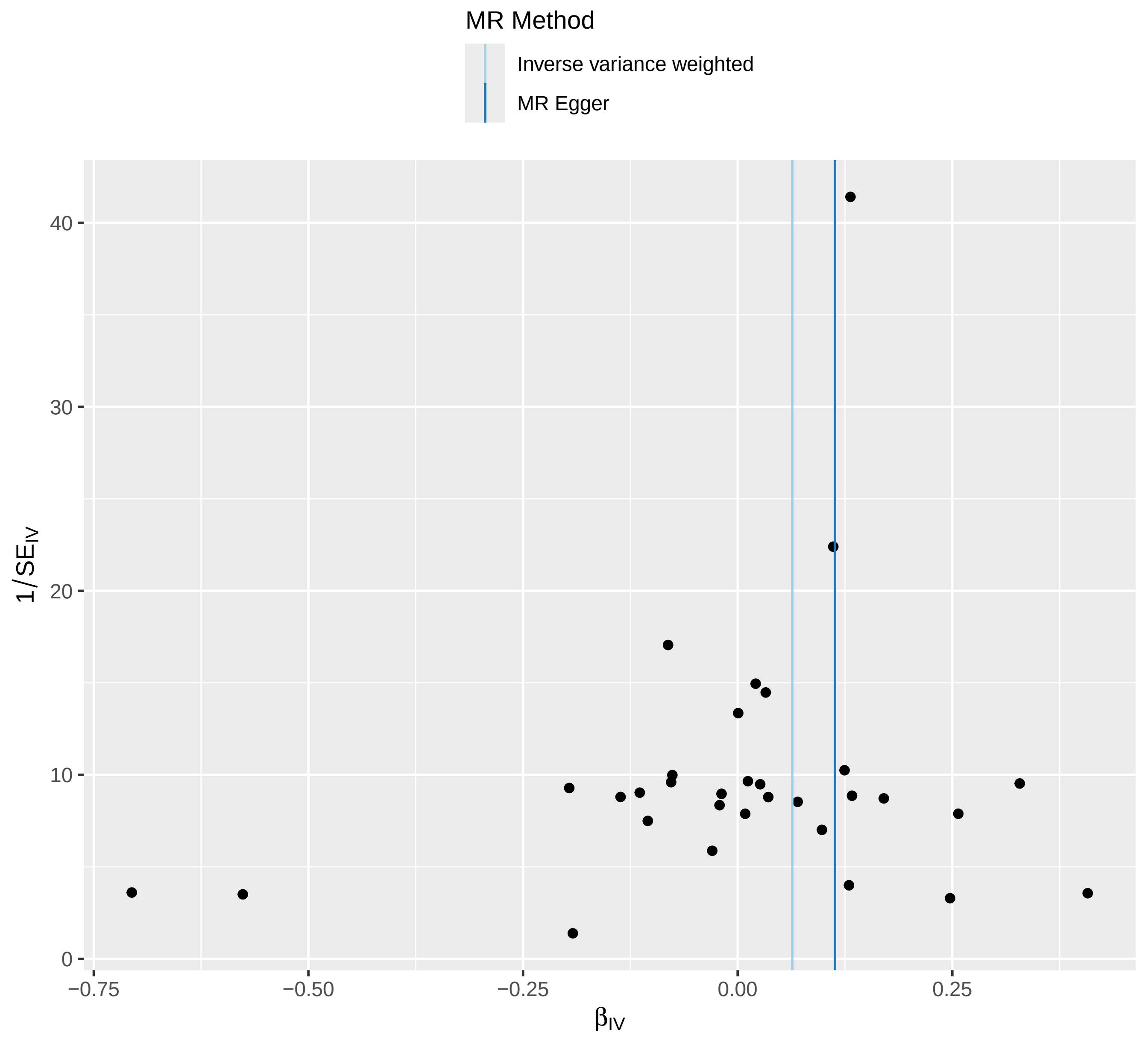
MR Method
Inverse variance weighted
MR Egger
1/SE_IV
β_IV
40
30
20
10
0
−0.75
−0.50
−0.25
0.00
0.25

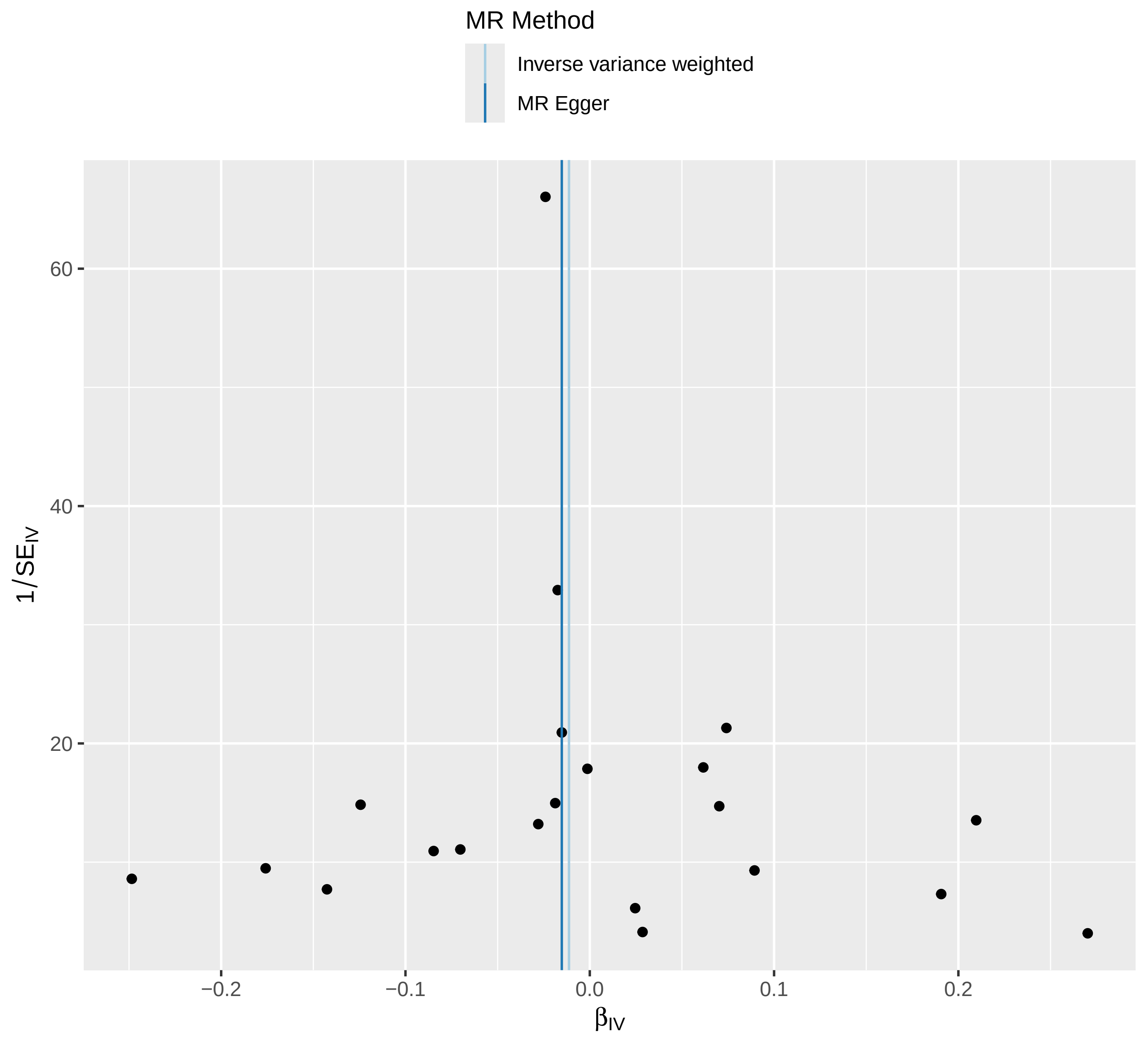
MR Method
Inverse variance weighted
MR Egger
1/SE_IV
60
40
20
−0.2
−0.1
0.0
0.1
0.2
β_IV

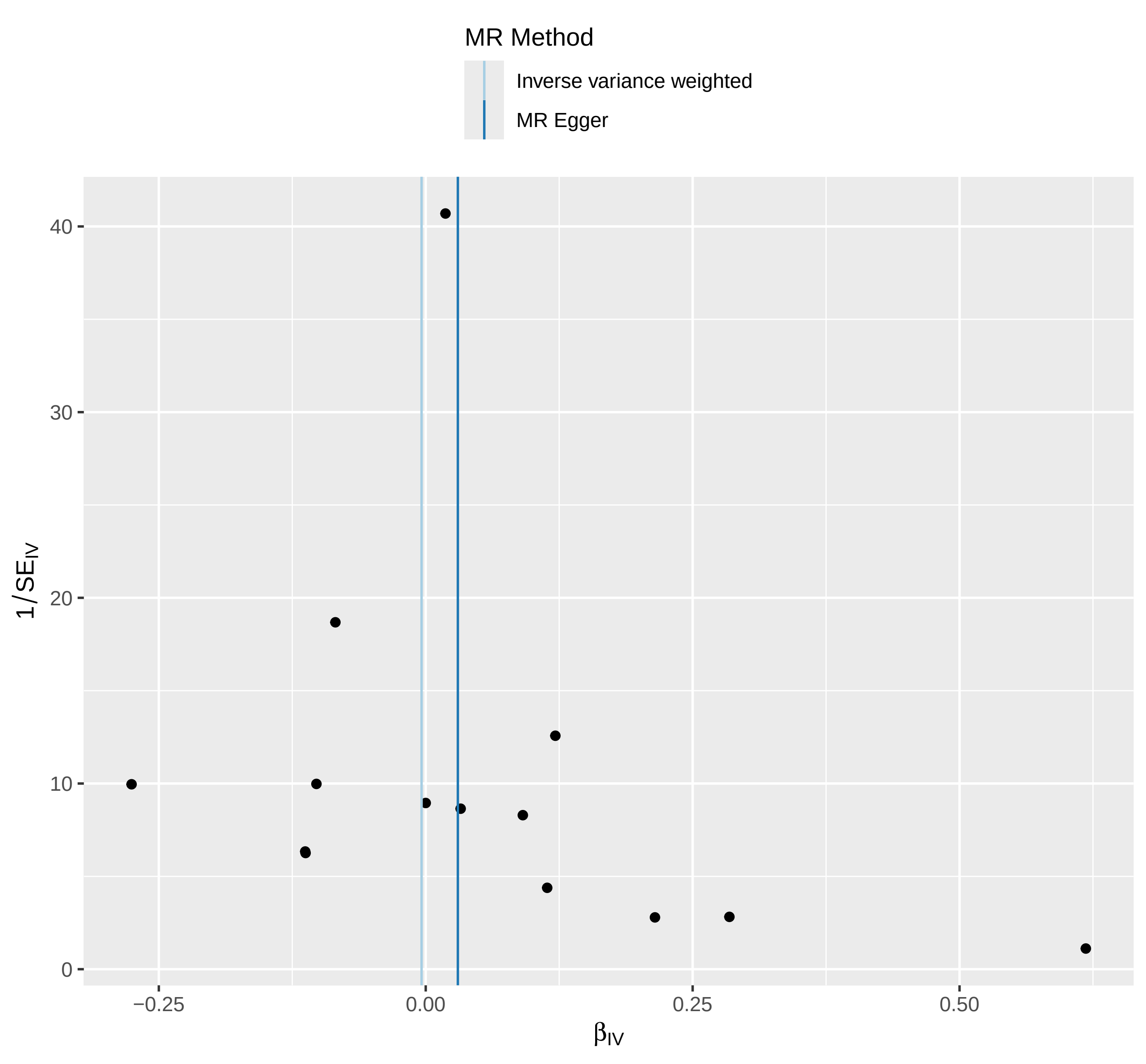

MR Method
Inverse variance weighted
MR Egger
40
30
20
10
0
1/SE_IV
−0.25
0.00
0.25
0.50
β_IV

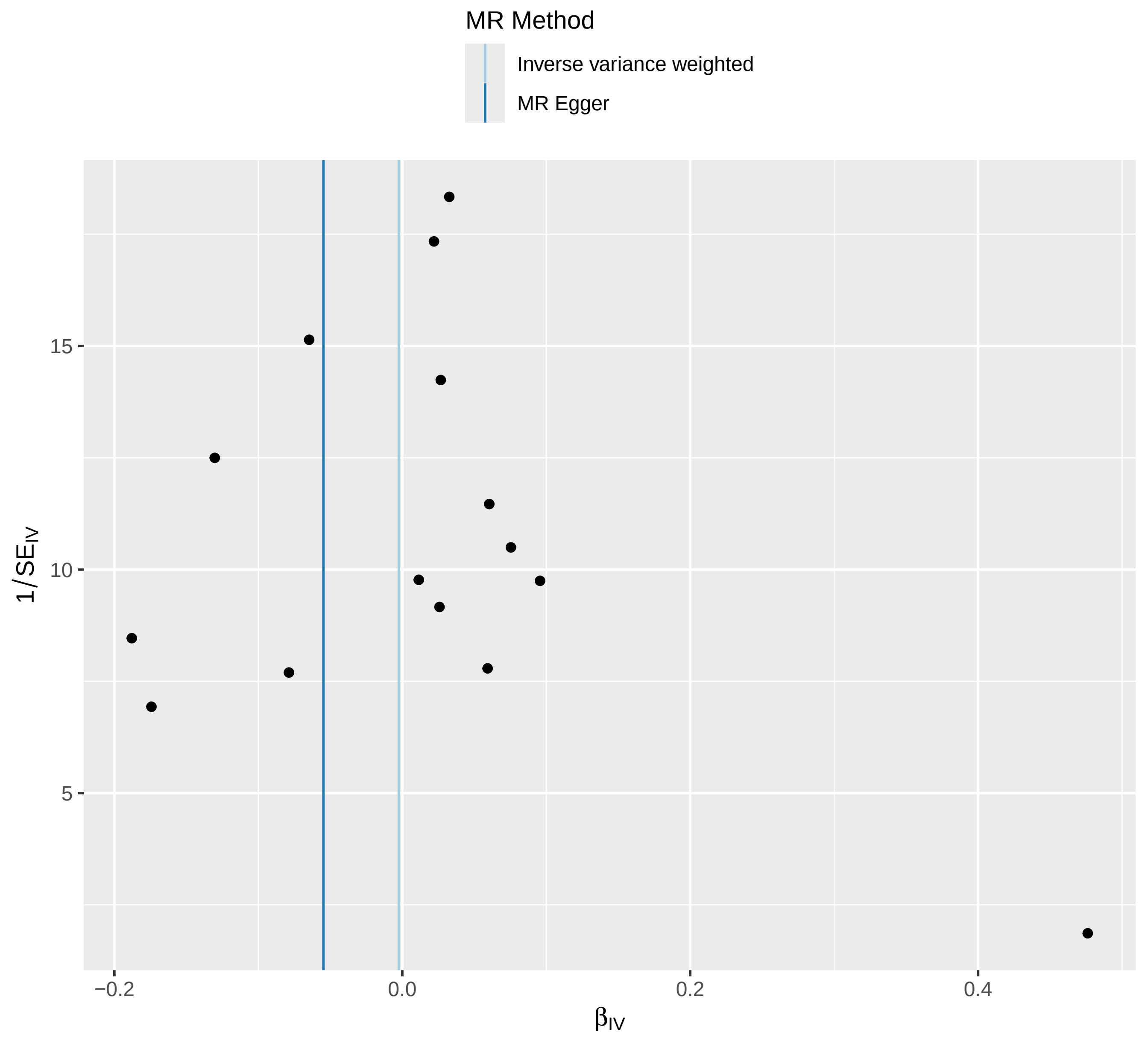
MR Method
Inverse variance weighted
MR Egger
15
10
5
$1/SE_{IV}$
−0.2
0.0
0.2
0.4
β_{IV}

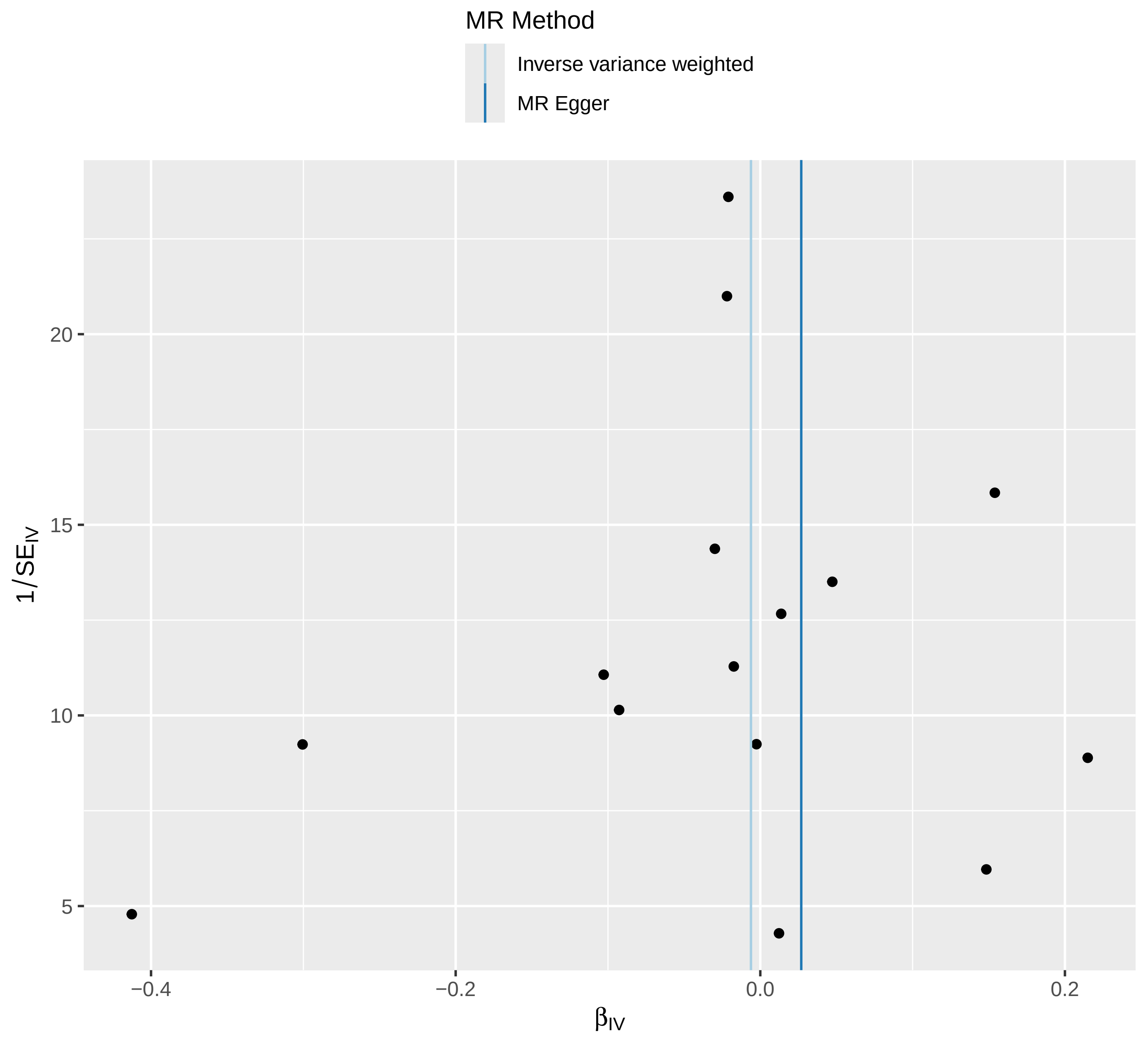
MR Method
Inverse variance weighted
MR Egger
20
15
10
5
1/SE_IV
−0.4
−0.2
0.0
0.2
β_IV

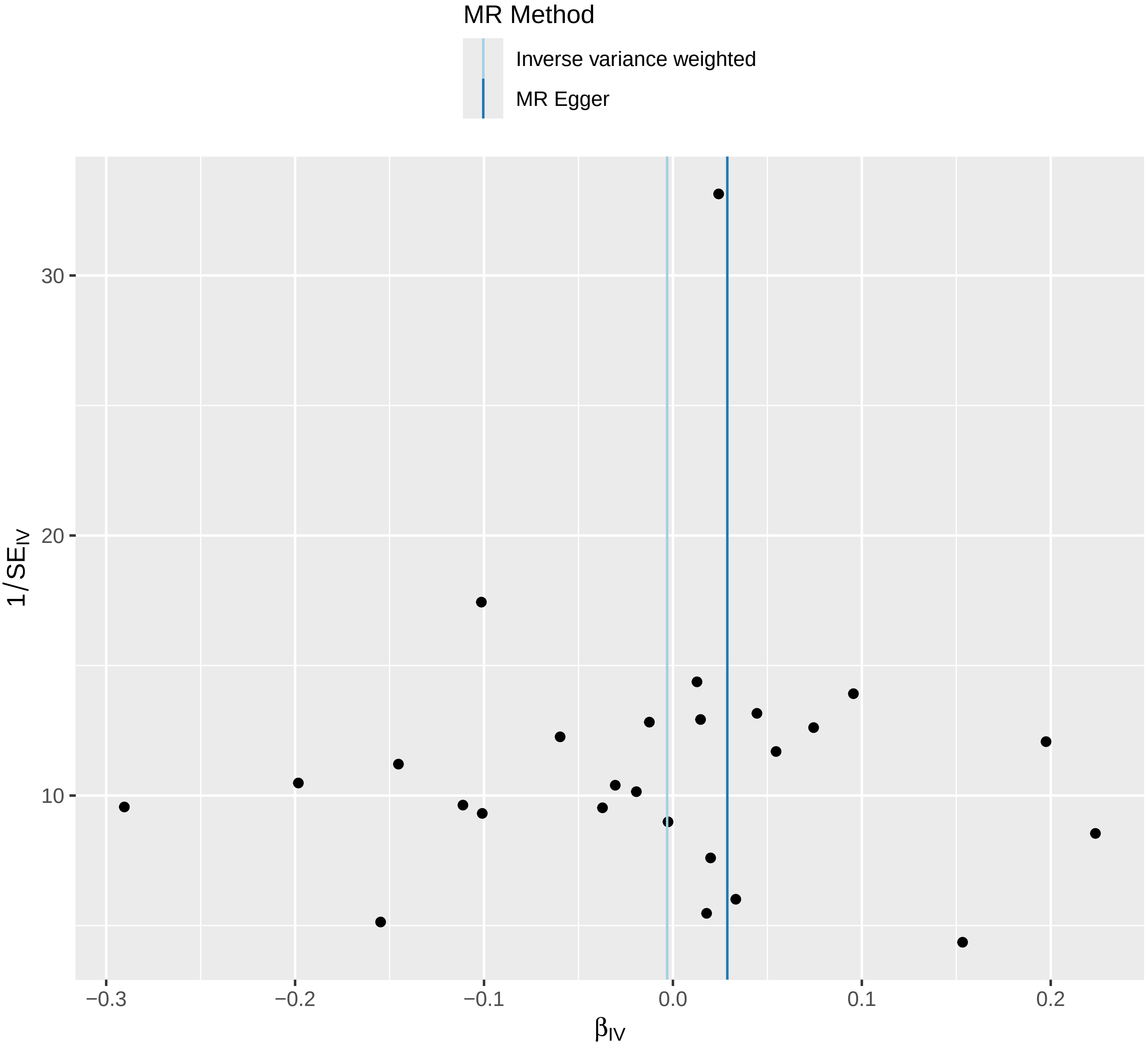

MR Method
Inverse variance weighted
MR Egger
30
20
10
$1/SE_{IV}$
−0.3
−0.2
−0.1
0.0
0.1
0.2
β_{IV}

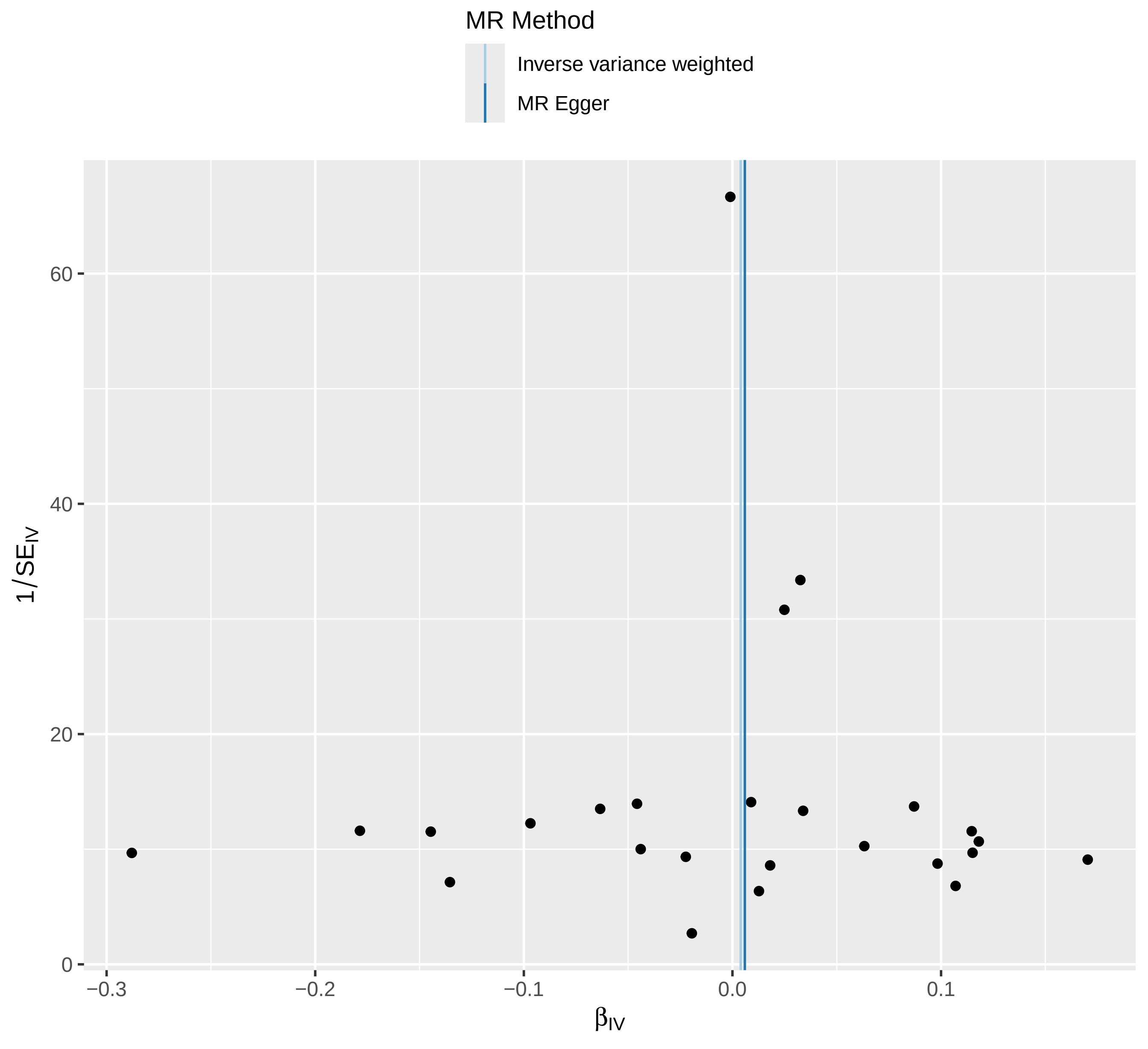
MR Method
Inverse variance weighted
MR Egger
1/SE$_{IV}$
60
40
20
0
−0.3
−0.2
−0.1
0.0
0.1
β$_{IV}$

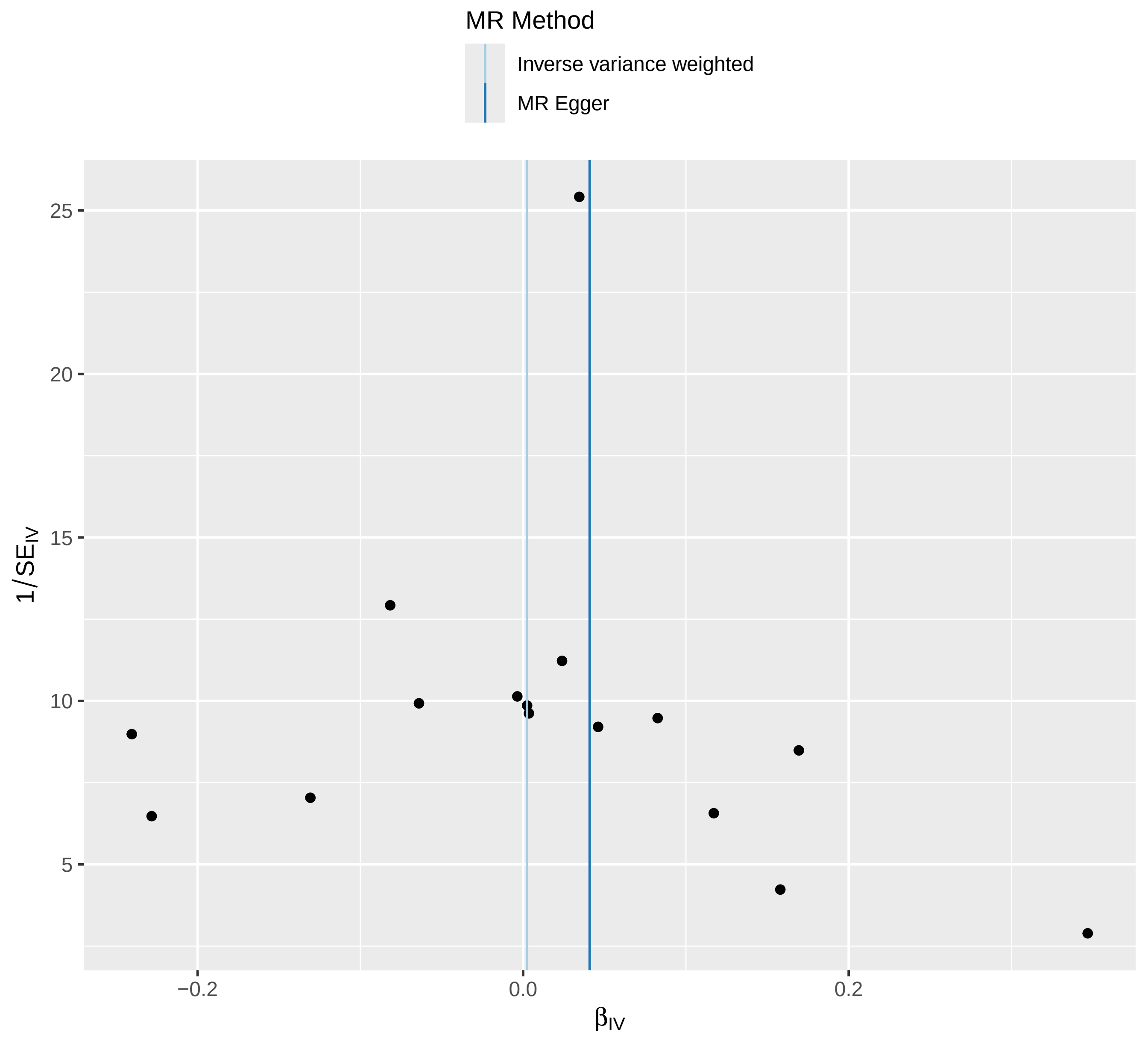
MR Method
Inverse variance weighted
MR Egger
25
20
15
10
5
1/SE$_{IV}$
−0.2
0.0
0.2
β$_{IV}$

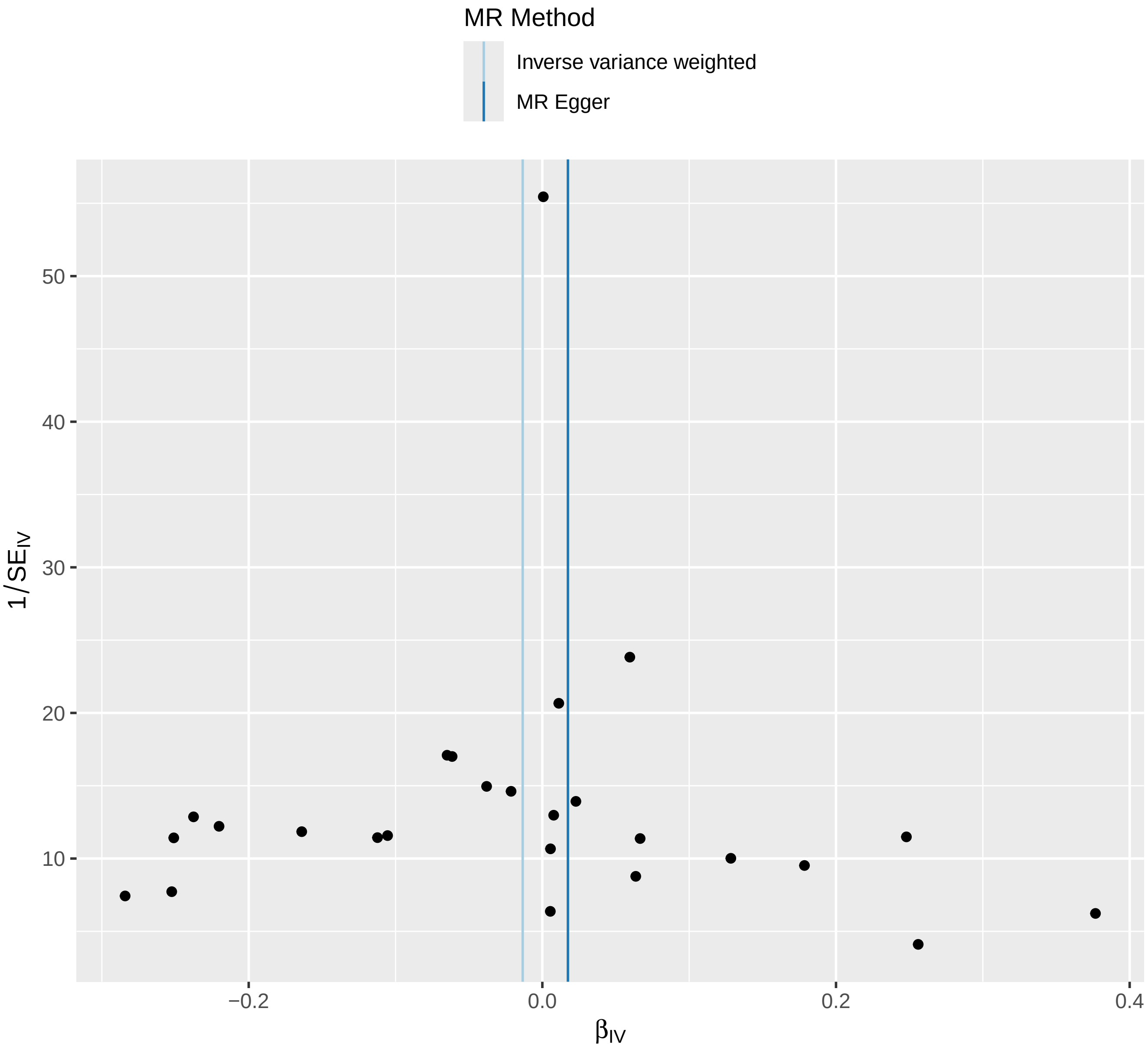
MR Method
Inverse variance weighted
MR Egger
1/SE$_{IV}$
50
40
30
20
10
−0.2
0.0
0.2
0.4
β$_{IV}$

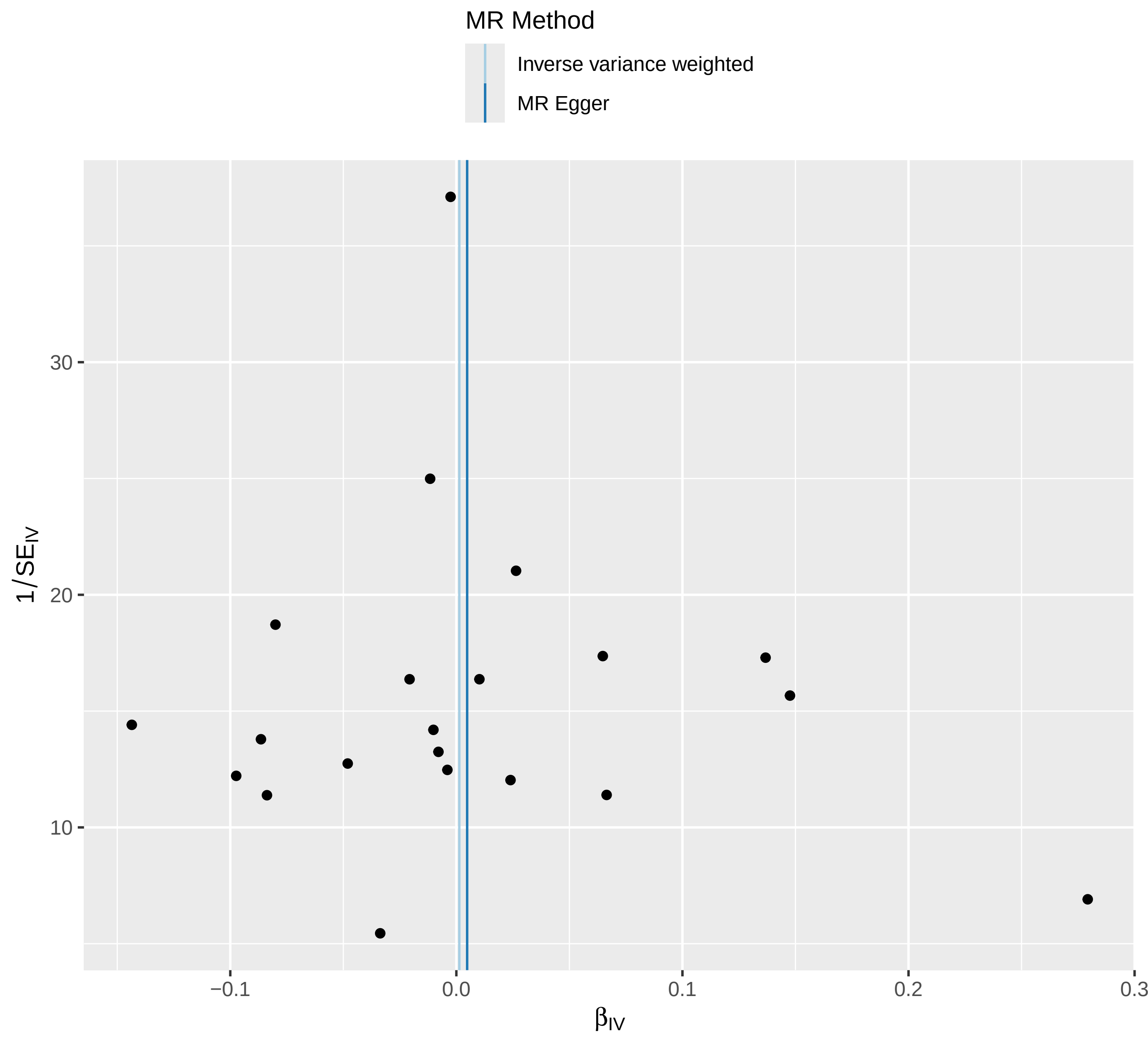
MR Method
Inverse variance weighted
MR Egger
1/SE_IV
30
20
10
−0.1
0.0
0.1
0.2
0.3
β_IV

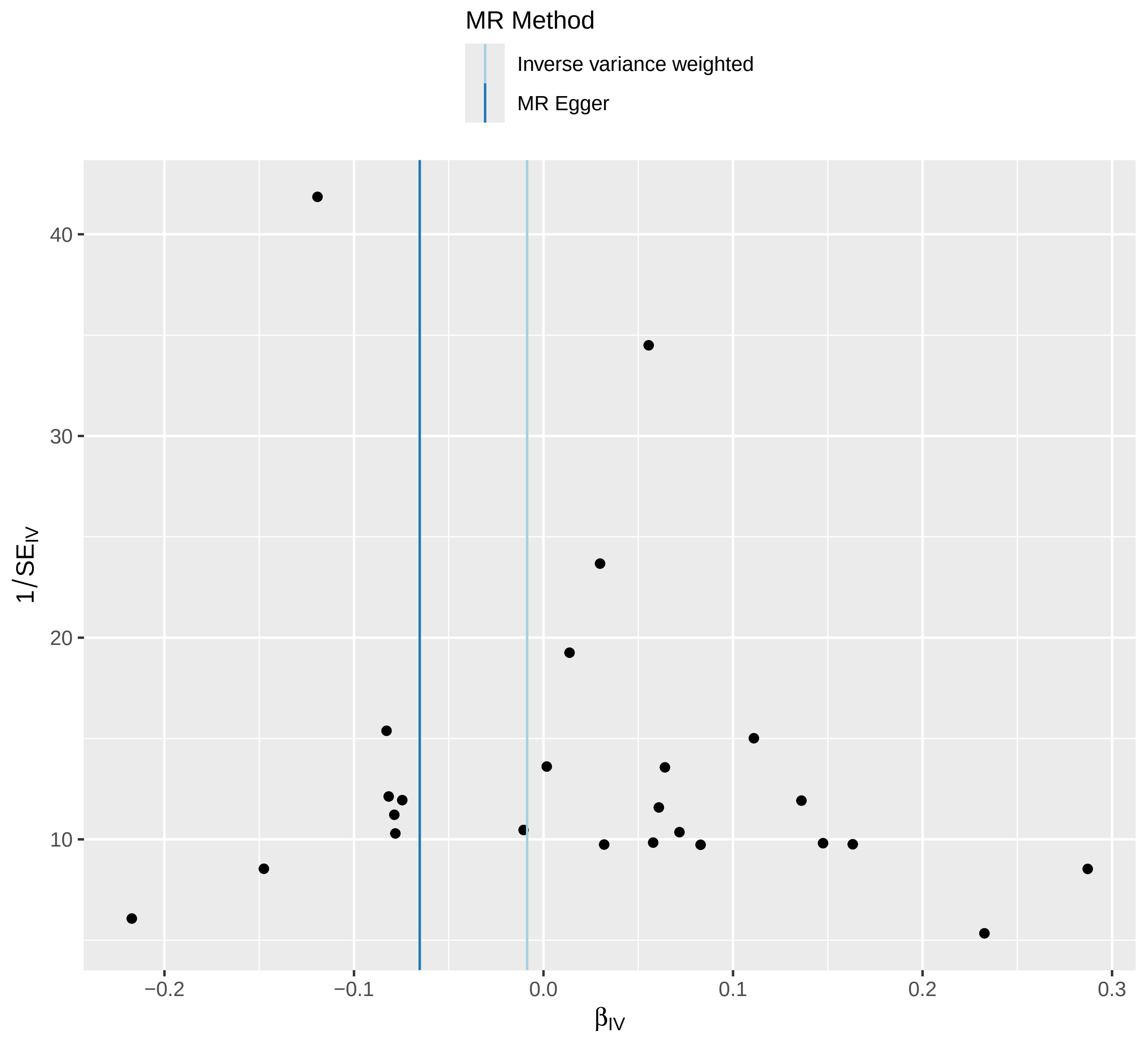

MR Method
Inverse variance weighted
MR Egger
40
30
20
10
1/SE$_{IV}$
−0.2
−0.1
0.0
0.1
0.2
0.3
β_{IV}

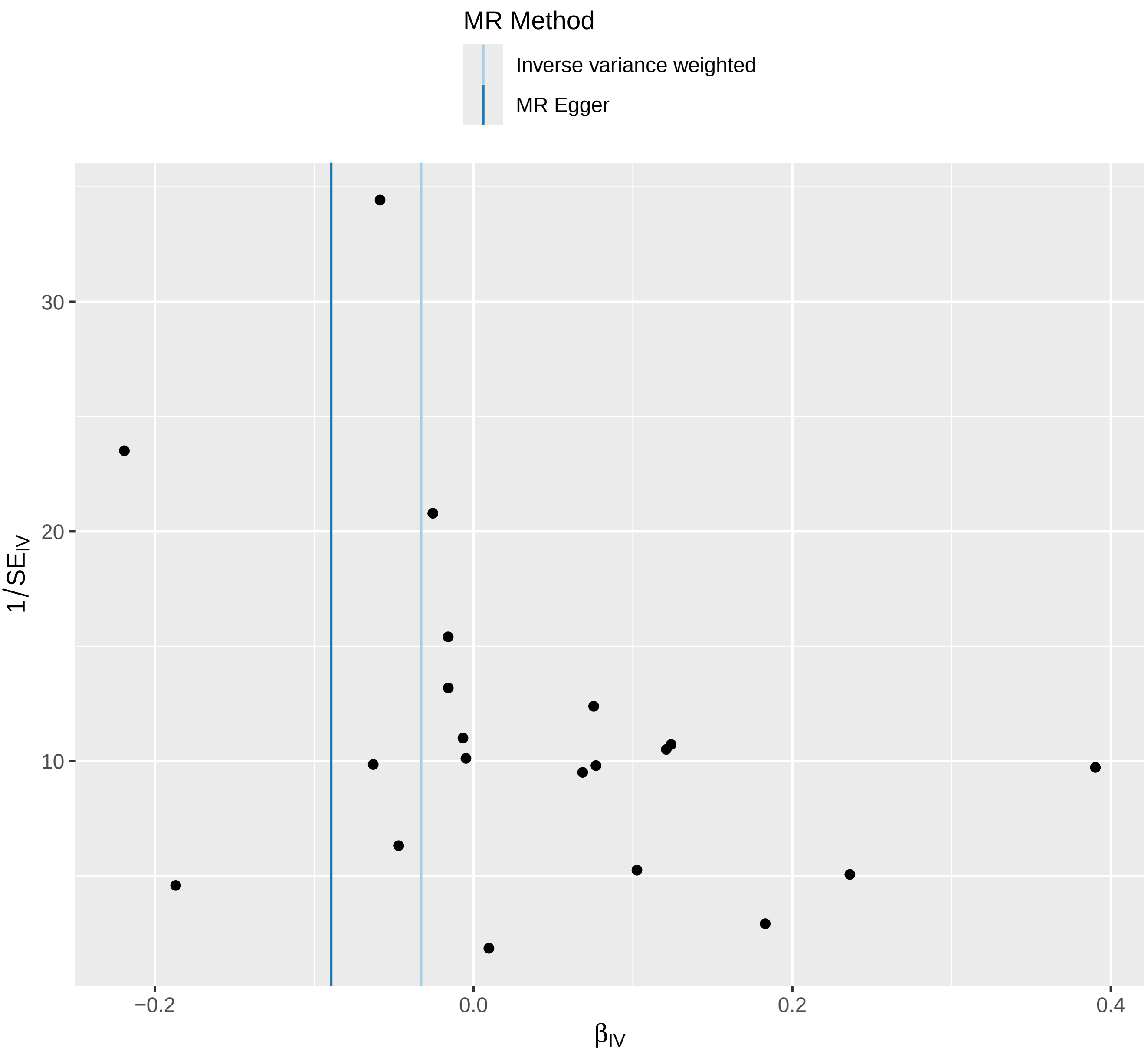
MR Method
Inverse variance weighted
MR Egger
30
20
10
1/SE$_{IV}$
−0.2
0.0
0.2
0.4
β$_{IV}$

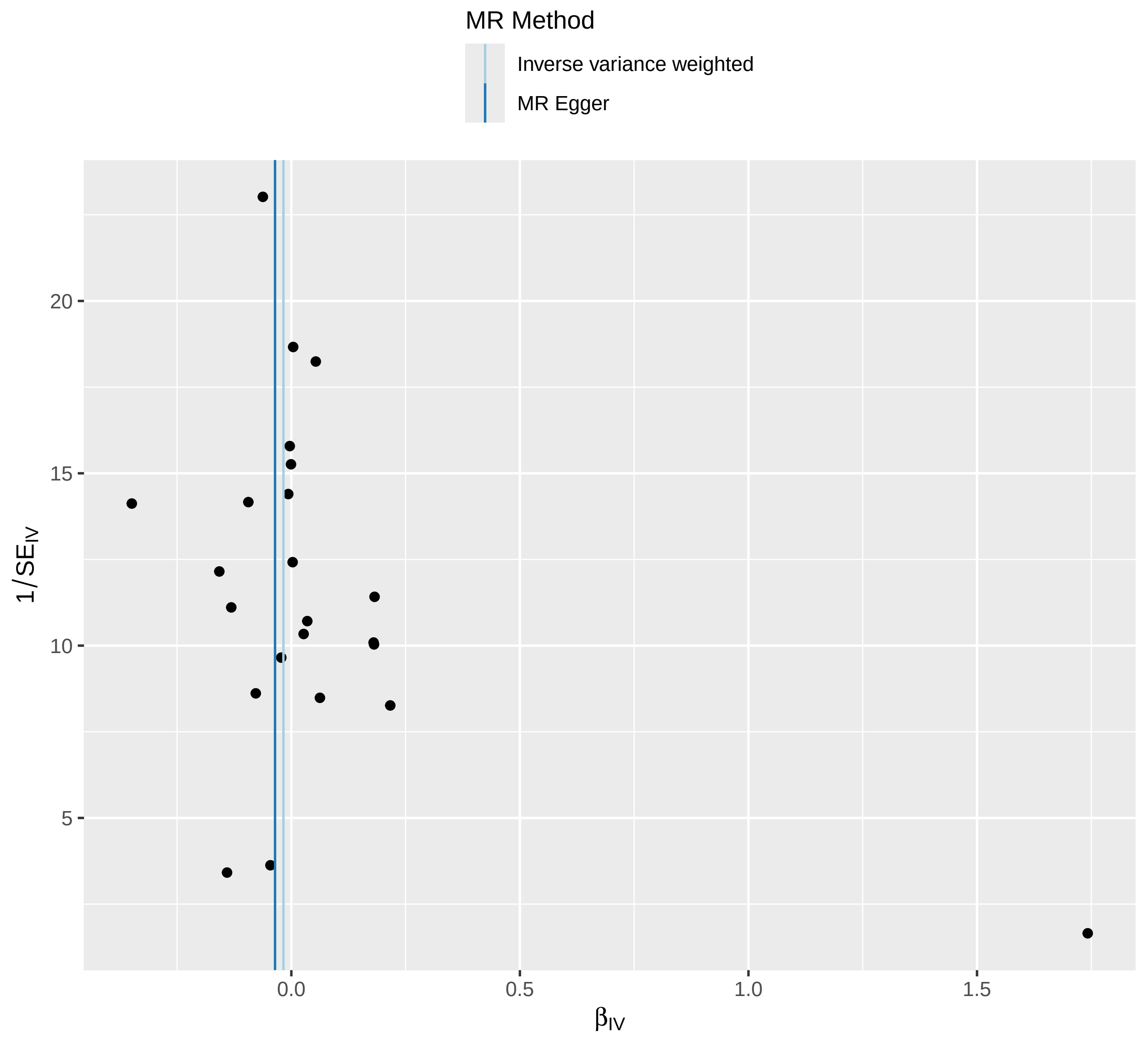
MR Method
Inverse variance weighted
MR Egger
20
15
10
5
$1/SE_{IV}$
0.0
0.5
1.0
1.5
β_{IV}

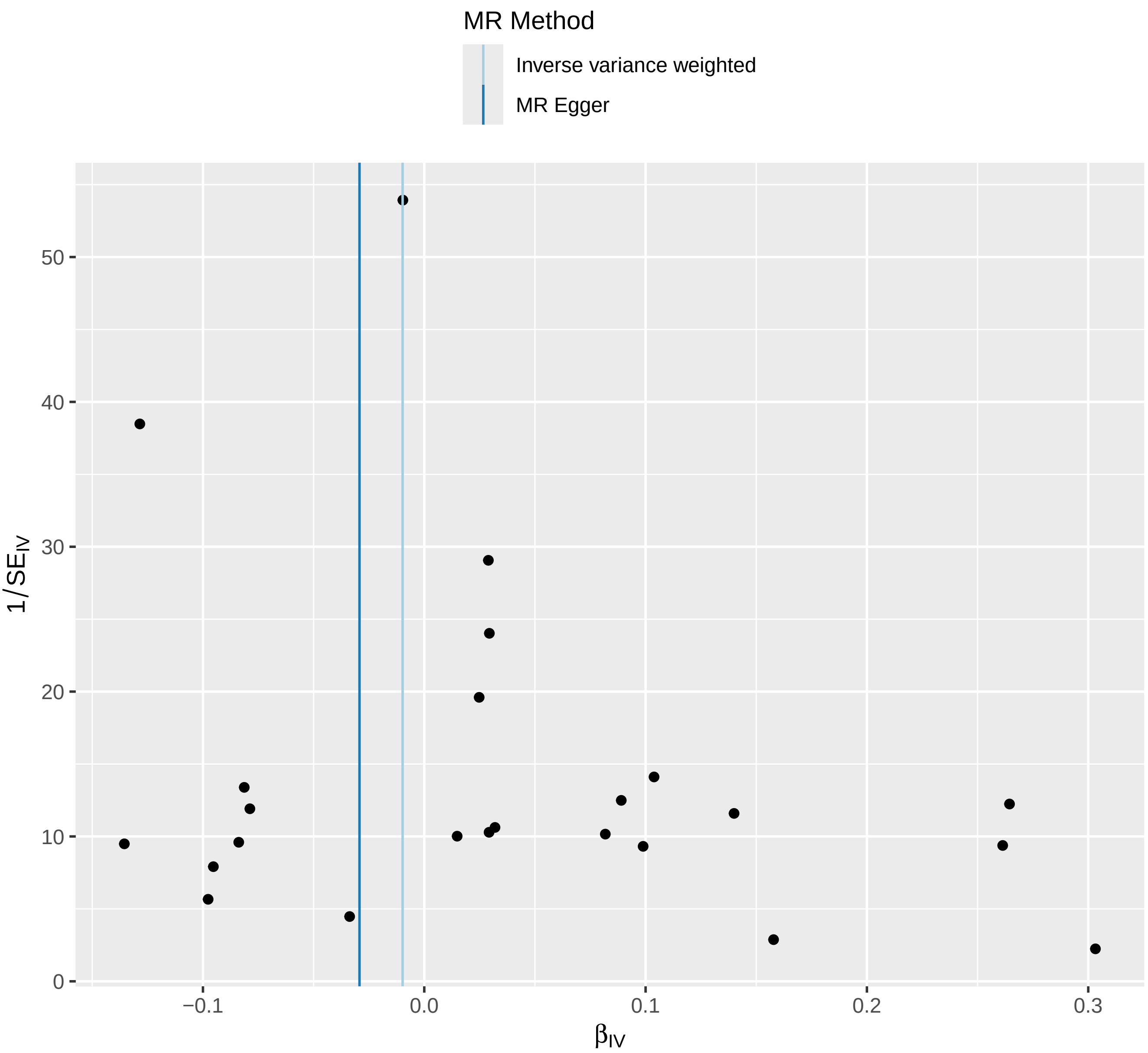
MR Method
Inverse variance weighted
MR Egger
1/SE_IV
β_IV
0
10
20
30
40
50
−0.1
0.0
0.1
0.2
0.3

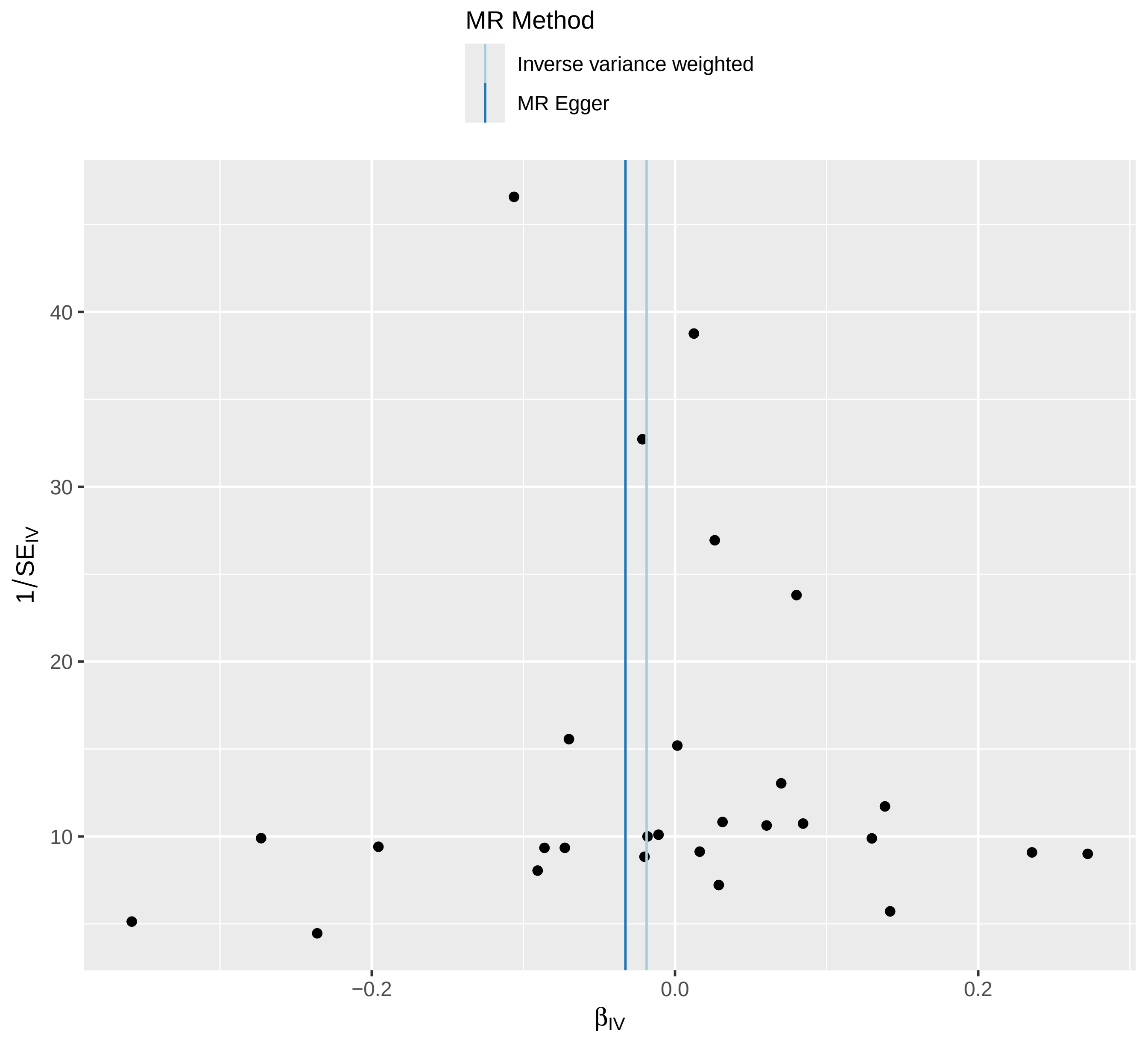
MR Method
Inverse variance weighted
MR Egger
40
30
20
10
1/SE_IV
−0.2
0.0
0.2
β_IV

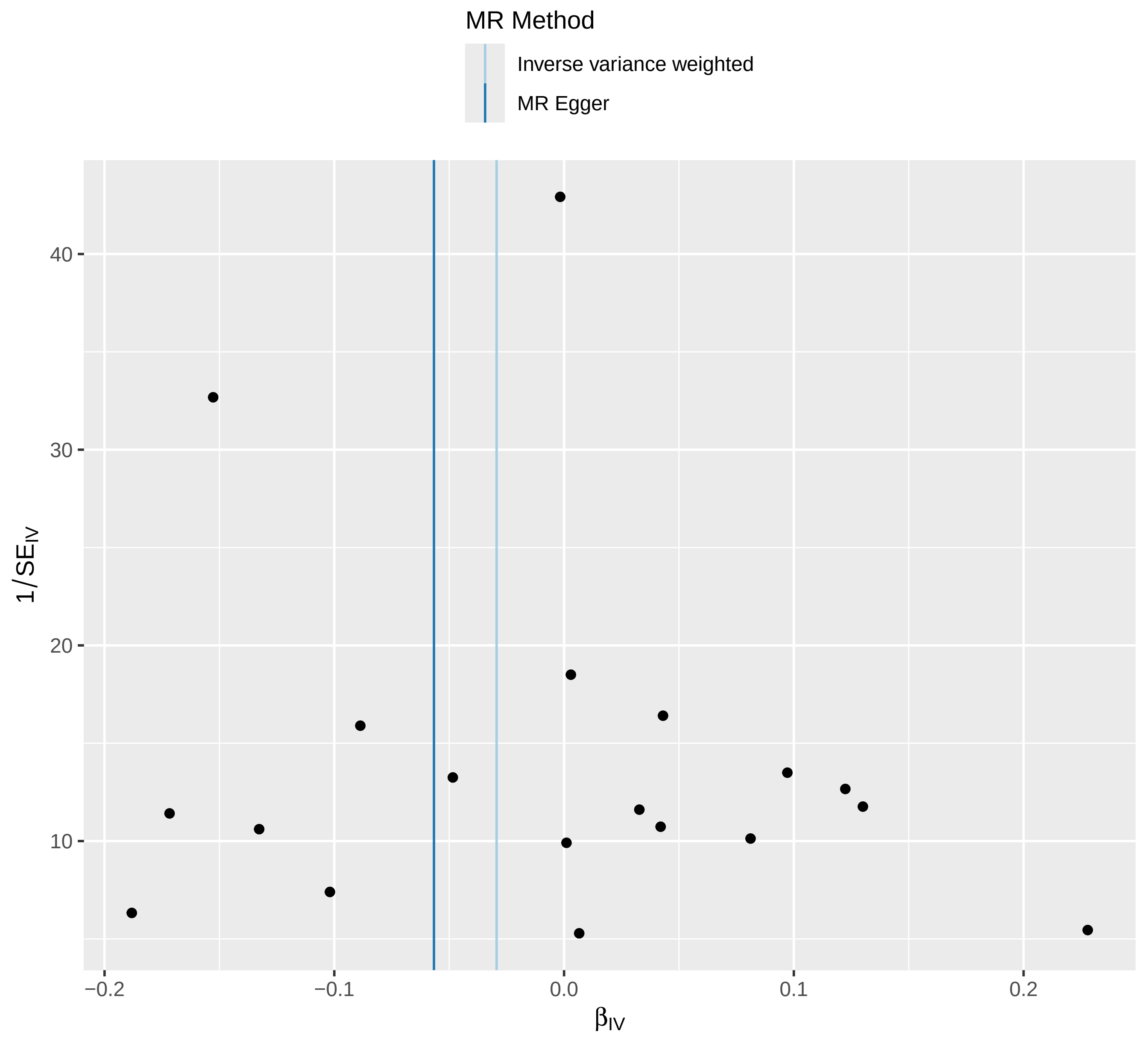
MR Method
Inverse variance weighted
MR Egger
40
30
20
10
1/SE_IV
−0.2
−0.1
0.0
0.1
0.2
β_IV

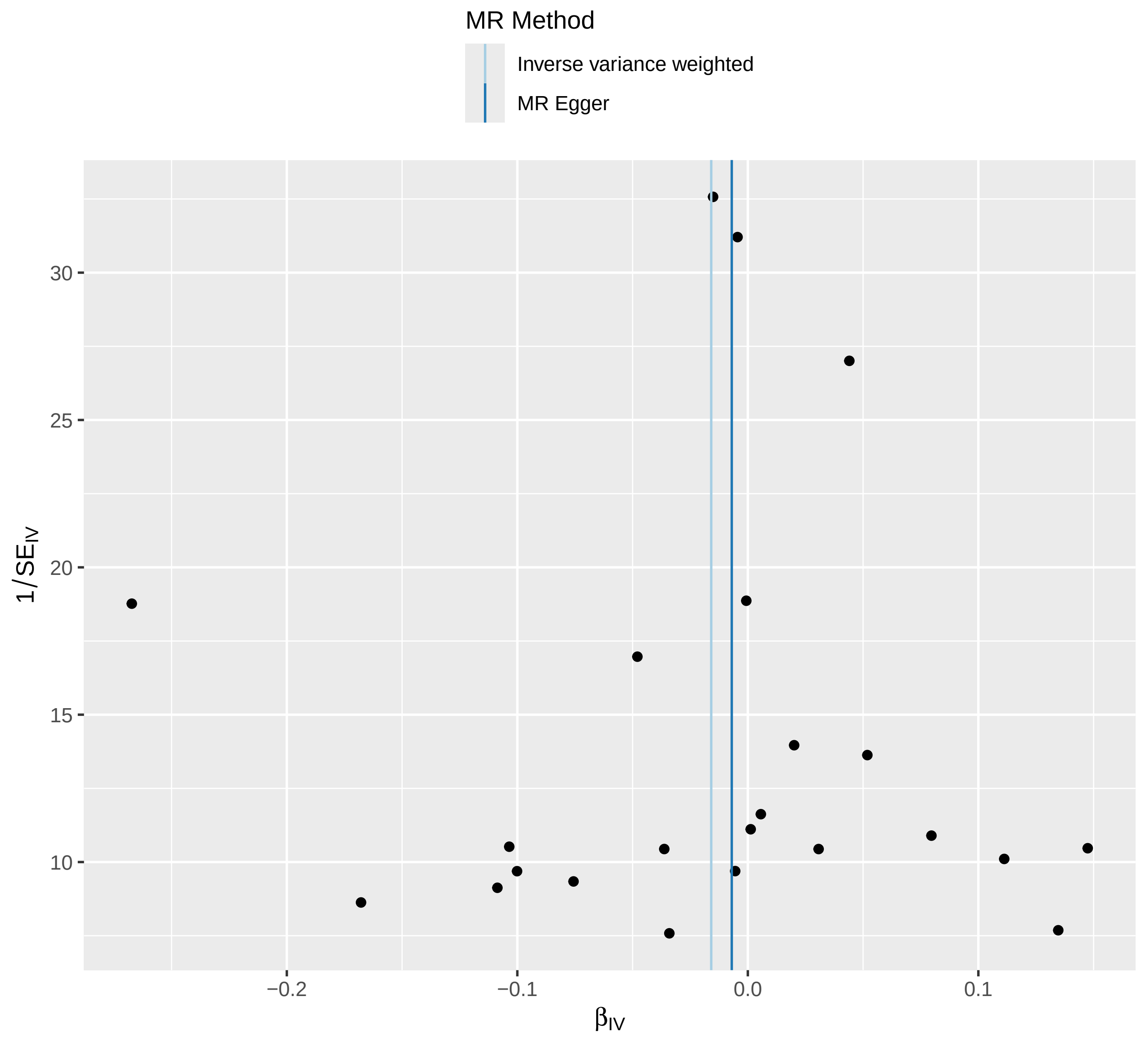
MR Method
Inverse variance weighted
MR Egger
1/SE$_{IV}$
β$_{IV}$
30
25
20
15
10
−0.2
−0.1
0.0
0.1

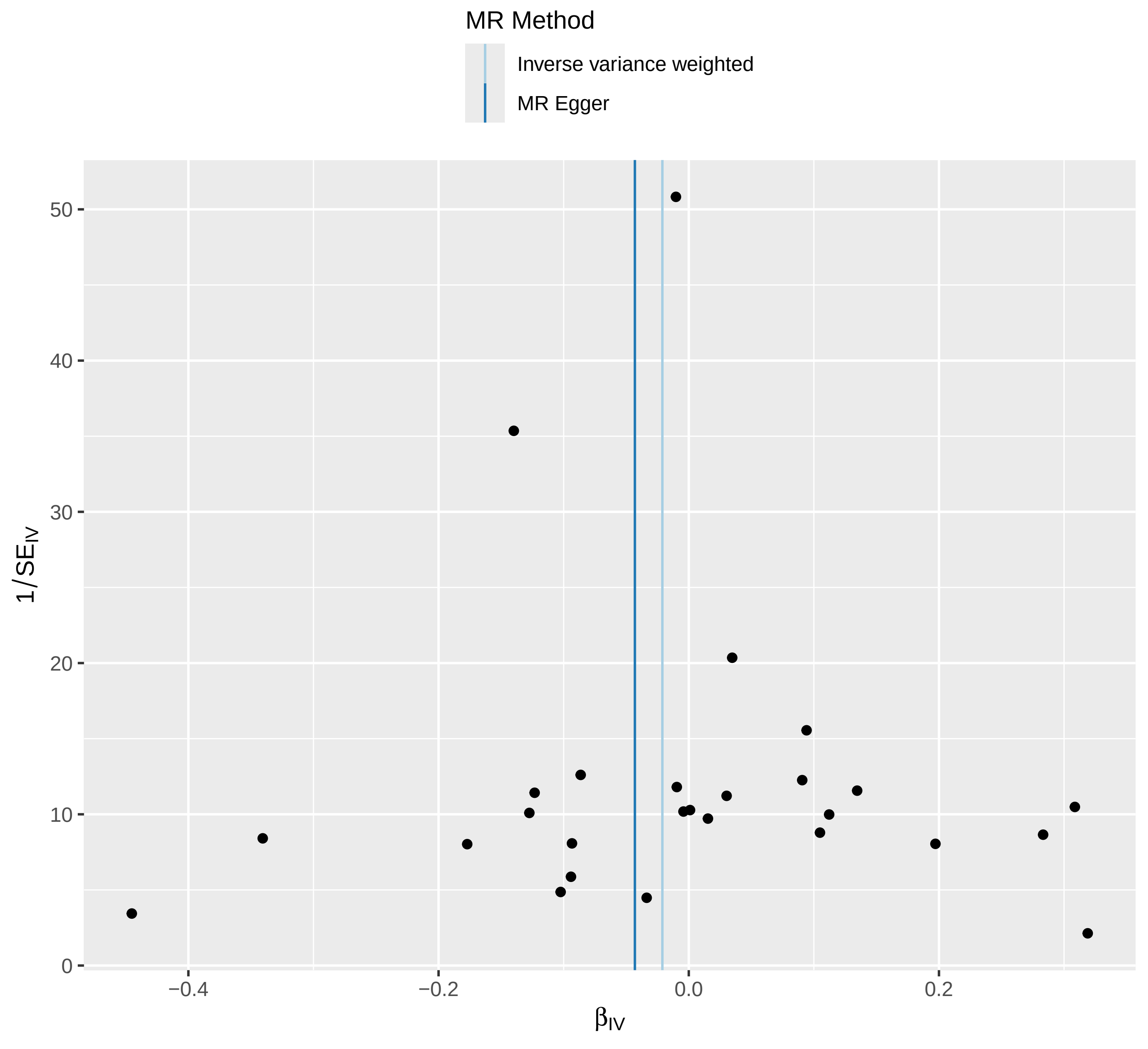
MR Method
Inverse variance weighted
MR Egger
50
40
30
20
10
0
1/SE_IV
−0.4
−0.2
0.0
0.2
β_IV

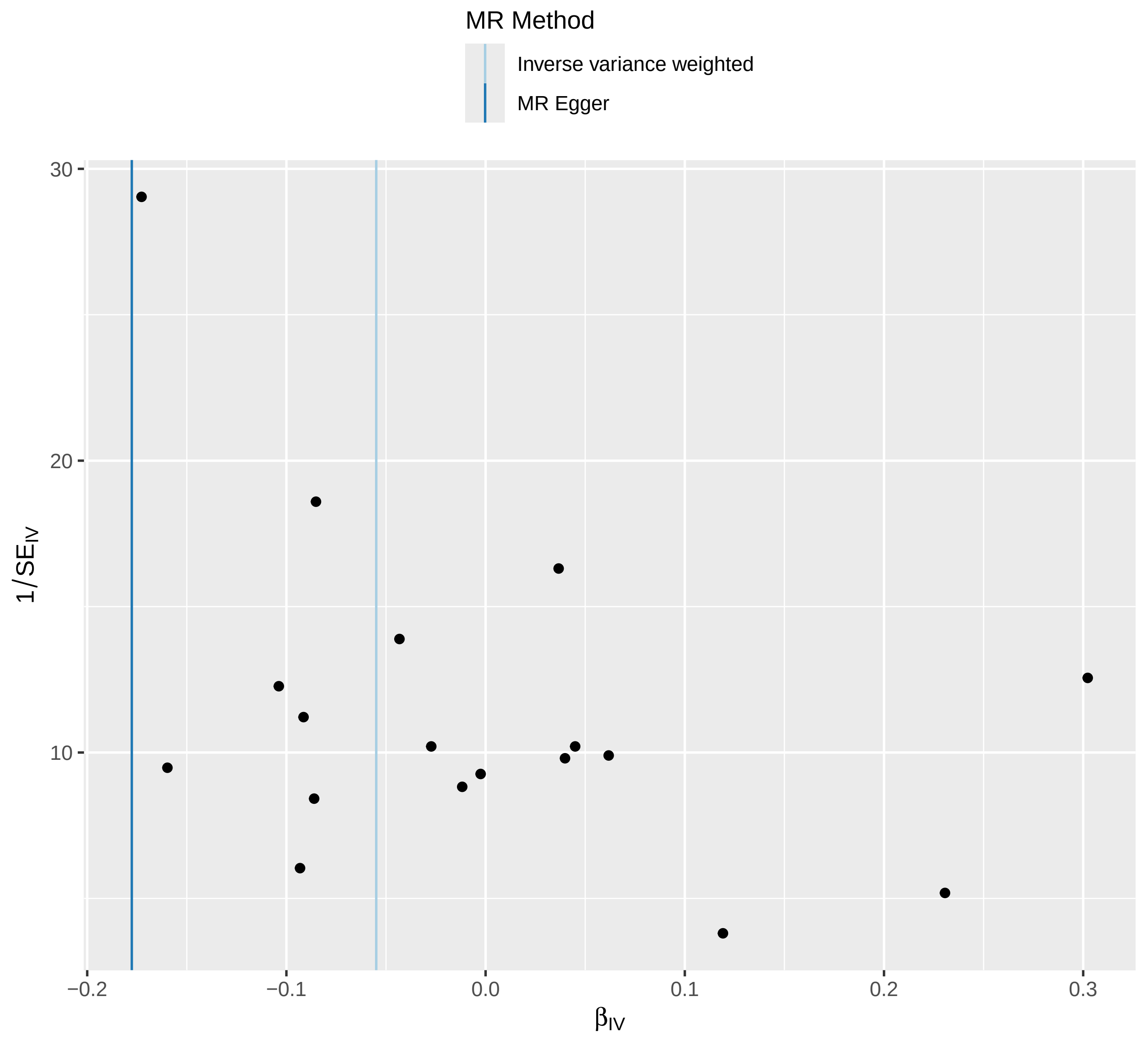
MR Method
Inverse variance weighted
MR Egger
30
20
10
1/SE$_{IV}$
−0.2
−0.1
0.0
0.1
0.2
0.3
β$_{IV}$

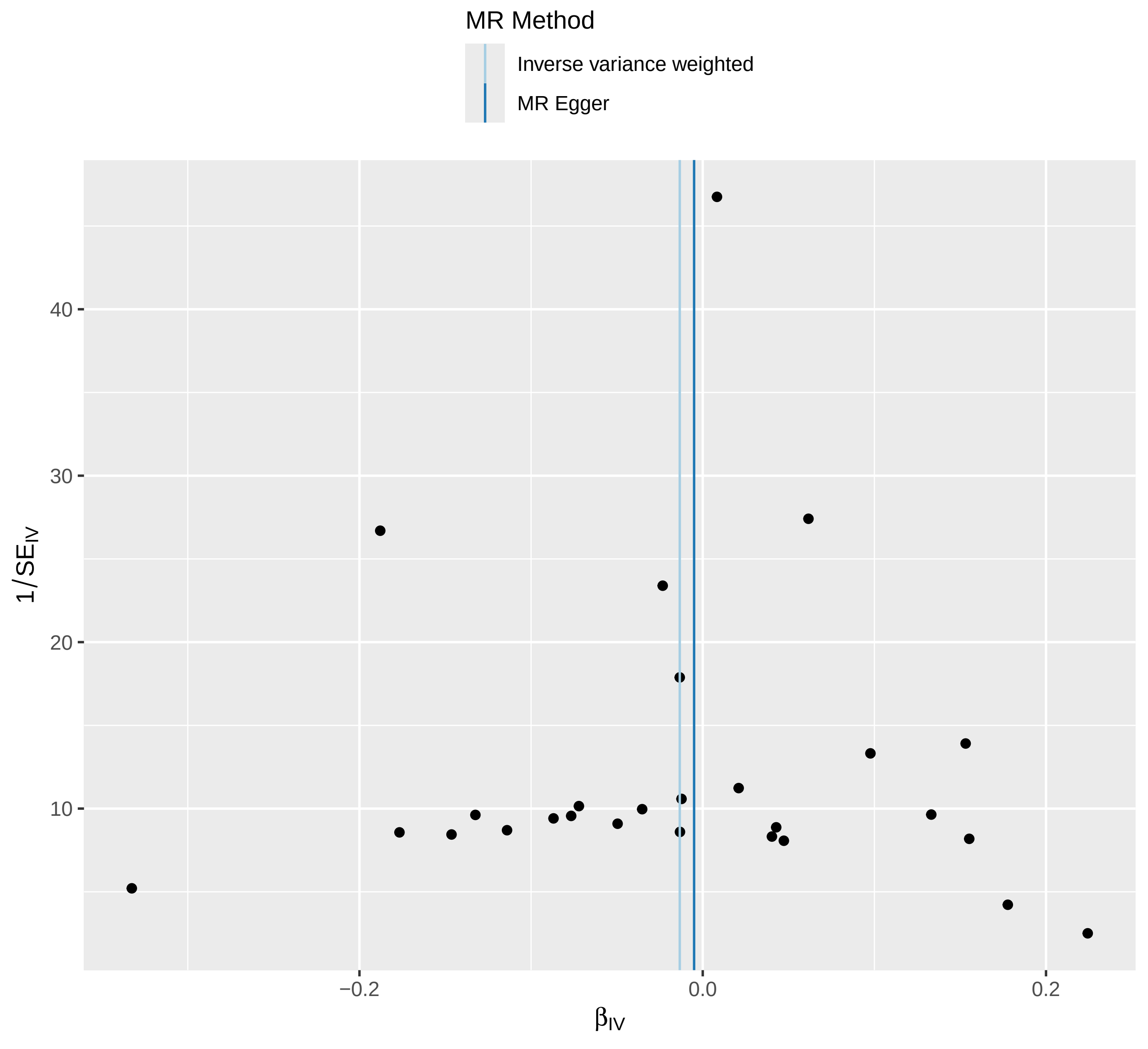
MR Method
Inverse variance weighted
MR Egger
40
30
20
10
1/SE_IV
−0.2
0.0
0.2
β_IV

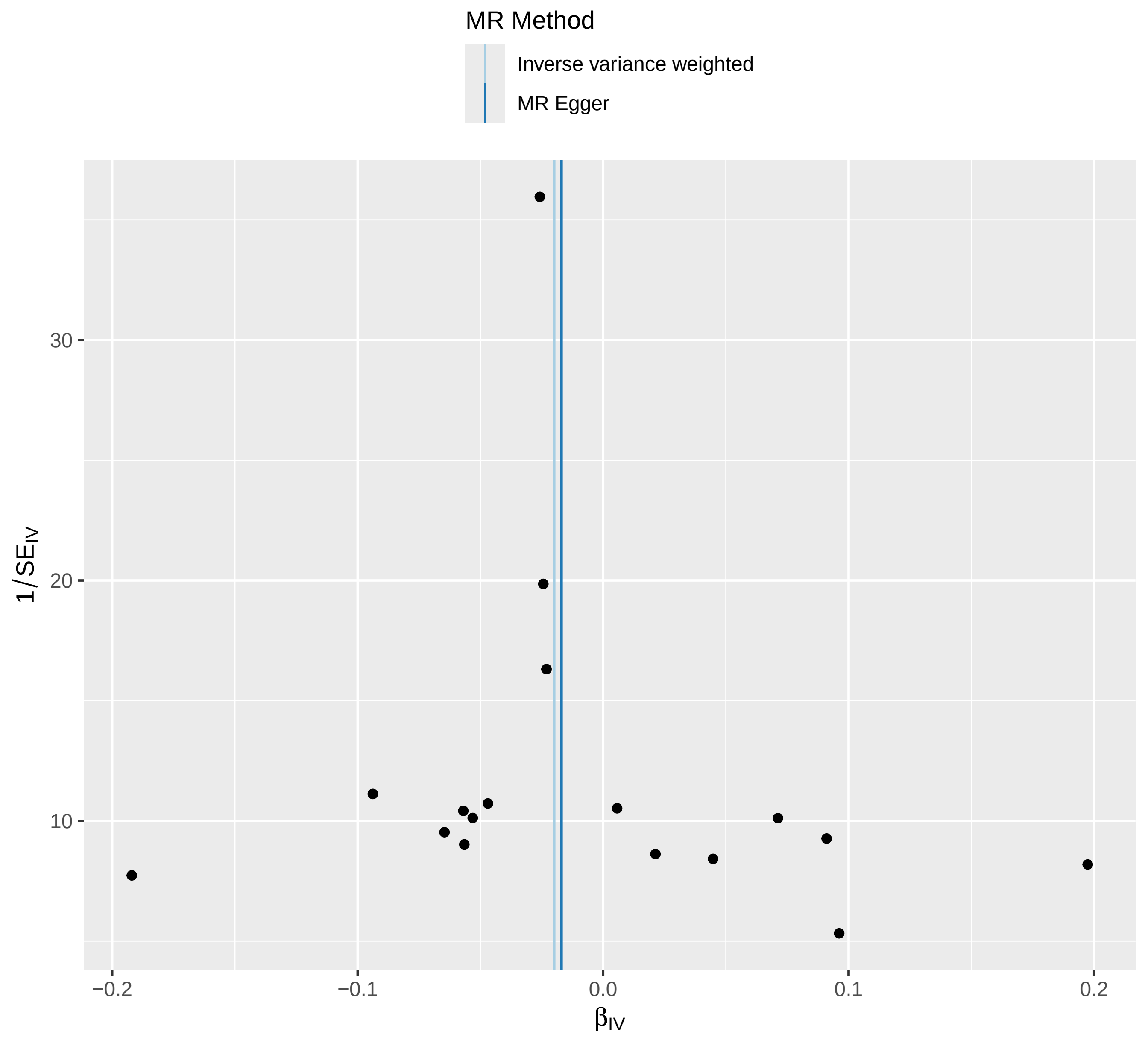

MR Method
Inverse variance weighted
MR Egger
30
20
10
1/SE_IV
−0.2
−0.1
0.0
0.1
0.2
β_IV

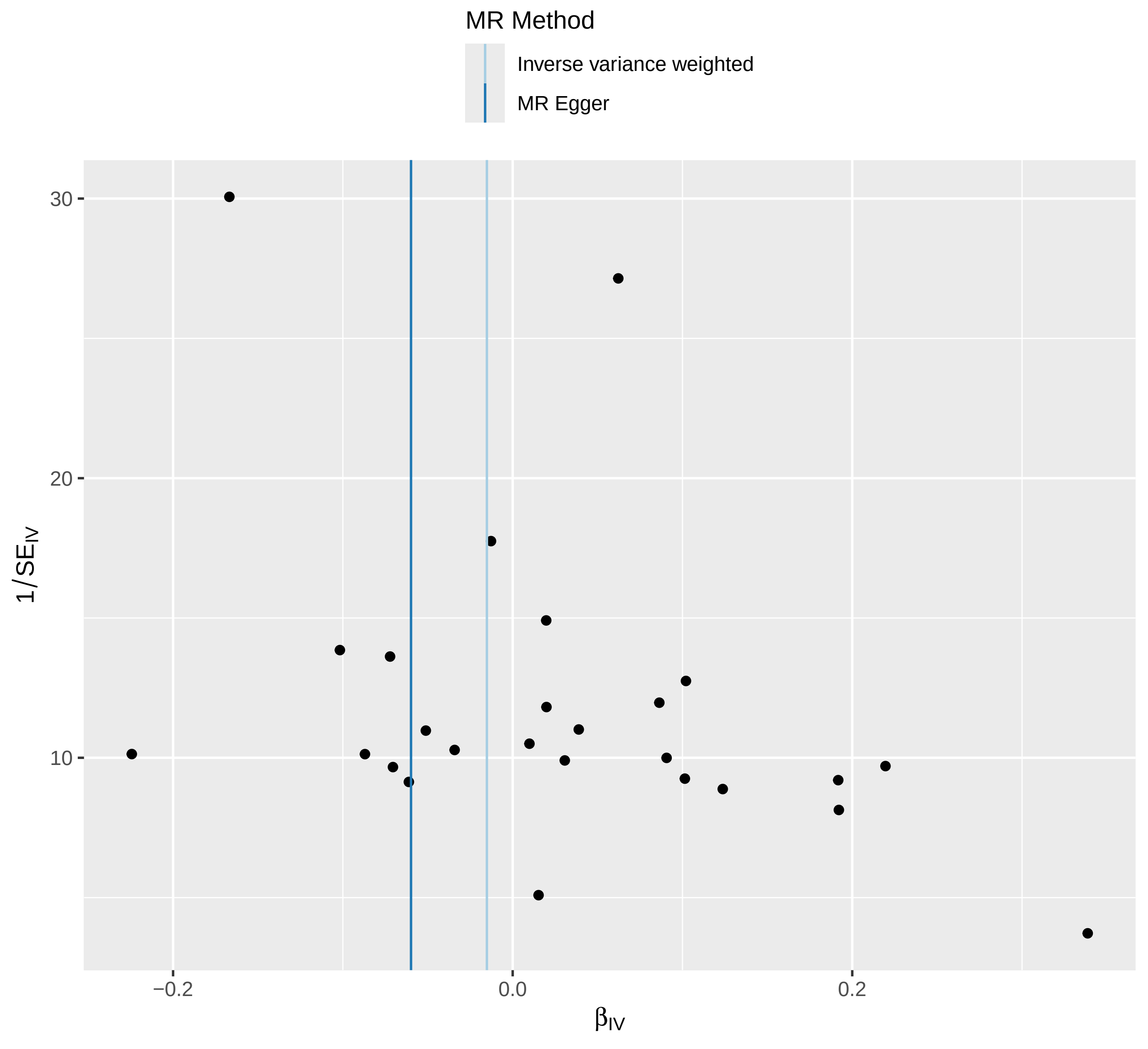
MR Method
Inverse variance weighted
MR Egger
30
20
10
1/SE_IV
−0.2
0.0
0.2
β_IV

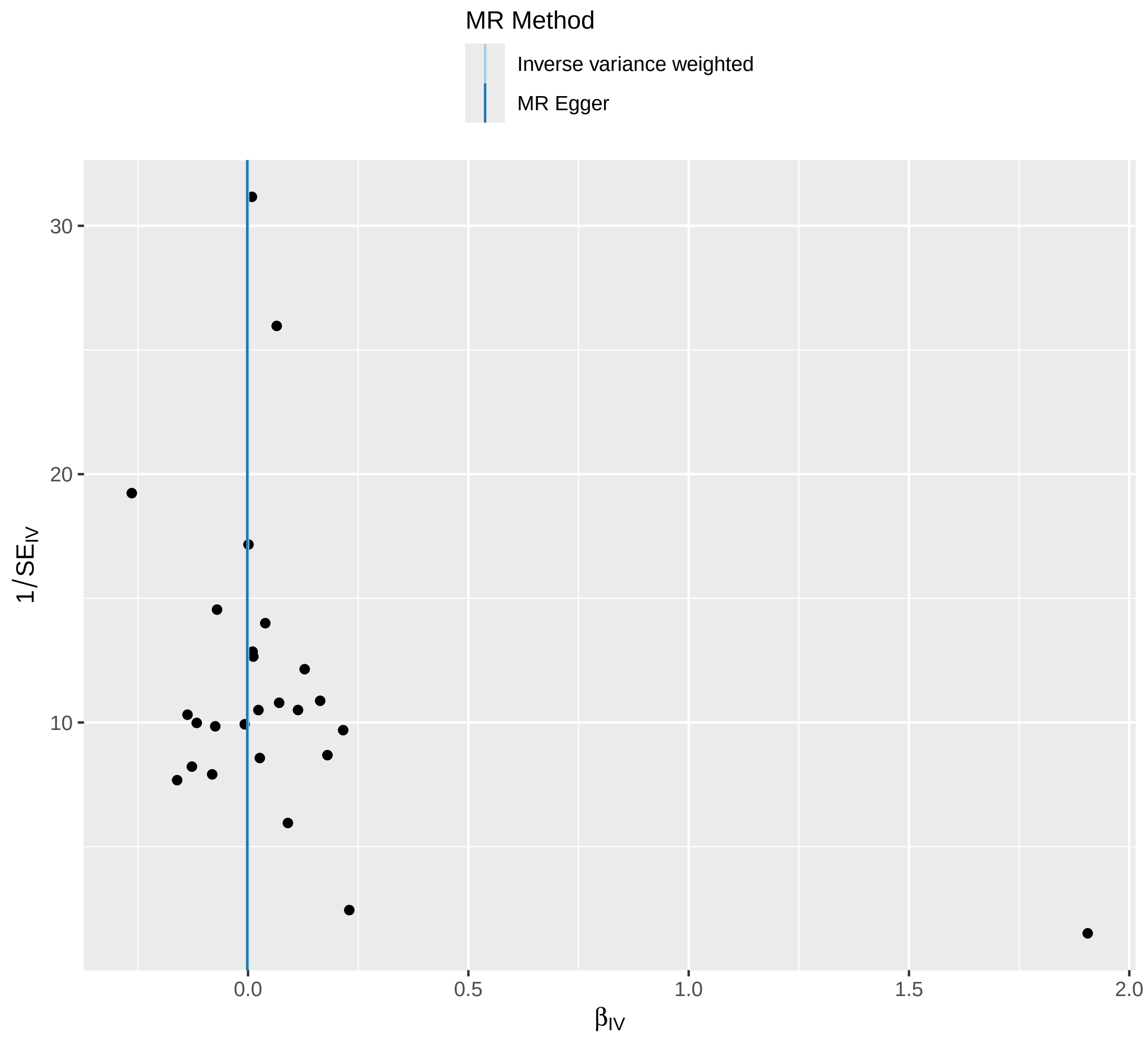
MR Method
Inverse variance weighted
MR Egger
30
20
10
1/SE$_{IV}$
0.0
0.5
1.0
1.5
2.0
β$_{IV}$

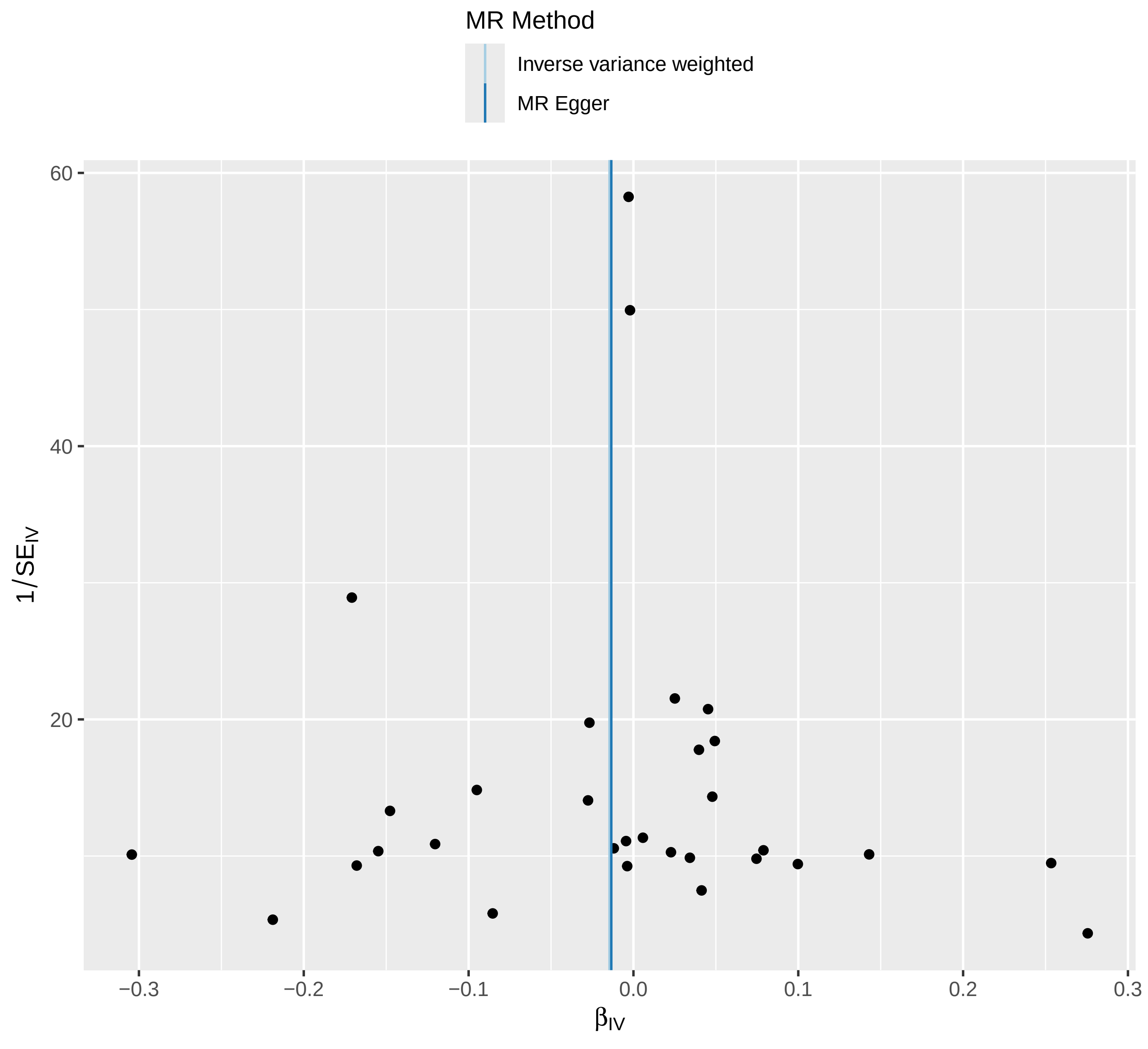

MR Method
Inverse variance weighted
MR Egger
60
40
20
$1/SE_{IV}$
−0.3
−0.2
−0.1
0.0
0.1
0.2
0.3
β_{IV}

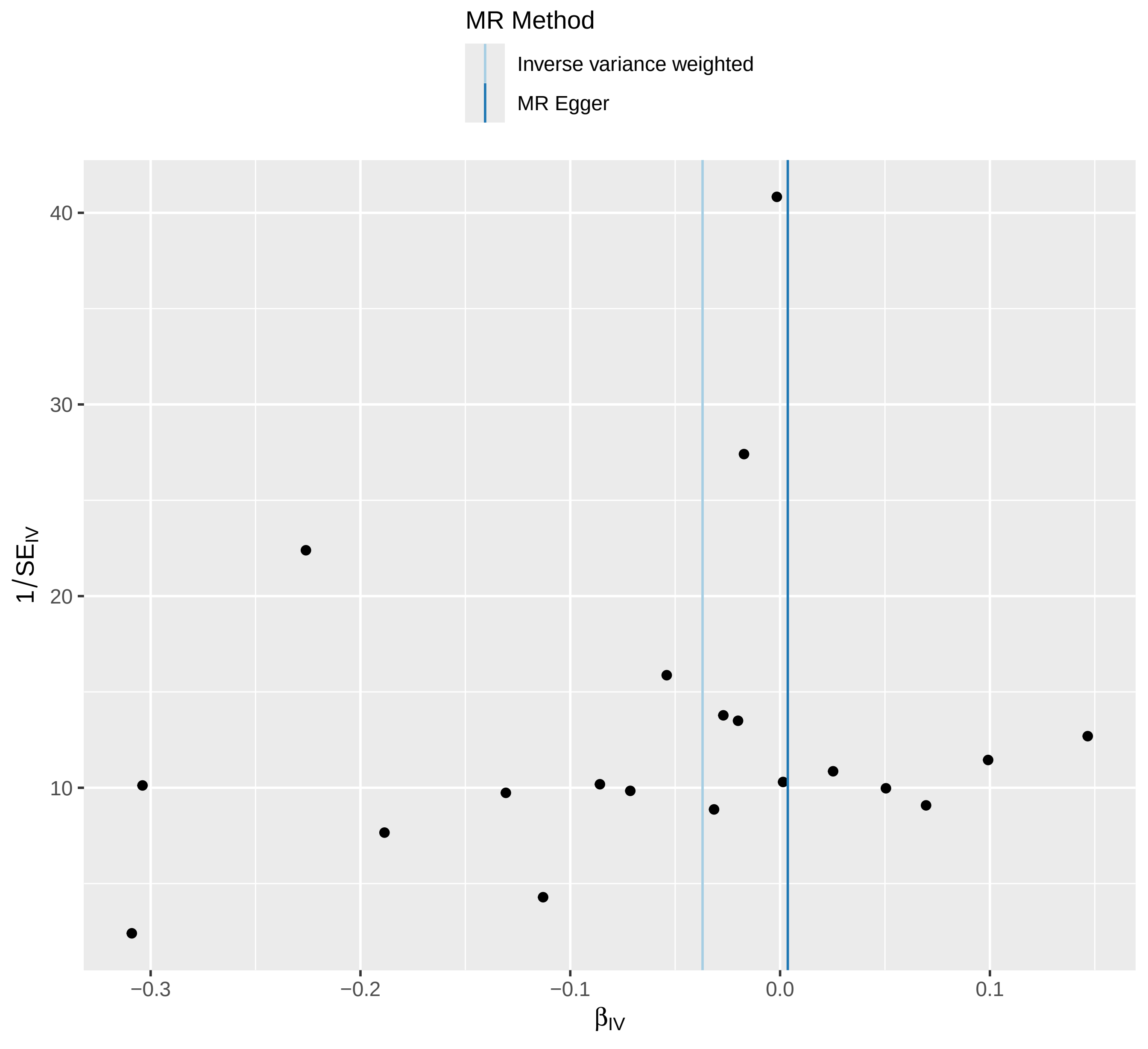

MR Method
Inverse variance weighted
MR Egger
40
30
20
10
1/SE_IV
−0.3
−0.2
−0.1
0.0
0.1
β_IV

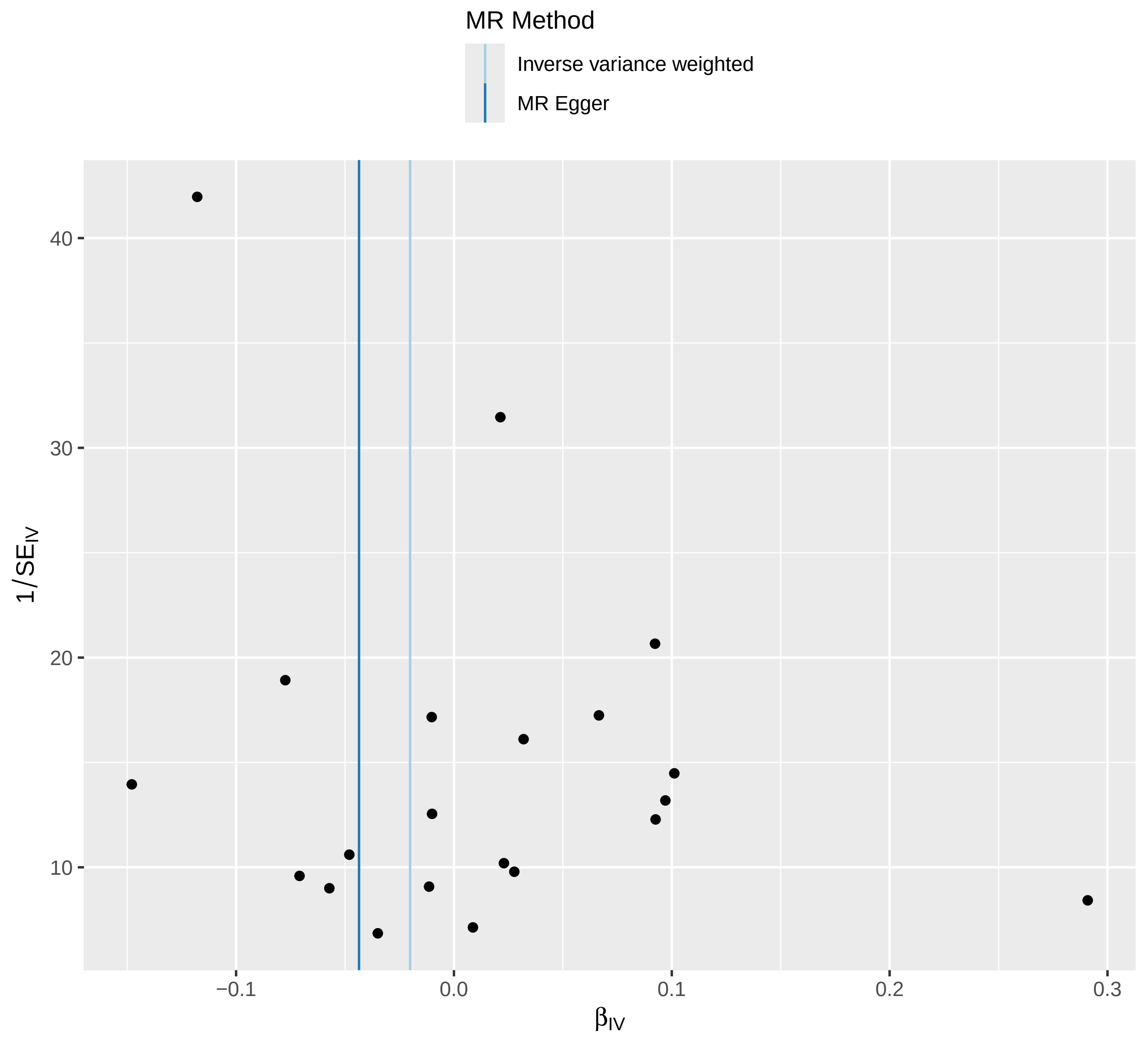
MR Method
Inverse variance weighted
MR Egger
40
30
20
10
1/SE_IV
−0.1
0.0
0.1
0.2
0.3
β_IV

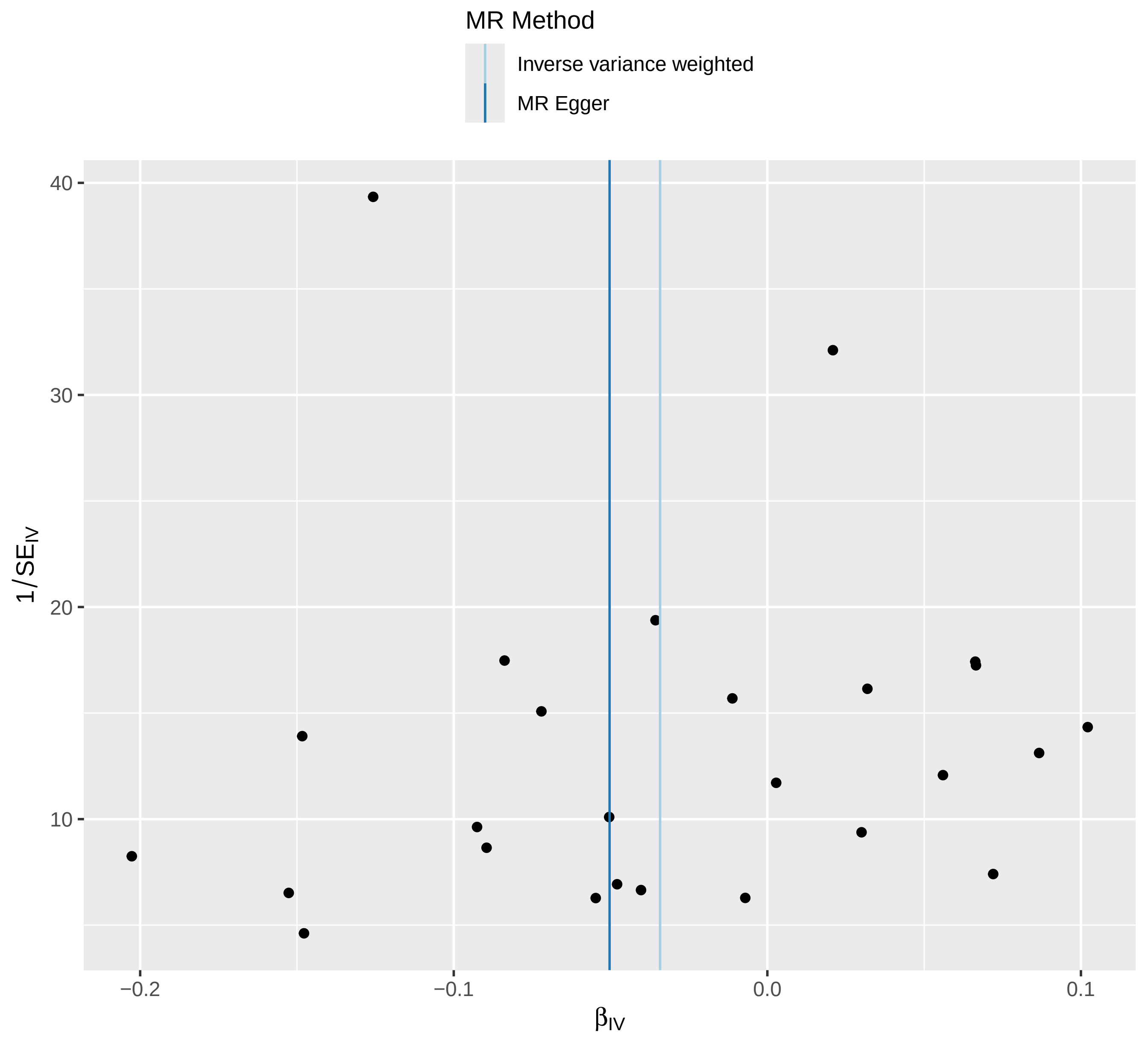

MR Method
Inverse variance weighted
MR Egger
1/SE_IV
40
30
20
10
−0.2
−0.1
0.0
0.1
β_IV

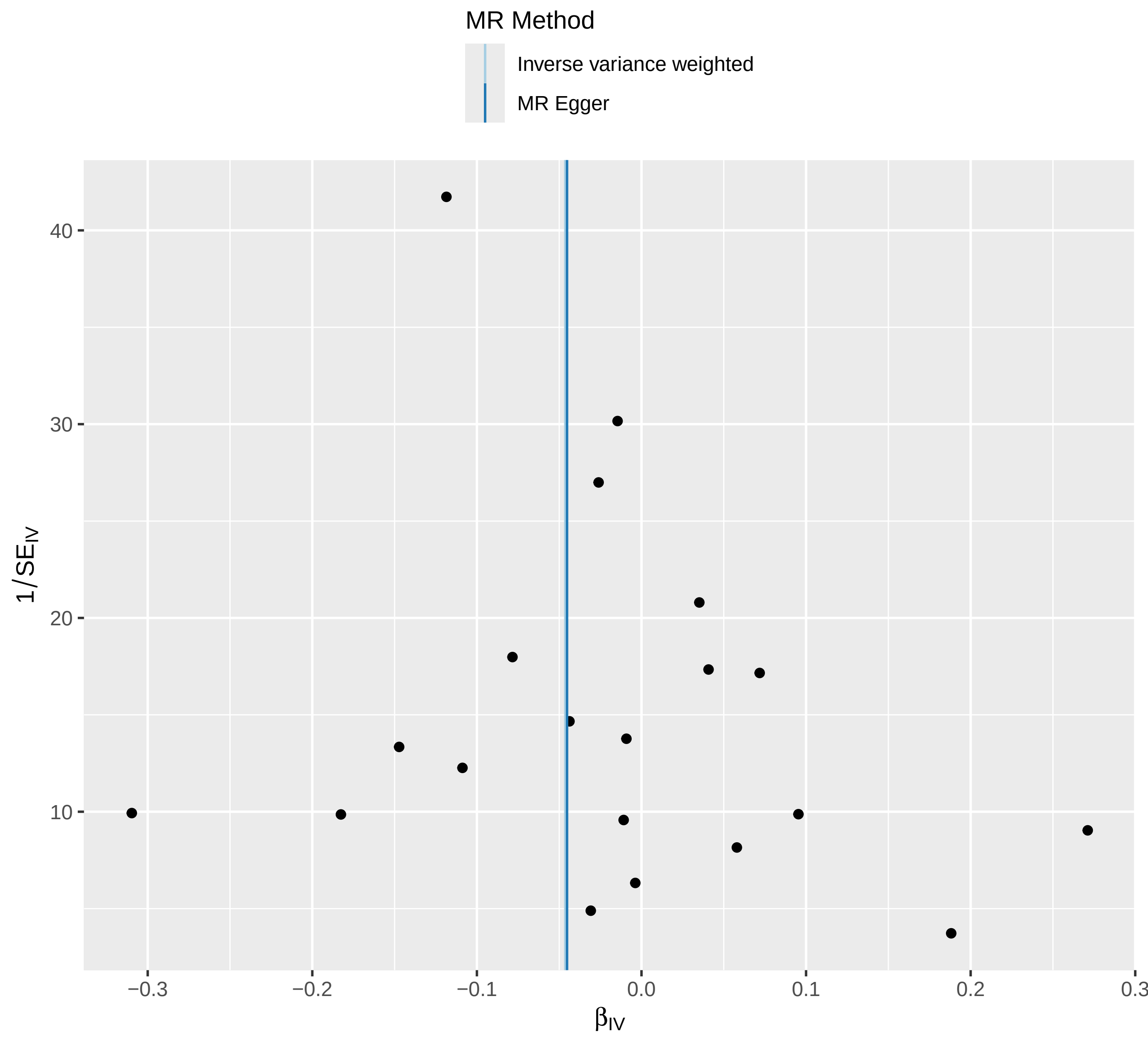
MR Method
Inverse variance weighted
MR Egger
1/SE_IV
40
30
20
10
−0.3
−0.2
−0.1
0.0
0.1
0.2
0.3
β_IV

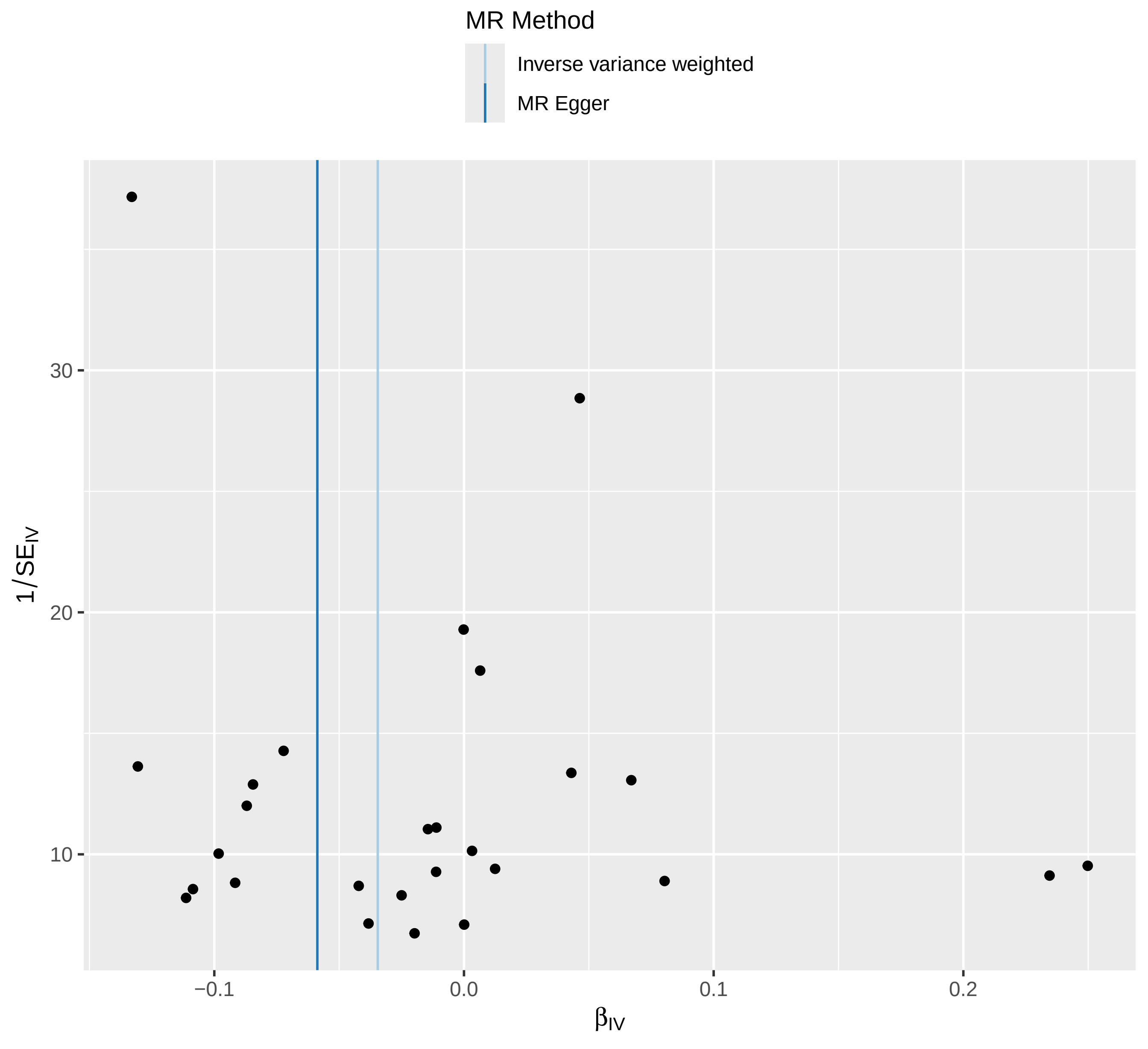
MR Method
Inverse variance weighted
MR Egger
30
20
10
1/SE_IV
−0.1
0.0
0.1
0.2
β_IV

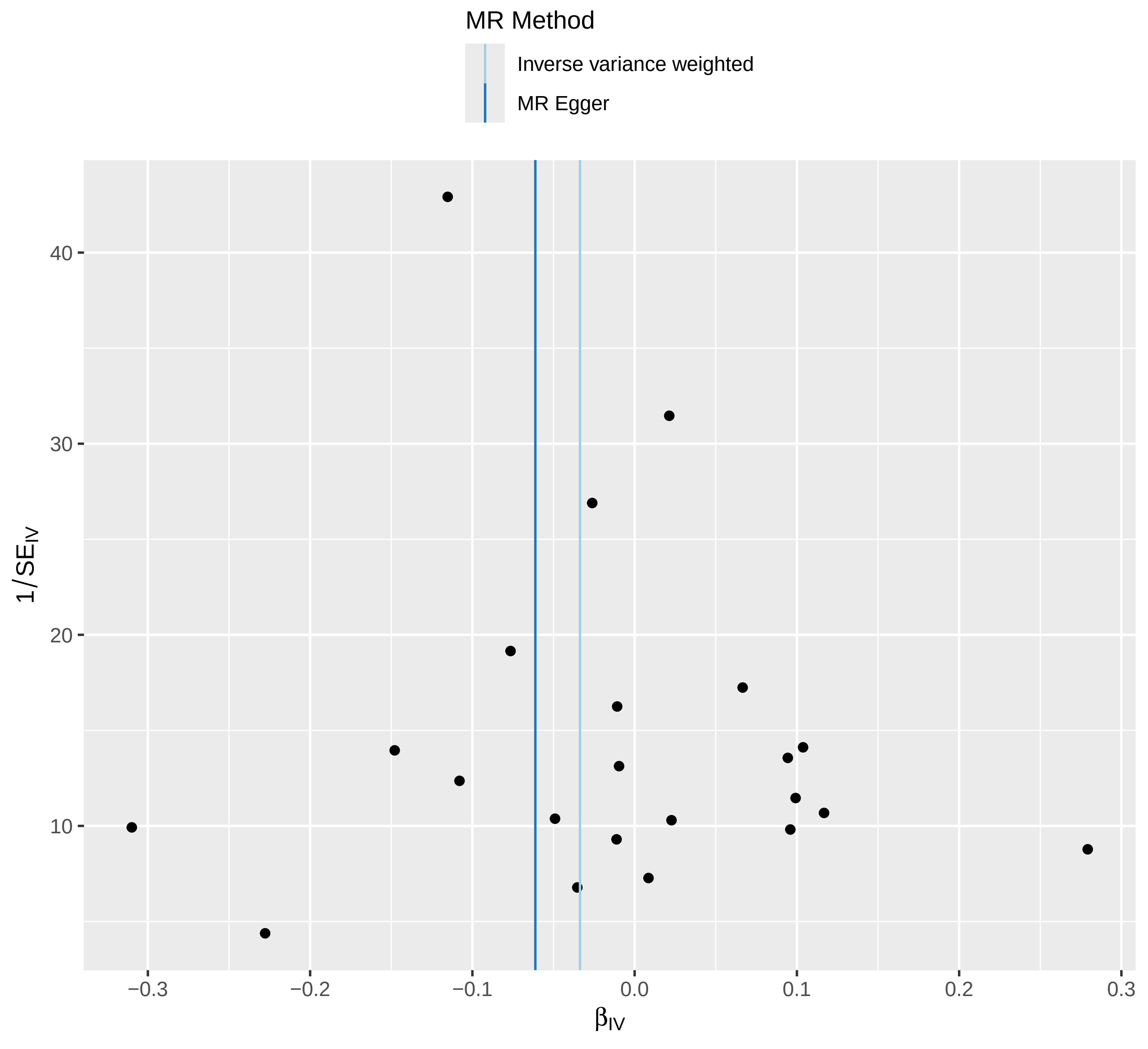

MR Method
Inverse variance weighted
MR Egger
40
30
20
10
1/SE_IV
−0.3
−0.2
−0.1
0.0
0.1
0.2
0.3
β_IV

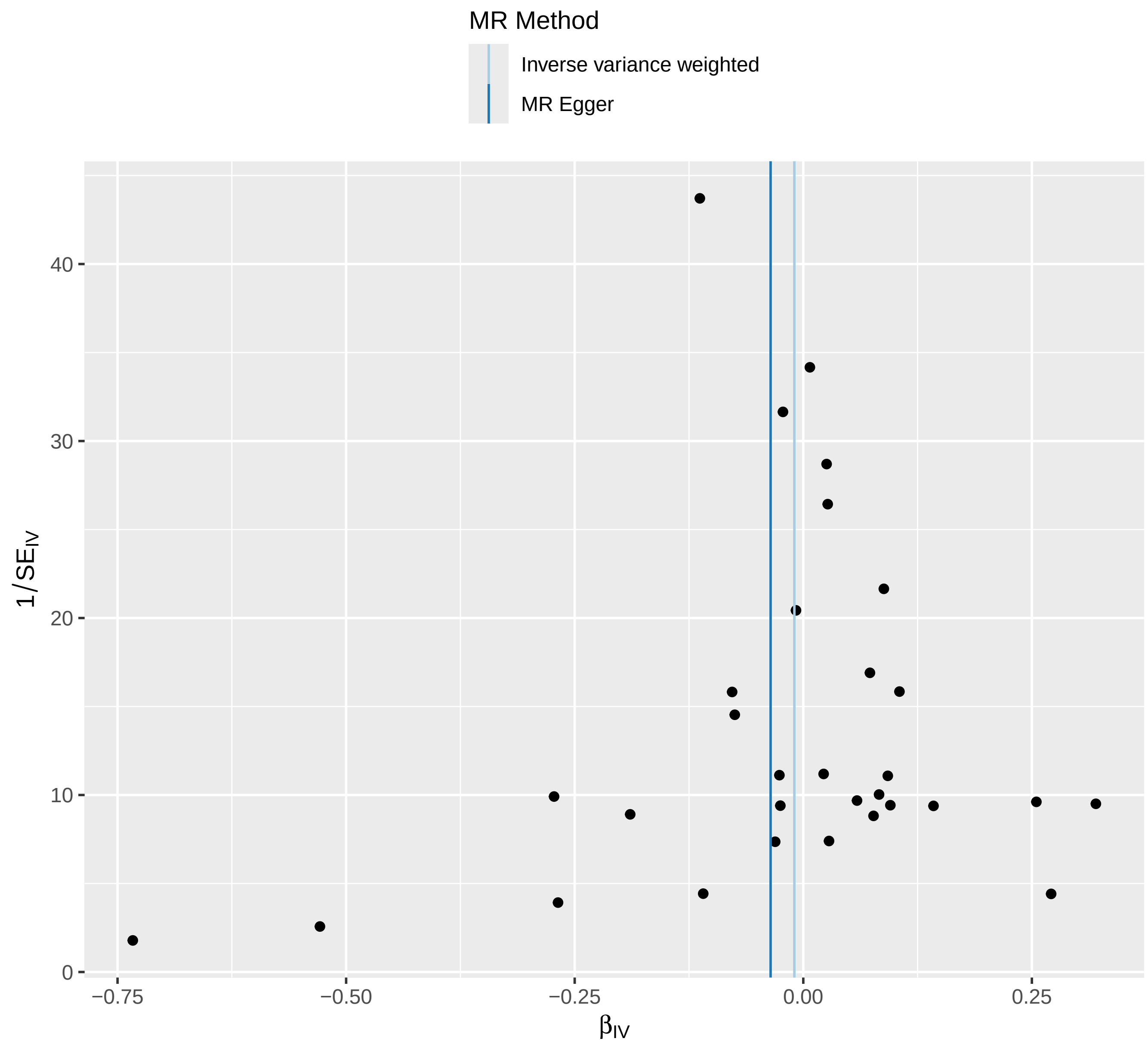
MR Method
Inverse variance weighted
MR Egger
1/SE_IV
40
30
20
10
0
−0.75
−0.50
−0.25
0.00
0.25
β_IV

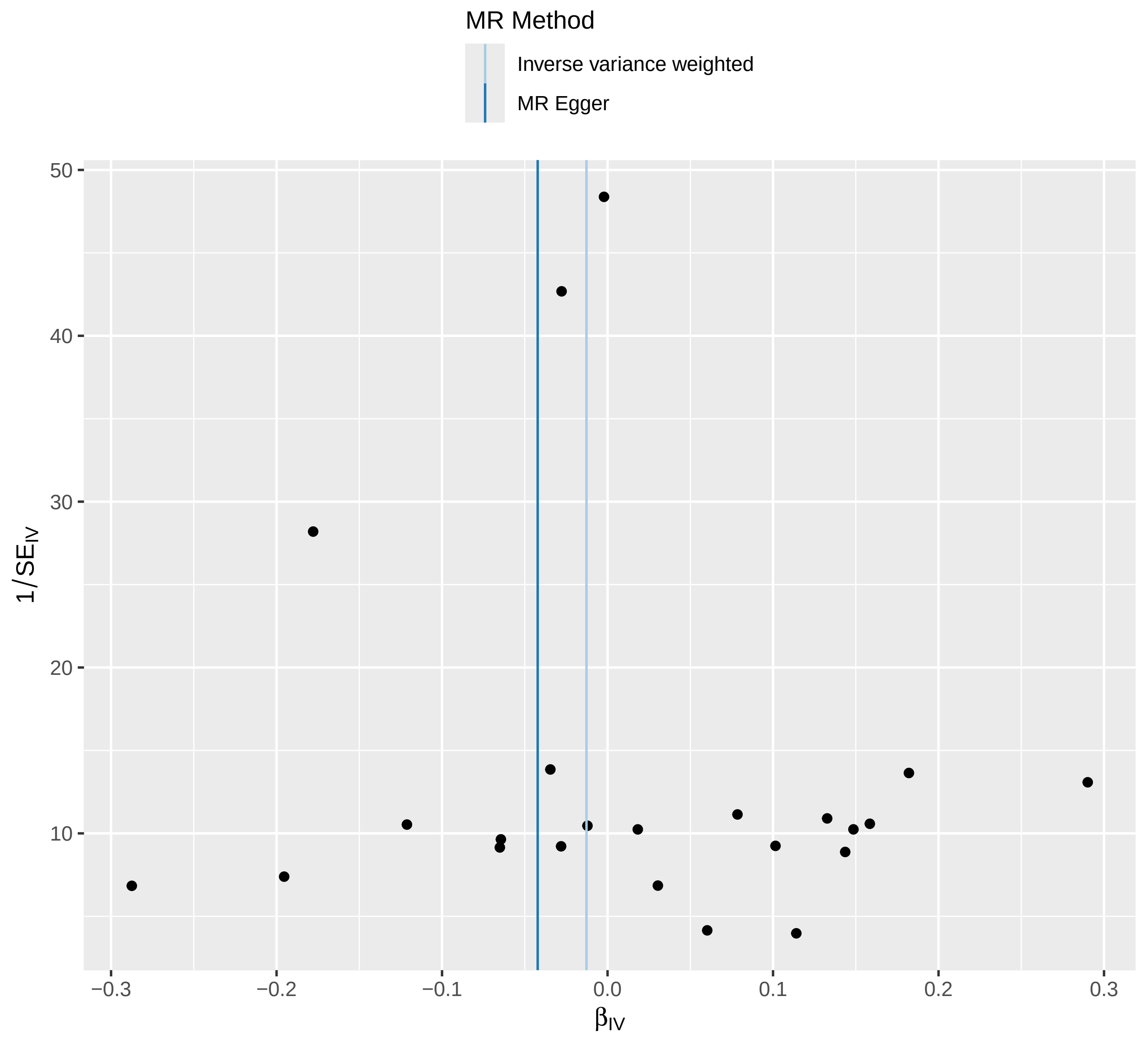
MR Method
Inverse variance weighted
MR Egger
50
40
30
20
10
1/SE_IV
−0.3
−0.2
−0.1
0.0
0.1
0.2
0.3
β_IV

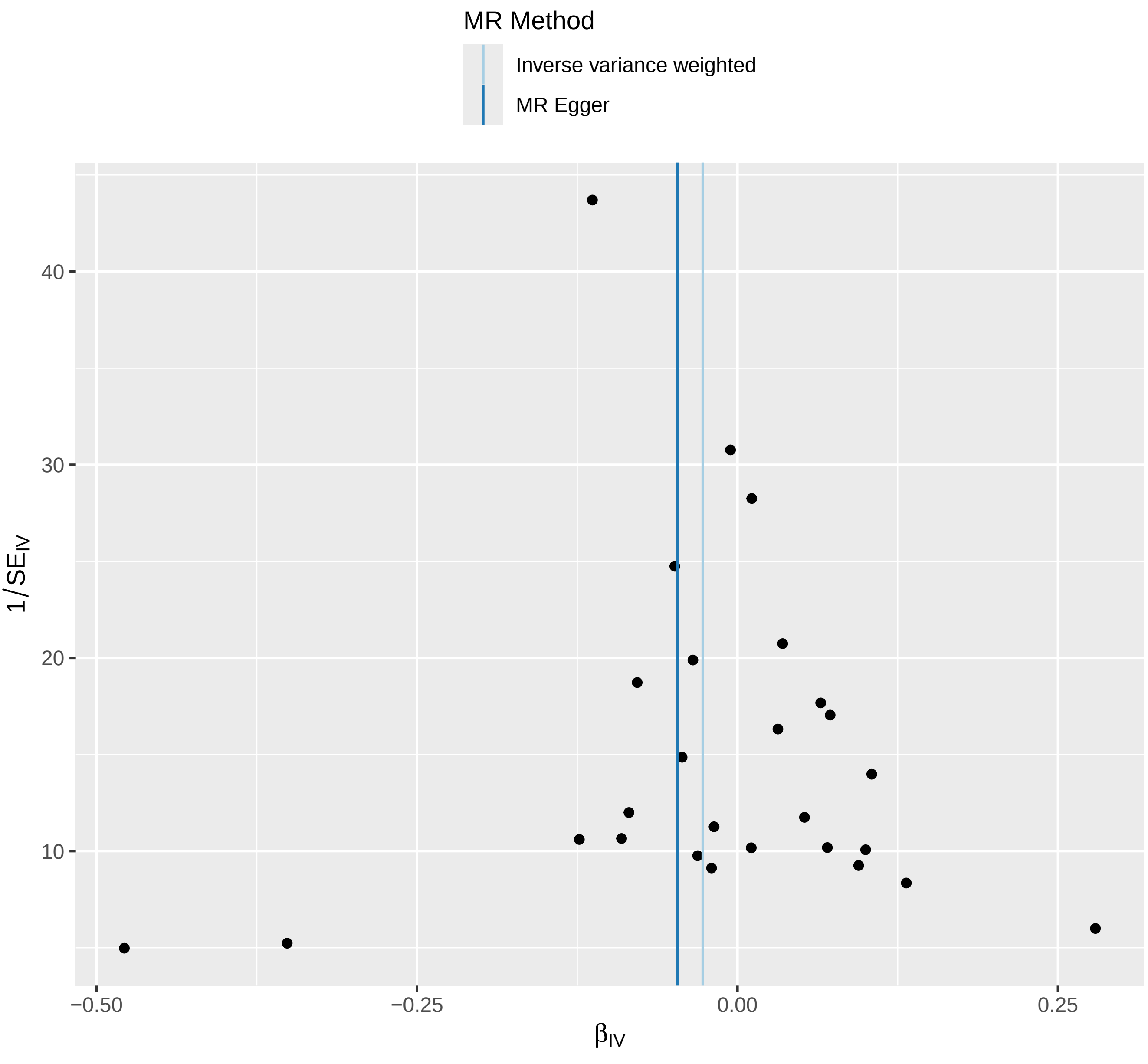
MR Method
Inverse variance weighted
MR Egger
40
30
20
10
1/SE$_{IV}$
−0.50
−0.25
0.00
0.25
β$_{IV}$

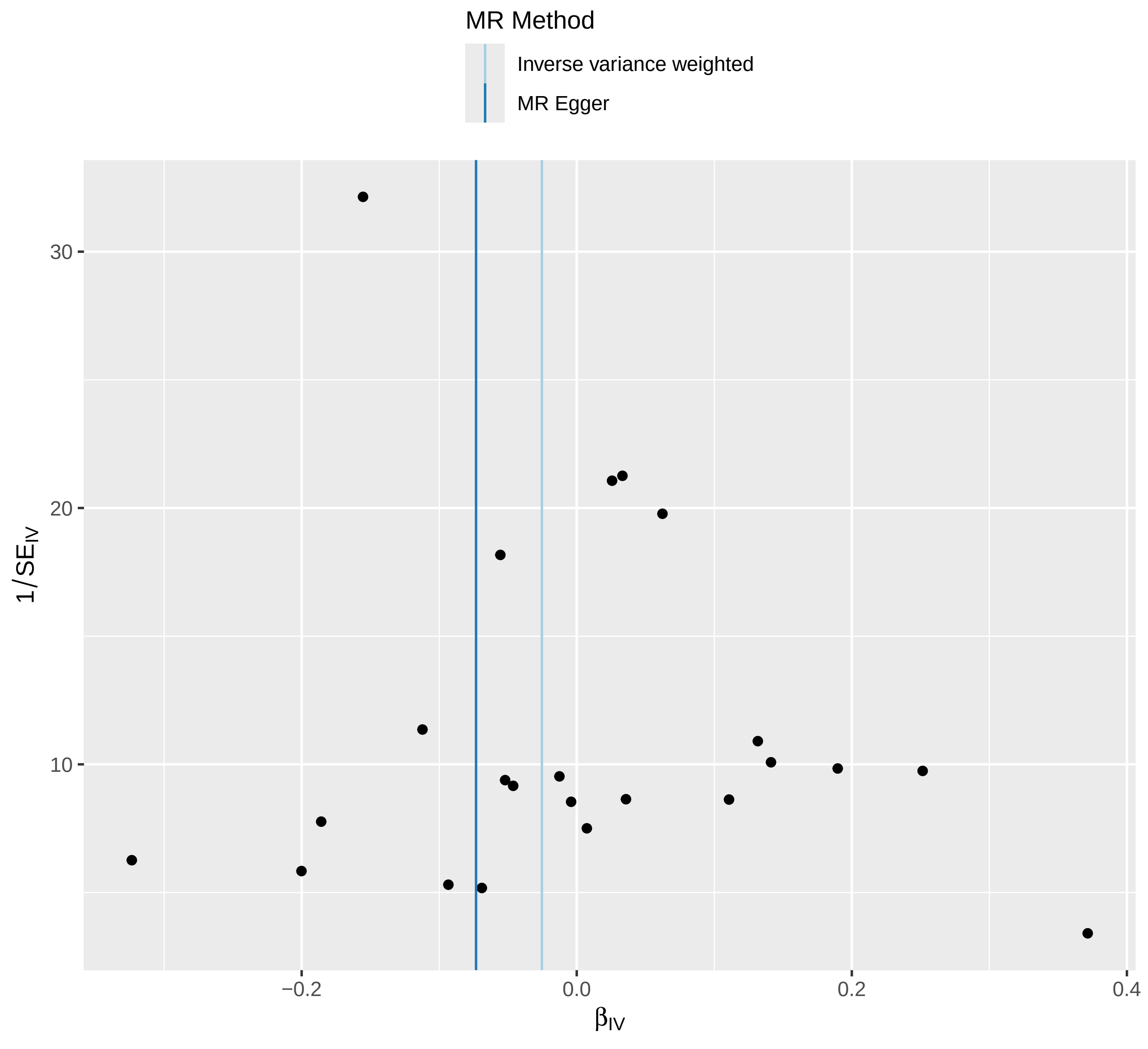
MR Method
Inverse variance weighted
MR Egger
30
20
10
1/SE_IV
−0.2
0.0
0.2
0.4
β_IV

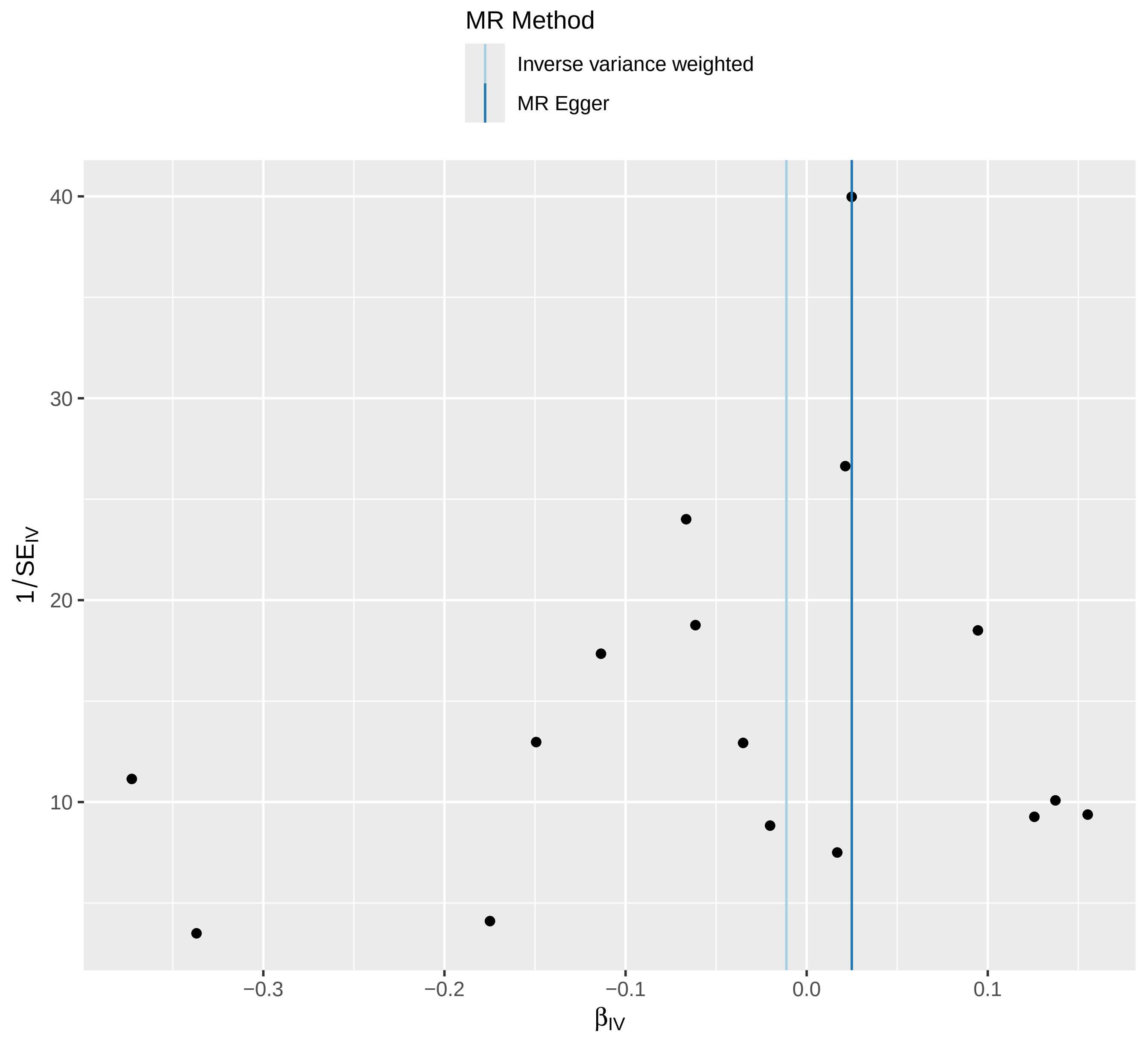
MR Method
Inverse variance weighted
MR Egger
40
30
20
10
1/SE_IV
−0.3
−0.2
−0.1
0.0
0.1
β_IV

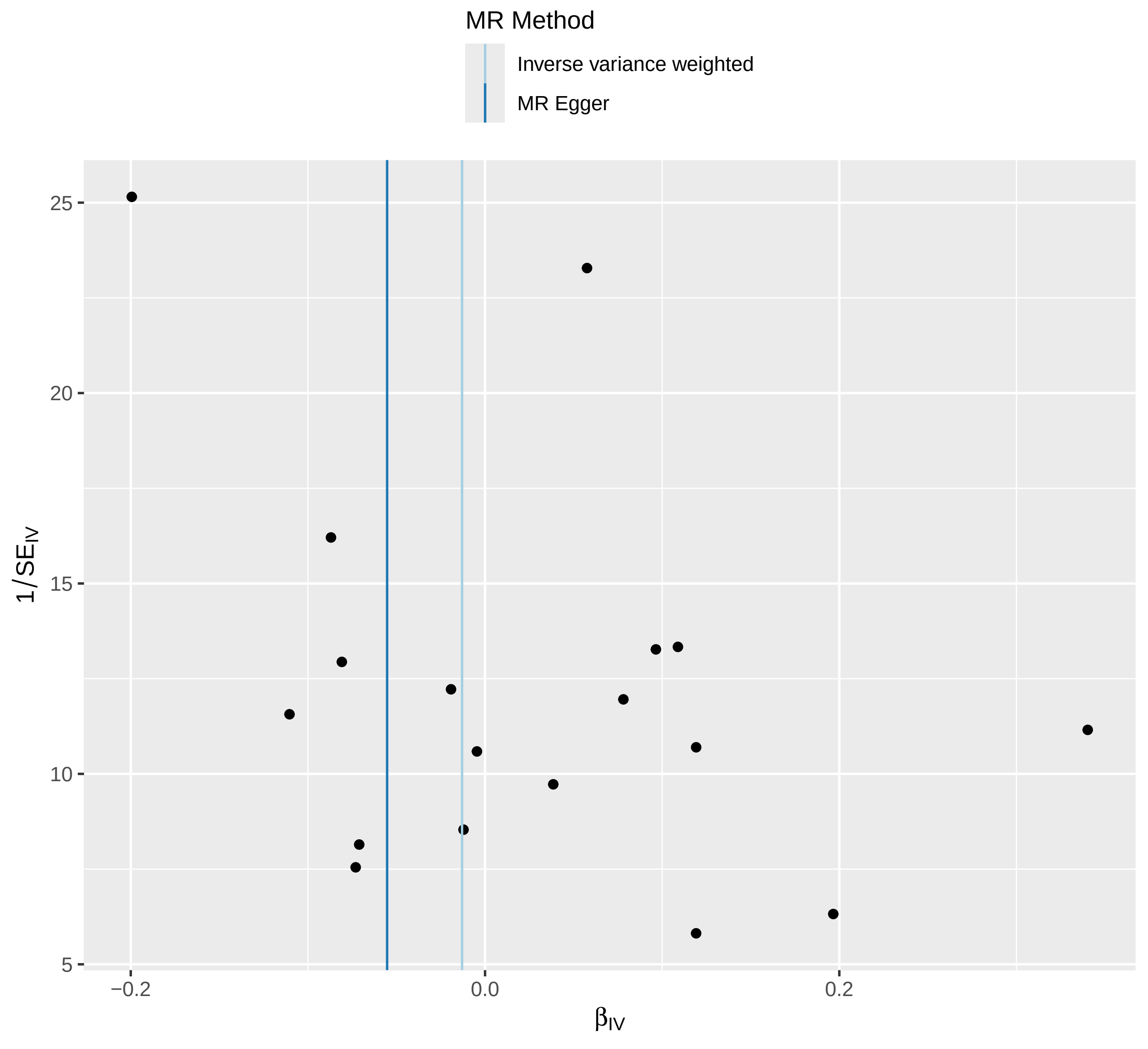
MR Method
Inverse variance weighted
MR Egger
25
20
15
10
5
1/SE_IV
−0.2
0.0
0.2
β_IV

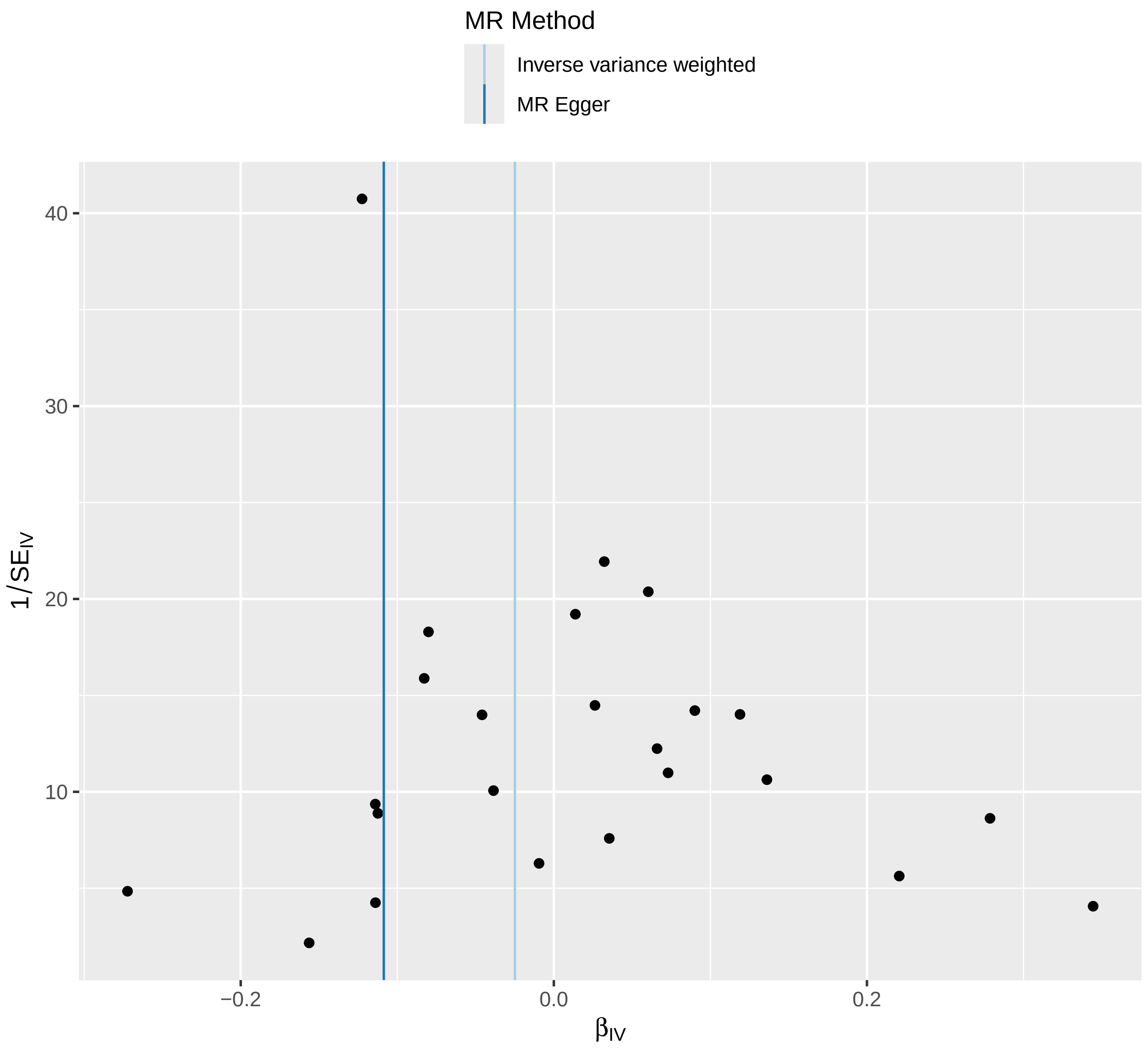

MR Method
Inverse variance weighted
MR Egger
40
30
20
10
−0.2
0.0
0.2
$1/SE_{IV}$
β_{IV}

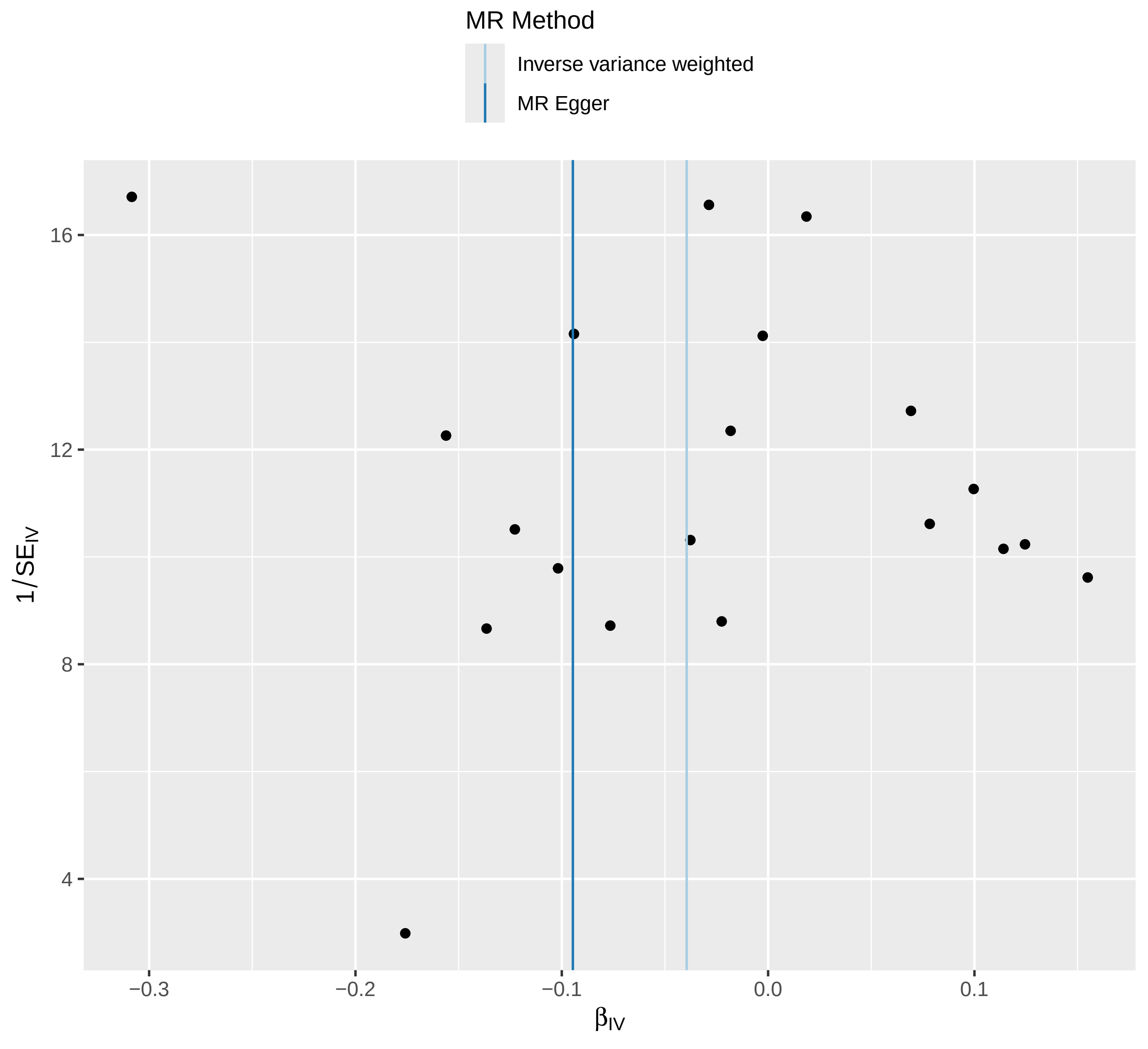

MR Method
Inverse variance weighted
MR Egger
1/SE$_{IV}$
16
12
8
4
−0.3
−0.2
−0.1
0.0
0.1
β_{IV}

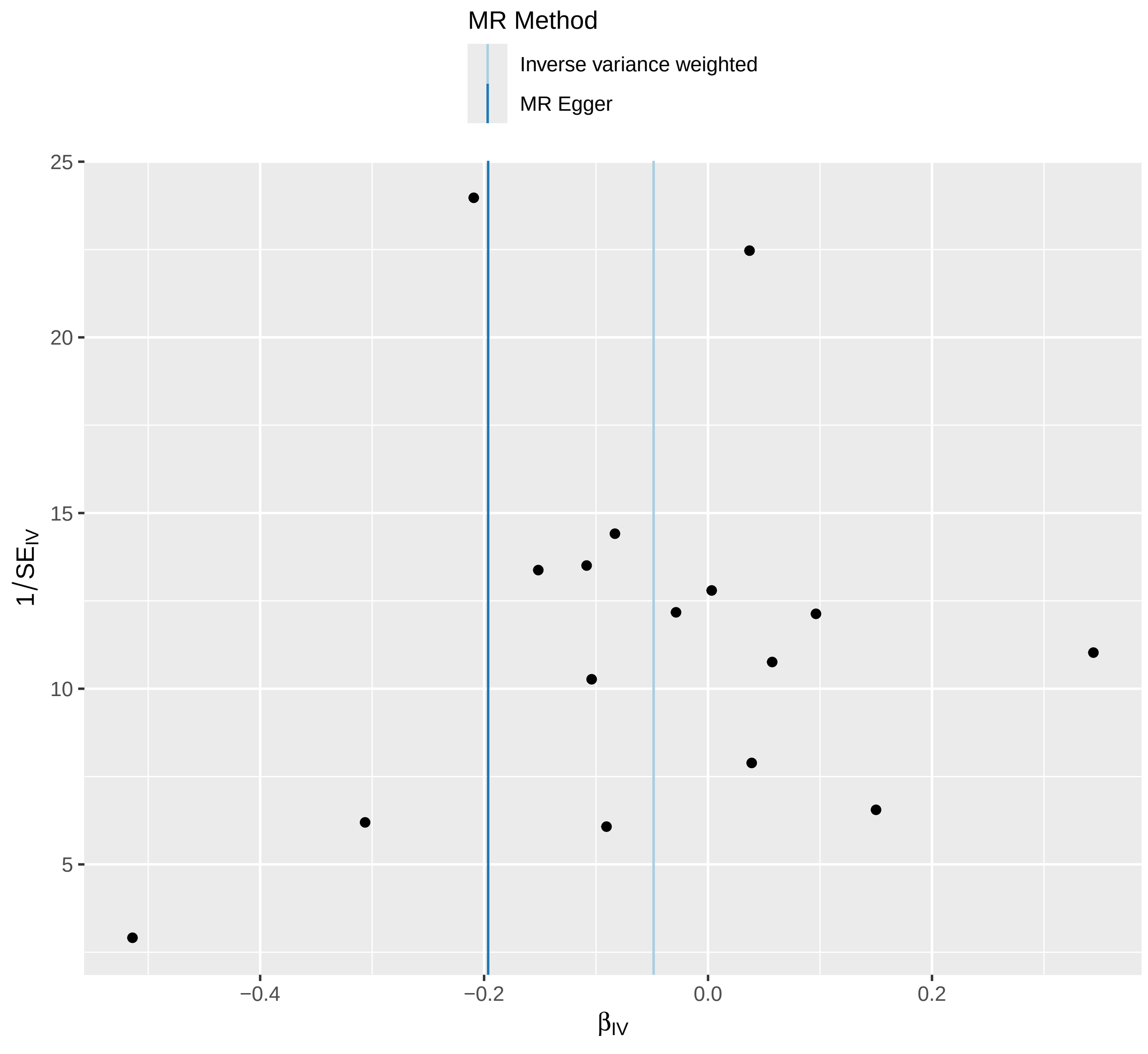
MR Method
Inverse variance weighted
MR Egger
25
20
15
10
5
1/SE_IV
−0.4
−0.2
0.0
0.2
β_IV

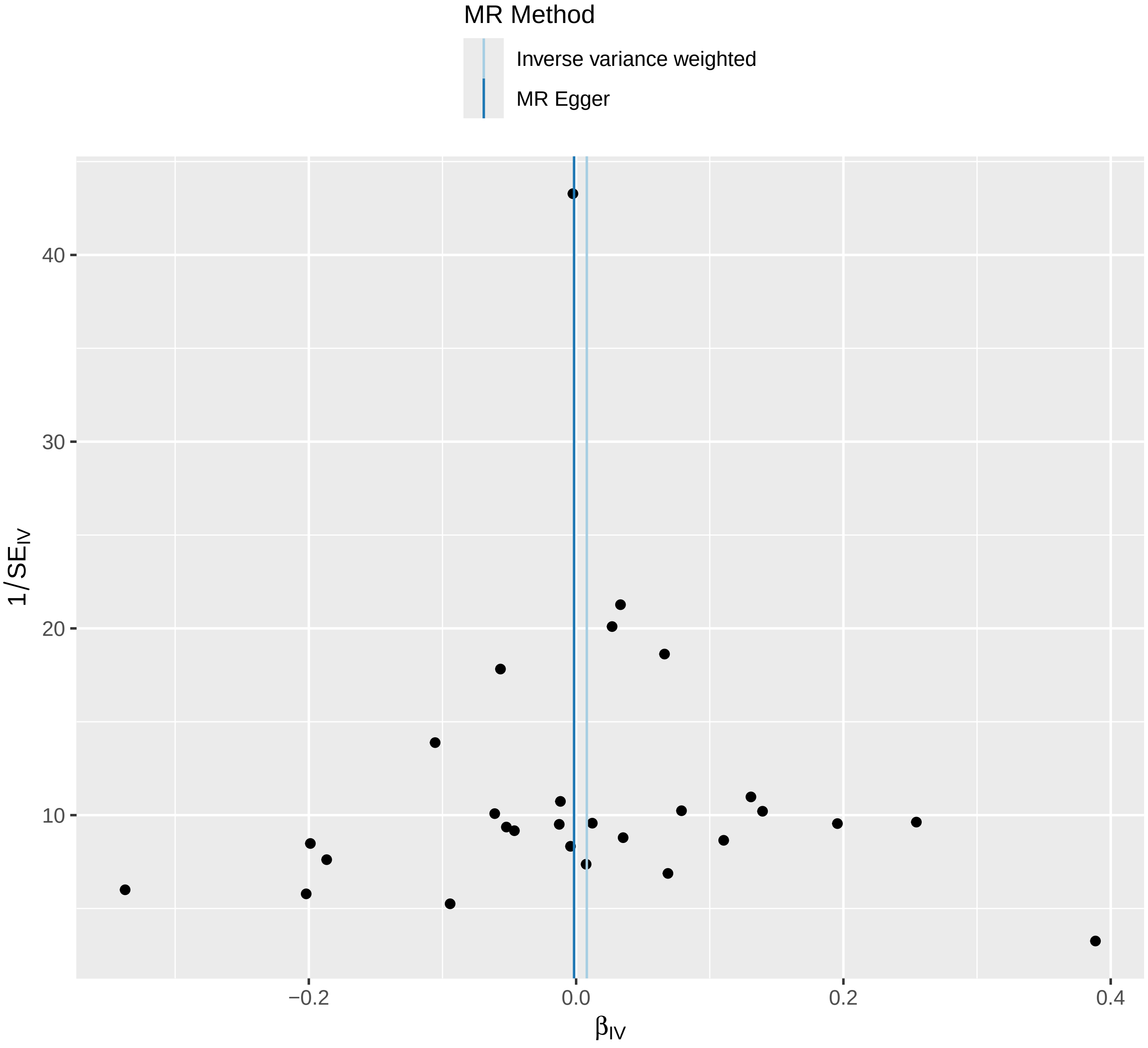
MR Method
Inverse variance weighted
MR Egger
1/SE_IV
40
30
20
10
−0.2
0.0
0.2
0.4
β_IV

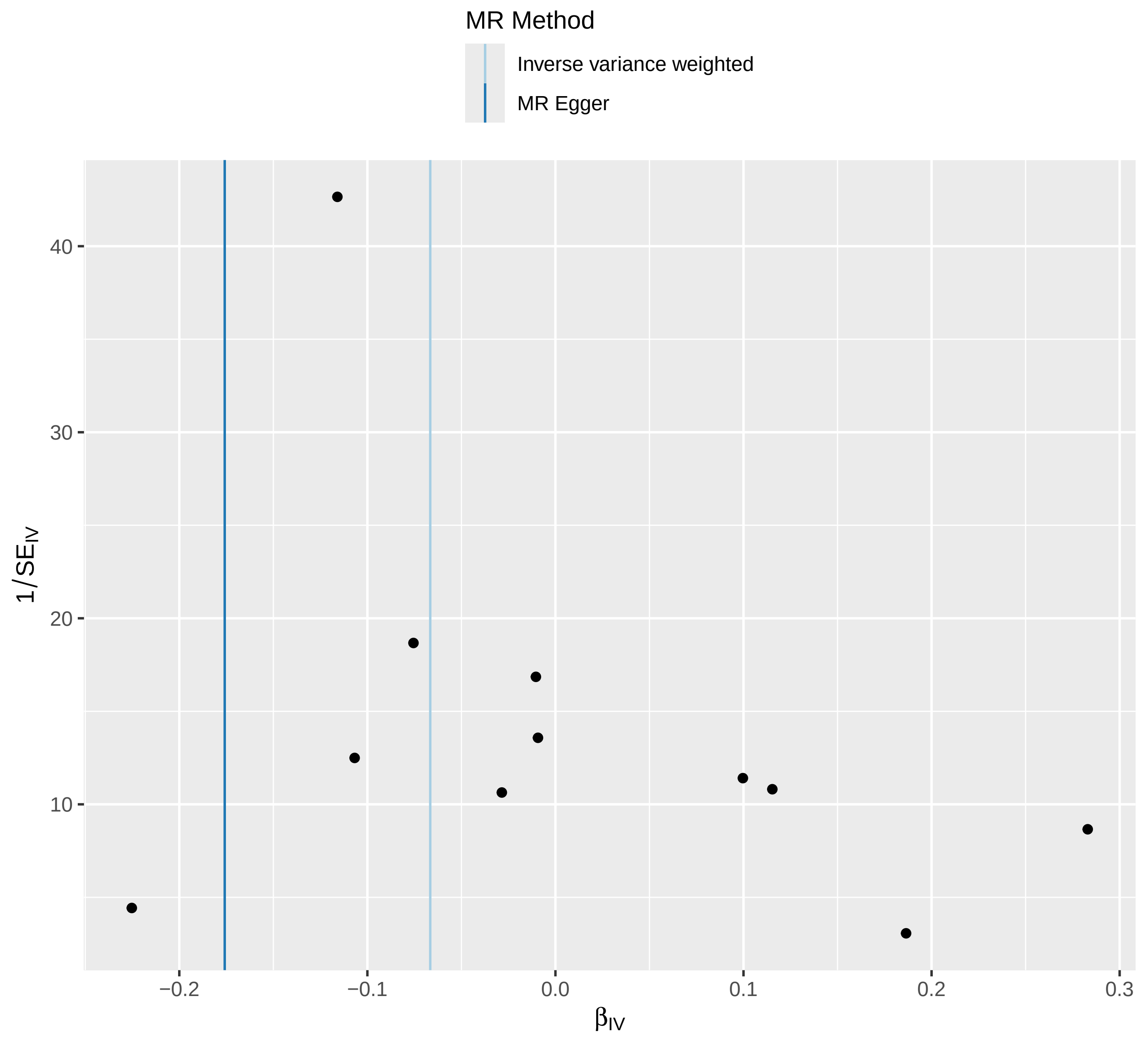
MR Method
Inverse variance weighted
MR Egger
40
30
20
10
1/SE_IV
−0.2
−0.1
0.0
0.1
0.2
0.3
β_IV

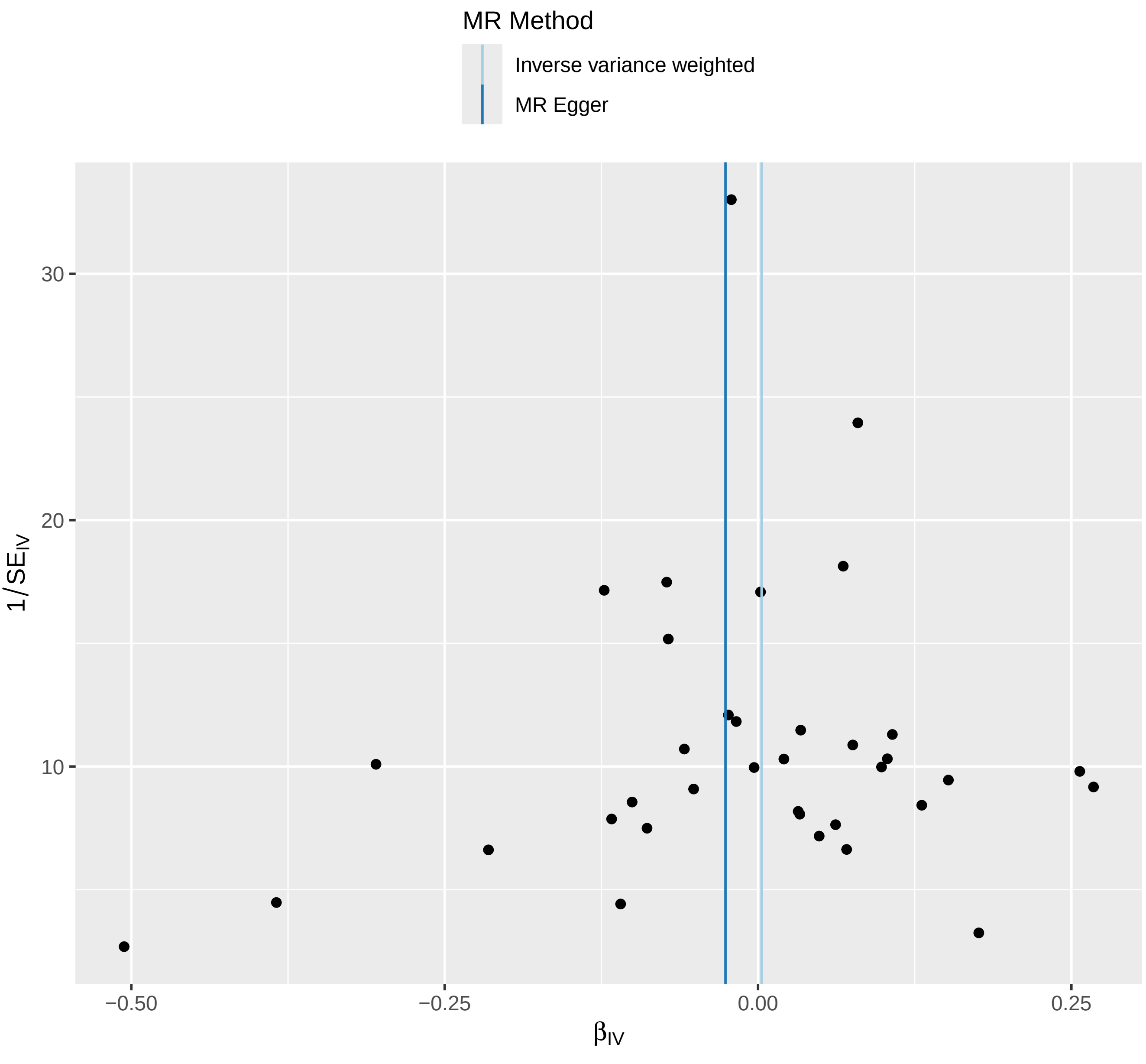
MR Method
Inverse variance weighted
MR Egger
30
20
10
1/SE_IV
−0.50
−0.25
0.00
0.25
β_IV

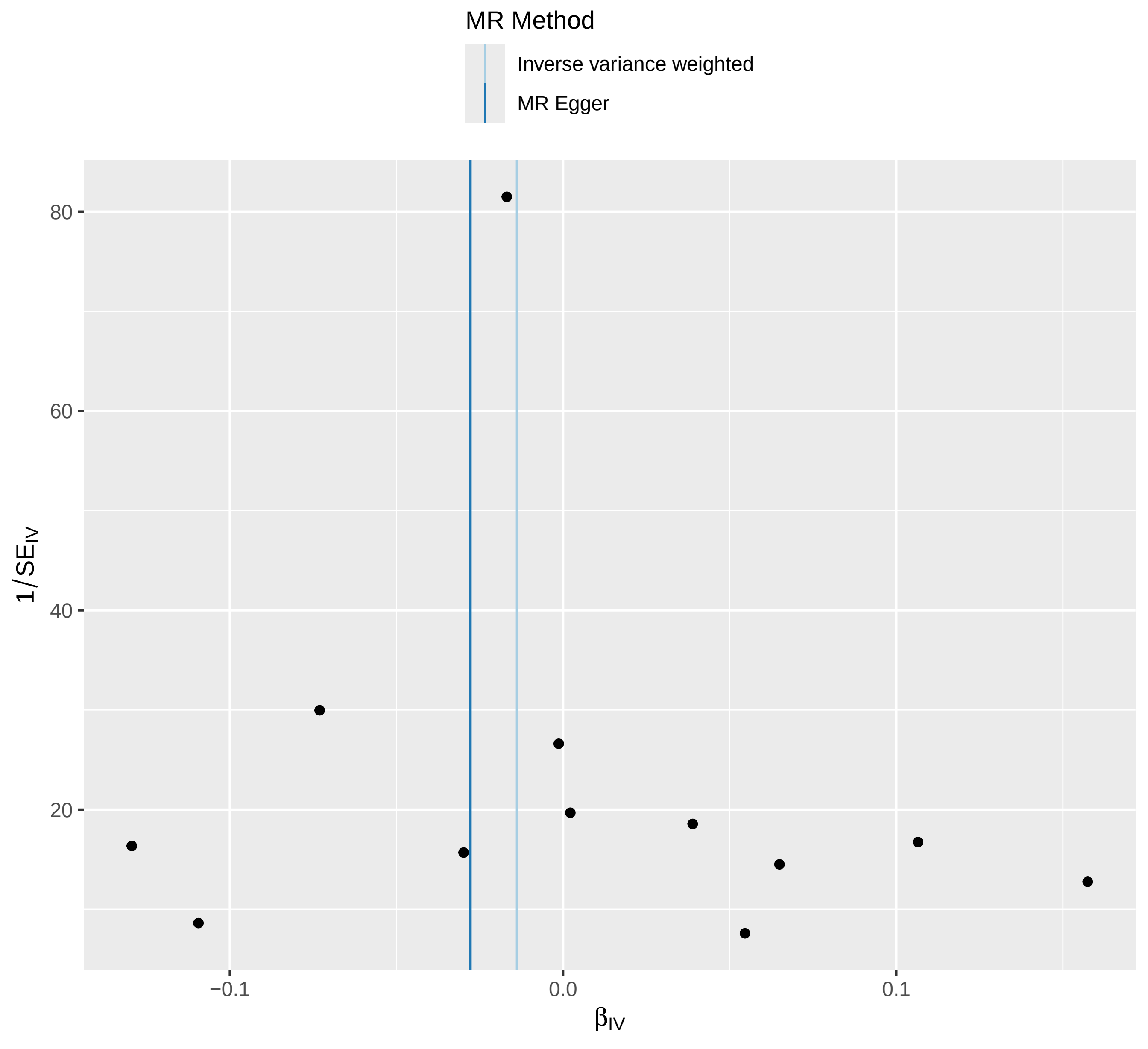

MR Method
Inverse variance weighted
MR Egger
1/SE_IV
80
60
40
20
−0.1
0.0
0.1
β_IV

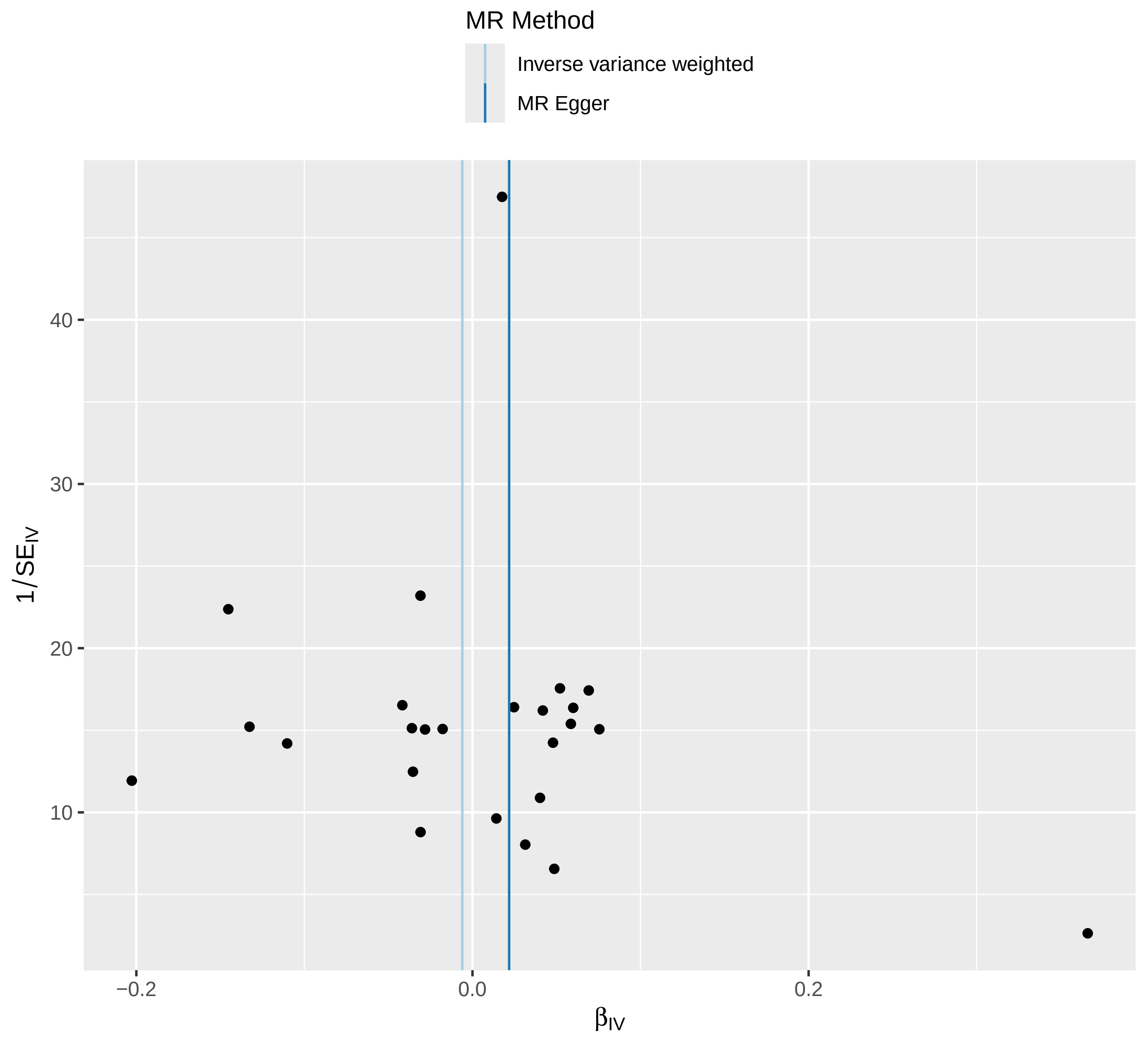
MR Method
Inverse variance weighted
MR Egger
1/SE_IV
40
30
20
10
−0.2
0.0
0.2
β_IV

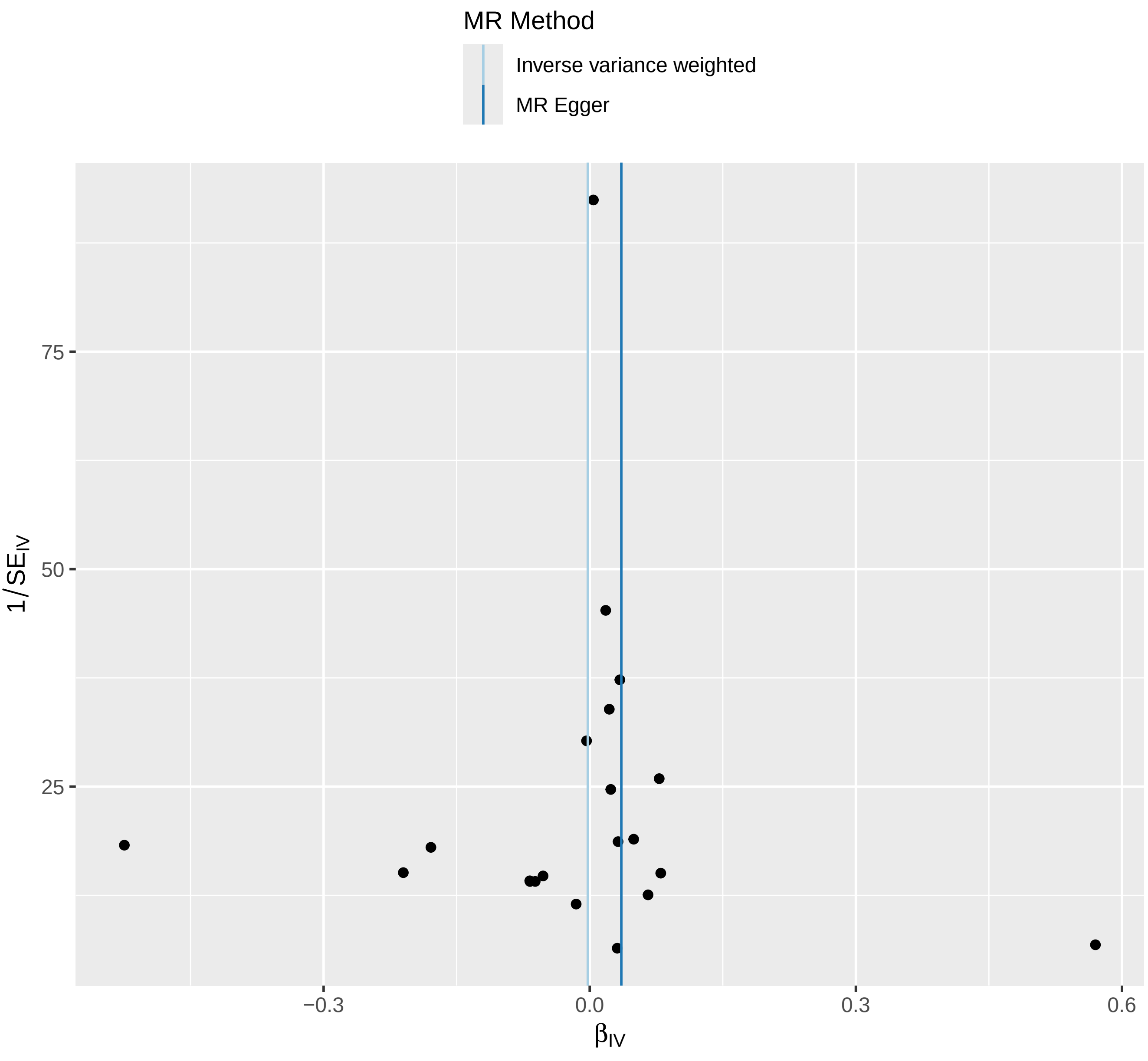
MR Method
Inverse variance weighted
MR Egger
75
50
25
1/SE_IV
−0.3
0.0
0.3
0.6
β_IV

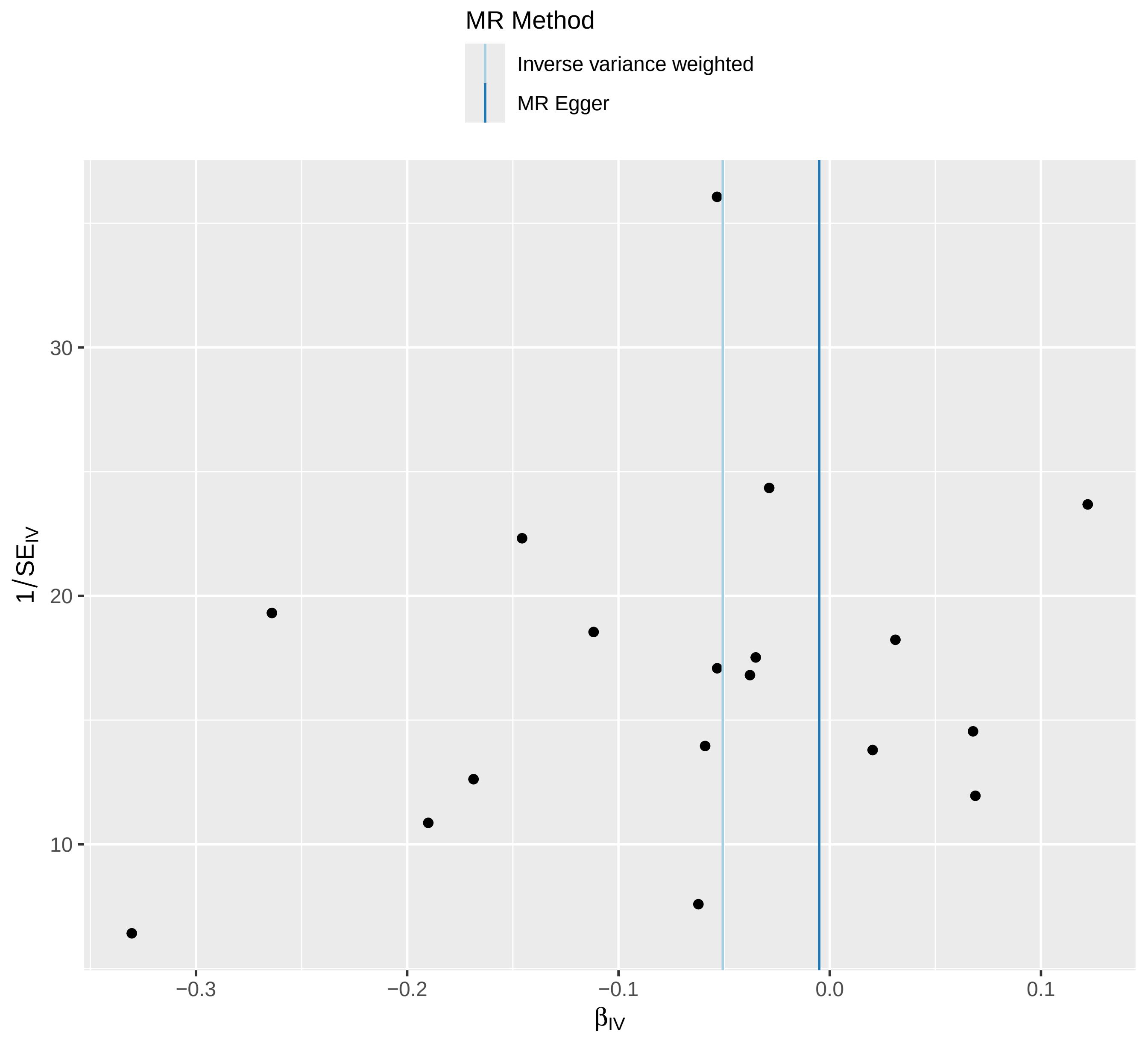
MR Method
Inverse variance weighted
MR Egger
30
20
10
1/SE$_{IV}$
−0.3
−0.2
−0.1
0.0
0.1
β_{IV}

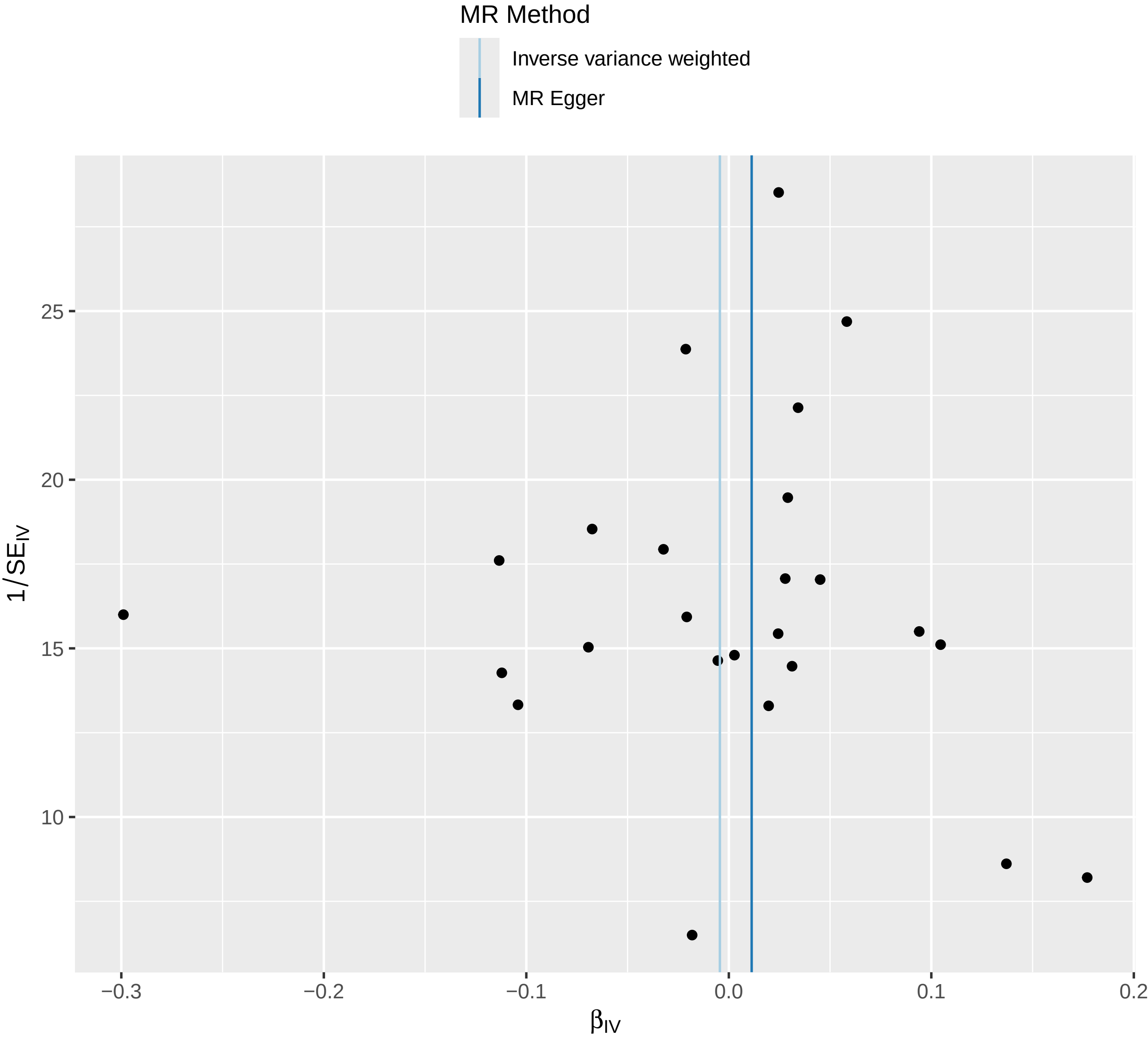
MR Method
Inverse variance weighted
MR Egger
25
20
15
10
$1/SE_{IV}$
−0.3
−0.2
−0.1
0.0
0.1
0.2
β_{IV}

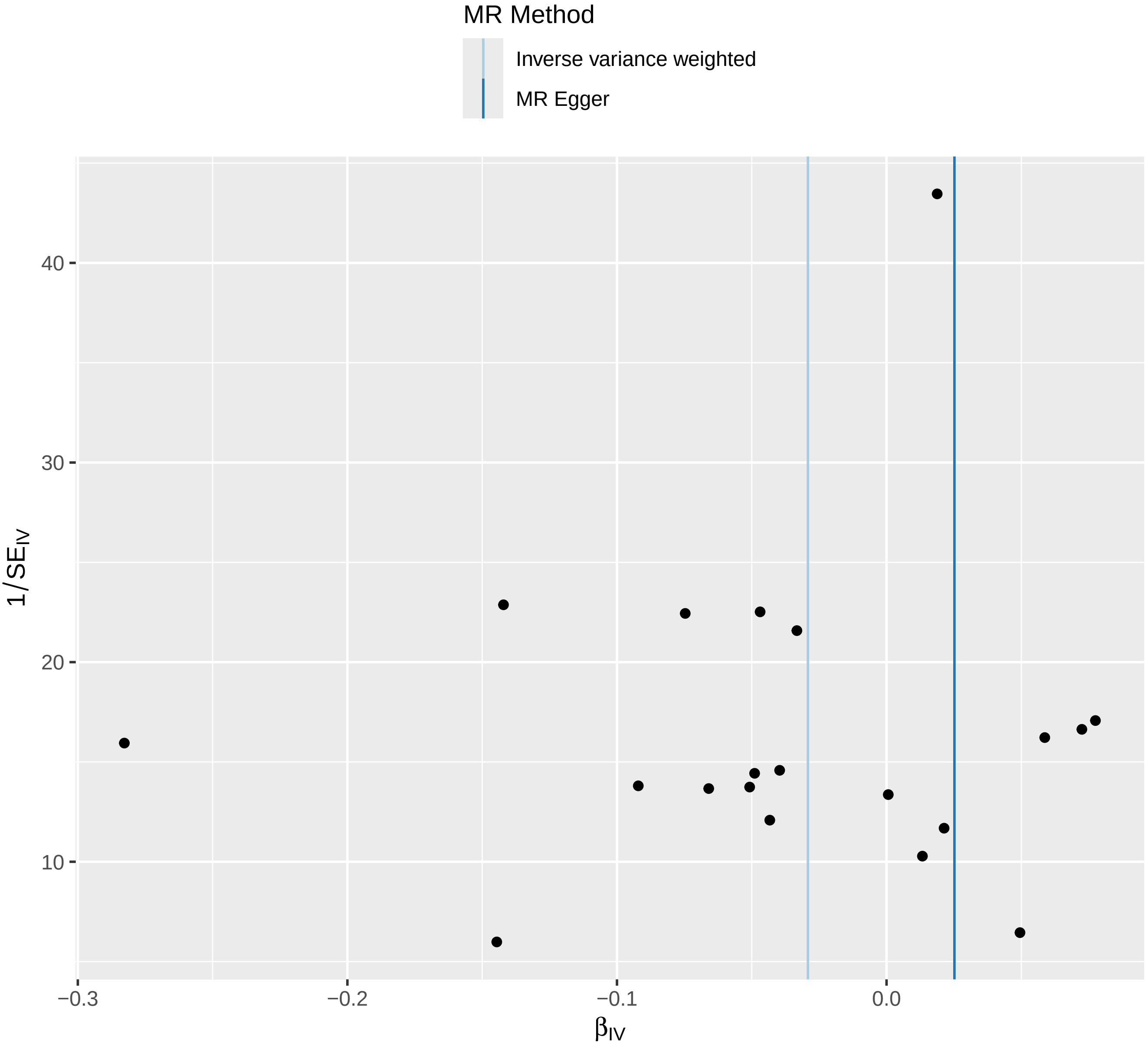
MR Method
Inverse variance weighted
MR Egger
40
30
20
10
1/SE$_{IV}$
−0.3
−0.2
−0.1
0.0
β$_{IV}$

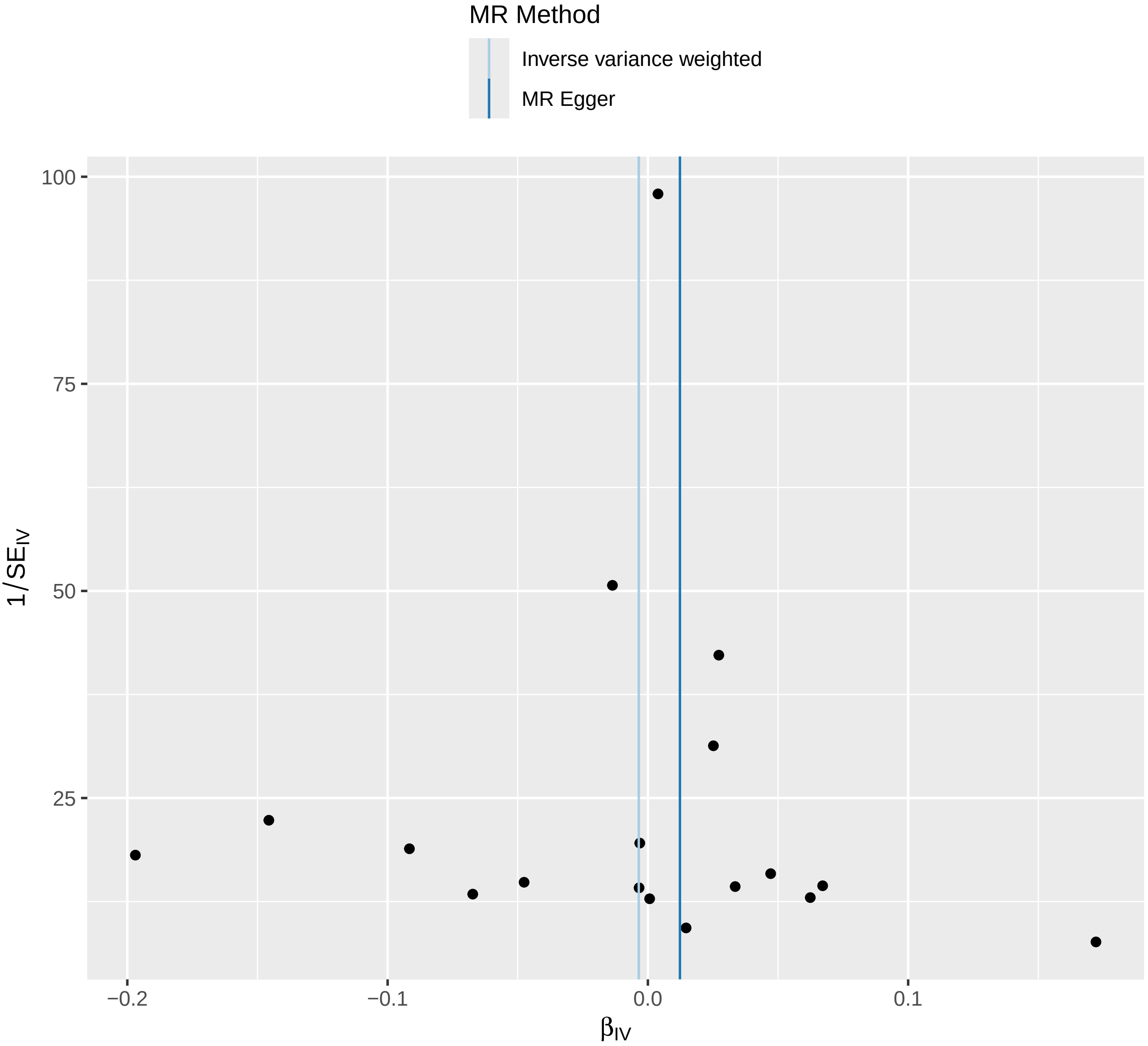
MR Method
Inverse variance weighted
MR Egger
100
75
50
25
1/SE_IV
−0.2
−0.1
0.0
0.1
β_IV

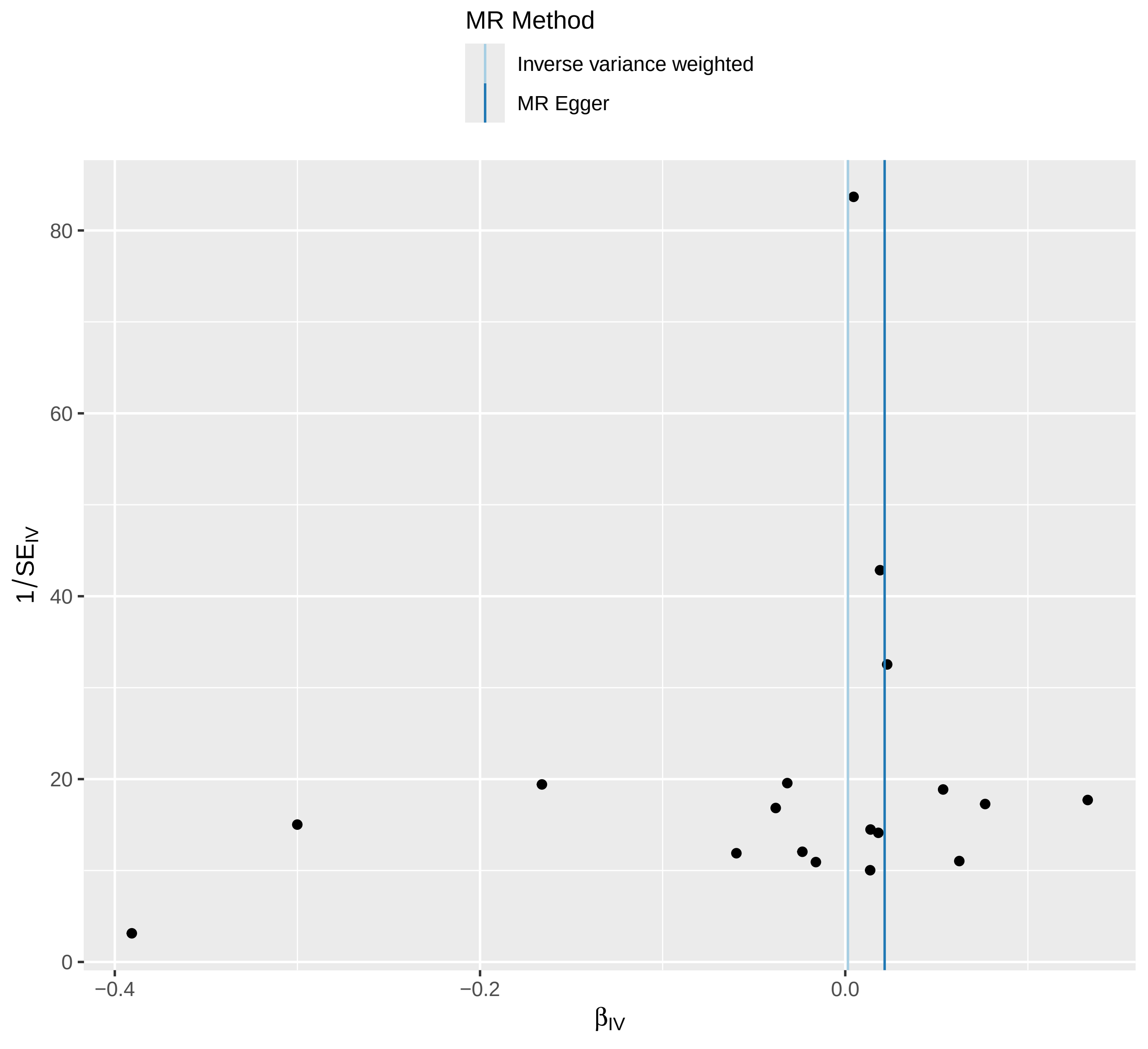

MR Method
Inverse variance weighted
MR Egger
80
60
40
20
0
1/SE$_{IV}$
−0.4
−0.2
0.0
β$_{IV}$

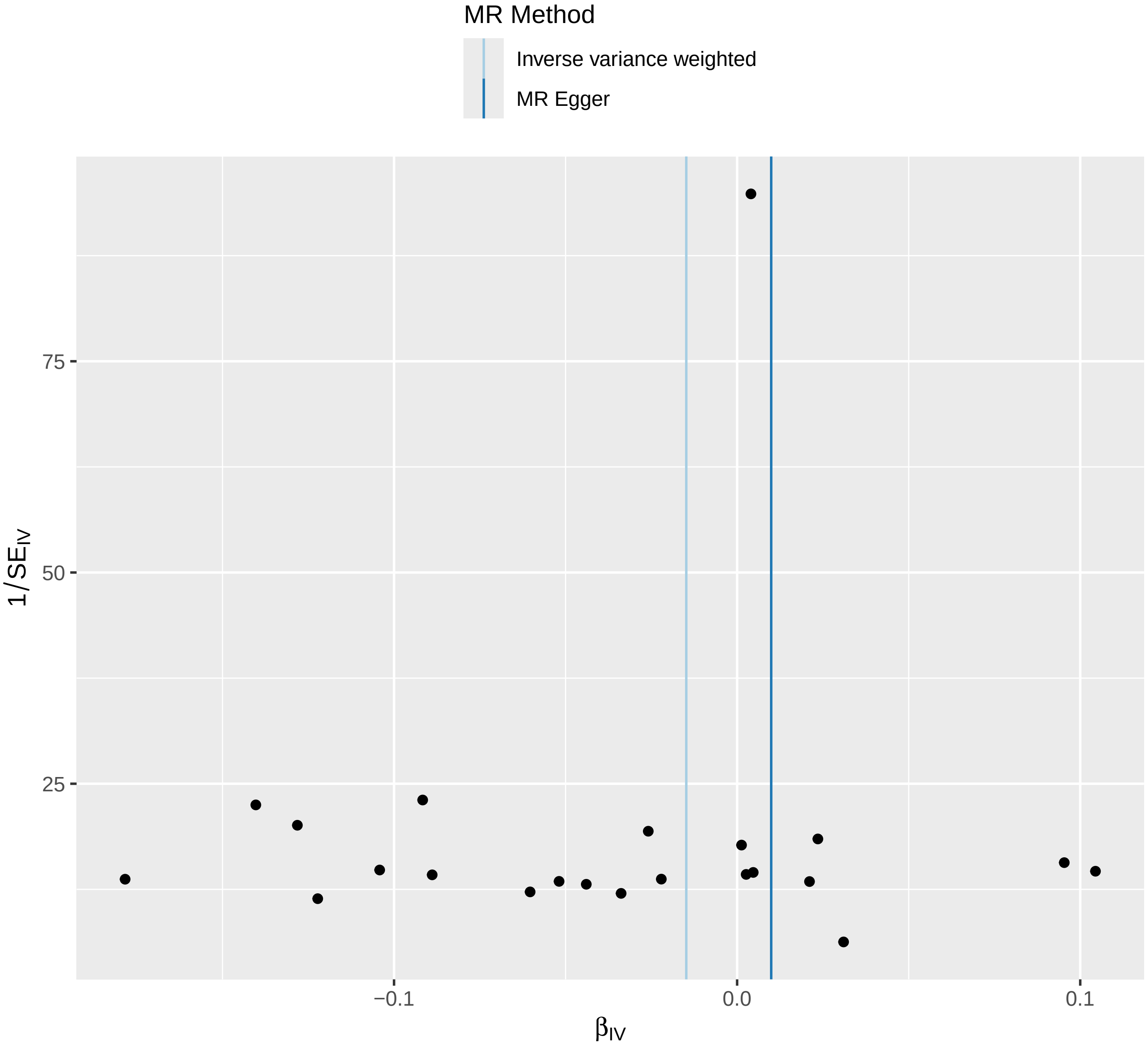
MR Method
Inverse variance weighted
MR Egger
75
50
25
1/SE_IV
−0.1
0.0
0.1
β_IV

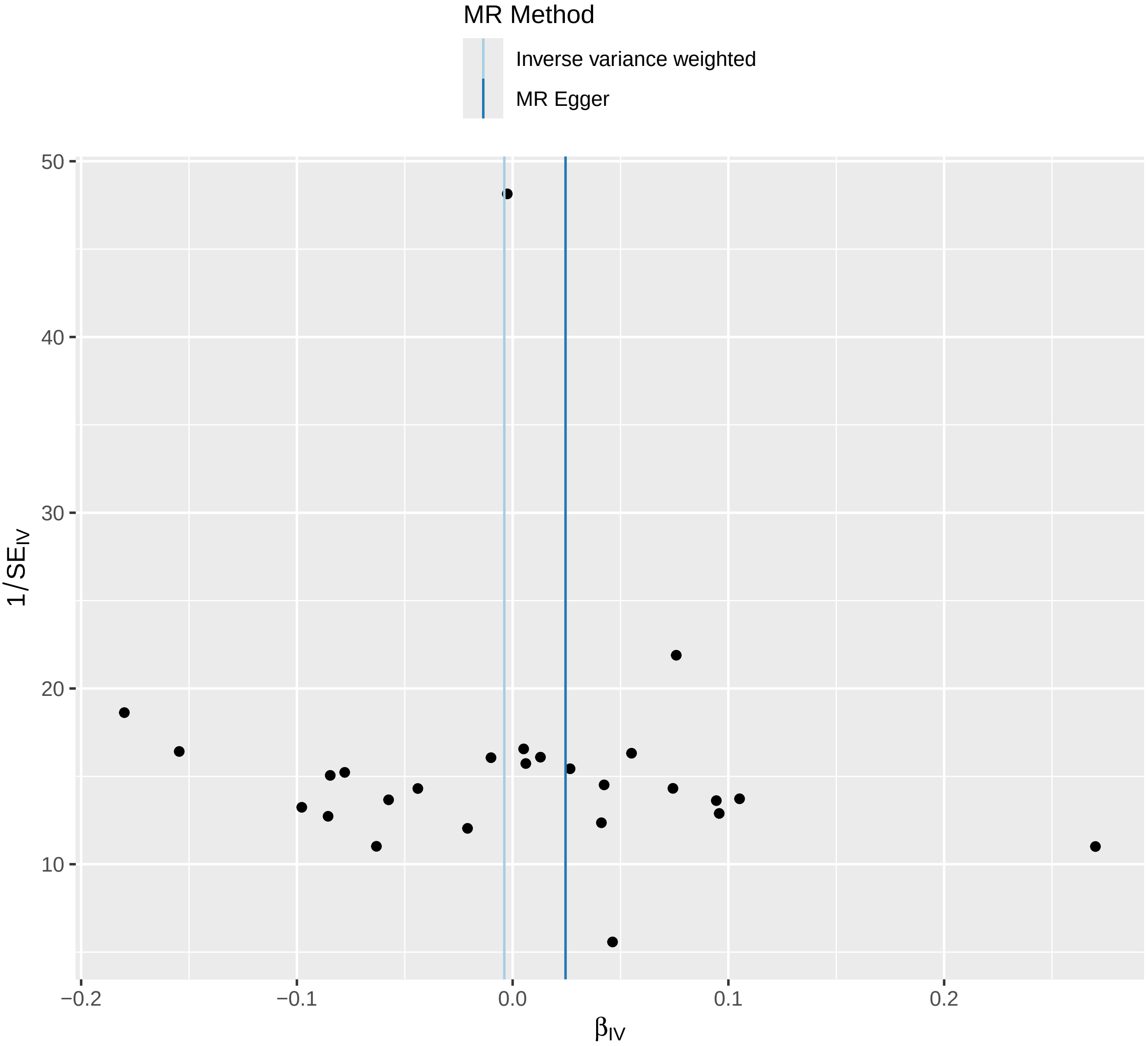
MR Method
Inverse variance weighted
MR Egger
50
40
30
20
10
1/SE$_{IV}$
−0.2
−0.1
0.0
0.1
0.2
β$_{IV}$

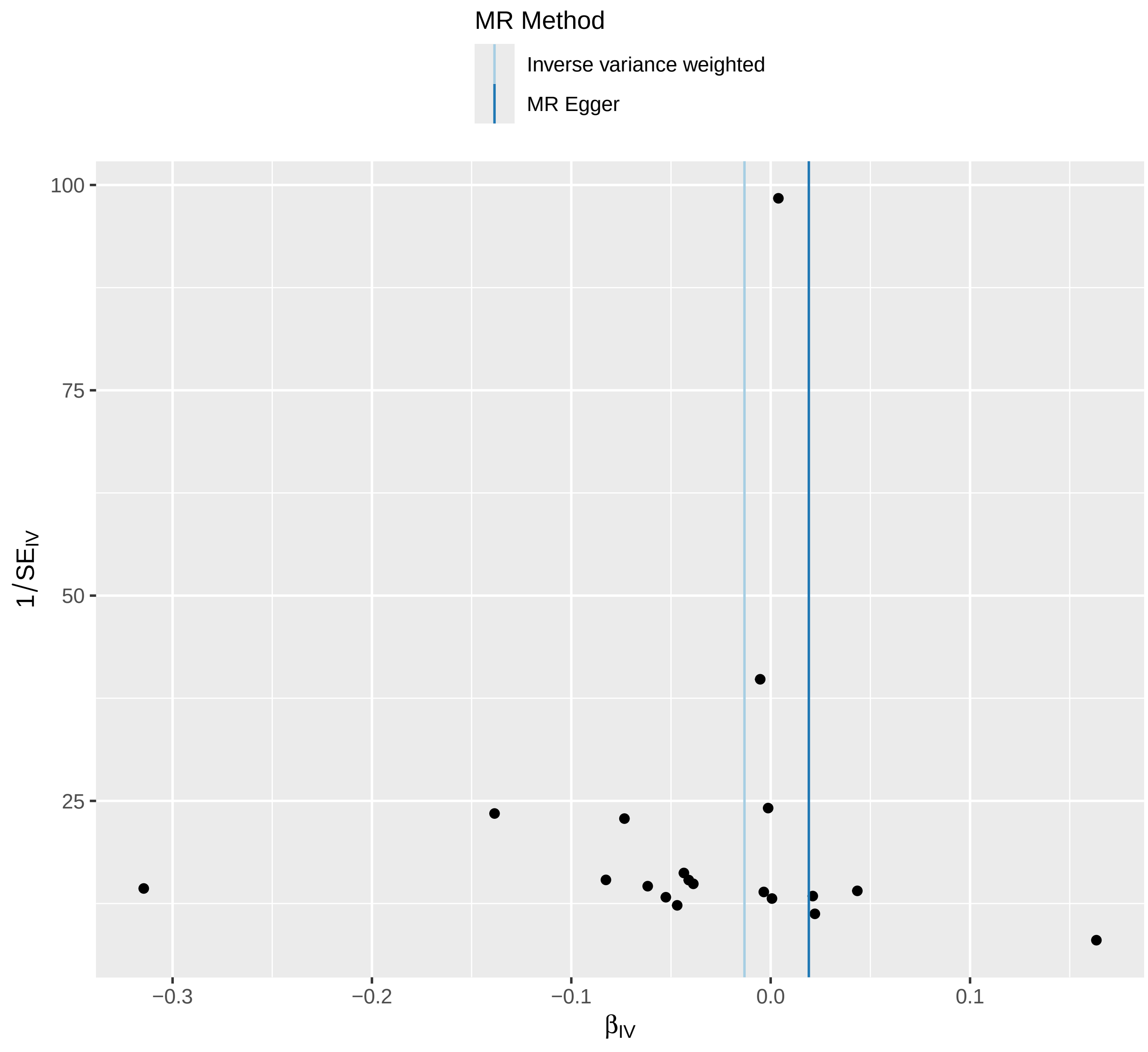
MR Method
Inverse variance weighted
MR Egger
100
75
50
25
1/SE_IV
−0.3
−0.2
−0.1
0.0
0.1
β_IV

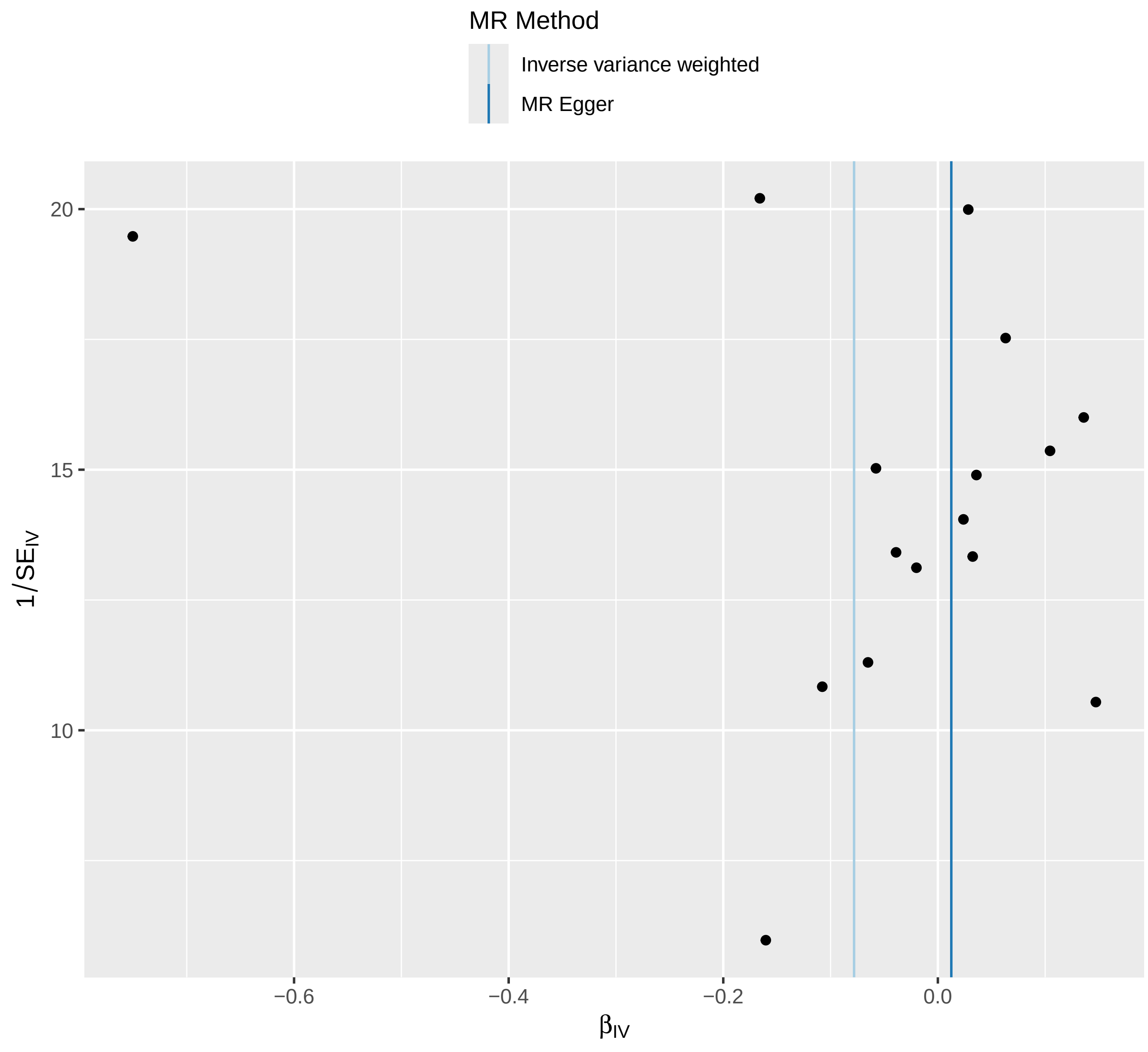
MR Method
Inverse variance weighted
MR Egger
20
15
10
1/SE_IV
−0.6
−0.4
−0.2
0.0
β_IV

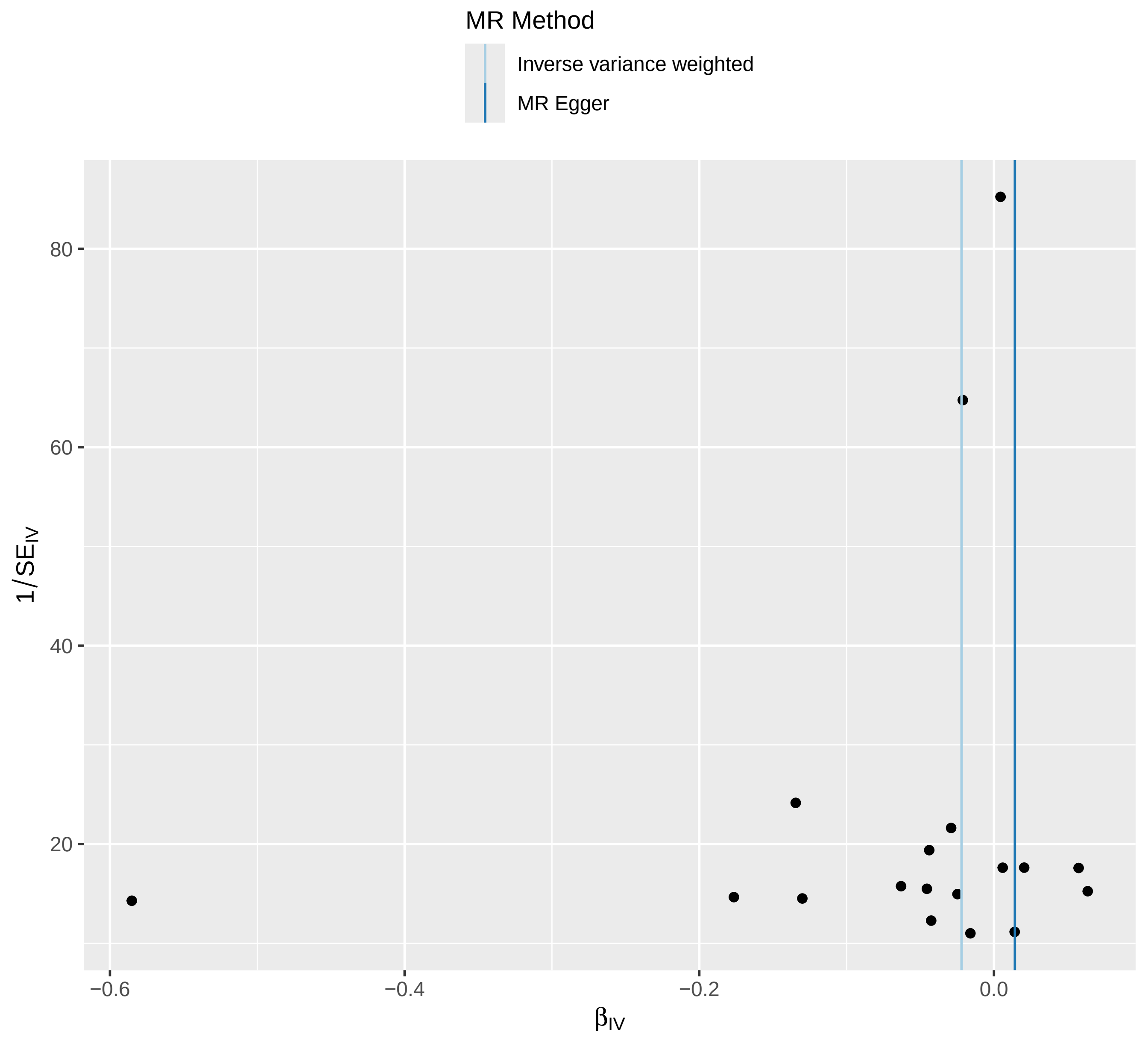

MR Method
Inverse variance weighted
MR Egger
80
60
40
20
1/SE$_{IV}$
−0.6
−0.4
−0.2
0.0
β$_{IV}$

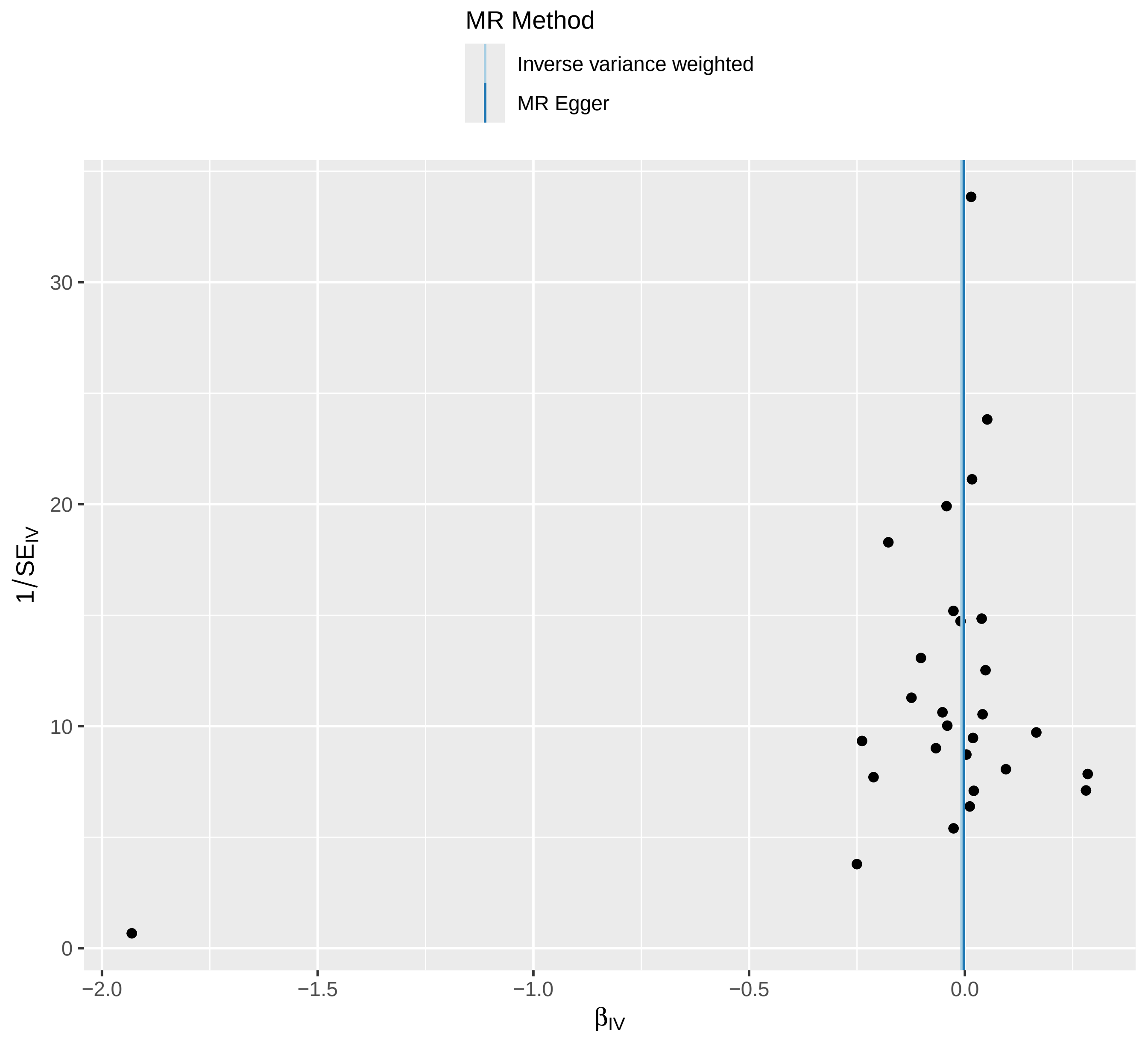
MR Method
Inverse variance weighted
MR Egger
30
20
10
0
1/SE_IV
−2.0
−1.5
−1.0
−0.5
0.0
β_IV

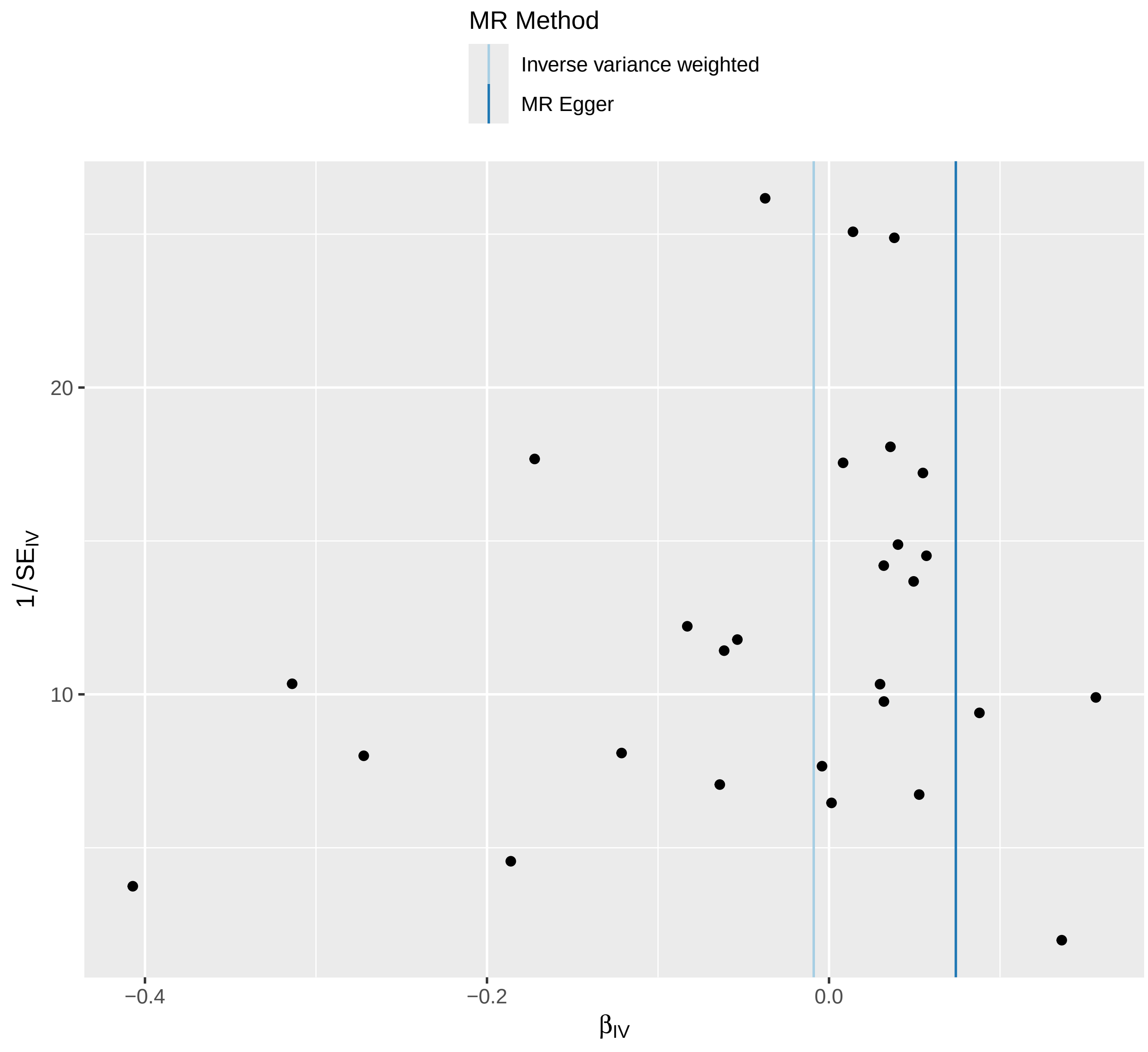
MR Method
Inverse variance weighted
MR Egger
20
10
1/SE$_{IV}$
−0.4
−0.2
0.0
β$_{IV}$

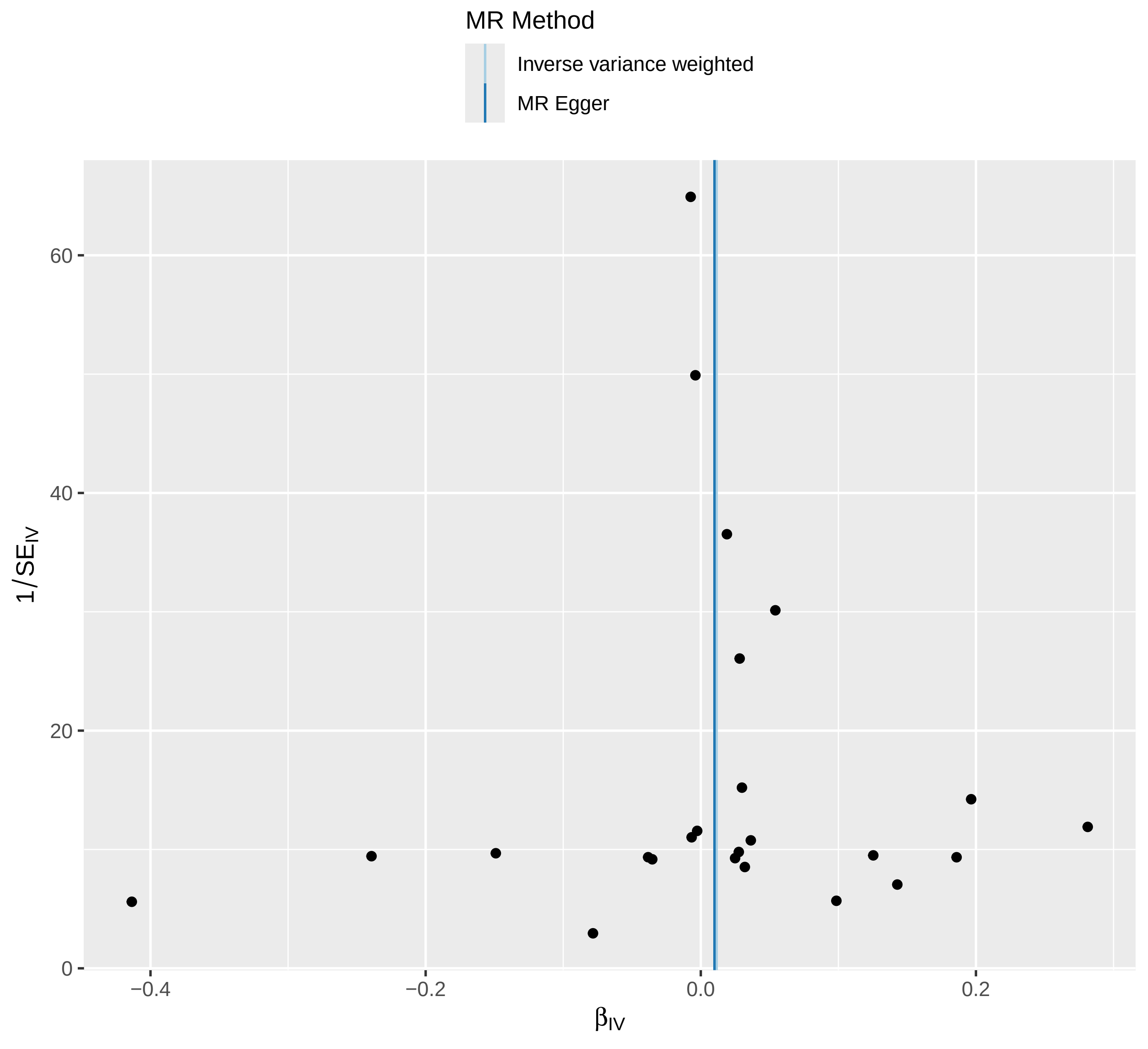
MR Method
Inverse variance weighted
MR Egger
60
40
20
0
1/SE_IV
−0.4
−0.2
0.0
0.2
β_IV

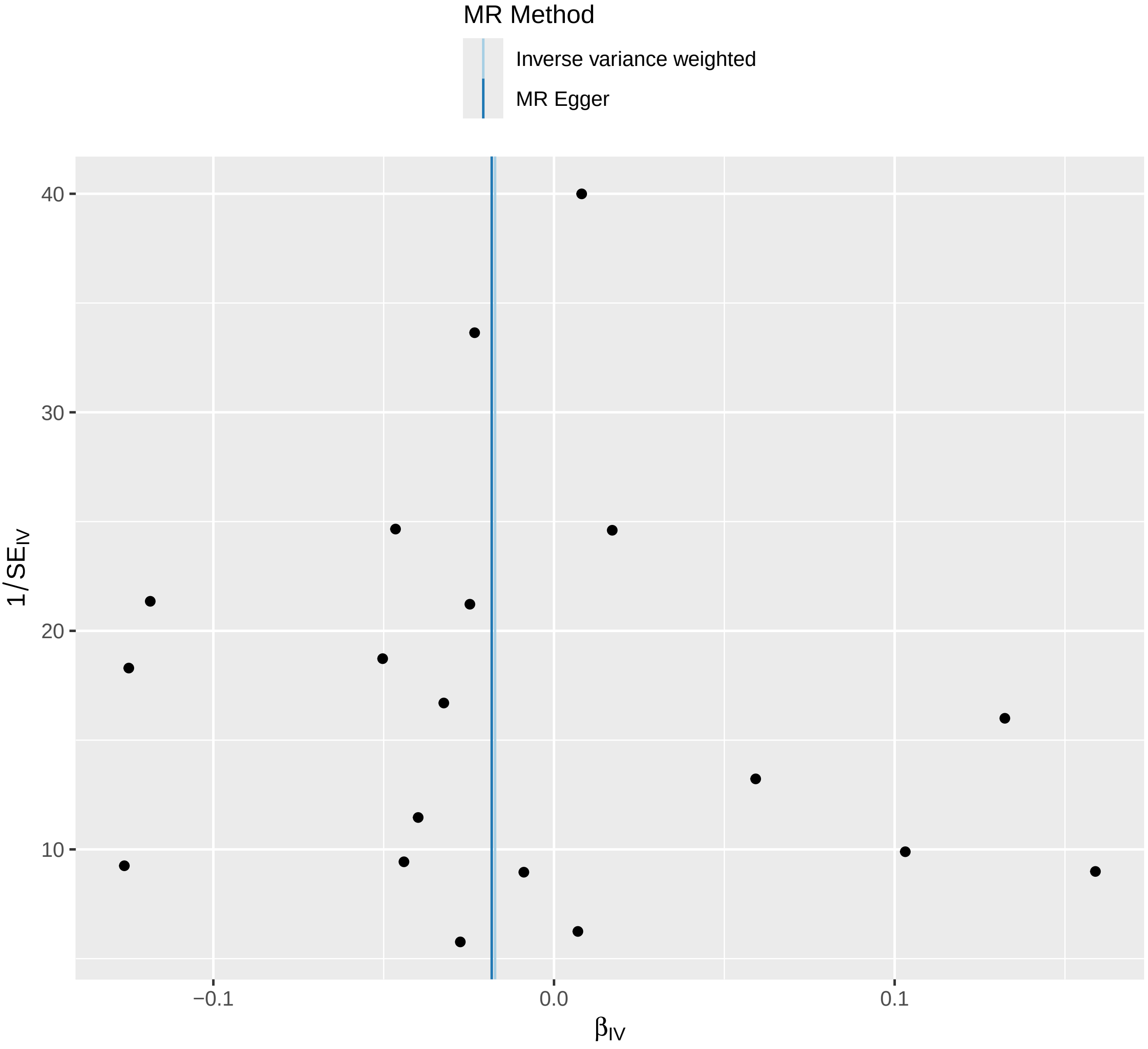
MR Method
Inverse variance weighted
MR Egger
40
30
20
10
$1/SE_{IV}$
−0.1
0.0
0.1
β_{IV}

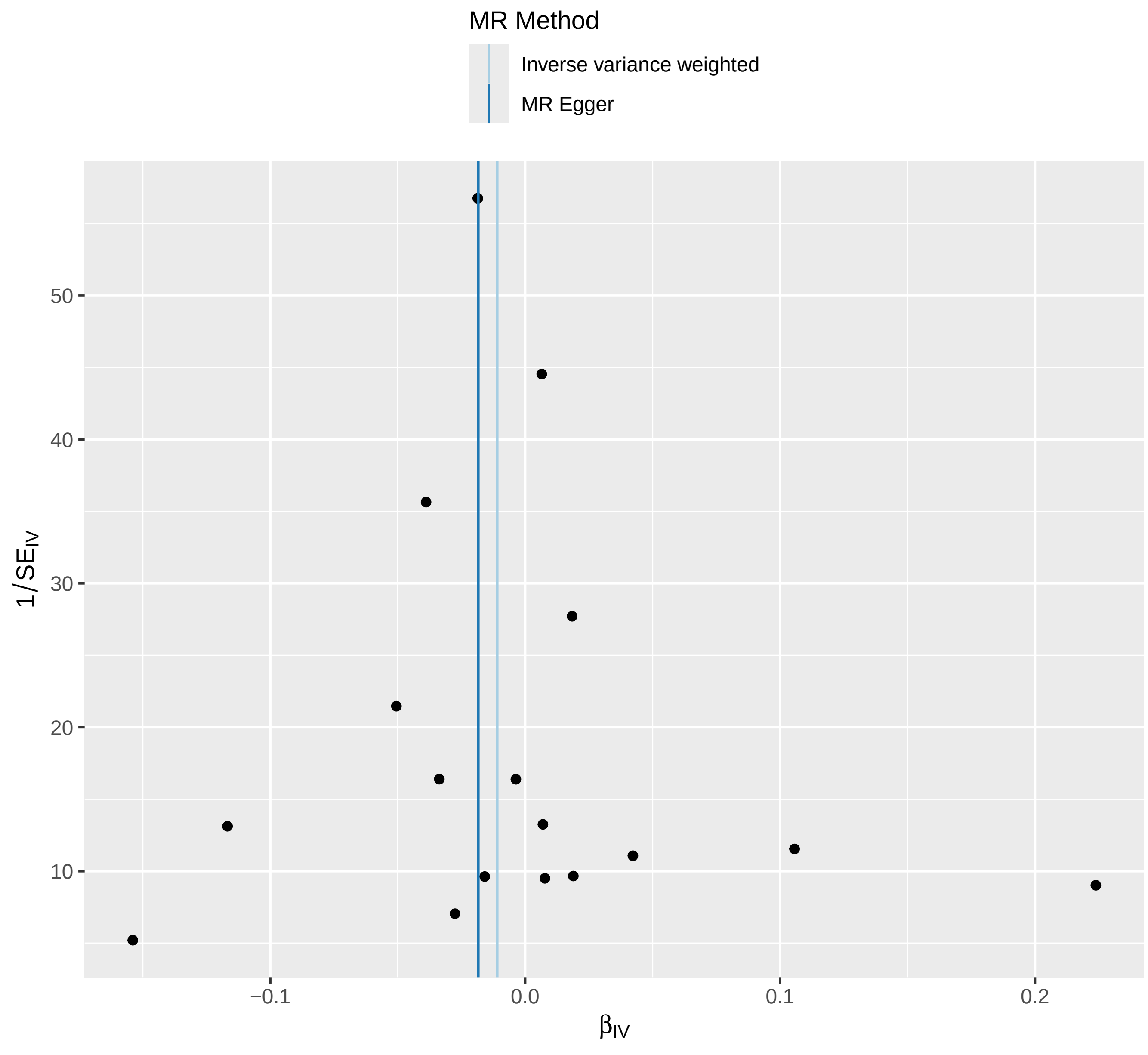
MR Method
Inverse variance weighted
MR Egger
50
40
30
20
10
1/SE_IV
−0.1
0.0
0.1
0.2
β_IV

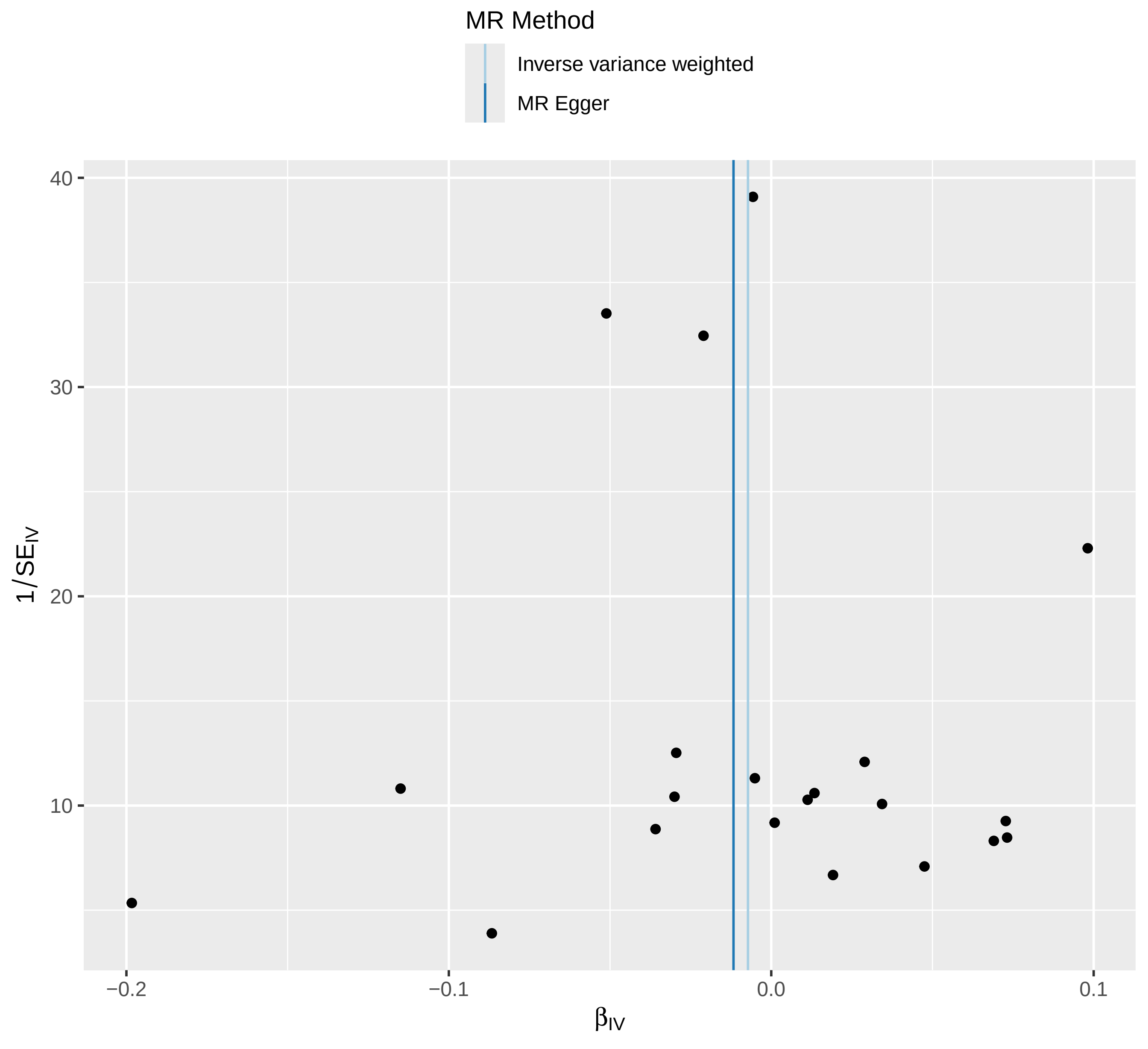
MR Method
Inverse variance weighted
MR Egger
40
30
20
10
$1/SE_{IV}$
−0.2
−0.1
0.0
0.1
β_{IV}

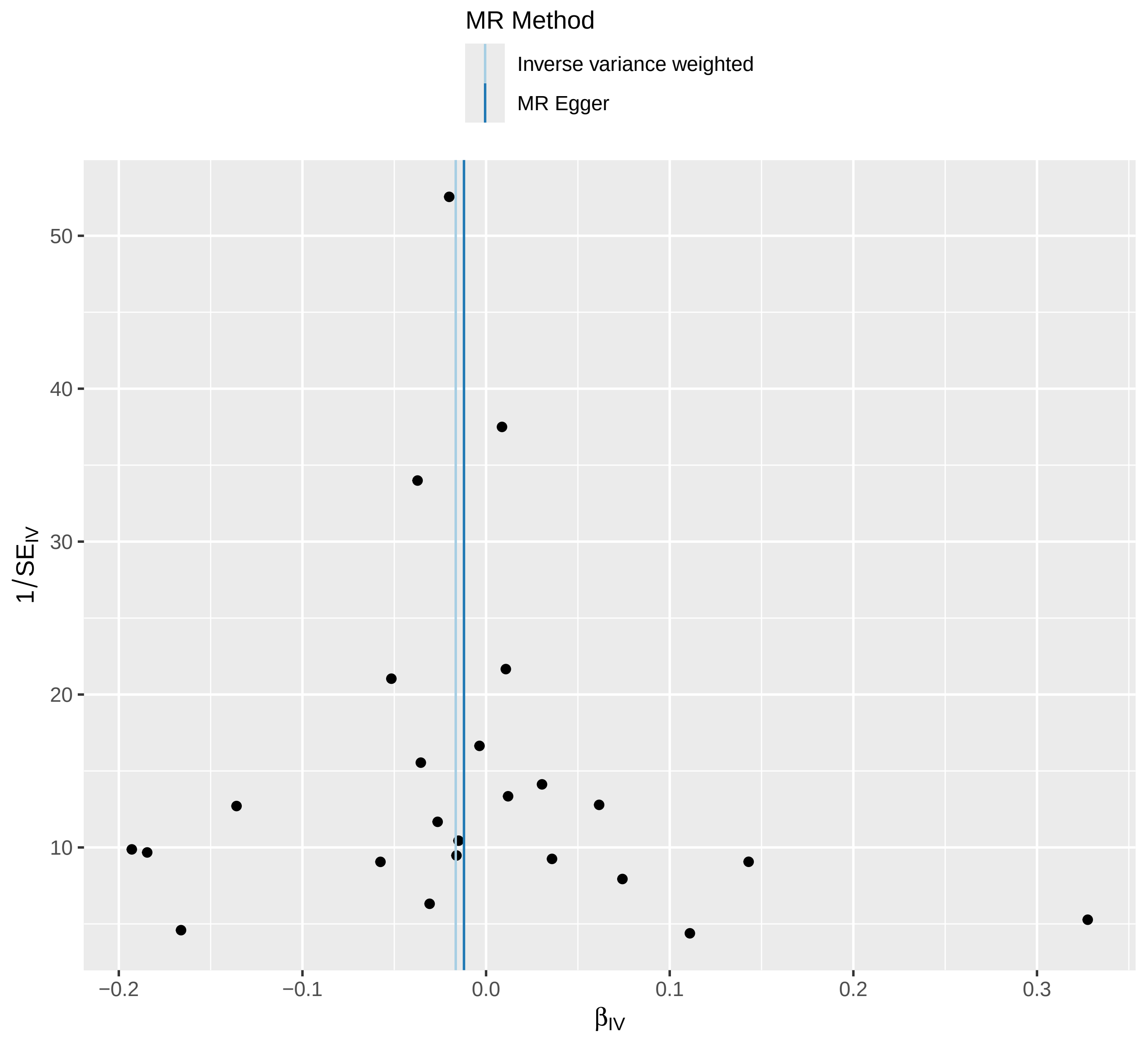
MR Method
Inverse variance weighted
MR Egger
50
40
30
20
10
1/SE_IV
−0.2
−0.1
0.0
0.1
0.2
0.3
β_IV

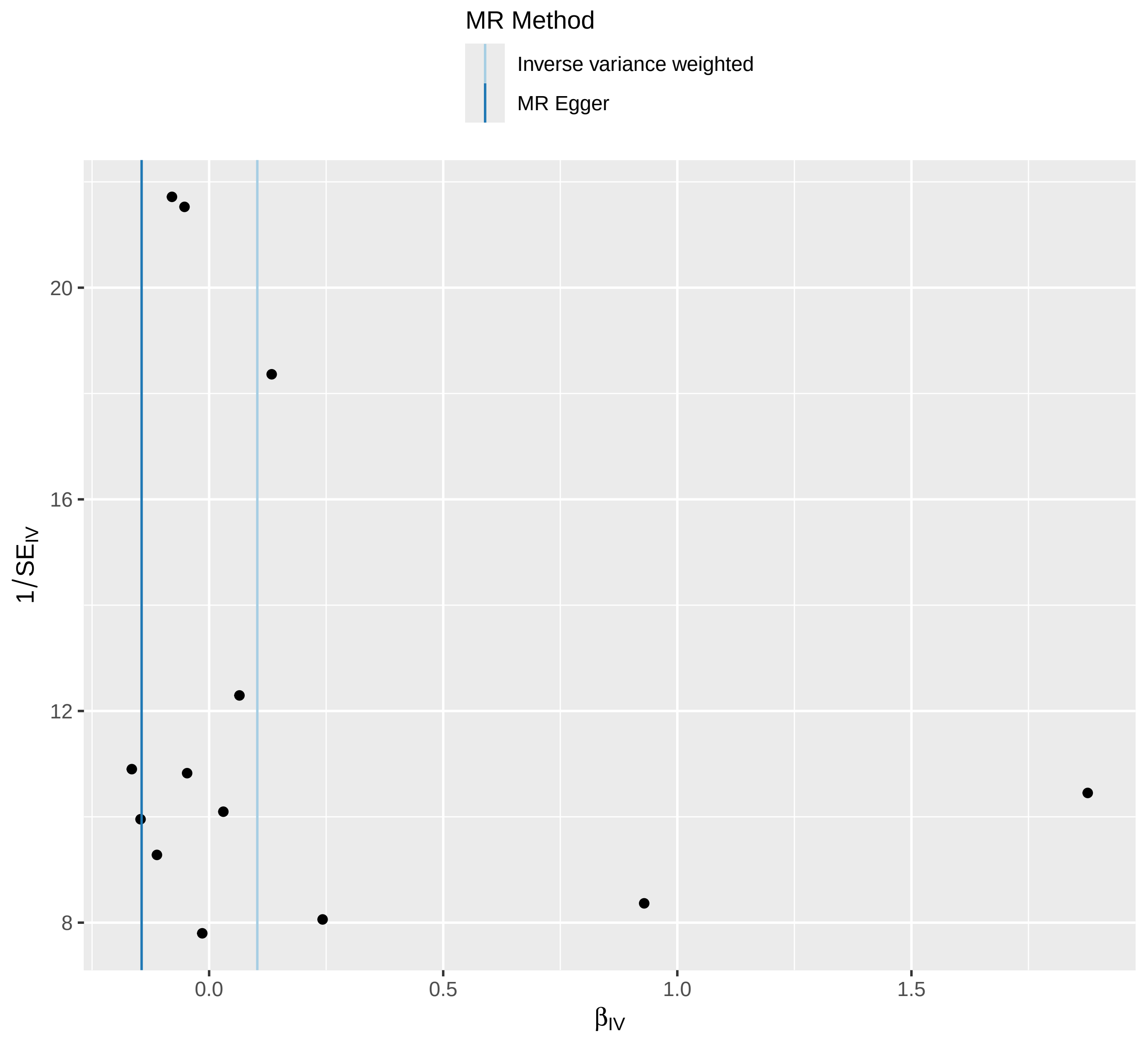
MR Method
Inverse variance weighted
MR Egger
20
16
12
8
1/SE_IV
0.0
0.5
1.0
1.5
β_IV

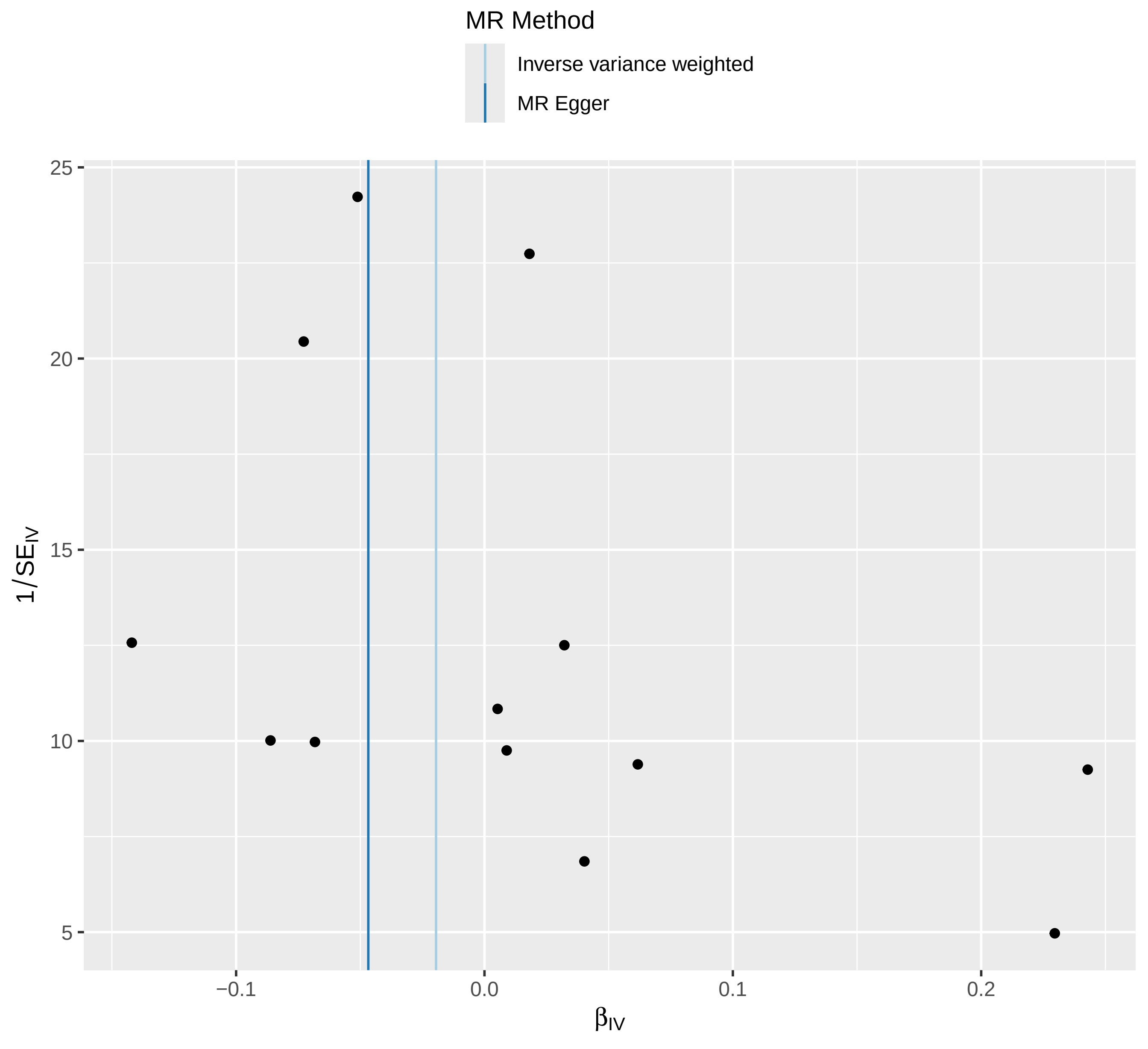
MR Method
Inverse variance weighted
MR Egger
25
20
15
10
5
$1/SE_{IV}$
−0.1
0.0
0.1
0.2
β_{IV}

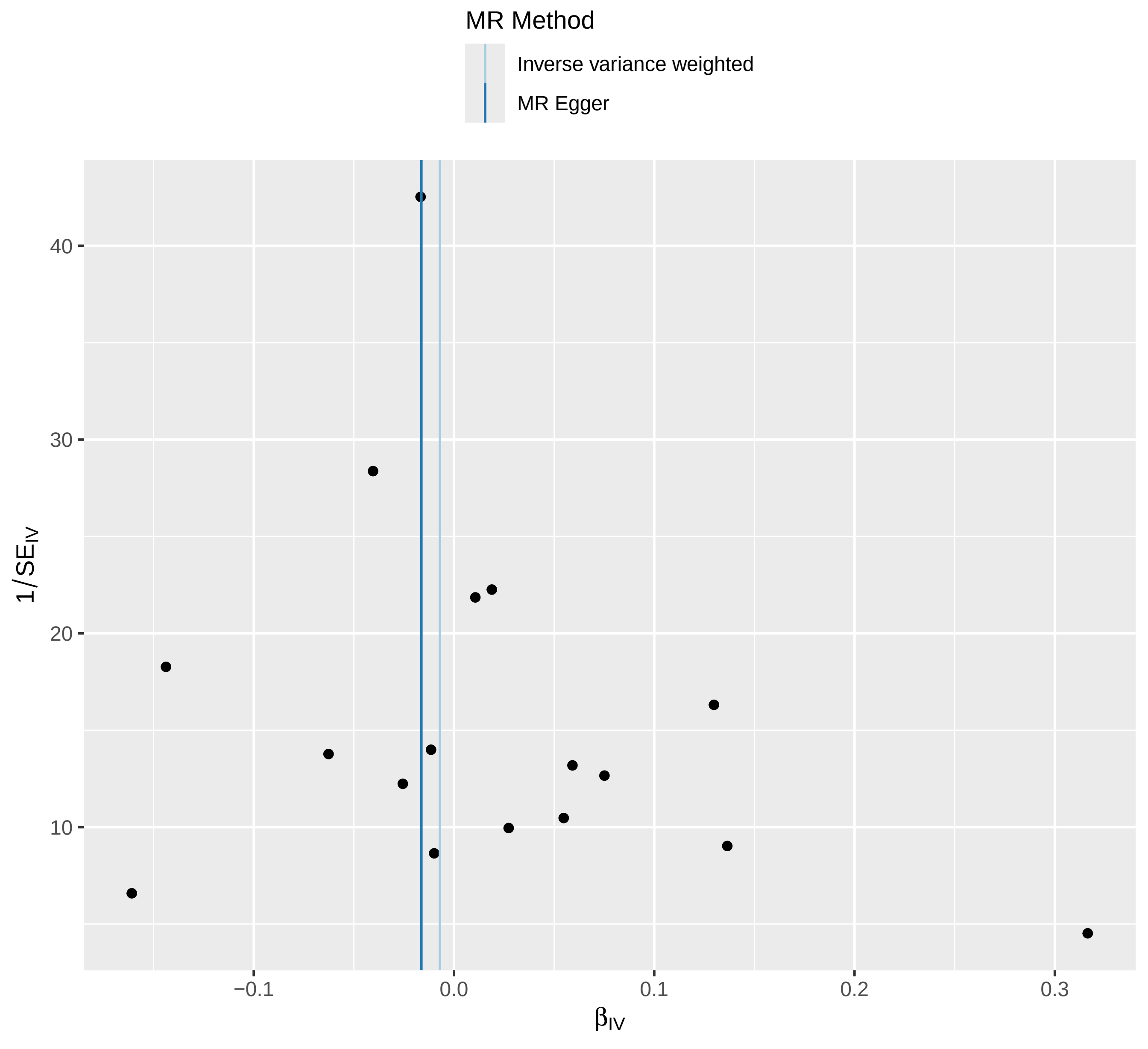
MR Method
Inverse variance weighted
MR Egger
40
30
20
10
1/SE_IV
−0.1
0.0
0.1
0.2
0.3
β_IV

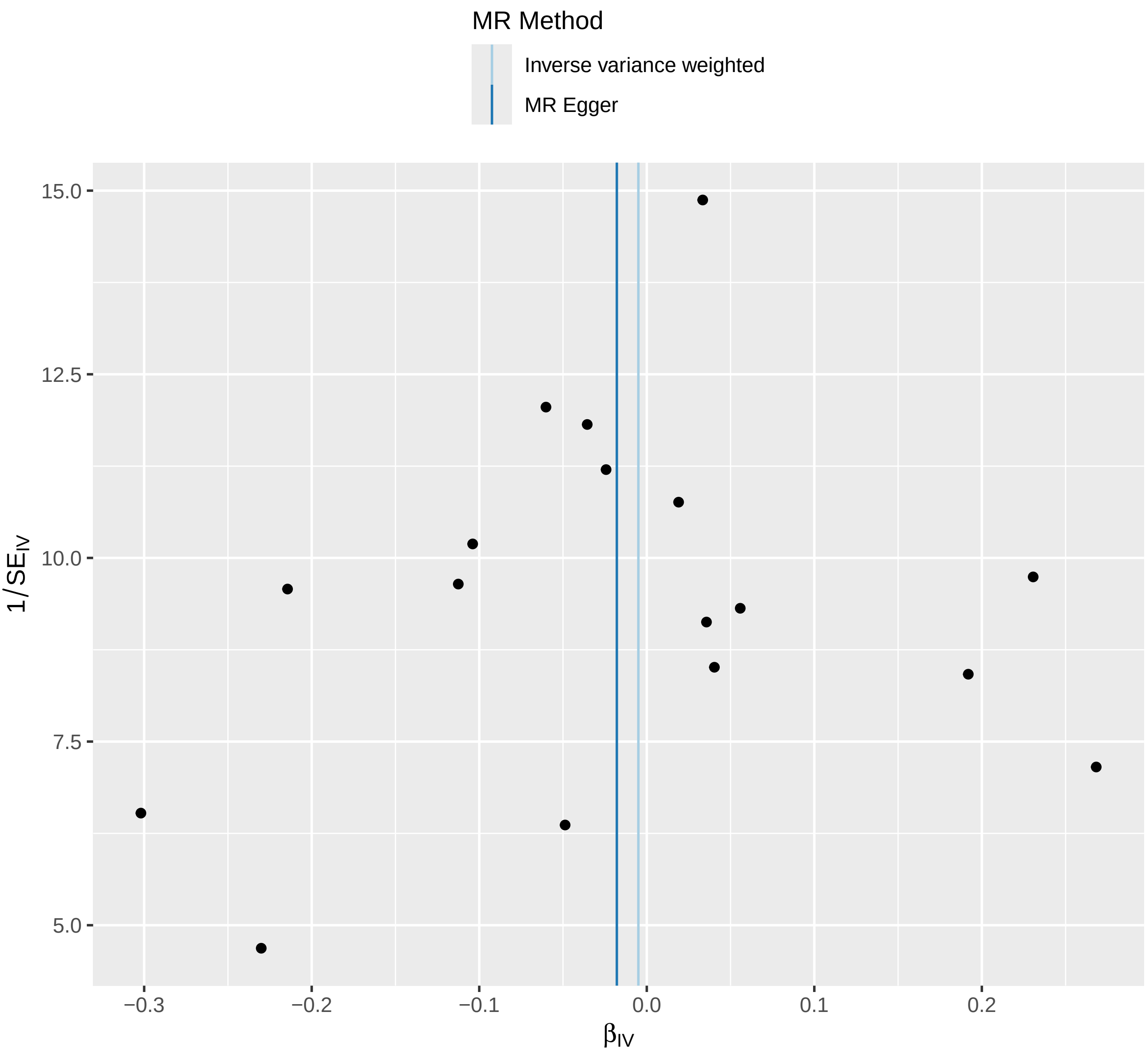
MR Method
Inverse variance weighted
MR Egger
15.0
12.5
10.0
7.5
5.0
$1/SE_{IV}$
−0.3
−0.2
−0.1
0.0
0.1
0.2
β_{IV}

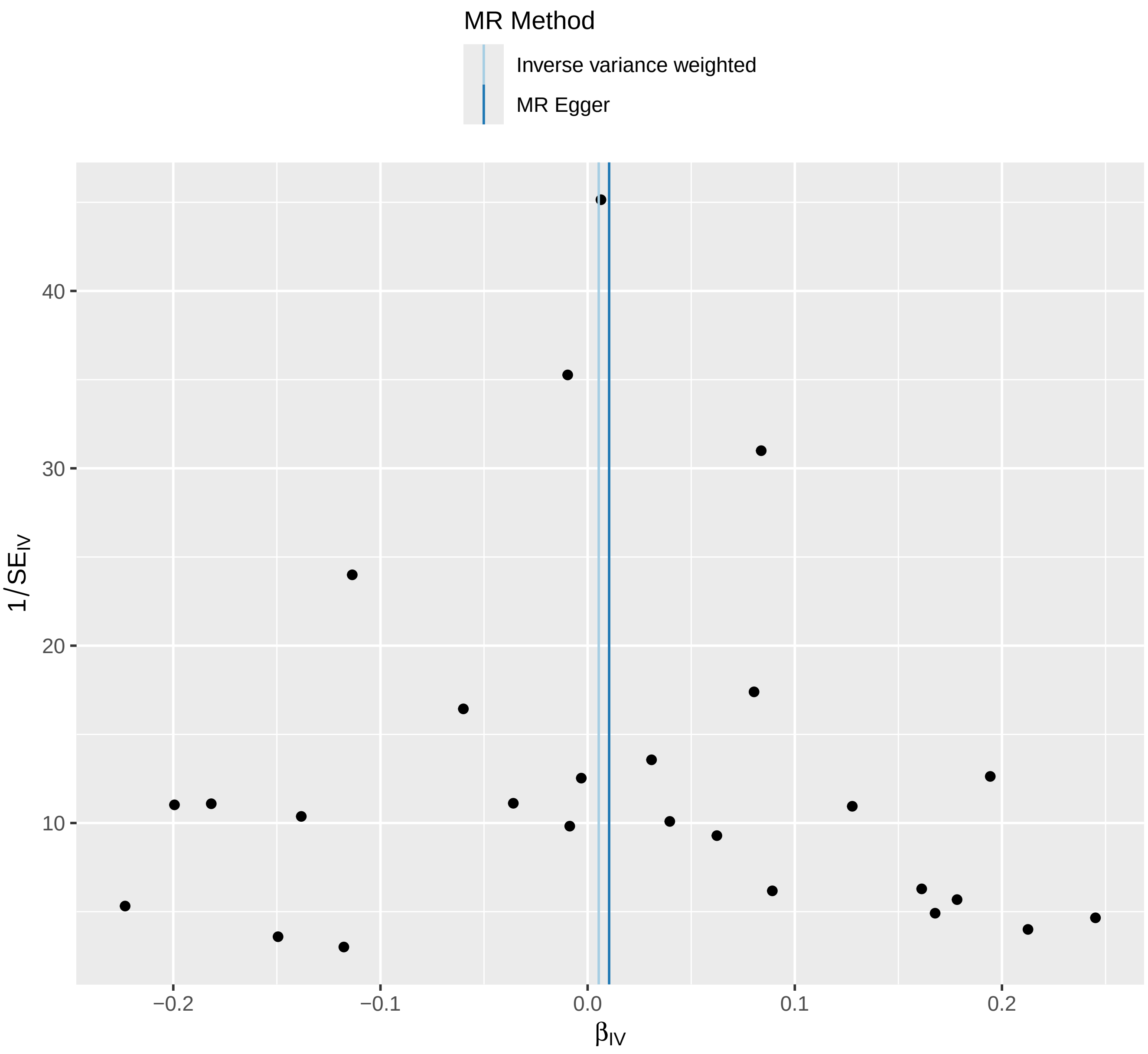
MR Method
Inverse variance weighted
MR Egger
40
30
20
10
1/SE_IV
−0.2
−0.1
0.0
0.1
0.2
β_IV

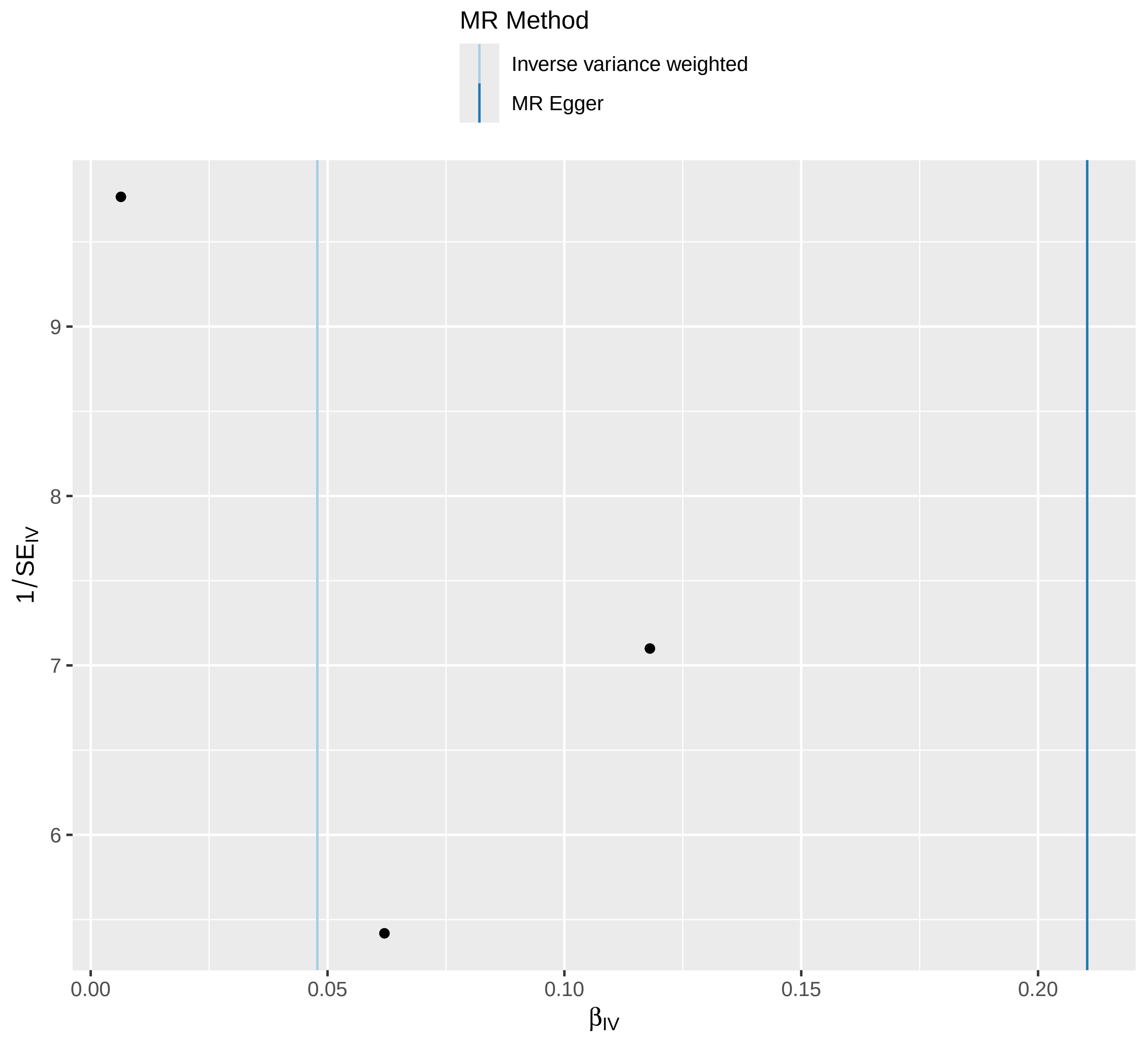

MR Method
Inverse variance weighted
MR Egger
9
8
7
6
1/SE_IV
0.00
0.05
0.10
0.15
0.20
β_IV

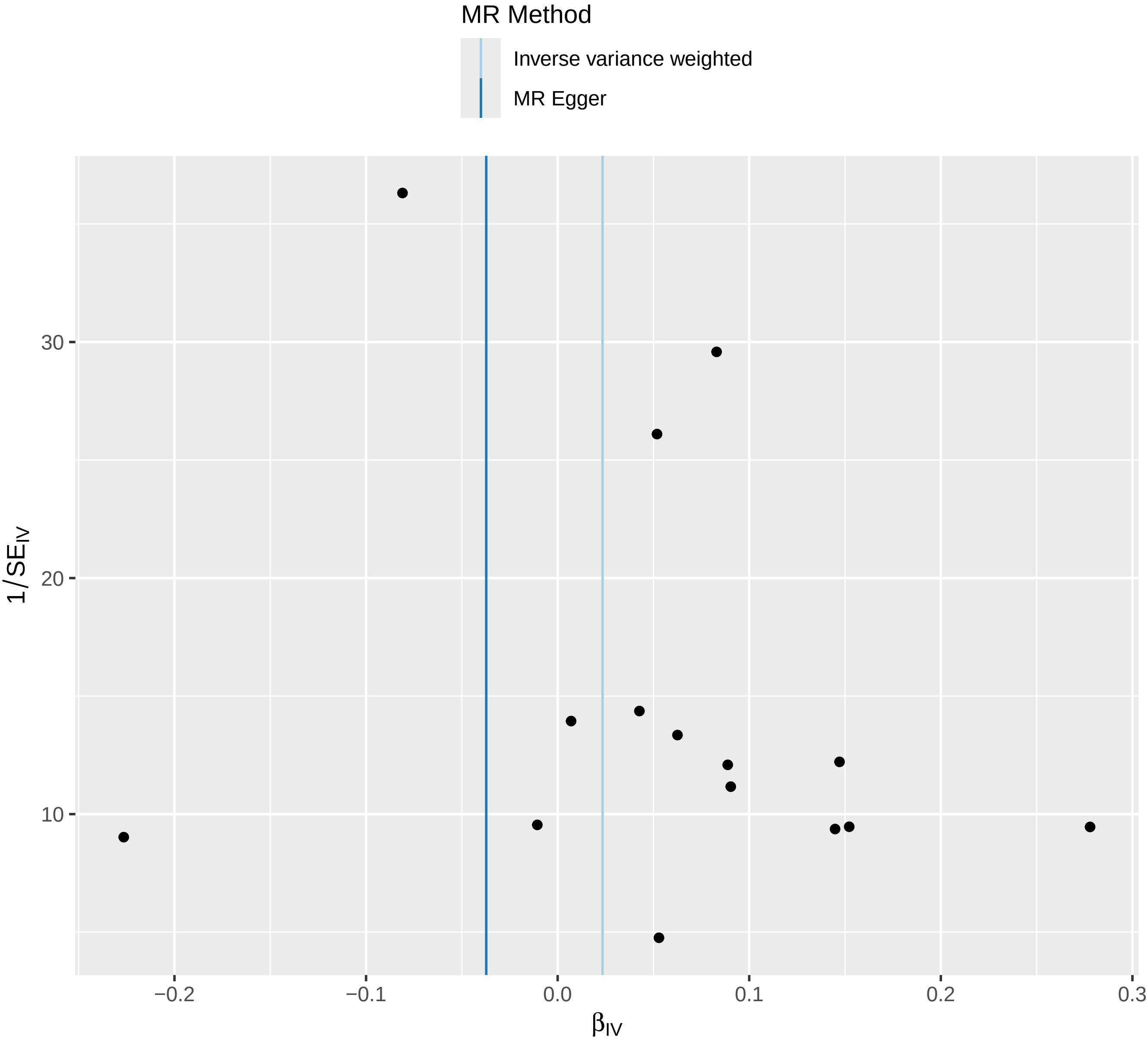
MR Method
Inverse variance weighted
MR Egger
1/SE_IV
β_IV
30
20
10
−0.2
−0.1
0.0
0.1
0.2
0.3

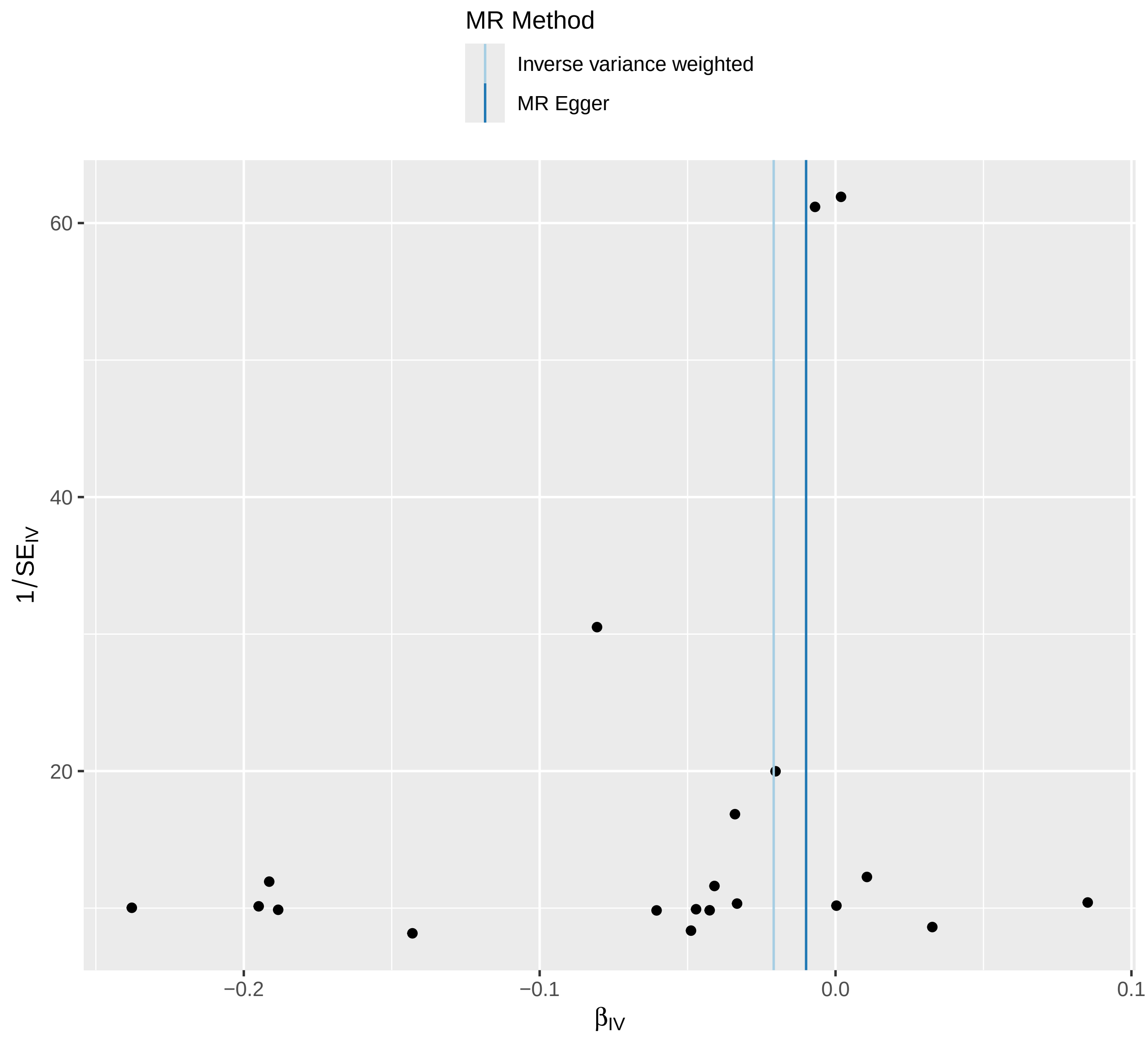
MR Method
Inverse variance weighted
MR Egger
60
40
20
1/SE$_{IV}$
−0.2
−0.1
0.0
0.1
β$_{IV}$

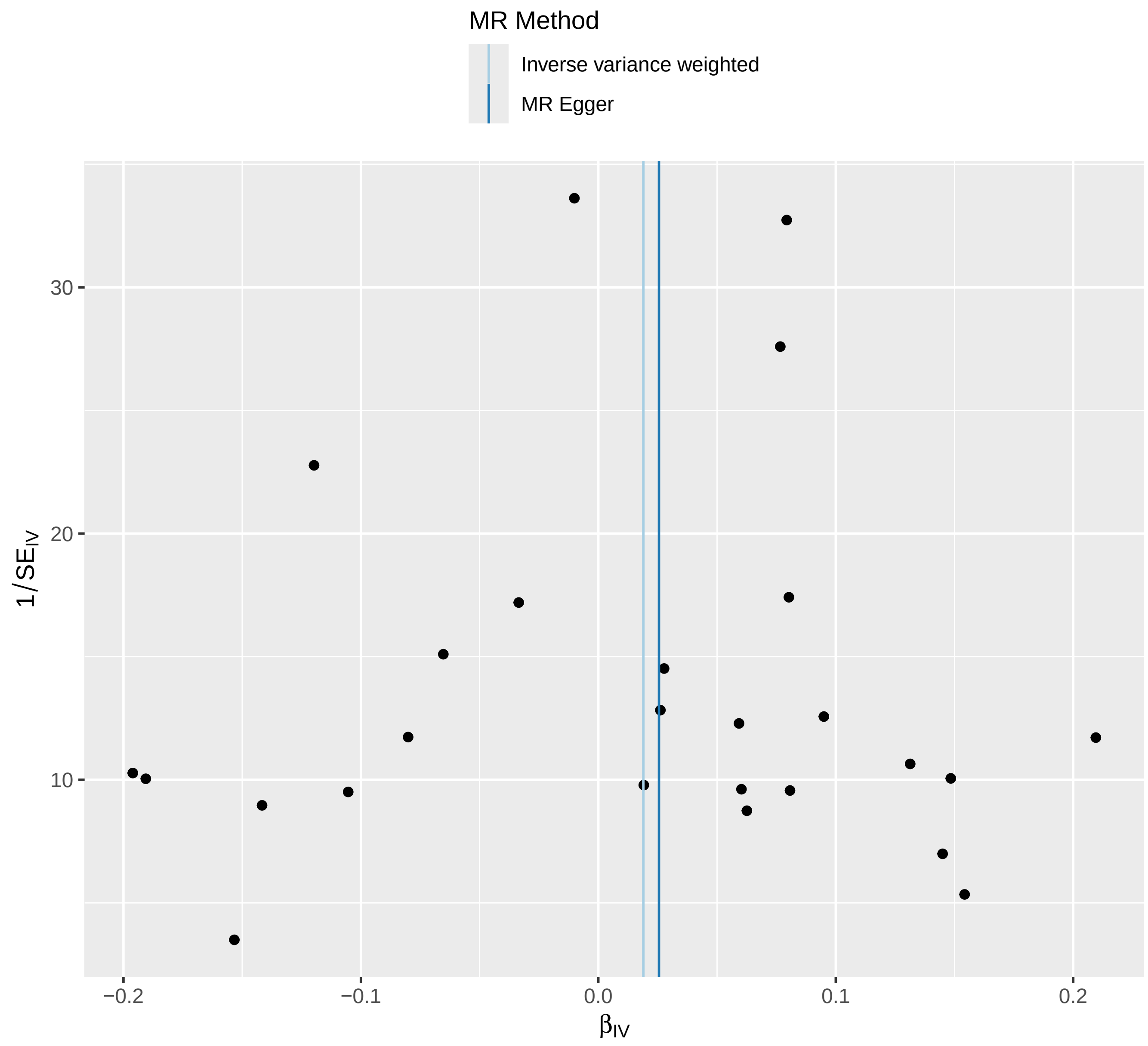
MR Method
Inverse variance weighted
MR Egger
30
20
10
1/SE$_{IV}$
−0.2
−0.1
0.0
0.1
0.2
β$_{IV}$

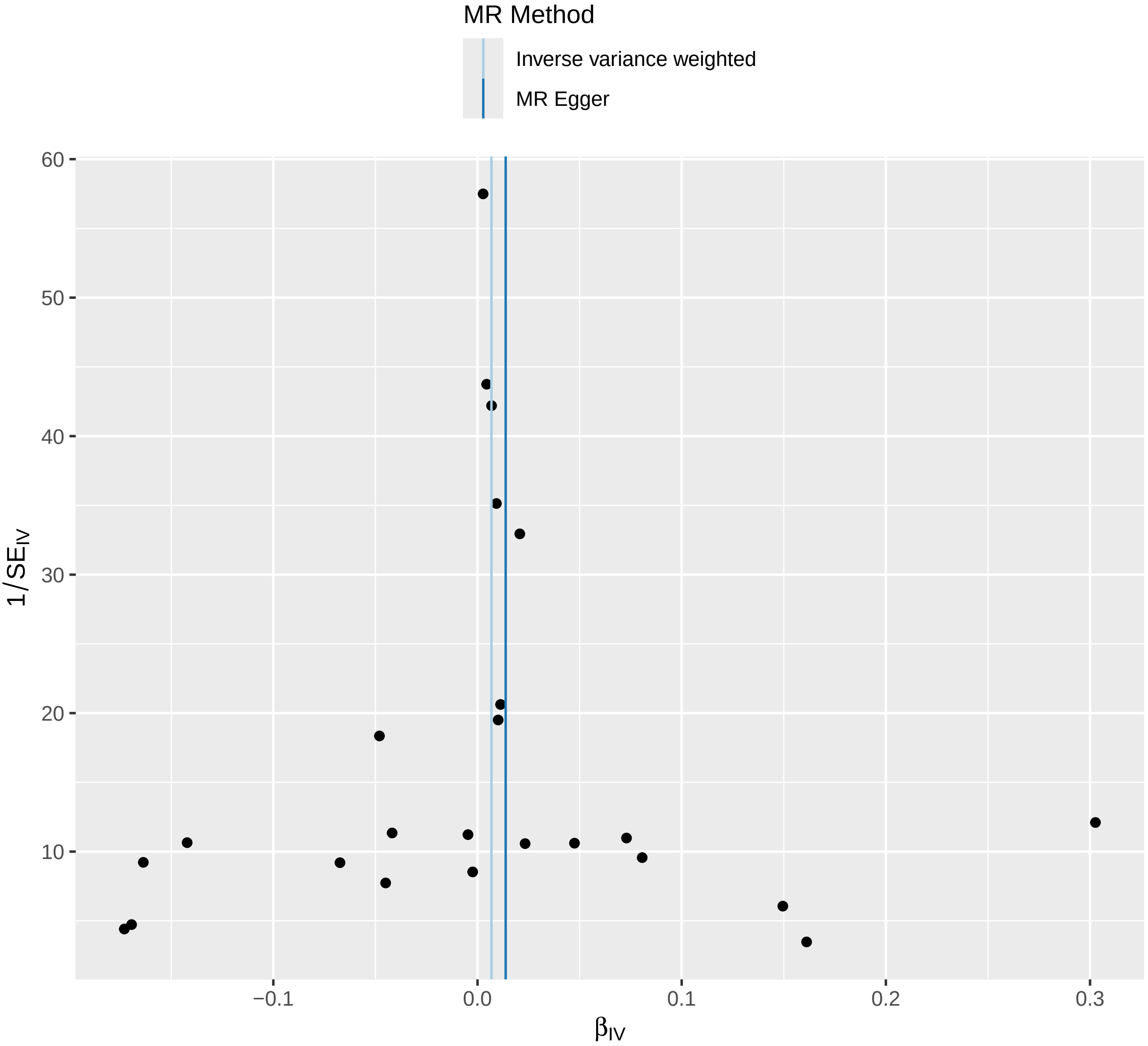
MR Method
Inverse variance weighted
MR Egger
60
50
40
30
20
10
$1/SE_{IV}$
−0.1
0.0
0.1
0.2
0.3
β_{IV}

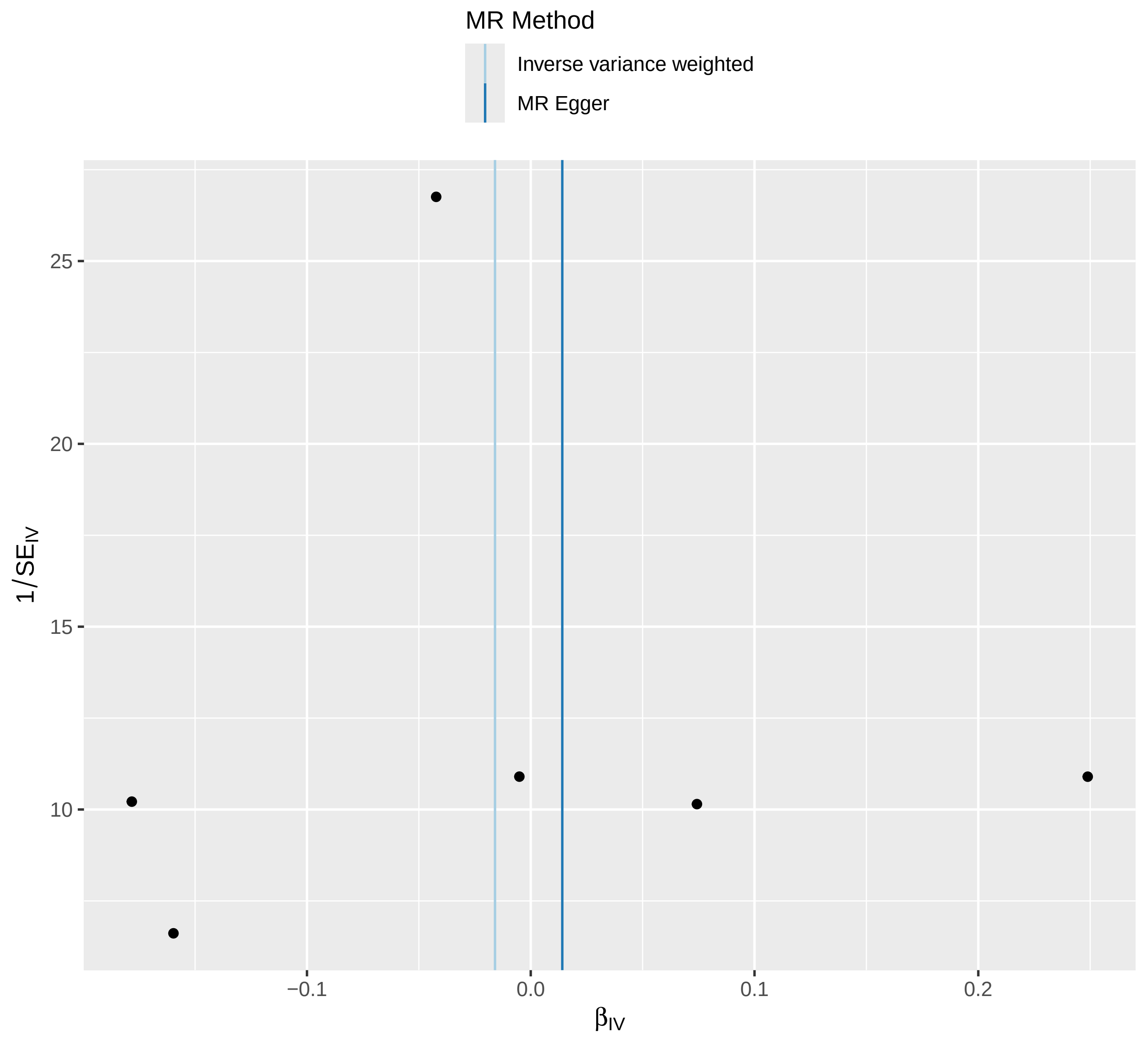
MR Method
Inverse variance weighted
MR Egger
25
20
15
10
1/SE_IV
−0.1
0.0
0.1
0.2
β_IV

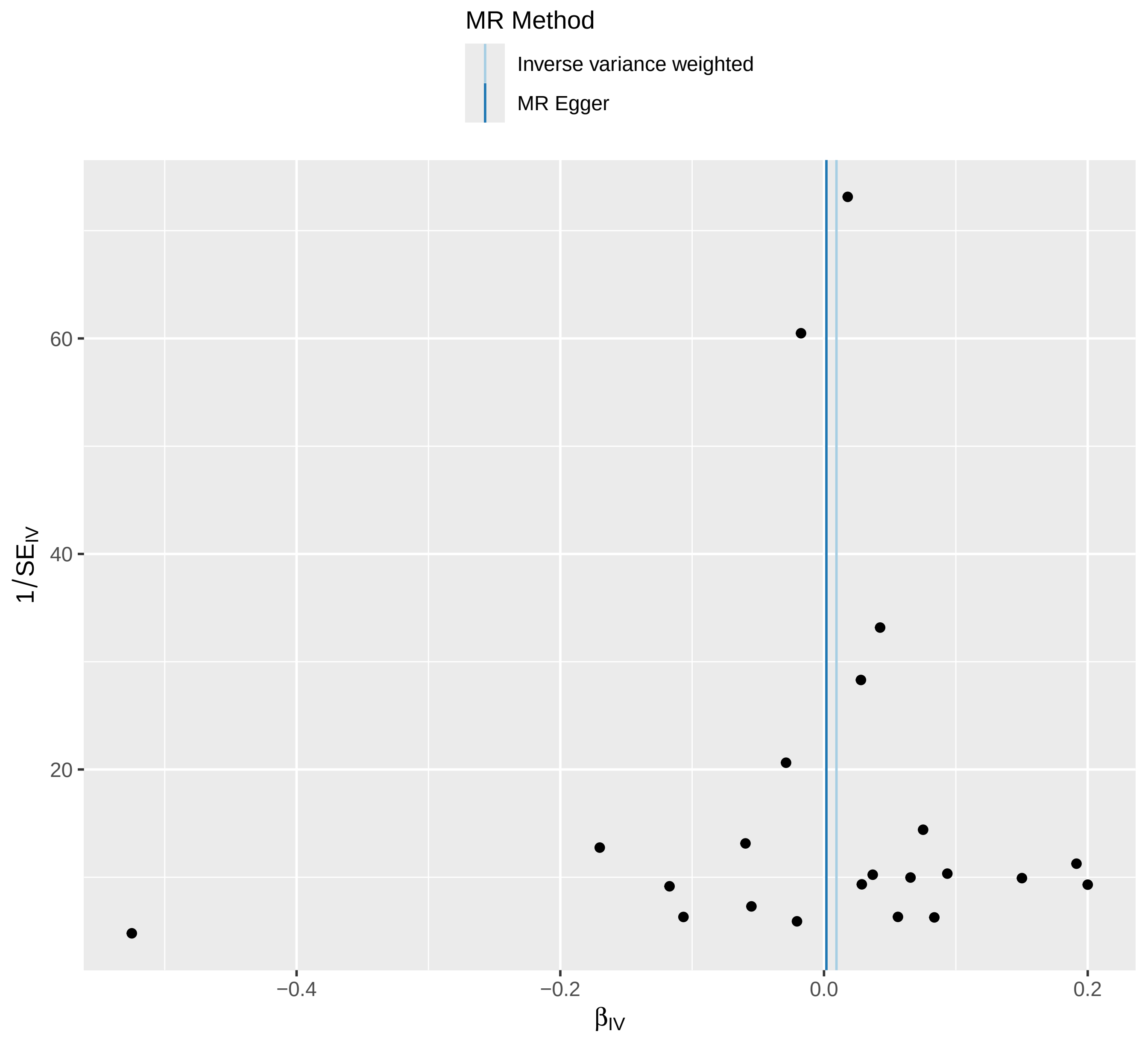
MR Method
Inverse variance weighted
MR Egger
60
40
20
1/SE_IV
−0.4
−0.2
0.0
0.2
β_IV

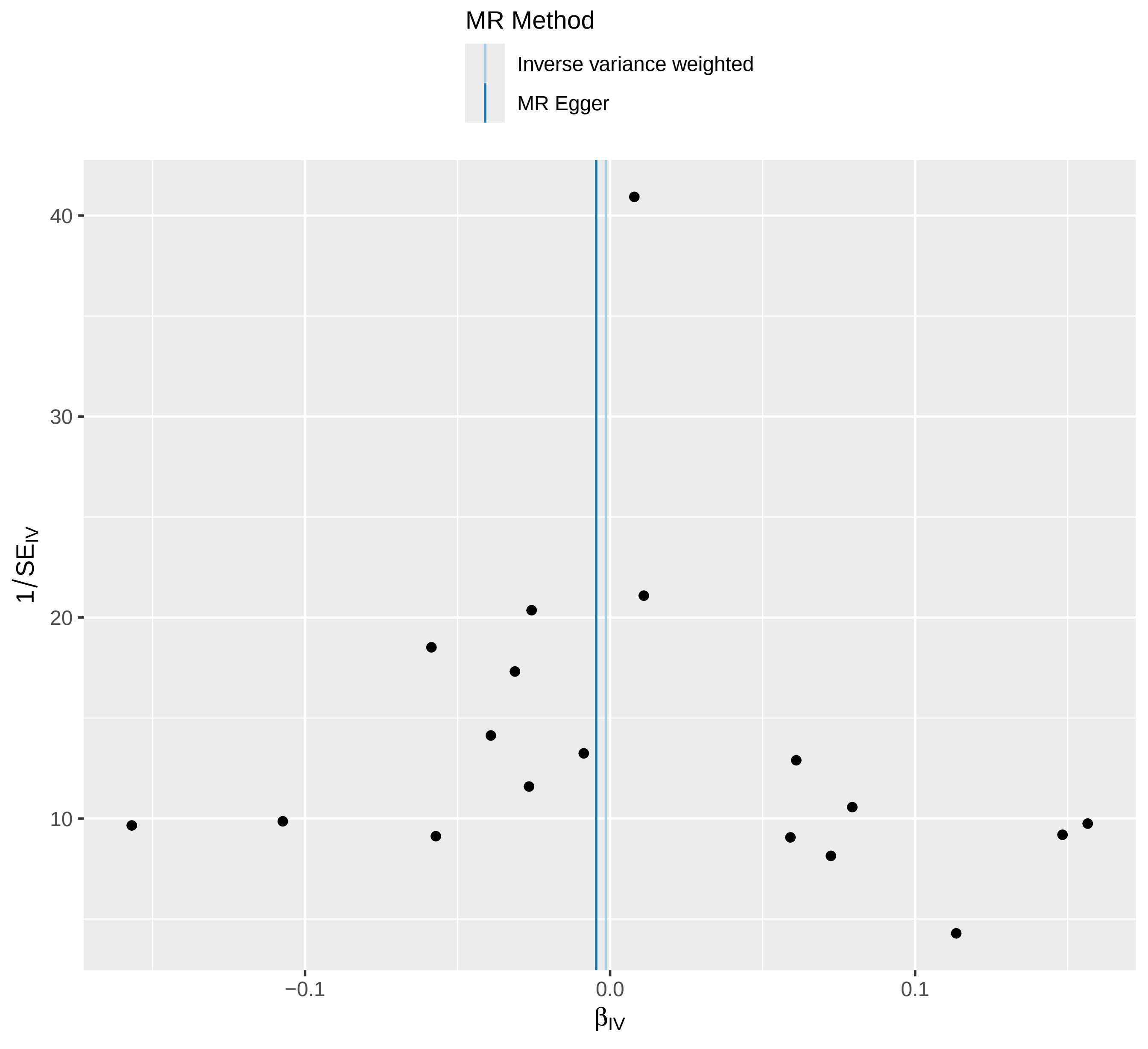
MR Method
Inverse variance weighted
MR Egger
40
30
20
10
1/SE$_{IV}$
−0.1
0.0
0.1
β$_{IV}$

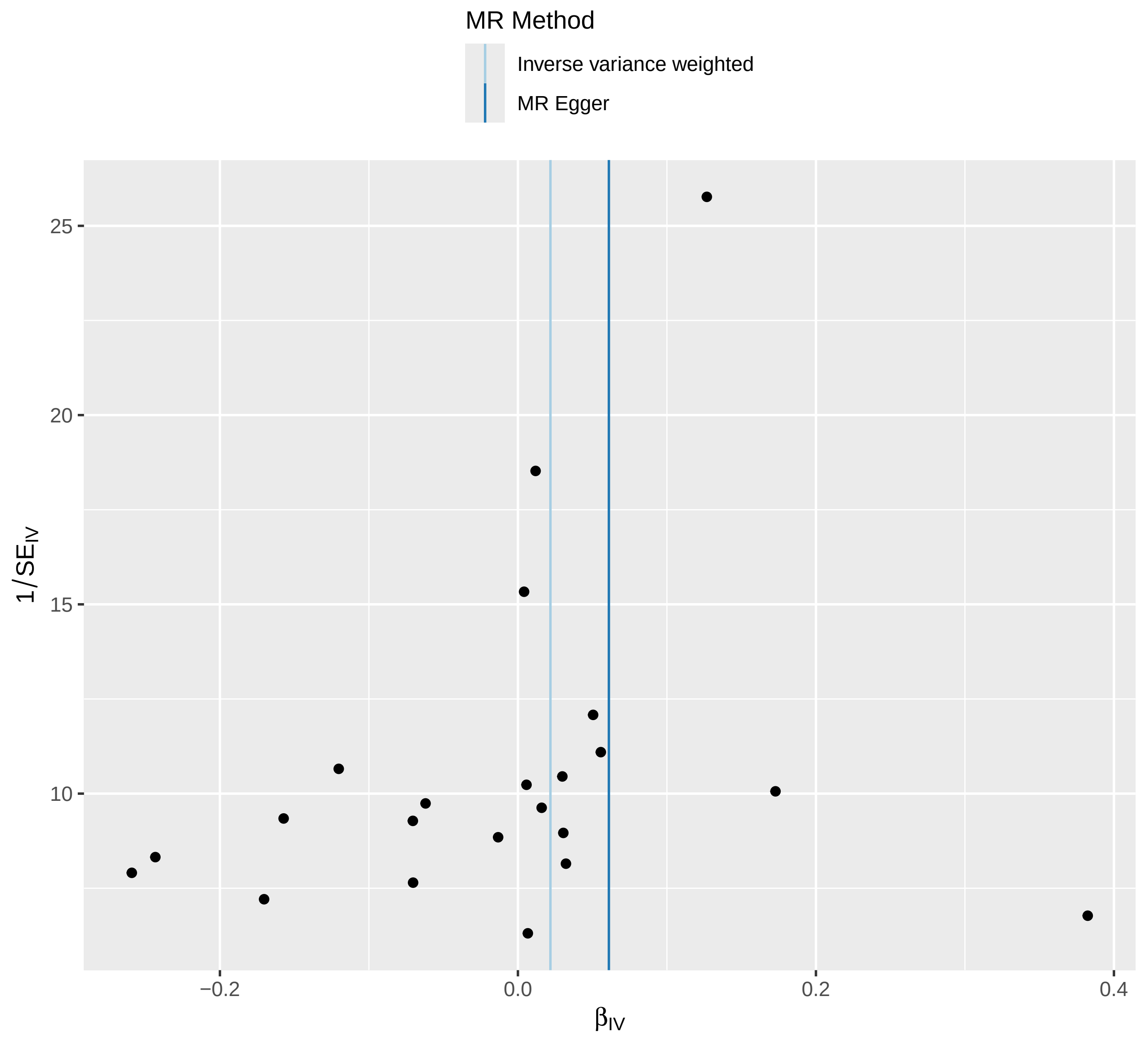
MR Method
Inverse variance weighted
MR Egger
25
20
15
10
$1/SE_{IV}$
−0.2
0.0
0.2
0.4
β_{IV}

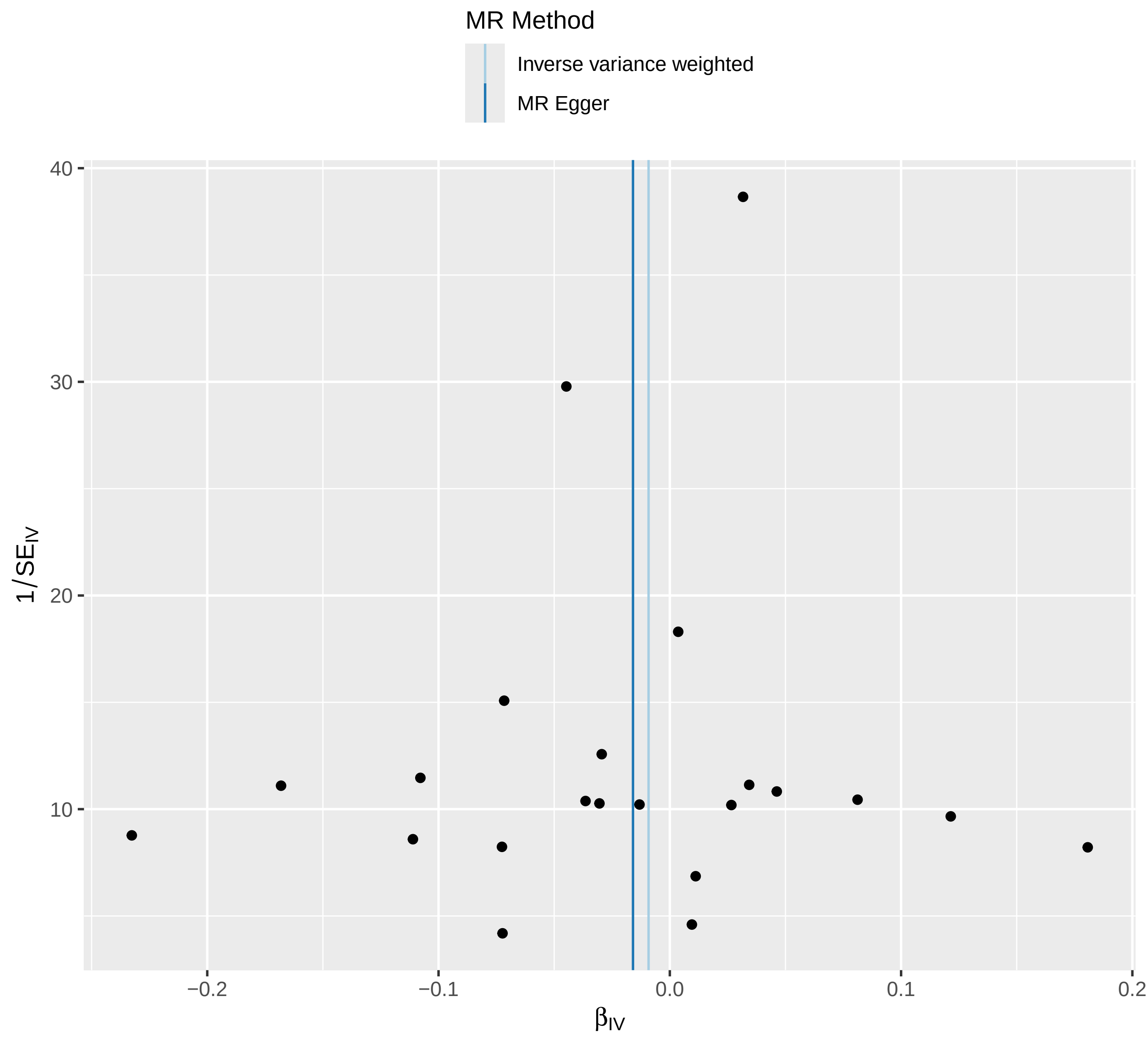
MR Method
Inverse variance weighted
MR Egger
40
30
20
10
1/SE_IV
−0.2
−0.1
0.0
0.1
0.2
β_IV

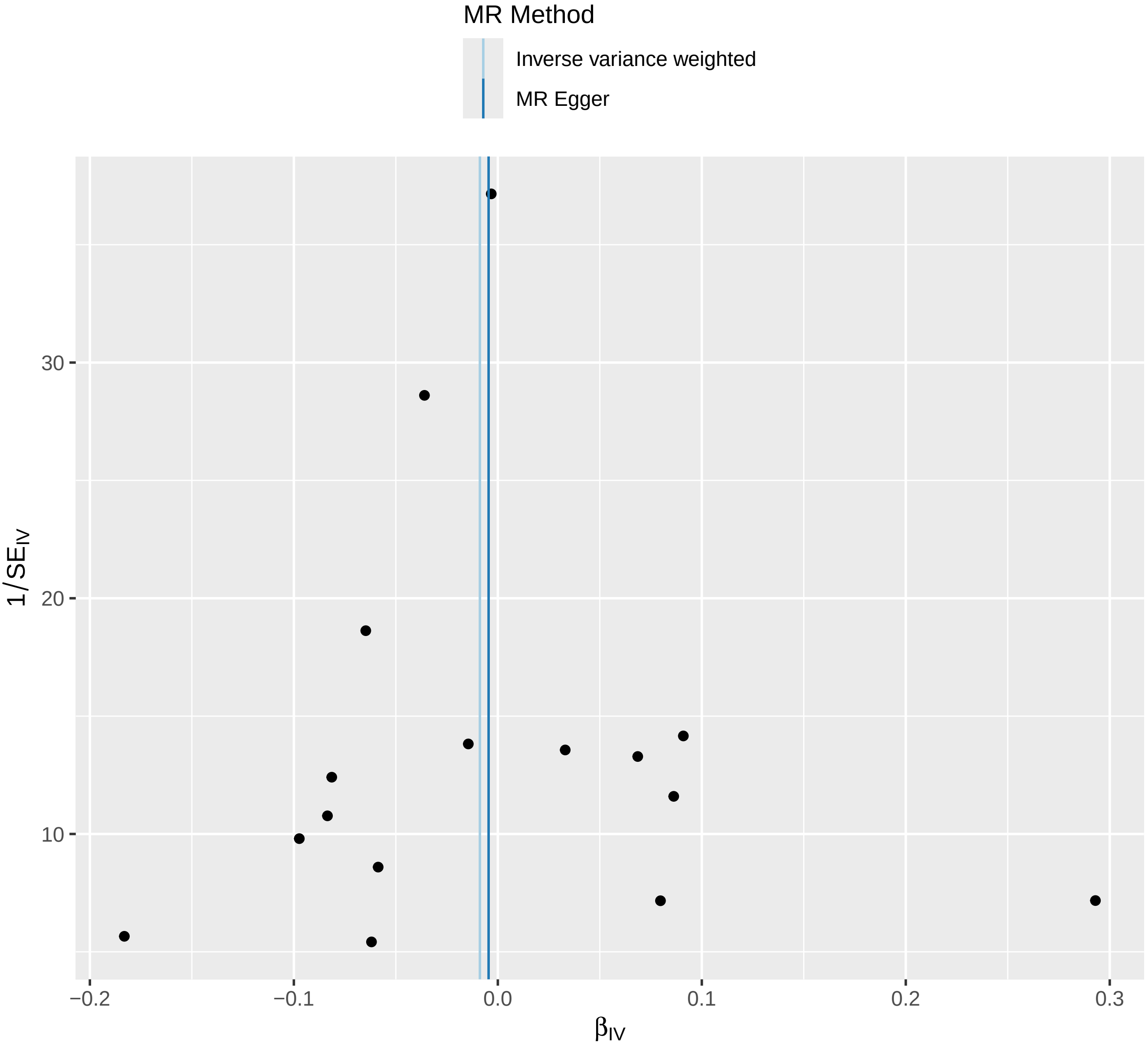
MR Method
Inverse variance weighted
MR Egger
1/SE$_{IV}$
30
20
10
−0.2
−0.1
0.0
0.1
0.2
0.3
β$_{IV}$

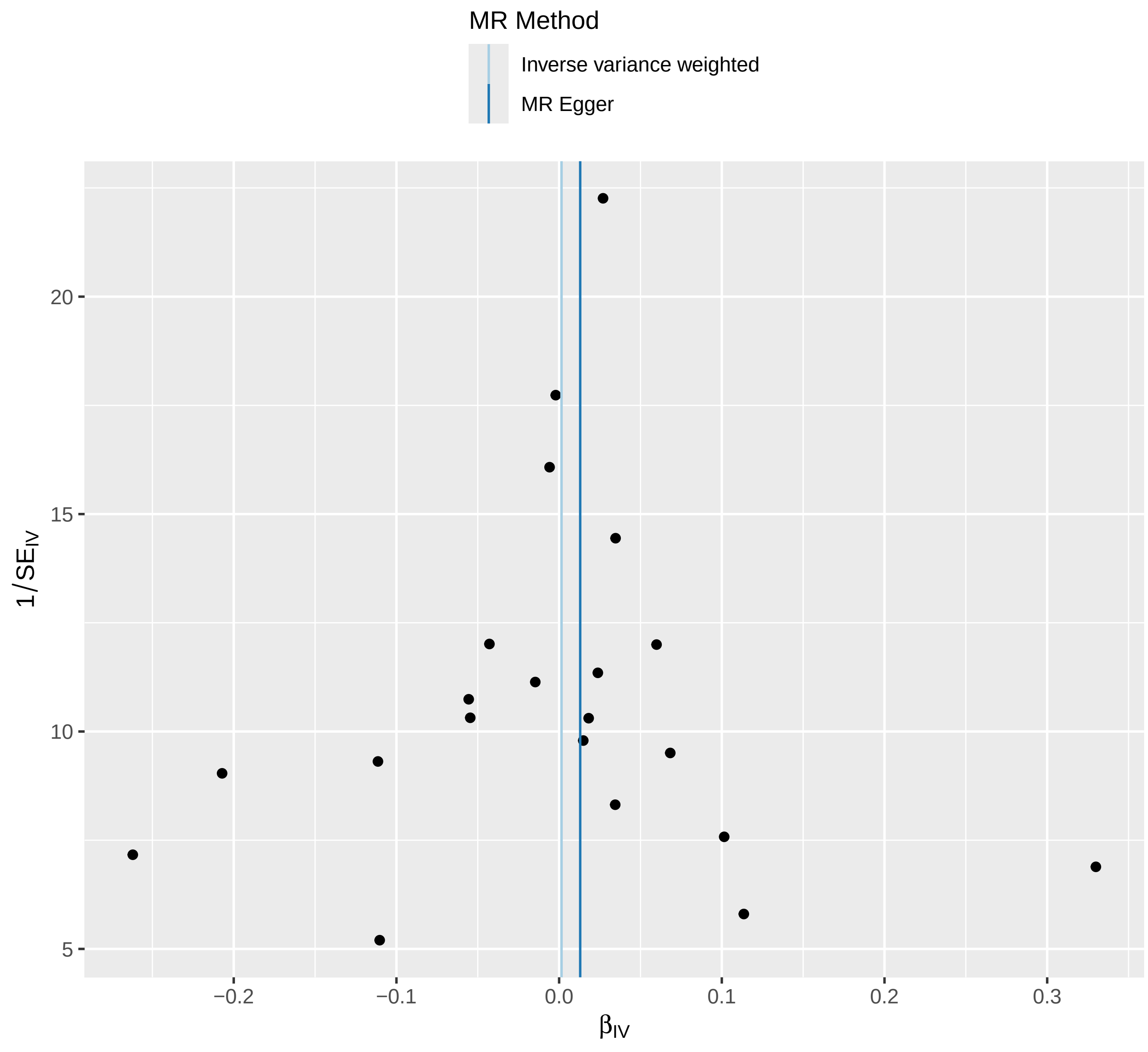

MR Method
Inverse variance weighted
MR Egger
20
15
10
5
1/SE_IV
−0.2
−0.1
0.0
0.1
0.2
0.3
β_IV

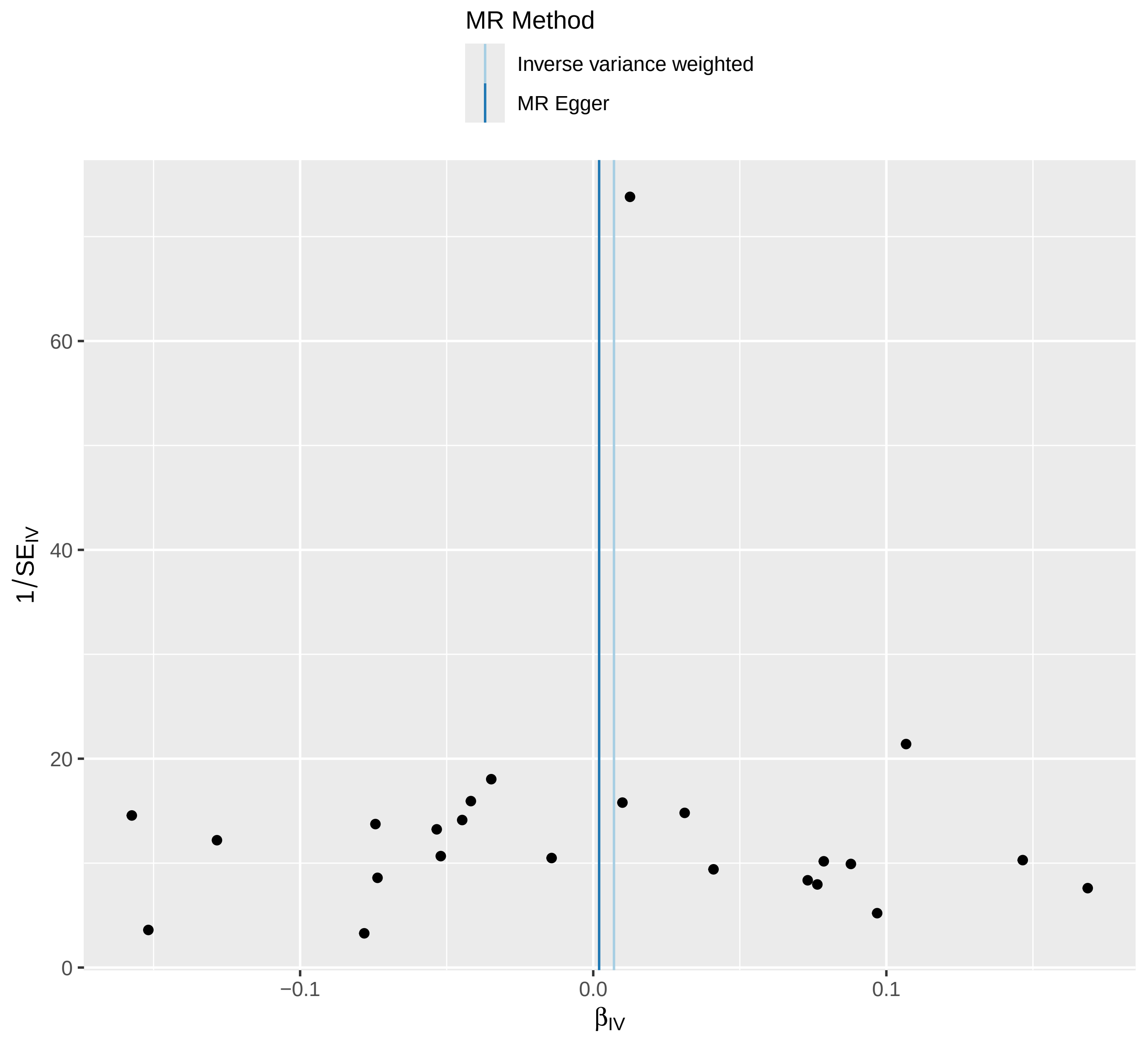
MR Method
Inverse variance weighted
MR Egger
60
40
20
0
1/SE$_{IV}$
−0.1
0.0
0.1
β$_{IV}$

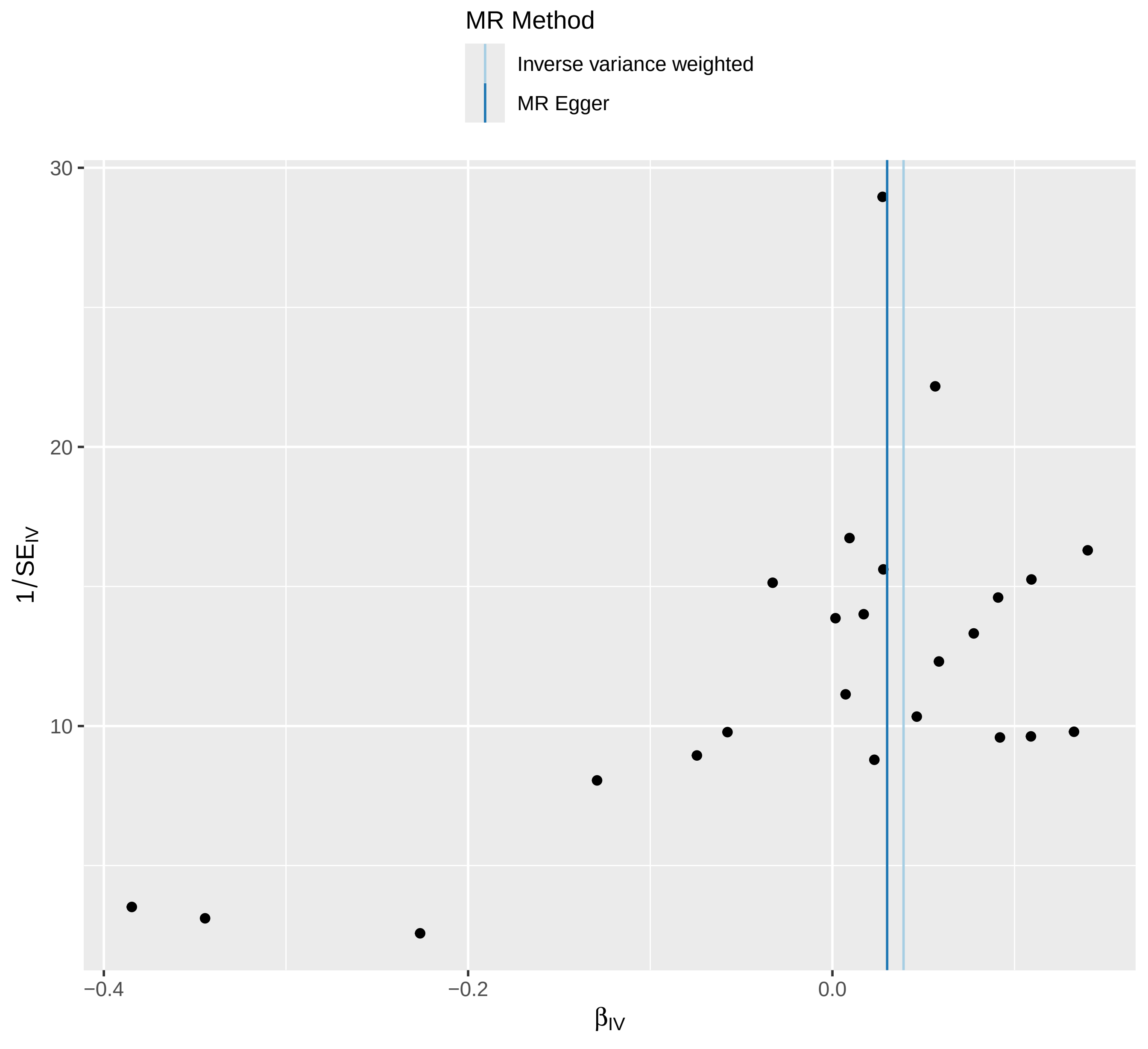

MR Method
Inverse variance weighted
MR Egger
30
20
10
1/SE$_{IV}$
−0.4
−0.2
0.0
β$_{IV}$

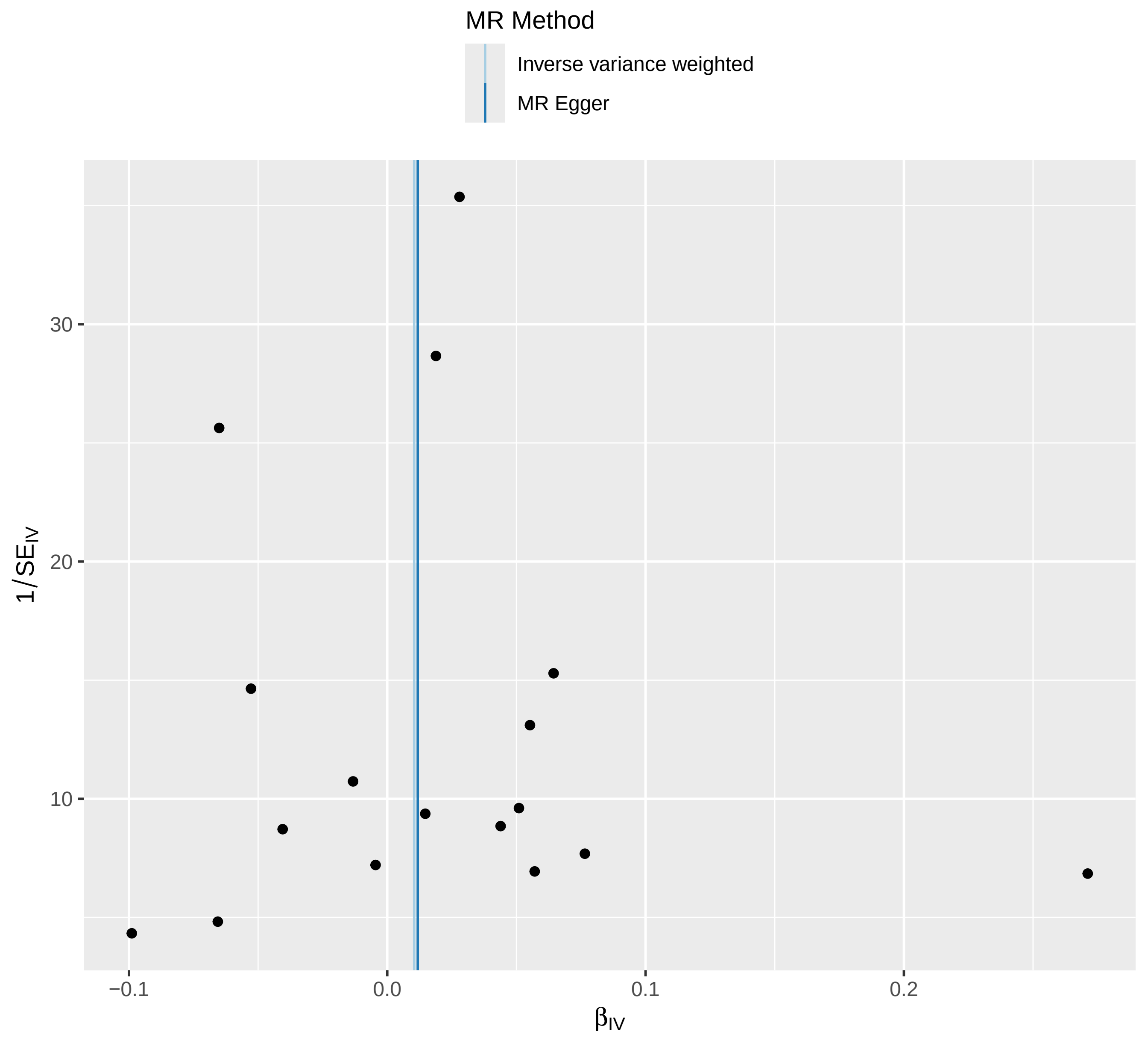
MR Method
Inverse variance weighted
MR Egger
30
20
10
−0.1
0.0
0.1
0.2
$1/SE_{IV}$
$β_{IV}$

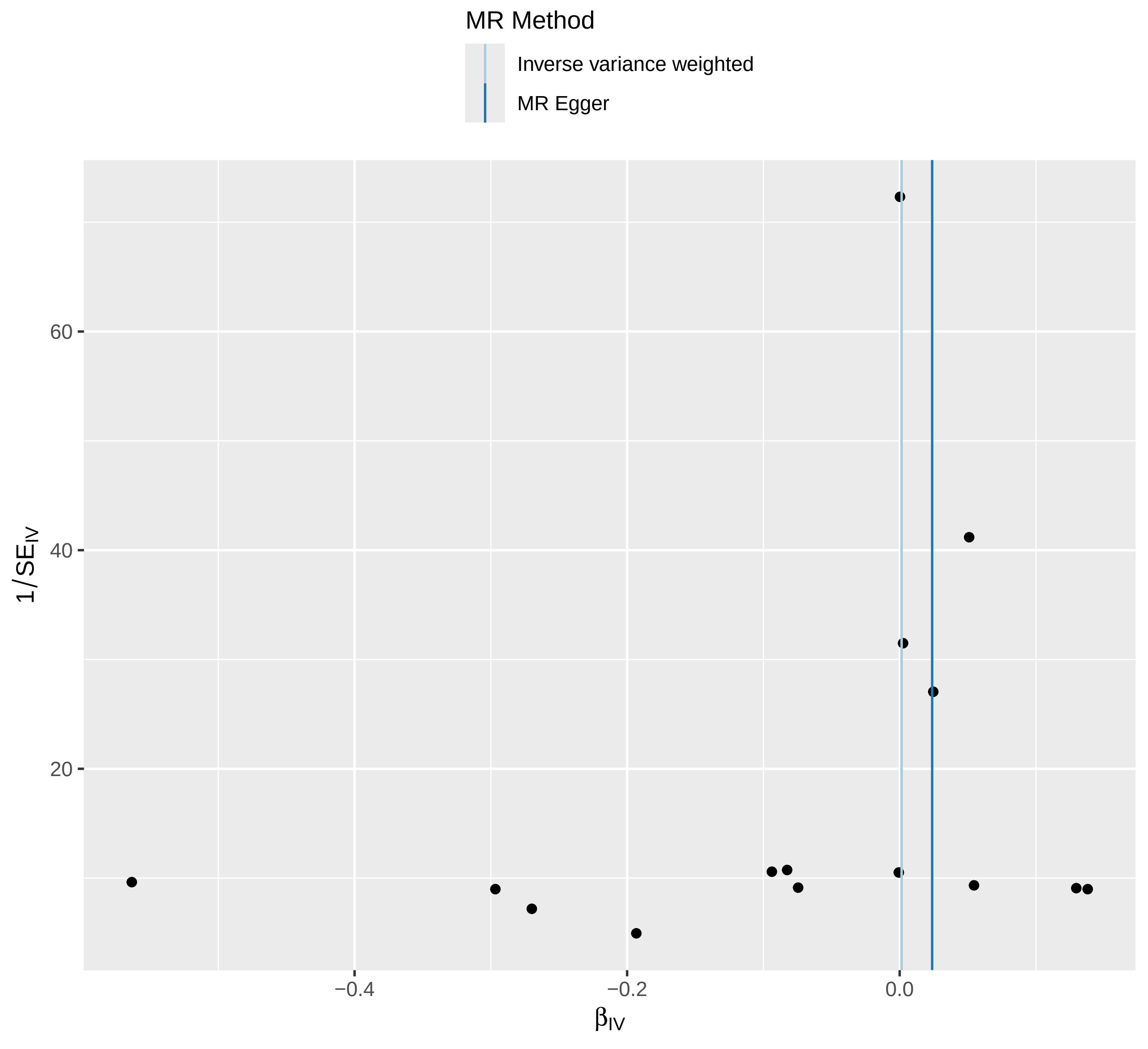
MR Method
Inverse variance weighted
MR Egger
60
40
20
1/SE_IV
−0.4
−0.2
0.0
β_IV

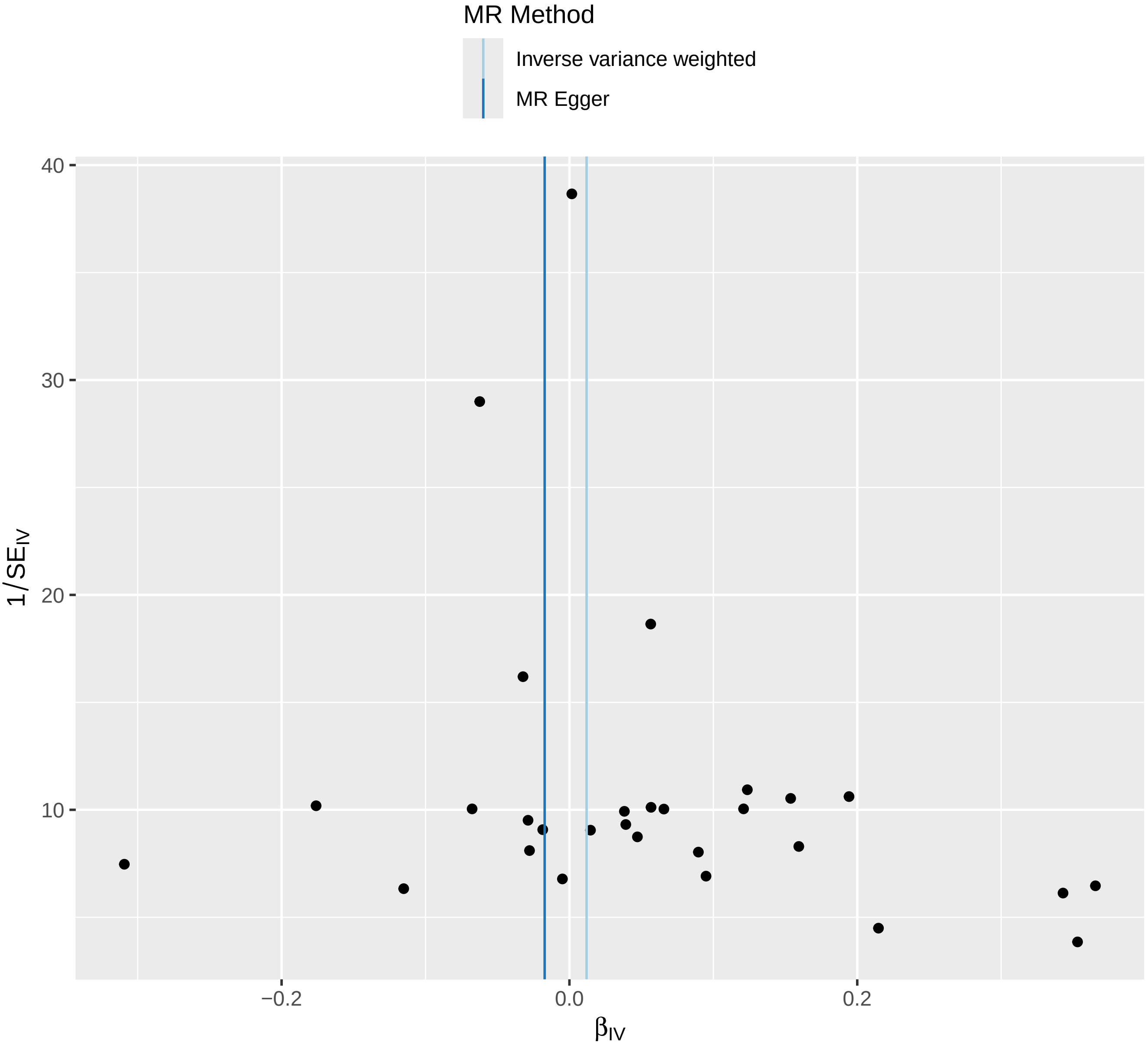
MR Method
Inverse variance weighted
MR Egger
40
30
20
10
1/SE_IV
−0.2
0.0
0.2
β_IV

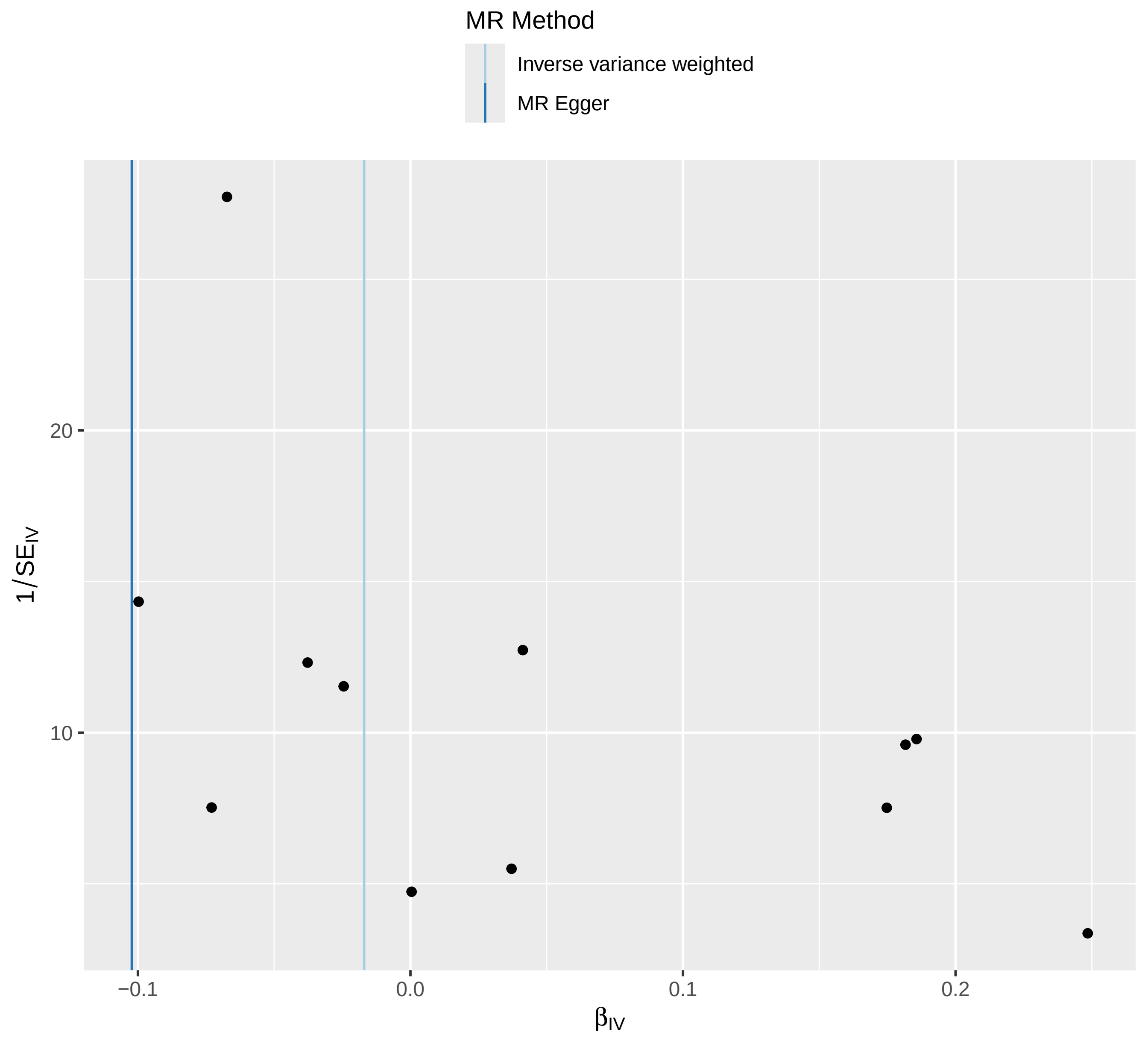
MR Method
Inverse variance weighted
MR Egger
20
10
1/SE_IV
−0.1
0.0
0.1
0.2
β_IV

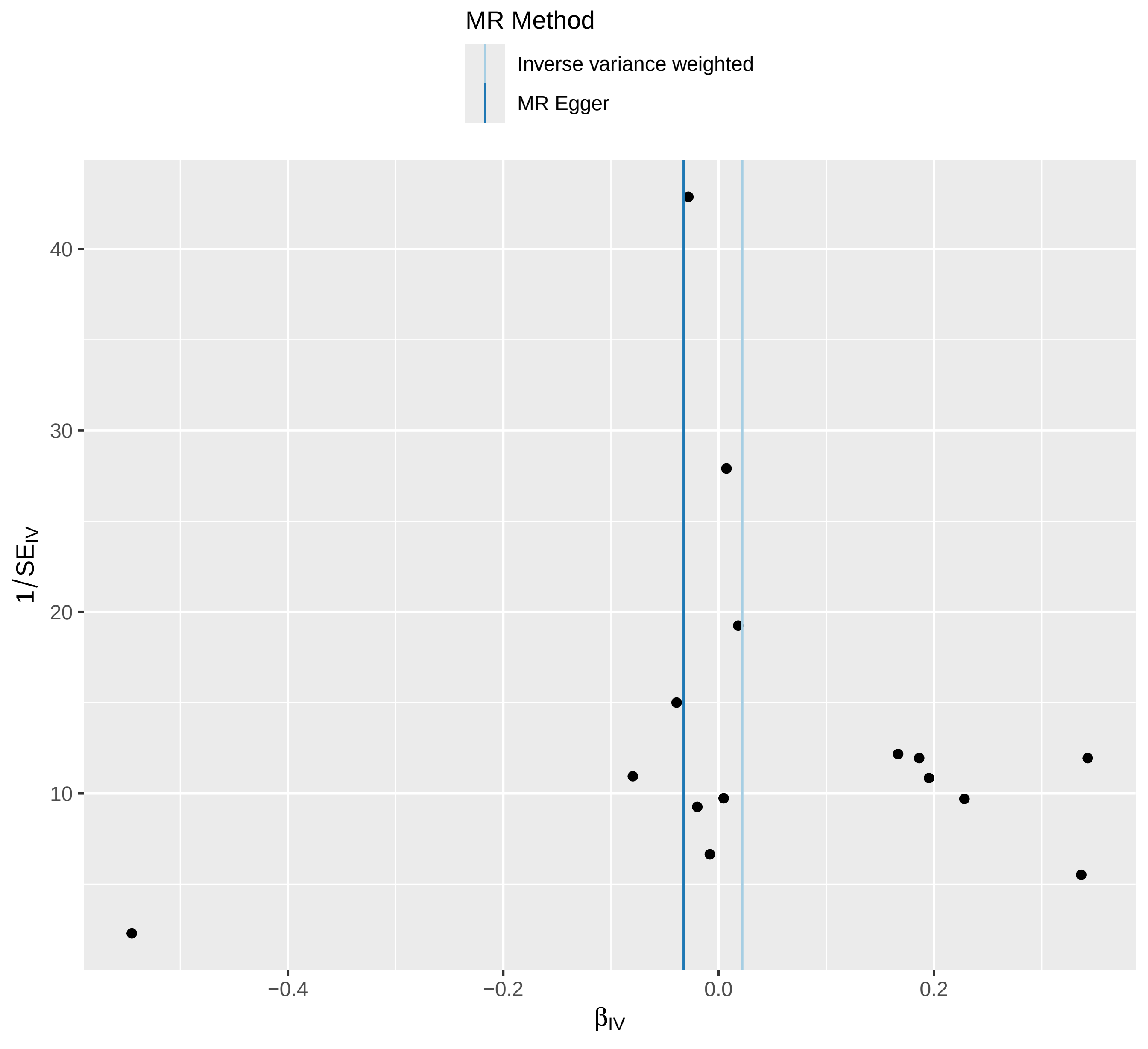
MR Method
Inverse variance weighted
MR Egger
40
30
20
10
1/SE_IV
−0.4
−0.2
0.0
0.2
β_IV

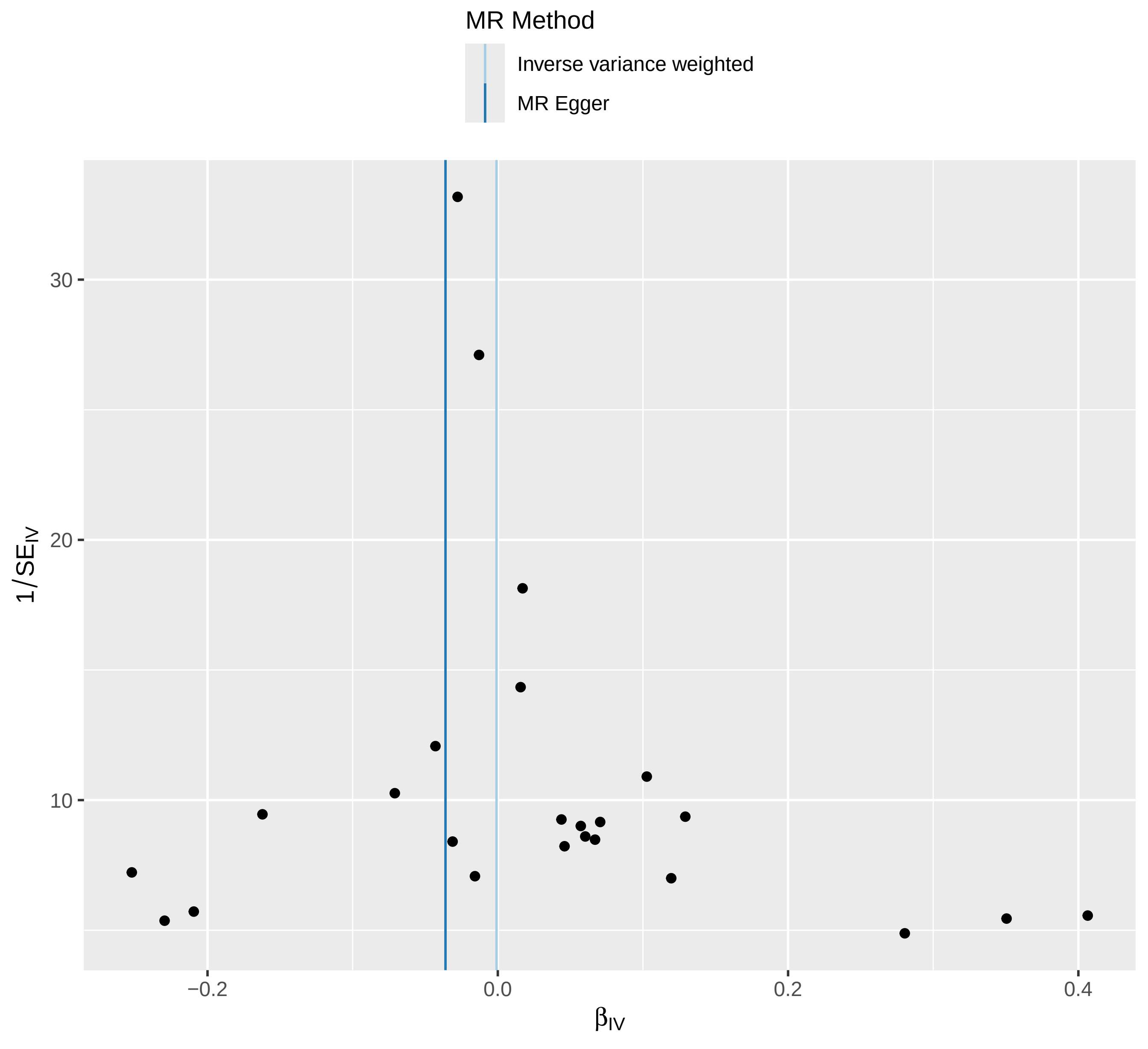
MR Method
Inverse variance weighted
MR Egger
30
20
10
1/SE_IV
−0.2
0.0
0.2
0.4
β_IV

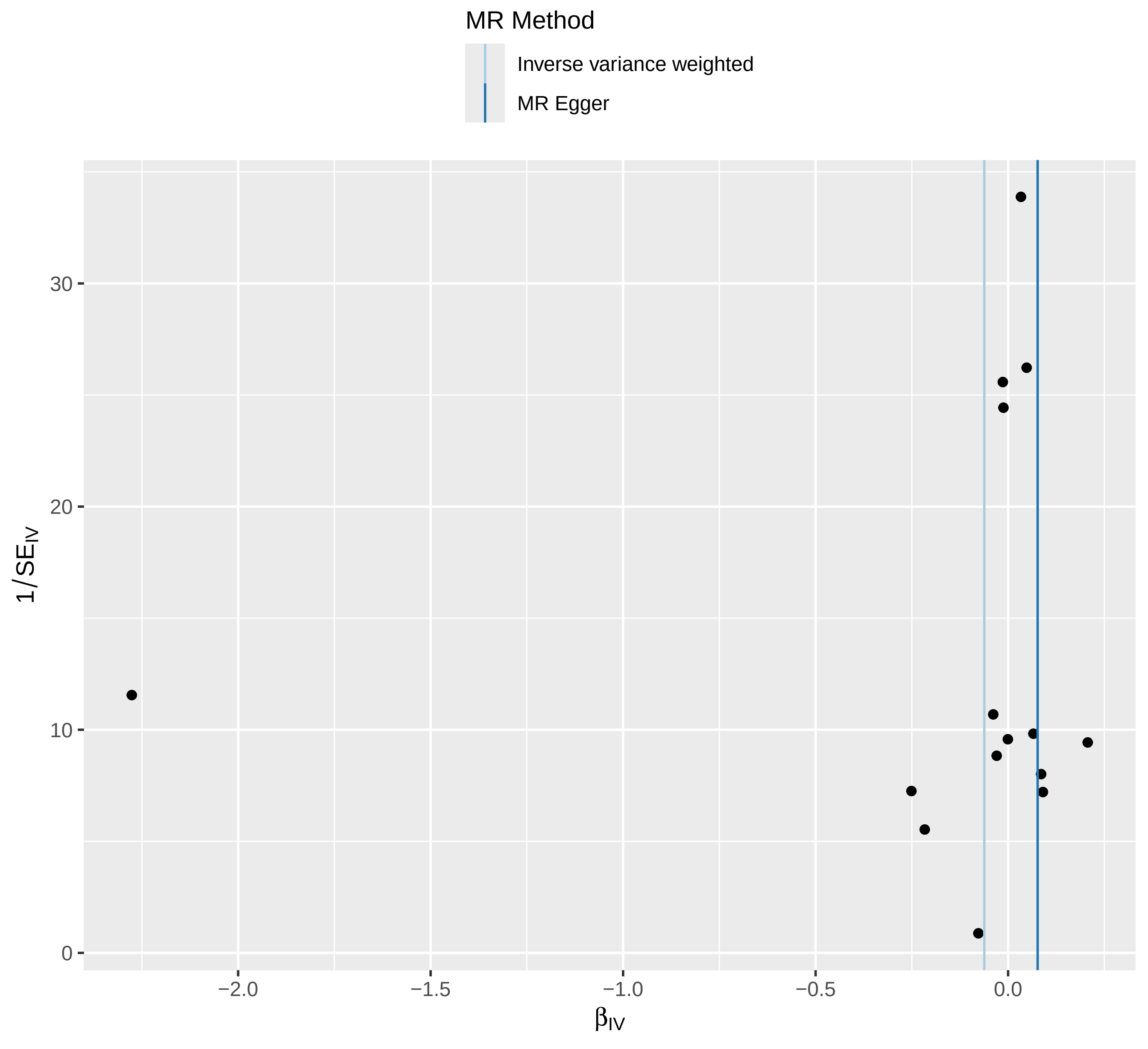
MR Method
Inverse variance weighted
MR Egger
30
20
10
0
1/SE$_{IV}$
−2.0
−1.5
−1.0
−0.5
0.0
β$_{IV}$

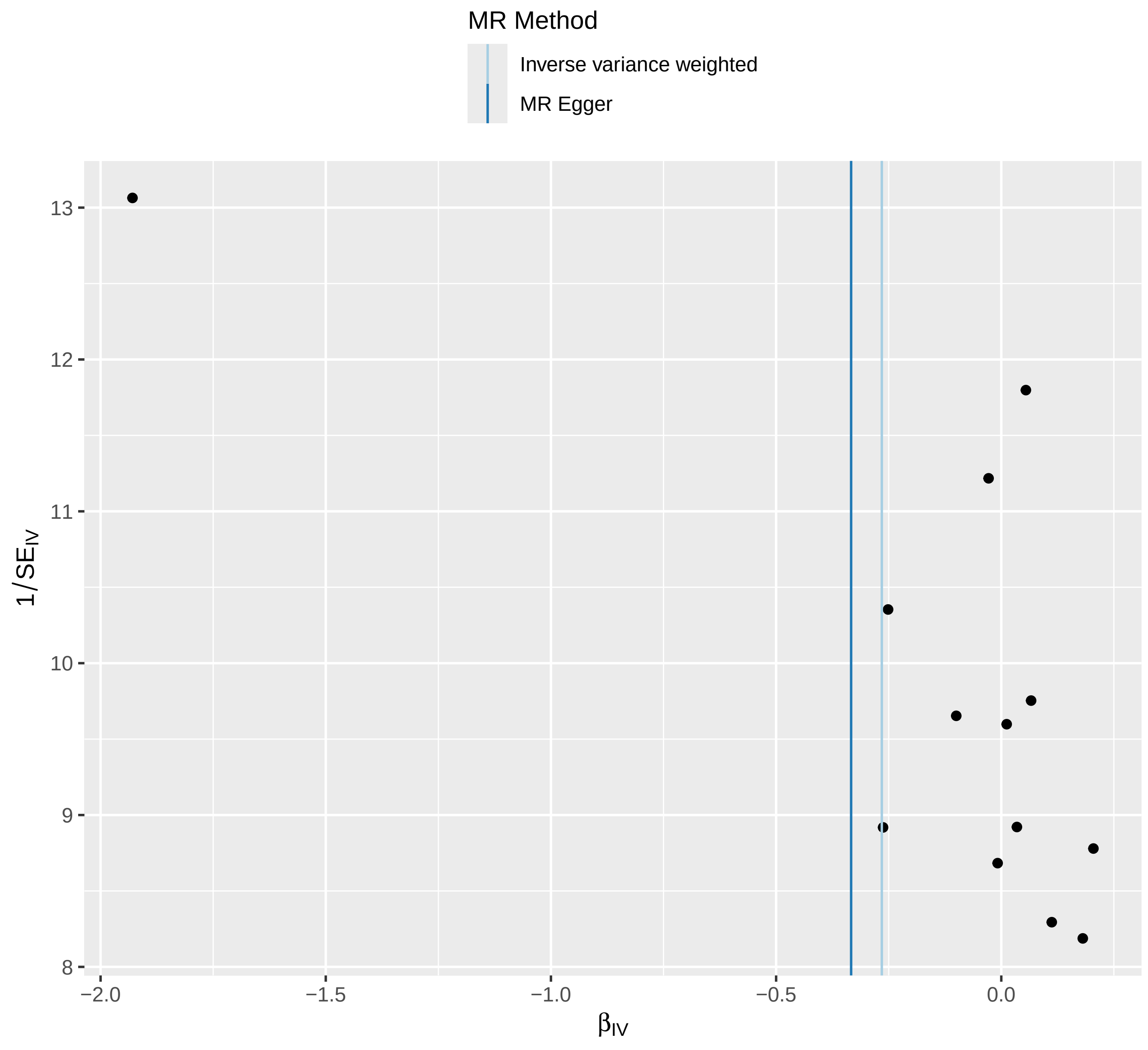
MR Method
Inverse variance weighted
MR Egger
13
12
11
10
9
8
1/SE_IV
−2.0
−1.5
−1.0
−0.5
0.0
β_IV

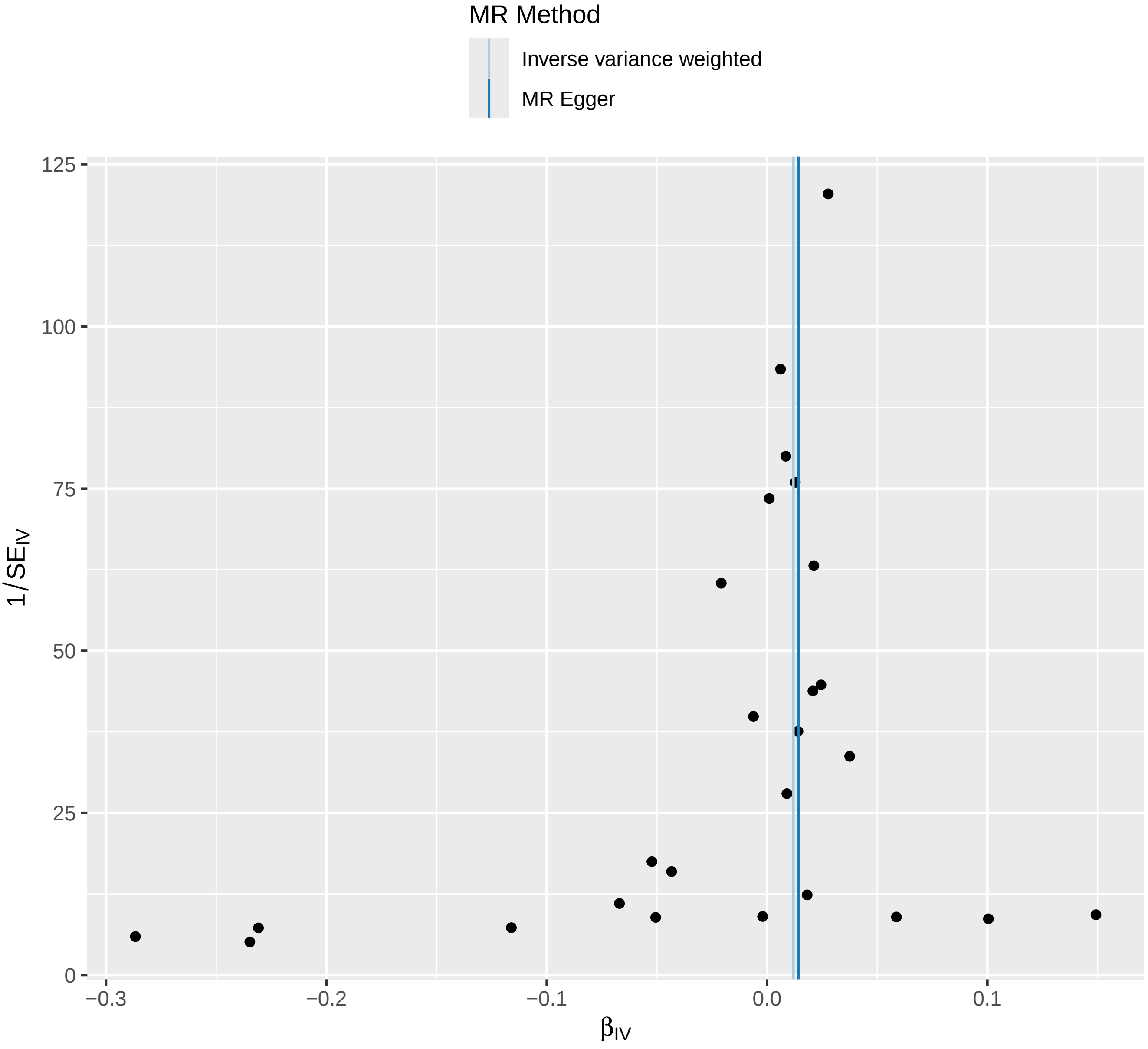
MR Method
Inverse variance weighted
MR Egger
125
100
75
50
25
0
1/SE$_{IV}$
−0.3
−0.2
−0.1
0.0
0.1
β$_{IV}$

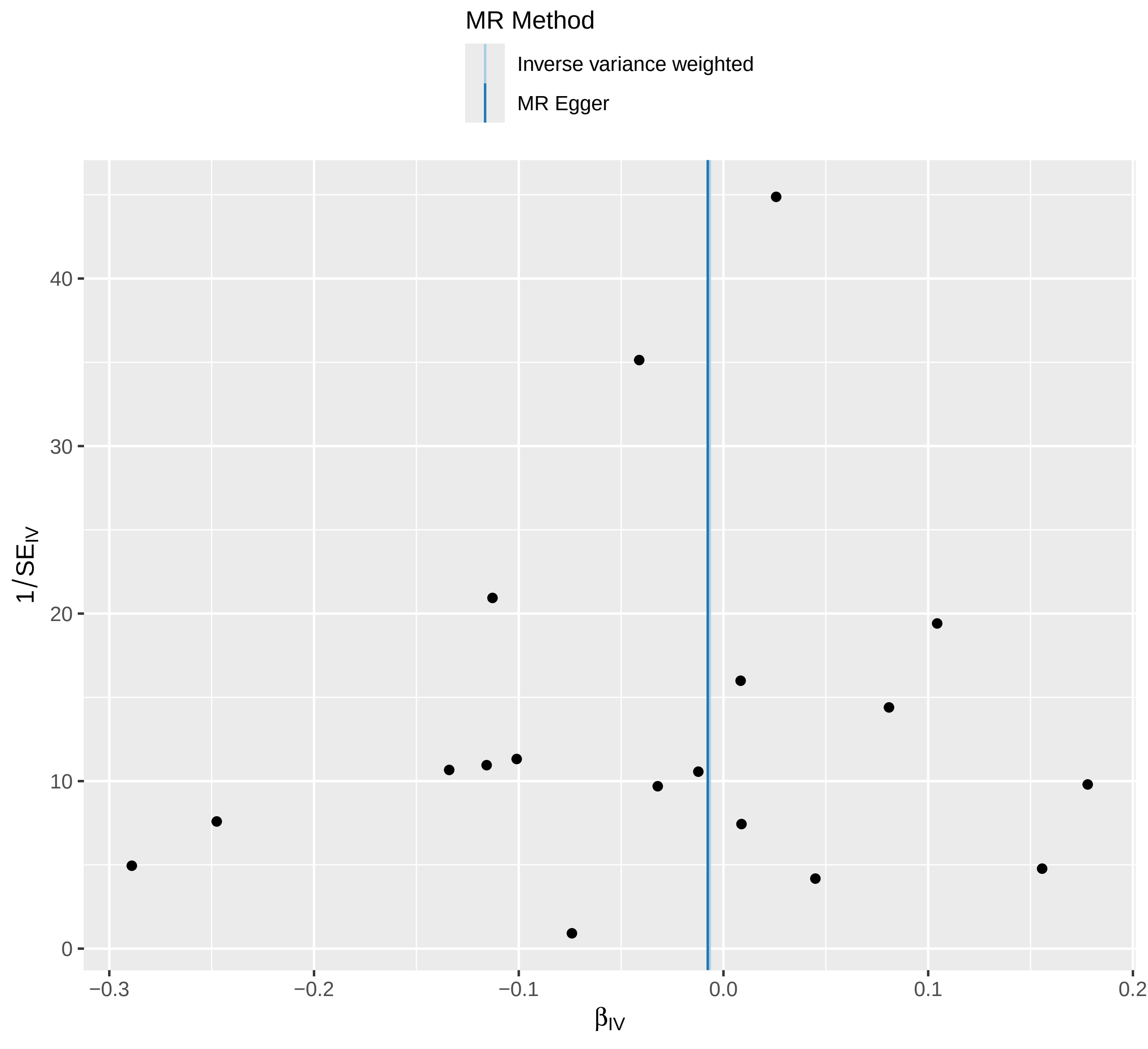

MR Method
Inverse variance weighted
MR Egger
40
30
20
10
0
1/SE$_{IV}$
−0.3
−0.2
−0.1
0.0
0.1
0.2
β$_{IV}$

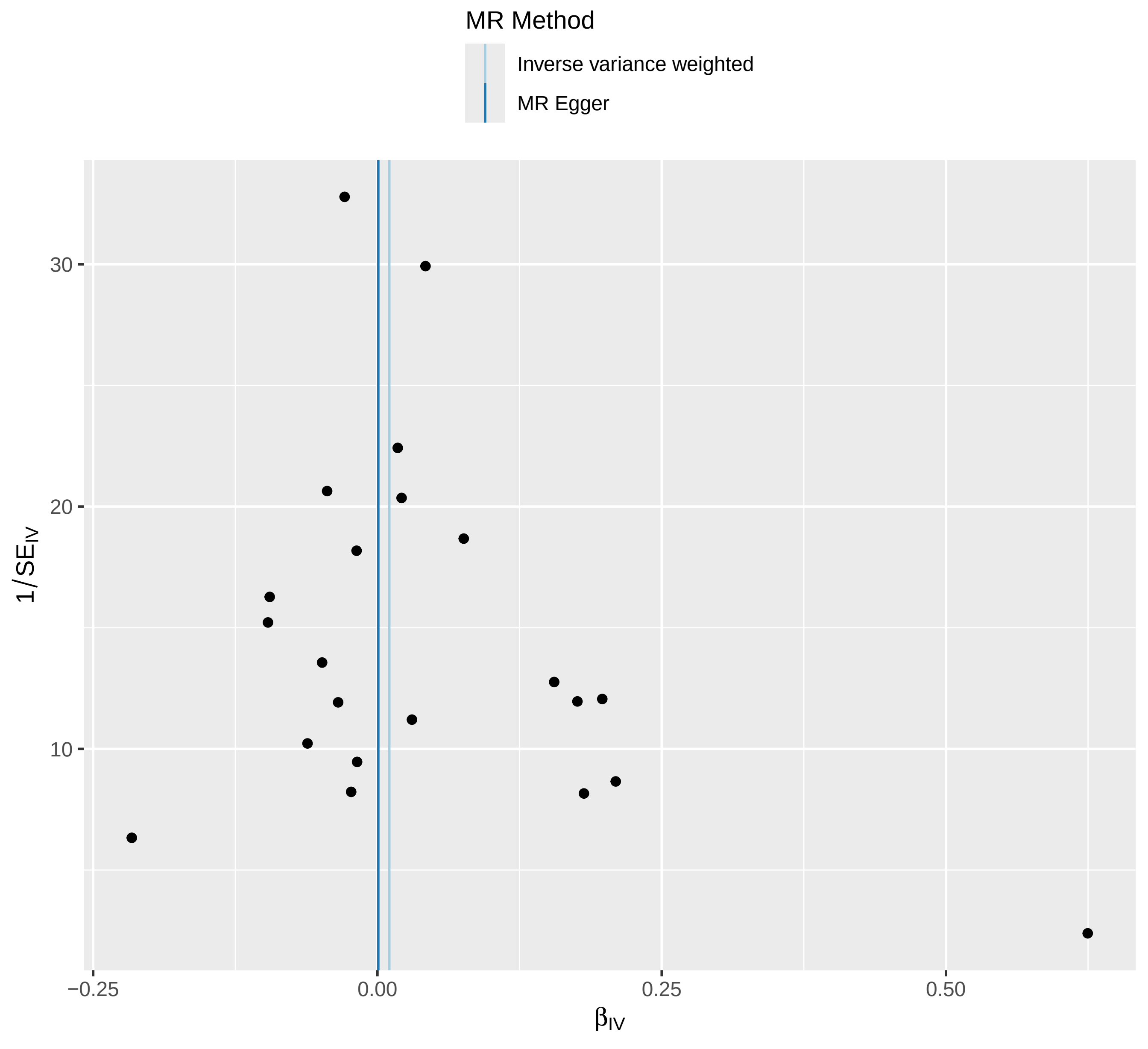
MR Method
Inverse variance weighted
MR Egger
30
20
10
1/SE_IV
−0.25
0.00
0.25
0.50
β_IV

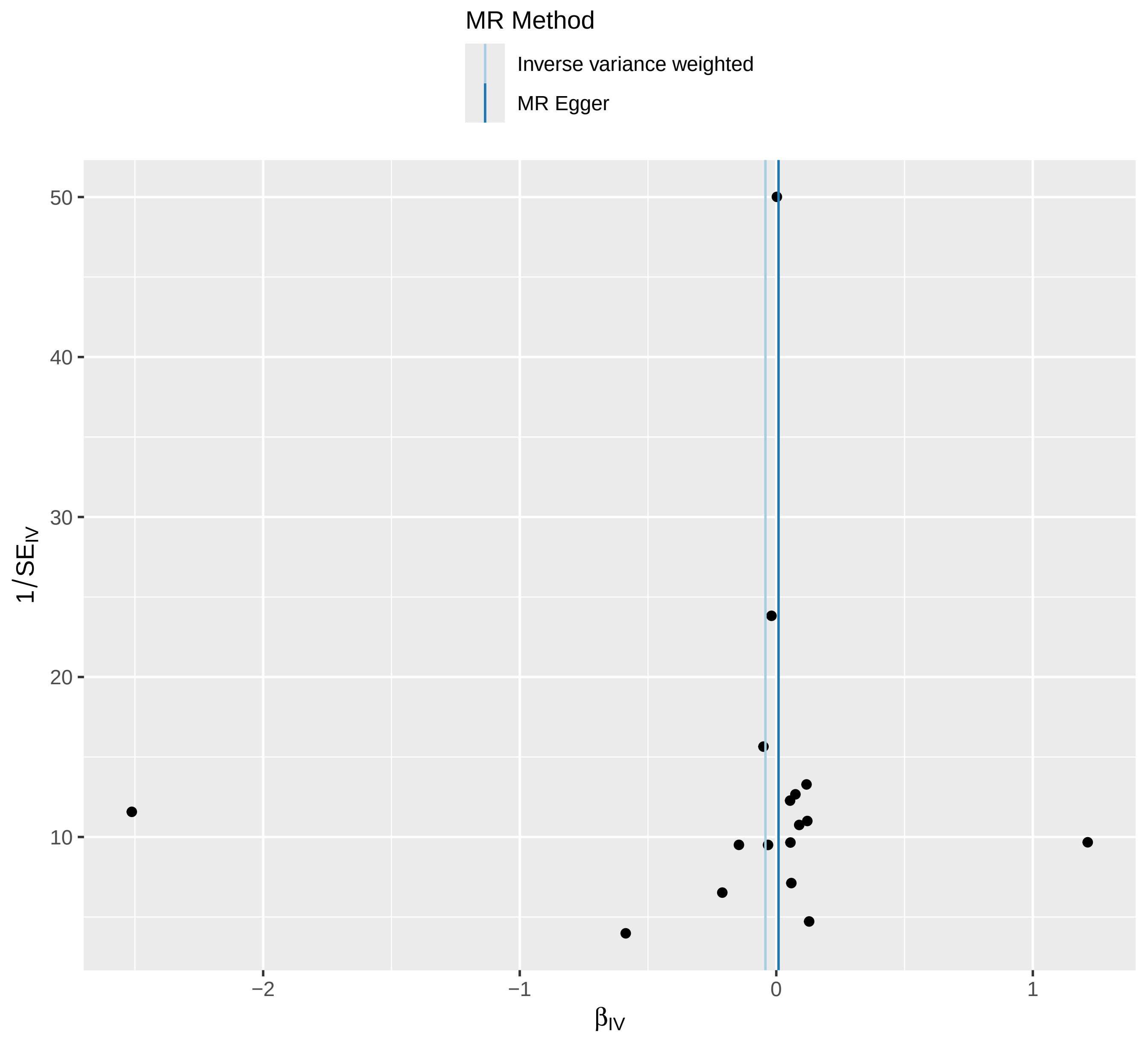
MR Method
Inverse variance weighted
MR Egger
1/SE_IV
50
40
30
20
10
−2
−1
0
1
β_IV

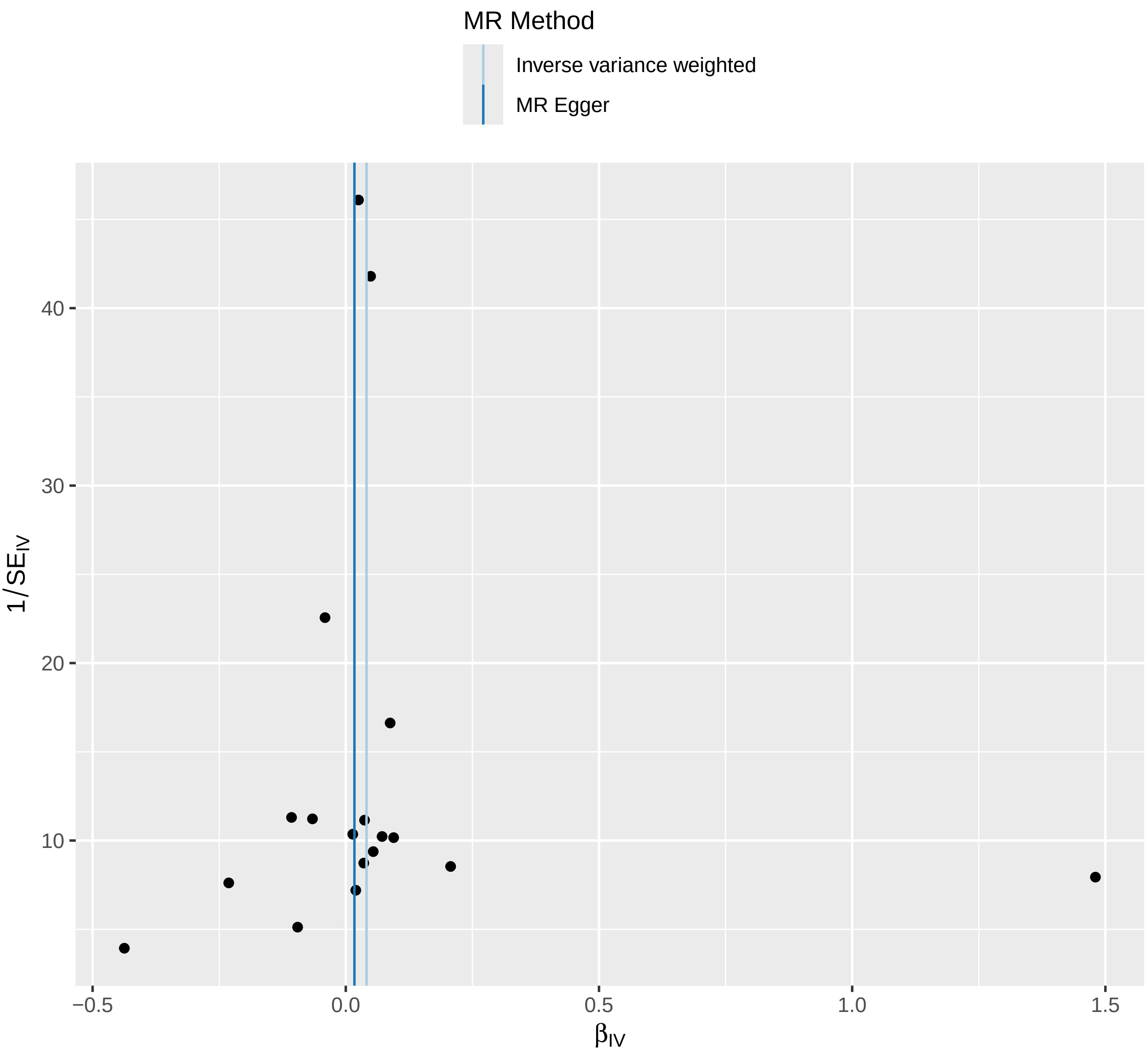

MR Method
Inverse variance weighted
MR Egger
40
30
20
10
1/SE$_{IV}$
−0.5
0.0
0.5
1.0
1.5
β$_{IV}$

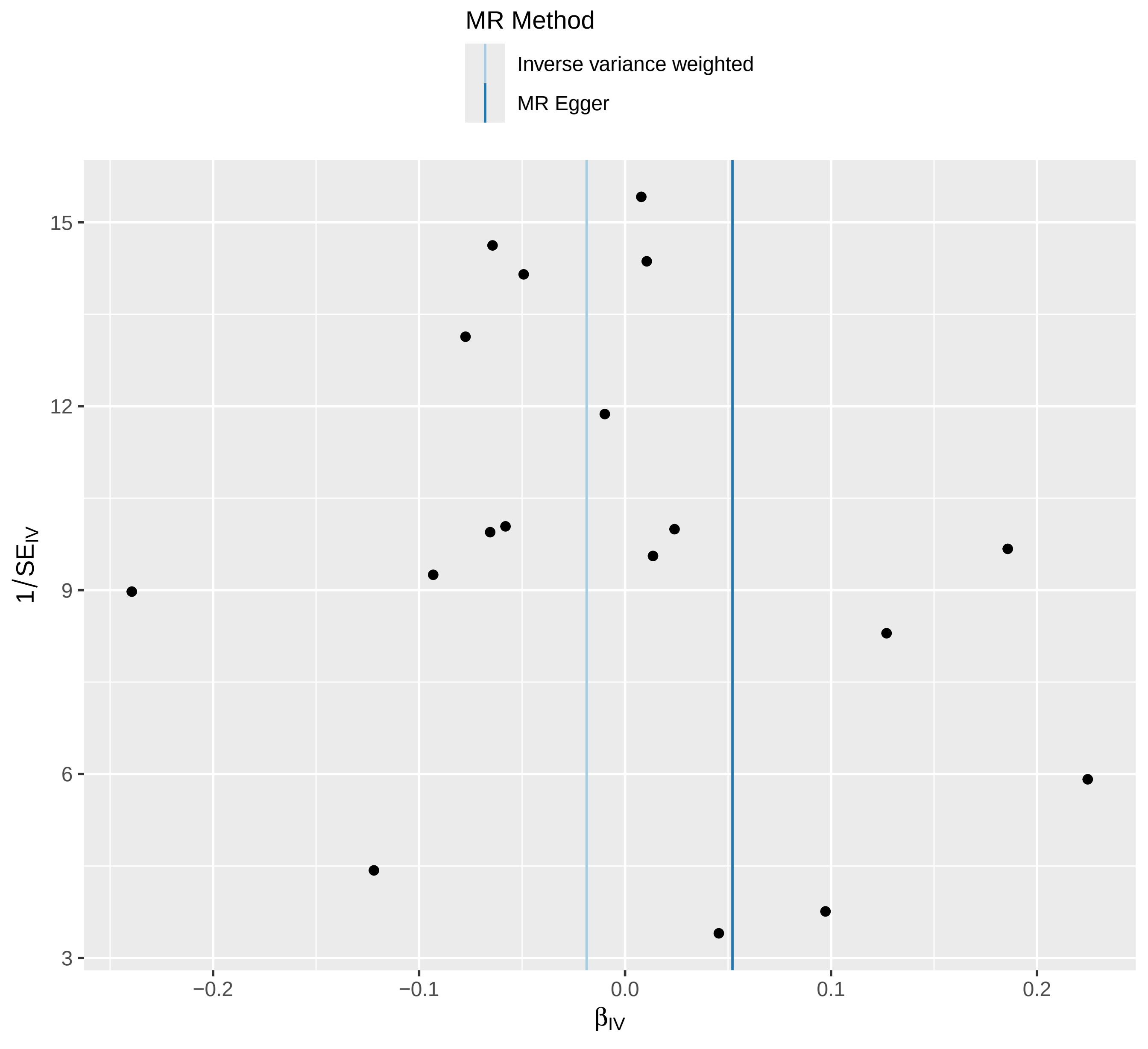

MR Method
Inverse variance weighted
MR Egger
15
12
9
6
3
1/SE_IV
−0.2
−0.1
0.0
0.1
0.2
β_IV

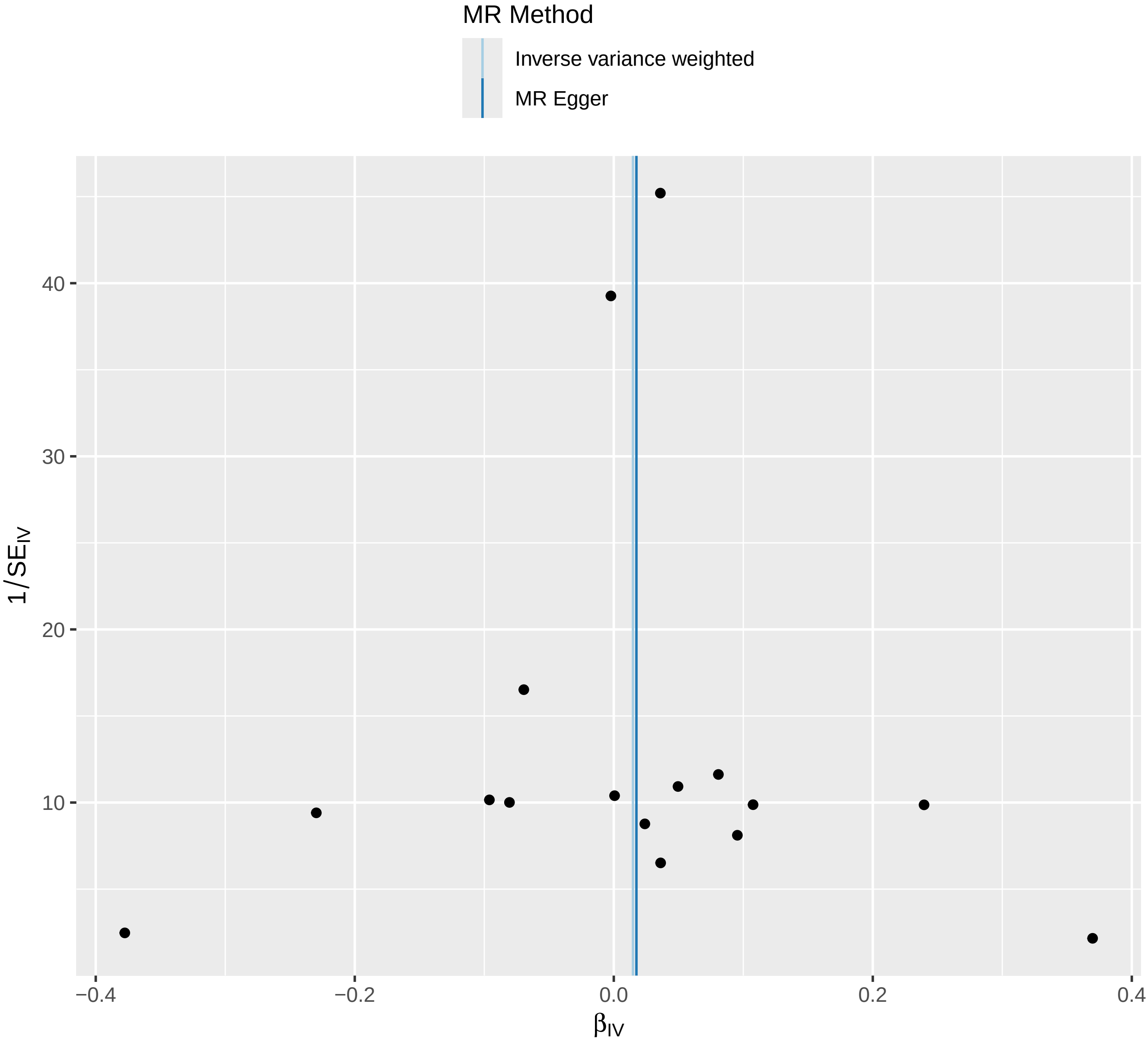
MR Method
Inverse variance weighted
MR Egger
40
30
20
10
1/SE_IV
−0.4
−0.2
0.0
0.2
0.4
β_IV

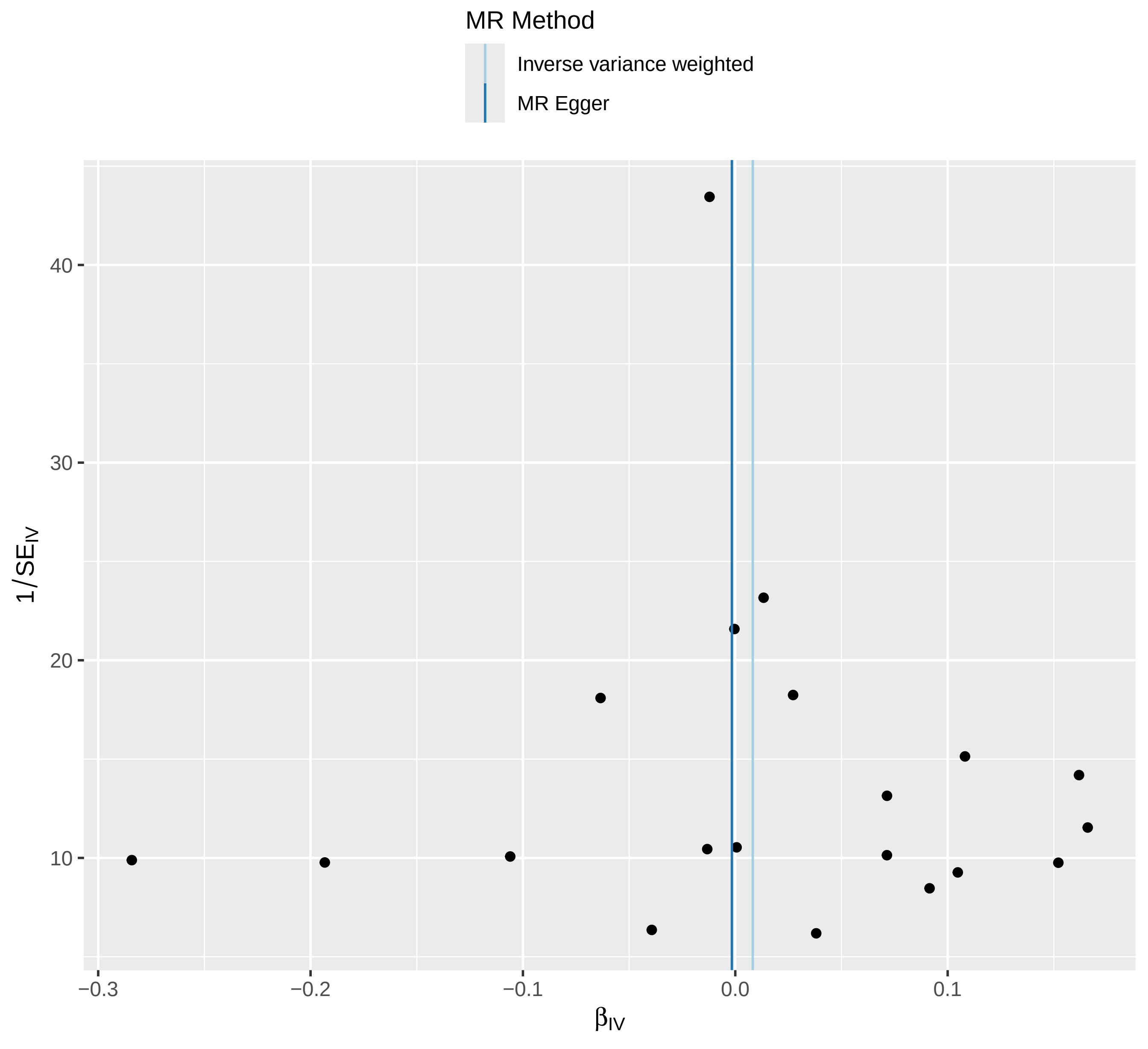
MR Method
Inverse variance weighted
MR Egger
40
30
20
10
−0.3
−0.2
−0.1
0.0
0.1
1/SE_IV
β_IV

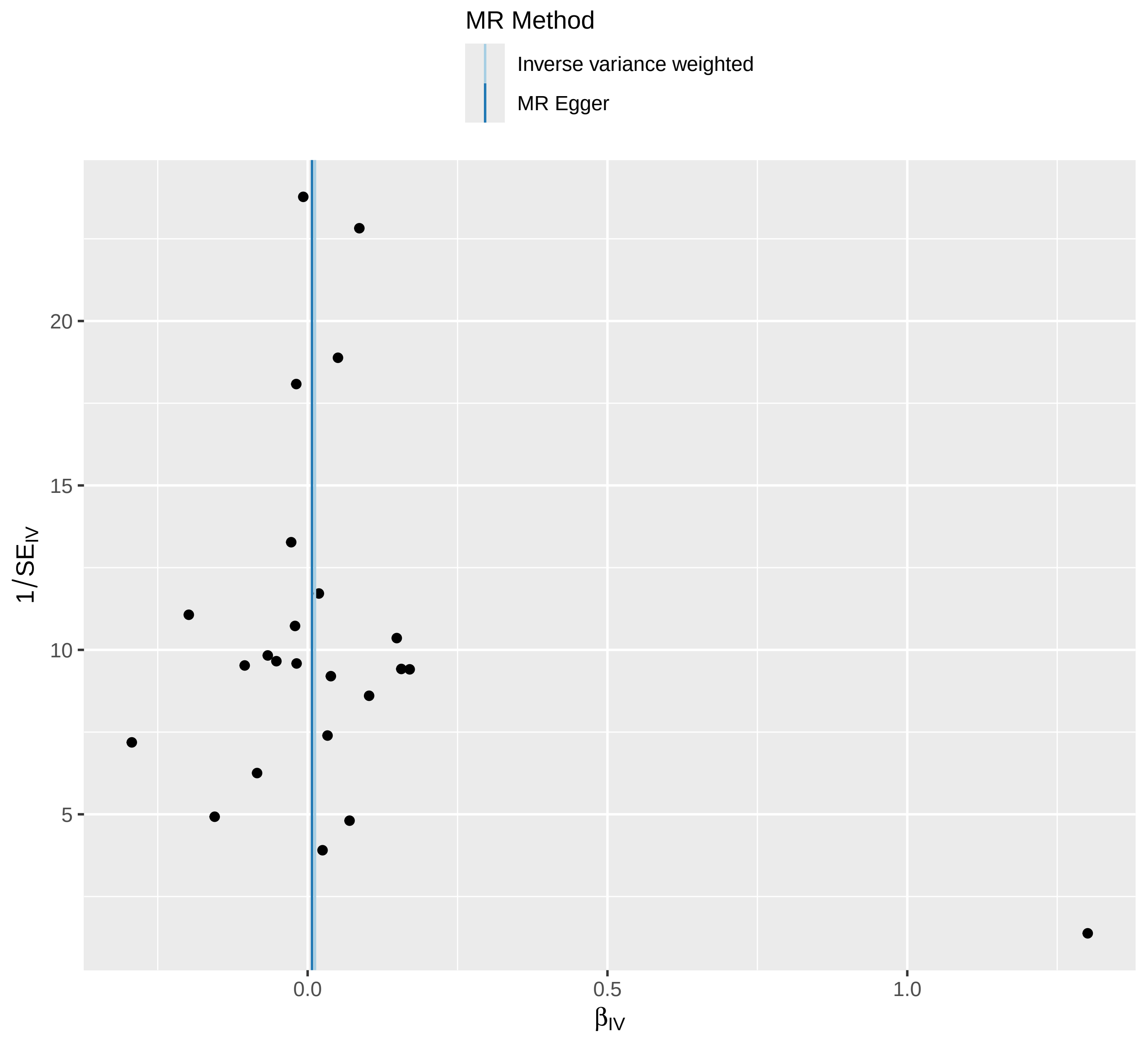
MR Method
Inverse variance weighted
MR Egger
20
15
10
5
0.0
0.5
1.0
$1/SE_{IV}$
β_{IV}

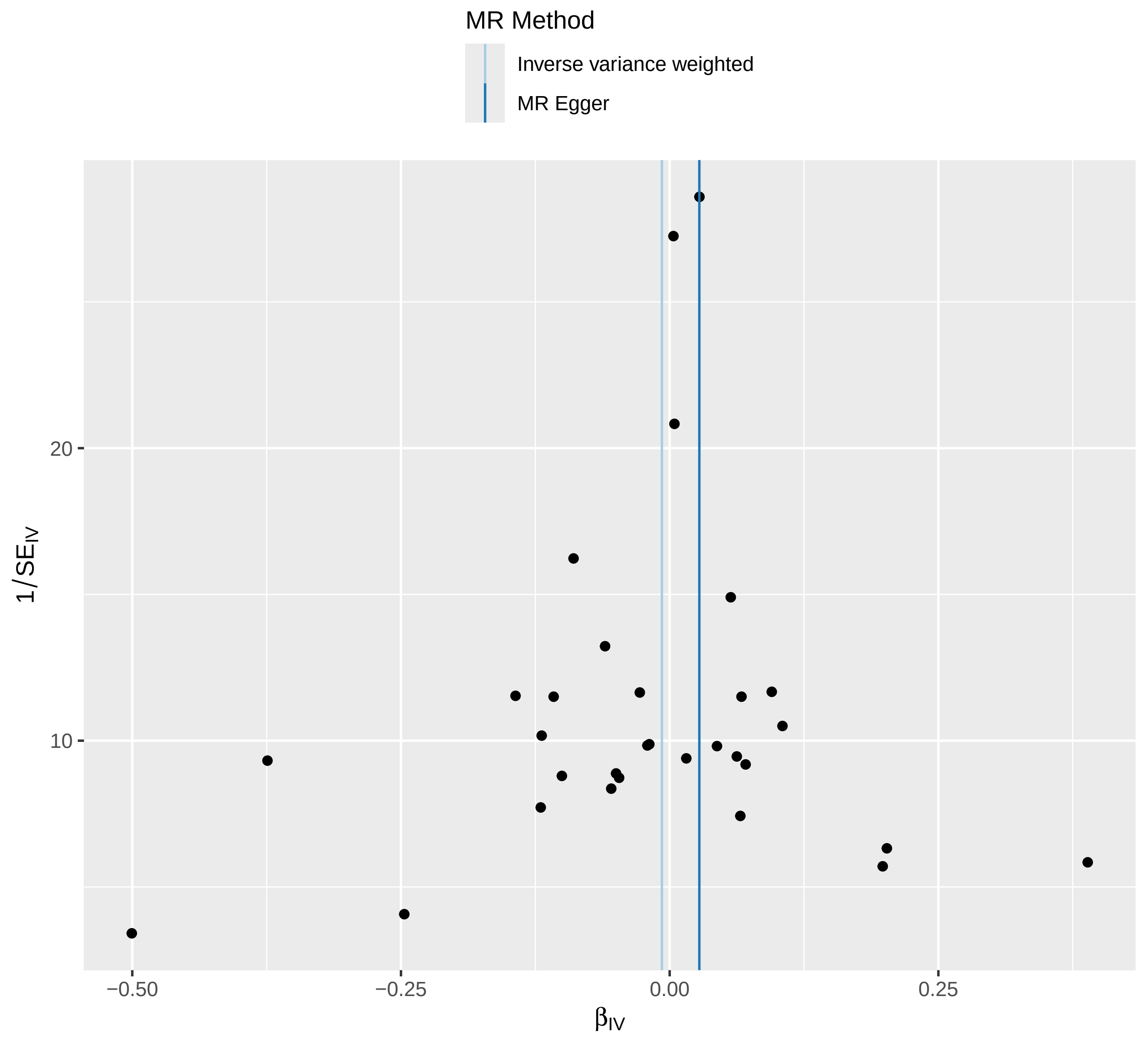
MR Method
Inverse variance weighted
MR Egger
1/SE$_{IV}$
20
10
−0.50
−0.25
0.00
0.25
β$_{IV}$

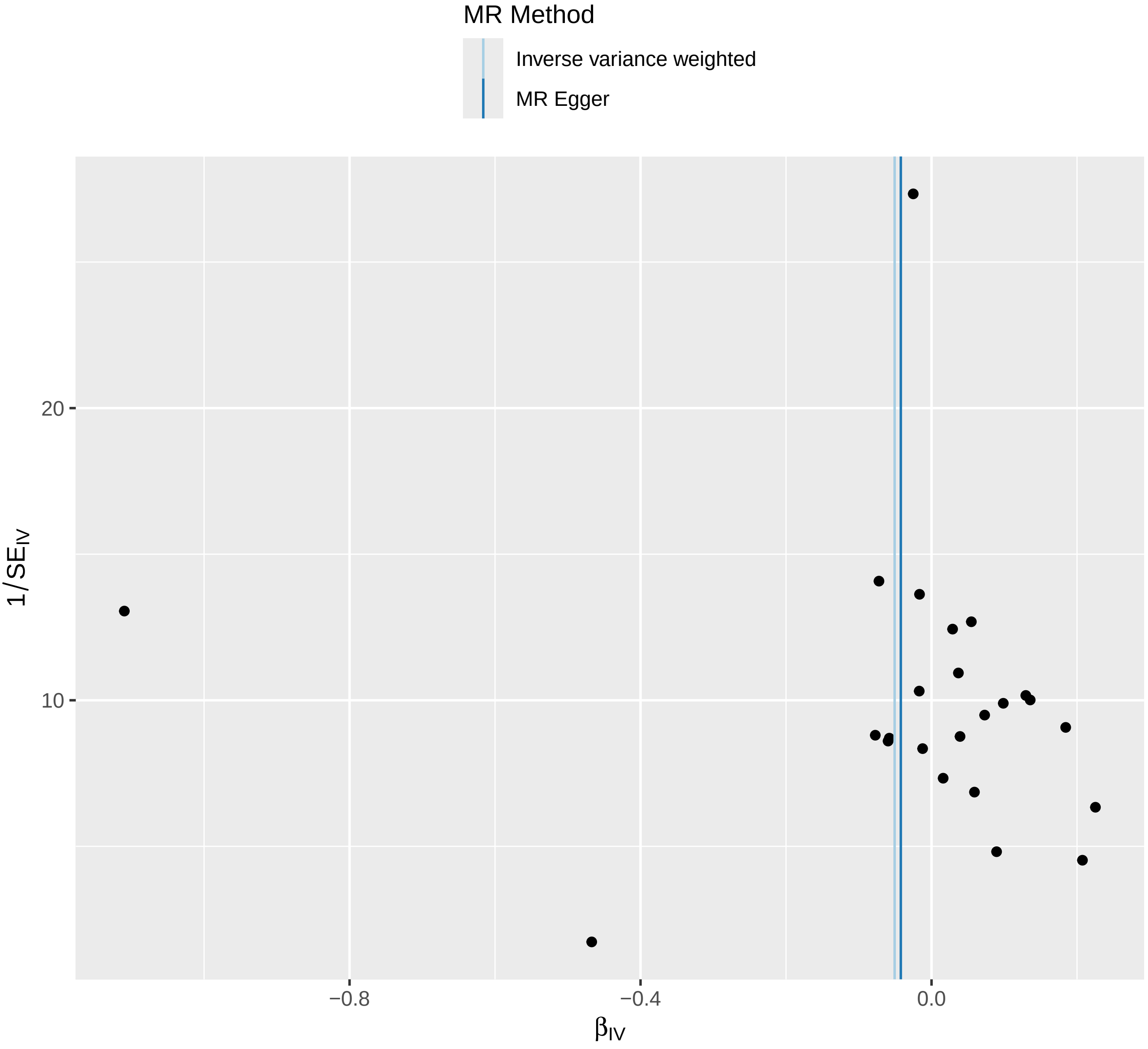
MR Method
Inverse variance weighted
MR Egger
20
10
1/SE$_{IV}$
−0.8
−0.4
0.0
β$_{IV}$

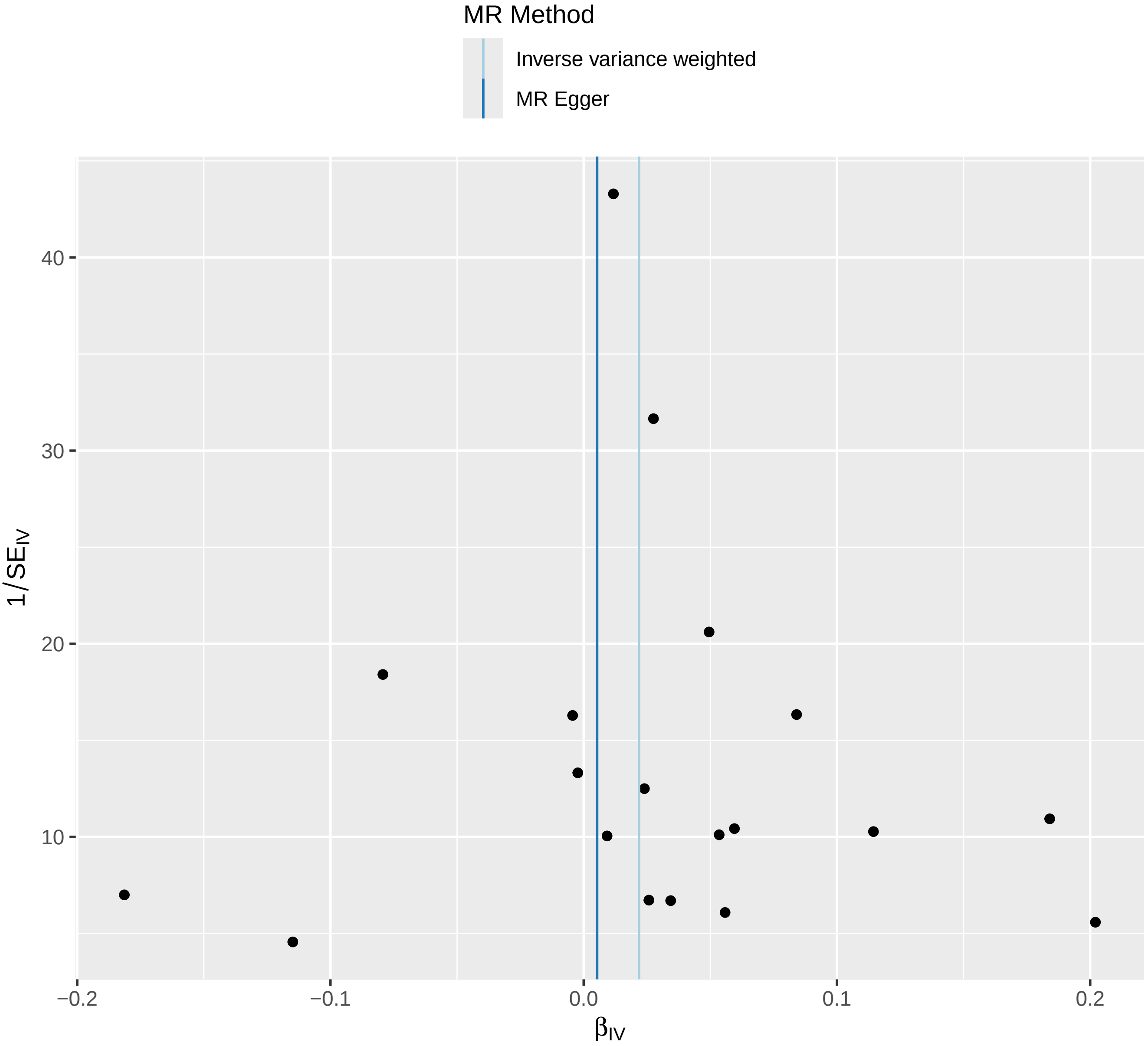
MR Method
Inverse variance weighted
MR Egger
40
30
20
10
1/SE_IV
−0.2
−0.1
0.0
0.1
0.2
β_IV

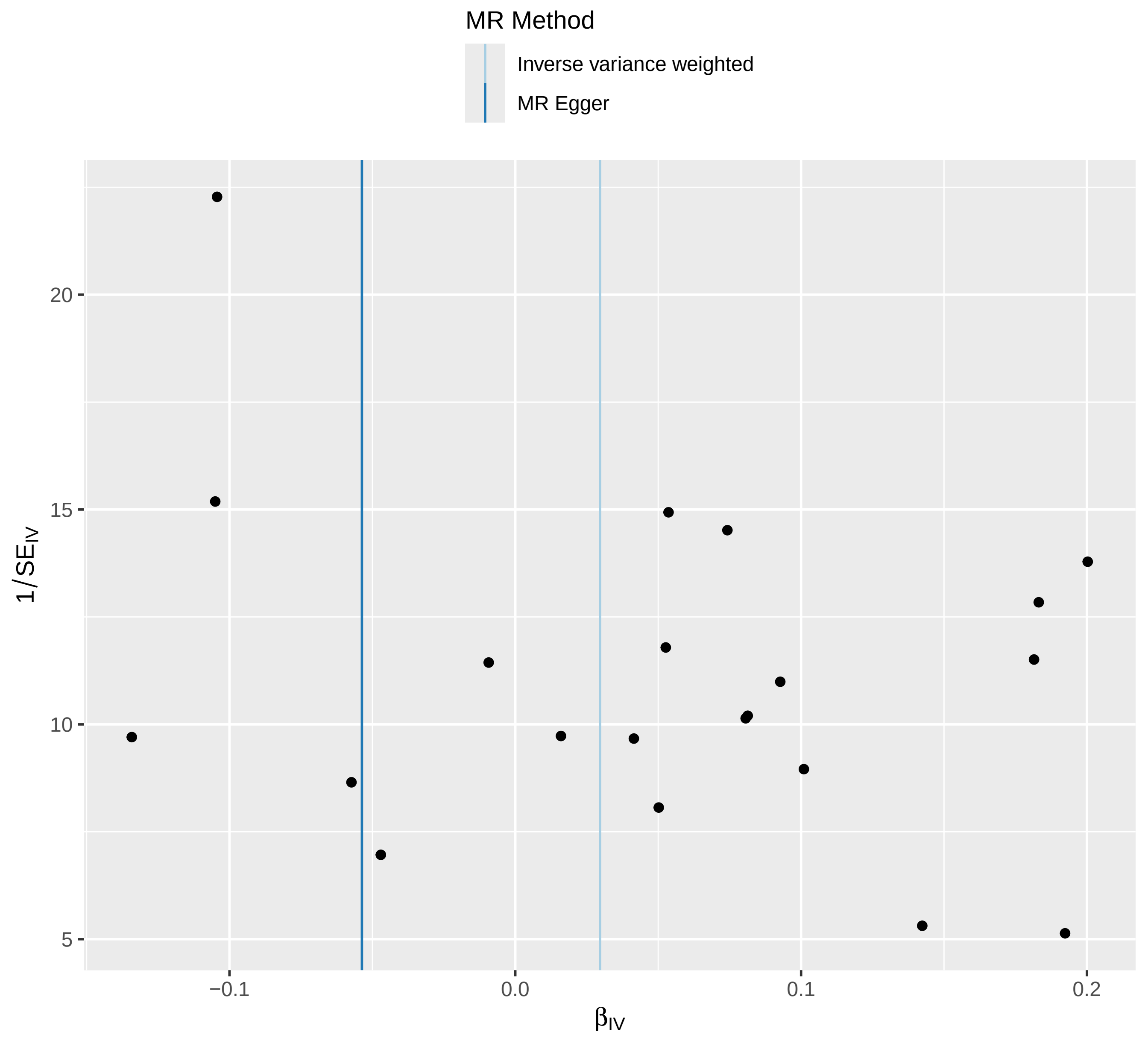
MR Method
Inverse variance weighted
MR Egger
20
15
10
5
−0.1
0.0
0.1
0.2
$1/SE_{IV}$
β_{IV}

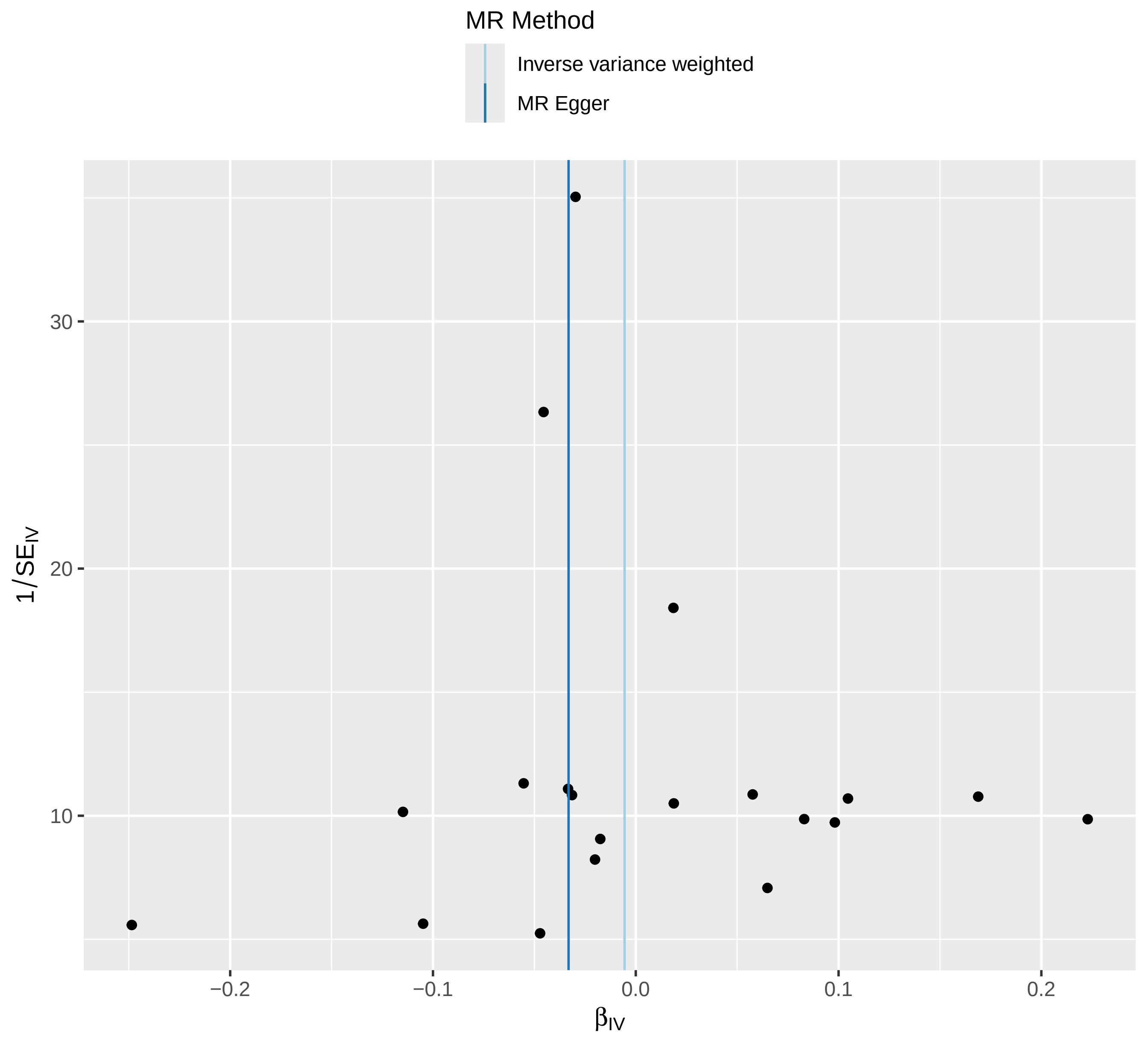
MR Method
Inverse variance weighted
MR Egger
30
20
10
−0.2
−0.1
0.0
0.1
0.2
$1/SE_{IV}$
β_{IV}

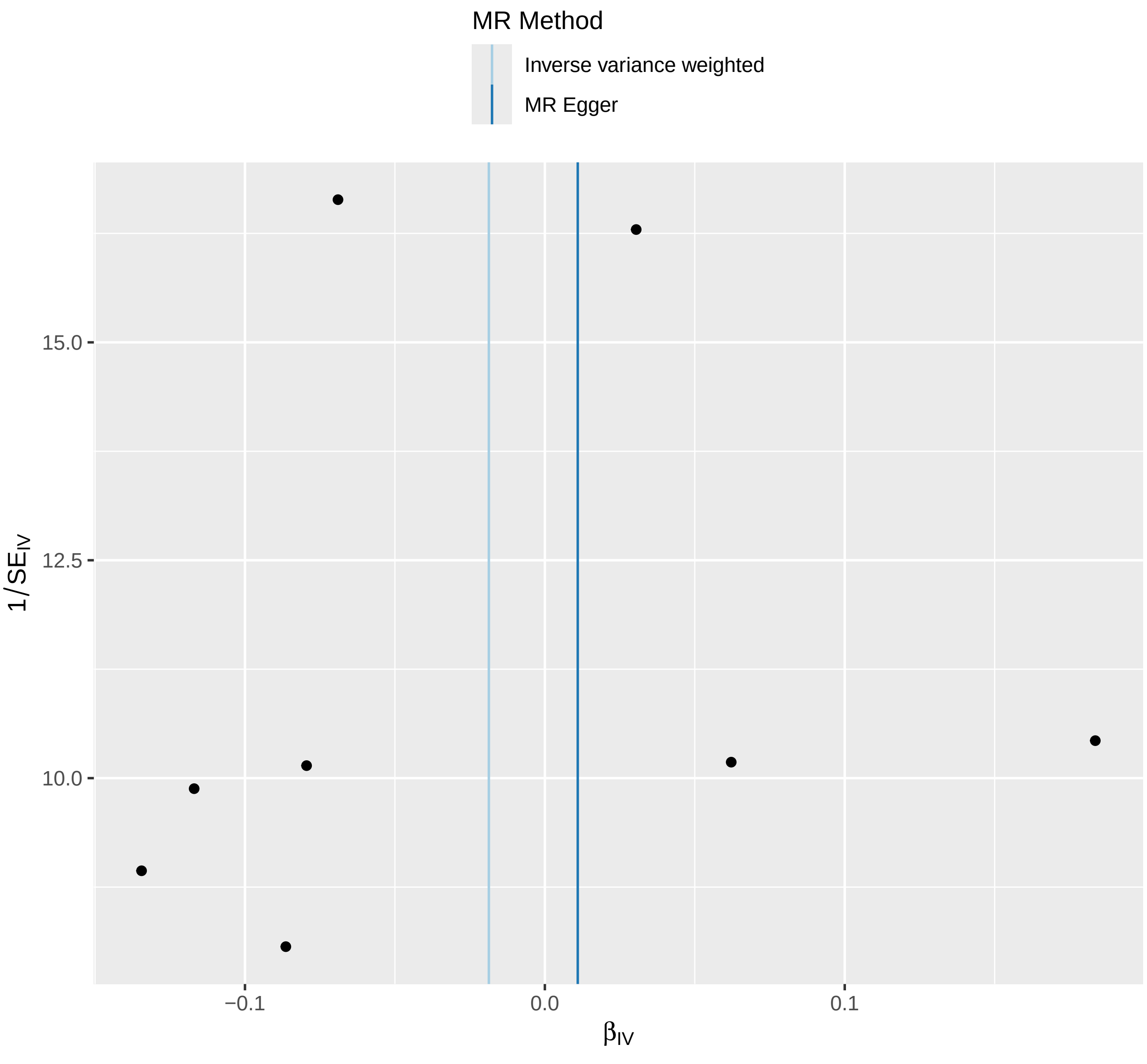

MR Method
Inverse variance weighted
MR Egger
15.0
12.5
10.0
1/SE_IV
−0.1
0.0
0.1
β_IV

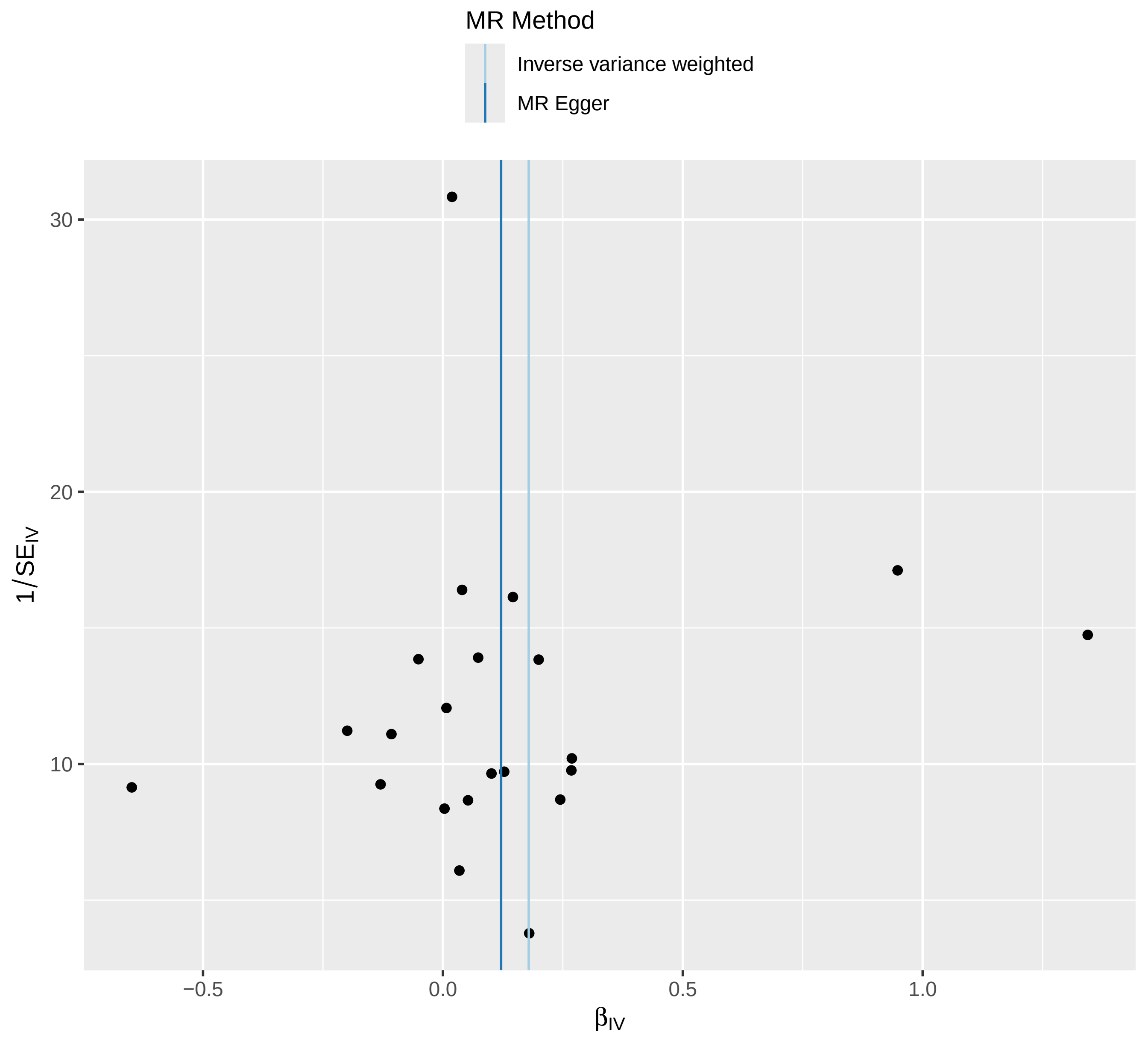
MR Method
Inverse variance weighted
MR Egger
30
20
10
1/SE_IV
−0.5
0.0
0.5
1.0
β_IV

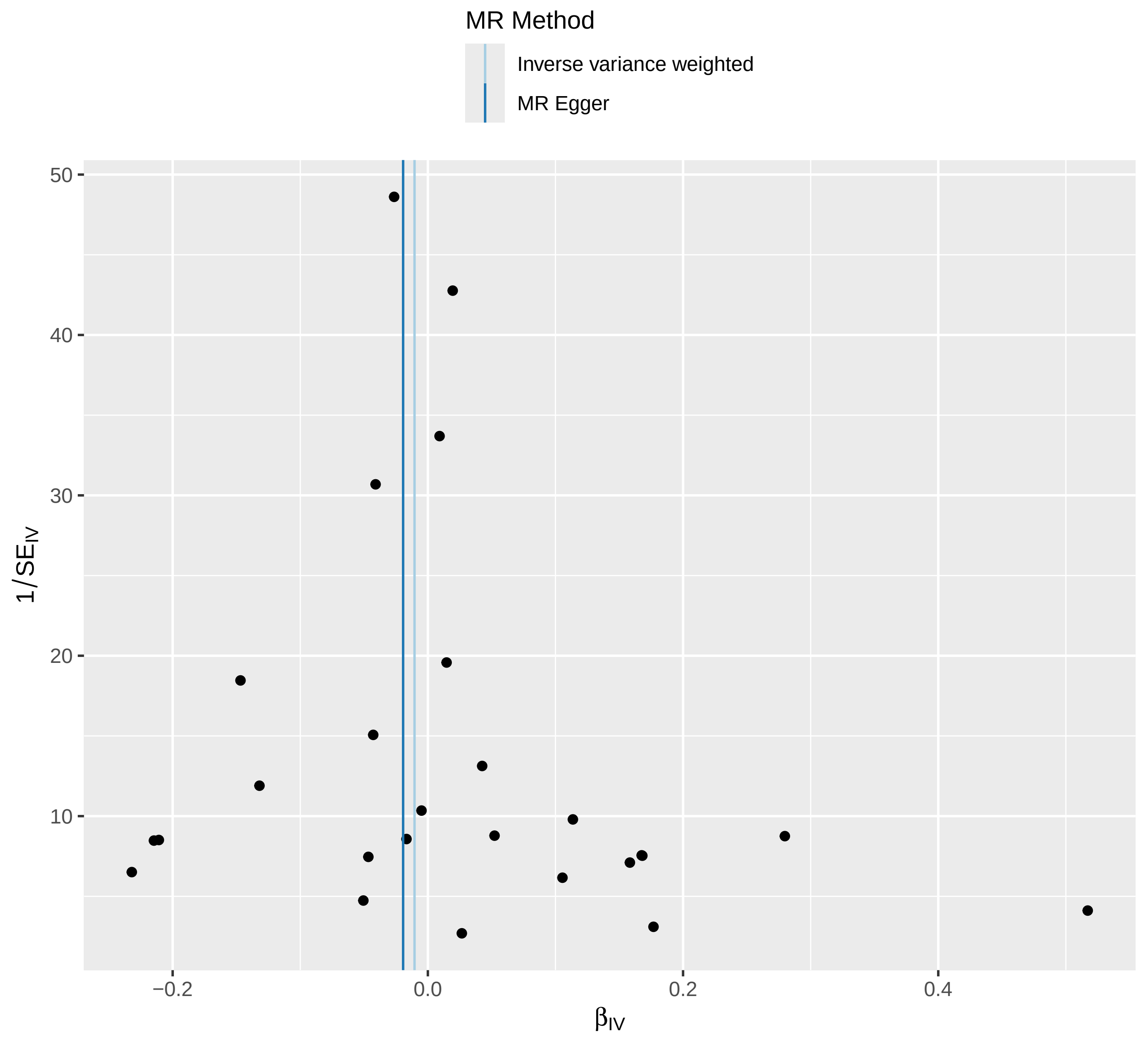
MR Method
Inverse variance weighted
MR Egger
50
40
30
20
10
1/SE_IV
−0.2
0.0
0.2
0.4
β_IV

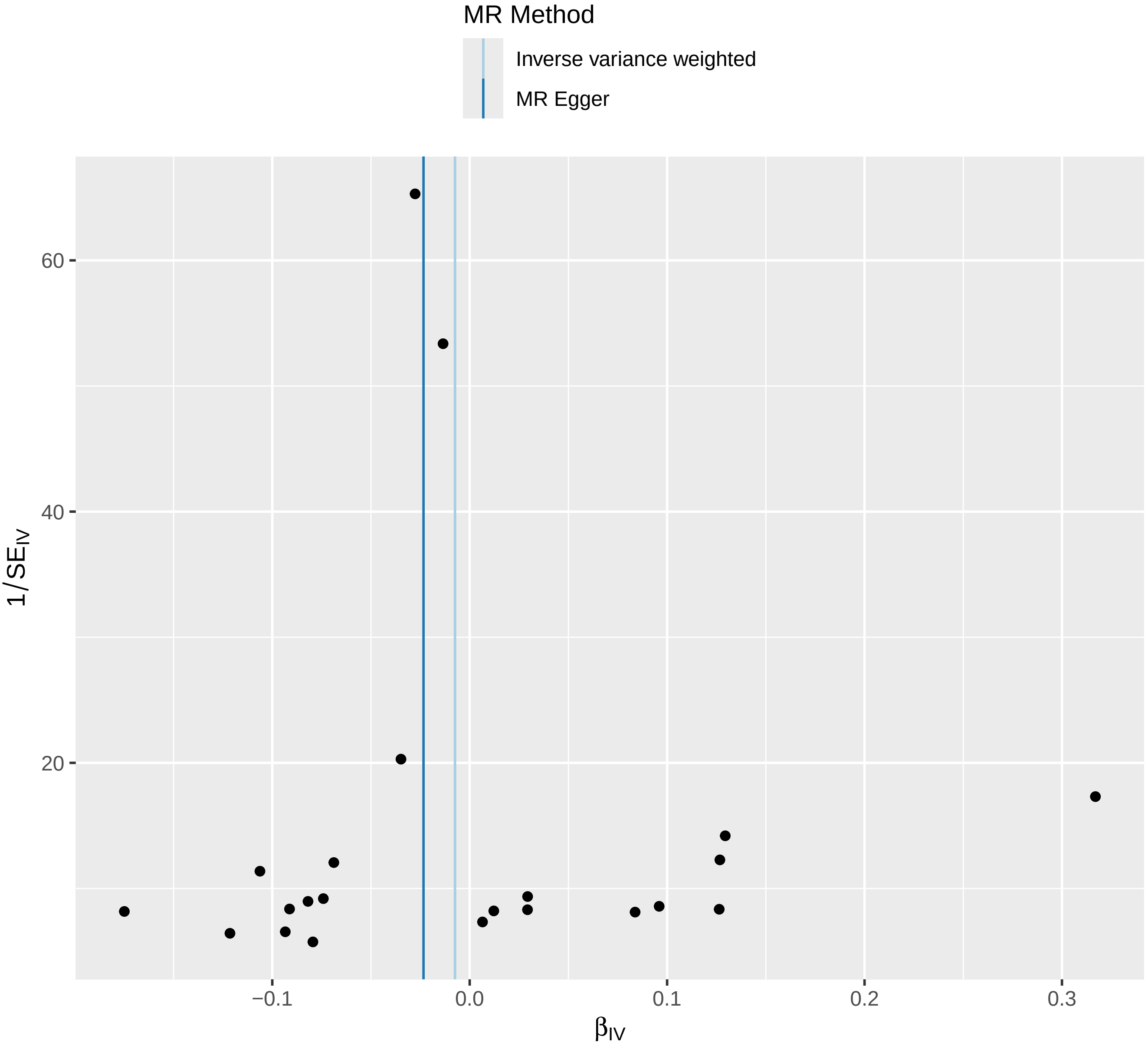
MR Method
Inverse variance weighted
MR Egger
60
40
20
1/SE$_{IV}$
−0.1
0.0
0.1
0.2
0.3
β$_{IV}$

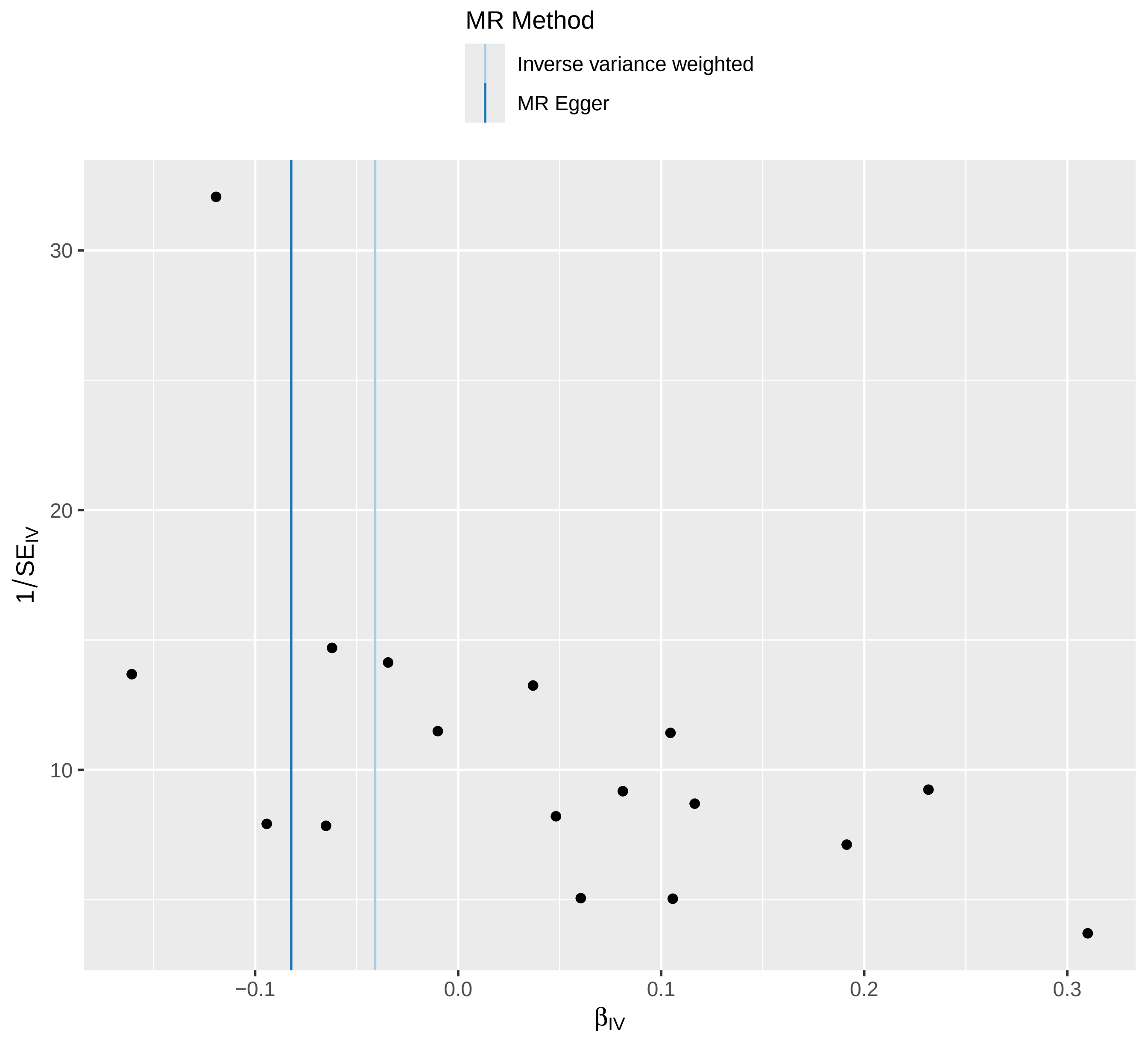

MR Method
Inverse variance weighted
MR Egger
30
20
10
1/SE$_{IV}$
−0.1
0.0
0.1
0.2
0.3
β$_{IV}$

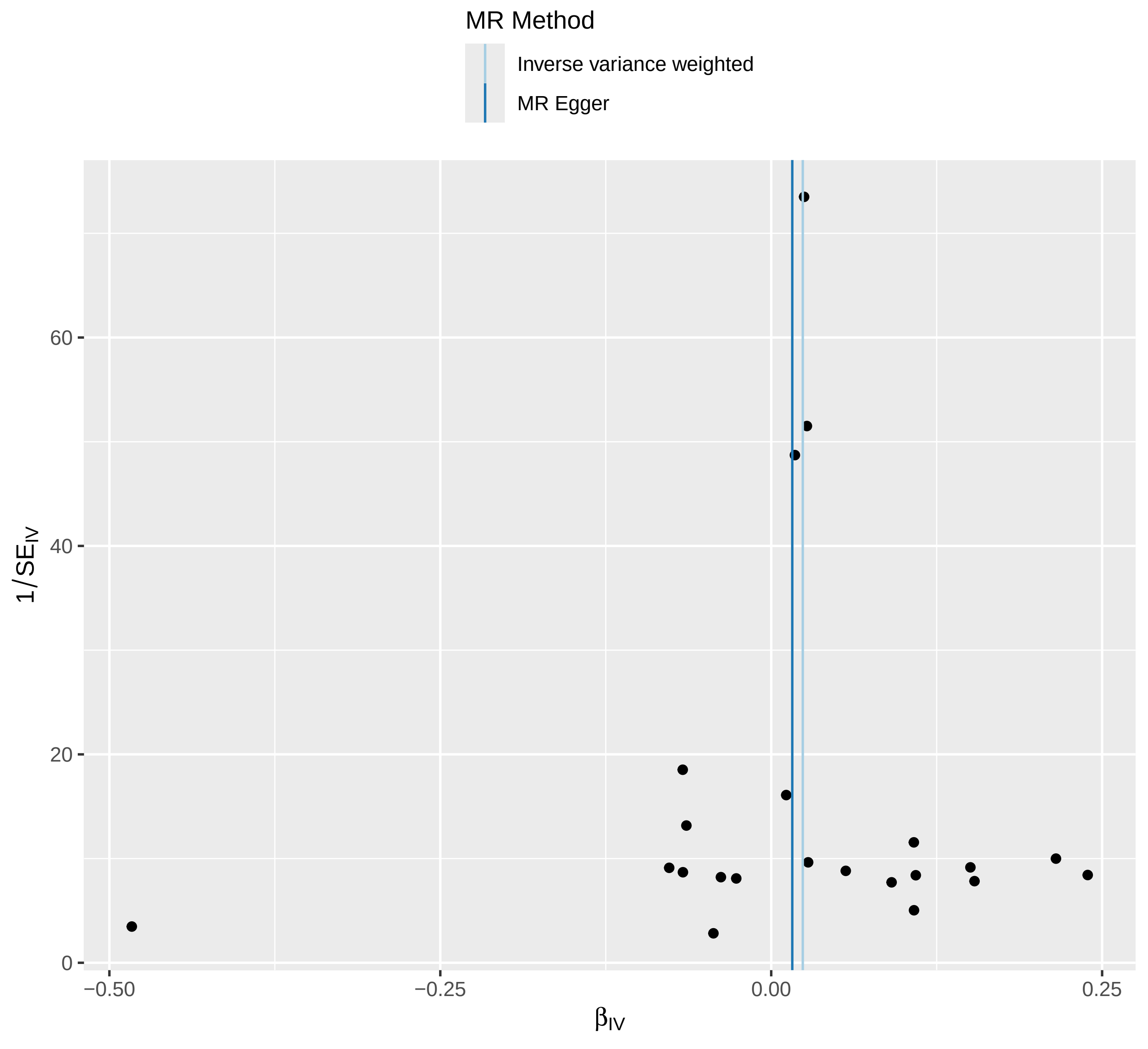

MR Method
Inverse variance weighted
MR Egger
1/SE_IV
60
40
20
0
−0.50
−0.25
0.00
0.25
β_IV

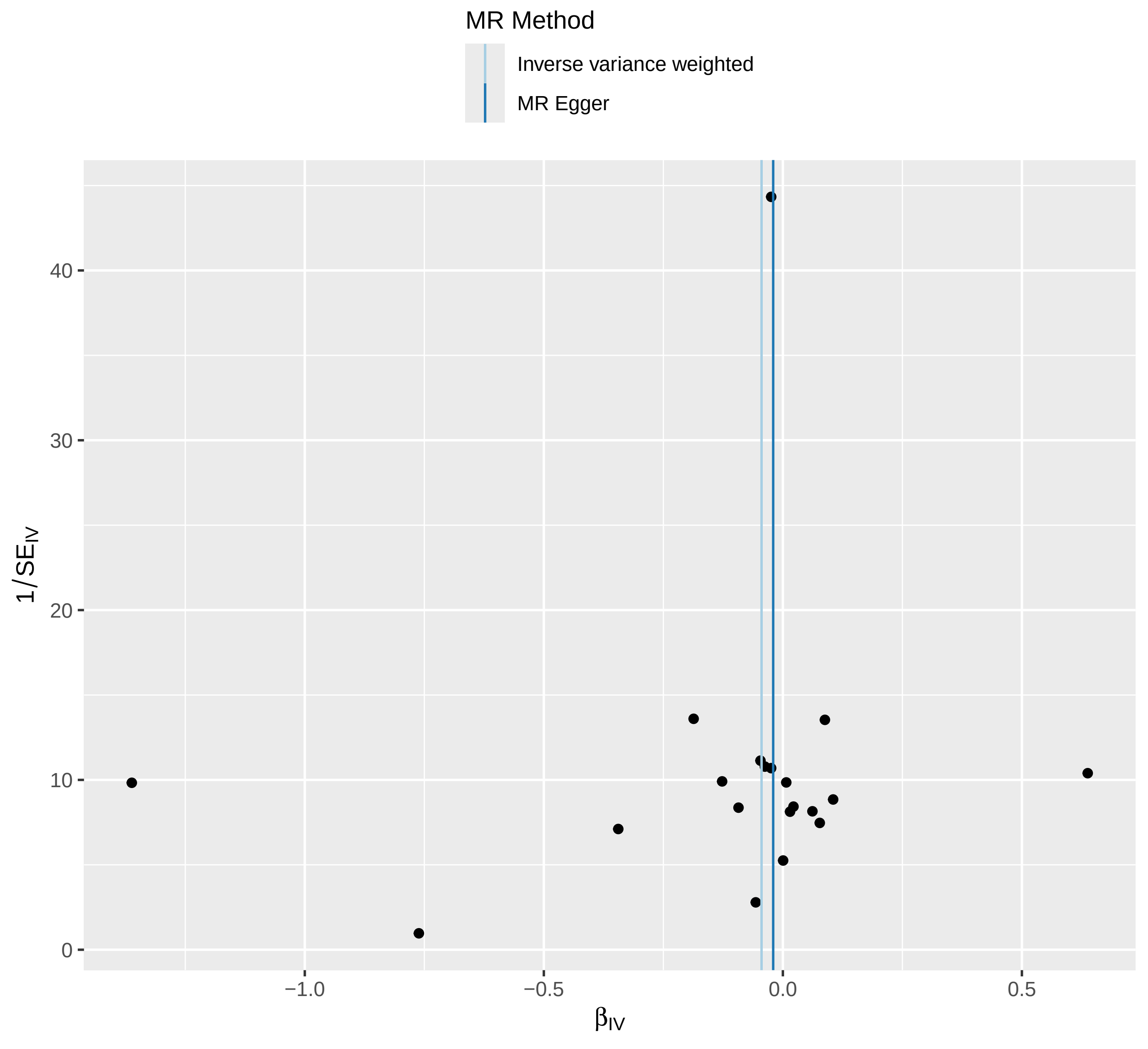

MR Method
Inverse variance weighted
MR Egger
40
30
20
10
0
1/SE$_{IV}$
−1.0
−0.5
0.0
0.5
β$_{IV}$

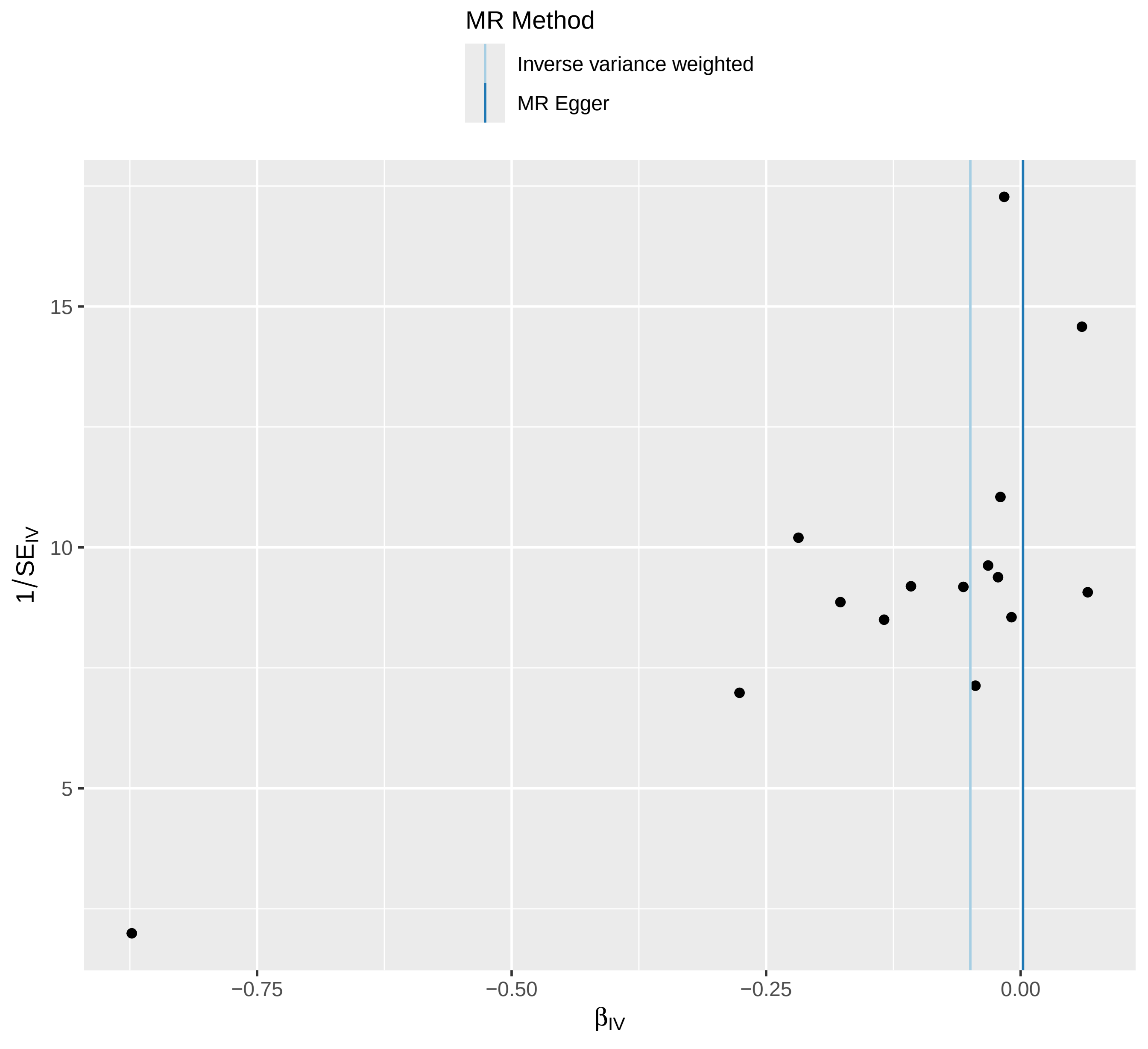
MR Method
Inverse variance weighted
MR Egger
1/SE_IV
β_IV
15
10
5
−0.75
−0.50
−0.25
0.00

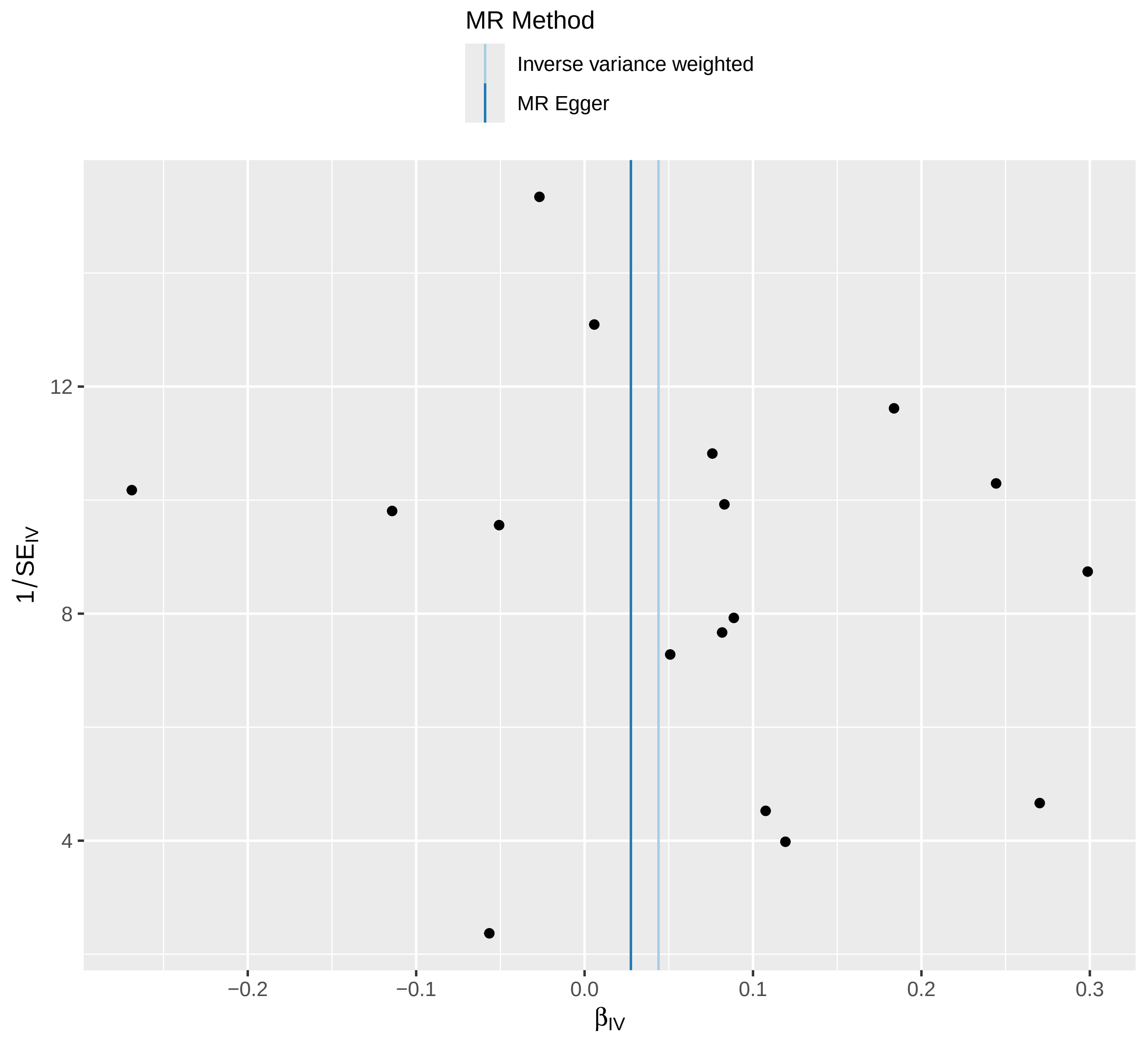

MR Method
Inverse variance weighted
MR Egger
1/SE$_{IV}$
12
8
4
−0.2
−0.1
0.0
0.1
0.2
0.3
β$_{IV}$

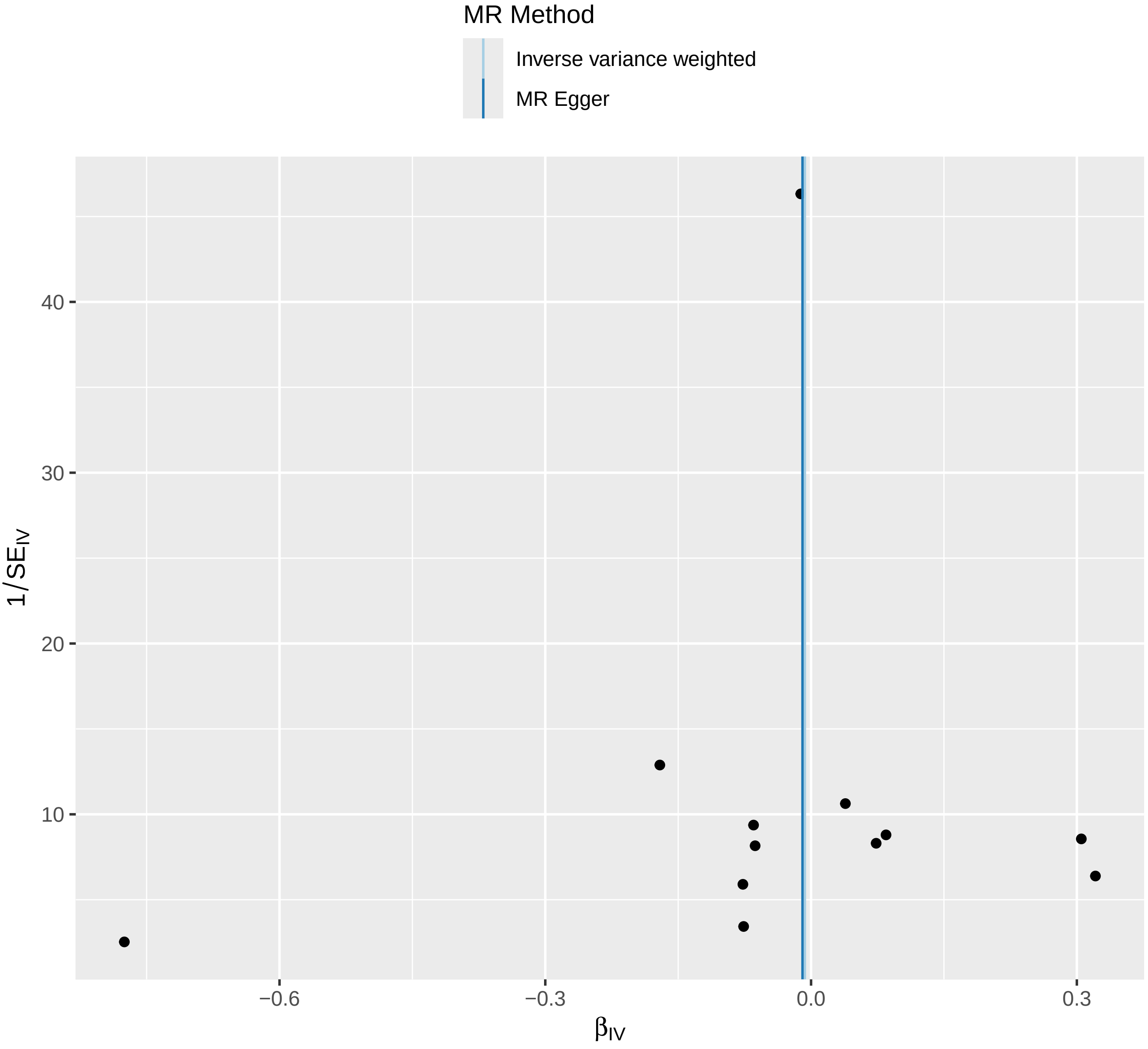
MR Method
Inverse variance weighted
MR Egger
40
30
20
10
1/SE_IV
−0.6
−0.3
0.0
0.3
β_IV

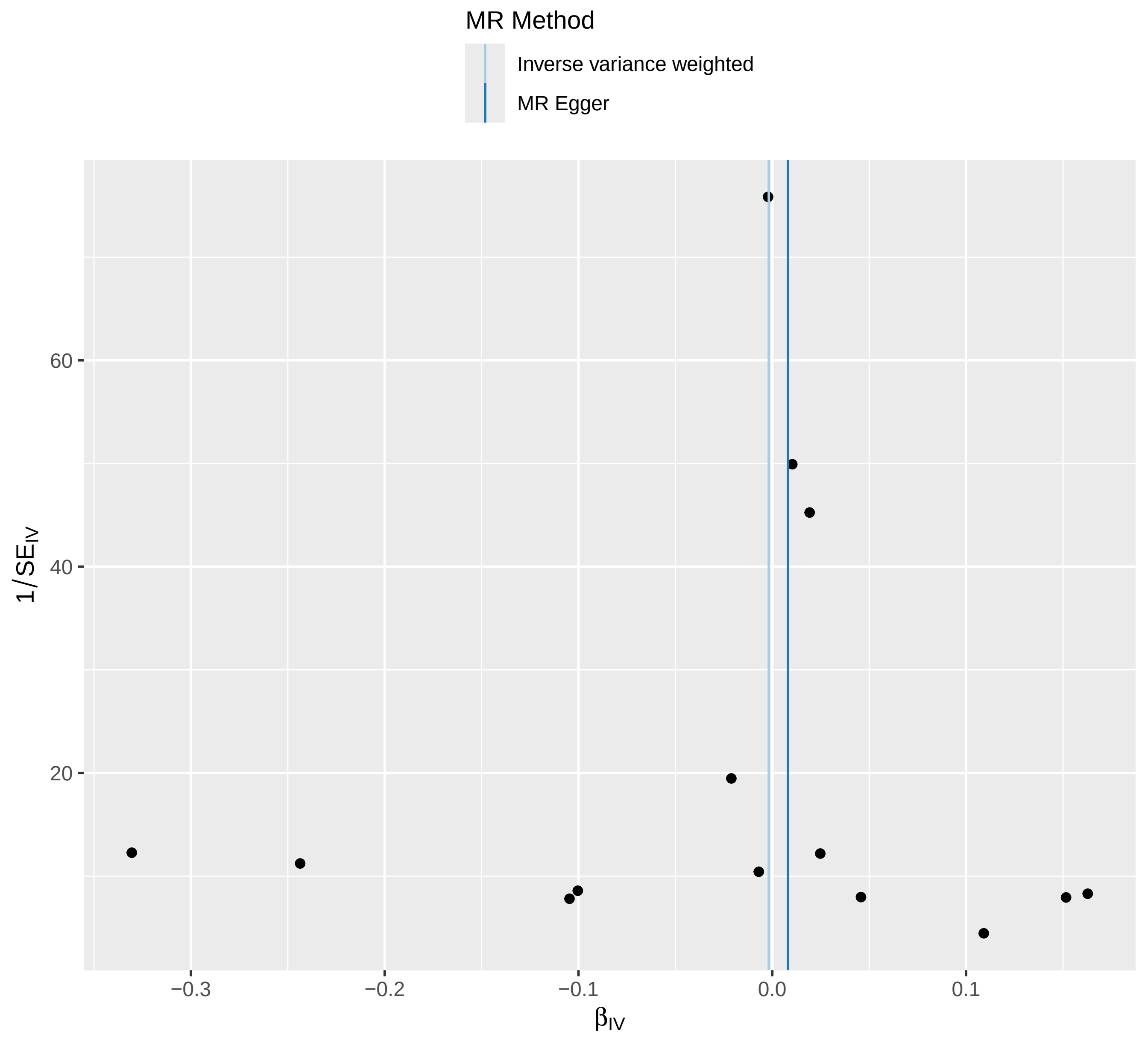

MR Method
Inverse variance weighted
MR Egger
60
40
20
1/SE_IV
−0.3
−0.2
−0.1
0.0
0.1
β_IV

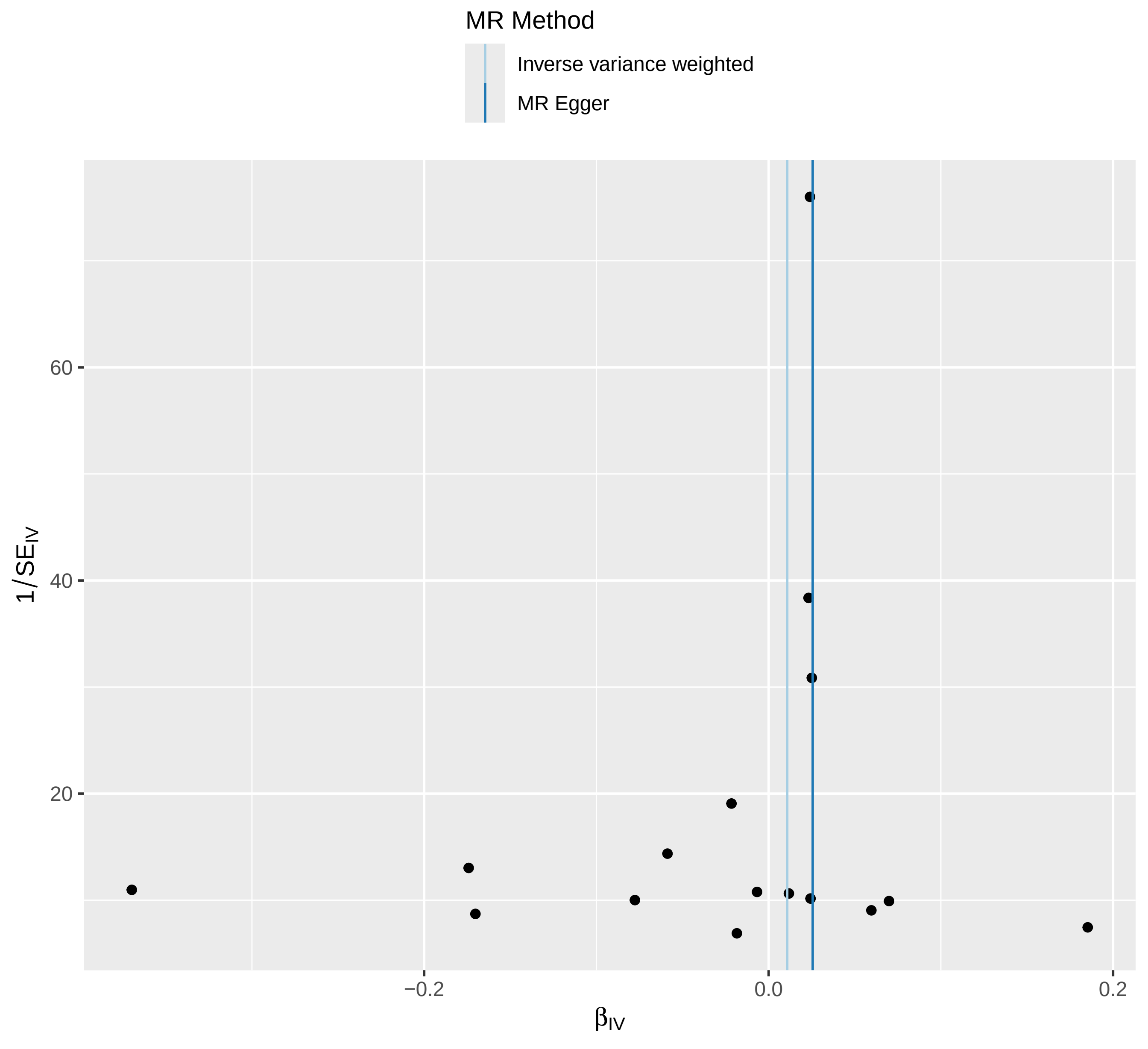

MR Method
Inverse variance weighted
MR Egger
60
40
20
$1/SE_{IV}$
−0.2
0.0
0.2
β_{IV}

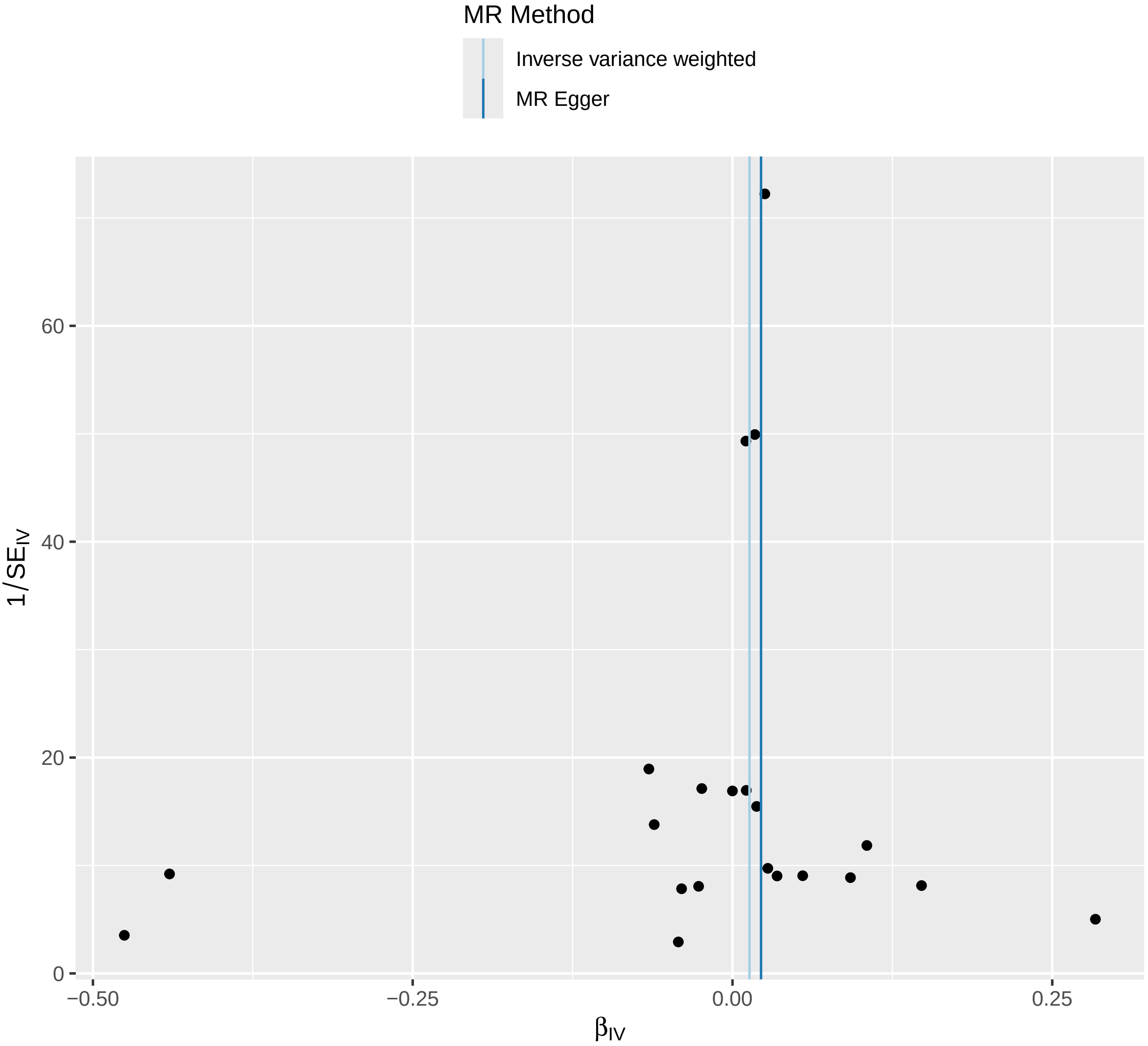
MR Method
Inverse variance weighted
MR Egger
60
40
20
0
1/SE_IV
−0.50
−0.25
0.00
0.25
β_IV

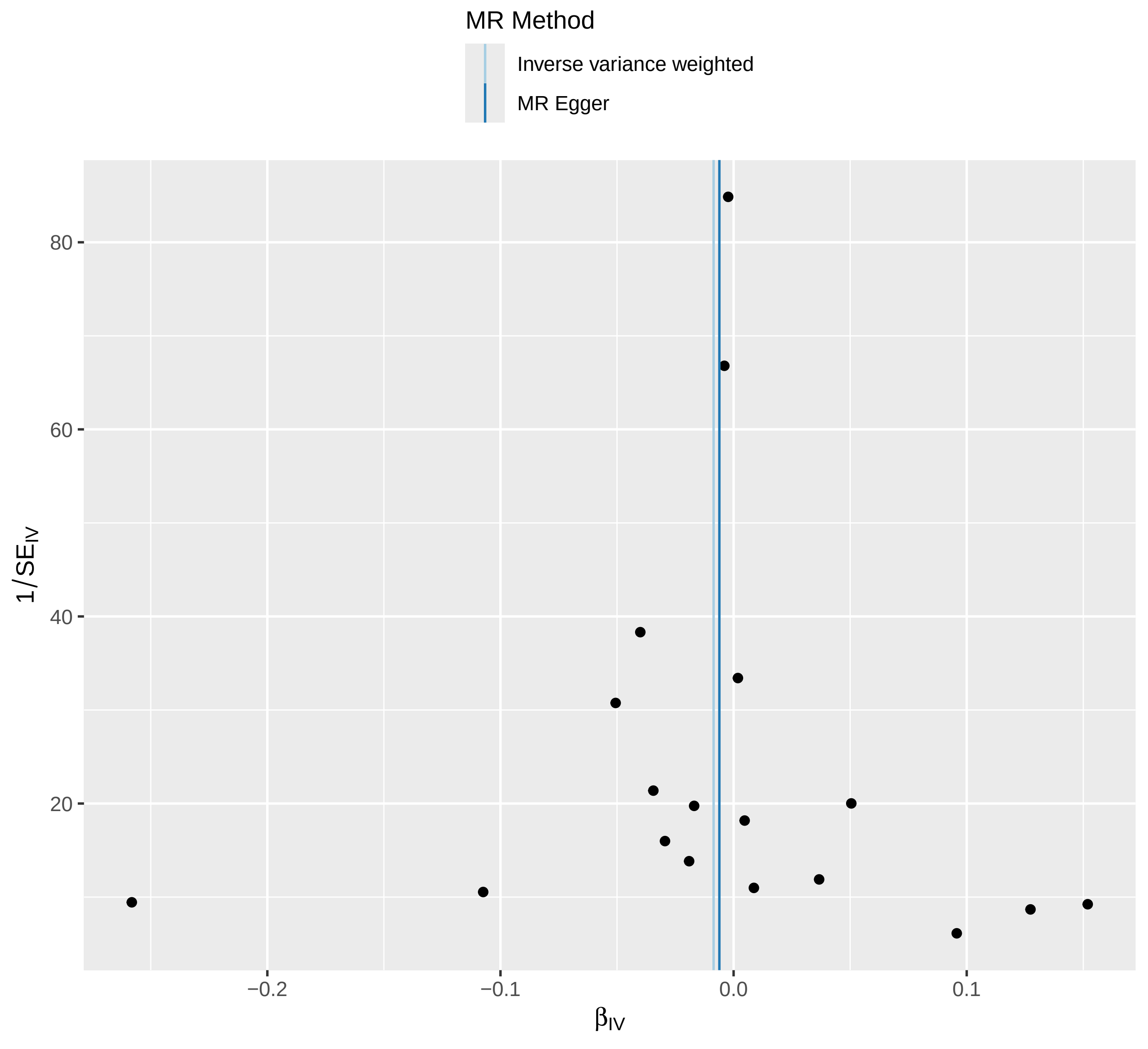
MR Method
Inverse variance weighted
MR Egger
80
60
40
20
1/SE_IV
−0.2
−0.1
0.0
0.1
β_IV

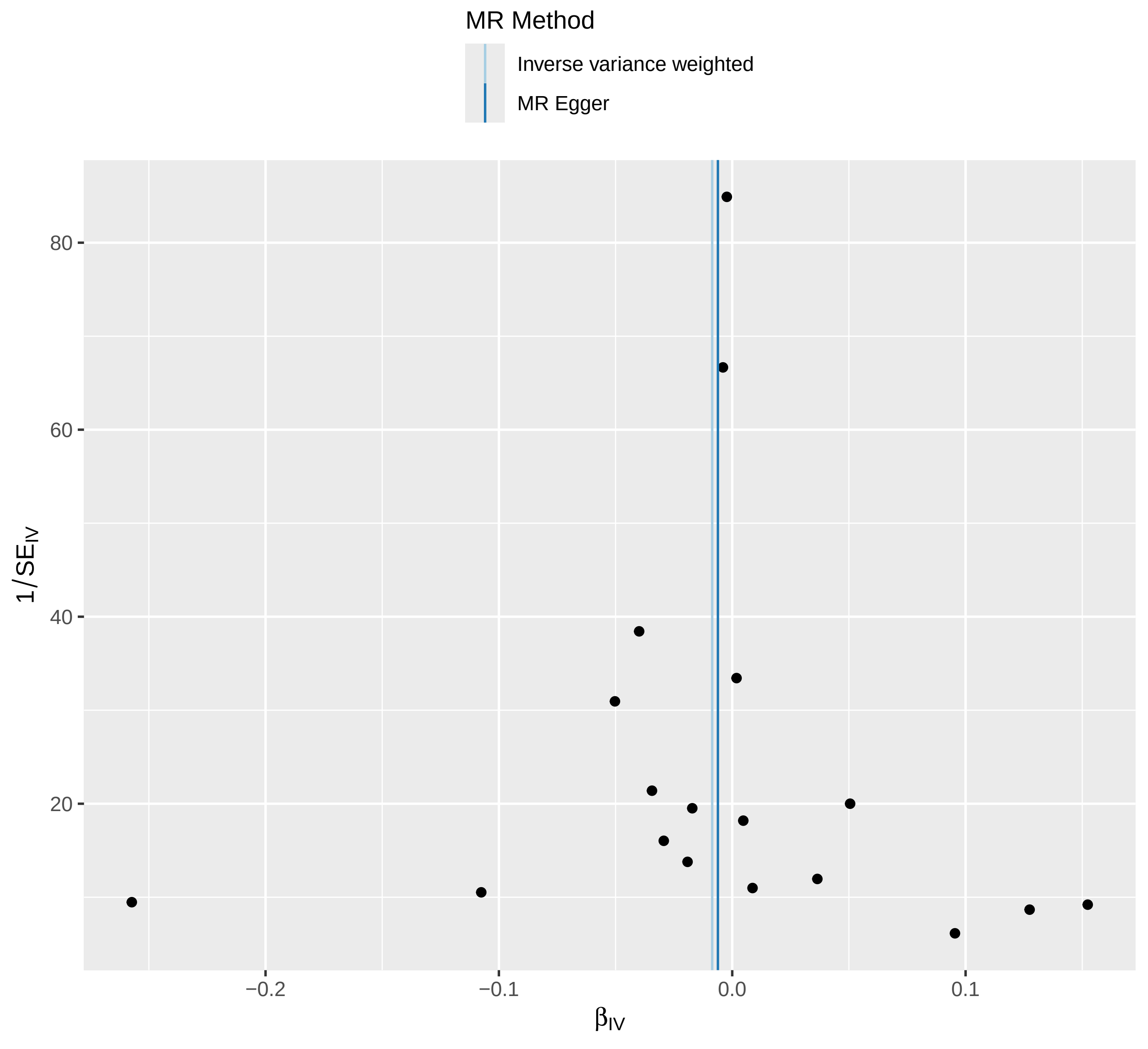
MR Method
Inverse variance weighted
MR Egger
80
60
40
20
1/SE_IV
−0.2
−0.1
0.0
0.1
β_IV

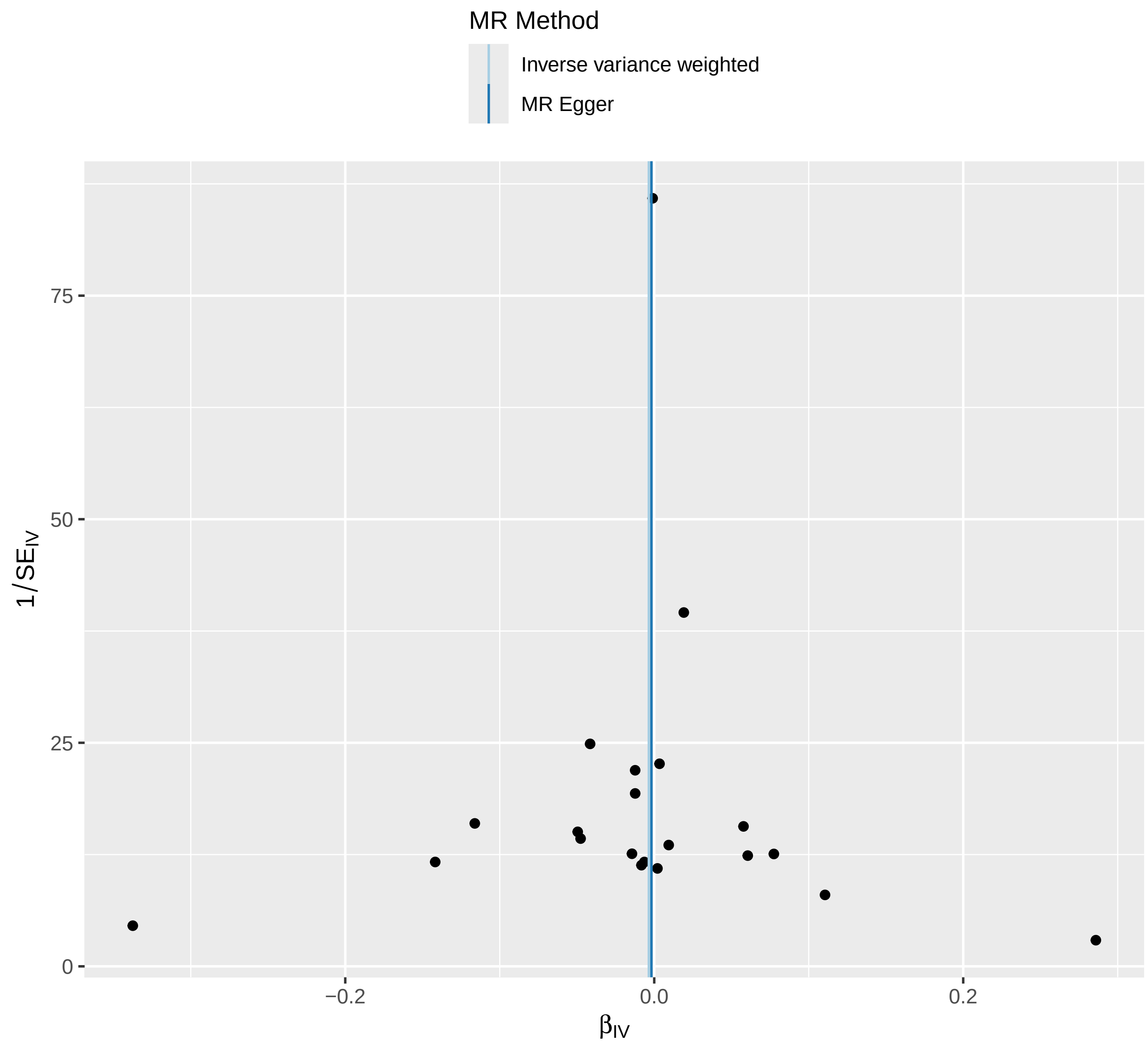
MR Method
Inverse variance weighted
MR Egger
1/SE$_{IV}$
75
50
25
0
−0.2
0.0
0.2
β$_{IV}$

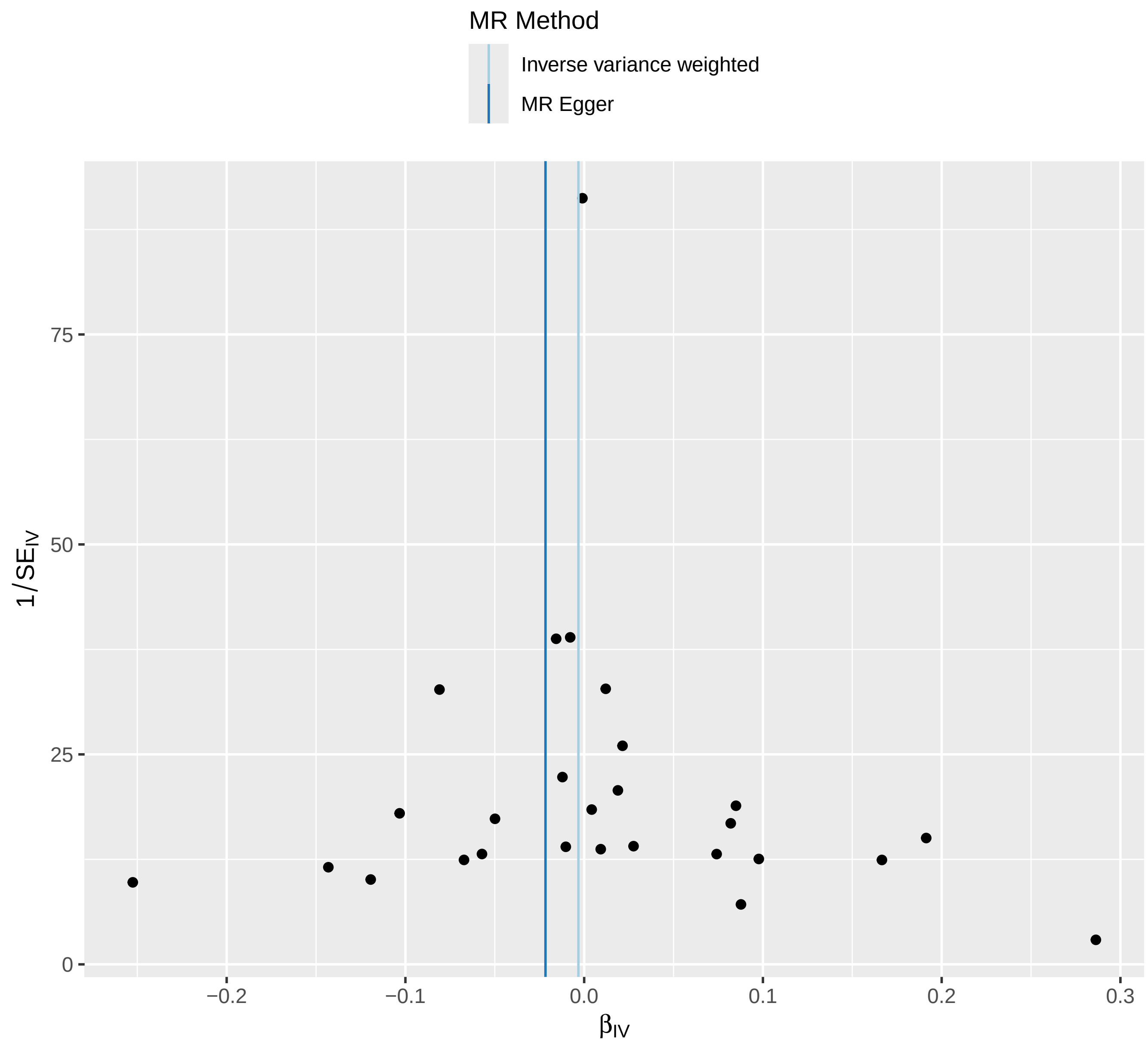
MR Method
Inverse variance weighted
MR Egger
75
50
25
0
1/SE_IV
−0.2
−0.1
0.0
0.1
0.2
0.3
β_IV

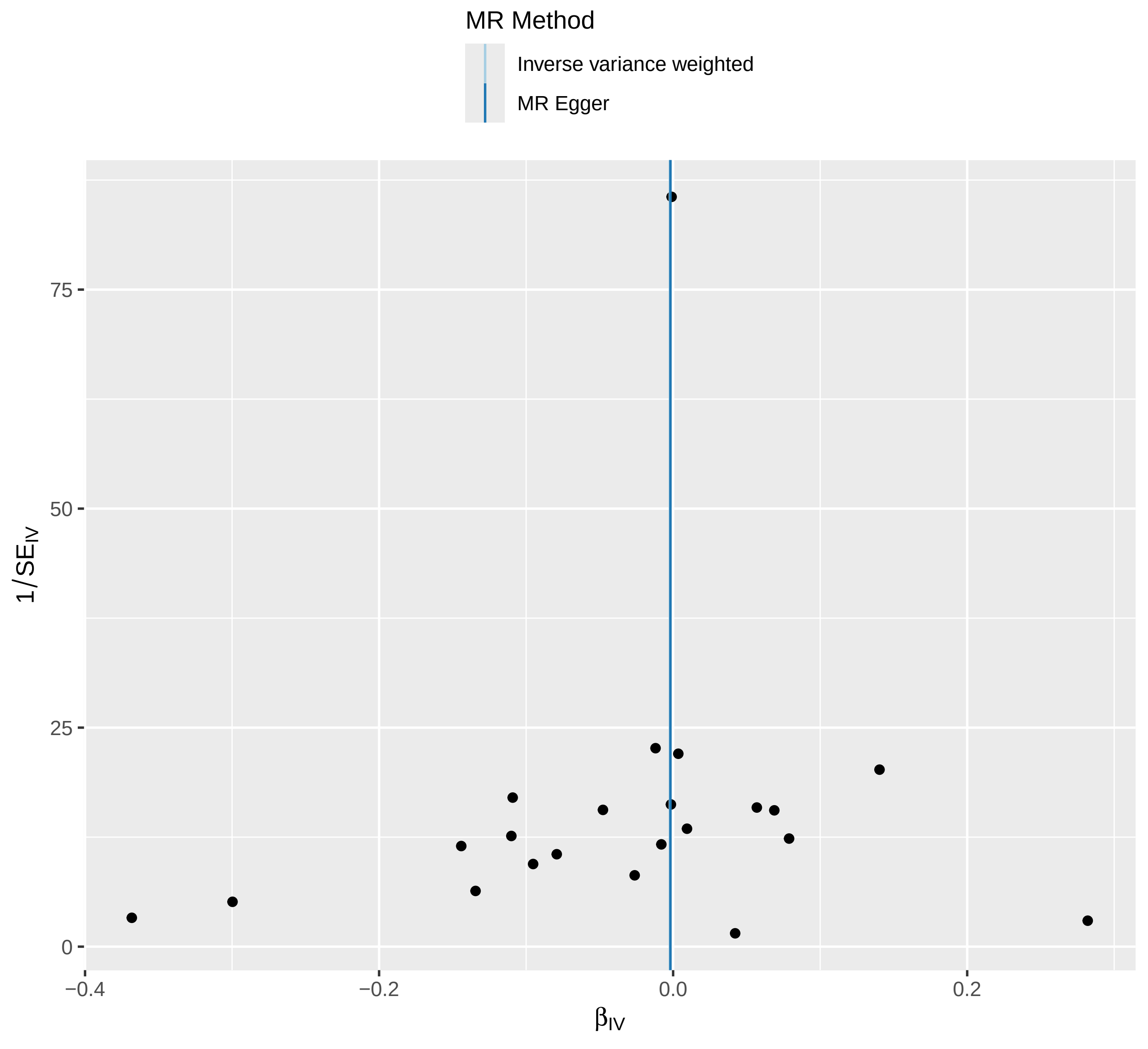

MR Method
Inverse variance weighted
MR Egger
75
50
25
0
1/SE_IV
−0.4
−0.2
0.0
0.2
β_IV

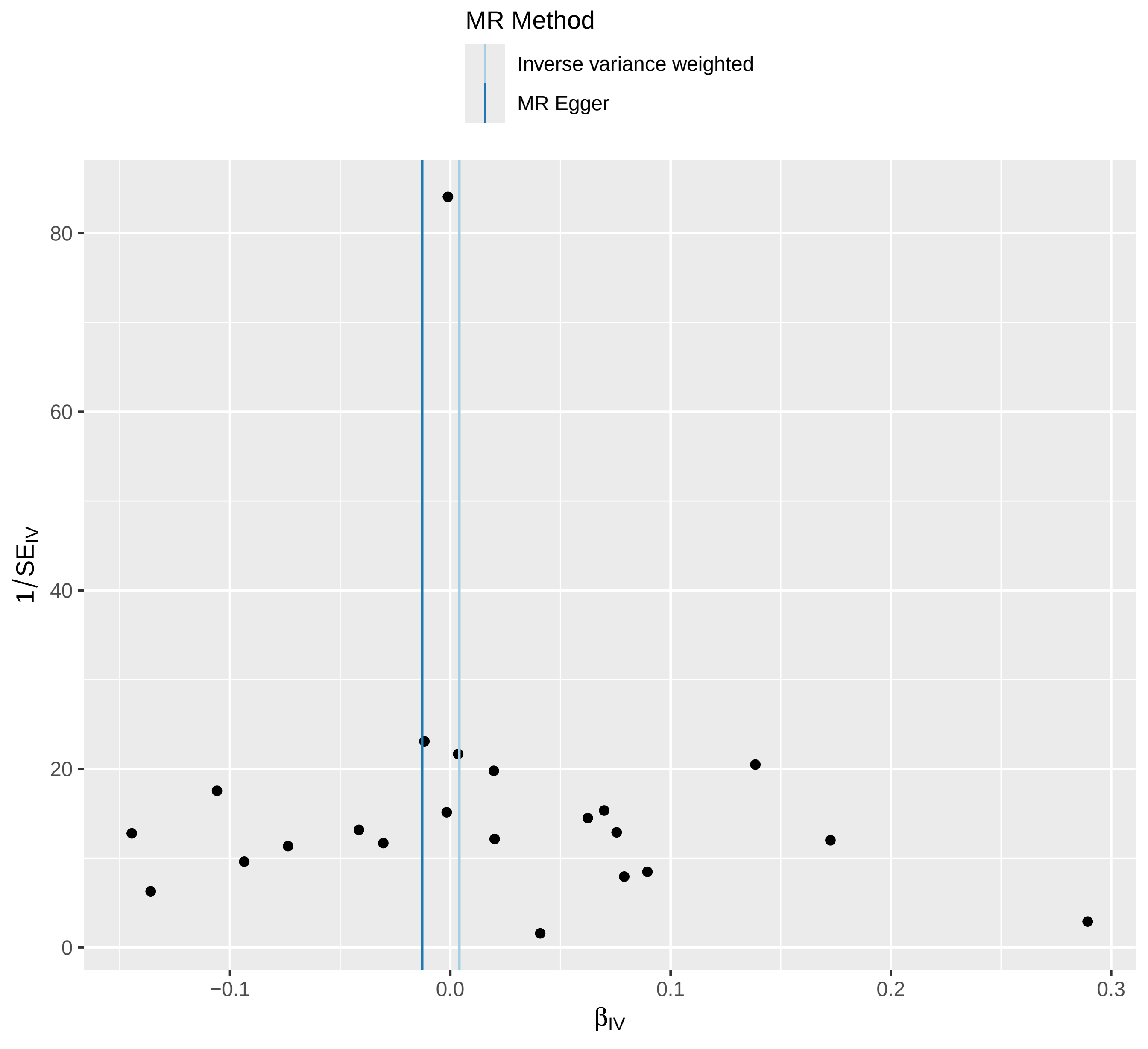

MR Method
Inverse variance weighted
MR Egger
80
60
40
20
0
1/SE_IV
−0.1
0.0
0.1
0.2
0.3
β_IV

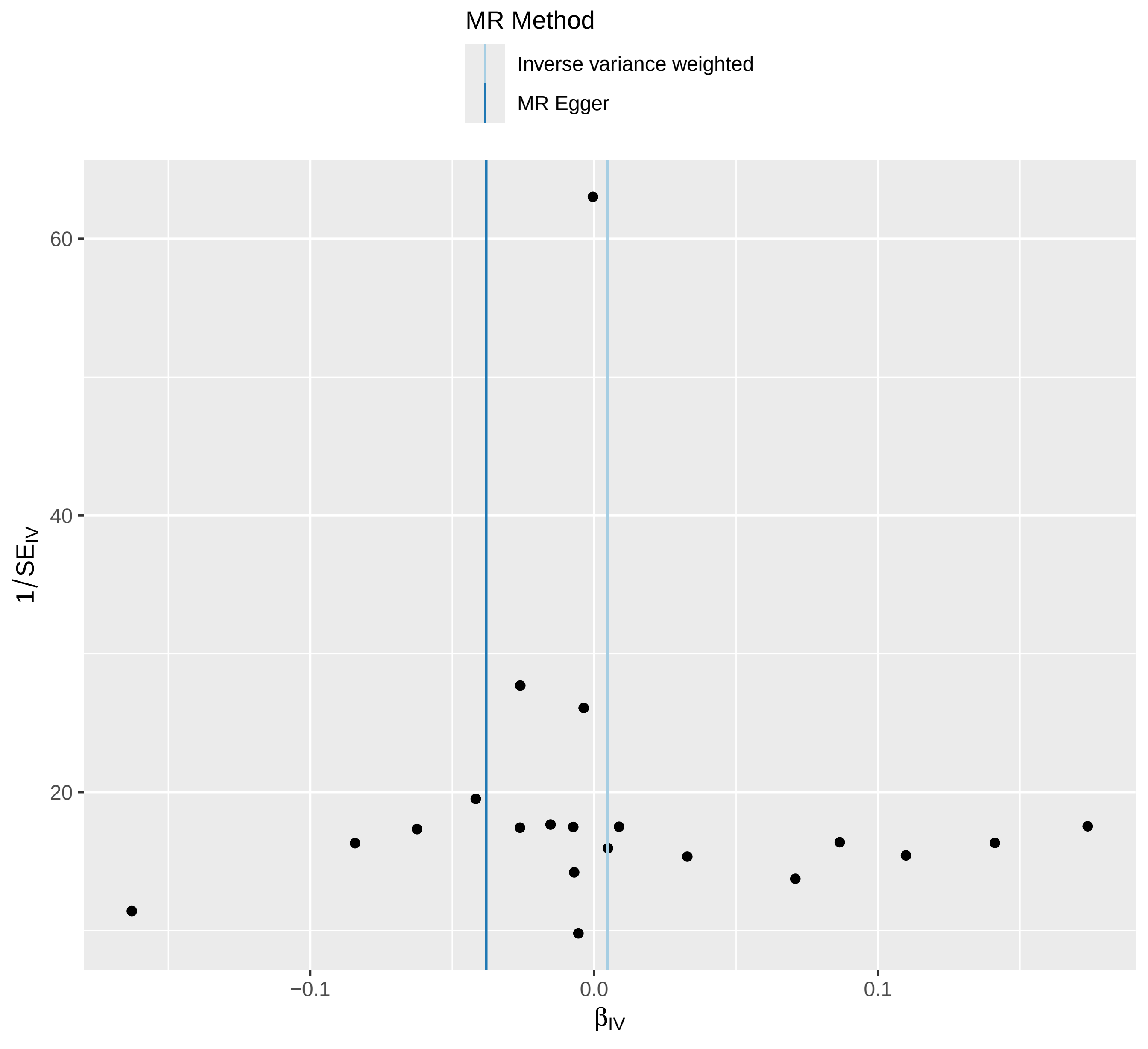
MR Method
Inverse variance weighted
MR Egger
60
40
20
1/SE$_{IV}$
−0.1
0.0
0.1
β$_{IV}$

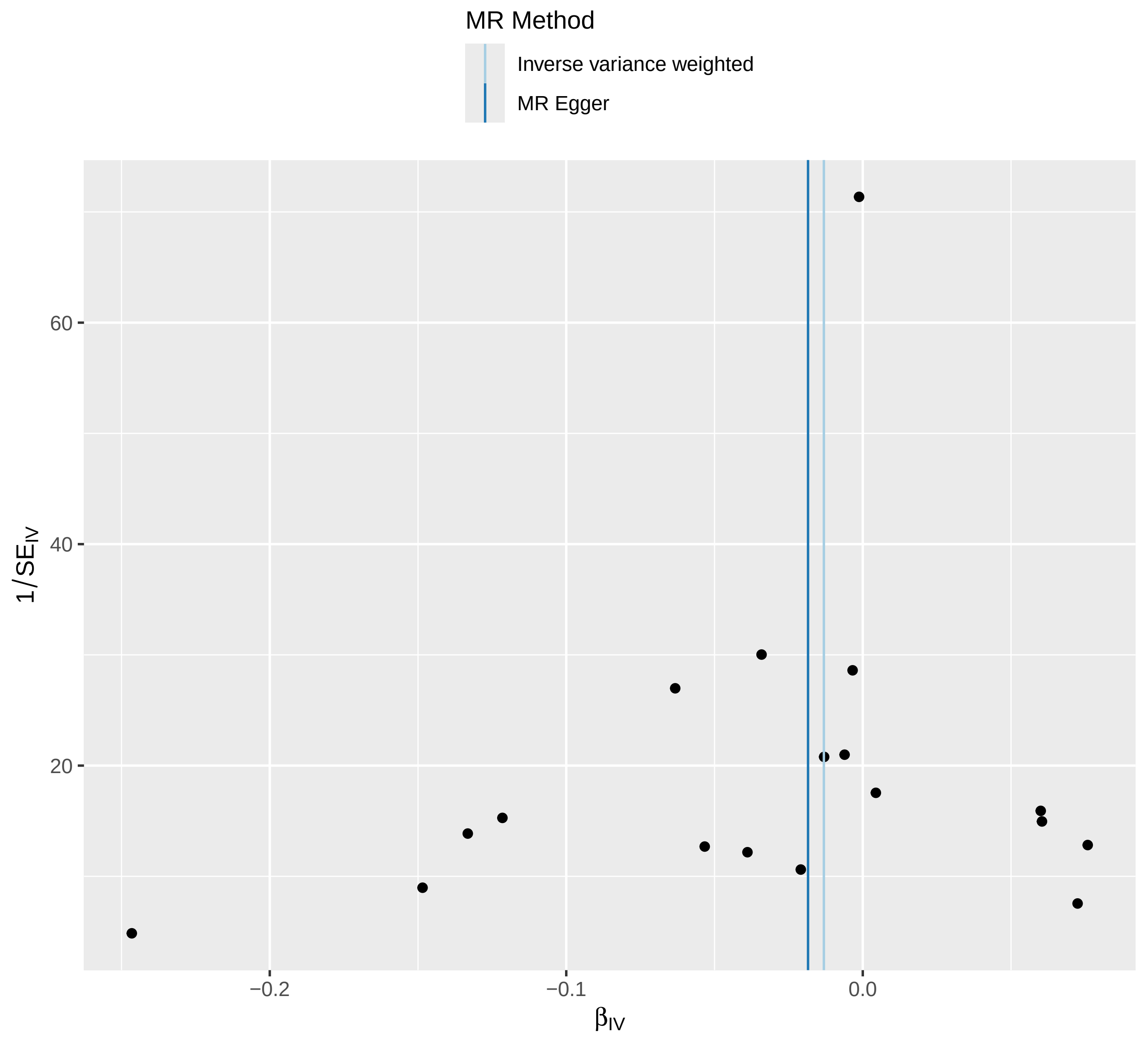
MR Method
Inverse variance weighted
MR Egger
60
40
20
1/SE_IV
−0.2
−0.1
0.0
β_IV

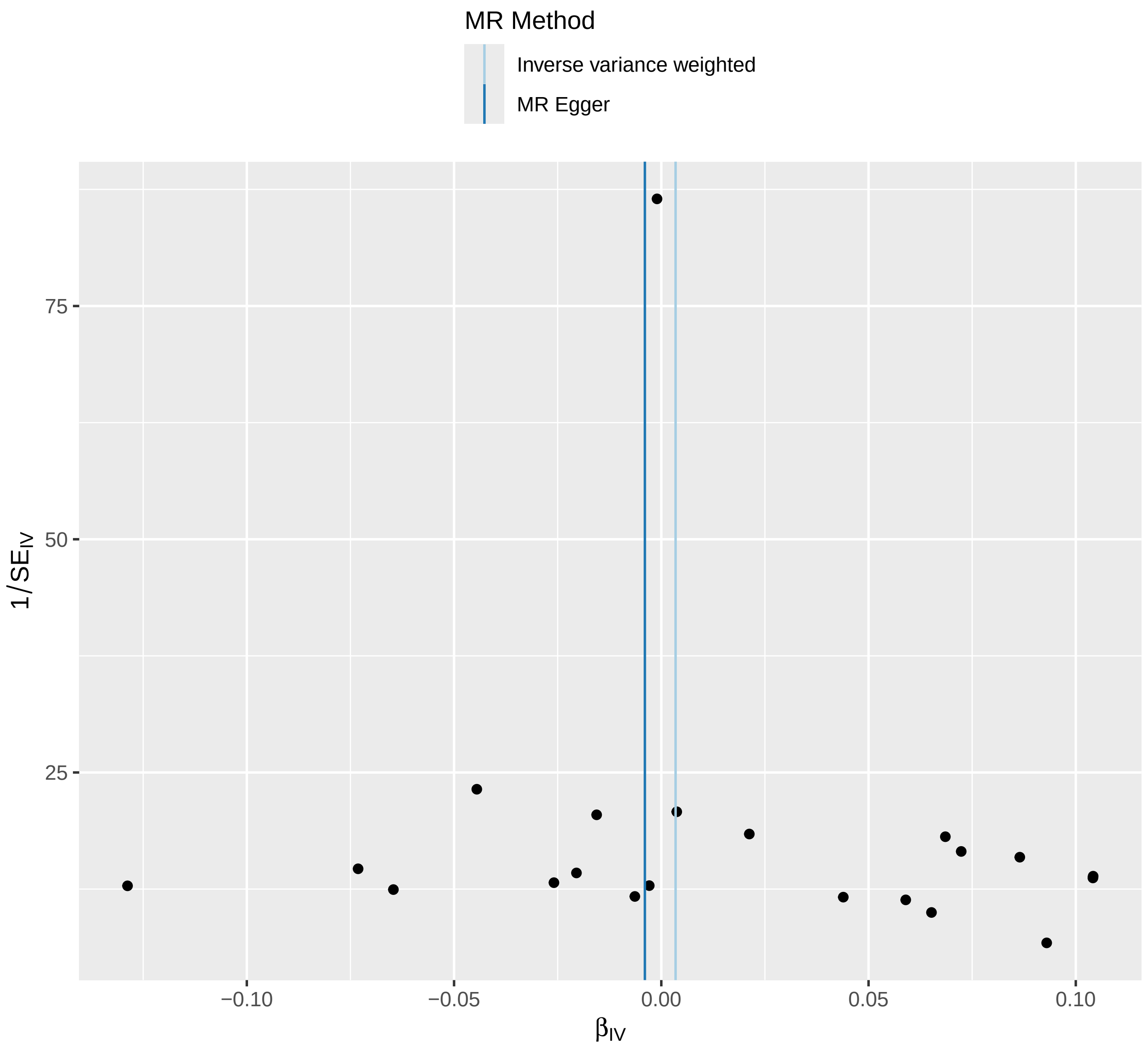
MR Method
Inverse variance weighted
MR Egger
75
50
25
1/SE_IV
−0.10
−0.05
0.00
0.05
0.10
β_IV

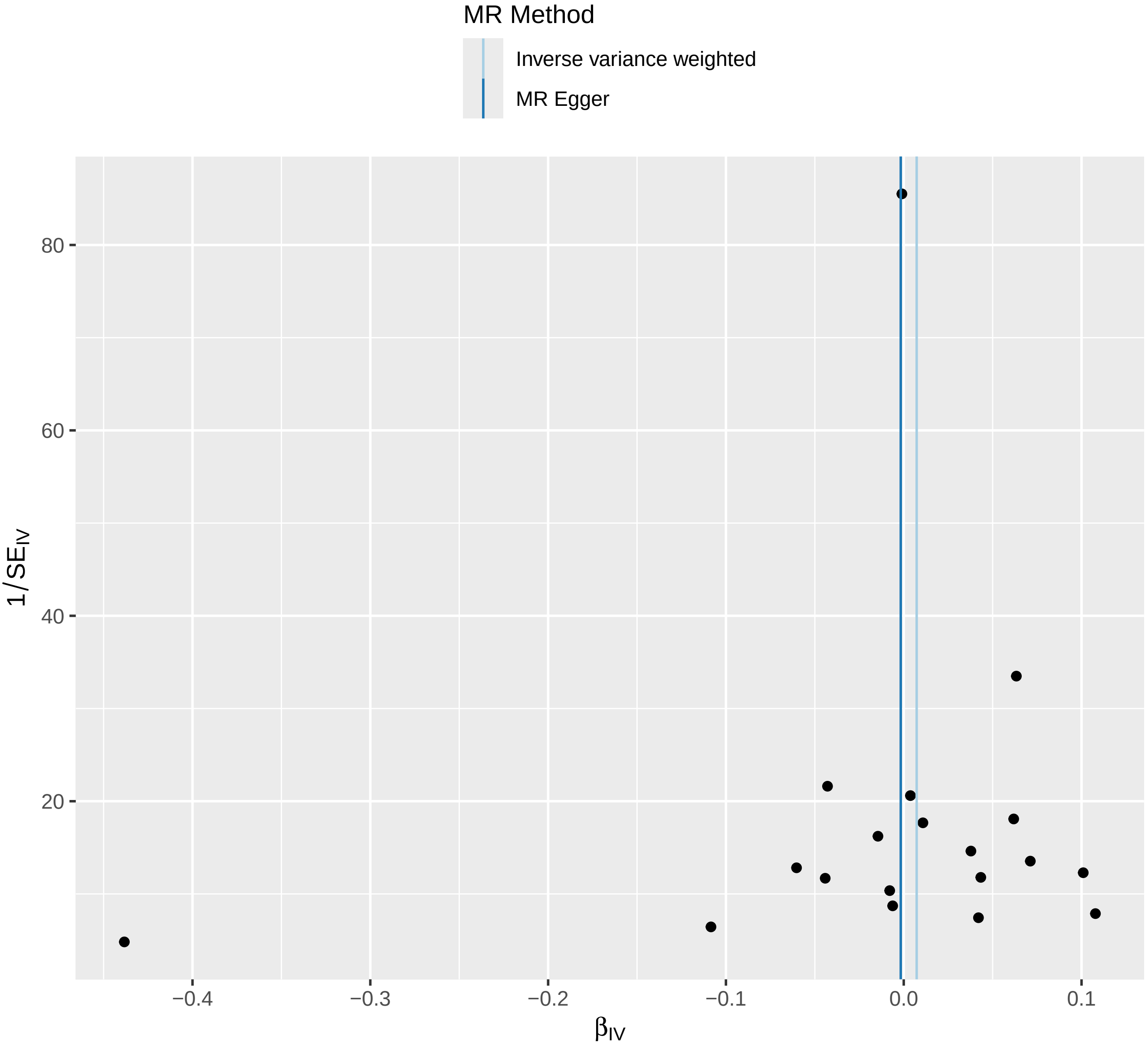
MR Method
Inverse variance weighted
MR Egger
80
60
40
20
1/SE_IV
−0.4
−0.3
−0.2
−0.1
0.0
0.1
β_IV

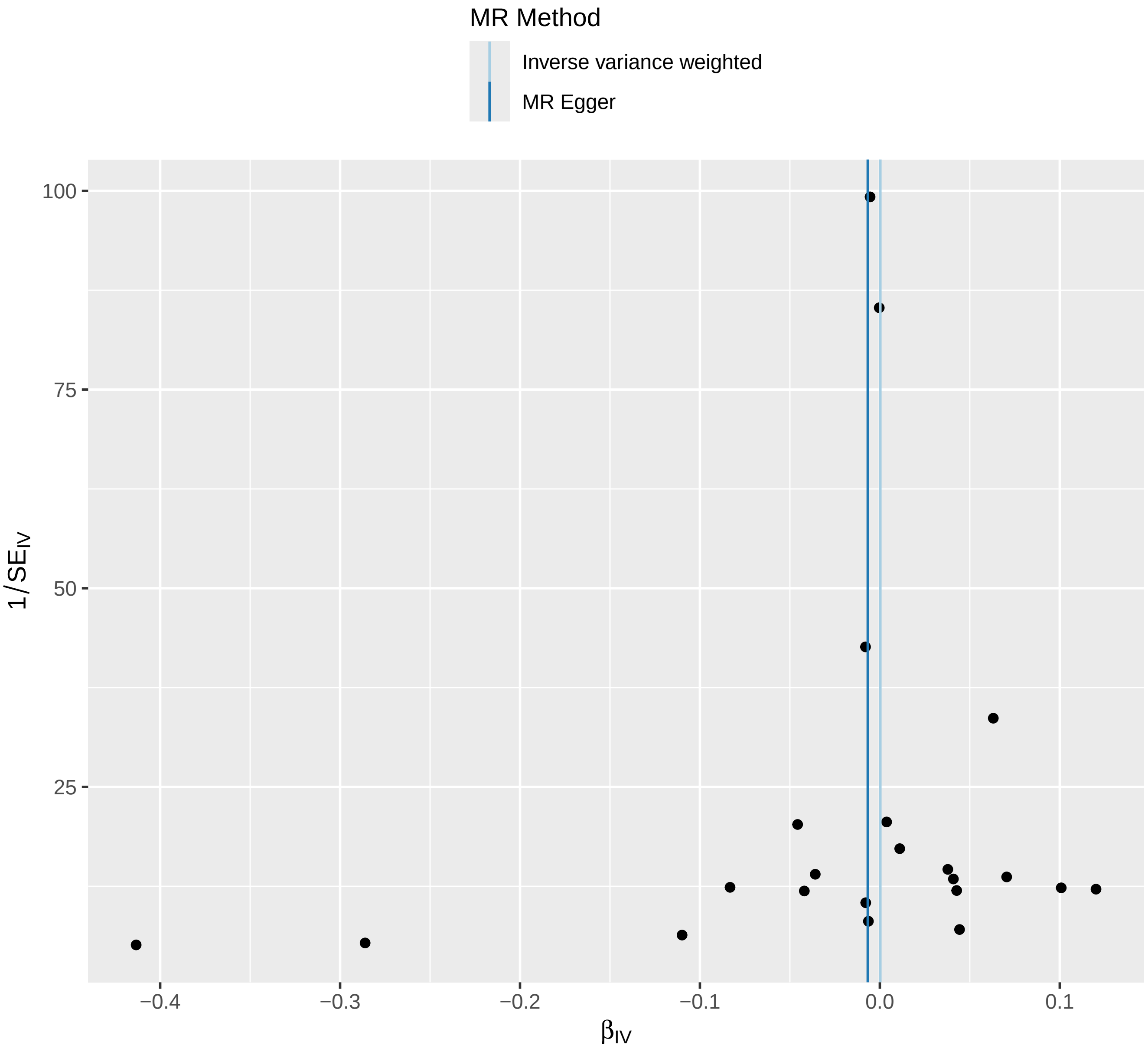

MR Method
Inverse variance weighted
MR Egger
100
75
50
25
1/SE$_{IV}$
−0.4
−0.3
−0.2
−0.1
0.0
0.1
β$_{IV}$

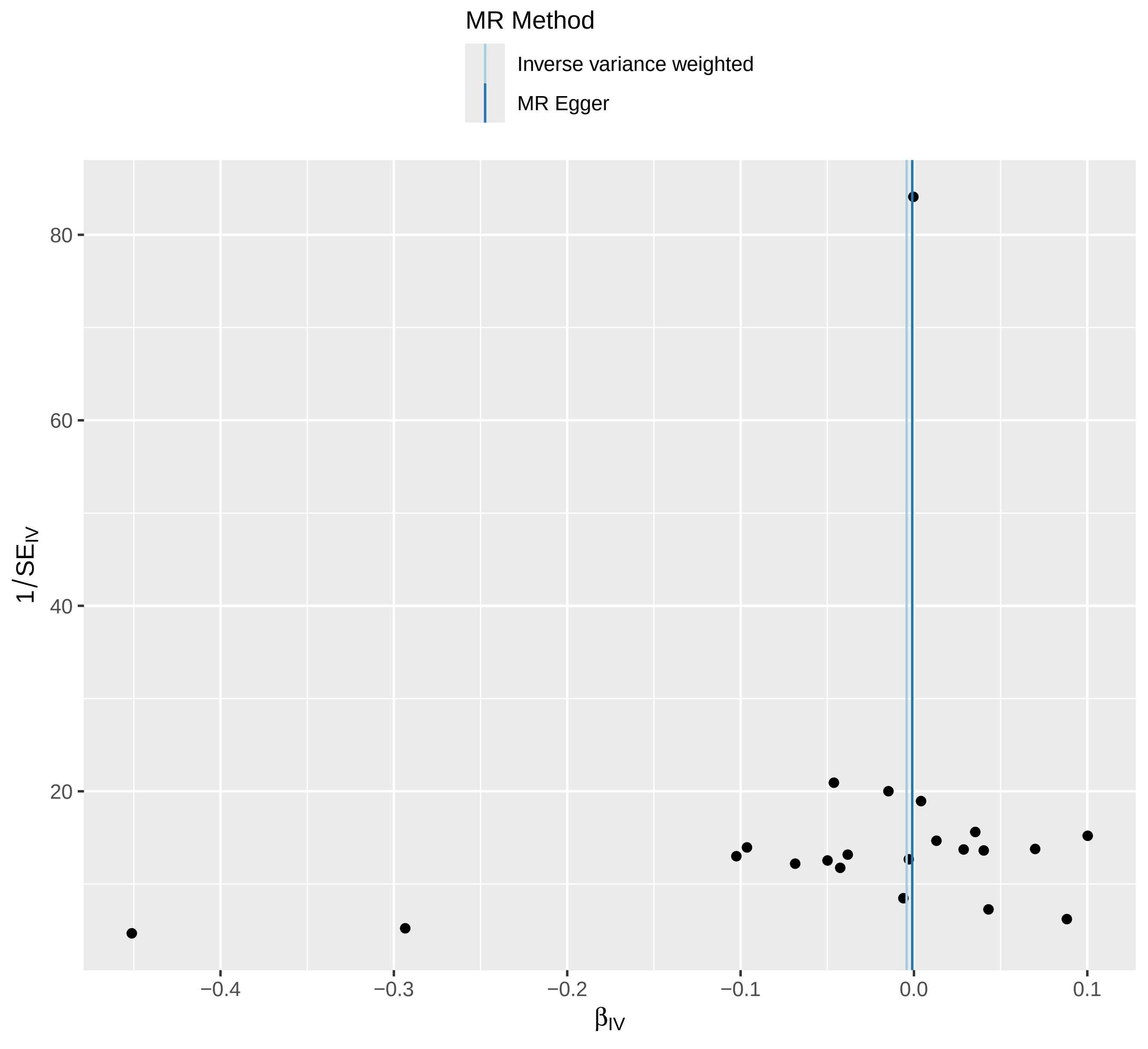
MR Method
Inverse variance weighted
MR Egger
1/SE_IV
80
60
40
20
−0.4
−0.3
−0.2
−0.1
0.0
0.1
β_IV

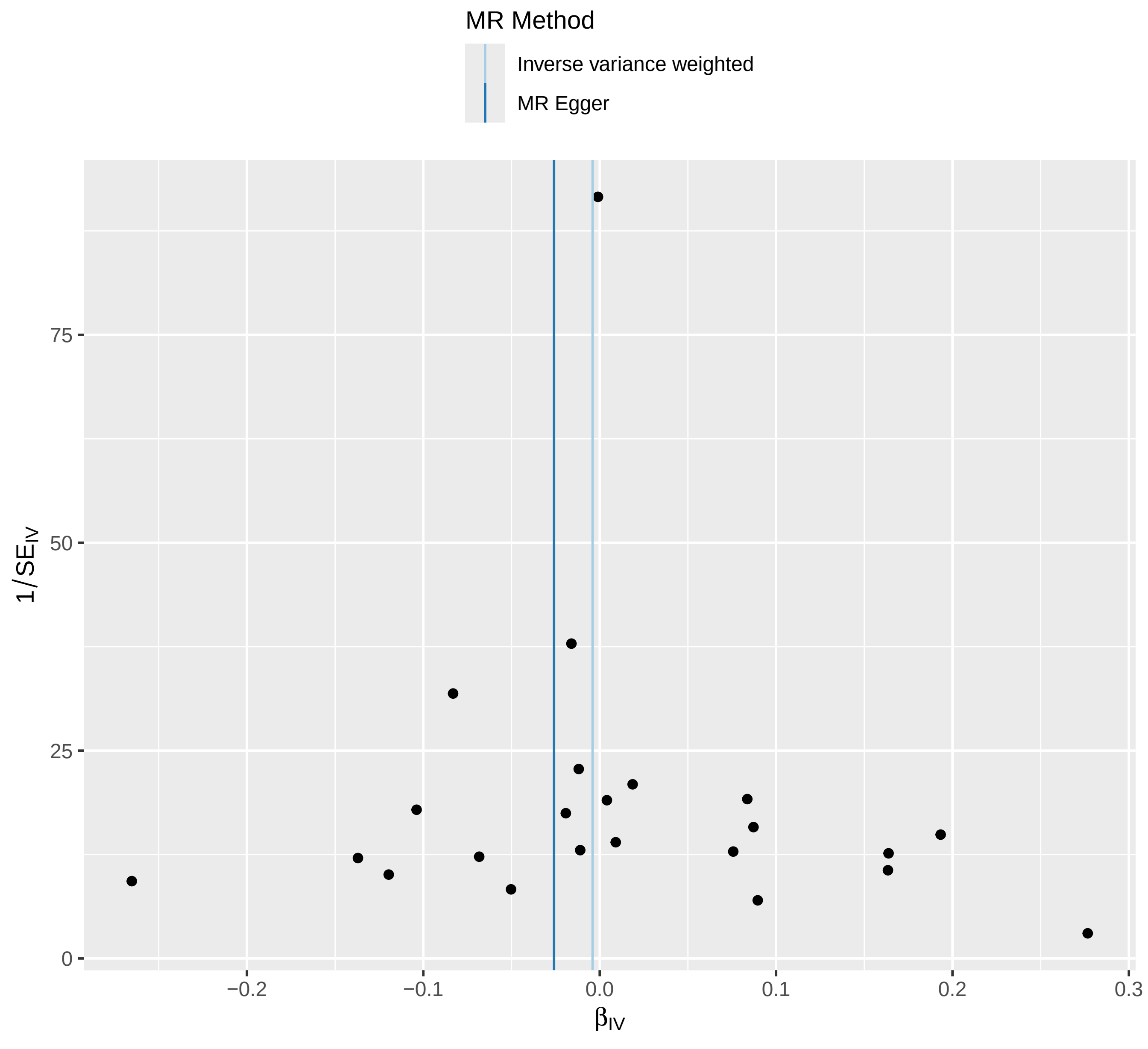
MR Method
Inverse variance weighted
MR Egger
1/SE_IV
75
50
25
0
−0.2
−0.1
0.0
0.1
0.2
0.3
β_IV

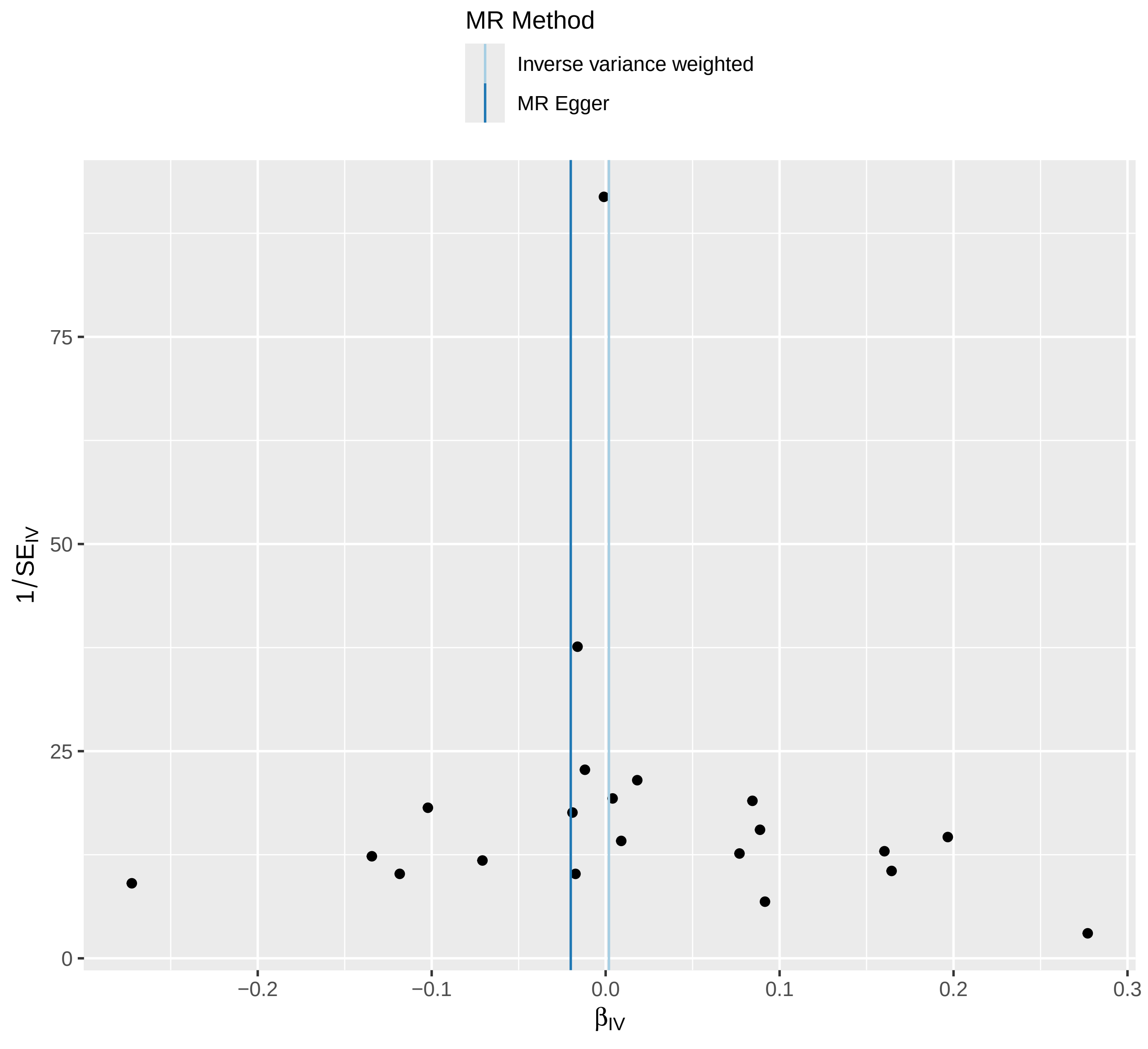
MR Method
Inverse variance weighted
MR Egger
1/SE_IV
75
50
25
0
−0.2
−0.1
0.0
0.1
0.2
0.3
β_IV

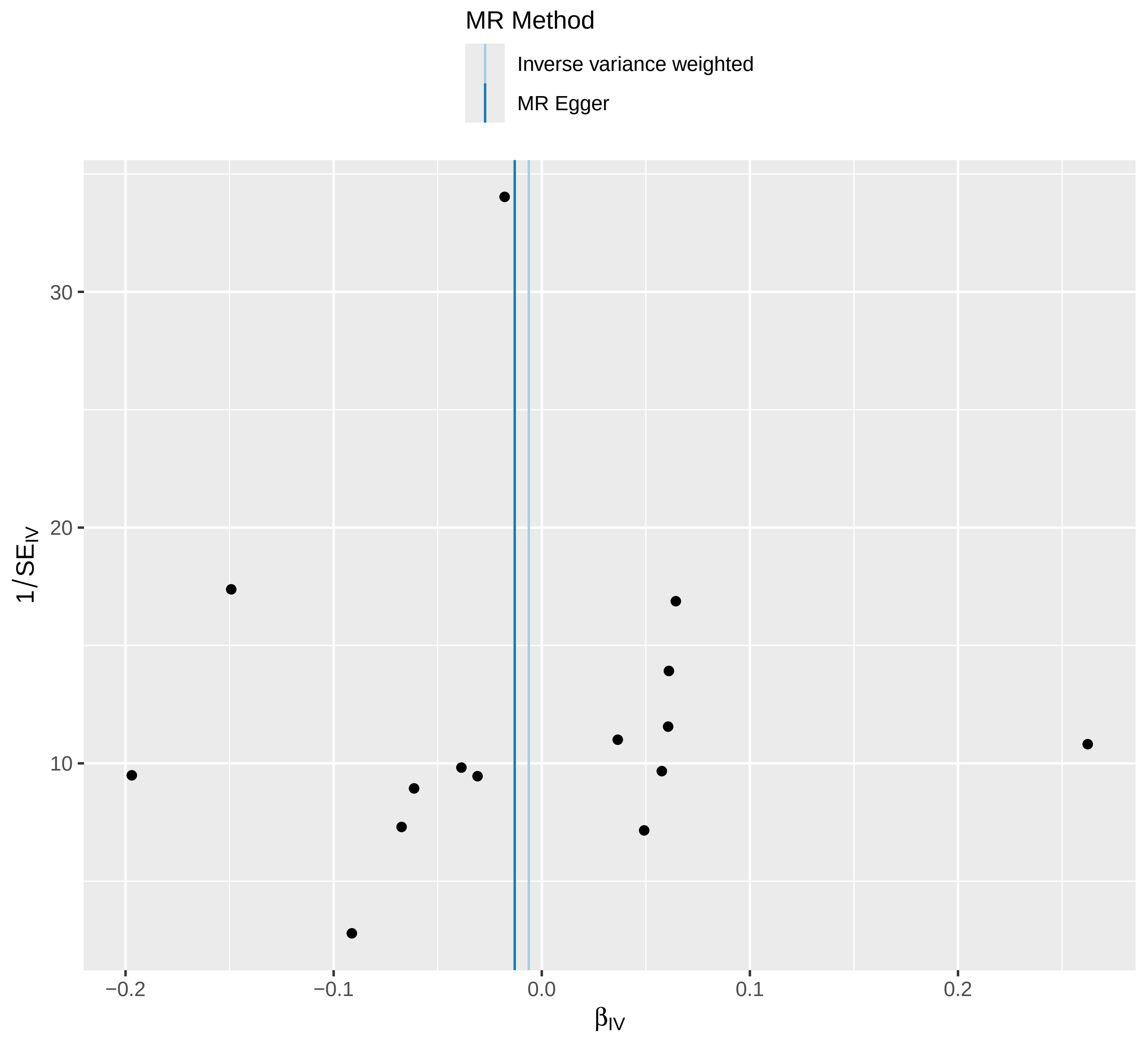

MR Method
Inverse variance weighted
MR Egger
30
20
10
1/SE$_{IV}$
−0.2
−0.1
0.0
0.1
0.2
β$_{IV}$

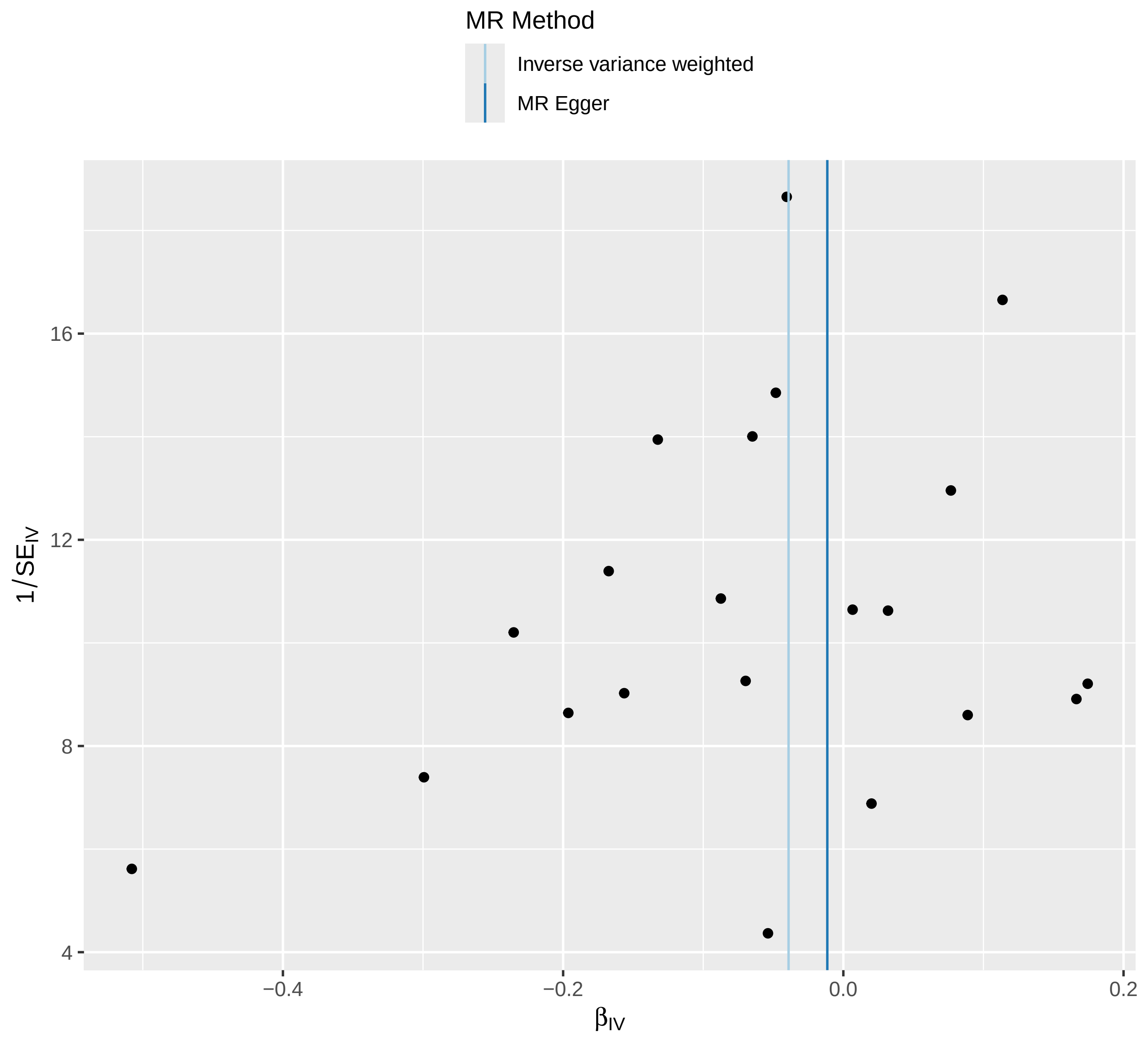
MR Method
Inverse variance weighted
MR Egger
16
12
8
4
1/SE$_{IV}$
−0.4
−0.2
0.0
0.2
β$_{IV}$

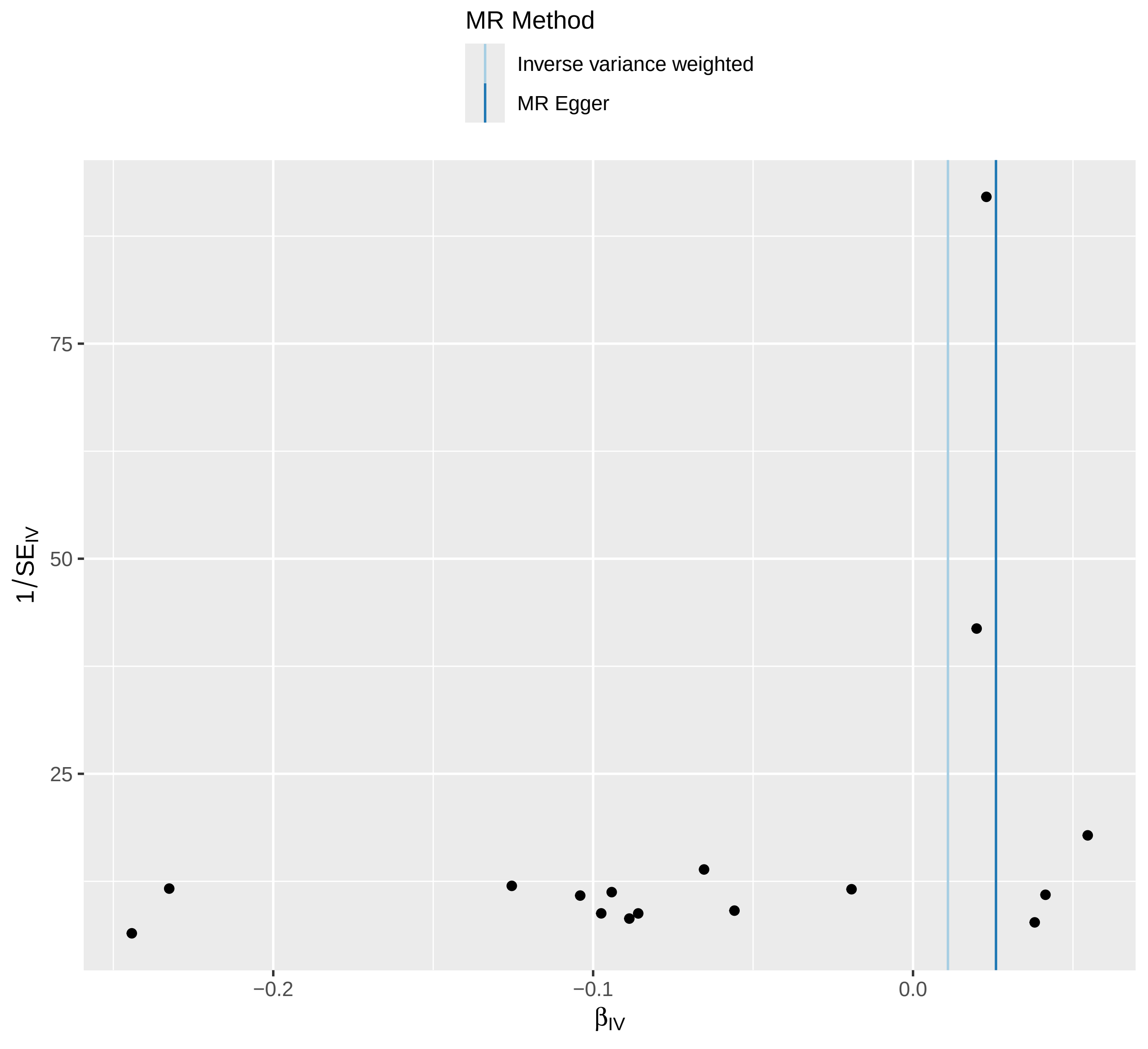

MR Method
Inverse variance weighted
MR Egger
1/SE$_{IV}$
75
50
25
−0.2
−0.1
0.0
β$_{IV}$

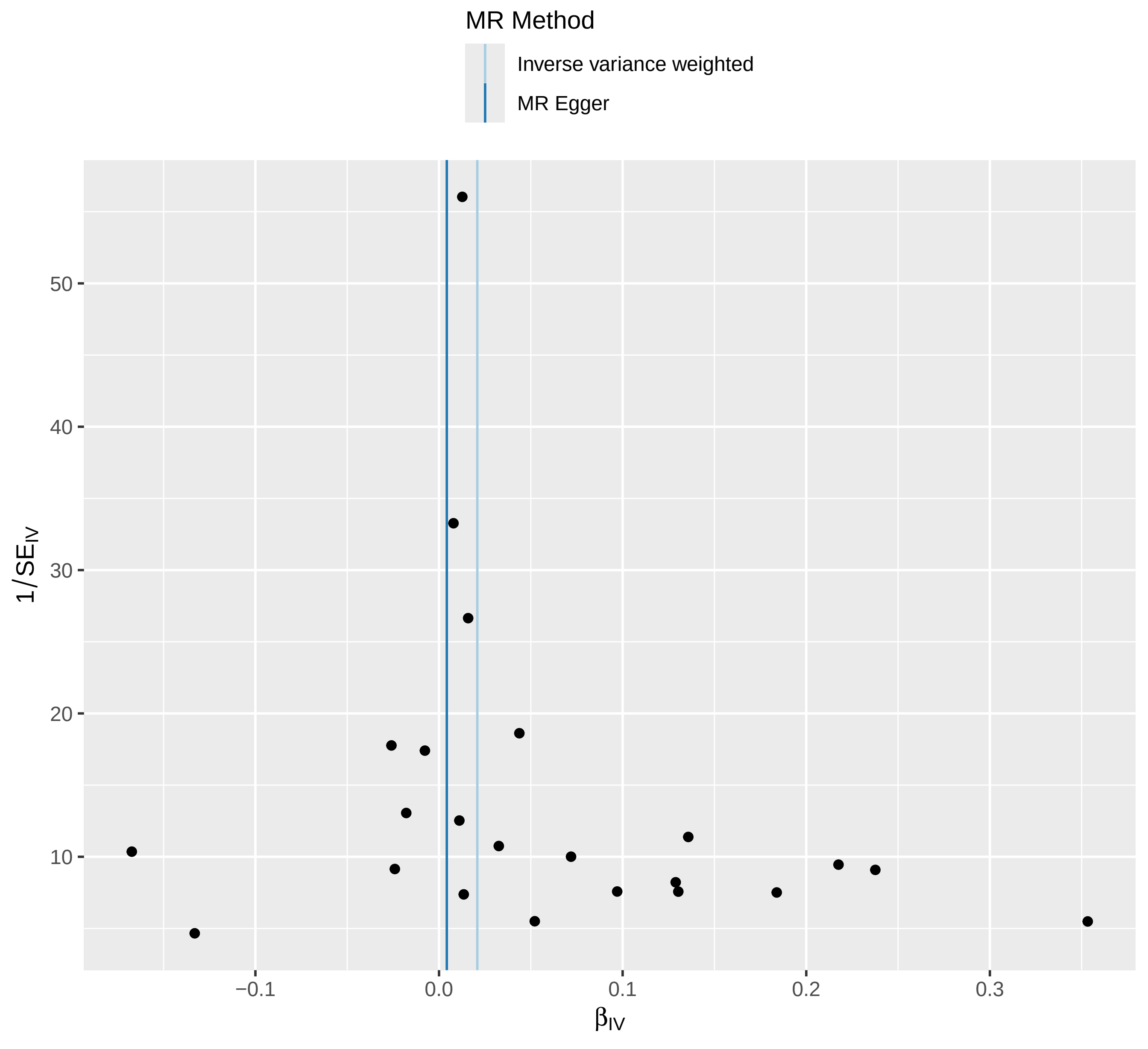

MR Method
Inverse variance weighted
MR Egger
50
40
30
20
10
1/SE_IV
−0.1
0.0
0.1
0.2
0.3
β_IV

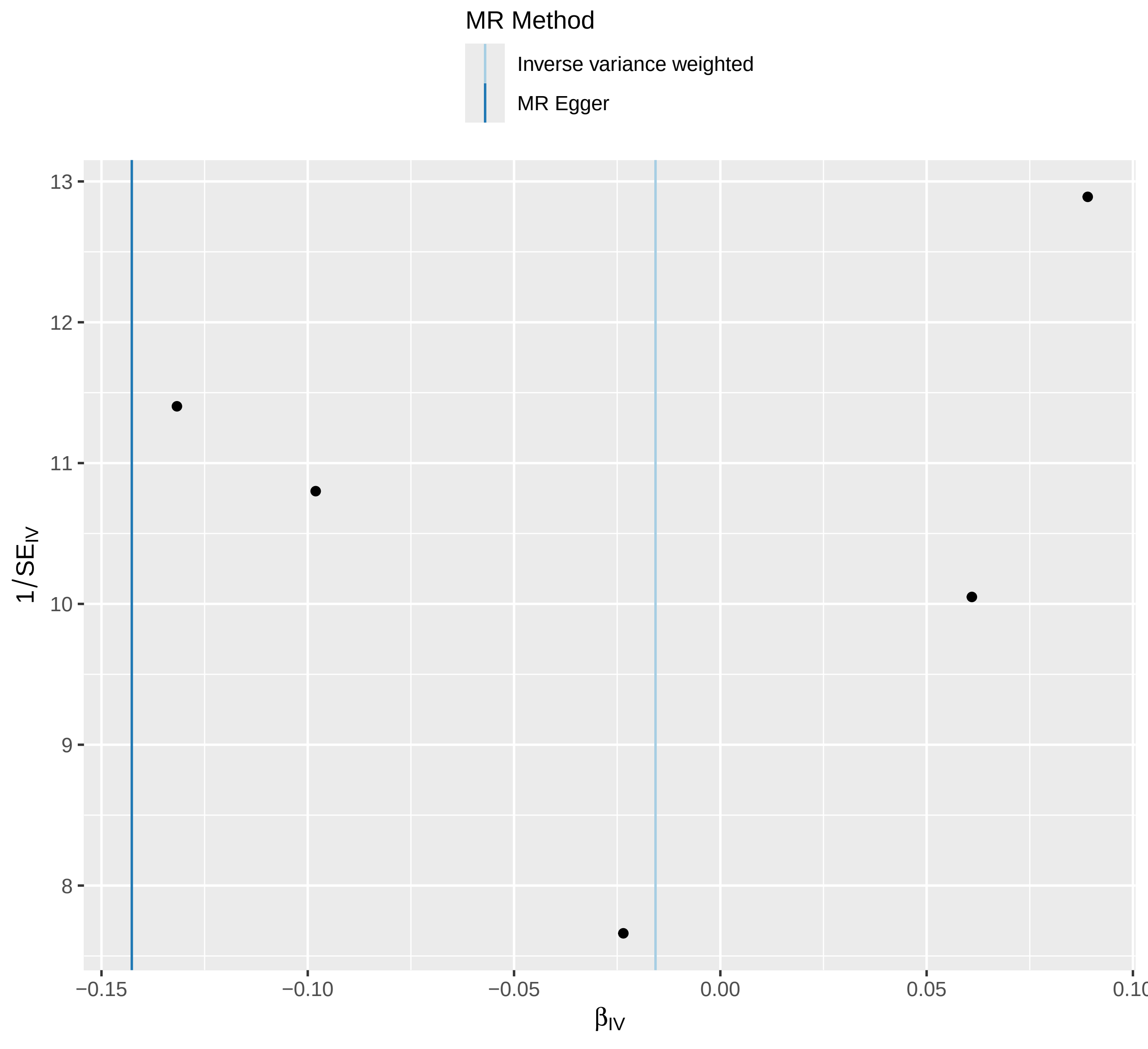
MR Method
Inverse variance weighted
MR Egger
13
12
11
10
9
8
$1/SE_{IV}$
−0.15
−0.10
−0.05
0.00
0.05
0.10
β_{IV}

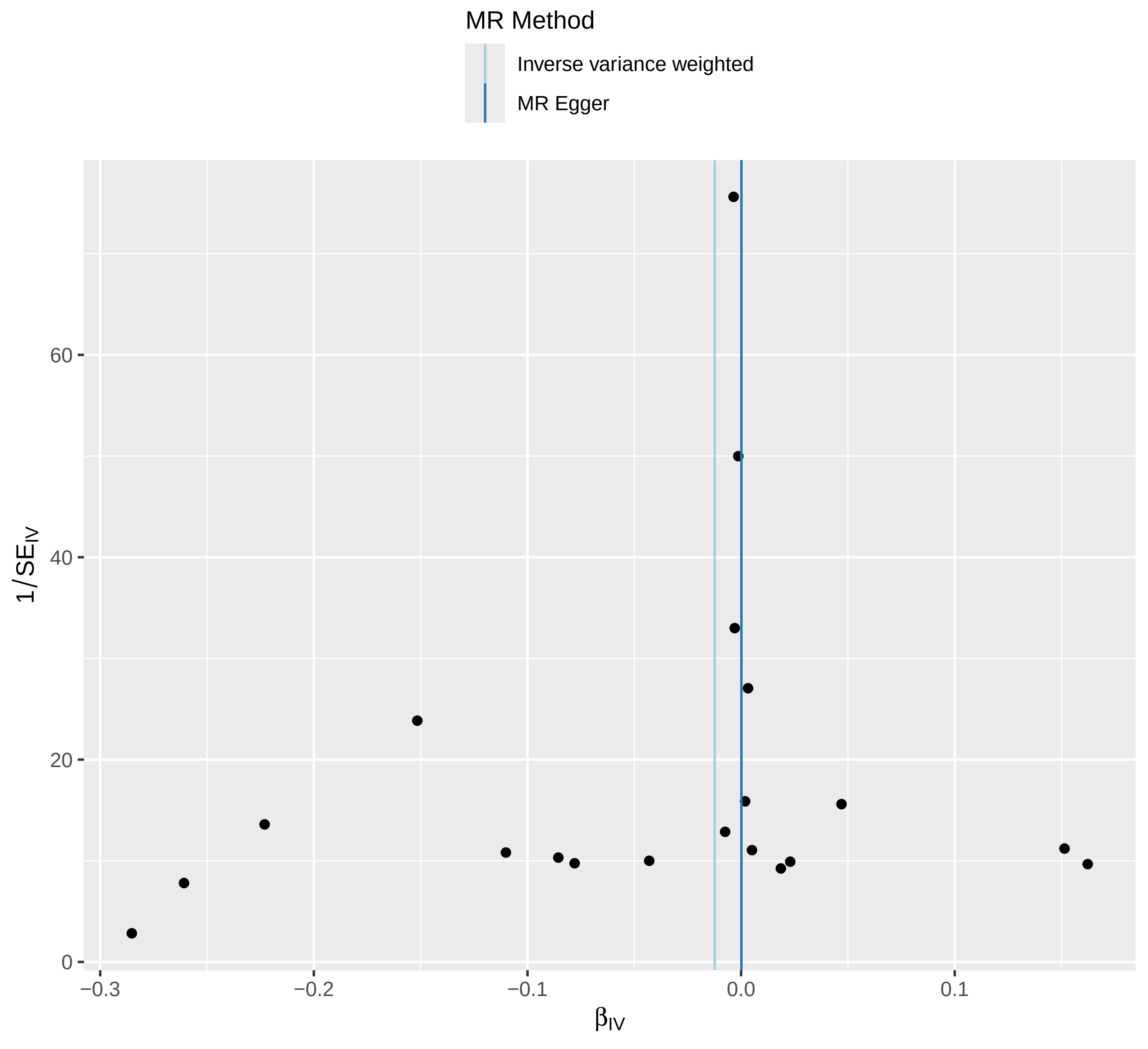

MR Method
Inverse variance weighted
MR Egger
1/SE$_{IV}$
60
40
20
0
−0.3
−0.2
−0.1
0.0
0.1
β$_{IV}$

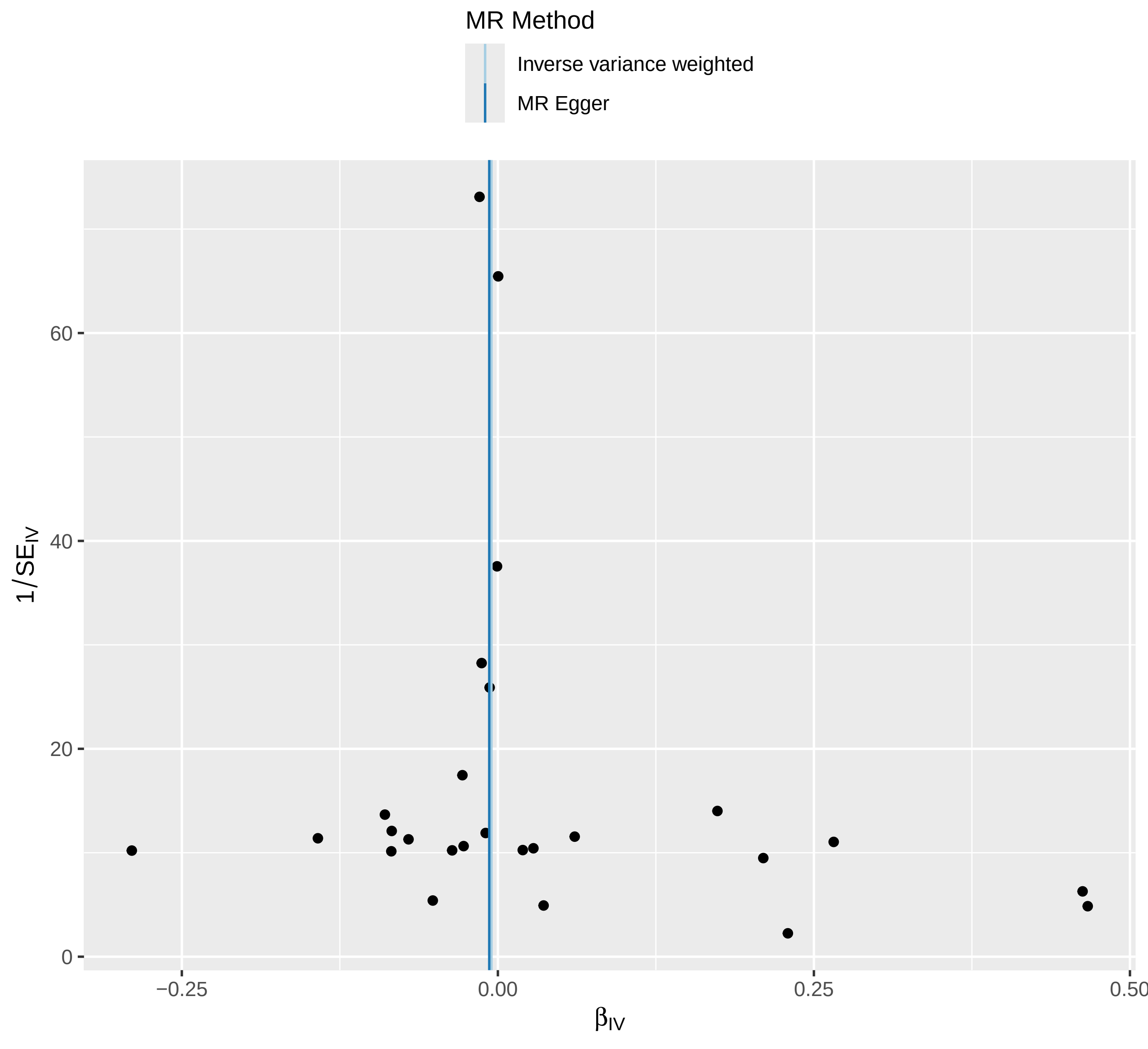
MR Method
Inverse variance weighted
MR Egger
1/SE$_{IV}$
0
20
40
60
−0.25
0.00
0.25
0.50
β$_{IV}$

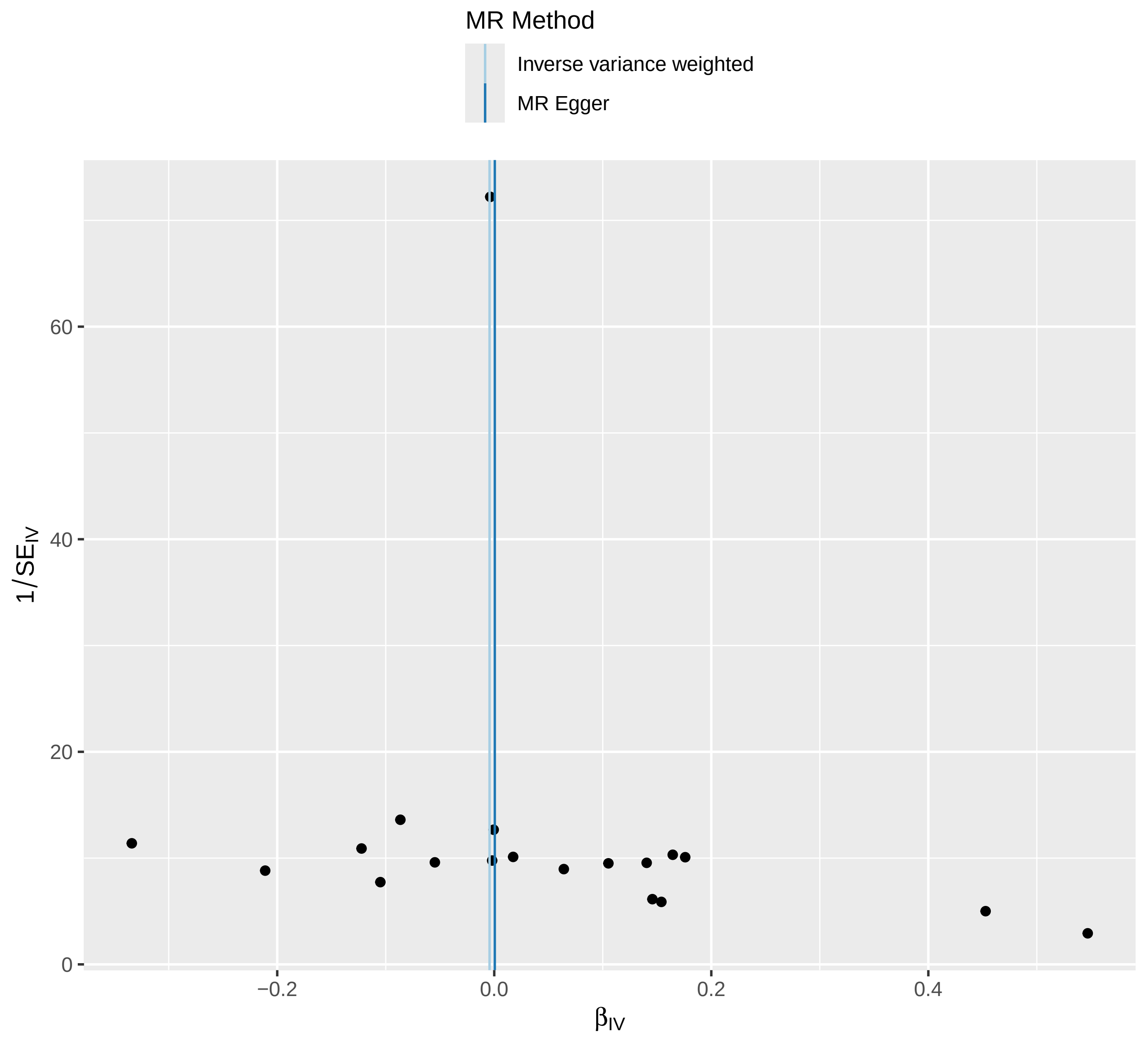

MR Method
Inverse variance weighted
MR Egger
1/SE$_{IV}$
60
40
20
0
−0.2
0.0
0.2
0.4
β$_{IV}$

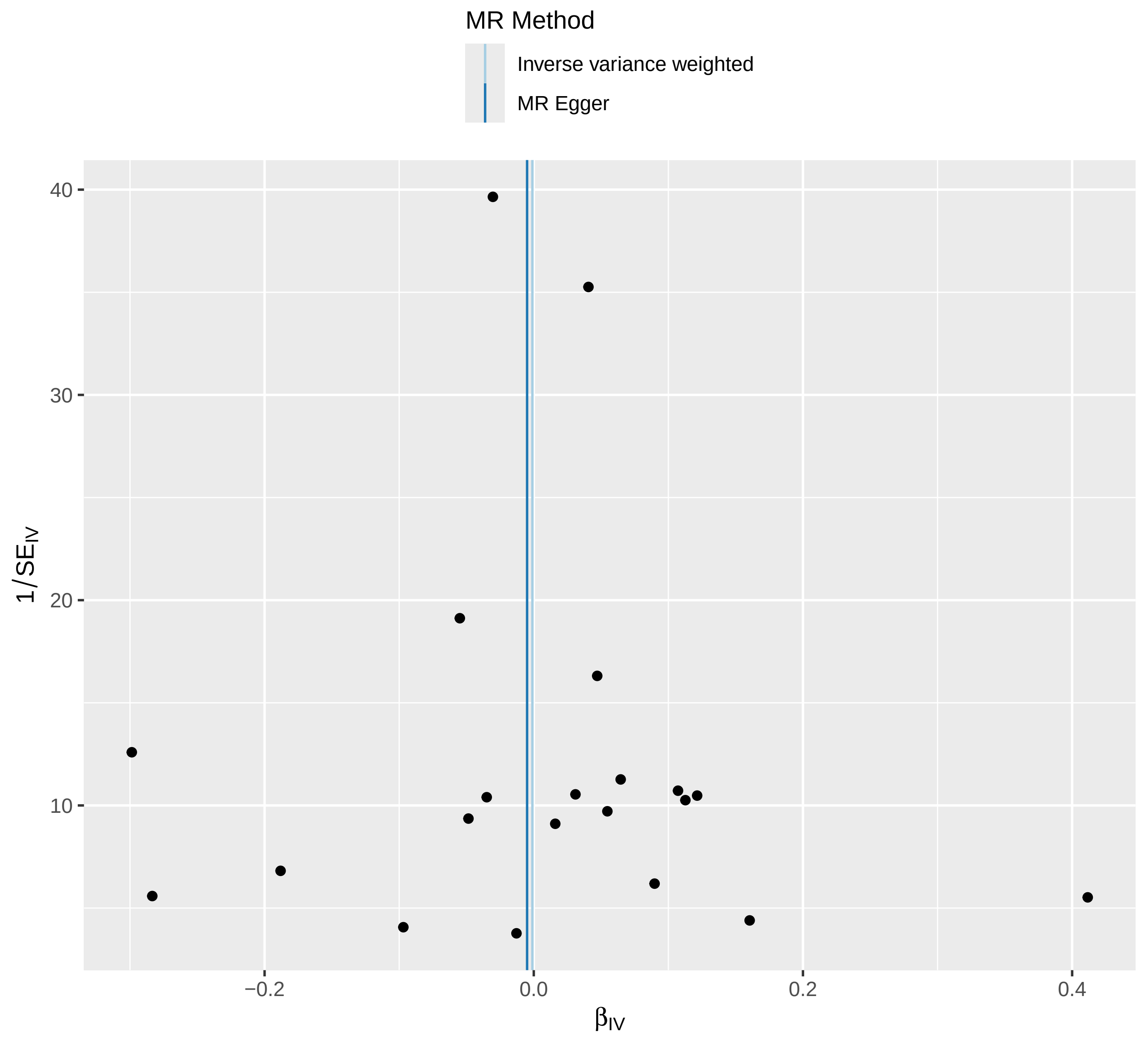

MR Method
Inverse variance weighted
MR Egger
1/SE_IV
β_IV
40
30
20
10
−0.2
0.0
0.2
0.4

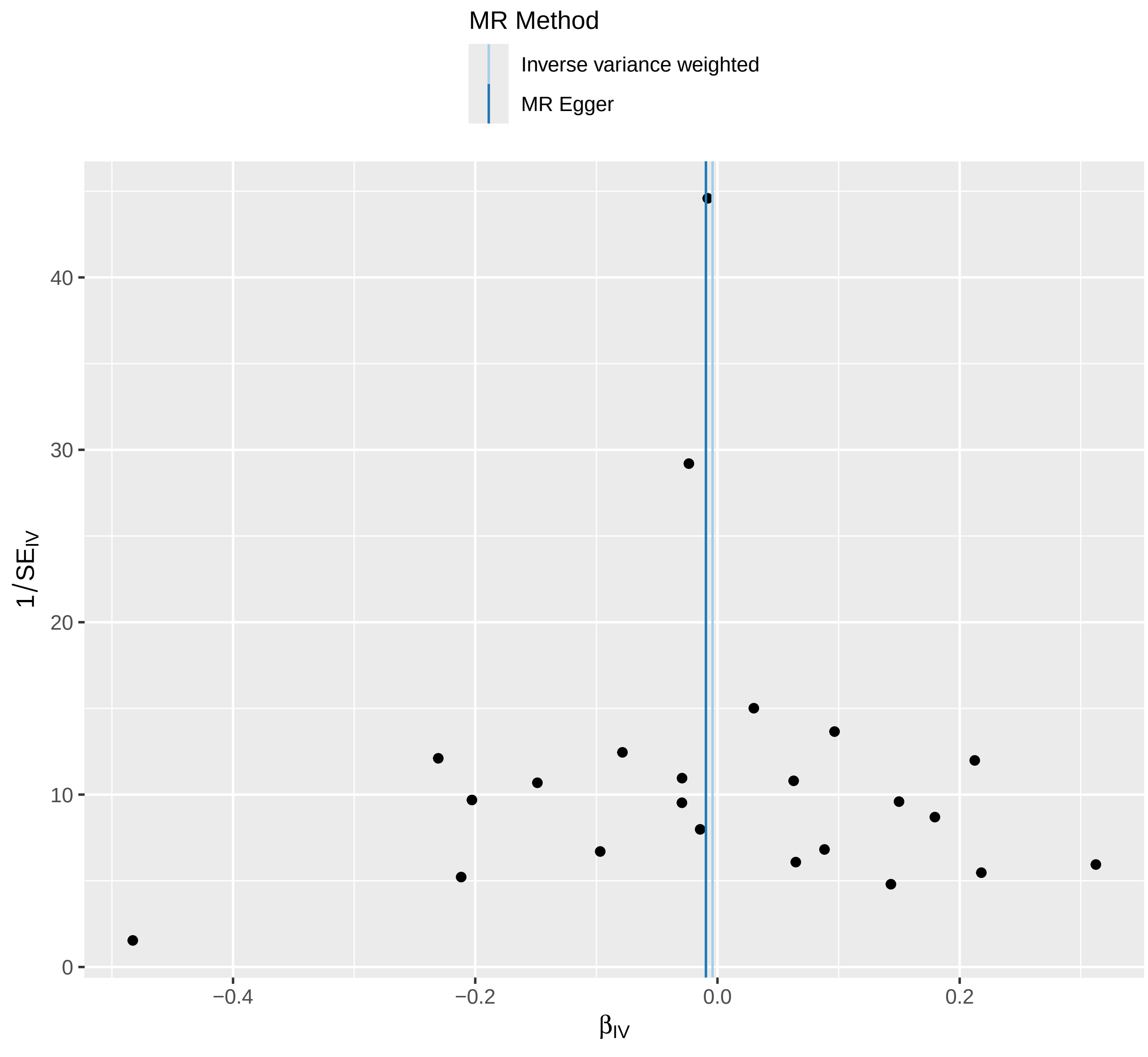
MR Method
Inverse variance weighted
MR Egger
1/SE$_{IV}$
40
30
20
10
0
−0.4
−0.2
0.0
0.2
β$_{IV}$

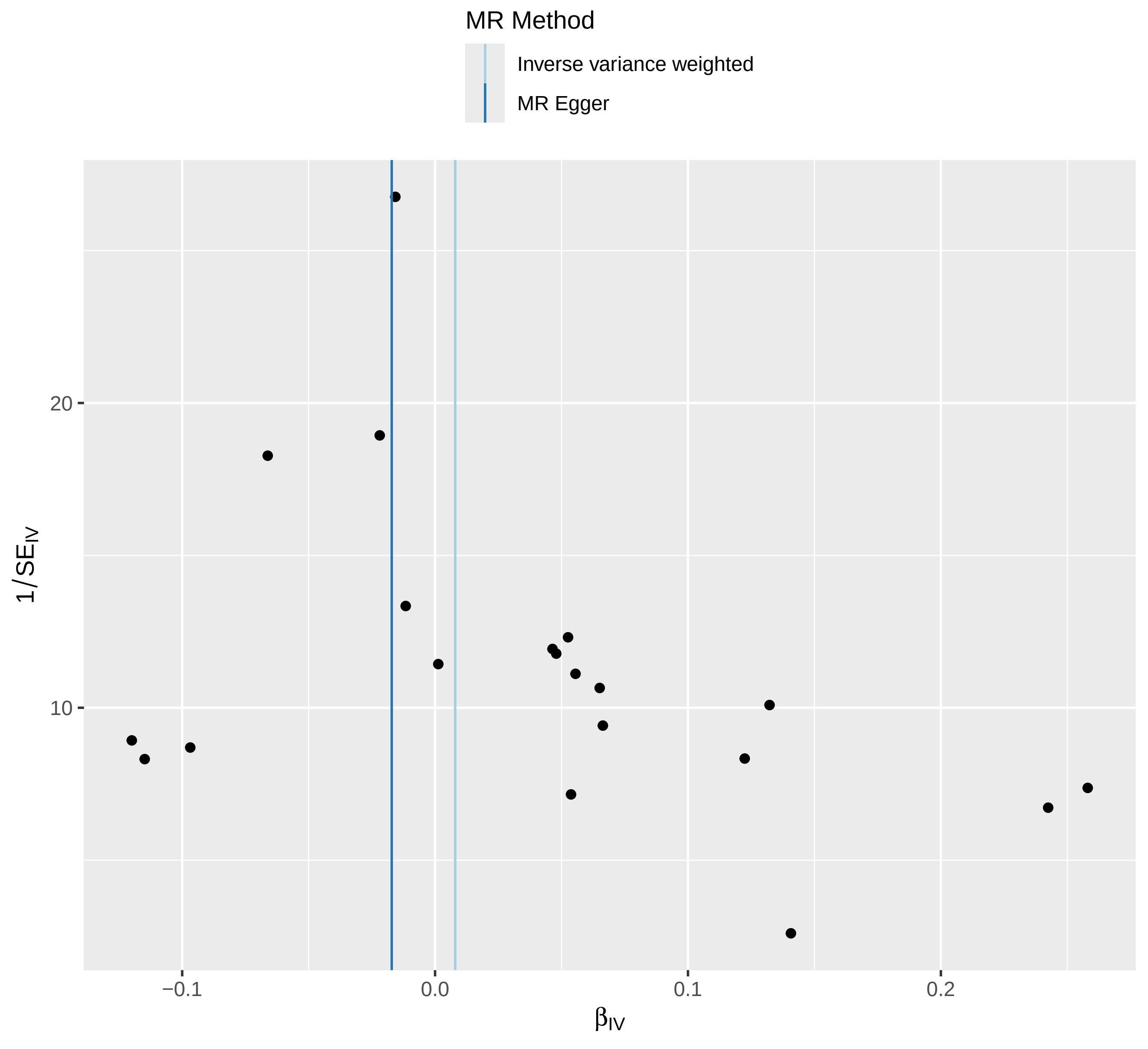

MR Method
Inverse variance weighted
MR Egger
20
10
1/SE$_{IV}$
−0.1
0.0
0.1
0.2
β$_{IV}$

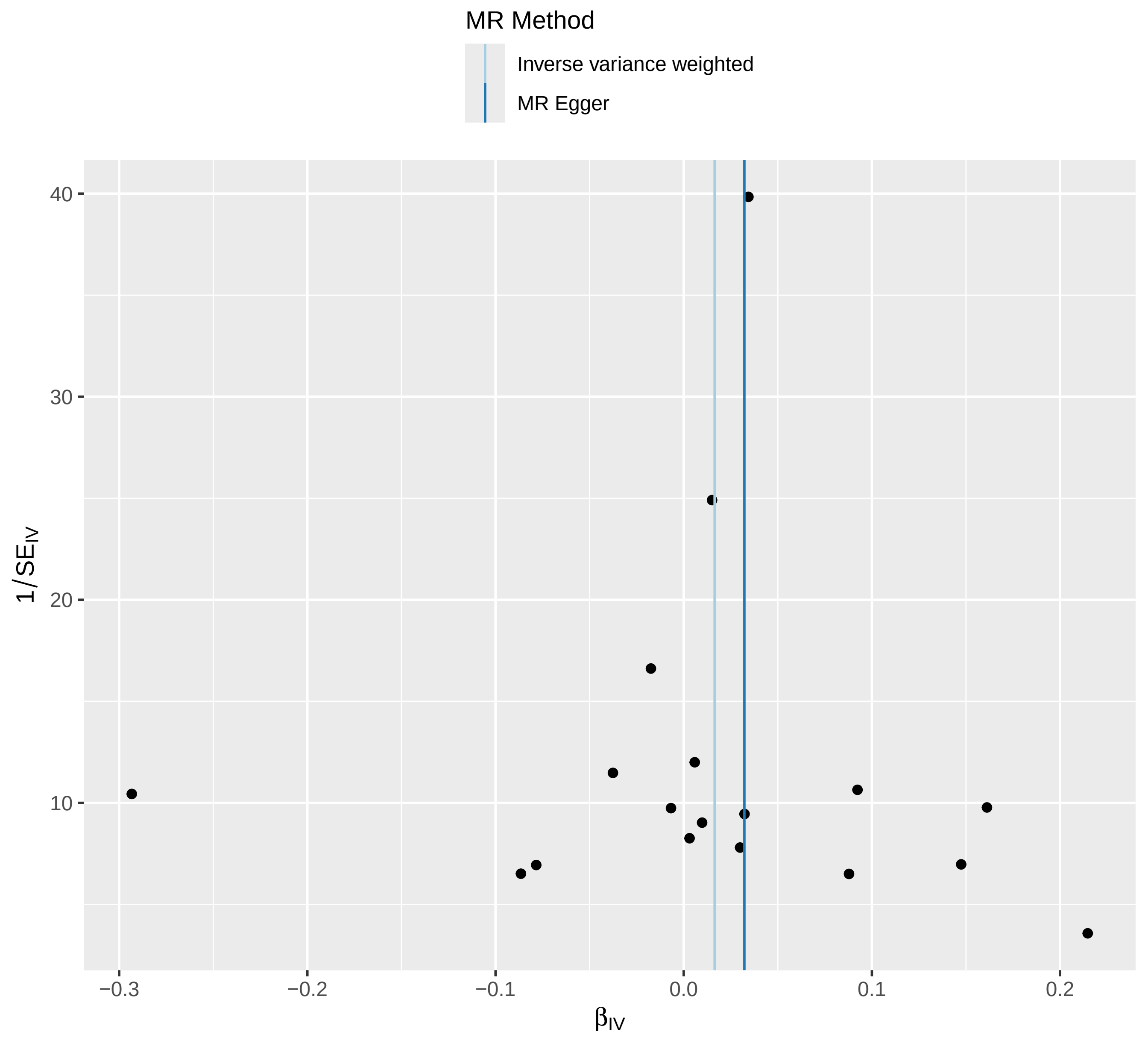

MR Method
Inverse variance weighted
MR Egger
40
30
20
10
1/SE_IV
−0.3
−0.2
−0.1
0.0
0.1
0.2
β_IV

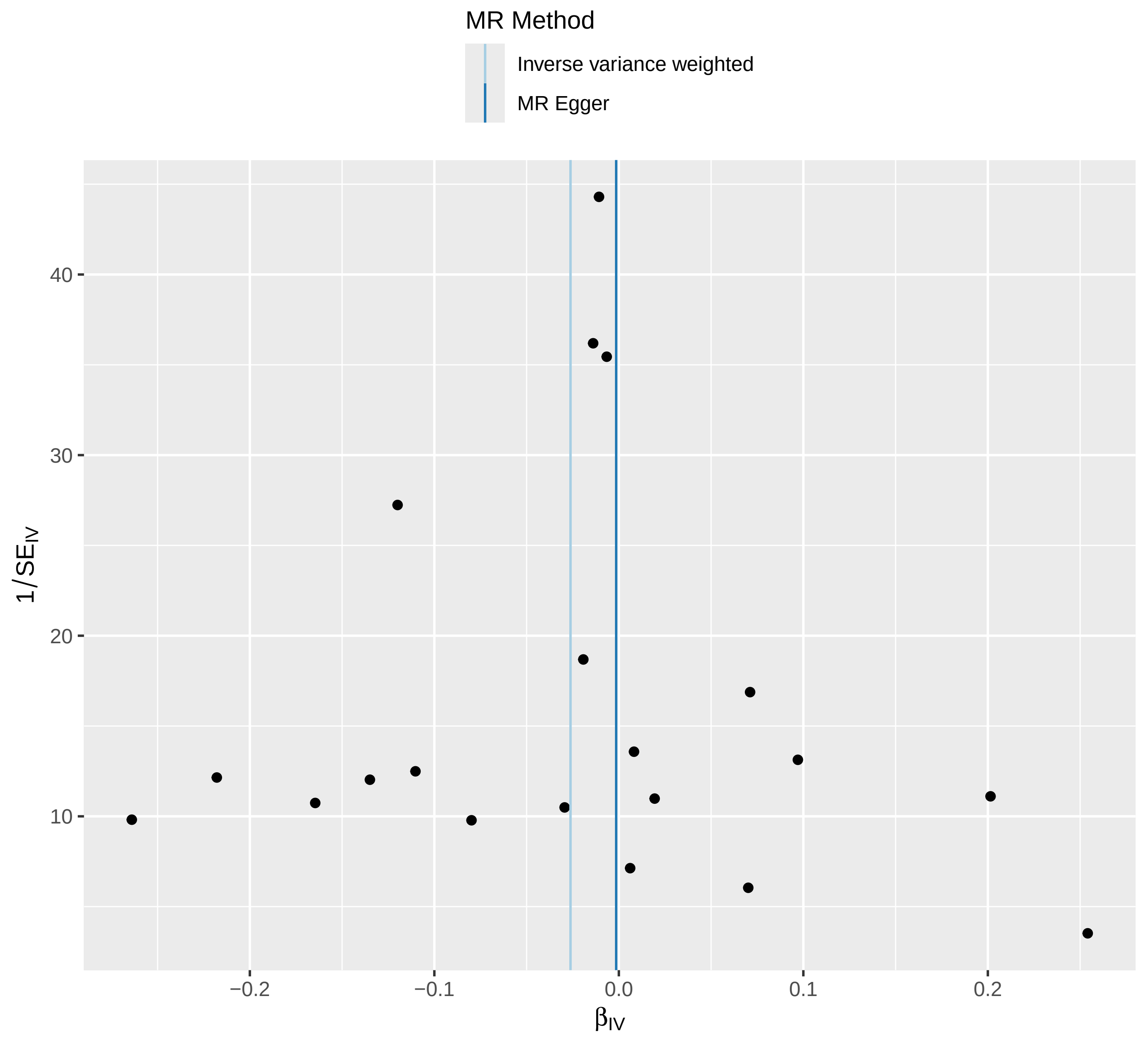

MR Method
Inverse variance weighted
MR Egger
40
30
20
10
1/SE$_{IV}$
−0.2
−0.1
0.0
0.1
0.2
β$_{IV}$

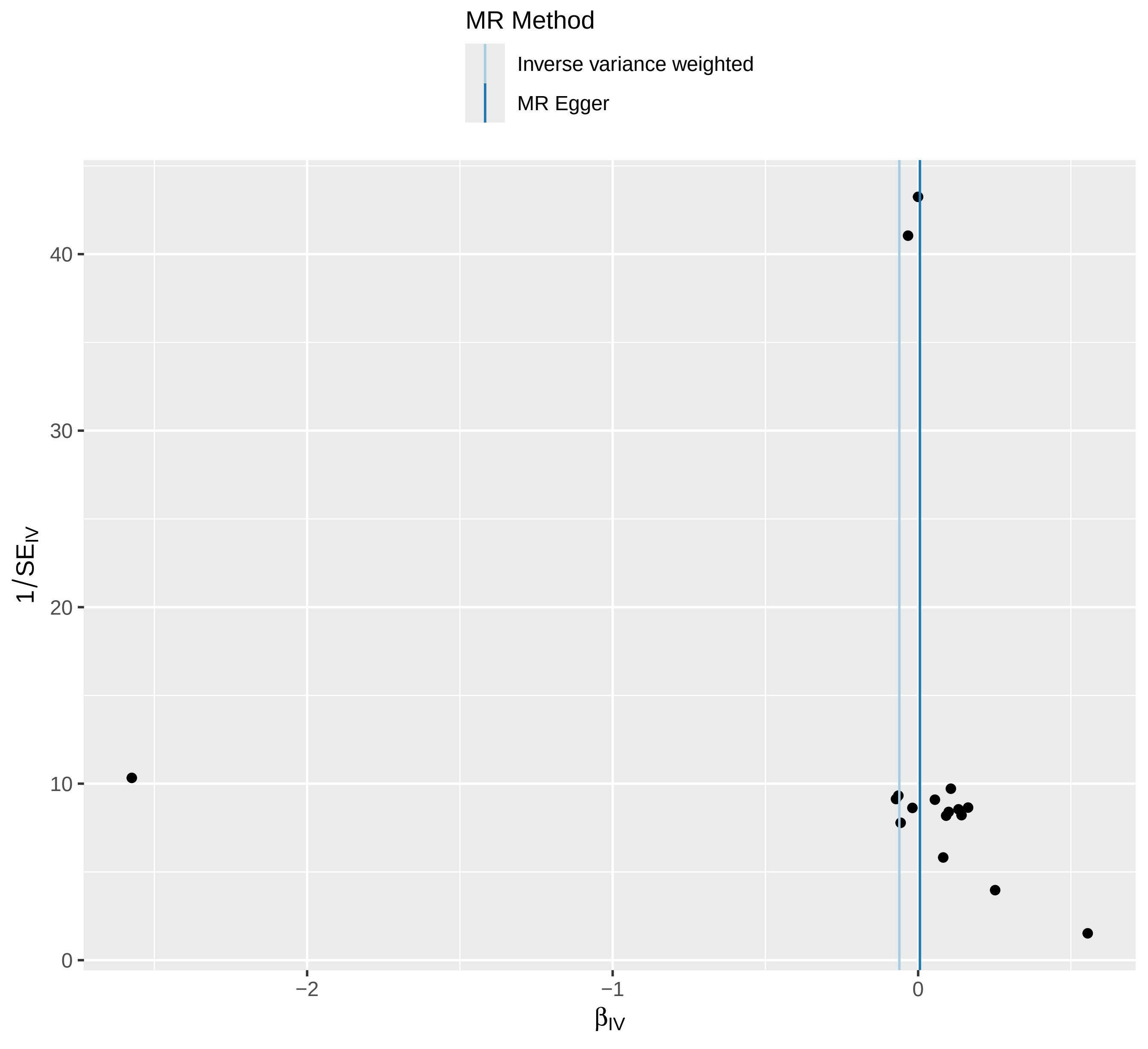
MR Method
Inverse variance weighted
MR Egger
1/SE$_{IV}$
40
30
20
10
0
−2
−1
0
β$_{IV}$

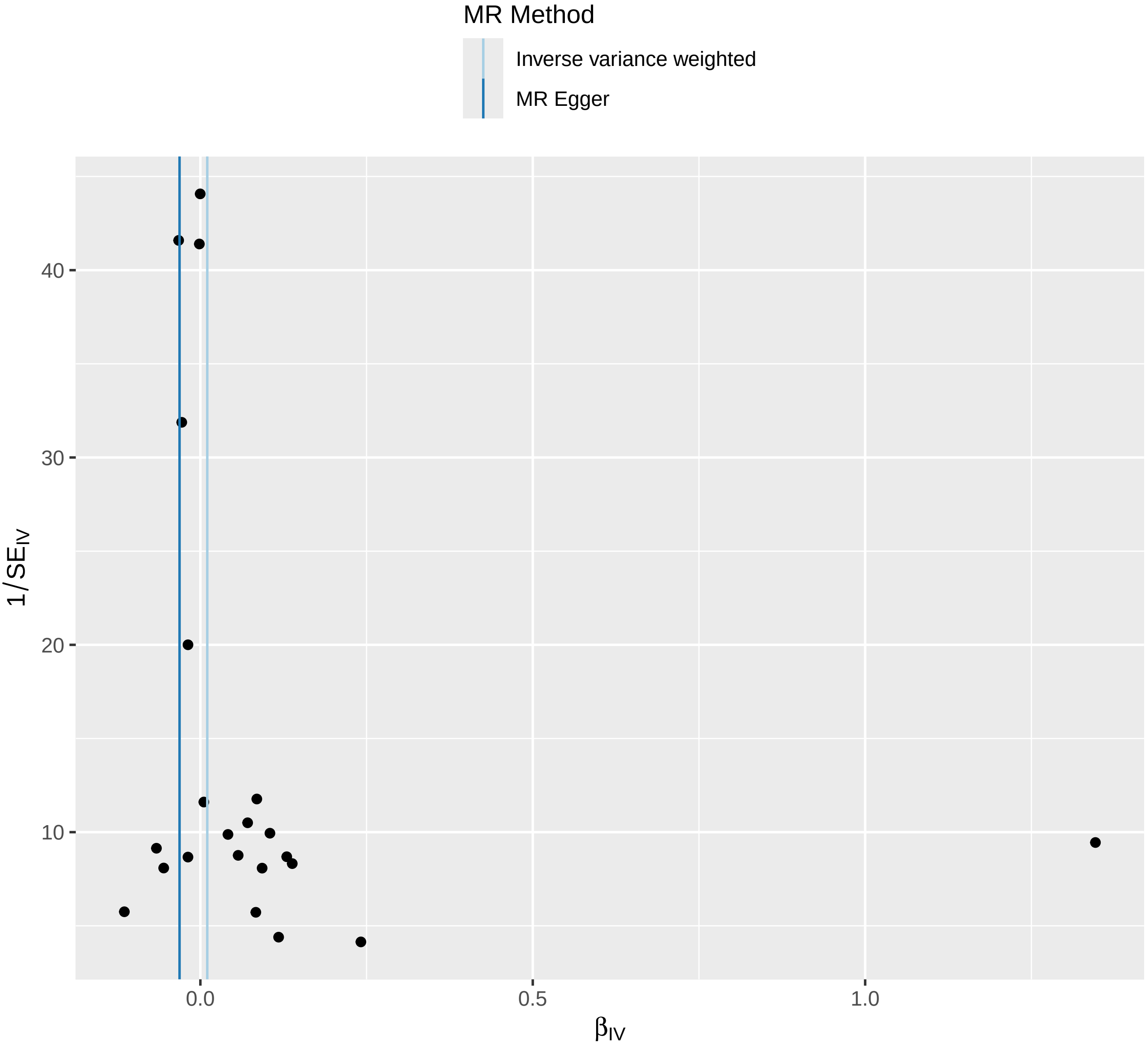
MR Method
Inverse variance weighted
MR Egger
40
30
20
10
0.0
0.5
1.0
1/SE_IV
β_IV

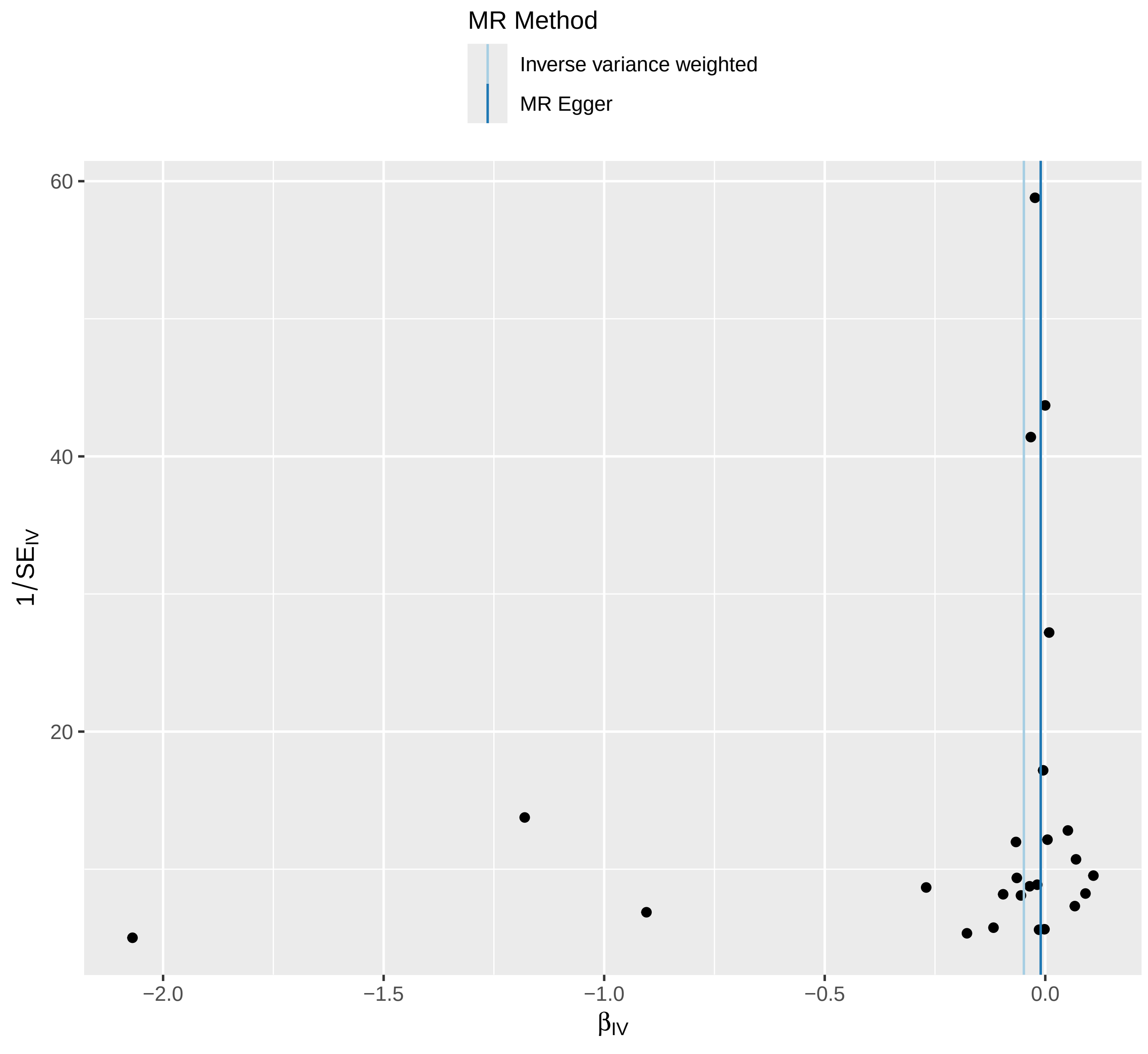
MR Method
Inverse variance weighted
MR Egger
60
40
20
1/SE$_{IV}$
−2.0
−1.5
−1.0
−0.5
0.0
β$_{IV}$

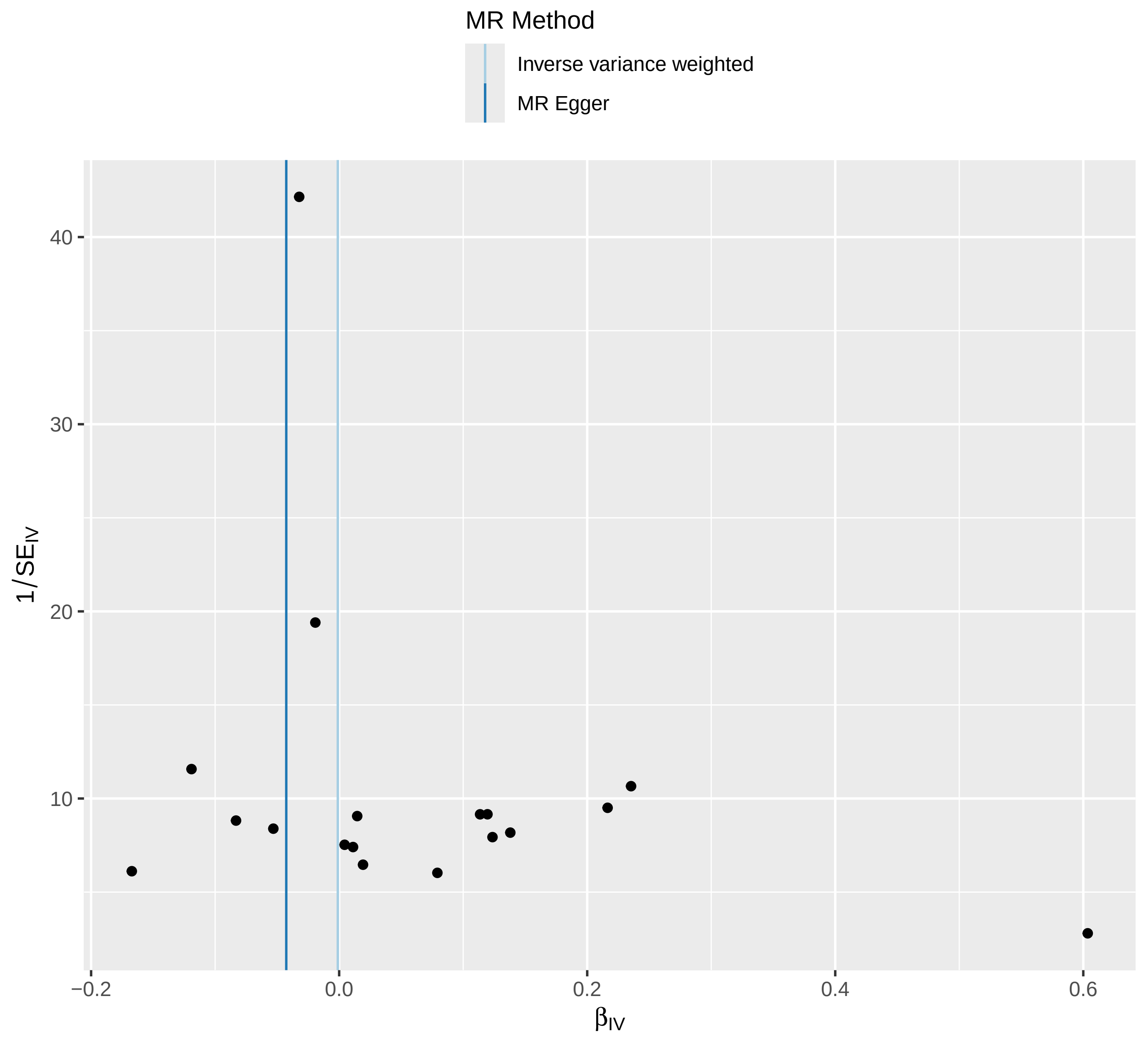

MR Method
Inverse variance weighted
MR Egger
40
30
20
10
1/SE_IV
−0.2
0.0
0.2
0.4
0.6
β_IV

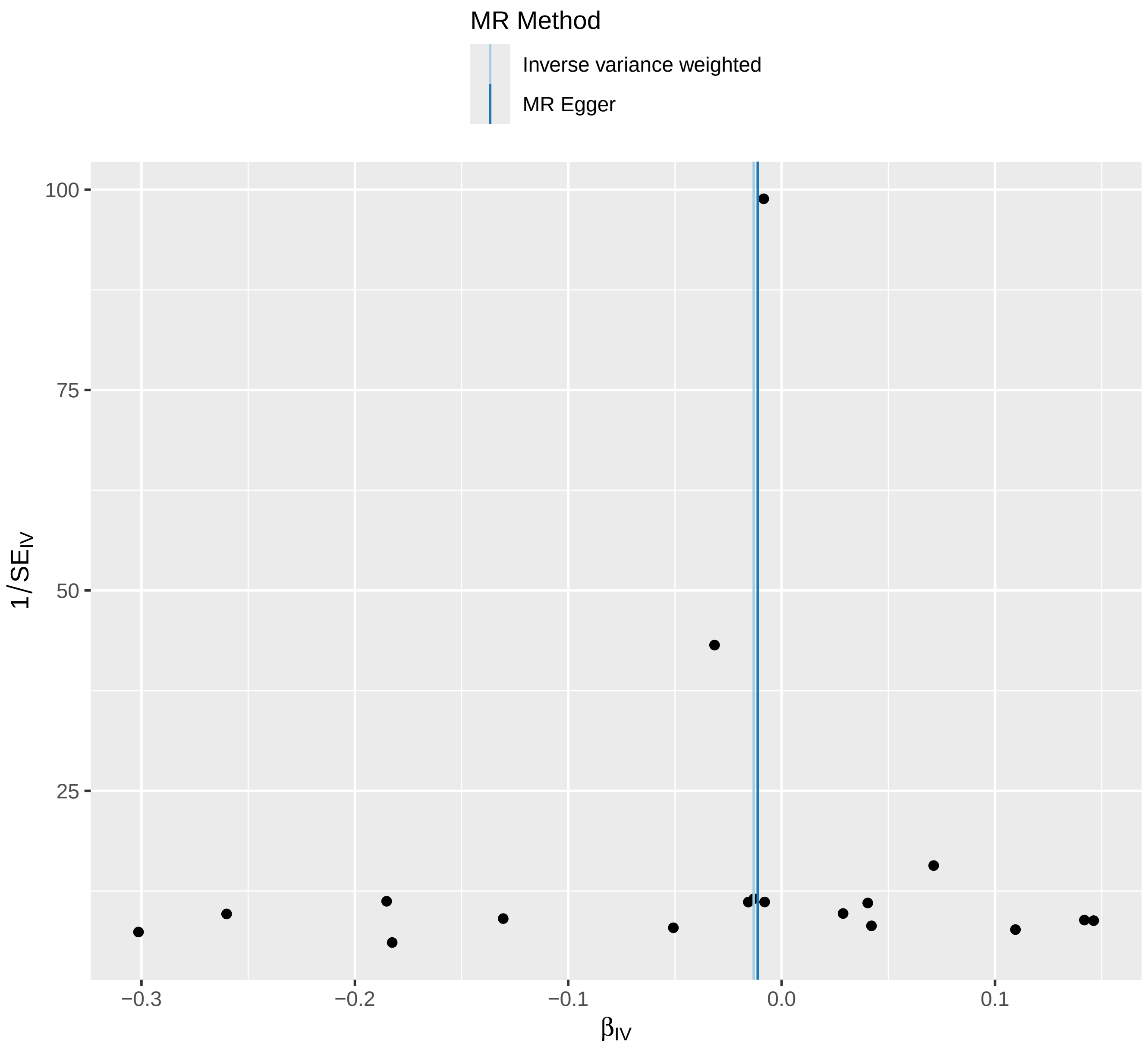
MR Method
Inverse variance weighted
MR Egger
100
75
50
25
1/SE_IV
−0.3
−0.2
−0.1
0.0
0.1
β_IV

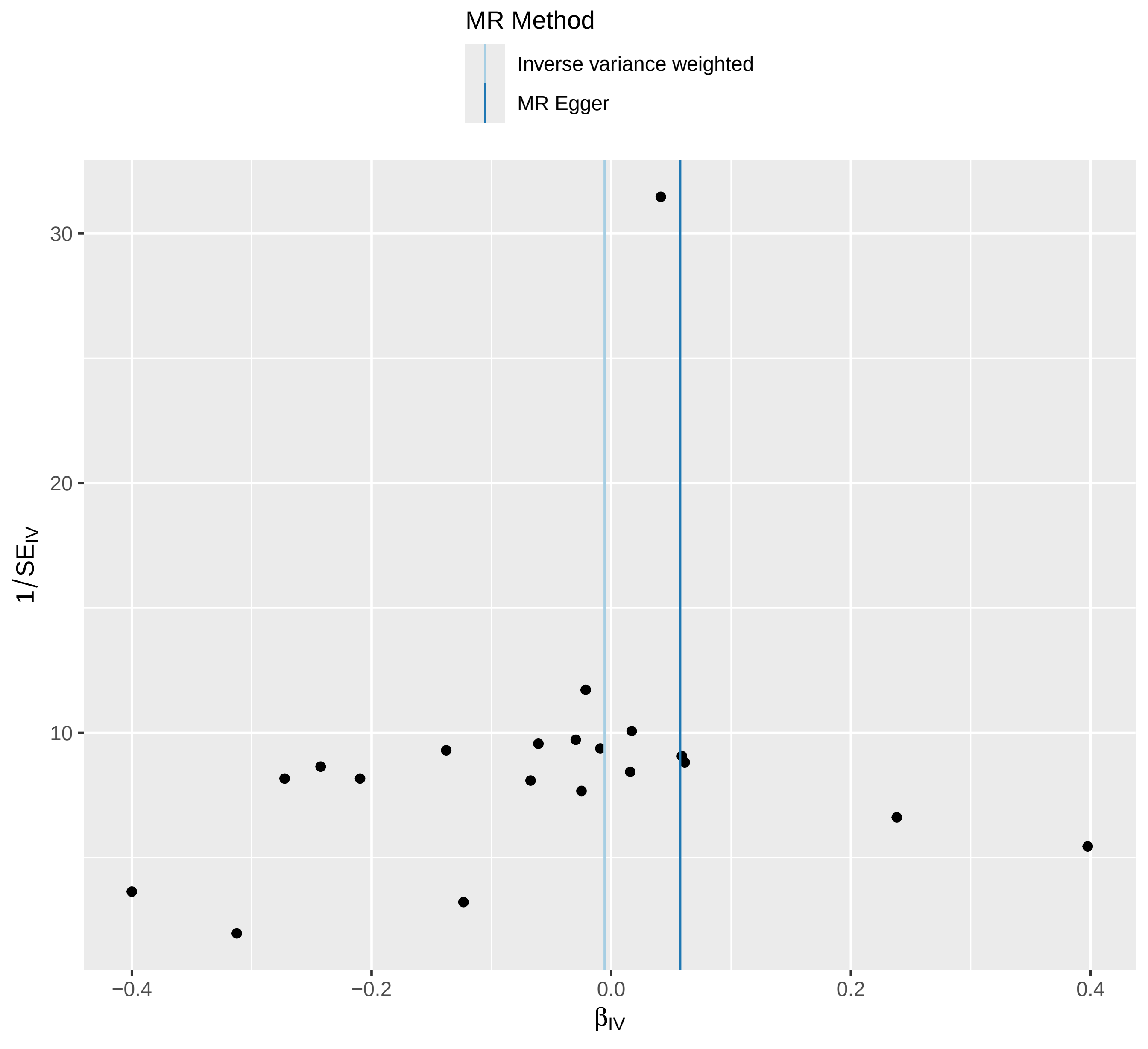
MR Method
Inverse variance weighted
MR Egger
30
20
10
1/SE_IV
−0.4
−0.2
0.0
0.2
0.4
β_IV

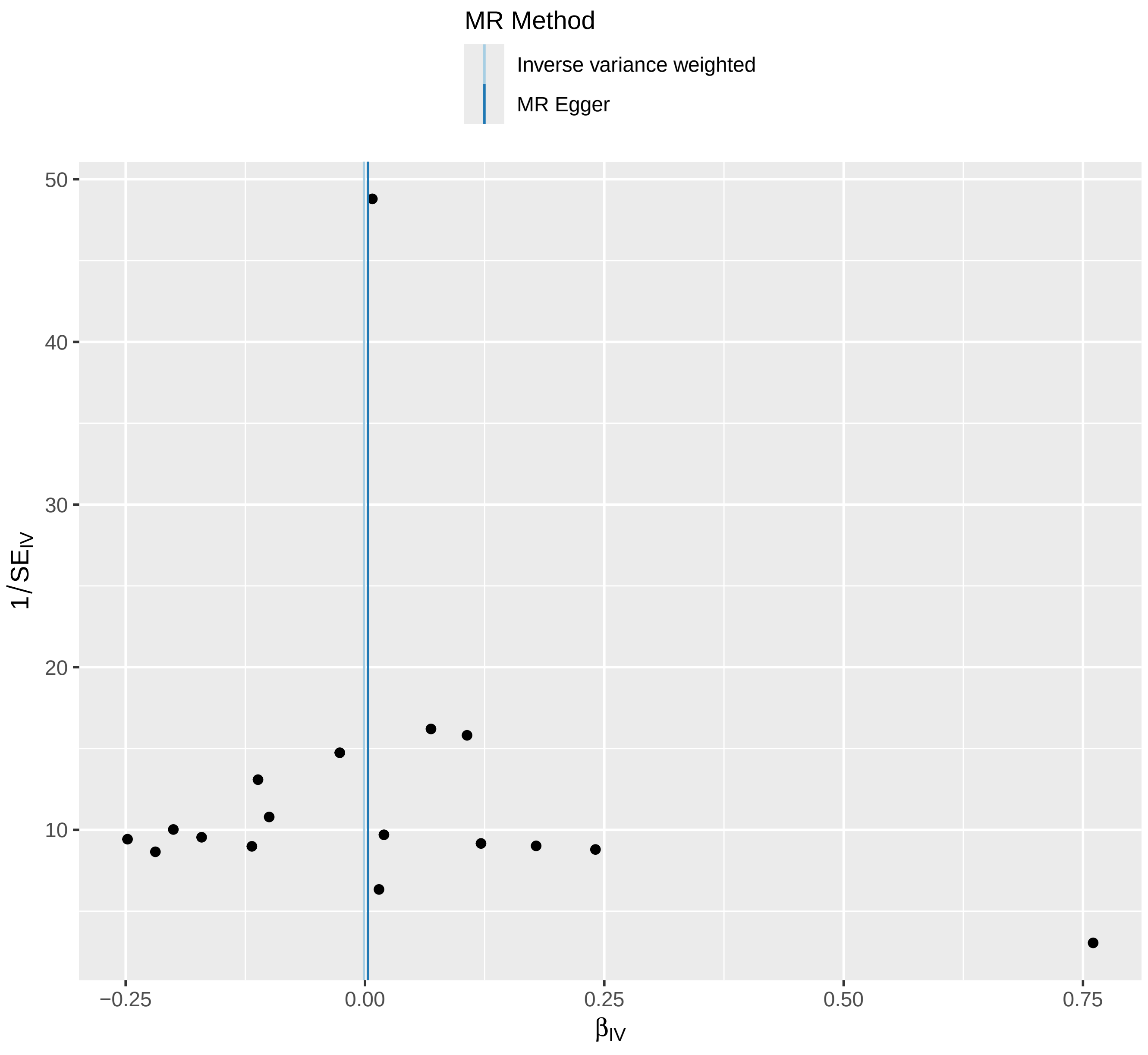

MR Method
Inverse variance weighted
MR Egger
1/SE_IV
βIV
50
40
30
20
10
−0.25
0.00
0.25
0.50
0.75

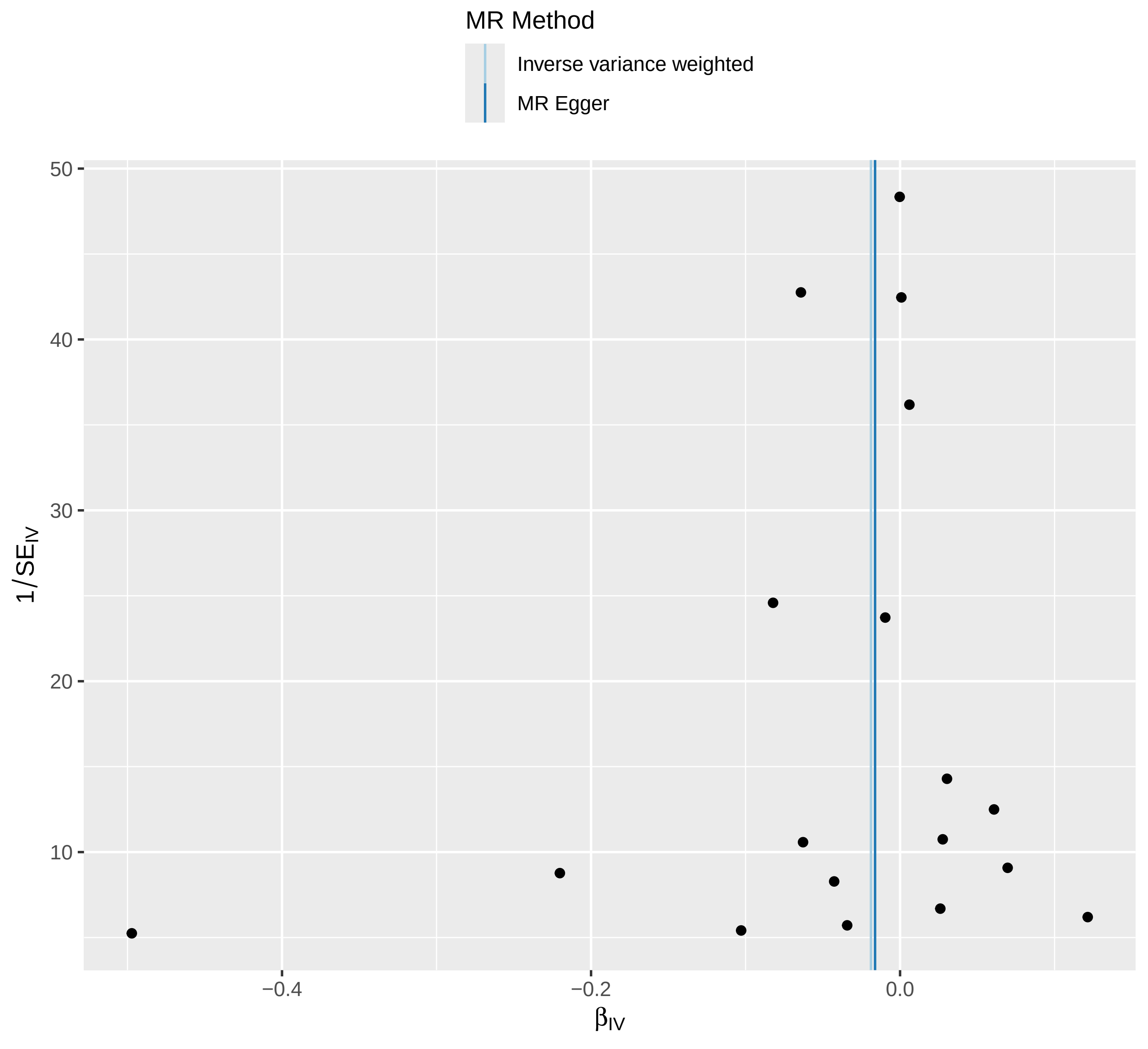
MR Method
Inverse variance weighted
MR Egger
50
40
30
20
10
1/SE$_{IV}$
−0.4
−0.2
0.0
β$_{IV}$

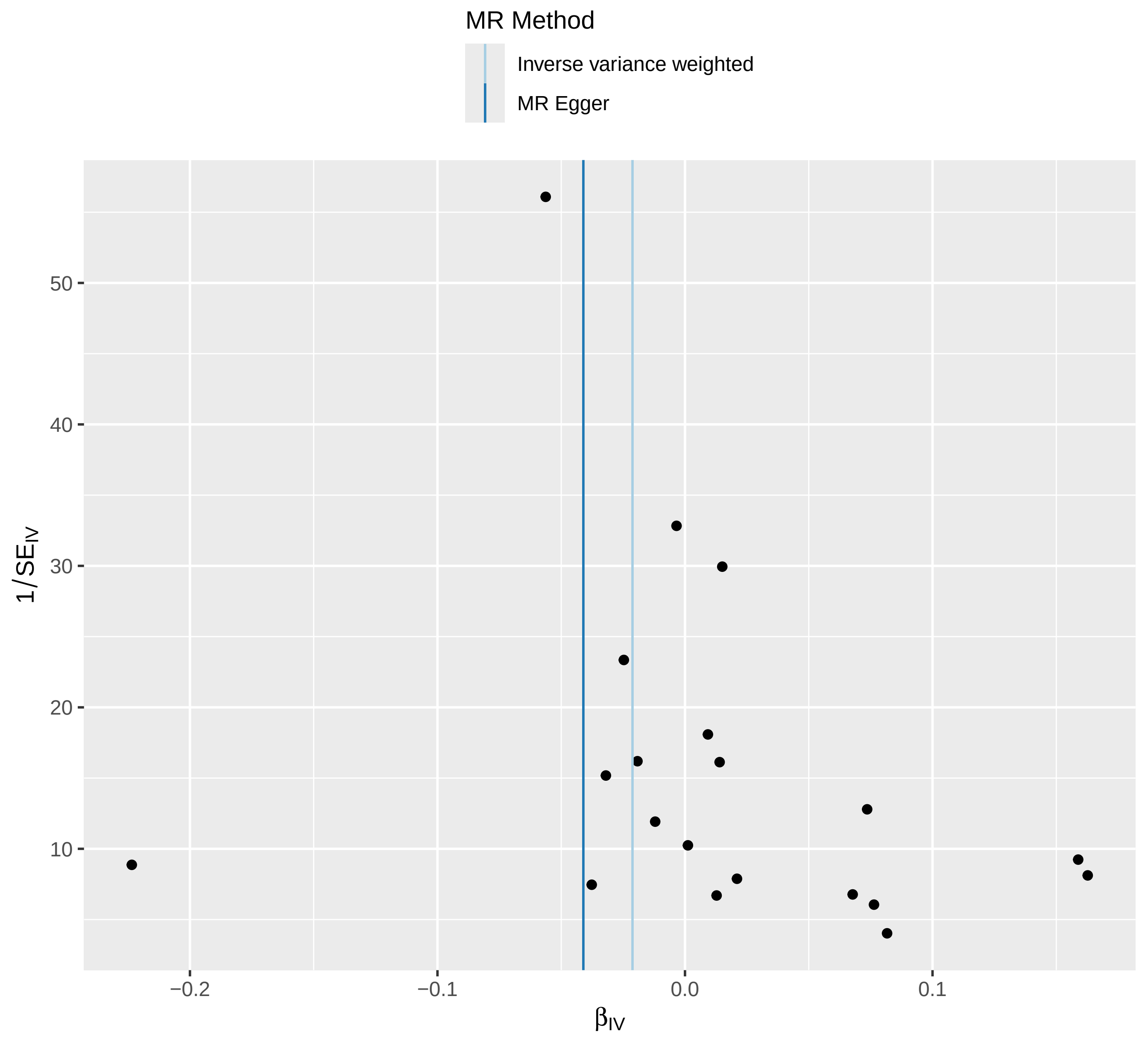
MR Method
Inverse variance weighted
MR Egger
50
40
30
20
10
1/SE_IV
−0.2
−0.1
0.0
0.1
β_IV

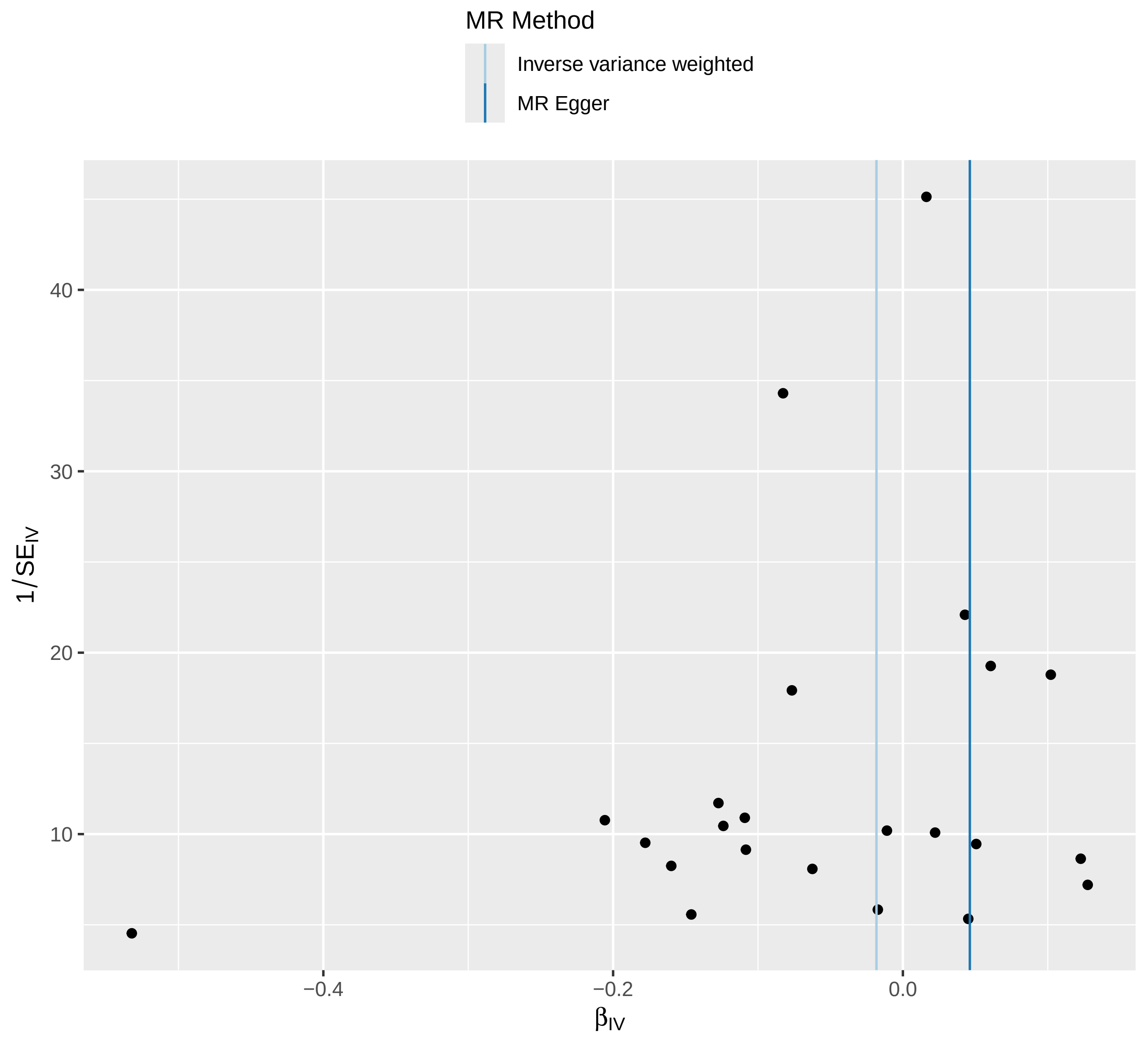

MR Method
Inverse variance weighted
MR Egger
40
30
20
10
1/SE_IV
−0.4
−0.2
0.0
β_IV

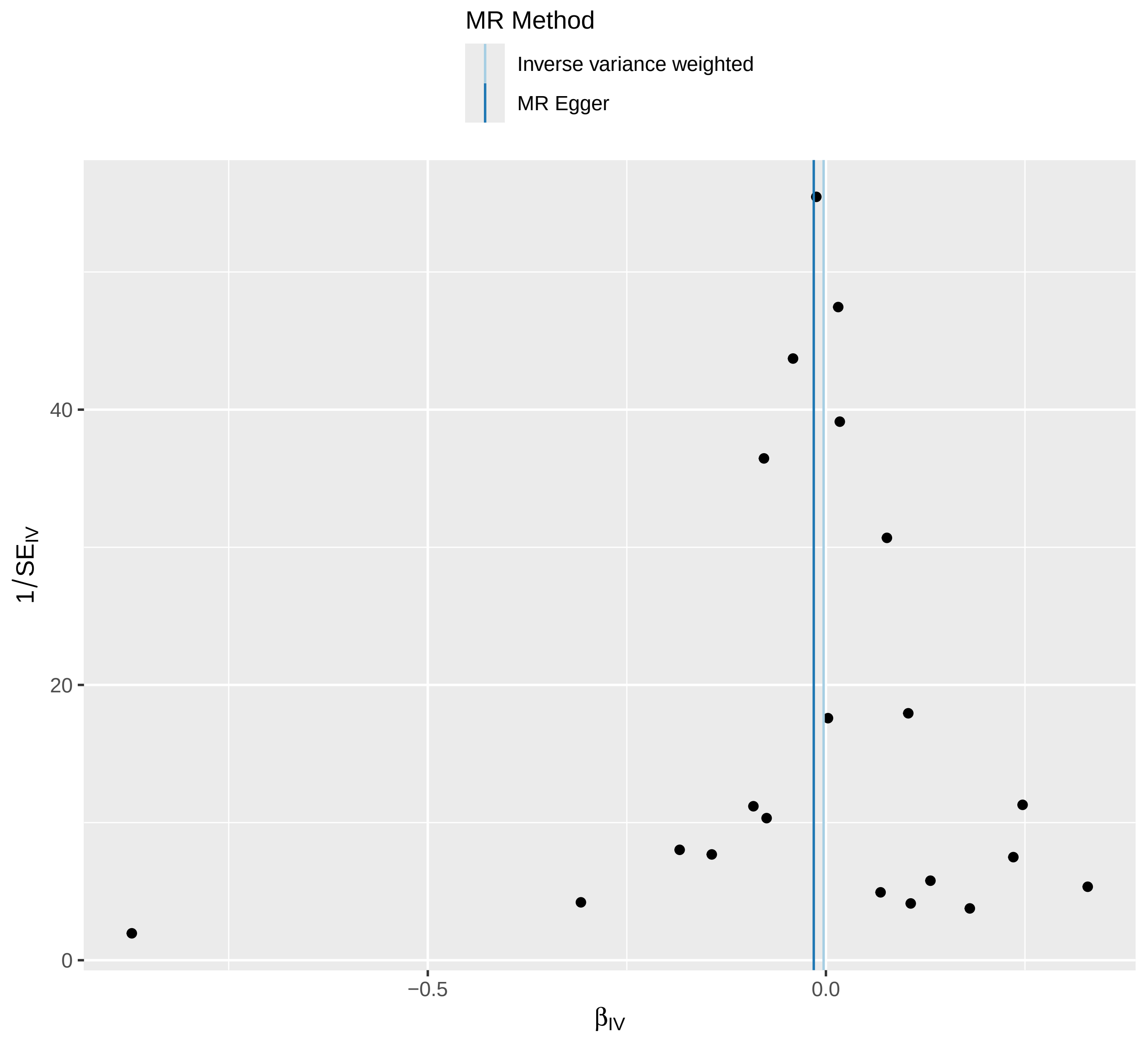
MR Method
Inverse variance weighted
MR Egger
1/SE_IV
β_IV
40
20
0
−0.5
0.0

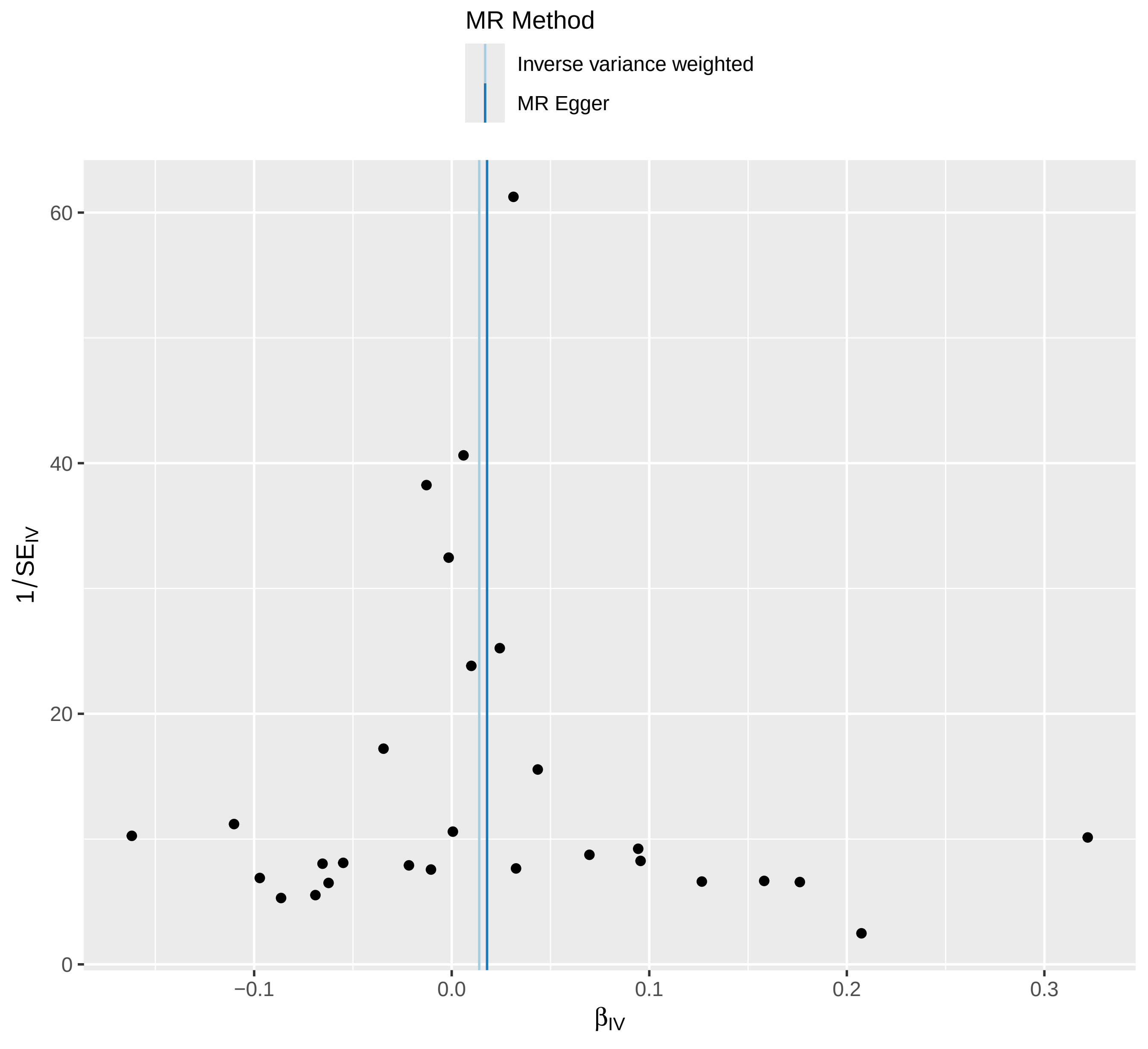
MR Method
Inverse variance weighted
MR Egger
60
40
20
0
1/SE_IV
−0.1
0.0
0.1
0.2
0.3
β_IV

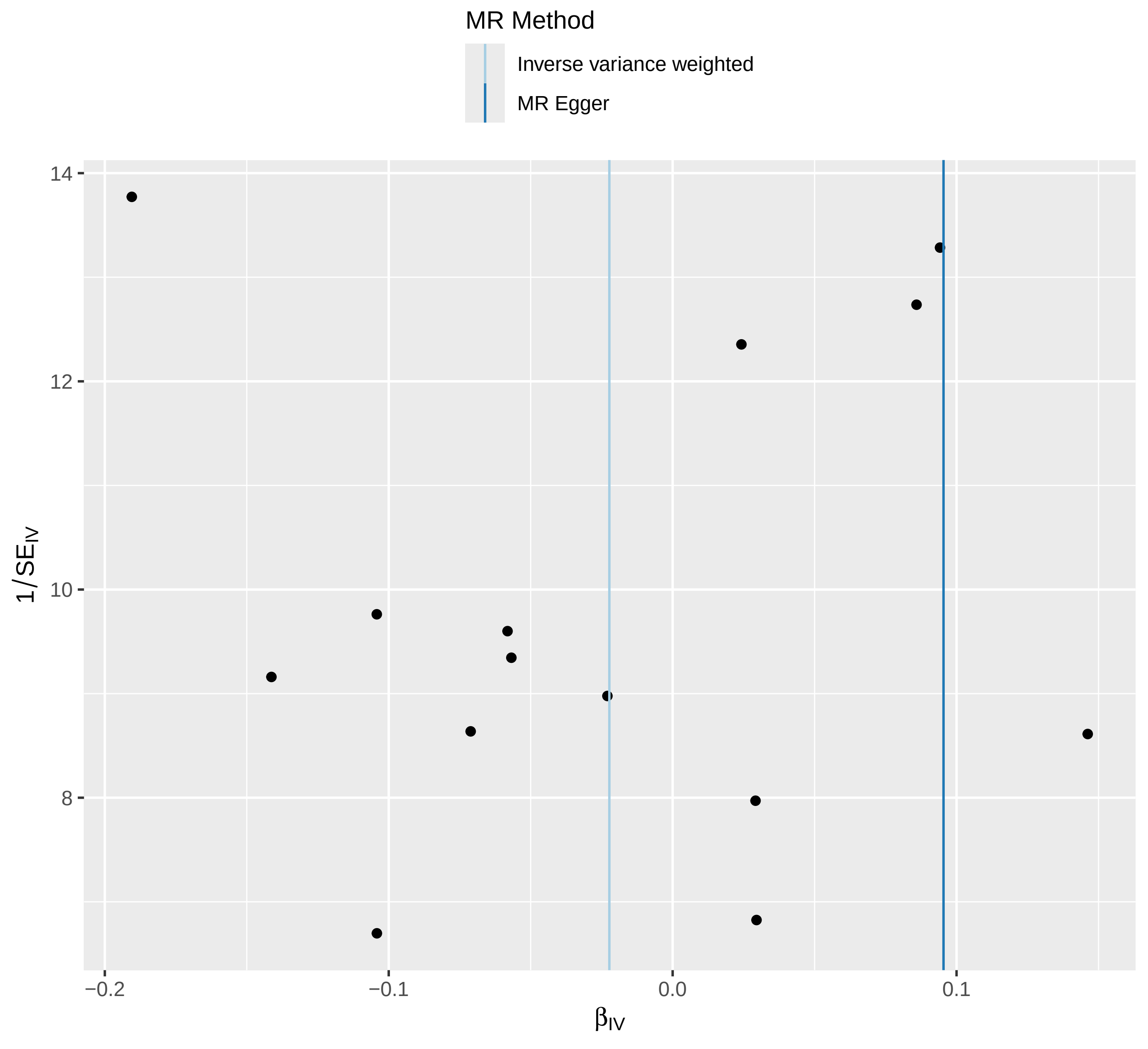
MR Method
Inverse variance weighted
MR Egger
14
12
10
8
1/SE$_{IV}$
−0.2
−0.1
0.0
0.1
β$_{IV}$

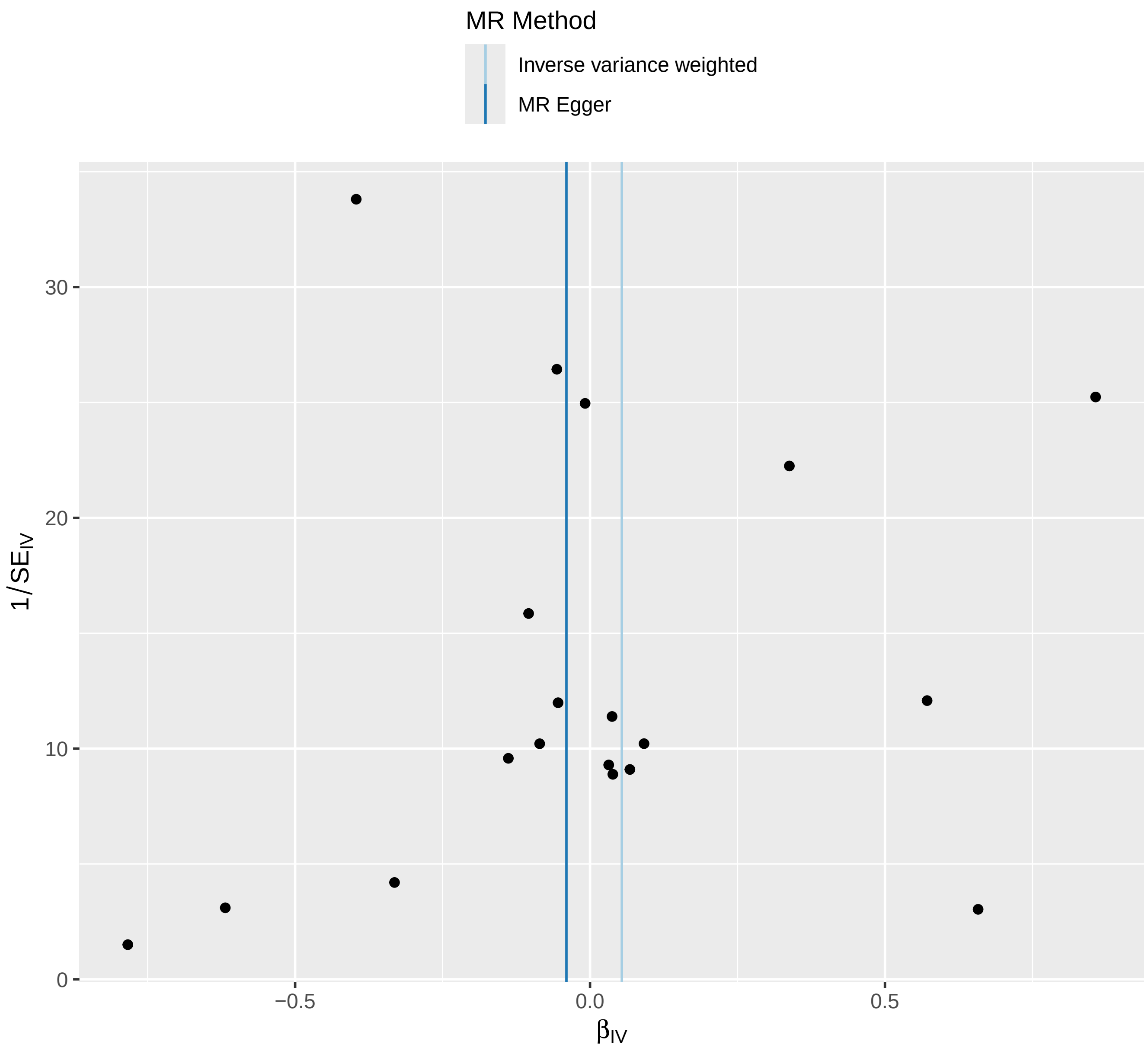
MR Method
Inverse variance weighted
MR Egger
30
20
10
0
−0.5
0.0
0.5
β_{IV}
$1/SE_{IV}$

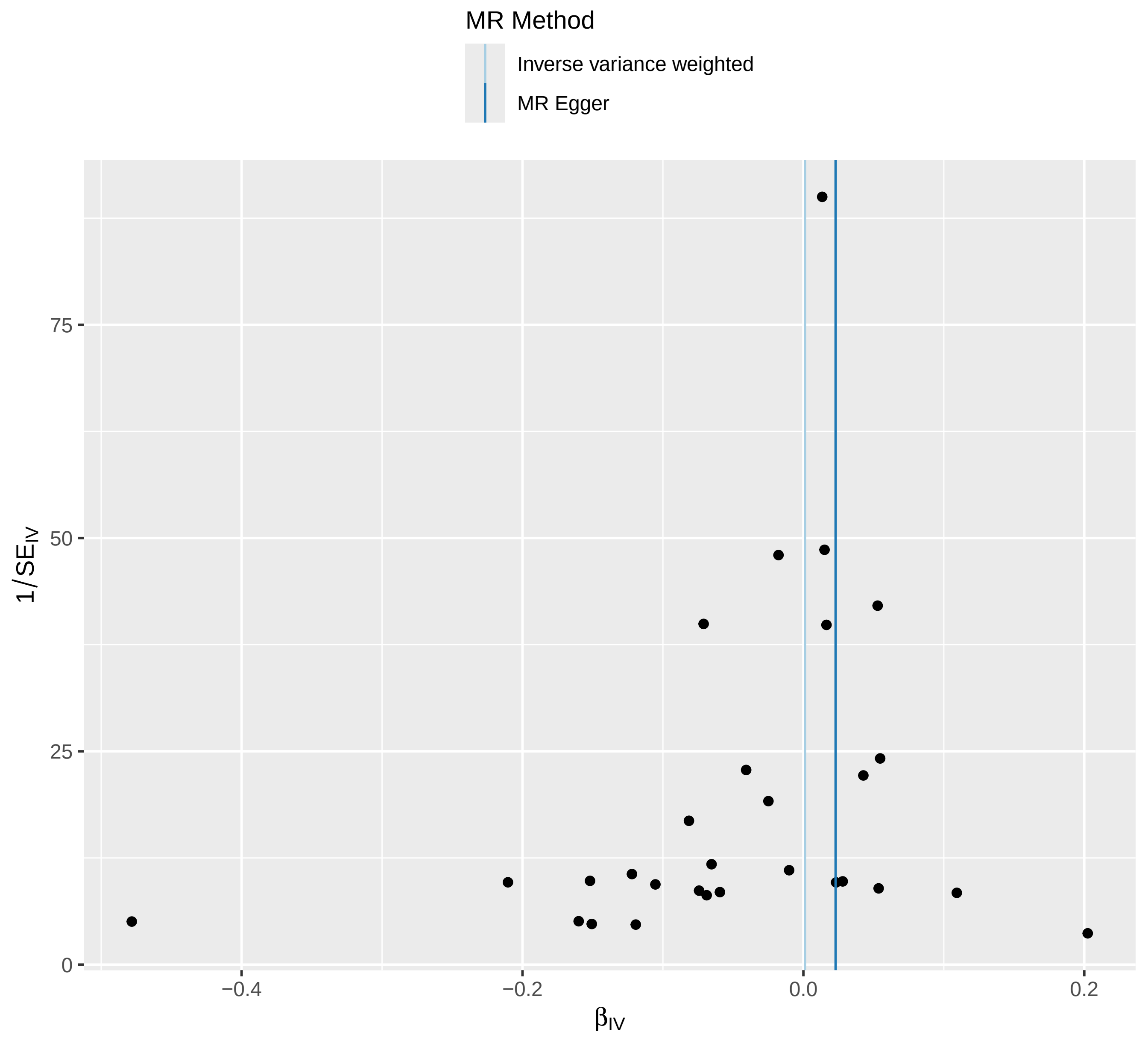
MR Method
Inverse variance weighted
MR Egger
1/SE_IV
75
50
25
0
−0.4
−0.2
0.0
0.2
β_IV

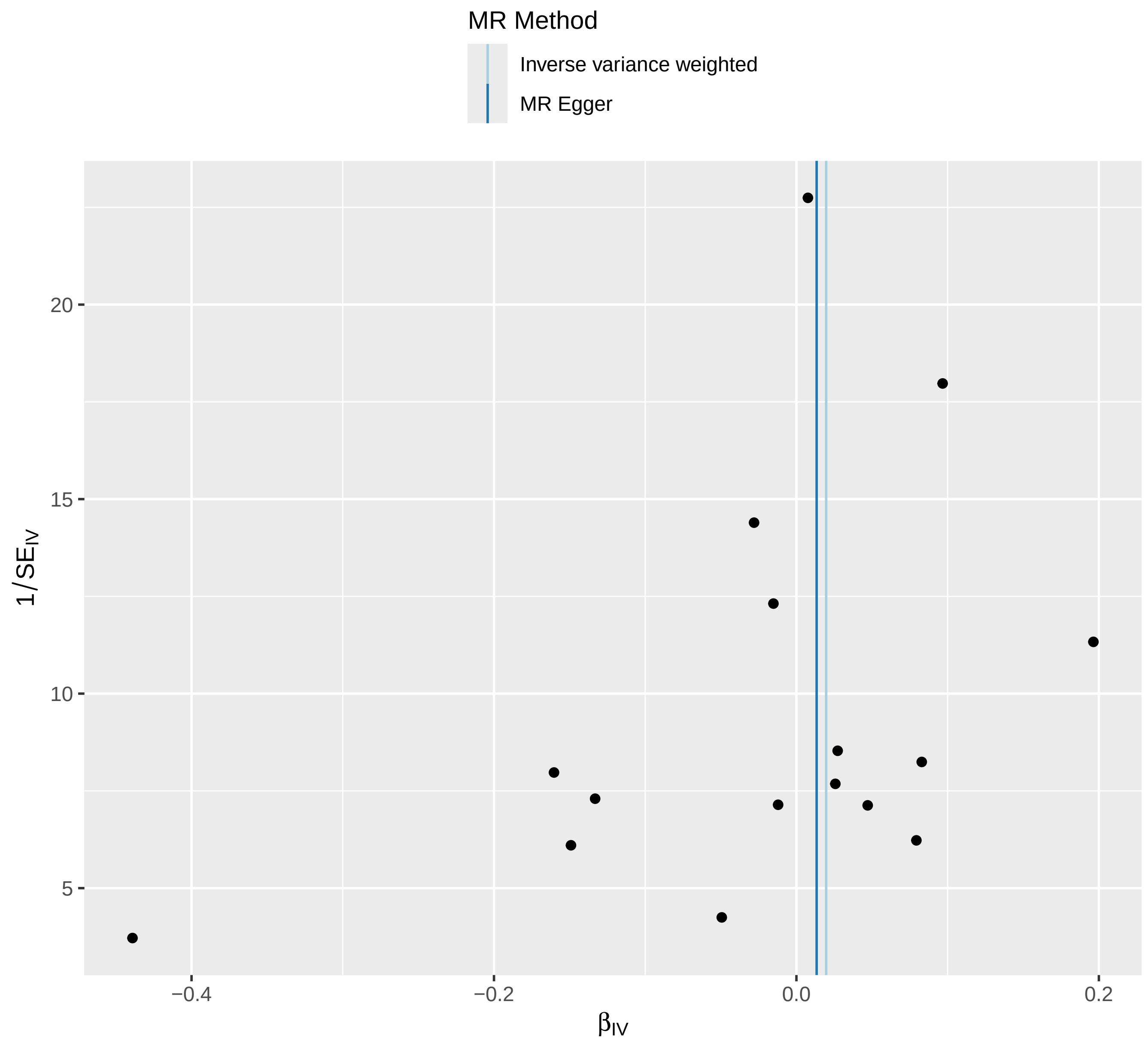
MR Method
Inverse variance weighted
MR Egger
20
15
10
5
1/SE_IV
−0.4
−0.2
0.0
0.2
β_IV

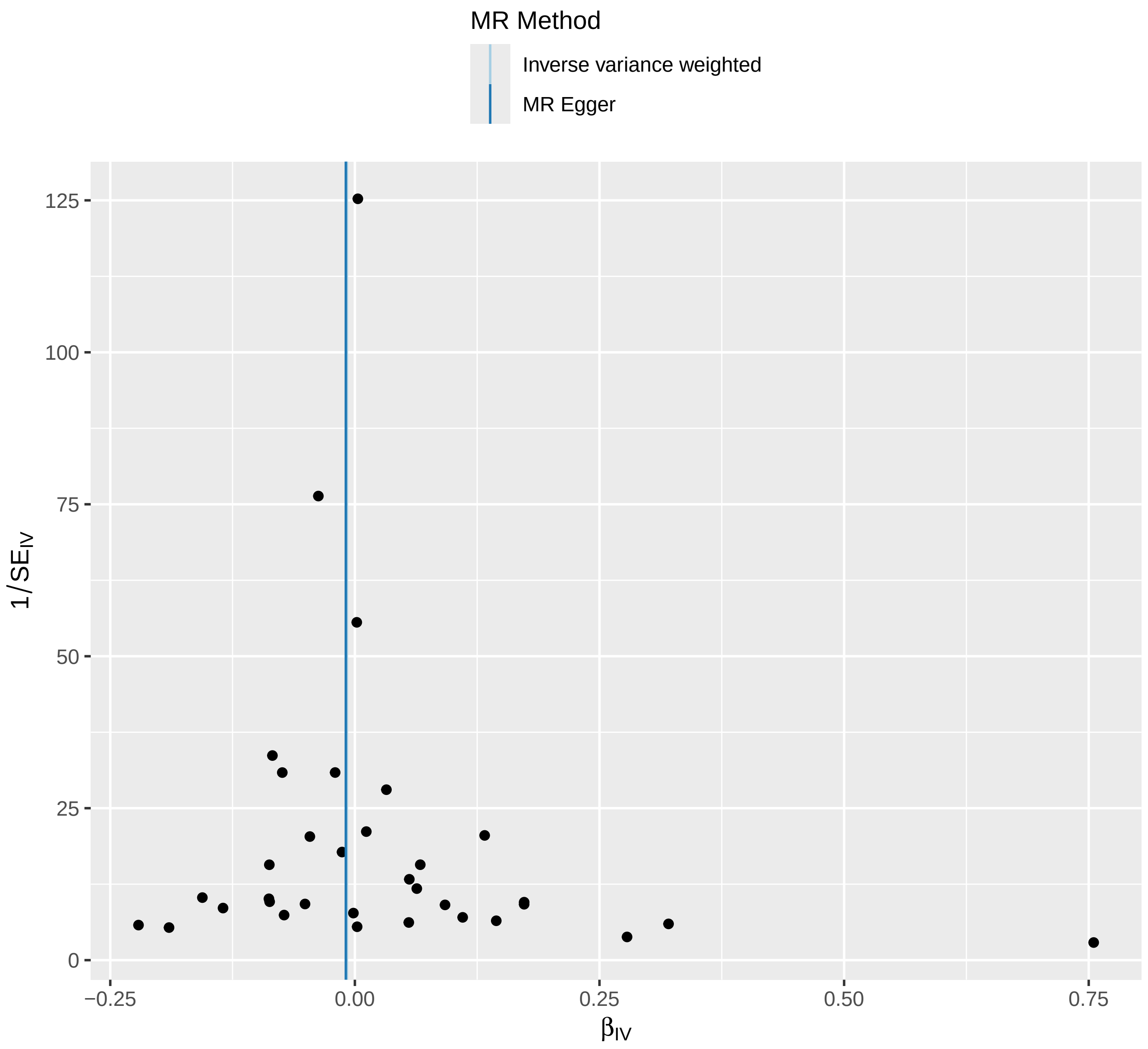

MR Method
Inverse variance weighted
MR Egger
125
100
75
50
25
0
1/SE$_{IV}$
−0.25
0.00
0.25
0.50
0.75
β$_{IV}$

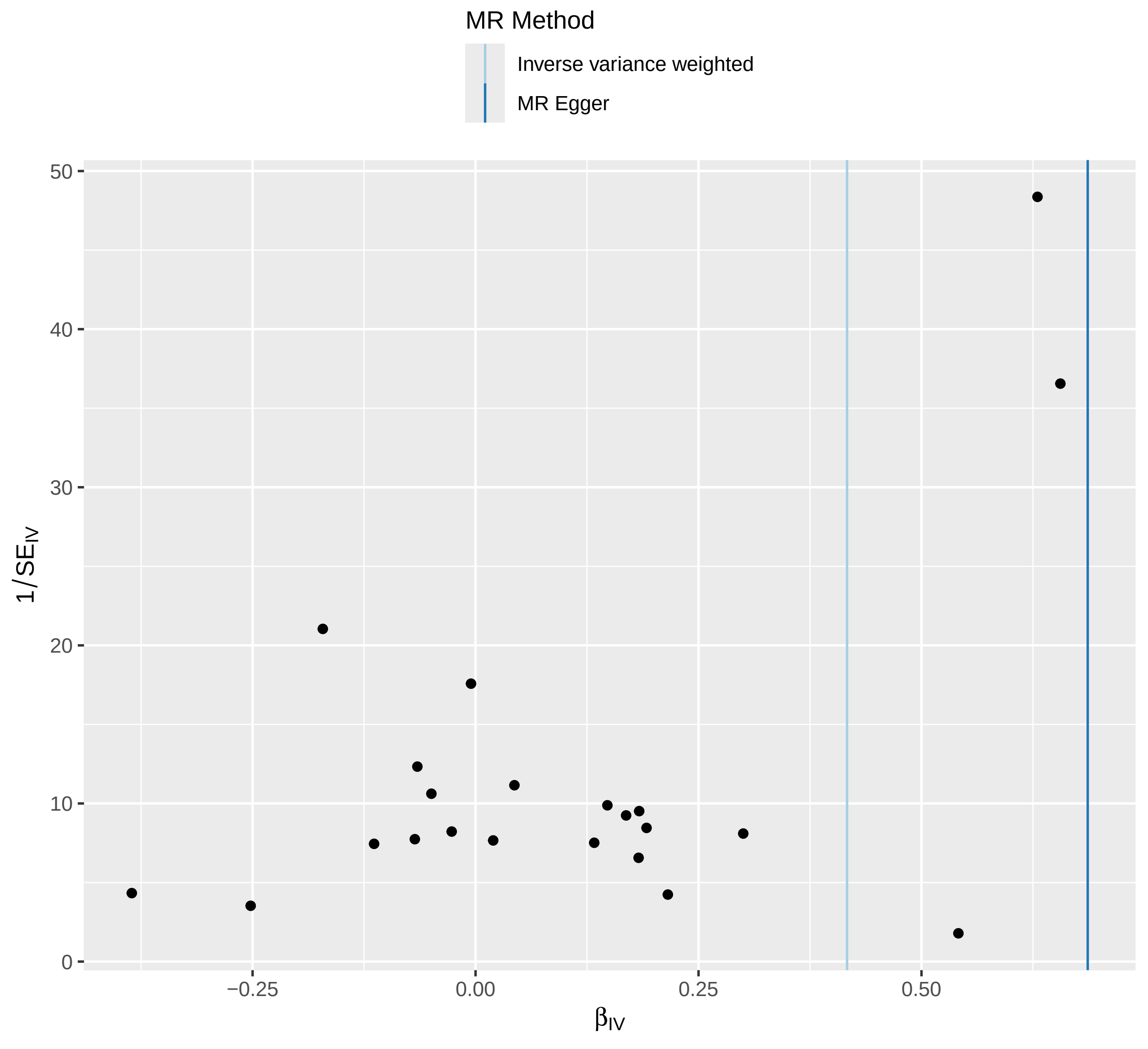

MR Method
Inverse variance weighted
MR Egger
50
40
30
20
10
0
1/SE_IV
−0.25
0.00
0.25
0.50
β_IV

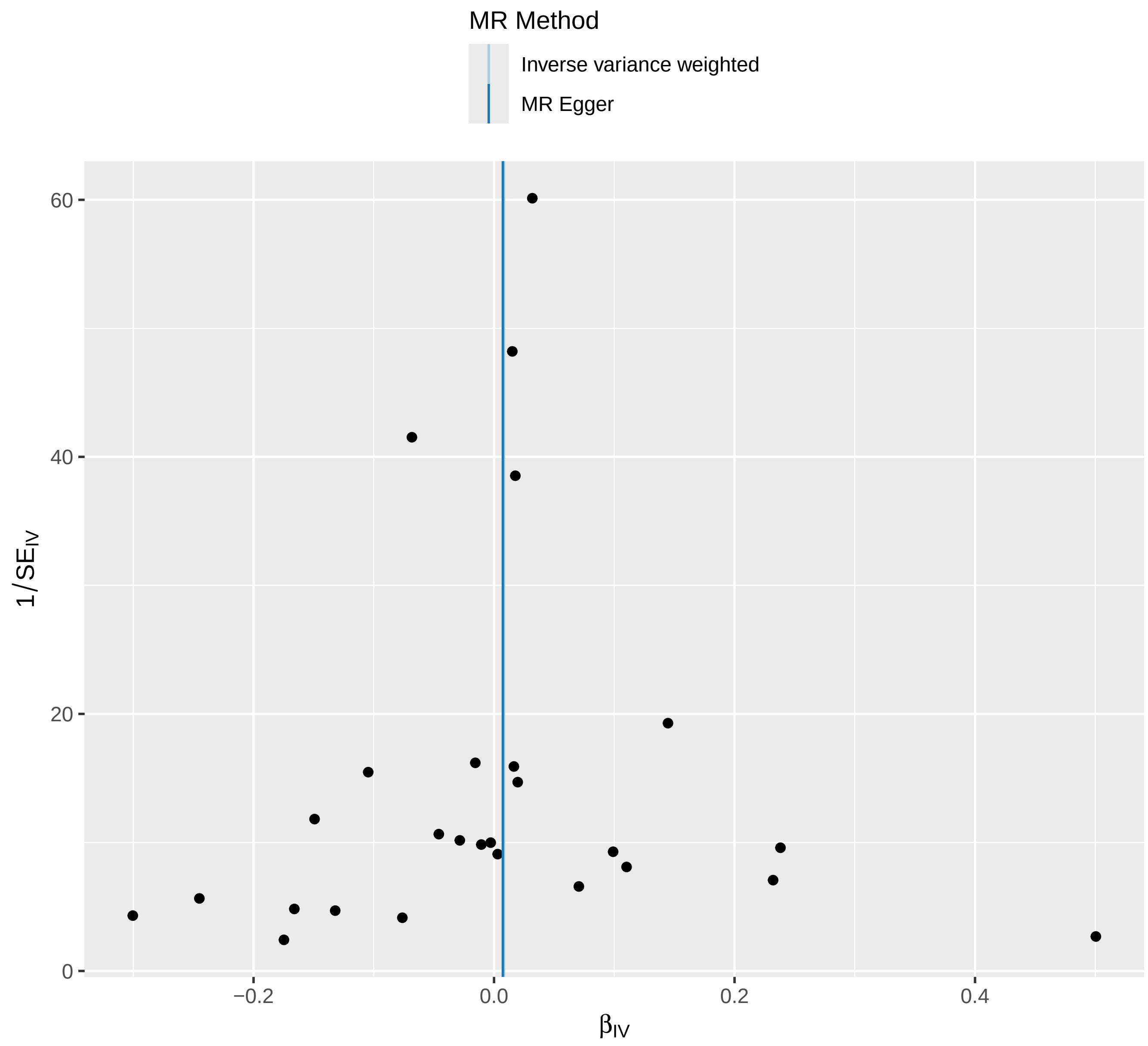

MR Method
Inverse variance weighted
MR Egger
60
40
20
0
−0.2
0.0
0.2
0.4
$1/SE_{IV}$
β_{IV}

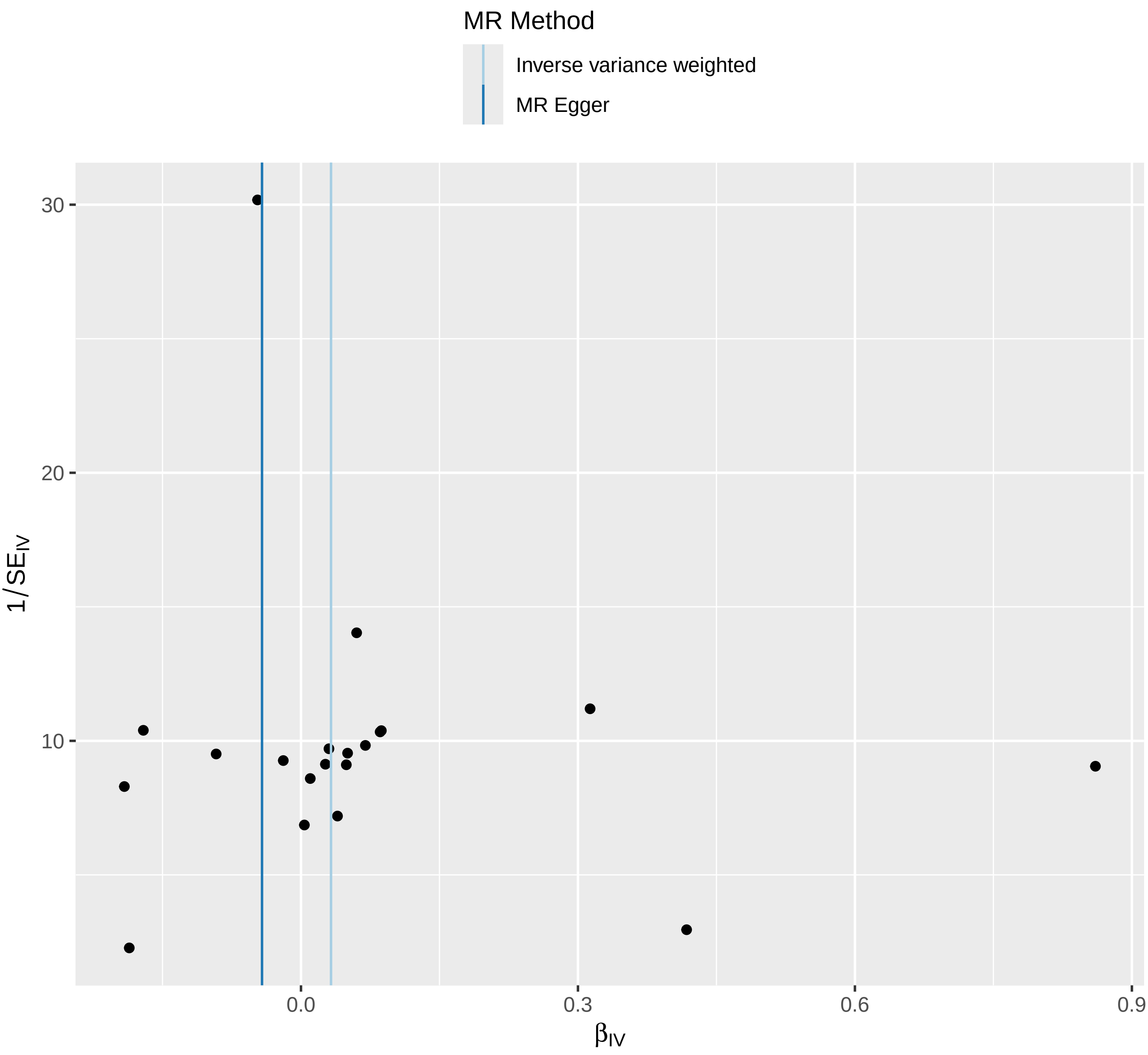
MR Method
Inverse variance weighted
MR Egger
30
20
10
0.0
0.3
0.6
0.9
1/SE_IV
β_IV

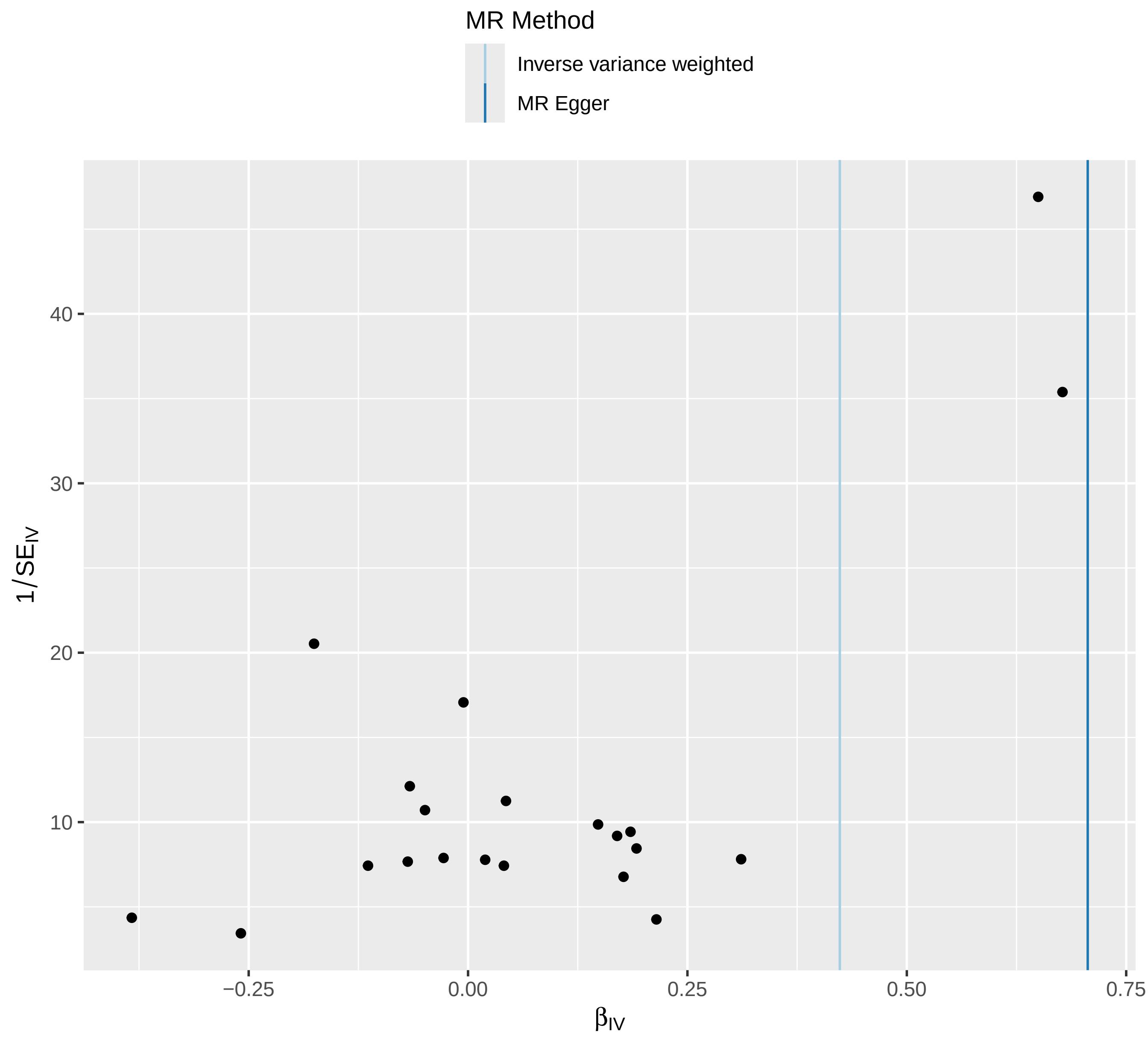
MR Method
Inverse variance weighted
MR Egger
40
30
20
10
1/SE$_{IV}$
−0.25
0.00
0.25
0.50
0.75
β$_{IV}$

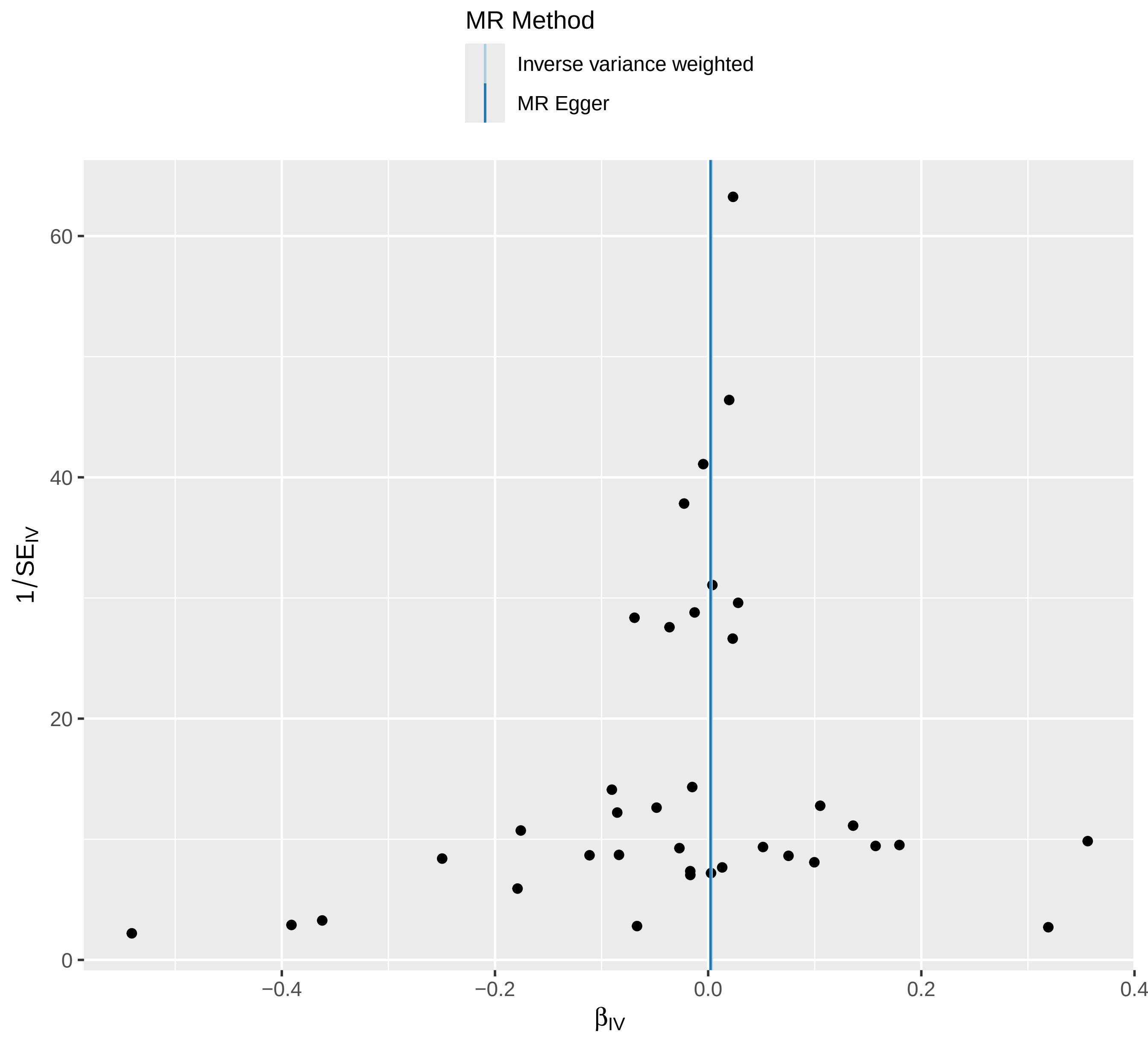
MR Method
Inverse variance weighted
MR Egger
1/SE_IV
60
40
20
0
−0.4
−0.2
0.0
0.2
0.4
β_IV

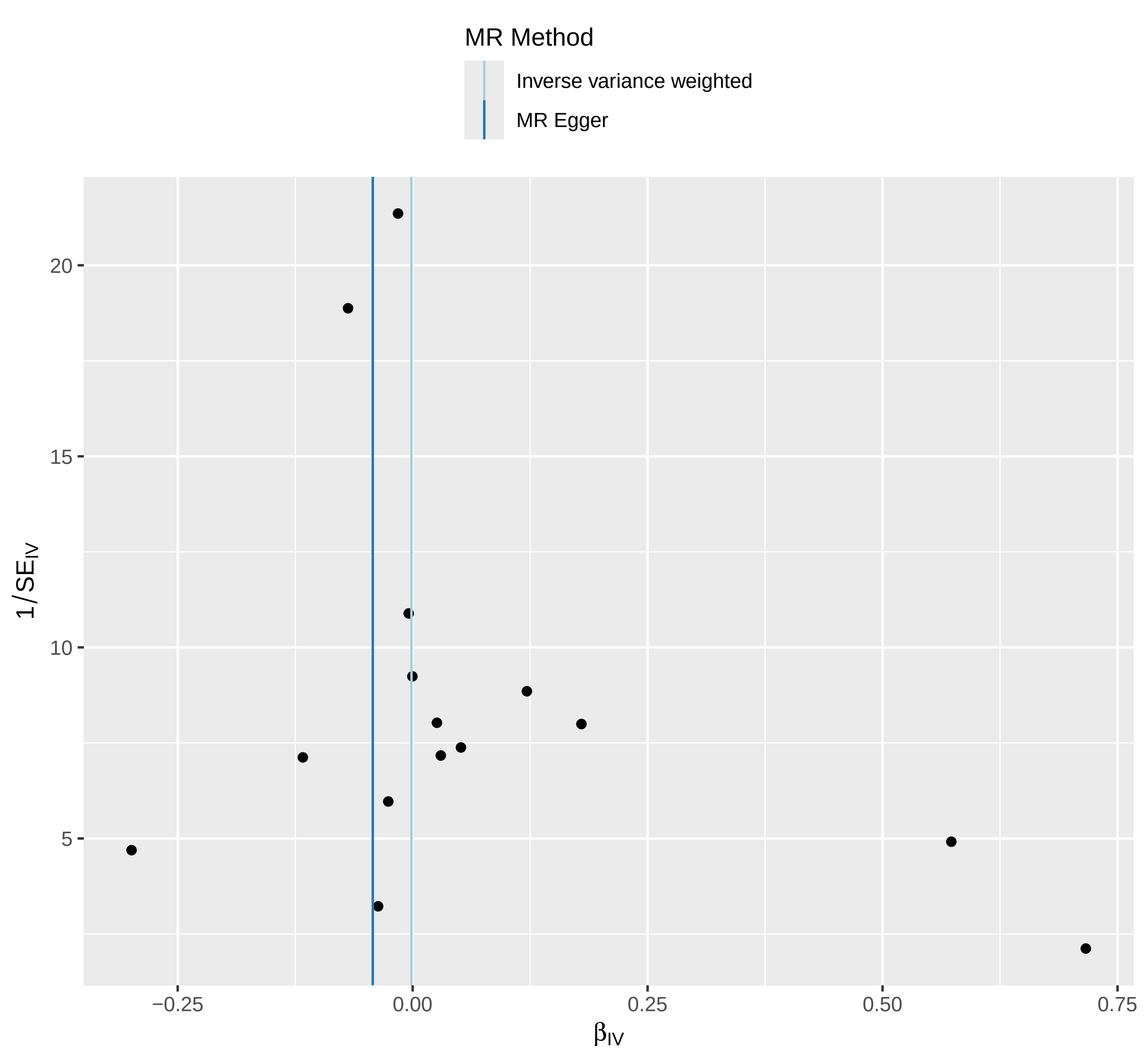
MR Method
Inverse variance weighted
MR Egger
1/SE_IV
20
15
10
5
−0.25
0.00
0.25
0.50
0.75
β_IV

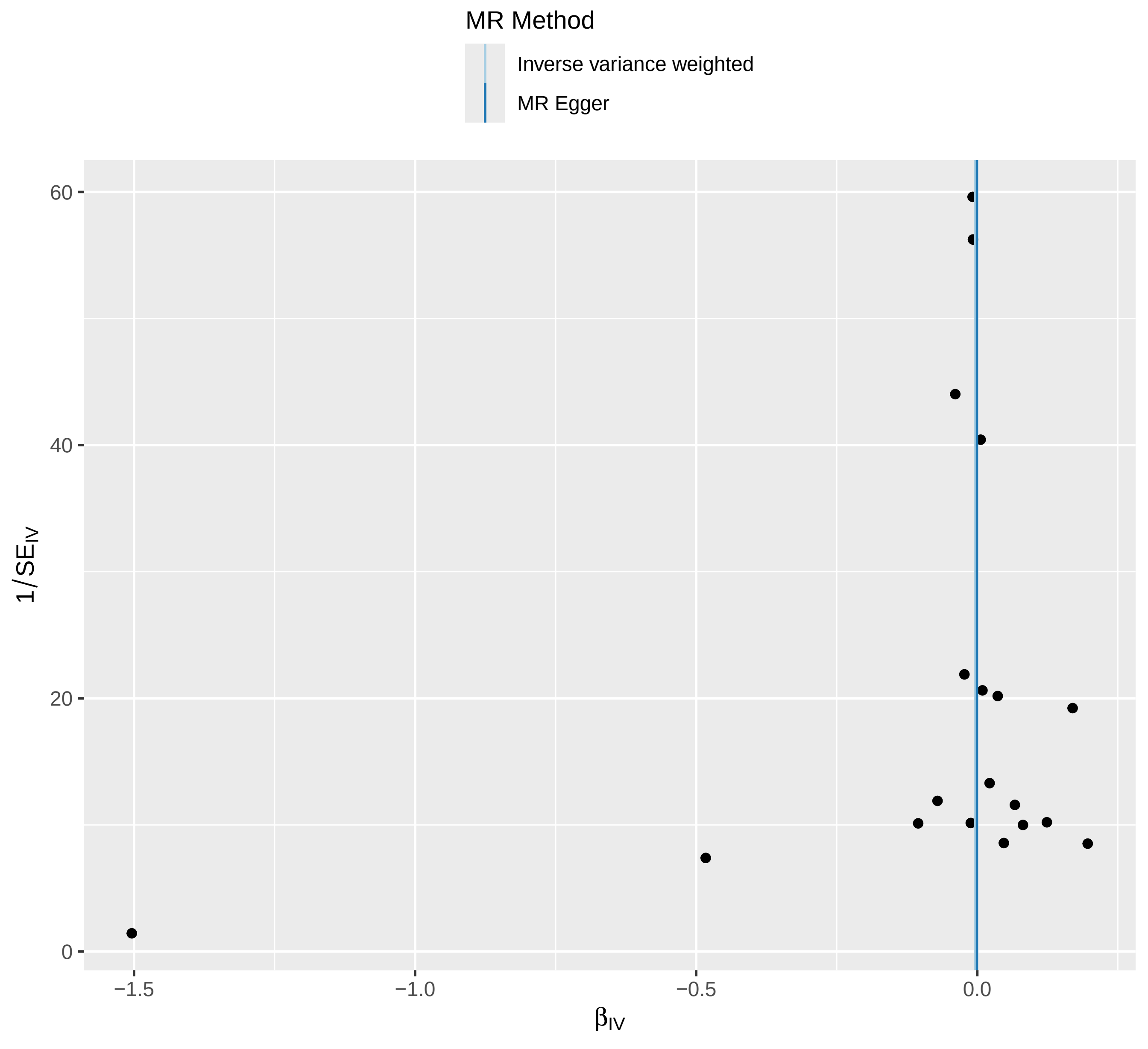
MR Method
Inverse variance weighted
MR Egger
60
40
20
0
1/SE_IV
−1.5
−1.0
−0.5
0.0
β_IV

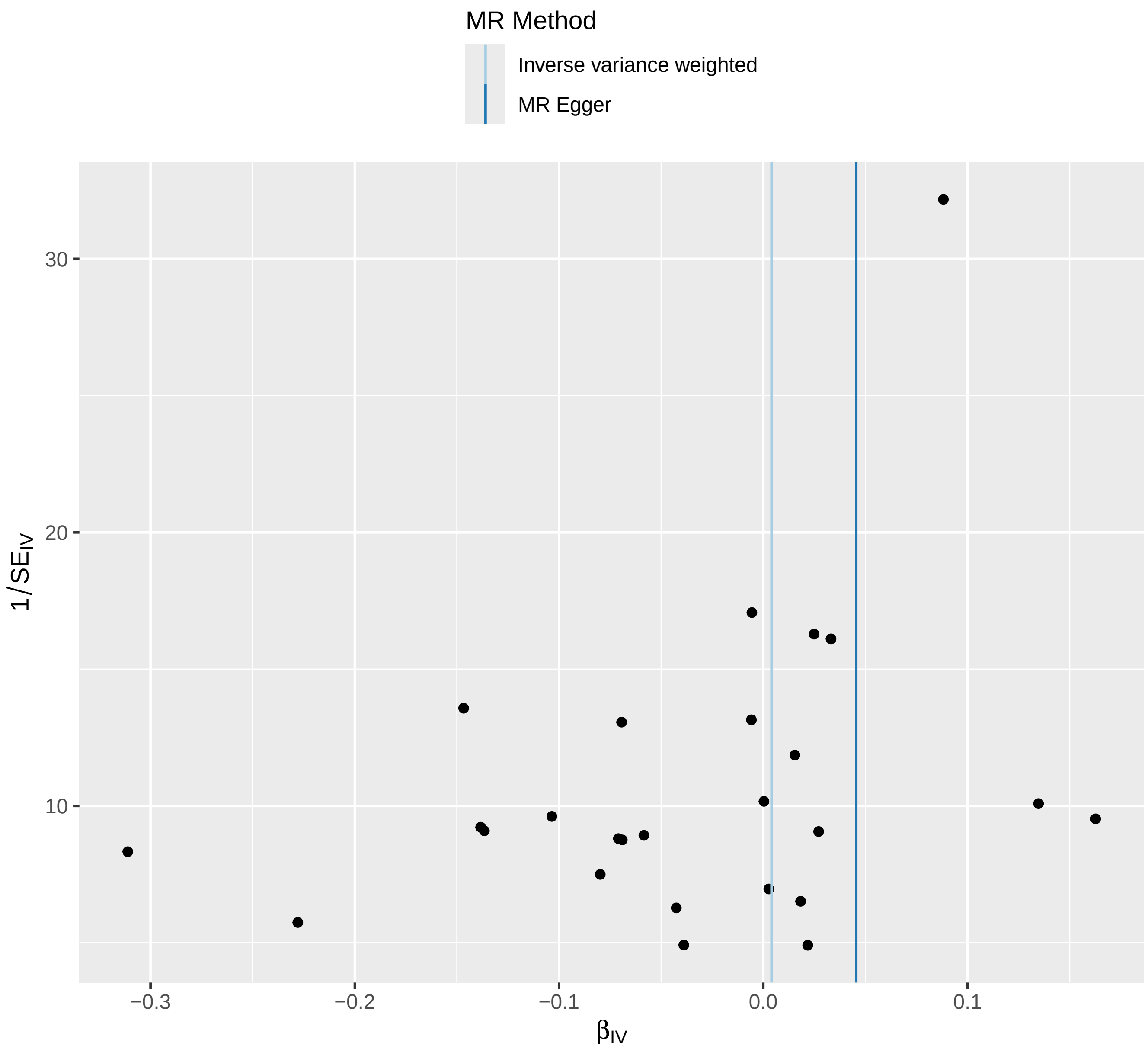
MR Method
Inverse variance weighted
MR Egger
30
20
10
1/SE_IV
−0.3
−0.2
−0.1
0.0
0.1
β_IV

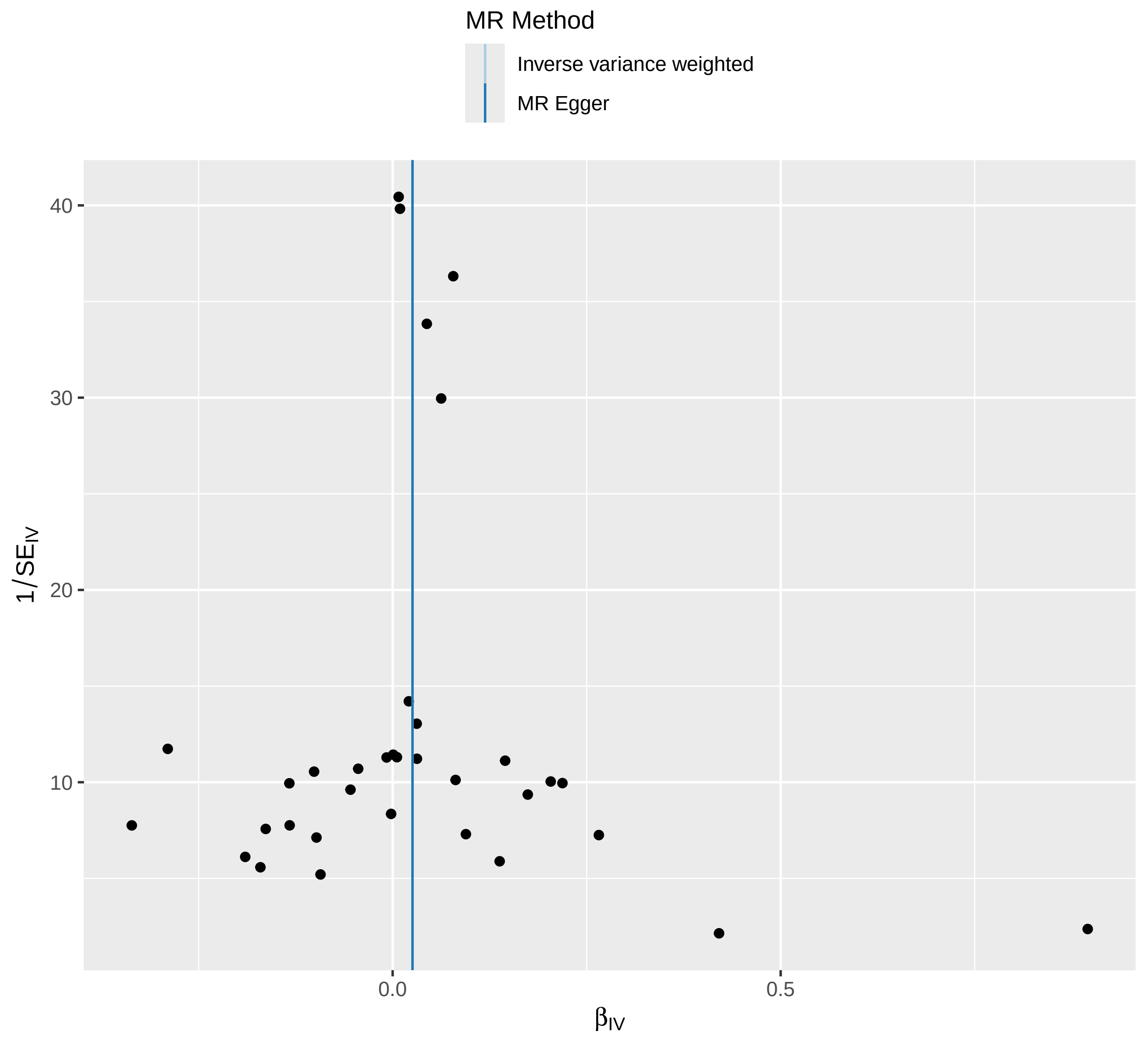
MR Method
Inverse variance weighted
MR Egger
40
30
20
10
0.0
0.5
1/SE_{IV}
β_{IV}

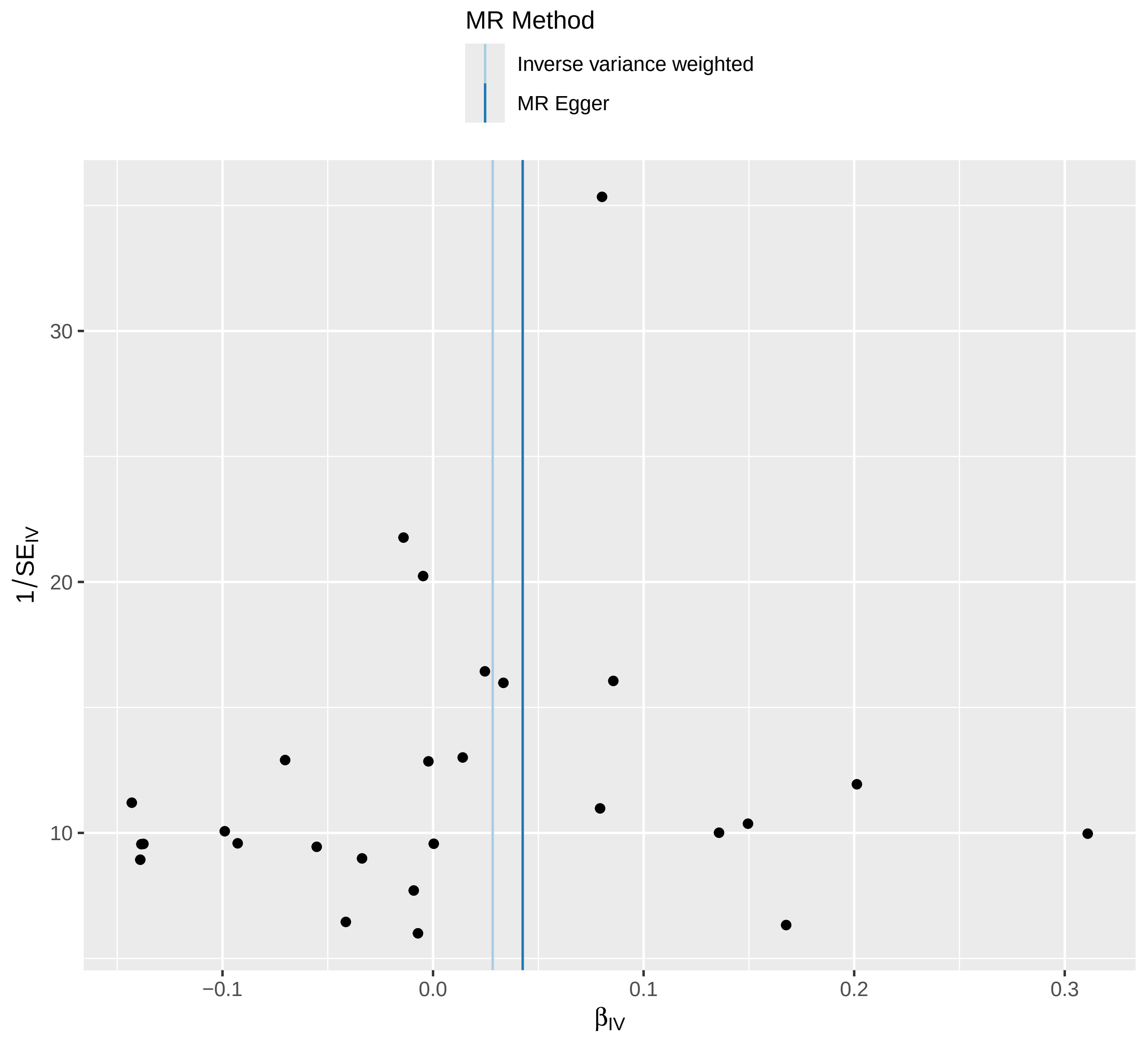

MR Method
Inverse variance weighted
MR Egger
30
20
10
1/SE$_{IV}$
−0.1
0.0
0.1
0.2
0.3
β$_{IV}$

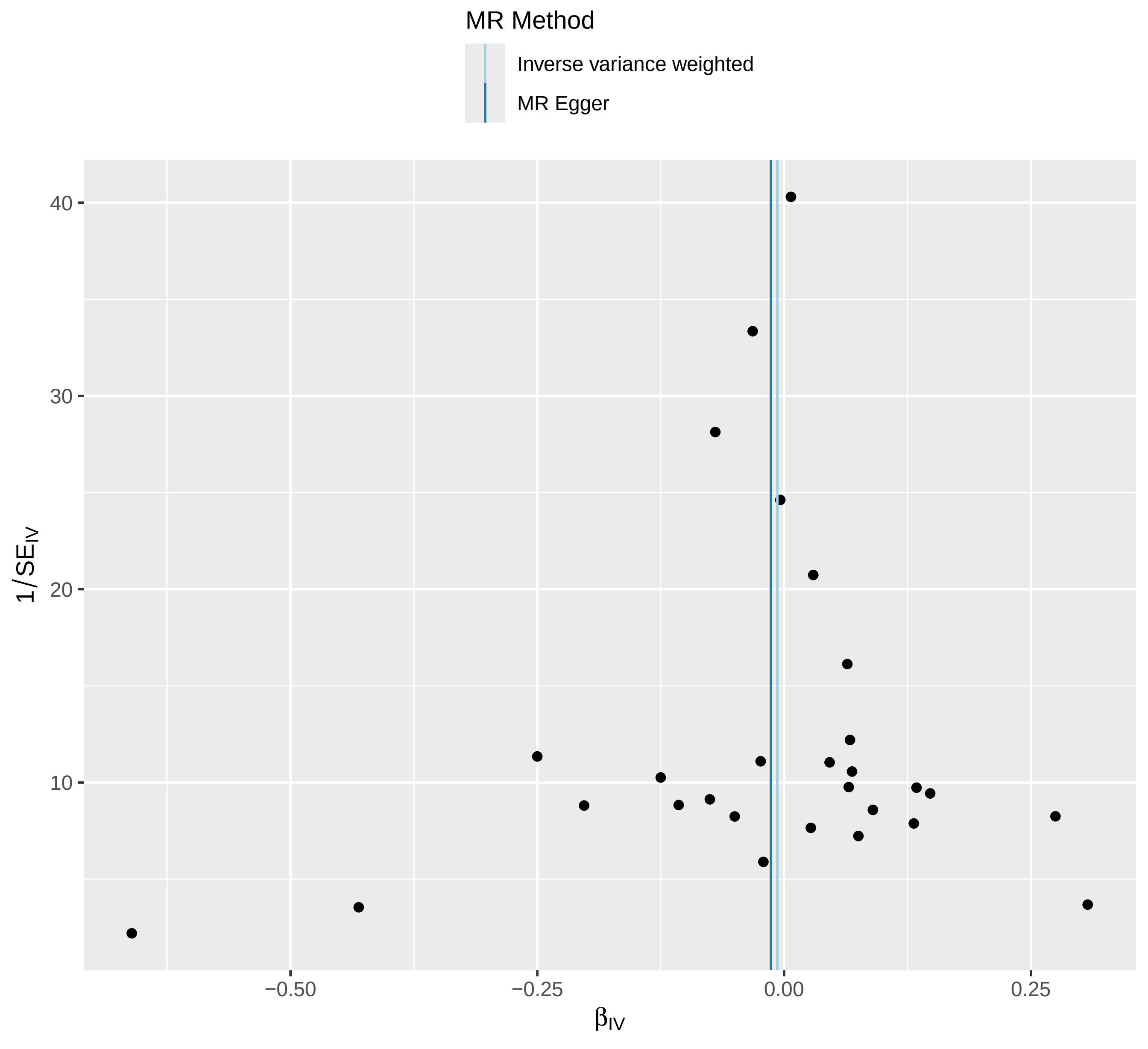

MR Method
Inverse variance weighted
MR Egger
40
30
20
10
$1/SE_{IV}$
−0.50
−0.25
0.00
0.25
β_{IV}

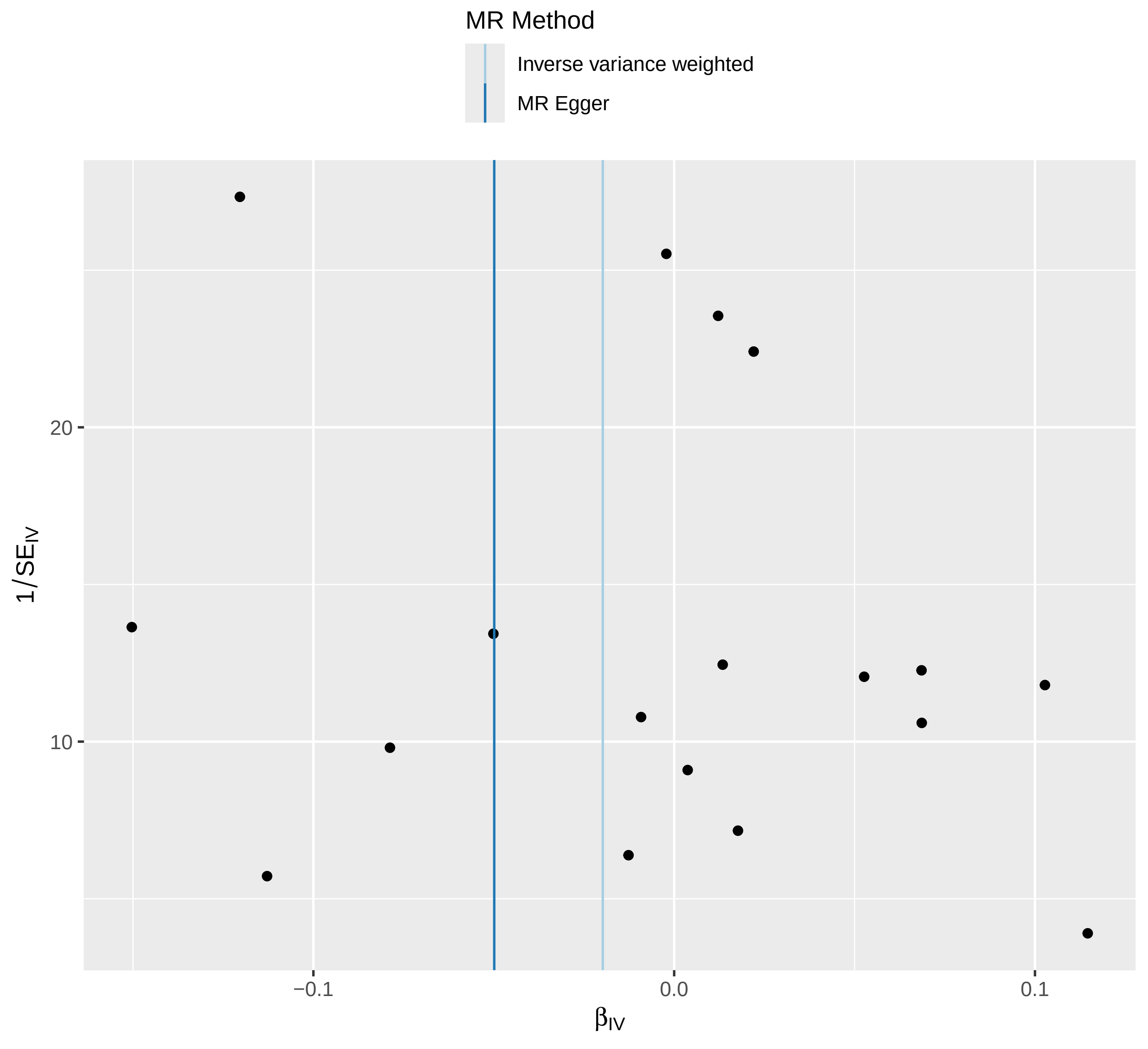
MR Method
Inverse variance weighted
MR Egger
20
10
$1/SE_{IV}$
−0.1
0.0
0.1
β_{IV}

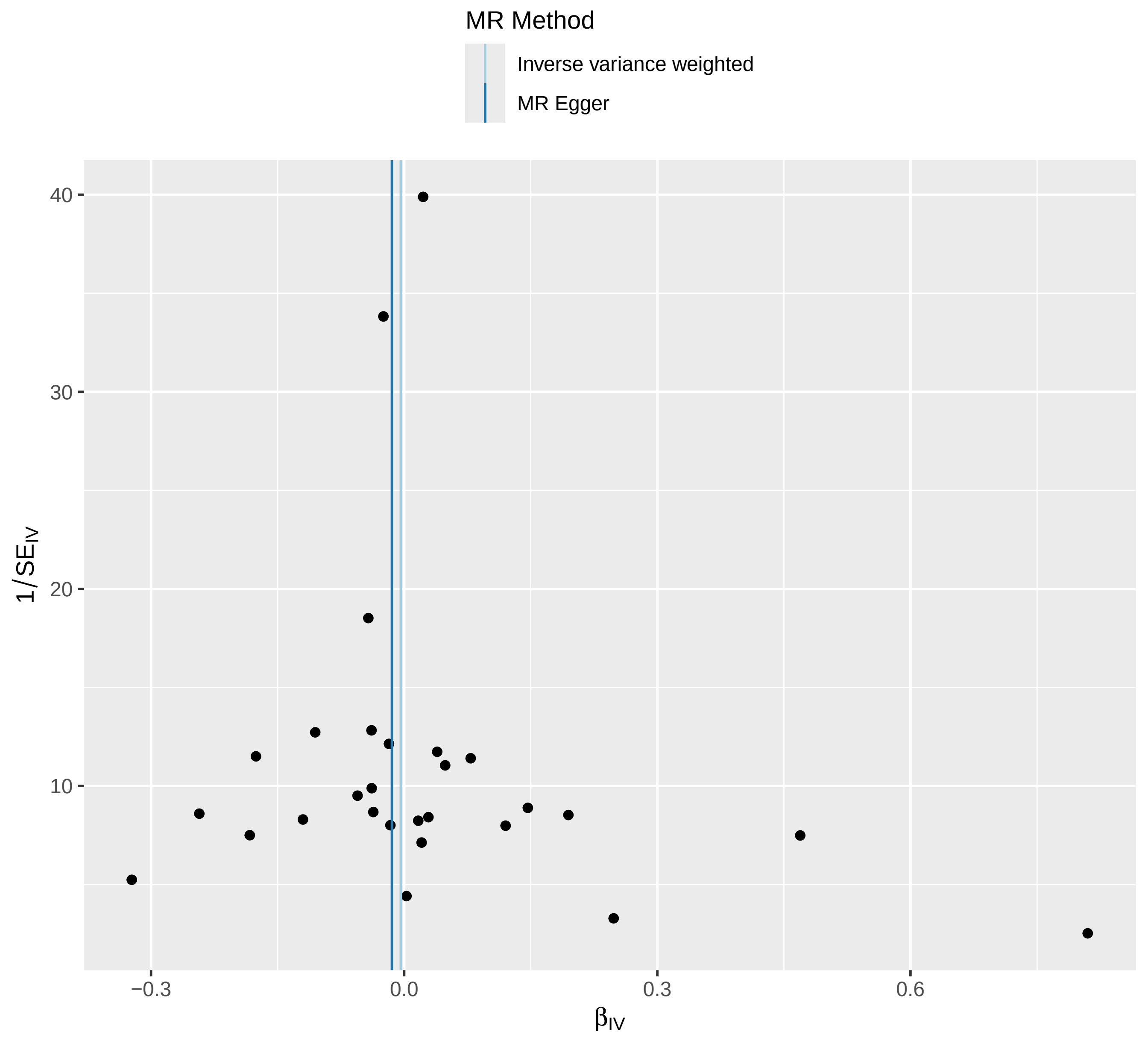
MR Method
Inverse variance weighted
MR Egger
40
30
20
10
1/SE_IV
−0.3
0.0
0.3
0.6
β_IV

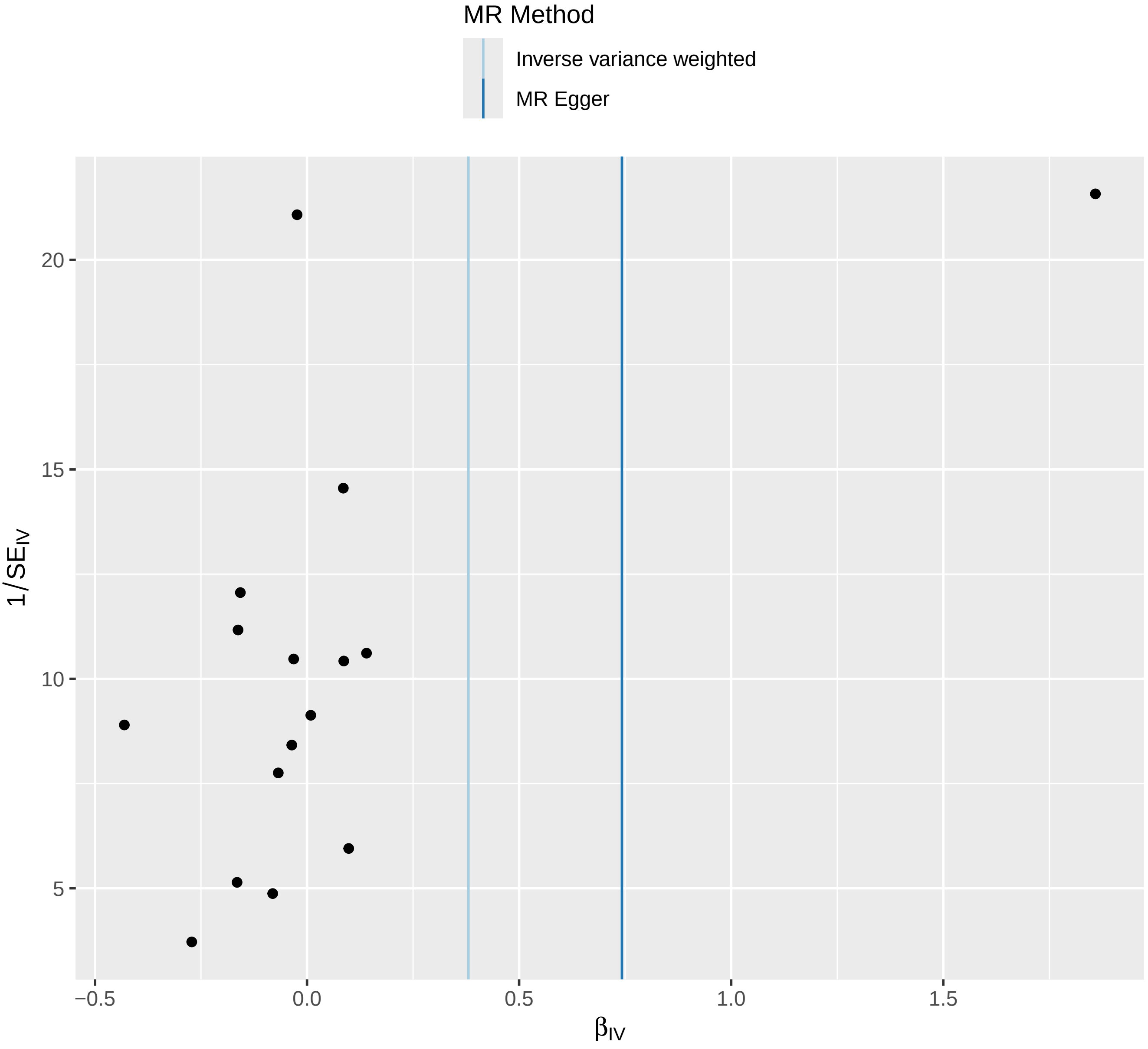
MR Method
Inverse variance weighted
MR Egger
20
15
10
5
$1/SE_{IV}$
−0.5
0.0
0.5
1.0
1.5
β_{IV}

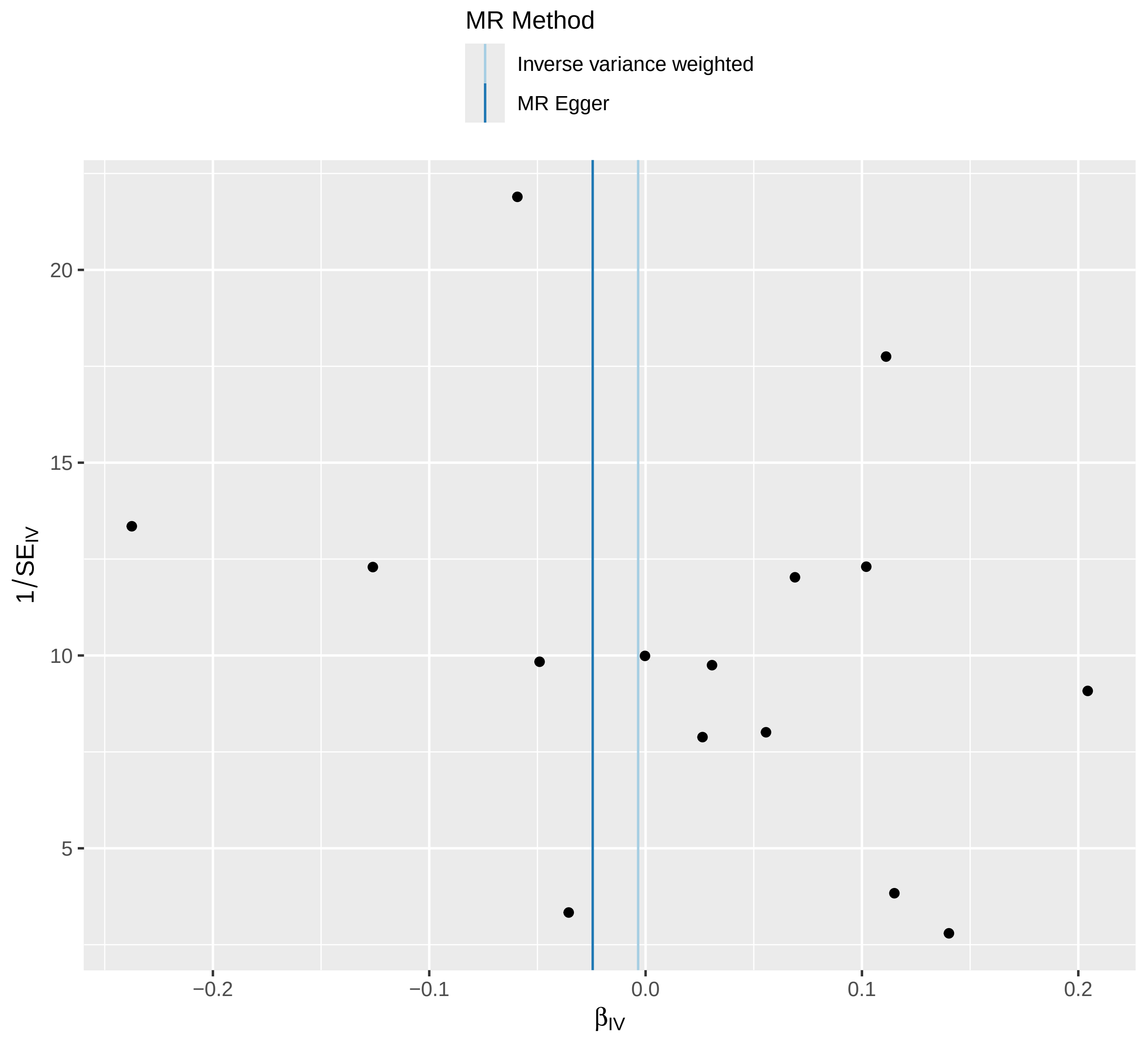
MR Method
Inverse variance weighted
MR Egger
20
15
10
5
1/SE_IV
−0.2
−0.1
0.0
0.1
0.2
β_IV

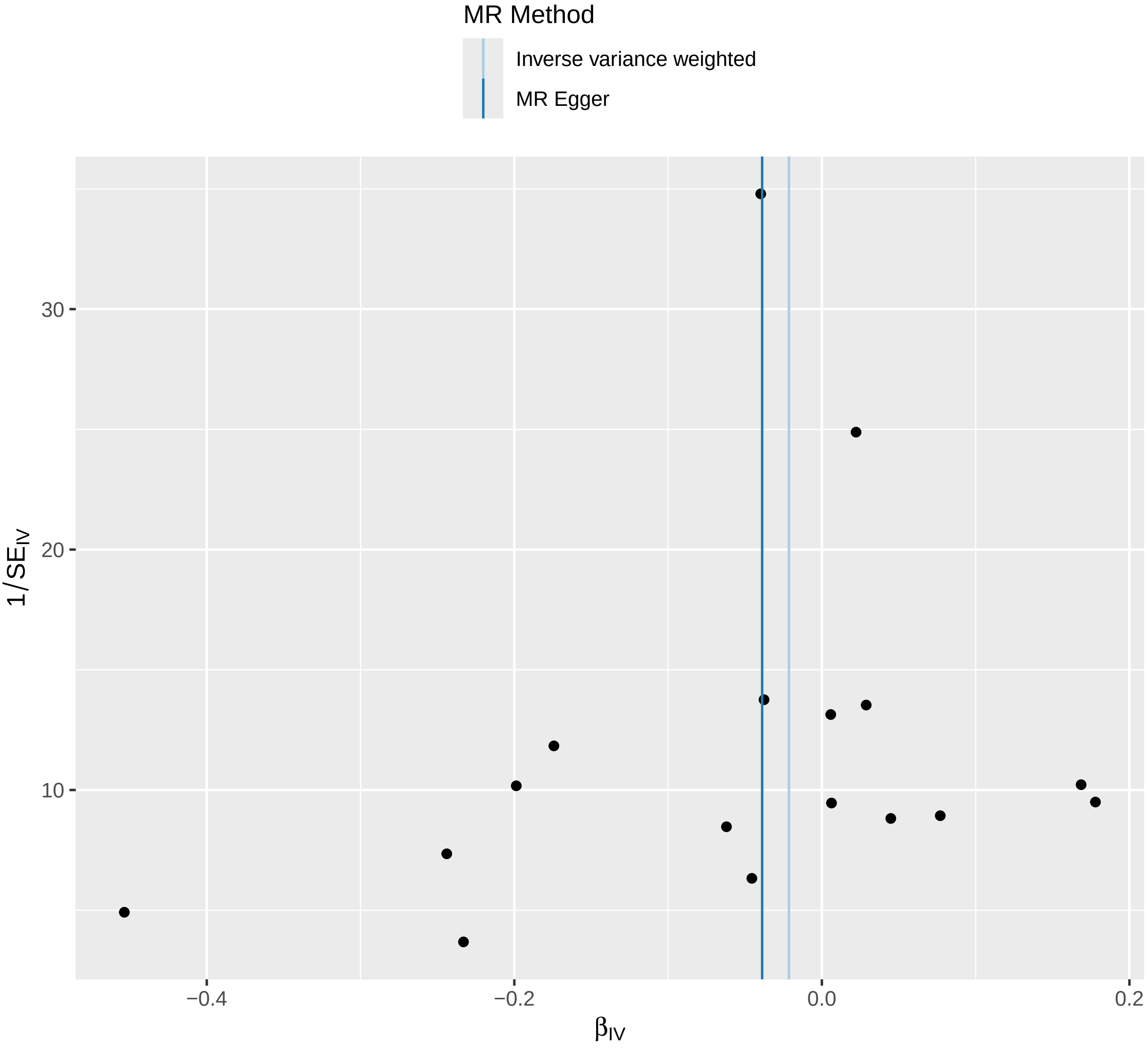

MR Method
Inverse variance weighted
MR Egger
30
20
10
1/SE_IV
−0.4
−0.2
0.0
0.2
β_IV

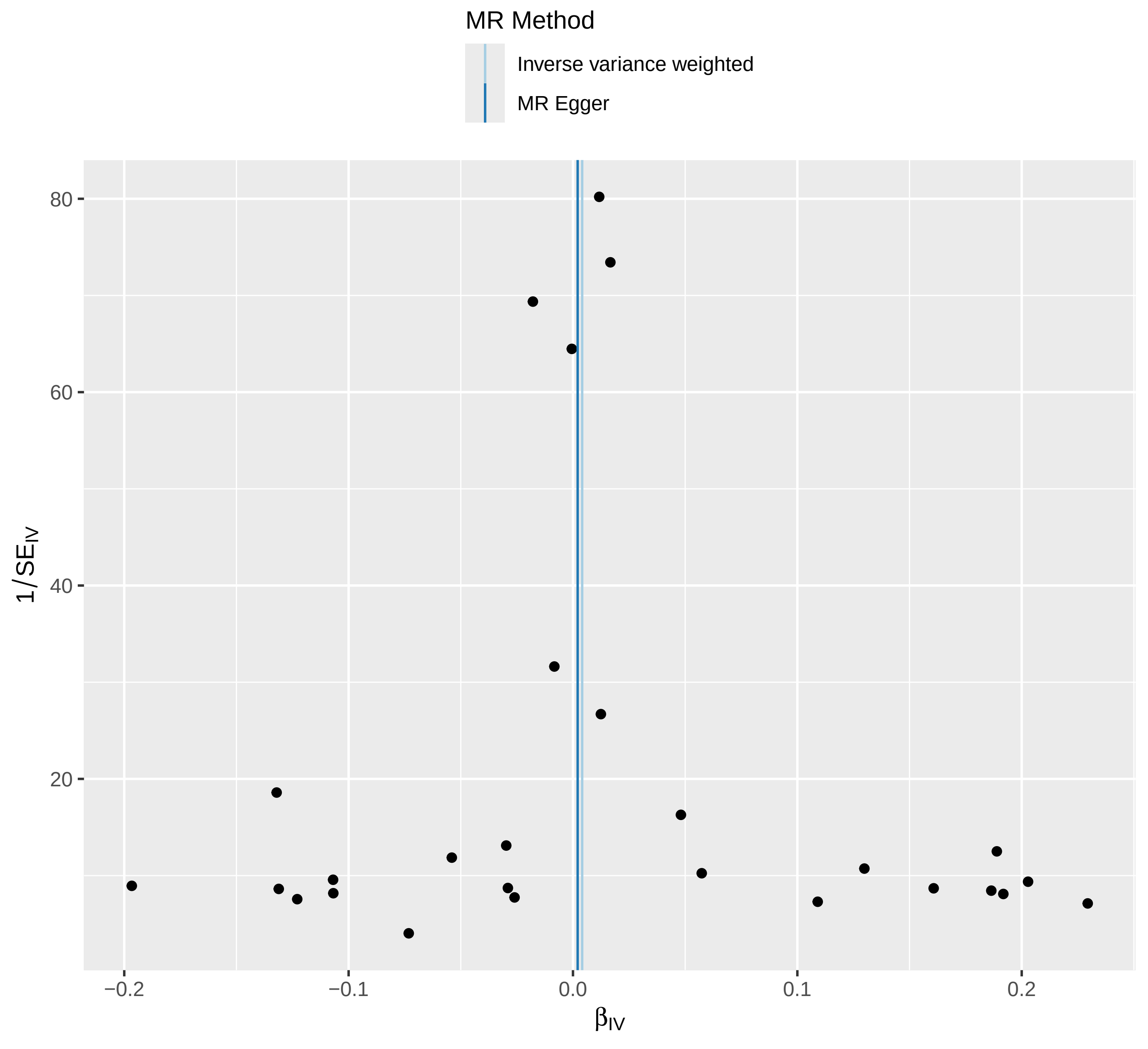
MR Method
Inverse variance weighted
MR Egger
80
60
40
20
1/SE$_{IV}$
−0.2
−0.1
0.0
0.1
0.2
β$_{IV}$

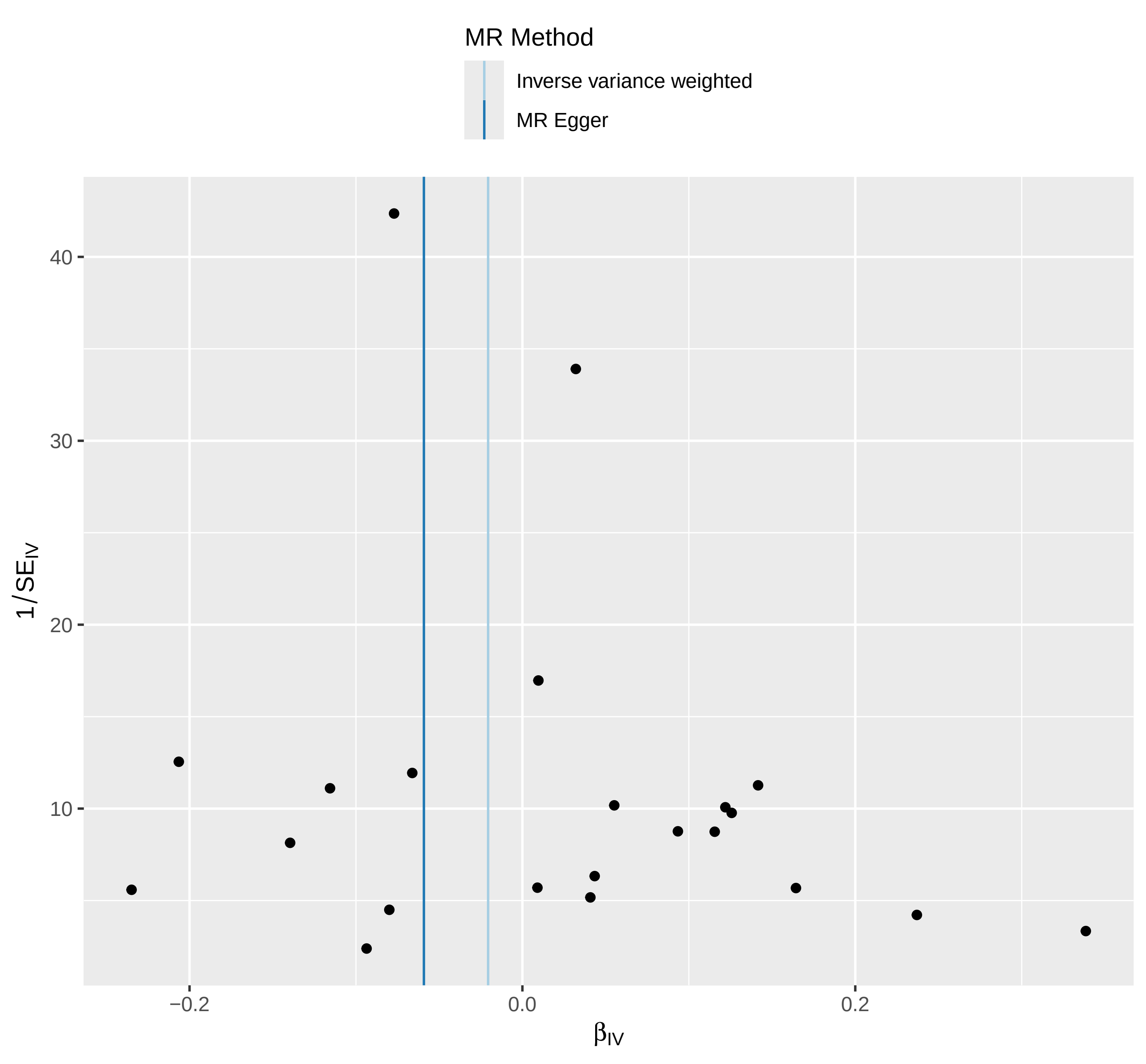

MR Method
Inverse variance weighted
MR Egger
40
30
20
10
1/SE$_{IV}$
−0.2
0.0
0.2
β$_{IV}$

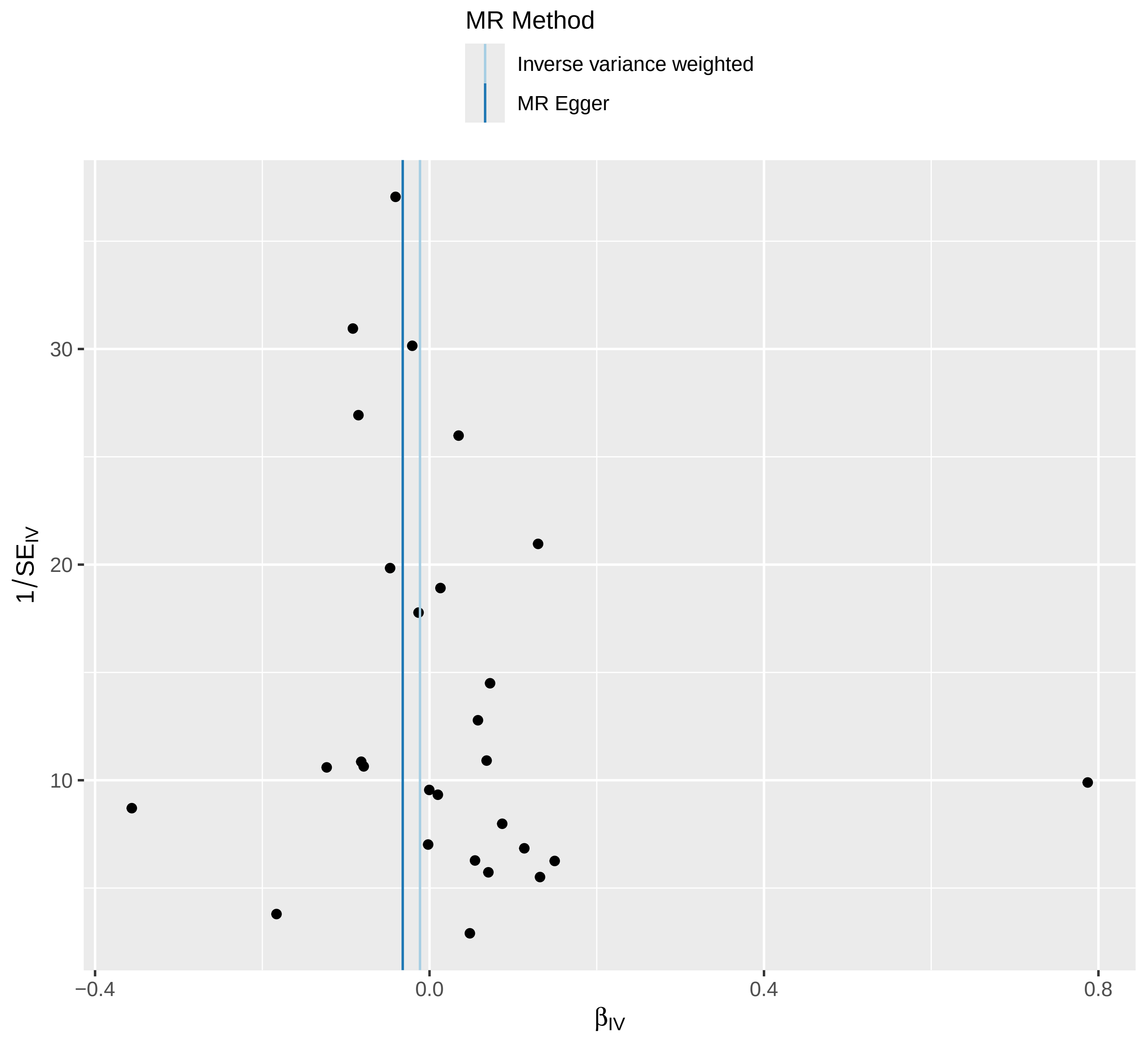
MR Method
Inverse variance weighted
MR Egger
1/SE_IV
30
20
10
−0.4
0.0
0.4
0.8
β_IV

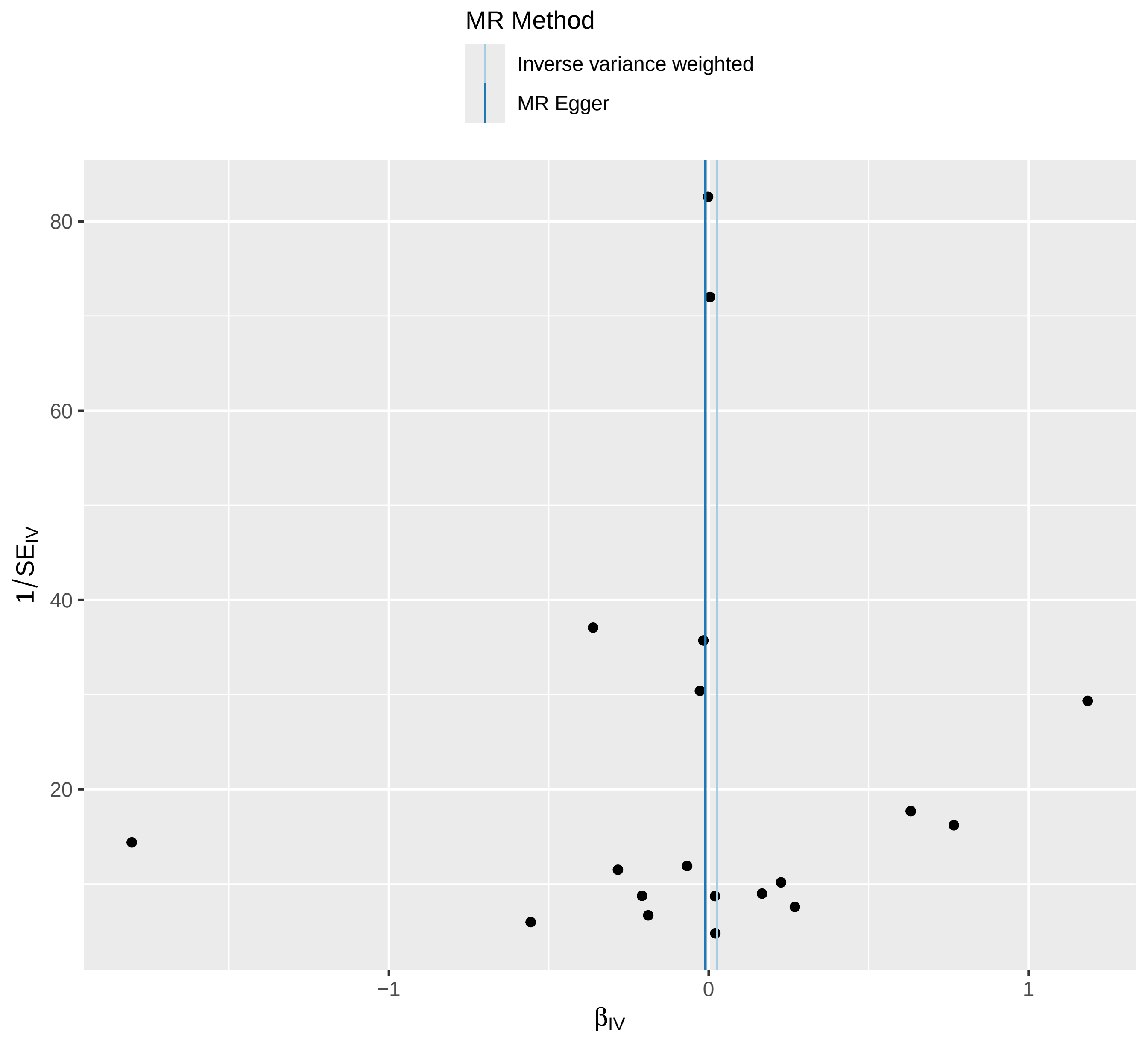

MR Method
Inverse variance weighted
MR Egger
80
60
40
20
1/SE_IV
−1
0
1
β_IV

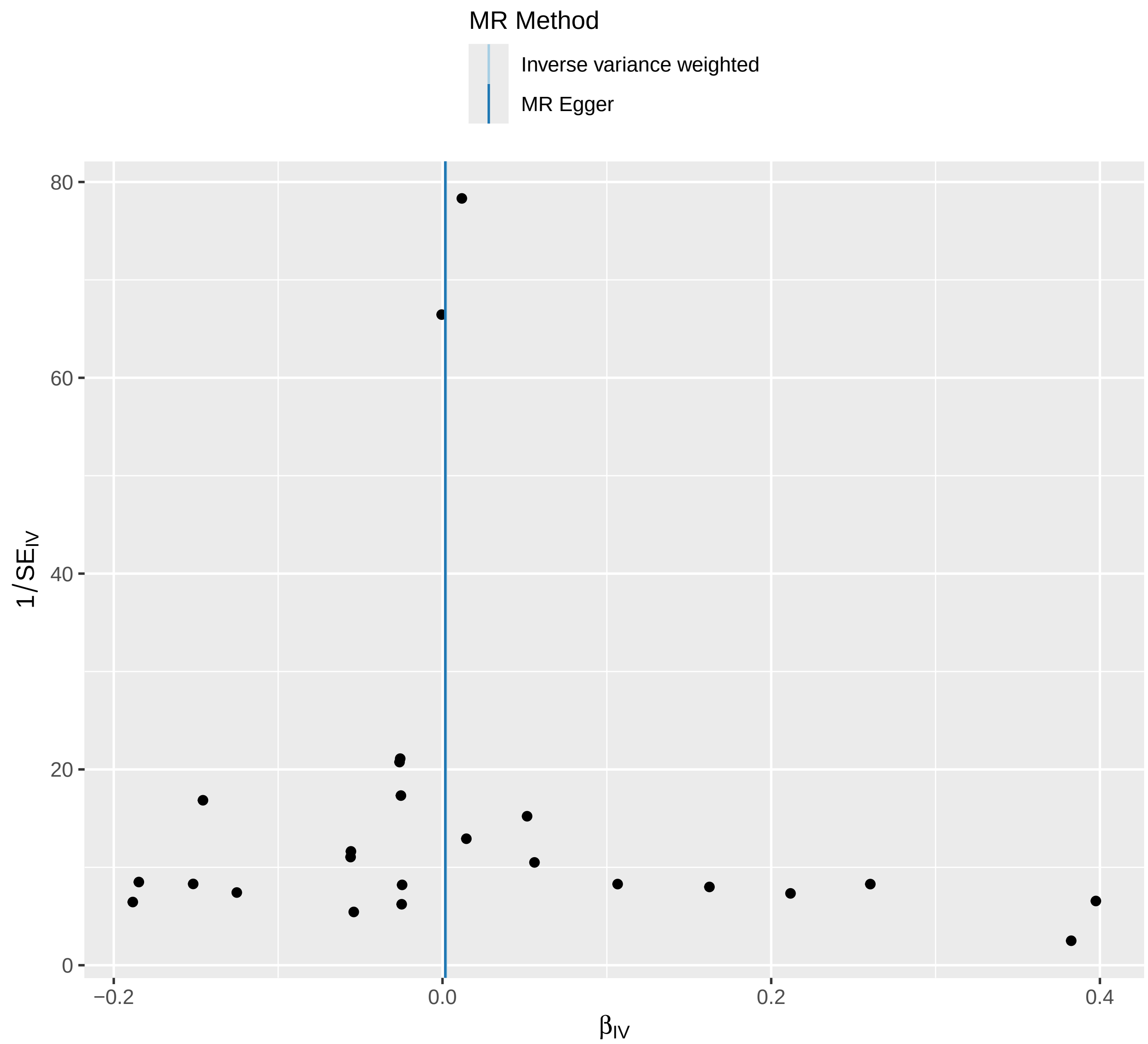
MR Method
Inverse variance weighted
MR Egger
80
60
40
20
0
1/SE_IV
−0.2
0.0
0.2
0.4
β_IV

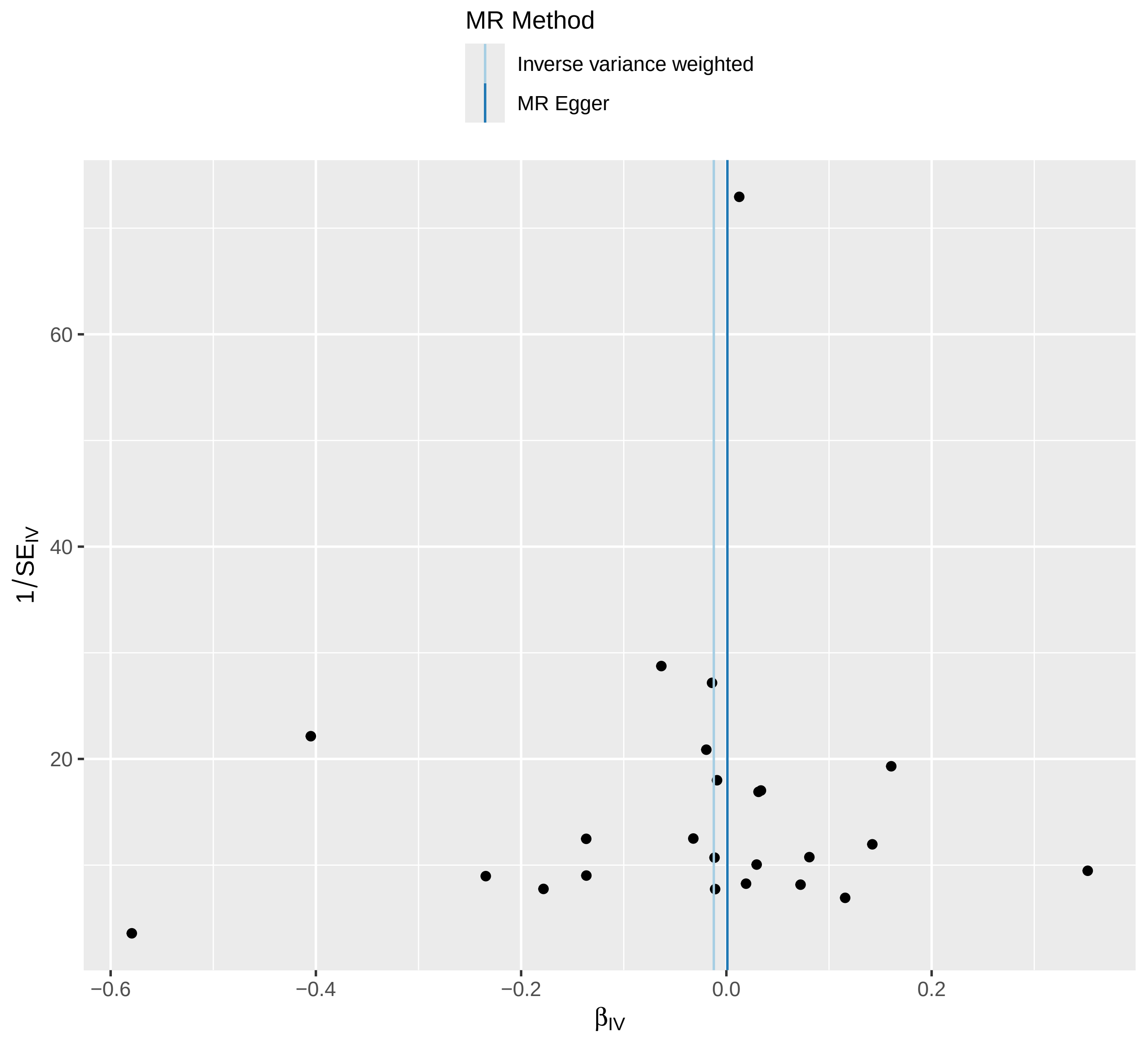
MR Method
Inverse variance weighted
MR Egger
60
40
20
1/SE_IV
−0.6
−0.4
−0.2
0.0
0.2
β_IV

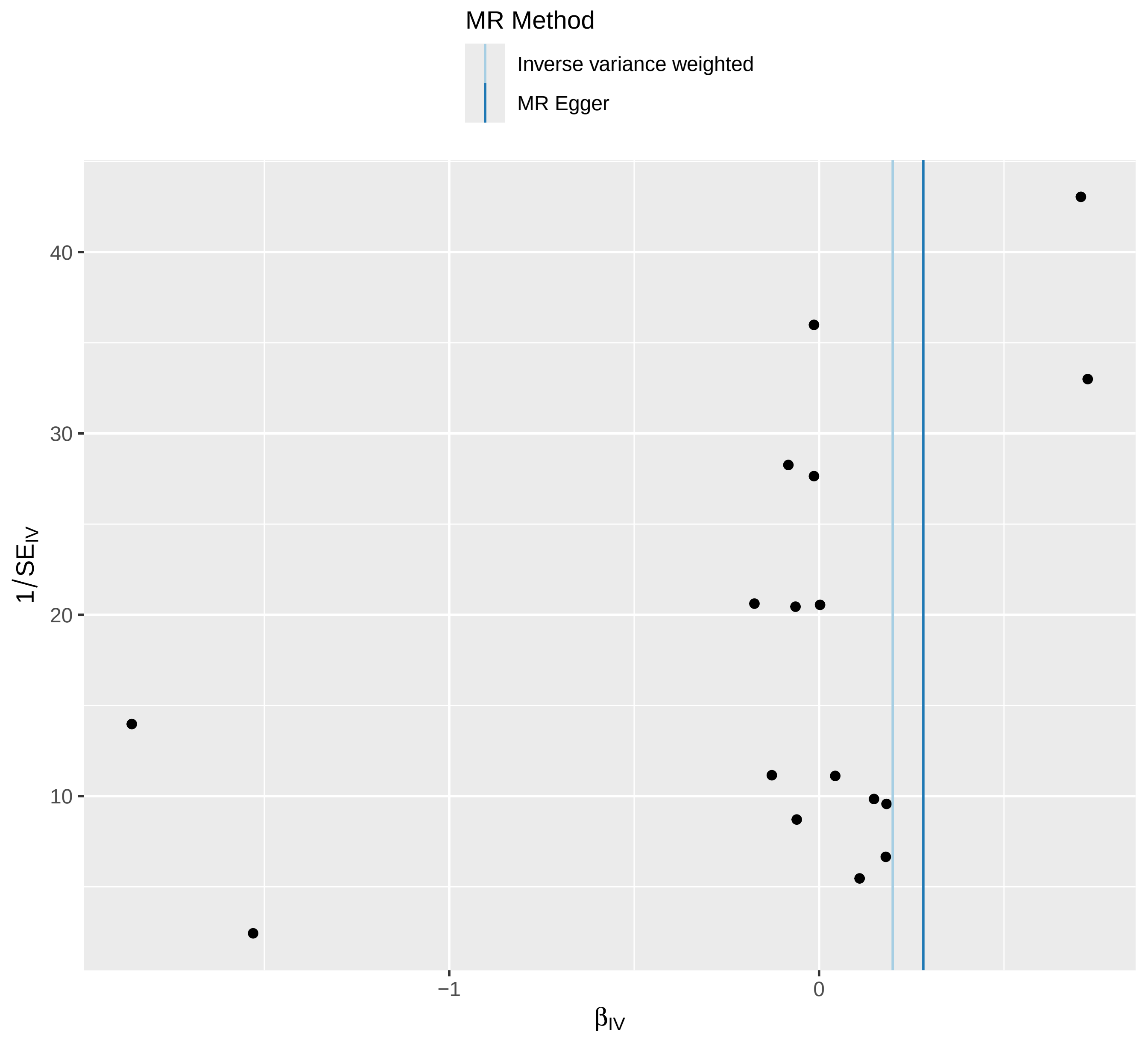

MR Method
Inverse variance weighted
MR Egger
40
30
20
10
1/SE$_{IV}$
−1
0
β$_{IV}$

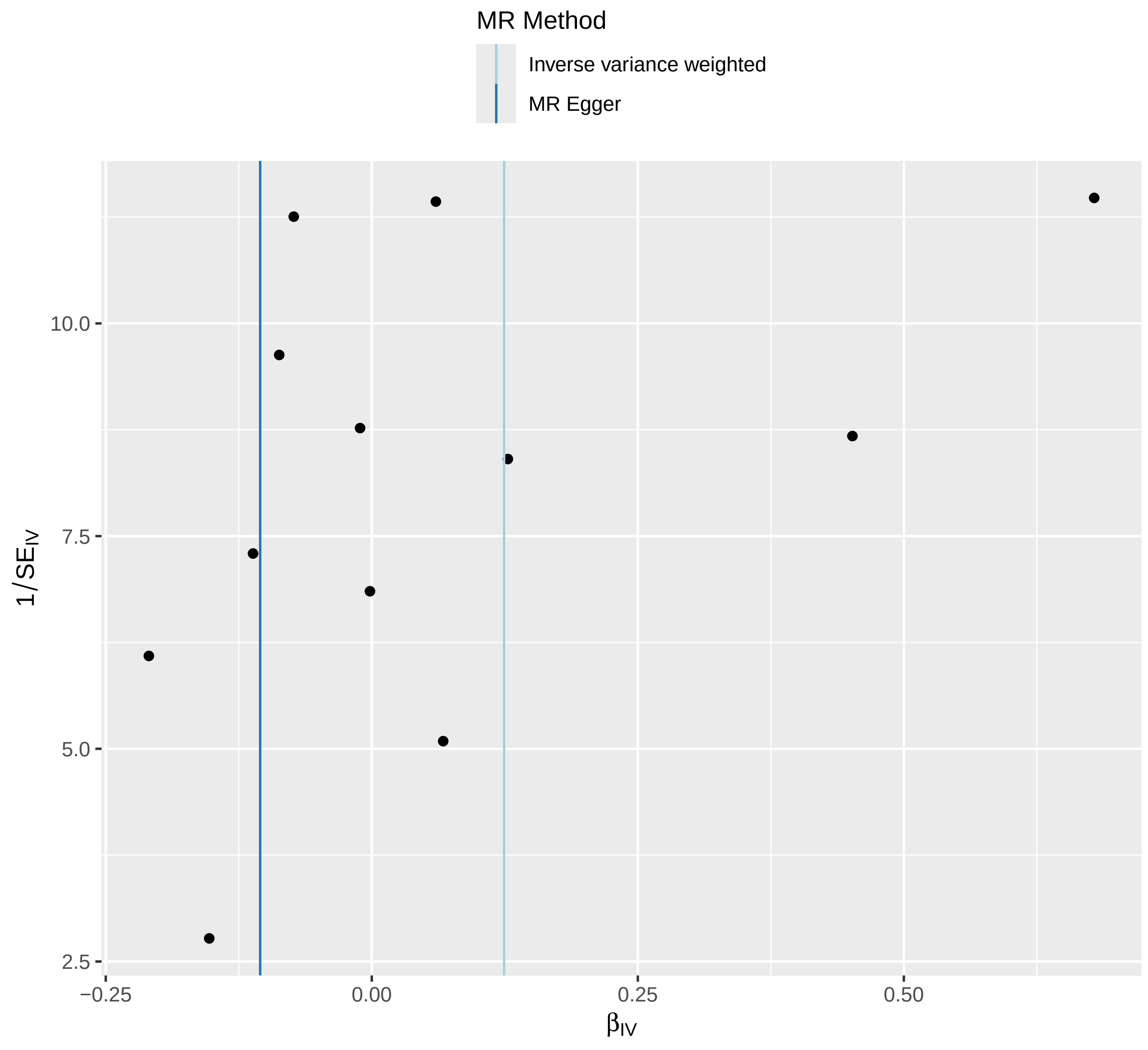
MR Method
Inverse variance weighted
MR Egger
10.0
7.5
5.0
2.5
$1/SE_{IV}$
−0.25
0.00
0.25
0.50
β_{IV}

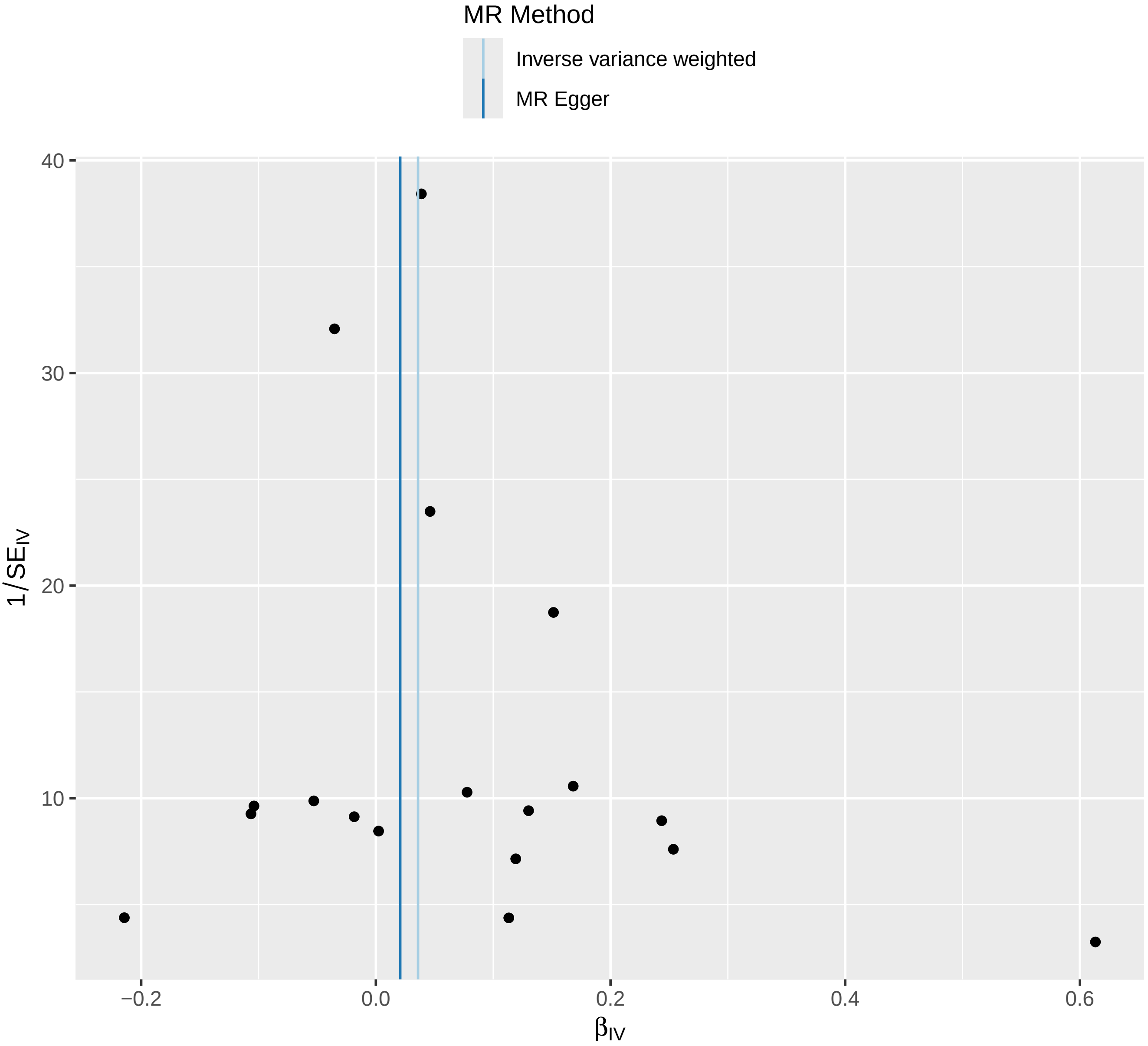
MR Method
Inverse variance weighted
MR Egger
40
30
20
10
1/SE_IV
−0.2
0.0
0.2
0.4
0.6
β_IV

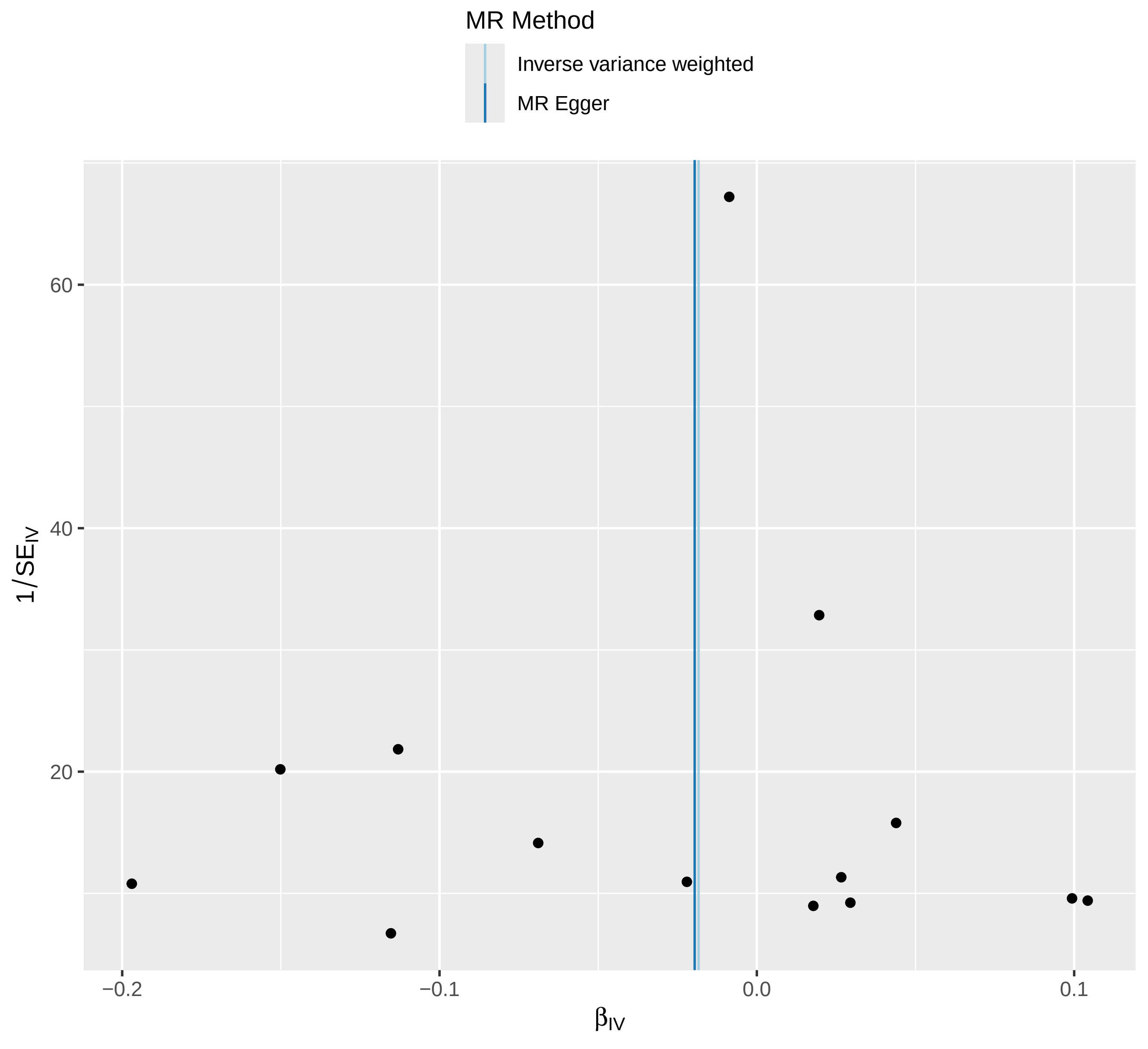
MR Method
Inverse variance weighted
MR Egger
60
40
20
1/SE_IV
−0.2
−0.1
0.0
0.1
β_IV

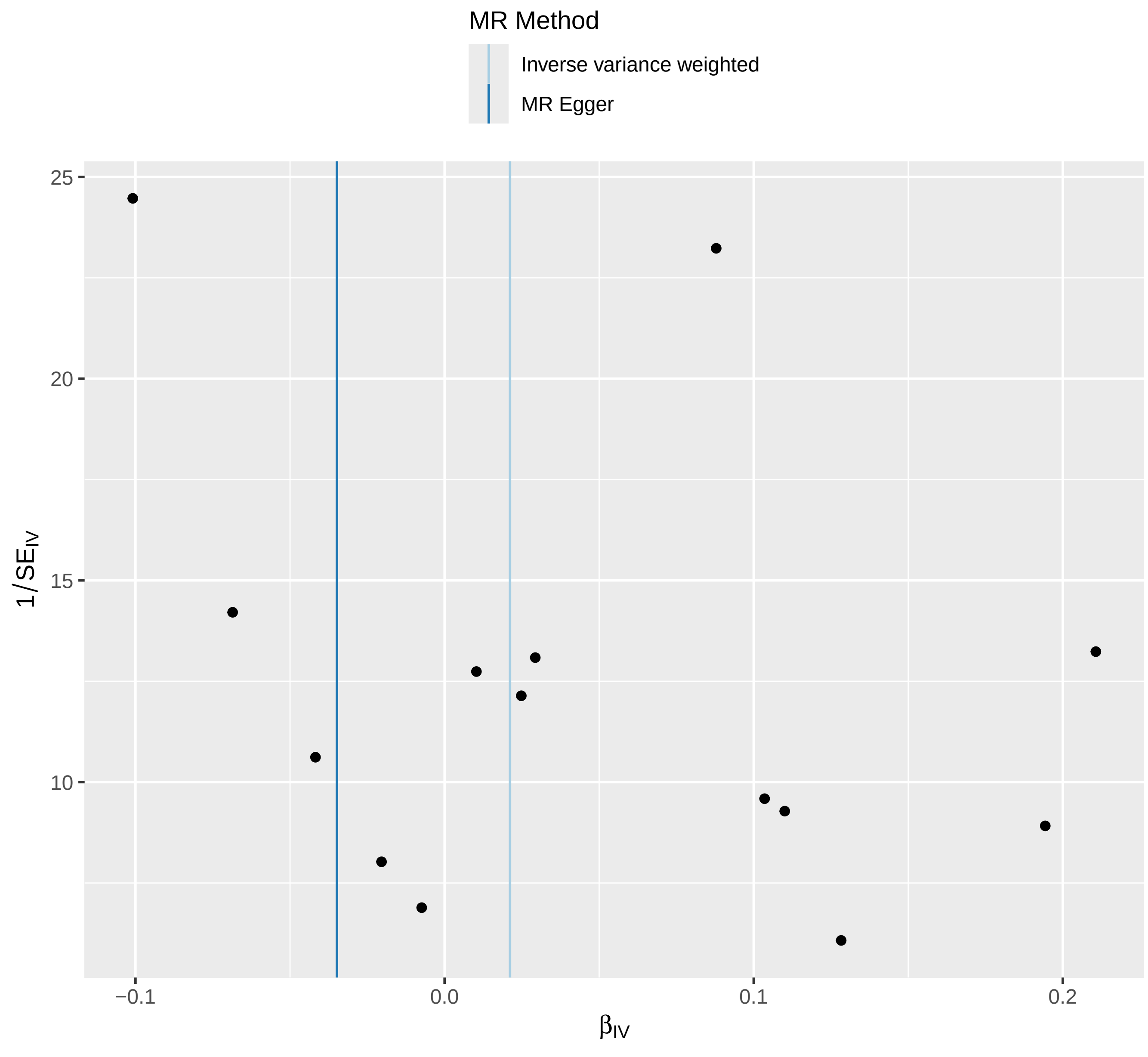
MR Method
Inverse variance weighted
MR Egger
25
20
15
10
1/SE$_{IV}$
−0.1
0.0
0.1
0.2
β_{IV}

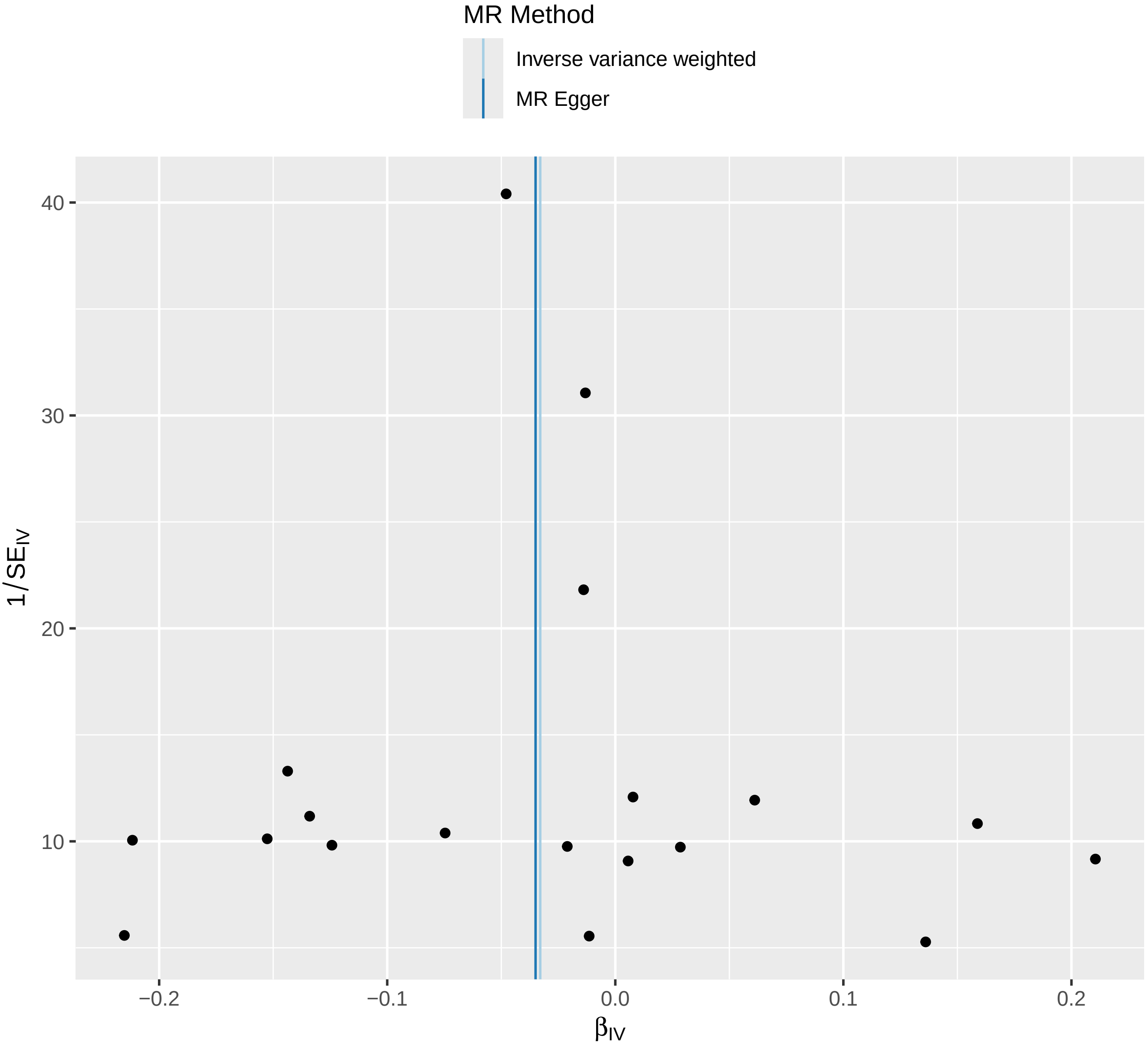
MR Method
Inverse variance weighted
MR Egger
40
30
20
10
−0.2
−0.1
0.0
0.1
0.2
1/SE_IV
β_IV

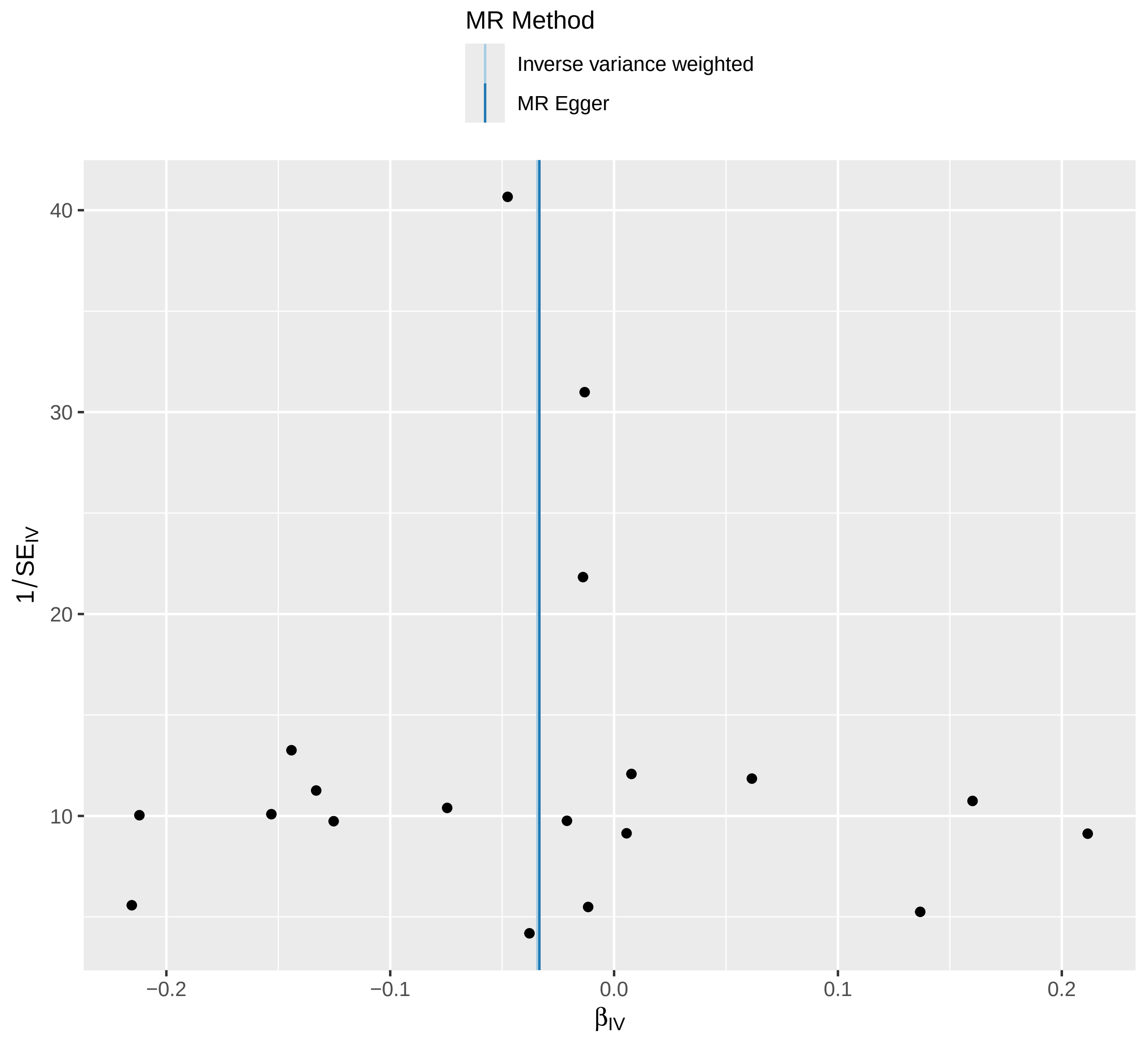
MR Method
Inverse variance weighted
MR Egger
40
30
20
10
1/SE$_{IV}$
−0.2
−0.1
0.0
0.1
0.2
β$_{IV}$

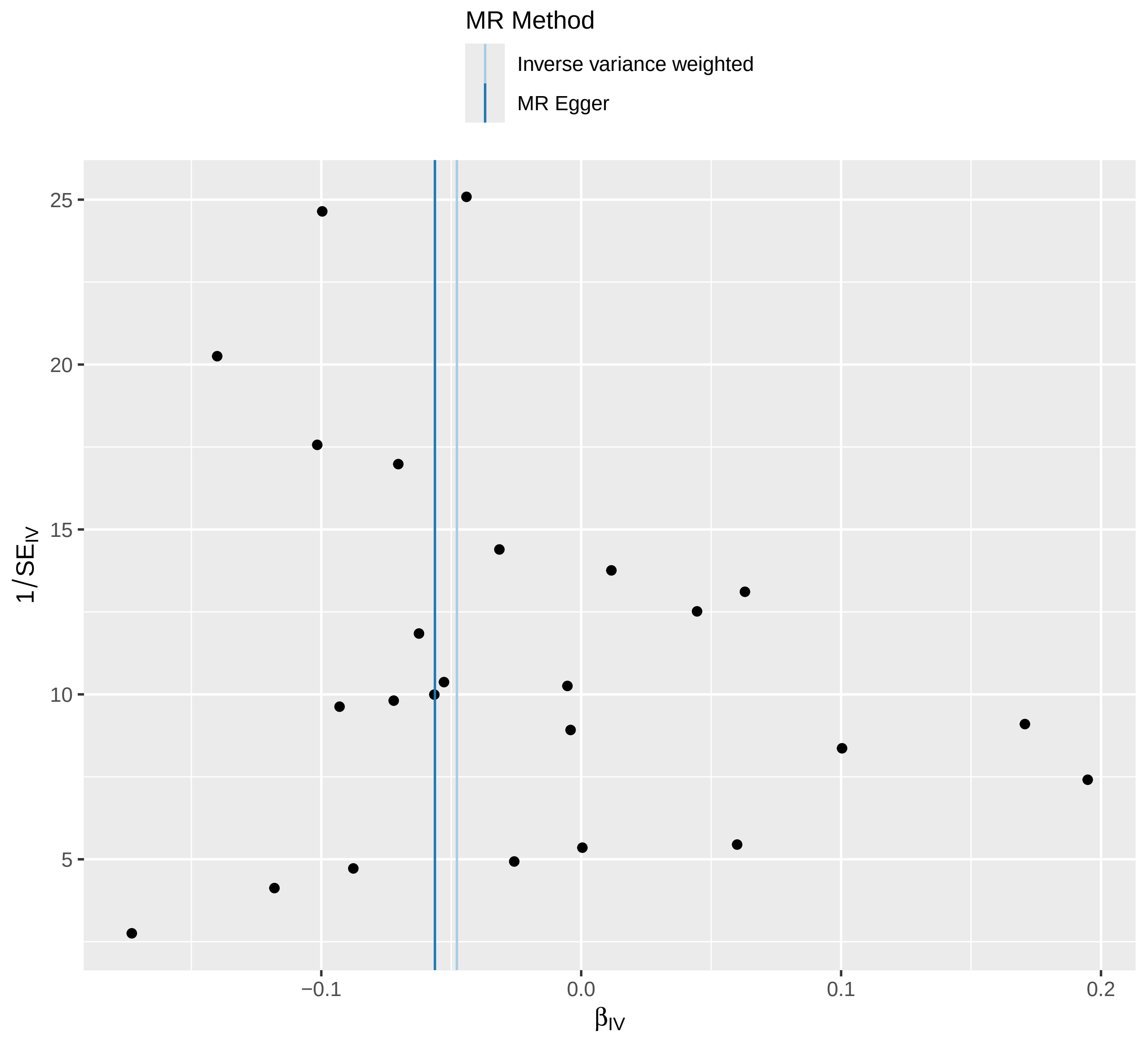
MR Method
Inverse variance weighted
MR Egger
25
20
15
10
5
1/SE$_{IV}$
−0.1
0.0
0.1
0.2
β$_{IV}$

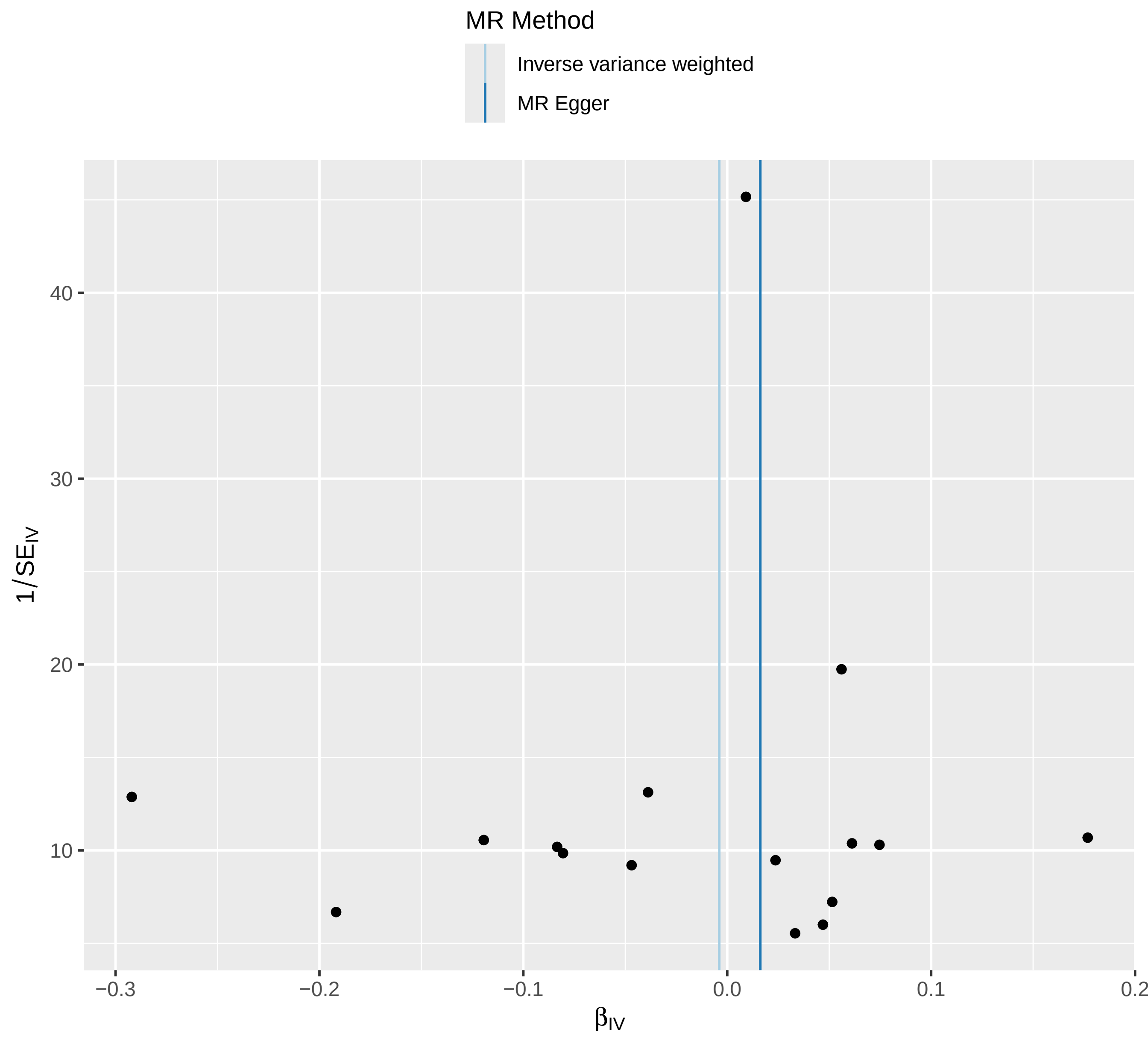
MR Method
Inverse variance weighted
MR Egger
40
30
20
10
−0.3
−0.2
−0.1
0.0
0.1
0.2
1/SE_IV
β_IV

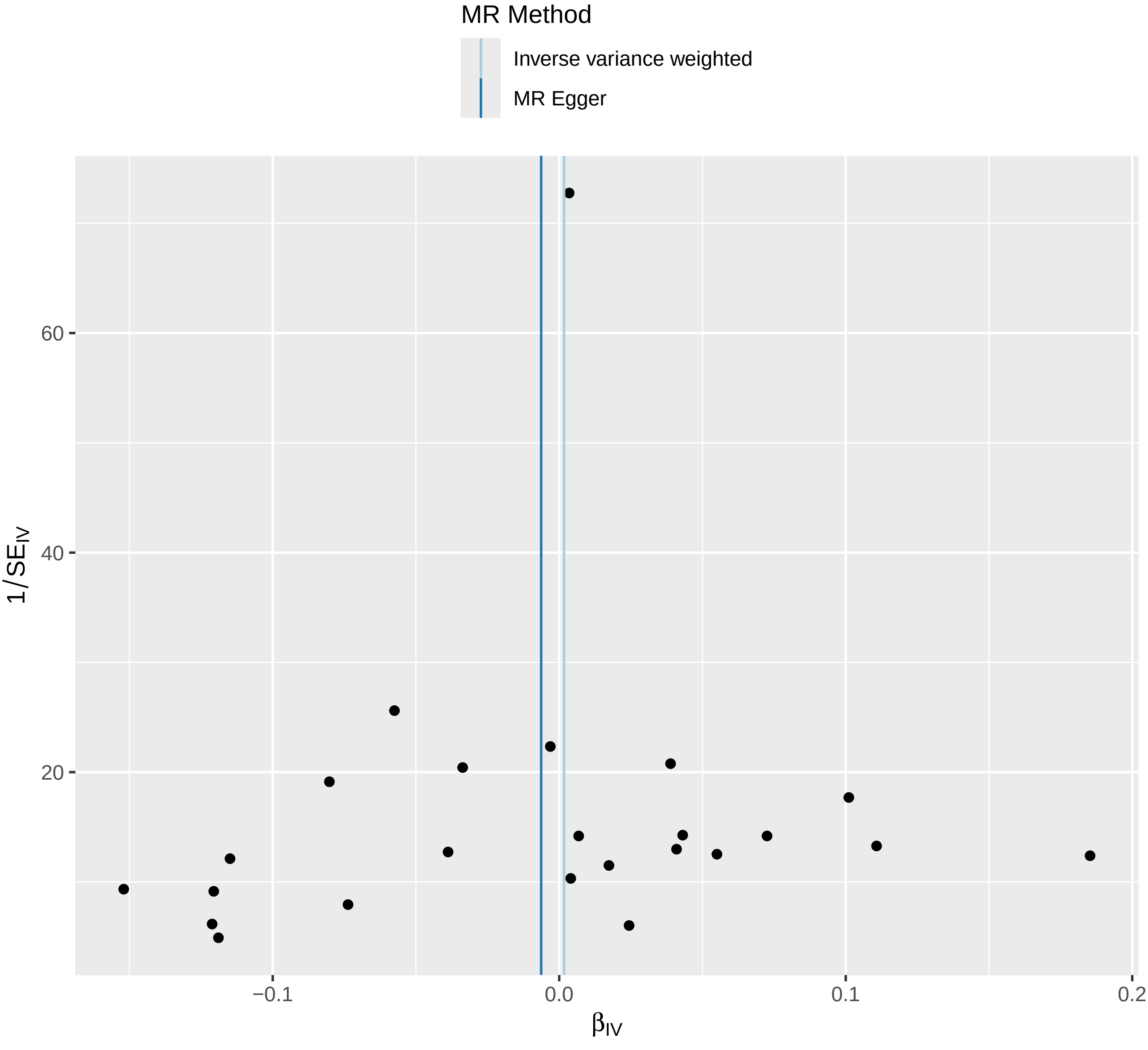
MR Method
Inverse variance weighted
MR Egger
60
40
20
1/SE_IV
−0.1
0.0
0.1
0.2
β_IV

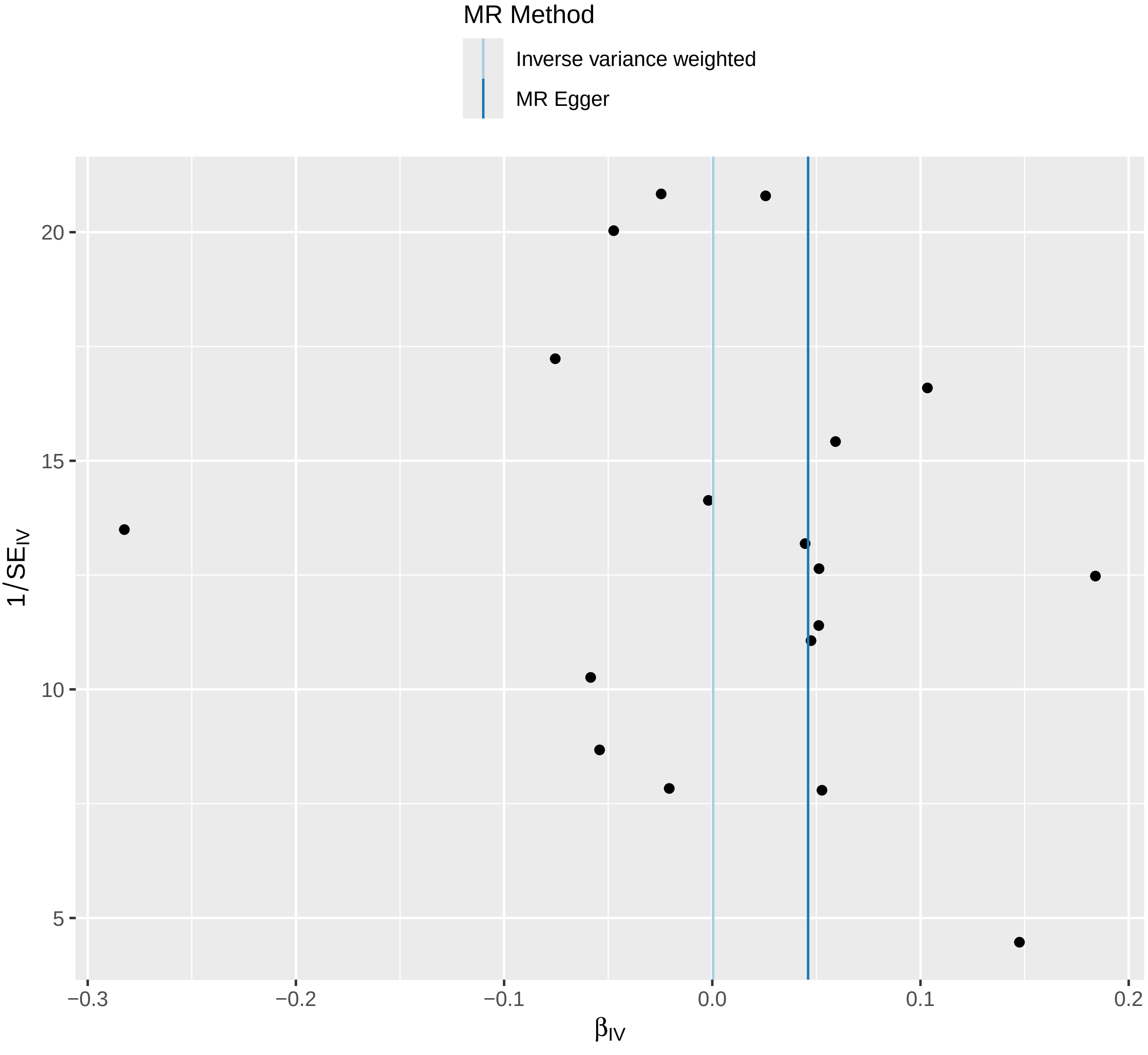

MR Method
Inverse variance weighted
MR Egger
20
15
10
5
1/SE_IV
−0.3
−0.2
−0.1
0.0
0.1
0.2
β_IV

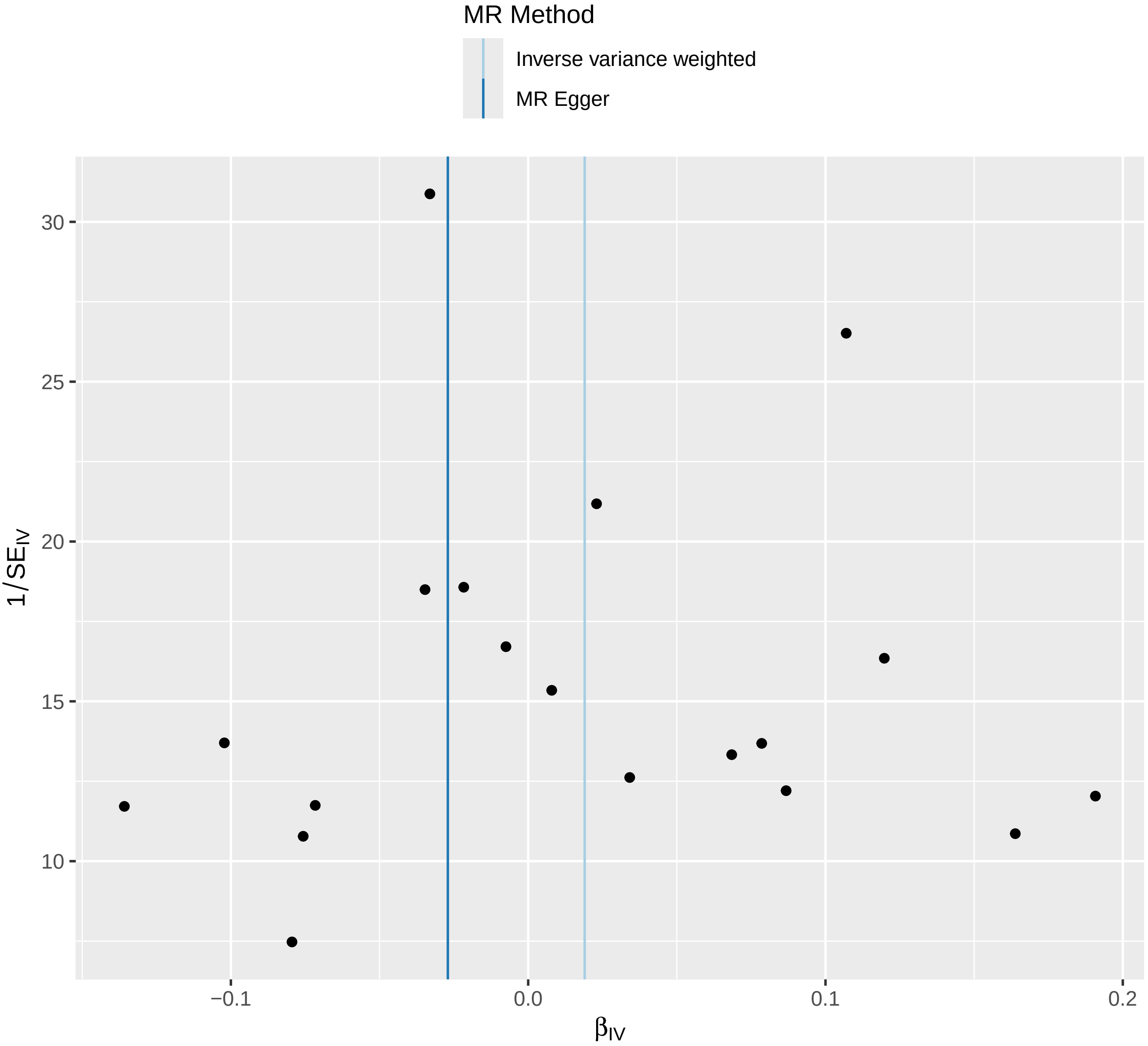
MR Method
Inverse variance weighted
MR Egger
1/SE_IV
30
25
20
15
10
−0.1
0.0
0.1
0.2
β_IV

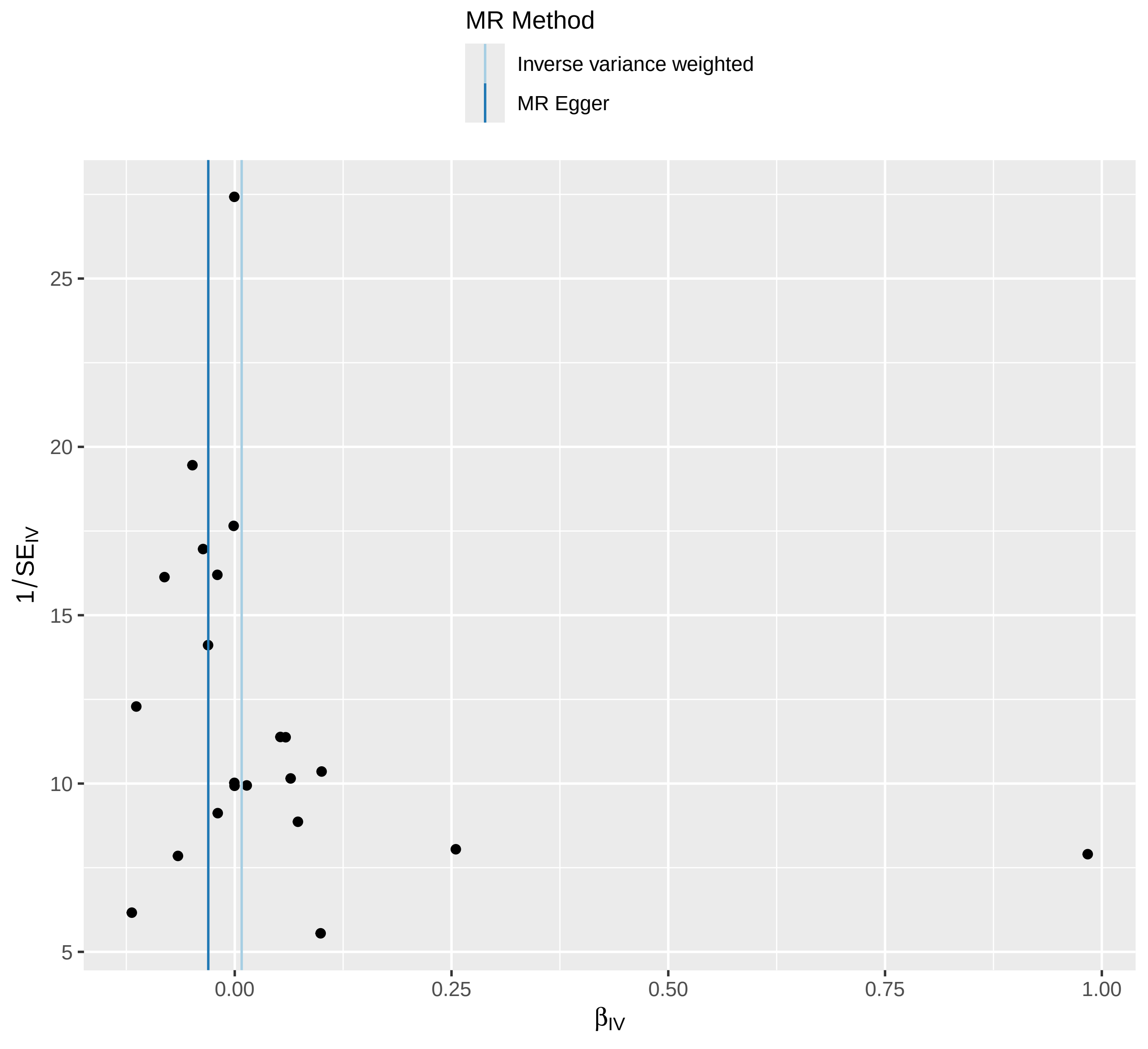

MR Method
Inverse variance weighted
MR Egger
1/SE_IV
25
20
15
10
5
0.00
0.25
0.50
0.75
1.00
β_IV

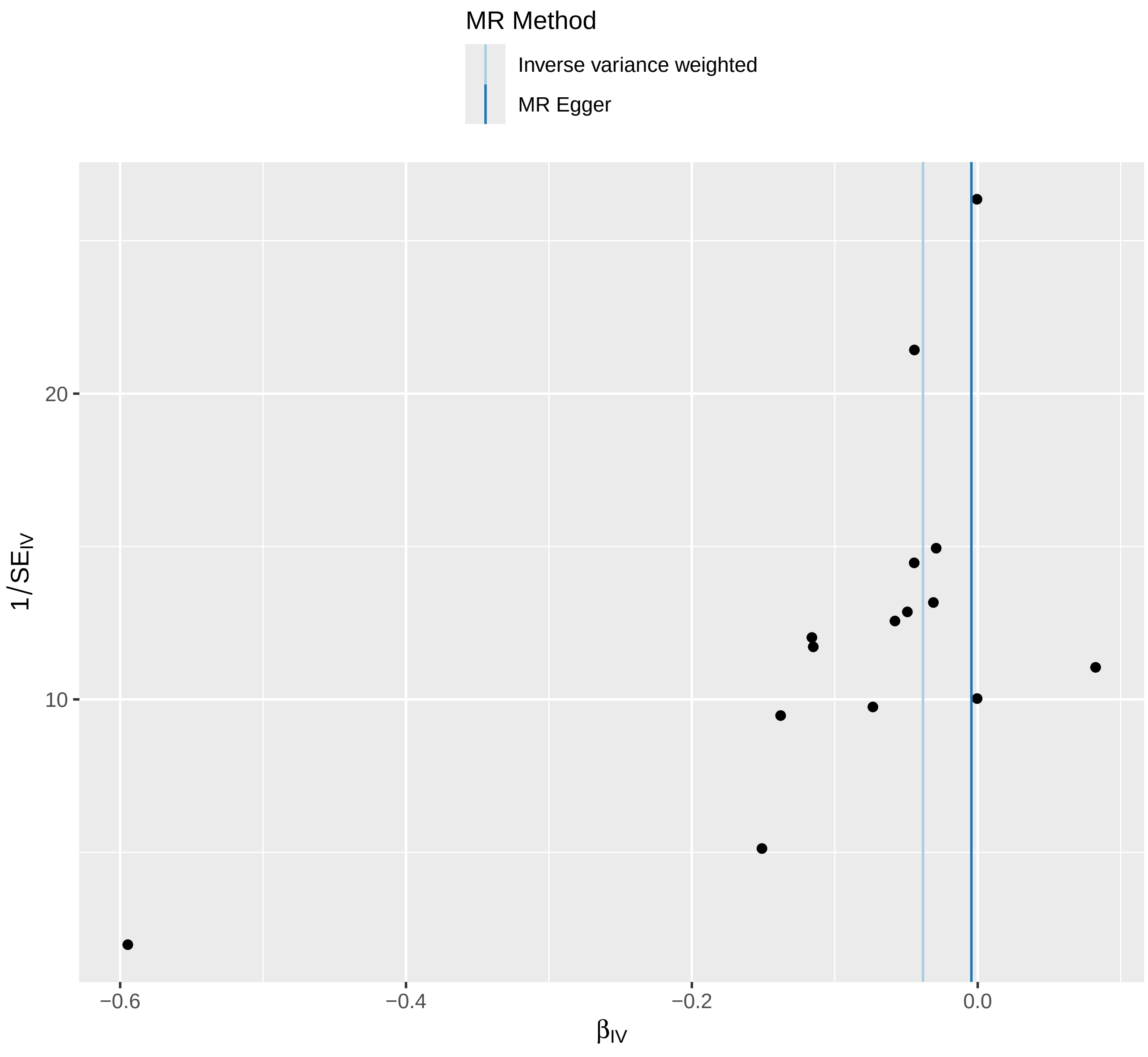
MR Method
Inverse variance weighted
MR Egger
1/SE$_{IV}$
20
10
−0.6
−0.4
−0.2
0.0
β$_{IV}$

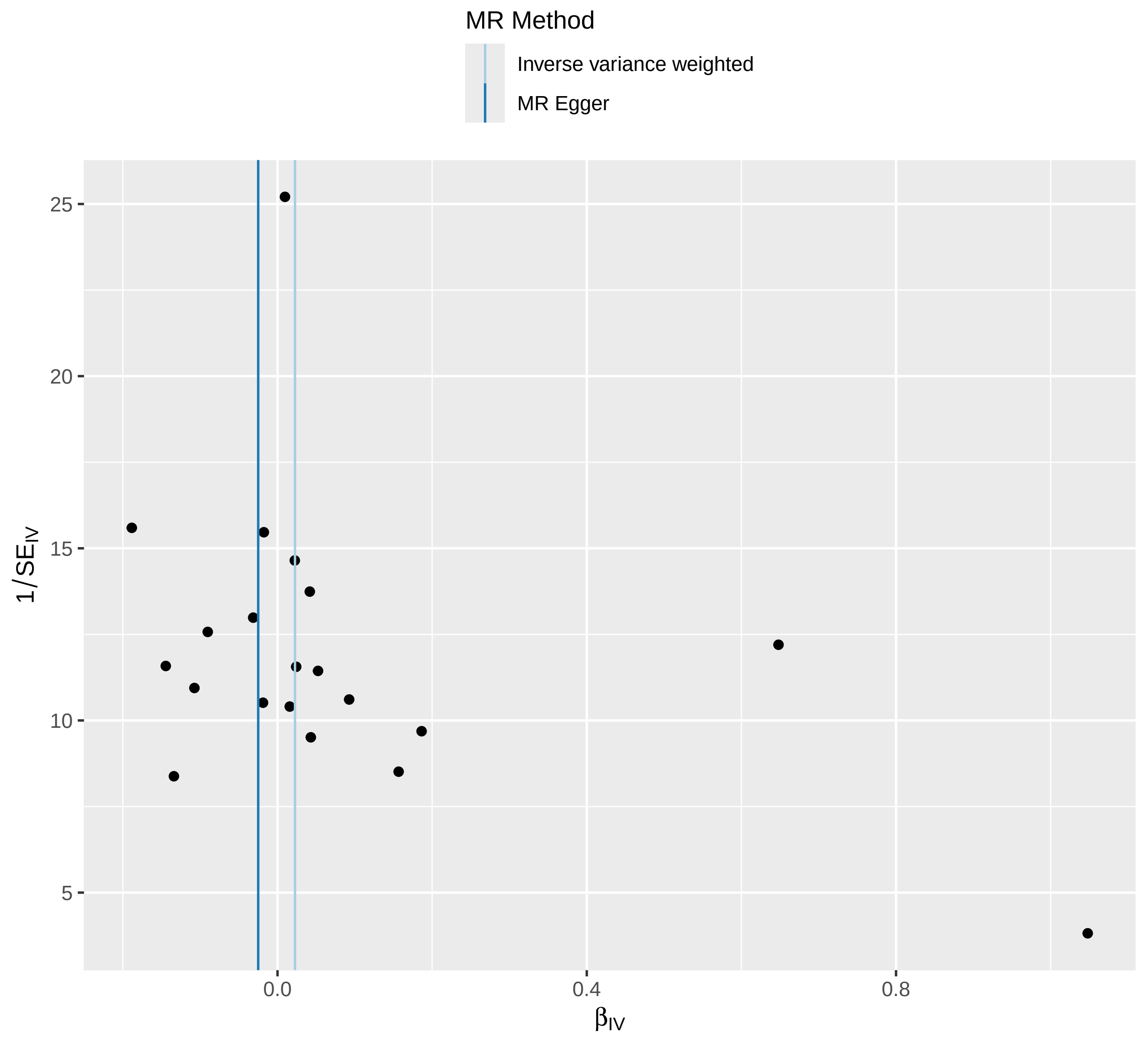
MR Method
Inverse variance weighted
MR Egger
1/SE_IV
25
20
15
10
5
0.0
0.4
0.8
β_IV

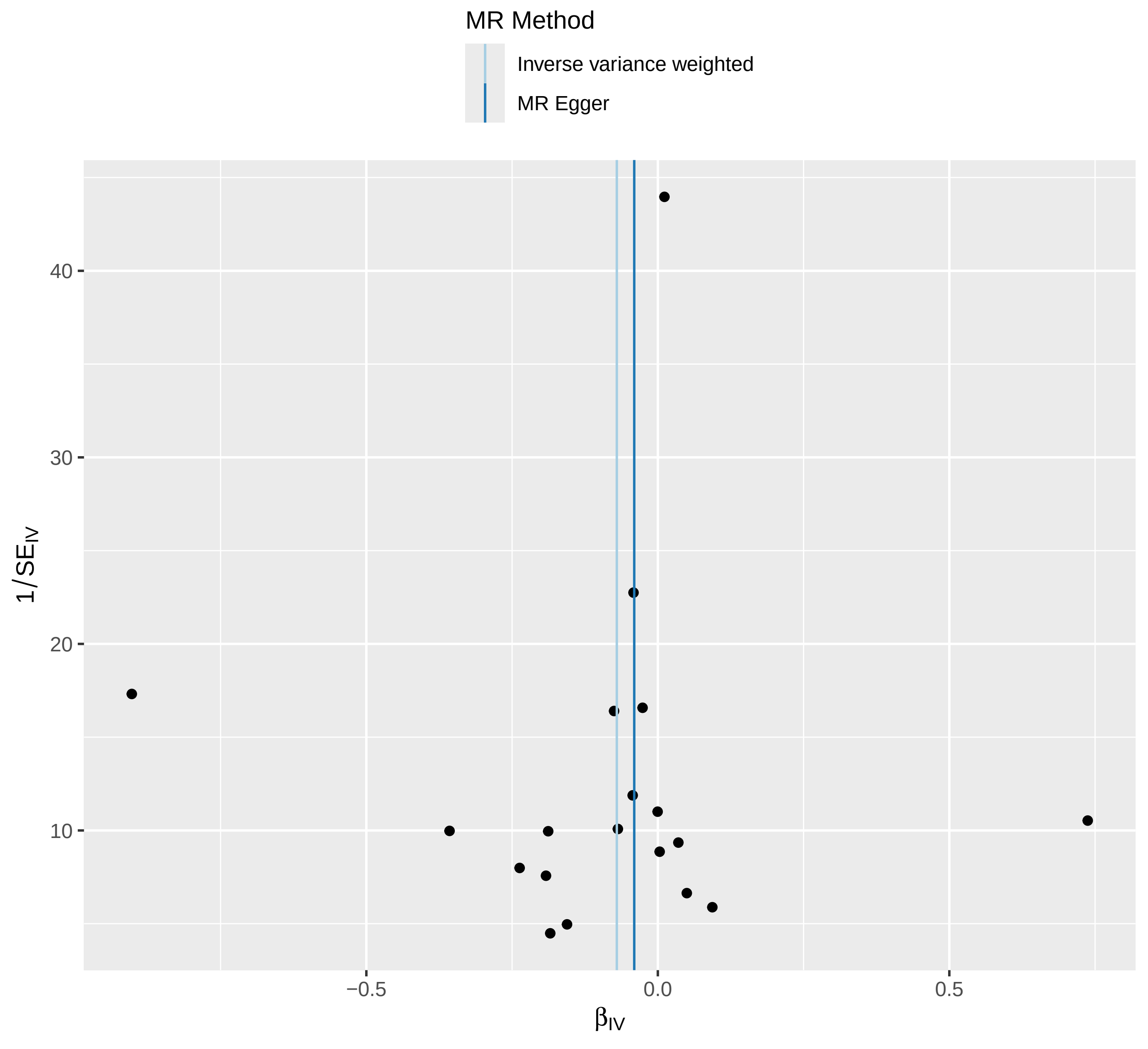
MR Method
Inverse variance weighted
MR Egger
40
30
20
10
1/SE_IV
−0.5
0.0
0.5
β_IV

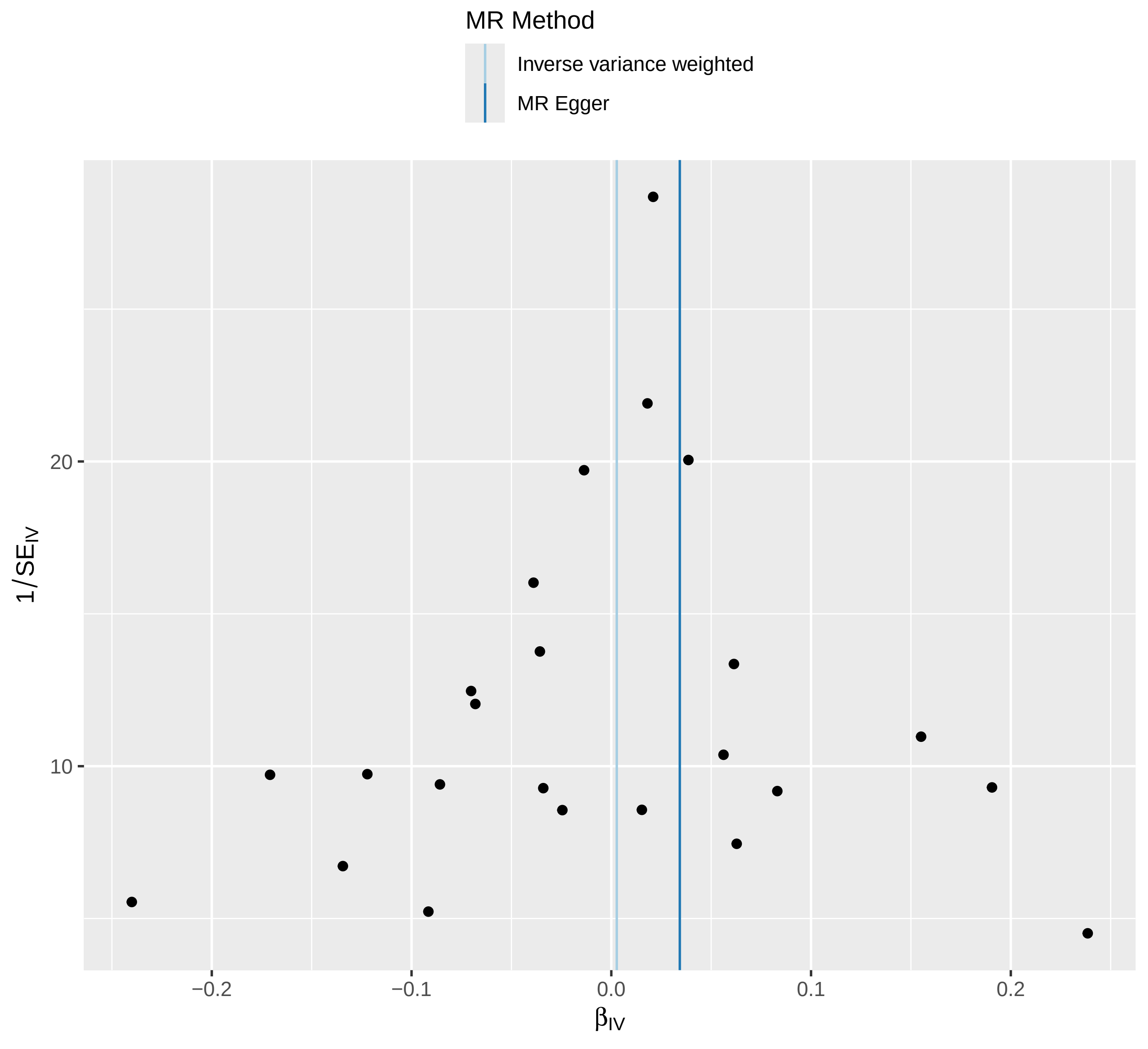

MR Method
Inverse variance weighted
MR Egger
20
10
1/SE$_{IV}$
−0.2
−0.1
0.0
0.1
0.2
β$_{IV}$

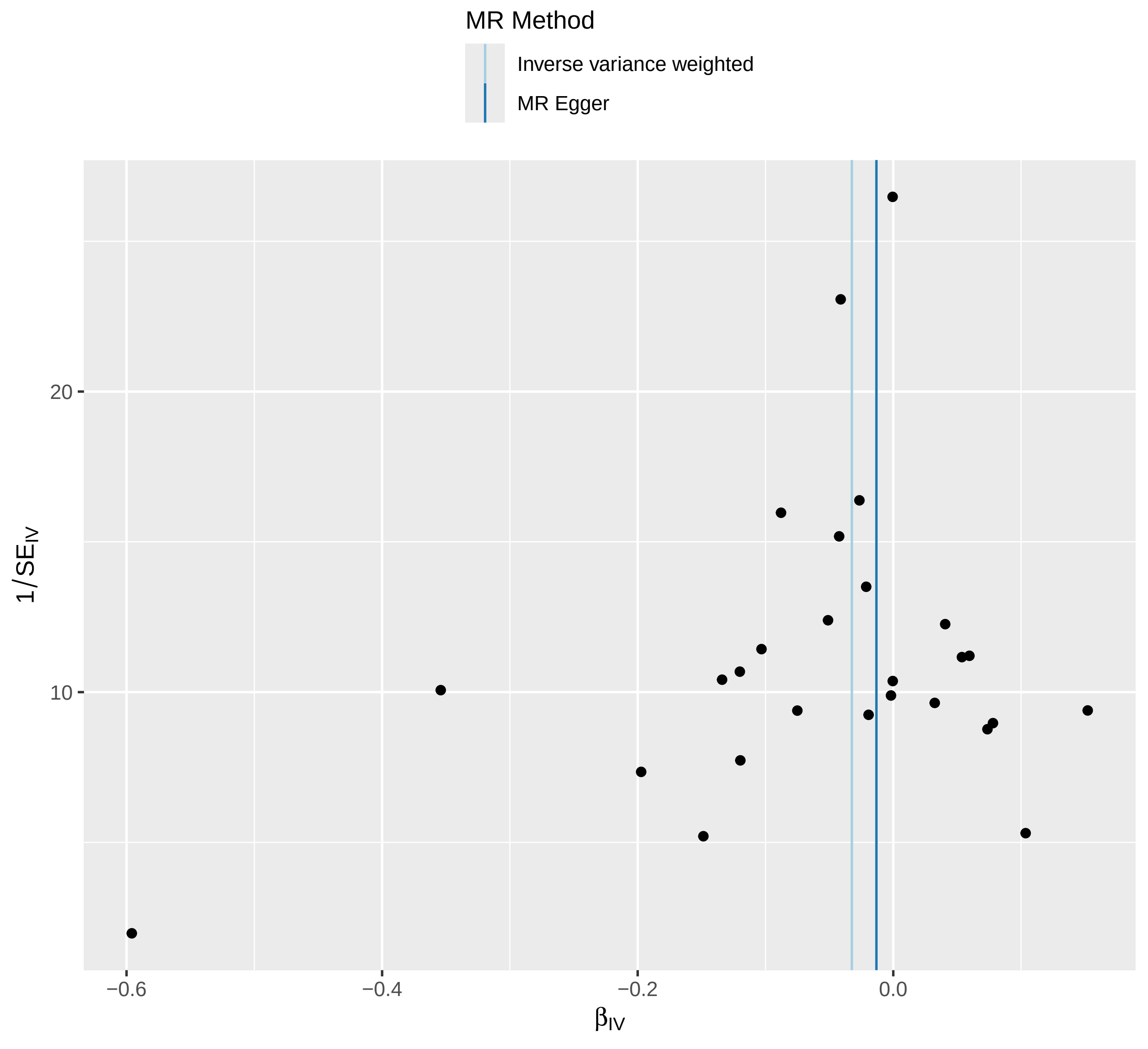
MR Method
Inverse variance weighted
MR Egger
20
10
1/SE_IV
−0.6
−0.4
−0.2
0.0
β_IV

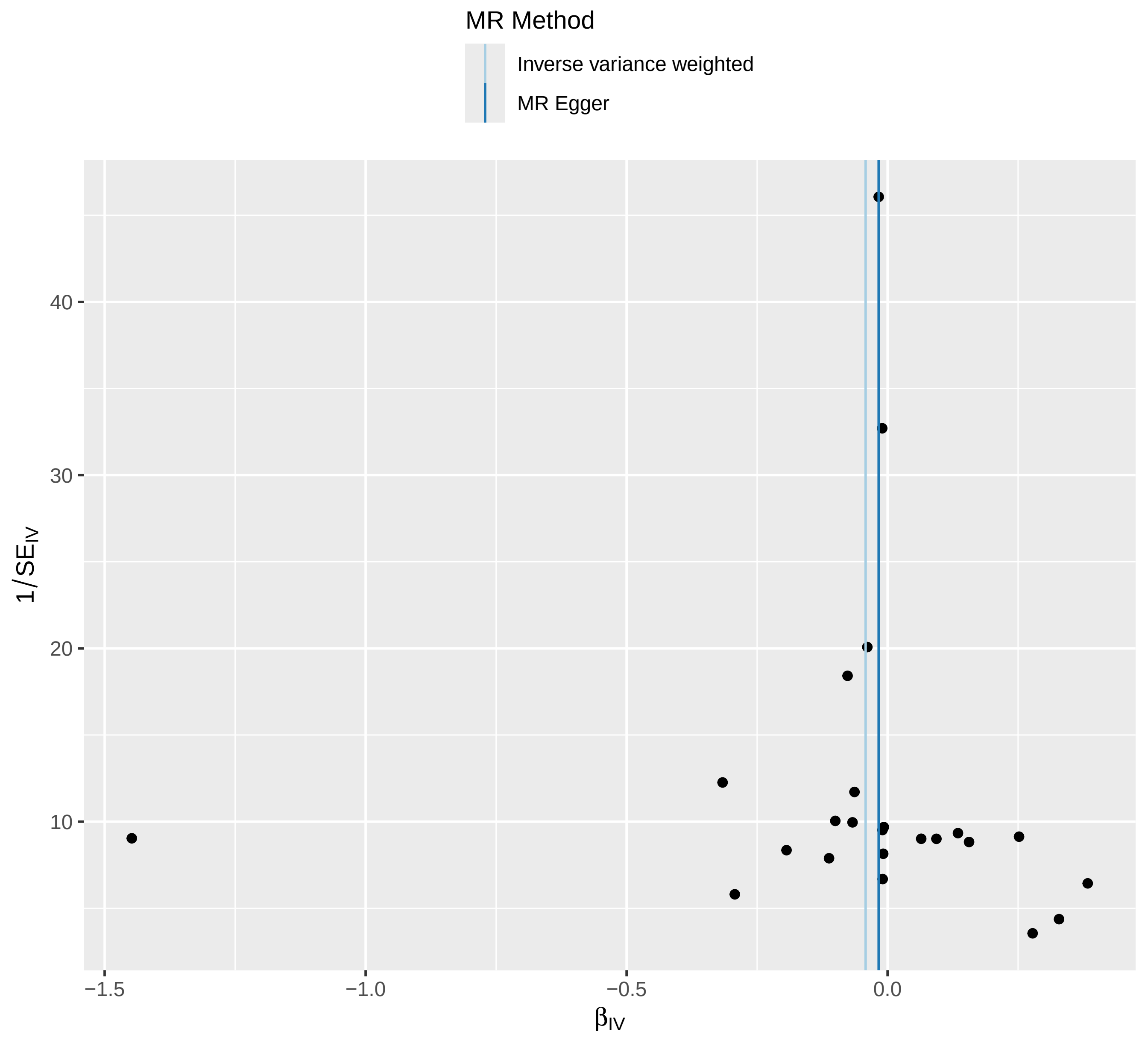
MR Method
Inverse variance weighted
MR Egger
40
30
20
10
1/SE$_{IV}$
−1.5
−1.0
−0.5
0.0
β$_{IV}$

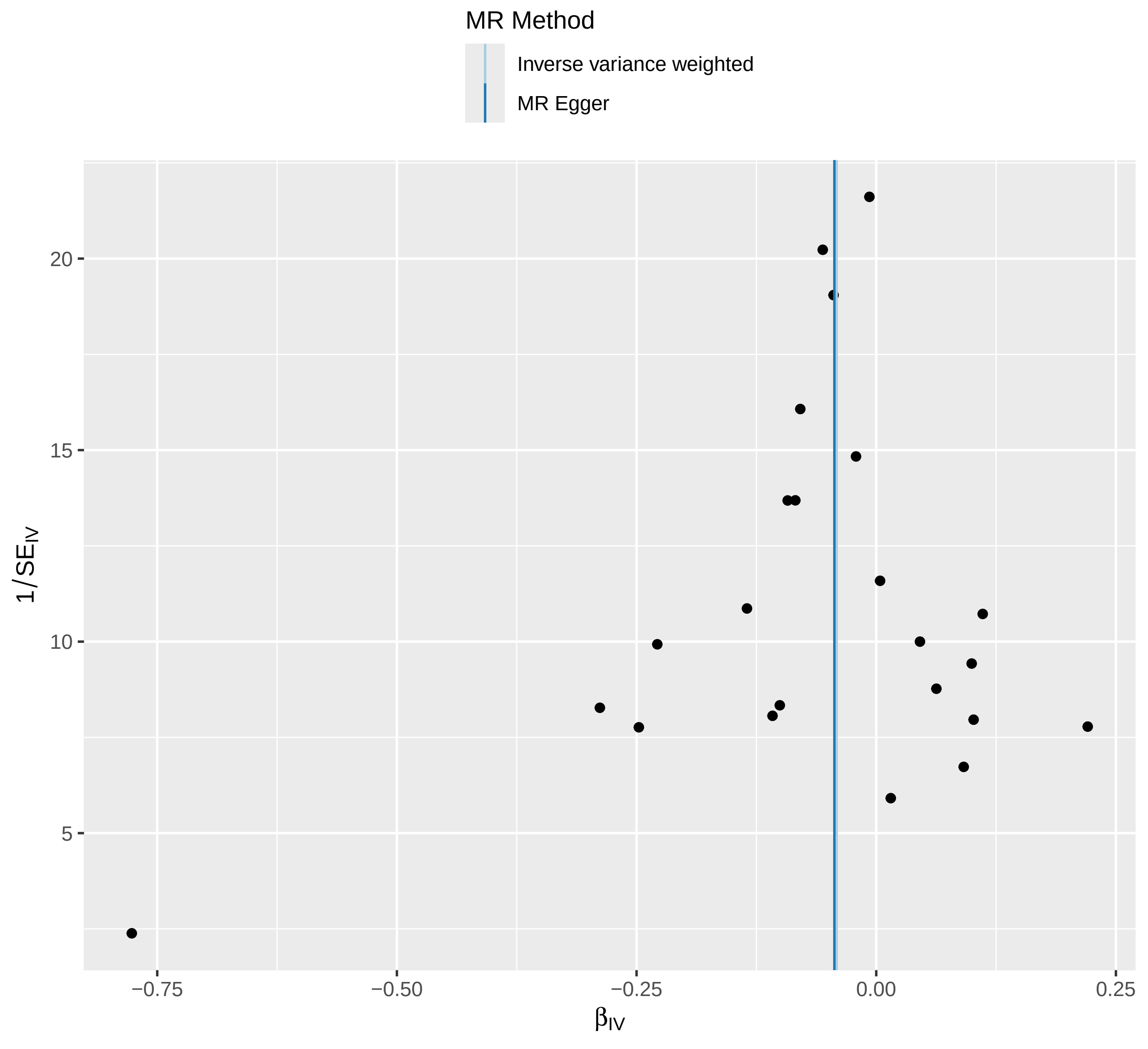
MR Method
Inverse variance weighted
MR Egger
$1/SE_{IV}$
20
15
10
5
−0.75
−0.50
−0.25
0.00
0.25
β_{IV}

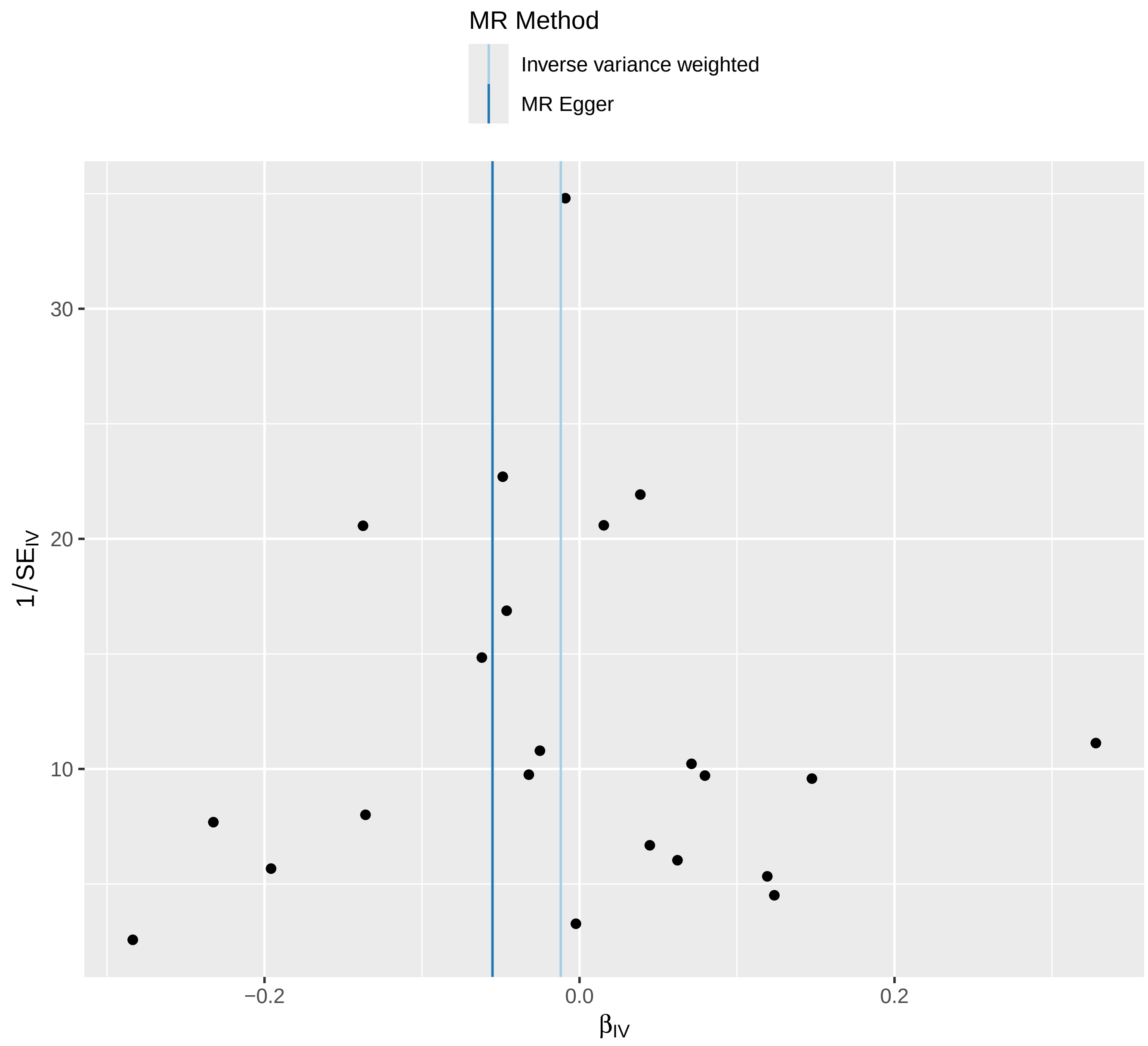
MR Method
Inverse variance weighted
MR Egger
30
20
10
1/SE_IV
−0.2
0.0
0.2
β_IV

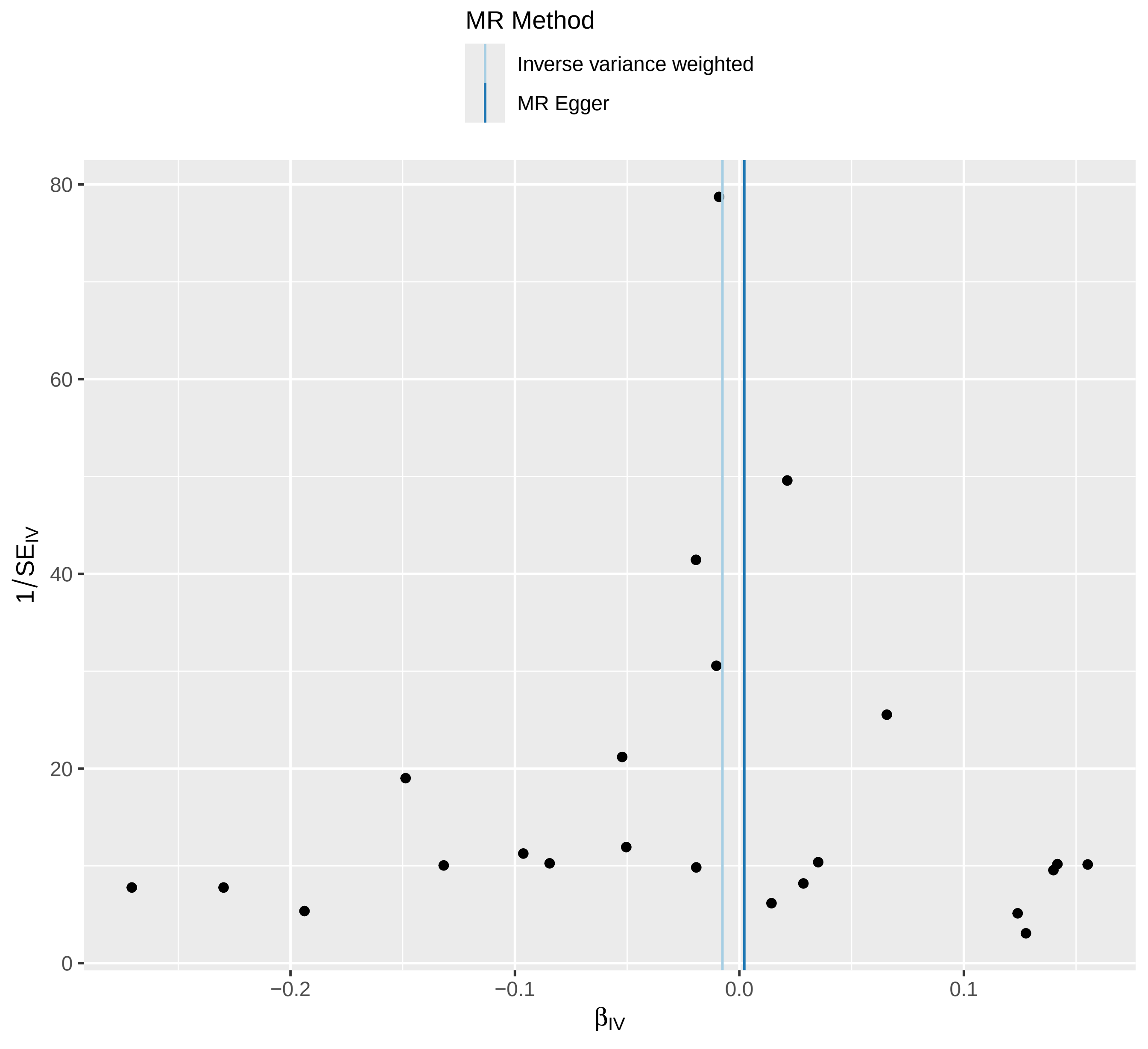

MR Method
Inverse variance weighted
MR Egger
1/SE_IV
80
60
40
20
0
−0.2
−0.1
0.0
0.1
β_IV

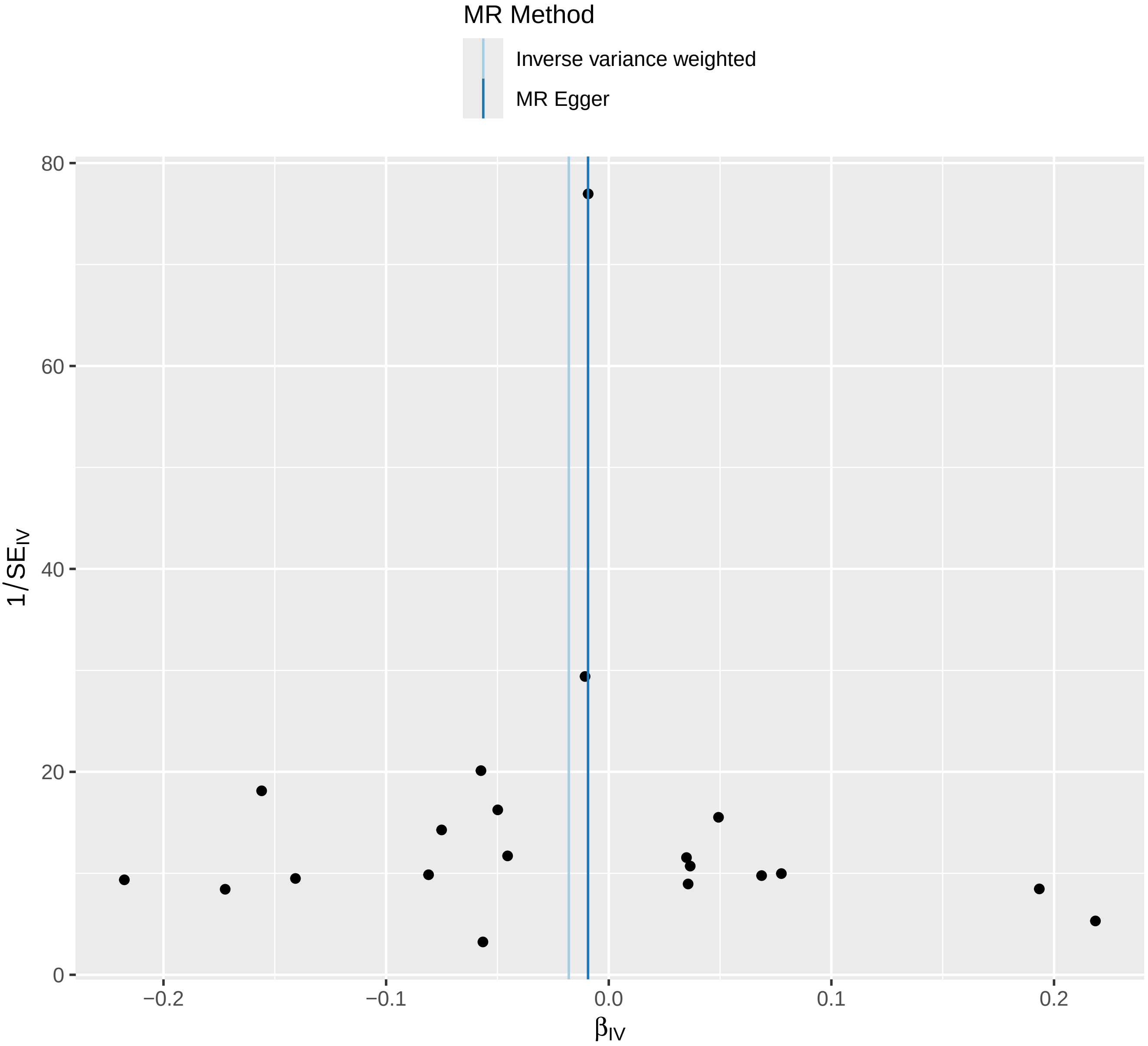
MR Method
Inverse variance weighted
MR Egger
80
60
40
20
0
1/SE_IV
−0.2
−0.1
0.0
0.1
0.2
β_IV

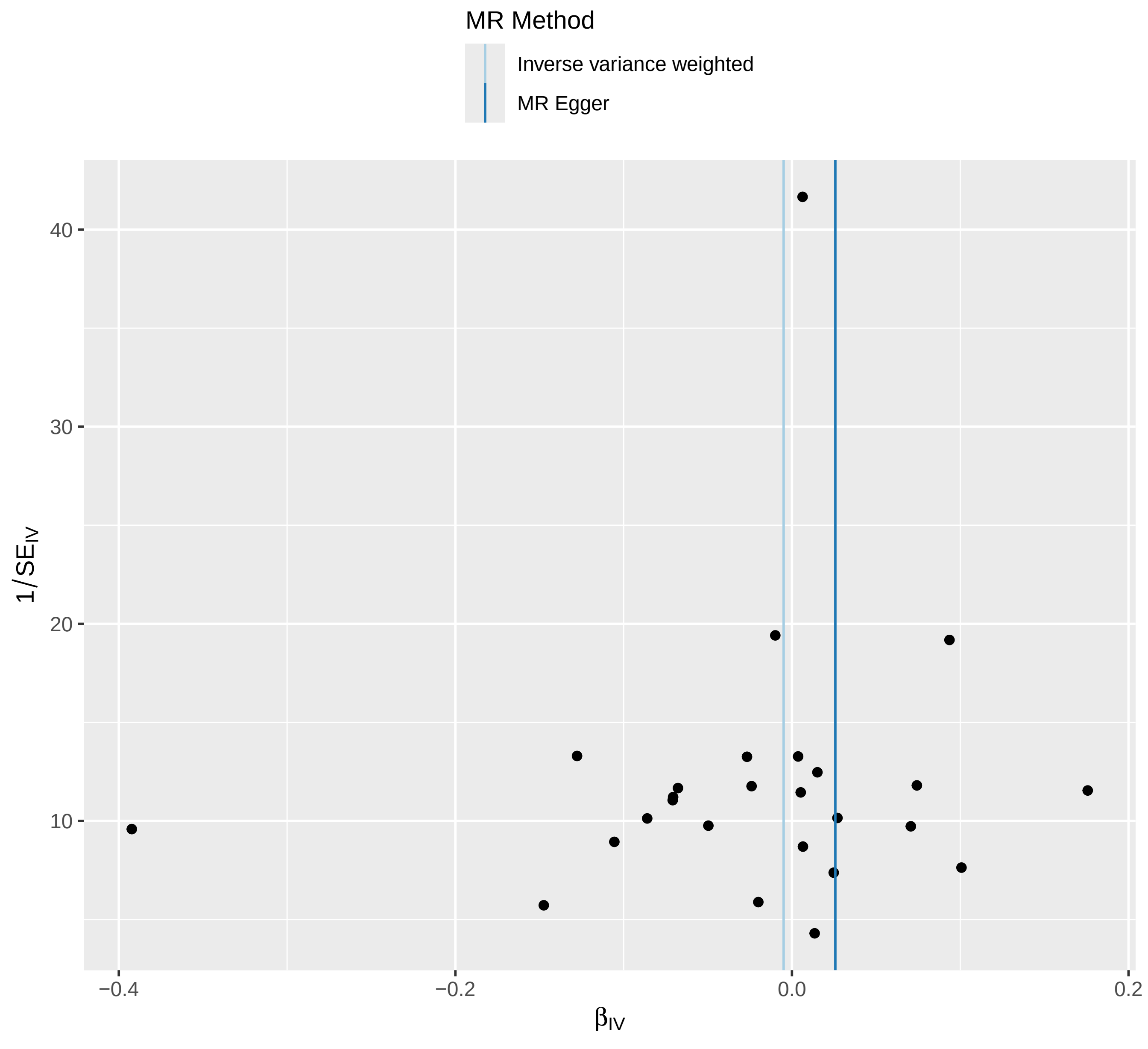

MR Method
Inverse variance weighted
MR Egger
40
30
20
10
1/SE_IV
−0.4
−0.2
0.0
0.2
β_IV

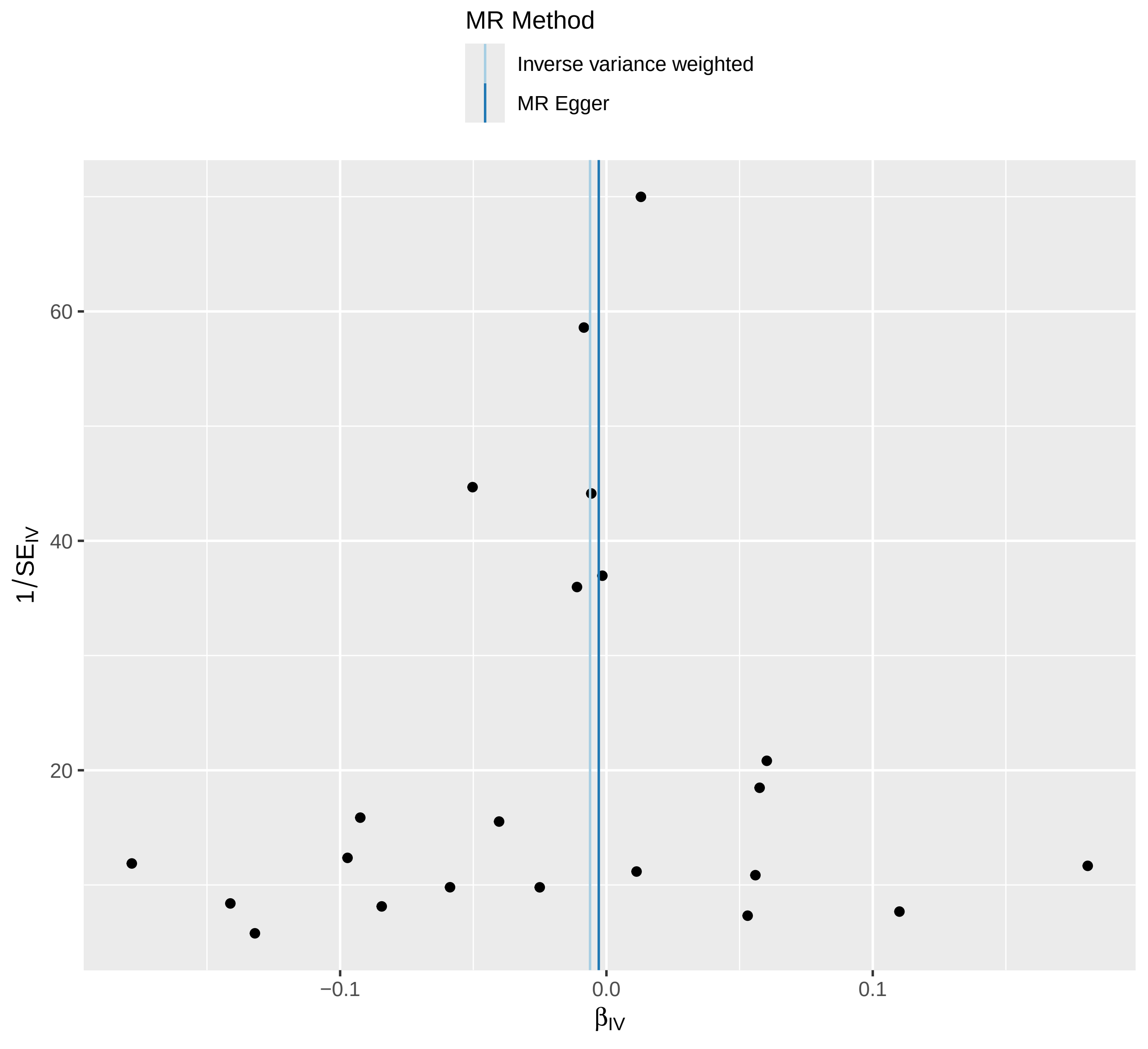

MR Method
Inverse variance weighted
MR Egger
60
40
20
1/SE$_{IV}$
−0.1
0.0
0.1
β$_{IV}$

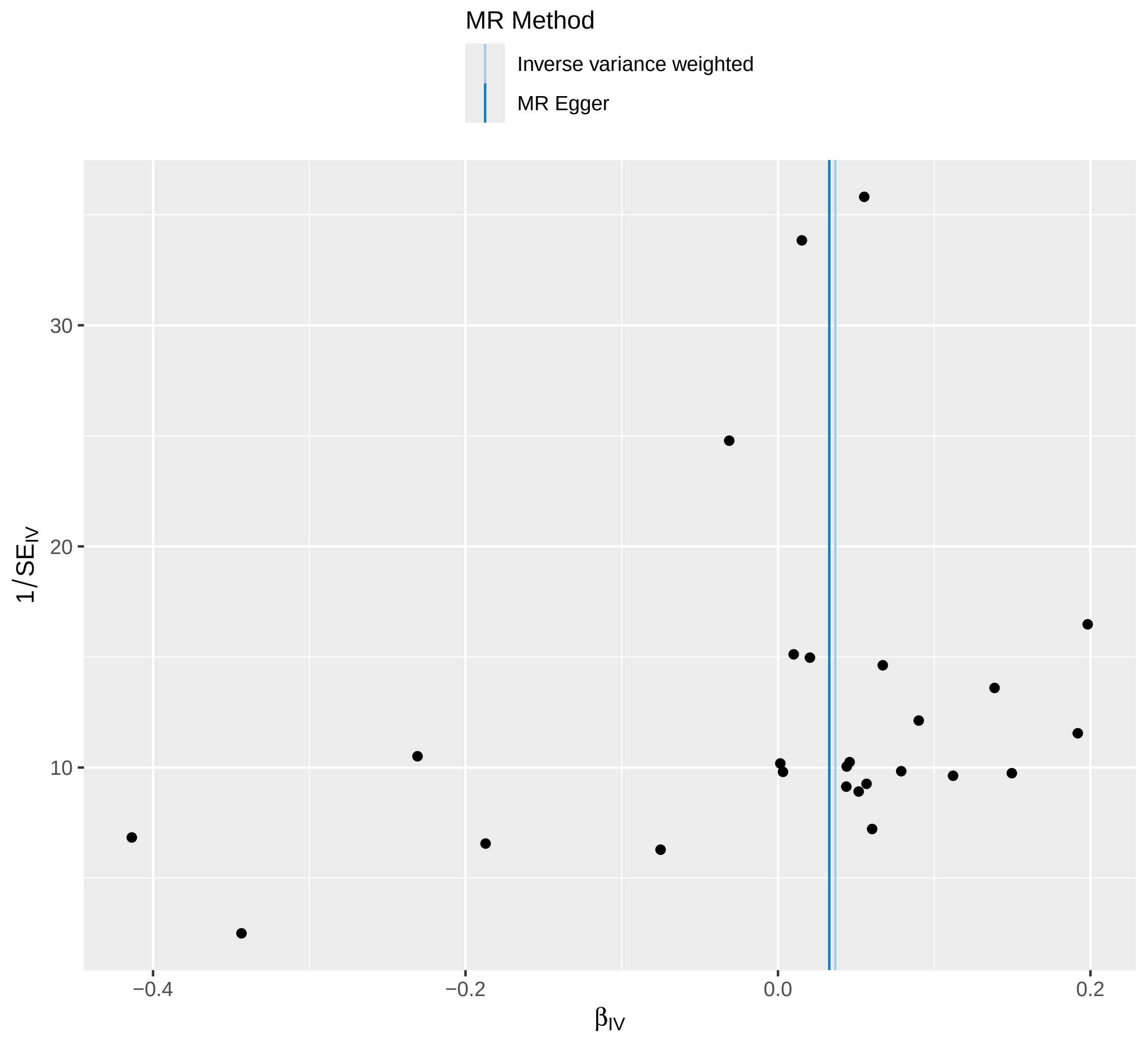
MR Method
Inverse variance weighted
MR Egger
30
20
10
1/SE_IV
−0.4
−0.2
0.0
0.2
β_IV

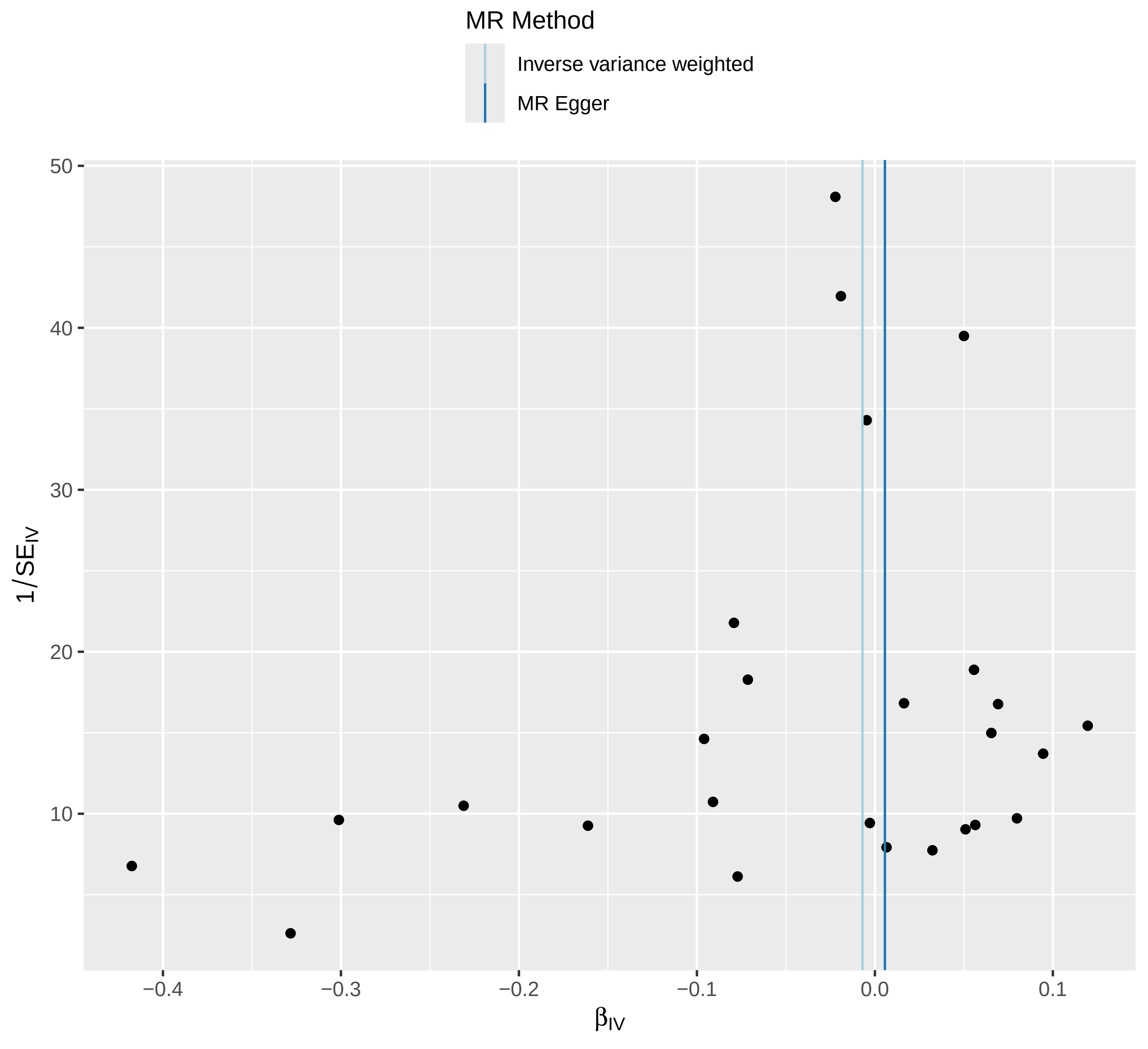

MR Method
Inverse variance weighted
MR Egger
50
40
30
20
10
$1/SE_{IV}$
−0.4
−0.3
−0.2
−0.1
0.0
0.1
β_{IV}

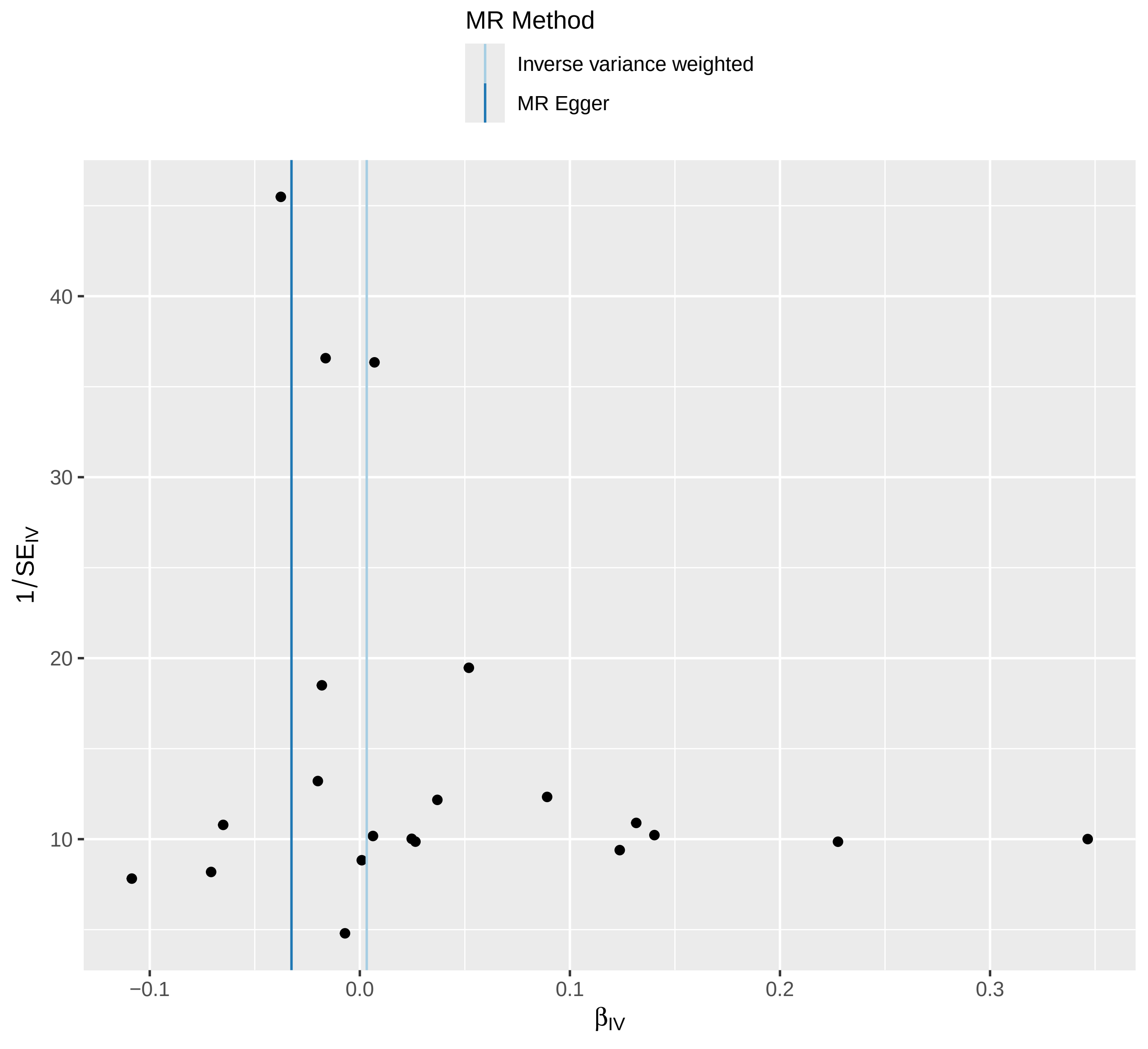

MR Method
Inverse variance weighted
MR Egger
40
30
20
10
1/SE$_{IV}$
−0.1
0.0
0.1
0.2
0.3
β$_{IV}$

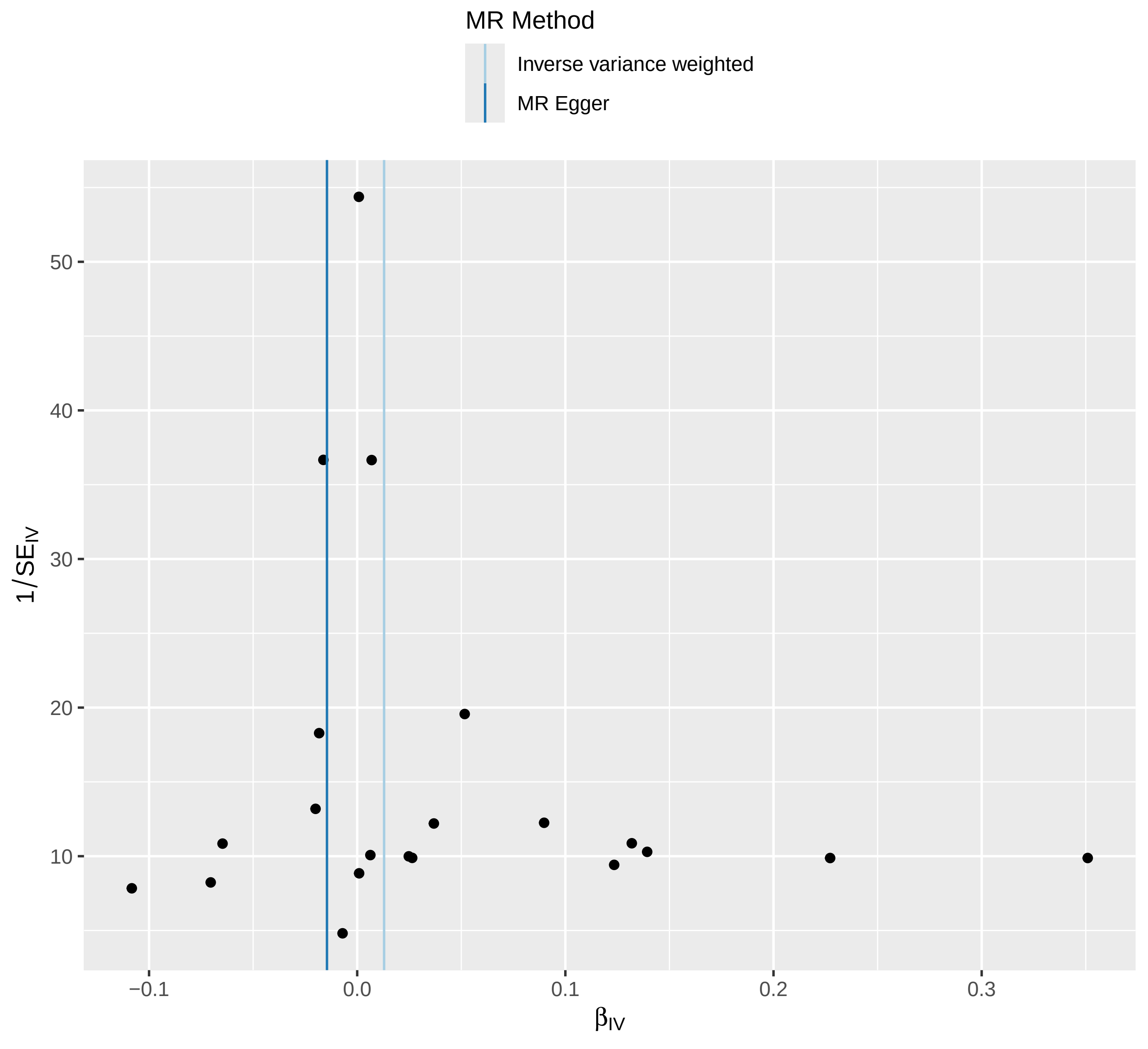
MR Method
Inverse variance weighted
MR Egger
1/SE_IV
50
40
30
20
10
−0.1
0.0
0.1
0.2
0.3
β_IV

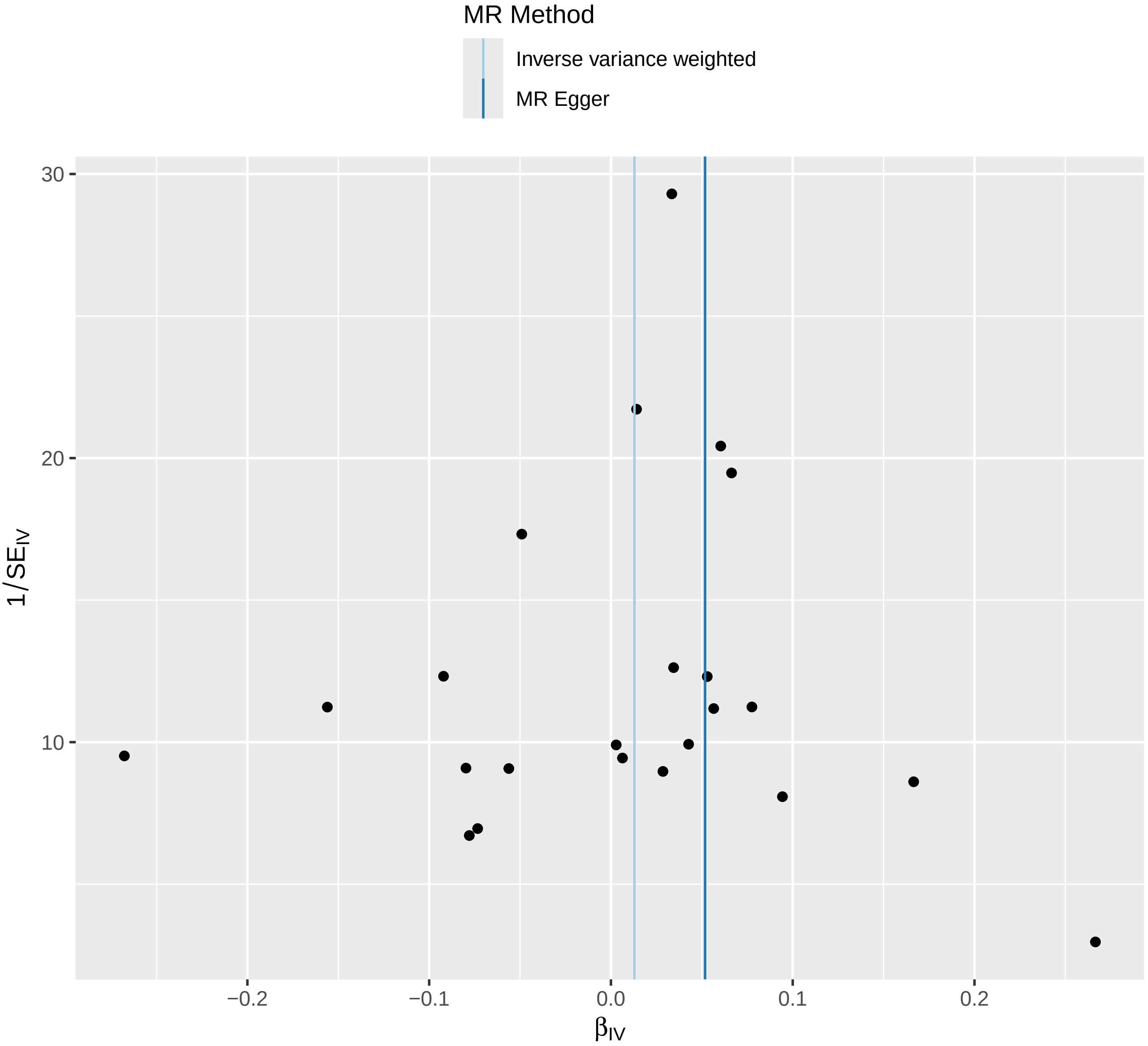
MR Method
Inverse variance weighted
MR Egger
30
20
10
1/SE$_{IV}$
−0.2
−0.1
0.0
0.1
0.2
β$_{IV}$

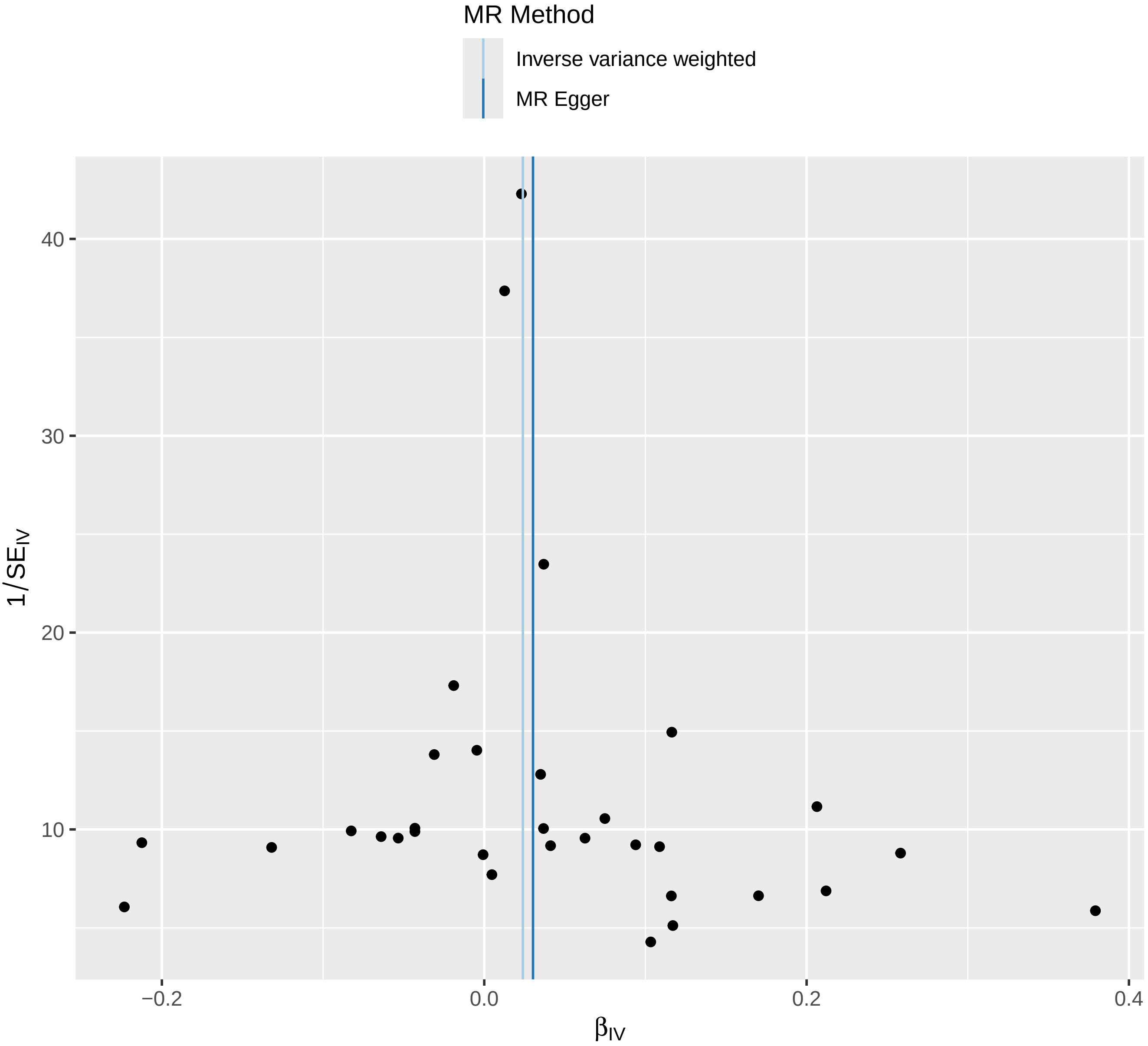

MR Method
Inverse variance weighted
MR Egger
40
30
20
10
1/SE_IV
−0.2
0.0
0.2
0.4
β_IV

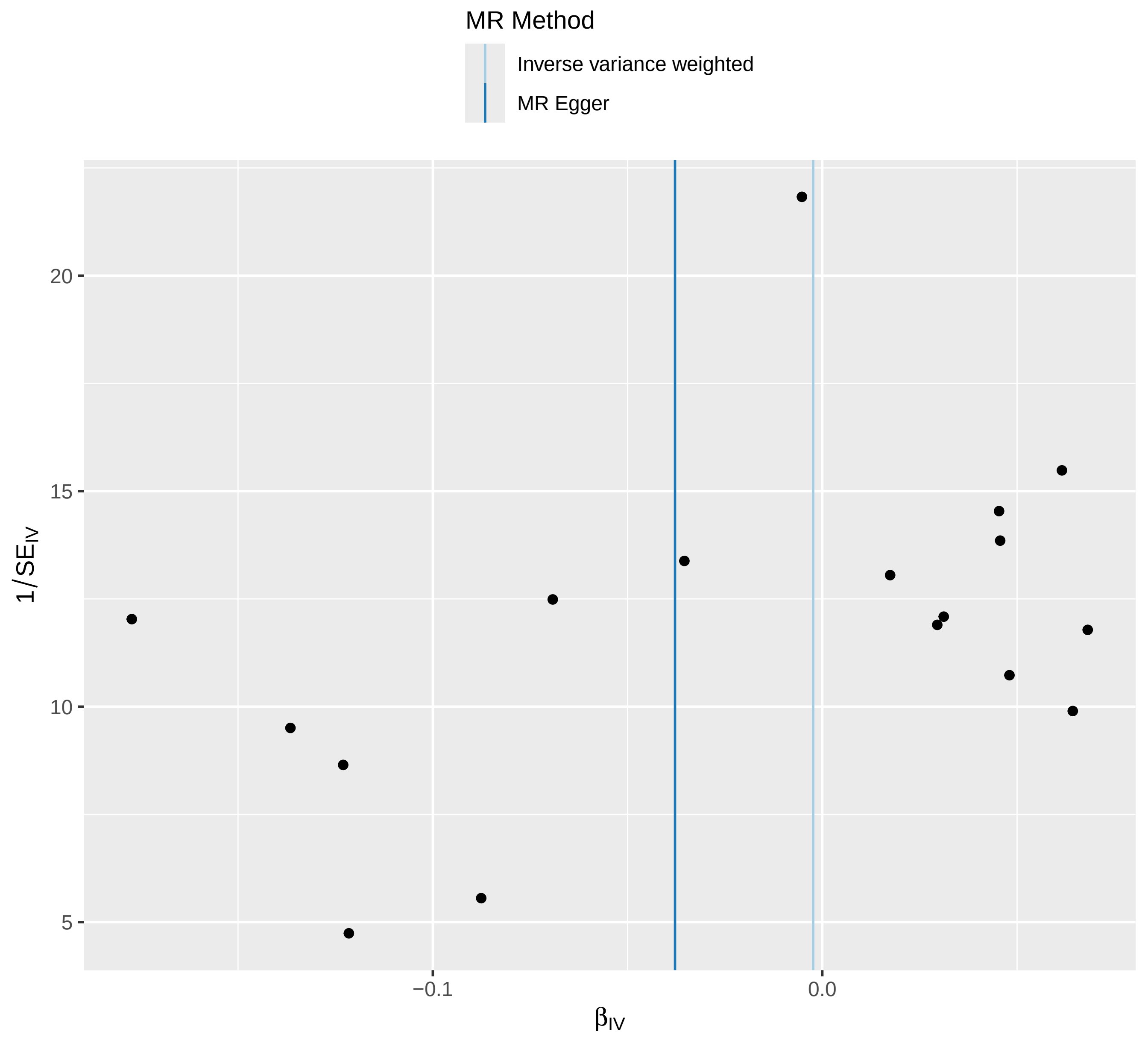

MR Method
Inverse variance weighted
MR Egger
20
15
10
5
1/SE$_{IV}$
−0.1
0.0
β$_{IV}$

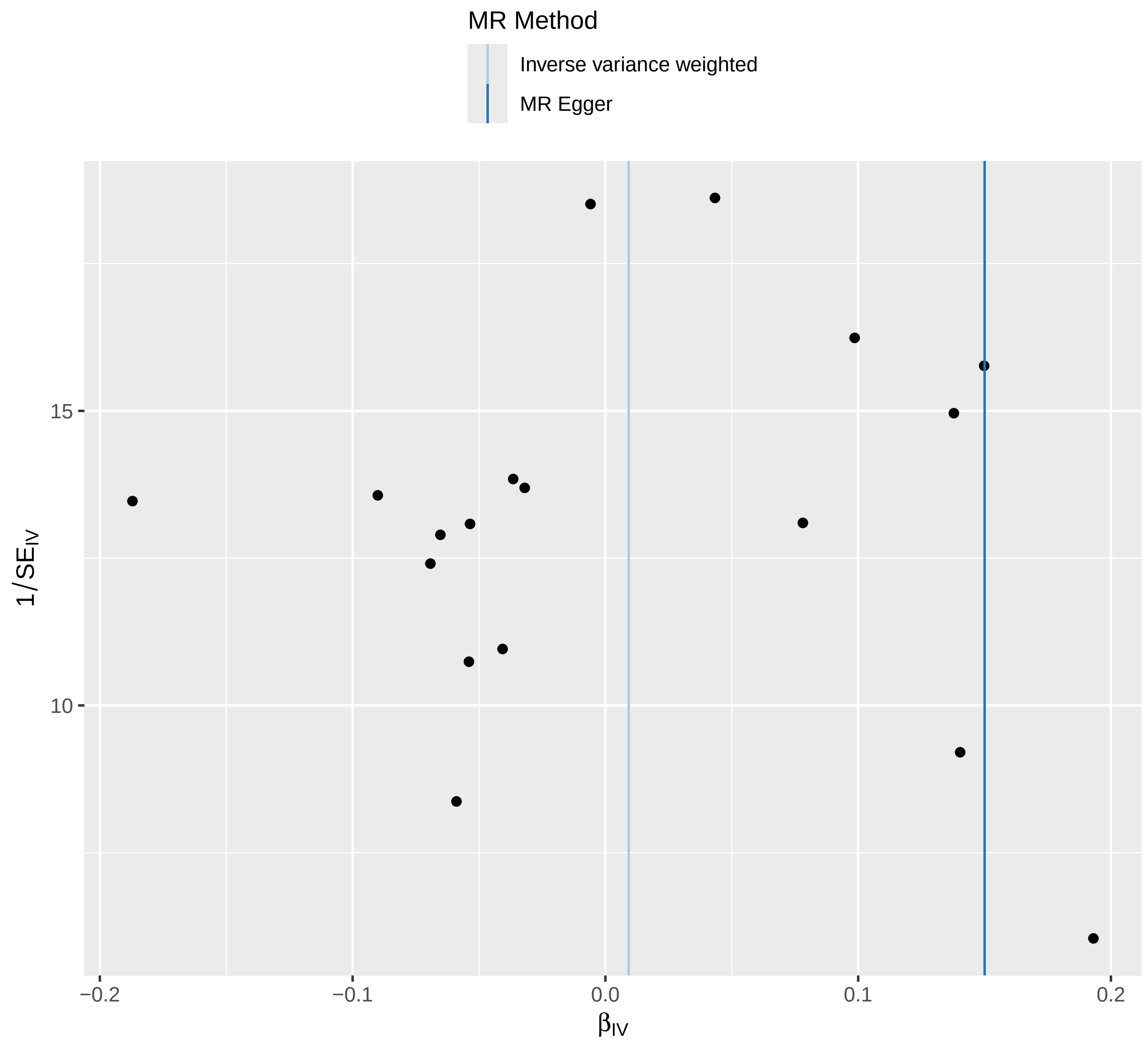
MR Method
Inverse variance weighted
MR Egger
15
10
1/SE_IV
−0.2
−0.1
0.0
0.1
0.2
β_IV

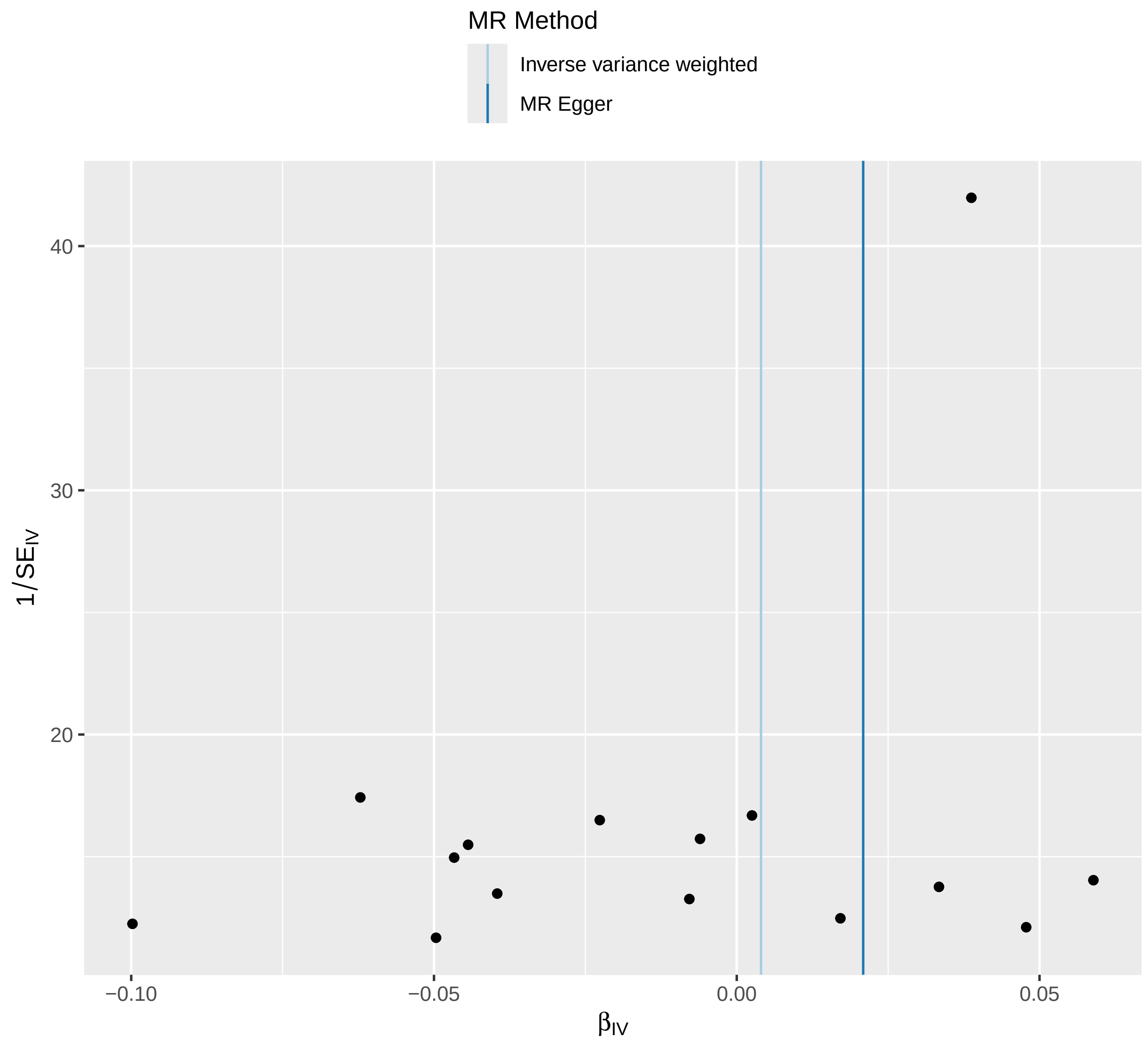
MR Method
Inverse variance weighted
MR Egger
40
30
20
$1/SE_{IV}$
−0.10
−0.05
0.00
0.05
β_{IV}

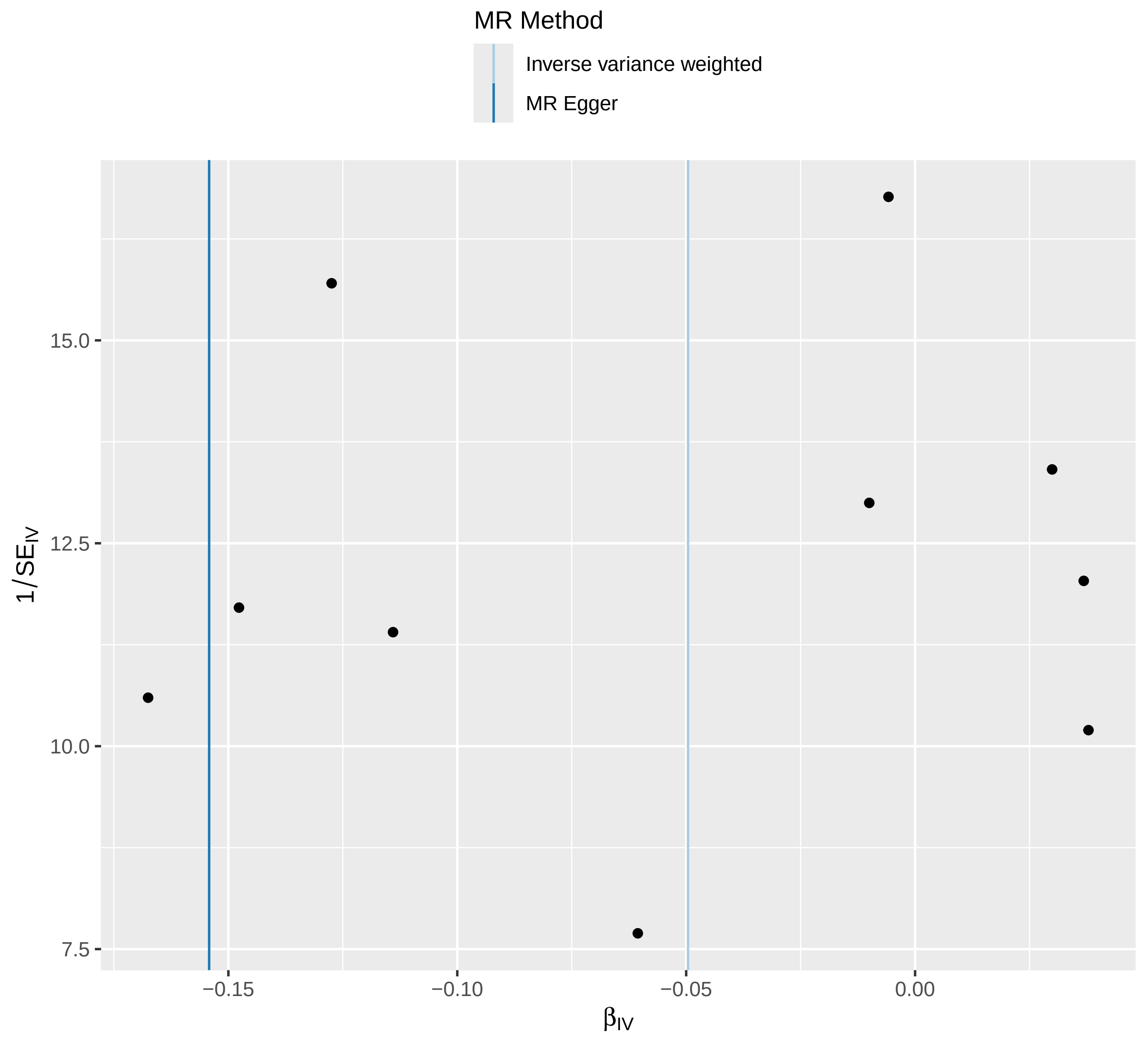

MR Method
Inverse variance weighted
MR Egger
15.0
12.5
10.0
7.5
1/SE$_{IV}$
−0.15
−0.10
−0.05
0.00
β$_{IV}$

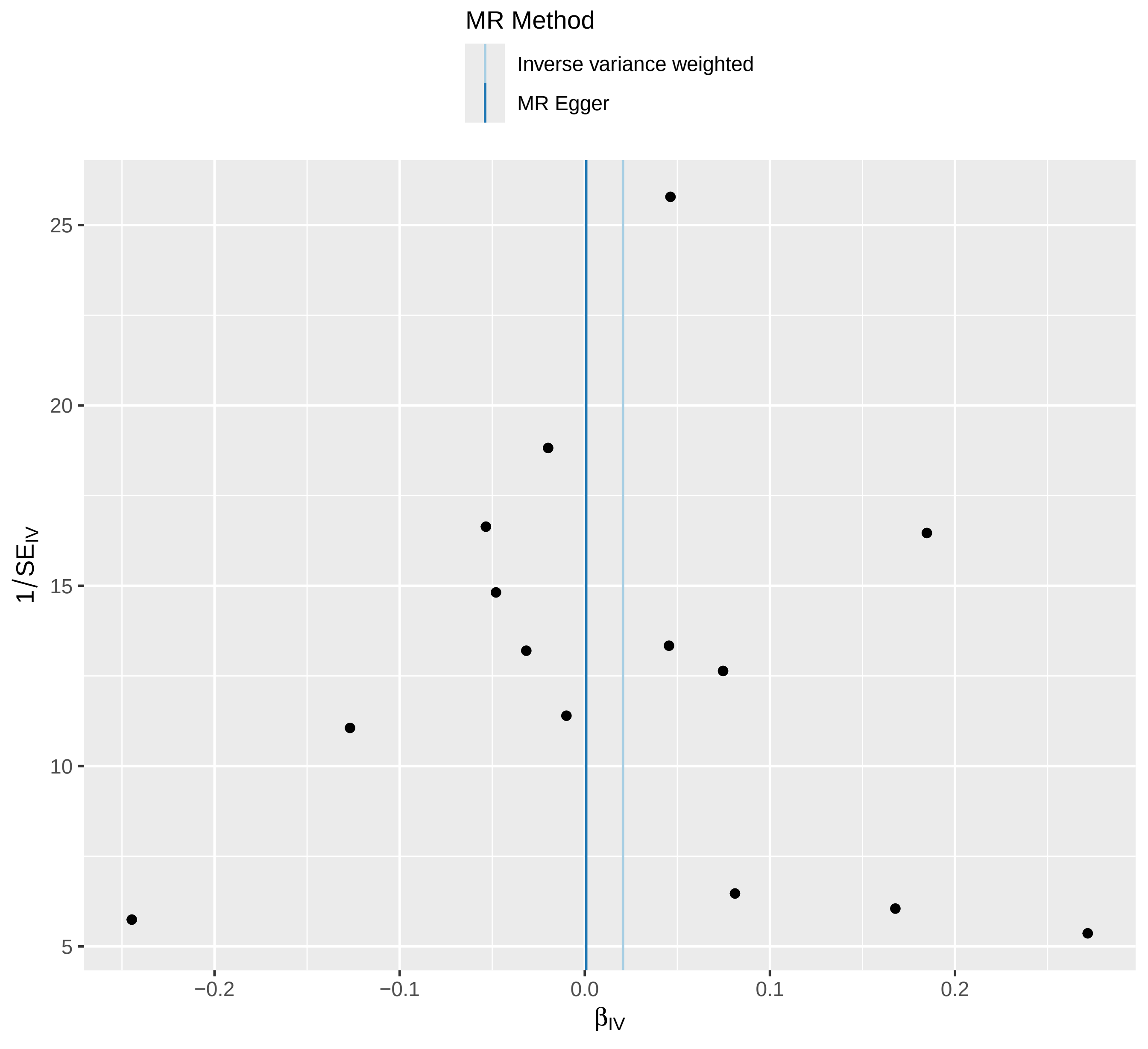
MR Method
Inverse variance weighted
MR Egger
25
20
15
10
5
1/SE_IV
−0.2
−0.1
0.0
0.1
0.2
β_IV

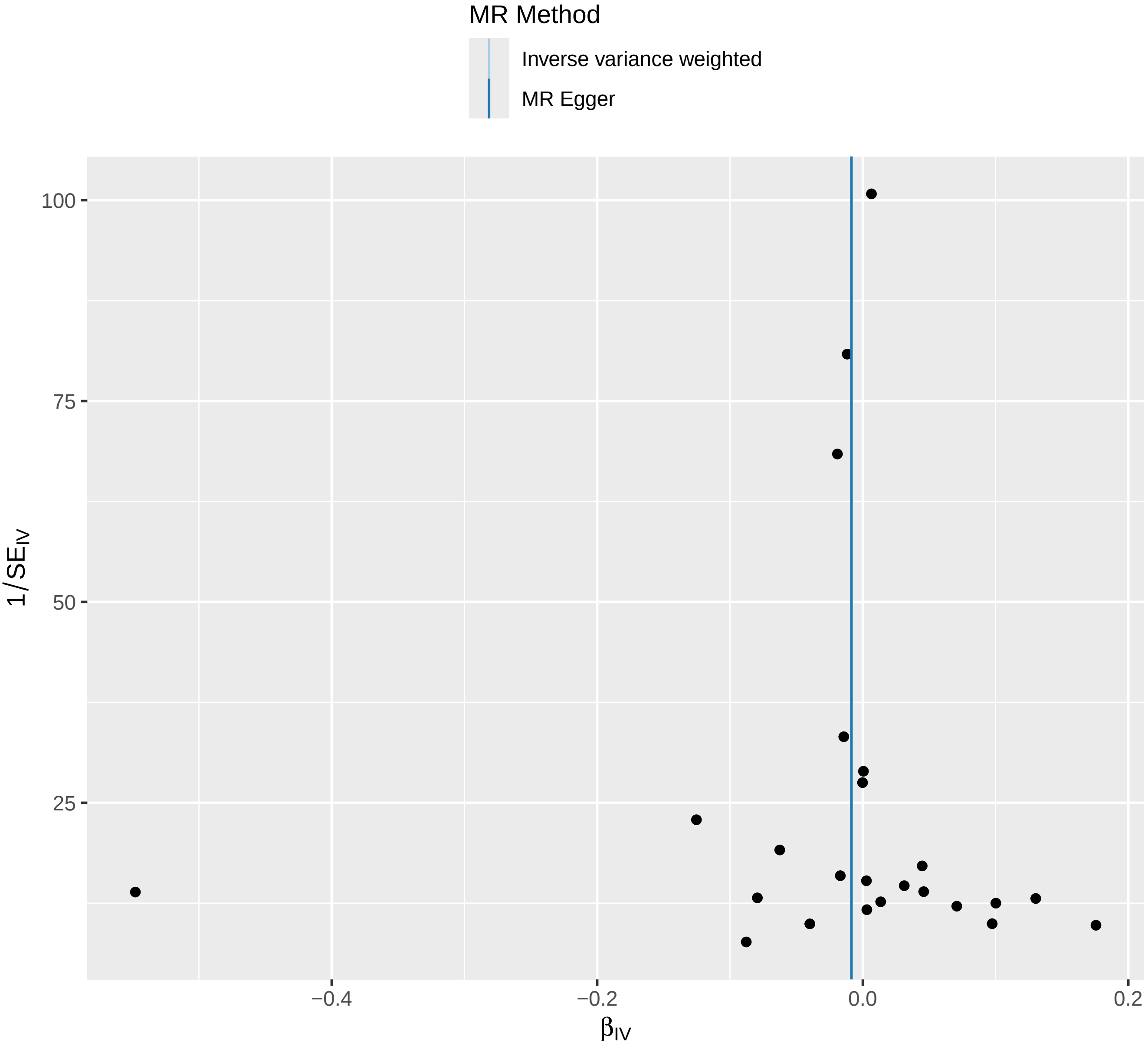
MR Method
Inverse variance weighted
MR Egger
100
75
50
25
1/SE$_{IV}$
−0.4
−0.2
0.0
0.2
β$_{IV}$

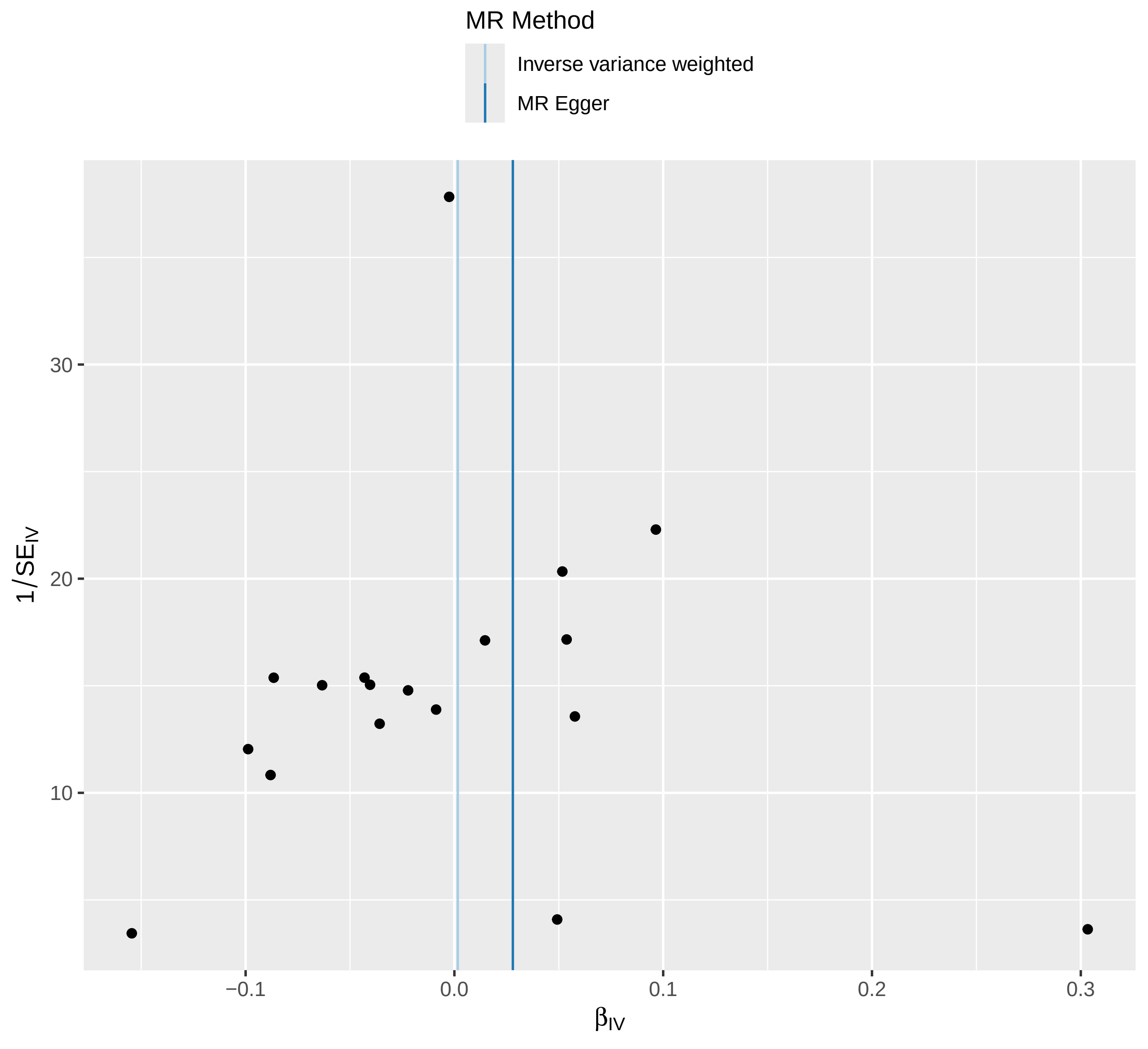
MR Method
Inverse variance weighted
MR Egger
1/SE_IV
30
20
10
−0.1
0.0
0.1
0.2
0.3
β_IV

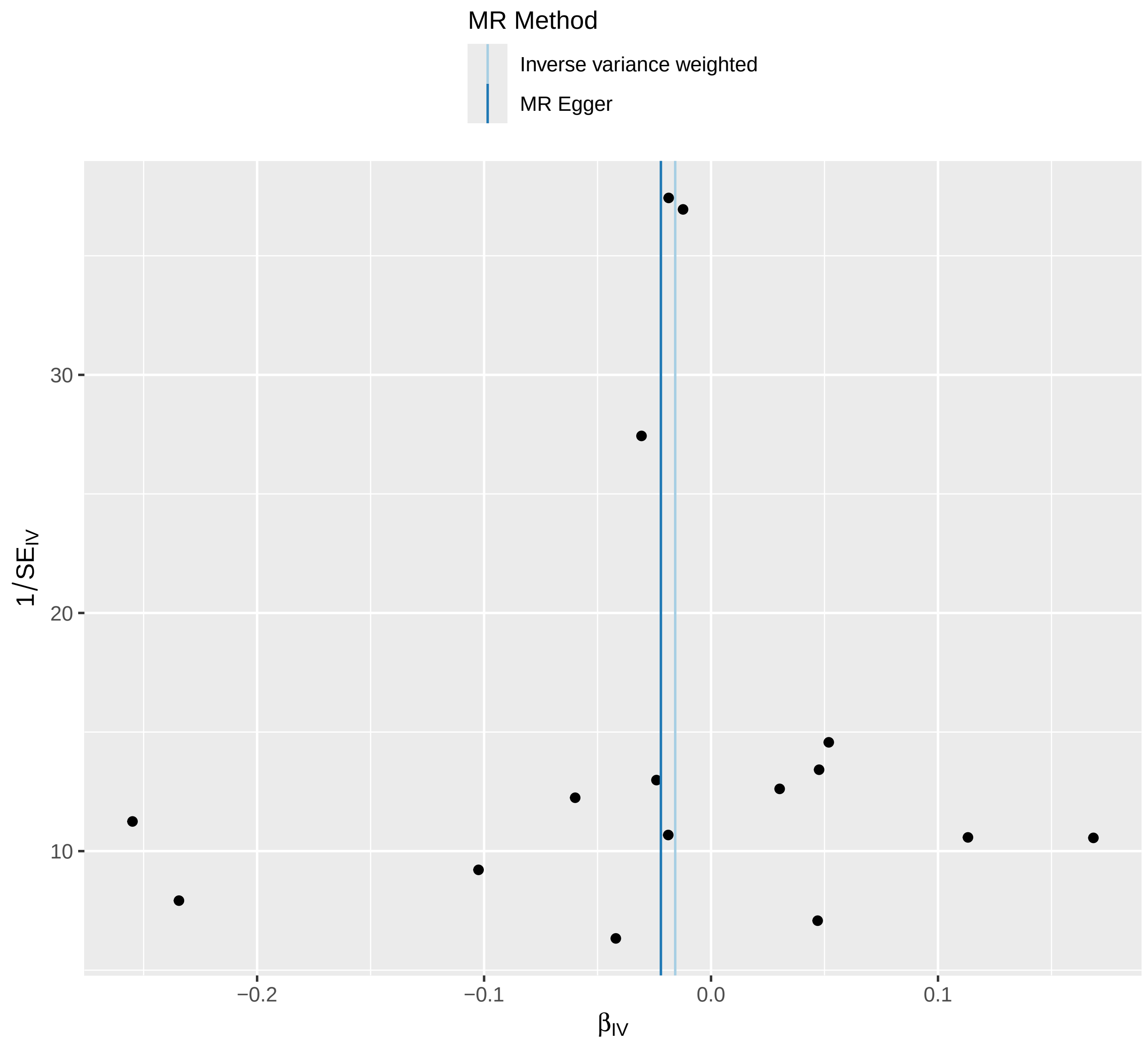
MR Method
Inverse variance weighted
MR Egger
1/SE_IV
30
20
10
−0.2
−0.1
0.0
0.1
β_IV

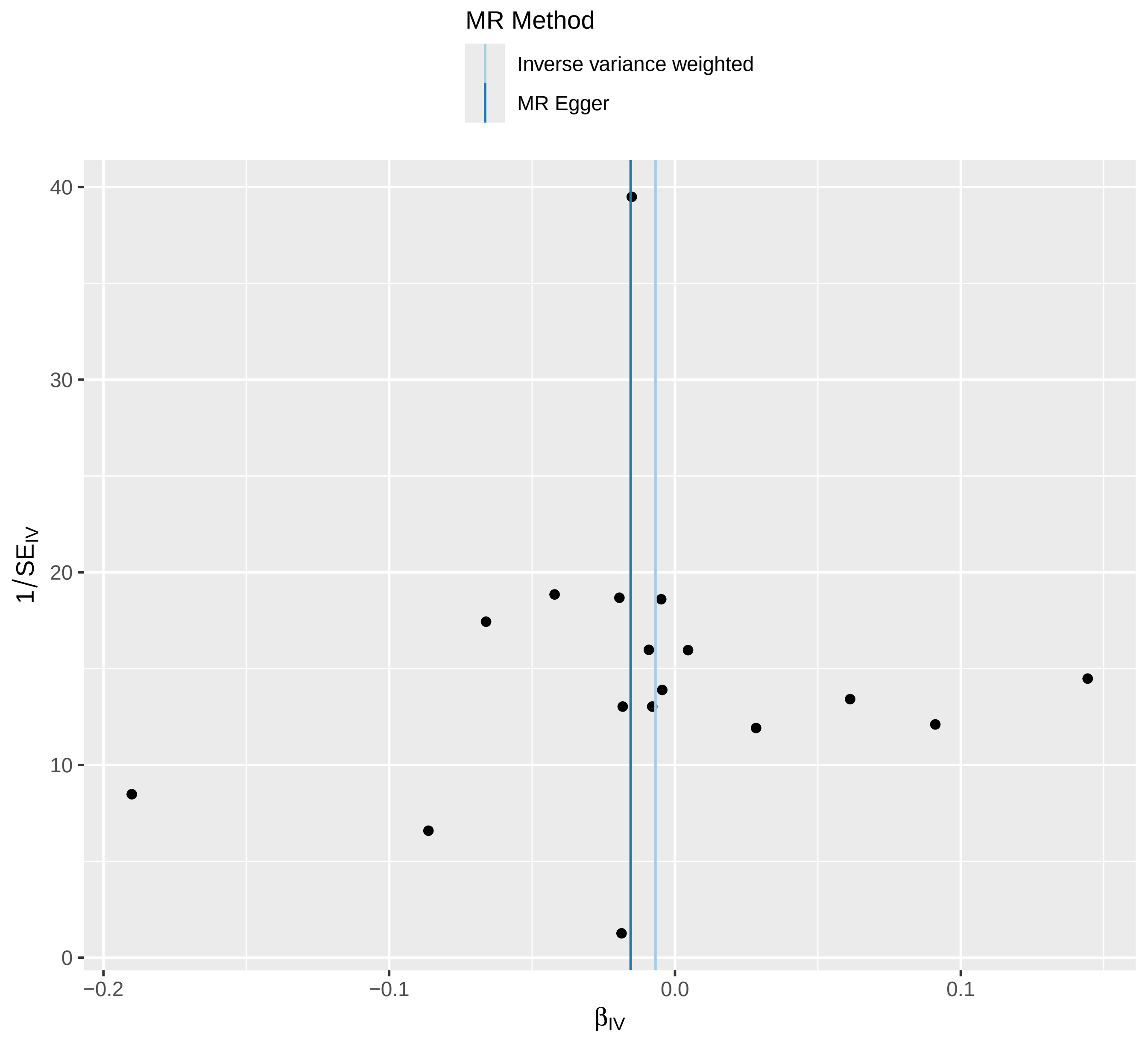
MR Method
Inverse variance weighted
MR Egger
40
30
20
10
0
$1/SE_{IV}$
−0.2
−0.1
0.0
0.1
β_{IV}

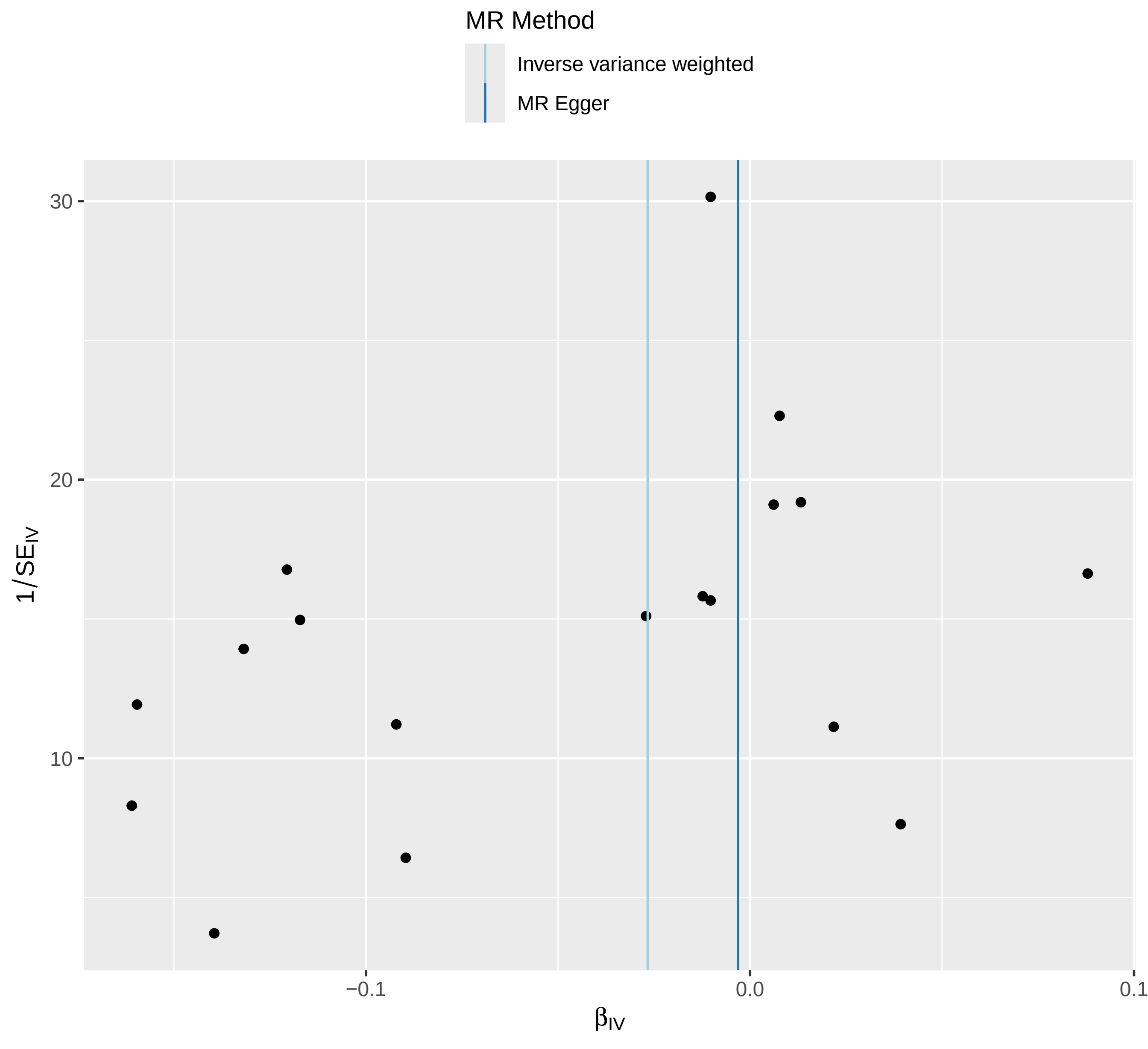
MR Method
Inverse variance weighted
MR Egger
30
20
10
1/SE_{IV}
−0.1
0.0
0.1
β_{IV}

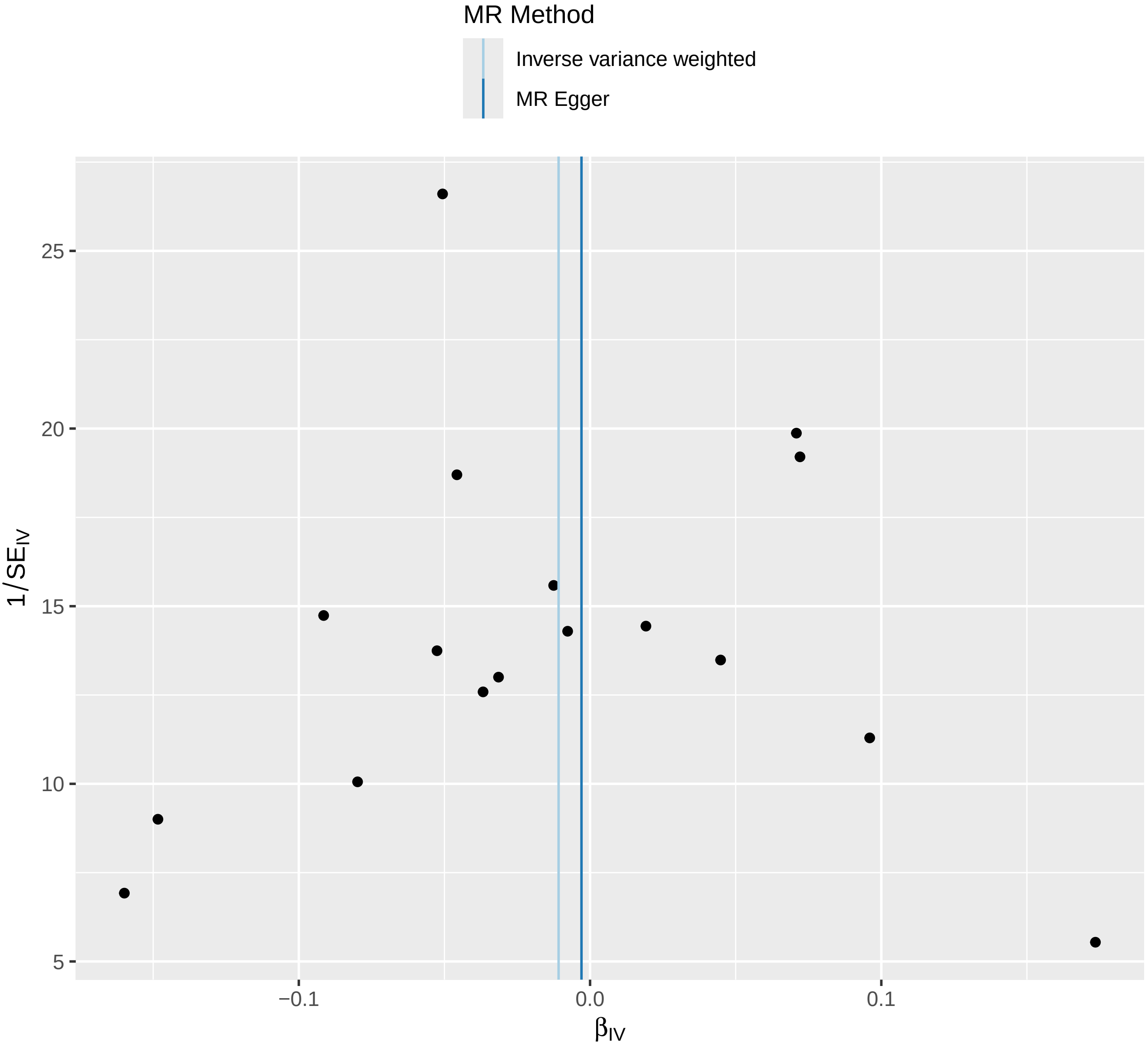

MR Method
Inverse variance weighted
MR Egger
25
20
15
10
5
1/SE$_{IV}$
−0.1
0.0
0.1
β$_{IV}$

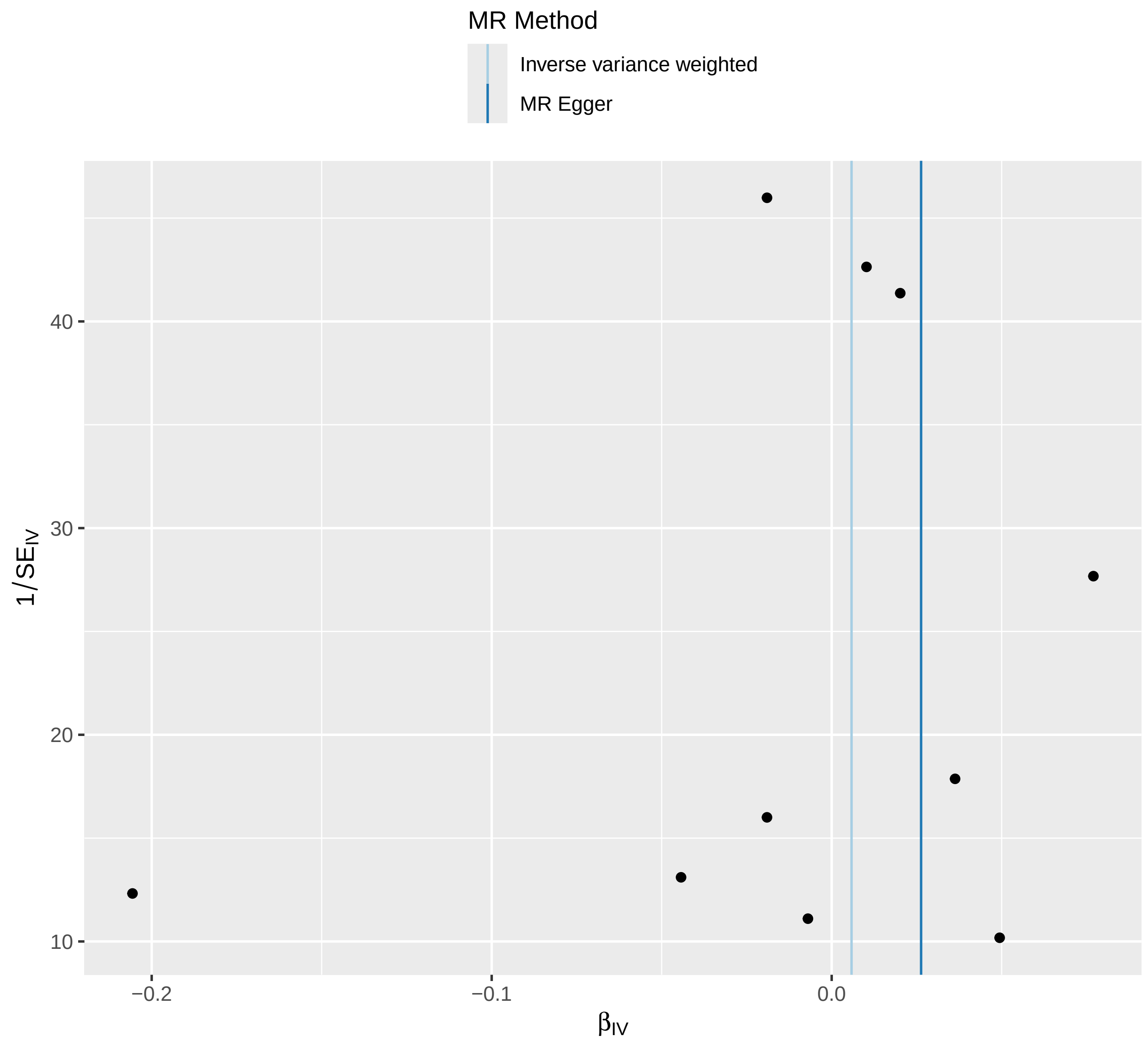
MR Method
Inverse variance weighted
MR Egger
40
30
20
10
−0.2
−0.1
0.0
$1/SE_{IV}$
β_{IV}

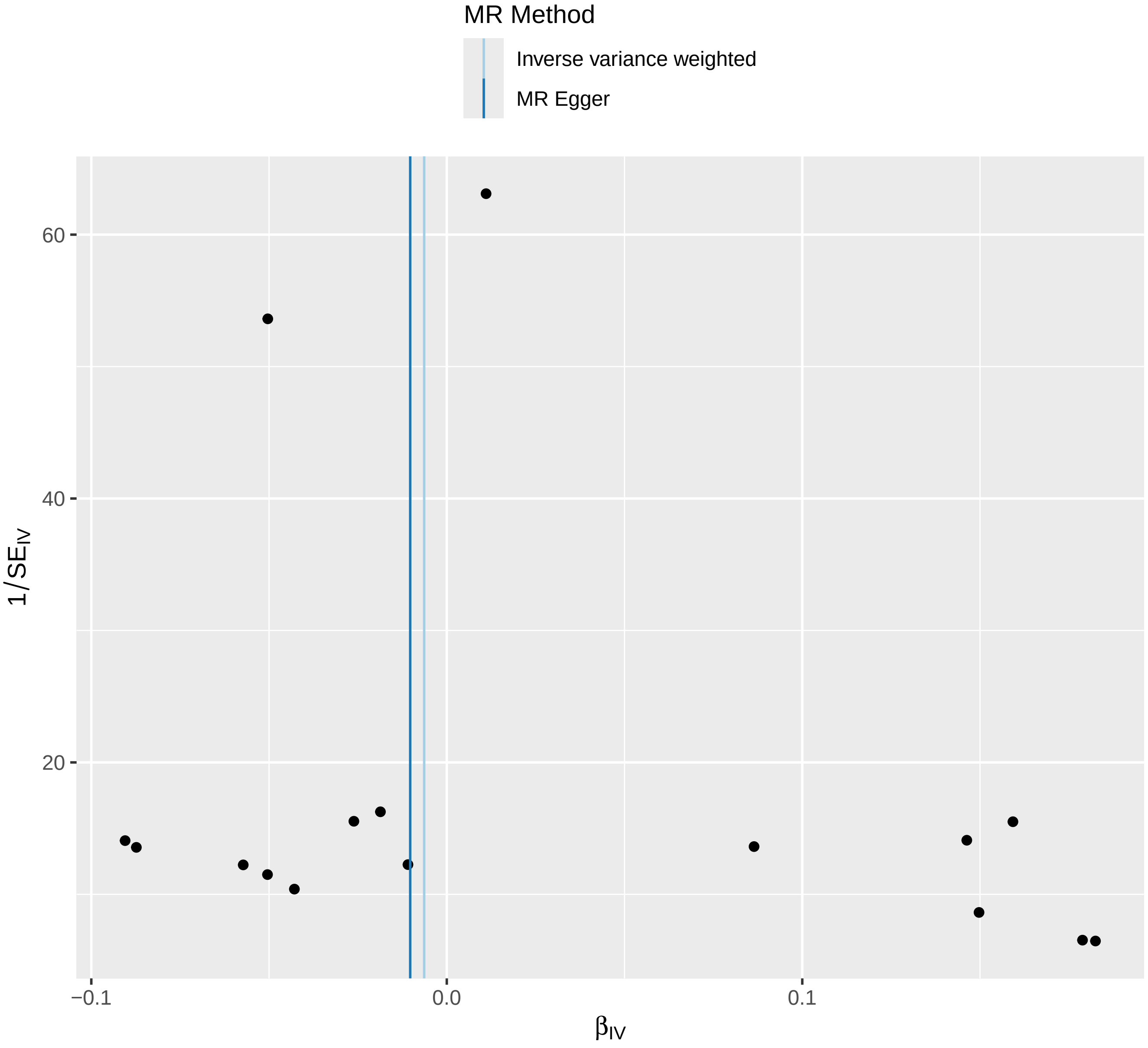

MR Method
Inverse variance weighted
MR Egger
60
40
20
1/SE_IV
−0.1
0.0
0.1
β_IV

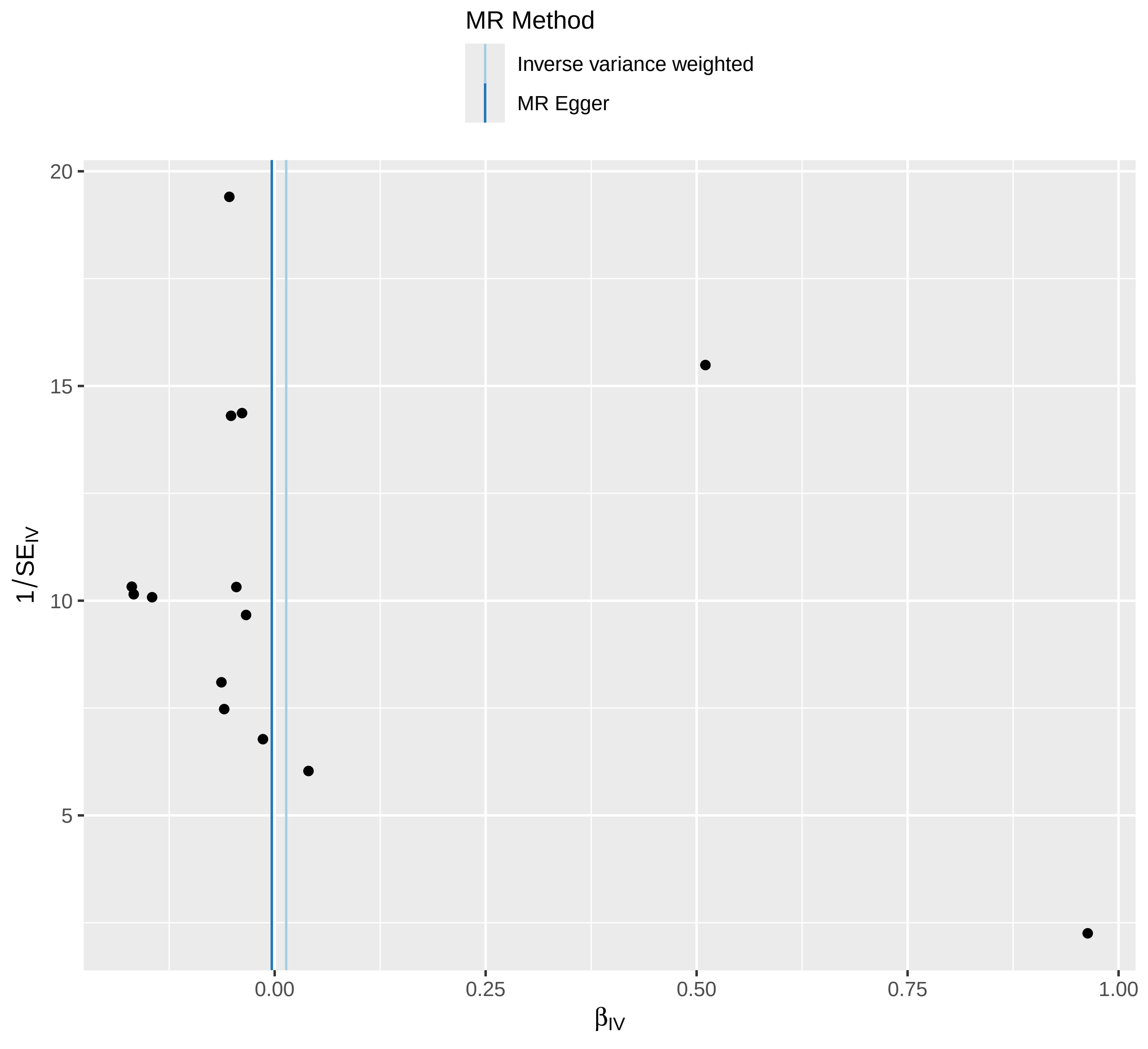
MR Method
Inverse variance weighted
MR Egger
20
15
10
5
0.00
0.25
0.50
0.75
1.00
$1/SE_{IV}$
β_{IV}

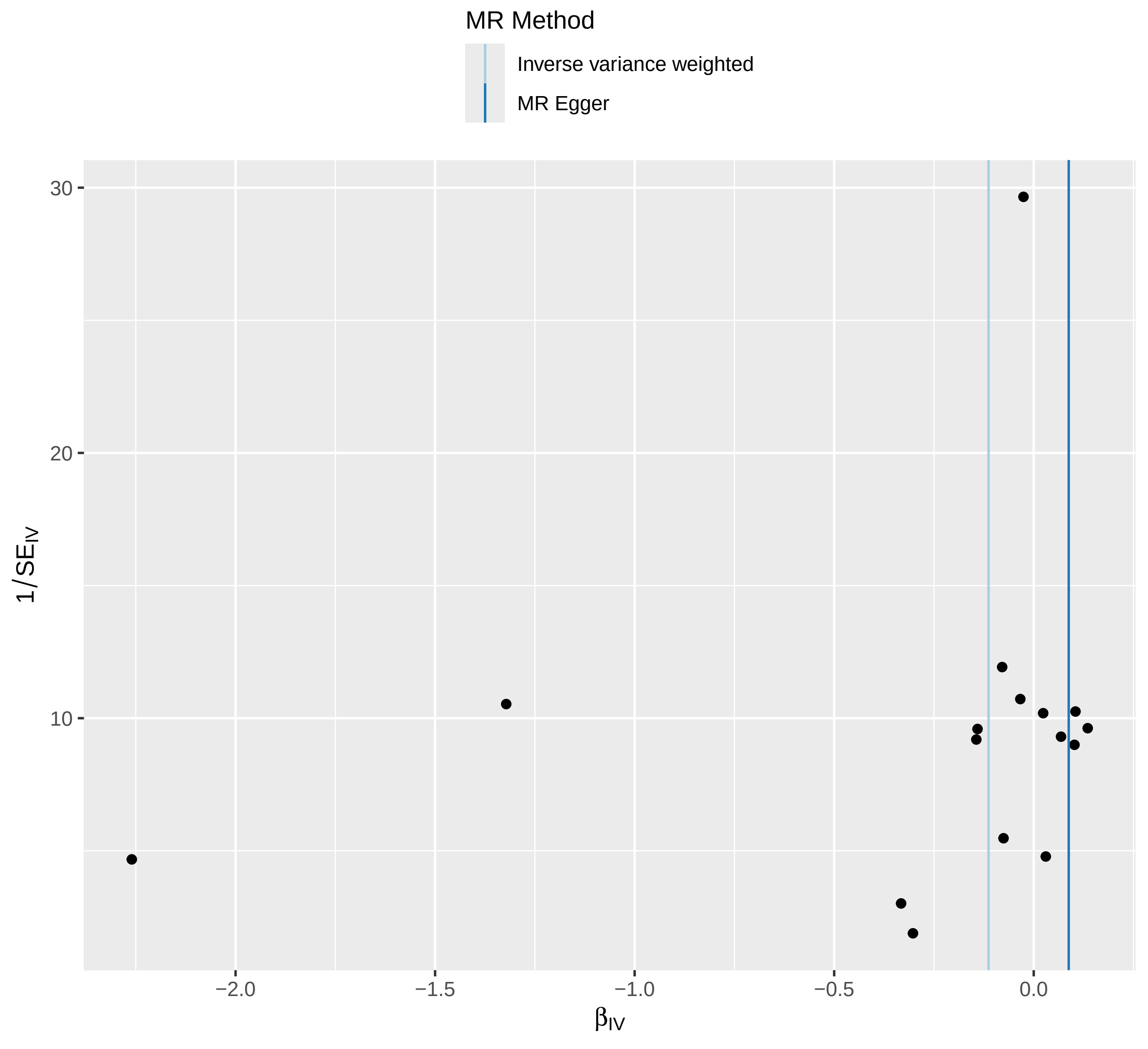
MR Method
Inverse variance weighted
MR Egger
30
20
10
1/SE_IV
−2.0
−1.5
−1.0
−0.5
0.0
β_IV

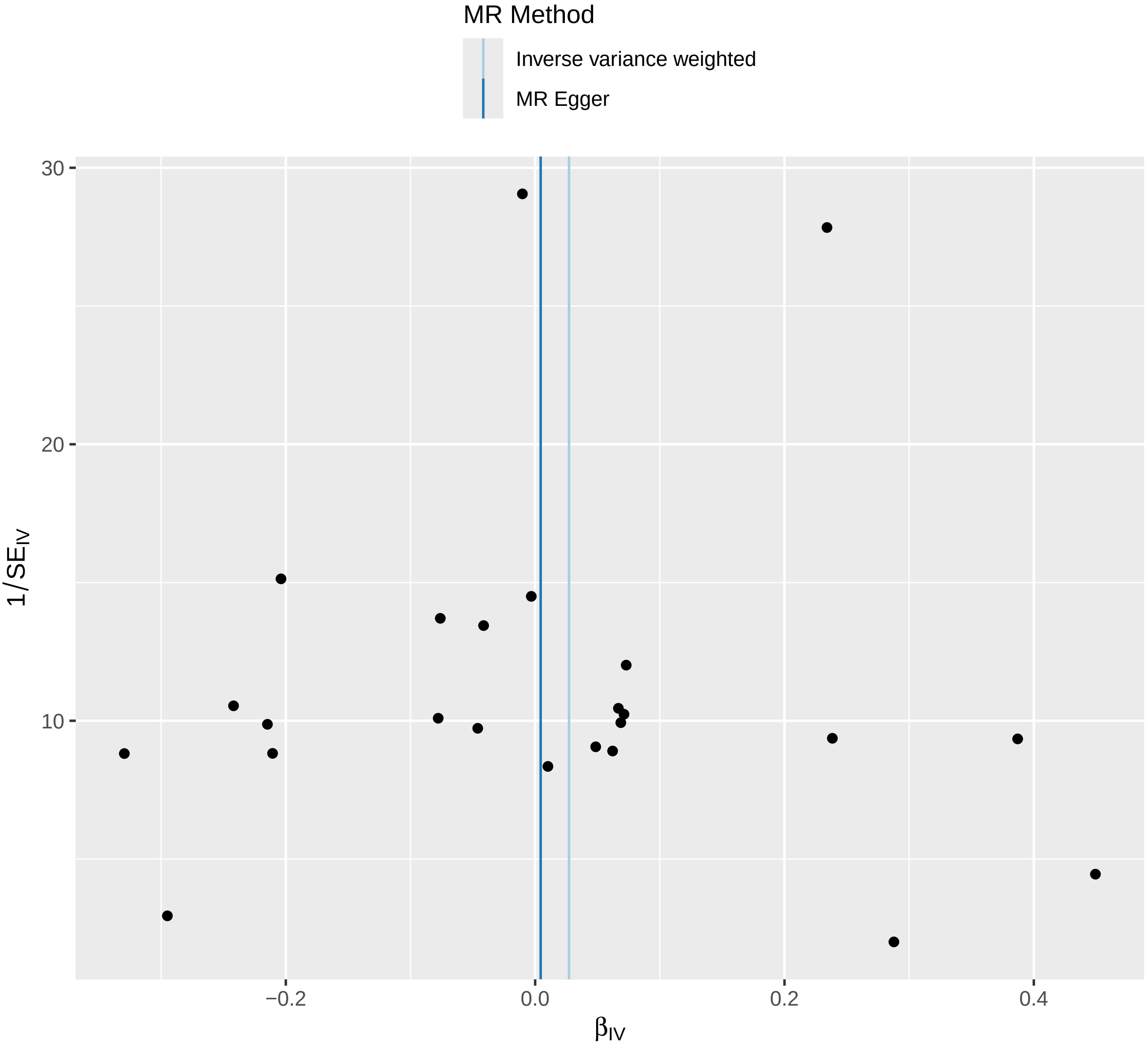
MR Method
Inverse variance weighted
MR Egger
30
20
10
1/SE$_{IV}$
−0.2
0.0
0.2
0.4
β$_{IV}$

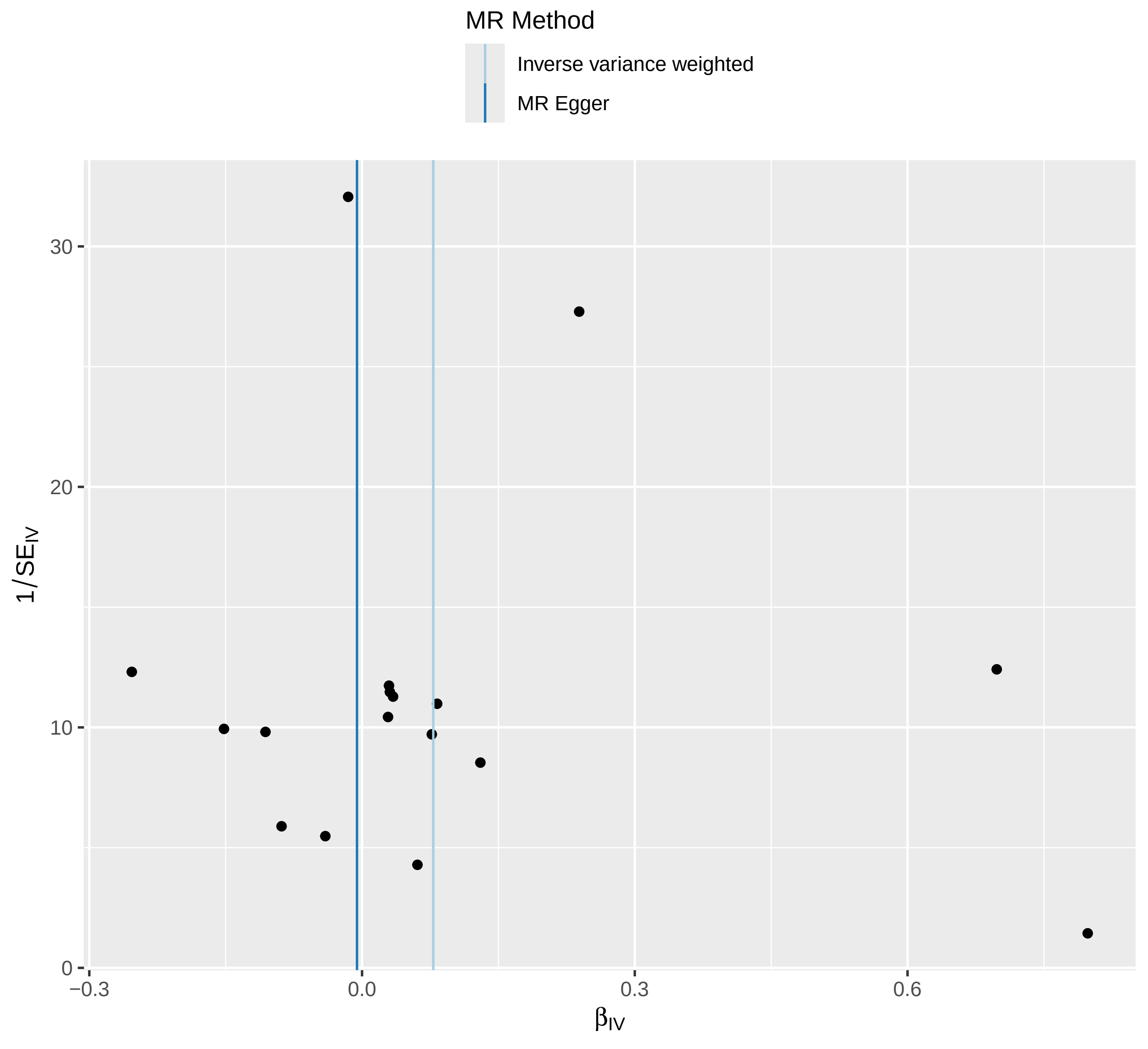

MR Method
Inverse variance weighted
MR Egger
1/SE_{IV}
30
20
10
0
−0.3
0.0
0.3
0.6
β_{IV}

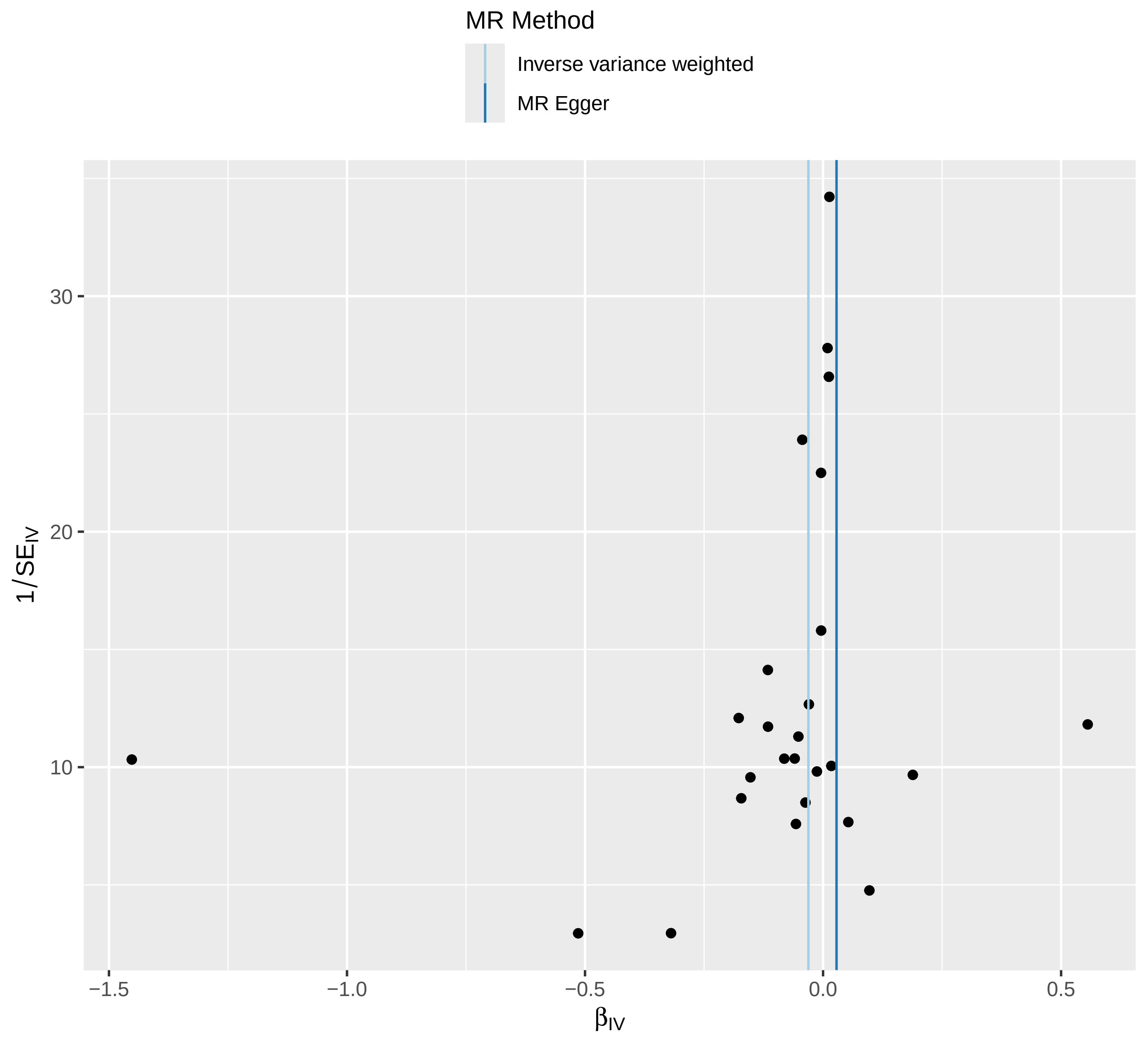

MR Method
Inverse variance weighted
MR Egger
30
20
10
1/SE_IV
−1.5
−1.0
−0.5
0.0
0.5
β_IV

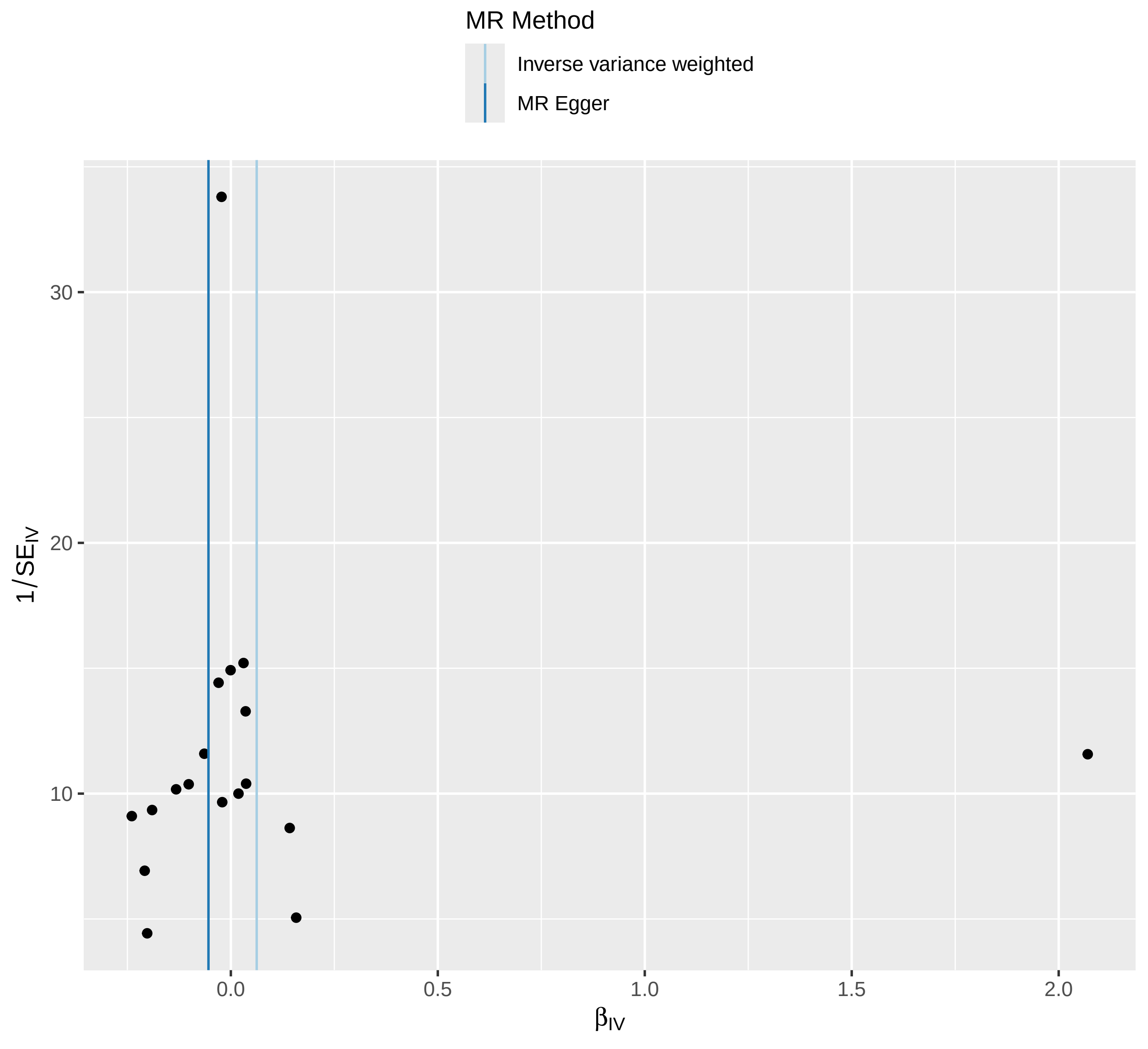
MR Method
Inverse variance weighted
MR Egger
30
20
10
1/SE$_{IV}$
0.0
0.5
1.0
1.5
2.0
β$_{IV}$

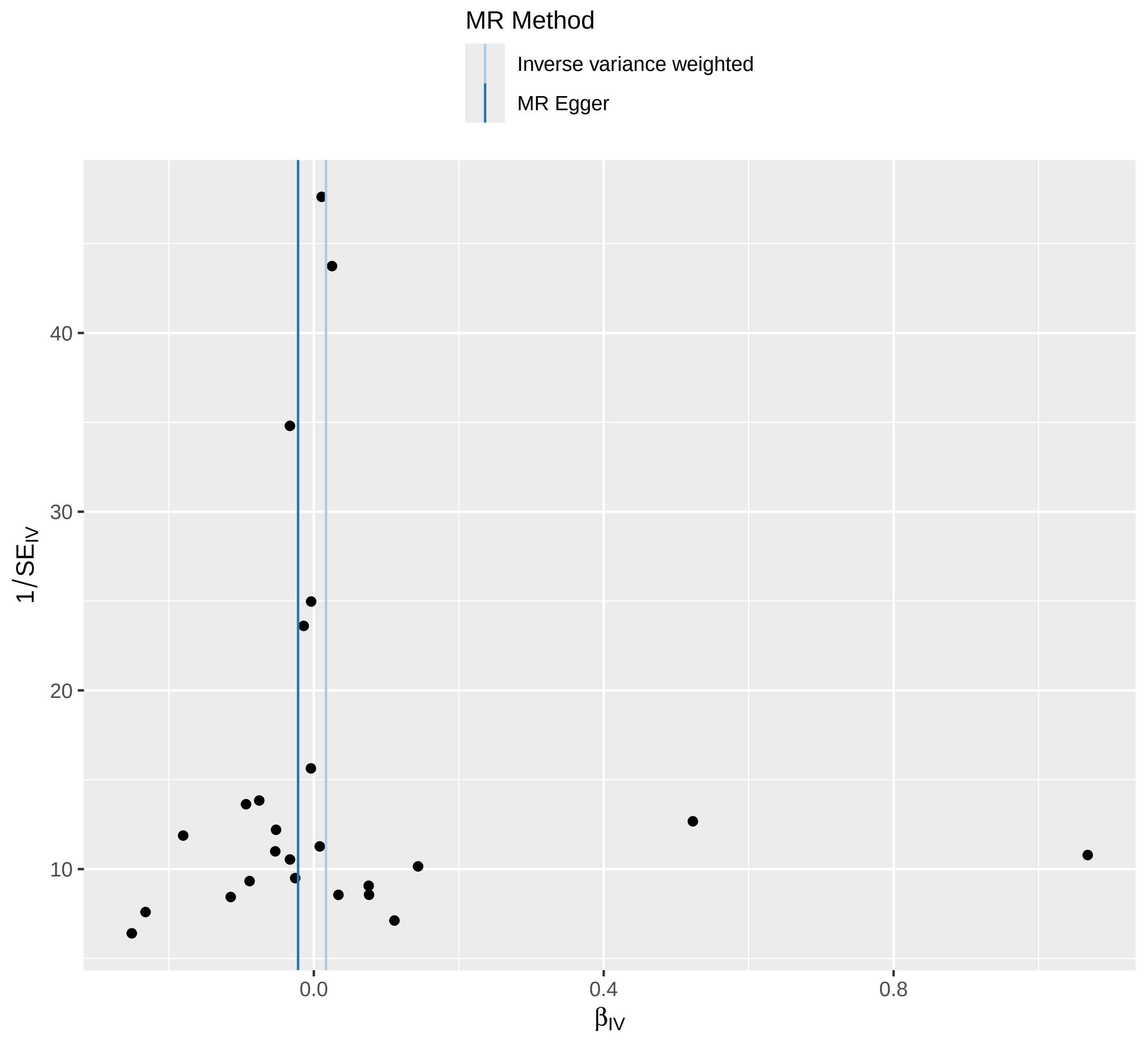
MR Method
Inverse variance weighted
MR Egger
40
30
20
10
1/SE_IV
0.0
0.4
0.8
β_IV

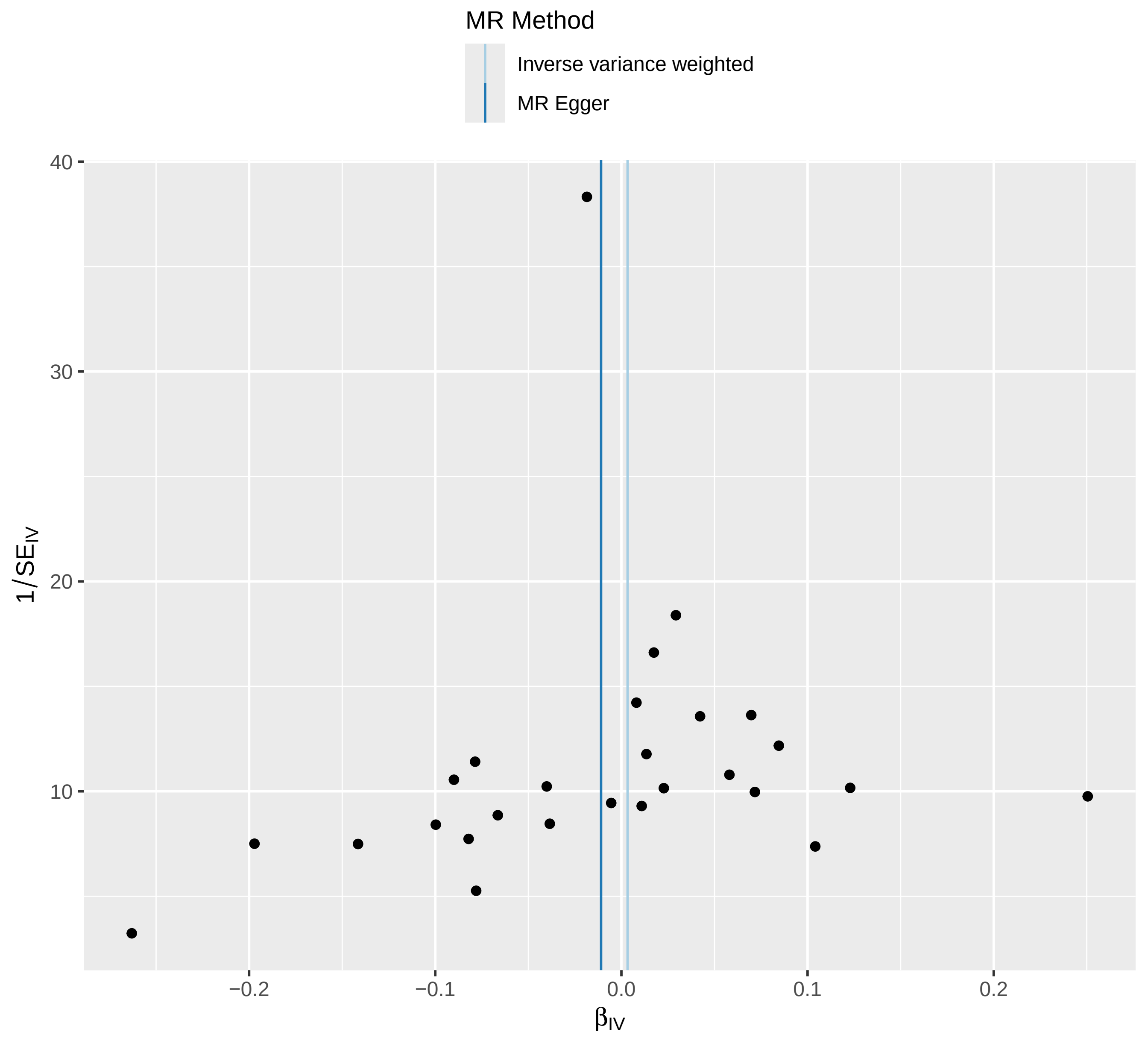

MR Method
Inverse variance weighted
MR Egger
40
30
20
10
1/SE_IV
−0.2
−0.1
0.0
0.1
0.2
β_IV

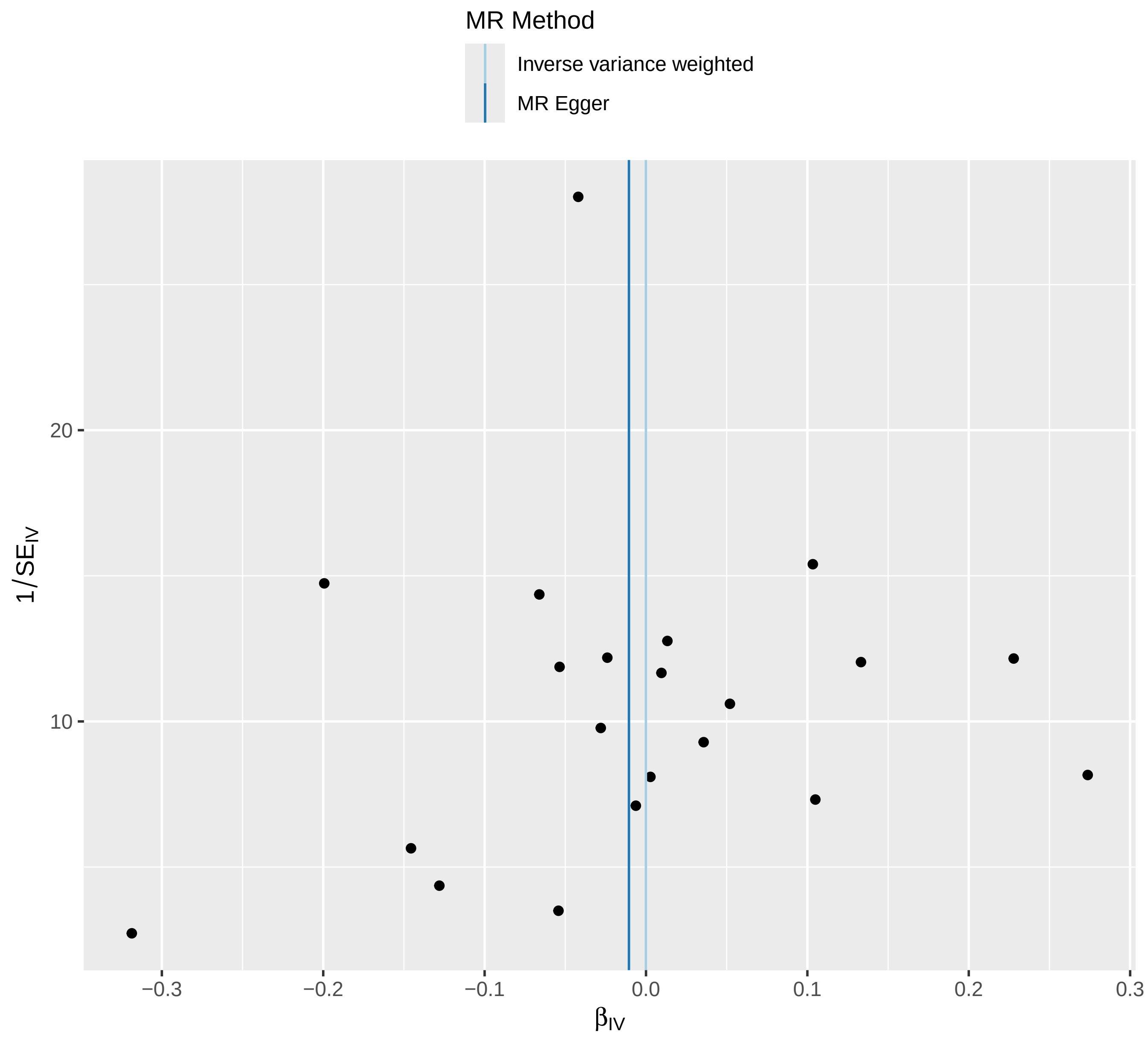
MR Method
Inverse variance weighted
MR Egger
20
10
$1/SE_{IV}$
−0.3
−0.2
−0.1
0.0
0.1
0.2
0.3
β_{IV}

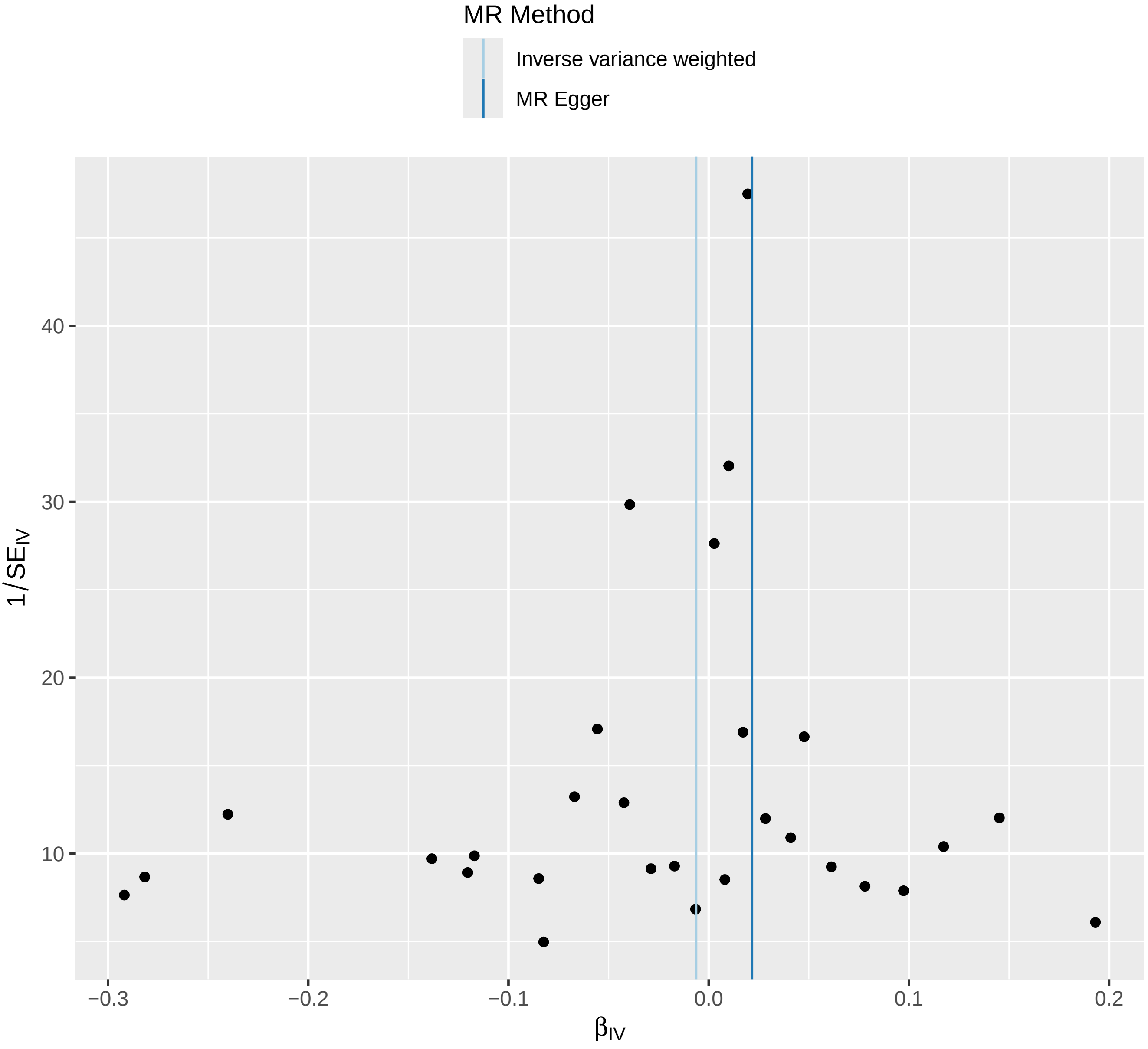
MR Method
Inverse variance weighted
MR Egger
1/SE_IV
40
30
20
10
−0.3
−0.2
−0.1
0.0
0.1
0.2
β_IV

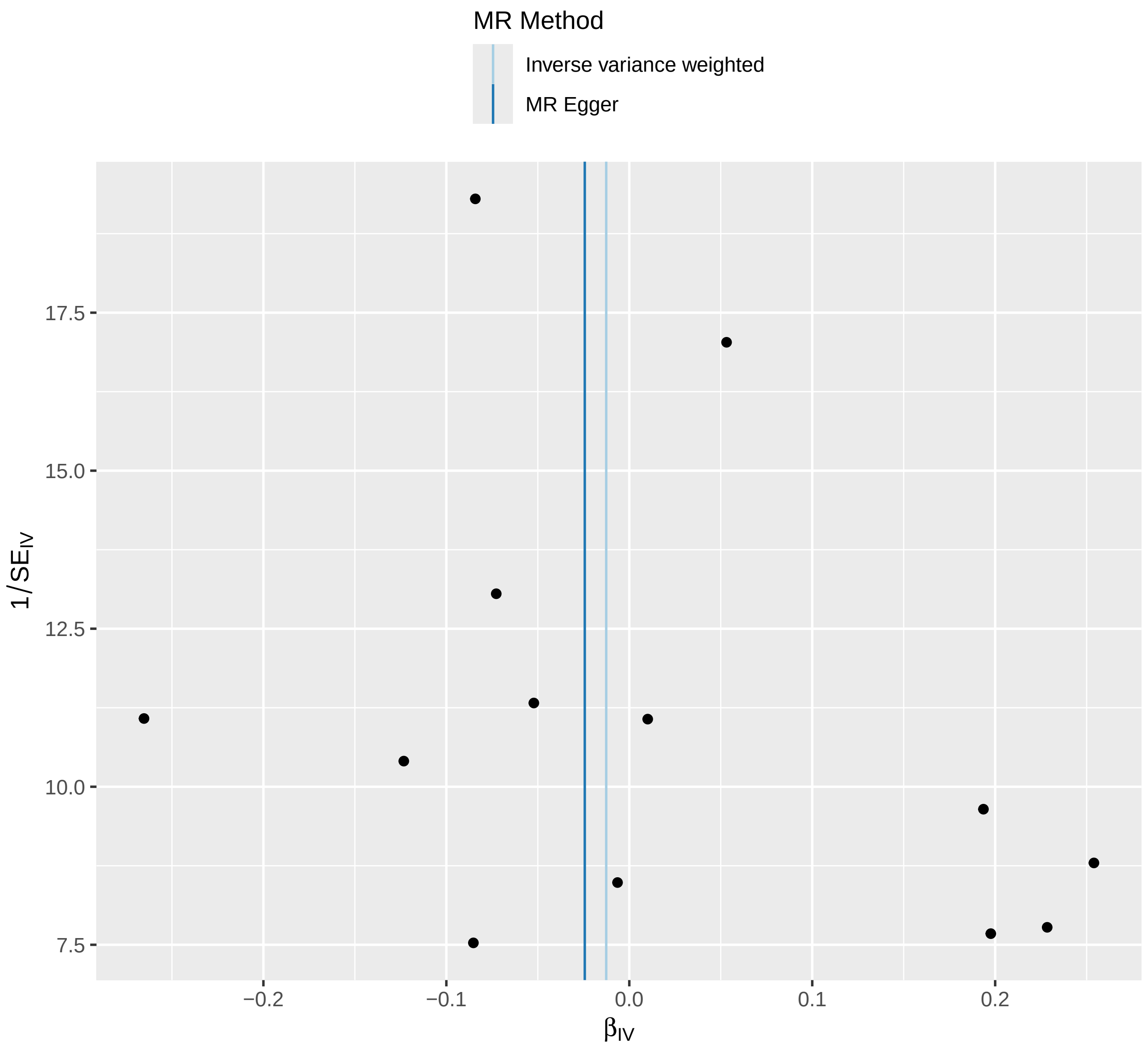
MR Method
Inverse variance weighted
MR Egger
17.5
15.0
12.5
10.0
7.5
1/SE$_{IV}$
−0.2
−0.1
0.0
0.1
0.2
β$_{IV}$

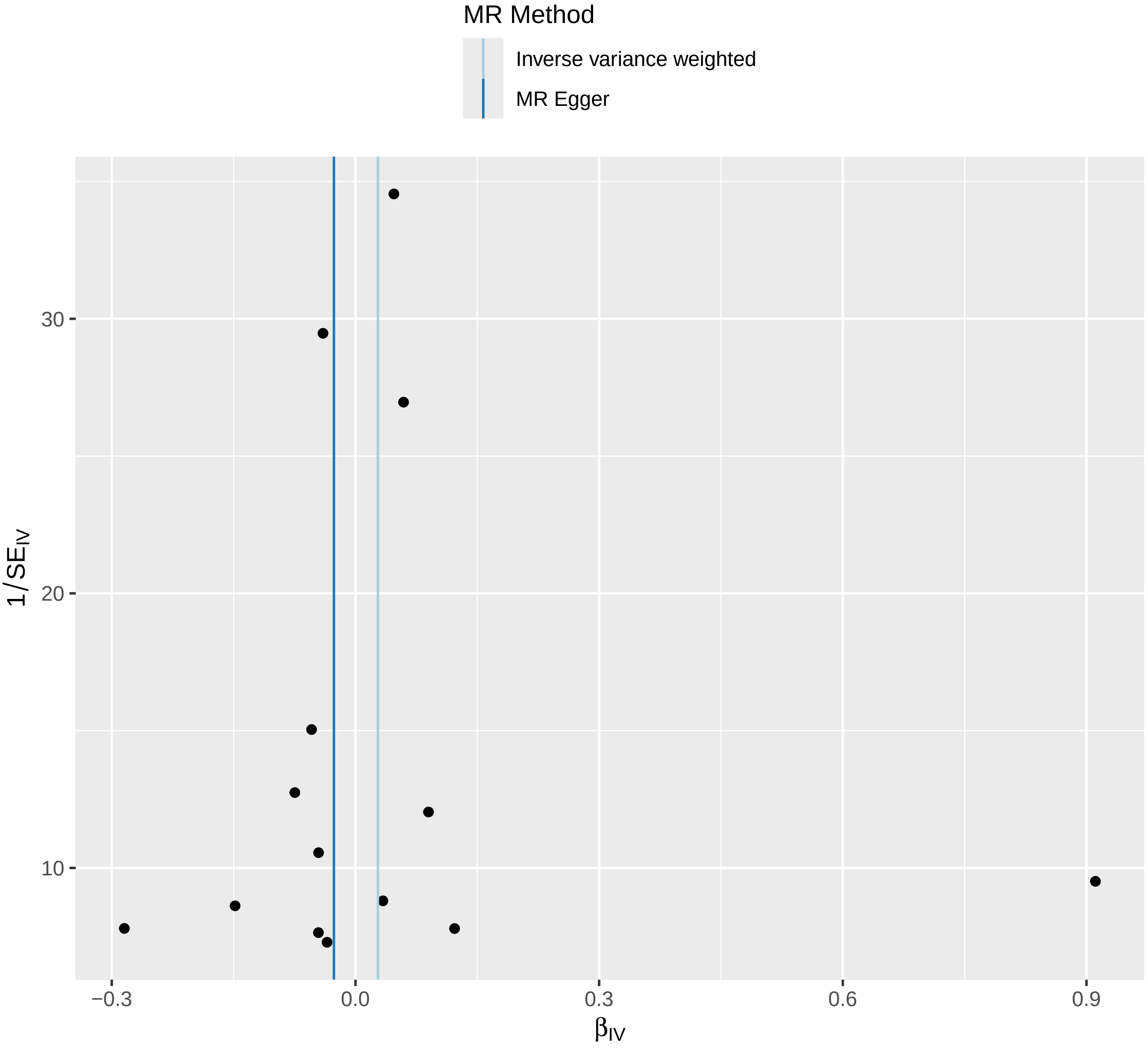
MR Method
Inverse variance weighted
MR Egger
30
20
10
1/SE_IV
−0.3
0.0
0.3
0.6
0.9
β_IV

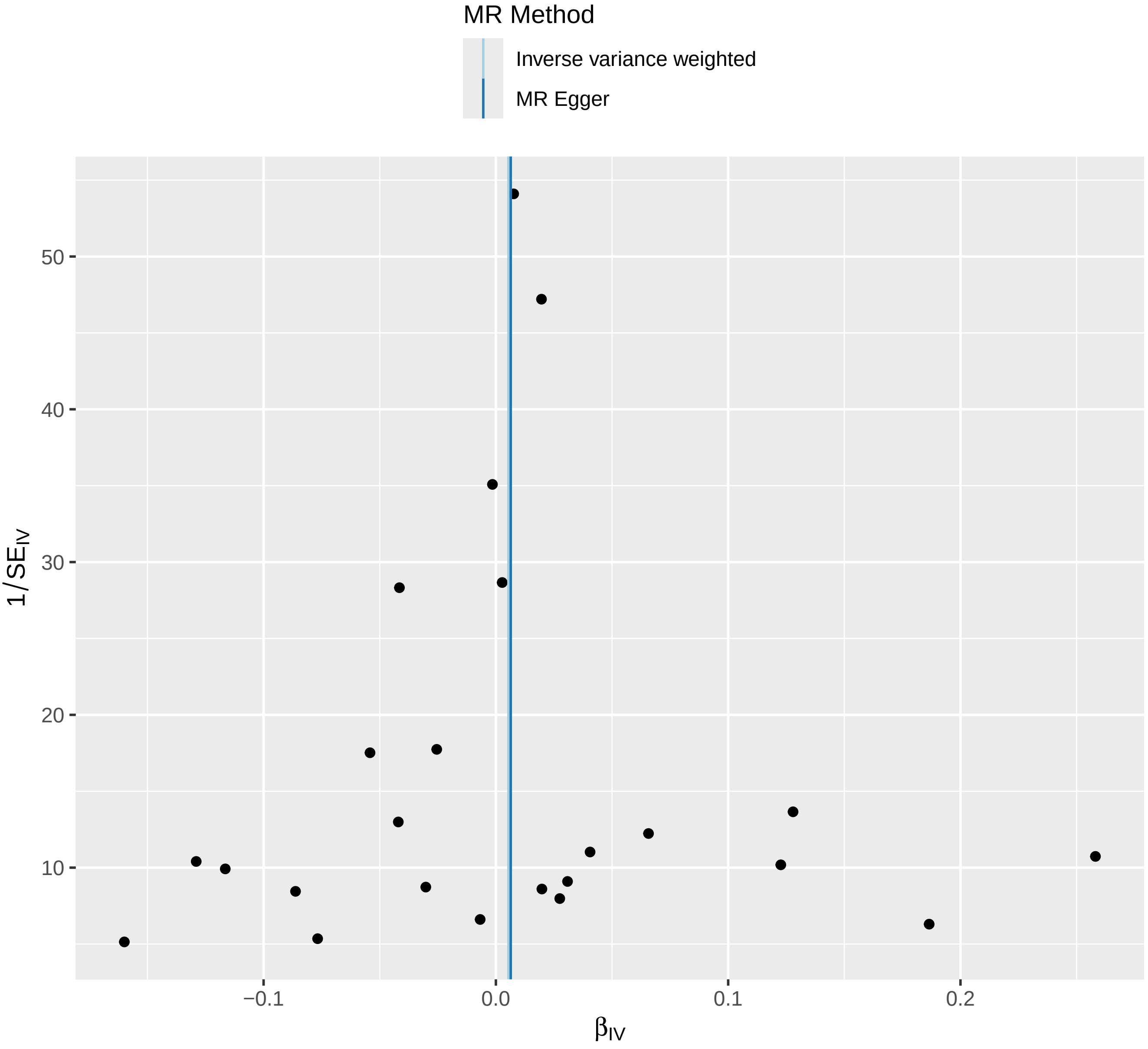

MR Method
Inverse variance weighted
MR Egger
50
40
30
20
10
1/SE$_{IV}$
−0.1
0.0
0.1
0.2
β$_{IV}$

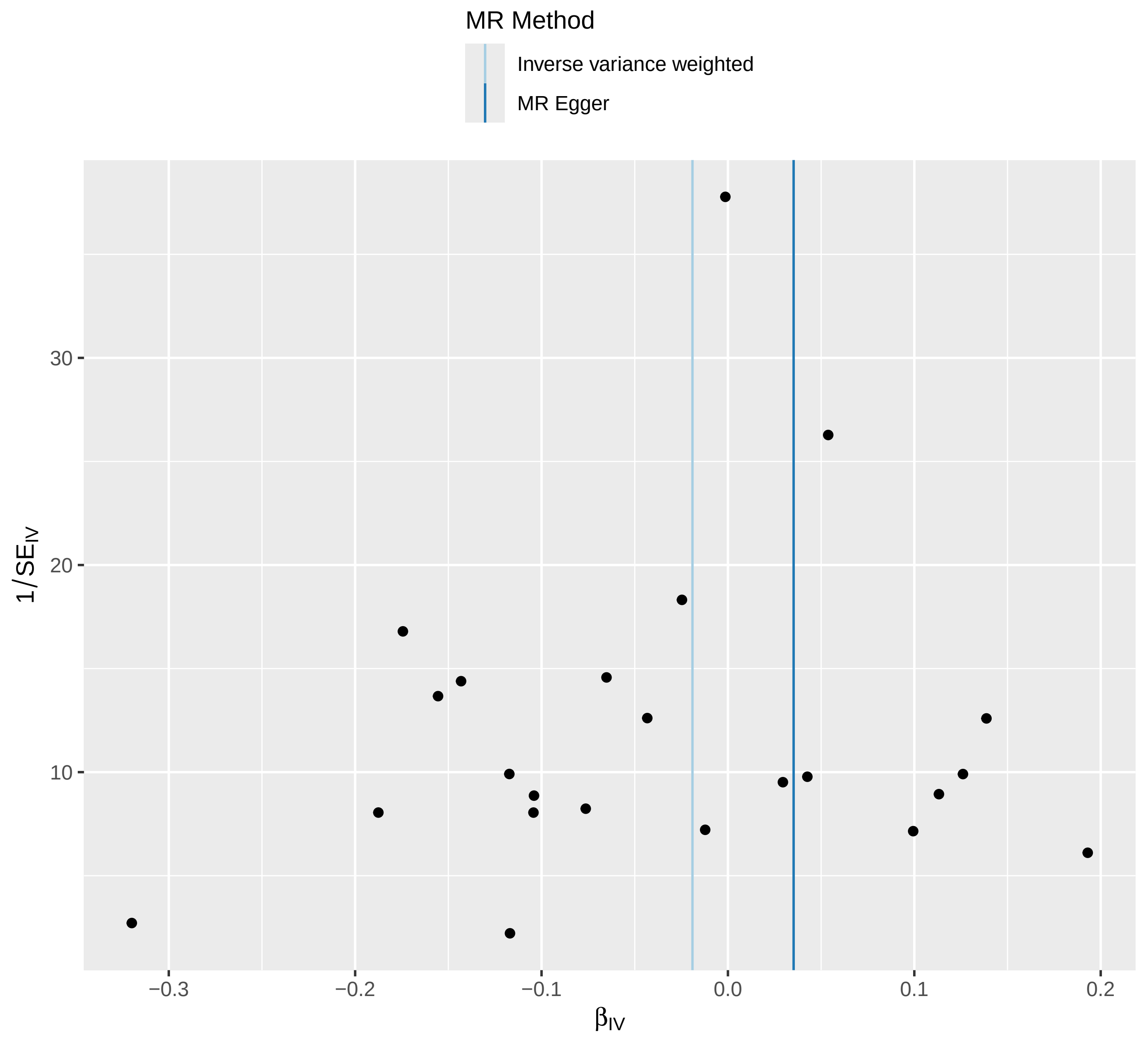
MR Method
Inverse variance weighted
MR Egger
1/SE_IV
30
20
10
−0.3
−0.2
−0.1
0.0
0.1
0.2
β_IV

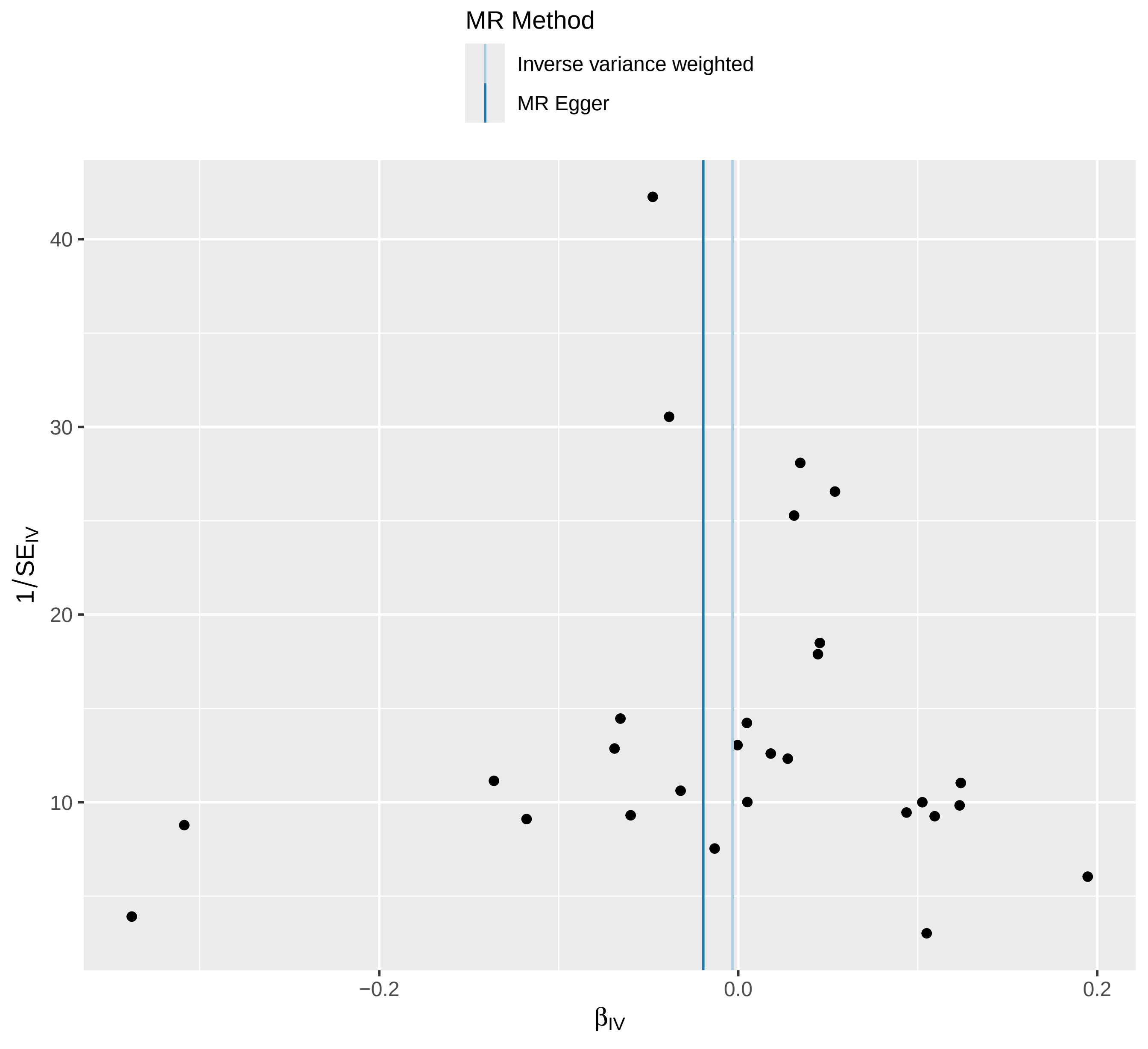
MR Method
Inverse variance weighted
MR Egger
40
30
20
10
1/SE_{IV}
−0.2
0.0
0.2
β_{IV}

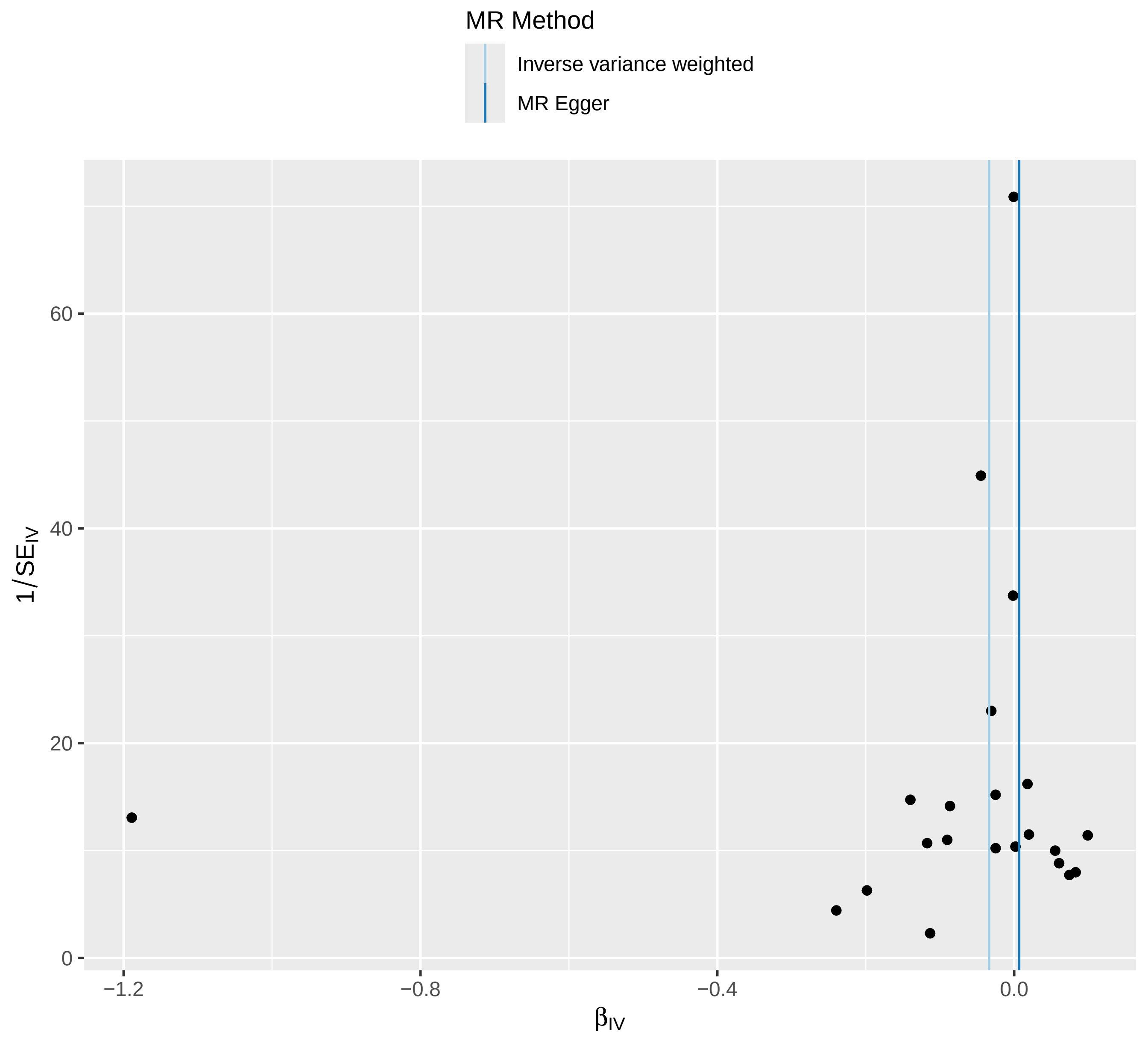
MR Method
Inverse variance weighted
MR Egger
60
40
20
0
1/SE_IV
−1.2
−0.8
−0.4
0.0
β_IV

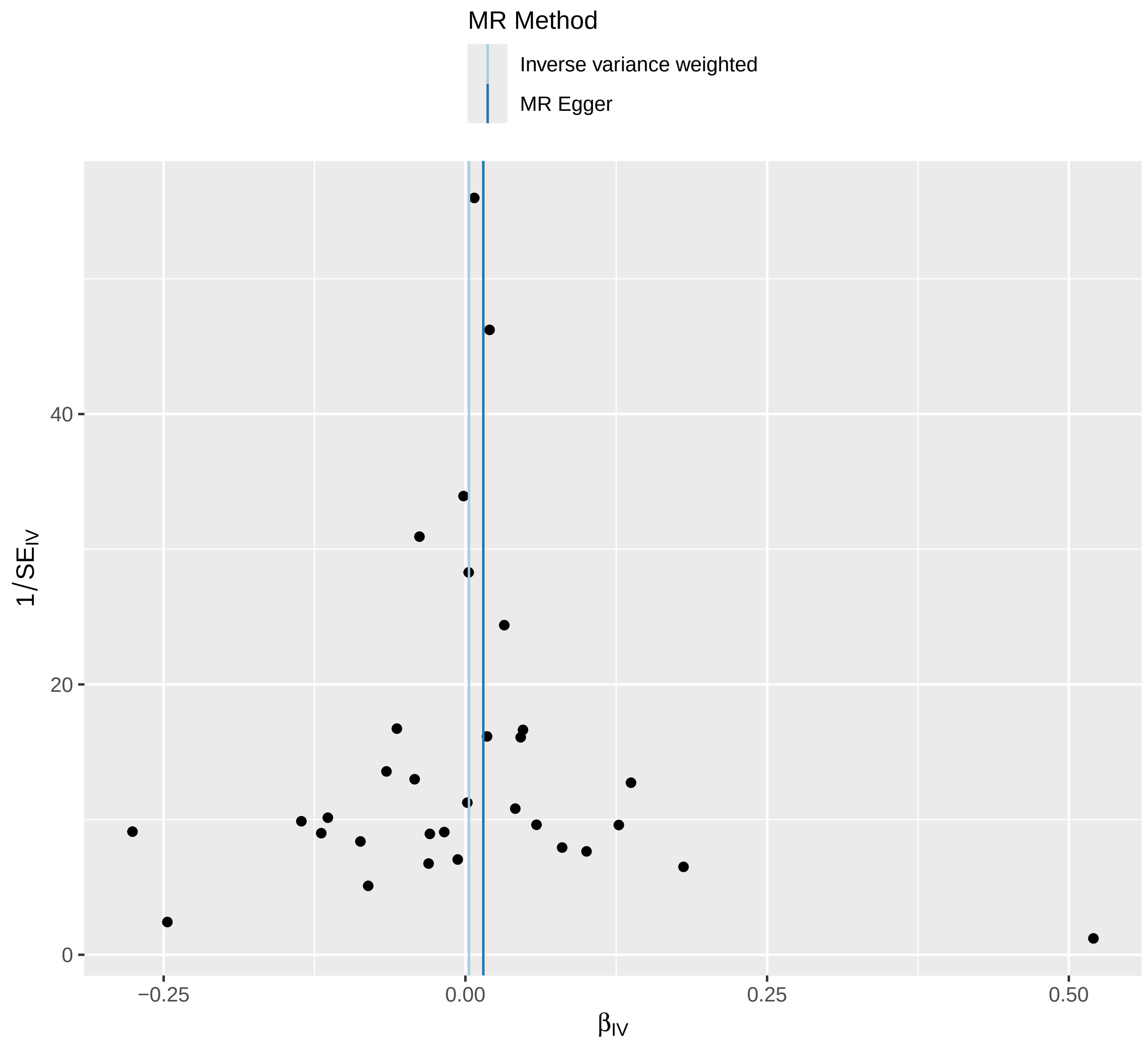

MR Method
Inverse variance weighted
MR Egger
40
20
0
1/SE$_{IV}$
−0.25
0.00
0.25
0.50
β$_{IV}$

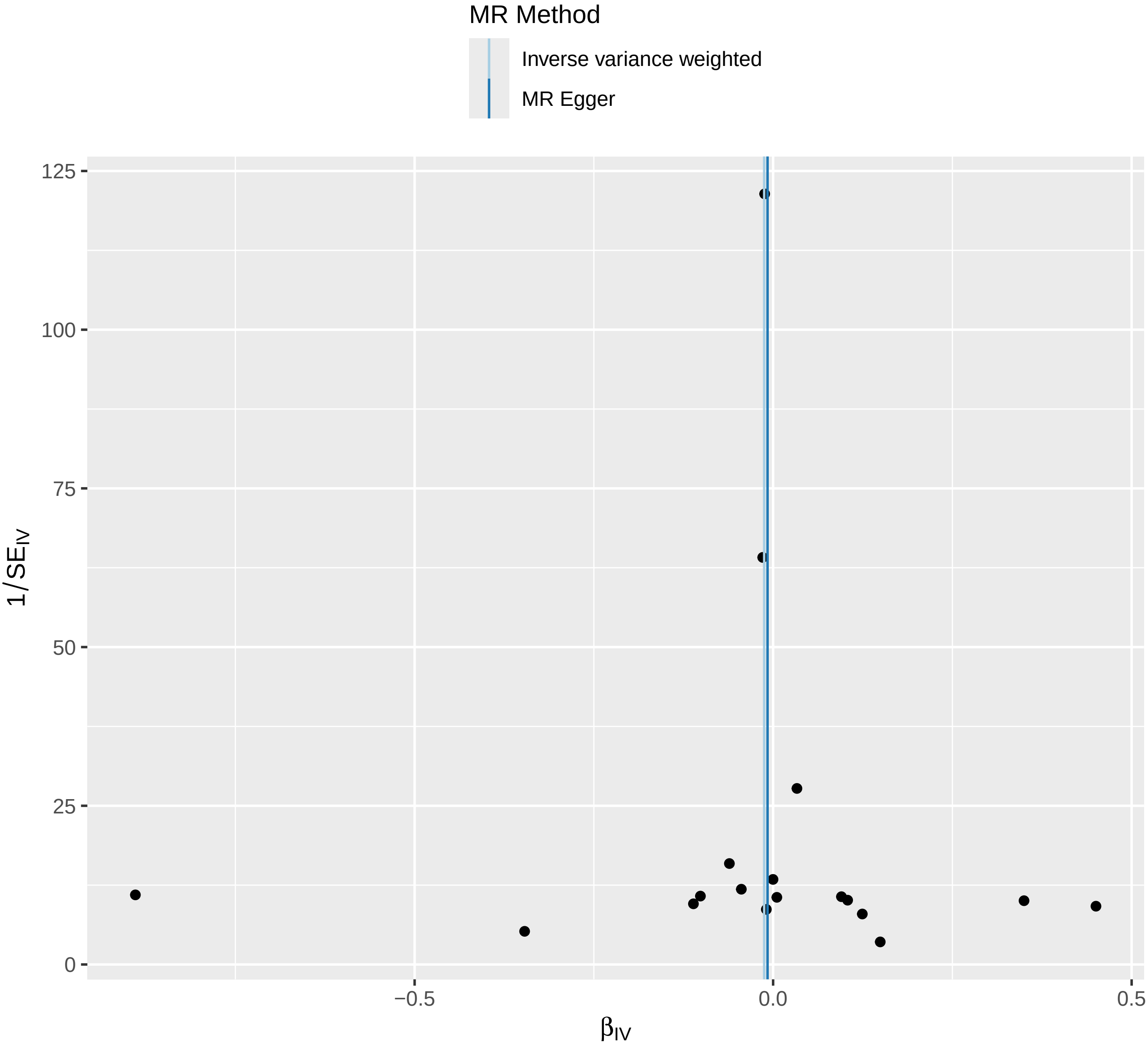

MR Method
Inverse variance weighted
MR Egger
125
100
75
50
25
0
1/SE$_{IV}$
−0.5
0.0
0.5
β$_{IV}$

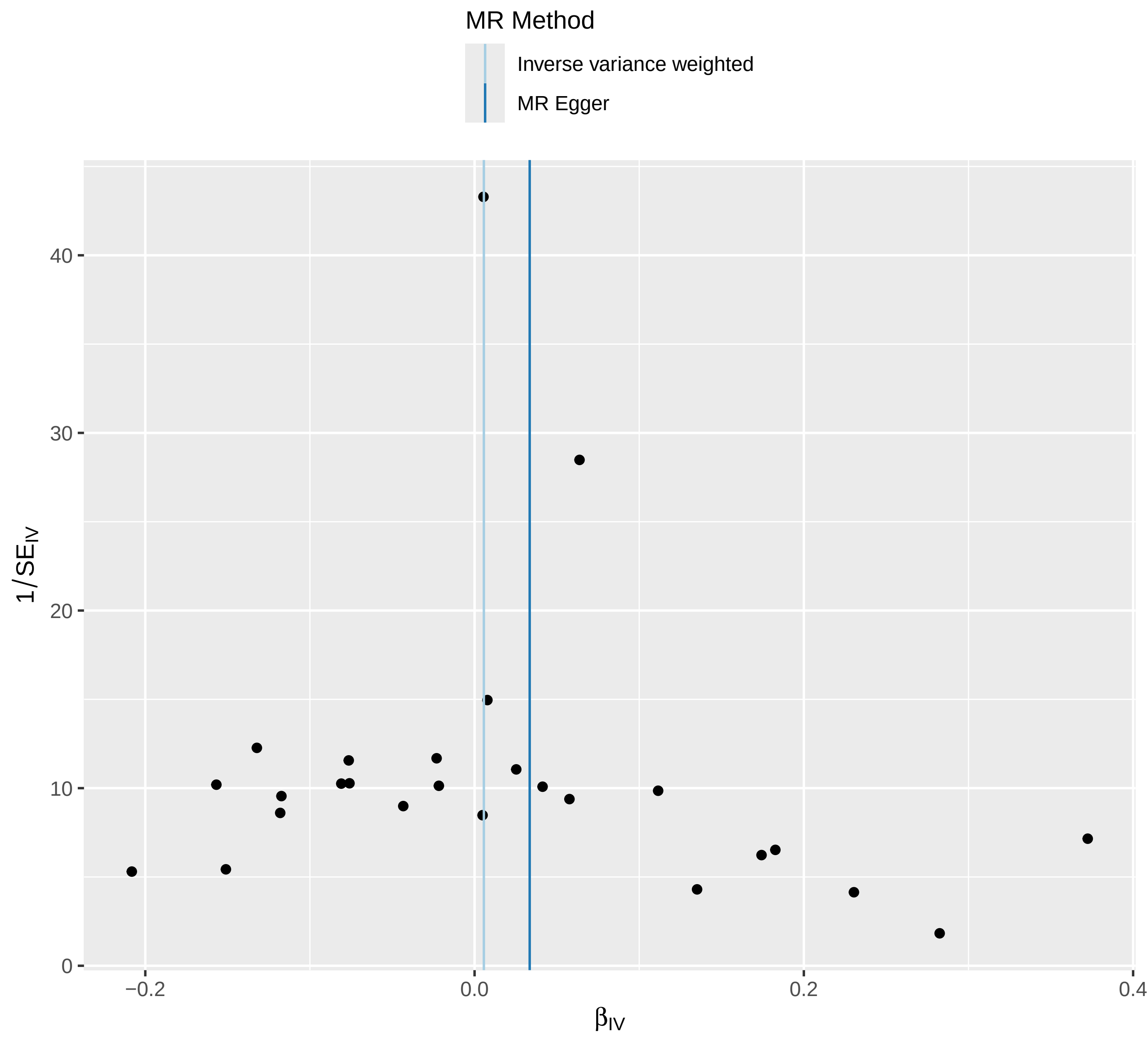

MR Method
Inverse variance weighted
MR Egger
40
30
20
10
0
1/SE_IV
−0.2
0.0
0.2
0.4
β_IV

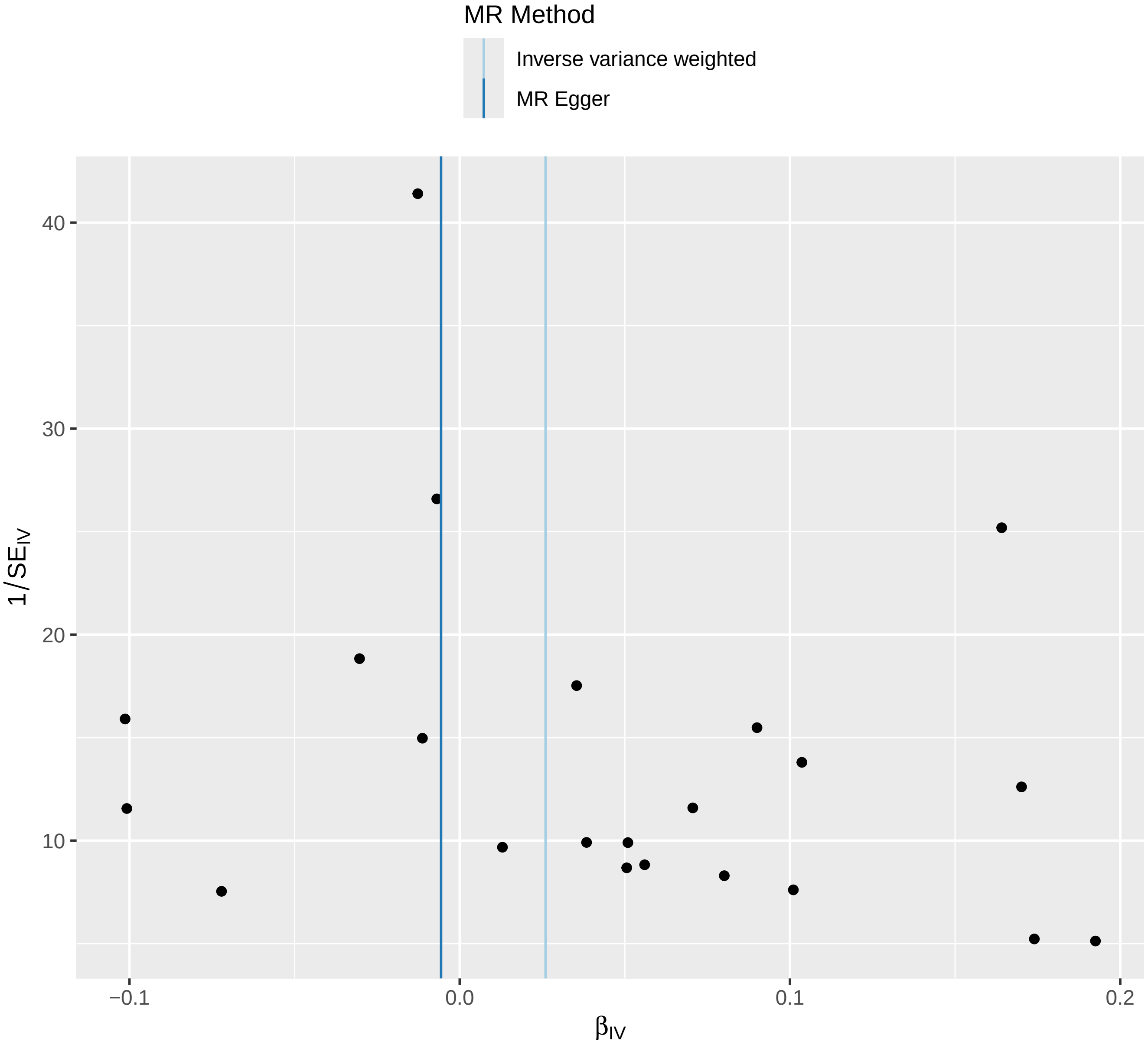
MR Method
Inverse variance weighted
MR Egger
40
30
20
10
1/SE$_{IV}$
−0.1
0.0
0.1
0.2
β$_{IV}$

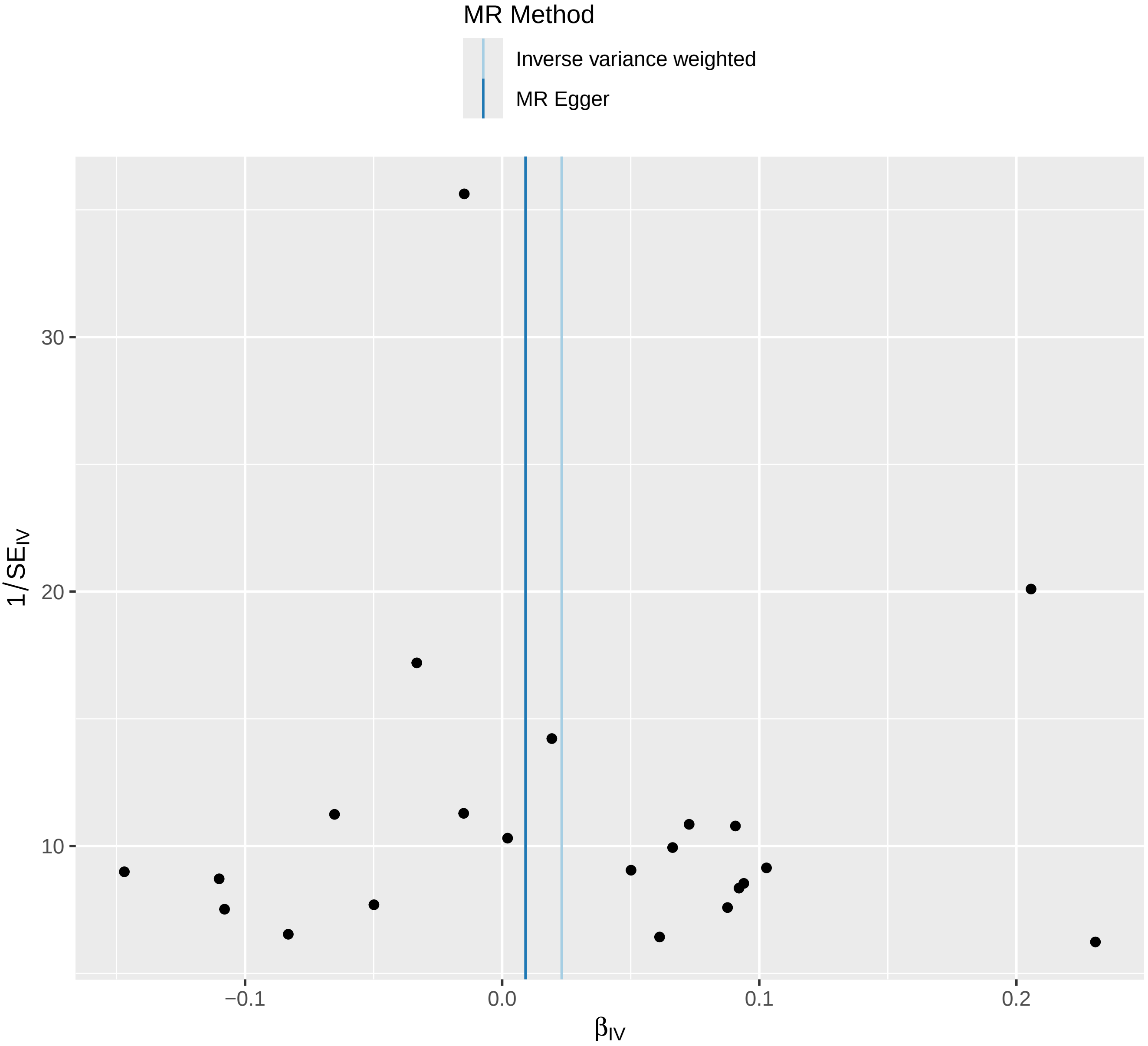
MR Method
Inverse variance weighted
MR Egger
1/SE_IV
30
20
10
−0.1
0.0
0.1
0.2
β_IV

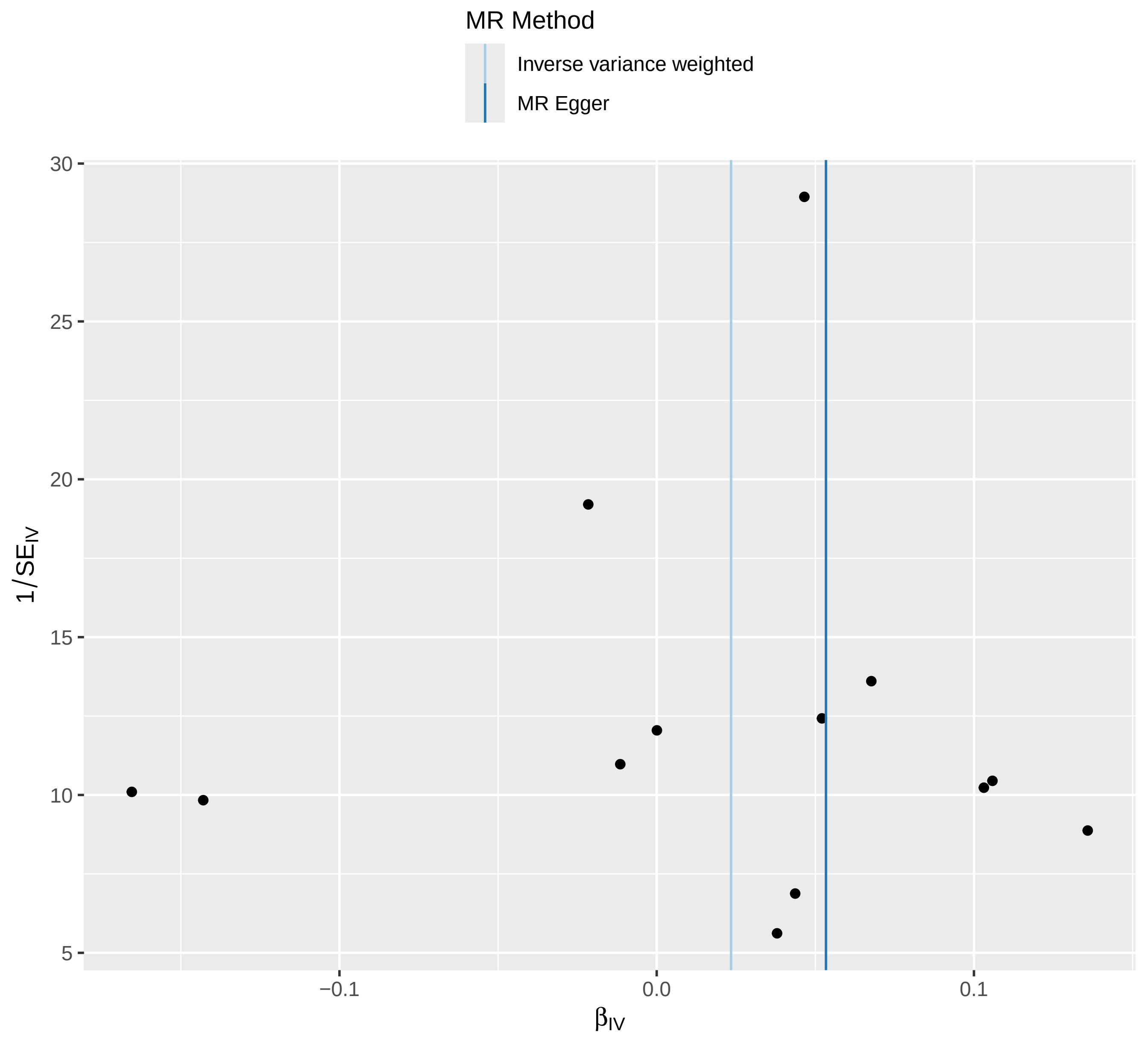

MR Method
Inverse variance weighted
MR Egger
30
25
20
15
10
5
−0.1
0.0
0.1
$1/SE_{IV}$
β_{IV}

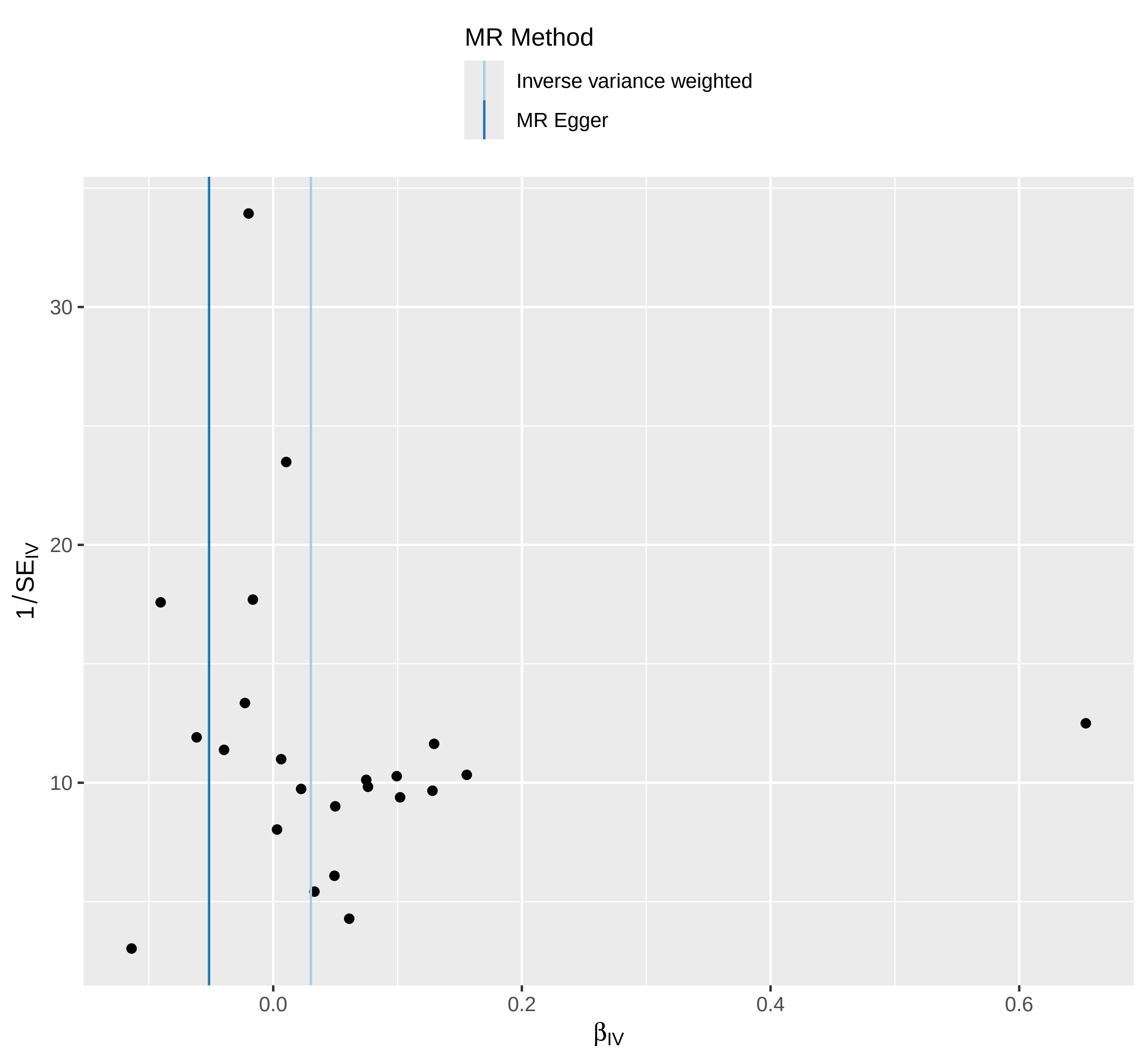

MR Method
Inverse variance weighted
MR Egger
30
20
10
1/SE$_{IV}$
0.0
0.2
0.4
0.6
β$_{IV}$

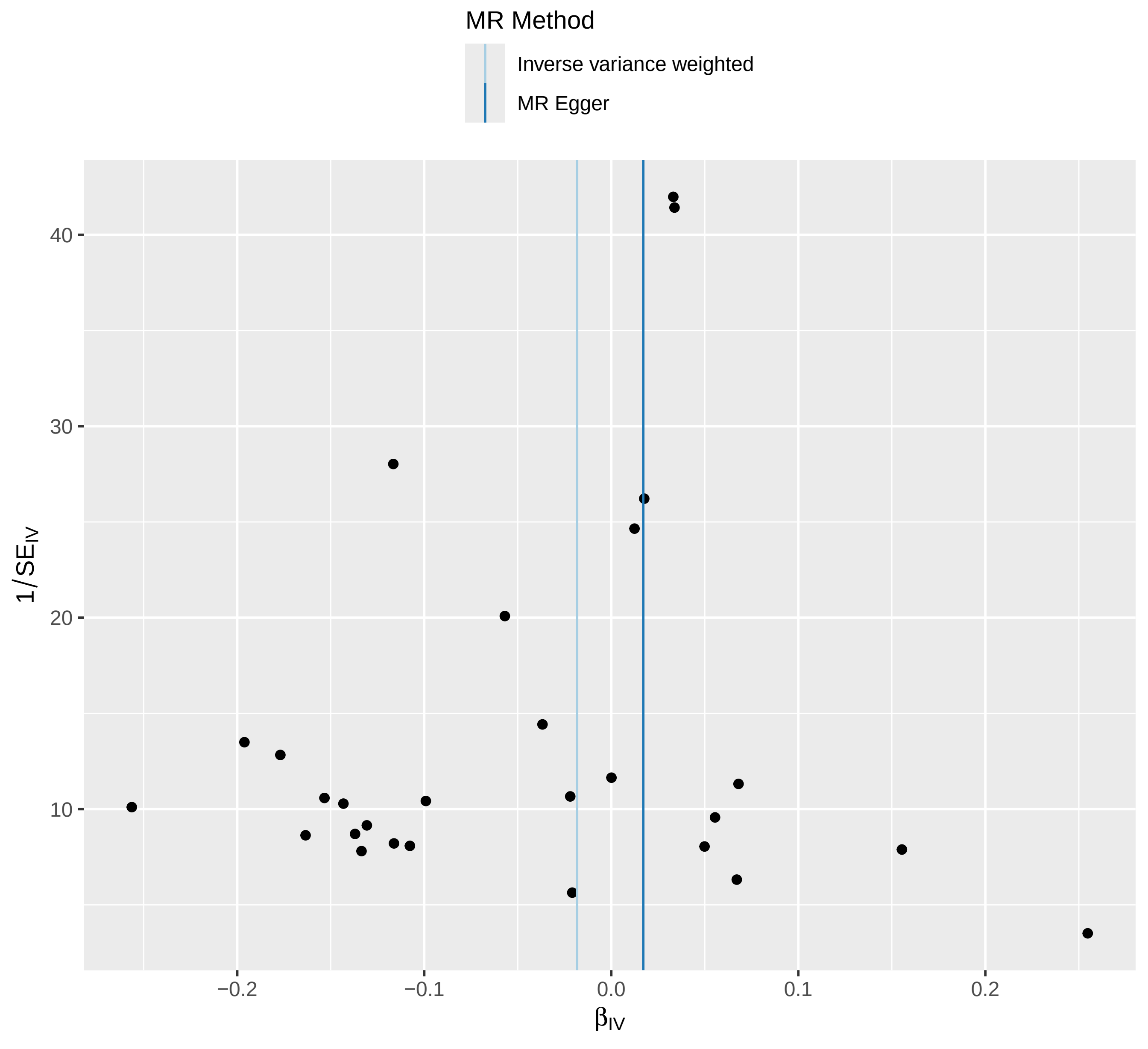
MR Method
Inverse variance weighted
MR Egger
1/SE_{IV}
40
30
20
10
−0.2
−0.1
0.0
0.1
0.2
β_{IV}

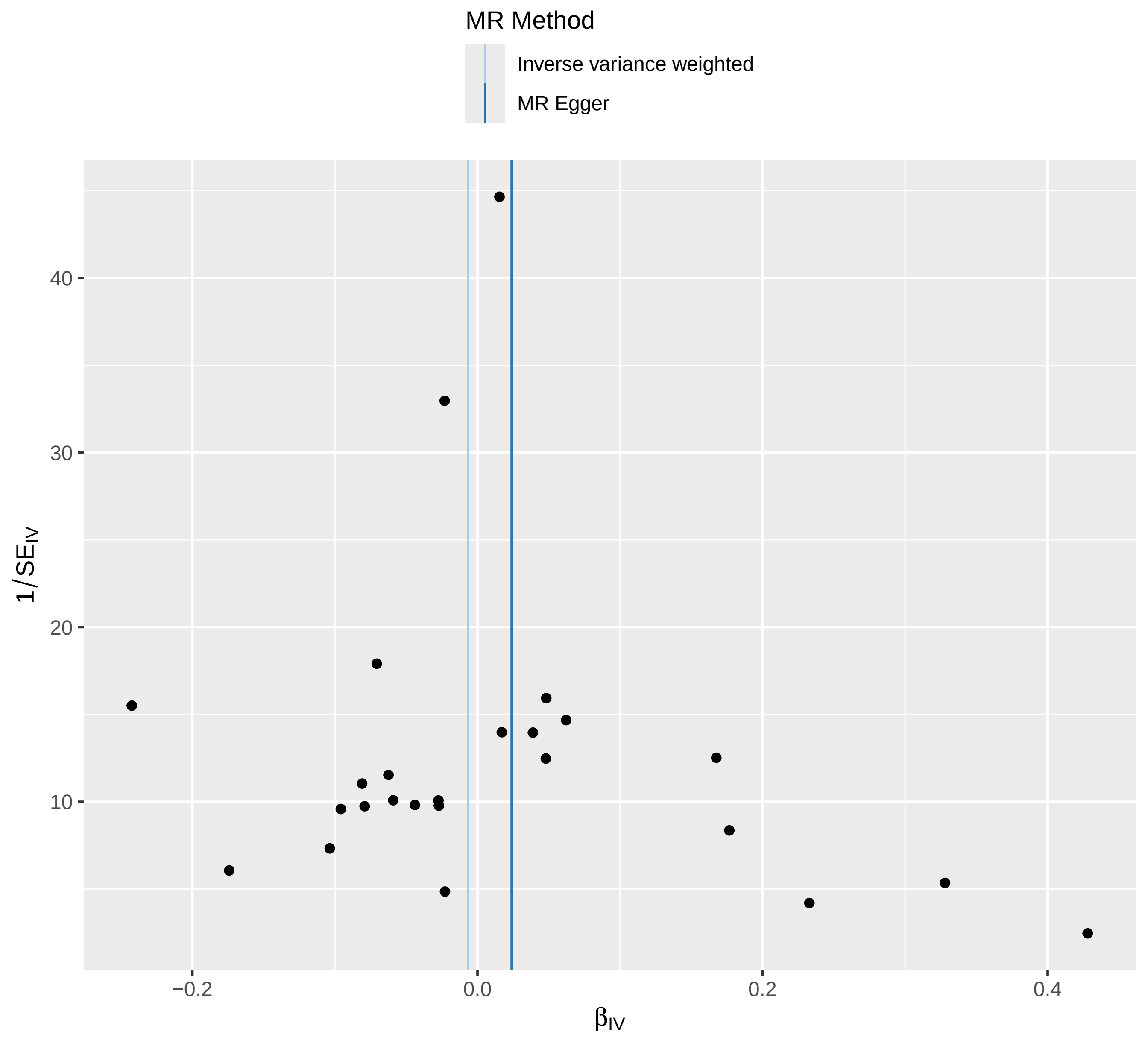
MR Method
Inverse variance weighted
MR Egger
40
30
20
10
1/SE_IV
−0.2
0.0
0.2
0.4
β_IV

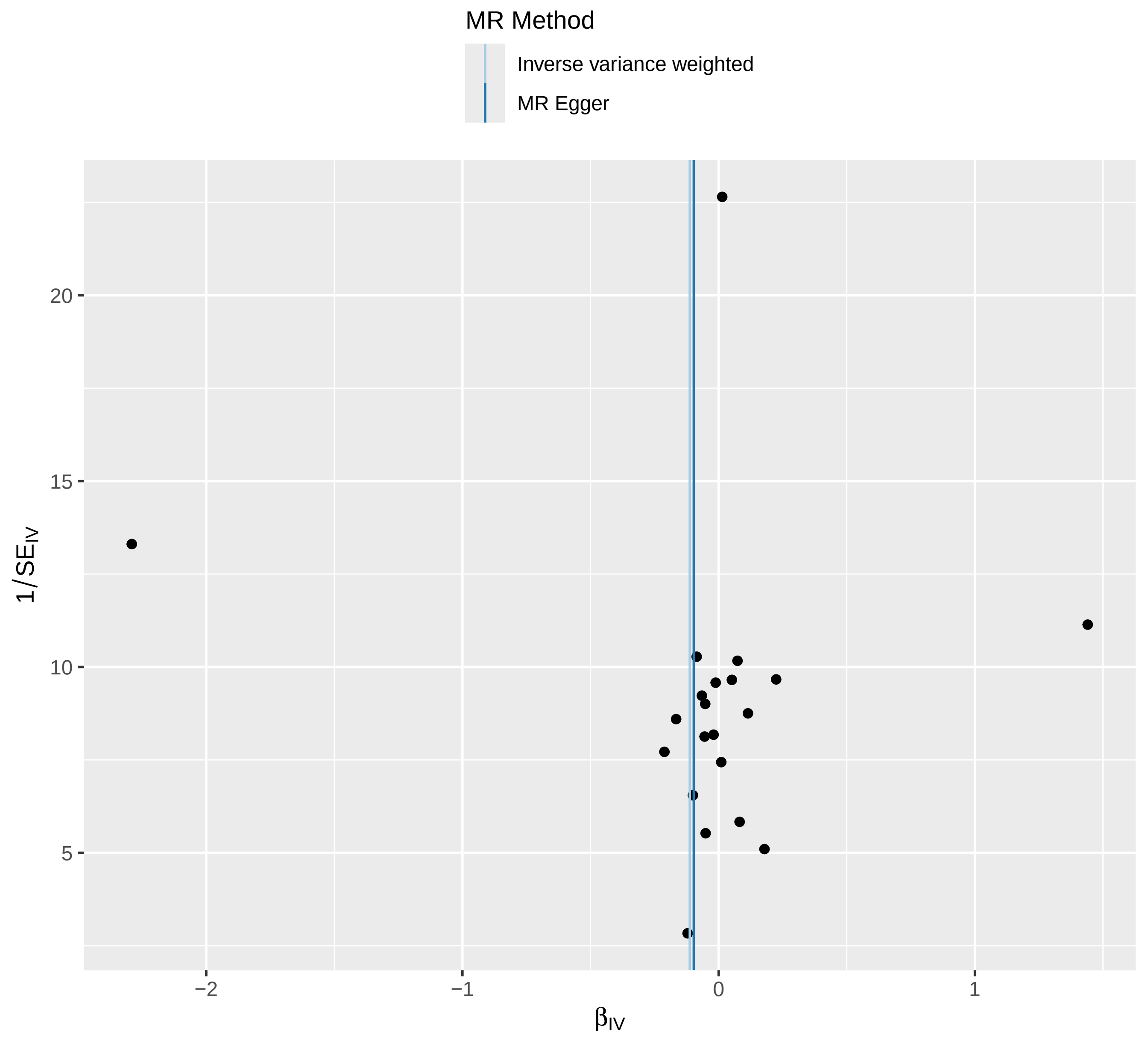

MR Method
Inverse variance weighted
MR Egger
20
15
10
5
1/SE_IV
−2
−1
0
1
β_IV

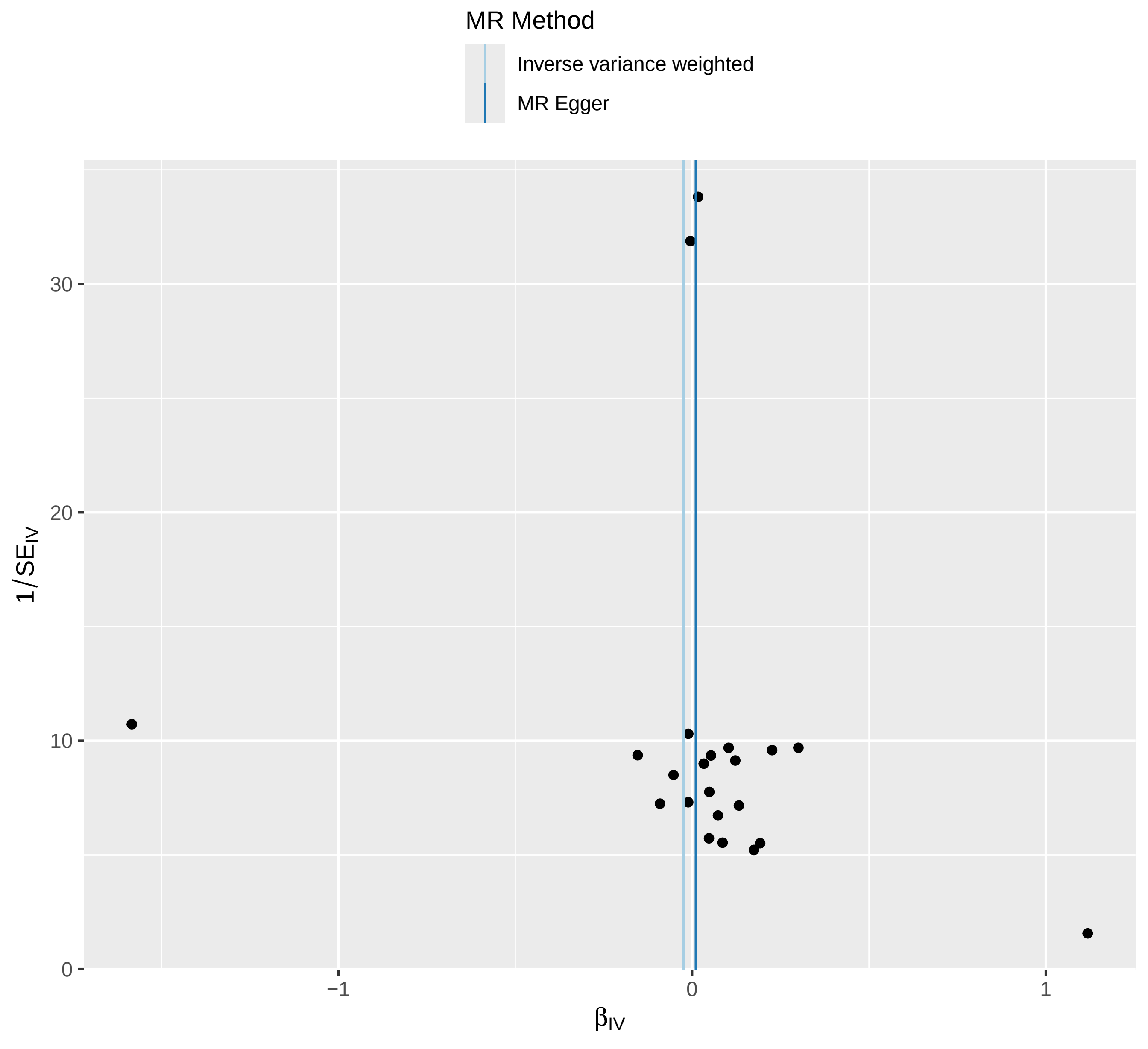

MR Method
Inverse variance weighted
MR Egger
30
20
10
0
1/SE_IV
−1
0
1
β_IV

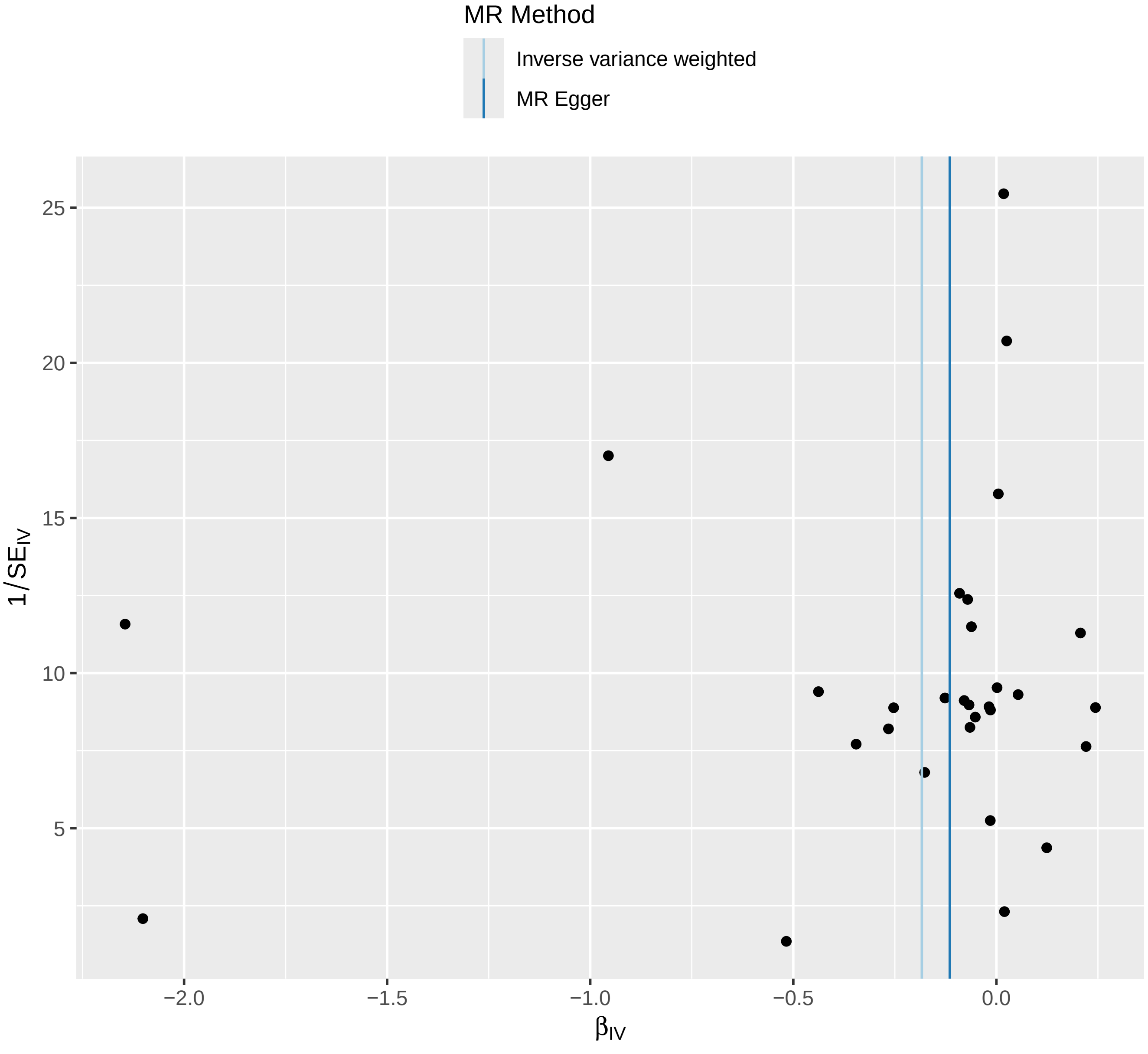
MR Method
Inverse variance weighted
MR Egger
25
20
15
10
5
$1/SE_{IV}$
−2.0
−1.5
−1.0
−0.5
0.0
β_{IV}

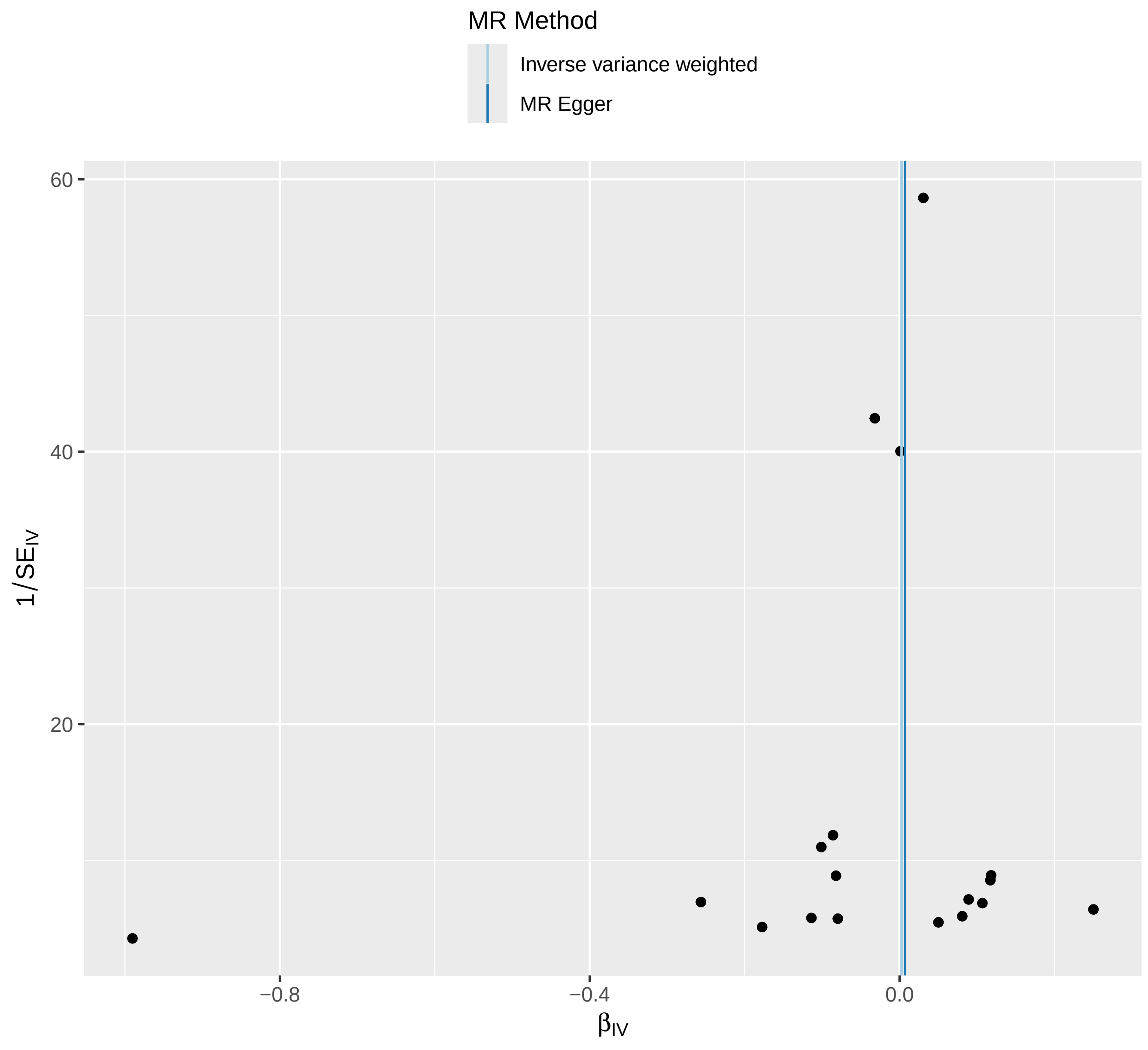
MR Method
Inverse variance weighted
MR Egger
60
40
20
1/SE$_{IV}$
−0.8
−0.4
0.0
β_{IV}

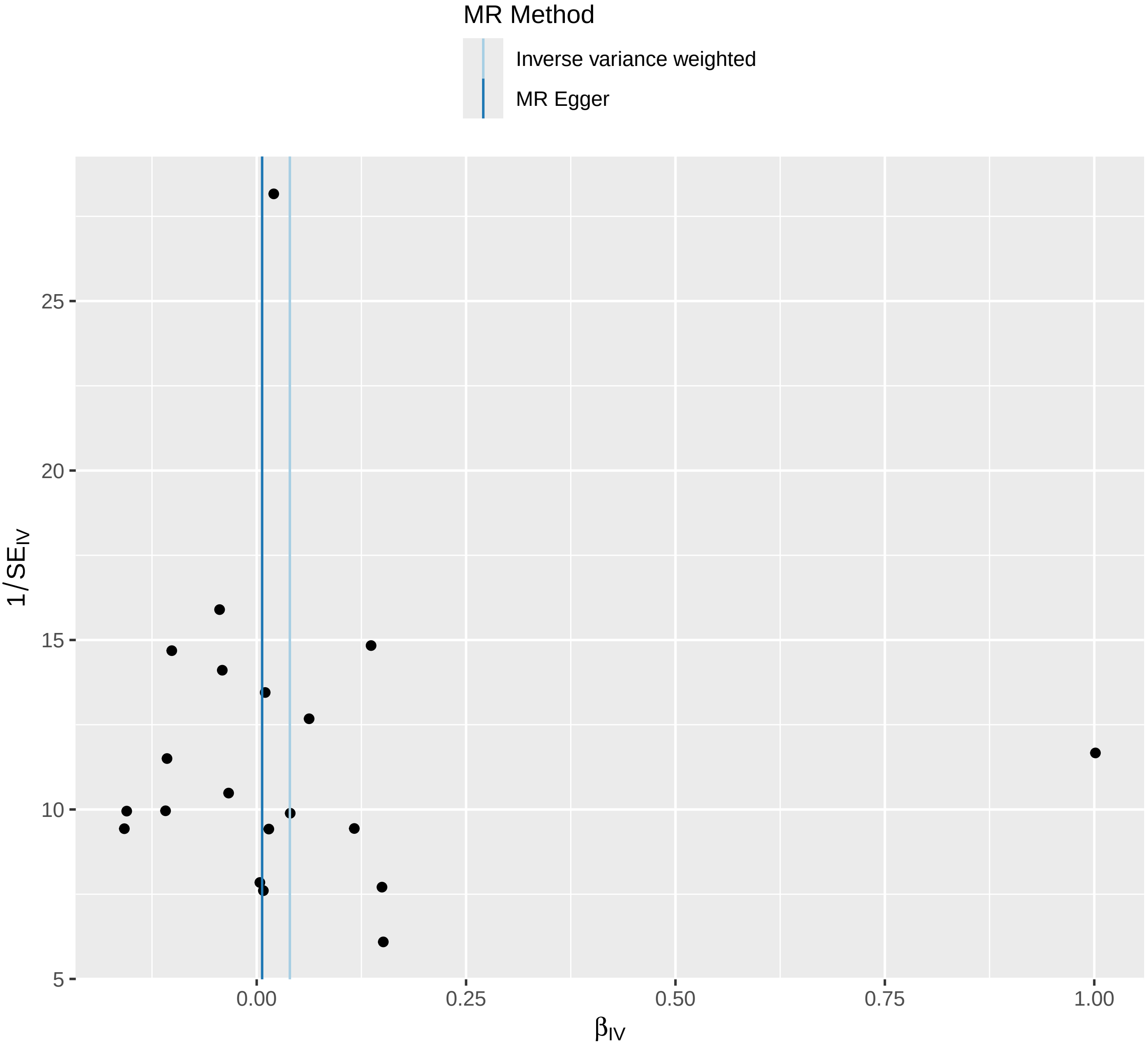
MR Method
Inverse variance weighted
MR Egger
1/SE_IV
β_IV
25
20
15
10
5
0.00
0.25
0.50
0.75
1.00

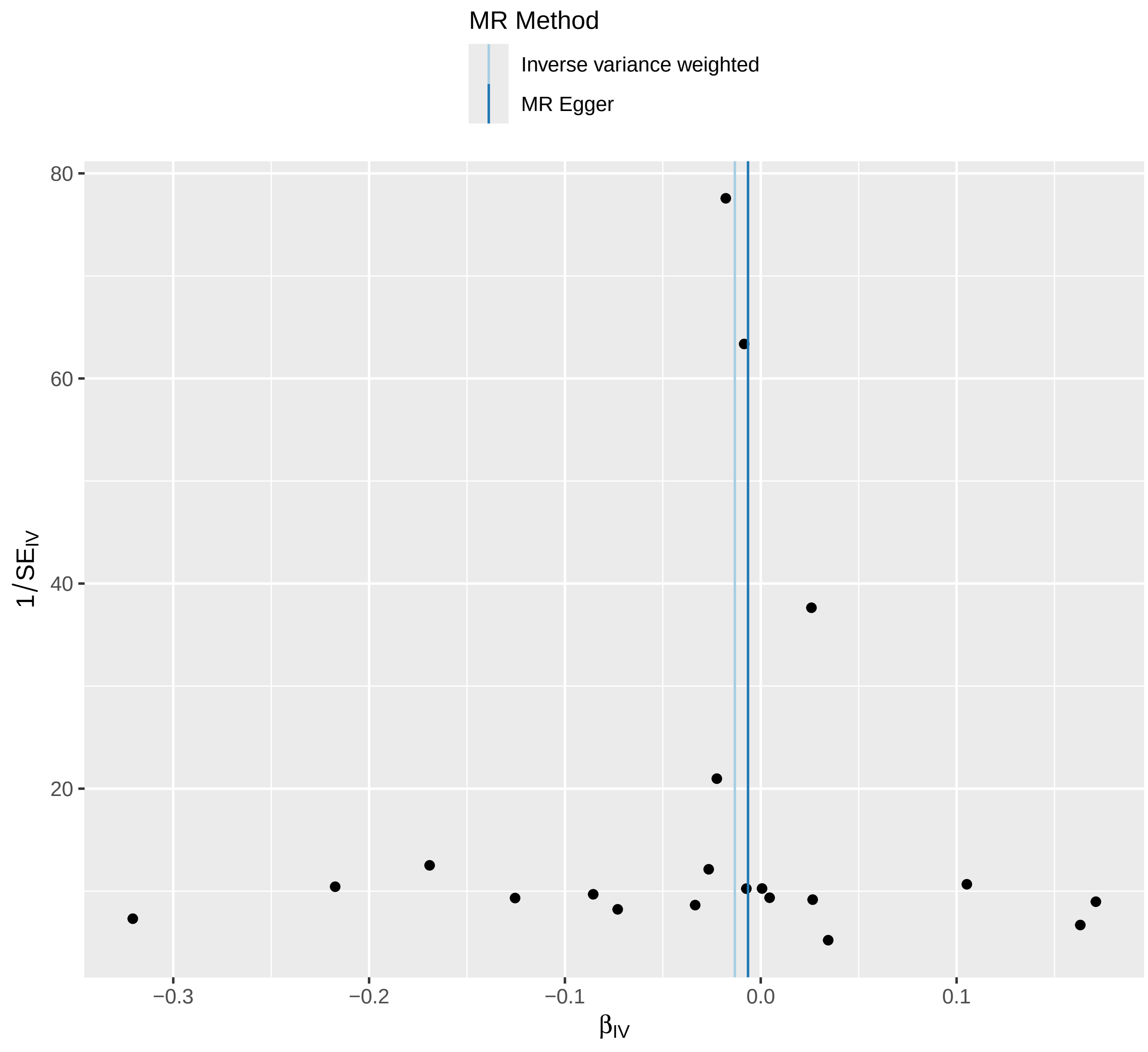

MR Method
Inverse variance weighted
MR Egger
80
60
40
20
1/SE$_{IV}$
−0.3
−0.2
−0.1
0.0
0.1
β$_{IV}$

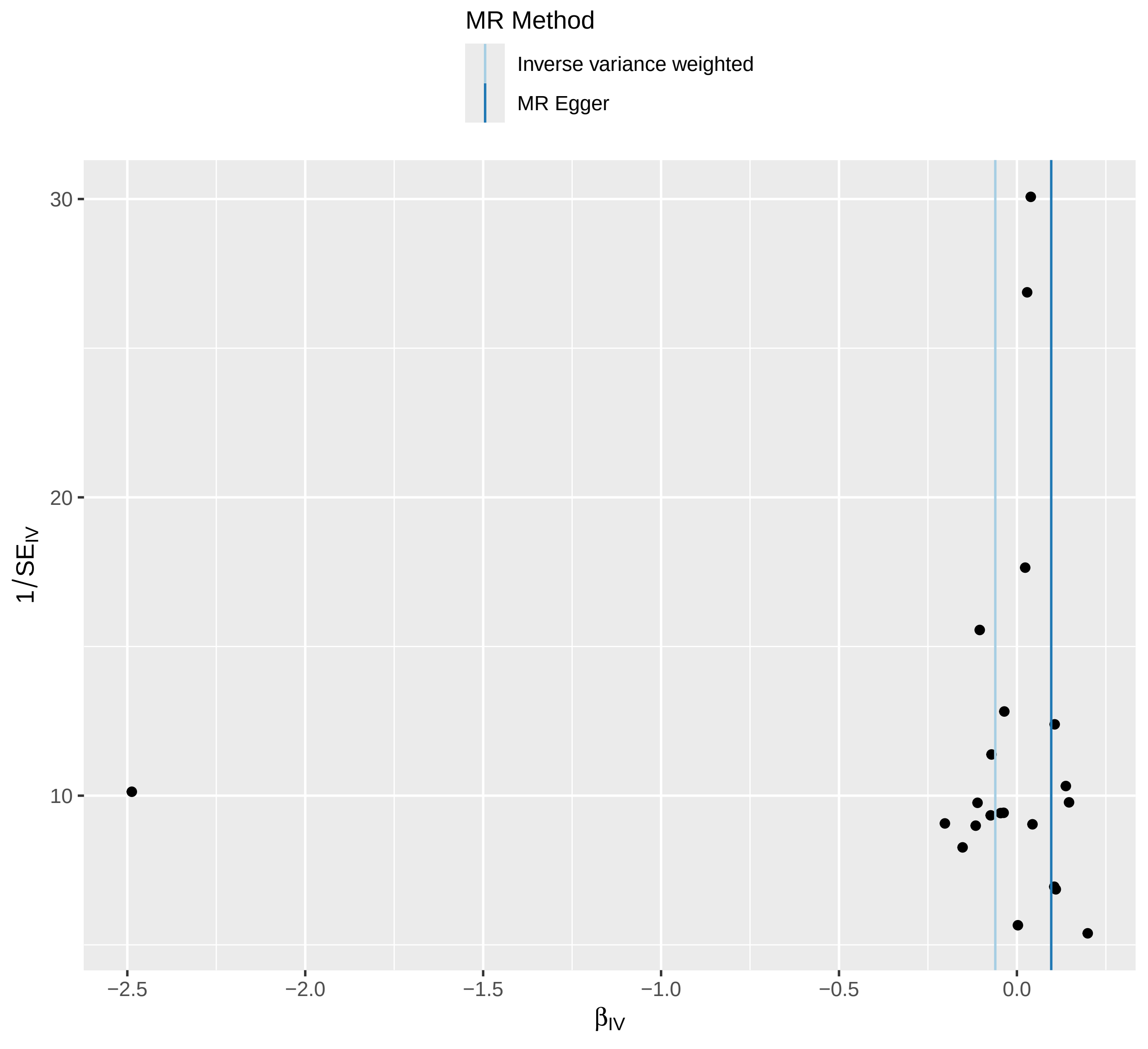
MR Method
Inverse variance weighted
MR Egger
30
20
10
1/SE$_{IV}$
−2.5
−2.0
−1.5
−1.0
−0.5
0.0
β$_{IV}$

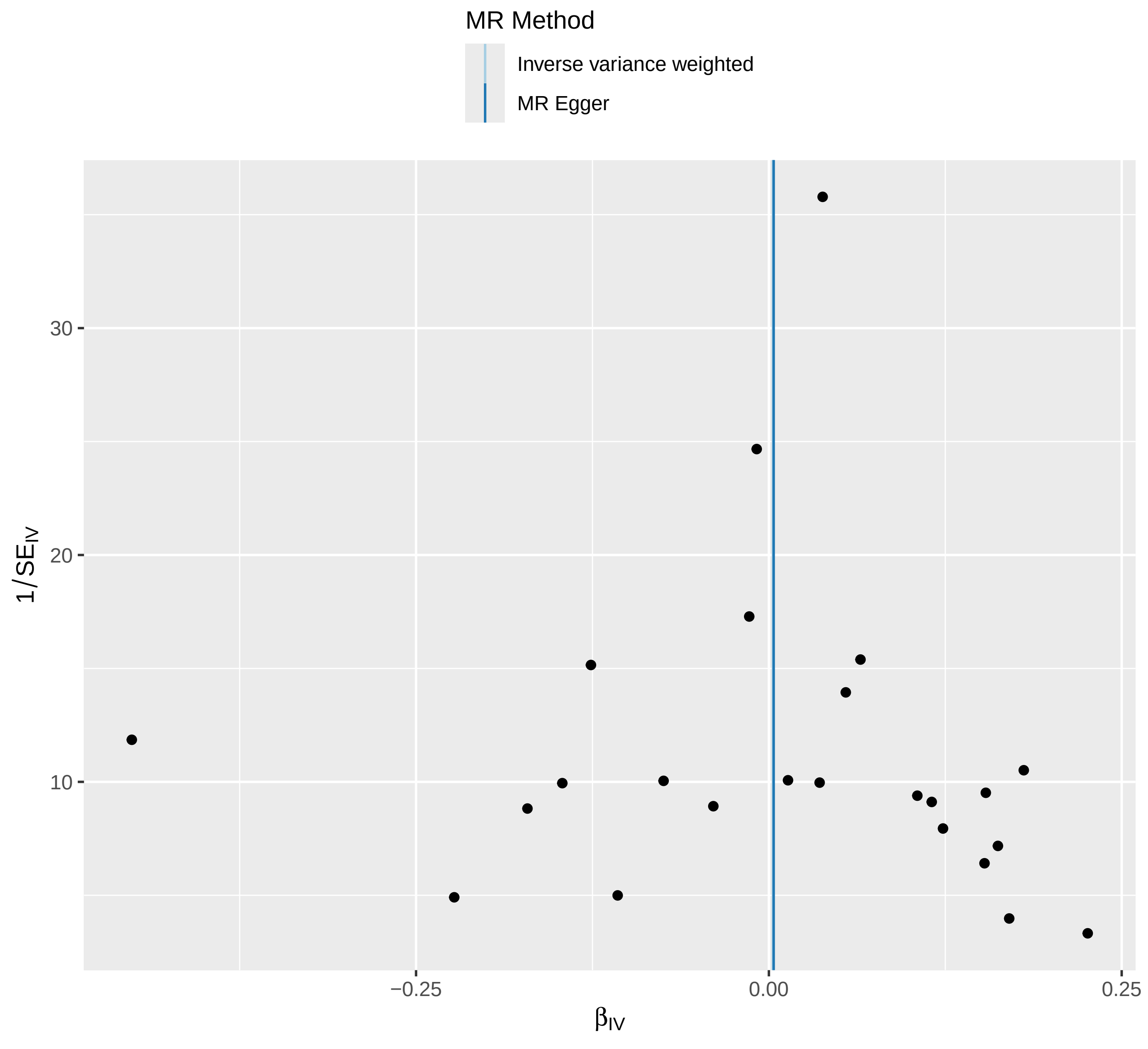
MR Method
Inverse variance weighted
MR Egger
30
20
10
1/SE_IV
−0.25
0.00
0.25
β_IV

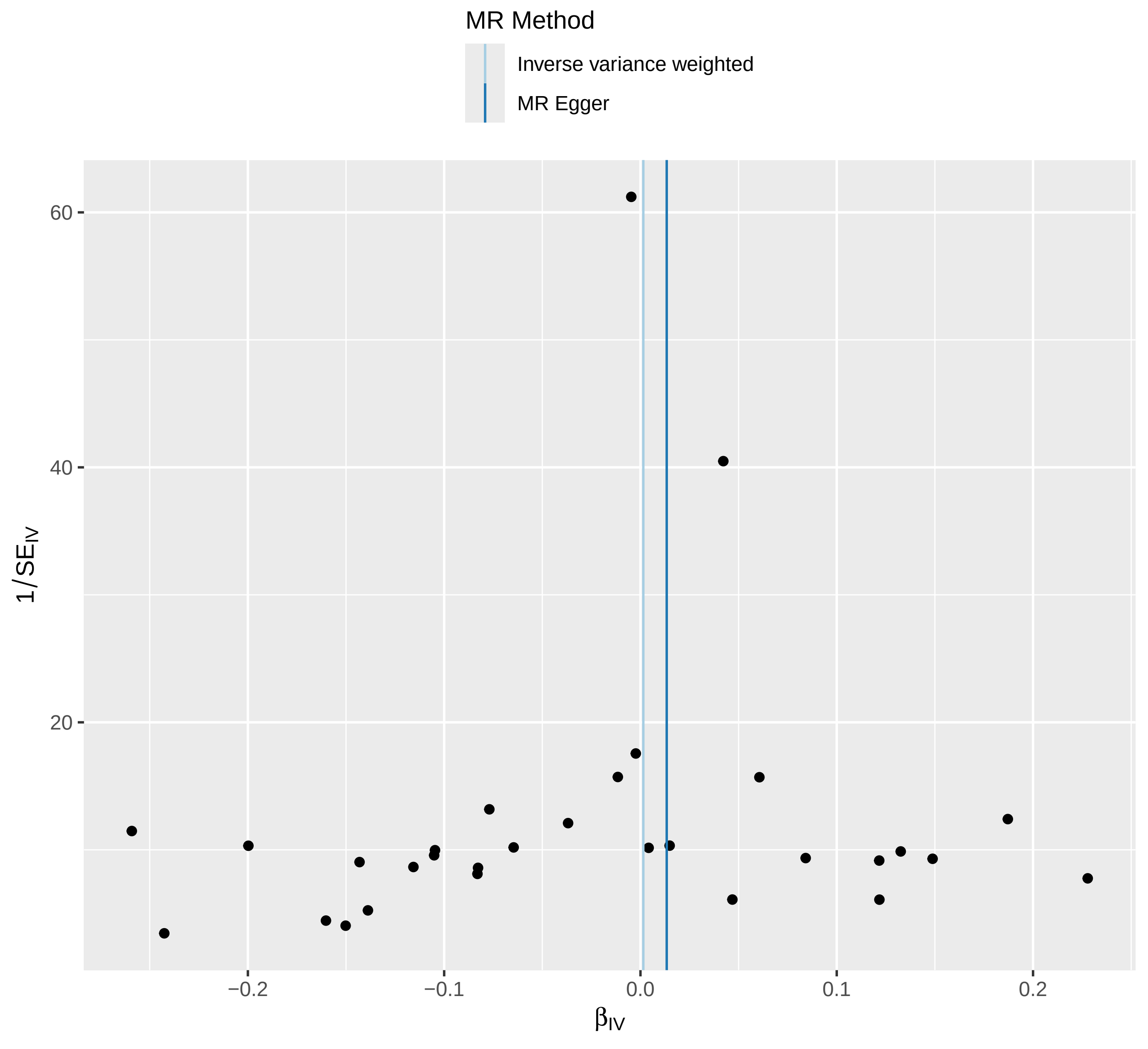
MR Method
Inverse variance weighted
MR Egger
60
40
20
1/SE_IV
−0.2
−0.1
0.0
0.1
0.2
β_IV

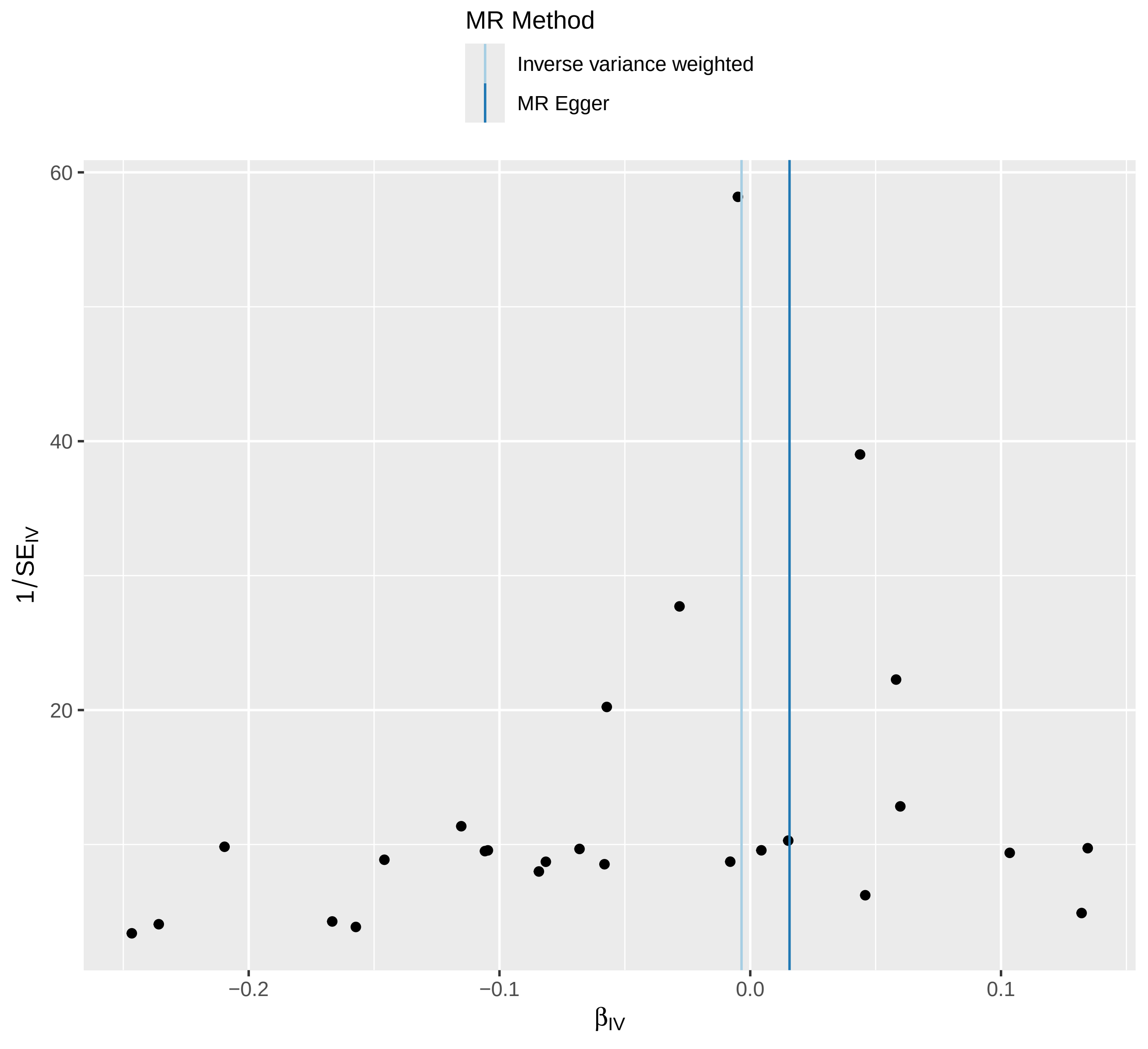
MR Method
Inverse variance weighted
MR Egger
60
40
20
1/SE_IV
−0.2
−0.1
0.0
0.1
β_IV

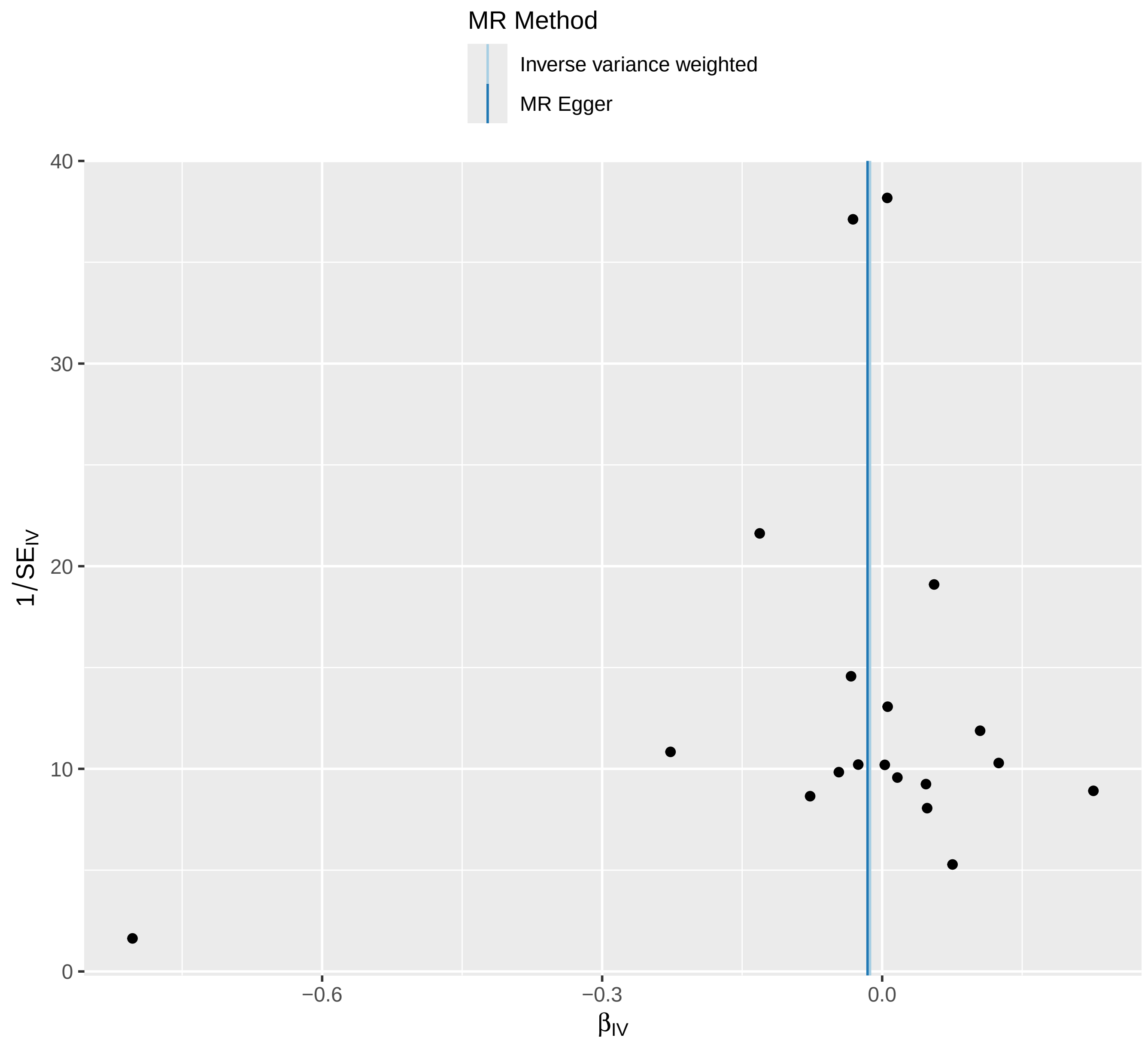

MR Method
Inverse variance weighted
MR Egger
40
30
20
10
0
1/SE_IV
−0.6
−0.3
0.0
β_IV

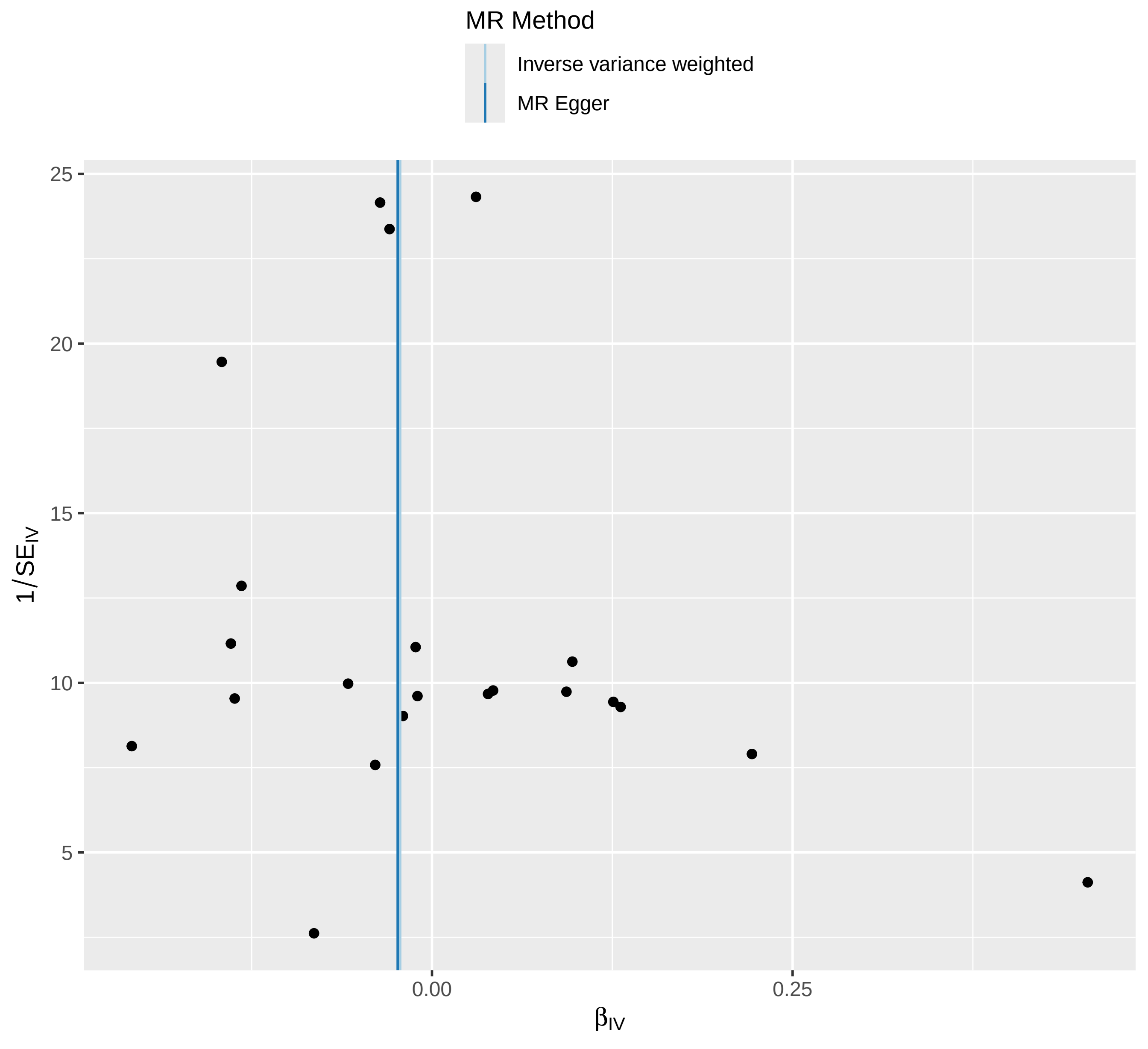

MR Method
Inverse variance weighted
MR Egger
25
20
15
10
5
1/SE_IV
0.00
0.25
β_IV

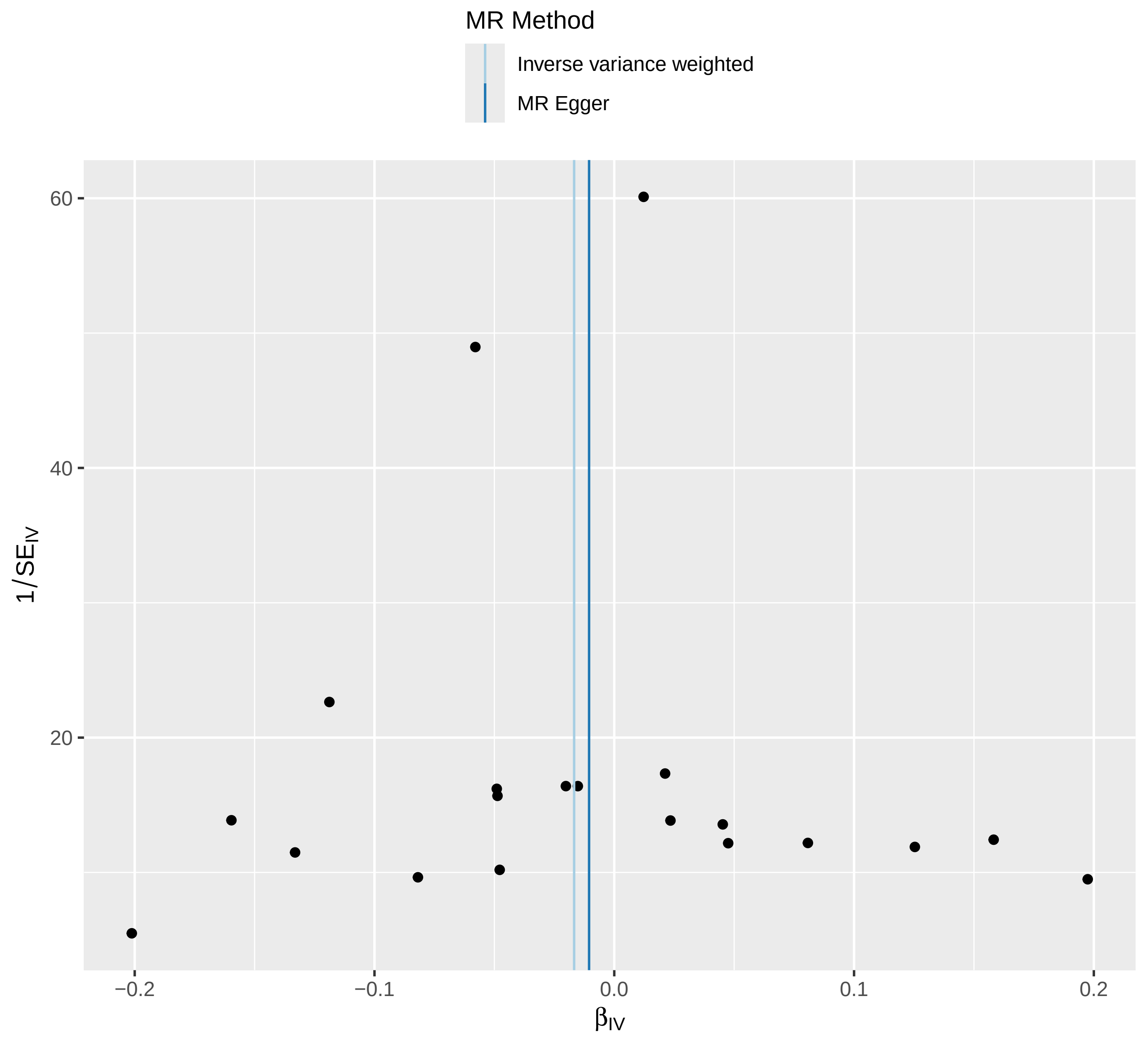
MR Method
Inverse variance weighted
MR Egger
60
40
20
1/SE_IV
−0.2
−0.1
0.0
0.1
0.2
β_IV

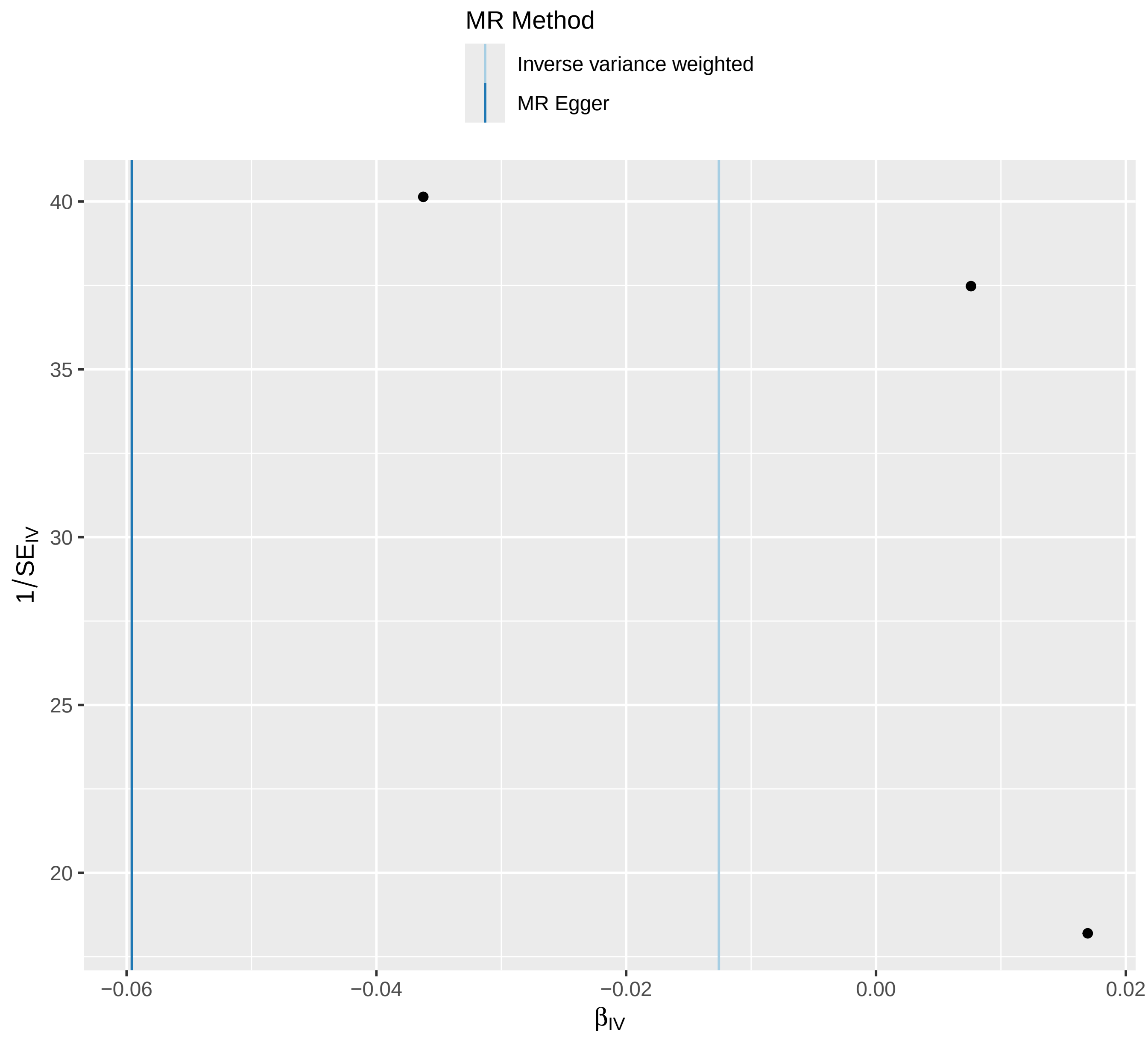

MR Method
Inverse variance weighted
MR Egger
40
35
30
25
20
1/SE$_{IV}$
−0.06
−0.04
−0.02
0.00
0.02
β$_{IV}$

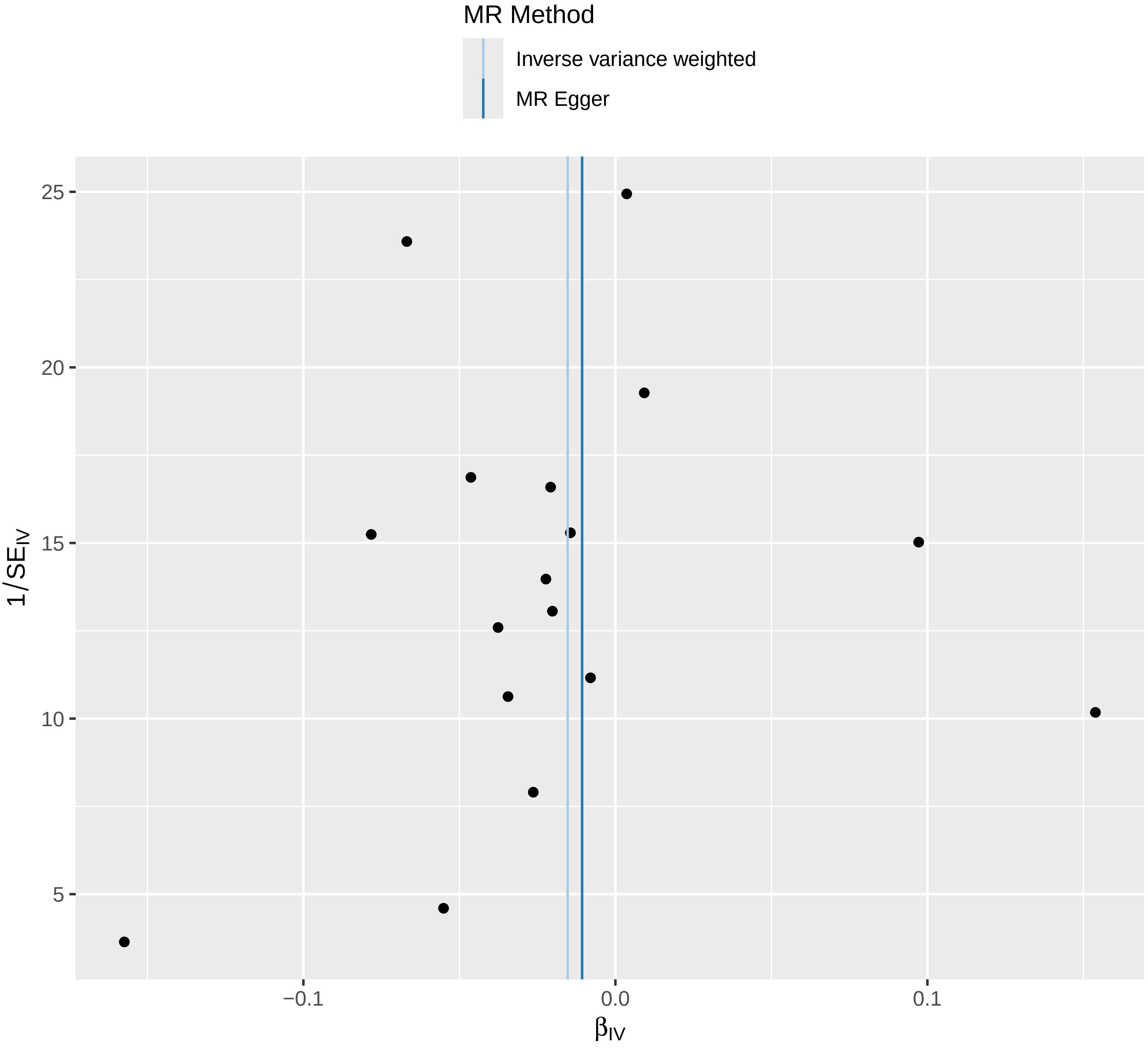
MR Method
Inverse variance weighted
MR Egger
1/SE$_{IV}$
25
20
15
10
5
−0.1
0.0
0.1
β$_{IV}$

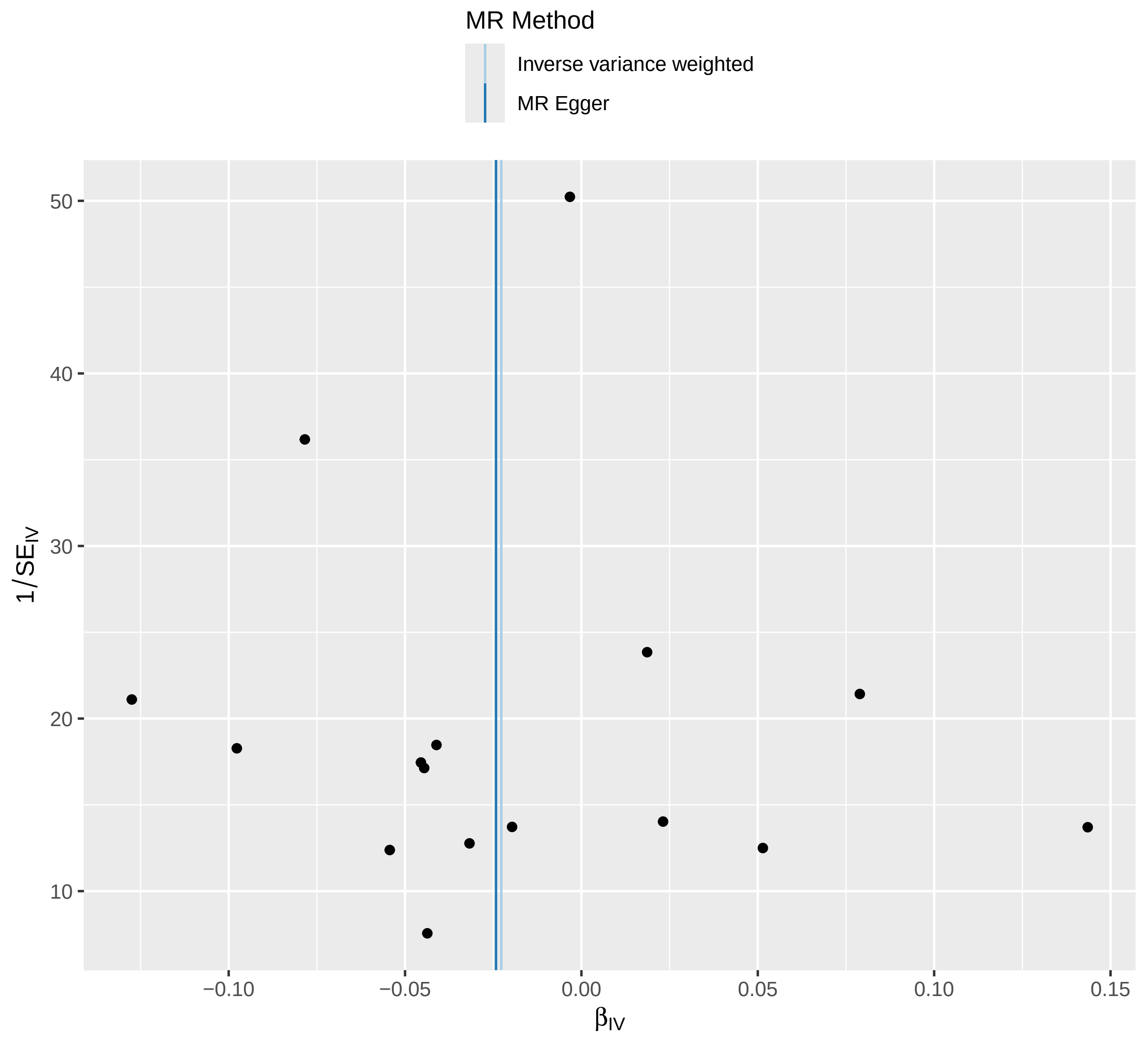
MR Method
Inverse variance weighted
MR Egger
50
40
30
20
10
1/SE_IV
−0.10
−0.05
0.00
0.05
0.10
0.15
β_IV

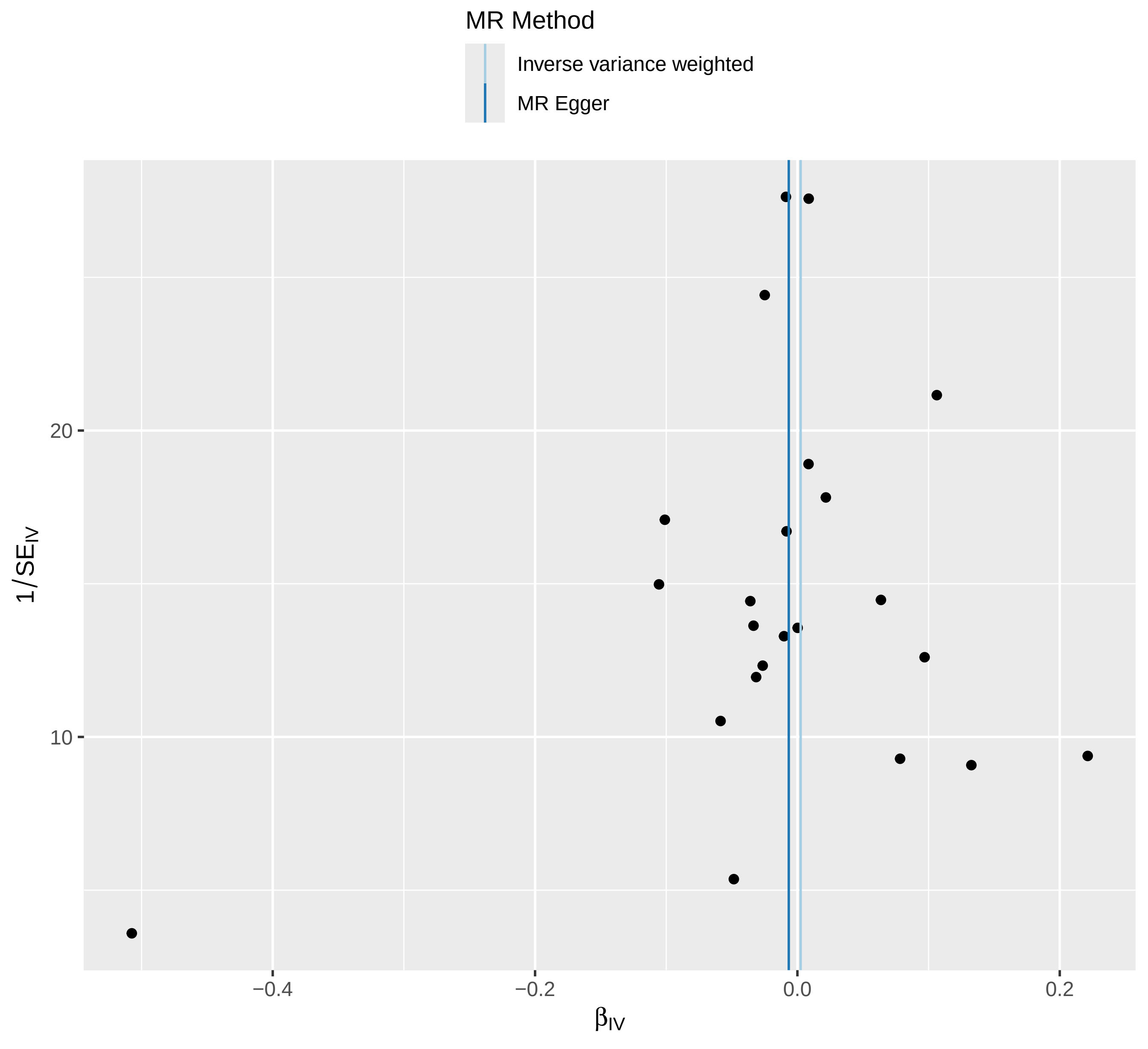
MR Method
Inverse variance weighted
MR Egger
20
10
1/SE$_{IV}$
−0.4
−0.2
0.0
0.2
β$_{IV}$

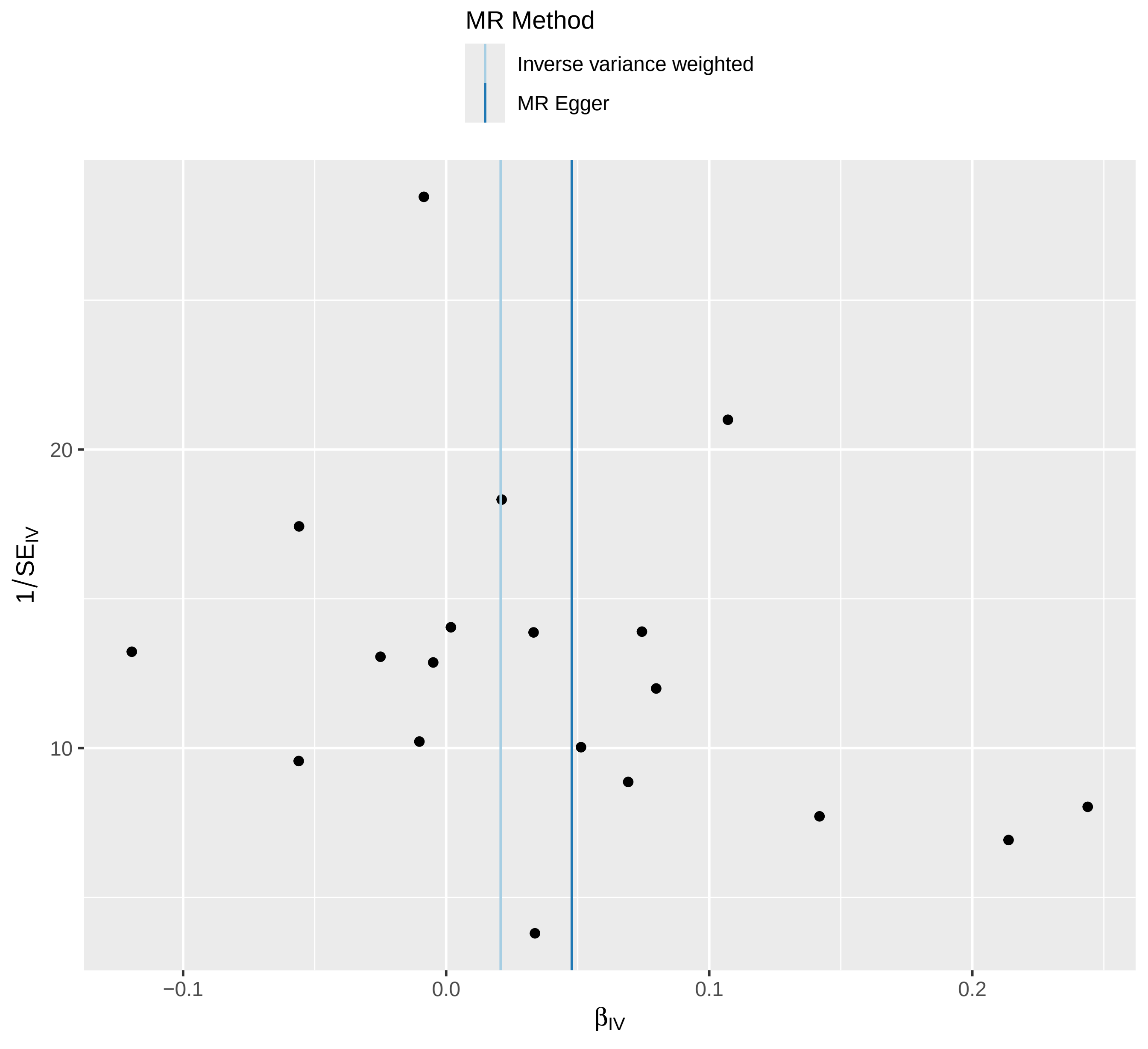

MR Method
Inverse variance weighted
MR Egger
20
10
1/SE_IV
−0.1
0.0
0.1
0.2
β_IV

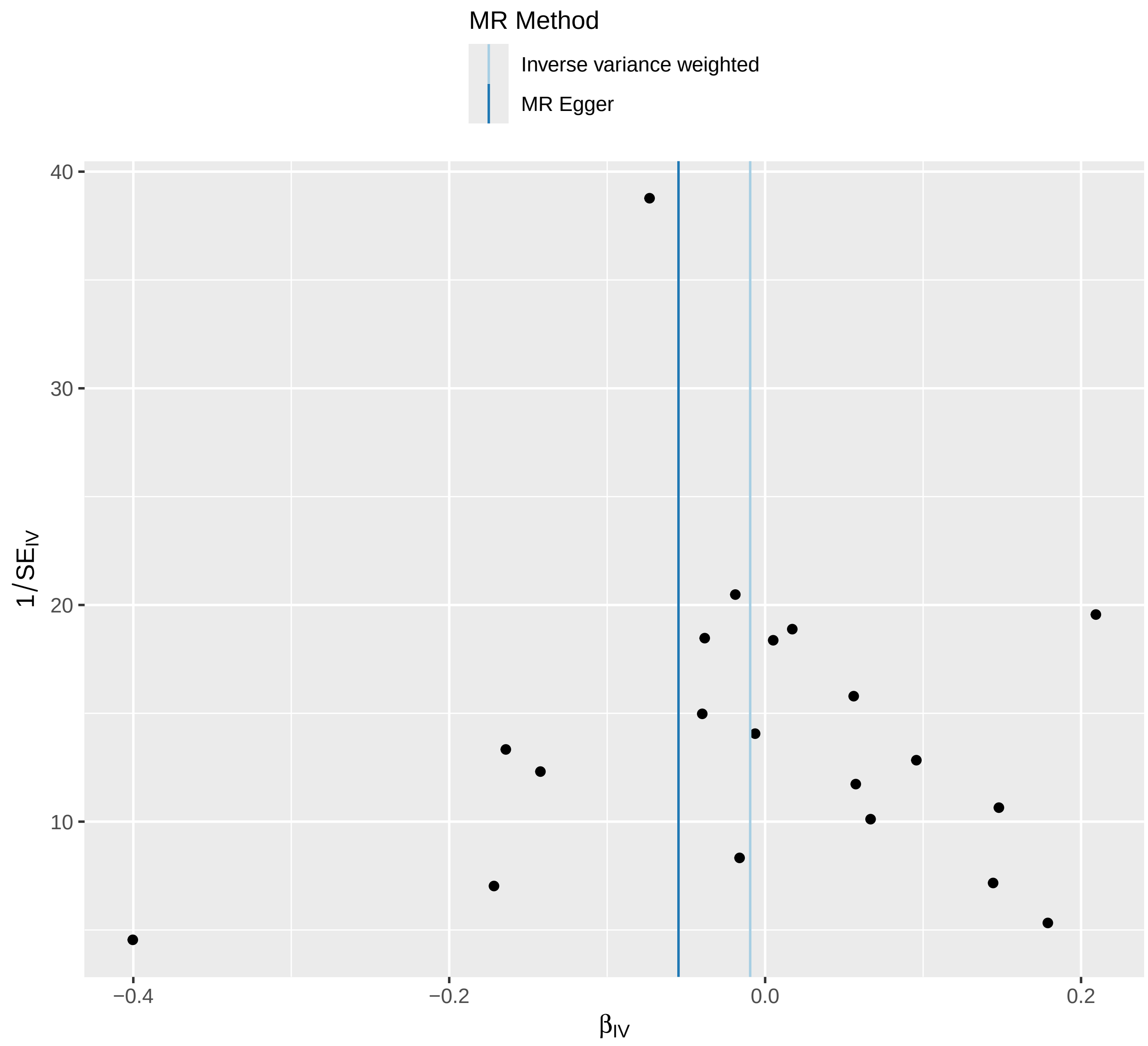
MR Method
Inverse variance weighted
MR Egger
40
30
20
10
$1/SE_{IV}$
−0.4
−0.2
0.0
0.2
β_{IV}

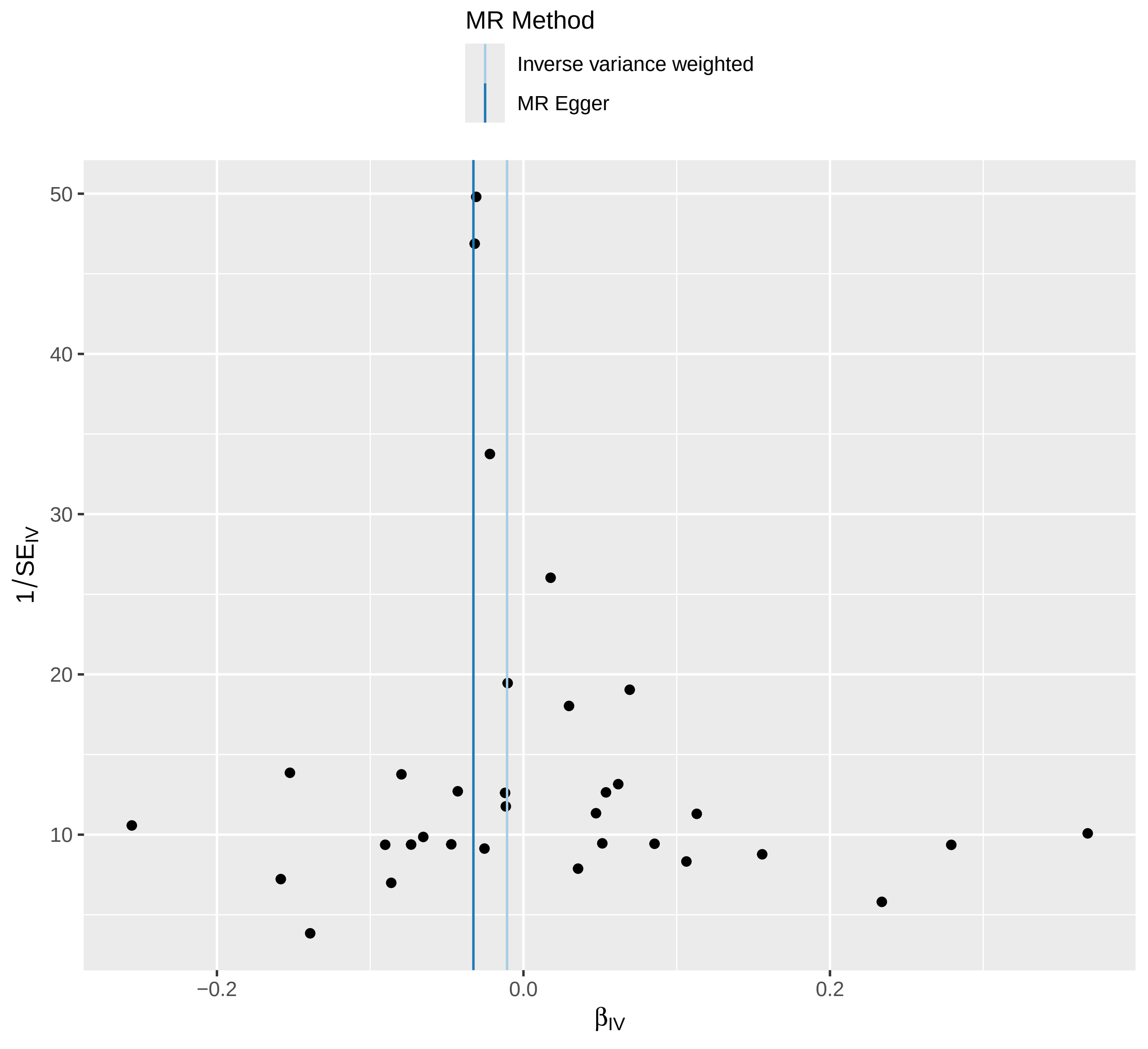
MR Method
Inverse variance weighted
MR Egger
50
40
30
20
10
$1/SE_{IV}$
−0.2
0.0
0.2
β_{IV}

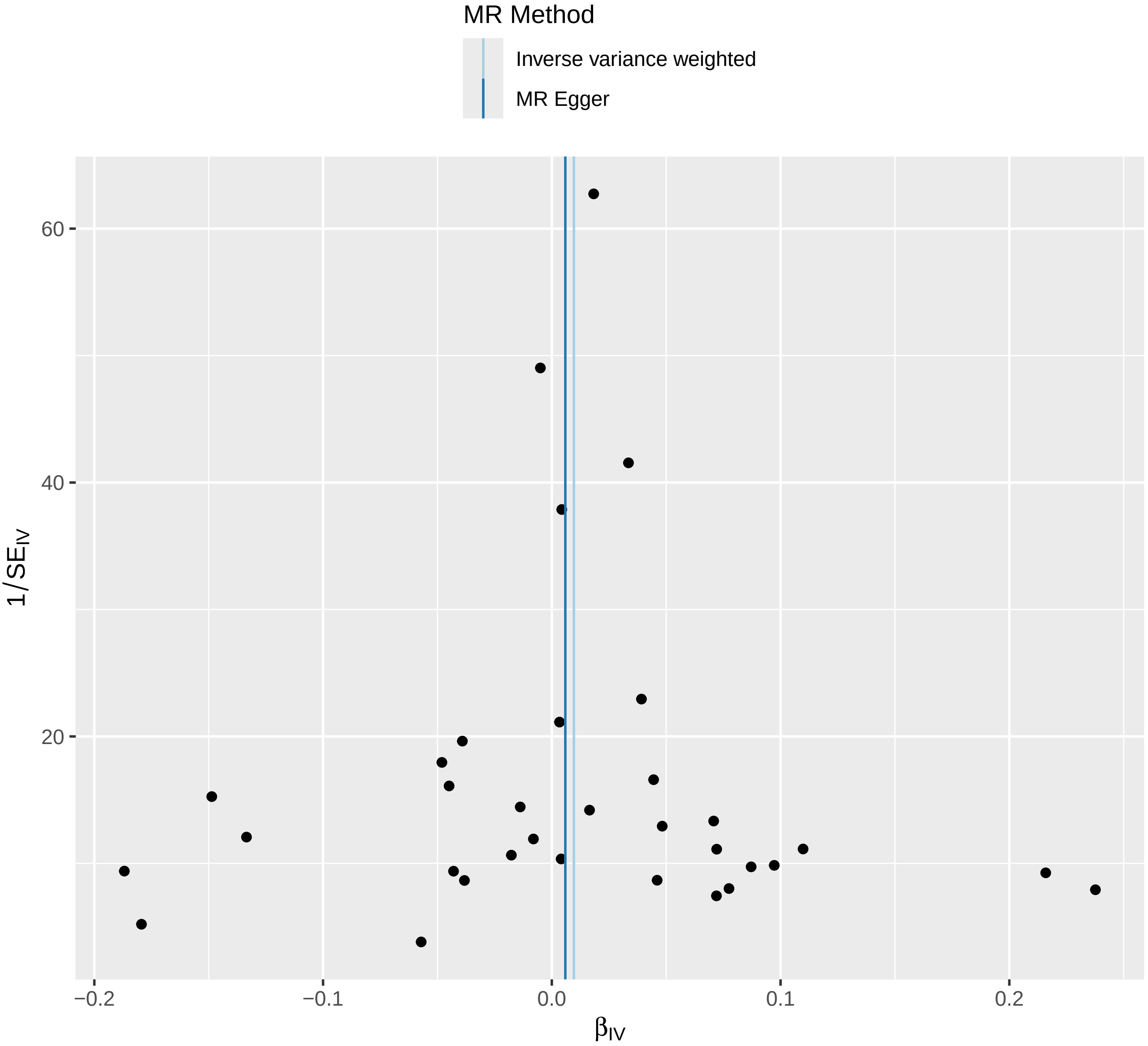
MR Method
Inverse variance weighted
MR Egger
60
40
20
1/SE$_{IV}$
−0.2
−0.1
0.0
0.1
0.2
β$_{IV}$

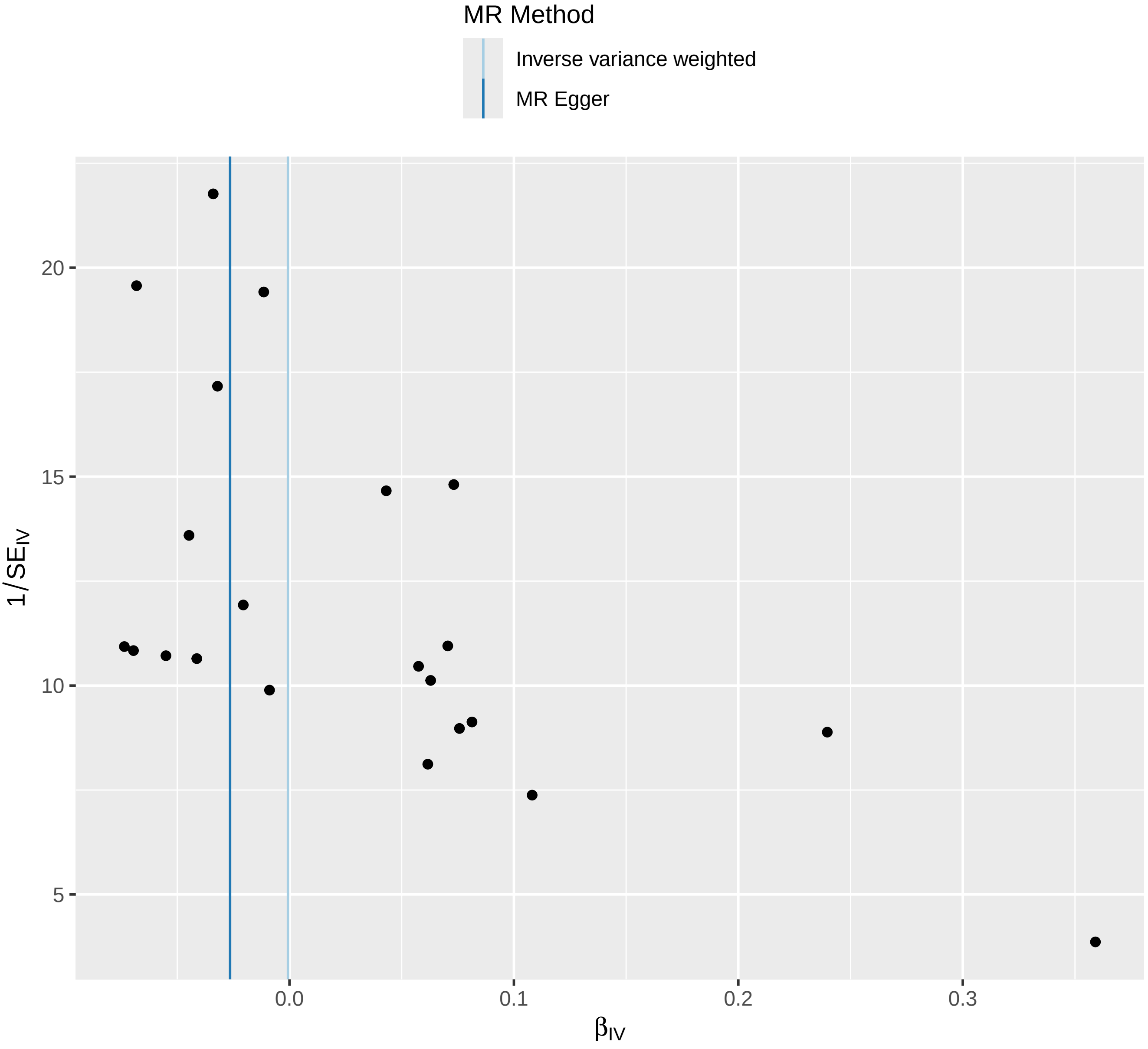

MR Method
Inverse variance weighted
MR Egger
20
15
10
5
$1/SE_{IV}$
0.0
0.1
0.2
0.3
β_{IV}

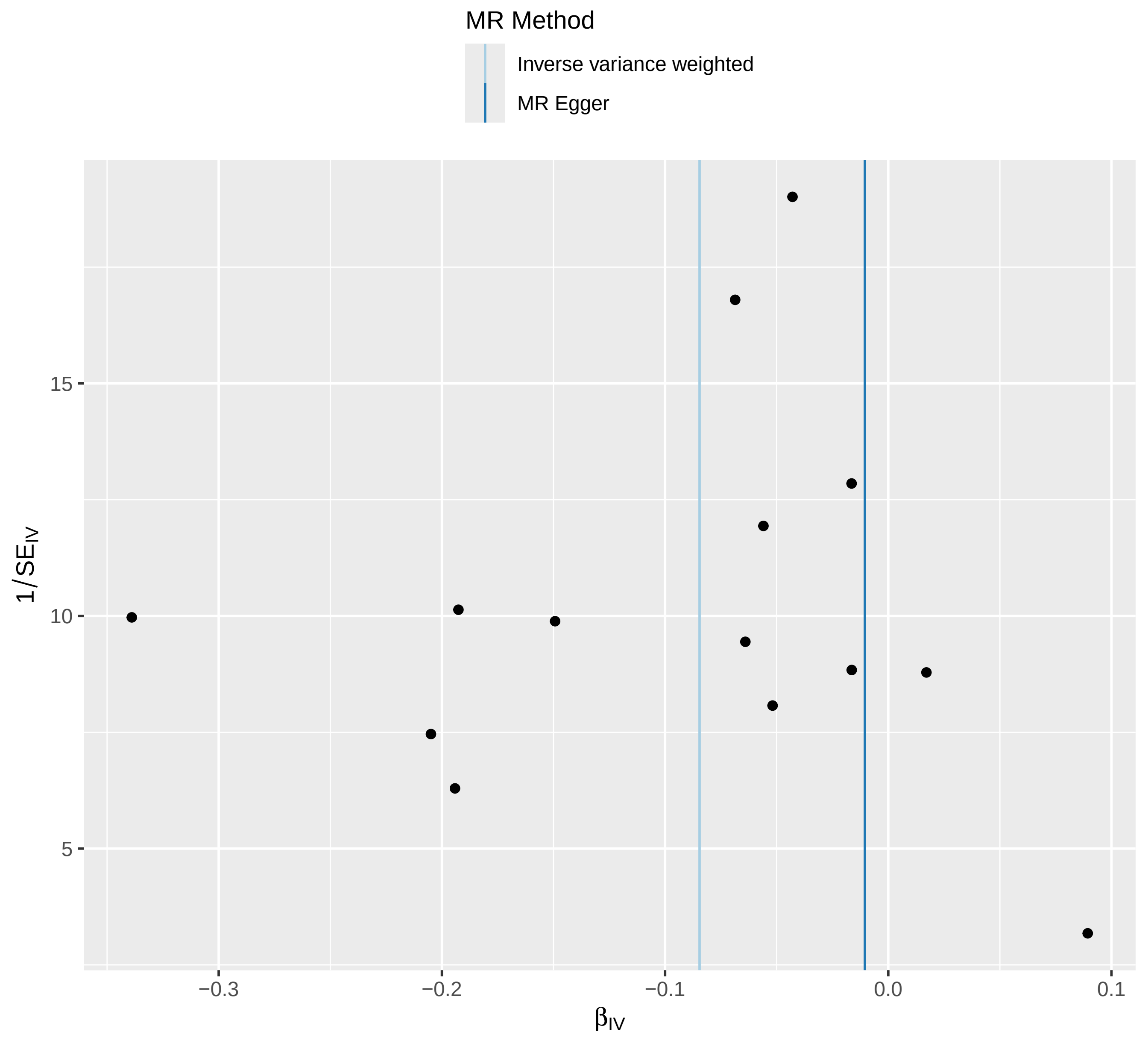
MR Method
Inverse variance weighted
MR Egger
15
10
5
1/SE_IV
−0.3
−0.2
−0.1
0.0
0.1
β_IV

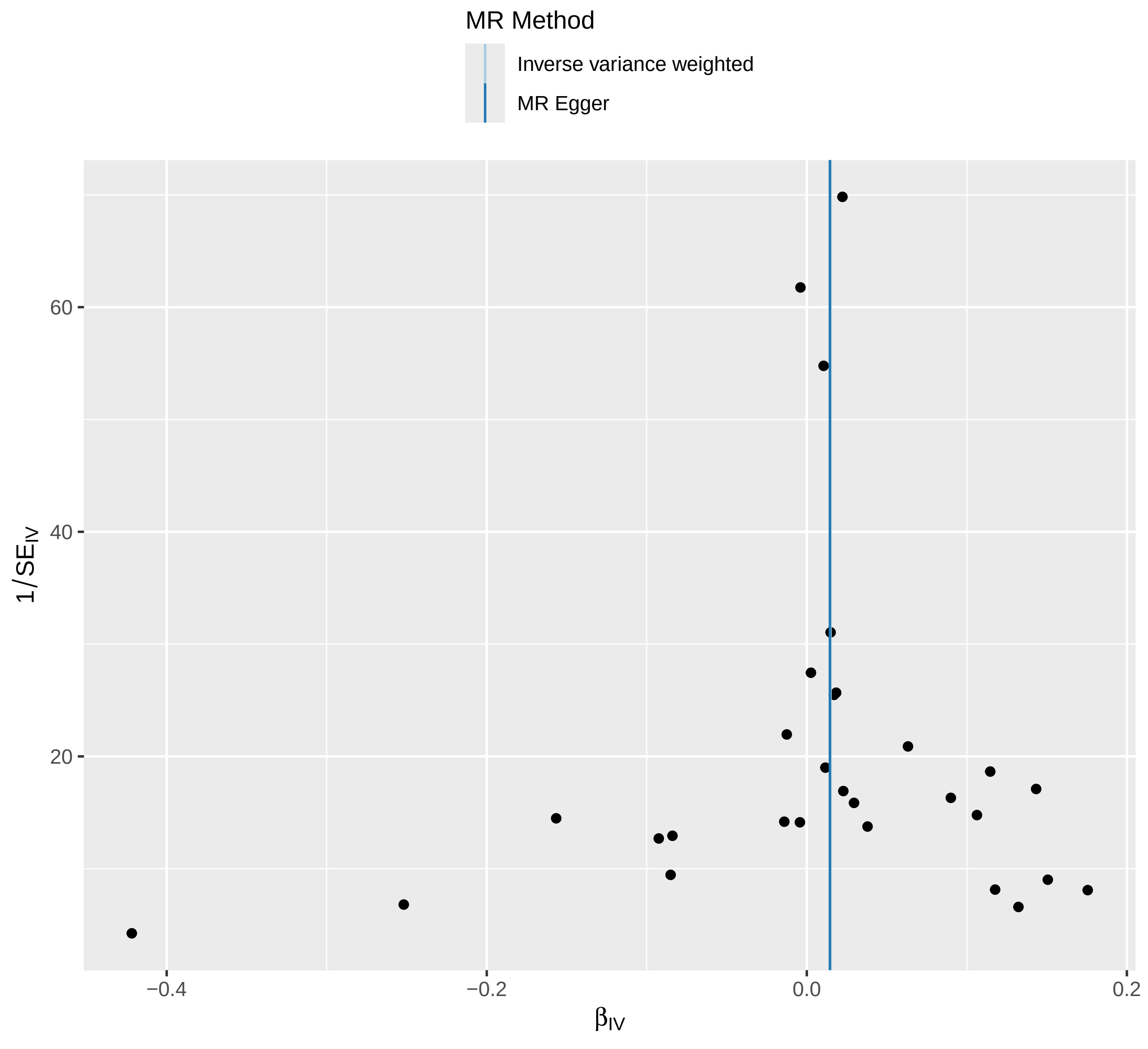
MR Method
Inverse variance weighted
MR Egger
60
40
20
1/SE$_{IV}$
−0.4
−0.2
0.0
0.2
β$_{IV}$

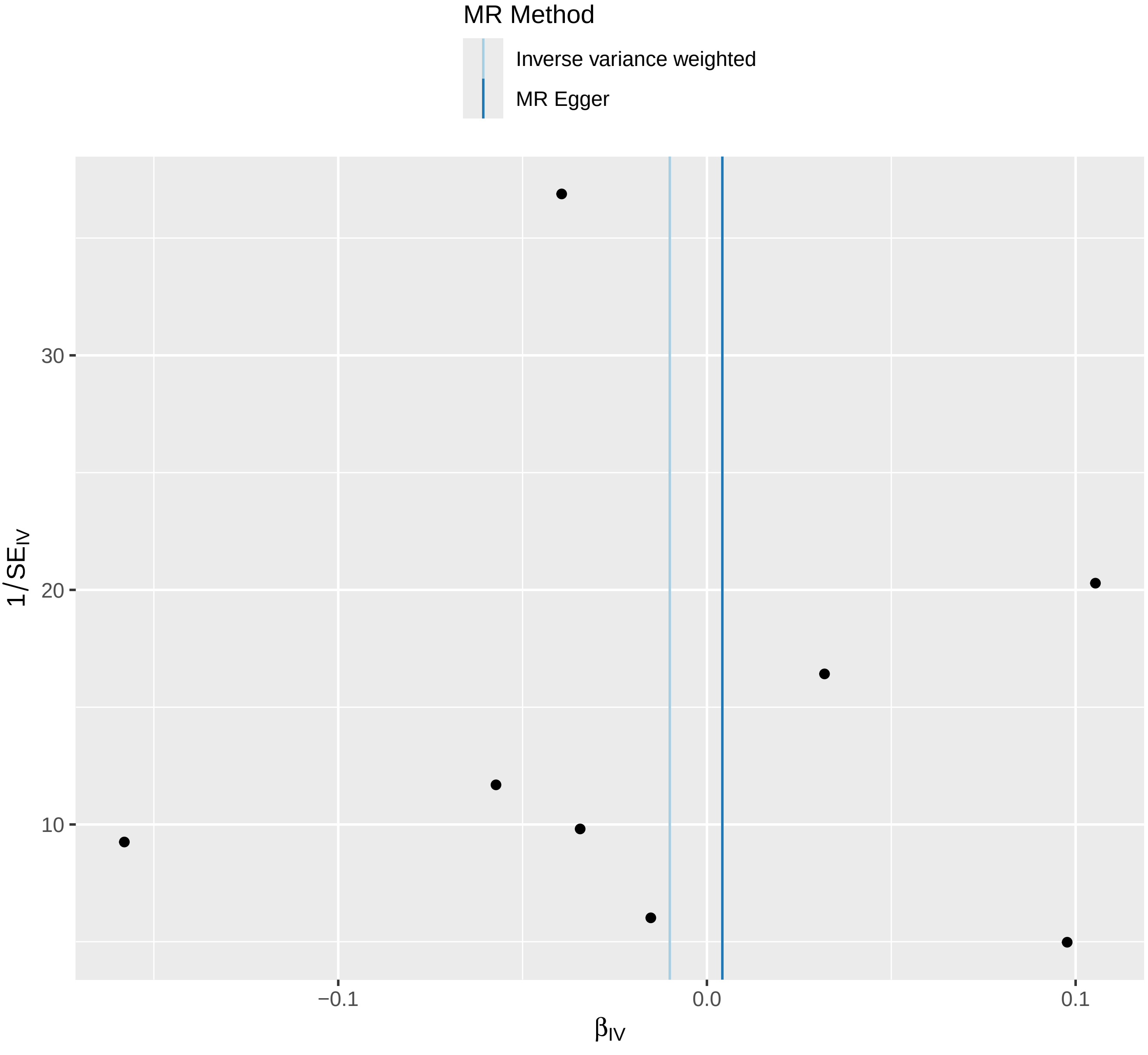

MR Method
Inverse variance weighted
MR Egger
30
20
10
1/SE_IV
−0.1
0.0
0.1
β_IV

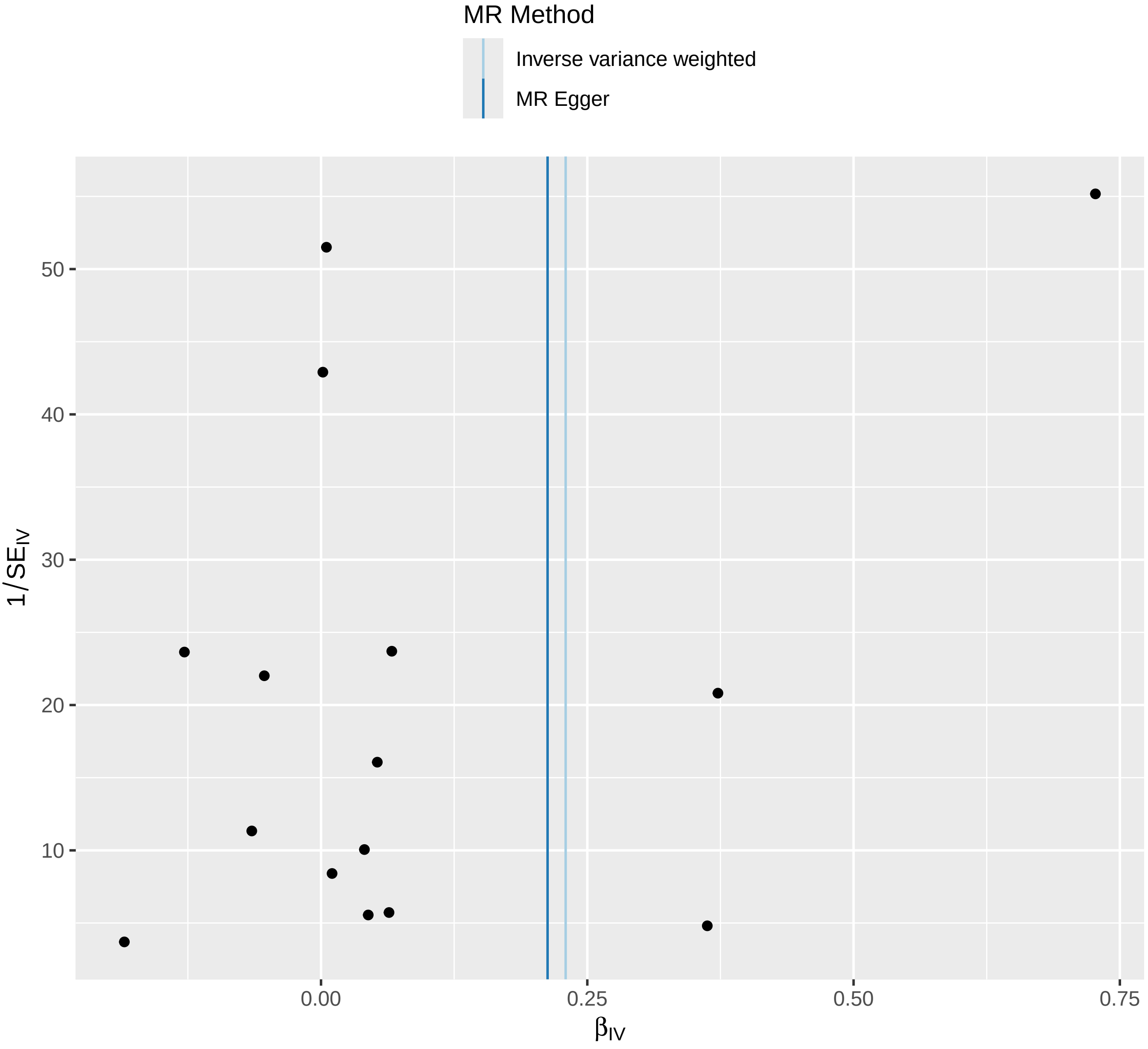
MR Method
Inverse variance weighted
MR Egger
50
40
30
20
10
1/SE_IV
0.00
0.25
0.50
0.75
β_IV

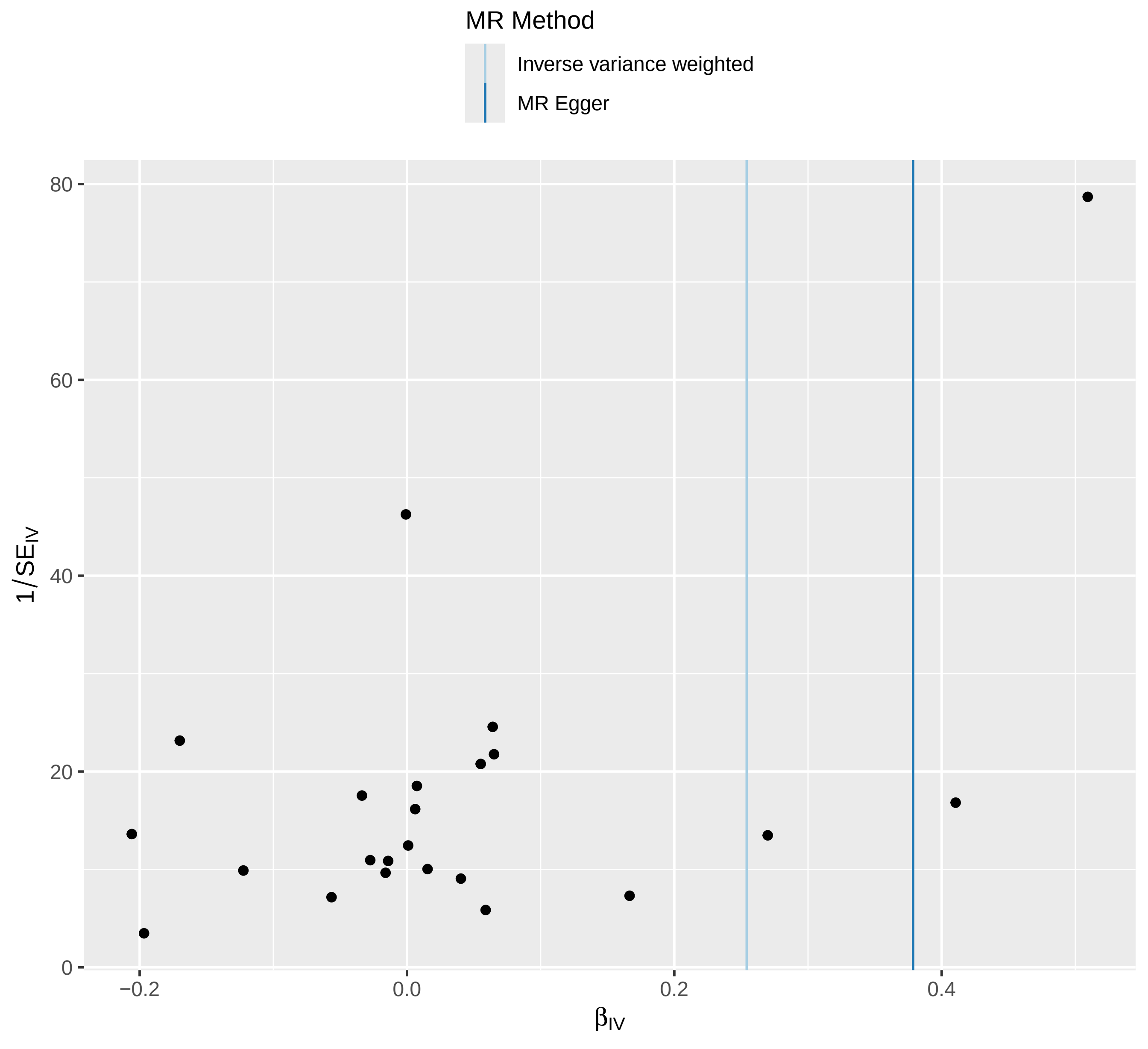
MR Method
Inverse variance weighted
MR Egger
80
60
40
20
0
1/SE_IV
−0.2
0.0
0.2
0.4
β_IV

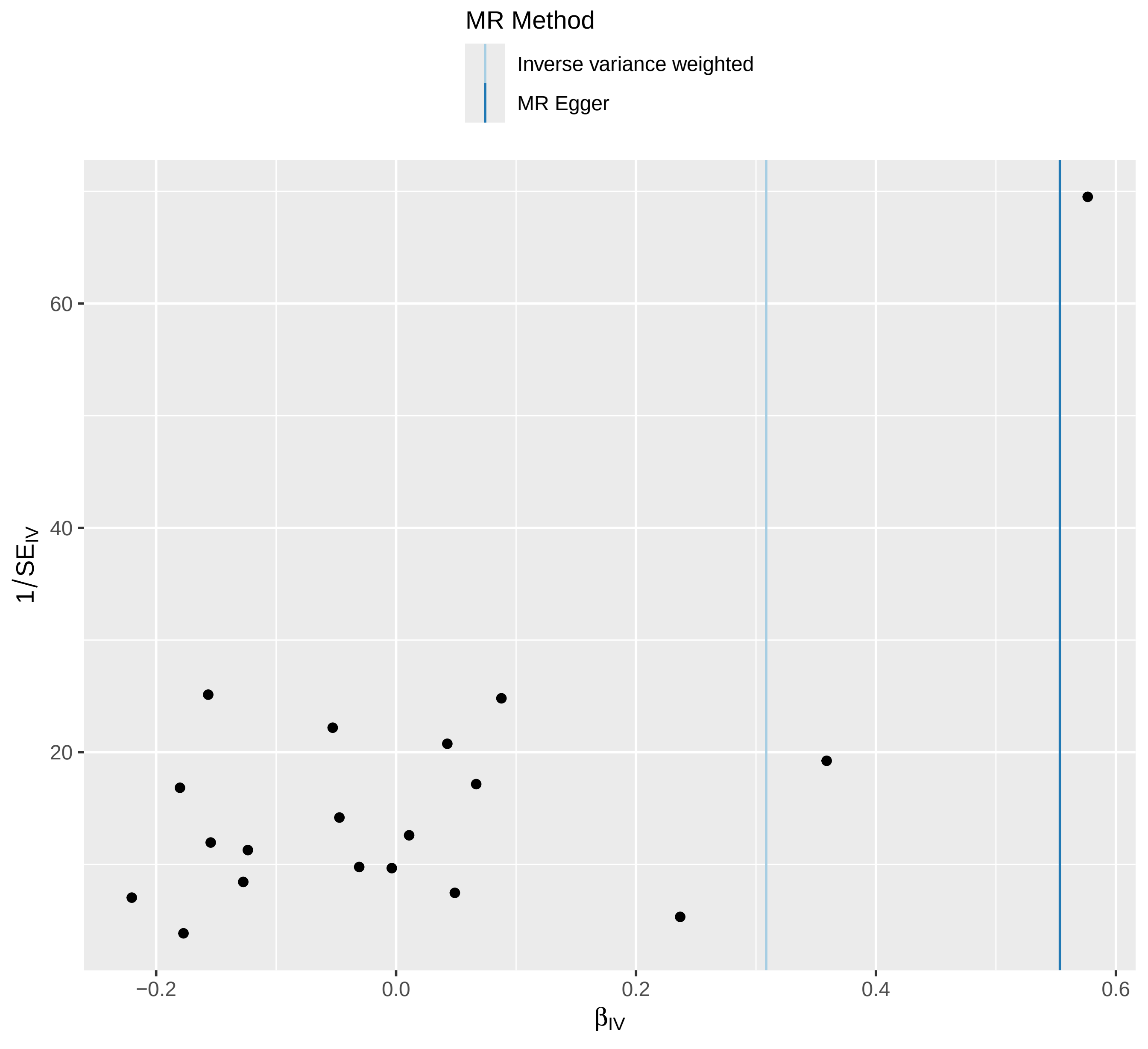

MR Method
Inverse variance weighted
MR Egger
60
40
20
1/SE$_{IV}$
−0.2
0.0
0.2
0.4
0.6
β$_{IV}$

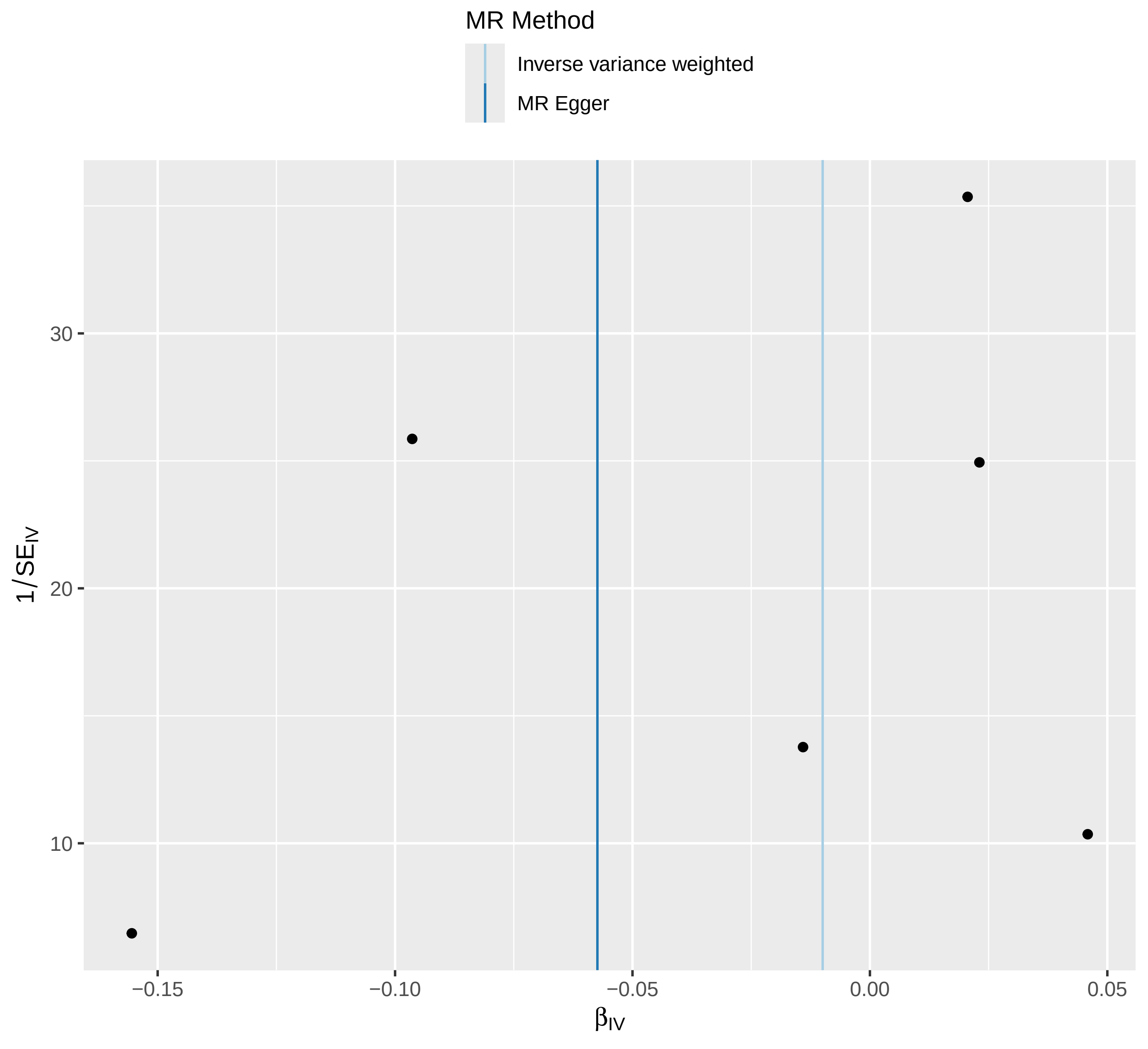
MR Method
Inverse variance weighted
MR Egger
30
20
10
1/SE_IV
−0.15
−0.10
−0.05
0.00
0.05
β_IV

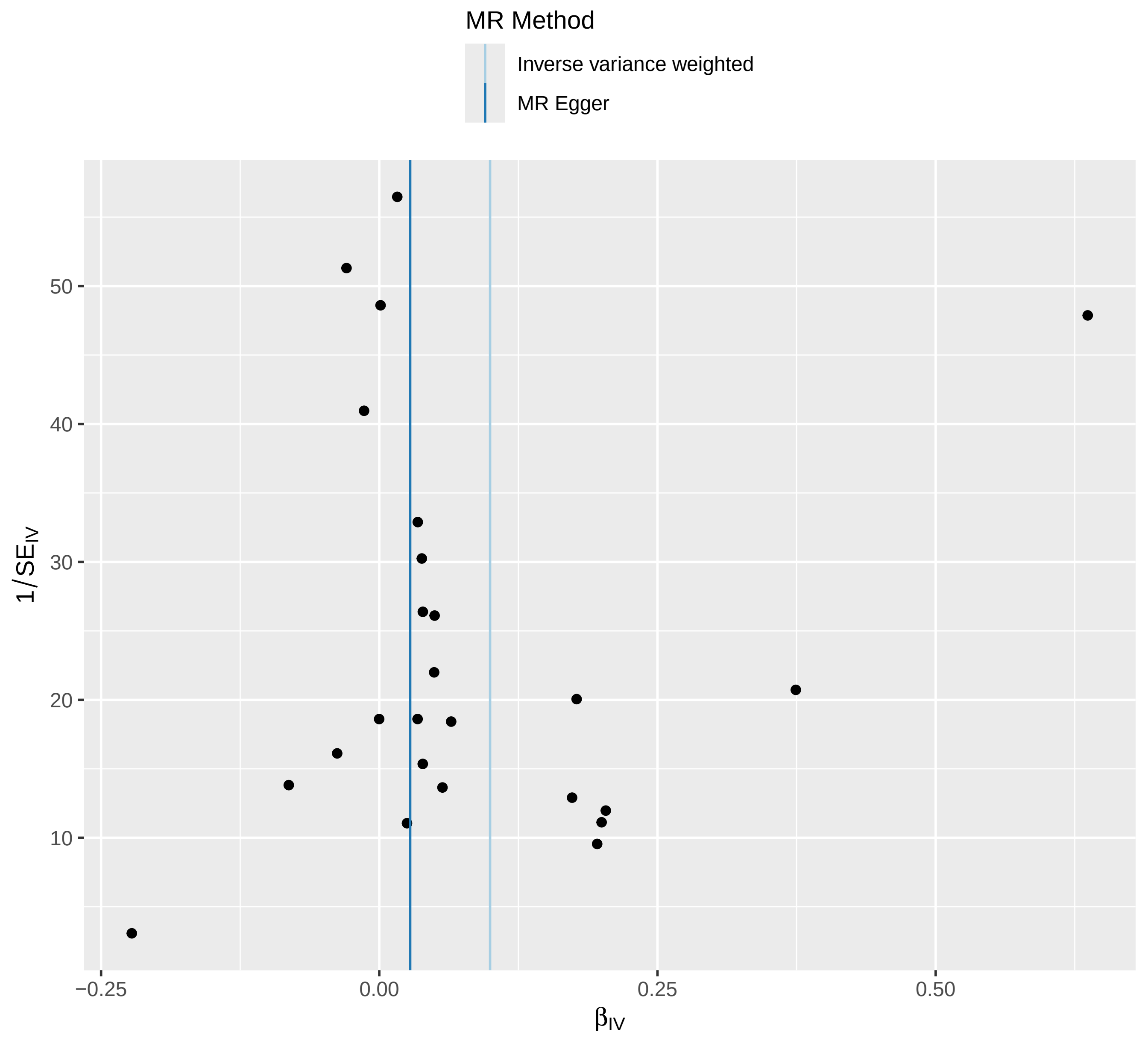
MR Method
Inverse variance weighted
MR Egger
1/SE_IV
50
40
30
20
10
−0.25
0.00
0.25
0.50
β_IV

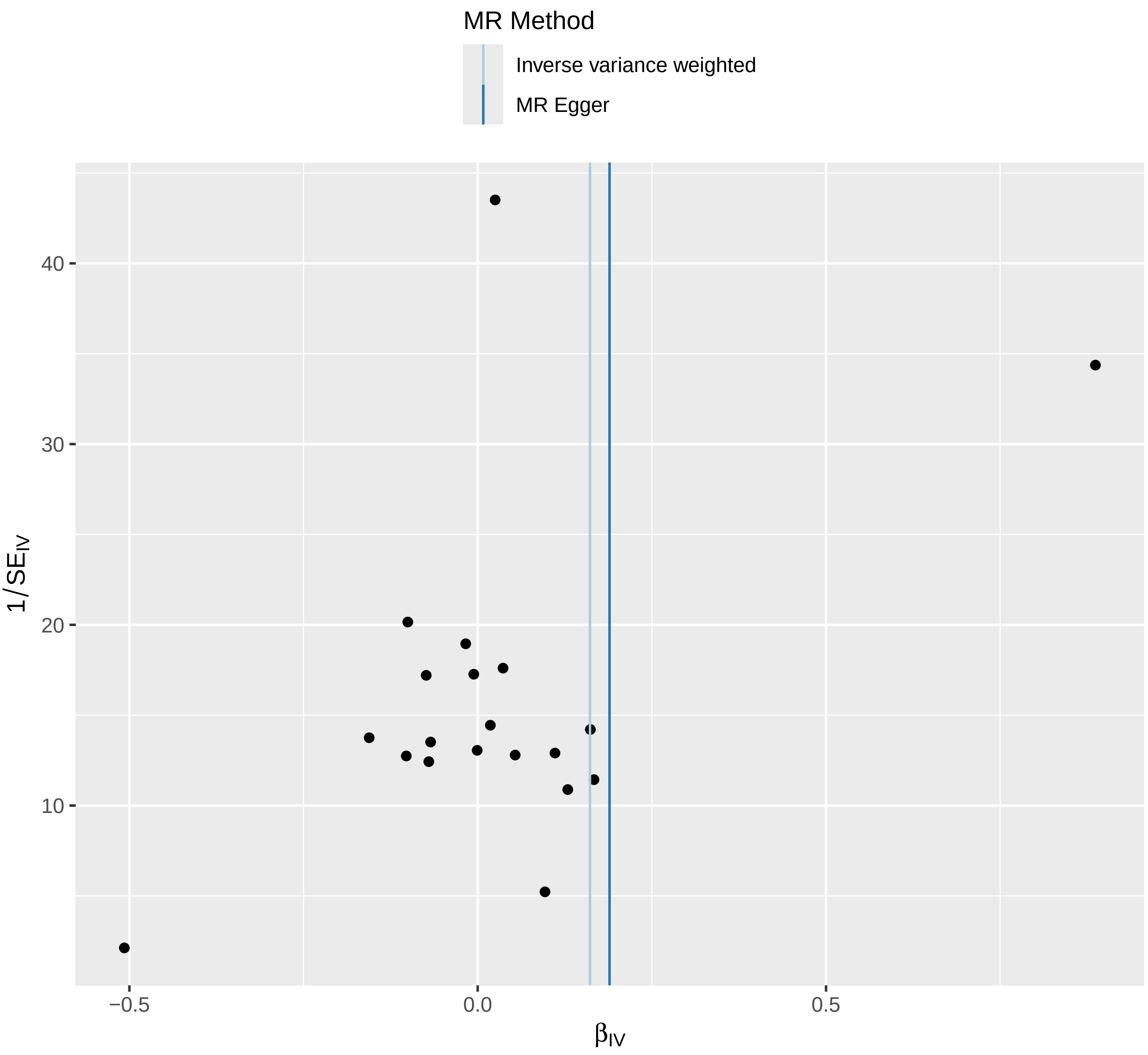
MR Method
Inverse variance weighted
MR Egger
40
30
20
10
−0.5
0.0
0.5
$1/SE_{IV}$
β_{IV}

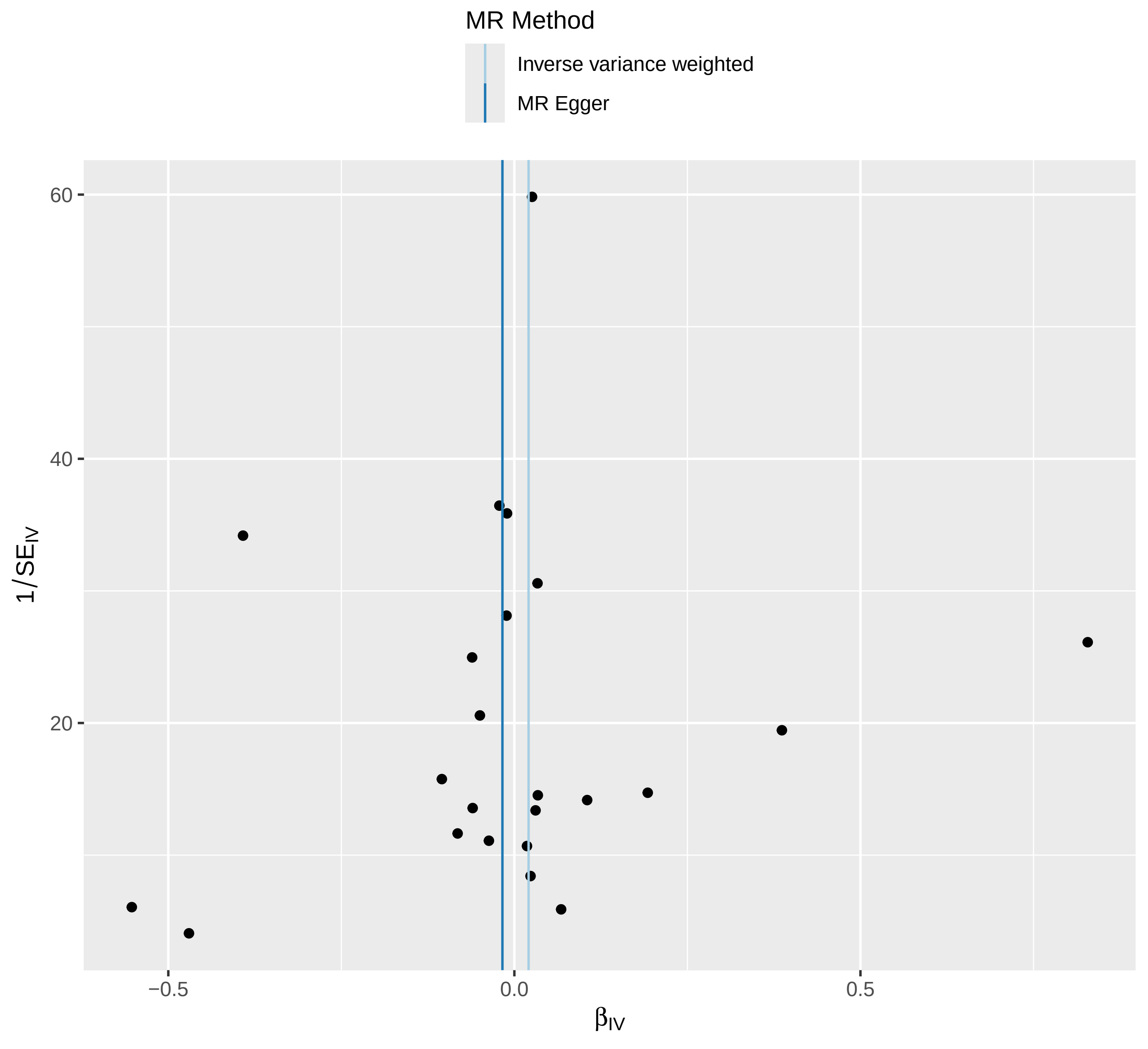
MR Method
Inverse variance weighted
MR Egger
60
40
20
1/SE_IV
−0.5
0.0
0.5
β_IV

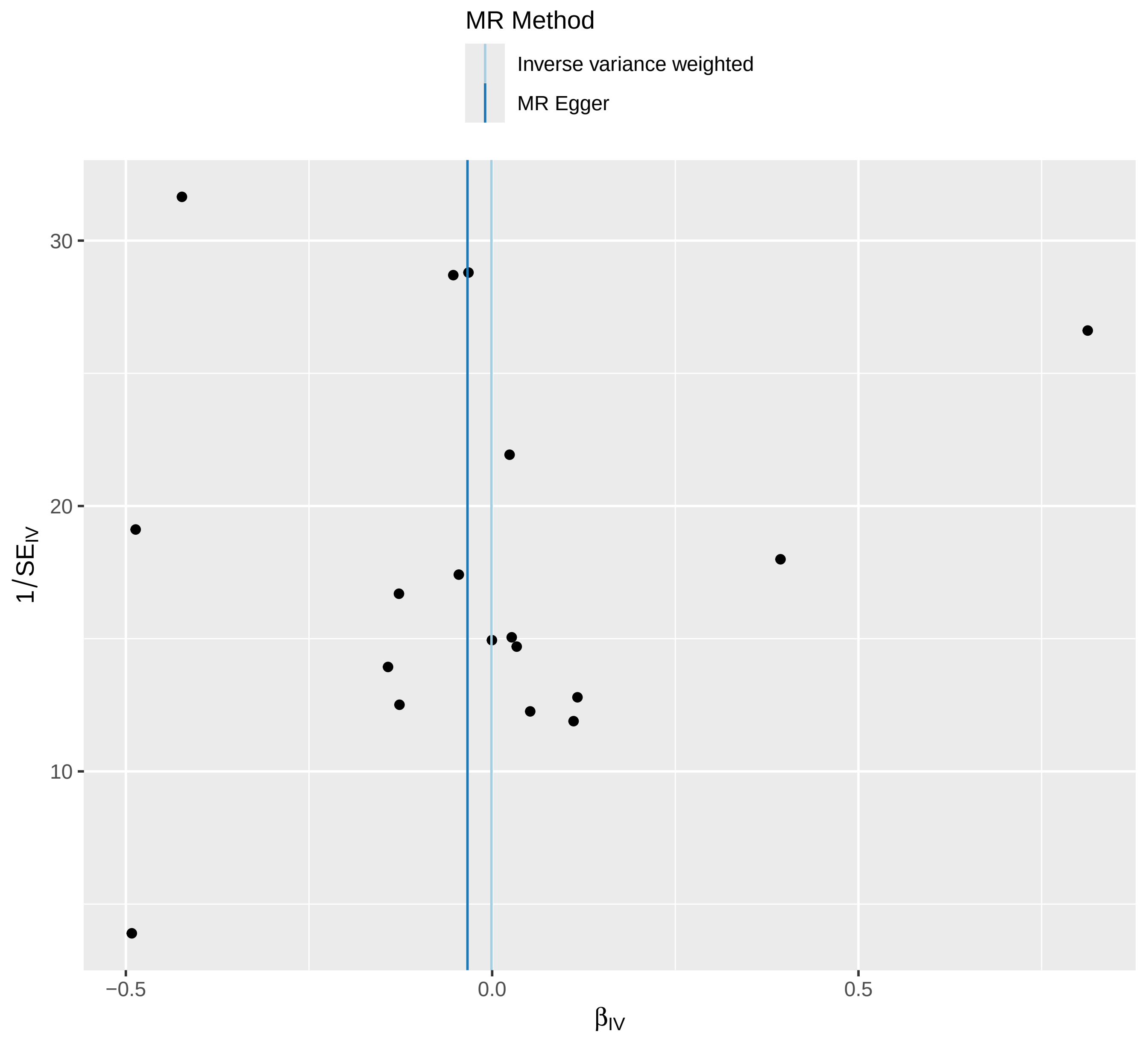
MR Method
Inverse variance weighted
MR Egger
30
20
10
1/SE$_{IV}$
−0.5
0.0
0.5
β$_{IV}$

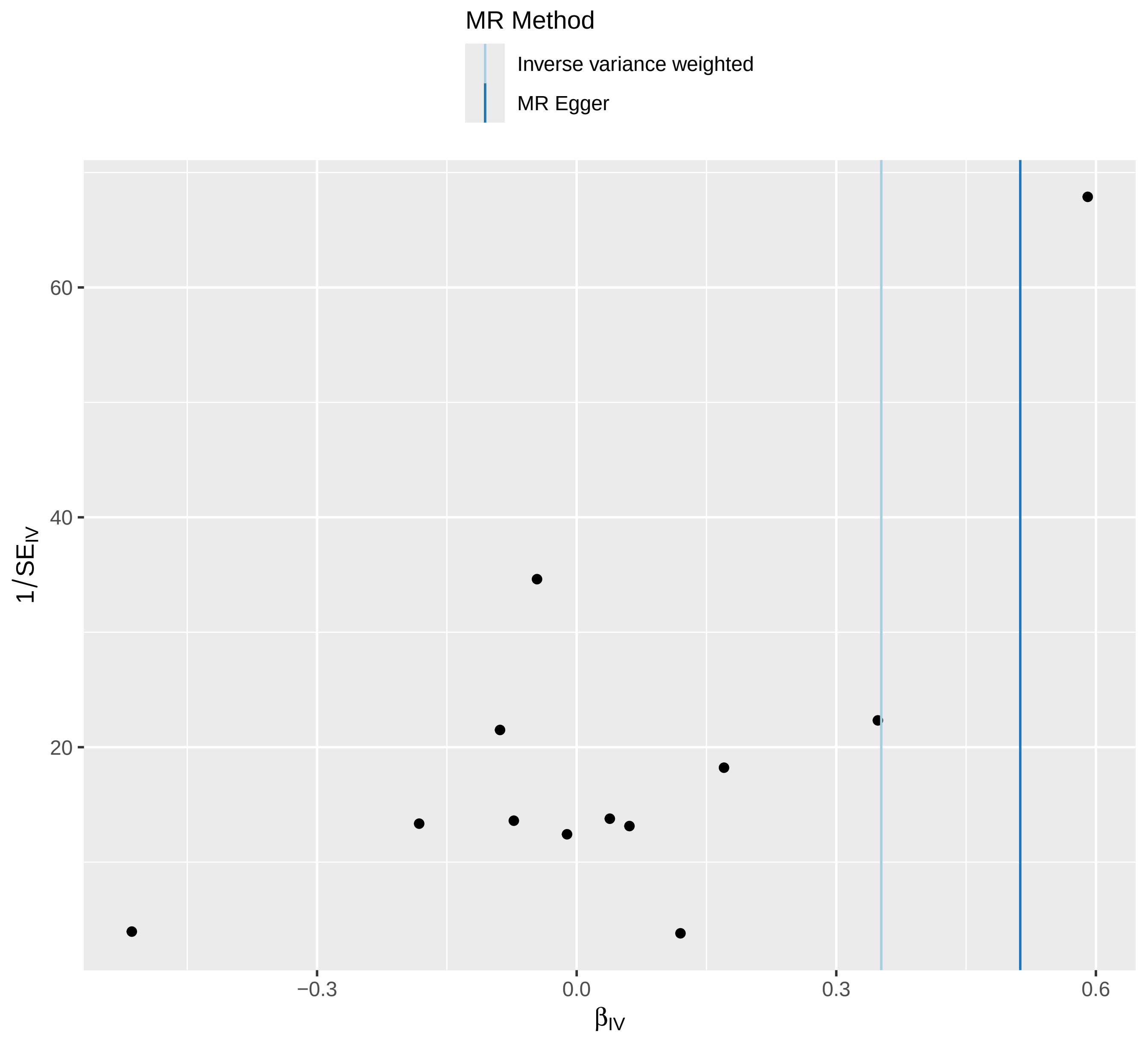
MR Method
Inverse variance weighted
MR Egger
60
40
20
1/SE$_{IV}$
−0.3
0.0
0.3
0.6
β$_{IV}$

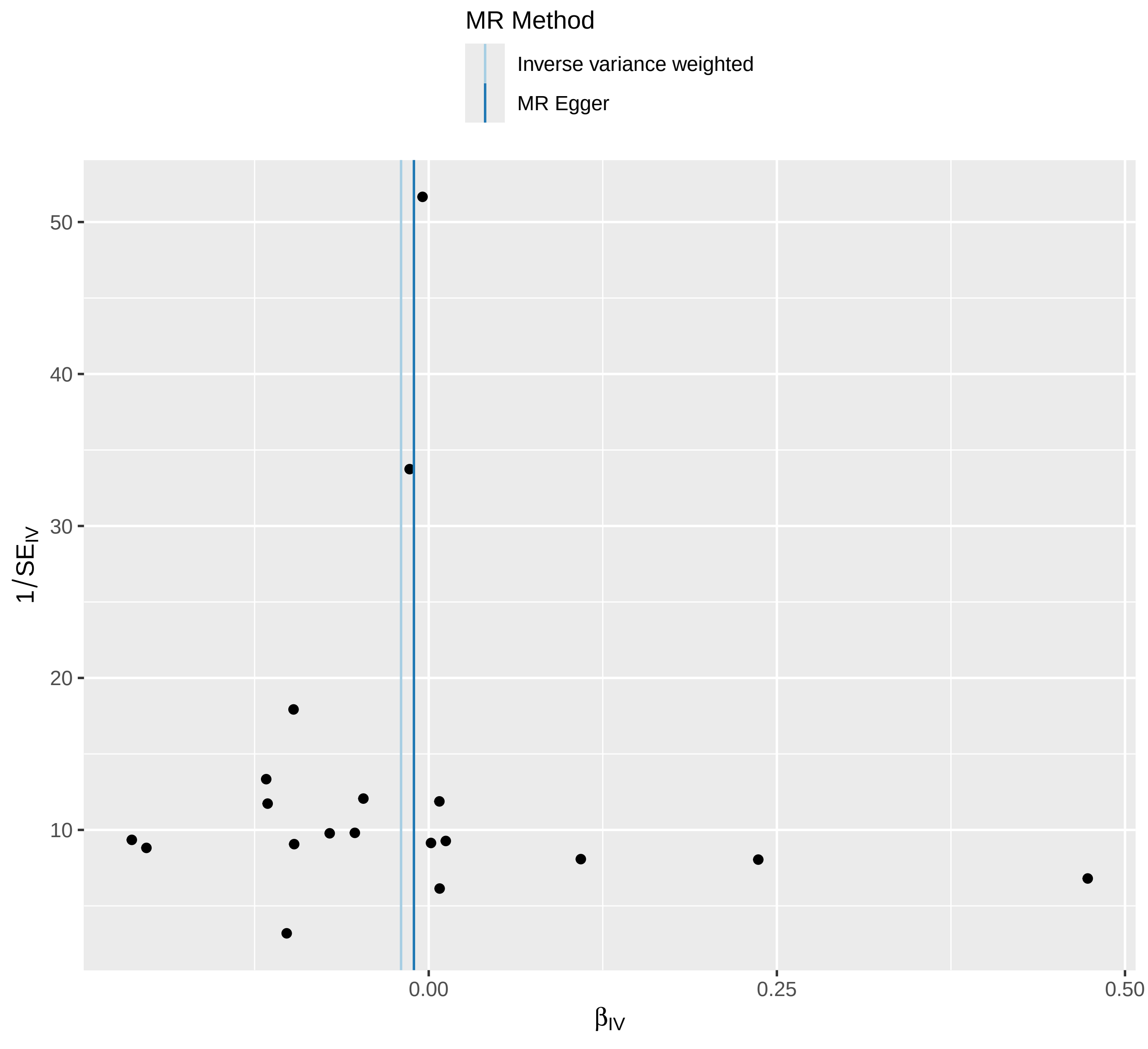
MR Method
Inverse variance weighted
MR Egger
1/SE_IV
50
40
30
20
10
0.00
0.25
0.50
β_IV

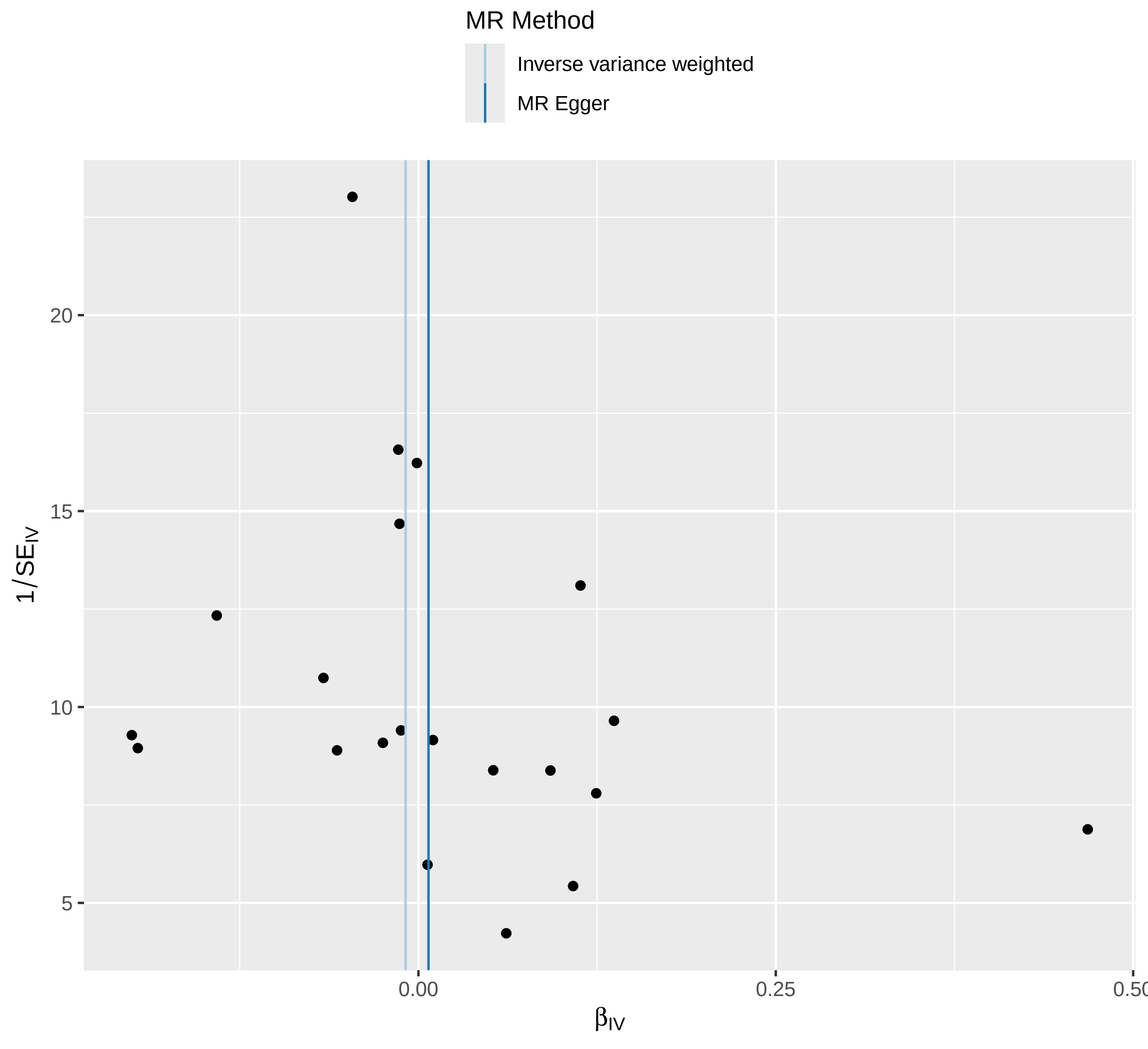
MR Method
Inverse variance weighted
MR Egger
1/SE_IV
20
15
10
5
0.00
0.25
0.50
β_IV

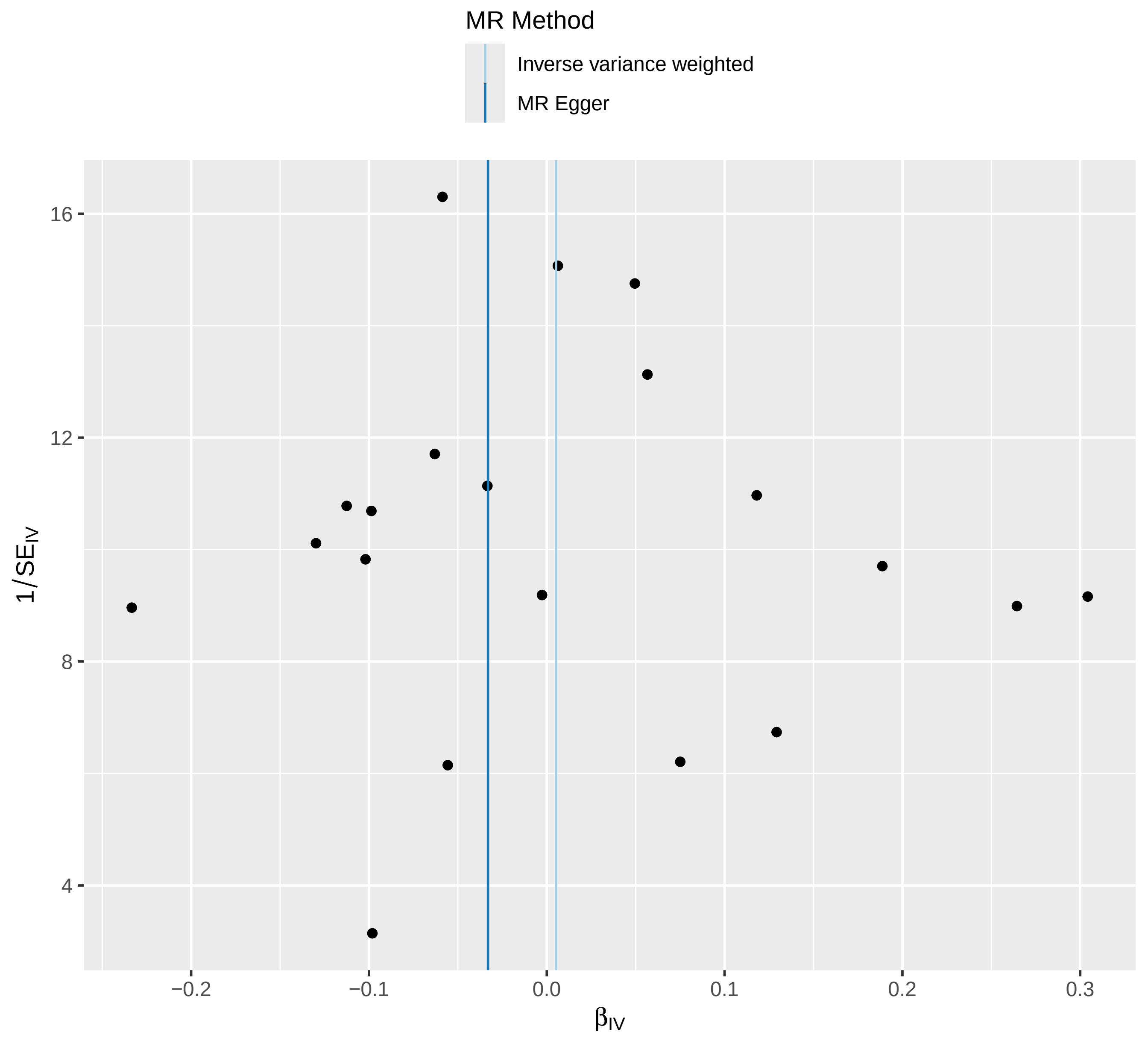
MR Method
Inverse variance weighted
MR Egger
16
12
8
4
1/SE$_{IV}$
−0.2
−0.1
0.0
0.1
0.2
0.3
β$_{IV}$

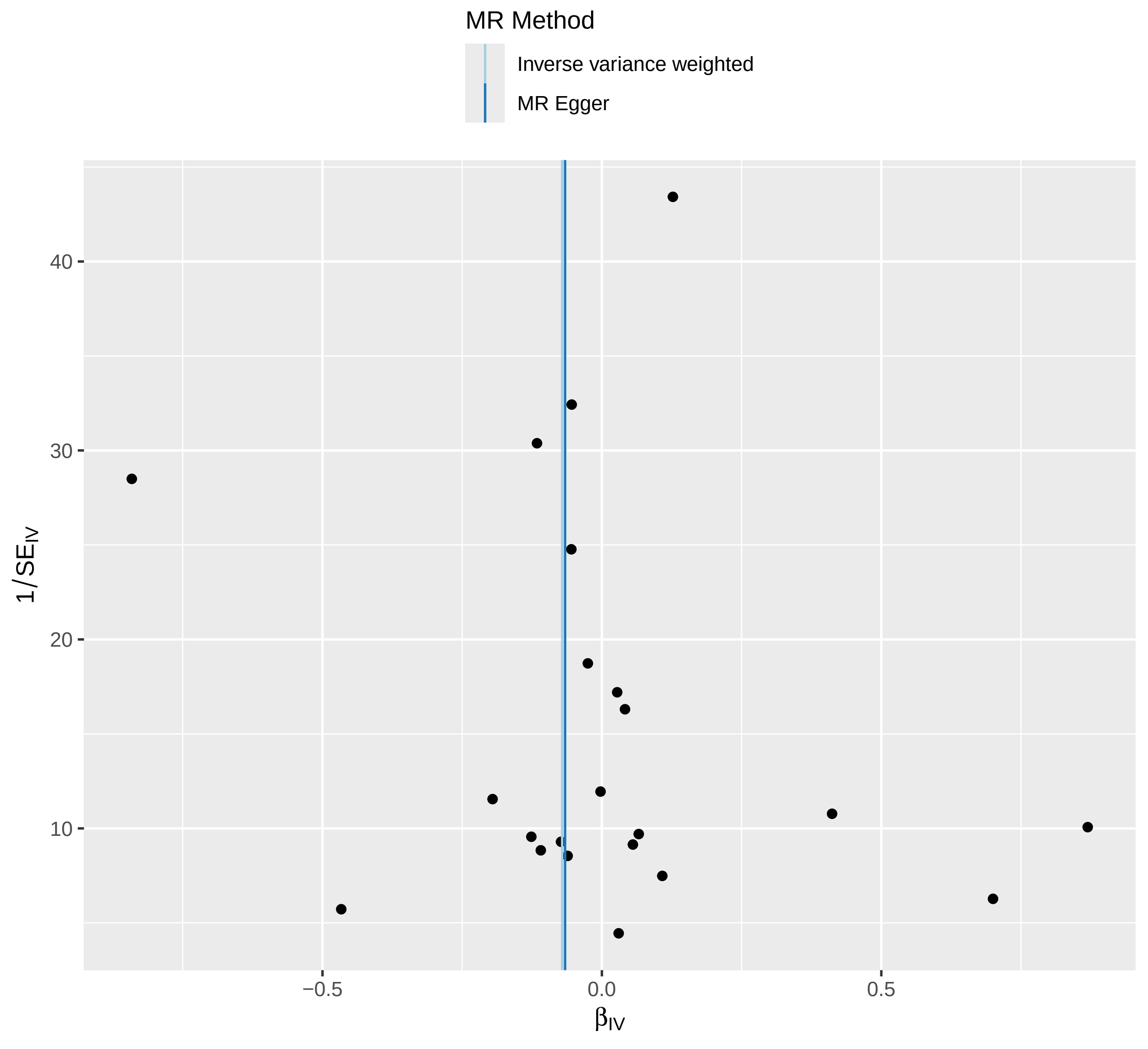
MR Method
Inverse variance weighted
MR Egger
40
30
20
10
1/SE_IV
−0.5
0.0
0.5
β_IV

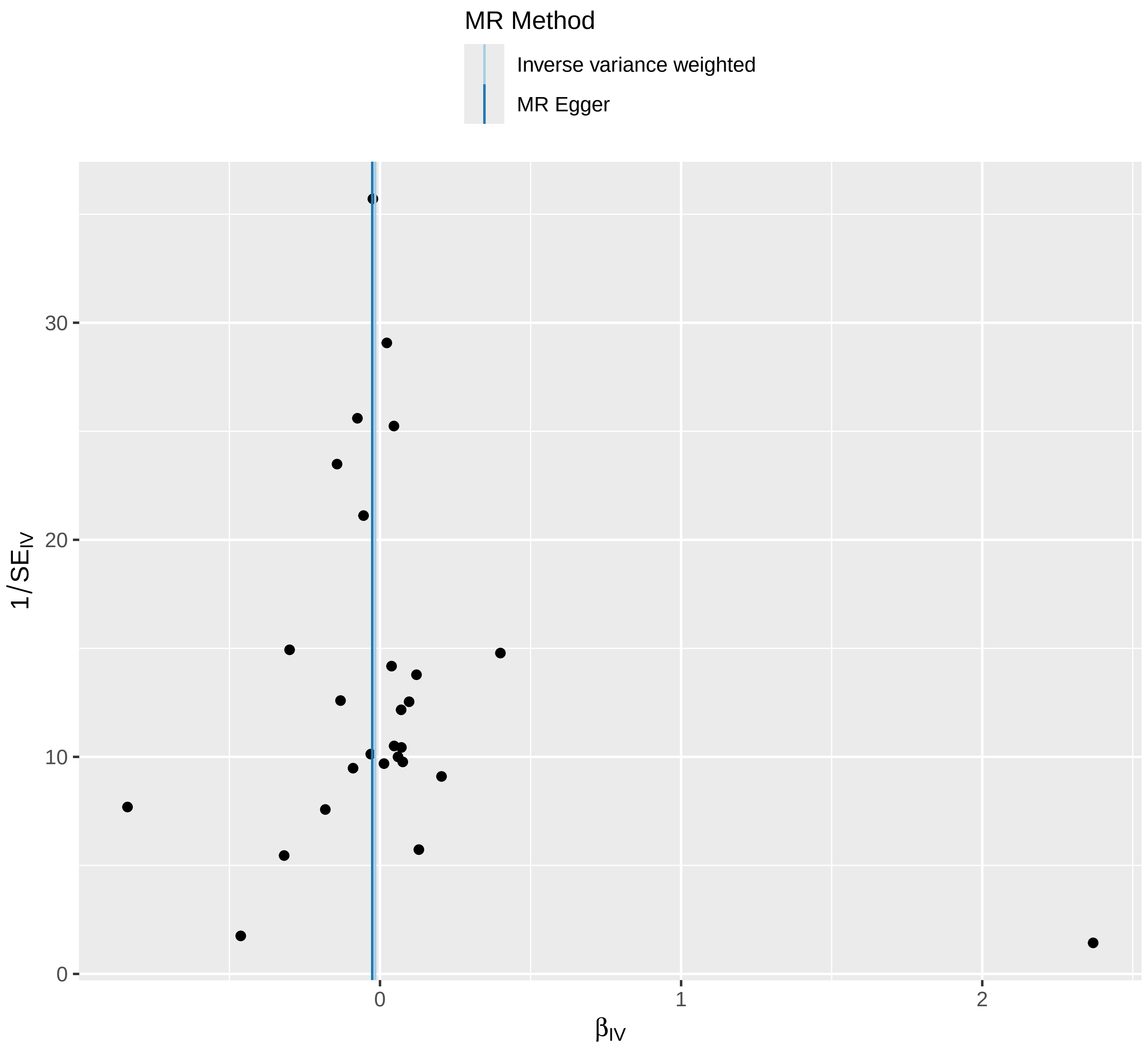

MR Method
Inverse variance weighted
MR Egger
30
20
10
0
$1/SE_{IV}$
0
1
2
β_{IV}

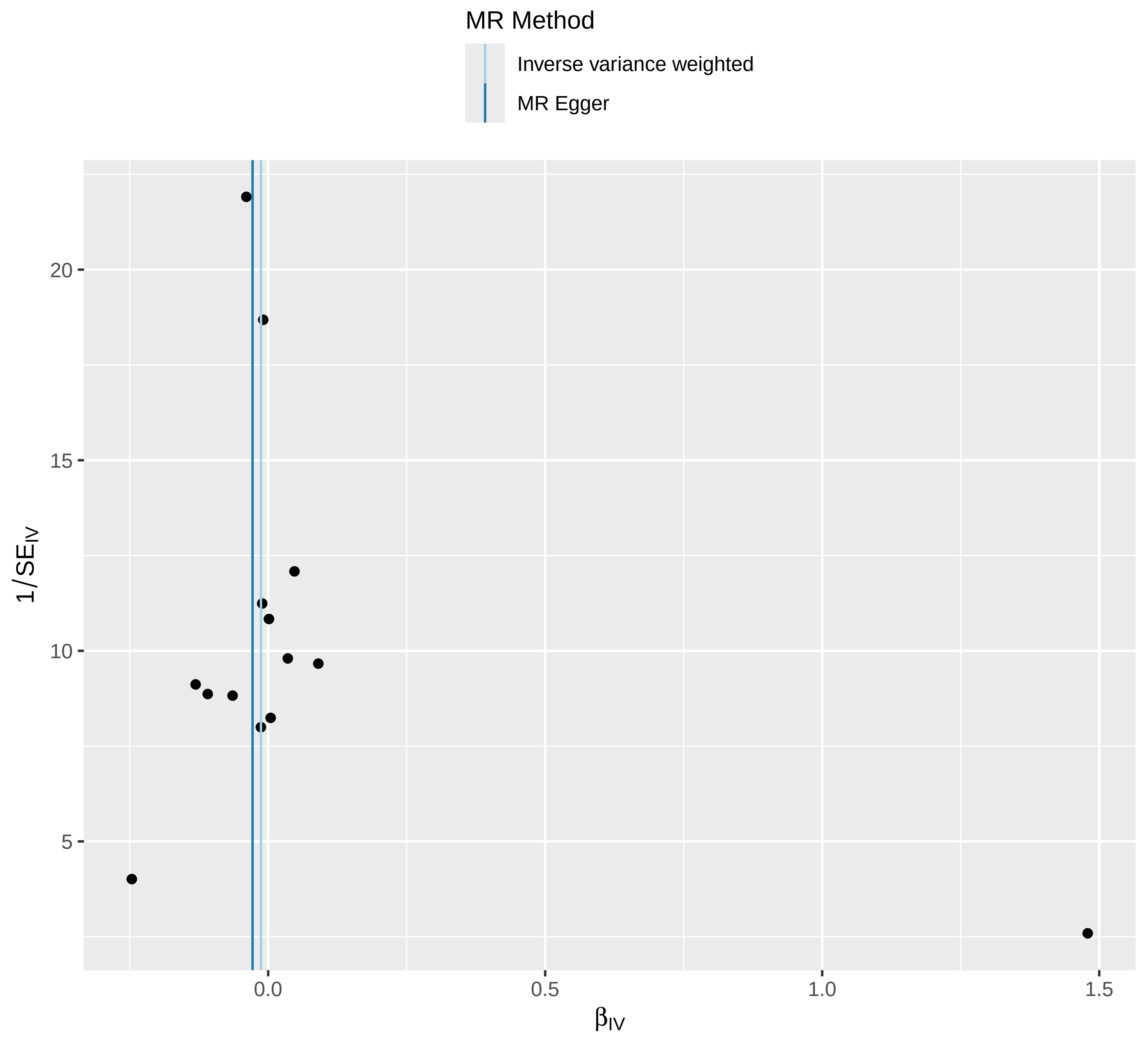

MR Method
Inverse variance weighted
MR Egger
1/SE_IV
20
15
10
5
0.0
0.5
1.0
1.5
β_IV

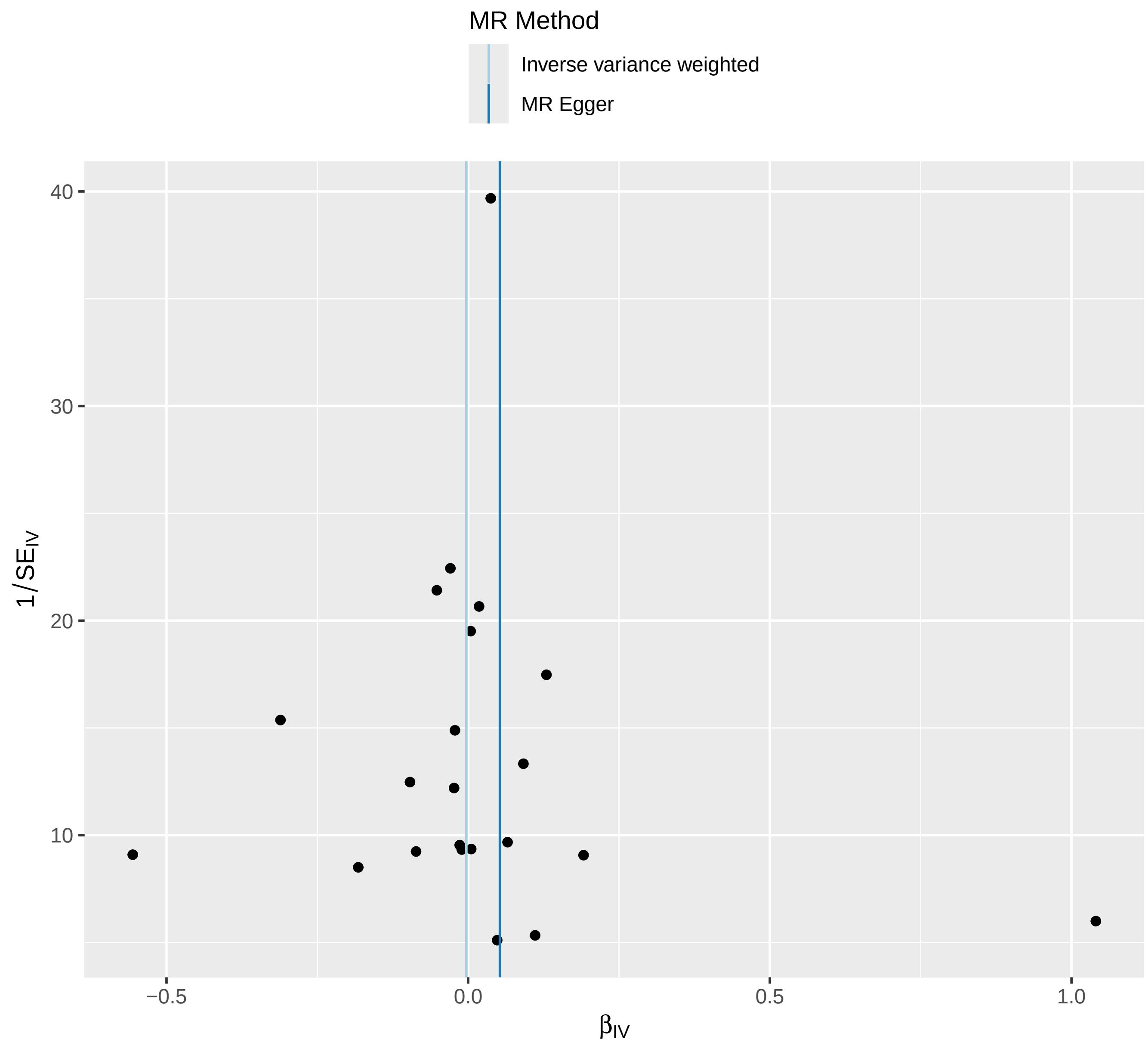
MR Method
Inverse variance weighted
MR Egger
40
30
20
10
1/SE_IV
−0.5
0.0
0.5
1.0
β_IV

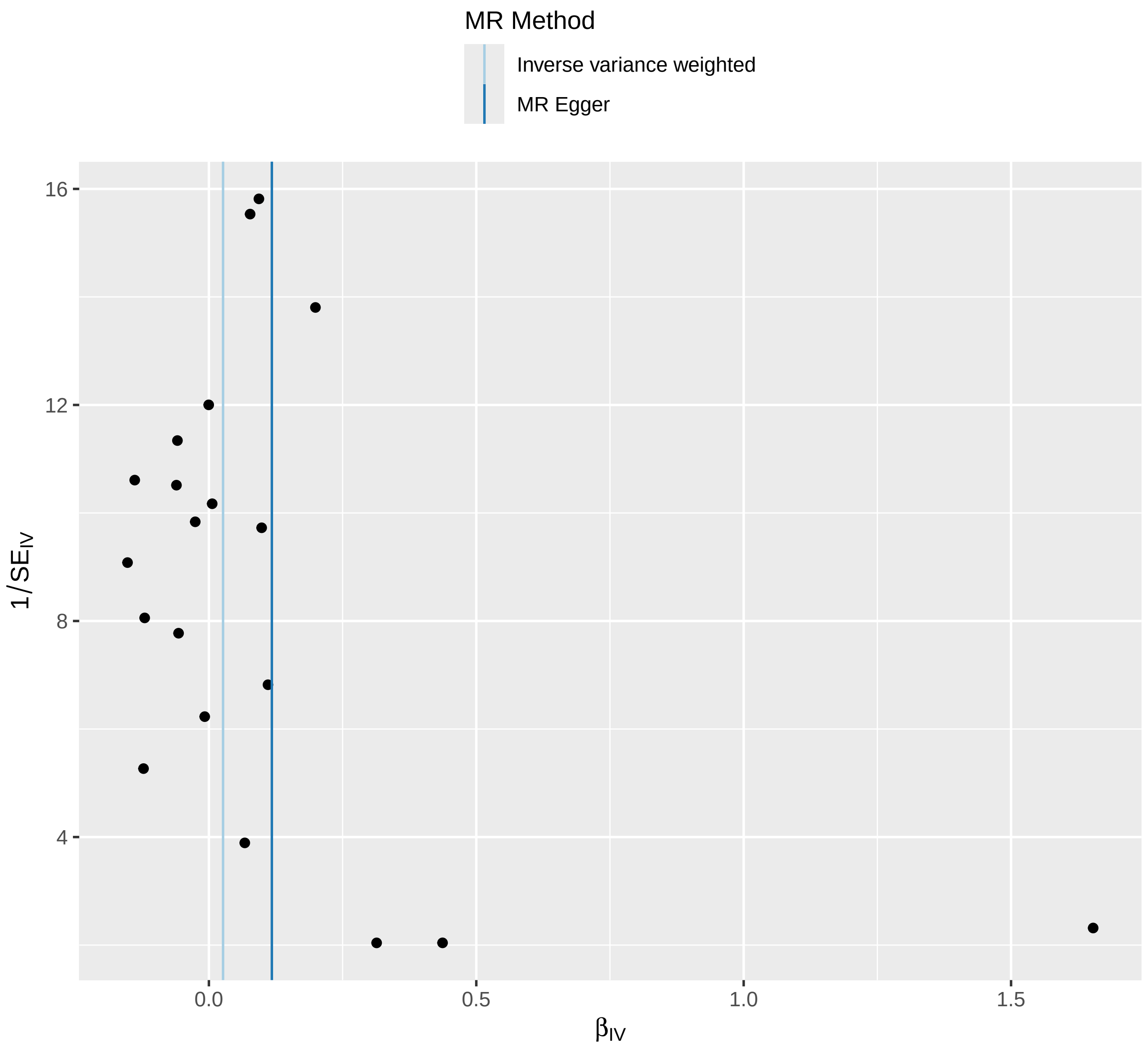
MR Method
Inverse variance weighted
MR Egger
16
12
8
4
1/SE$_{IV}$
0.0
0.5
1.0
1.5
β$_{IV}$

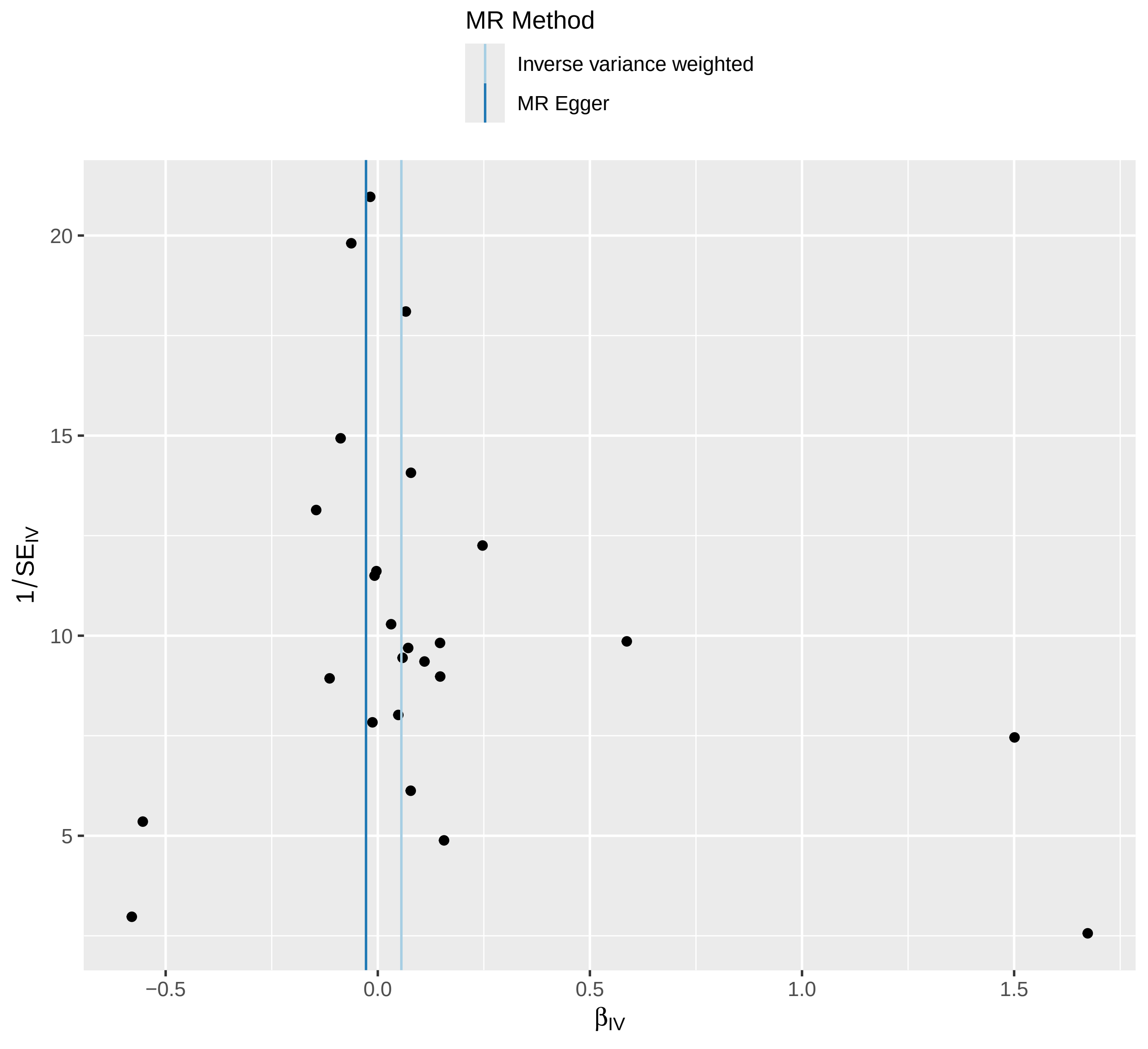
MR Method
Inverse variance weighted
MR Egger
20
15
10
5
1/SE_IV
−0.5
0.0
0.5
1.0
1.5
β_IV

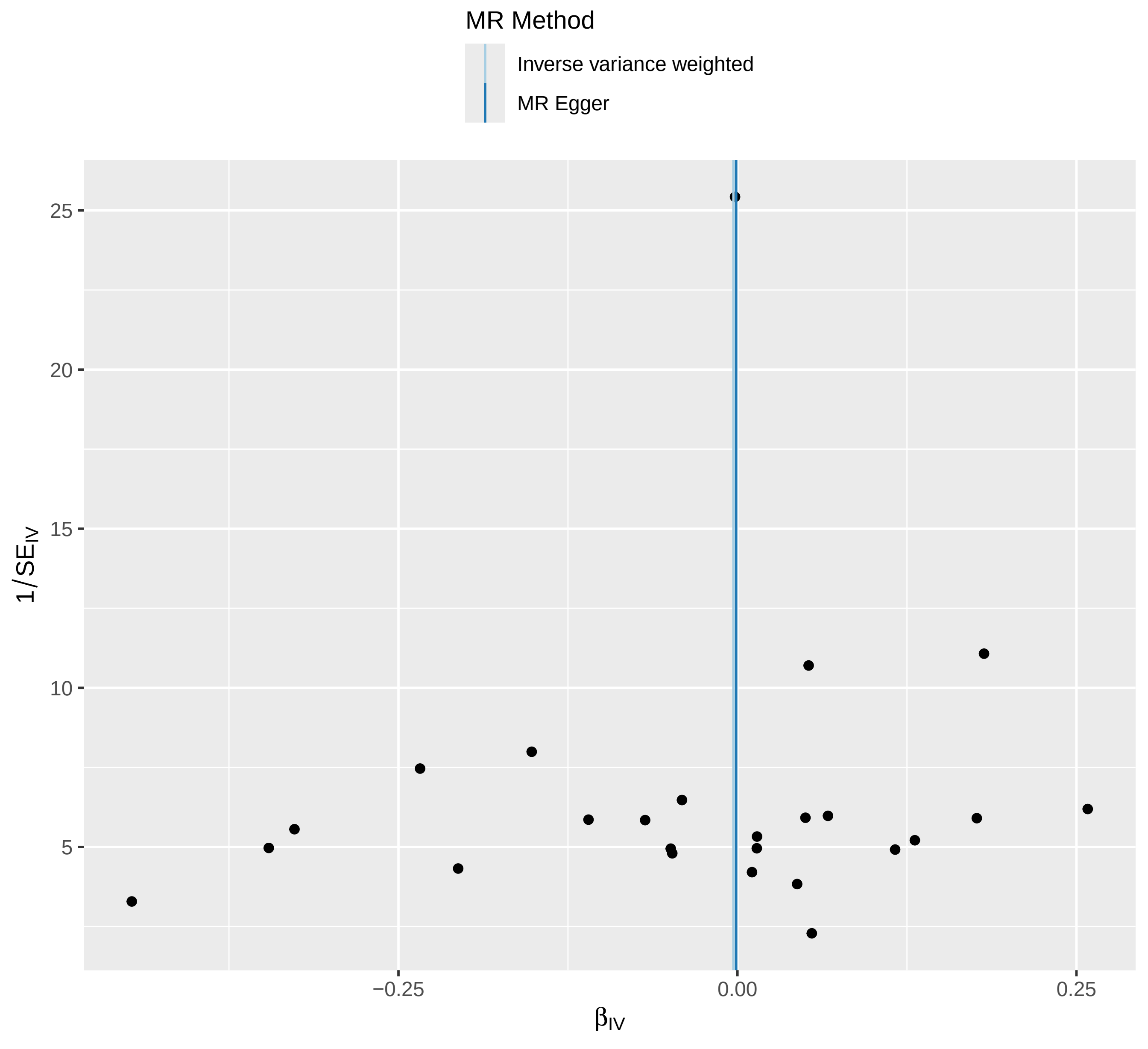
MR Method
Inverse variance weighted
MR Egger
25
20
15
10
5
1/SE_IV
−0.25
0.00
0.25
β_IV

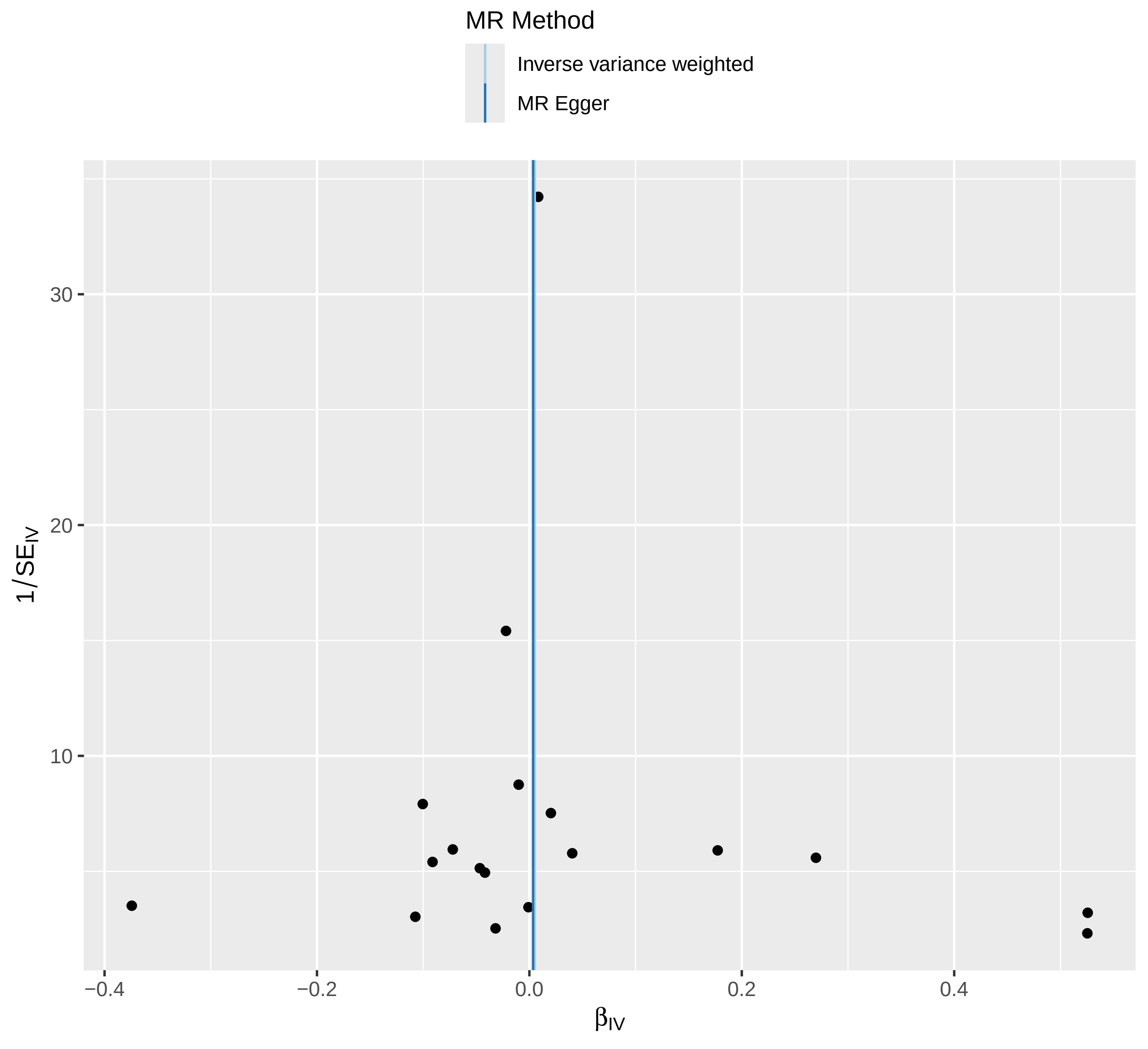
MR Method
Inverse variance weighted
MR Egger
30
20
10
1/SE_IV
−0.4
−0.2
0.0
0.2
0.4
β_IV

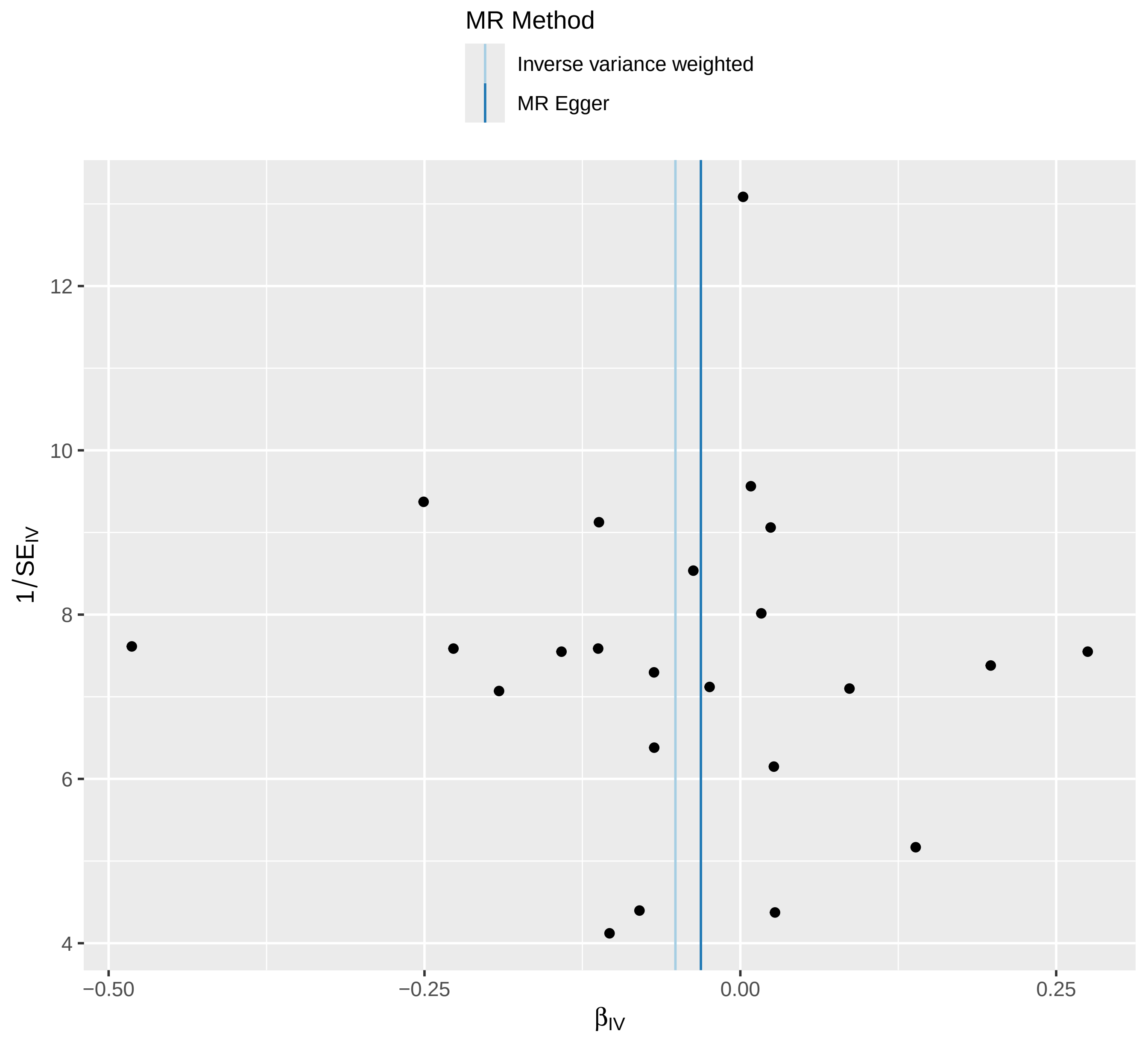
MR Method
Inverse variance weighted
MR Egger
12
10
8
6
4
1/SE_IV
−0.50
−0.25
0.00
0.25
β_IV

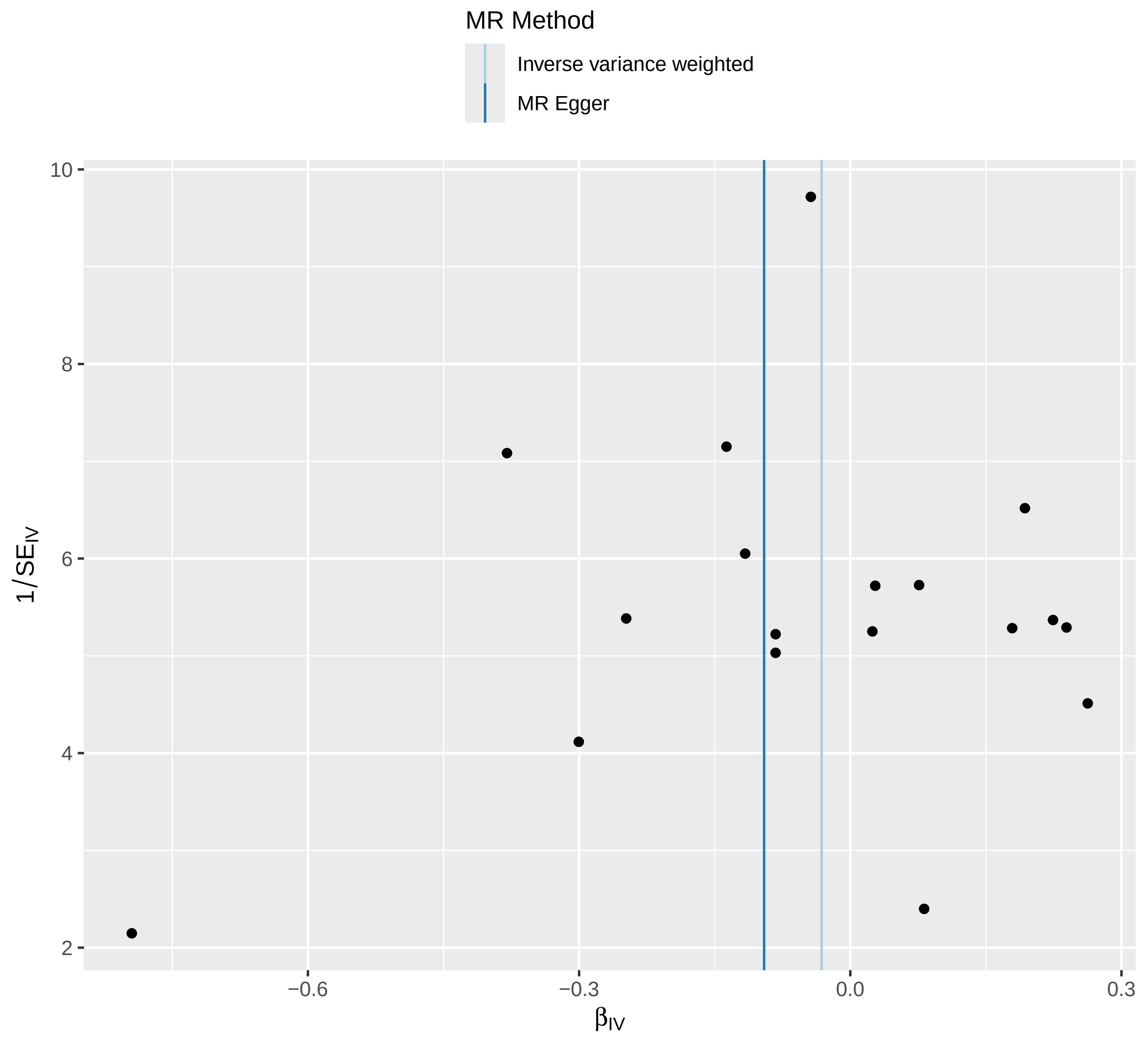
MR Method
Inverse variance weighted
MR Egger
10
8
6
4
2
1/SE$_{IV}$
−0.6
−0.3
0.0
0.3
β$_{IV}$

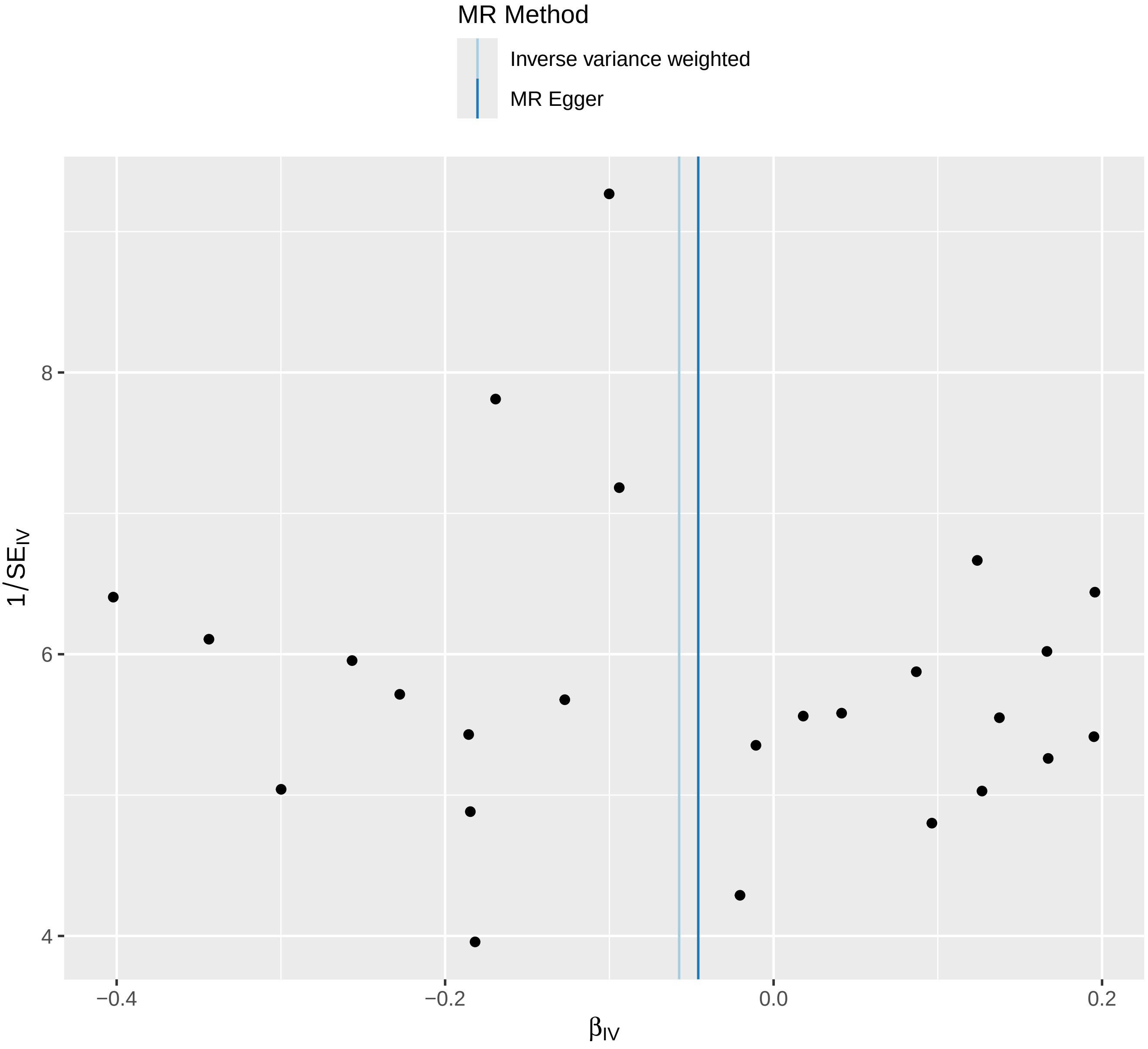

MR Method
Inverse variance weighted
MR Egger
8
6
4
1/SE_IV
−0.4
−0.2
0.0
0.2
β_IV

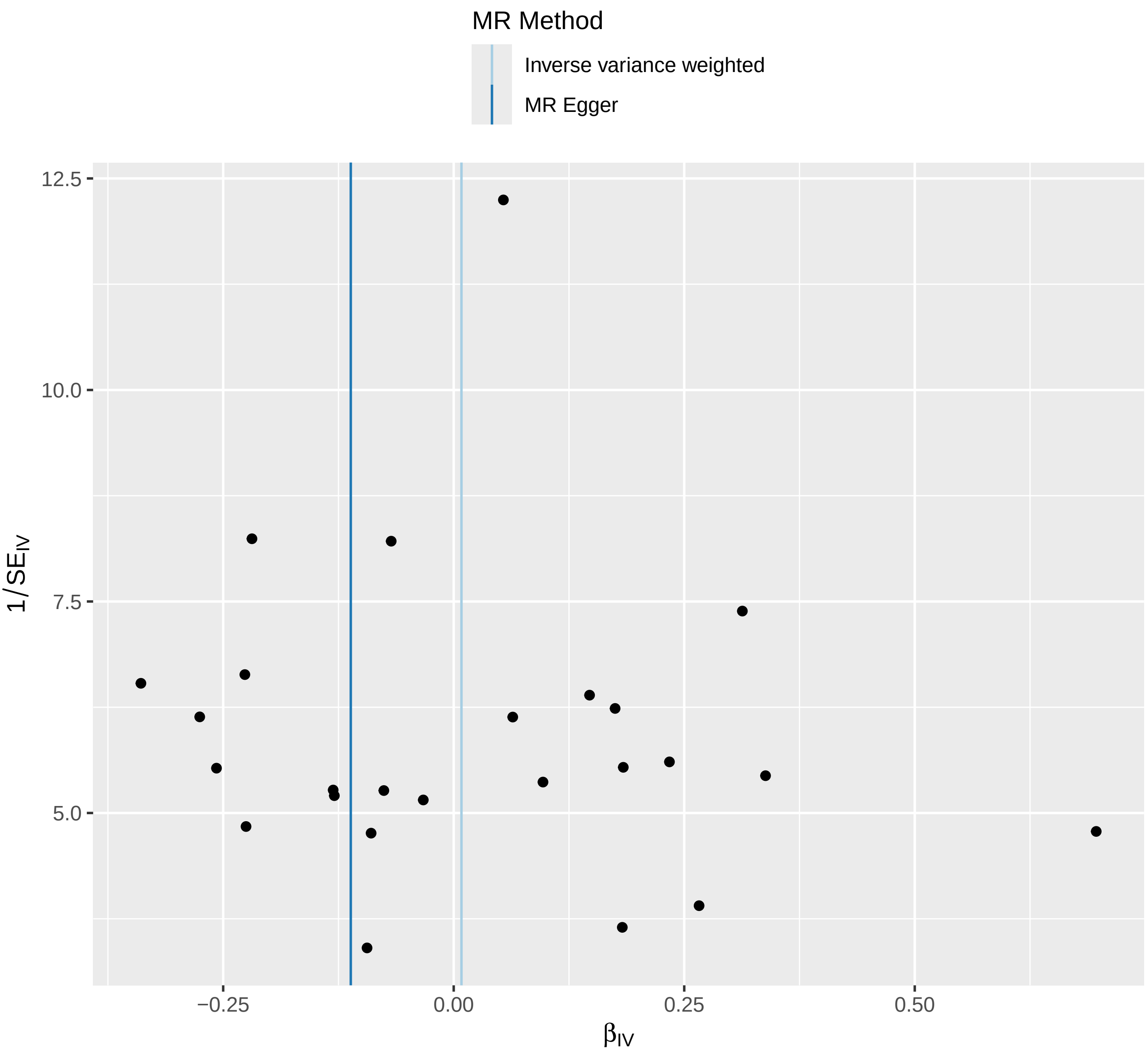
MR Method
Inverse variance weighted
MR Egger
12.5
10.0
7.5
5.0
1/SE_IV
−0.25
0.00
0.25
0.50
β_IV

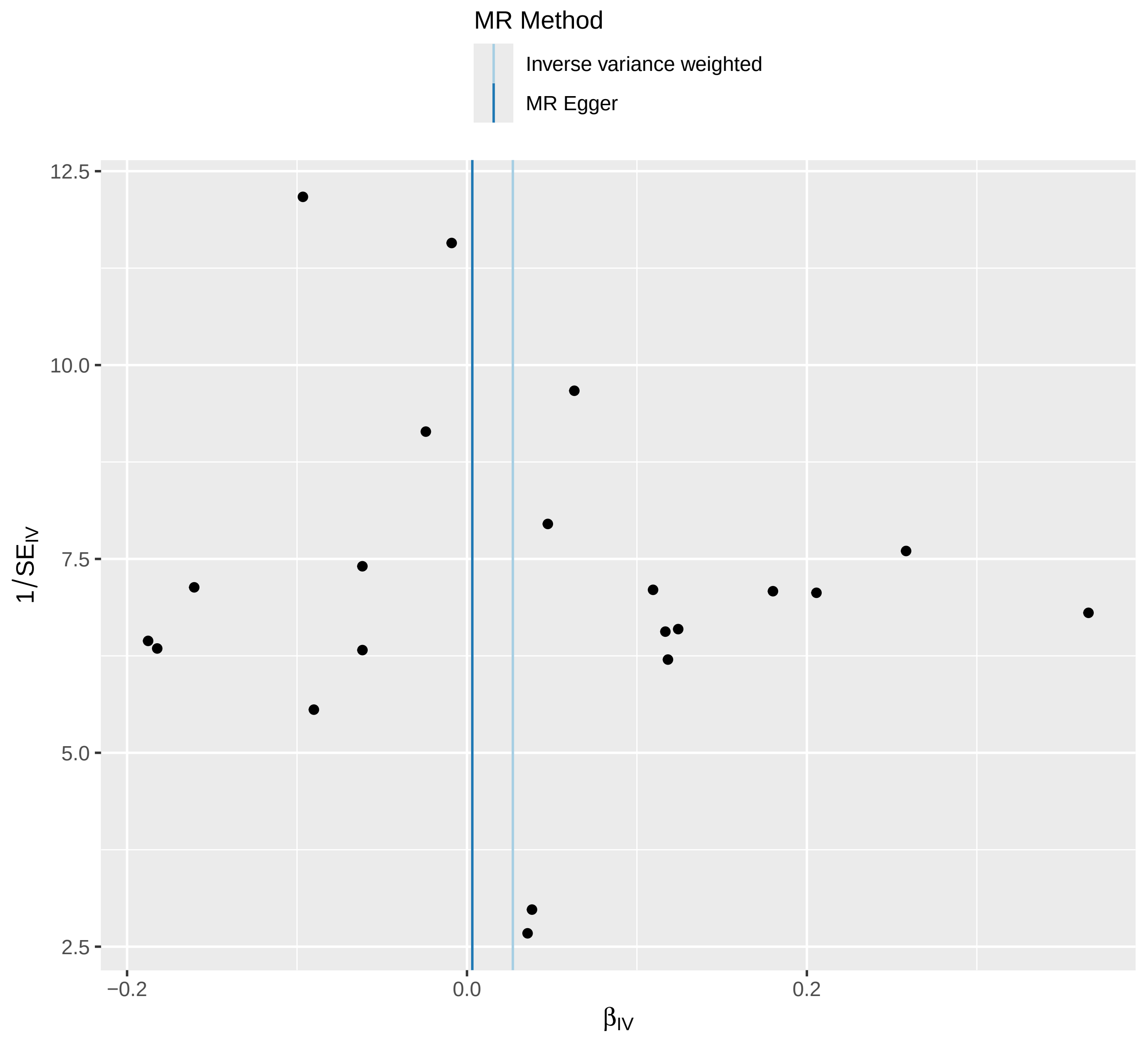
MR Method
Inverse variance weighted
MR Egger
12.5
10.0
7.5
5.0
2.5
1/SE_IV
−0.2
0.0
0.2
β_IV

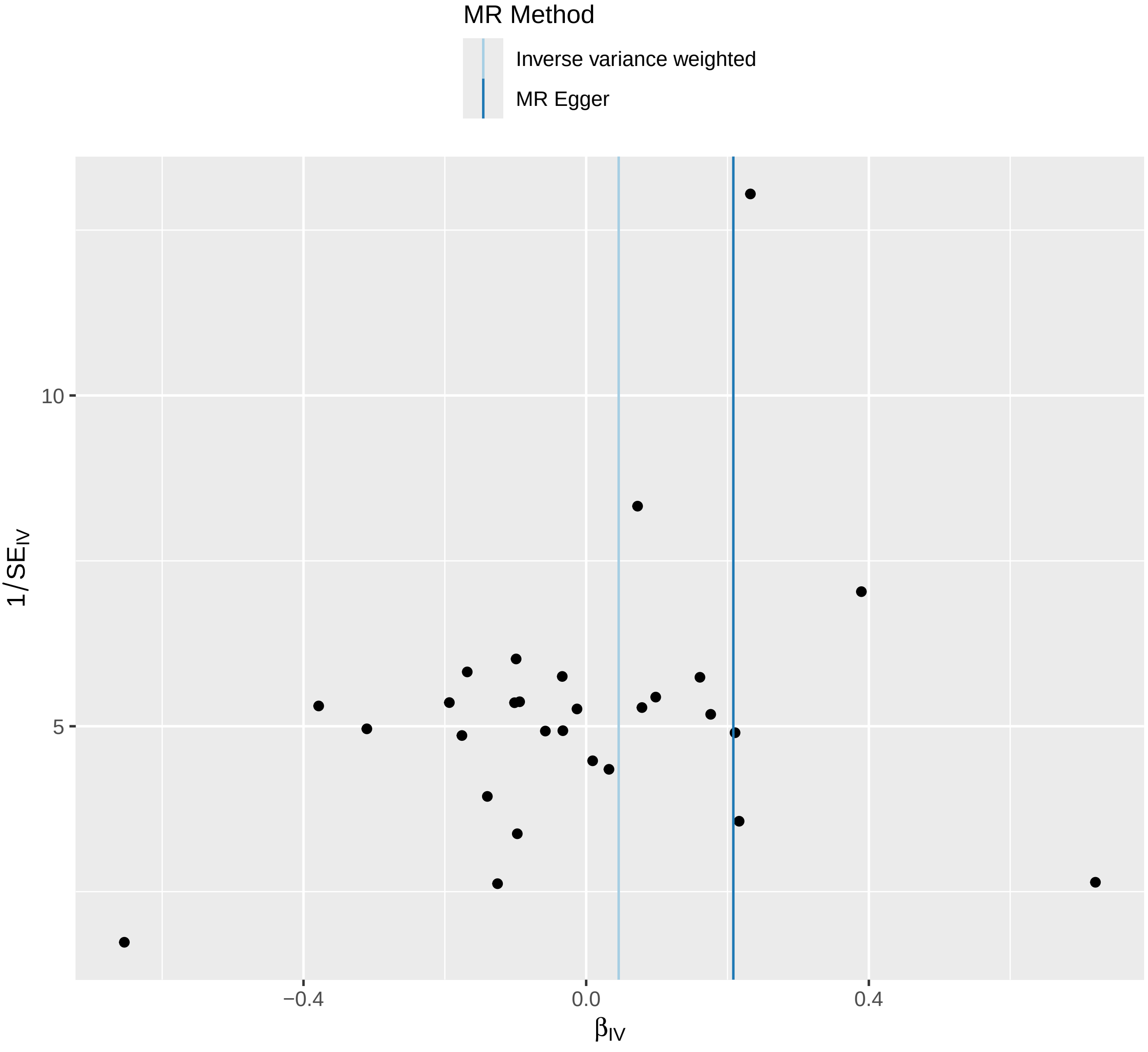
MR Method
Inverse variance weighted
MR Egger
10
5
1/SE_IV
−0.4
0.0
0.4
β_IV

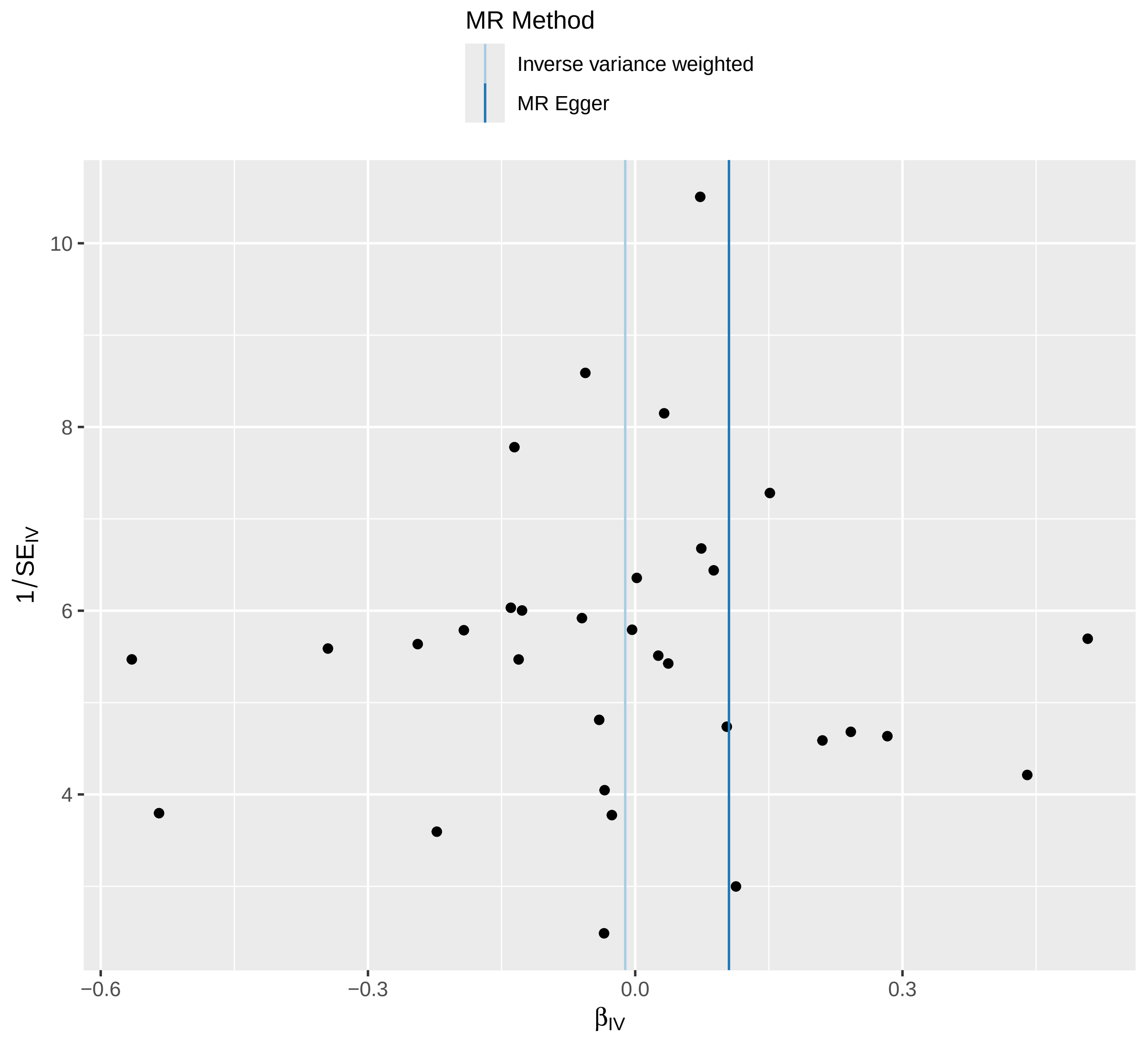
MR Method
Inverse variance weighted
MR Egger
1/SE_IV
10
8
6
4
−0.6
−0.3
0.0
0.3
β_IV

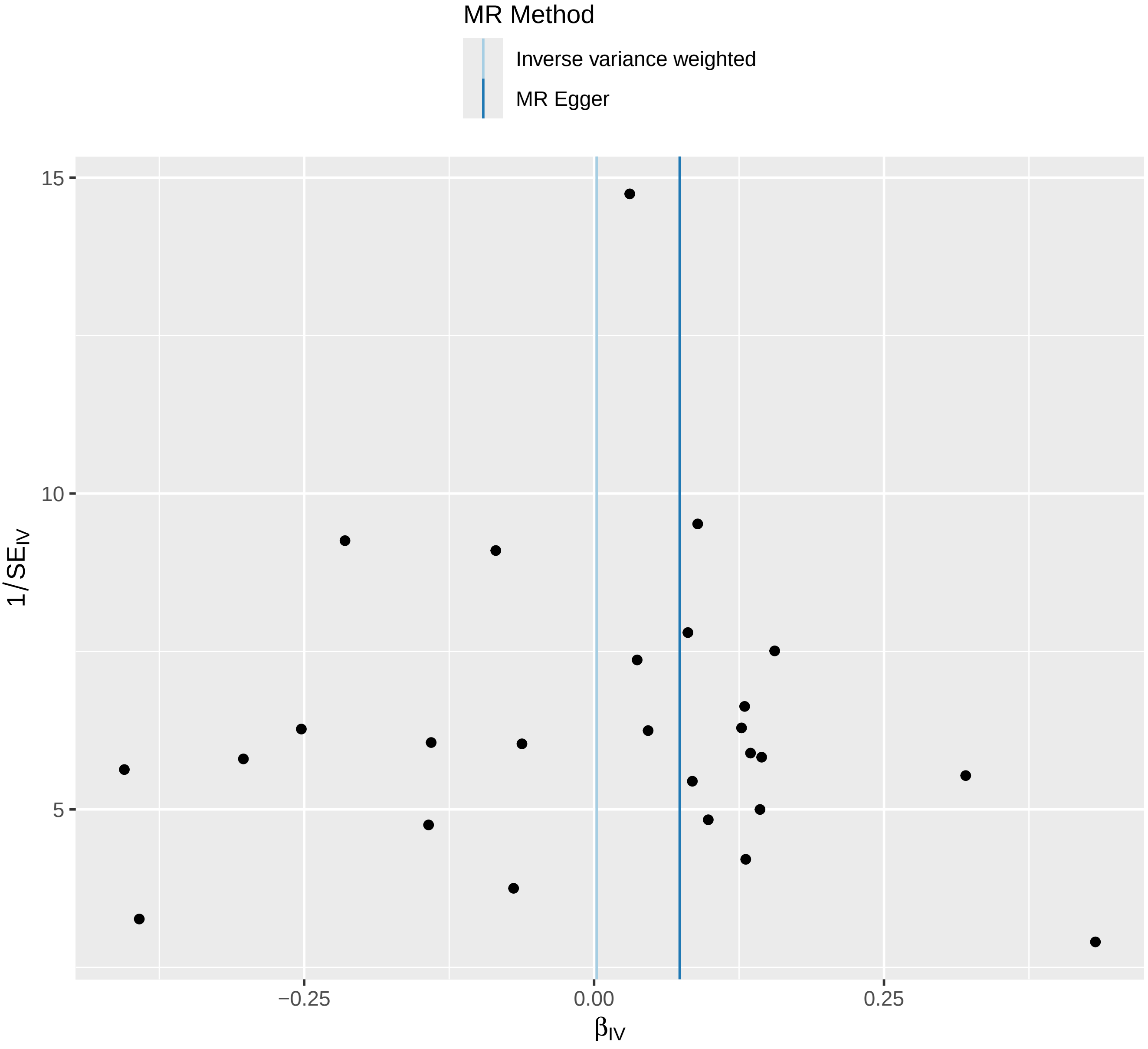
MR Method
Inverse variance weighted
MR Egger
15
10
5
$1/SE_{IV}$
−0.25
0.00
0.25
β_{IV}

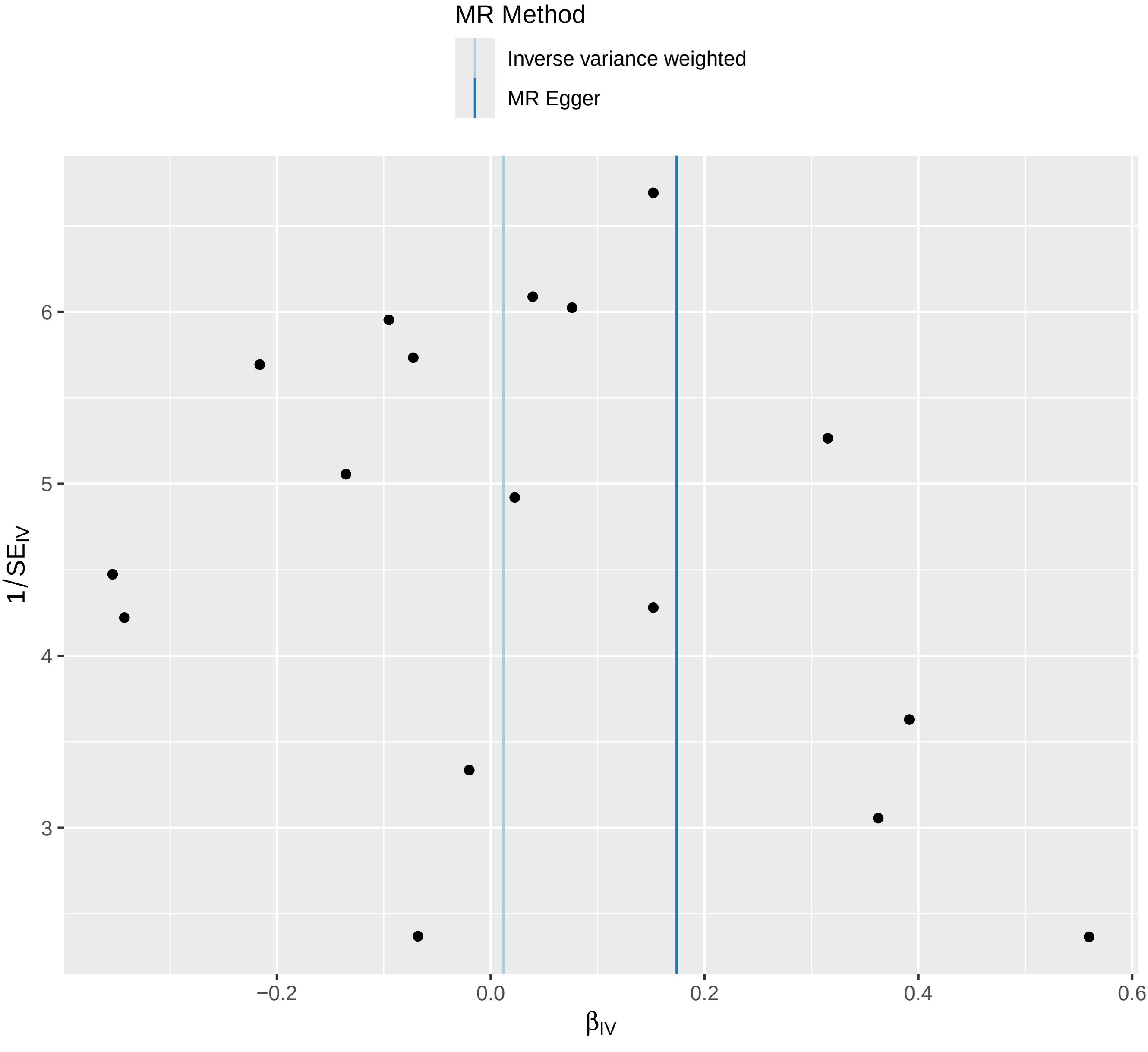
MR Method
Inverse variance weighted
MR Egger
1/SE_IV
6
5
4
3
−0.2
0.0
0.2
0.4
0.6
β_IV

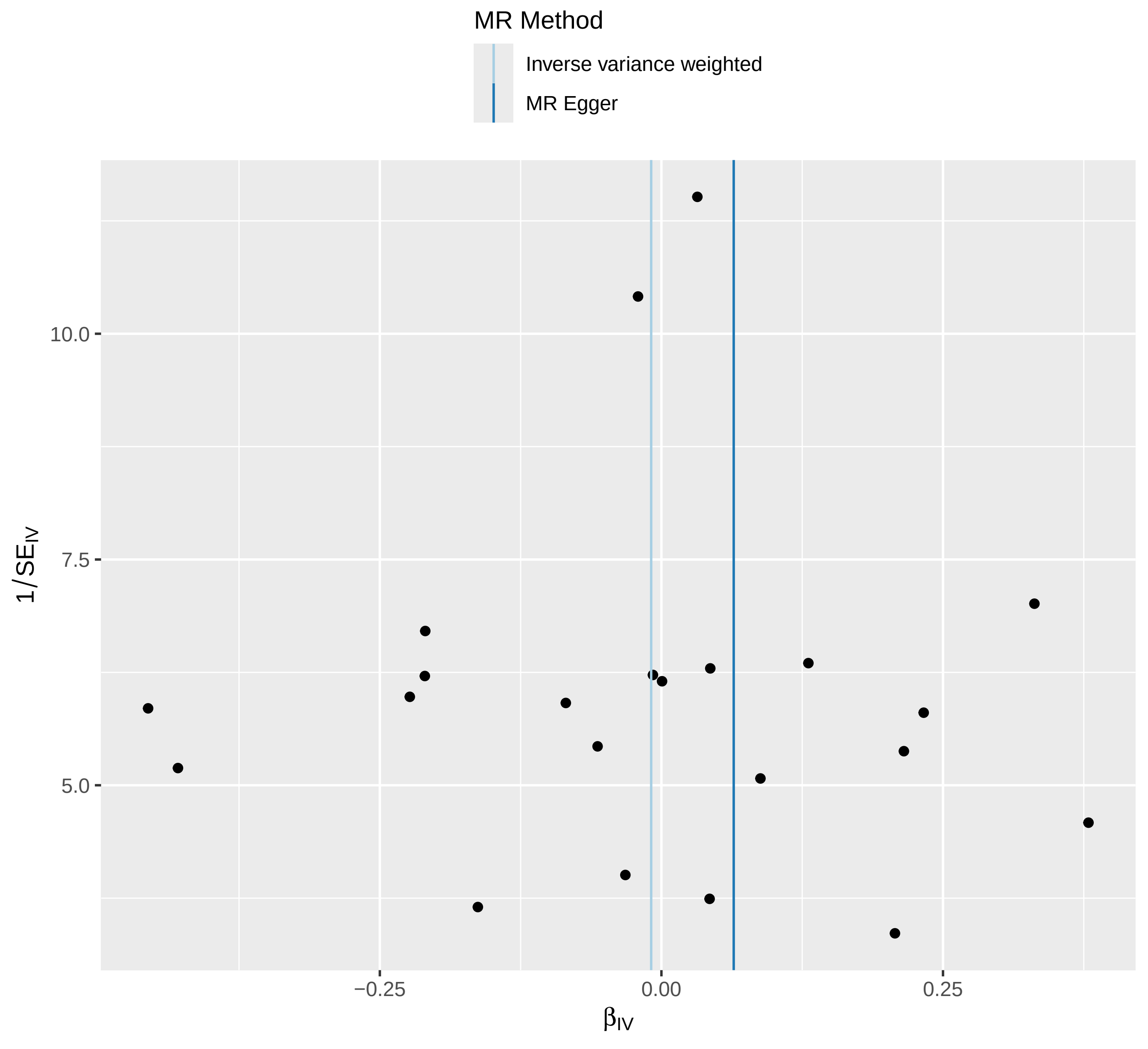
MR Method
Inverse variance weighted
MR Egger
1/SE$_{IV}$
10.0
7.5
5.0
−0.25
0.00
0.25
β$_{IV}$

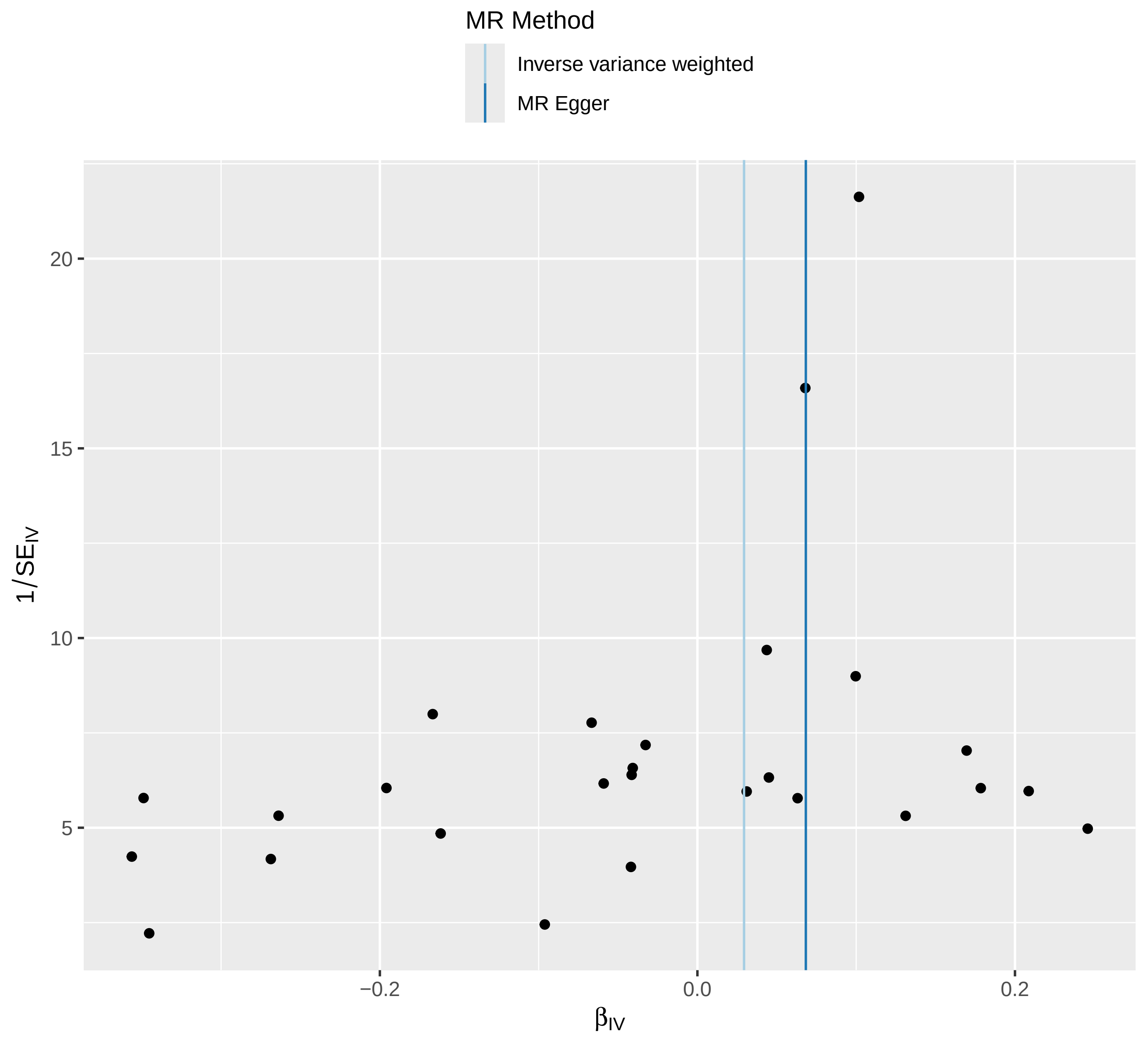
MR Method
Inverse variance weighted
MR Egger
1/SE$_{IV}$
20
15
10
5
β$_{IV}$
−0.2
0.0
0.2

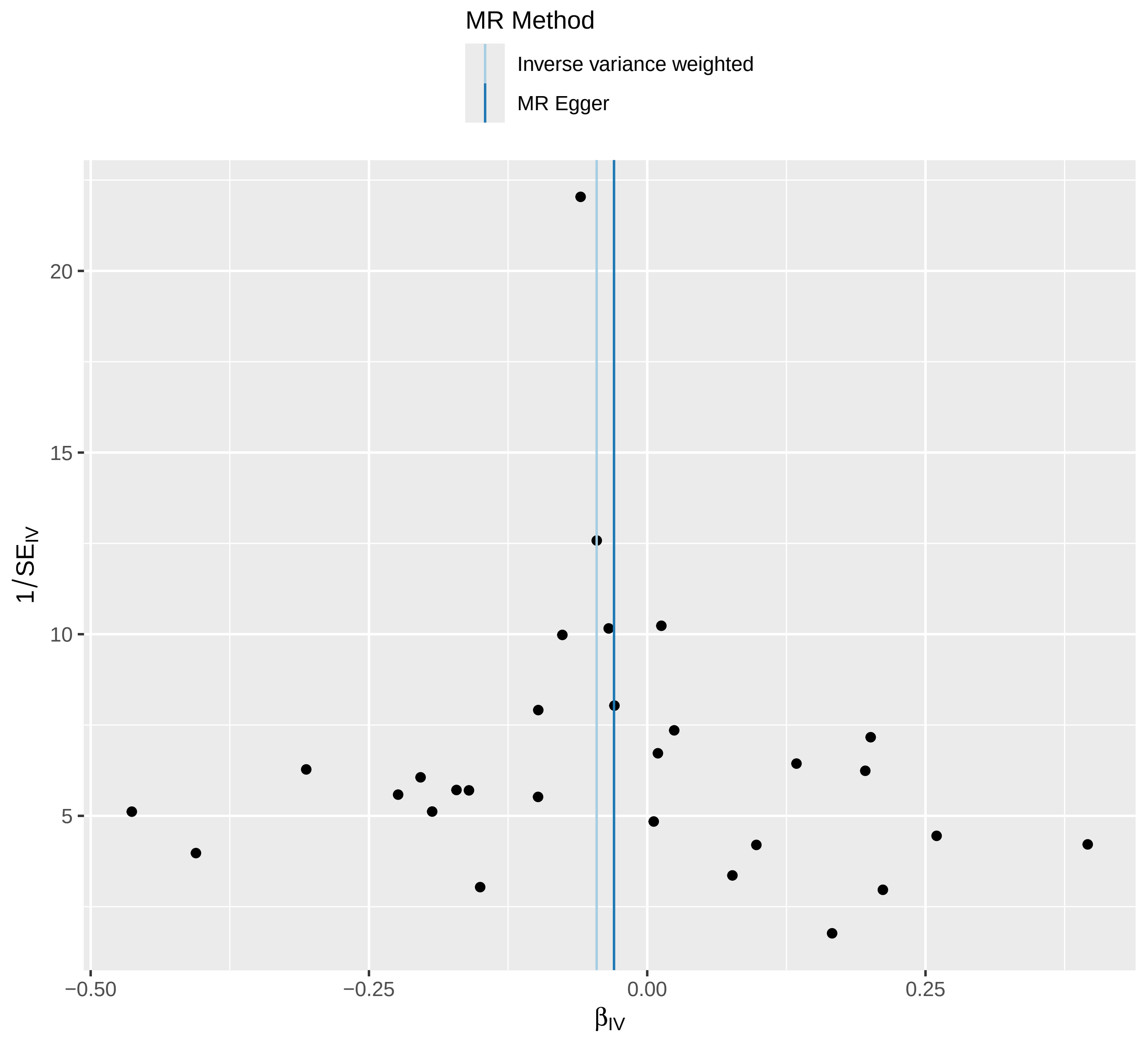
MR Method
Inverse variance weighted
MR Egger
1/SE_IV
20
15
10
5
−0.50
−0.25
0.00
0.25
β_IV

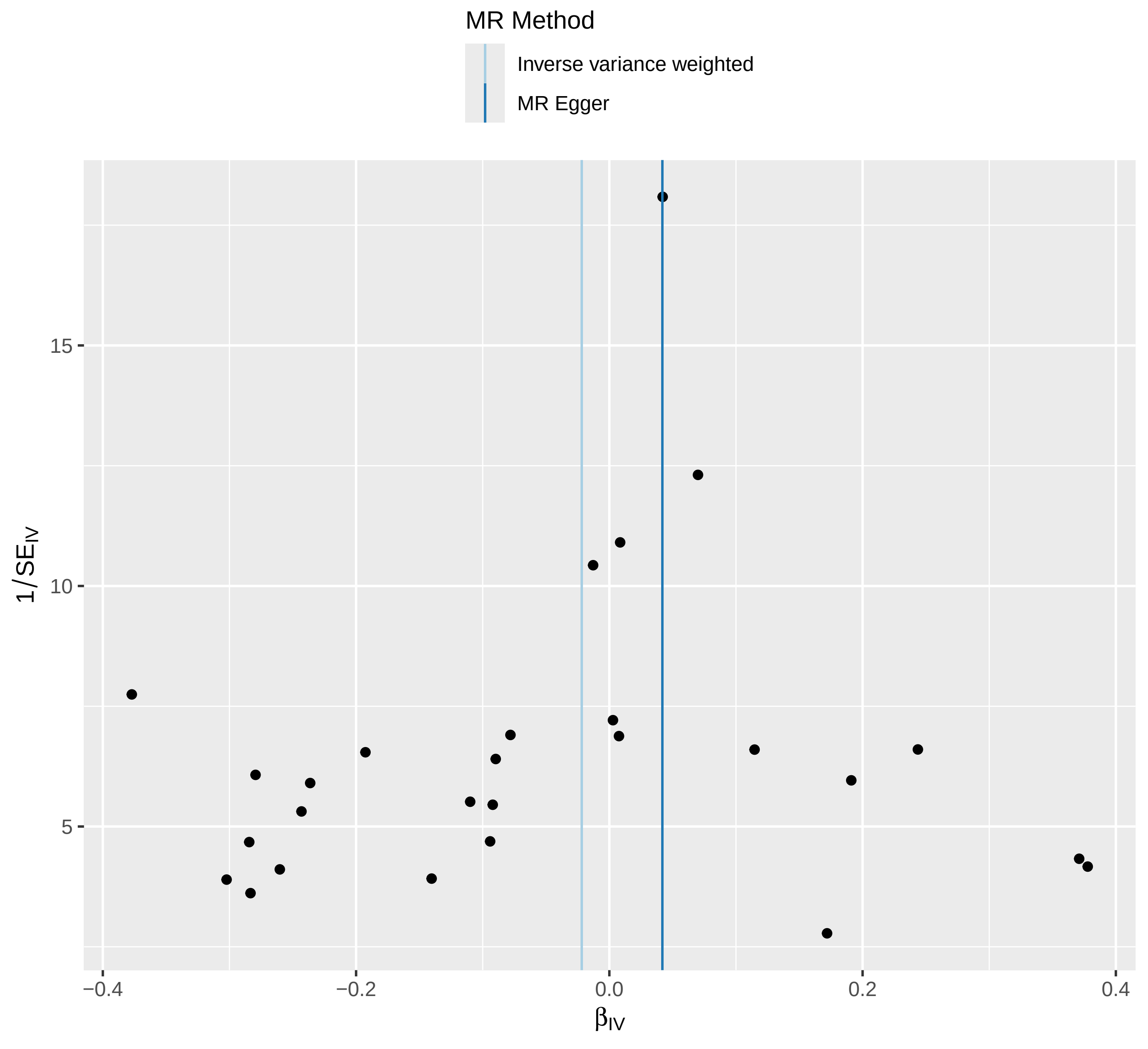

MR Method
Inverse variance weighted
MR Egger
15
10
5
1/SE_IV
−0.4
−0.2
0.0
0.2
0.4
β_IV

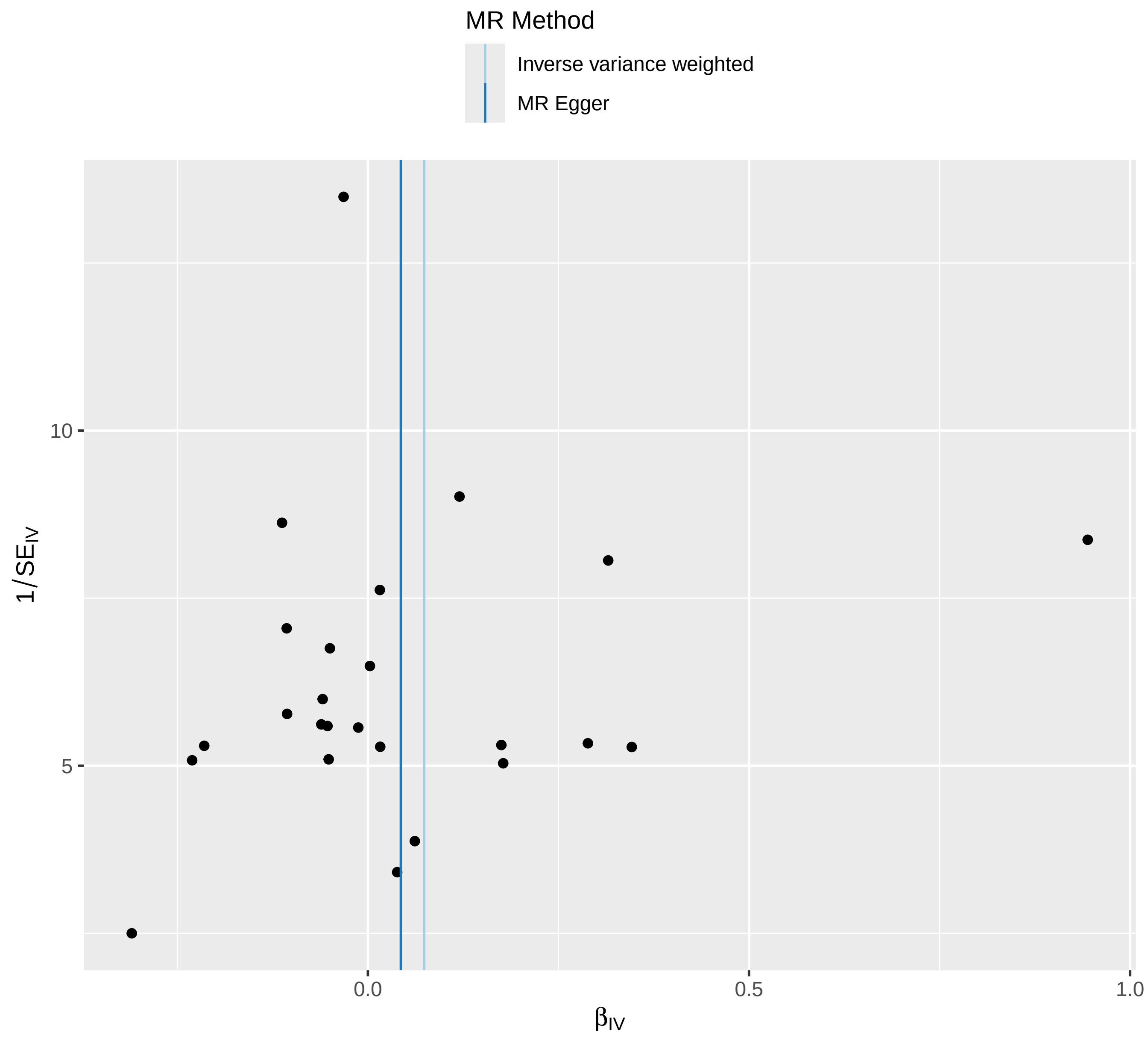

MR Method
Inverse variance weighted
MR Egger
1/SE_IV
10
5
0.0
0.5
1.0
β_IV

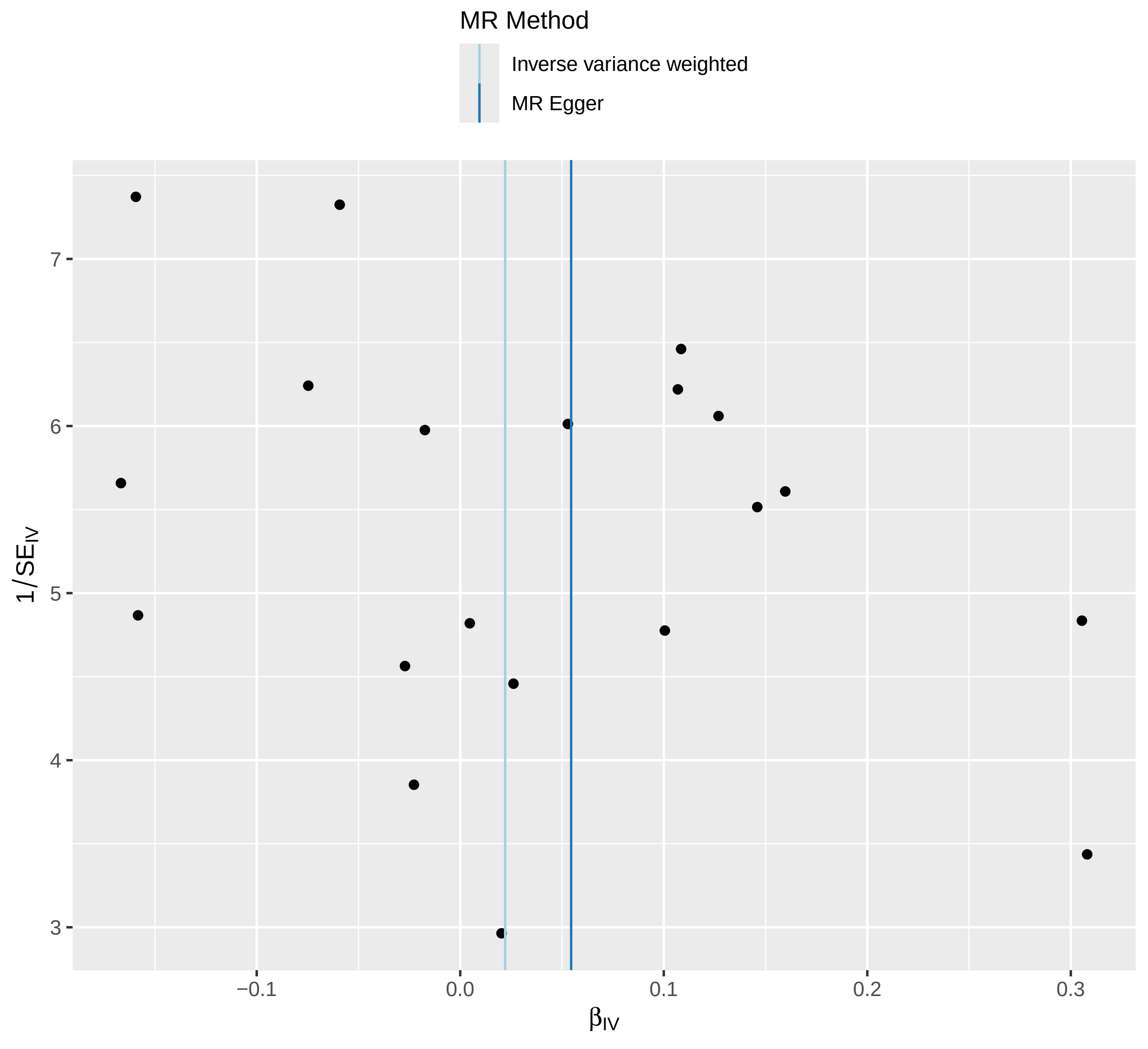
MR Method
Inverse variance weighted
MR Egger
7
6
5
4
3
1/SE_IV
−0.1
0.0
0.1
0.2
0.3
β_IV

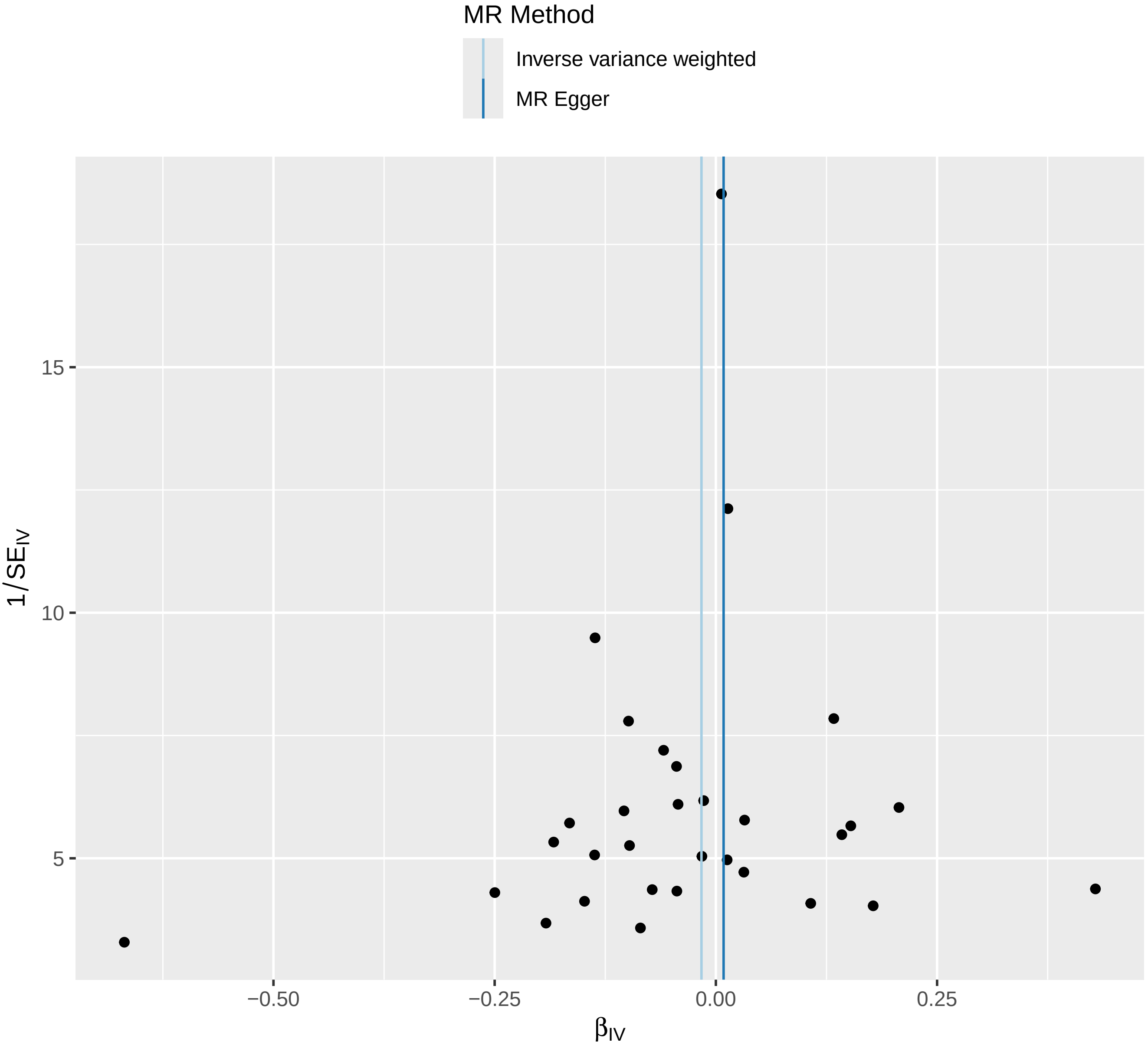
MR Method
Inverse variance weighted
MR Egger
15
10
5
1/SE_IV
−0.50
−0.25
0.00
0.25
β_IV

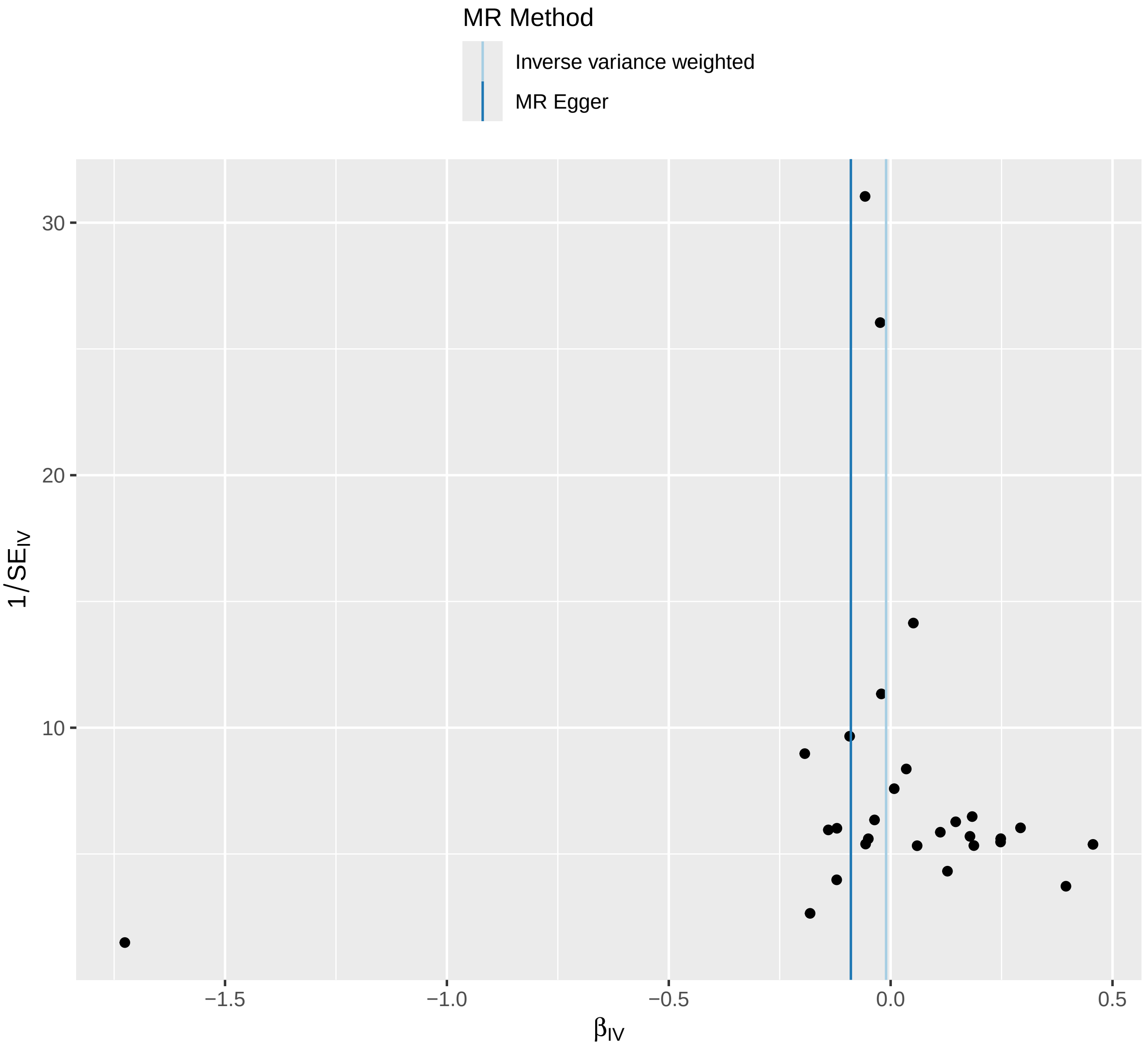
MR Method
Inverse variance weighted
MR Egger
30
20
10
1/SE_IV
−1.5
−1.0
−0.5
0.0
0.5
β_IV

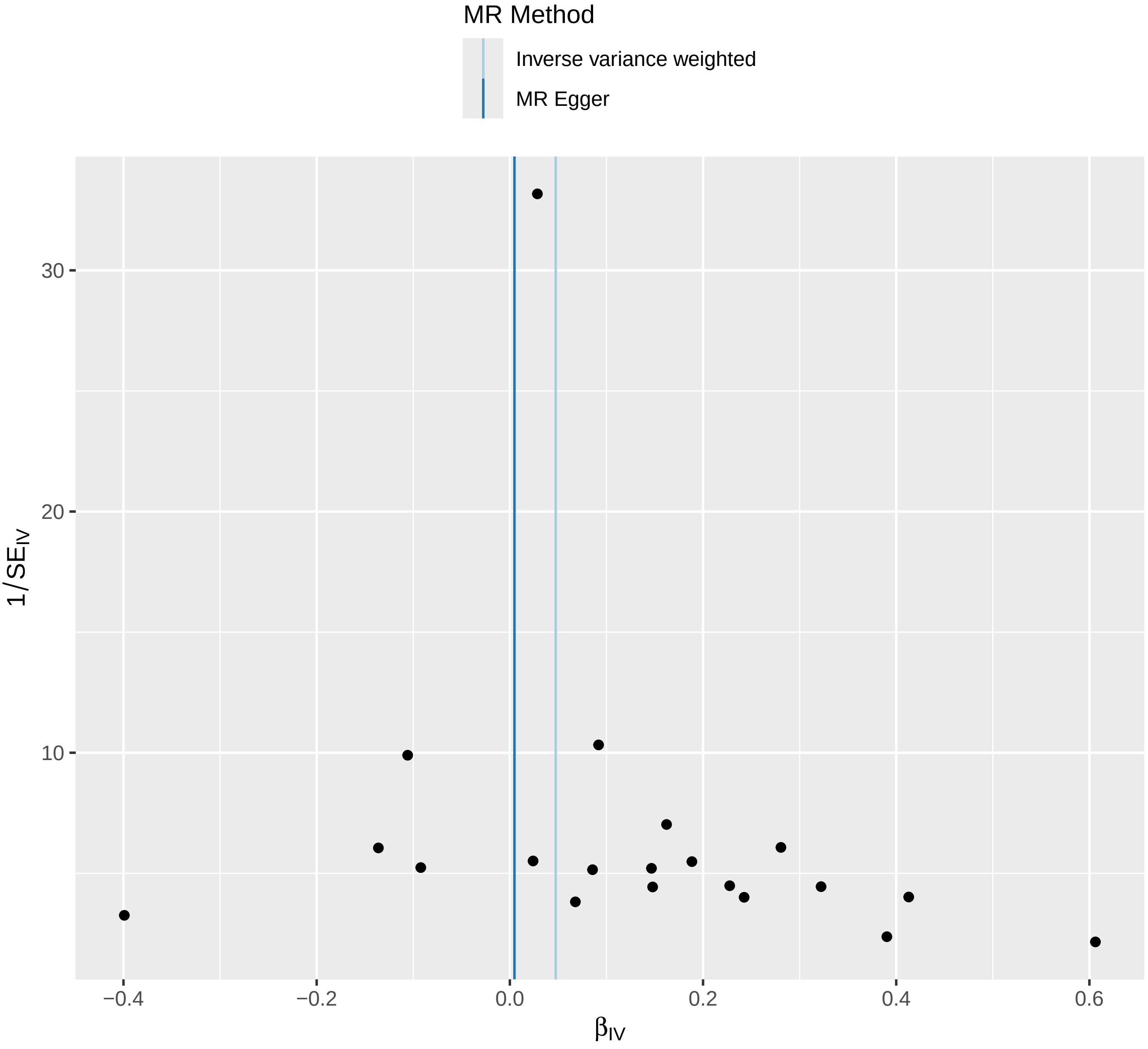

MR Method
Inverse variance weighted
MR Egger
30
20
10
$1/SE_{IV}$
−0.4
−0.2
0.0
0.2
0.4
0.6
β_{IV}

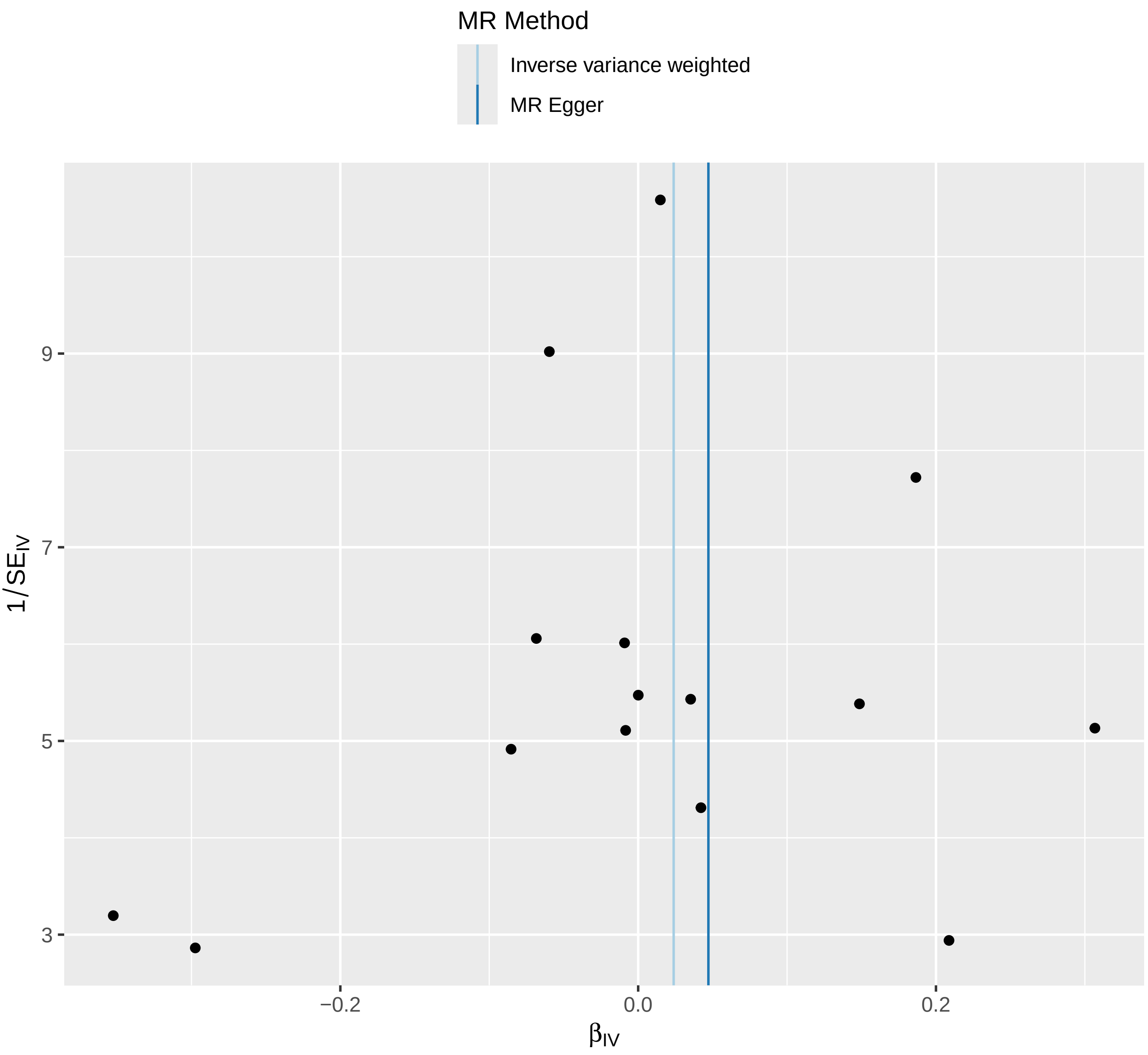
MR Method
Inverse variance weighted
MR Egger
1/SE_IV
9
7
5
3
−0.2
0.0
0.2
β_IV

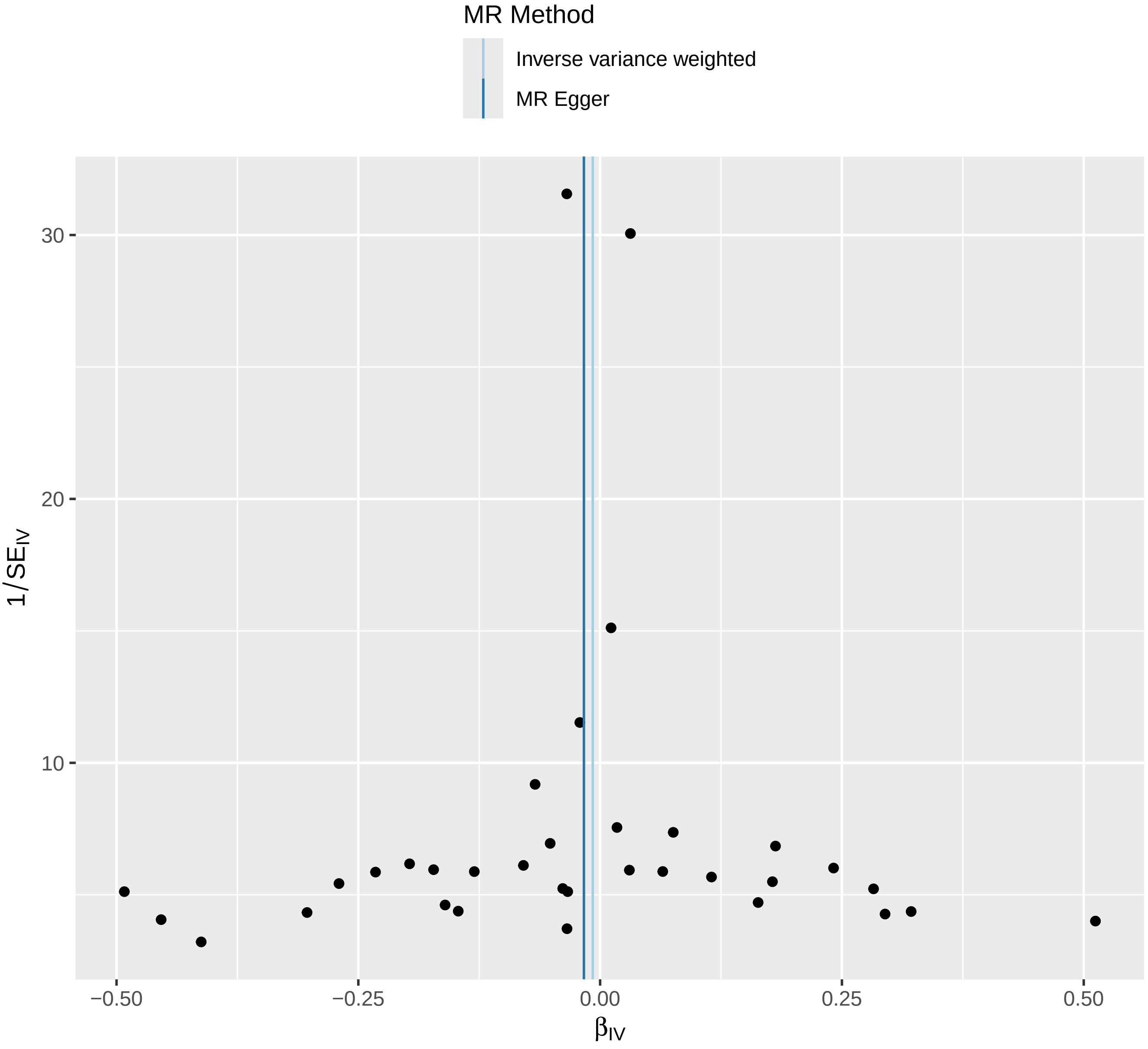
MR Method
Inverse variance weighted
MR Egger
30
20
10
1/SE_IV
−0.50
−0.25
0.00
0.25
0.50
β_IV

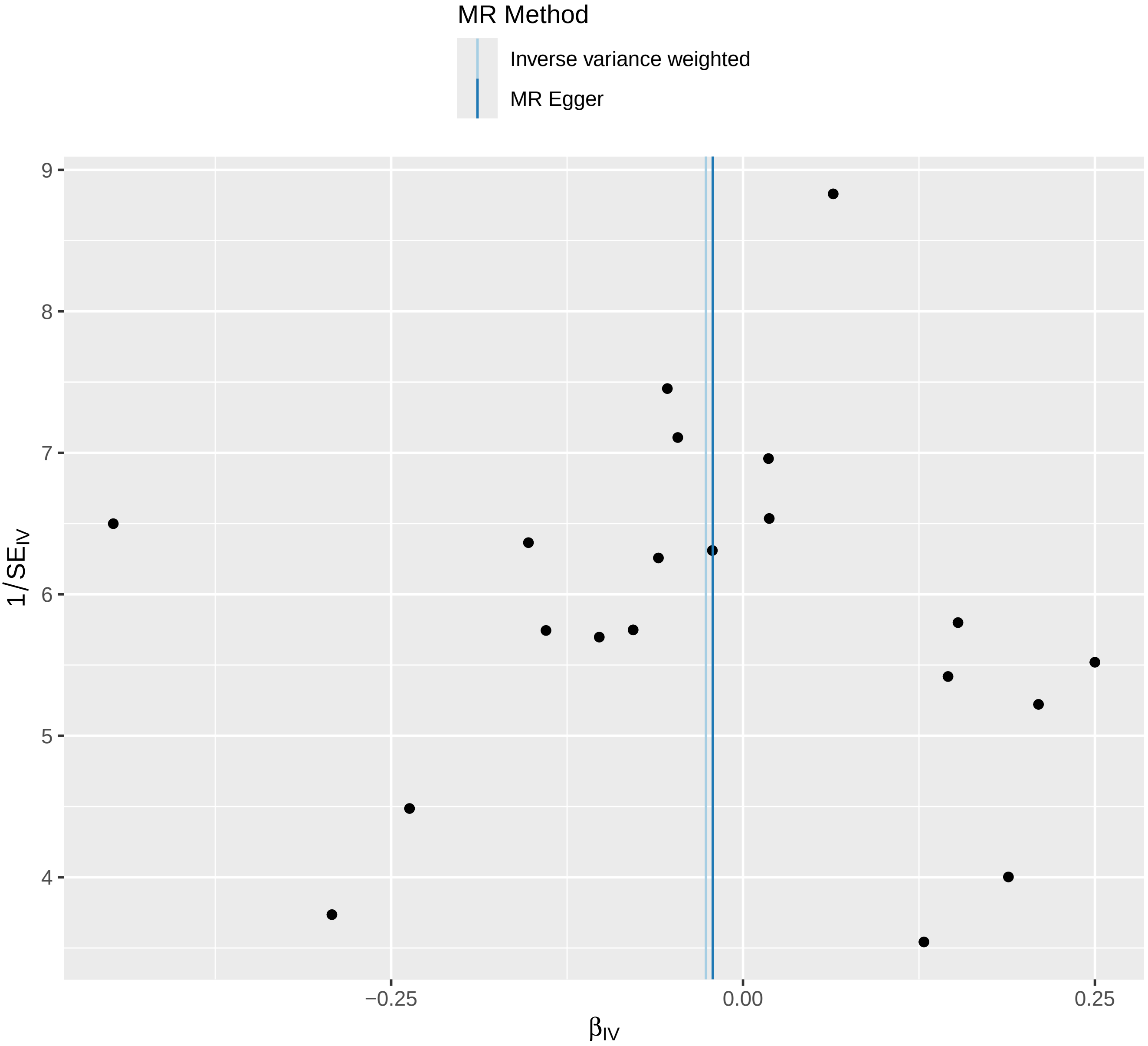
MR Method
Inverse variance weighted
MR Egger
9
8
7
6
5
4
$1/SE_{IV}$
−0.25
0.00
0.25
β_{IV}

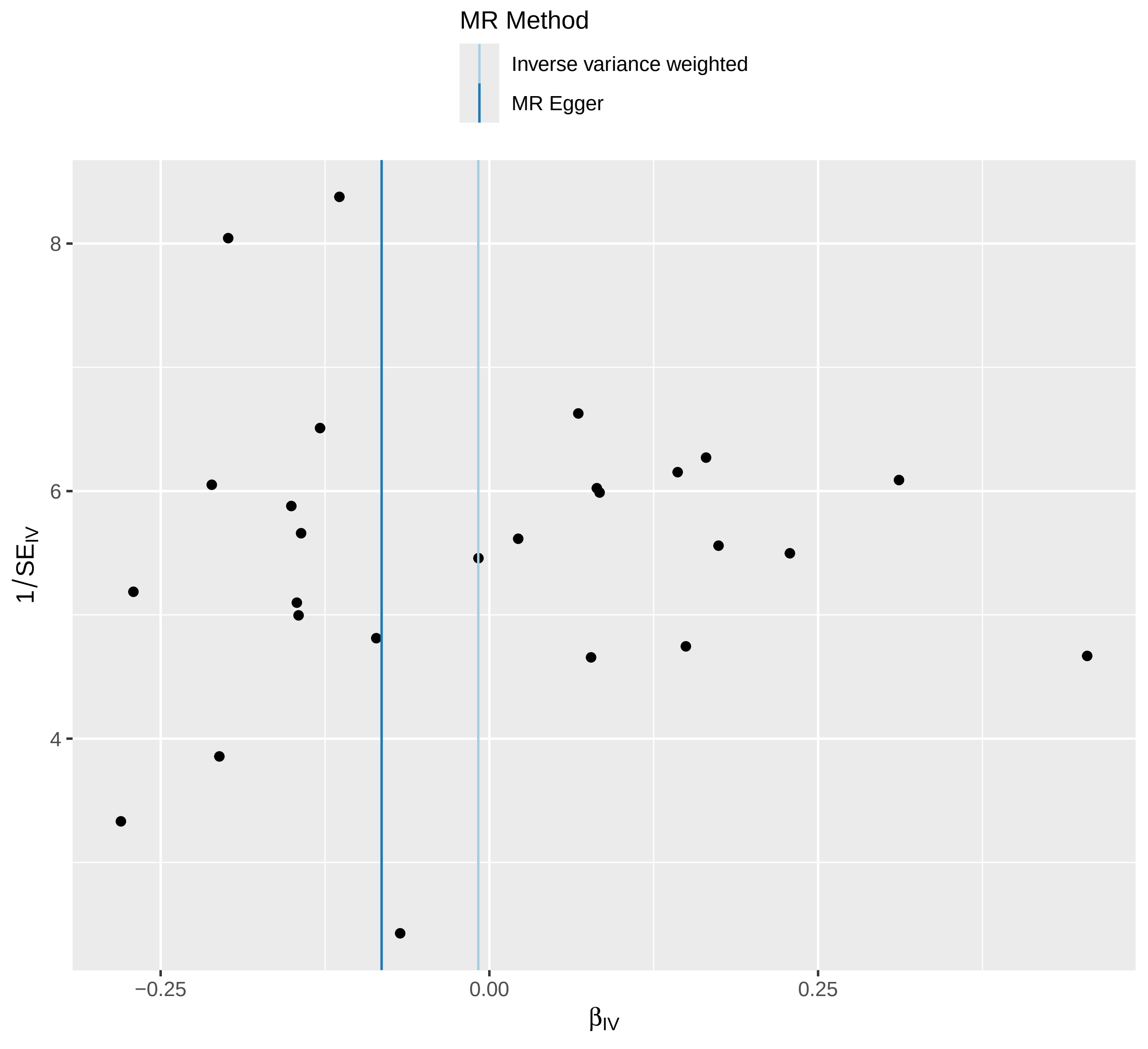
MR Method
Inverse variance weighted
MR Egger
8
6
4
1/SE$_{IV}$
−0.25
0.00
0.25
β$_{IV}$

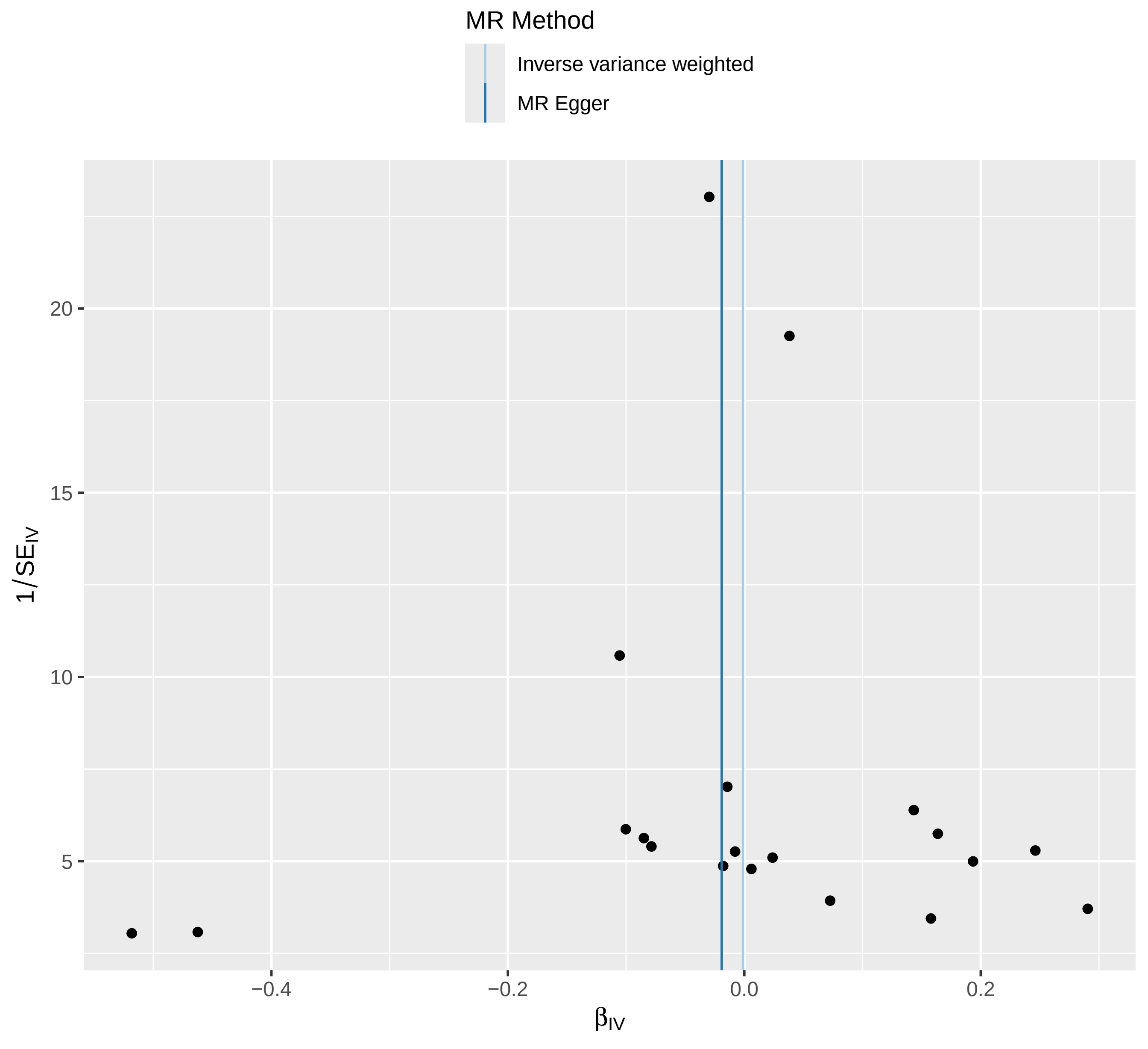
MR Method
Inverse variance weighted
MR Egger
20
15
10
5
−0.4
−0.2
0.0
0.2
$1/SE_{IV}$
β_{IV}

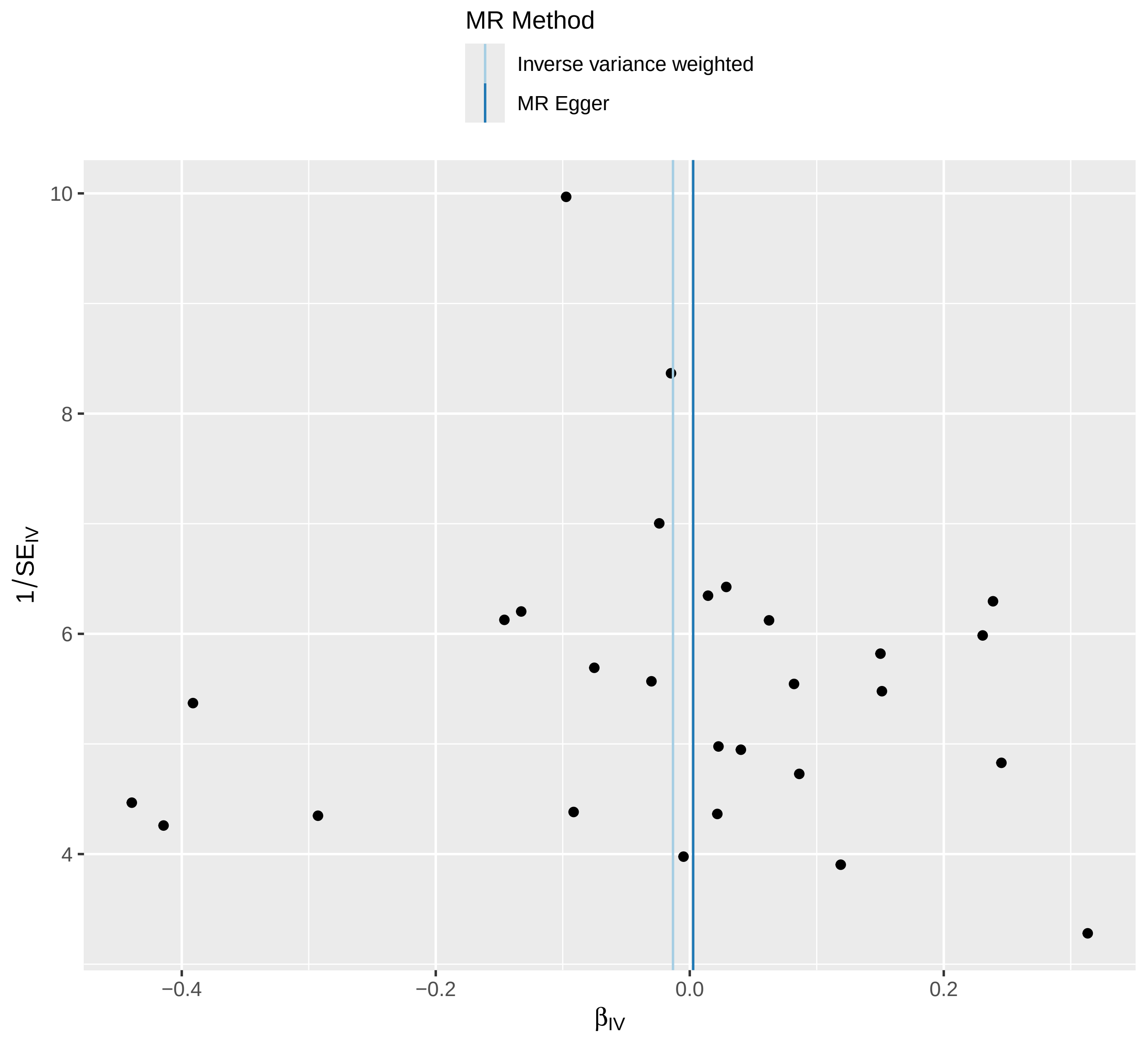

MR Method
Inverse variance weighted
MR Egger
10
8
6
4
1/SE$_{IV}$
−0.4
−0.2
0.0
0.2
β$_{IV}$

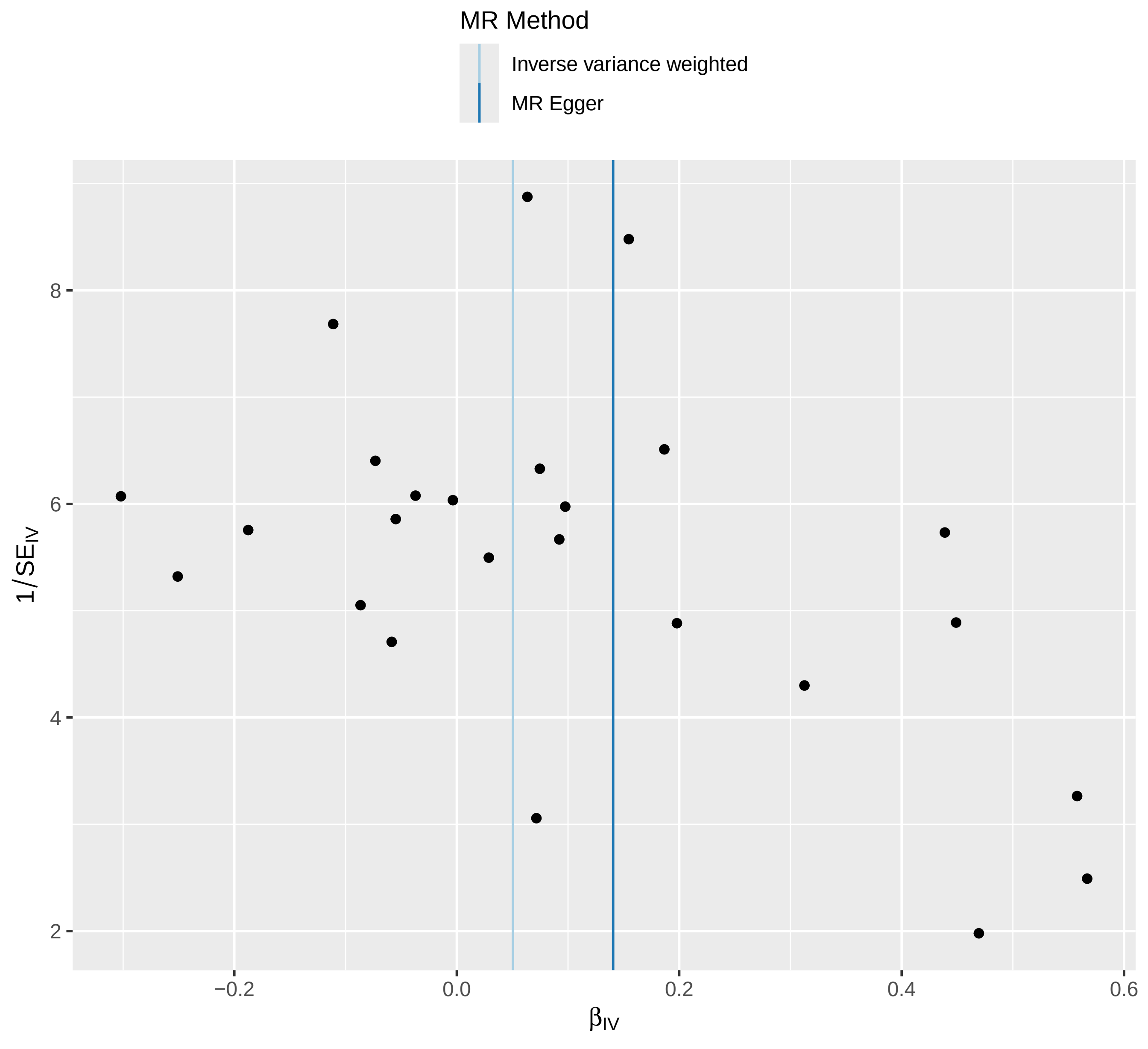

MR Method
Inverse variance weighted
MR Egger
8
6
4
2
1/SE_IV
−0.2
0.0
0.2
0.4
0.6
β_IV

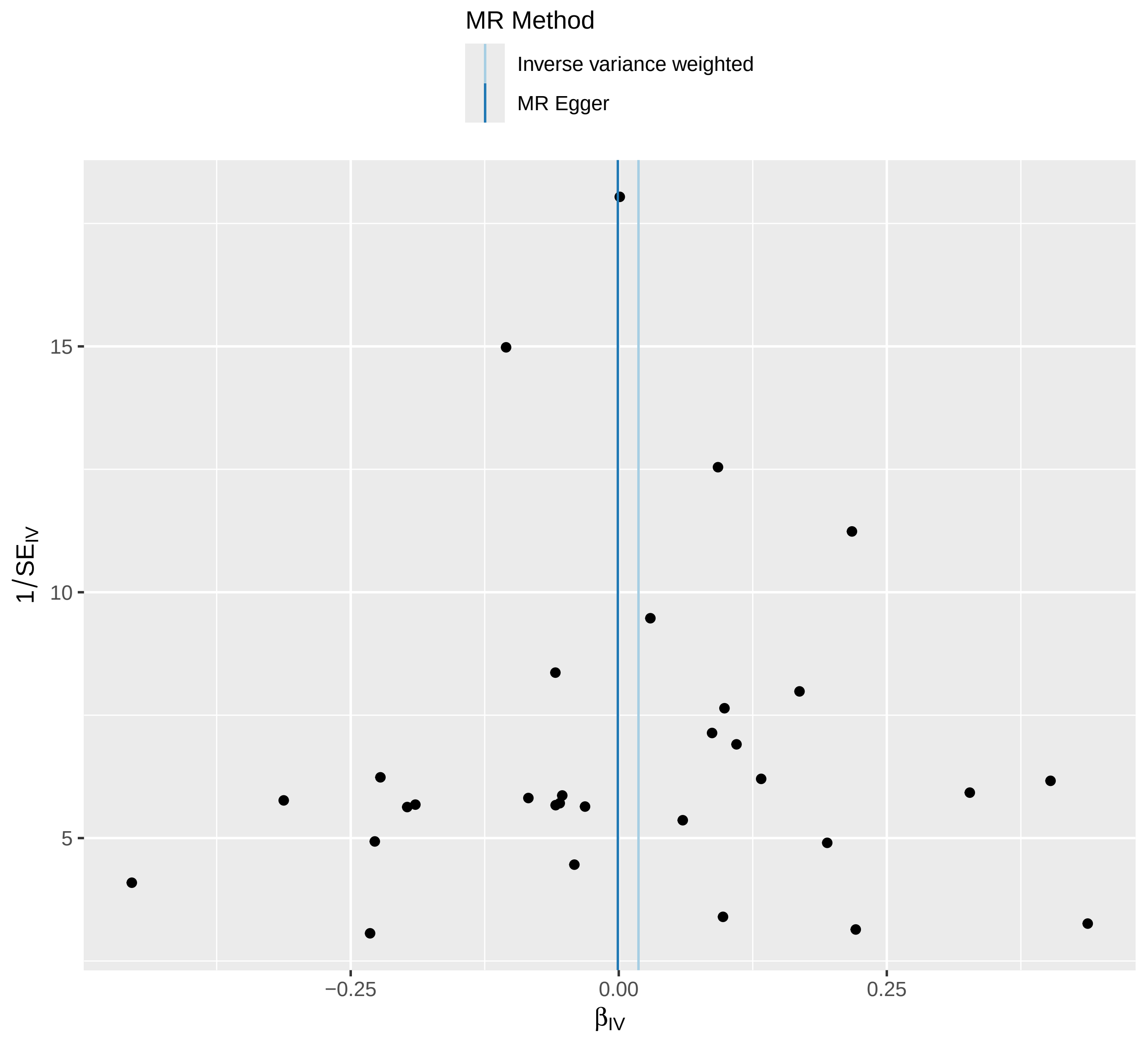

MR Method
Inverse variance weighted
MR Egger
1/SE_IV
15
10
5
−0.25
0.00
0.25
β_IV

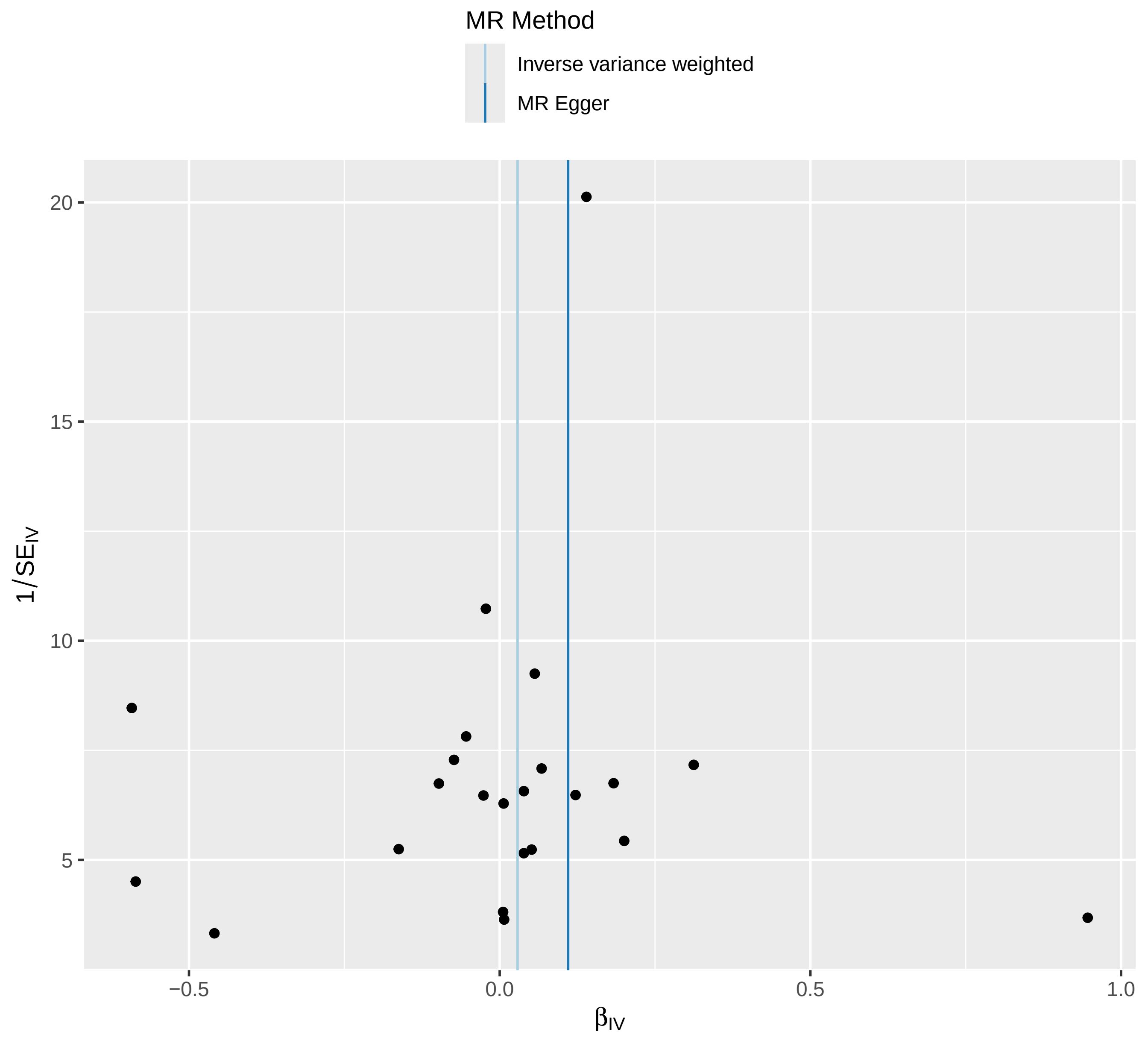
MR Method
Inverse variance weighted
MR Egger
1/SE$_{IV}$
20
15
10
5
−0.5
0.0
0.5
1.0
β$_{IV}$

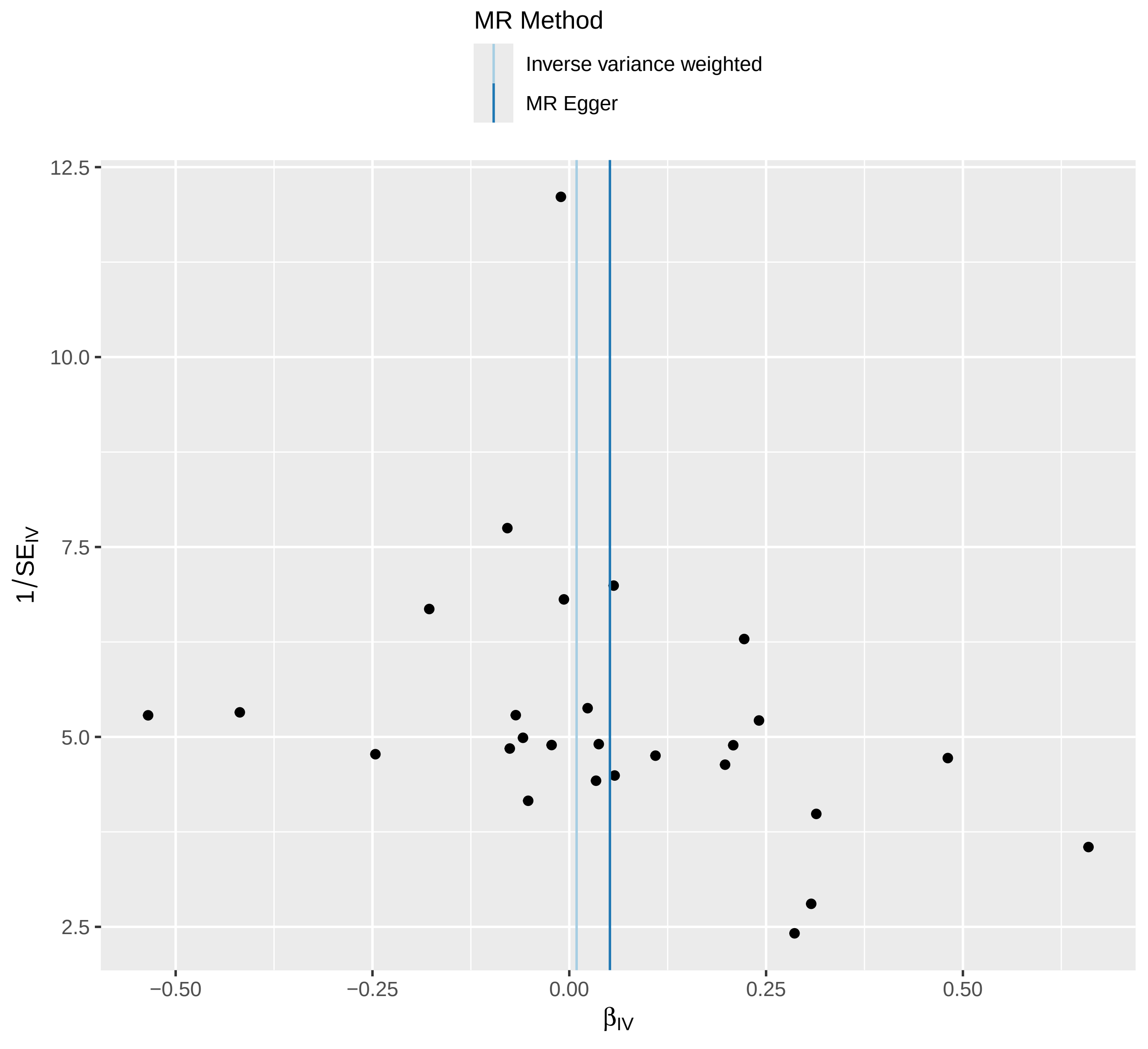
MR Method
Inverse variance weighted
MR Egger
12.5
10.0
7.5
5.0
2.5
1/SE$_{IV}$
−0.50
−0.25
0.00
0.25
0.50
β$_{IV}$

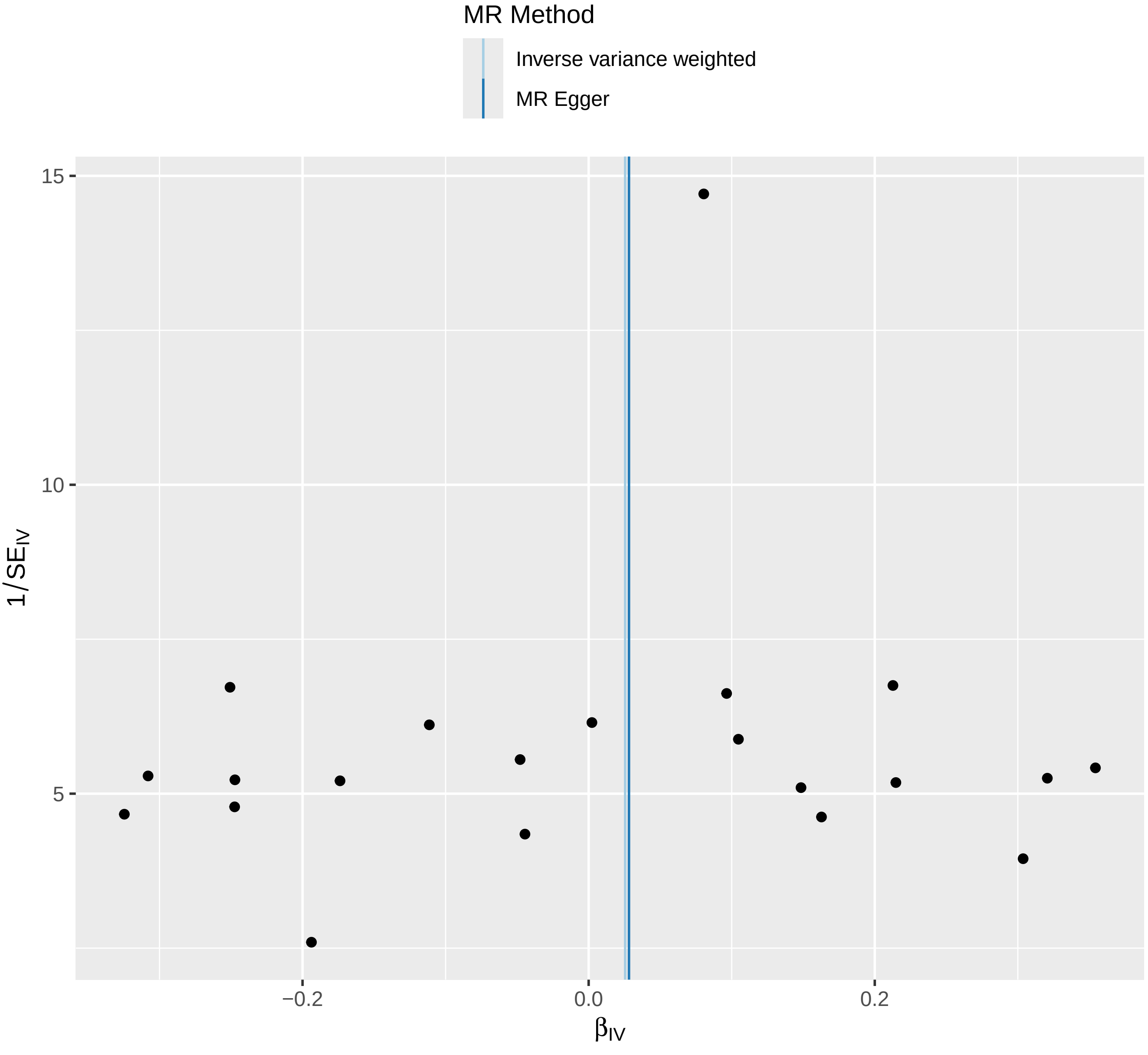

MR Method
Inverse variance weighted
MR Egger
15
10
5
1/SE_IV
−0.2
0.0
0.2
β_IV

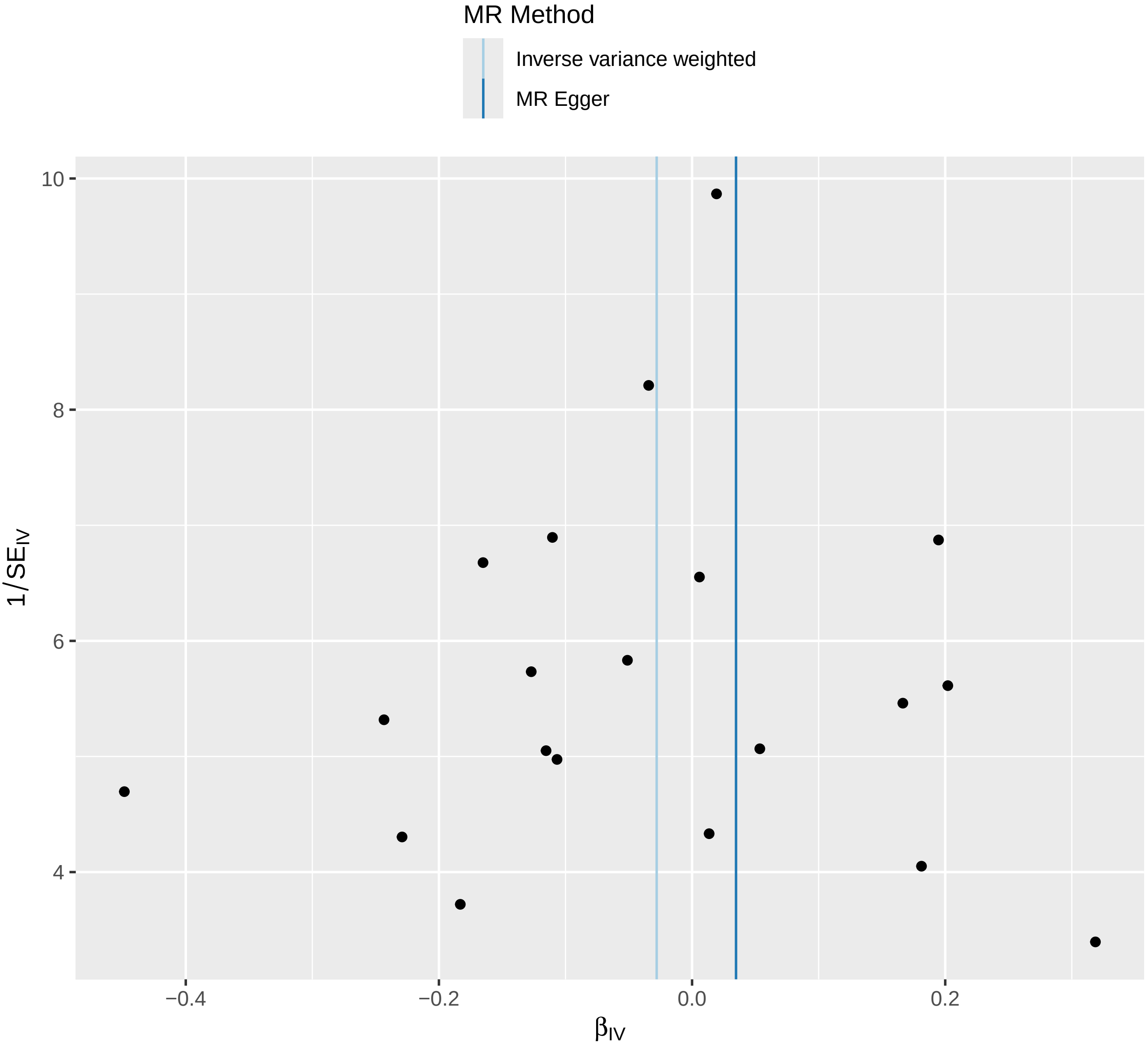
MR Method
Inverse variance weighted
MR Egger
10
8
6
4
1/SE_IV
−0.4
−0.2
0.0
0.2
β_IV

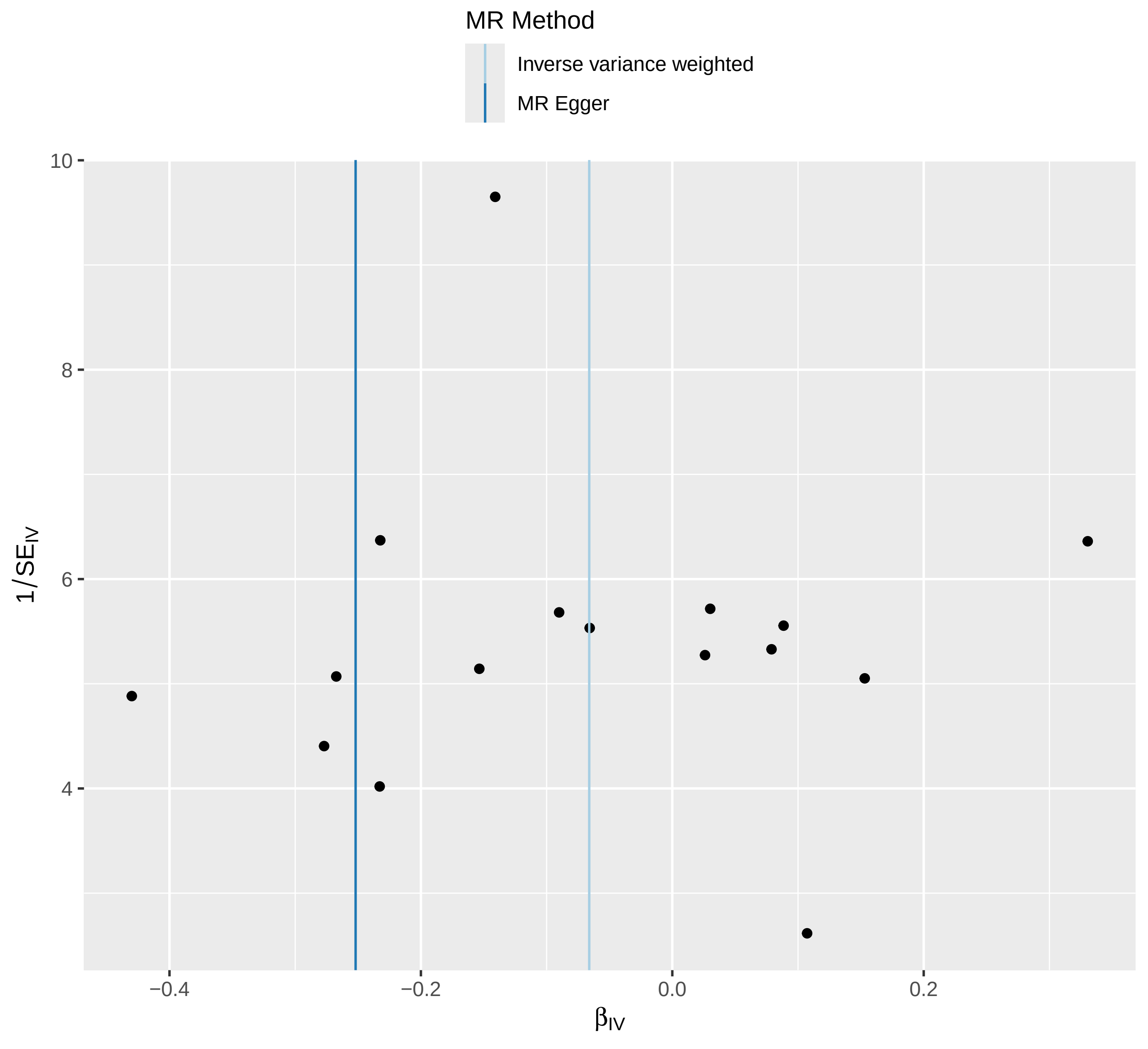

MR Method
Inverse variance weighted
MR Egger
10
8
6
4
1/SE_IV
−0.4
−0.2
0.0
0.2
β_IV

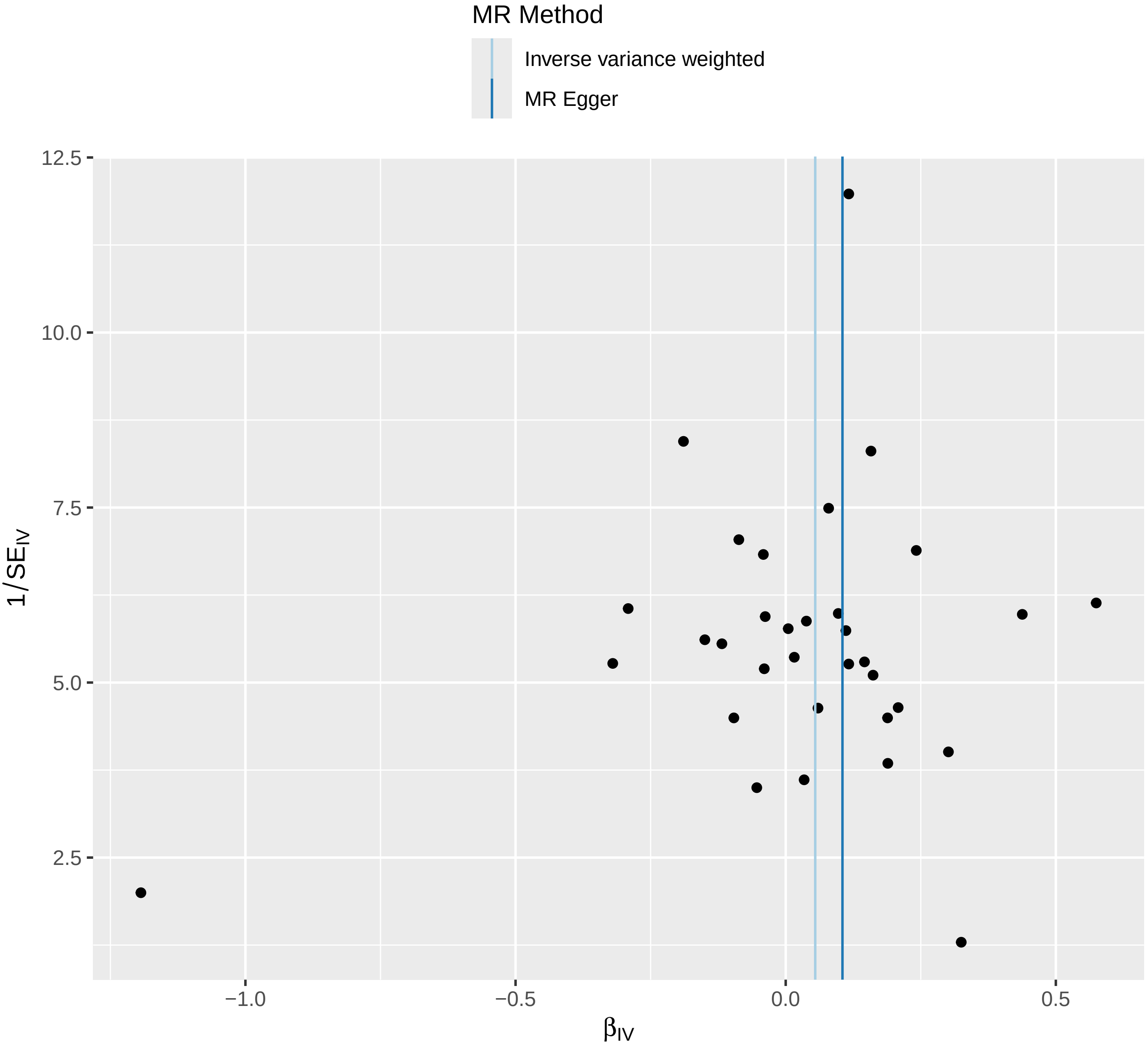
MR Method
Inverse variance weighted
MR Egger
12.5
10.0
7.5
5.0
2.5
$1/SE_{IV}$
−1.0
−0.5
0.0
0.5
β_{IV}

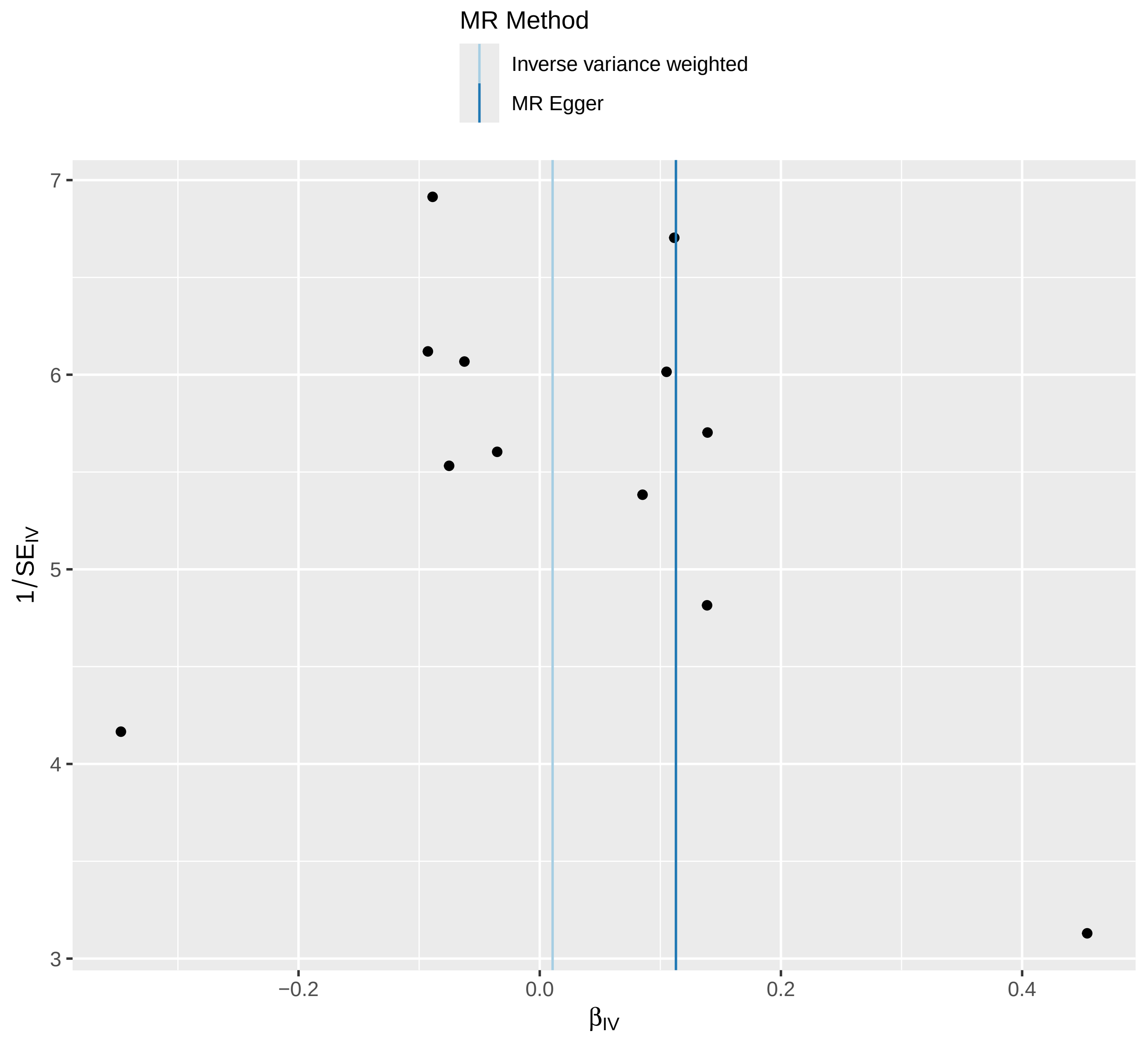

MR Method
Inverse variance weighted
MR Egger
7
6
5
4
3
1/SE_IV
−0.2
0.0
0.2
0.4
β_IV

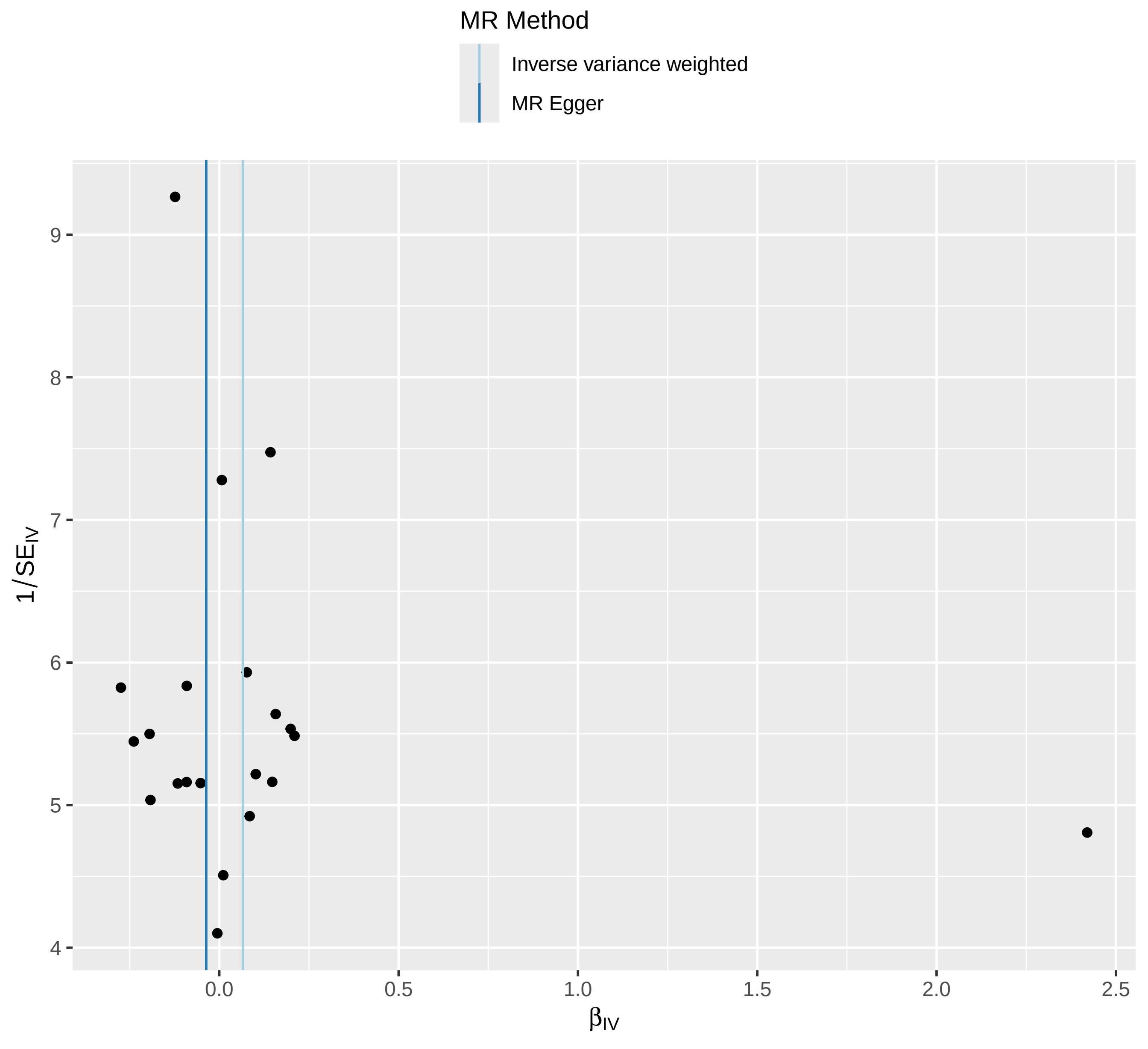

MR Method
Inverse variance weighted
MR Egger
9
8
7
6
5
4
1/SE$_{IV}$
0.0
0.5
1.0
1.5
2.0
2.5
β$_{IV}$

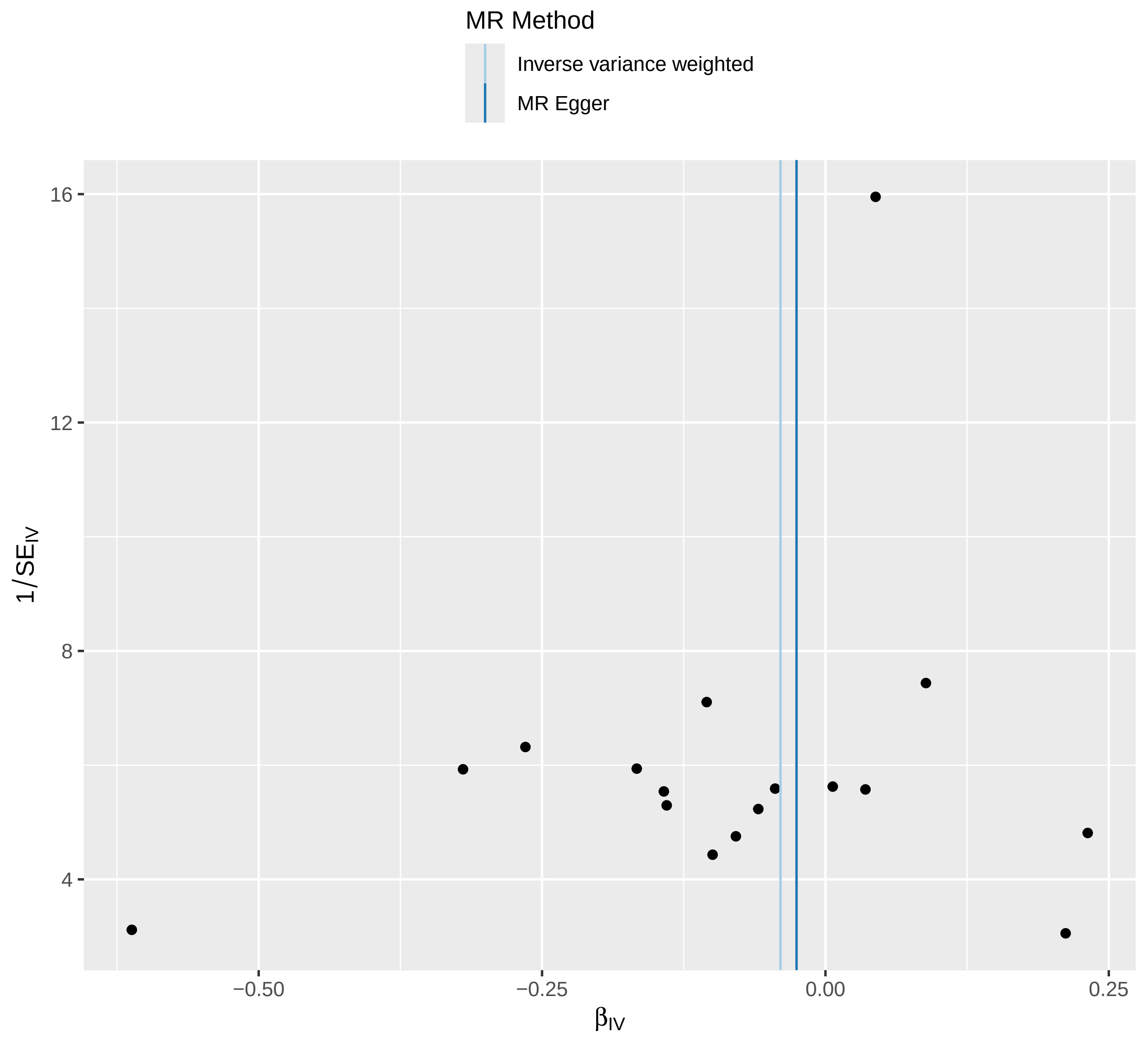
MR Method
Inverse variance weighted
MR Egger
16
12
8
4
$1/SE_{IV}$
−0.50
−0.25
0.00
0.25
β_{IV}

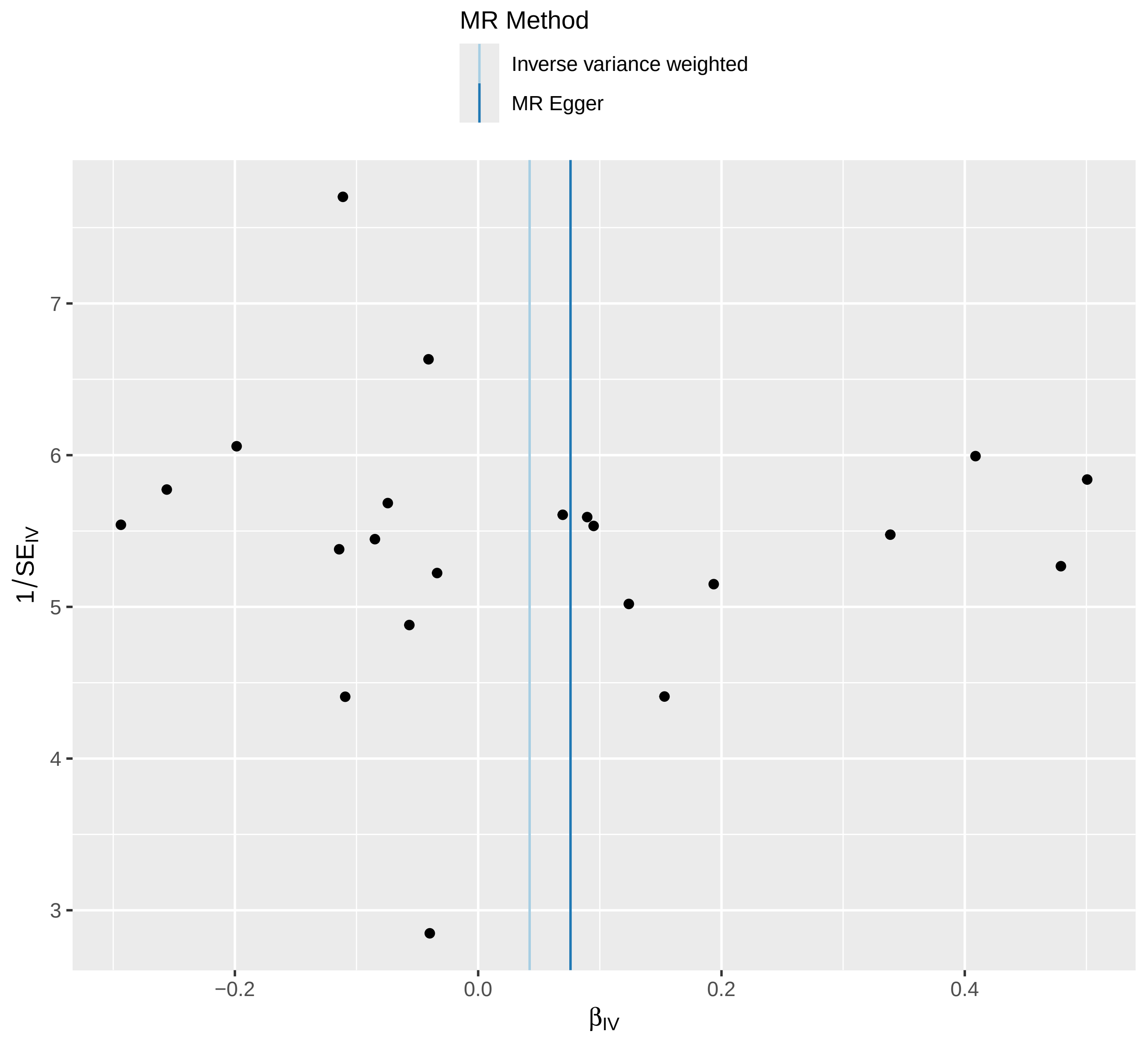
MR Method
Inverse variance weighted
MR Egger
1/SE$_{IV}$
7
6
5
4
3
−0.2
0.0
0.2
0.4
β$_{IV}$

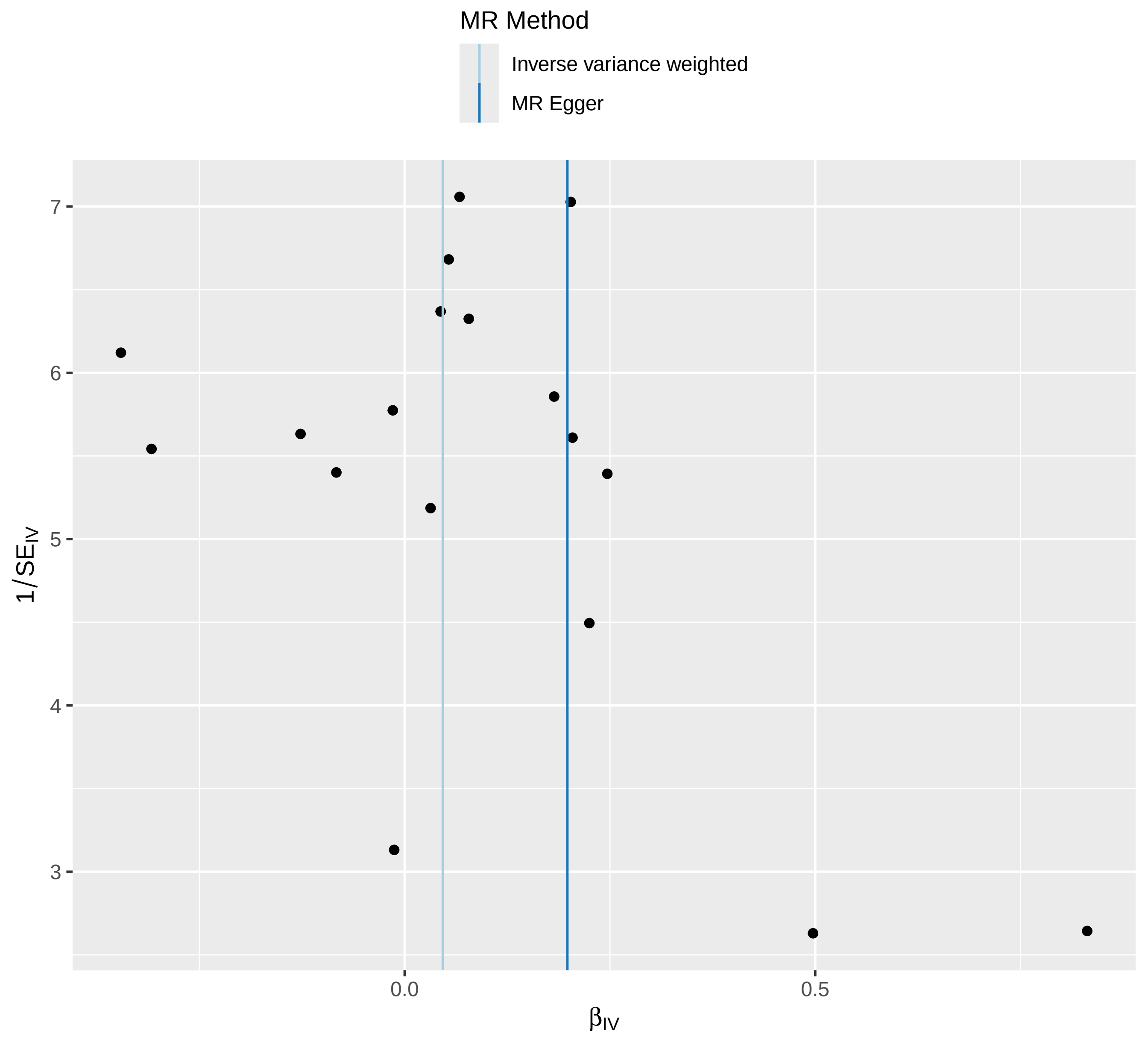
MR Method
Inverse variance weighted
MR Egger
1/SE$_{IV}$
7
6
5
4
3
0.0
0.5
β$_{IV}$

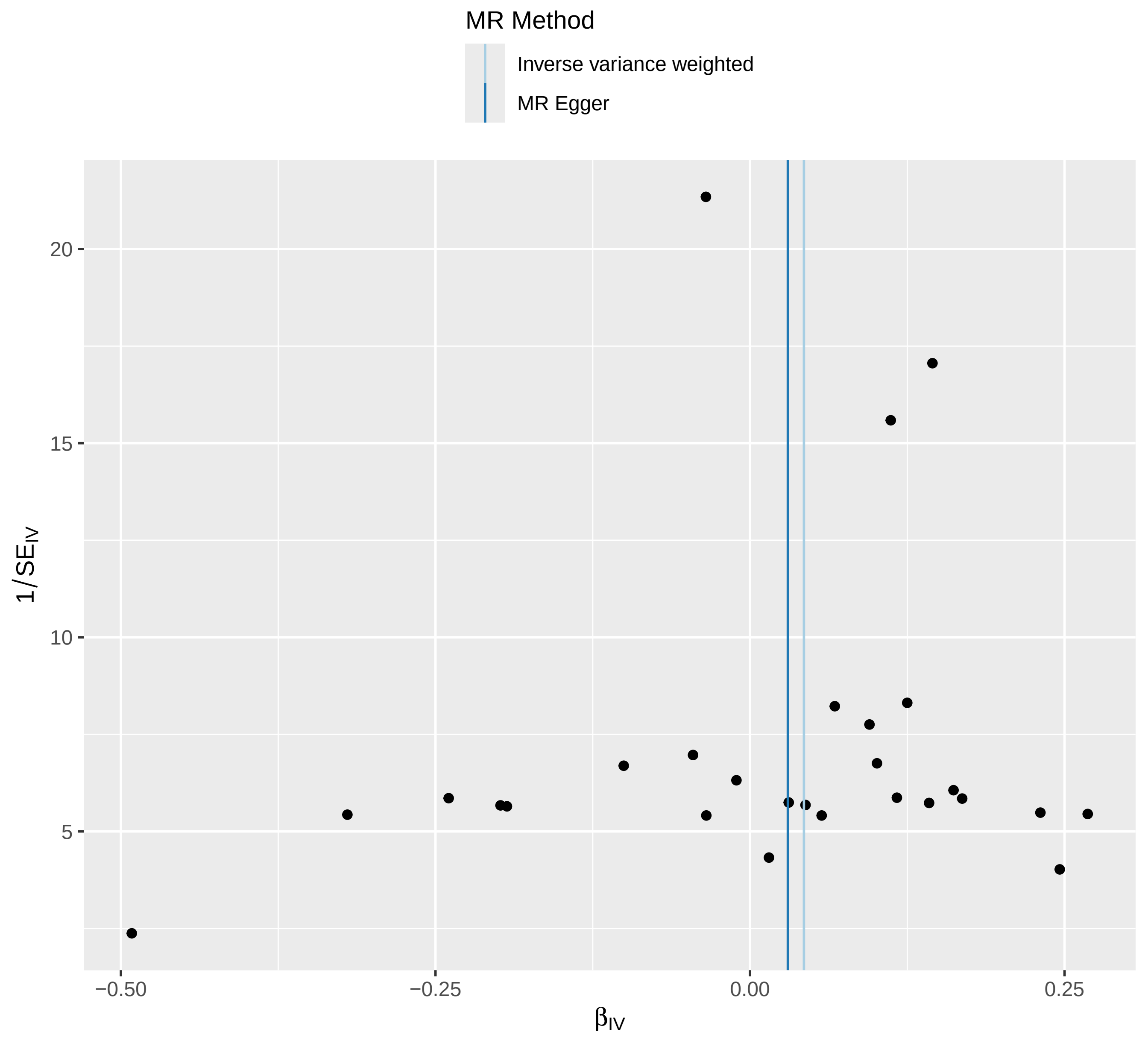
MR Method
Inverse variance weighted
MR Egger
20
15
10
5
$1/SE_{IV}$
−0.50
−0.25
0.00
0.25
β_{IV}

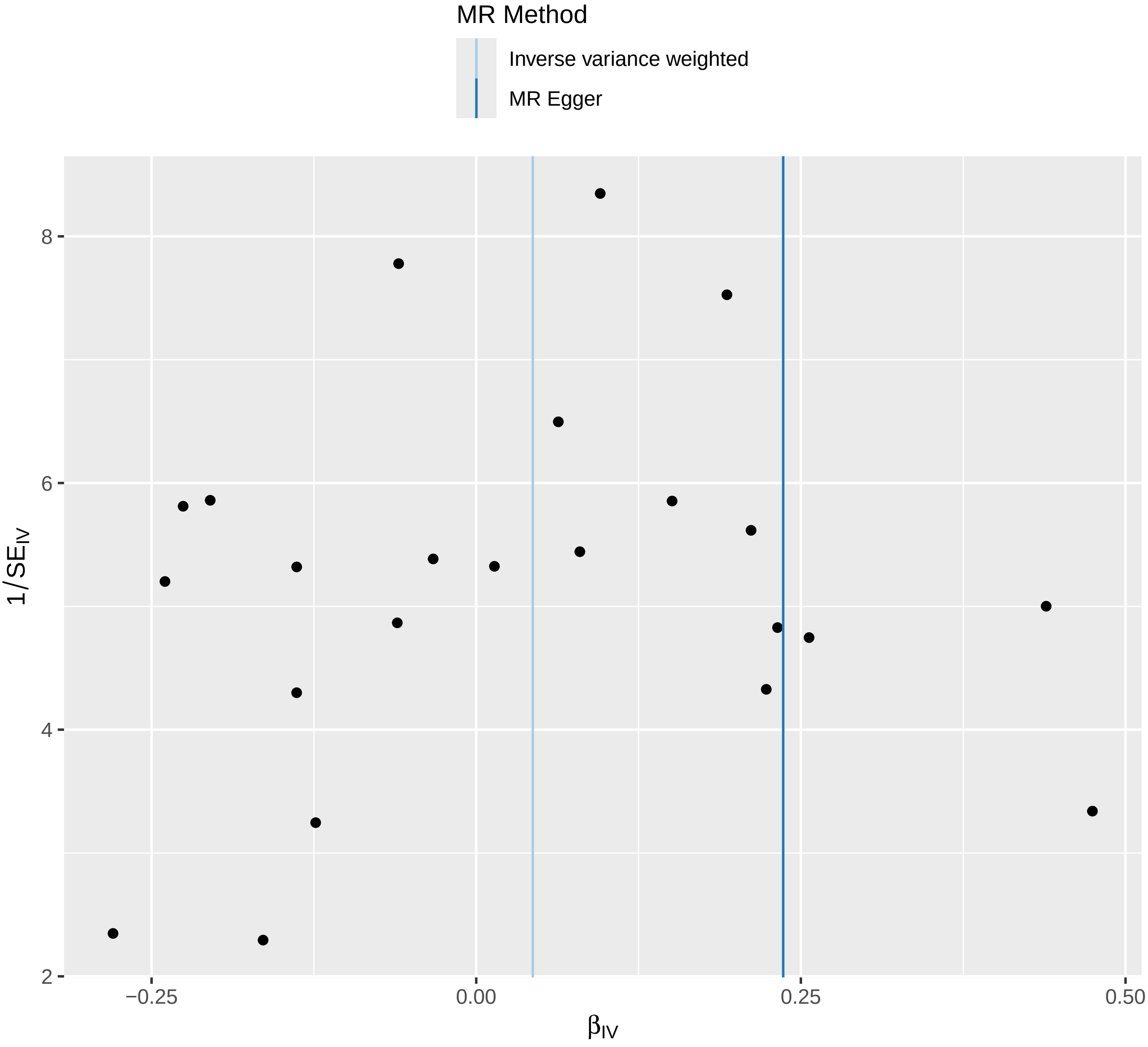

MR Method
Inverse variance weighted
MR Egger
8
6
4
2
1/SE$_{IV}$
−0.25
0.00
0.25
0.50
β$_{IV}$

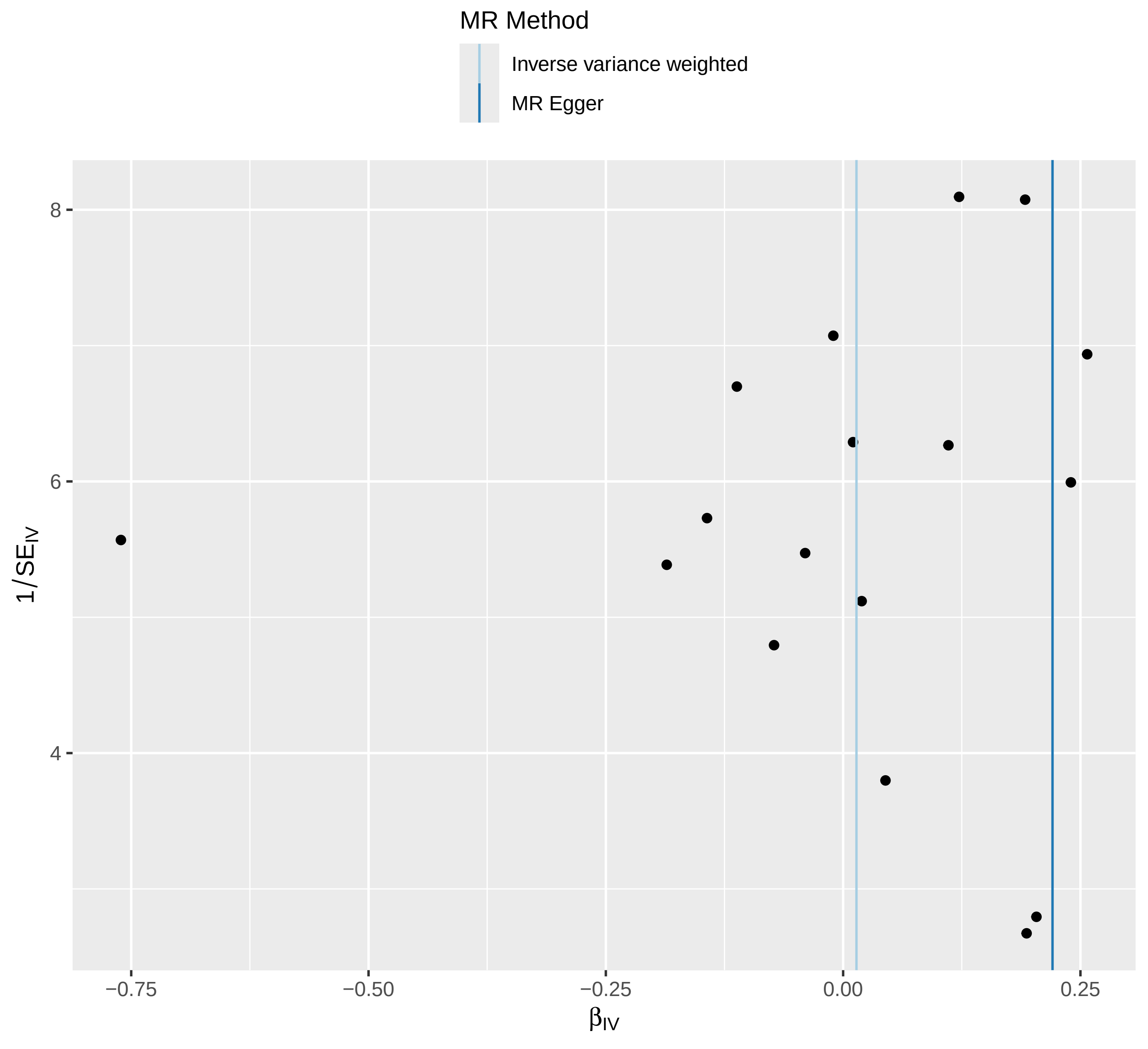
MR Method
Inverse variance weighted
MR Egger
8
6
4
$1/SE_{IV}$
−0.75
−0.50
−0.25
0.00
0.25
β_{IV}

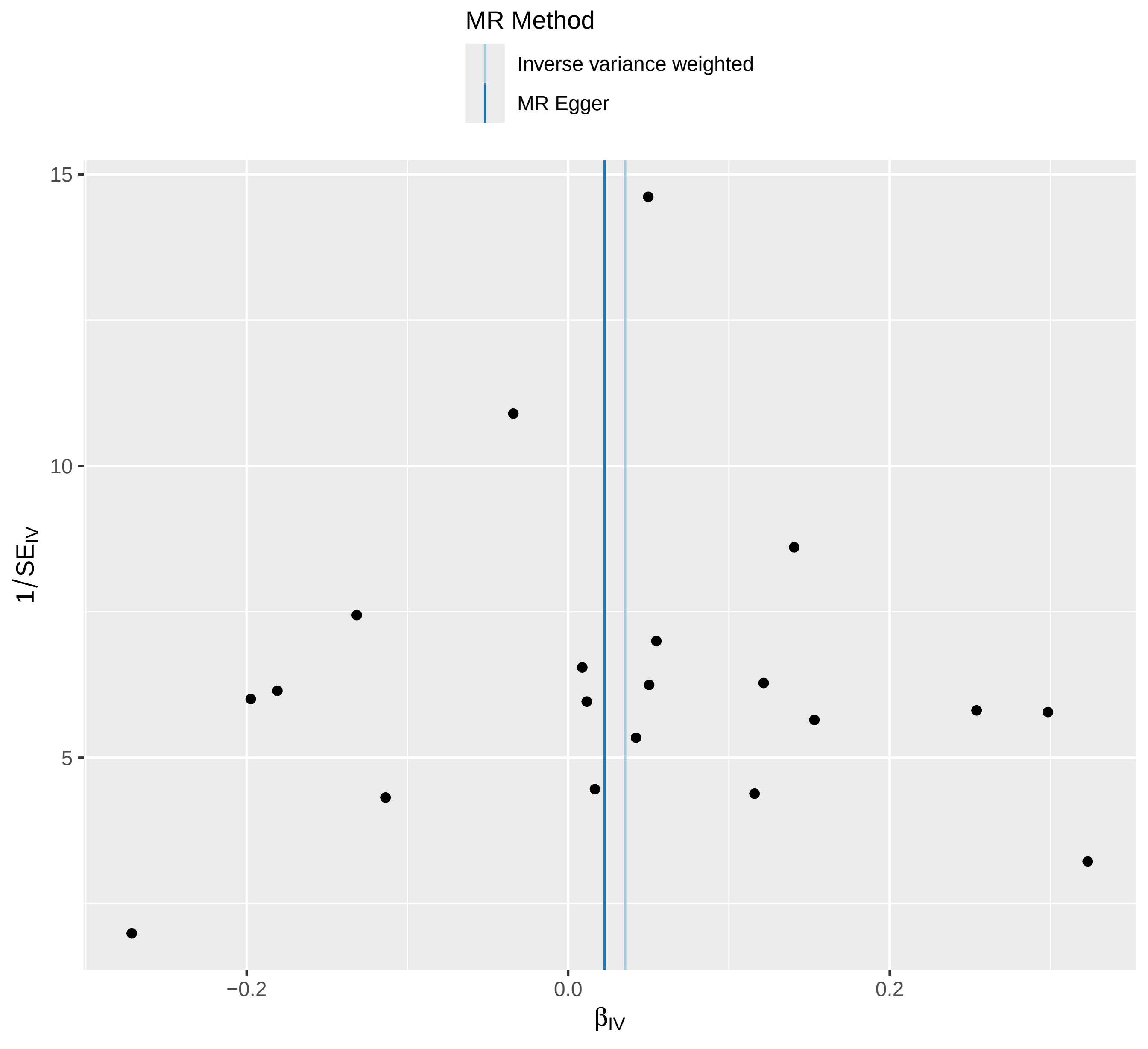
MR Method
Inverse variance weighted
MR Egger
15
10
5
1/SE_IV
−0.2
0.0
0.2
β_IV

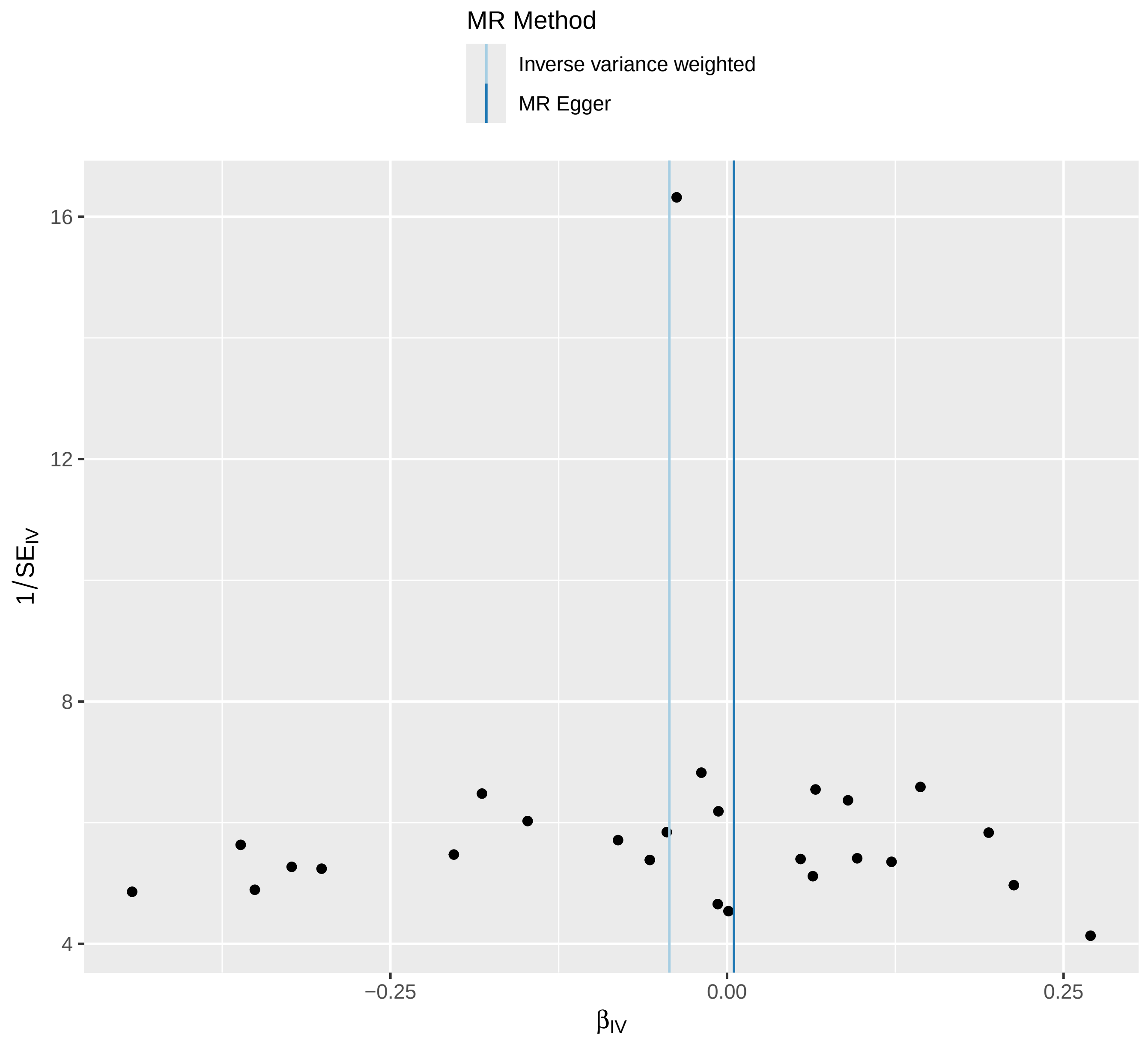
MR Method
Inverse variance weighted
MR Egger
16
12
8
4
1/SE$_{IV}$
−0.25
0.00
0.25
β$_{IV}$

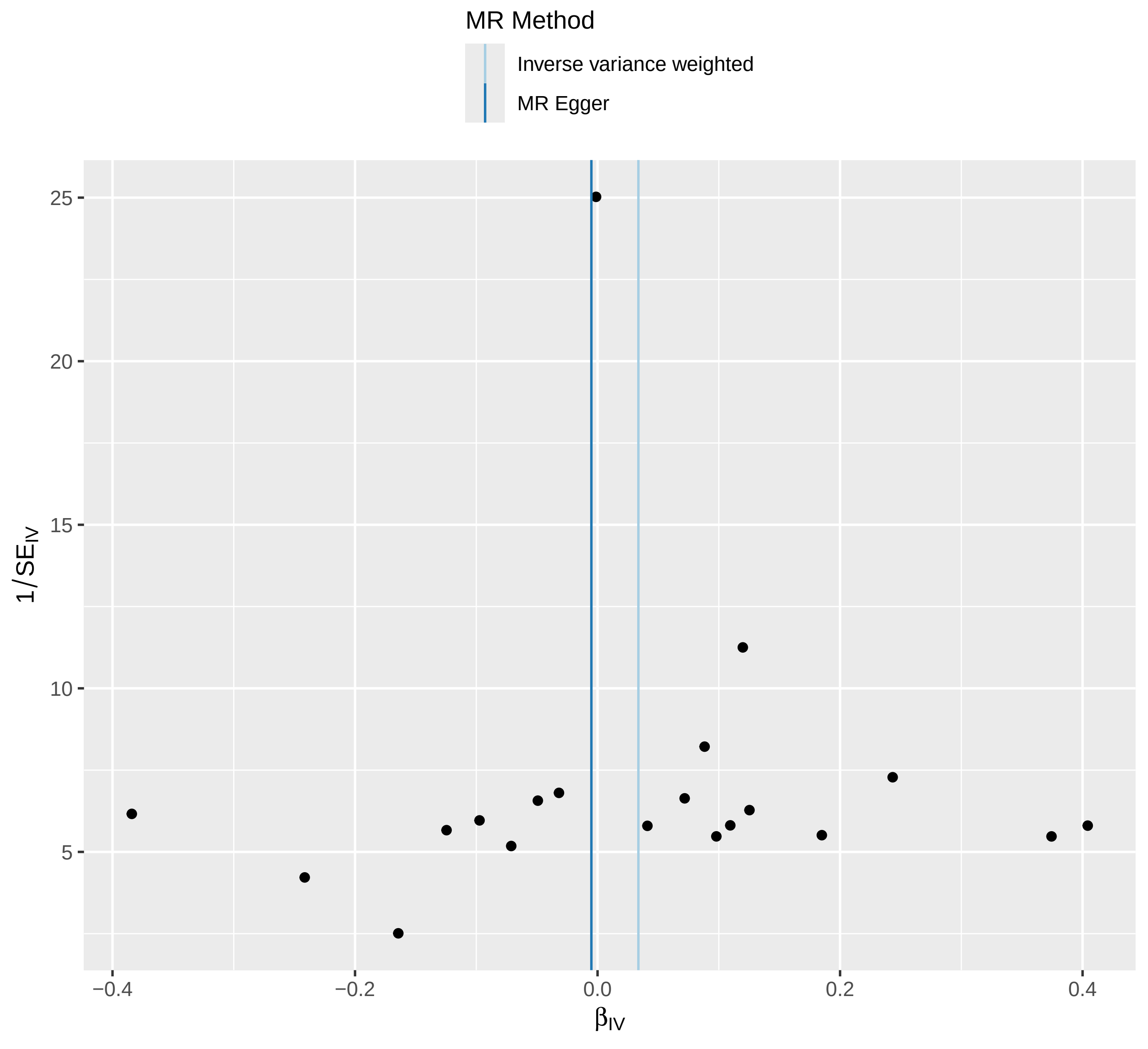
MR Method
Inverse variance weighted
MR Egger
1/SE$_{IV}$
25
20
15
10
5
−0.4
−0.2
0.0
0.2
0.4
β_{IV}

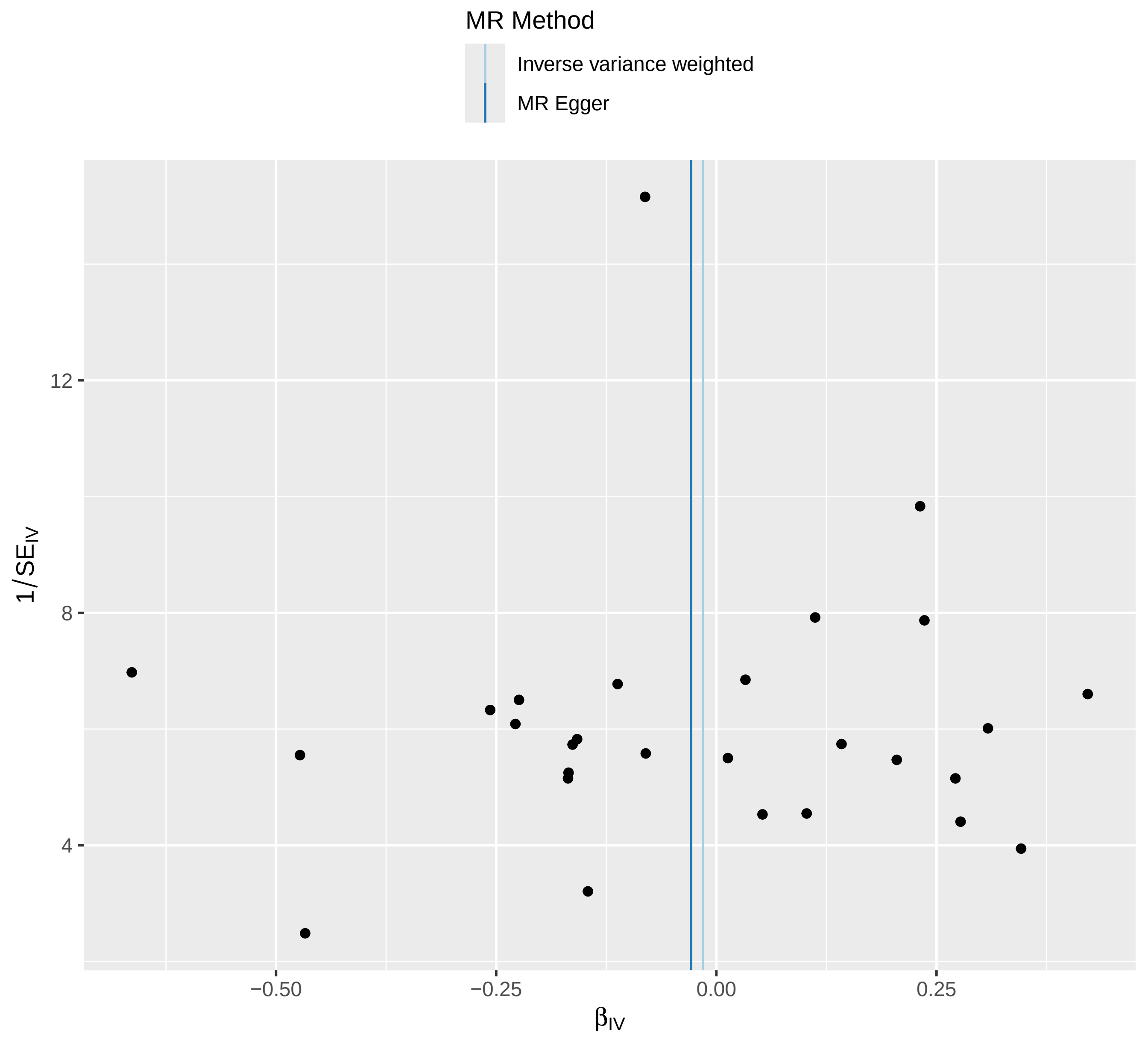
MR Method
Inverse variance weighted
MR Egger
12
8
4
1/SE_{IV}
−0.50
−0.25
0.00
0.25
β_{IV}

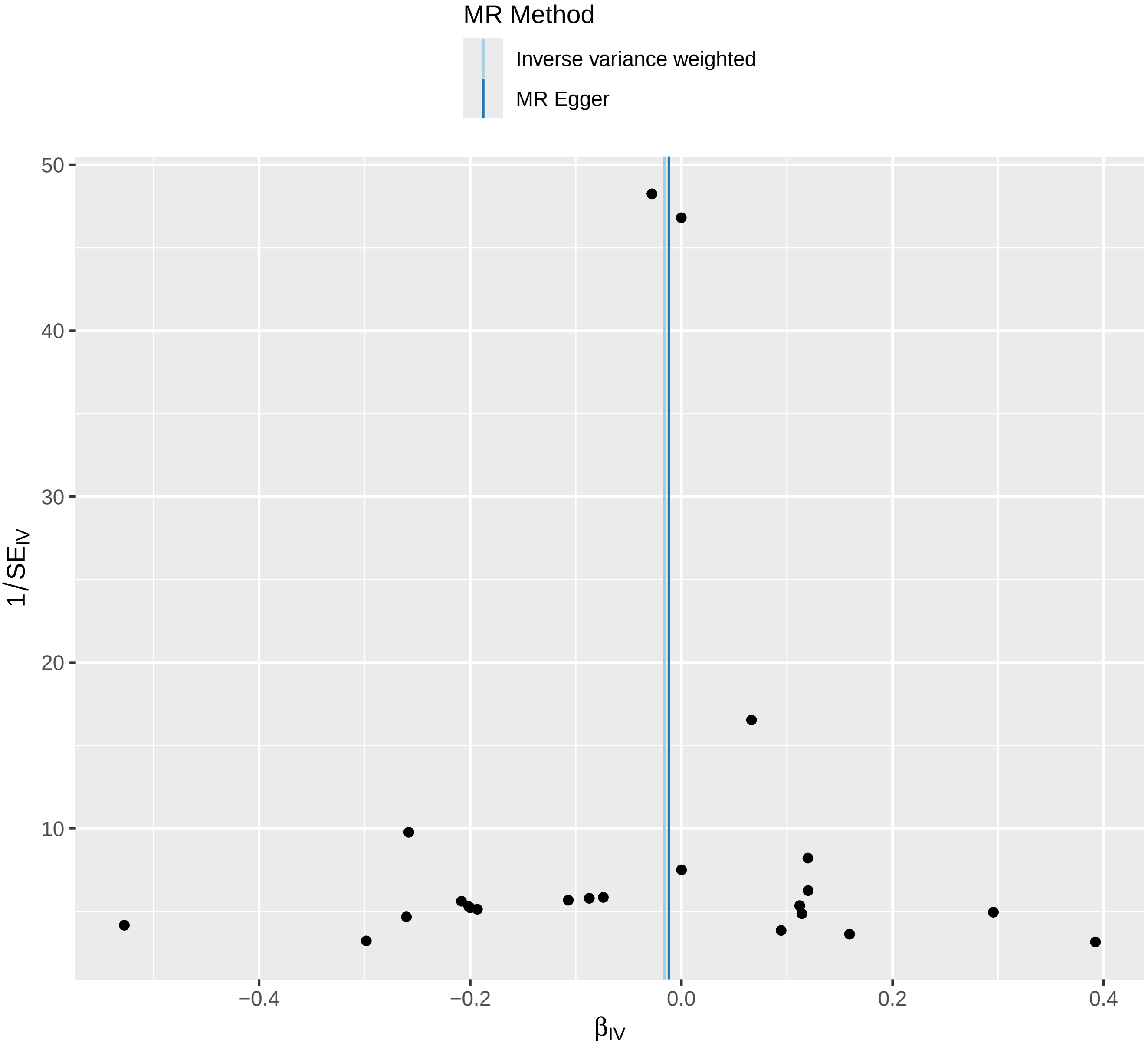
MR Method
Inverse variance weighted
MR Egger
50
40
30
20
10
1/SE_IV
−0.4
−0.2
0.0
0.2
0.4
β_IV

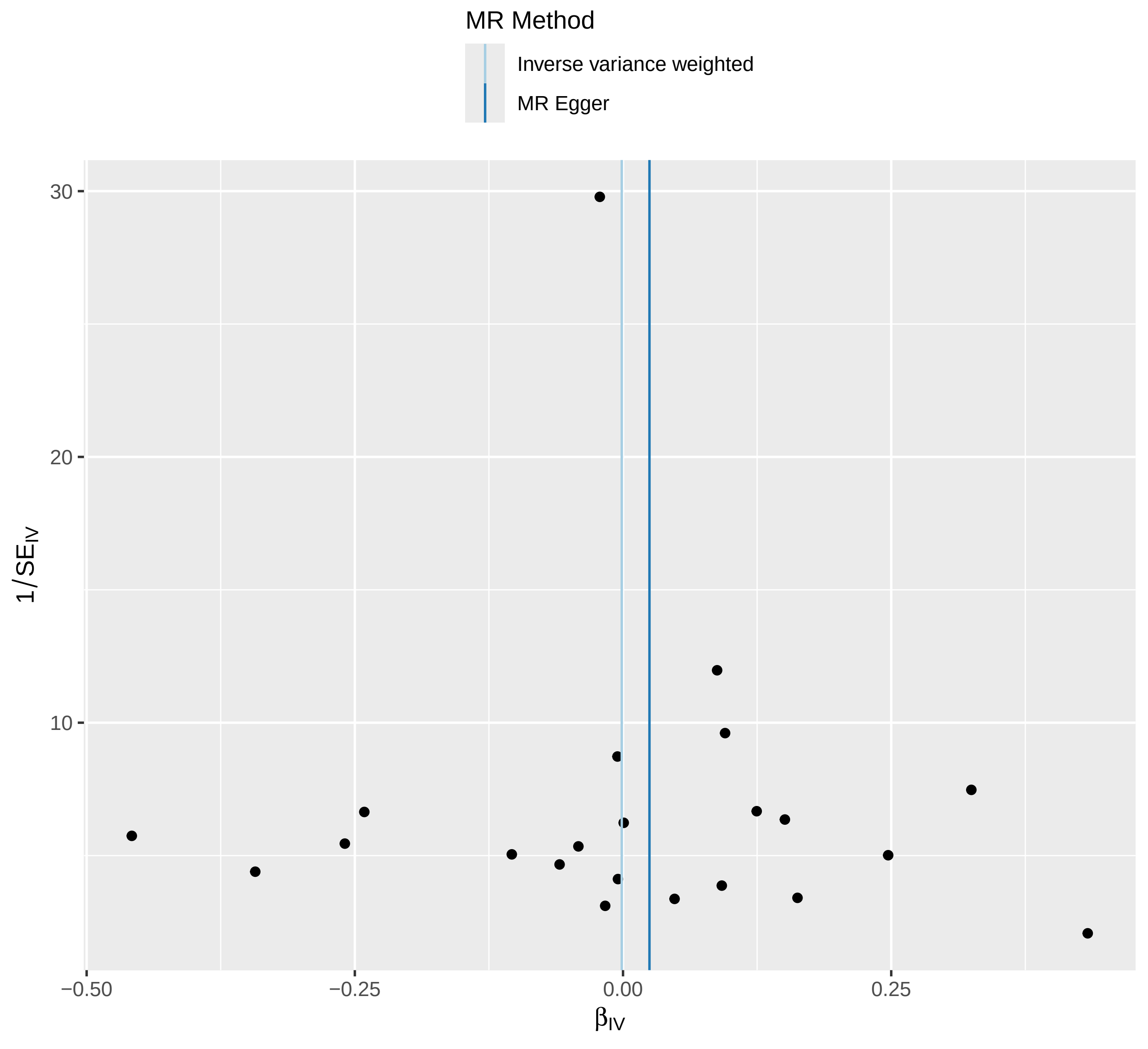

MR Method
Inverse variance weighted
MR Egger
30
20
10
1/SE$_{IV}$
−0.50
−0.25
0.00
0.25
β$_{IV}$

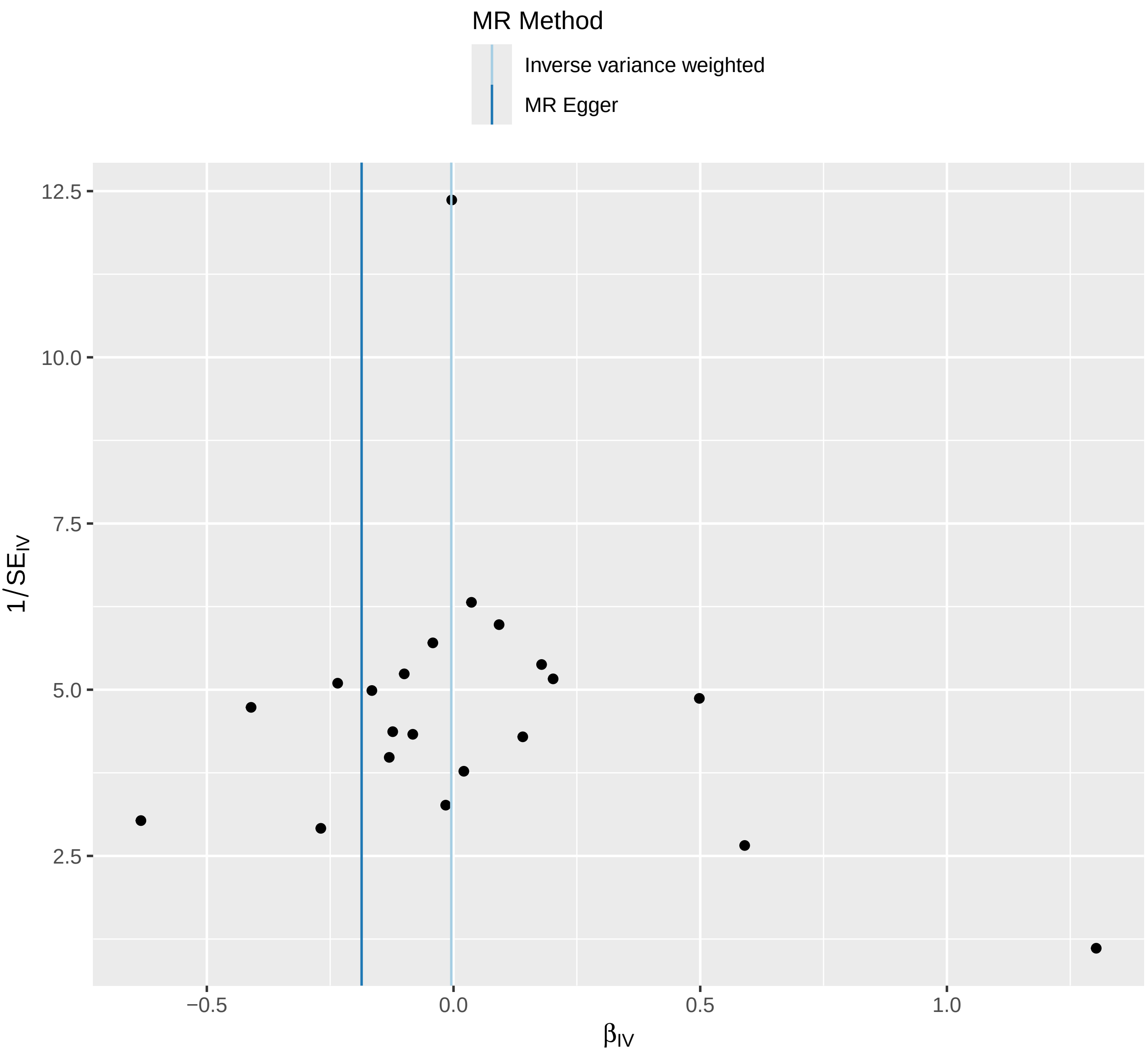

MR Method
Inverse variance weighted
MR Egger
12.5
10.0
7.5
5.0
2.5
$1/SE_{IV}$
−0.5
0.0
0.5
1.0
β_{IV}

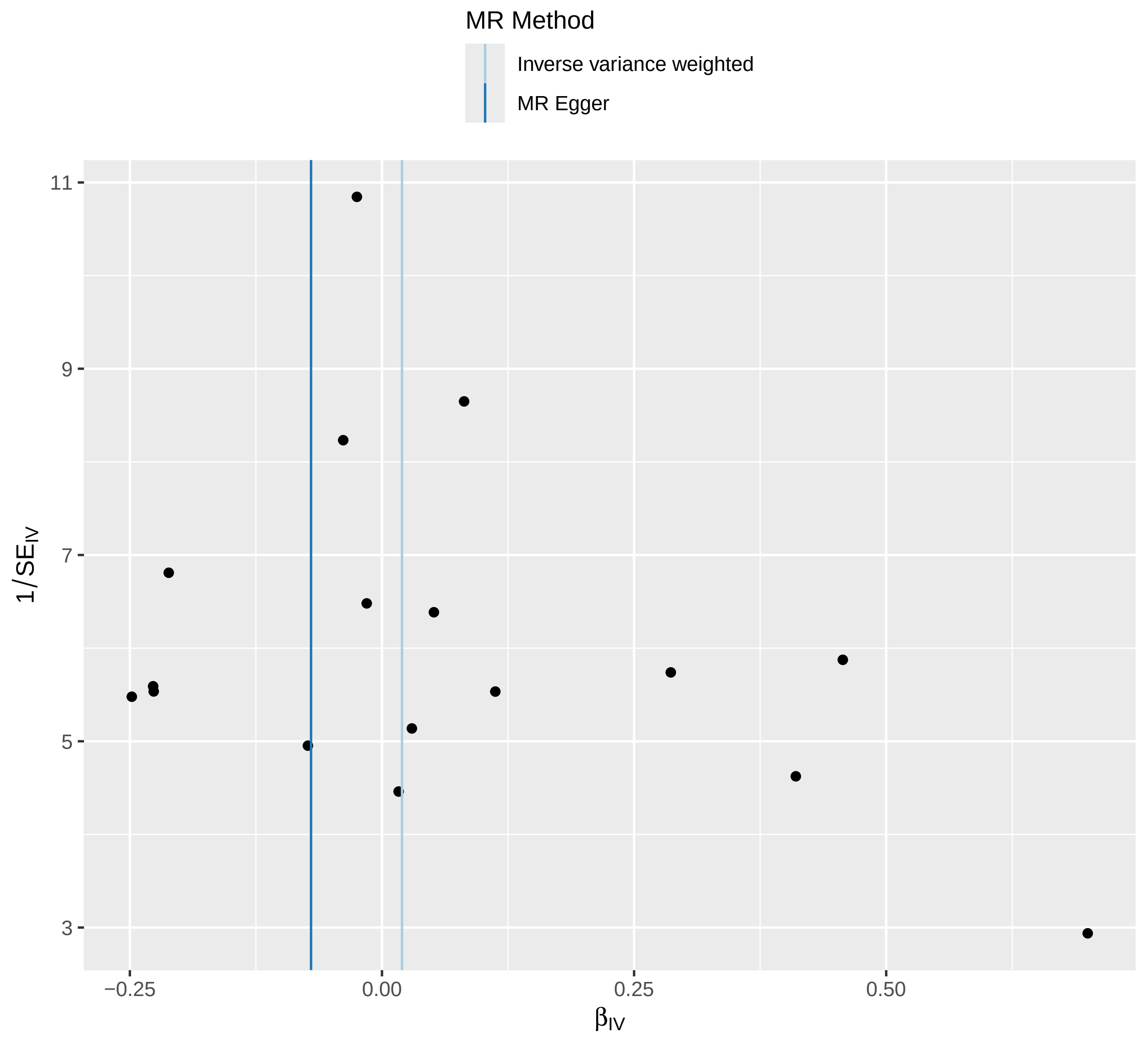
MR Method
Inverse variance weighted
MR Egger
11
9
7
5
3
$1/SE_{IV}$
−0.25
0.00
0.25
0.50
β_{IV}

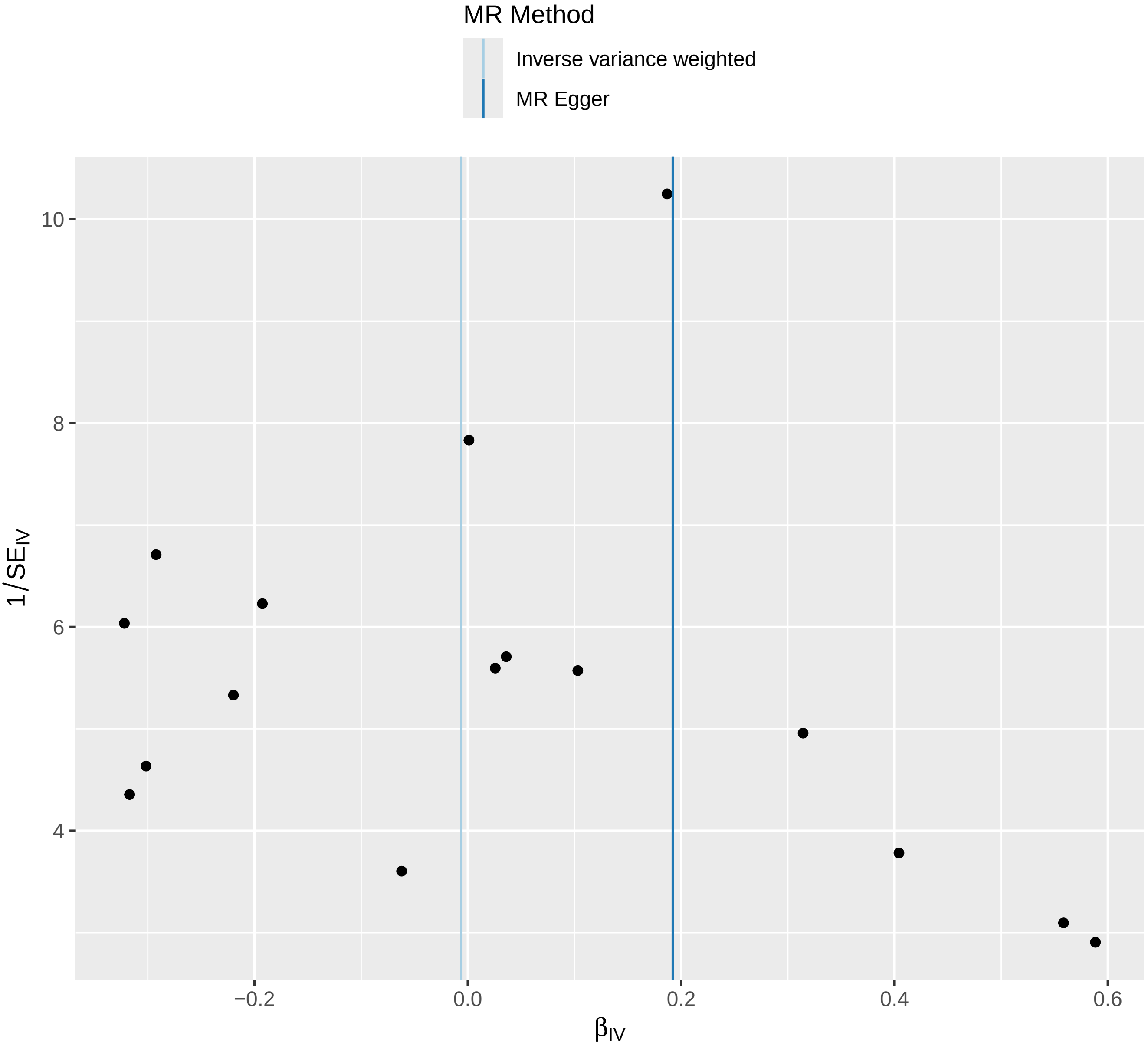
MR Method
Inverse variance weighted
MR Egger
10
8
6
4
−0.2
0.0
0.2
0.4
0.6
1/SE_IV
β_IV

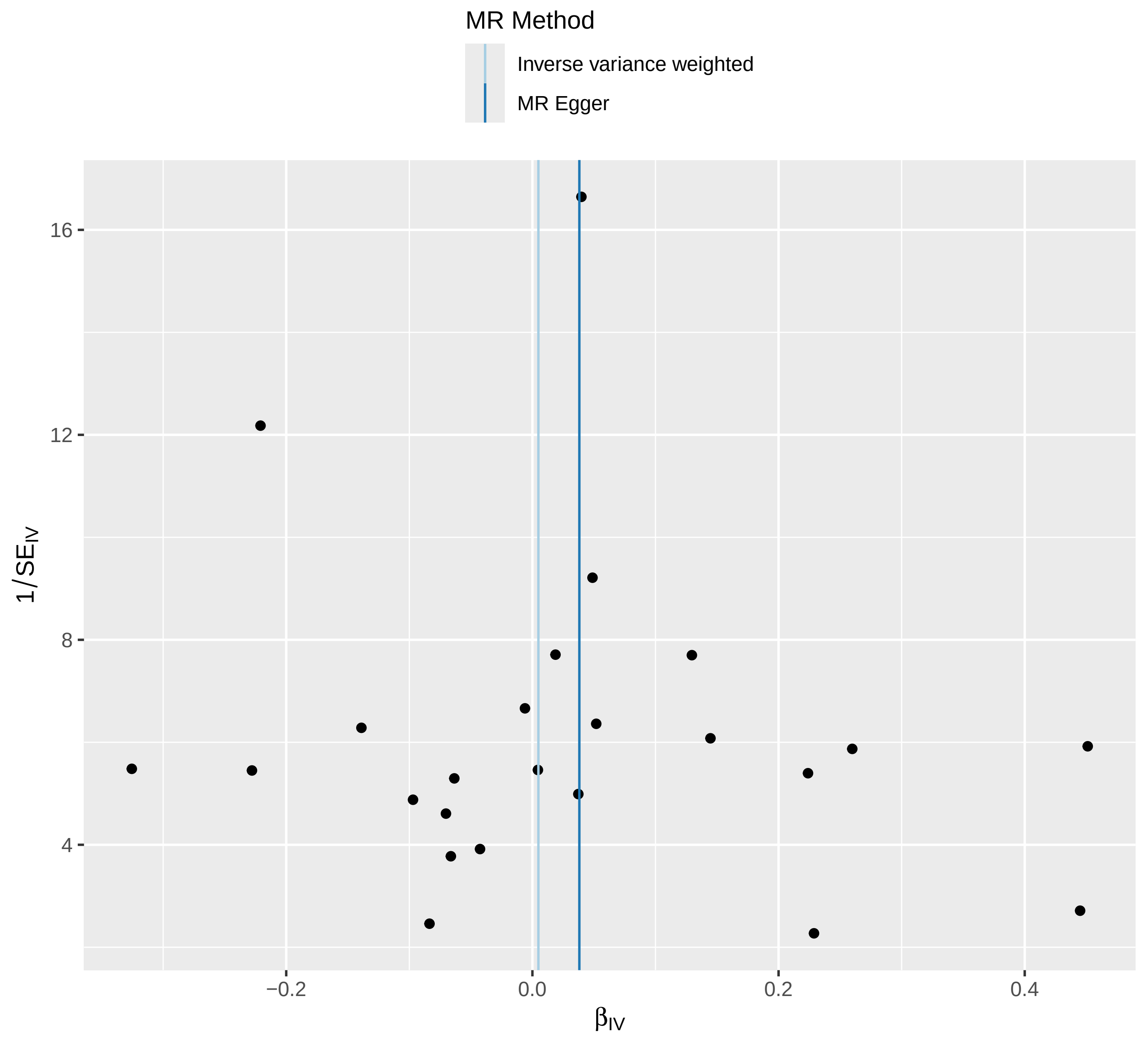
MR Method
Inverse variance weighted
MR Egger
16
12
8
4
1/SE_IV
−0.2
0.0
0.2
0.4
β_IV

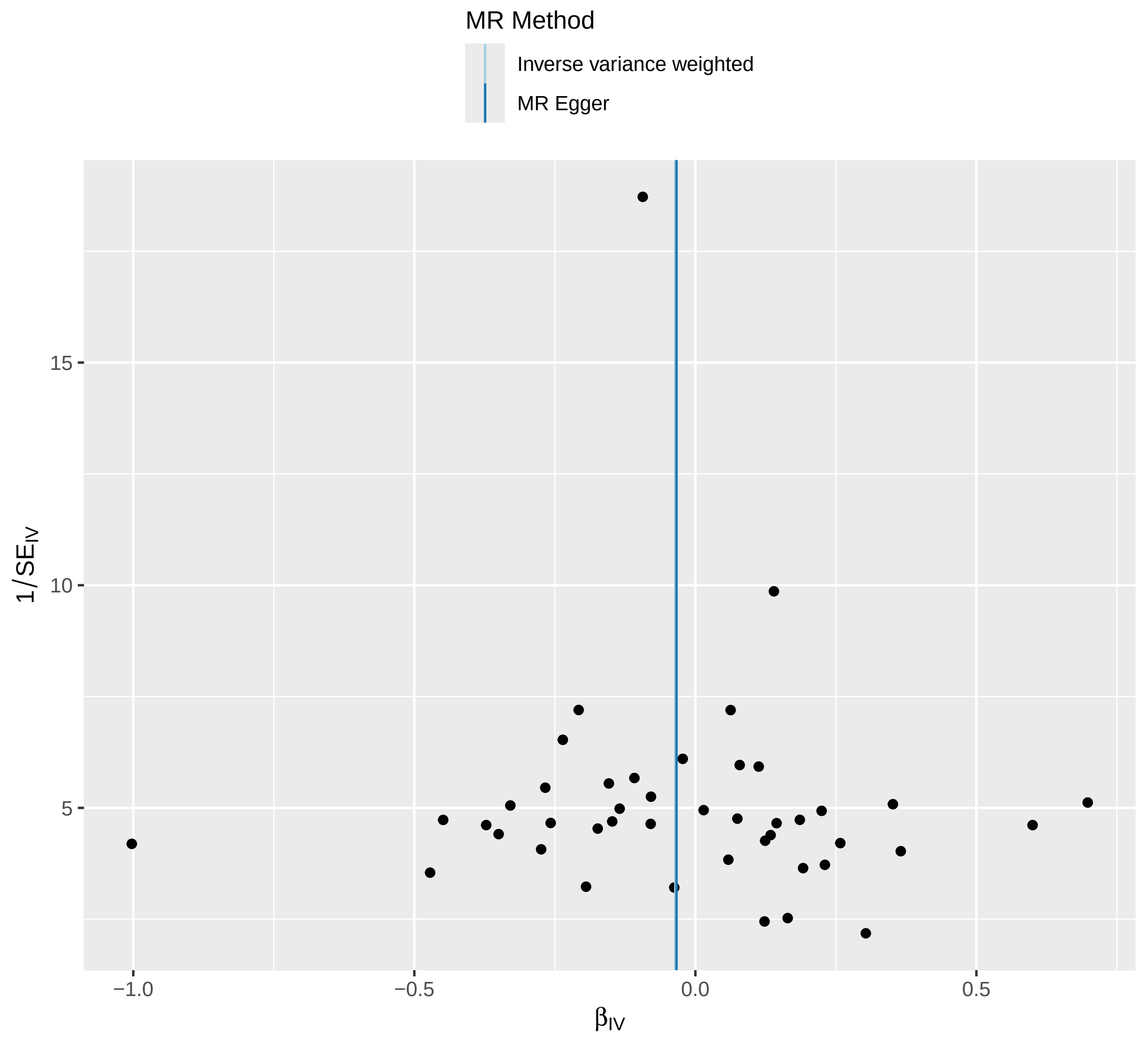
MR Method
Inverse variance weighted
MR Egger
15
10
5
1/SE_IV
−1.0
−0.5
0.0
0.5
β_IV

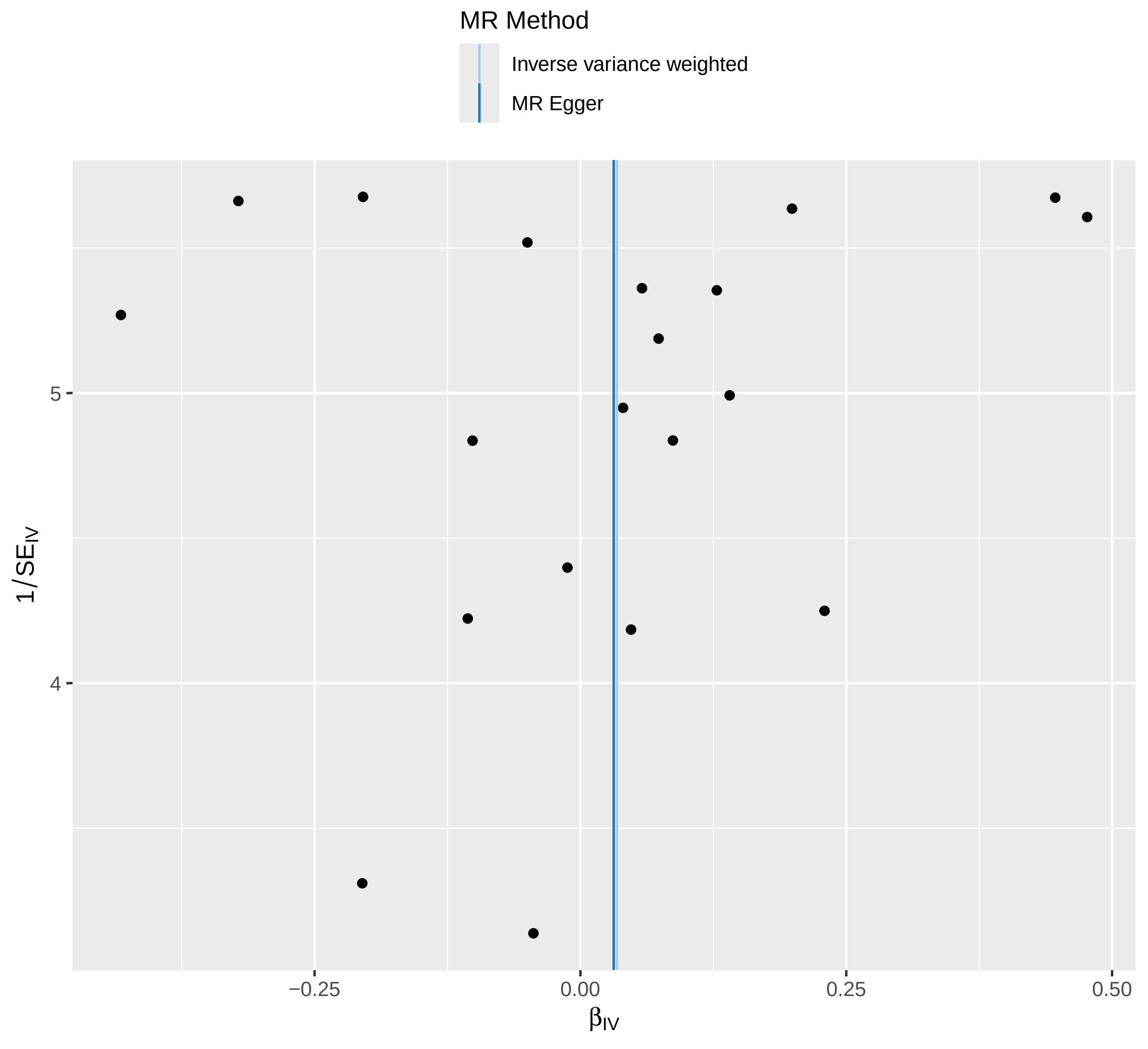
MR Method
Inverse variance weighted
MR Egger
5
4
1/SE_IV
−0.25
0.00
0.25
0.50
β_IV

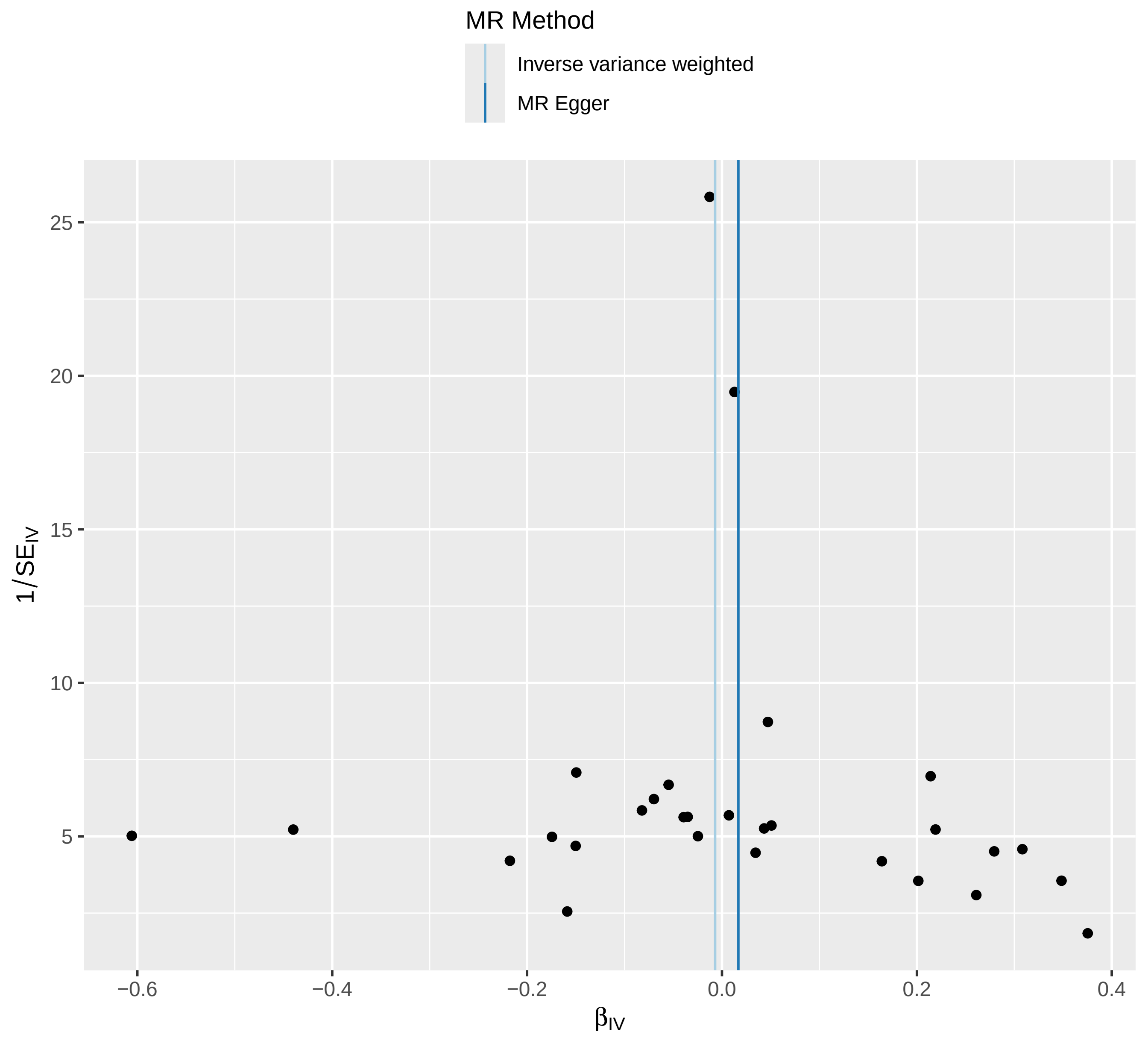

MR Method
Inverse variance weighted
MR Egger
25
20
15
10
5
1/SE$_{IV}$
−0.6
−0.4
−0.2
0.0
0.2
0.4
β$_{IV}$

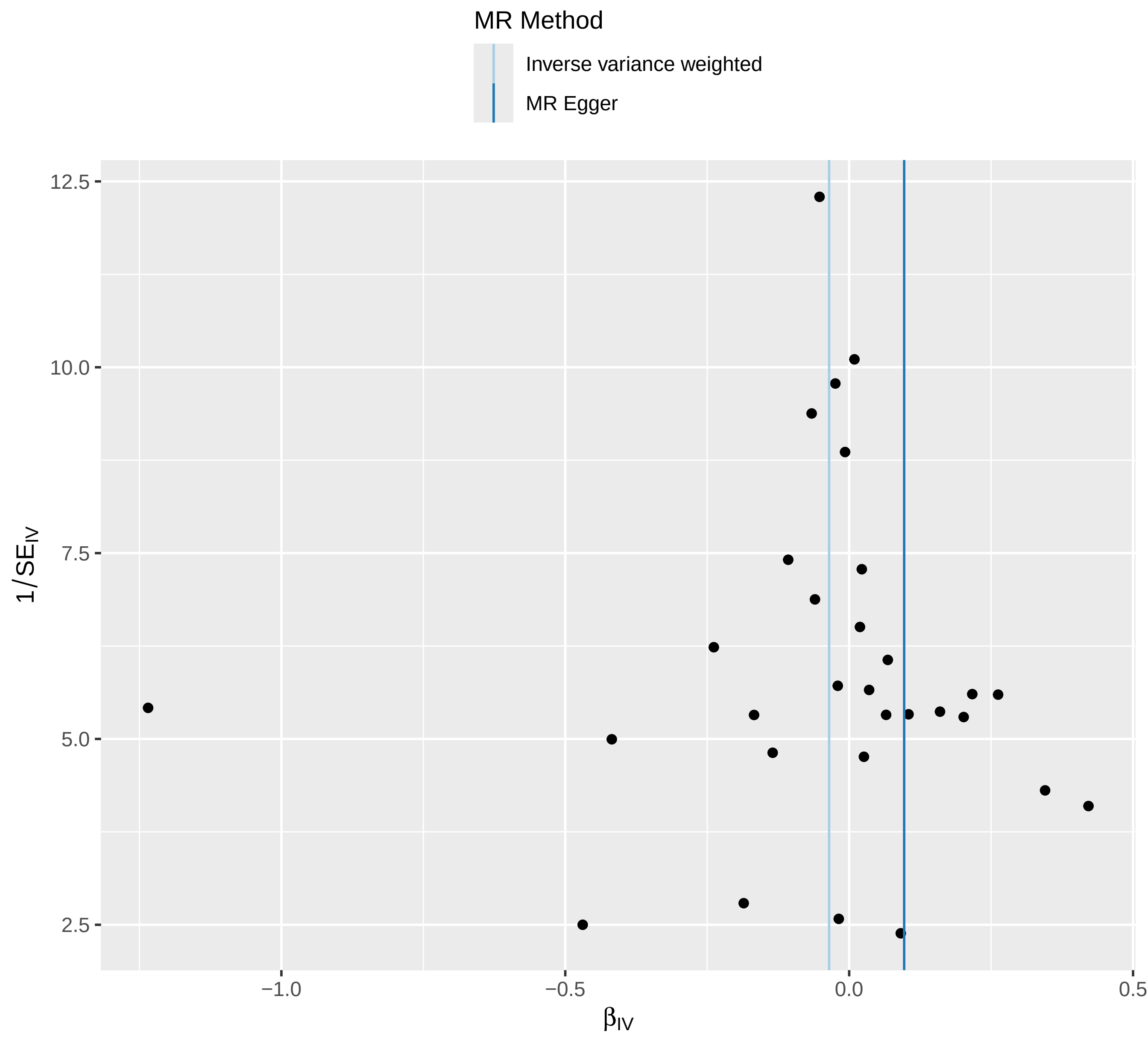

MR Method
Inverse variance weighted
MR Egger
12.5
10.0
7.5
5.0
2.5
1/SE_IV
−1.0
−0.5
0.0
0.5
β_IV

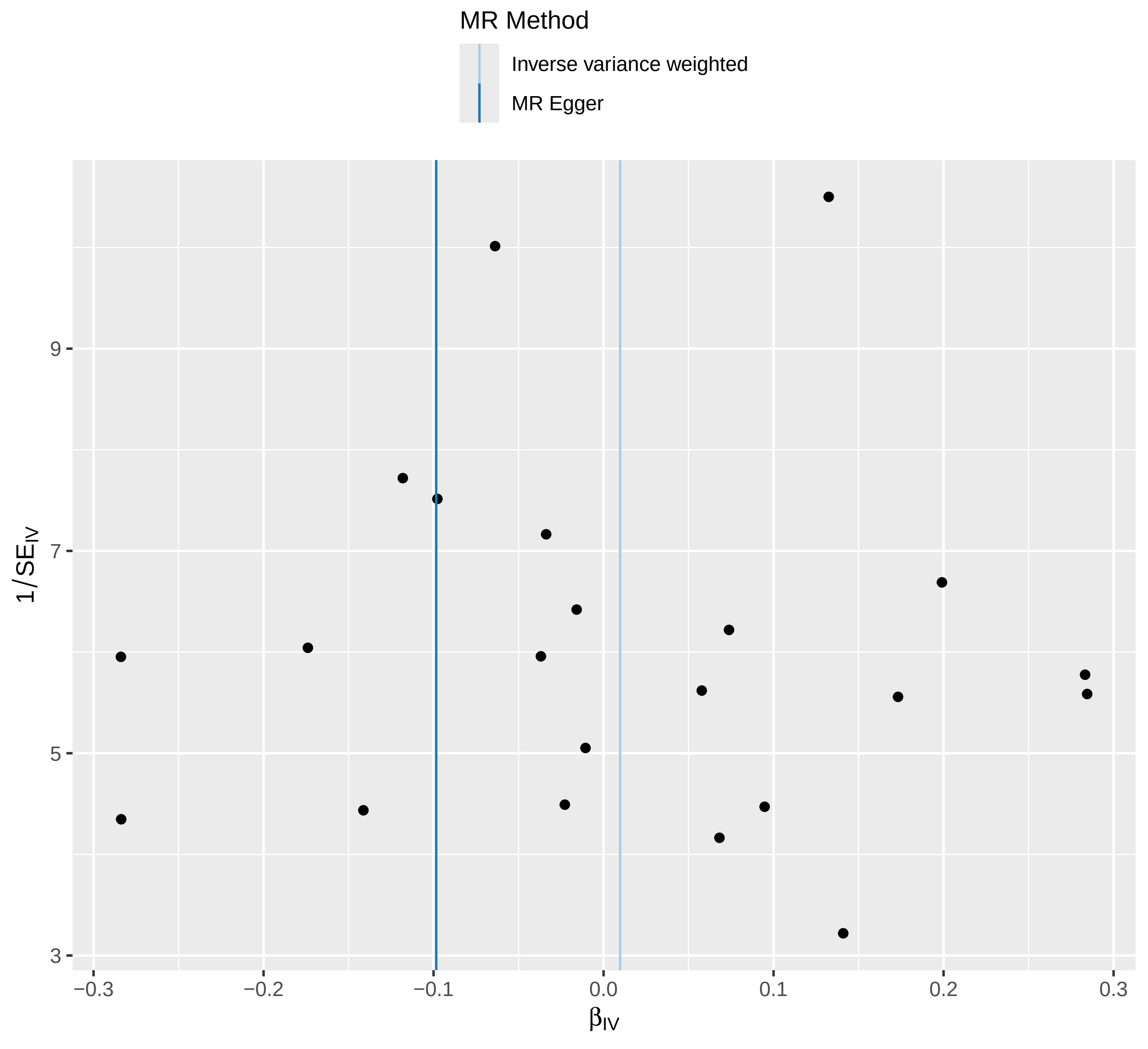

MR Method
Inverse variance weighted
MR Egger
9
7
5
3
1/SE_IV
−0.3
−0.2
−0.1
0.0
0.1
0.2
0.3
β_IV

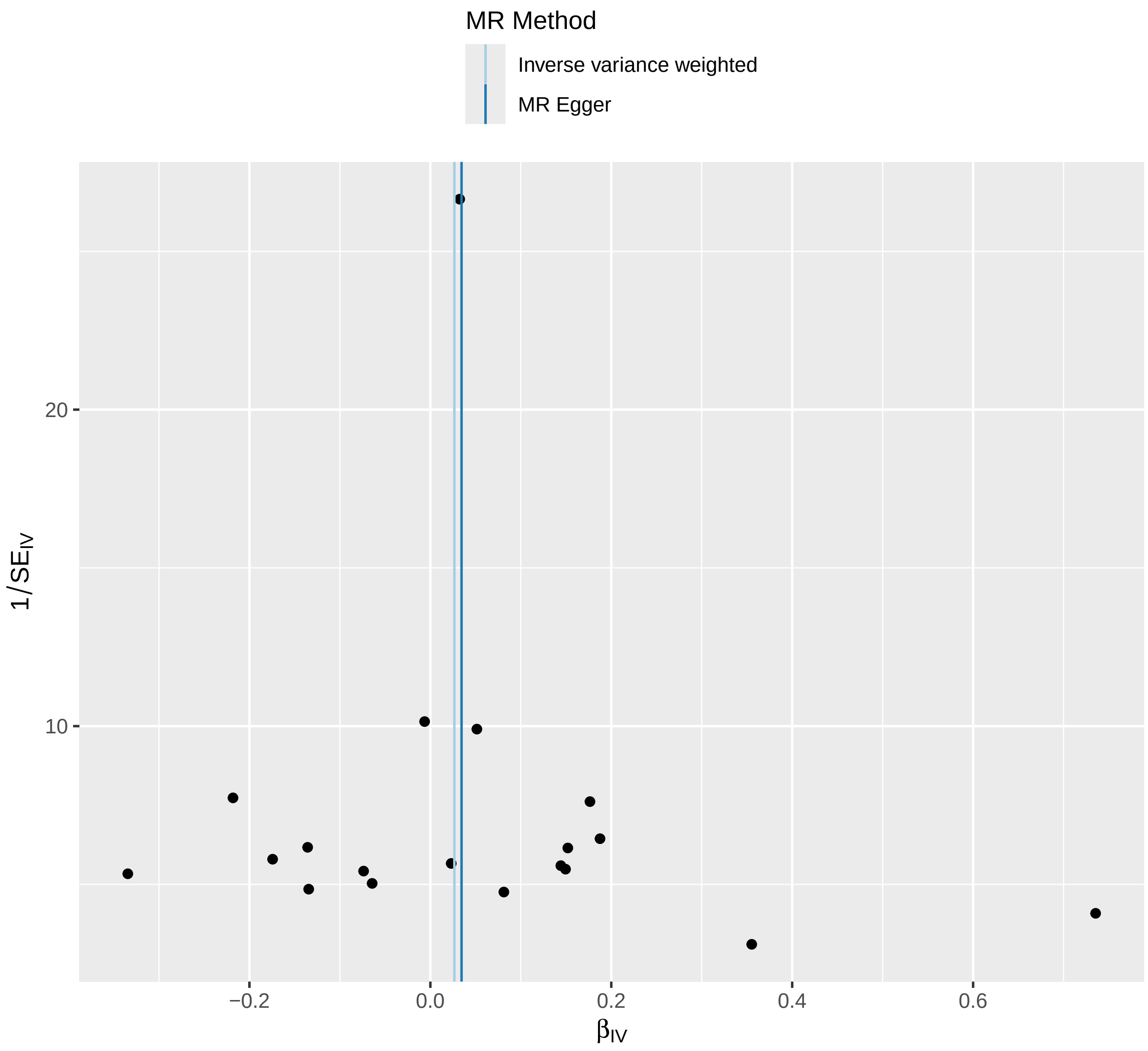

MR Method
Inverse variance weighted
MR Egger
20
10
1/SE_IV
−0.2
0.0
0.2
0.4
0.6
β_IV

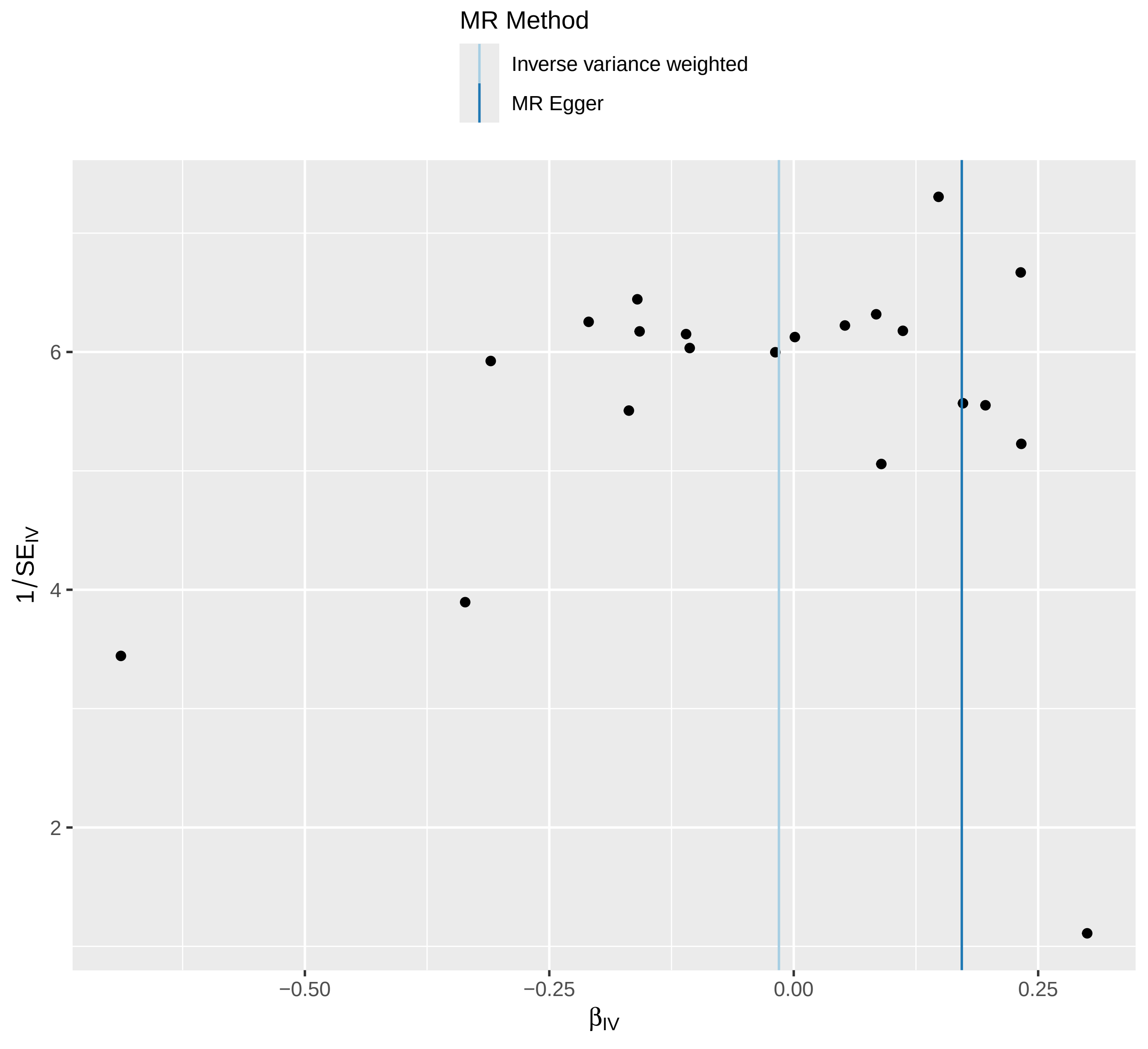
MR Method
Inverse variance weighted
MR Egger
6
4
2
1/SE$_{IV}$
−0.50
−0.25
0.00
0.25
β$_{IV}$

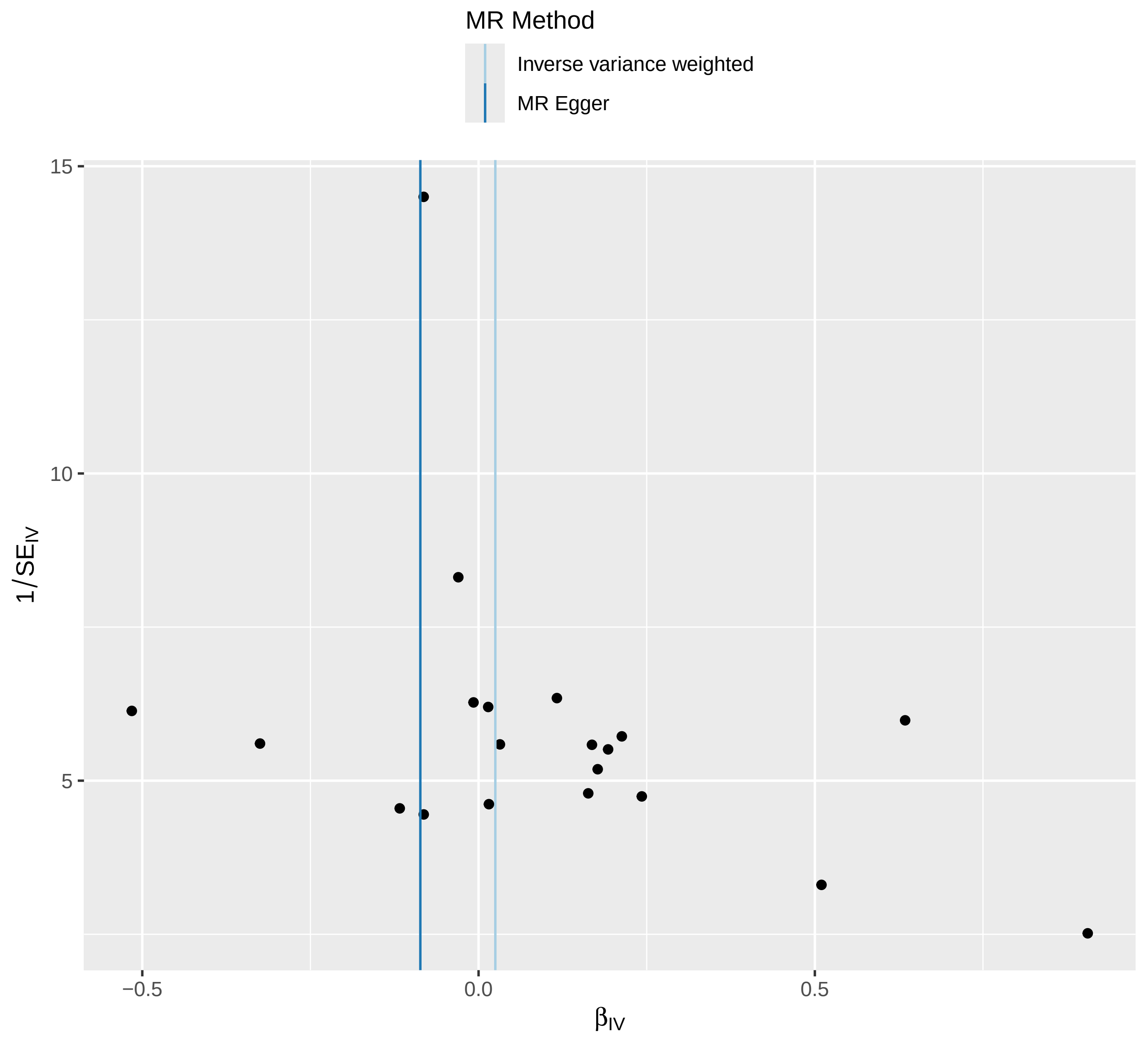

MR Method
Inverse variance weighted
MR Egger
15
10
5
1/SE_IV
−0.5
0.0
0.5
β_IV

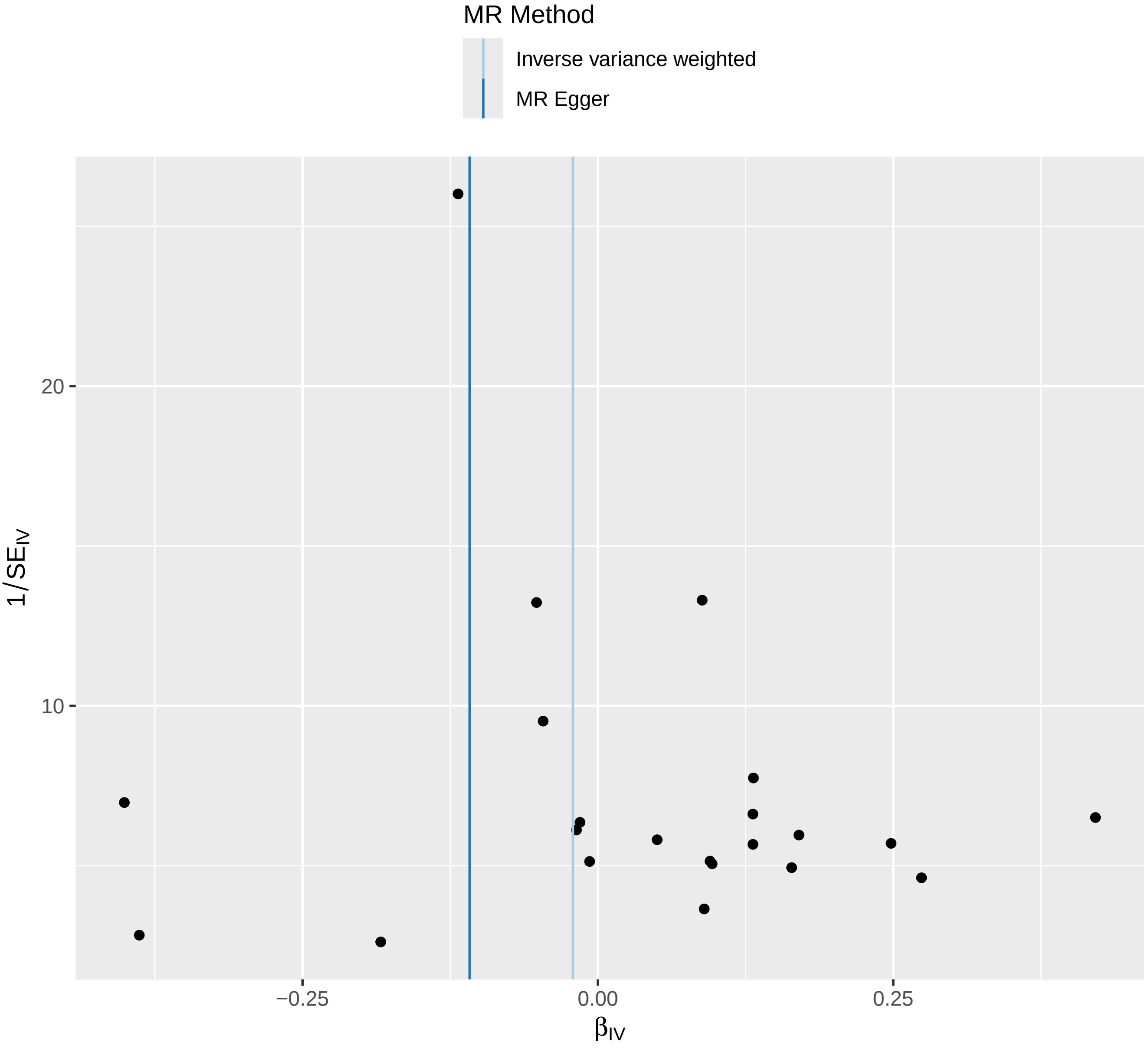
MR Method
Inverse variance weighted
MR Egger
20
10
1/SE$_{IV}$
−0.25
0.00
0.25
β$_{IV}$

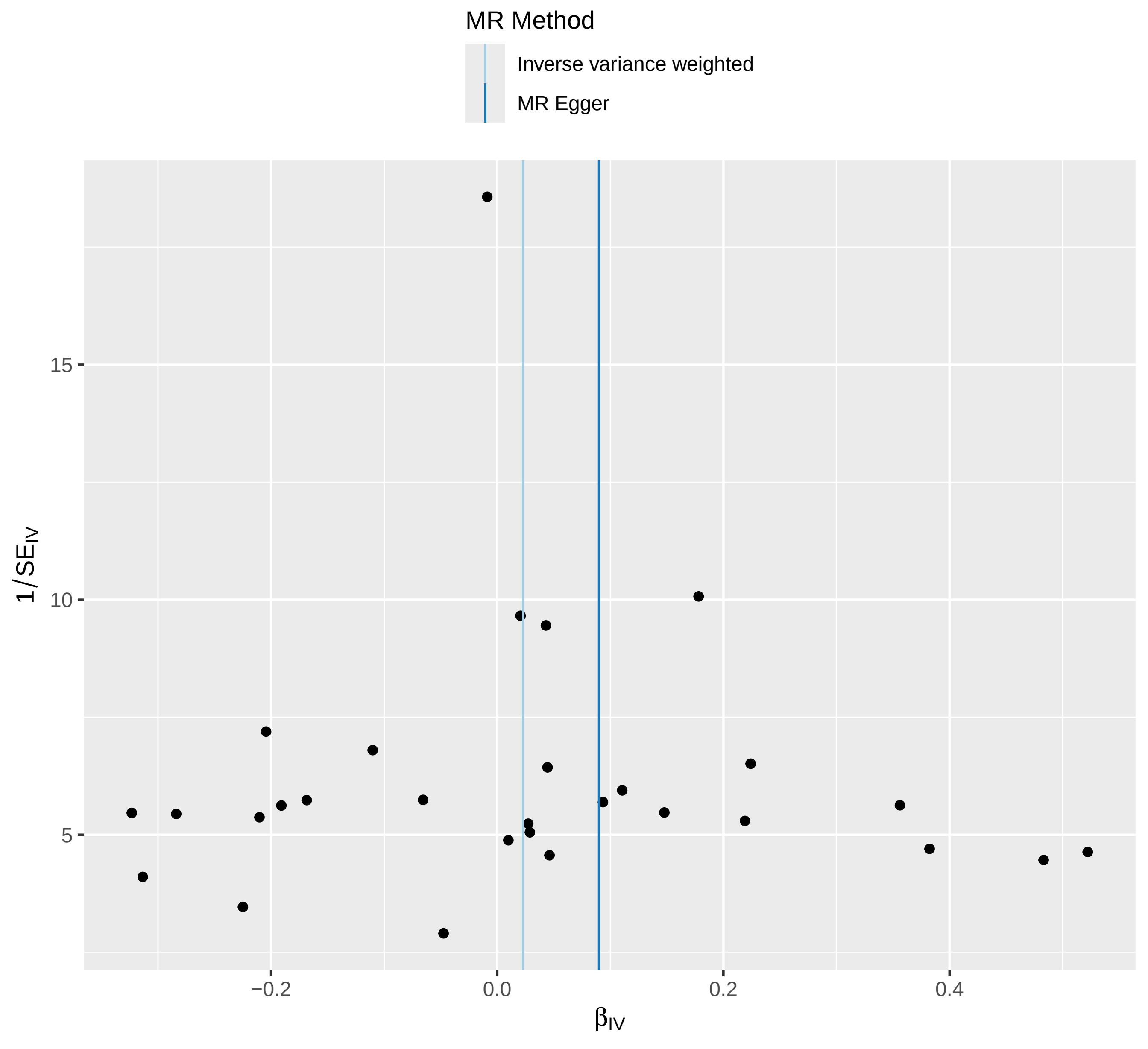

MR Method
Inverse variance weighted
MR Egger
15
10
5
1/SE$_{IV}$
−0.2
0.0
0.2
0.4
β$_{IV}$

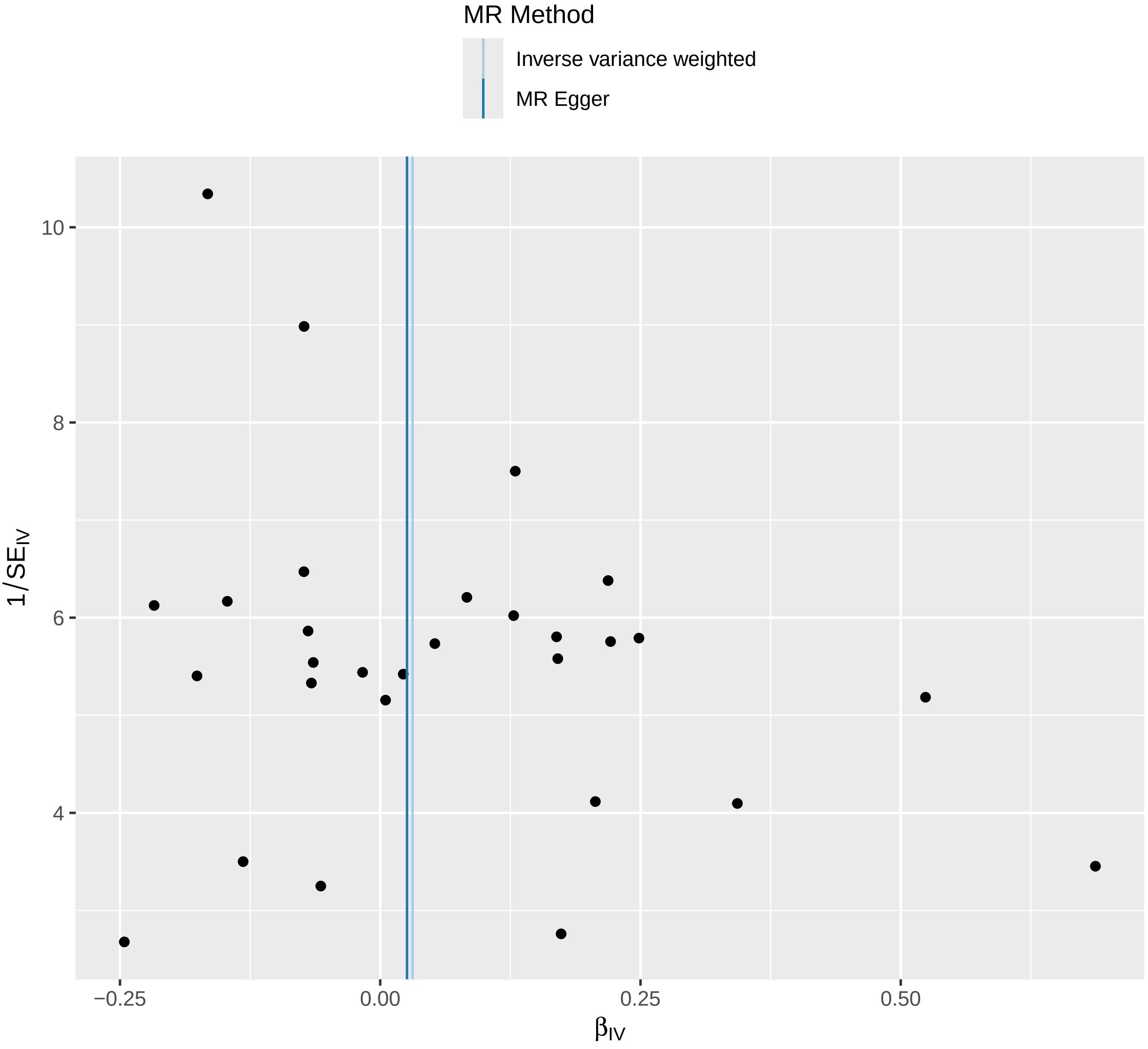
MR Method
Inverse variance weighted
MR Egger
1/SE_IV
10
8
6
4
−0.25
0.00
0.25
0.50
β_IV

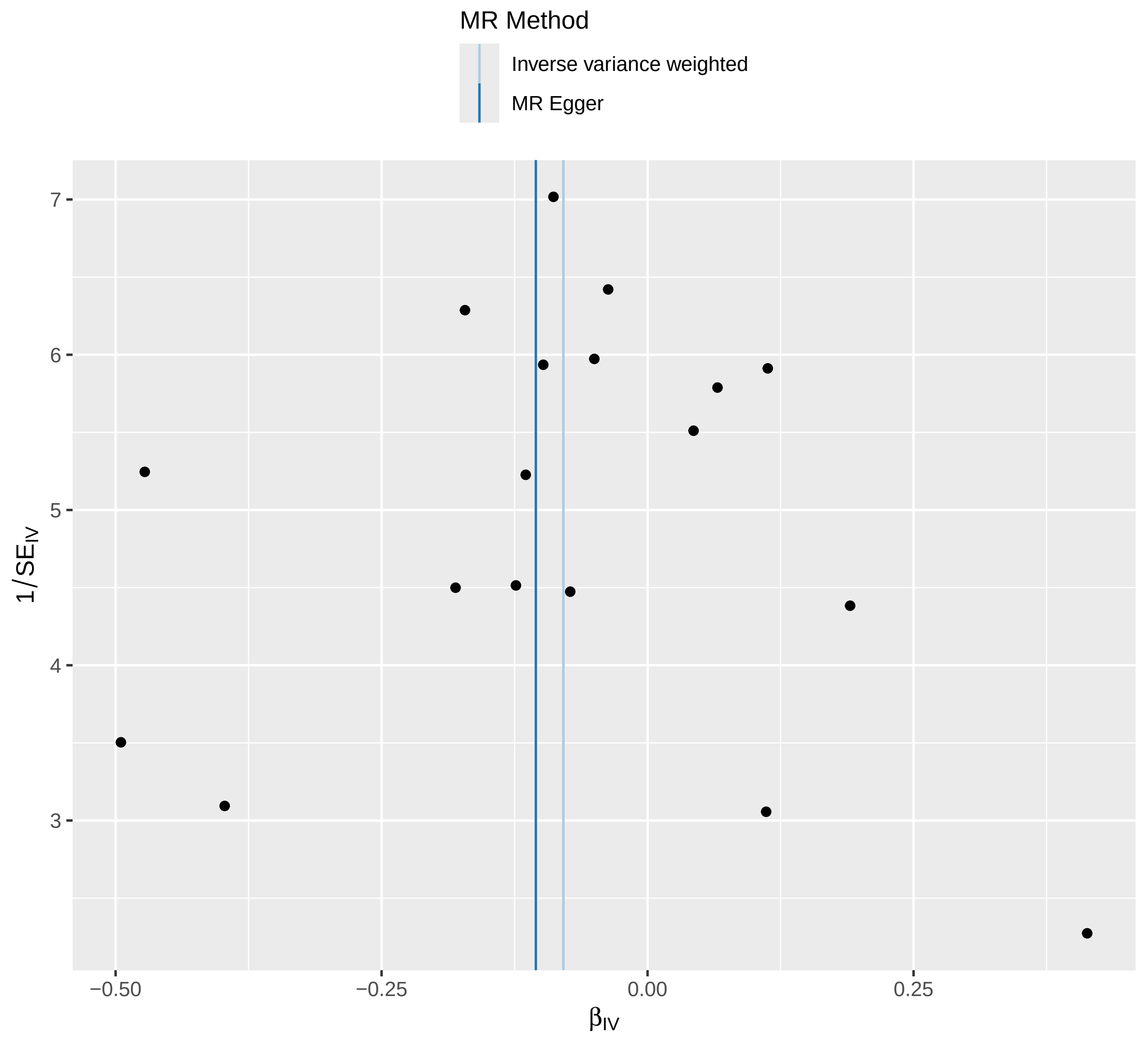

MR Method
Inverse variance weighted
MR Egger
7
6
5
4
3
1/SE$_{IV}$
−0.50
−0.25
0.00
0.25
β$_{IV}$

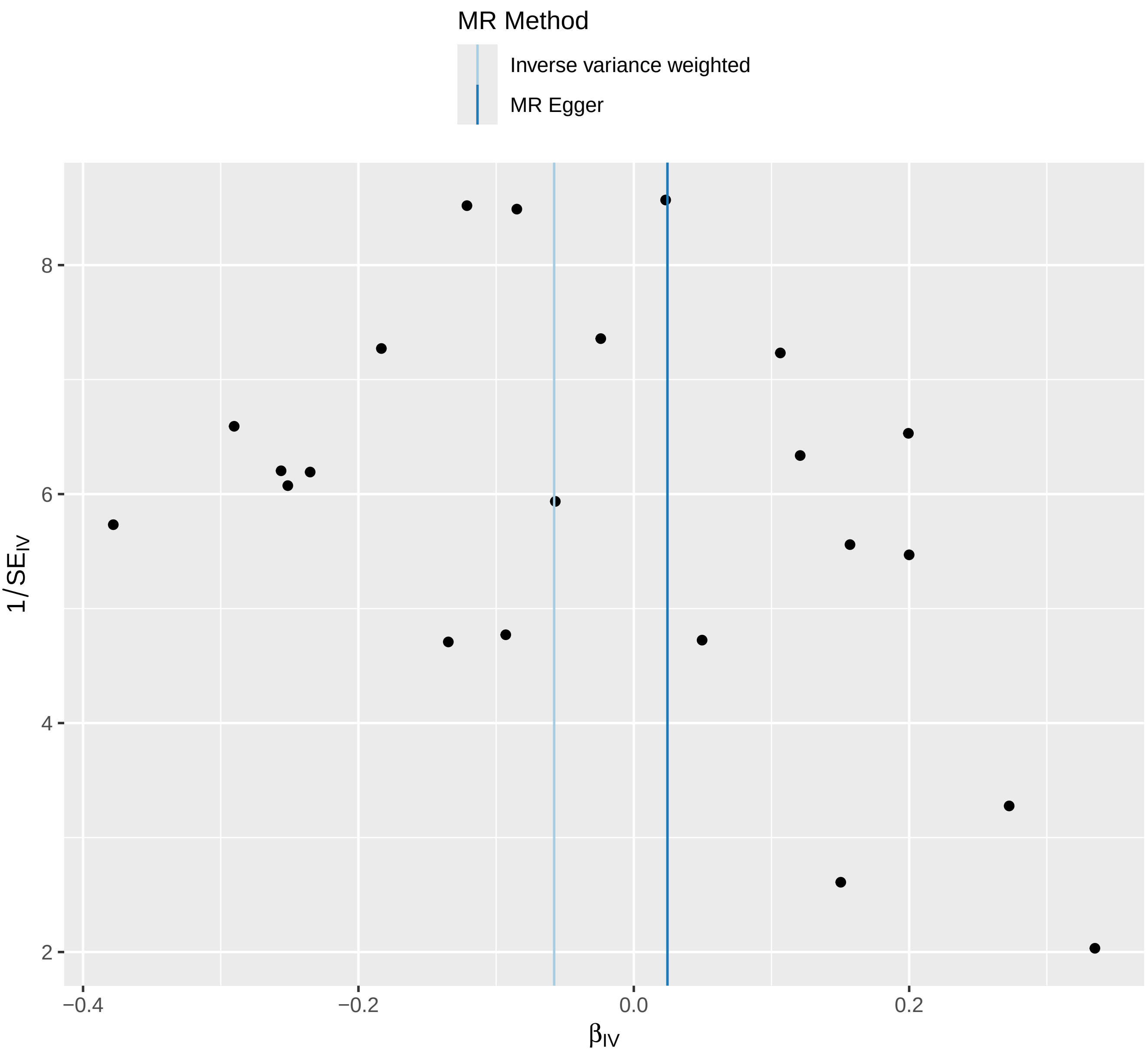

MR Method
Inverse variance weighted
MR Egger
8
6
4
2
$1/SE_{IV}$
−0.4
−0.2
0.0
0.2
β_{IV}

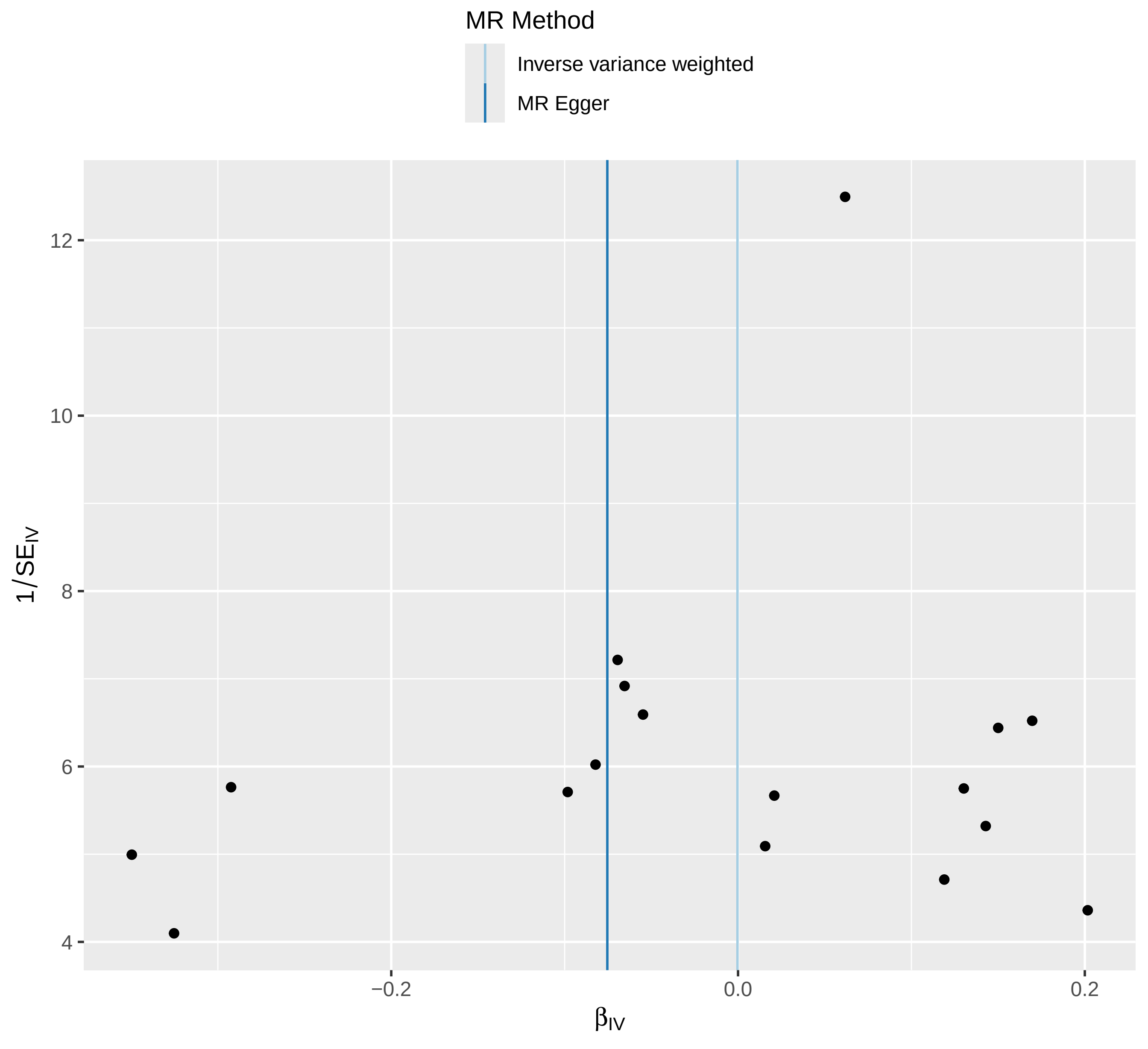
MR Method
Inverse variance weighted
MR Egger
12
10
8
6
4
1/SE_IV
−0.2
0.0
0.2
β_IV

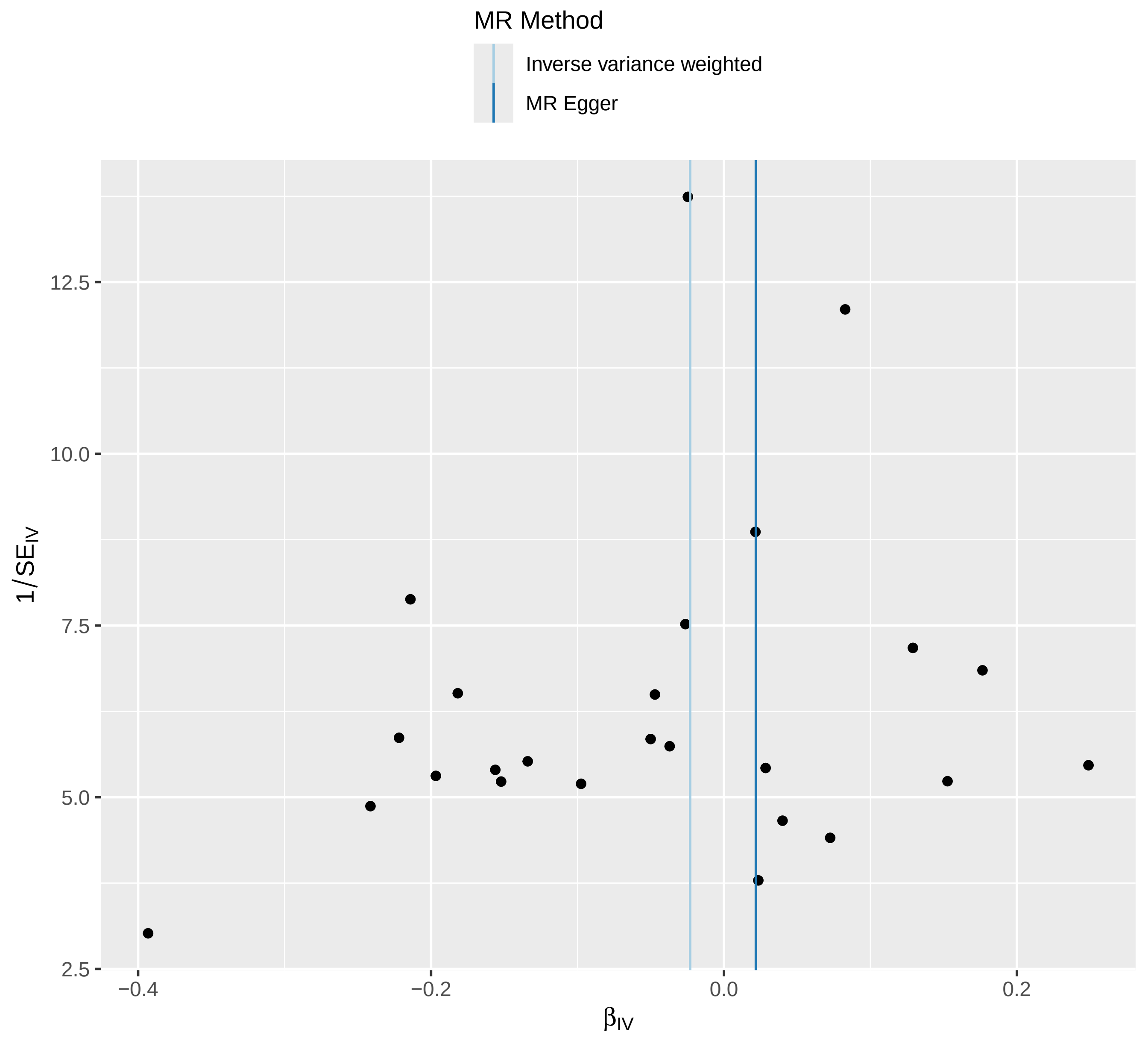
MR Method
Inverse variance weighted
MR Egger
1/SE_IV
β_IV
12.5
10.0
7.5
5.0
2.5
−0.4
−0.2
0.0
0.2

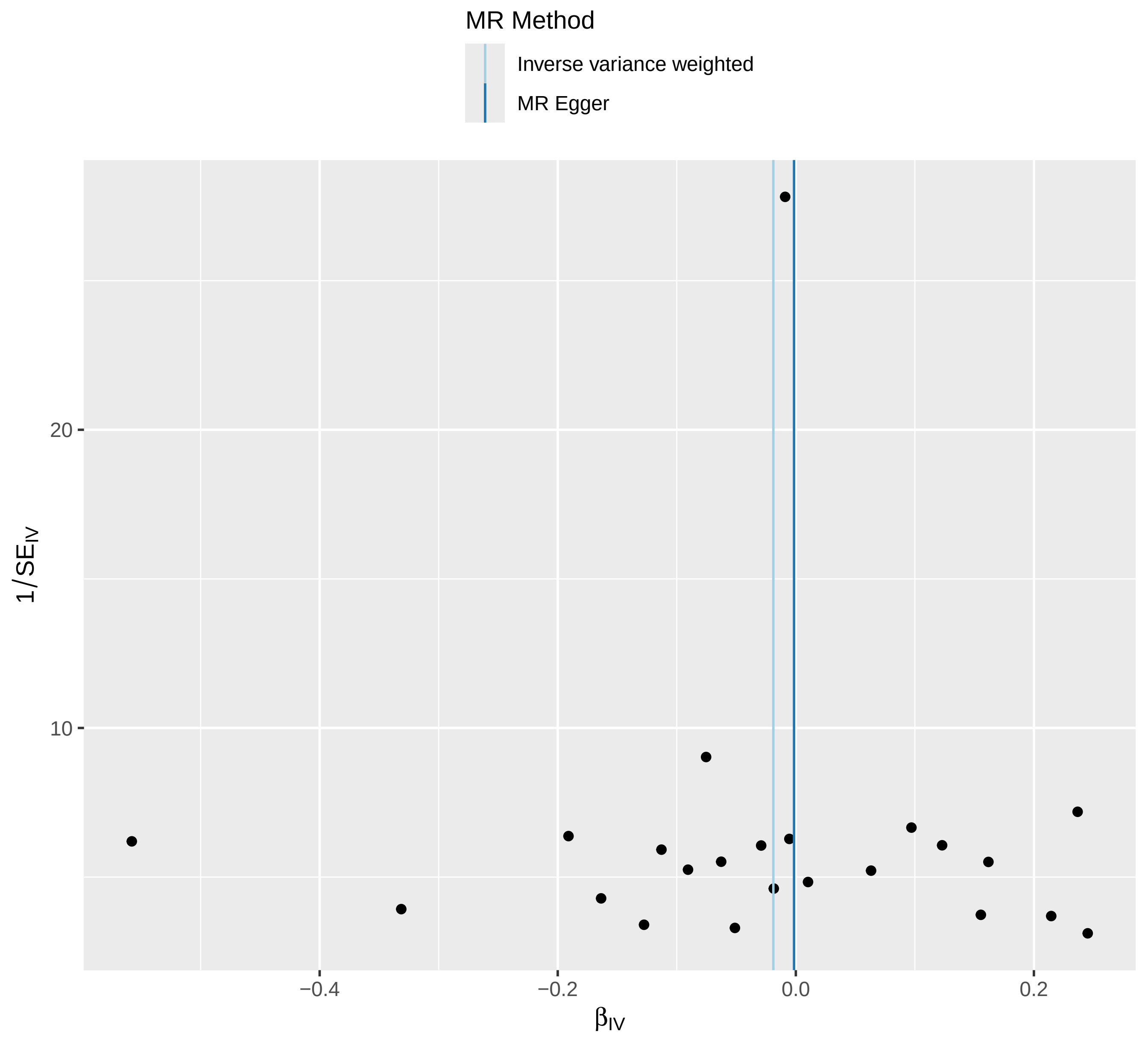

MR Method
Inverse variance weighted
MR Egger
20
10
1/SE$_{IV}$
−0.4
−0.2
0.0
0.2
β$_{IV}$

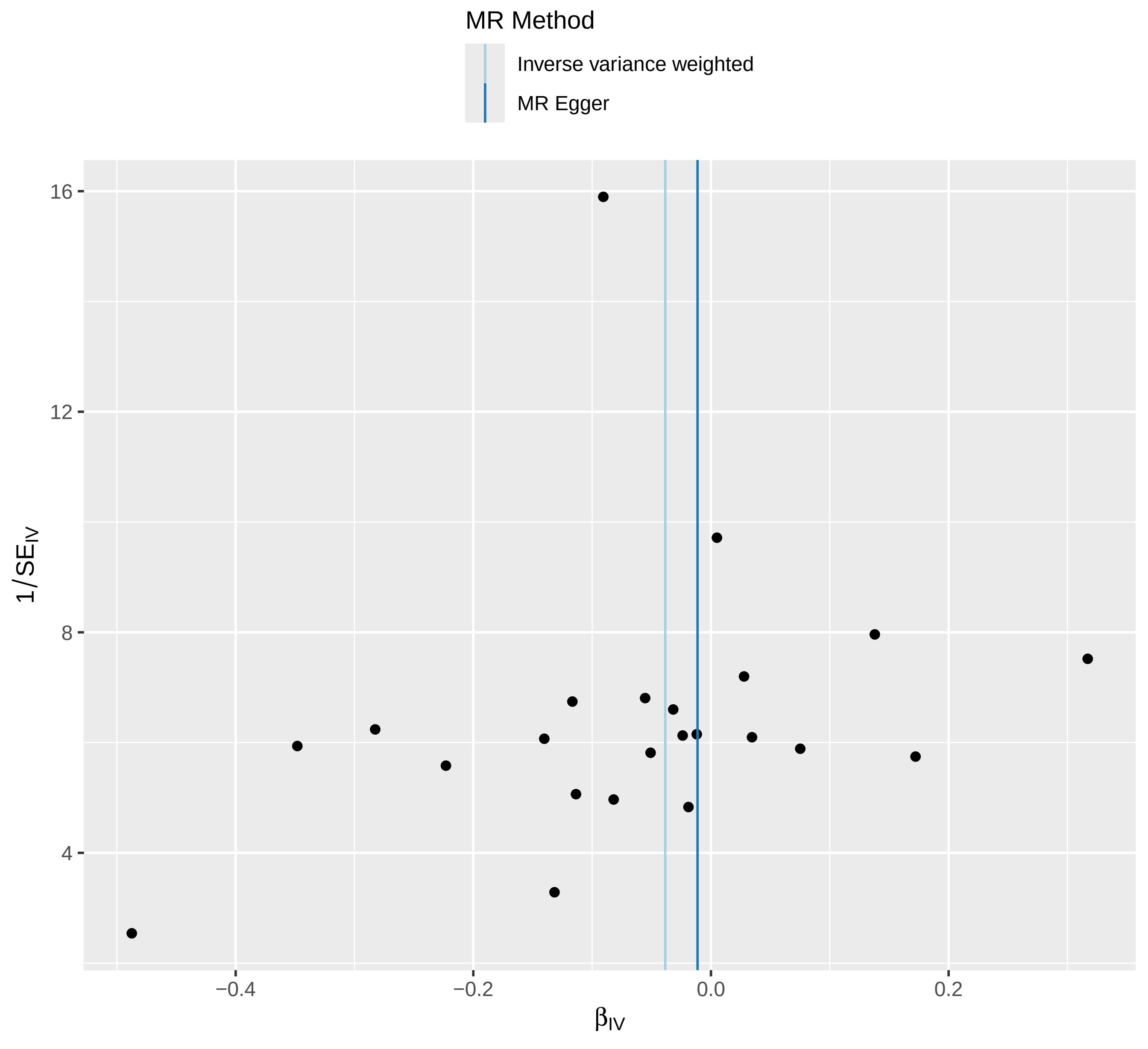

MR Method
Inverse variance weighted
MR Egger
16
12
8
4
1/SE_IV
−0.4
−0.2
0.0
0.2
β_IV

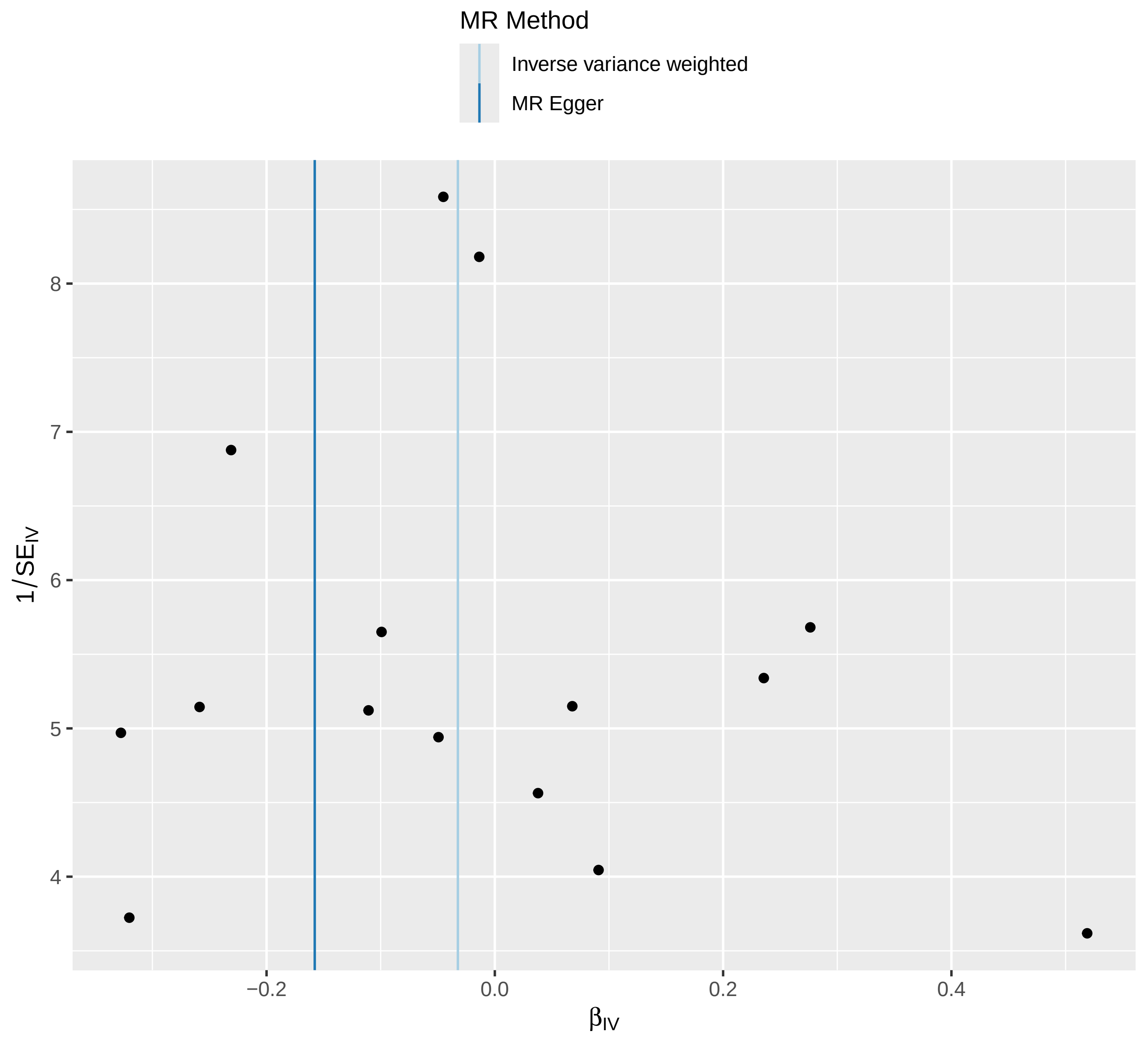
MR Method
Inverse variance weighted
MR Egger
8
7
6
5
4
1/SE_IV
−0.2
0.0
0.2
0.4
β_IV

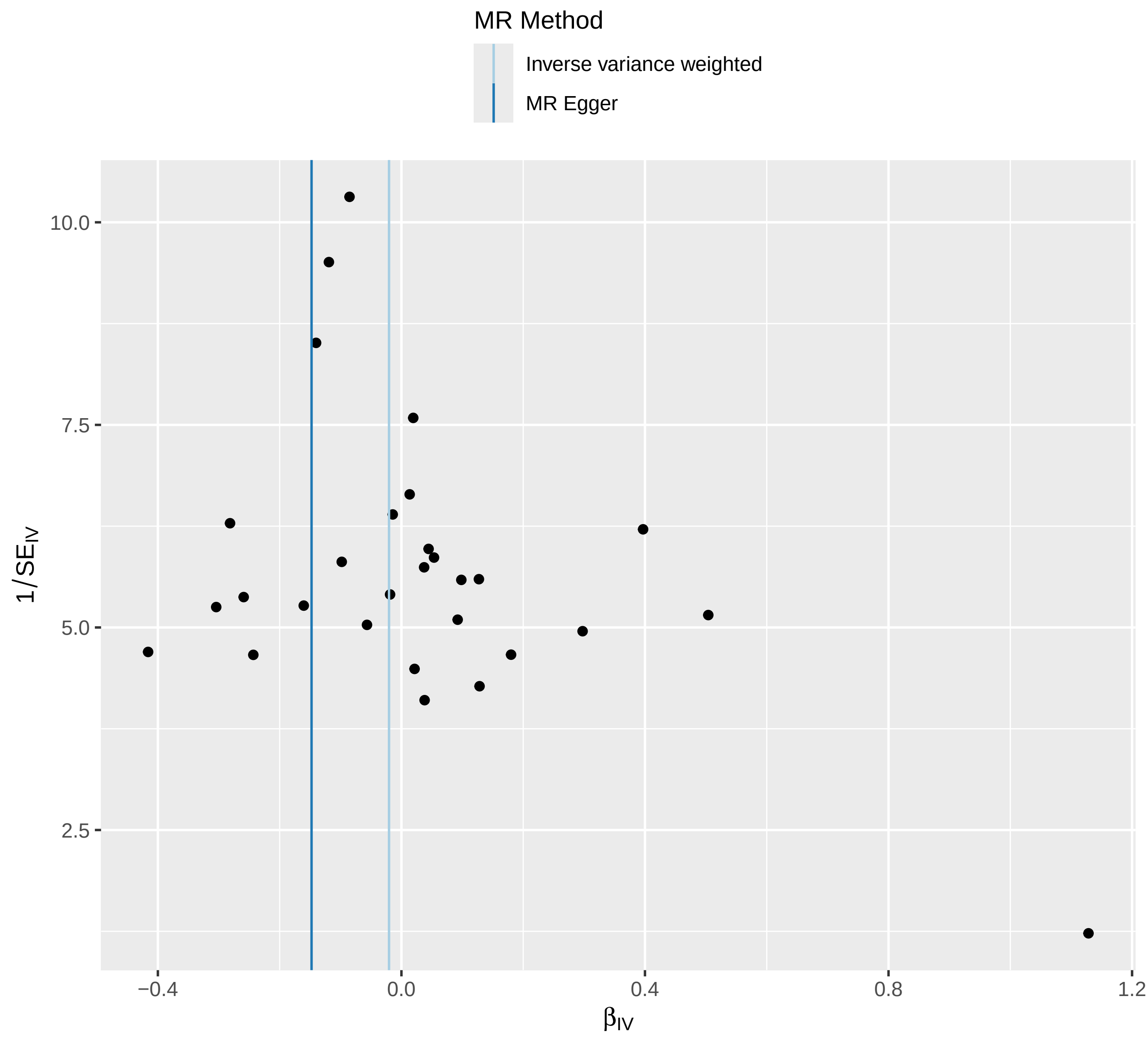
MR Method
Inverse variance weighted
MR Egger
10.0
7.5
5.0
2.5
1/SE_IV
−0.4
0.0
0.4
0.8
1.2
β_IV

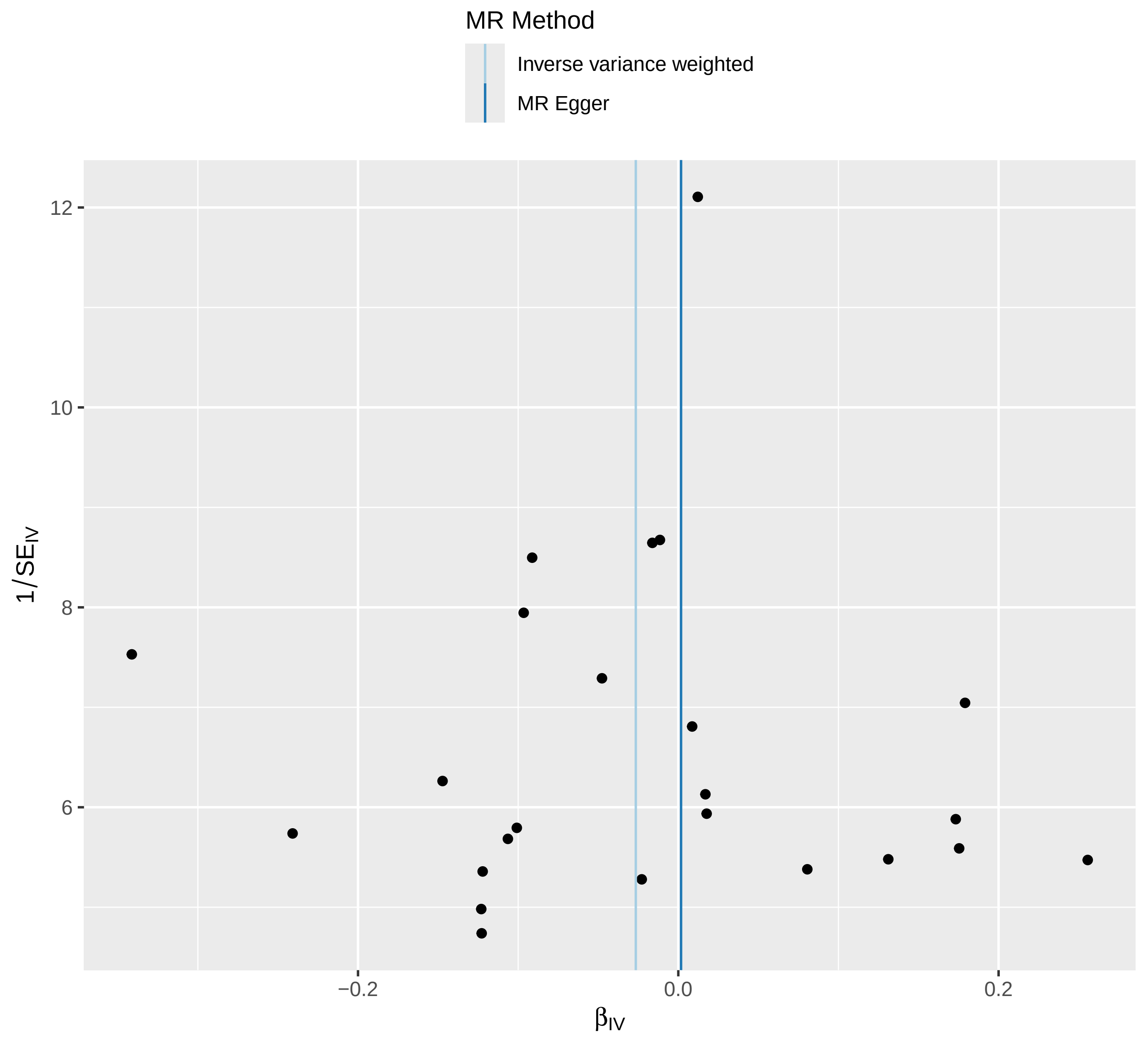

MR Method
Inverse variance weighted
MR Egger
12
10
8
6
−0.2
0.0
0.2
$1/SE_{IV}$
β_{IV}

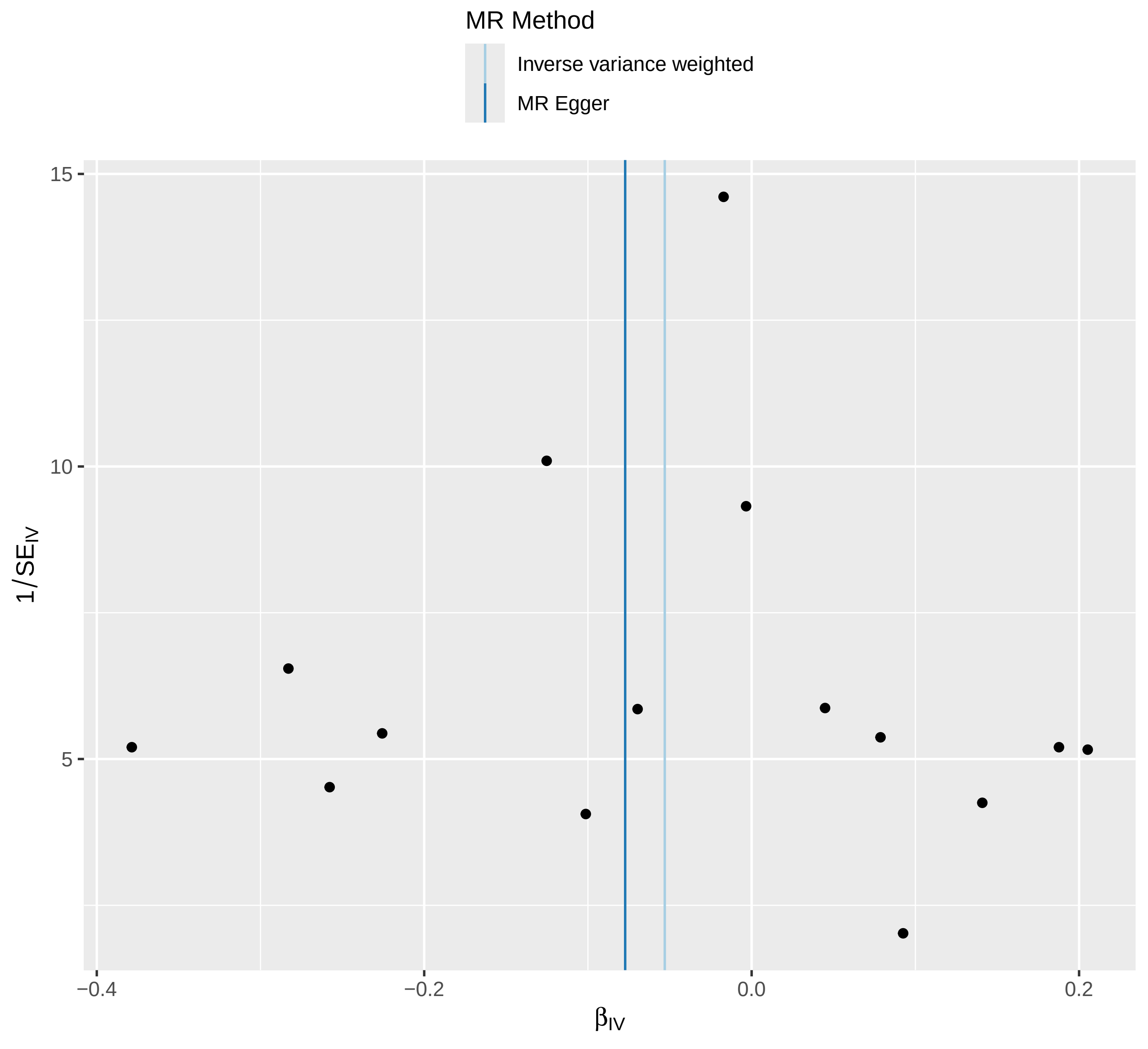

MR Method
Inverse variance weighted
MR Egger
15
10
5
$1/SE_{IV}$
−0.4
−0.2
0.0
0.2
β_{IV}

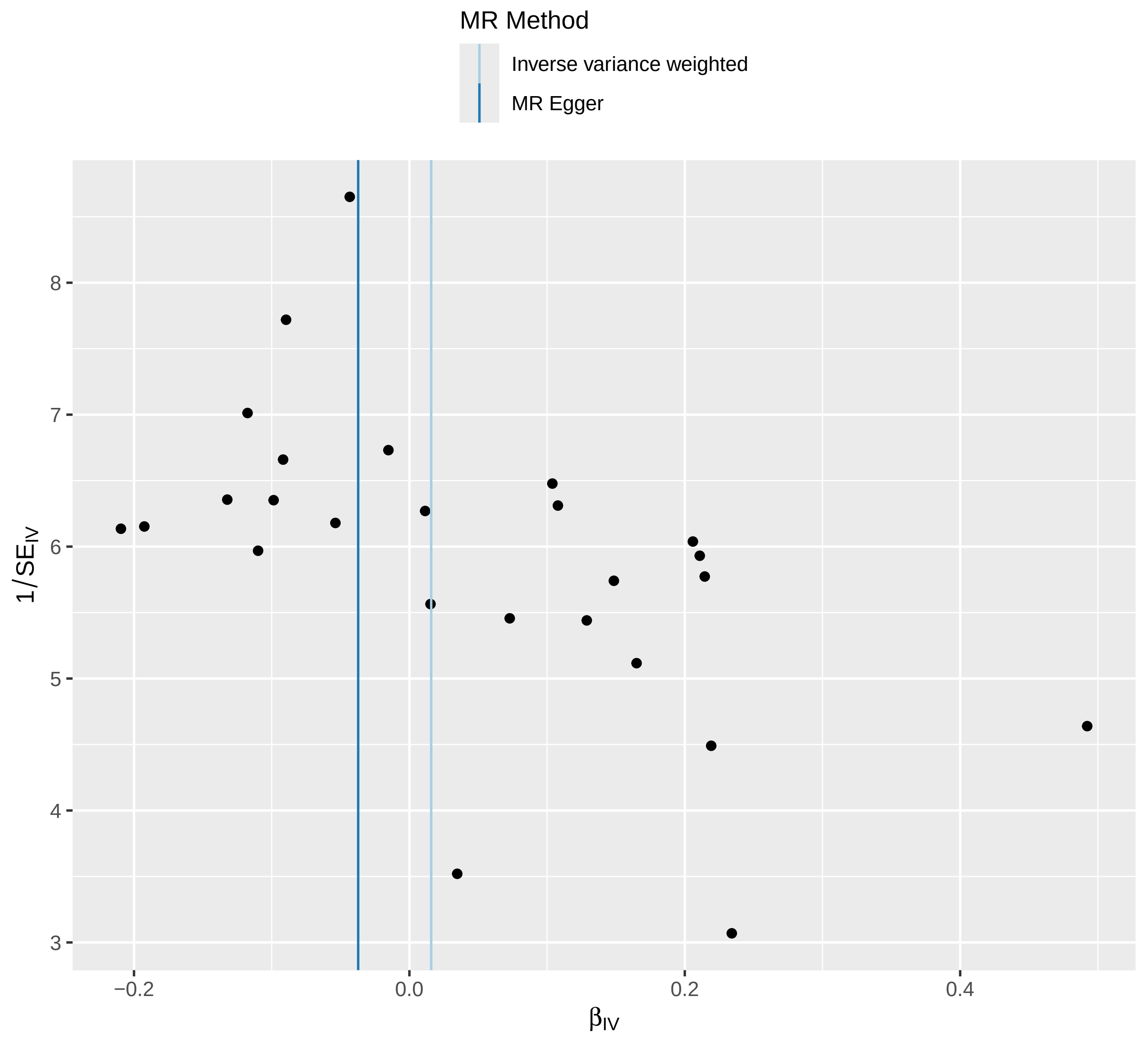
MR Method
Inverse variance weighted
MR Egger
1/SE_IV
β_IV
8
7
6
5
4
3
−0.2
0.0
0.2
0.4

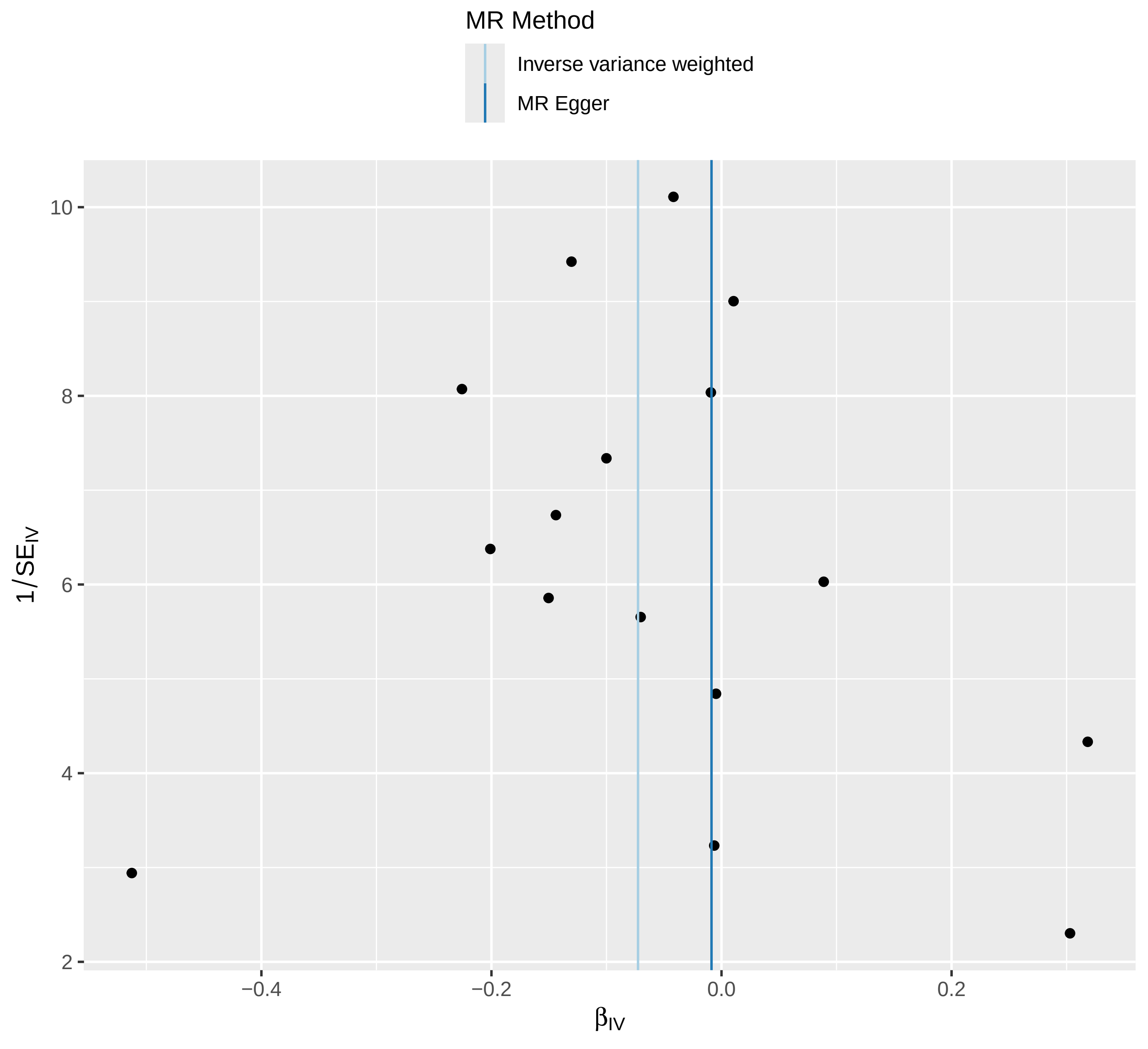
MR Method
Inverse variance weighted
MR Egger
1/SE_IV
10
8
6
4
2
−0.4
−0.2
0.0
0.2
β_IV

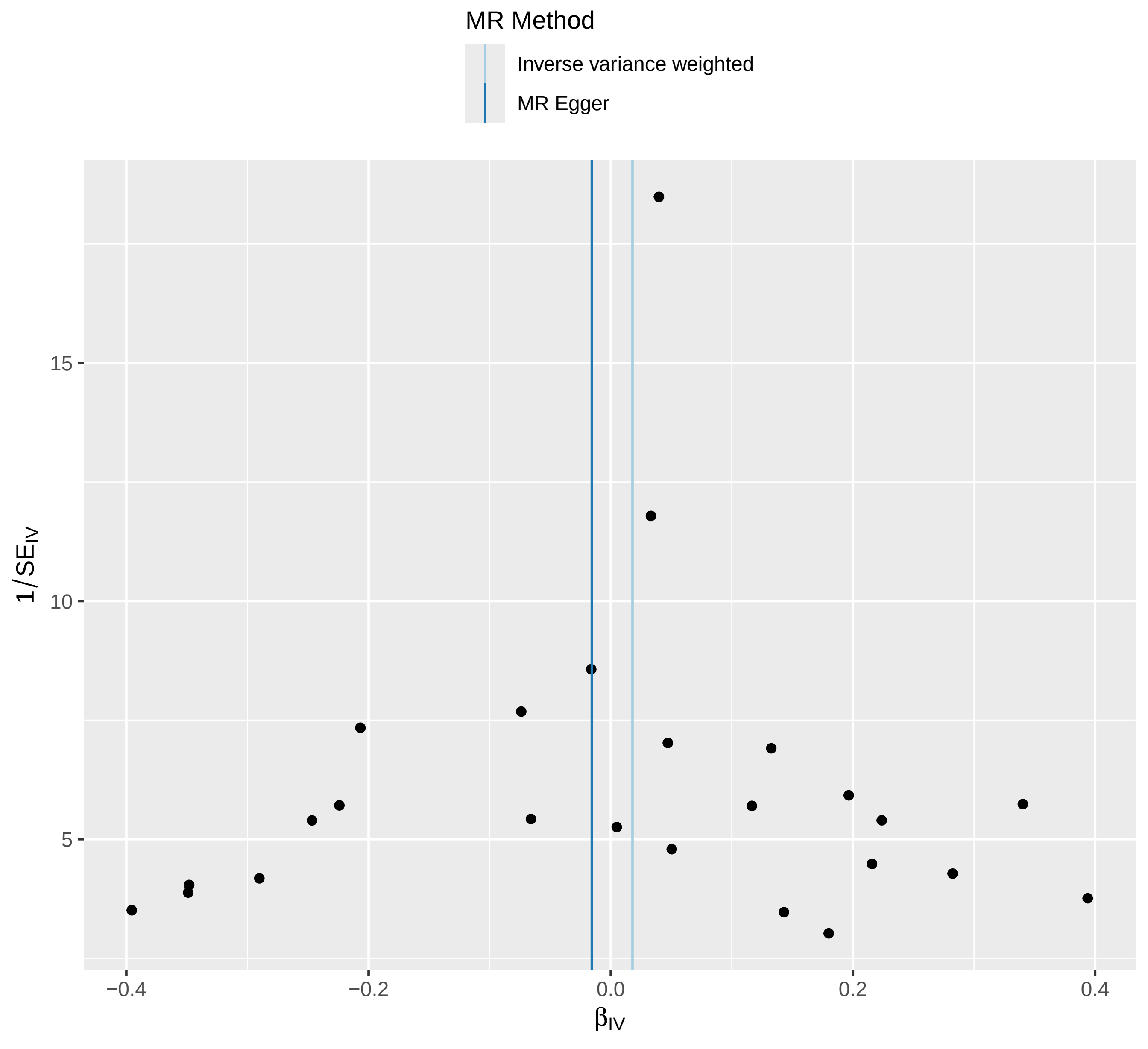

MR Method
Inverse variance weighted
MR Egger
15
10
5
1/SE_IV
−0.4
−0.2
0.0
0.2
0.4
β_IV

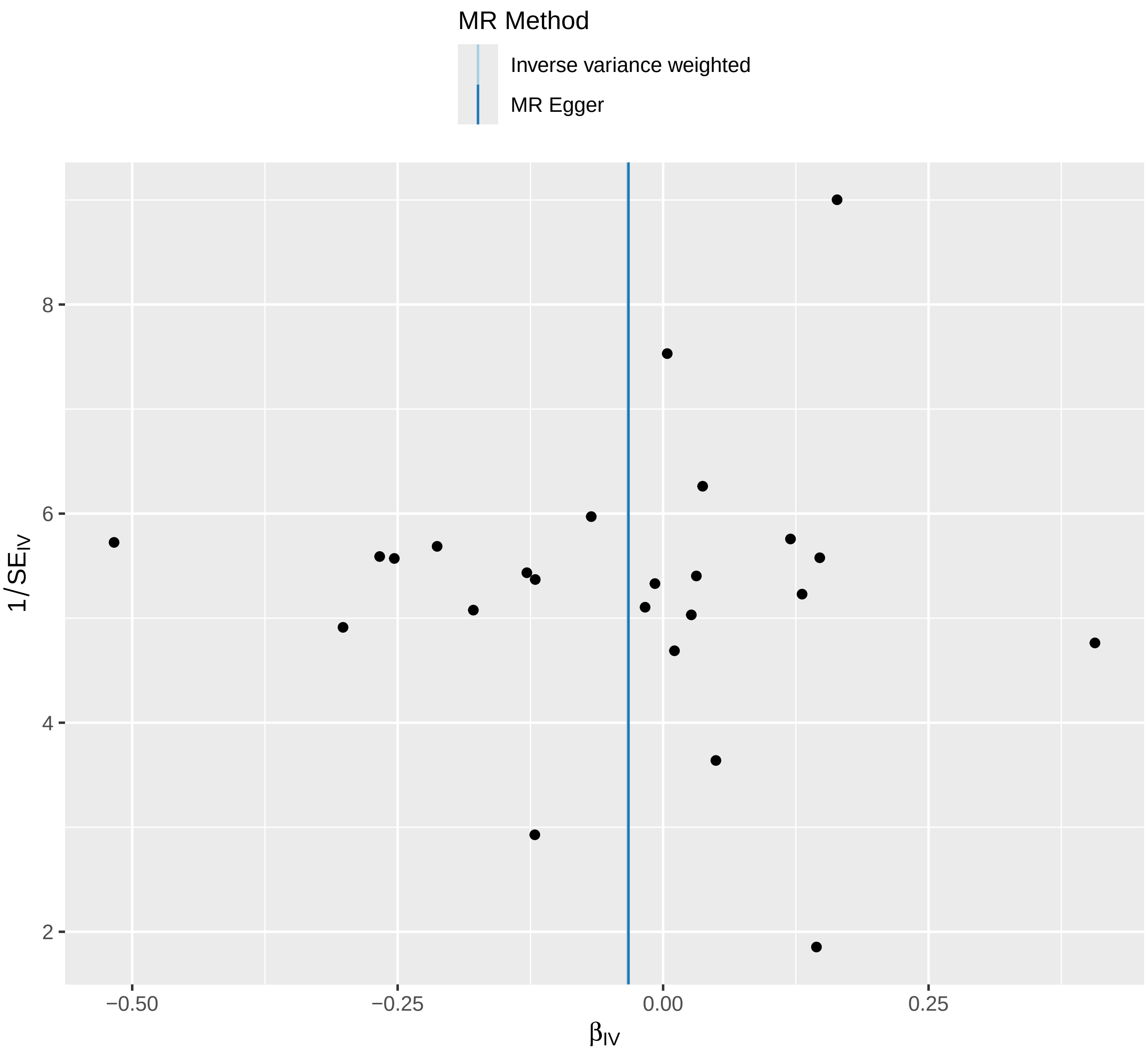
MR Method
Inverse variance weighted
MR Egger
8
6
4
2
1/SE_IV
−0.50
−0.25
0.00
0.25
β_IV

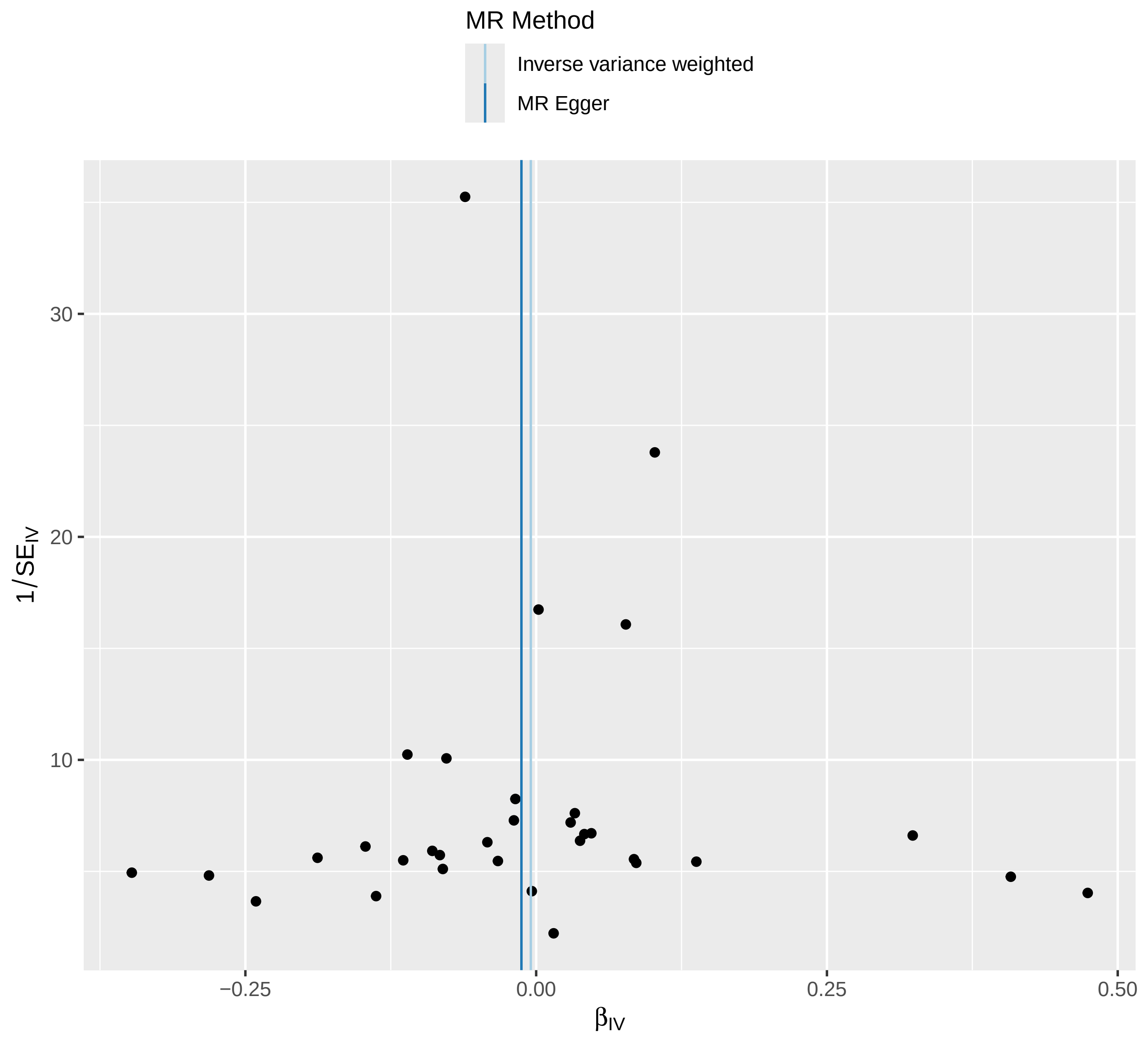
MR Method
Inverse variance weighted
MR Egger
30
20
10
1/SE_IV
−0.25
0.00
0.25
0.50
β_IV

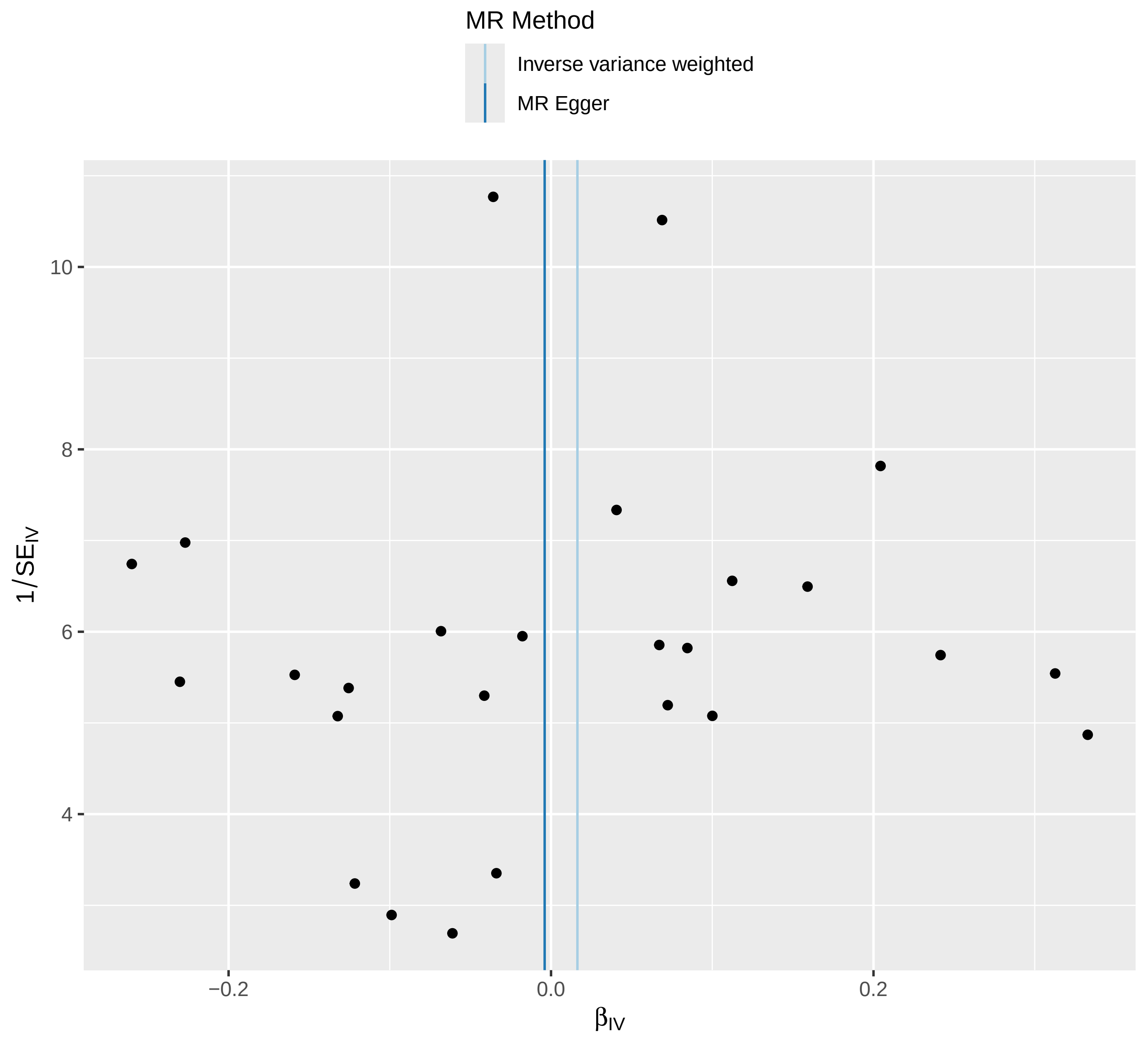
MR Method
Inverse variance weighted
MR Egger
1/SE_IV
10
8
6
4
−0.2
0.0
0.2
β_IV

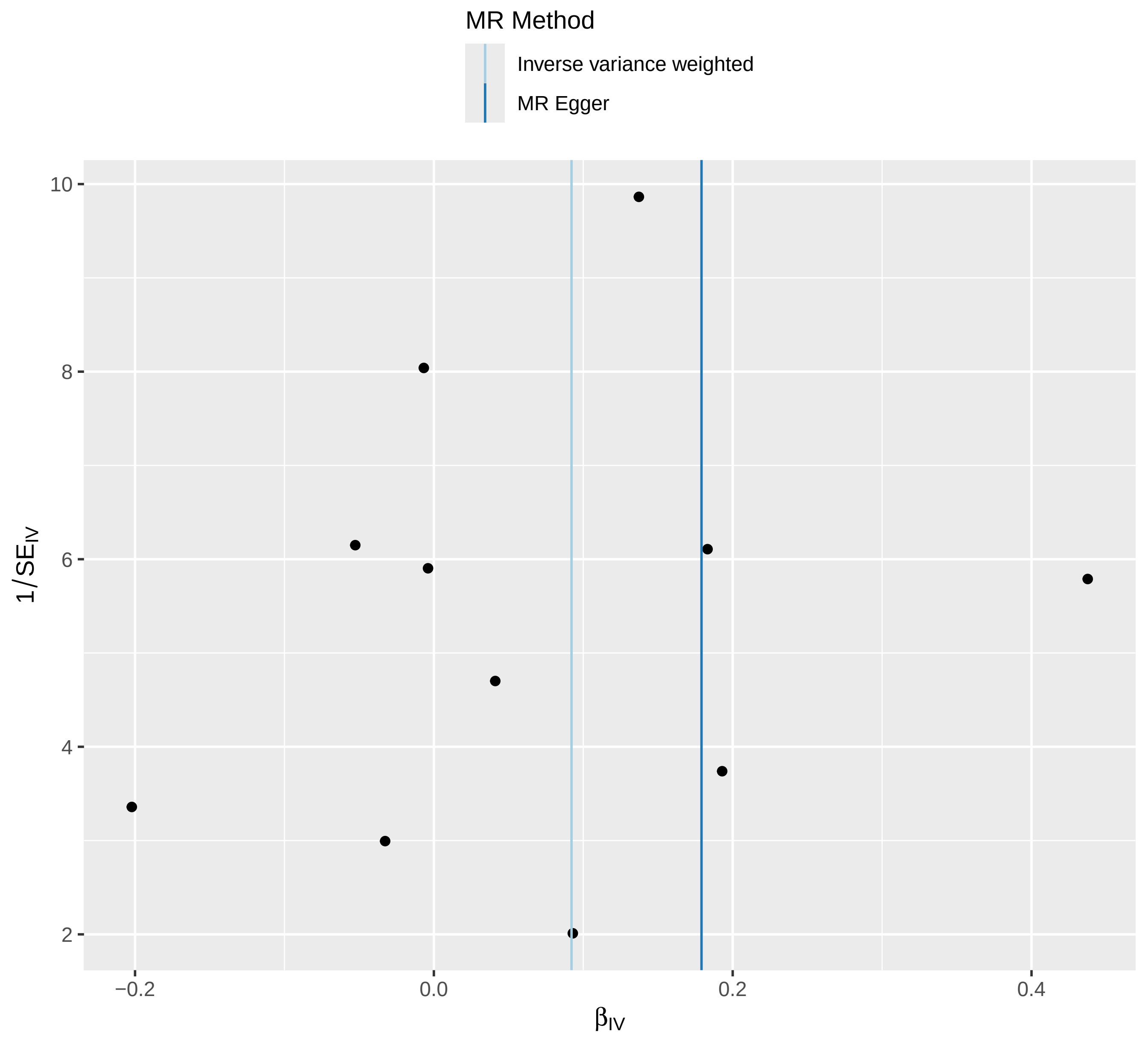

MR Method
Inverse variance weighted
MR Egger
10
8
6
4
2
1/SE$_{IV}$
−0.2
0.0
0.2
0.4
β$_{IV}$

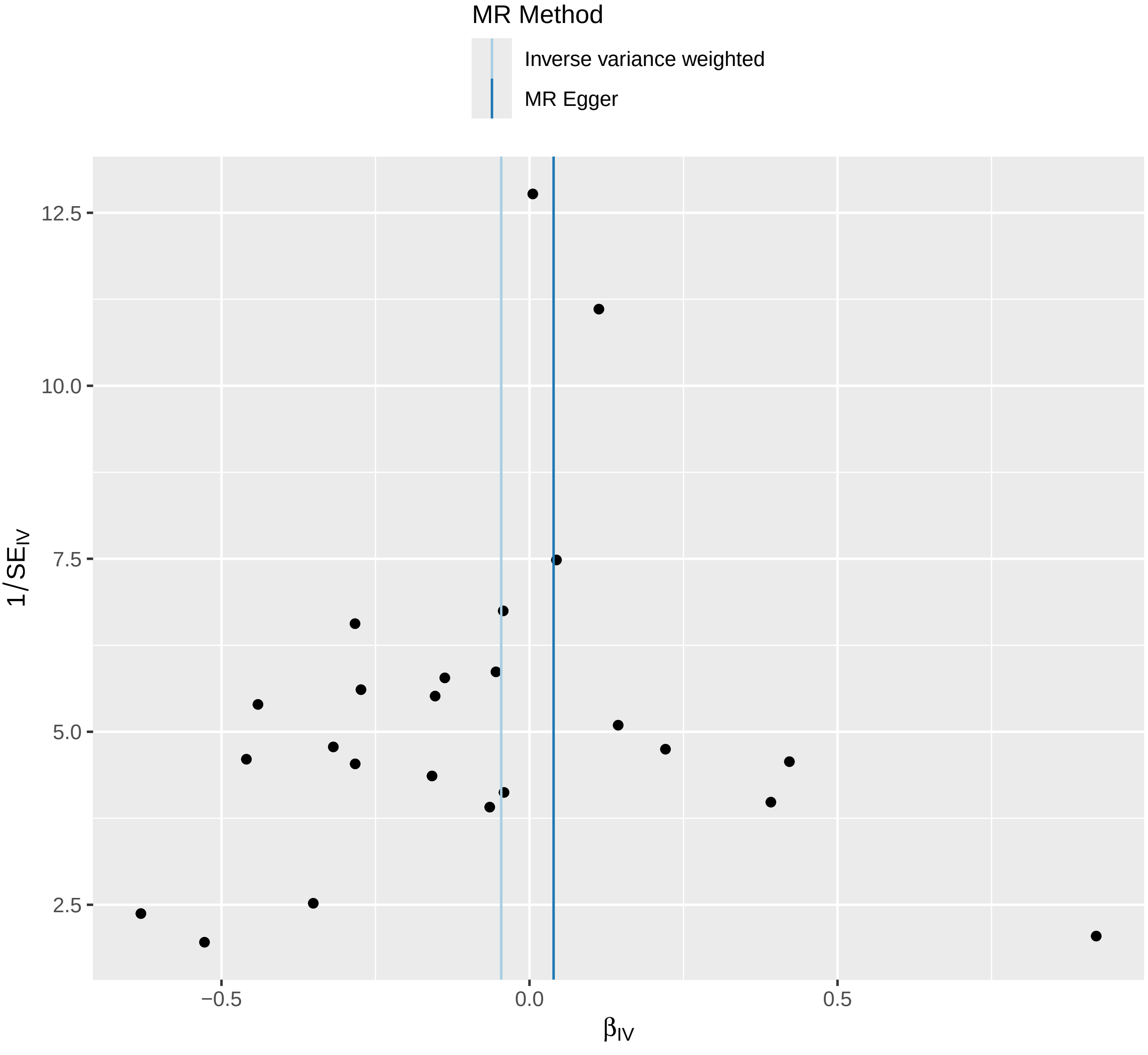

MR Method
Inverse variance weighted
MR Egger
12.5
10.0
7.5
5.0
2.5
1/SE_IV
−0.5
0.0
0.5
β_IV

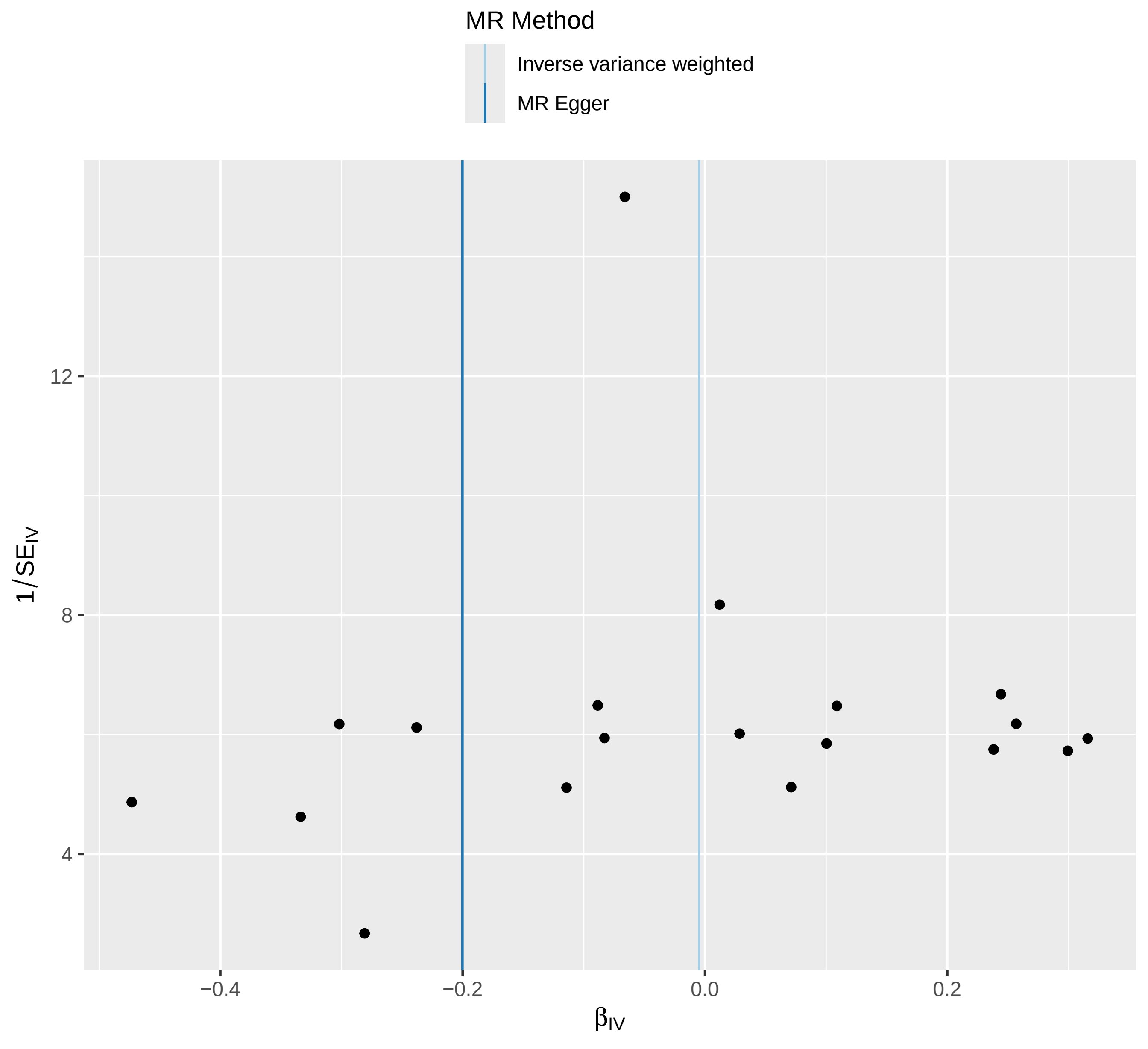

MR Method
Inverse variance weighted
MR Egger
12
8
4
1/SE$_{IV}$
−0.4
−0.2
0.0
0.2
β$_{IV}$

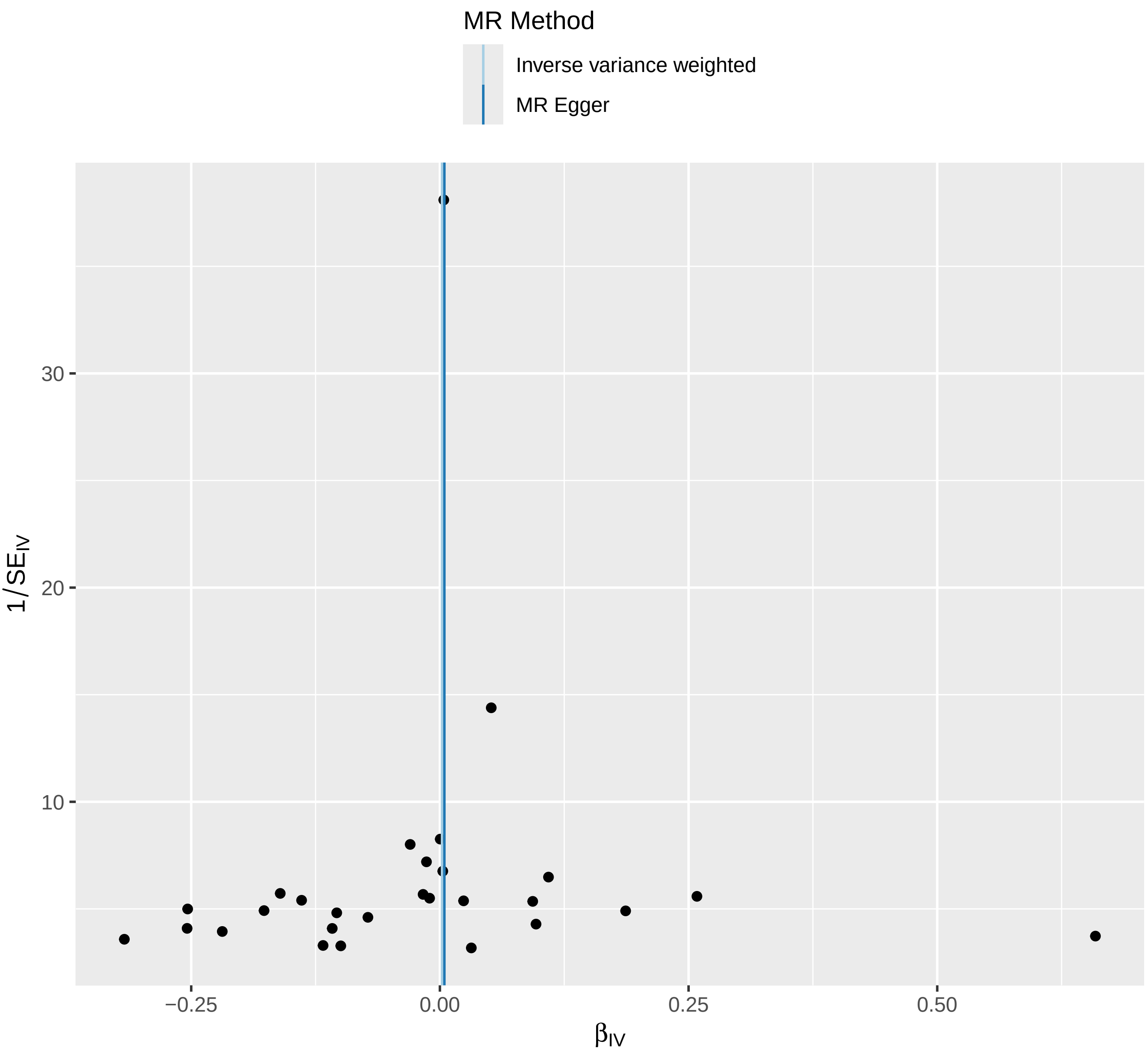
MR Method
Inverse variance weighted
MR Egger
1/SE_IV
30
20
10
−0.25
0.00
0.25
0.50
β_IV

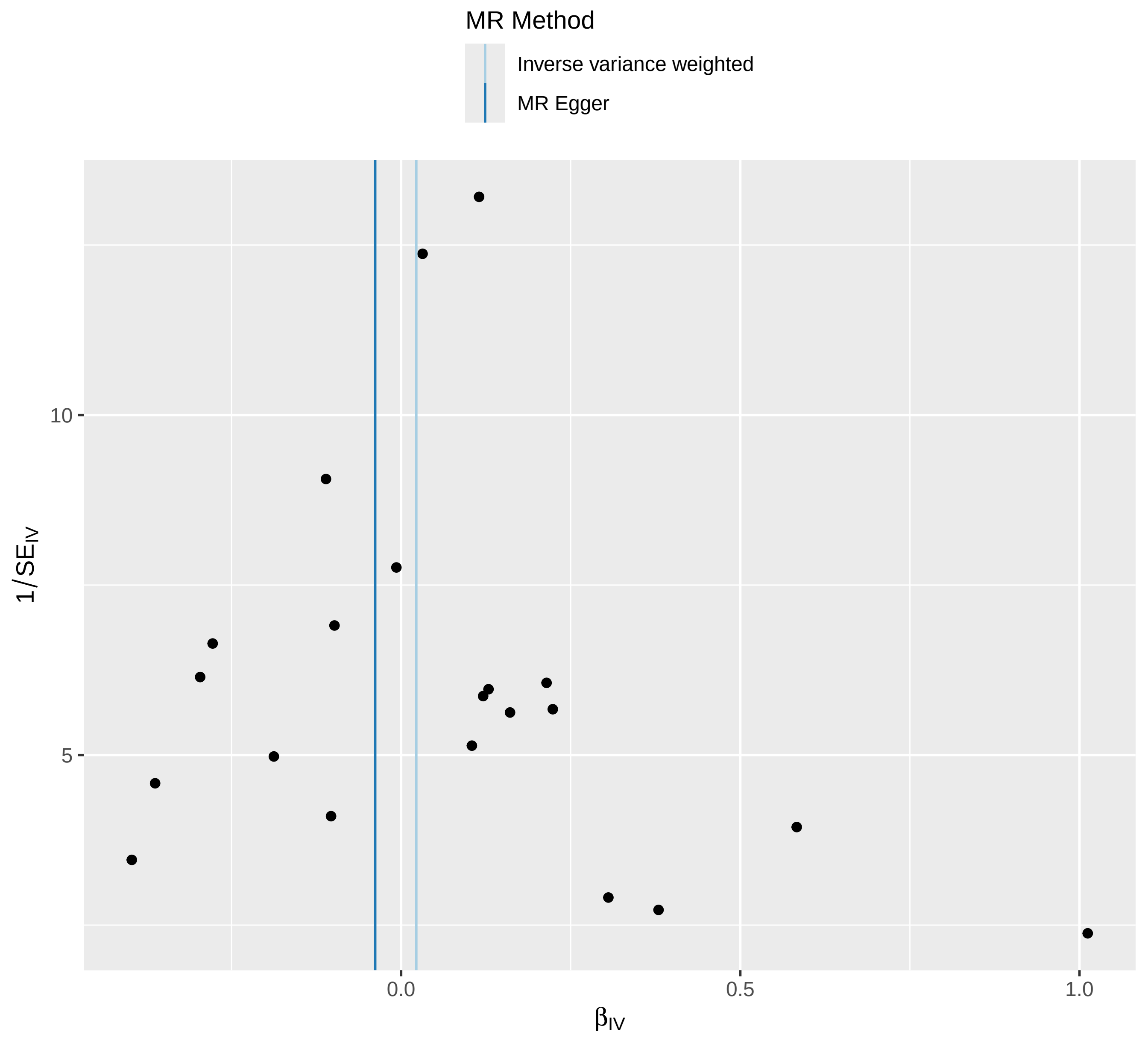
MR Method
Inverse variance weighted
MR Egger
10
5
1/SE$_{IV}$
0.0
0.5
1.0
β$_{IV}$

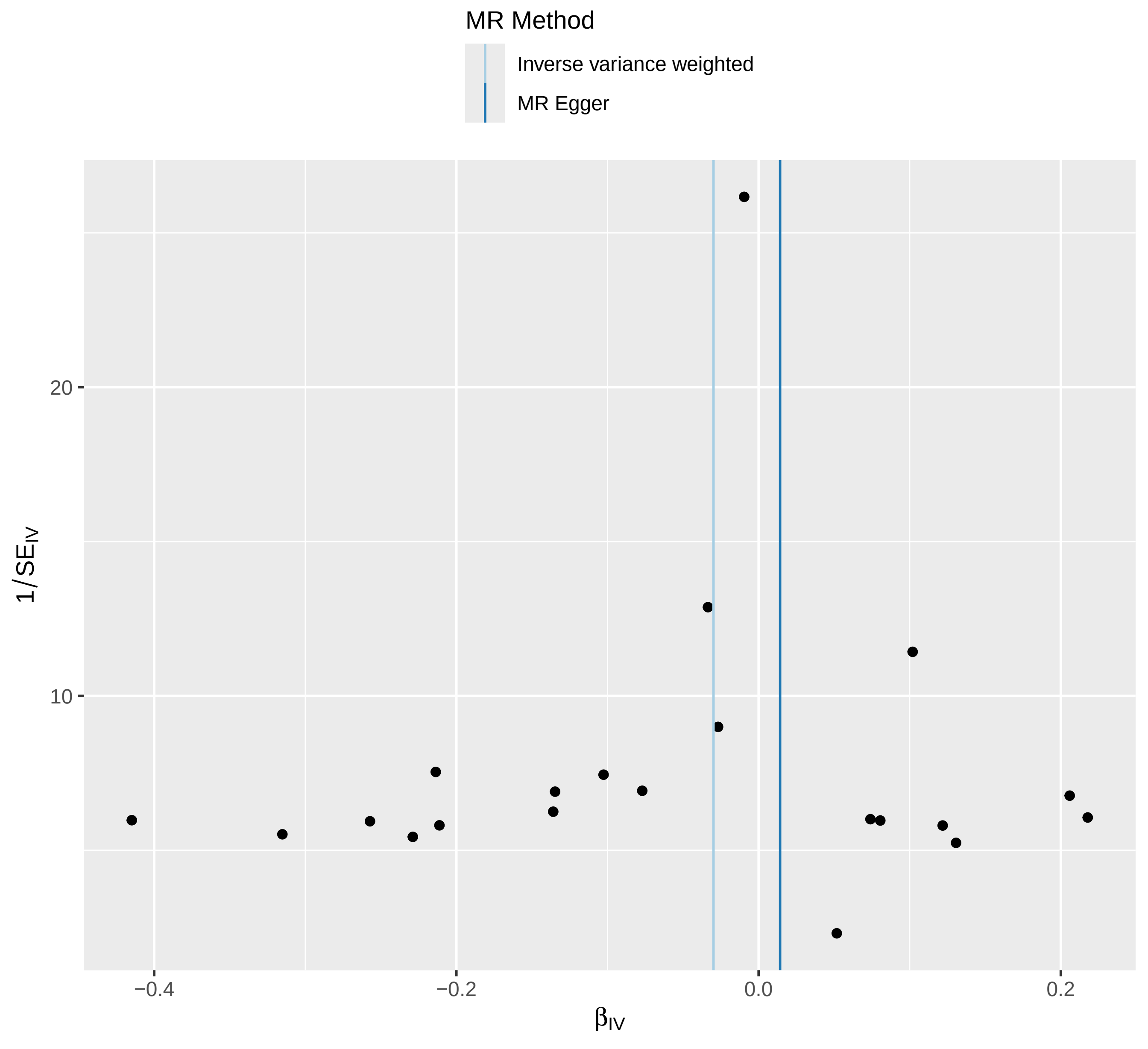

MR Method
Inverse variance weighted
MR Egger
20
10
1/SE_IV
−0.4
−0.2
0.0
0.2
β_IV

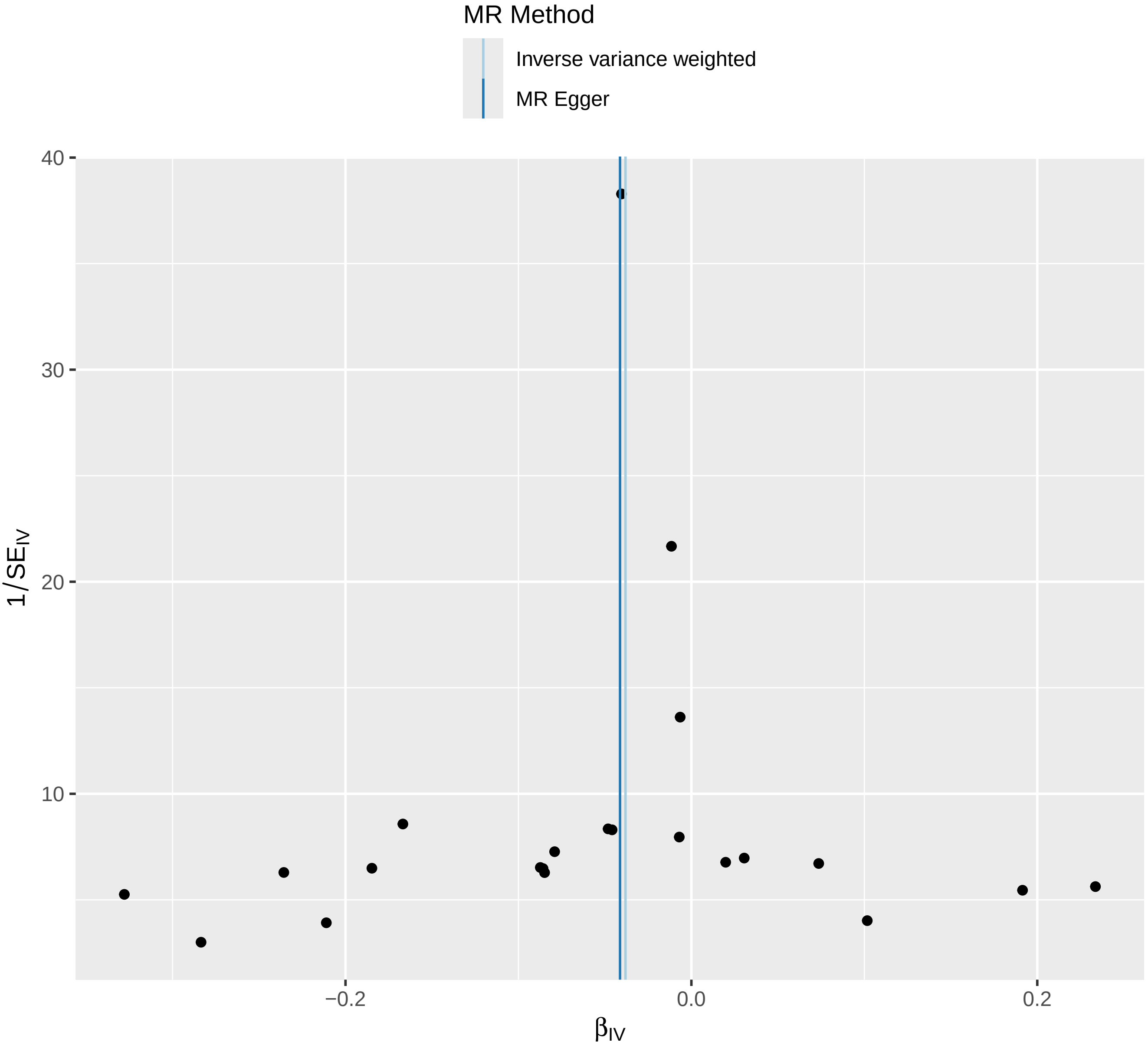

MR Method
Inverse variance weighted
MR Egger
40
30
20
10
1/SE_IV
−0.2
0.0
0.2
β_IV

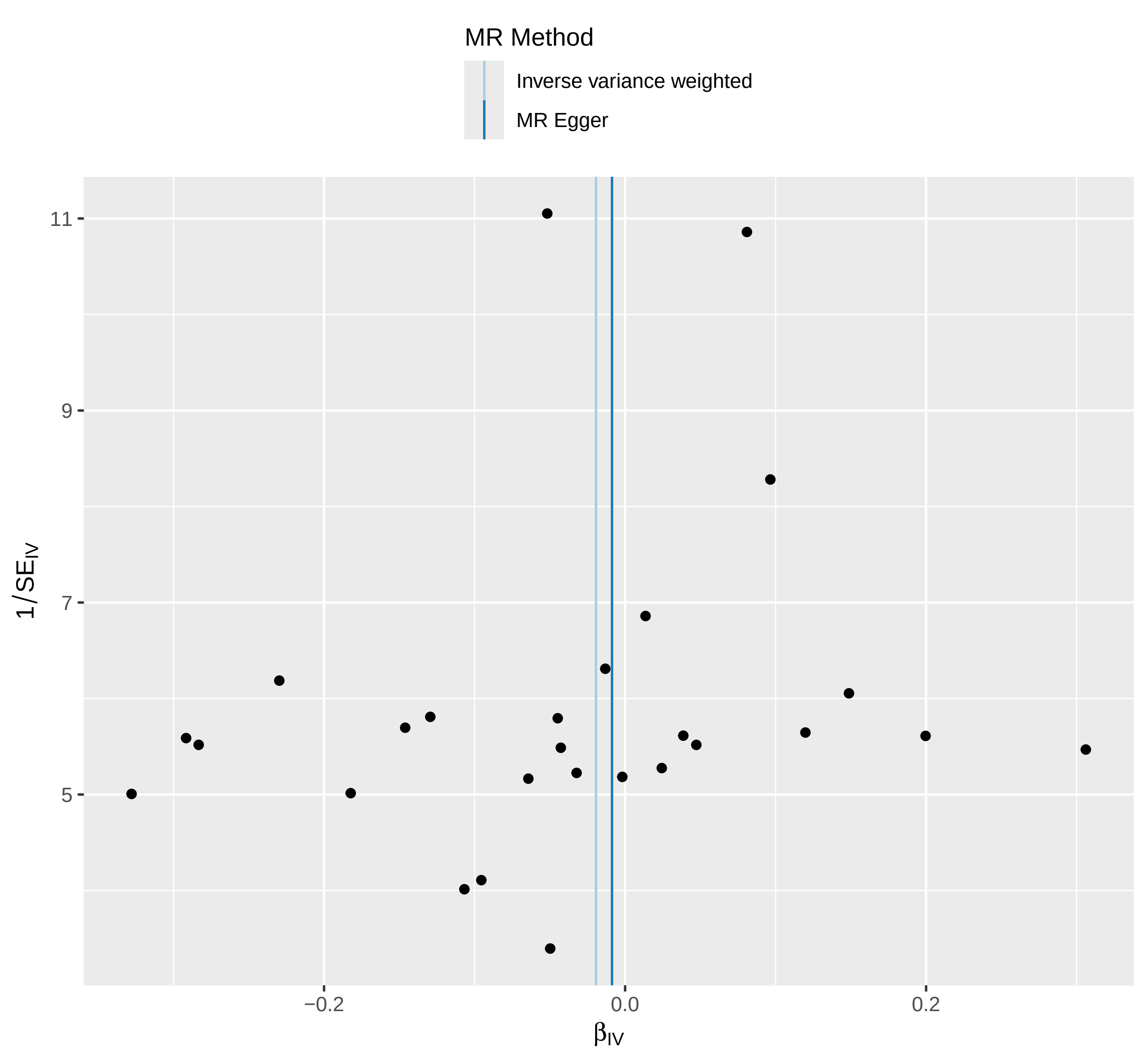
MR Method
Inverse variance weighted
MR Egger
1/SE_IV
11
9
7
5
−0.2
0.0
0.2
β_IV

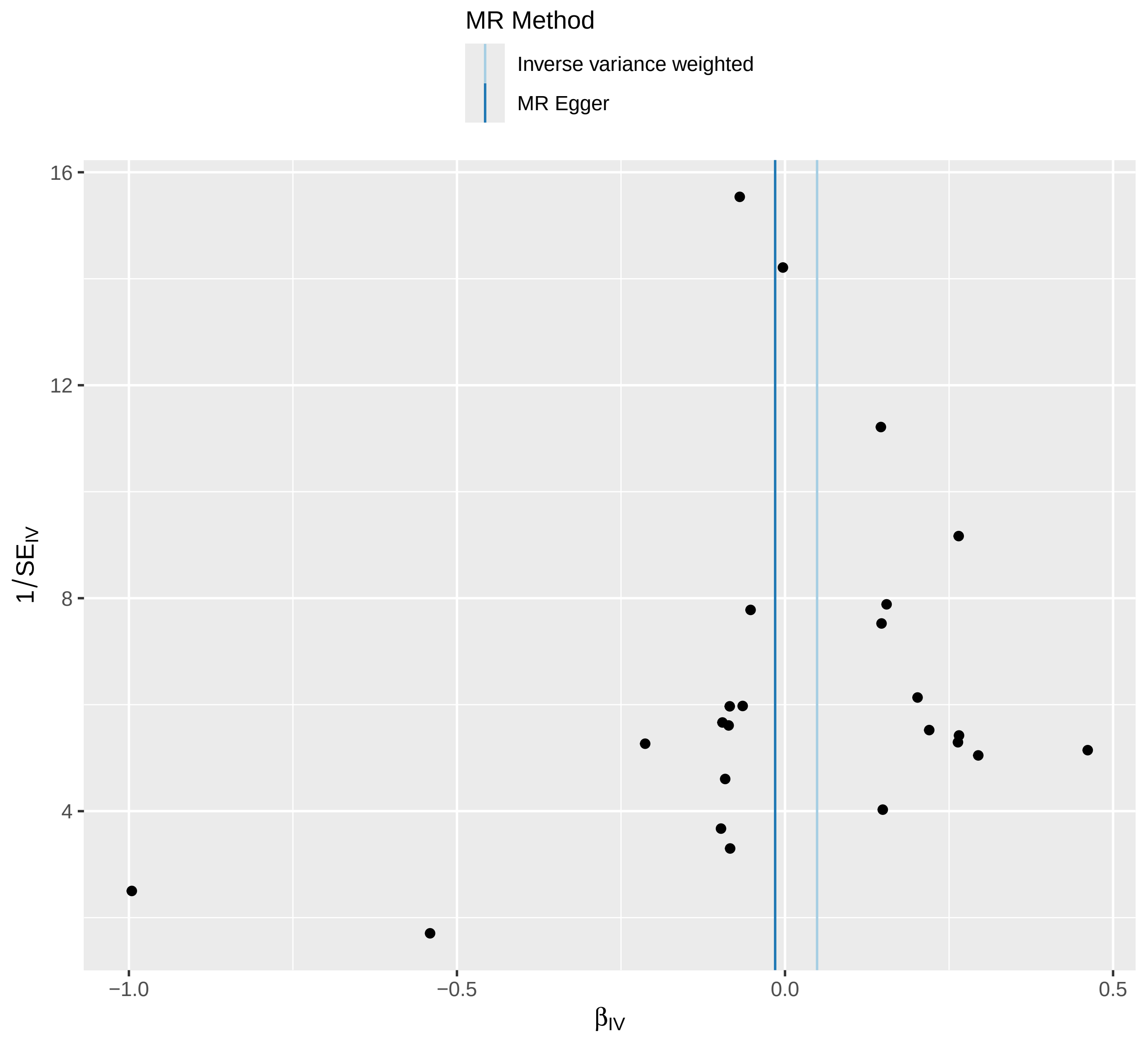
MR Method
Inverse variance weighted
MR Egger
16
12
8
4
1/SE$_{IV}$
−1.0
−0.5
0.0
0.5
β$_{IV}$

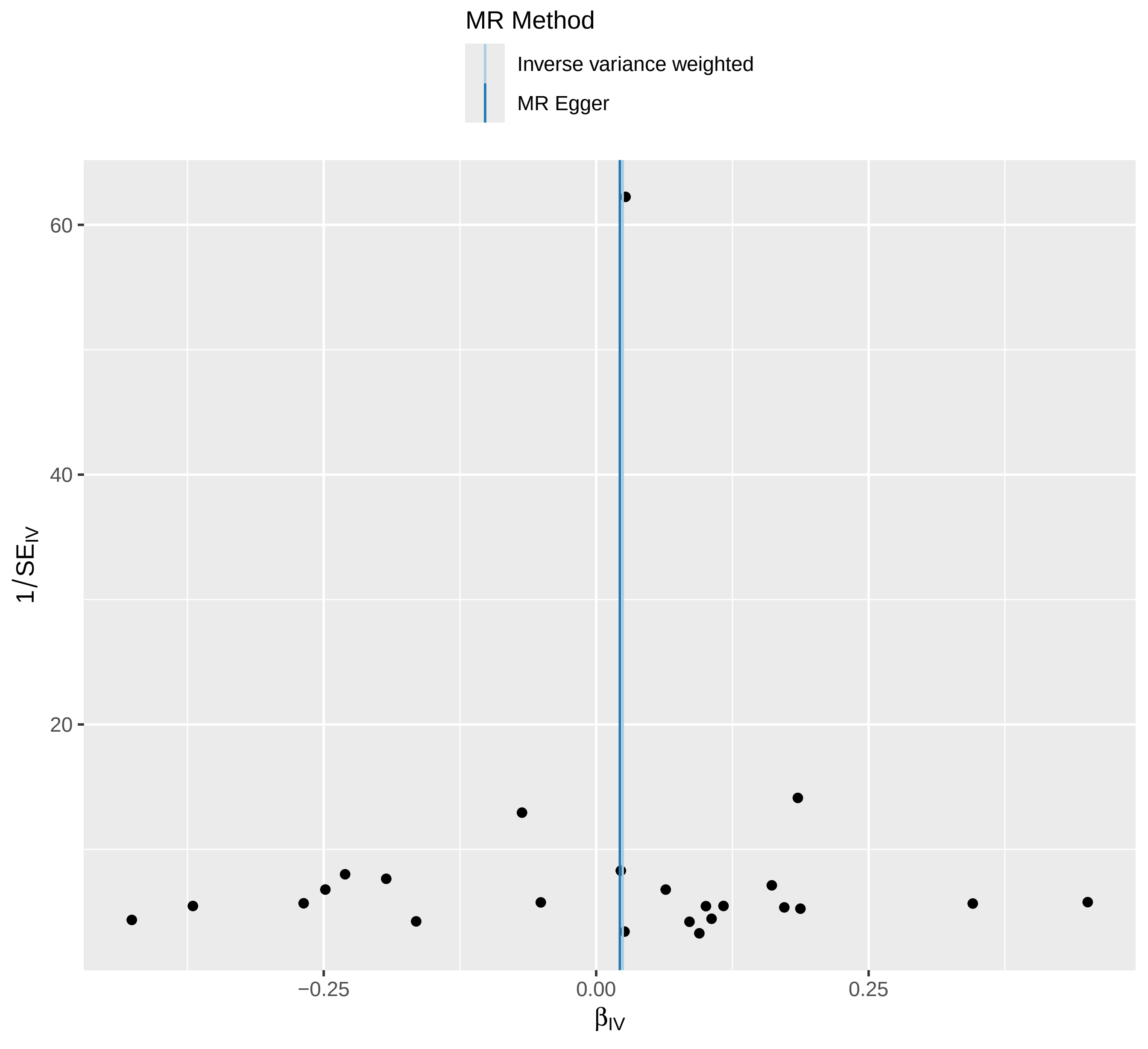

MR Method
Inverse variance weighted
MR Egger
60
40
20
1/SE_IV
−0.25
0.00
0.25
β_IV

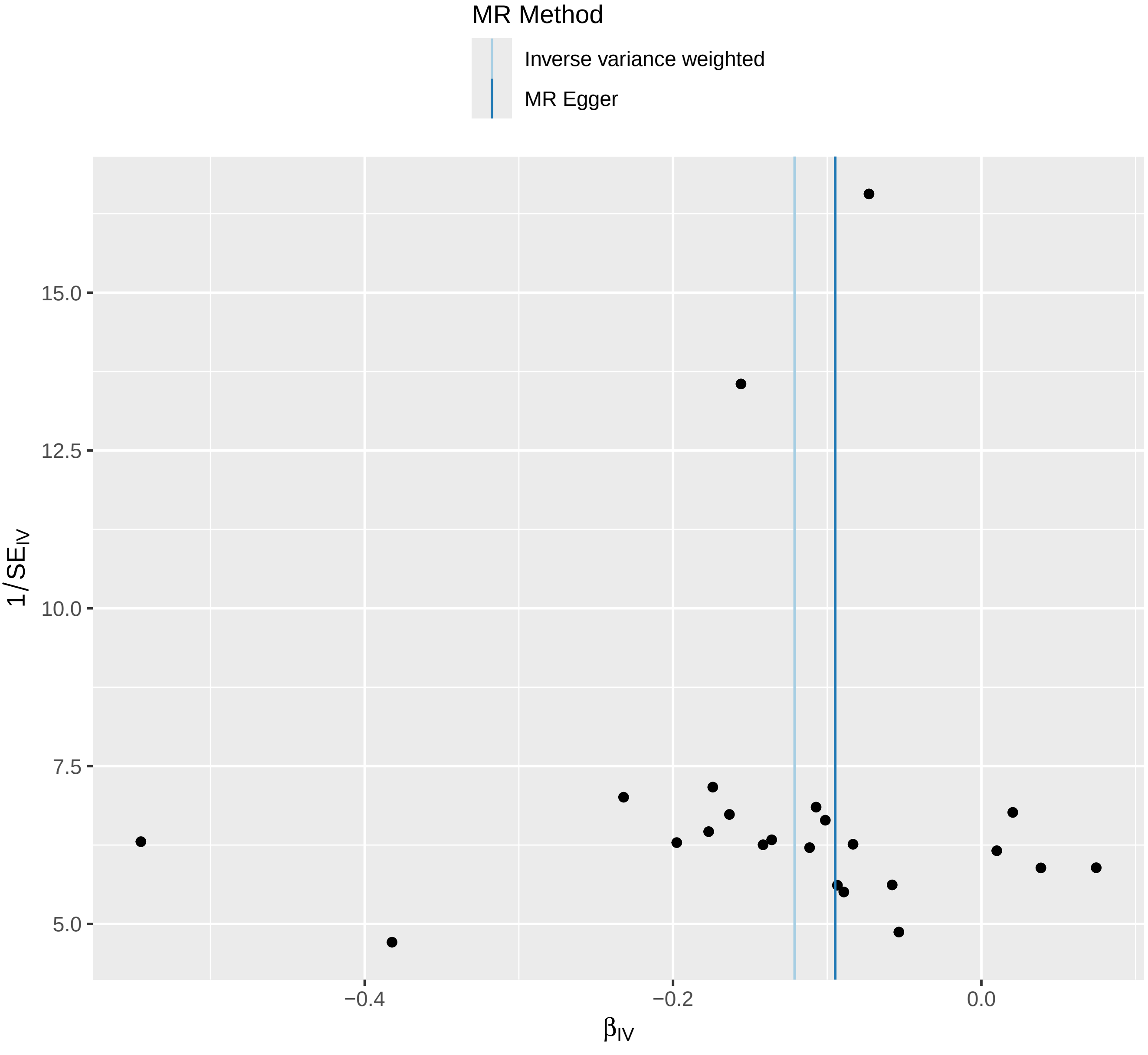
MR Method
Inverse variance weighted
MR Egger
15.0
12.5
10.0
7.5
5.0
$1/SE_{IV}$
−0.4
−0.2
0.0
β_{IV}

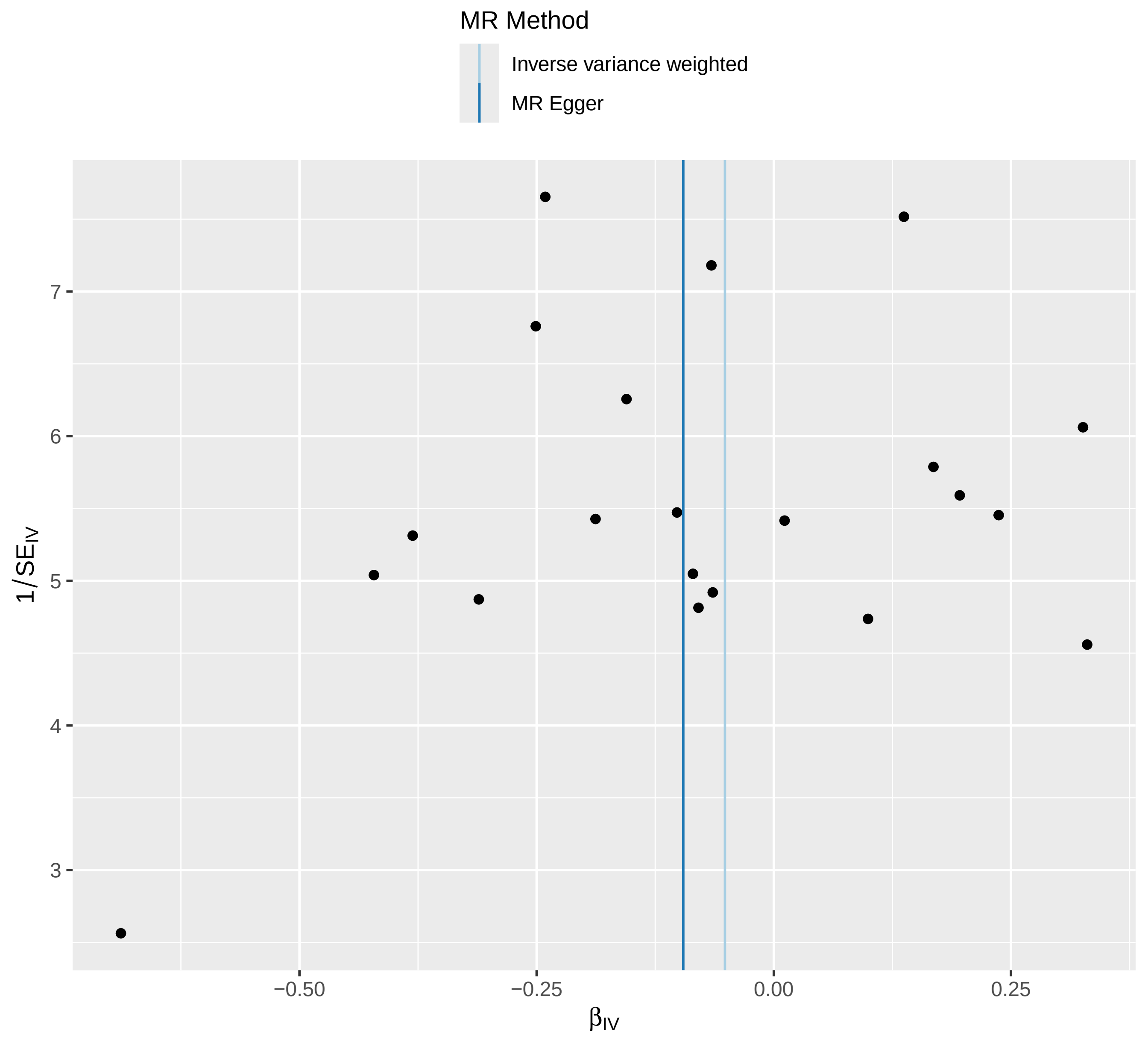

MR Method
Inverse variance weighted
MR Egger
1/SE_IV
7
6
5
4
3
−0.50
−0.25
0.00
0.25
β_IV

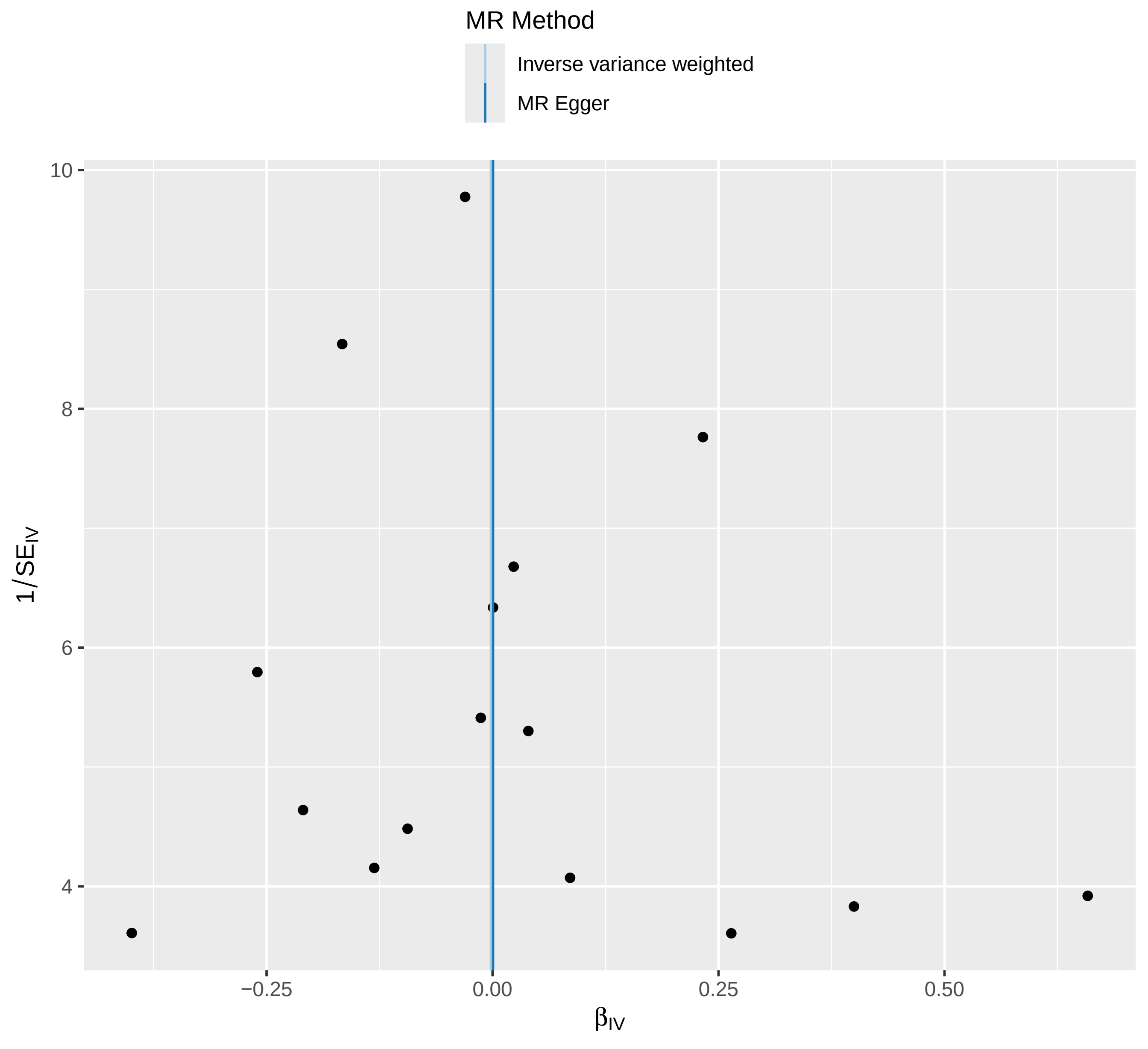

MR Method
Inverse variance weighted
MR Egger
10
8
6
4
1/SE_IV
−0.25
0.00
0.25
0.50
β_IV

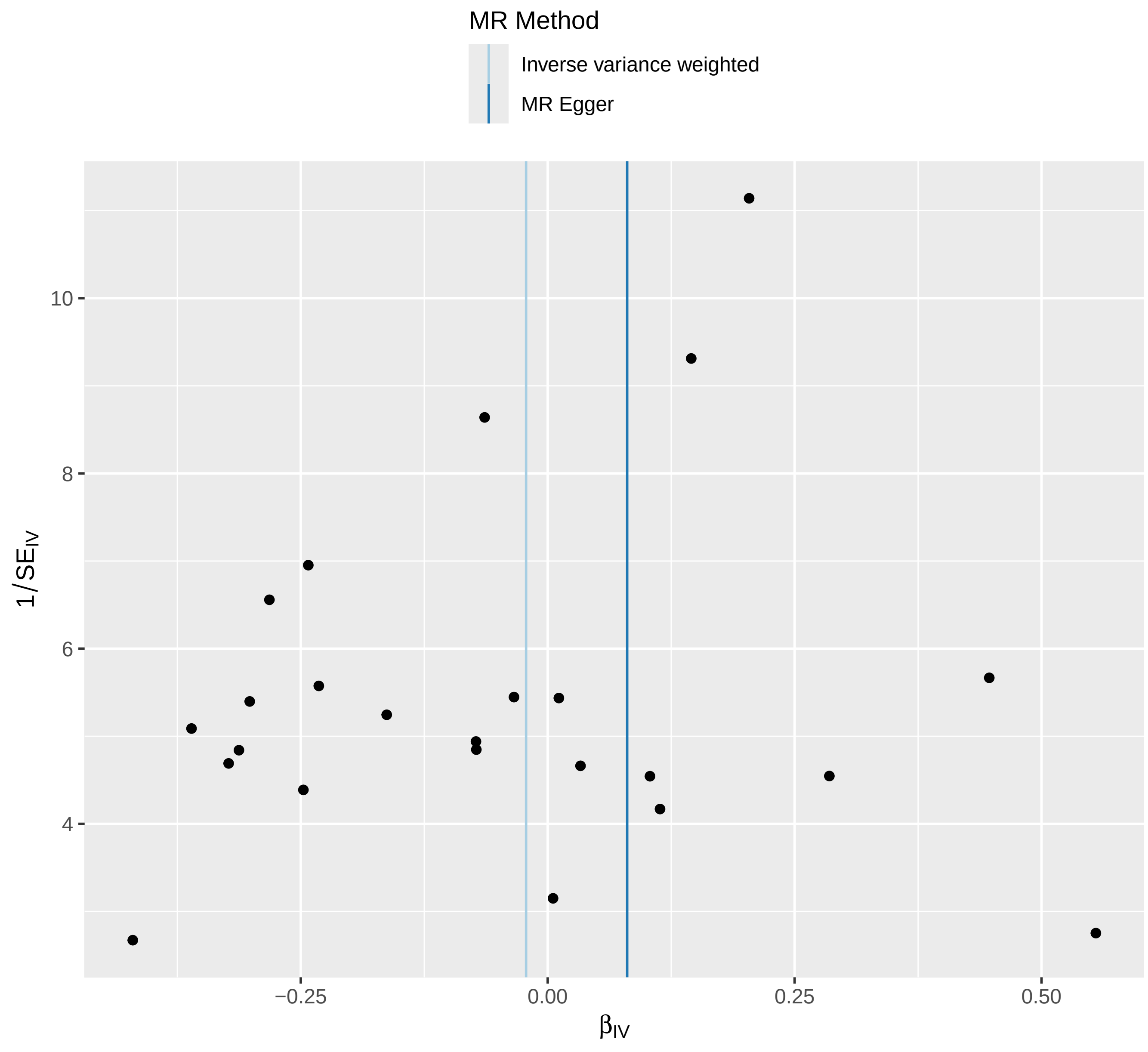
MR Method
Inverse variance weighted
MR Egger
10
8
6
4
$1/SE_{IV}$
−0.25
0.00
0.25
0.50
β_{IV}

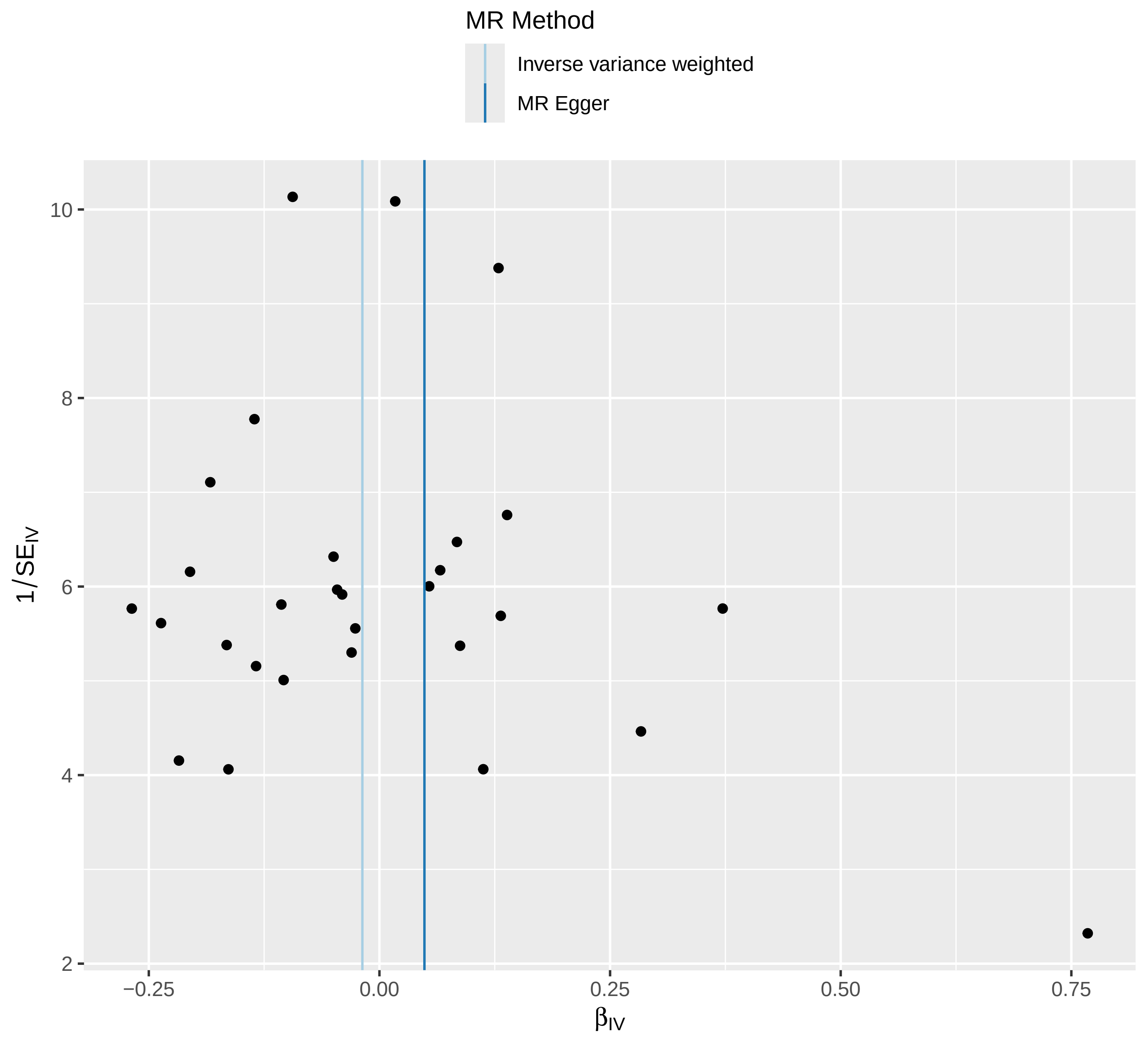

MR Method
Inverse variance weighted
MR Egger
10
8
6
4
2
$1/SE_{IV}$
−0.25
0.00
0.25
0.50
0.75
β_{IV}

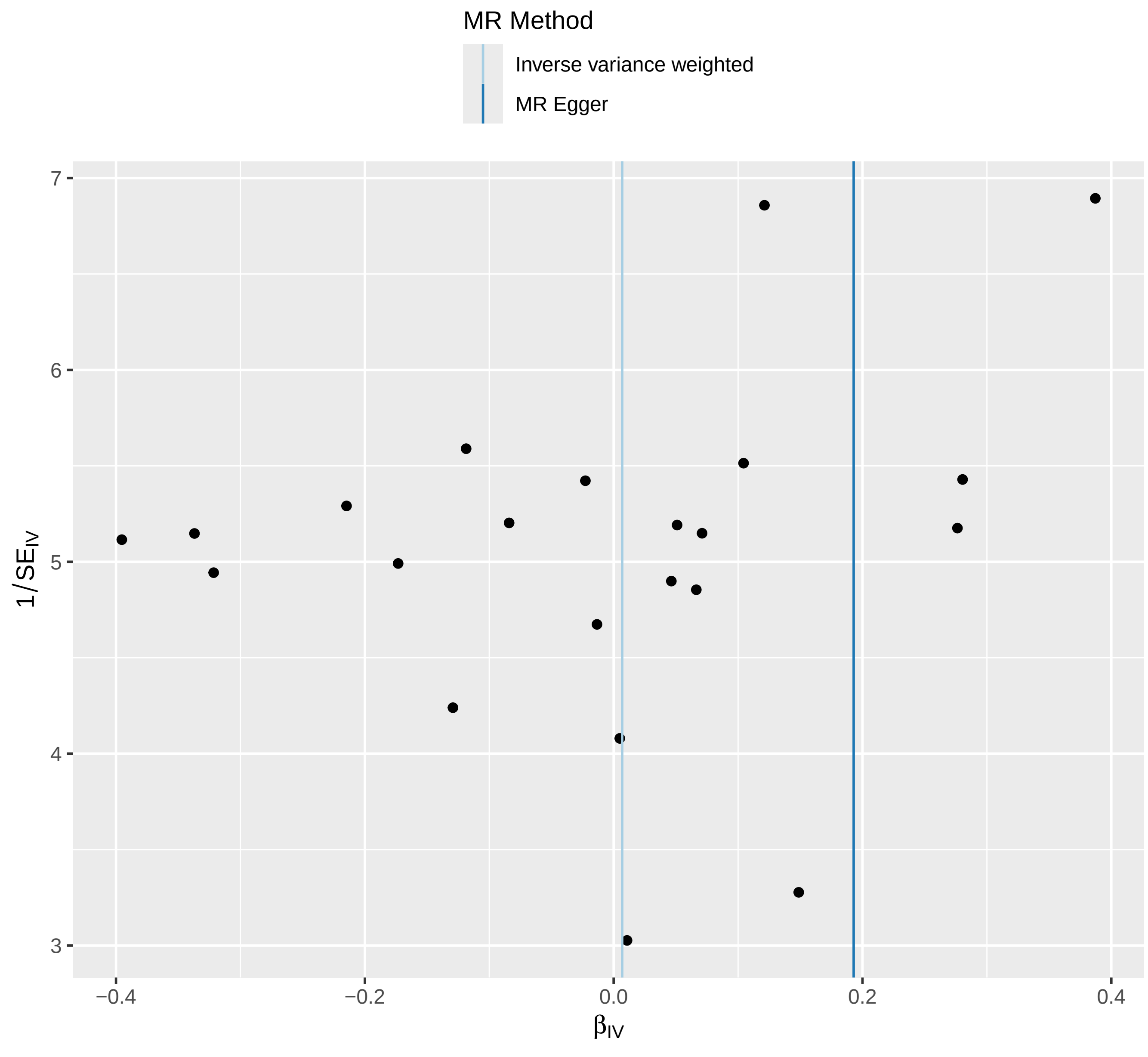

MR Method
Inverse variance weighted
MR Egger
7
6
5
4
3
1/SE_IV
−0.4
−0.2
0.0
0.2
0.4
β_IV

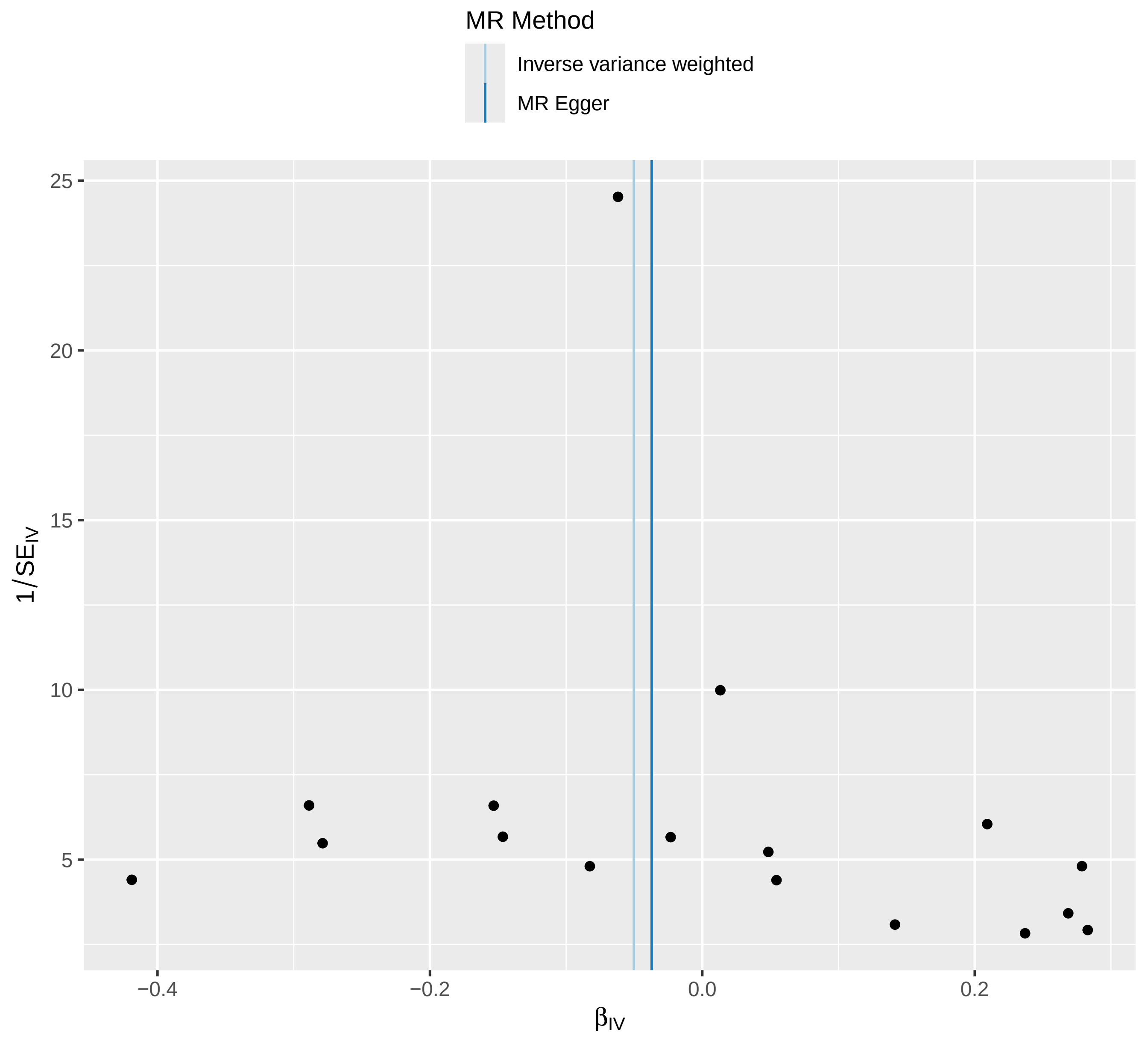
MR Method
Inverse variance weighted
MR Egger
25
20
15
10
5
1/SE_IV
−0.4
−0.2
0.0
0.2
β_IV

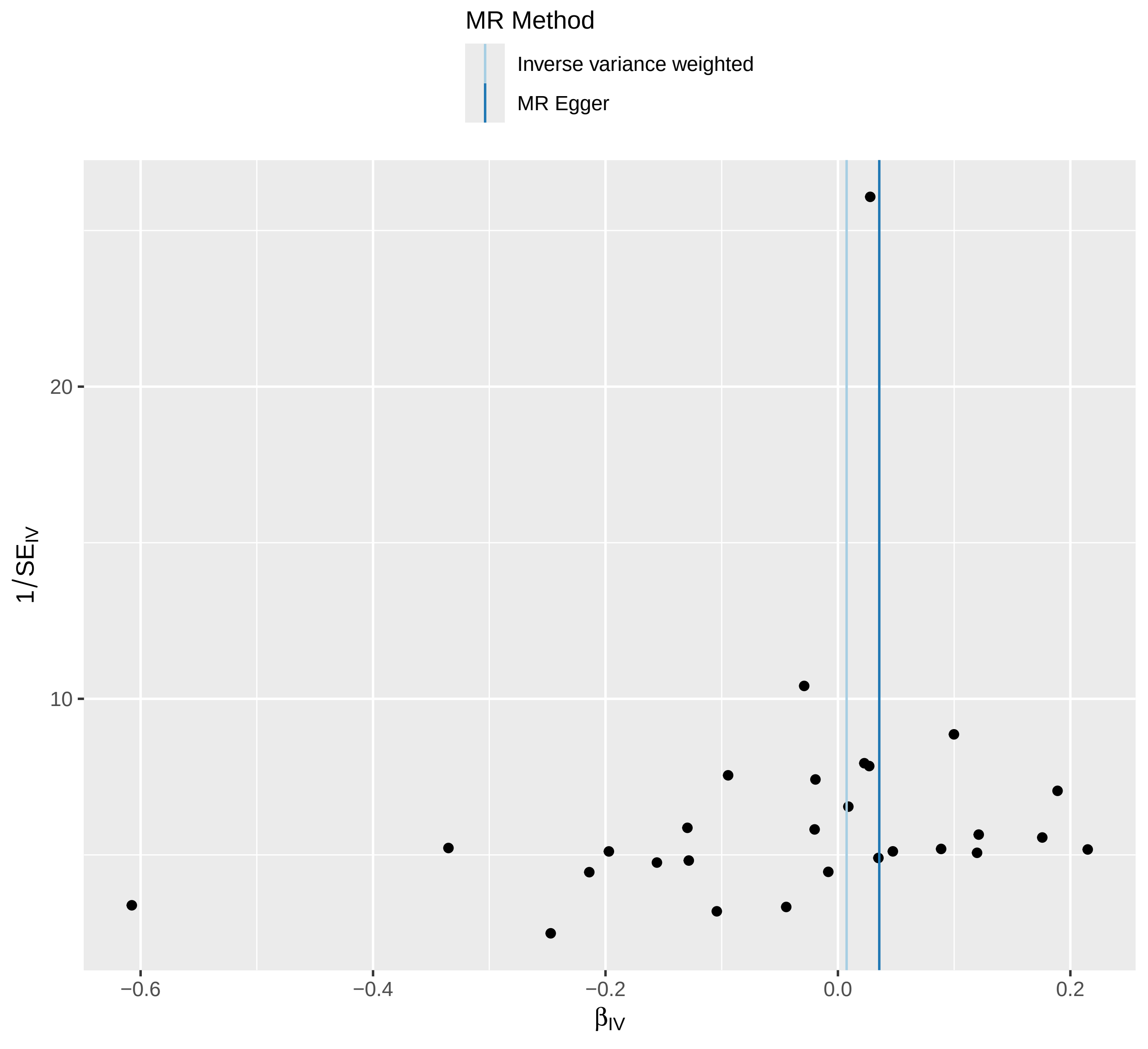

MR Method
Inverse variance weighted
MR Egger
20
10
1/SE_IV
−0.6
−0.4
−0.2
0.0
0.2
β_IV

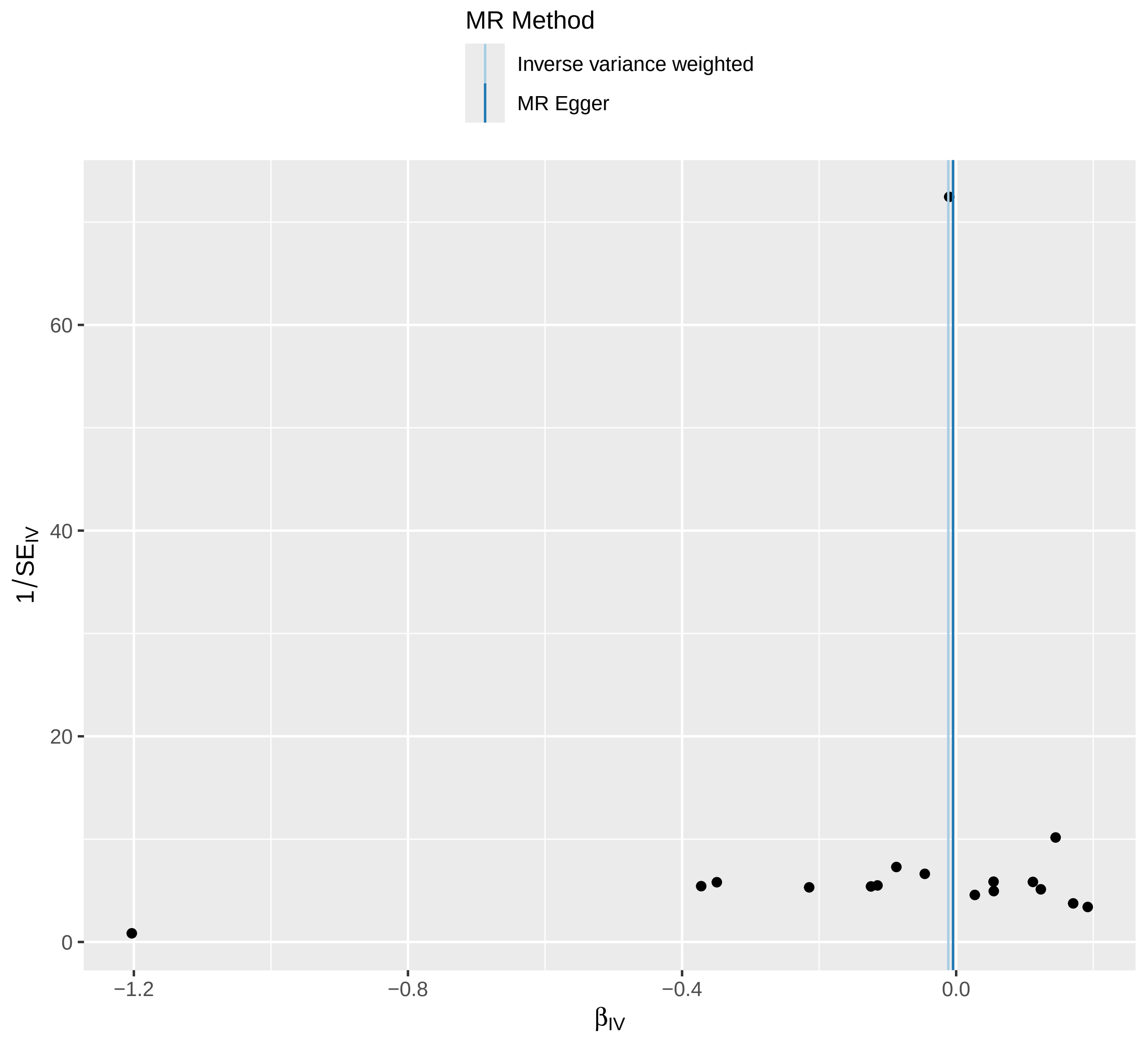
MR Method
Inverse variance weighted
MR Egger
60
40
20
0
1/SE_IV
−1.2
−0.8
−0.4
0.0
β_IV

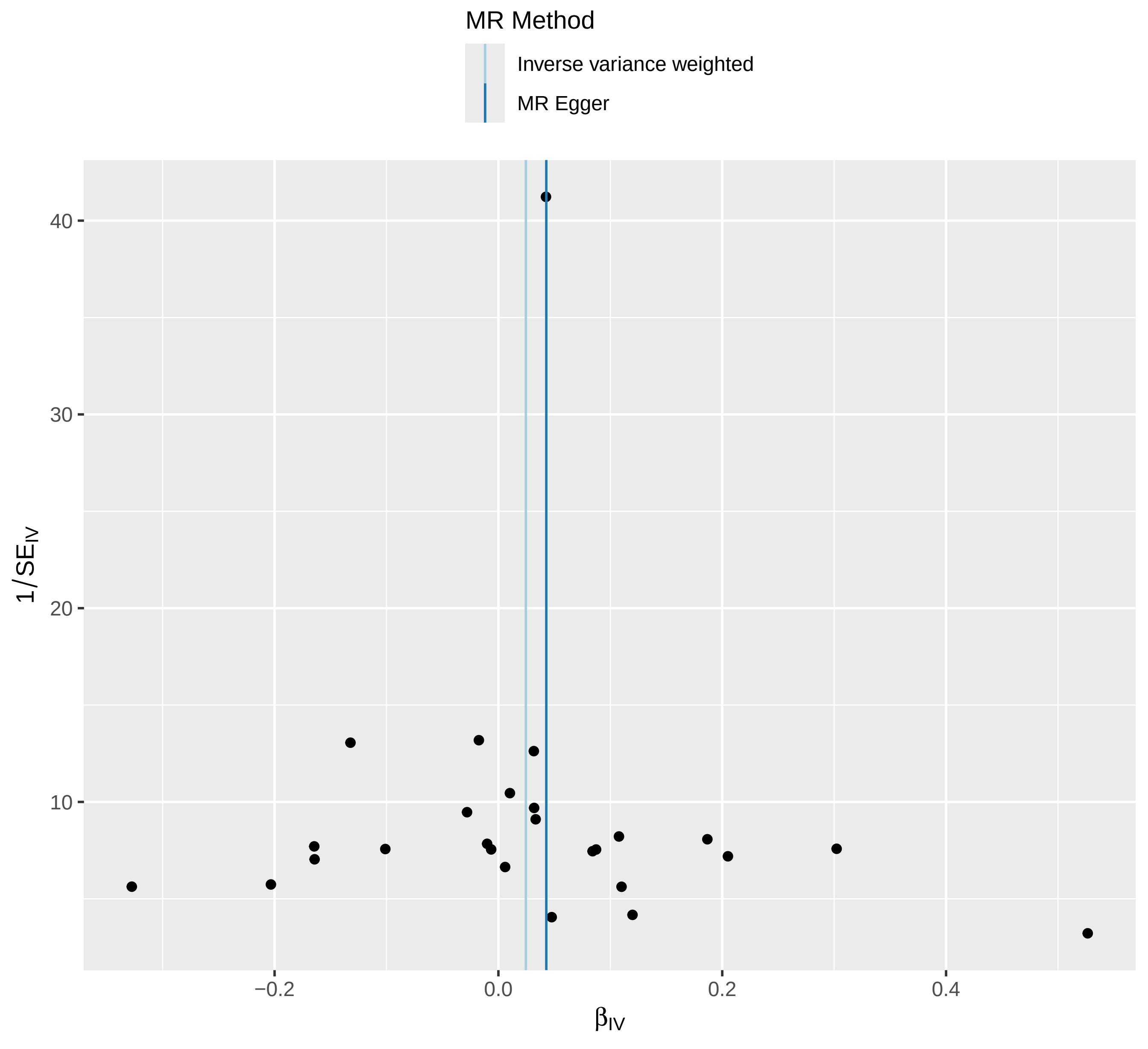
MR Method
Inverse variance weighted
MR Egger
1/SE_IV
β_IV
40
30
20
10
−0.2
0.0
0.2
0.4

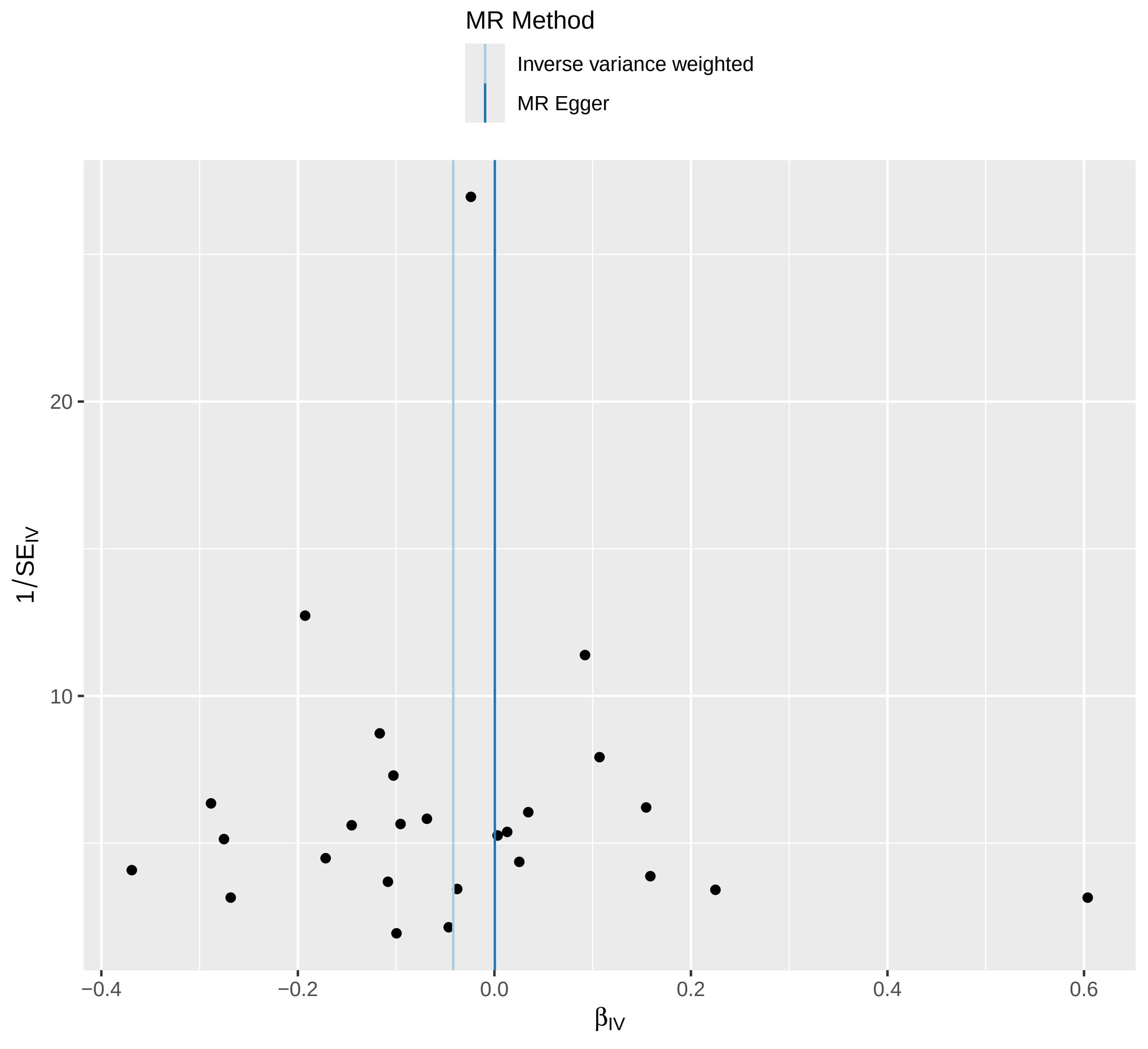

MR Method
Inverse variance weighted
MR Egger
20
10
1/SE_IV
−0.4
−0.2
0.0
0.2
0.4
0.6
β_IV

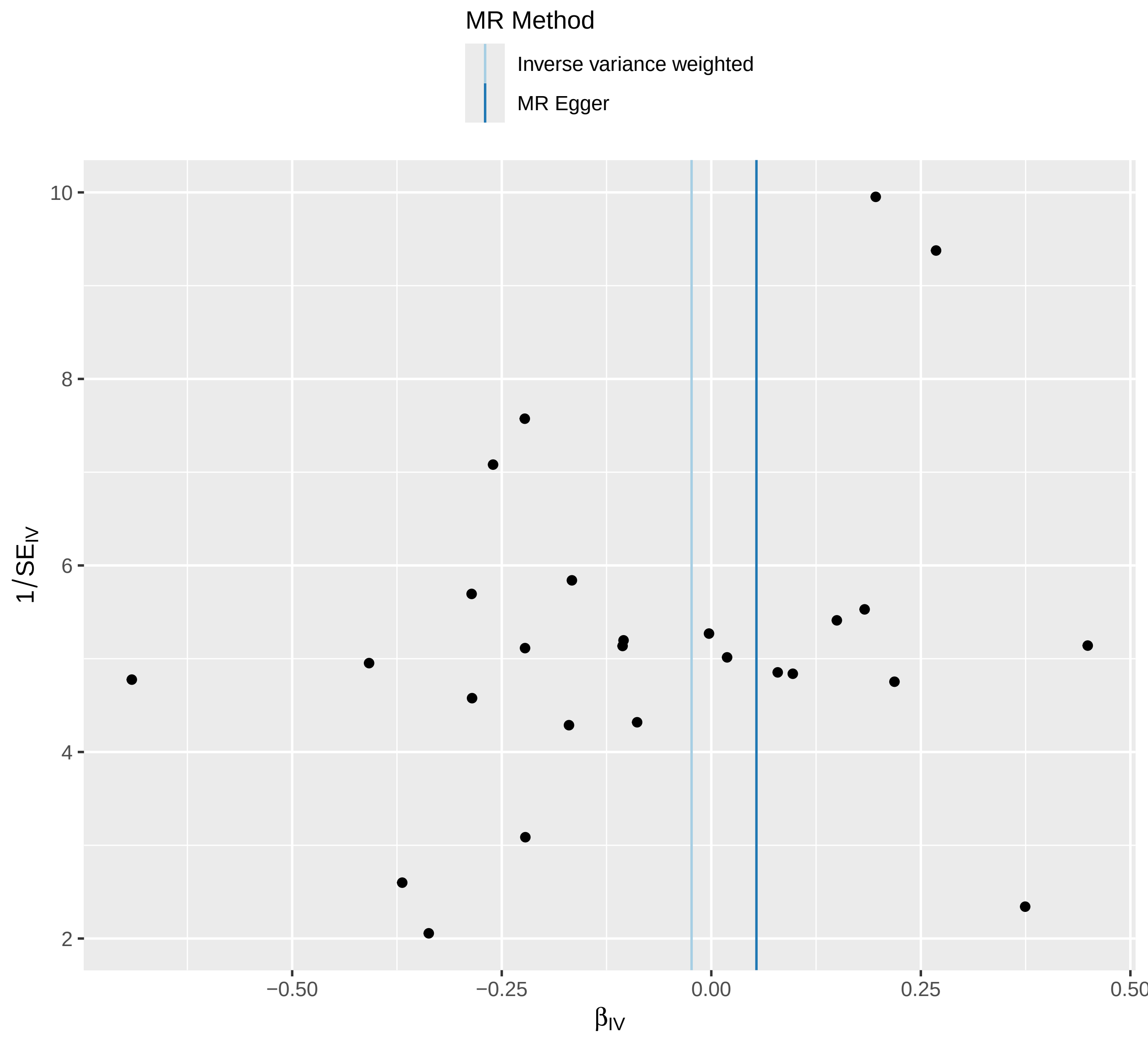

MR Method
Inverse variance weighted
MR Egger
1/SE_IV
10
8
6
4
2
−0.50
−0.25
0.00
0.25
0.50
β_IV

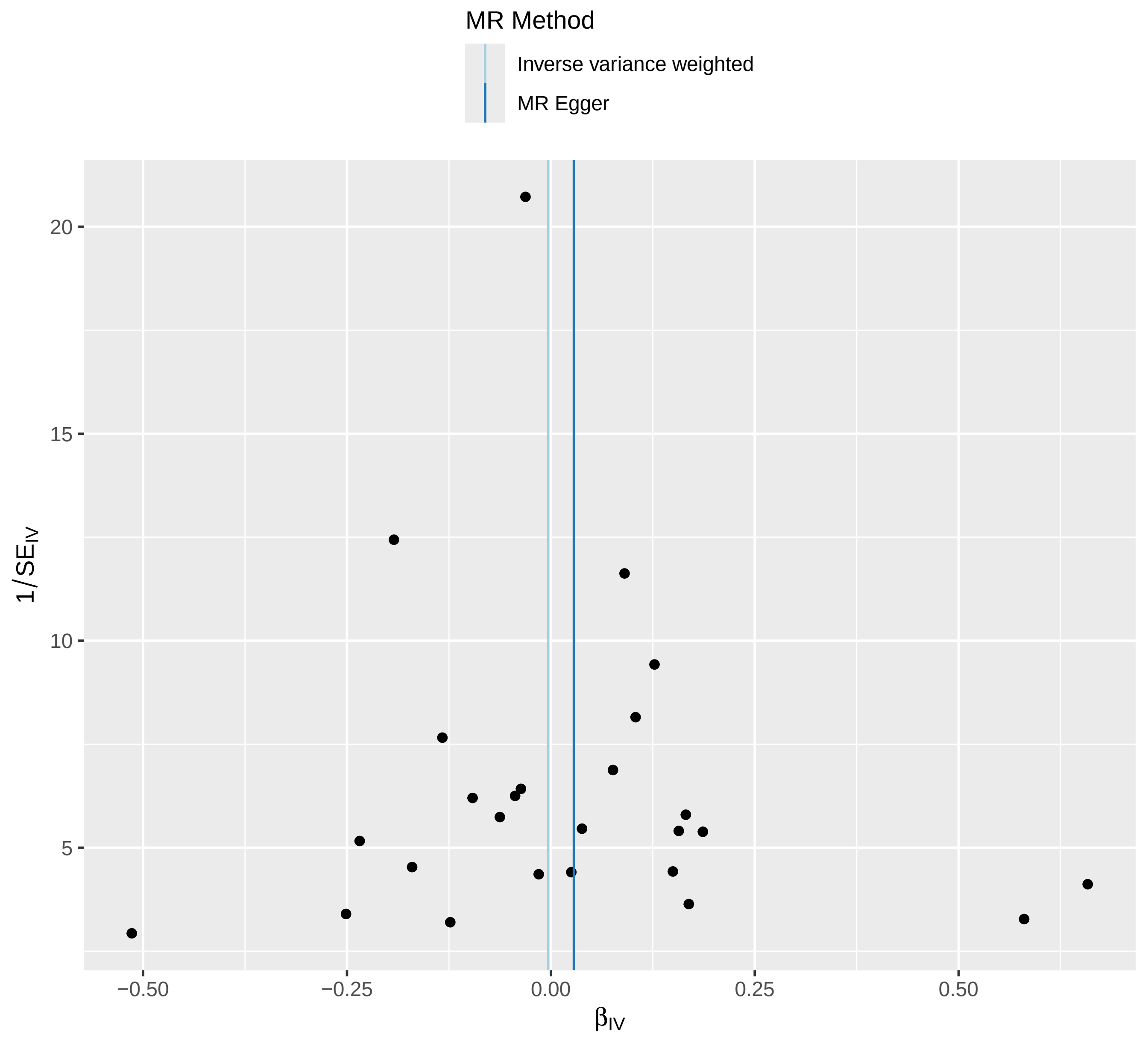

MR Method
Inverse variance weighted
MR Egger
20
15
10
5
$1/SE_{IV}$
−0.50
−0.25
0.00
0.25
0.50
β_{IV}

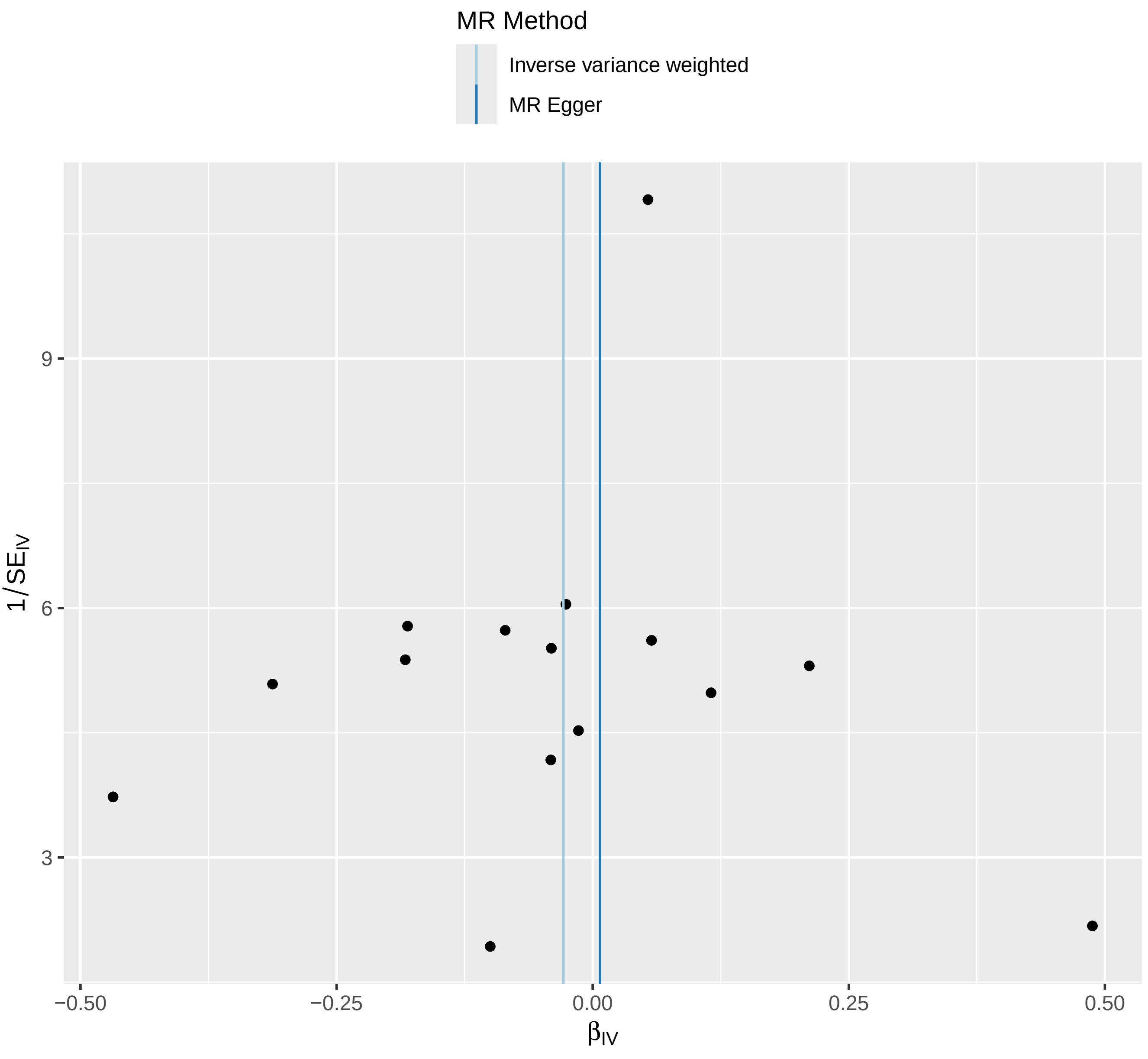
MR Method
Inverse variance weighted
MR Egger
1/SE_IV
9
6
3
−0.50
−0.25
0.00
0.25
0.50
β_IV

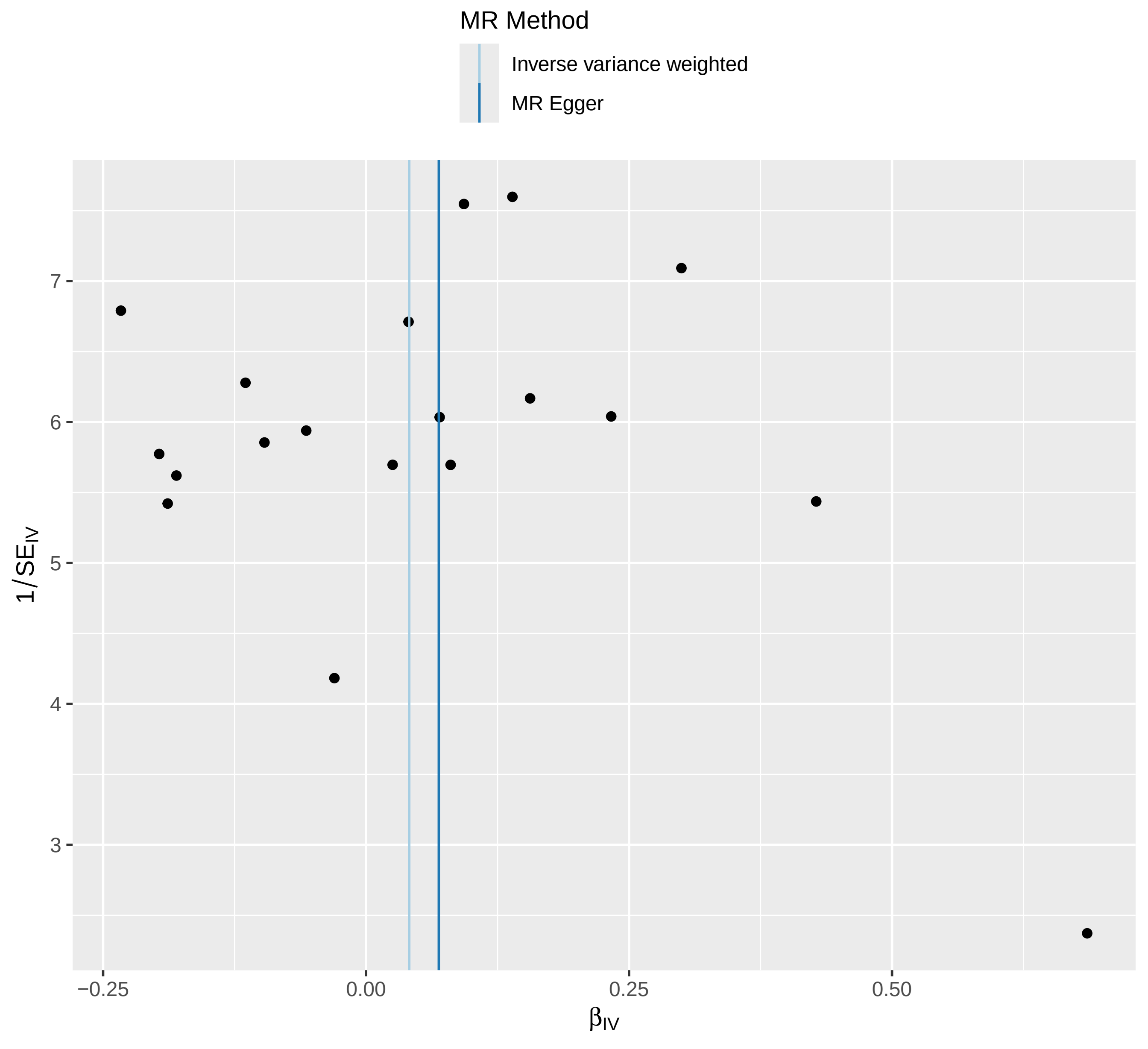

MR Method
Inverse variance weighted
MR Egger
1/SE_IV
7
6
5
4
3
−0.25
0.00
0.25
0.50
β_IV

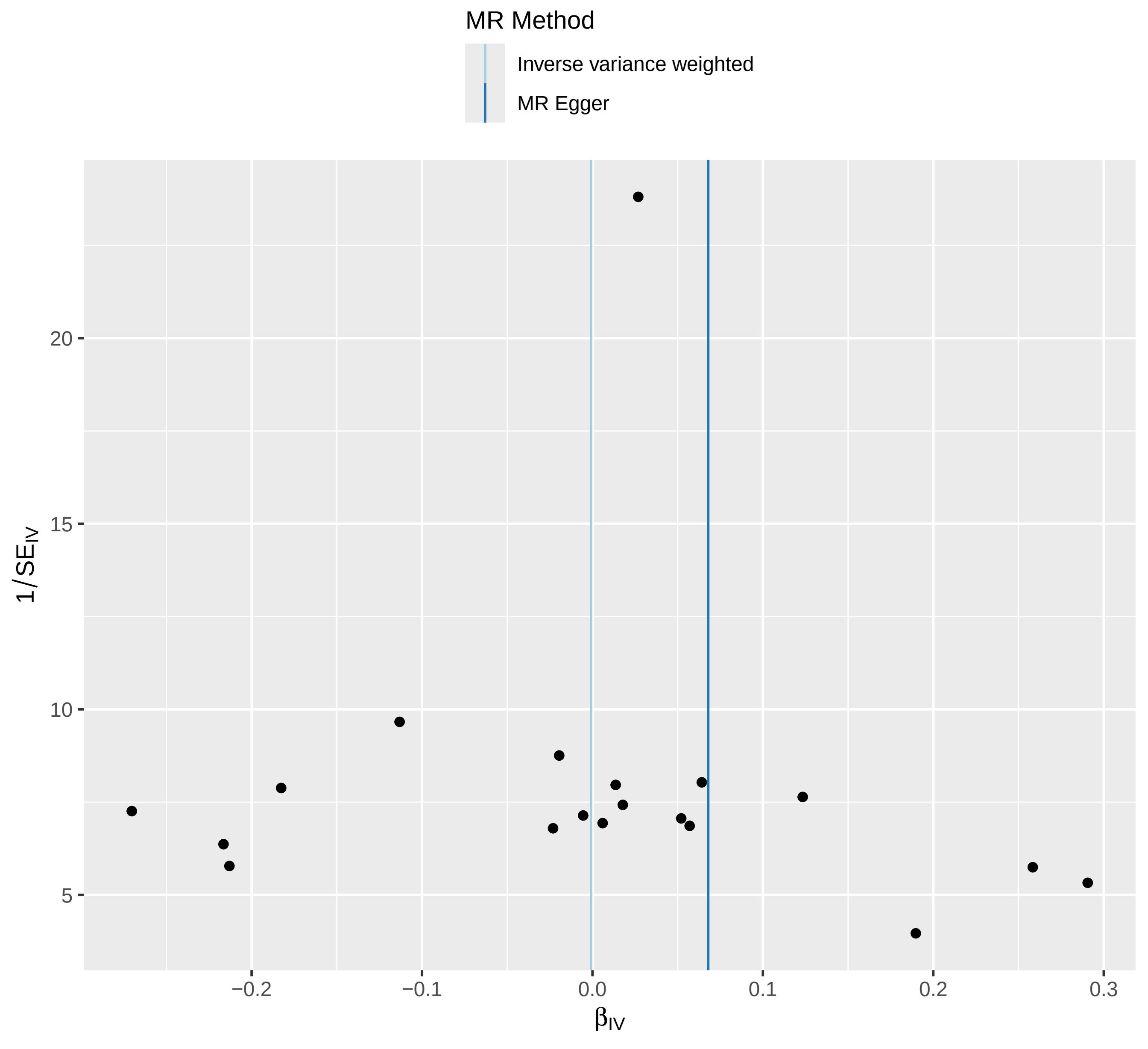

MR Method
Inverse variance weighted
MR Egger
20
15
10
5
1/SE_IV
−0.2
−0.1
0.0
0.1
0.2
0.3
β_IV

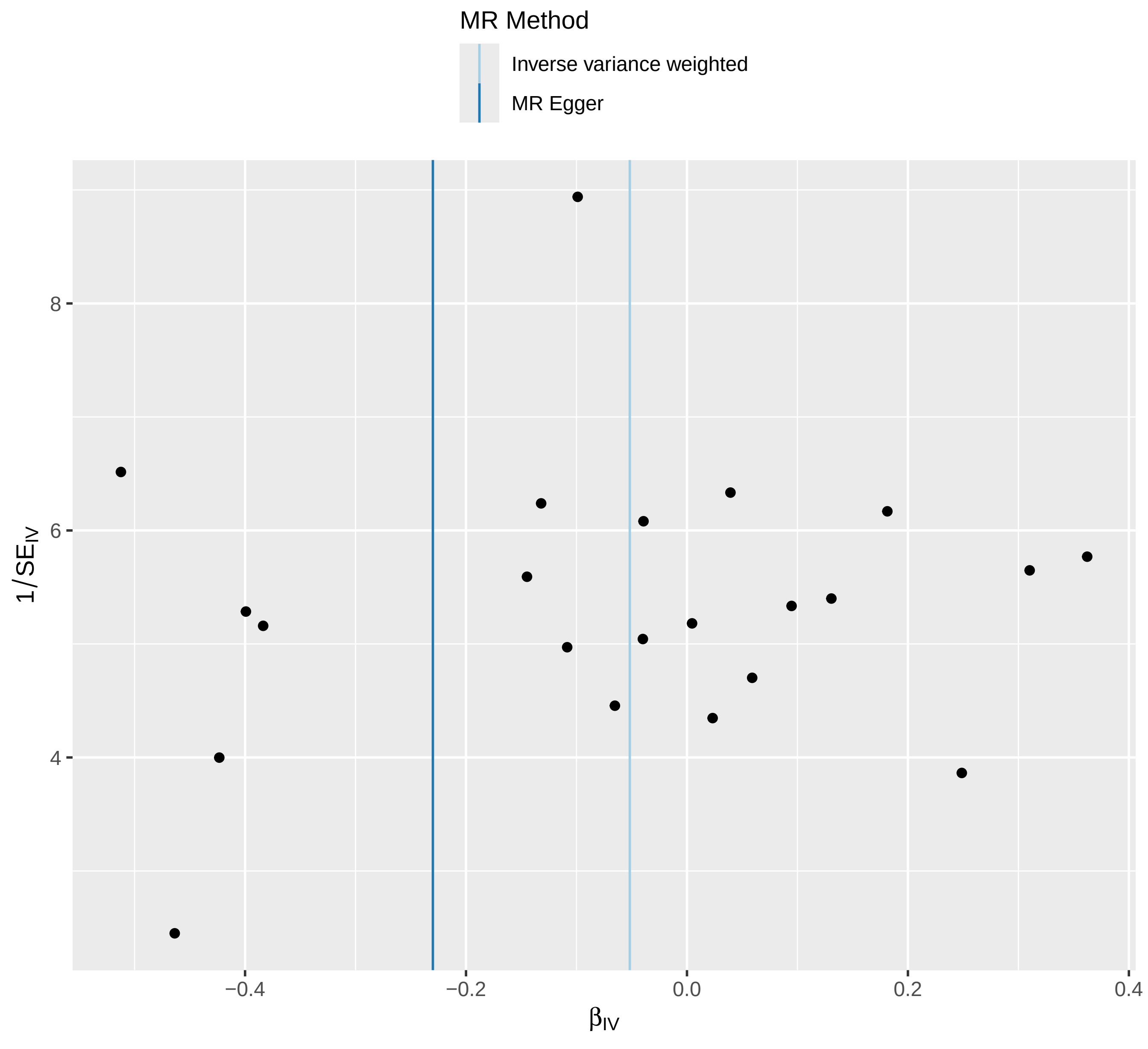

MR Method
Inverse variance weighted
MR Egger
8
6
4
1/SE_IV
−0.4
−0.2
0.0
0.2
0.4
β_IV

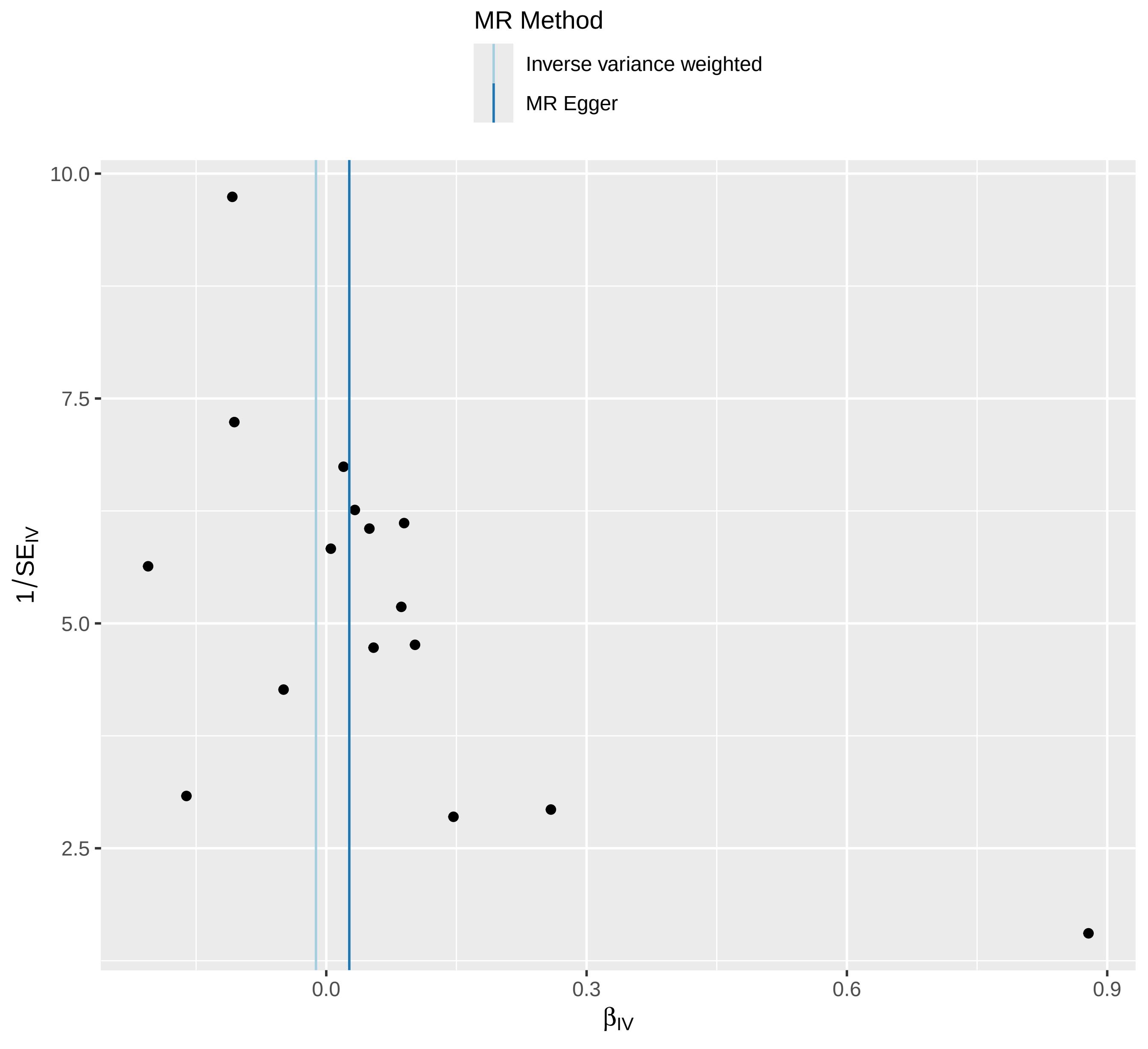

MR Method
Inverse variance weighted
MR Egger
10.0
7.5
5.0
2.5
1/SE$_{IV}$
0.0
0.3
0.6
0.9
β$_{IV}$

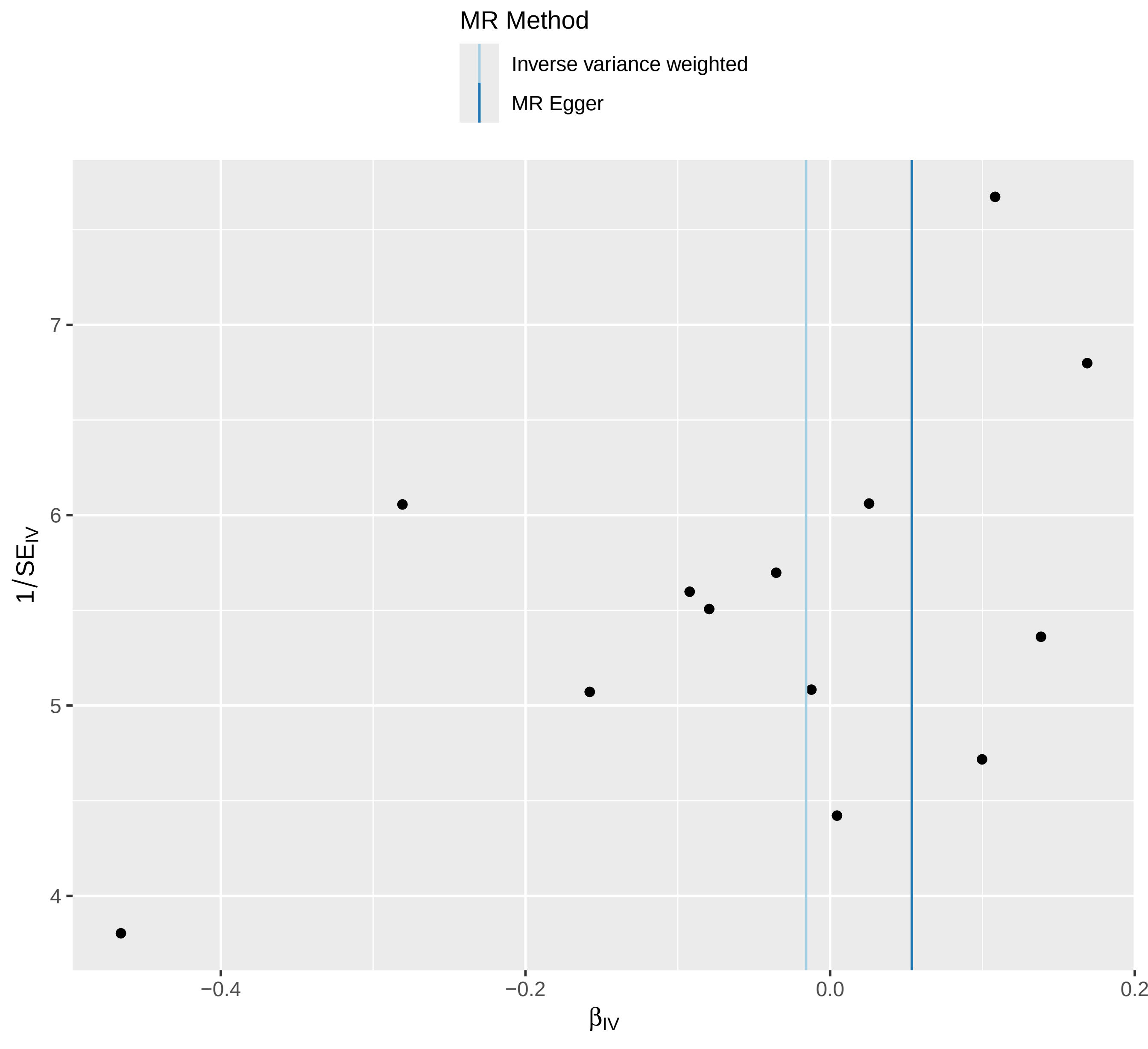

MR Method
Inverse variance weighted
MR Egger
7
6
5
4
1/SE_IV
−0.4
−0.2
0.0
0.2
β_IV

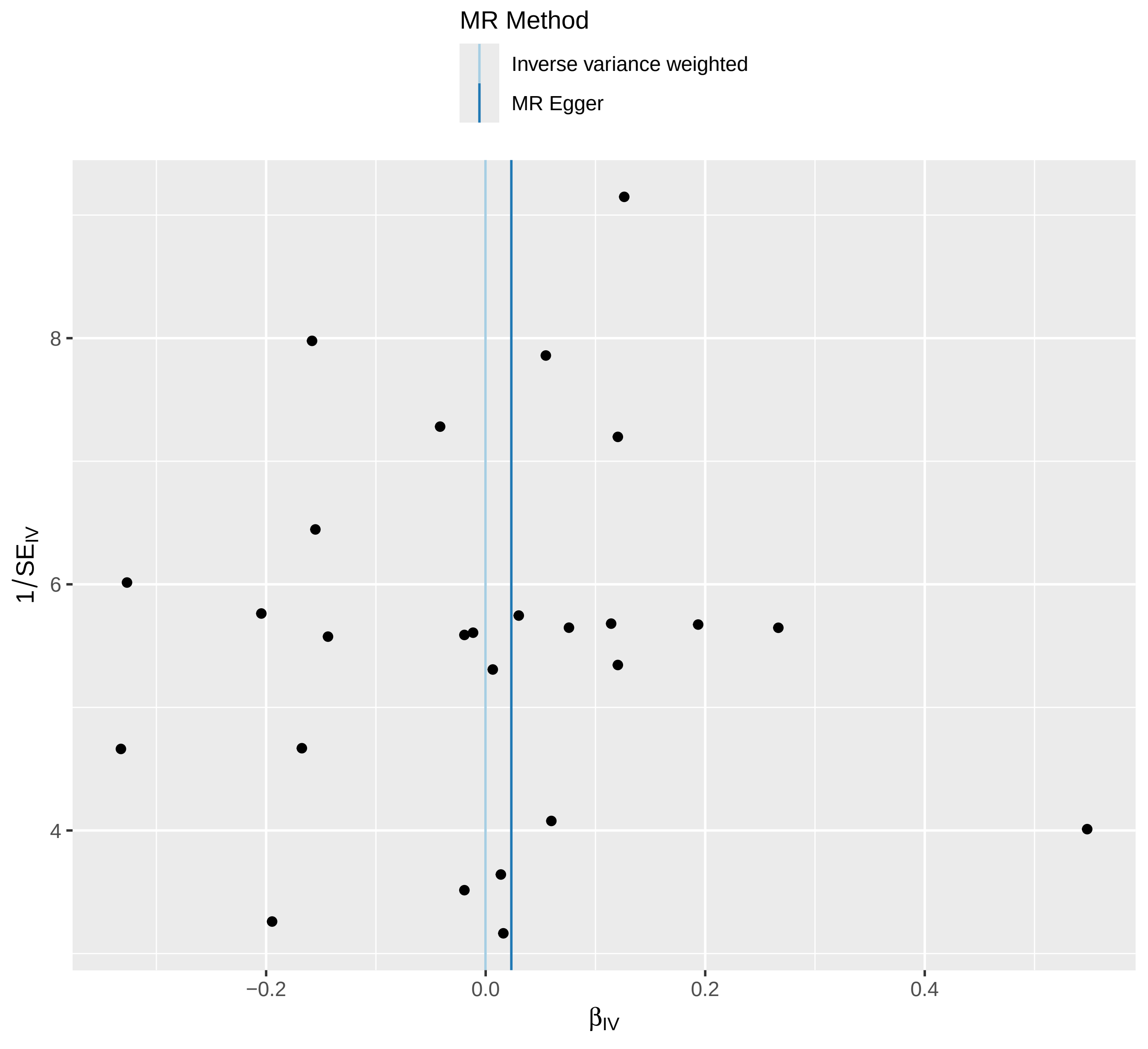
MR Method
Inverse variance weighted
MR Egger
8
6
4
1/SE_IV
−0.2
0.0
0.2
0.4
β_IV

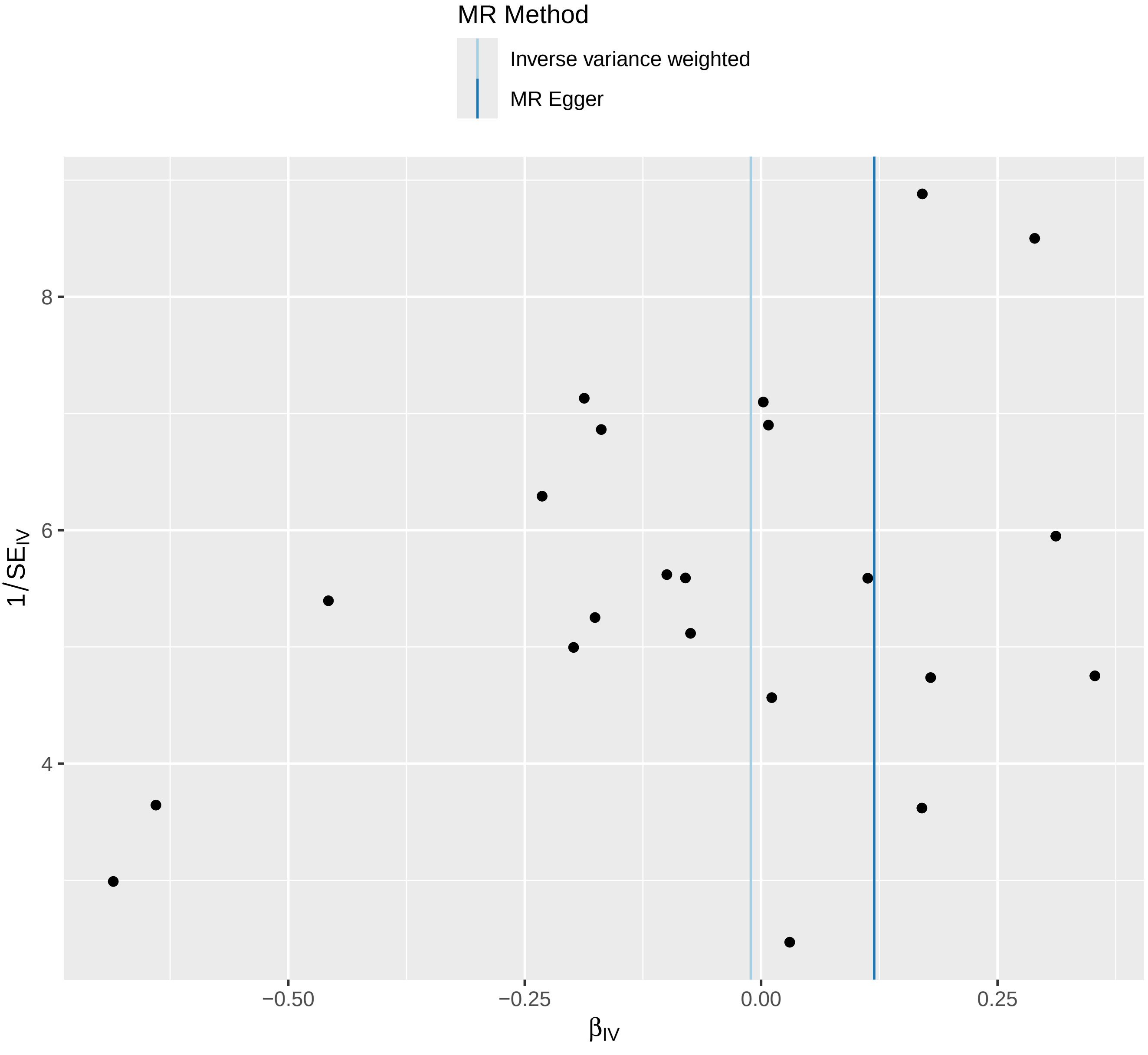
MR Method
Inverse variance weighted
MR Egger
8
6
4
1/SE_IV
−0.50
−0.25
0.00
0.25
β_IV

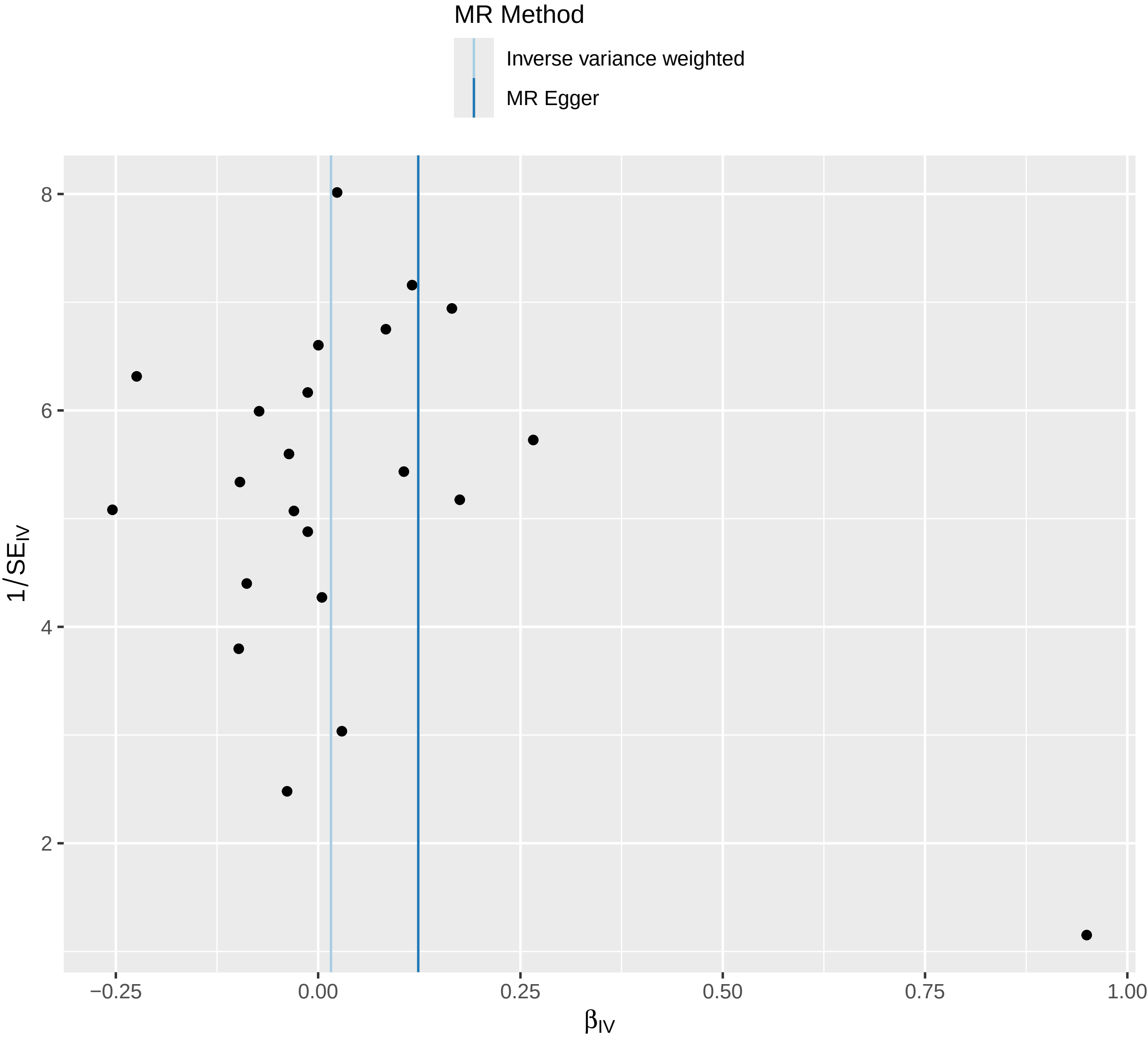
MR Method
Inverse variance weighted
MR Egger
1/SE_IV
8
6
4
2
β_IV
−0.25
0.00
0.25
0.50
0.75
1.00

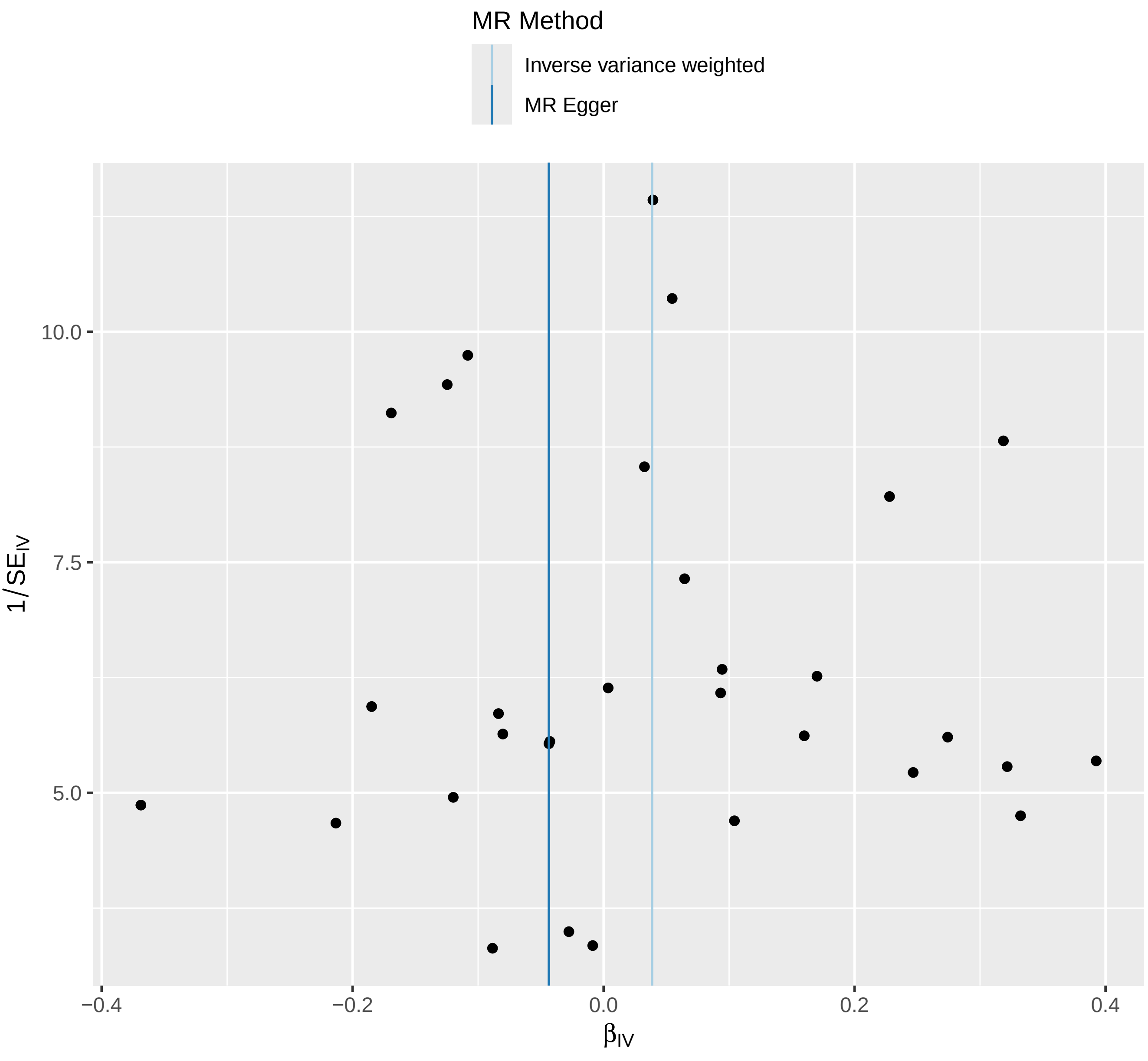
MR Method
Inverse variance weighted
MR Egger
10.0
7.5
5.0
$1/SE_{IV}$
−0.4
−0.2
0.0
0.2
0.4
β_{IV}

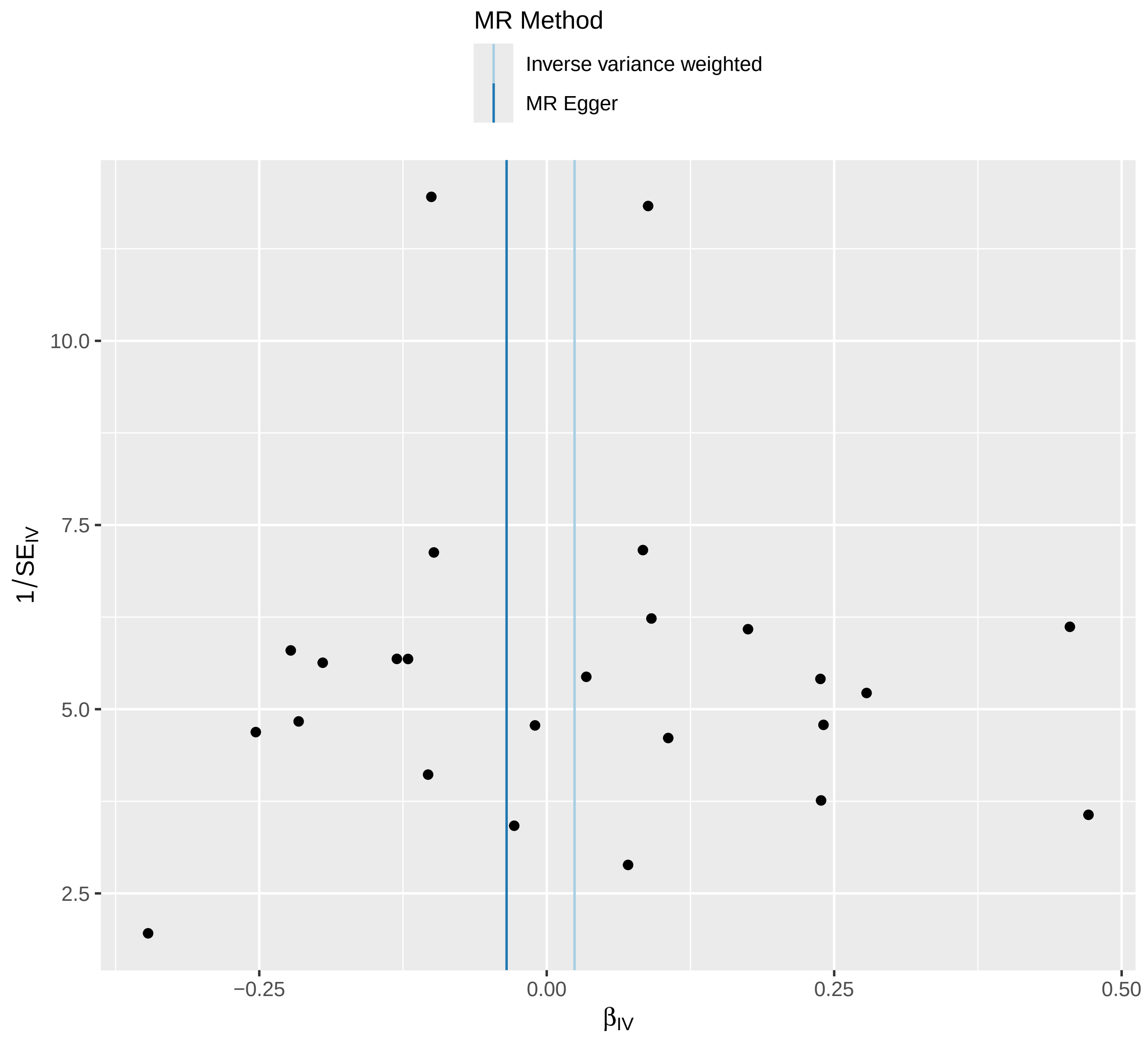
MR Method
Inverse variance weighted
MR Egger
10.0
7.5
5.0
2.5
1/SE_IV
−0.25
0.00
0.25
0.50
β_IV

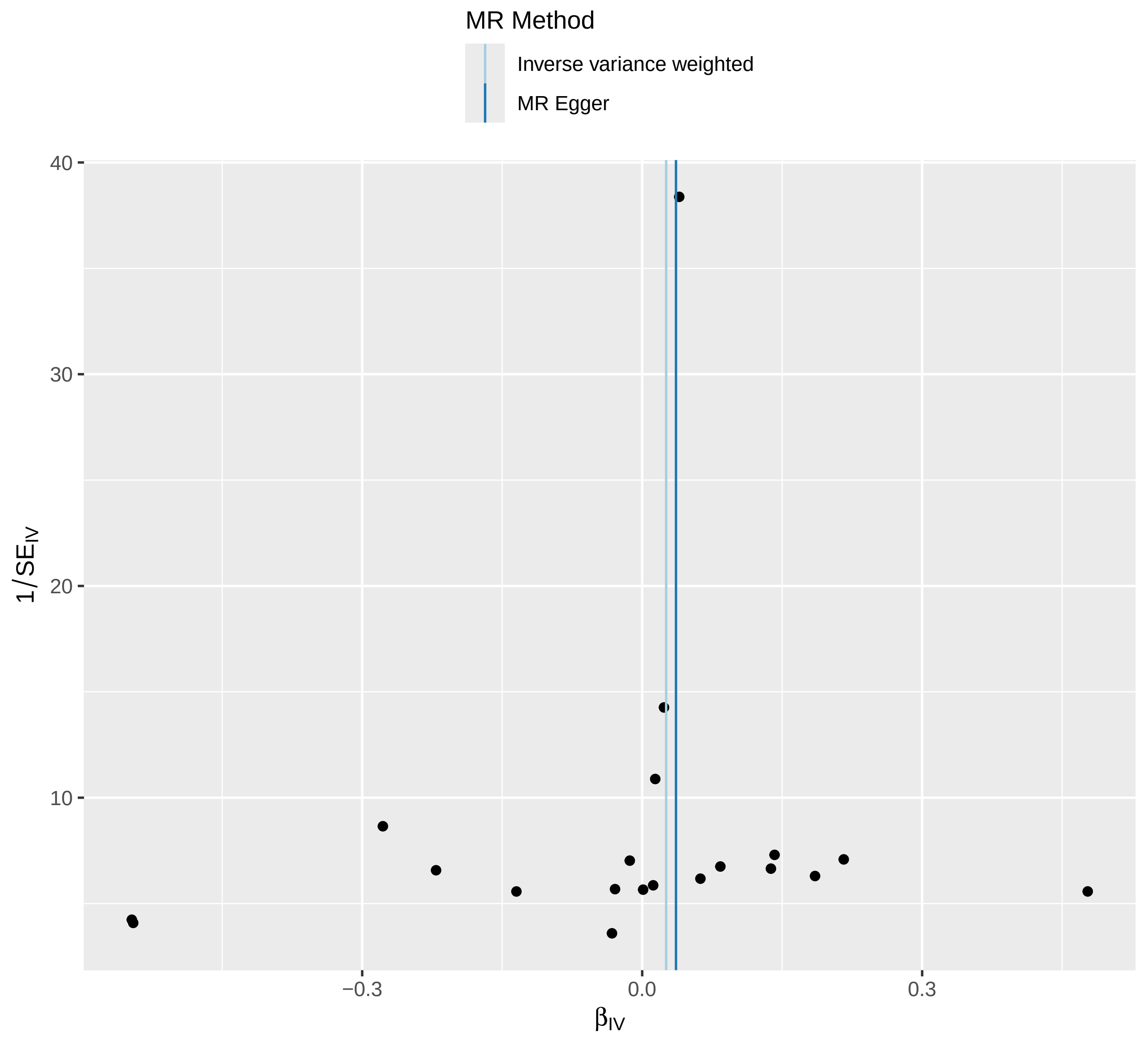
MR Method
Inverse variance weighted
MR Egger
40
30
20
10
1/SE$_{IV}$
−0.3
0.0
0.3
β$_{IV}$

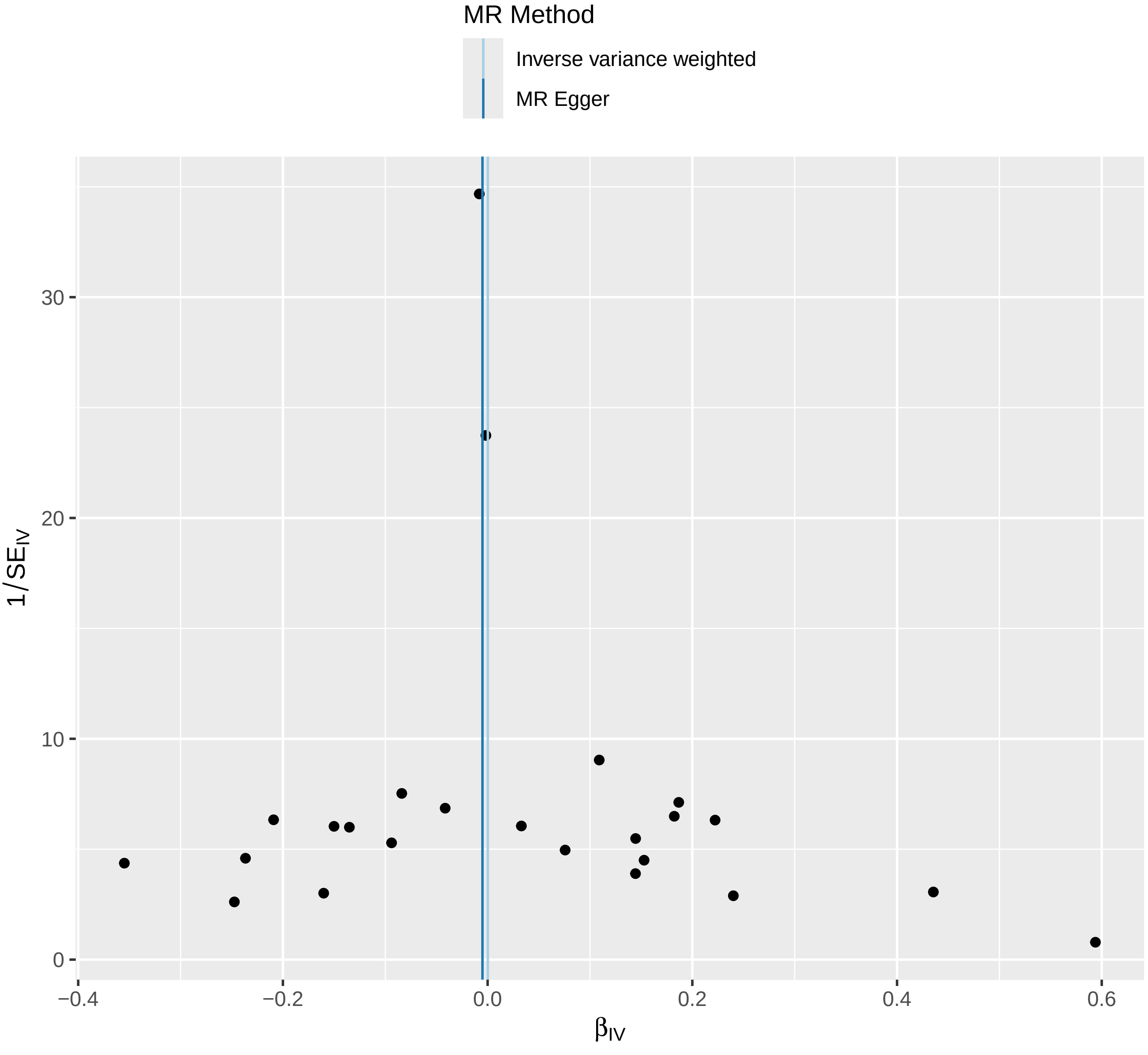
MR Method
Inverse variance weighted
MR Egger
1/SE_IV
β_IV
0
10
20
30
−0.4
−0.2
0.0
0.2
0.4
0.6

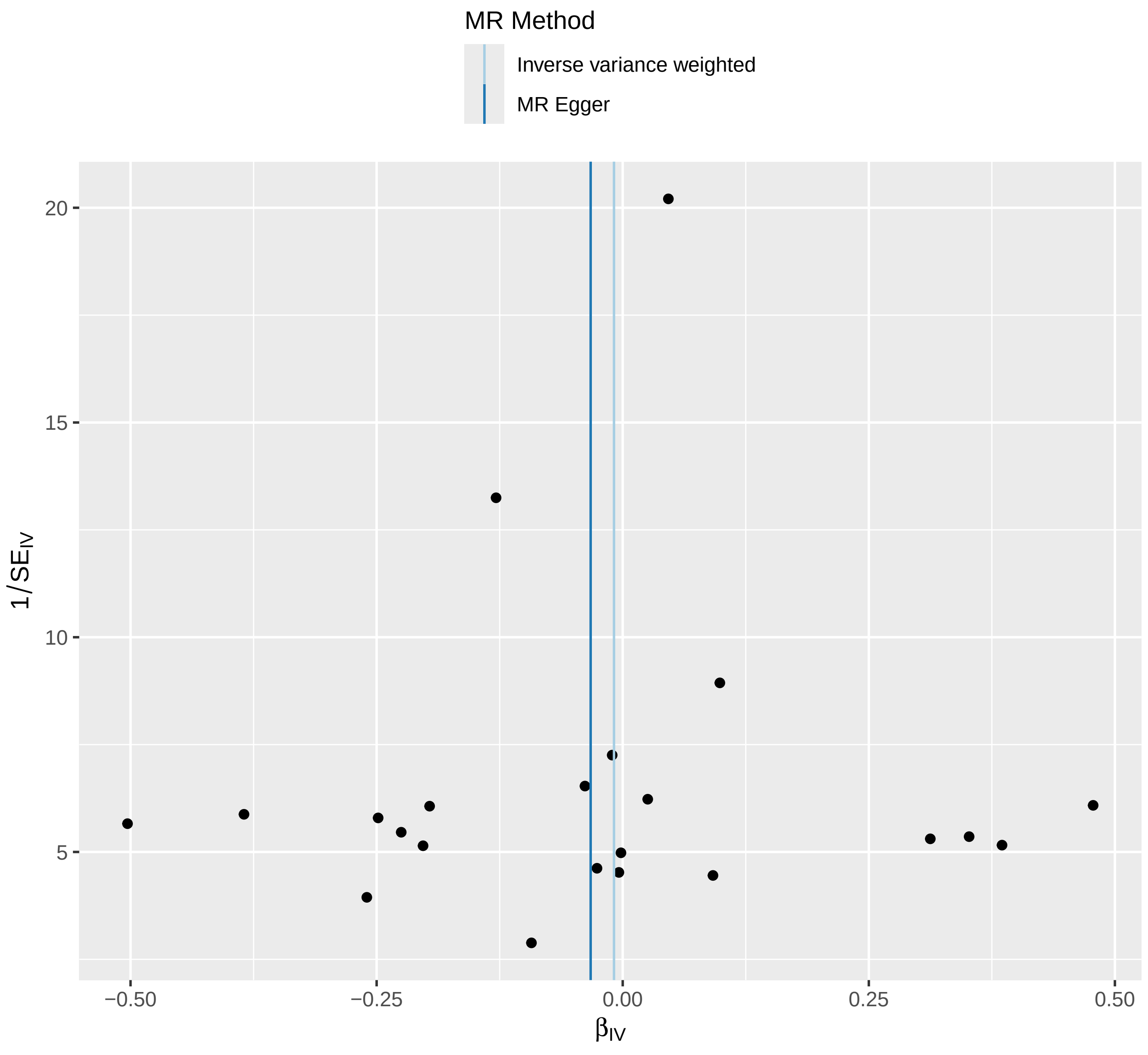
MR Method
Inverse variance weighted
MR Egger
20
15
10
5
$1/SE_{IV}$
−0.50
−0.25
0.00
0.25
0.50
β_{IV}

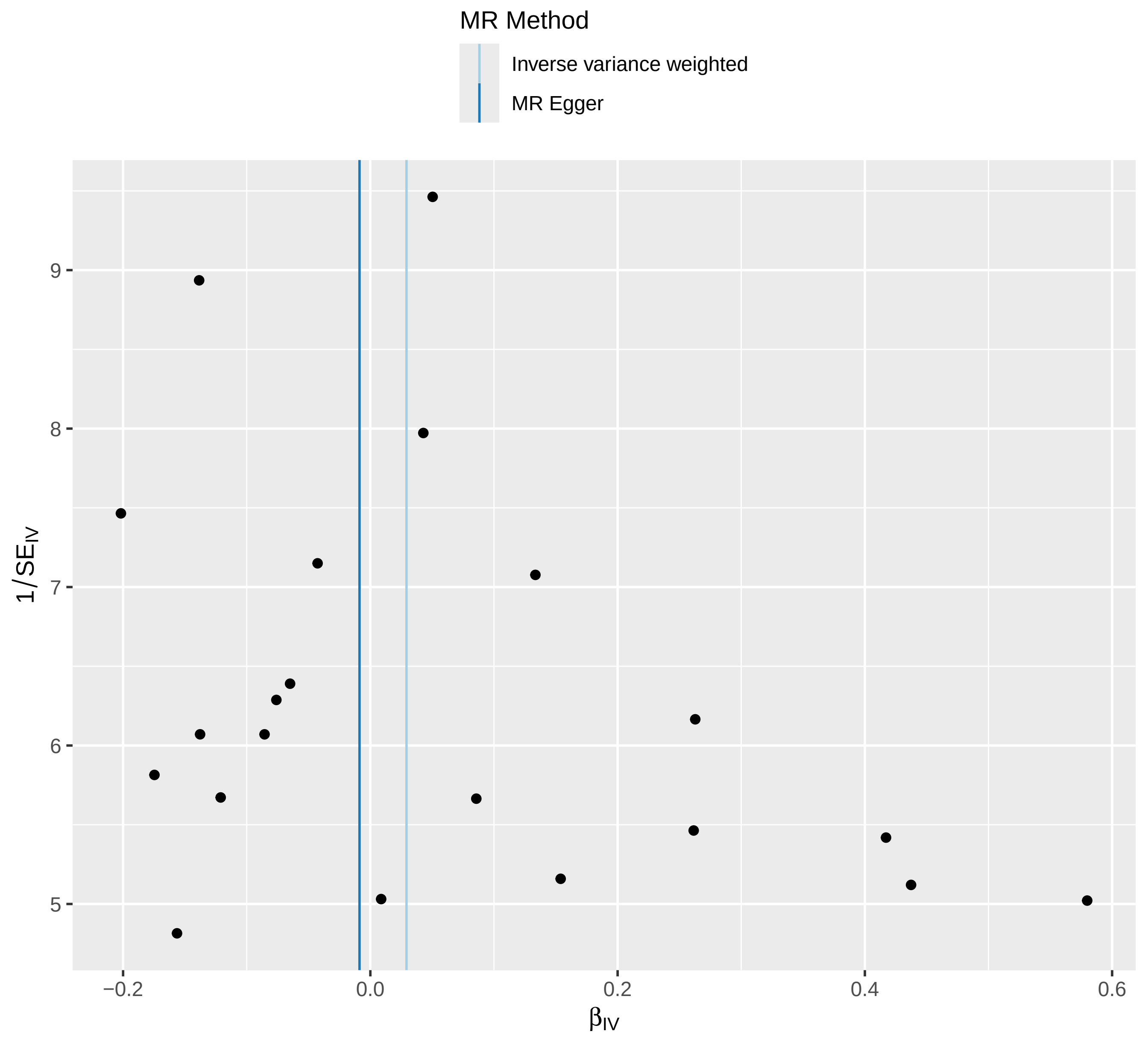
MR Method
Inverse variance weighted
MR Egger
1/SE_IV
β_IV
9
8
7
6
5
−0.2
0.0
0.2
0.4
0.6

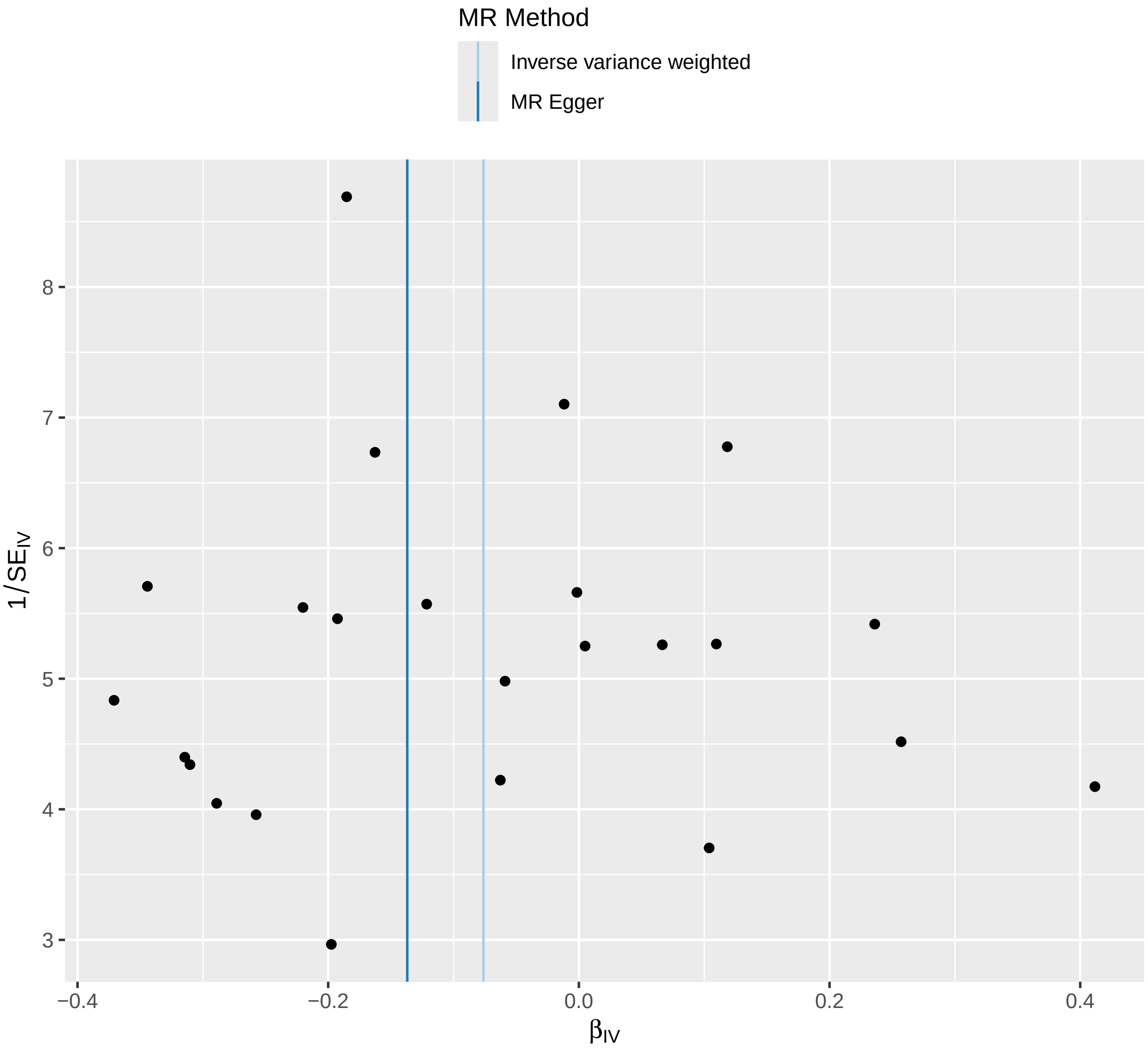

MR Method
Inverse variance weighted
MR Egger
8
7
6
5
4
3
1/SE$_{IV}$
−0.4
−0.2
0.0
0.2
0.4
β$_{IV}$

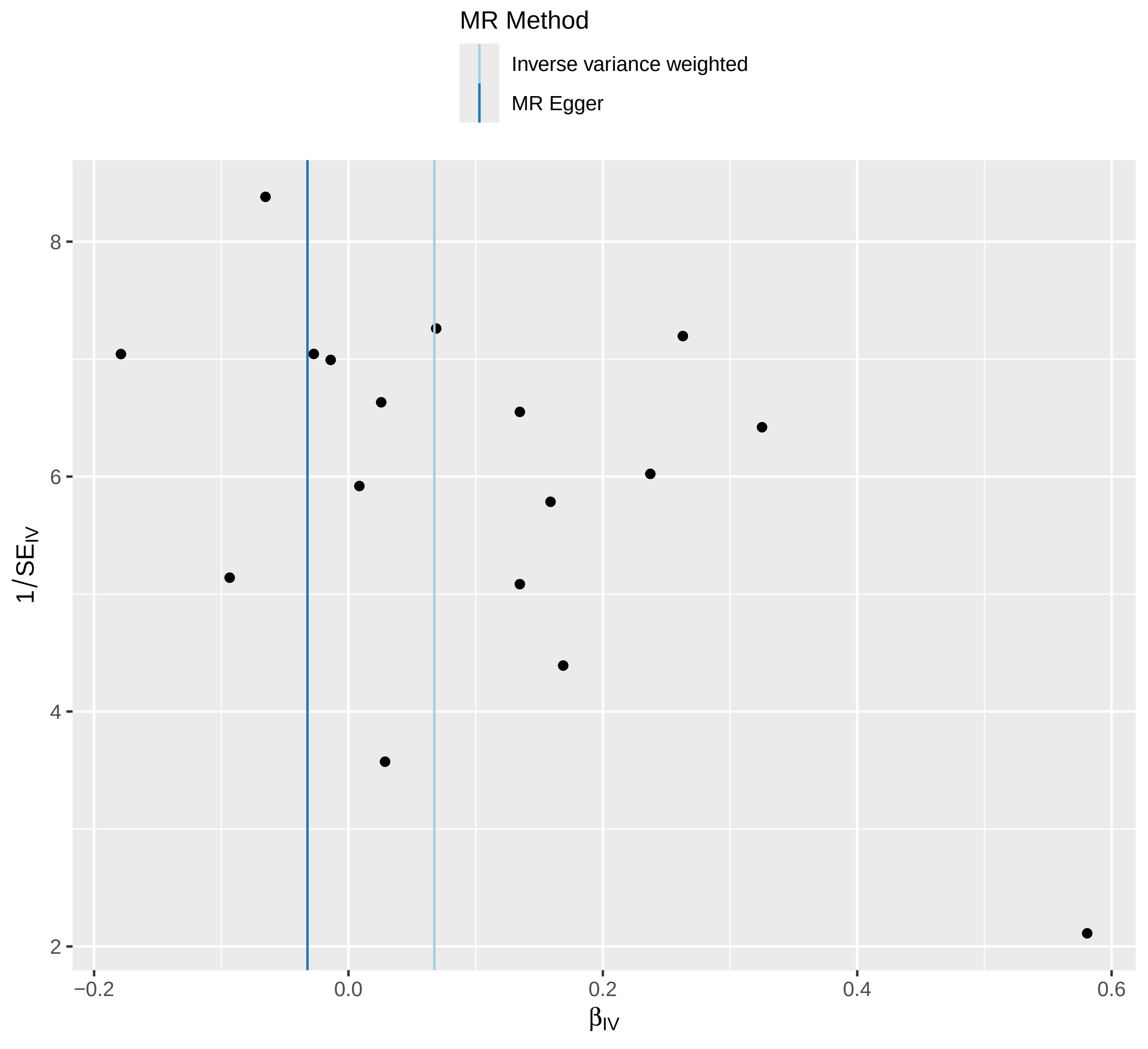
MR Method
Inverse variance weighted
MR Egger
8
6
4
2
1/SE_IV
−0.2
0.0
0.2
0.4
0.6
β_IV

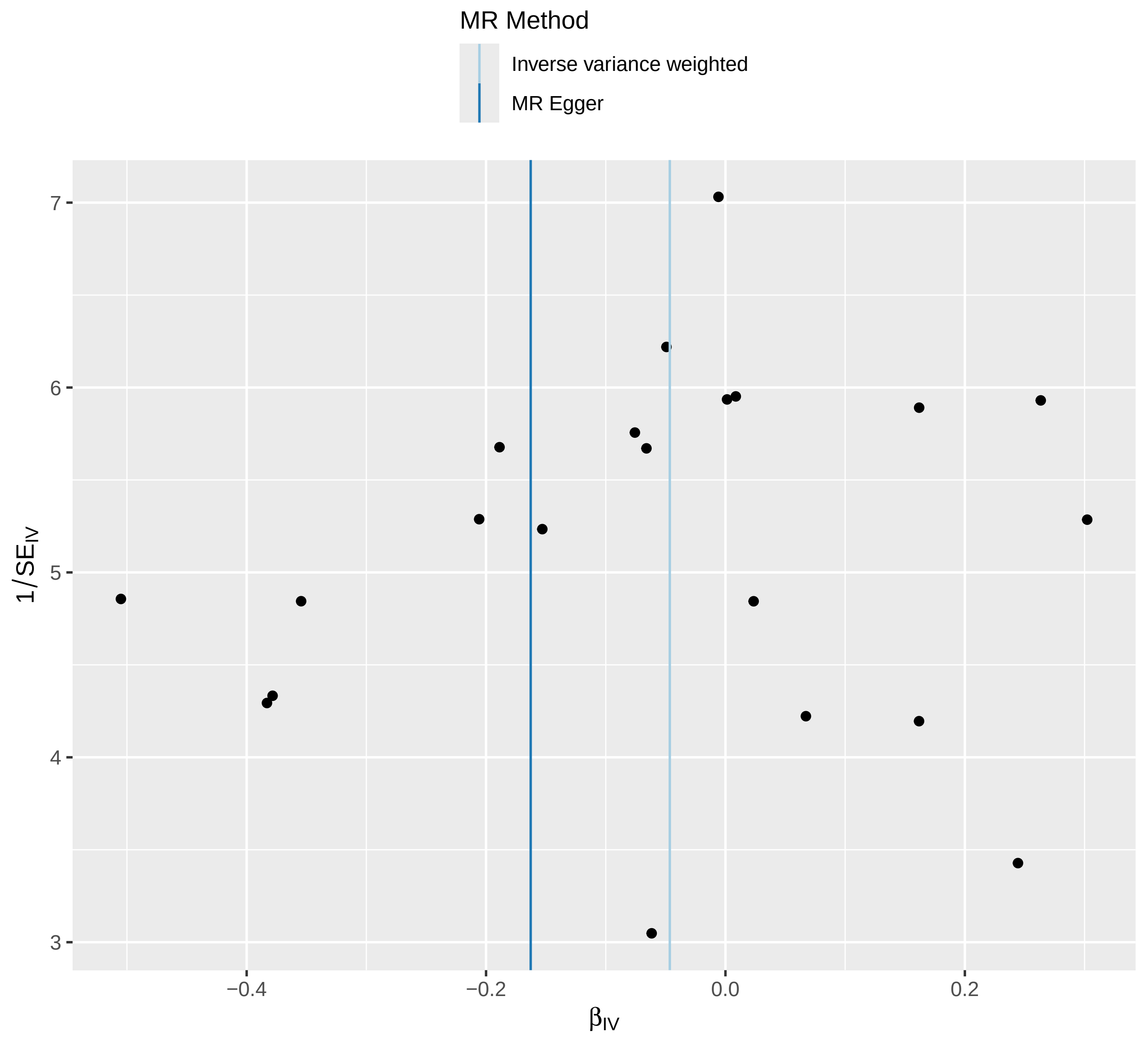

MR Method
Inverse variance weighted
MR Egger
7
6
5
4
3
1/SE_IV
−0.4
−0.2
0.0
0.2
β_IV

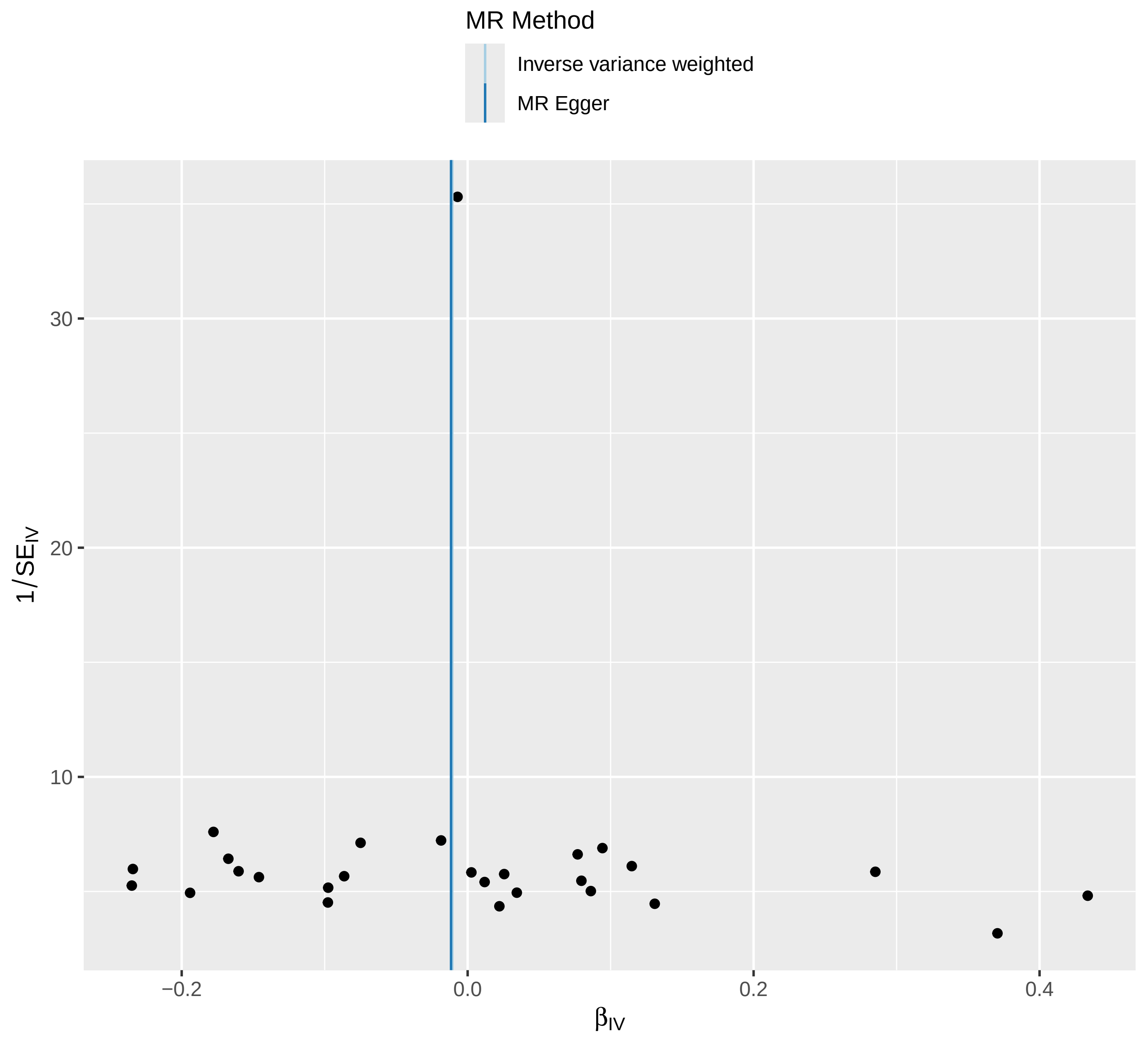
MR Method
Inverse variance weighted
MR Egger
30
20
10
$1/SE_{IV}$
−0.2
0.0
0.2
0.4
β_{IV}

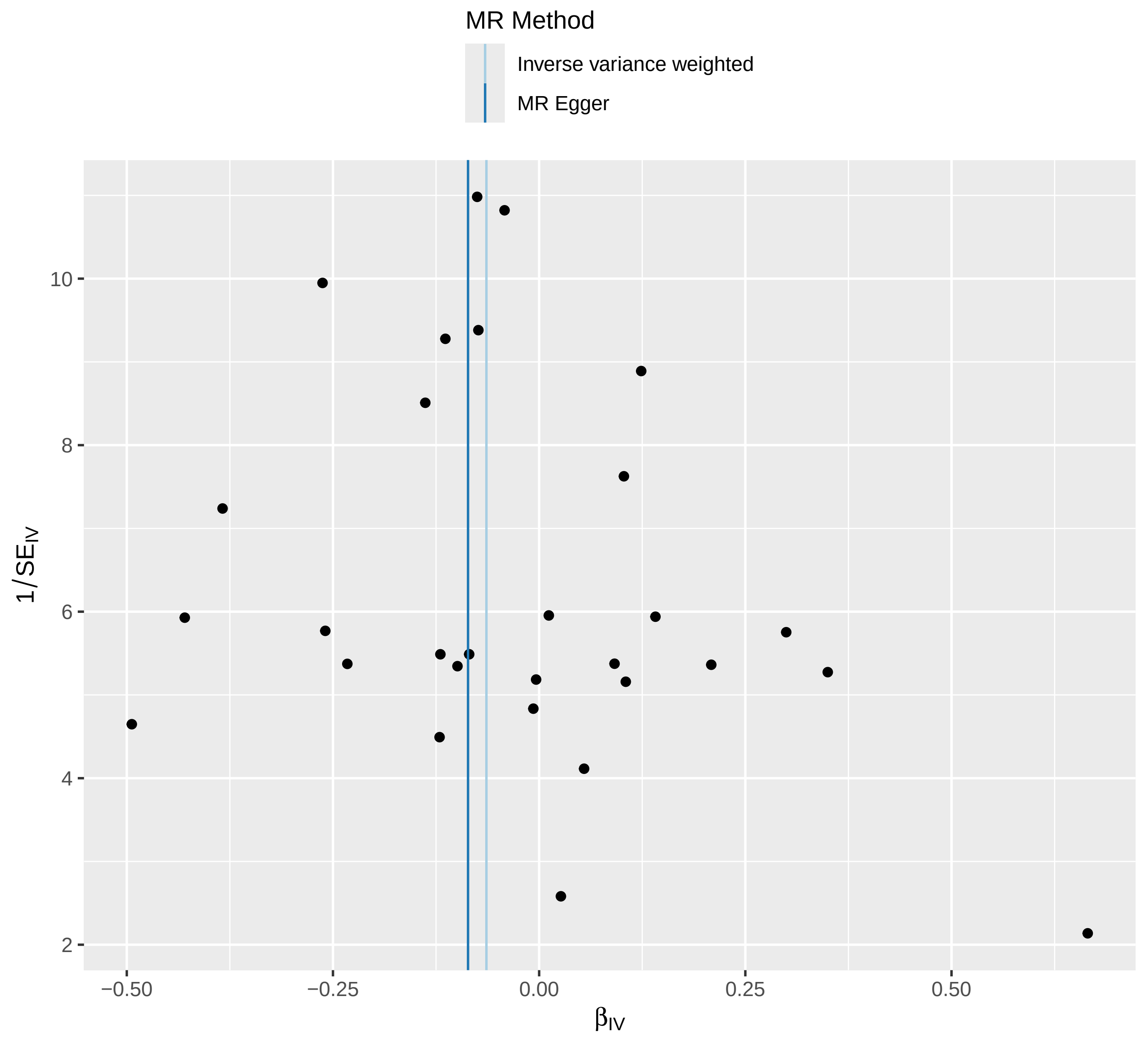
MR Method
Inverse variance weighted
MR Egger
1/SE_IV
β_IV
10
8
6
4
2
−0.50
−0.25
0.00
0.25
0.50

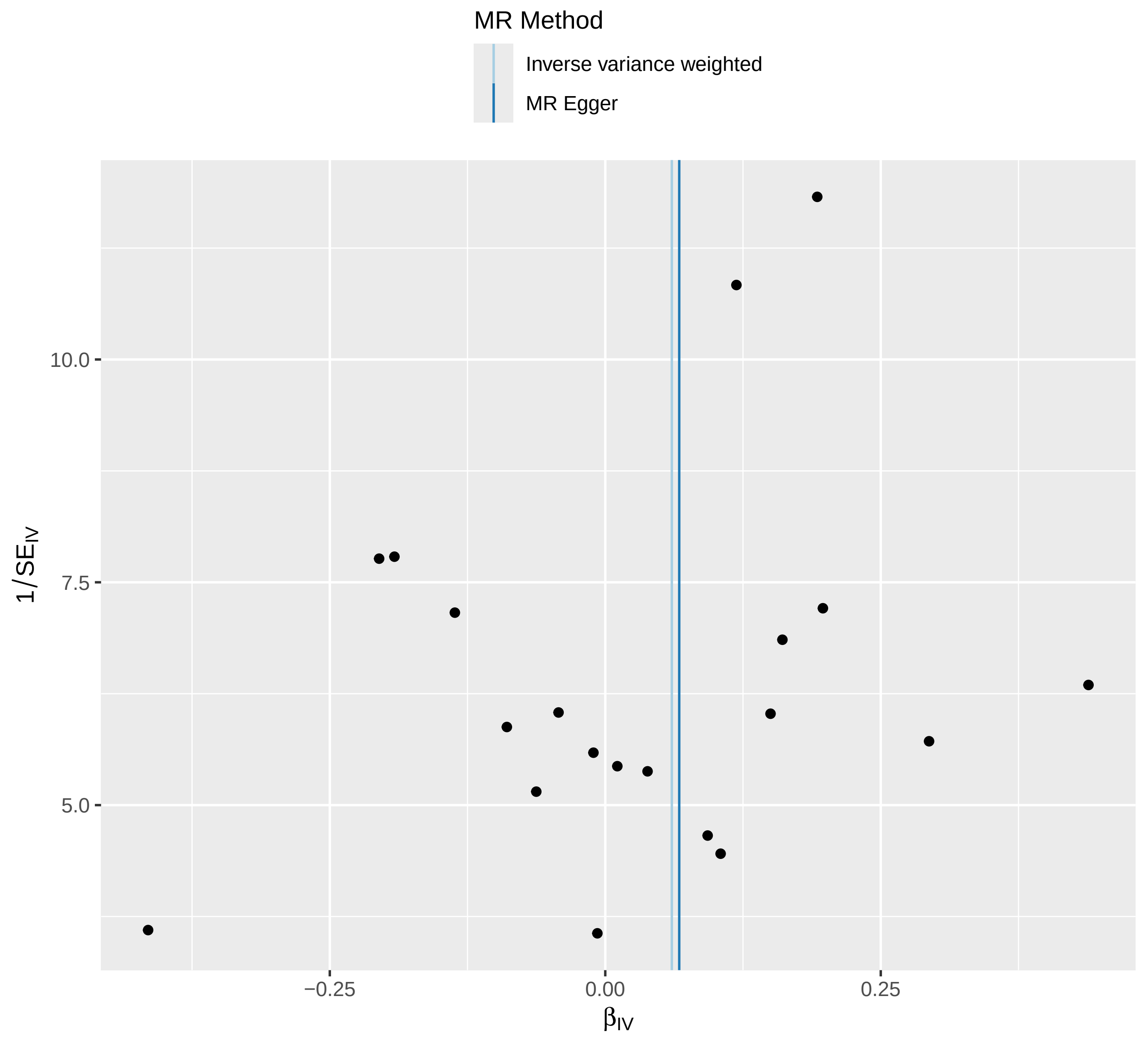

MR Method
Inverse variance weighted
MR Egger
10.0
7.5
5.0
1/SE_IV
−0.25
0.00
0.25
β_IV

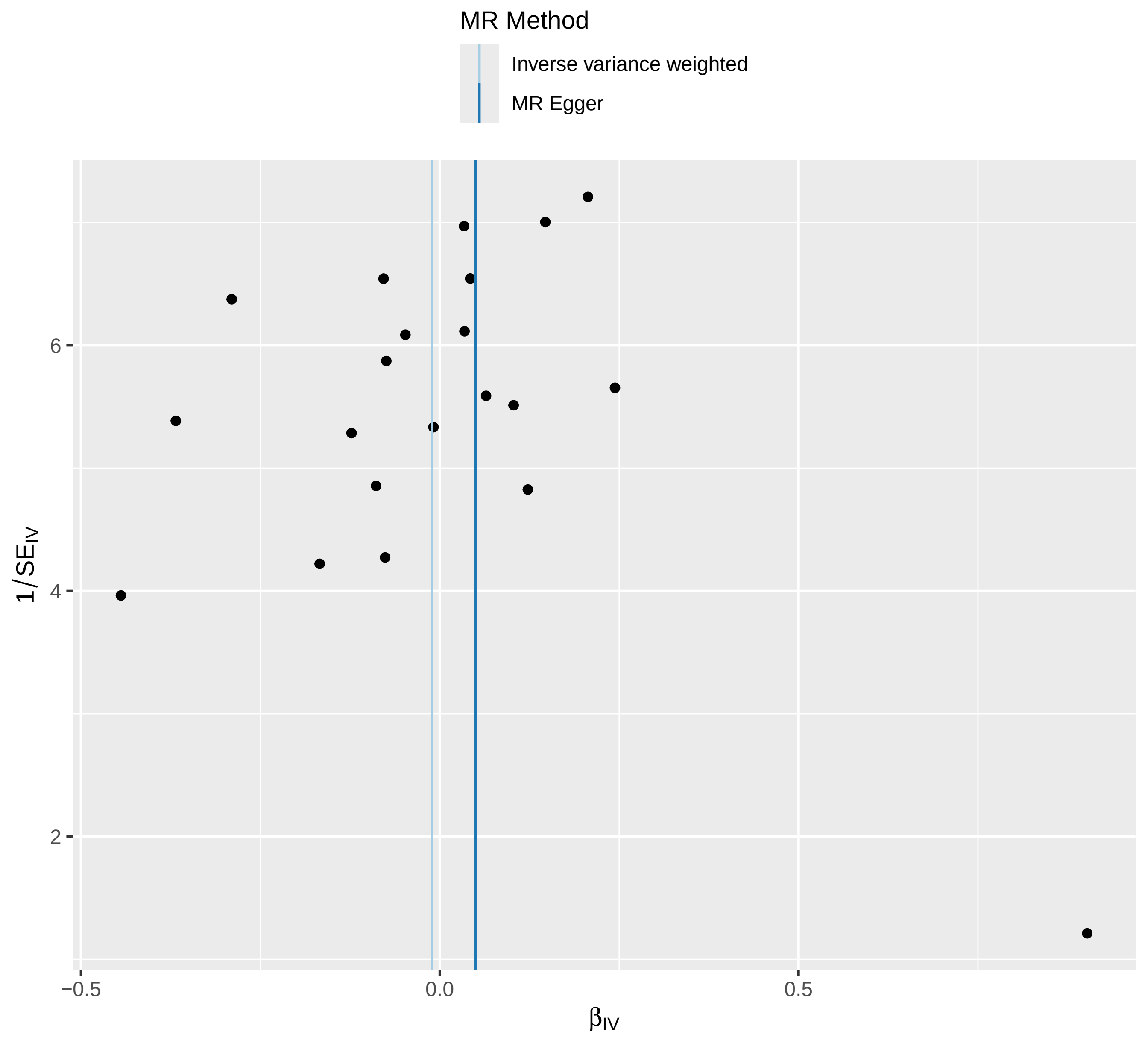

MR Method
Inverse variance weighted
MR Egger
1/SE_IV
6
4
2
−0.5
0.0
0.5
β_IV

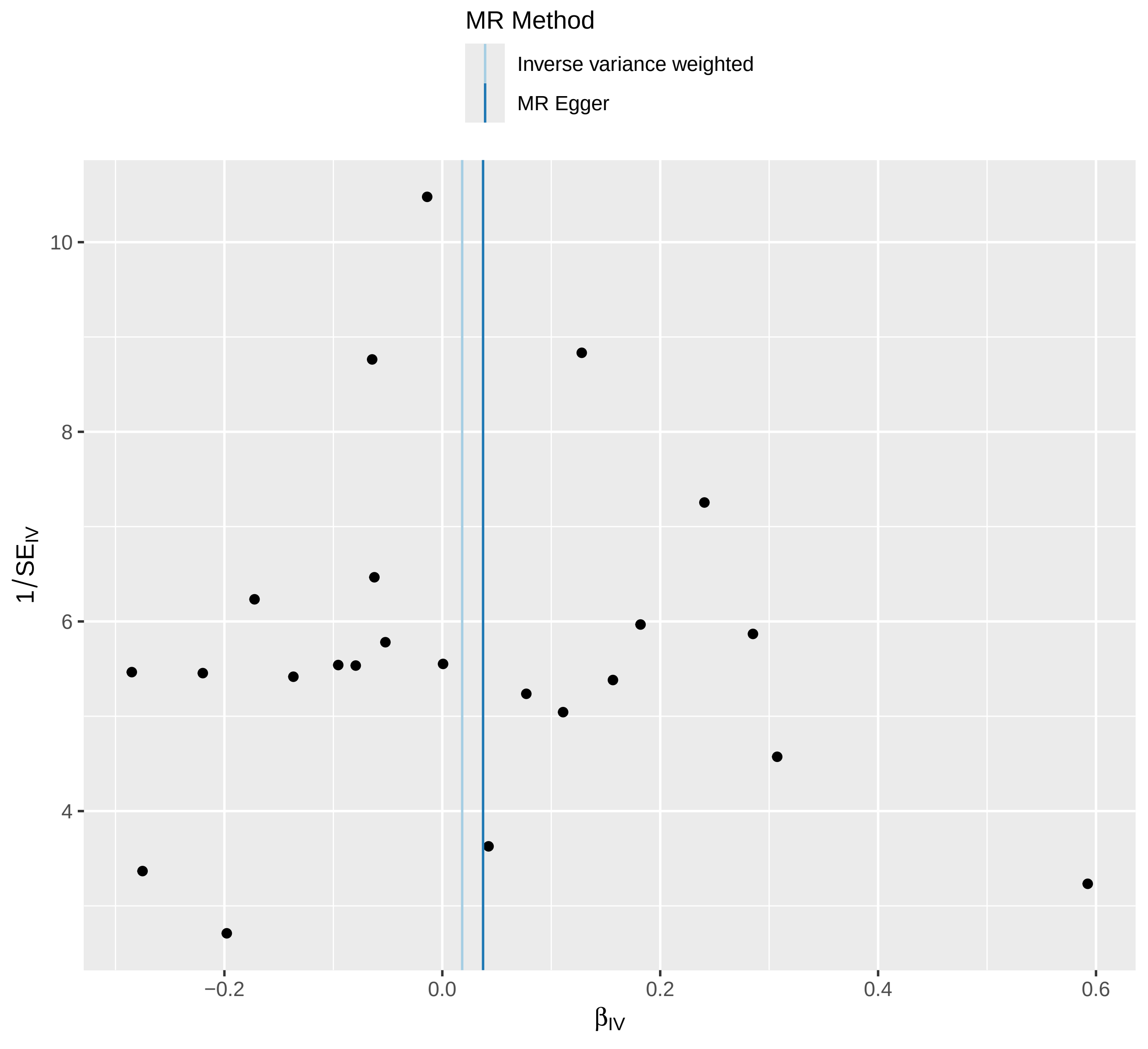
MR Method
Inverse variance weighted
MR Egger
1/SE_IV
β_IV
10
8
6
4
−0.2
0.0
0.2
0.4
0.6

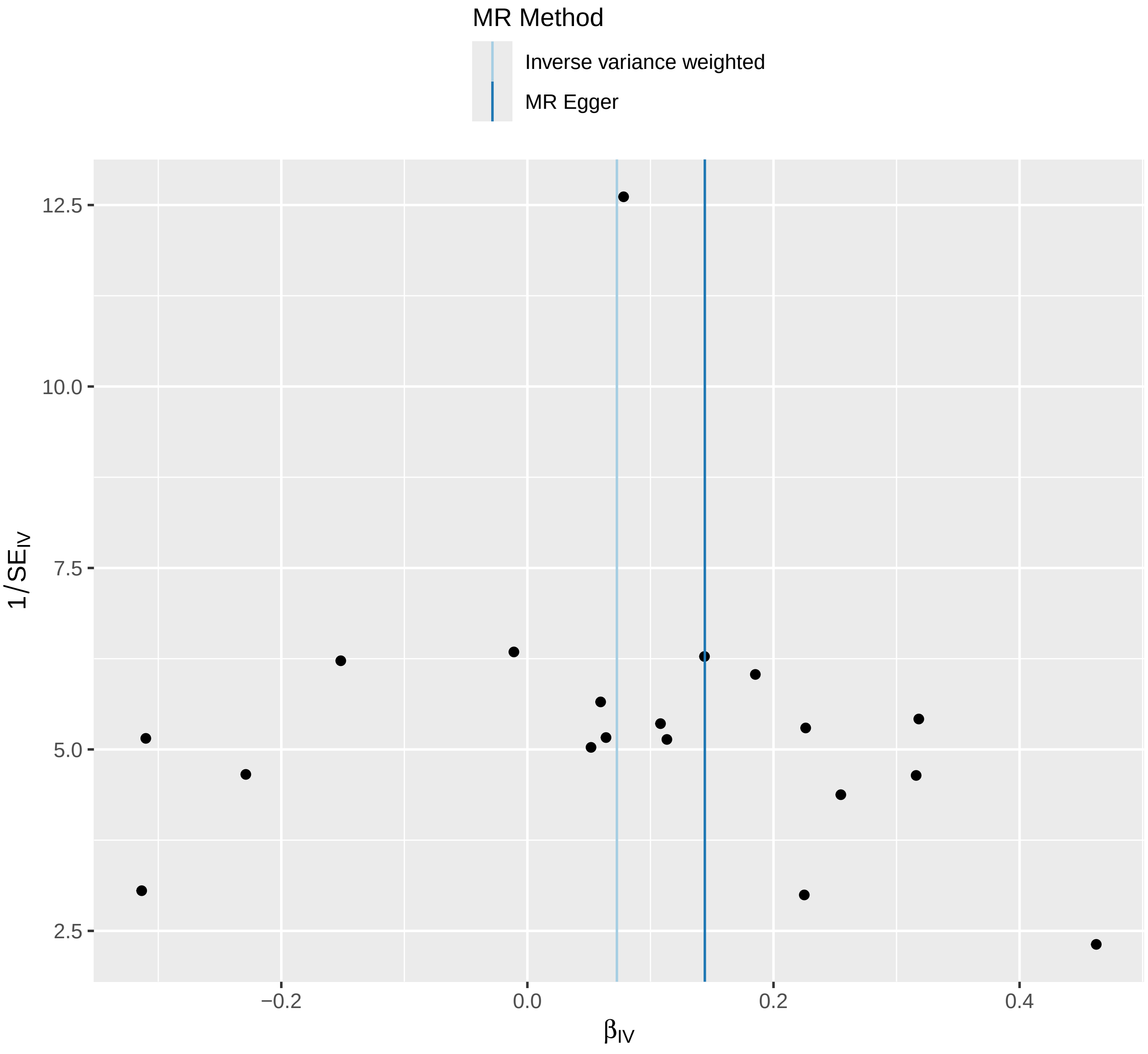

MR Method
Inverse variance weighted
MR Egger
12.5
10.0
7.5
5.0
2.5
$1/SE_{IV}$
−0.2
0.0
0.2
0.4
β_{IV}

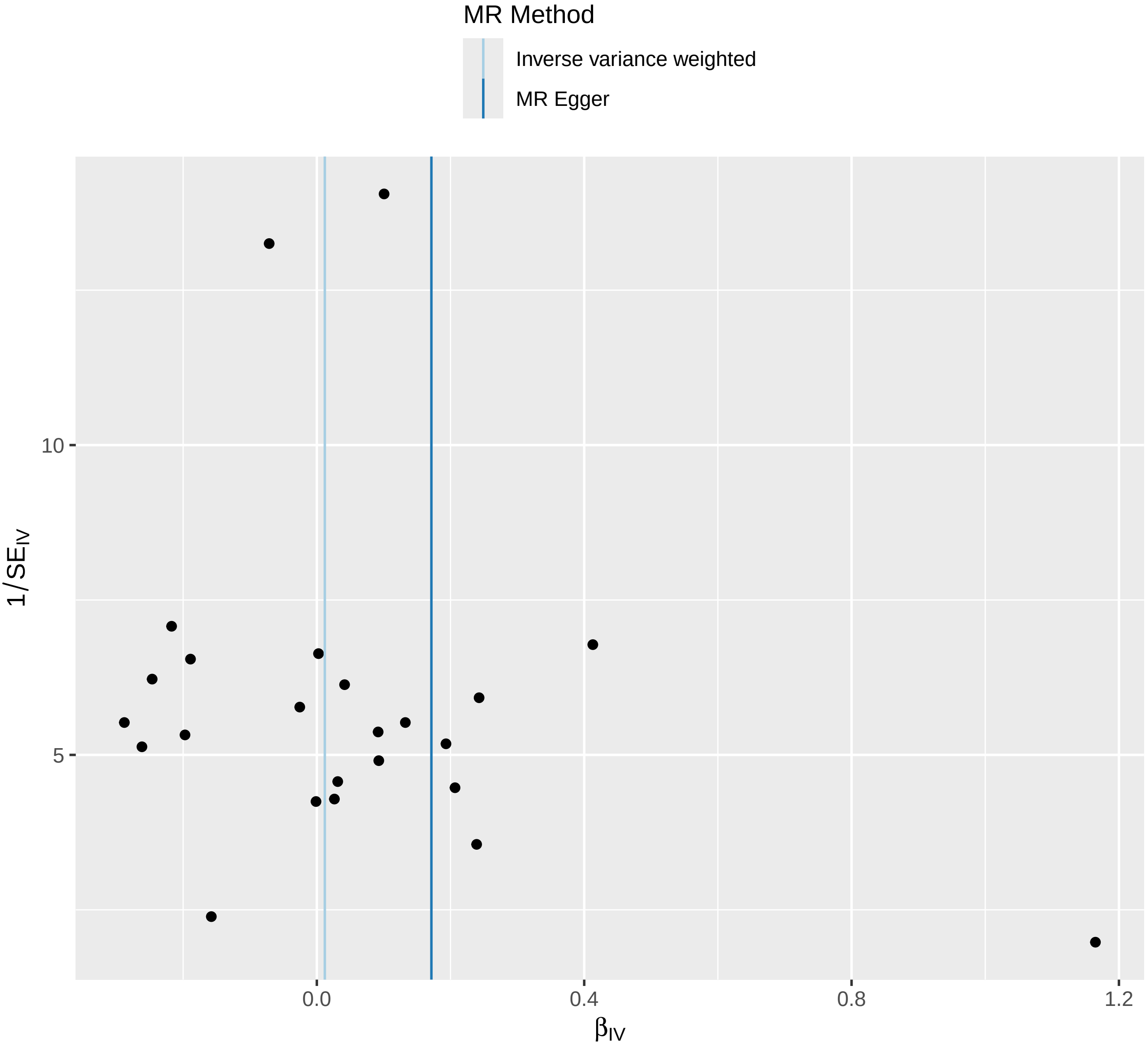
MR Method
Inverse variance weighted
MR Egger
10
5
1/SE_IV
0.0
0.4
0.8
1.2
β_IV

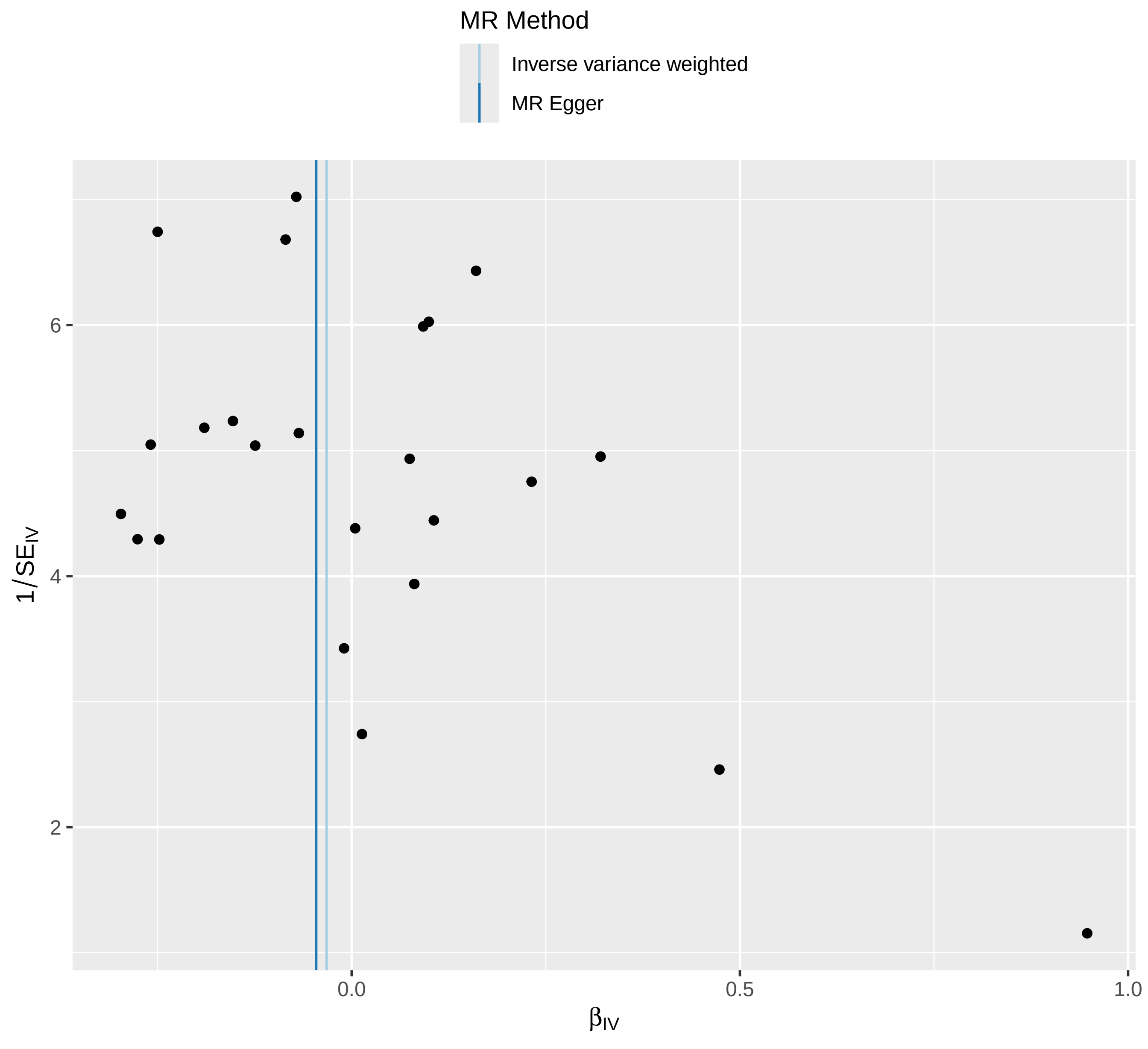
MR Method
Inverse variance weighted
MR Egger
6
4
2
1/SE_IV
0.0
0.5
1.0
β_IV

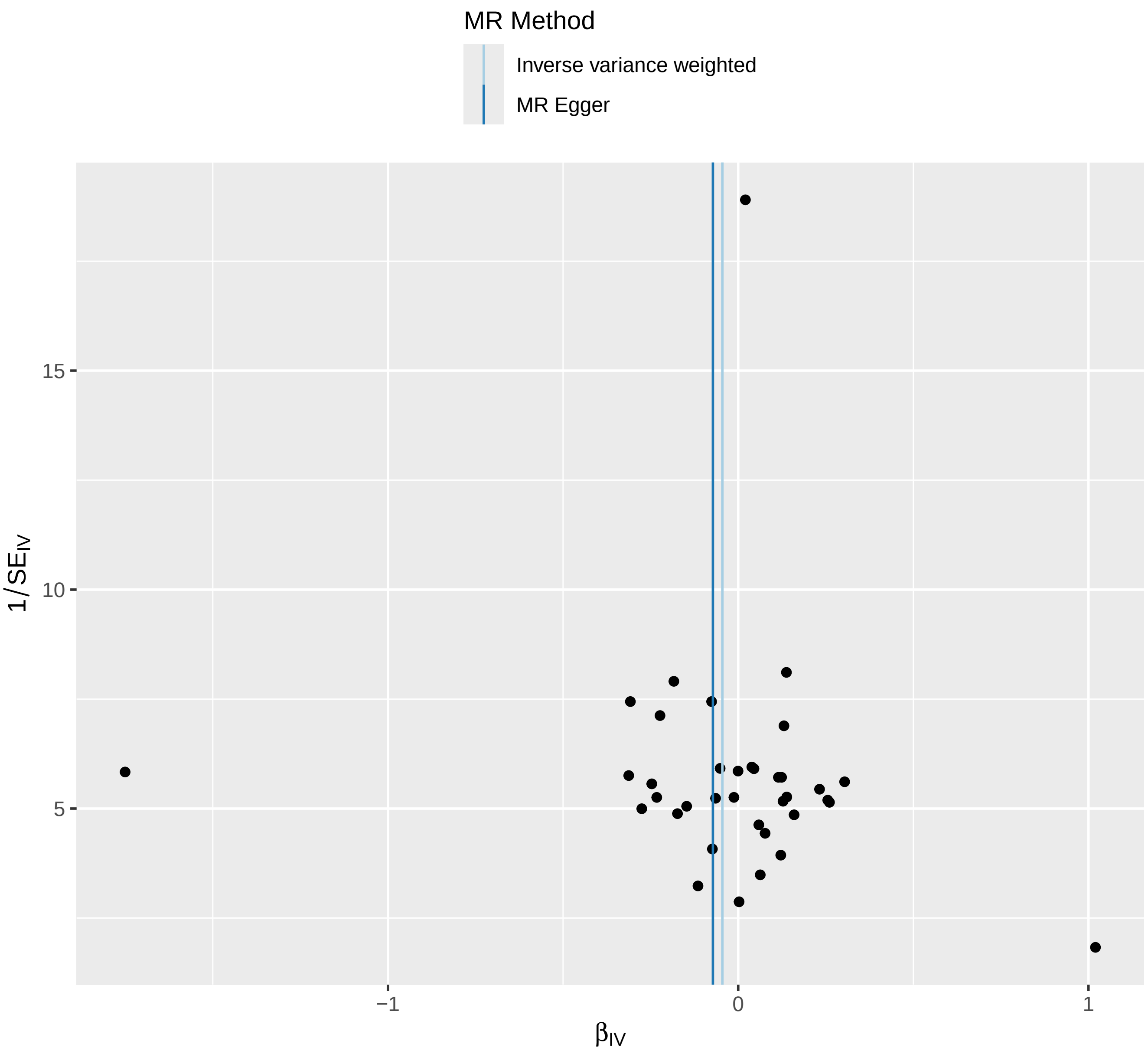
MR Method
Inverse variance weighted
MR Egger
15
10
5
1/SE$_{IV}$
−1
0
1
β$_{IV}$

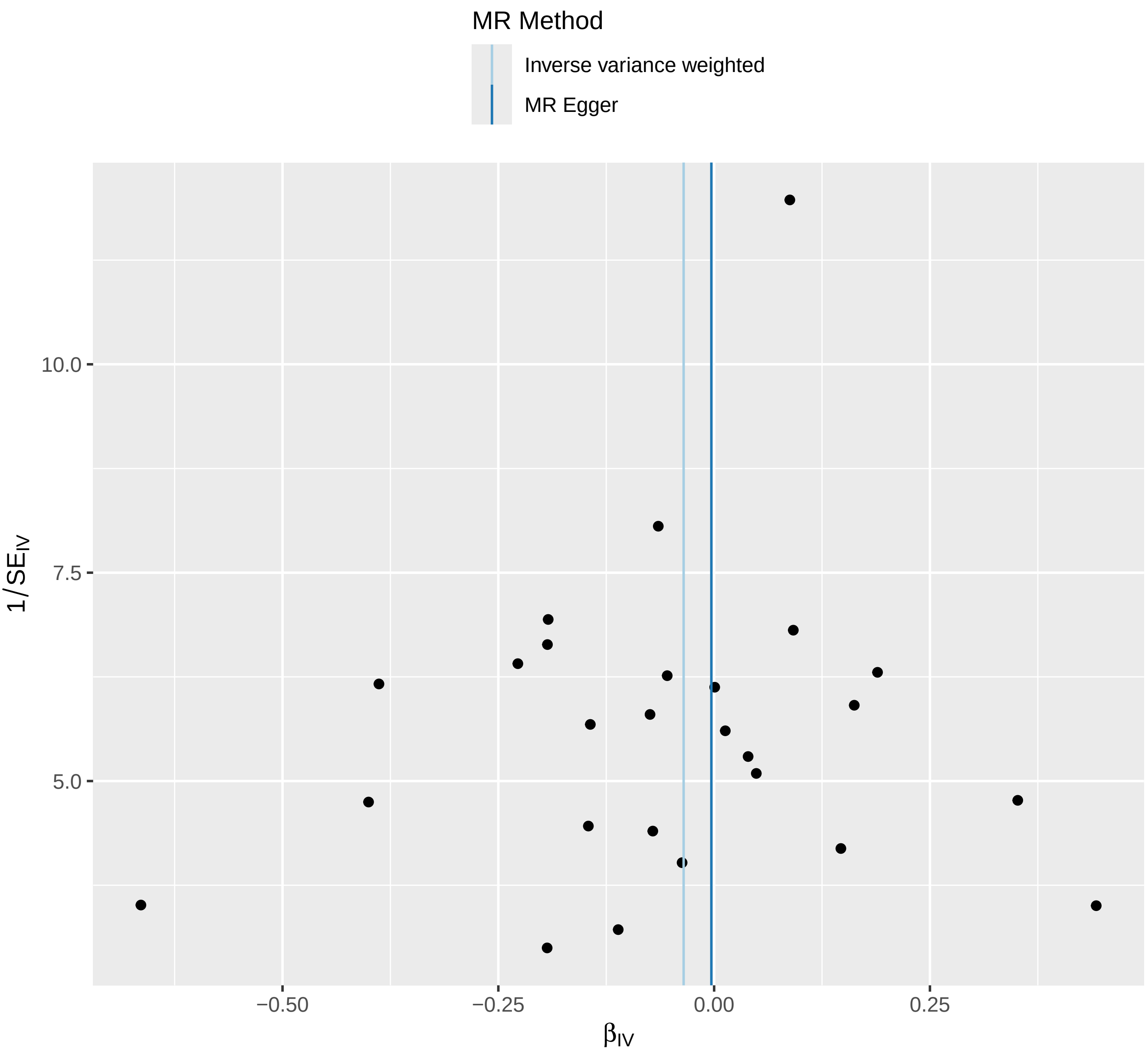

MR Method
Inverse variance weighted
MR Egger
10.0
7.5
5.0
1/SE_IV
−0.50
−0.25
0.00
0.25
β_IV

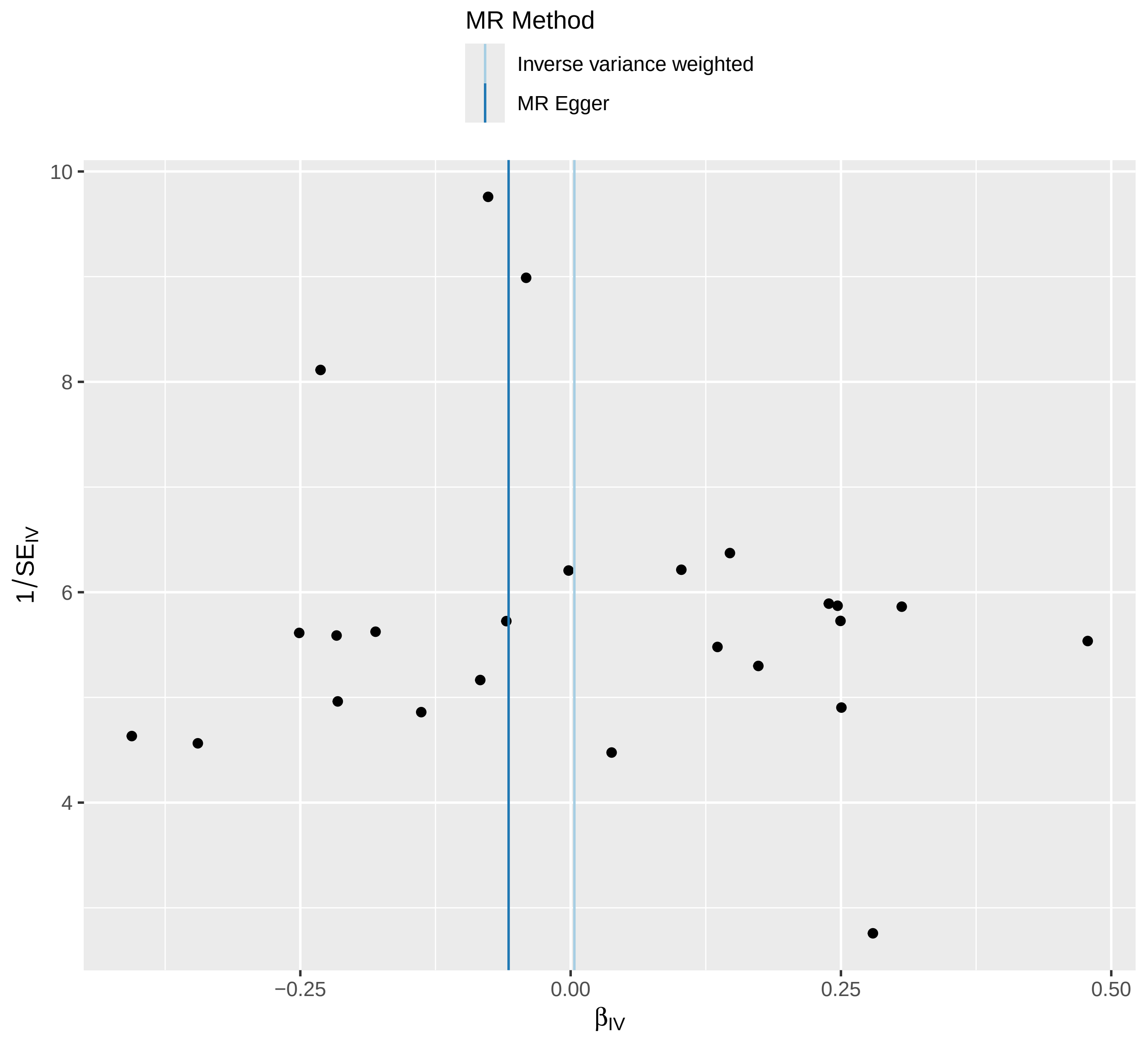
MR Method
Inverse variance weighted
MR Egger
10
8
6
4
−0.25
0.00
0.25
0.50
$1/SE_{IV}$
β_{IV}

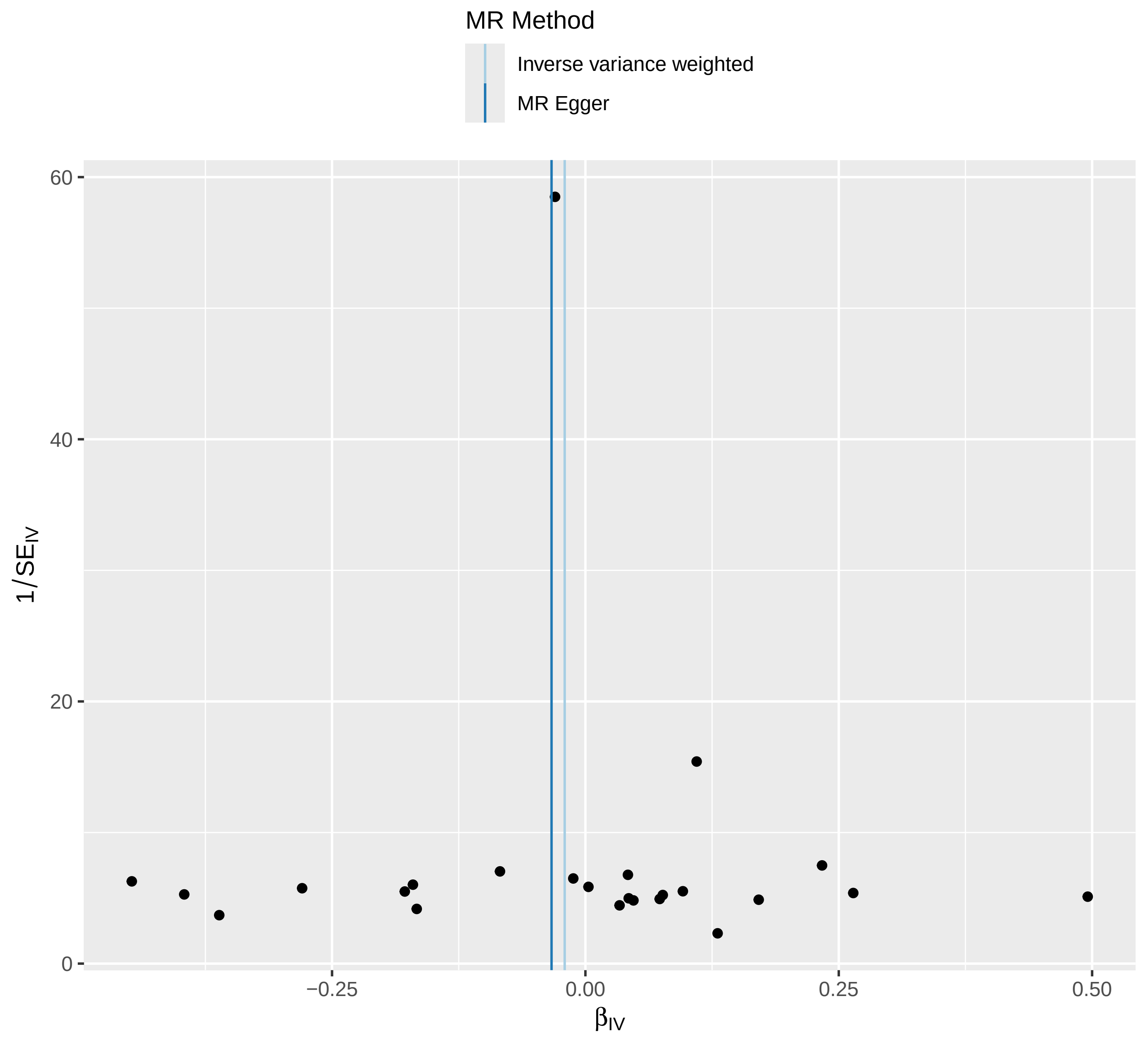

MR Method
Inverse variance weighted
MR Egger
60
40
20
0
$1/SE_{IV}$
−0.25
0.00
0.25
0.50
β_{IV}

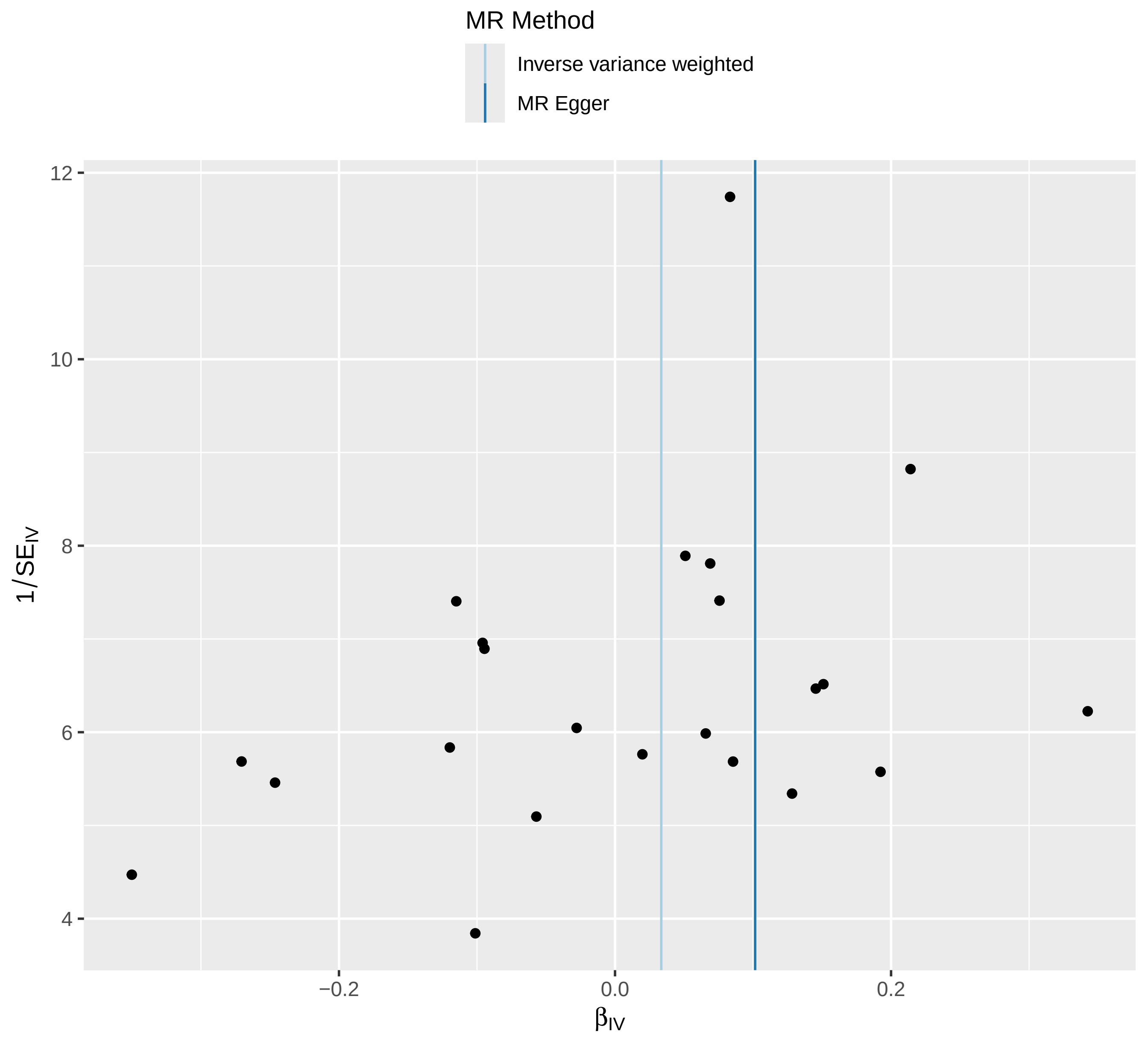
MR Method
Inverse variance weighted
MR Egger
12
10
8
6
4
1/SE_IV
−0.2
0.0
0.2
β_IV

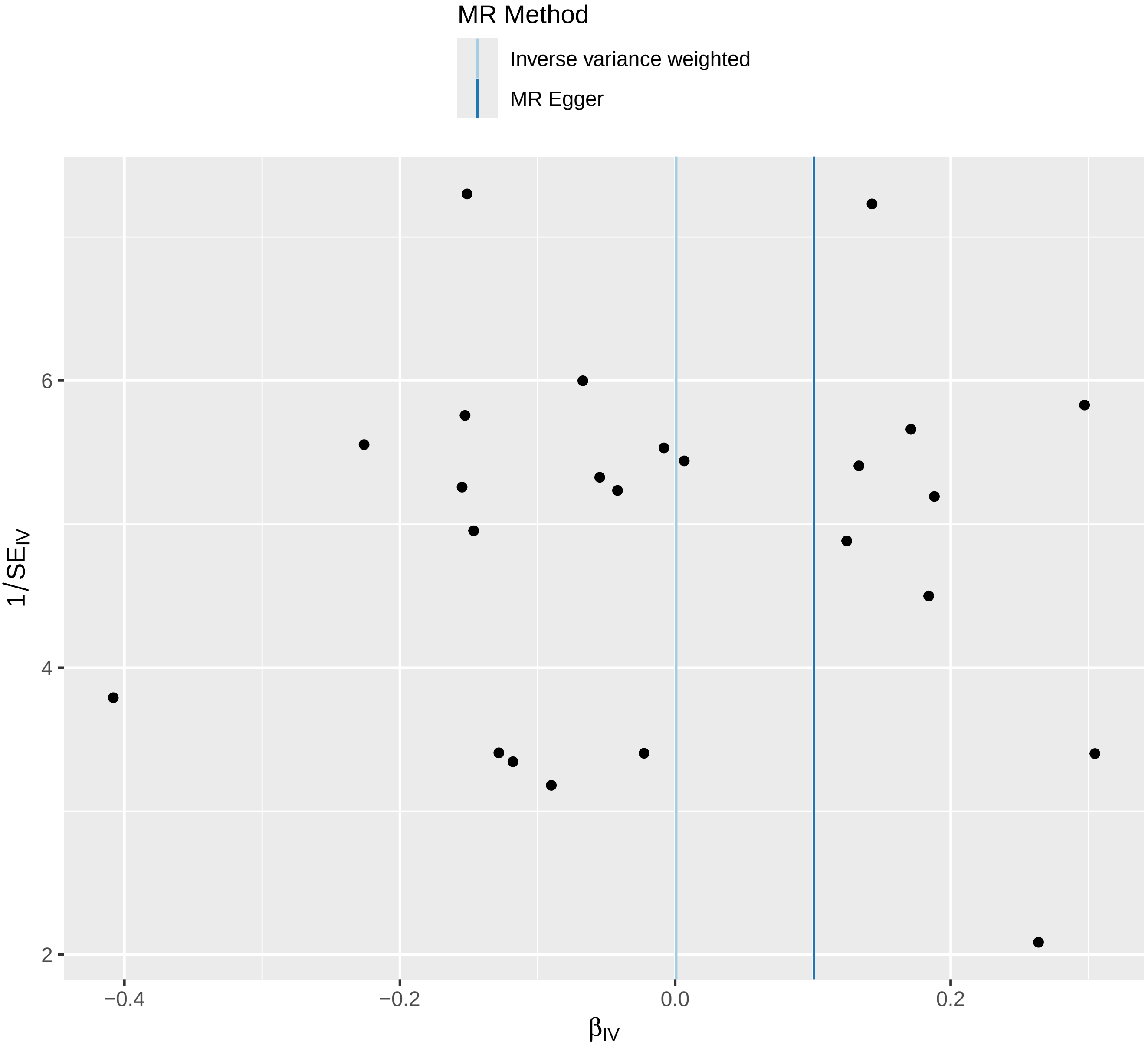
MR Method
Inverse variance weighted
MR Egger
1/SE$_{IV}$
6
4
2
−0.4
−0.2
0.0
0.2
β$_{IV}$

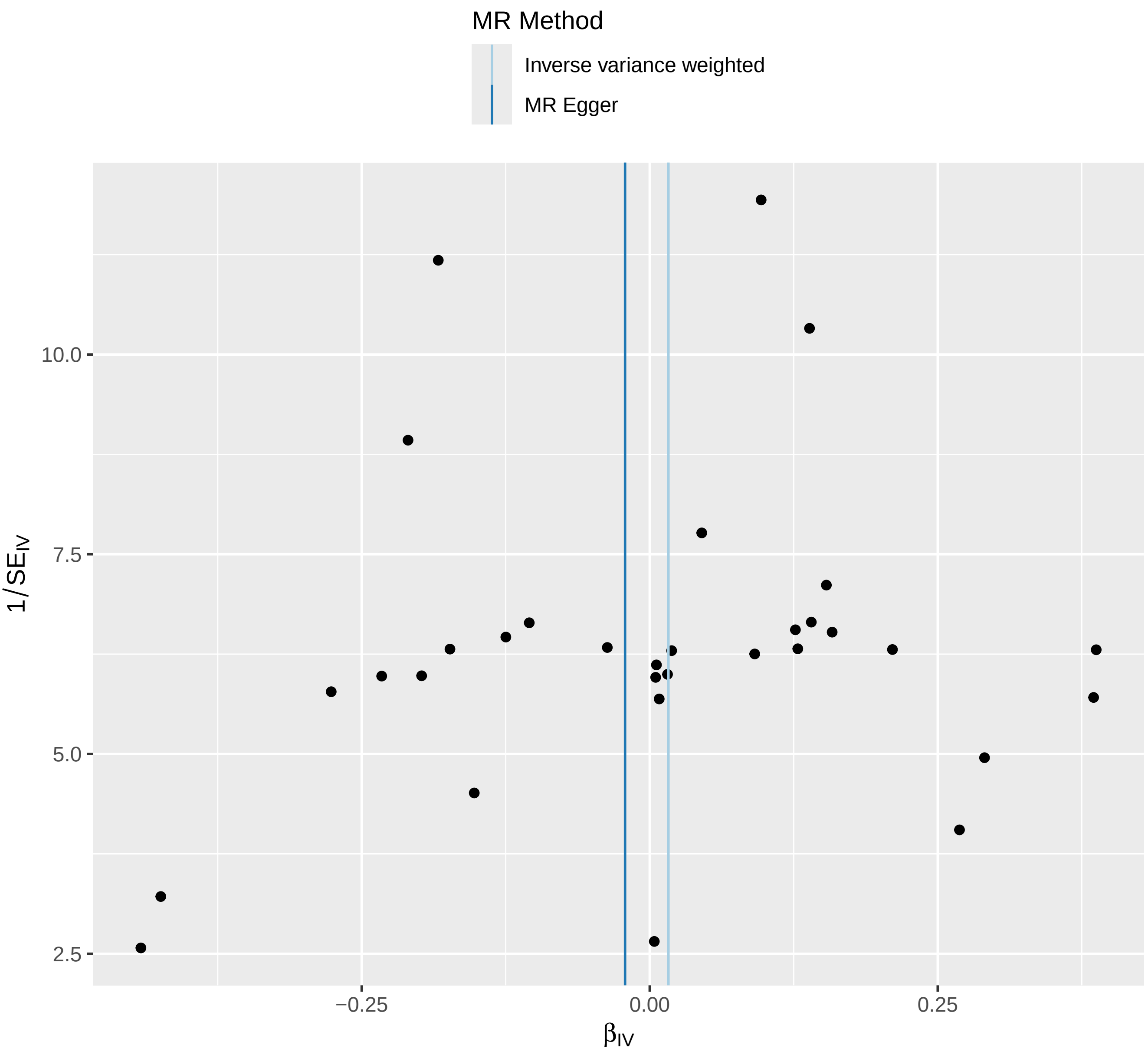
MR Method
Inverse variance weighted
MR Egger
10.0
7.5
5.0
2.5
1/SE$_{IV}$
−0.25
0.00
0.25
β_{IV}

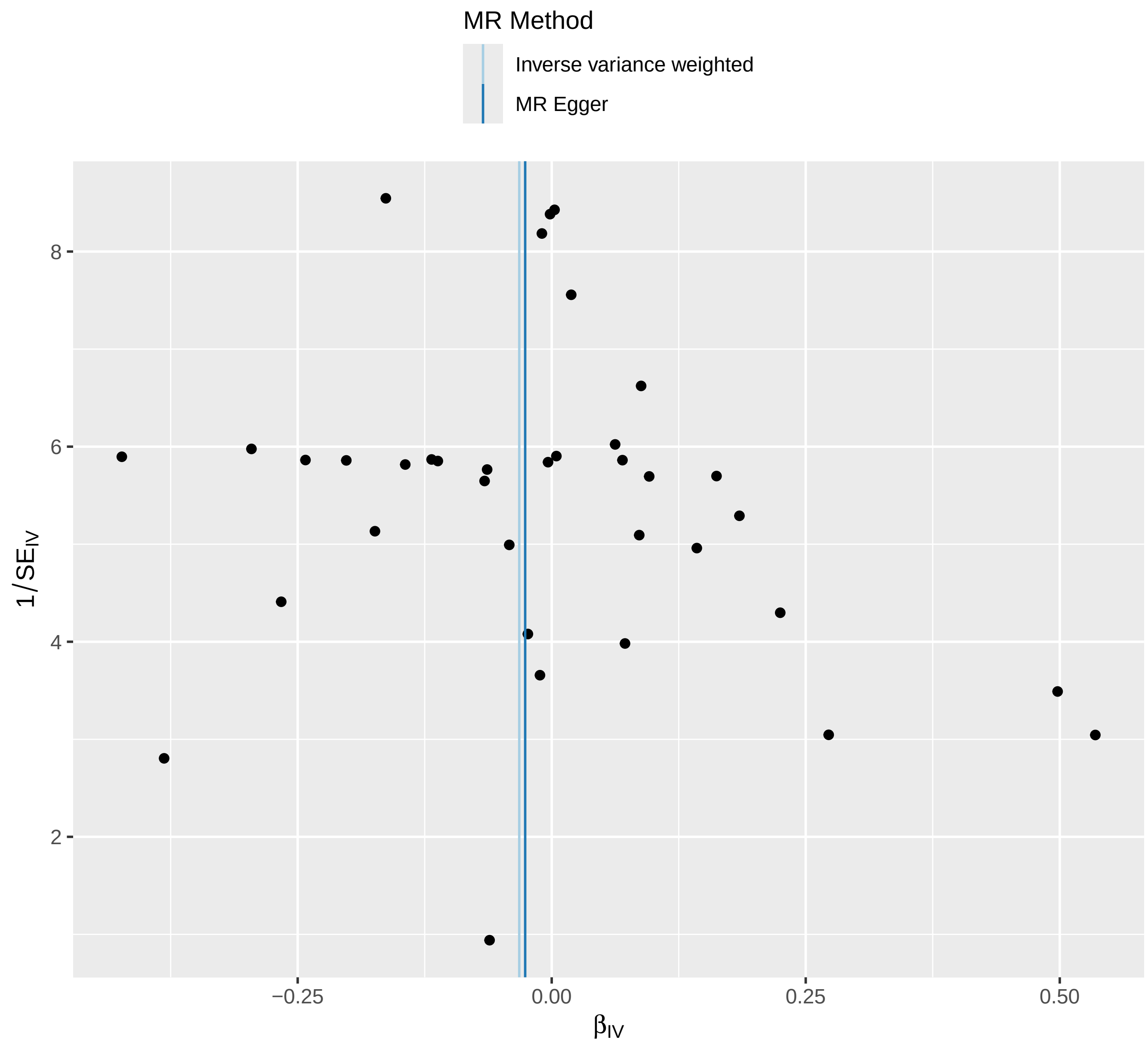
MR Method
Inverse variance weighted
MR Egger
8
6
4
2
1/SE$_{IV}$
−0.25
0.00
0.25
0.50
β$_{IV}$

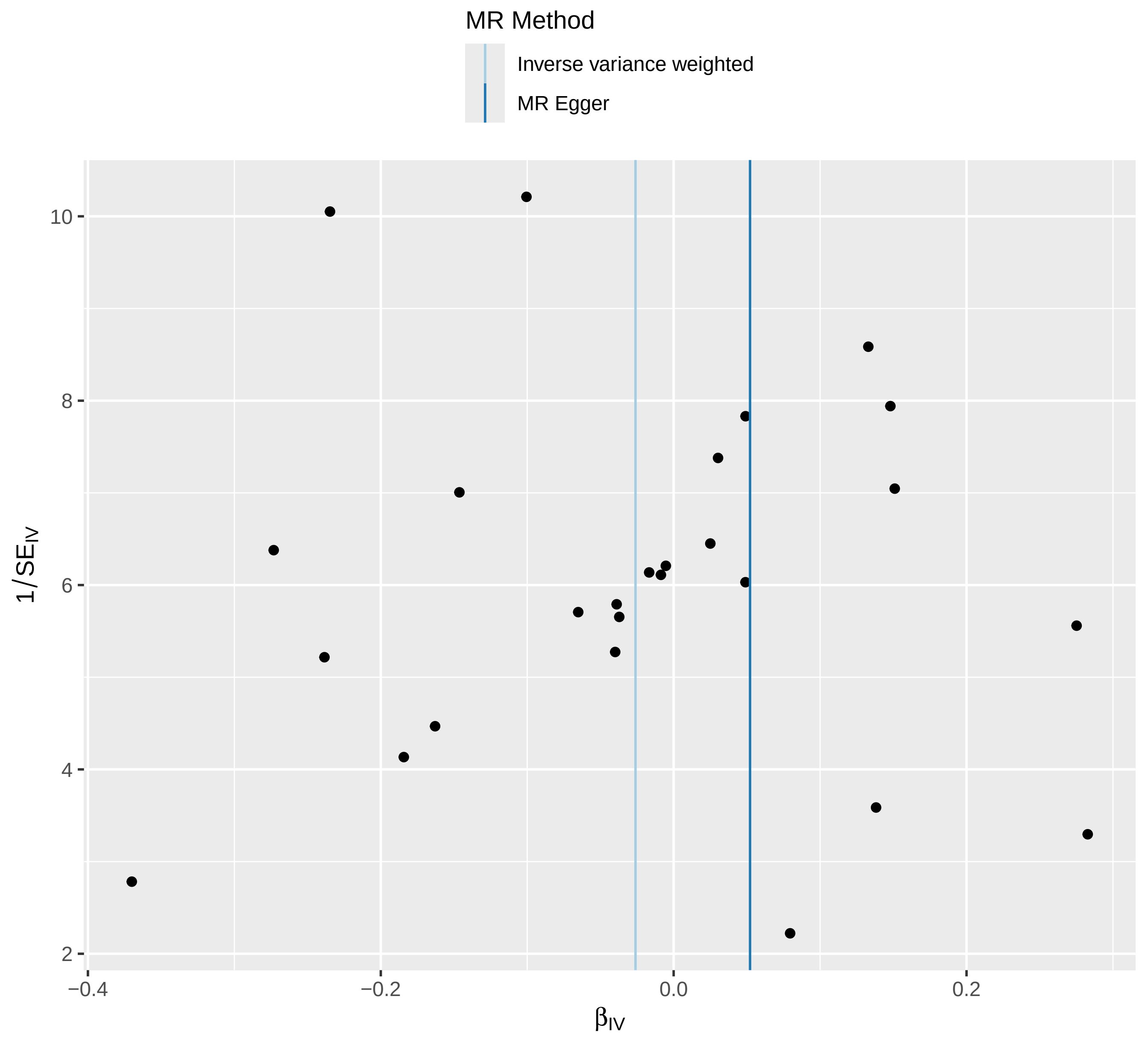
MR Method
Inverse variance weighted
MR Egger
10
8
6
4
2
−0.4
−0.2
0.0
0.2
$1/SE_{IV}$
β_{IV}

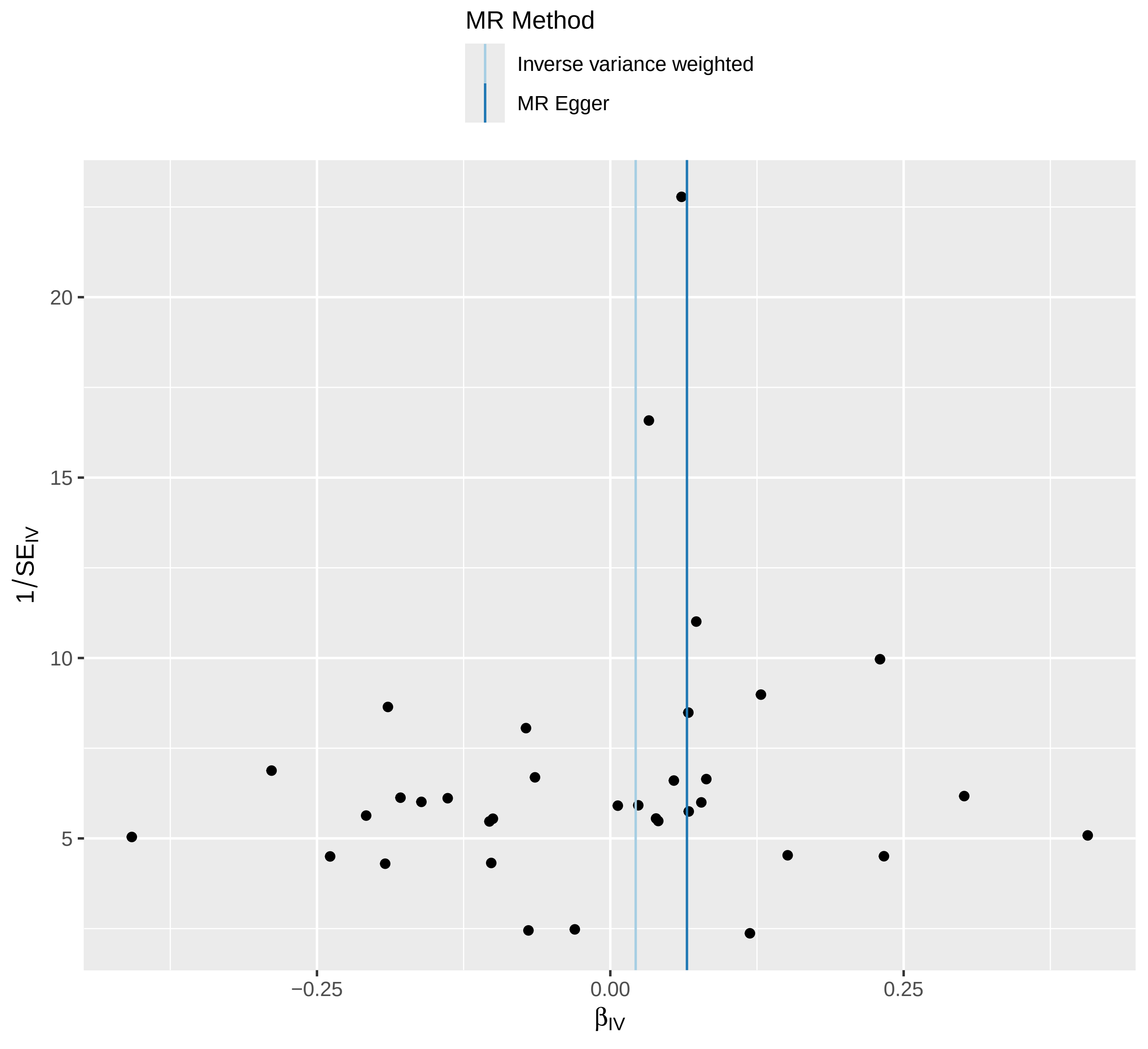
MR Method
Inverse variance weighted
MR Egger
20
15
10
5
1/SE_IV
−0.25
0.00
0.25
β_IV

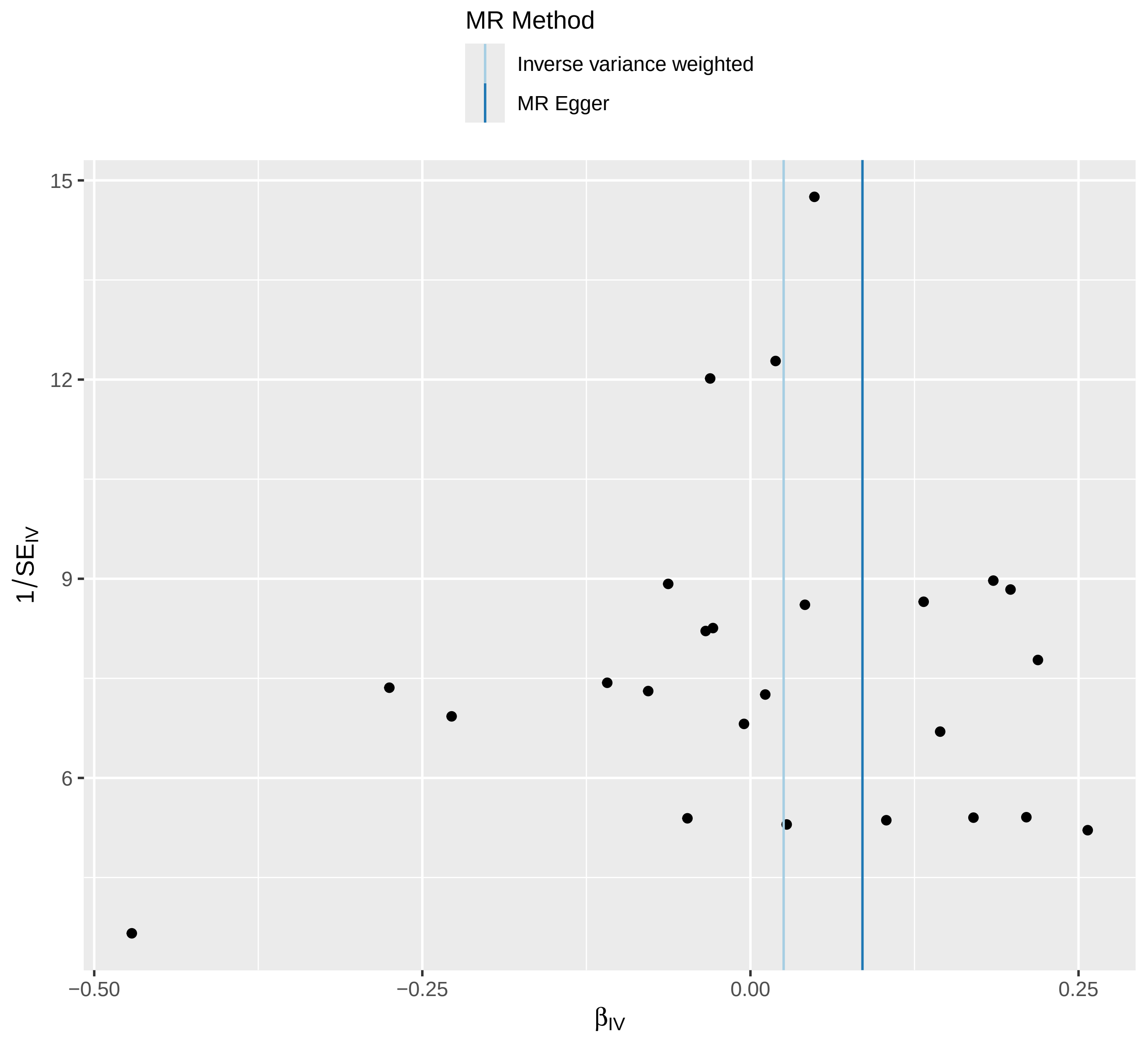

MR Method
Inverse variance weighted
MR Egger
15
12
9
6
1/SE_IV
−0.50
−0.25
0.00
0.25
β_IV

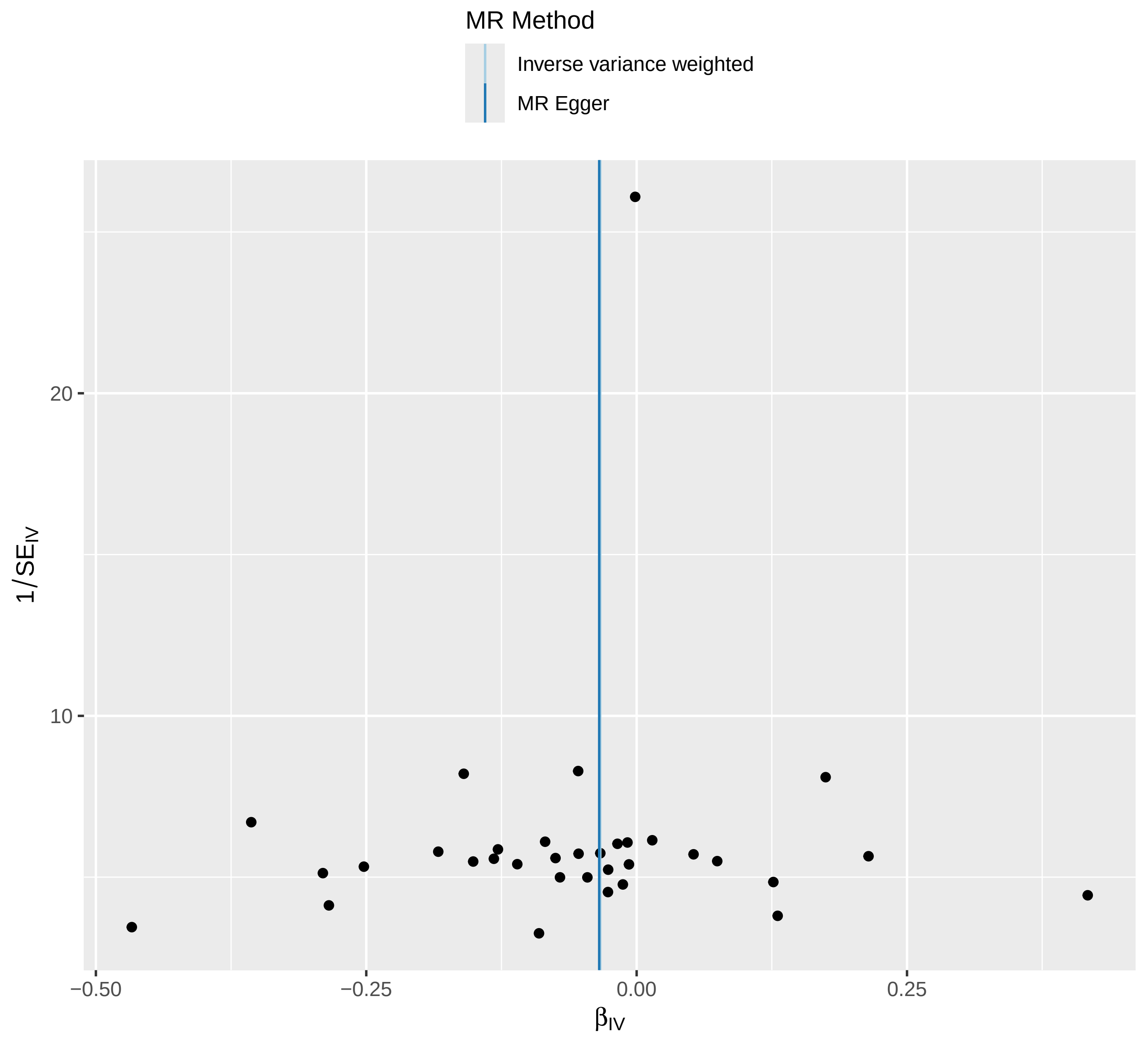
MR Method
Inverse variance weighted
MR Egger
20
10
1/SE_{IV}
−0.50
−0.25
0.00
0.25
β_{IV}

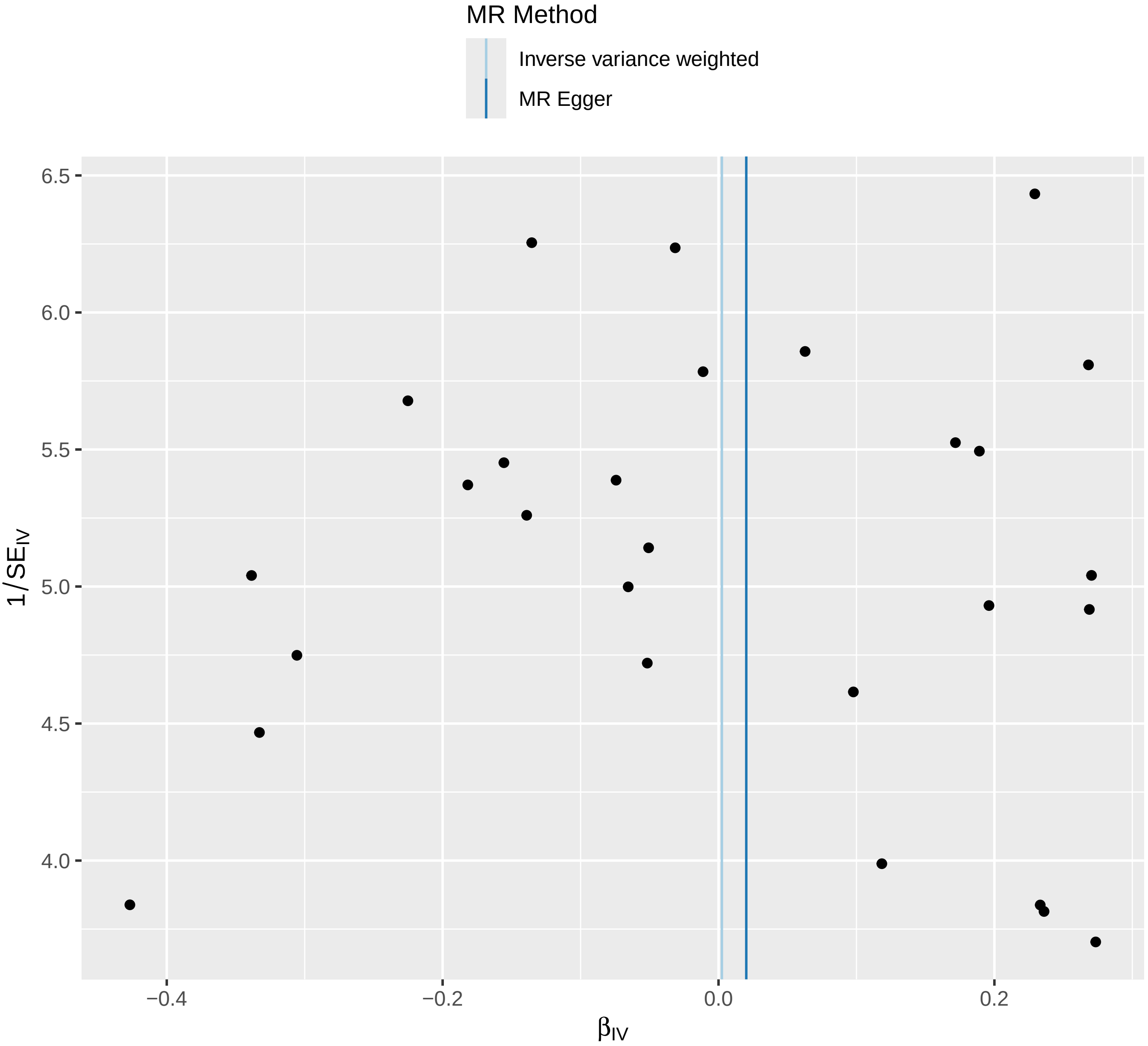
MR Method
Inverse variance weighted
MR Egger
6.5
6.0
5.5
5.0
4.5
4.0
$1/SE_{IV}$
−0.4
−0.2
0.0
0.2
β_{IV}

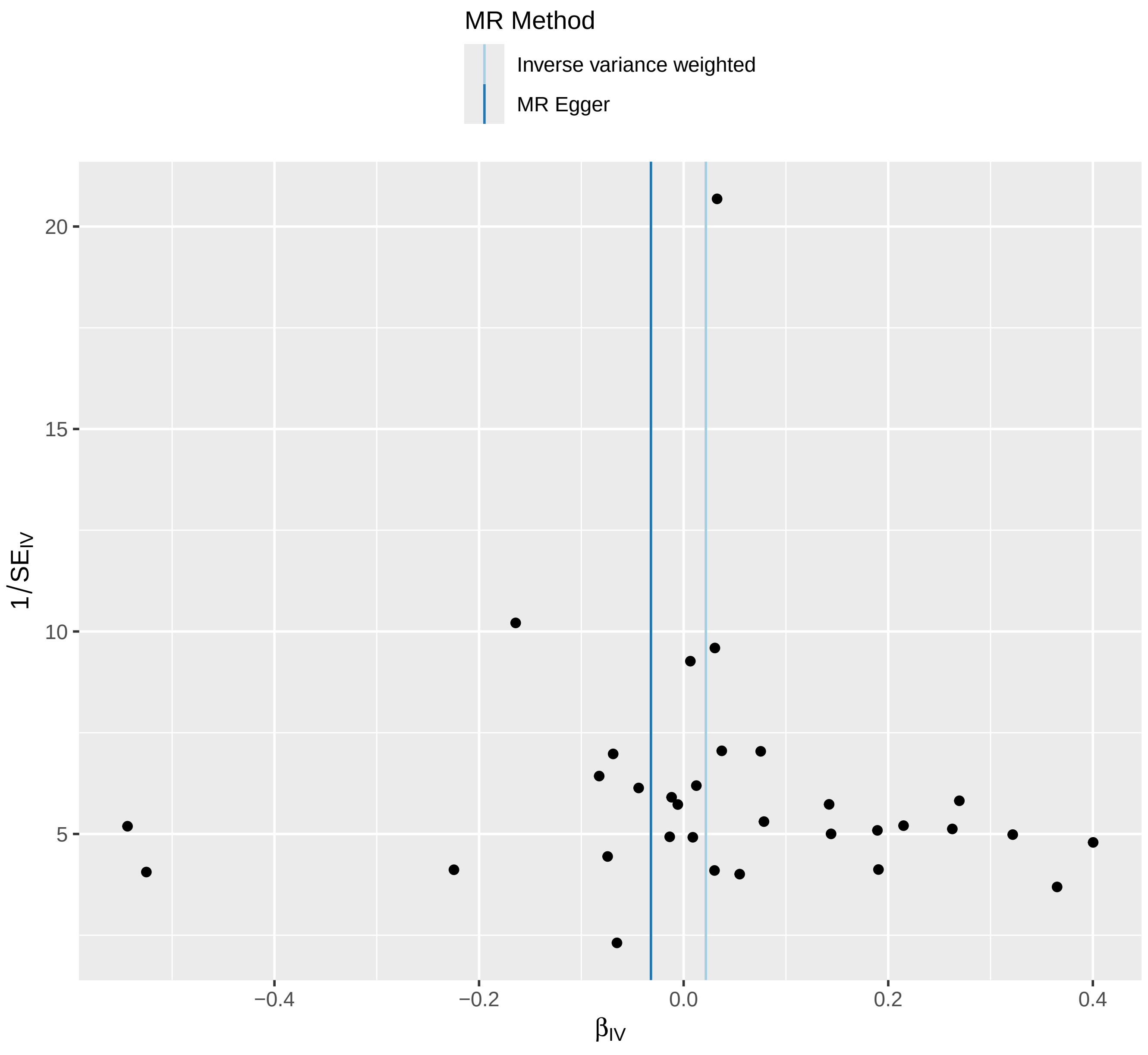
MR Method
Inverse variance weighted
MR Egger
20
15
10
5
1/SE_IV
−0.4
−0.2
0.0
0.2
0.4
β_IV

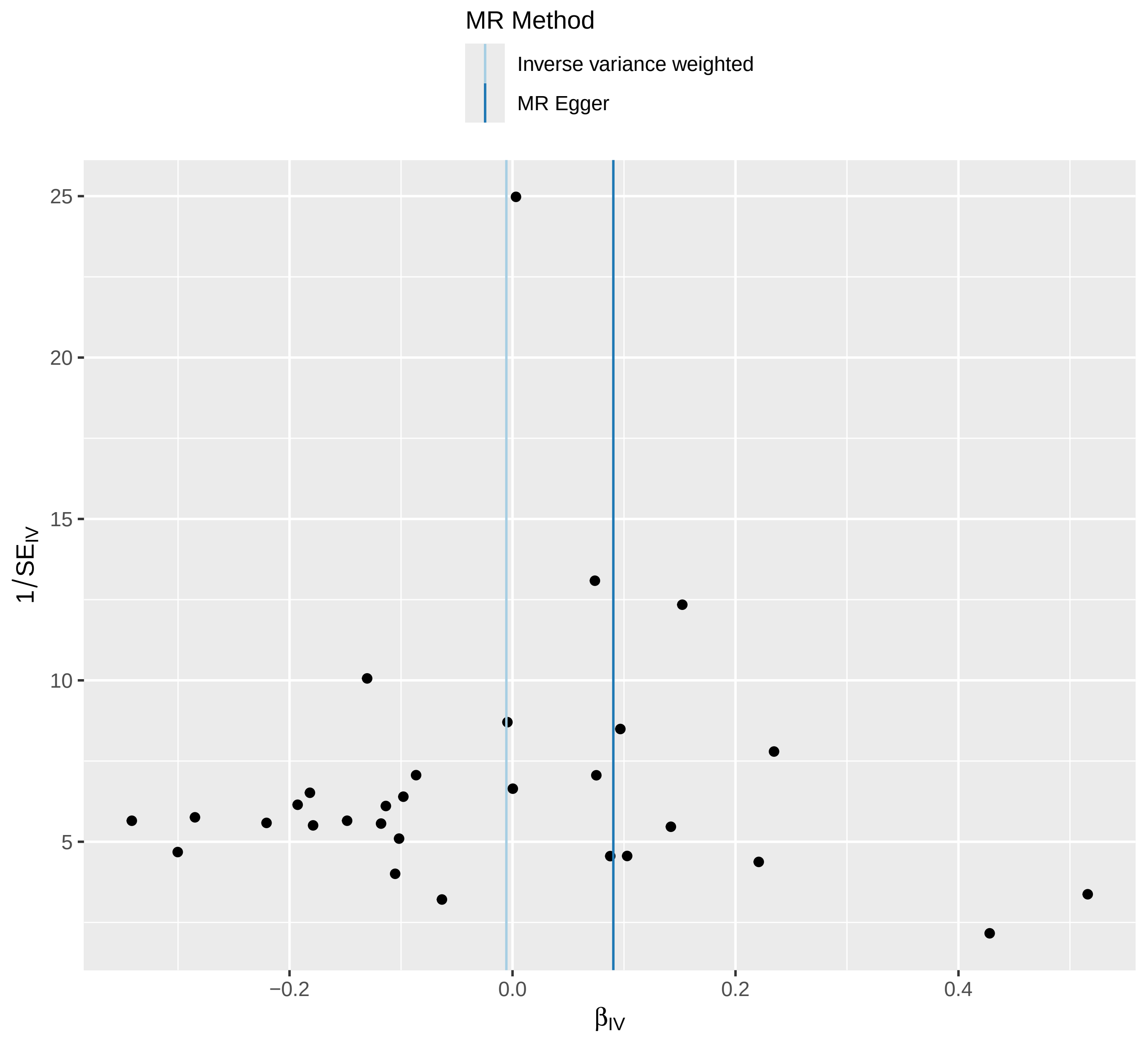

MR Method
Inverse variance weighted
MR Egger
25
20
15
10
5
1/SE_IV
−0.2
0.0
0.2
0.4
β_IV

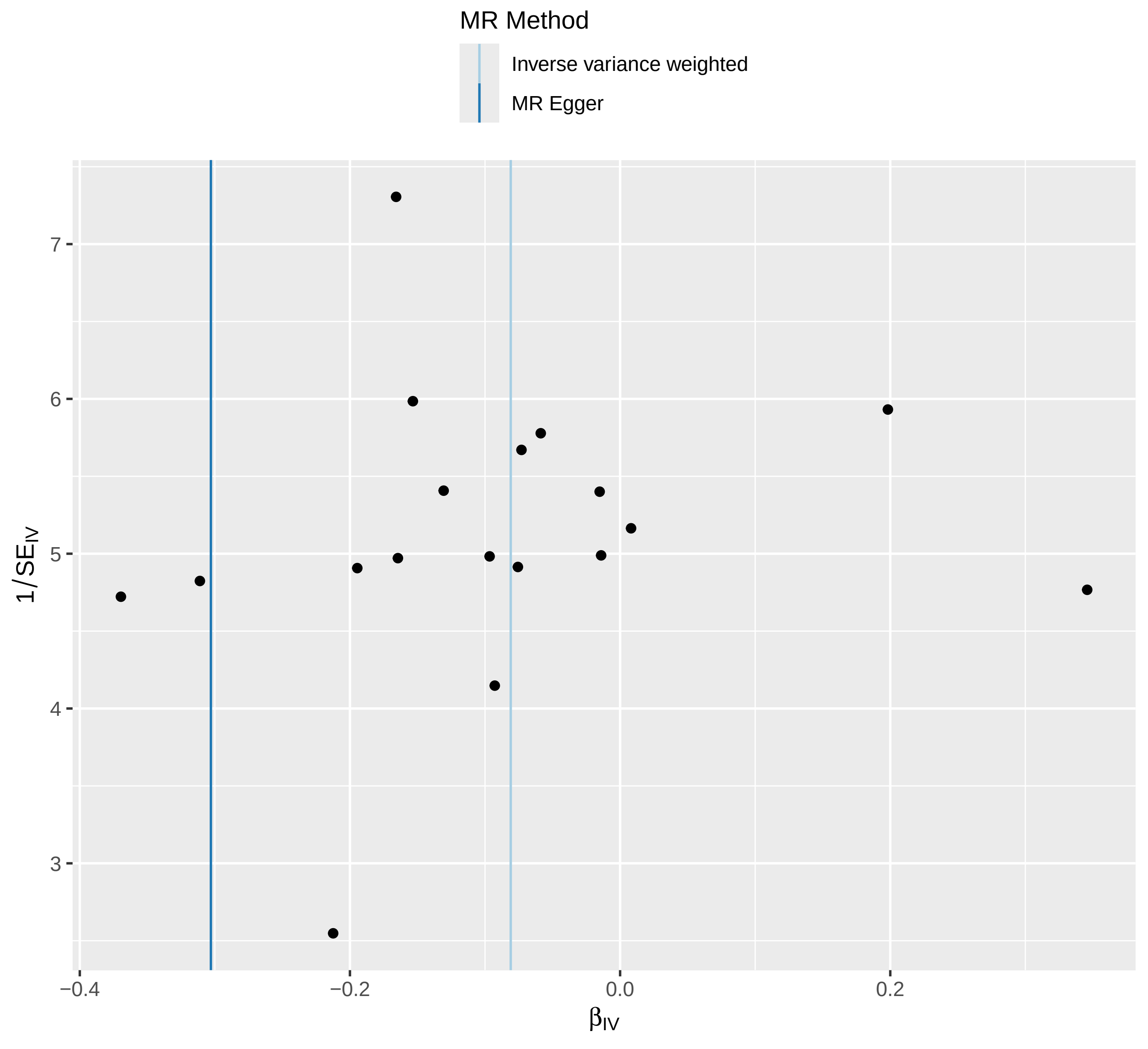
MR Method
Inverse variance weighted
MR Egger
7
6
5
4
3
$1/SE_{IV}$
−0.4
−0.2
0.0
0.2
β_{IV}

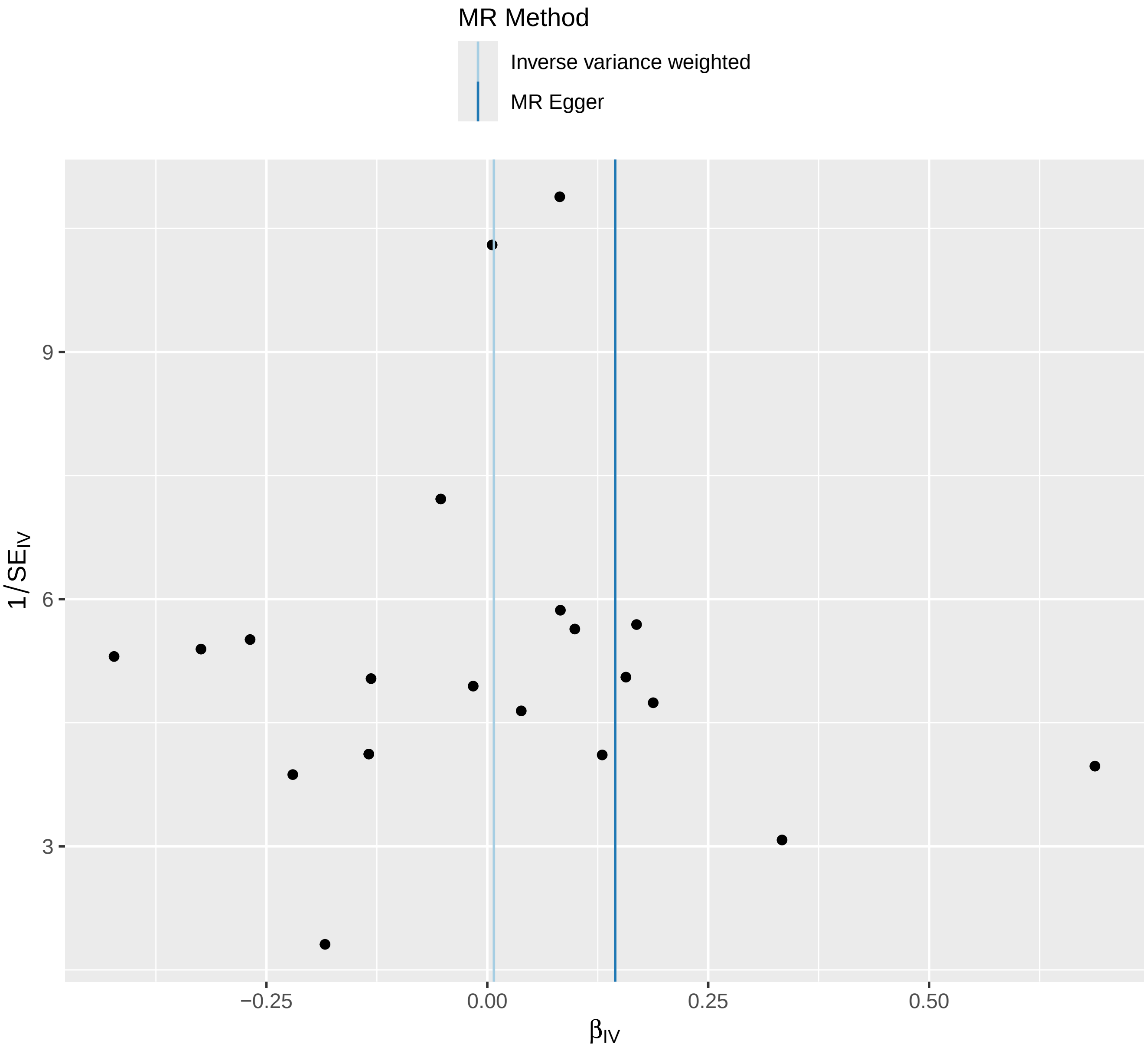

MR Method
Inverse variance weighted
MR Egger
9
6
3
$1/SE_{IV}$
−0.25
0.00
0.25
0.50
β_{IV}

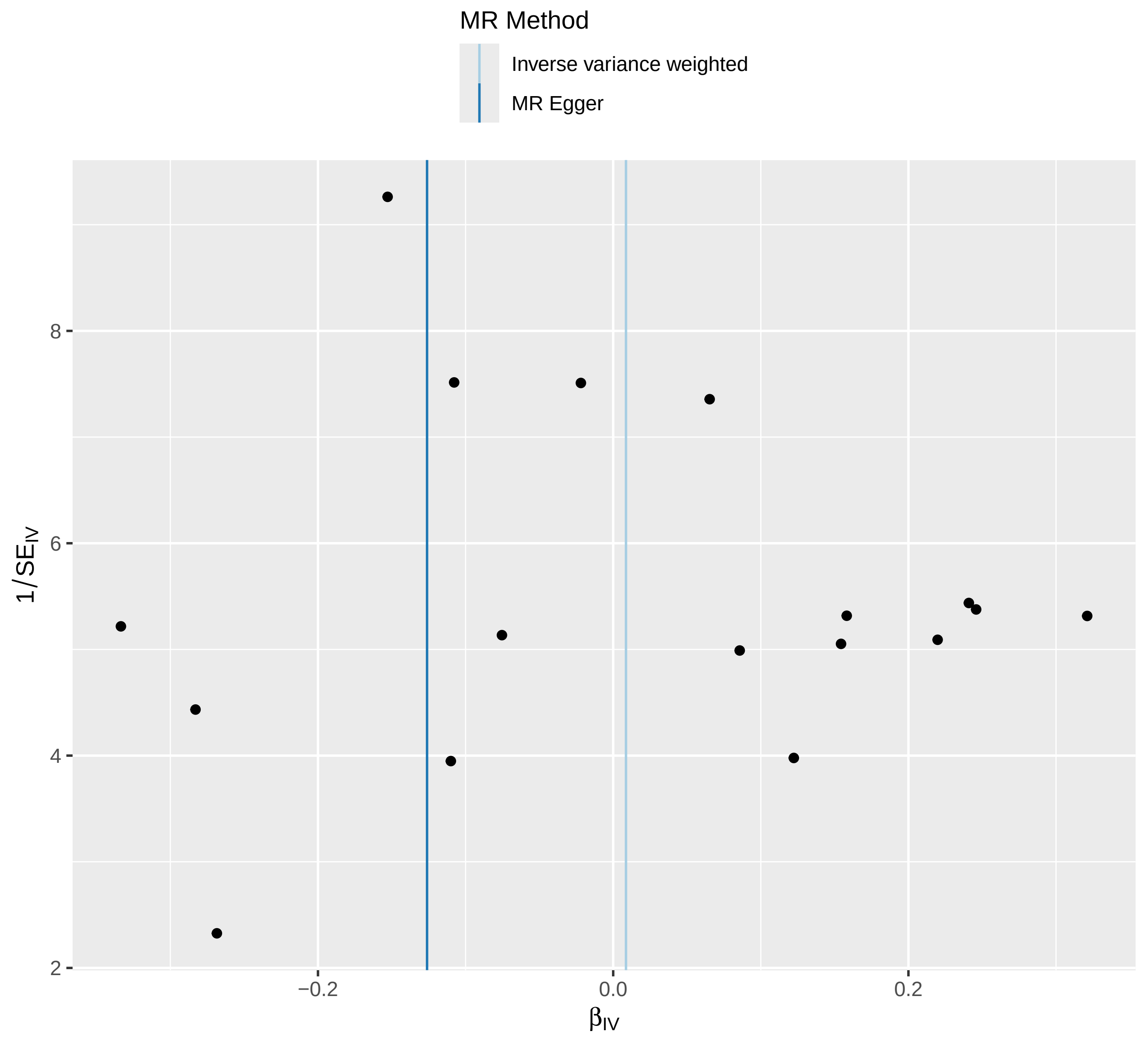

MR Method
Inverse variance weighted
MR Egger
8
6
4
2
1/SE$_{IV}$
−0.2
0.0
0.2
β$_{IV}$

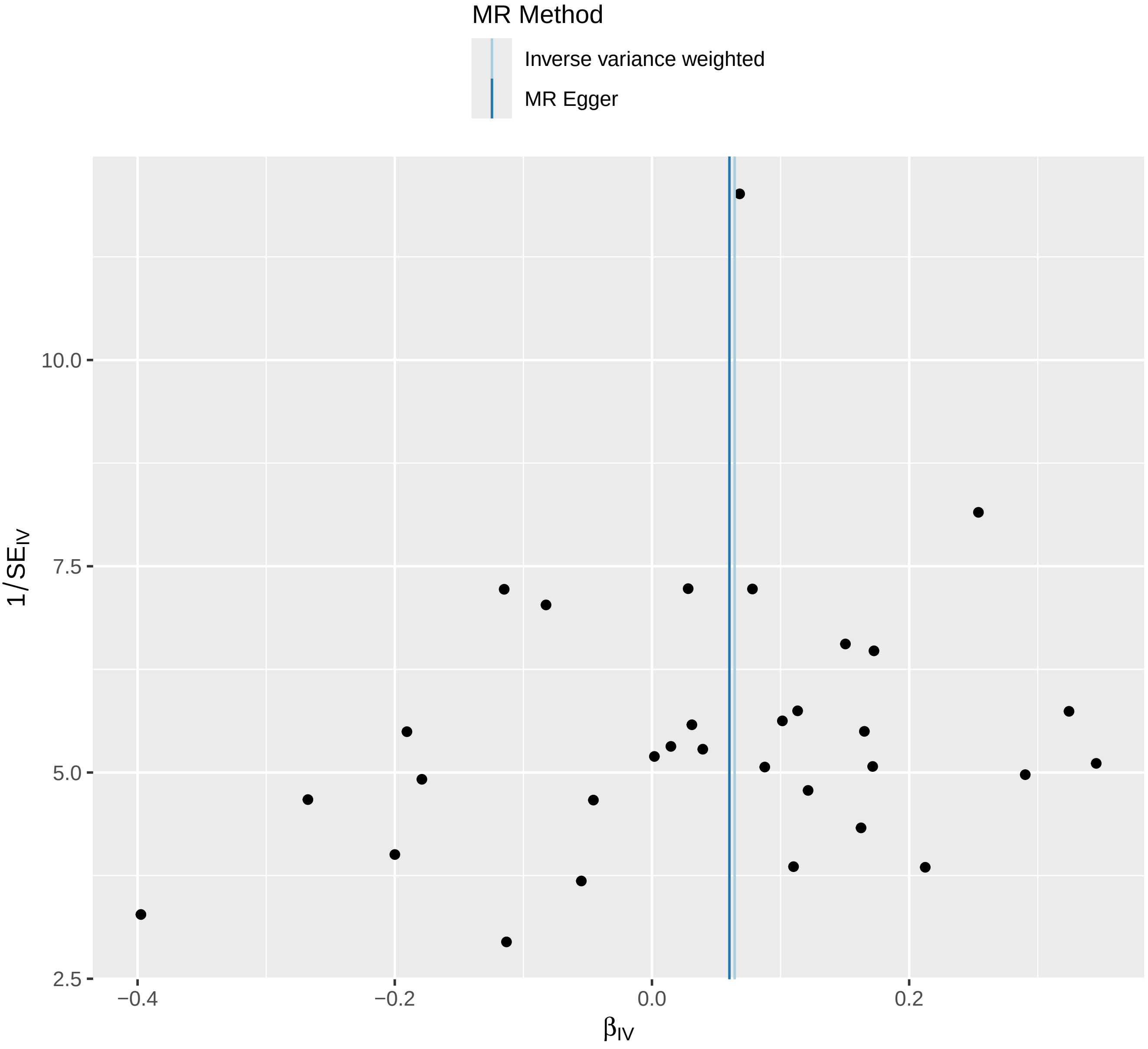

MR Method
Inverse variance weighted
MR Egger
10.0
7.5
5.0
2.5
−0.4
−0.2
0.0
0.2
$1/SE_{IV}$
β_{IV}

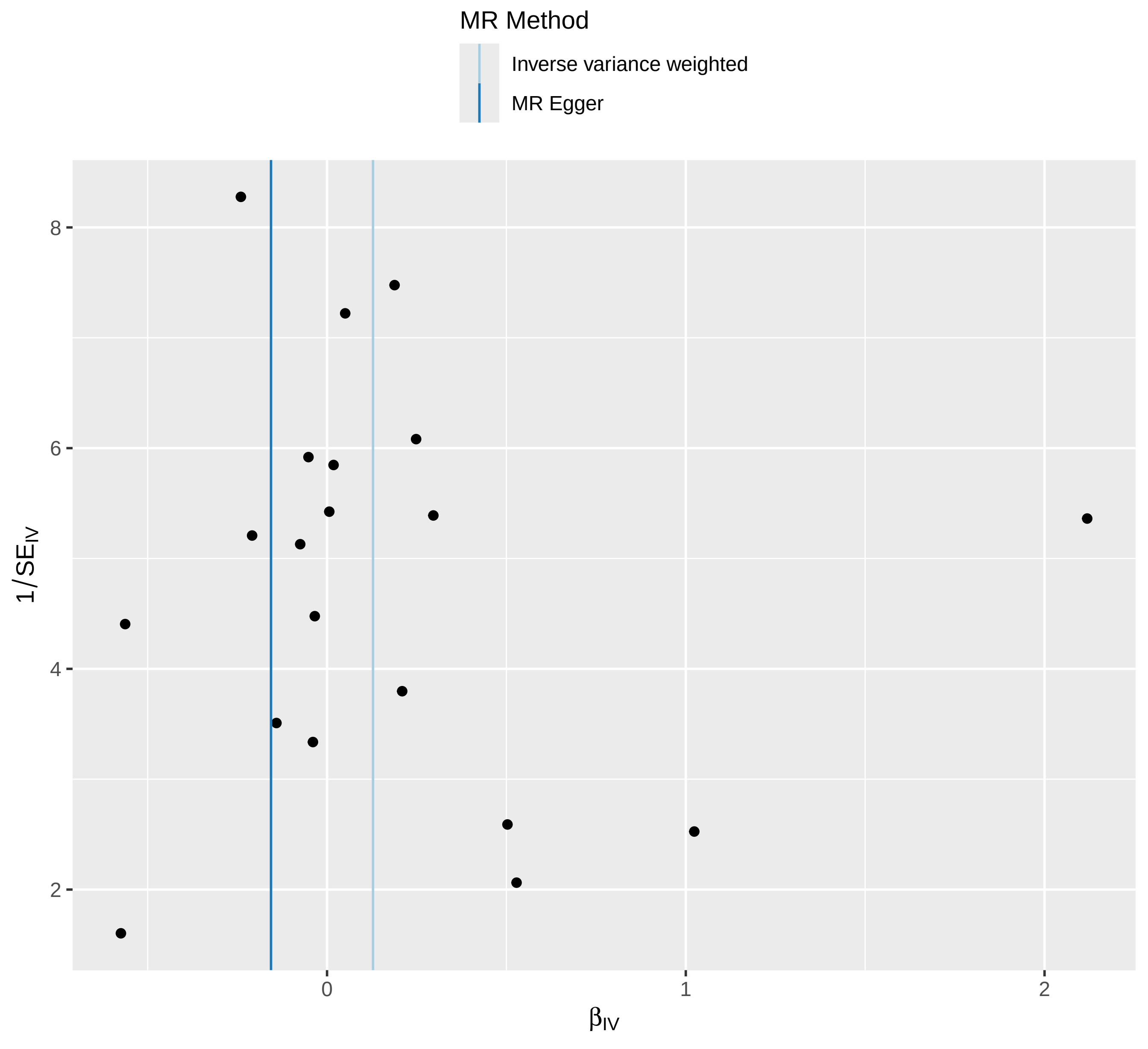
MR Method
Inverse variance weighted
MR Egger
8
6
4
2
1/SE_IV
0
1
2
β_IV

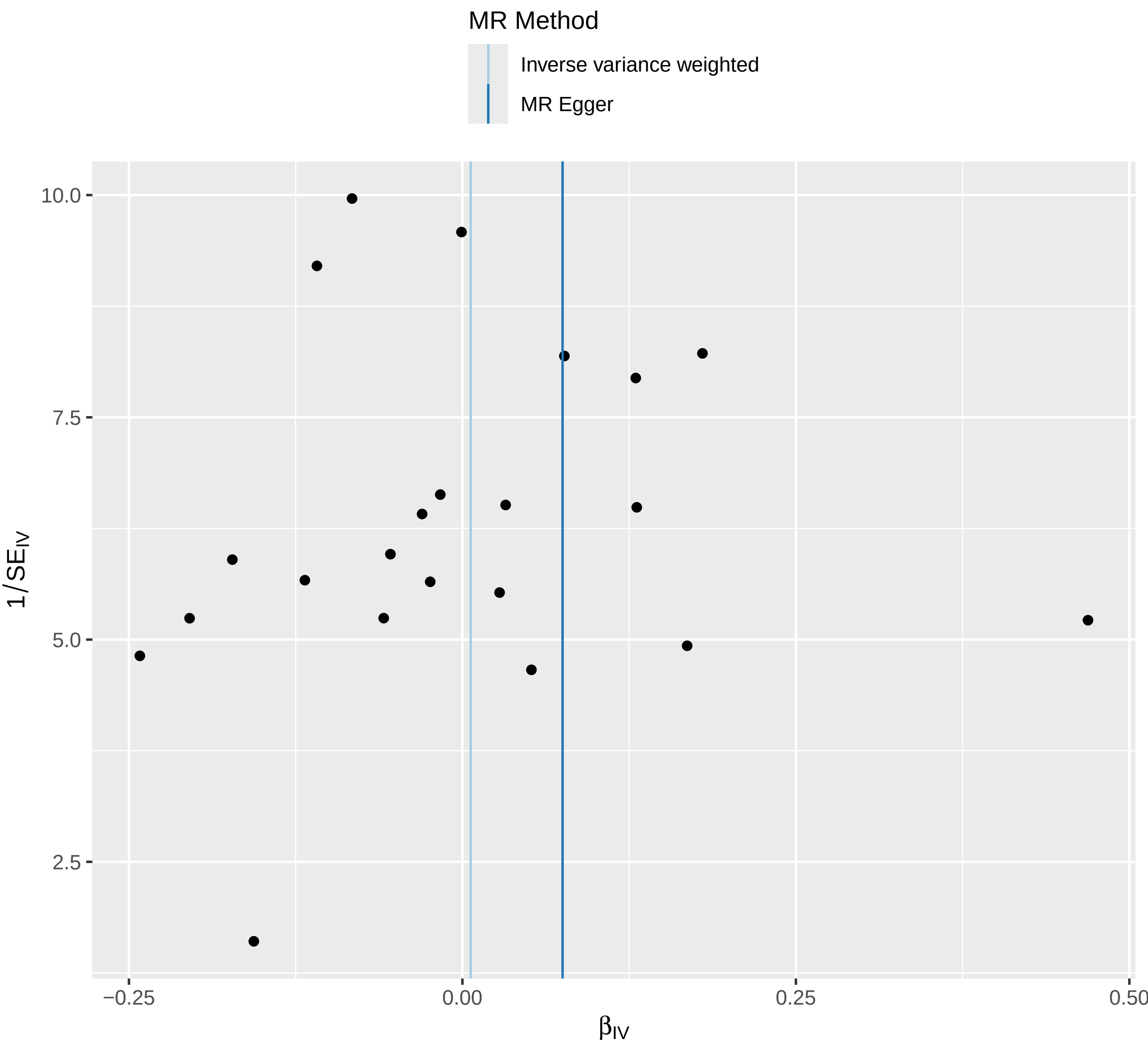
MR Method
Inverse variance weighted
MR Egger
10.0
7.5
5.0
2.5
$1/SE_{IV}$
−0.25
0.00
0.25
0.50
β_{IV}

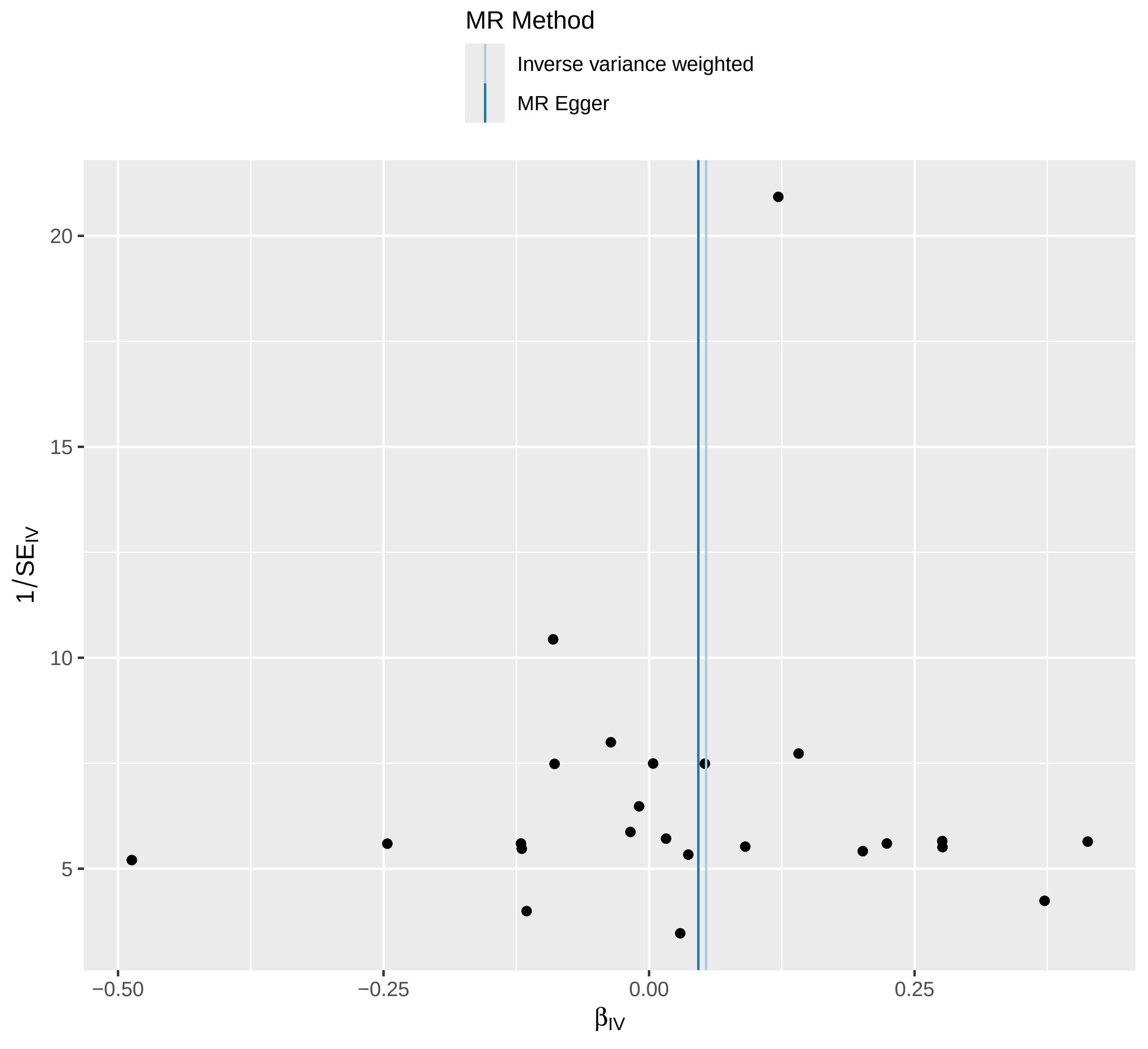
MR Method
Inverse variance weighted
MR Egger
1/SE_IV
20
15
10
5
−0.50
−0.25
0.00
0.25
β_IV

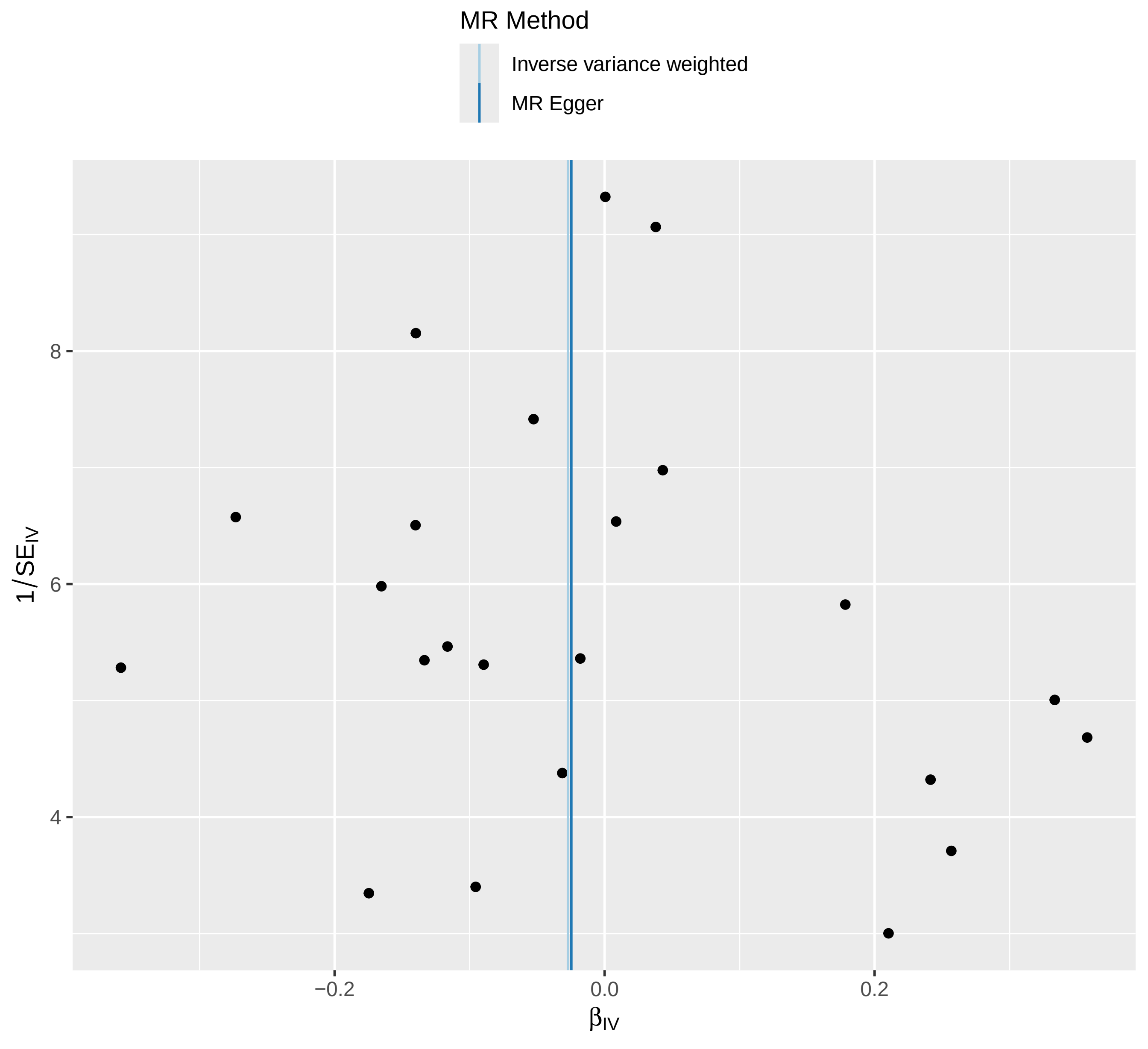
MR Method
Inverse variance weighted
MR Egger
8
6
4
1/SE_IV
−0.2
0.0
0.2
β_IV

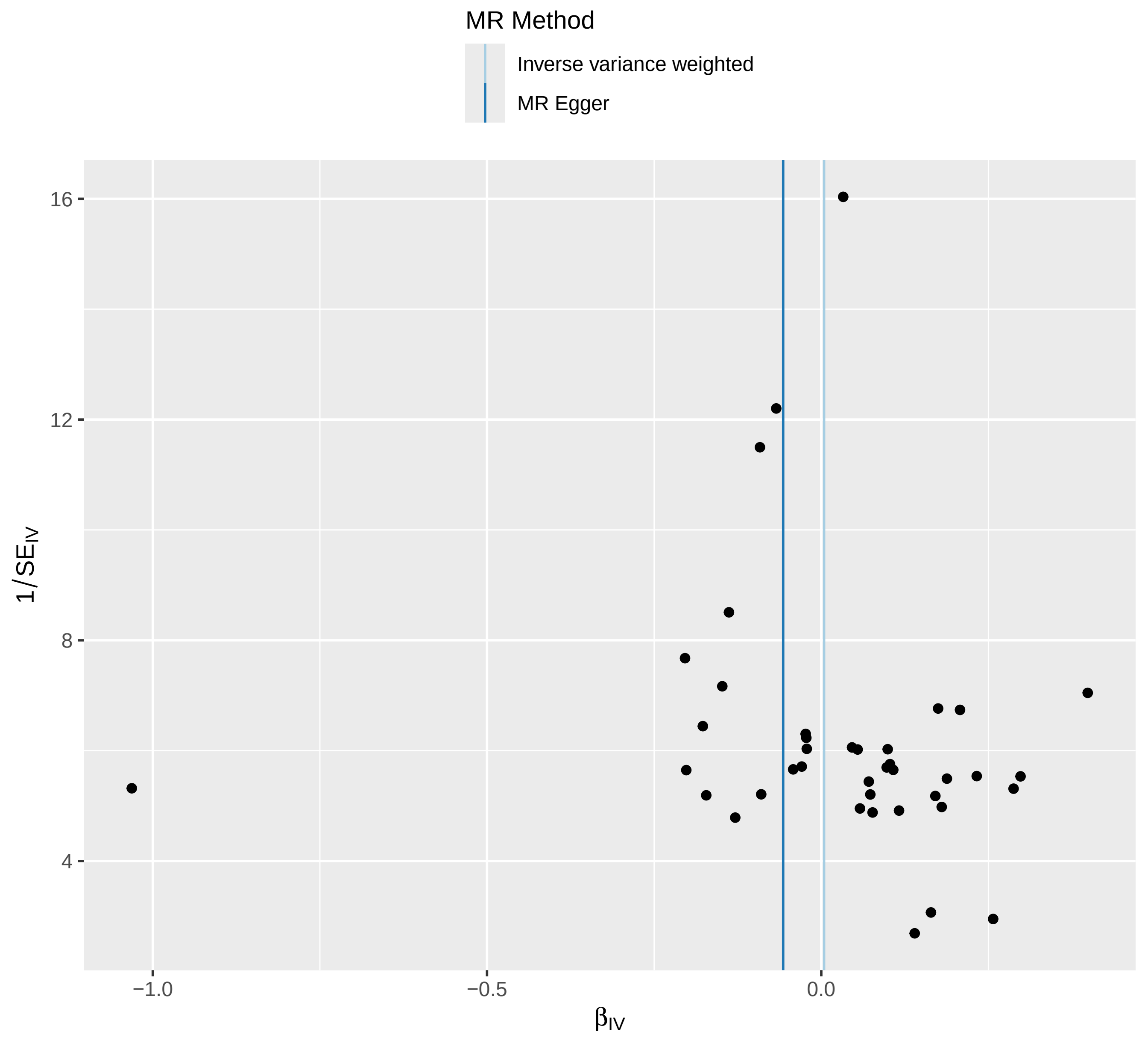
MR Method
Inverse variance weighted
MR Egger
16
12
8
4
$1/SE_{IV}$
−1.0
−0.5
0.0
β_{IV}

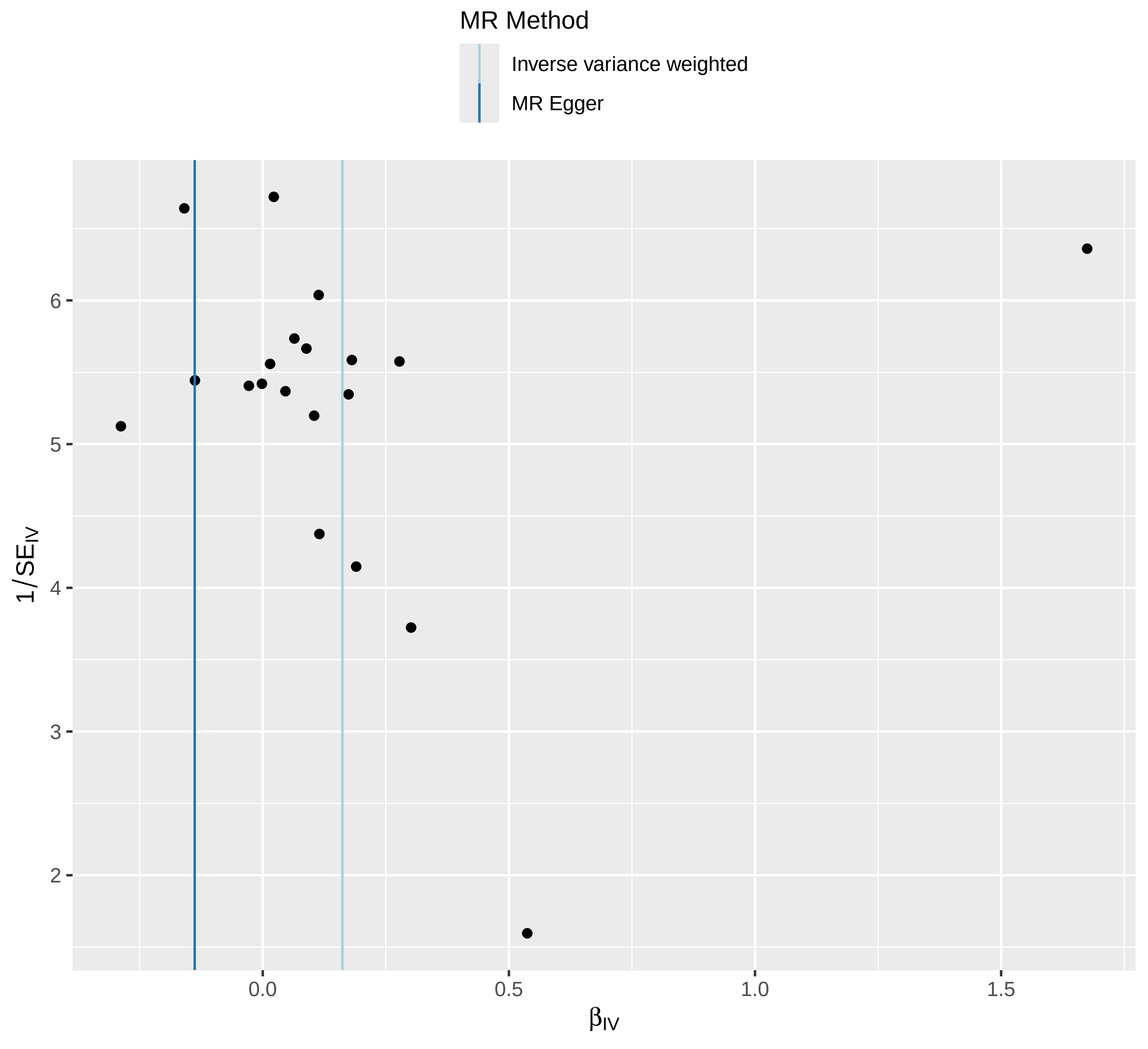

MR Method
Inverse variance weighted
MR Egger
6
5
4
3
2
1/SE_IV
0.0
0.5
1.0
1.5
β_IV

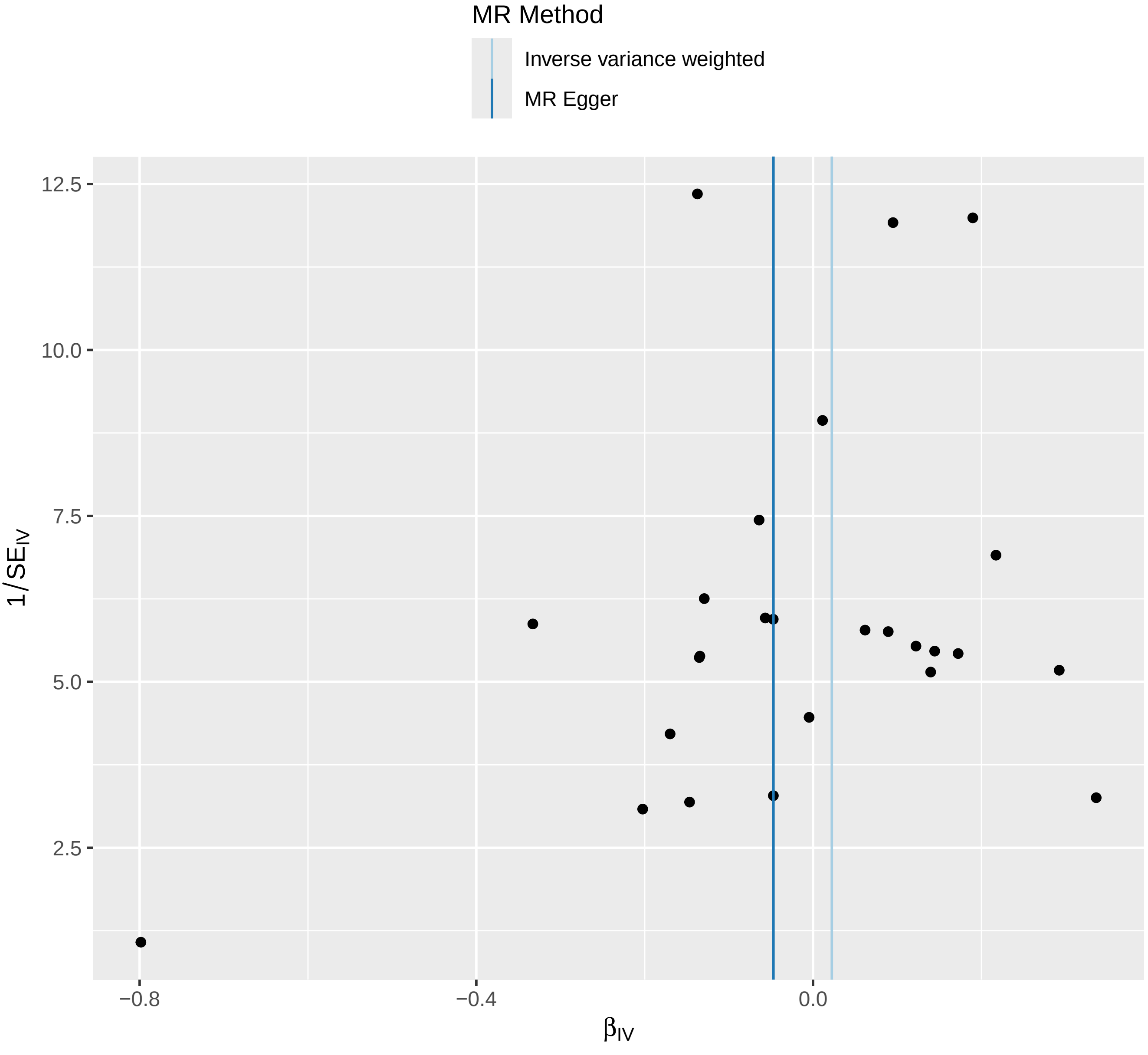
MR Method
Inverse variance weighted
MR Egger
12.5
10.0
7.5
5.0
2.5
1/SE$_{IV}$
−0.8
−0.4
0.0
β$_{IV}$

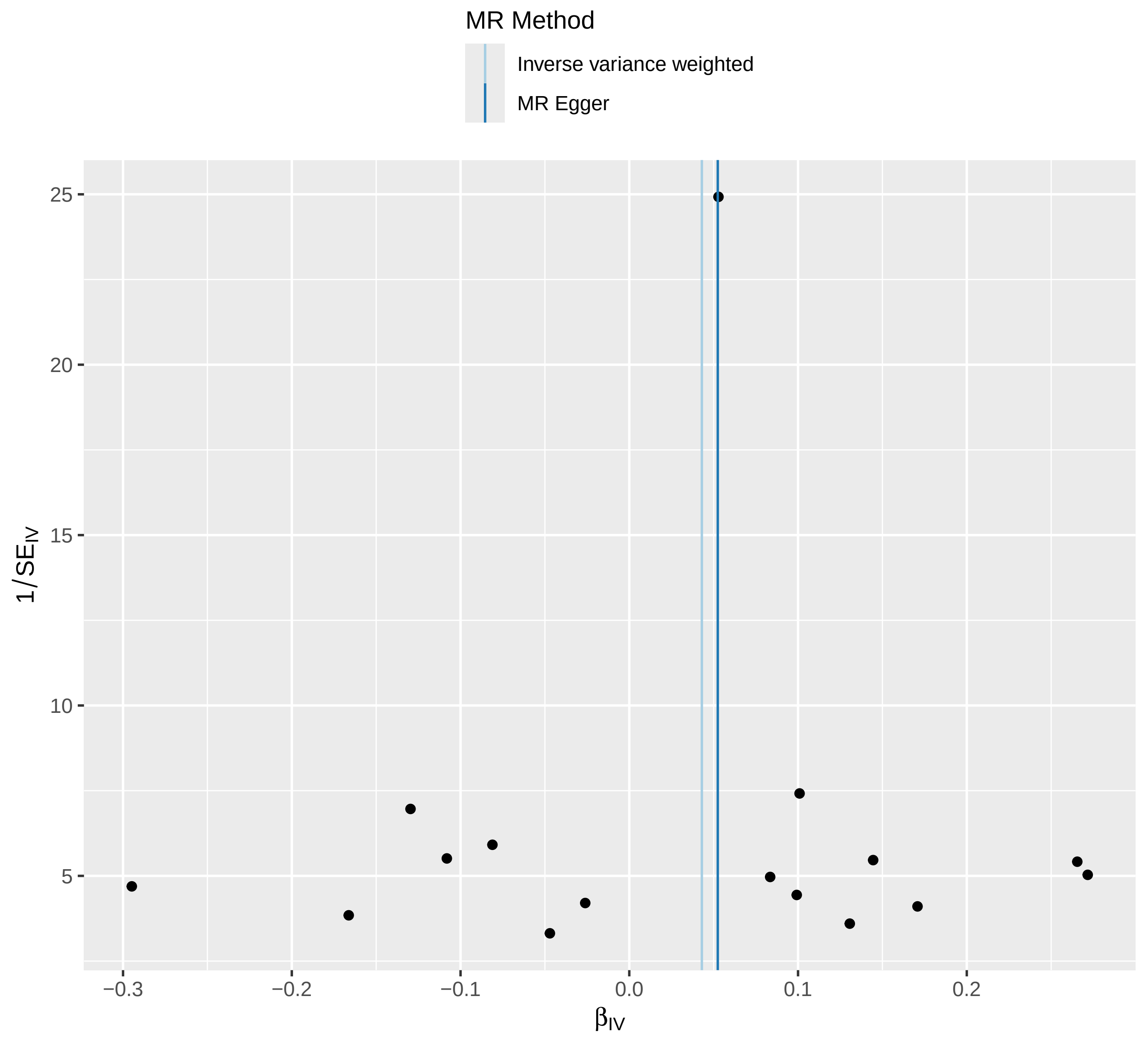
MR Method
Inverse variance weighted
MR Egger
1/SE_IV
25
20
15
10
5
−0.3
−0.2
−0.1
0.0
0.1
0.2
β_IV

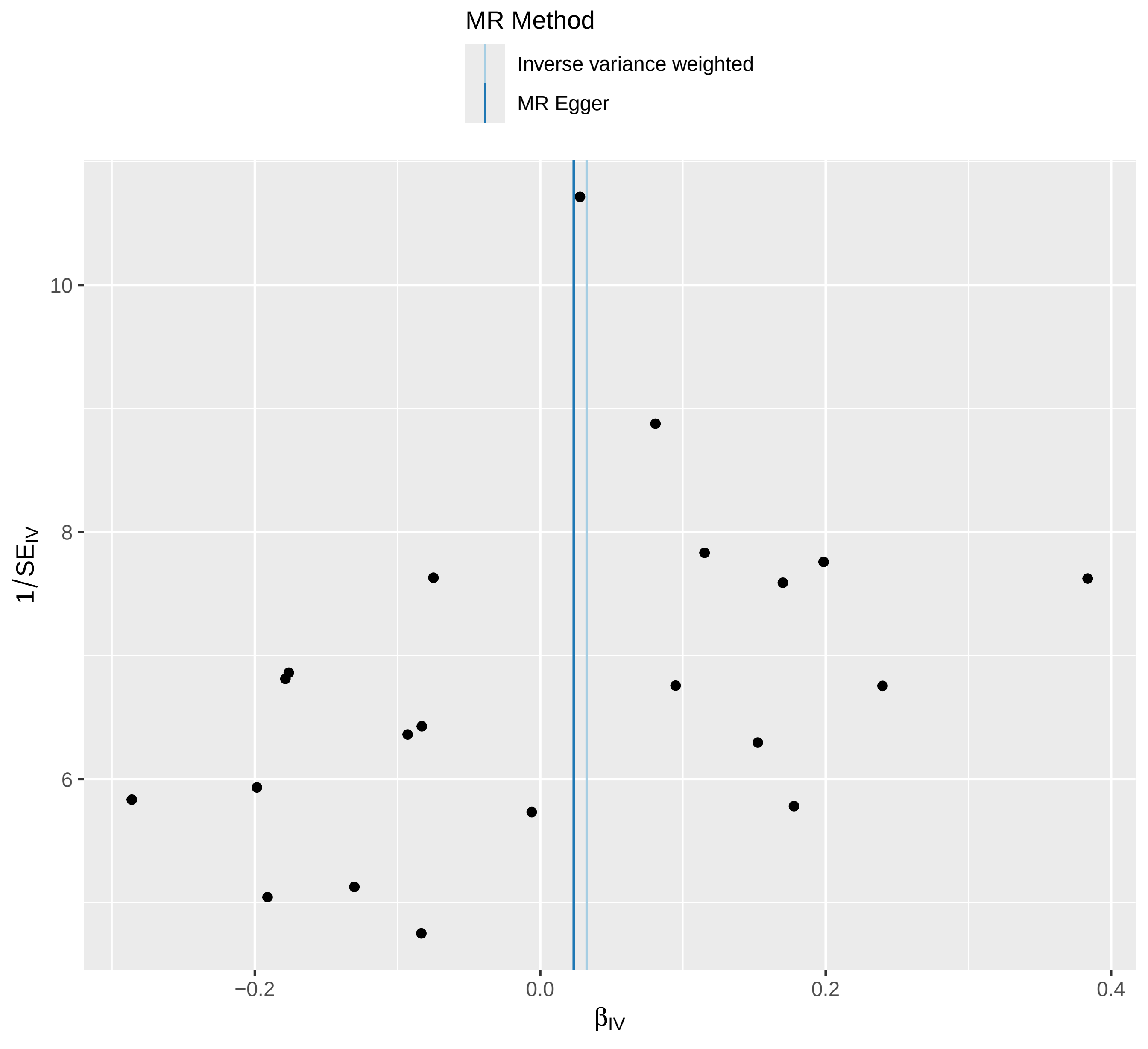
MR Method
Inverse variance weighted
MR Egger
10
8
6
1/SE_IV
−0.2
0.0
0.2
0.4
β_IV

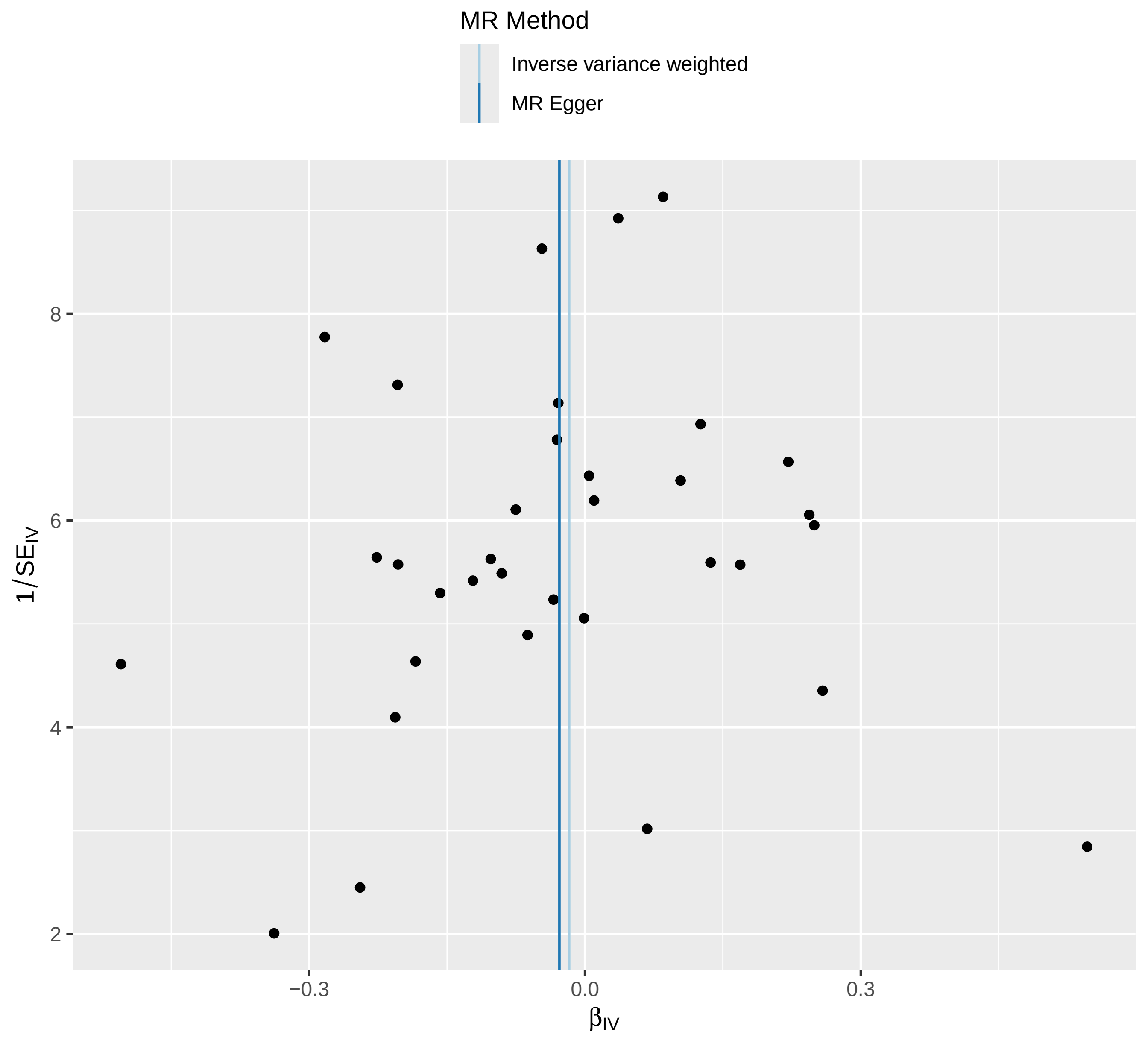
MR Method
Inverse variance weighted
MR Egger
8
6
4
2
1/SE_IV
−0.3
0.0
0.3
β_IV

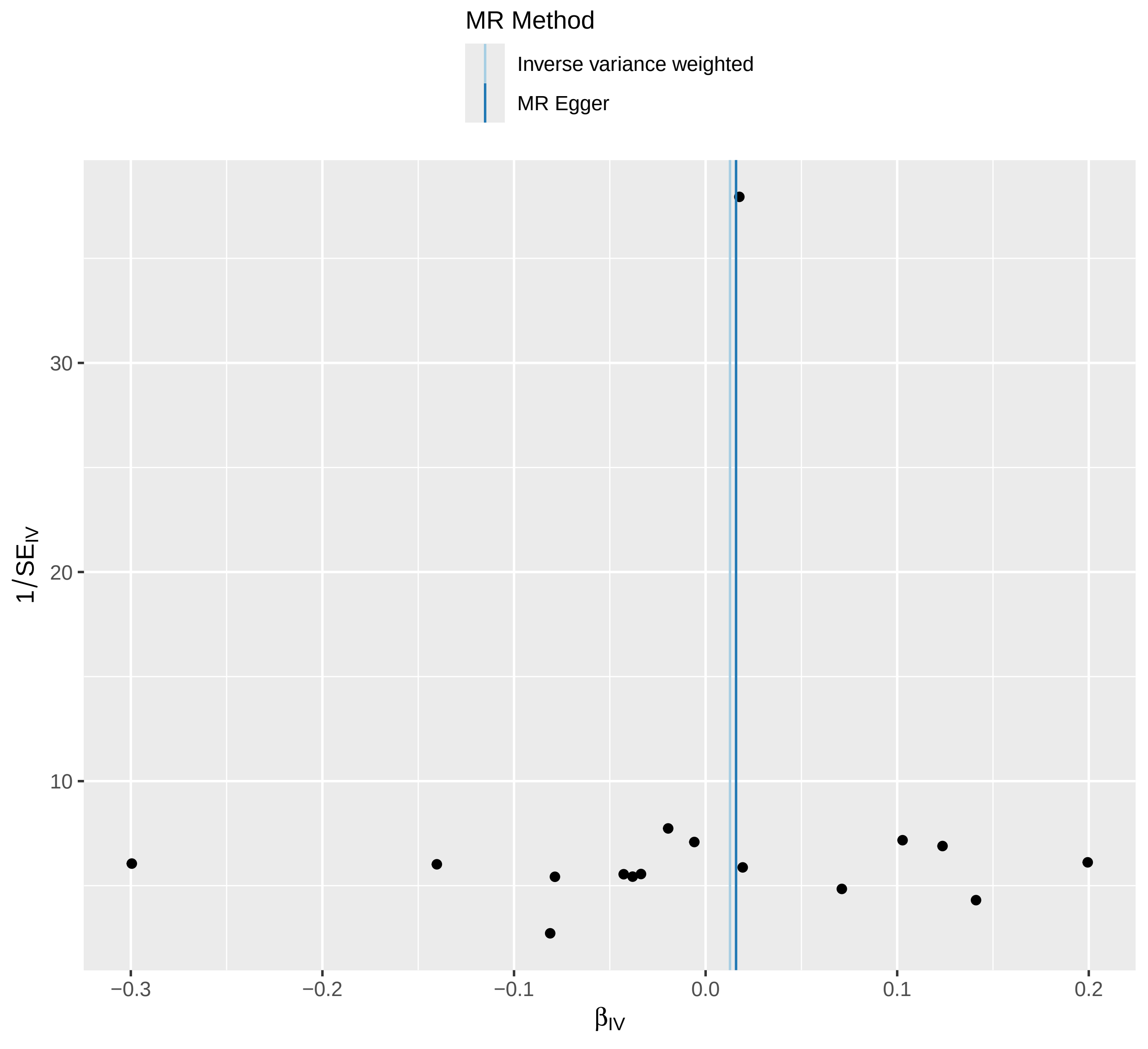

MR Method
Inverse variance weighted
MR Egger
1/SE_IV
30
20
10
−0.3
−0.2
−0.1
0.0
0.1
0.2
β_IV

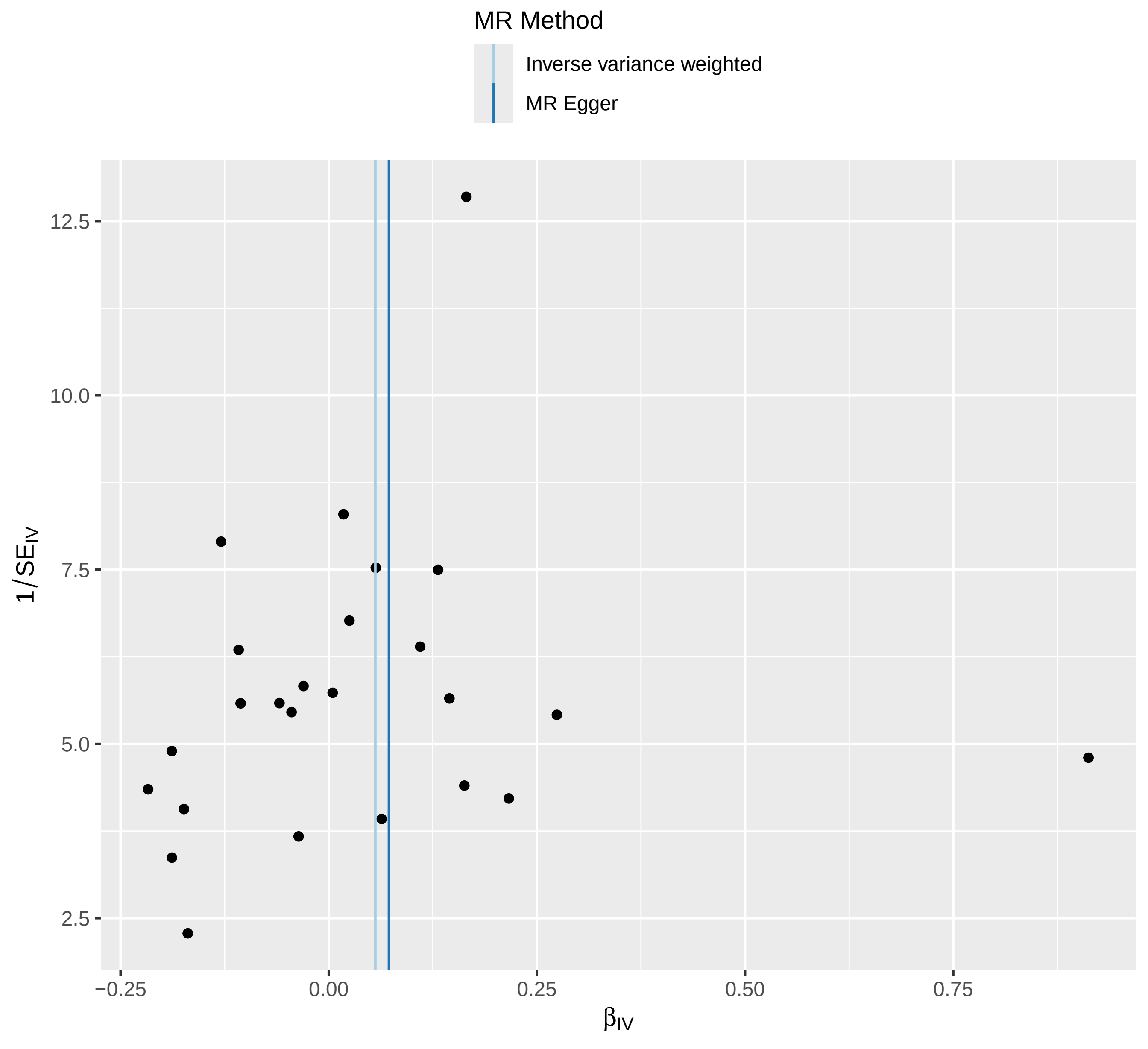

MR Method
Inverse variance weighted
MR Egger
12.5
10.0
7.5
5.0
2.5
1/SE_IV
−0.25
0.00
0.25
0.50
0.75
β_IV

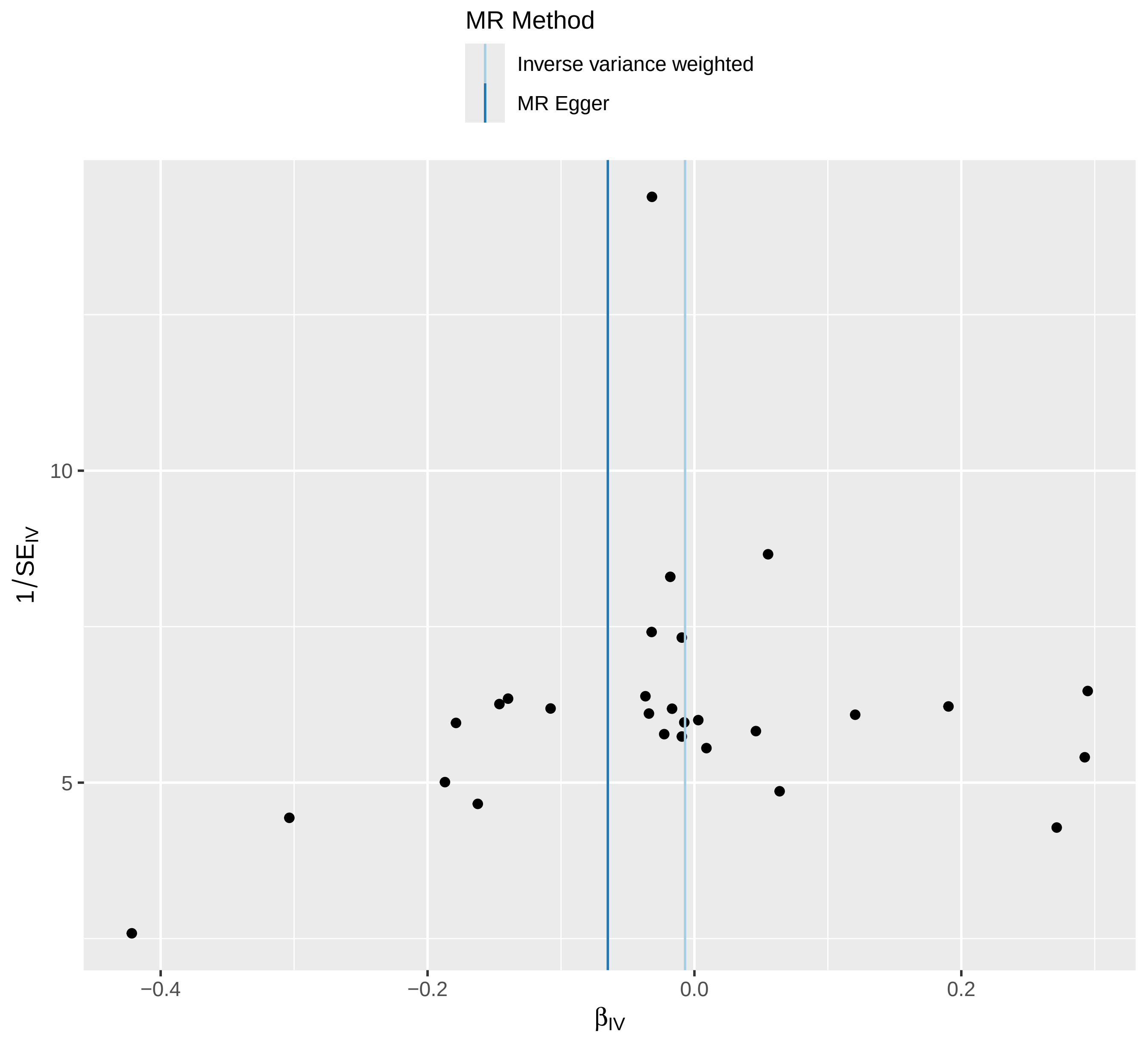
MR Method
Inverse variance weighted
MR Egger
1/SE_IV
10
5
−0.4
−0.2
0.0
0.2
β_IV

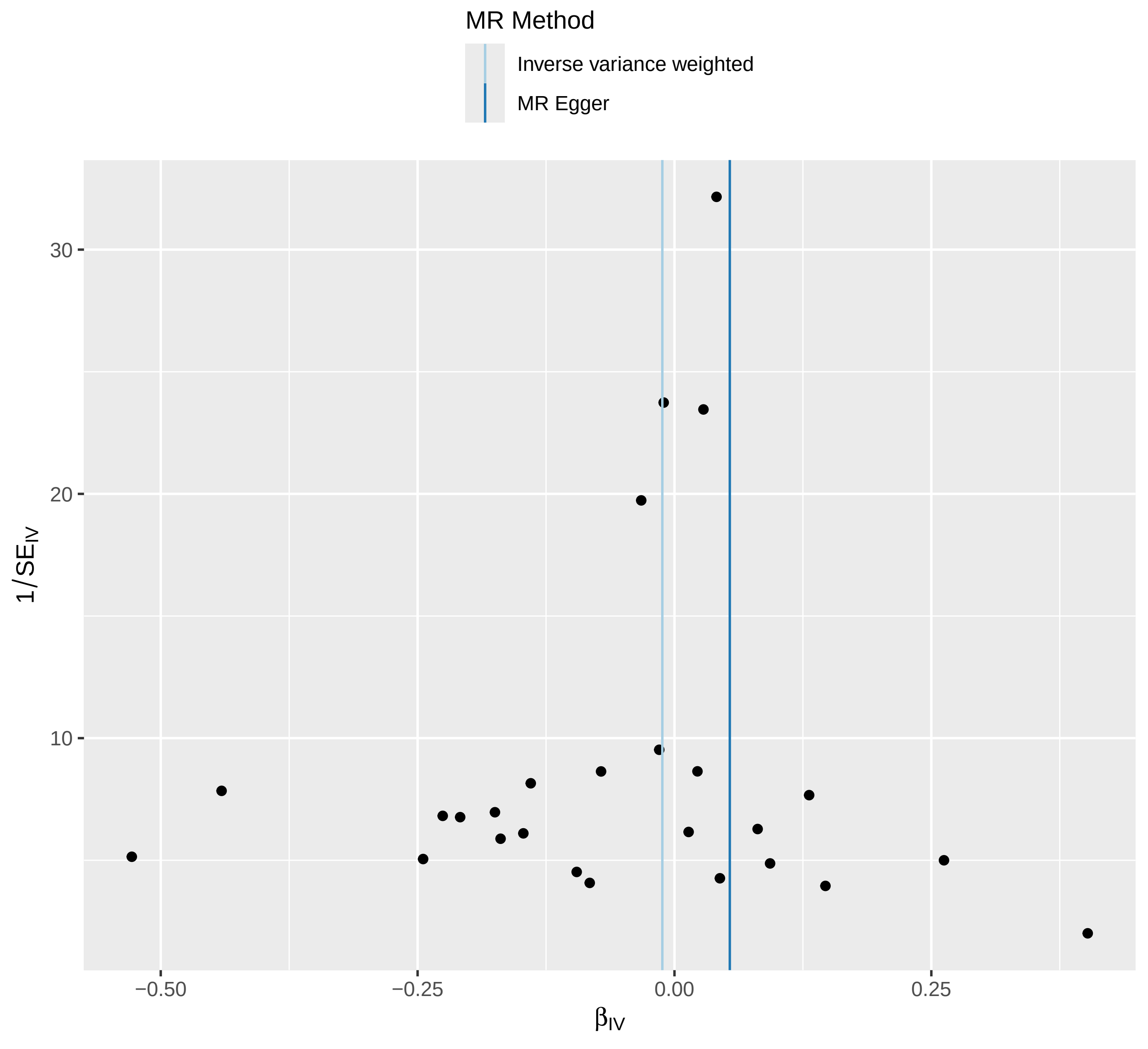
MR Method
Inverse variance weighted
MR Egger
30
20
10
1/SE$_{IV}$
−0.50
−0.25
0.00
0.25
β$_{IV}$

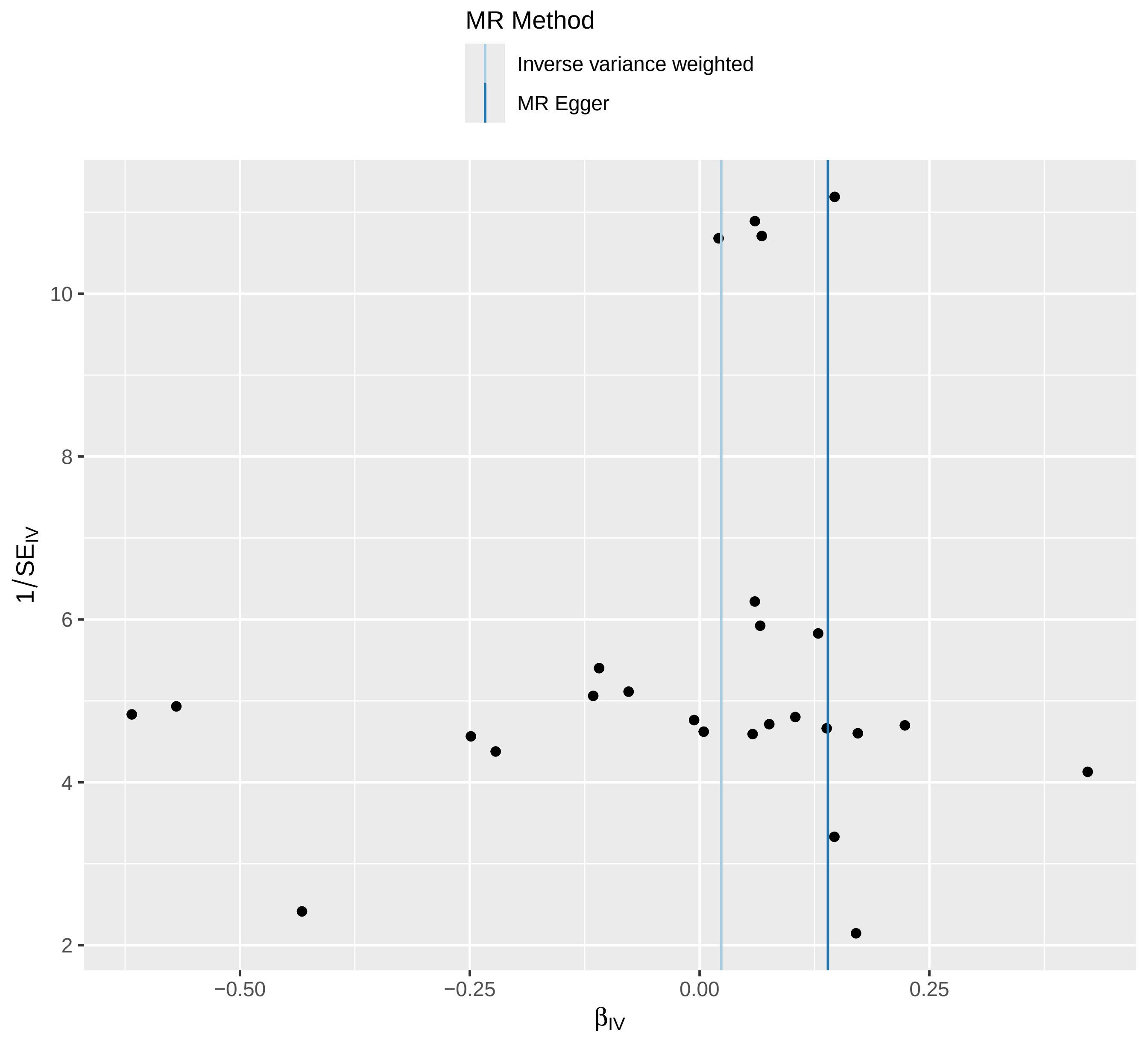
MR Method
Inverse variance weighted
MR Egger
10
8
6
4
2
$1/SE_{IV}$
−0.50
−0.25
0.00
0.25
β_{IV}

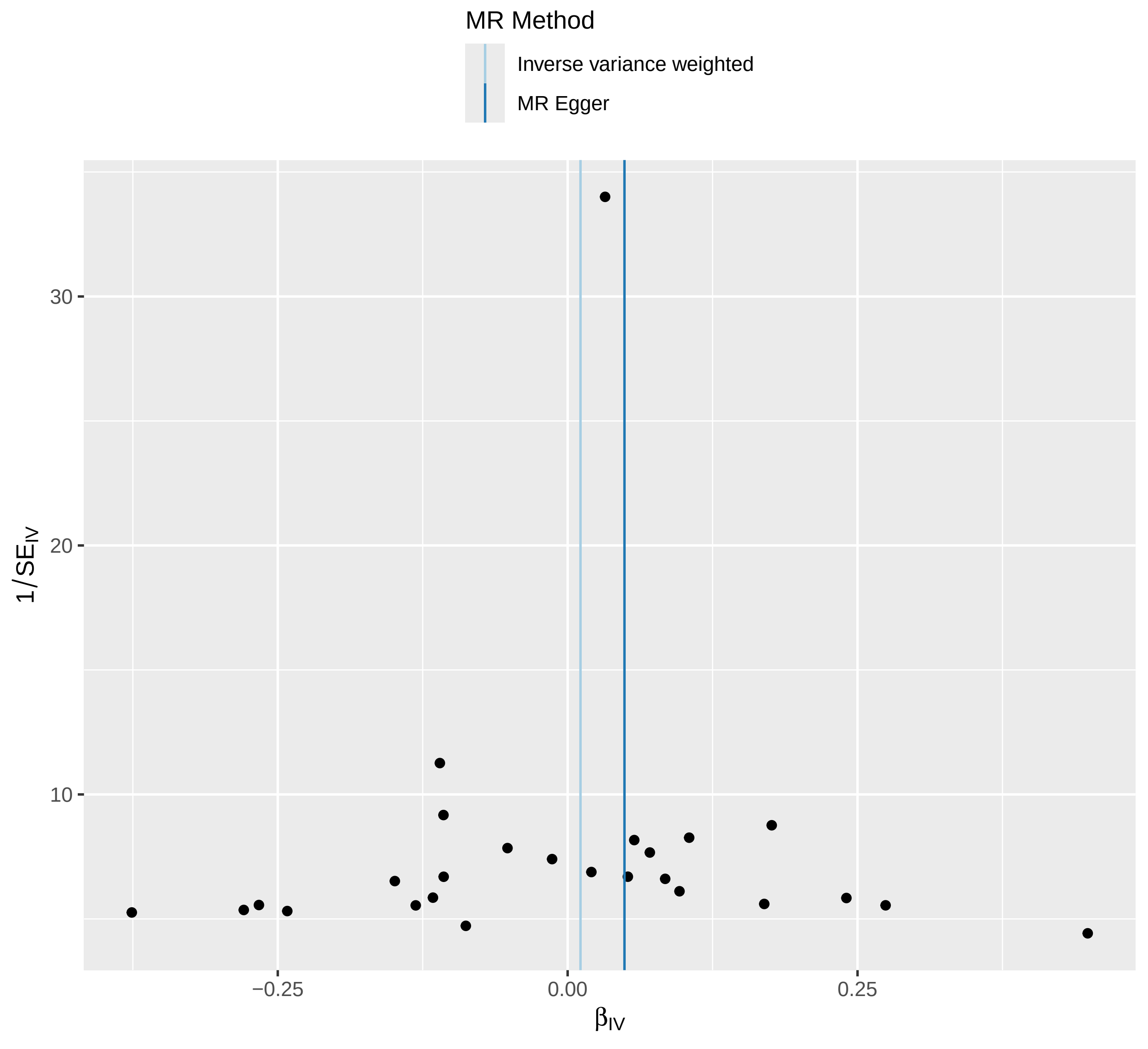

MR Method
Inverse variance weighted
MR Egger
30
20
10
1/SE_IV
−0.25
0.00
0.25
β_IV

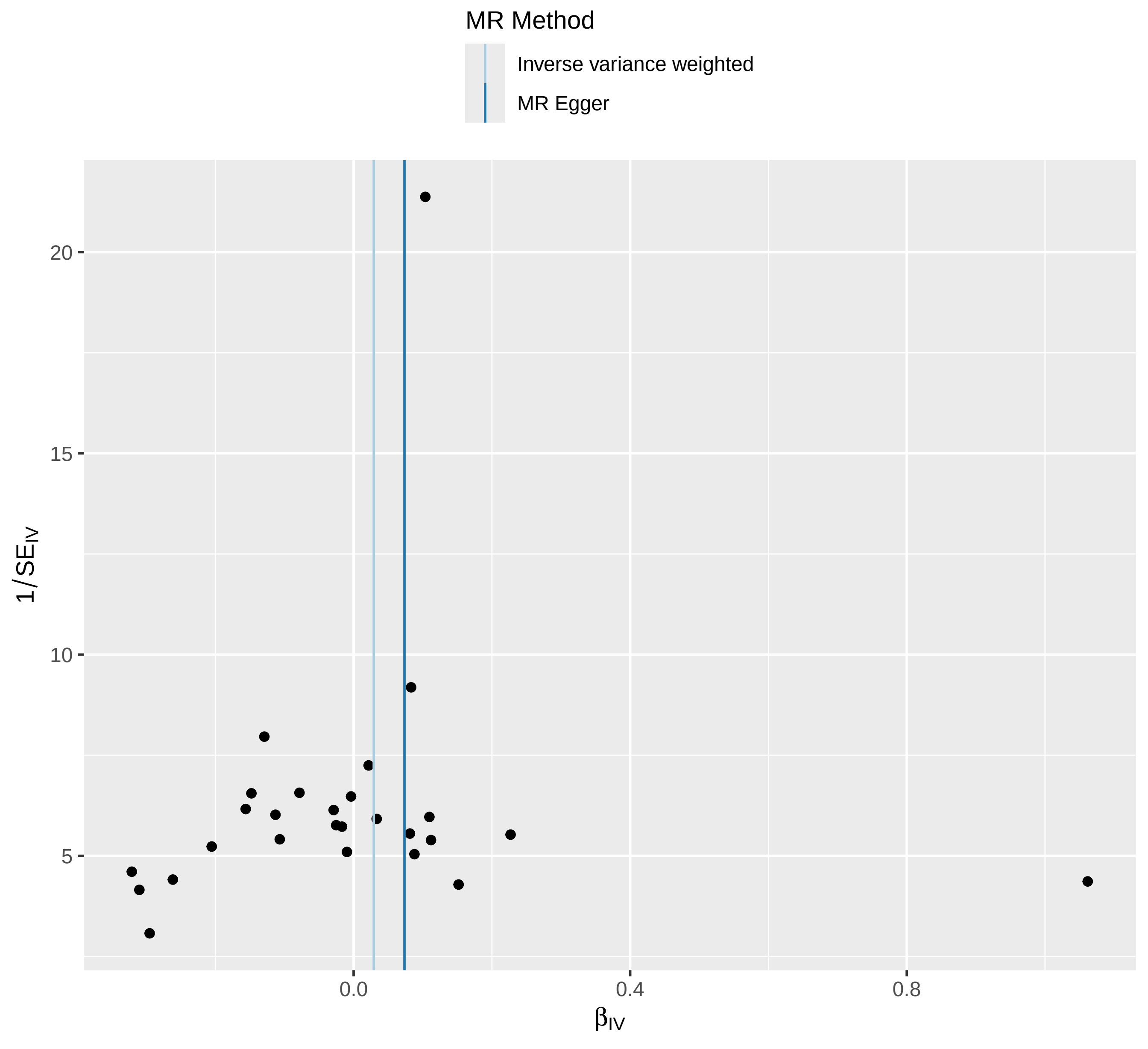
MR Method
Inverse variance weighted
MR Egger
1/SE_IV
β_IV
20
15
10
5
0.0
0.4
0.8

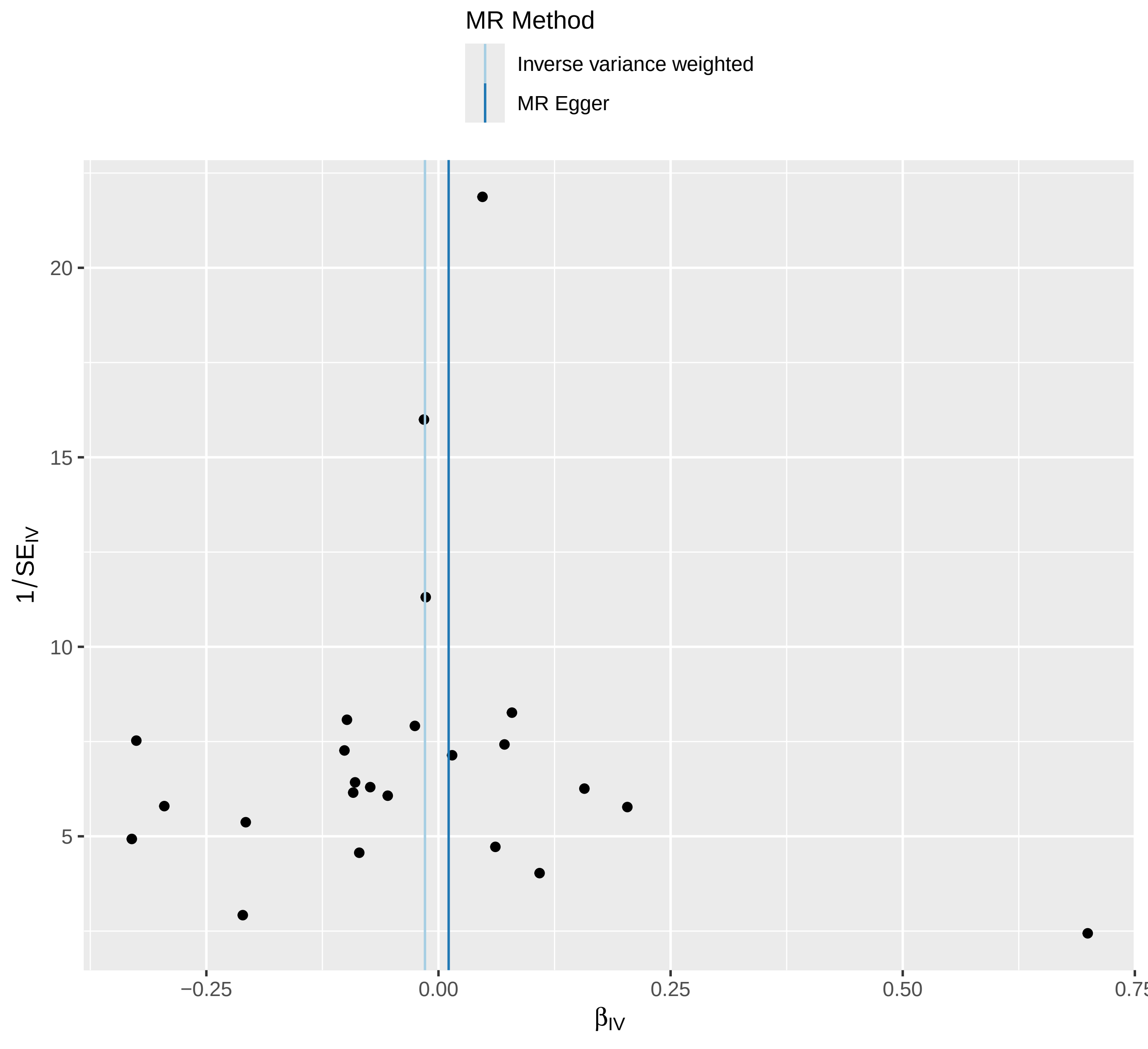
MR Method
Inverse variance weighted
MR Egger
20
15
10
5
1/SE_IV
−0.25
0.00
0.25
0.50
0.75
β_IV

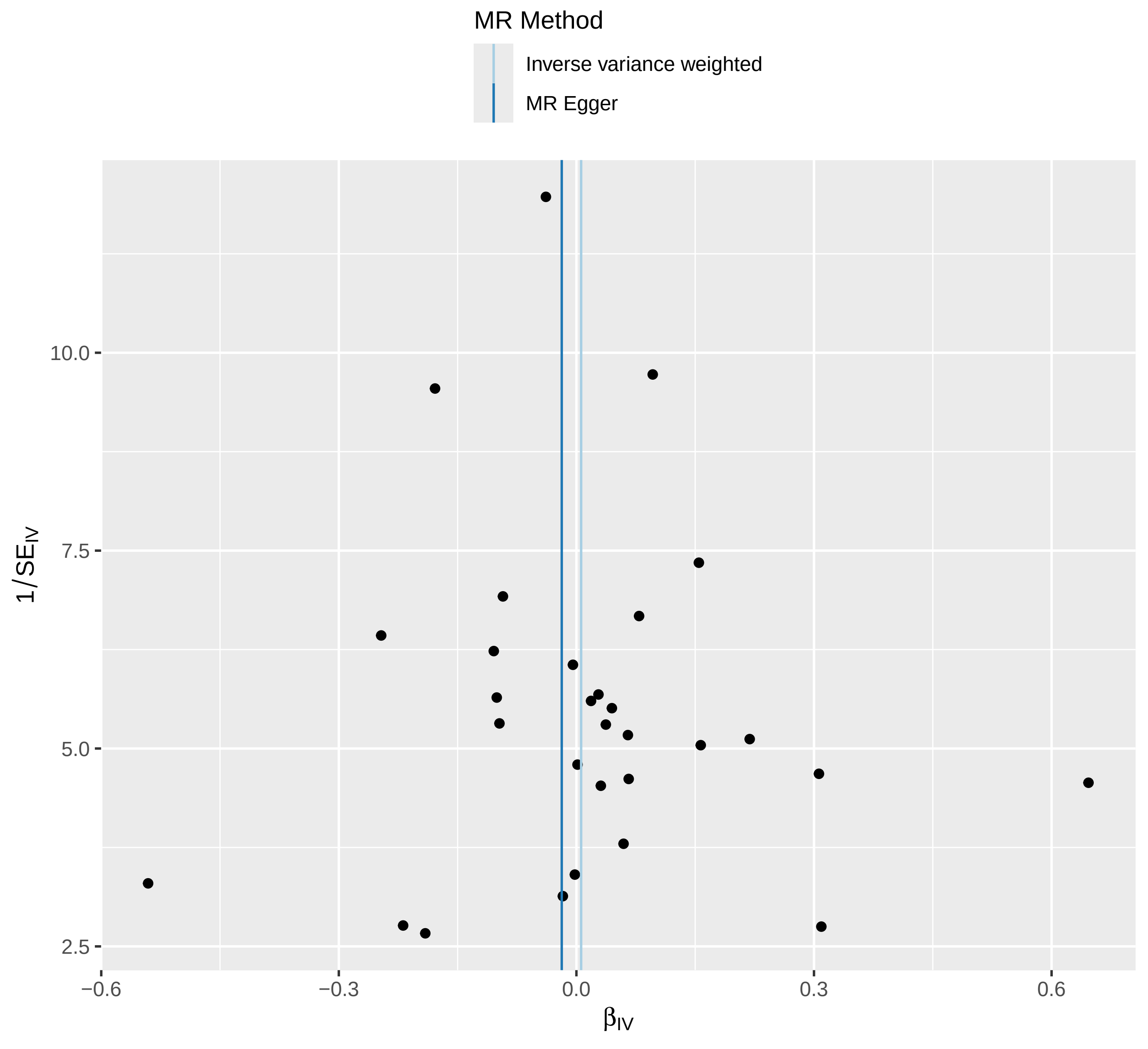
MR Method
Inverse variance weighted
MR Egger
10.0
7.5
5.0
2.5
$1/SE_{IV}$
−0.6
−0.3
0.0
0.3
0.6
β_{IV}

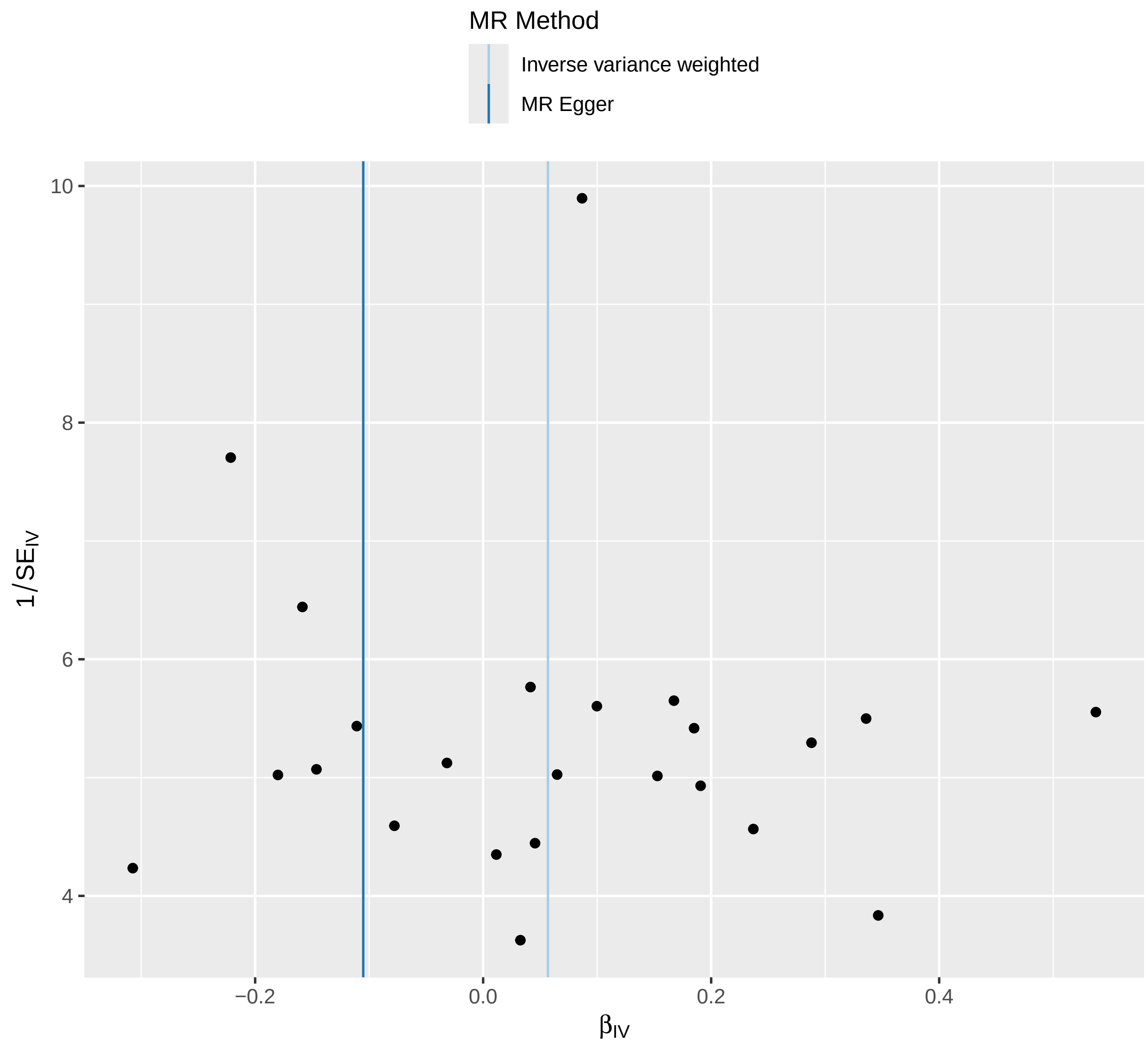
MR Method
Inverse variance weighted
MR Egger
10
8
6
4
$1/SE_{IV}$
−0.2
0.0
0.2
0.4
β_{IV}

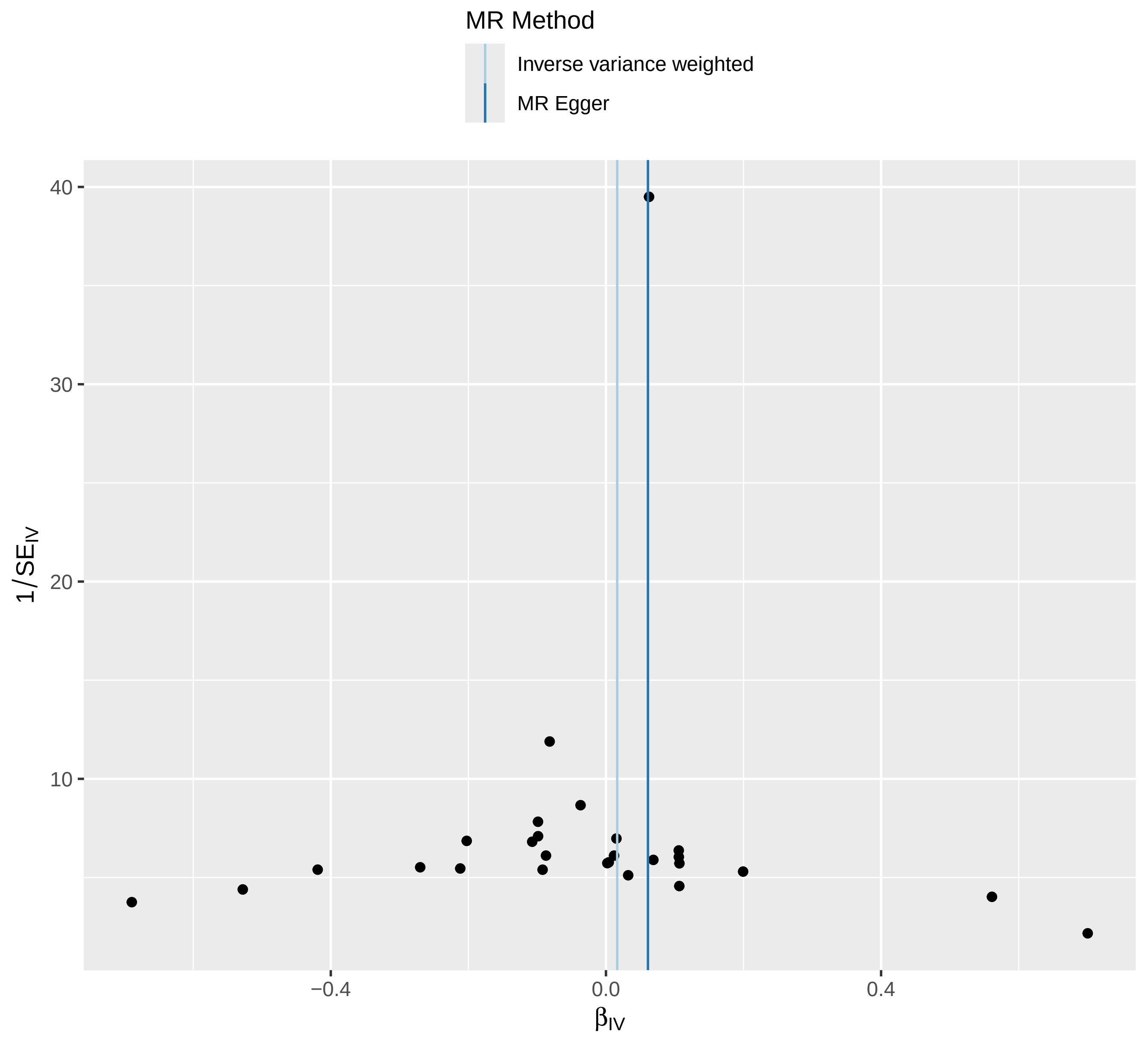

MR Method
Inverse variance weighted
MR Egger
40
30
20
10
1/SE$_{IV}$
−0.4
0.0
0.4
β$_{IV}$

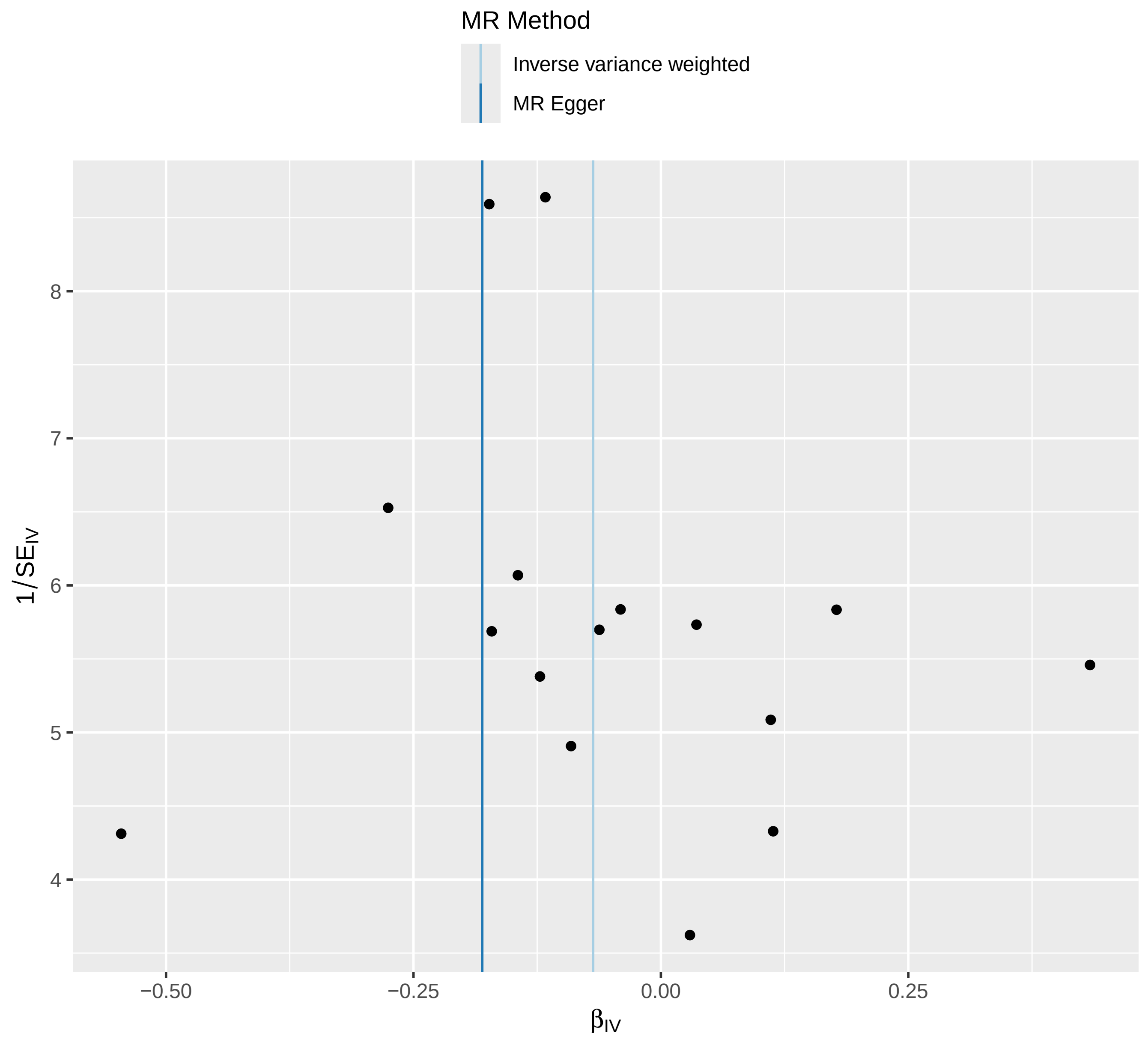

MR Method
Inverse variance weighted
MR Egger
8
7
6
5
4
1/SE_IV
−0.50
−0.25
0.00
0.25
β_IV

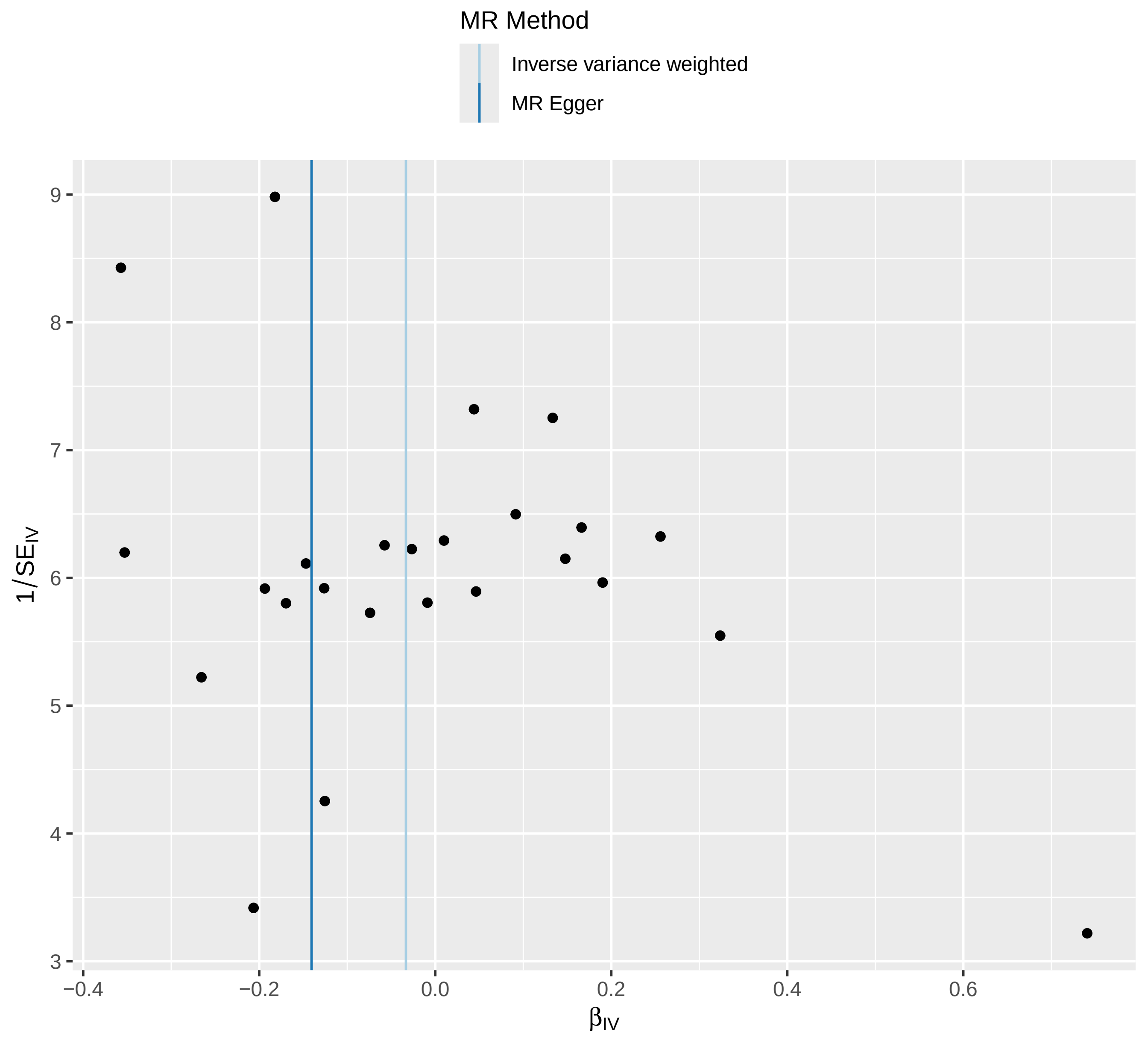

MR Method
Inverse variance weighted
MR Egger
1/SE_IV
β_IV
9
8
7
6
5
4
3
−0.4
−0.2
0.0
0.2
0.4
0.6

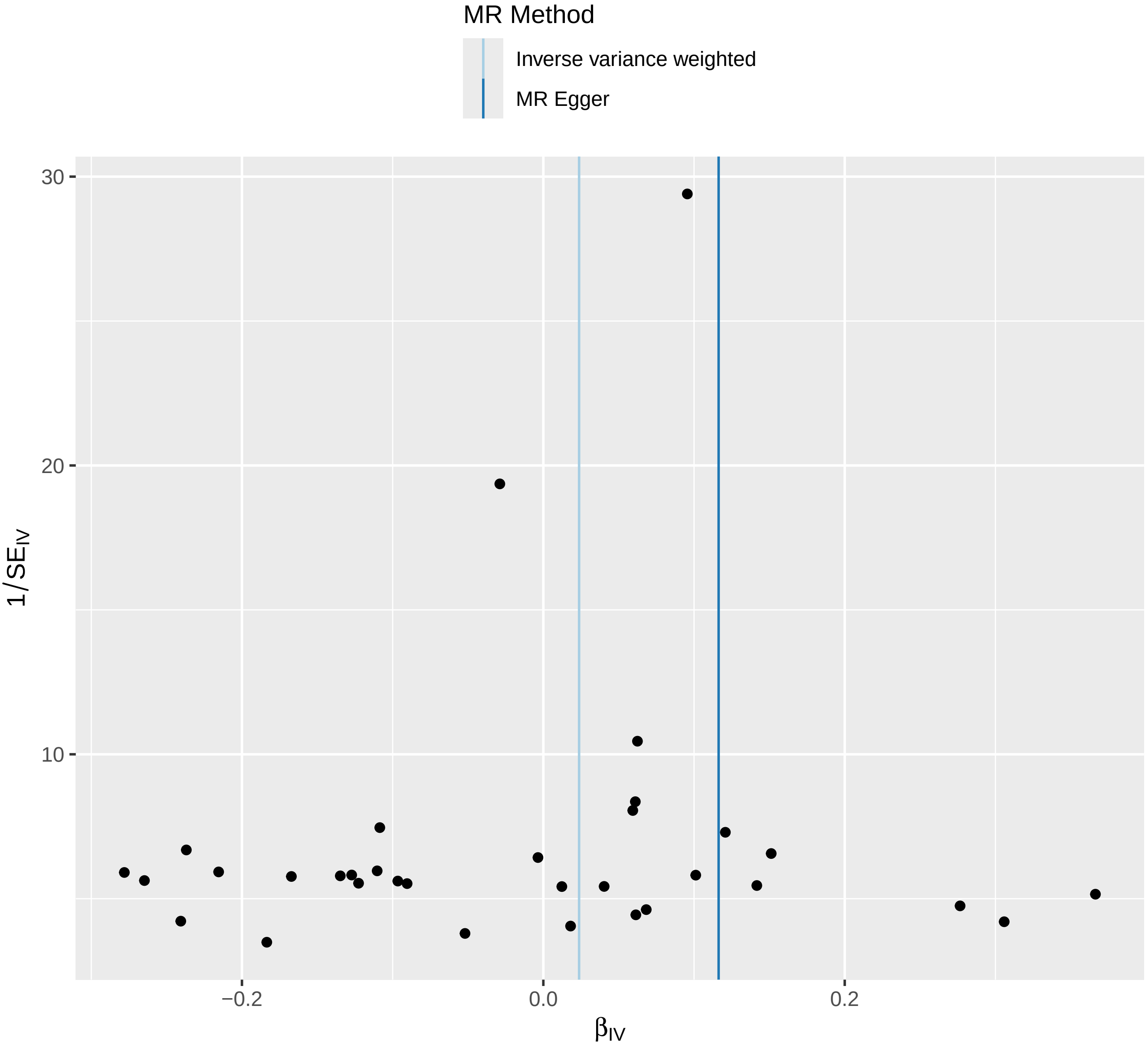
MR Method
Inverse variance weighted
MR Egger
30
20
10
1/SE_IV
−0.2
0.0
0.2
β_IV

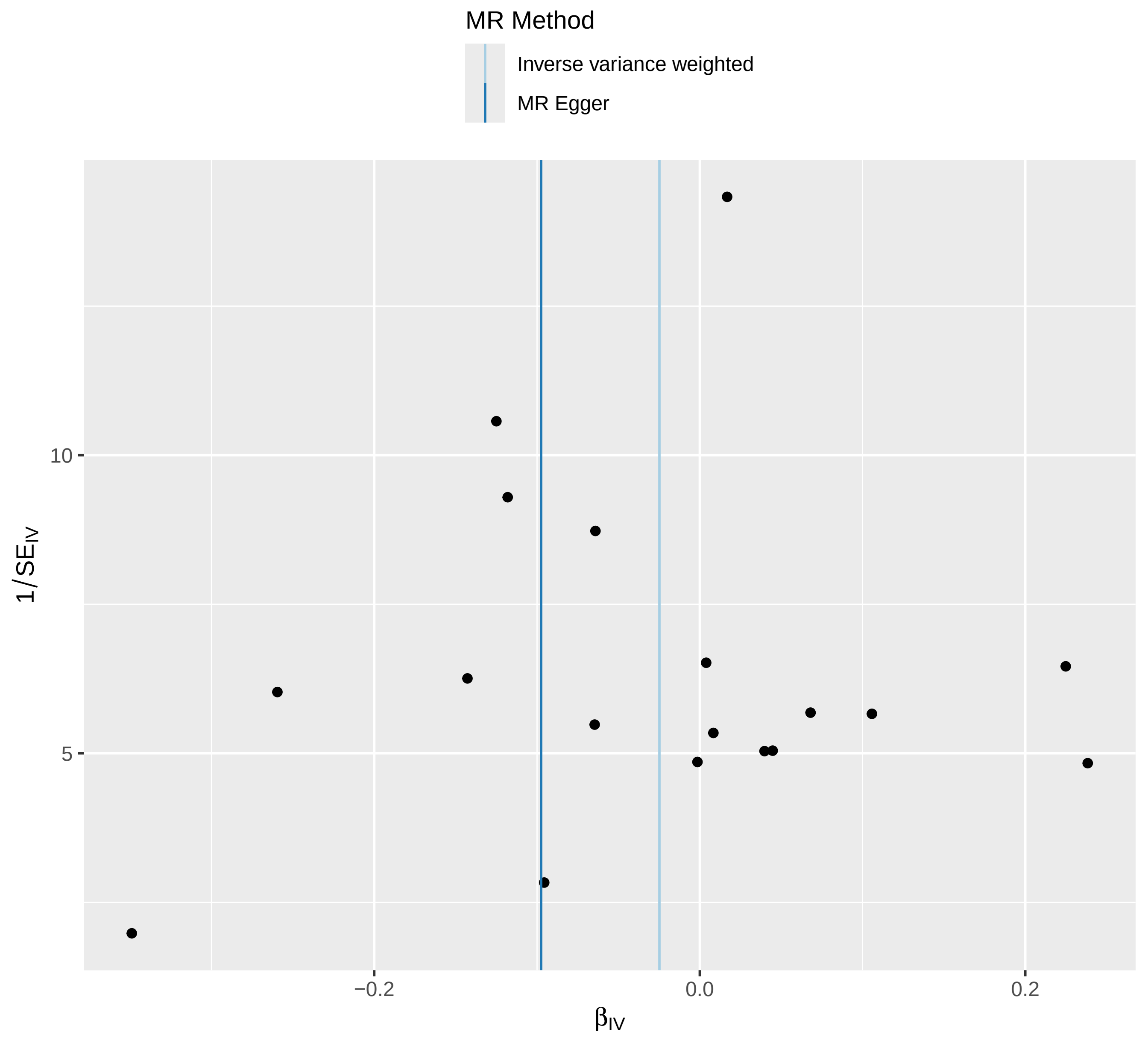
MR Method
Inverse variance weighted
MR Egger
10
5
1/SE_IV
−0.2
0.0
0.2
β_IV

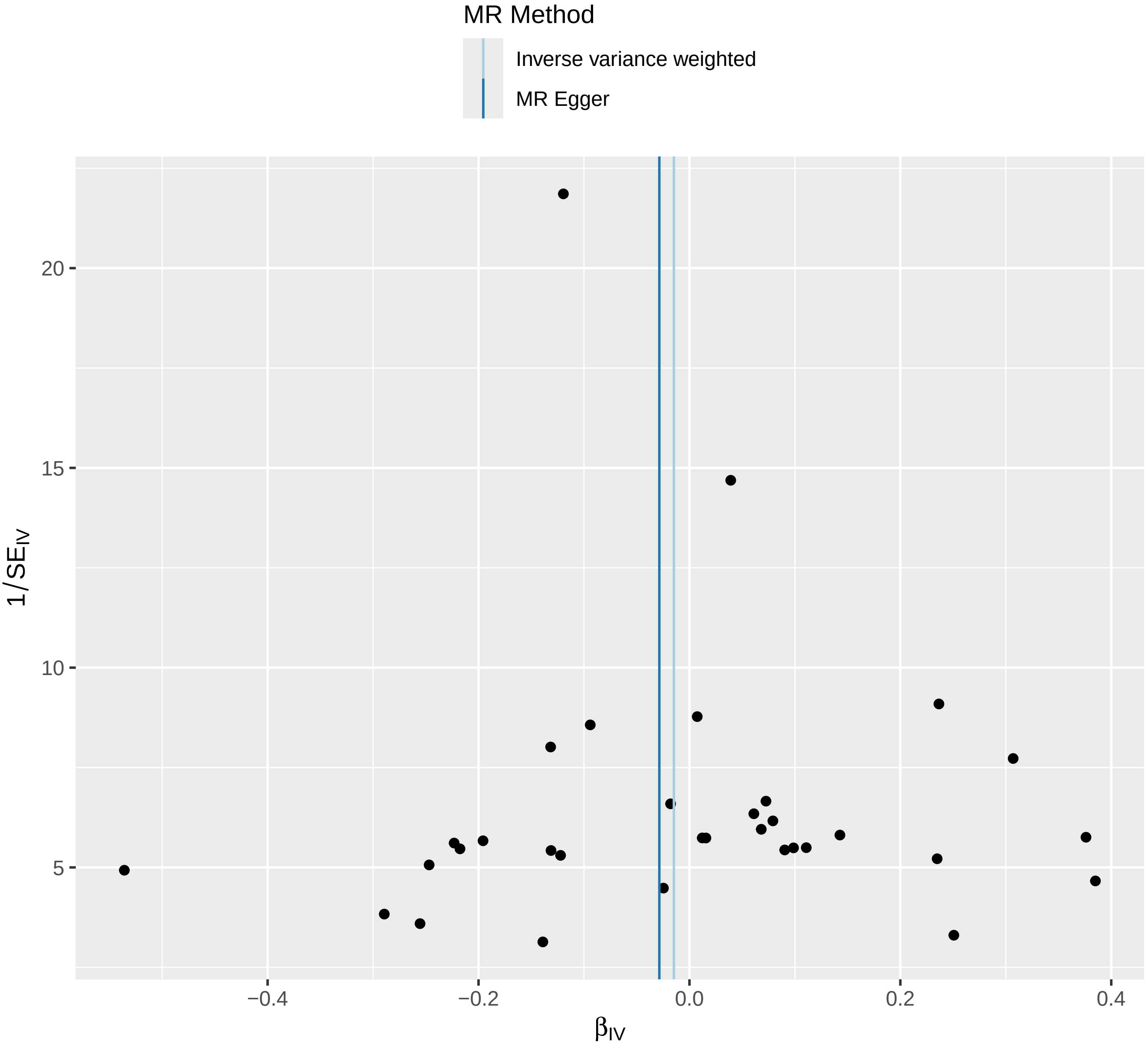
MR Method
Inverse variance weighted
MR Egger
1/SE_IV
20
15
10
5
−0.4
−0.2
0.0
0.2
0.4
β_IV

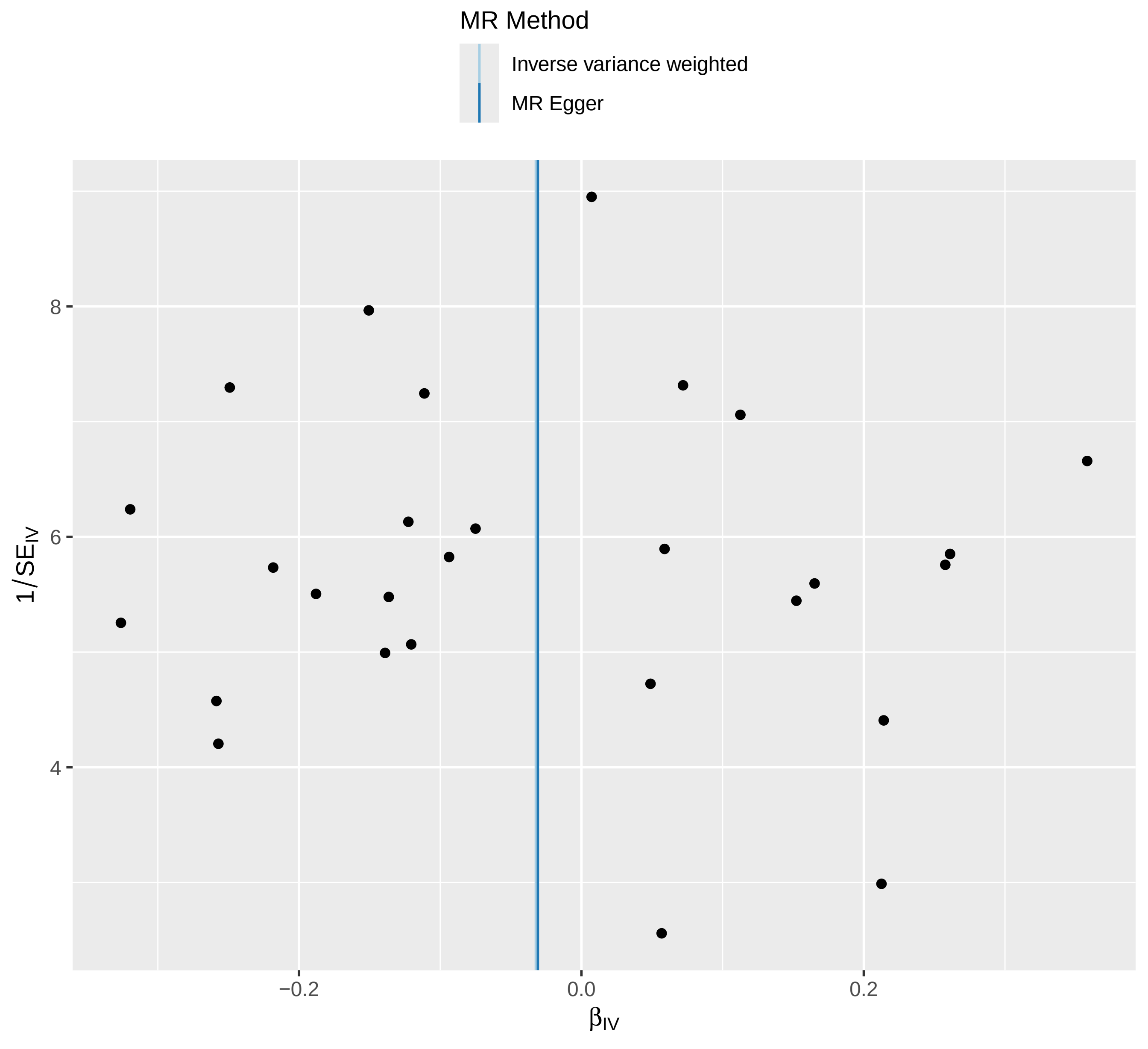

MR Method
Inverse variance weighted
MR Egger
8
6
4
1/SE$_{IV}$
−0.2
0.0
0.2
β$_{IV}$

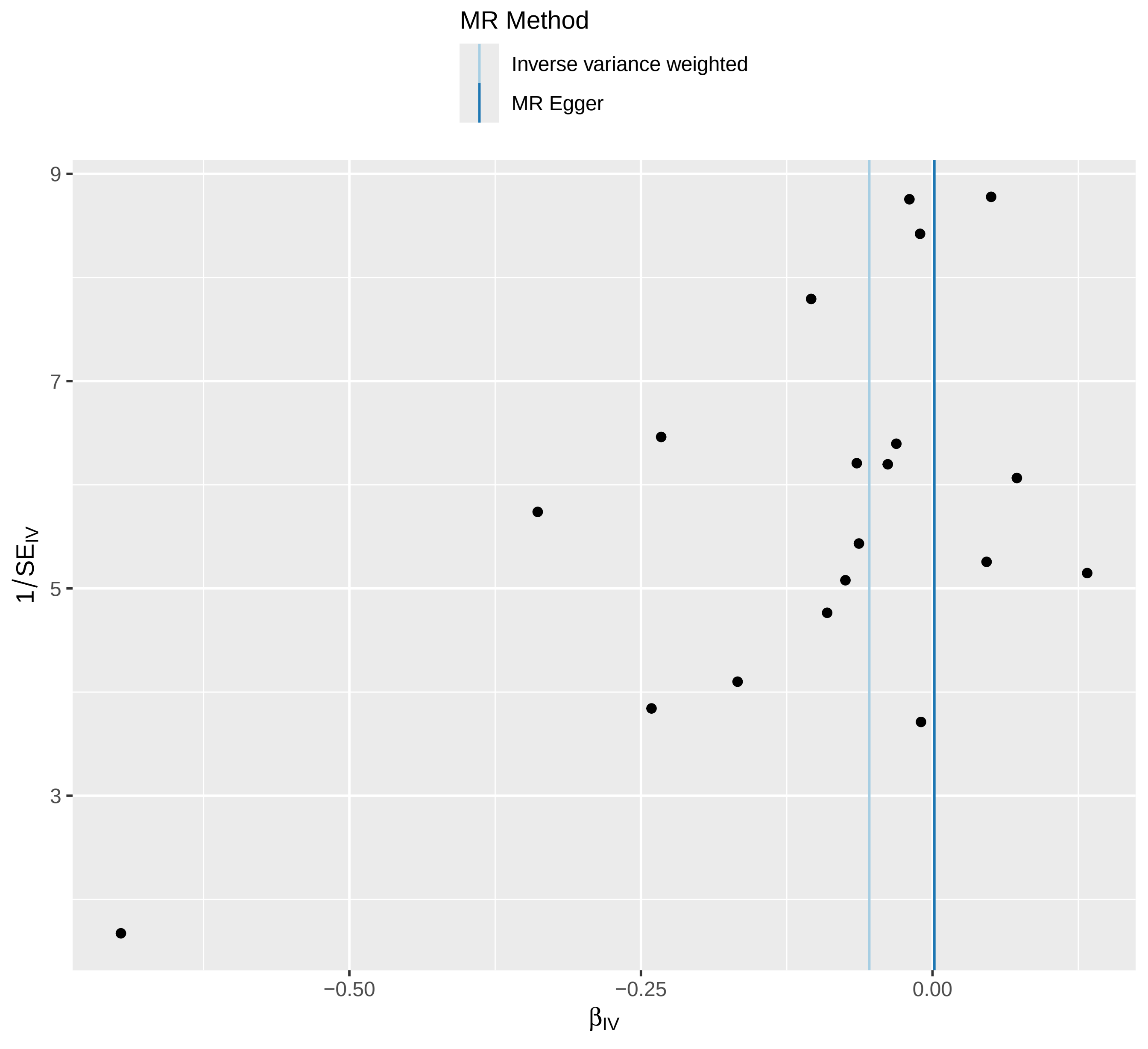
MR Method
Inverse variance weighted
MR Egger
9
7
5
3
1/SE_IV
−0.50
−0.25
0.00
β_IV

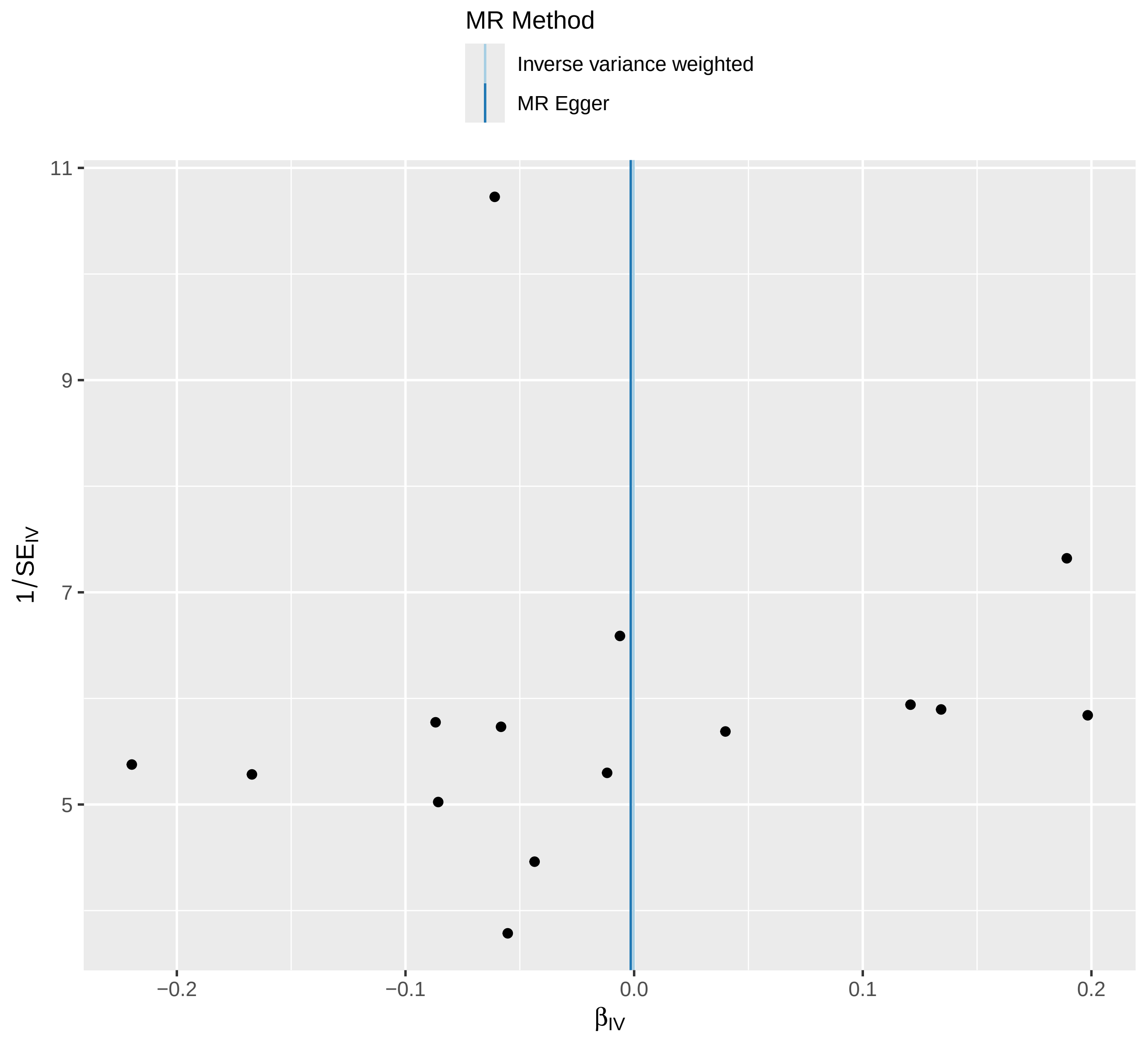
MR Method
Inverse variance weighted
MR Egger
11
9
7
5
1/SE_IV
−0.2
−0.1
0.0
0.1
0.2
β_IV

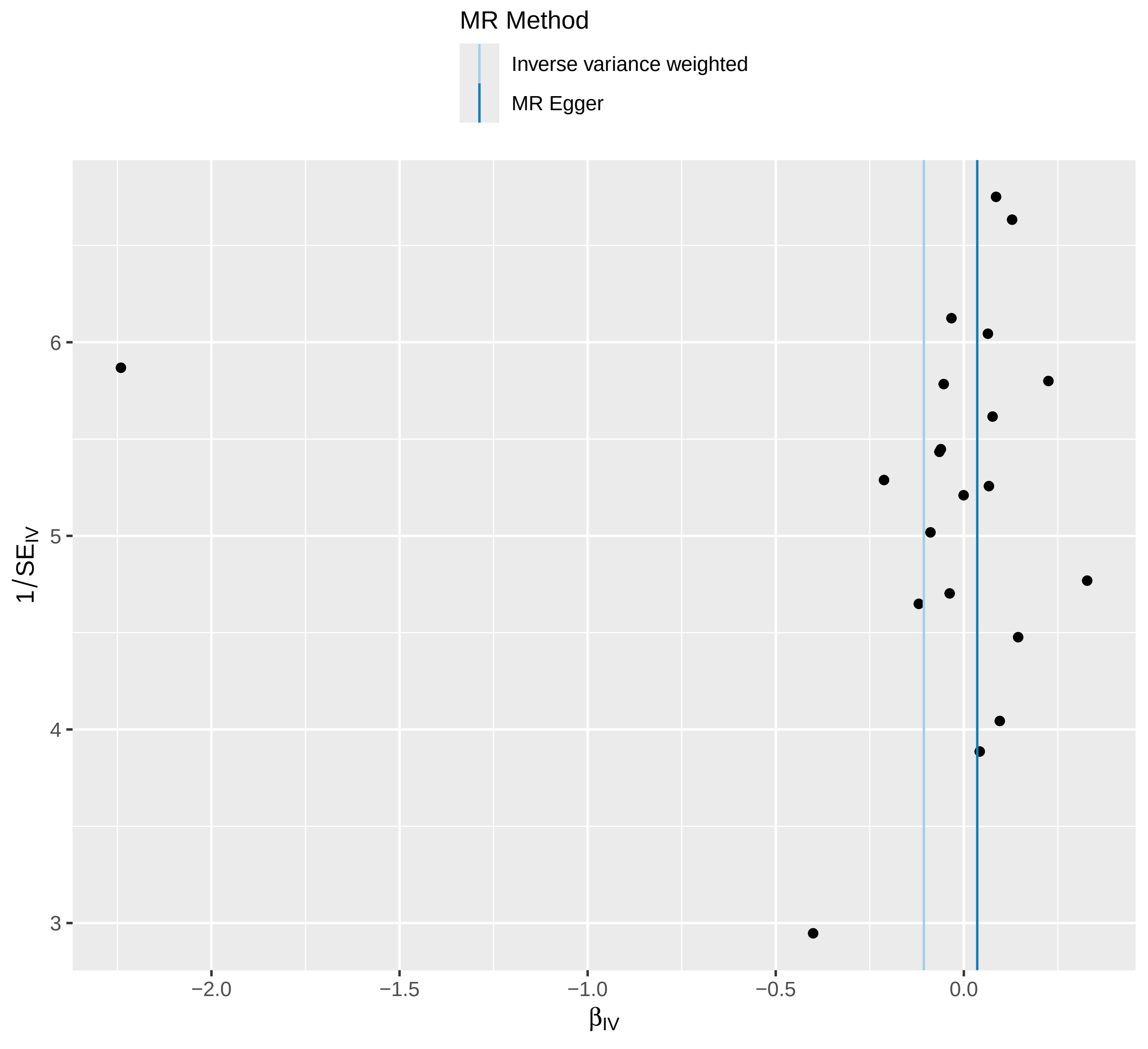

MR Method
Inverse variance weighted
MR Egger
6
5
4
3
1/SE_IV
−2.0
−1.5
−1.0
−0.5
0.0
β_IV

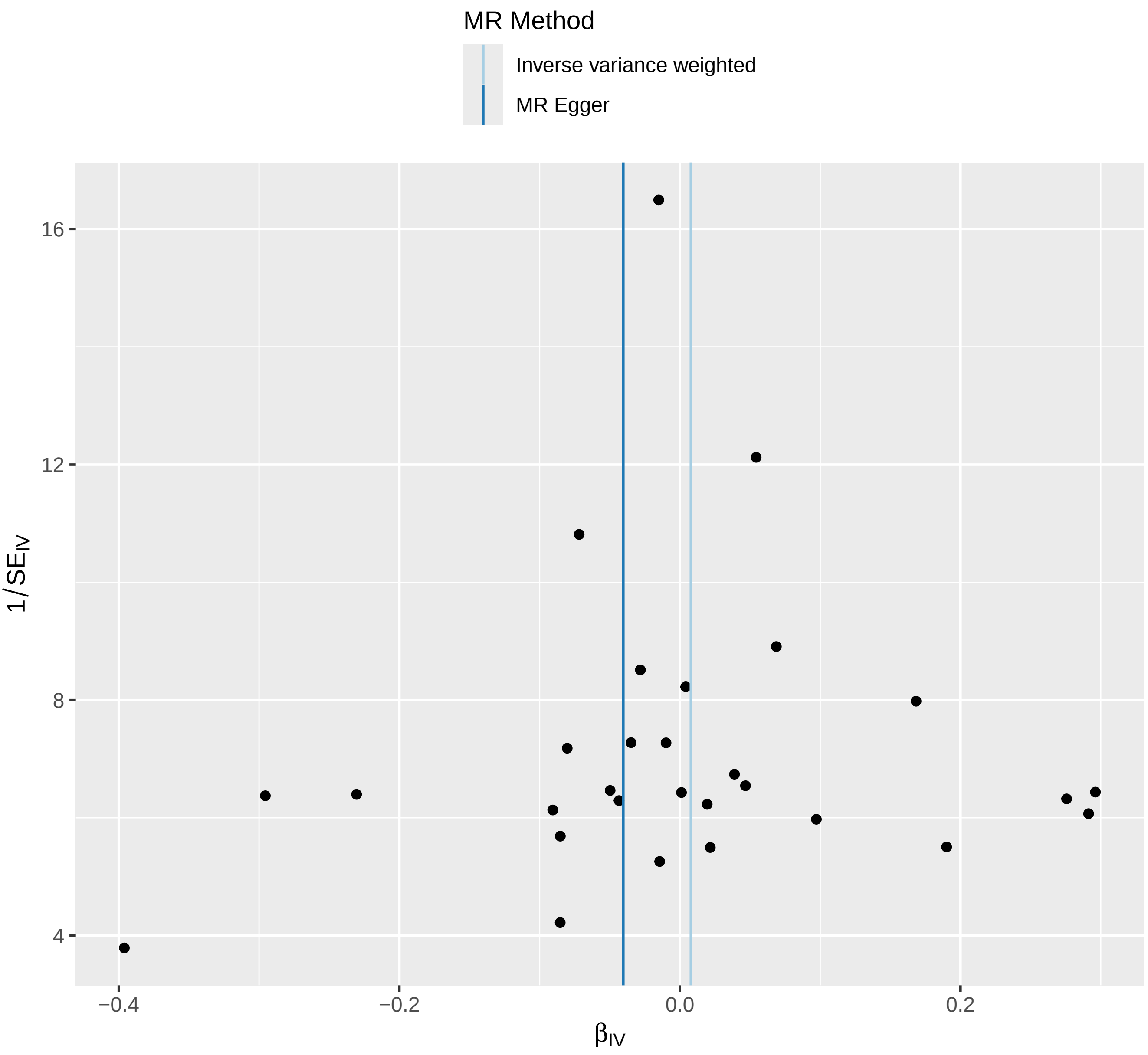
MR Method
Inverse variance weighted
MR Egger
16
12
8
4
$1/SE_{IV}$
−0.4
−0.2
0.0
0.2
β_{IV}

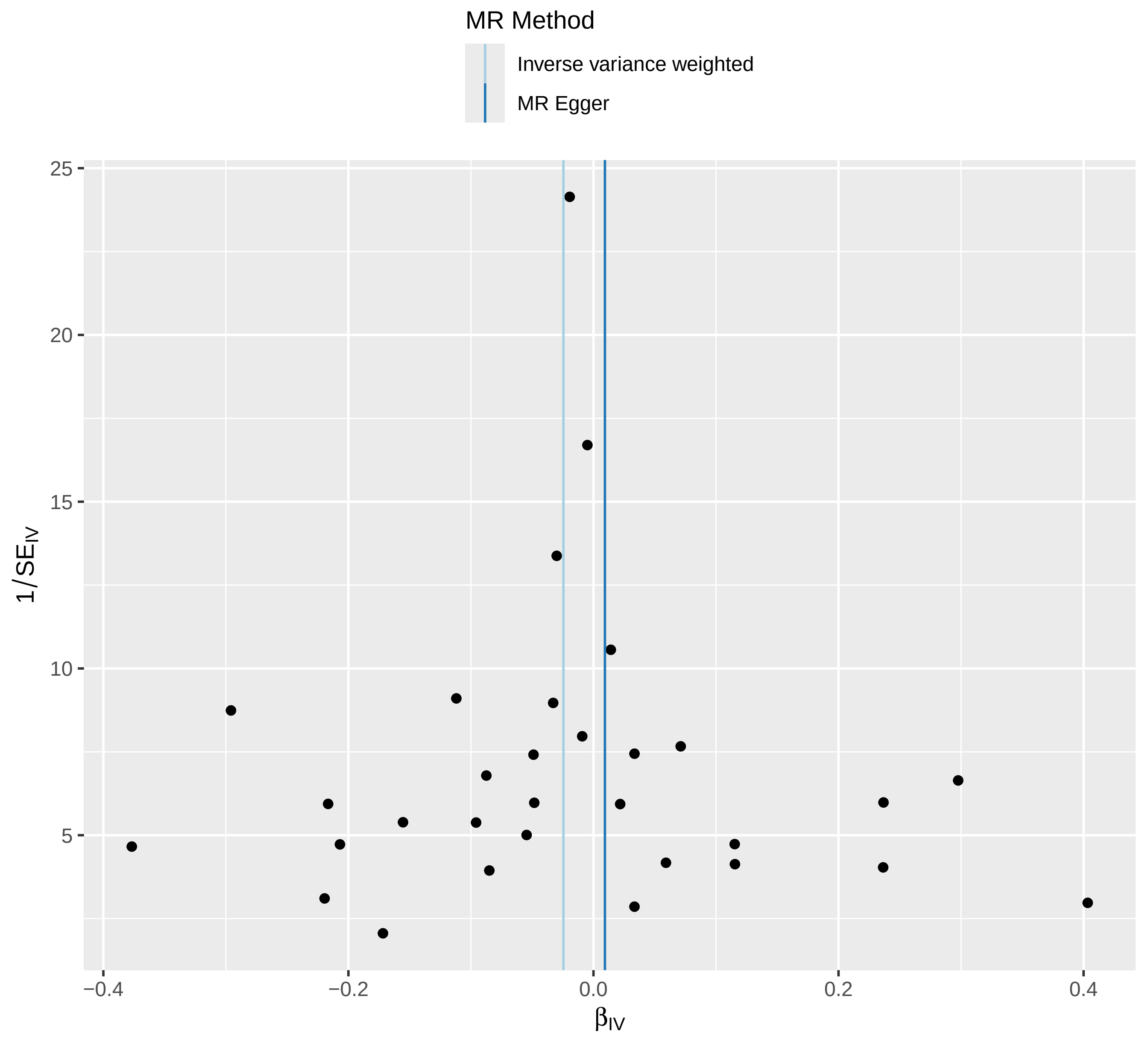
MR Method
Inverse variance weighted
MR Egger
25
20
15
10
5
1/SE_IV
−0.4
−0.2
0.0
0.2
0.4
β_IV

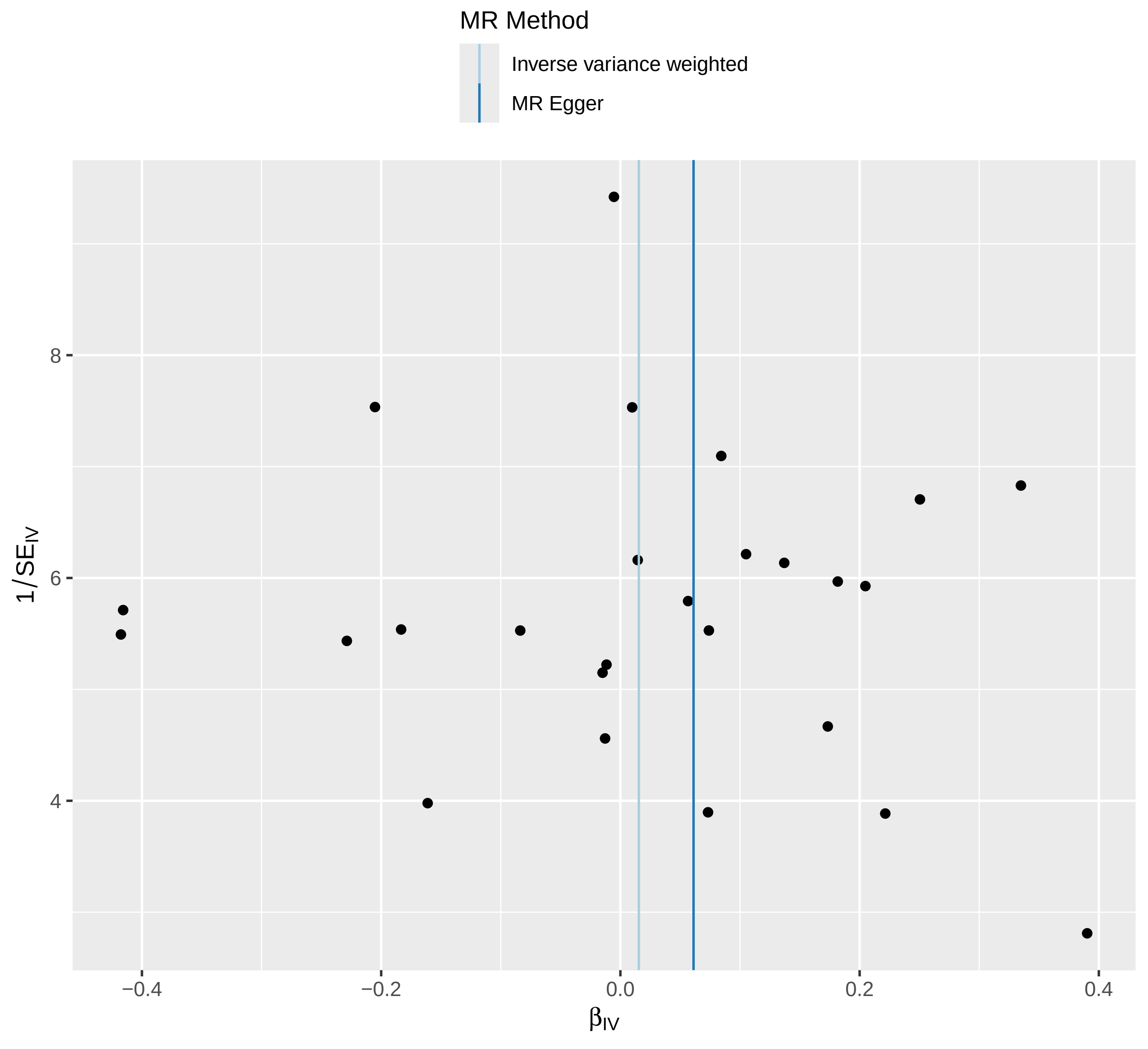

MR Method
Inverse variance weighted
MR Egger
8
6
4
1/SE_IV
−0.4
−0.2
0.0
0.2
0.4
β_IV

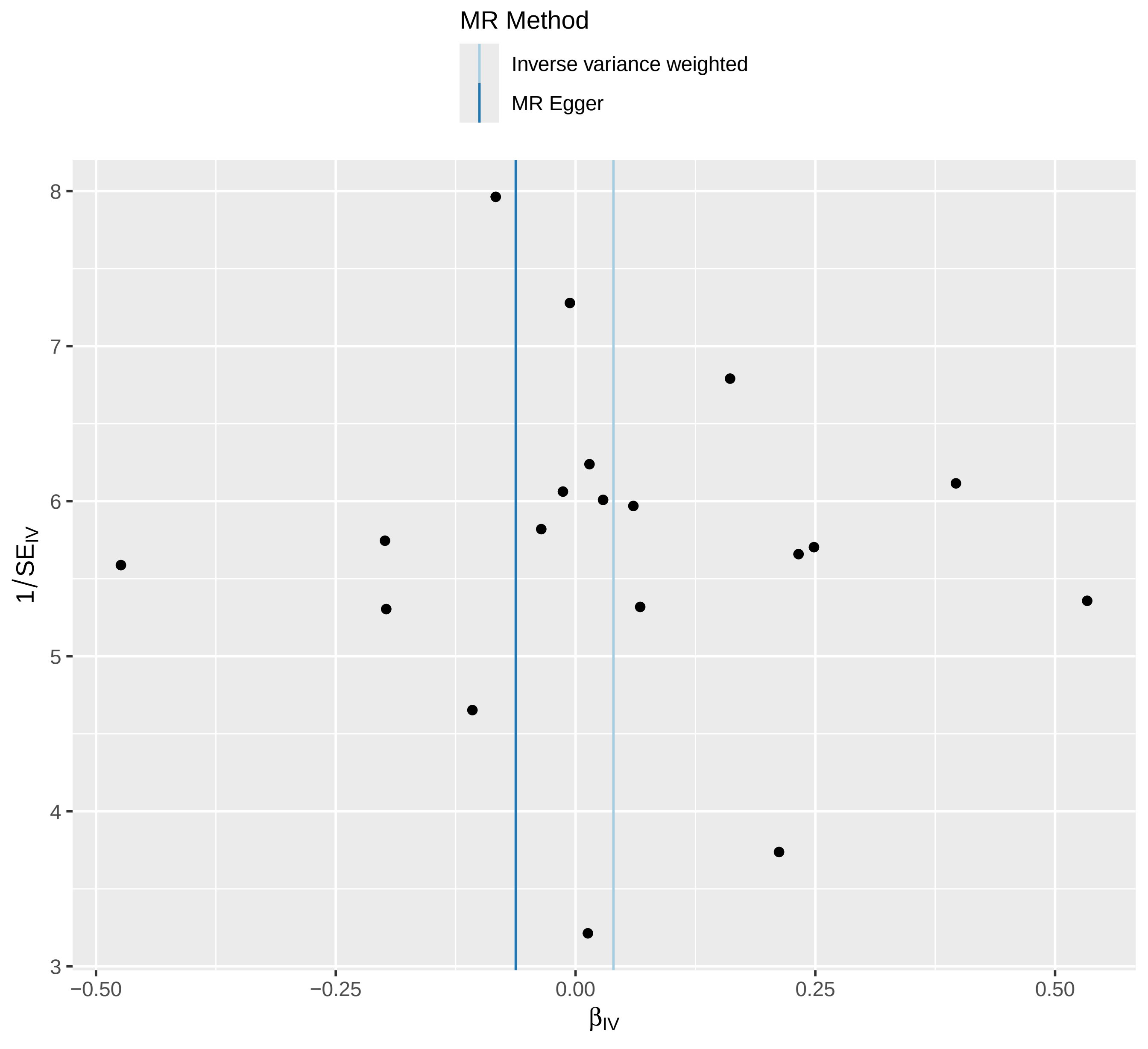
MR Method
Inverse variance weighted
MR Egger
8
7
6
5
4
3
1/SE_IV
−0.50
−0.25
0.00
0.25
0.50
β_IV

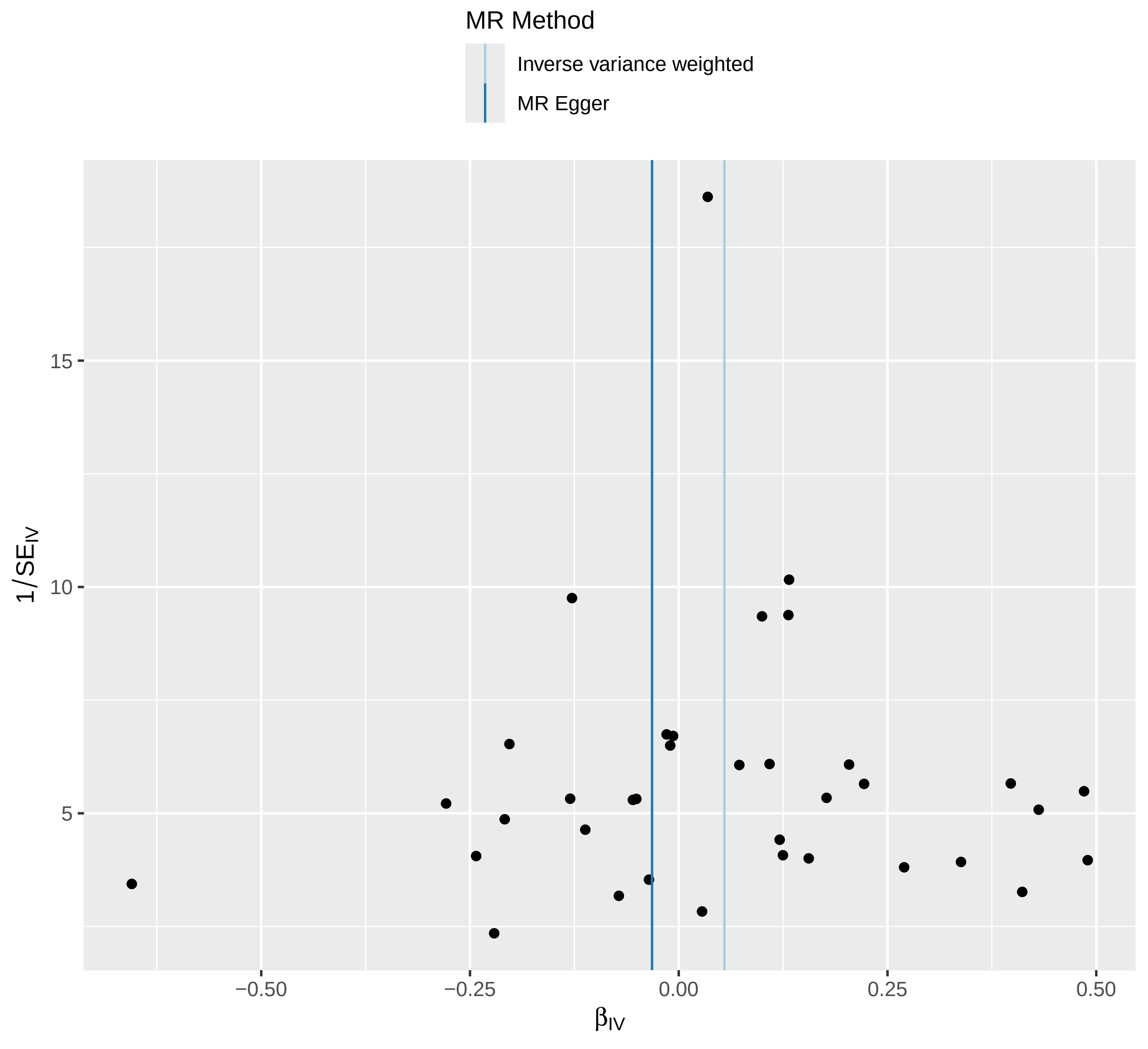
MR Method
Inverse variance weighted
MR Egger
1/SE_IV
15
10
5
−0.50
−0.25
0.00
0.25
0.50
β_IV

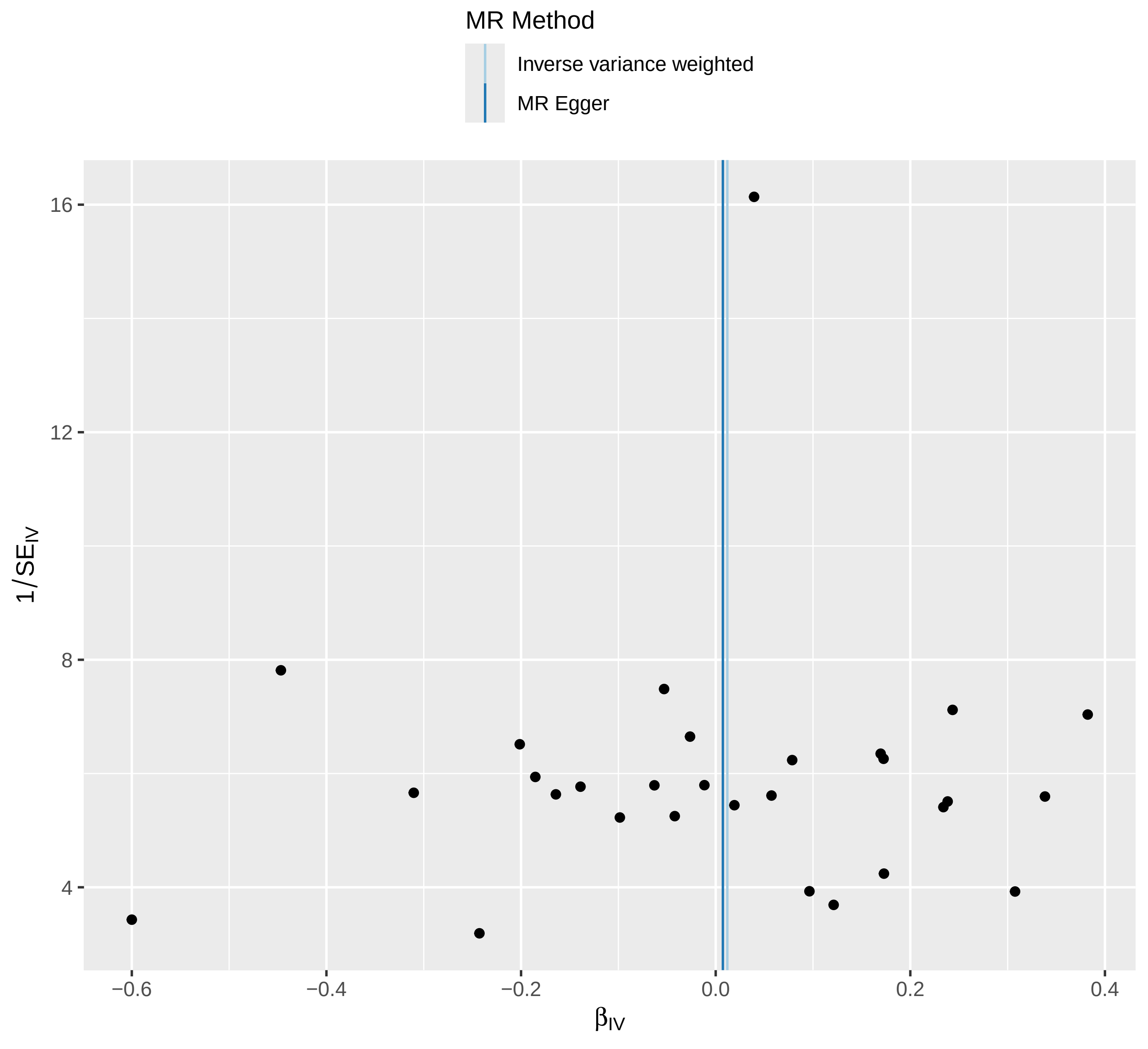
MR Method
Inverse variance weighted
MR Egger
1/SE$_{IV}$
16
12
8
4
−0.6
−0.4
−0.2
0.0
0.2
0.4
β_{IV}

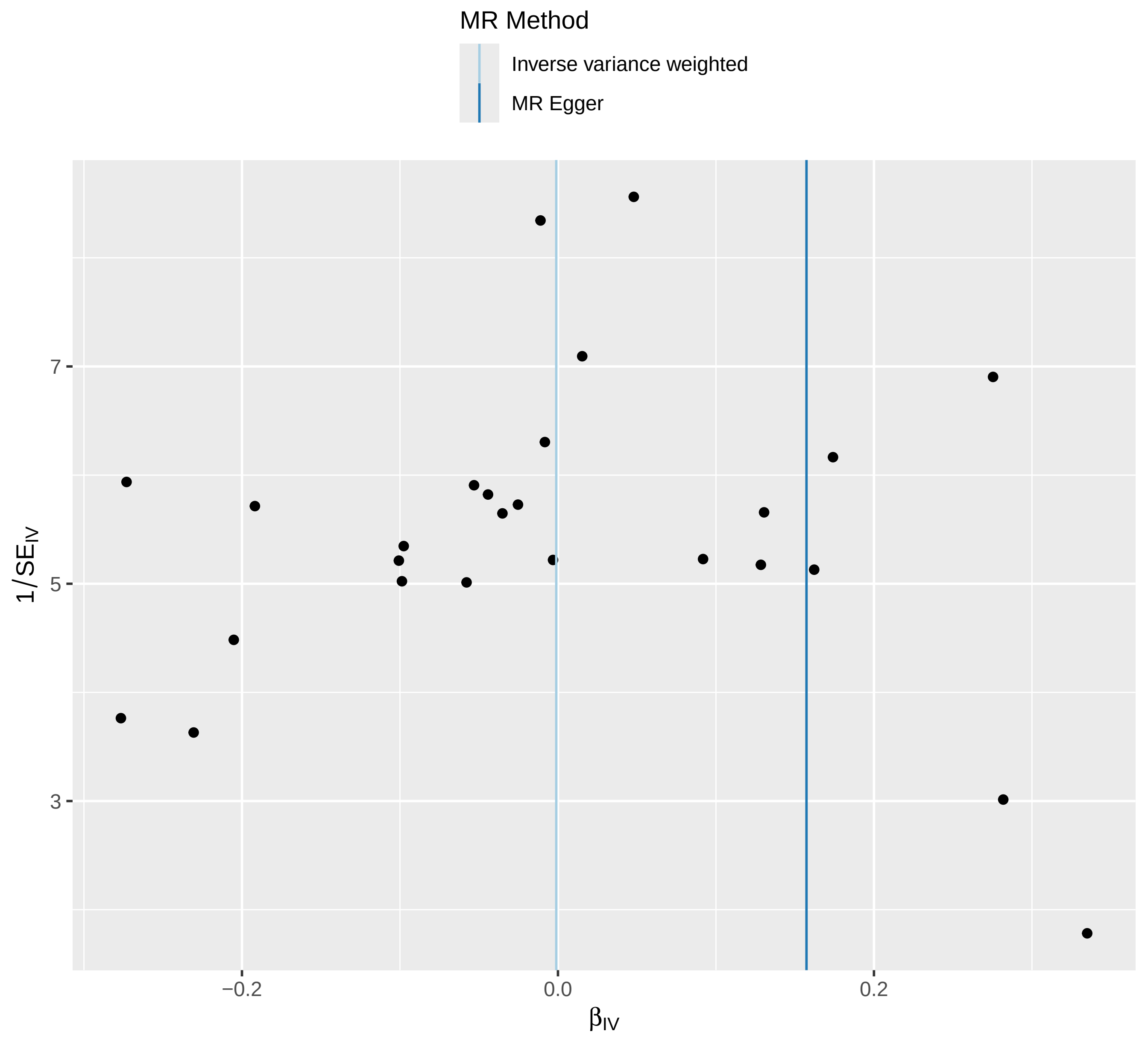
MR Method
Inverse variance weighted
MR Egger
7
5
3
1/SE$_{IV}$
−0.2
0.0
0.2
β$_{IV}$

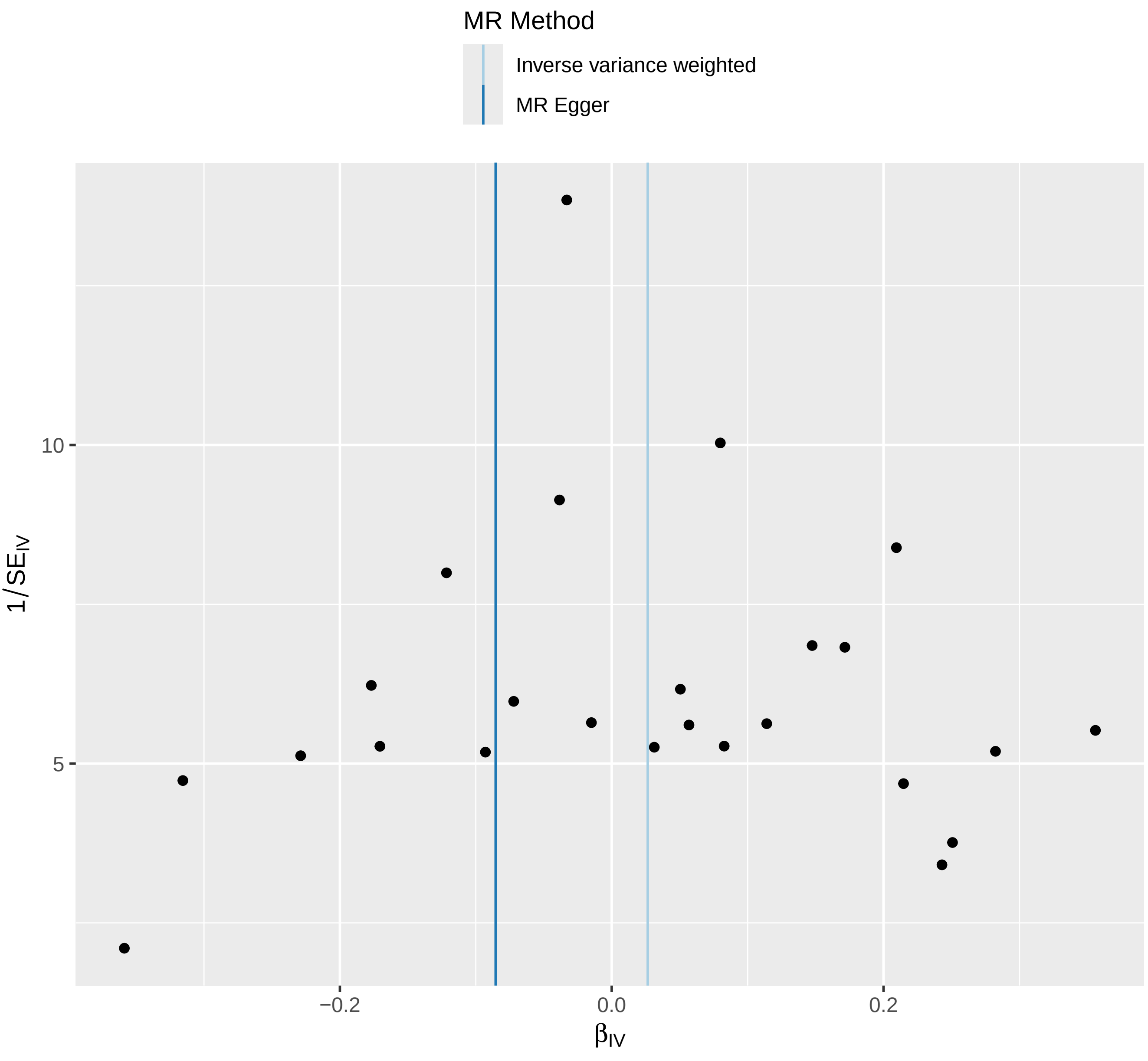

MR Method
Inverse variance weighted
MR Egger
10
5
1/SE_IV
−0.2
0.0
0.2
β_IV

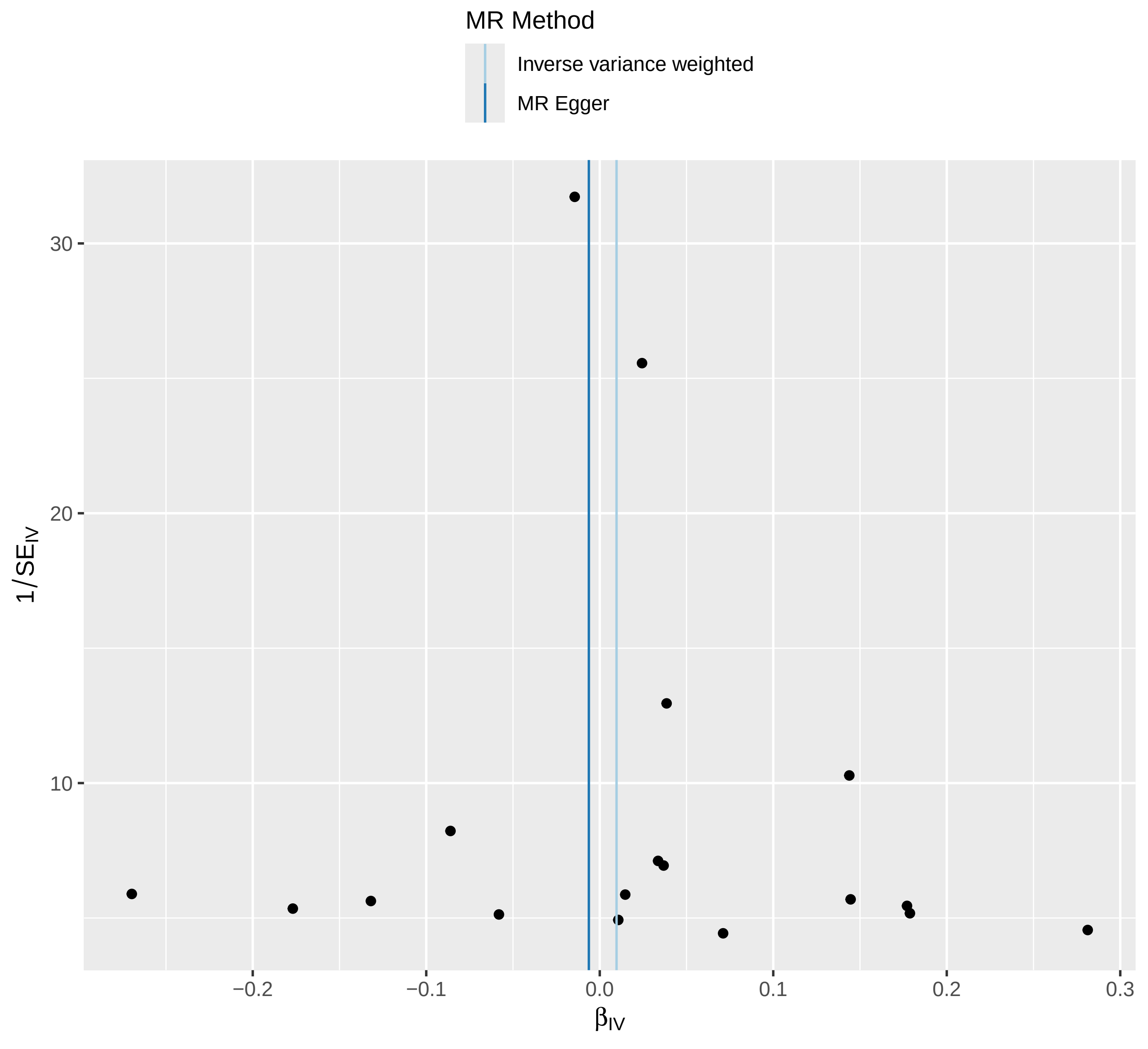
MR Method
Inverse variance weighted
MR Egger
30
20
10
1/SE_IV
−0.2
−0.1
0.0
0.1
0.2
0.3
β_IV

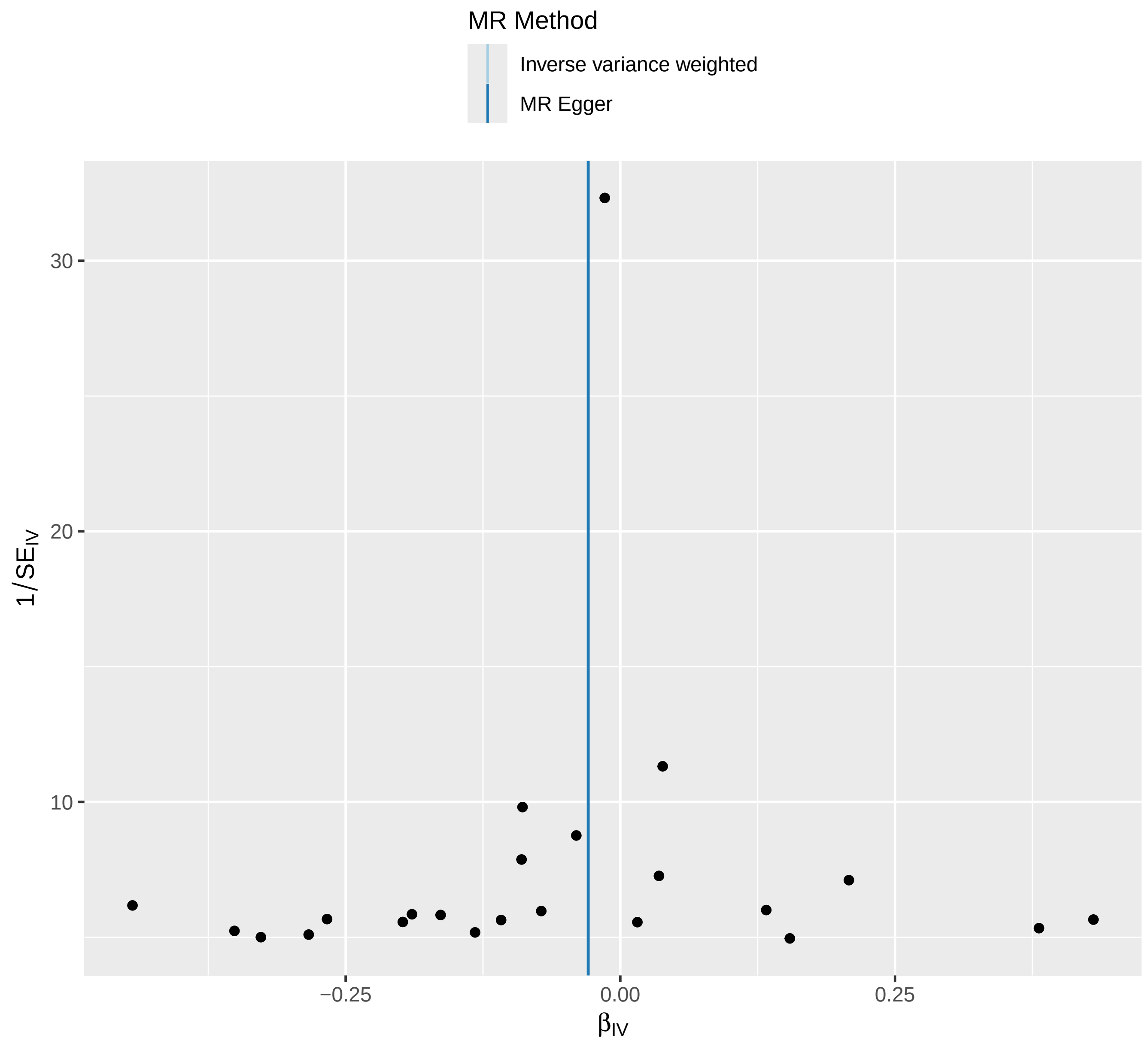

MR Method
Inverse variance weighted
MR Egger
30
20
10
1/SE$_{IV}$
−0.25
0.00
0.25
β$_{IV}$

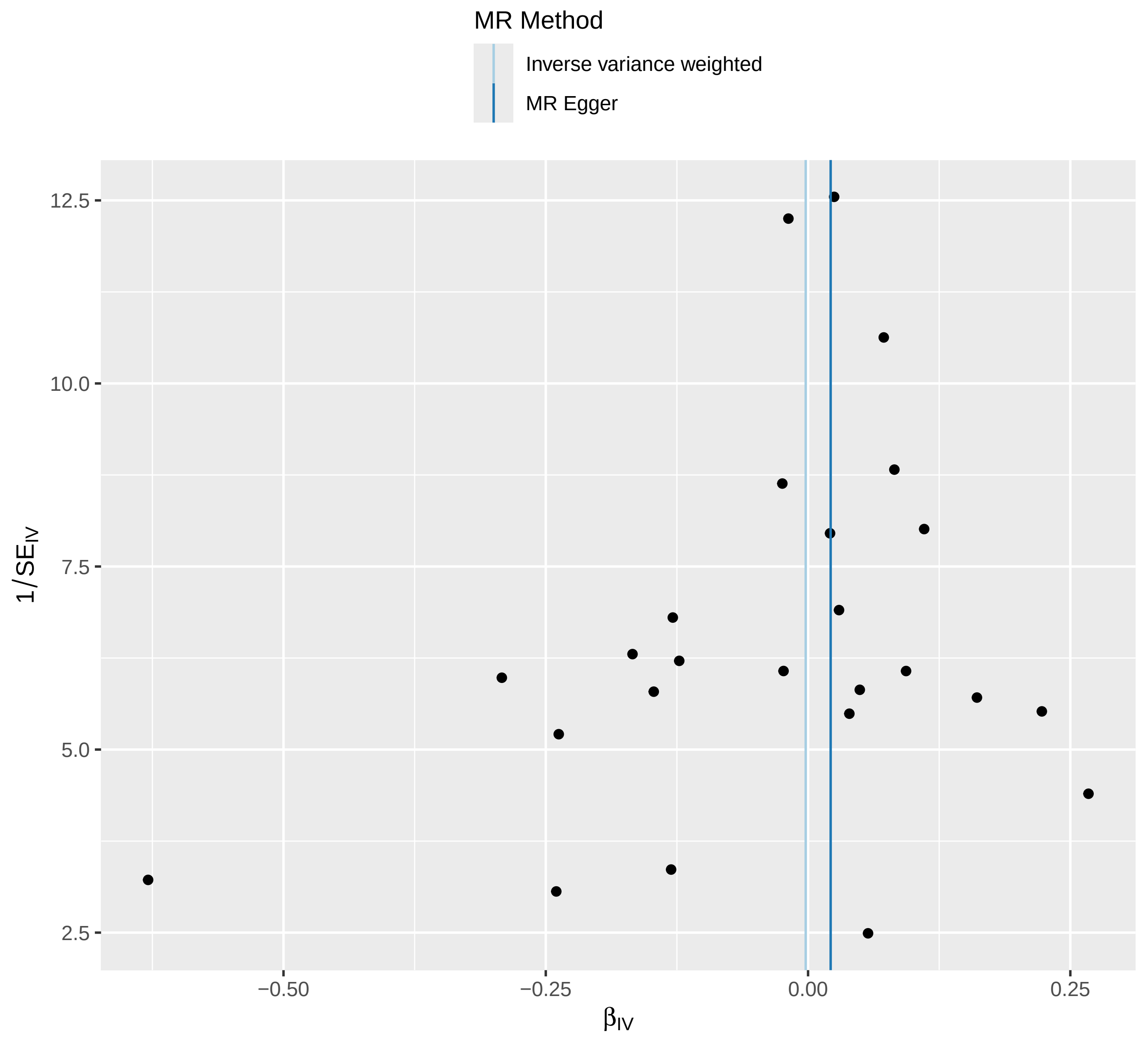
MR Method
Inverse variance weighted
MR Egger
12.5
10.0
7.5
5.0
2.5
1/SE_IV
−0.50
−0.25
0.00
0.25
β_IV

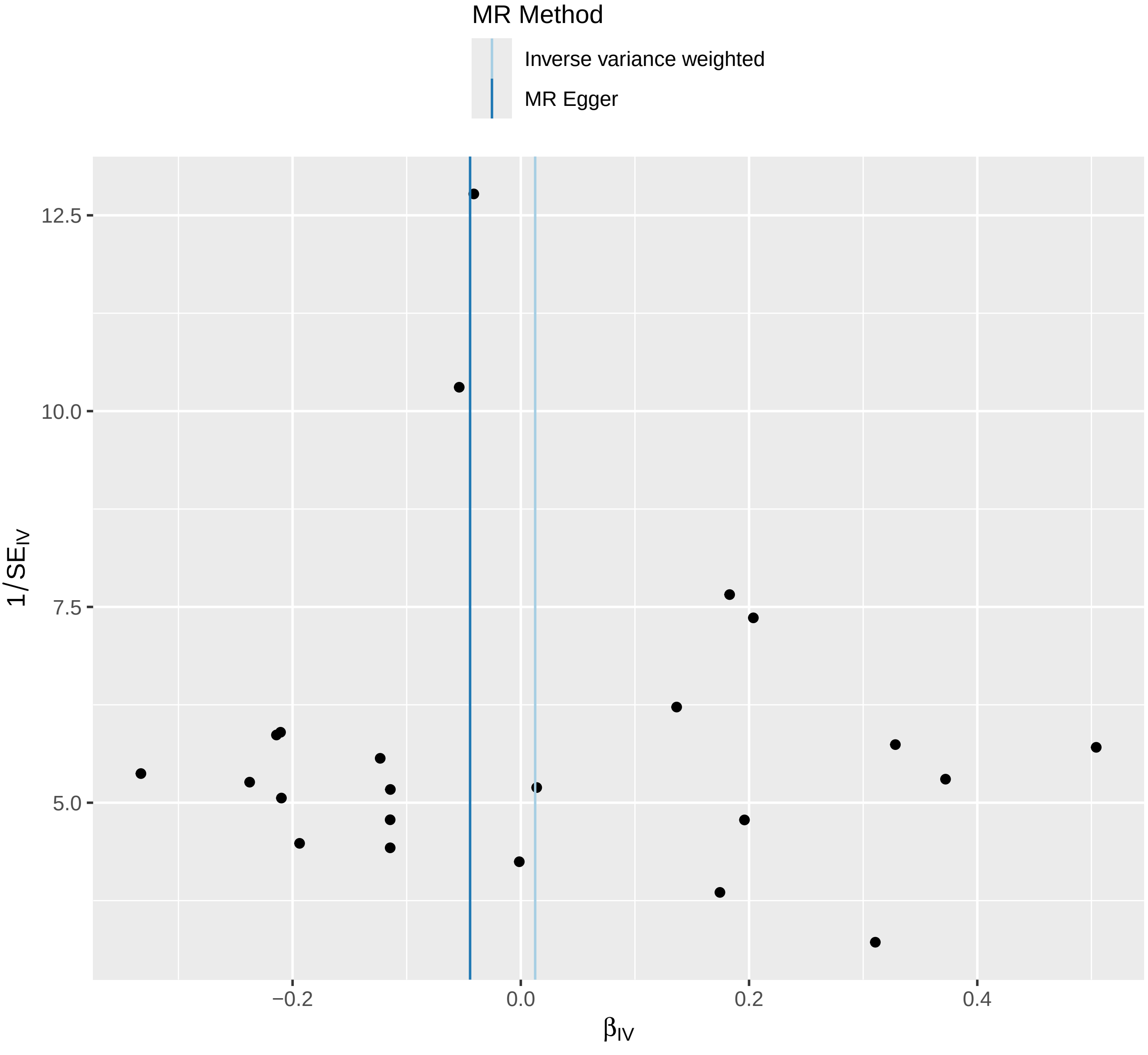
MR Method
Inverse variance weighted
MR Egger
12.5
10.0
7.5
5.0
$1/SE_{IV}$
−0.2
0.0
0.2
0.4
β_{IV}

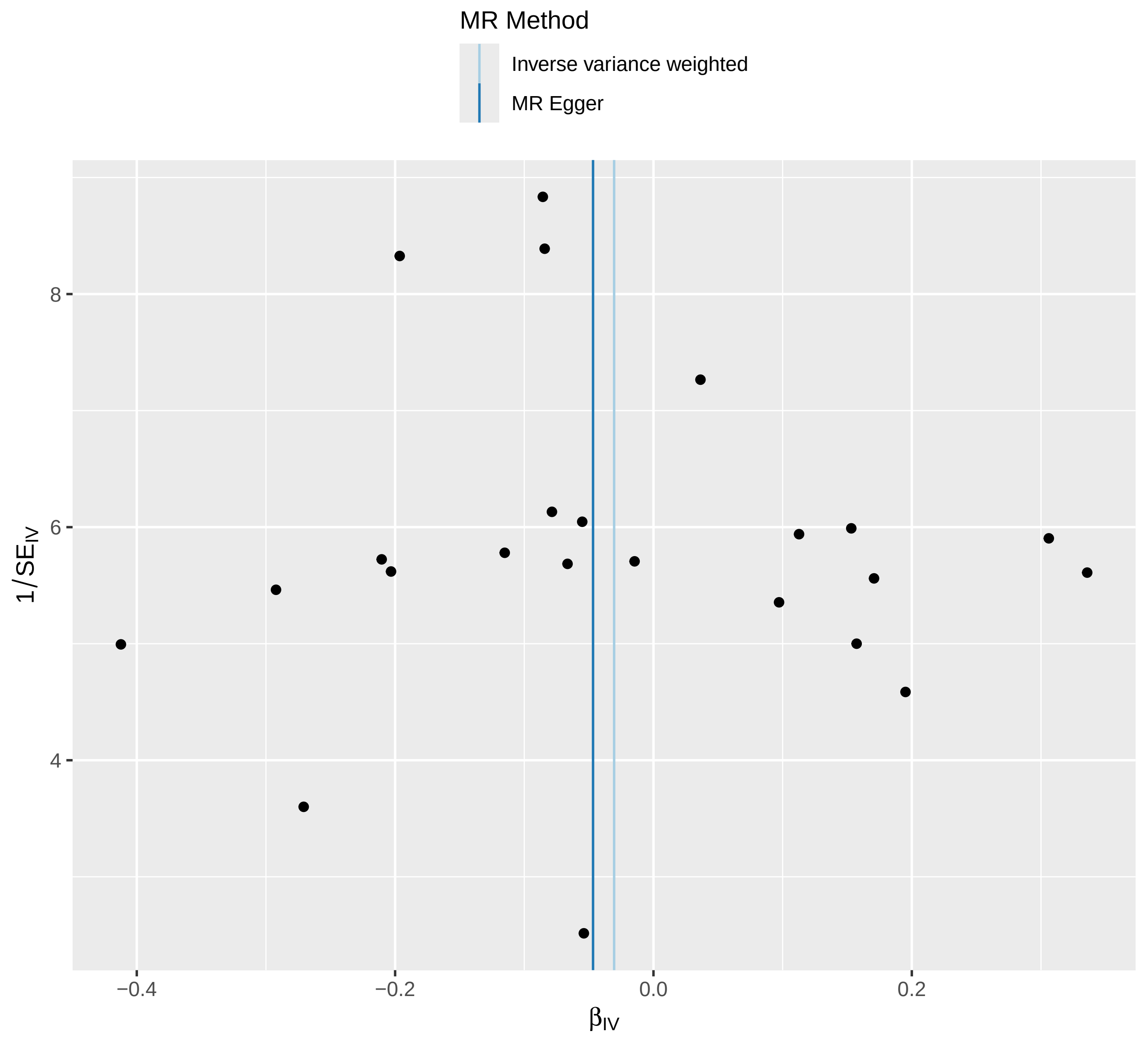
MR Method
Inverse variance weighted
MR Egger
8
6
4
1/SE_IV
−0.4
−0.2
0.0
0.2
β_IV

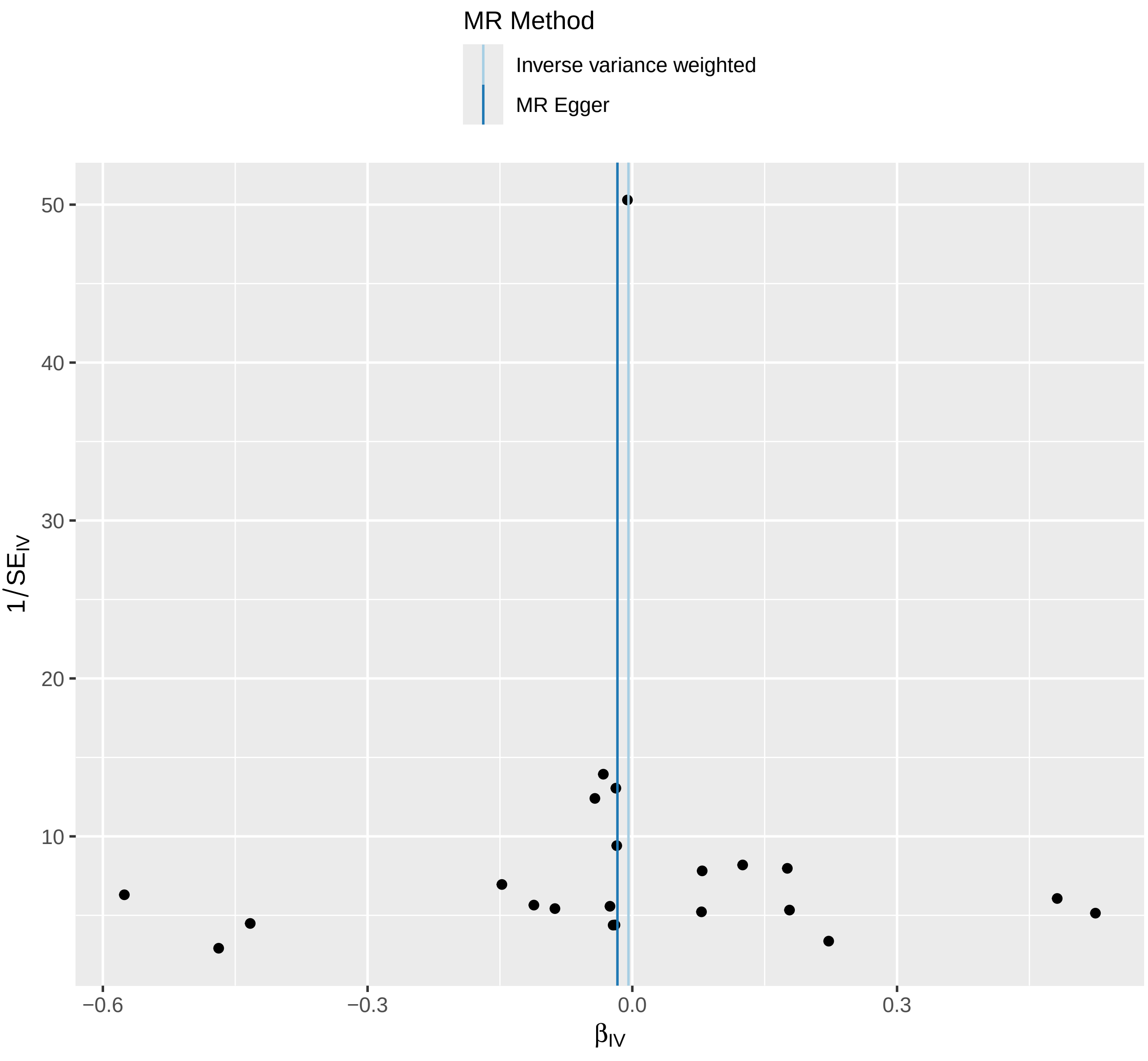

MR Method
Inverse variance weighted
MR Egger
50
40
30
20
10
1/SE_{IV}
−0.6
−0.3
0.0
0.3
β_{IV}

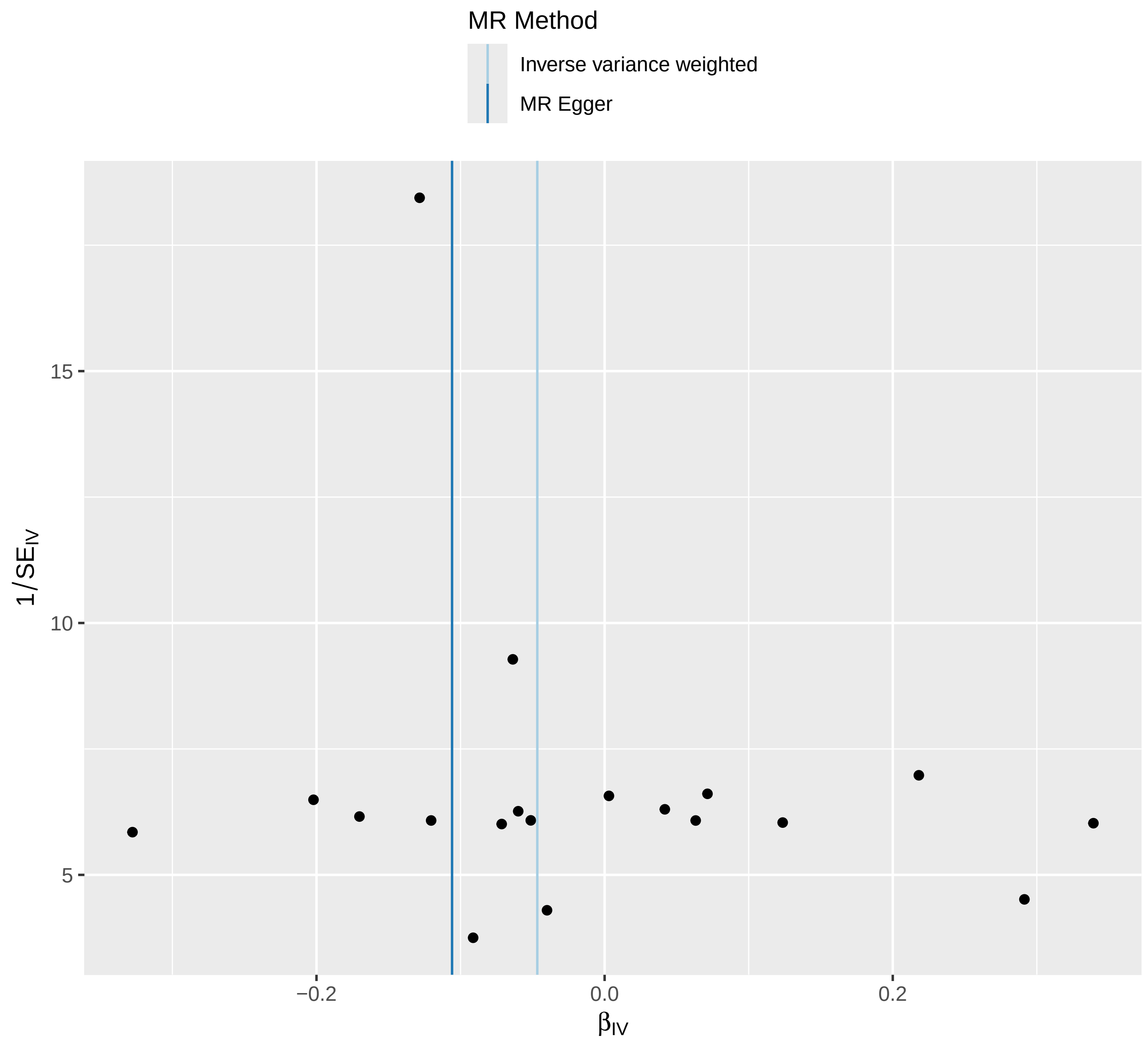
MR Method
Inverse variance weighted
MR Egger
1/SE_IV
15
10
5
−0.2
0.0
0.2
β_IV

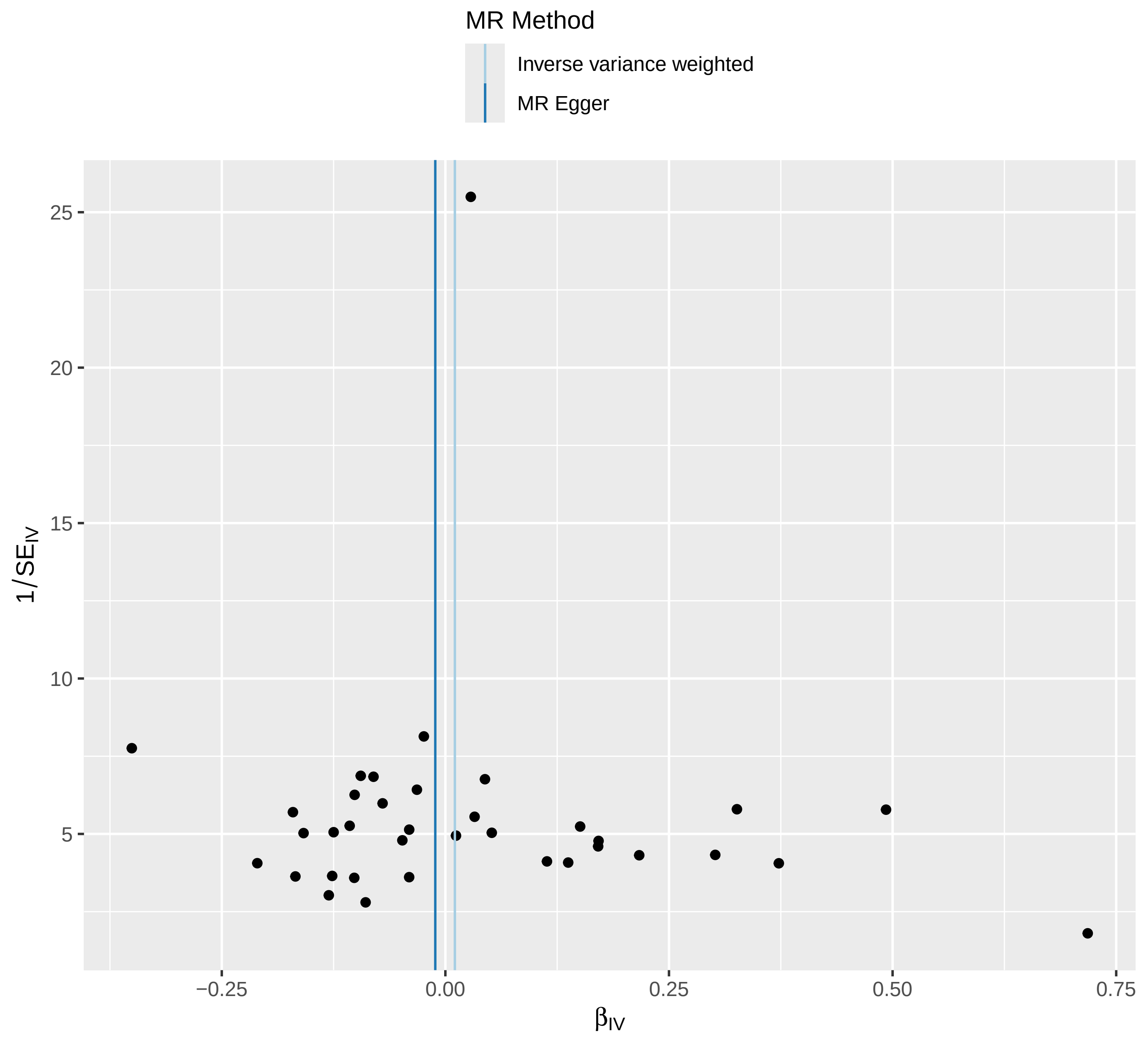

MR Method
Inverse variance weighted
MR Egger
25
20
15
10
5
$1/SE_{IV}$
−0.25
0.00
0.25
0.50
0.75
β_{IV}

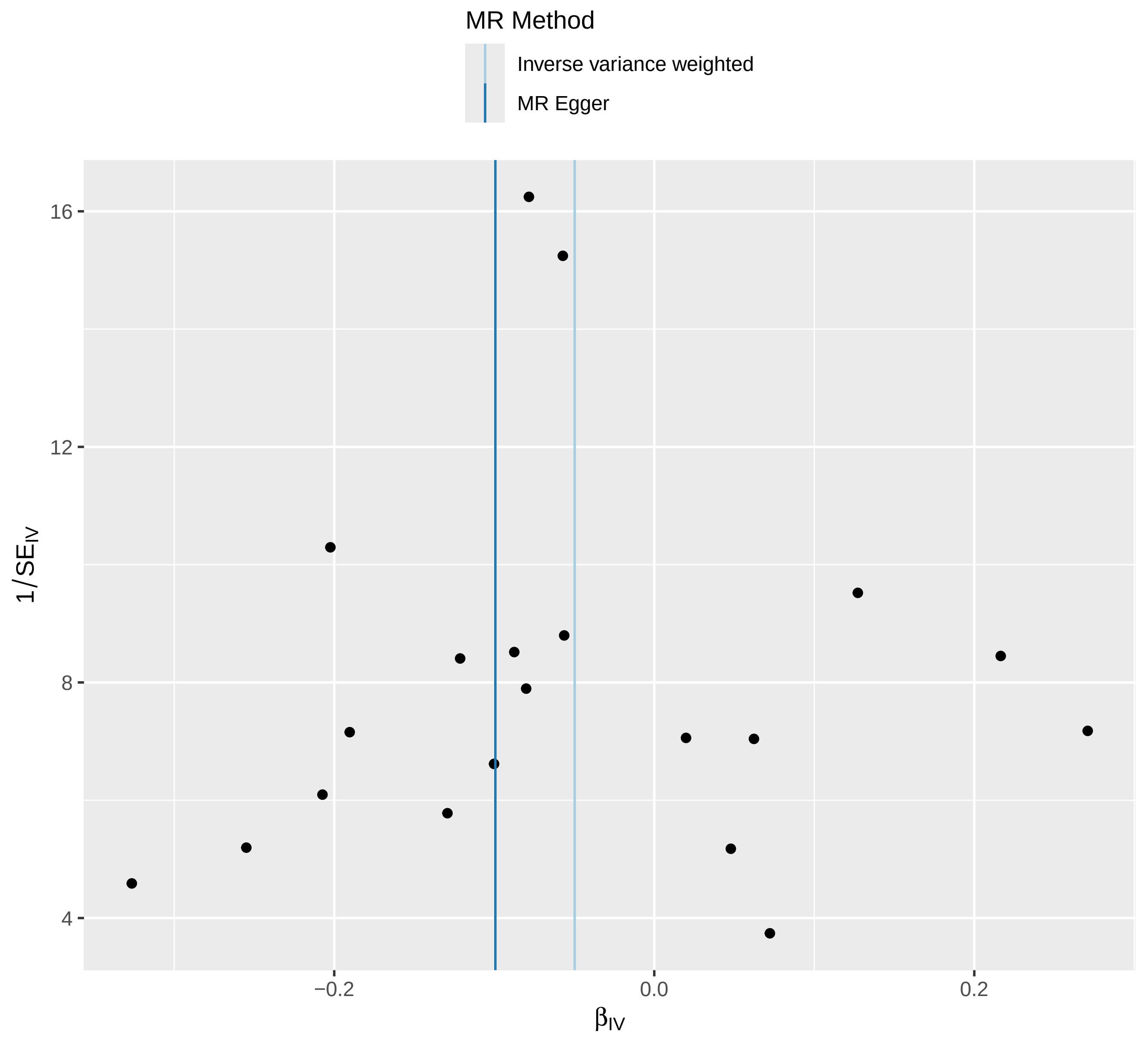
MR Method
Inverse variance weighted
MR Egger
16
12
8
4
1/SE_IV
−0.2
0.0
0.2
β_IV

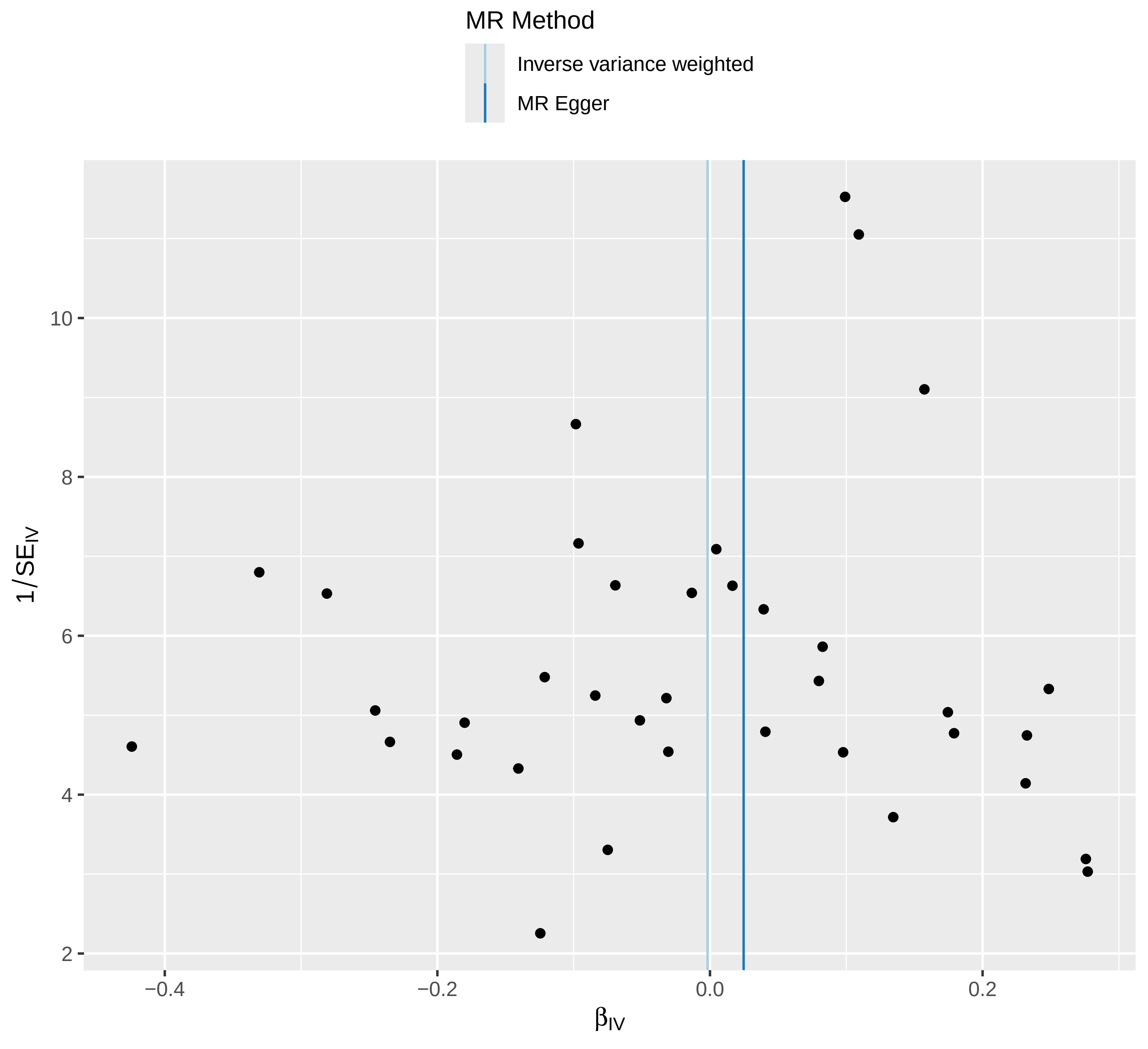

MR Method
Inverse variance weighted
MR Egger
1/SE_IV
β_IV
10
8
6
4
2
−0.4
−0.2
0.0
0.2

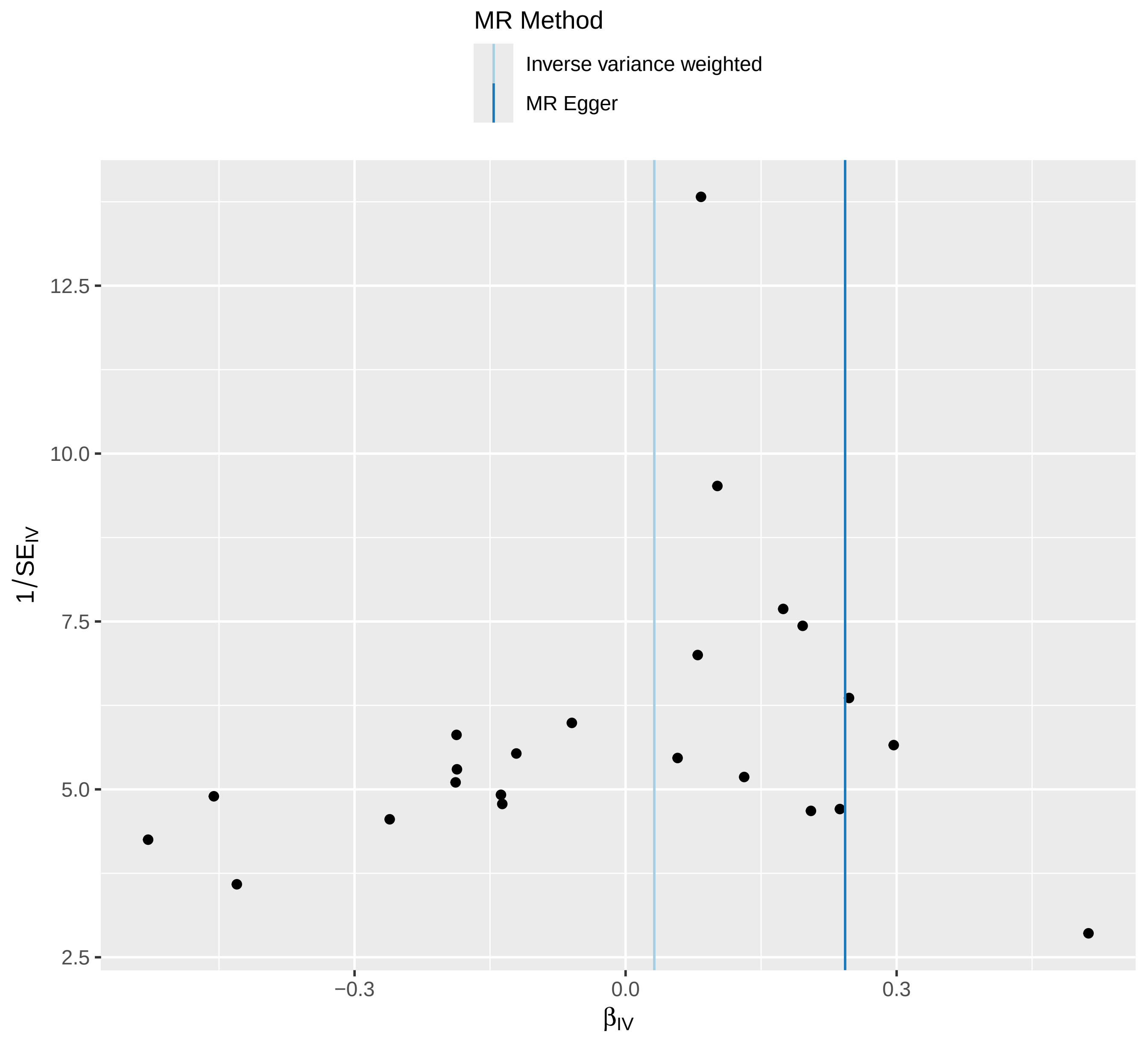
MR Method
Inverse variance weighted
MR Egger
1/SE_IV
12.5
10.0
7.5
5.0
2.5
−0.3
0.0
0.3
β_IV

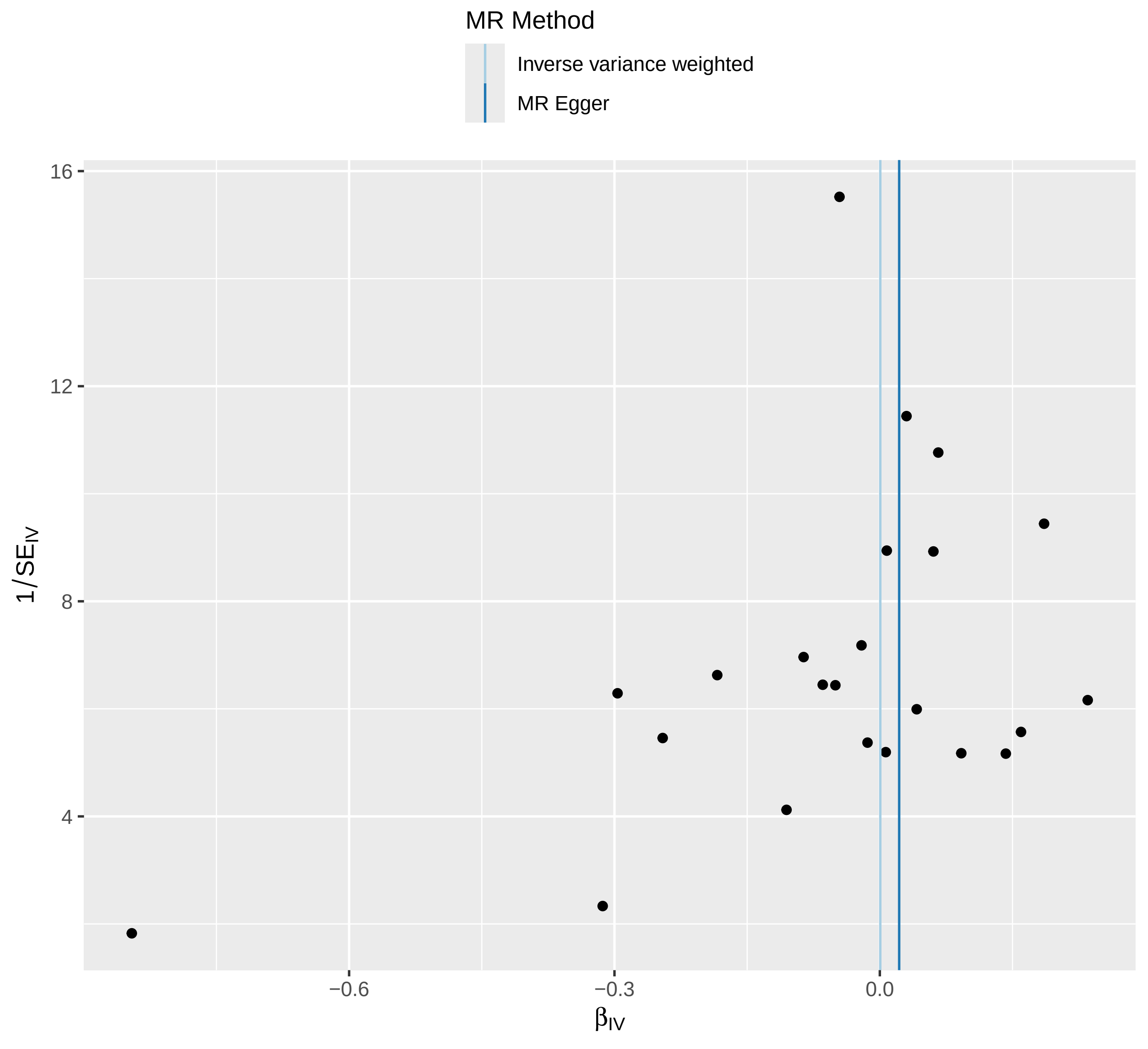
MR Method
Inverse variance weighted
MR Egger
16
12
8
4
1/SE_IV
−0.6
−0.3
0.0
β_IV

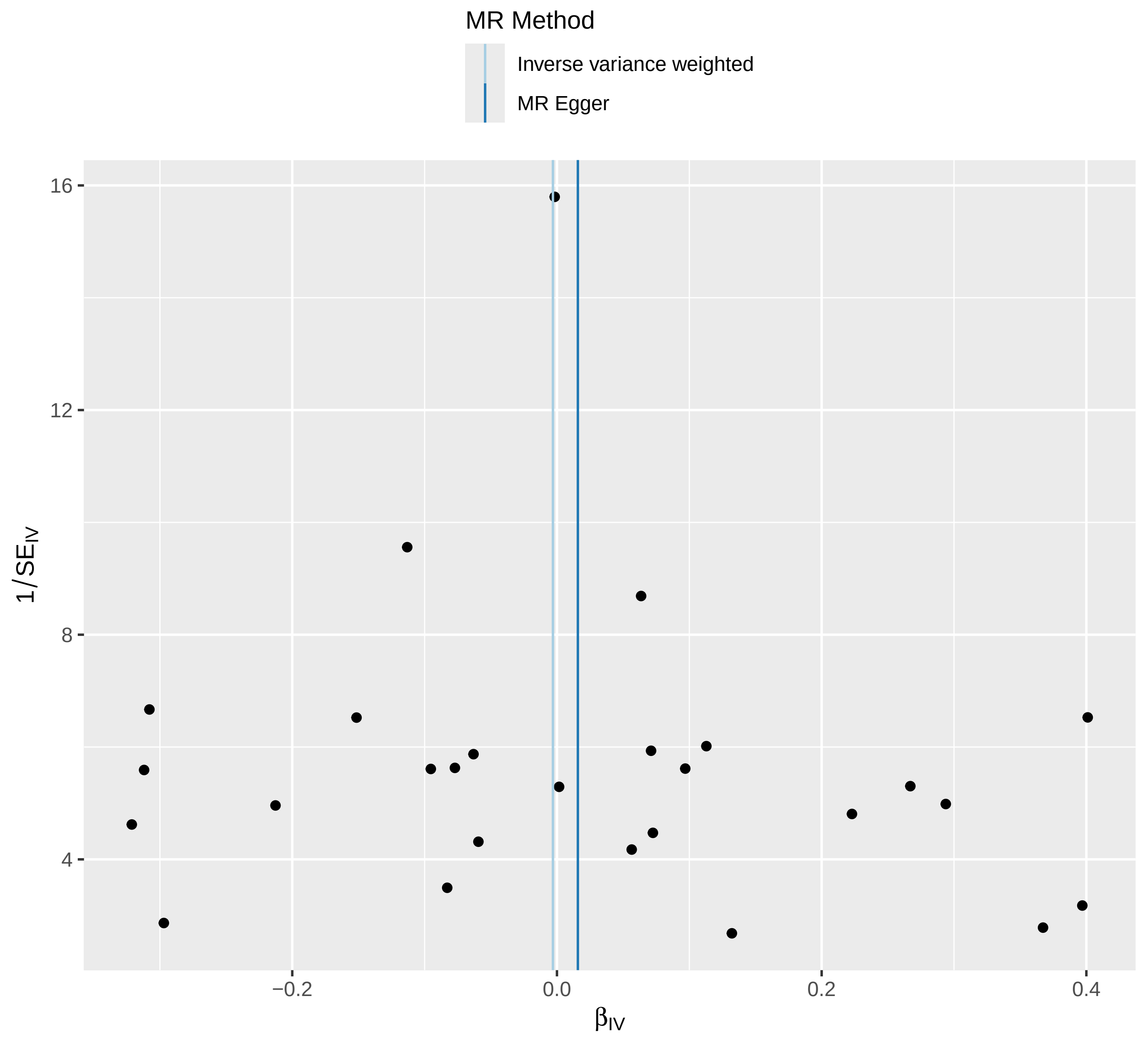
MR Method
Inverse variance weighted
MR Egger
16
12
8
4
$1/SE_{IV}$
−0.2
0.0
0.2
0.4
β_{IV}

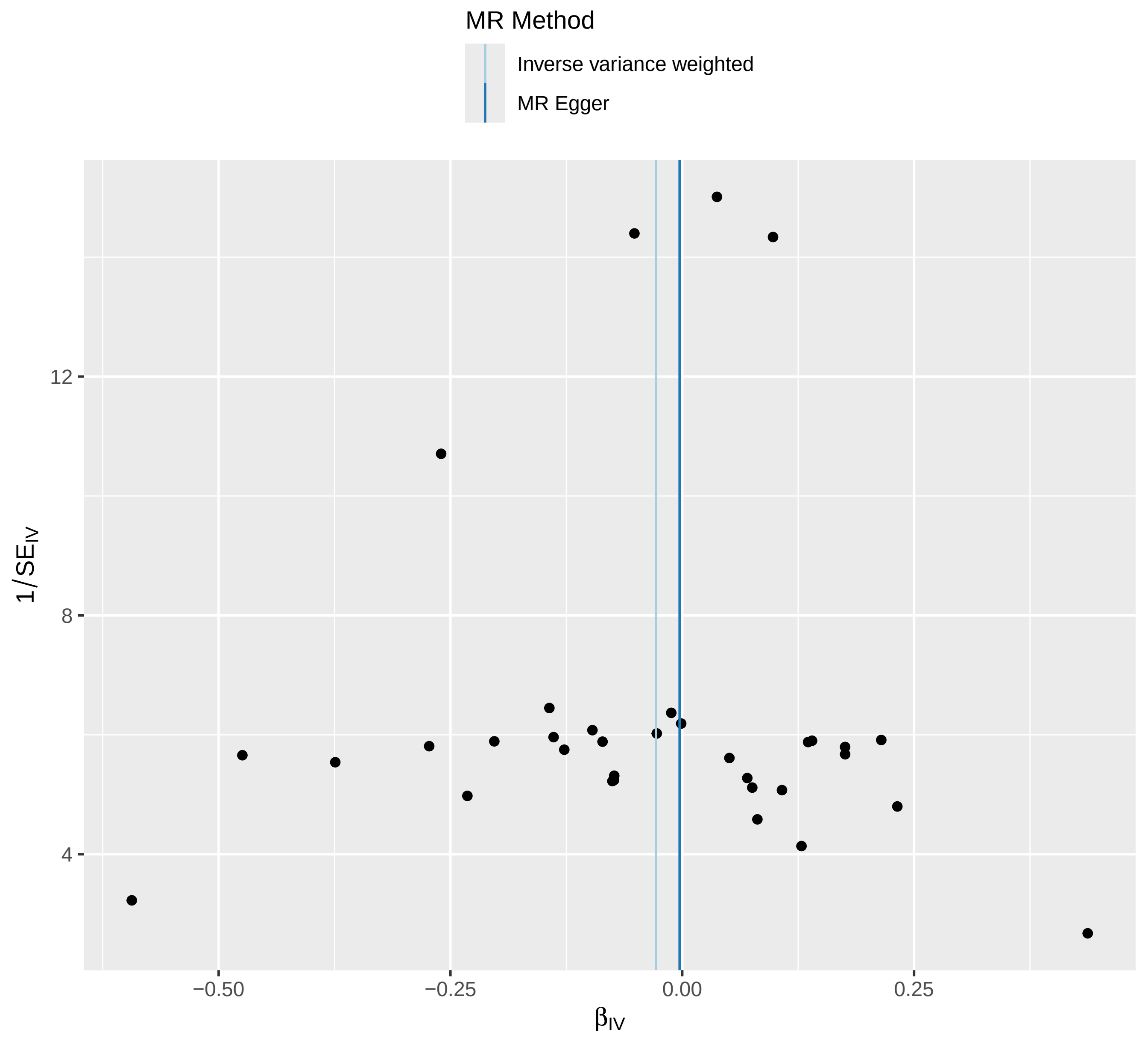
MR Method
Inverse variance weighted
MR Egger
12
8
4
1/SE_IV
−0.50
−0.25
0.00
0.25
β_IV

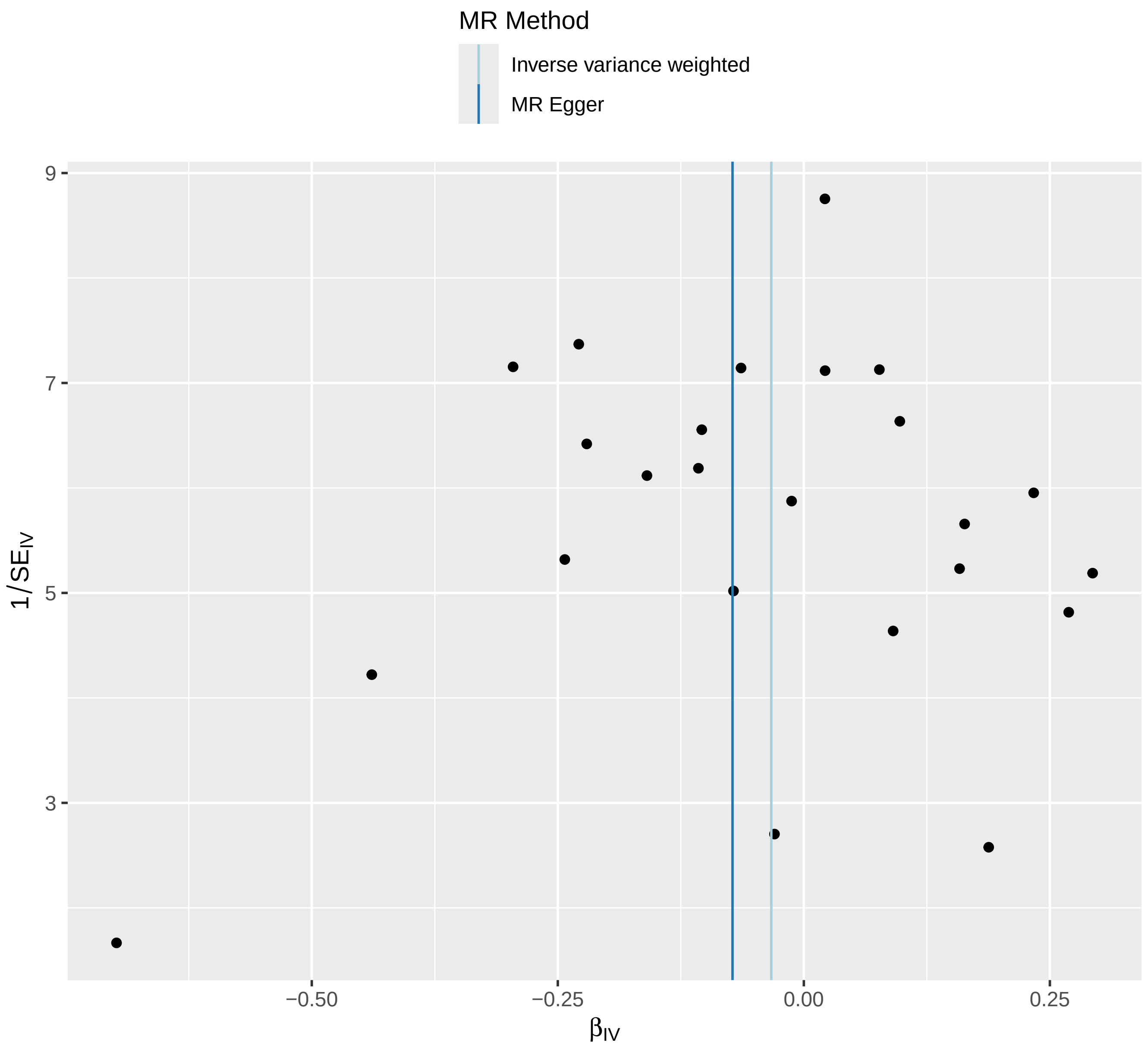
MR Method
Inverse variance weighted
MR Egger
9
7
5
3
1/SE_IV
−0.50
−0.25
0.00
0.25
β_IV

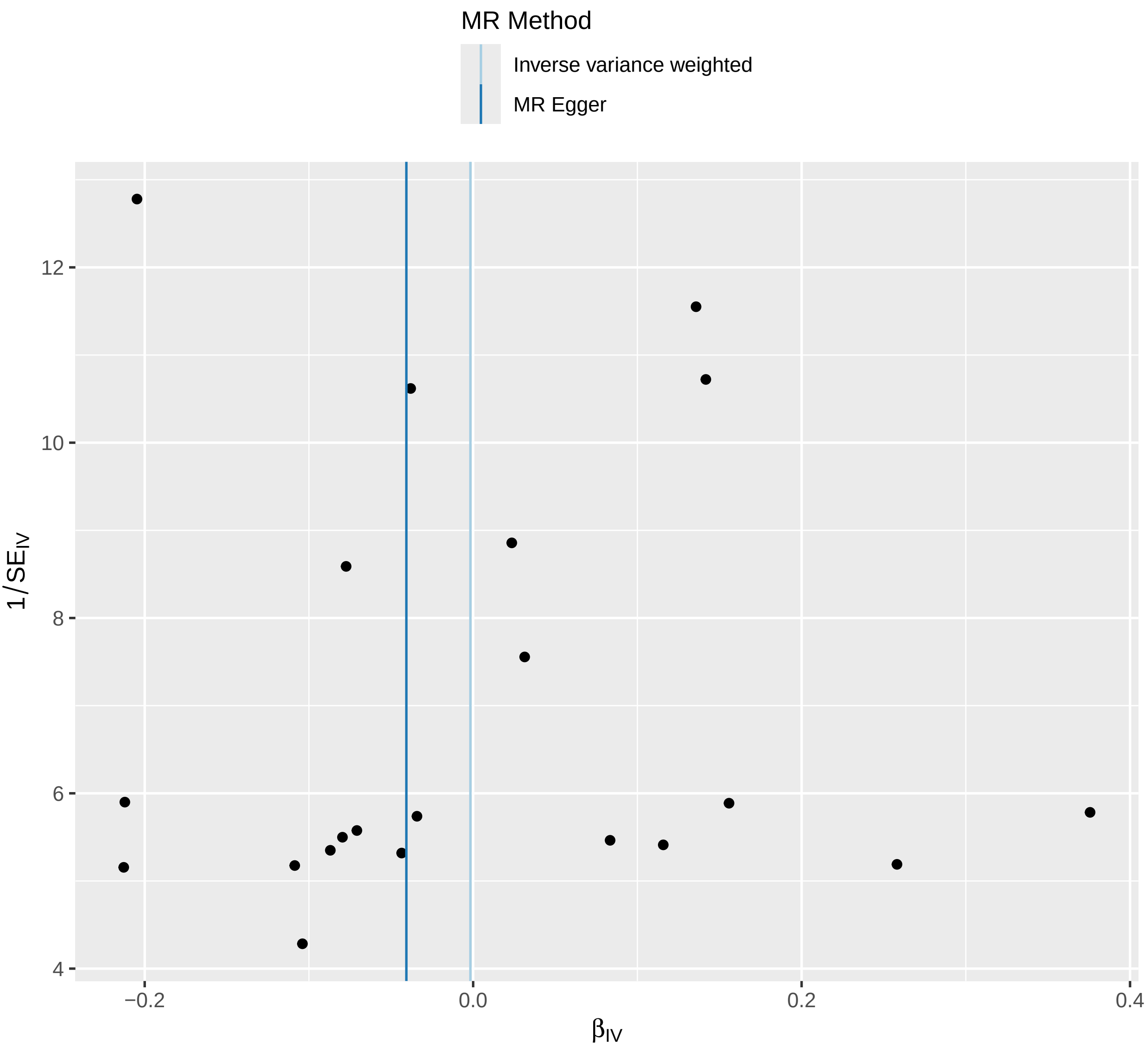

MR Method
Inverse variance weighted
MR Egger
1/SE_IV
12
10
8
6
4
−0.2
0.0
0.2
0.4
β_IV

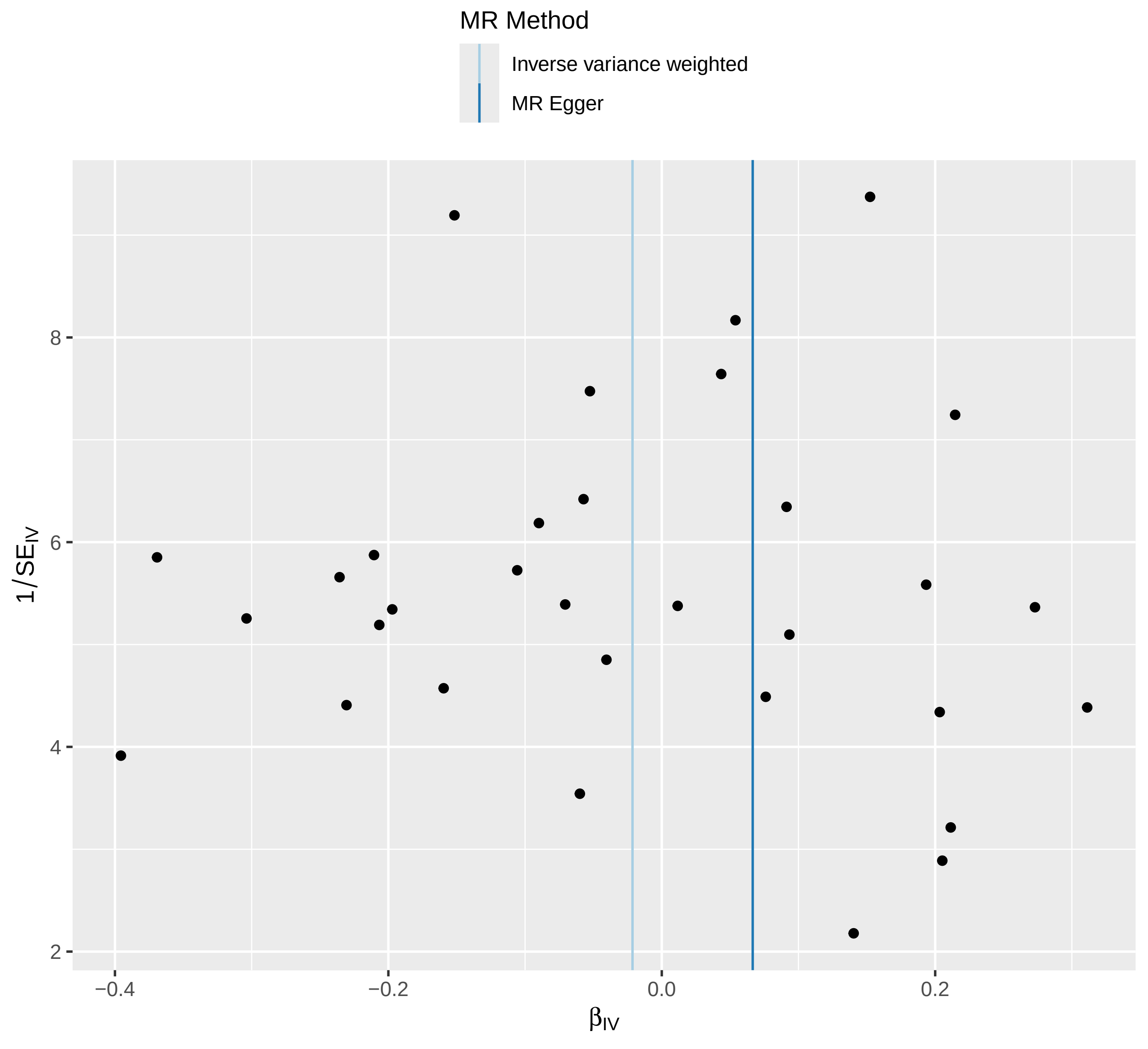
MR Method
Inverse variance weighted
MR Egger
1/SE_IV
8
6
4
2
−0.4
−0.2
0.0
0.2
β_IV

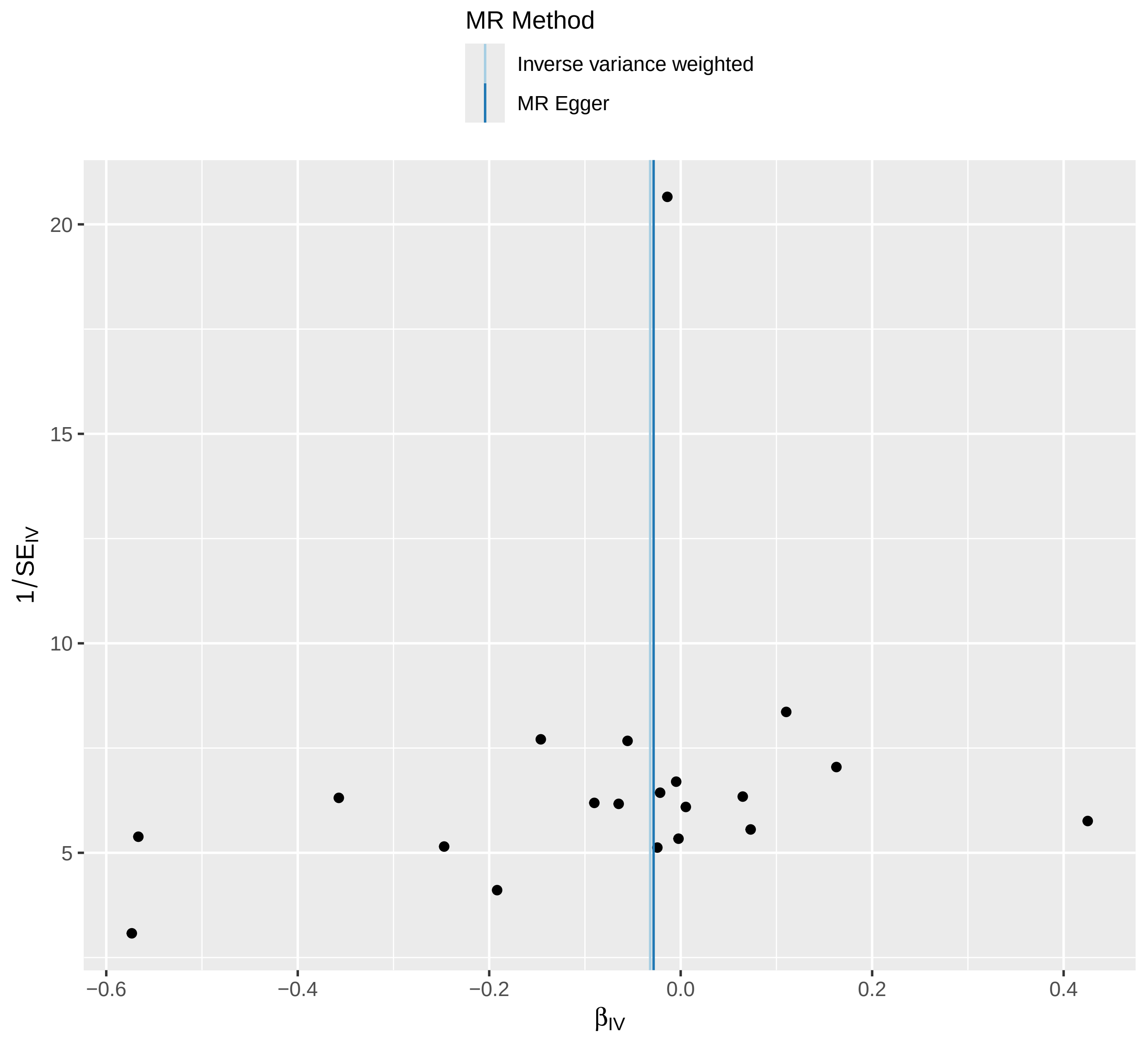

MR Method
Inverse variance weighted
MR Egger
20
15
10
5
1/SE$_{IV}$
−0.6
−0.4
−0.2
0.0
0.2
0.4
β$_{IV}$

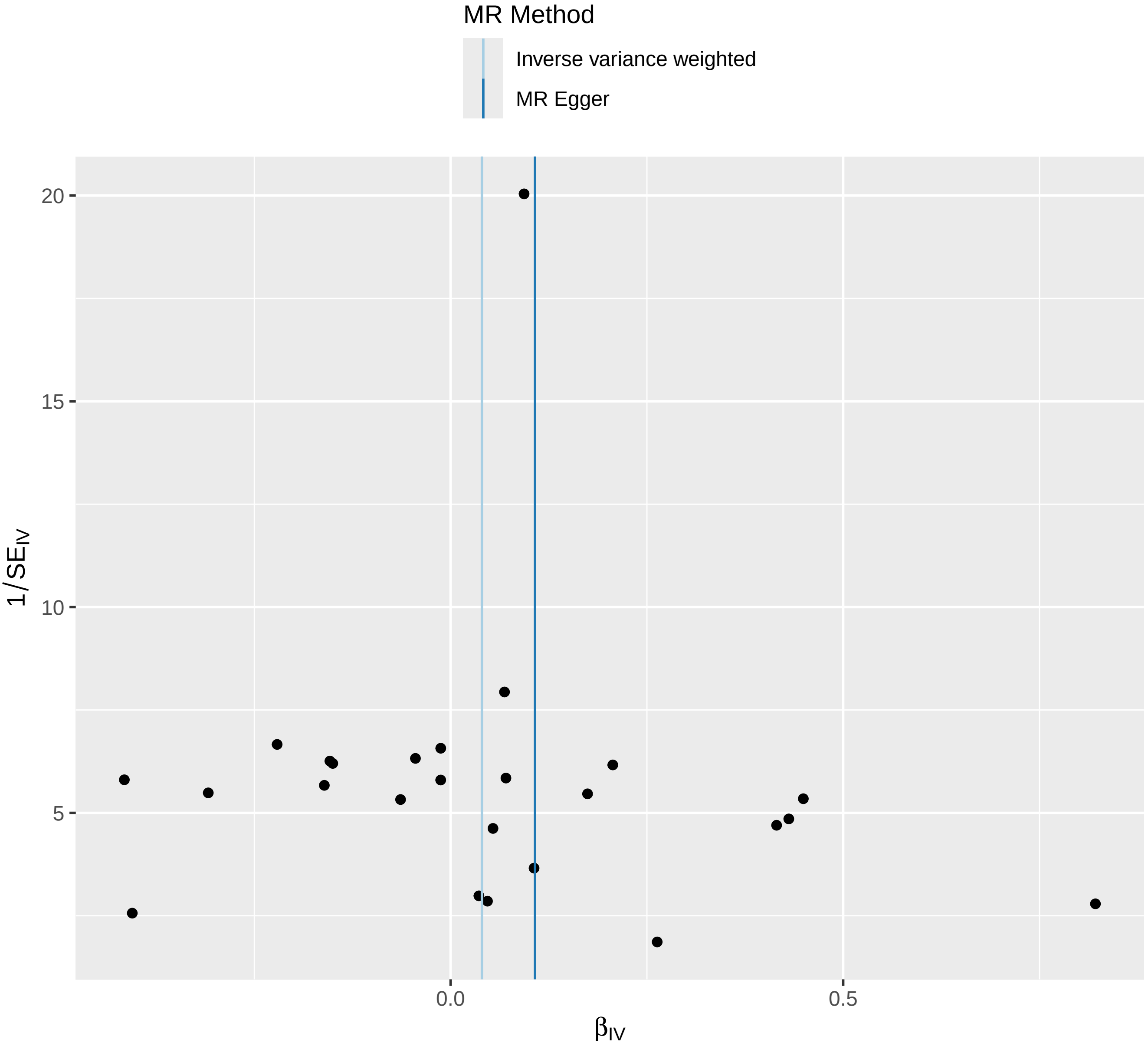

MR Method
Inverse variance weighted
MR Egger
20
15
10
5
1/SE_IV
0.0
0.5
β_IV

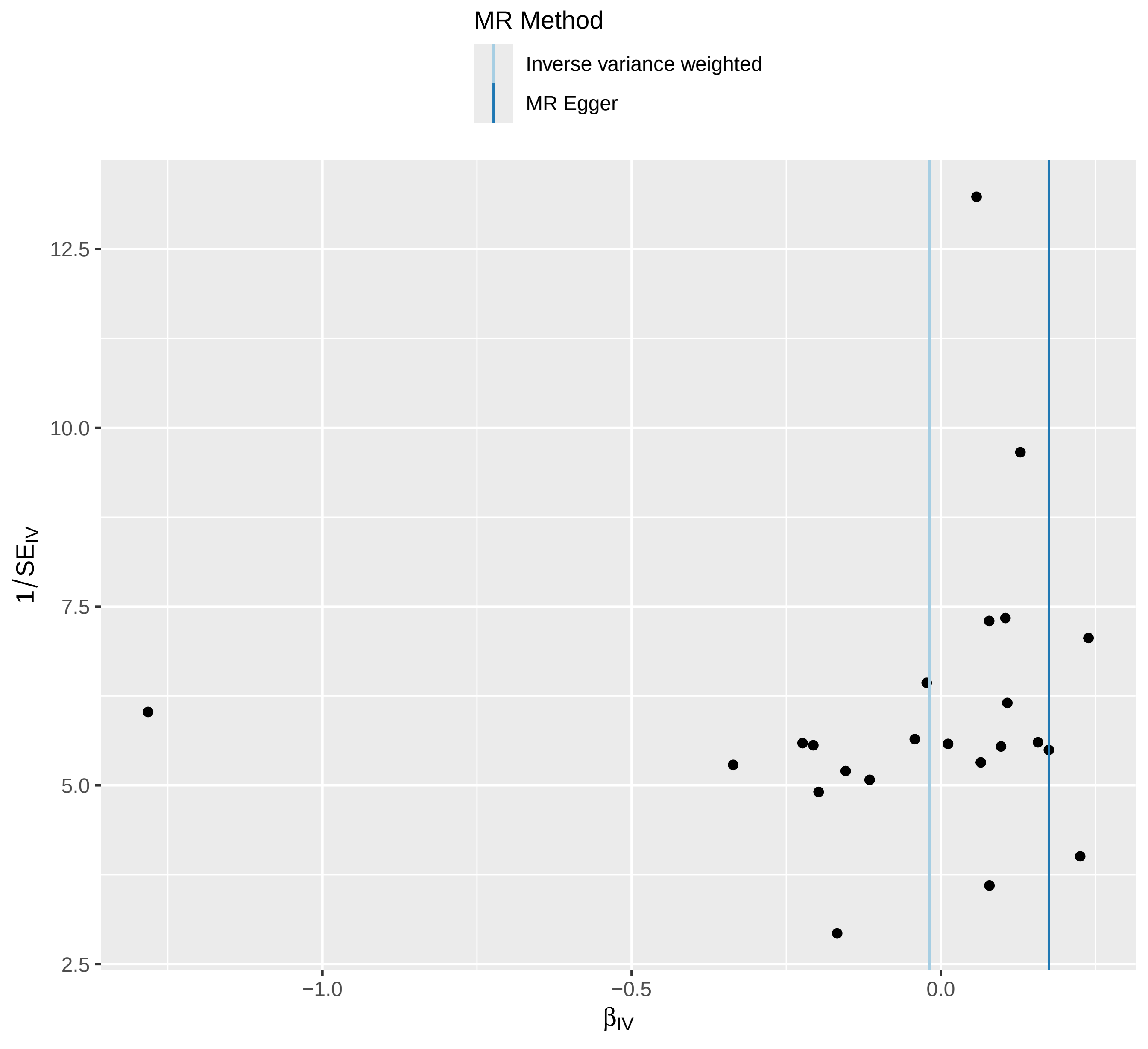

MR Method
Inverse variance weighted
MR Egger
12.5
10.0
7.5
5.0
2.5
1/SE_IV
−1.0
−0.5
0.0
β_IV

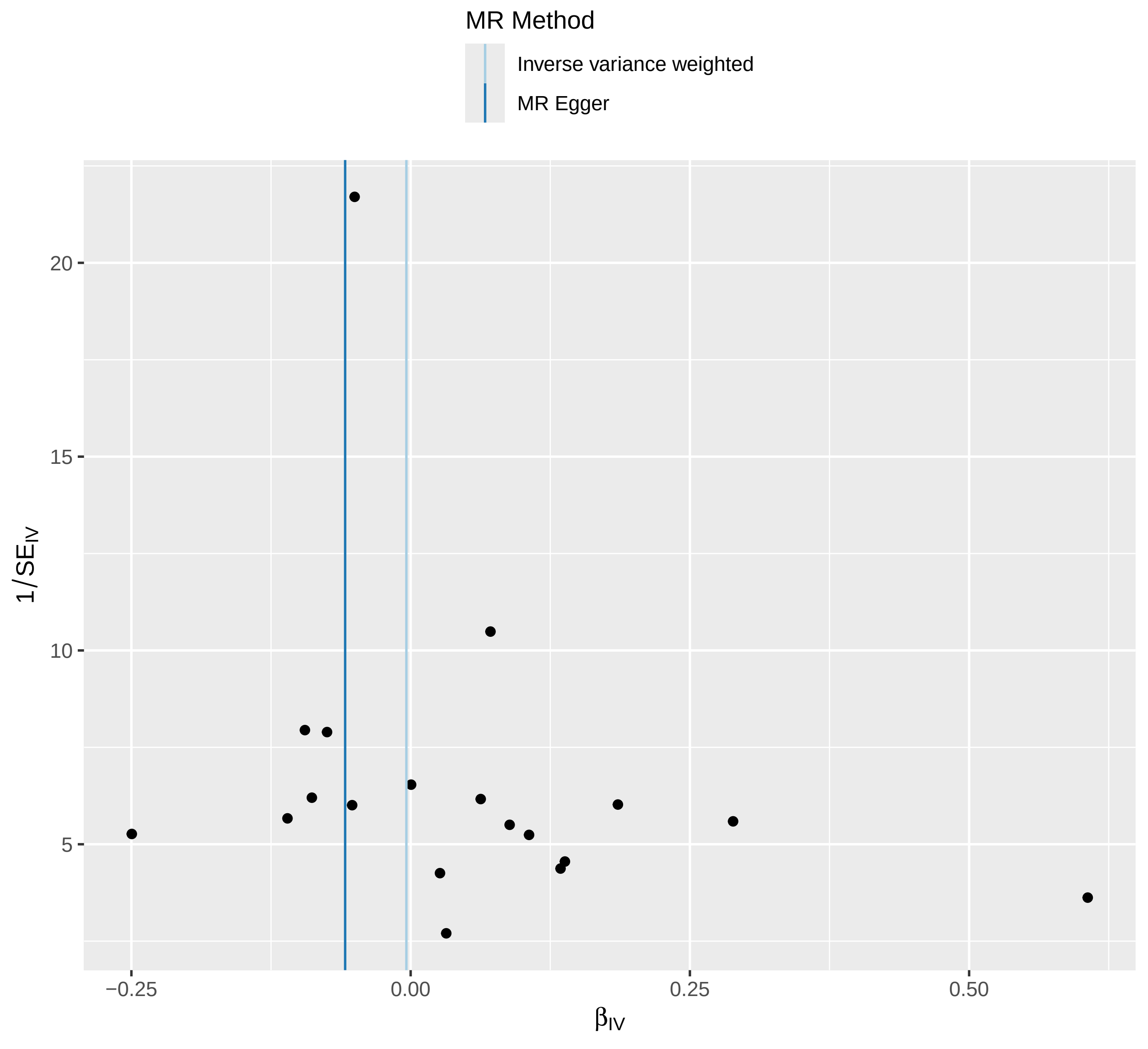
MR Method
Inverse variance weighted
MR Egger
20
15
10
5
1/SE_IV
−0.25
0.00
0.25
0.50
β_IV

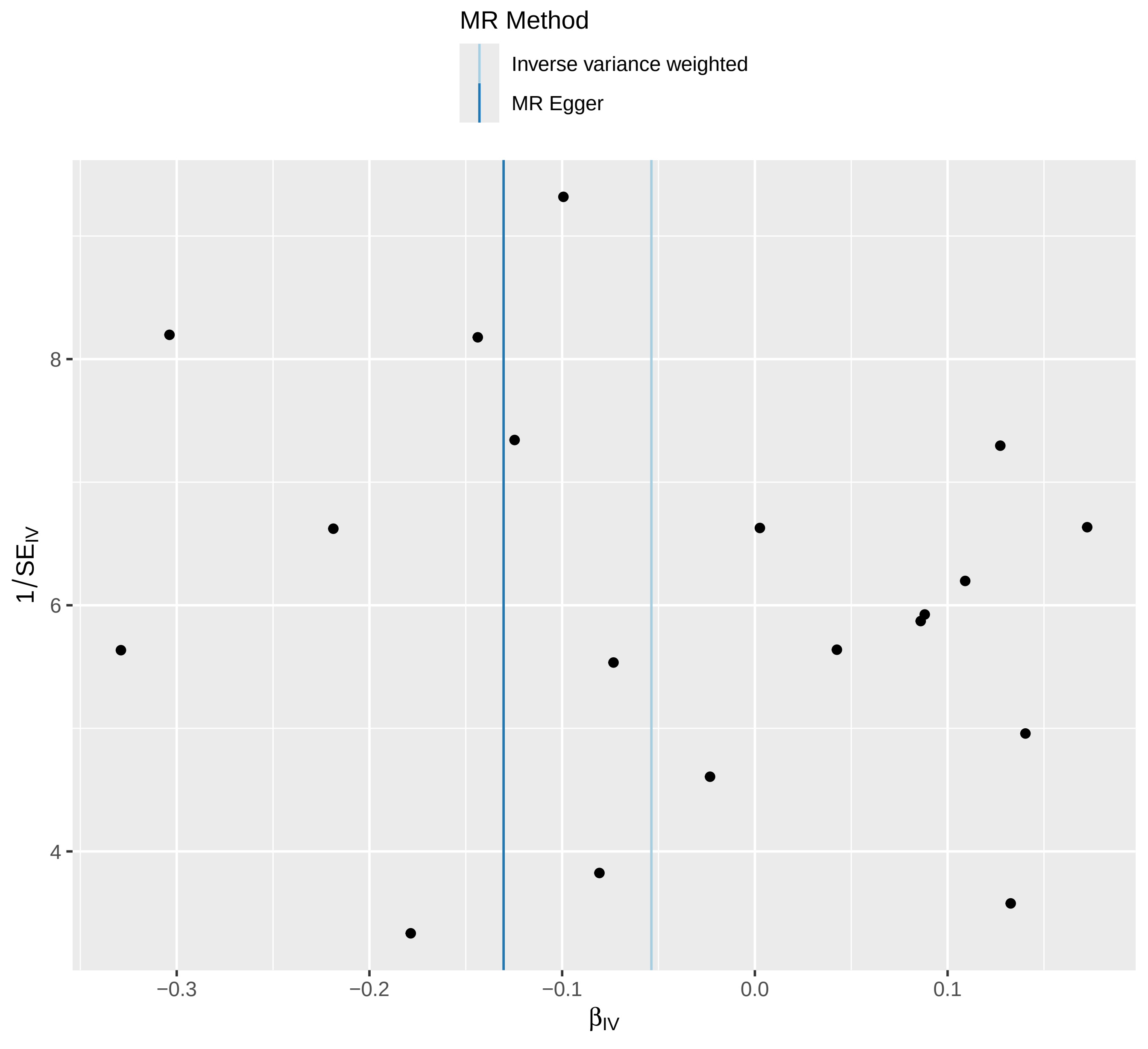

MR Method
Inverse variance weighted
MR Egger
8
6
4
1/SE$_{IV}$
−0.3
−0.2
−0.1
0.0
0.1
β_{IV}

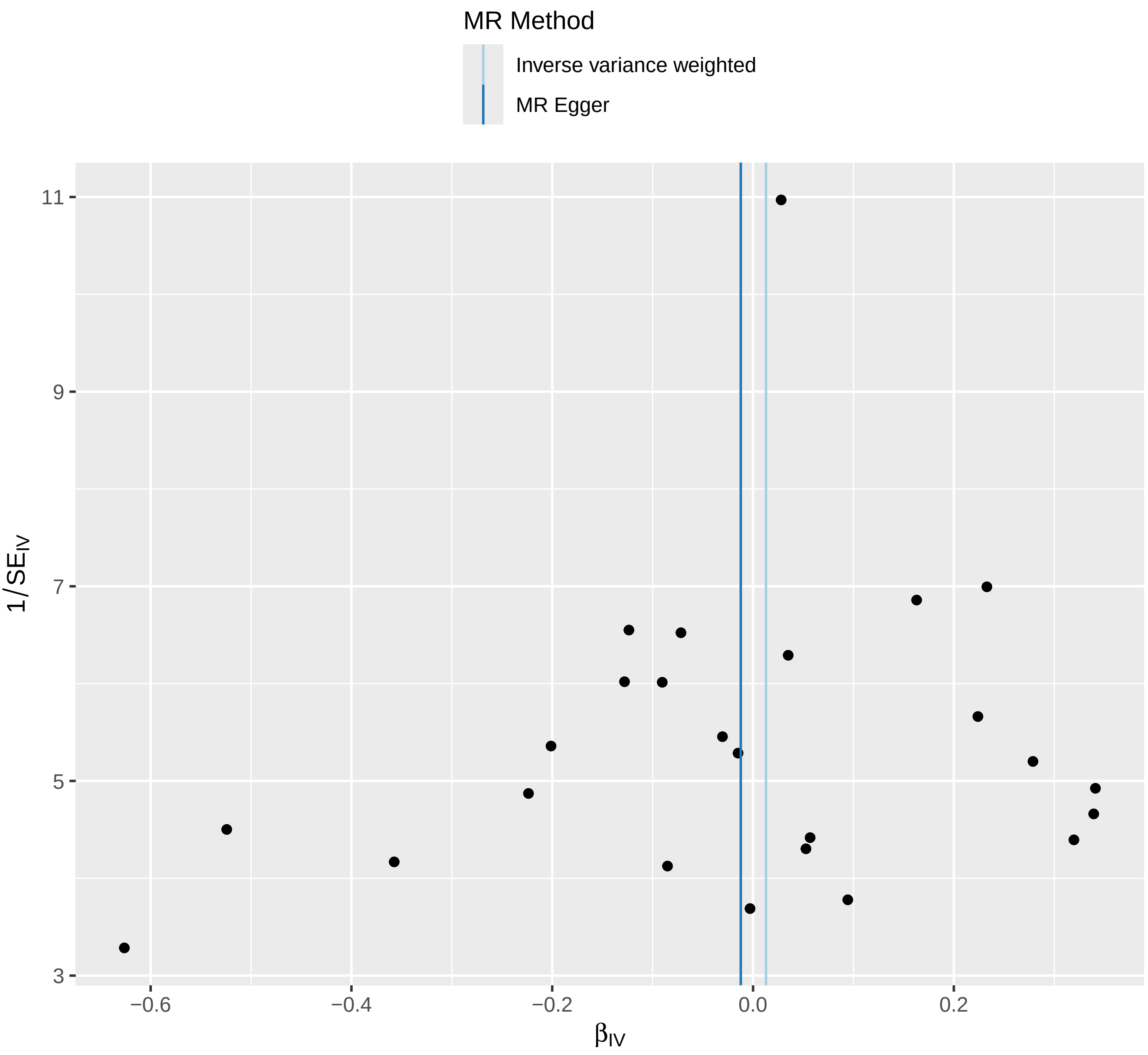

MR Method
Inverse variance weighted
MR Egger
11
9
7
5
3
1/SE$_{IV}$
−0.6
−0.4
−0.2
0.0
0.2
β$_{IV}$

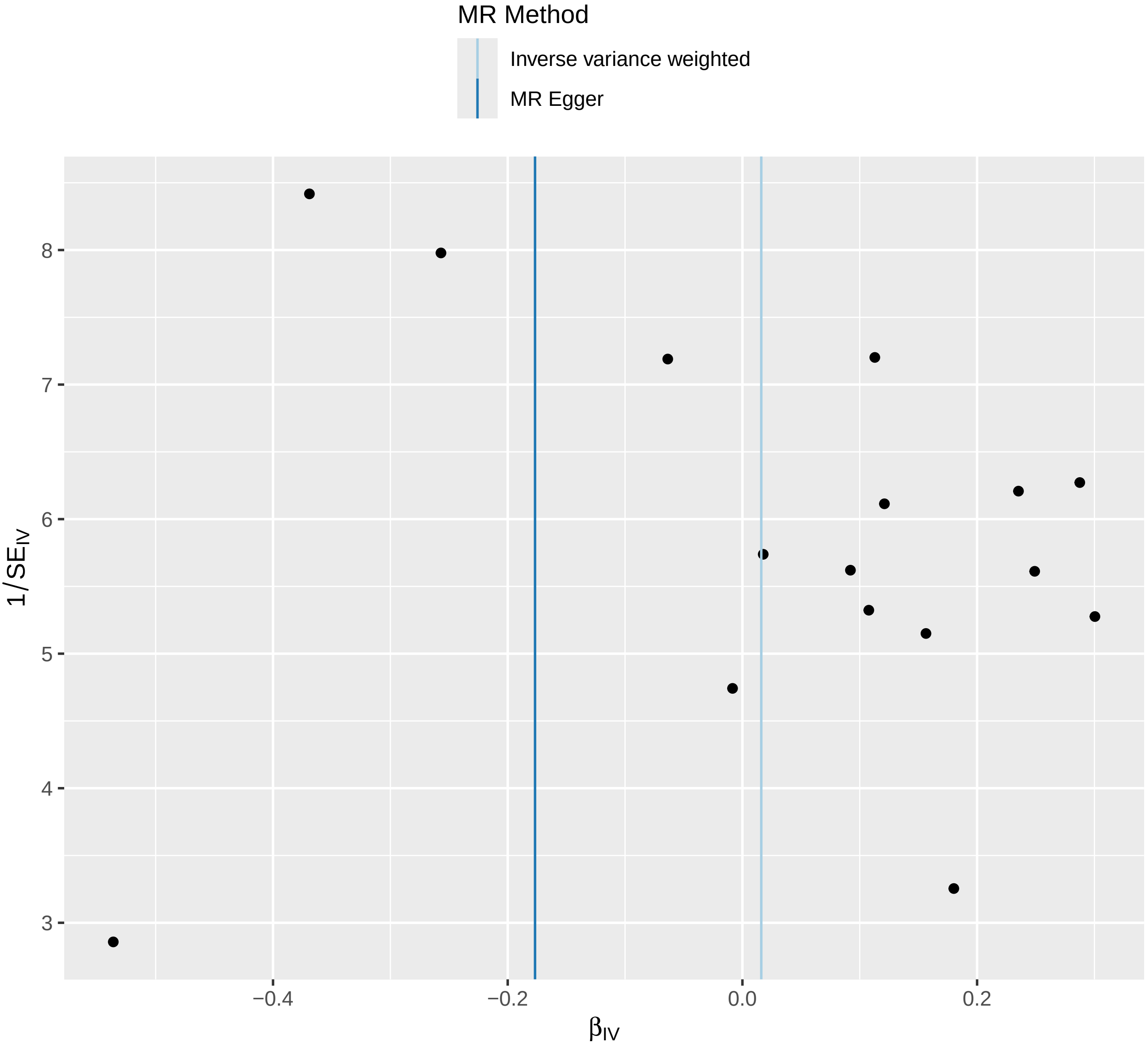
MR Method
Inverse variance weighted
MR Egger
1/SE_{IV}
β_{IV}
8
7
6
5
4
3
−0.4
−0.2
0.0
0.2

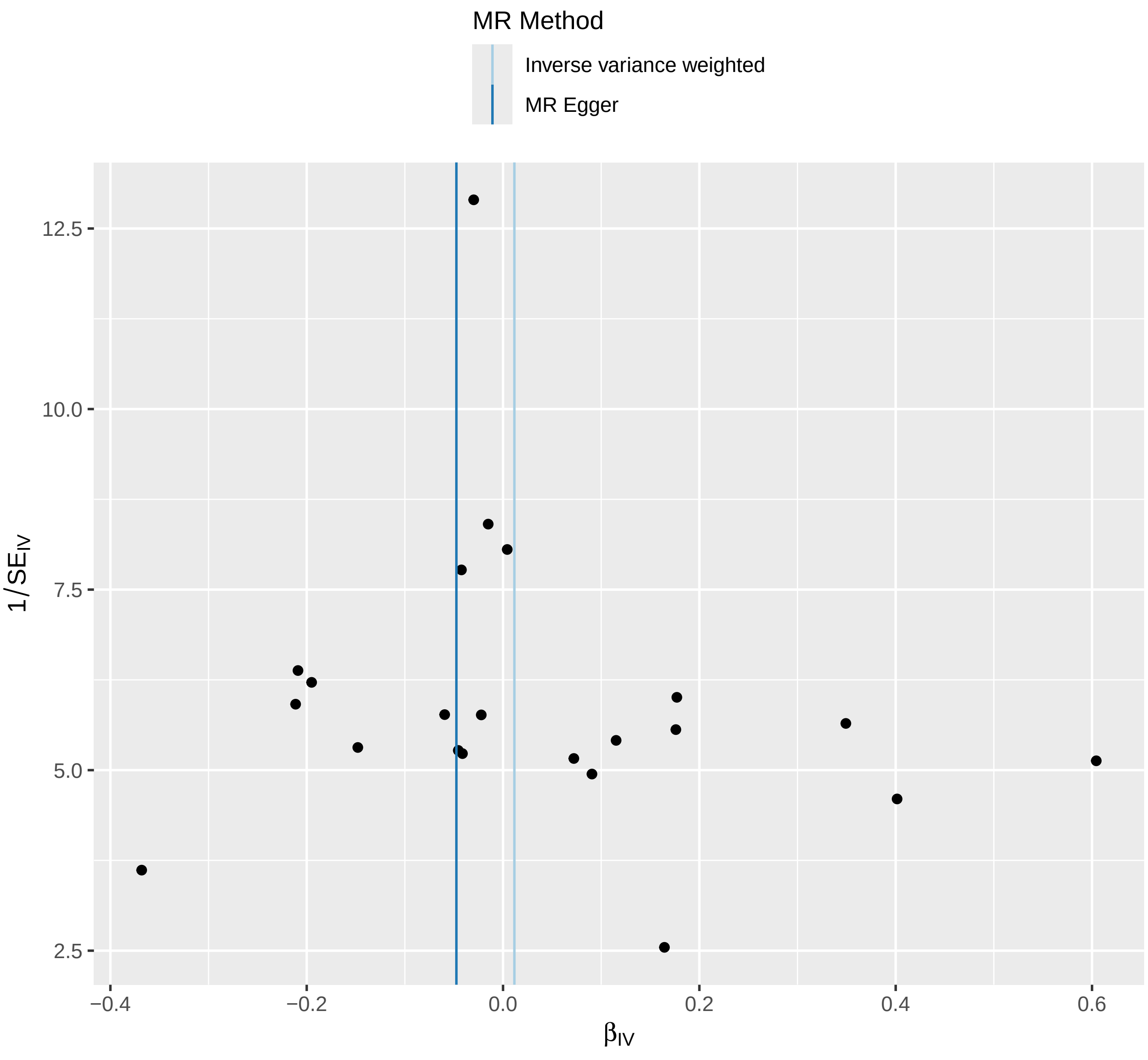
MR Method
Inverse variance weighted
MR Egger
12.5
10.0
7.5
5.0
2.5
1/SE_IV
−0.4
−0.2
0.0
0.2
0.4
0.6
β_IV

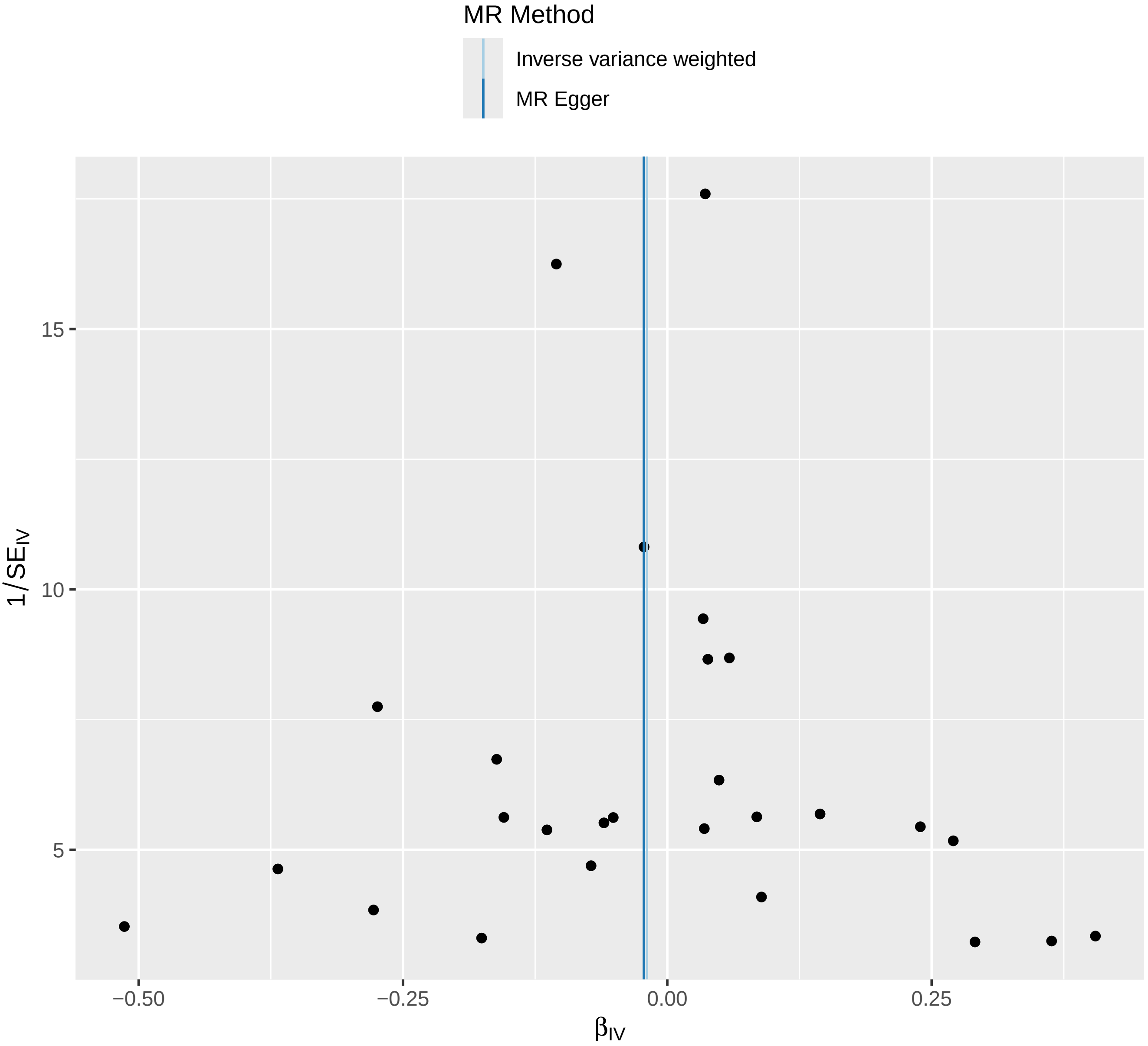
MR Method
Inverse variance weighted
MR Egger
15
10
5
1/SE_IV
−0.50
−0.25
0.00
0.25
β_IV

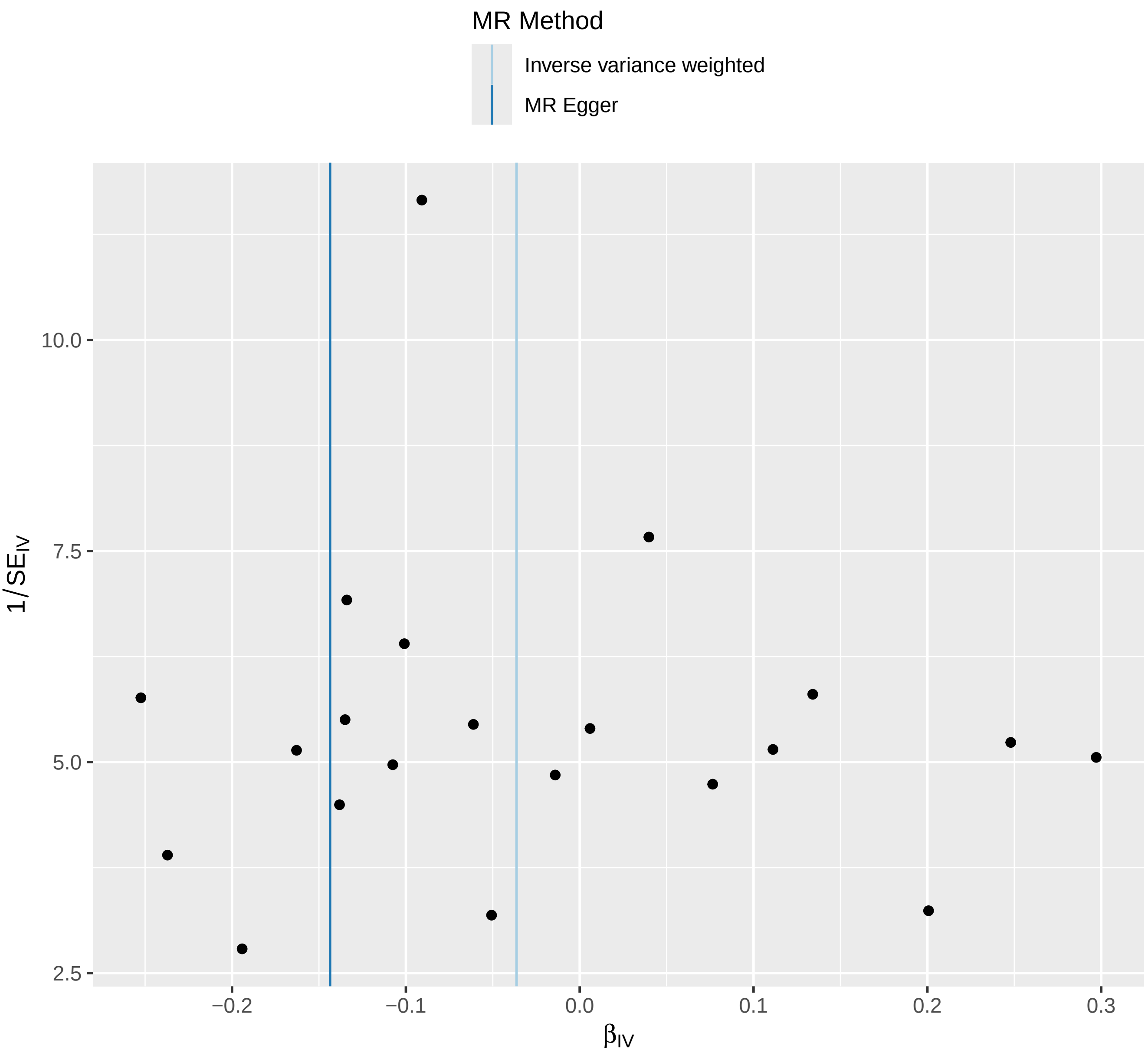
MR Method
Inverse variance weighted
MR Egger
1/SE_IV
10.0
7.5
5.0
2.5
β_IV
−0.2
−0.1
0.0
0.1
0.2
0.3

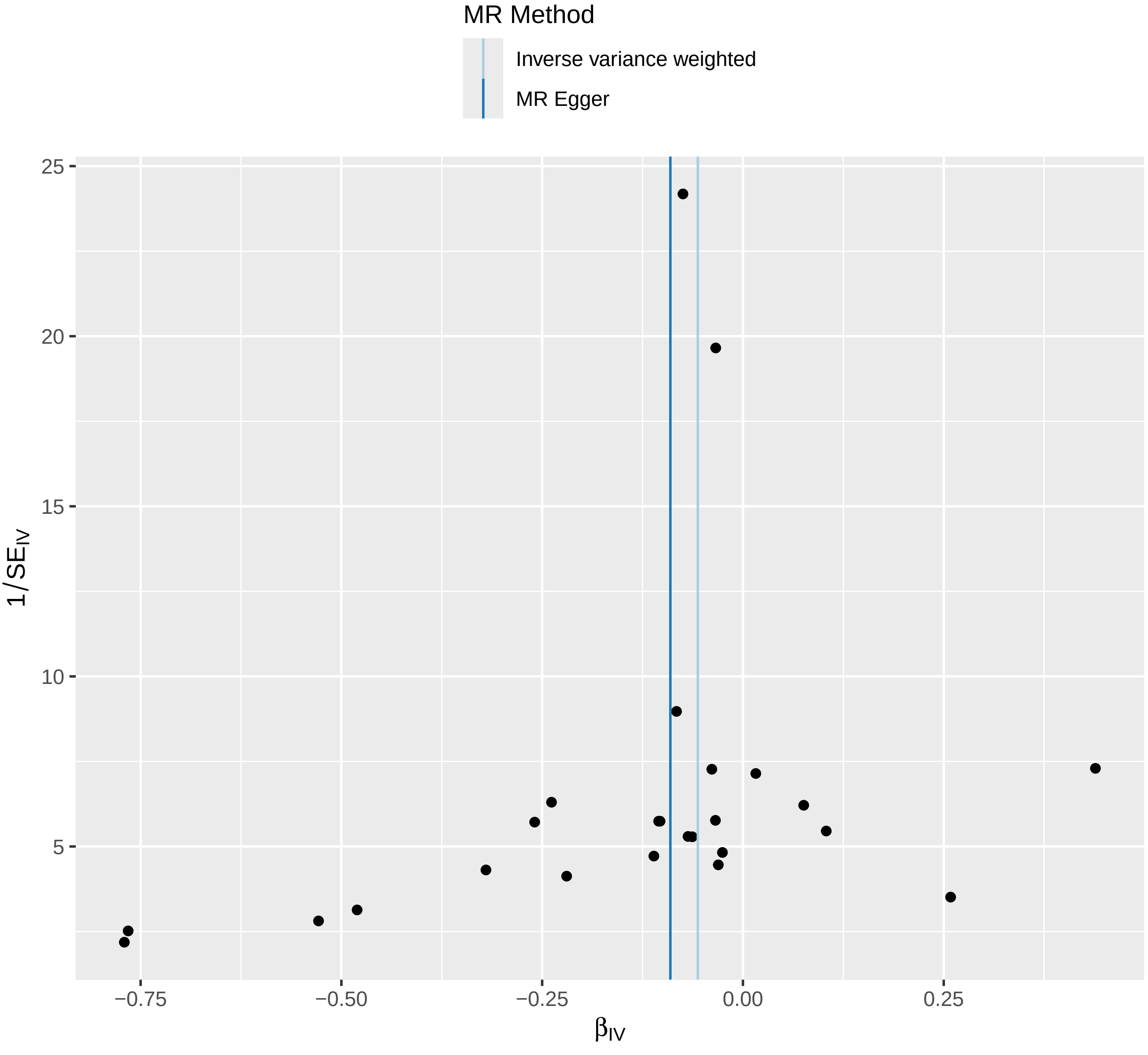

MR Method
Inverse variance weighted
MR Egger
25
20
15
10
5
1/SE_IV
−0.75
−0.50
−0.25
0.00
0.25
β_IV

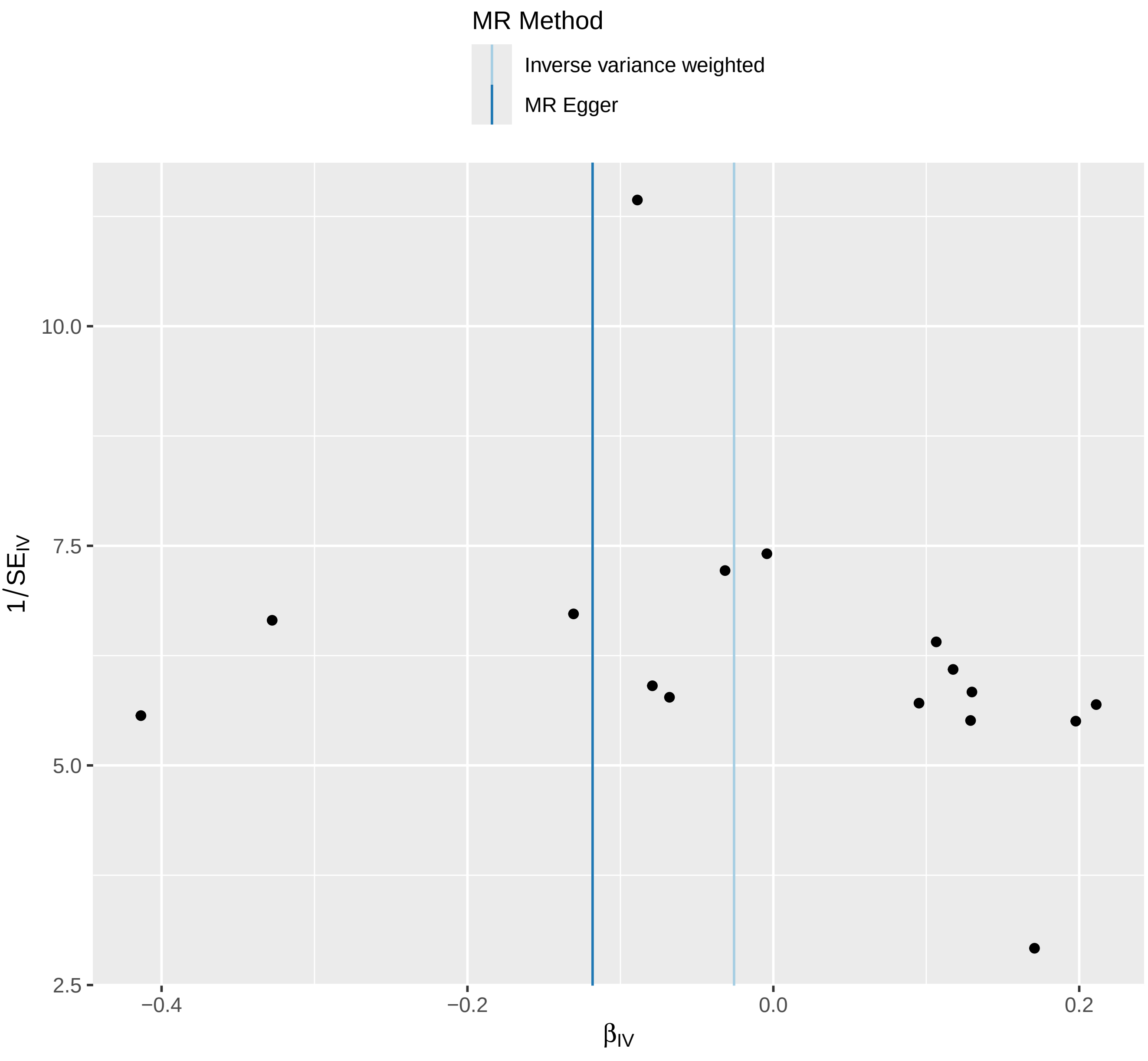

MR Method
Inverse variance weighted
MR Egger
10.0
7.5
5.0
2.5
−0.4
−0.2
0.0
0.2
β_{IV}
$1/SE_{IV}$

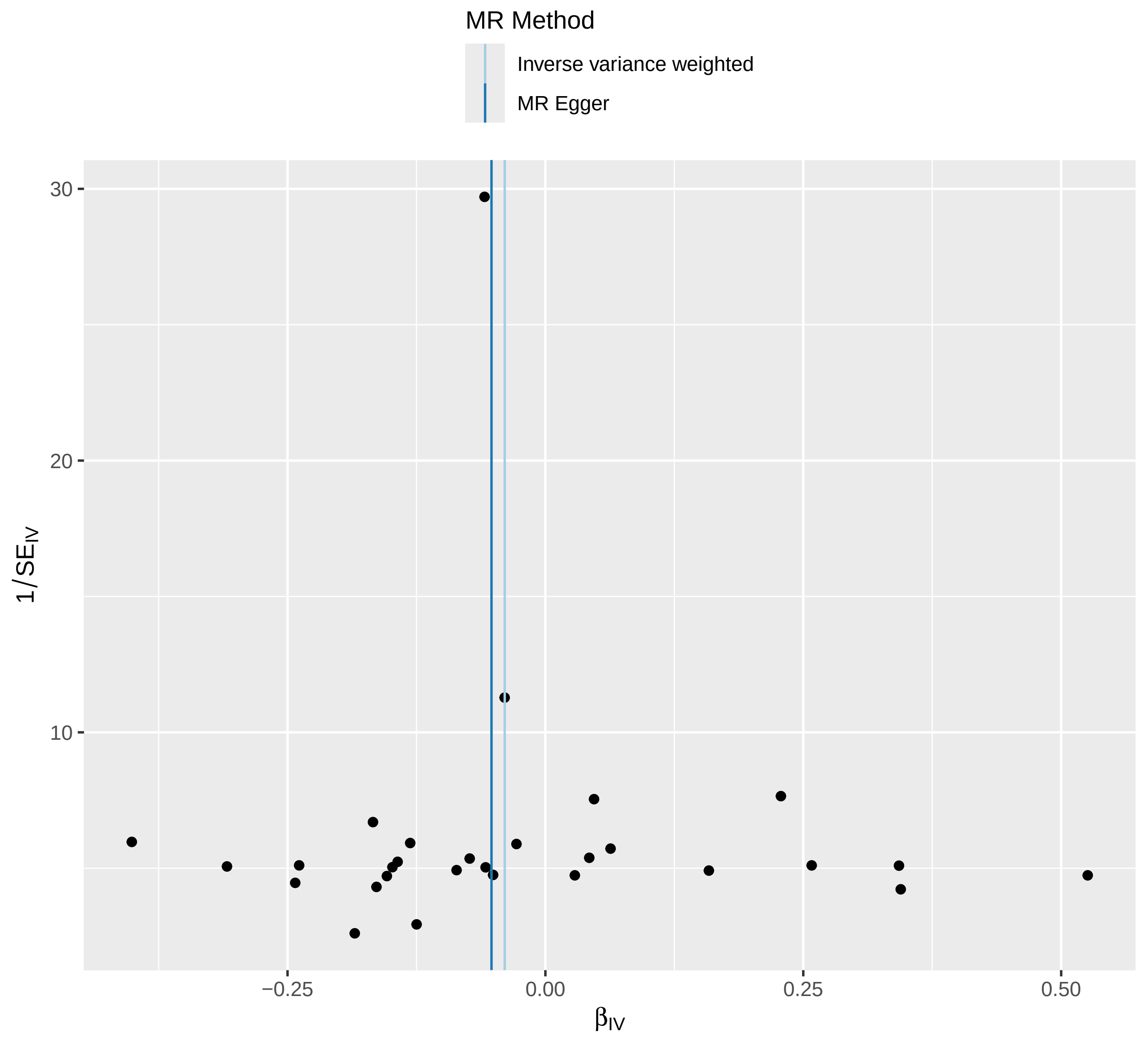
MR Method
Inverse variance weighted
MR Egger
30
20
10
1/SE$_{IV}$
−0.25
0.00
0.25
0.50
β$_{IV}$

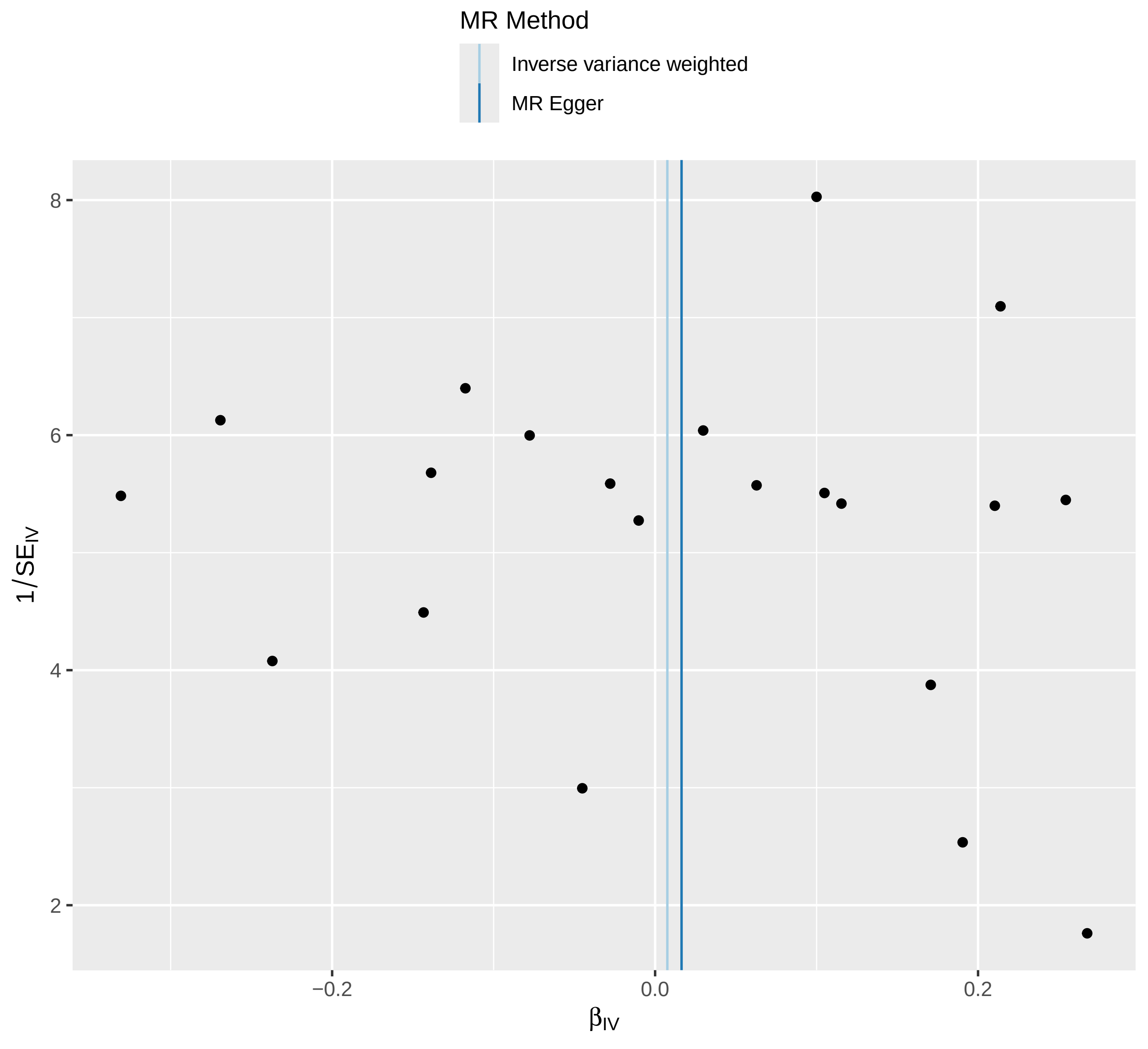
MR Method
Inverse variance weighted
MR Egger
8
6
4
2
1/SE_IV
−0.2
0.0
0.2
β_IV

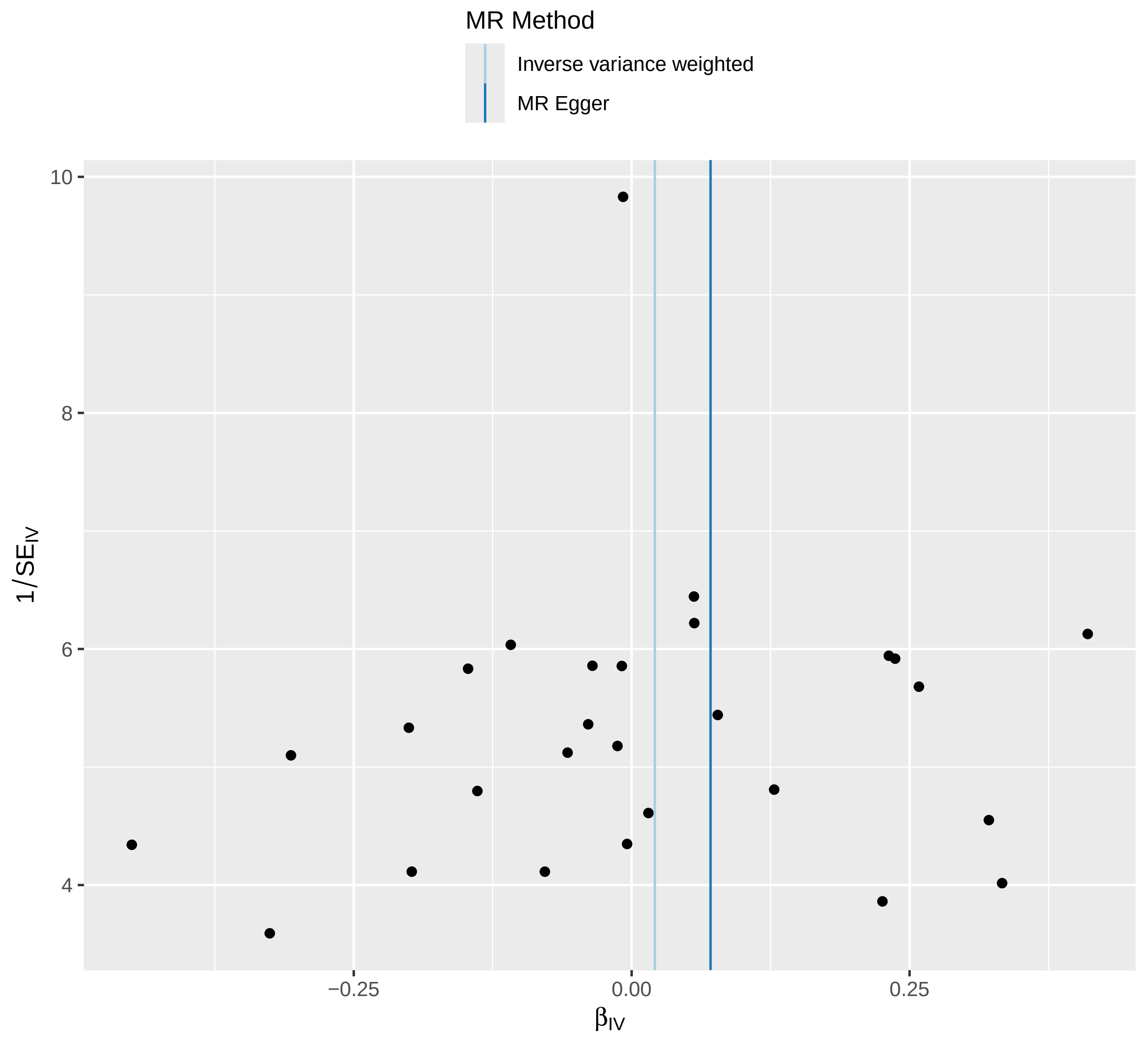
MR Method
Inverse variance weighted
MR Egger
10
8
6
4
1/SE_IV
−0.25
0.00
0.25
β_IV

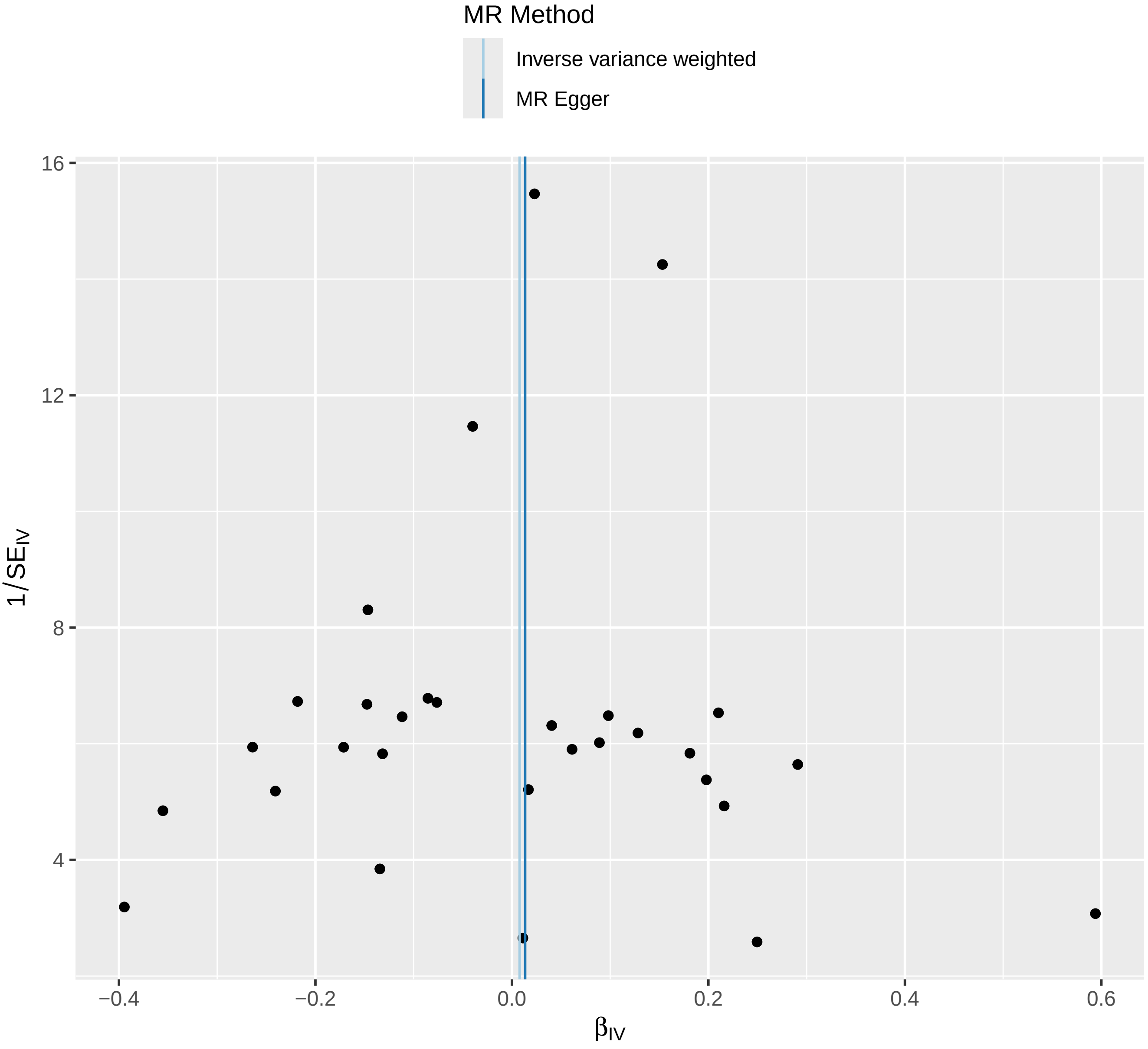

MR Method
Inverse variance weighted
MR Egger
16
12
8
4
$1/SE_{IV}$
−0.4
−0.2
0.0
0.2
0.4
0.6
β_{IV}

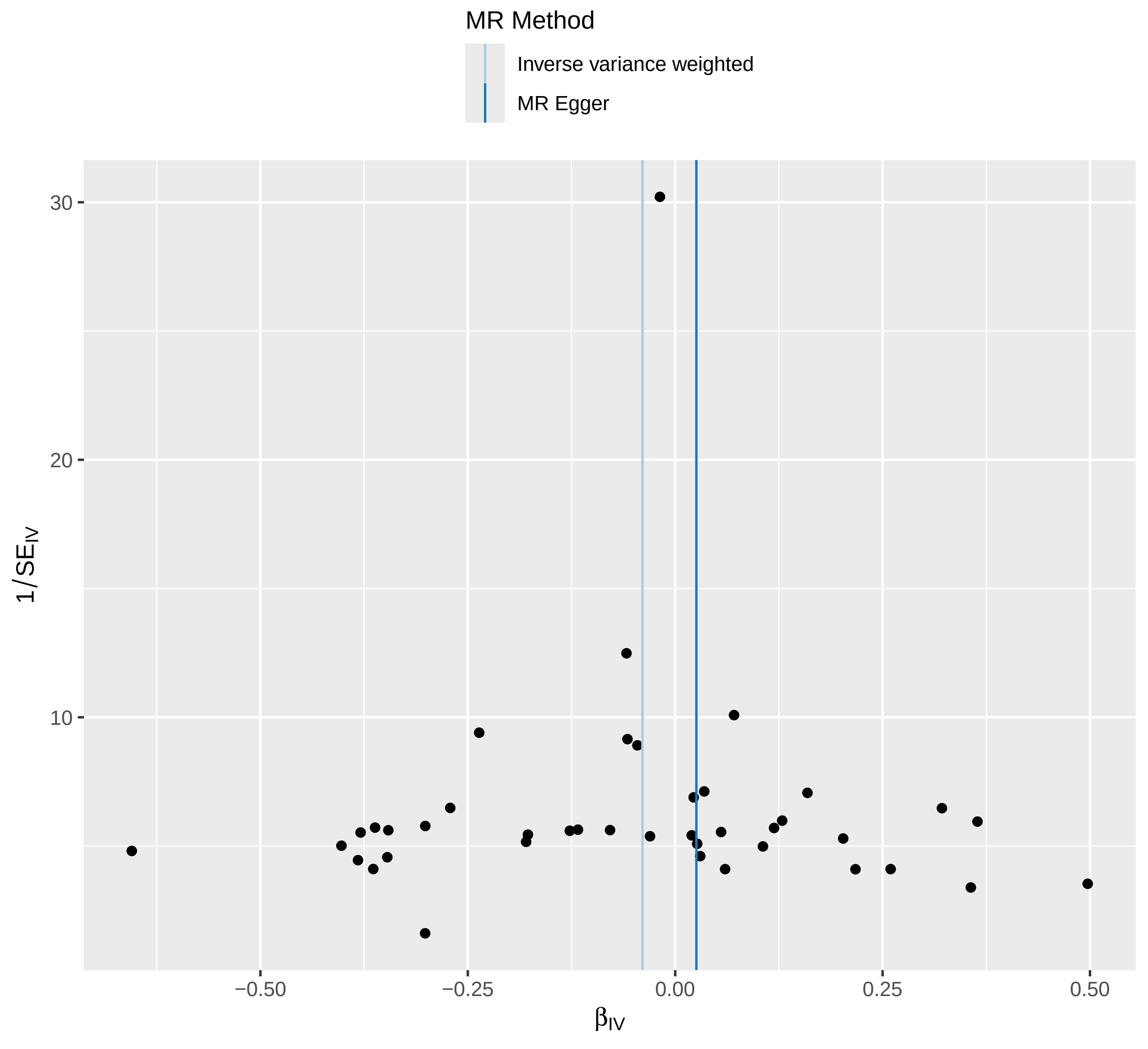

MR Method
Inverse variance weighted
MR Egger
30
20
10
1/SE_IV
−0.50
−0.25
0.00
0.25
0.50
β_IV

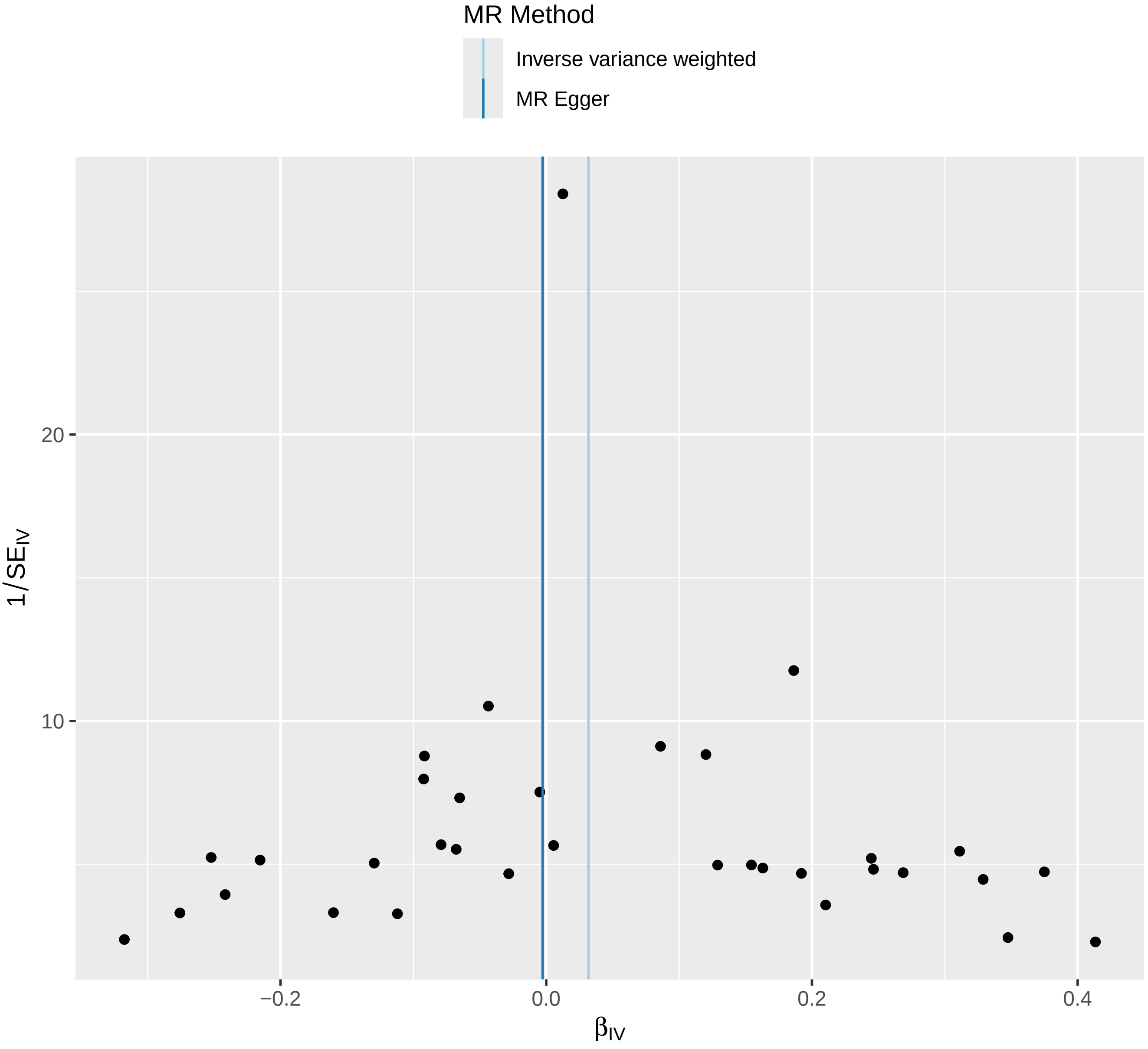
MR Method
Inverse variance weighted
MR Egger
20
10
1/SE_IV
−0.2
0.0
0.2
0.4
β_IV

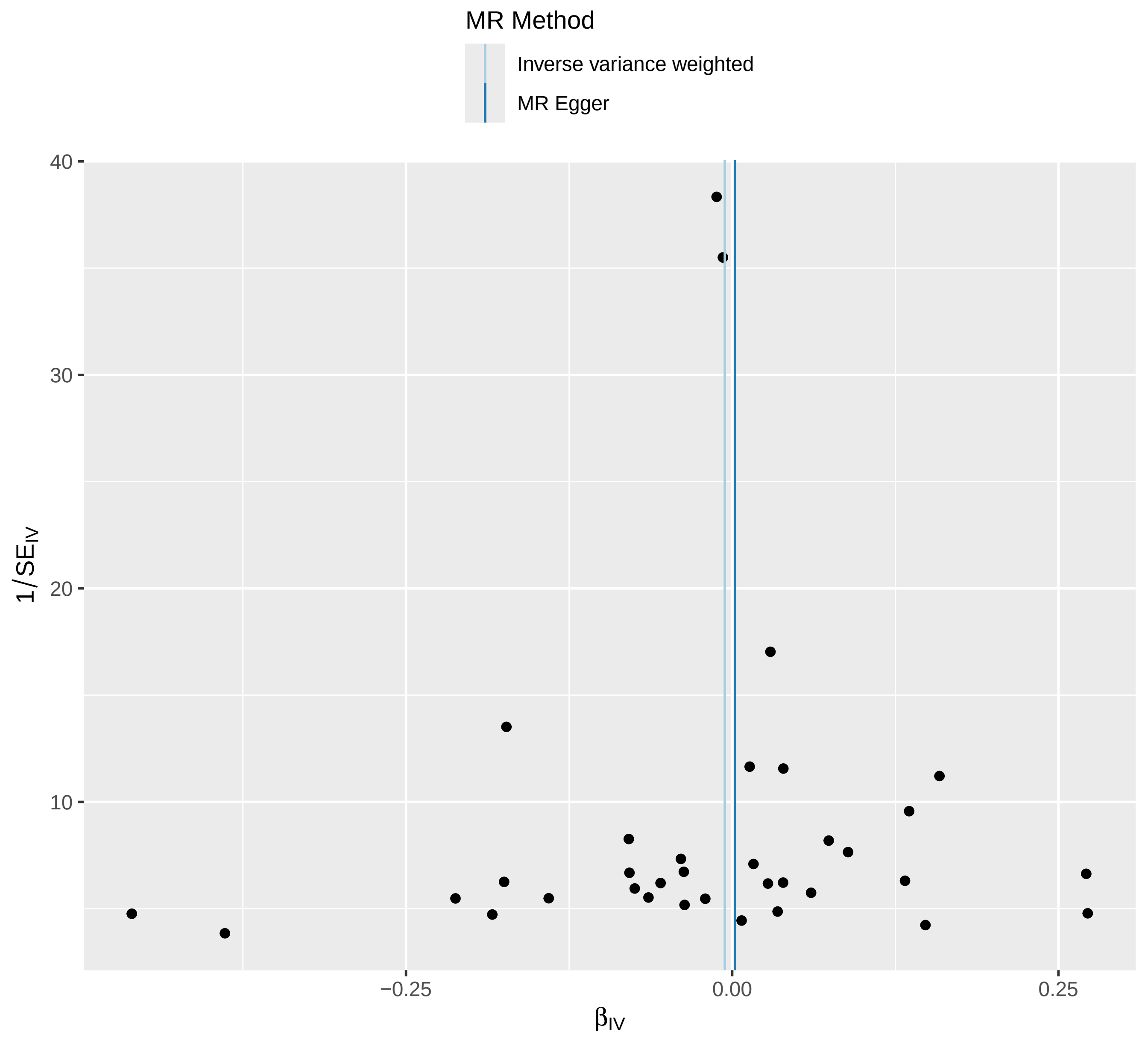
MR Method
Inverse variance weighted
MR Egger
40
30
20
10
−0.25
0.00
0.25
1/SE_IV
β_IV

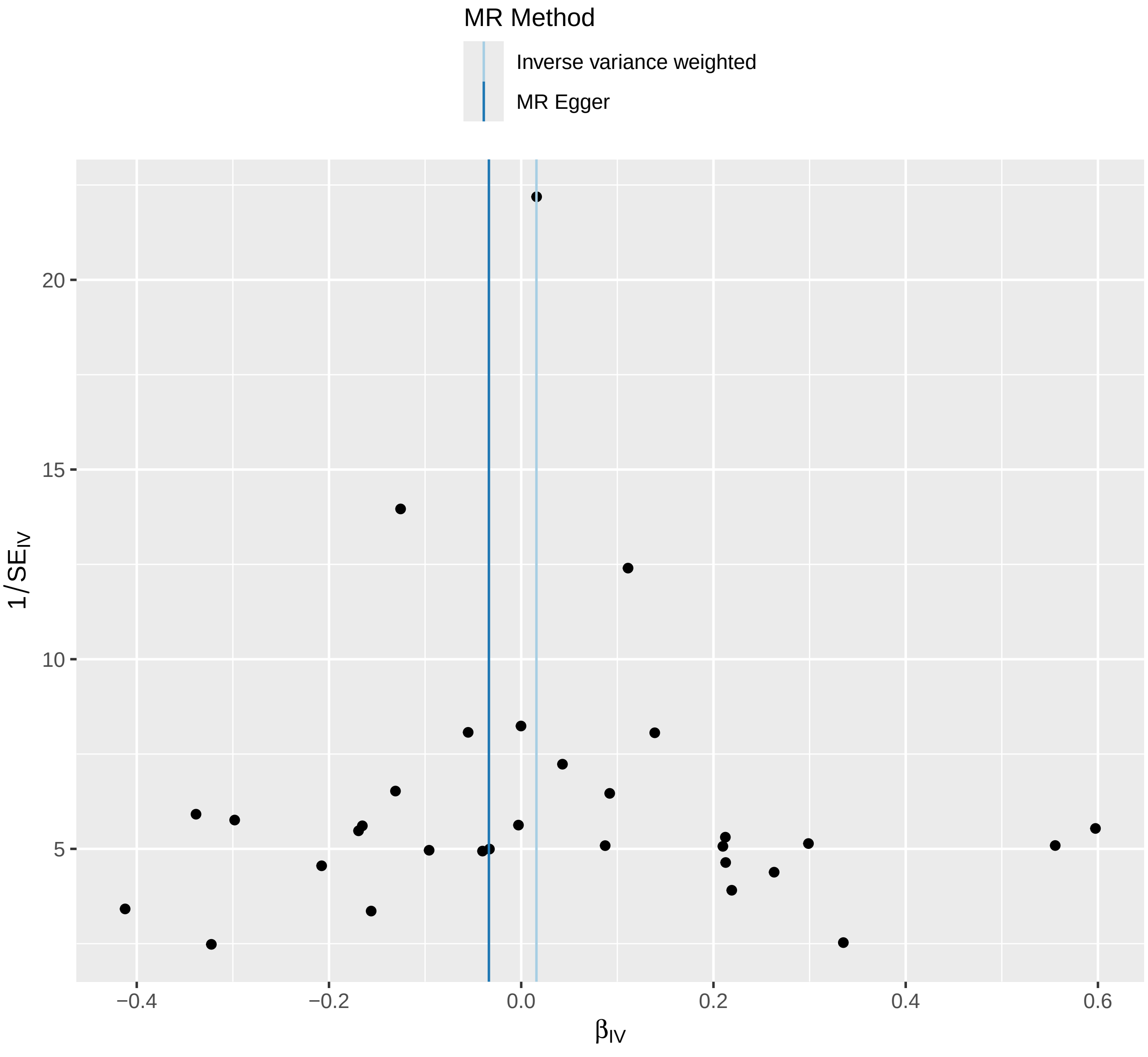

MR Method
Inverse variance weighted
MR Egger
20
15
10
5
−0.4
−0.2
0.0
0.2
0.4
0.6
$1/SE_{IV}$
β_{IV}

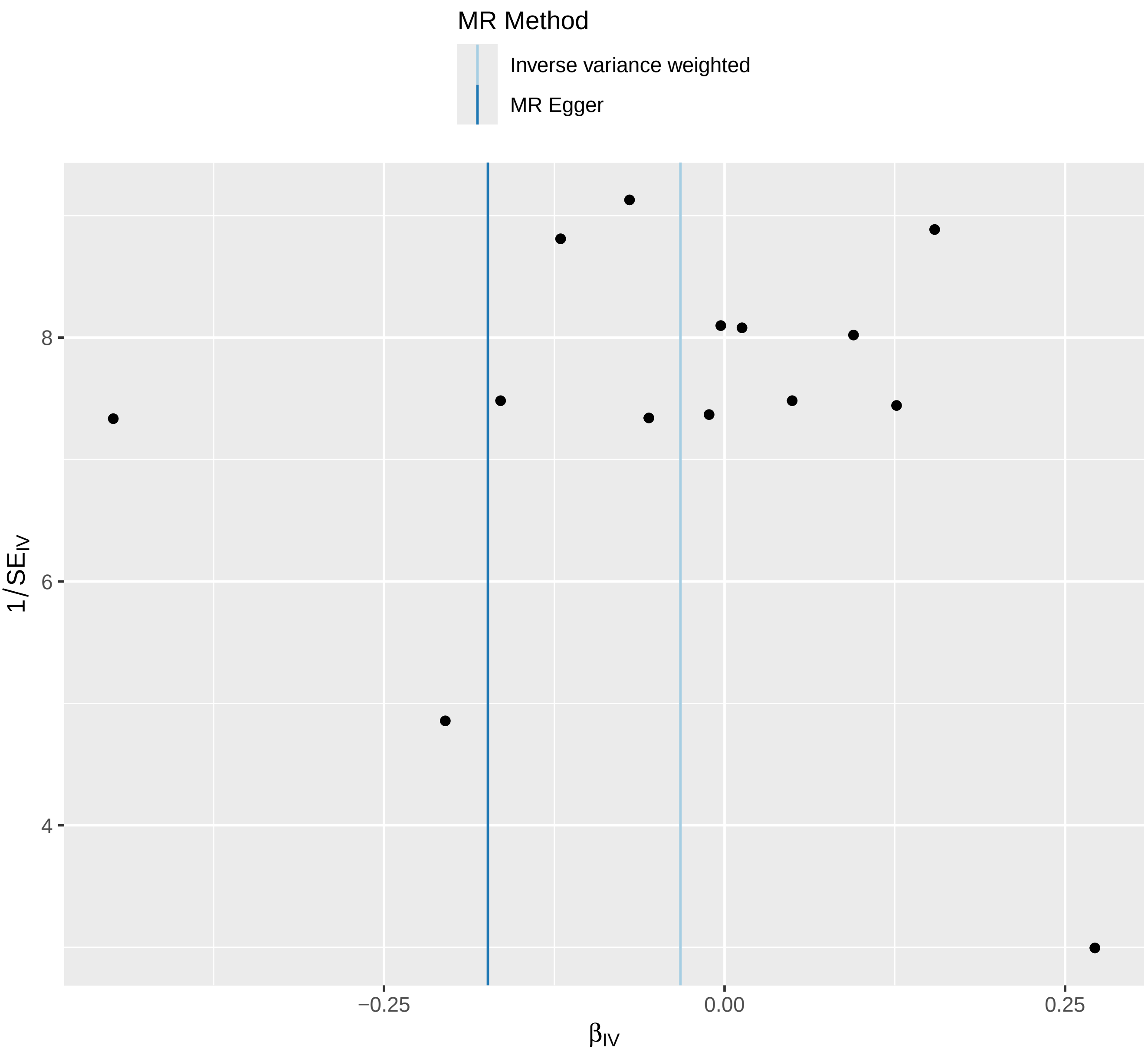
MR Method
Inverse variance weighted
MR Egger
8
6
4
1/SE$_{IV}$
−0.25
0.00
0.25
β$_{IV}$

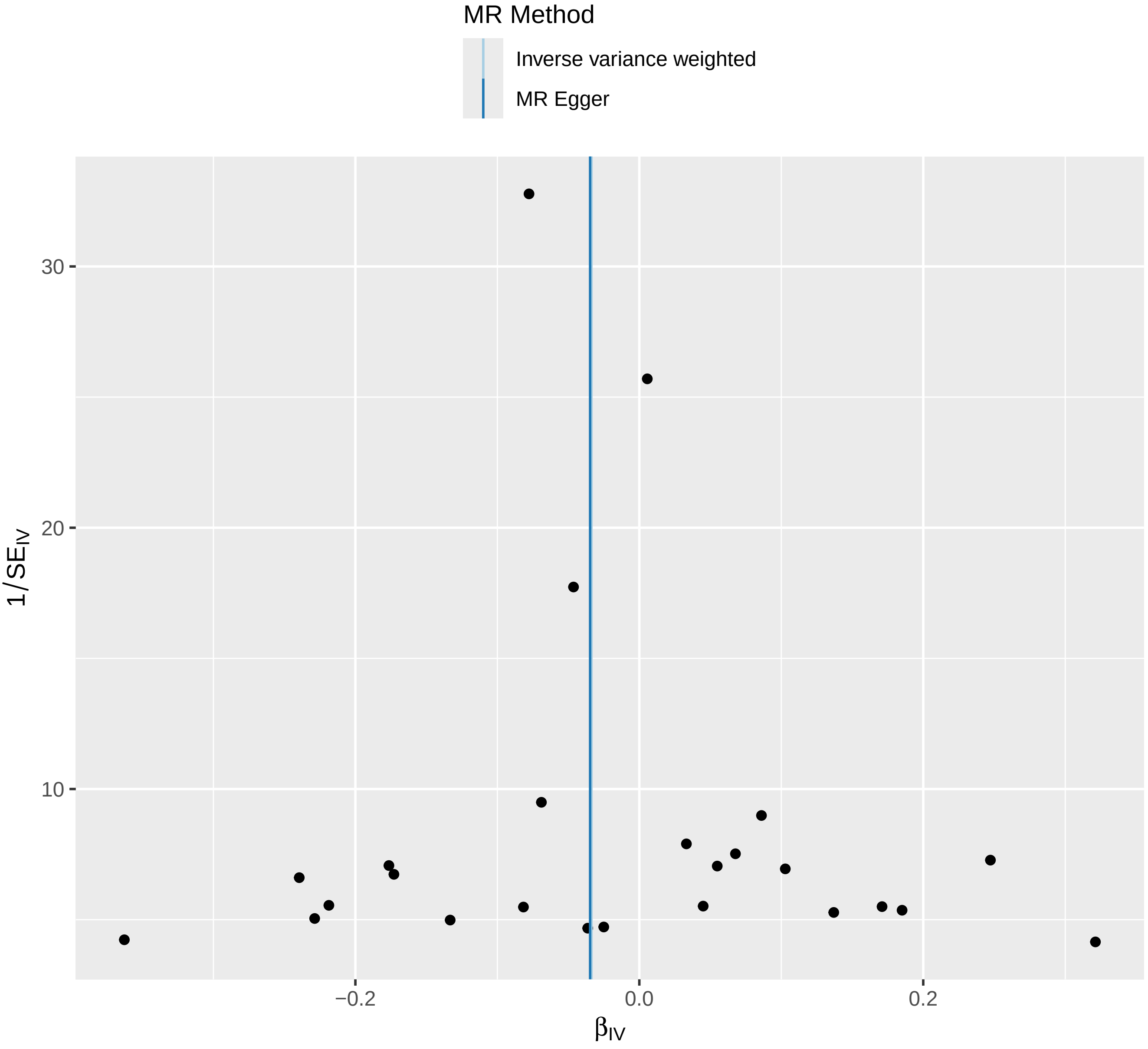

MR Method
Inverse variance weighted
MR Egger
30
20
10
1/SE_IV
−0.2
0.0
0.2
β_IV

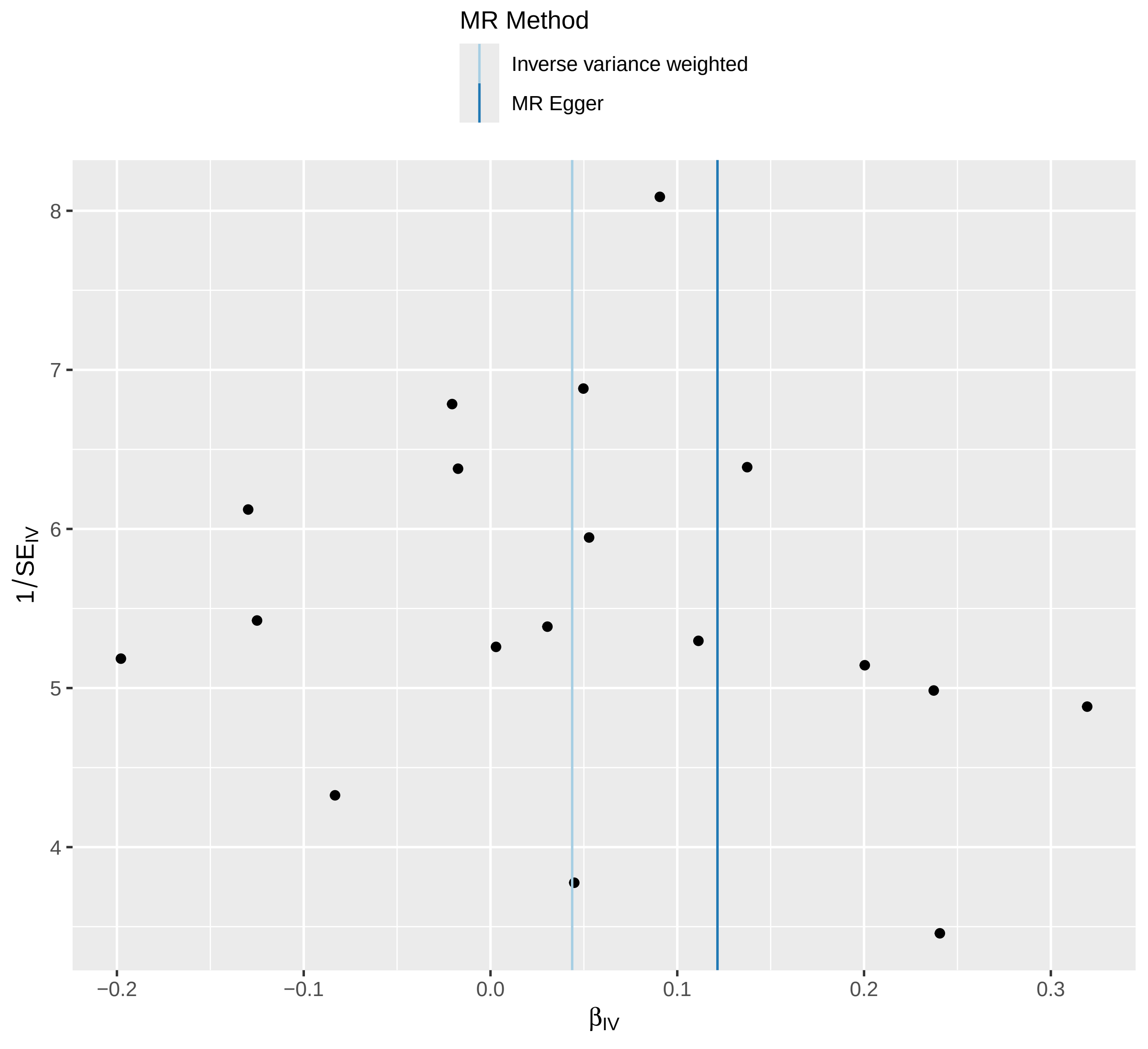

MR Method
Inverse variance weighted
MR Egger
8
7
6
5
4
1/SE_IV
−0.2
−0.1
0.0
0.1
0.2
0.3
β_IV

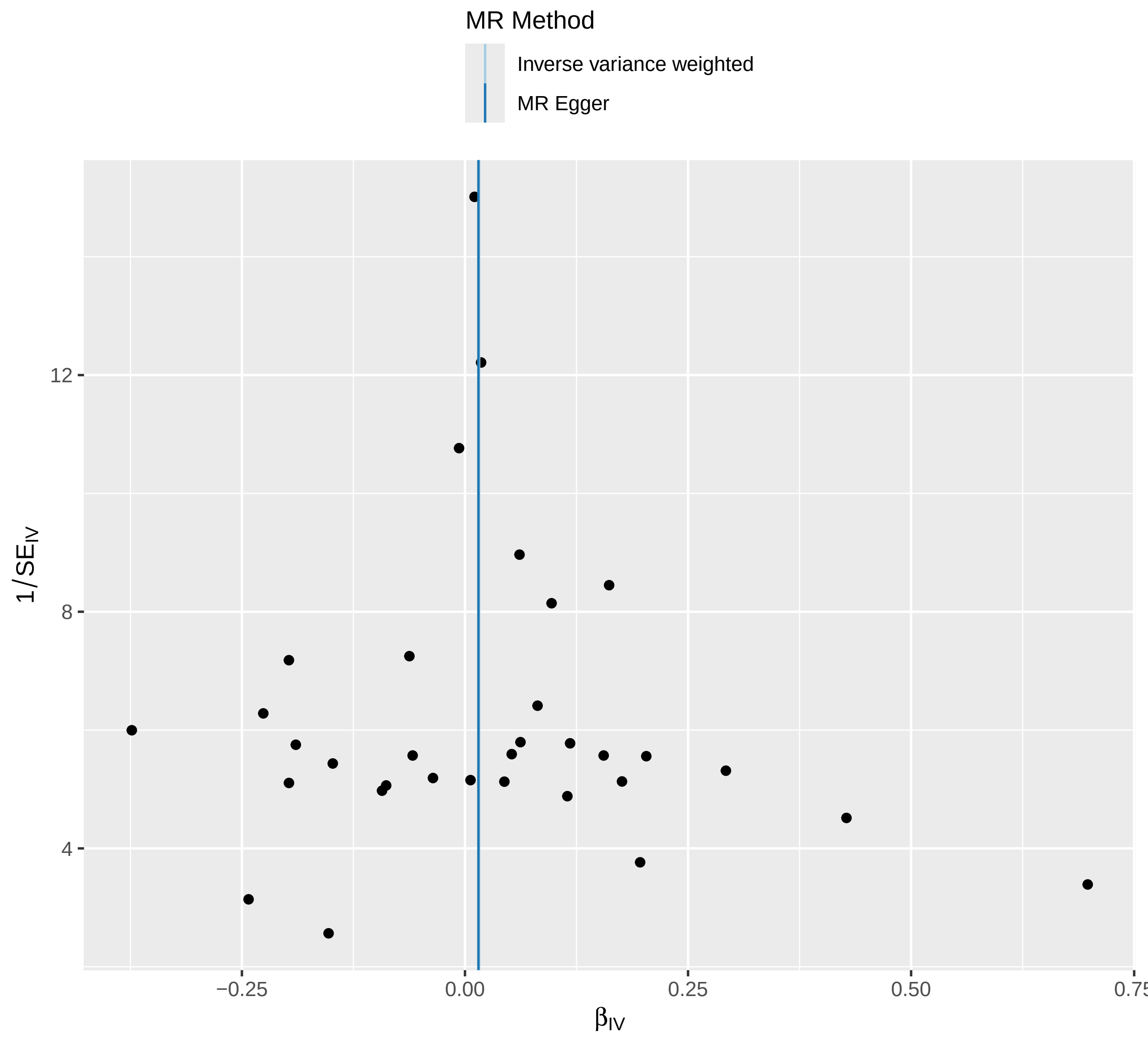

MR Method
Inverse variance weighted
MR Egger
1/SE_IV
12
8
4
−0.25
0.00
0.25
0.50
0.75
β_IV

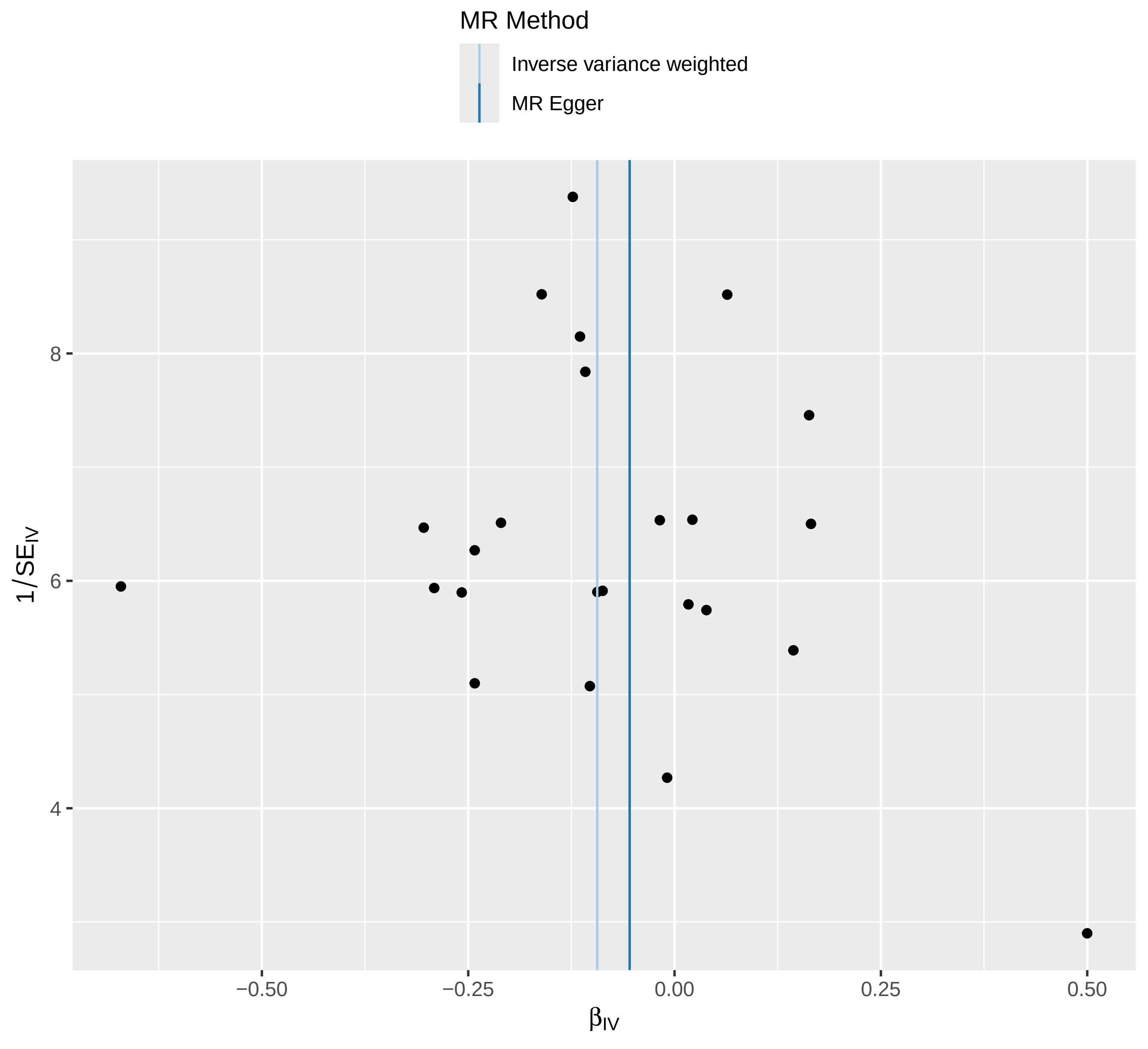
MR Method
Inverse variance weighted
MR Egger
8
6
4
1/SE_IV
−0.50
−0.25
0.00
0.25
0.50
β_IV

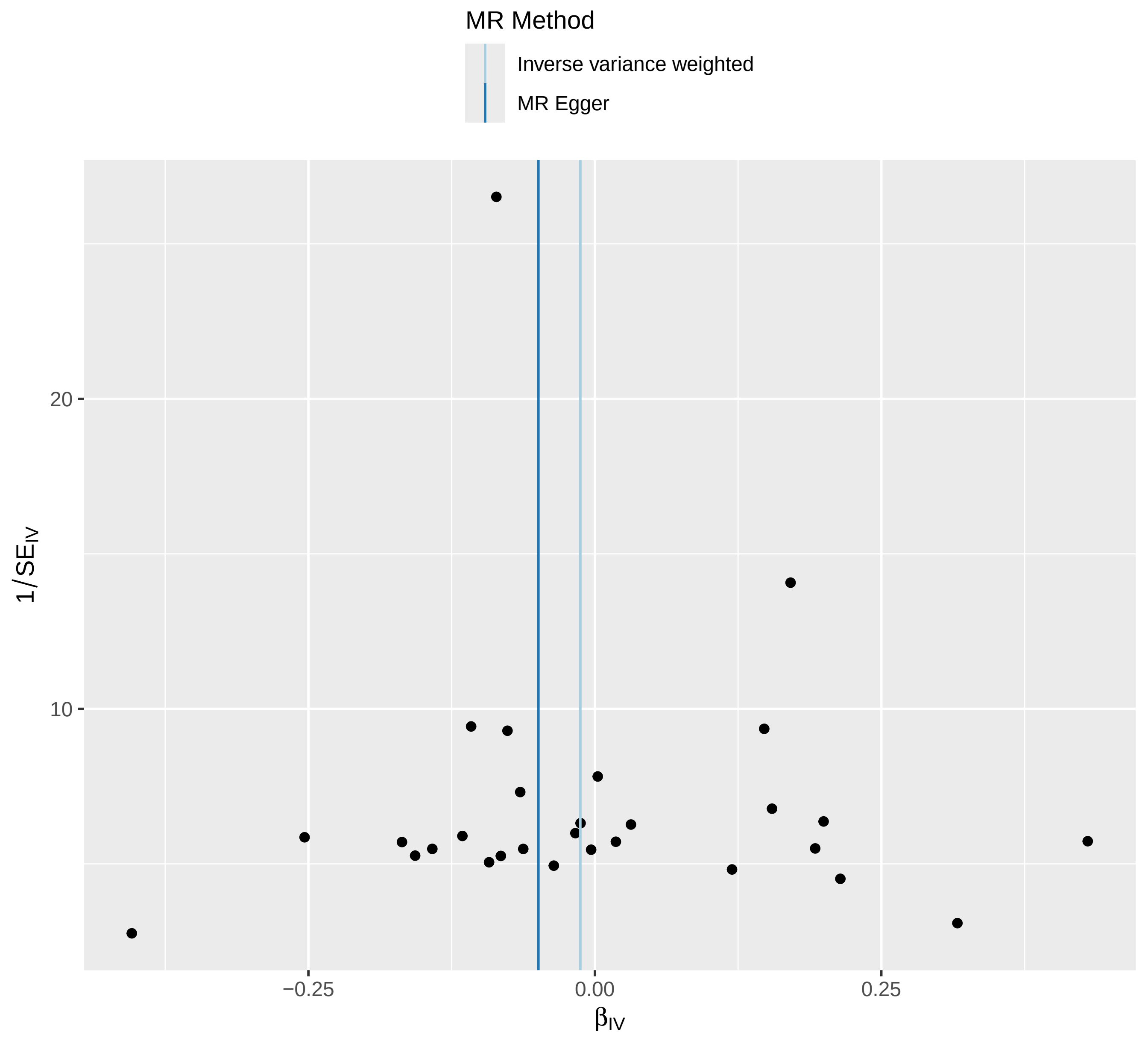
MR Method
Inverse variance weighted
MR Egger
20
10
1/SE_IV
−0.25
0.00
0.25
β_IV

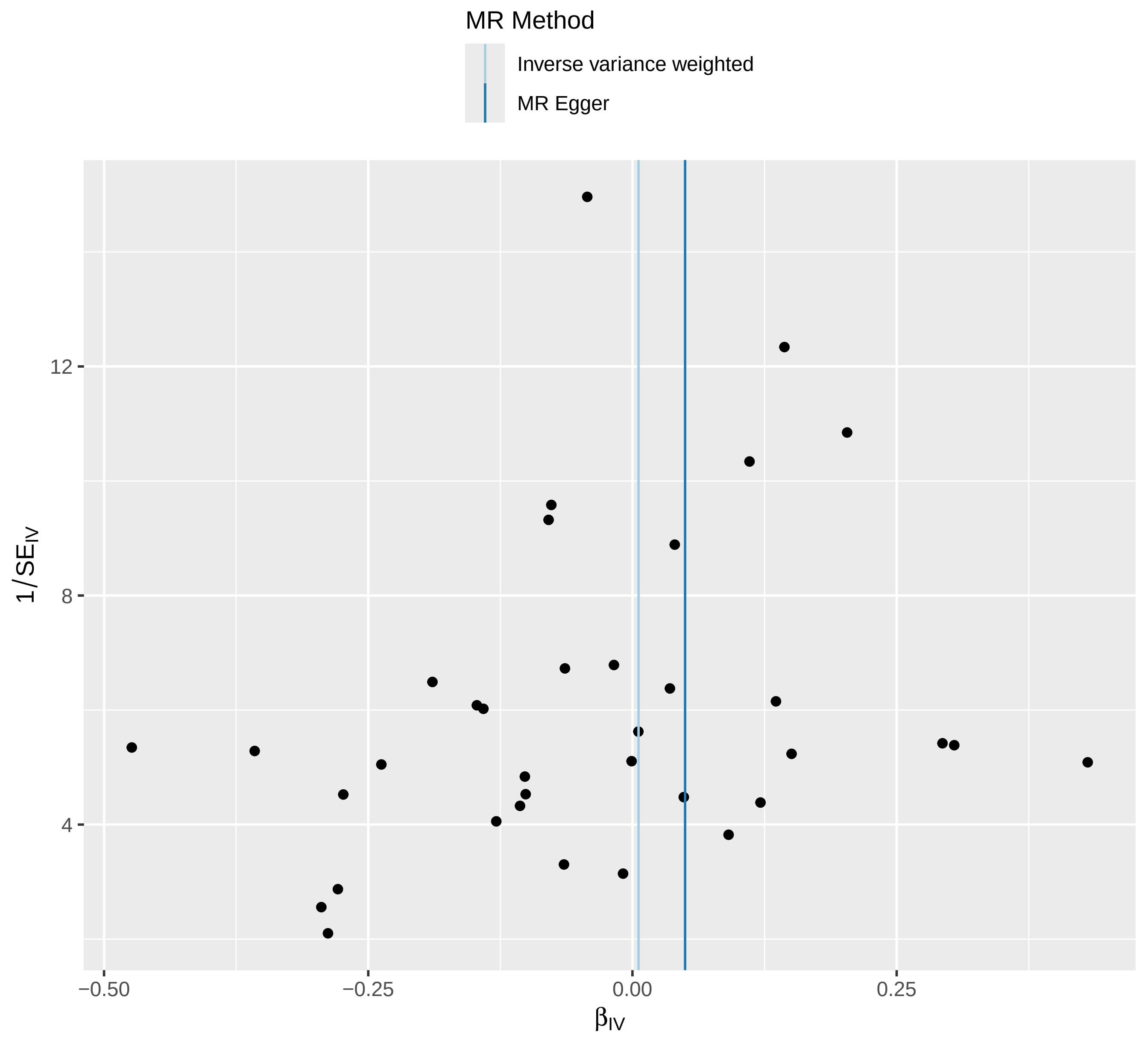

MR Method
Inverse variance weighted
MR Egger
12
8
4
1/SE_IV
−0.50
−0.25
0.00
0.25
β_IV

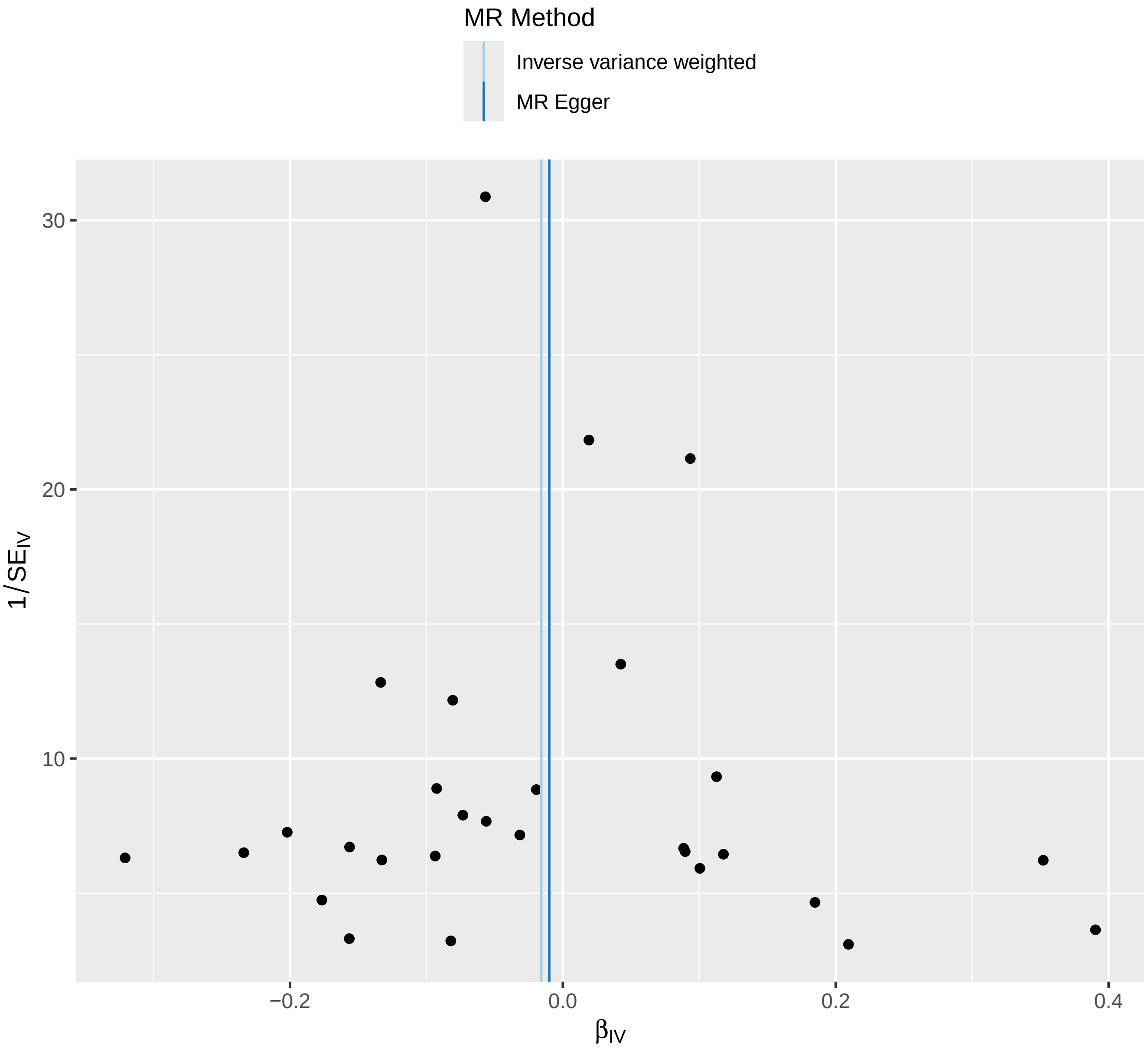
MR Method
Inverse variance weighted
MR Egger
30
20
10
$1/SE_{IV}$
−0.2
0.0
0.2
0.4
β_{IV}

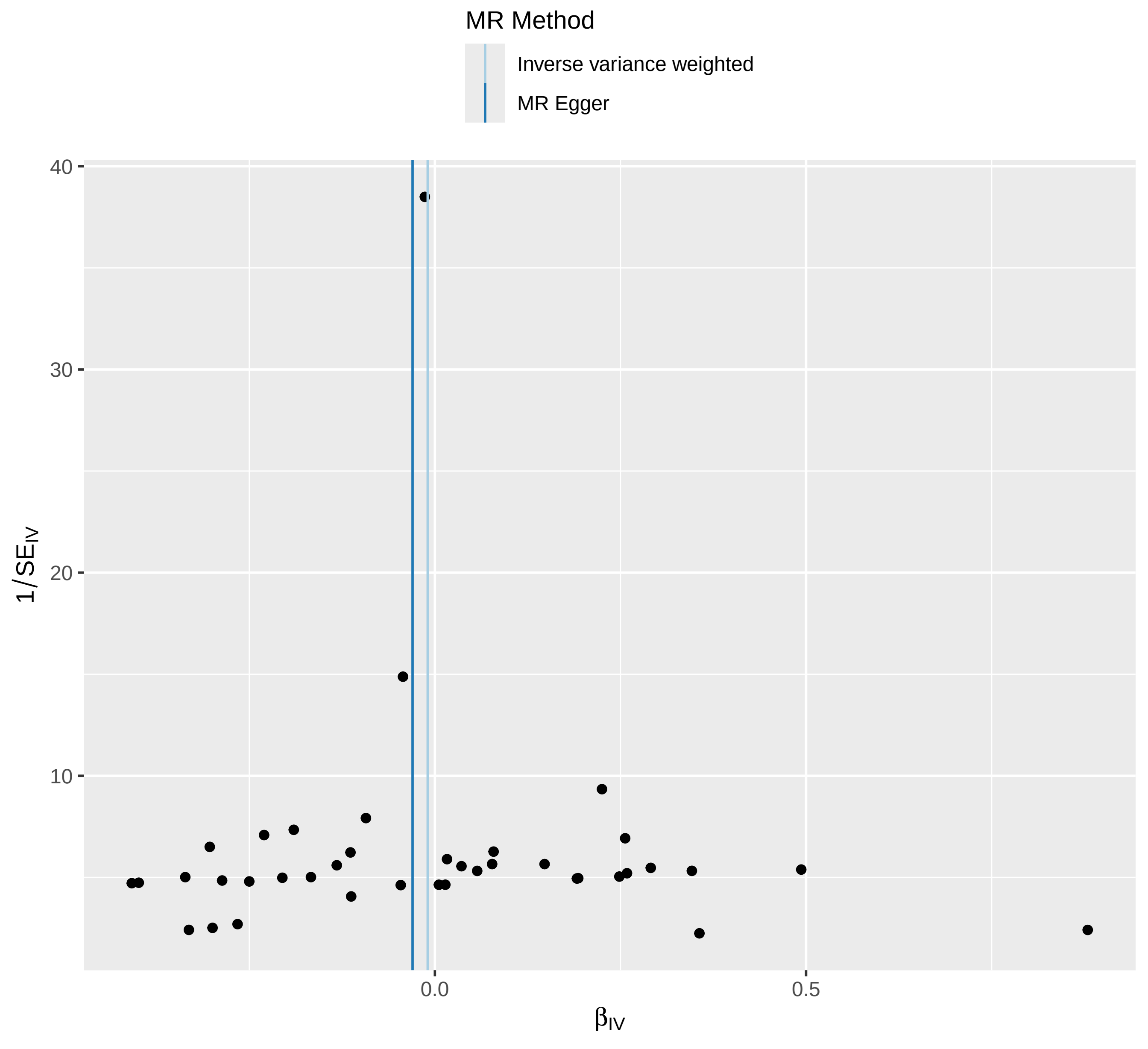

MR Method
Inverse variance weighted
MR Egger
40
30
20
10
0.0
0.5
$1/SE_{IV}$
β_{IV}

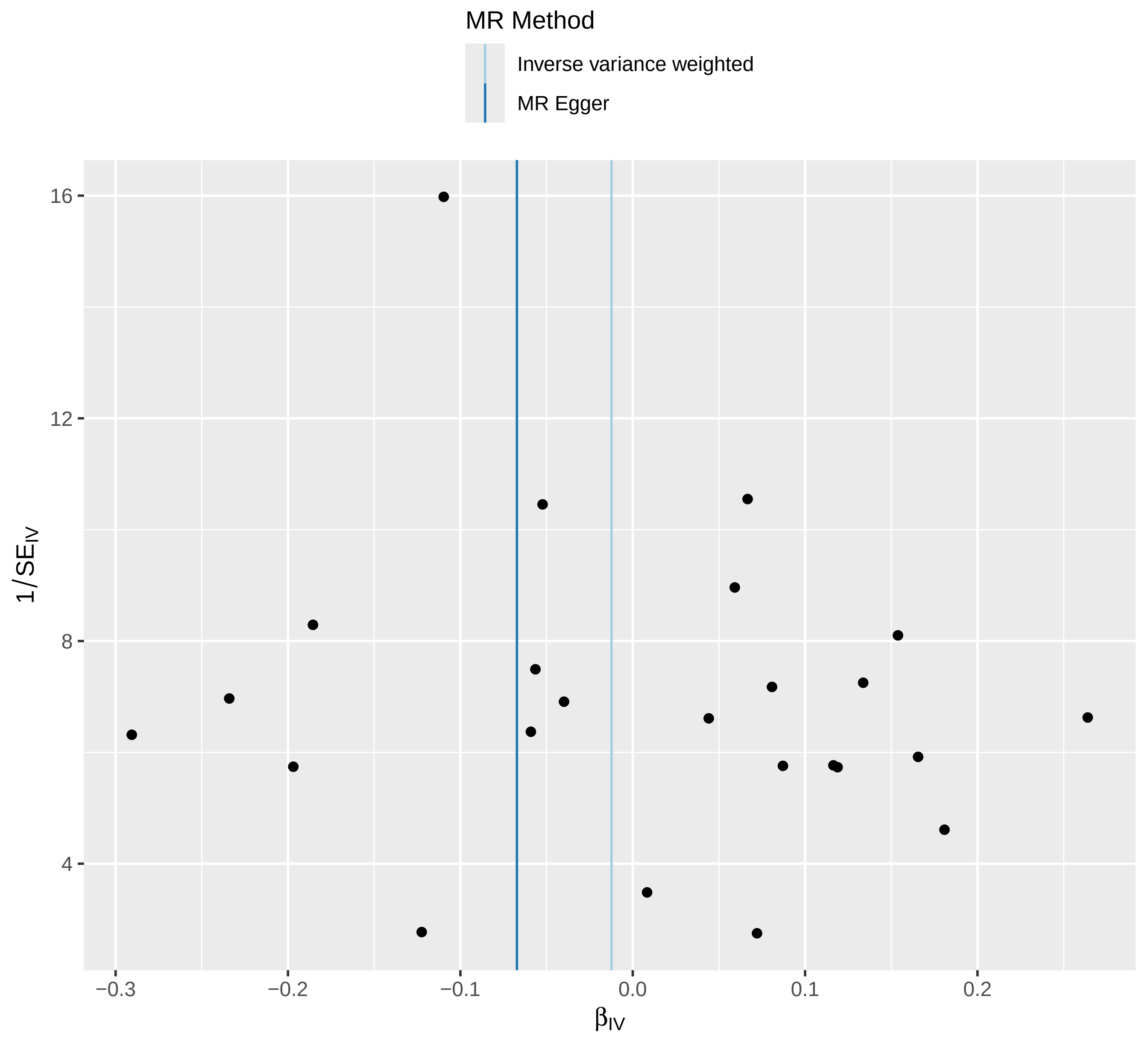
MR Method
Inverse variance weighted
MR Egger
16
12
8
4
1/SE_IV
−0.3
−0.2
−0.1
0.0
0.1
0.2
β_IV

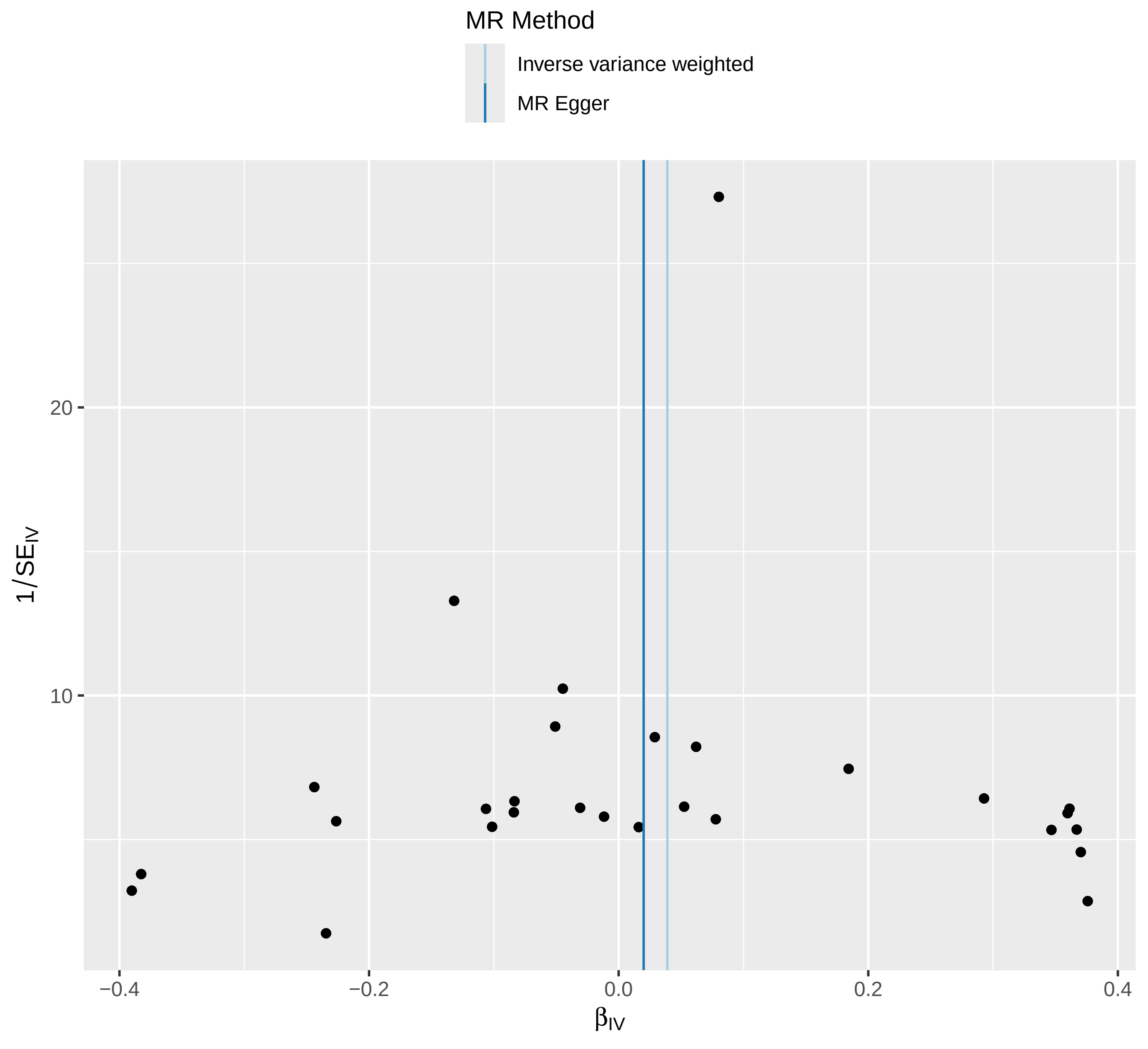
MR Method
Inverse variance weighted
MR Egger
20
10
1/SE_IV
−0.4
−0.2
0.0
0.2
0.4
β_IV

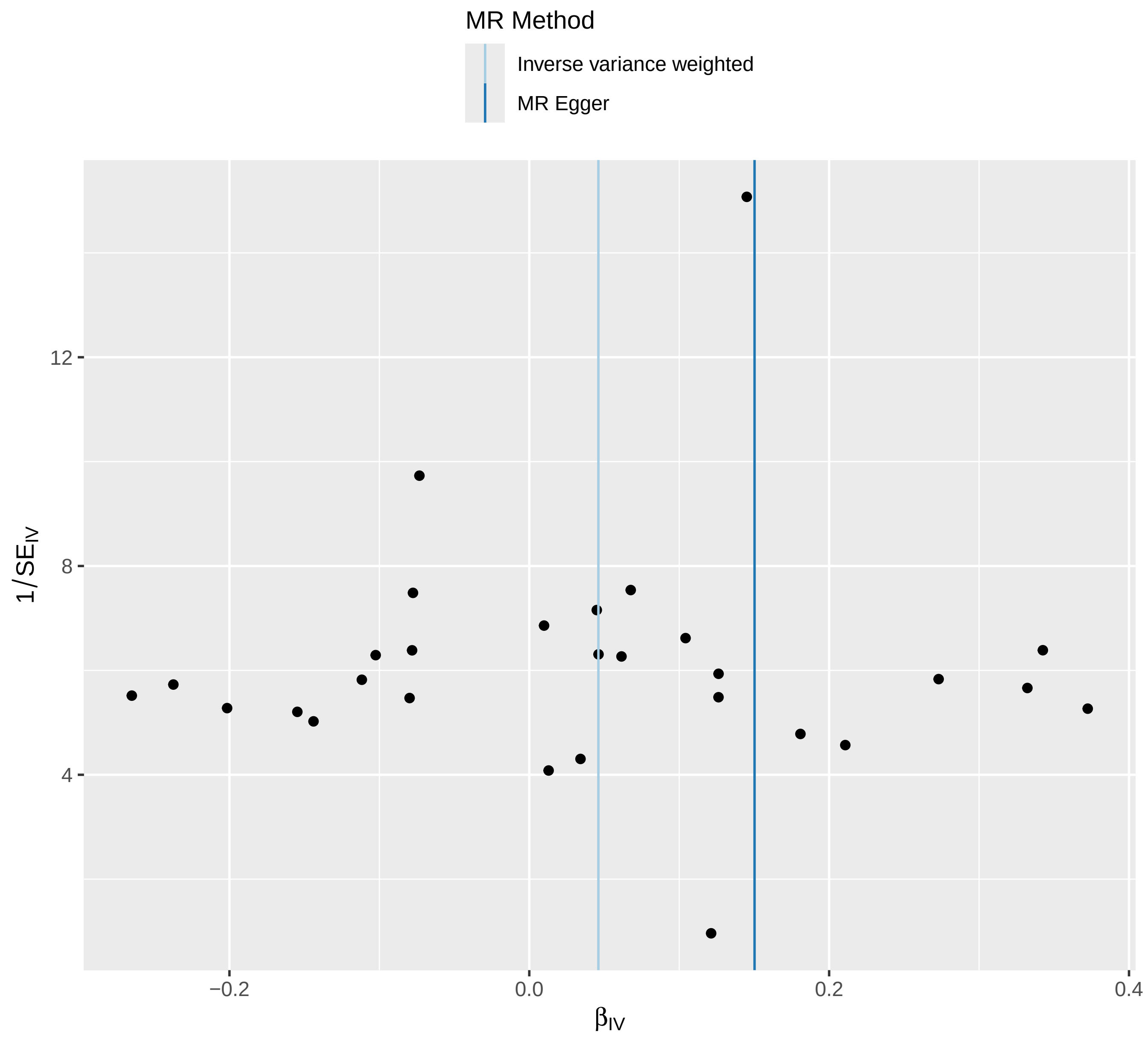
MR Method
Inverse variance weighted
MR Egger
1/SE_IV
12
8
4
−0.2
0.0
0.2
0.4
β_IV

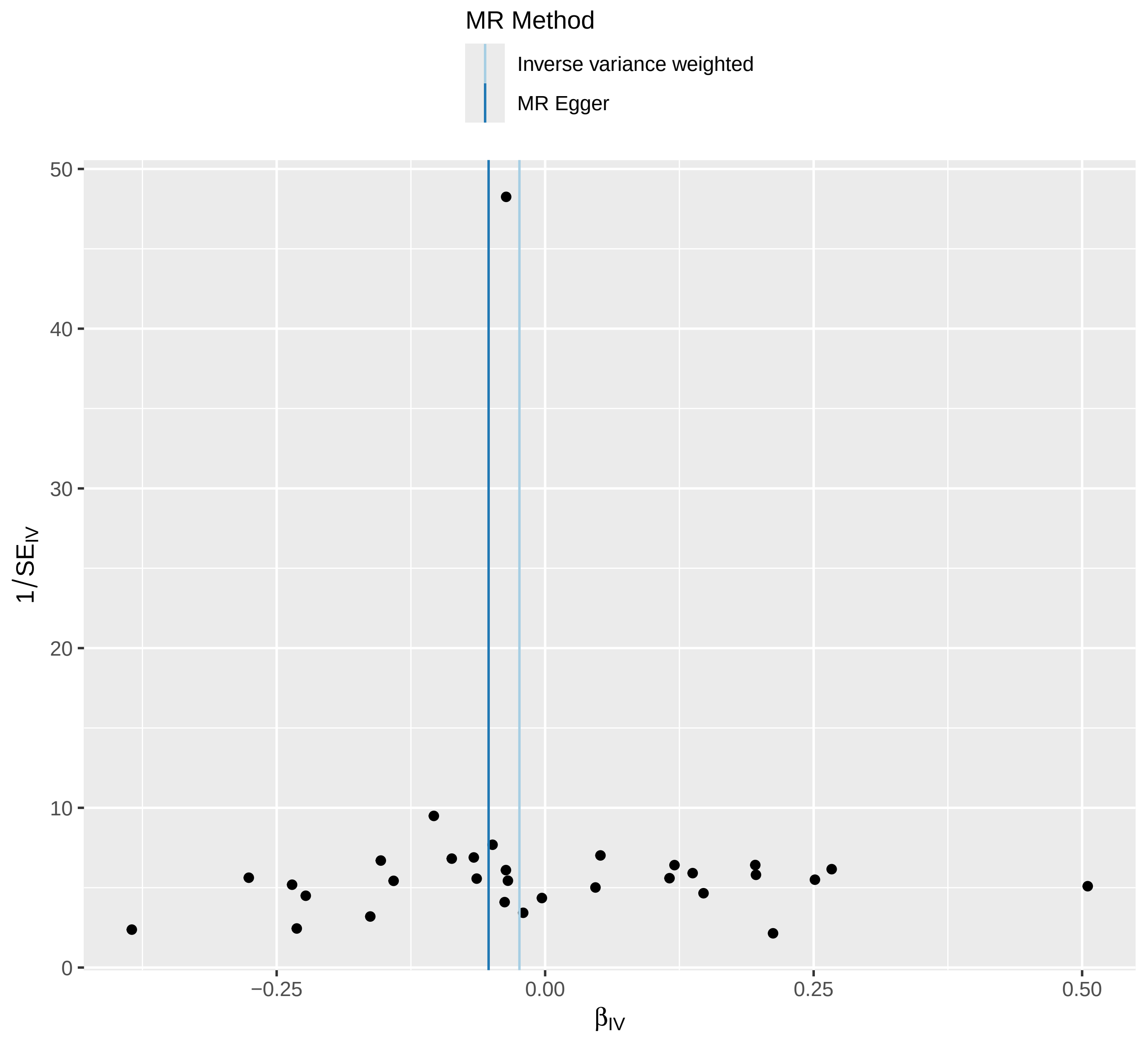

MR Method
Inverse variance weighted
MR Egger
50
40
30
20
10
0
−0.25
0.00
0.25
0.50
$1/SE_{IV}$
β_{IV}

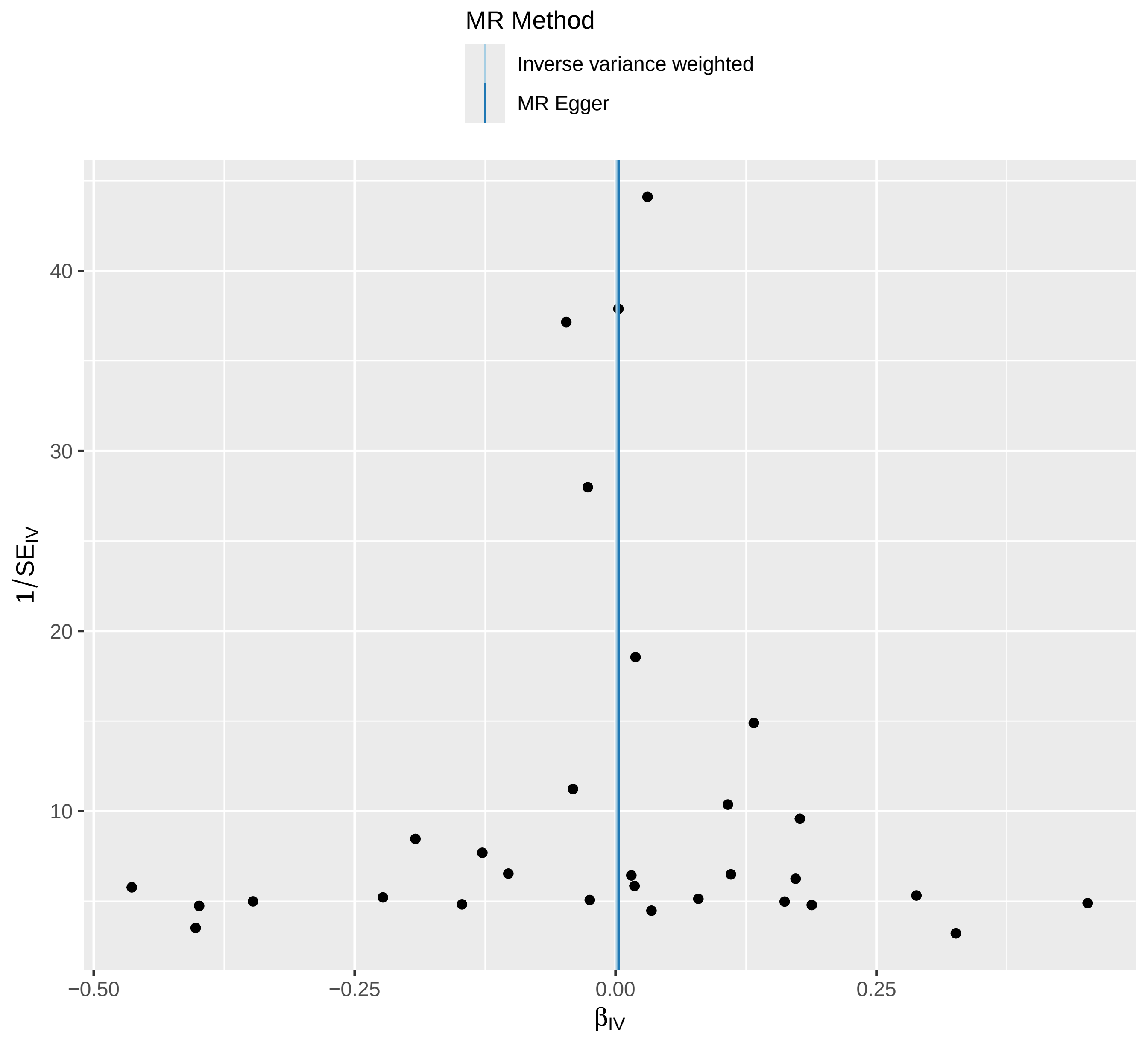
MR Method
Inverse variance weighted
MR Egger
40
30
20
10
1/SE_IV
−0.50
−0.25
0.00
0.25
β_IV

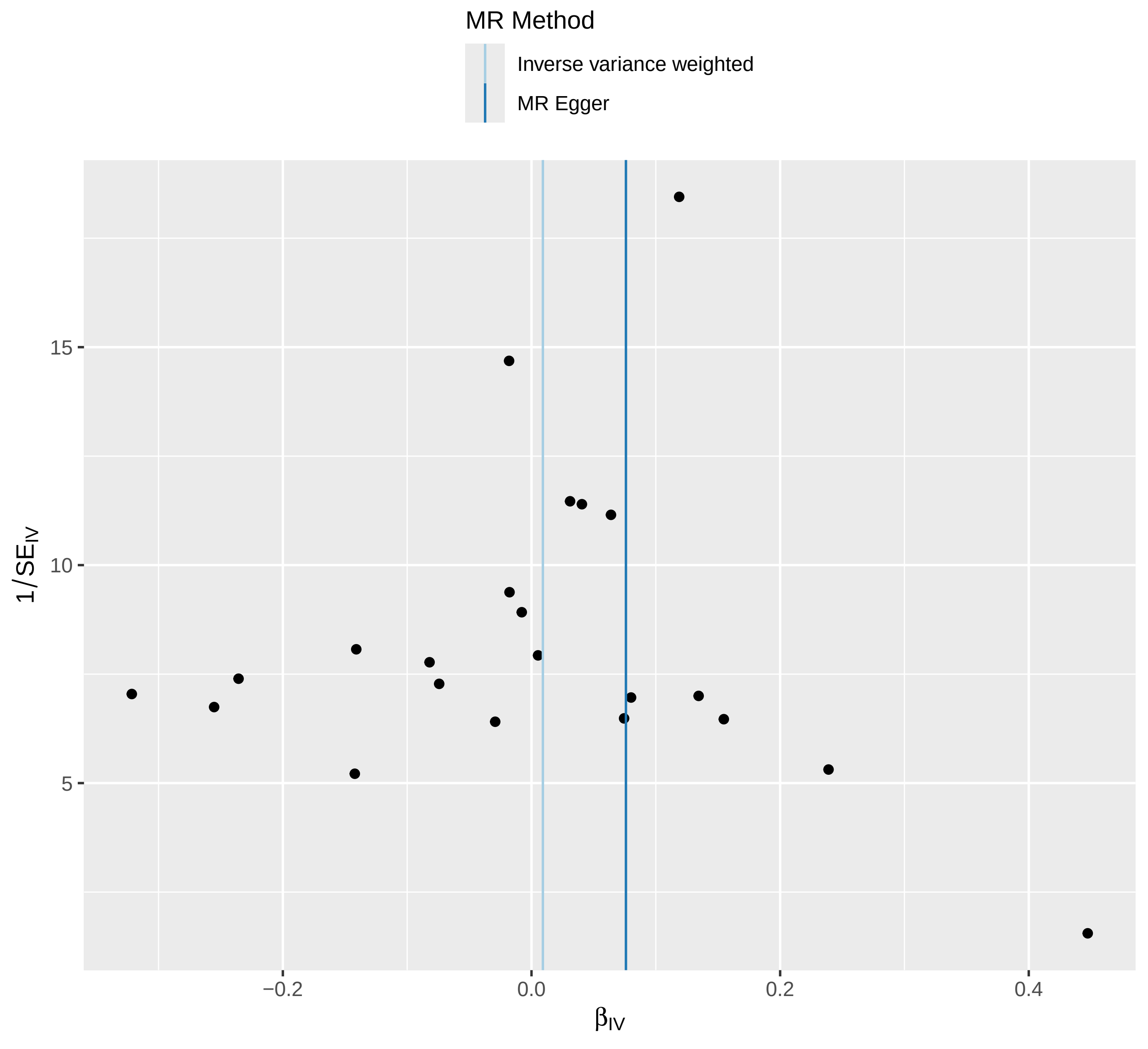
MR Method
Inverse variance weighted
MR Egger
1/SE_IV
15
10
5
−0.2
0.0
0.2
0.4
β_IV

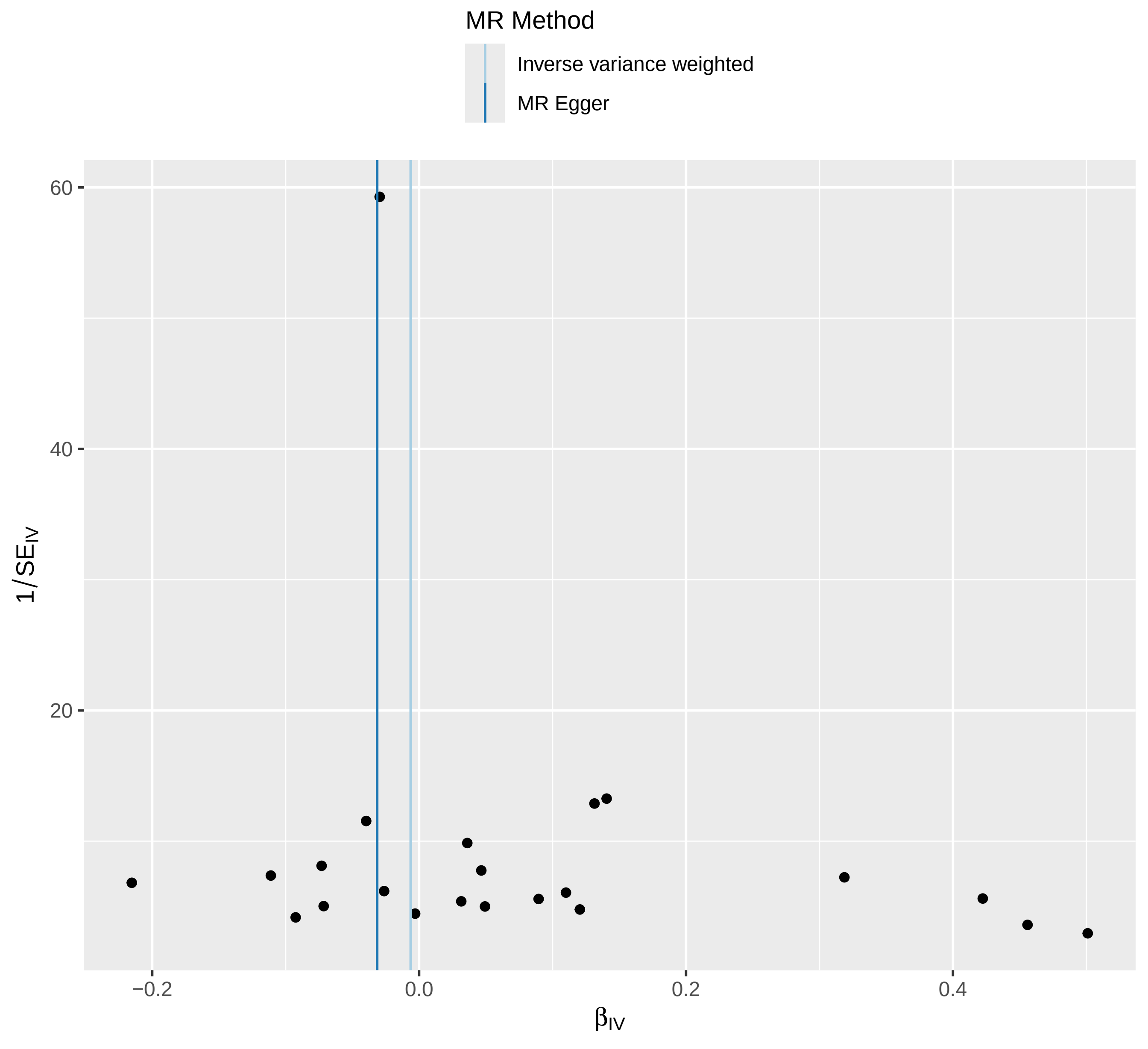
MR Method
Inverse variance weighted
MR Egger
60
40
20
1/SE$_{IV}$
−0.2
0.0
0.2
0.4
β$_{IV}$

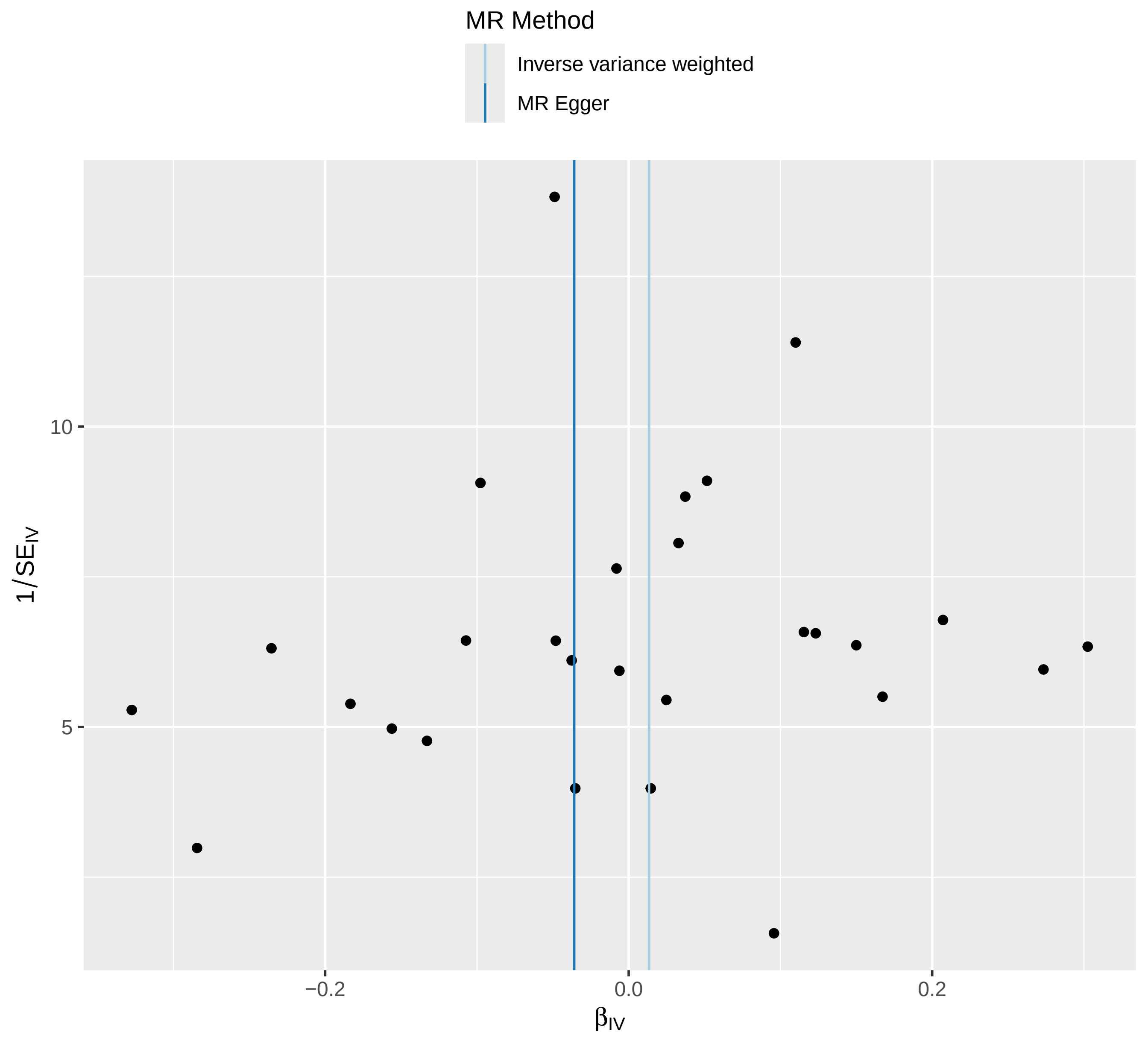

MR Method
Inverse variance weighted
MR Egger
10
5
1/SE_IV
−0.2
0.0
0.2
β_IV

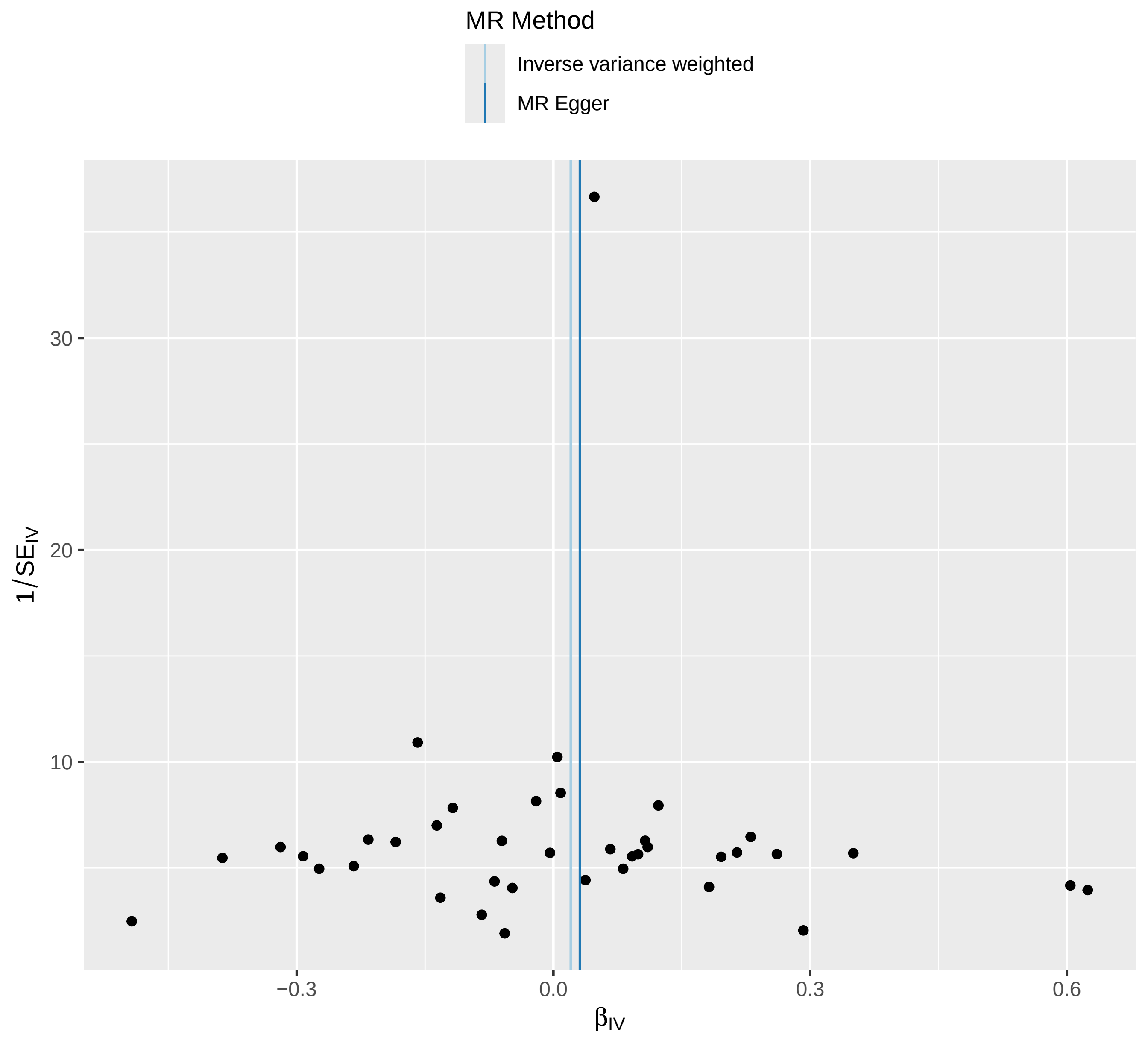
MR Method
Inverse variance weighted
MR Egger
30
20
10
1/SE_IV
−0.3
0.0
0.3
0.6
β_IV

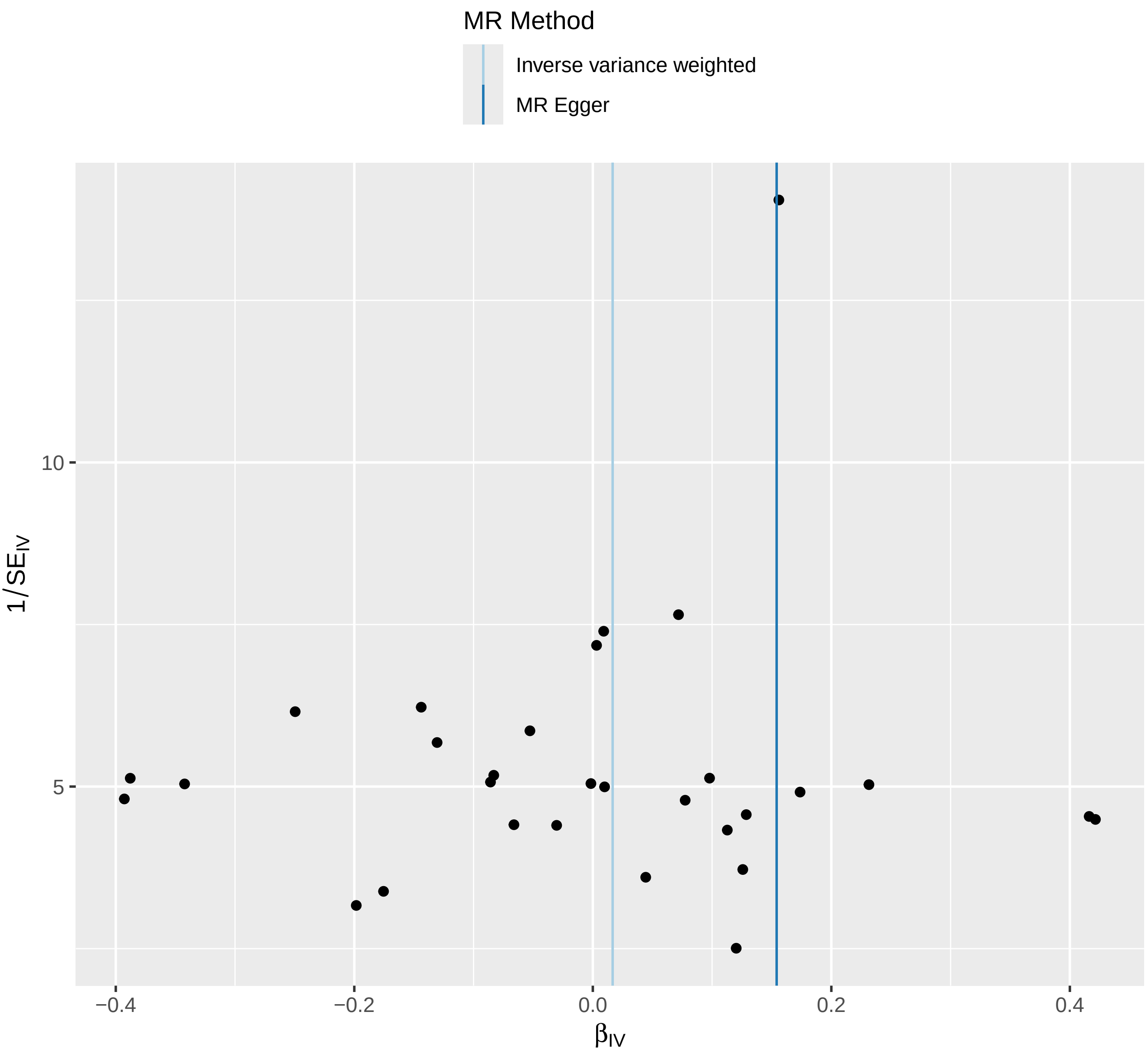

MR Method
Inverse variance weighted
MR Egger
10
5
$1/SE_{IV}$
−0.4
−0.2
0.0
0.2
0.4
β_{IV}

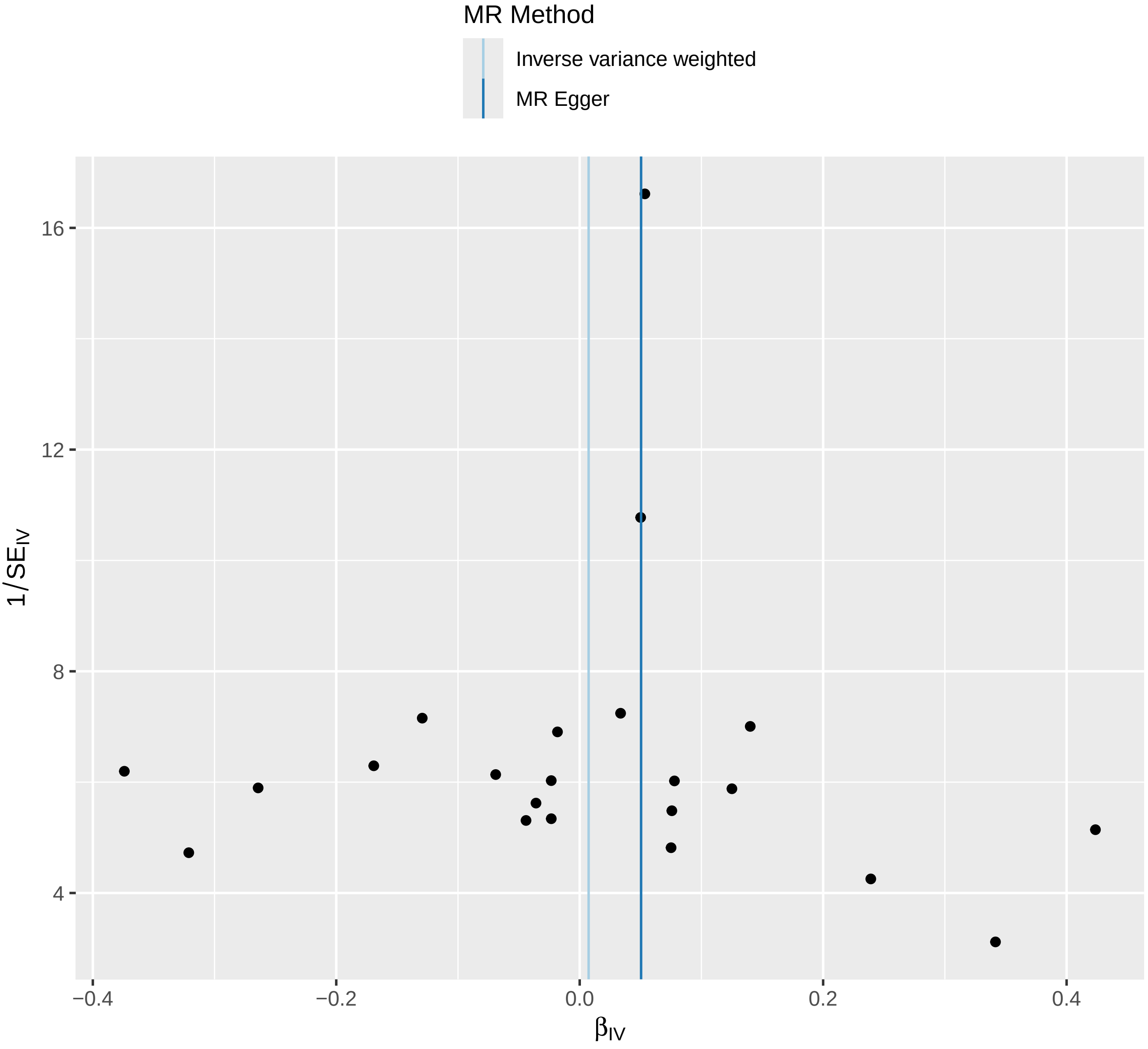
MR Method
Inverse variance weighted
MR Egger
16
12
8
4
1/SE_IV
−0.4
−0.2
0.0
0.2
0.4
β_IV

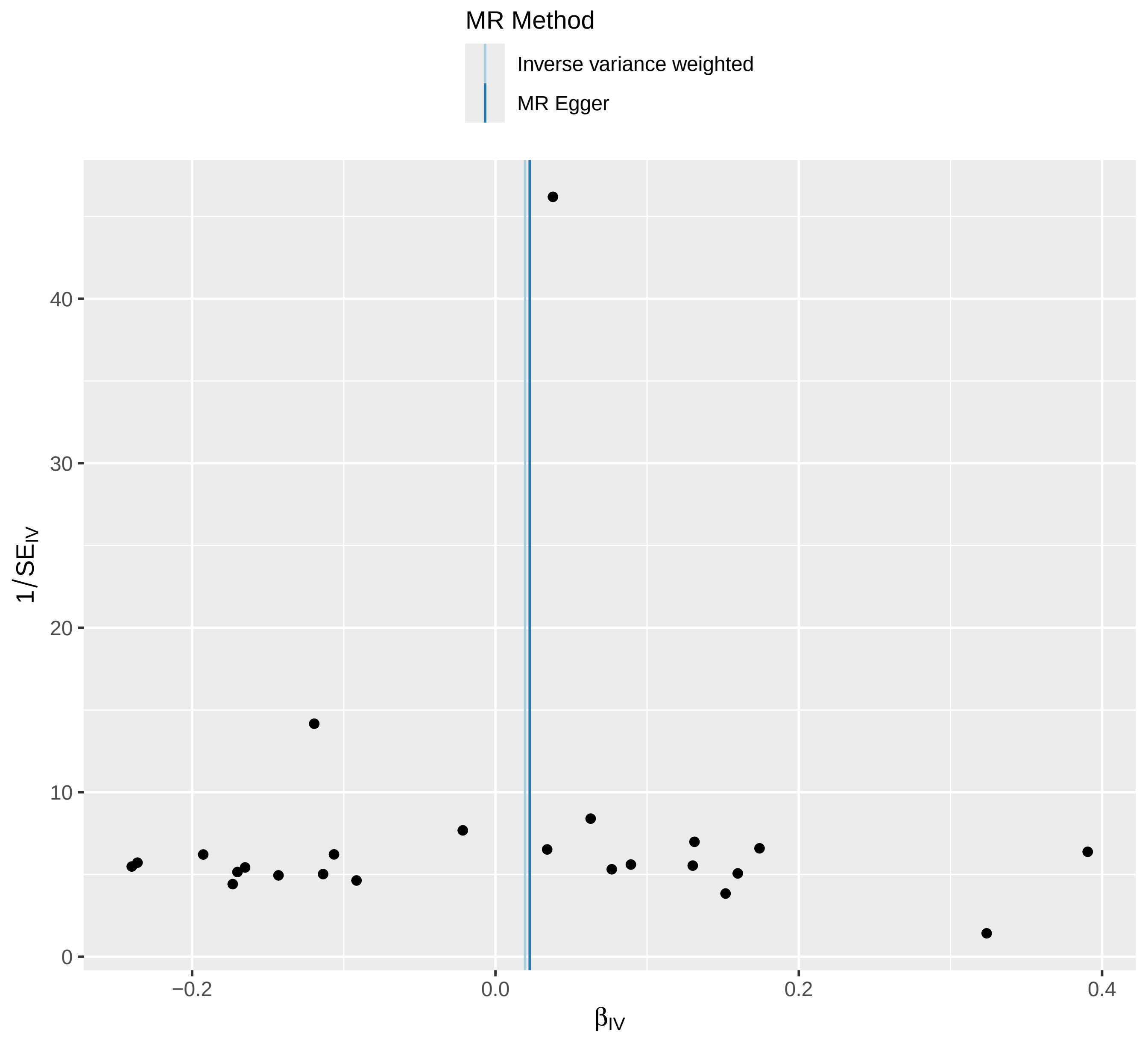

MR Method
Inverse variance weighted
MR Egger
$1/SE_{IV}$
β_{IV}
0
10
20
30
40
−0.2
0.0
0.2
0.4

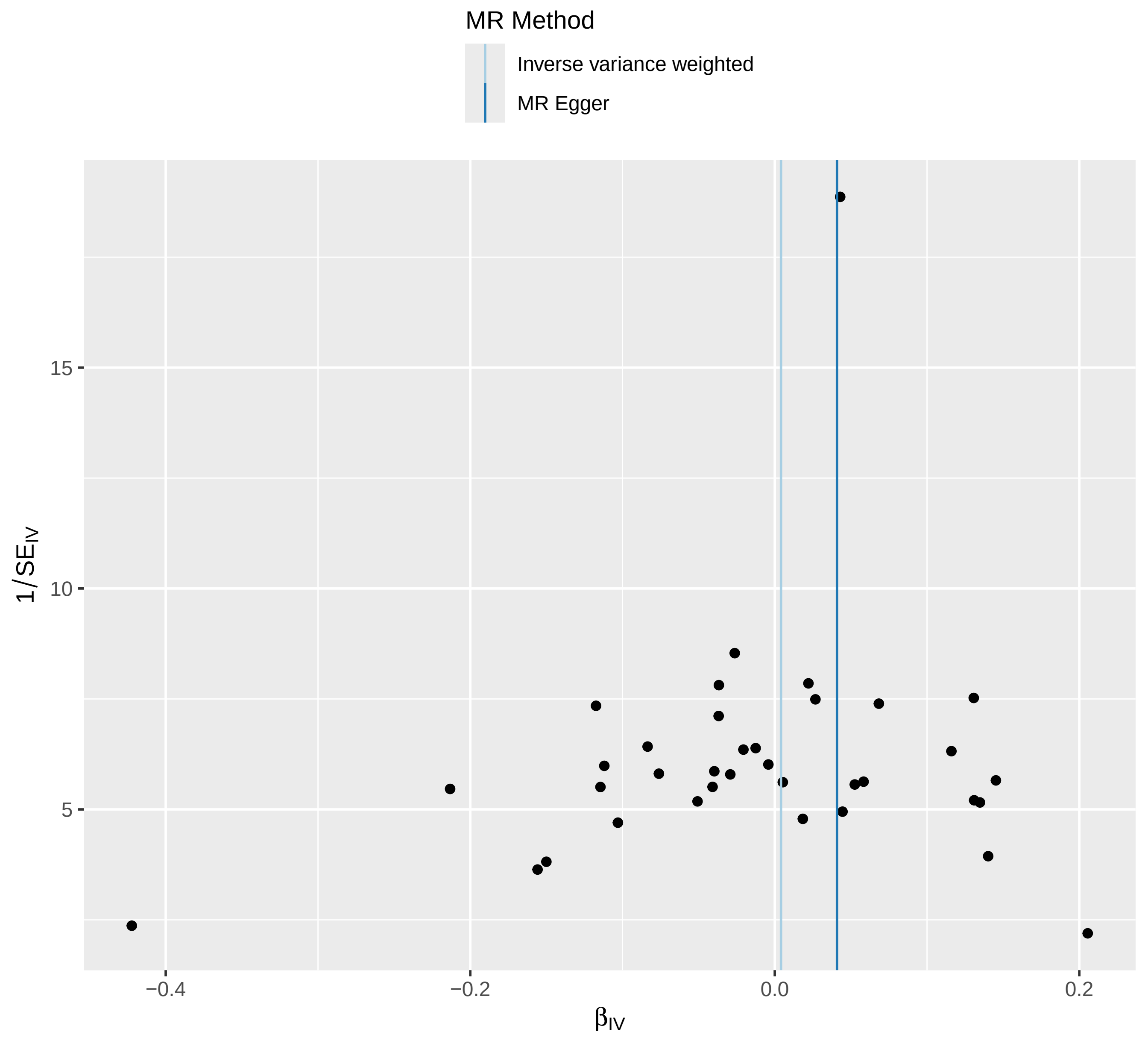
MR Method
Inverse variance weighted
MR Egger
15
10
5
1/SE_IV
−0.4
−0.2
0.0
0.2
β_IV

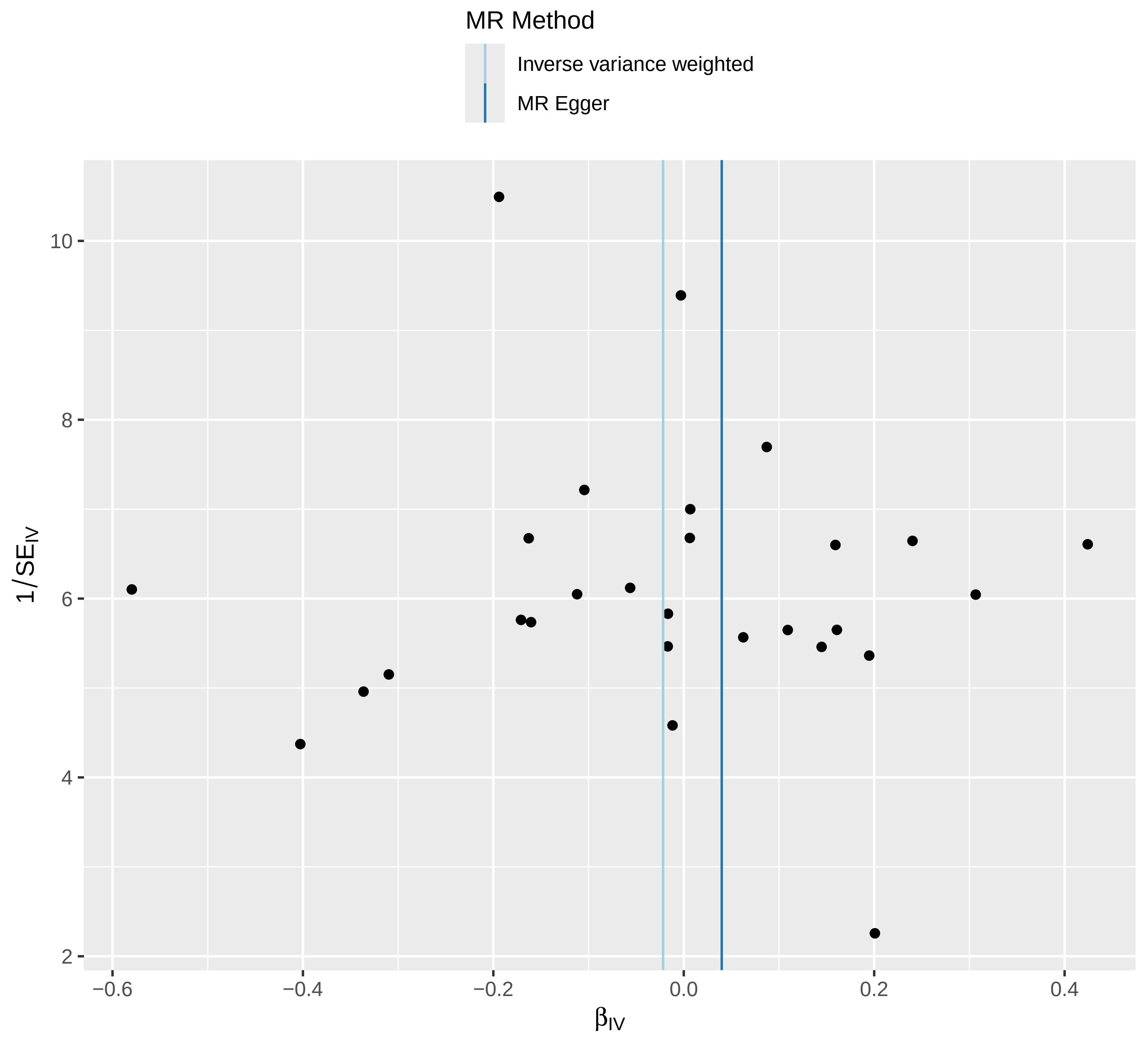

MR Method
Inverse variance weighted
MR Egger
1/SE_IV
10
8
6
4
2
−0.6
−0.4
−0.2
0.0
0.2
0.4
β_IV

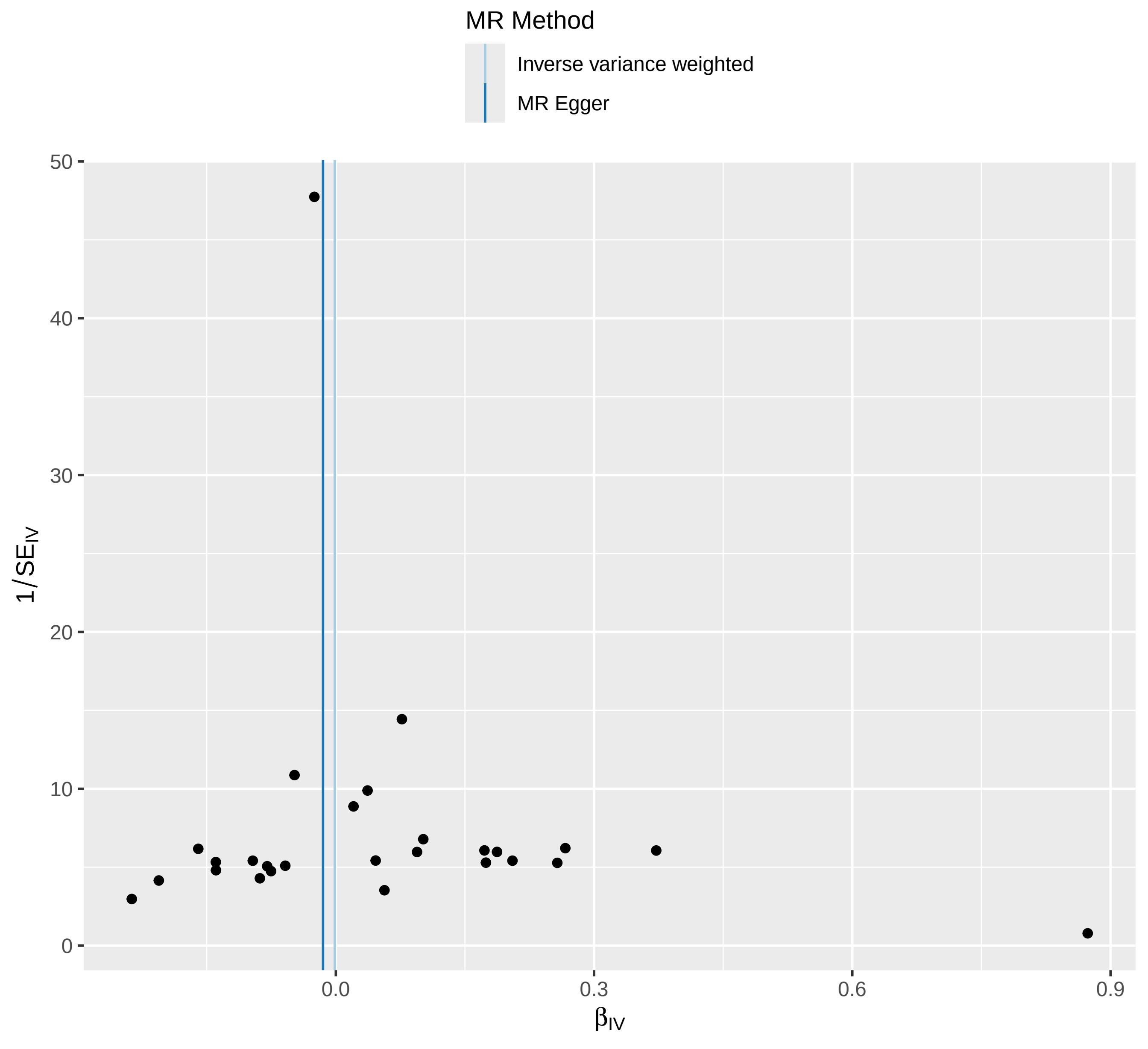
MR Method
Inverse variance weighted
MR Egger
$1/SE_{IV}$
β_{IV}
0
10
20
30
40
50
0.0
0.3
0.6
0.9

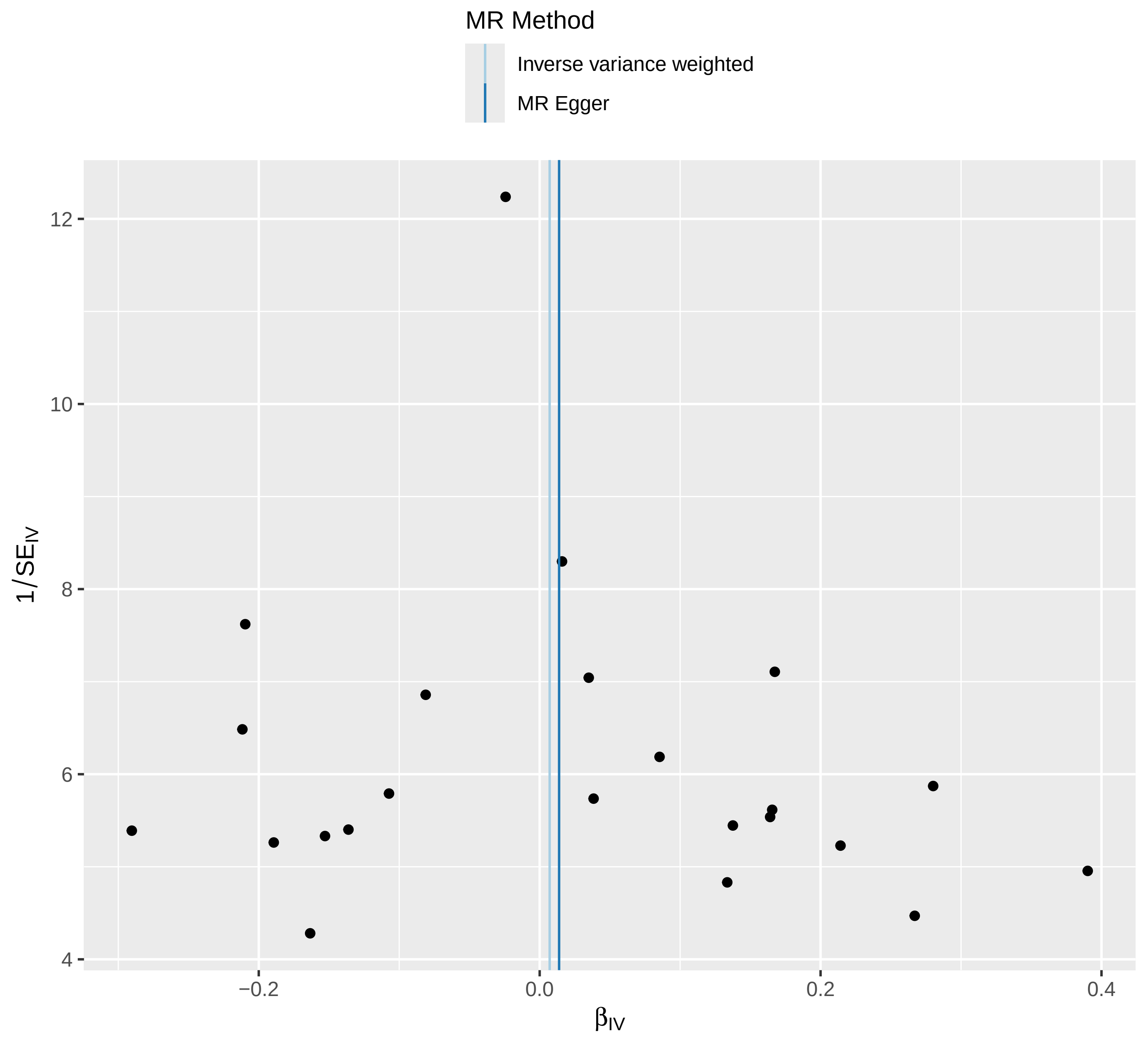
MR Method
Inverse variance weighted
MR Egger
12
10
8
6
4
1/SE_IV
−0.2
0.0
0.2
0.4
β_IV

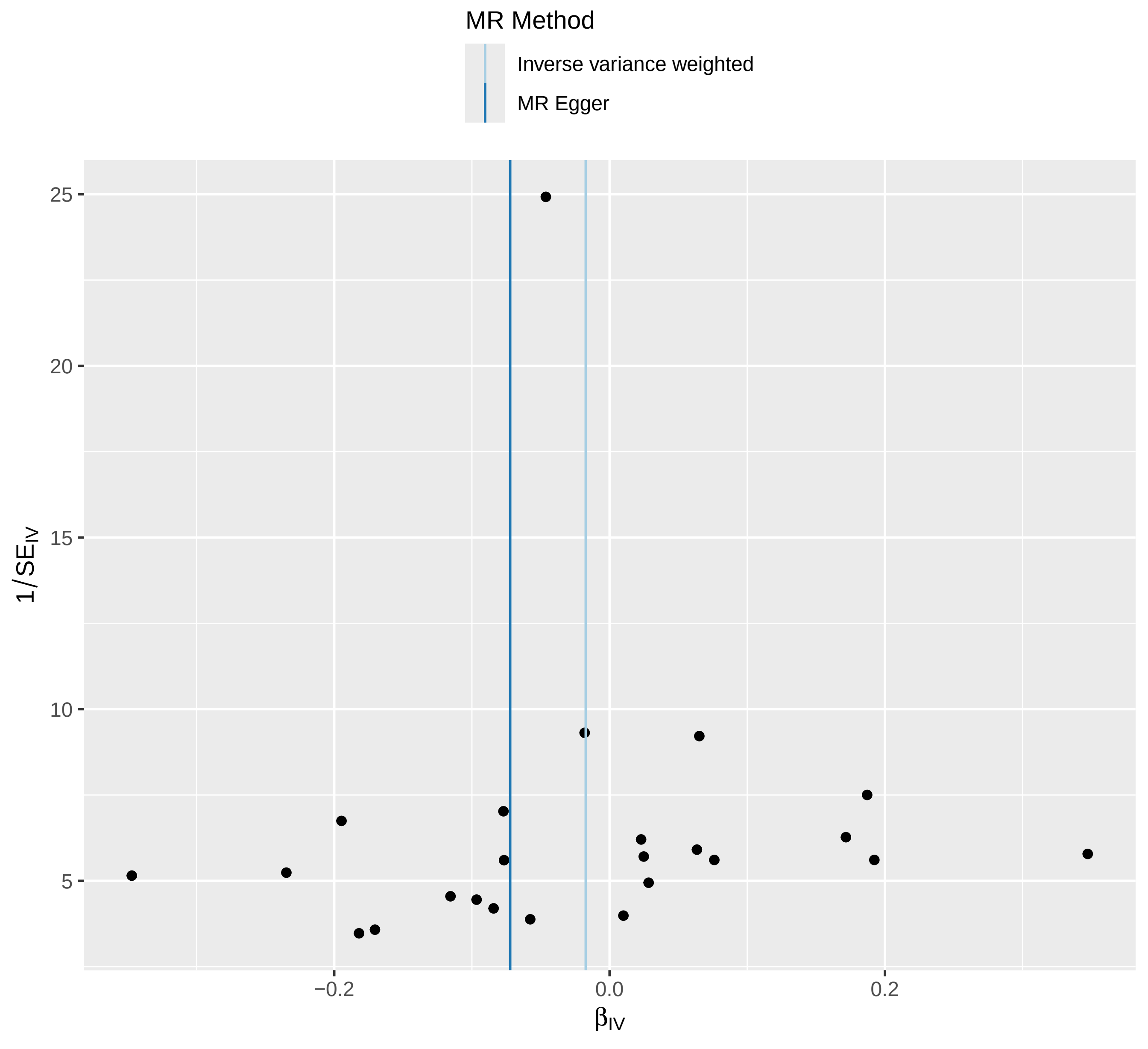
MR Method
Inverse variance weighted
MR Egger
25
20
15
10
5
$1/SE_{IV}$
−0.2
0.0
0.2
β_{IV}

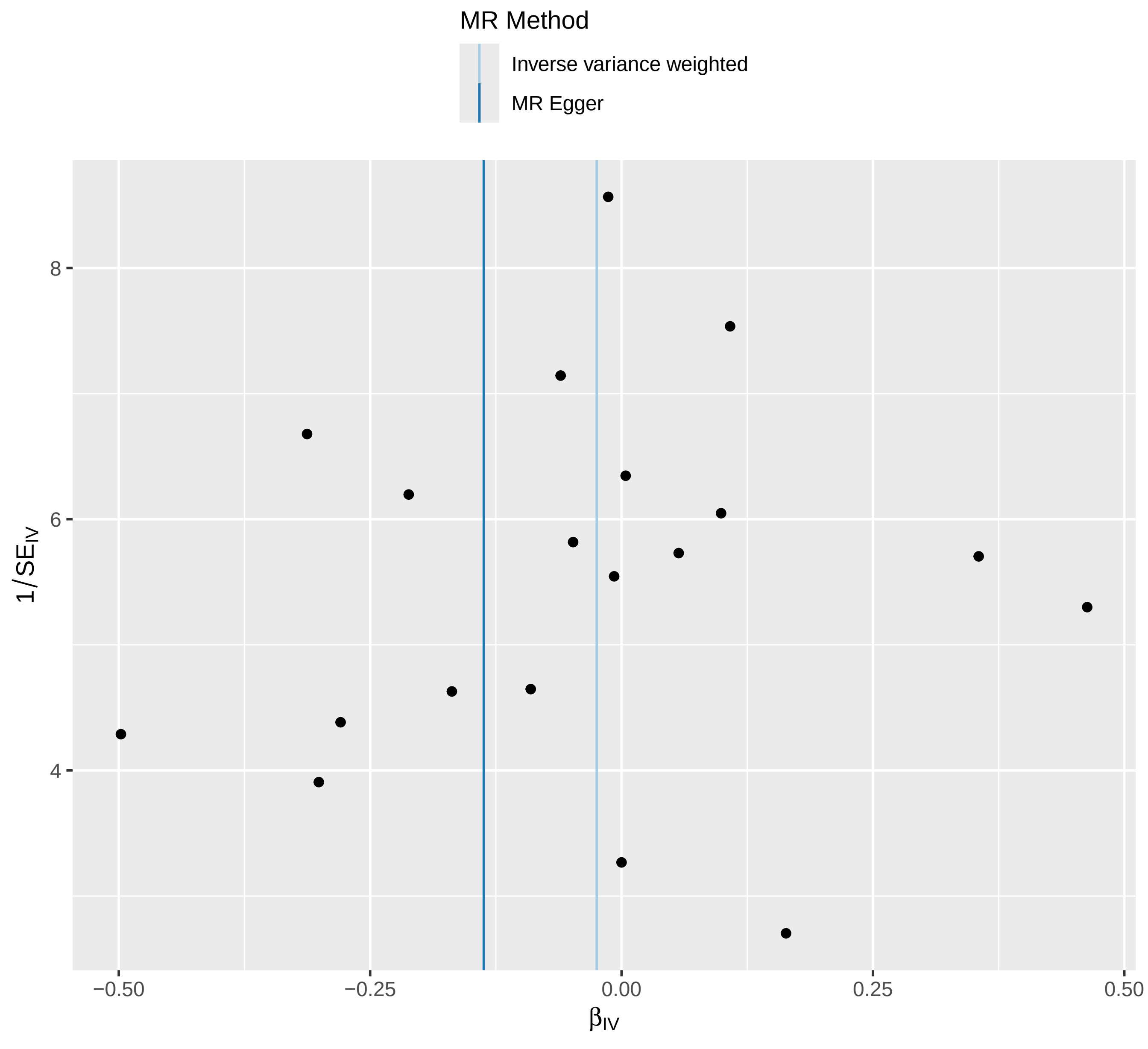

MR Method
Inverse variance weighted
MR Egger
8
6
4
1/SE_IV
−0.50
−0.25
0.00
0.25
0.50
β_IV

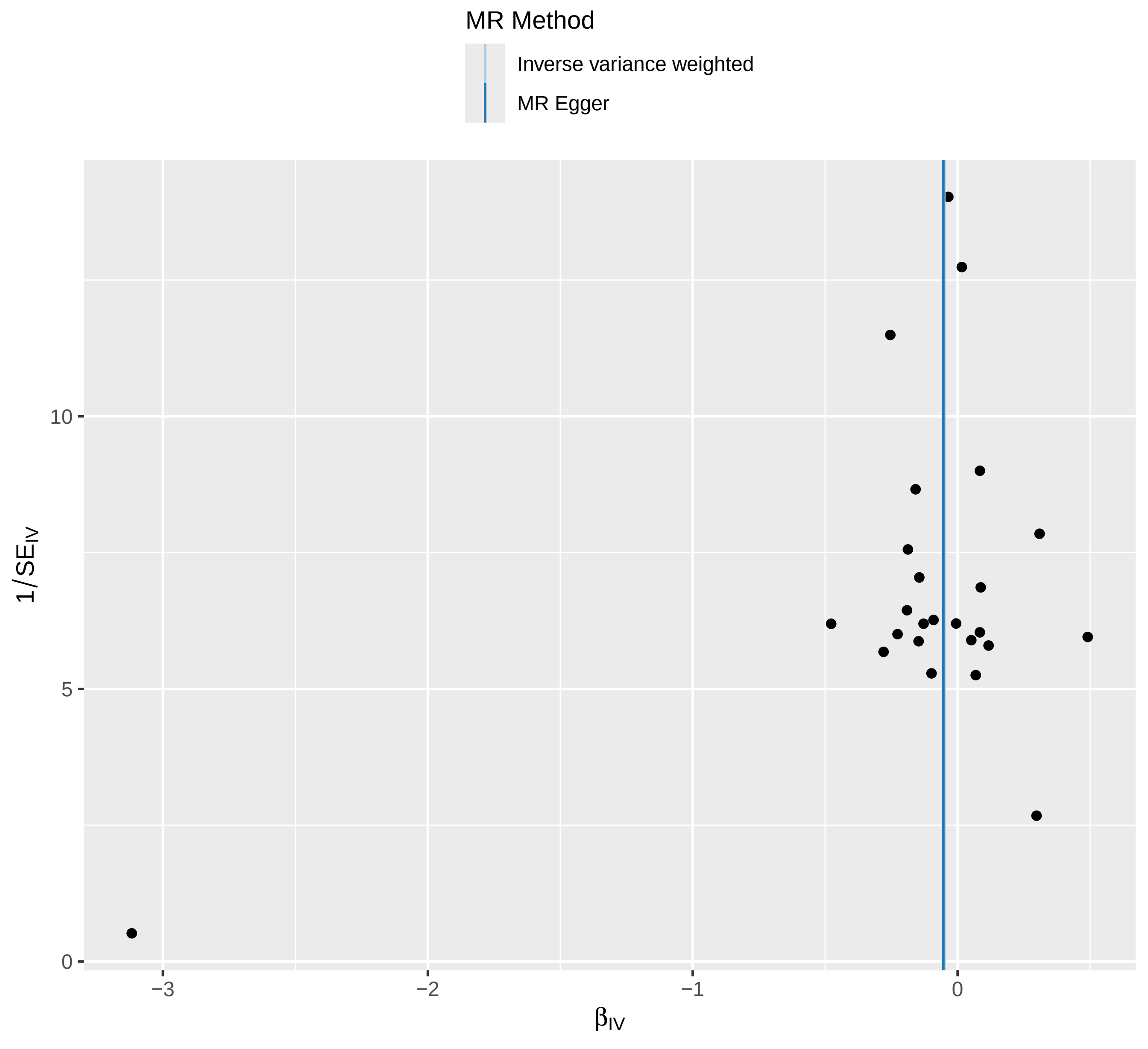

MR Method
Inverse variance weighted
MR Egger
1/SE_IV
10
5
0
−3
−2
−1
0
β_IV

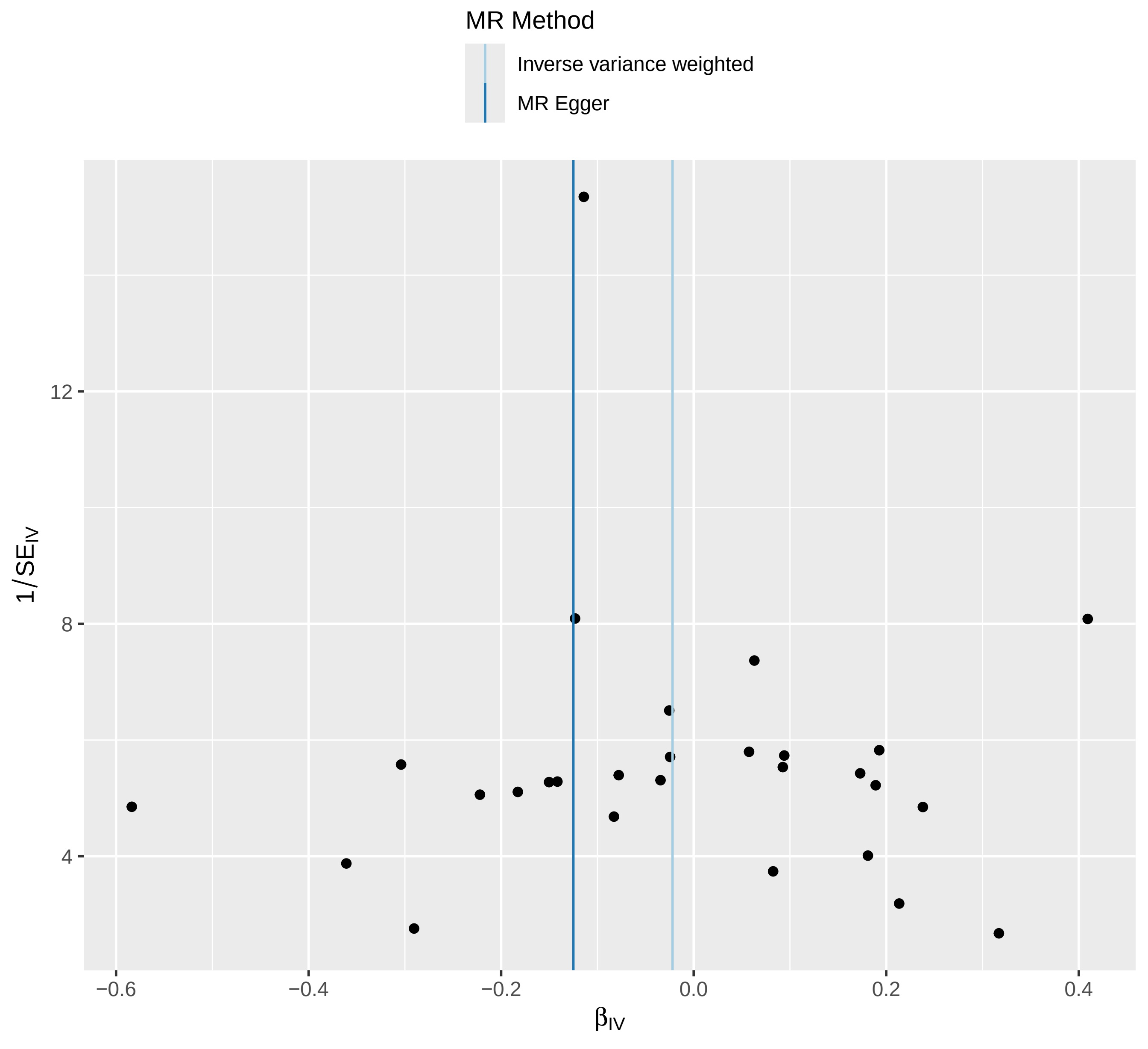

MR Method
Inverse variance weighted
MR Egger
12
8
4
1/SE_IV
−0.6
−0.4
−0.2
0.0
0.2
0.4
β_IV

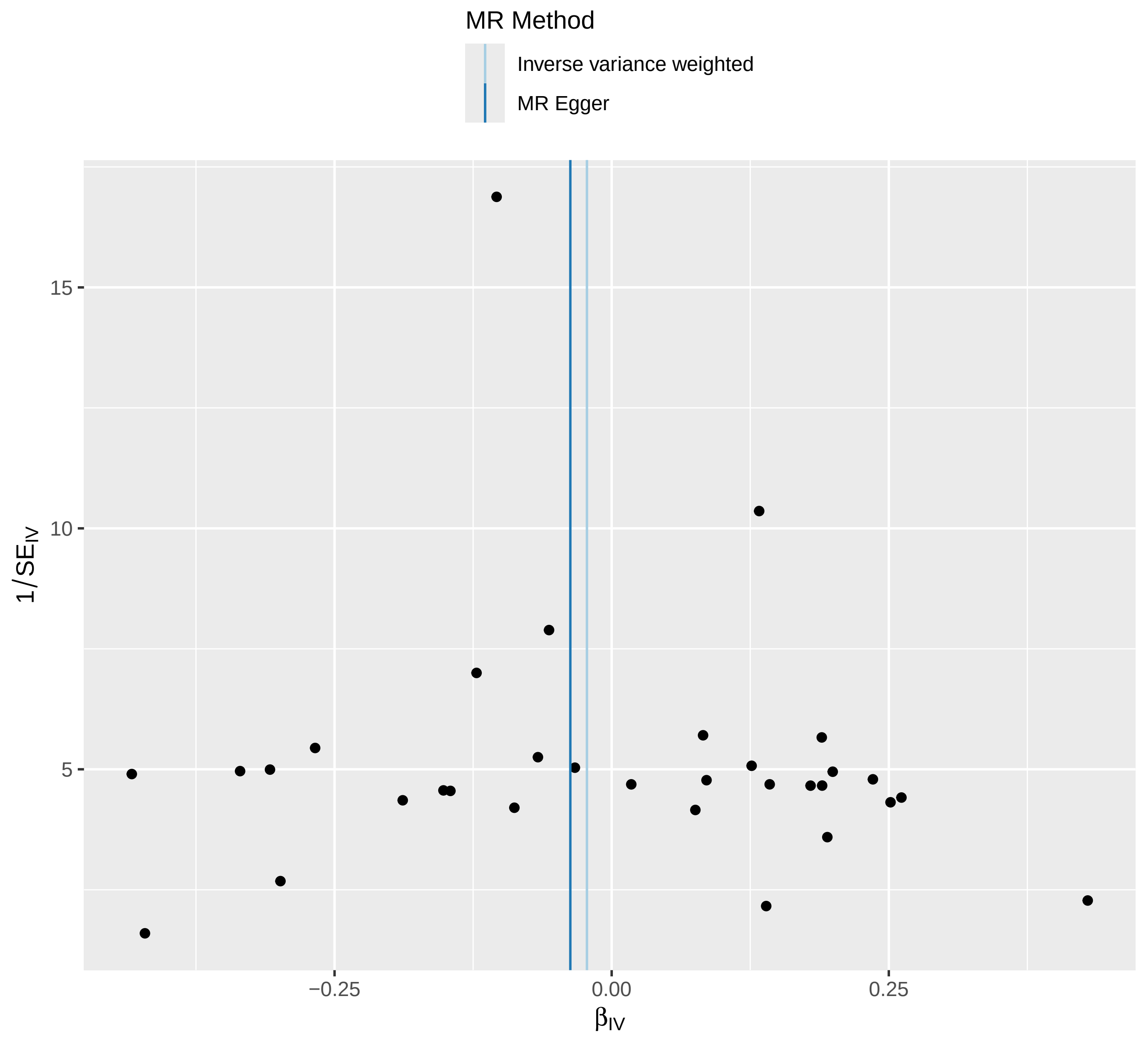
MR Method
Inverse variance weighted
MR Egger
15
10
5
−0.25
0.00
0.25
$1/SE_{IV}$
β_{IV}

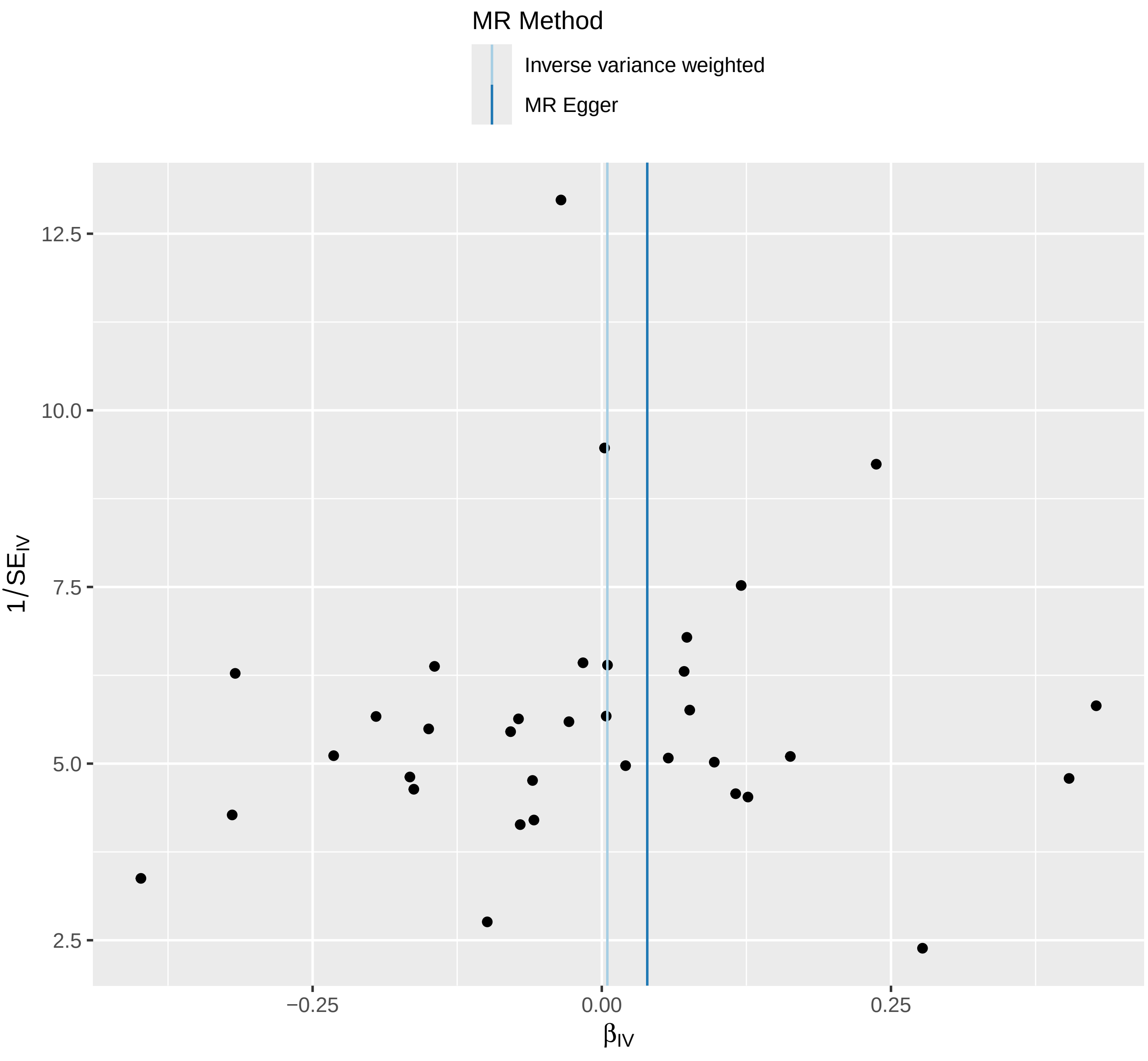
MR Method
Inverse variance weighted
MR Egger
12.5
10.0
7.5
5.0
2.5
$1/SE_{IV}$
−0.25
0.00
0.25
β_{IV}

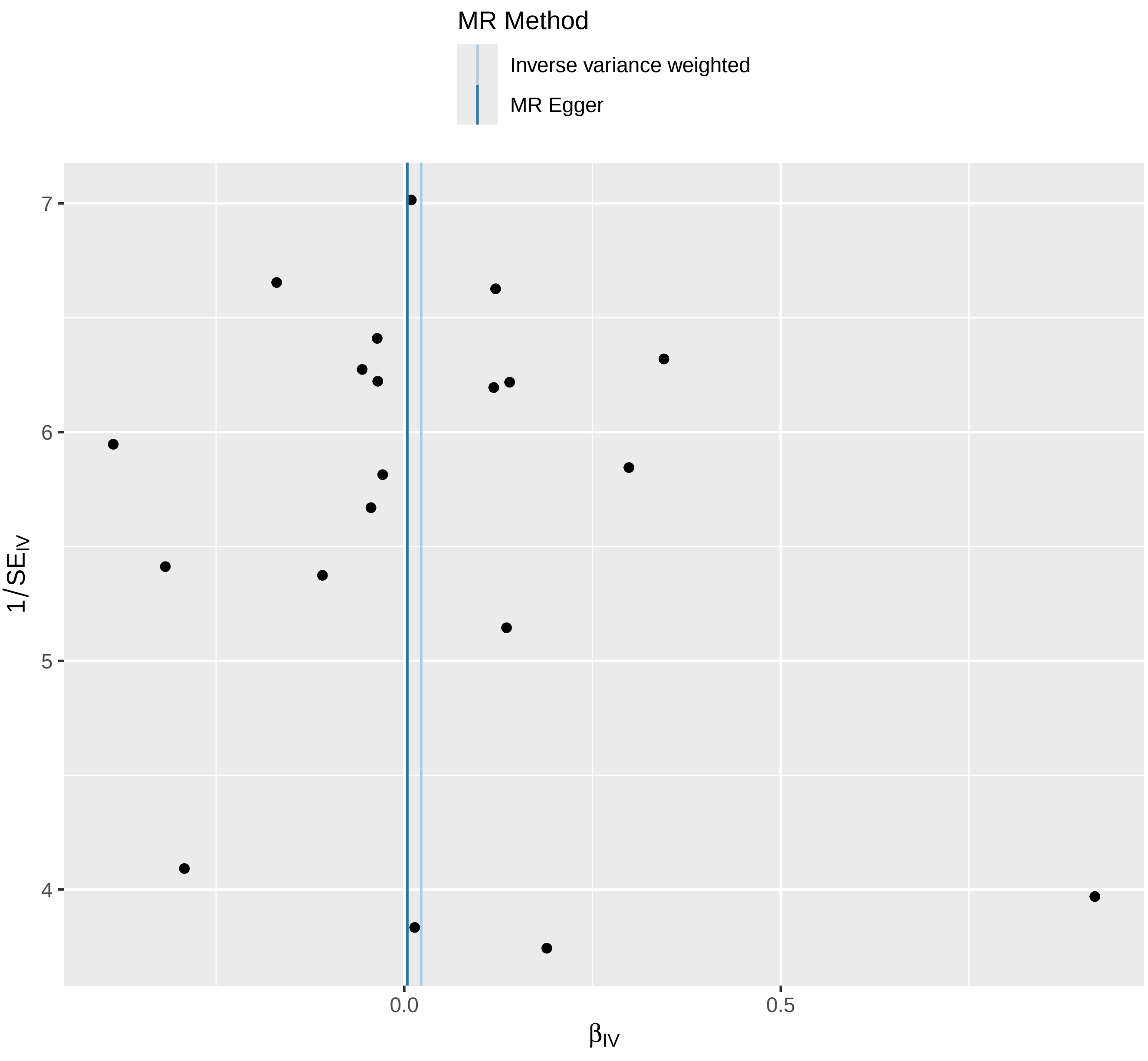
MR Method
Inverse variance weighted
MR Egger
7
6
5
4
1/SE_IV
0.0
0.5
β_IV

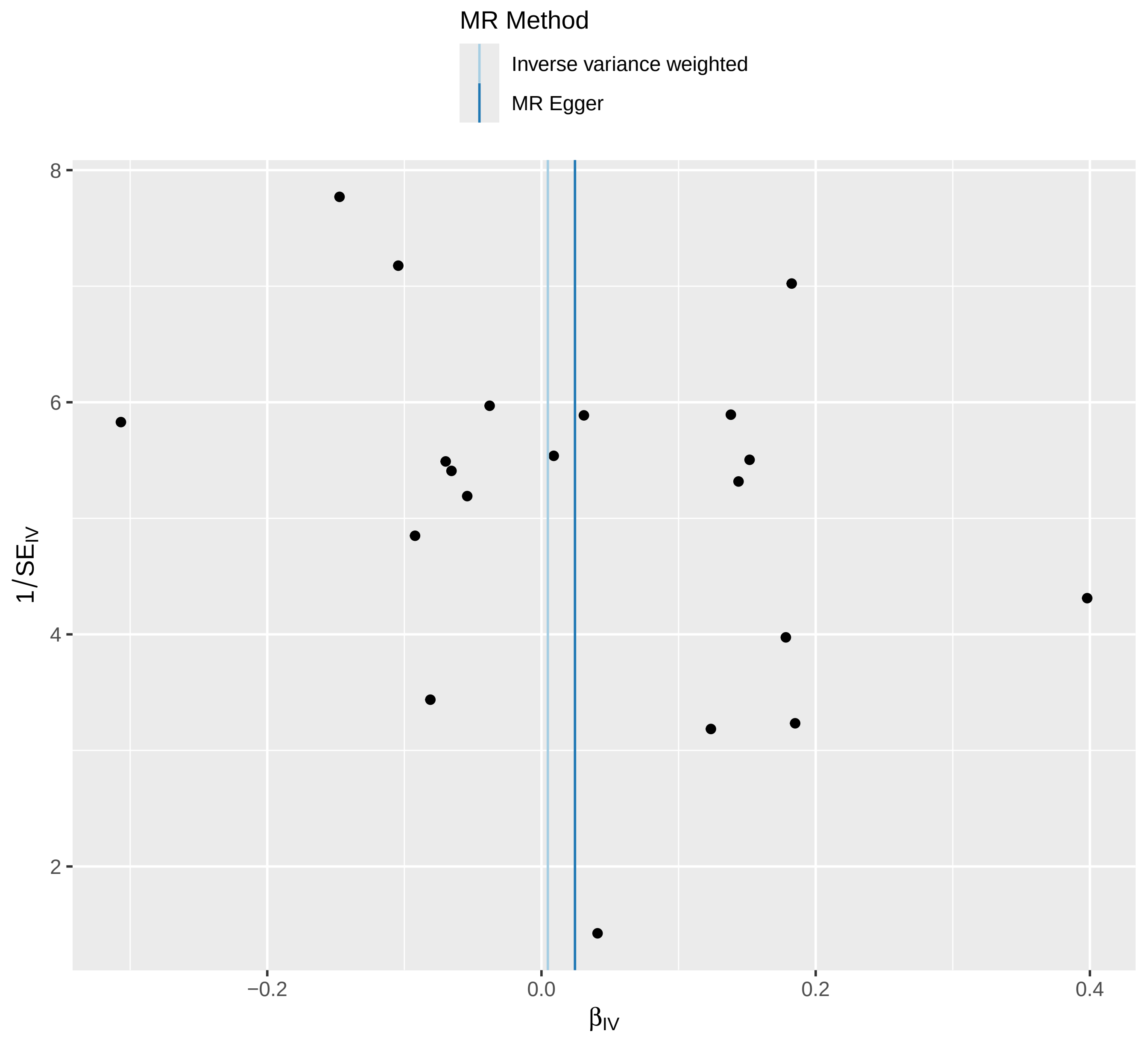
MR Method
Inverse variance weighted
MR Egger
1/SE_IV
β_IV
8
6
4
2
−0.2
0.0
0.2
0.4

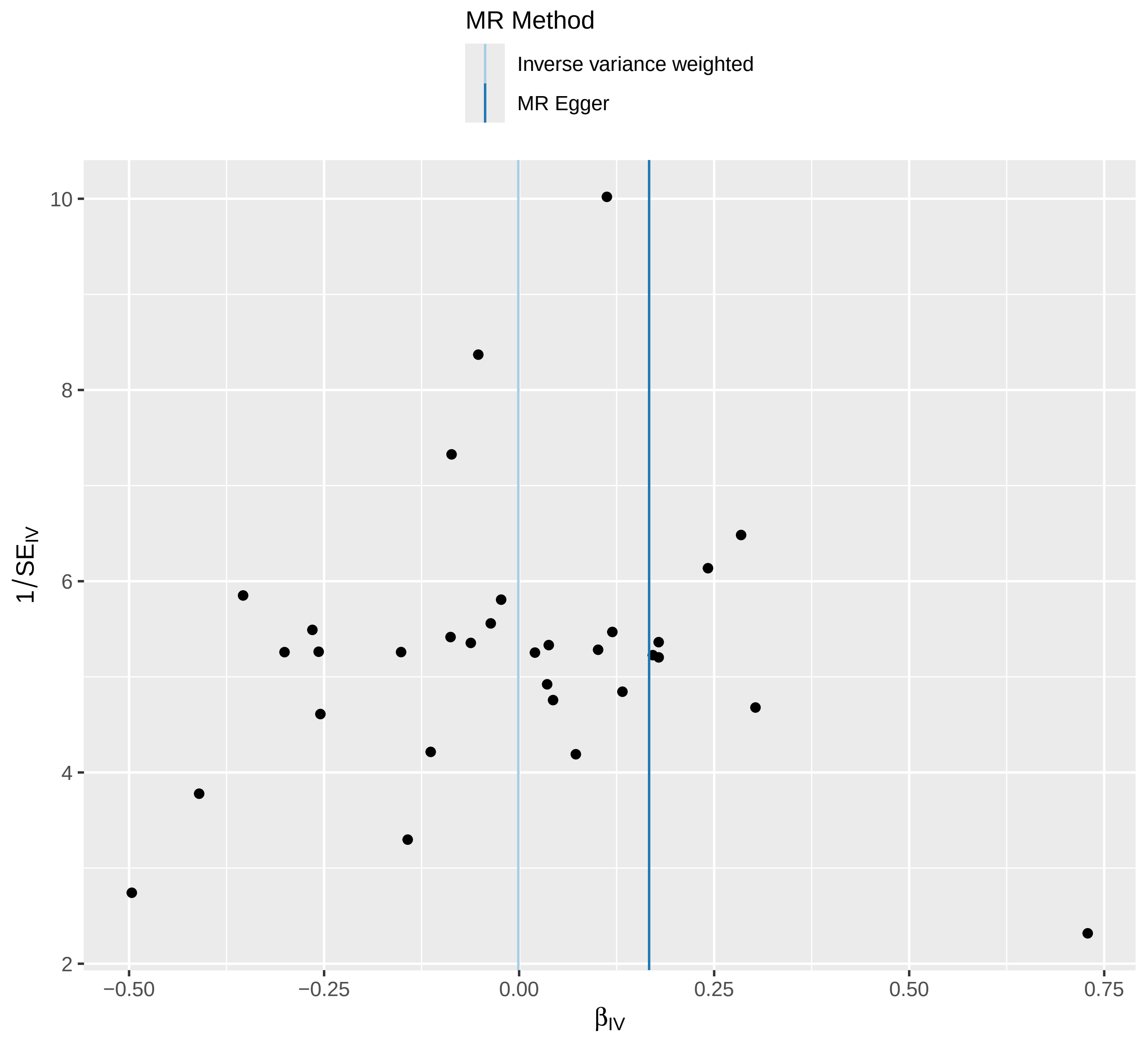

MR Method
Inverse variance weighted
MR Egger
10
8
6
4
2
$1/SE_{IV}$
−0.50
−0.25
0.00
0.25
0.50
0.75
β_{IV}

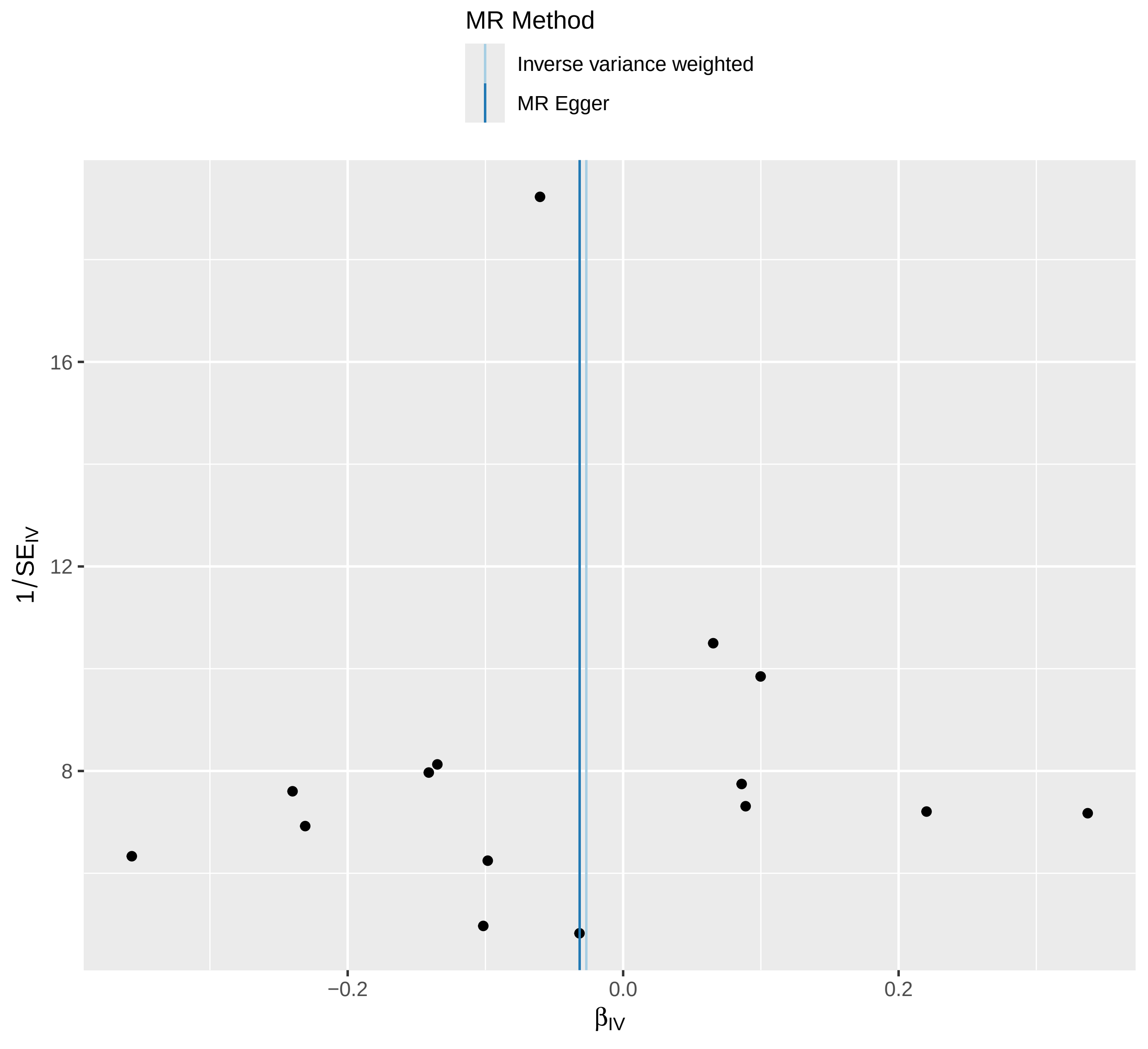
MR Method
Inverse variance weighted
MR Egger
16
12
8
1/SE_IV
−0.2
0.0
0.2
β_IV

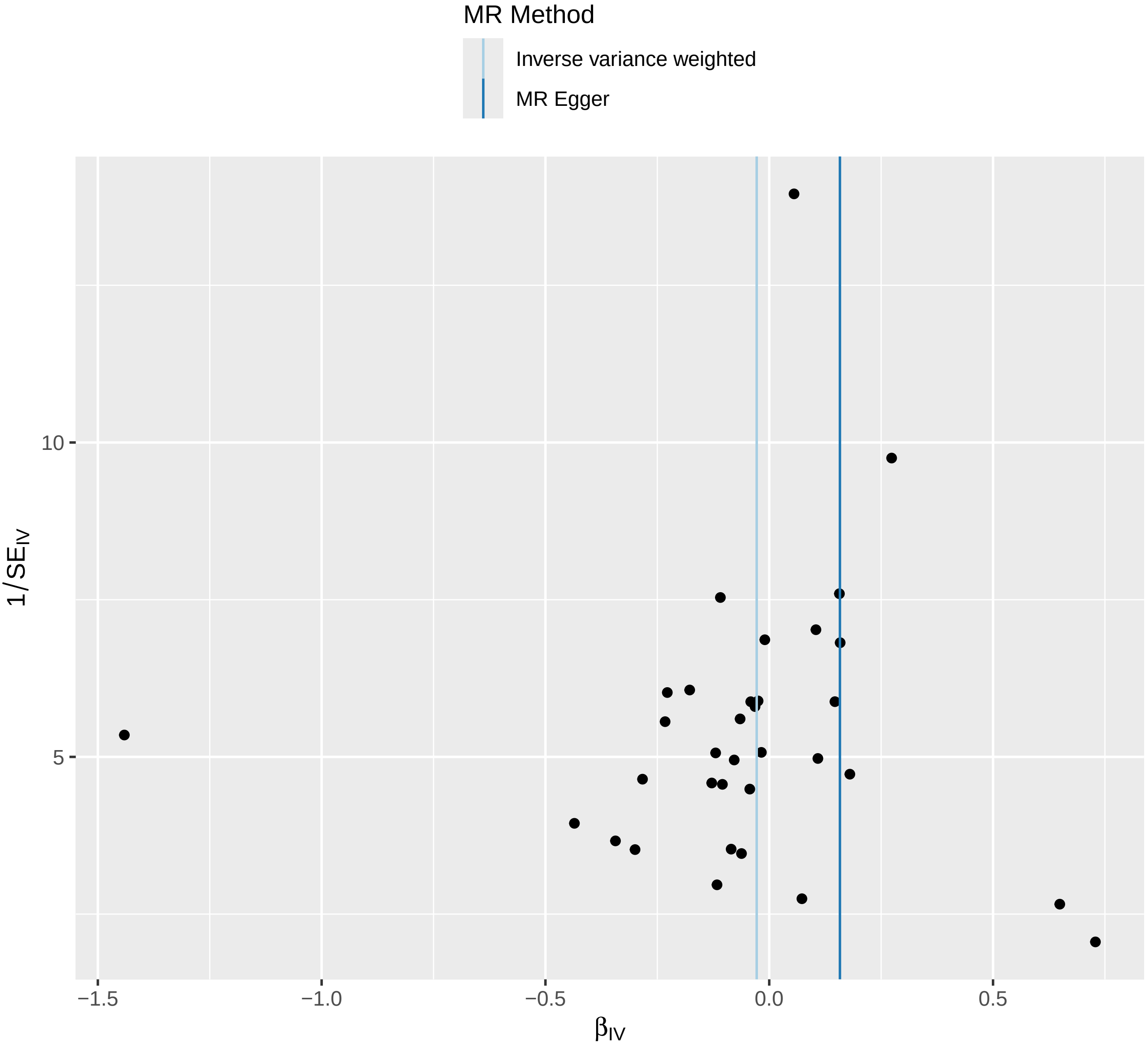

MR Method
Inverse variance weighted
MR Egger
10
5
1/SE$_{IV}$
−1.5
−1.0
−0.5
0.0
0.5
β$_{IV}$

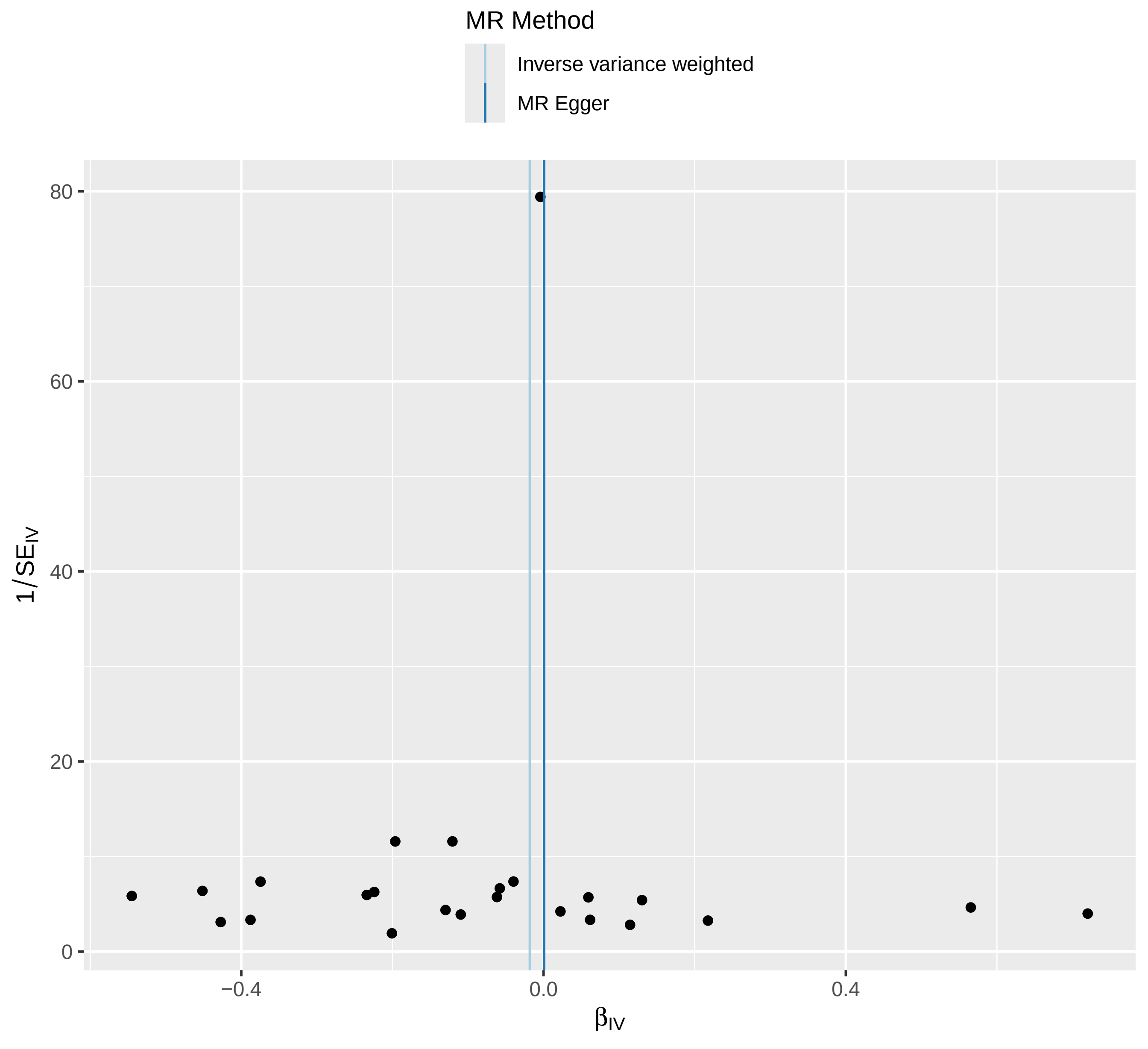
MR Method
Inverse variance weighted
MR Egger
80
60
40
20
0
1/SE_IV
−0.4
0.0
0.4
β_IV

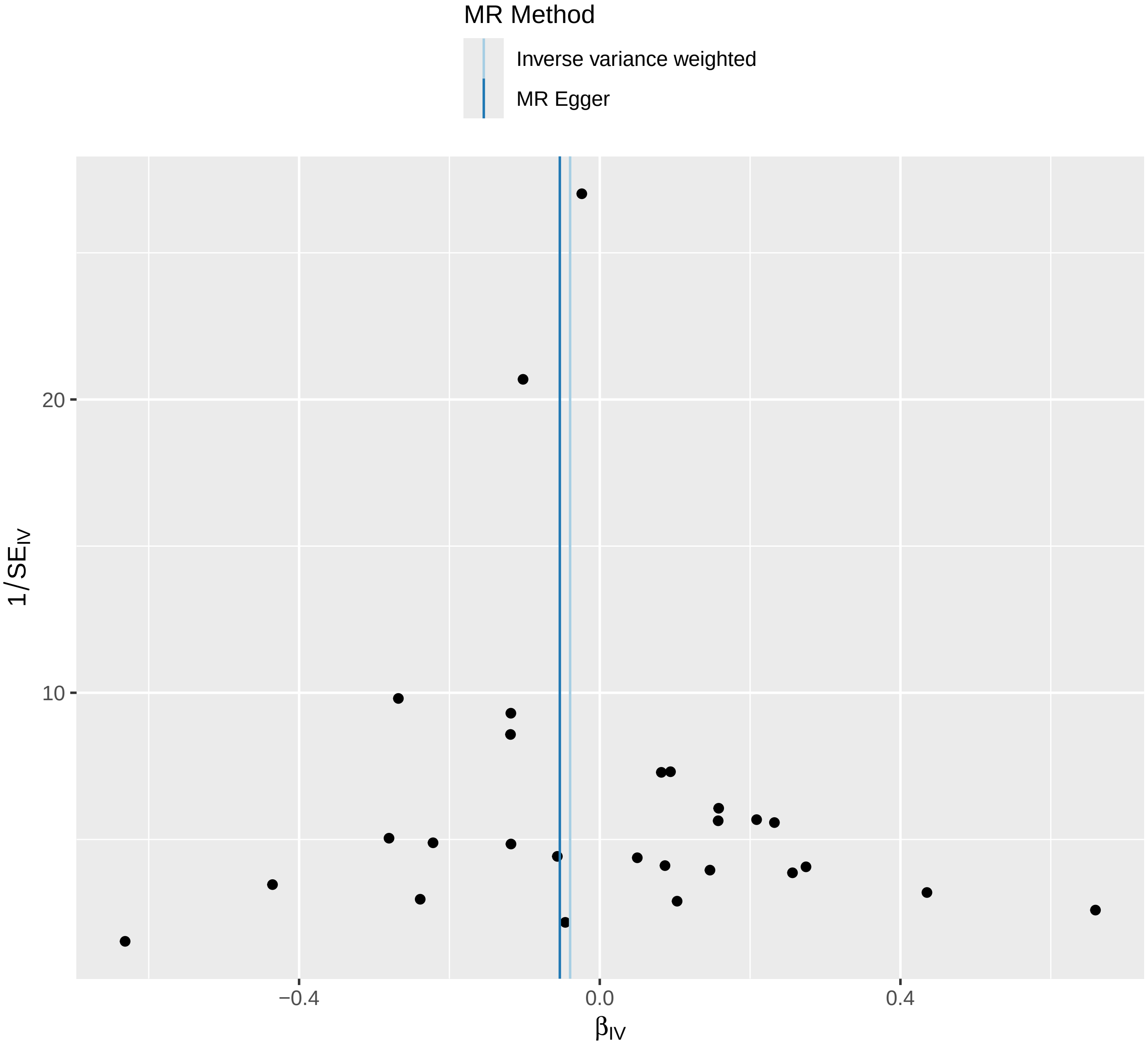
MR Method
Inverse variance weighted
MR Egger
20
10
1/SE$_{IV}$
−0.4
0.0
0.4
β$_{IV}$

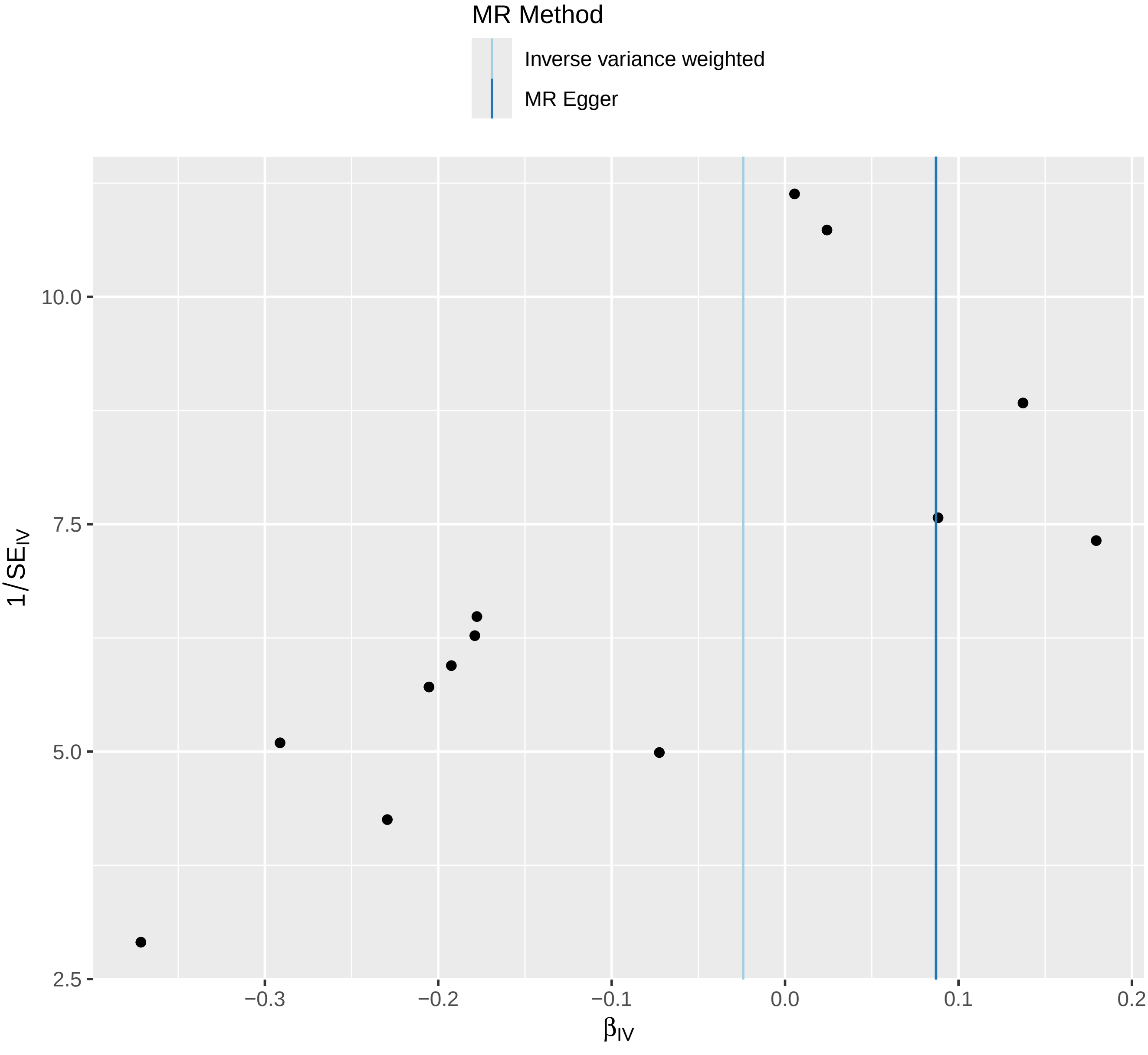

MR Method
Inverse variance weighted
MR Egger
10.0
7.5
5.0
2.5
$1/SE_{IV}$
−0.3
−0.2
−0.1
0.0
0.1
0.2
β_{IV}

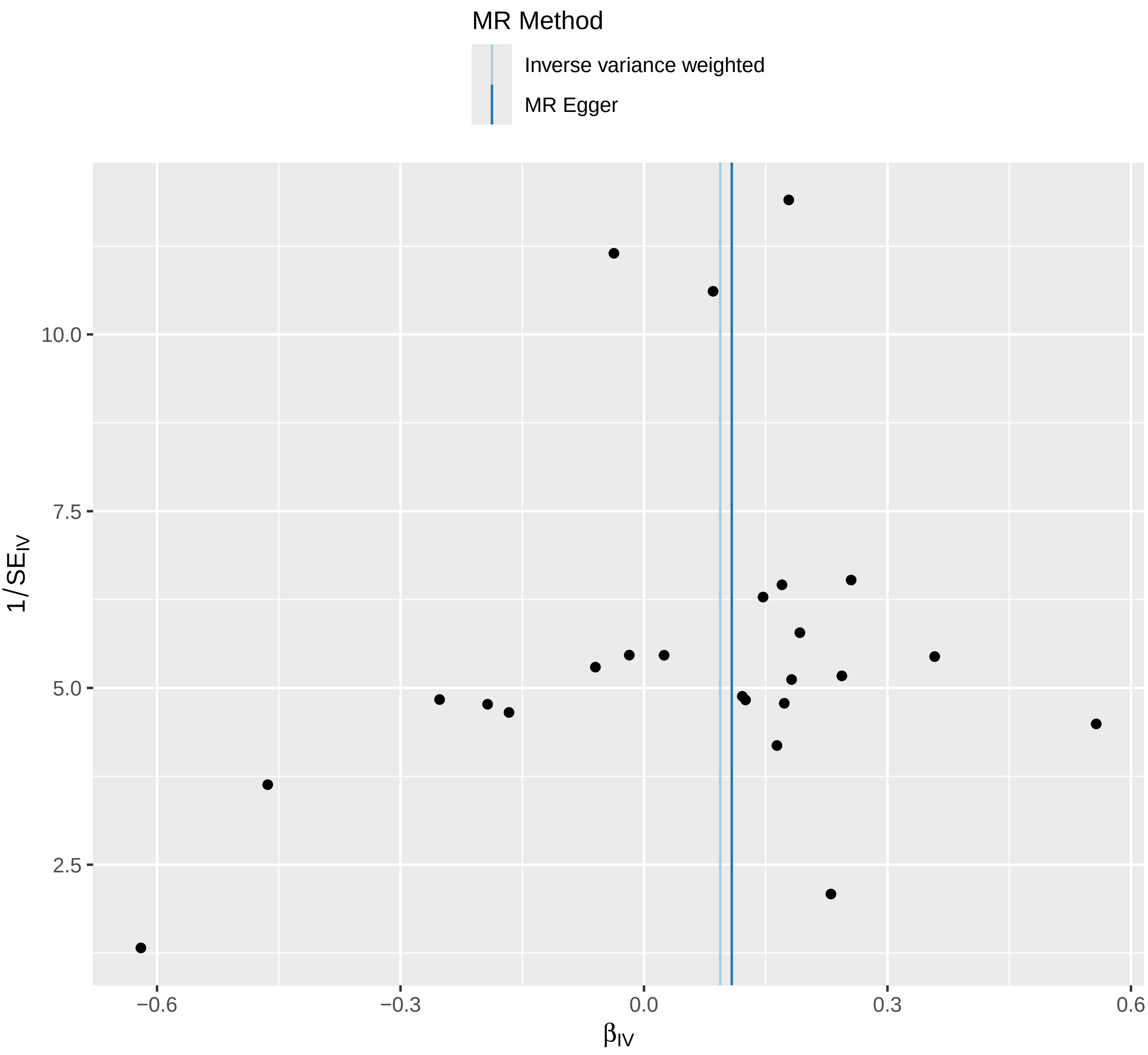
MR Method
Inverse variance weighted
MR Egger
10.0
7.5
5.0
2.5
$1/SE_{IV}$
−0.6
−0.3
0.0
0.3
0.6
β_{IV}

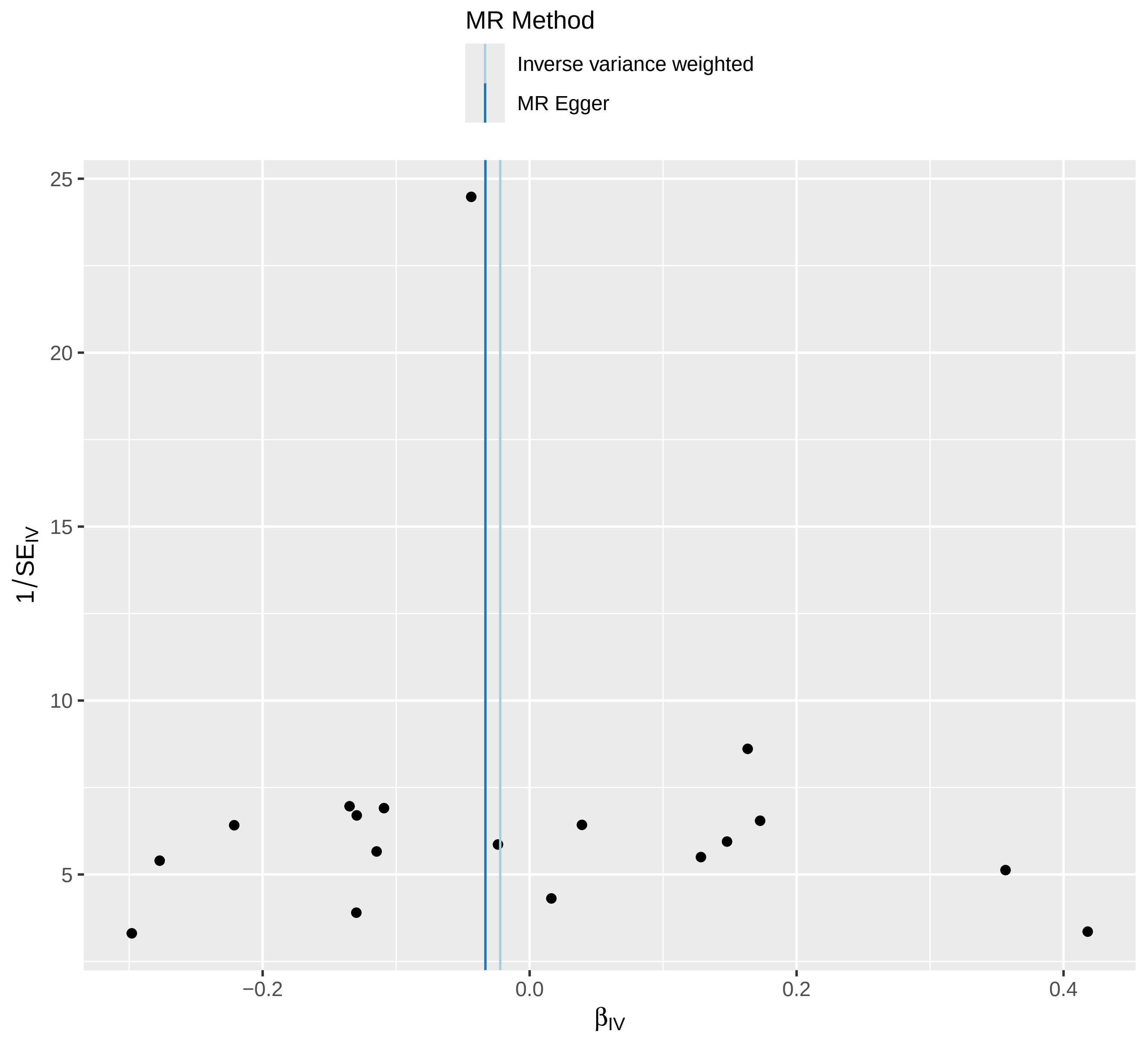
MR Method
Inverse variance weighted
MR Egger
25
20
15
10
5
1/SE_IV
−0.2
0.0
0.2
0.4
β_IV

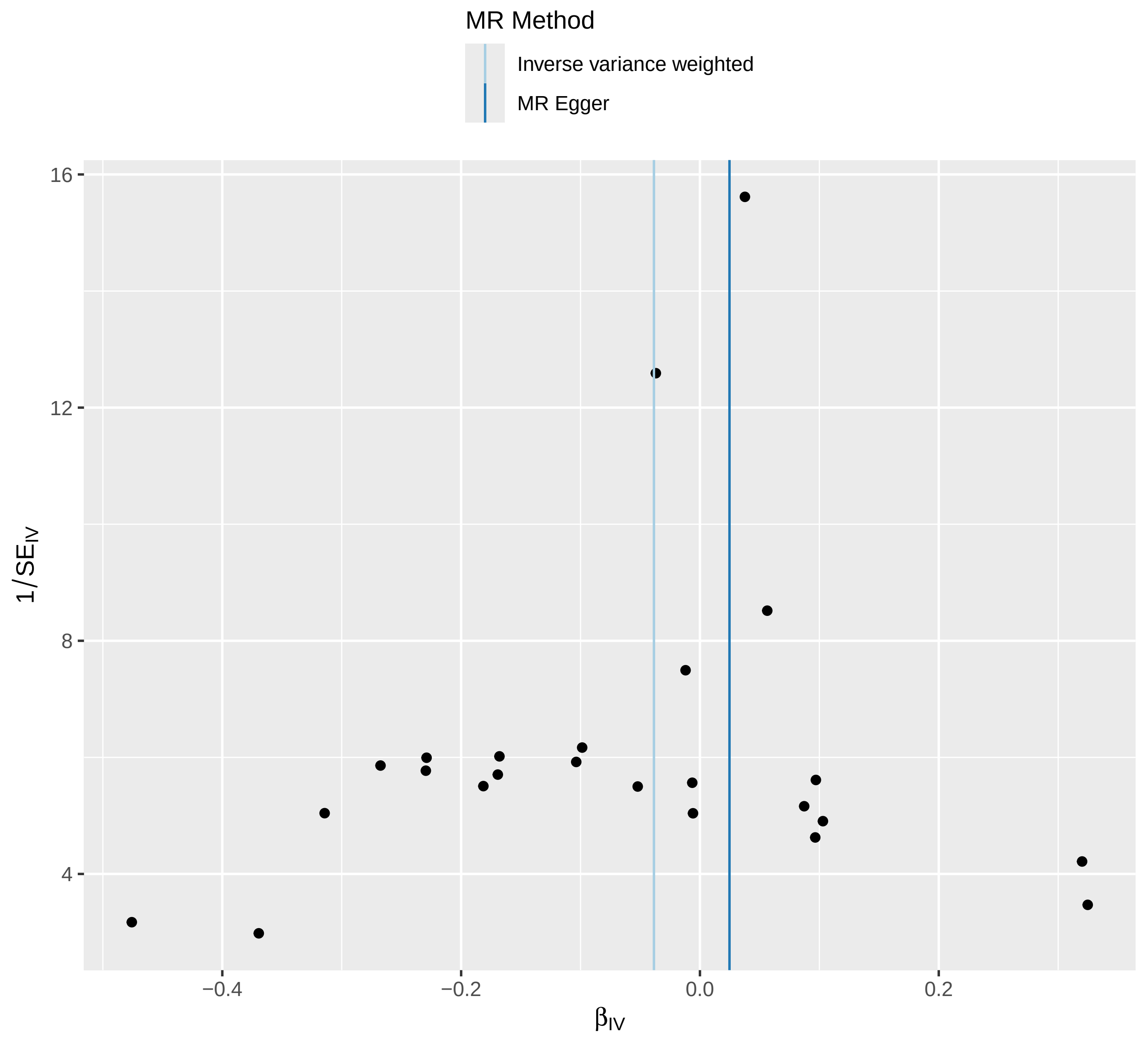
MR Method
Inverse variance weighted
MR Egger
16
12
8
4
1/SE_IV
−0.4
−0.2
0.0
0.2
β_IV

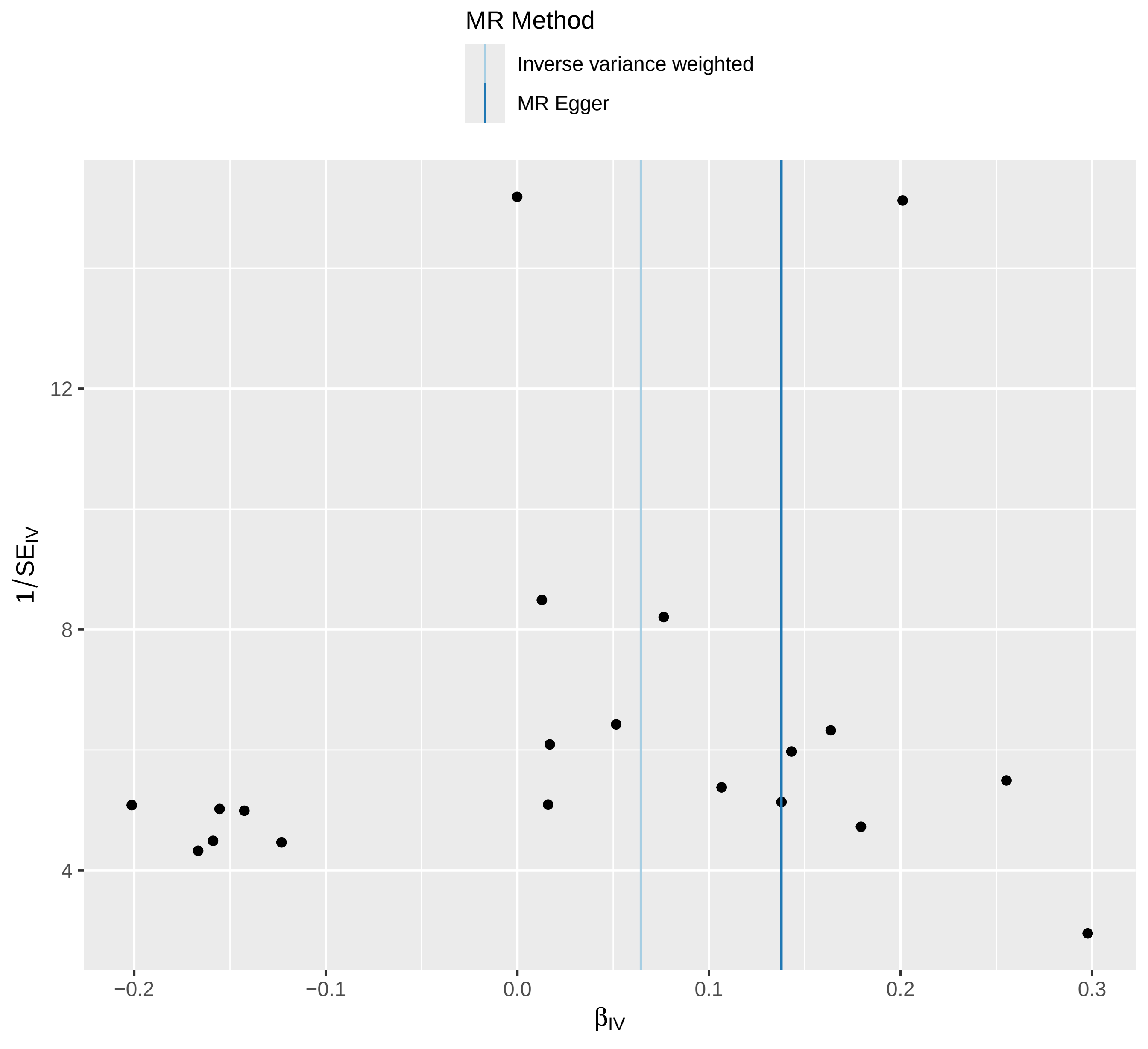
MR Method
Inverse variance weighted
MR Egger
12
8
4
1/SE_IV
−0.2
−0.1
0.0
0.1
0.2
0.3
β_IV

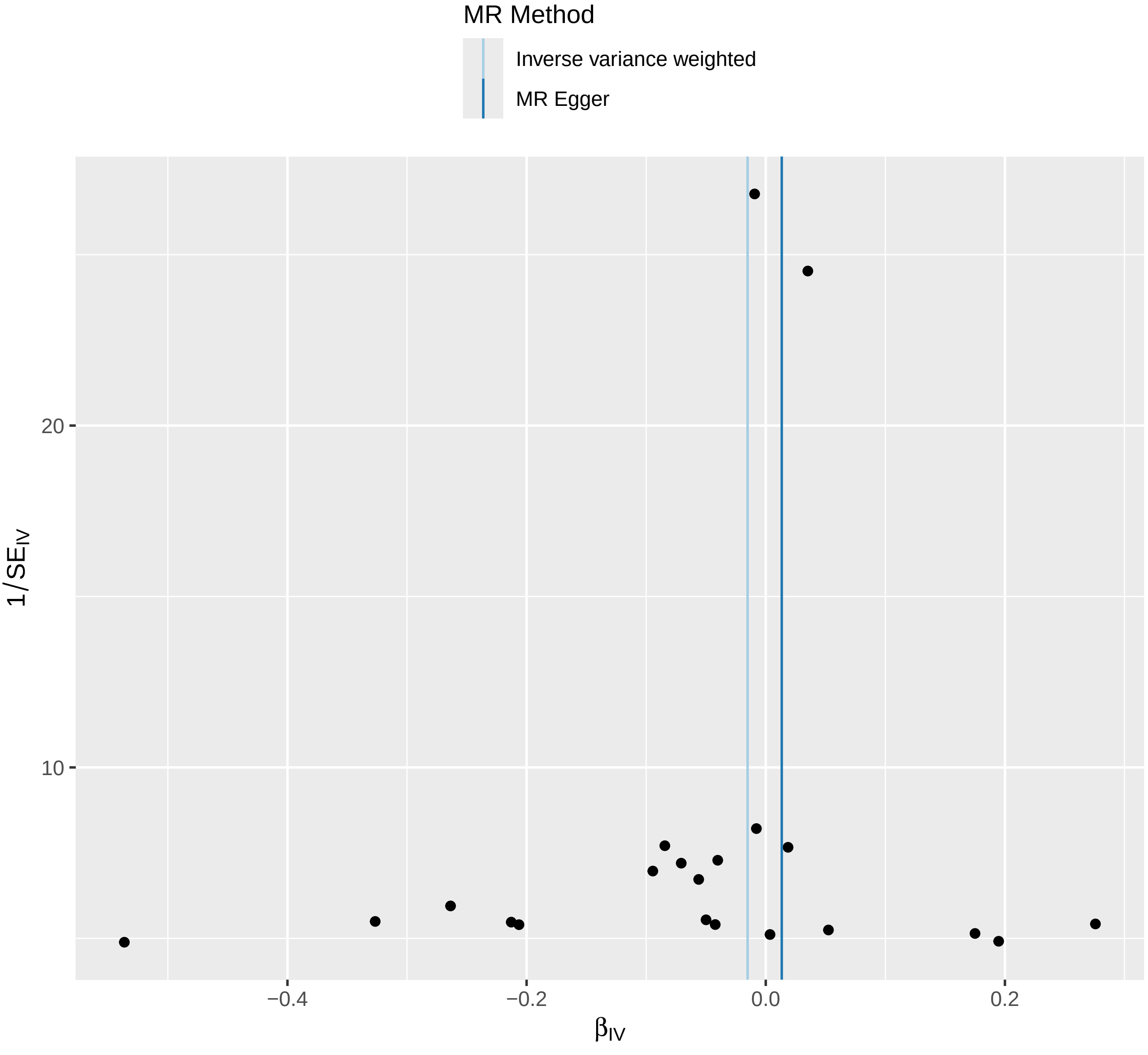
MR Method
Inverse variance weighted
MR Egger
20
10
1/SE_IV
−0.4
−0.2
0.0
0.2
β_IV

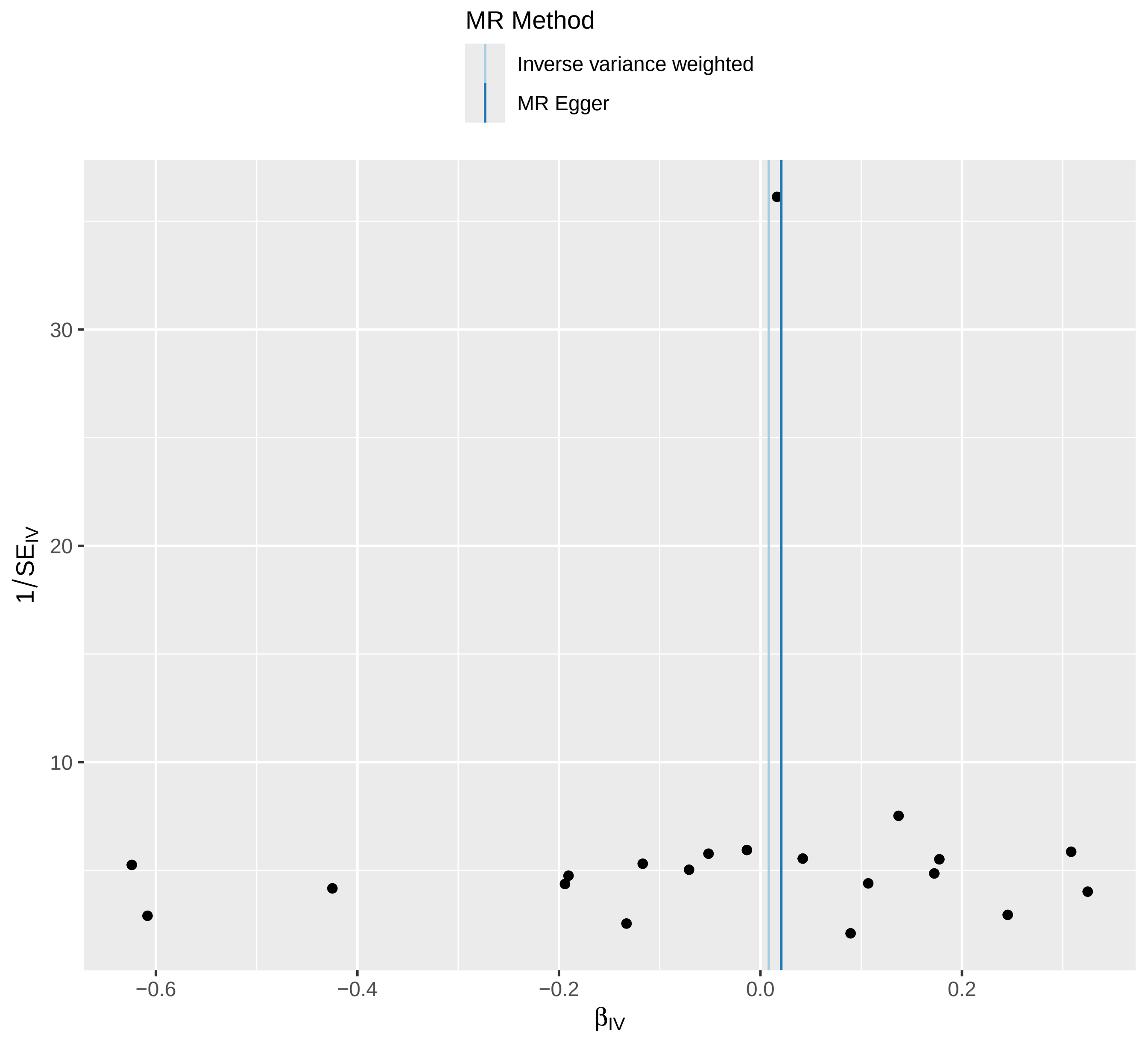

MR Method
Inverse variance weighted
MR Egger
30
20
10
1/SE_IV
−0.6
−0.4
−0.2
0.0
0.2
β_IV

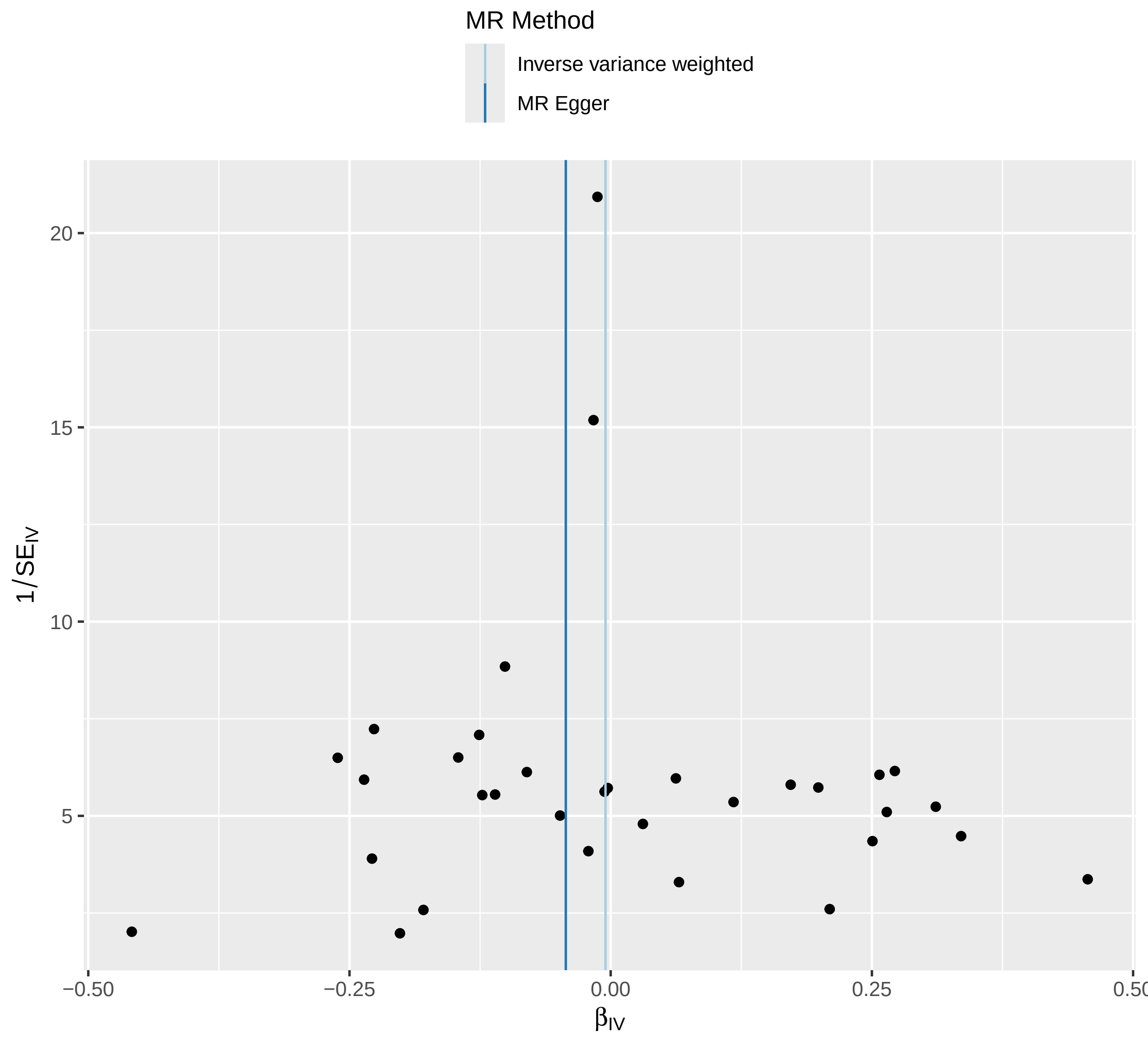

MR Method
Inverse variance weighted
MR Egger
20
15
10
5
1/SE_IV
−0.50
−0.25
0.00
0.25
0.50
β_IV

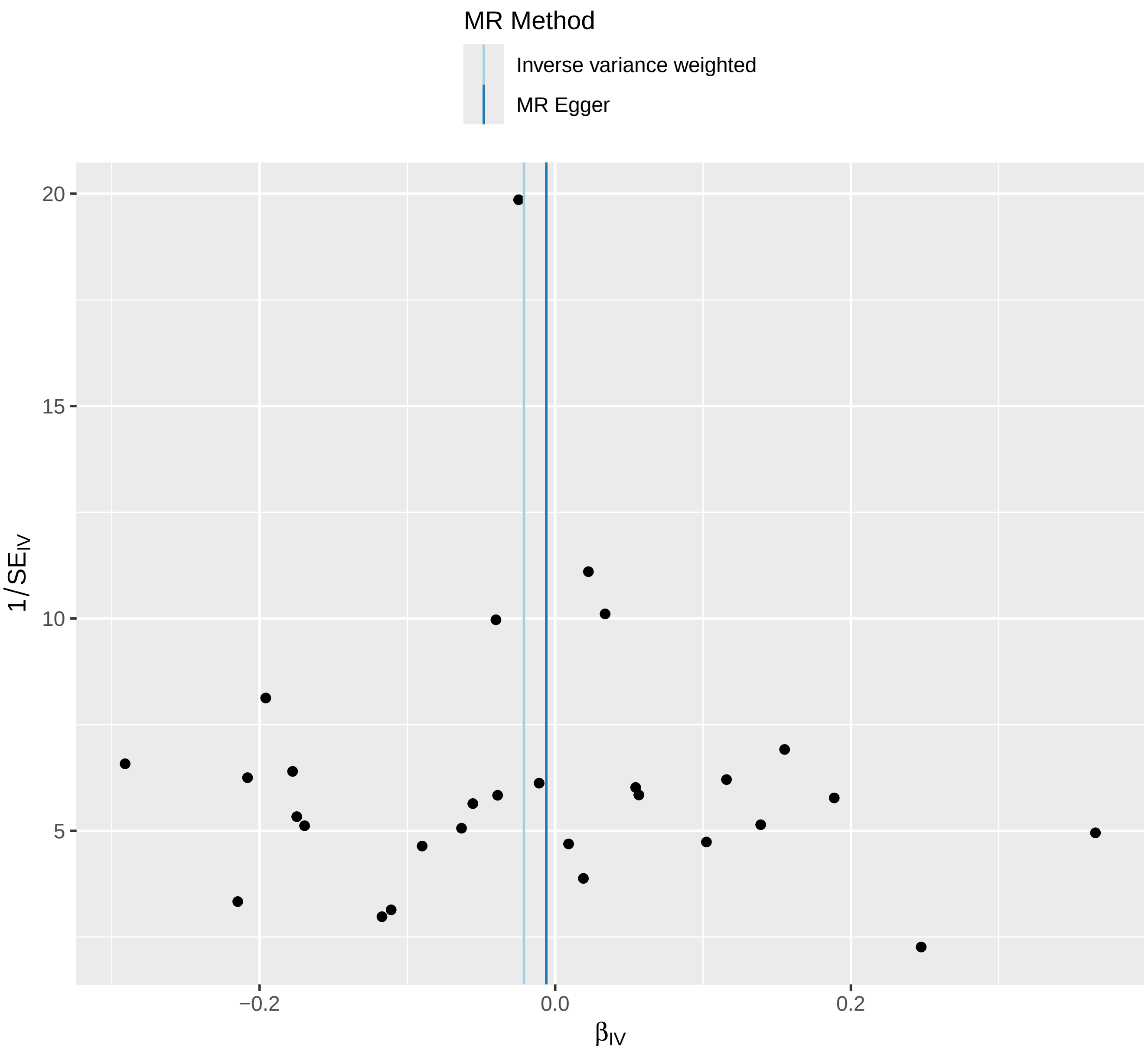

MR Method
Inverse variance weighted
MR Egger
20
15
10
5
1/SE_IV
−0.2
0.0
0.2
β_IV

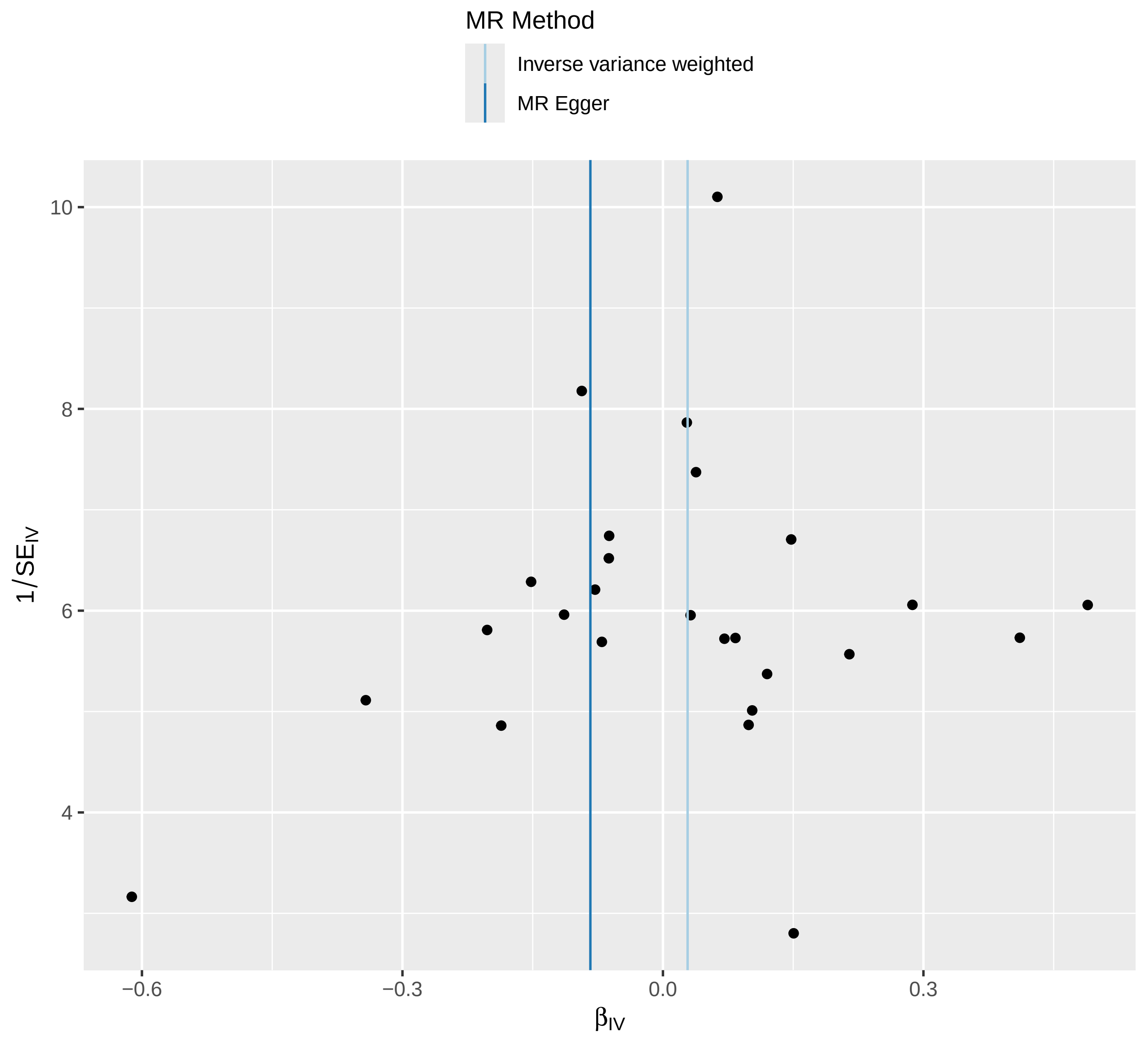
MR Method
Inverse variance weighted
MR Egger
10
8
6
4
1/SE_IV
−0.6
−0.3
0.0
0.3
β_IV

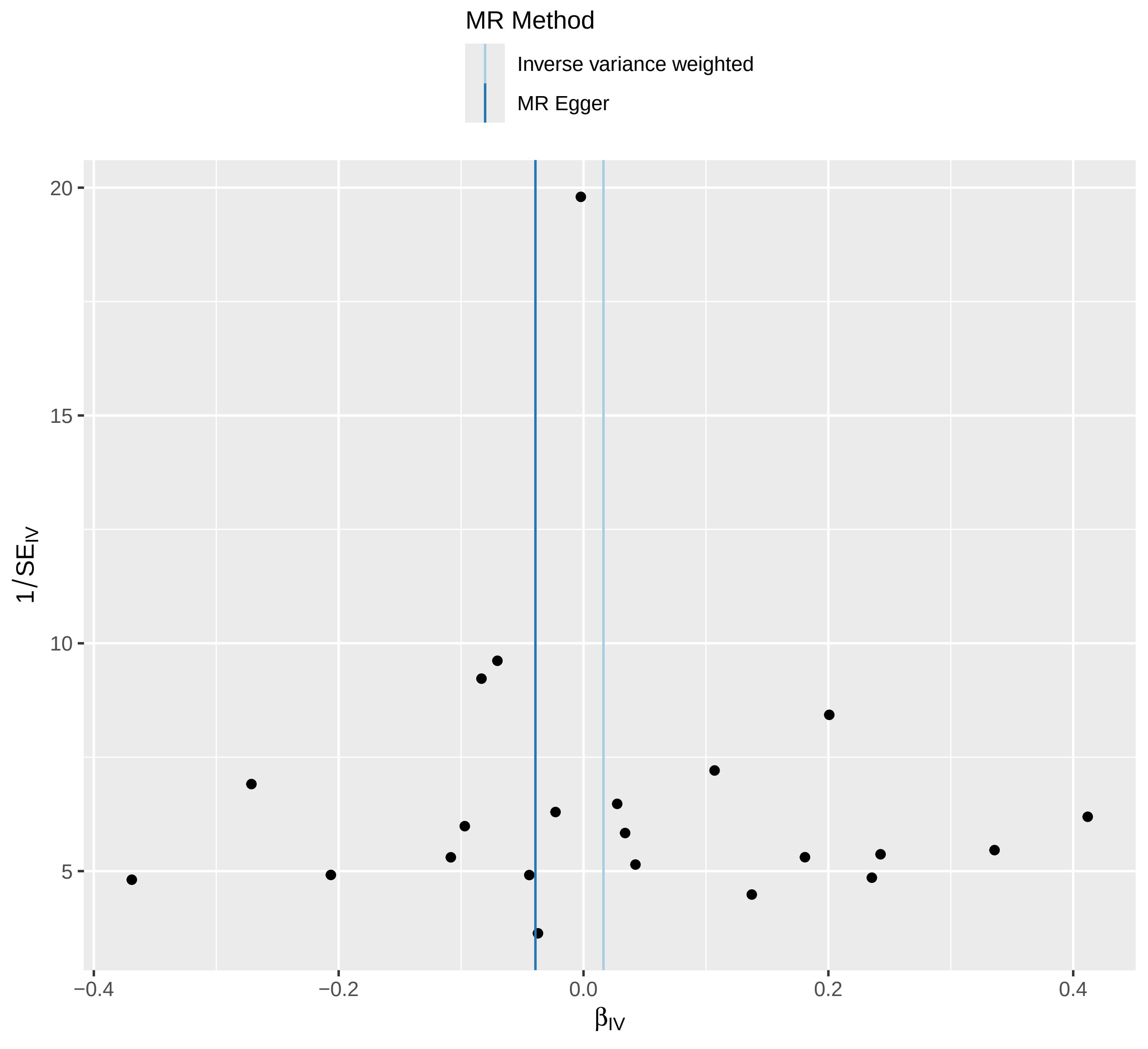
MR Method
Inverse variance weighted
MR Egger
20
15
10
5
1/SE_IV
−0.4
−0.2
0.0
0.2
0.4
β_IV

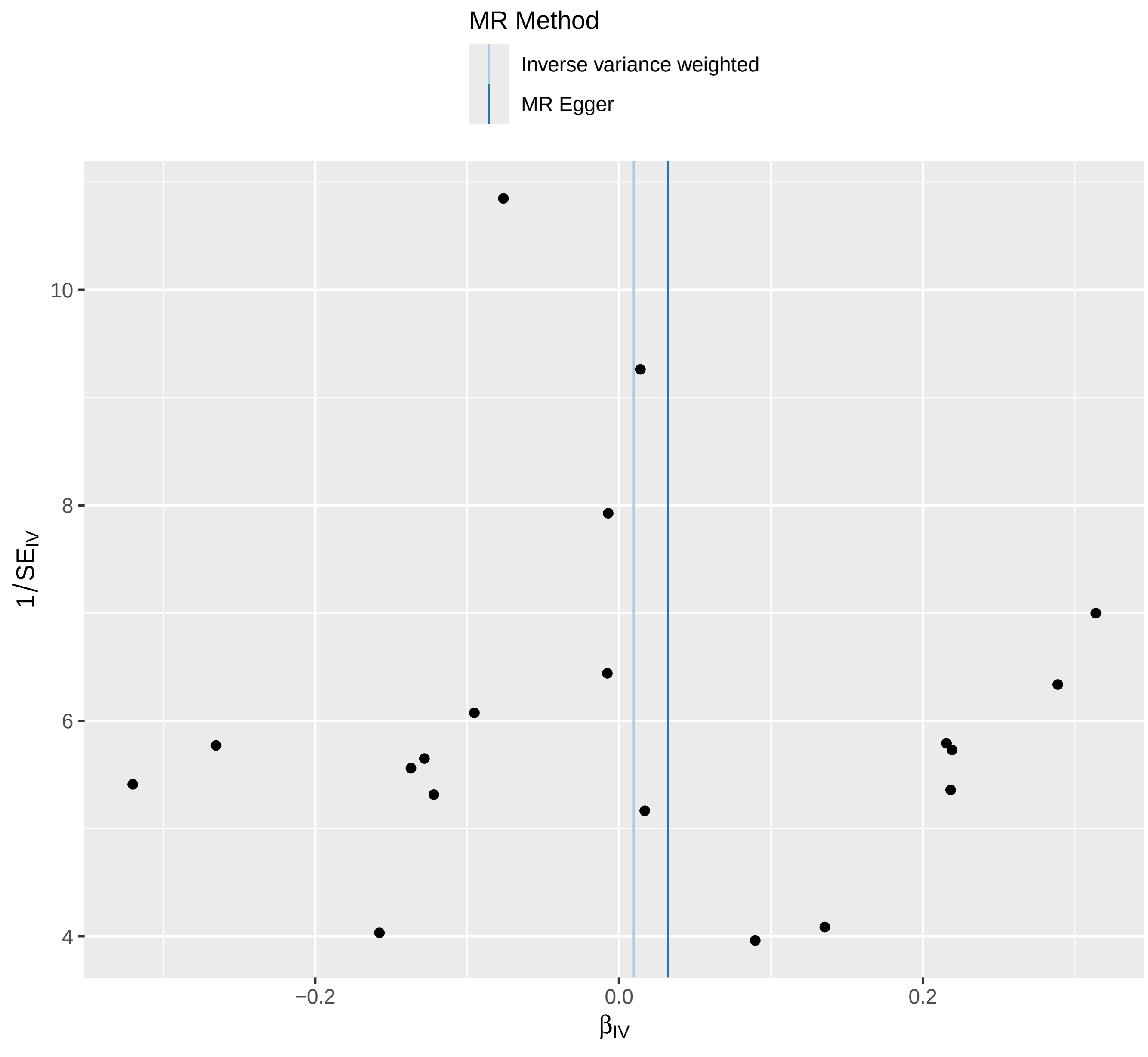
MR Method
Inverse variance weighted
MR Egger
1/SE$_{IV}$
10
8
6
4
−0.2
0.0
0.2
β$_{IV}$

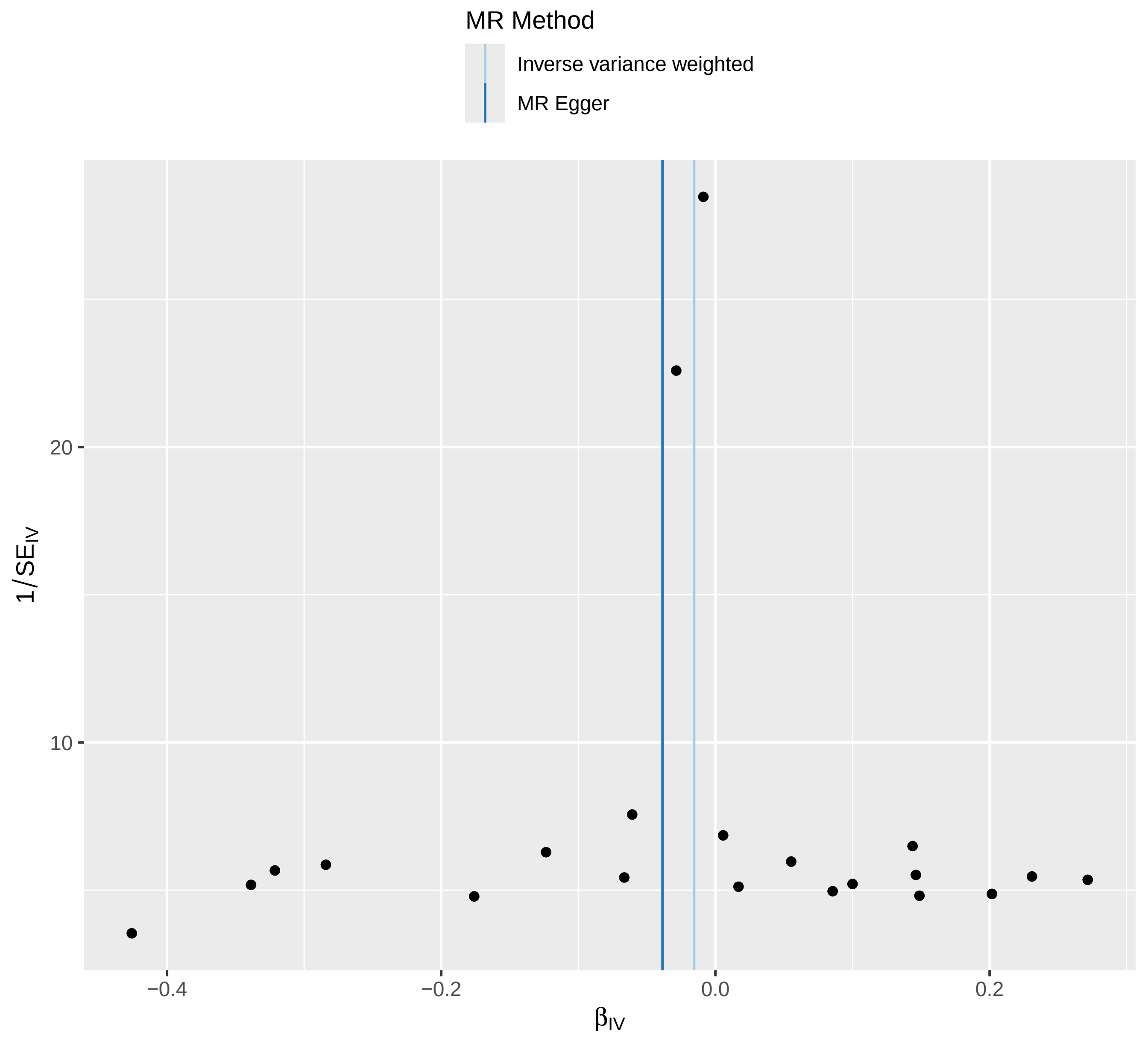
MR Method
Inverse variance weighted
MR Egger
20
10
1/SE_IV
−0.4
−0.2
0.0
0.2
β_IV

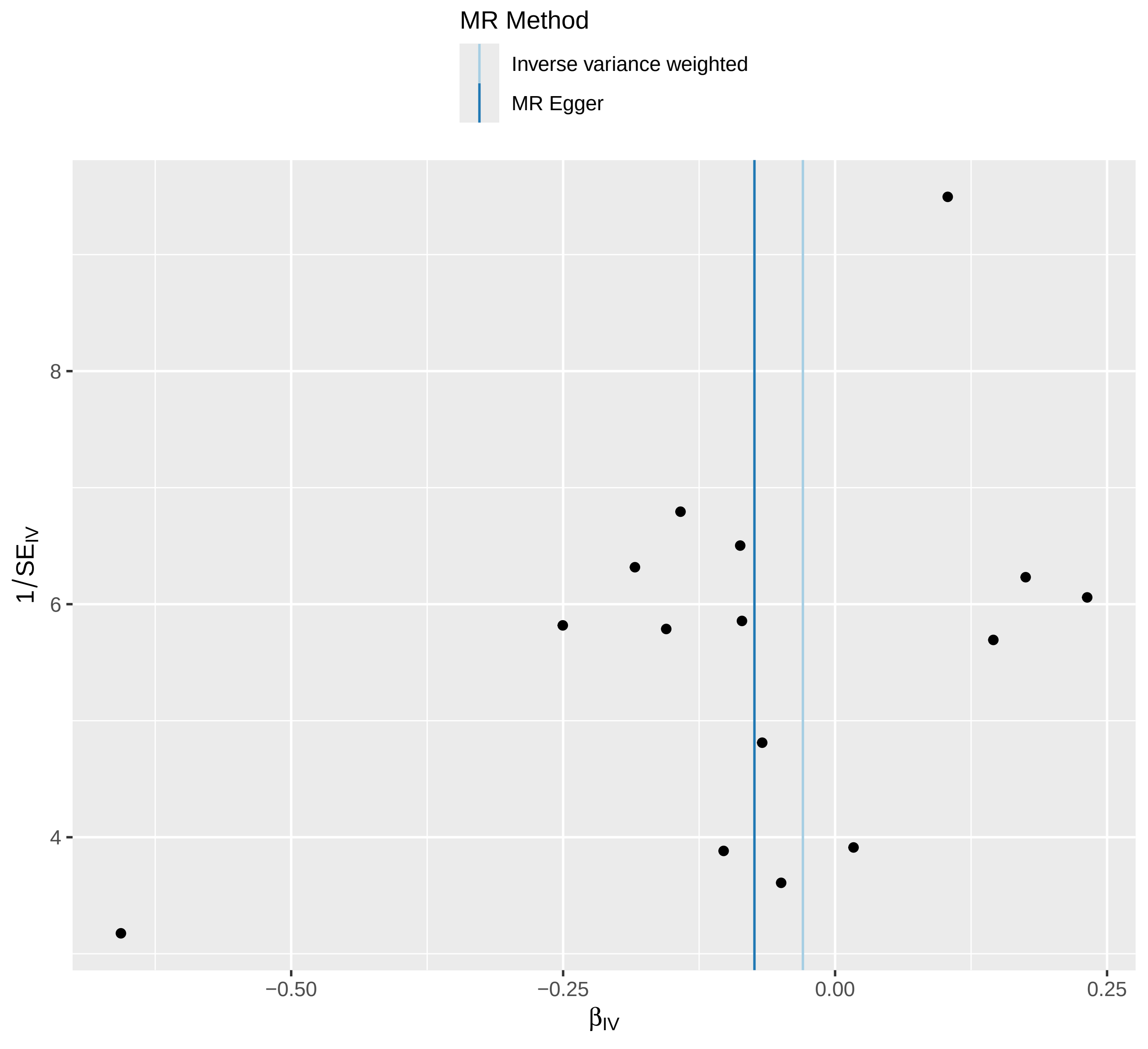

MR Method
Inverse variance weighted
MR Egger
1/SE$_{IV}$
8
6
4
−0.50
−0.25
0.00
0.25
β$_{IV}$

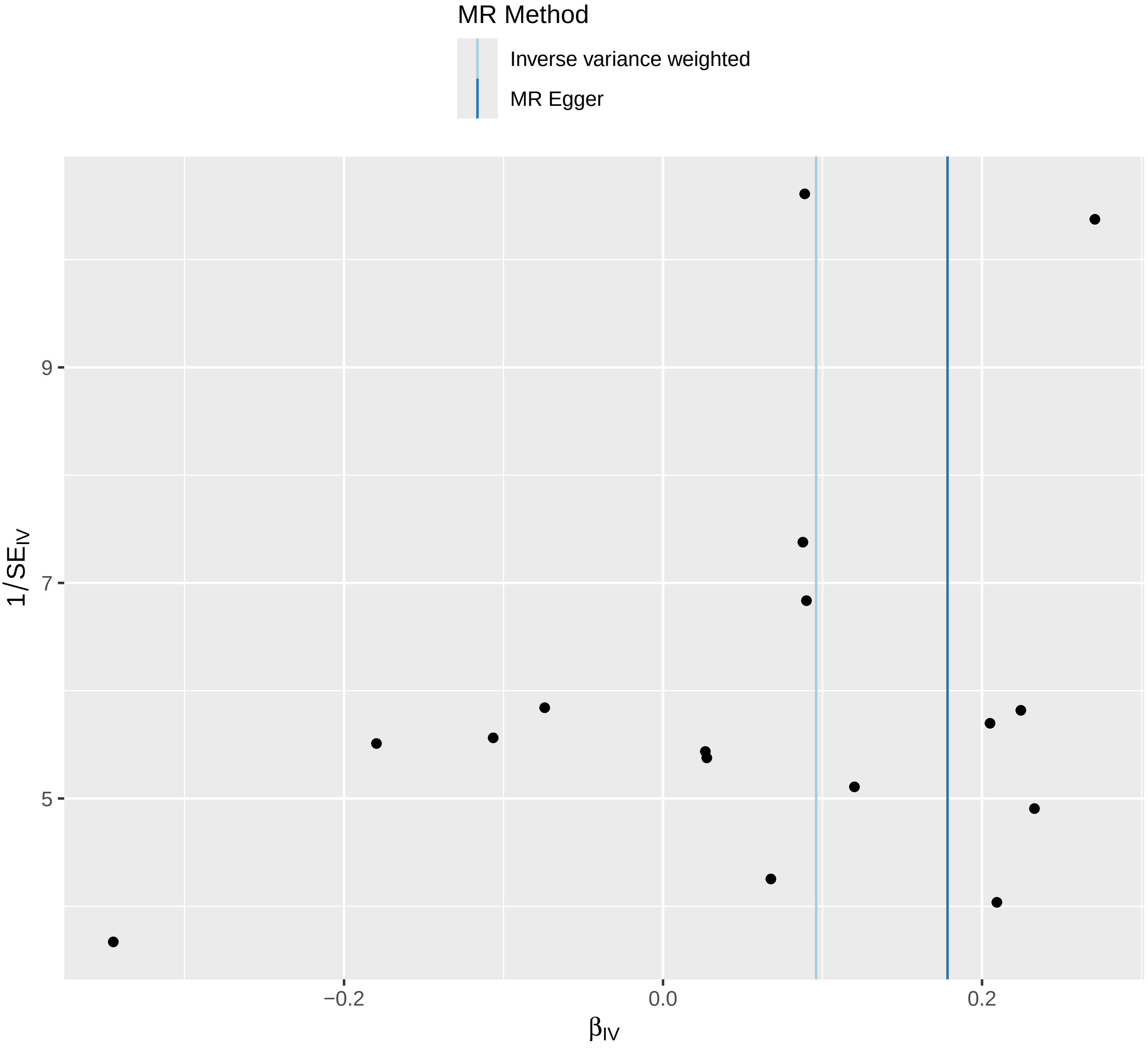

MR Method
Inverse variance weighted
MR Egger
1/SE$_{IV}$
9
7
5
−0.2
0.0
0.2
β$_{IV}$

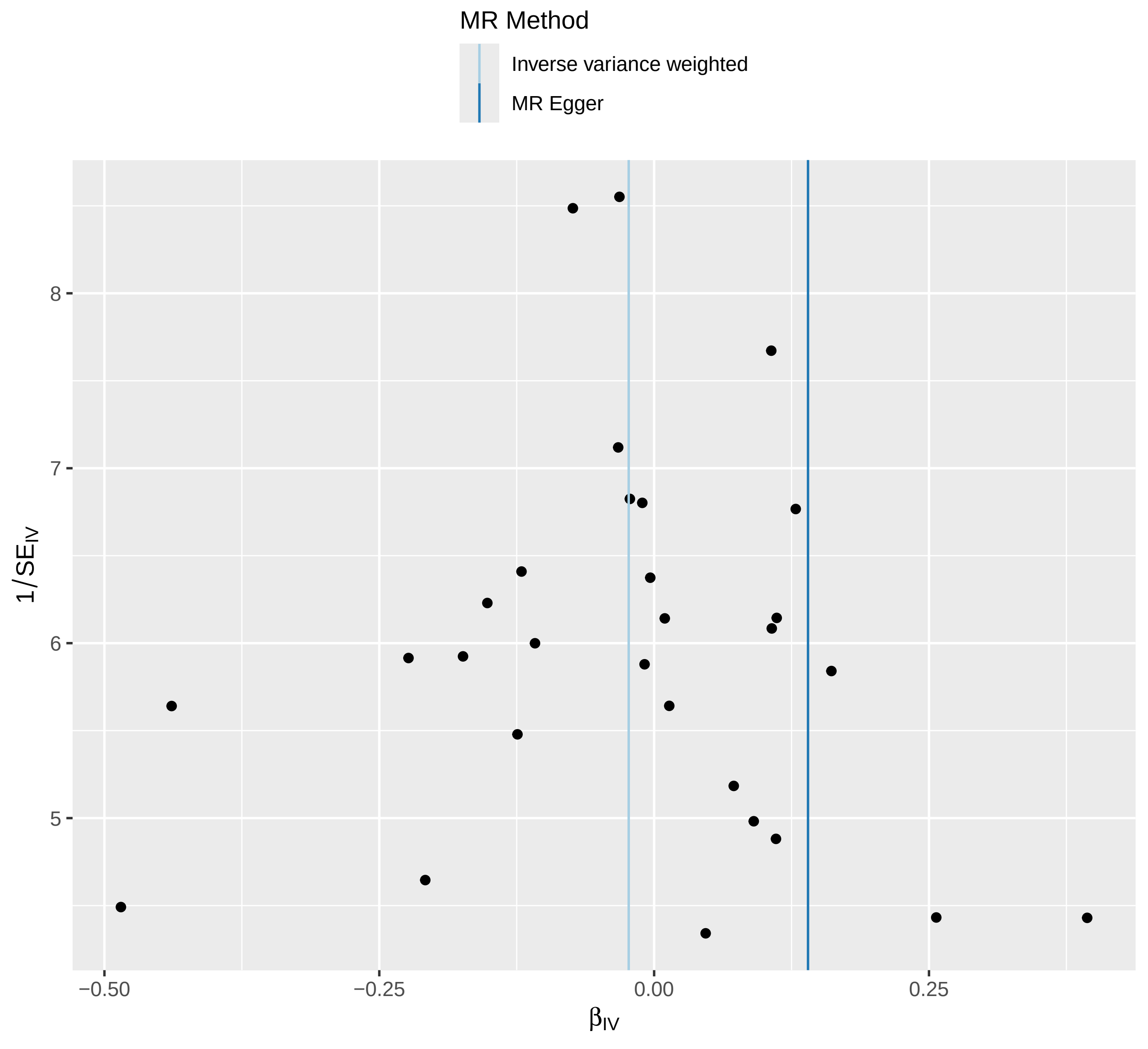
MR Method
Inverse variance weighted
MR Egger
1/SE_IV
β_IV
8
7
6
5
−0.50
−0.25
0.00
0.25

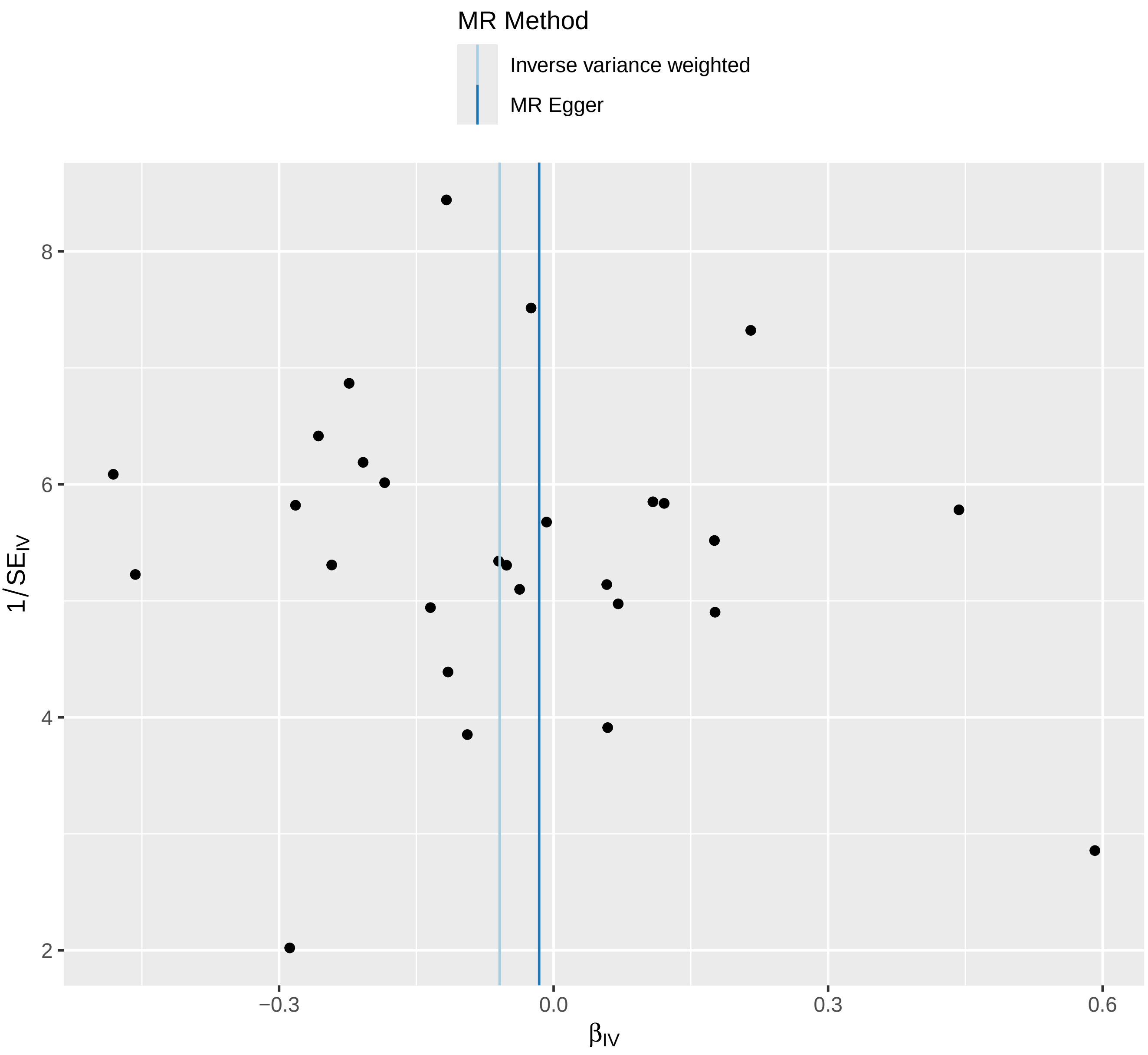
MR Method
Inverse variance weighted
MR Egger
1/SE_IV
8
6
4
2
−0.3
0.0
0.3
0.6
β_IV

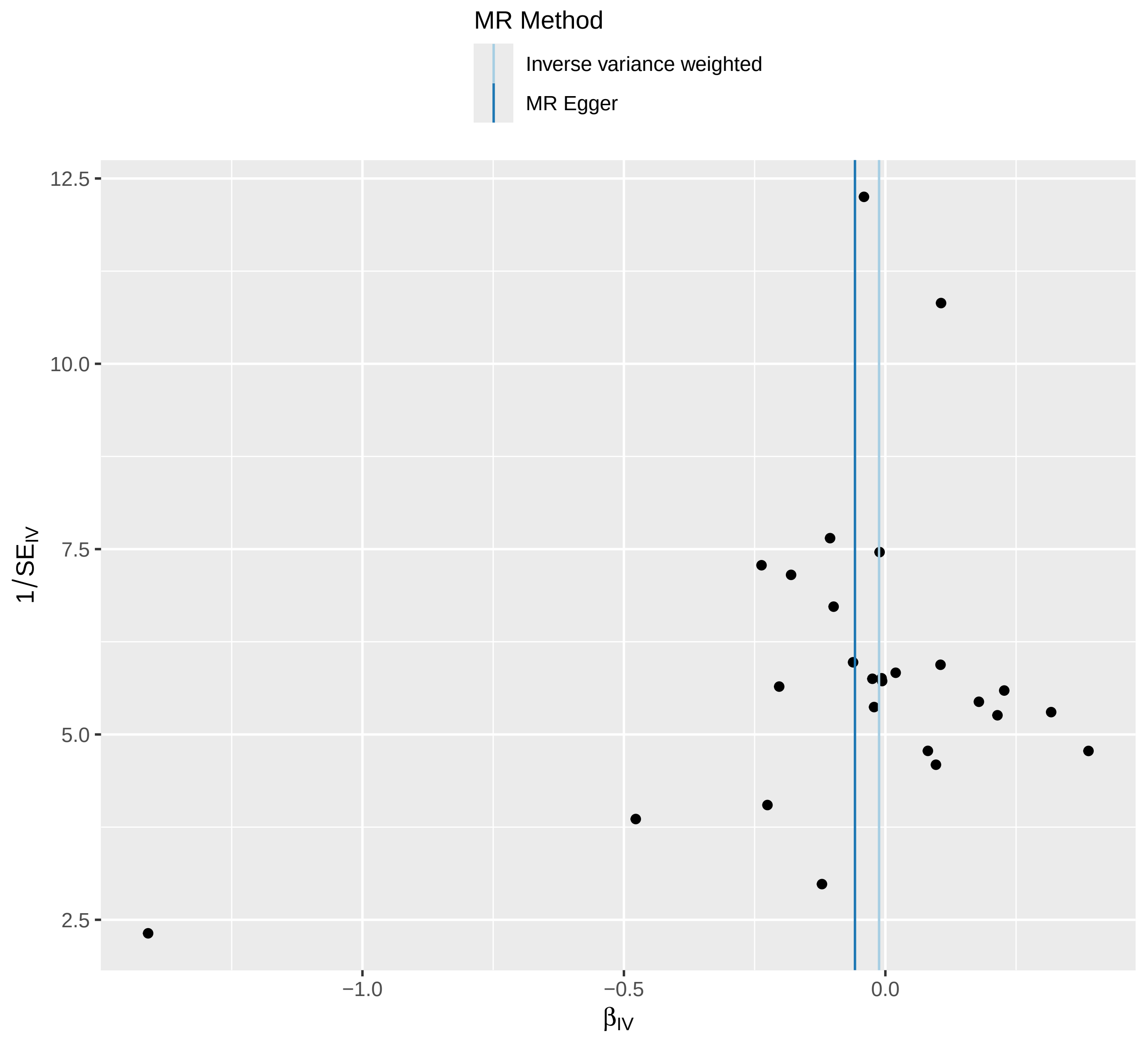
MR Method
Inverse variance weighted
MR Egger
12.5
10.0
7.5
5.0
2.5
1/SE_IV
−1.0
−0.5
0.0
β_IV

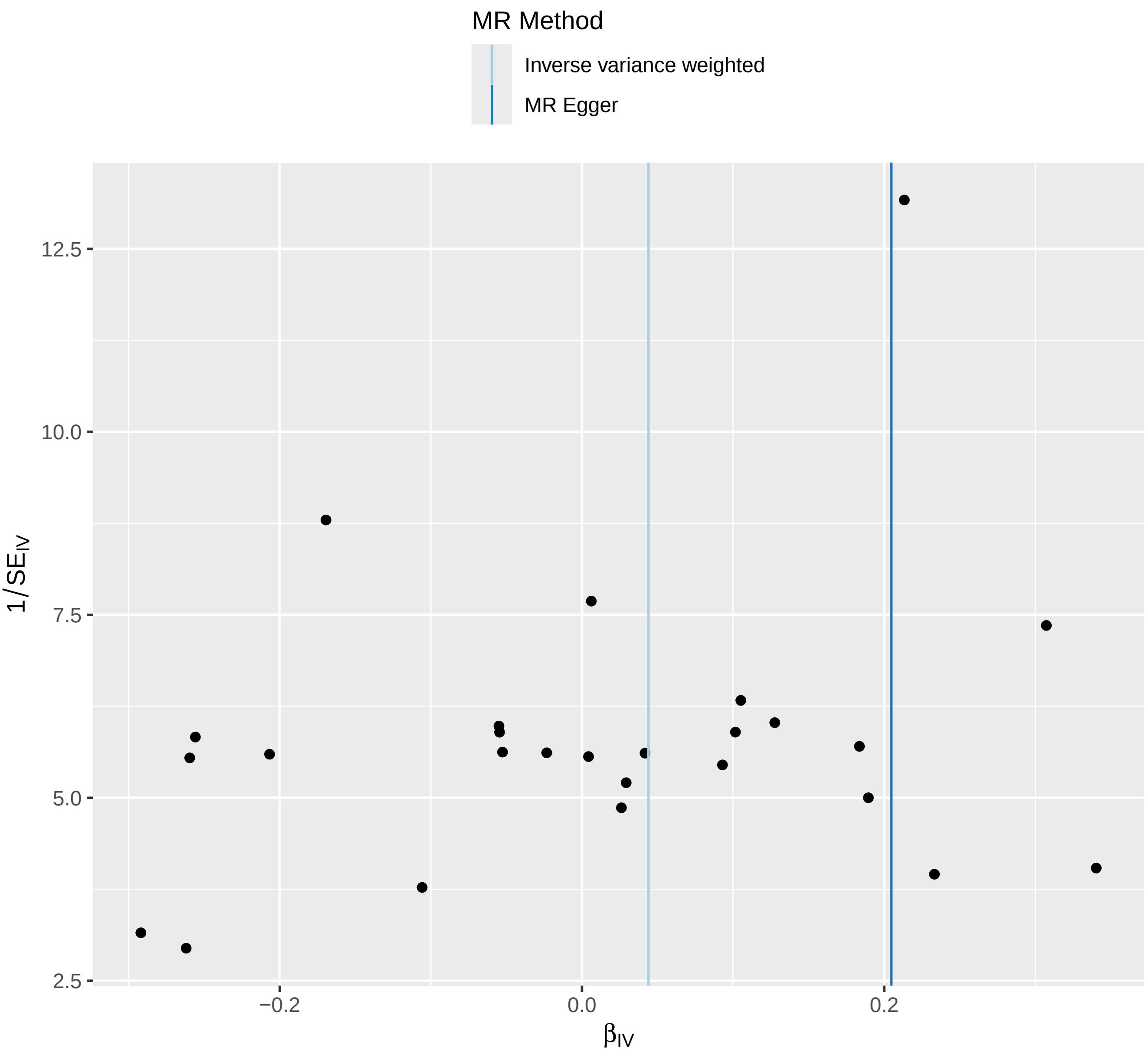

MR Method
Inverse variance weighted
MR Egger
12.5
10.0
7.5
5.0
2.5
1/SE_IV
−0.2
0.0
0.2
β_IV

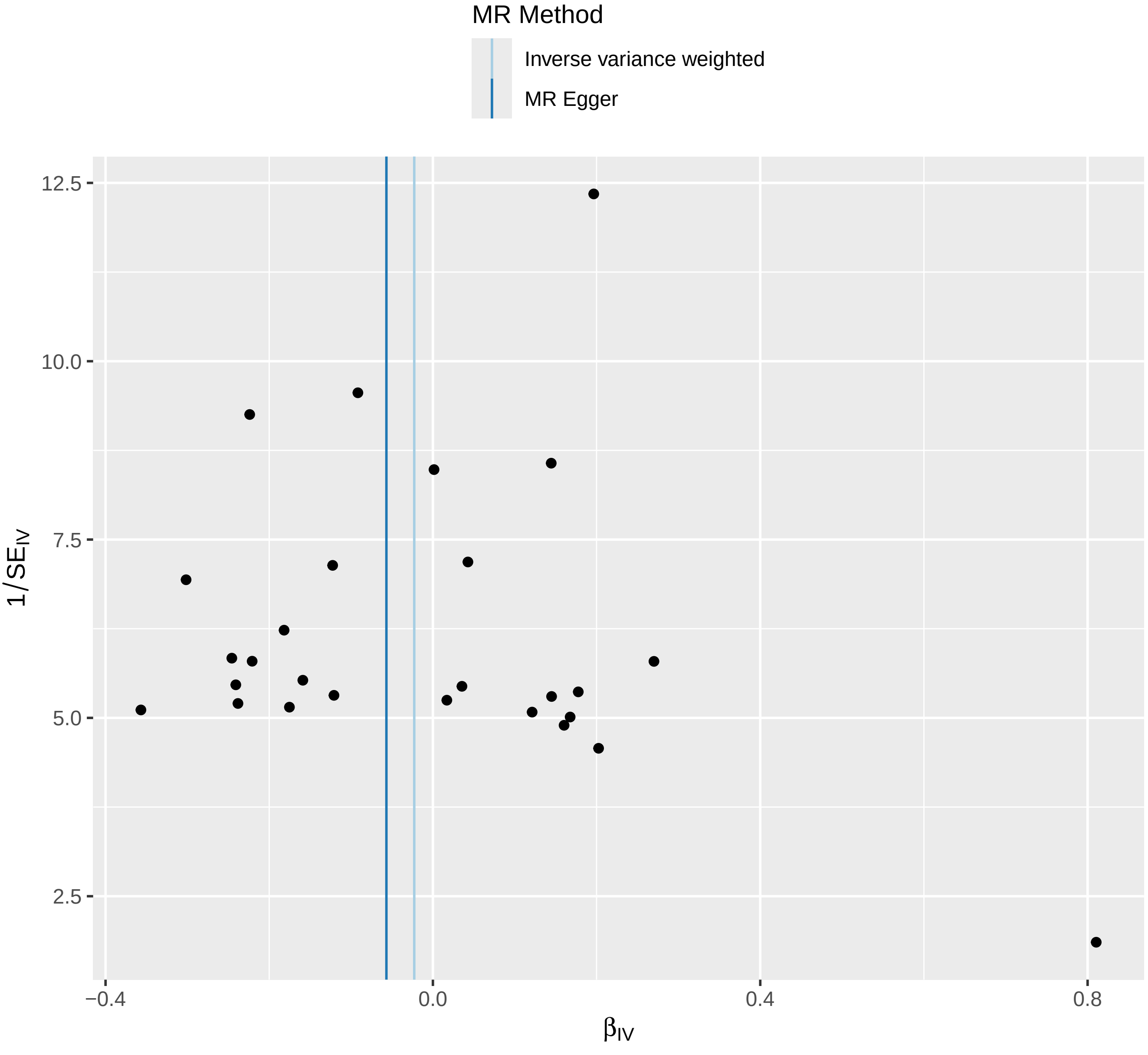
MR Method
Inverse variance weighted
MR Egger
12.5
10.0
7.5
5.0
2.5
1/SE$_{IV}$
−0.4
0.0
0.4
0.8
β$_{IV}$

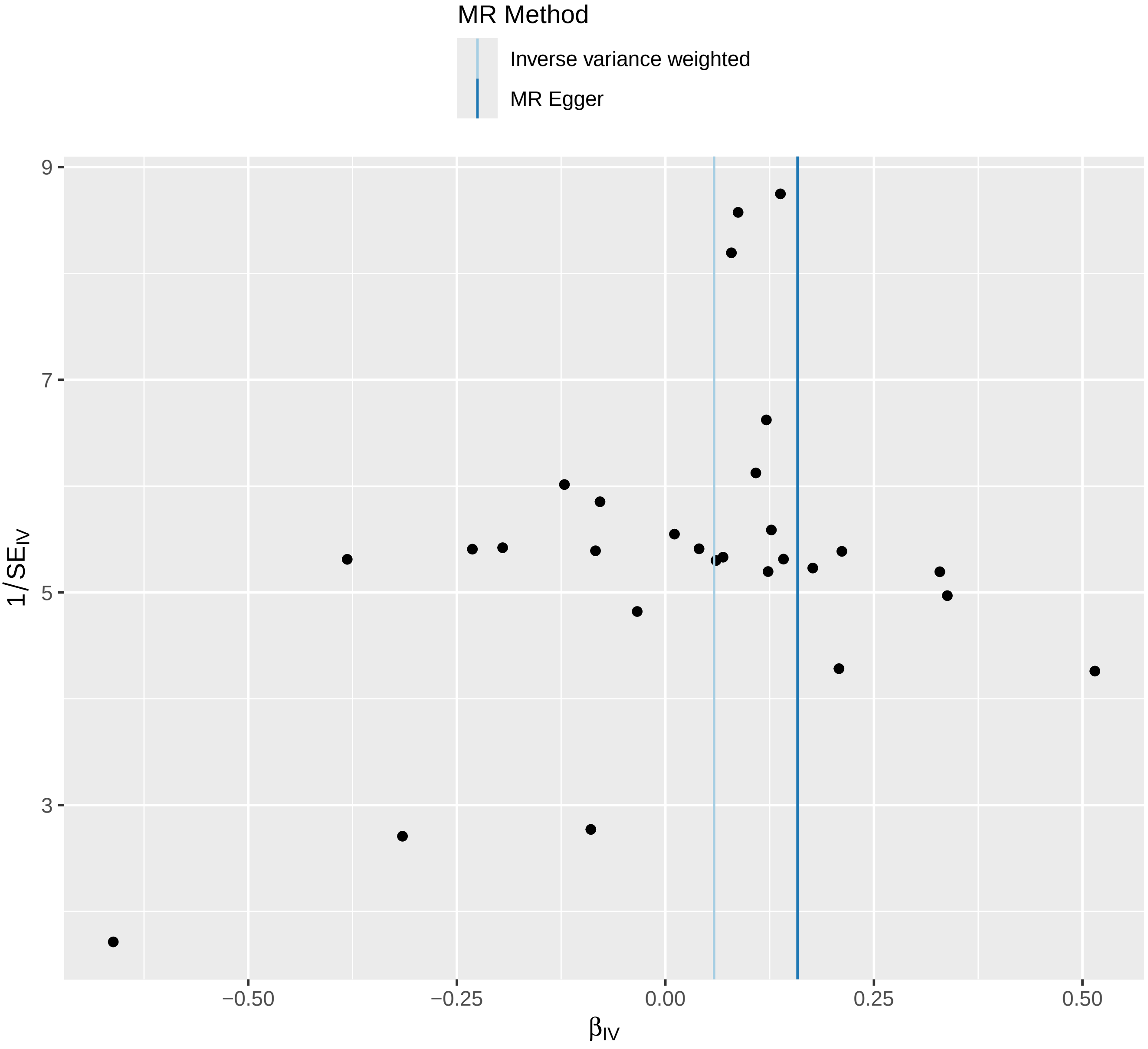

MR Method
Inverse variance weighted
MR Egger
9
7
5
3
$1/SE_{IV}$
−0.50
−0.25
0.00
0.25
0.50
β_{IV}

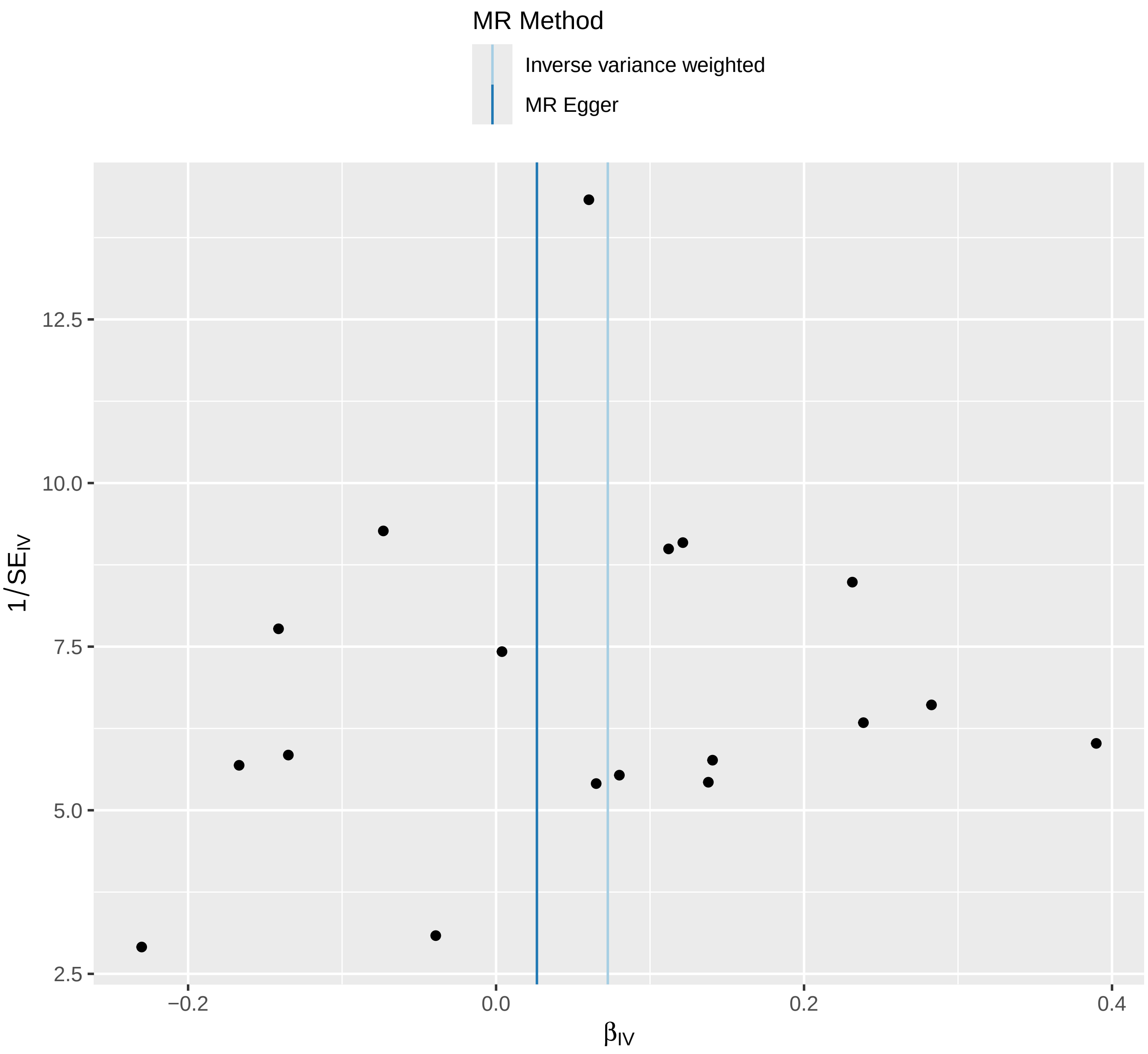
MR Method
Inverse variance weighted
MR Egger
12.5
10.0
7.5
5.0
2.5
−0.2
0.0
0.2
0.4
$1/SE_{IV}$
β_{IV}

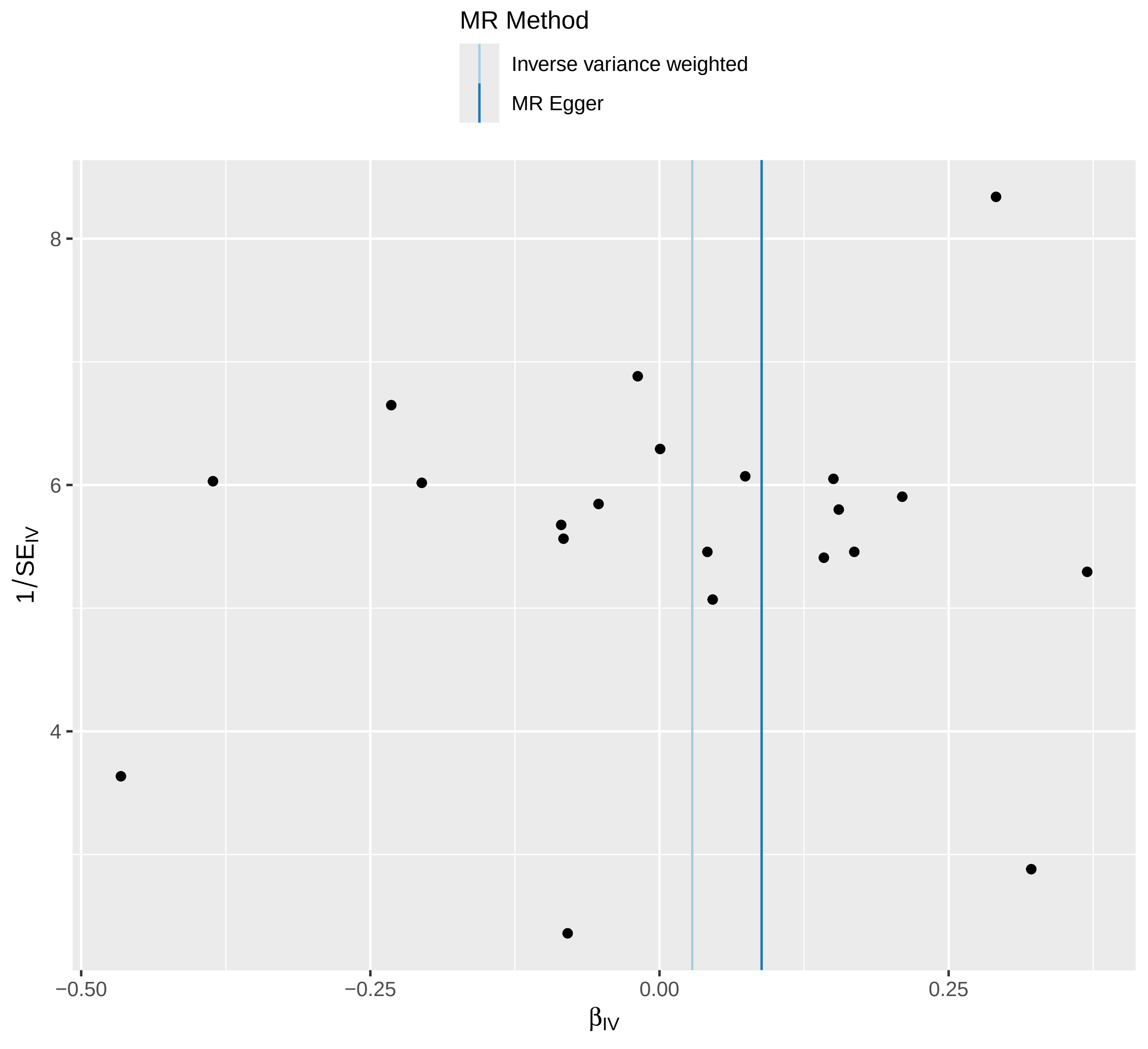
MR Method
Inverse variance weighted
MR Egger
1/SE_IV
8
6
4
β_IV
−0.50
−0.25
0.00
0.25

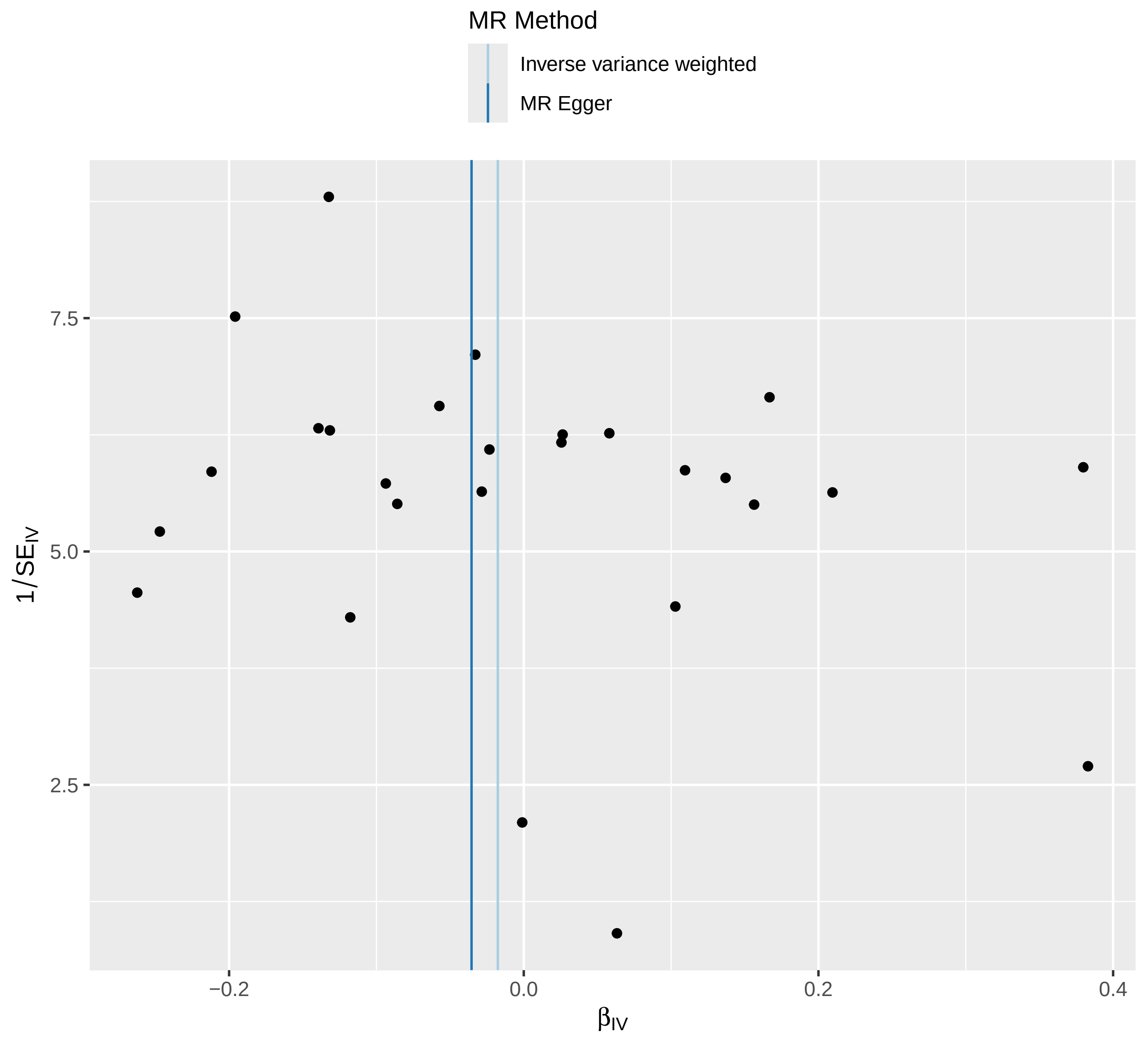

MR Method
Inverse variance weighted
MR Egger
7.5
5.0
2.5
1/SE_IV
−0.2
0.0
0.2
0.4
β_IV

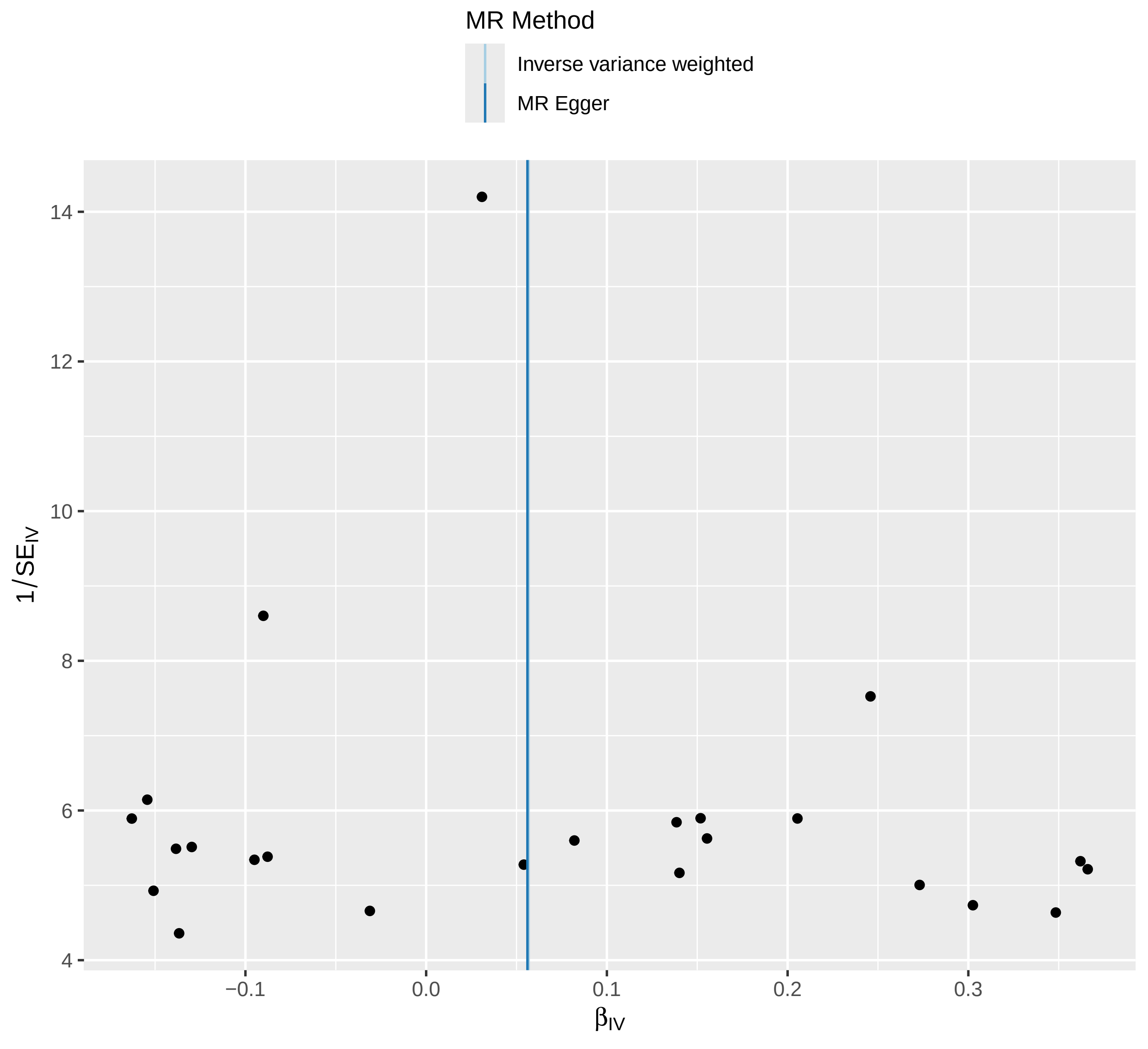

MR Method
Inverse variance weighted
MR Egger
1/SE_IV
β_IV
14
12
10
8
6
4
−0.1
0.0
0.1
0.2
0.3

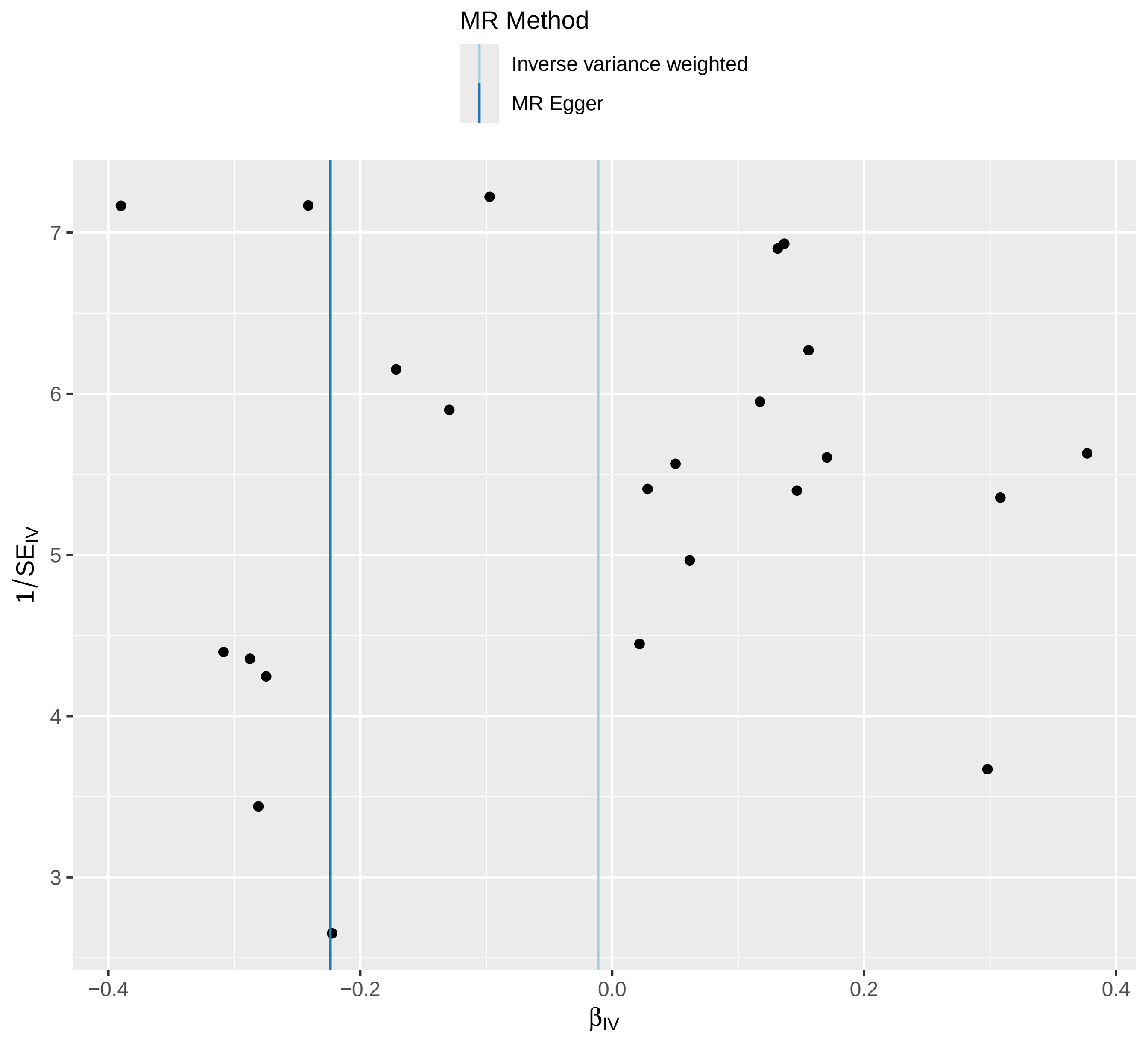
MR Method
Inverse variance weighted
MR Egger
1/SE_IV
β_IV
−0.4
−0.2
0.0
0.2
0.4
3
4
5
6
7

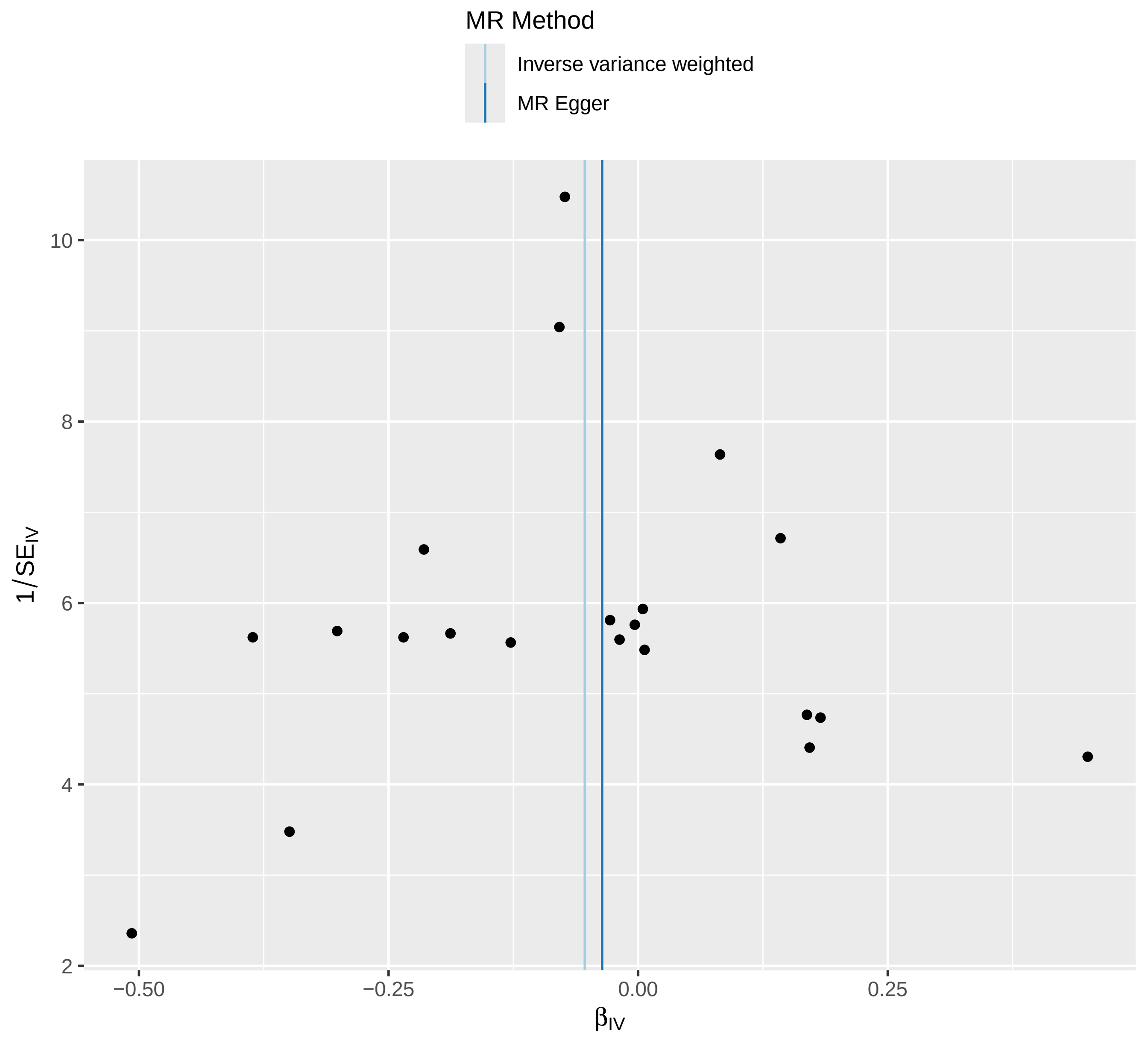

MR Method
Inverse variance weighted
MR Egger
10
8
6
4
2
1/SE_IV
−0.50
−0.25
0.00
0.25
β_IV

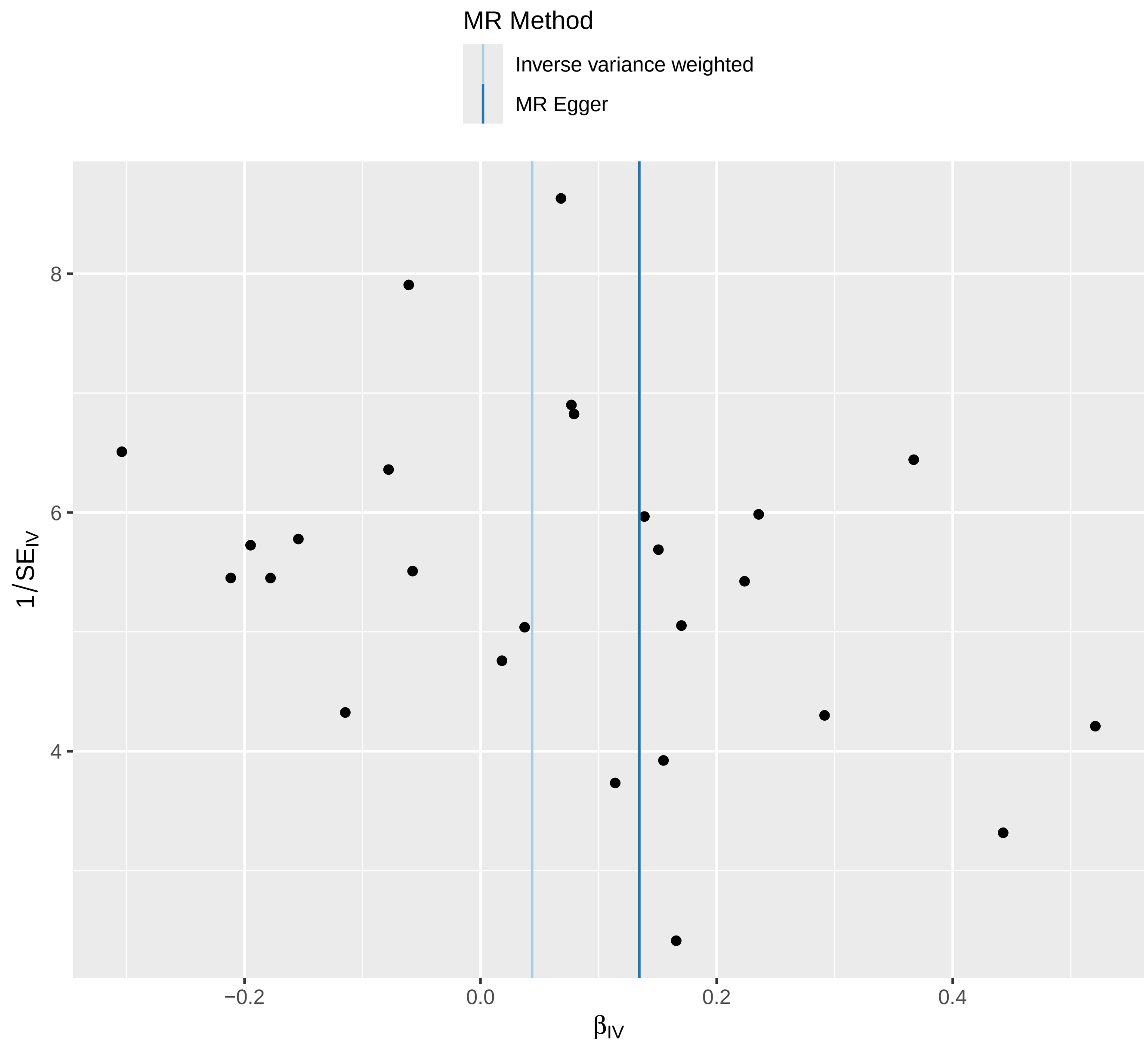

MR Method
Inverse variance weighted
MR Egger
8
6
4
$1/SE_{IV}$
−0.2
0.0
0.2
0.4
β_{IV}

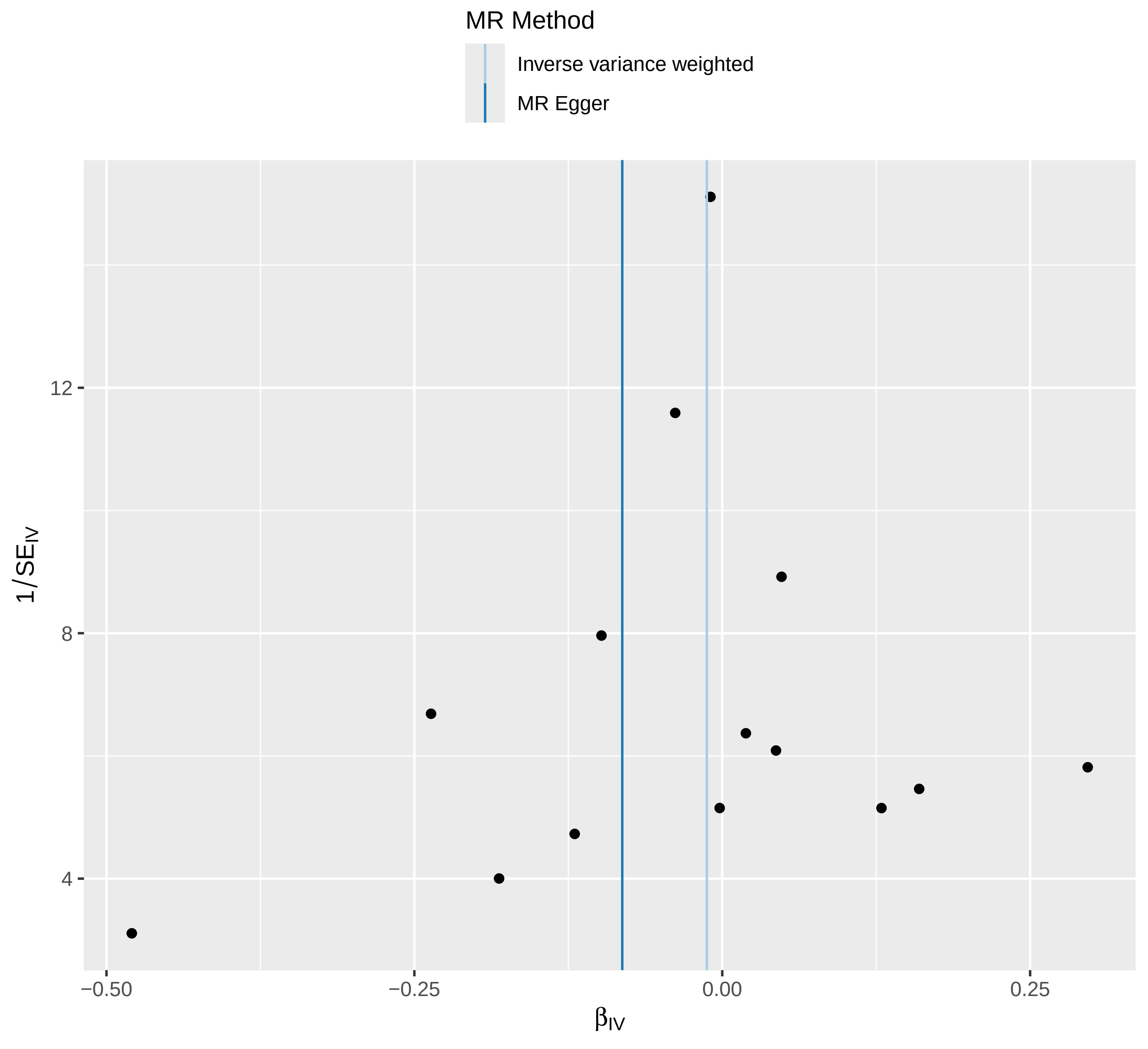
MR Method
Inverse variance weighted
MR Egger
1/SE_IV
12
8
4
−0.50
−0.25
0.00
0.25
β_IV

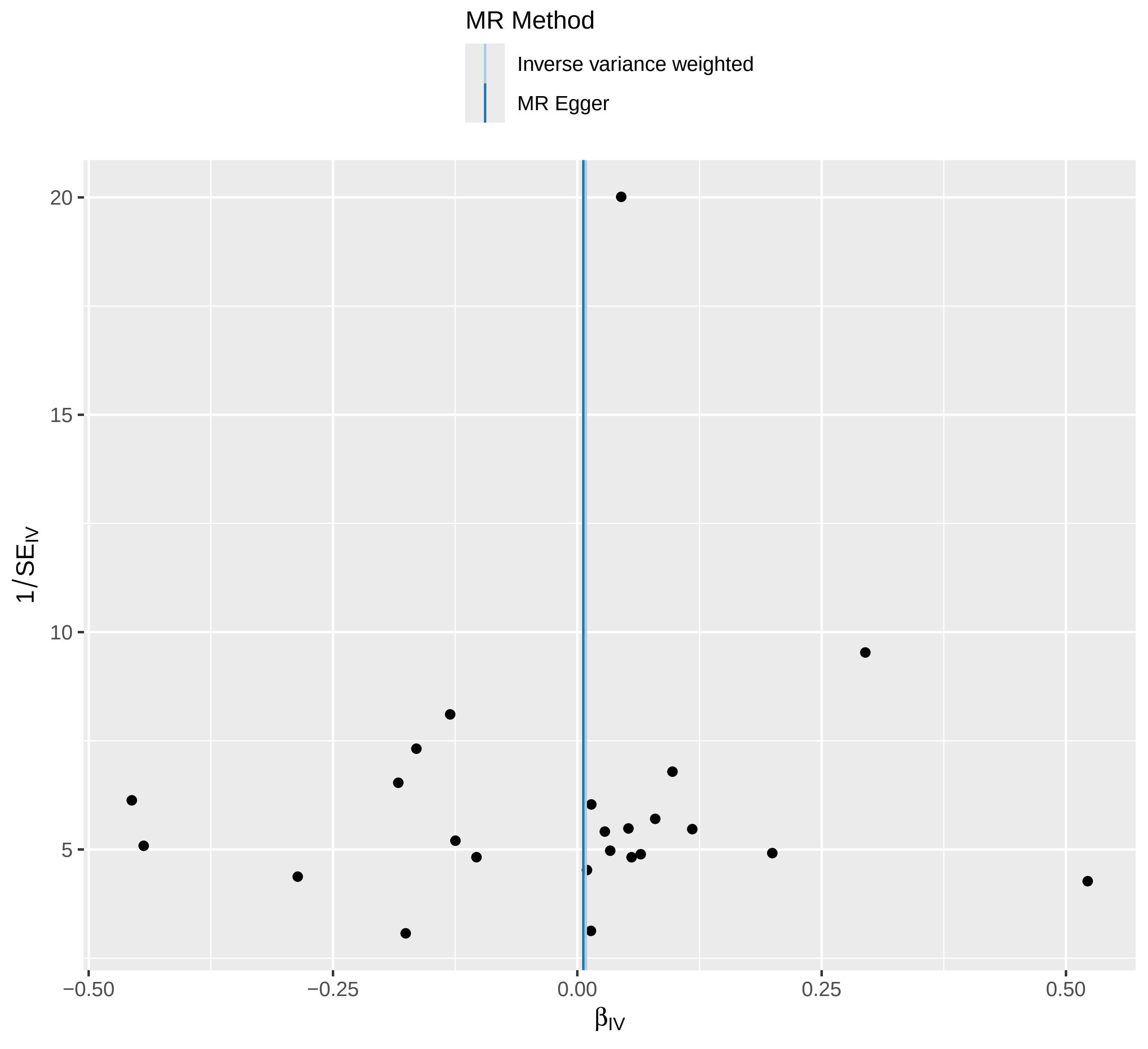
MR Method
Inverse variance weighted
MR Egger
20
15
10
5
−0.50
−0.25
0.00
0.25
0.50
$1/SE_{IV}$
β_{IV}

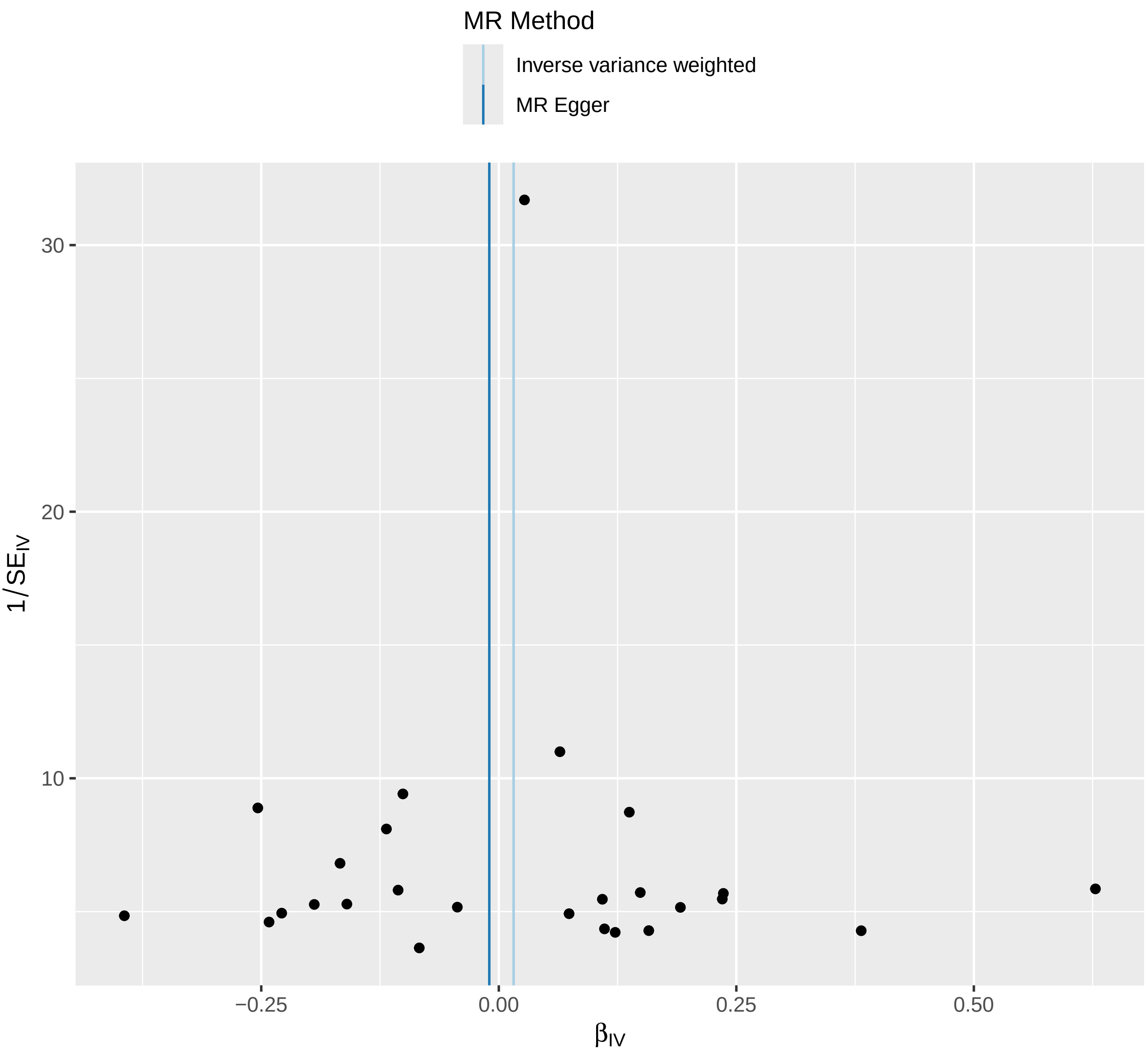
MR Method
Inverse variance weighted
MR Egger
30
20
10
1/SE_{IV}
−0.25
0.00
0.25
0.50
β_{IV}

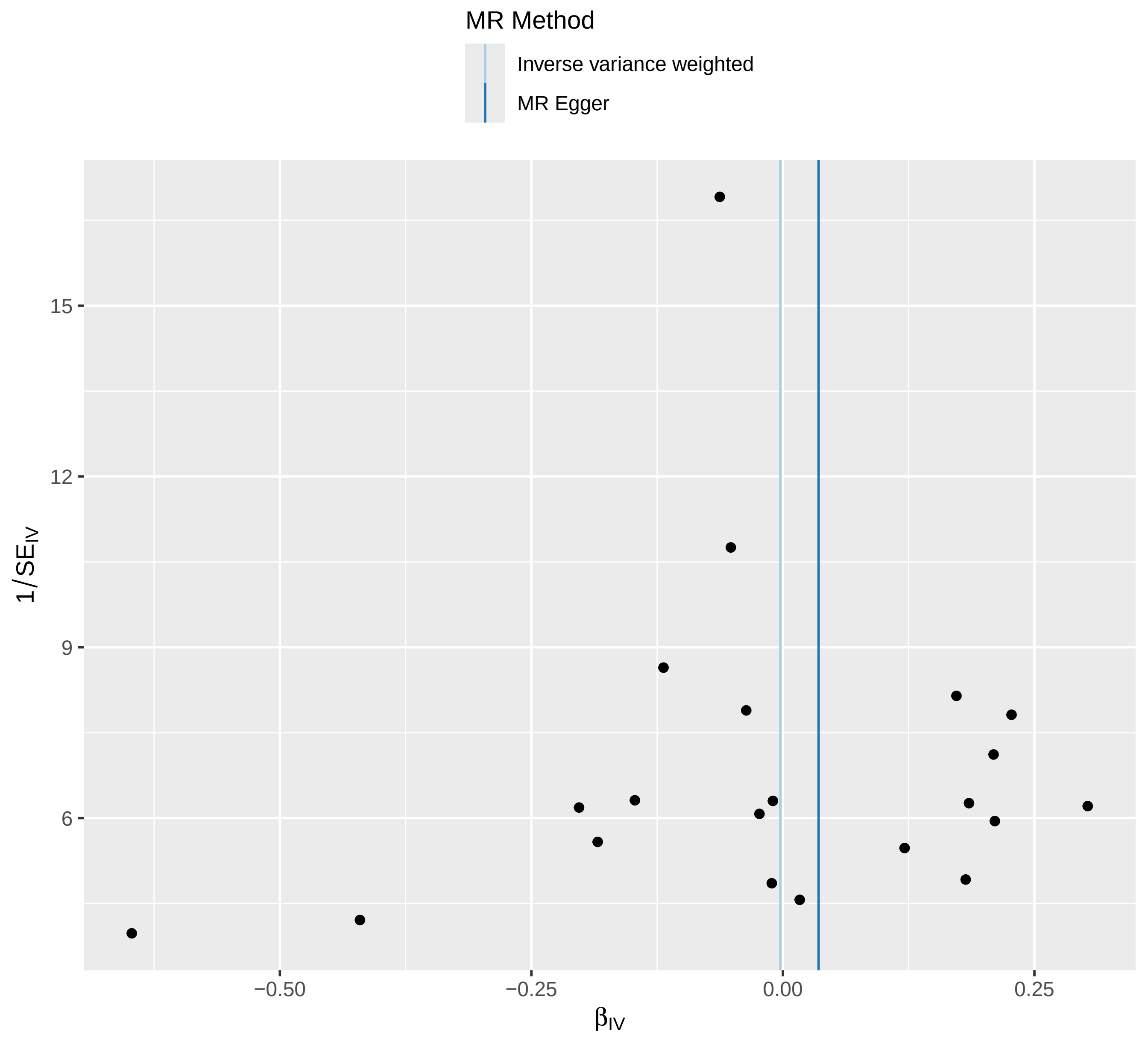

MR Method
Inverse variance weighted
MR Egger
15
12
9
6
1/SE_IV
−0.50
−0.25
0.00
0.25
β_IV

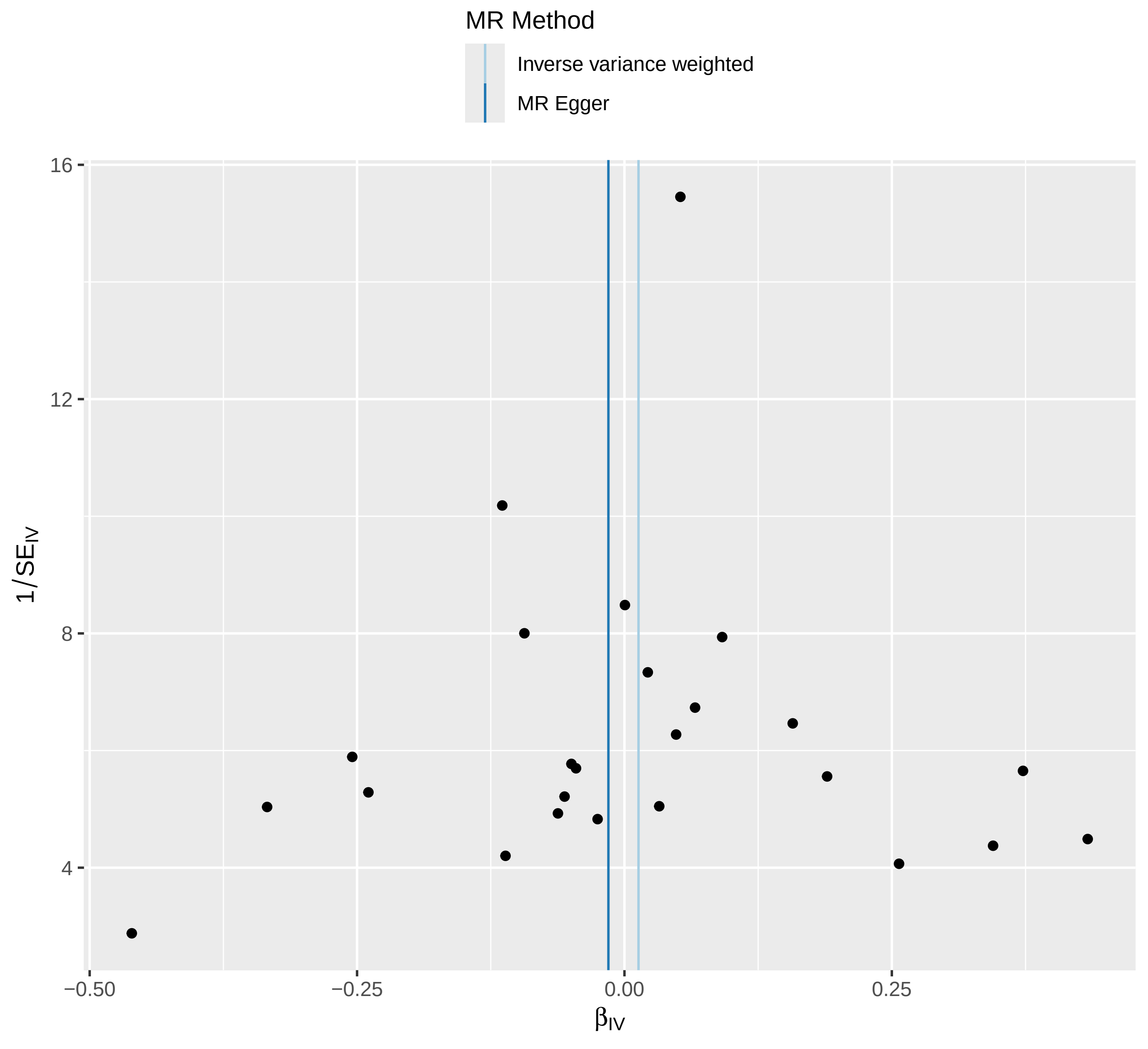
MR Method
Inverse variance weighted
MR Egger
16
12
8
4
1/SE_IV
−0.50
−0.25
0.00
0.25
β_IV

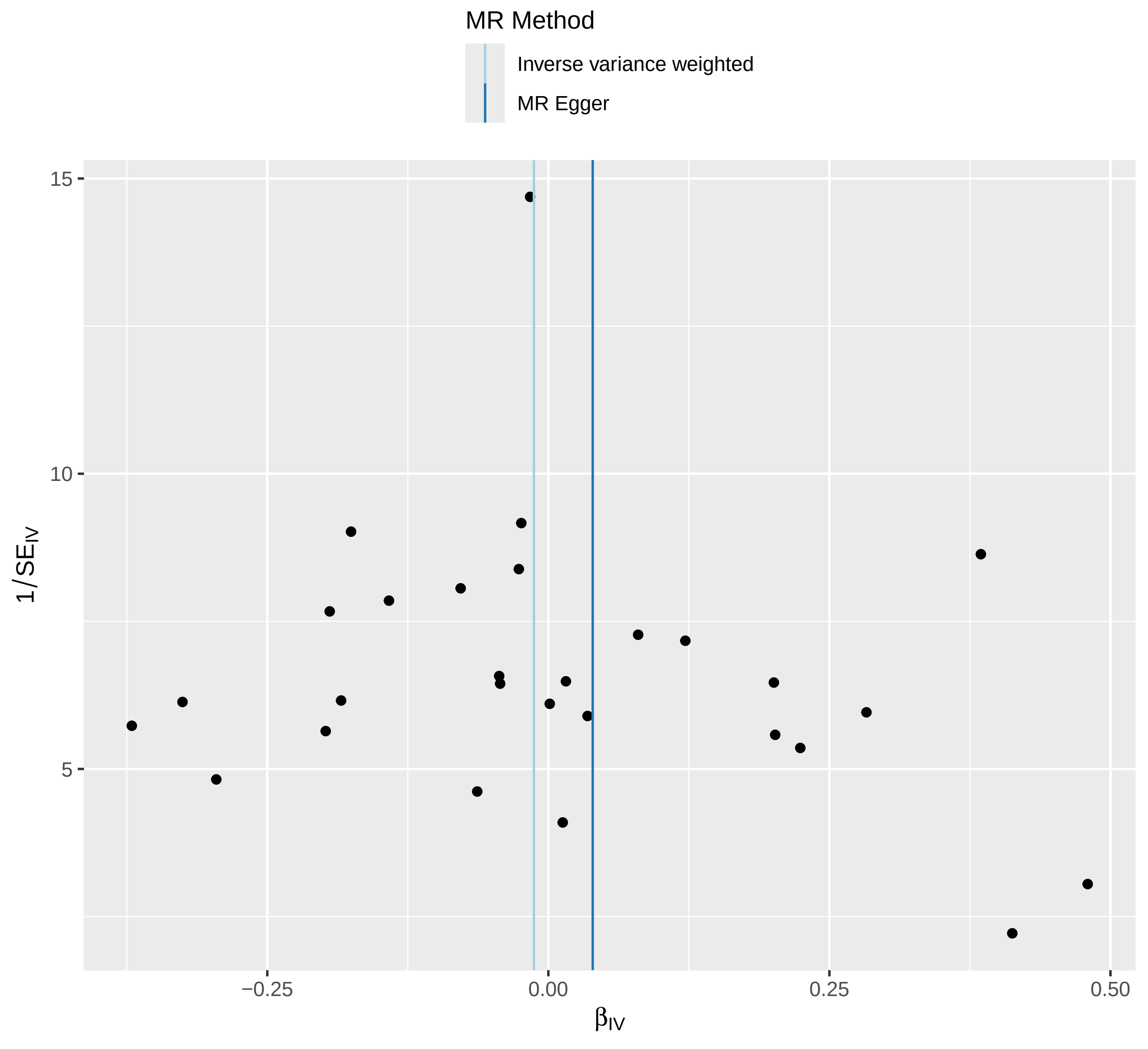
MR Method
Inverse variance weighted
MR Egger
15
10
5
1/SE_IV
−0.25
0.00
0.25
0.50
β_IV

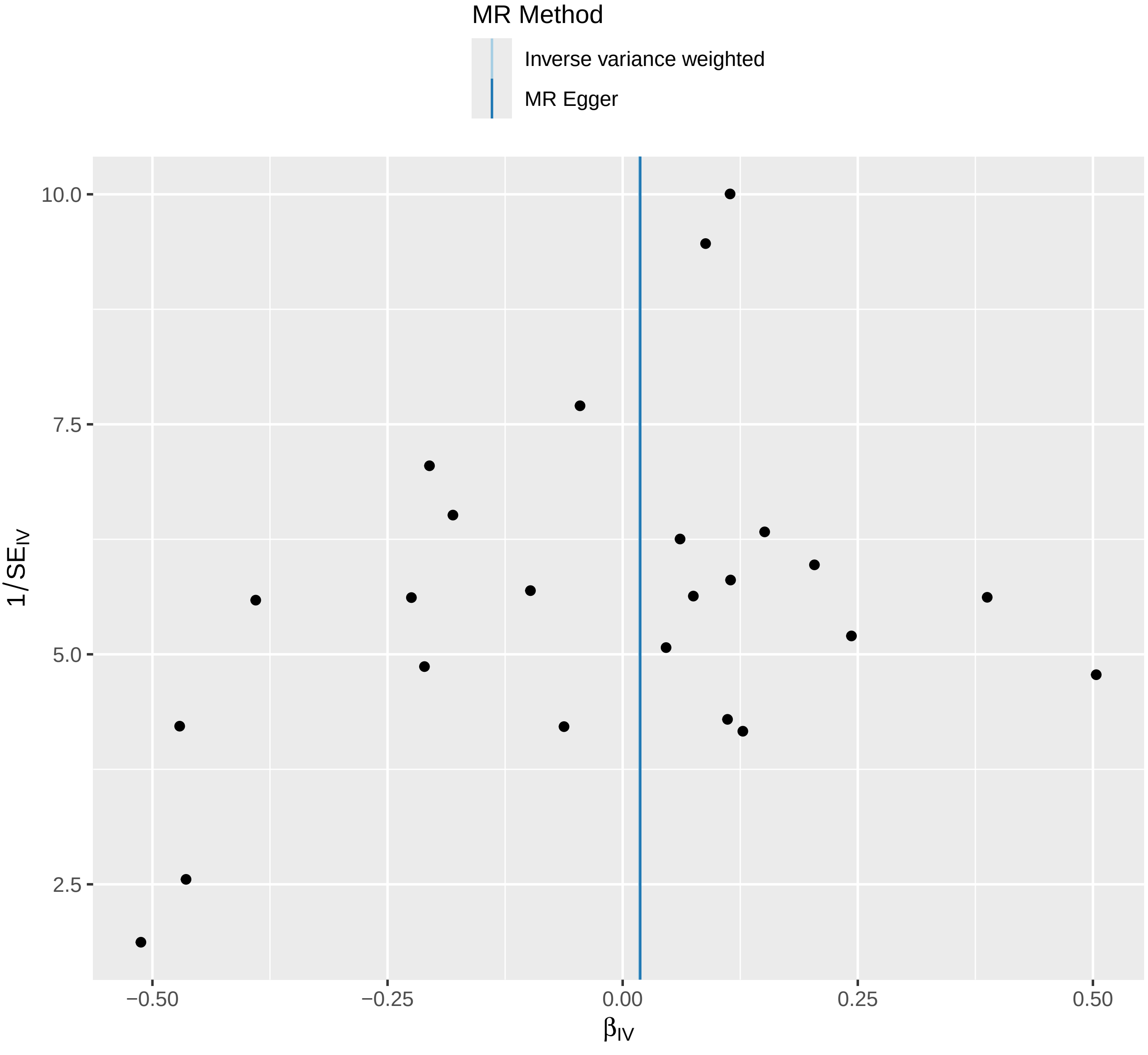
MR Method
Inverse variance weighted
MR Egger
10.0
7.5
5.0
2.5
1/SE_IV
−0.50
−0.25
0.00
0.25
0.50
β_IV

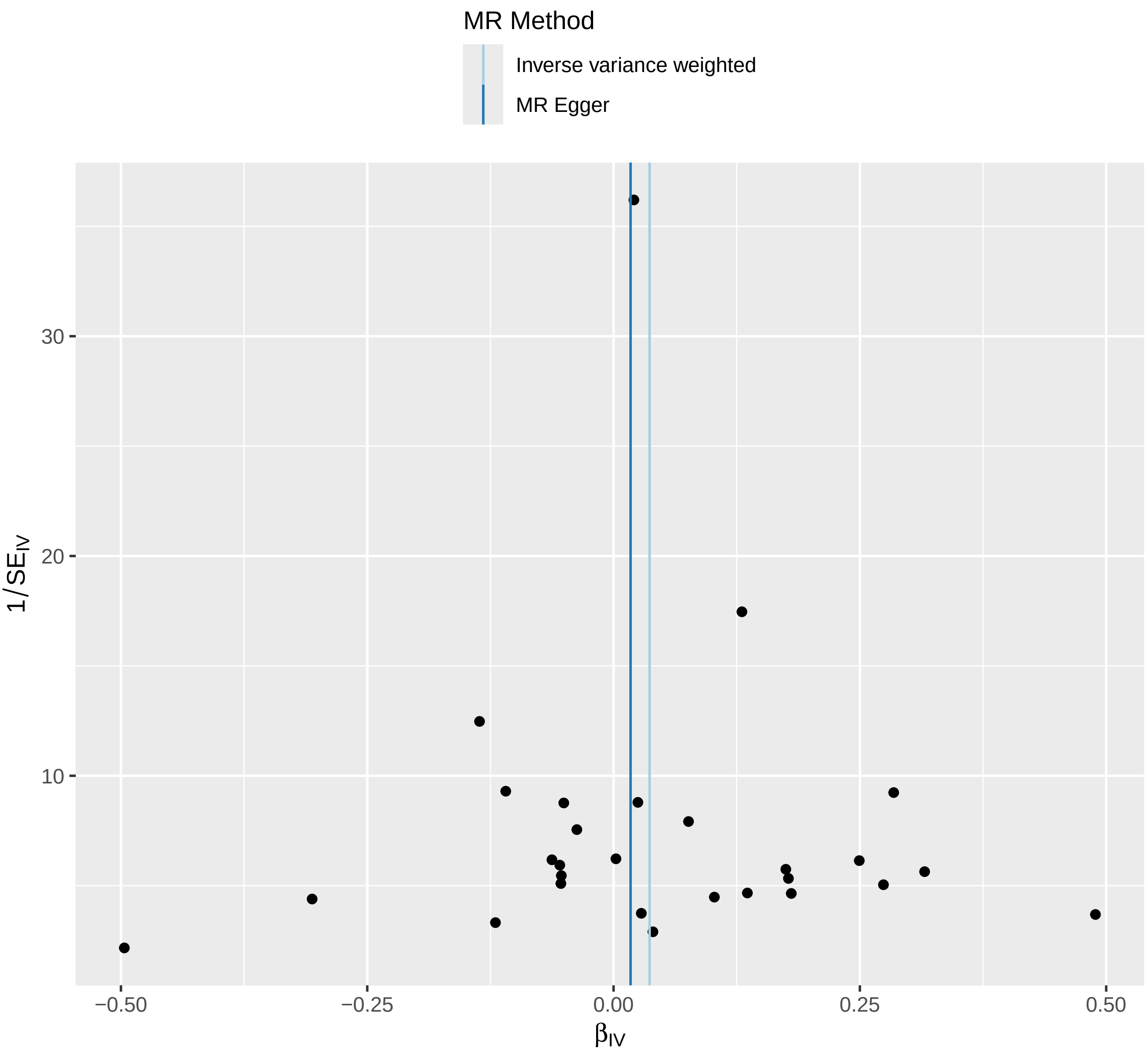
MR Method
Inverse variance weighted
MR Egger
30
20
10
$1/SE_{IV}$
−0.50
−0.25
0.00
0.25
0.50
β_{IV}

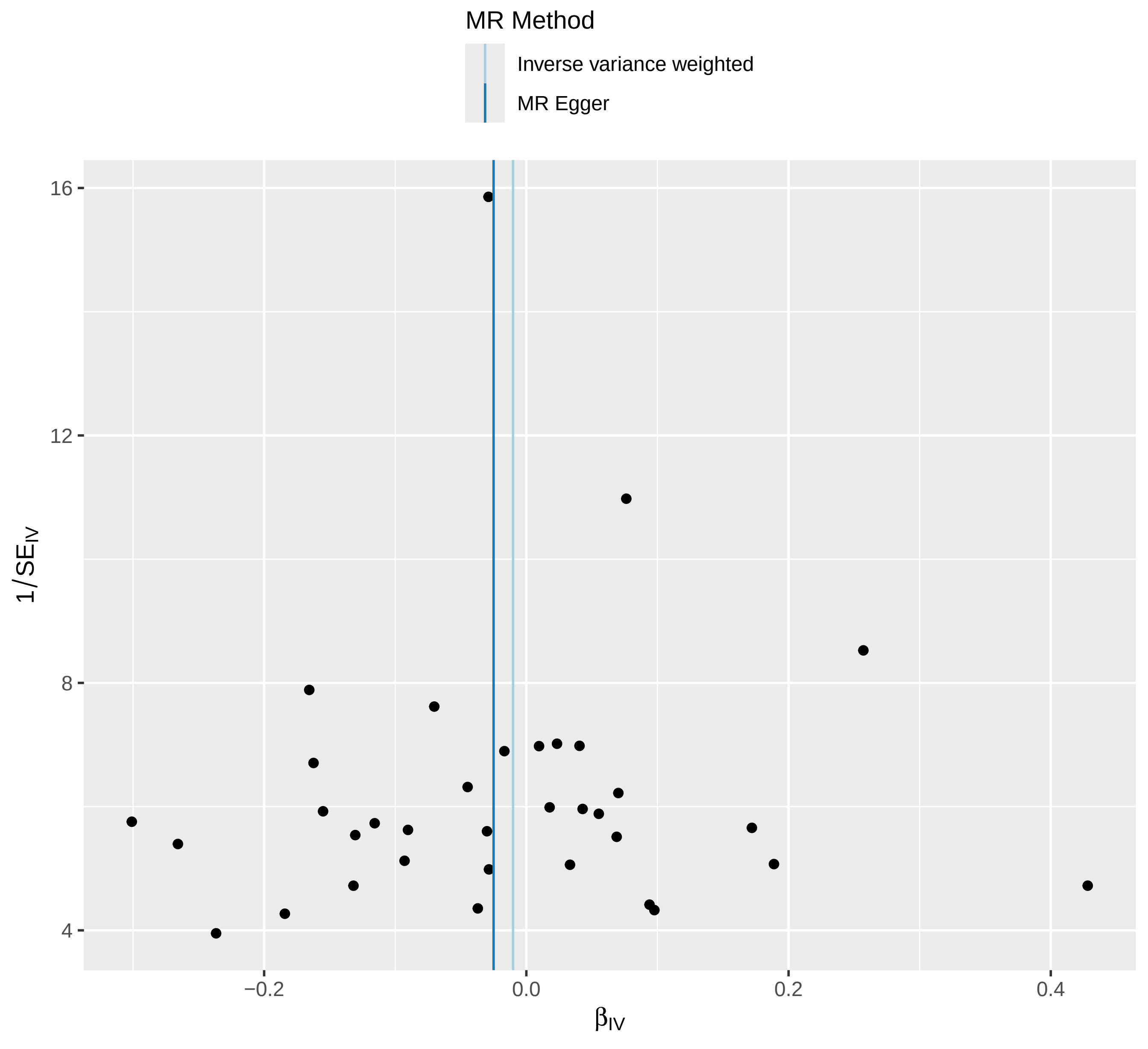
MR Method
Inverse variance weighted
MR Egger
16
12
8
4
$1/SE_{IV}$
−0.2
0.0
0.2
0.4
β_{IV}

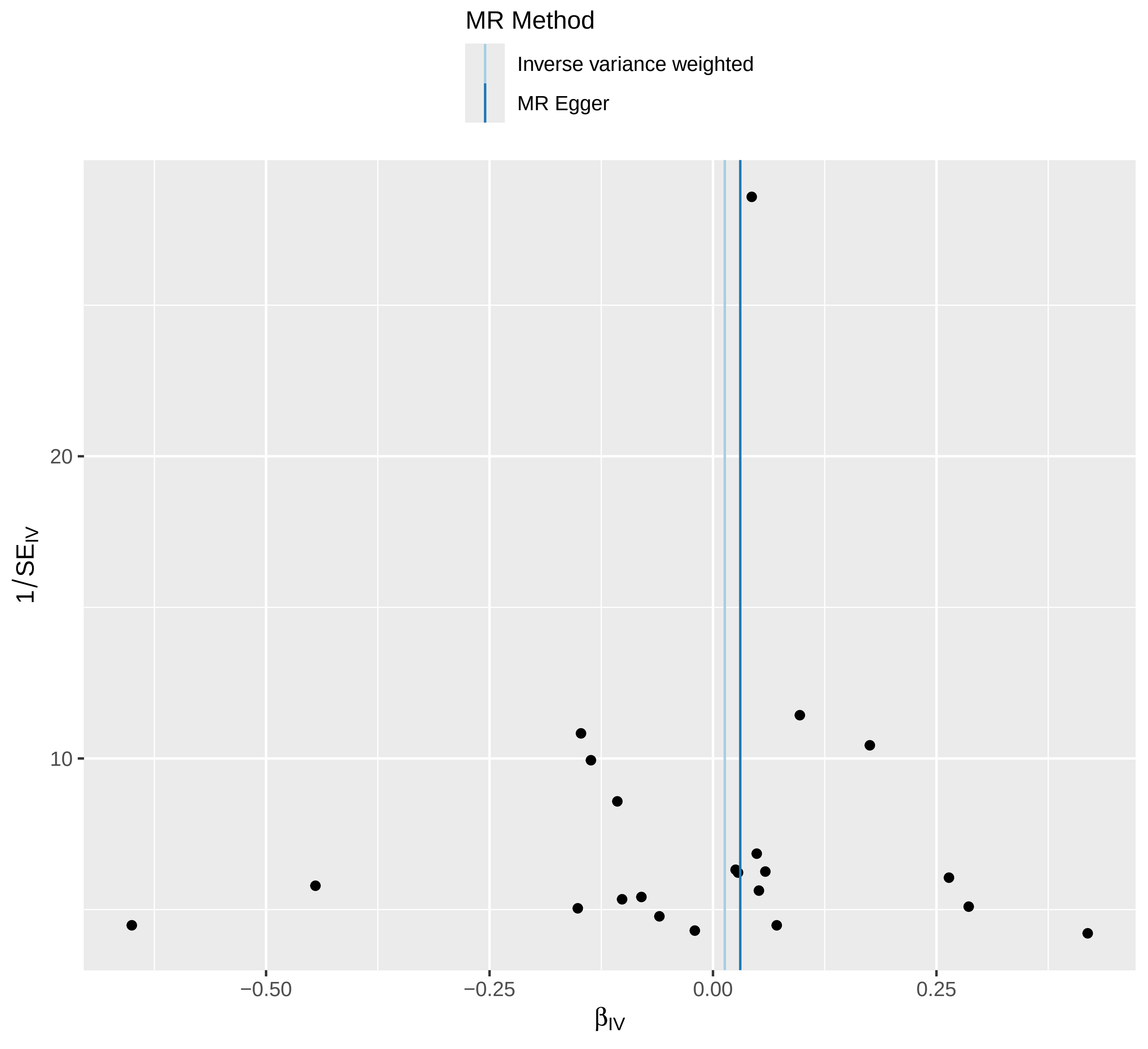
MR Method
Inverse variance weighted
MR Egger
20
10
1/SE$_{IV}$
−0.50
−0.25
0.00
0.25
β$_{IV}$

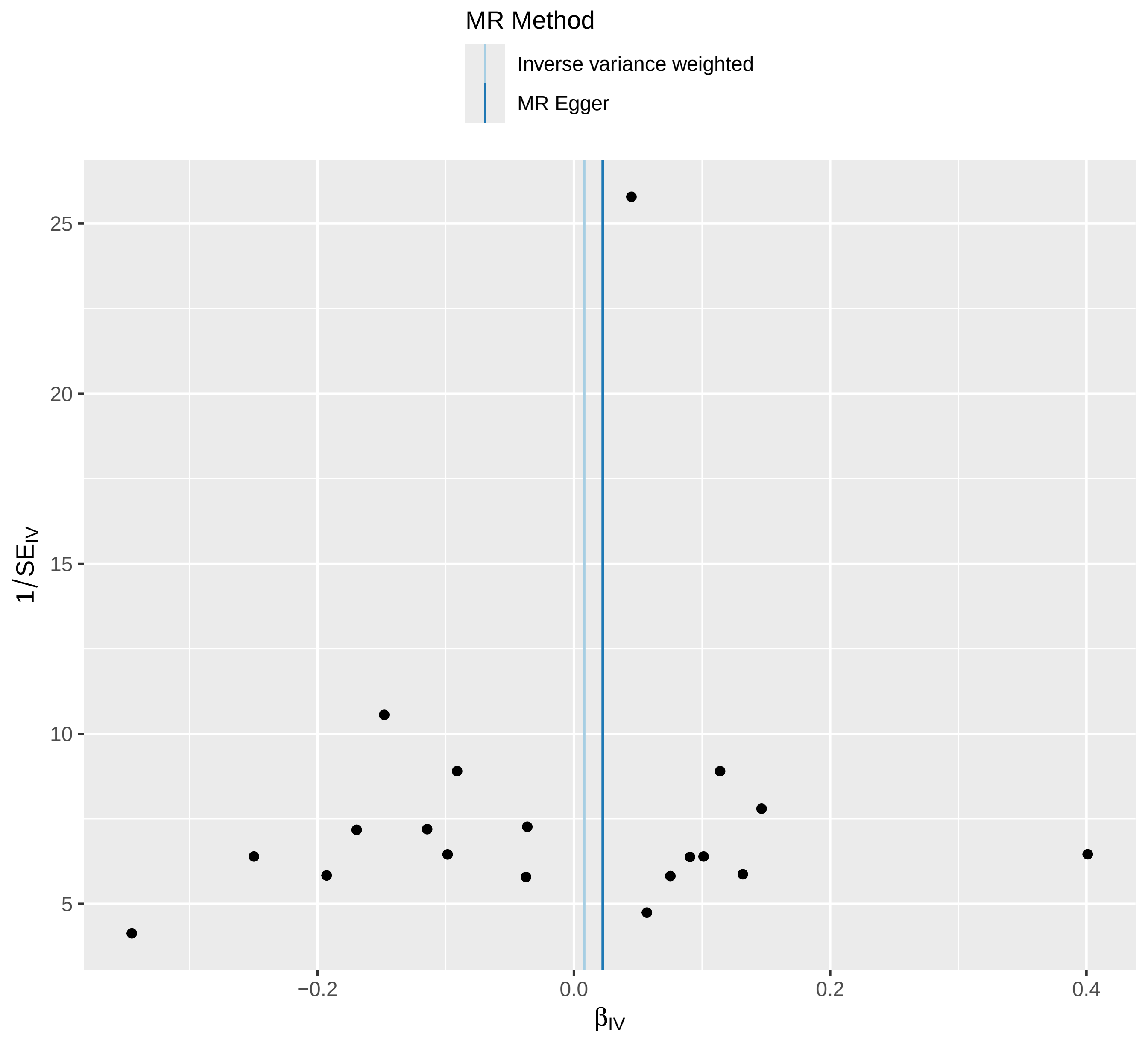
MR Method
Inverse variance weighted
MR Egger
25
20
15
10
5
1/SE_IV
−0.2
0.0
0.2
0.4
β_IV

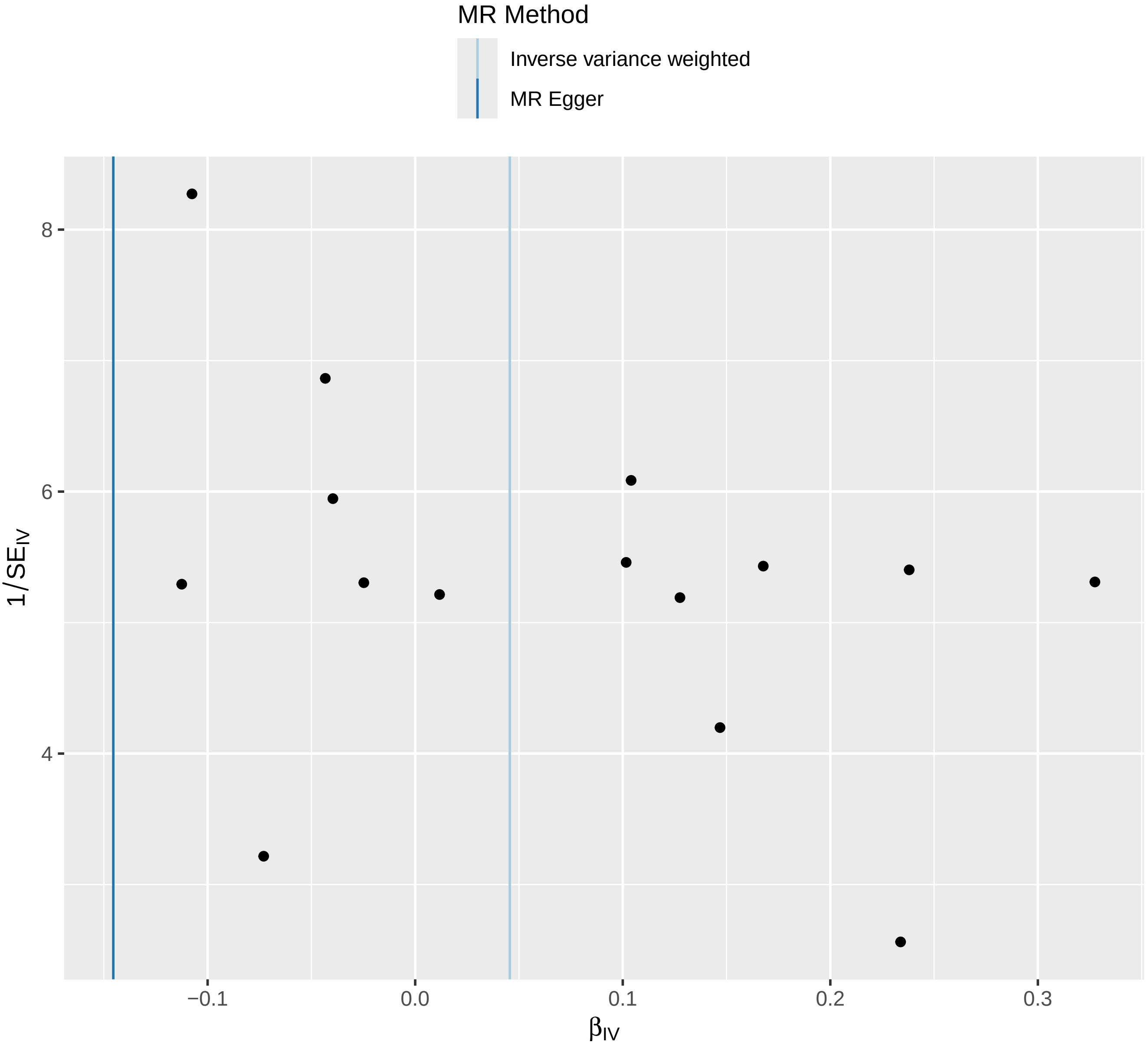
MR Method
Inverse variance weighted
MR Egger
8
6
4
1/SE$_{IV}$
−0.1
0.0
0.1
0.2
0.3
β$_{IV}$

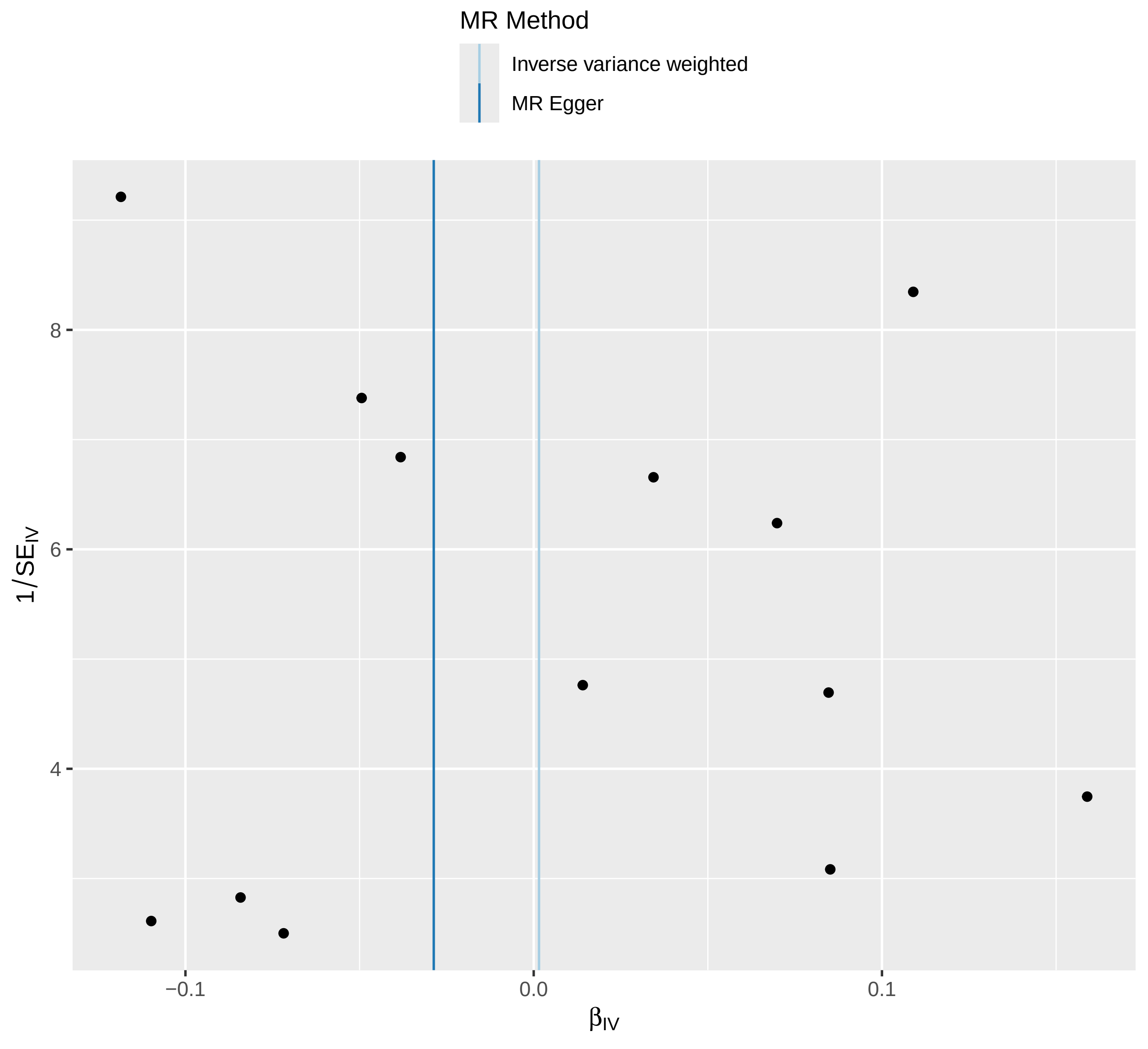

MR Method
Inverse variance weighted
MR Egger
8
6
4
1/SE_IV
−0.1
0.0
0.1
β_IV

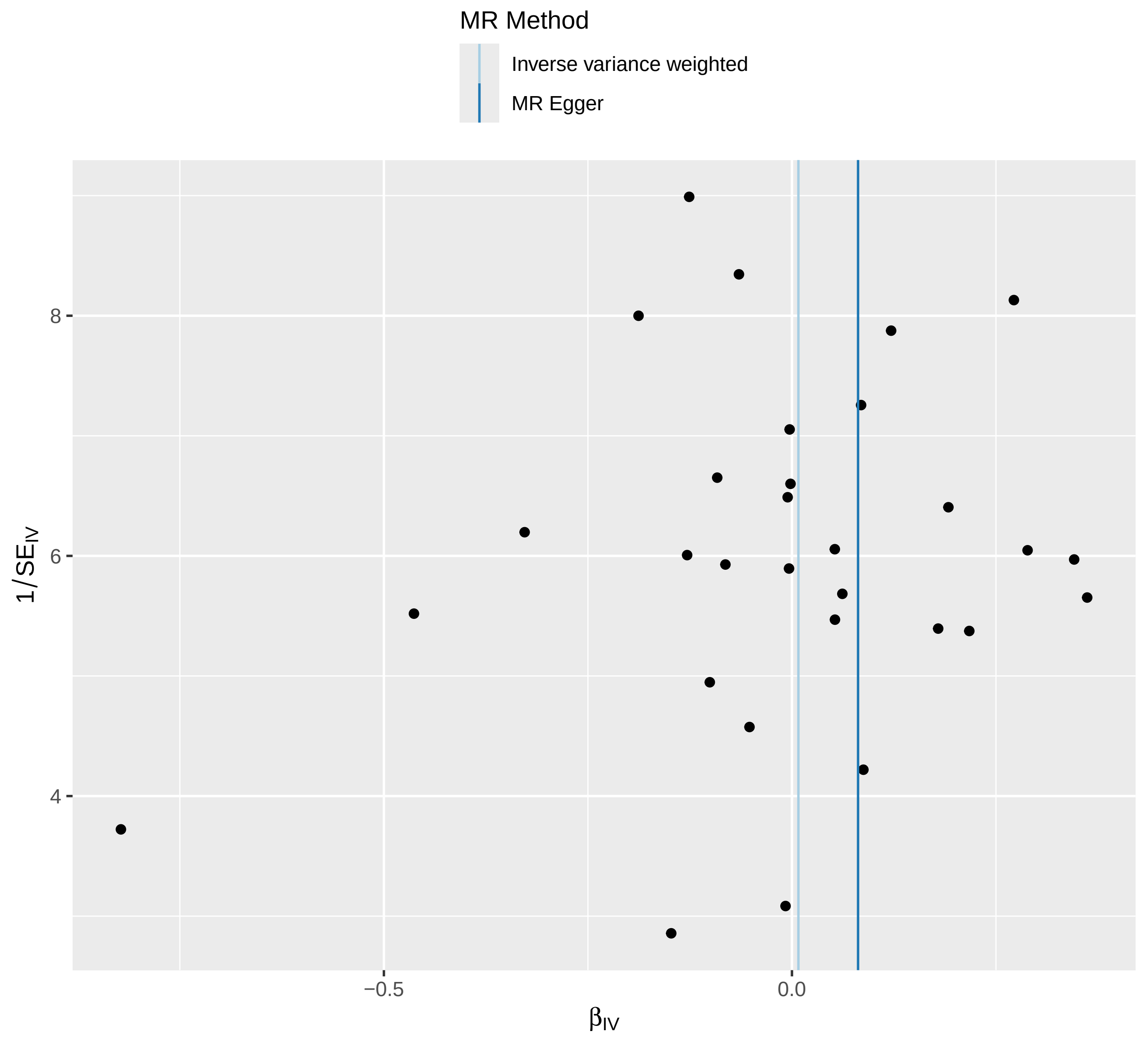
MR Method
Inverse variance weighted
MR Egger
8
6
4
1/SE$_{IV}$
−0.5
0.0
β$_{IV}$

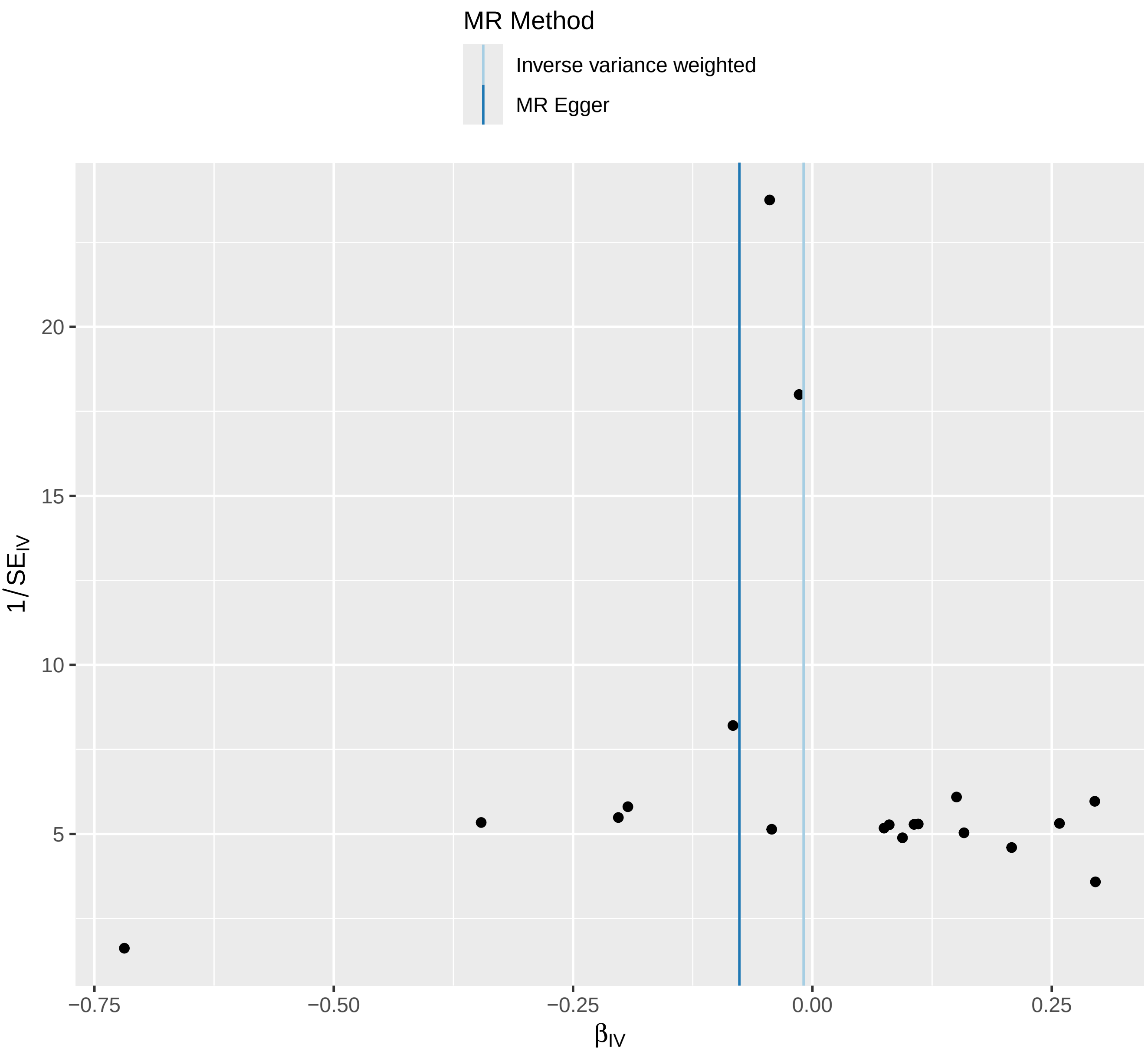
MR Method
Inverse variance weighted
MR Egger
20
15
10
5
−0.75
−0.50
−0.25
0.00
0.25
$1/SE_{IV}$
β_{IV}

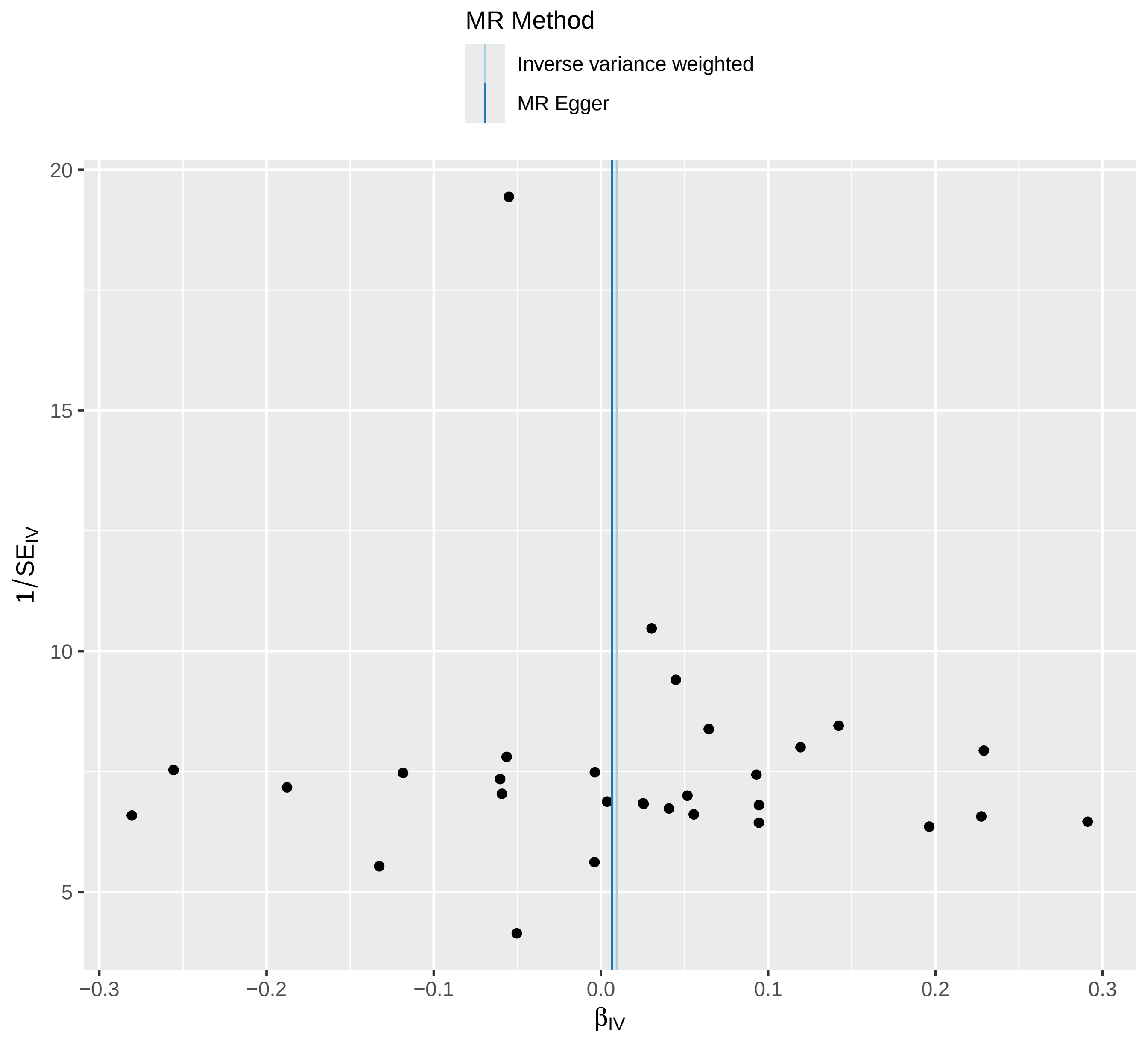

MR Method
Inverse variance weighted
MR Egger
20
15
10
5
1/SE_IV
−0.3
−0.2
−0.1
0.0
0.1
0.2
0.3
β_IV

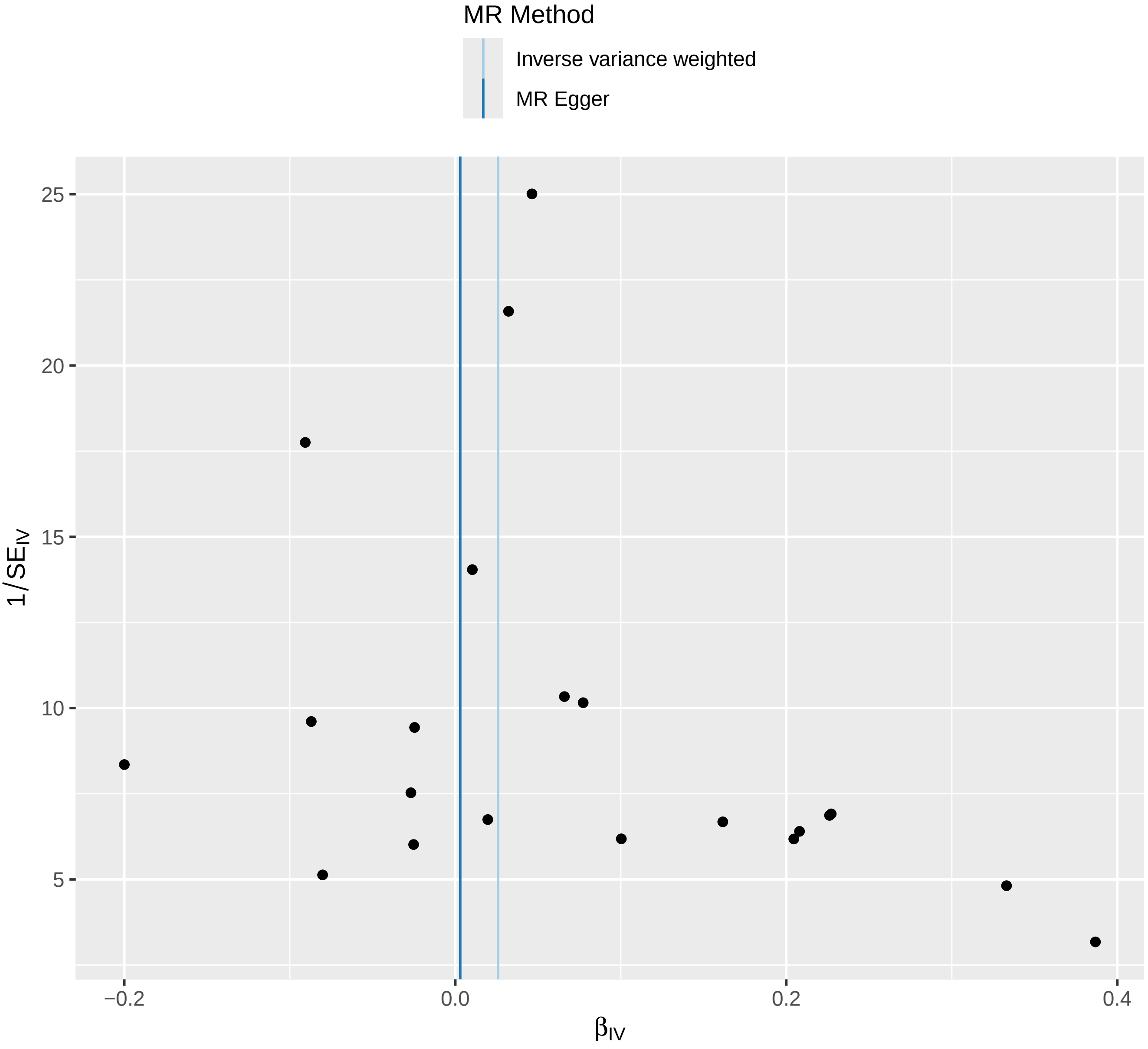
MR Method
Inverse variance weighted
MR Egger
25
20
15
10
5
1/SE_IV
−0.2
0.0
0.2
0.4
β_IV

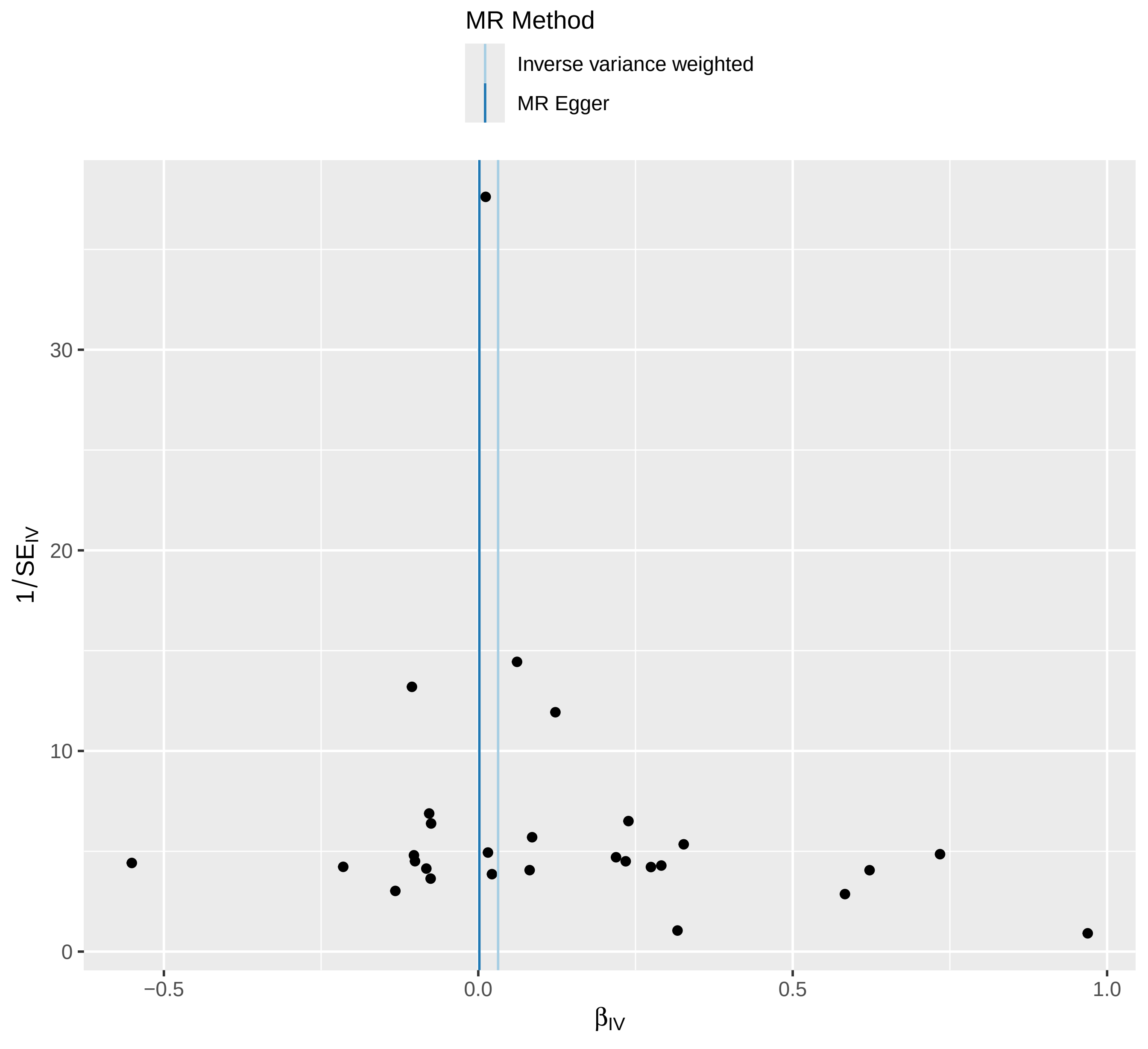
MR Method
Inverse variance weighted
MR Egger
30
20
10
0
1/SE_IV
−0.5
0.0
0.5
1.0
β_IV

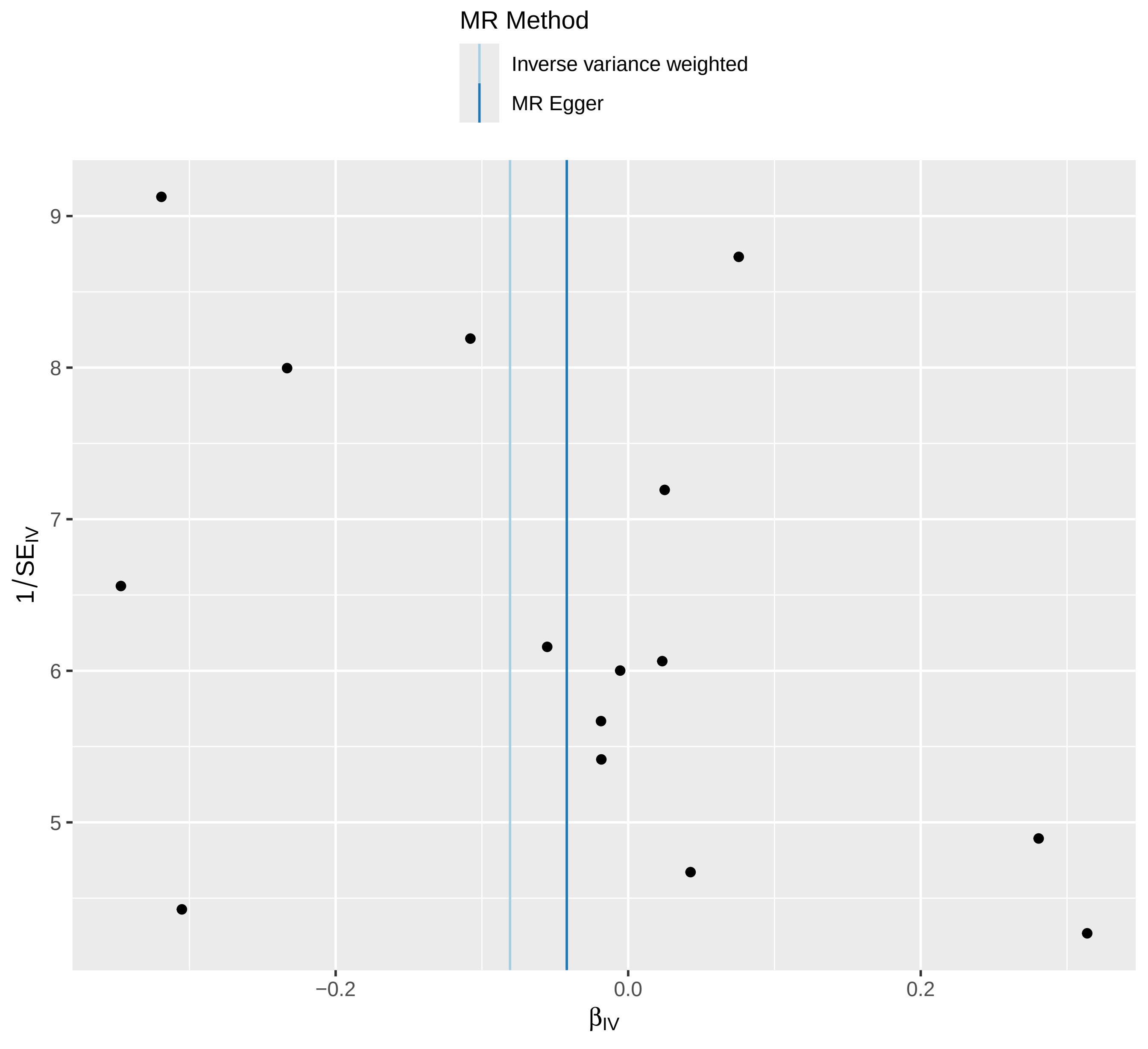
MR Method
Inverse variance weighted
MR Egger
9
8
7
6
5
1/SE$_{IV}$
−0.2
0.0
0.2
β$_{IV}$

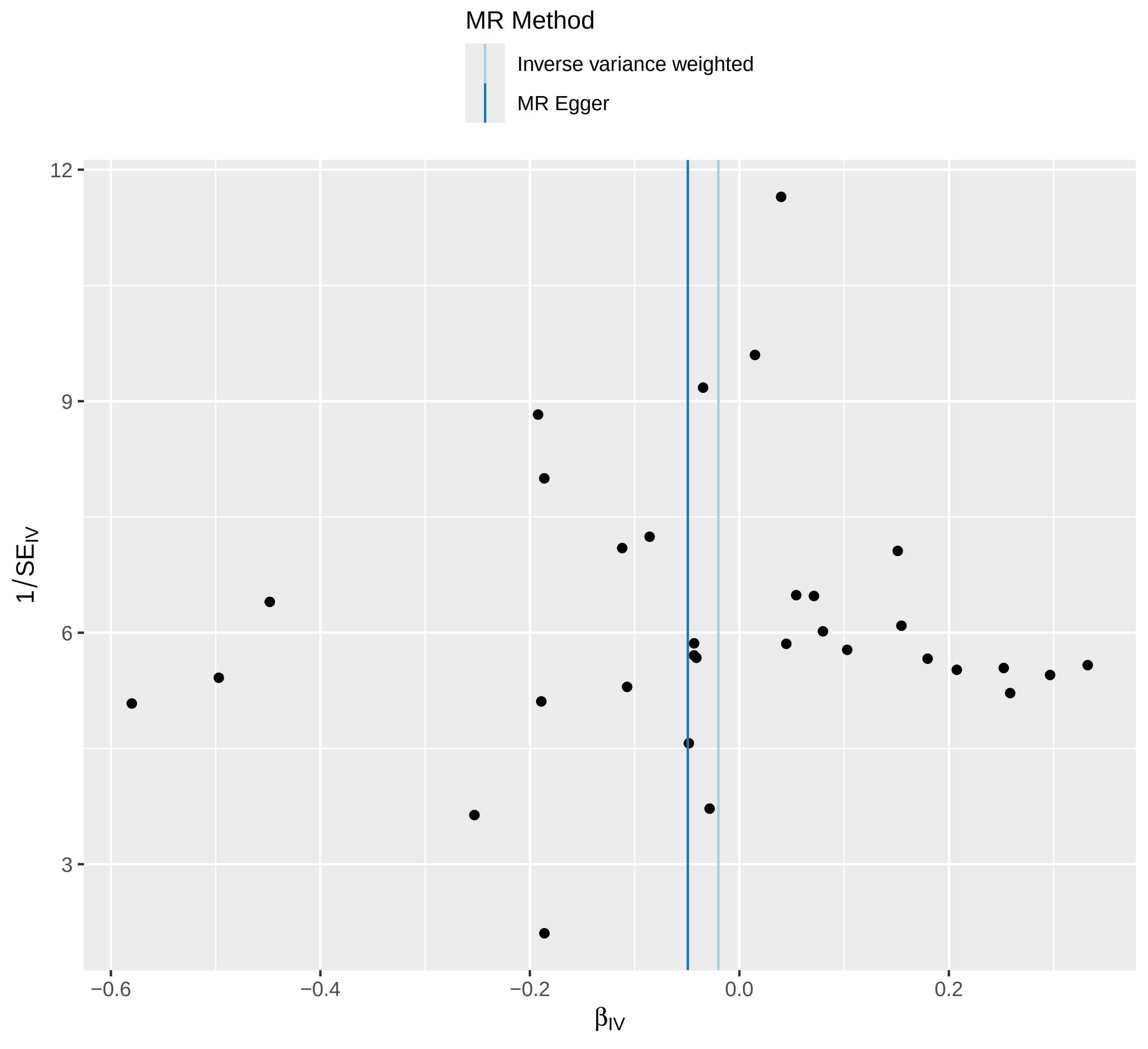
MR Method
Inverse variance weighted
MR Egger
1/SE$_{IV}$
12
9
6
3
−0.6
−0.4
−0.2
0.0
0.2
β$_{IV}$

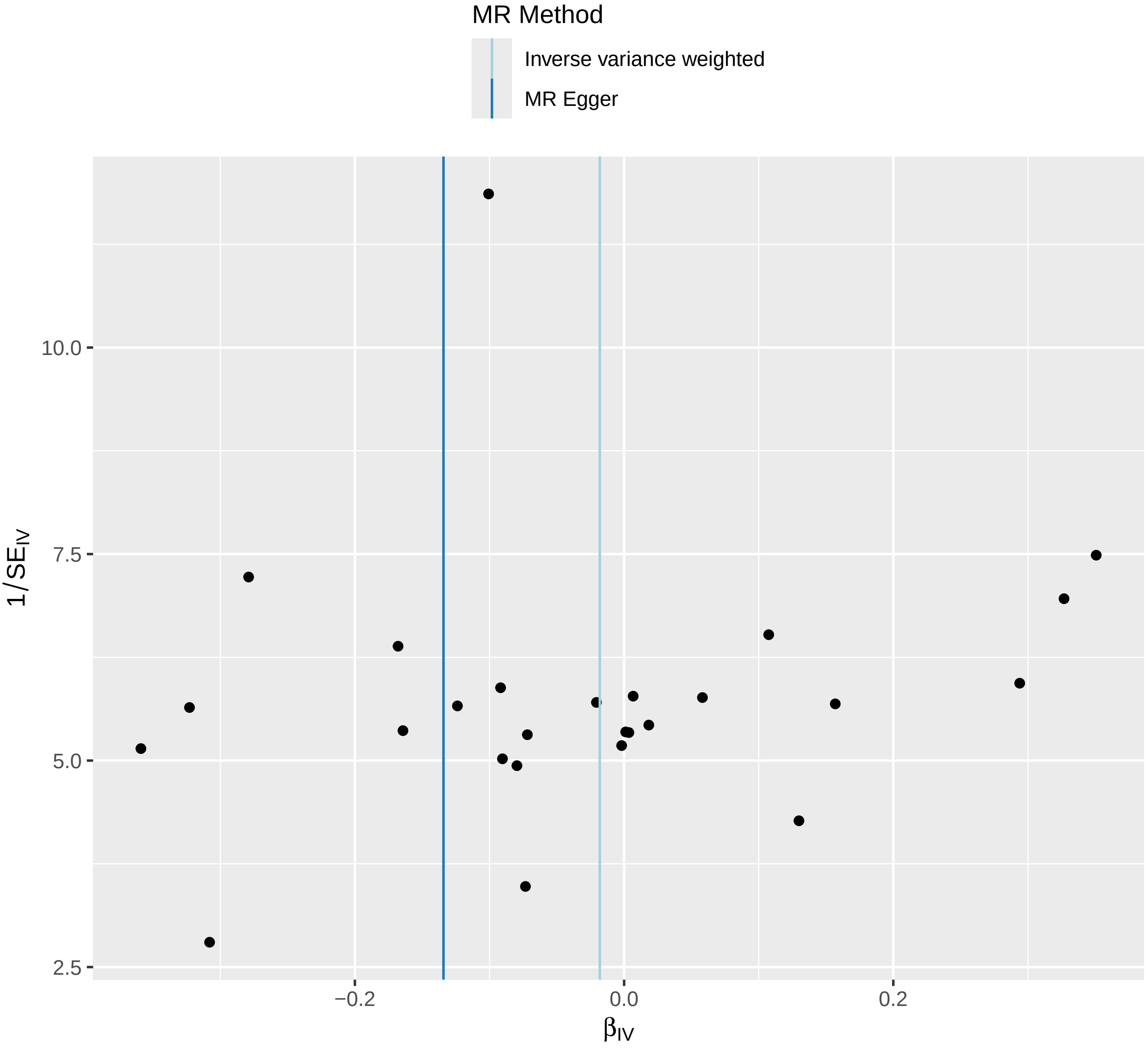

MR Method
Inverse variance weighted
MR Egger
10.0
7.5
5.0
2.5
1/SE_IV
−0.2
0.0
0.2
β_IV

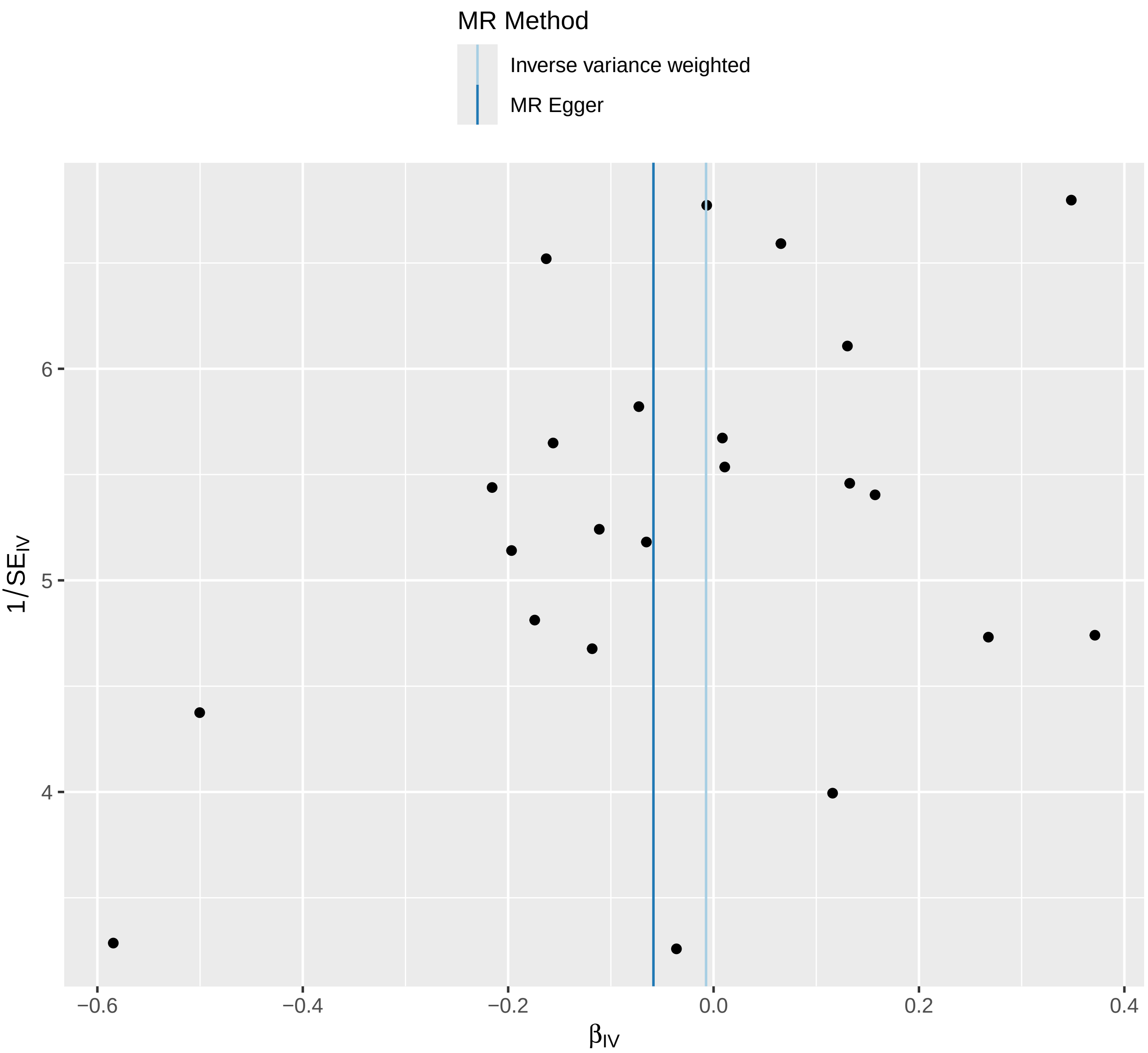
MR Method
Inverse variance weighted
MR Egger
6
5
4
1/SE_IV
−0.6
−0.4
−0.2
0.0
0.2
0.4
β_IV

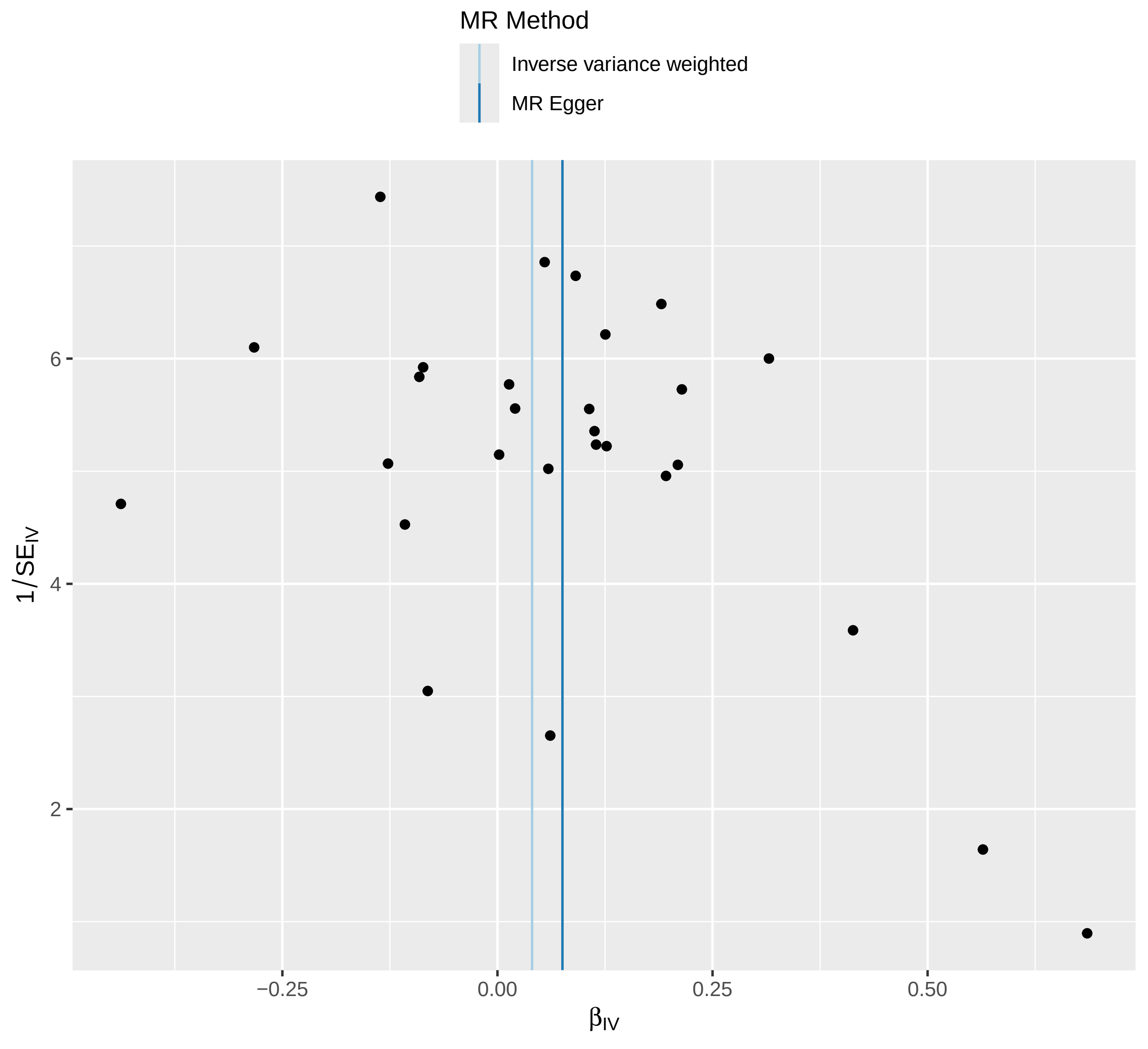
MR Method
Inverse variance weighted
MR Egger
6
4
2
$1/SE_{IV}$
−0.25
0.00
0.25
0.50
β_{IV}

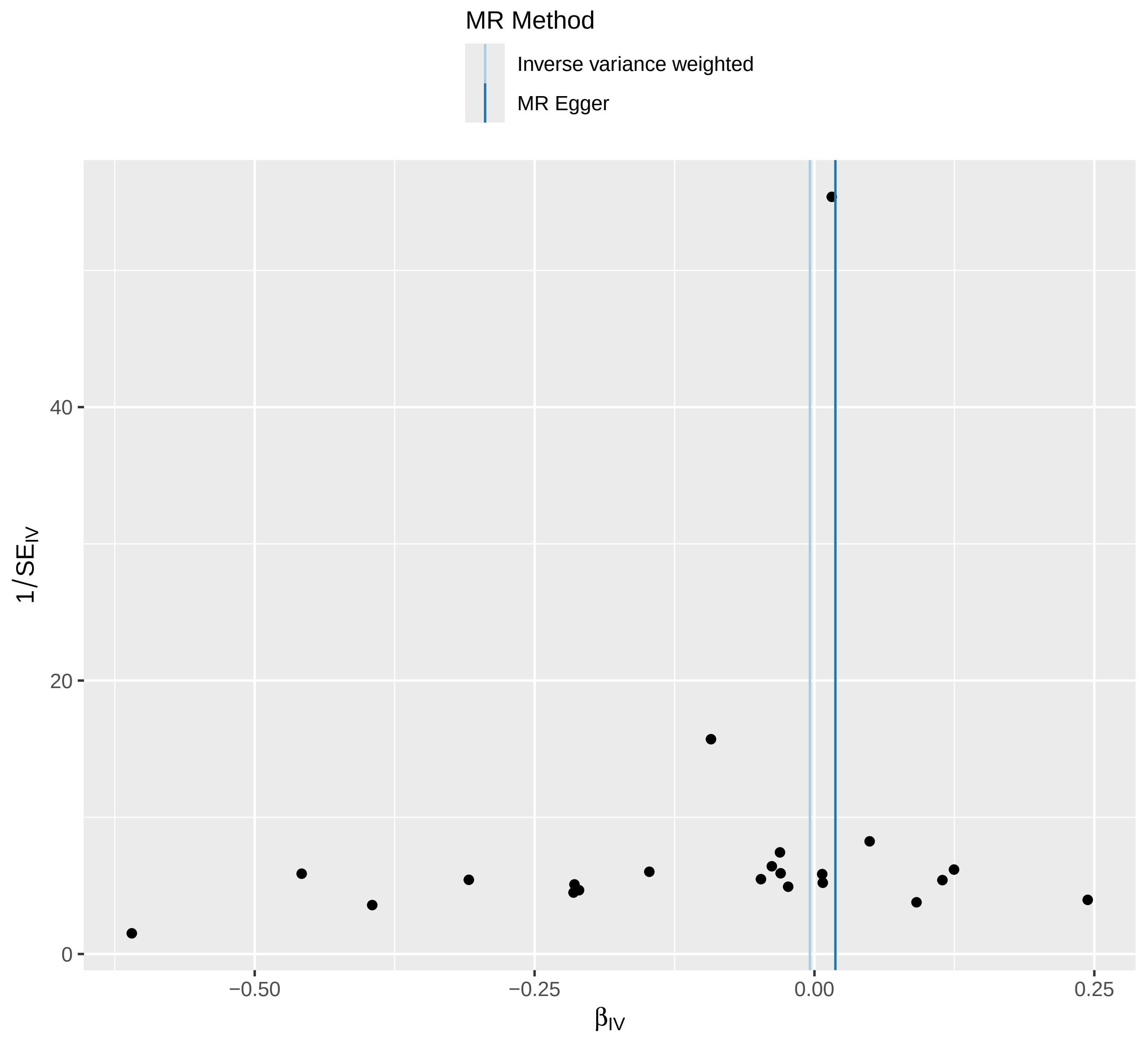
MR Method
Inverse variance weighted
MR Egger
1/SE_IV
40
20
0
−0.50
−0.25
0.00
0.25
β_IV

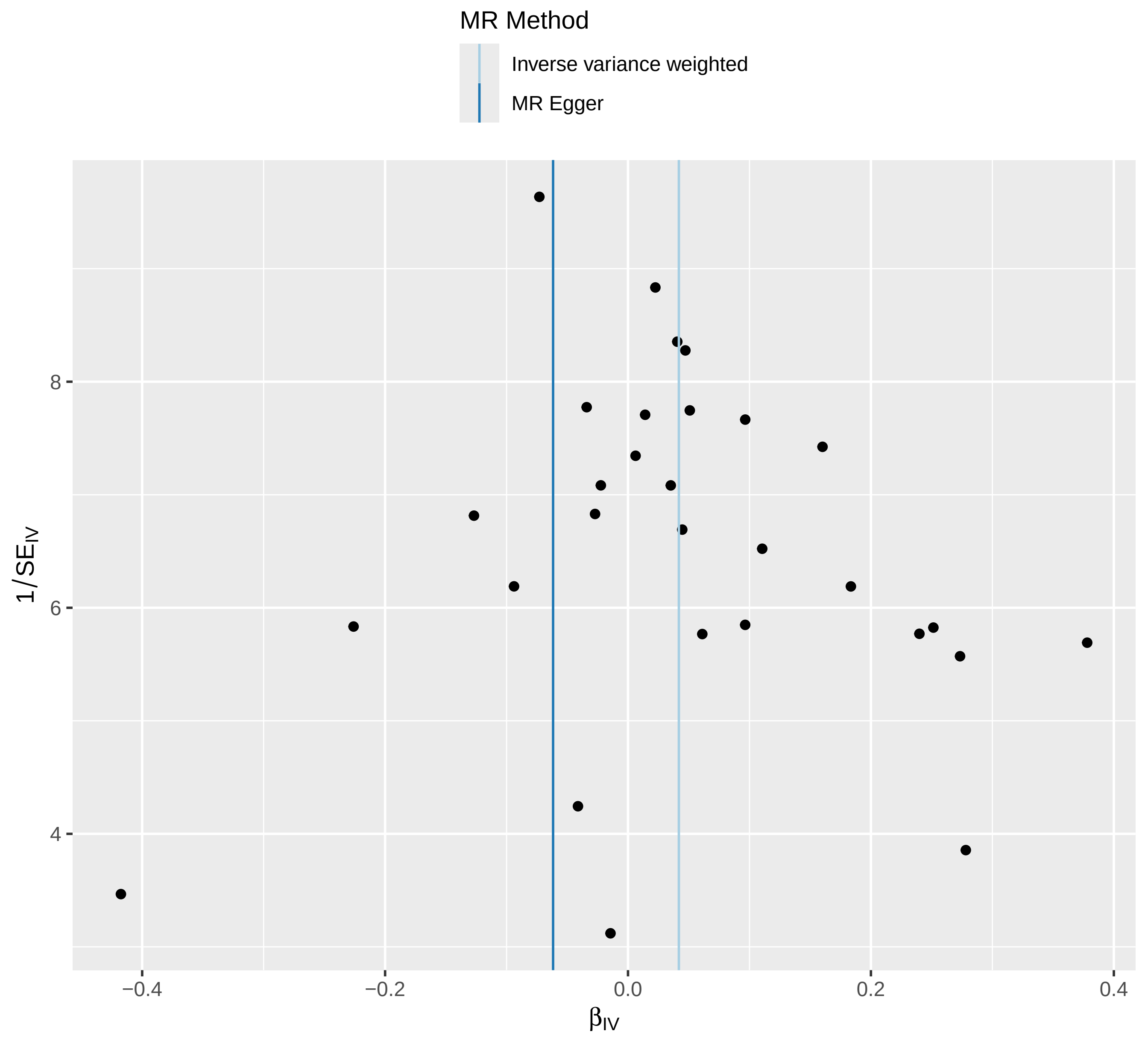

MR Method
Inverse variance weighted
MR Egger
8
6
4
1/SE$_{IV}$
−0.4
−0.2
0.0
0.2
0.4
β$_{IV}$

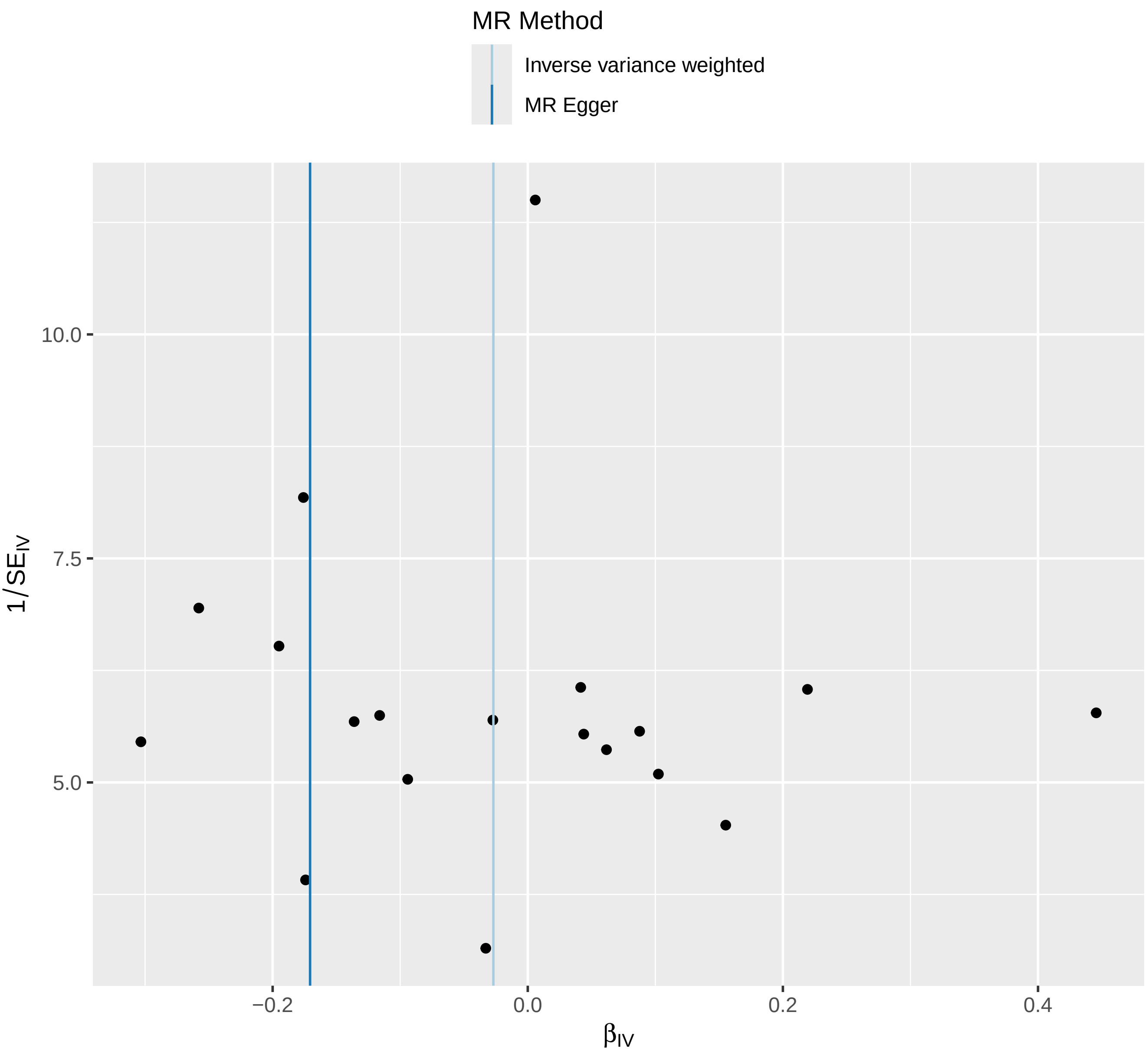
MR Method
Inverse variance weighted
MR Egger
10.0
7.5
5.0
$1/SE_{IV}$
−0.2
0.0
0.2
0.4
β_{IV}

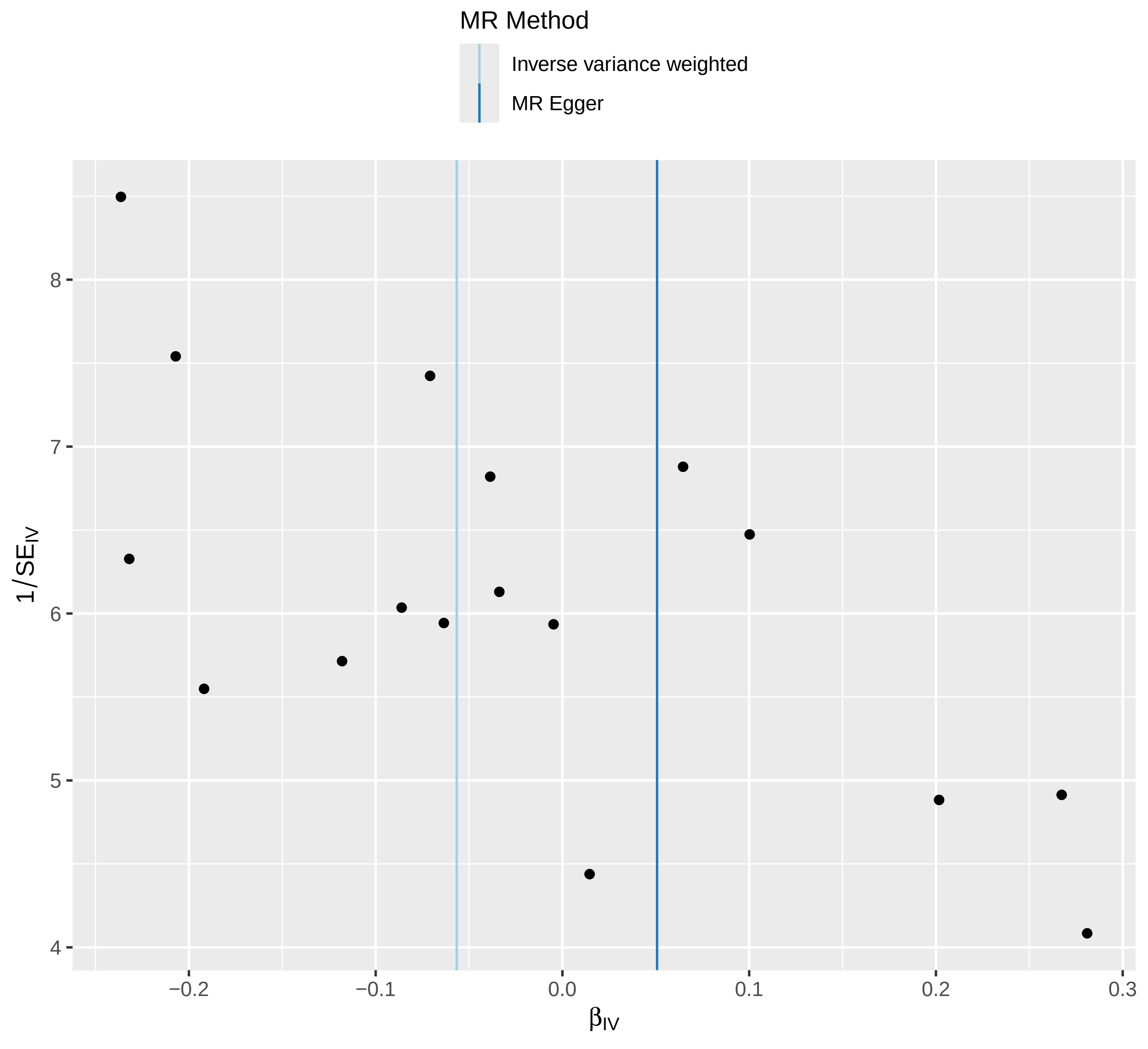
MR Method
Inverse variance weighted
MR Egger
1/SE_IV
8
7
6
5
4
−0.2
−0.1
0.0
0.1
0.2
0.3
β_IV

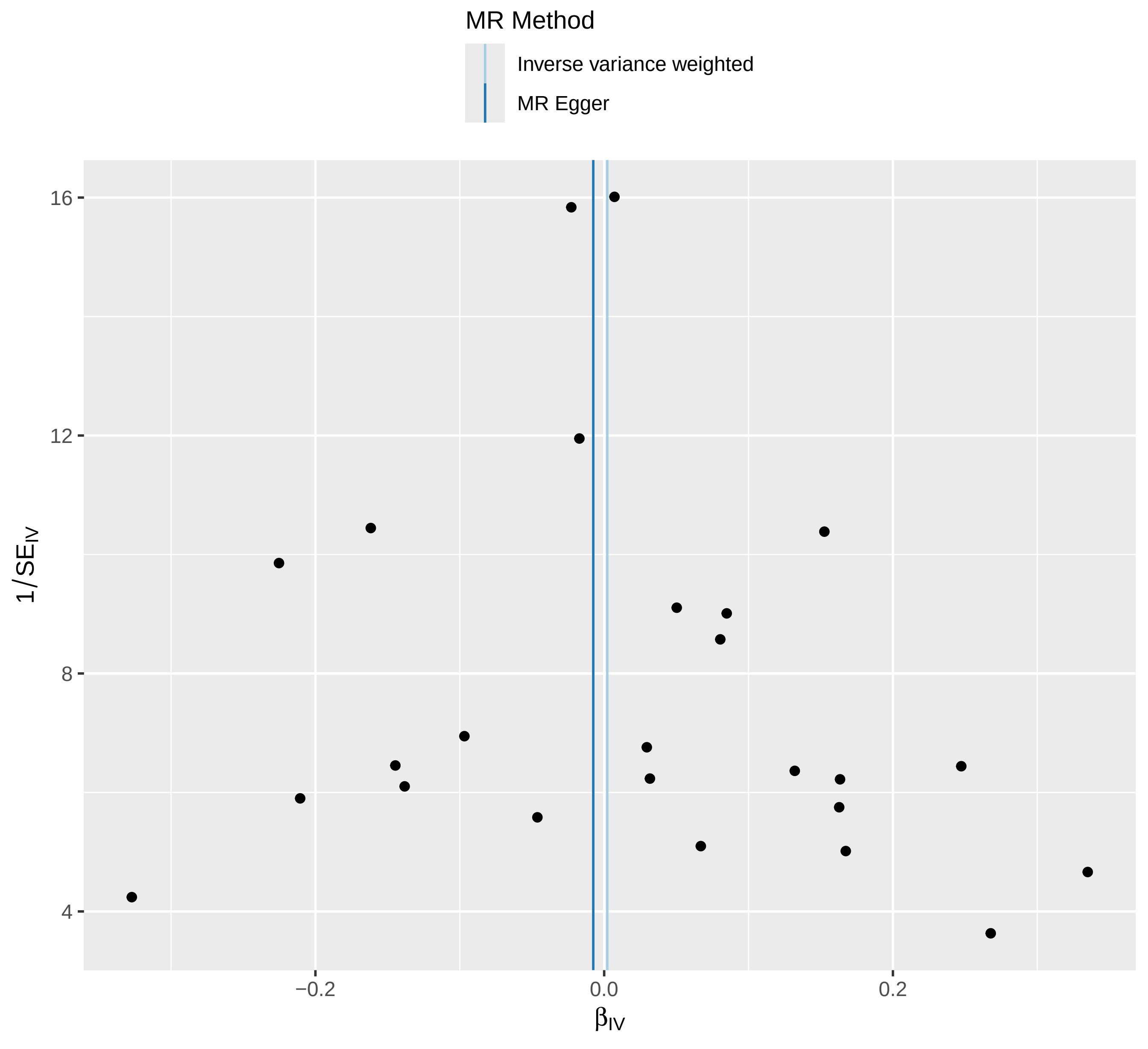
MR Method
Inverse variance weighted
MR Egger
16
12
8
4
1/SE_IV
−0.2
0.0
0.2
β_IV

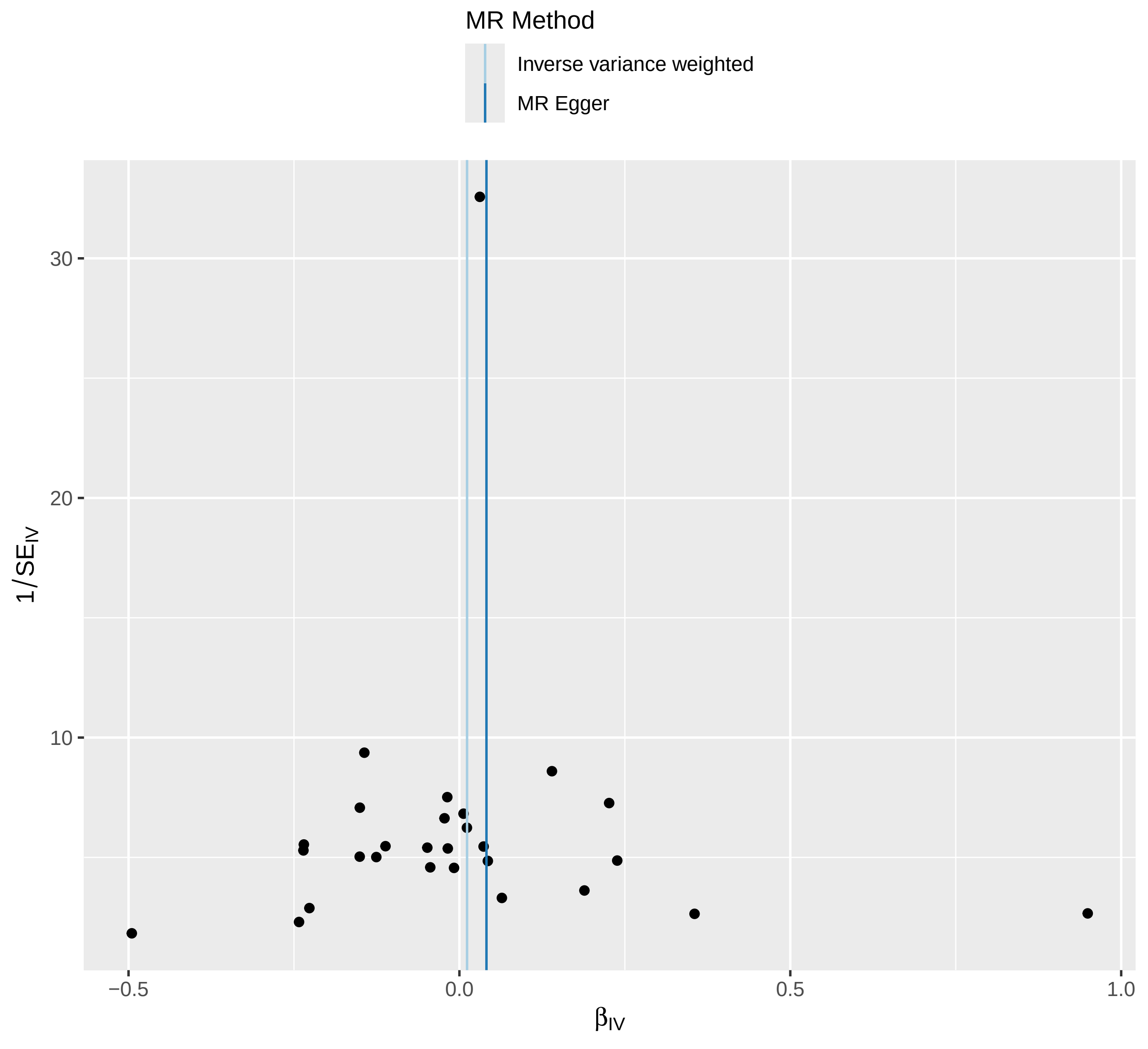

MR Method
Inverse variance weighted
MR Egger
30
20
10
1/SE$_{IV}$
−0.5
0.0
0.5
1.0
β$_{IV}$

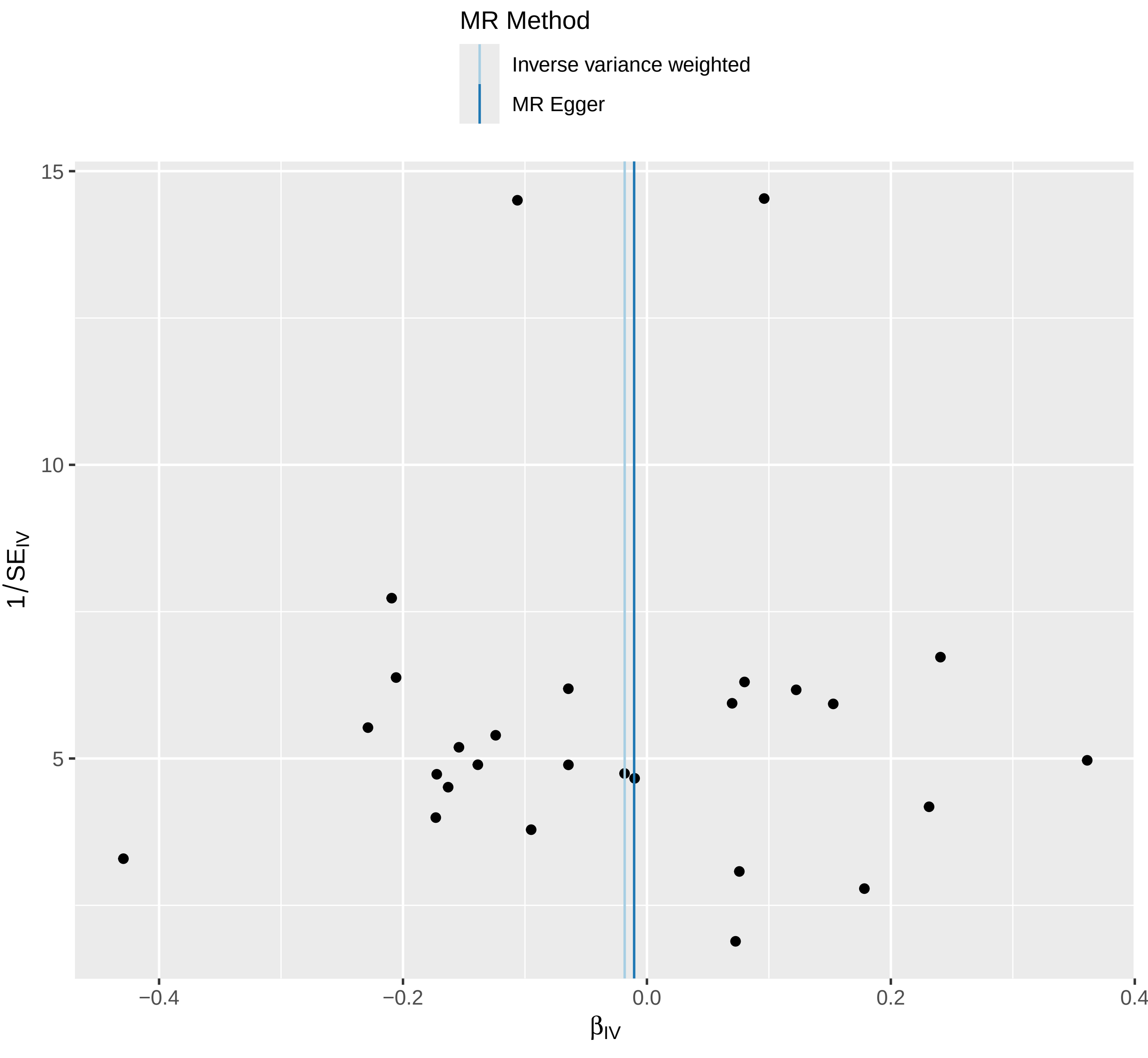
MR Method
Inverse variance weighted
MR Egger
15
10
5
1/SE_IV
−0.4
−0.2
0.0
0.2
0.4
β_IV

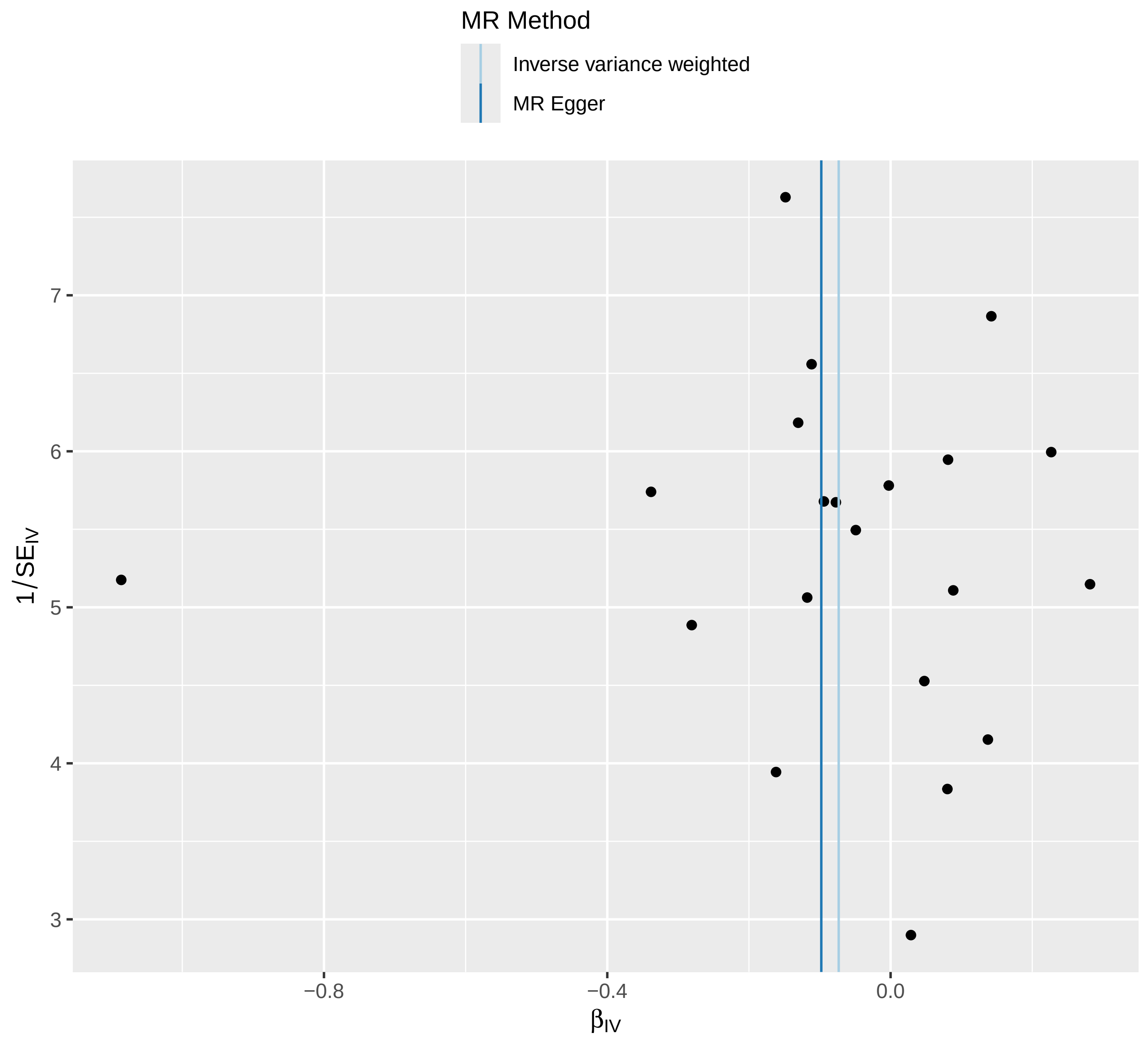

MR Method
Inverse variance weighted
MR Egger
7
6
5
4
3
1/SE_IV
−0.8
−0.4
0.0
β_IV

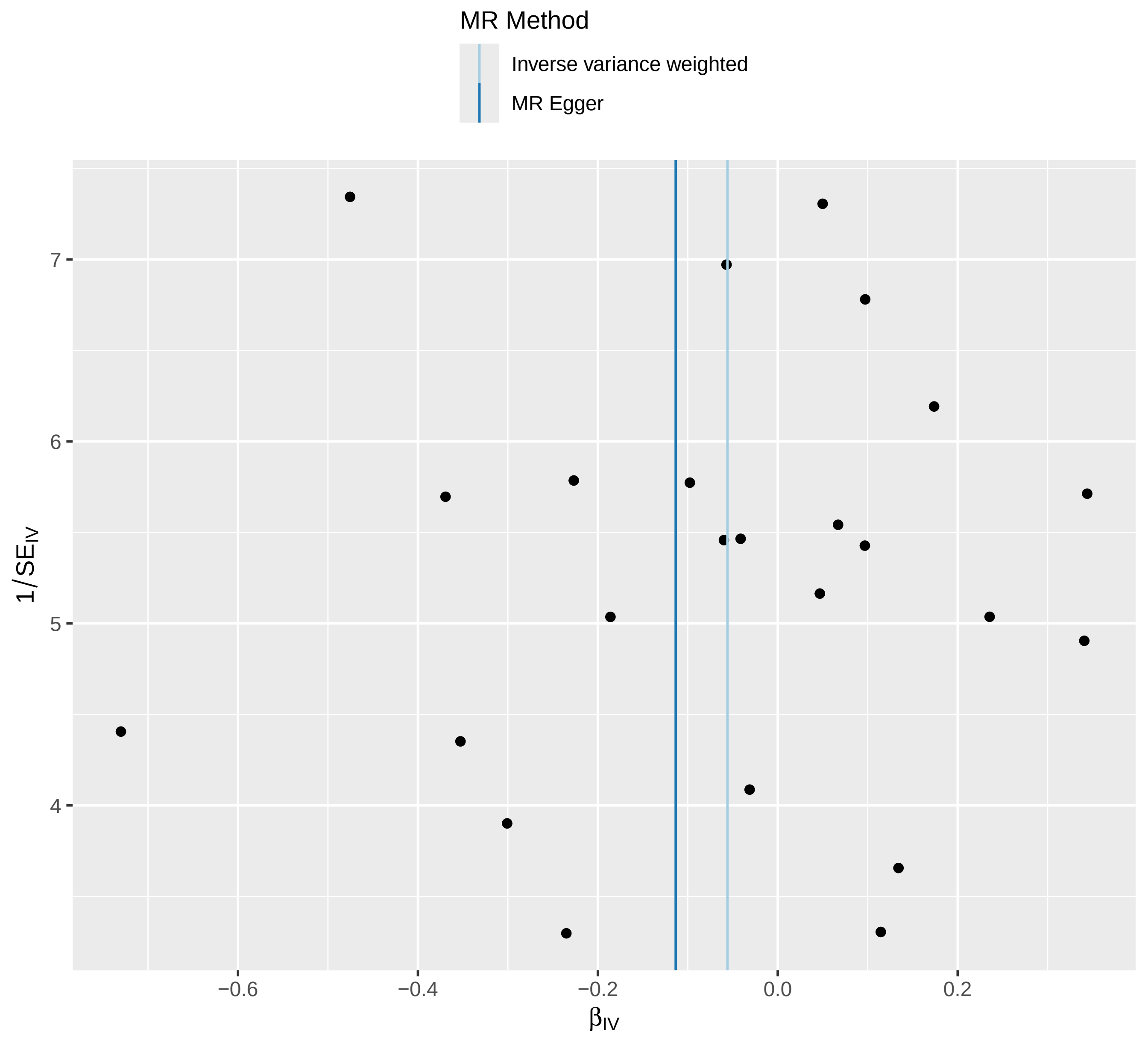
MR Method
Inverse variance weighted
MR Egger
1/SE_IV
β_IV
7
6
5
4
−0.6
−0.4
−0.2
0.0
0.2

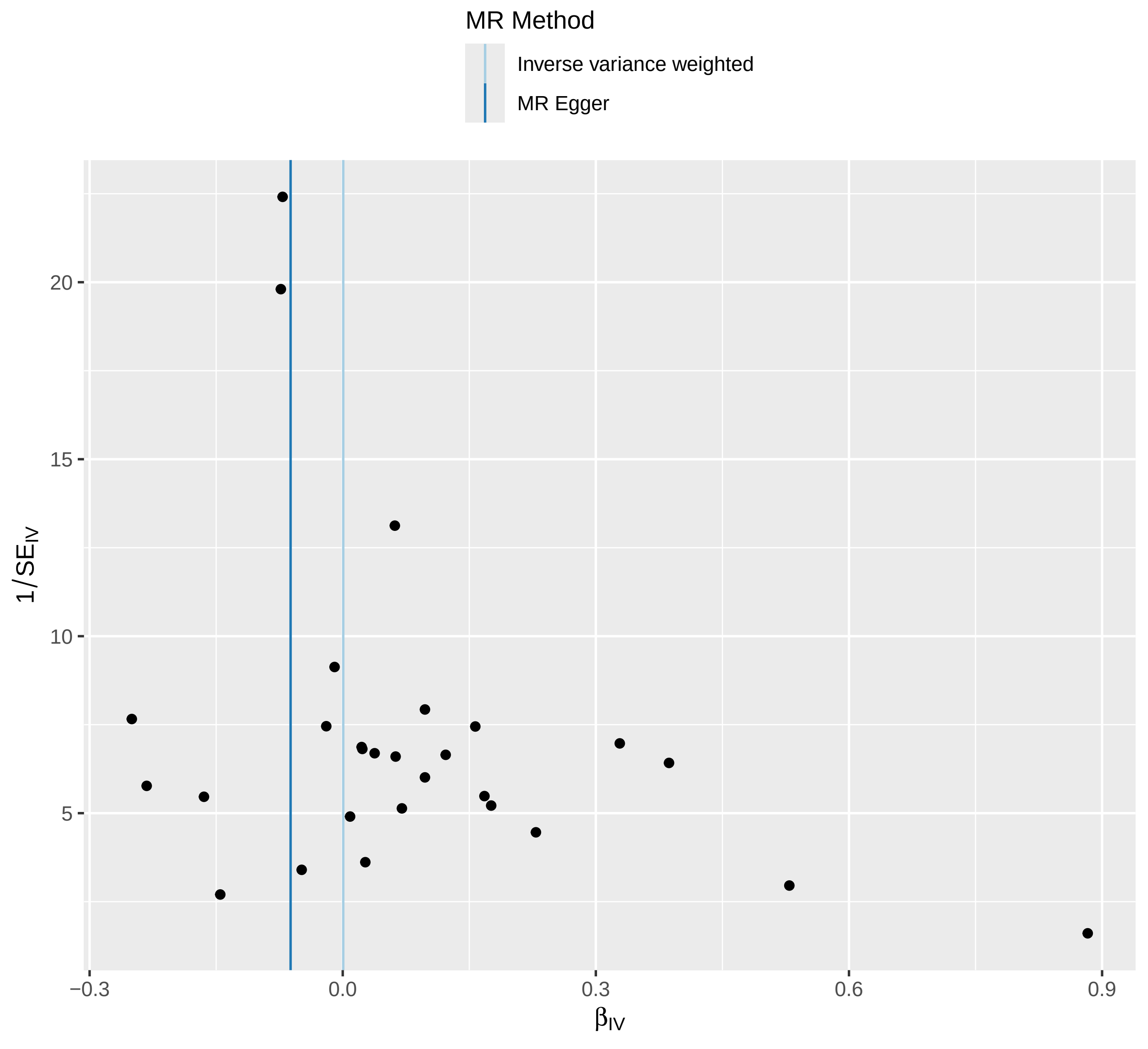

MR Method
Inverse variance weighted
MR Egger
20
15
10
5
1/SE_IV
−0.3
0.0
0.3
0.6
0.9
β_IV

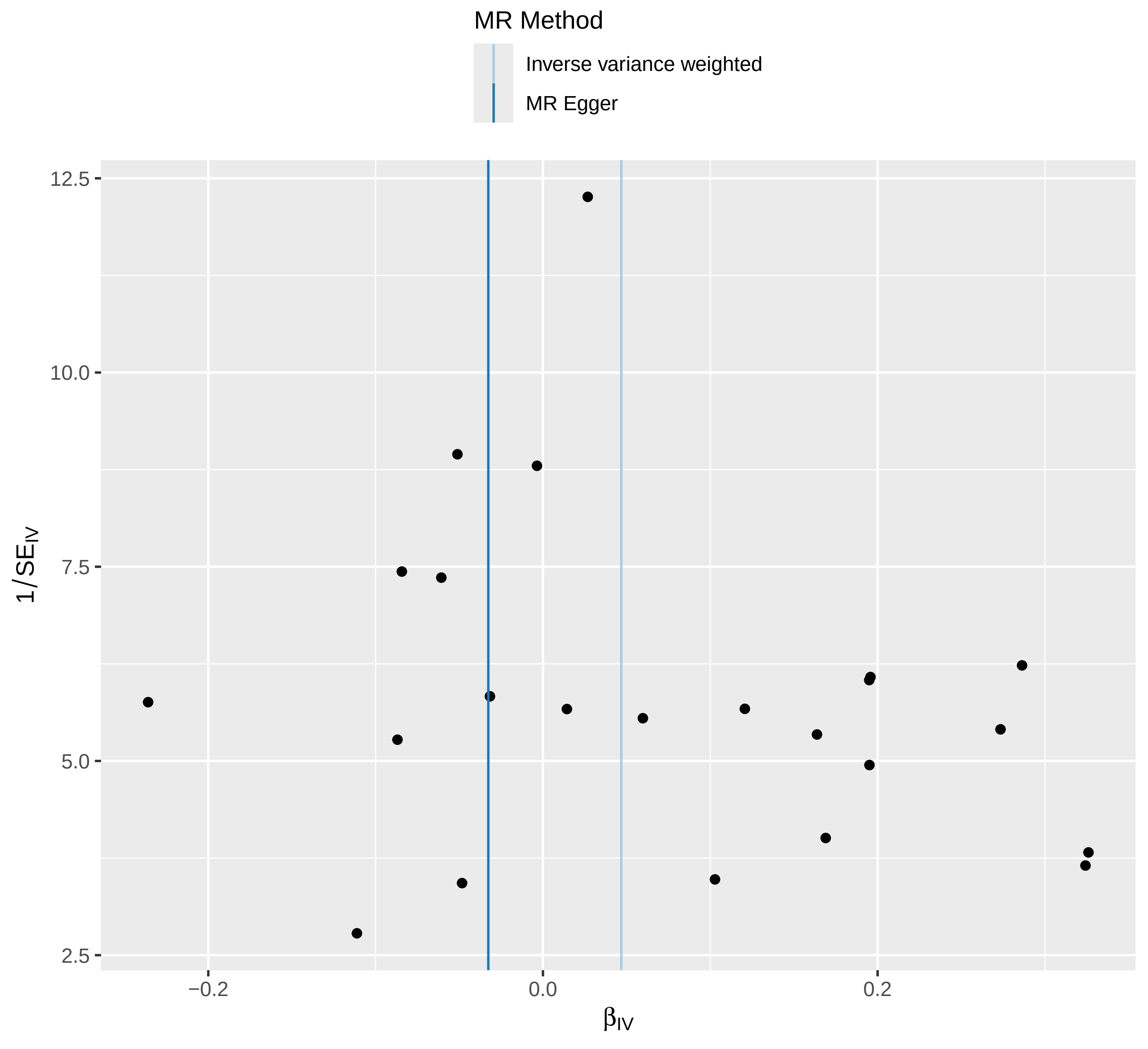

MR Method
Inverse variance weighted
MR Egger
12.5
10.0
7.5
5.0
2.5
1/SE_IV
−0.2
0.0
0.2
β_IV

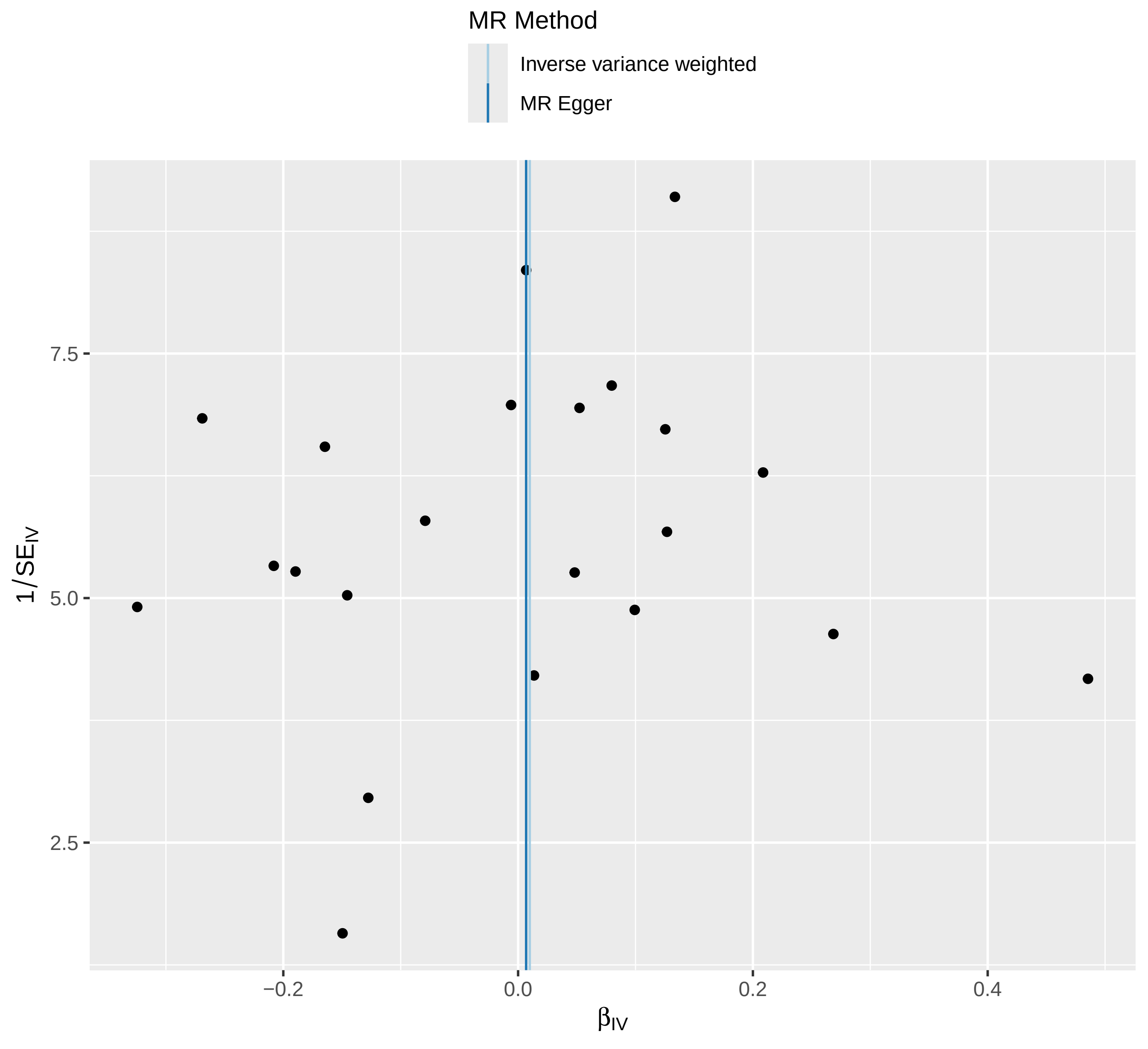

MR Method
Inverse variance weighted
MR Egger
1/SE_IV
7.5
5.0
2.5
−0.2
0.0
0.2
0.4
β_IV

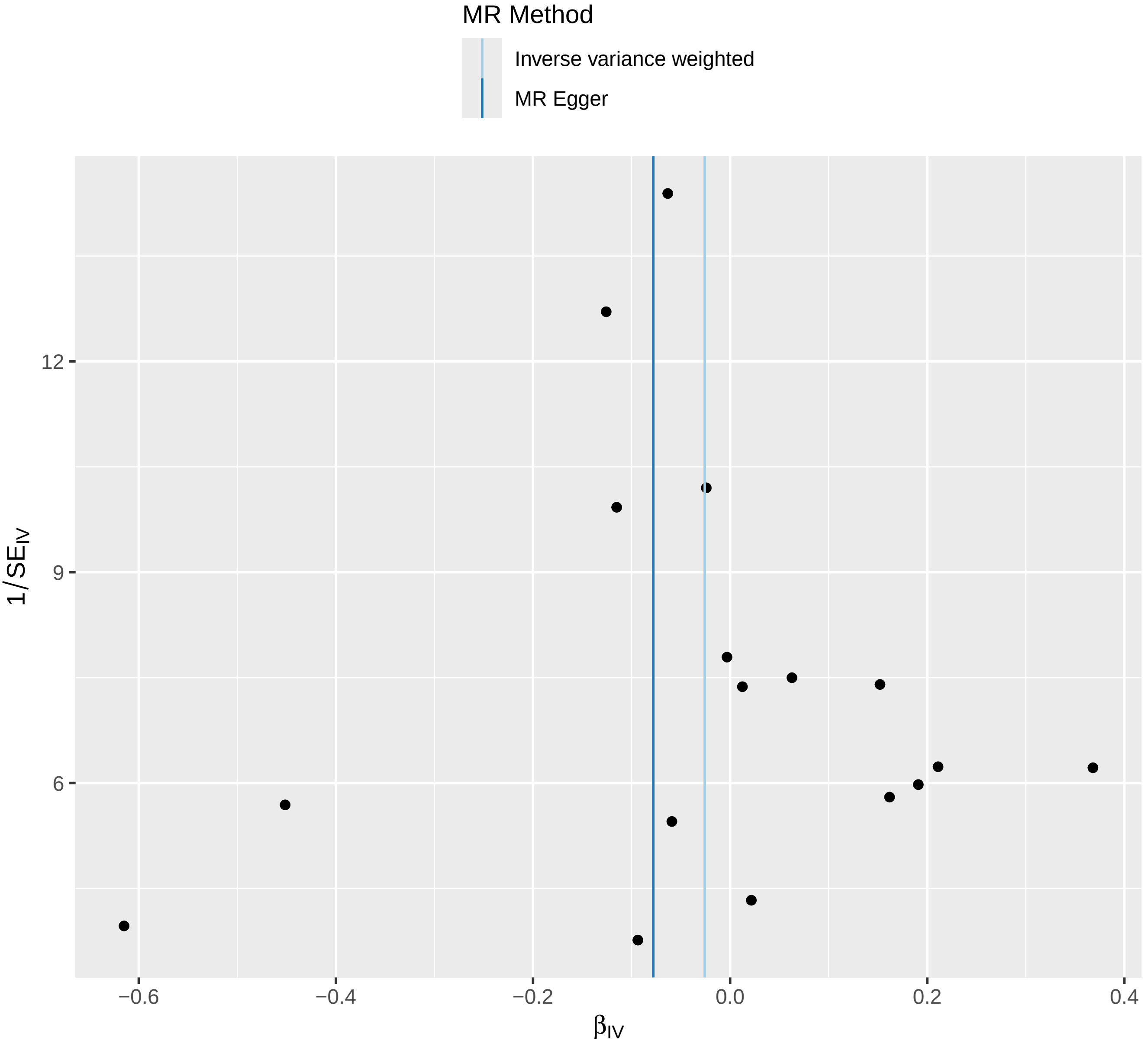
MR Method
Inverse variance weighted
MR Egger
12
9
6
1/SE_IV
−0.6
−0.4
−0.2
0.0
0.2
0.4
β_IV

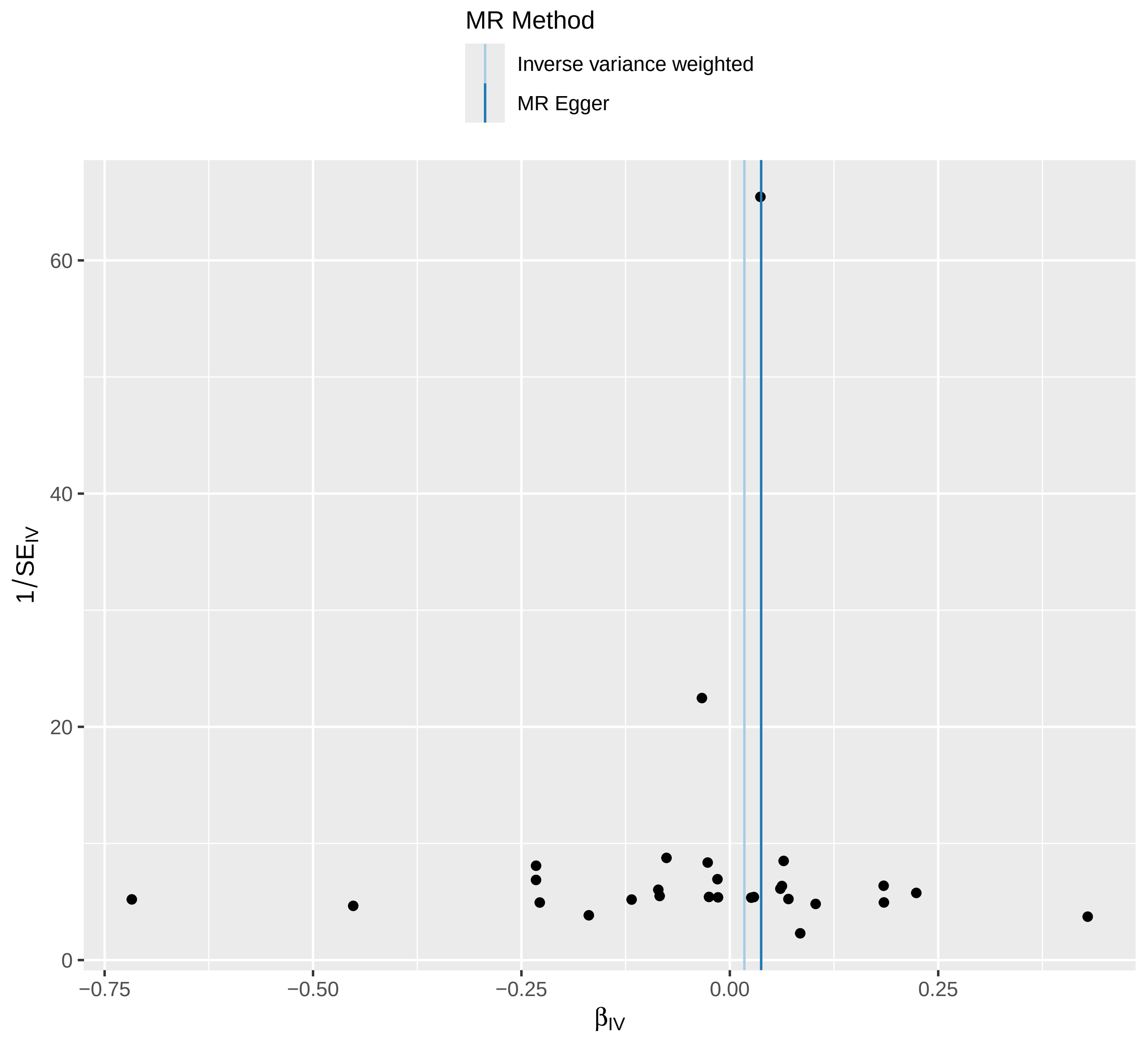
MR Method
Inverse variance weighted
MR Egger
60
40
20
0
1/SE_IV
−0.75
−0.50
−0.25
0.00
0.25
β_IV

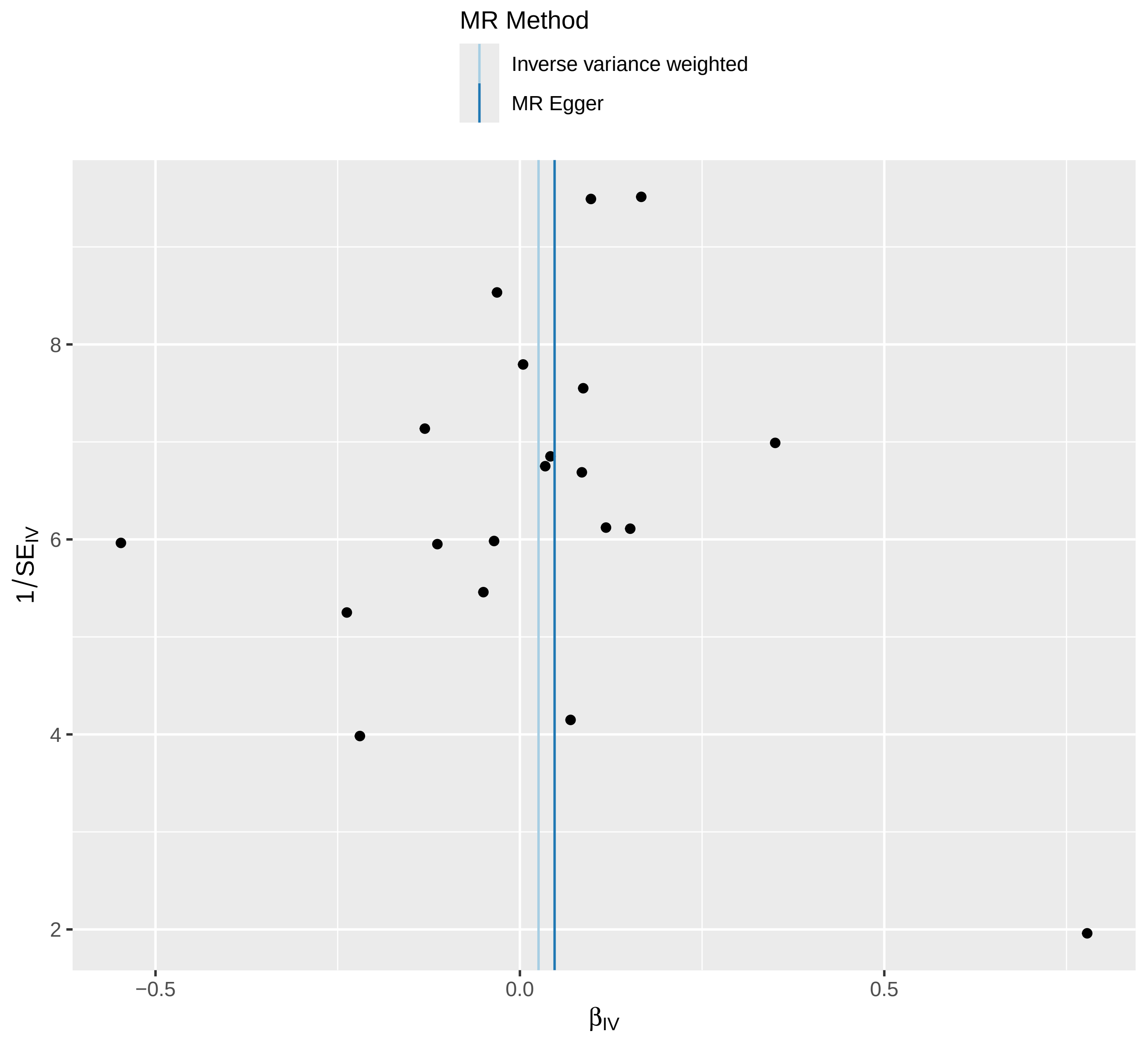
MR Method
Inverse variance weighted
MR Egger
1/SE_IV
8
6
4
2
−0.5
0.0
0.5
β_IV

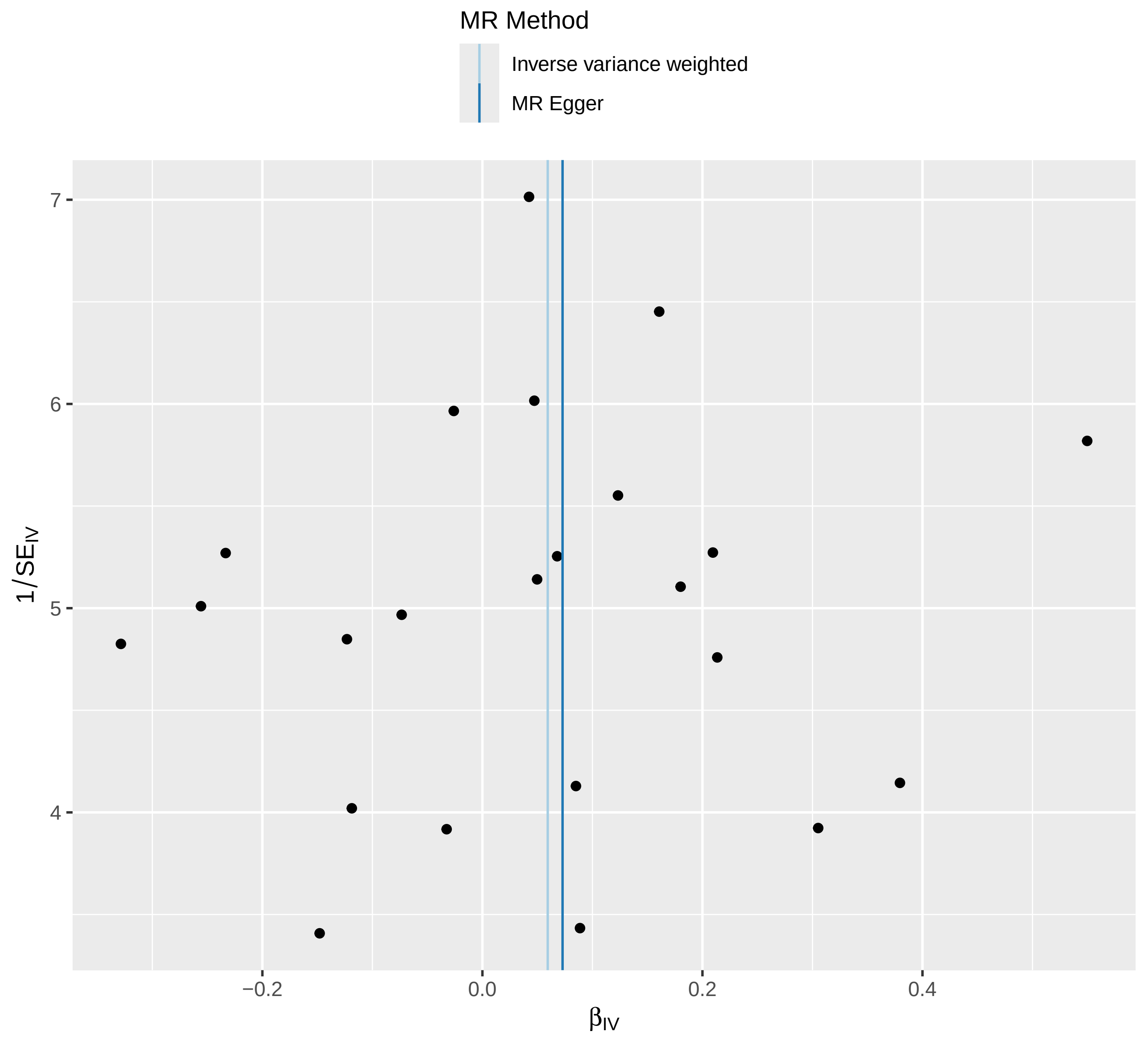
MR Method
Inverse variance weighted
MR Egger
7
6
5
4
−0.2
0.0
0.2
0.4
$1/SE_{IV}$
β_{IV}

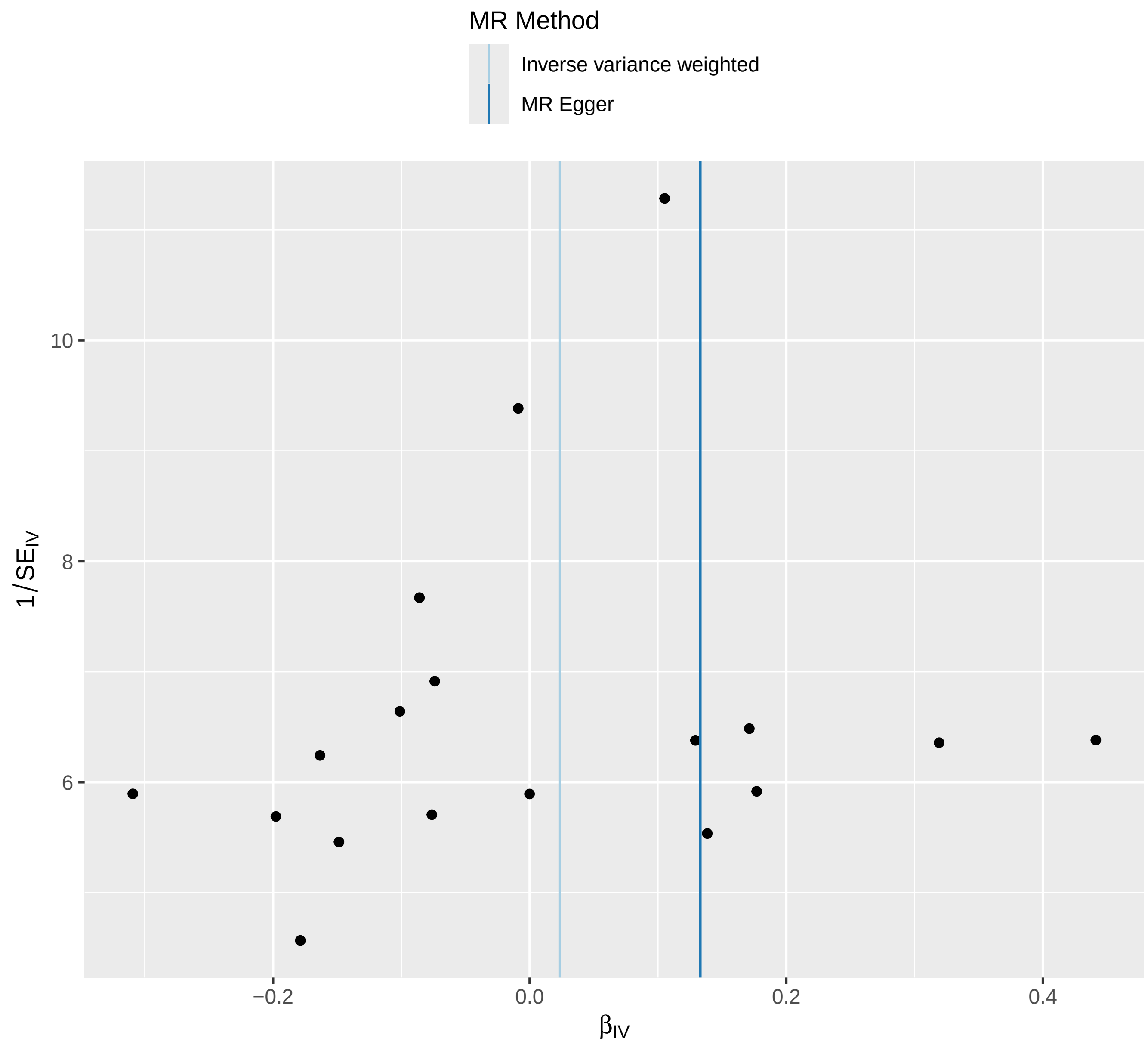
MR Method
Inverse variance weighted
MR Egger
$1/SE_{IV}$
10
8
6
−0.2
0.0
0.2
0.4
β_{IV}

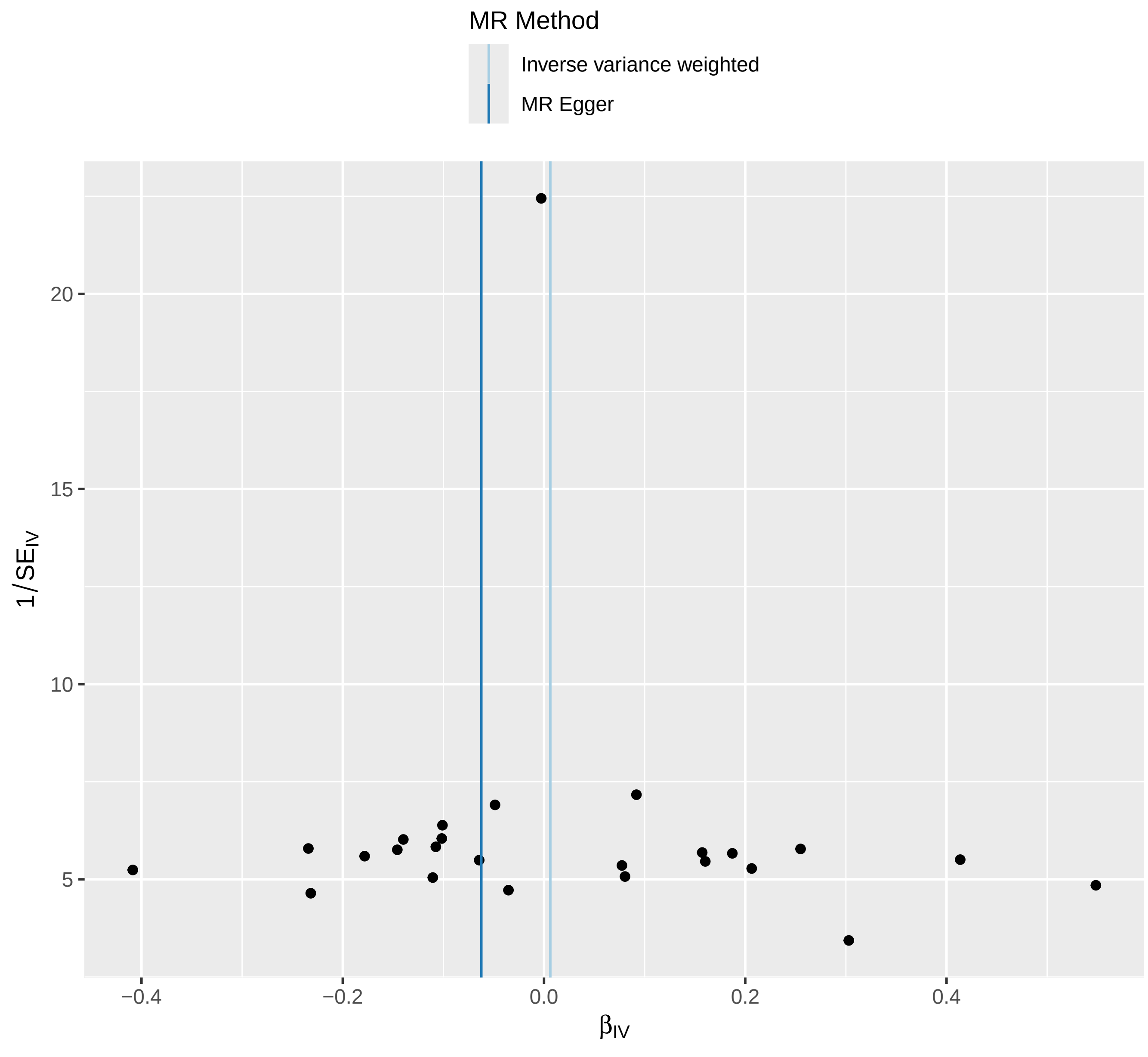
MR Method
Inverse variance weighted
MR Egger
20
15
10
5
1/SE_IV
−0.4
−0.2
0.0
0.2
0.4
β_IV

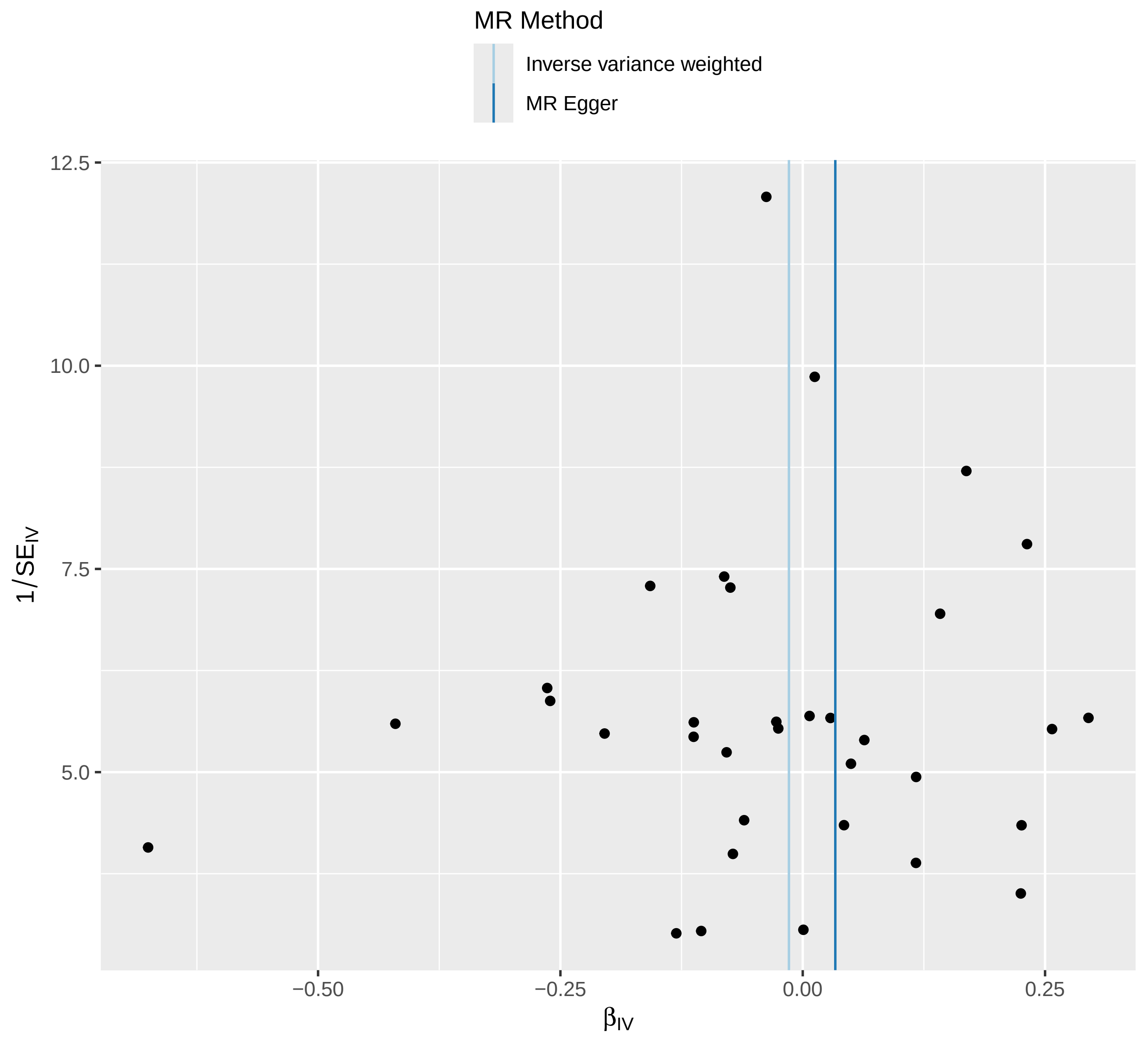

MR Method
Inverse variance weighted
MR Egger
12.5
10.0
7.5
5.0
1/SE_IV
−0.50
−0.25
0.00
0.25
β_IV

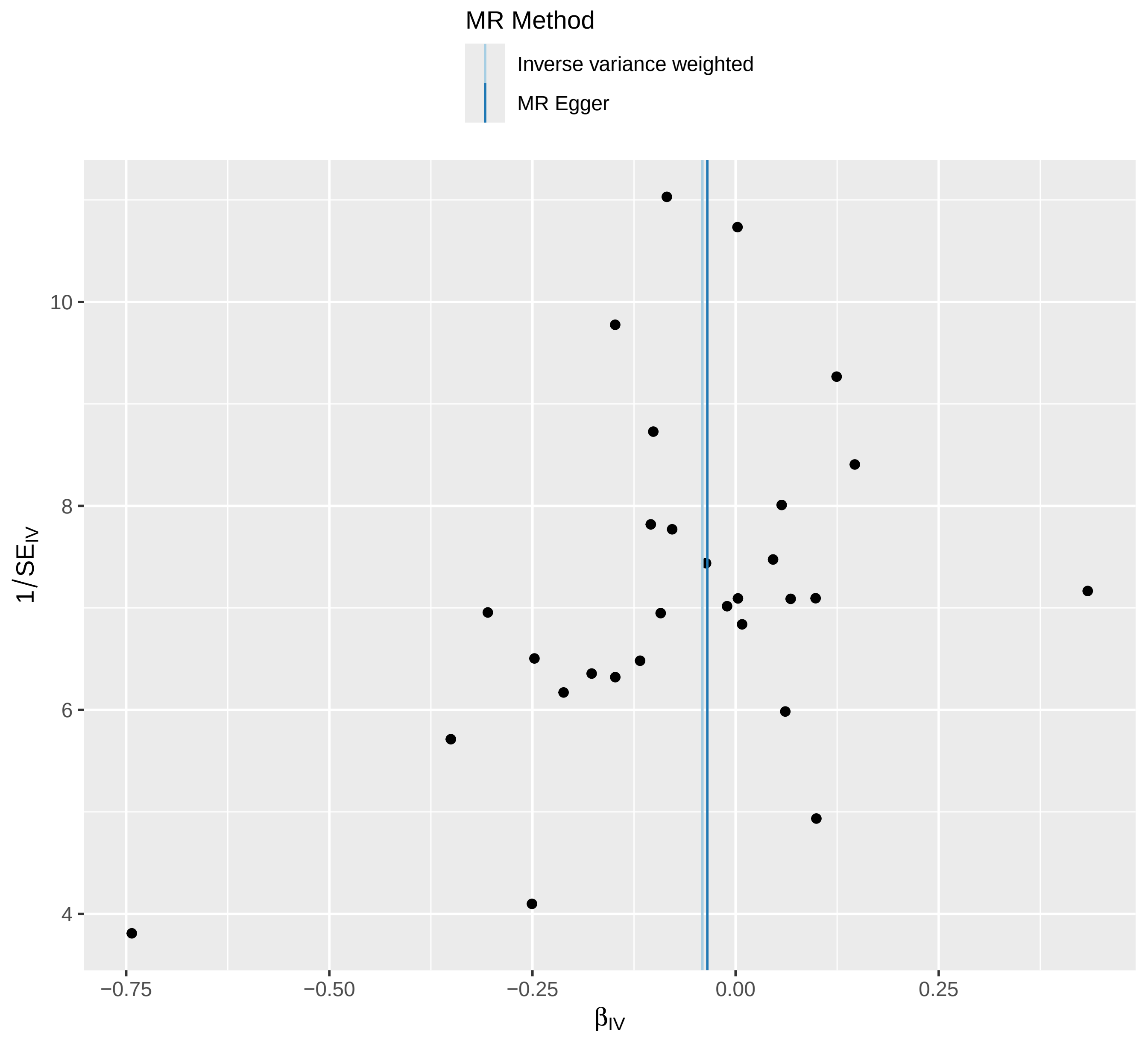
MR Method
Inverse variance weighted
MR Egger
1/SE_IV
β_IV
10
8
6
4
−0.75
−0.50
−0.25
0.00
0.25

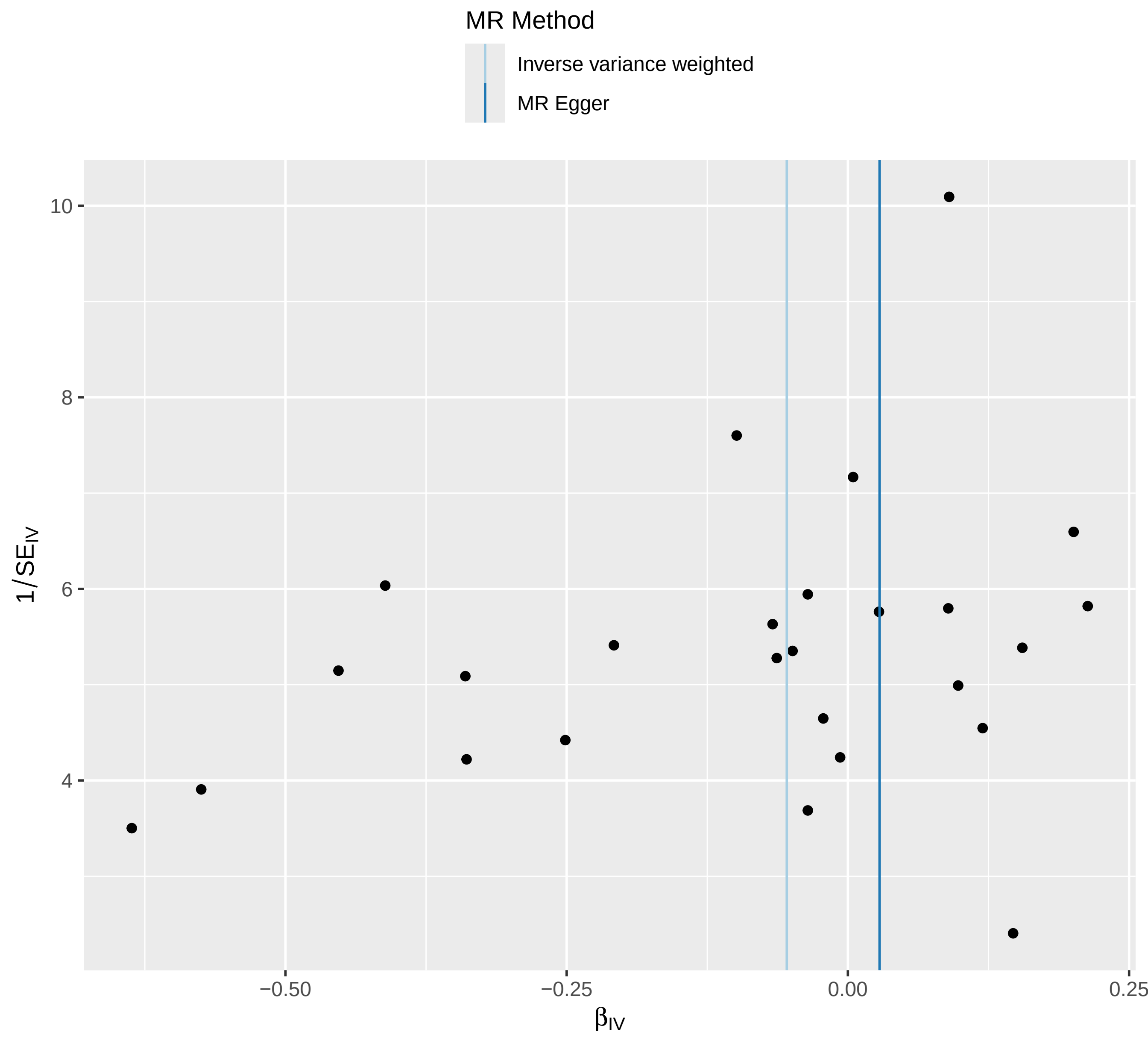
MR Method
Inverse variance weighted
MR Egger
10
8
6
4
1/SE_IV
−0.50
−0.25
0.00
0.25
β_IV

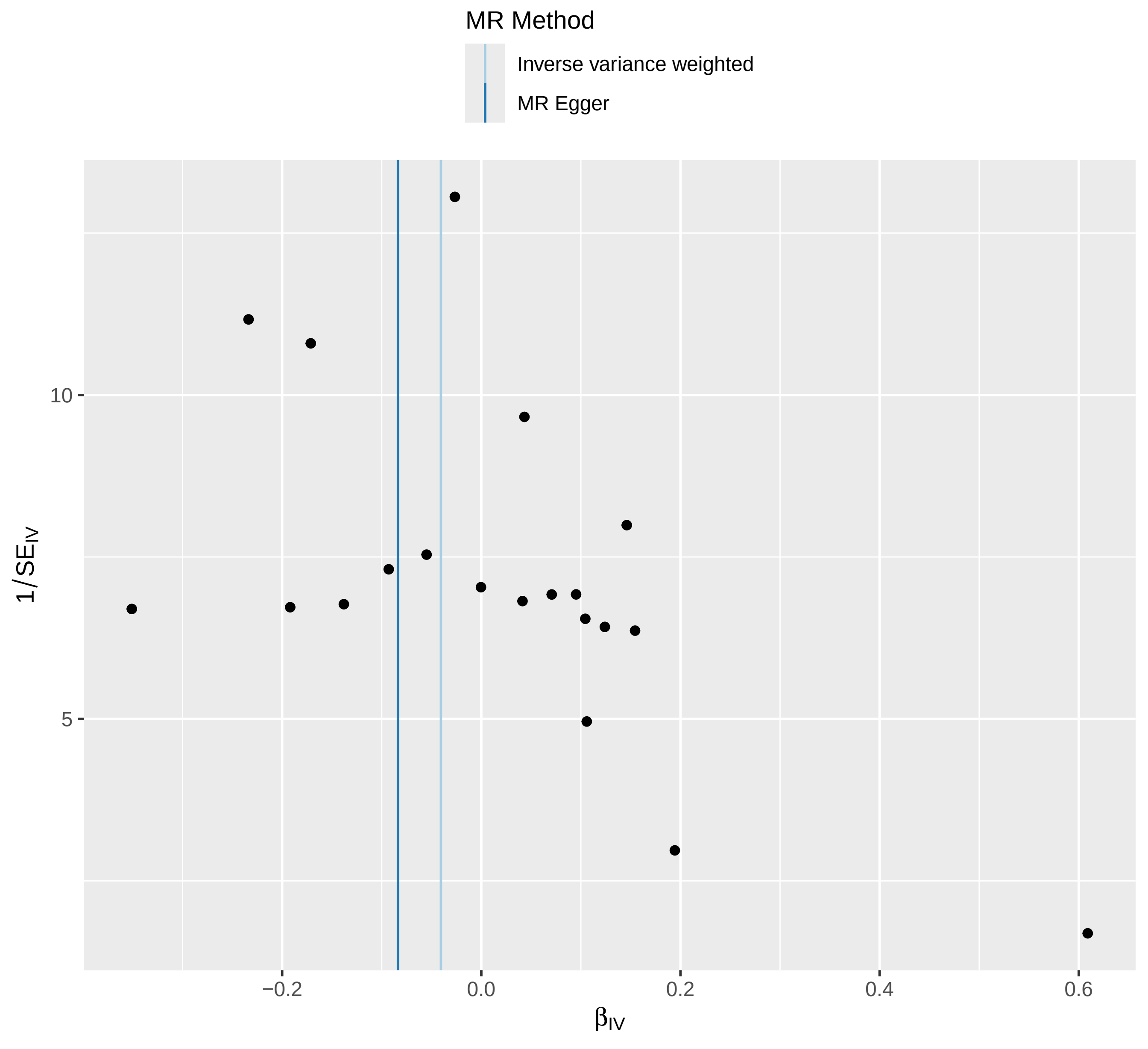
MR Method
Inverse variance weighted
MR Egger
10
5
1/SE_IV
−0.2
0.0
0.2
0.4
0.6
β_IV

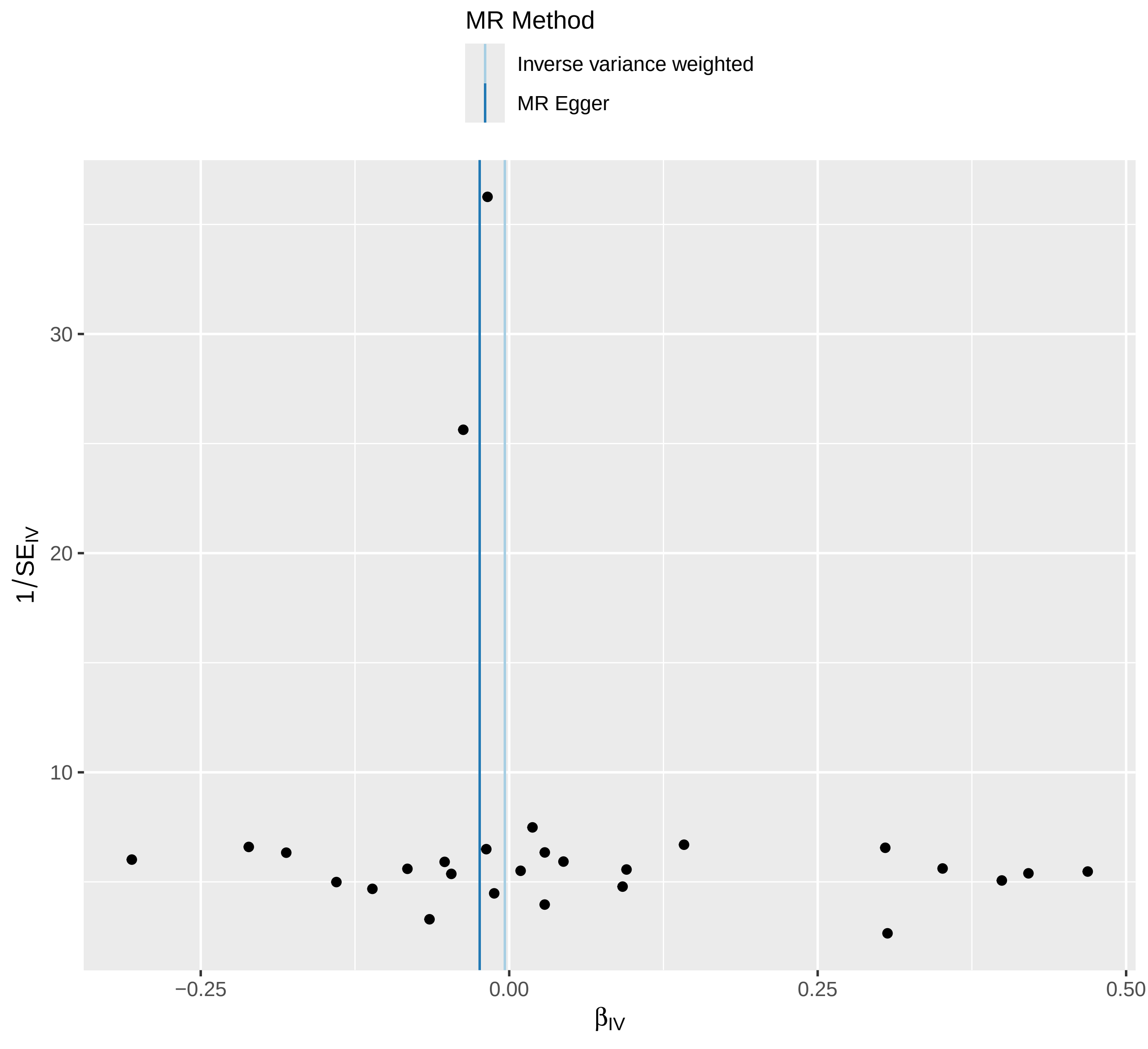
MR Method
Inverse variance weighted
MR Egger
30
20
10
1/SE_IV
−0.25
0.00
0.25
0.50
β_IV

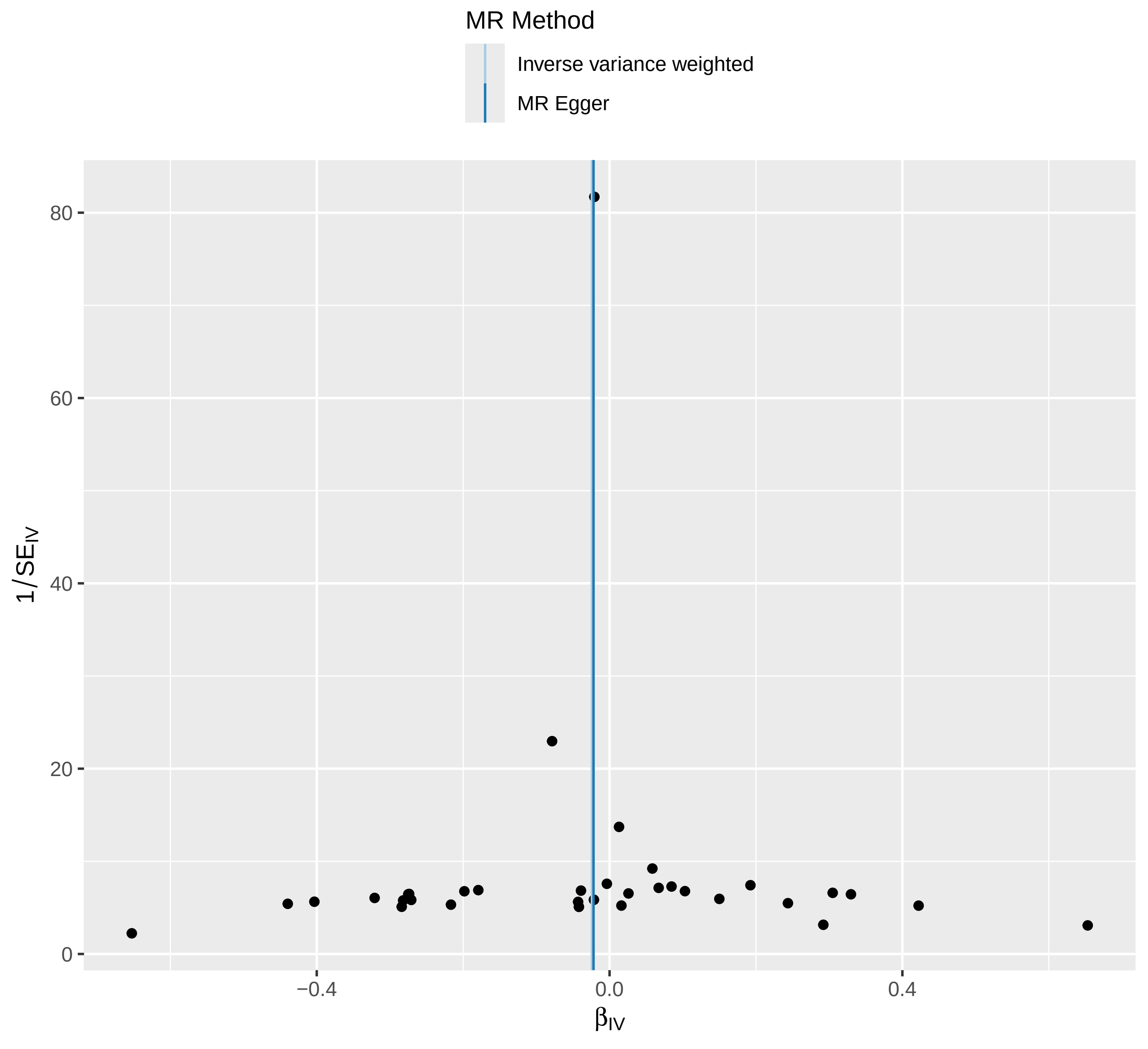

MR Method
Inverse variance weighted
MR Egger
80
60
40
20
0
1/SE$_{IV}$
−0.4
0.0
0.4
β$_{IV}$

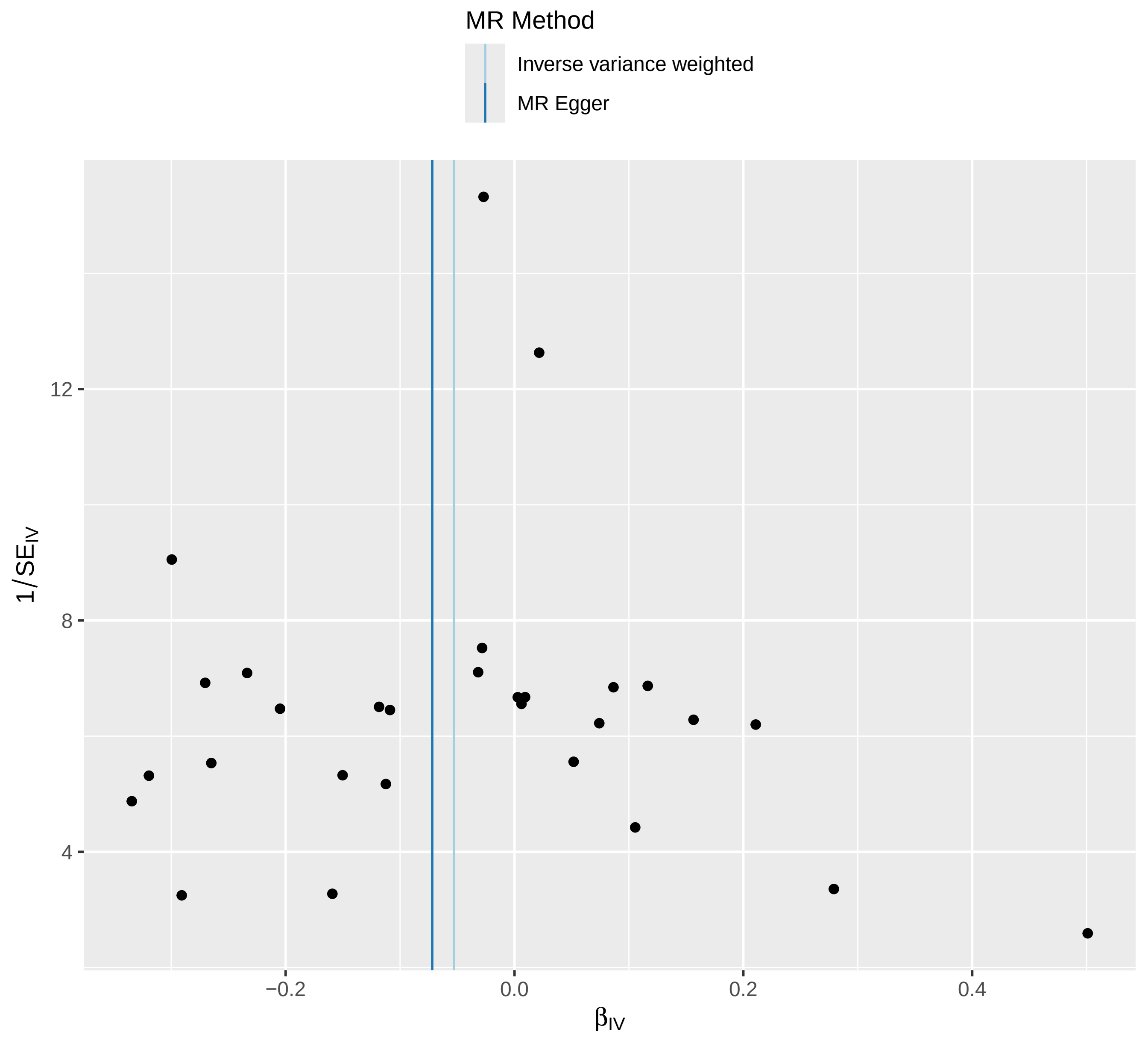
MR Method
Inverse variance weighted
MR Egger
12
8
4
1/SE$_{IV}$
−0.2
0.0
0.2
0.4
β$_{IV}$

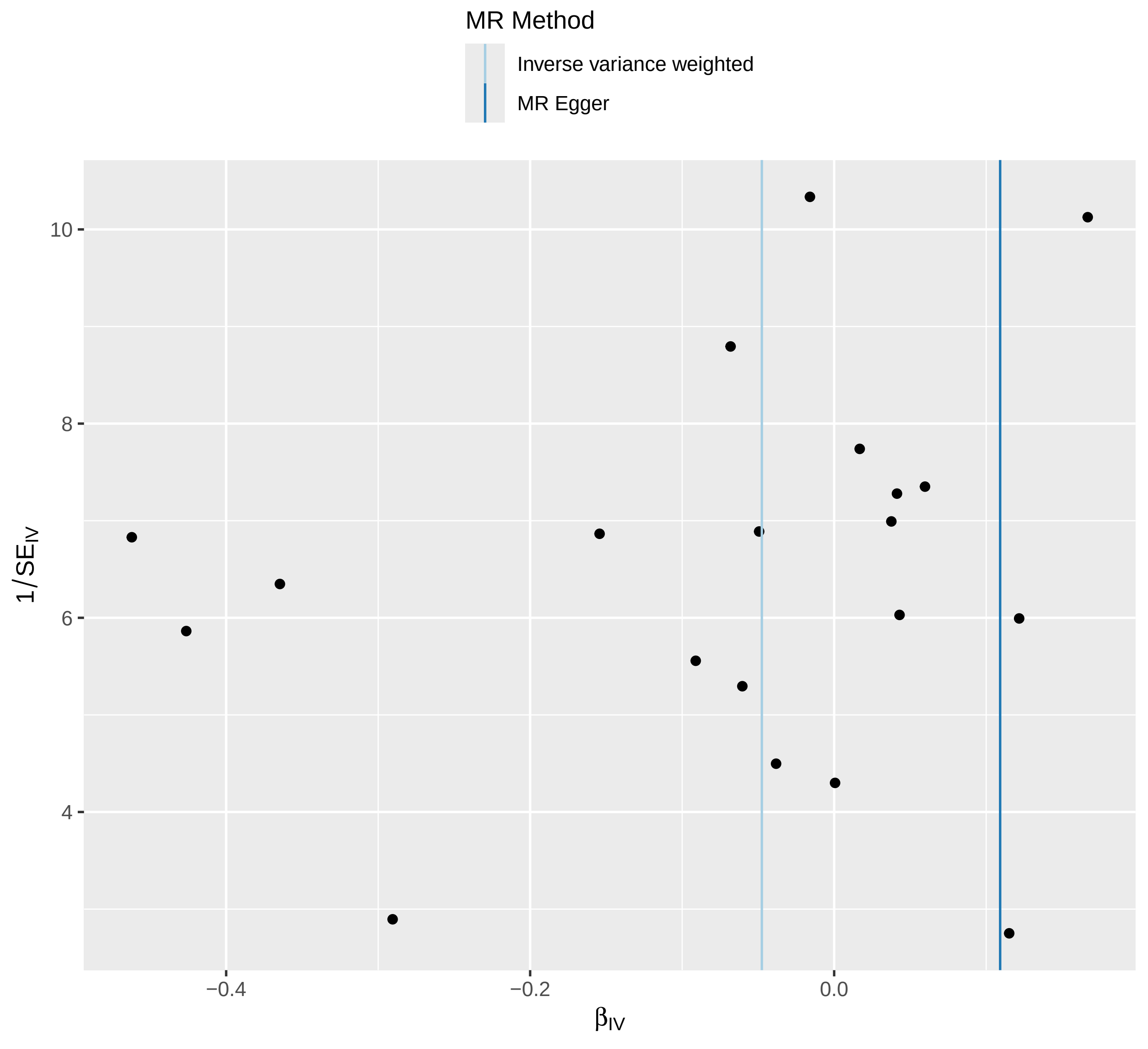
MR Method
Inverse variance weighted
MR Egger
10
8
6
4
$1/SE_{IV}$
−0.4
−0.2
0.0
β_{IV}

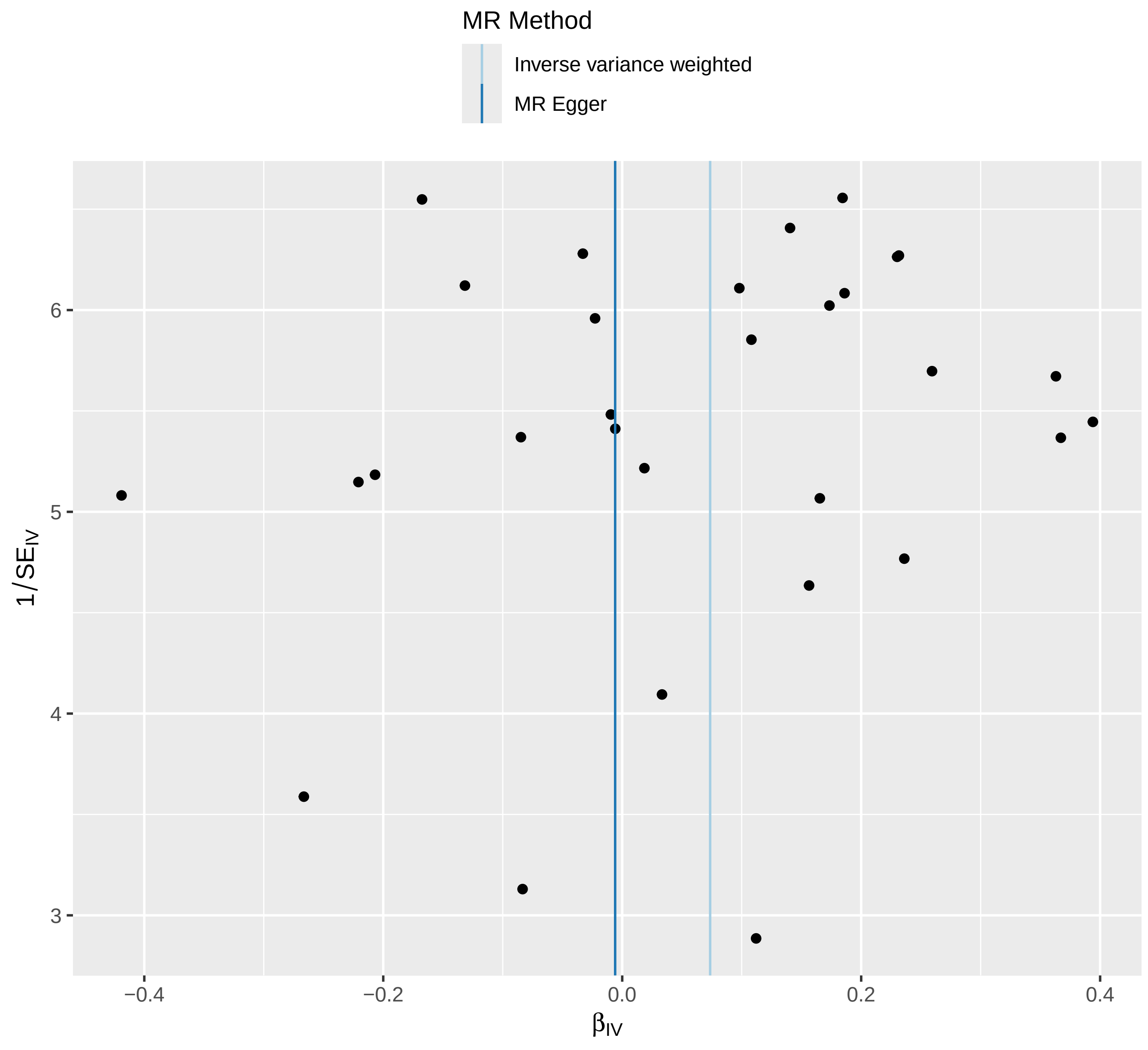

MR Method
Inverse variance weighted
MR Egger
1/SE_IV
6
5
4
3
−0.4
−0.2
0.0
0.2
0.4
β_IV

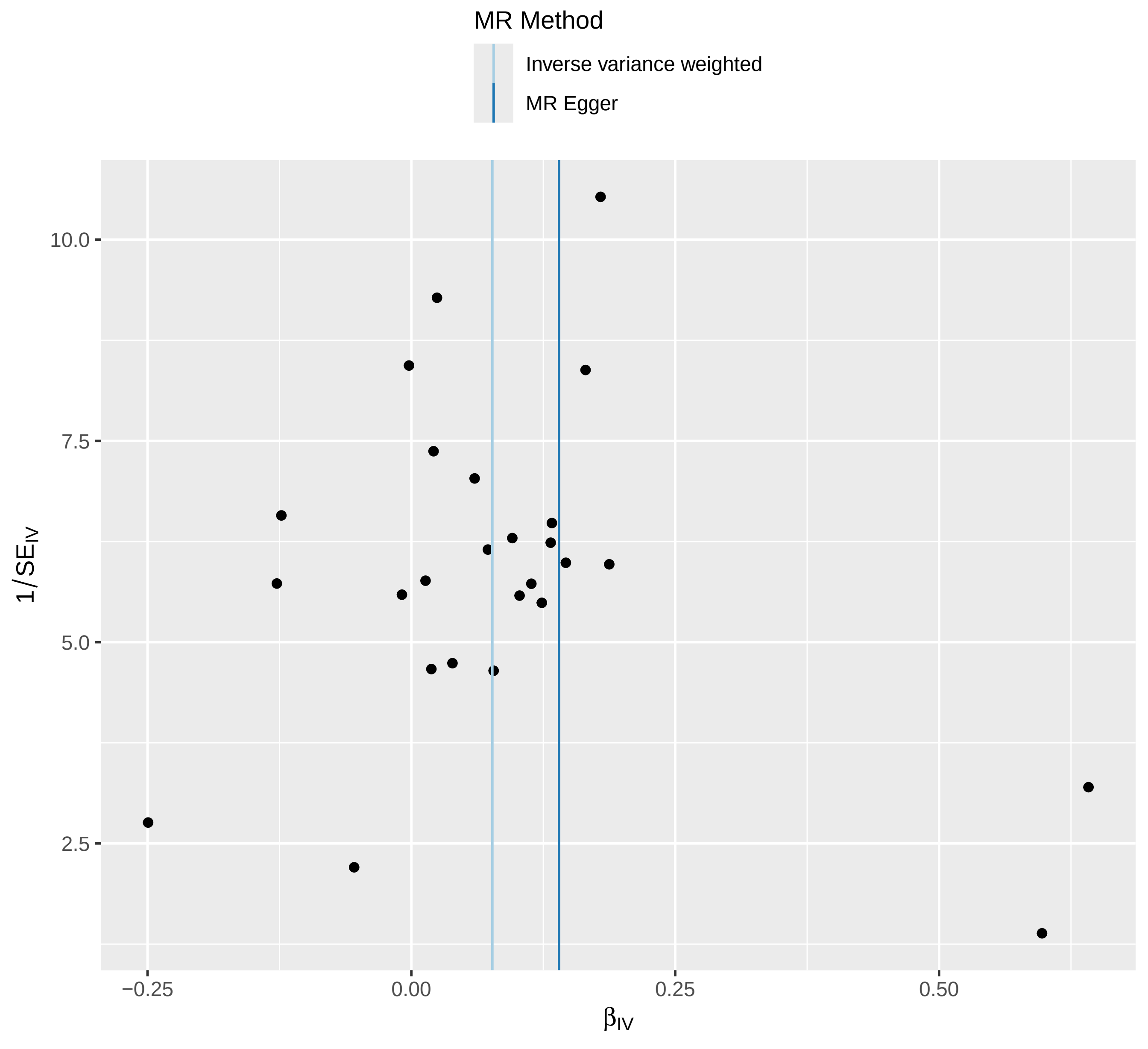
MR Method
Inverse variance weighted
MR Egger
1/SE$_{IV}$
10.0
7.5
5.0
2.5
−0.25
0.00
0.25
0.50
β$_{IV}$

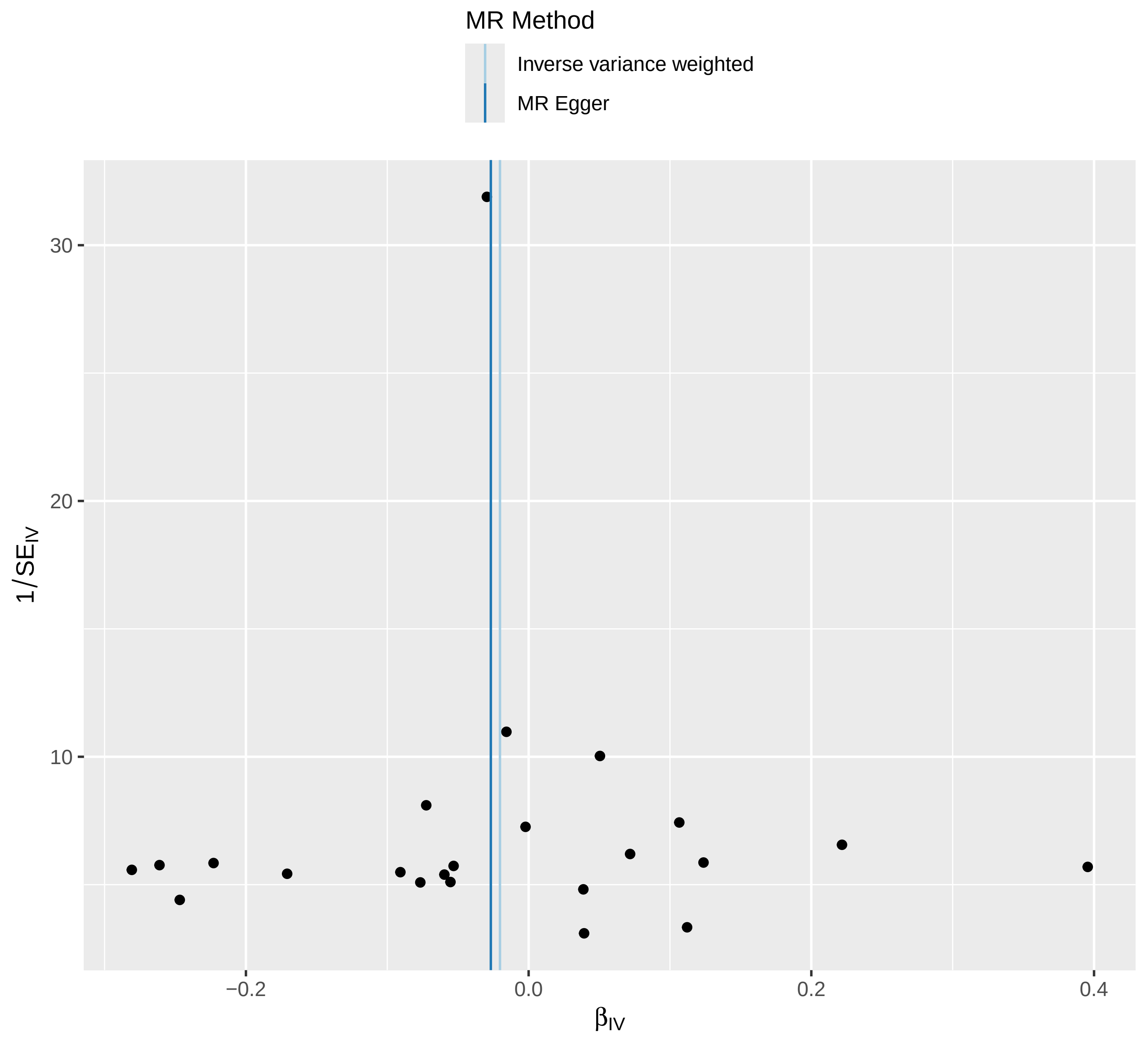
MR Method
Inverse variance weighted
MR Egger
30
20
10
1/SE$_{IV}$
−0.2
0.0
0.2
0.4
β$_{IV}$

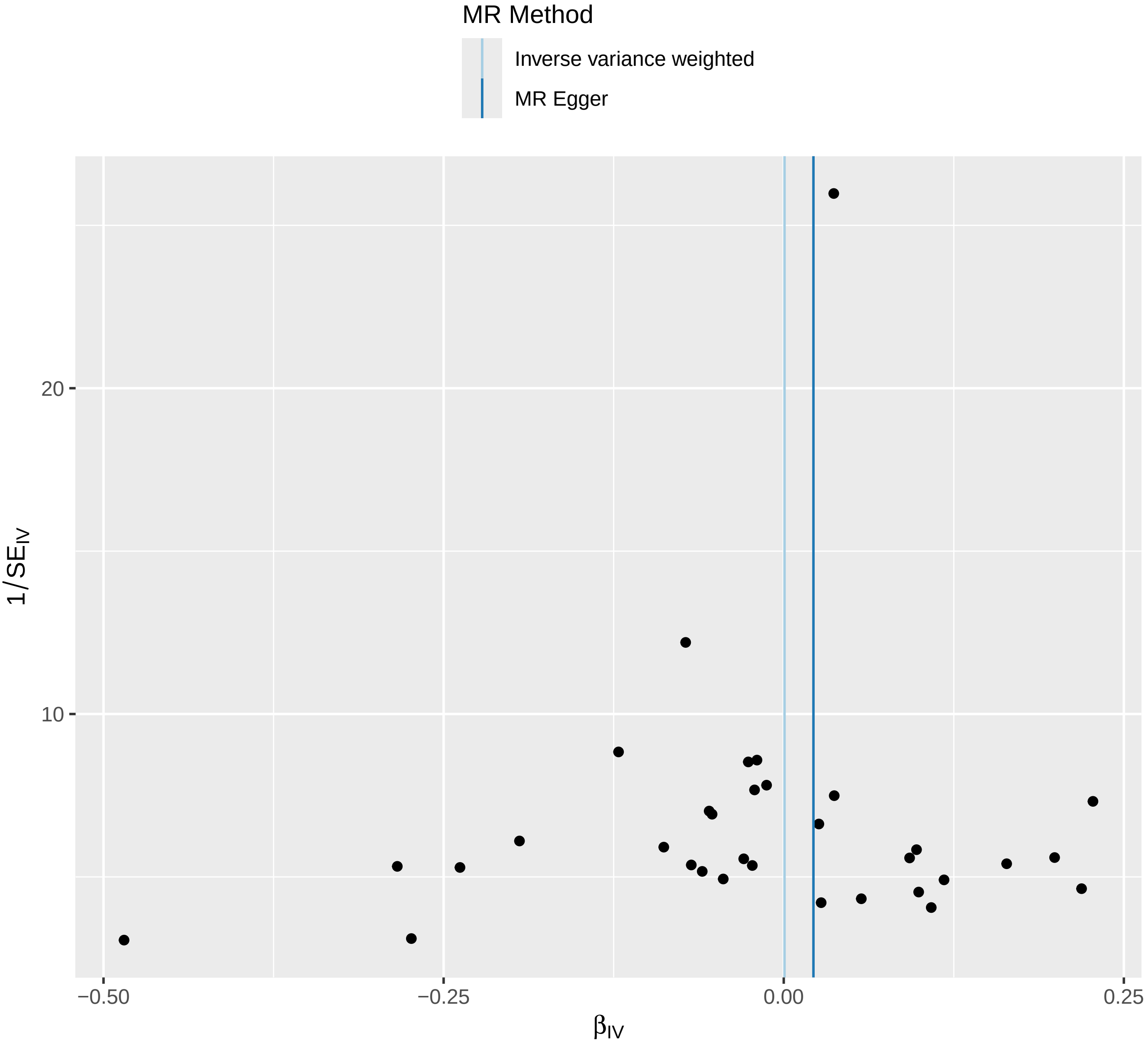
MR Method
Inverse variance weighted
MR Egger
20
10
1/SE_IV
−0.50
−0.25
0.00
0.25
β_IV

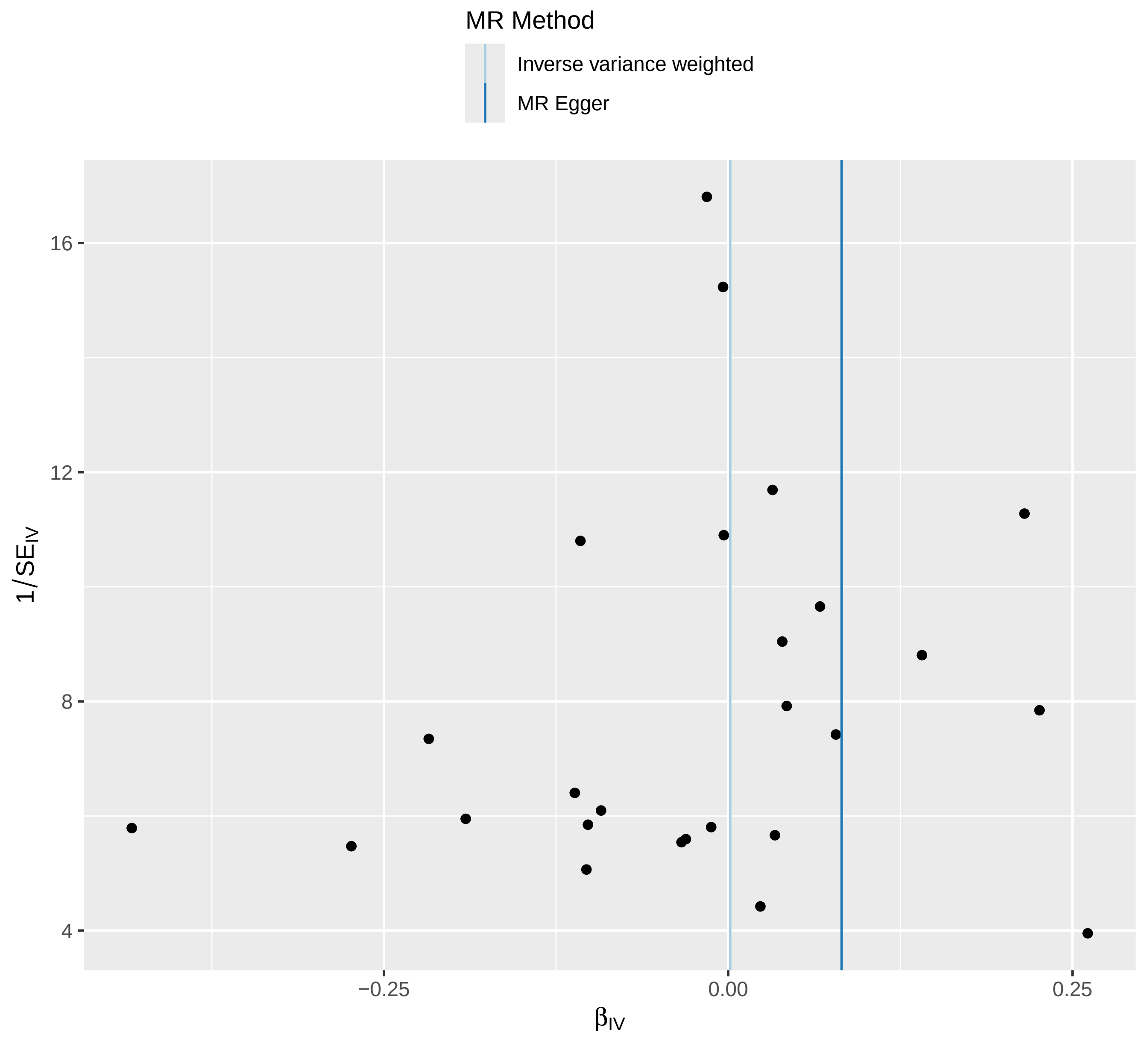

MR Method
Inverse variance weighted
MR Egger
16
12
8
4
1/SE_IV
−0.25
0.00
0.25
β_IV

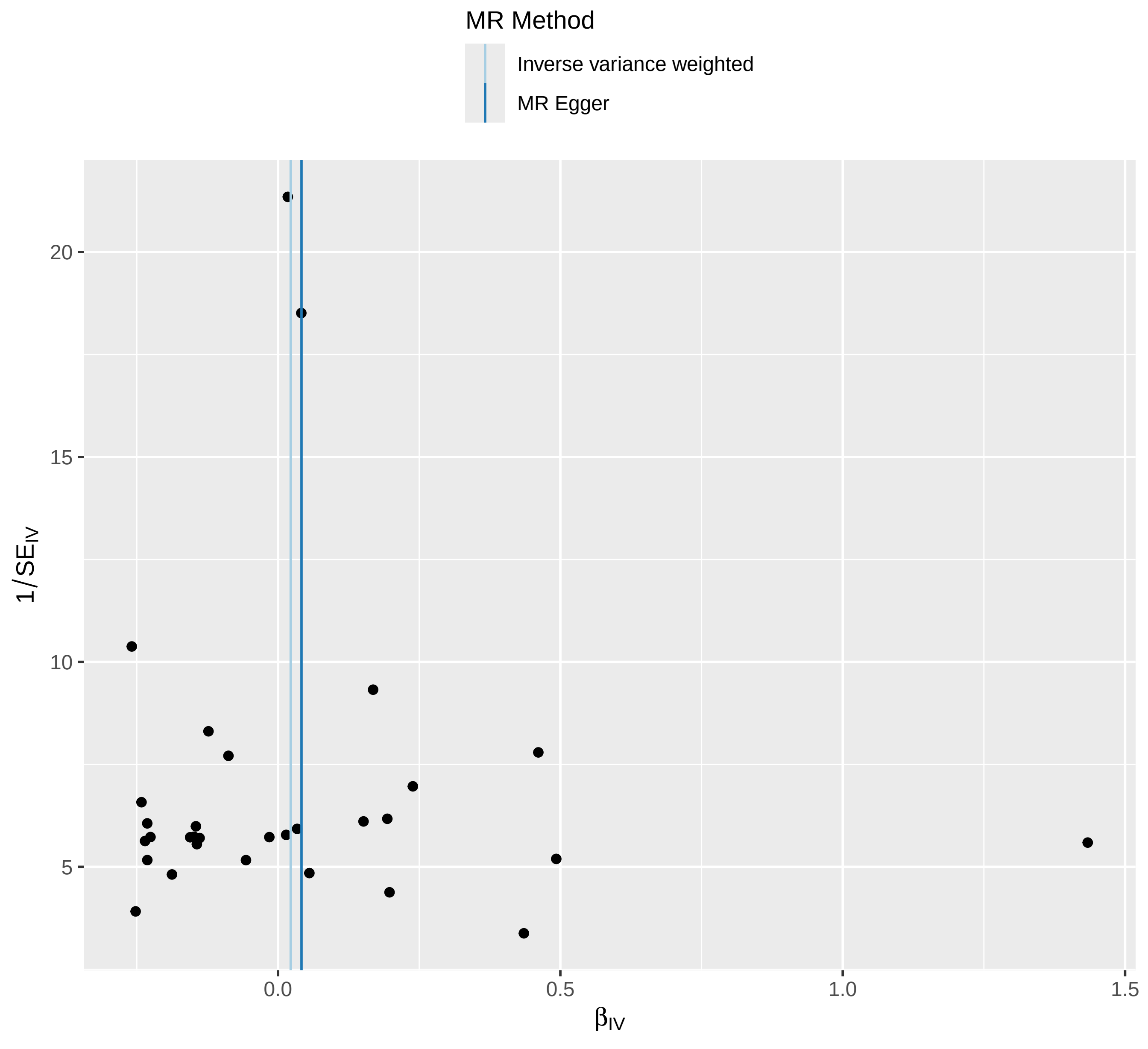

MR Method
Inverse variance weighted
MR Egger
20
15
10
5
1/SE_IV
0.0
0.5
1.0
1.5
β_IV

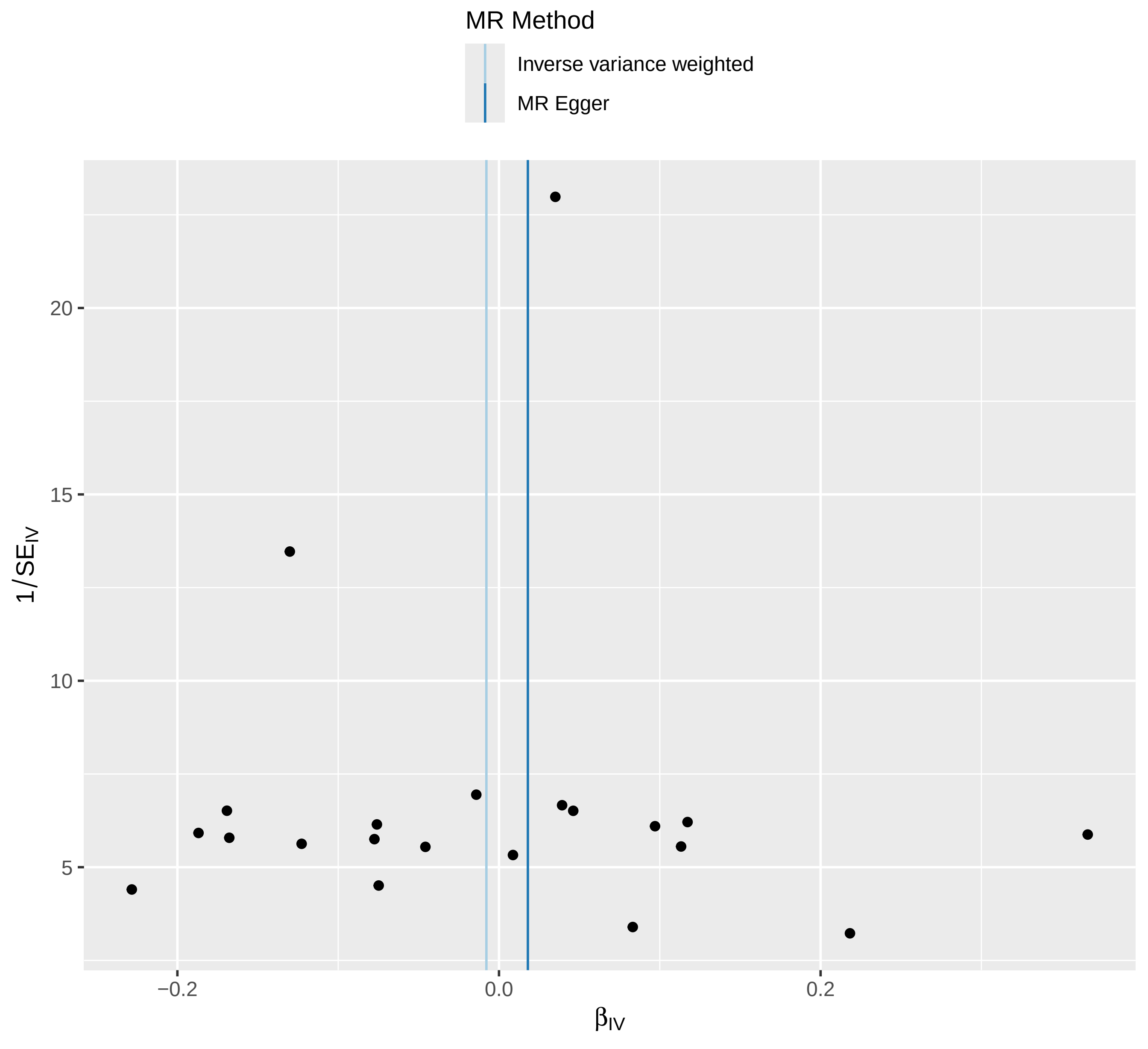
MR Method
Inverse variance weighted
MR Egger
1/SE_IV
20
15
10
5
−0.2
0.0
0.2
β_IV

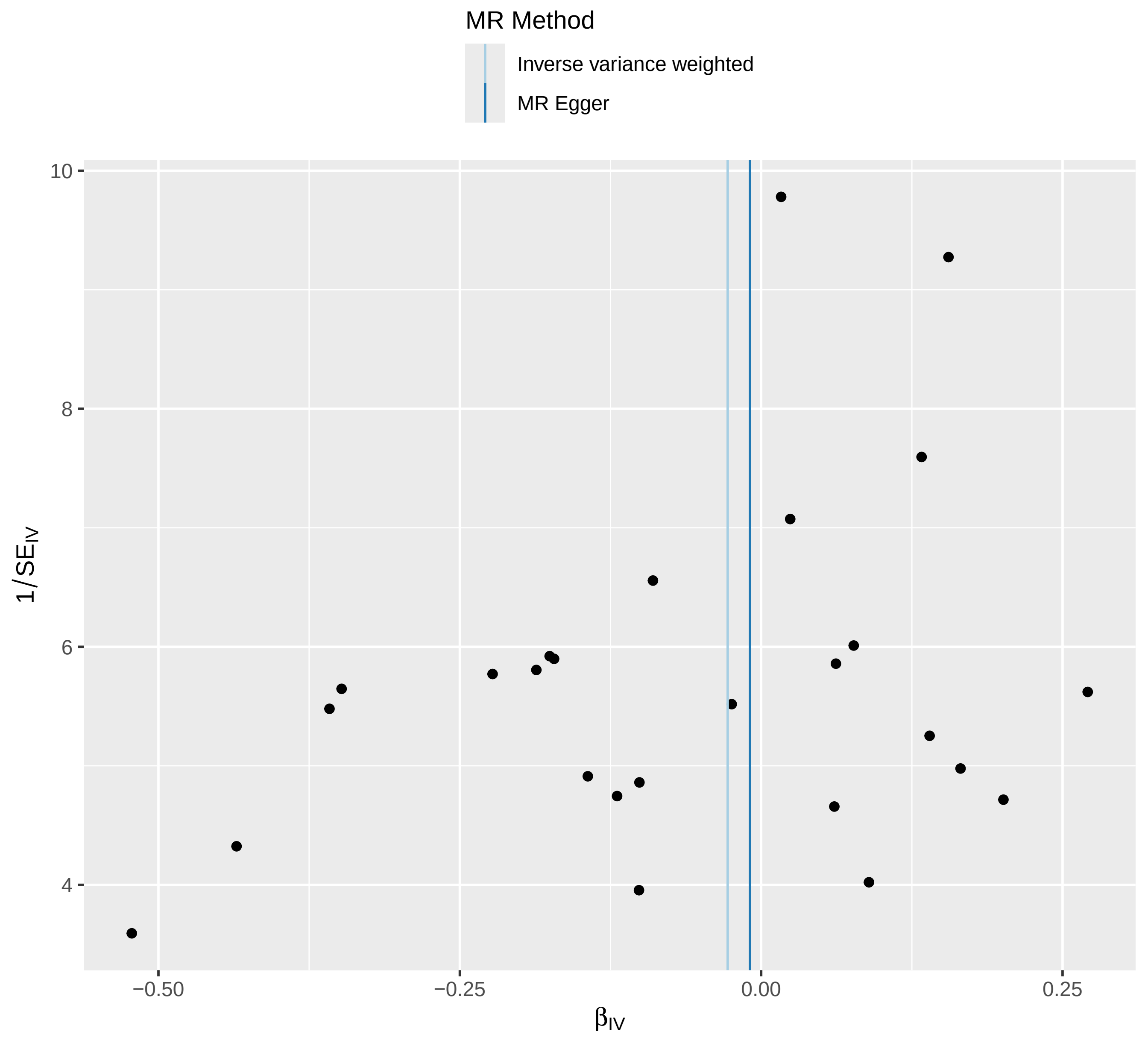

MR Method
Inverse variance weighted
MR Egger
10
8
6
4
1/SE_IV
−0.50
−0.25
0.00
0.25
β_IV

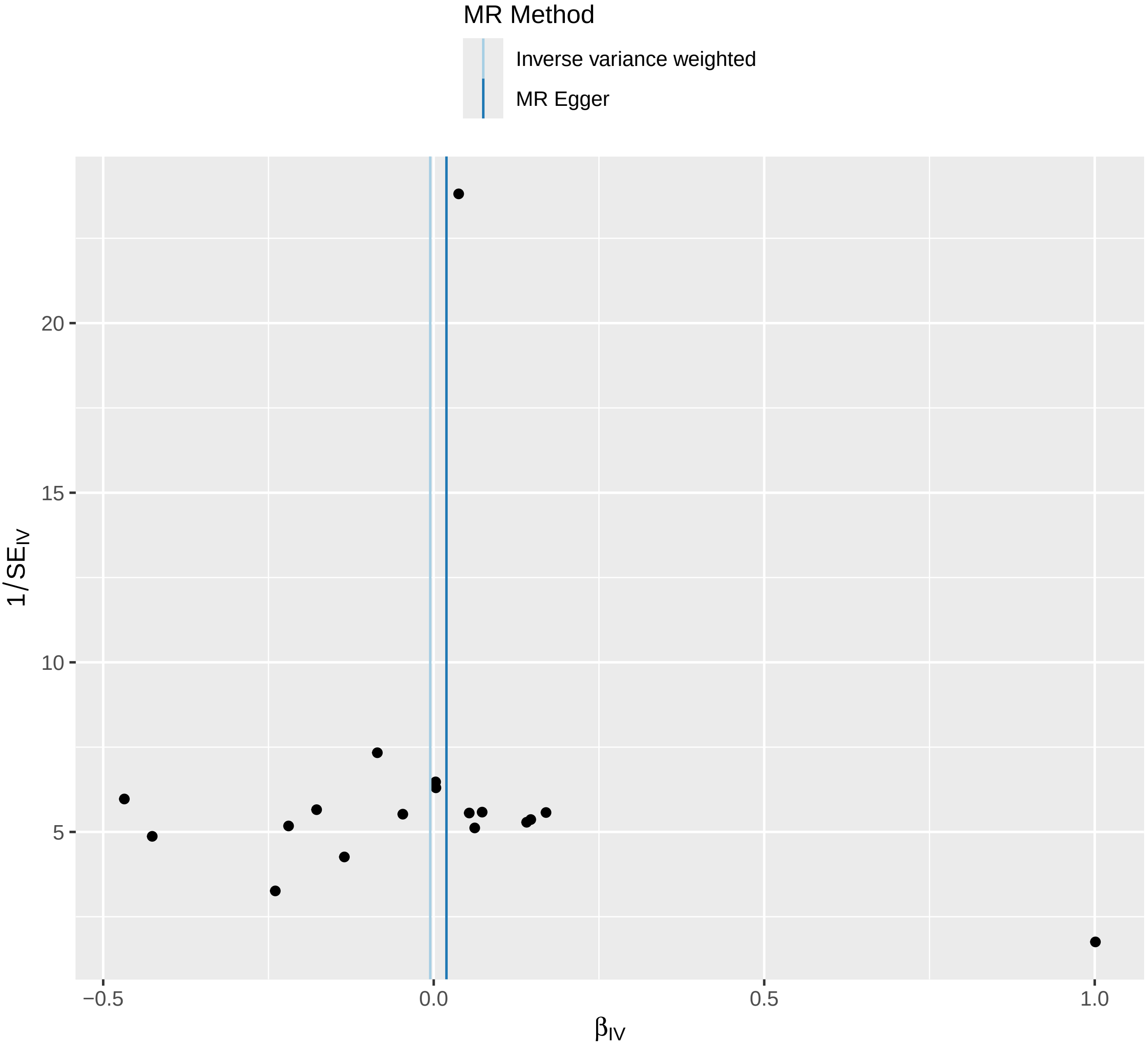
MR Method
Inverse variance weighted
MR Egger
20
15
10
5
1/SE_IV
−0.5
0.0
0.5
1.0
β_IV

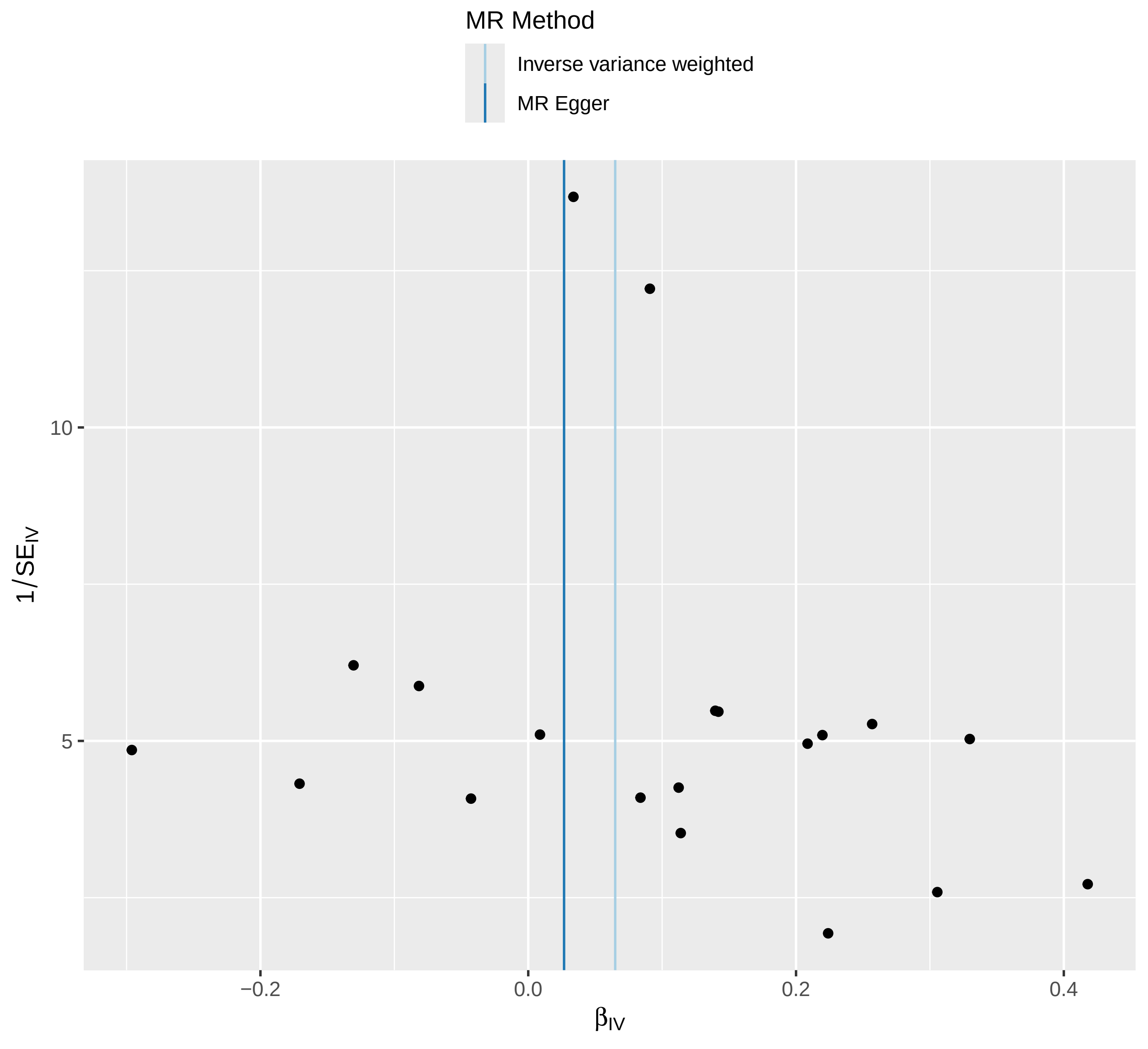
MR Method
Inverse variance weighted
MR Egger
10
5
1/SE_IV
−0.2
0.0
0.2
0.4
β_IV

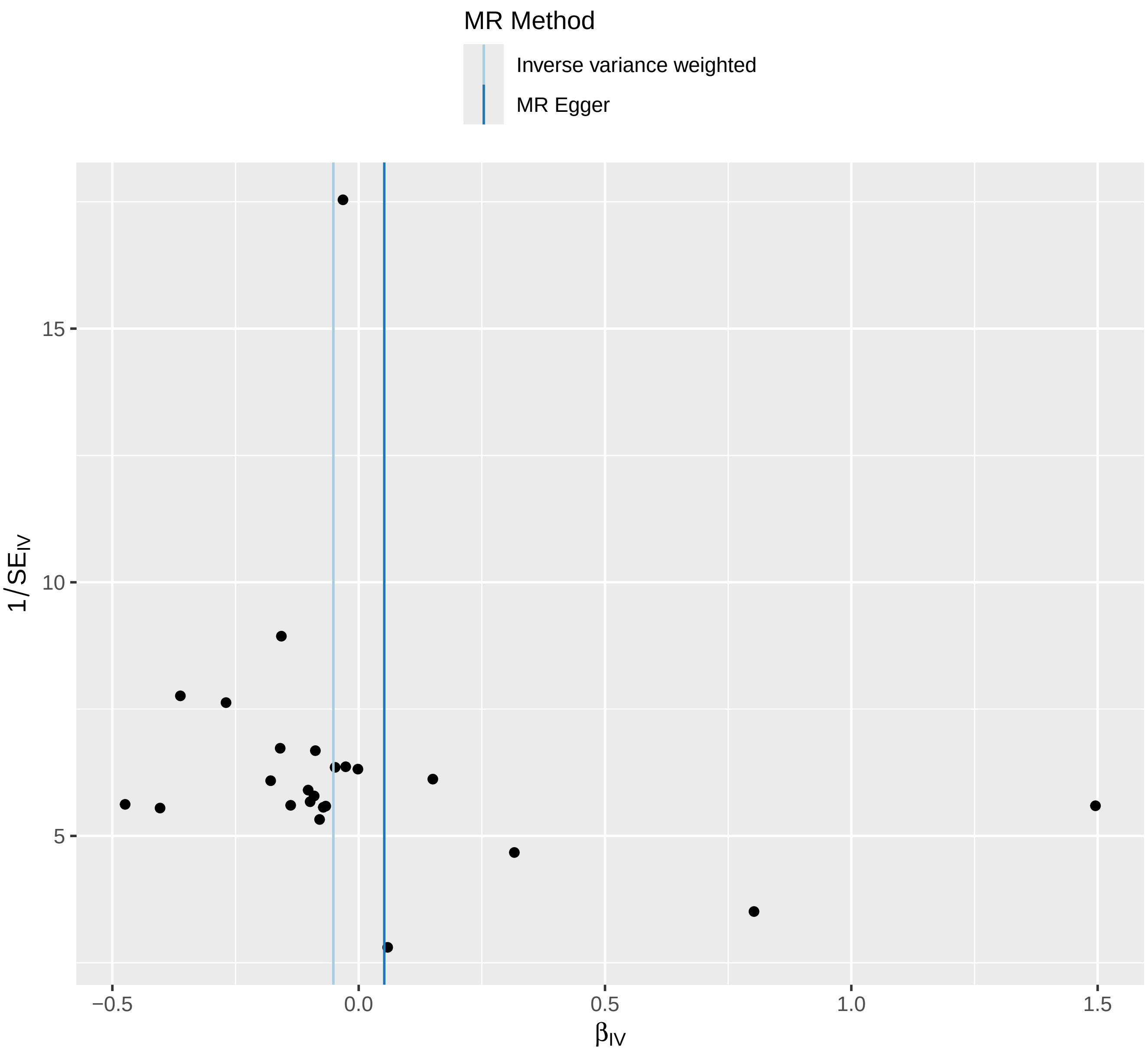
MR Method
Inverse variance weighted
MR Egger
1/SE_IV
15
10
5
−0.5
0.0
0.5
1.0
1.5
β_IV

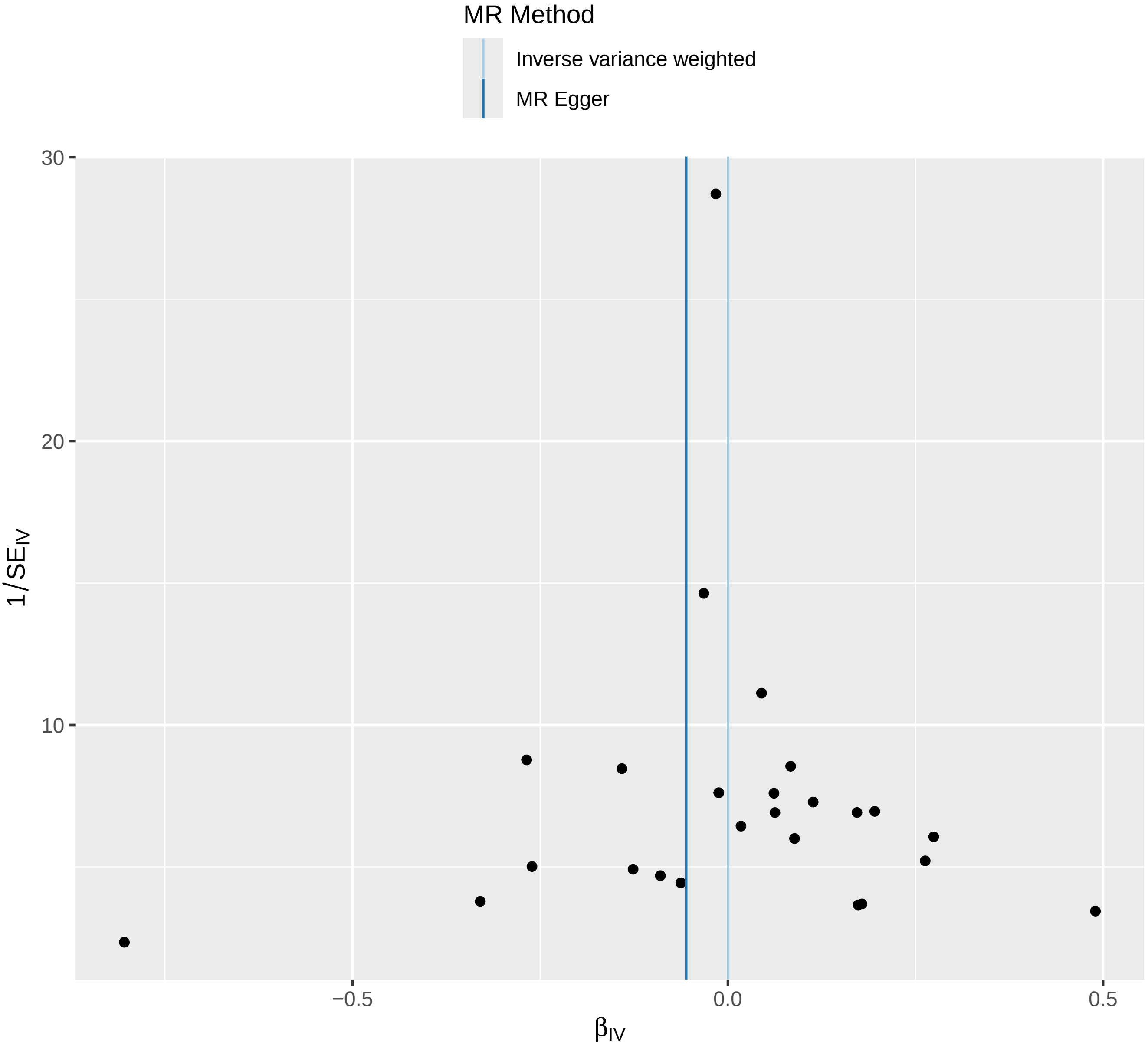

MR Method
Inverse variance weighted
MR Egger
30
20
10
1/SE_IV
−0.5
0.0
0.5
β_IV

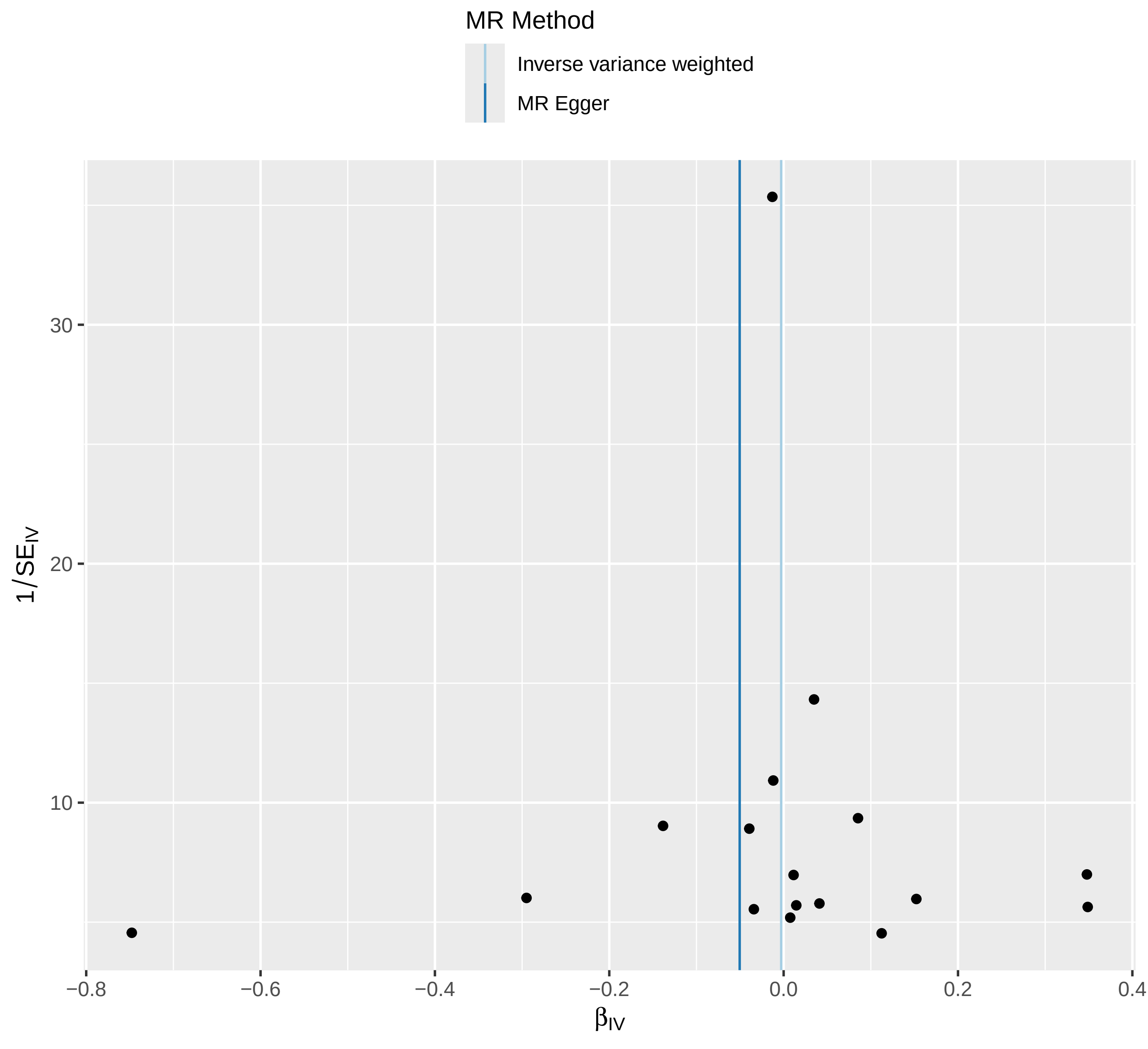

MR Method
Inverse variance weighted
MR Egger
30
20
10
1/SE$_{IV}$
−0.8
−0.6
−0.4
−0.2
0.0
0.2
0.4
β$_{IV}$

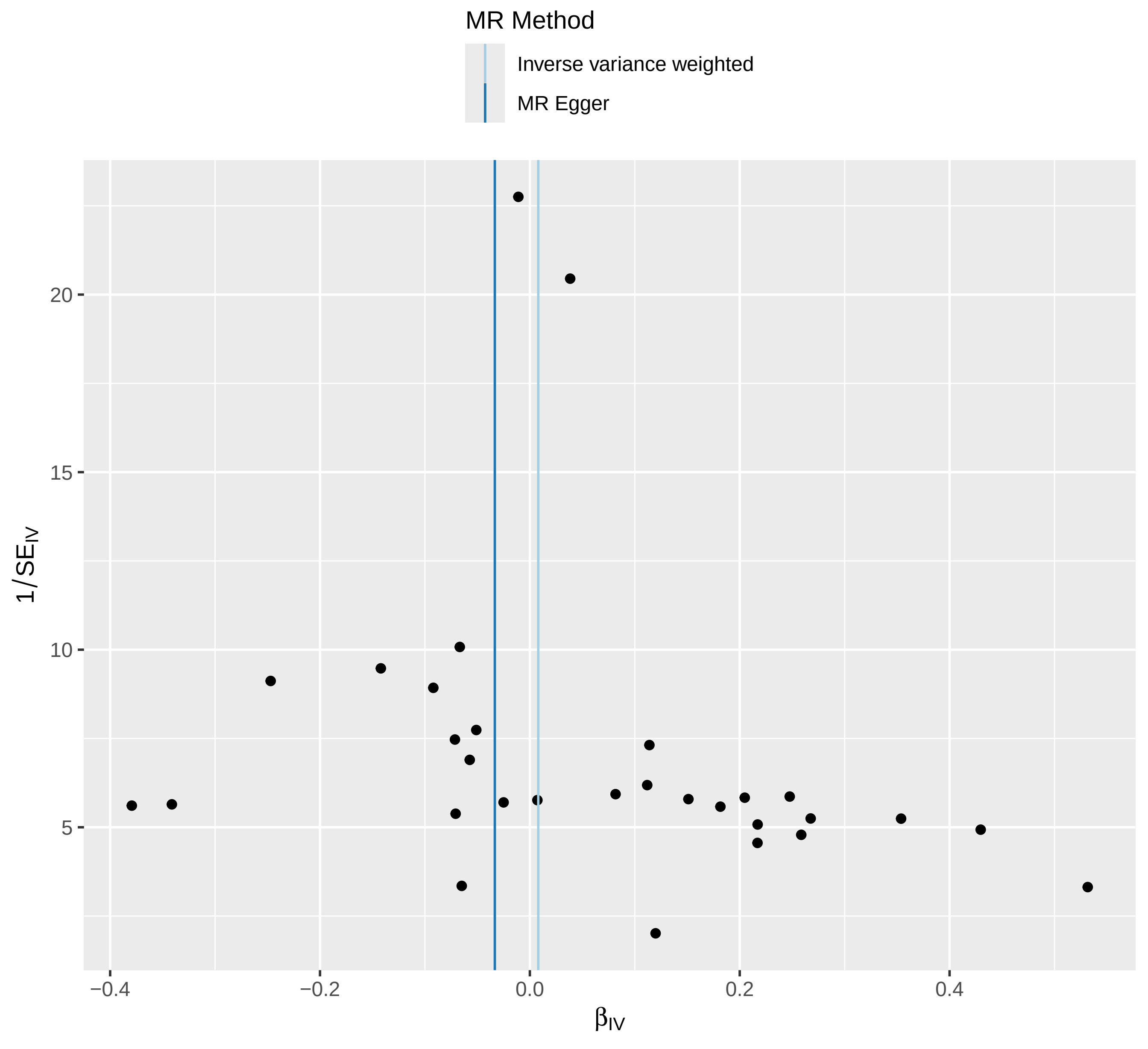

MR Method
Inverse variance weighted
MR Egger
1/SE_IV
20
15
10
5
−0.4
−0.2
0.0
0.2
0.4
β_IV

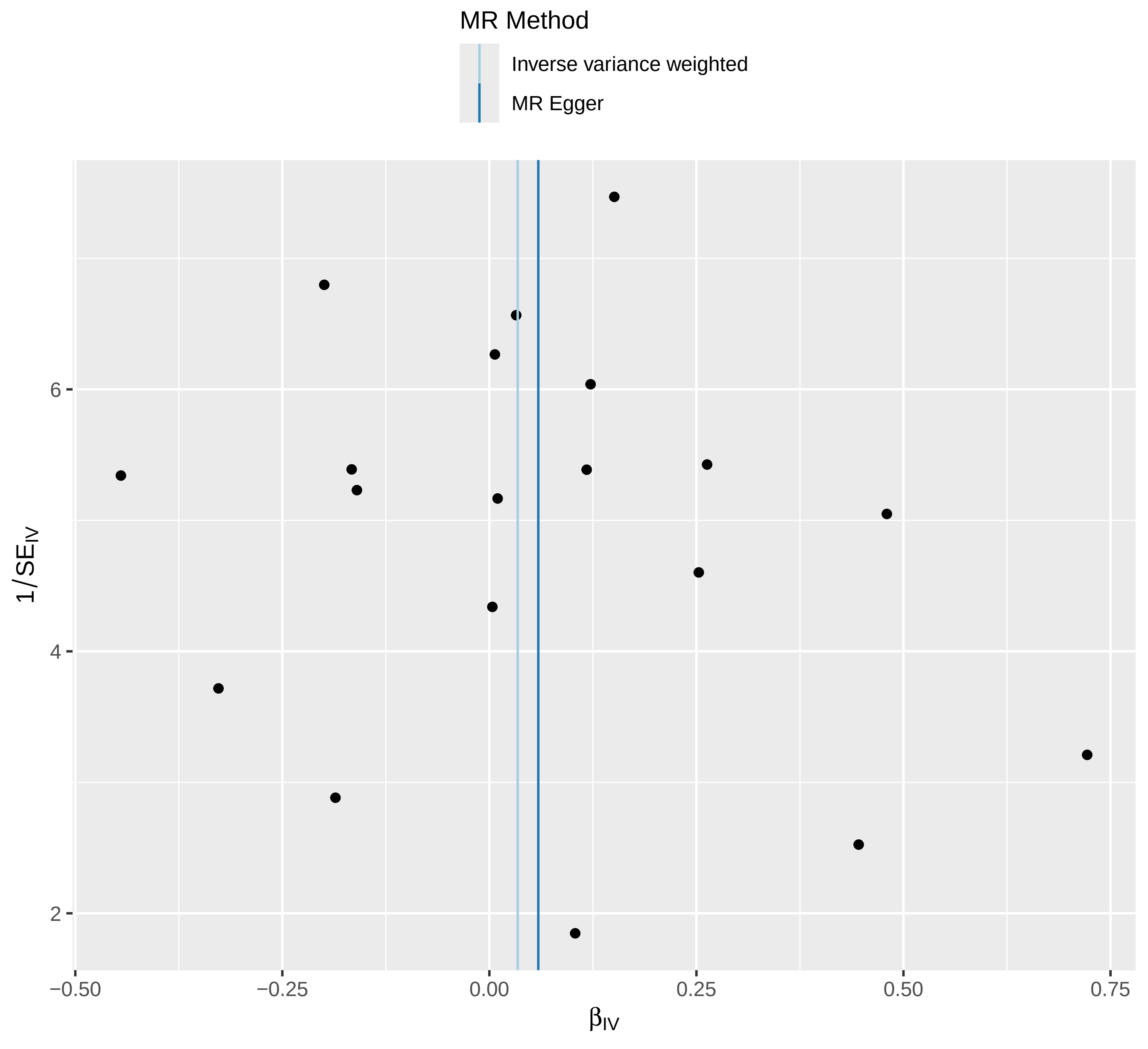
MR Method
Inverse variance weighted
MR Egger
$1/SE_{IV}$
6
4
2
β_{IV}
−0.50
−0.25
0.00
0.25
0.50
0.75

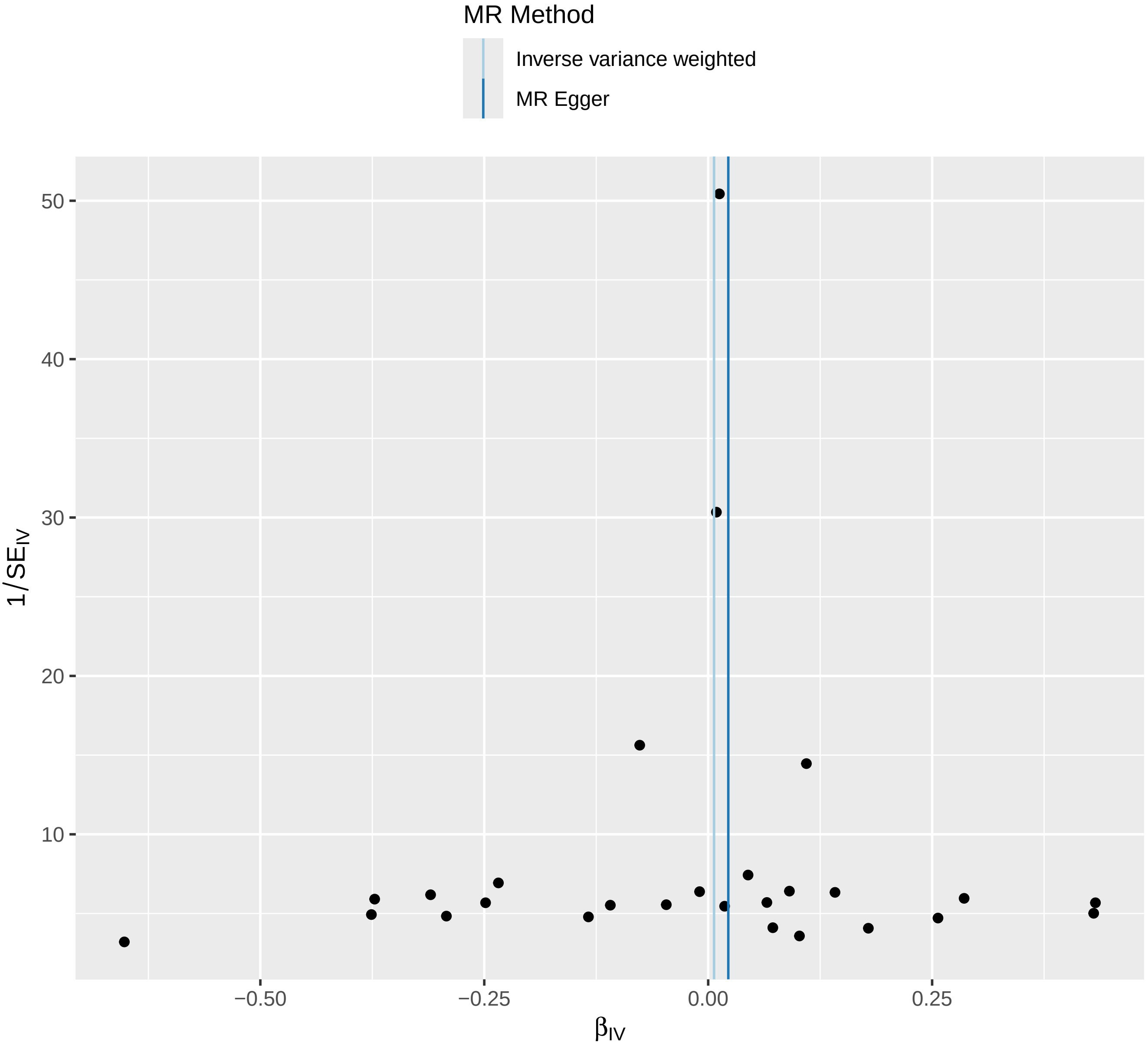
MR Method
Inverse variance weighted
MR Egger
50
40
30
20
10
$1/SE_{IV}$
−0.50
−0.25
0.00
0.25
β_{IV}

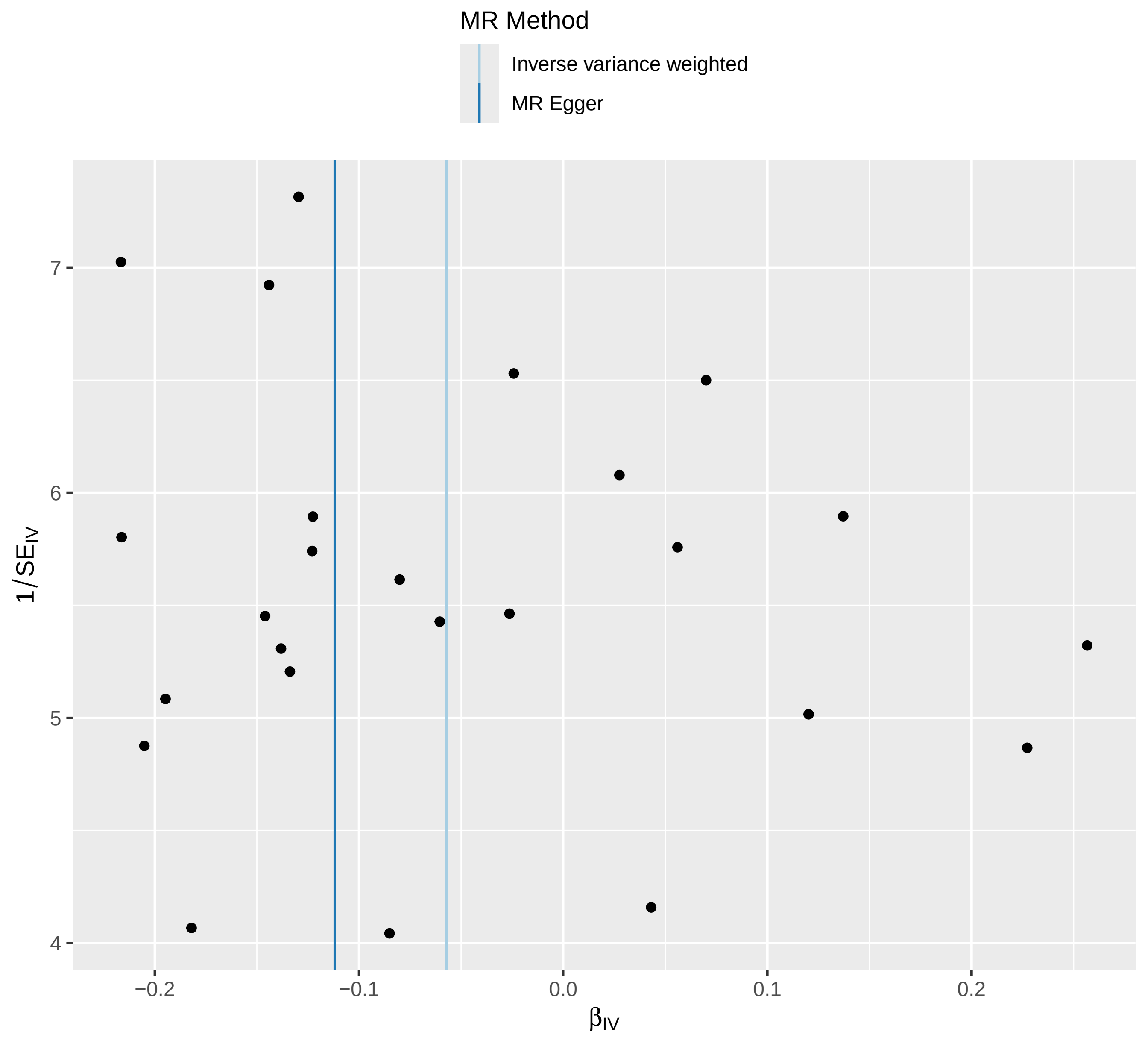

MR Method
Inverse variance weighted
MR Egger
7
6
5
4
1/SE_IV
−0.2
−0.1
0.0
0.1
0.2
β_IV

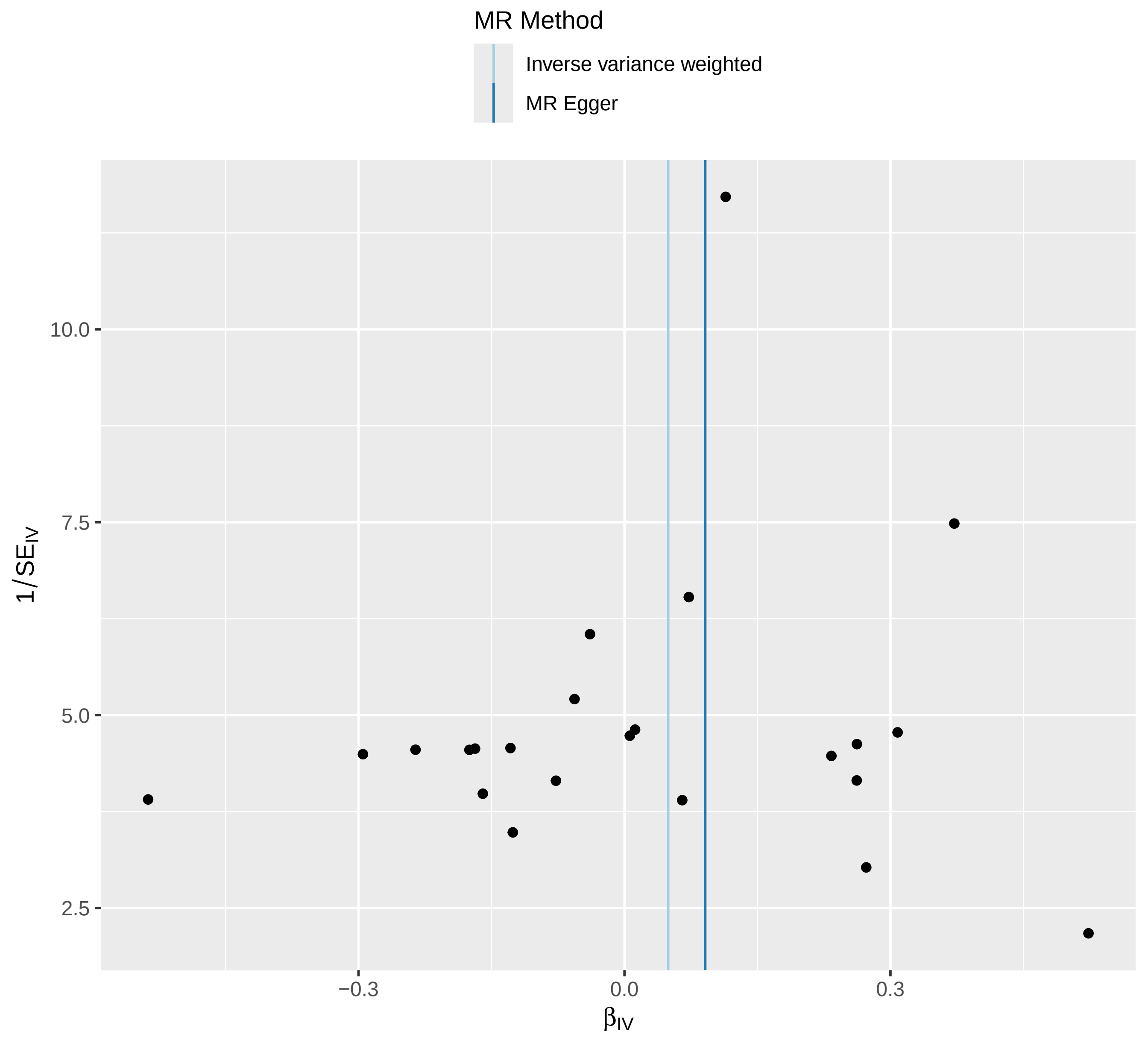
MR Method
Inverse variance weighted
MR Egger
10.0
7.5
5.0
2.5
1/SE$_{IV}$
−0.3
0.0
0.3
β$_{IV}$

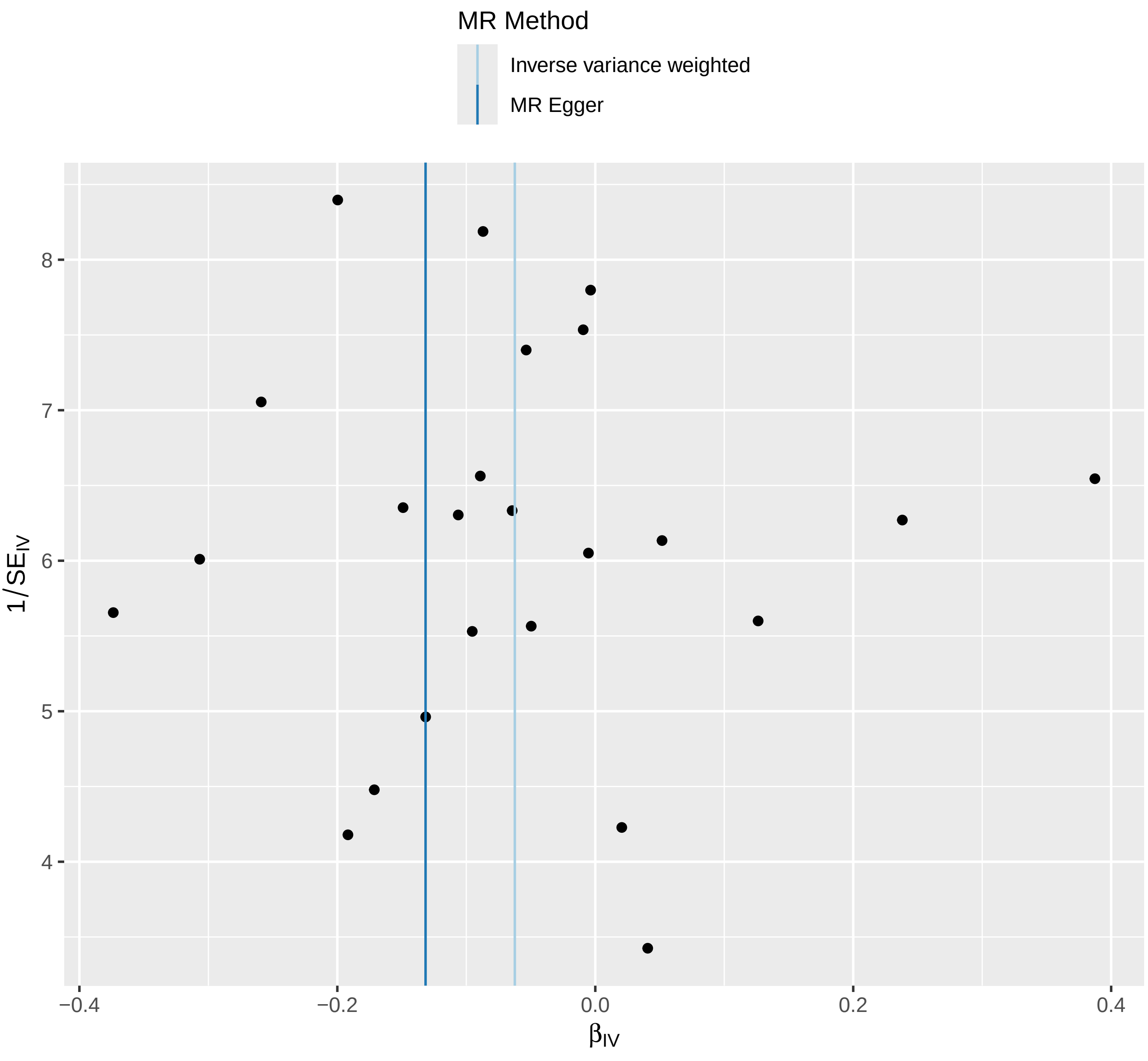
MR Method
Inverse variance weighted
MR Egger
1/SE_IV
8
7
6
5
4
−0.4
−0.2
0.0
0.2
0.4
β_IV

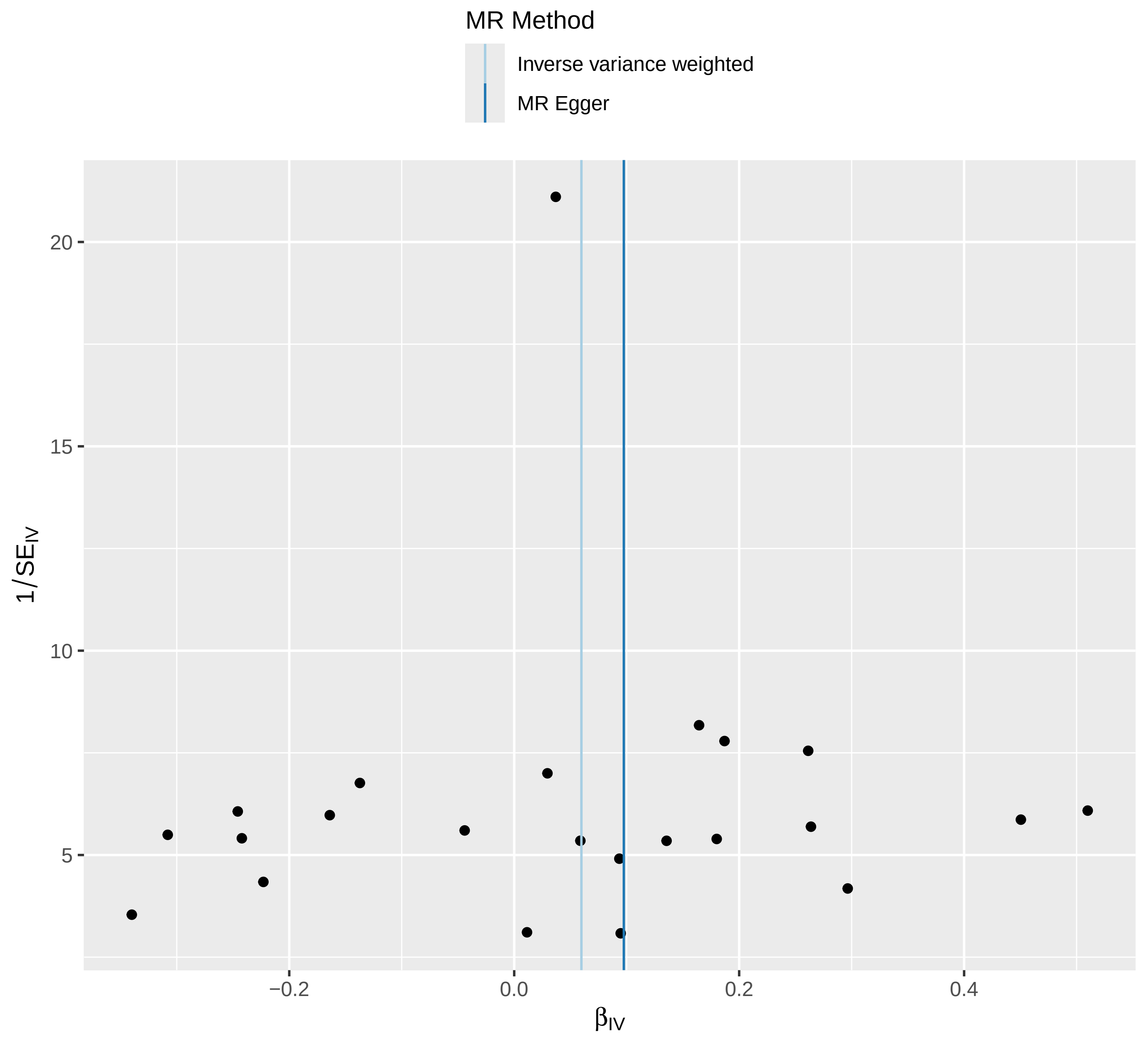

MR Method
Inverse variance weighted
MR Egger
20
15
10
5
1/SE_IV
−0.2
0.0
0.2
0.4
β_IV

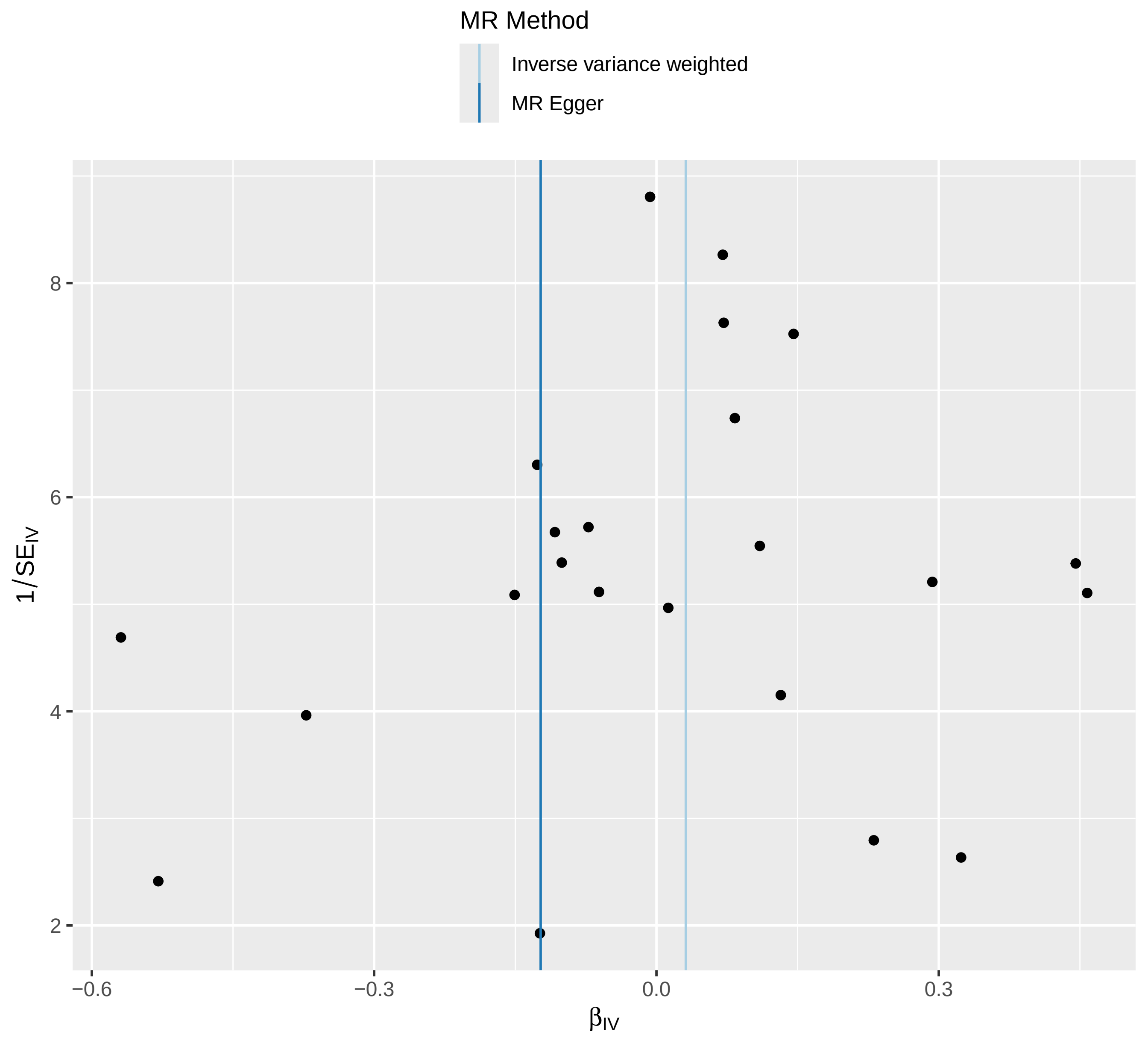
MR Method
Inverse variance weighted
MR Egger
1/SE_IV
8
6
4
2
−0.6
−0.3
0.0
0.3
β_IV

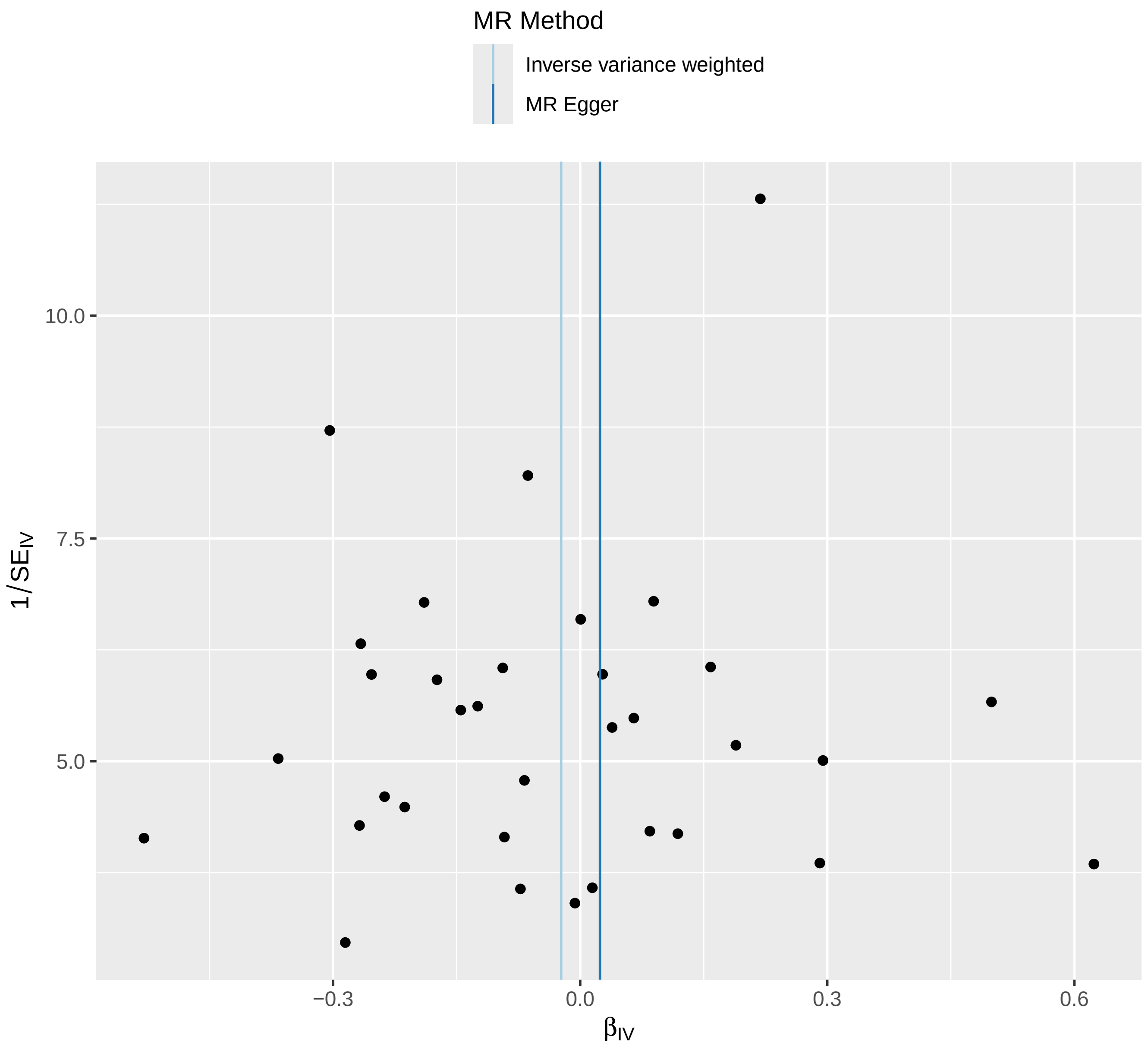
MR Method
Inverse variance weighted
MR Egger
10.0
7.5
5.0
1/SE_IV
−0.3
0.0
0.3
0.6
β_IV

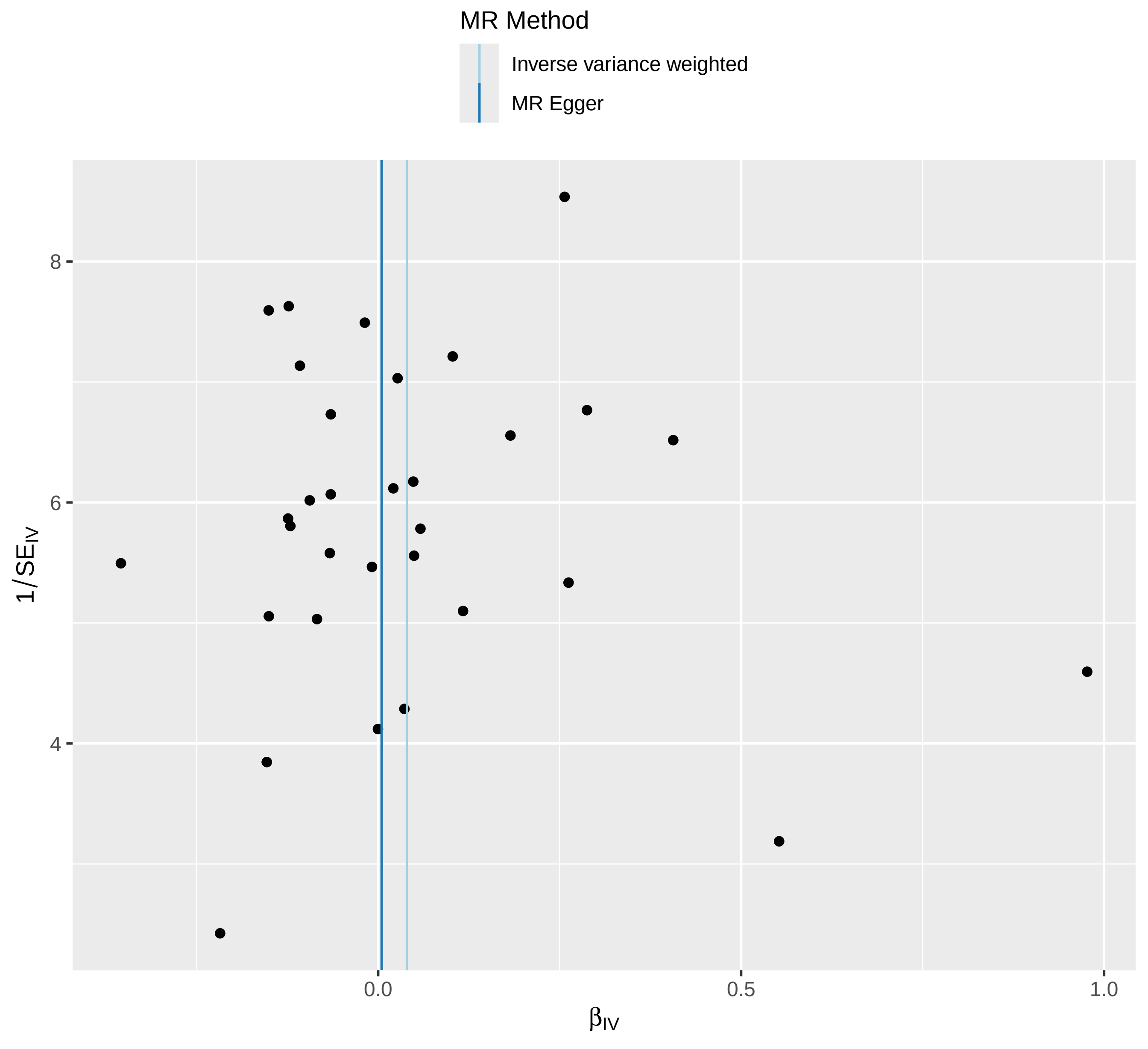

MR Method
Inverse variance weighted
MR Egger
8
6
4
1/SE$_{IV}$
0.0
0.5
1.0
β$_{IV}$

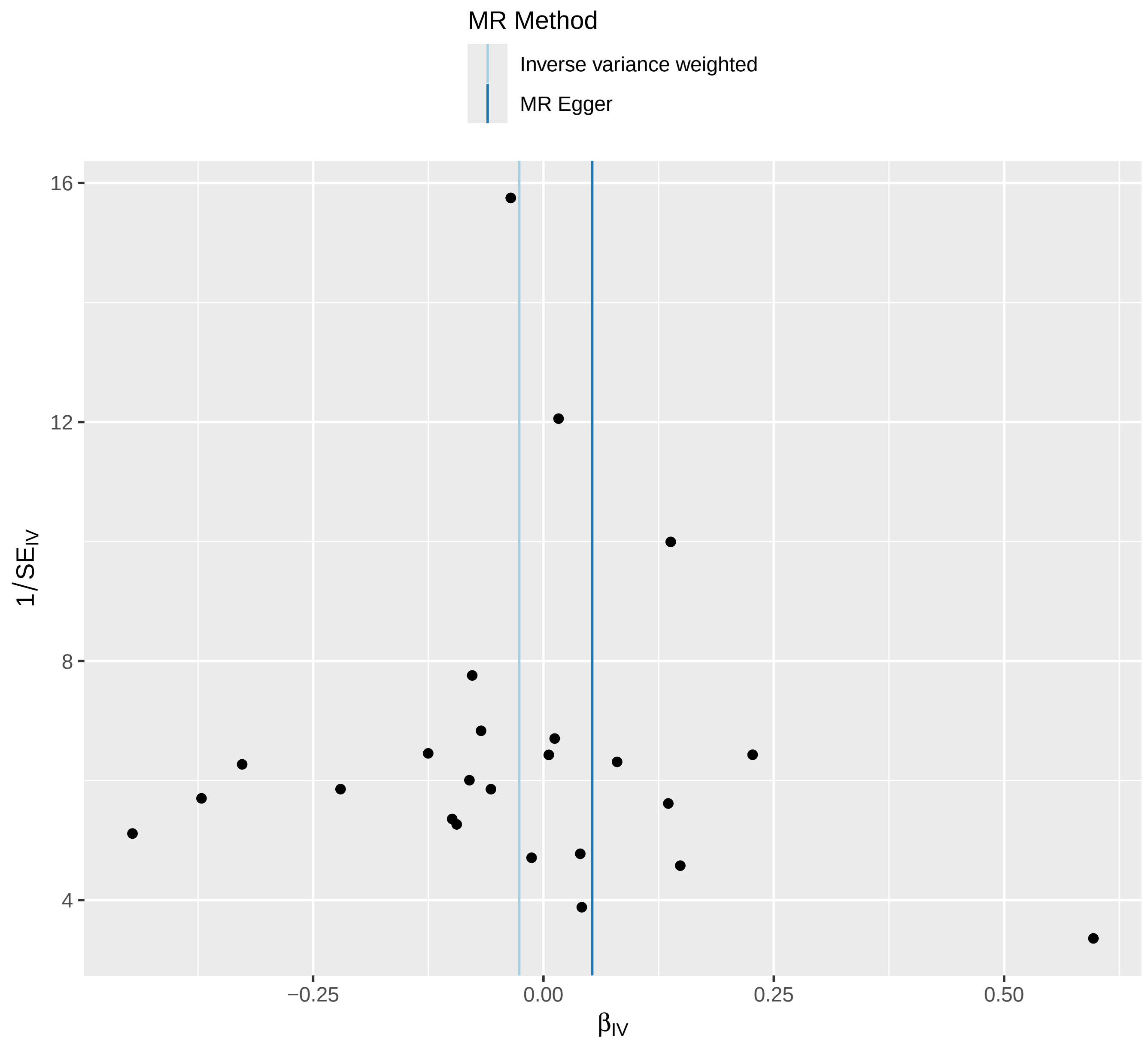

MR Method
Inverse variance weighted
MR Egger
16
12
8
4
1/SE_IV
−0.25
0.00
0.25
0.50
β_IV

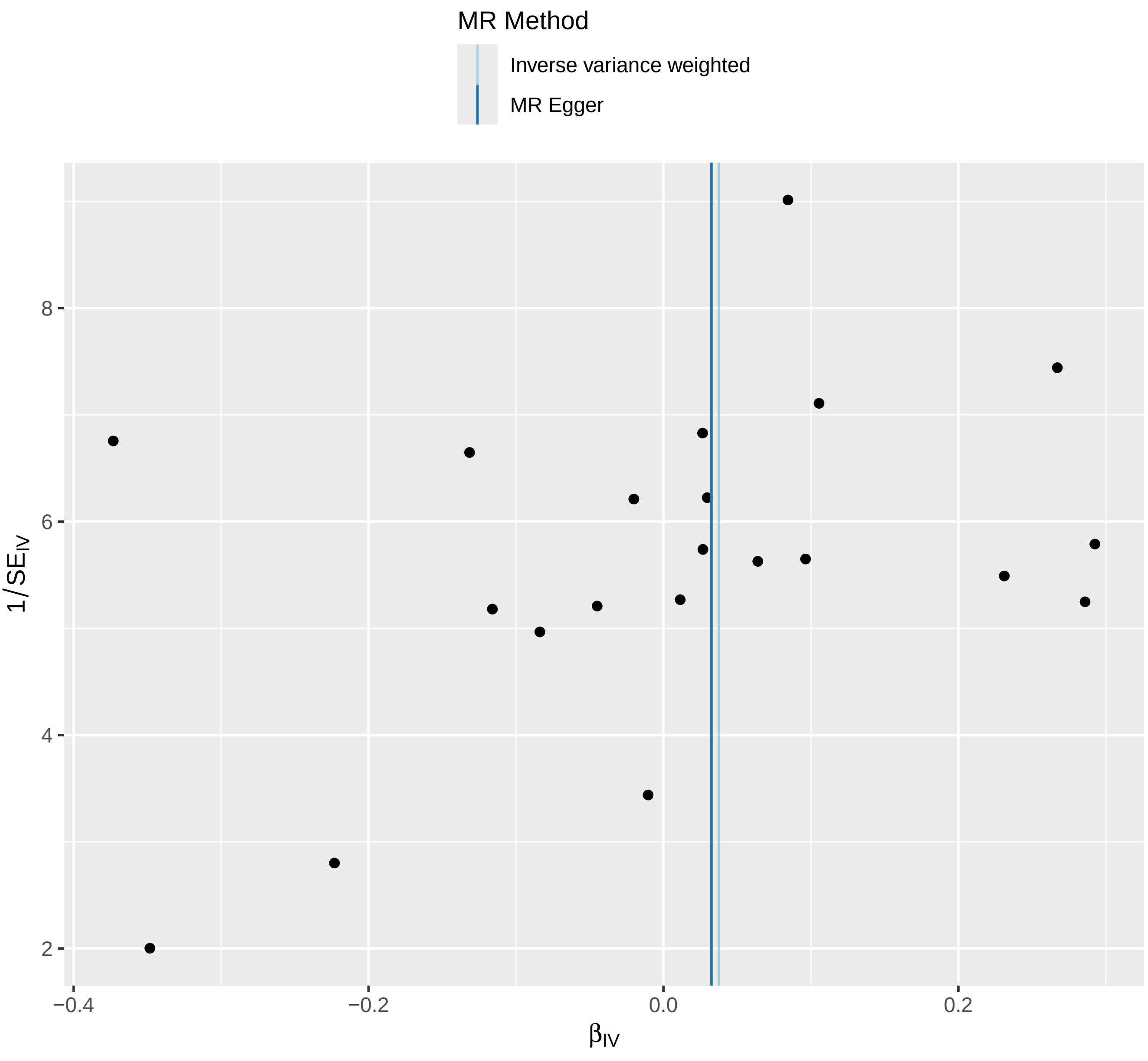
MR Method
Inverse variance weighted
MR Egger
1/SE$_{IV}$
β$_{IV}$
8
6
4
2
−0.4
−0.2
0.0
0.2

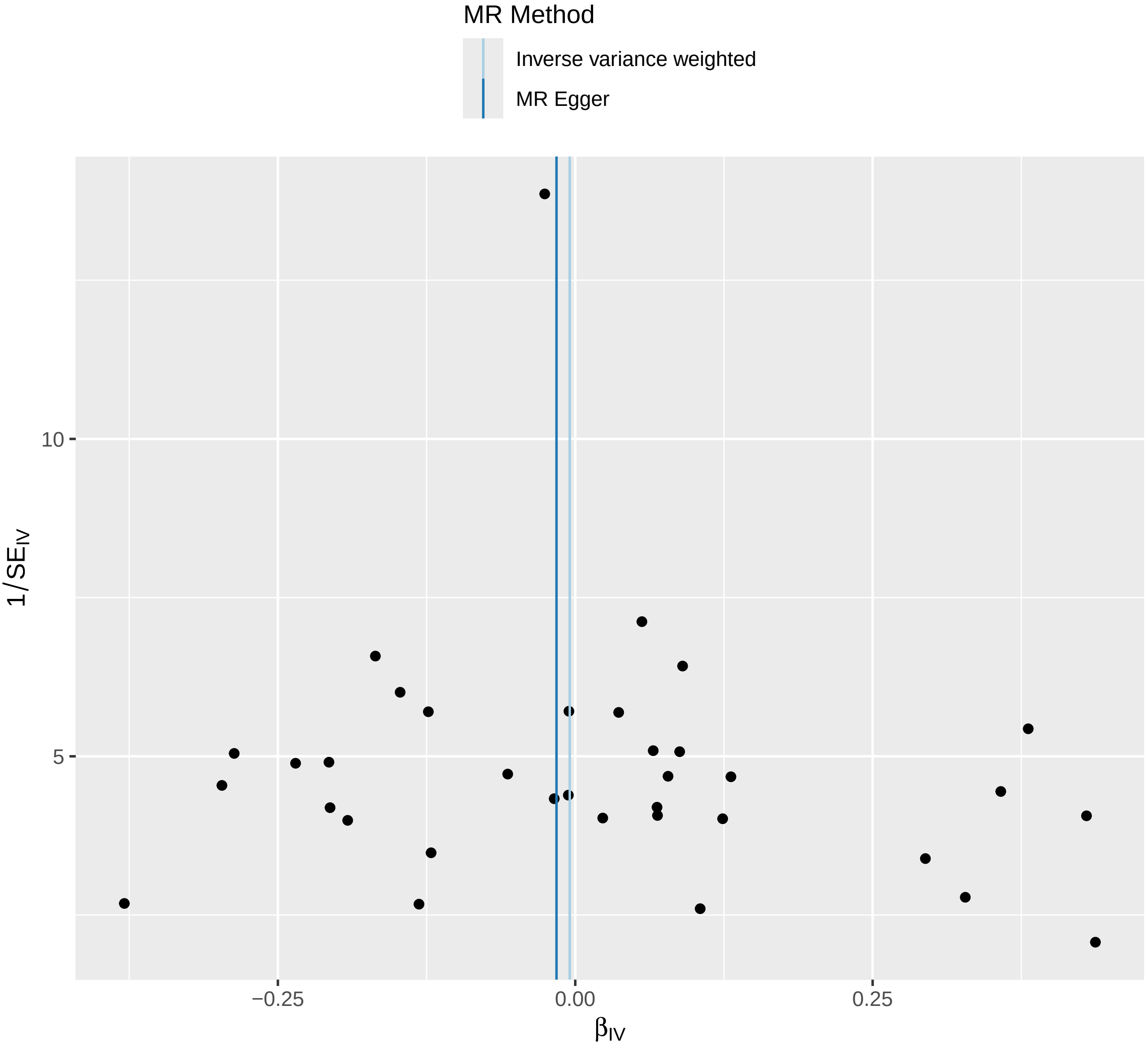
MR Method
Inverse variance weighted
MR Egger
10
5
1/SE_IV
−0.25
0.00
0.25
β_IV

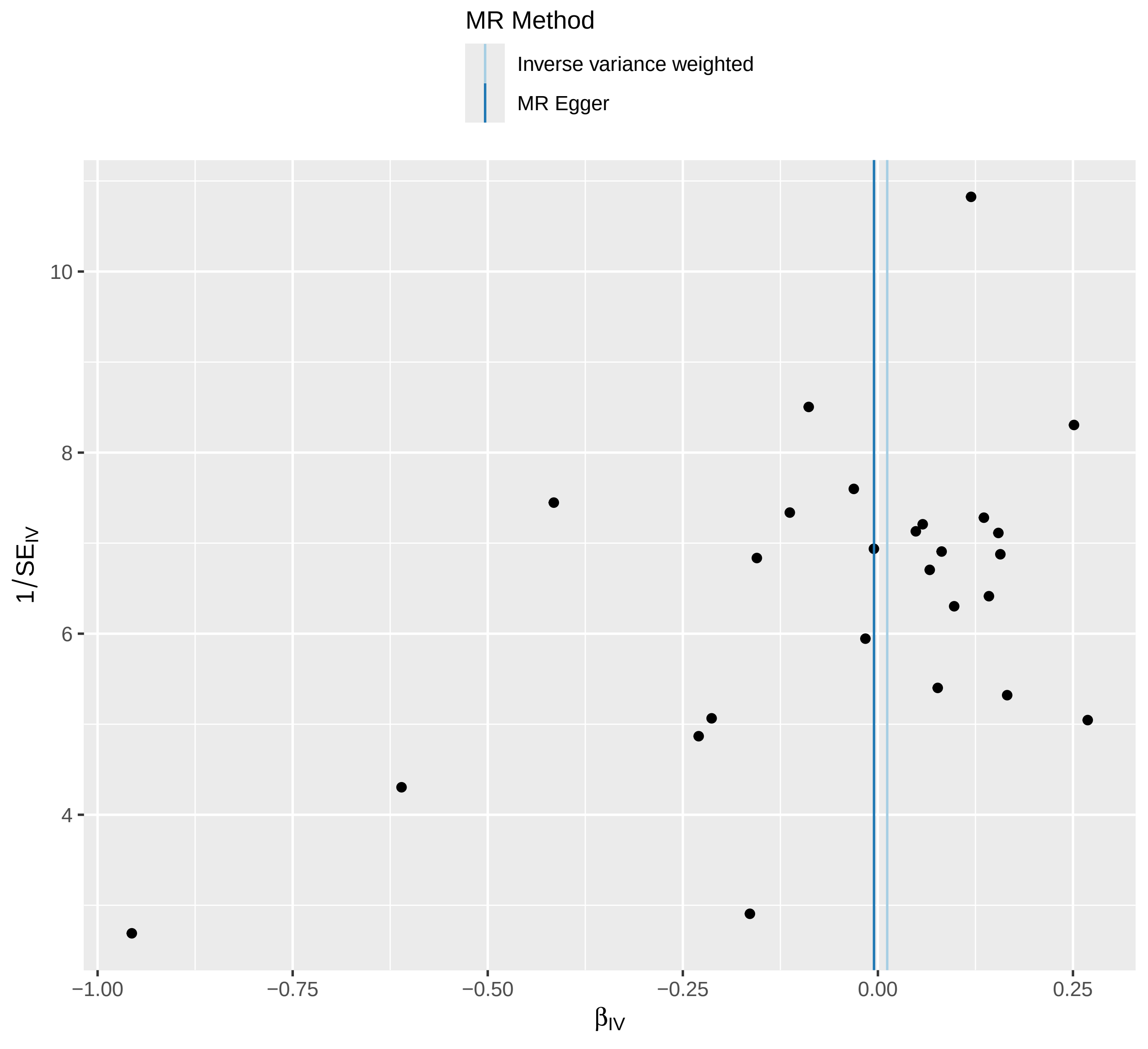
MR Method
Inverse variance weighted
MR Egger
10
8
6
4
1/SE_IV
−1.00
−0.75
−0.50
−0.25
0.00
0.25
β_IV

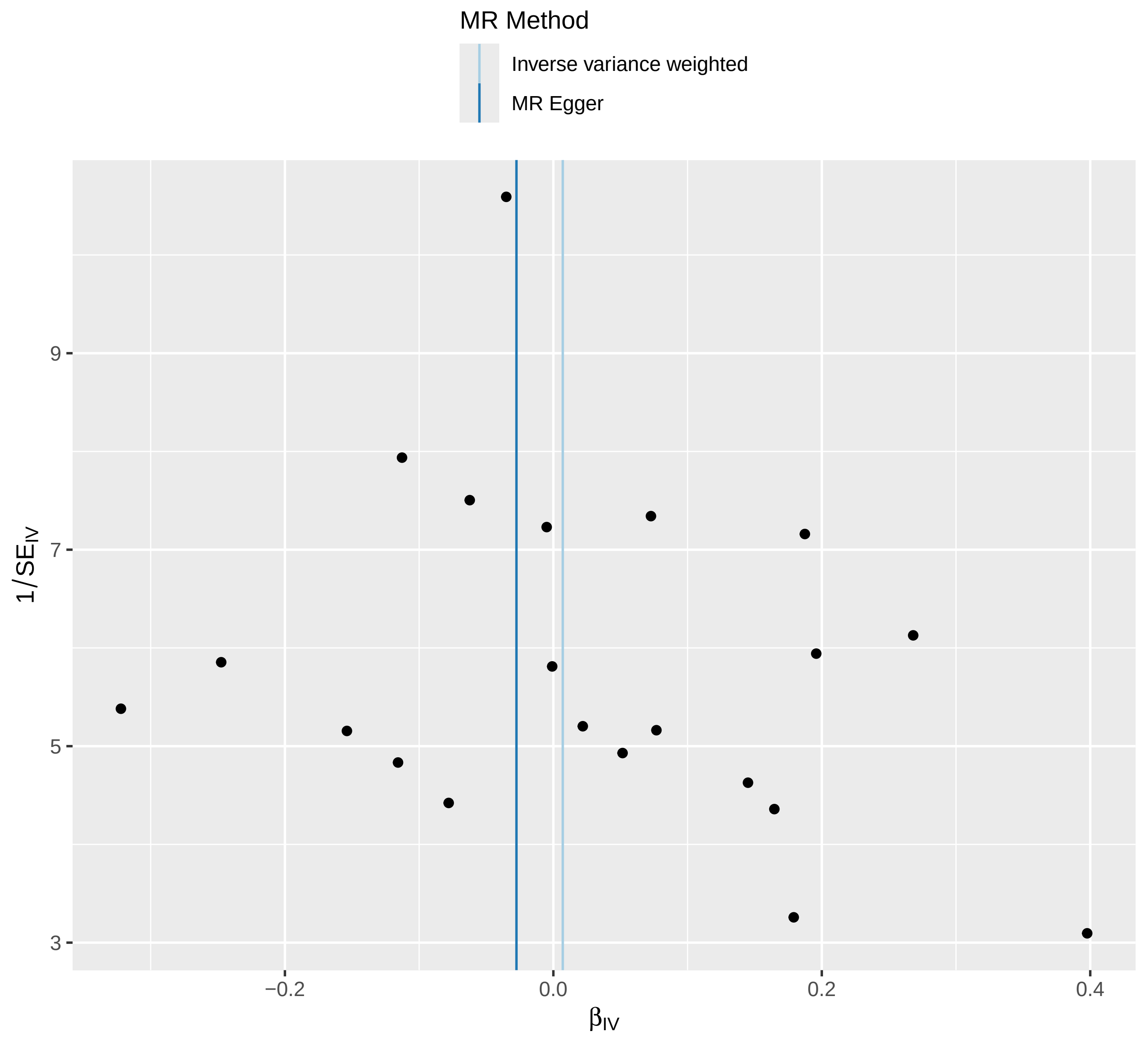

MR Method
Inverse variance weighted
MR Egger
9
7
5
3
1/SE$_{IV}$
−0.2
0.0
0.2
0.4
β$_{IV}$

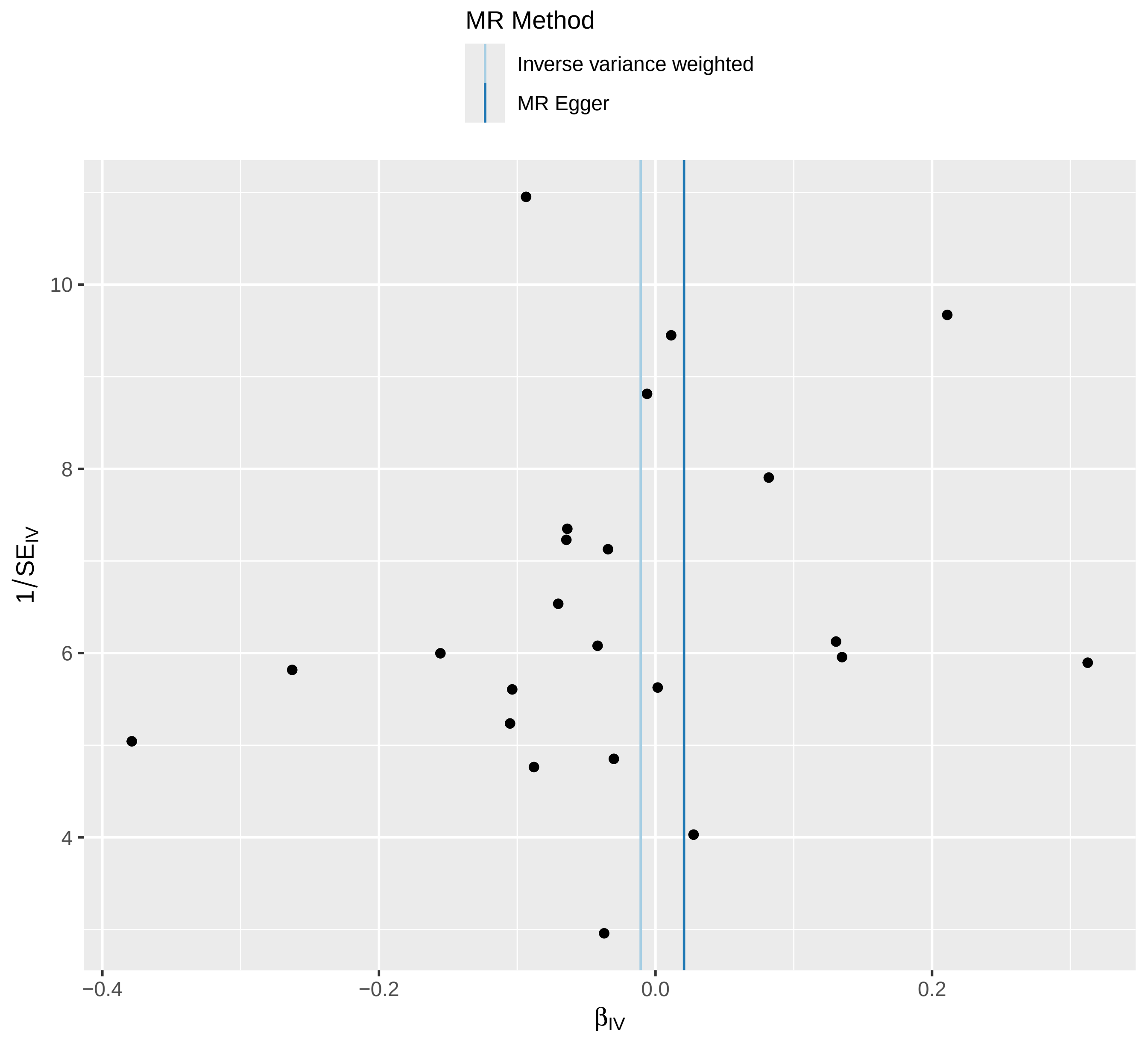
MR Method
Inverse variance weighted
MR Egger
10
8
6
4
$1/SE_{IV}$
−0.4
−0.2
0.0
0.2
β_{IV}

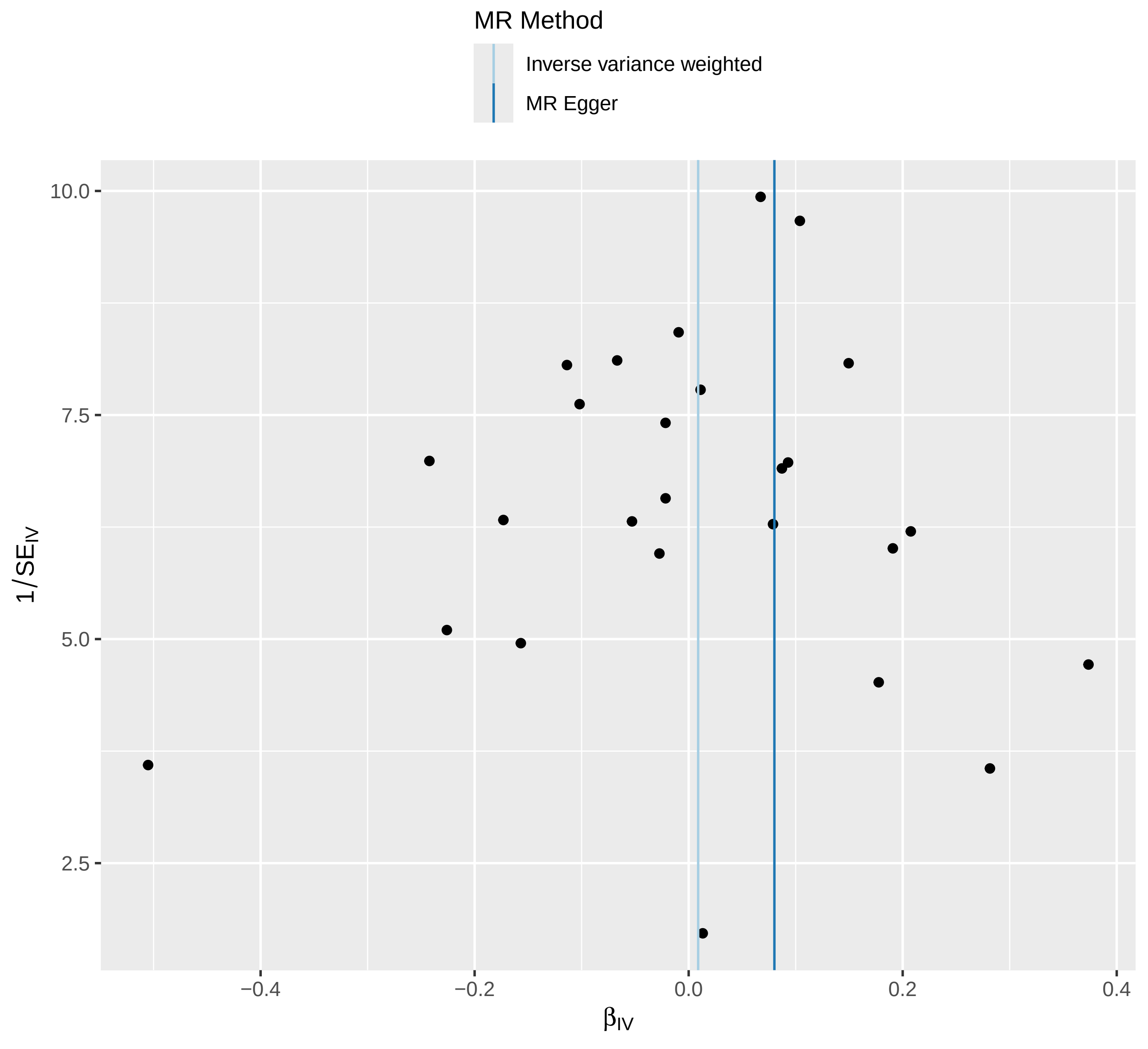
MR Method
Inverse variance weighted
MR Egger
10.0
7.5
5.0
2.5
1/SE$_{IV}$
−0.4
−0.2
0.0
0.2
0.4
β$_{IV}$

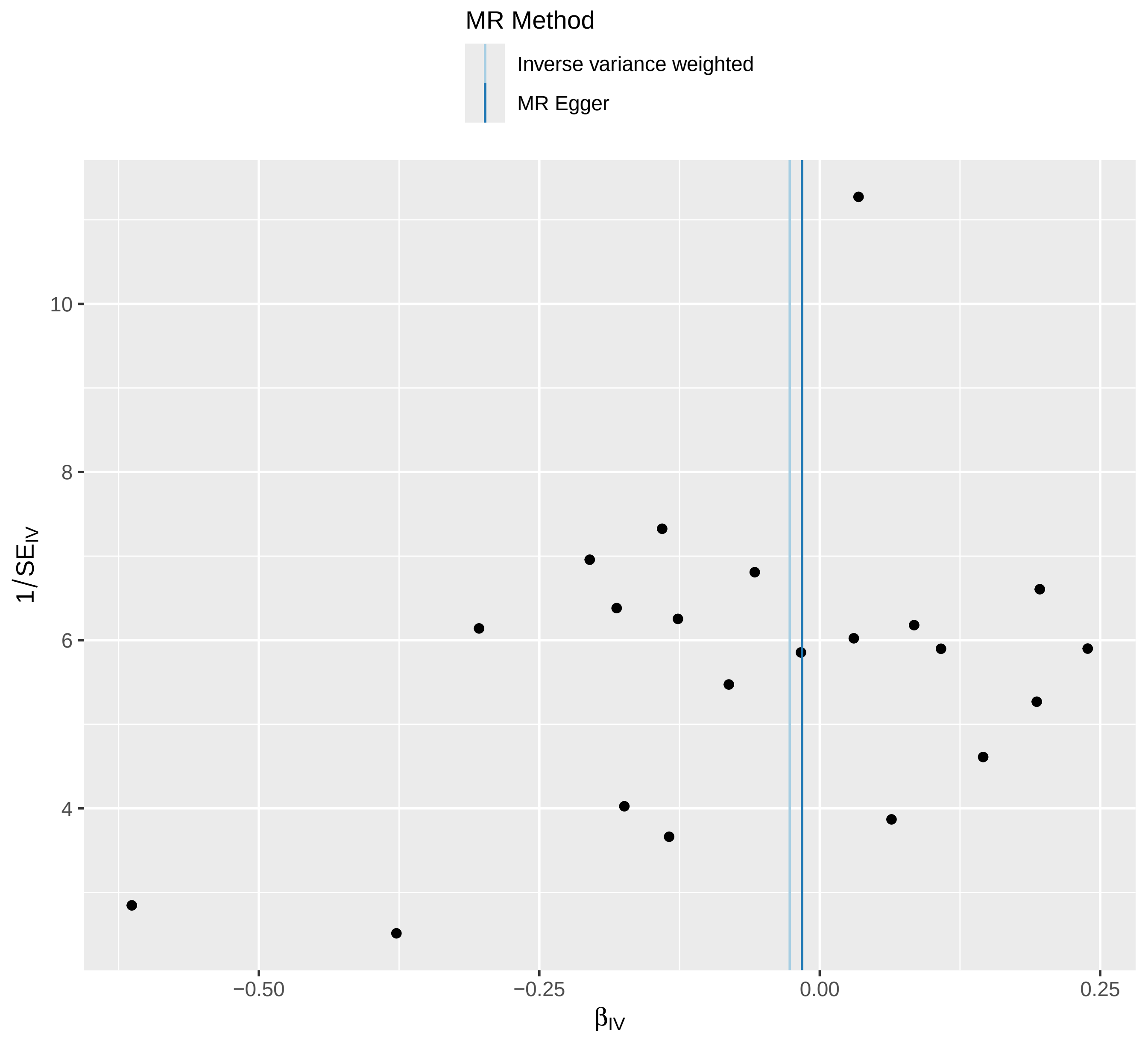
MR Method
Inverse variance weighted
MR Egger
10
8
6
4
1/SE_IV
−0.50
−0.25
0.00
0.25
β_IV

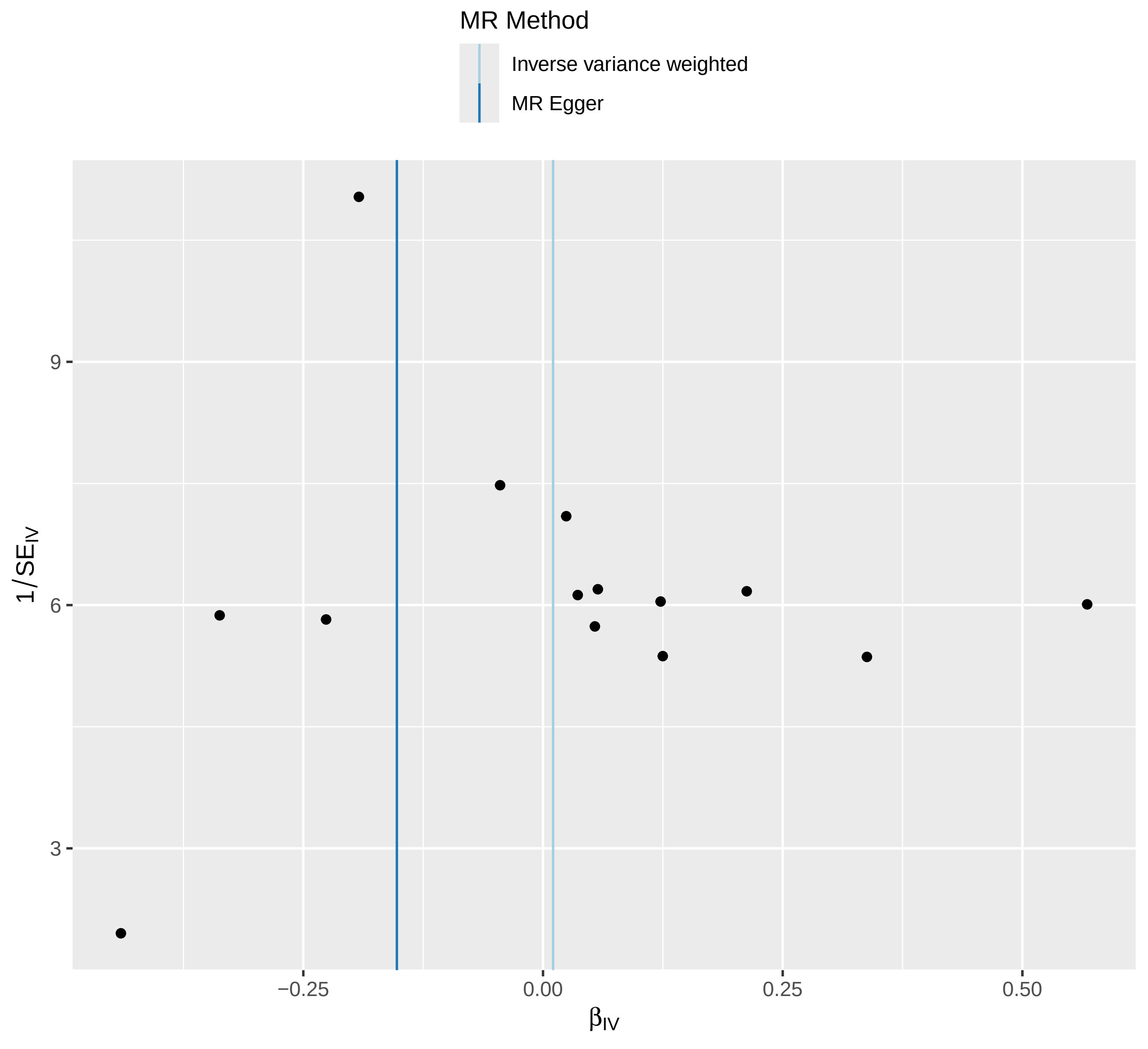
MR Method
Inverse variance weighted
MR Egger
9
6
3
$1/SE_{IV}$
−0.25
0.00
0.25
0.50
β_{IV}

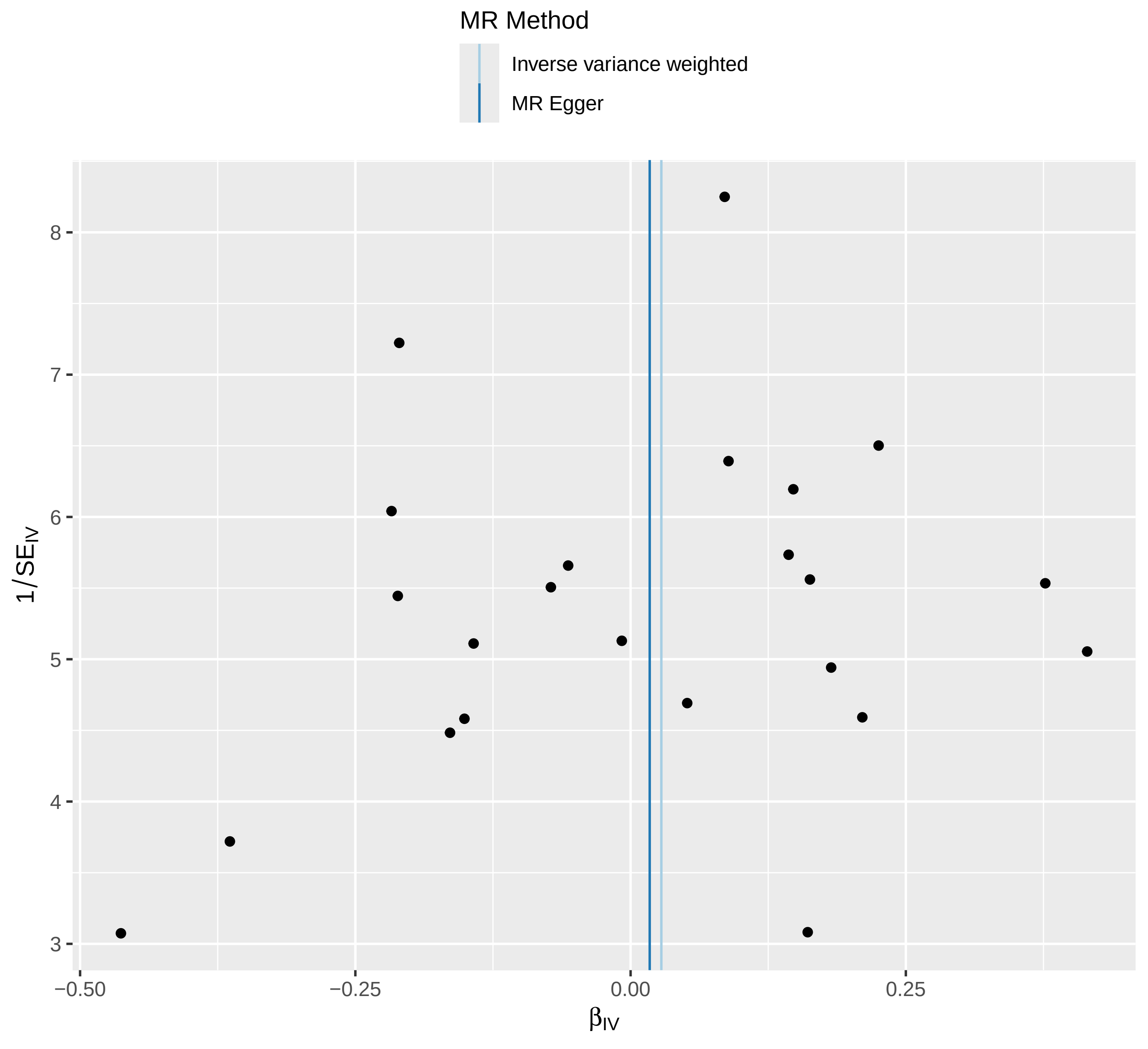
MR Method
Inverse variance weighted
MR Egger
$1/SE_{IV}$
8
7
6
5
4
3
−0.50
−0.25
0.00
0.25
β_{IV}

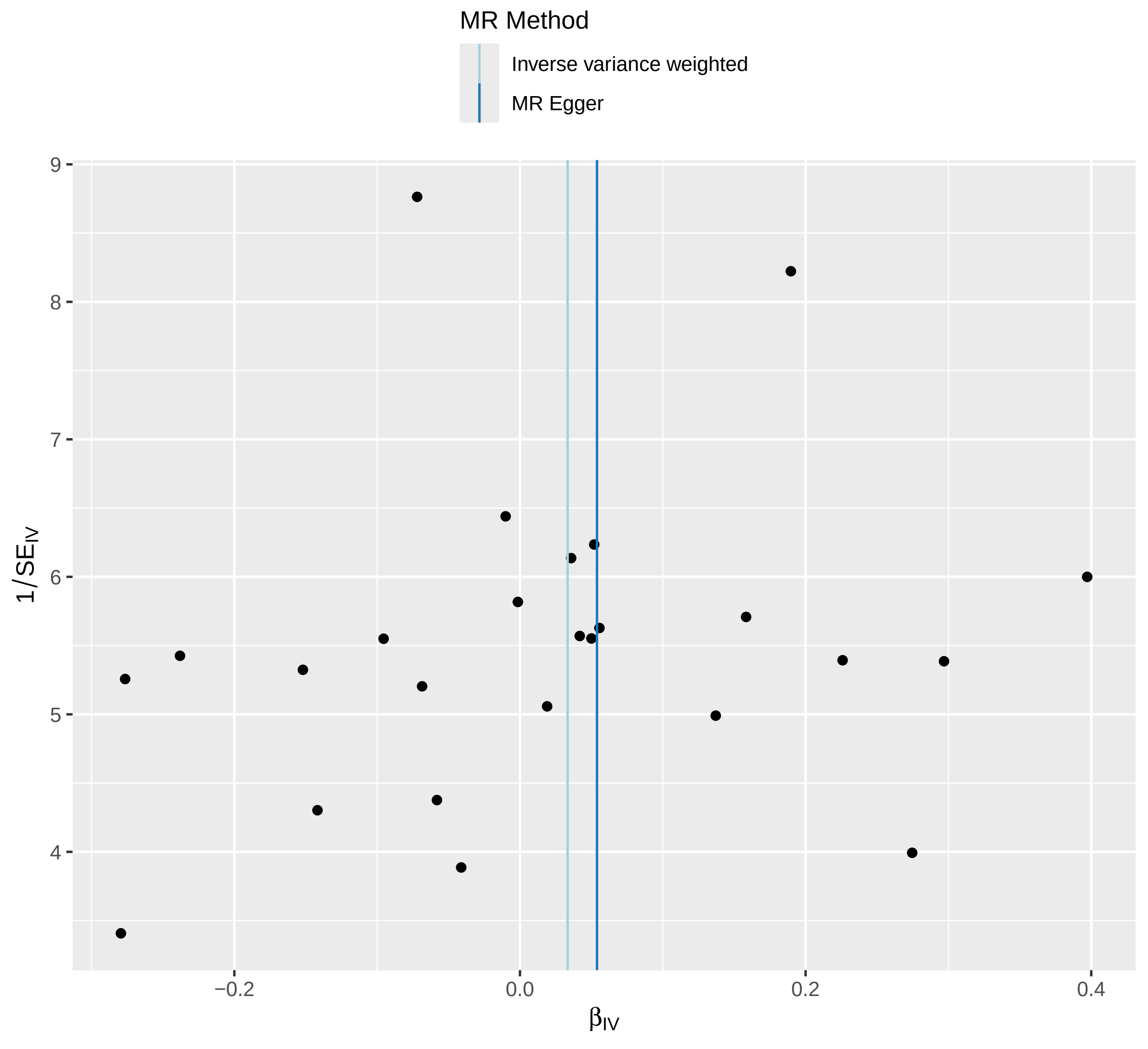

MR Method
Inverse variance weighted
MR Egger
9
8
7
6
5
4
1/SE_IV
−0.2
0.0
0.2
0.4
β_IV

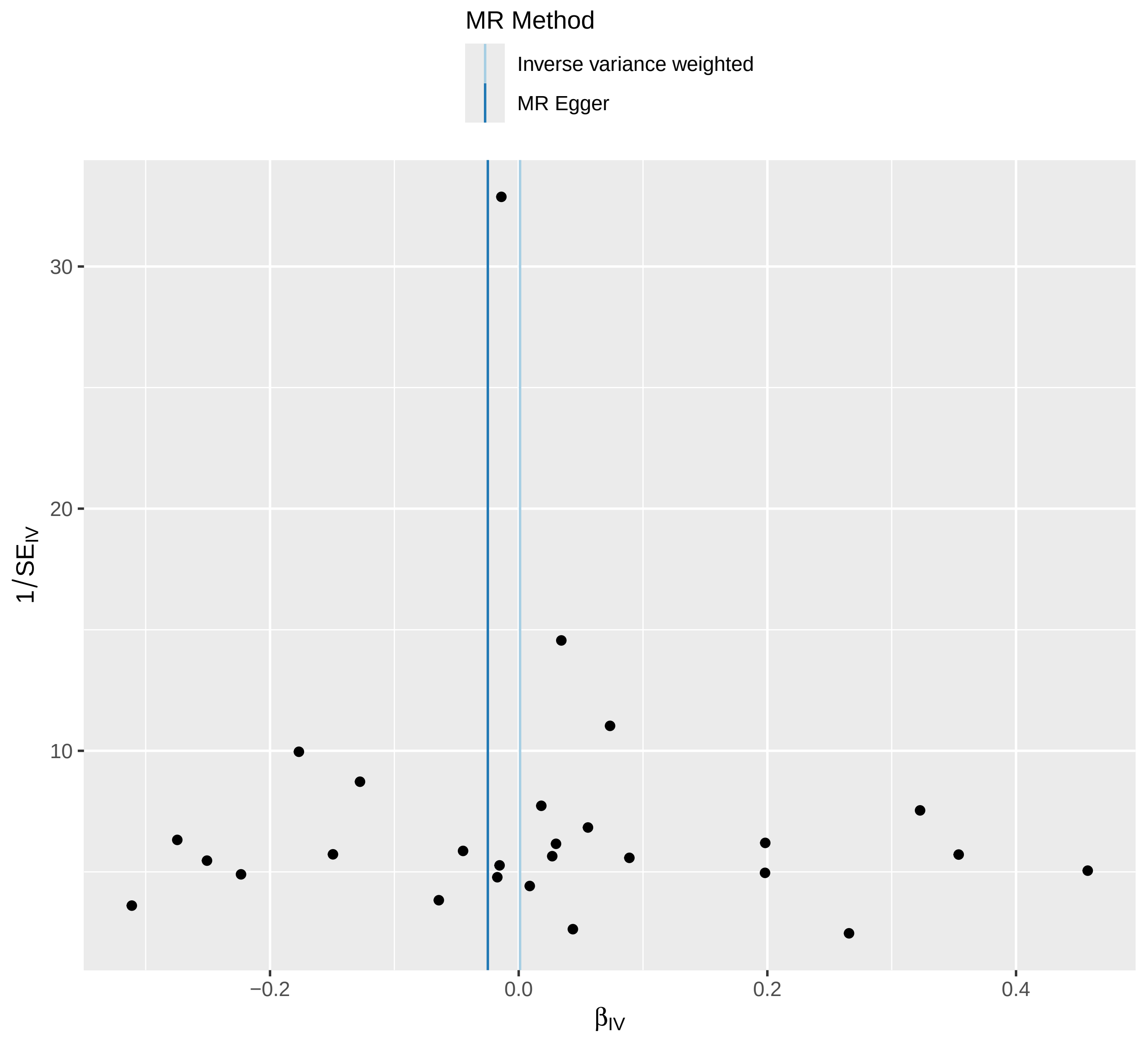
MR Method
Inverse variance weighted
MR Egger
30
20
10
1/SE_IV
−0.2
0.0
0.2
0.4
β_IV

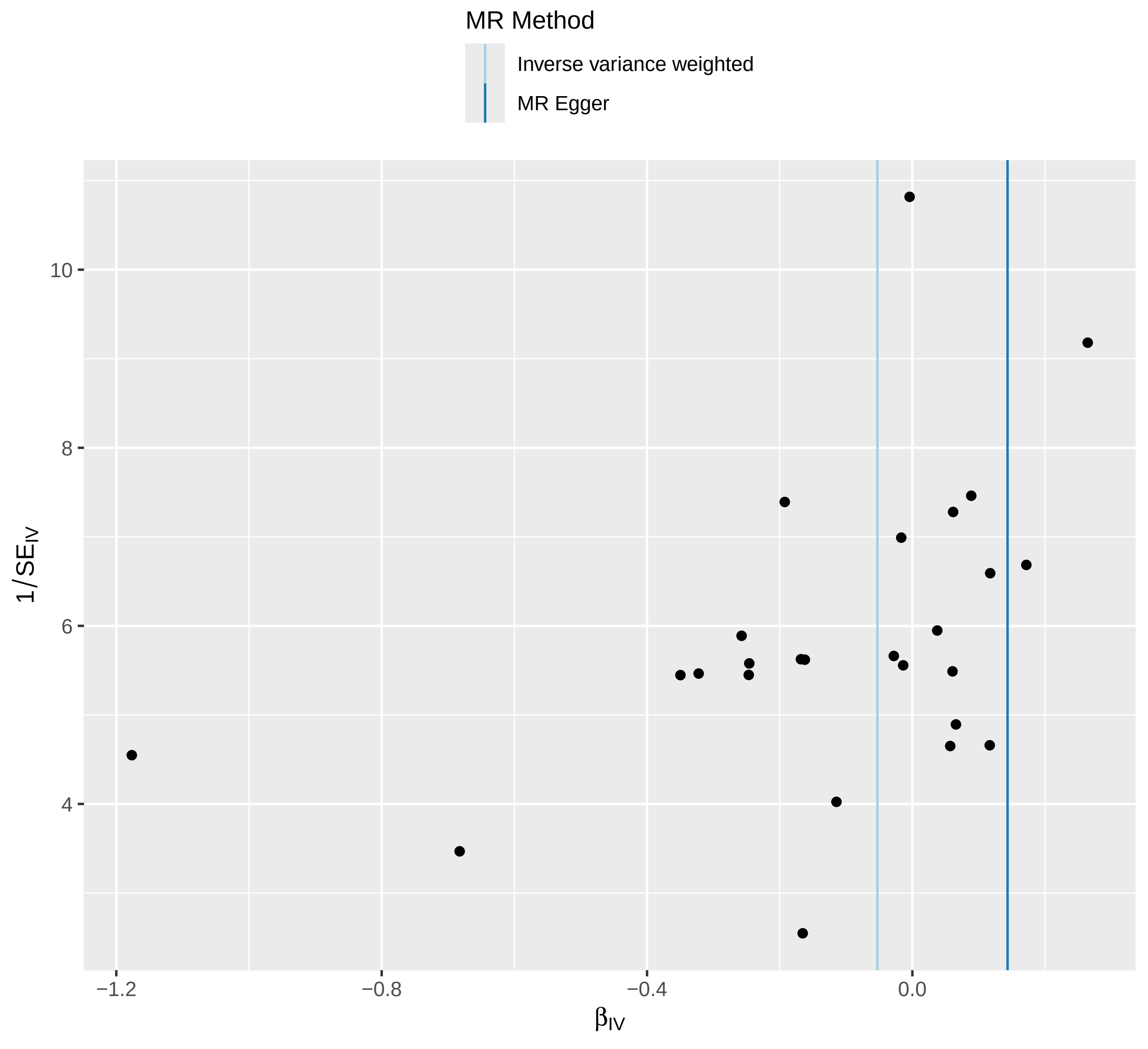

MR Method
Inverse variance weighted
MR Egger
10
8
6
4
1/SE_IV
−1.2
−0.8
−0.4
0.0
β_IV

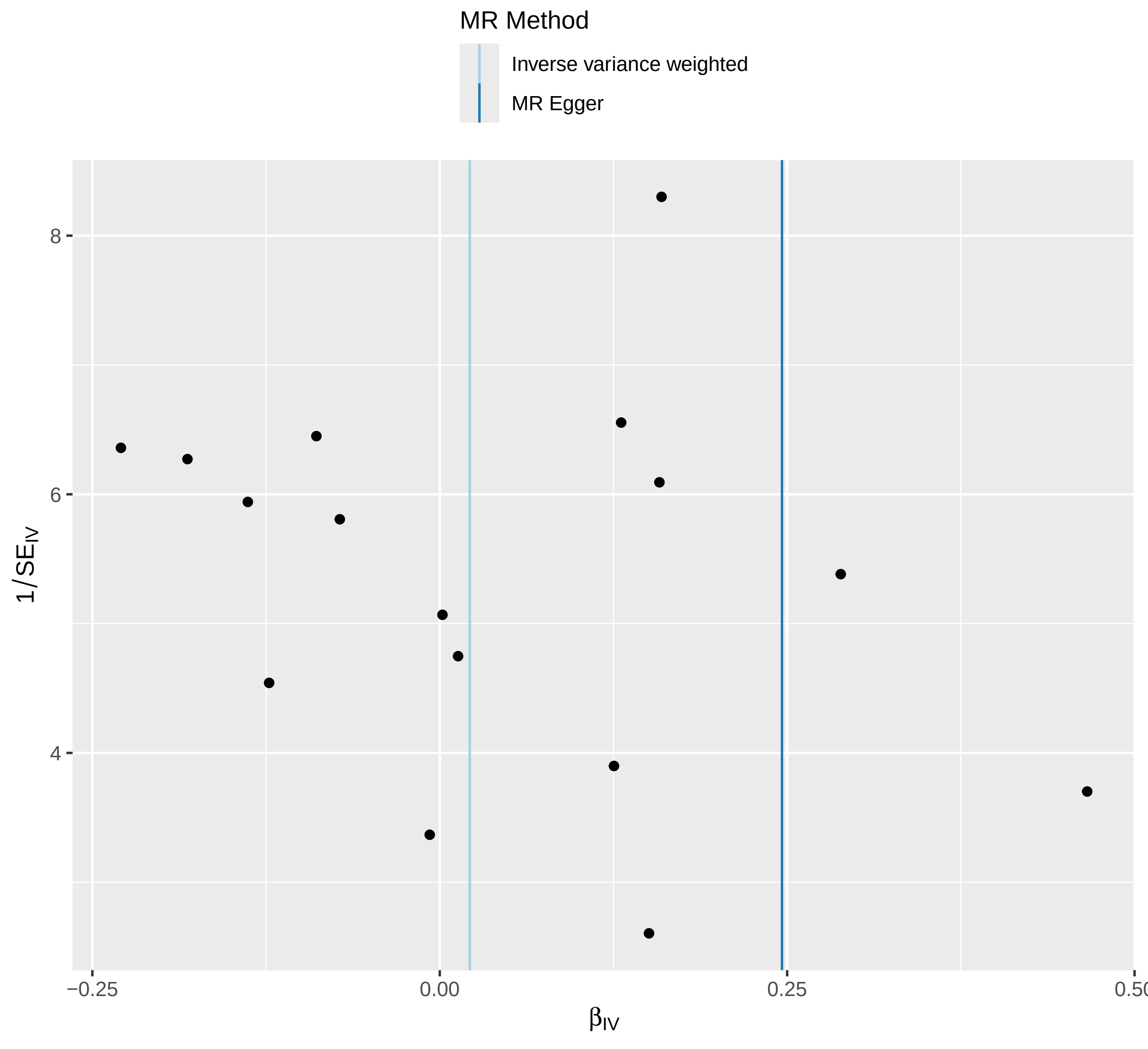
MR Method
Inverse variance weighted
MR Egger
8
6
4
1/SE$_{IV}$
−0.25
0.00
0.25
0.50
β$_{IV}$

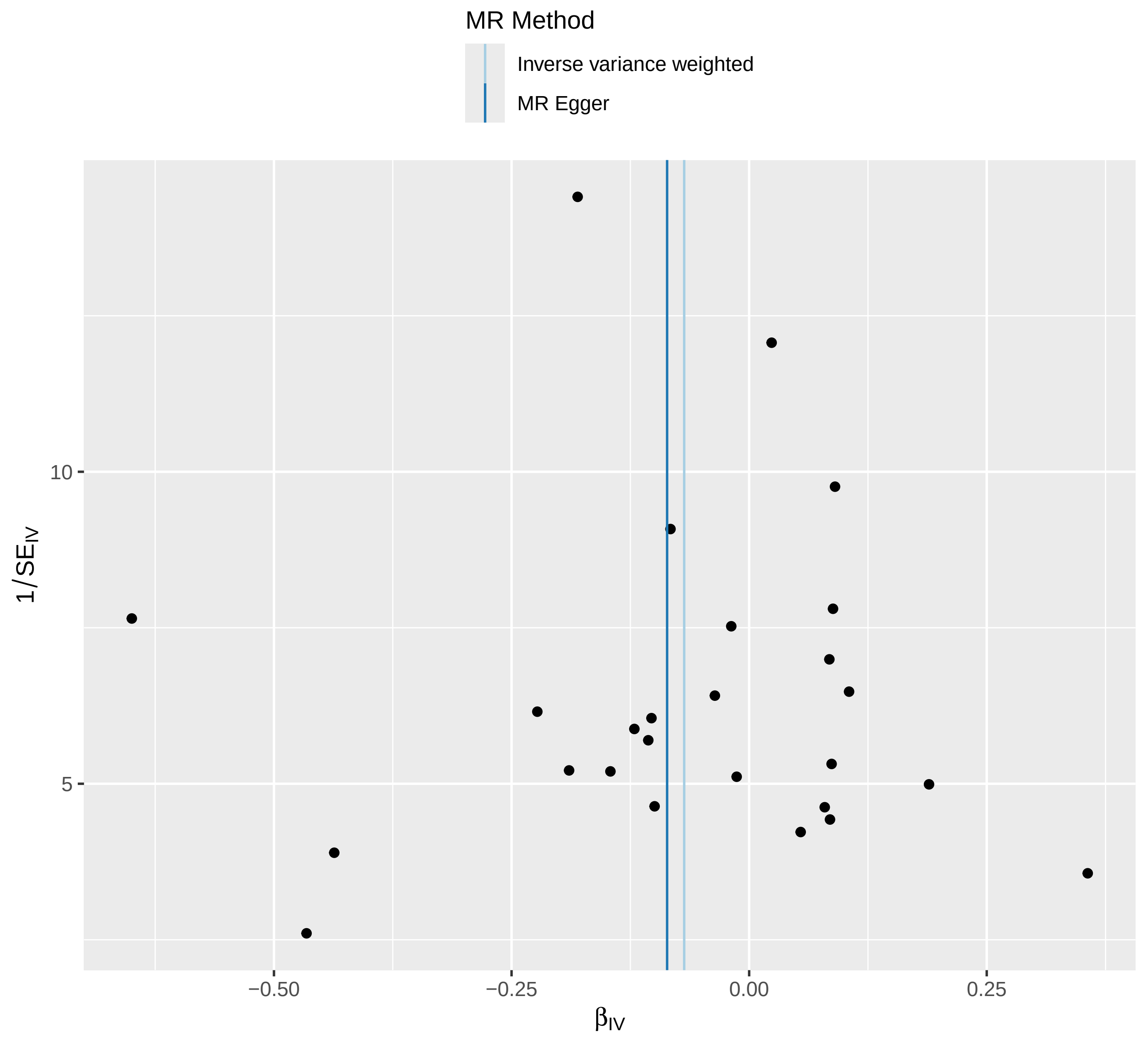

MR Method
Inverse variance weighted
MR Egger
10
5
1/SE$_{IV}$
−0.50
−0.25
0.00
0.25
β$_{IV}$

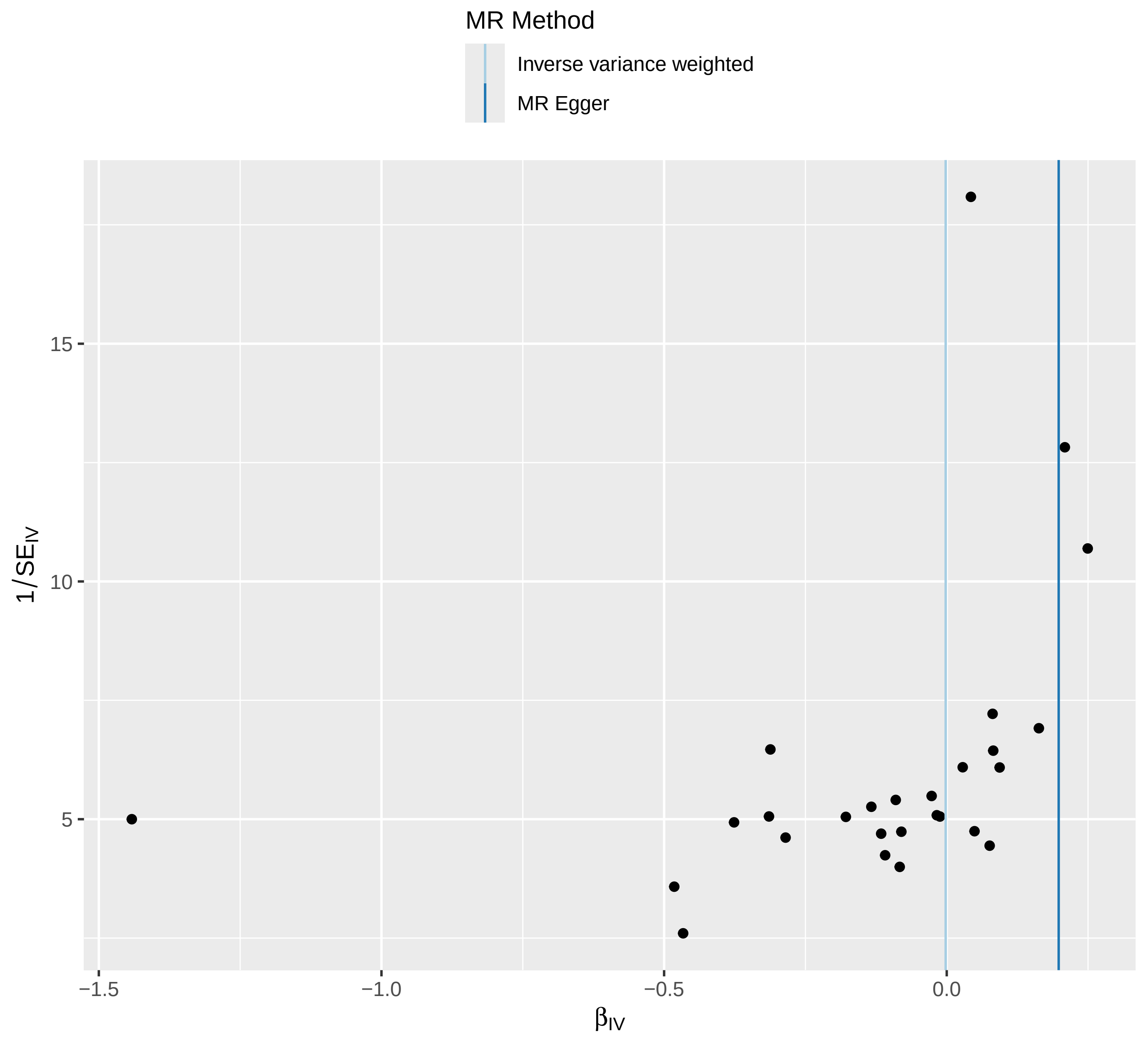
MR Method
Inverse variance weighted
MR Egger
15
10
5
1/SE_IV
−1.5
−1.0
−0.5
0.0
β_IV

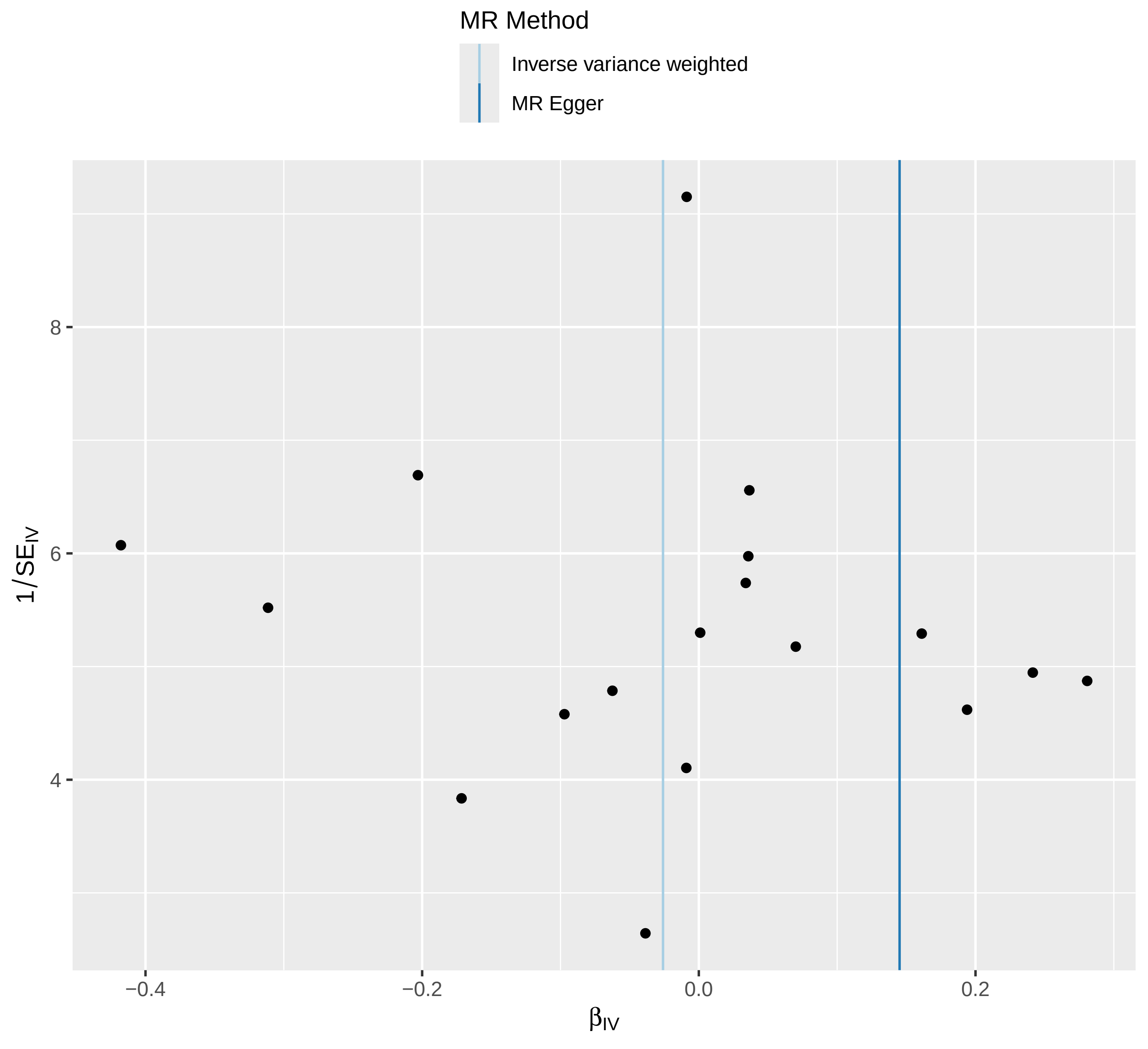
MR Method
Inverse variance weighted
MR Egger
8
6
4
1/SE$_{IV}$
−0.4
−0.2
0.0
0.2
β$_{IV}$

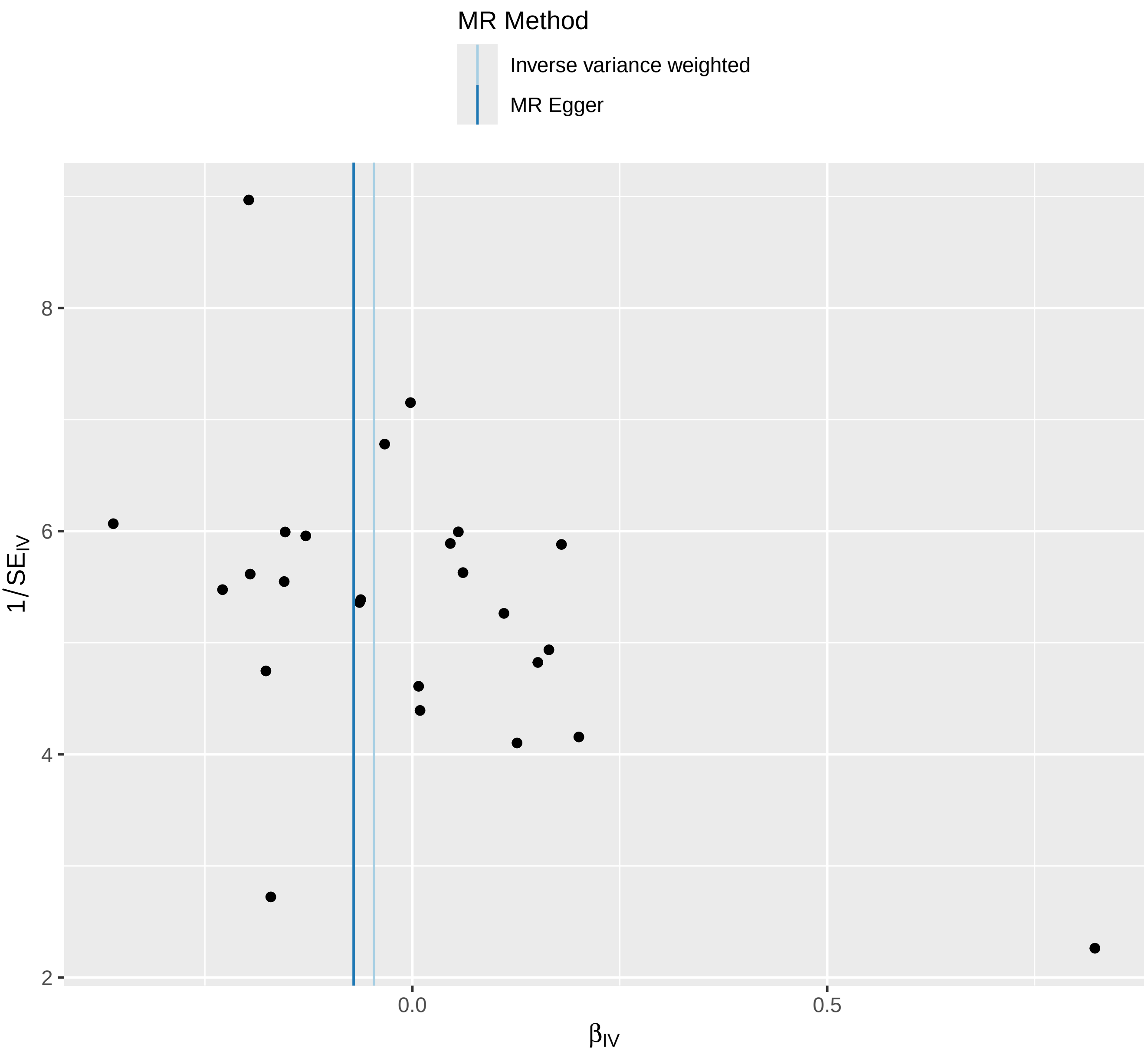
MR Method
Inverse variance weighted
MR Egger
8
6
4
2
1/SE$_{IV}$
0.0
0.5
β$_{IV}$

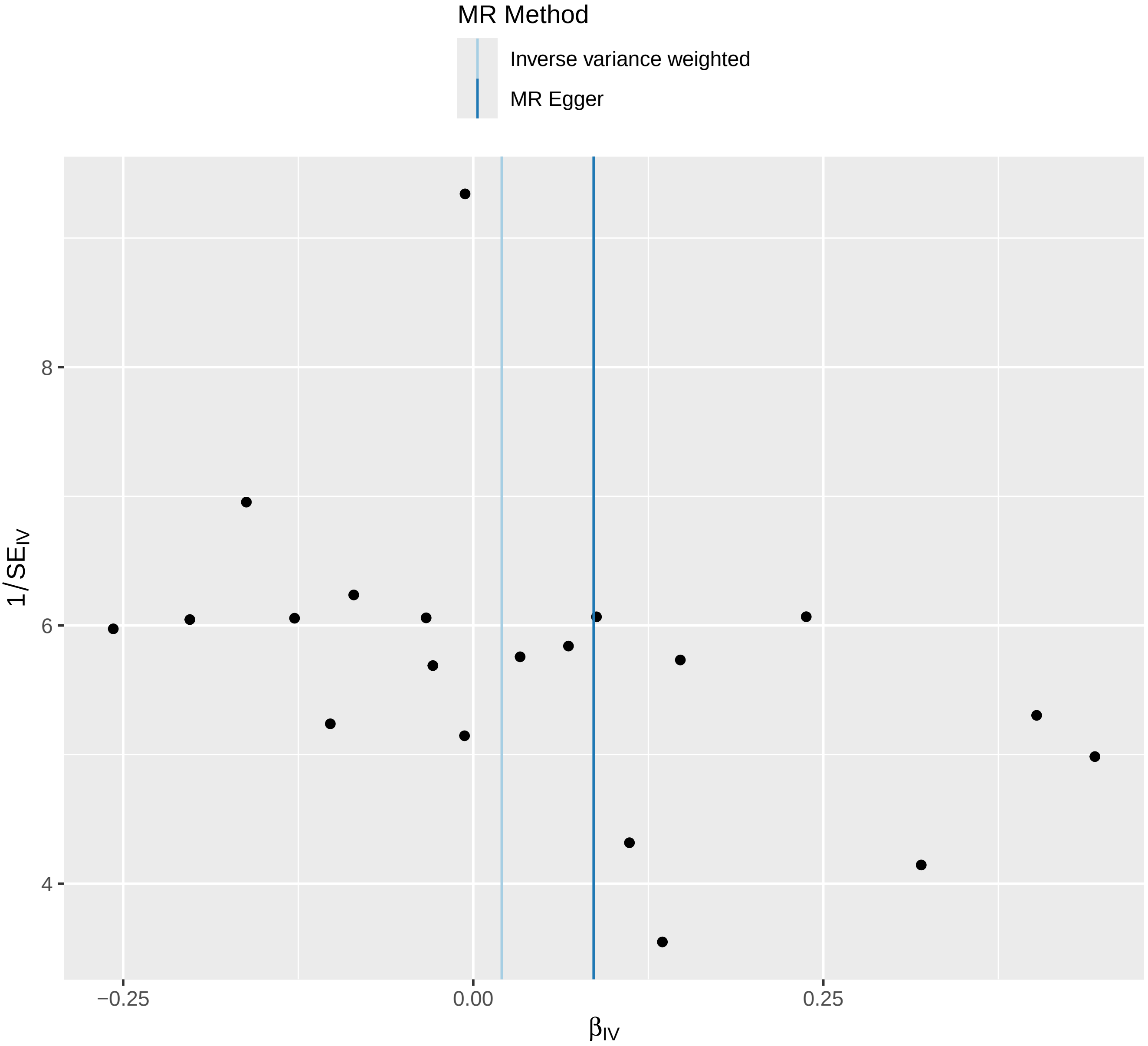

MR Method
Inverse variance weighted
MR Egger
8
6
4
1/SE_IV
−0.25
0.00
0.25
β_IV

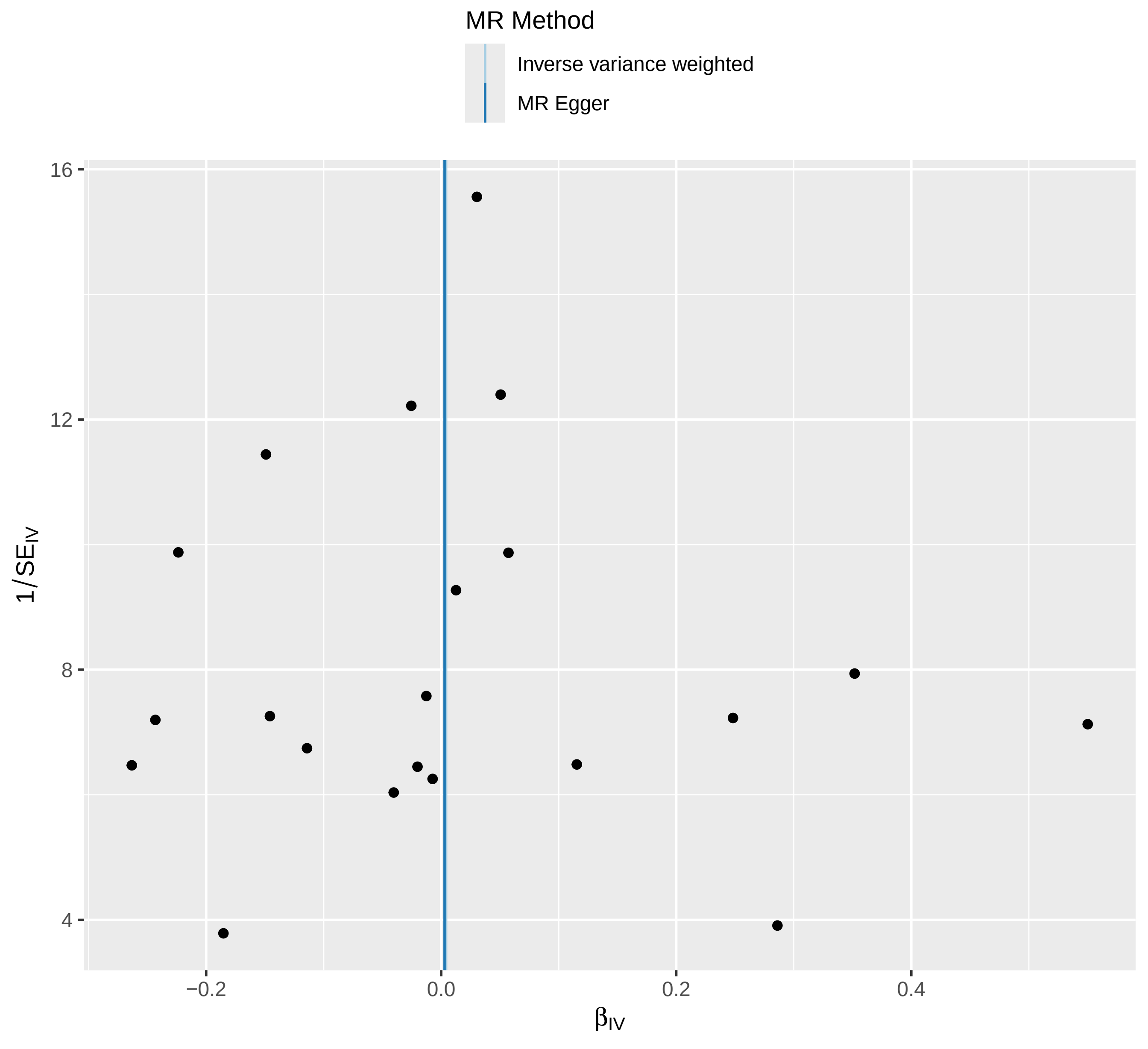
MR Method
Inverse variance weighted
MR Egger
16
12
8
4
1/SE$_{IV}$
−0.2
0.0
0.2
0.4
β$_{IV}$

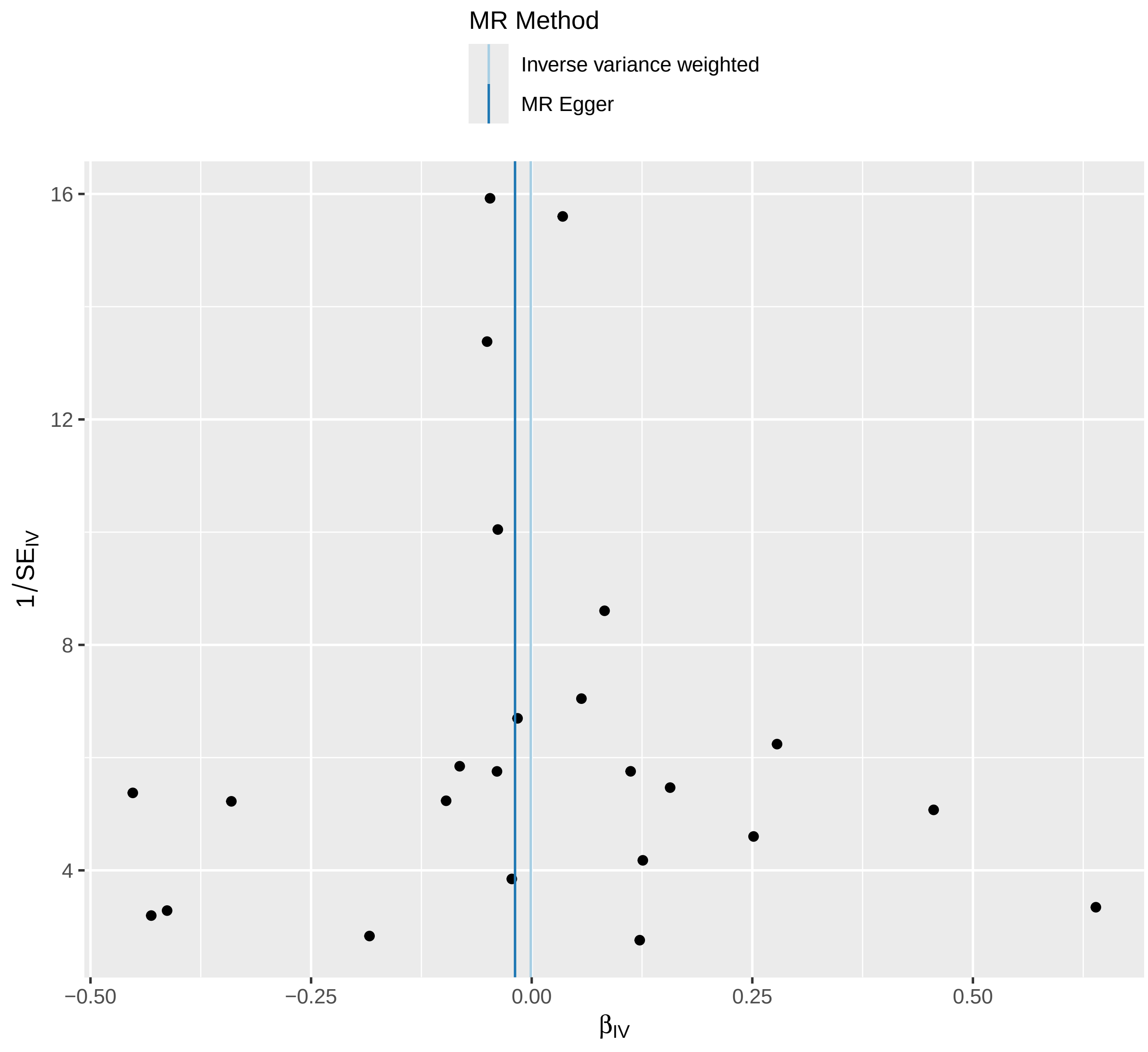
MR Method
Inverse variance weighted
MR Egger
16
12
8
4
1/SE$_{IV}$
−0.50
−0.25
0.00
0.25
0.50
β$_{IV}$

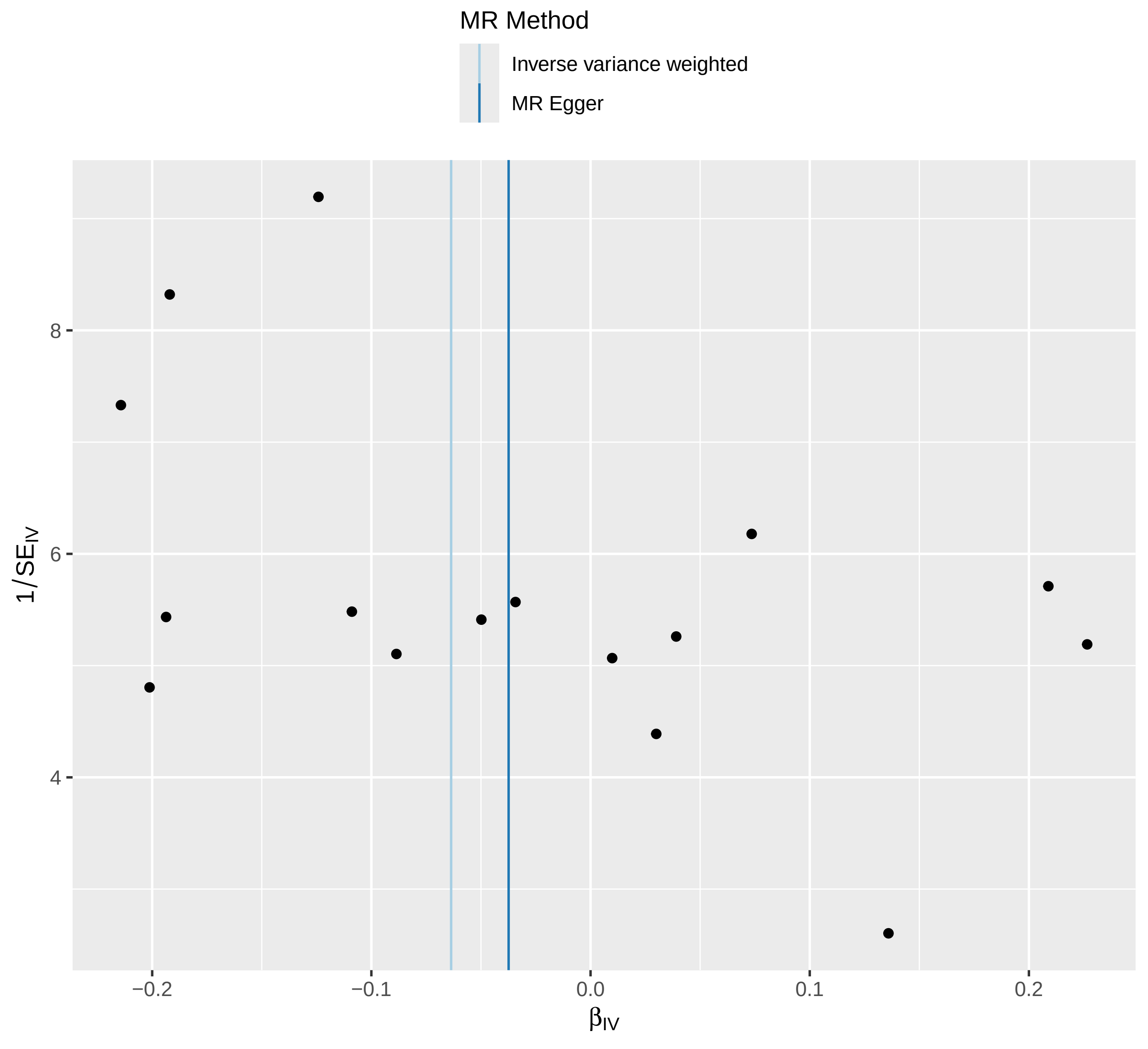
MR Method
Inverse variance weighted
MR Egger
8
6
4
1/SE_IV
−0.2
−0.1
0.0
0.1
0.2
β_IV

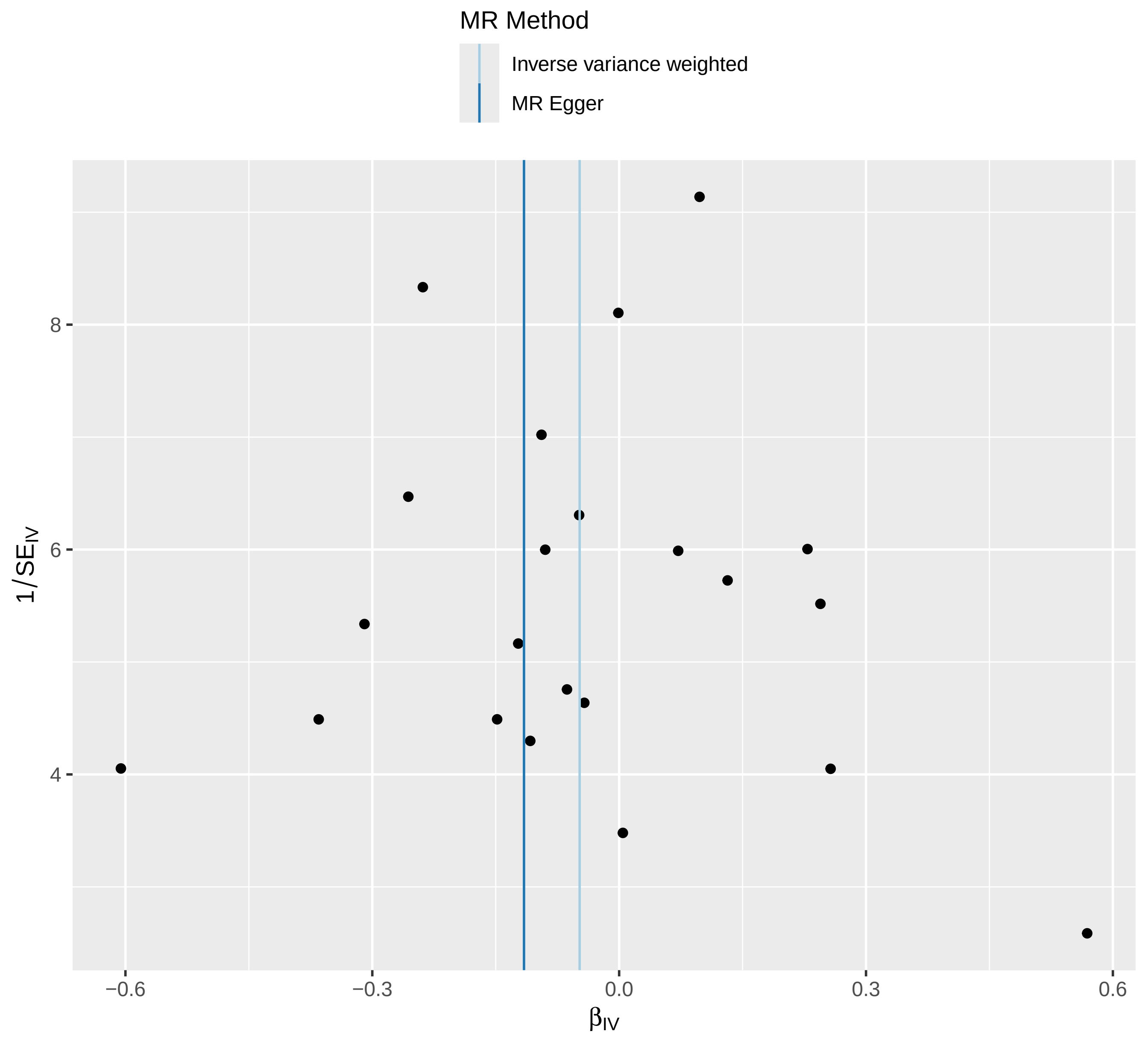
MR Method
Inverse variance weighted
MR Egger
8
6
4
1/SE_IV
−0.6
−0.3
0.0
0.3
0.6
β_IV

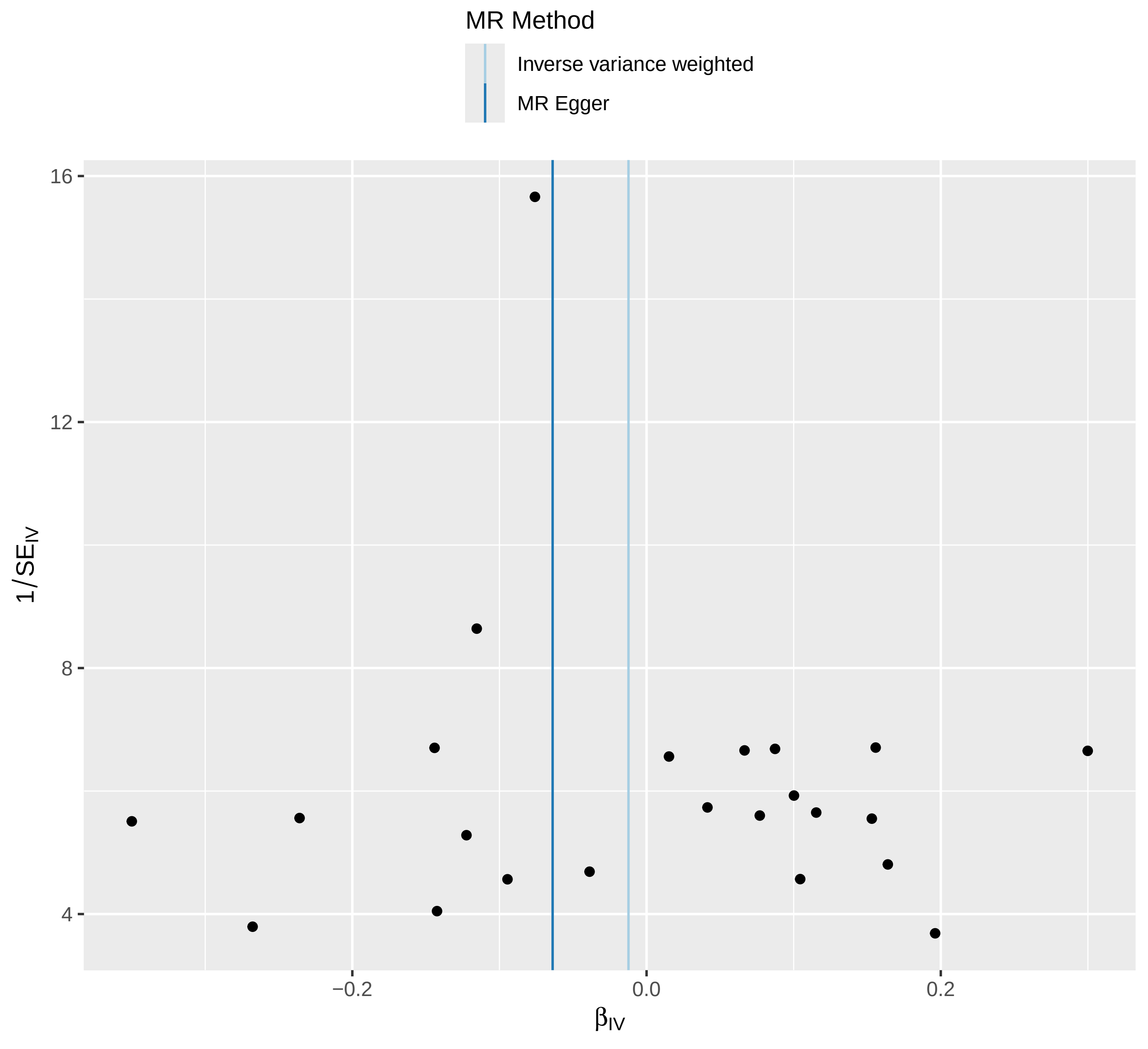

MR Method
Inverse variance weighted
MR Egger
16
12
8
4
−0.2
0.0
0.2
$1/SE_{IV}$
β_{IV}

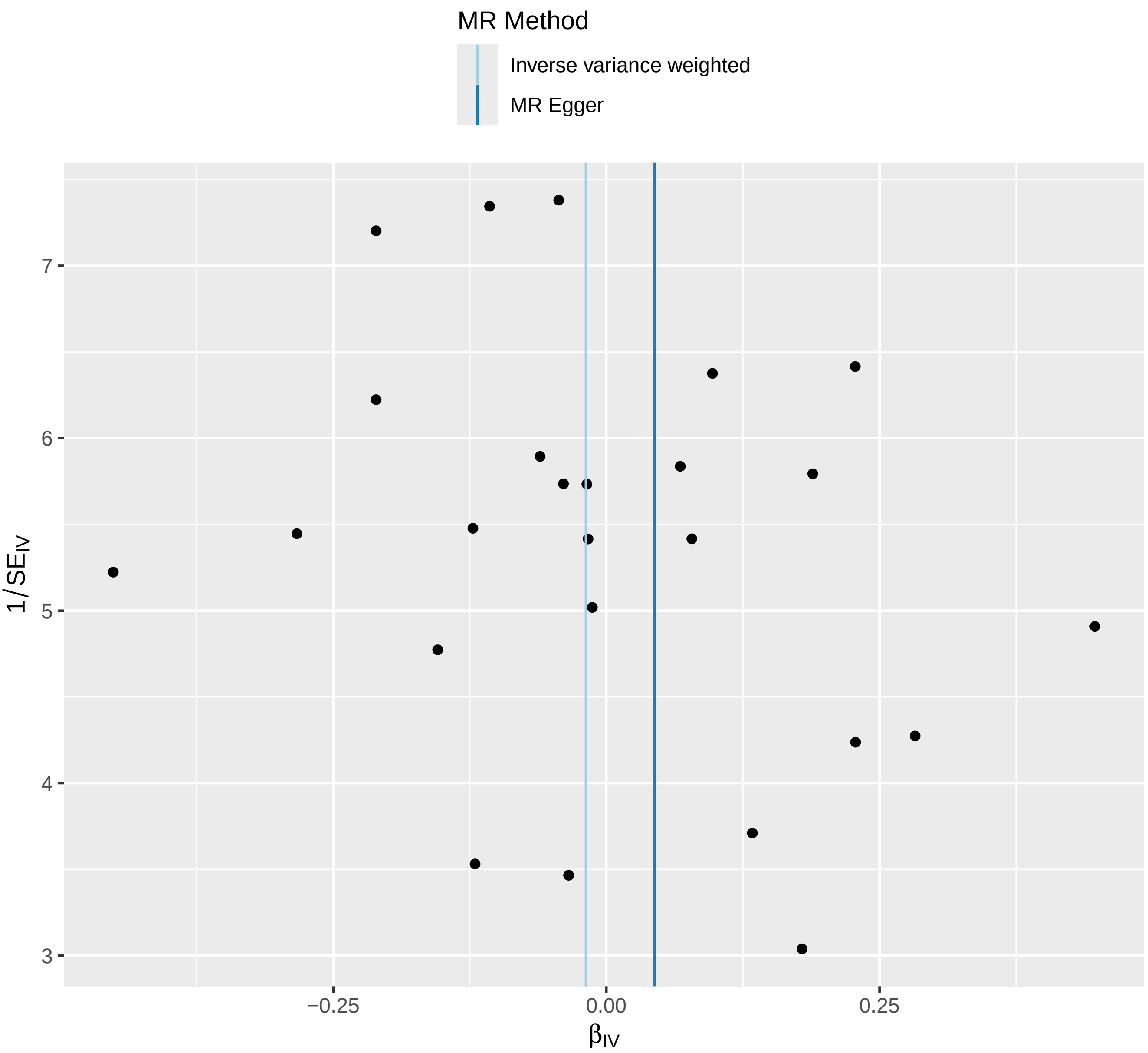
MR Method
Inverse variance weighted
MR Egger
7
6
5
4
3
1/SE_IV
−0.25
0.00
0.25
β_IV

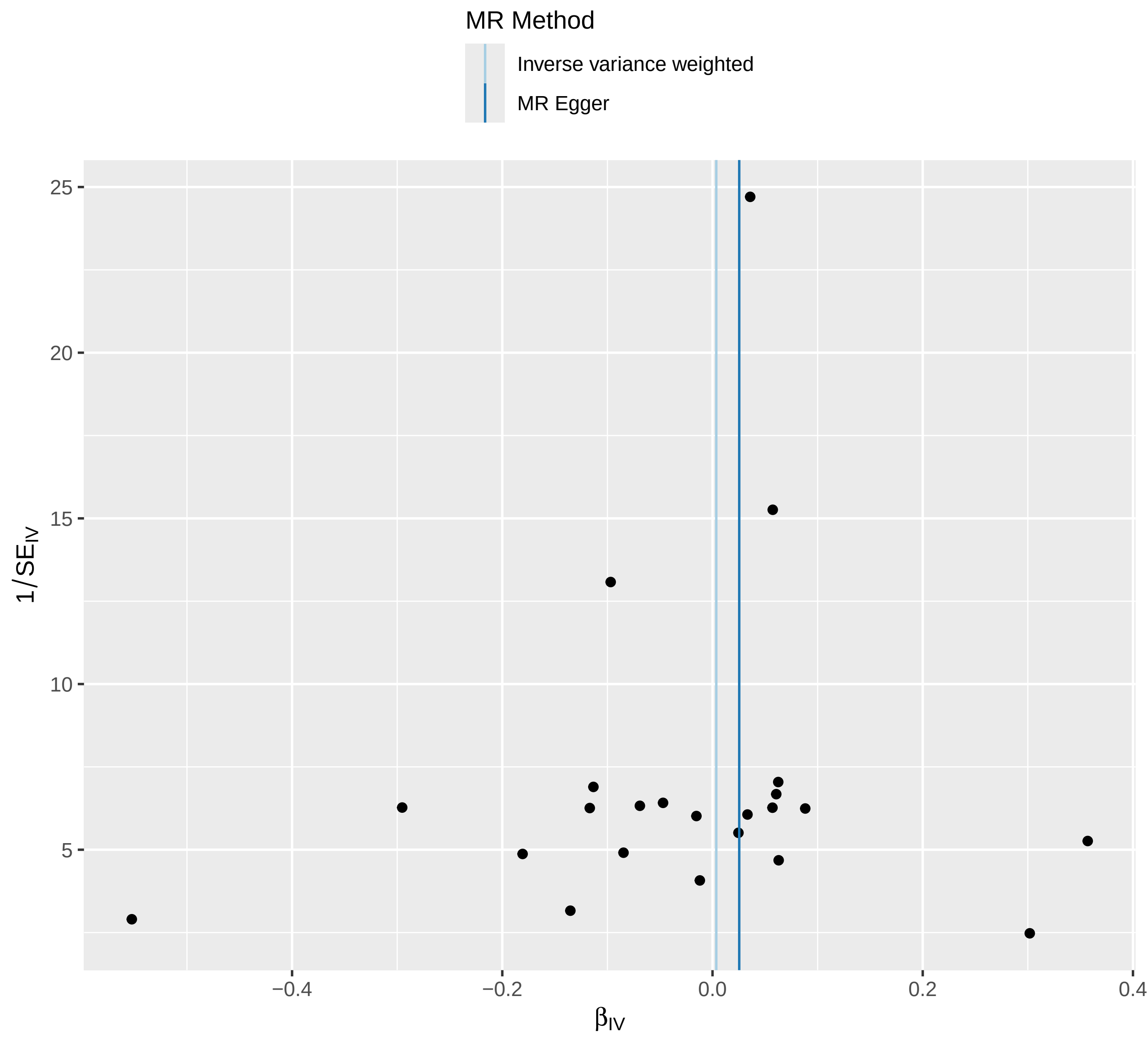
MR Method
Inverse variance weighted
MR Egger
25
20
15
10
5
1/SE_IV
−0.4
−0.2
0.0
0.2
0.4
β_IV

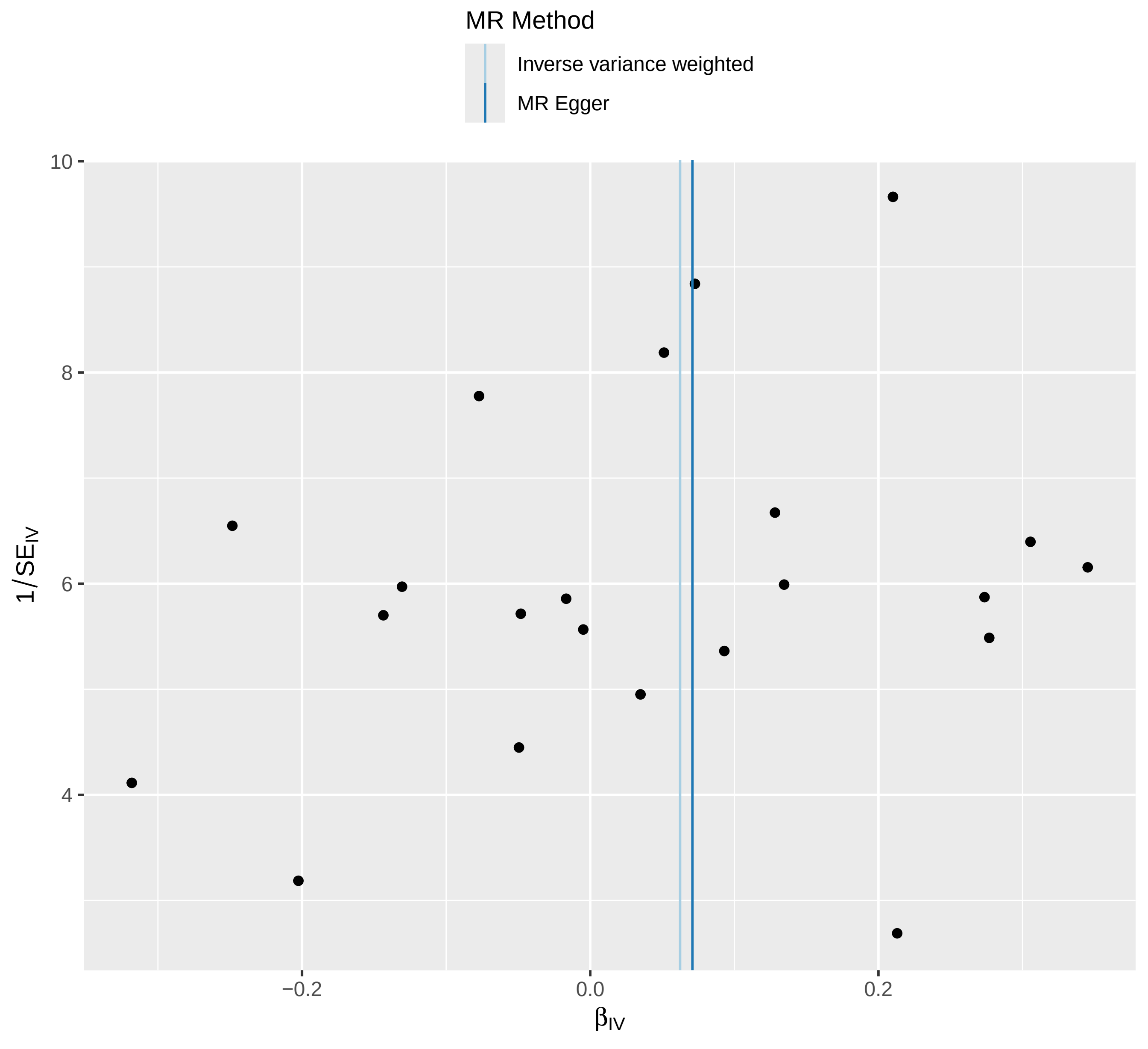

MR Method
Inverse variance weighted
MR Egger
10
8
6
4
$1/SE_{IV}$
−0.2
0.0
0.2
β_{IV}

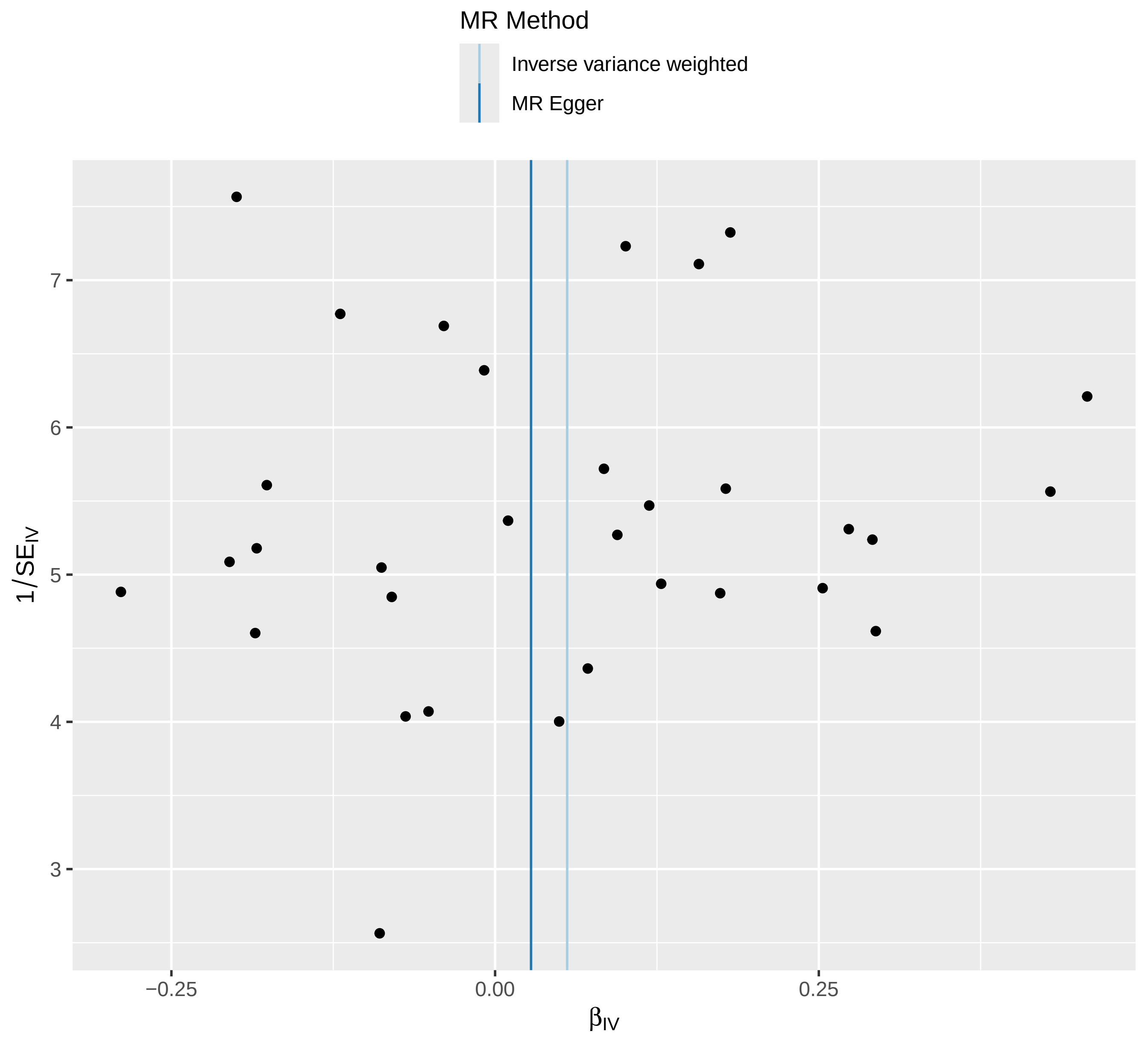

MR Method
Inverse variance weighted
MR Egger
1/SE$_{IV}$
β$_{IV}$
−0.25
0.00
0.25
3
4
5
6
7

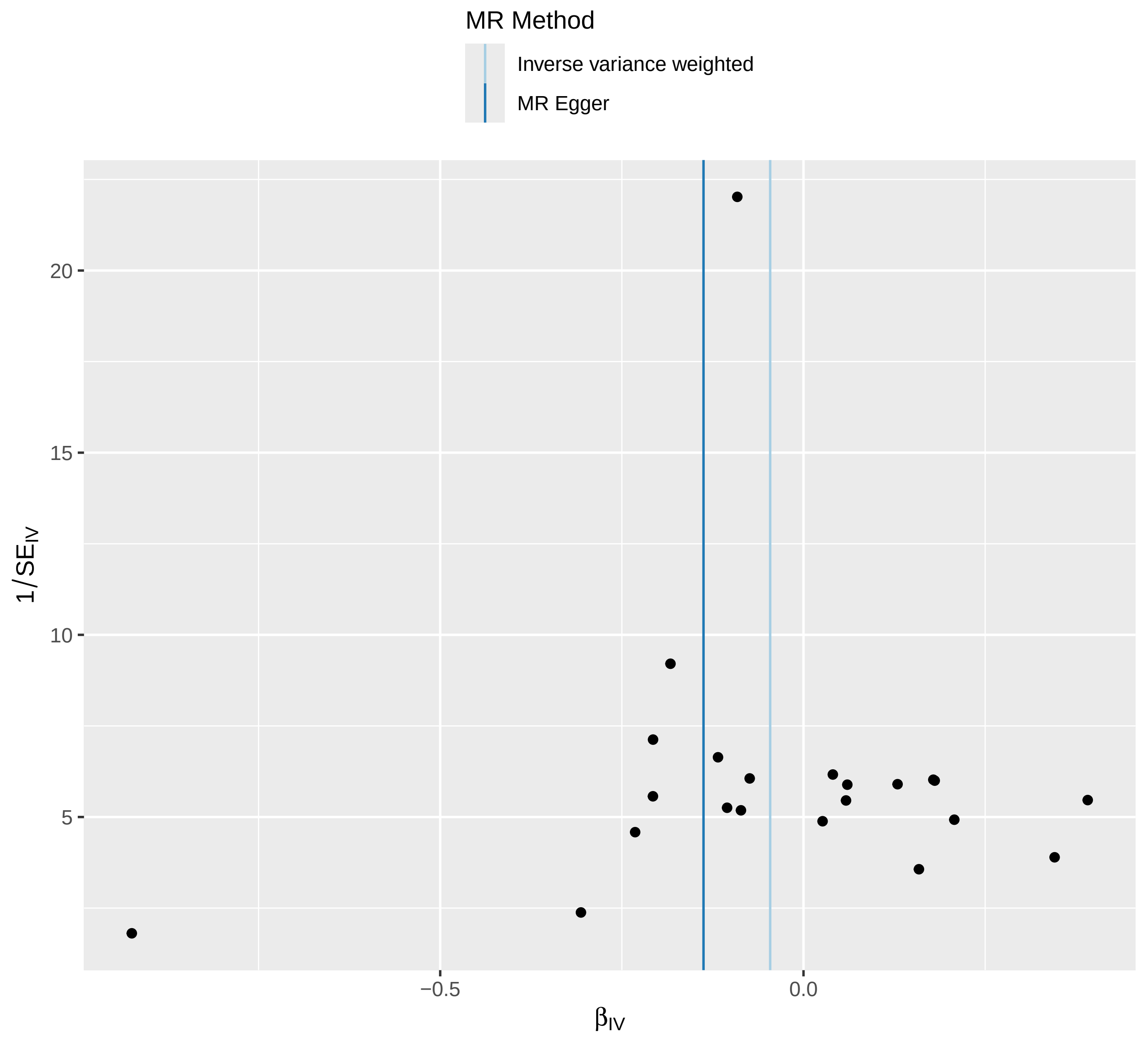
MR Method
Inverse variance weighted
MR Egger
20
15
10
5
1/SE_IV
−0.5
0.0
β_IV

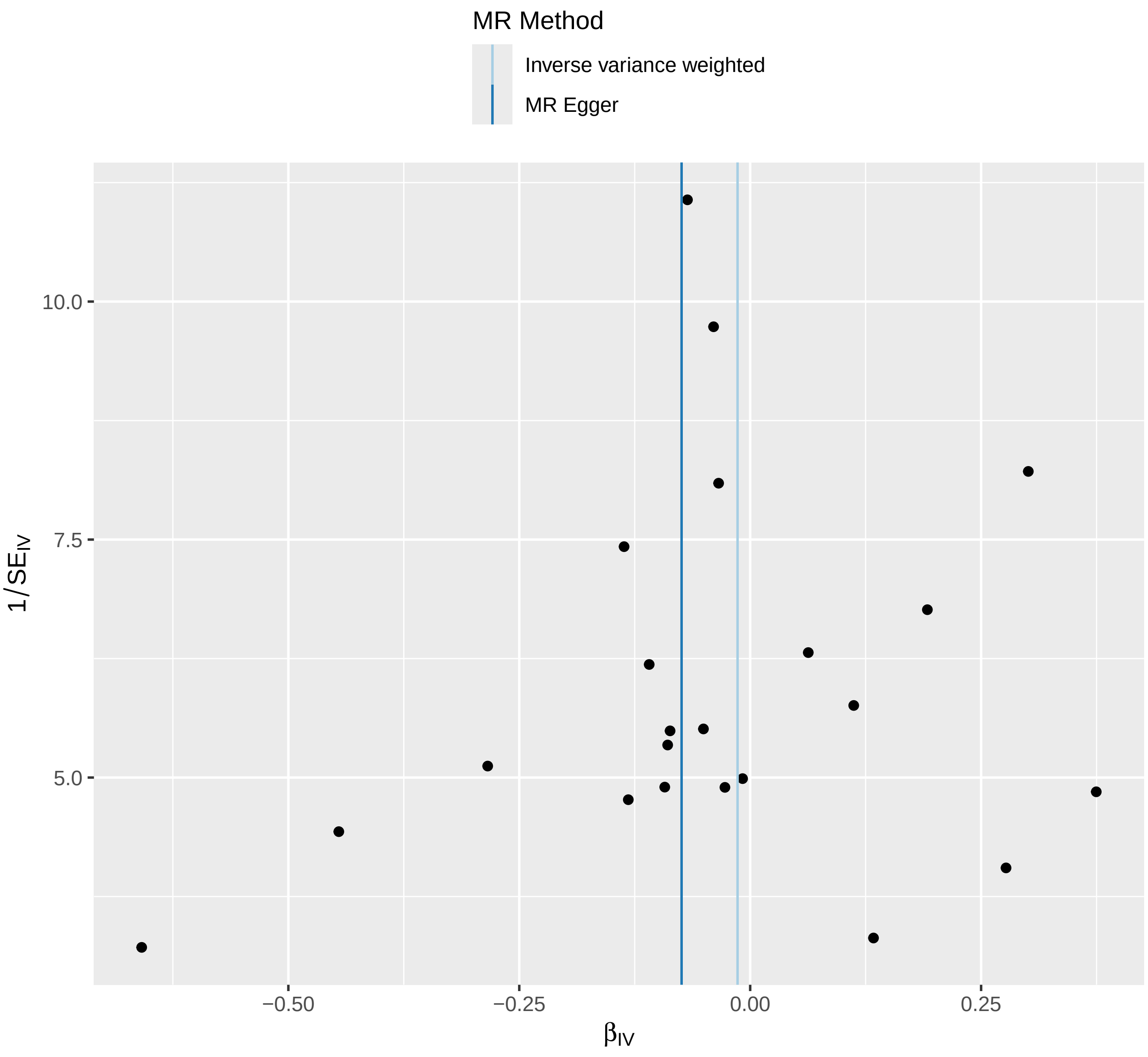

MR Method
Inverse variance weighted
MR Egger
10.0
7.5
5.0
$1/SE_{IV}$
−0.50
−0.25
0.00
0.25
β_{IV}

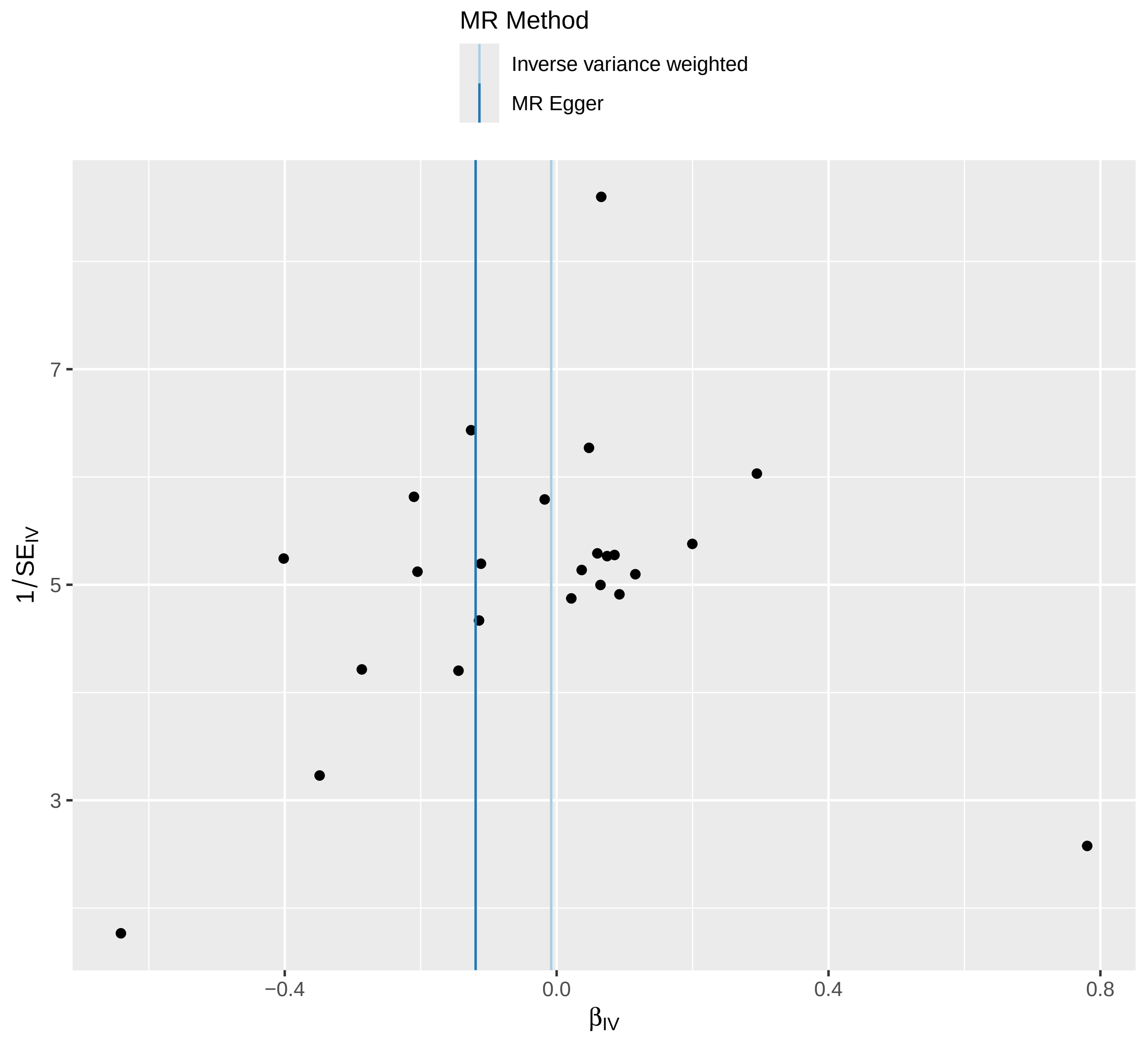
MR Method
Inverse variance weighted
MR Egger
1/SE_IV
7
5
3
−0.4
0.0
0.4
0.8
β_IV

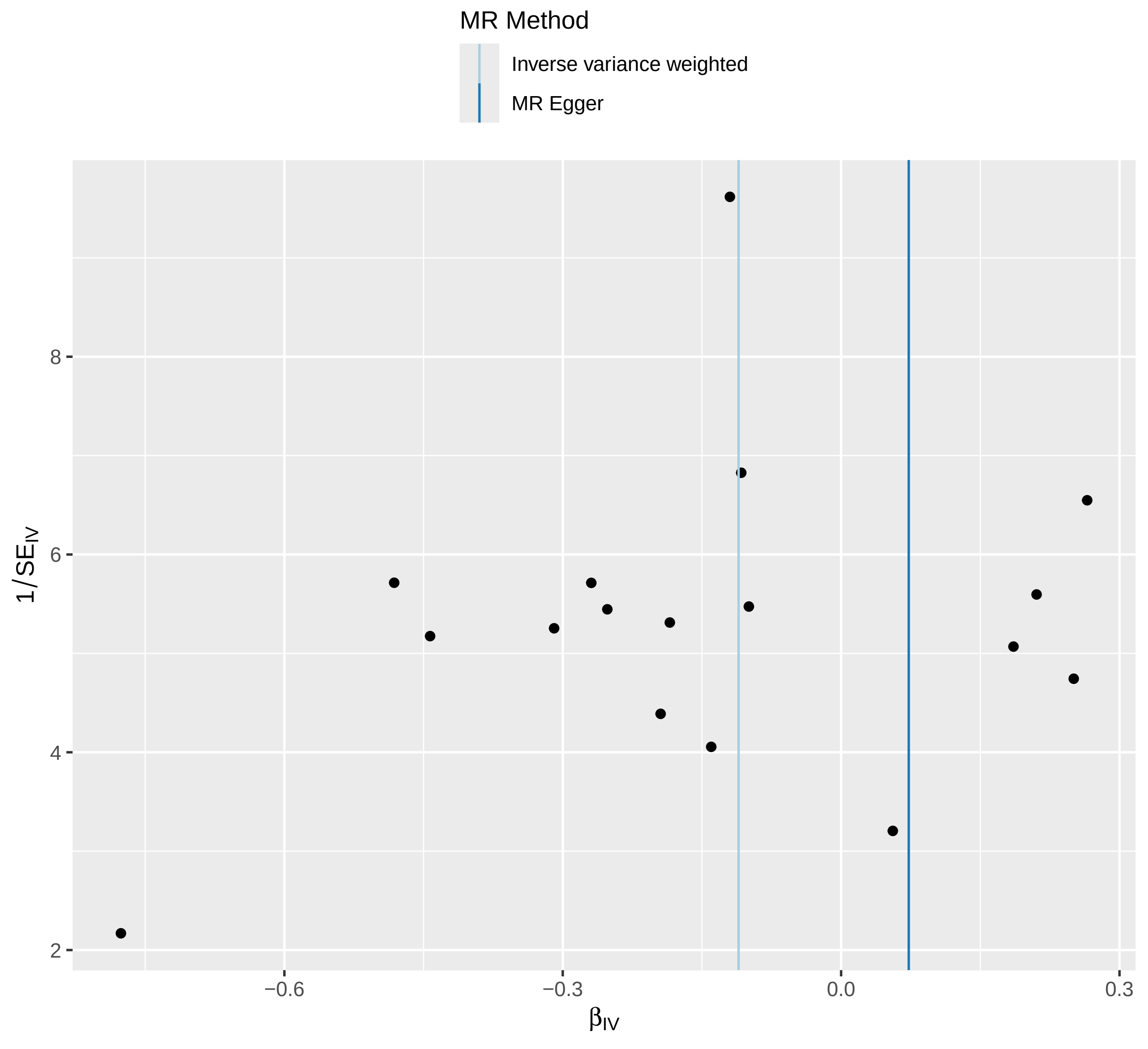

MR Method
Inverse variance weighted
MR Egger
1/SE_IV
8
6
4
2
−0.6
−0.3
0.0
0.3
β_IV

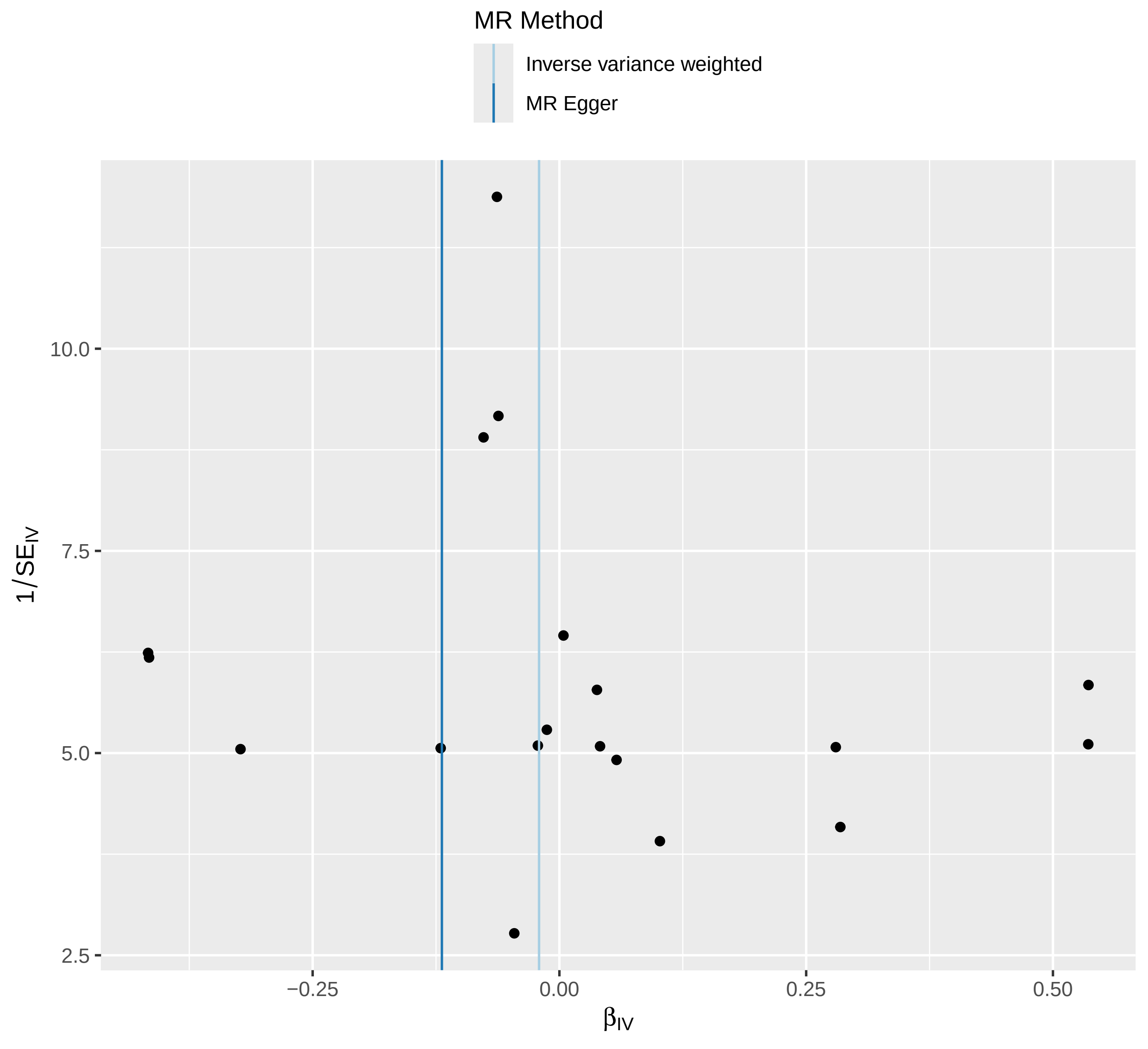
MR Method
Inverse variance weighted
MR Egger
10.0
7.5
5.0
2.5
$1/SE_{IV}$
−0.25
0.00
0.25
0.50
β_{IV}

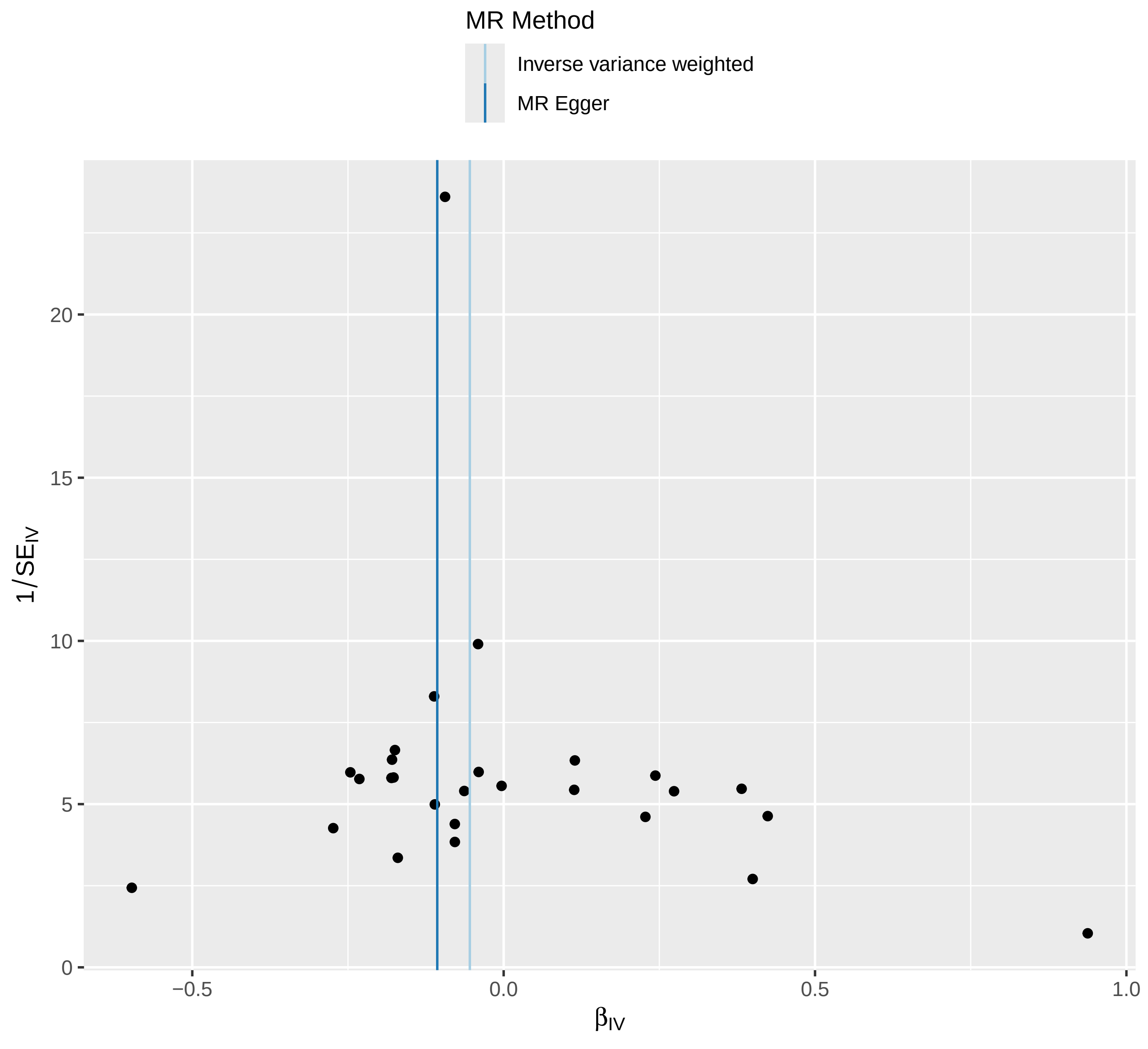

MR Method
Inverse variance weighted
MR Egger
20
15
10
5
0
−0.5
0.0
0.5
1.0
$1/SE_{IV}$
β_{IV}

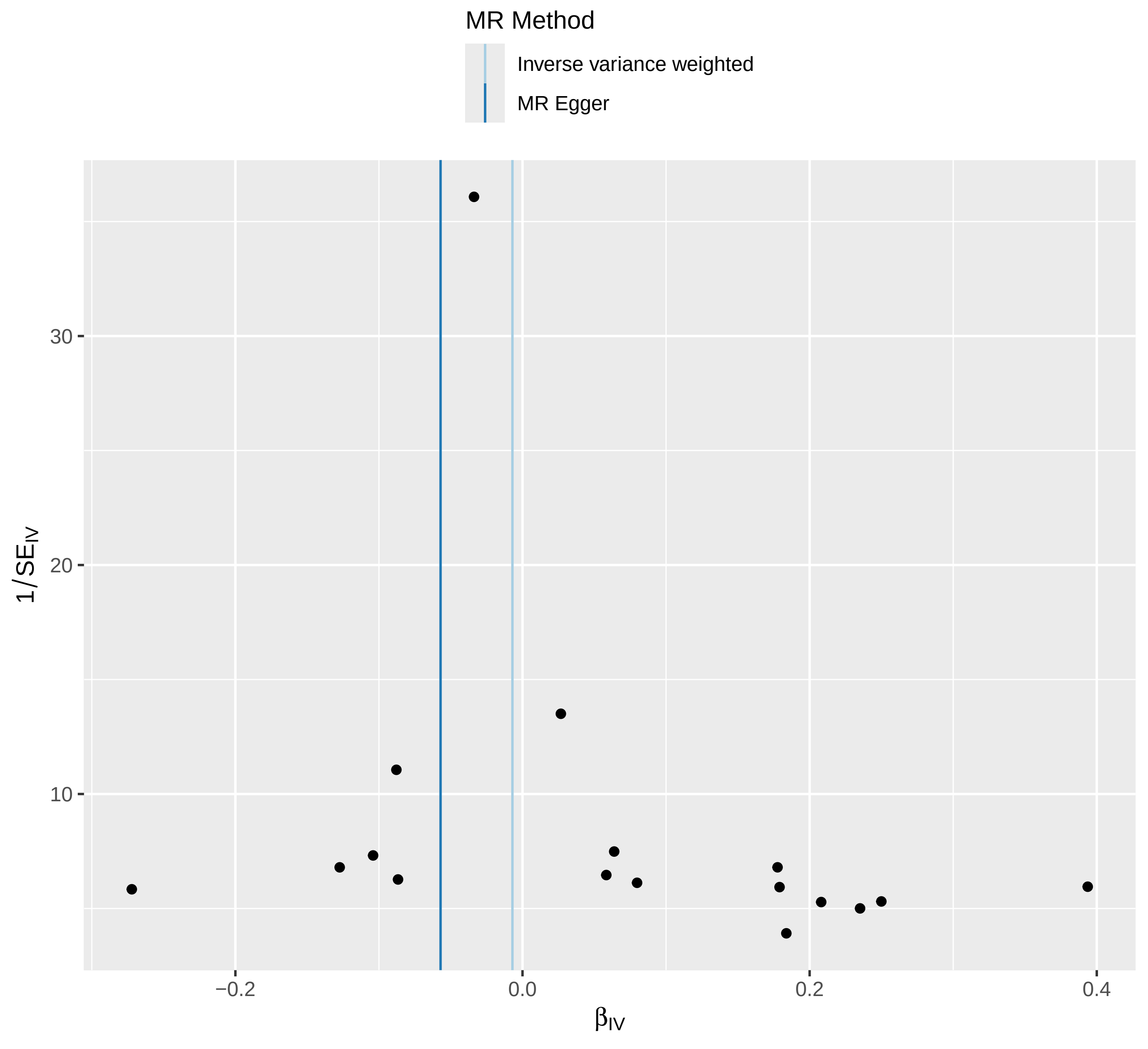
MR Method
Inverse variance weighted
MR Egger
30
20
10
−0.2
0.0
0.2
0.4
$1/SE_{IV}$
β_{IV}

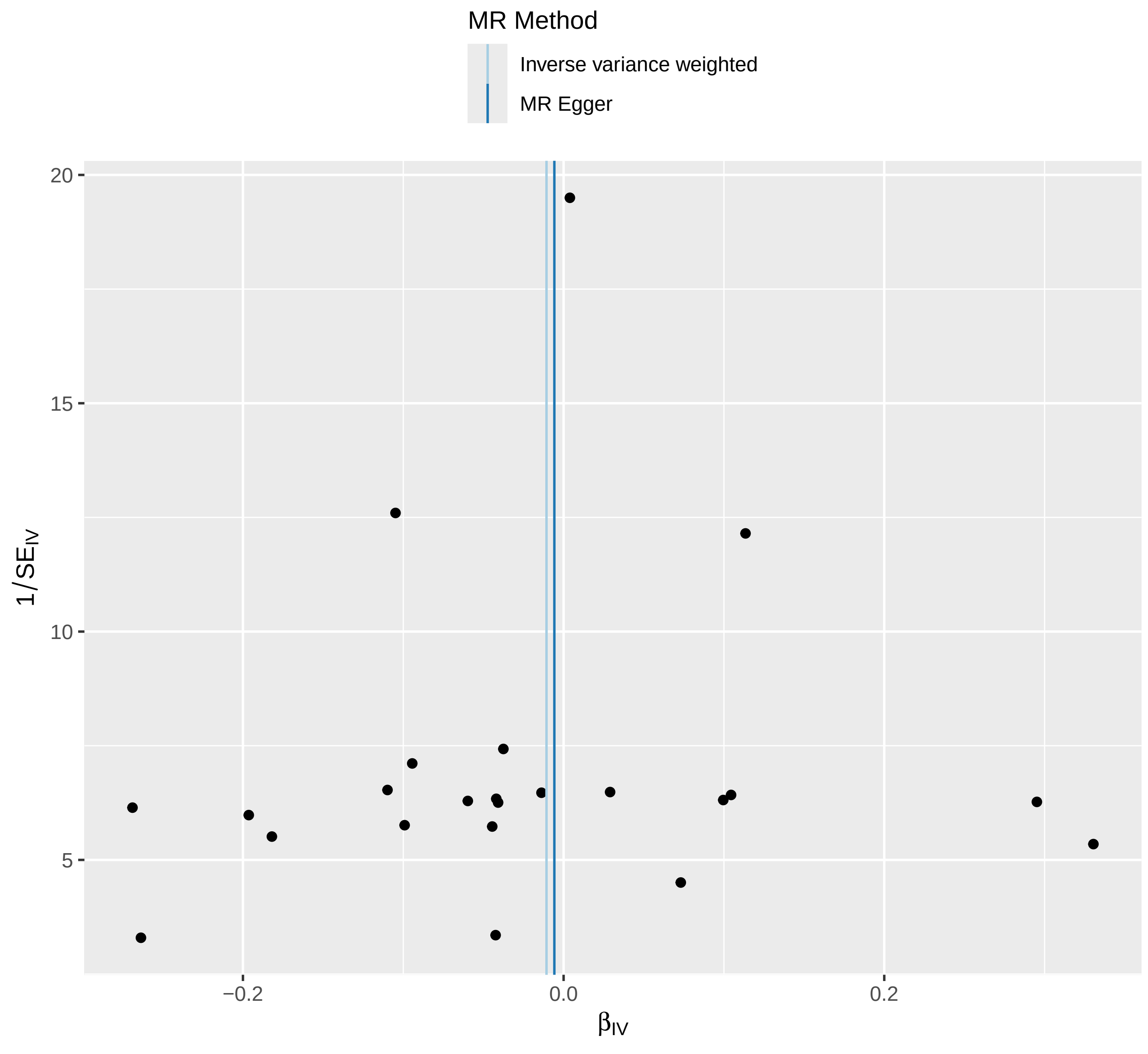
MR Method
Inverse variance weighted
MR Egger
20
15
10
5
1/SE_IV
−0.2
0.0
0.2
β_IV

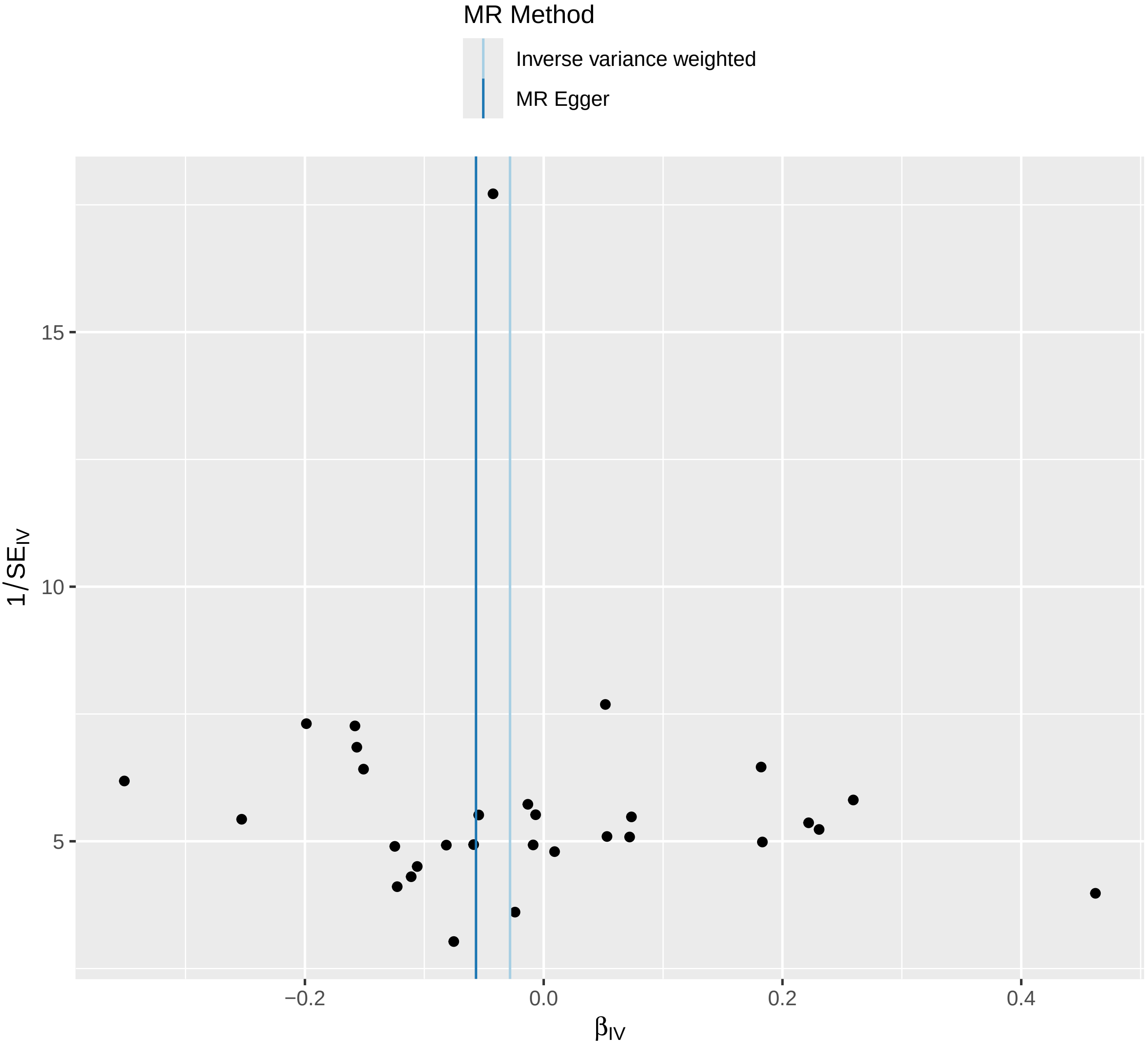
MR Method
Inverse variance weighted
MR Egger
1/SE_IV
15
10
5
−0.2
0.0
0.2
0.4
β_IV

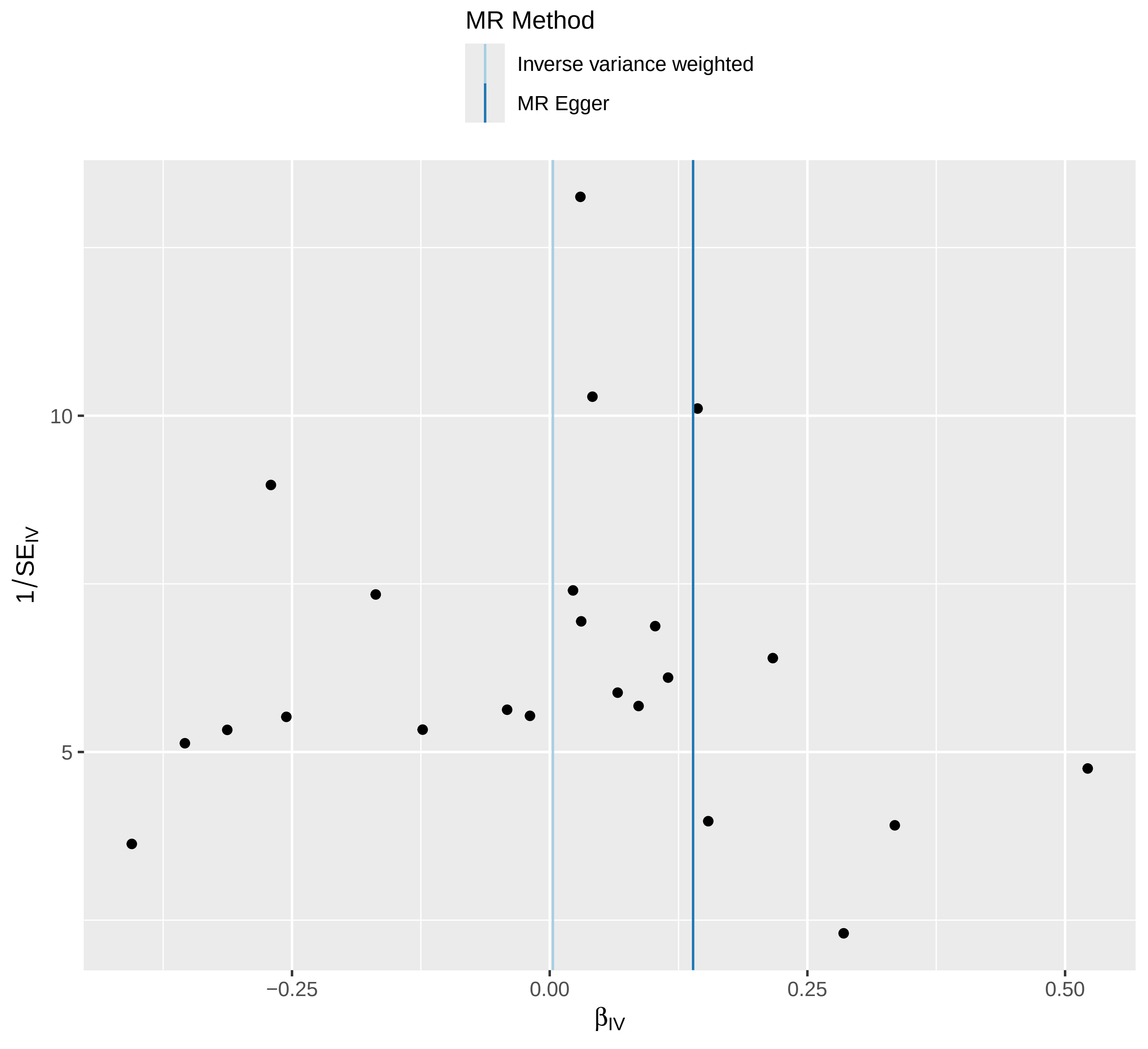
MR Method
Inverse variance weighted
MR Egger
10
5
1/SE_IV
−0.25
0.00
0.25
0.50
β_IV

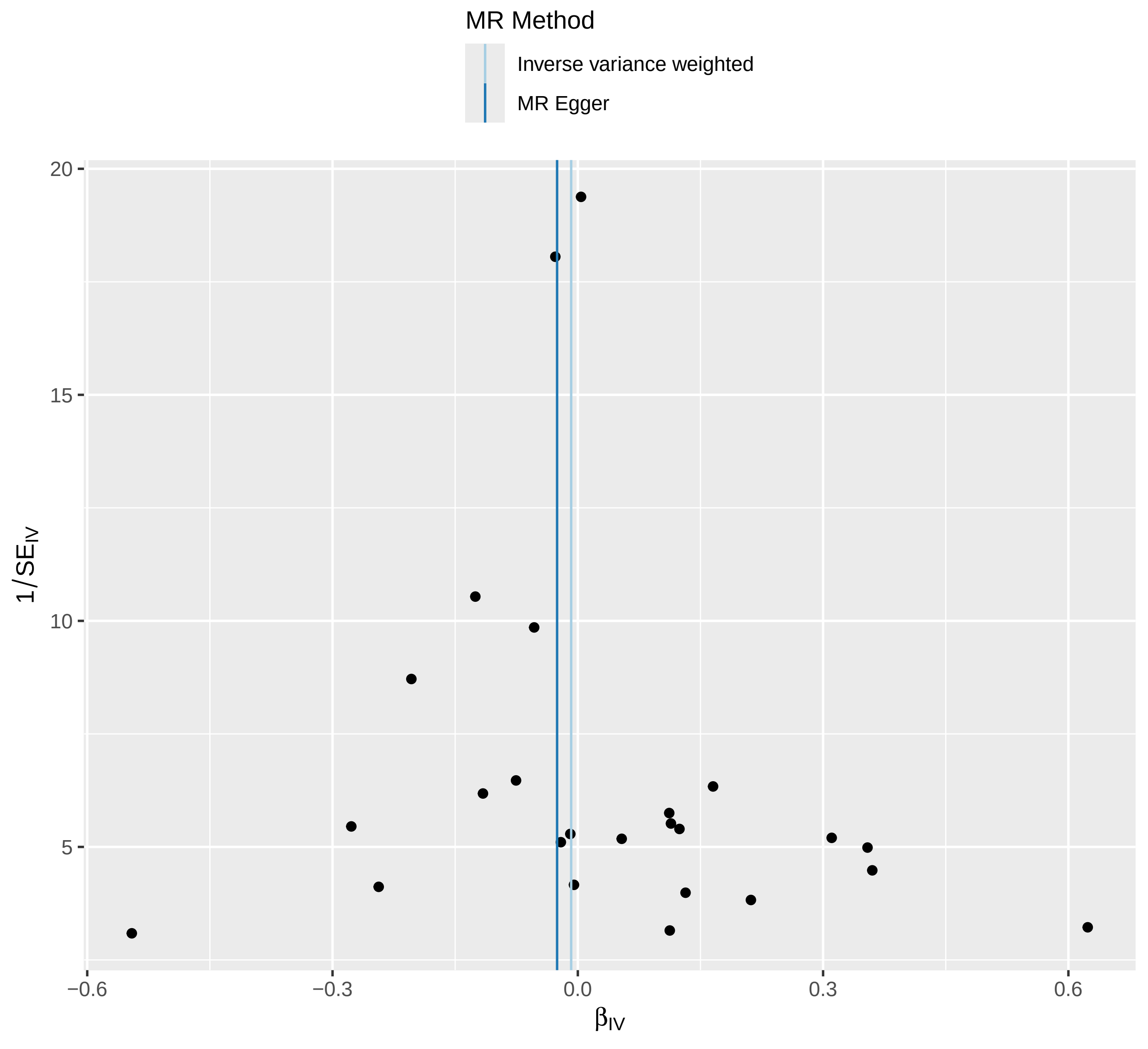
MR Method
Inverse variance weighted
MR Egger
20
15
10
5
1/SE_IV
−0.6
−0.3
0.0
0.3
0.6
β_IV

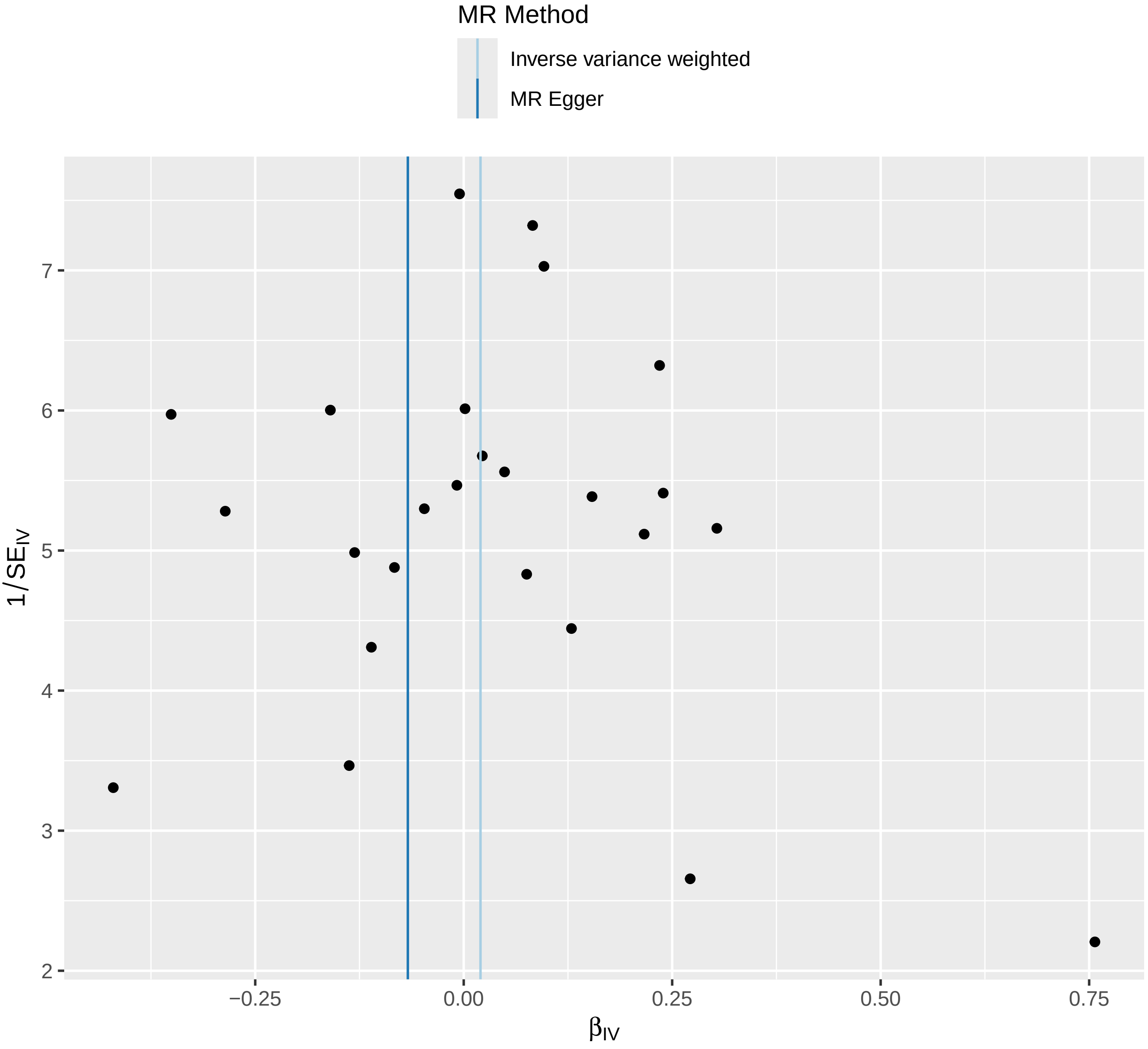

MR Method
Inverse variance weighted
MR Egger
7
6
5
4
3
2
1/SE_IV
−0.25
0.00
0.25
0.50
0.75
β_IV

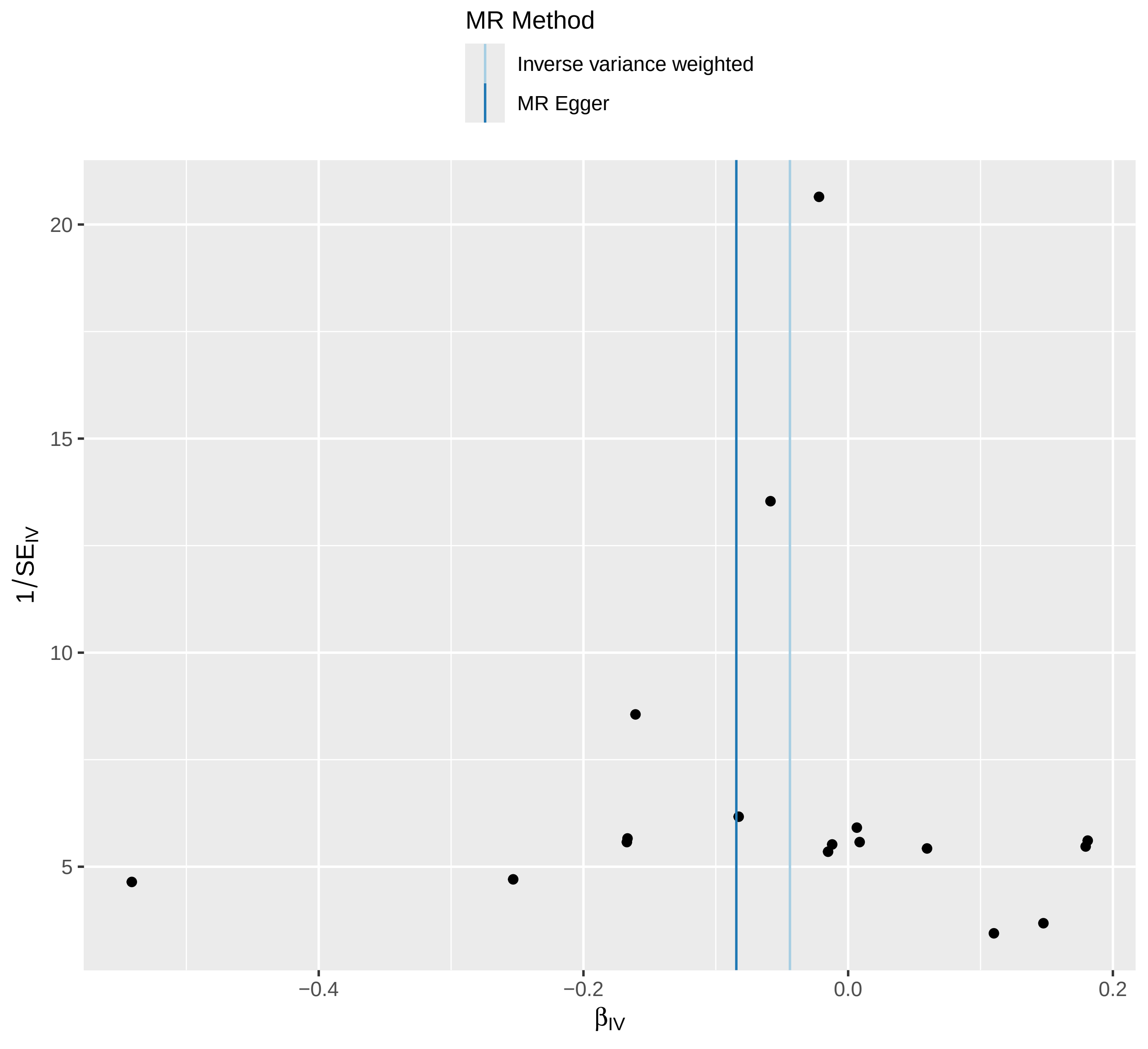

MR Method
Inverse variance weighted
MR Egger
20
15
10
5
1/SE$_{IV}$
−0.4
−0.2
0.0
0.2
β$_{IV}$

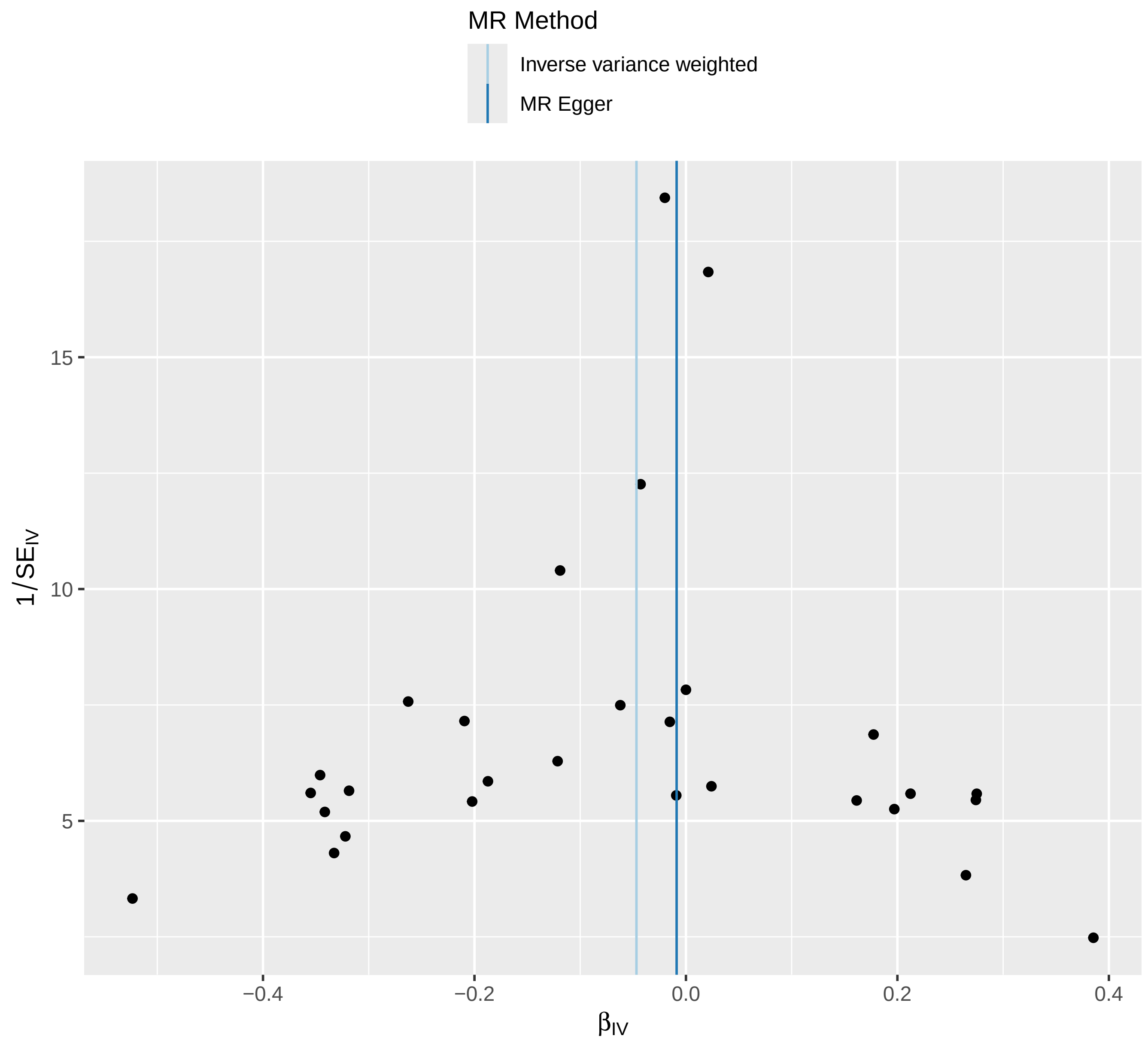
MR Method
Inverse variance weighted
MR Egger
15
10
5
1/SE_IV
−0.4
−0.2
0.0
0.2
0.4
β_IV

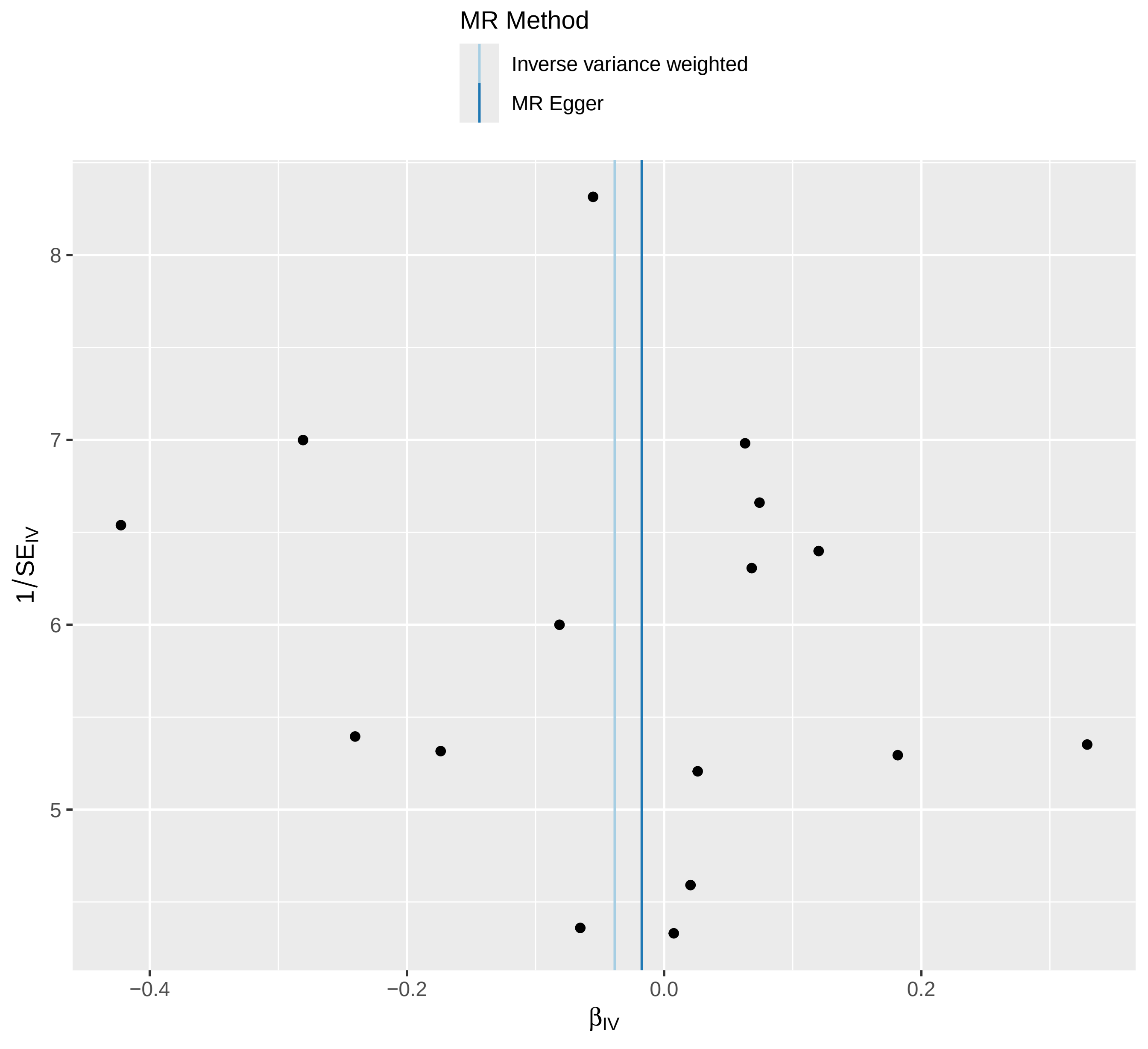
MR Method
Inverse variance weighted
MR Egger
8
7
6
5
1/SE_IV
−0.4
−0.2
0.0
0.2
β_IV

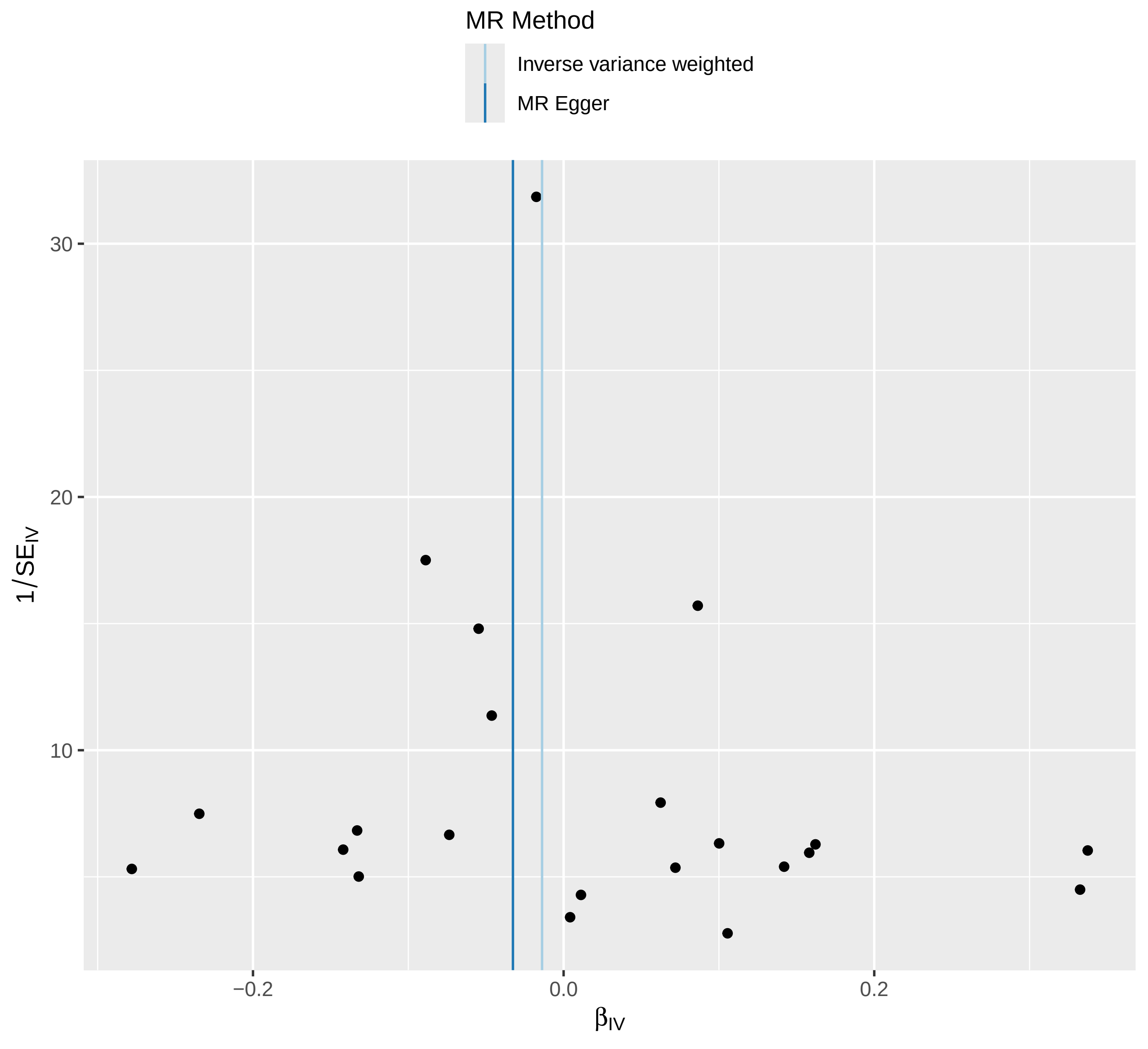
MR Method
Inverse variance weighted
MR Egger
30
20
10
1/SE$_{IV}$
−0.2
0.0
0.2
β$_{IV}$

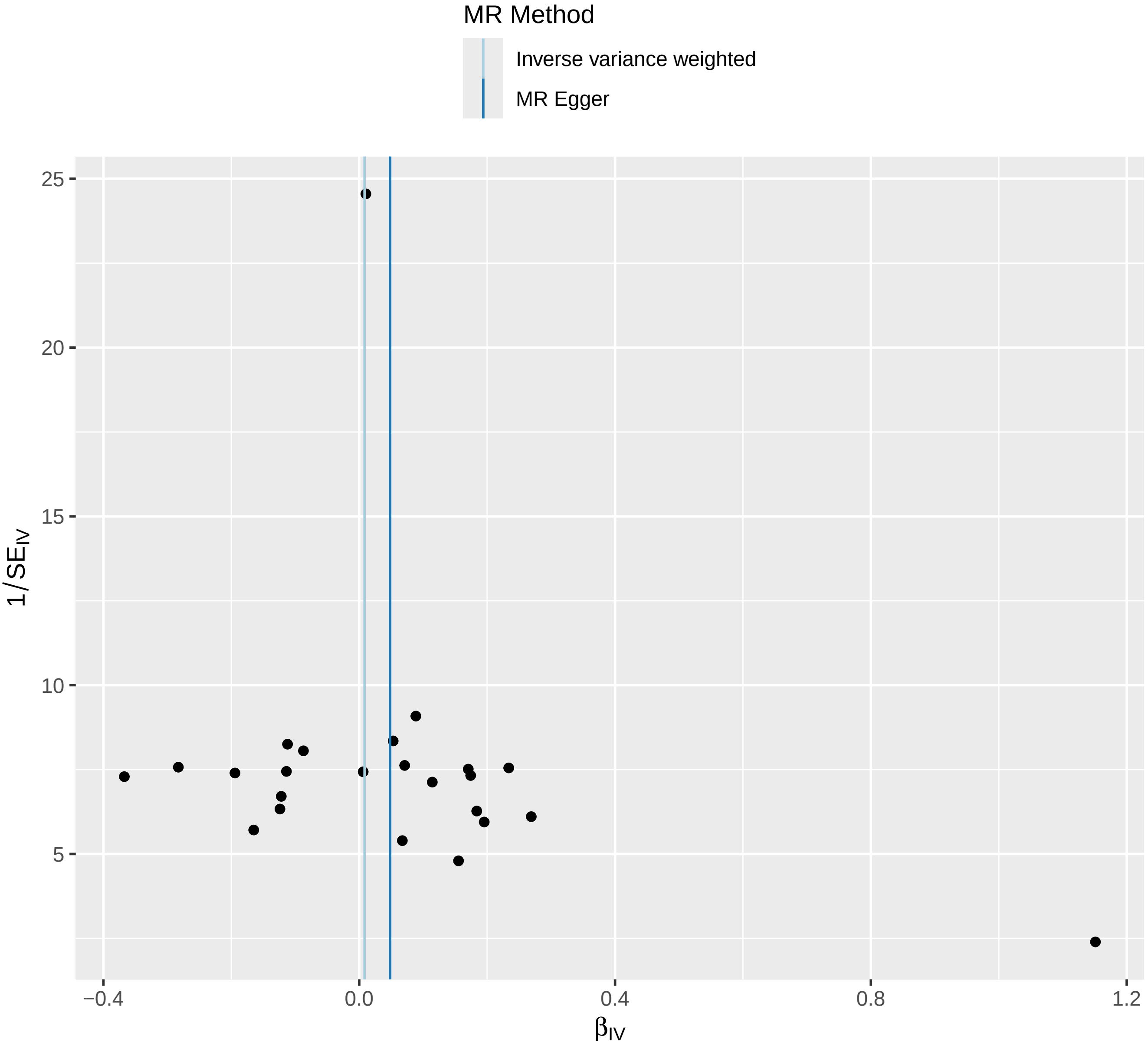

MR Method
Inverse variance weighted
MR Egger
25
20
15
10
5
1/SE_IV
−0.4
0.0
0.4
0.8
1.2
β_IV

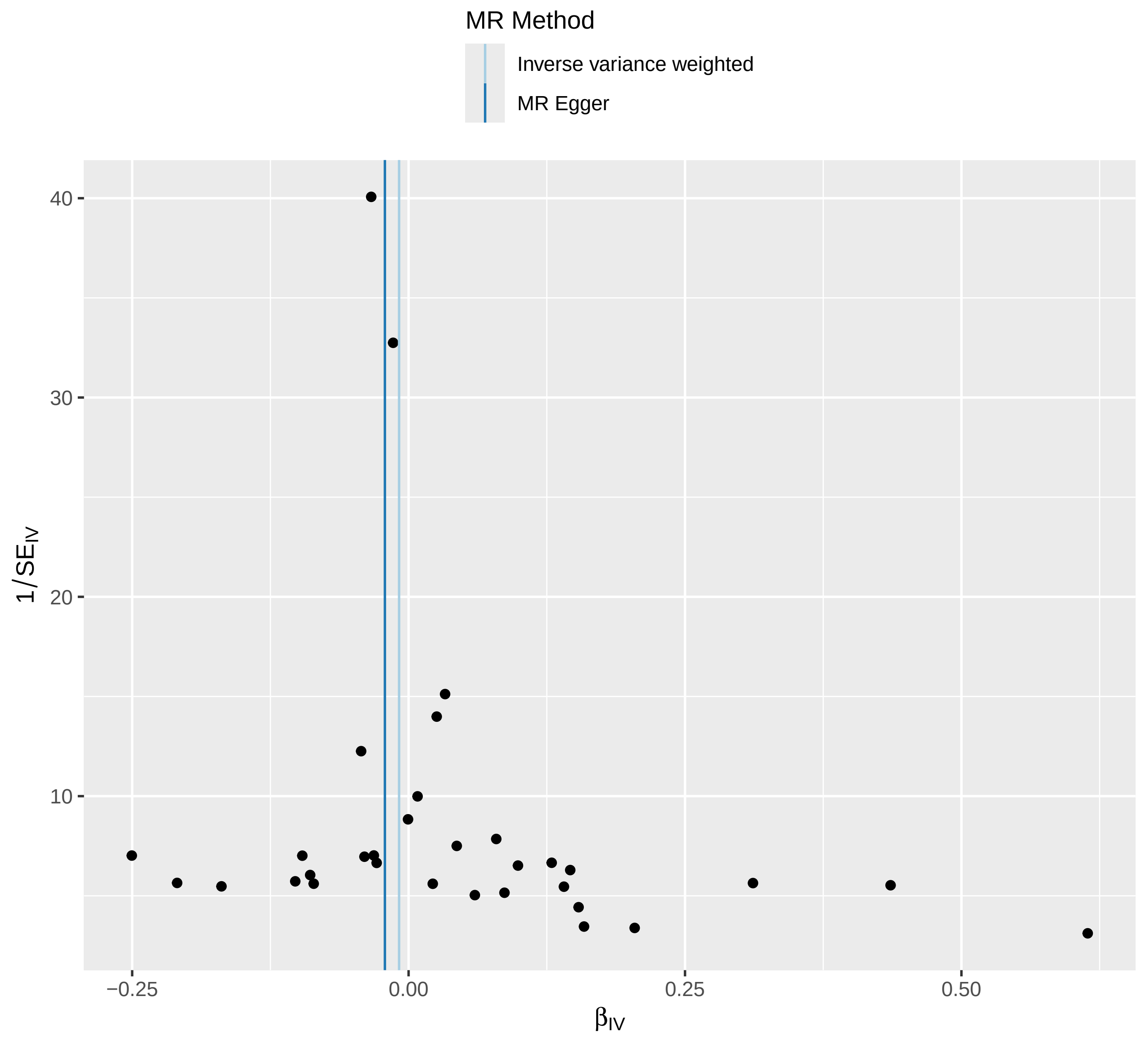
MR Method
Inverse variance weighted
MR Egger
40
30
20
10
1/SE$_{IV}$
−0.25
0.00
0.25
0.50
β$_{IV}$

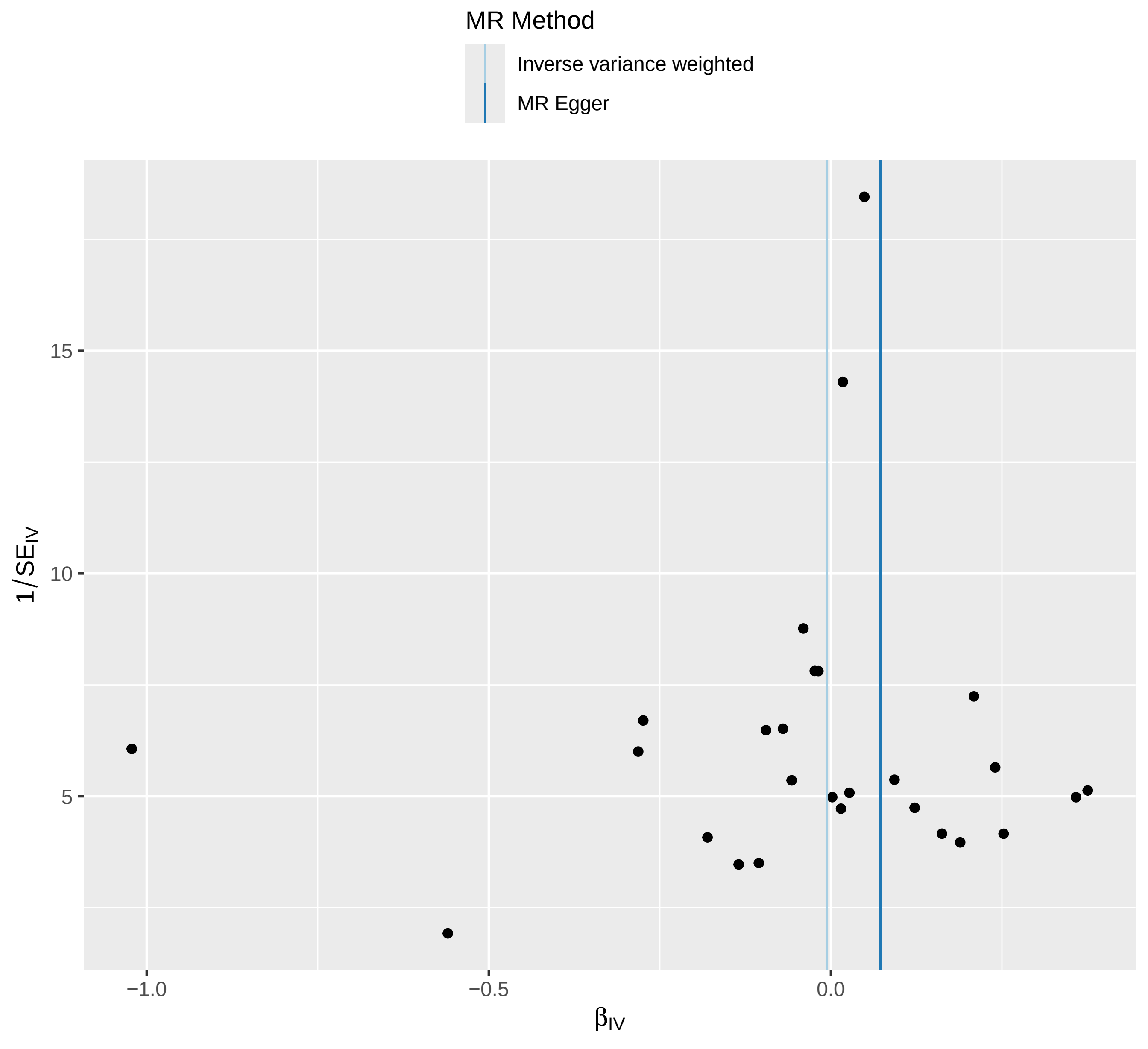
MR Method
Inverse variance weighted
MR Egger
1/SE$_{IV}$
15
10
5
−1.0
−0.5
0.0
β$_{IV}$

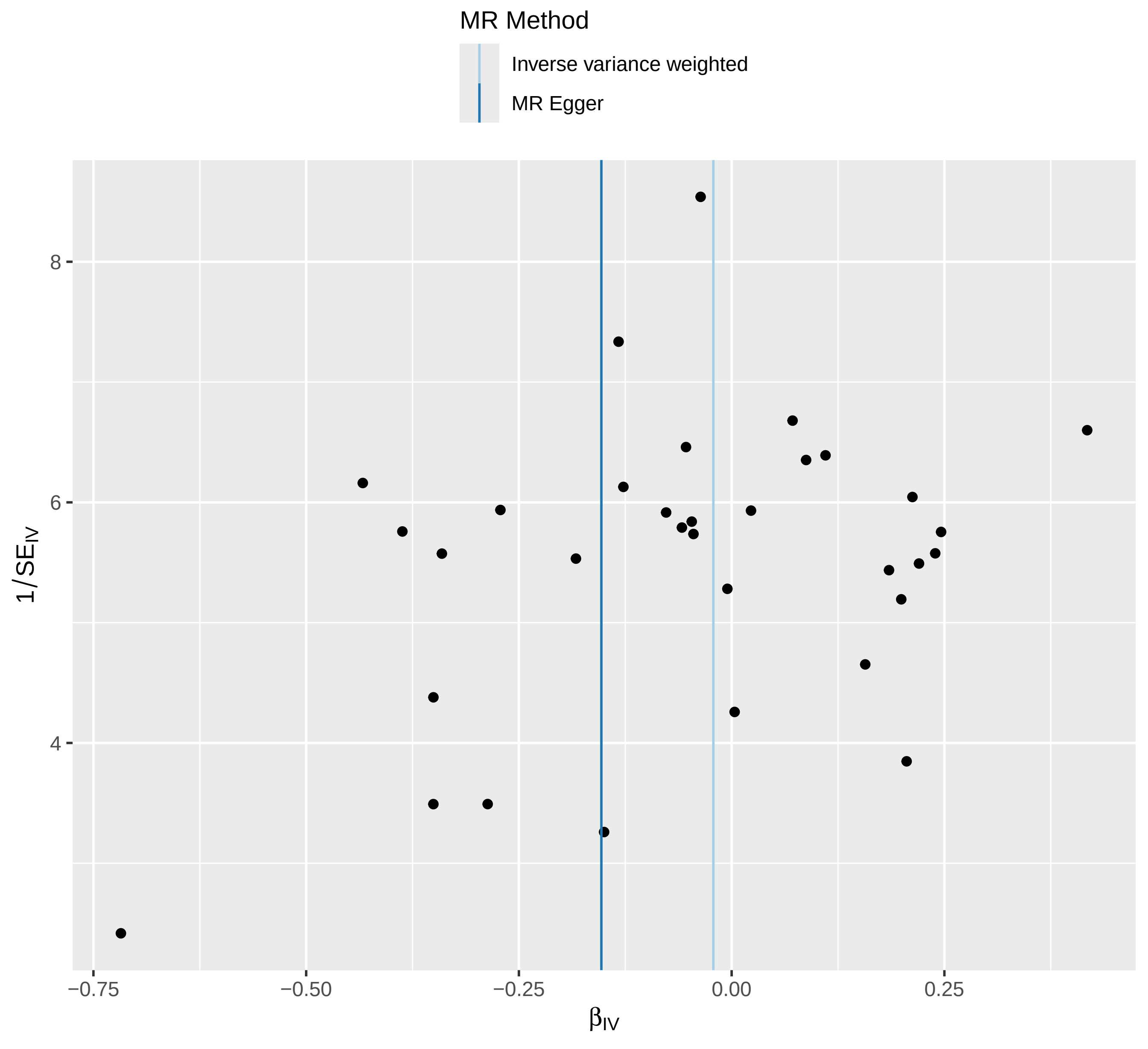
MR Method
Inverse variance weighted
MR Egger
8
6
4
1/SE_IV
−0.75
−0.50
−0.25
0.00
0.25
β_IV

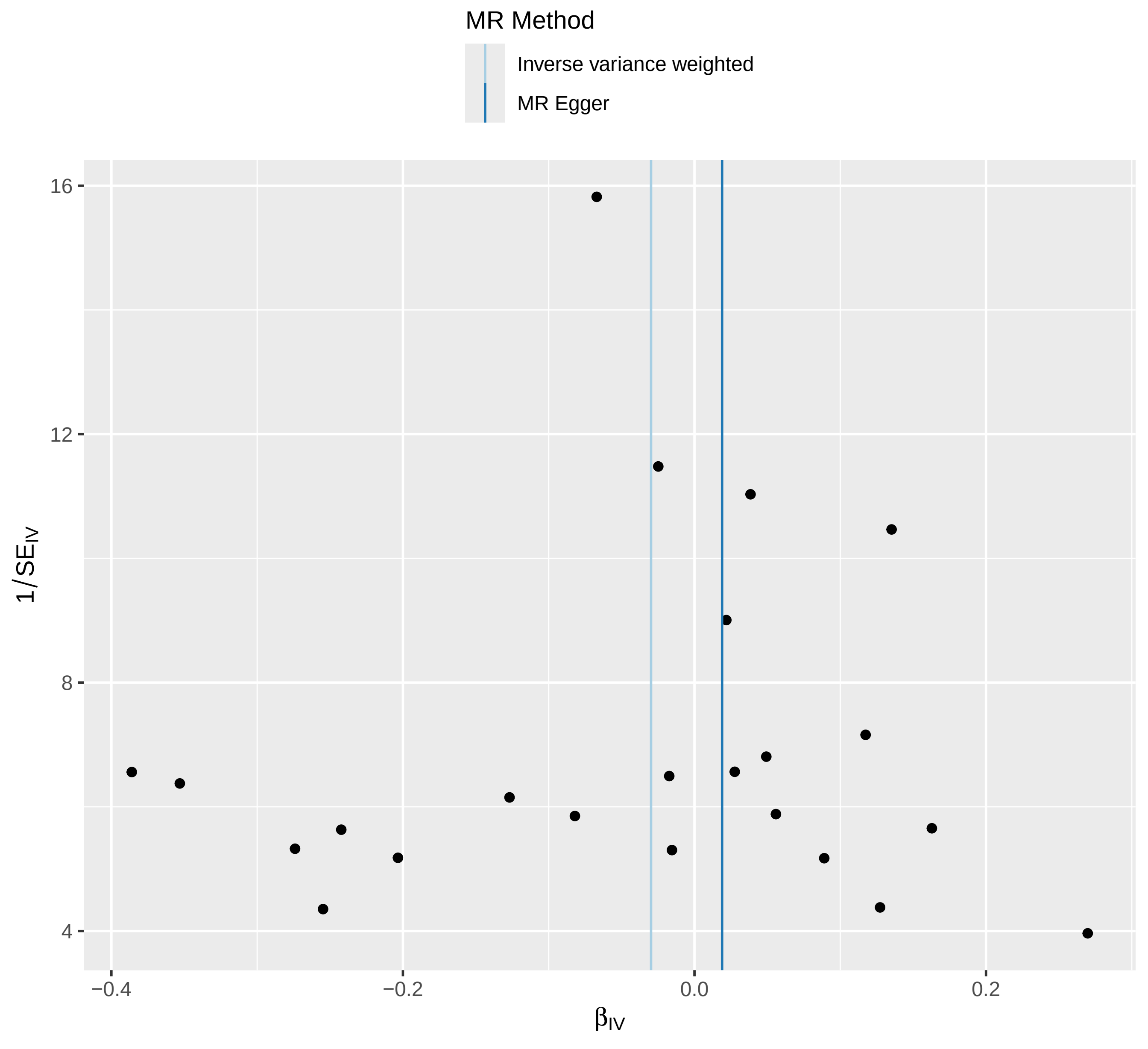
MR Method
Inverse variance weighted
MR Egger
16
12
8
4
1/SE_IV
−0.4
−0.2
0.0
0.2
β_IV

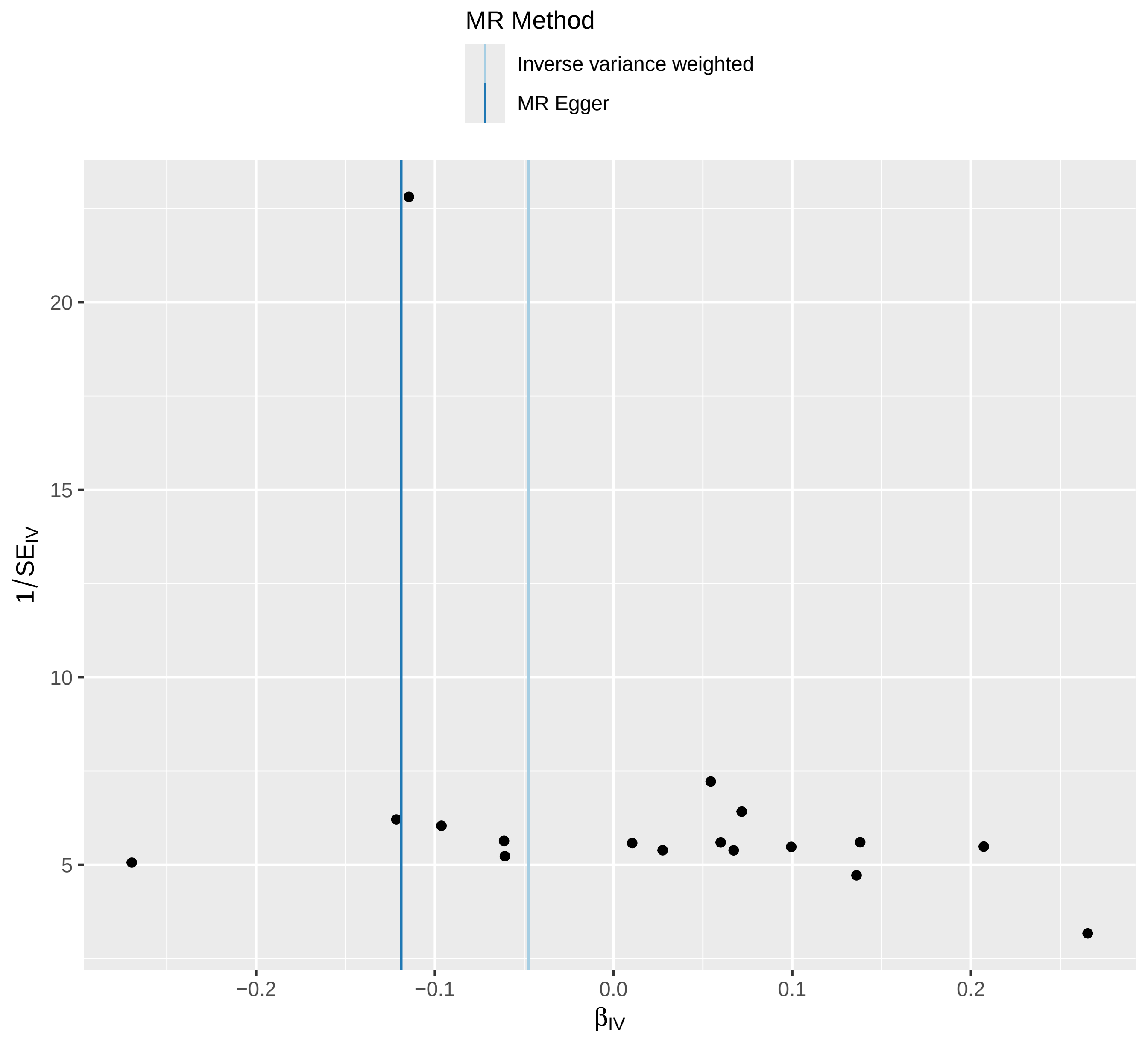
MR Method
Inverse variance weighted
MR Egger
20
15
10
5
1/SE$_{IV}$
−0.2
−0.1
0.0
0.1
0.2
β$_{IV}$

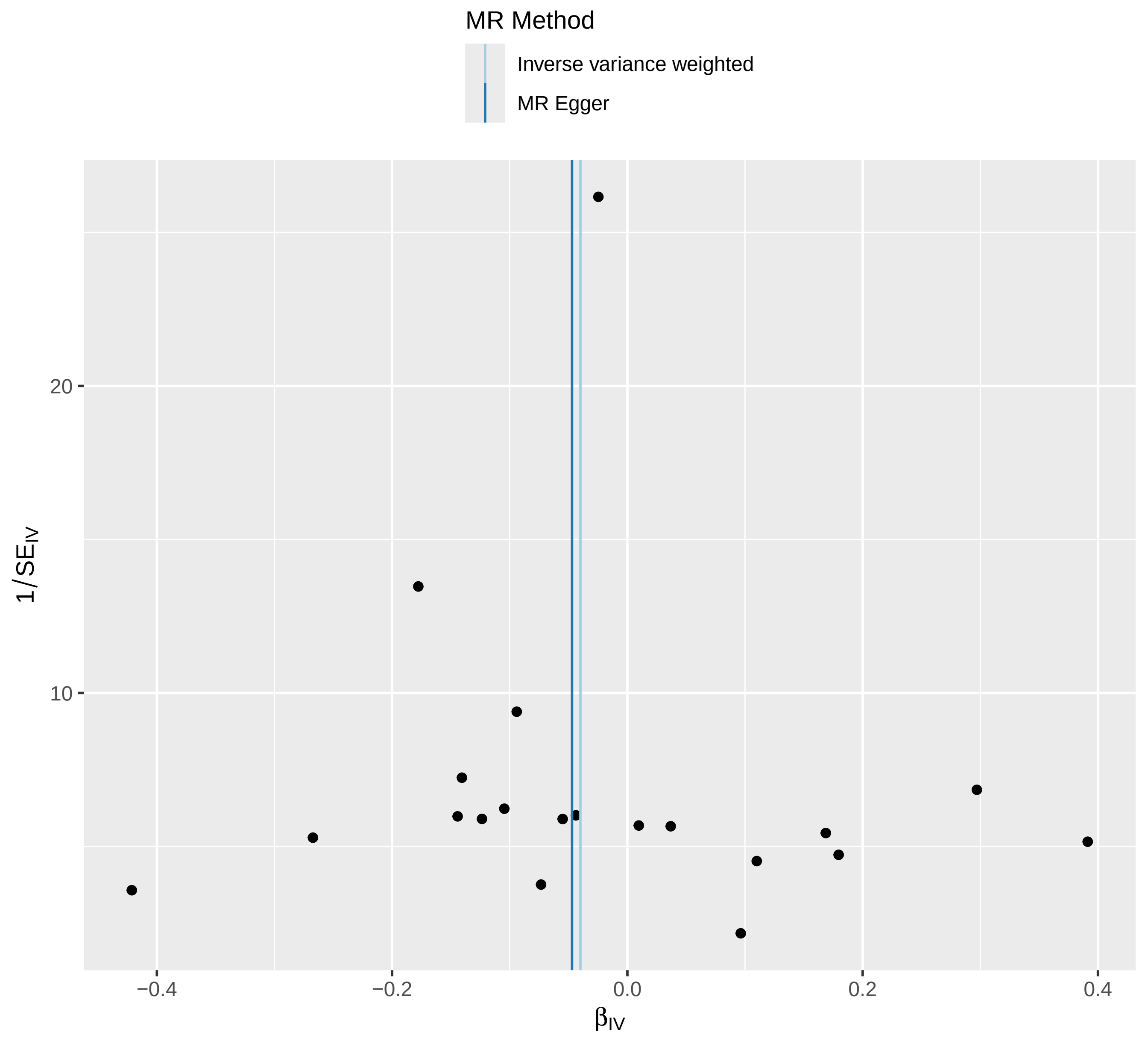
MR Method
Inverse variance weighted
MR Egger
20
10
1/SE_IV
−0.4
−0.2
0.0
0.2
0.4
β_IV

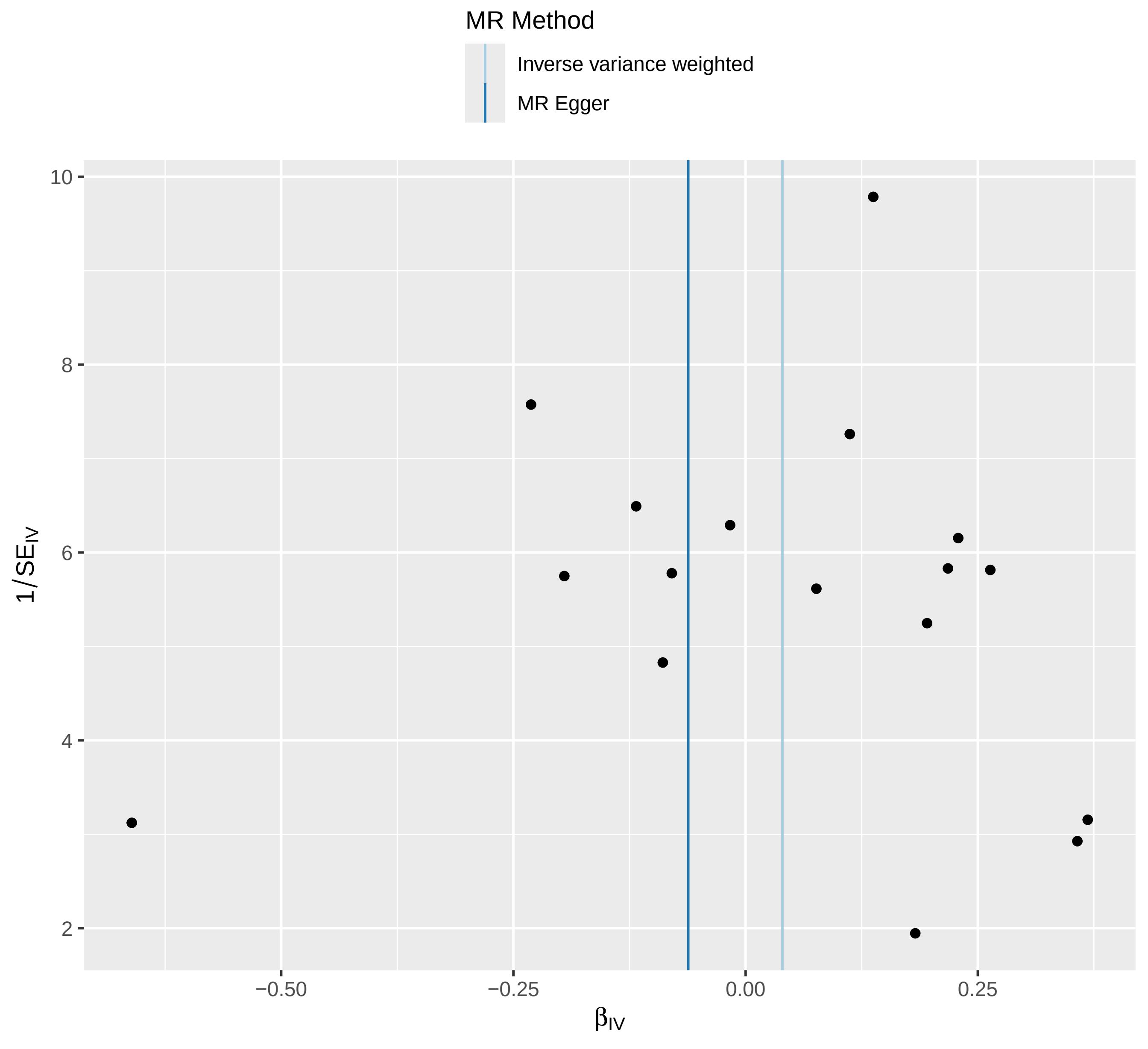

MR Method
Inverse variance weighted
MR Egger
10
8
6
4
2
−0.50
−0.25
0.00
0.25
1/SE_IV
β_IV

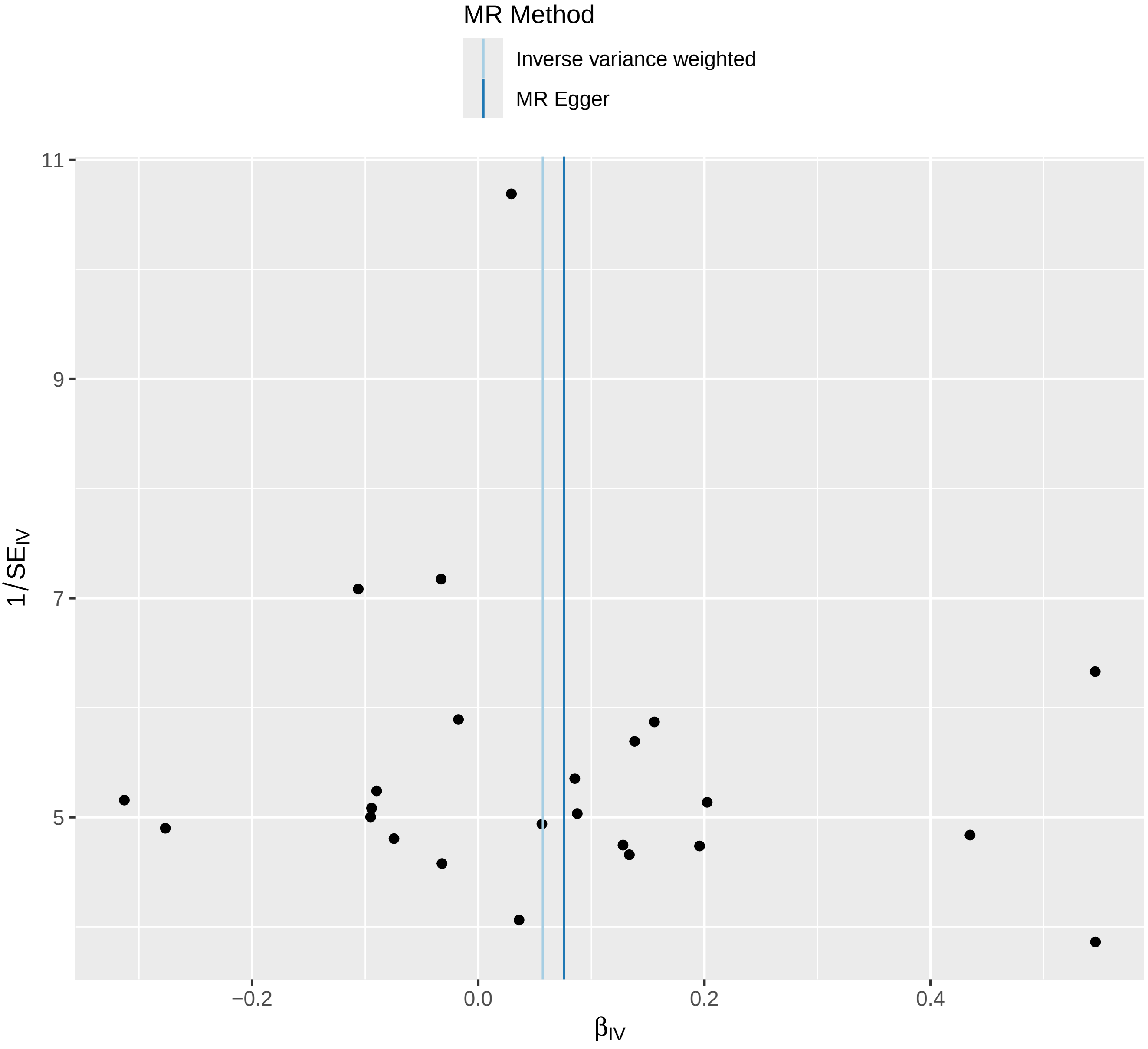

MR Method
Inverse variance weighted
MR Egger
11
9
7
5
1/SE$_{IV}$
−0.2
0.0
0.2
0.4
β$_{IV}$

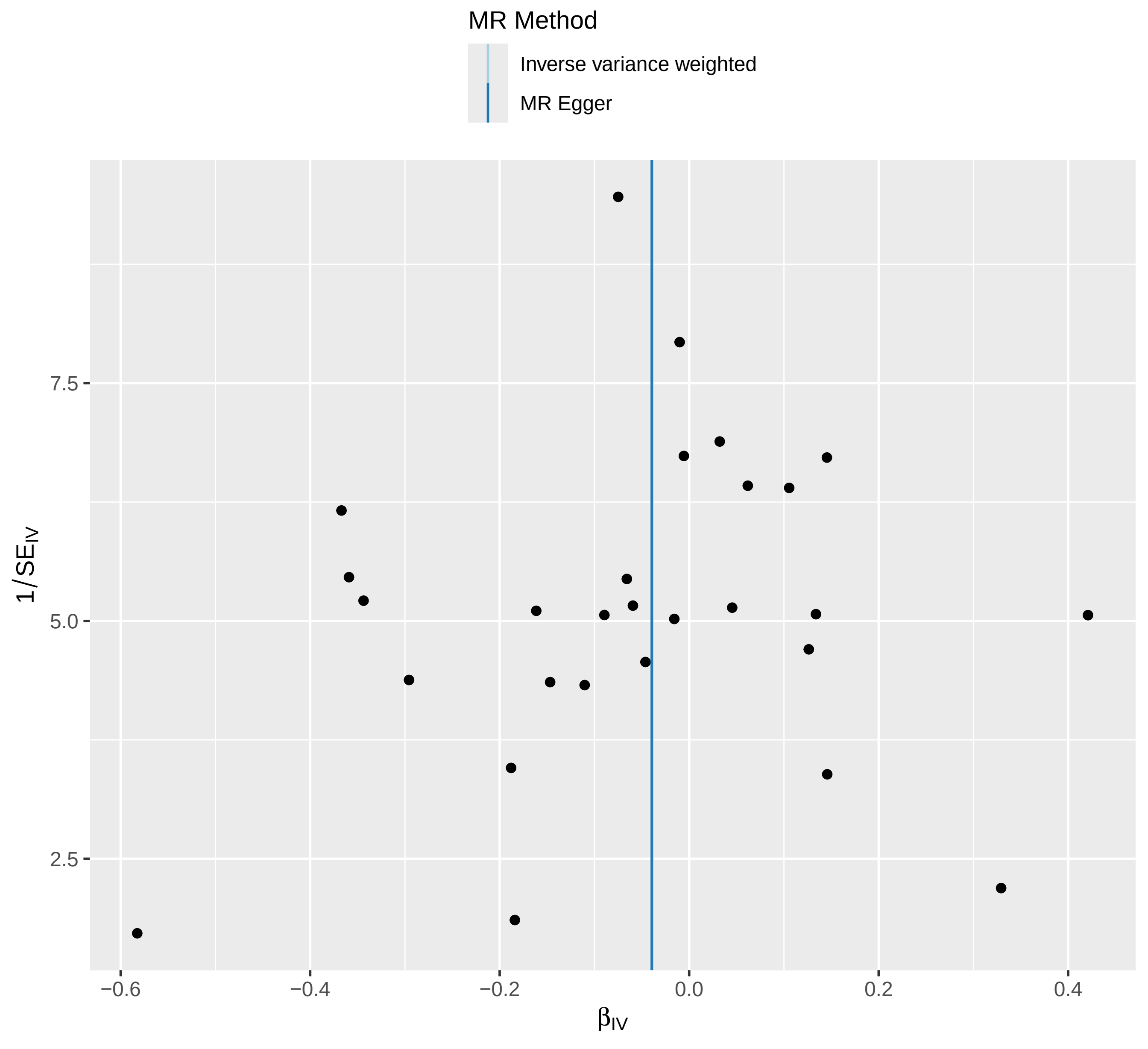

MR Method
Inverse variance weighted
MR Egger
7.5
5.0
2.5
1/SE_IV
−0.6
−0.4
−0.2
0.0
0.2
0.4
β_IV

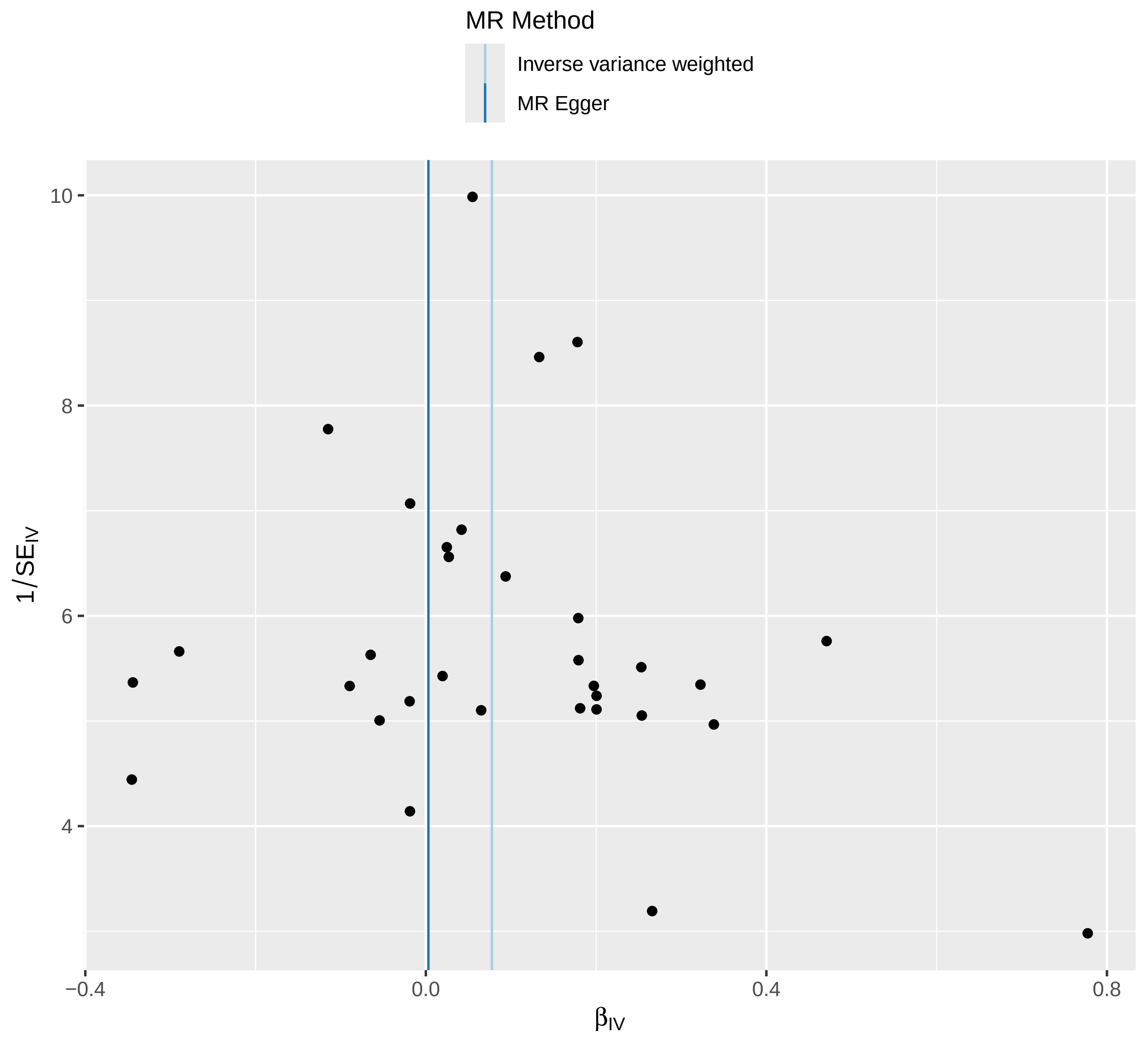
MR Method
Inverse variance weighted
MR Egger
10
8
6
4
1/SE_IV
−0.4
0.0
0.4
0.8
β_IV

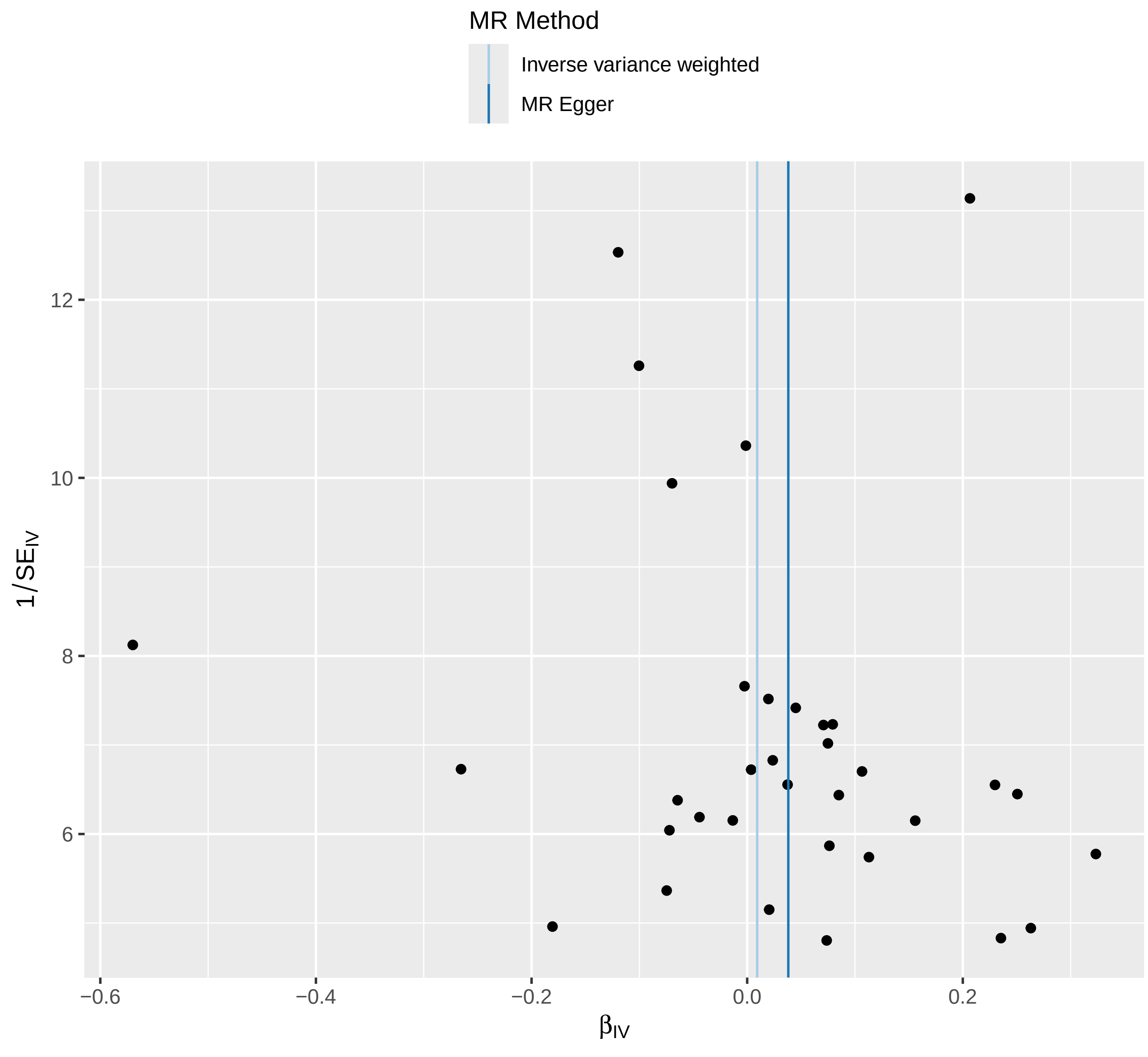

MR Method
Inverse variance weighted
MR Egger
12
10
8
6
1/SE_IV
−0.6
−0.4
−0.2
0.0
0.2
β_IV

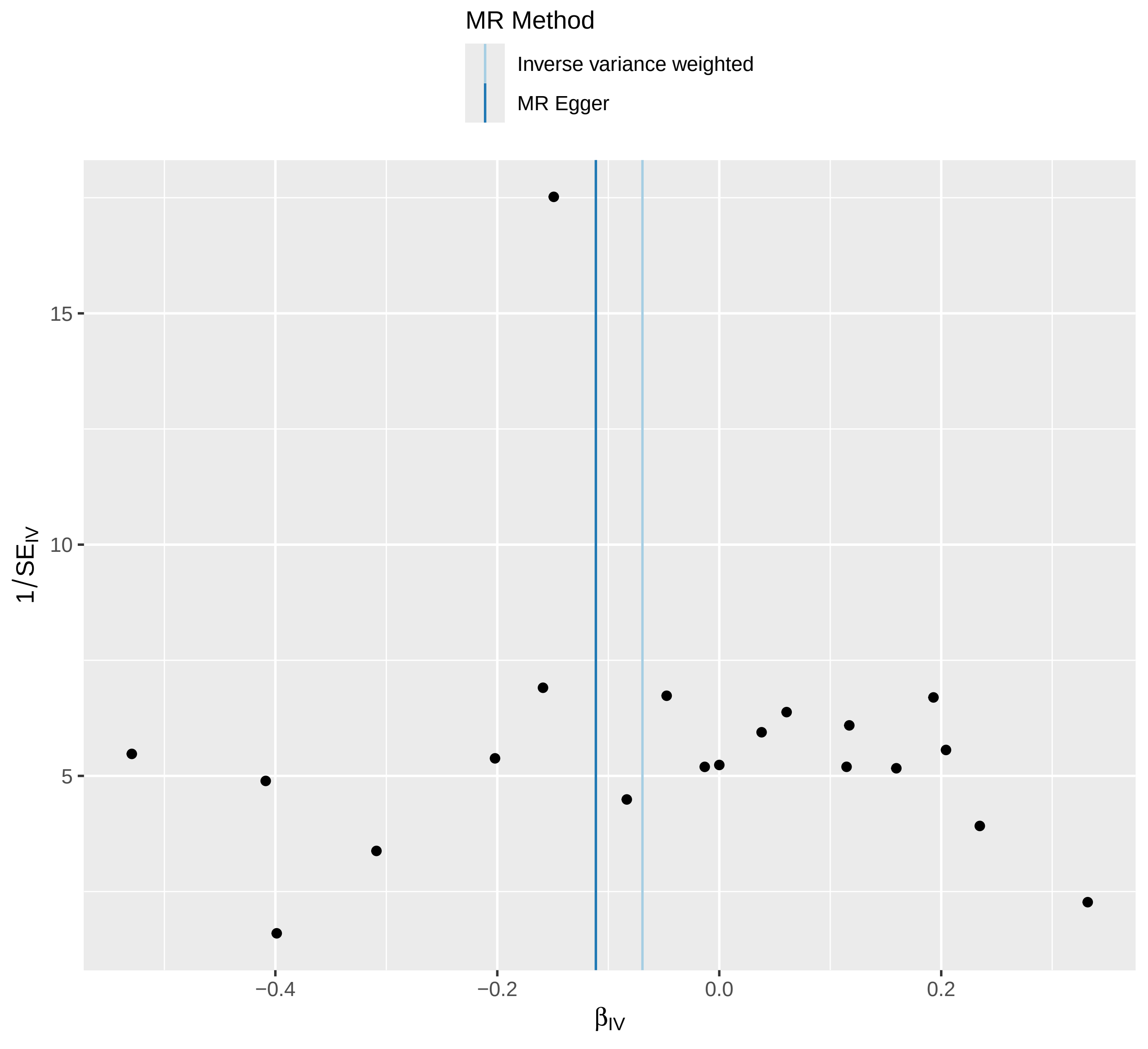
MR Method
Inverse variance weighted
MR Egger
15
10
5
1/SE$_{IV}$
−0.4
−0.2
0.0
0.2
β$_{IV}$

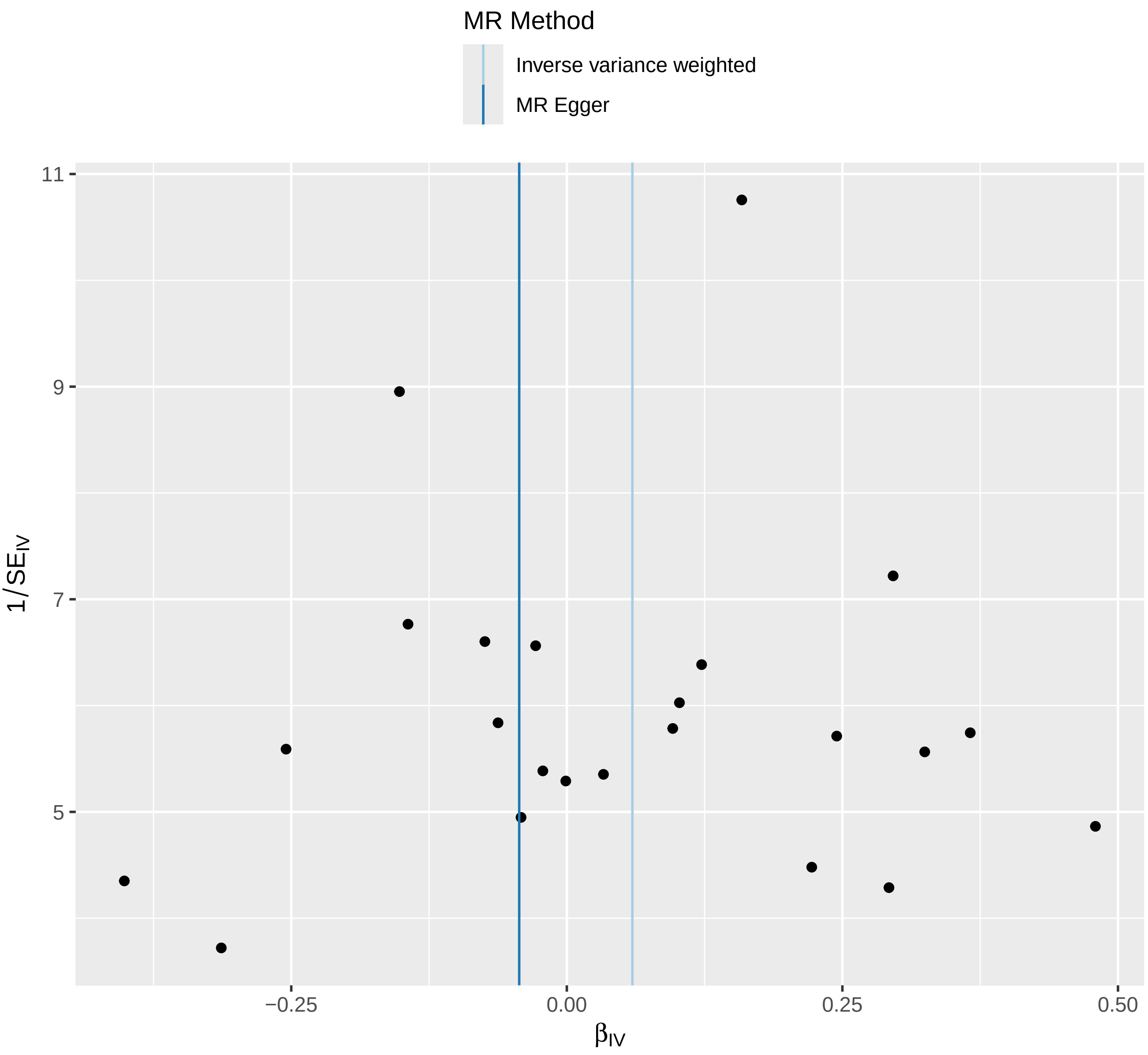
MR Method
Inverse variance weighted
MR Egger
11
9
7
5
$1/SE_{IV}$
−0.25
0.00
0.25
0.50
β_{IV}

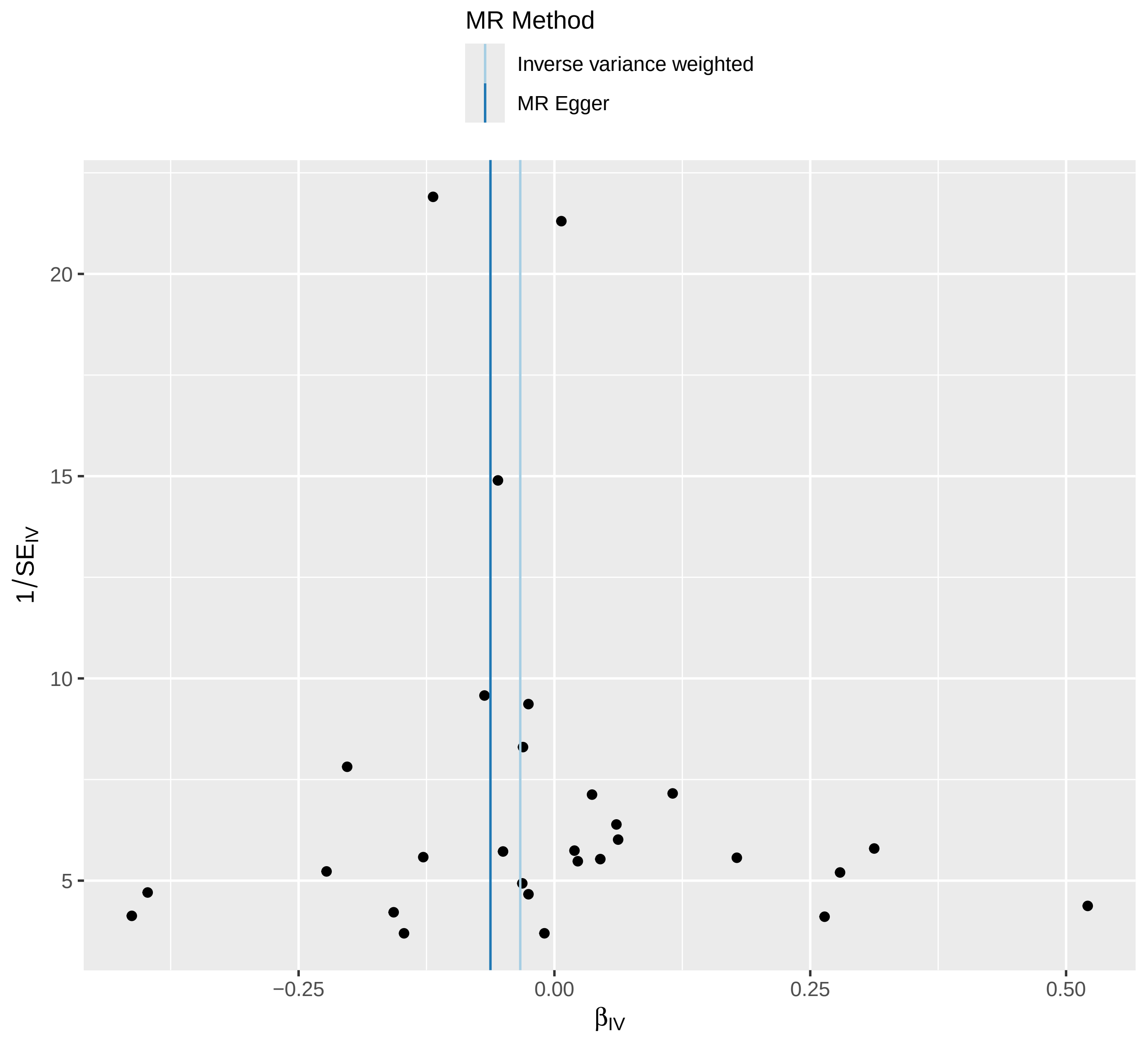
MR Method
Inverse variance weighted
MR Egger
20
15
10
5
1/SE_IV
−0.25
0.00
0.25
0.50
β_IV

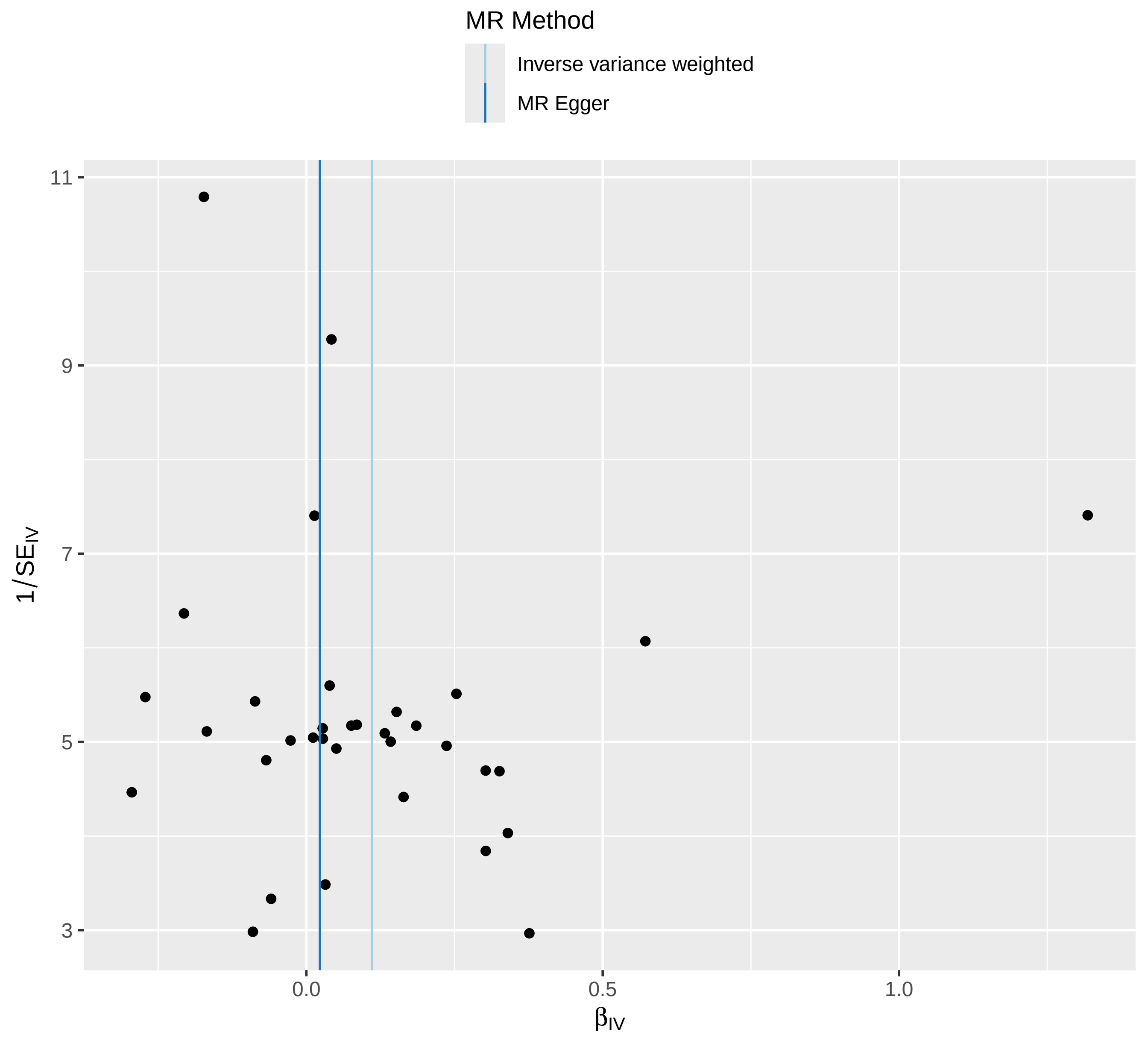
MR Method
Inverse variance weighted
MR Egger
11
9
7
5
3
$1/SE_{IV}$
0.0
0.5
1.0
β_{IV}

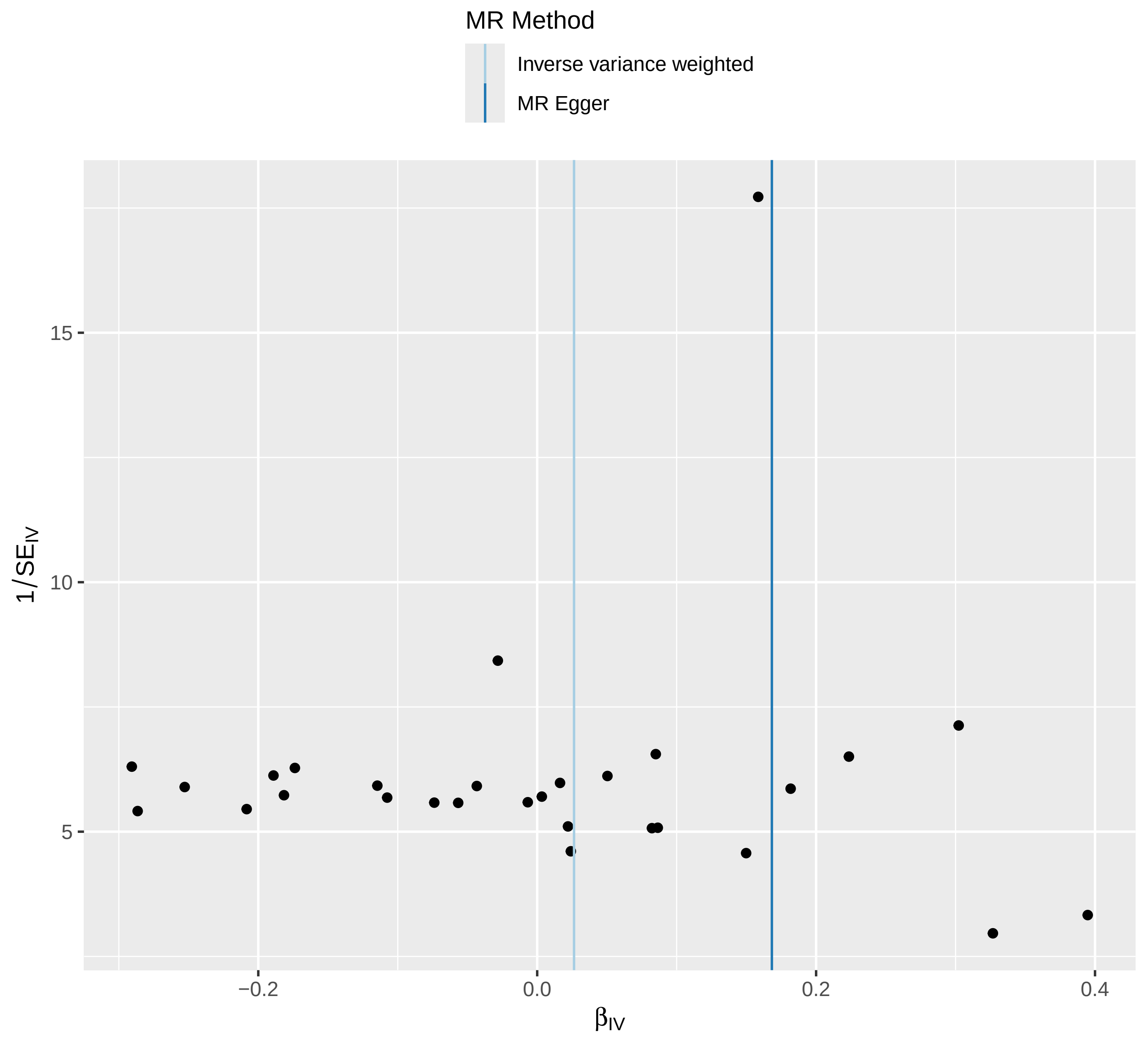
MR Method
Inverse variance weighted
MR Egger
1/SE_IV
15
10
5
−0.2
0.0
0.2
0.4
β_IV

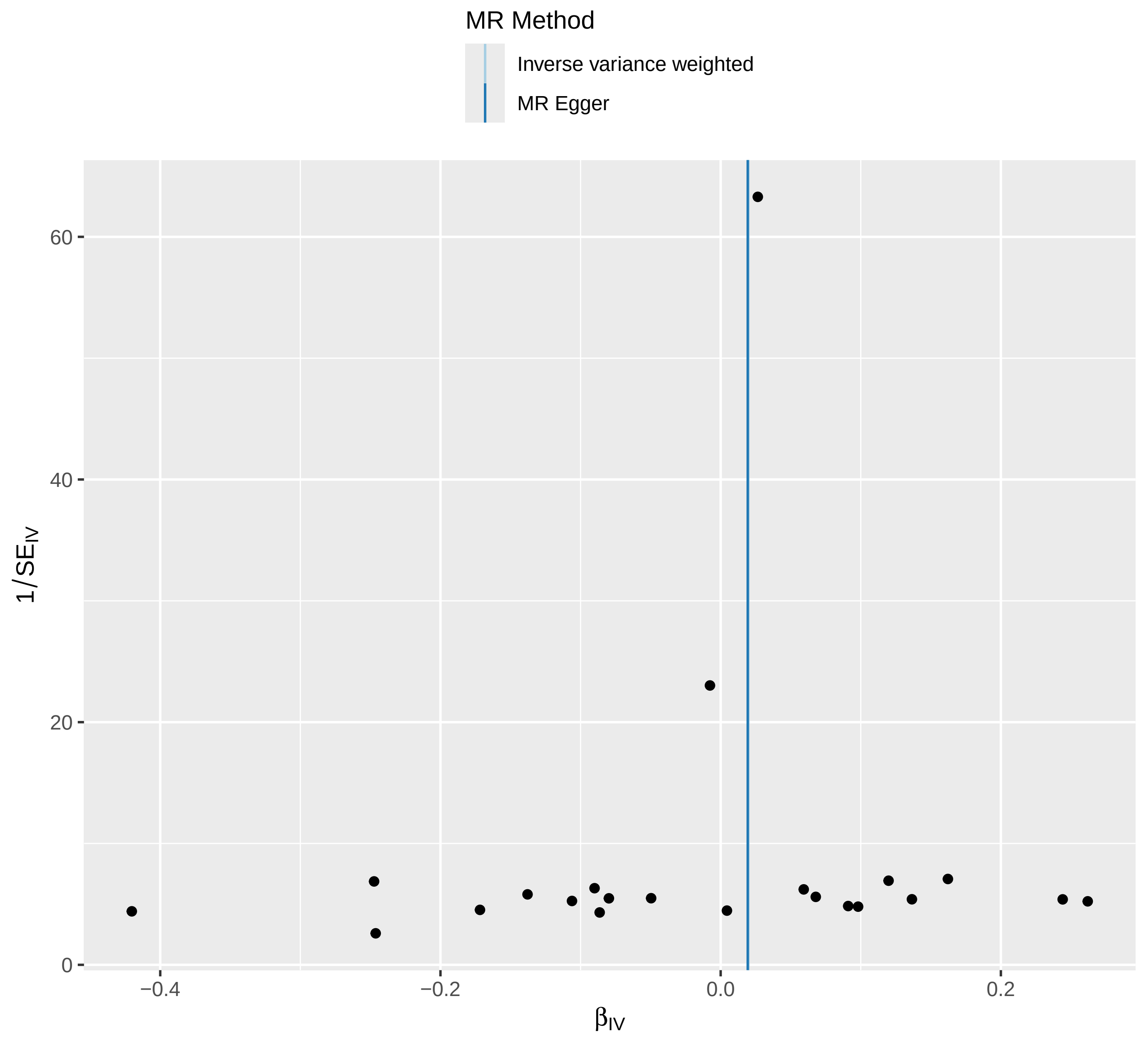

MR Method
Inverse variance weighted
MR Egger
60
40
20
0
−0.4
−0.2
0.0
0.2
$1/SE_{IV}$
β_{IV}

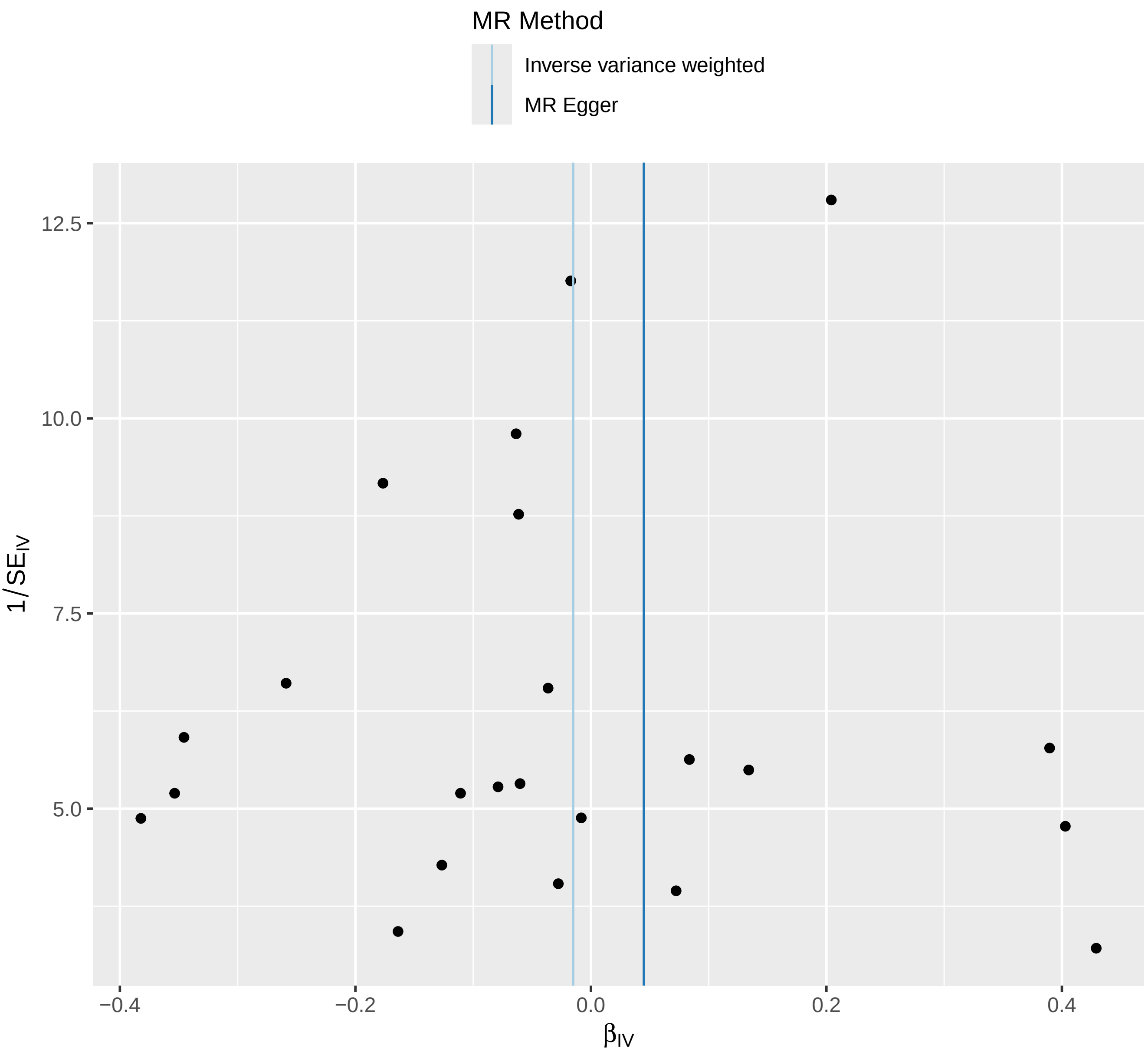
MR Method
Inverse variance weighted
MR Egger
12.5
10.0
7.5
5.0
1/SE_IV
−0.4
−0.2
0.0
0.2
0.4
β_IV

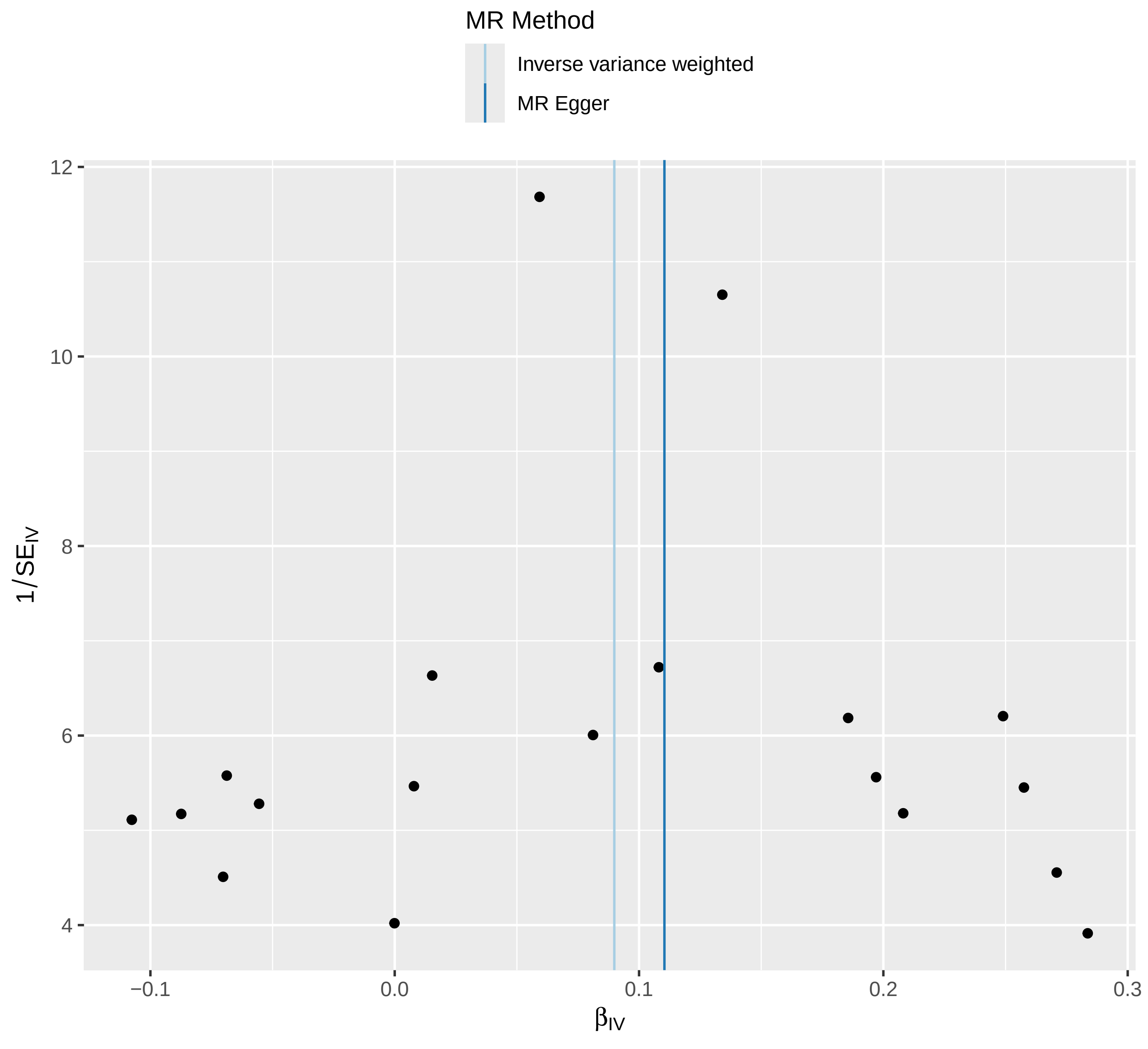
MR Method
Inverse variance weighted
MR Egger
12
10
8
6
4
1/SE$_{IV}$
−0.1
0.0
0.1
0.2
0.3
β$_{IV}$

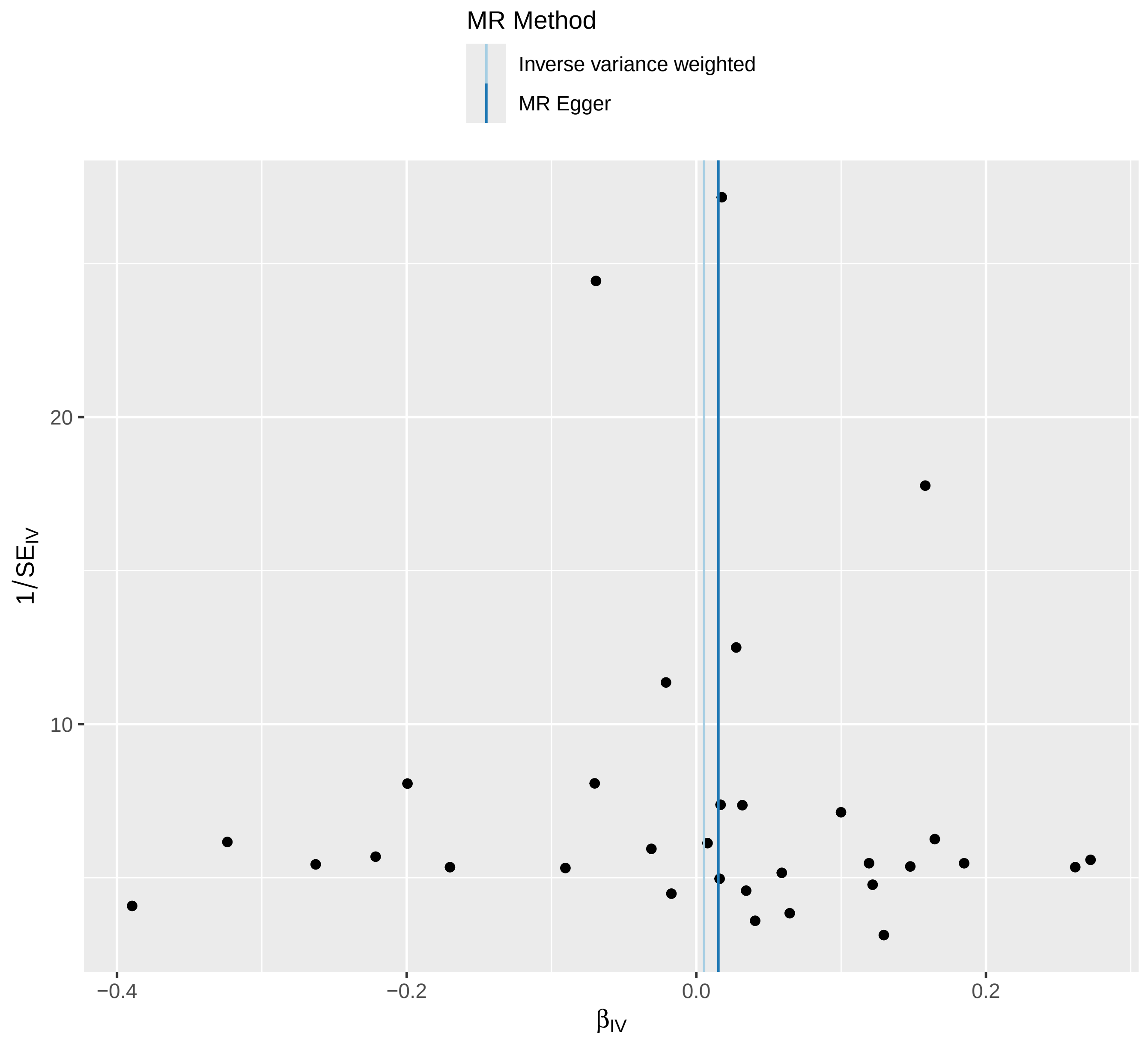
MR Method
Inverse variance weighted
MR Egger
20
10
1/SE_IV
−0.4
−0.2
0.0
0.2
β_IV

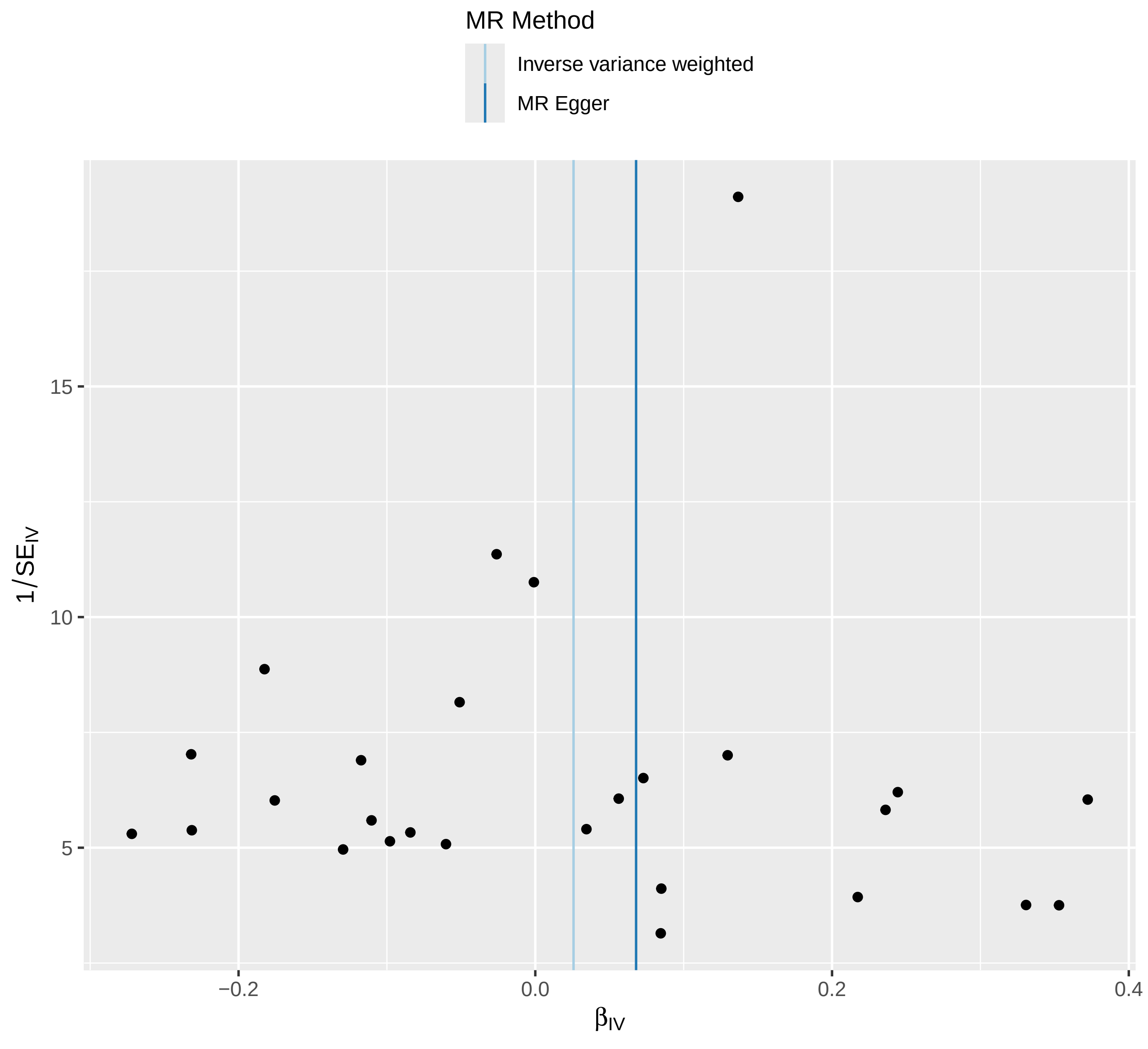
MR Method
Inverse variance weighted
MR Egger
15
10
5
1/SE_IV
−0.2
0.0
0.2
0.4
β_IV

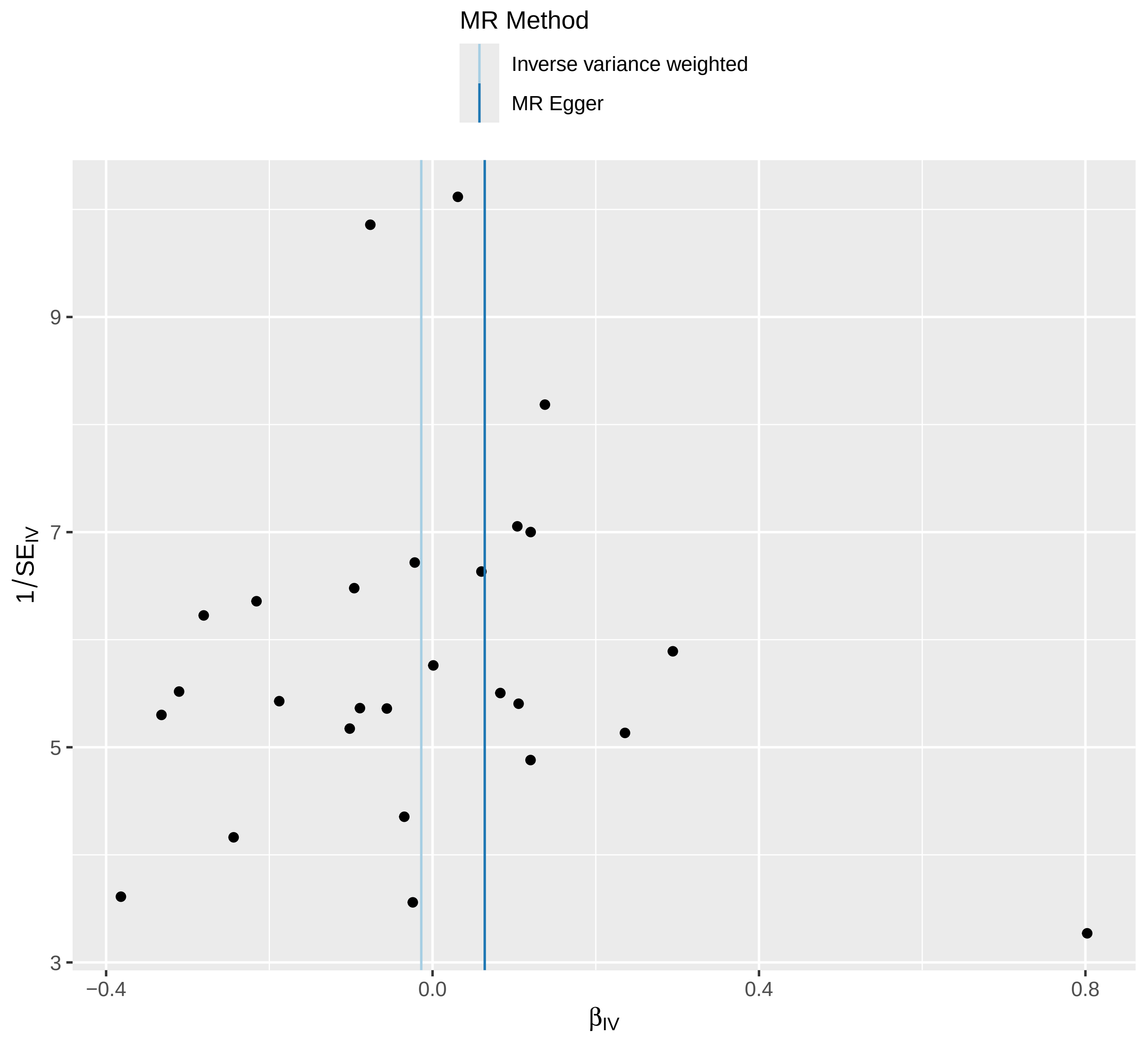

MR Method
Inverse variance weighted
MR Egger
1/SE_{IV}
9
7
5
3
−0.4
0.0
0.4
0.8
β_{IV}

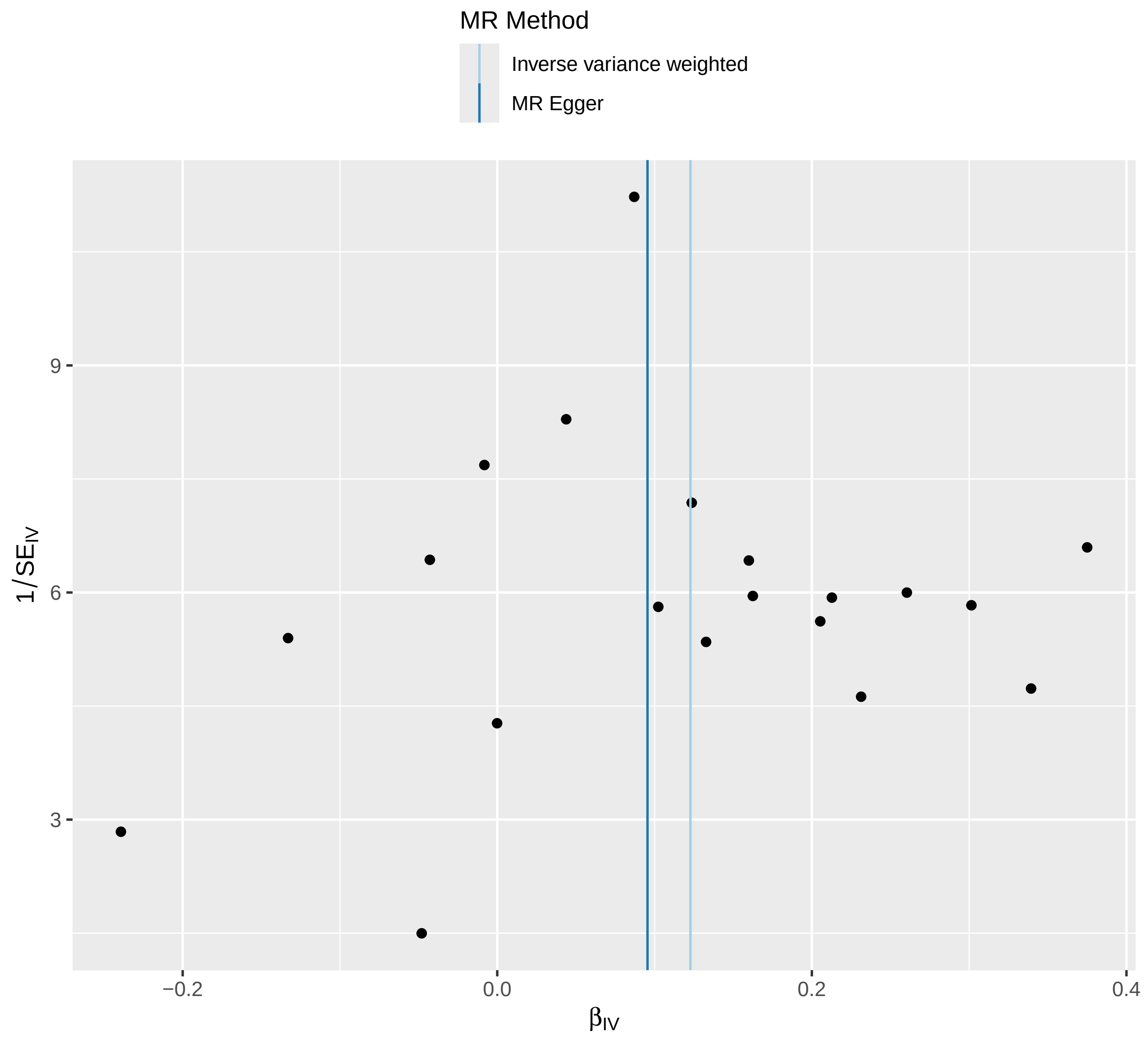

MR Method
Inverse variance weighted
MR Egger
9
6
3
−0.2
0.0
0.2
0.4
1/SE_IV
β_IV

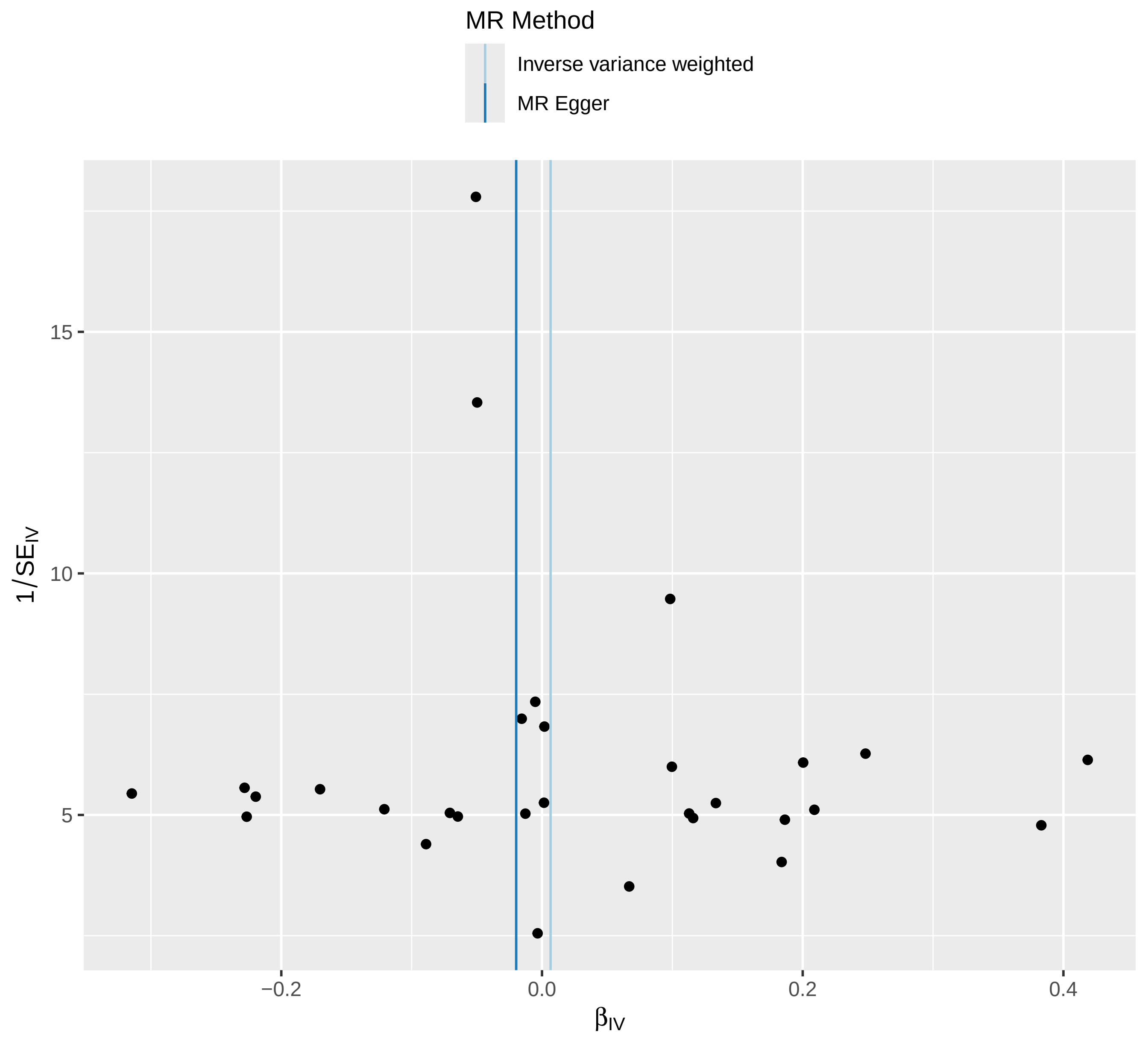
MR Method
Inverse variance weighted
MR Egger
1/SE_IV
15
10
5
−0.2
0.0
0.2
0.4
β_IV

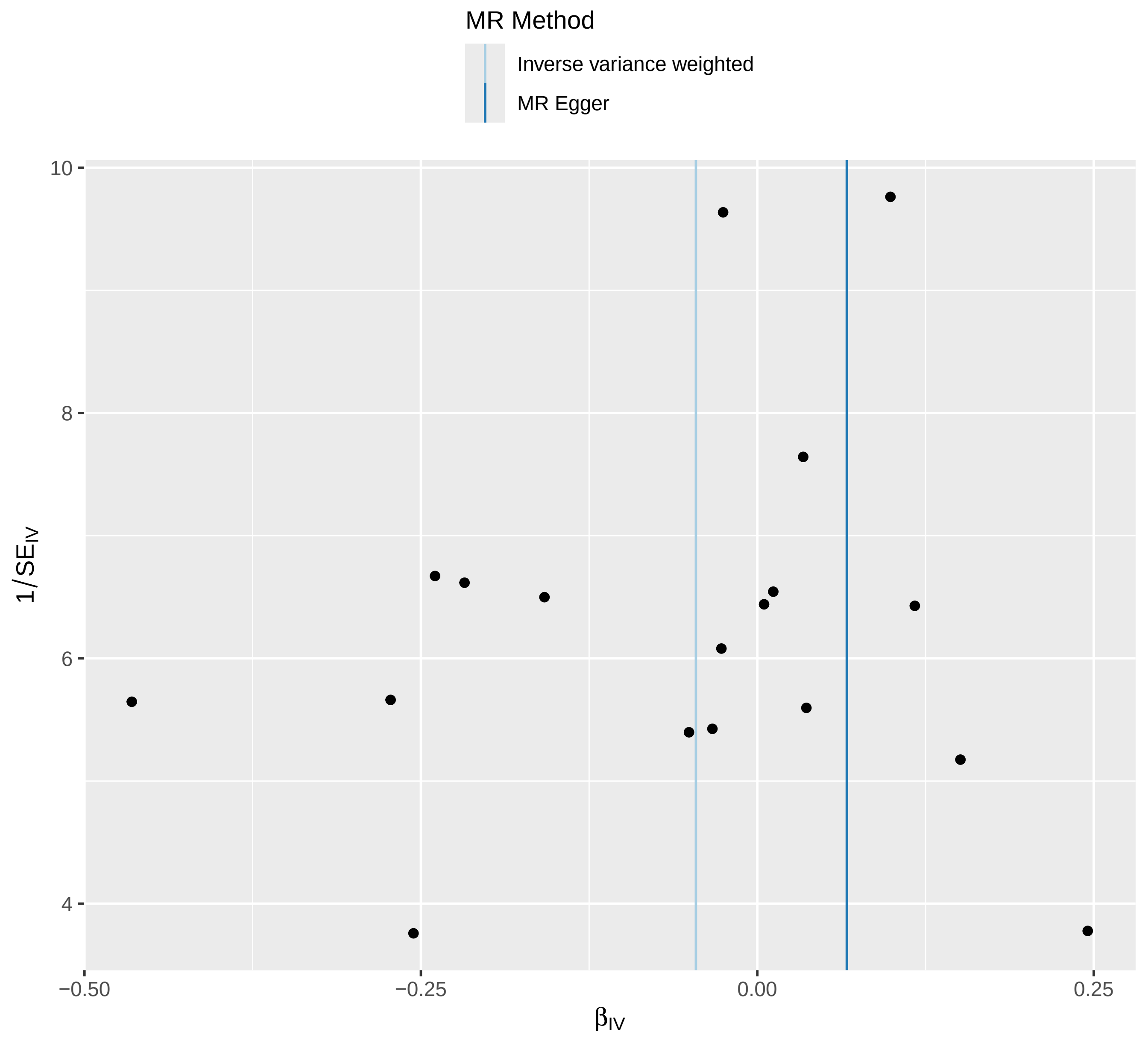
MR Method
Inverse variance weighted
MR Egger
10
8
6
4
1/SE_IV
−0.50
−0.25
0.00
0.25
β_IV

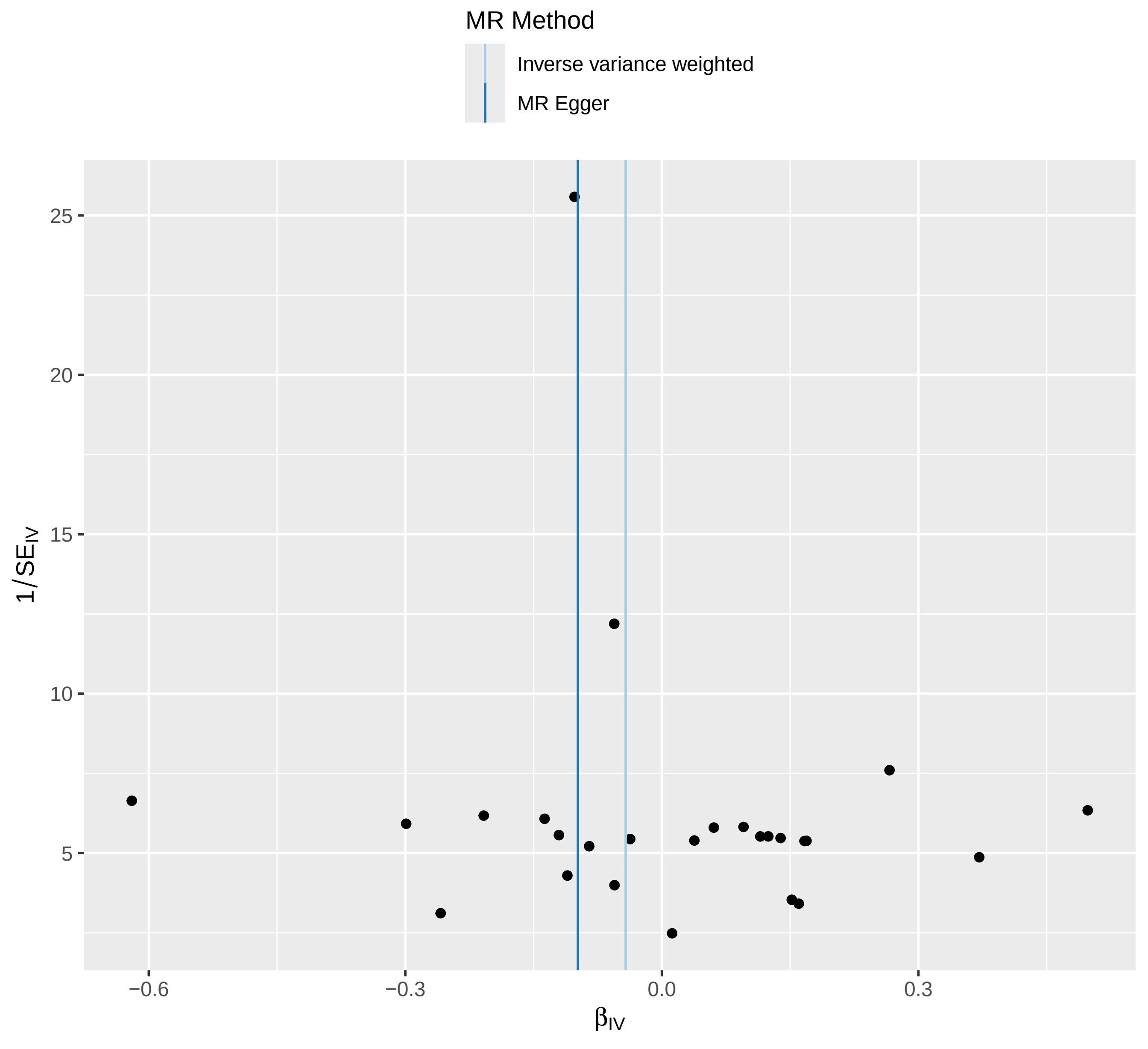
MR Method
Inverse variance weighted
MR Egger
25
20
15
10
5
$1/SE_{IV}$
−0.6
−0.3
0.0
0.3
β_{IV}

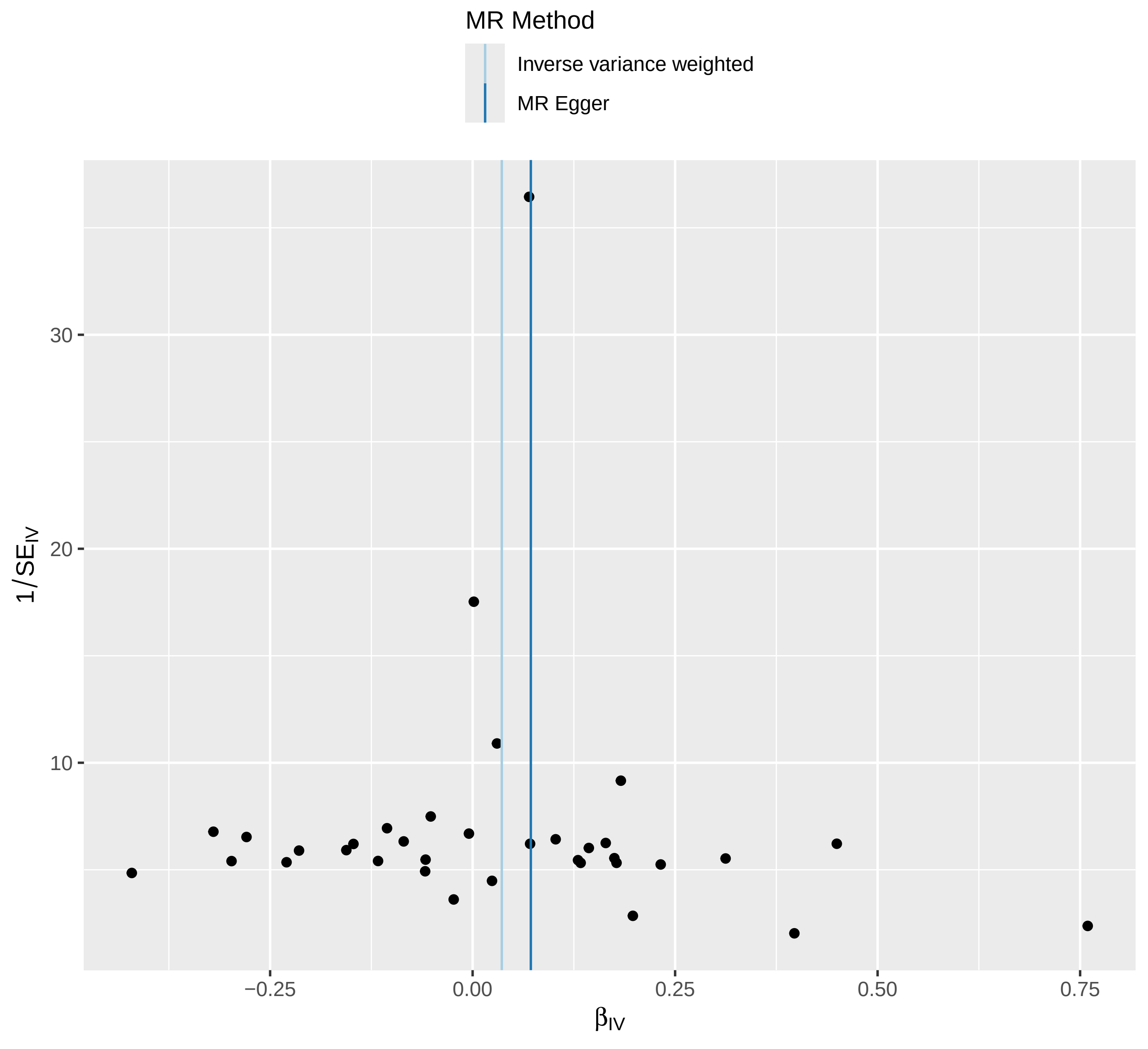
MR Method
Inverse variance weighted
MR Egger
1/SE_IV
30
20
10
β_IV
−0.25
0.00
0.25
0.50
0.75

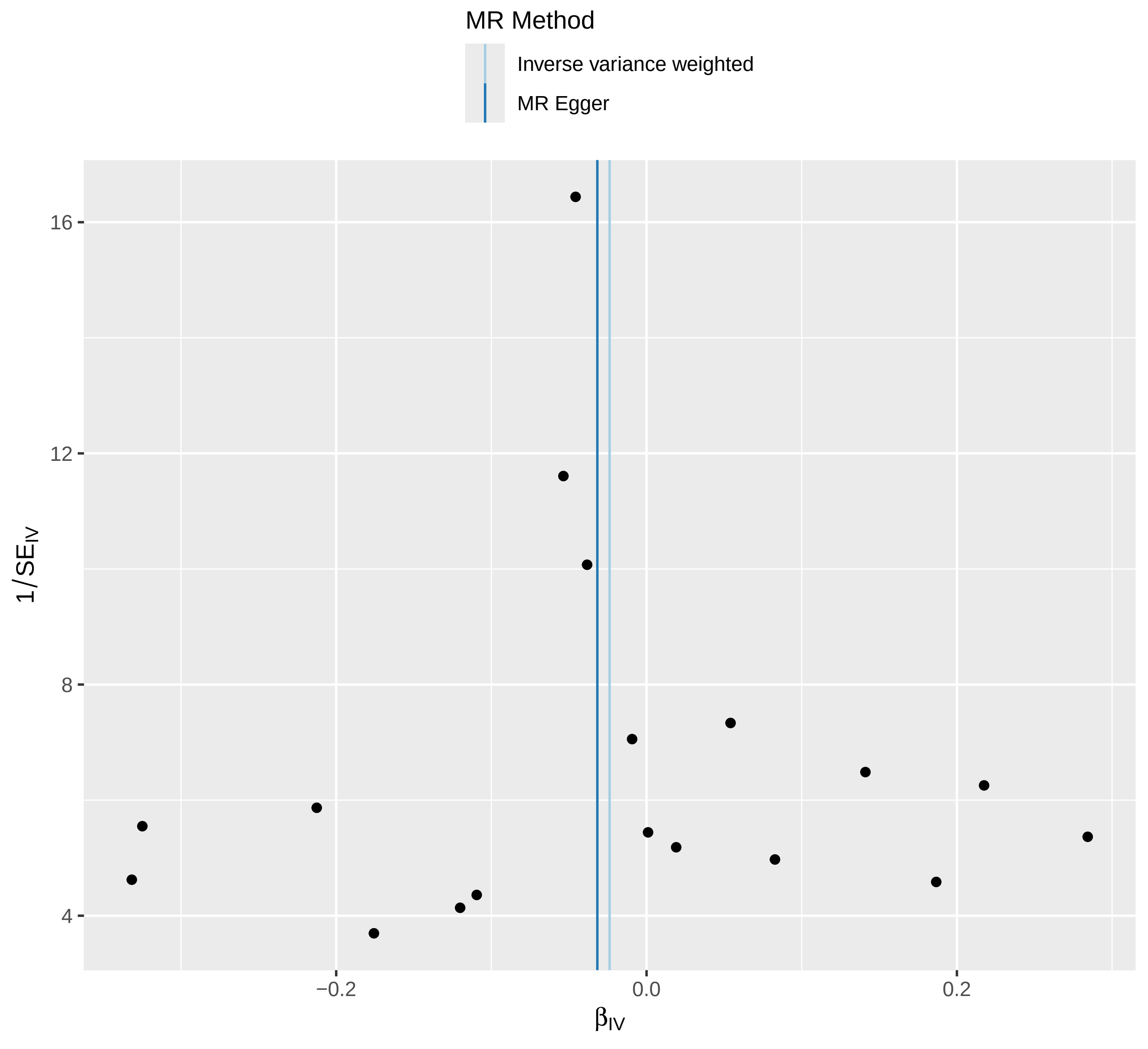

MR Method
Inverse variance weighted
MR Egger
16
12
8
4
1/SE_IV
−0.2
0.0
0.2
β_IV

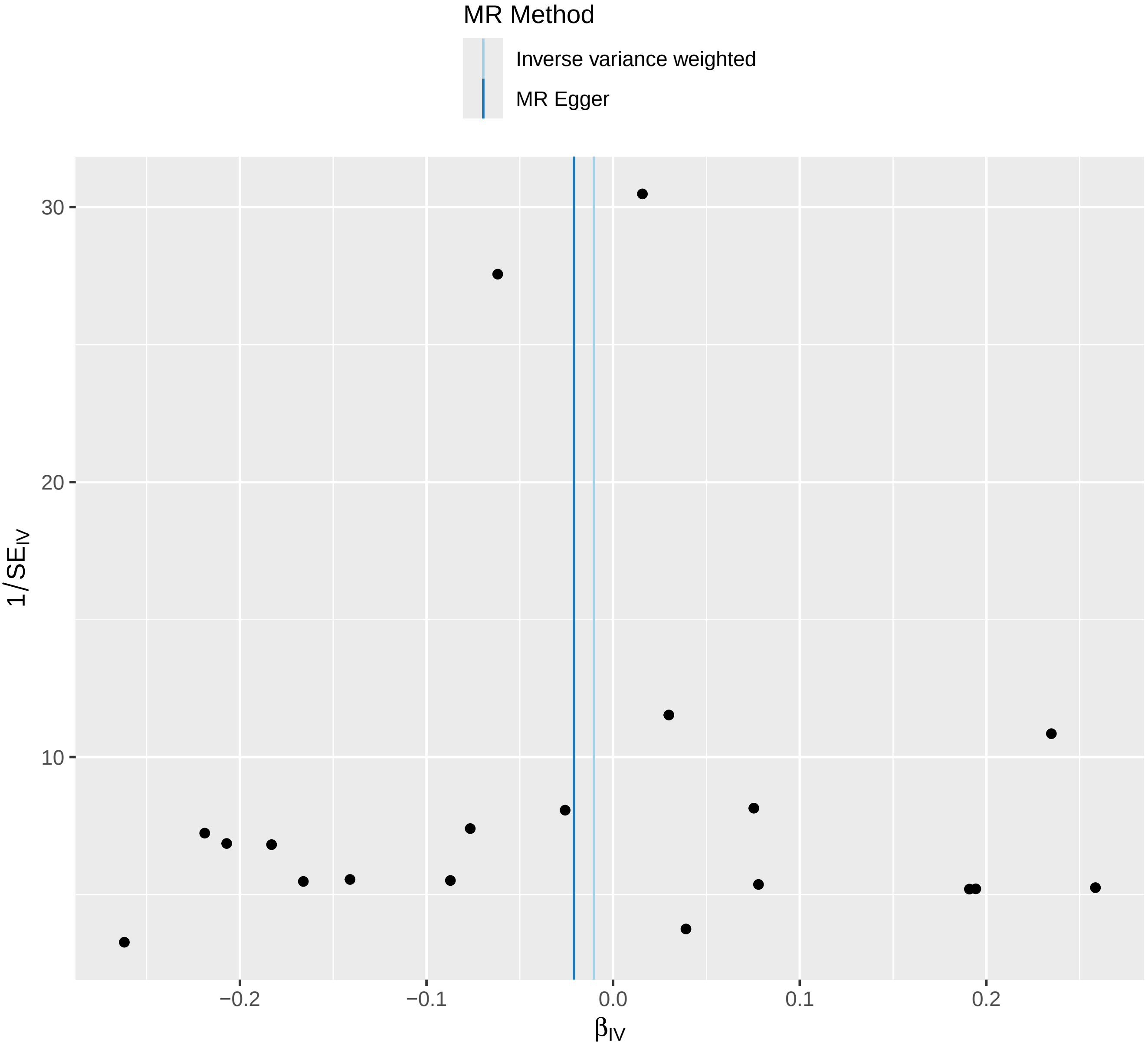
MR Method
Inverse variance weighted
MR Egger
30
20
10
$1/SE_{IV}$
−0.2
−0.1
0.0
0.1
0.2
β_{IV}

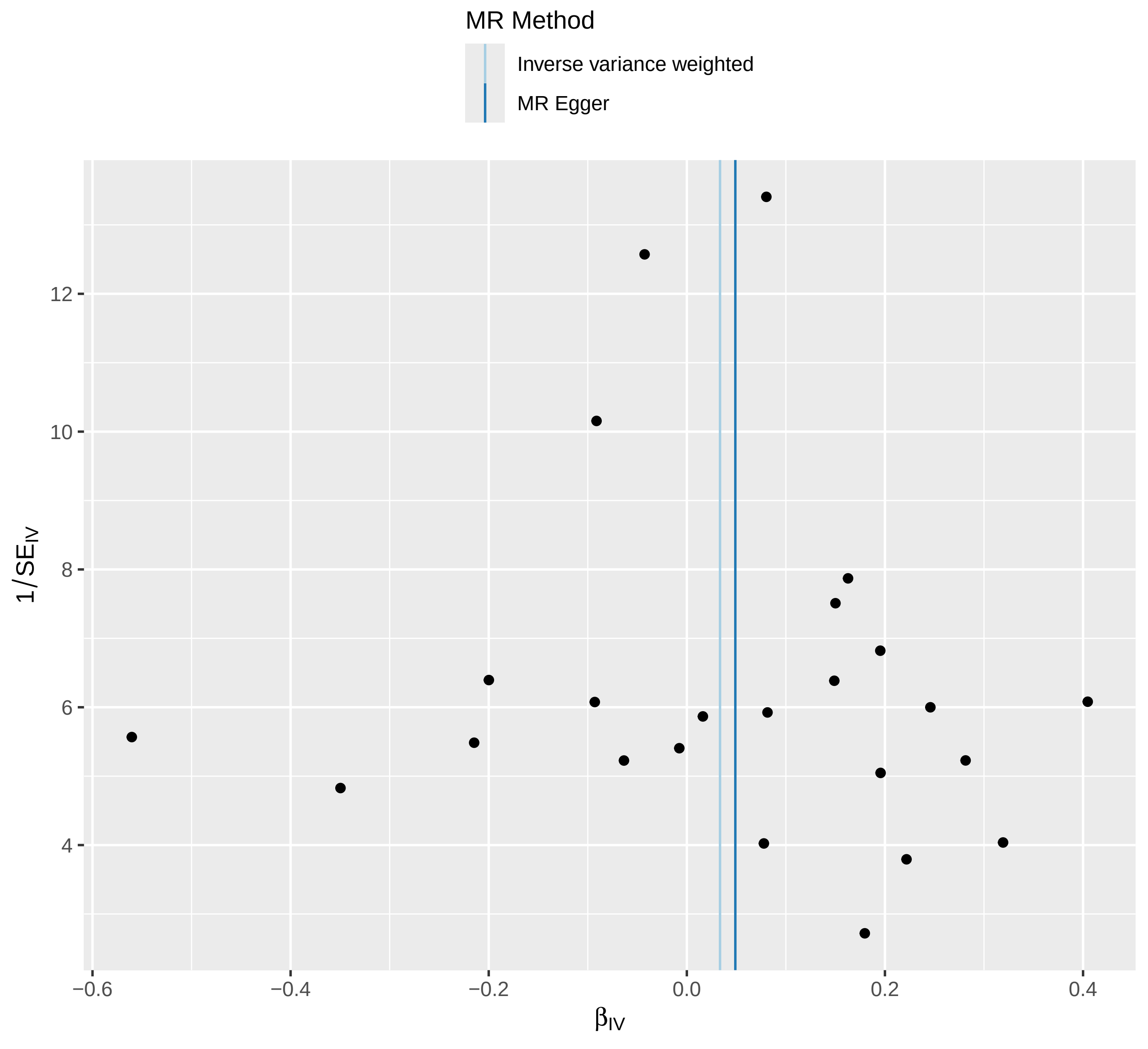

MR Method
Inverse variance weighted
MR Egger
1/SE_IV
β_IV
12
10
8
6
4
−0.6
−0.4
−0.2
0.0
0.2
0.4

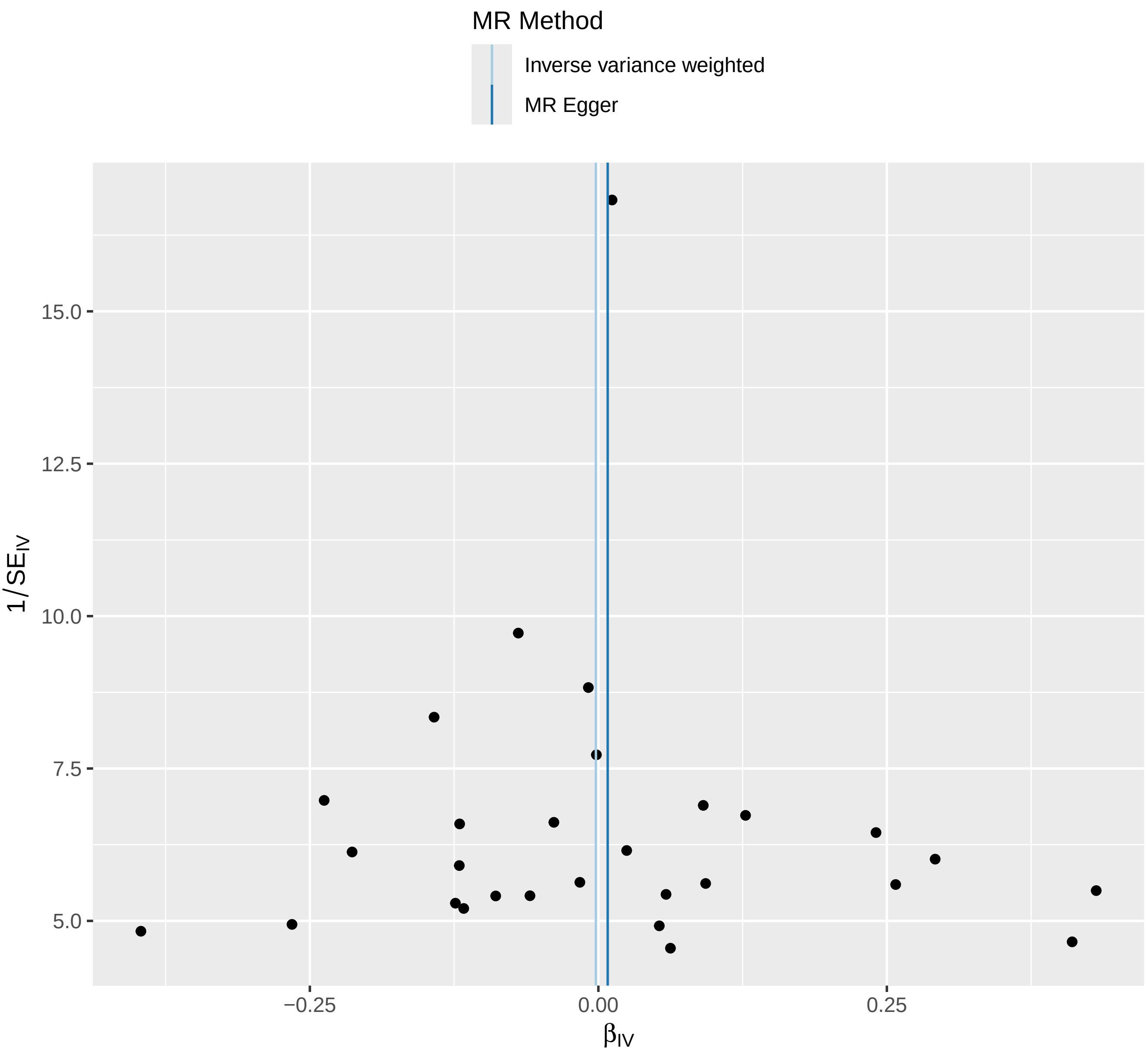
MR Method
Inverse variance weighted
MR Egger
15.0
12.5
10.0
7.5
5.0
1/SE_IV
−0.25
0.00
0.25
β_IV

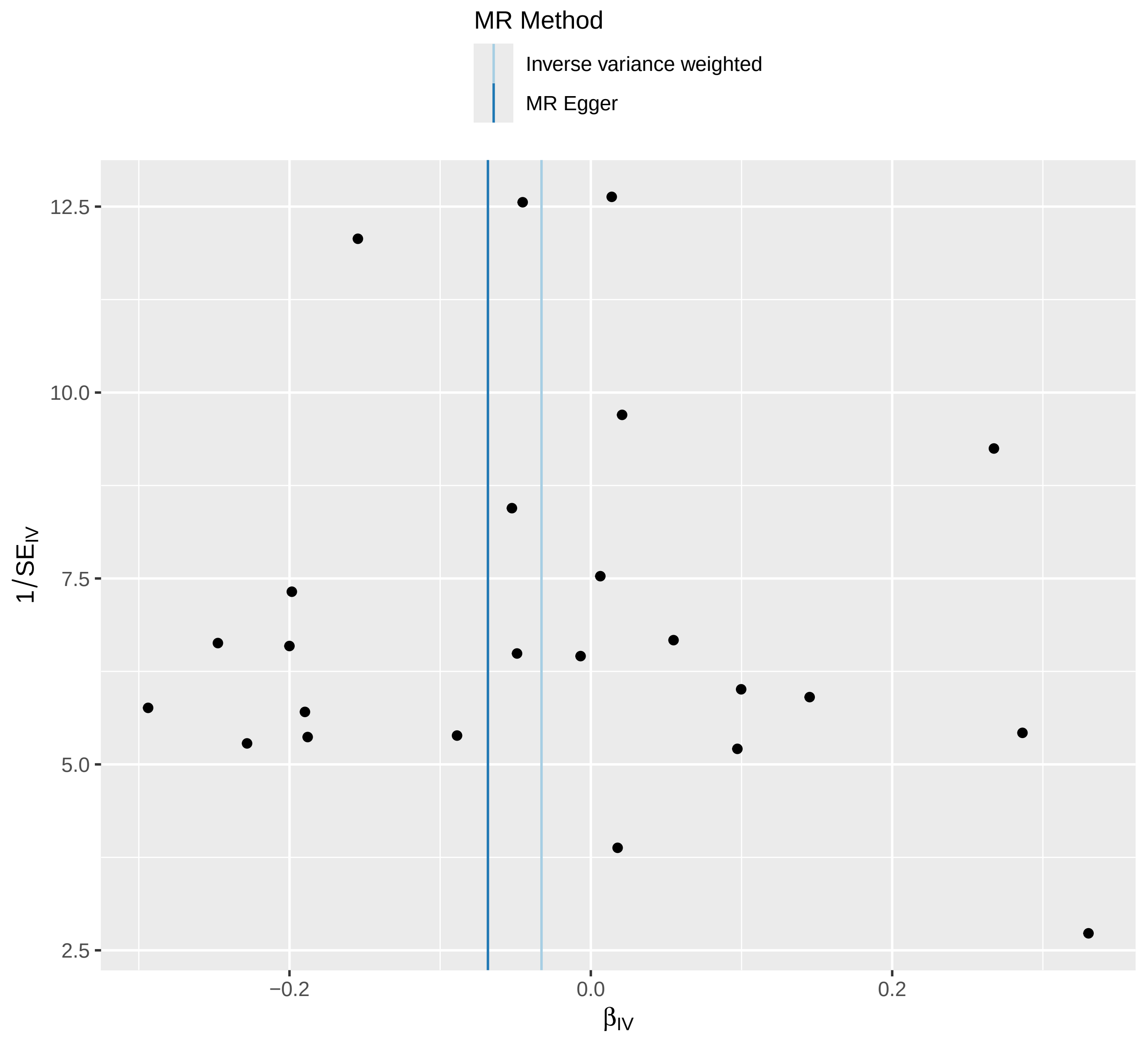
MR Method
Inverse variance weighted
MR Egger
12.5
10.0
7.5
5.0
2.5
$1/SE_{IV}$
−0.2
0.0
0.2
β_{IV}

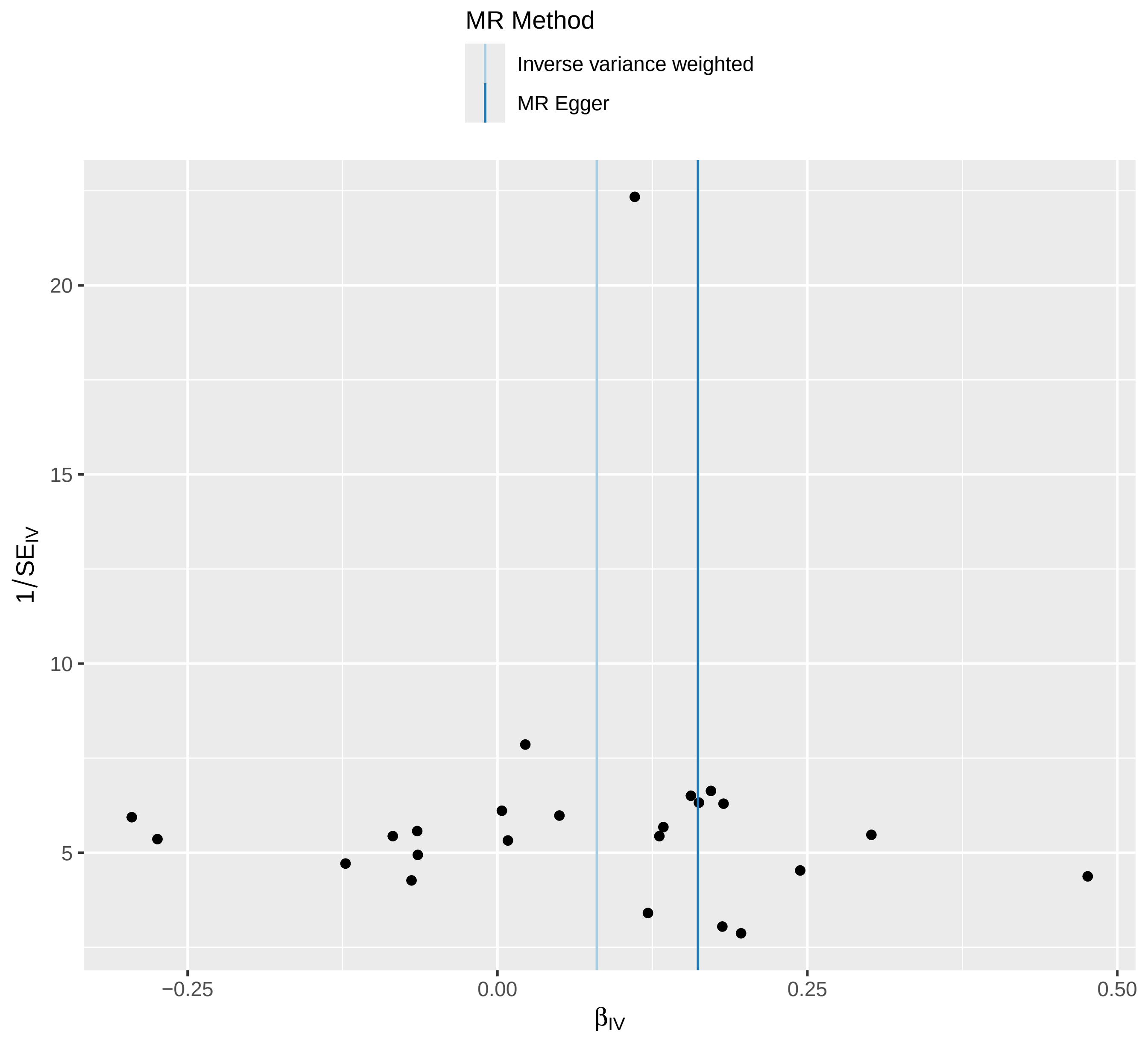
MR Method
Inverse variance weighted
MR Egger
20
15
10
5
1/SE_IV
−0.25
0.00
0.25
0.50
β_IV

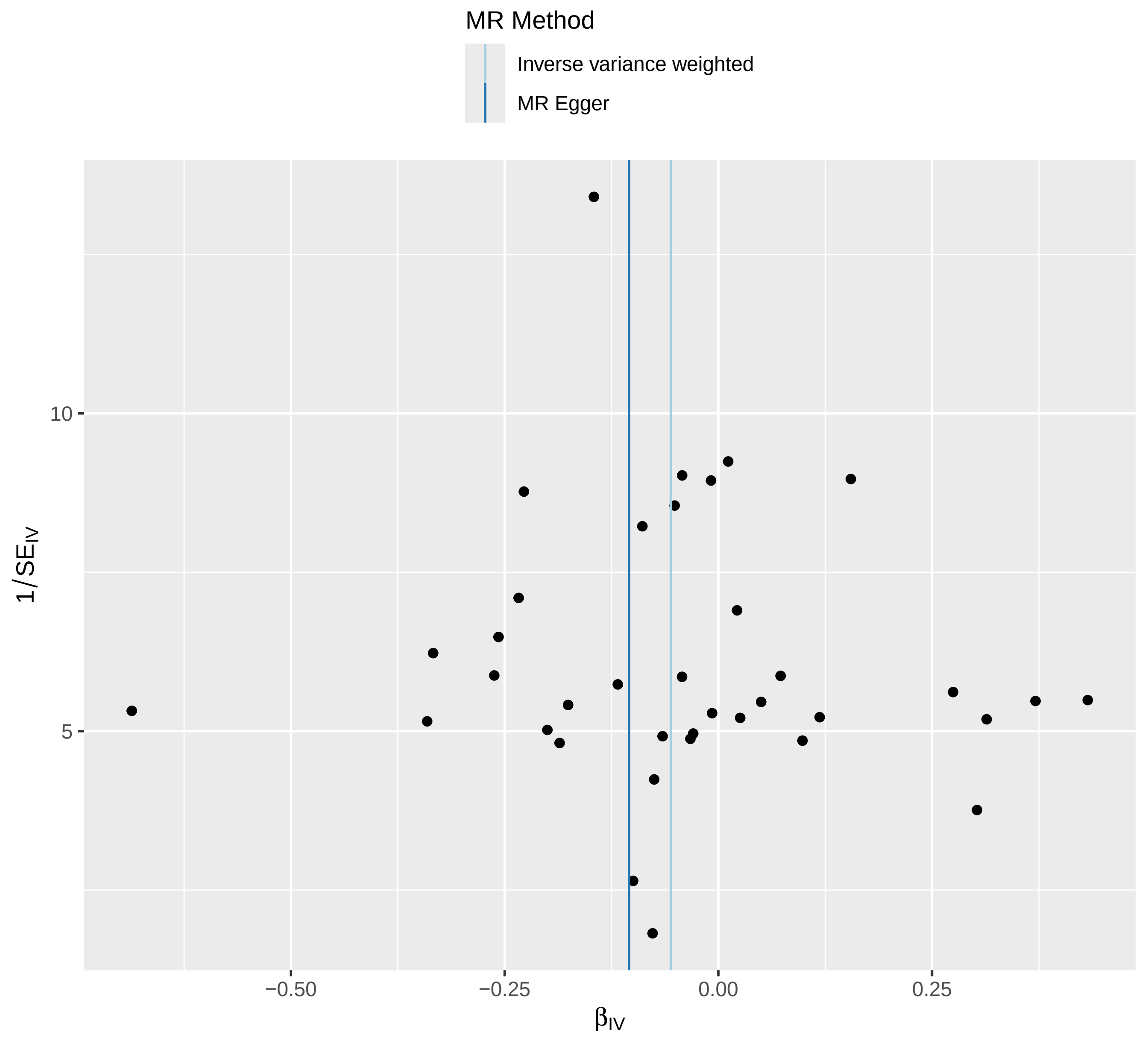
MR Method
Inverse variance weighted
MR Egger
10
5
1/SE$_{IV}$
−0.50
−0.25
0.00
0.25
β$_{IV}$

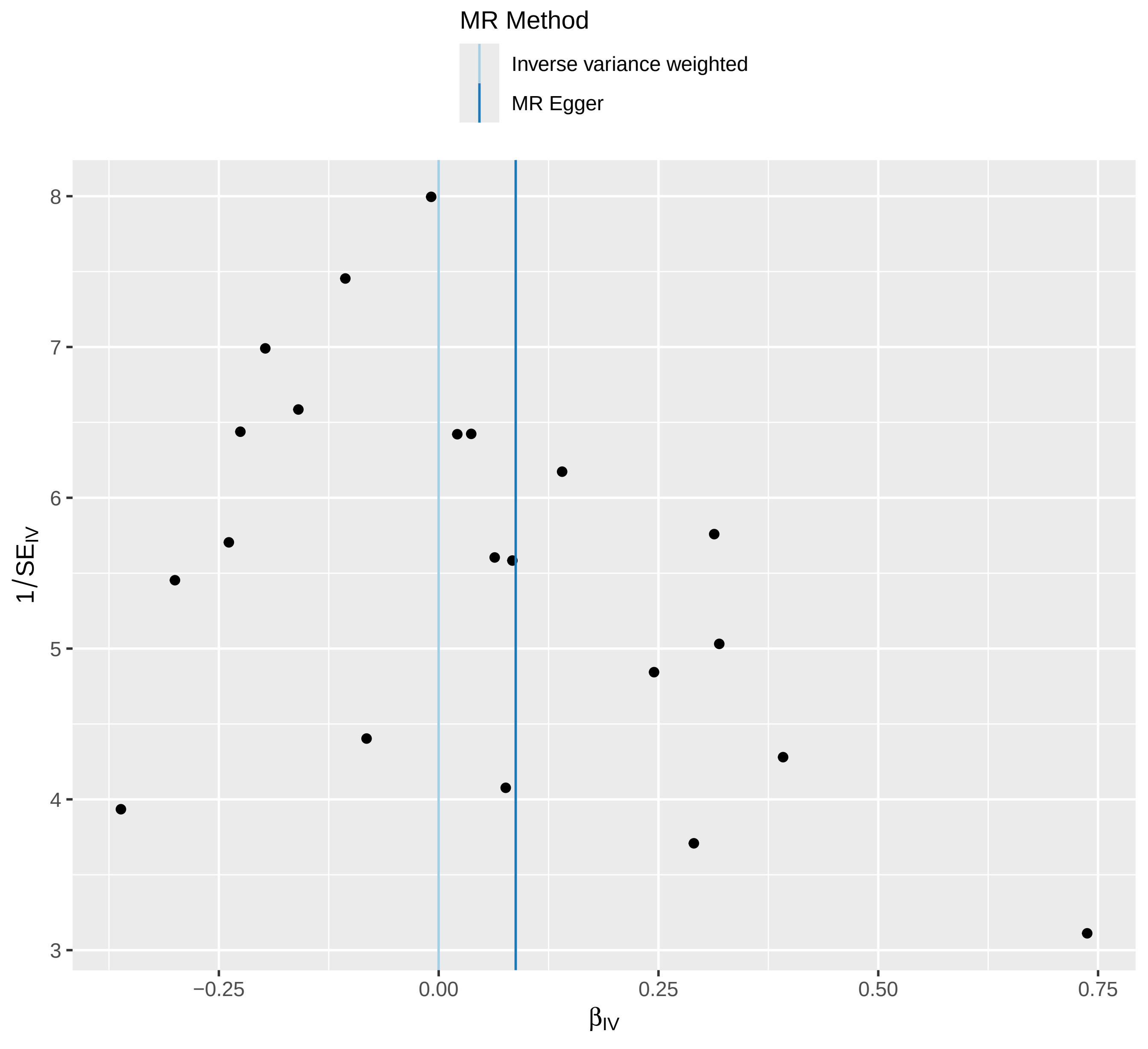
MR Method
Inverse variance weighted
MR Egger
8
7
6
5
4
3
1/SE_IV
−0.25
0.00
0.25
0.50
0.75
β_IV

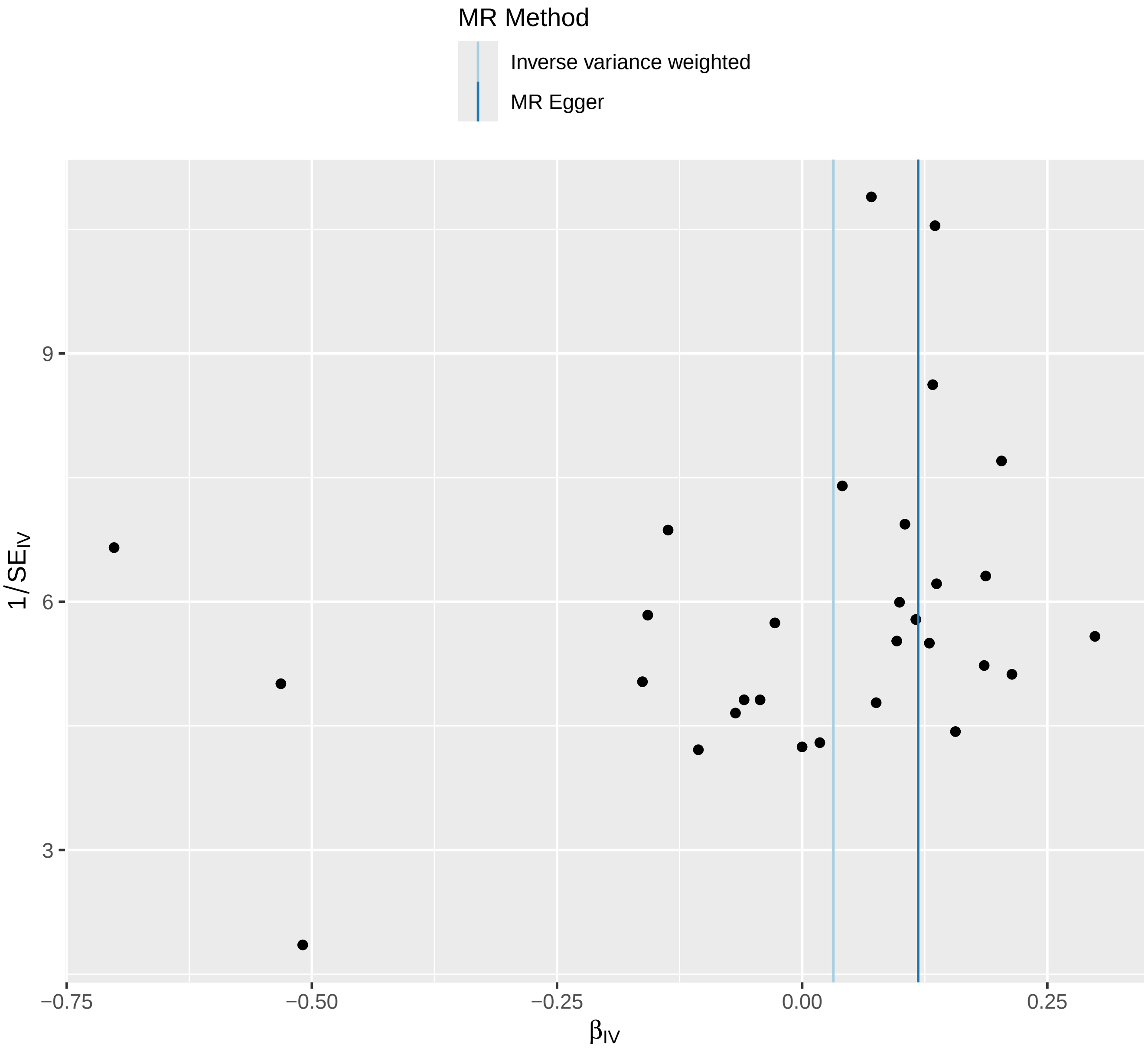
MR Method
Inverse variance weighted
MR Egger
9
6
3
1/SE_IV
−0.75
−0.50
−0.25
0.00
0.25
β_IV

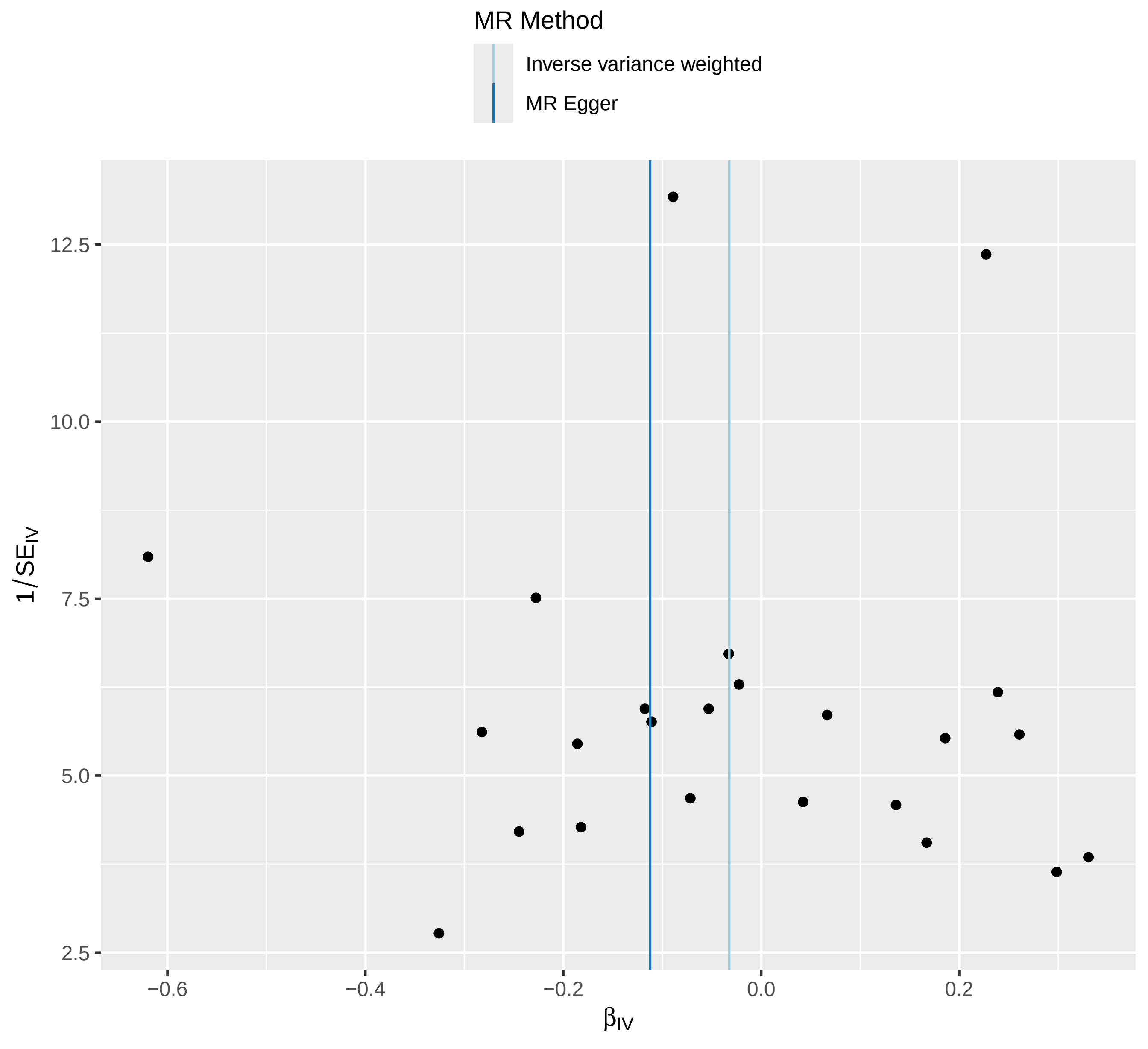
MR Method
Inverse variance weighted
MR Egger
12.5
10.0
7.5
5.0
2.5
1/SE_IV
−0.6
−0.4
−0.2
0.0
0.2
β_IV

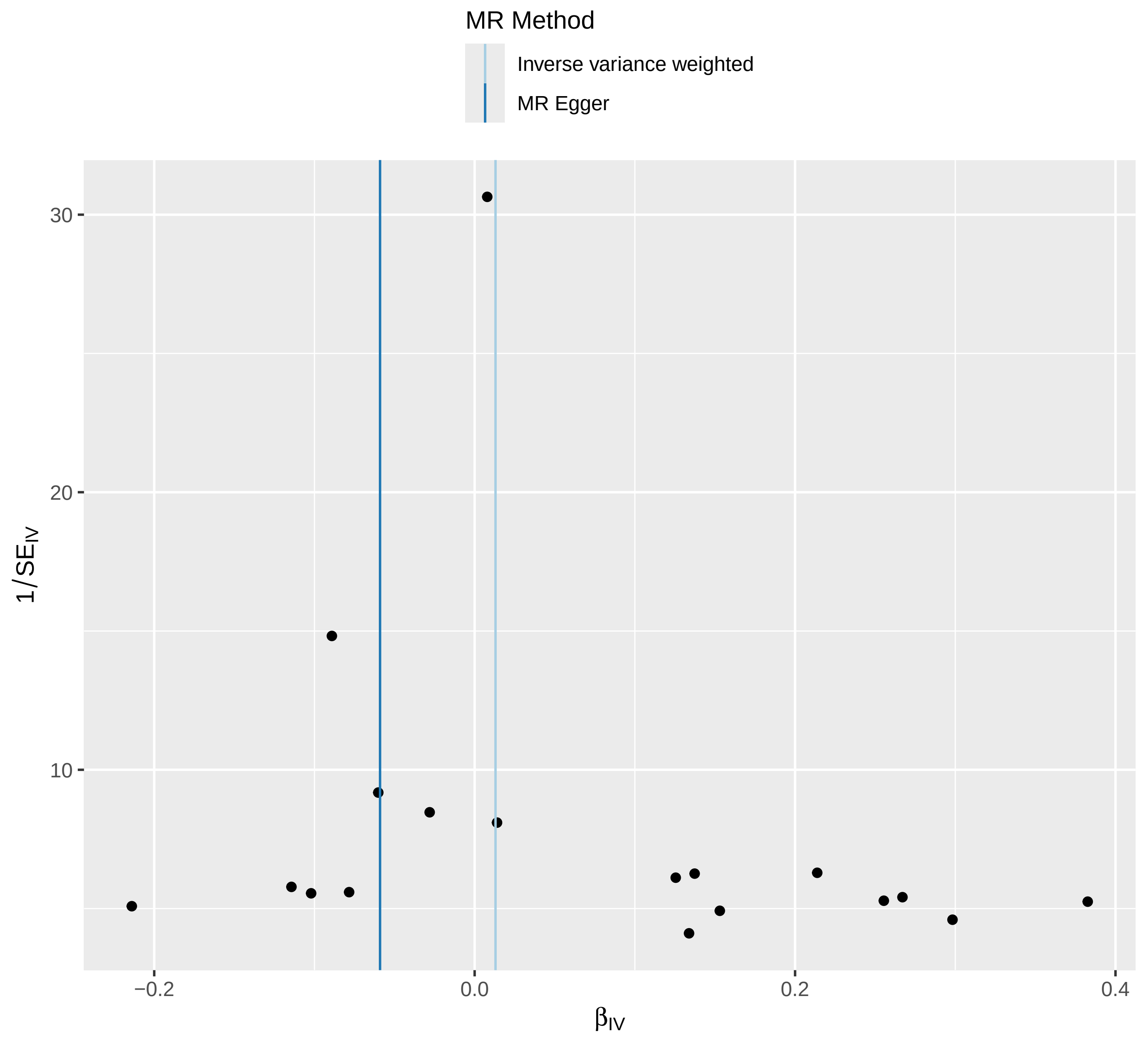
MR Method
Inverse variance weighted
MR Egger
30
20
10
1/SE$_{IV}$
−0.2
0.0
0.2
0.4
β$_{IV}$

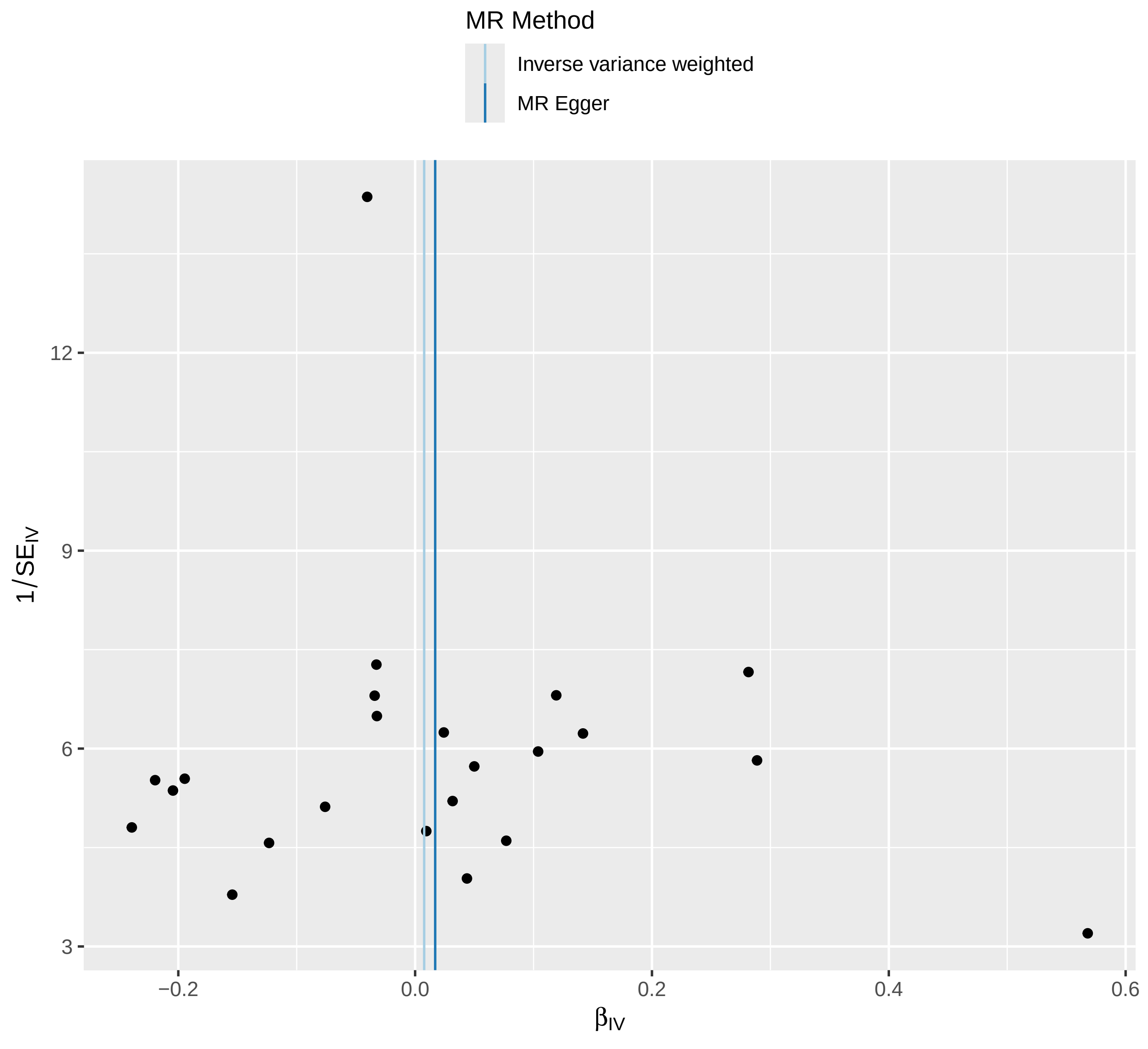
MR Method
Inverse variance weighted
MR Egger
1/SE_IV
12
9
6
3
−0.2
0.0
0.2
0.4
0.6
β_IV

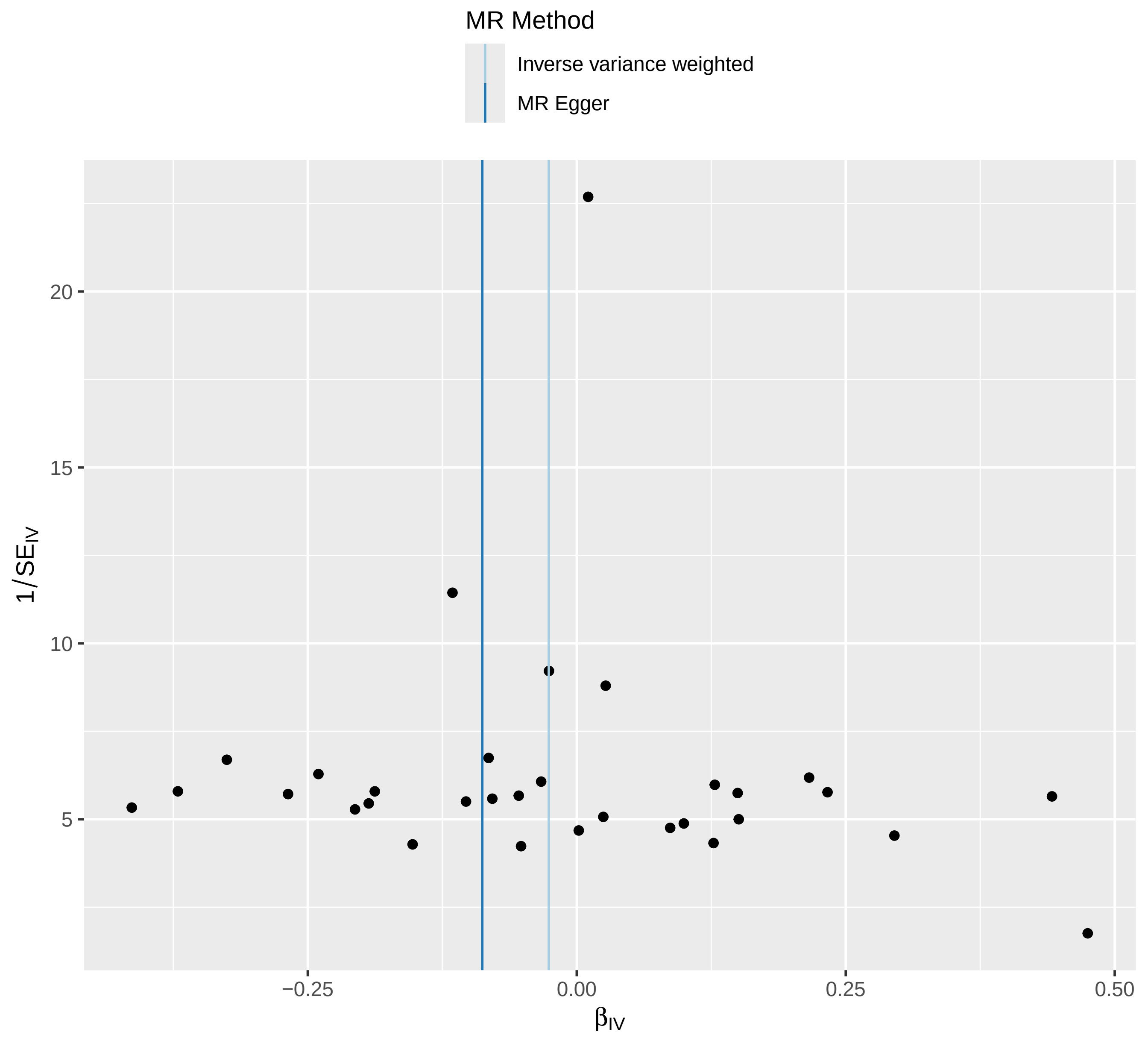

MR Method
Inverse variance weighted
MR Egger
20
15
10
5
1/SE$_{IV}$
−0.25
0.00
0.25
0.50
β$_{IV}$

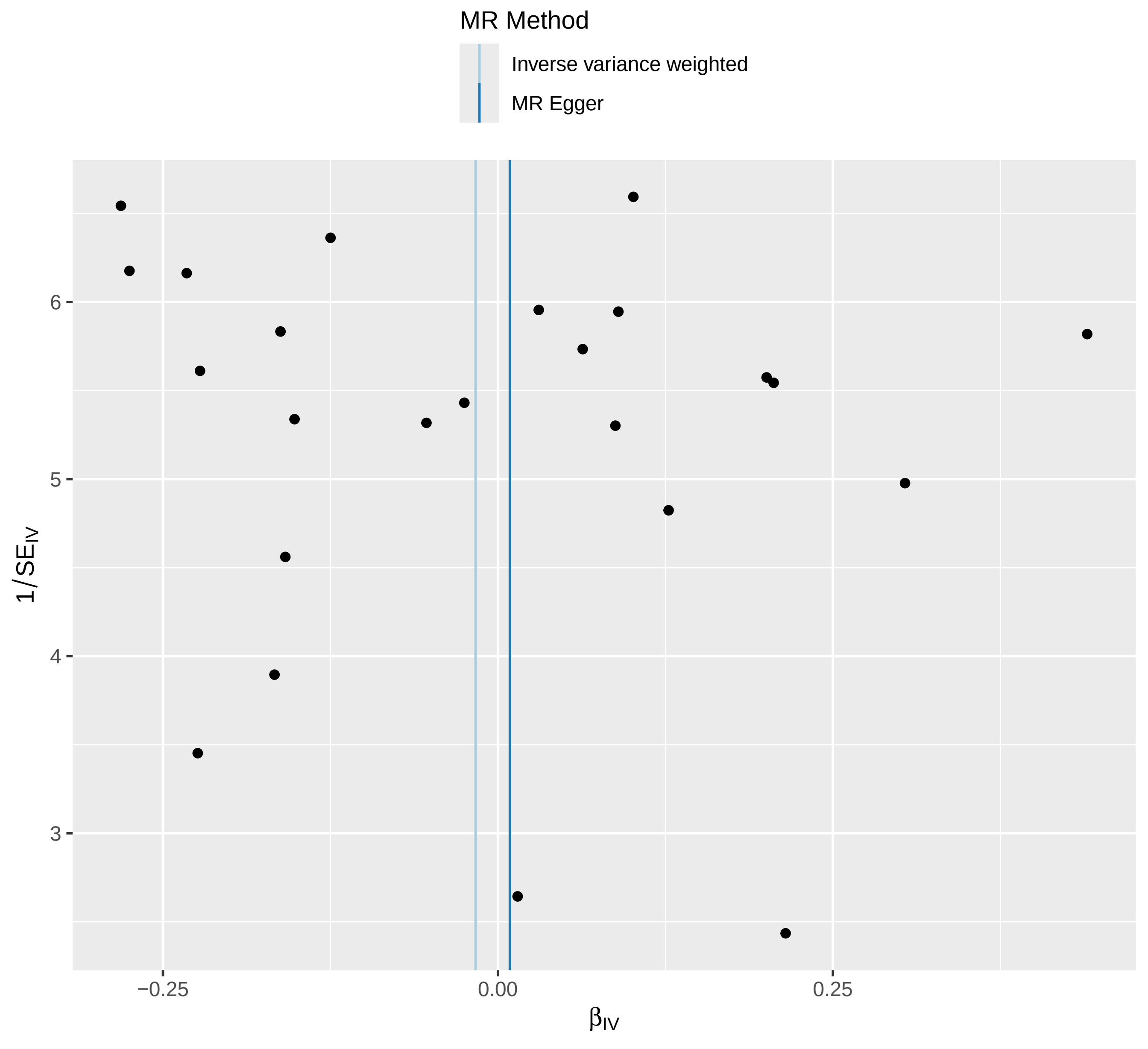
MR Method
Inverse variance weighted
MR Egger
1/SE_IV
6
5
4
3
−0.25
0.00
0.25
β_IV

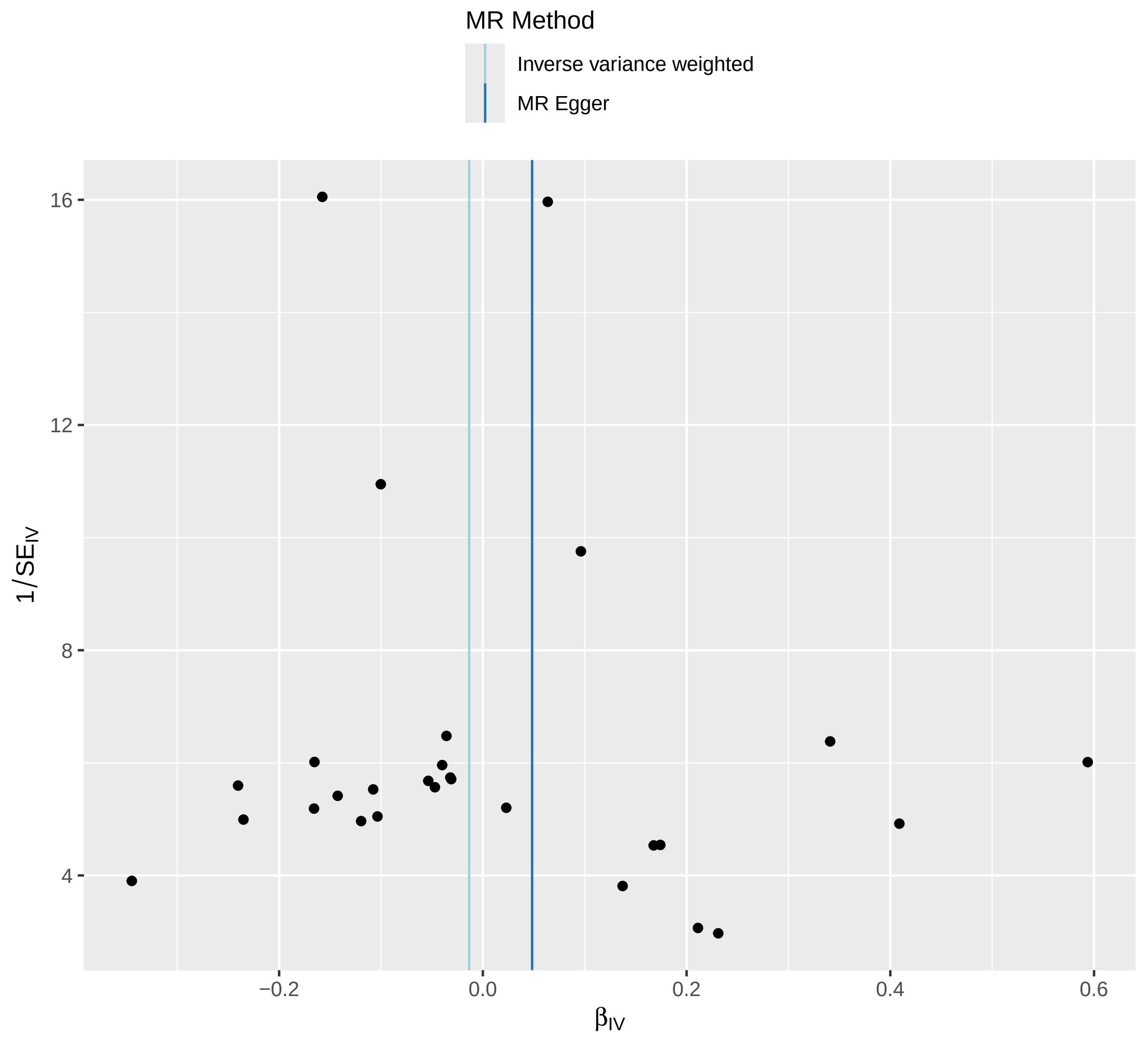
MR Method
Inverse variance weighted
MR Egger
16
12
8
4
1/SE_IV
−0.2
0.0
0.2
0.4
0.6
β_IV

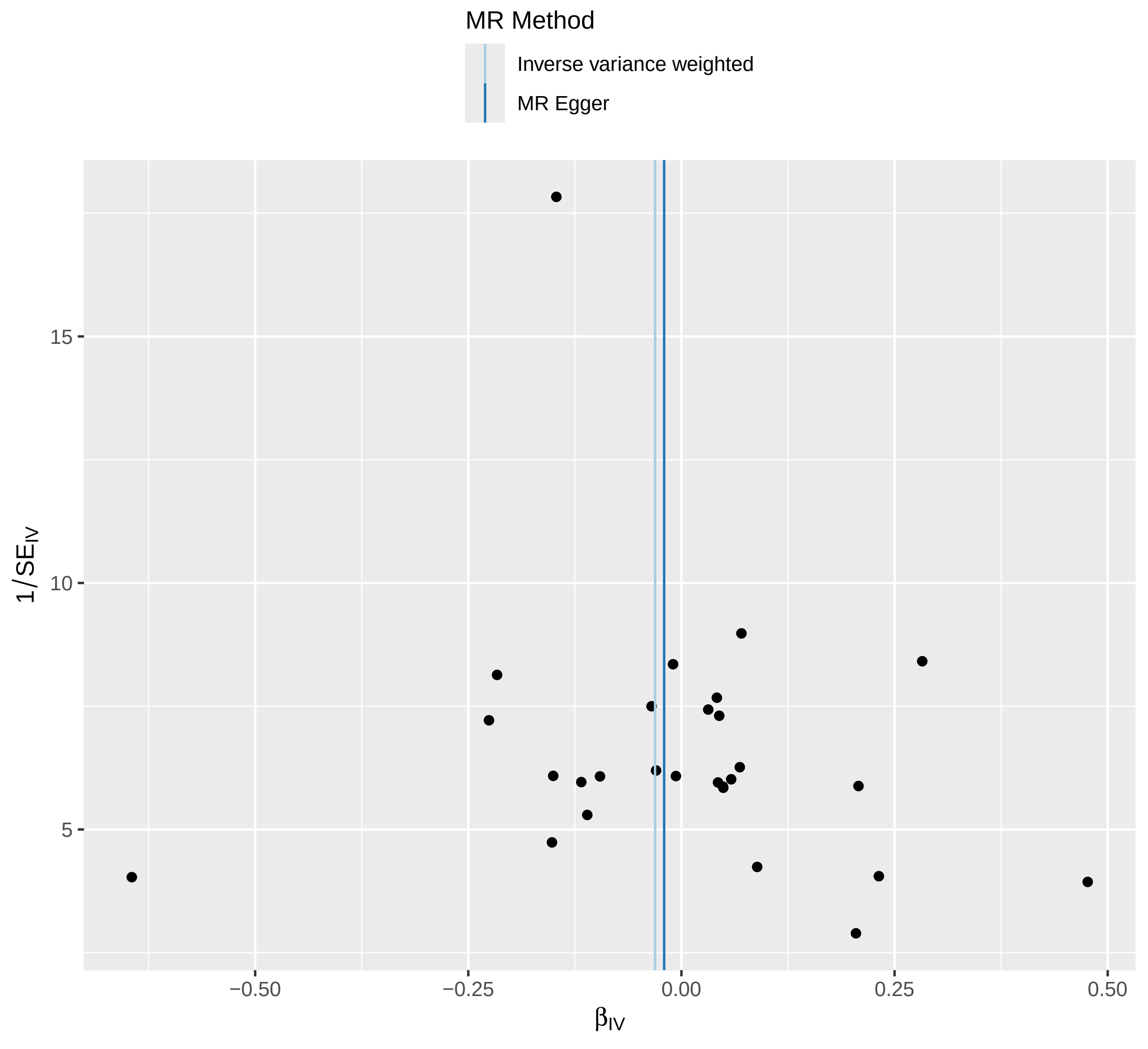

MR Method
Inverse variance weighted
MR Egger
15
10
5
1/SE$_{IV}$
−0.50
−0.25
0.00
0.25
0.50
β$_{IV}$

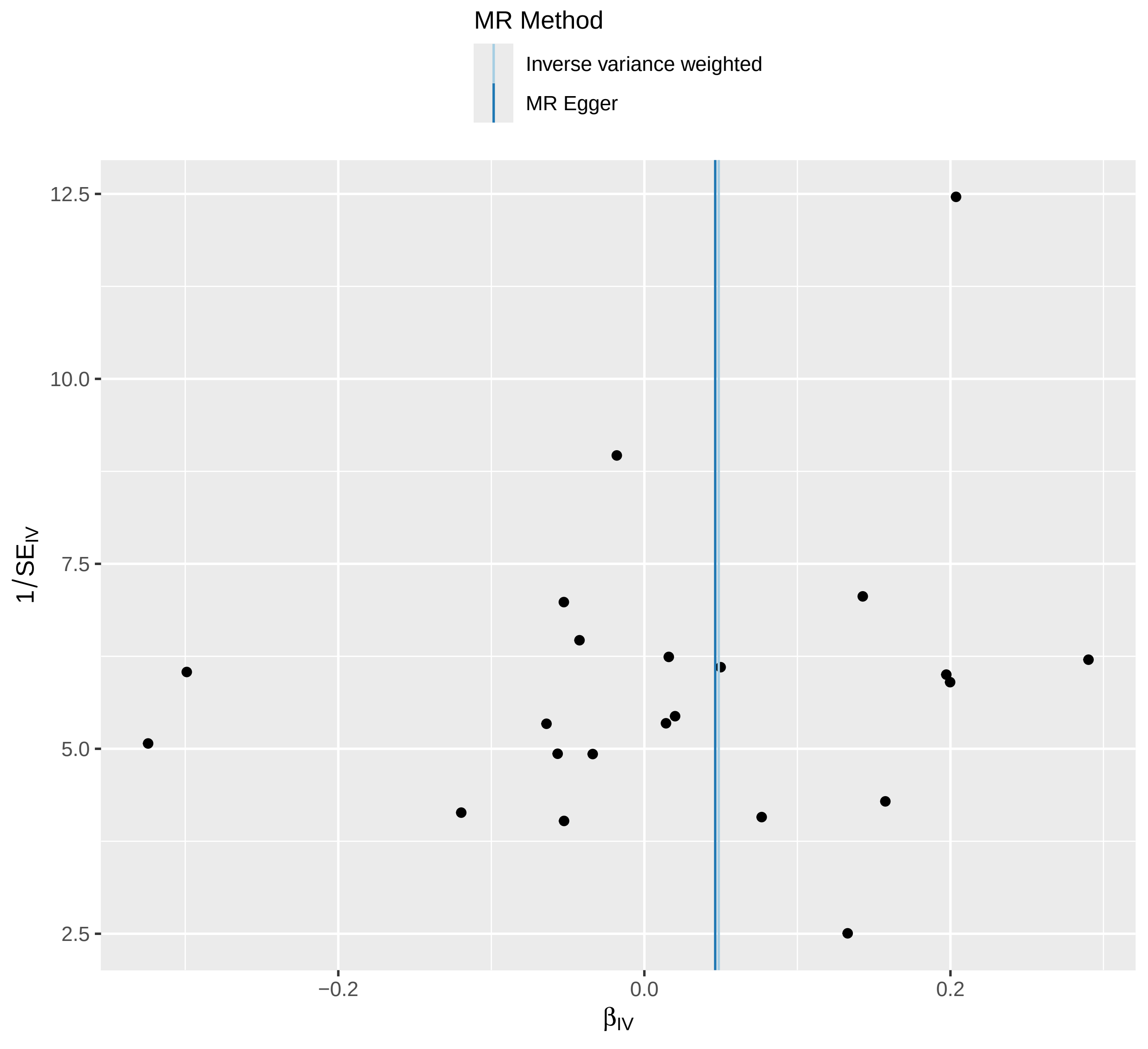

MR Method
Inverse variance weighted
MR Egger
12.5
10.0
7.5
5.0
2.5
$1/SE_{IV}$
−0.2
0.0
0.2
β_{IV}

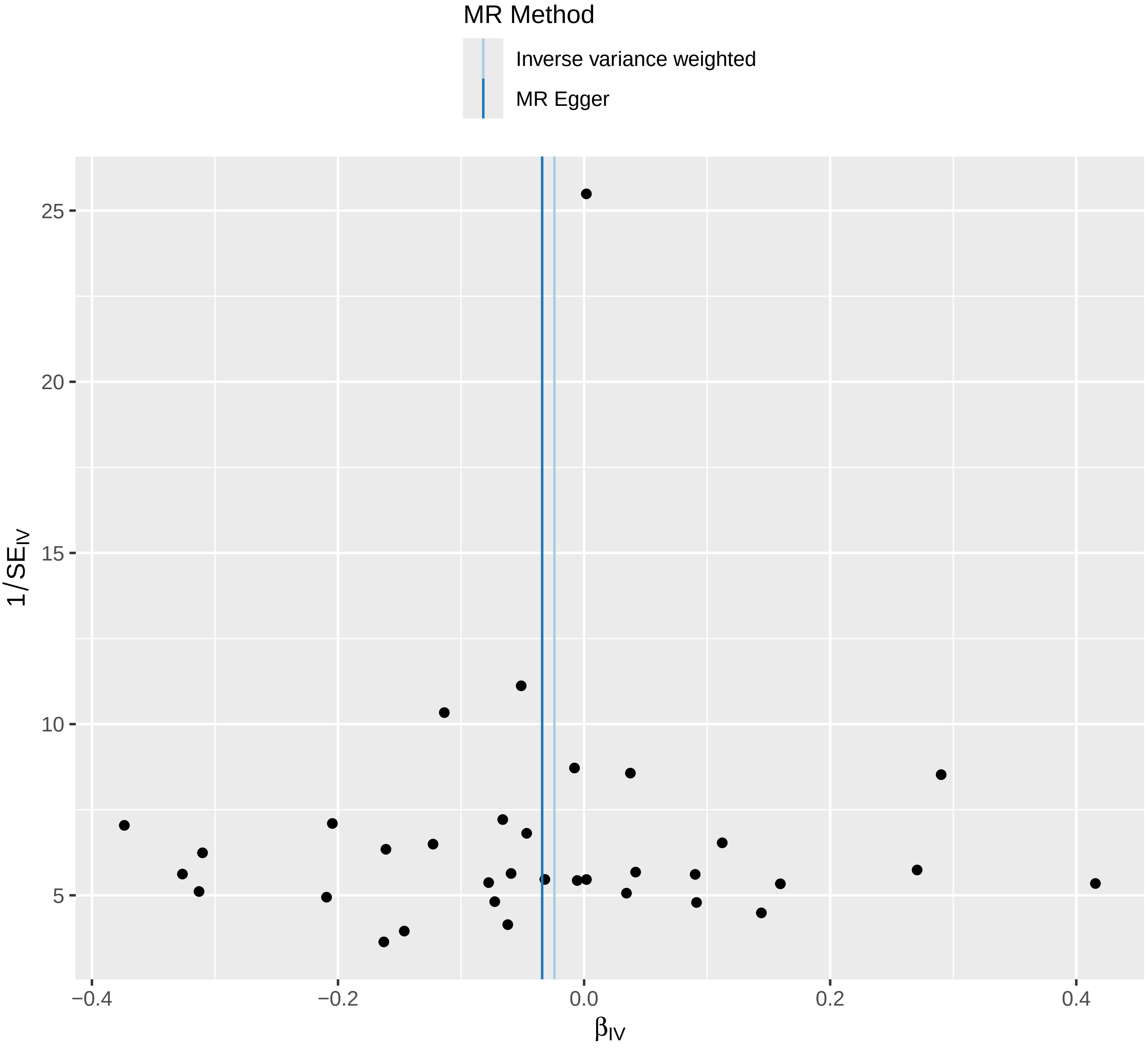

MR Method
Inverse variance weighted
MR Egger
1/SE_IV
25
20
15
10
5
−0.4
−0.2
0.0
0.2
0.4
β_IV

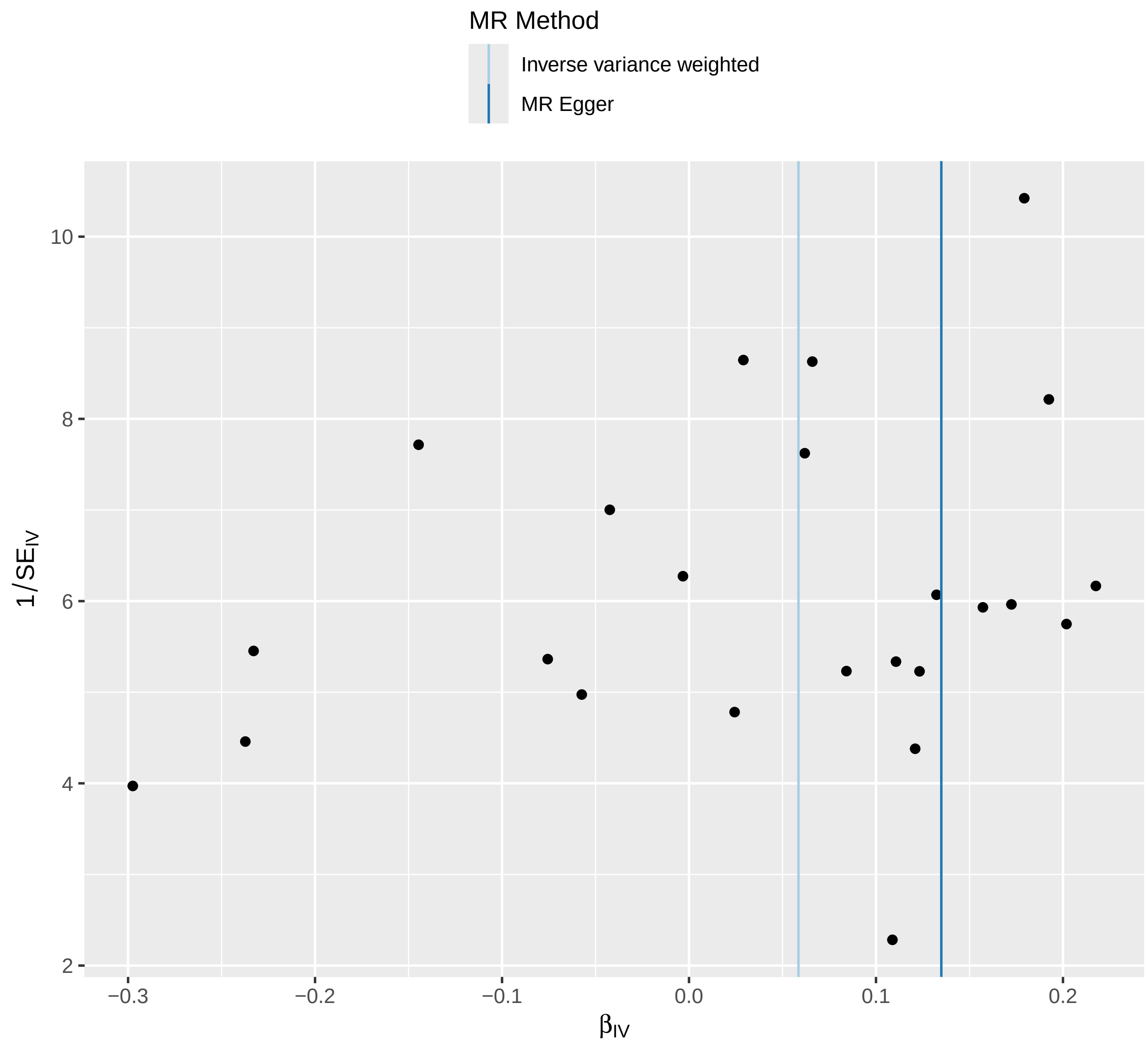
MR Method
Inverse variance weighted
MR Egger
10
8
6
4
2
1/SE_IV
−0.3
−0.2
−0.1
0.0
0.1
0.2
β_IV

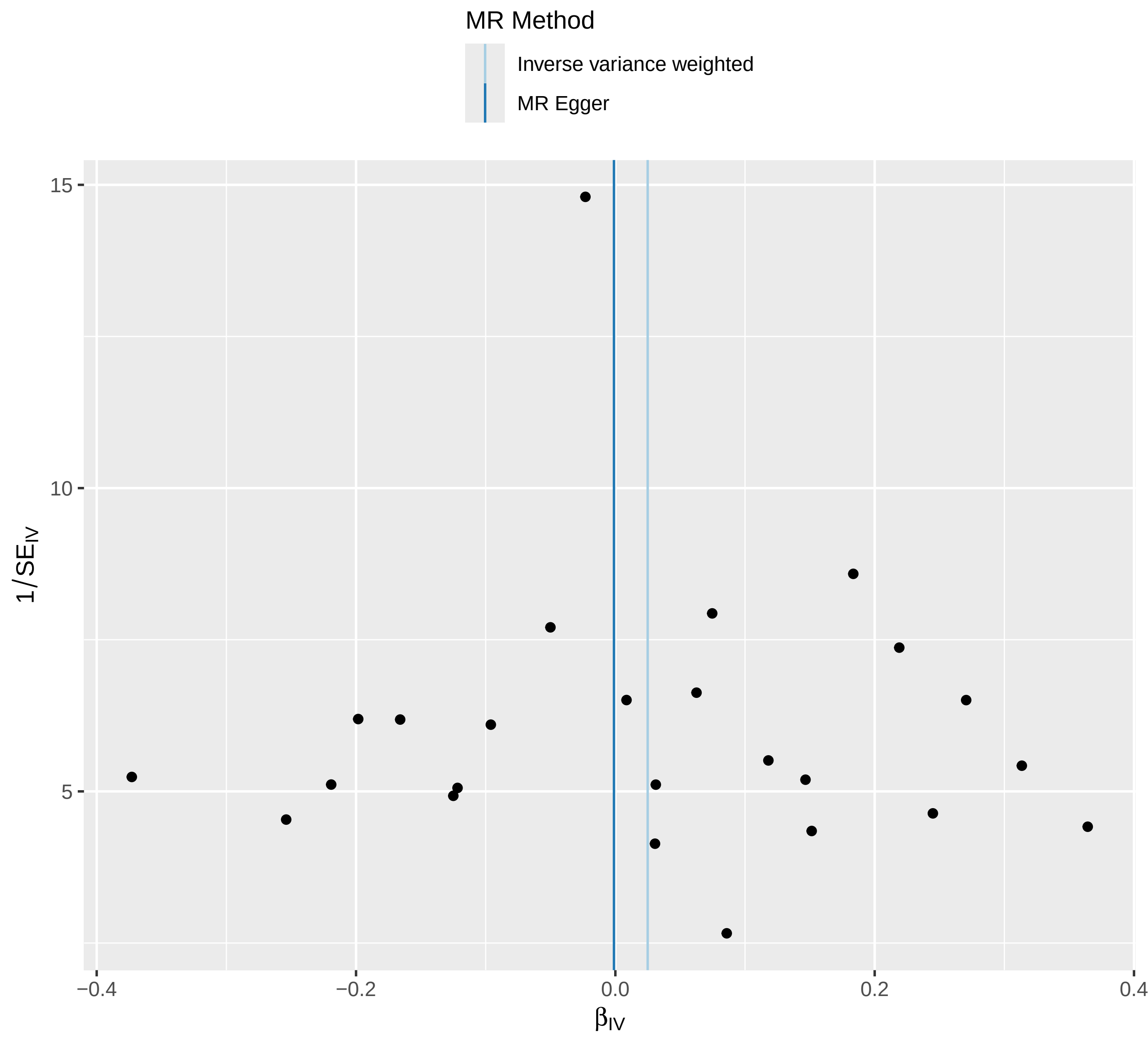
MR Method
Inverse variance weighted
MR Egger
1/SE_IV
15
10
5
β_IV
−0.4
−0.2
0.0
0.2
0.4

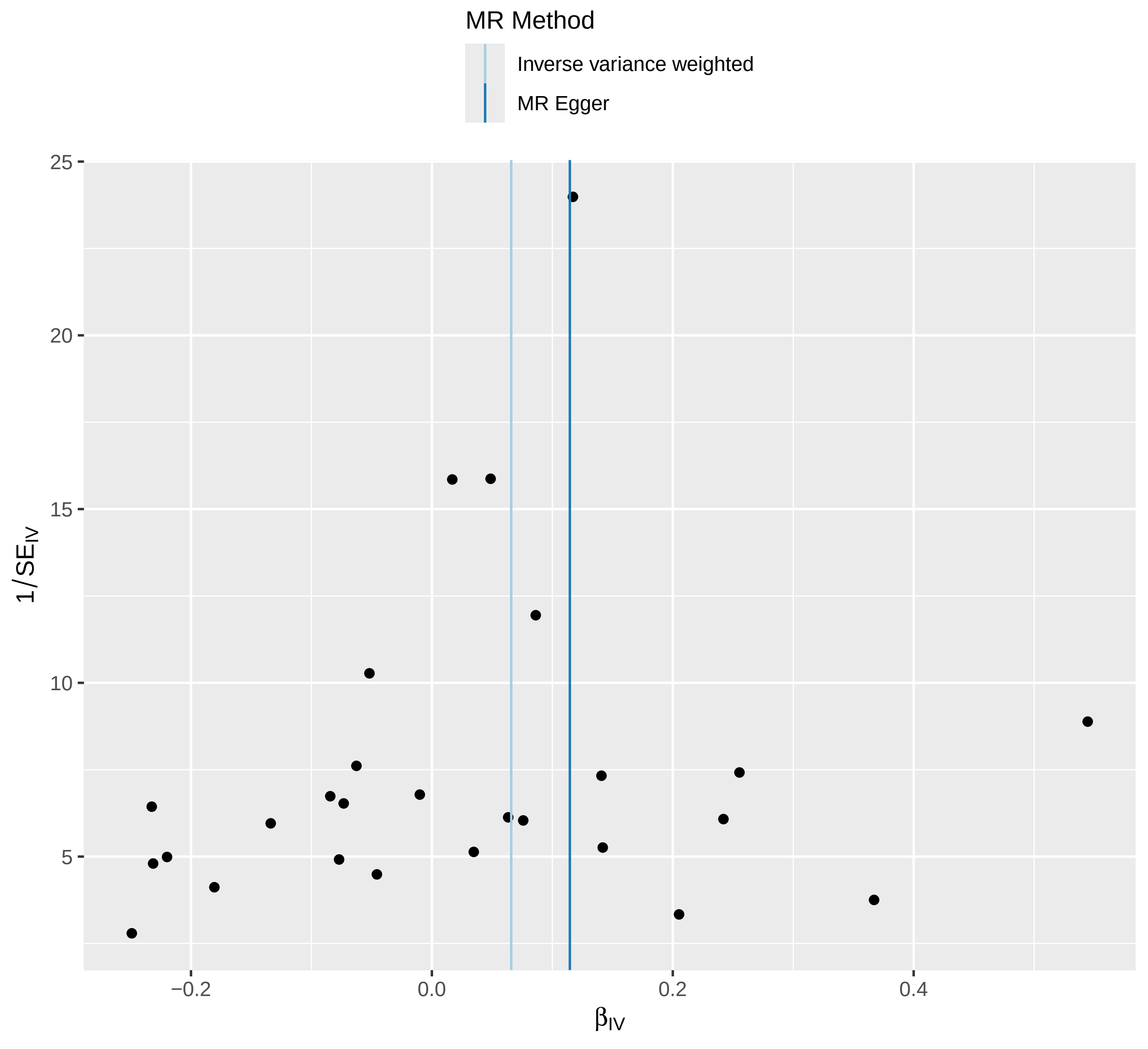
MR Method
Inverse variance weighted
MR Egger
25
20
15
10
5
1/SE$_{IV}$
−0.2
0.0
0.2
0.4
β$_{IV}$

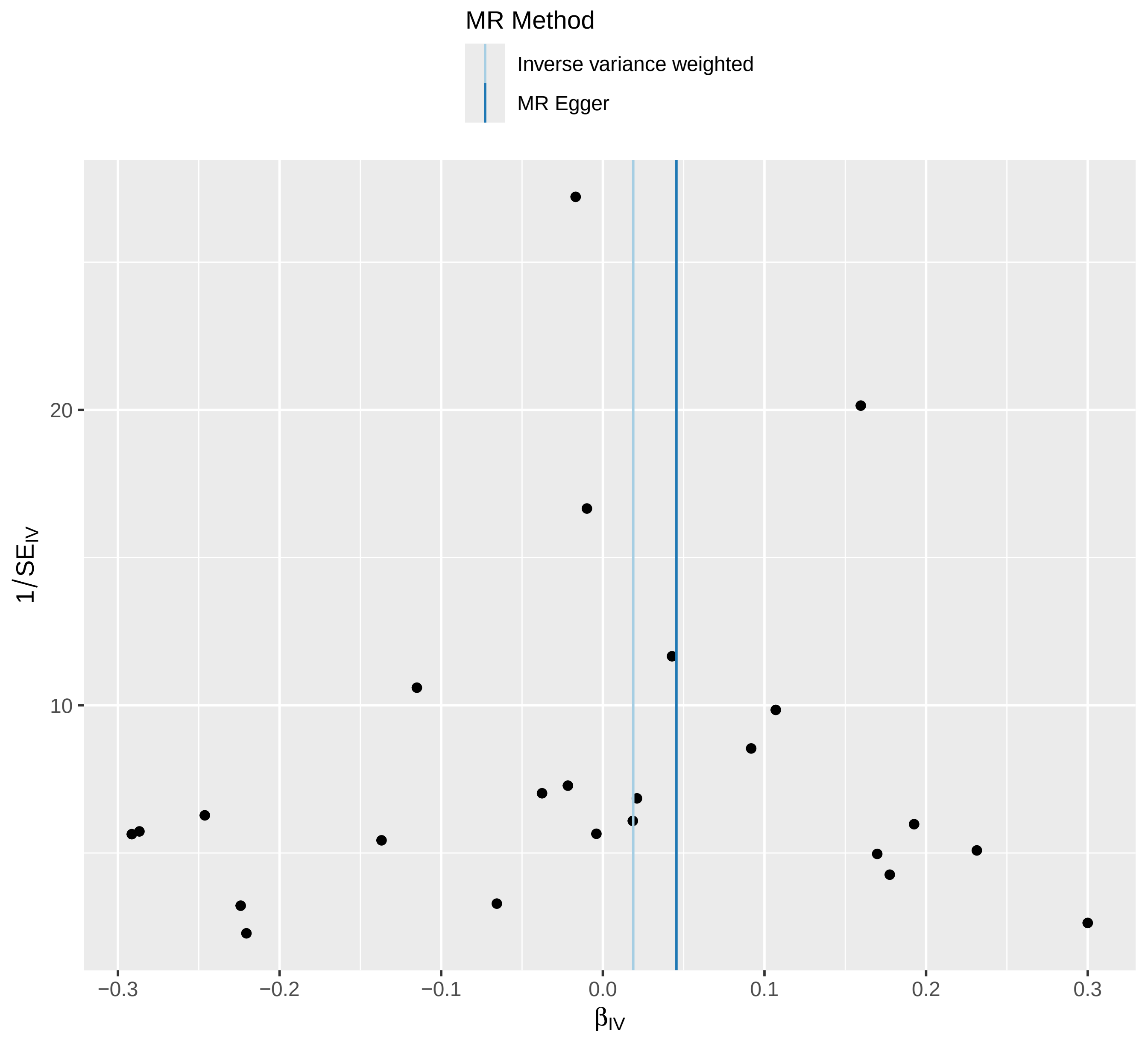
MR Method
Inverse variance weighted
MR Egger
20
10
−0.3
−0.2
−0.1
0.0
0.1
0.2
0.3
$1/SE_{IV}$
β_{IV}

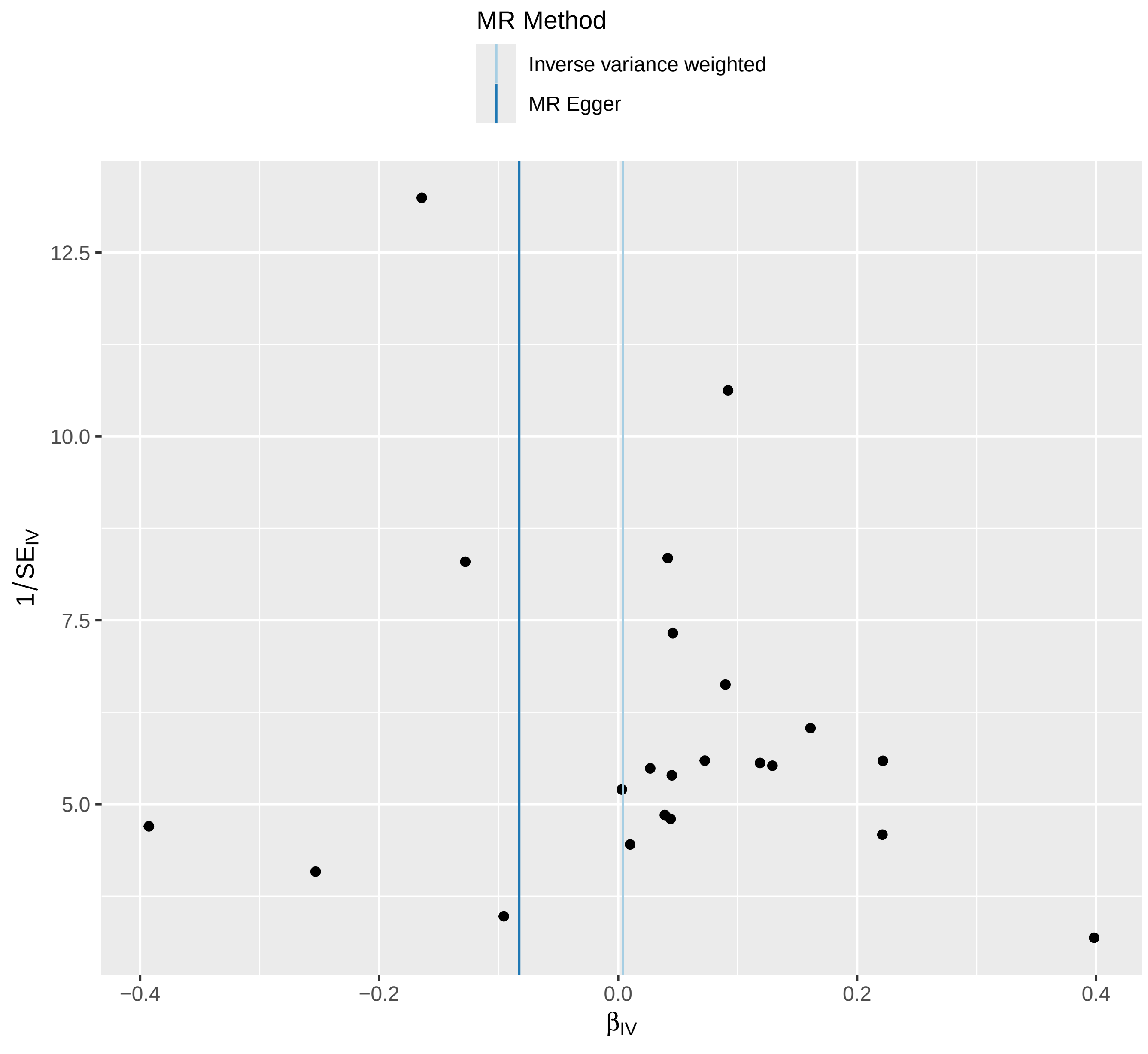
MR Method
Inverse variance weighted
MR Egger
12.5
10.0
7.5
5.0
$1/SE_{IV}$
−0.4
−0.2
0.0
0.2
0.4
β_{IV}

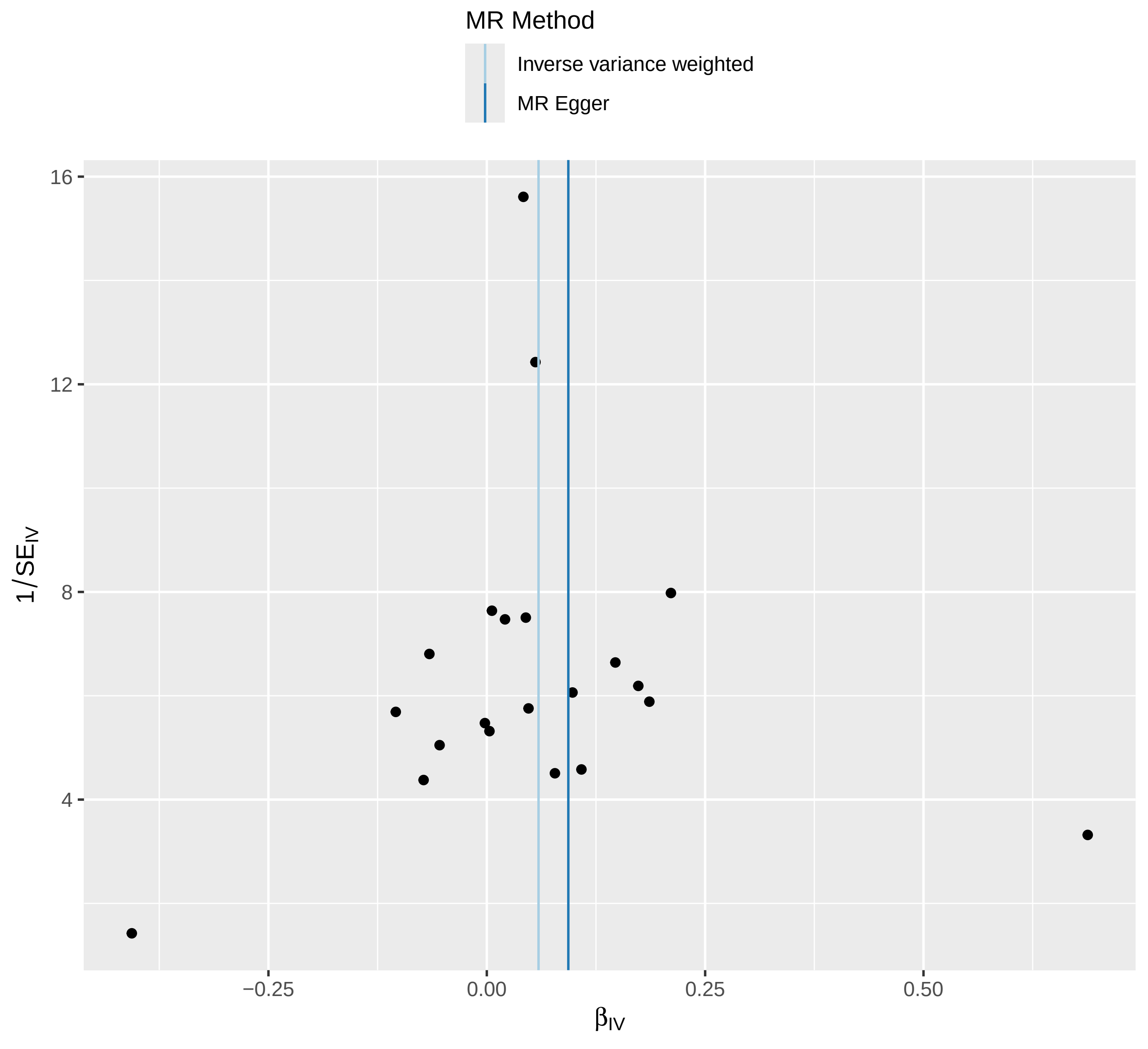
MR Method
Inverse variance weighted
MR Egger
16
12
8
4
1/SE_IV
−0.25
0.00
0.25
0.50
β_IV

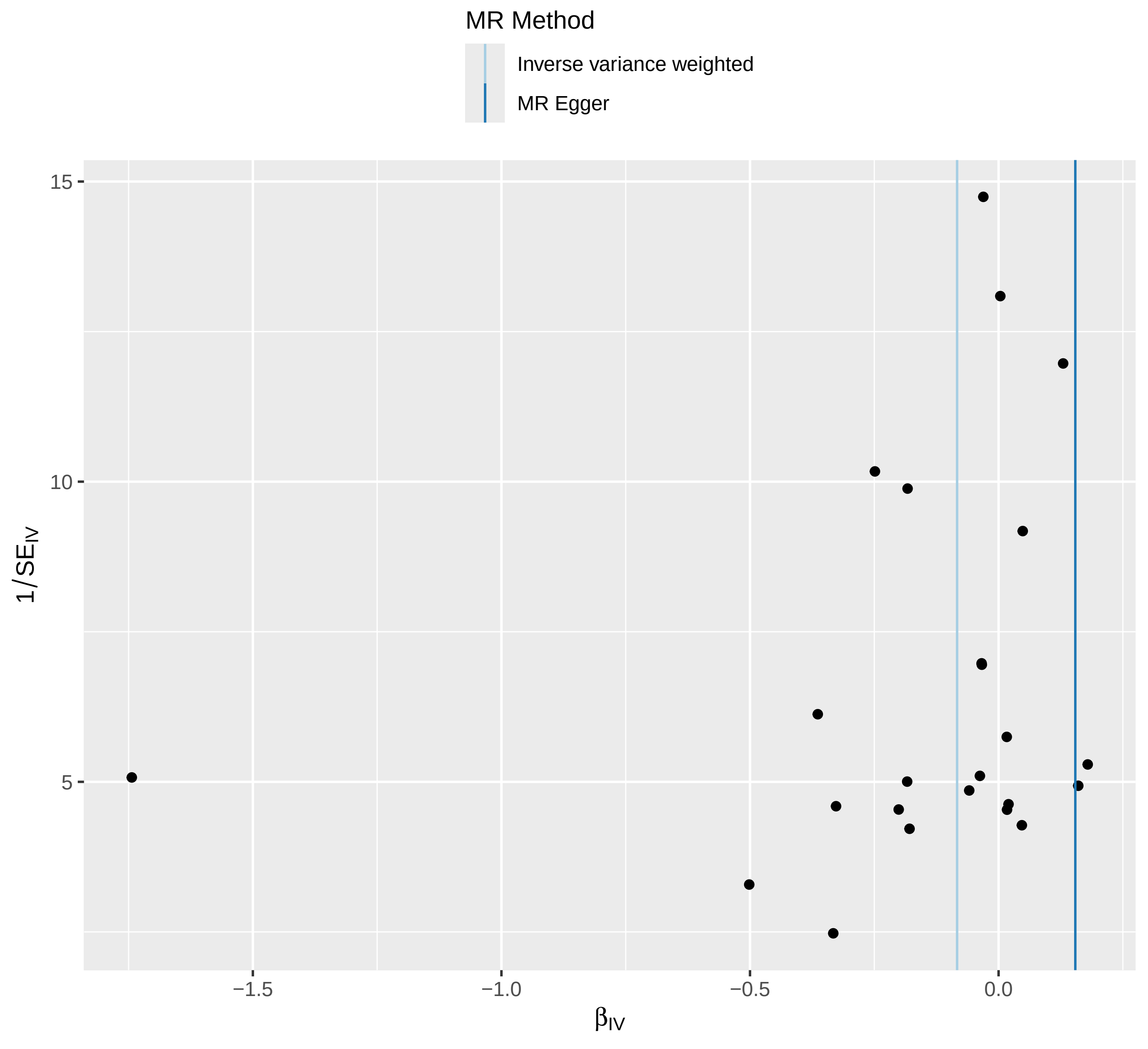

MR Method
Inverse variance weighted
MR Egger
15
10
5
1/SE_IV
−1.5
−1.0
−0.5
0.0
β_IV

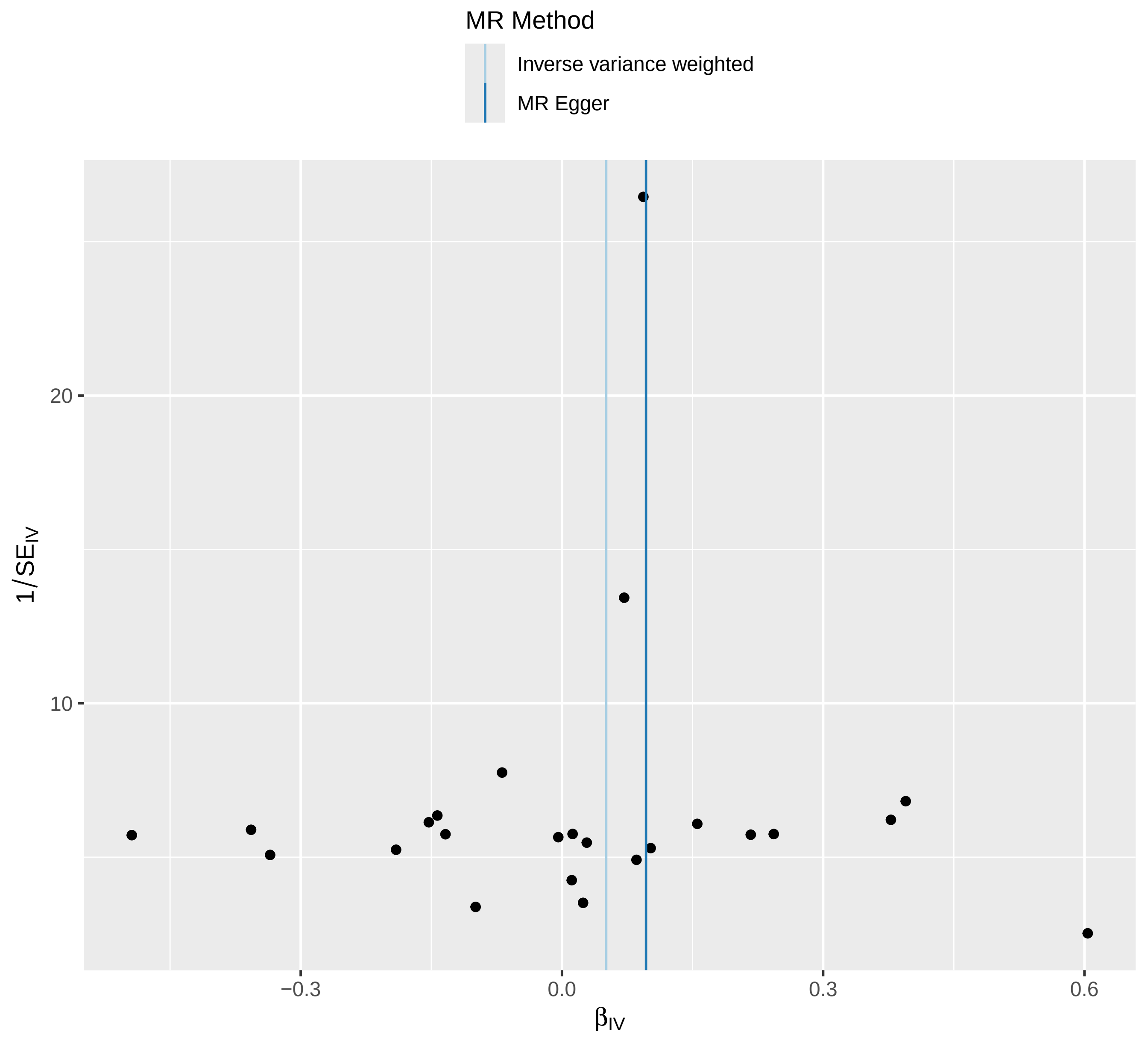
MR Method
Inverse variance weighted
MR Egger
20
10
1/SE_IV
−0.3
0.0
0.3
0.6
β_IV

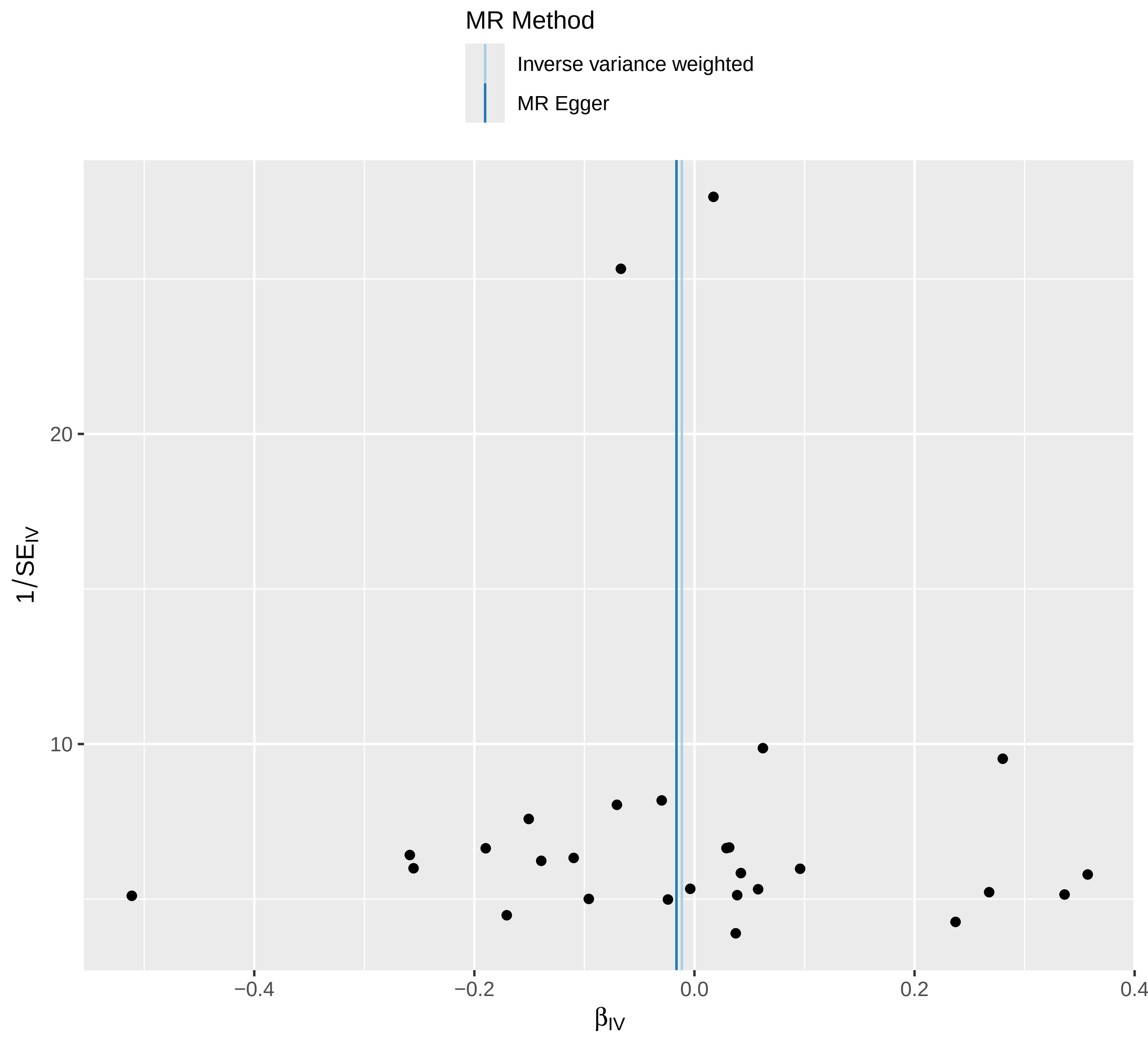
MR Method
Inverse variance weighted
MR Egger
20
10
1/SE$_{IV}$
−0.4
−0.2
0.0
0.2
0.4
β$_{IV}$

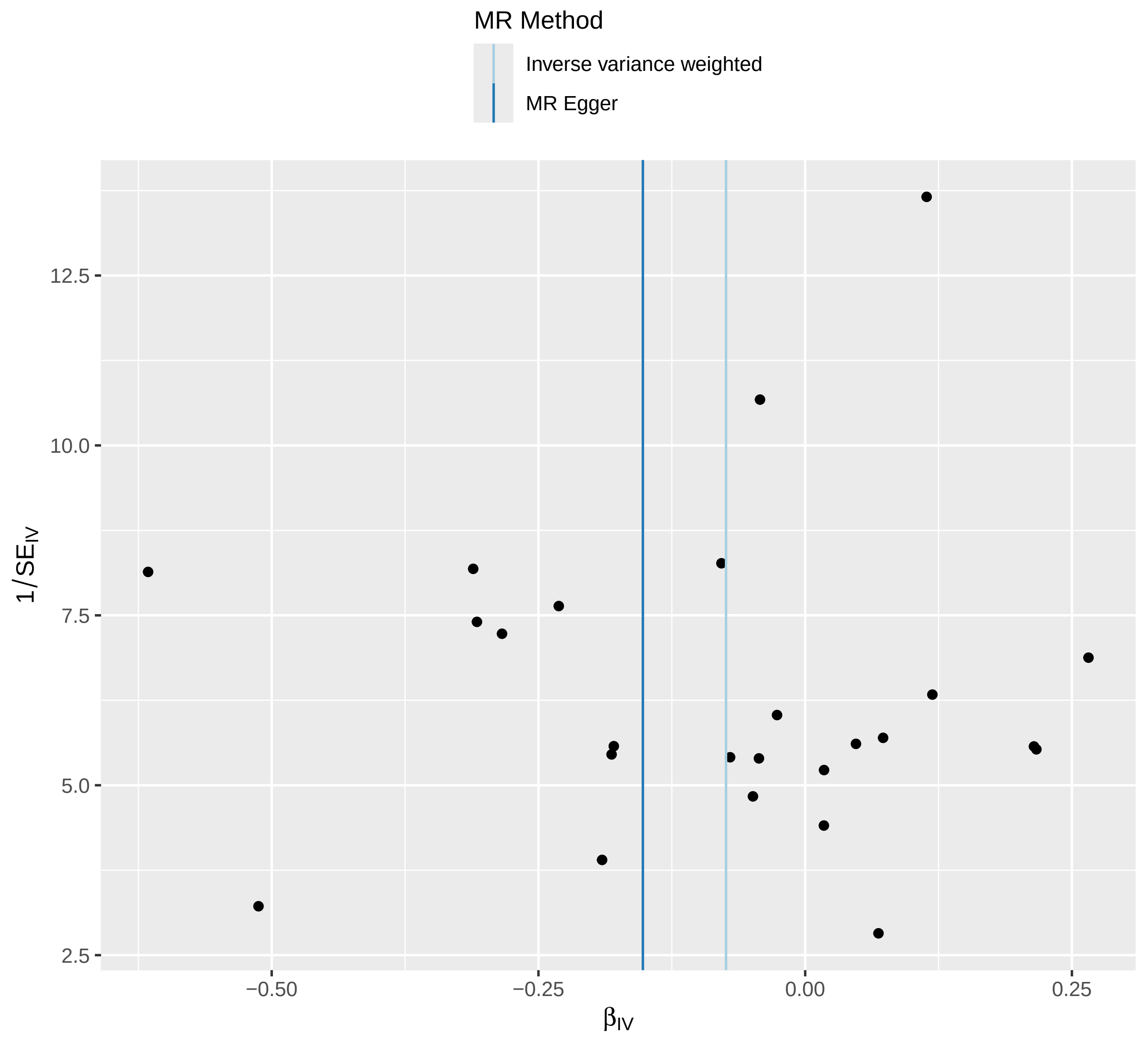

MR Method
Inverse variance weighted
MR Egger
12.5
10.0
7.5
5.0
2.5
1/SE$_{IV}$
−0.50
−0.25
0.00
0.25
β$_{IV}$

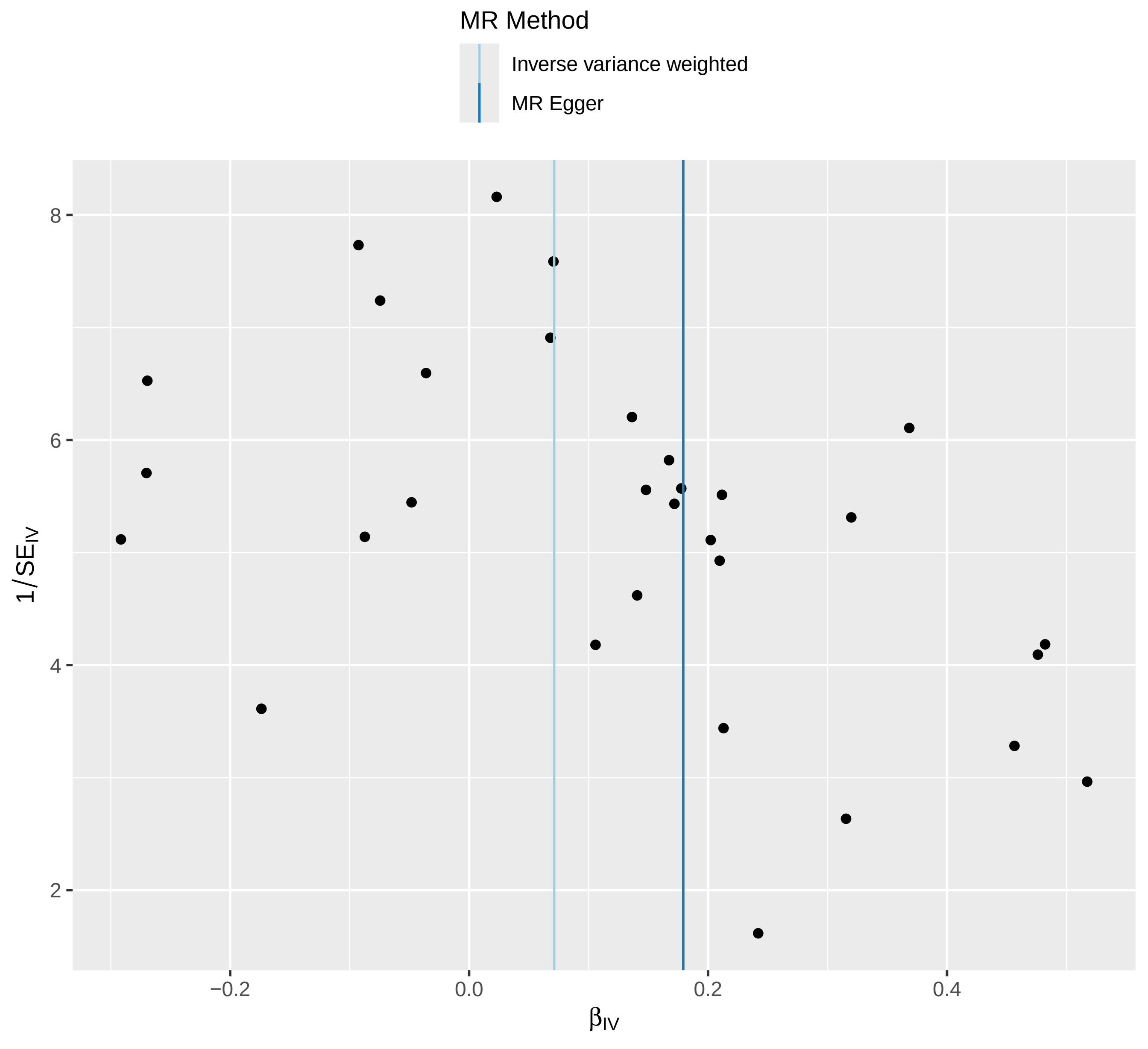
MR Method
Inverse variance weighted
MR Egger
8
6
4
2
1/SE_IV
−0.2
0.0
0.2
0.4
β_IV

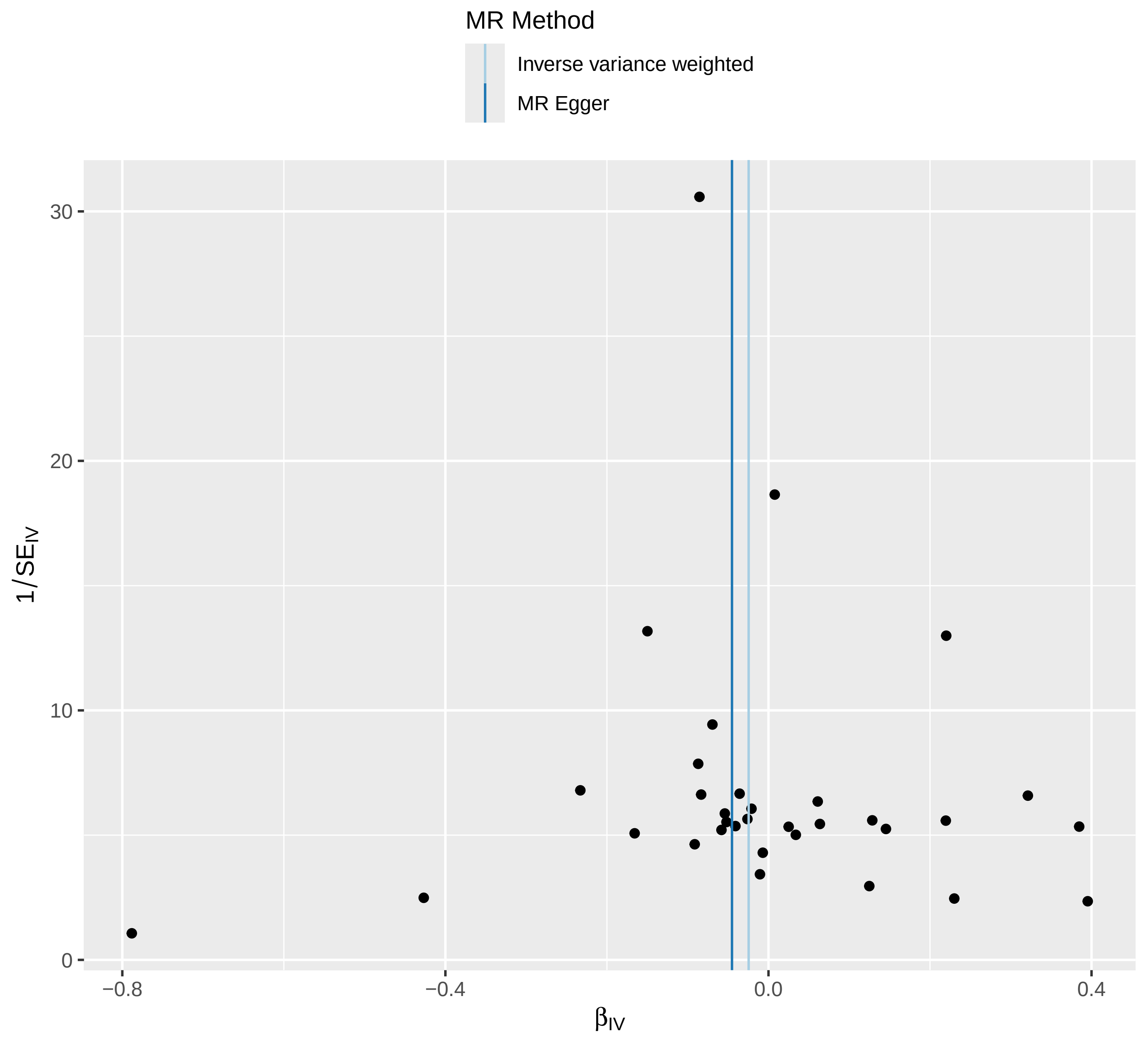
MR Method
Inverse variance weighted
MR Egger
30
20
10
0
−0.8
−0.4
0.0
0.4
$1/SE_{IV}$
β_{IV}

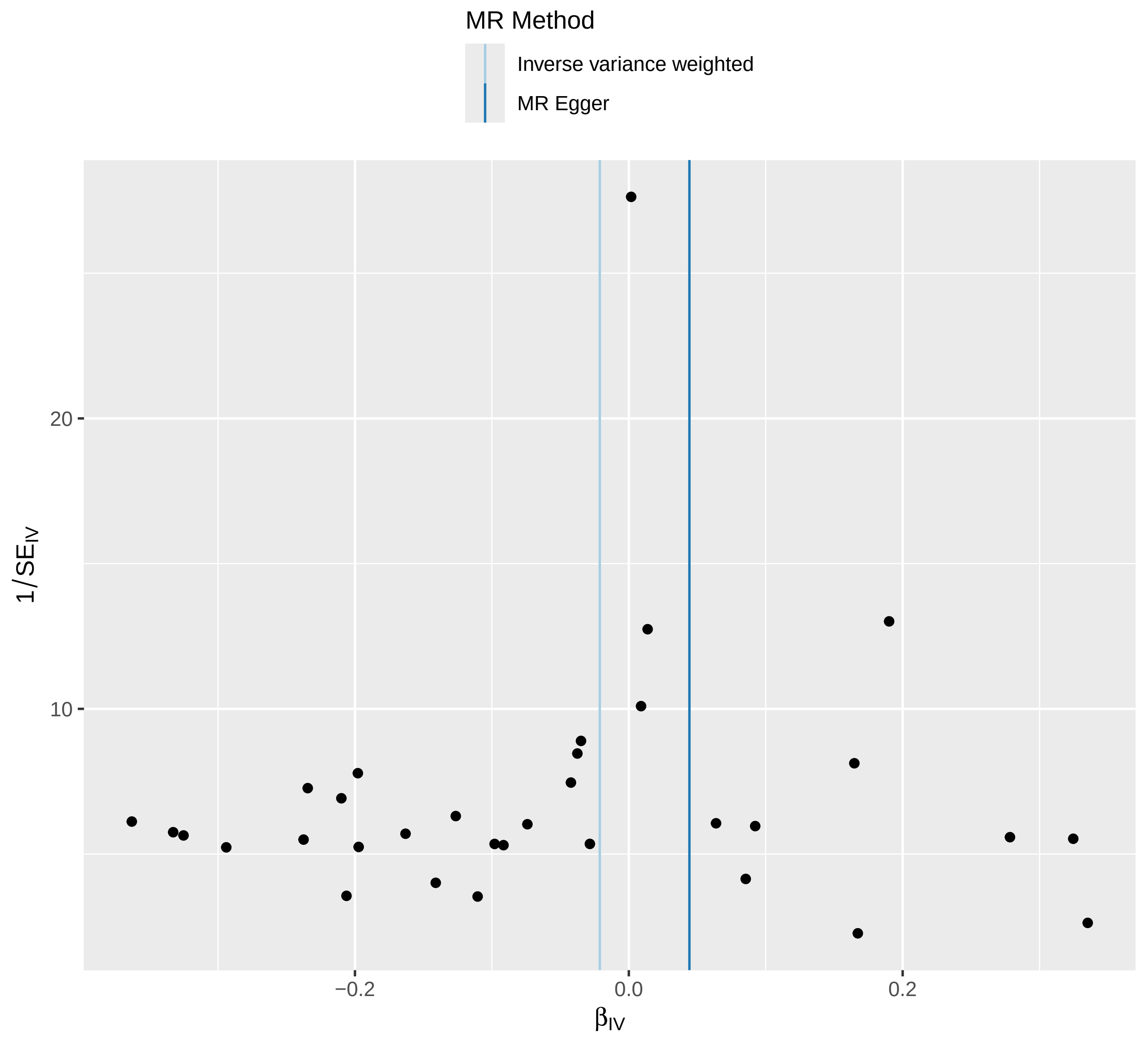

MR Method
Inverse variance weighted
MR Egger
20
10
1/SE_IV
−0.2
0.0
0.2
β_IV

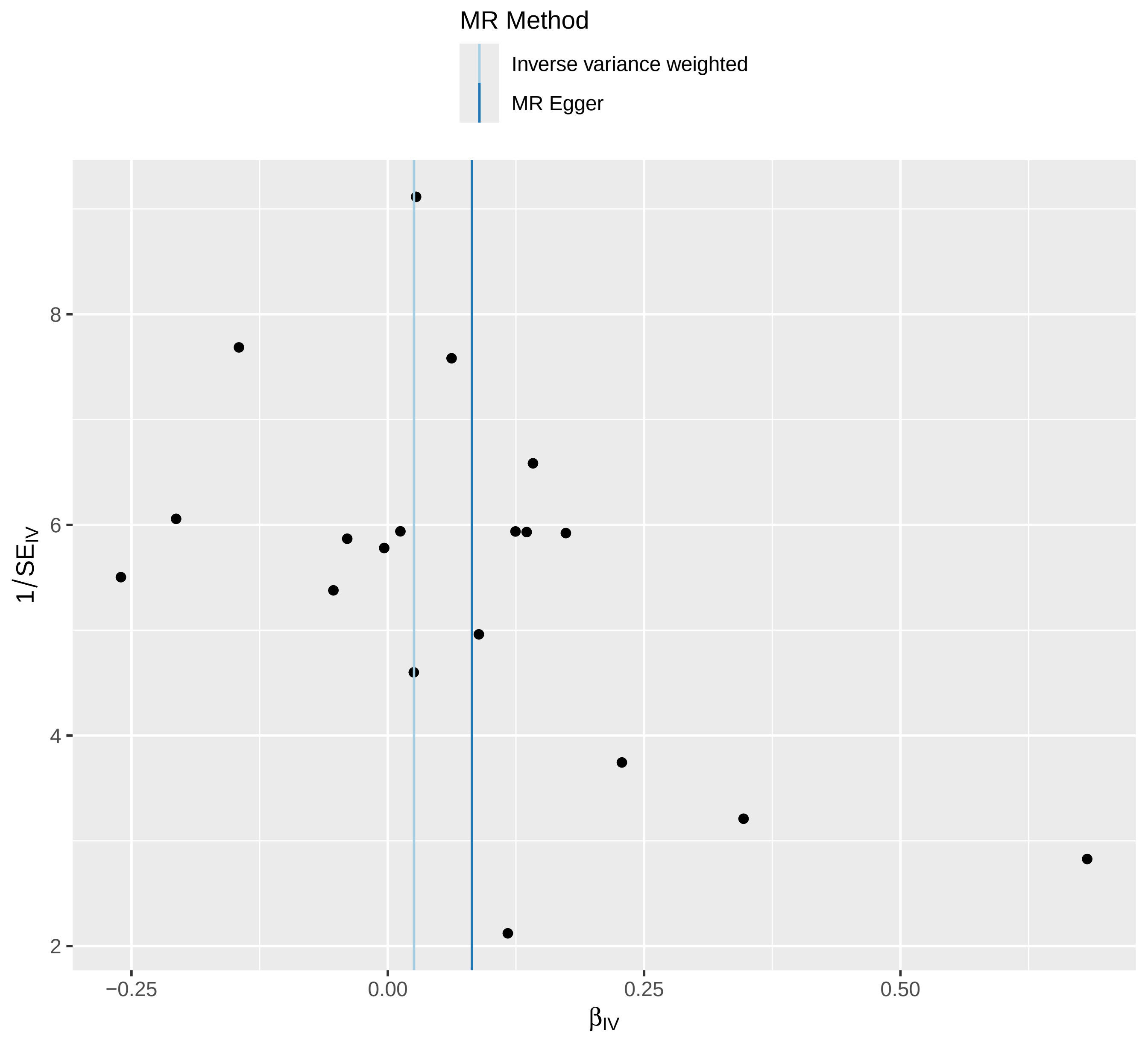
MR Method
Inverse variance weighted
MR Egger
8
6
4
2
1/SE$_{IV}$
−0.25
0.00
0.25
0.50
β$_{IV}$

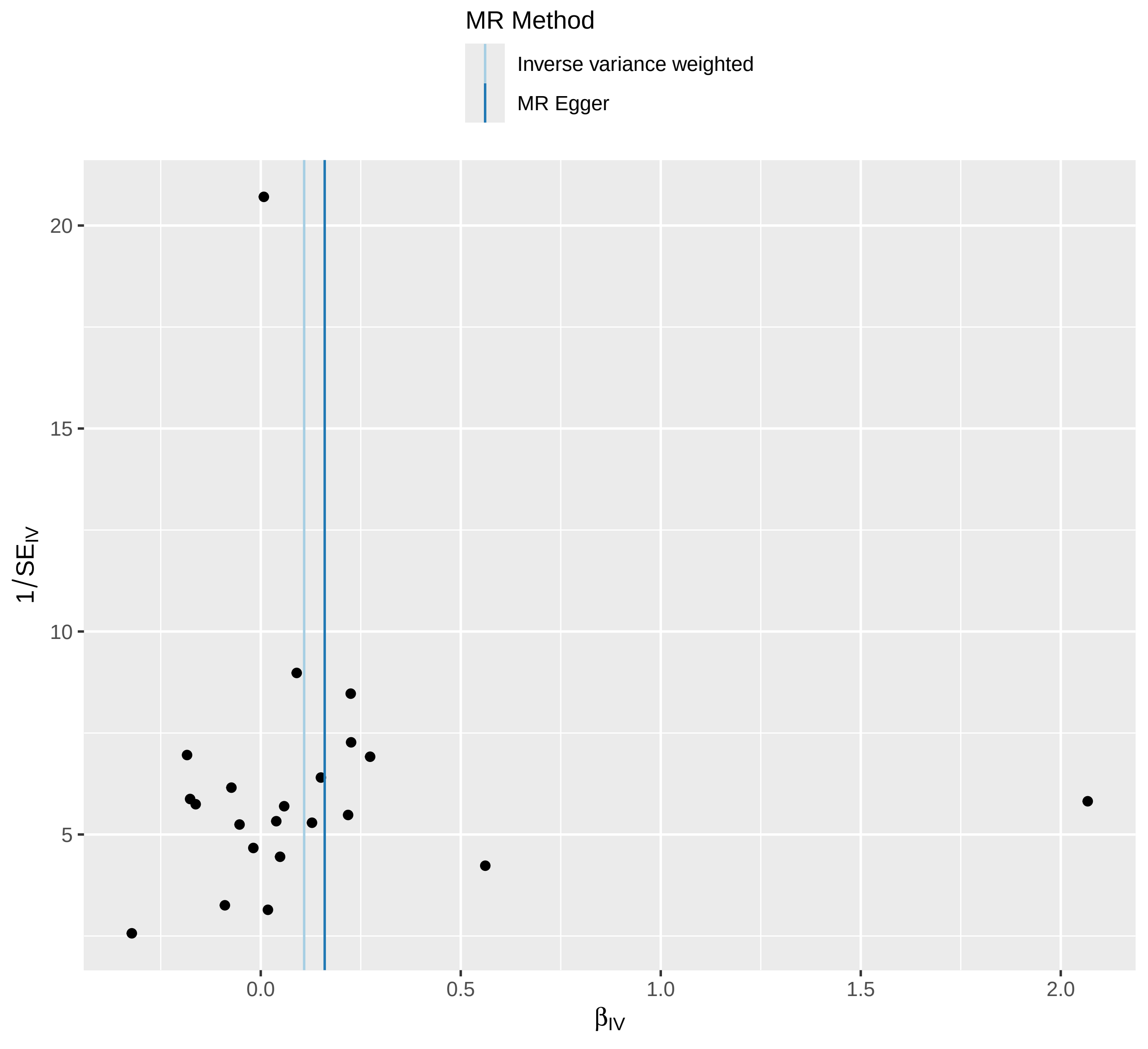
MR Method
Inverse variance weighted
MR Egger
20
15
10
5
$1/SE_{IV}$
0.0
0.5
1.0
1.5
2.0
β_{IV}

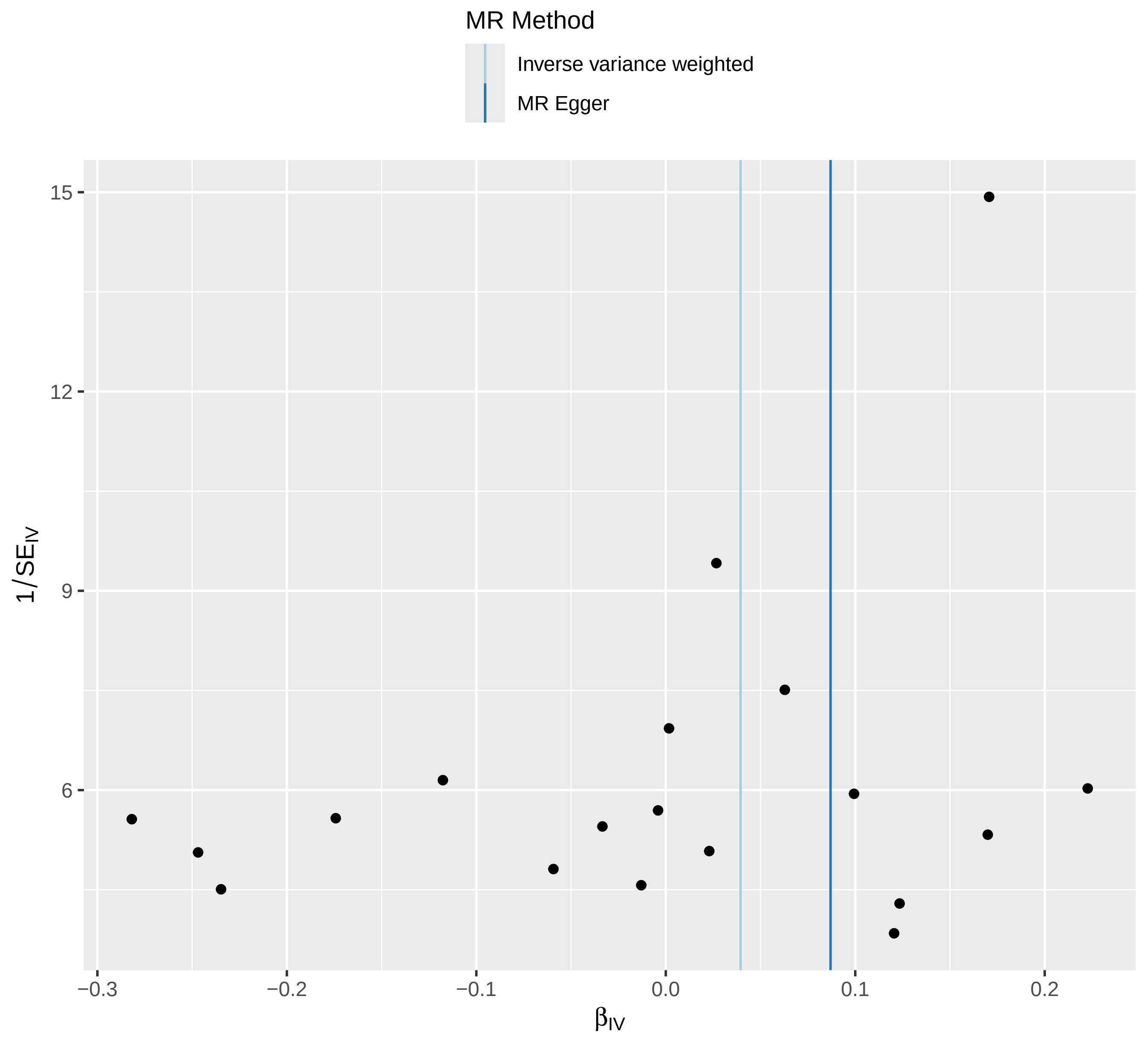
MR Method
Inverse variance weighted
MR Egger
15
12
9
6
−0.3
−0.2
−0.1
0.0
0.1
0.2
$1/SE_{IV}$
β_{IV}

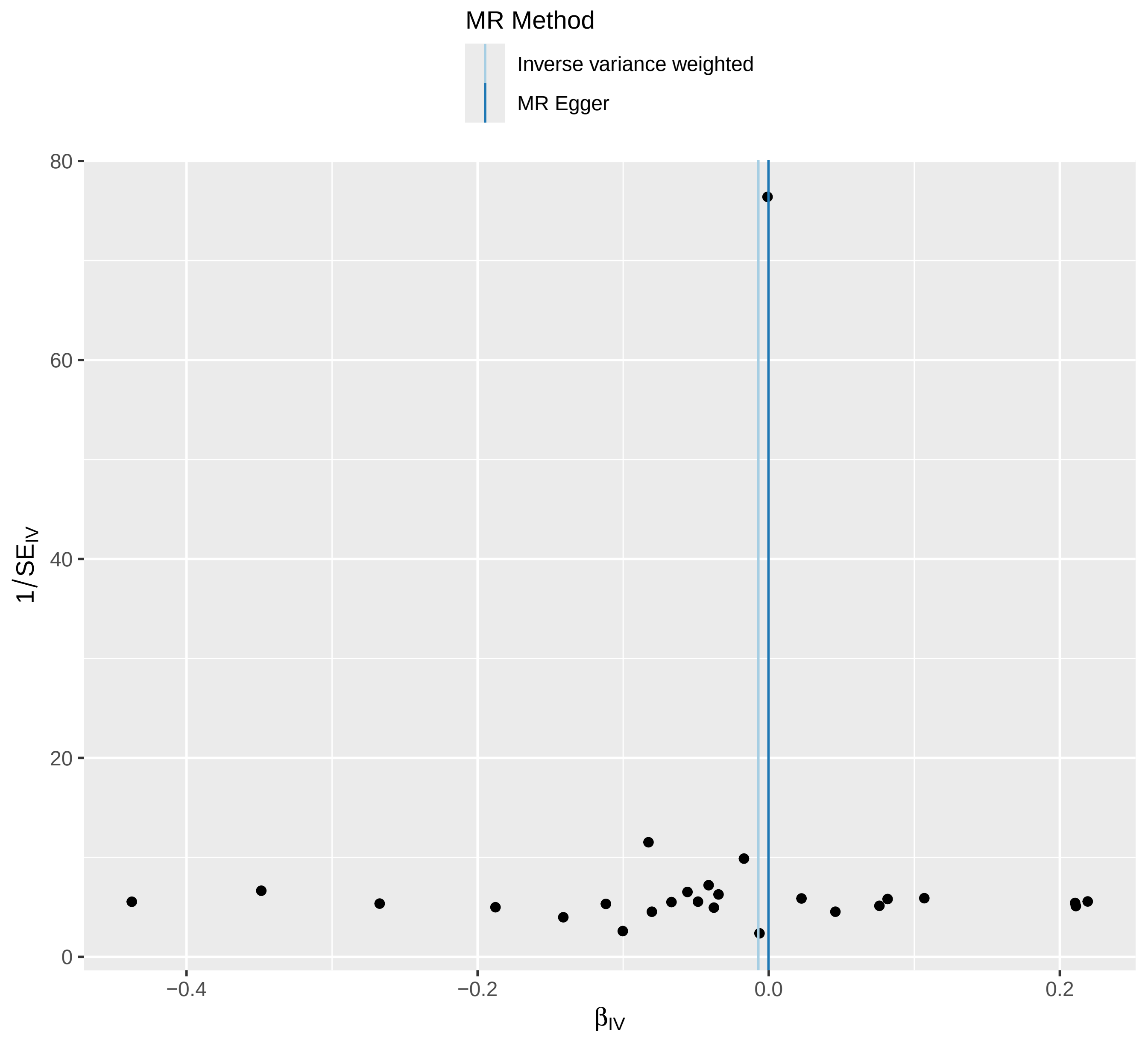
MR Method
Inverse variance weighted
MR Egger
80
60
40
20
0
1/SE_{IV}
−0.4
−0.2
0.0
0.2
β_{IV}

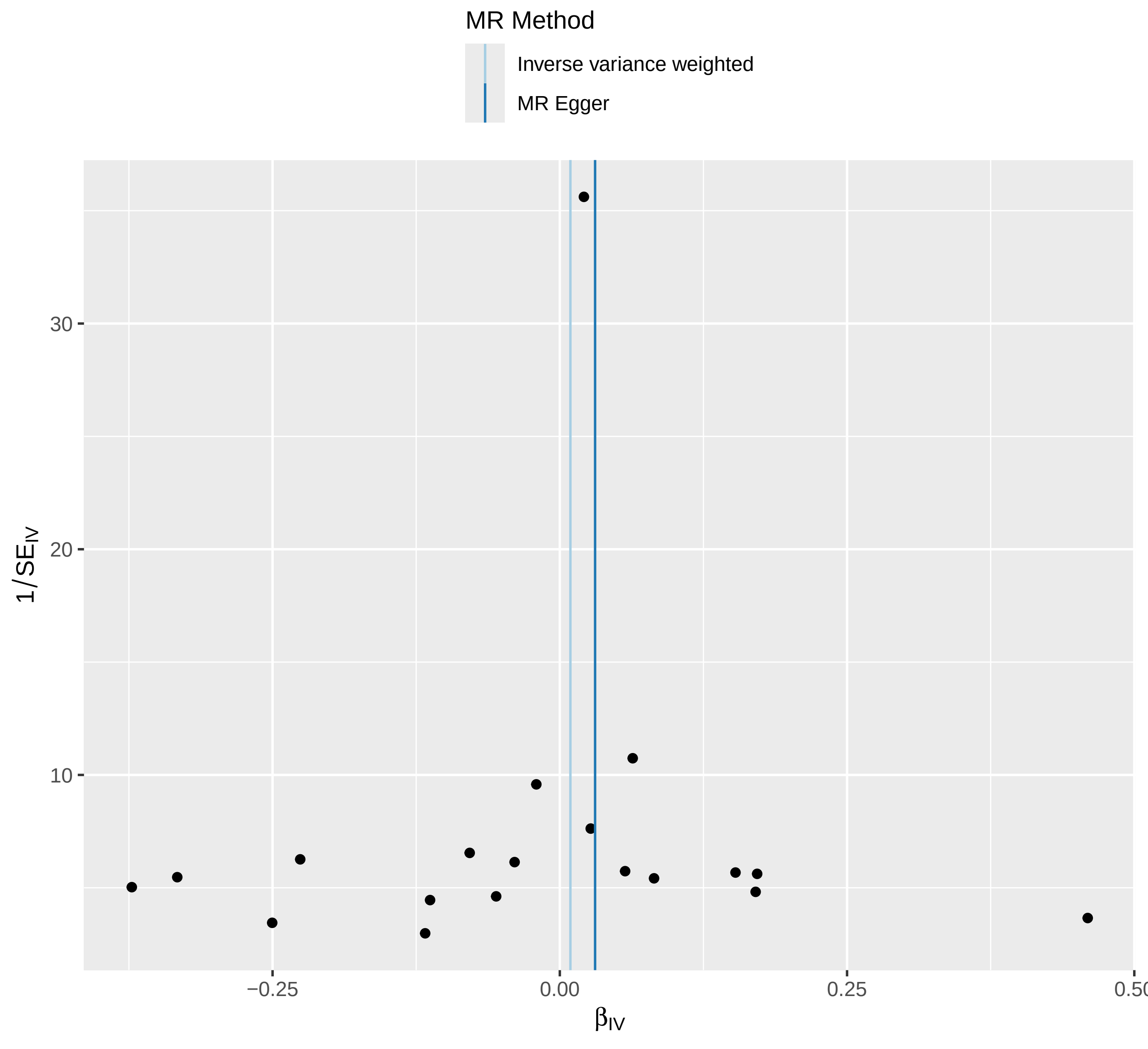

MR Method
Inverse variance weighted
MR Egger
30
20
10
1/SE$_{IV}$
−0.25
0.00
0.25
0.50
β$_{IV}$

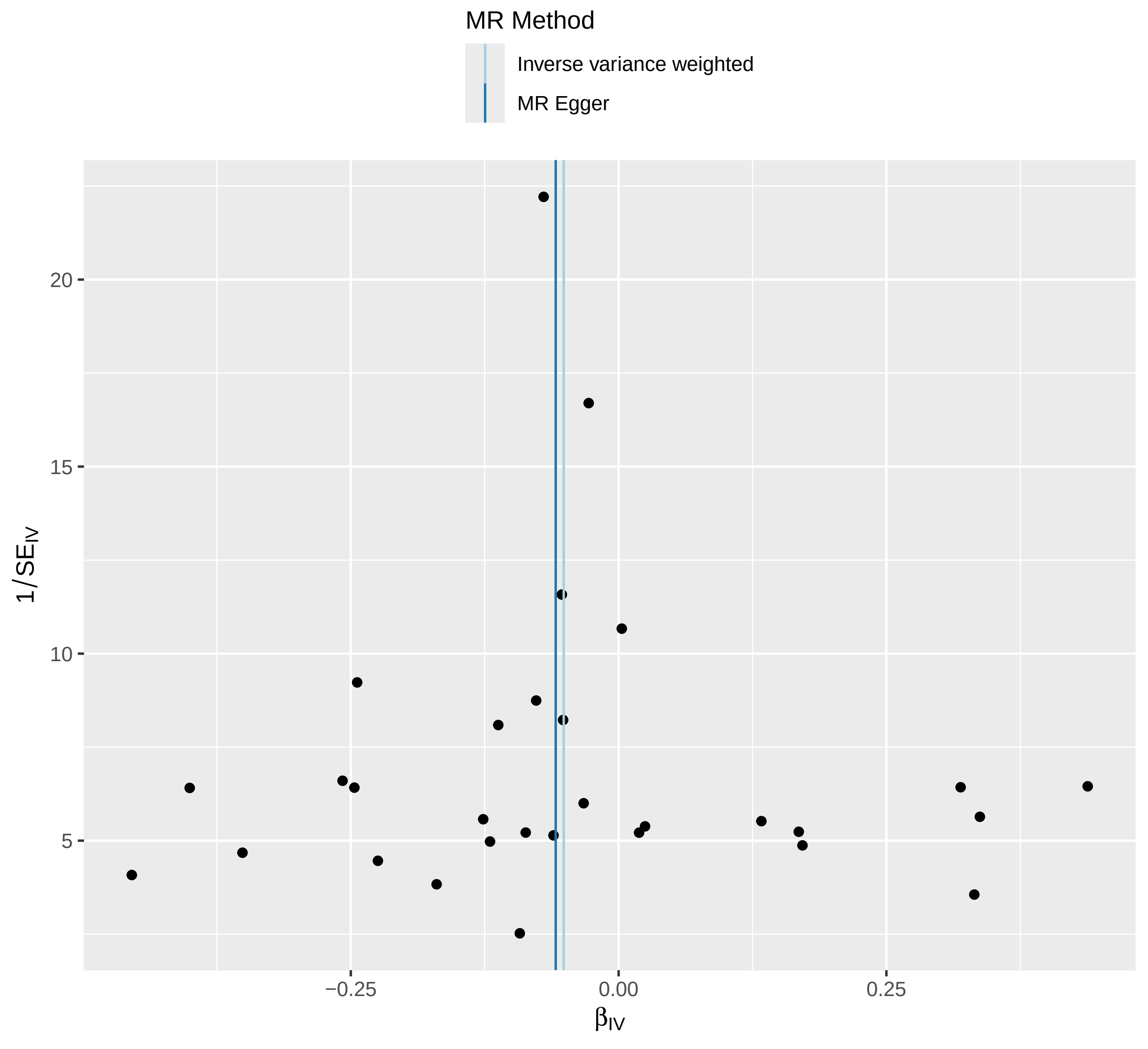
MR Method
Inverse variance weighted
MR Egger
20
15
10
5
1/SE_IV
−0.25
0.00
0.25
β_IV

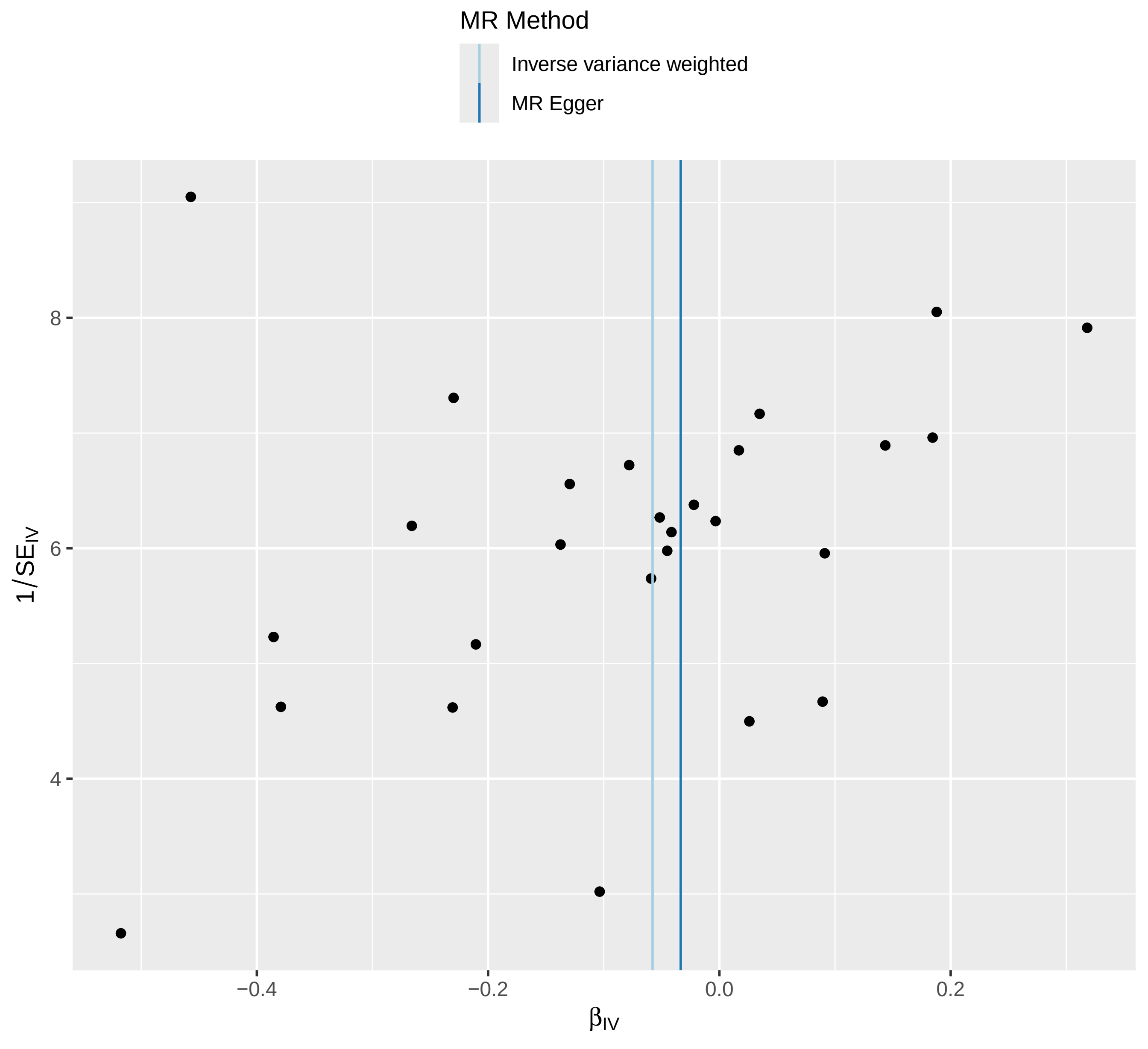
MR Method
Inverse variance weighted
MR Egger
1/SE_IV
8
6
4
−0.4
−0.2
0.0
0.2
β_IV

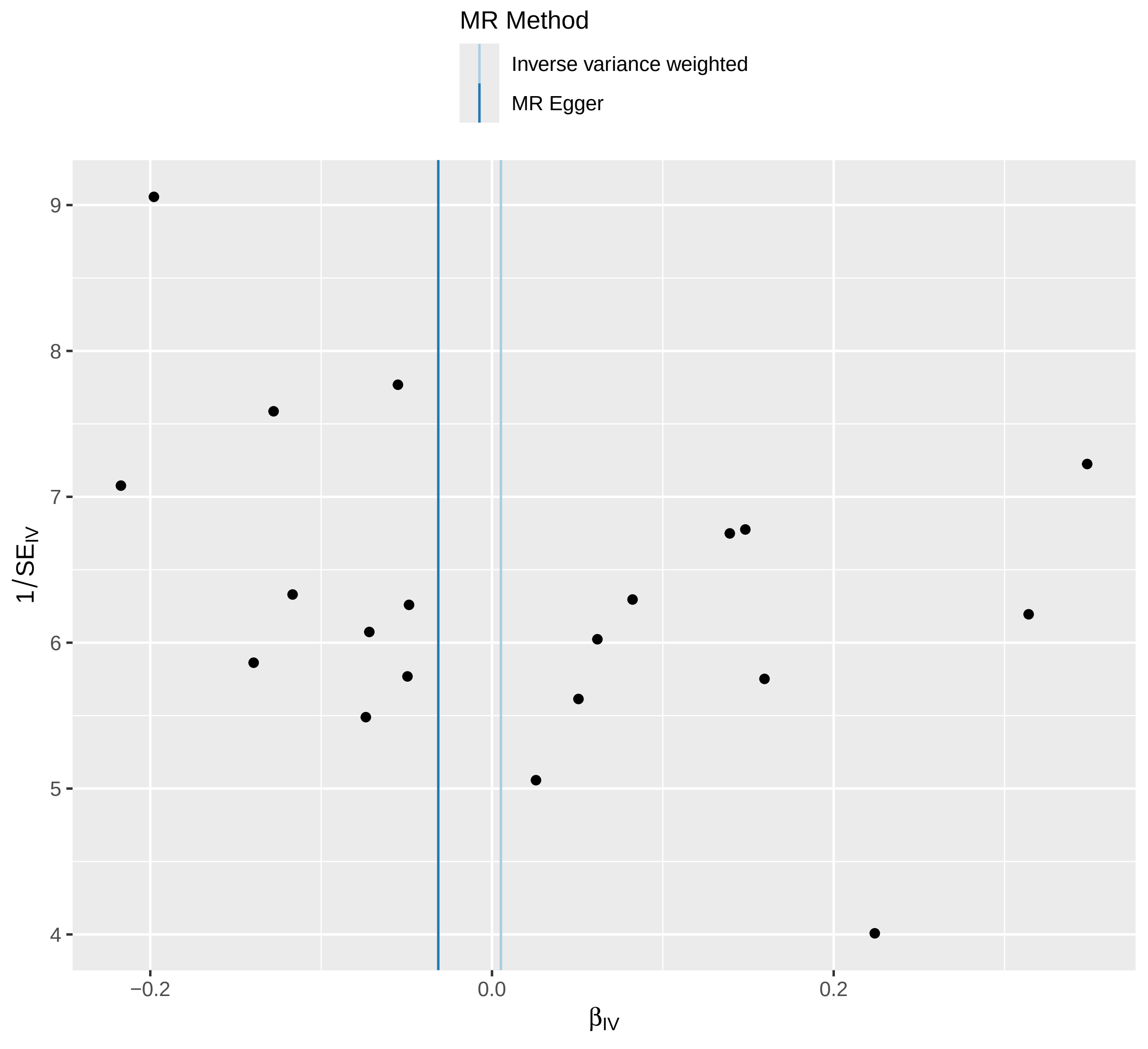
MR Method
Inverse variance weighted
MR Egger
9
8
7
6
5
4
1/SE$_{IV}$
−0.2
0.0
0.2
β$_{IV}$

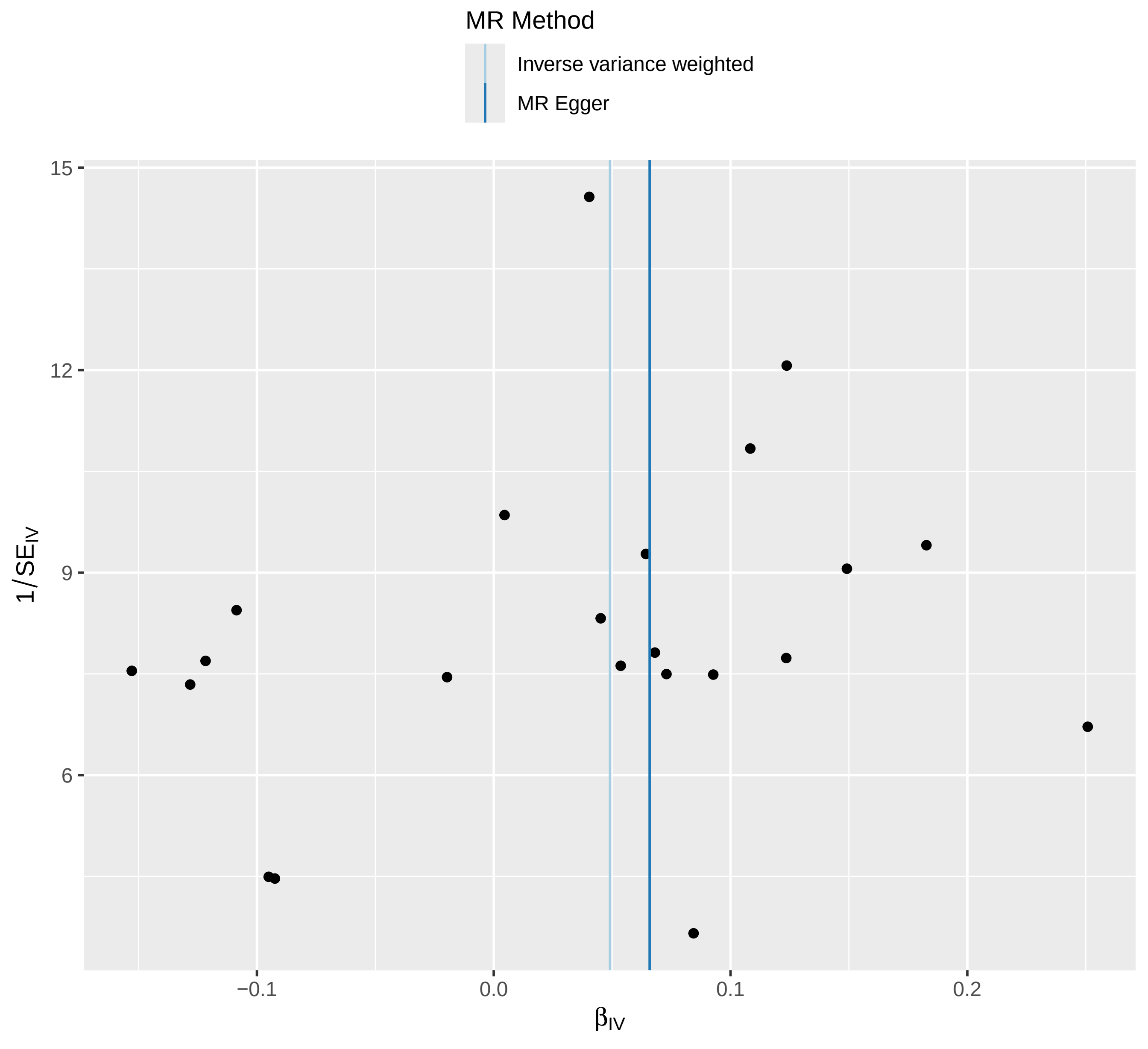
MR Method
Inverse variance weighted
MR Egger
15
12
9
6
1/SE_IV
−0.1
0.0
0.1
0.2
β_IV

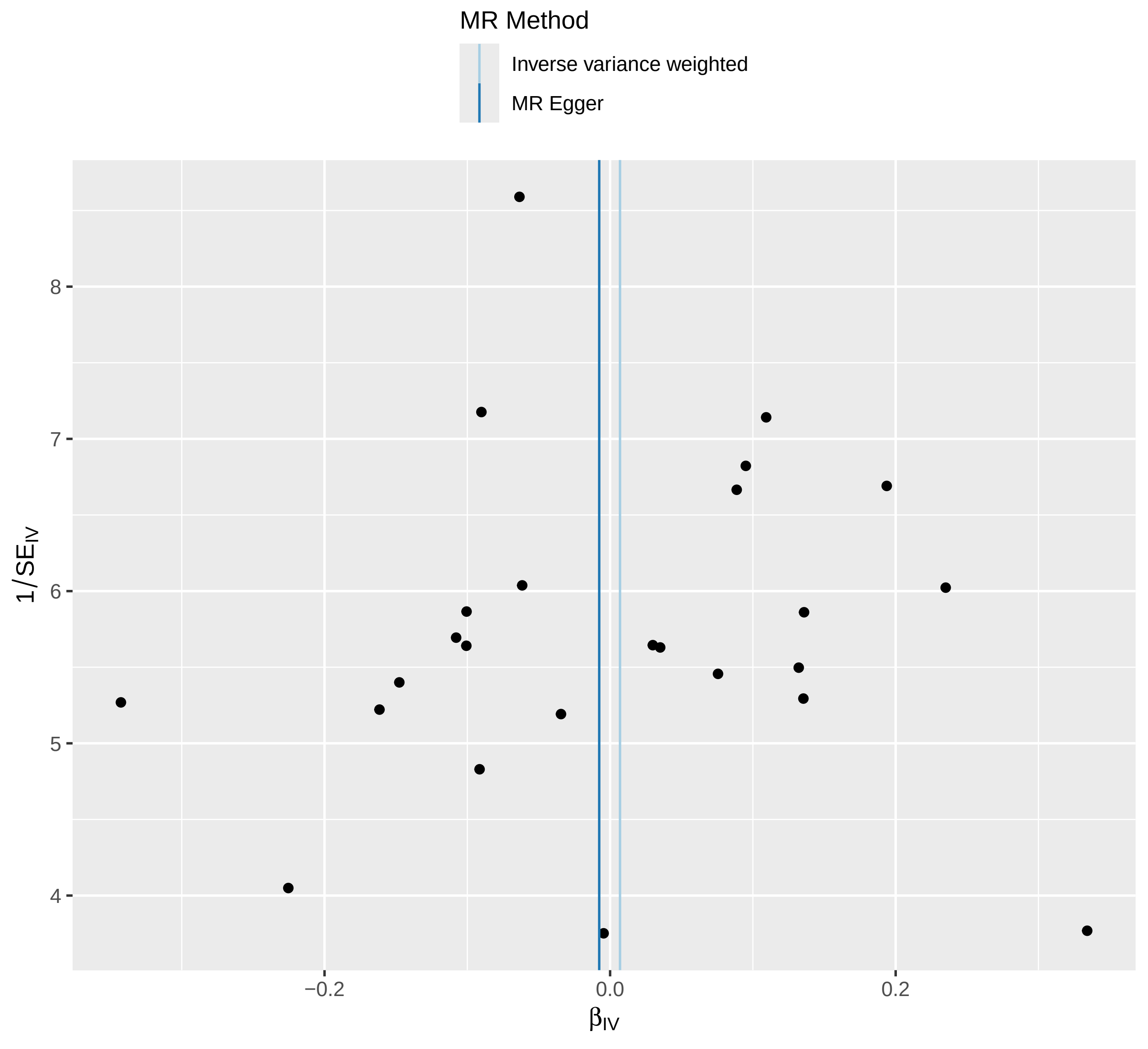
MR Method
Inverse variance weighted
MR Egger
1/SE_IV
8
7
6
5
4
−0.2
0.0
0.2
β_IV

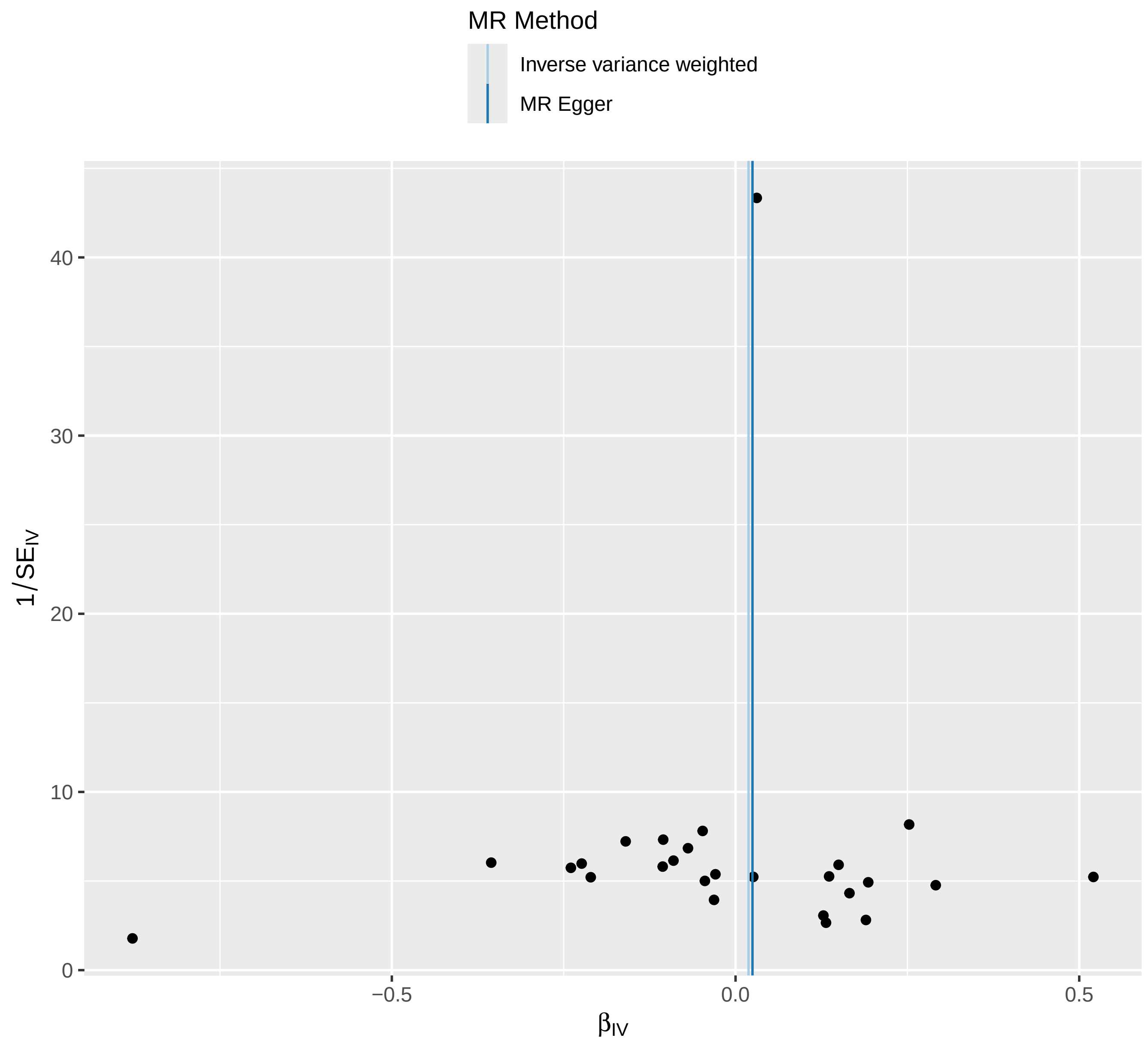
MR Method
Inverse variance weighted
MR Egger
1/SE_IV
40
30
20
10
0
−0.5
0.0
0.5
β_IV

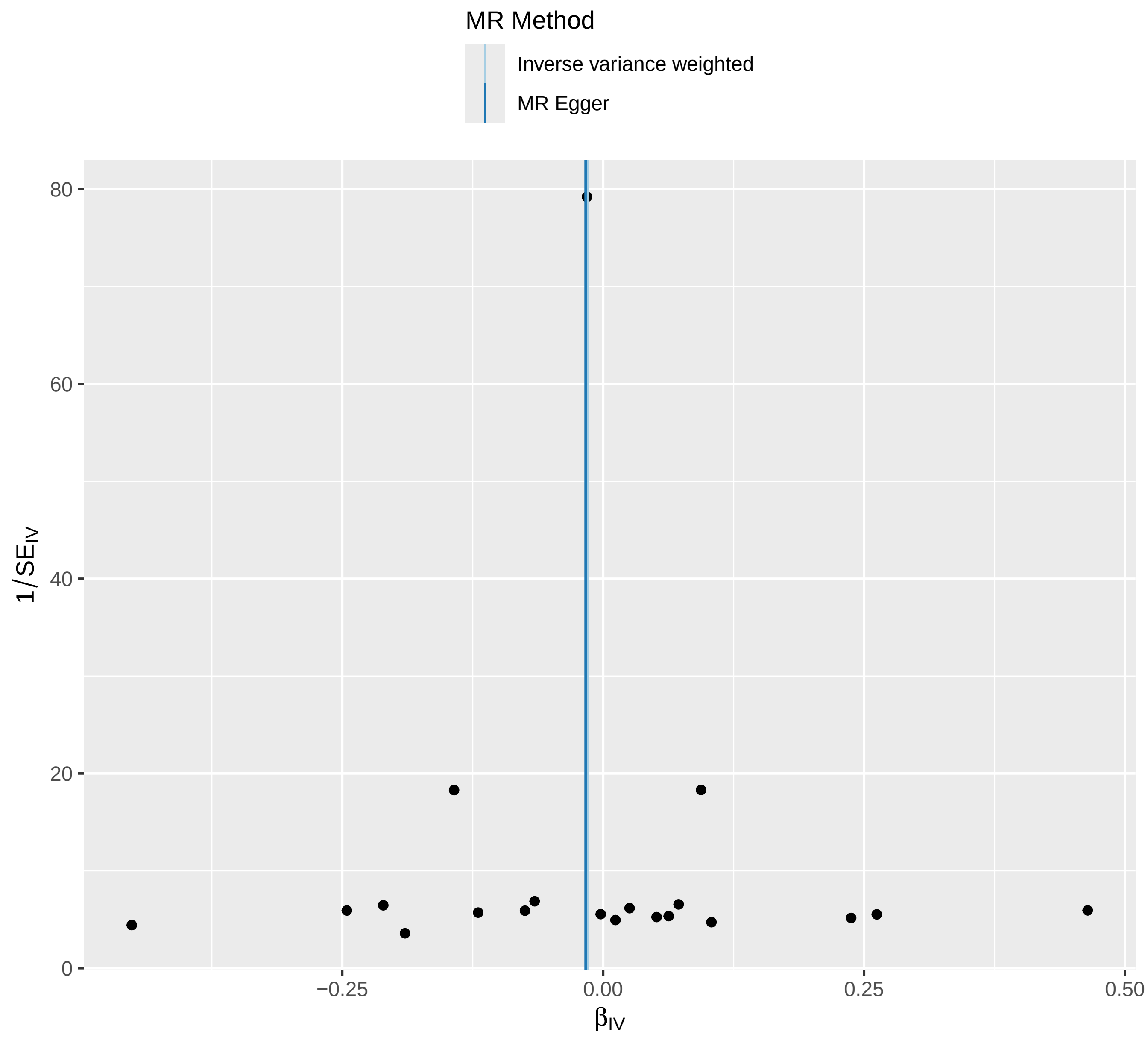

MR Method
Inverse variance weighted
MR Egger
80
60
40
20
0
1/SE_IV
−0.25
0.00
0.25
0.50
β_IV

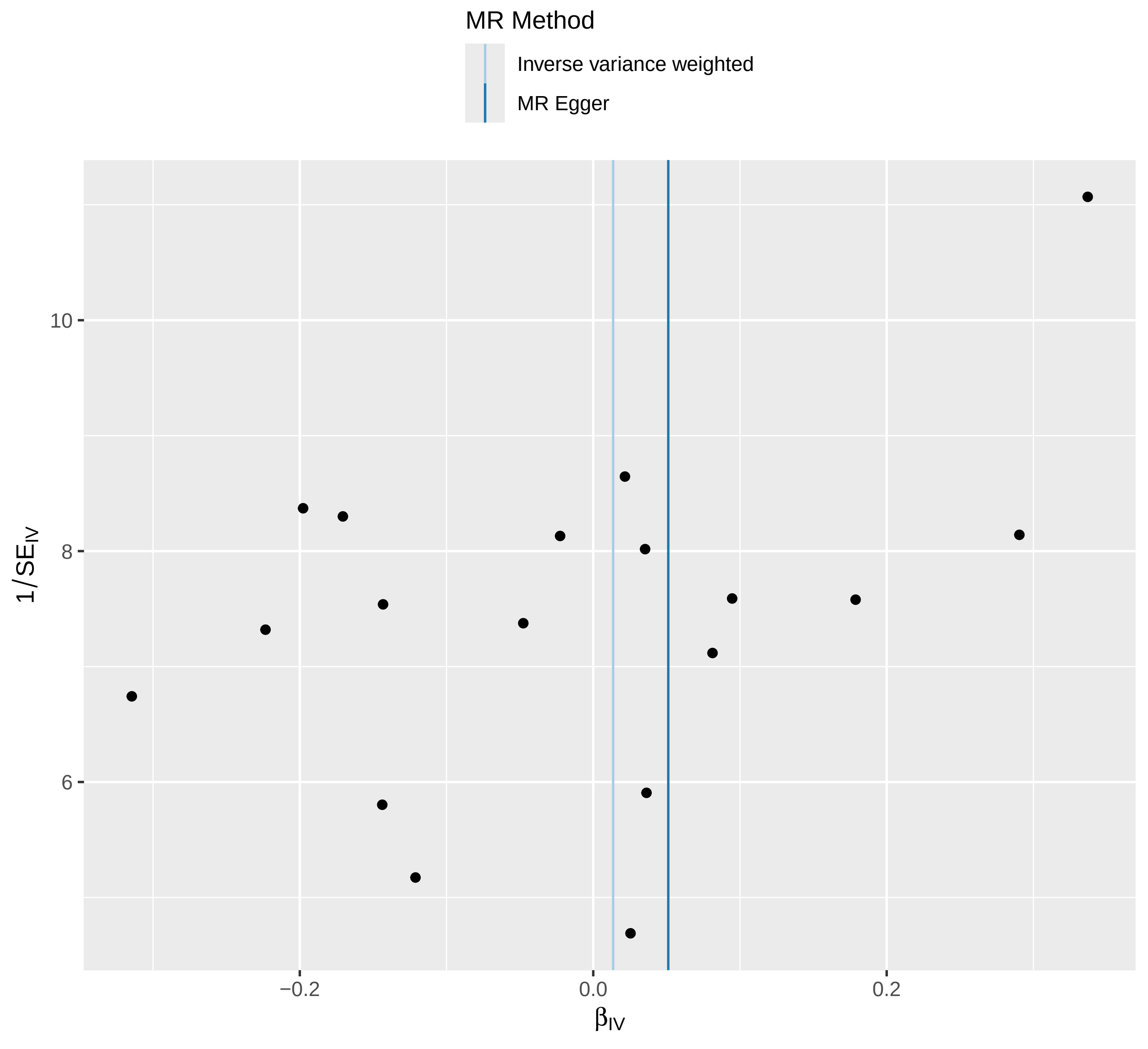
MR Method
Inverse variance weighted
MR Egger
10
8
6
1/SE_IV
−0.2
0.0
0.2
β_IV

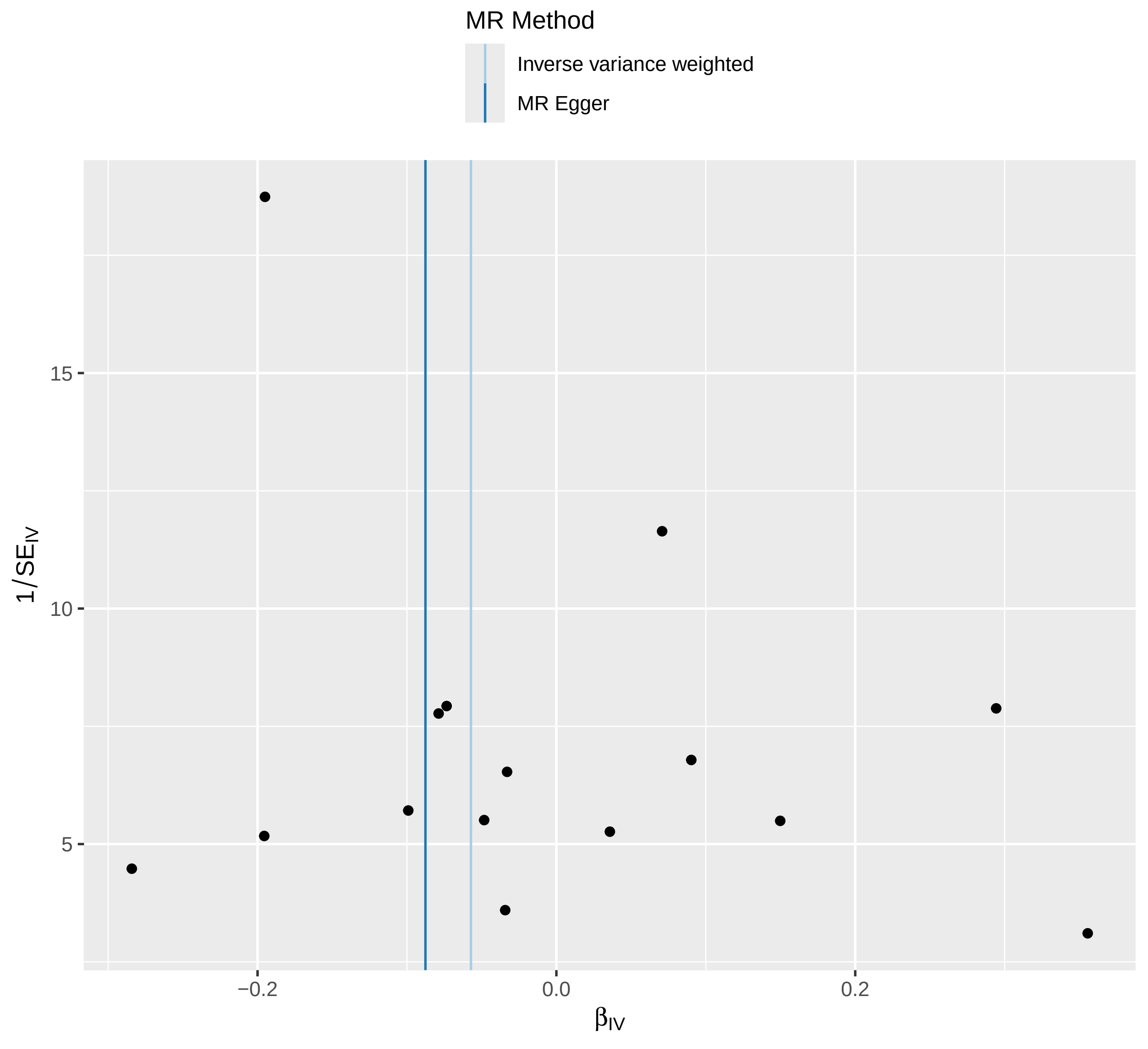
MR Method
Inverse variance weighted
MR Egger
15
10
5
1/SE_IV
−0.2
0.0
0.2
β_IV

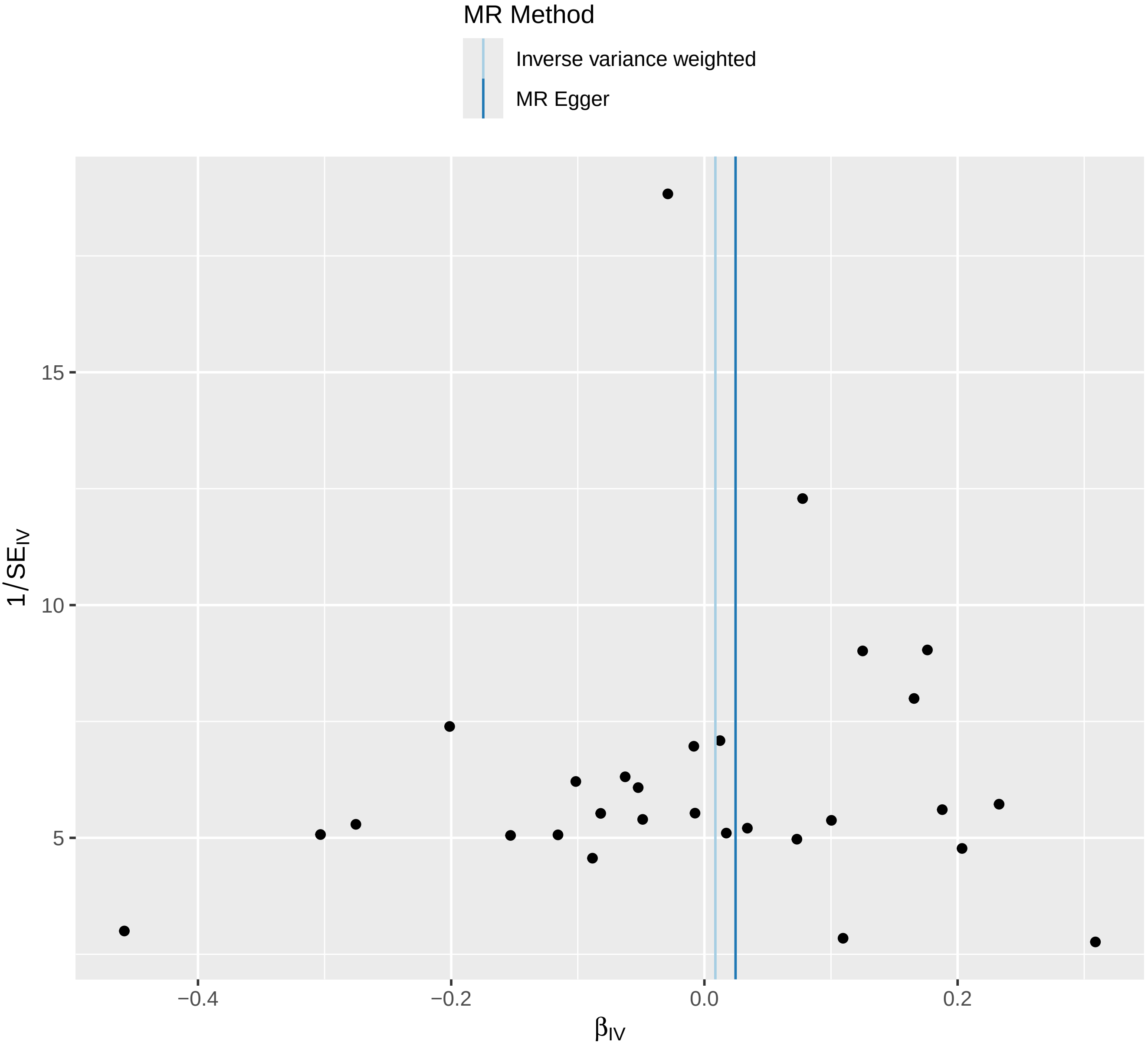

MR Method
Inverse variance weighted
MR Egger
15
10
5
1/SE_IV
−0.4
−0.2
0.0
0.2
β_IV

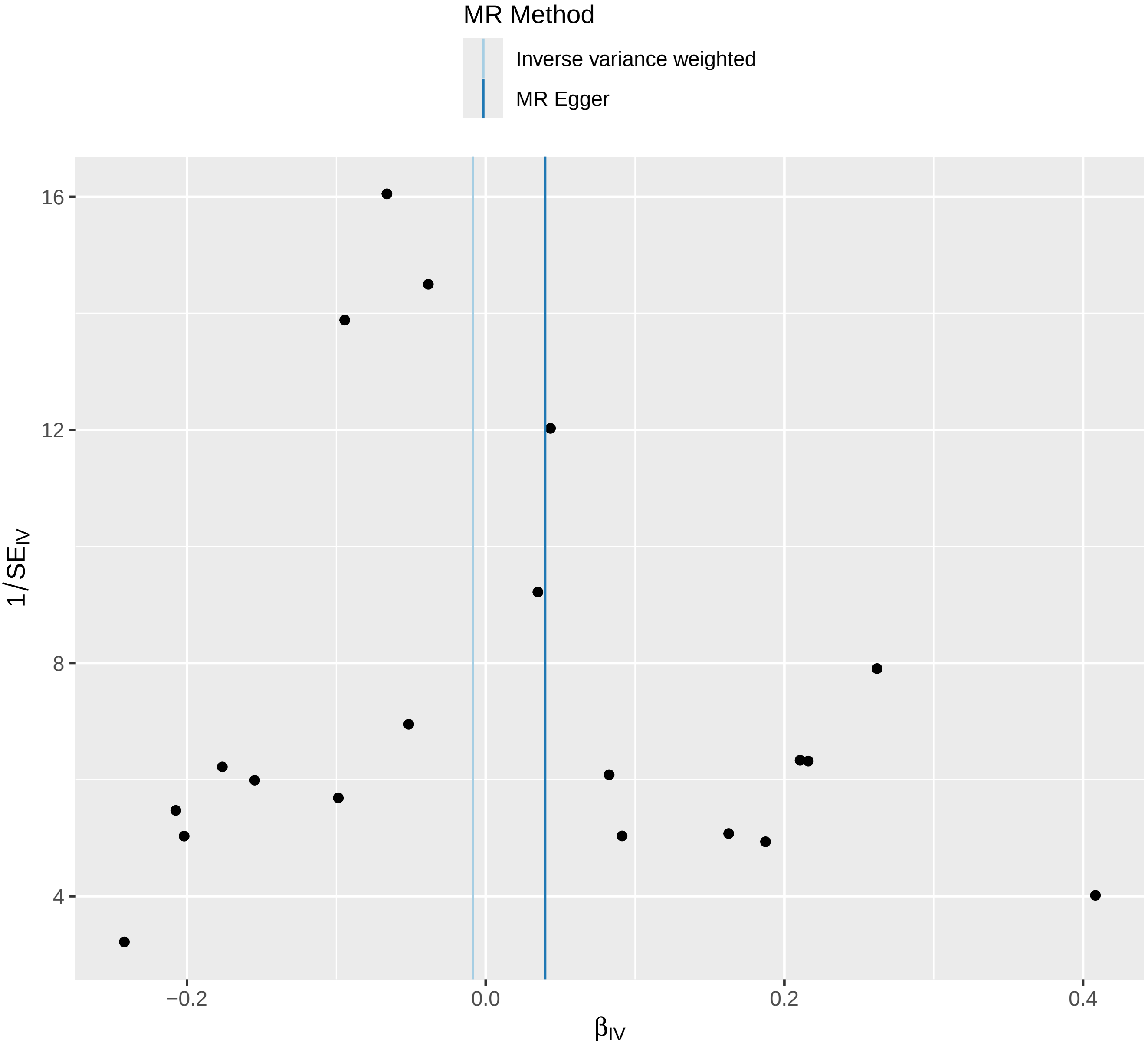
MR Method
Inverse variance weighted
MR Egger
16
12
8
4
1/SE_IV
−0.2
0.0
0.2
0.4
β_IV

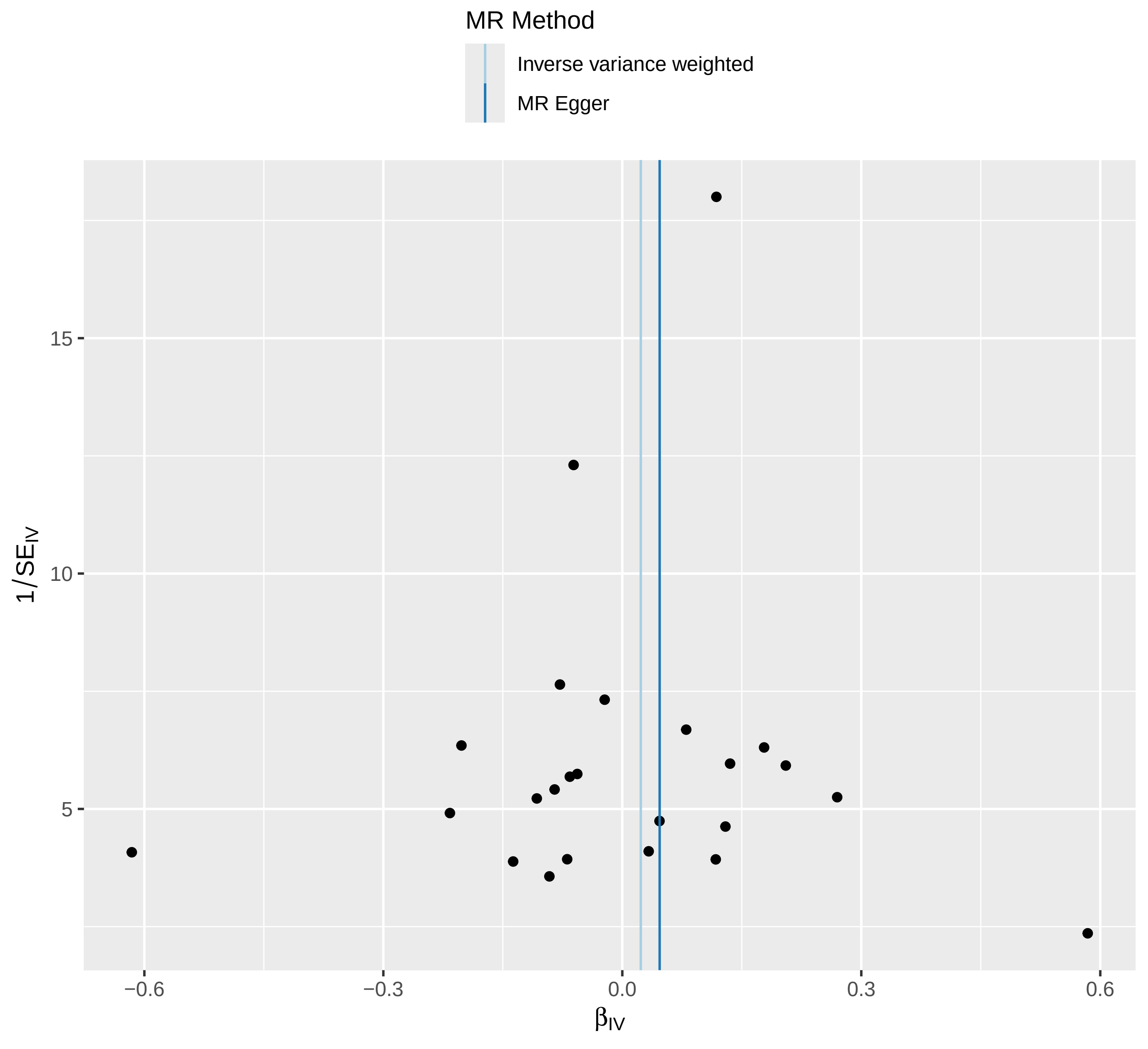

MR Method
Inverse variance weighted
MR Egger
15
10
5
1/SE$_{IV}$
−0.6
−0.3
0.0
0.3
0.6
β$_{IV}$

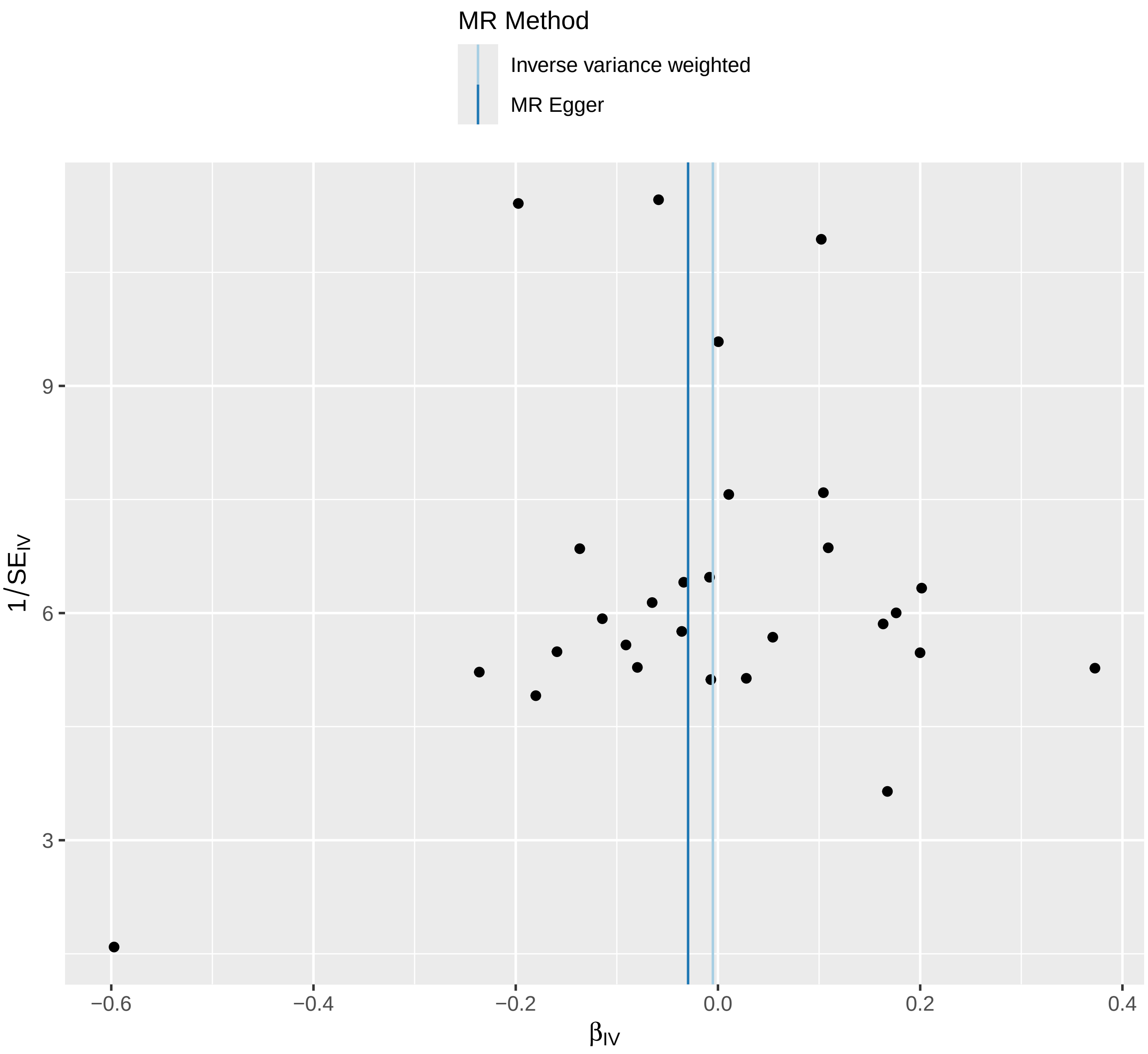
MR Method
Inverse variance weighted
MR Egger
1/SE_IV
9
6
3
−0.6
−0.4
−0.2
0.0
0.2
0.4
β_IV

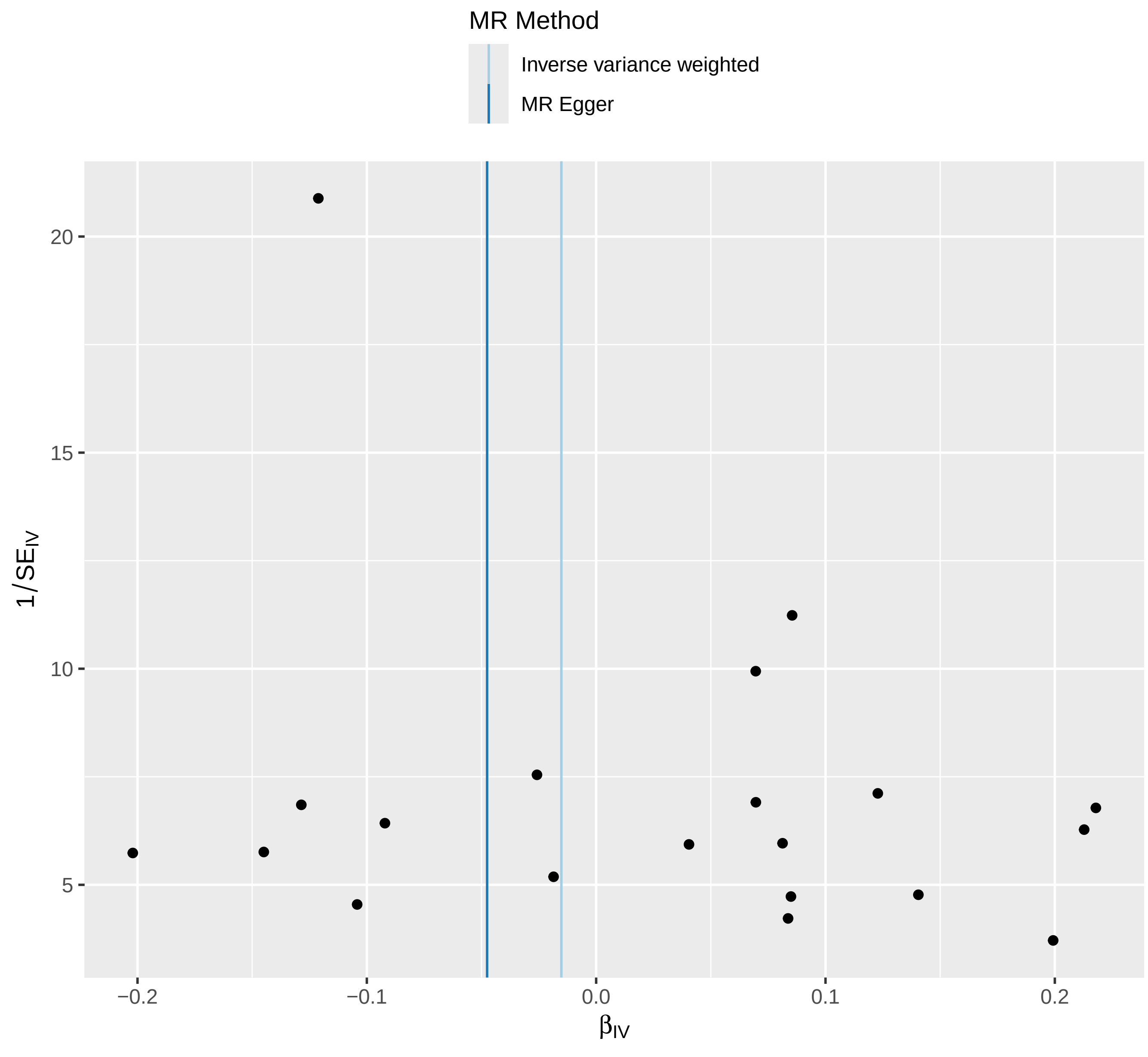

MR Method
Inverse variance weighted
MR Egger
20
15
10
5
1/SE$_{IV}$
−0.2
−0.1
0.0
0.1
0.2
β$_{IV}$

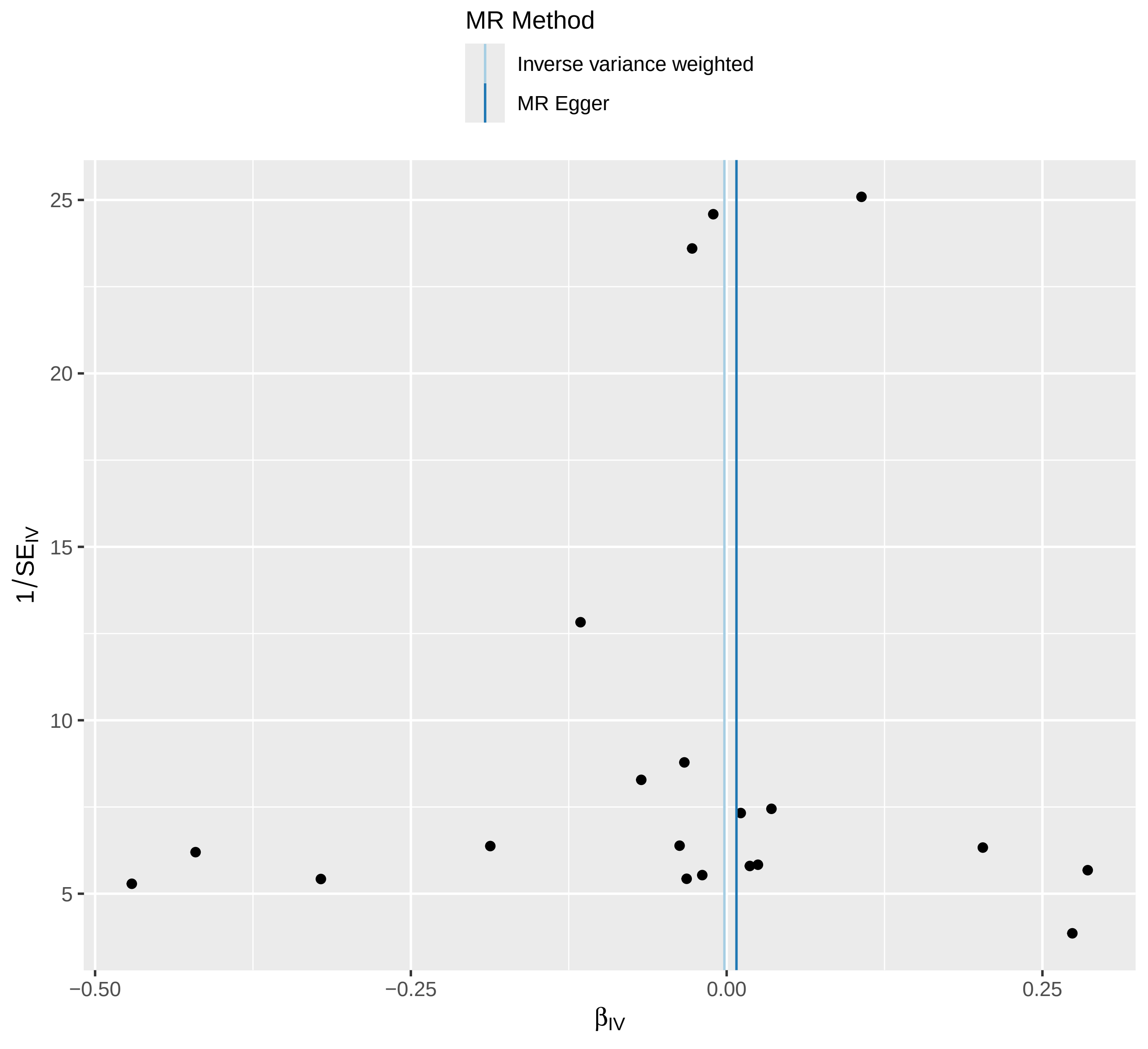
MR Method
Inverse variance weighted
MR Egger
25
20
15
10
5
1/SE_IV
−0.50
−0.25
0.00
0.25
β_IV

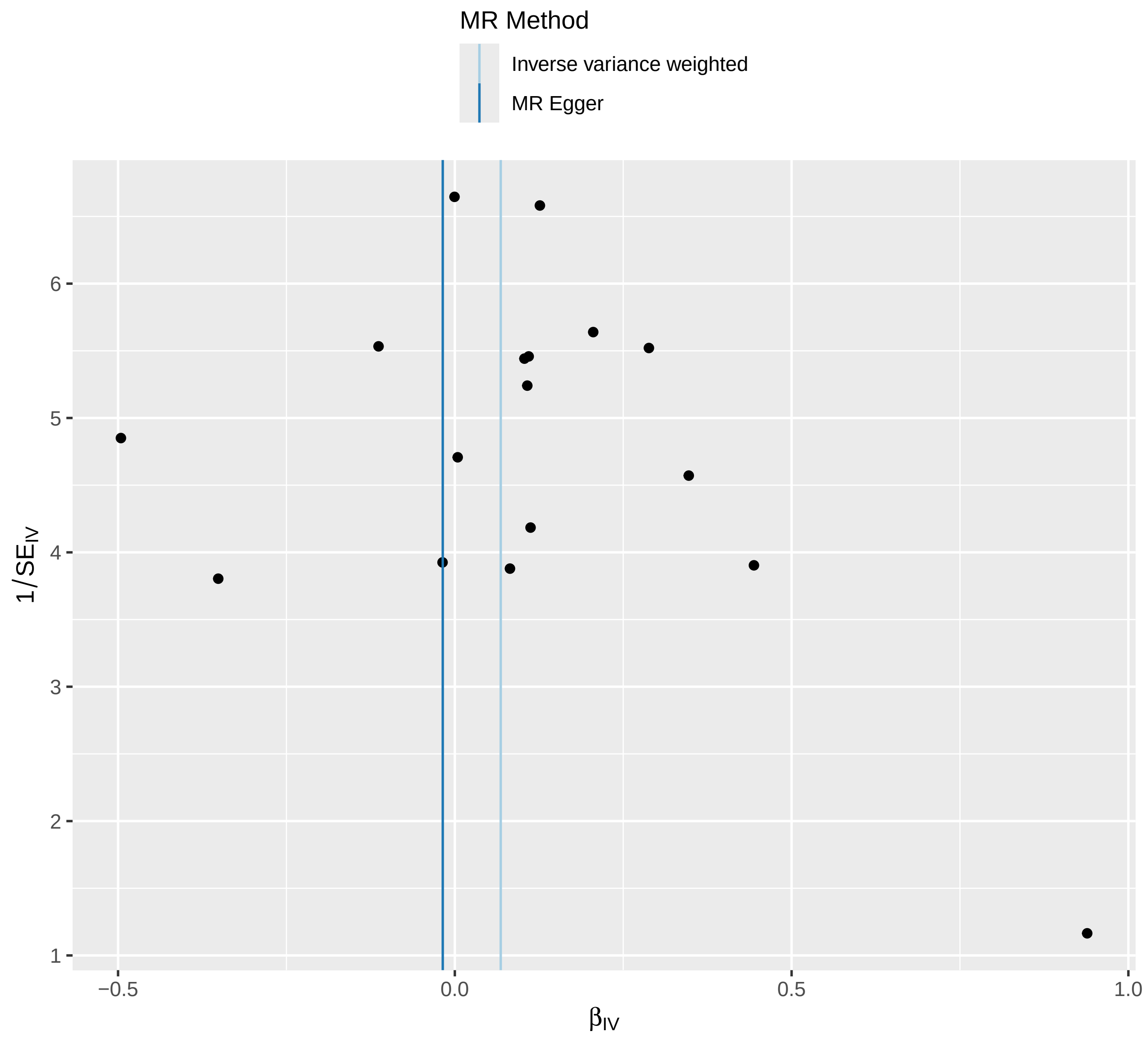
MR Method
Inverse variance weighted
MR Egger
1/SE_IV
β_IV
6
5
4
3
2
1
−0.5
0.0
0.5
1.0

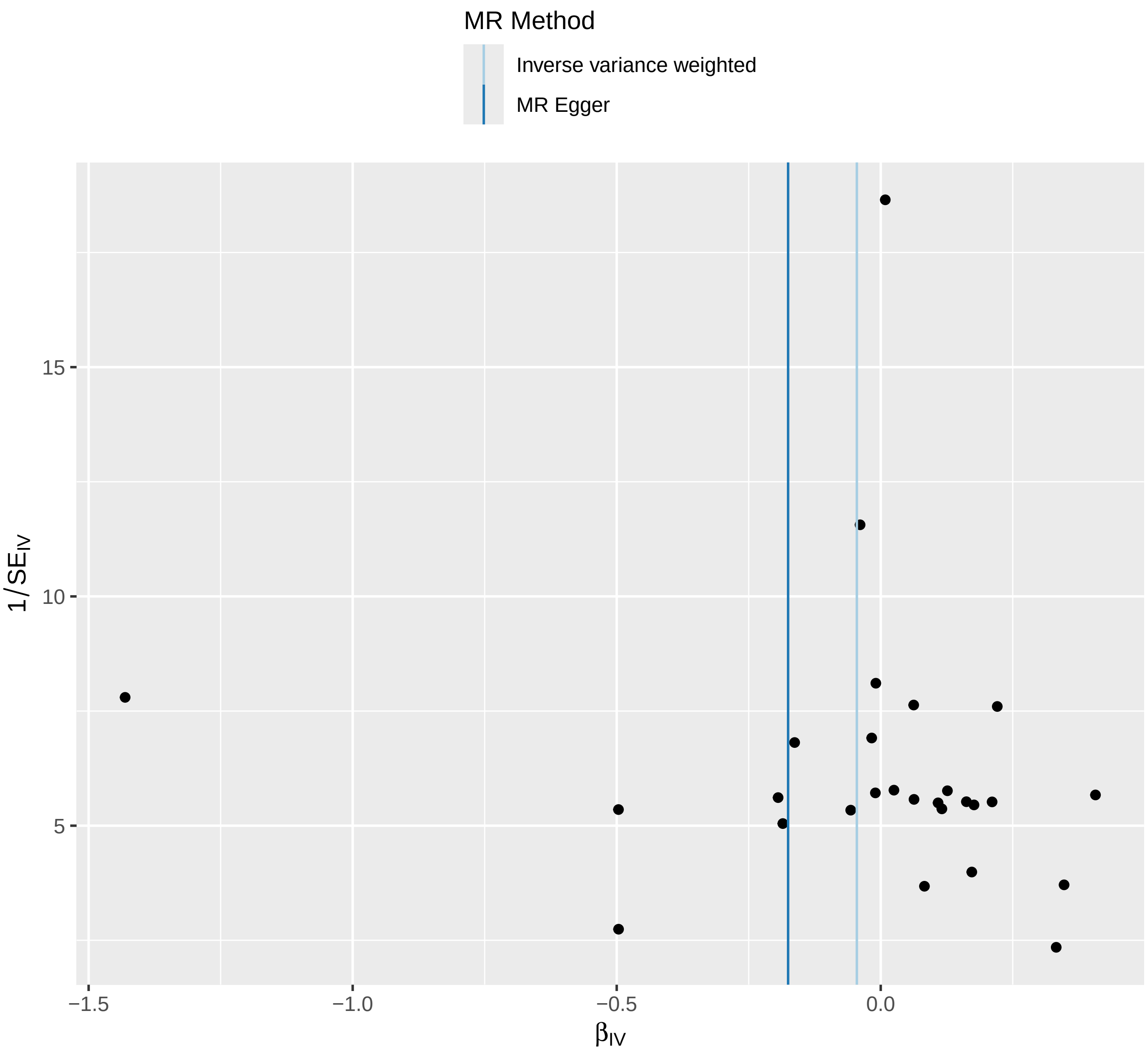
MR Method
Inverse variance weighted
MR Egger
15
10
5
$1/SE_{IV}$
−1.5
−1.0
−0.5
0.0
β_{IV}

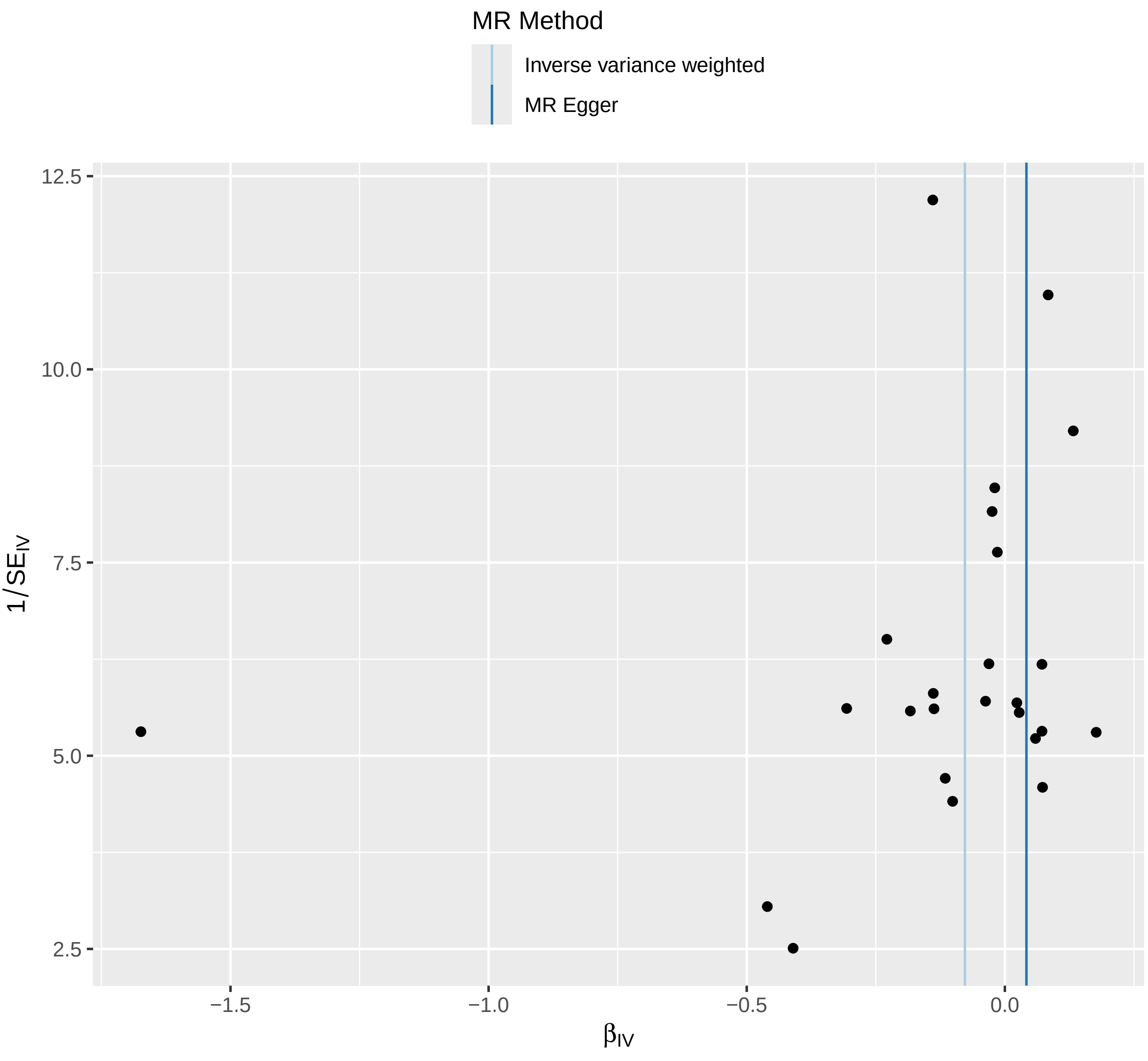
MR Method
Inverse variance weighted
MR Egger
12.5
10.0
7.5
5.0
2.5
1/SE$_{IV}$
−1.5
−1.0
−0.5
0.0
β$_{IV}$

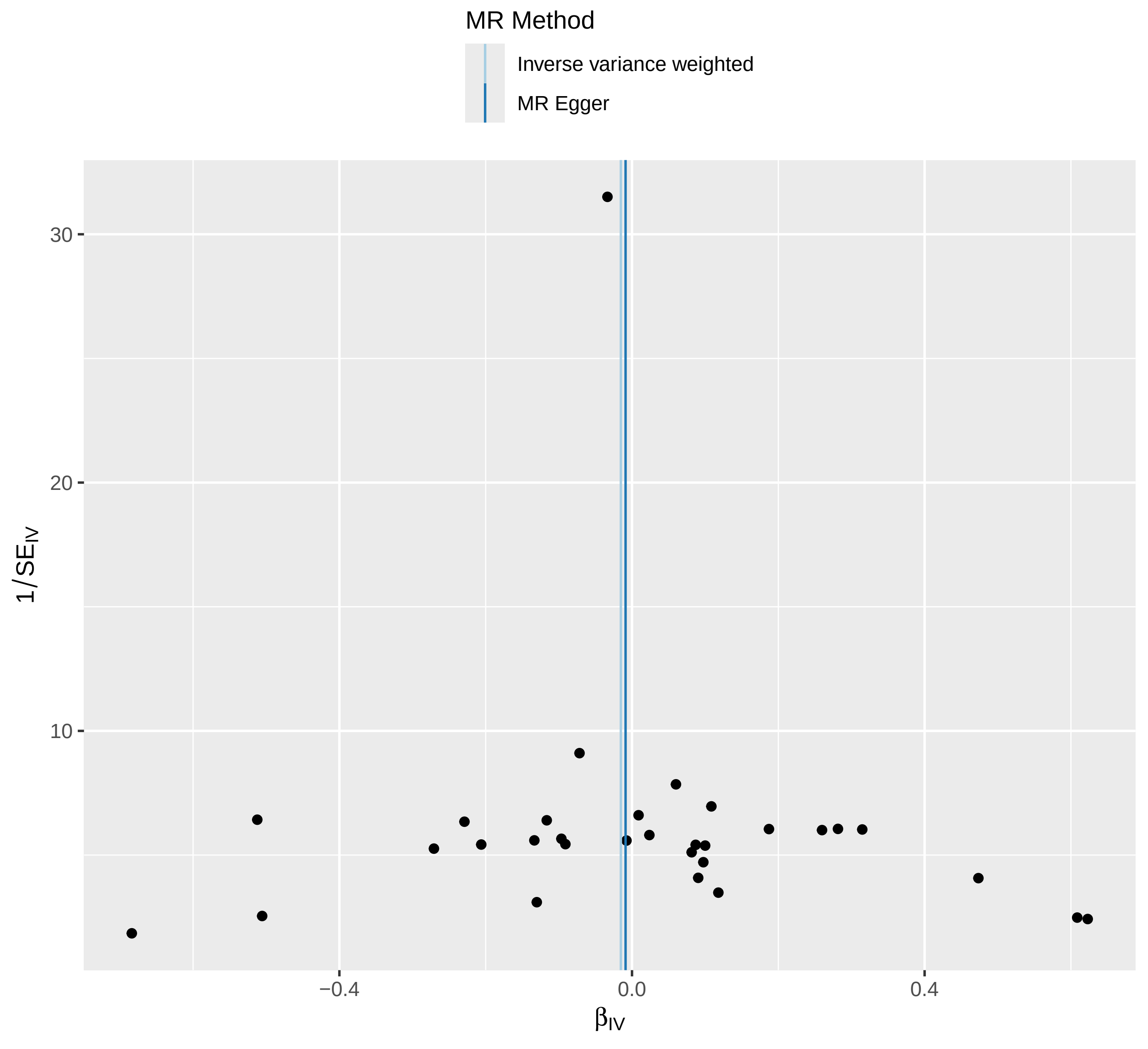
MR Method
Inverse variance weighted
MR Egger
30
20
10
1/SE$_{IV}$
−0.4
0.0
0.4
β$_{IV}$

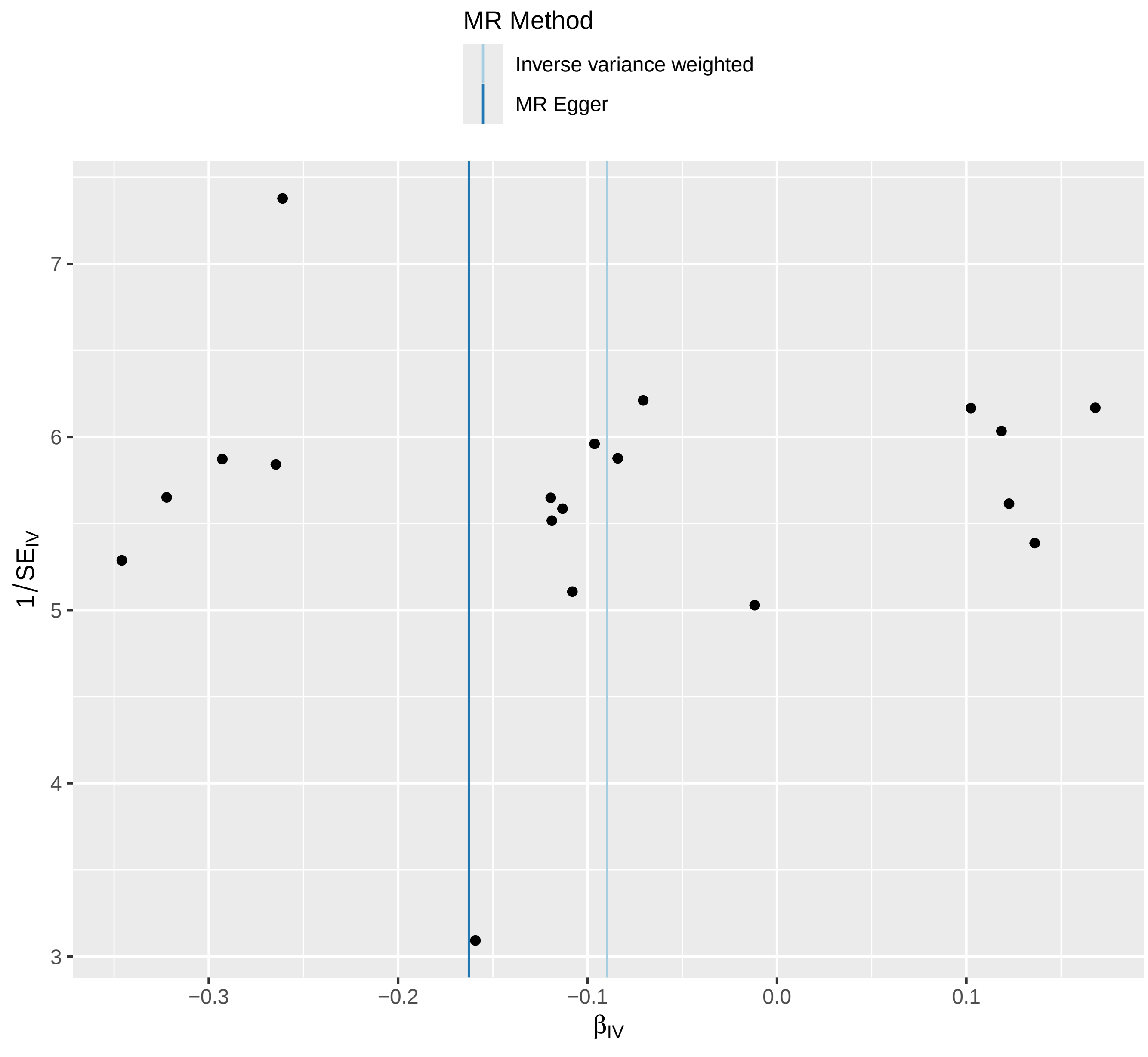

MR Method
Inverse variance weighted
MR Egger
1/SE_IV
7
6
5
4
3
−0.3
−0.2
−0.1
0.0
0.1
β_IV

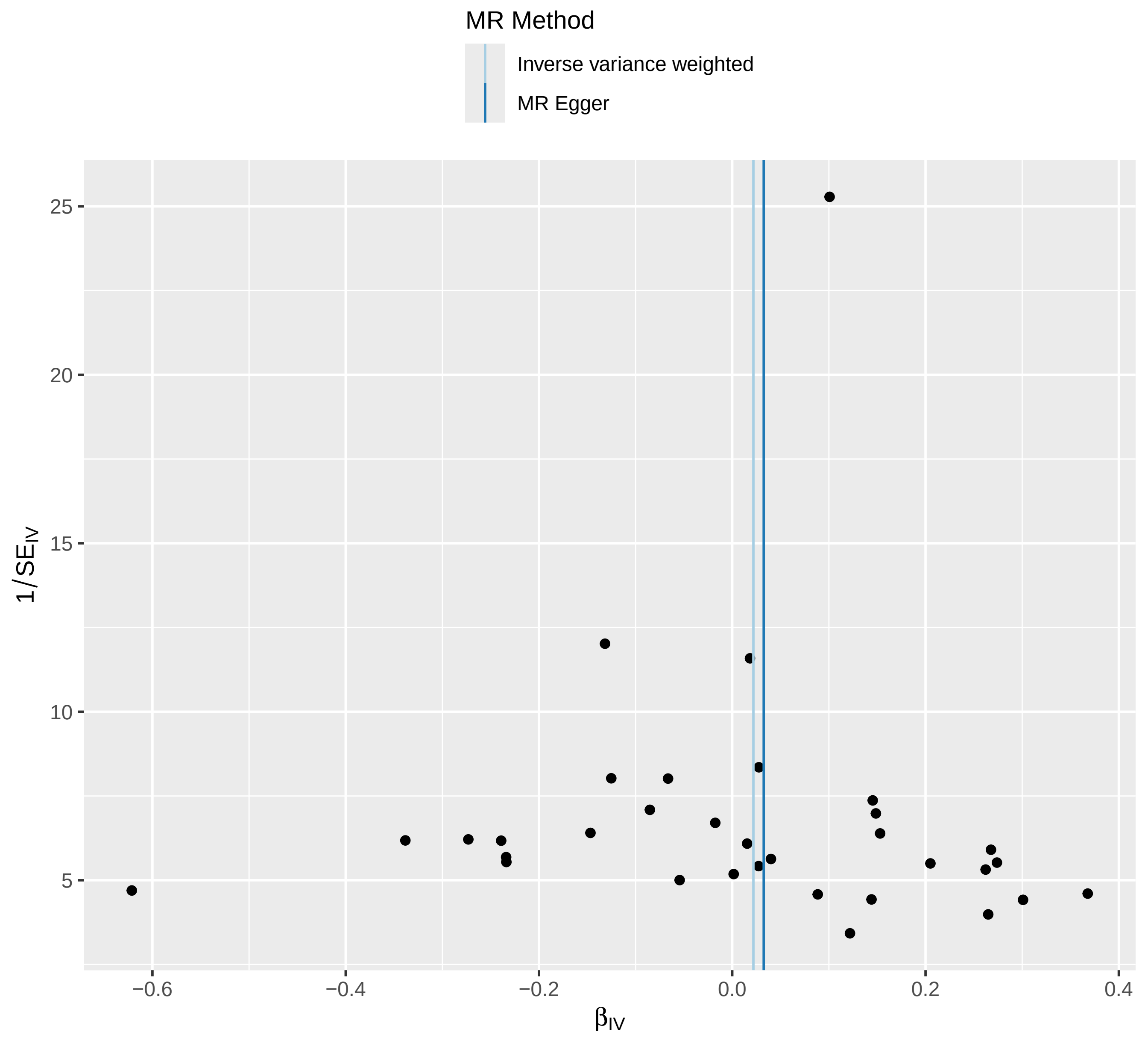
MR Method
Inverse variance weighted
MR Egger
25
20
15
10
5
$1/SE_{IV}$
−0.6
−0.4
−0.2
0.0
0.2
0.4
β_{IV}

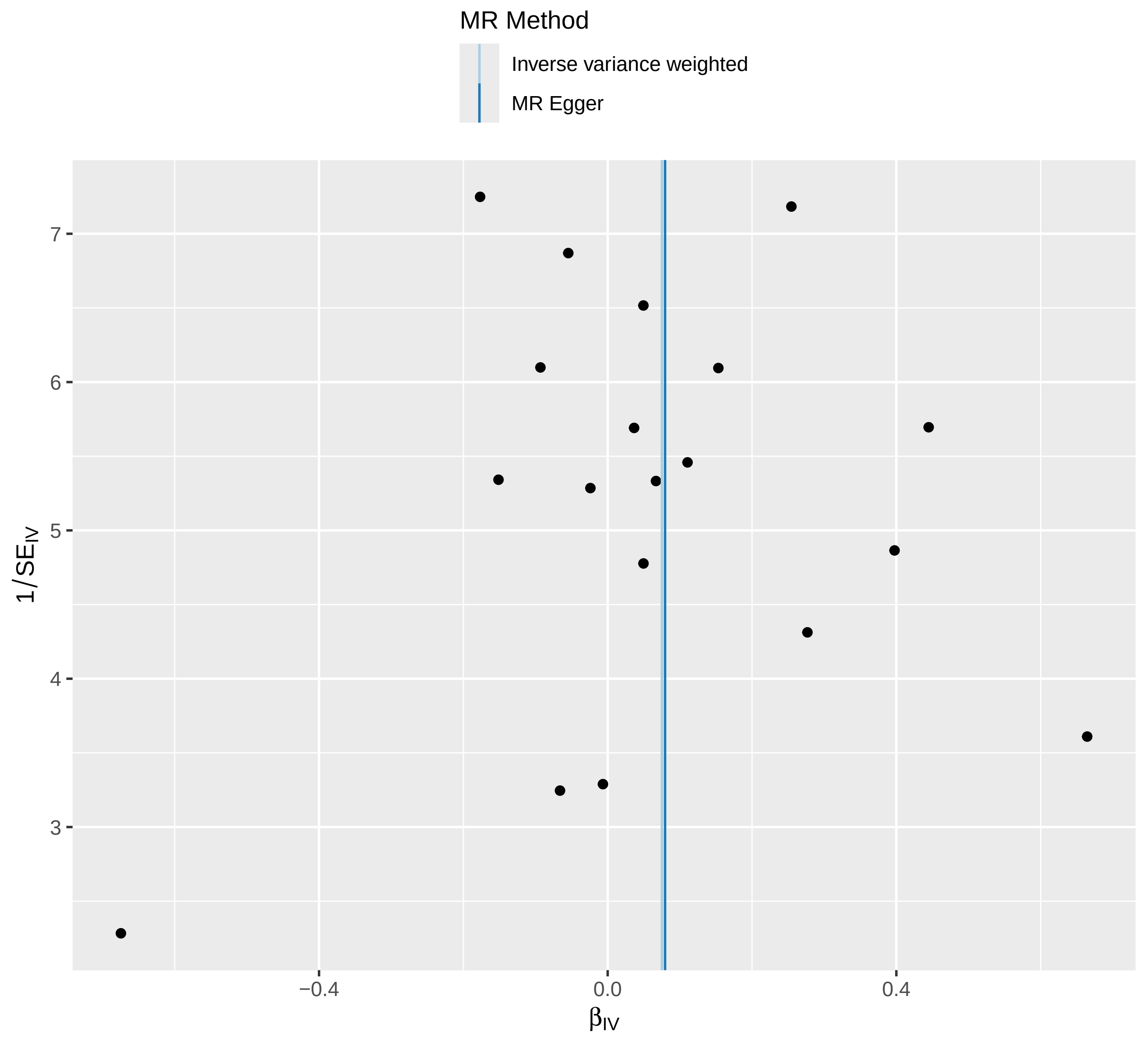

MR Method
Inverse variance weighted
MR Egger
1/SE$_{IV}$
7
6
5
4
3
β$_{IV}$
−0.4
0.0
0.4

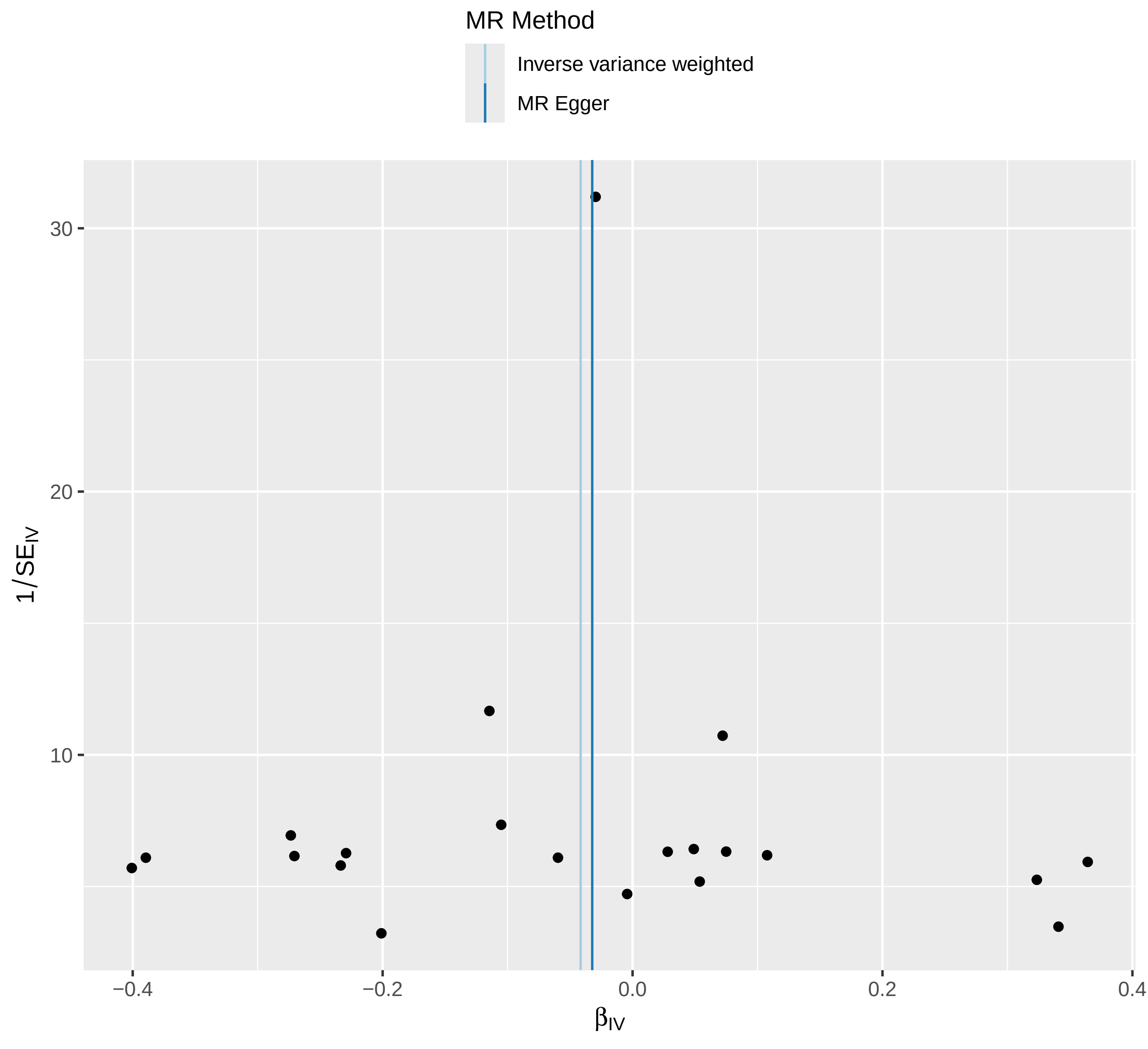

MR Method
Inverse variance weighted
MR Egger
30
20
10
1/SE$_{IV}$
−0.4
−0.2
0.0
0.2
0.4
β$_{IV}$

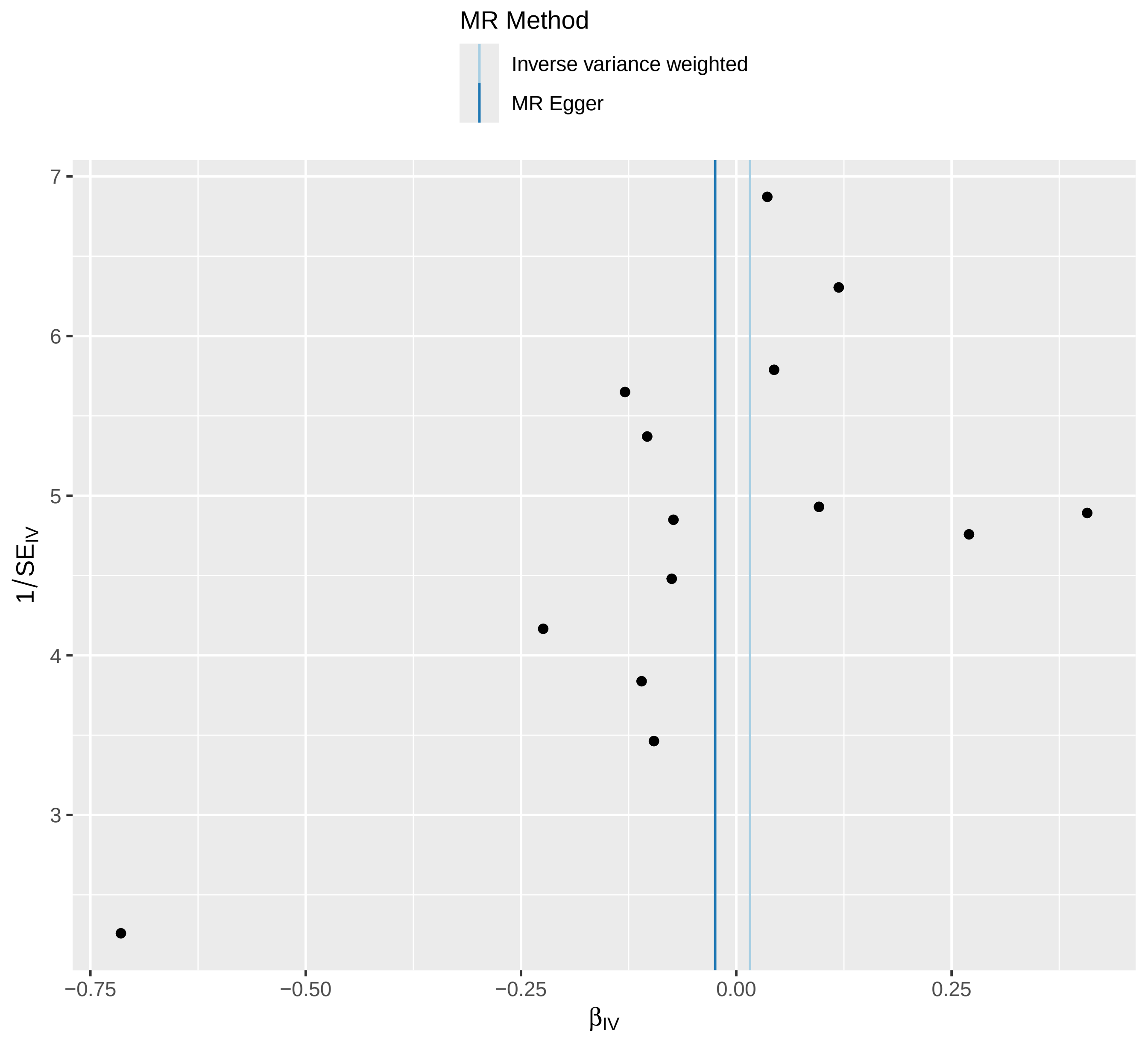

MR Method
Inverse variance weighted
MR Egger
7
6
5
4
3
1/SE_IV
−0.75
−0.50
−0.25
0.00
0.25
β_IV

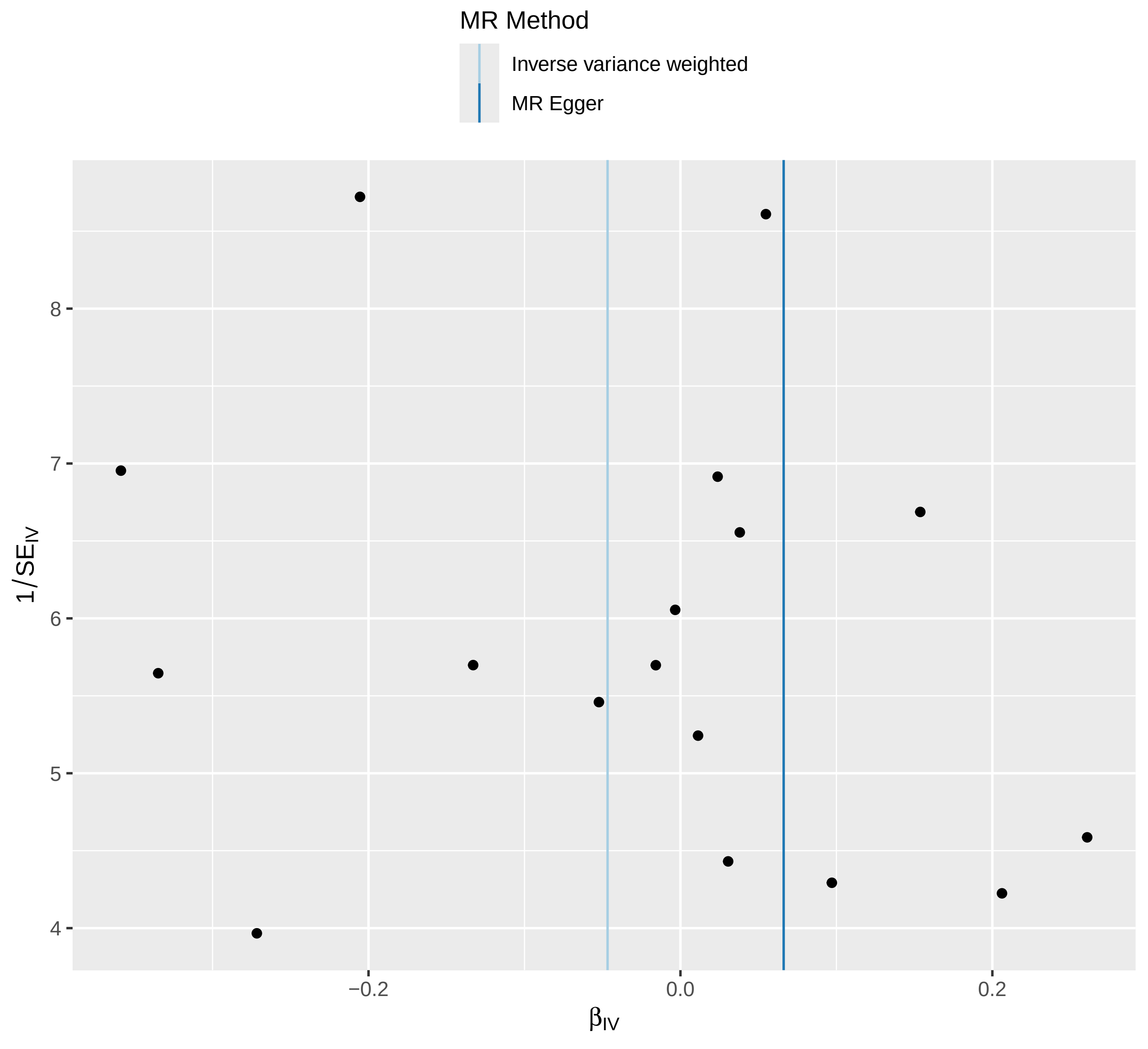
MR Method
Inverse variance weighted
MR Egger
8
7
6
5
4
1/SE_IV
−0.2
0.0
0.2
β_IV

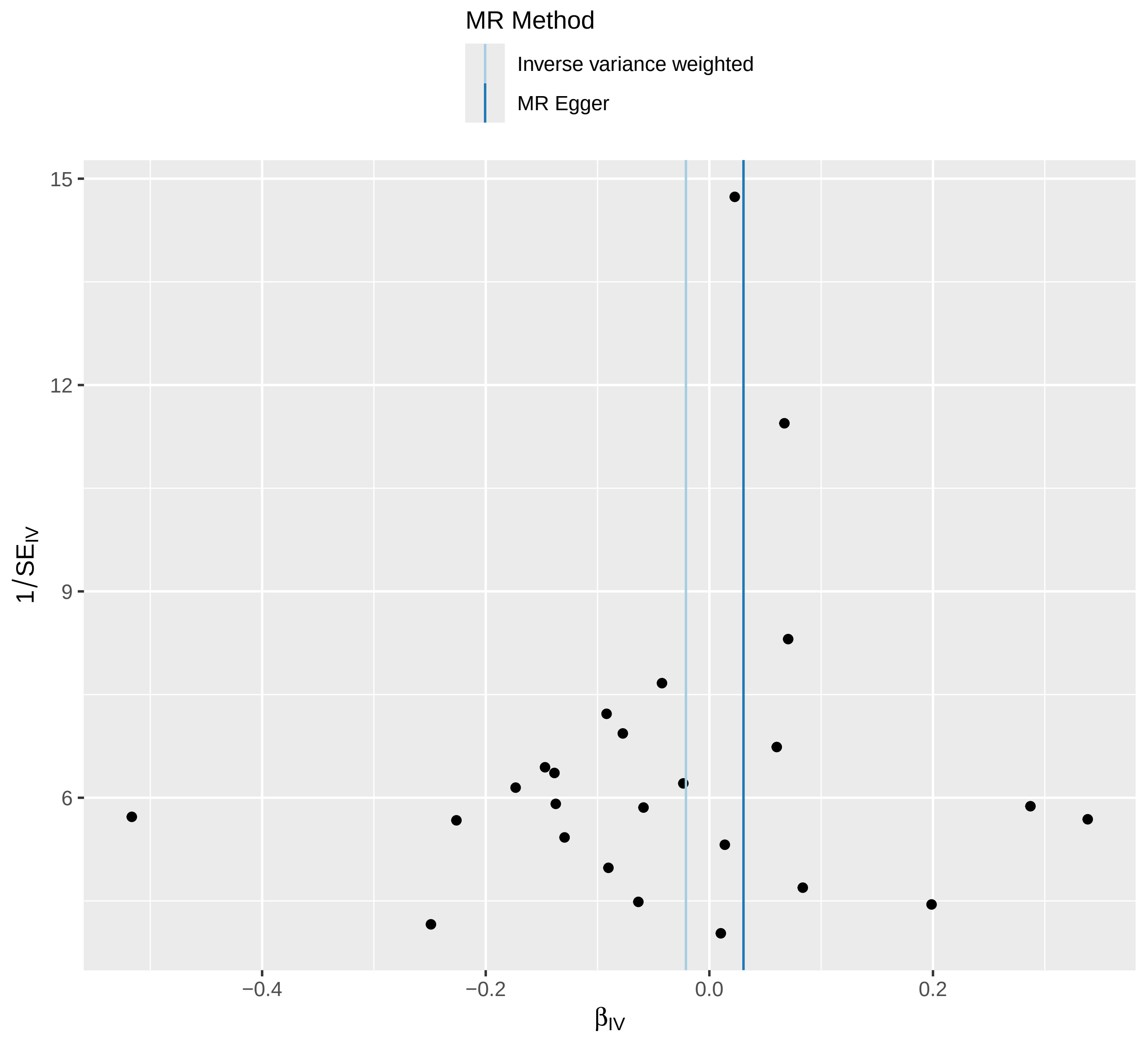

MR Method
Inverse variance weighted
MR Egger
15
12
9
6
1/SE_IV
−0.4
−0.2
0.0
0.2
β_IV

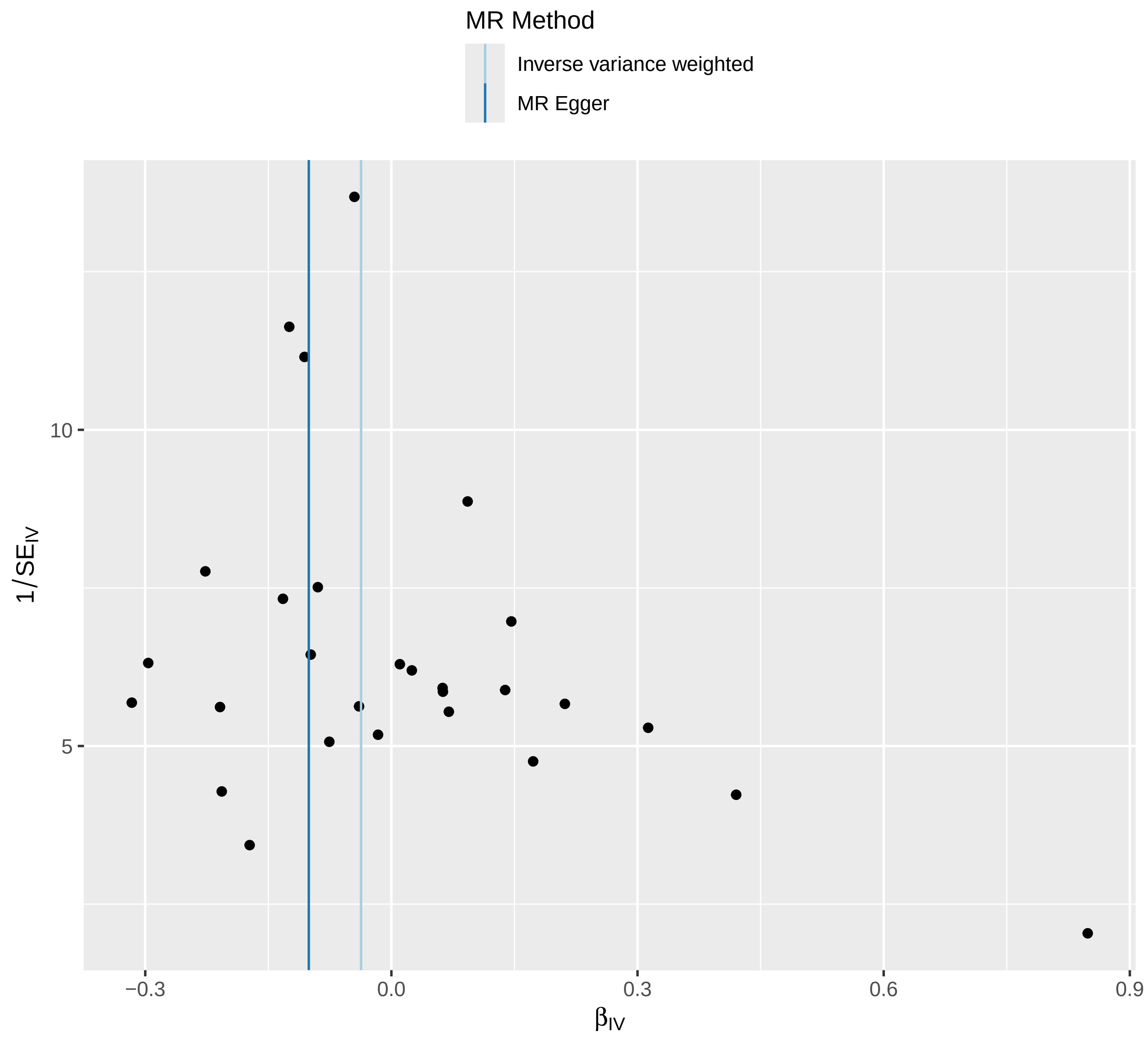
MR Method
Inverse variance weighted
MR Egger
10
5
1/SE$_{IV}$
−0.3
0.0
0.3
0.6
0.9
β$_{IV}$

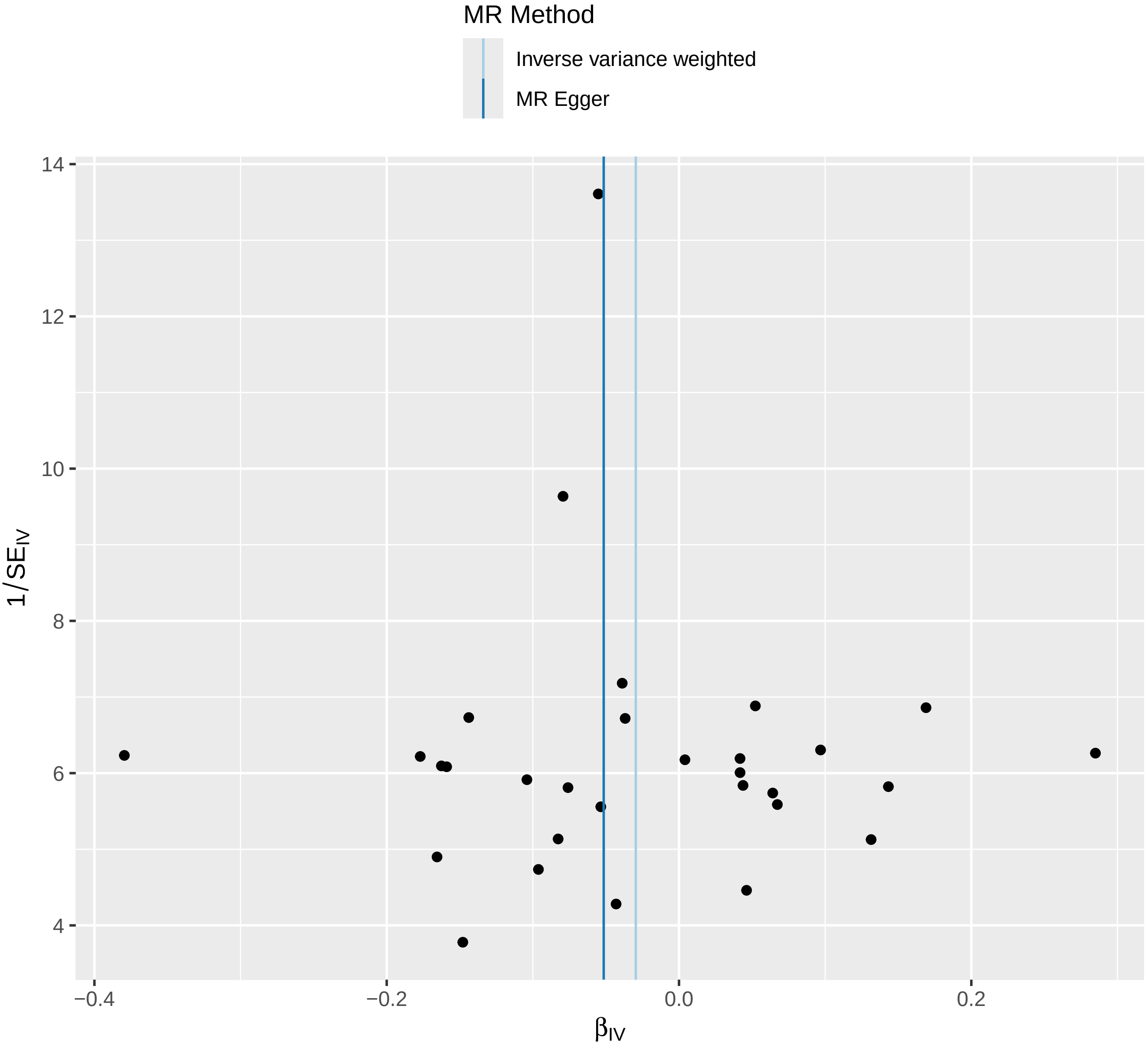
MR Method
Inverse variance weighted
MR Egger
14
12
10
8
6
4
$1/SE_{IV}$
−0.4
−0.2
0.0
0.2
β_{IV}

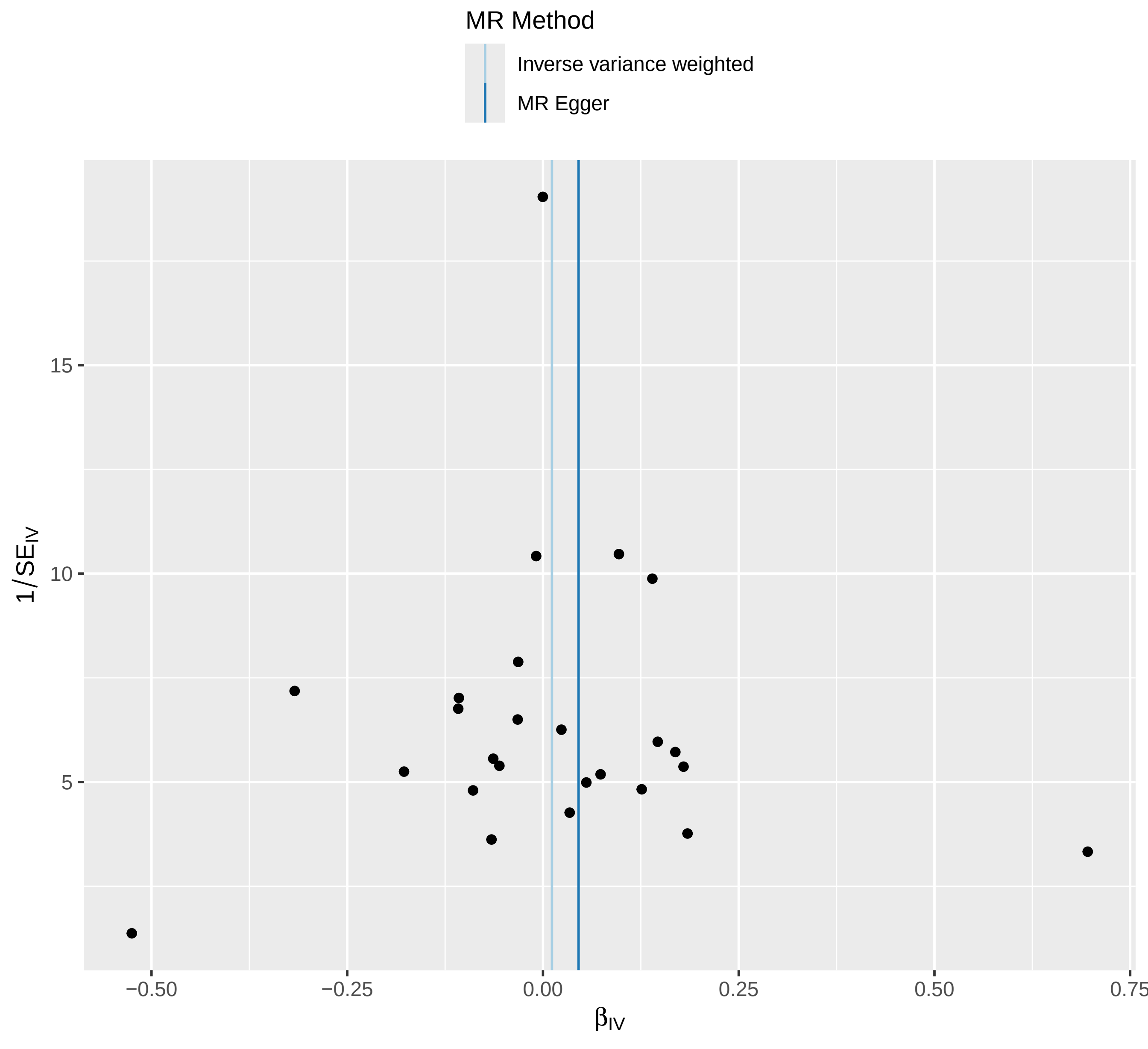

MR Method
Inverse variance weighted
MR Egger
15
10
5
1/SE_IV
−0.50
−0.25
0.00
0.25
0.50
0.75
β_IV

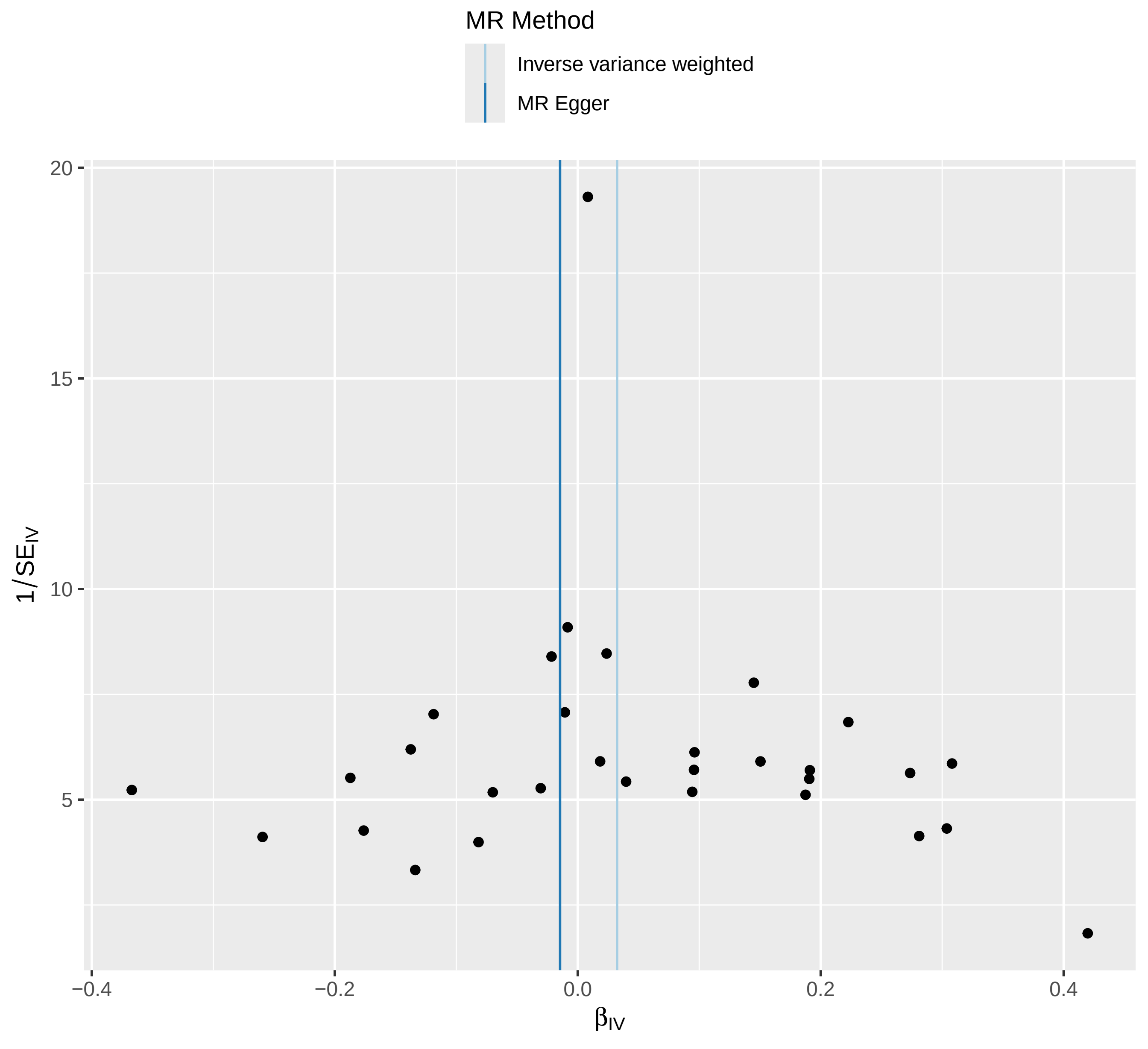
MR Method
Inverse variance weighted
MR Egger
20
15
10
5
1/SE_IV
−0.4
−0.2
0.0
0.2
0.4
β_IV

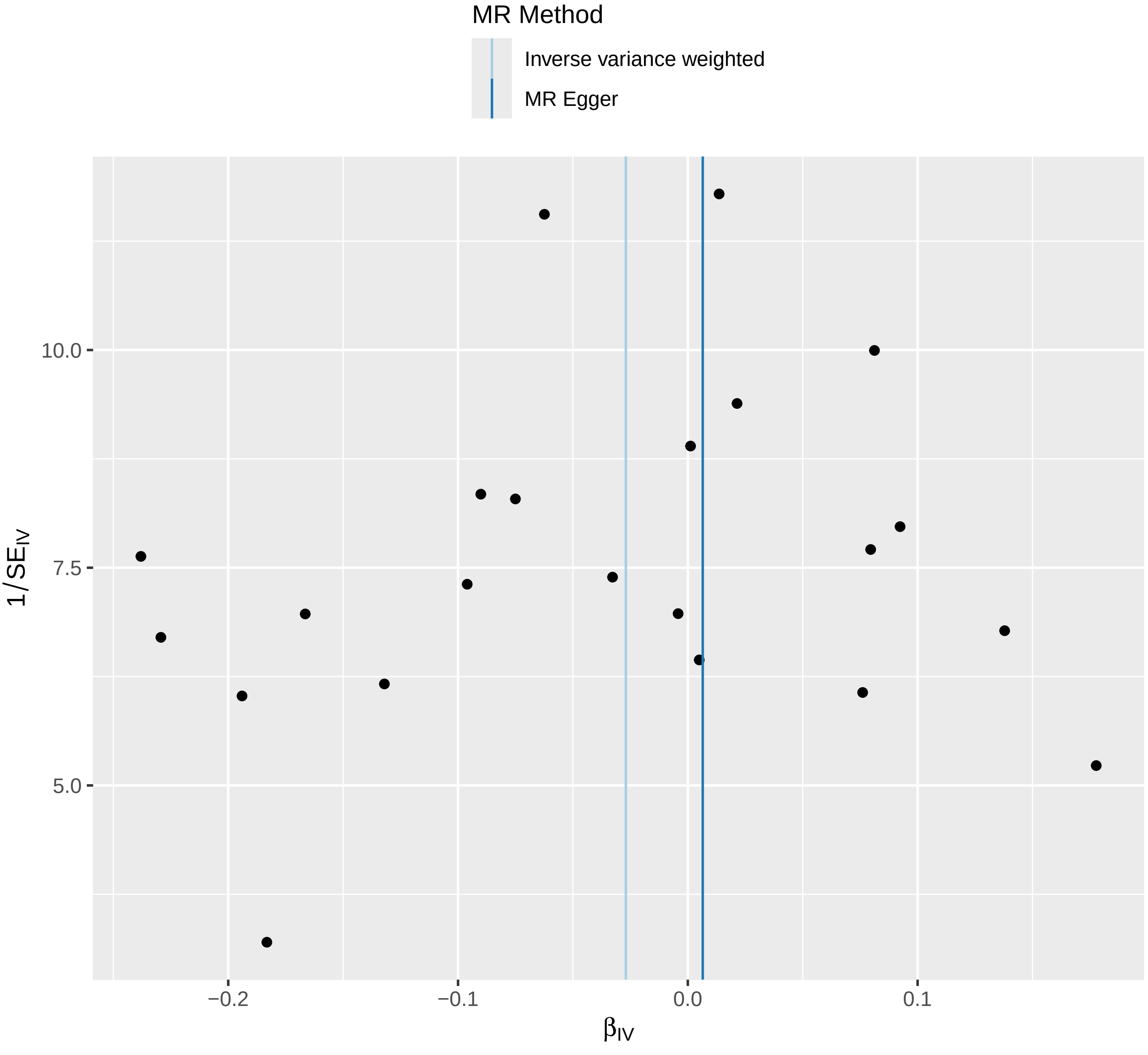

MR Method
Inverse variance weighted
MR Egger
10.0
7.5
5.0
1/SE$_{IV}$
−0.2
−0.1
0.0
0.1
β$_{IV}$

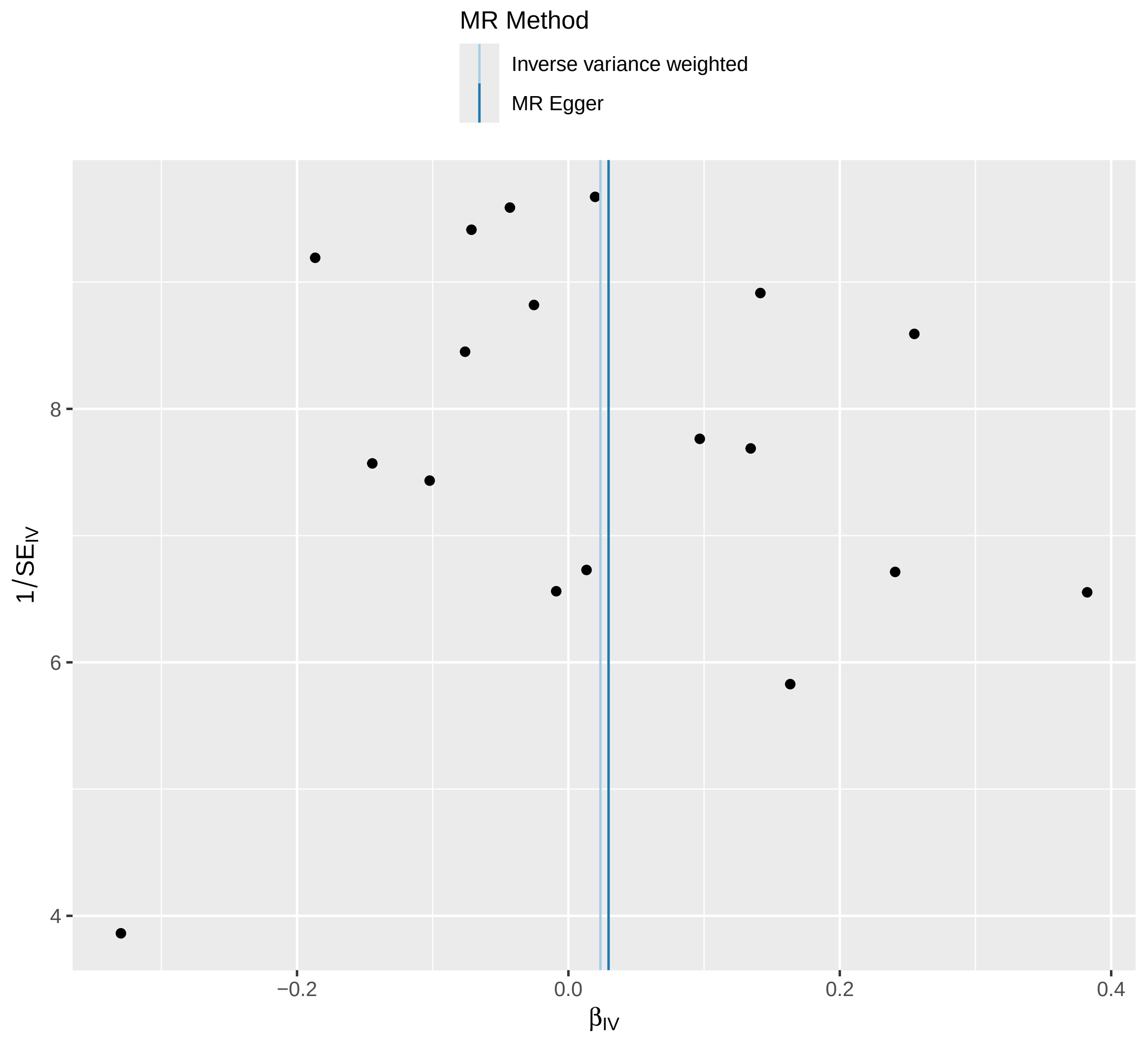
MR Method
Inverse variance weighted
MR Egger
$1/SE_{IV}$
8
6
4
−0.2
0.0
0.2
0.4
β_{IV}

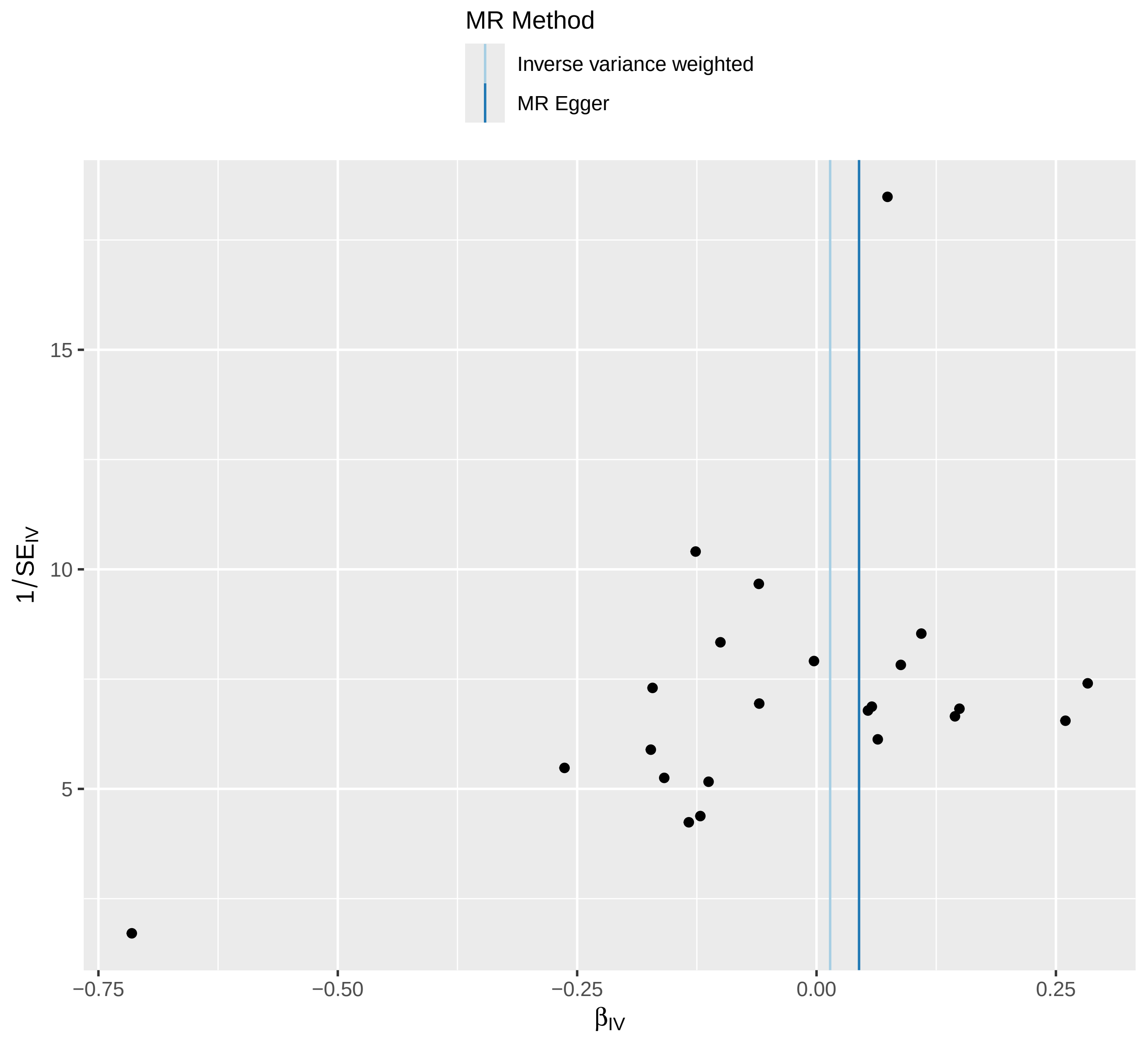

MR Method
Inverse variance weighted
MR Egger
15
10
5
1/SE$_{IV}$
−0.75
−0.50
−0.25
0.00
0.25
β$_{IV}$

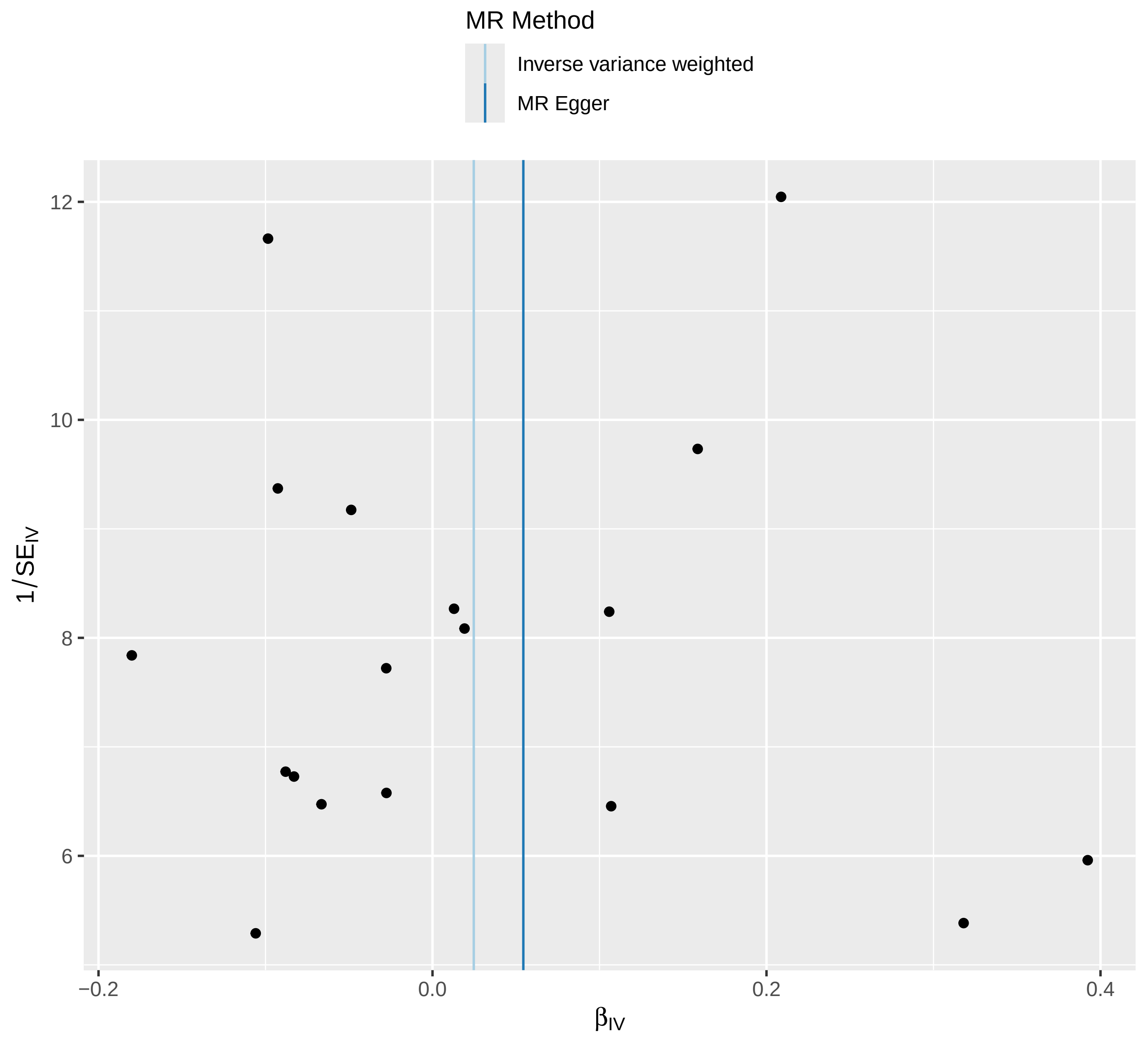
MR Method
Inverse variance weighted
MR Egger
12
10
8
6
1/SE_IV
−0.2
0.0
0.2
0.4
β_IV

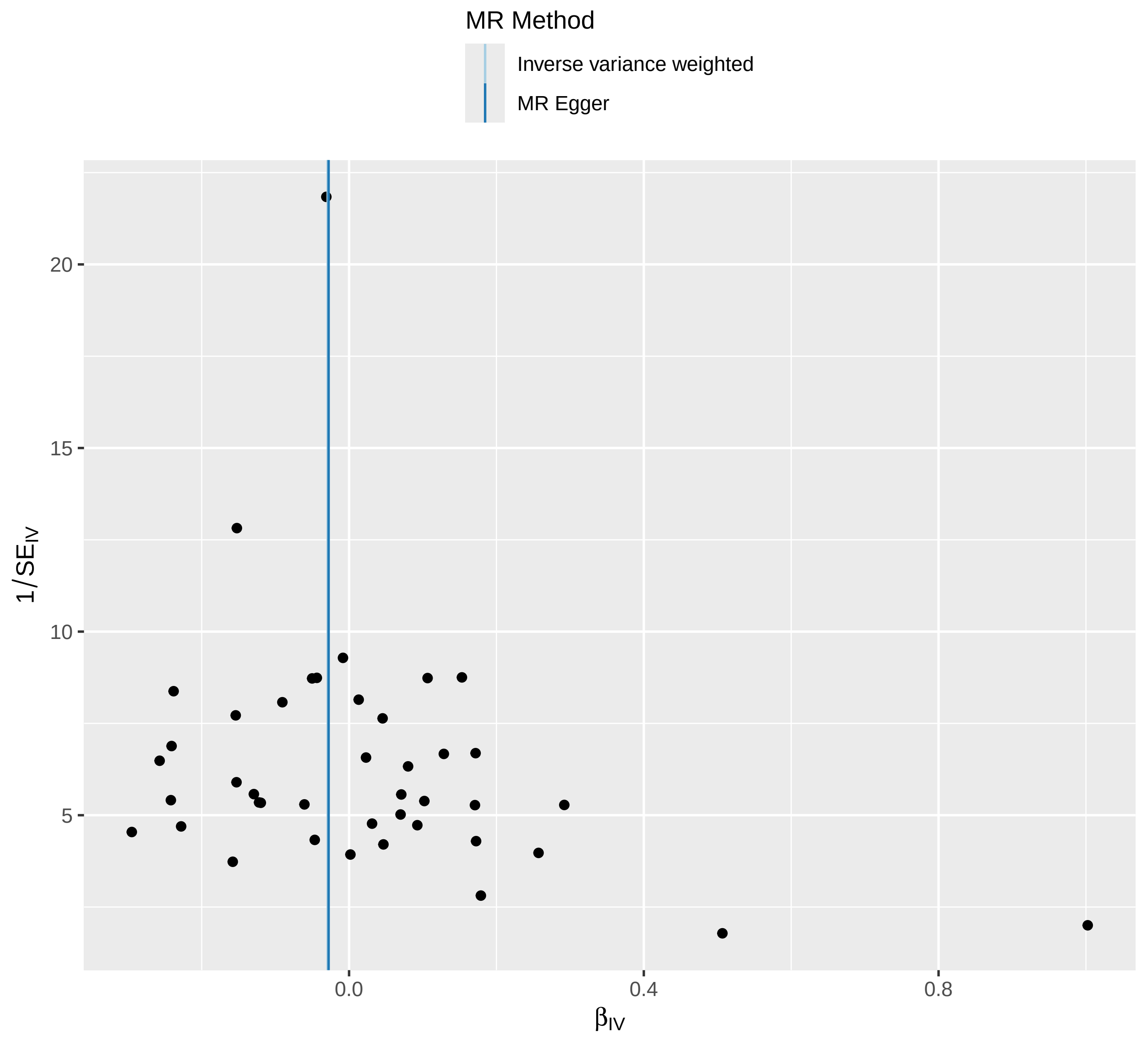

MR Method
Inverse variance weighted
MR Egger
20
15
10
5
1/SE$_{IV}$
0.0
0.4
0.8
β$_{IV}$

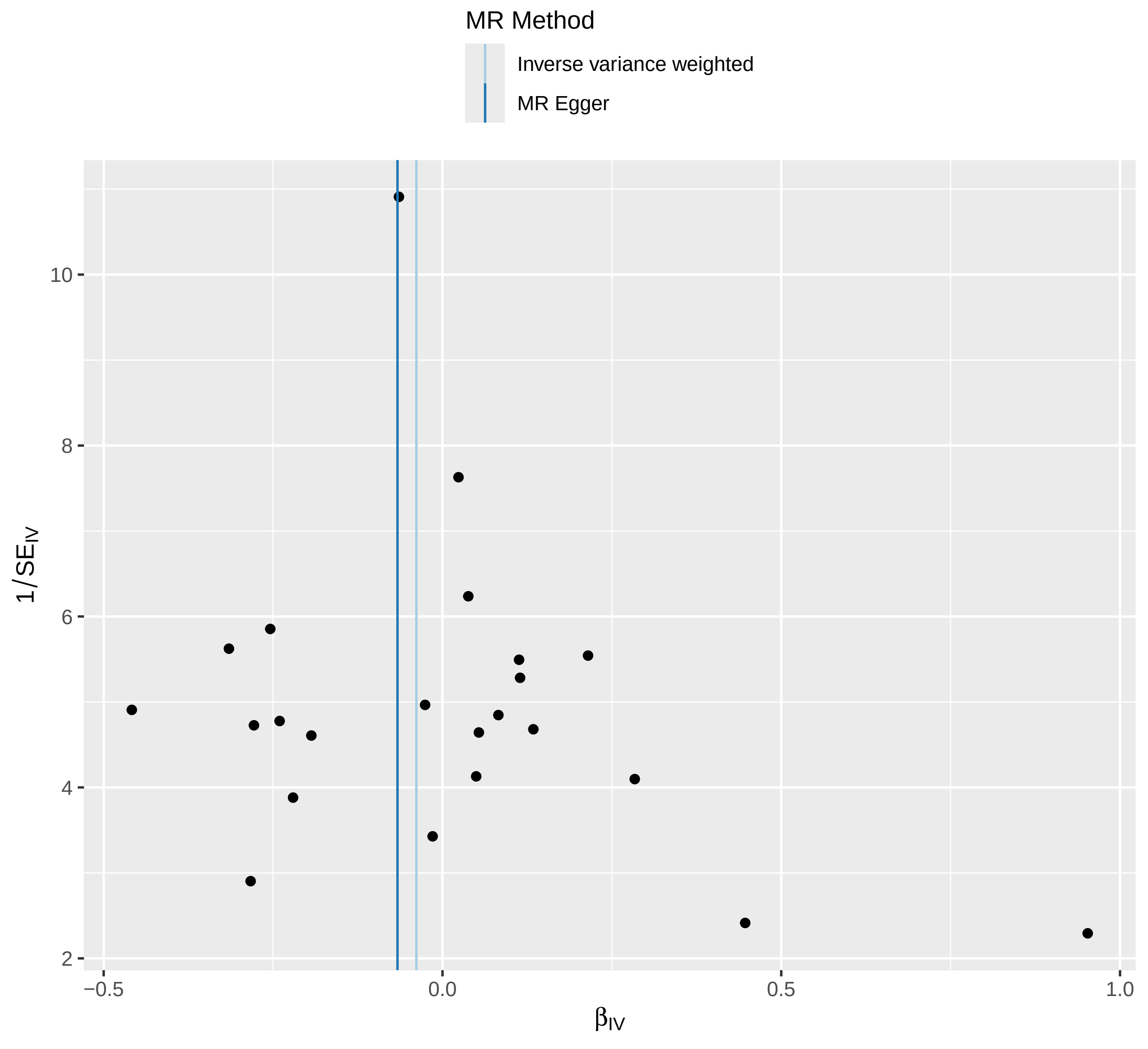

MR Method
Inverse variance weighted
MR Egger
10
8
6
4
2
−0.5
0.0
0.5
1.0
$1/SE_{IV}$
β_{IV}

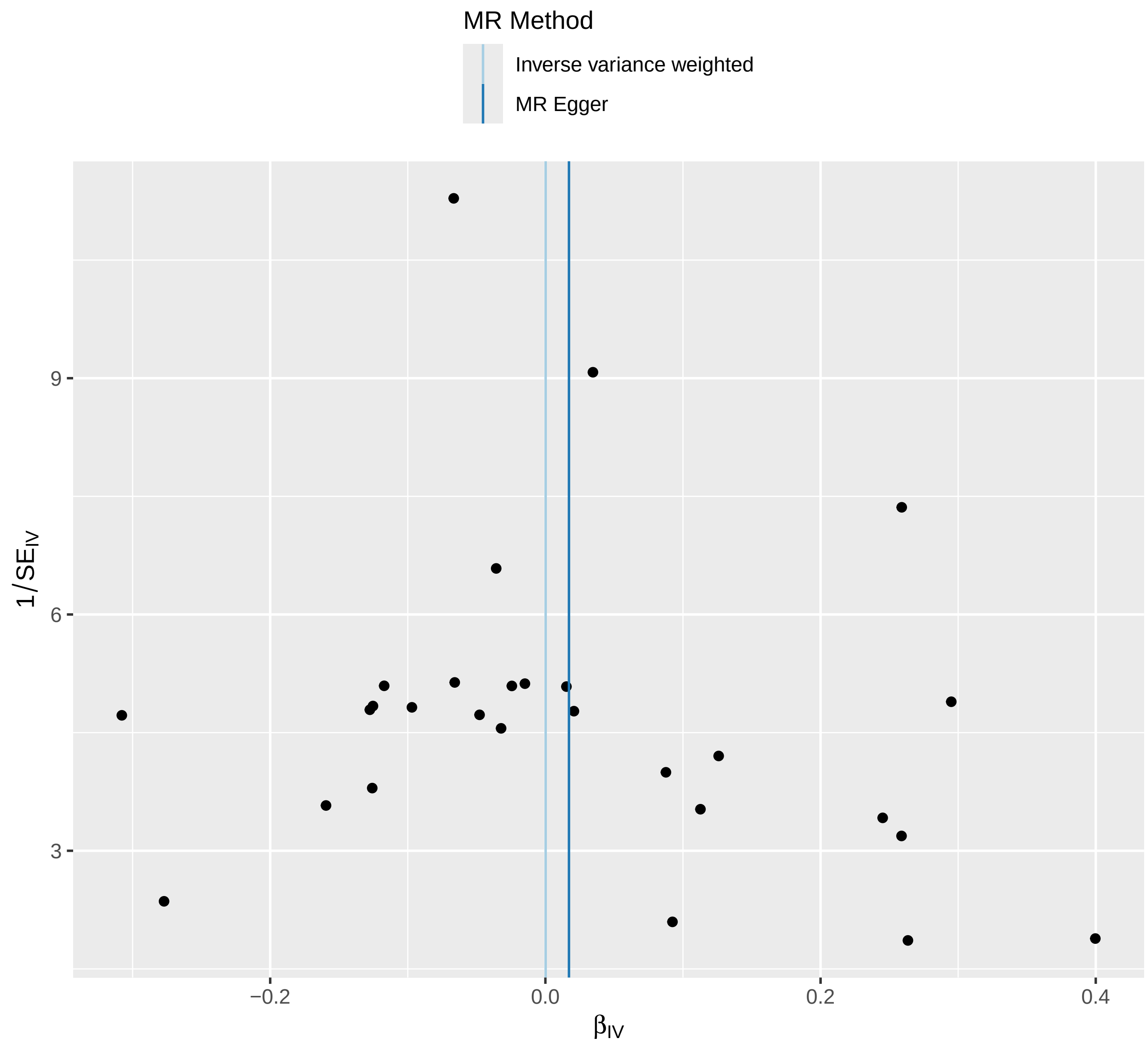

MR Method
Inverse variance weighted
MR Egger
1/SE_IV
9
6
3
−0.2
0.0
0.2
0.4
β_IV

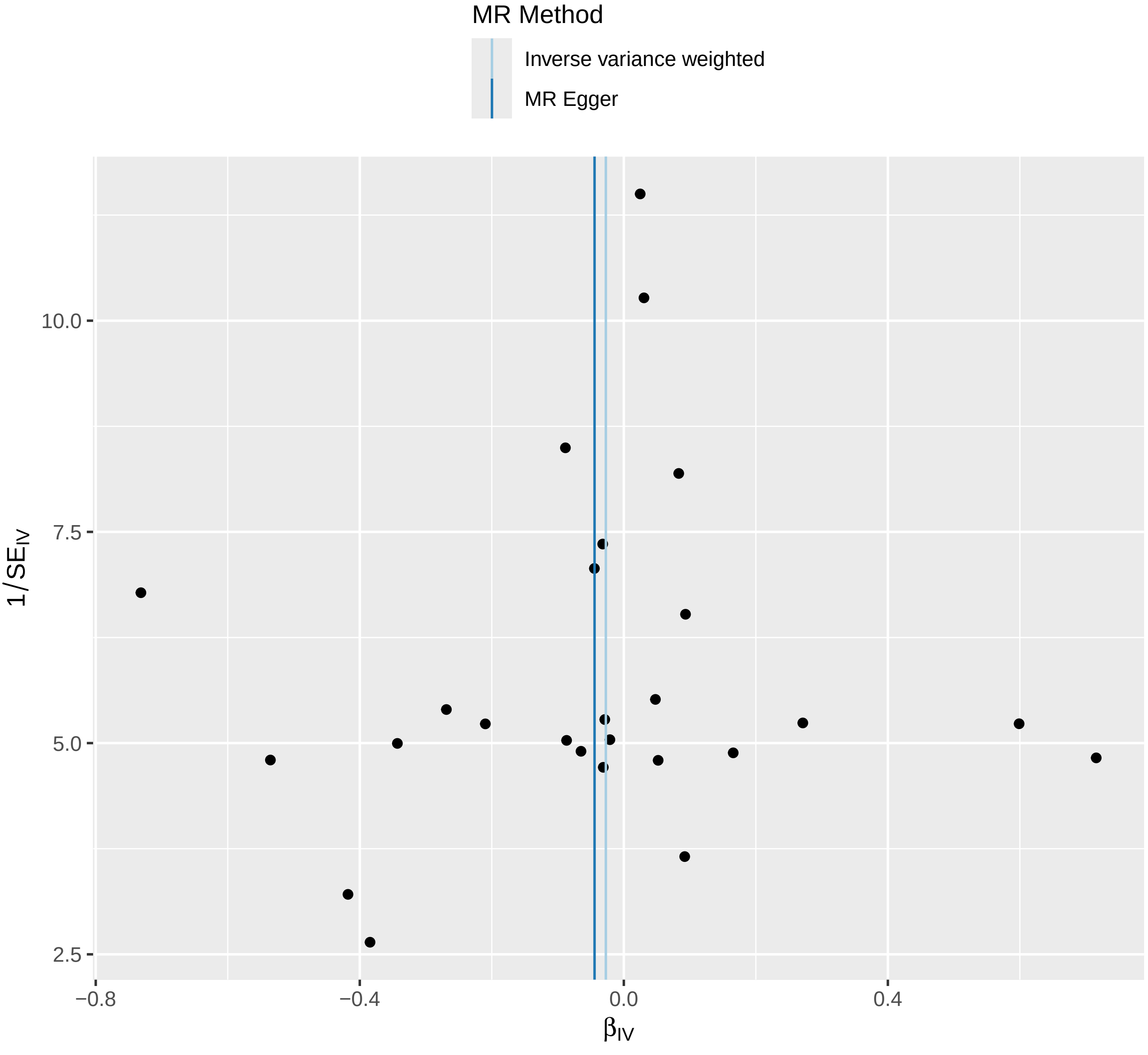
MR Method
Inverse variance weighted
MR Egger
10.0
7.5
5.0
2.5
1/SE$_{IV}$
−0.8
−0.4
0.0
0.4
β$_{IV}$

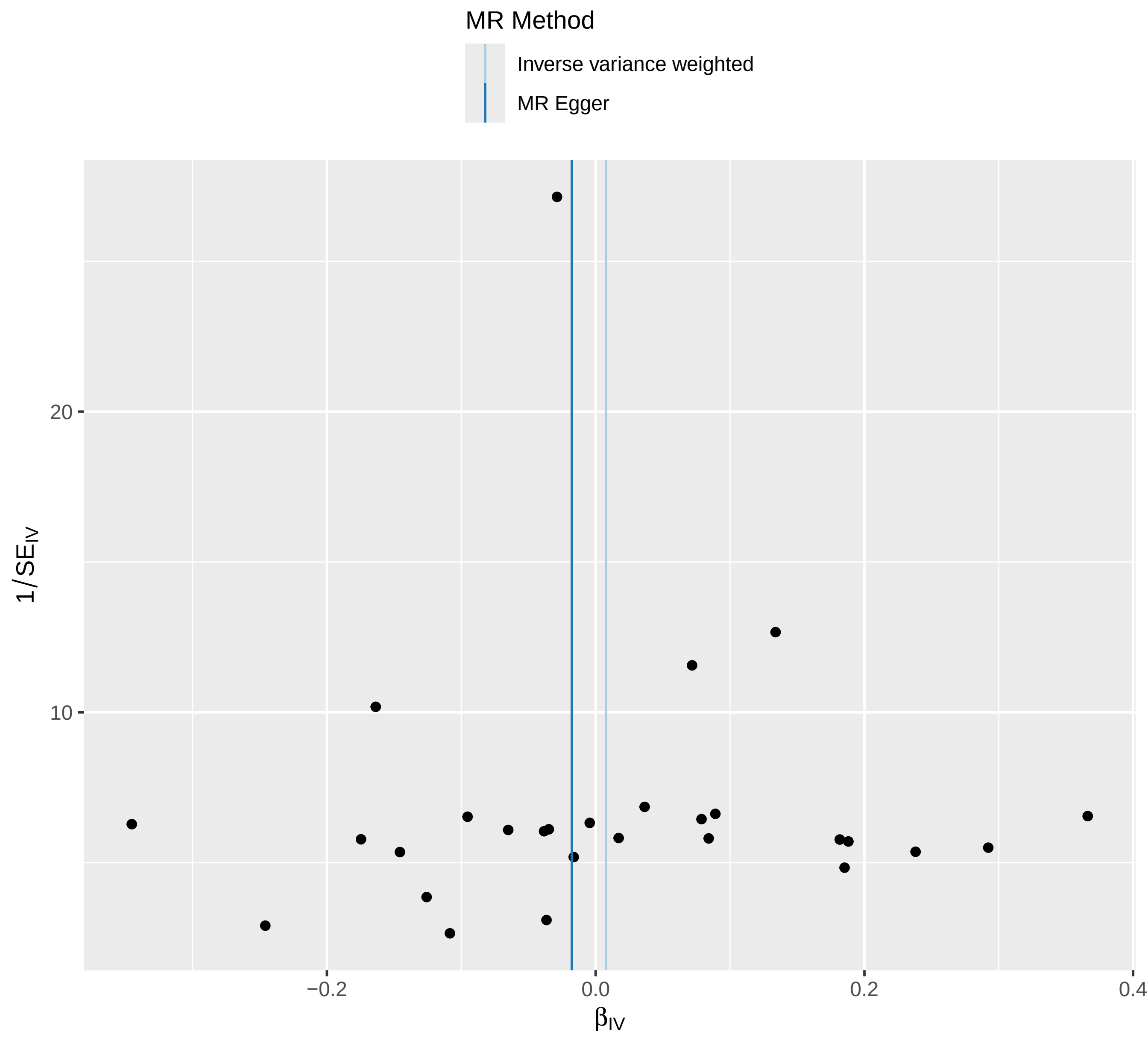
MR Method
Inverse variance weighted
MR Egger
20
10
1/SE_{IV}
−0.2
0.0
0.2
0.4
β_{IV}

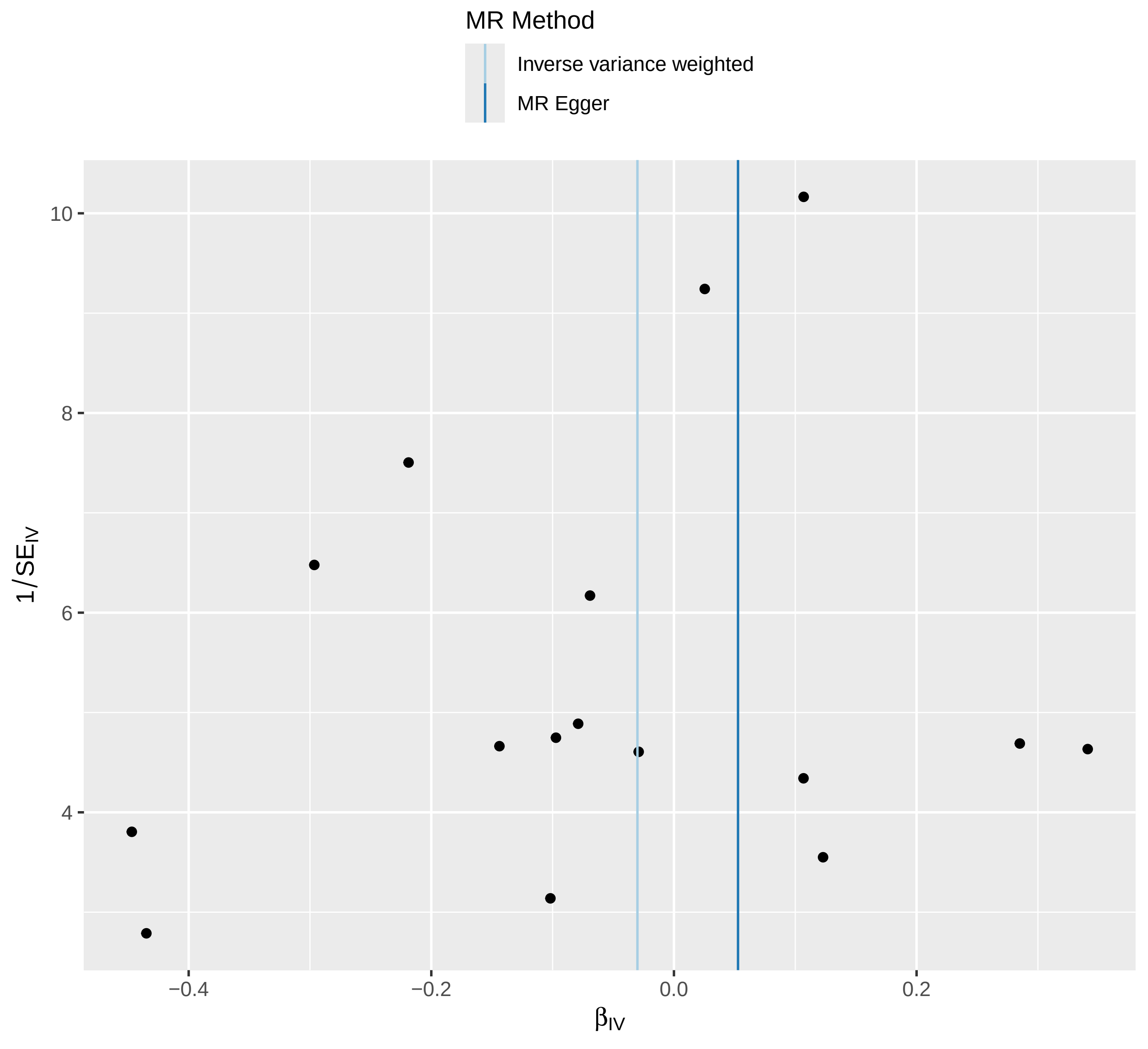

MR Method
Inverse variance weighted
MR Egger
10
8
6
4
1/SE_IV
−0.4
−0.2
0.0
0.2
β_IV

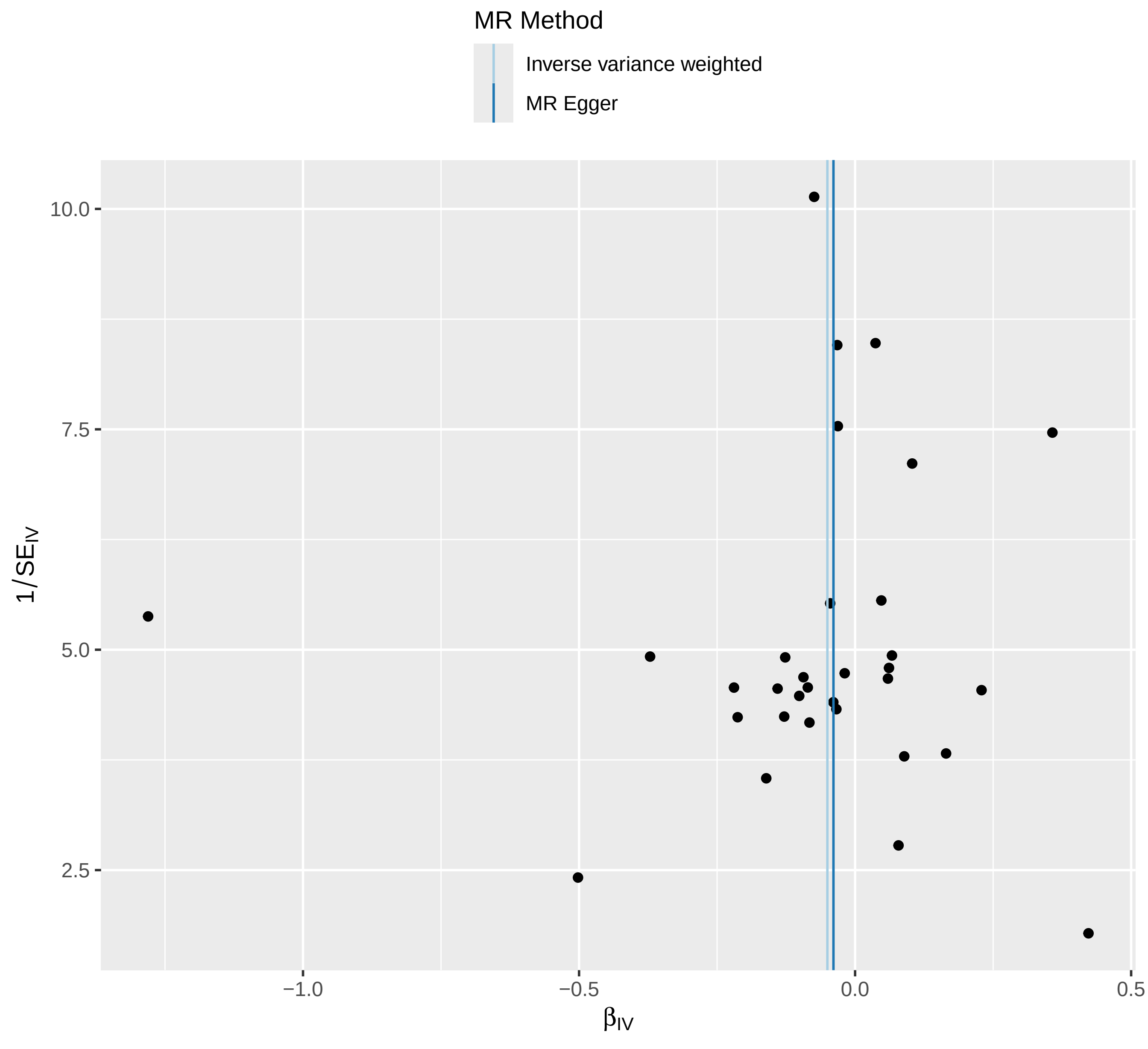

MR Method
Inverse variance weighted
MR Egger
10.0
7.5
5.0
2.5
1/SE_IV
−1.0
−0.5
0.0
0.5
β_IV

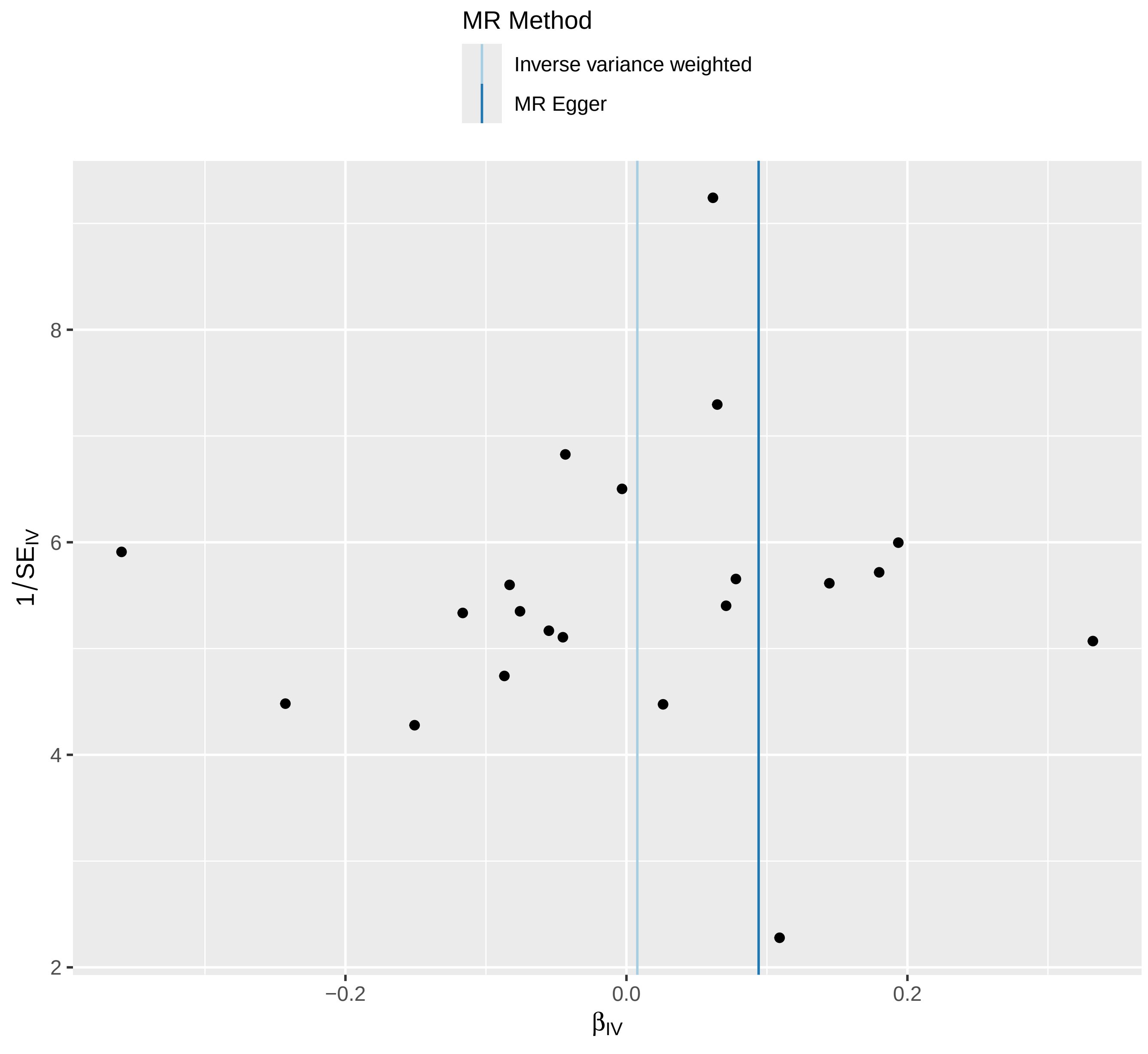

MR Method
Inverse variance weighted
MR Egger
8
6
4
2
1/SE$_{IV}$
−0.2
0.0
0.2
β$_{IV}$

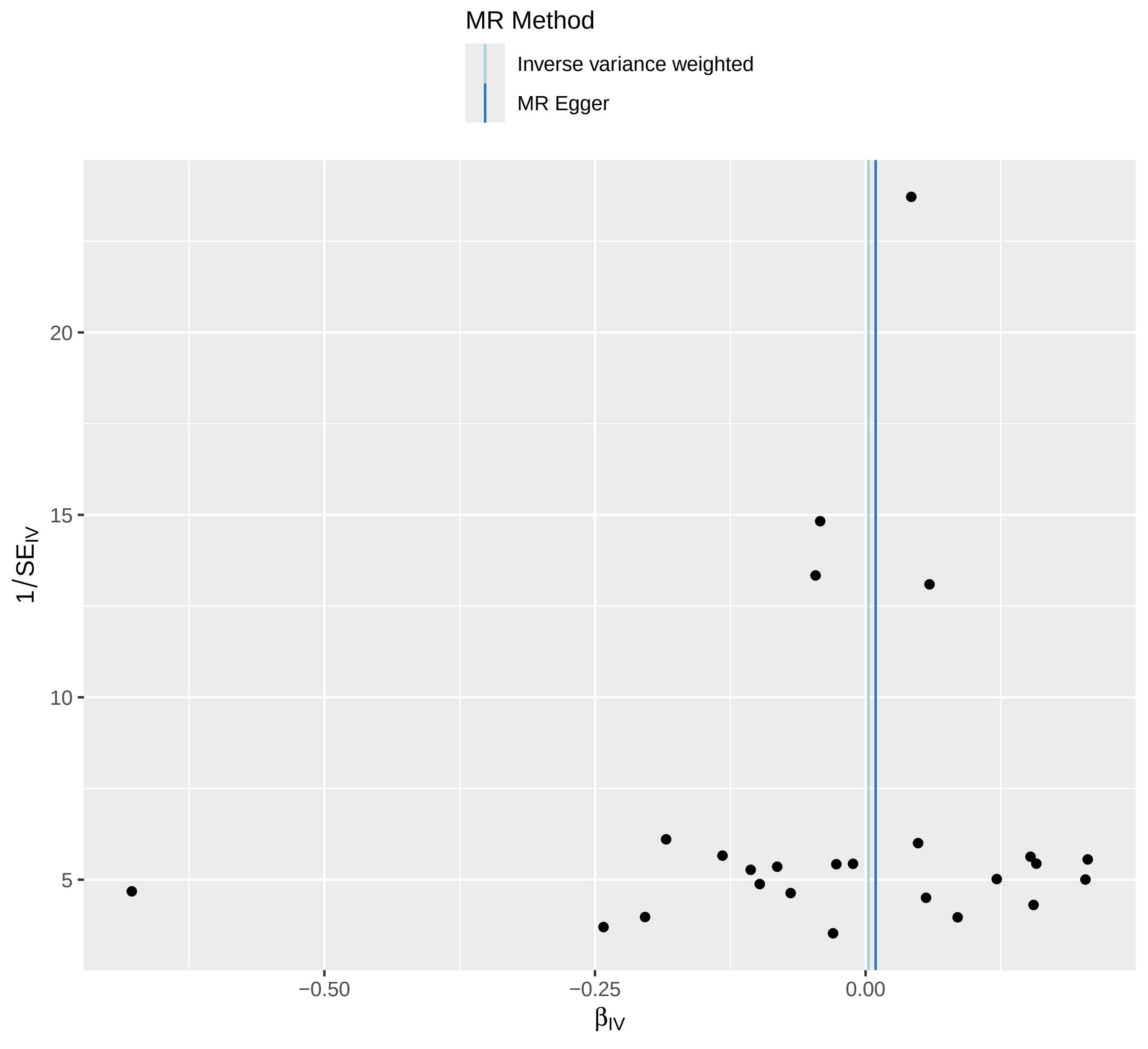

MR Method
Inverse variance weighted
MR Egger
1/SE_IV
20
15
10
5
−0.50
−0.25
0.00
β_IV

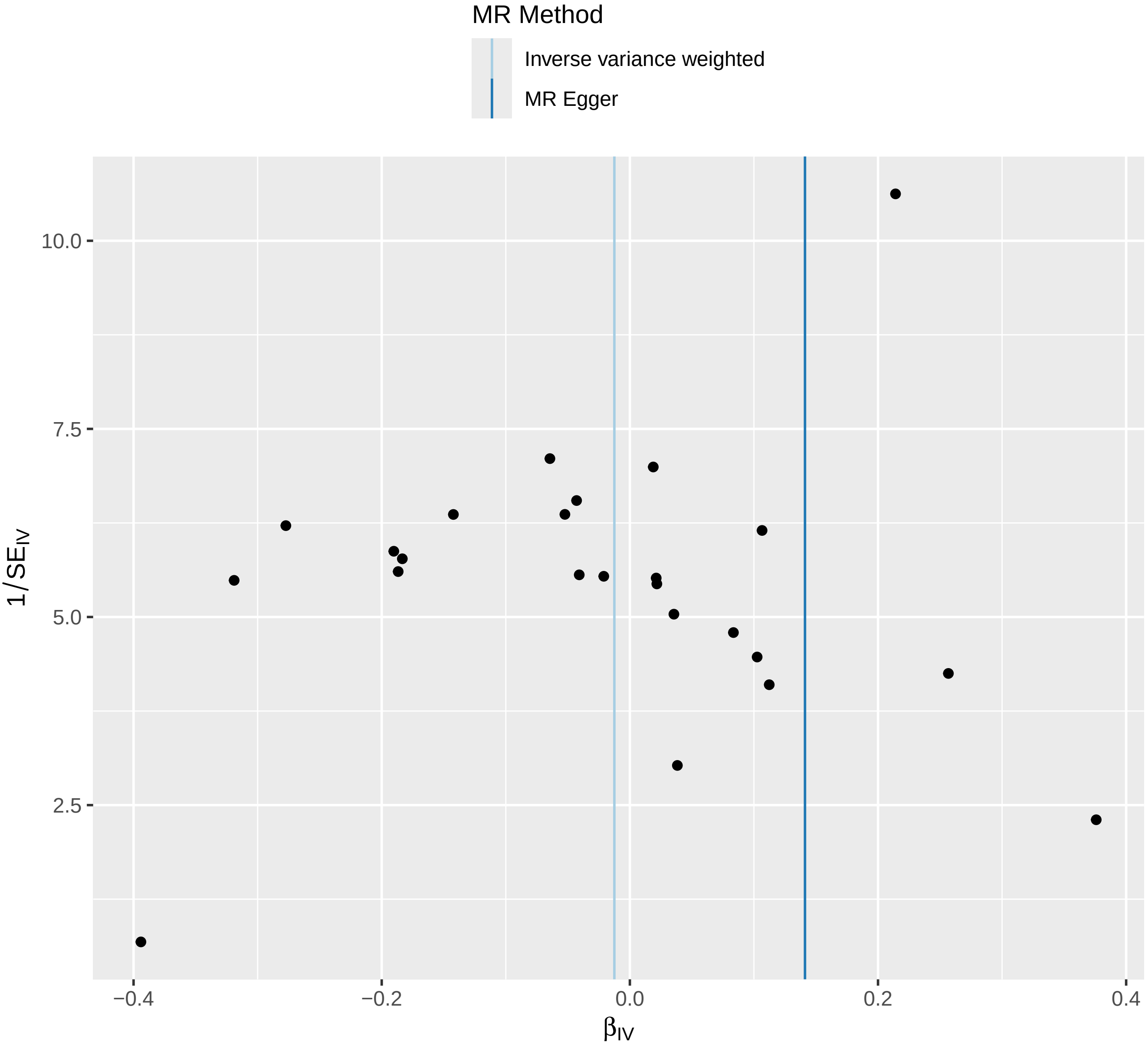
MR Method
Inverse variance weighted
MR Egger
10.0
7.5
5.0
2.5
1/SE_IV
−0.4
−0.2
0.0
0.2
0.4
β_IV

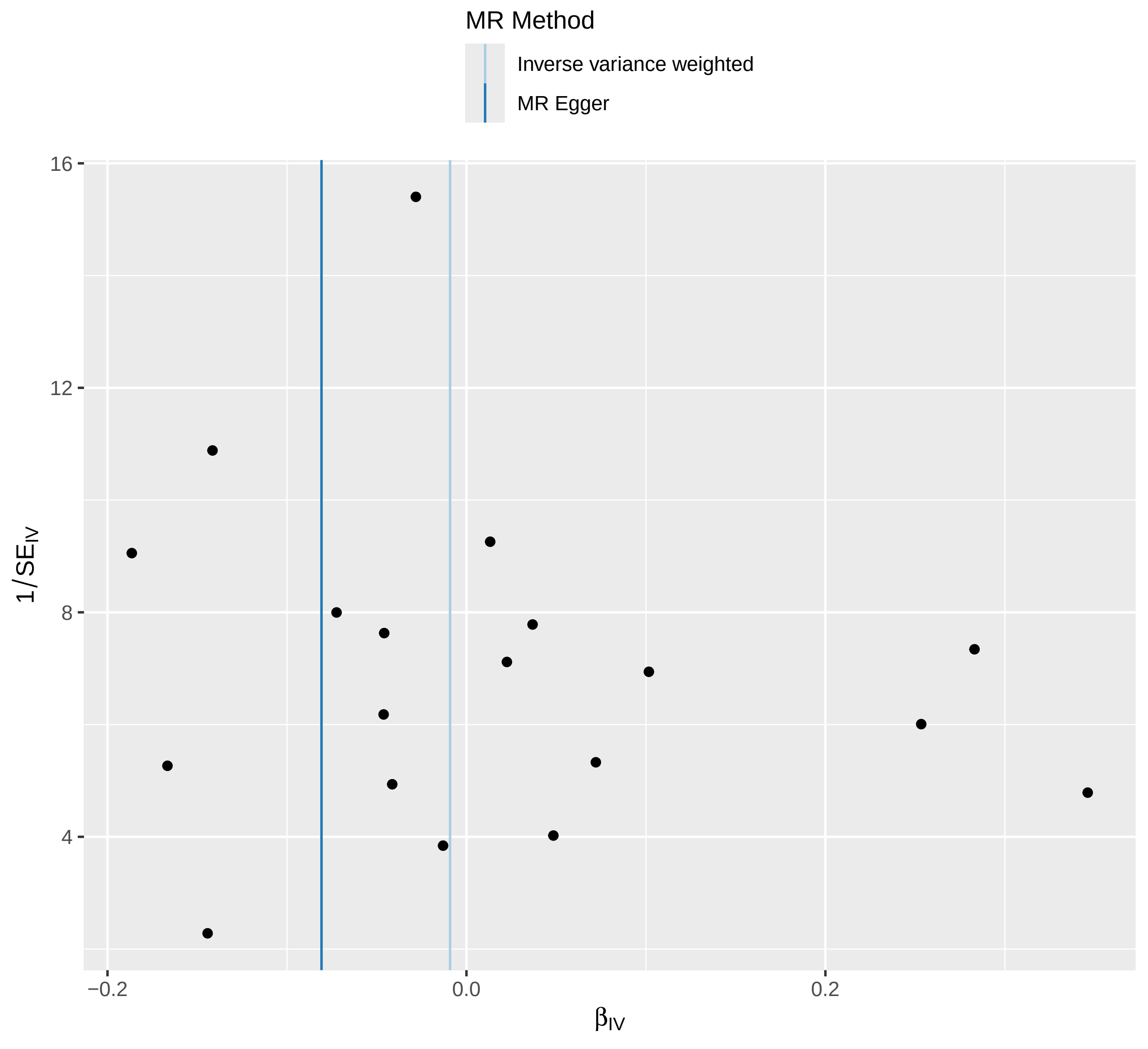
MR Method
Inverse variance weighted
MR Egger
16
12
8
4
1/SE_IV
−0.2
0.0
0.2
β_IV

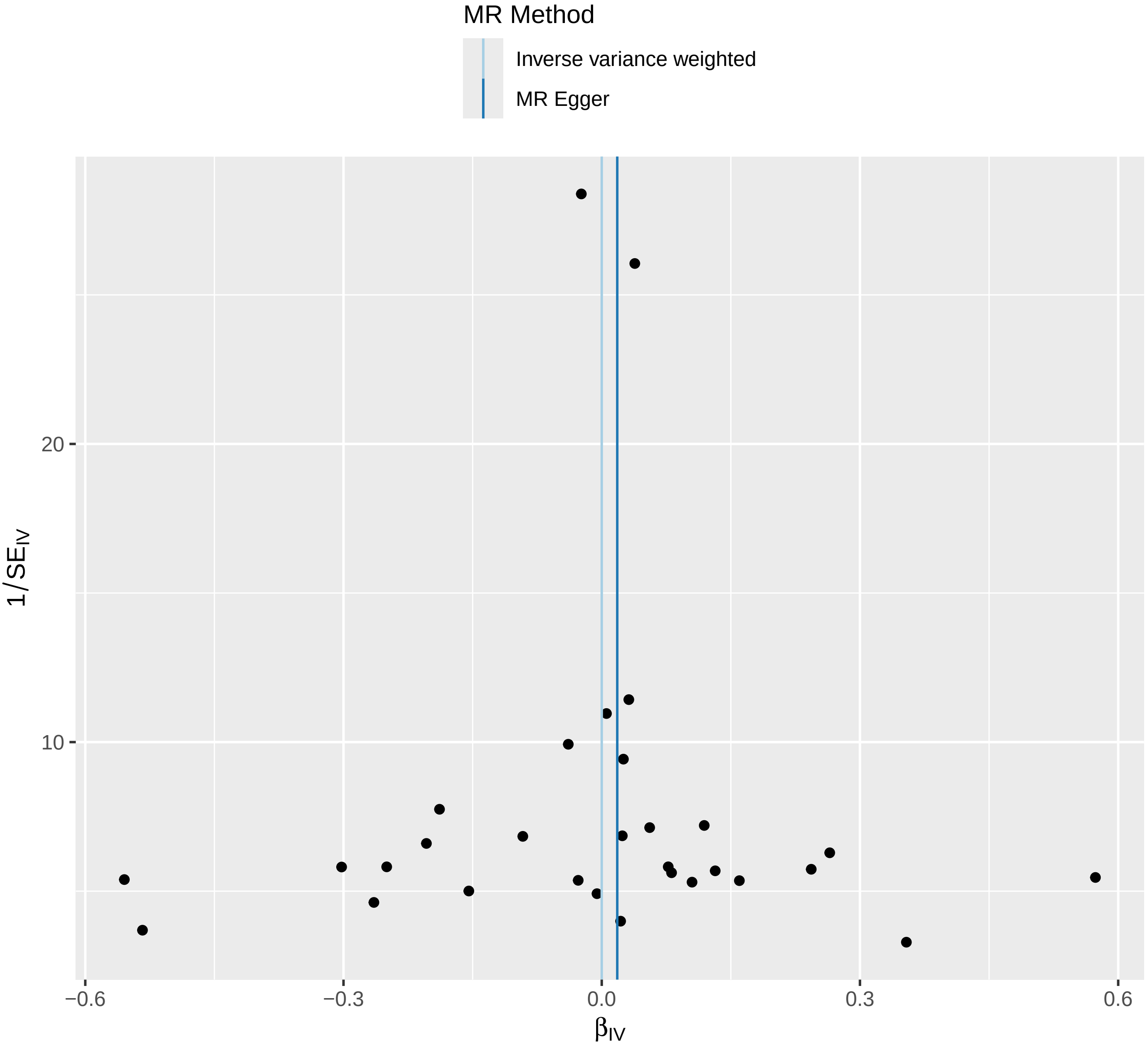

MR Method
Inverse variance weighted
MR Egger
20
10
1/SE$_{IV}$
−0.6
−0.3
0.0
0.3
0.6
β$_{IV}$

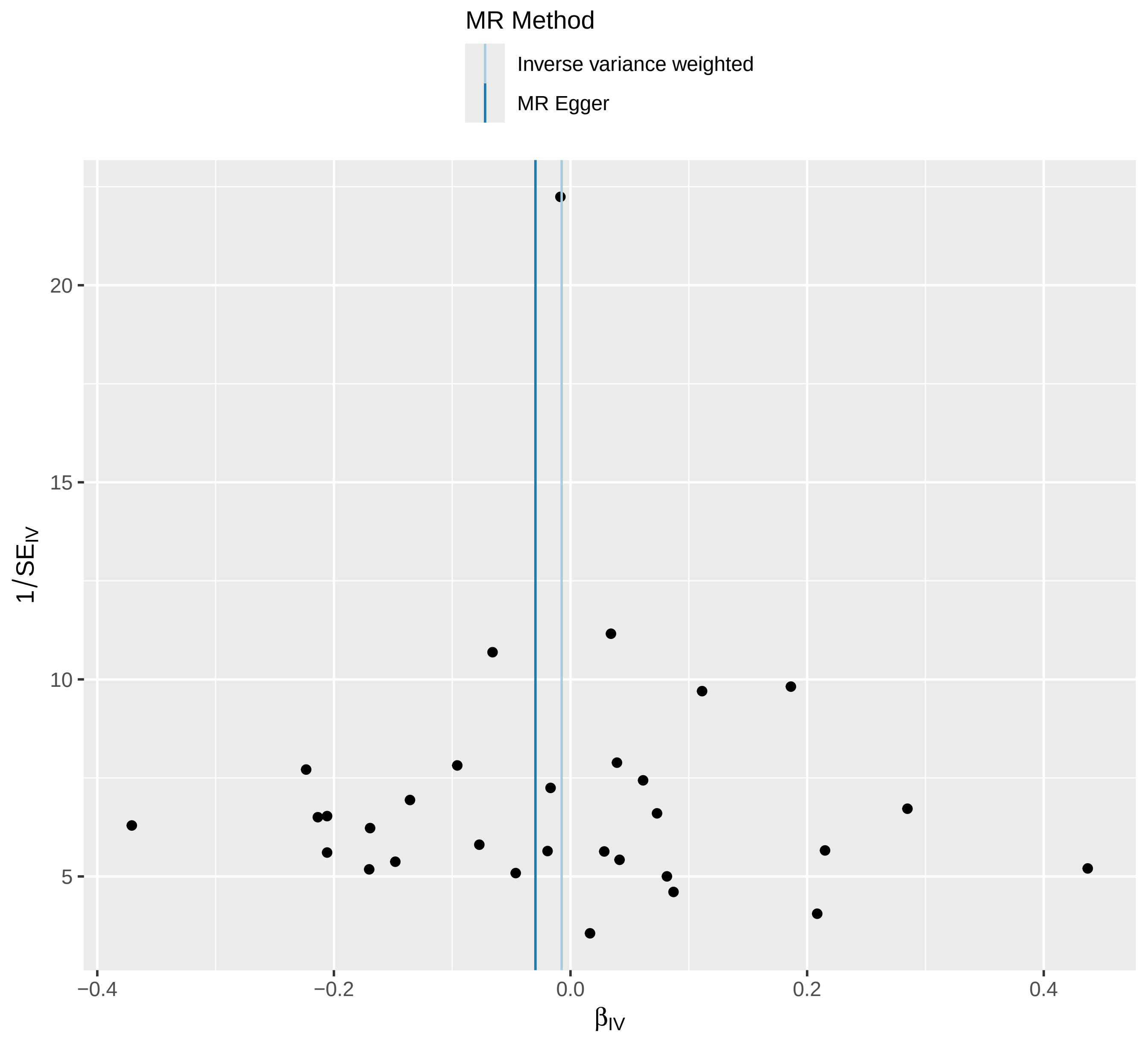
MR Method
Inverse variance weighted
MR Egger
20
15
10
5
1/SE_IV
−0.4
−0.2
0.0
0.2
0.4
β_IV

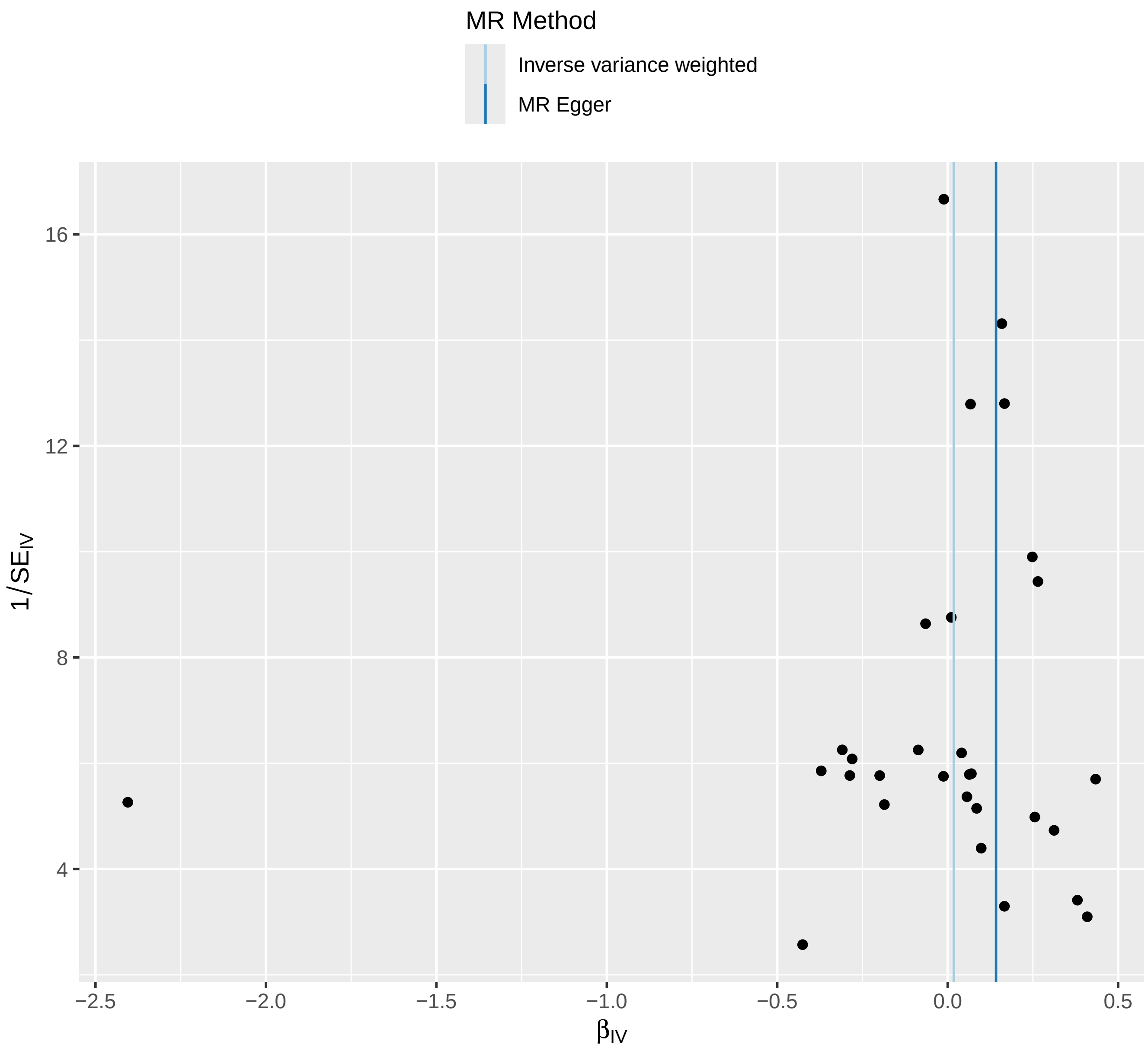
MR Method
Inverse variance weighted
MR Egger
16
12
8
4
$1/SE_{IV}$
−2.5
−2.0
−1.5
−1.0
−0.5
0.0
0.5
β_{IV}

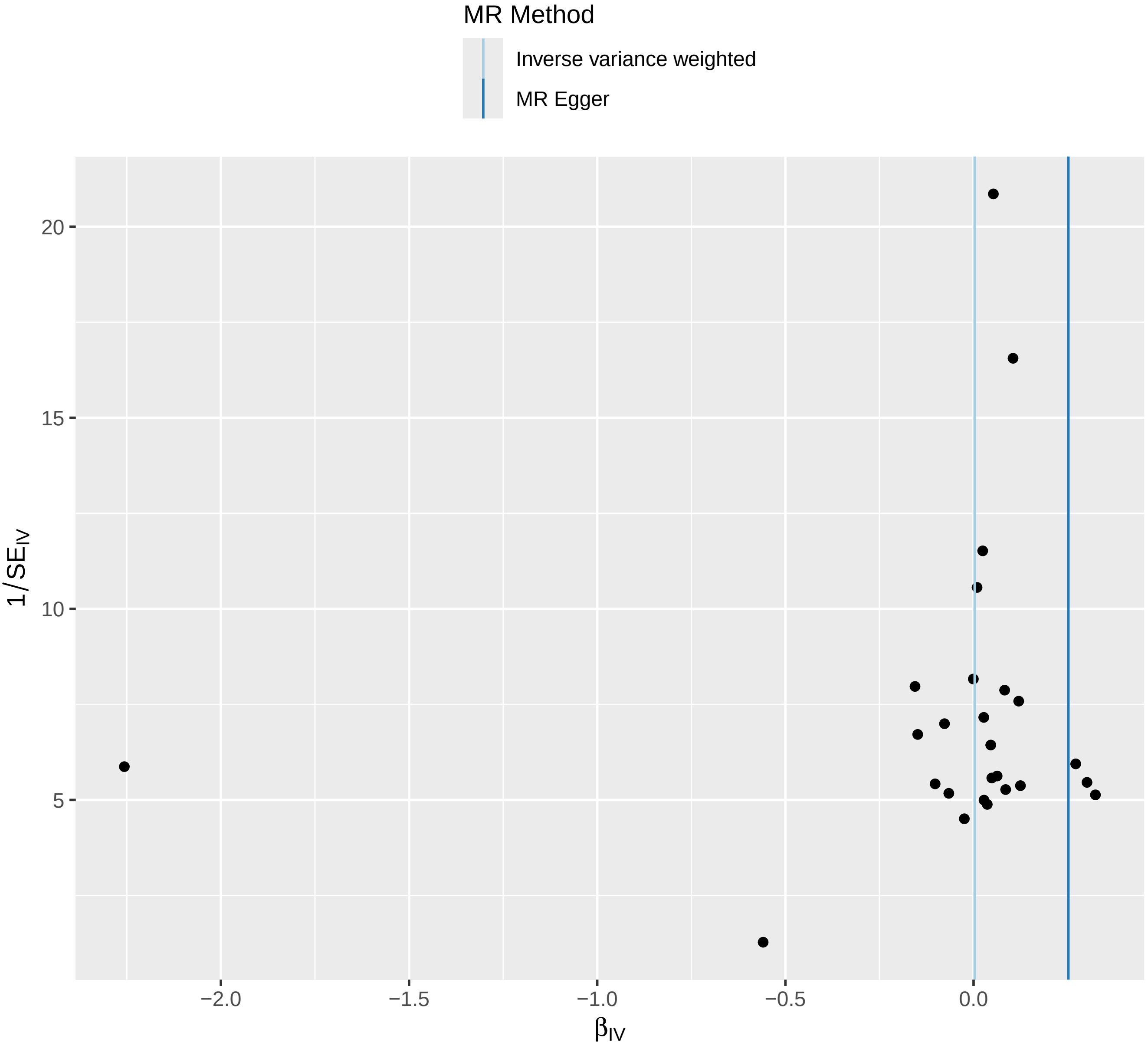
MR Method
Inverse variance weighted
MR Egger
20
15
10
5
1/SE_IV
−2.0
−1.5
−1.0
−0.5
0.0
β_IV

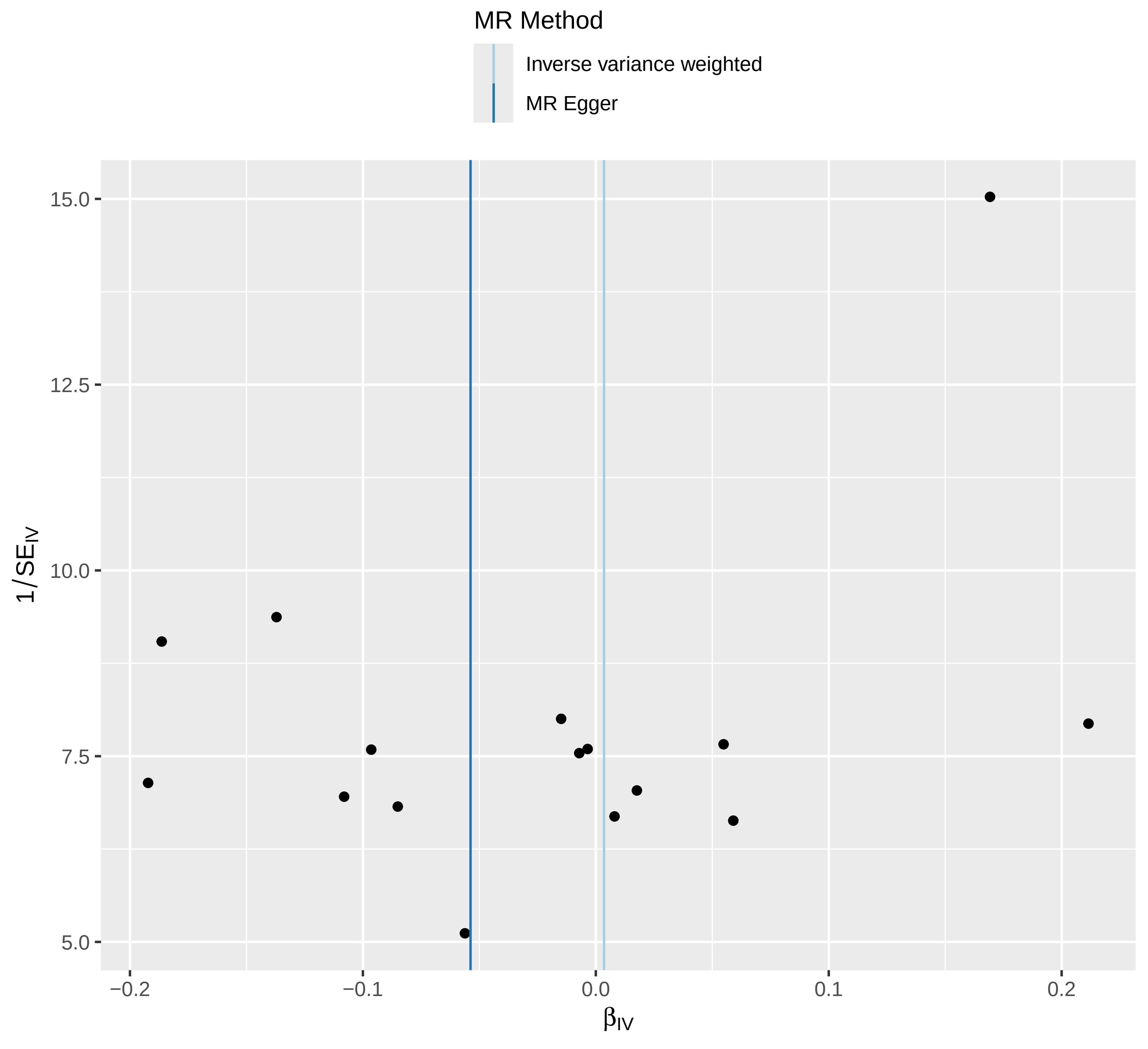

MR Method
Inverse variance weighted
MR Egger
15.0
12.5
10.0
7.5
5.0
1/SE_IV
−0.2
−0.1
0.0
0.1
0.2
β_IV

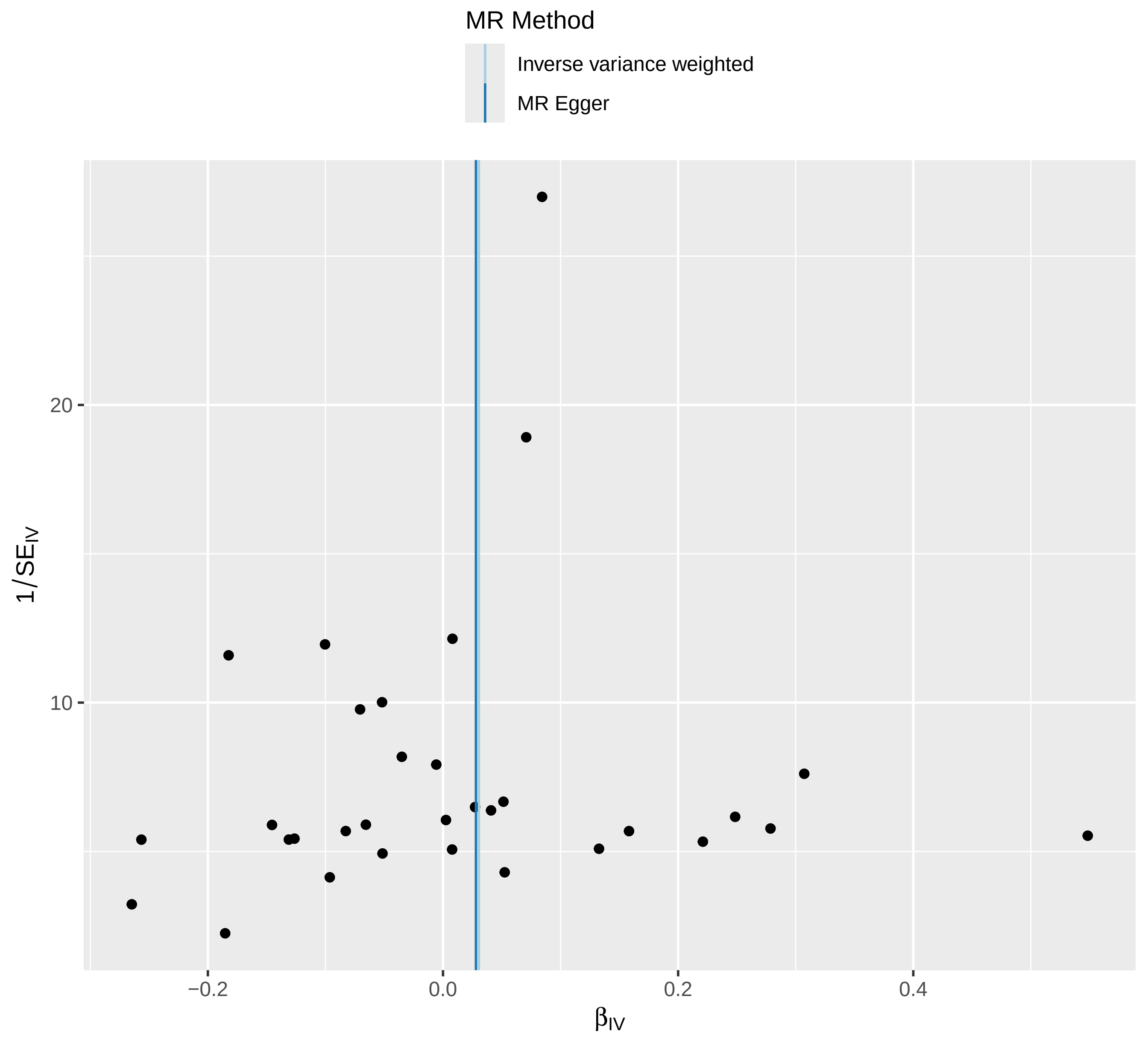

MR Method
Inverse variance weighted
MR Egger
20
10
1/SE_IV
−0.2
0.0
0.2
0.4
β_IV

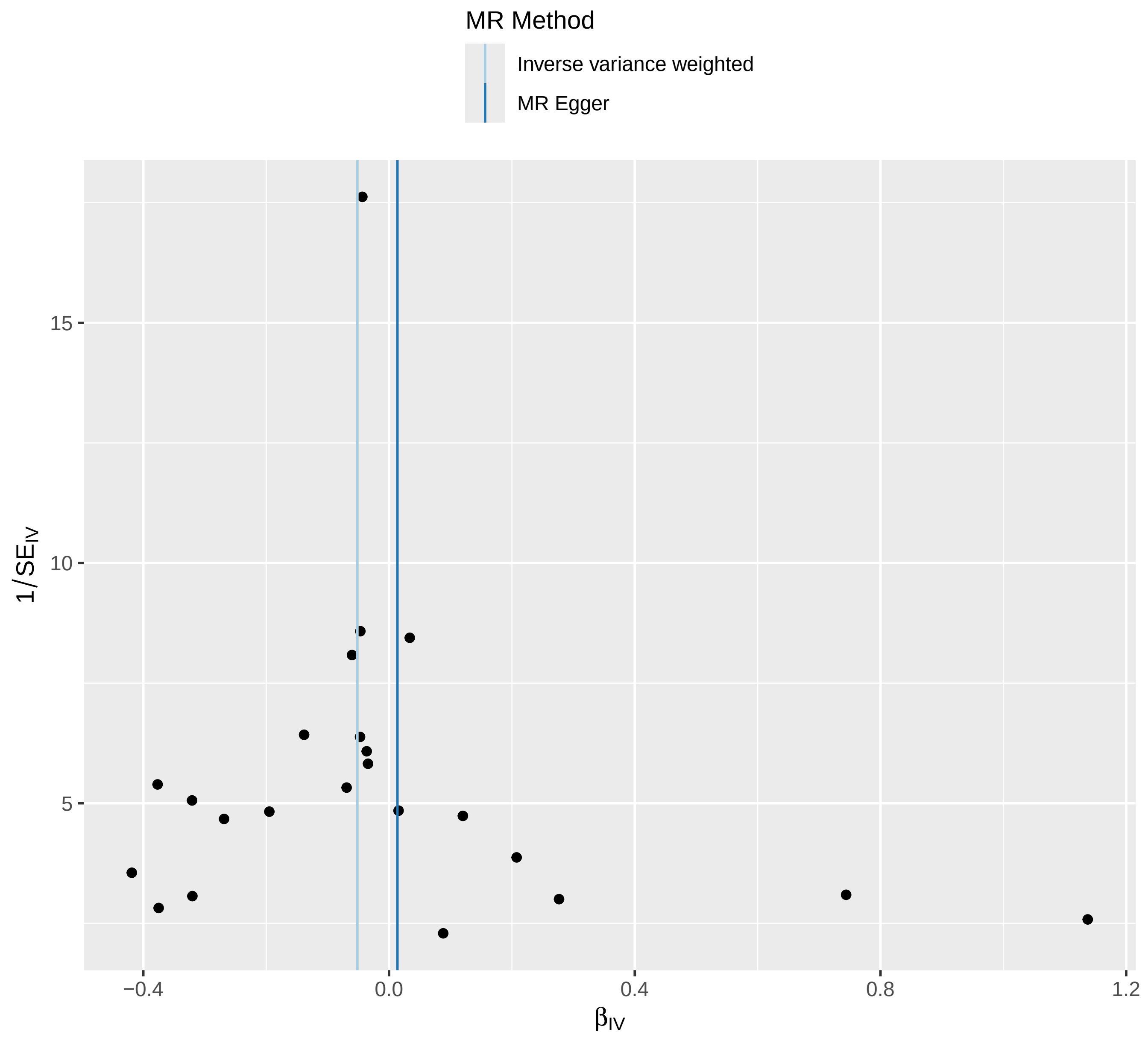
MR Method
Inverse variance weighted
MR Egger
1/SE_IV
15
10
5
−0.4
0.0
0.4
0.8
1.2
β_IV

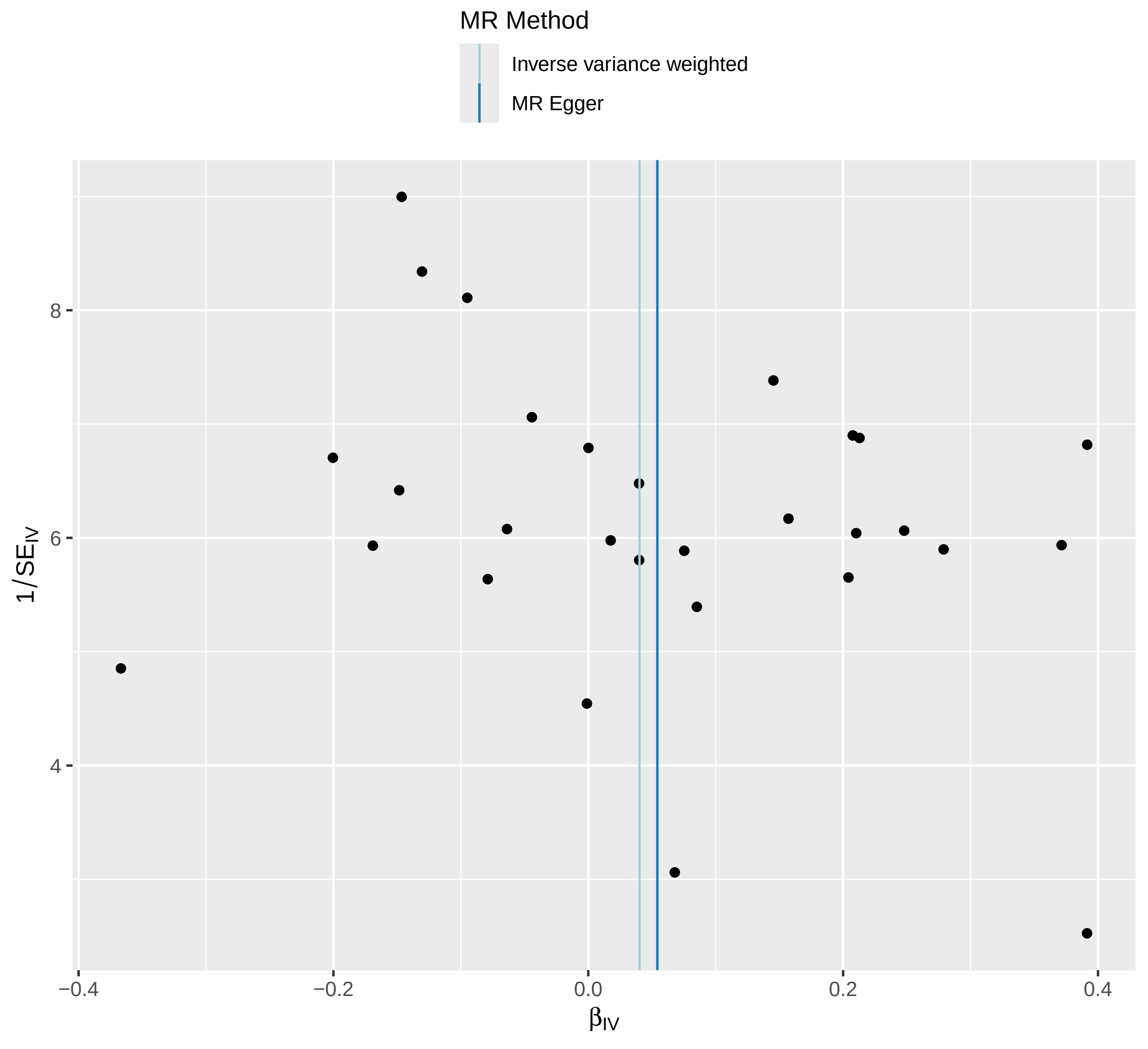
MR Method
Inverse variance weighted
MR Egger
8
6
4
1/SE$_{IV}$
−0.4
−0.2
0.0
0.2
0.4
β$_{IV}$

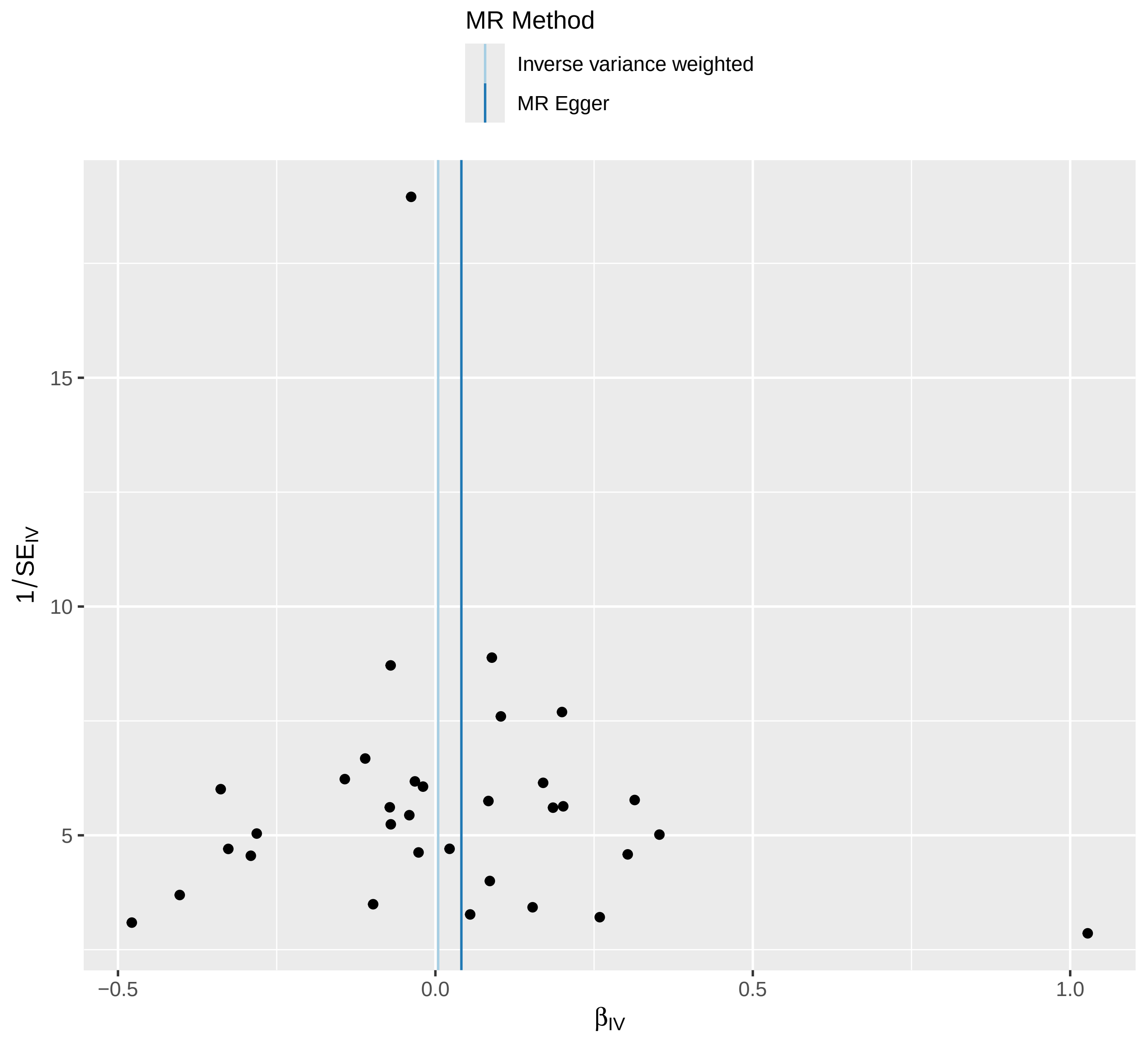
MR Method
Inverse variance weighted
MR Egger
1/SE_IV
15
10
5
−0.5
0.0
0.5
1.0
β_IV

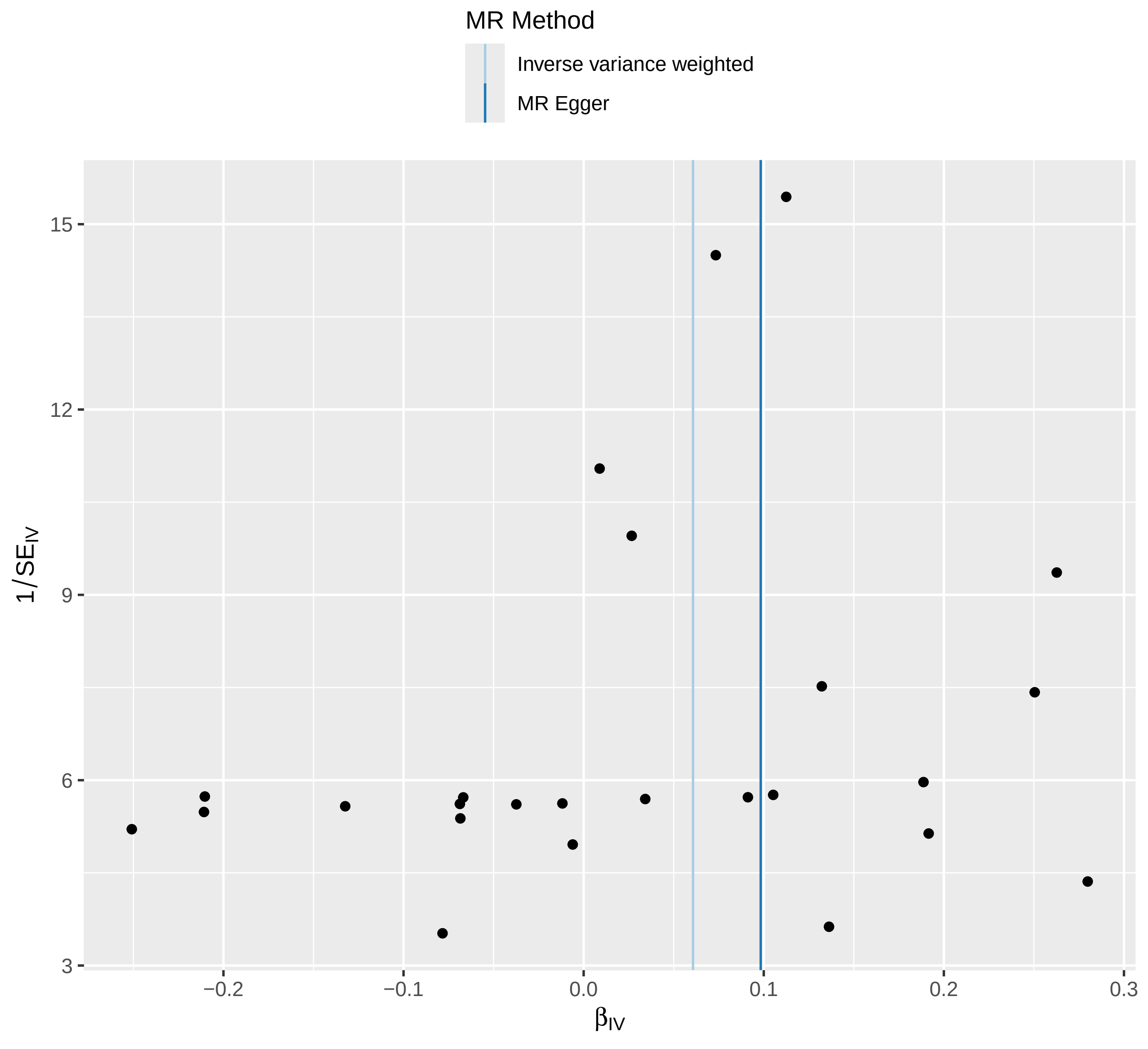
MR Method
Inverse variance weighted
MR Egger
15
12
9
6
3
1/SE_IV
−0.2
−0.1
0.0
0.1
0.2
0.3
β_IV

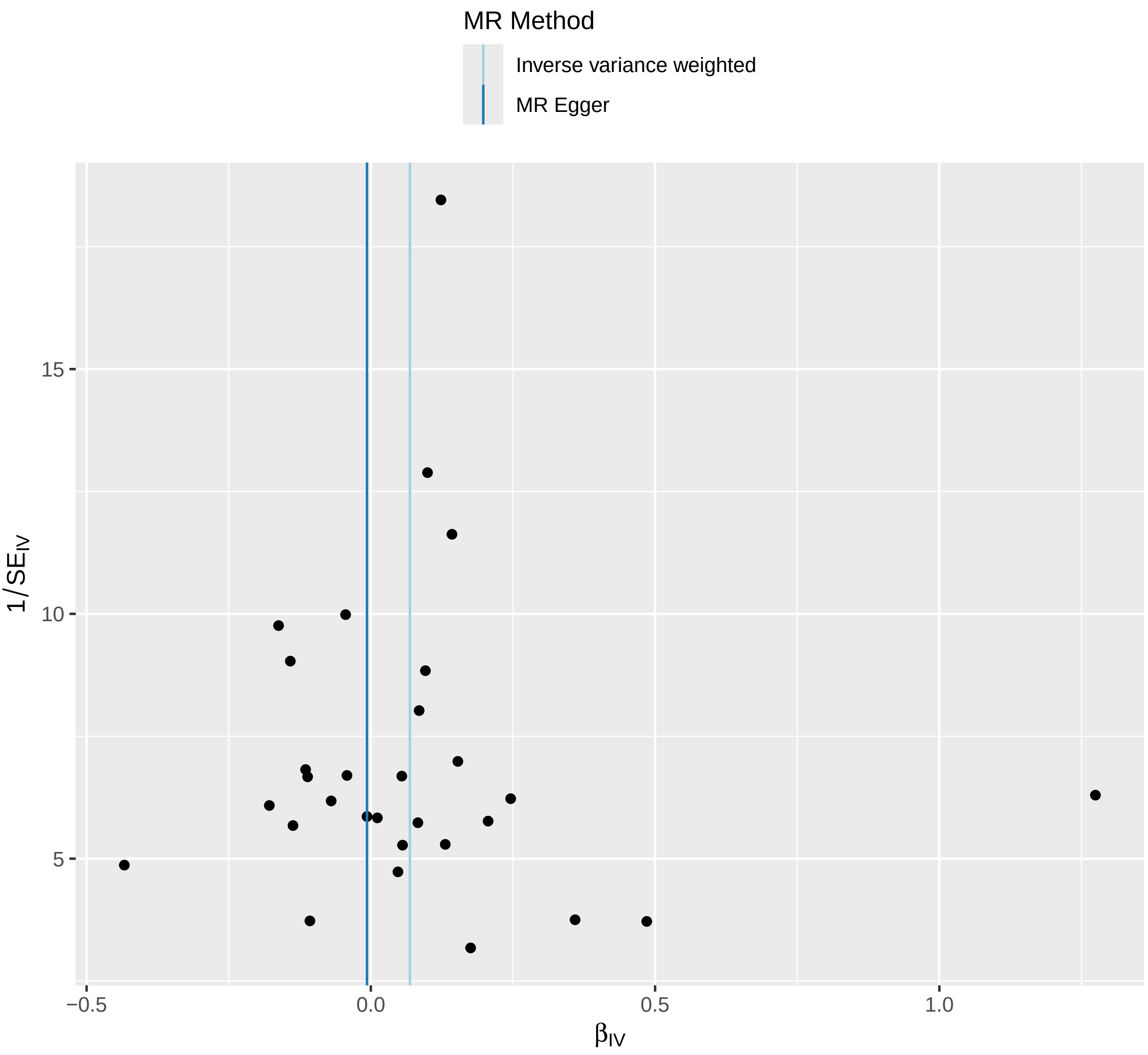
MR Method
Inverse variance weighted
MR Egger
15
10
5
1/SE_{IV}
−0.5
0.0
0.5
1.0
β_{IV}

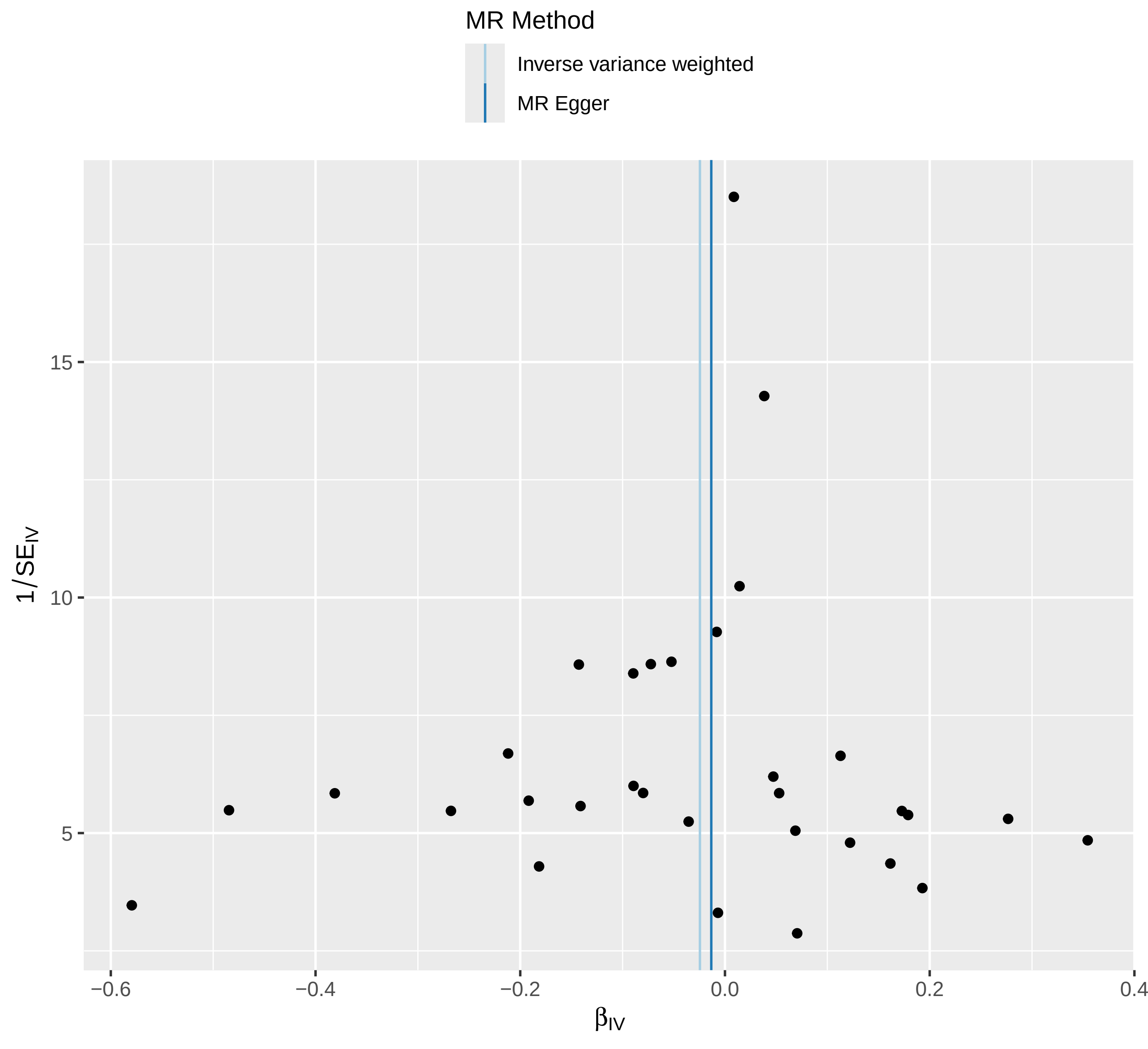
MR Method
Inverse variance weighted
MR Egger
1/SE_IV
15
10
5
−0.6
−0.4
−0.2
0.0
0.2
0.4
β_IV

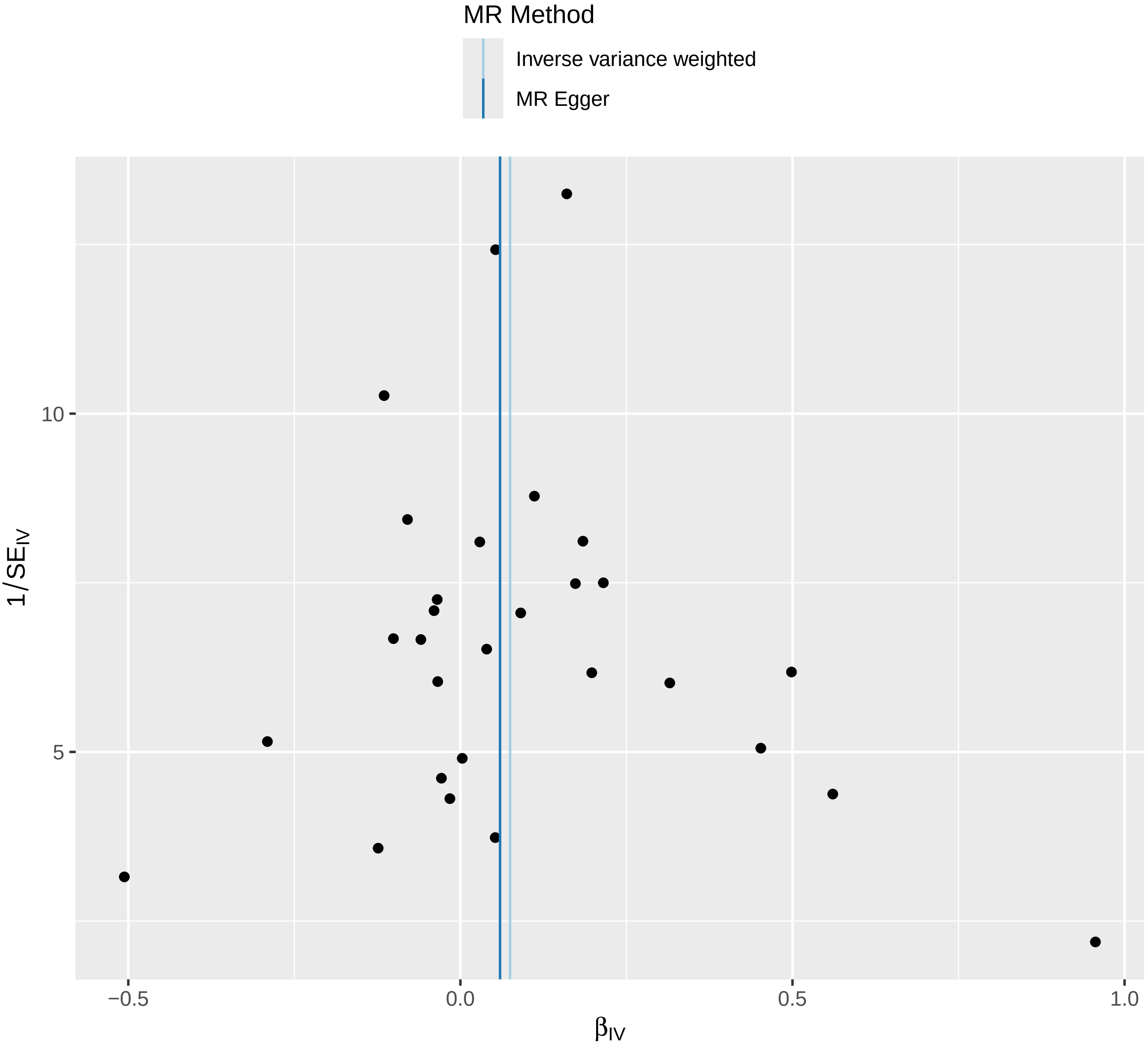
MR Method
Inverse variance weighted
MR Egger
10
5
1/SE_IV
−0.5
0.0
0.5
1.0
β_IV

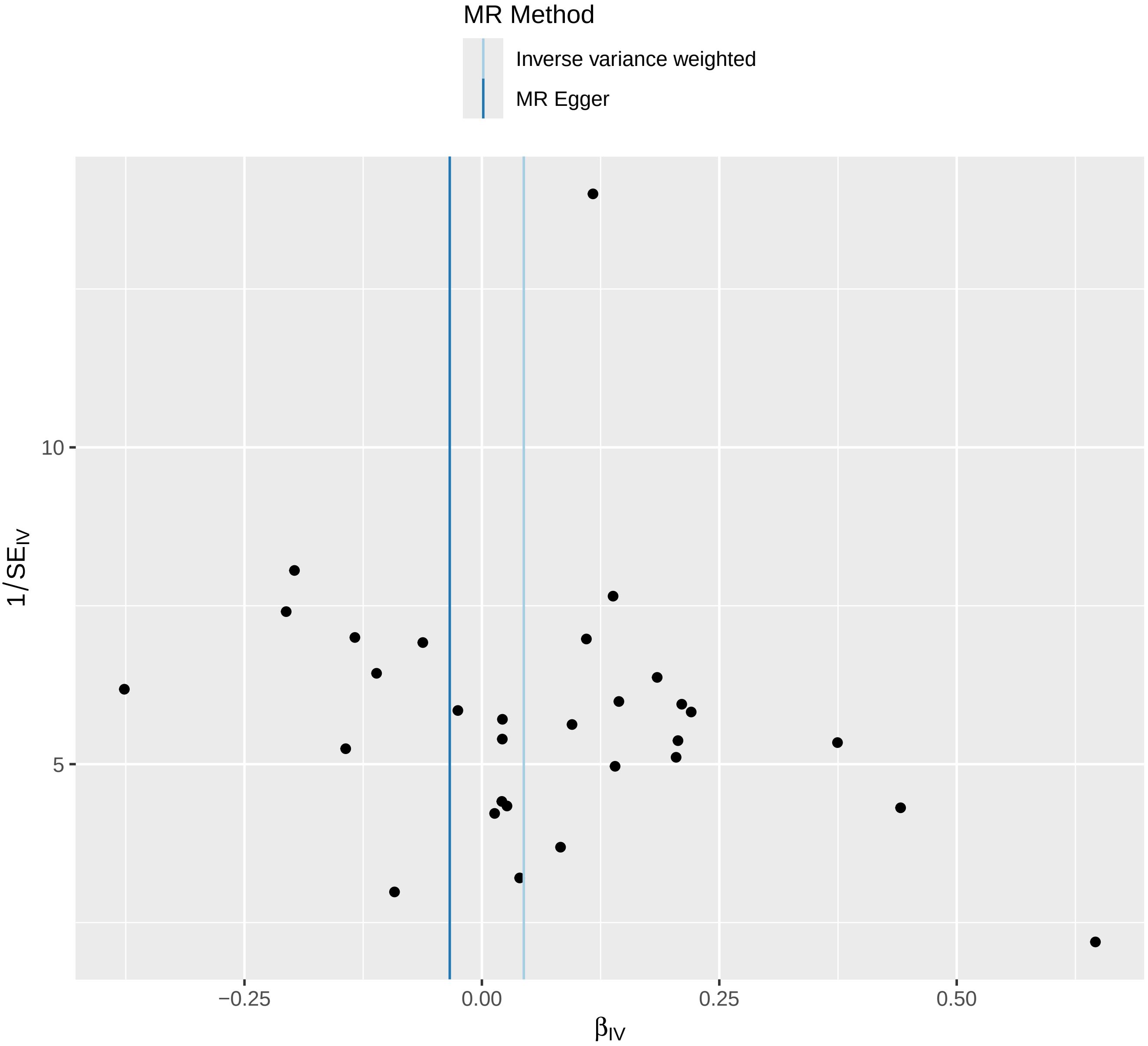

MR Method
Inverse variance weighted
MR Egger
10
5
1/SE_IV
−0.25
0.00
0.25
0.50
β_IV

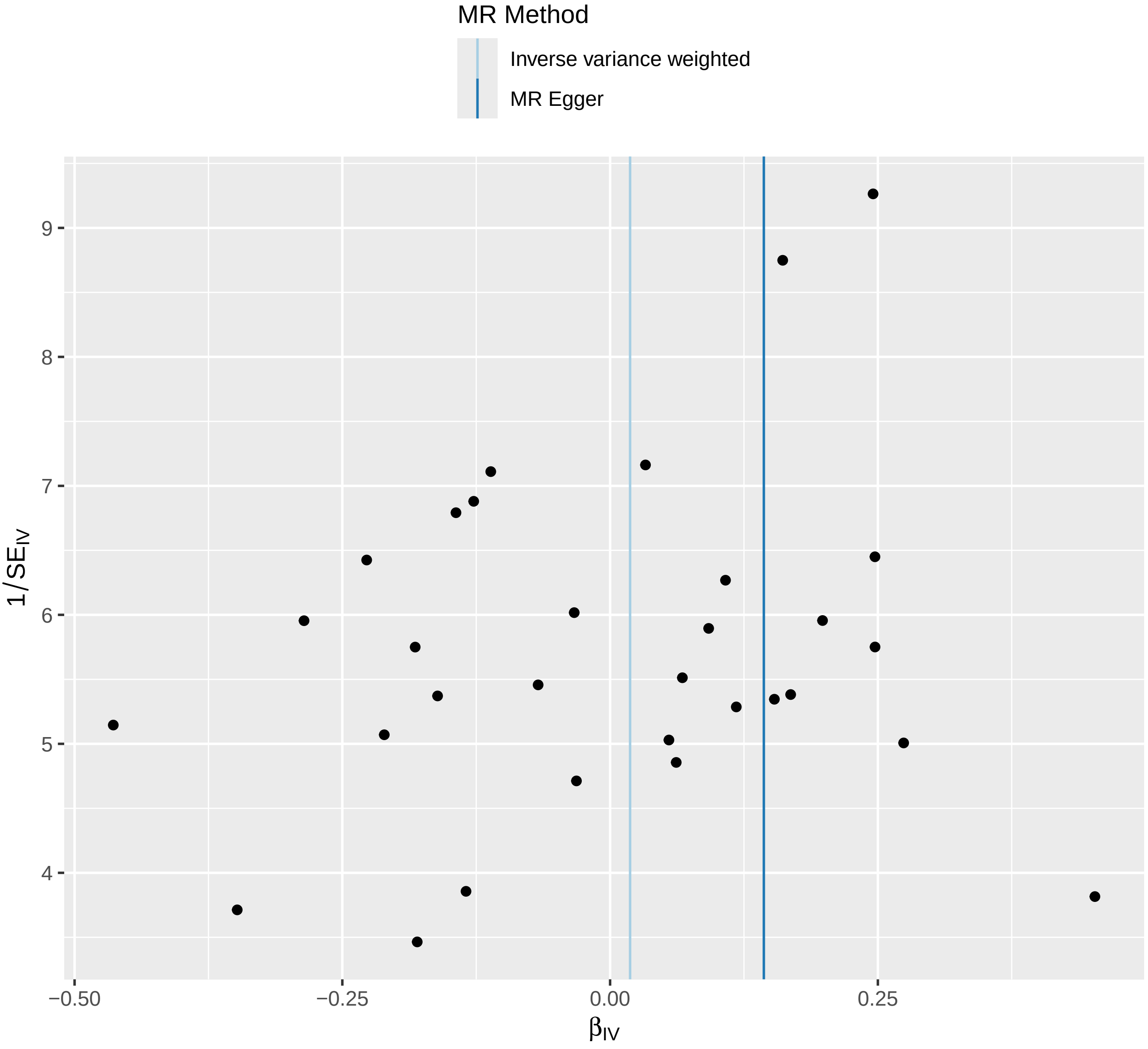

MR Method
Inverse variance weighted
MR Egger
9
8
7
6
5
4
1/SE$_{IV}$
−0.50
−0.25
0.00
0.25
β$_{IV}$

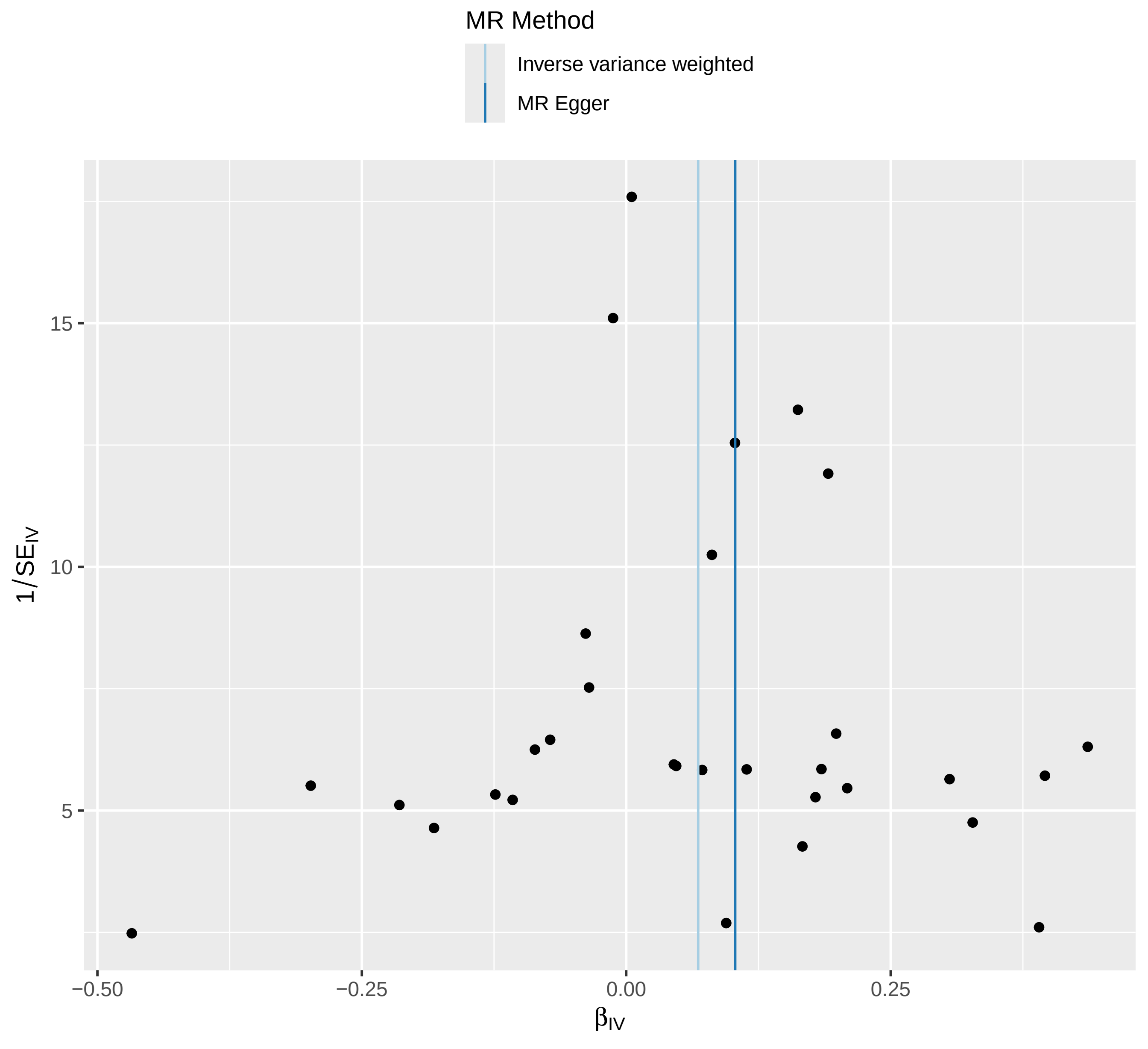

MR Method
Inverse variance weighted
MR Egger
15
10
5
1/SE_IV
−0.50
−0.25
0.00
0.25
β_IV

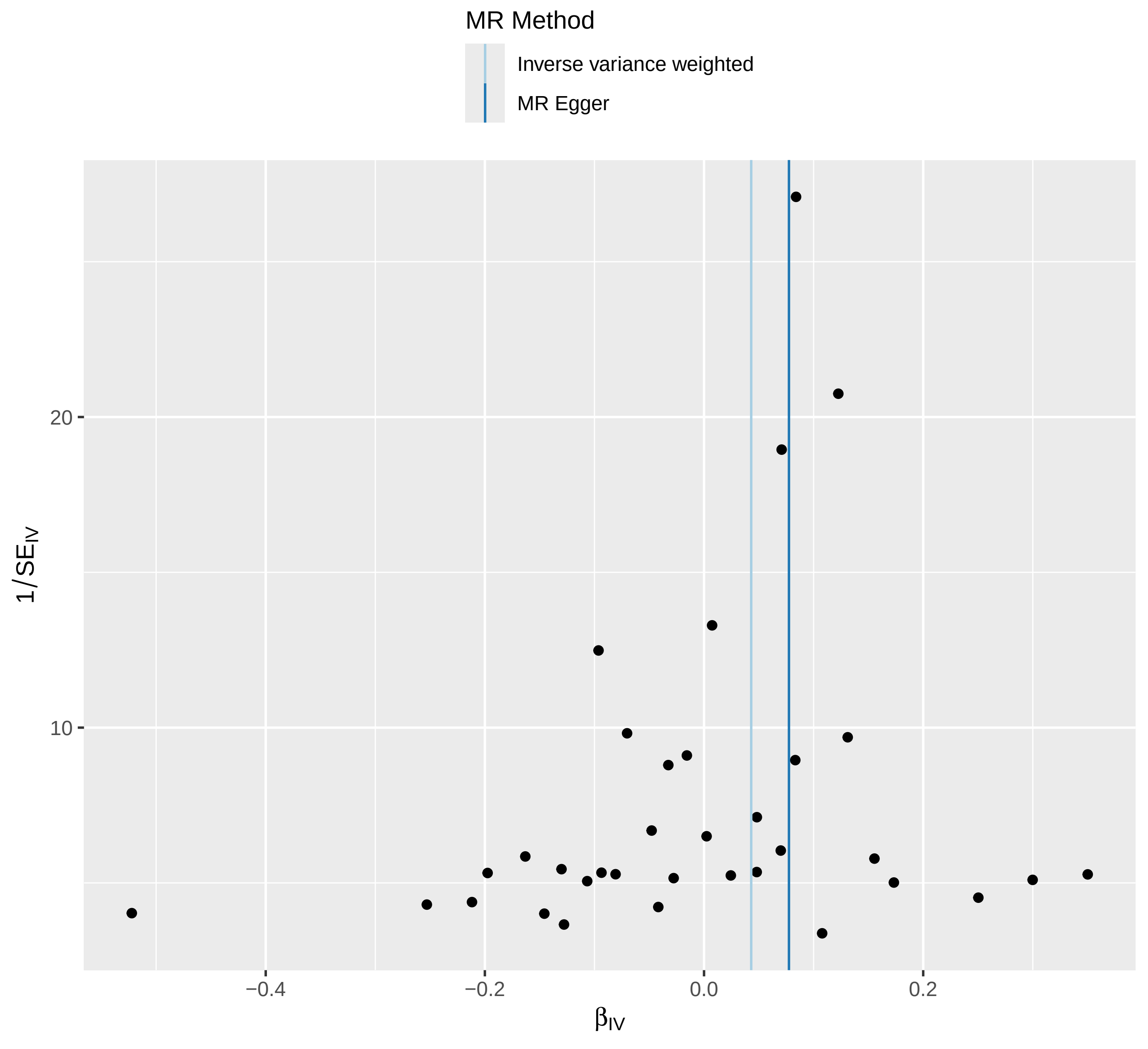
MR Method
Inverse variance weighted
MR Egger
20
10
1/SE$_{IV}$
−0.4
−0.2
0.0
0.2
β$_{IV}$

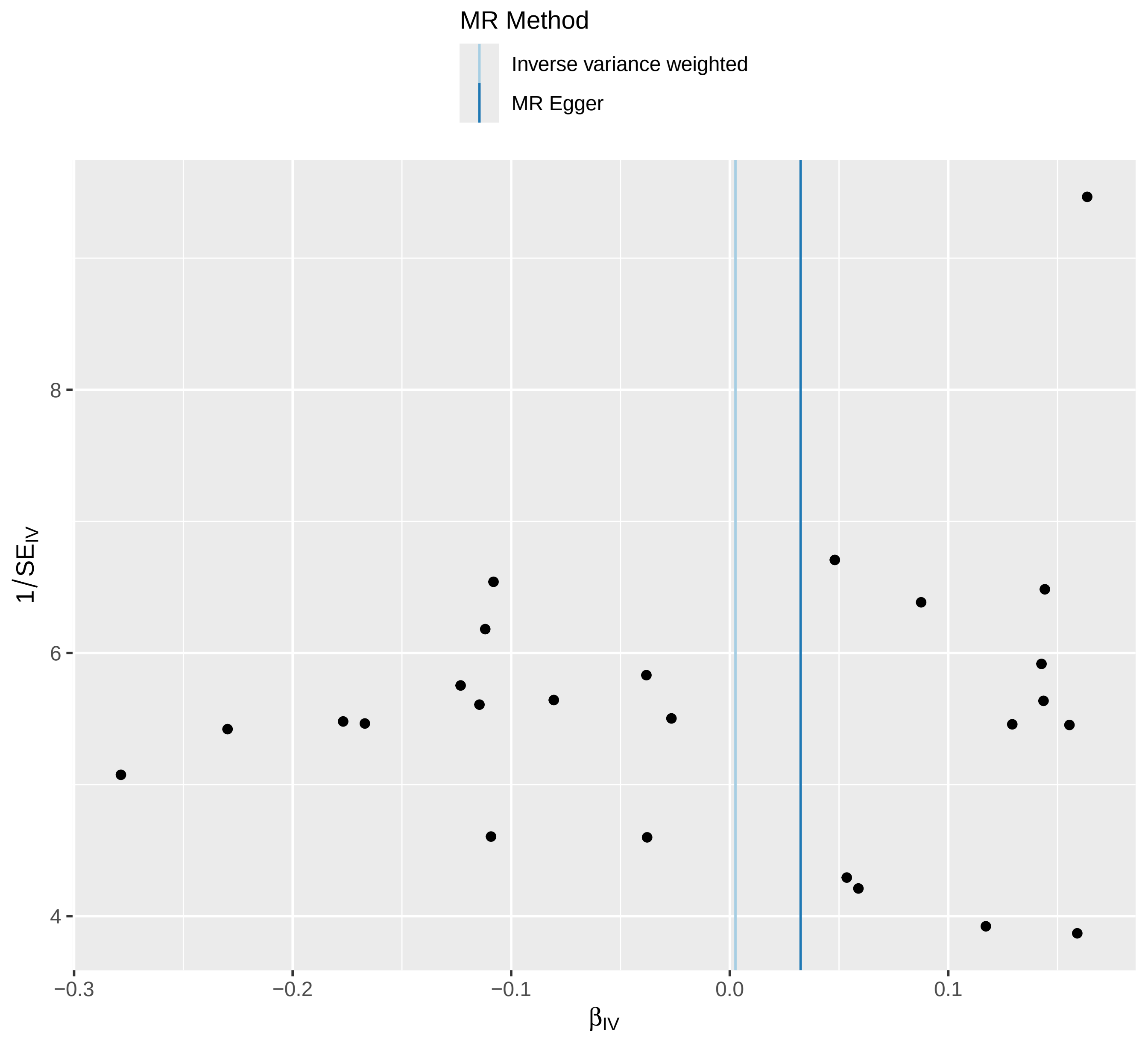
MR Method
Inverse variance weighted
MR Egger
8
6
4
−0.3
−0.2
−0.1
0.0
0.1
$1/SE_{IV}$
β_{IV}

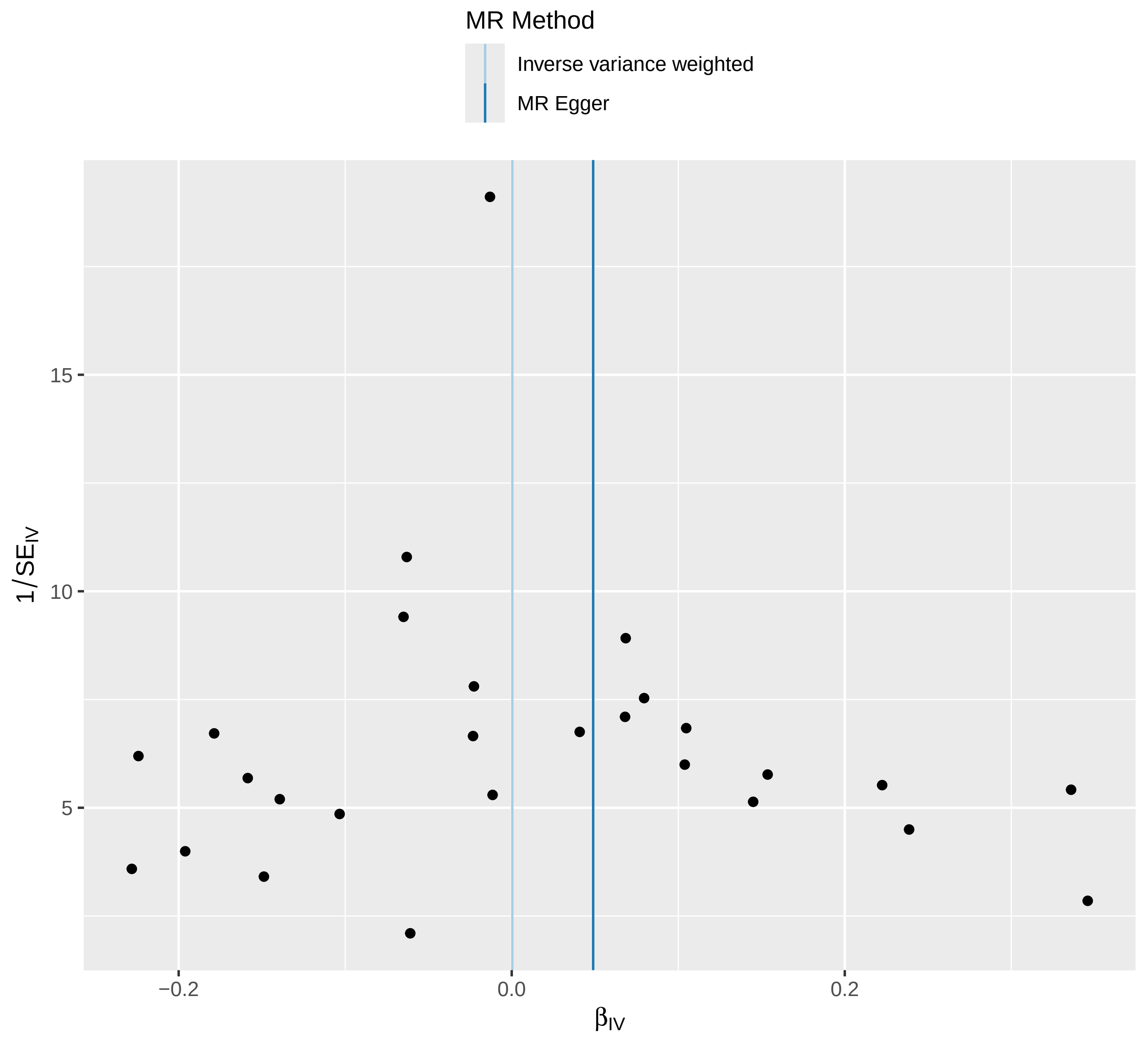

MR Method
Inverse variance weighted
MR Egger
1/SE_IV
15
10
5
−0.2
0.0
0.2
β_IV

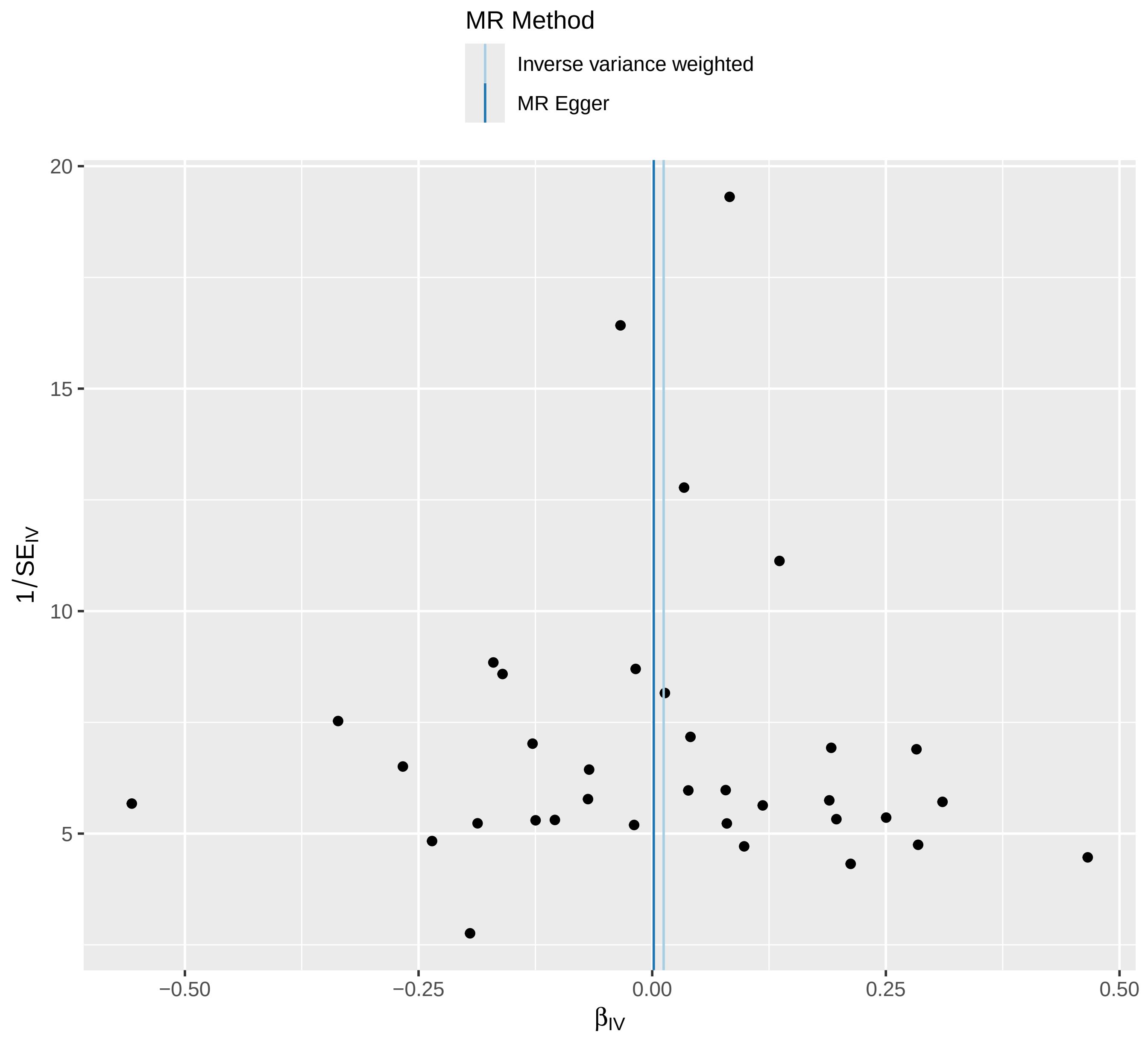

MR Method
Inverse variance weighted
MR Egger
20
15
10
5
−0.50
−0.25
0.00
0.25
0.50
$1/SE_{IV}$
β_{IV}

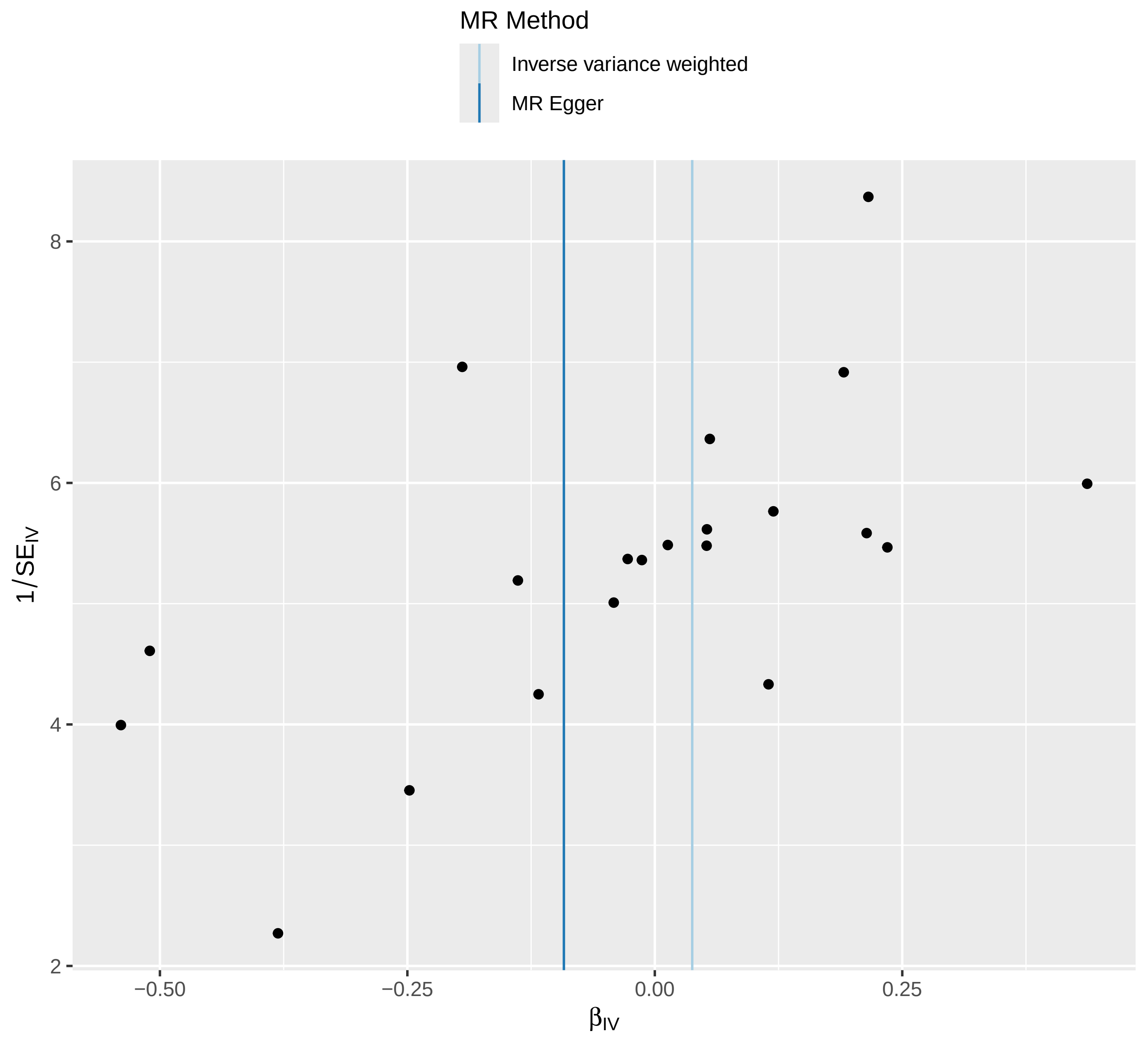

MR Method
Inverse variance weighted
MR Egger
1/SE_IV
β_IV
8
6
4
2
−0.50
−0.25
0.00
0.25

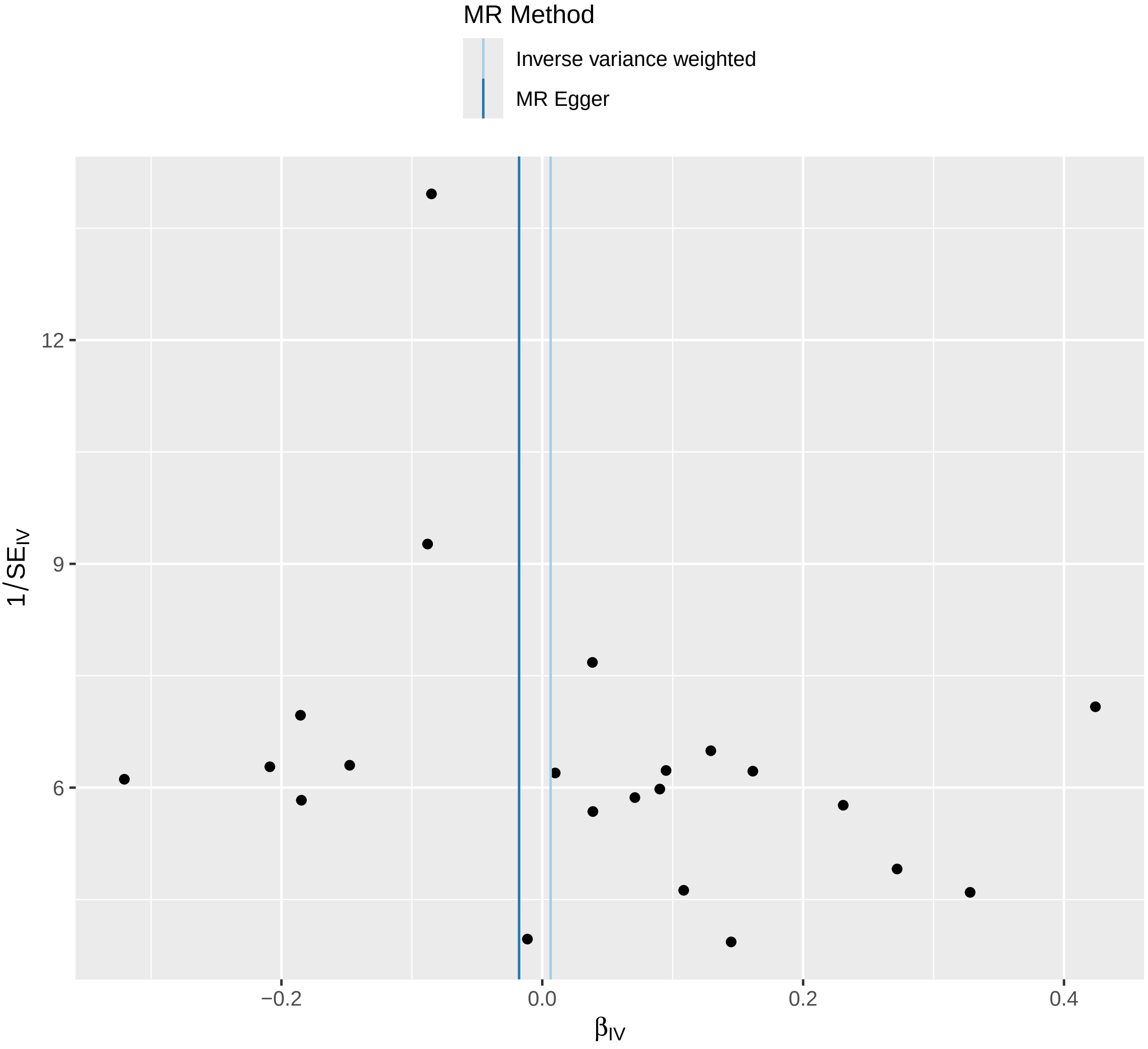
MR Method
Inverse variance weighted
MR Egger
1/SE_IV
12
9
6
β_IV
−0.2
0.0
0.2
0.4

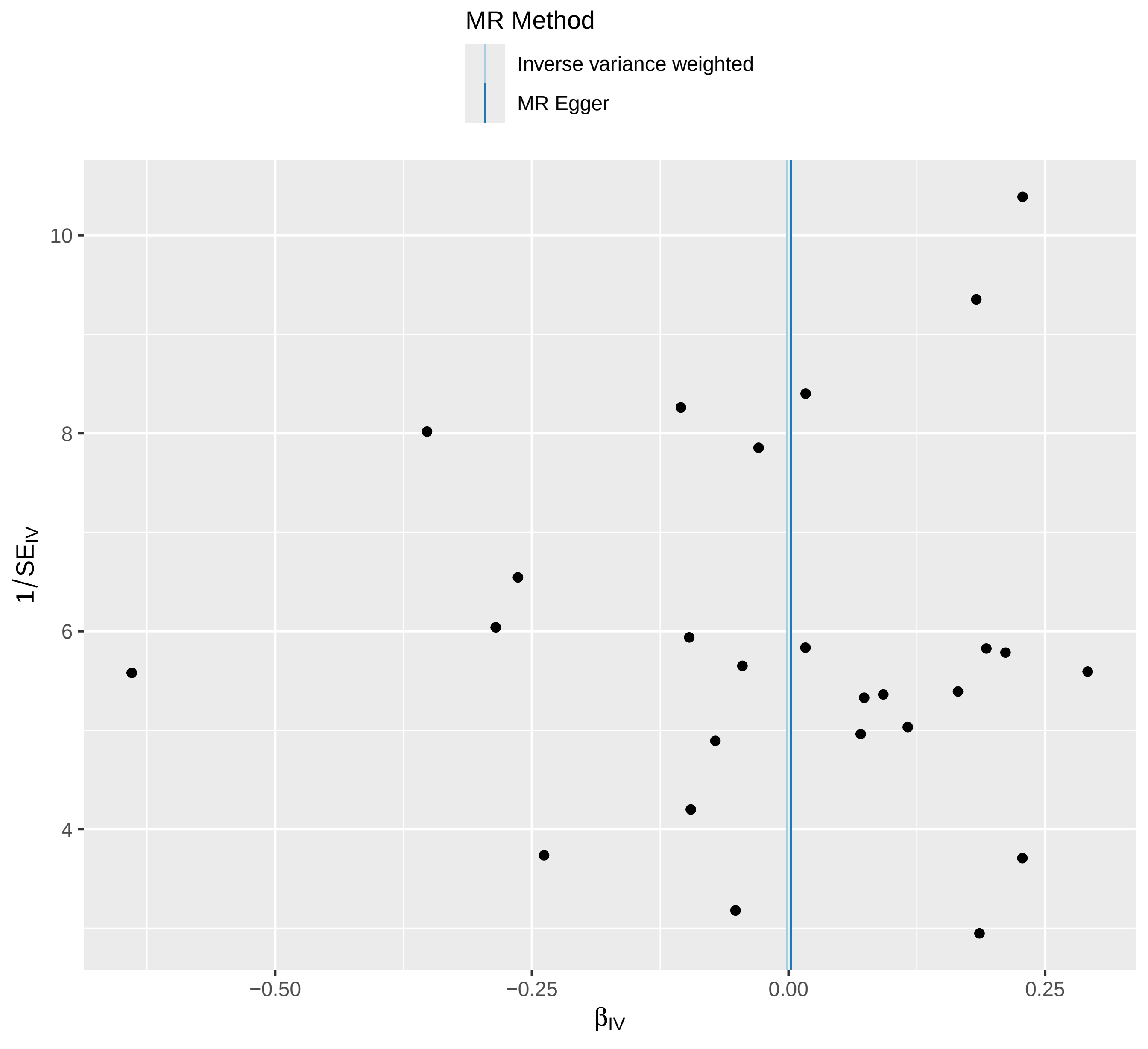

MR Method
Inverse variance weighted
MR Egger
10
8
6
4
1/SE$_{IV}$
−0.50
−0.25
0.00
0.25
β$_{IV}$

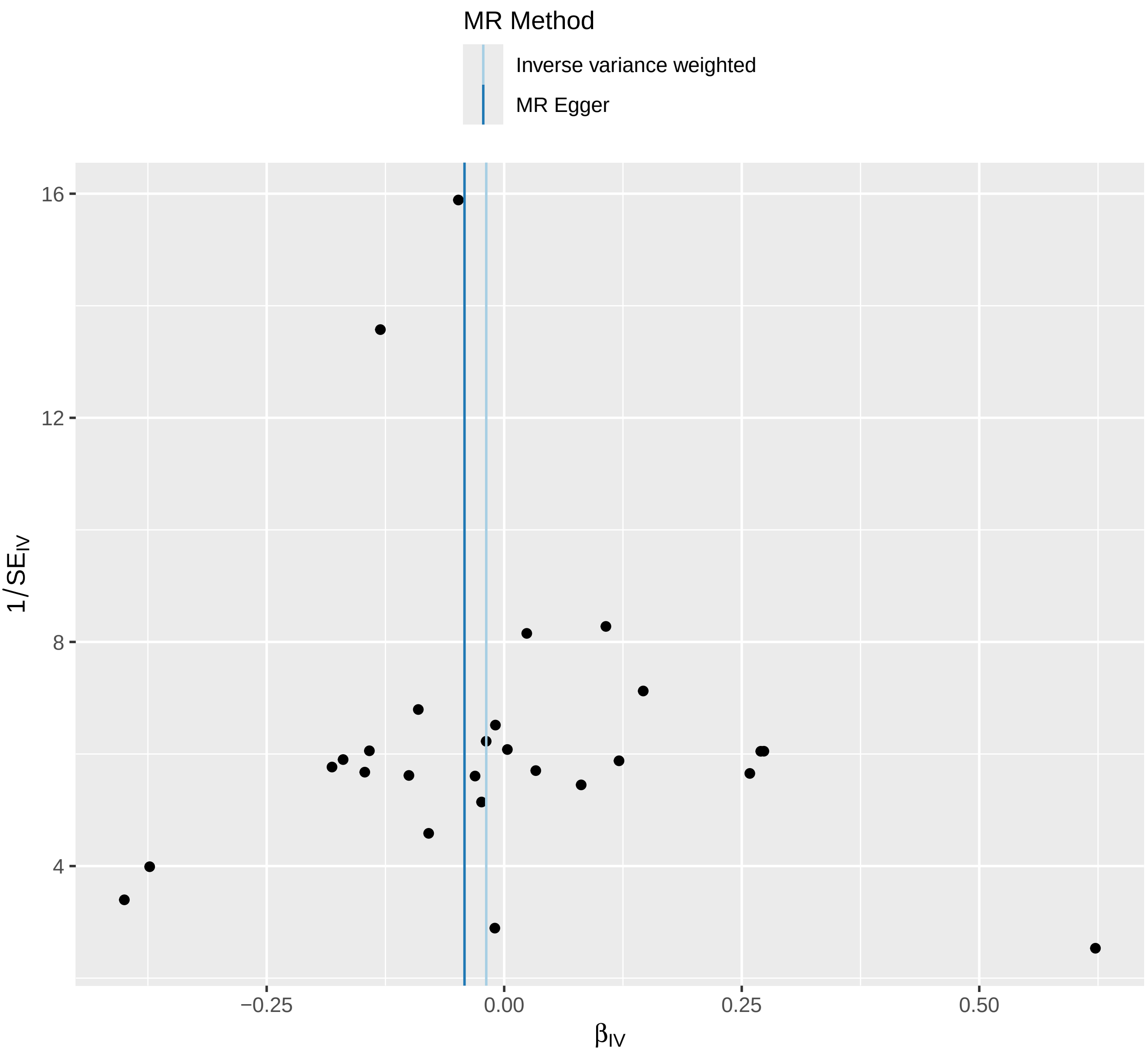
MR Method
Inverse variance weighted
MR Egger
16
12
8
4
1/SE$_{IV}$
−0.25
0.00
0.25
0.50
β$_{IV}$

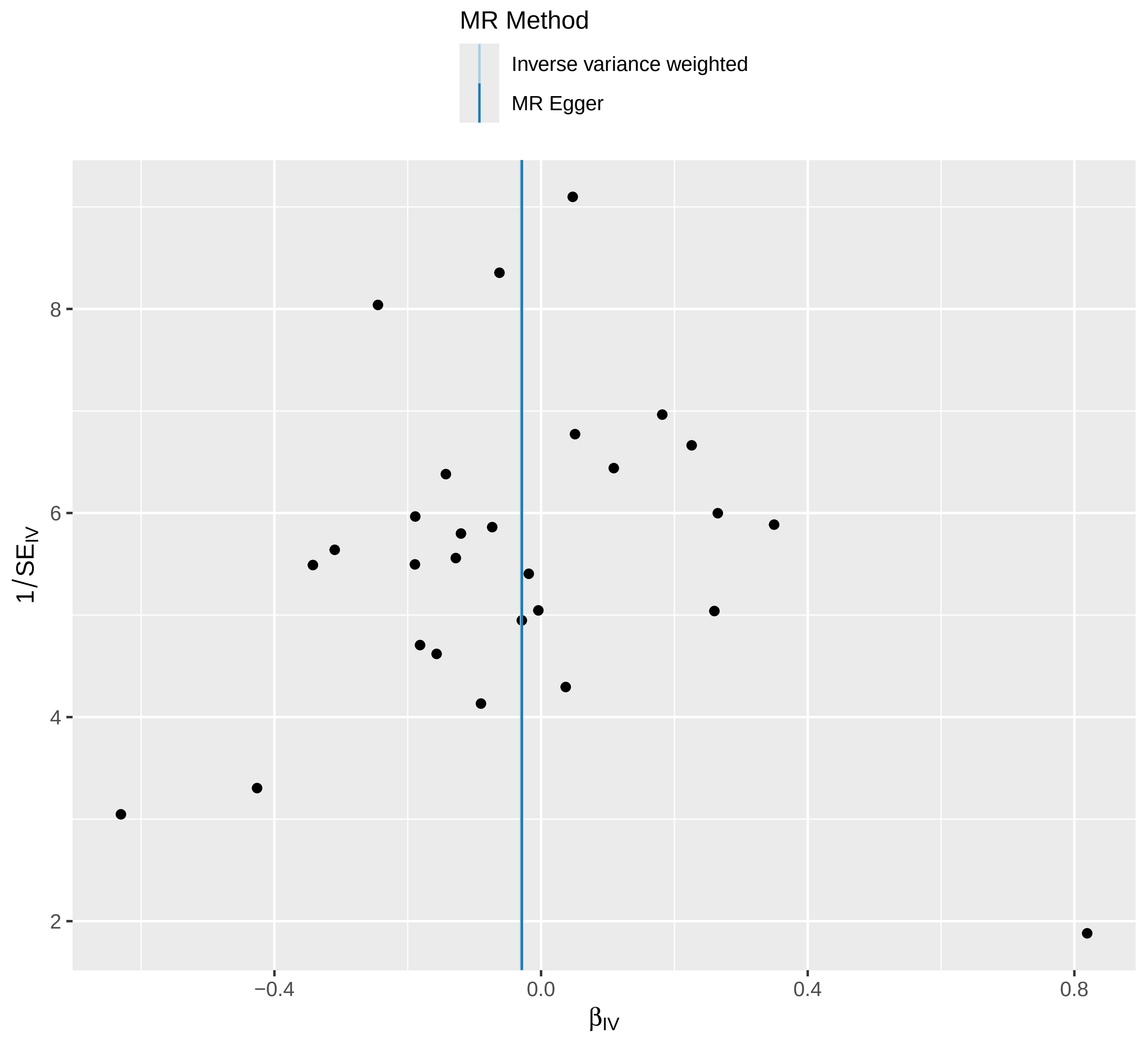

MR Method
Inverse variance weighted
MR Egger
8
6
4
2
1/SE$_{IV}$
−0.4
0.0
0.4
0.8
β$_{IV}$

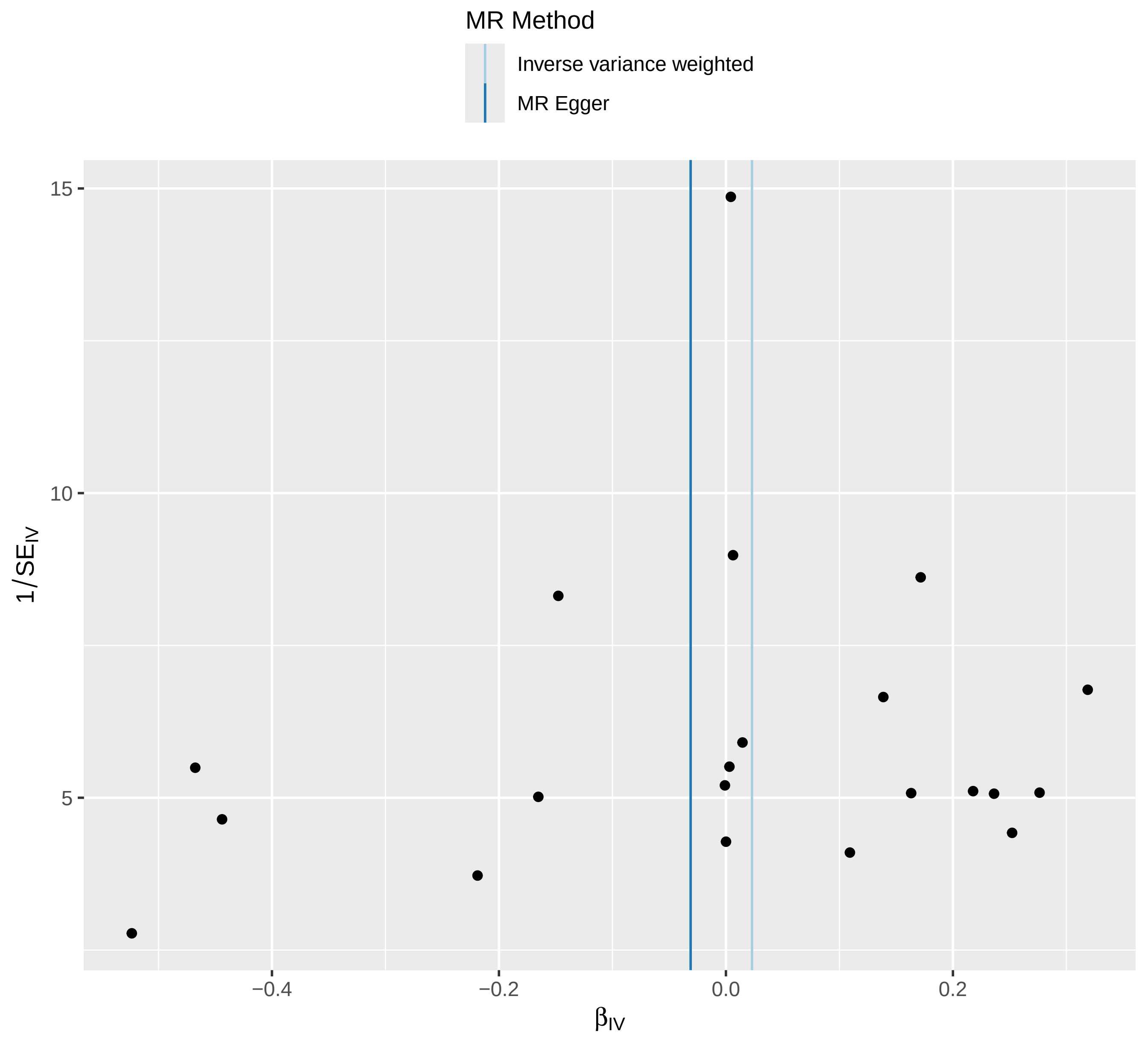
MR Method
Inverse variance weighted
MR Egger
15
10
5
1/SE_IV
−0.4
−0.2
0.0
0.2
β_IV

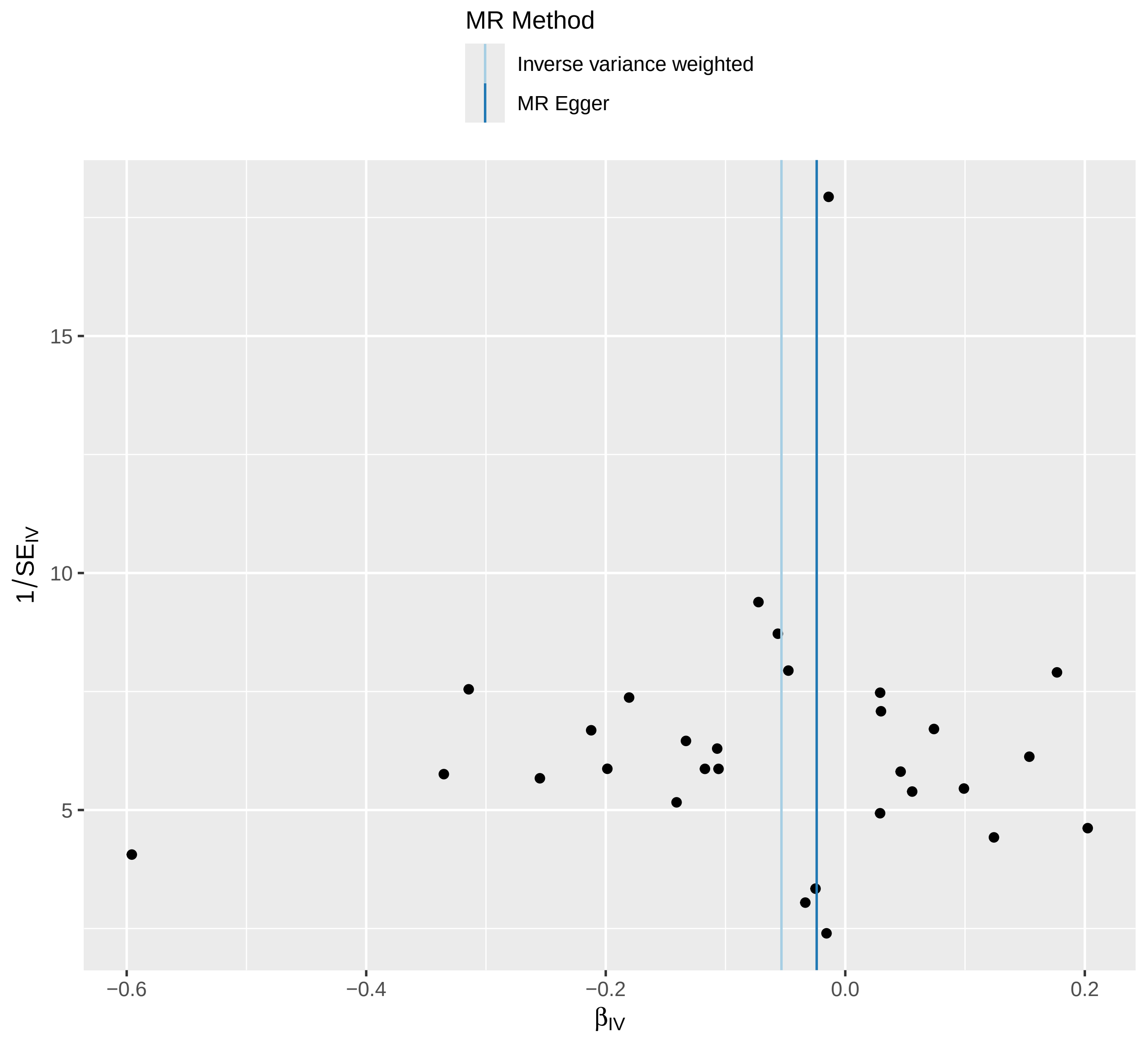
MR Method
Inverse variance weighted
MR Egger
15
10
5
1/SE_IV
−0.6
−0.4
−0.2
0.0
0.2
β_IV

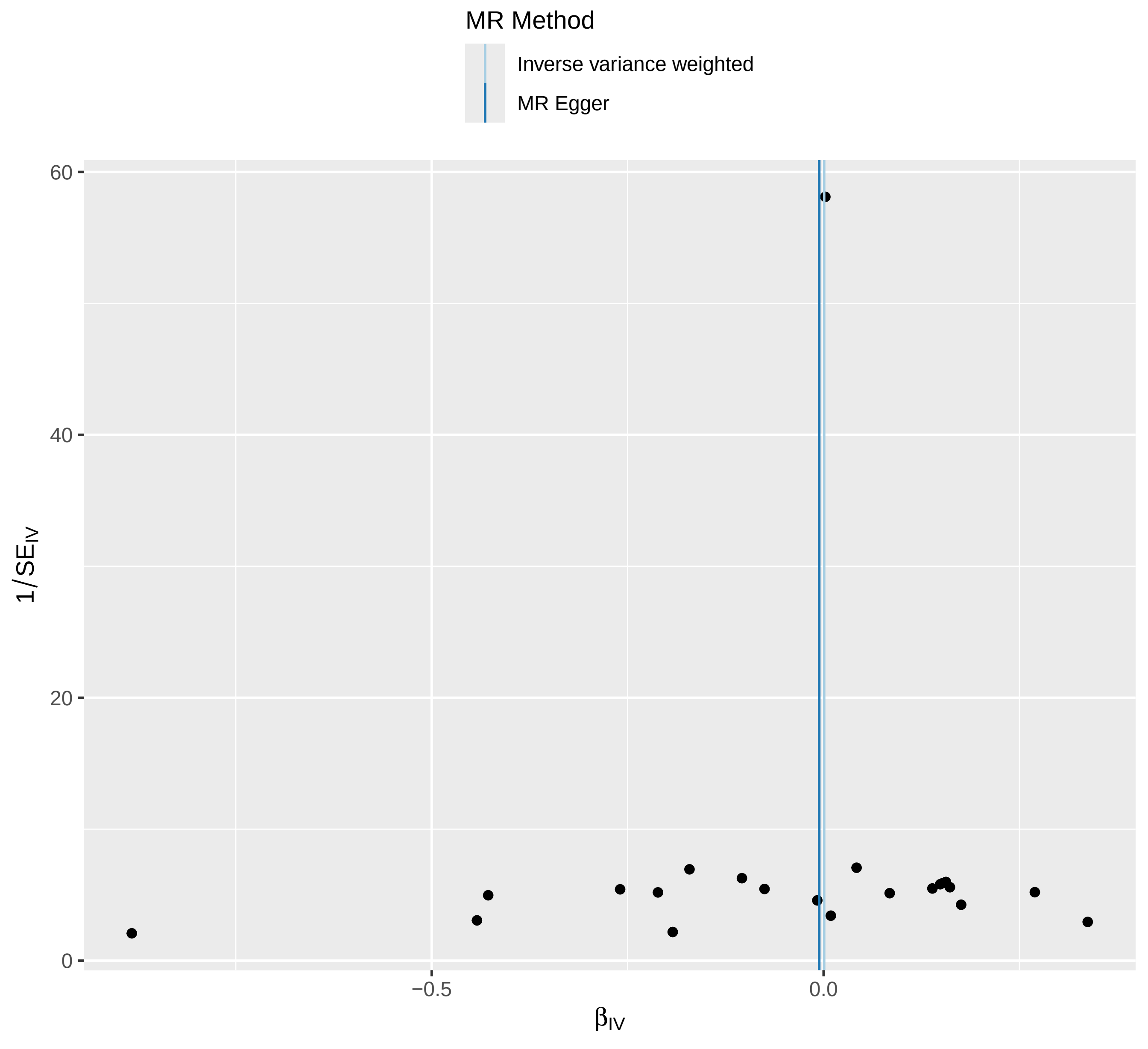

MR Method
Inverse variance weighted
MR Egger
60
40
20
0
1/SE_IV
−0.5
0.0
β_IV

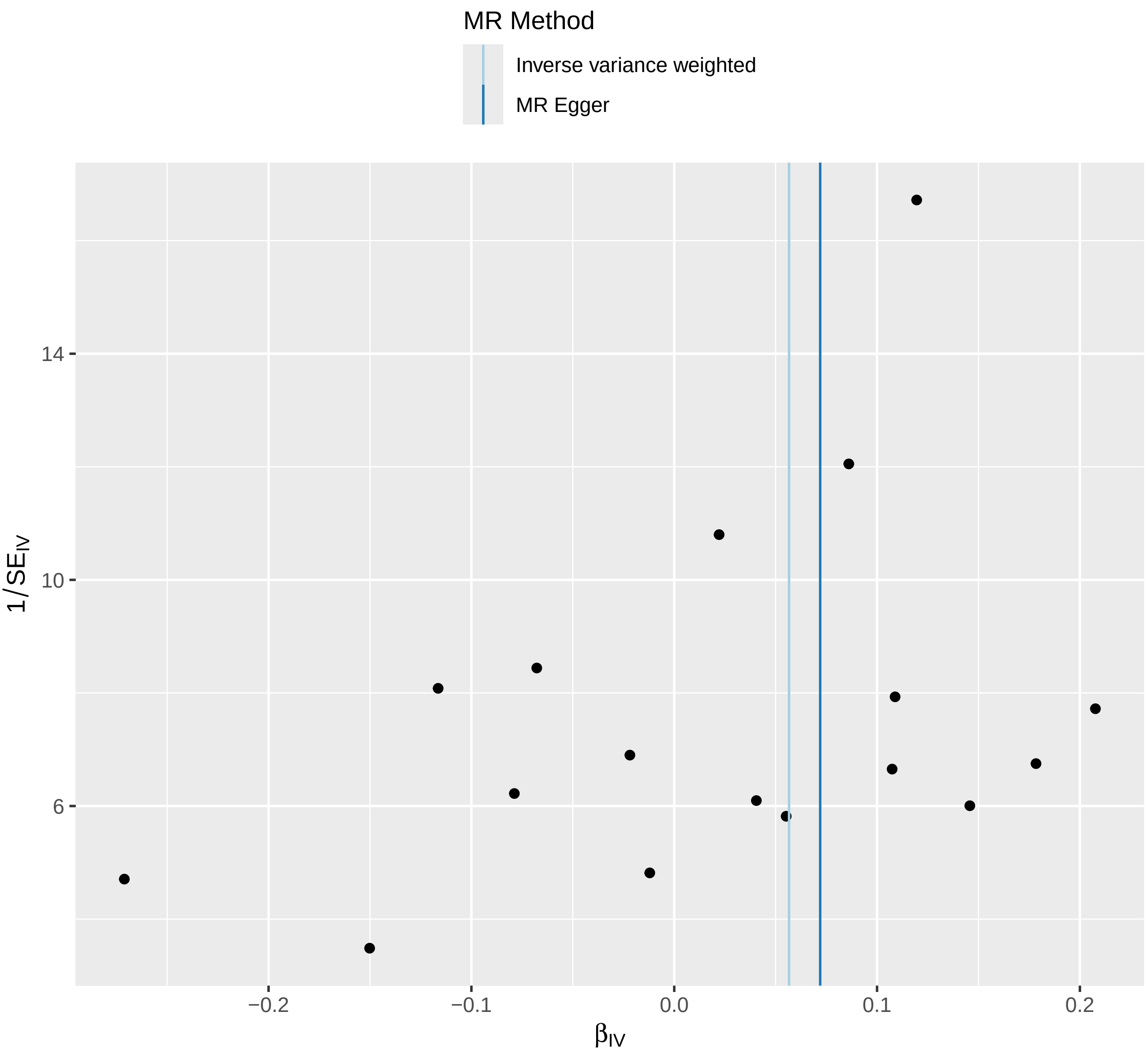
MR Method
Inverse variance weighted
MR Egger
1/SE_IV
14
10
6
−0.2
−0.1
0.0
0.1
0.2
β_IV

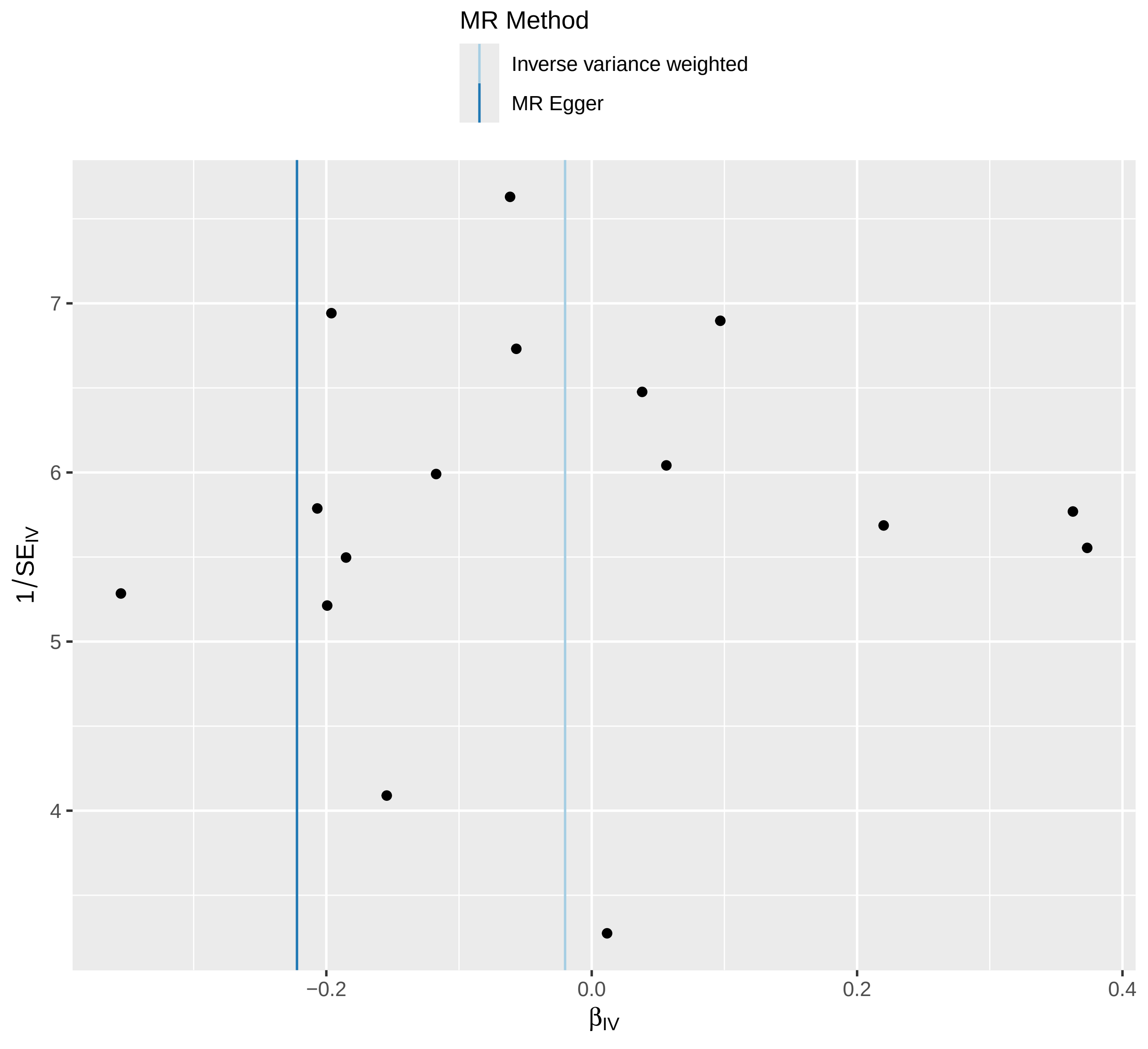

MR Method
Inverse variance weighted
MR Egger
7
6
5
4
1/SE_IV
−0.2
0.0
0.2
0.4
β_IV

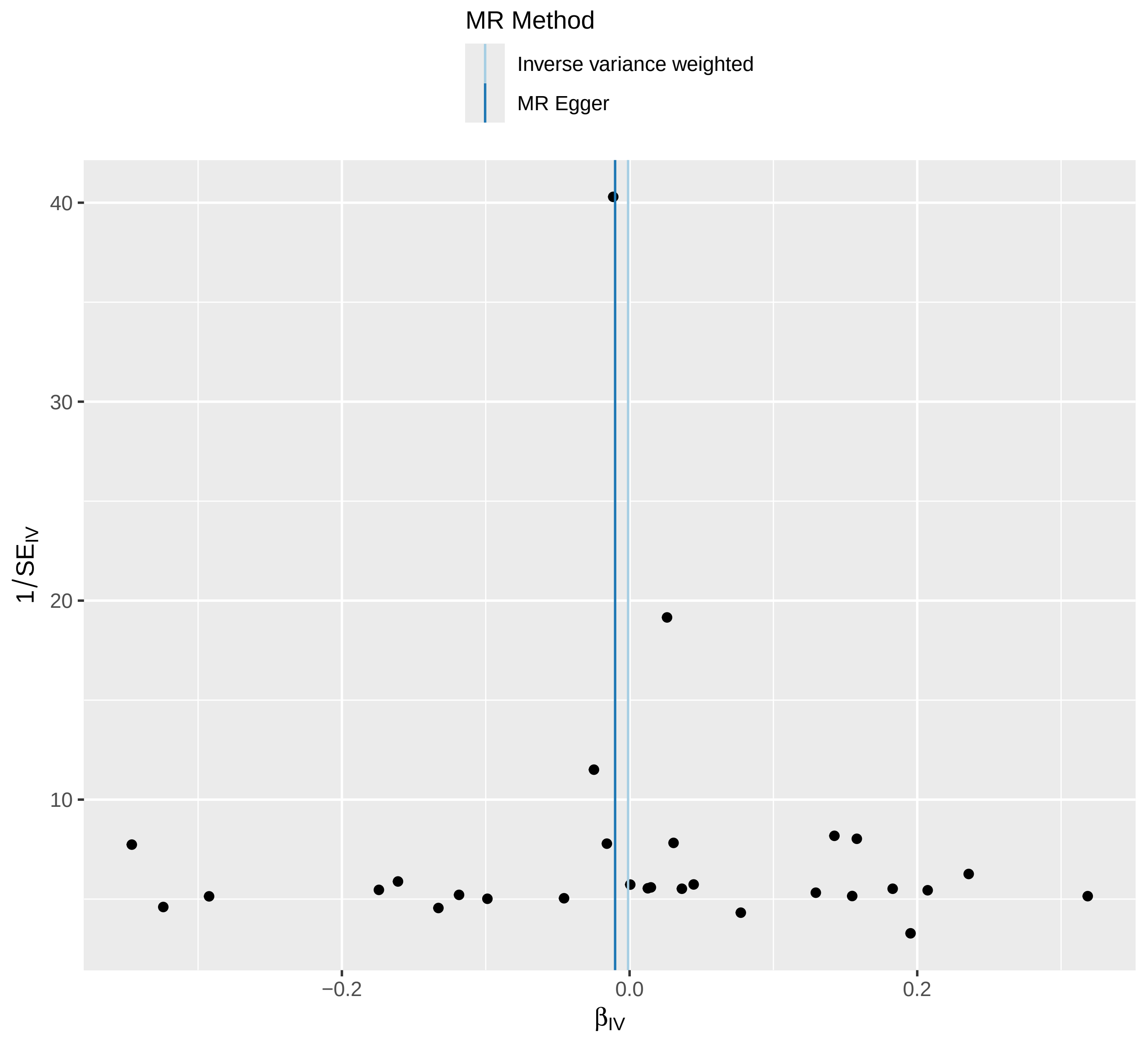
MR Method
Inverse variance weighted
MR Egger
1/SE_IV
40
30
20
10
−0.2
0.0
0.2
β_IV

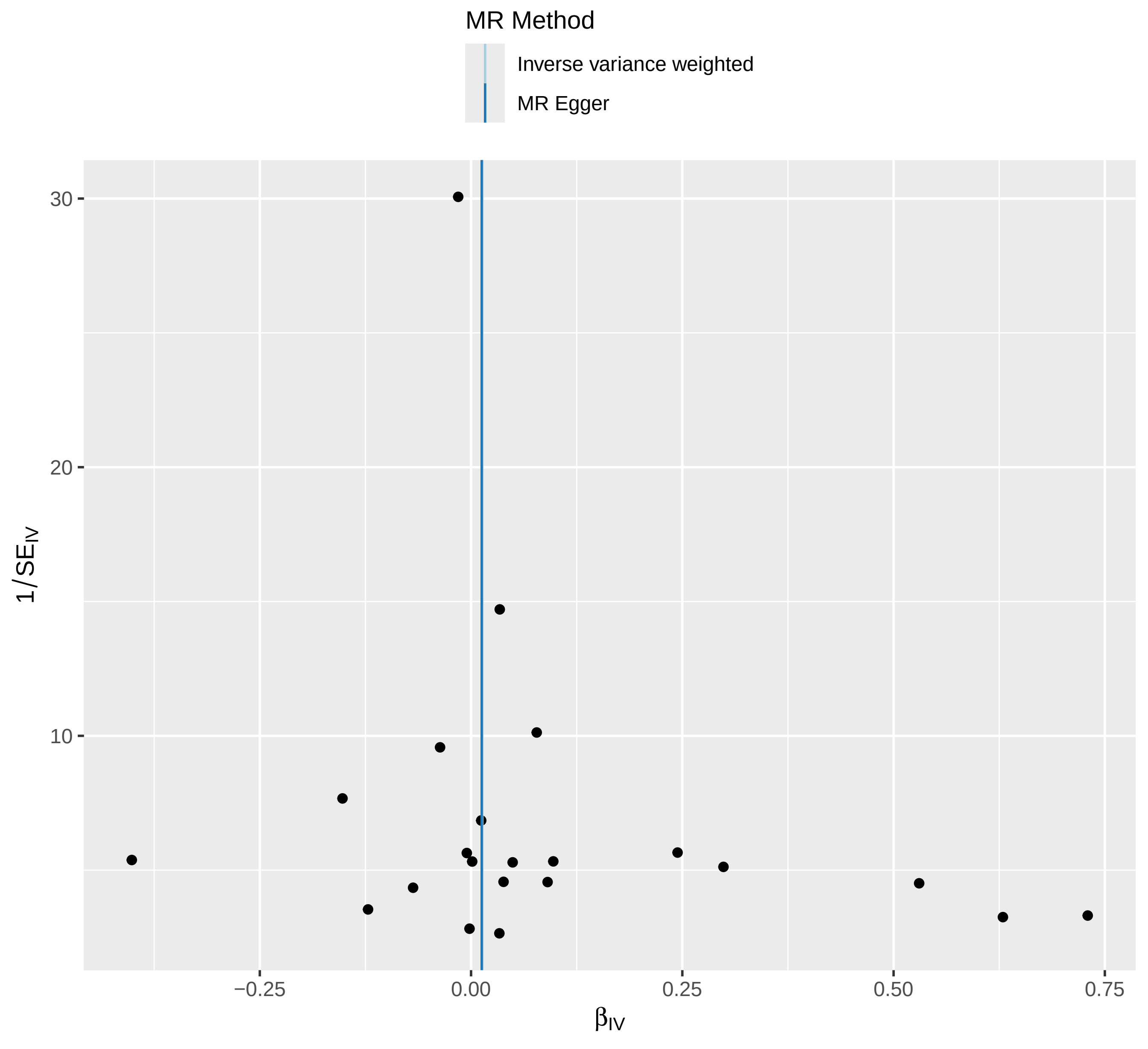

MR Method
Inverse variance weighted
MR Egger
30
20
10
1/SE_IV
−0.25
0.00
0.25
0.50
0.75
β_IV

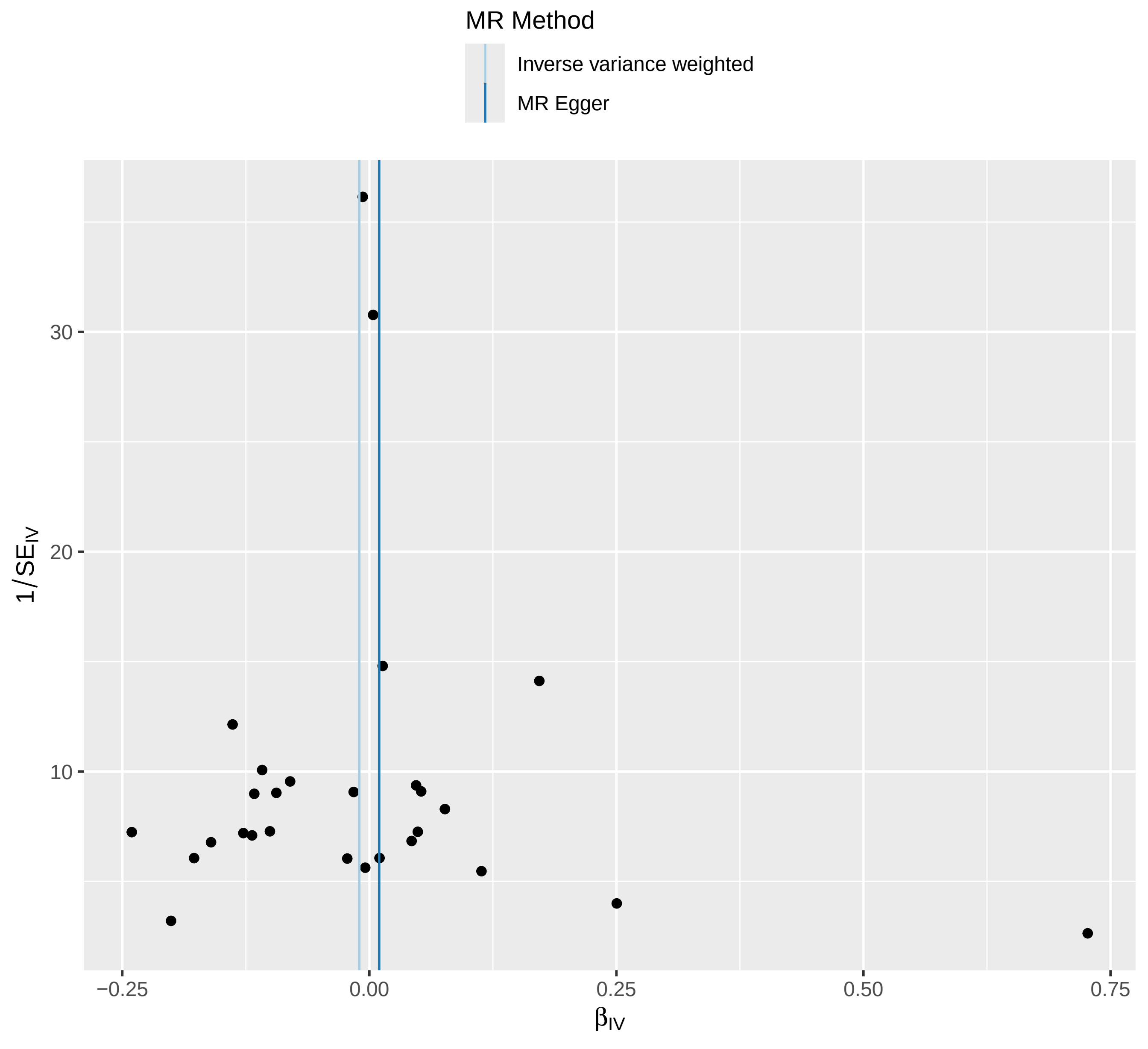
MR Method
Inverse variance weighted
MR Egger
30
20
10
1/SE$_{IV}$
−0.25
0.00
0.25
0.50
0.75
β$_{IV}$

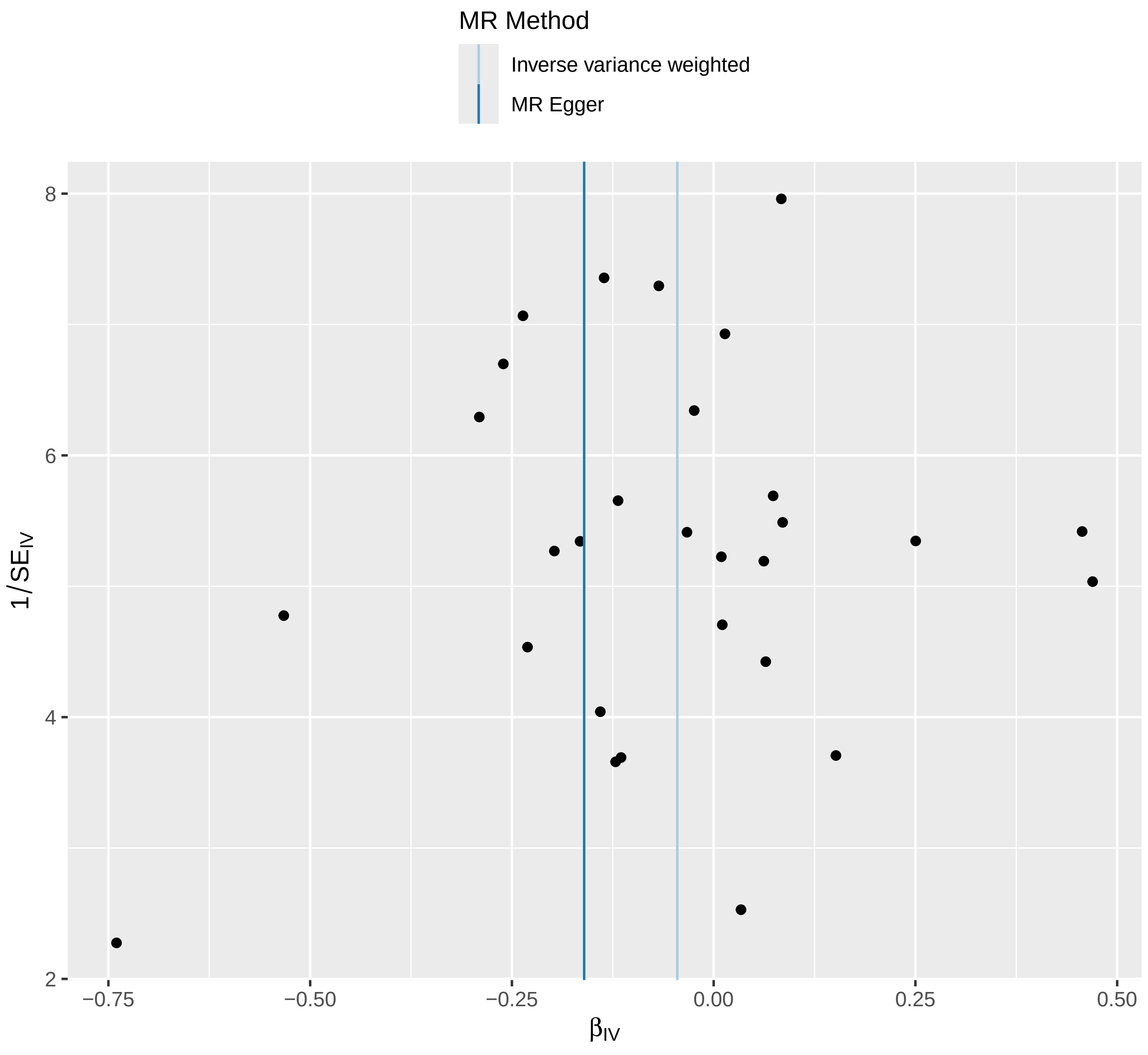
MR Method
Inverse variance weighted
MR Egger
8
6
4
2
−0.75
−0.50
−0.25
0.00
0.25
0.50
$1/SE_{IV}$
β_{IV}

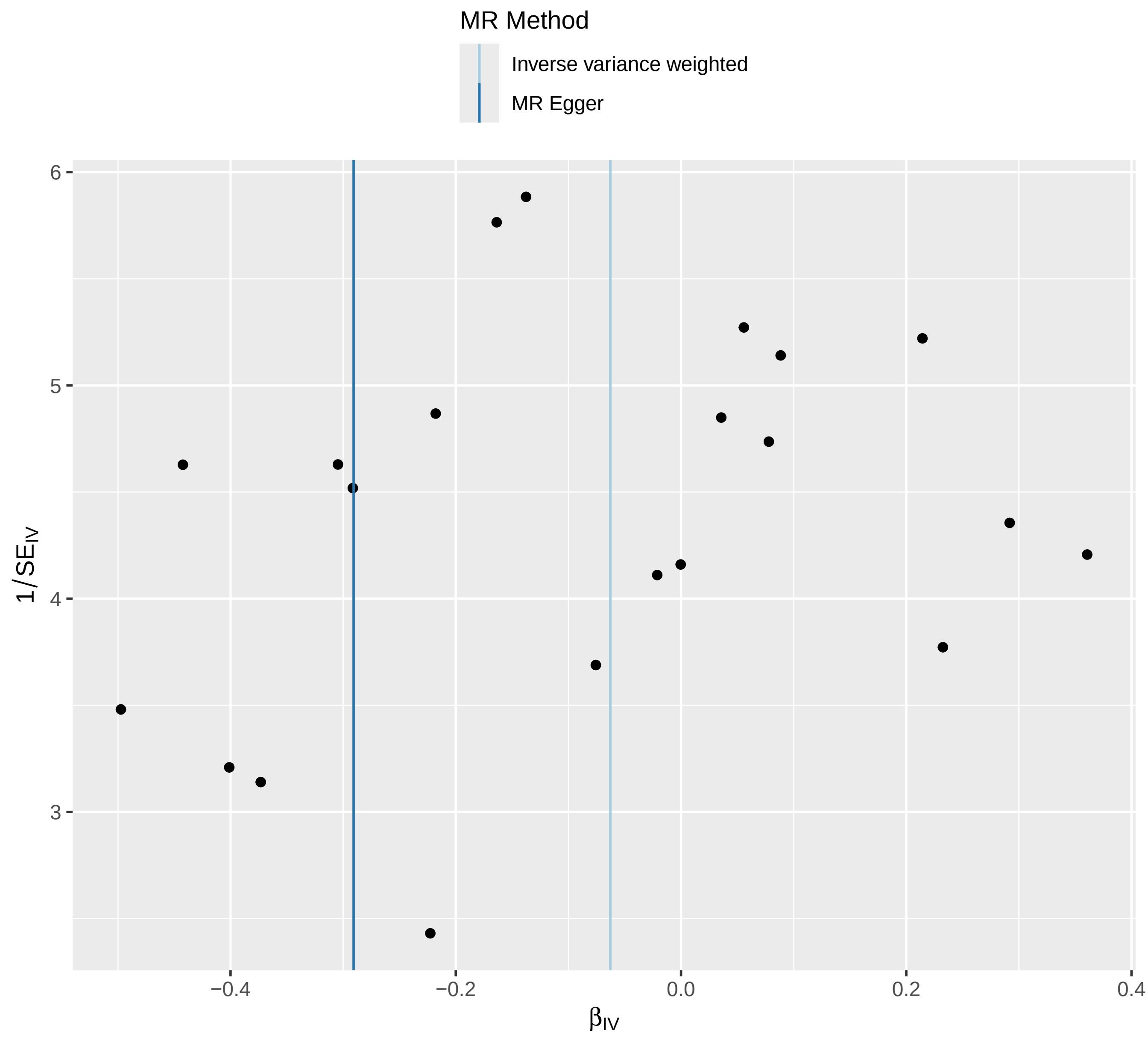
MR Method
Inverse variance weighted
MR Egger
1/SE$_{IV}$
6
5
4
3
−0.4
−0.2
0.0
0.2
0.4
β$_{IV}$

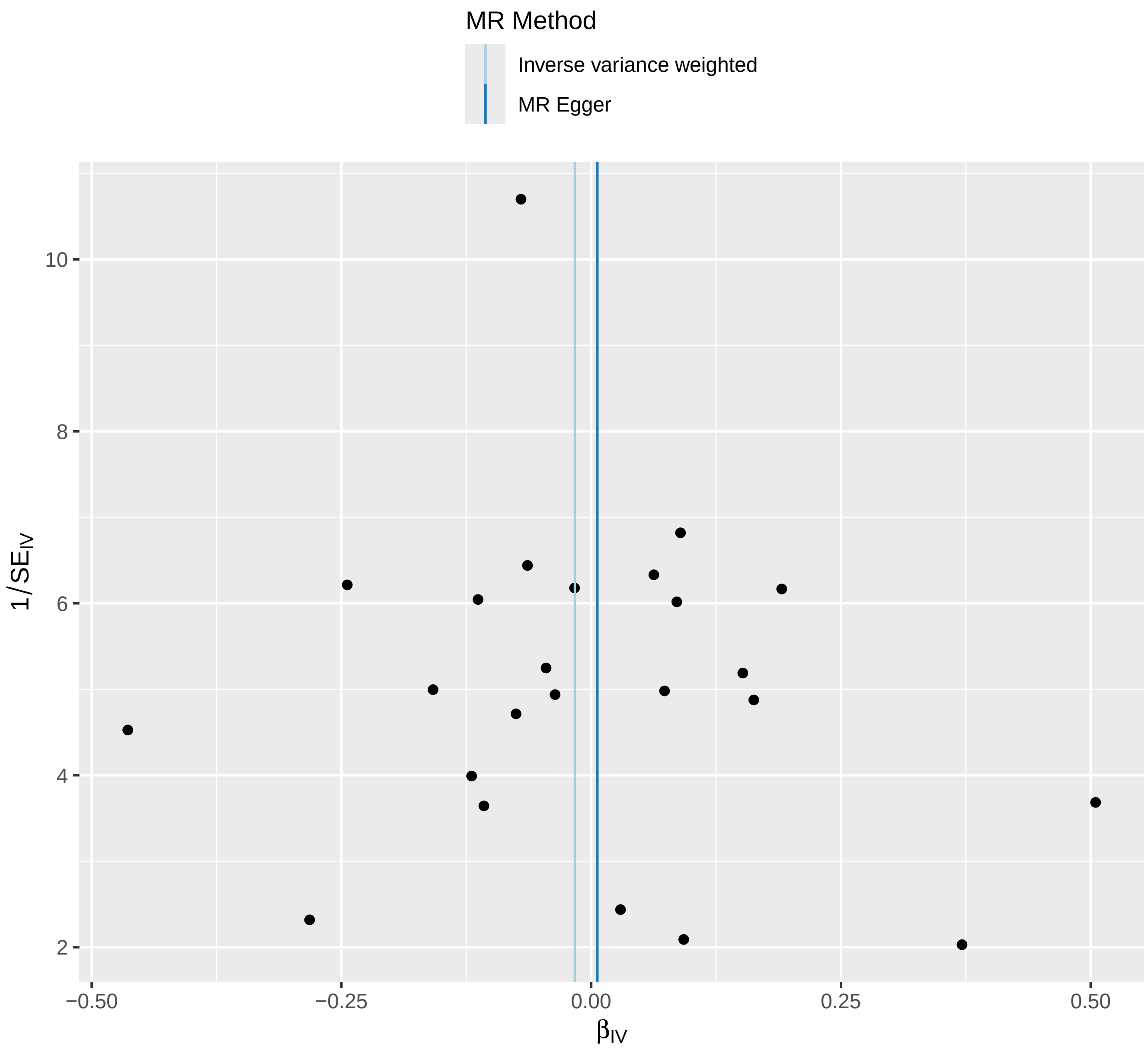

MR Method
Inverse variance weighted
MR Egger
10
8
6
4
2
1/SE_IV
−0.50
−0.25
0.00
0.25
0.50
β_IV

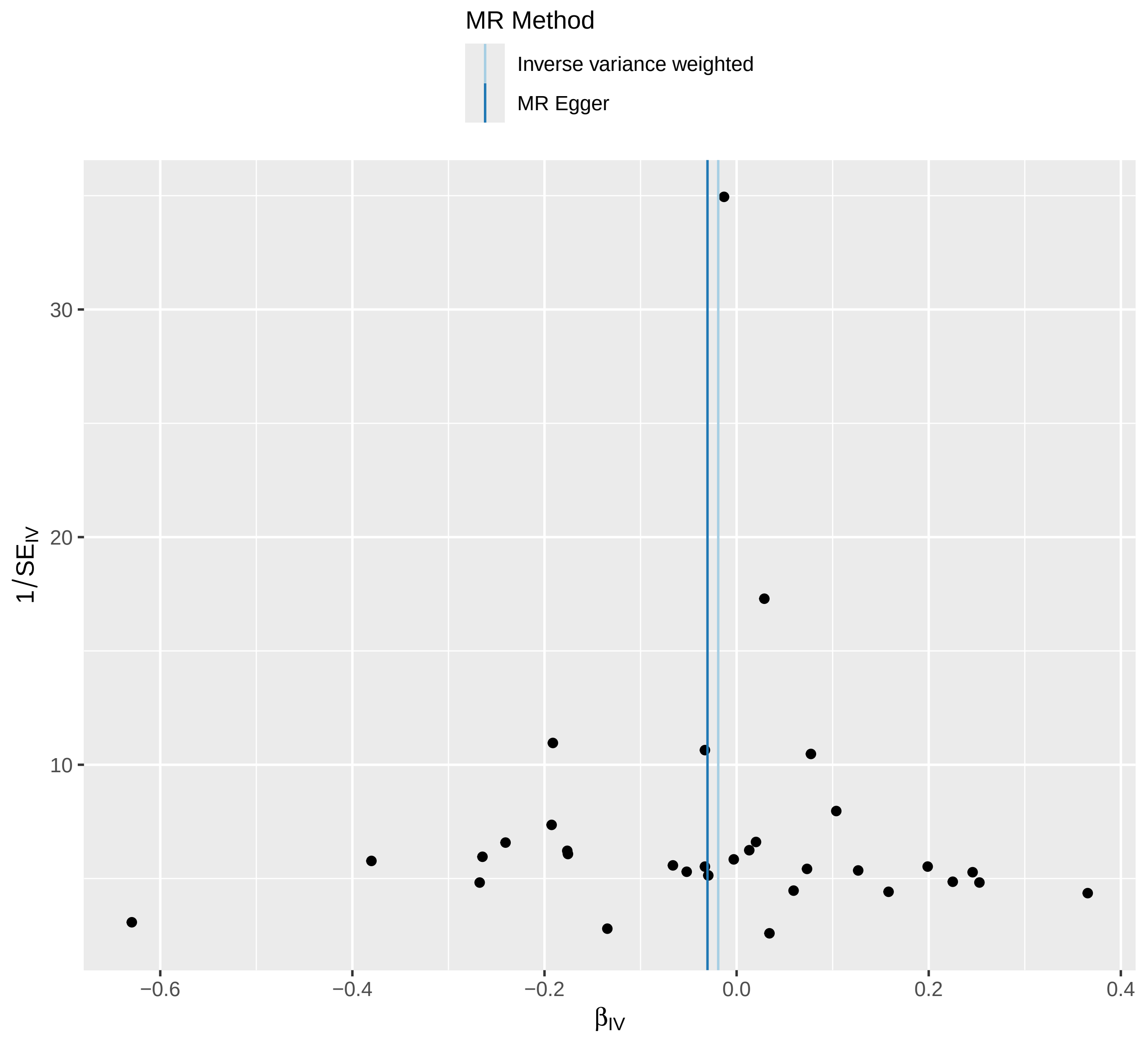
MR Method
Inverse variance weighted
MR Egger
30
20
10
$1/SE_{IV}$
−0.6
−0.4
−0.2
0.0
0.2
0.4
β_{IV}

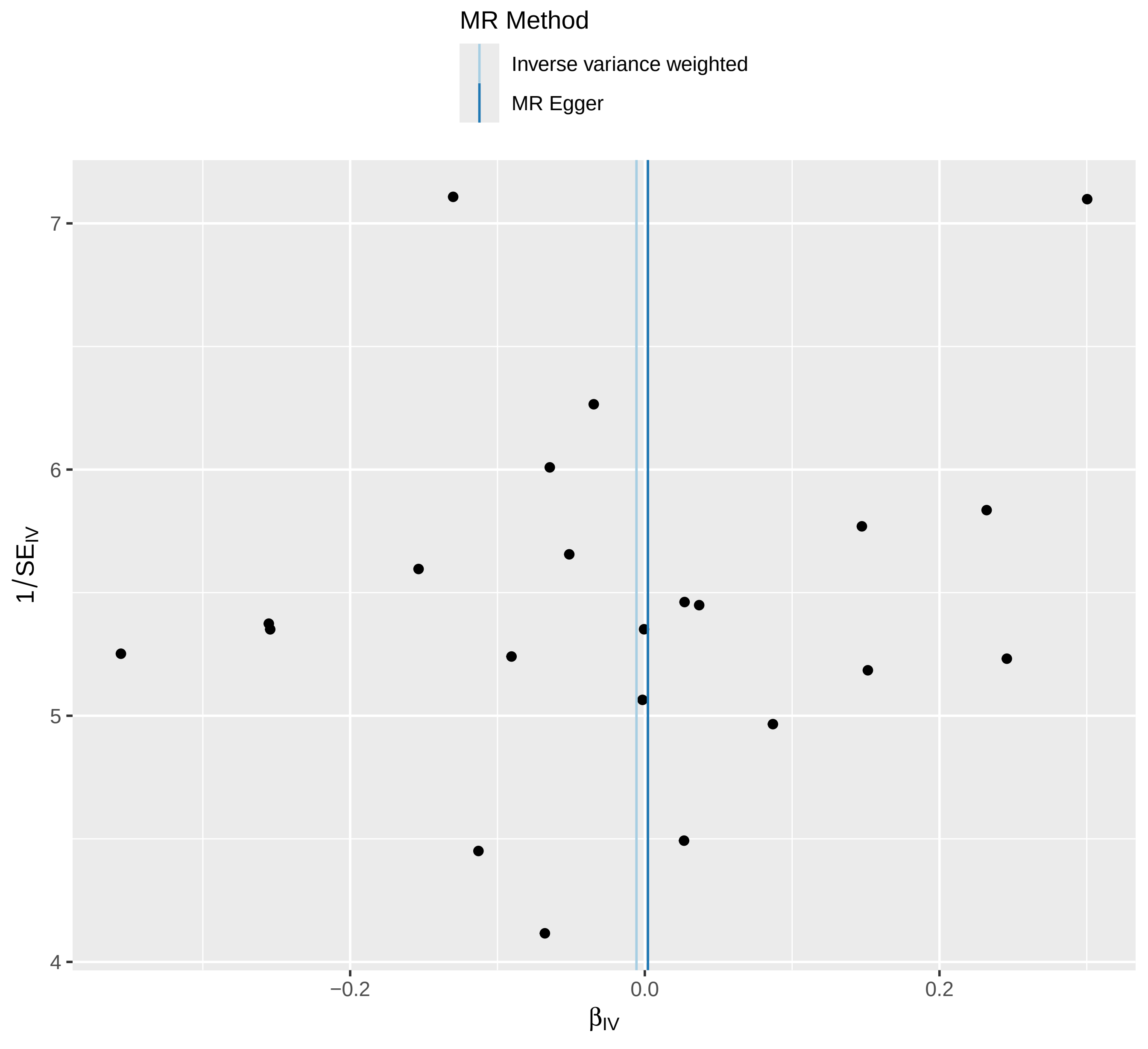
MR Method
Inverse variance weighted
MR Egger
7
6
5
4
1/SE_IV
−0.2
0.0
0.2
β_IV

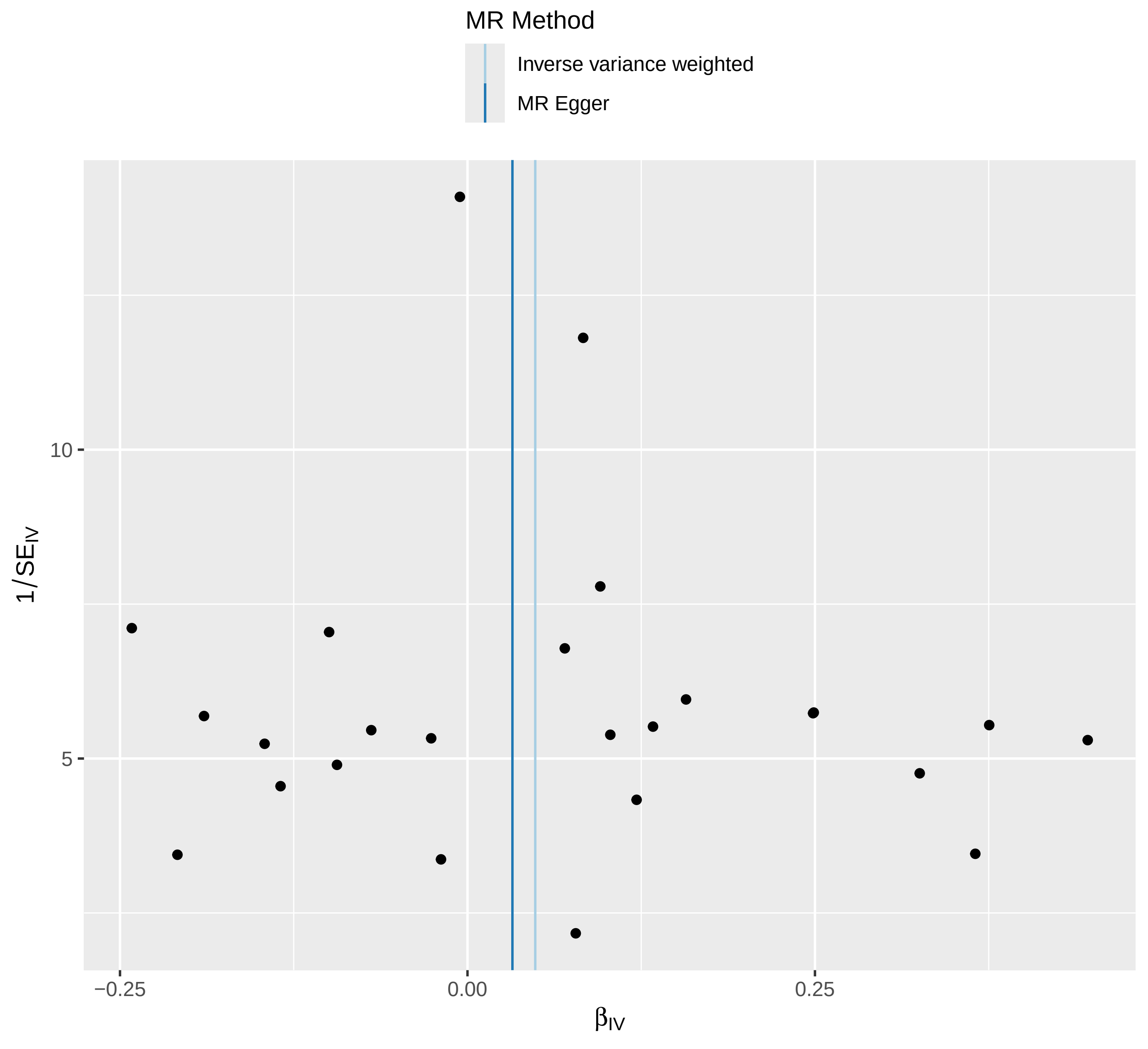
MR Method
Inverse variance weighted
MR Egger
10
5
1/SE$_{IV}$
−0.25
0.00
0.25
β$_{IV}$

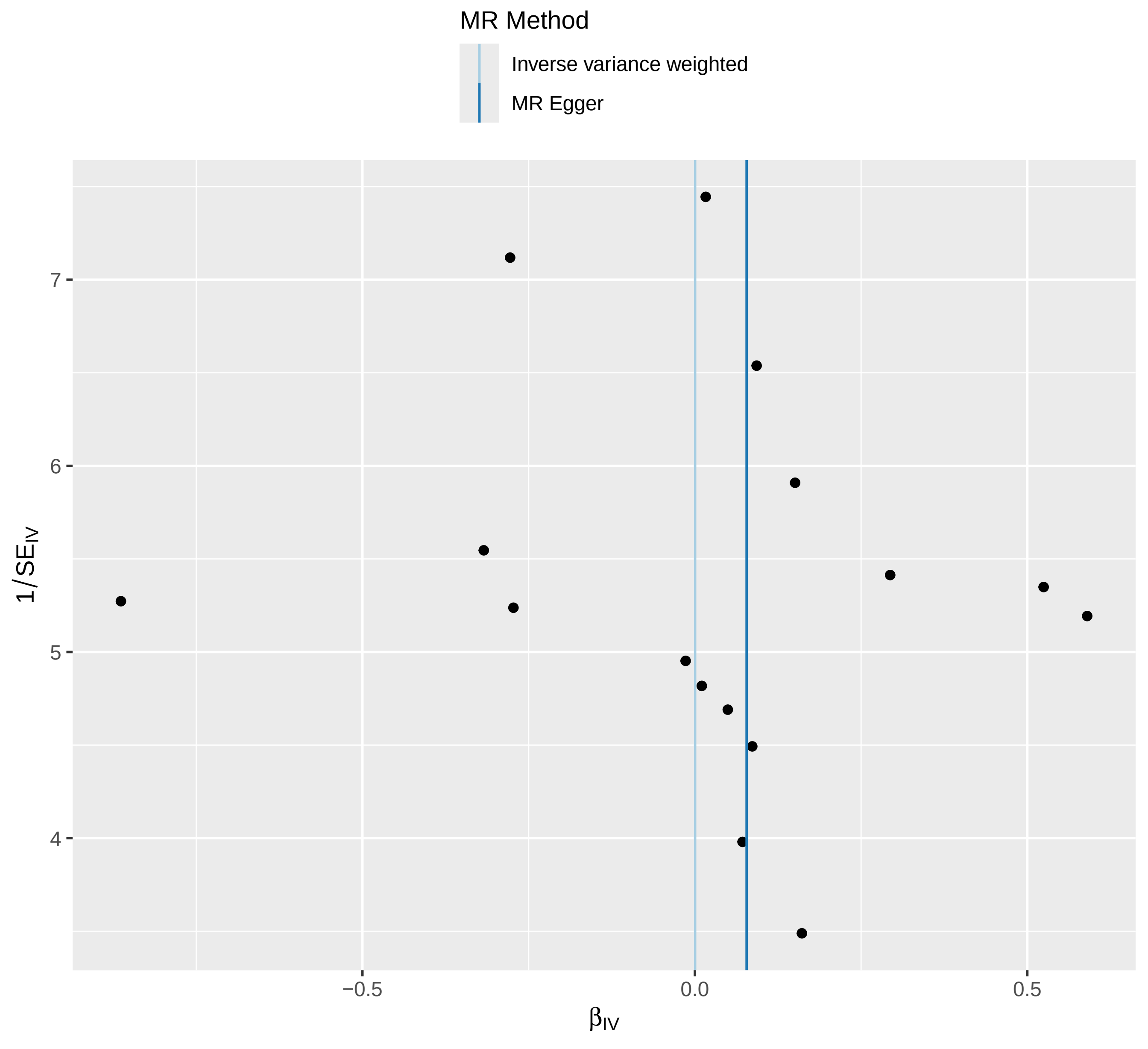
MR Method
Inverse variance weighted
MR Egger
7
6
5
4
1/SE_IV
−0.5
0.0
0.5
β_IV

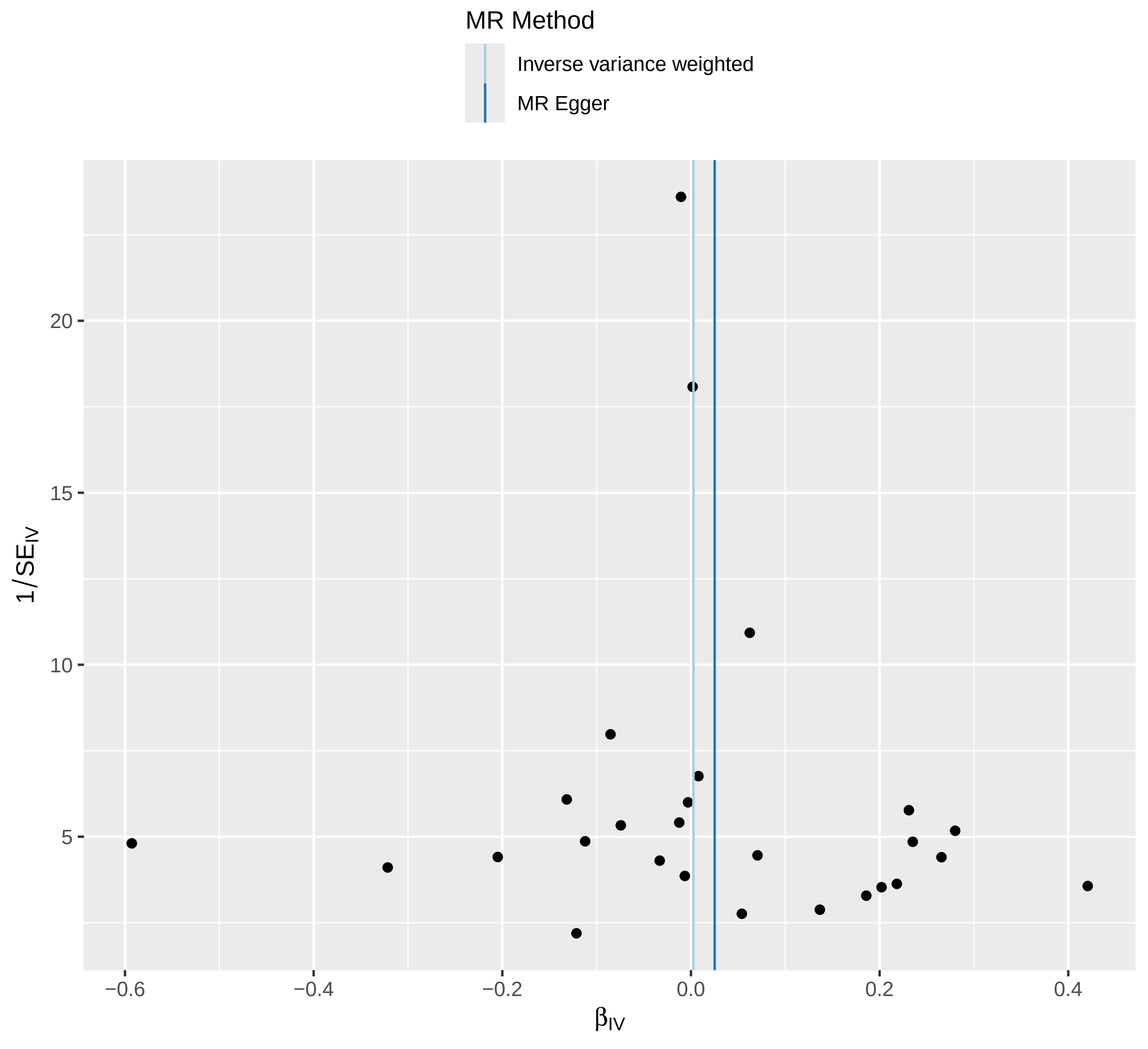
MR Method
Inverse variance weighted
MR Egger
1/SE_IV
20
15
10
5
−0.6
−0.4
−0.2
0.0
0.2
0.4
β_IV

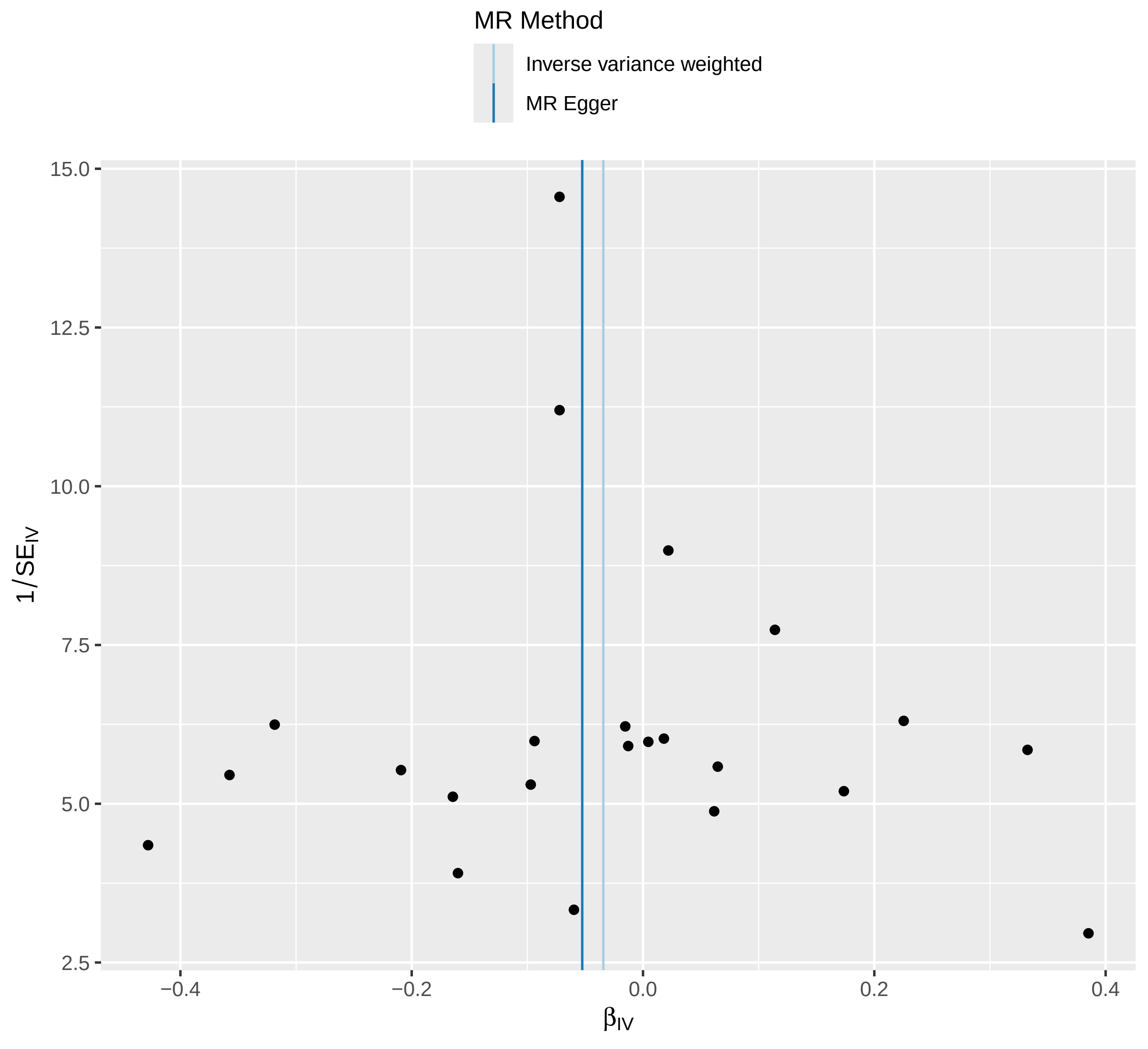
MR Method
Inverse variance weighted
MR Egger
15.0
12.5
10.0
7.5
5.0
2.5
1/SE$_{IV}$
−0.4
−0.2
0.0
0.2
0.4
β$_{IV}$

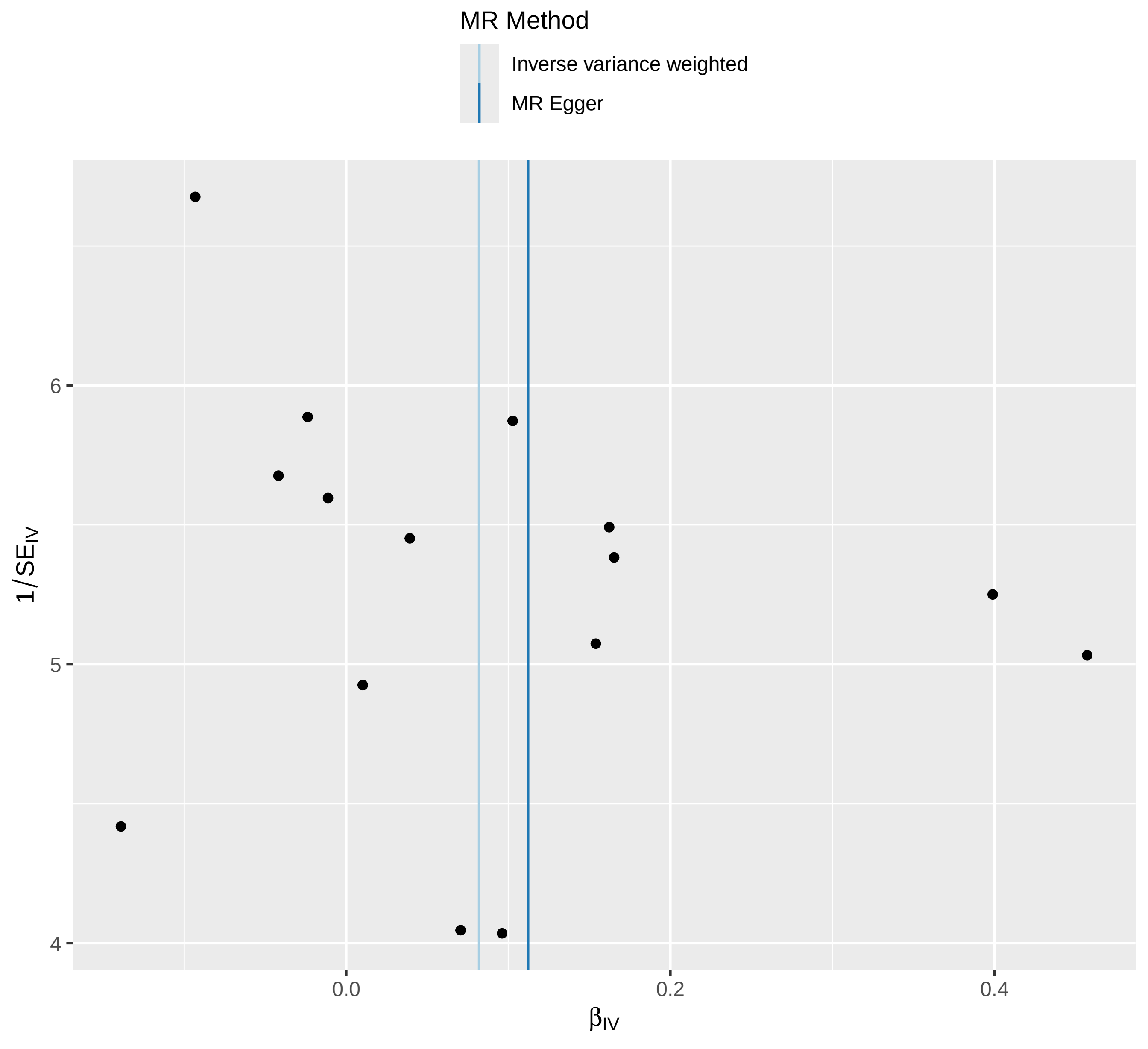

MR Method
Inverse variance weighted
MR Egger
1/SE_IV
6
5
4
0.0
0.2
0.4
β_IV

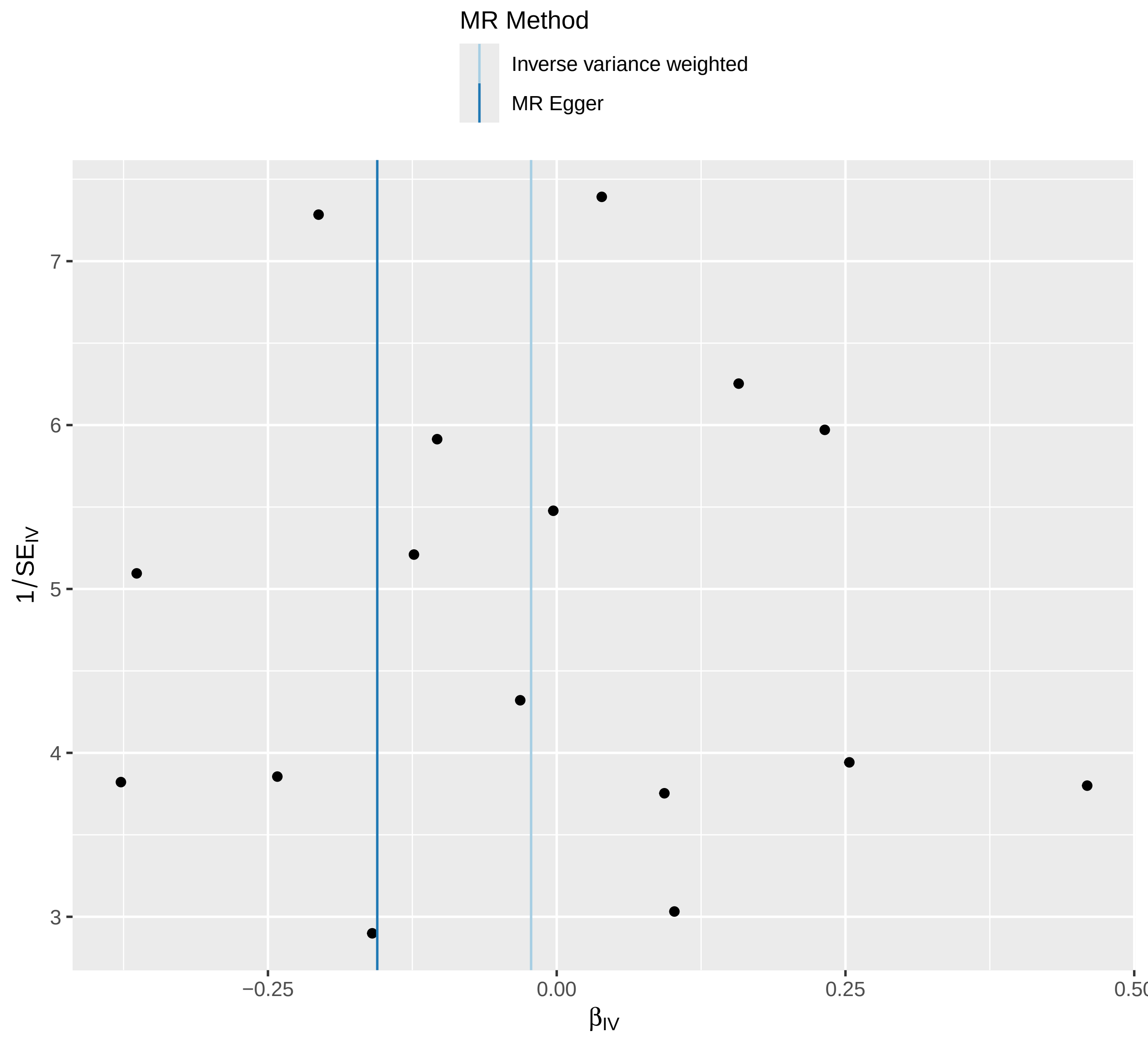
MR Method
Inverse variance weighted
MR Egger
7
6
5
4
3
1/SE$_{IV}$
−0.25
0.00
0.25
0.50
β$_{IV}$

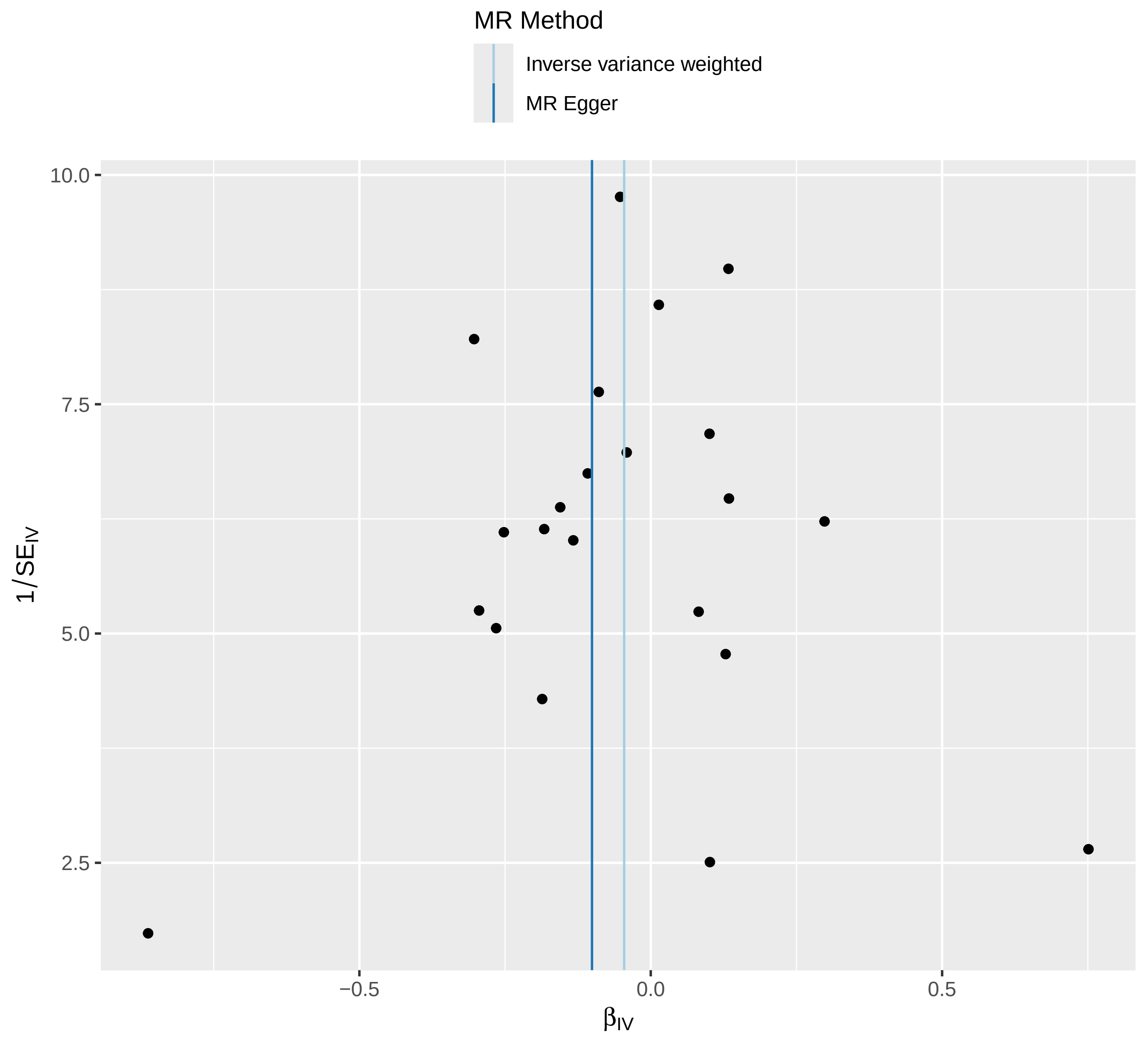
MR Method
Inverse variance weighted
MR Egger
10.0
7.5
5.0
2.5
1/SE_IV
−0.5
0.0
0.5
β_IV

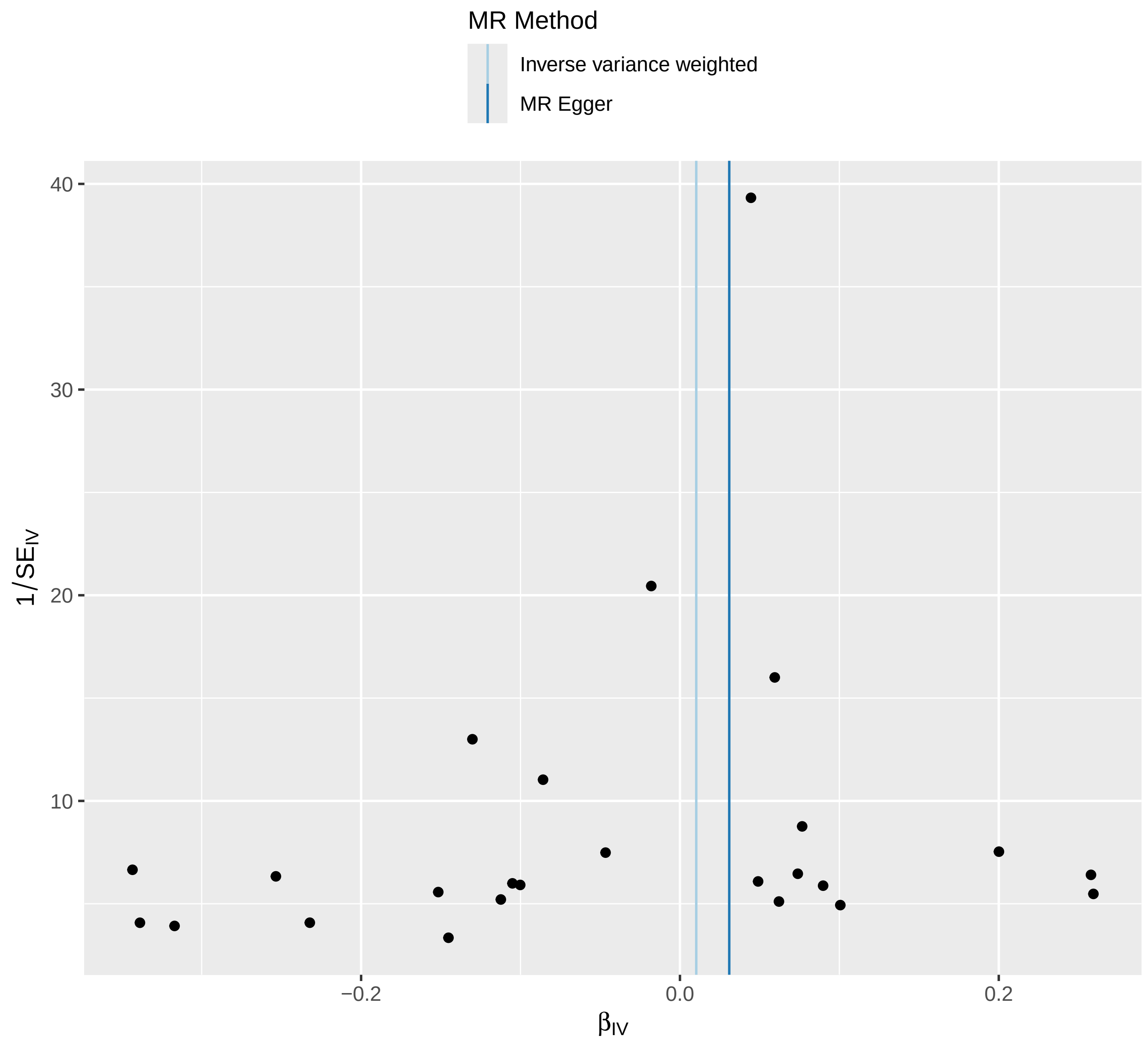

MR Method
Inverse variance weighted
MR Egger
40
30
20
10
1/SE_IV
−0.2
0.0
0.2
β_IV

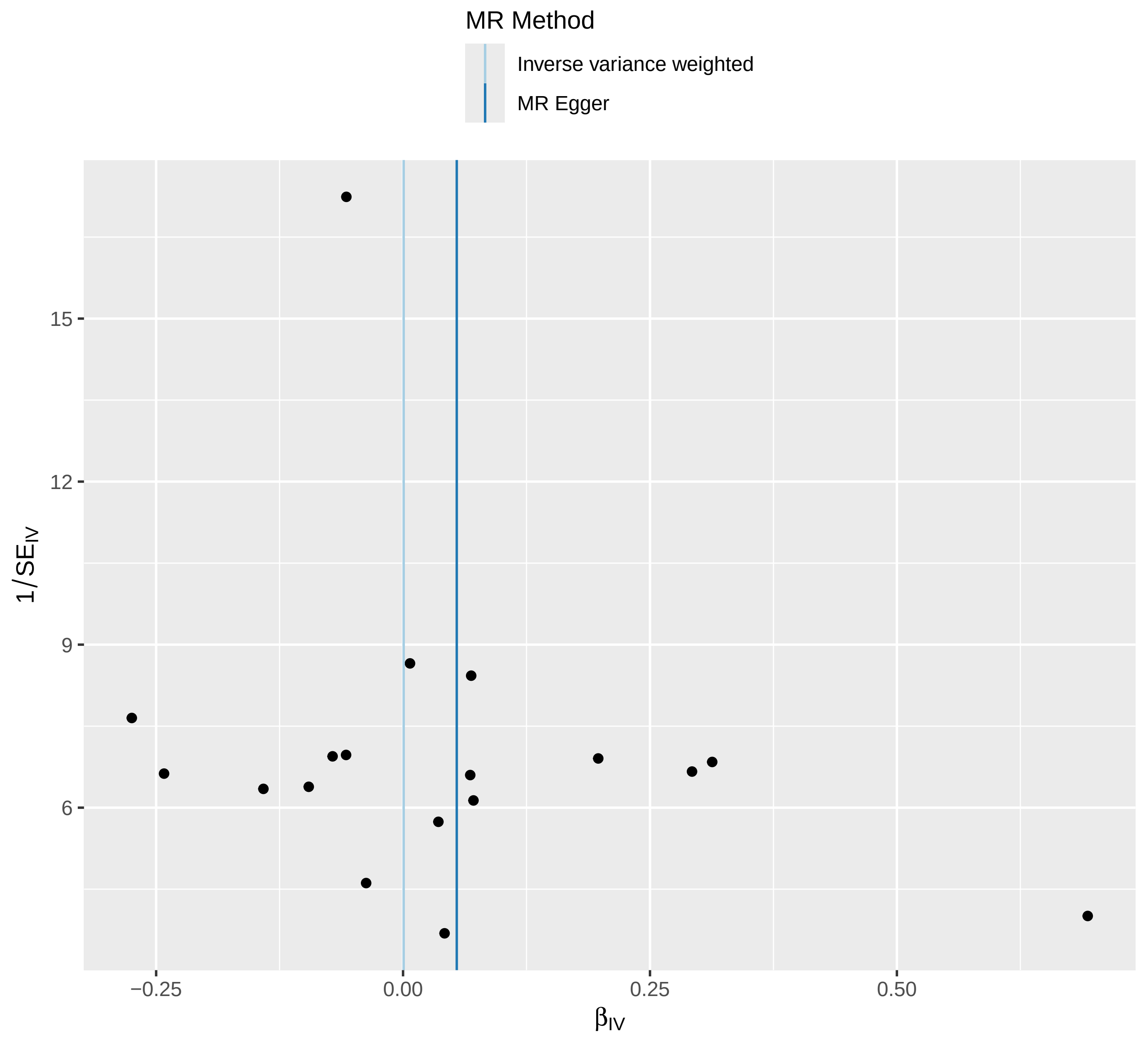
MR Method
Inverse variance weighted
MR Egger
15
12
9
6
$1/SE_{IV}$
−0.25
0.00
0.25
0.50
β_{IV}

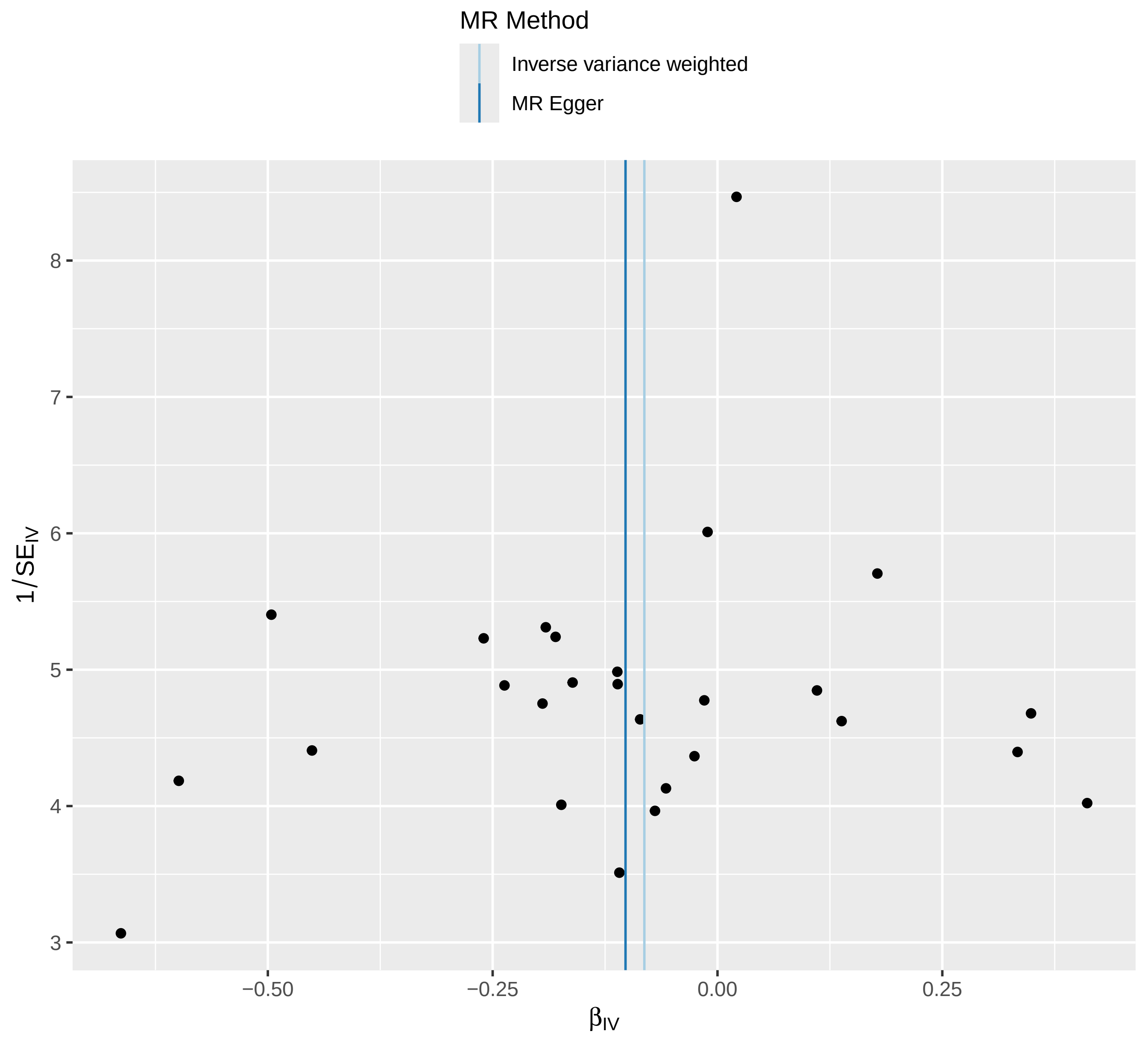

MR Method
Inverse variance weighted
MR Egger
8
7
6
5
4
3
1/SE$_{IV}$
−0.50
−0.25
0.00
0.25
β$_{IV}$

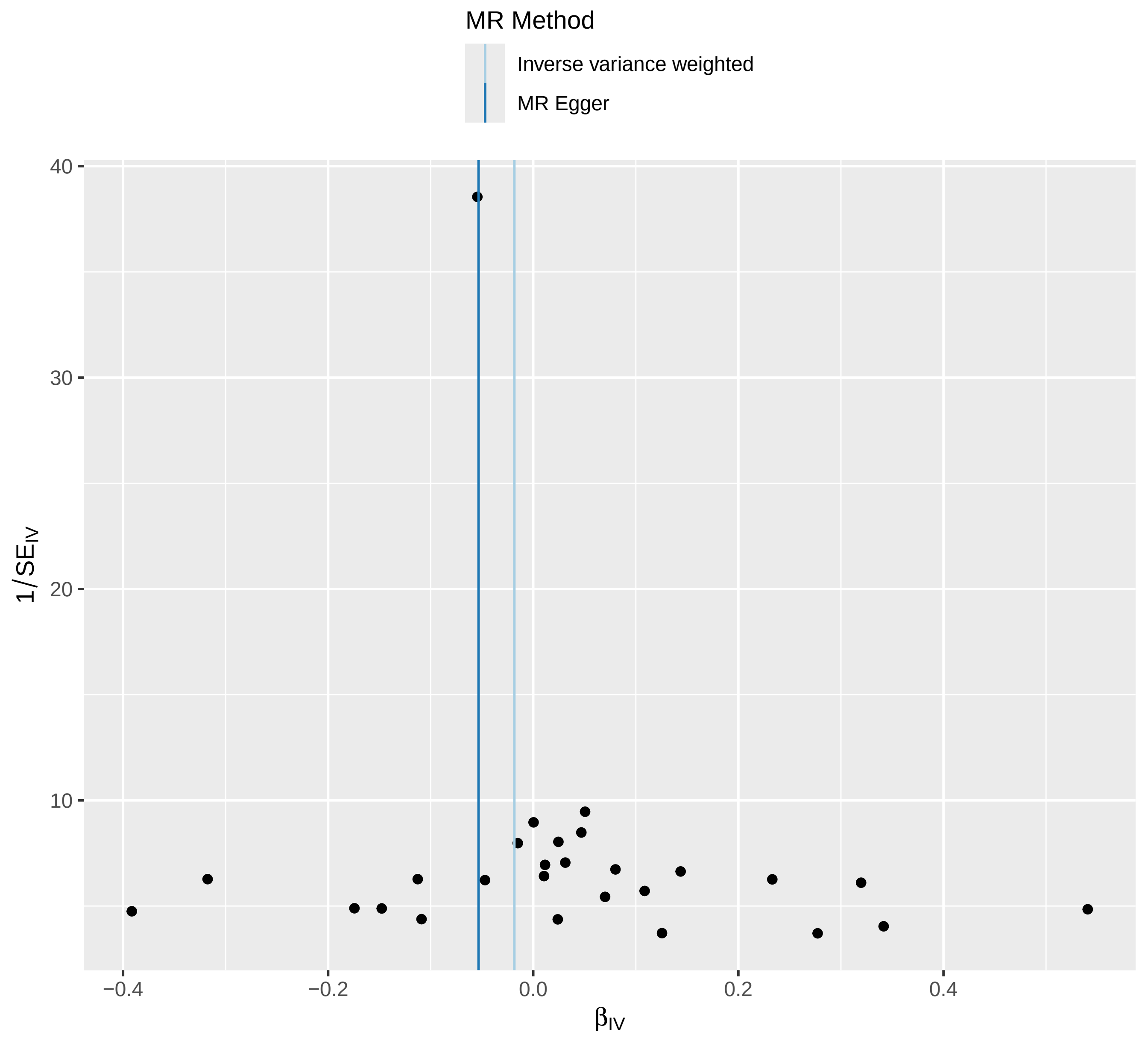
MR Method
Inverse variance weighted
MR Egger
40
30
20
10
1/SE_IV
−0.4
−0.2
0.0
0.2
0.4
β_IV

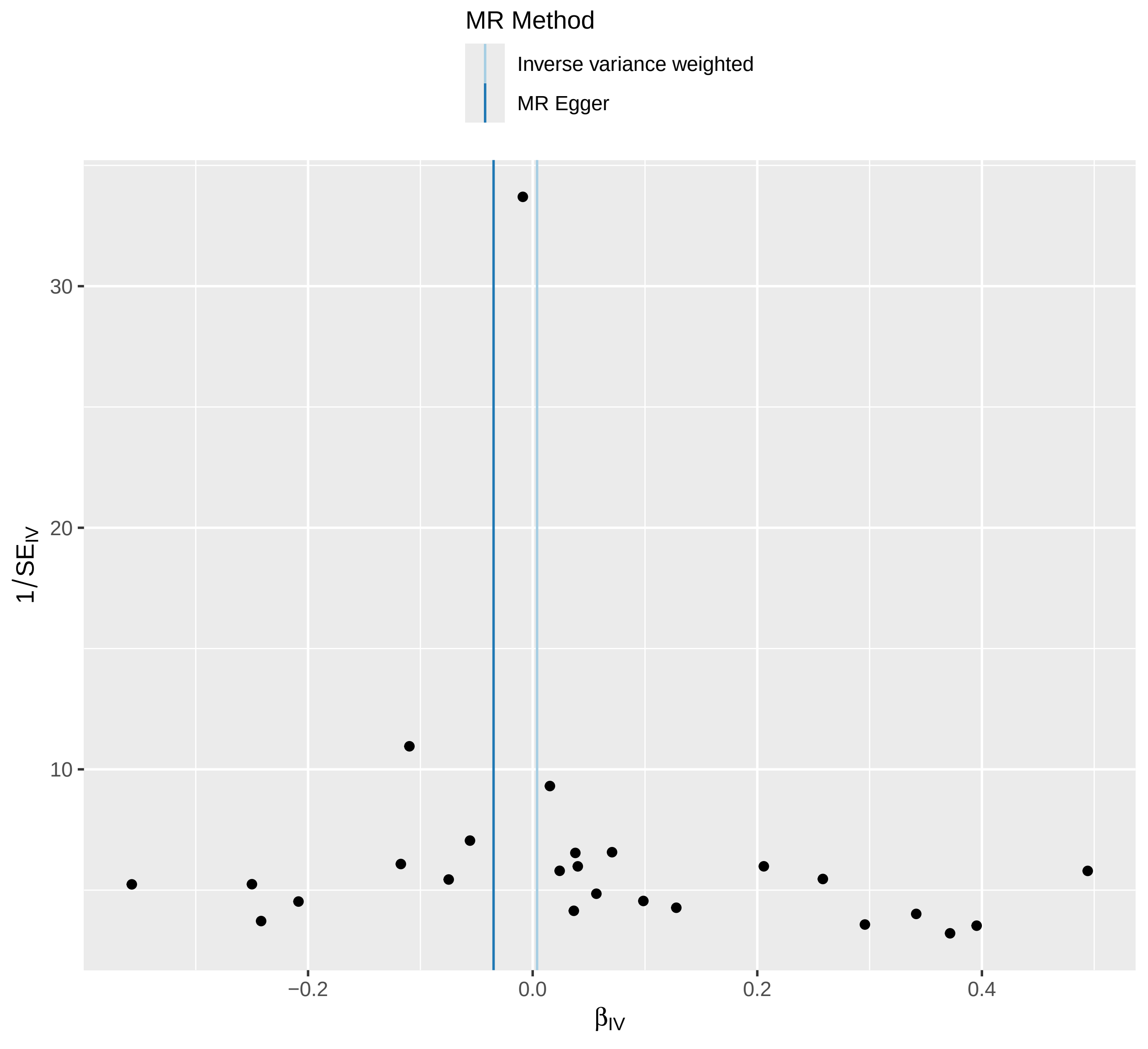
MR Method
Inverse variance weighted
MR Egger
30
20
10
1/SE_IV
−0.2
0.0
0.2
0.4
β_IV

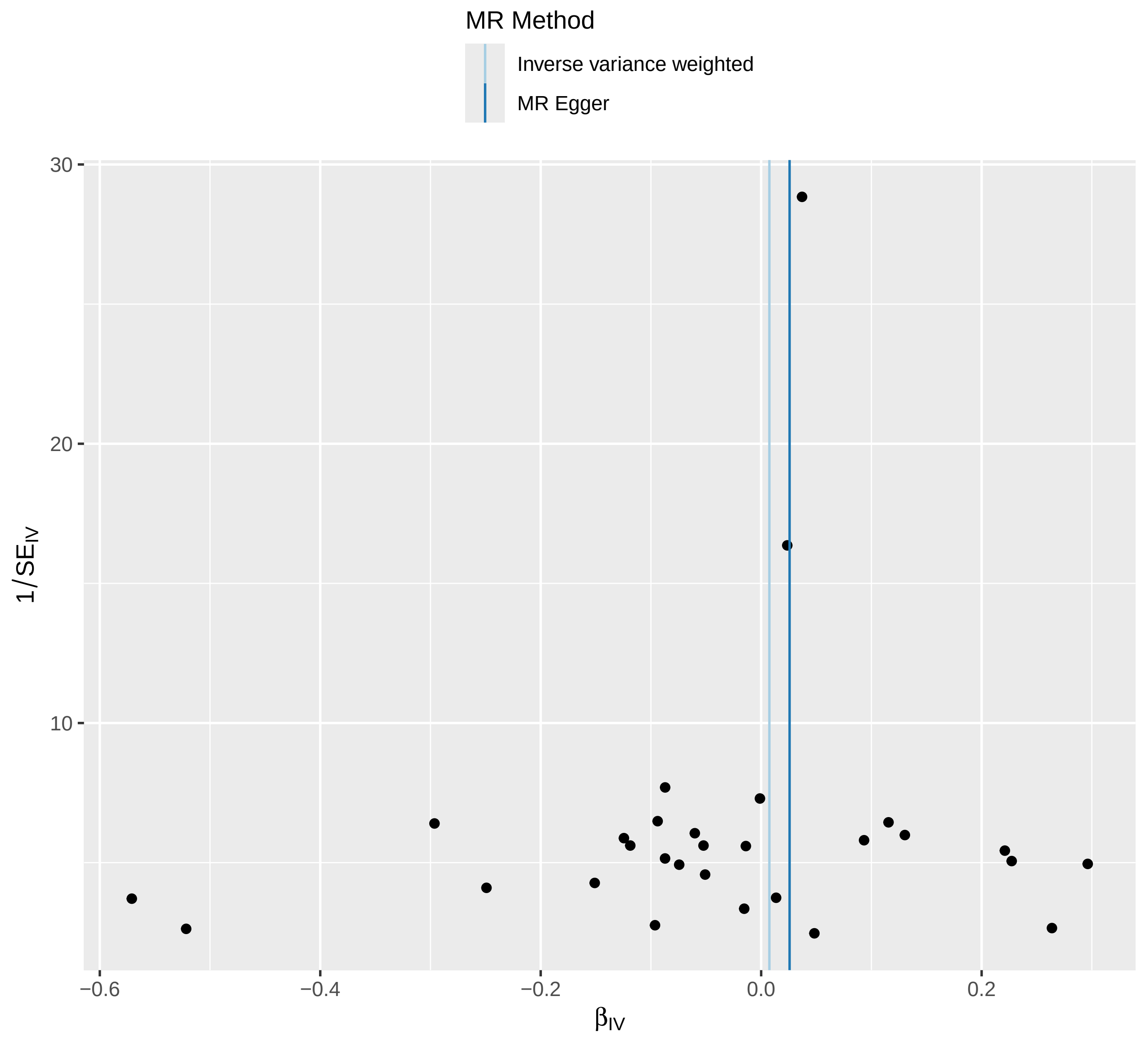
MR Method
Inverse variance weighted
MR Egger
30
20
10
1/SE_IV
−0.6
−0.4
−0.2
0.0
0.2
β_IV

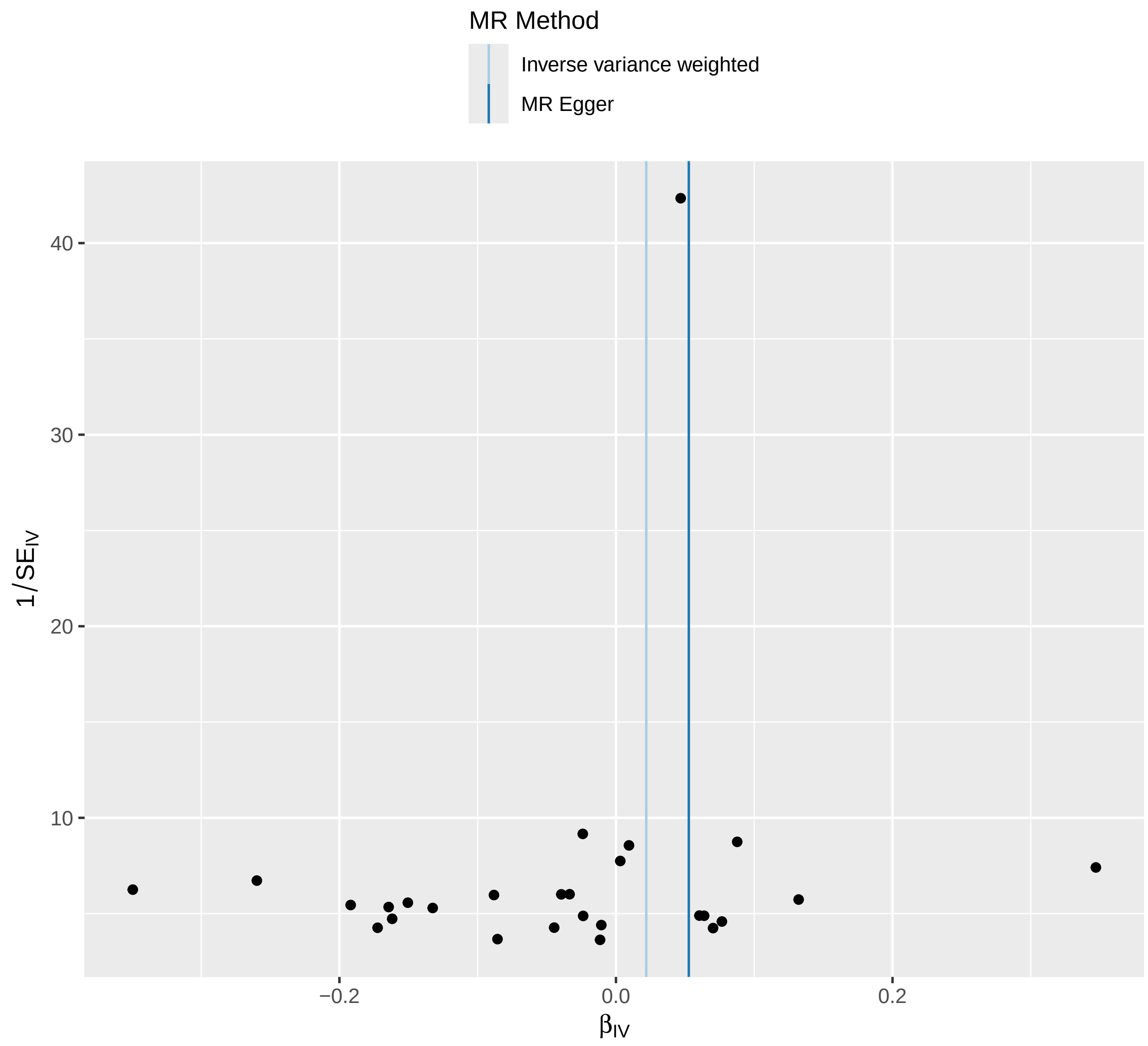

MR Method
Inverse variance weighted
MR Egger
40
30
20
10
$1/SE_{IV}$
−0.2
0.0
0.2
β_{IV}

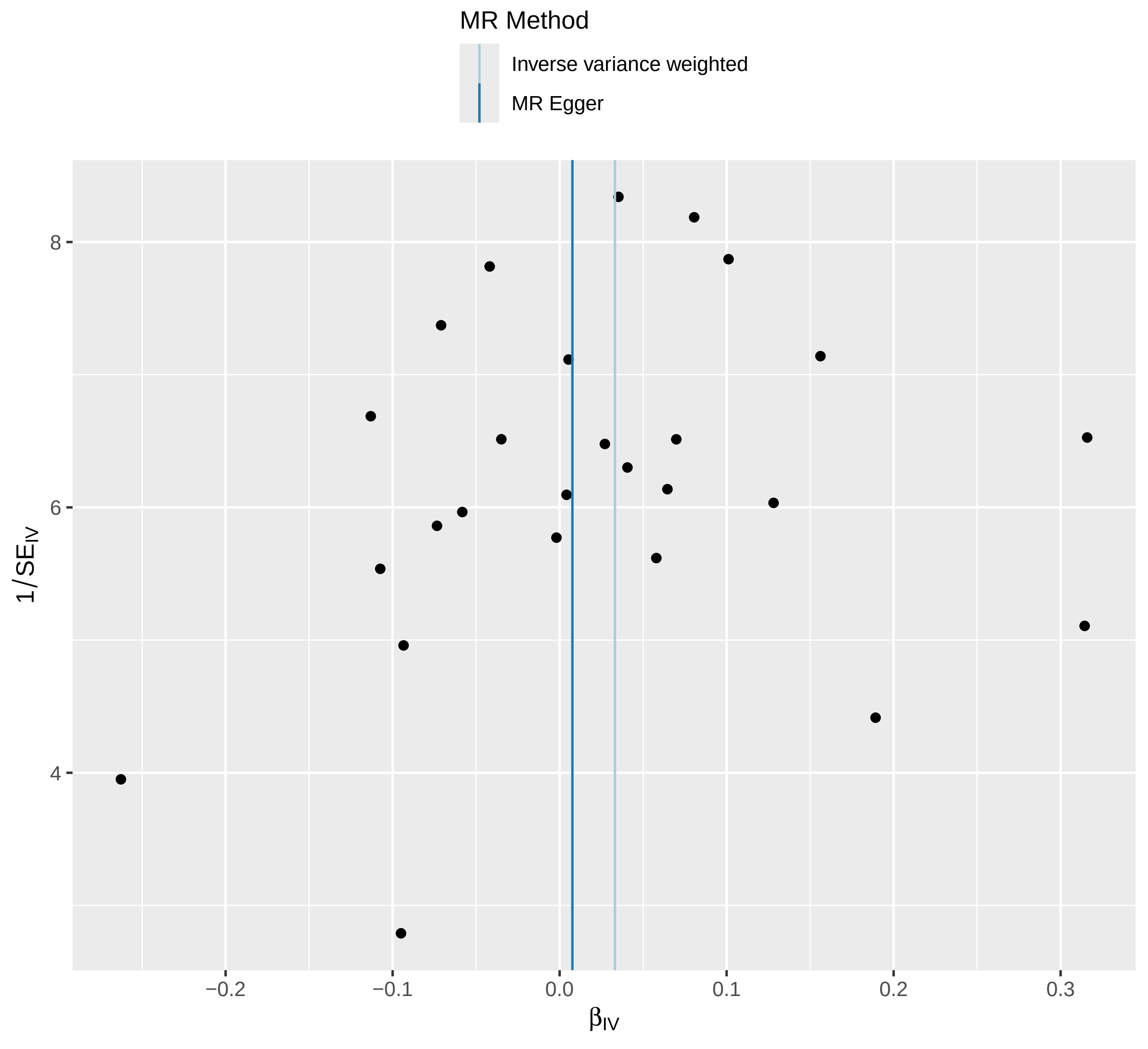

MR Method
Inverse variance weighted
MR Egger
8
6
4
1/SE_IV
−0.2
−0.1
0.0
0.1
0.2
0.3
β_IV

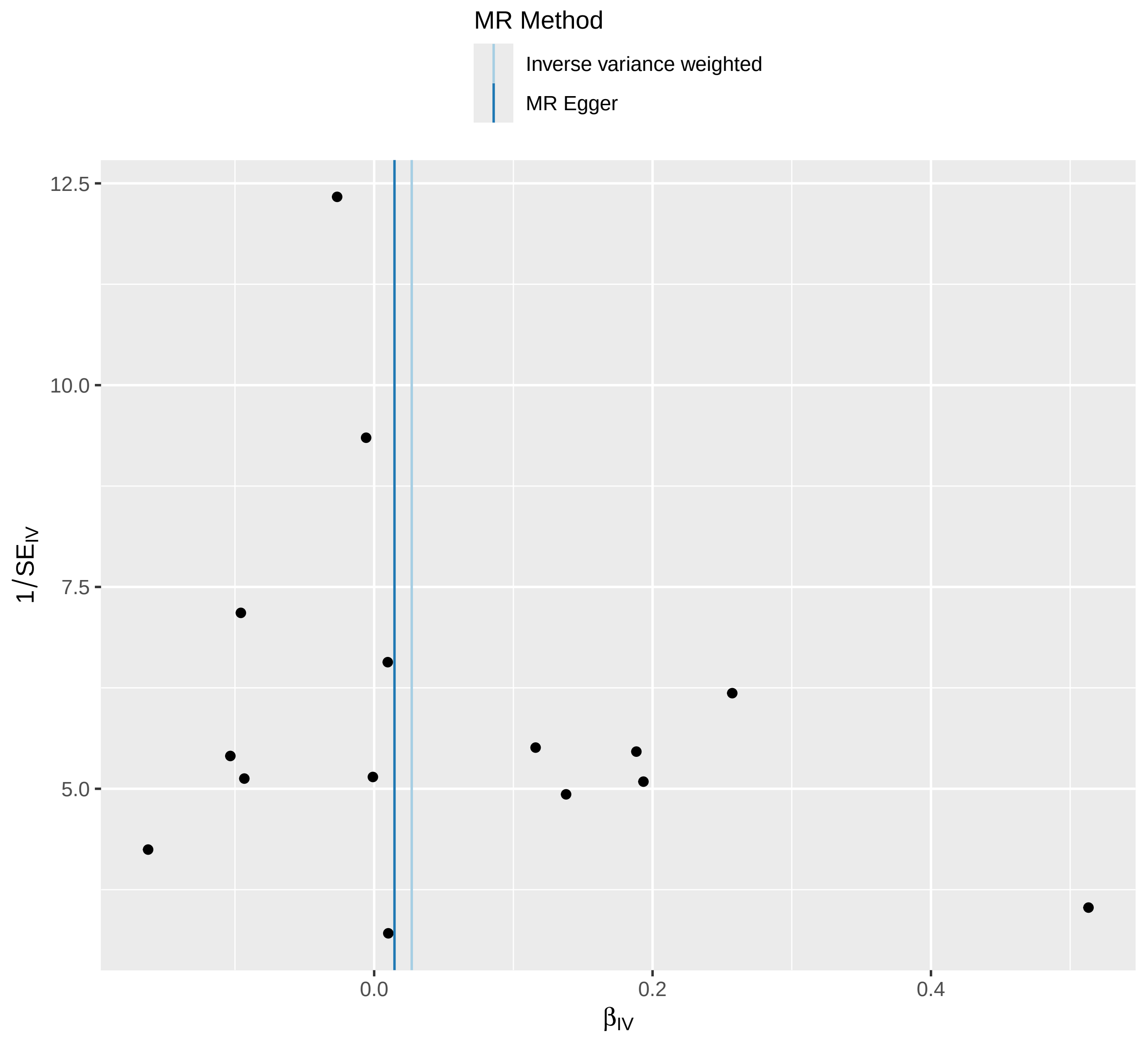
MR Method
Inverse variance weighted
MR Egger
12.5
10.0
7.5
5.0
1/SE_IV
0.0
0.2
0.4
β_IV

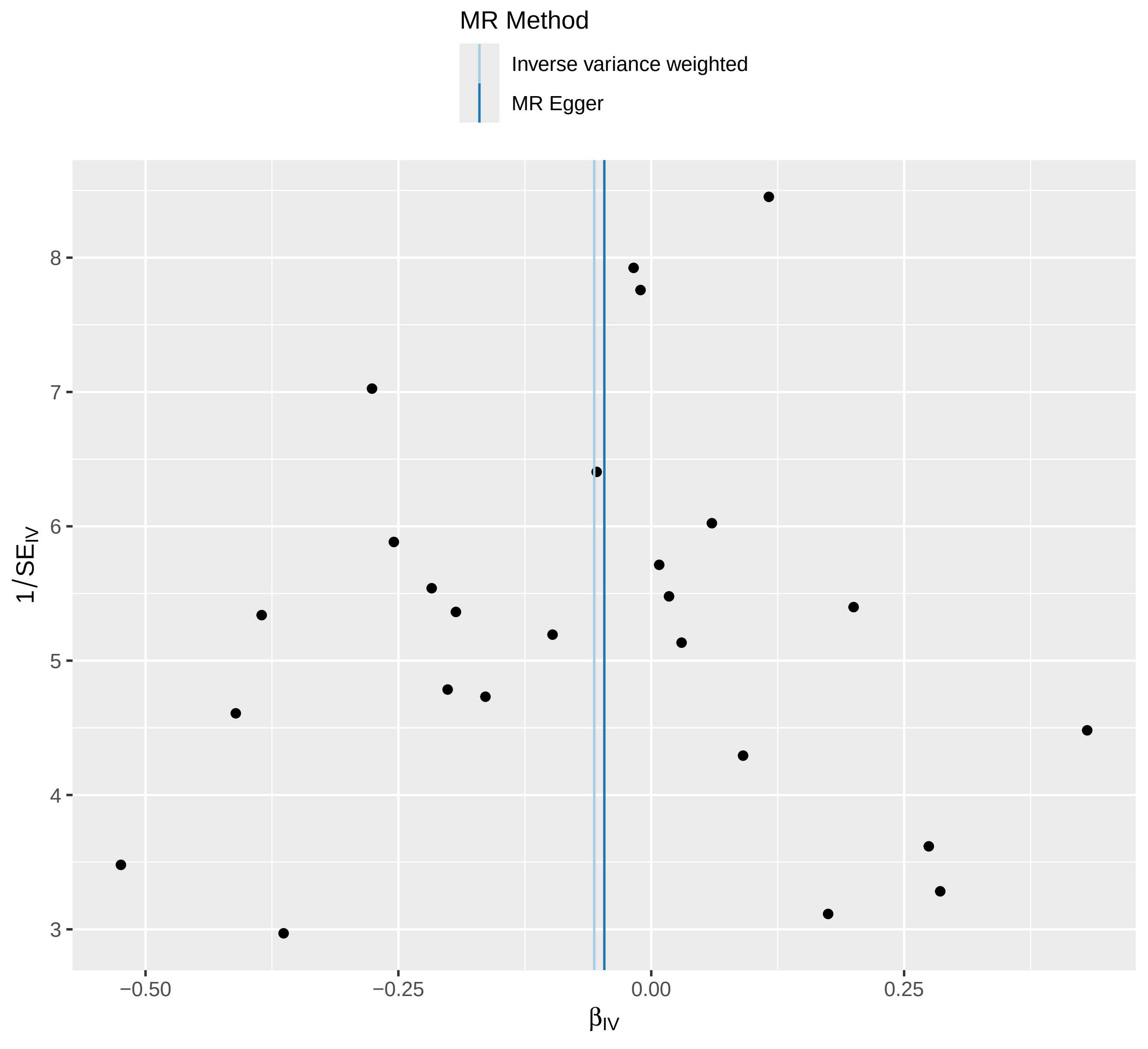
MR Method
Inverse variance weighted
MR Egger
8
7
6
5
4
3
$1/SE_{IV}$
−0.50
−0.25
0.00
0.25
β_{IV}

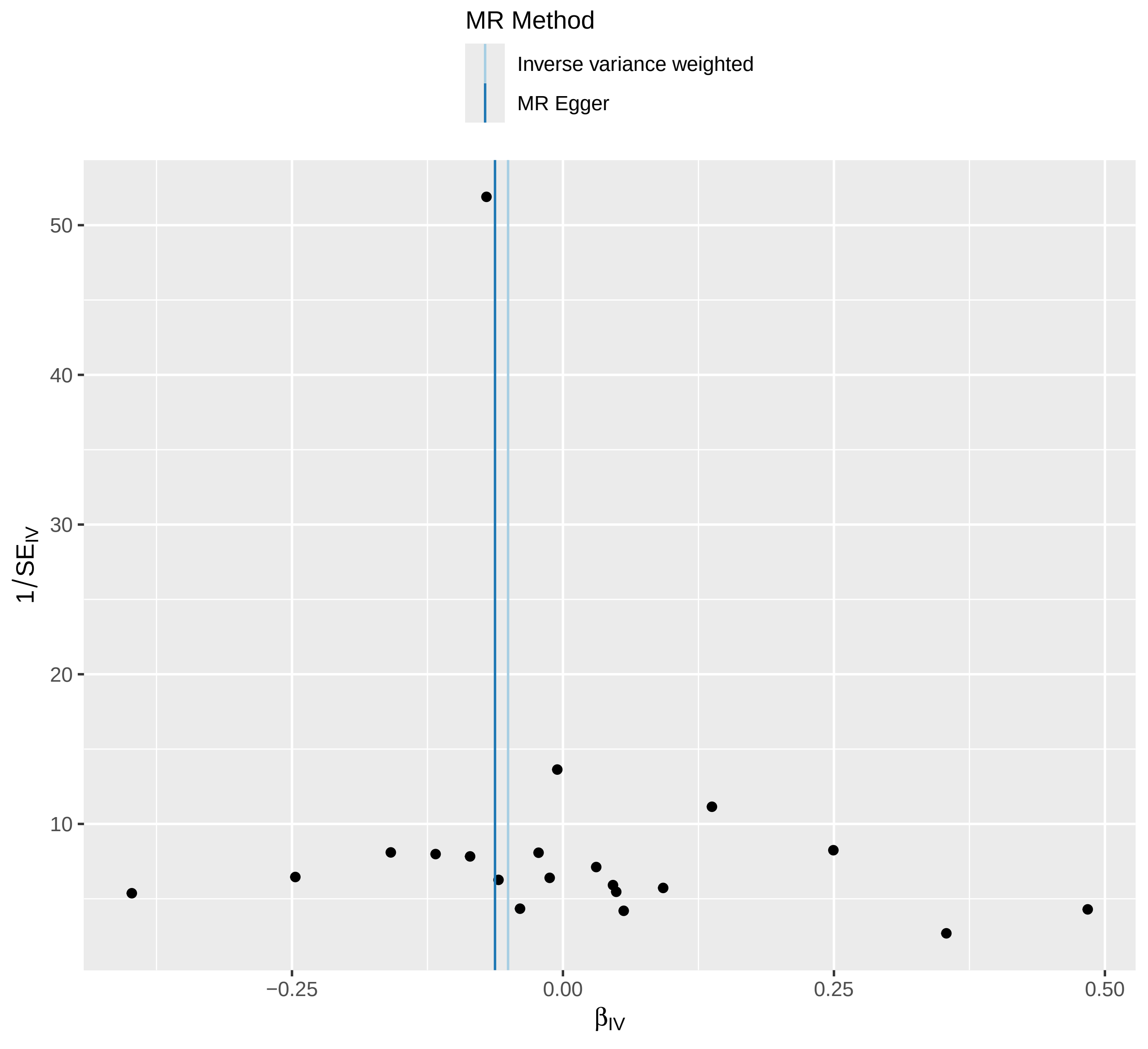

MR Method
Inverse variance weighted
MR Egger
50
40
30
20
10
1/SE_IV
−0.25
0.00
0.25
0.50
β_IV

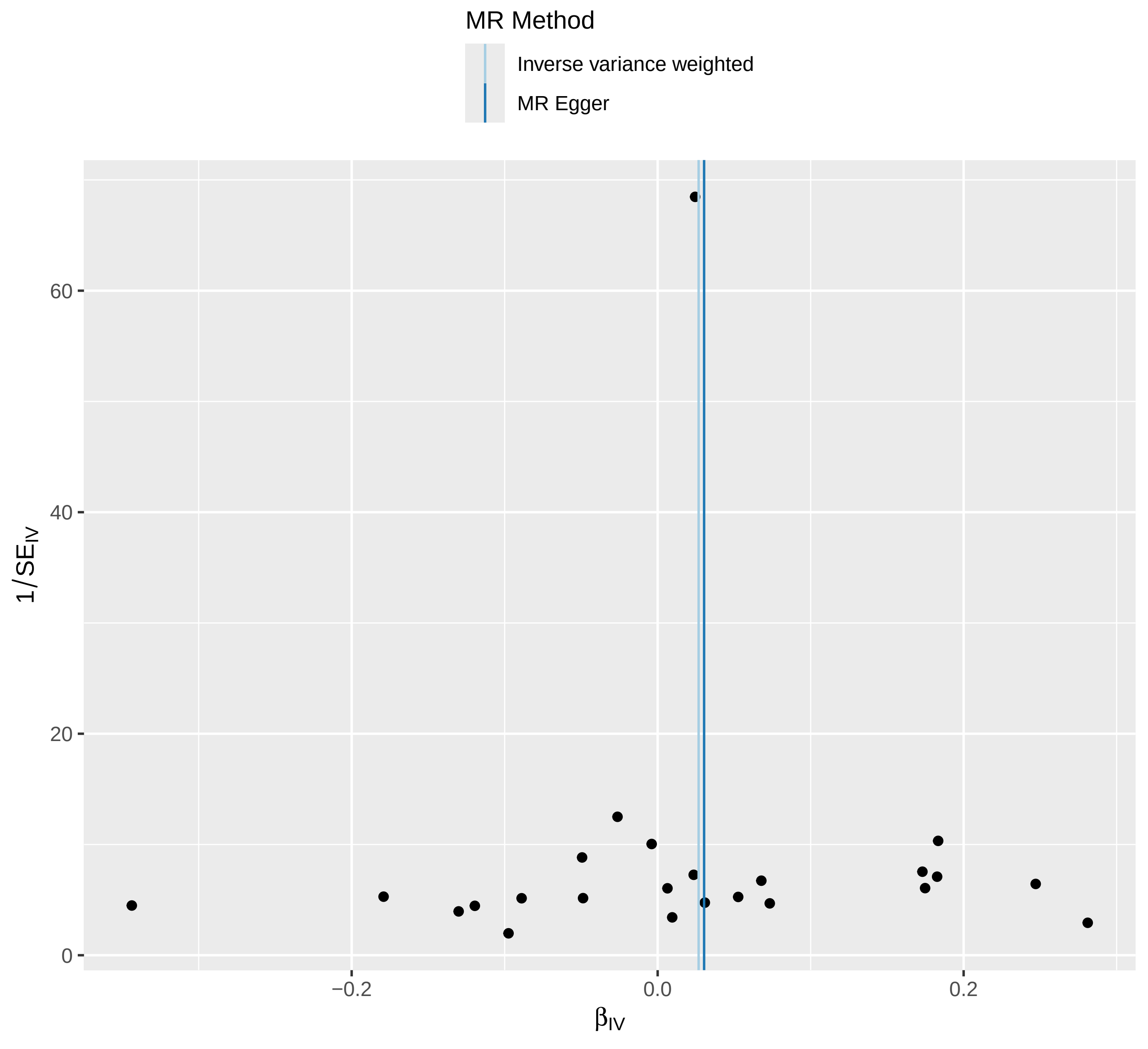
MR Method
Inverse variance weighted
MR Egger
60
40
20
0
1/SE_IV
−0.2
0.0
0.2
β_IV

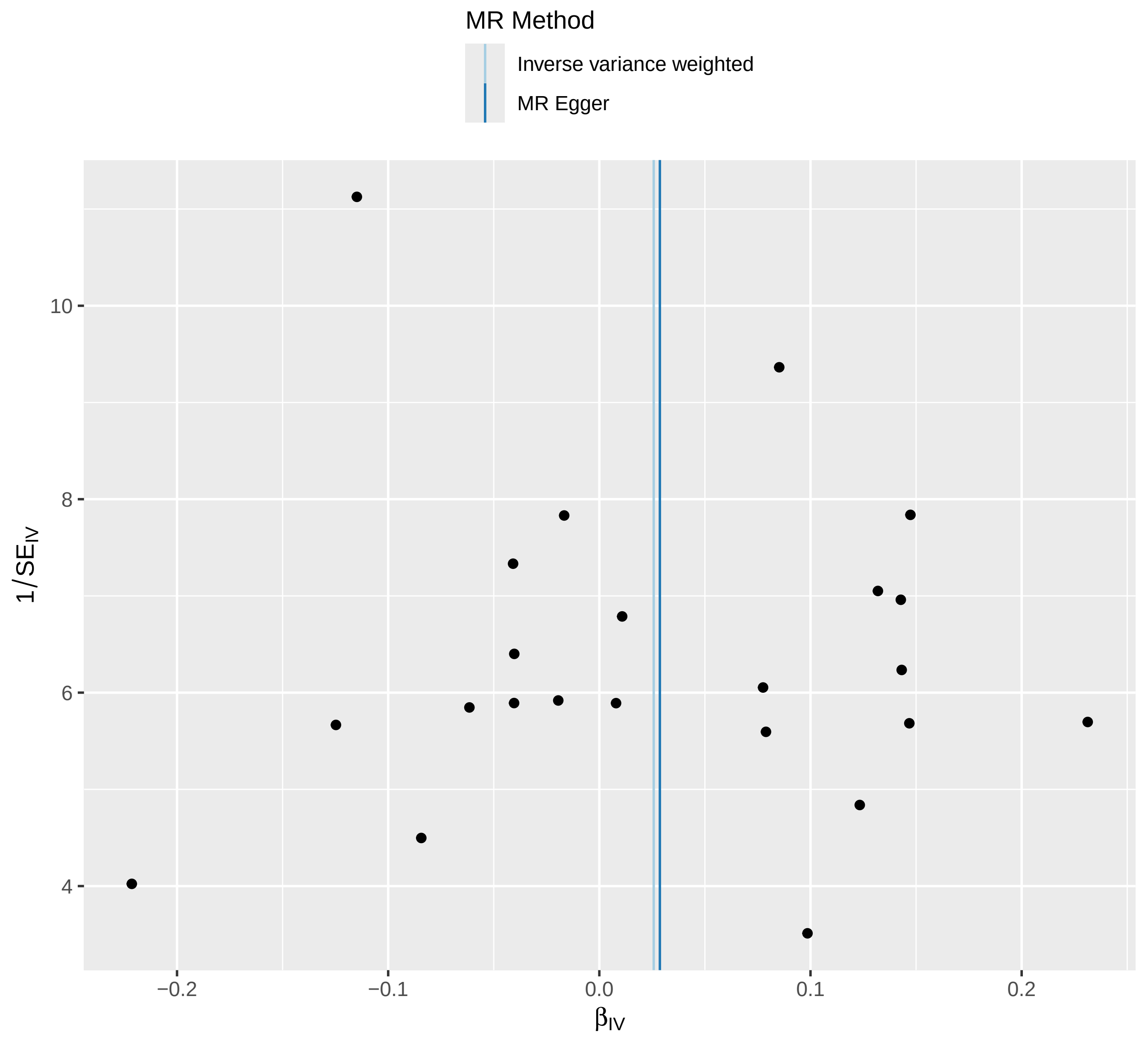

MR Method
Inverse variance weighted
MR Egger
10
8
6
4
1/SE$_{IV}$
−0.2
−0.1
0.0
0.1
0.2
β$_{IV}$

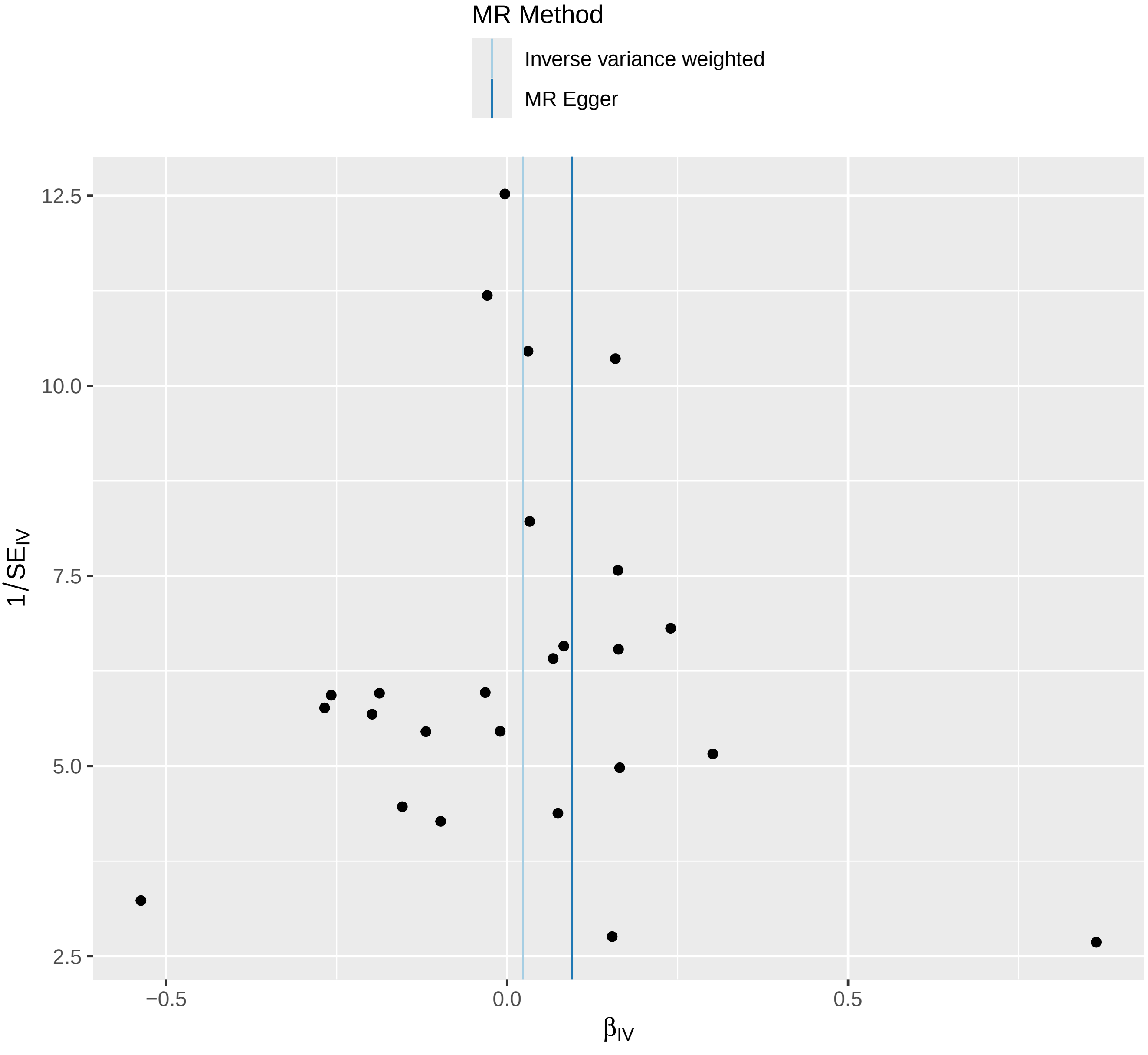
MR Method
Inverse variance weighted
MR Egger
12.5
10.0
7.5
5.0
2.5
$1/SE_{IV}$
−0.5
0.0
0.5
β_{IV}

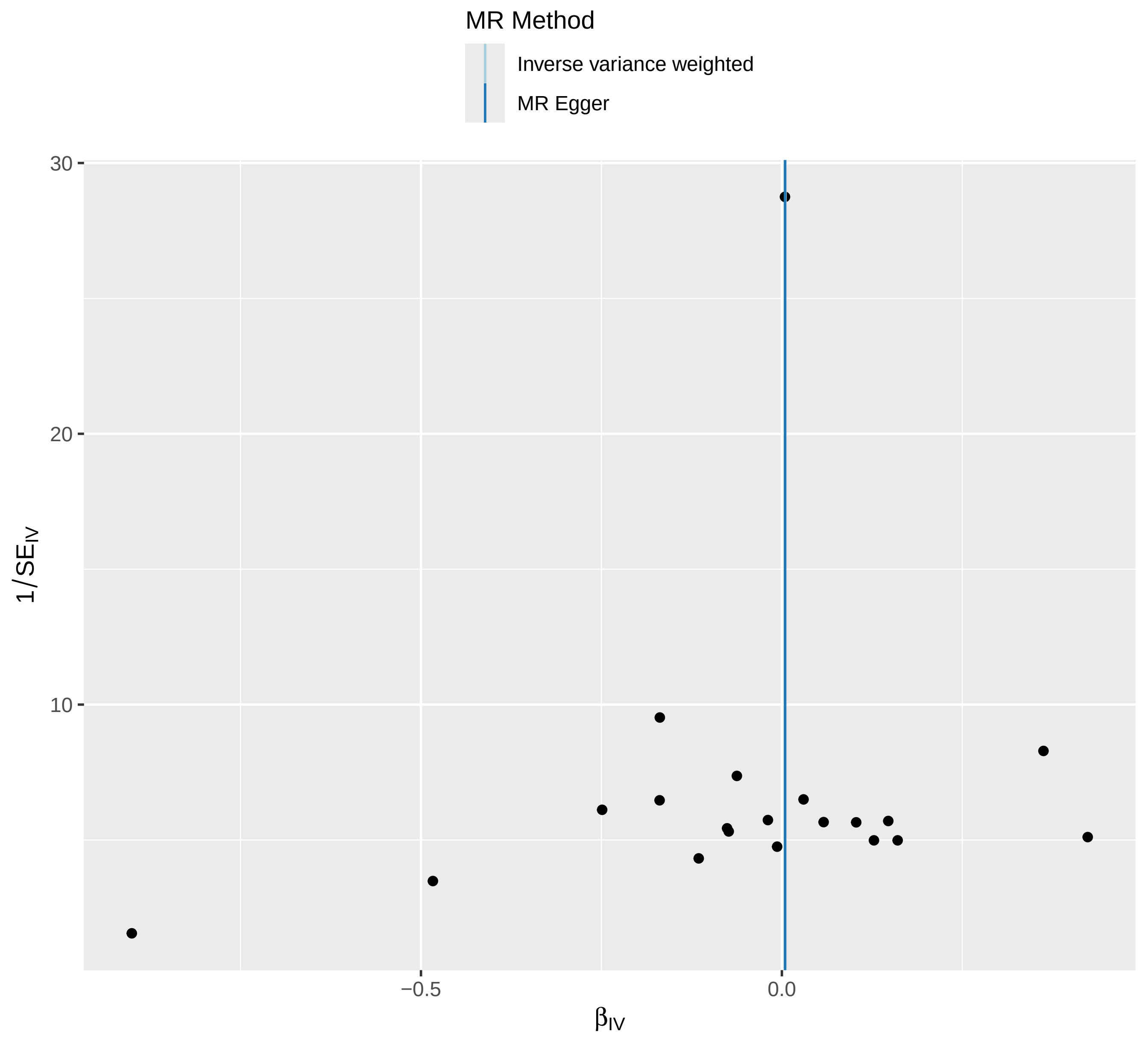
MR Method
Inverse variance weighted
MR Egger
30
20
10
1/SE_IV
−0.5
0.0
β_IV

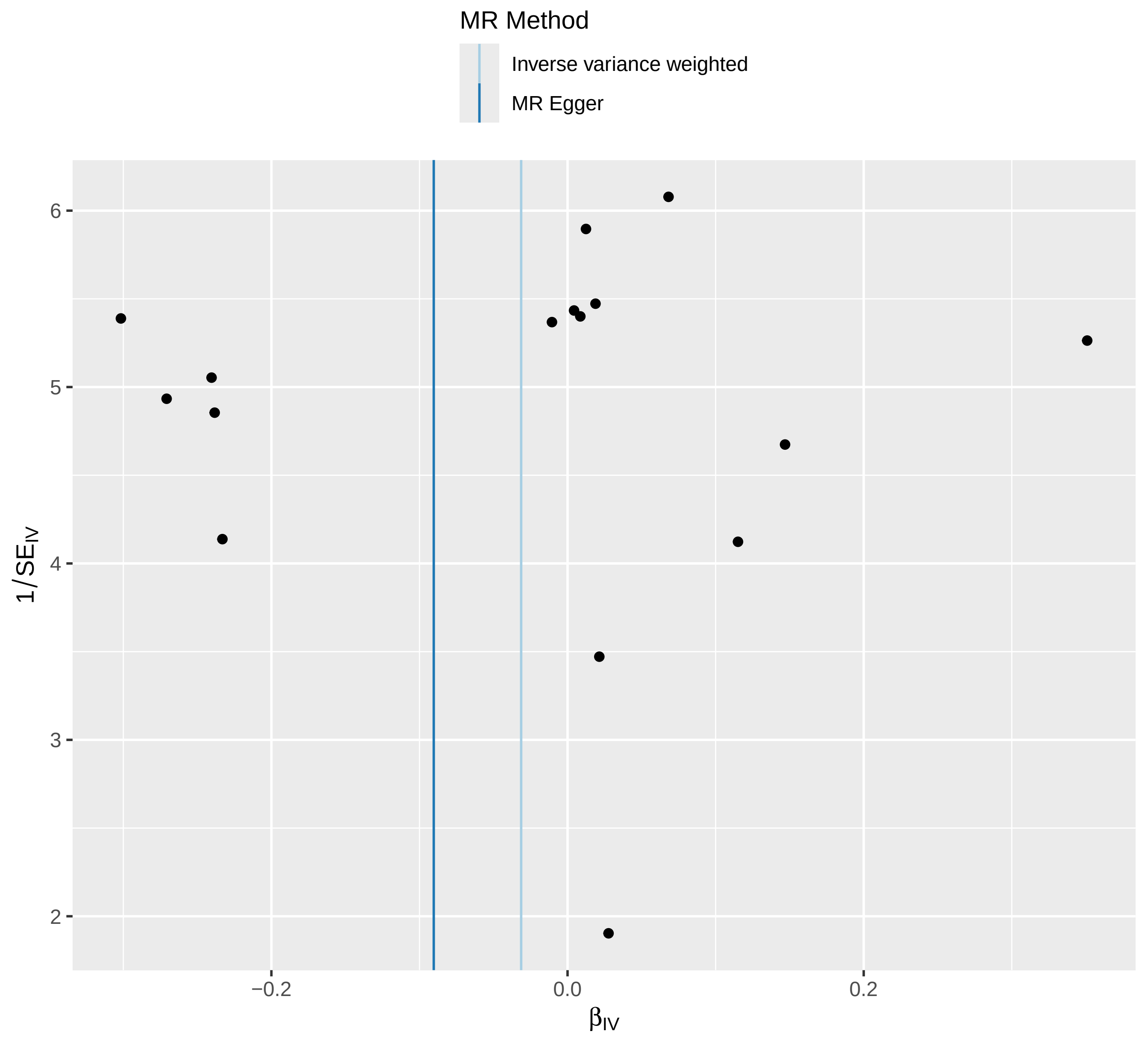

MR Method
Inverse variance weighted
MR Egger
6
5
4
3
2
1/SE_IV
−0.2
0.0
0.2
β_IV

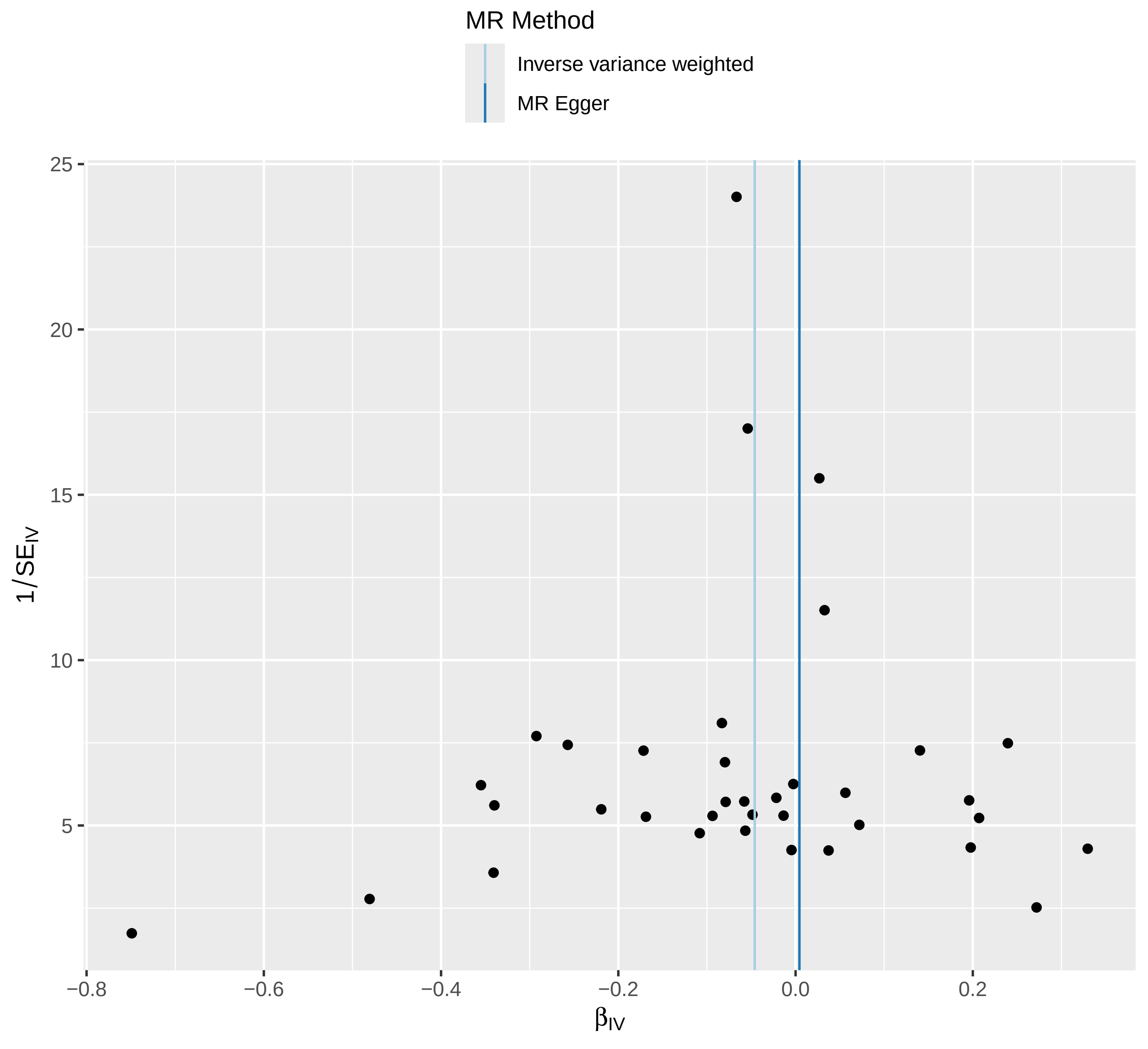
MR Method
Inverse variance weighted
MR Egger
25
20
15
10
5
1/SE_IV
−0.8
−0.6
−0.4
−0.2
0.0
0.2
β_IV

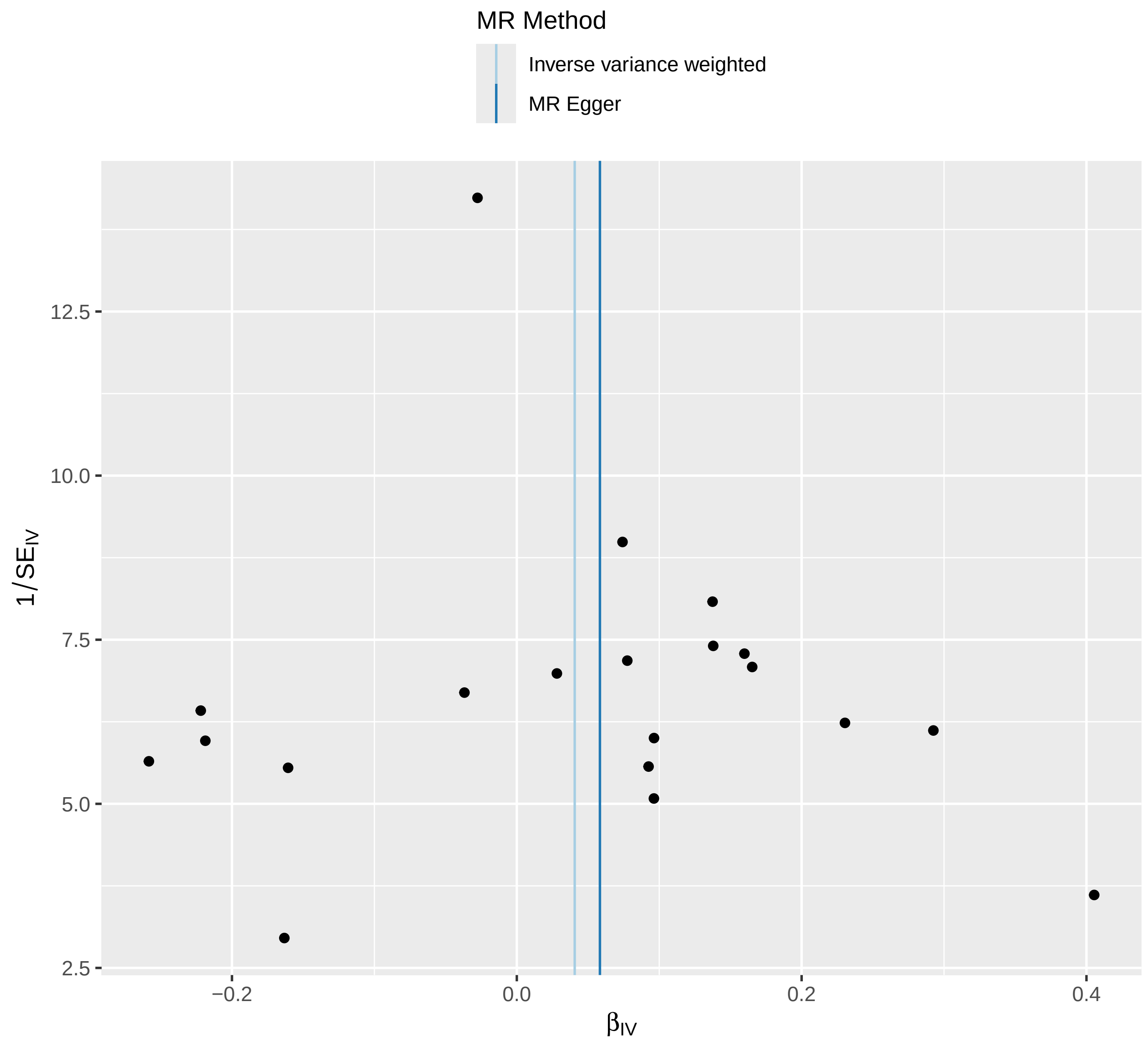
MR Method
Inverse variance weighted
MR Egger
12.5
10.0
7.5
5.0
2.5
−0.2
0.0
0.2
0.4
1/SE_IV
β_IV

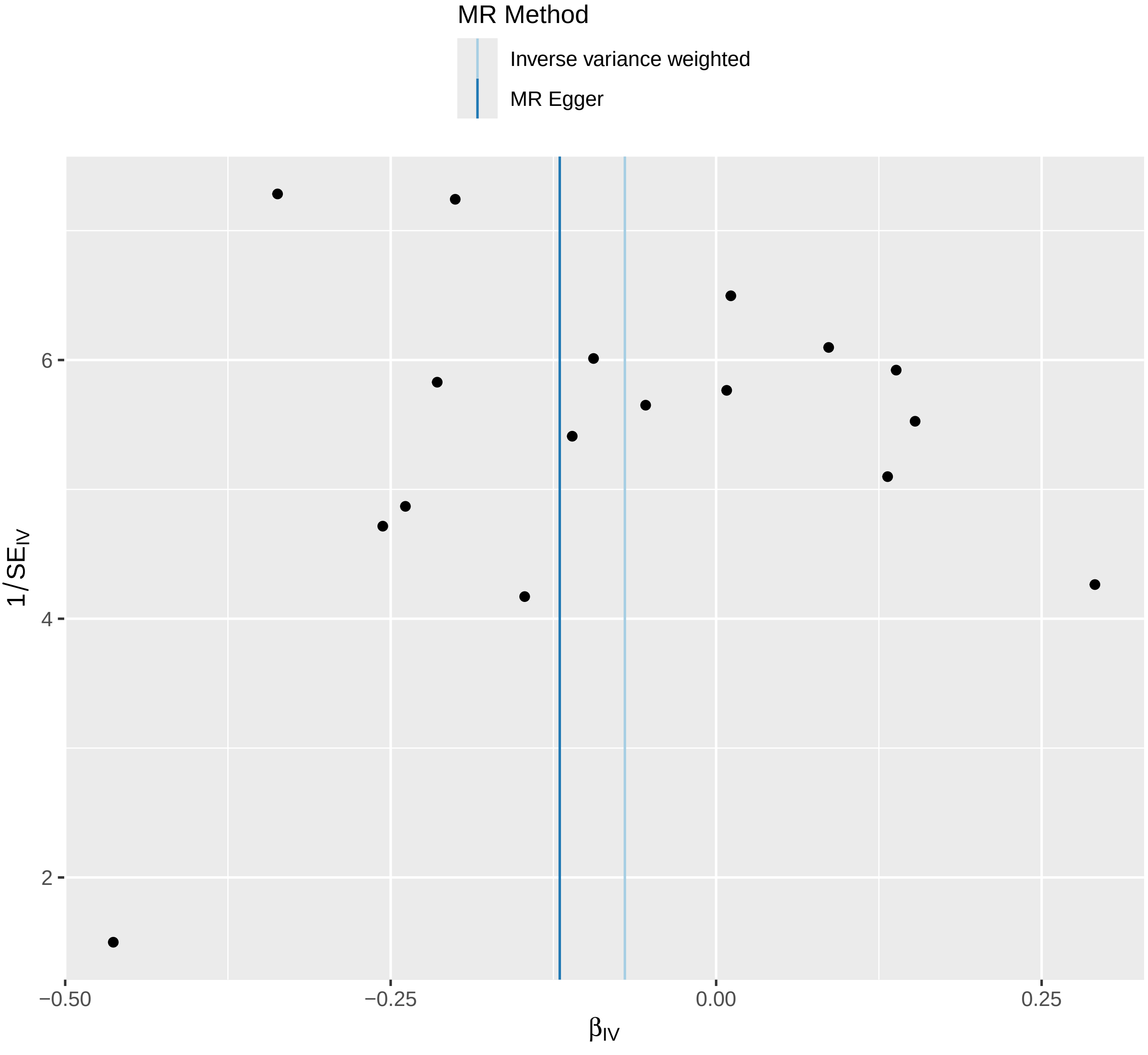

MR Method
Inverse variance weighted
MR Egger
6
4
2
1/SE_IV
−0.50
−0.25
0.00
0.25
β_IV

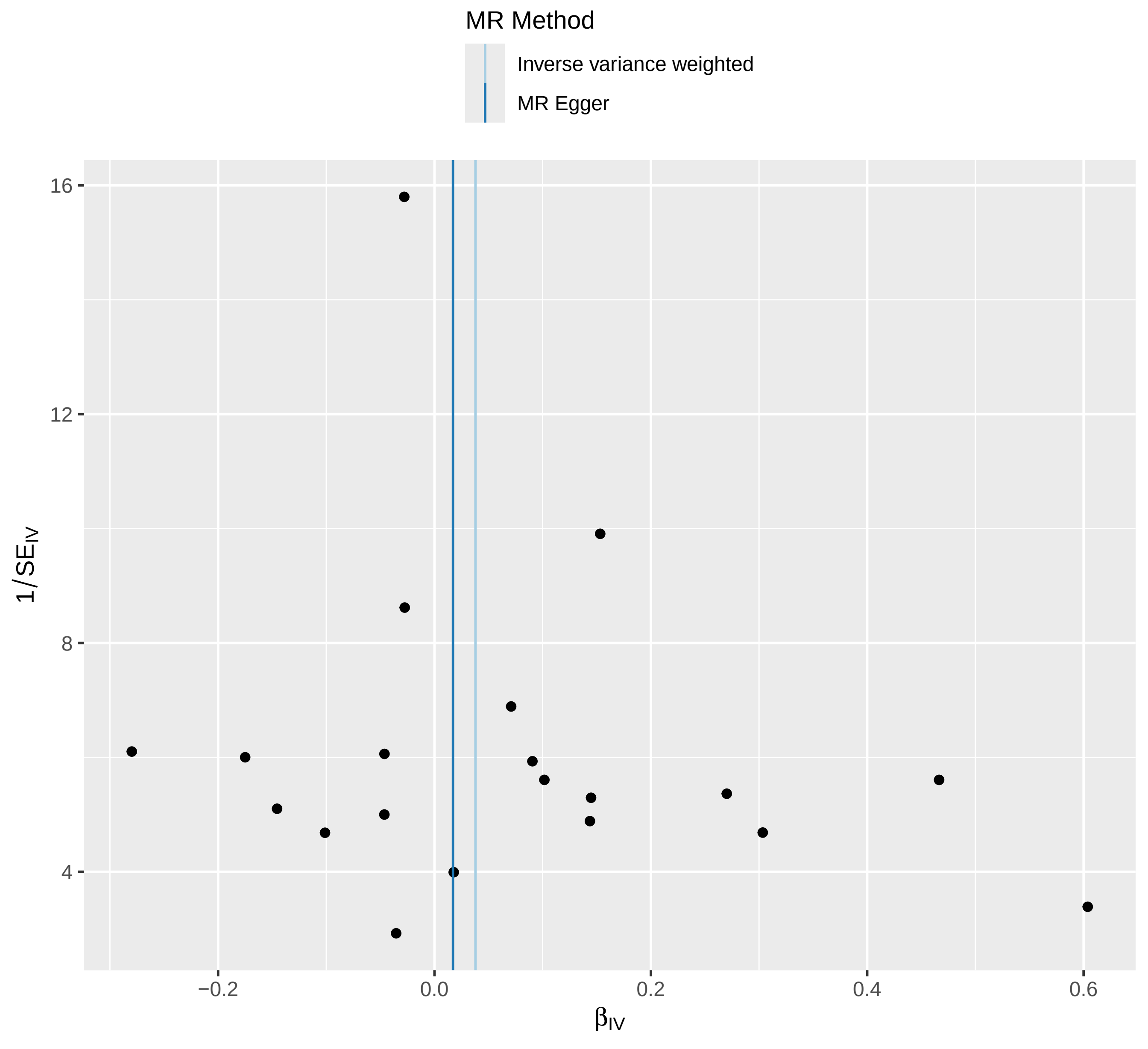

MR Method
Inverse variance weighted
MR Egger
16
12
8
4
1/SE_IV
−0.2
0.0
0.2
0.4
0.6
β_IV

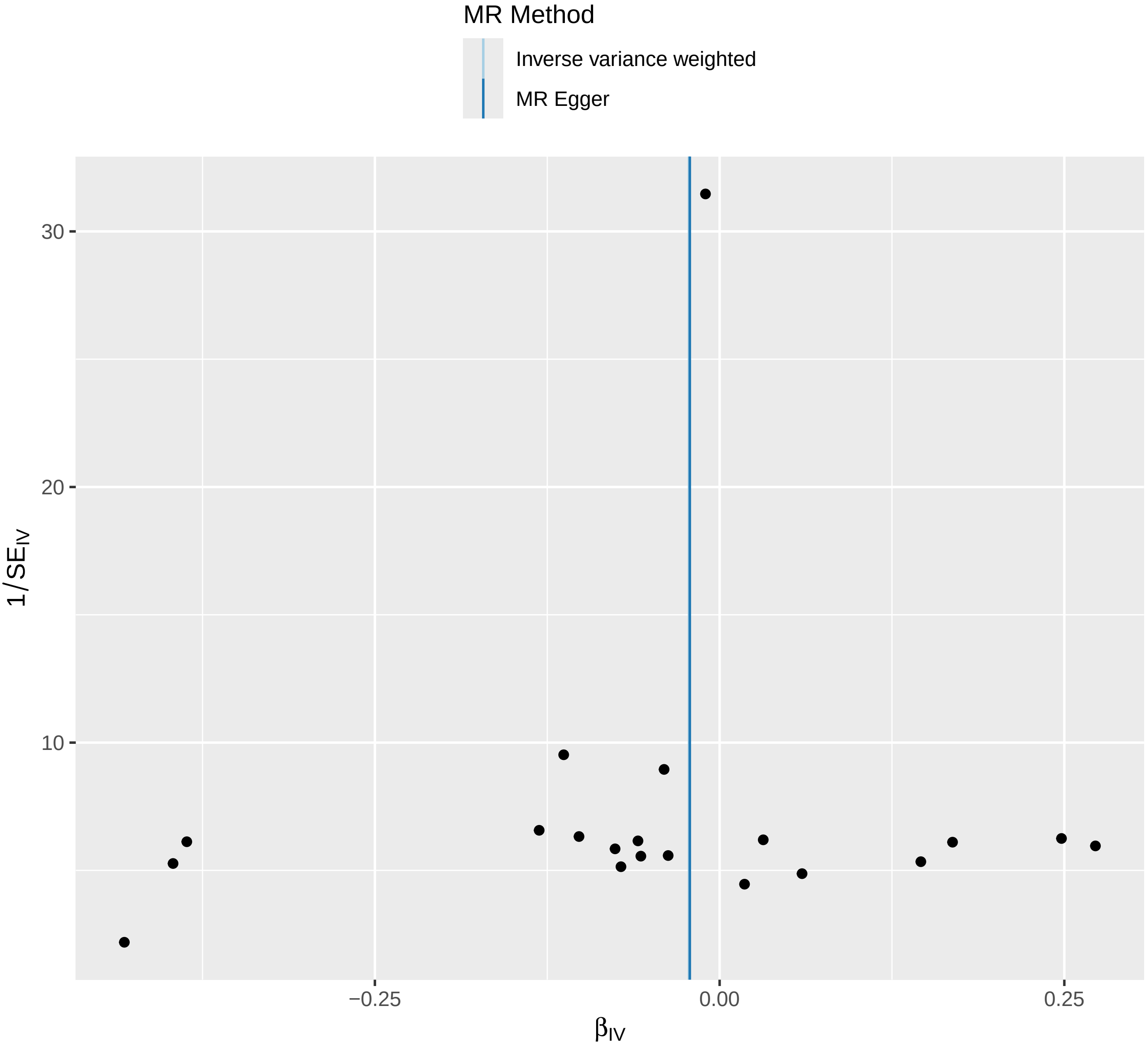
MR Method
Inverse variance weighted
MR Egger
30
20
10
1/SE$_{IV}$
−0.25
0.00
0.25
β$_{IV}$

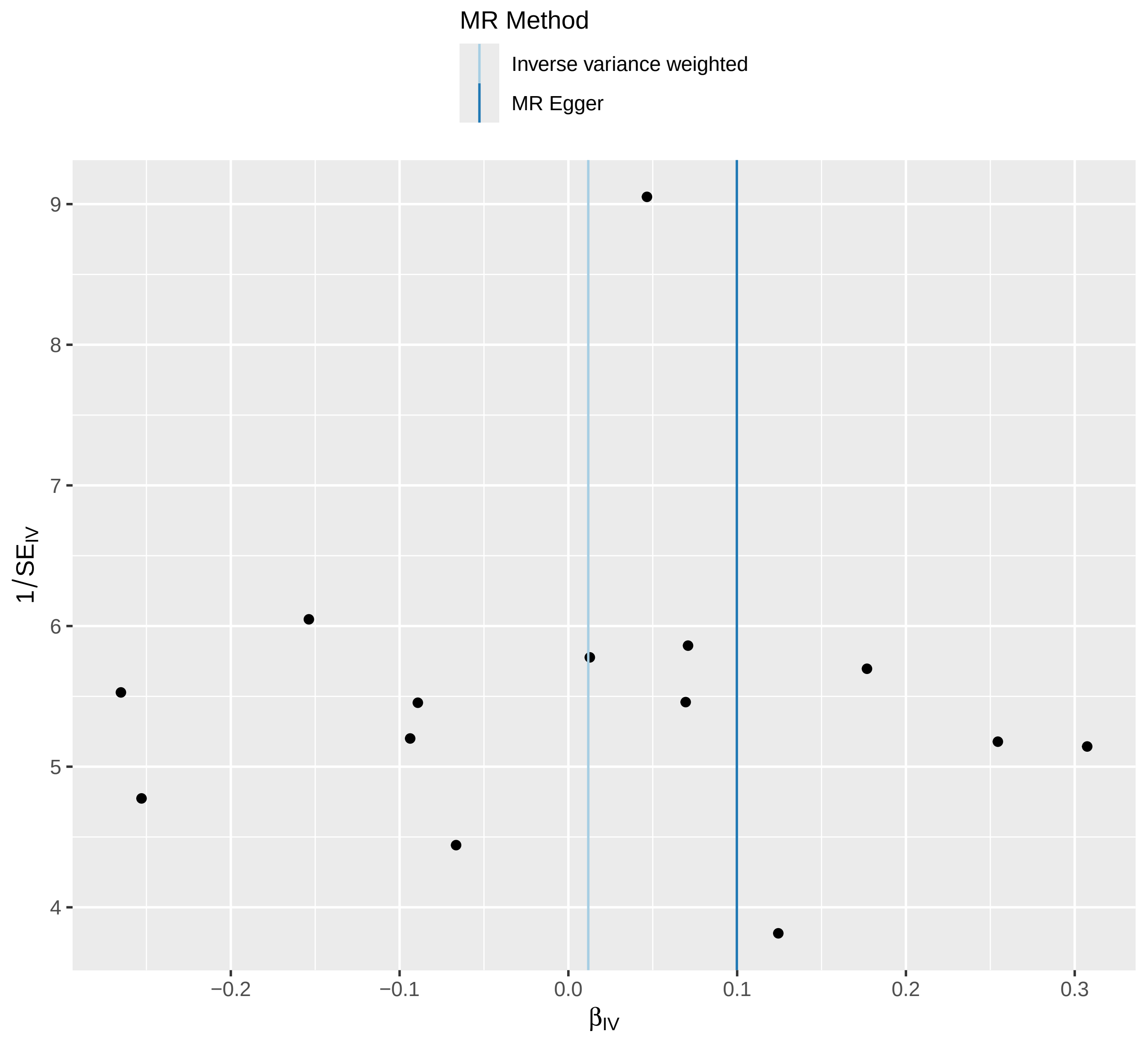

MR Method
Inverse variance weighted
MR Egger
9
8
7
6
5
4
1/SE_IV
−0.2
−0.1
0.0
0.1
0.2
0.3
β_IV

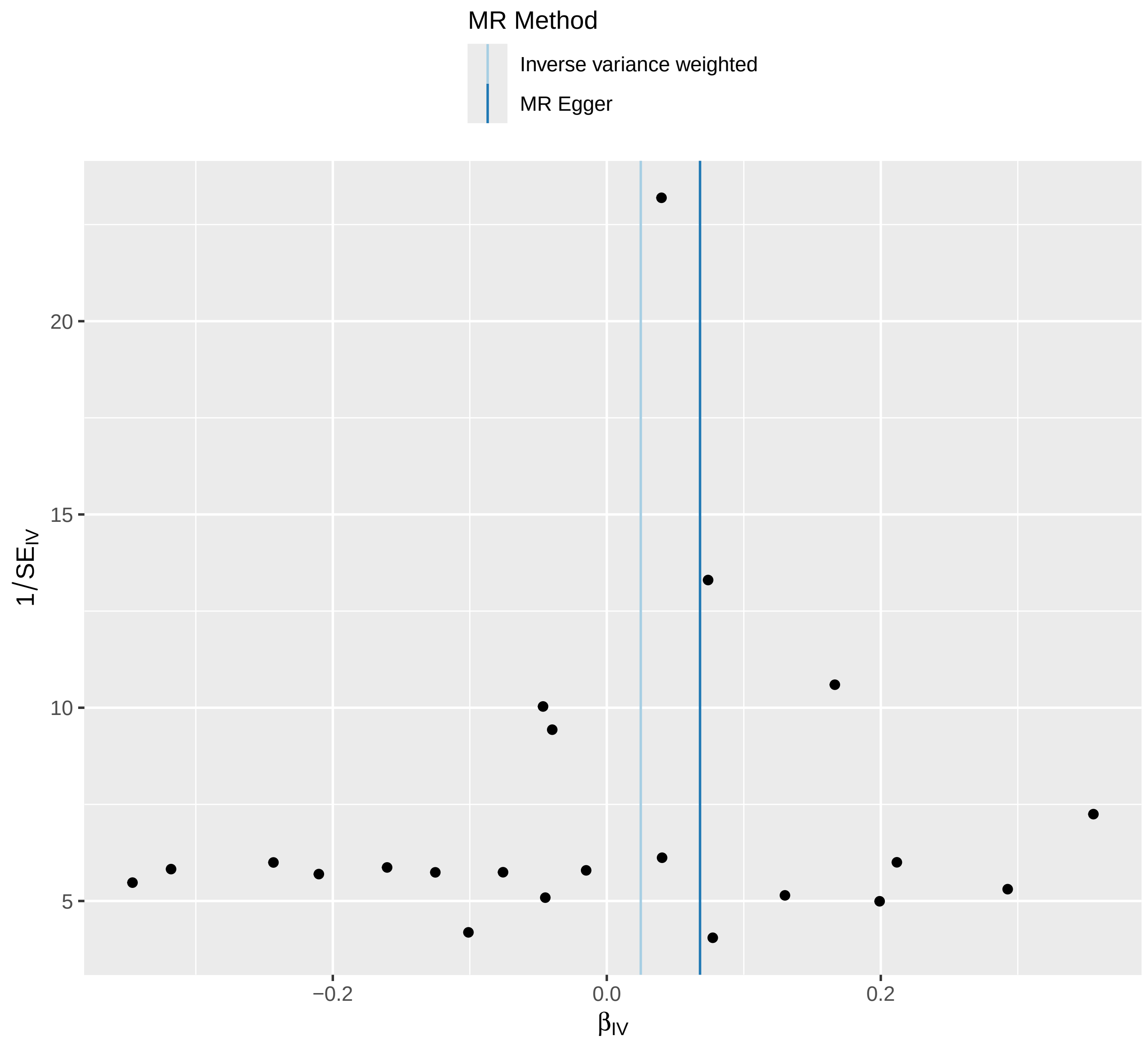

MR Method
Inverse variance weighted
MR Egger
20
15
10
5
1/SE_IV
−0.2
0.0
0.2
β_IV

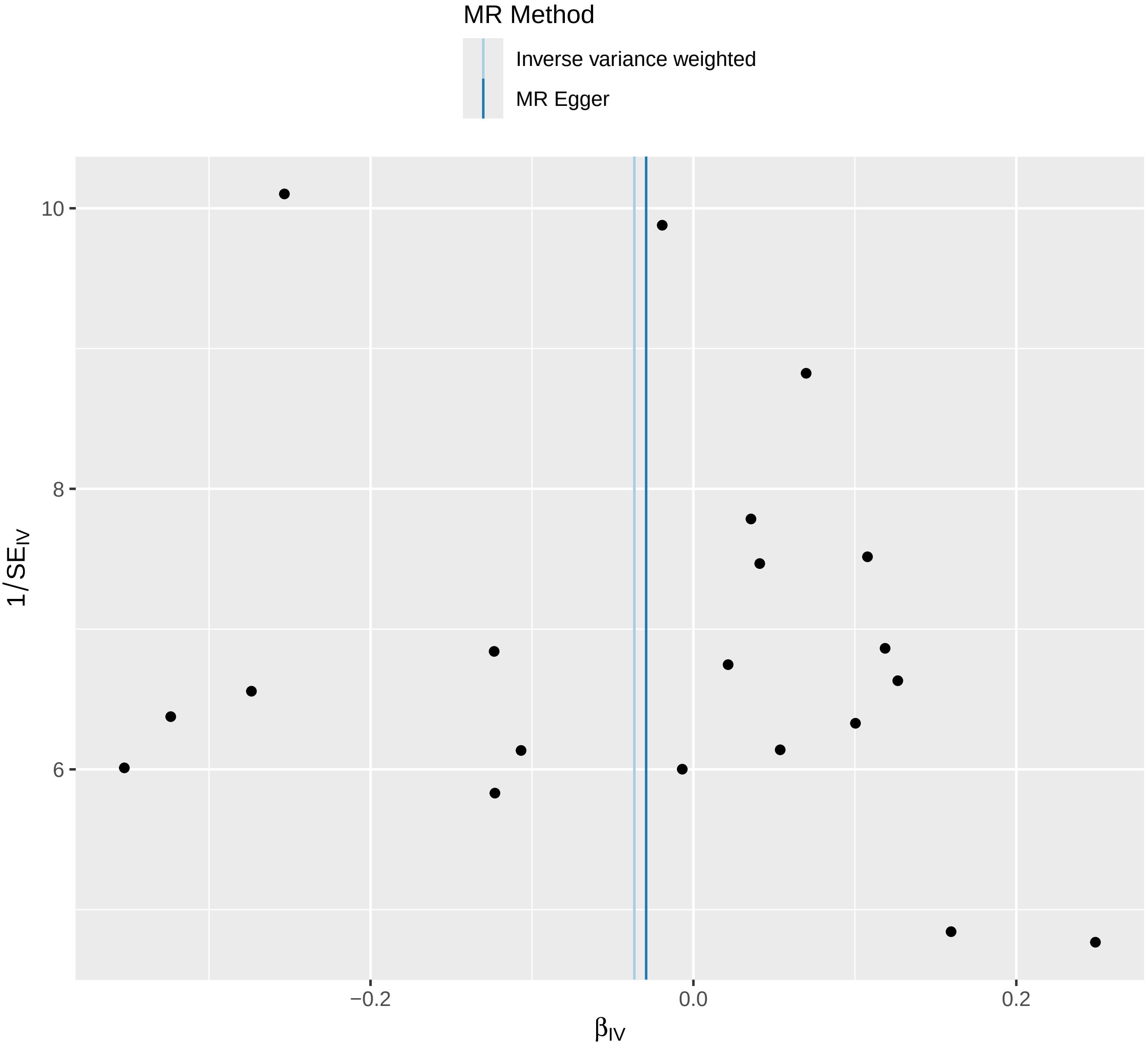
MR Method
Inverse variance weighted
MR Egger
10
8
6
−0.2
0.0
0.2
$1/SE_{IV}$
β_{IV}

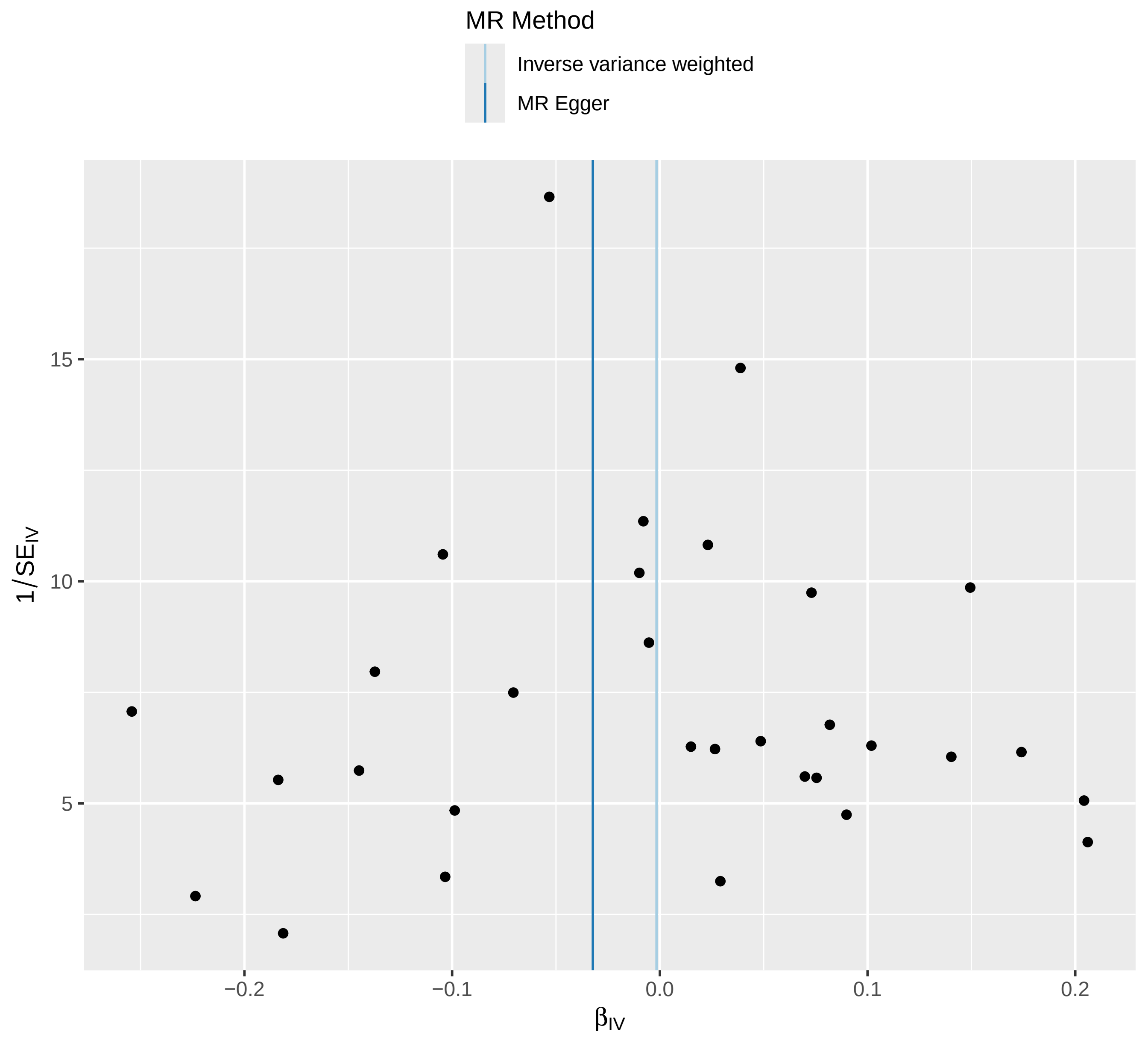
MR Method
Inverse variance weighted
MR Egger
15
10
5
$1/SE_{IV}$
−0.2
−0.1
0.0
0.1
0.2
β_{IV}

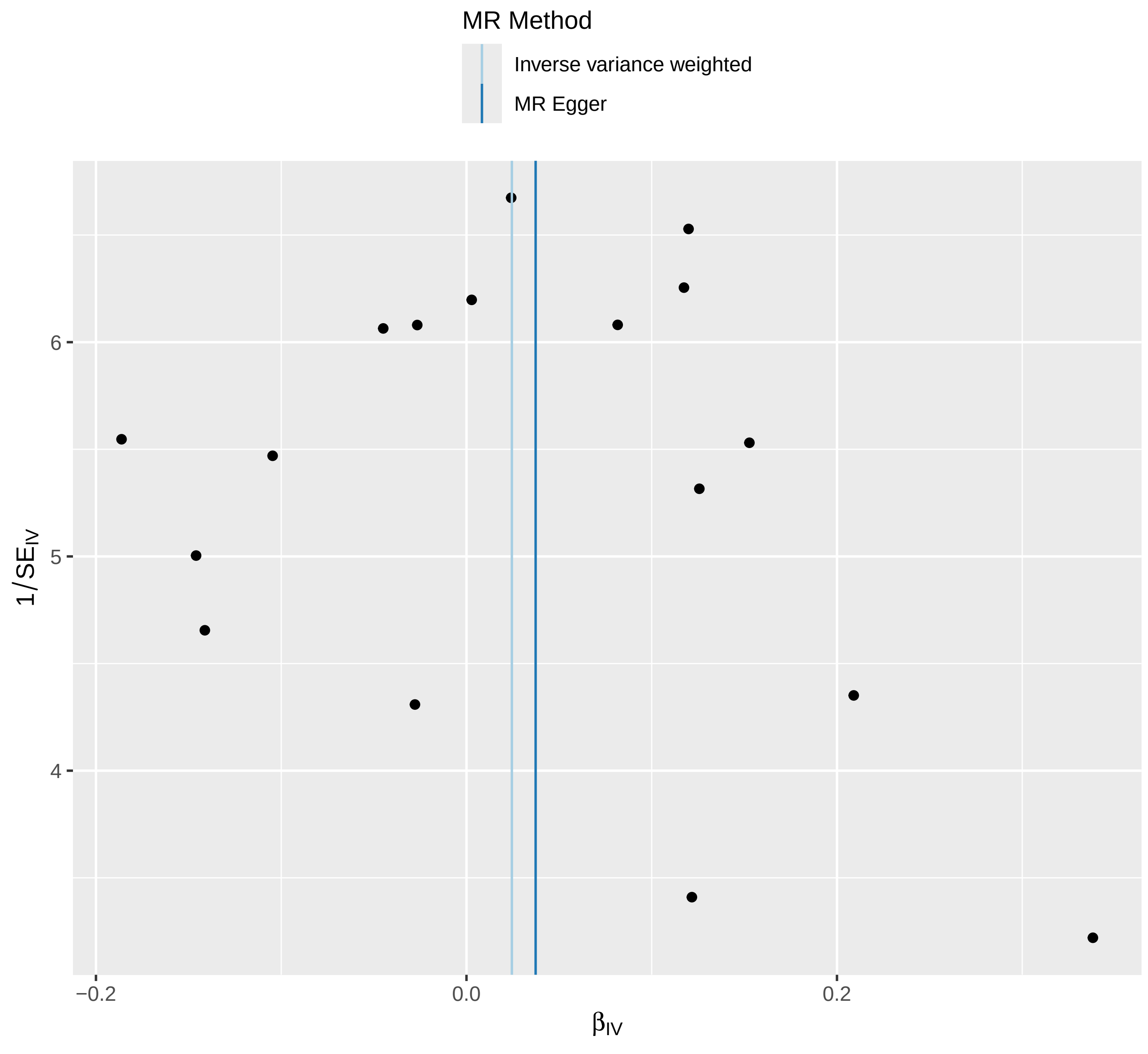
MR Method
Inverse variance weighted
MR Egger
1/SE_IV
6
5
4
−0.2
0.0
0.2
β_IV

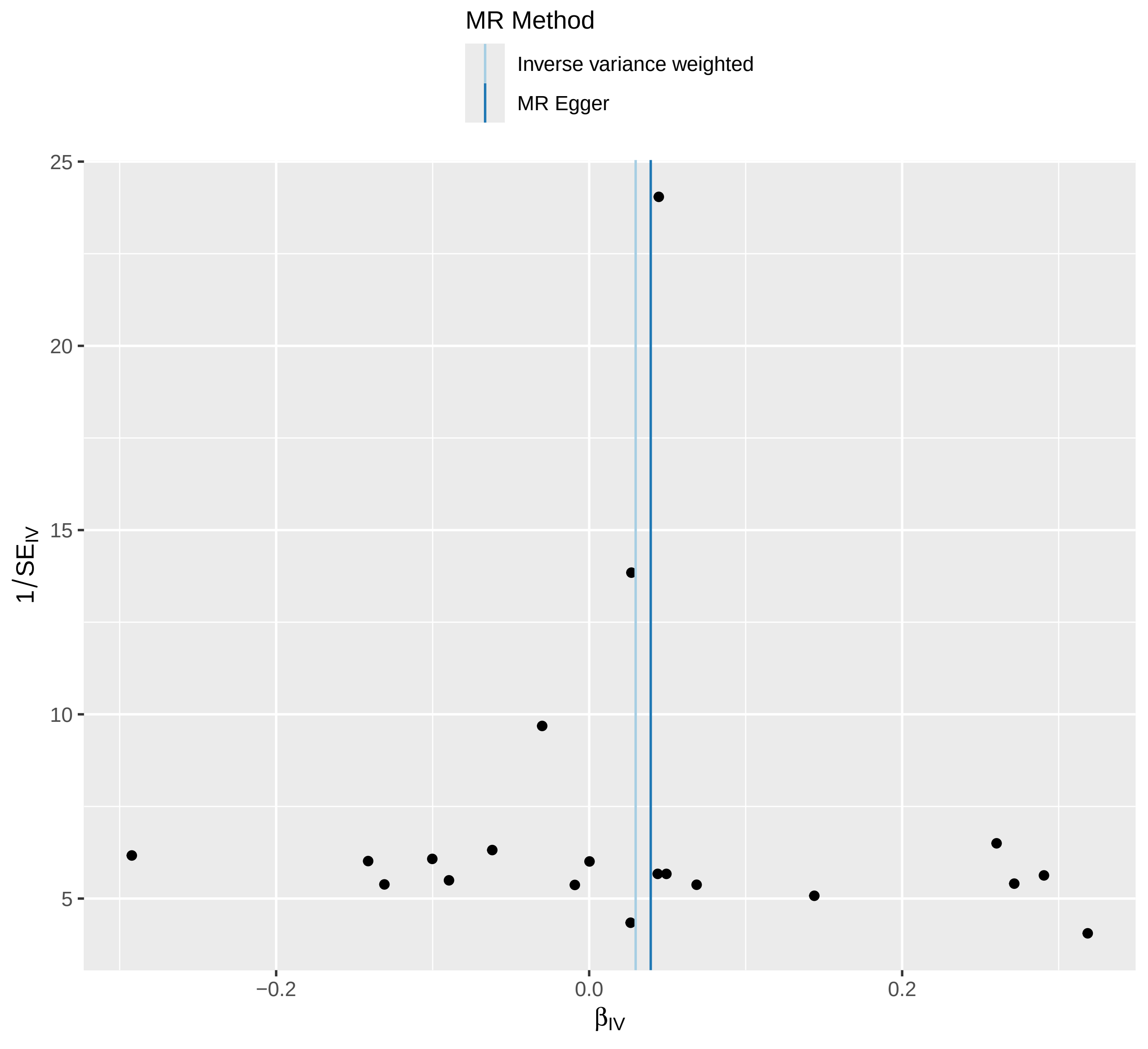
MR Method
Inverse variance weighted
MR Egger
25
20
15
10
5
1/SE_IV
−0.2
0.0
0.2
β_IV

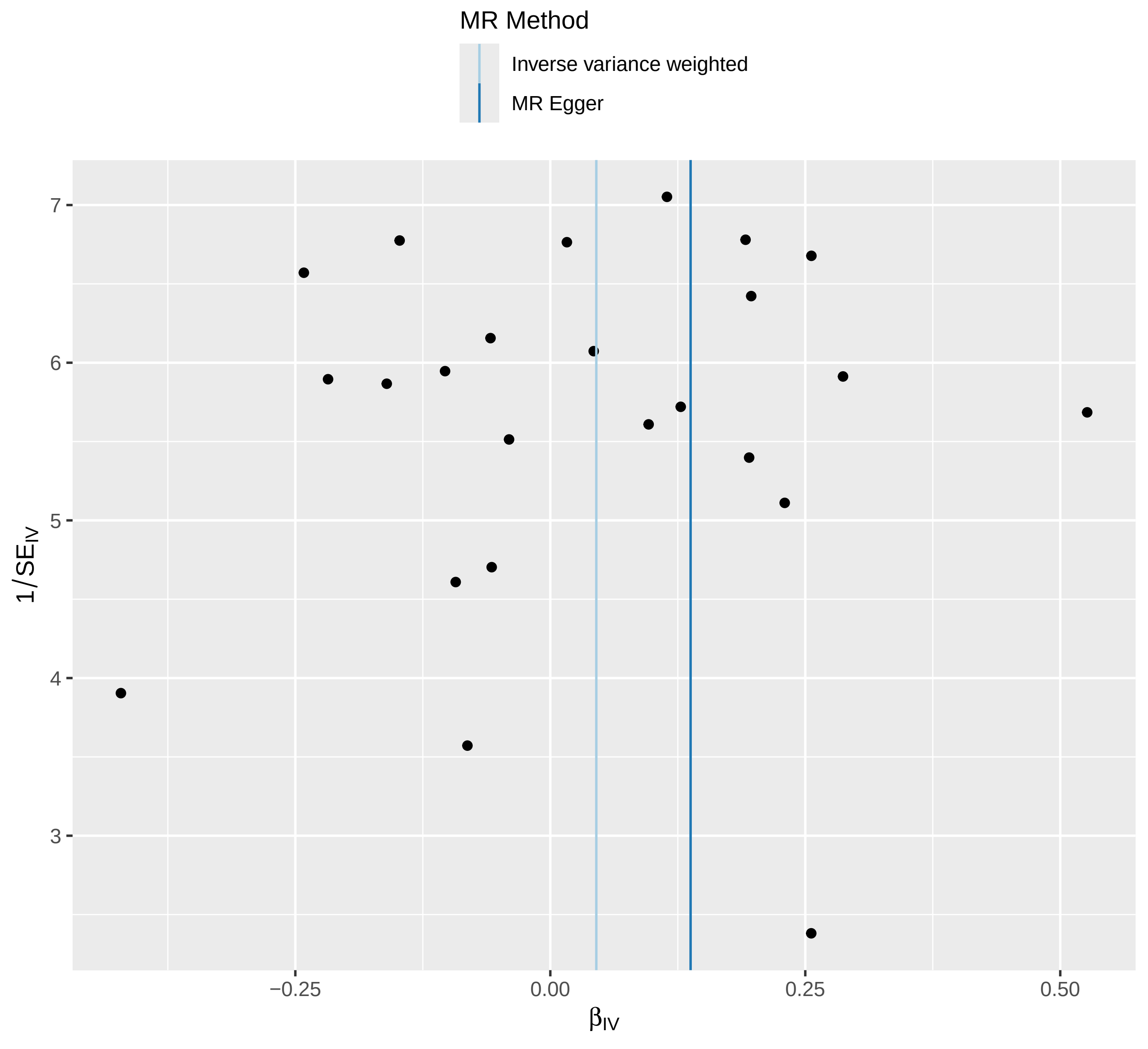

MR Method
Inverse variance weighted
MR Egger
7
6
5
4
3
1/SE_IV
−0.25
0.00
0.25
0.50
β_IV

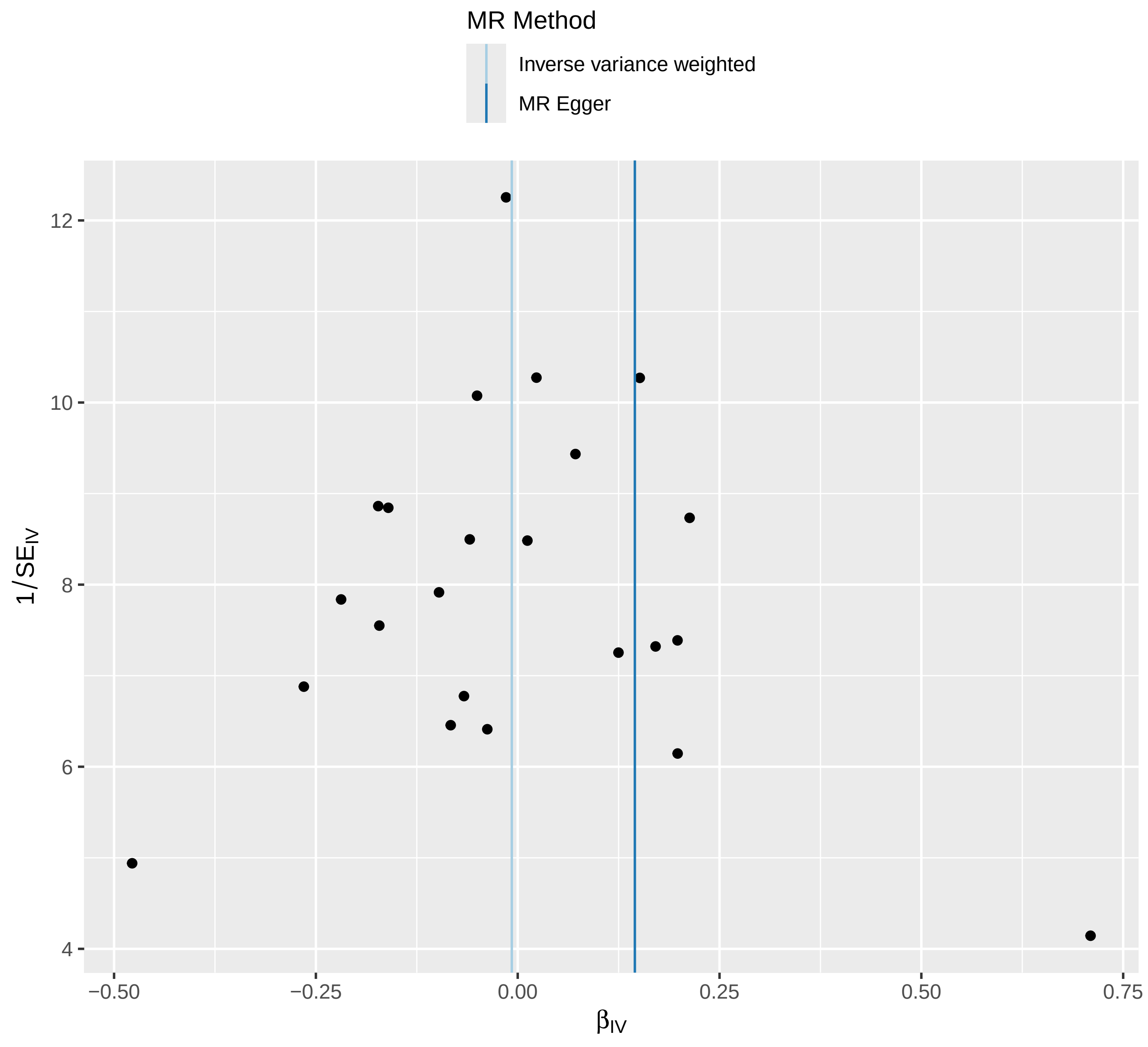

MR Method
Inverse variance weighted
MR Egger
12
10
8
6
4
1/SE_IV
−0.50
−0.25
0.00
0.25
0.50
0.75
β_IV

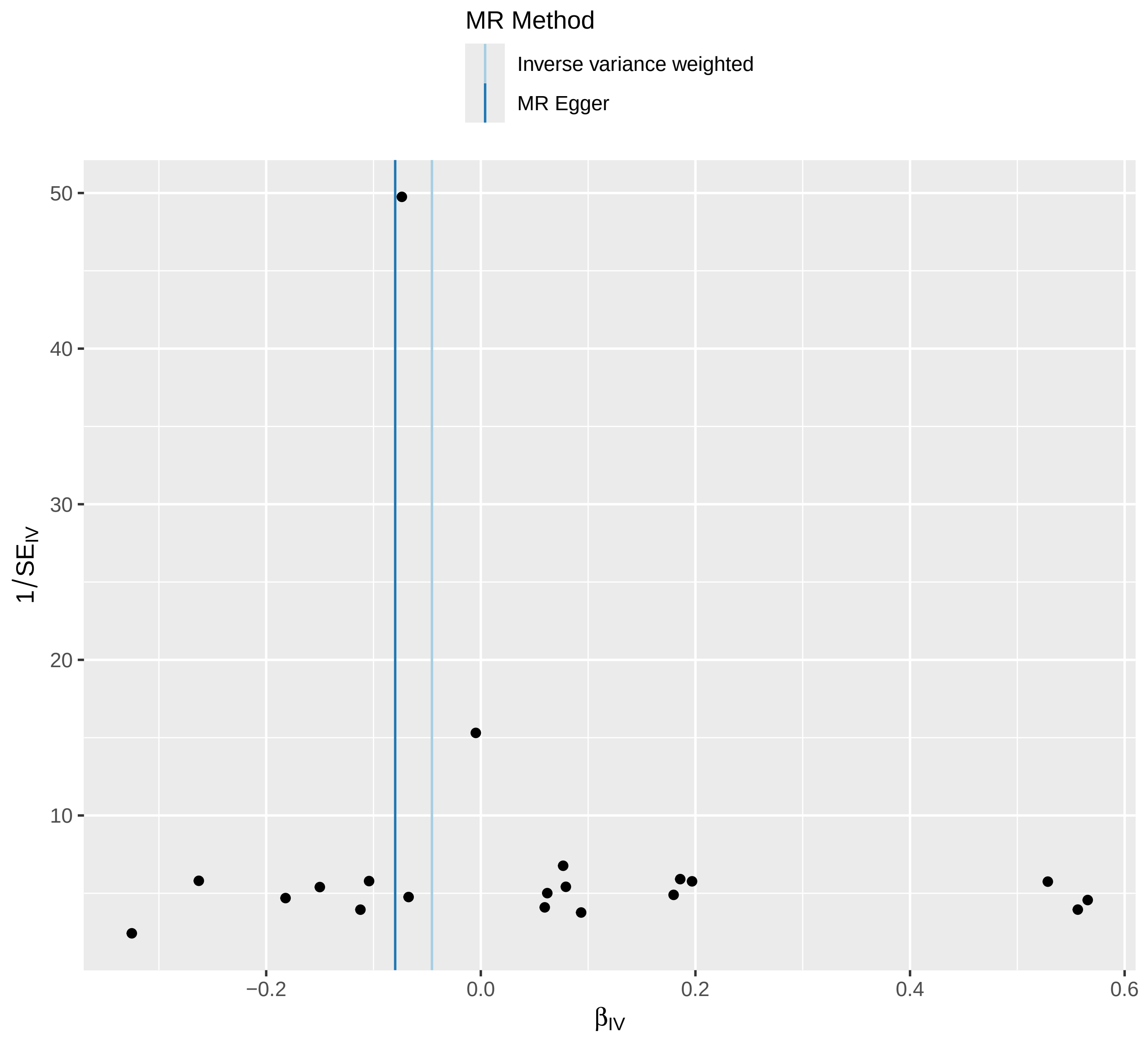

MR Method
Inverse variance weighted
MR Egger
50
40
30
20
10
1/SE_IV
−0.2
0.0
0.2
0.4
0.6
β_IV

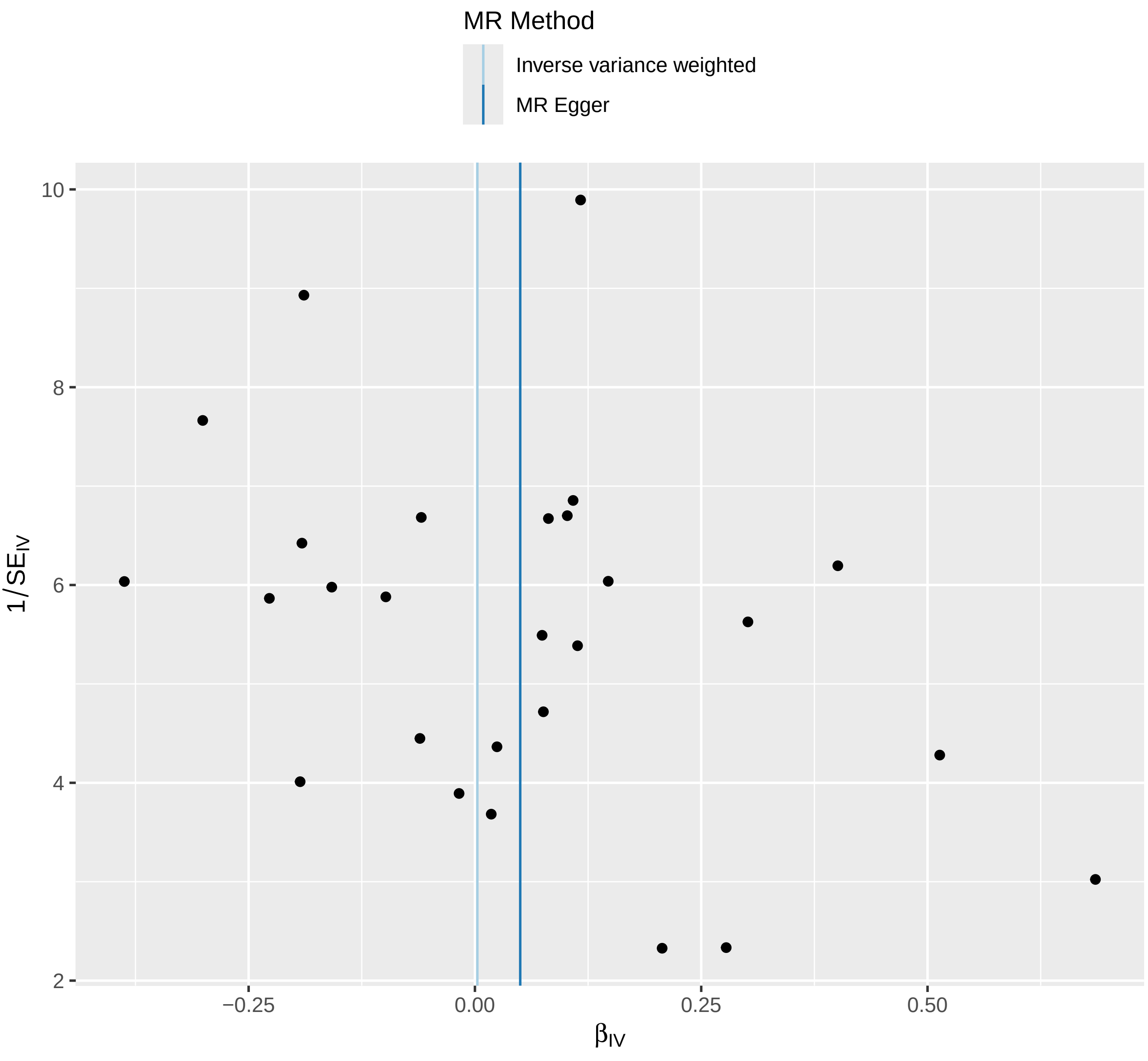
MR Method
Inverse variance weighted
MR Egger
10
8
6
4
2
1/SE$_{IV}$
−0.25
0.00
0.25
0.50
β$_{IV}$

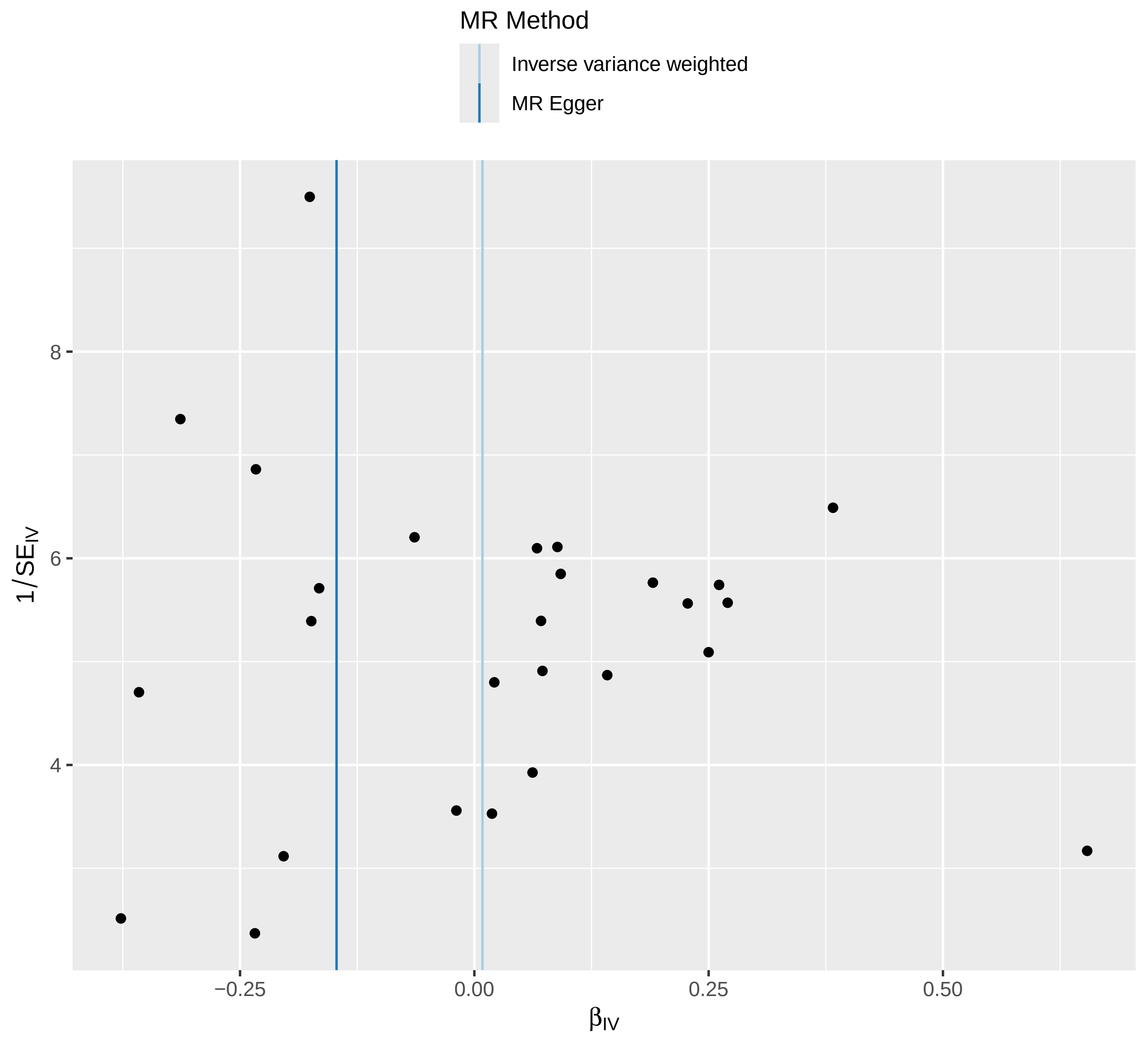
MR Method
Inverse variance weighted
MR Egger
8
6
4
1/SE_IV
−0.25
0.00
0.25
0.50
β_IV

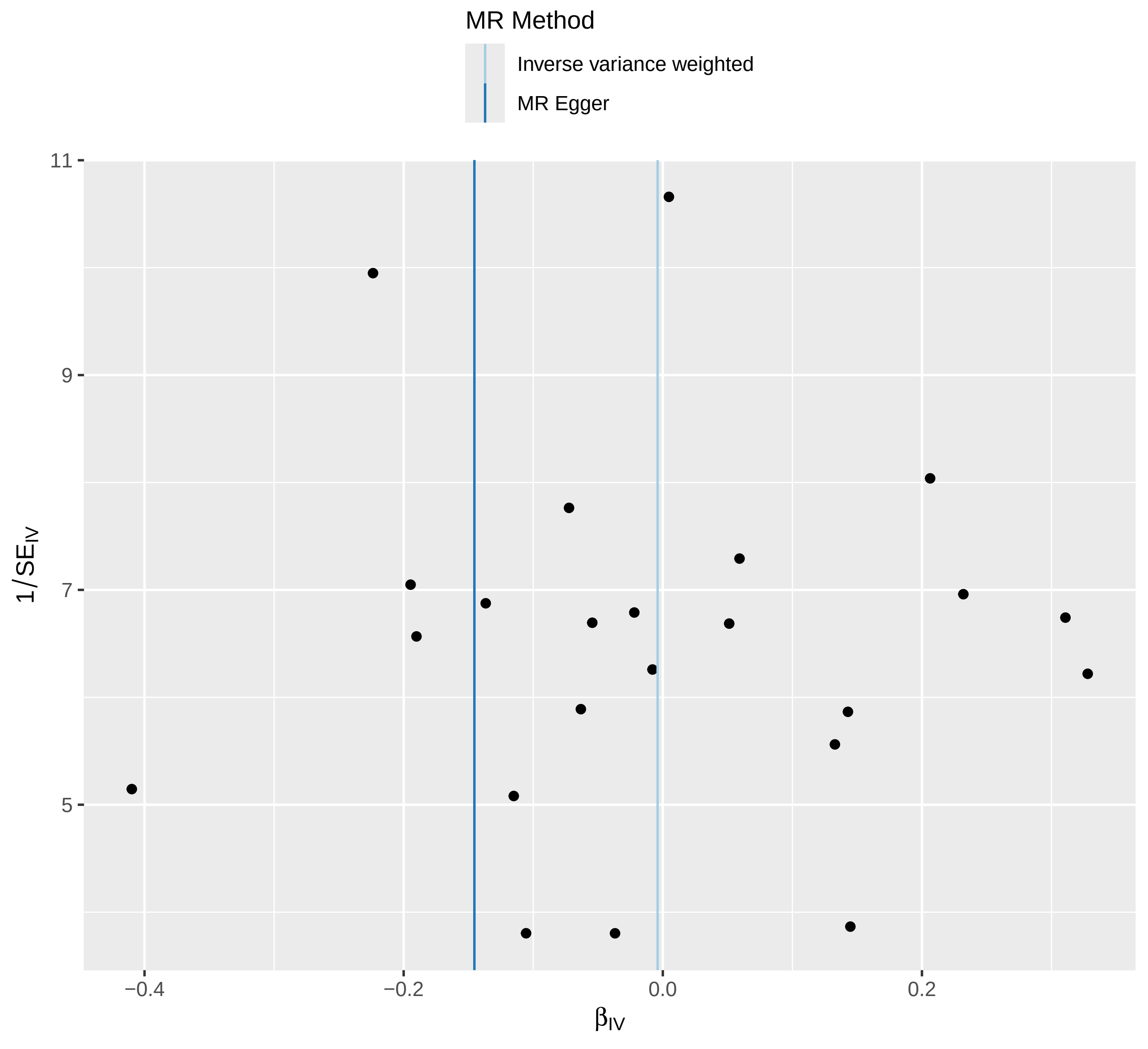
MR Method
Inverse variance weighted
MR Egger
11
9
7
5
1/SE_IV
−0.4
−0.2
0.0
0.2
β_IV

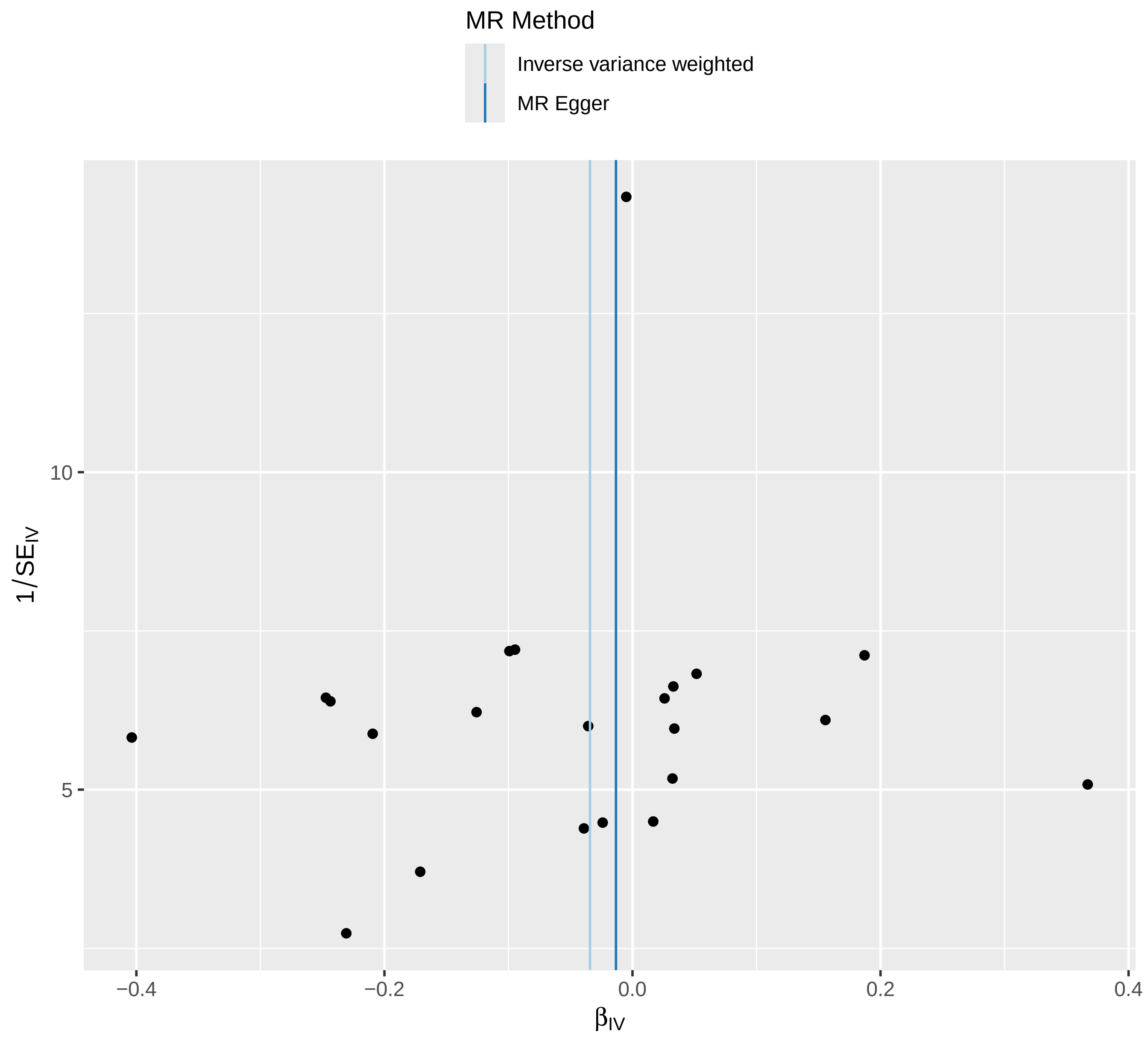

MR Method
Inverse variance weighted
MR Egger
10
5
1/SE_IV
−0.4
−0.2
0.0
0.2
0.4
β_IV

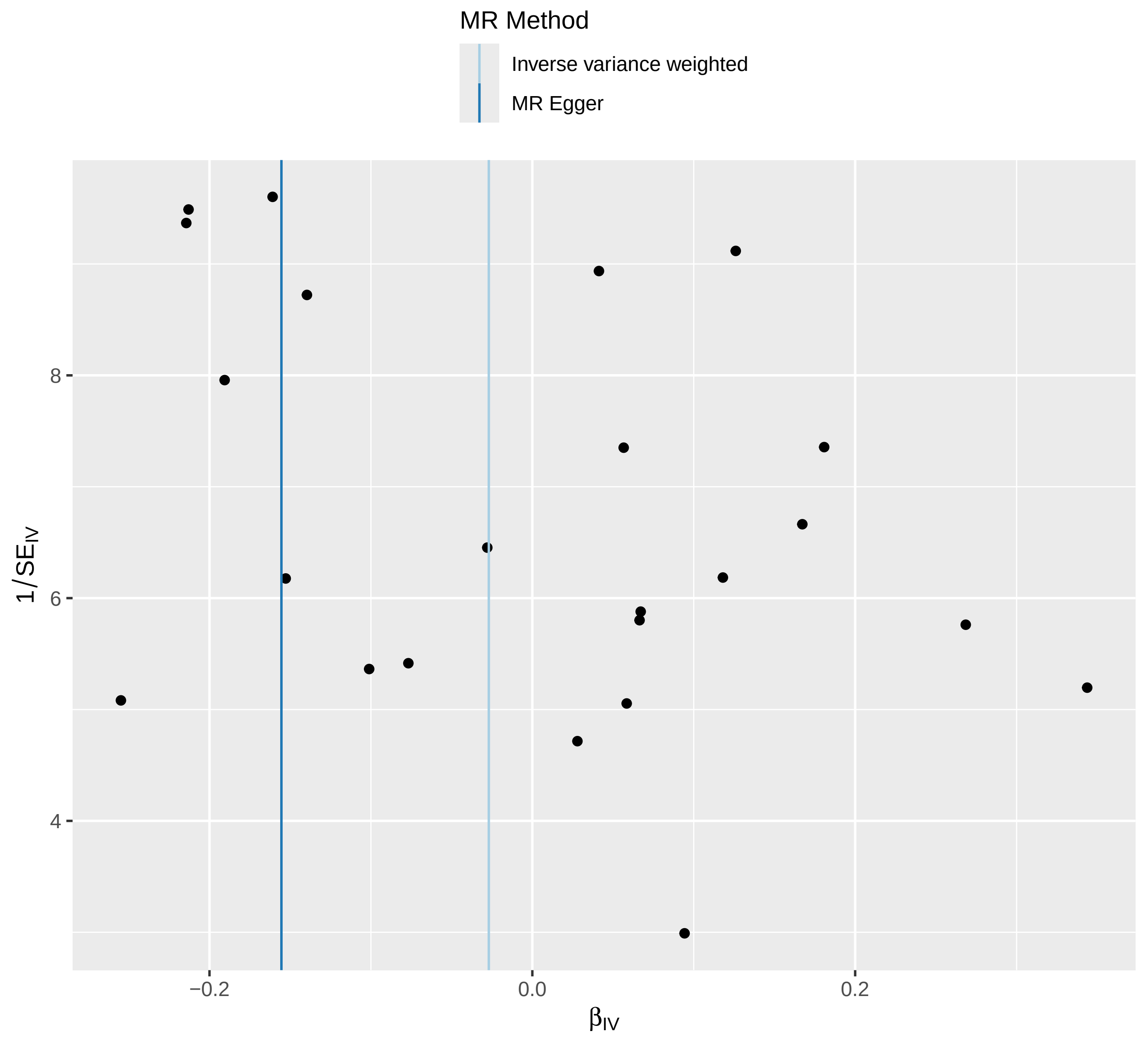

MR Method
Inverse variance weighted
MR Egger
8
6
4
1/SE$_{IV}$
−0.2
0.0
0.2
β$_{IV}$

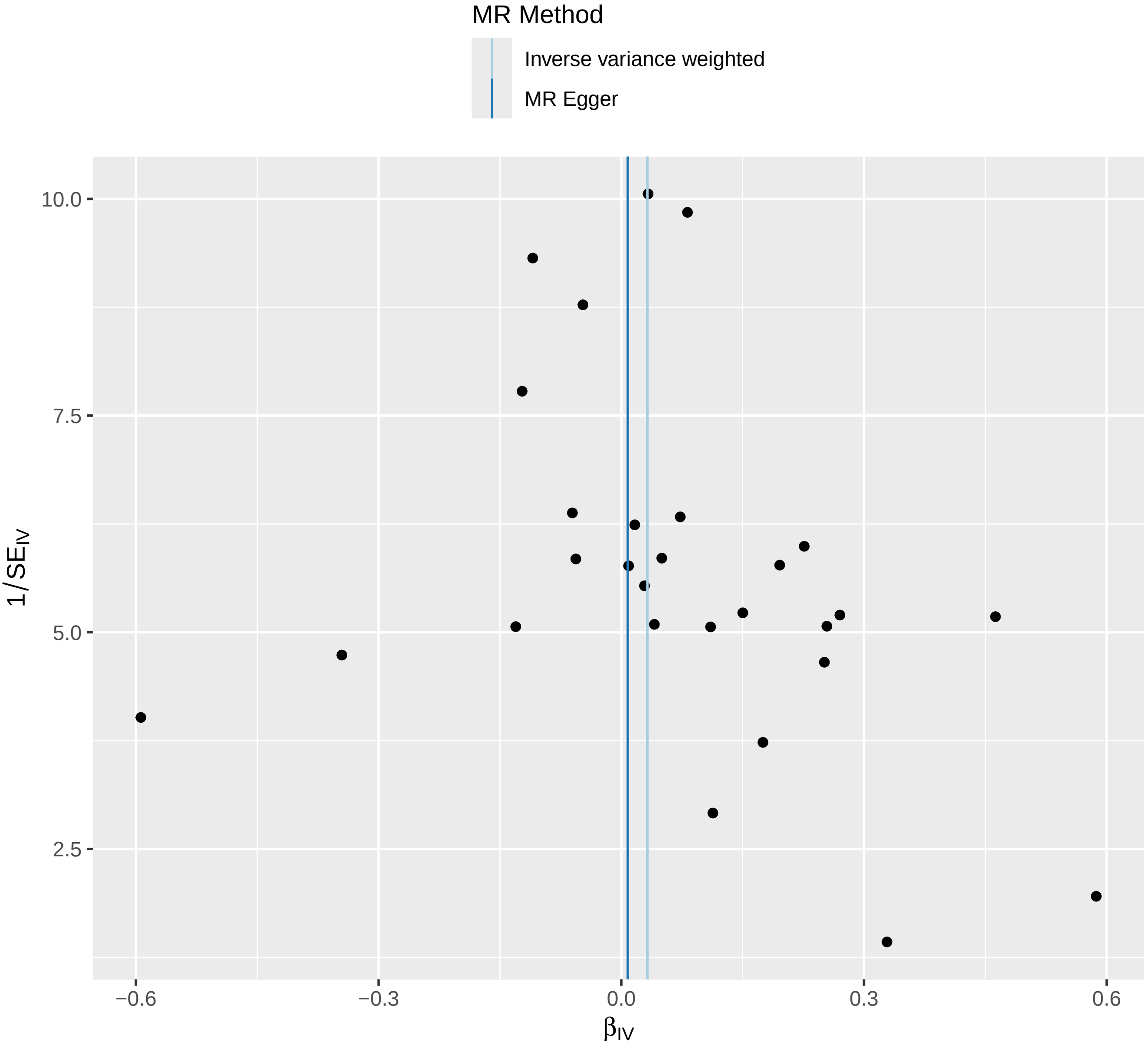

MR Method
Inverse variance weighted
MR Egger
10.0
7.5
5.0
2.5
1/SE_IV
−0.6
−0.3
0.0
0.3
0.6
β_IV

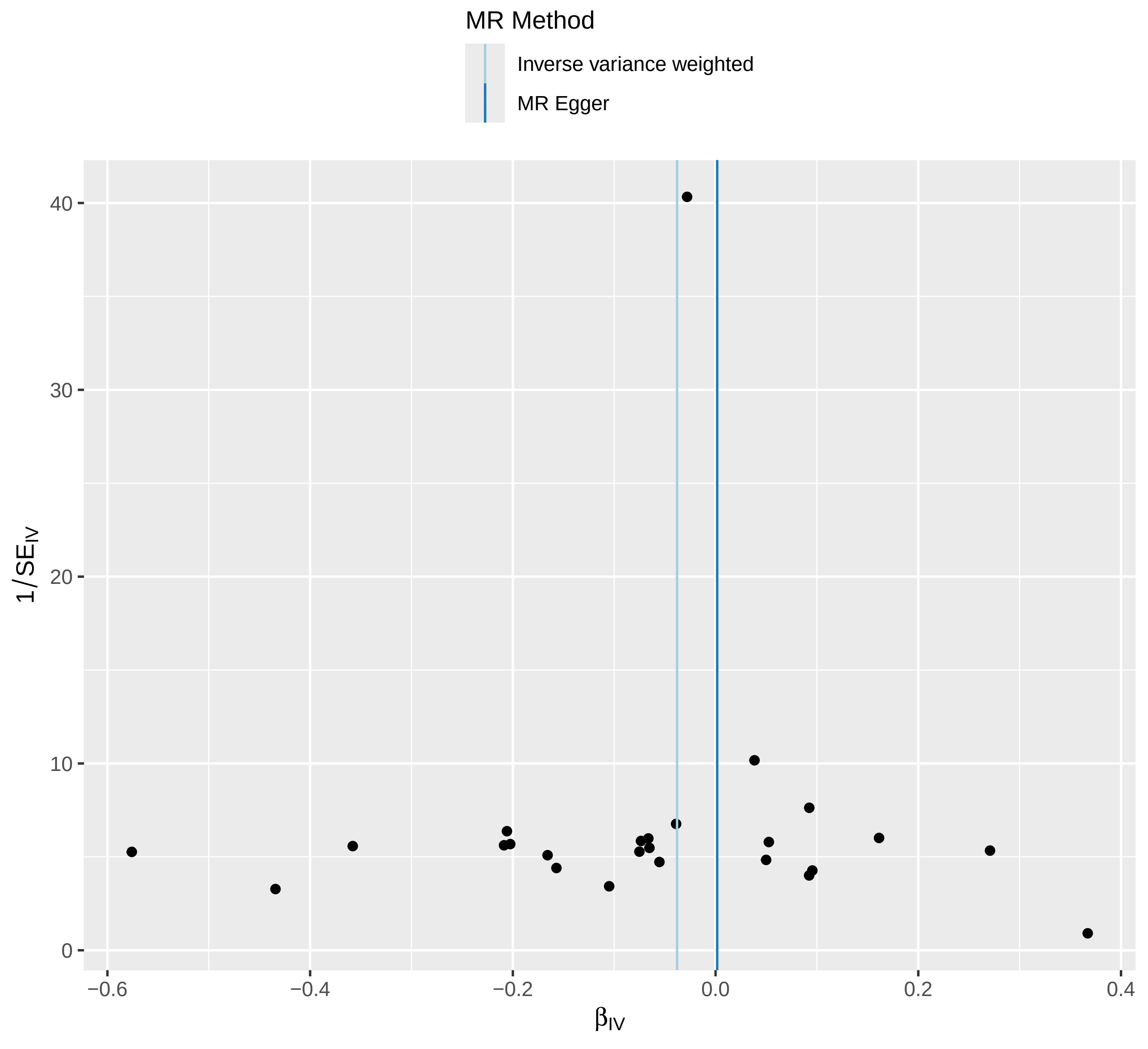
MR Method
Inverse variance weighted
MR Egger
1/SE_IV
40
30
20
10
0
β_IV
−0.6
−0.4
−0.2
0.0
0.2
0.4

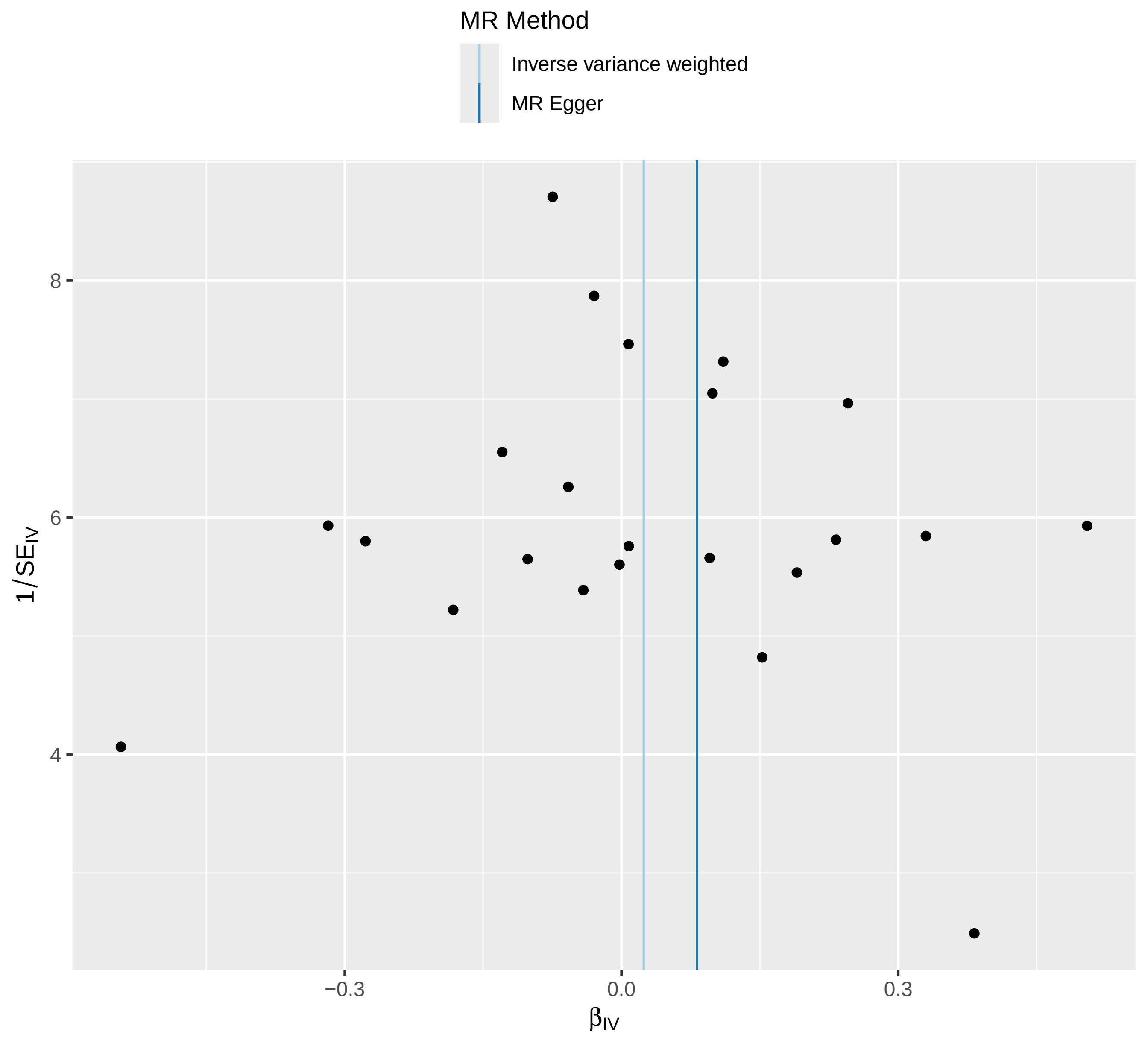

MR Method
Inverse variance weighted
MR Egger
8
6
4
1/SE_IV
−0.3
0.0
0.3
β_IV

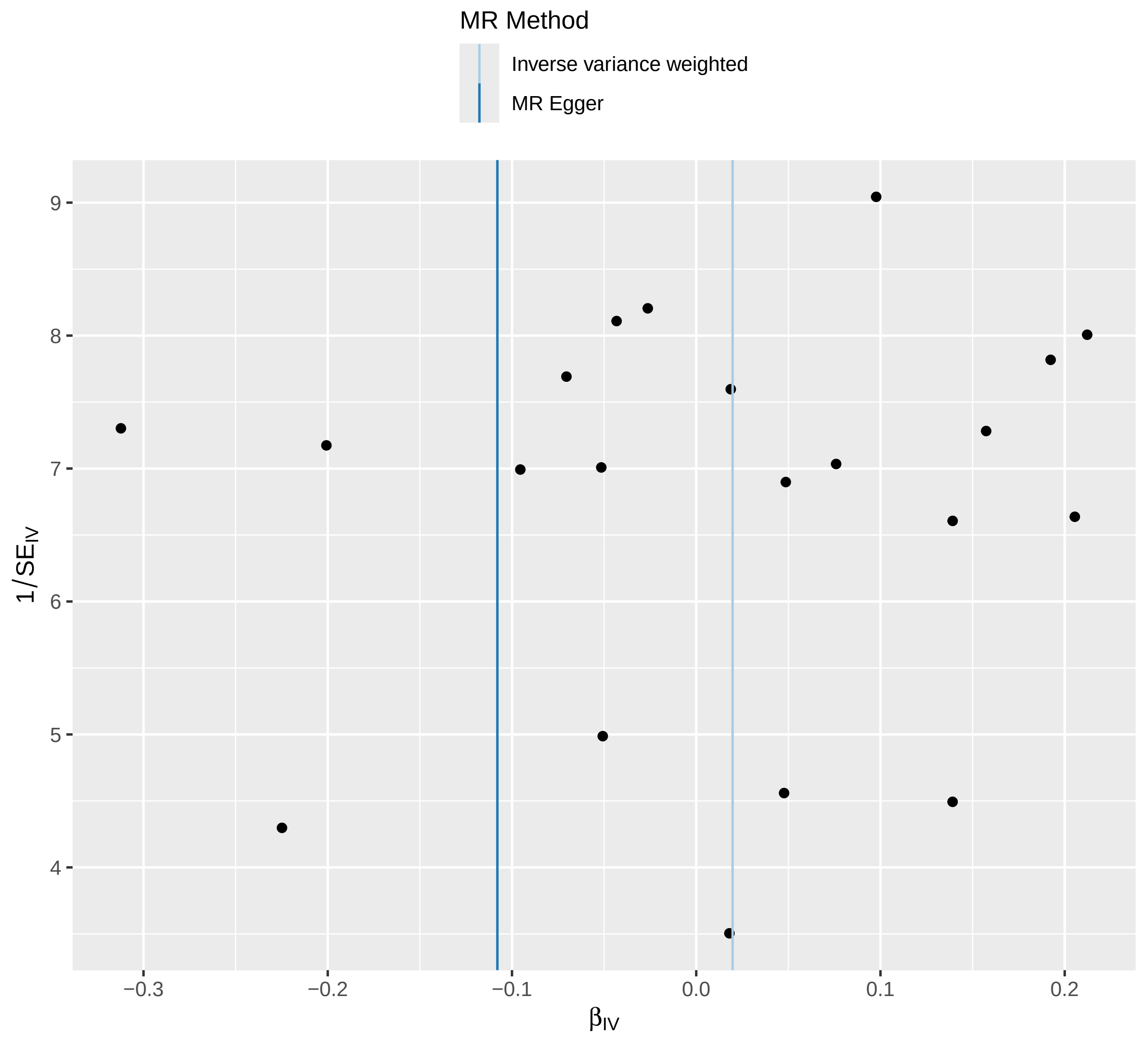

MR Method
Inverse variance weighted
MR Egger
9
8
7
6
5
4
1/SE_IV
−0.3
−0.2
−0.1
0.0
0.1
0.2
β_IV

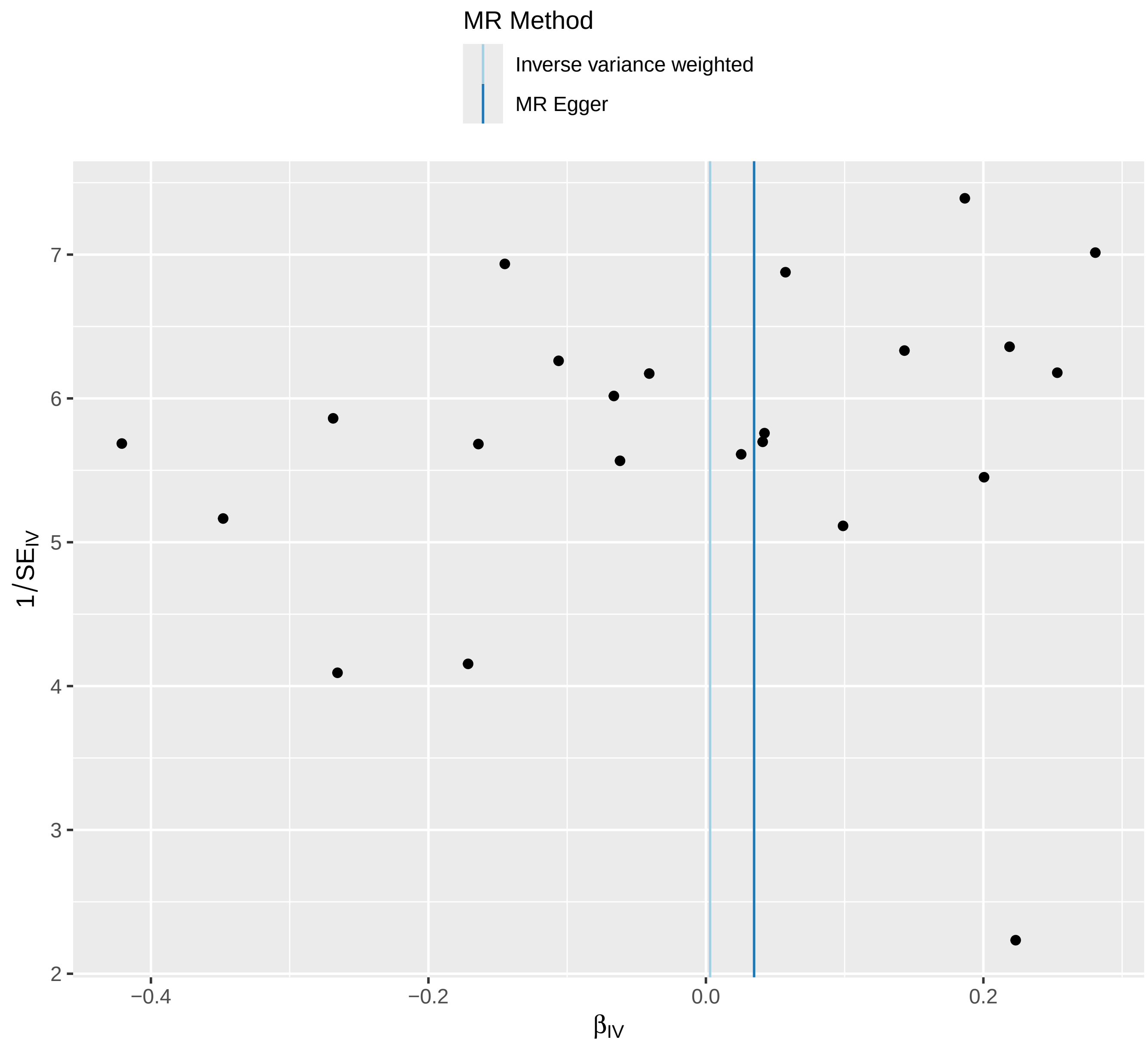
MR Method
Inverse variance weighted
MR Egger
7
6
5
4
3
2
1/SE_IV
−0.4
−0.2
0.0
0.2
β_IV

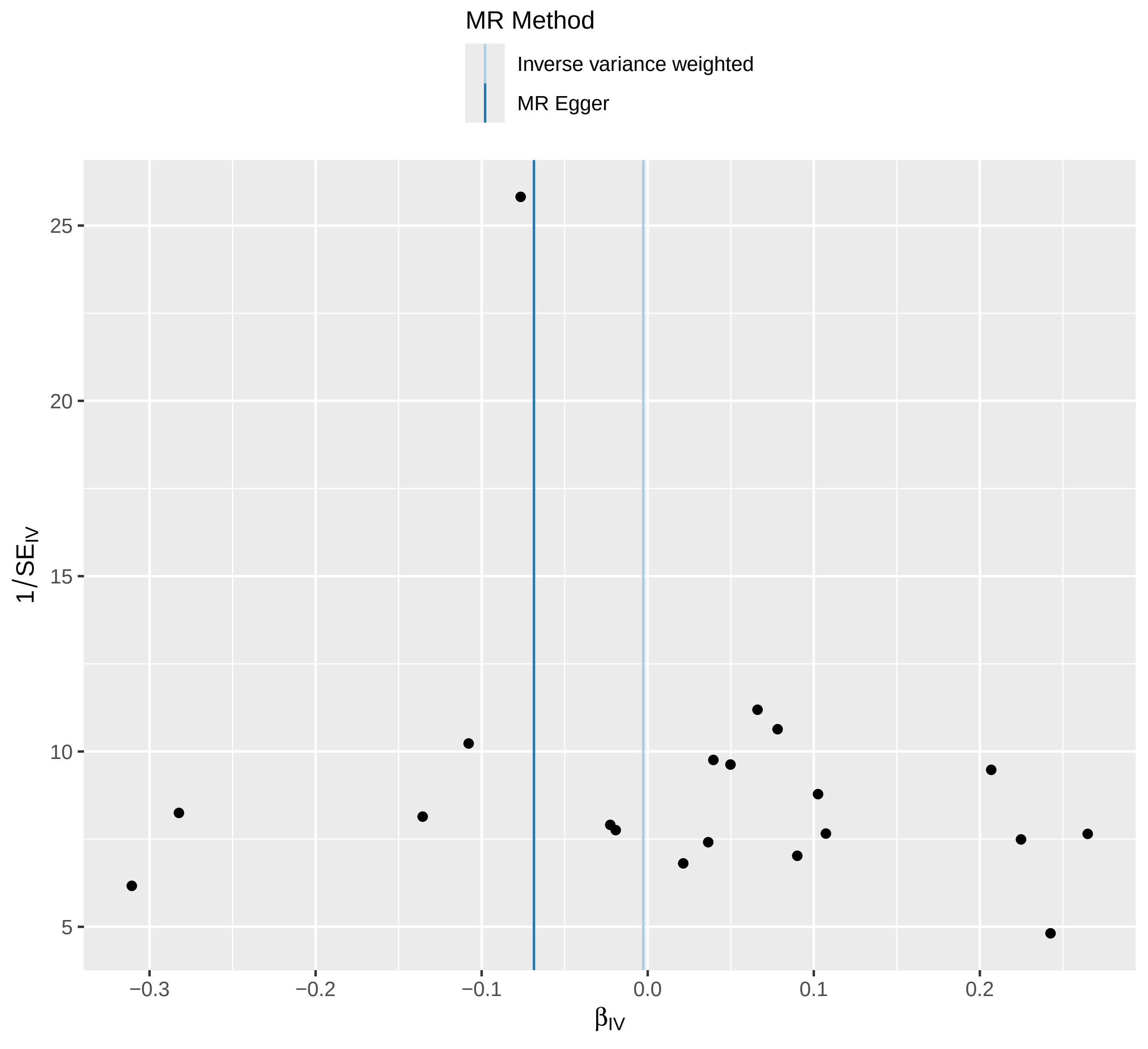
MR Method
Inverse variance weighted
MR Egger
1/SE_IV
β_IV
25
20
15
10
5
−0.3
−0.2
−0.1
0.0
0.1
0.2

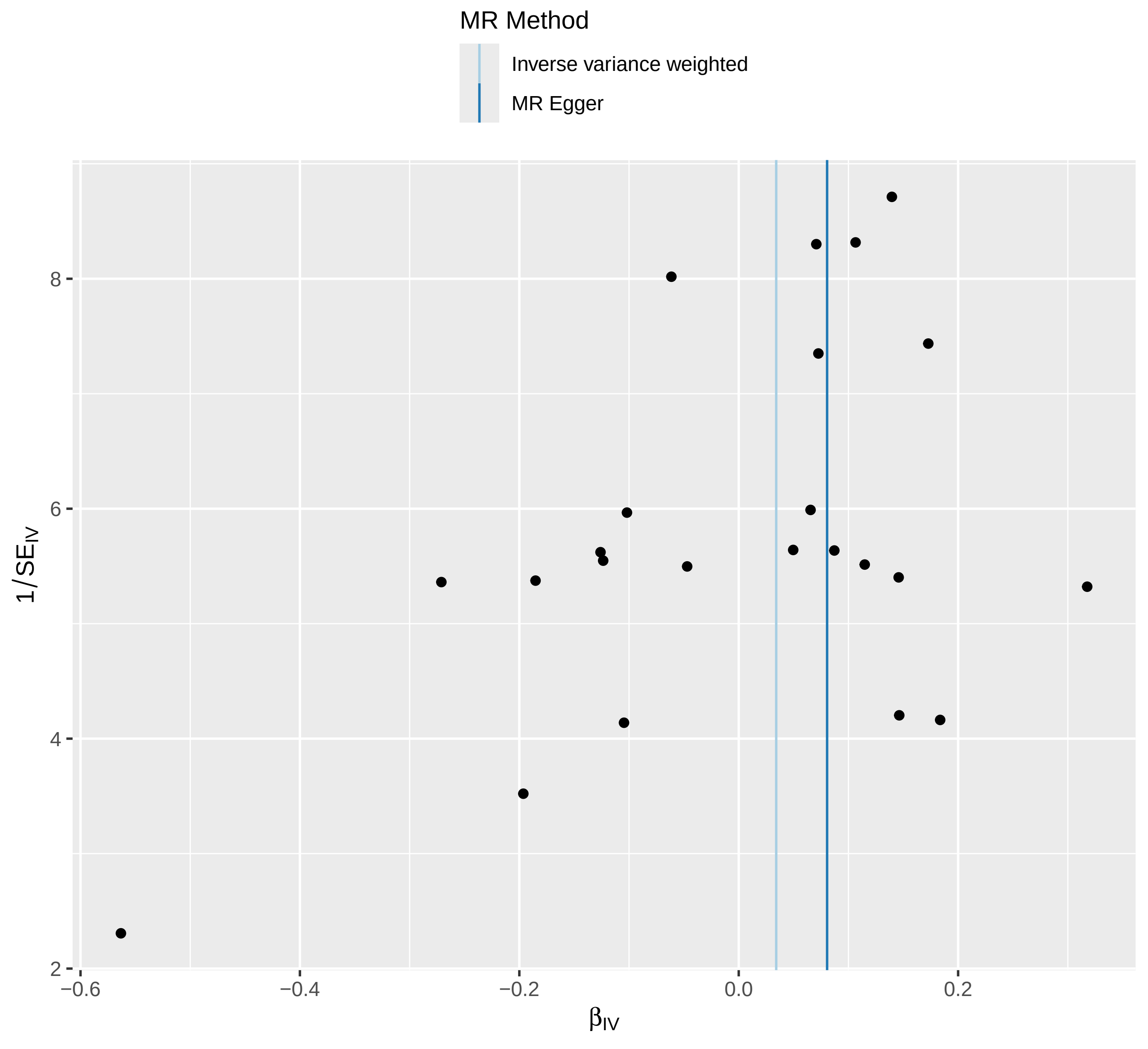

MR Method
Inverse variance weighted
MR Egger
8
6
4
2
1/SE_IV
−0.6
−0.4
−0.2
0.0
0.2
β_IV

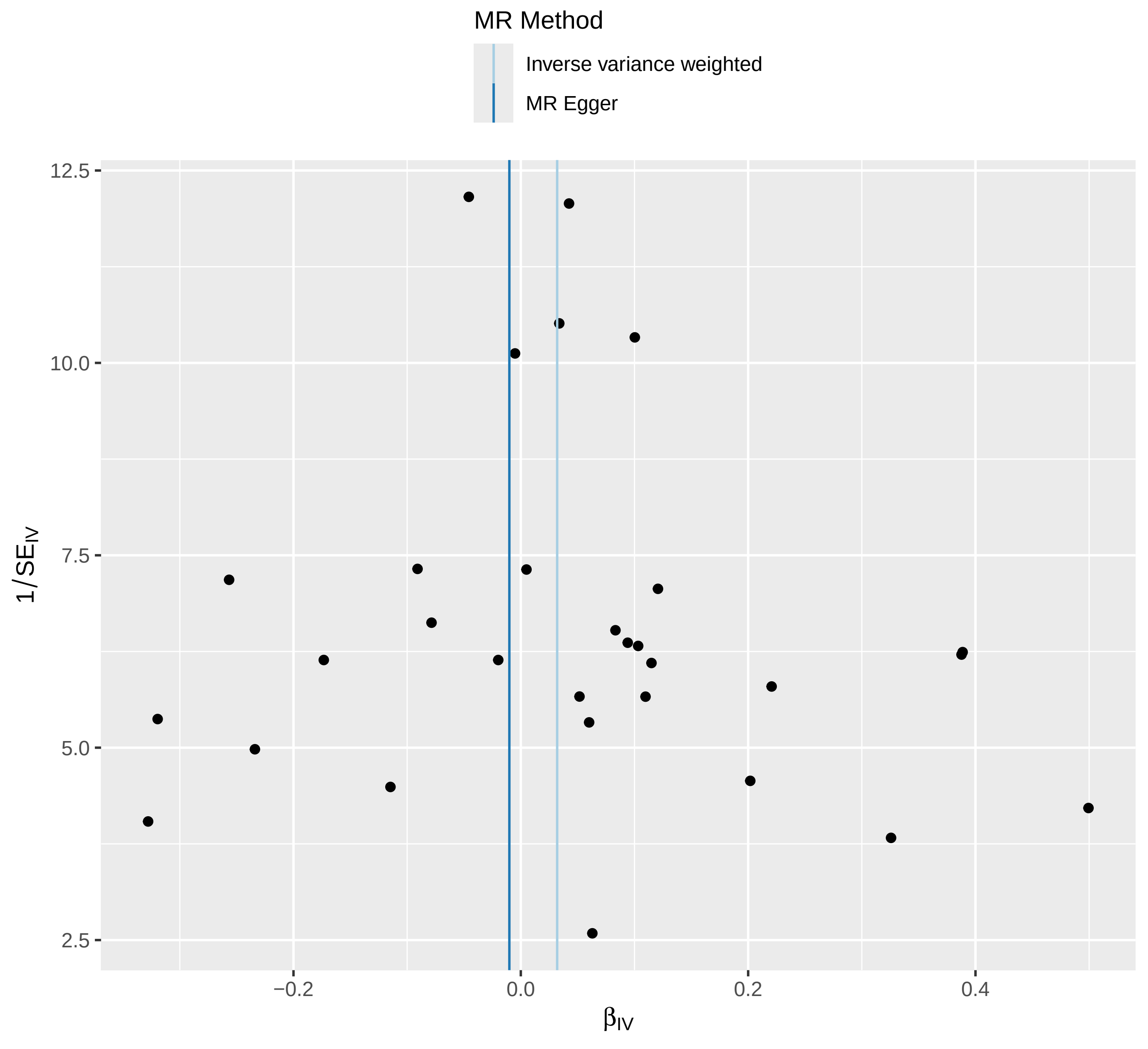
MR Method
Inverse variance weighted
MR Egger
12.5
10.0
7.5
5.0
2.5
1/SE_IV
−0.2
0.0
0.2
0.4
β_IV

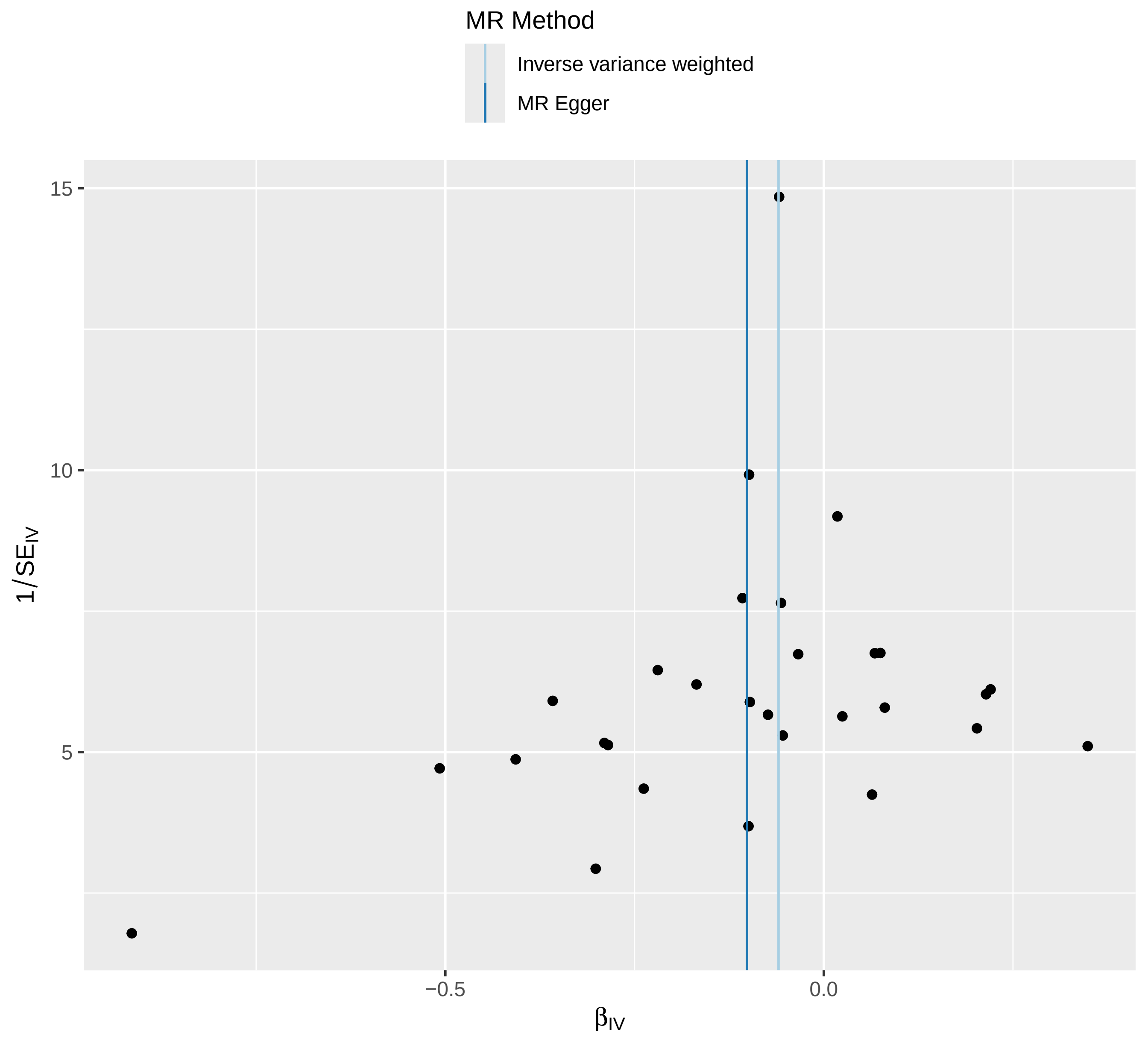
MR Method
Inverse variance weighted
MR Egger
15
10
5
1/SE_IV
−0.5
0.0
β_IV

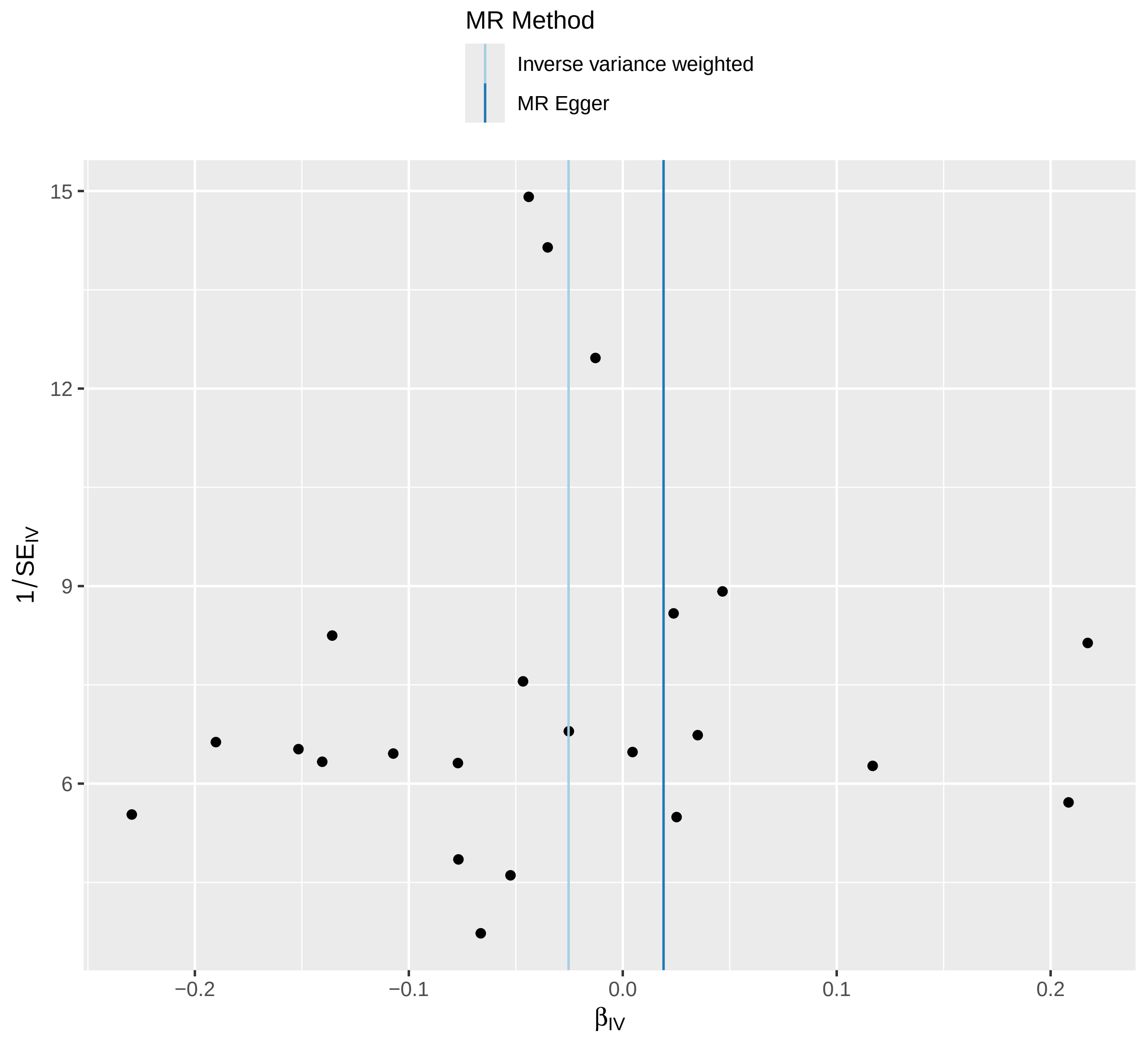
MR Method
Inverse variance weighted
MR Egger
15
12
9
6
1/SE_IV
−0.2
−0.1
0.0
0.1
0.2
β_IV

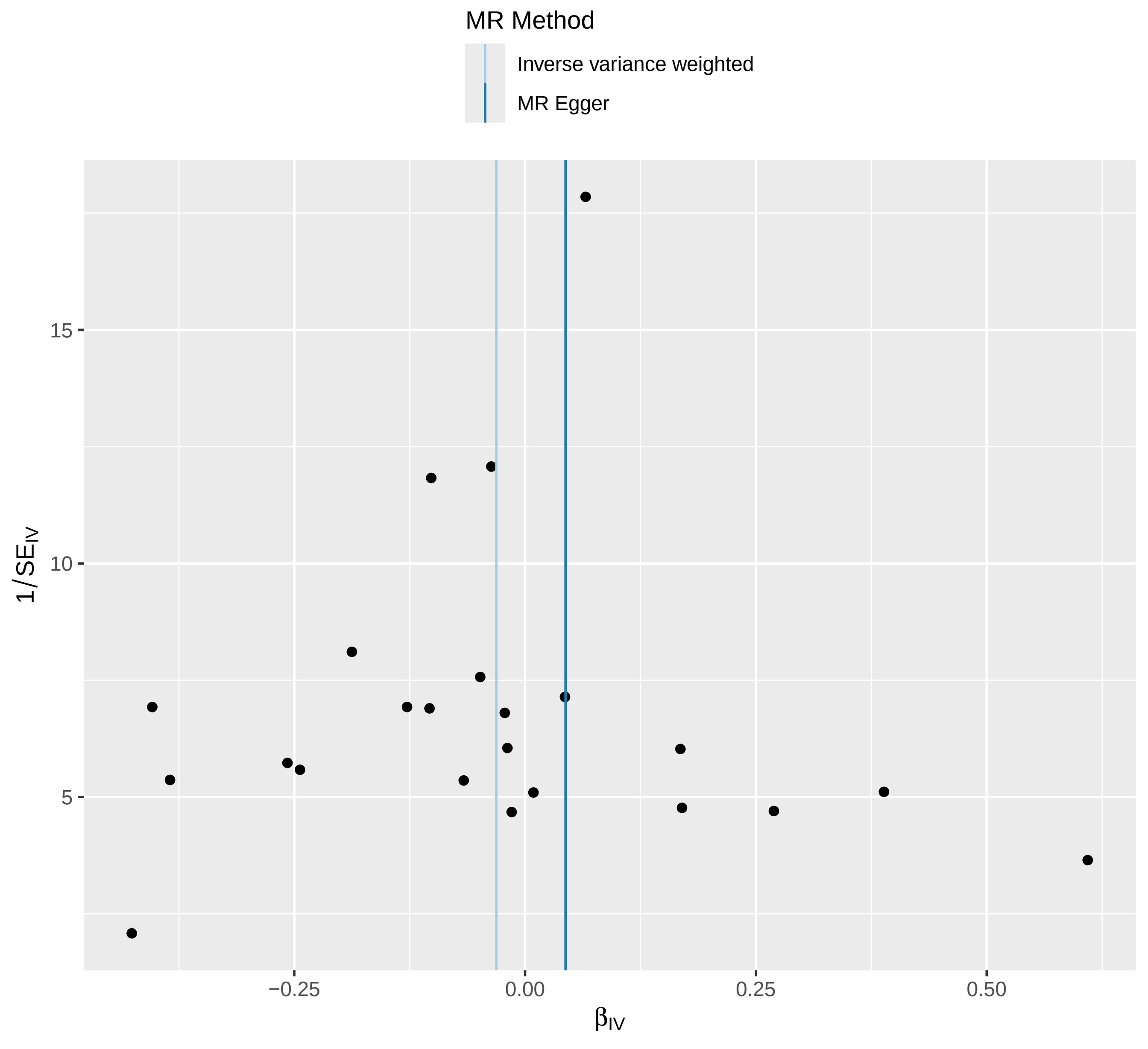

MR Method
Inverse variance weighted
MR Egger
1/SE$_{IV}$
15
10
5
−0.25
0.00
0.25
0.50
β$_{IV}$

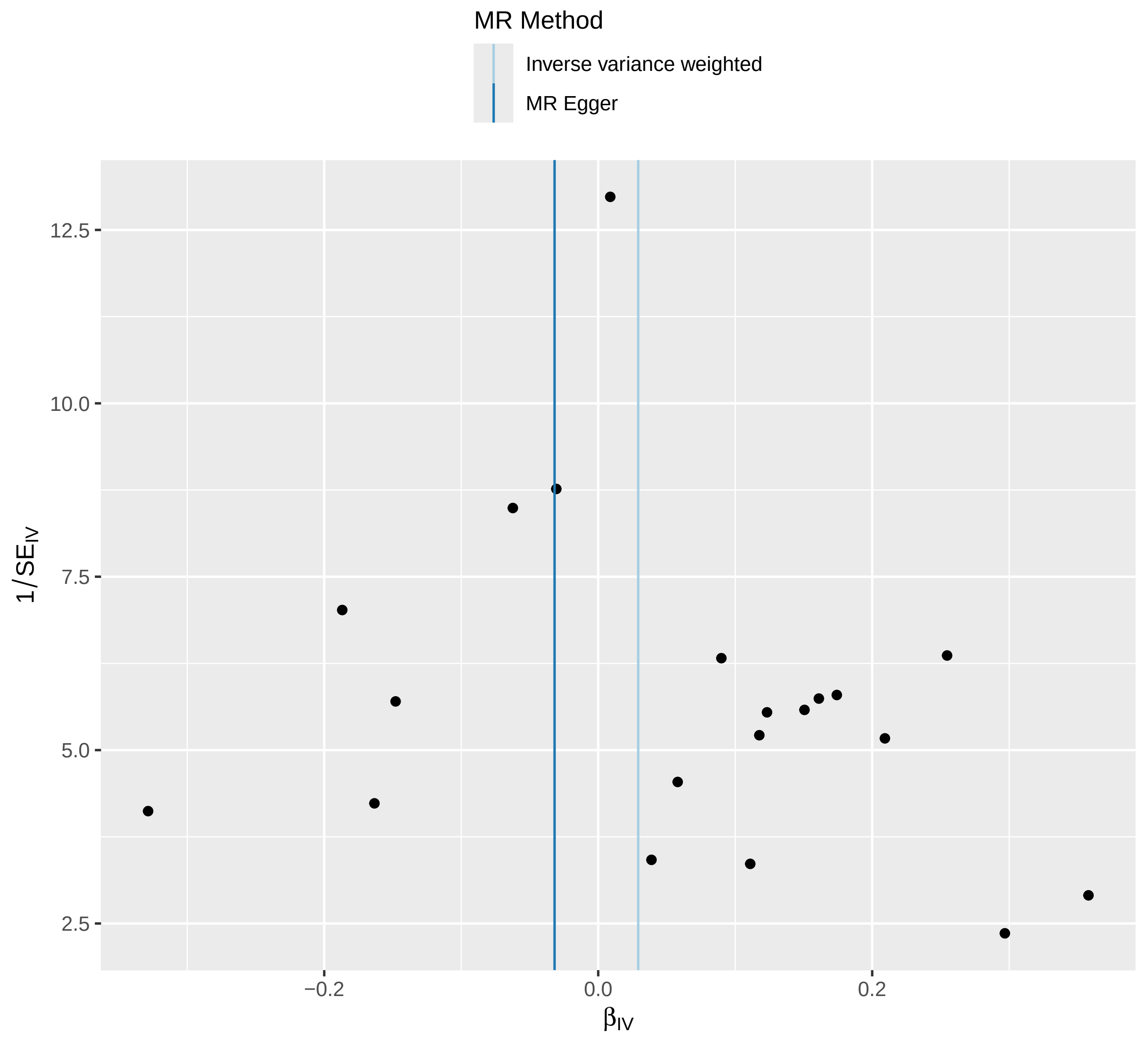
MR Method
Inverse variance weighted
MR Egger
12.5
10.0
7.5
5.0
2.5
1/SE_IV
−0.2
0.0
0.2
β_IV

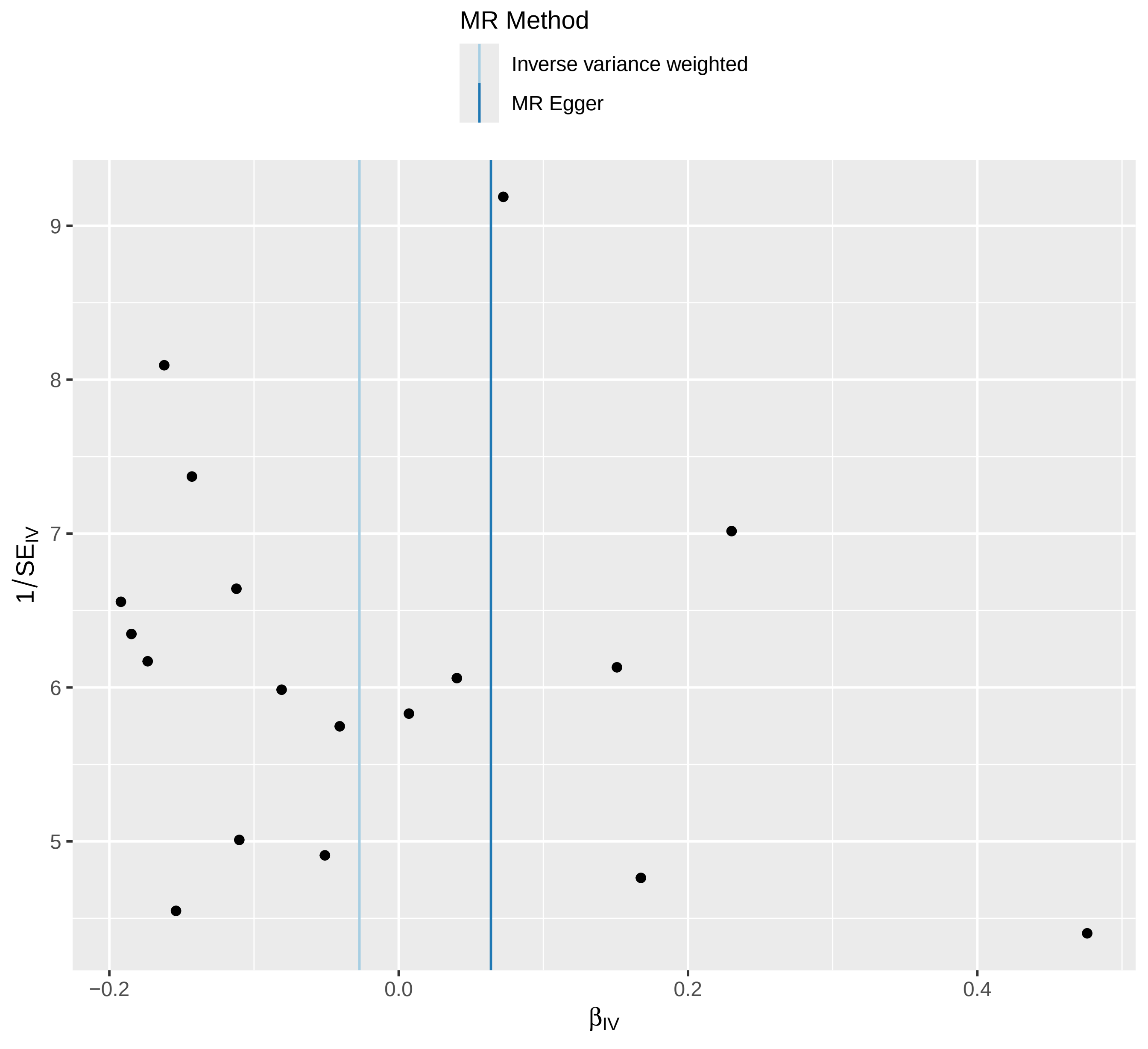
MR Method
Inverse variance weighted
MR Egger
9
8
7
6
5
1/SE_IV
−0.2
0.0
0.2
0.4
β_IV

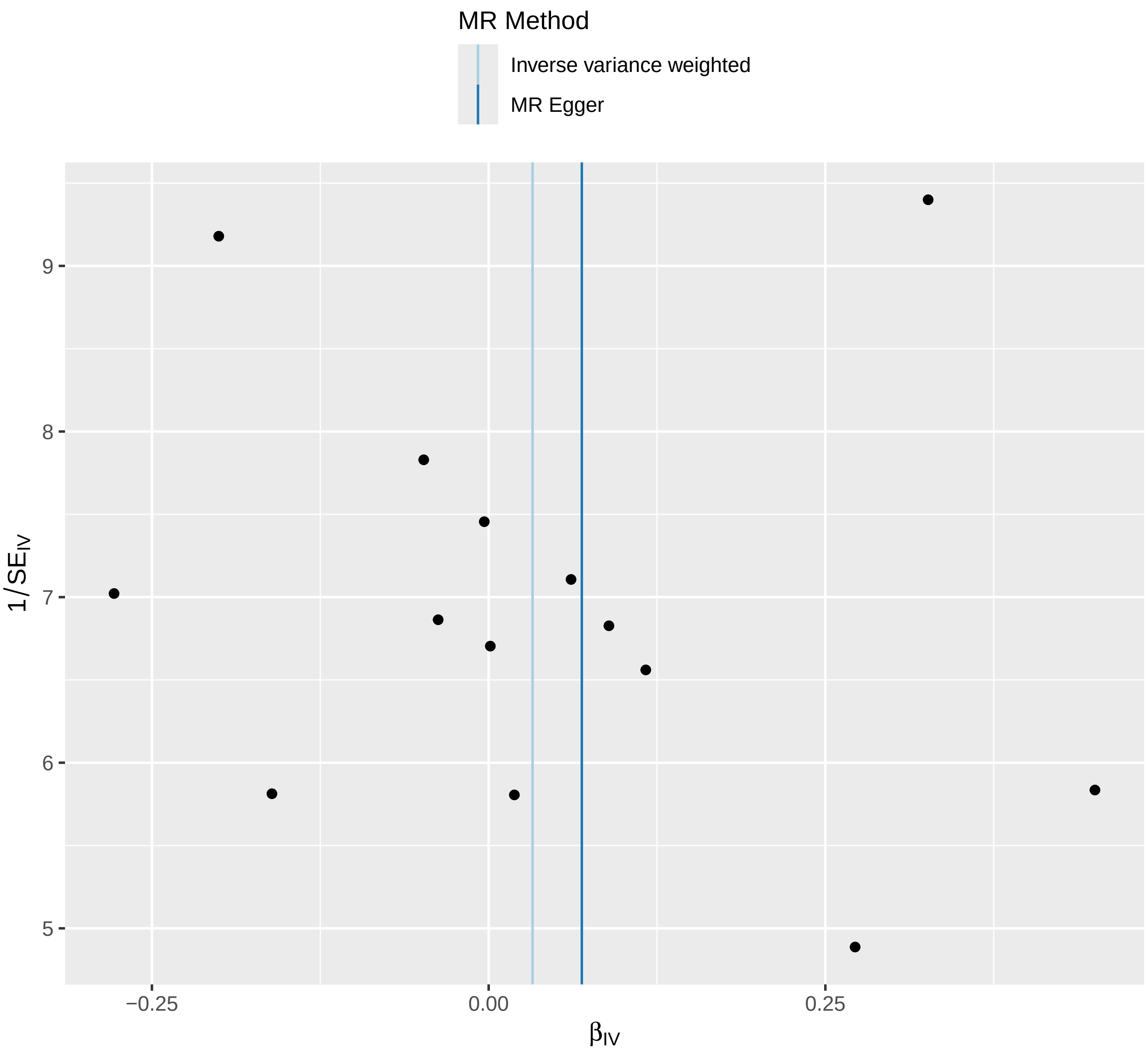
MR Method
Inverse variance weighted
MR Egger
9
8
7
6
5
1/SE_IV
−0.25
0.00
0.25
β_IV

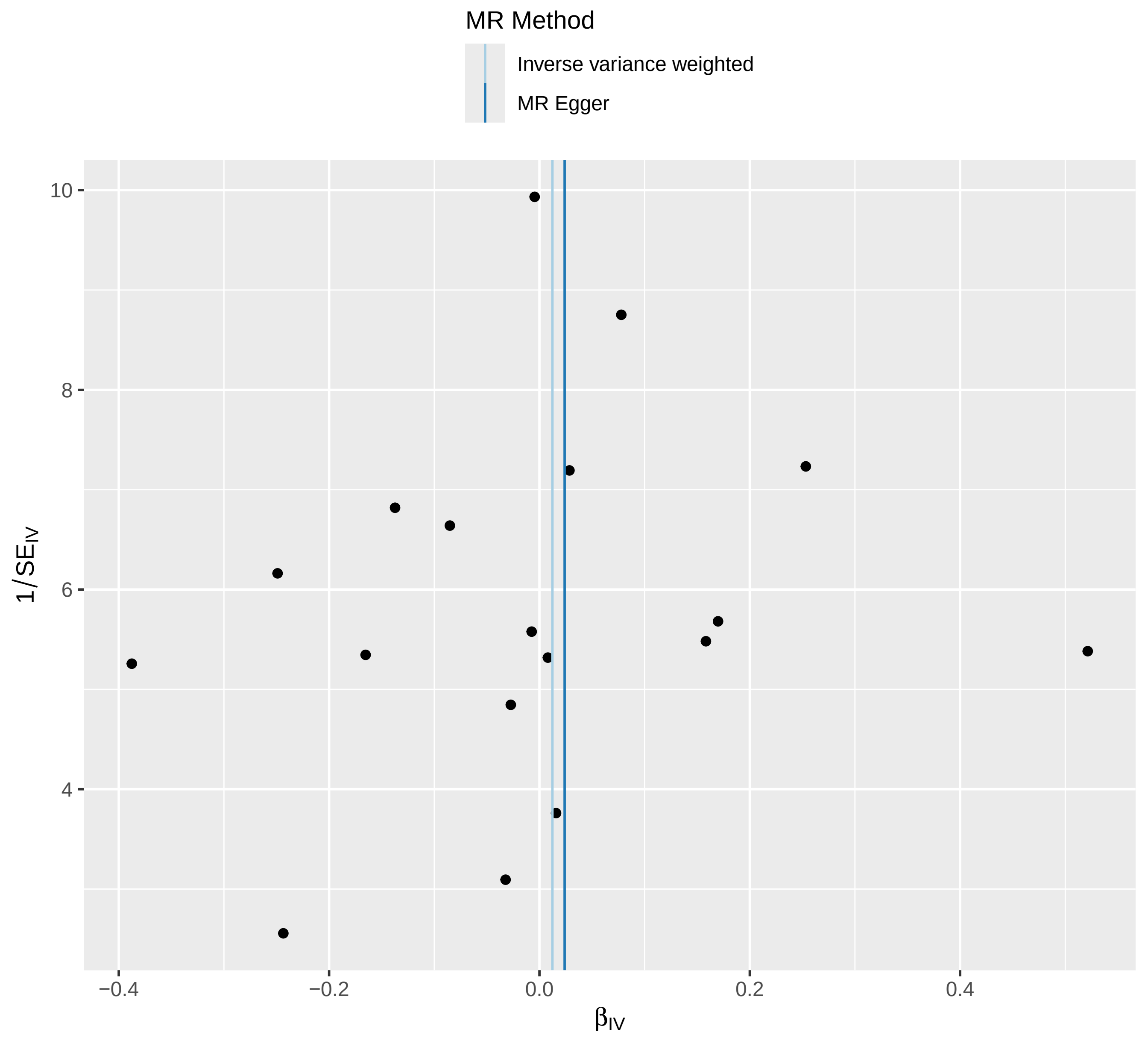

MR Method
Inverse variance weighted
MR Egger
10
8
6
4
1/SE_IV
−0.4
−0.2
0.0
0.2
0.4
β_IV

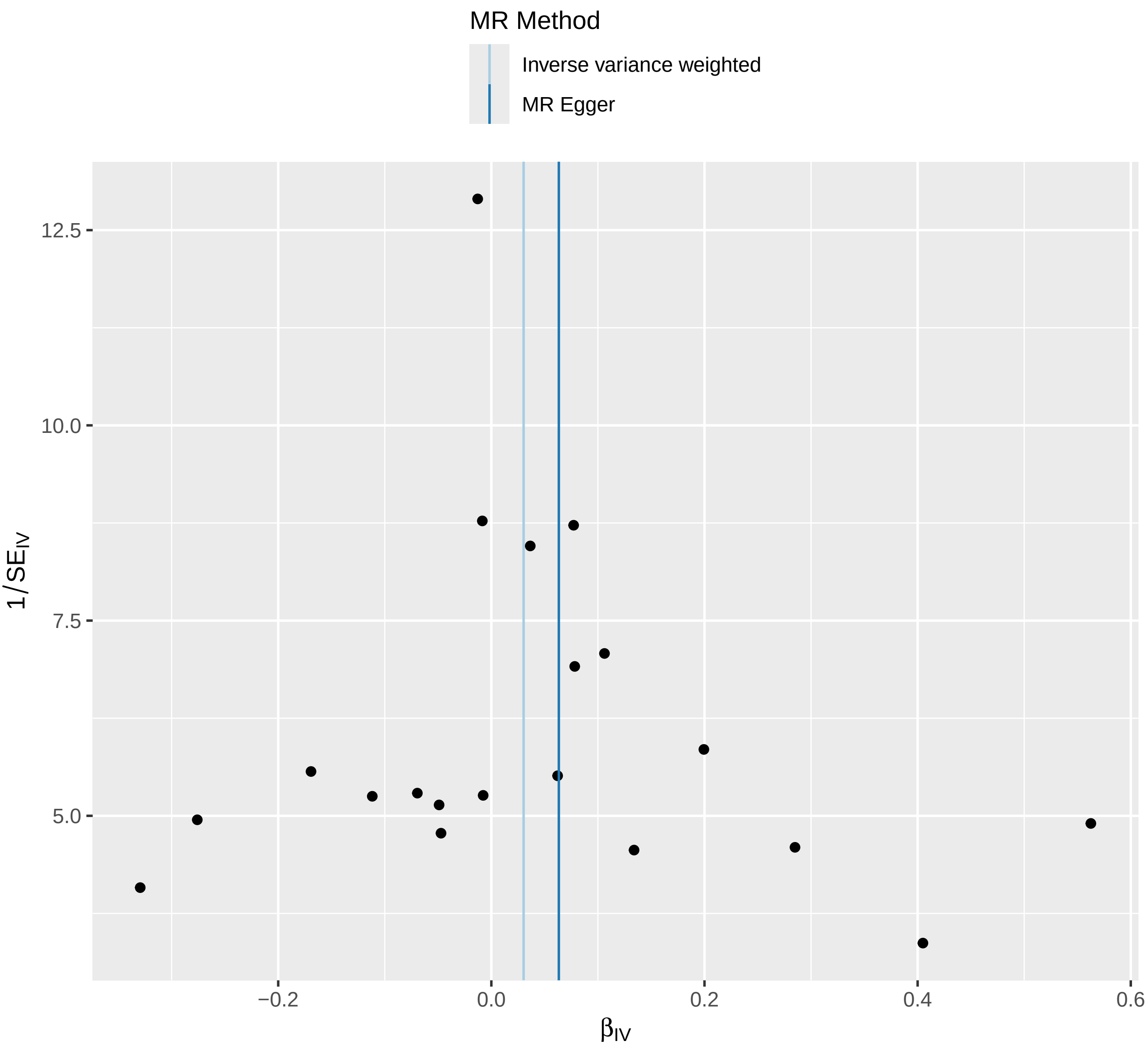
MR Method
Inverse variance weighted
MR Egger
12.5
10.0
7.5
5.0
1/SE_IV
−0.2
0.0
0.2
0.4
0.6
β_IV

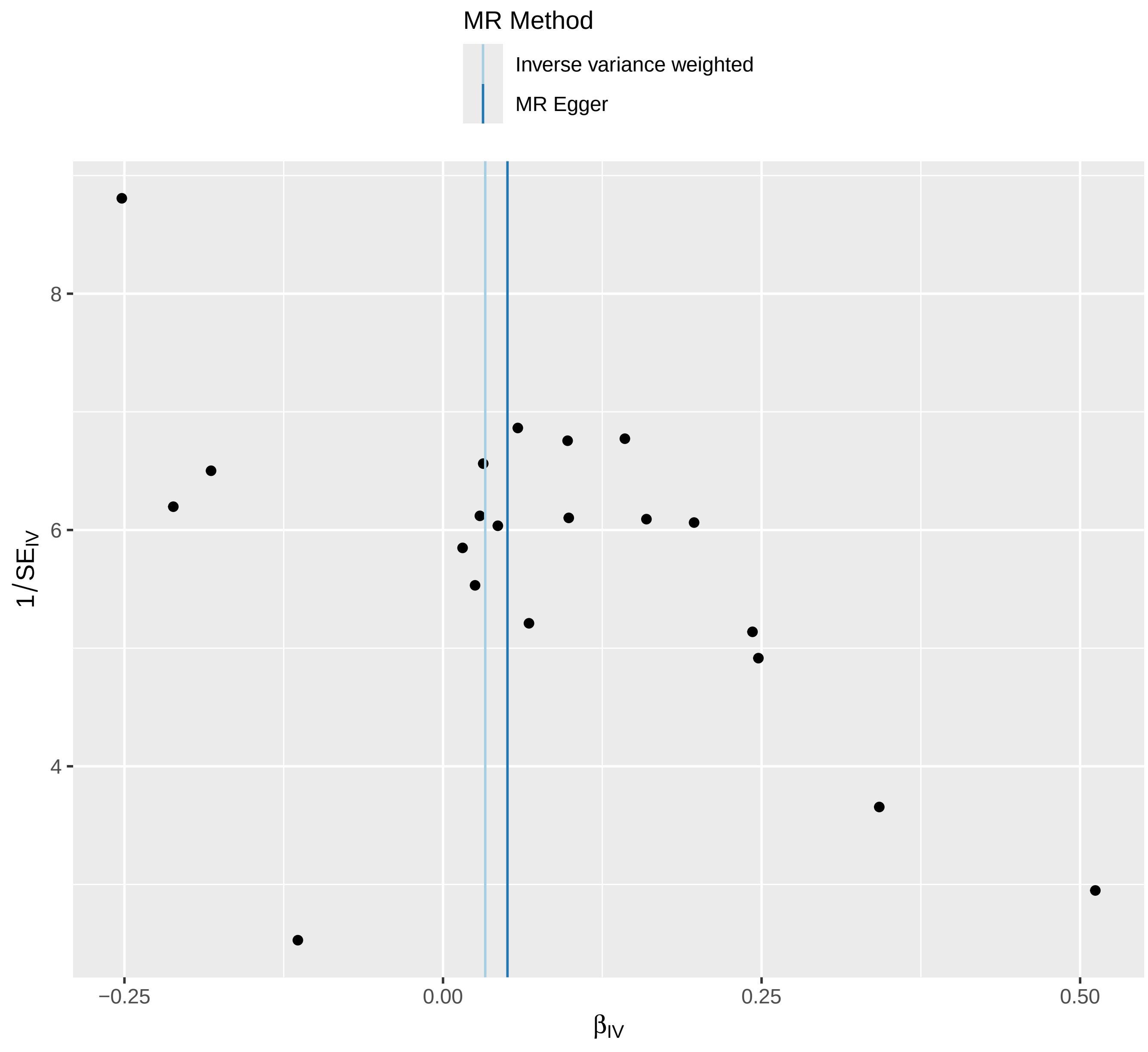
MR Method
Inverse variance weighted
MR Egger
8
6
4
$1/SE_{IV}$
−0.25
0.00
0.25
0.50
β_{IV}

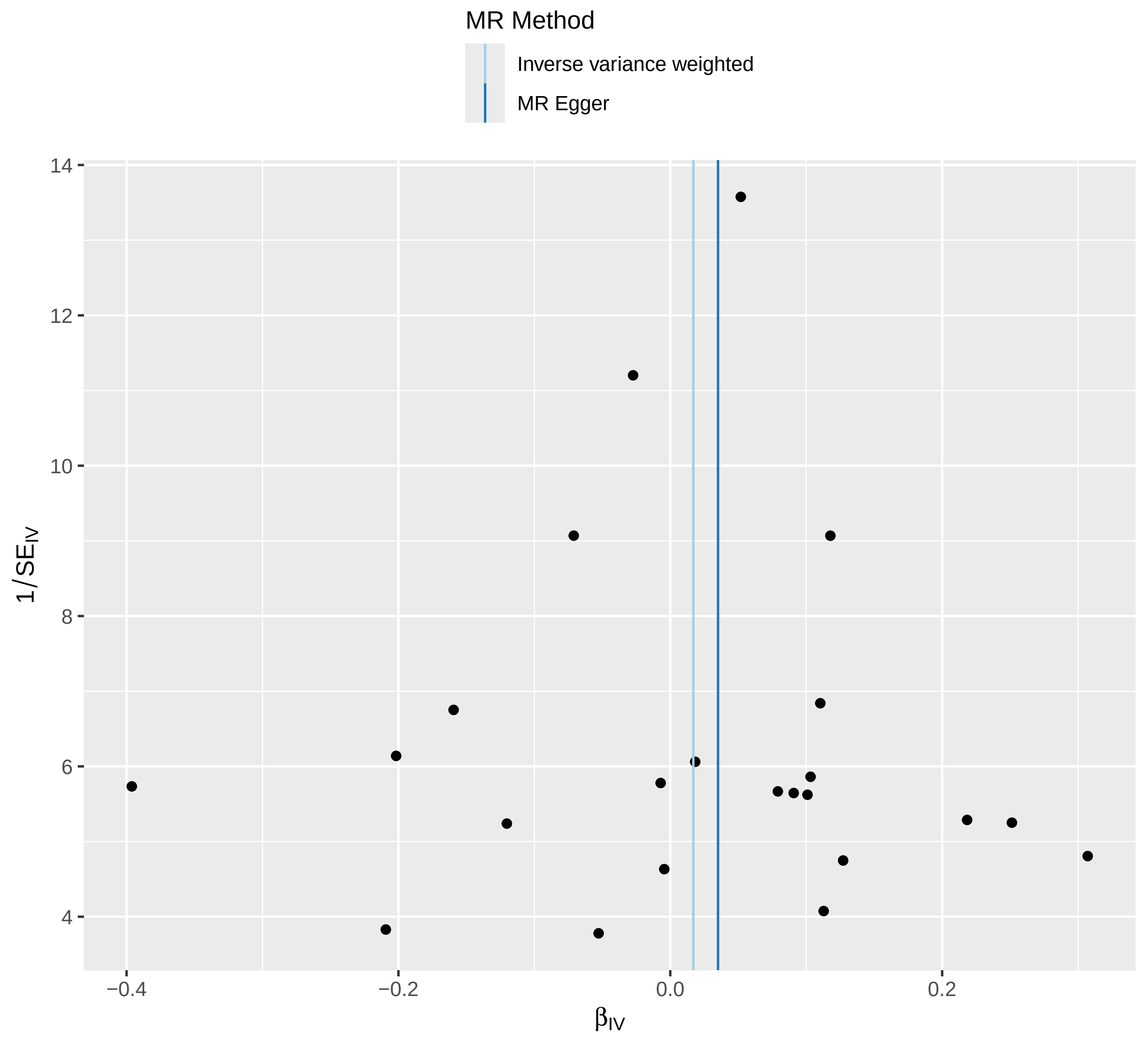
MR Method
Inverse variance weighted
MR Egger
14
12
10
8
6
4
1/SE$_{IV}$
−0.4
−0.2
0.0
0.2
β$_{IV}$

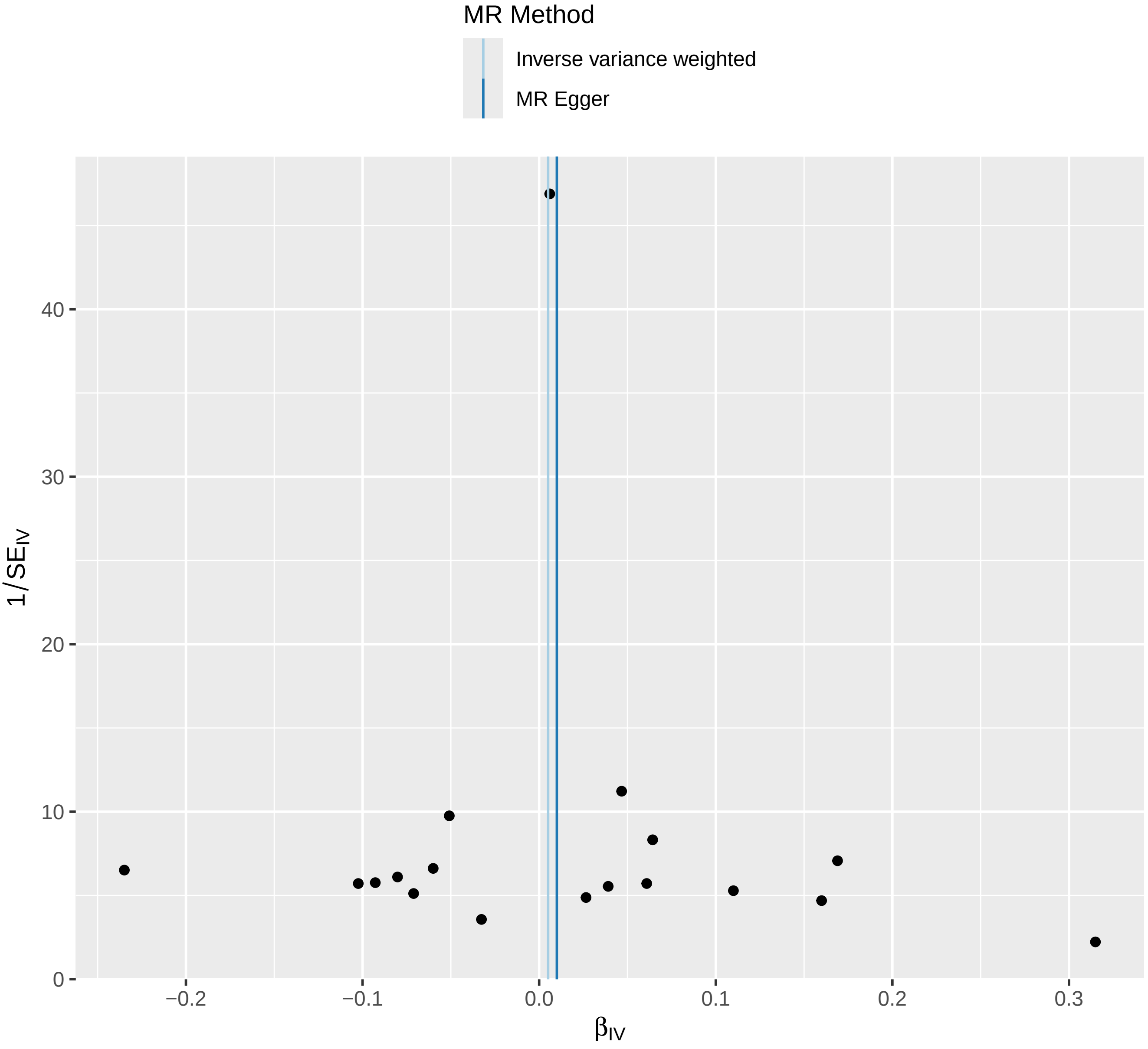

MR Method
Inverse variance weighted
MR Egger
1/SE$_{IV}$
β$_{IV}$
0
10
20
30
40
−0.2
−0.1
0.0
0.1
0.2
0.3

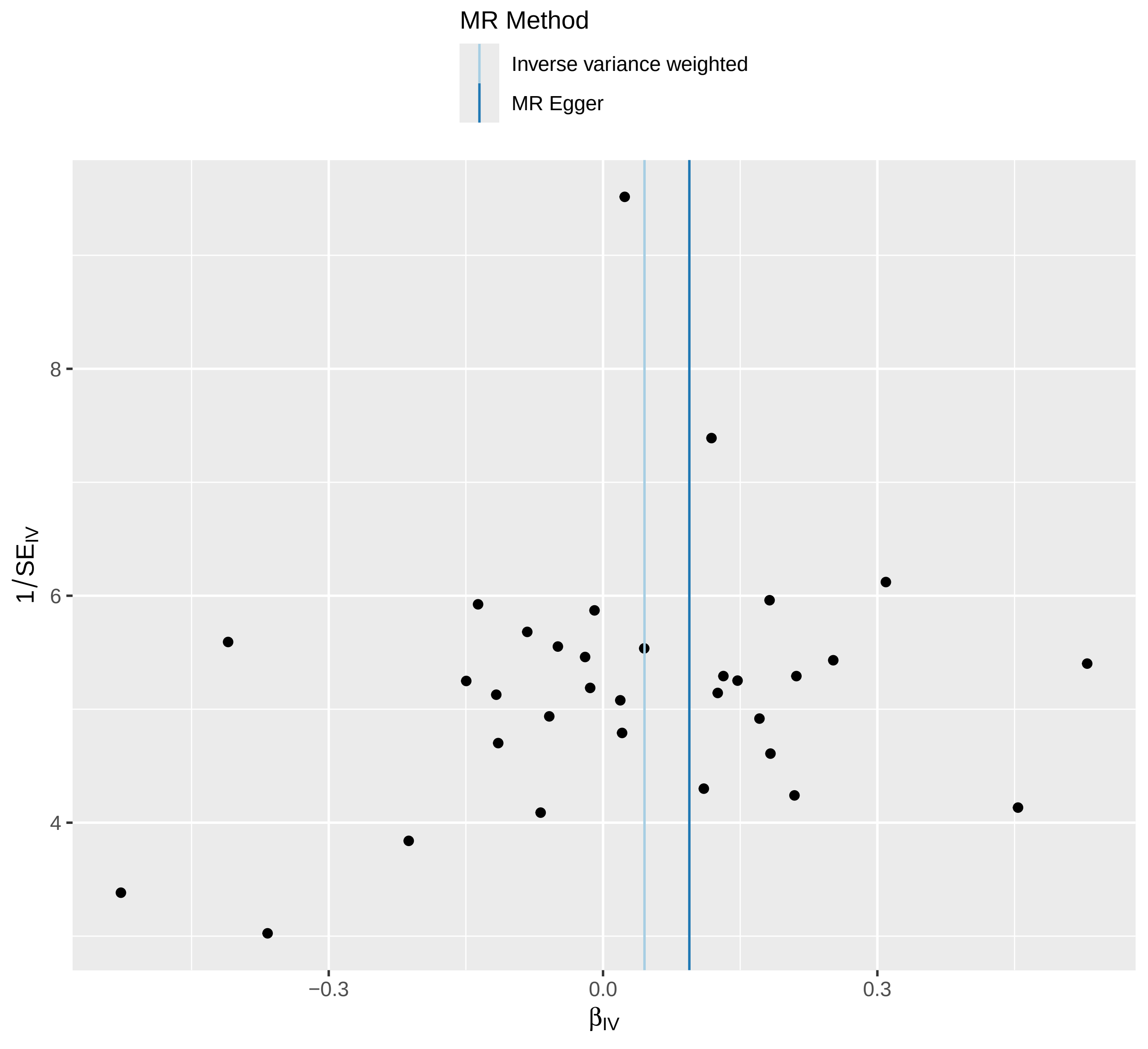
MR Method
Inverse variance weighted
MR Egger
$1/SE_{IV}$
8
6
4
−0.3
0.0
0.3
β_{IV}

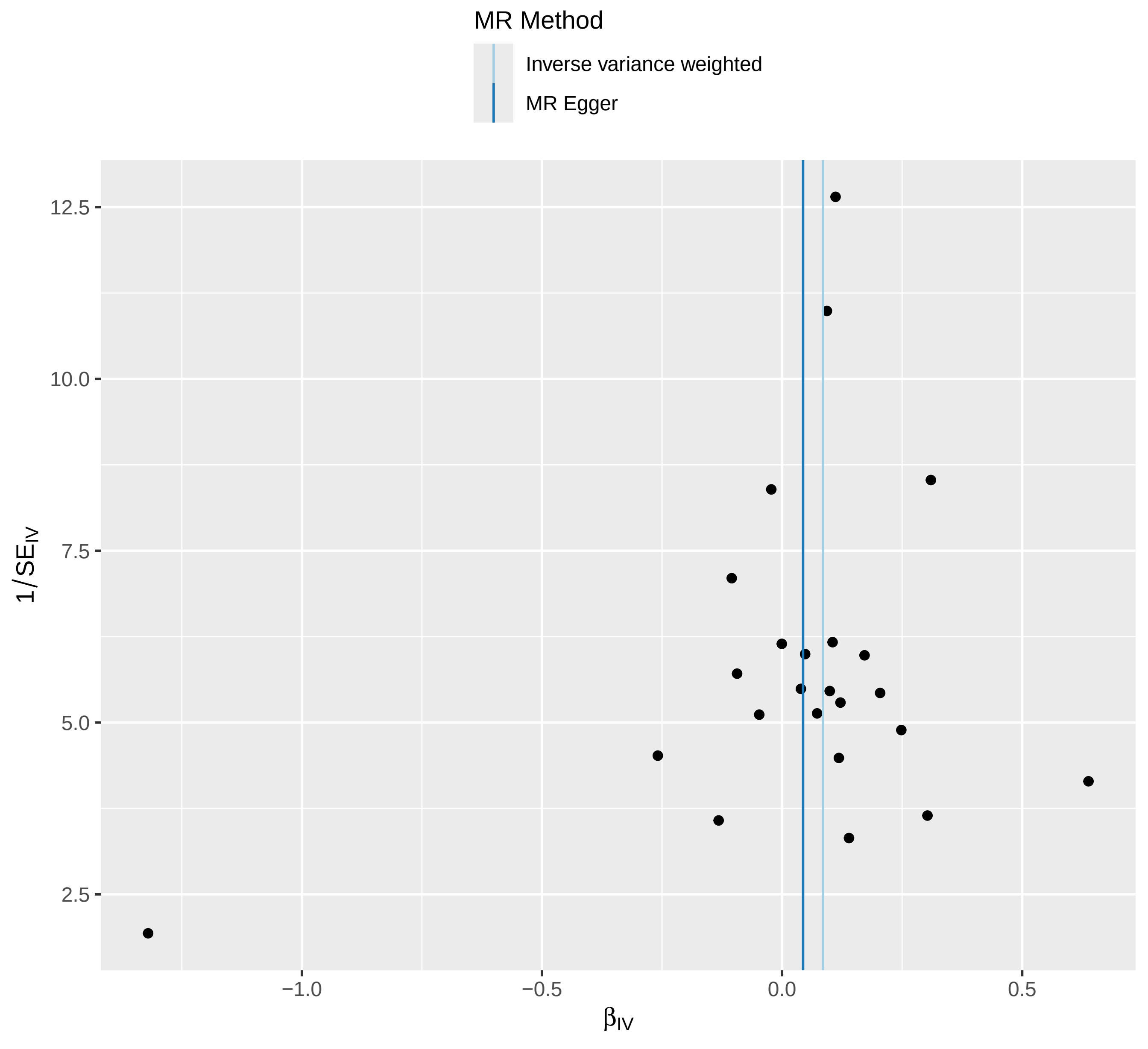
MR Method
Inverse variance weighted
MR Egger
1/SE_IV
12.5
10.0
7.5
5.0
2.5
−1.0
−0.5
0.0
0.5
β_IV

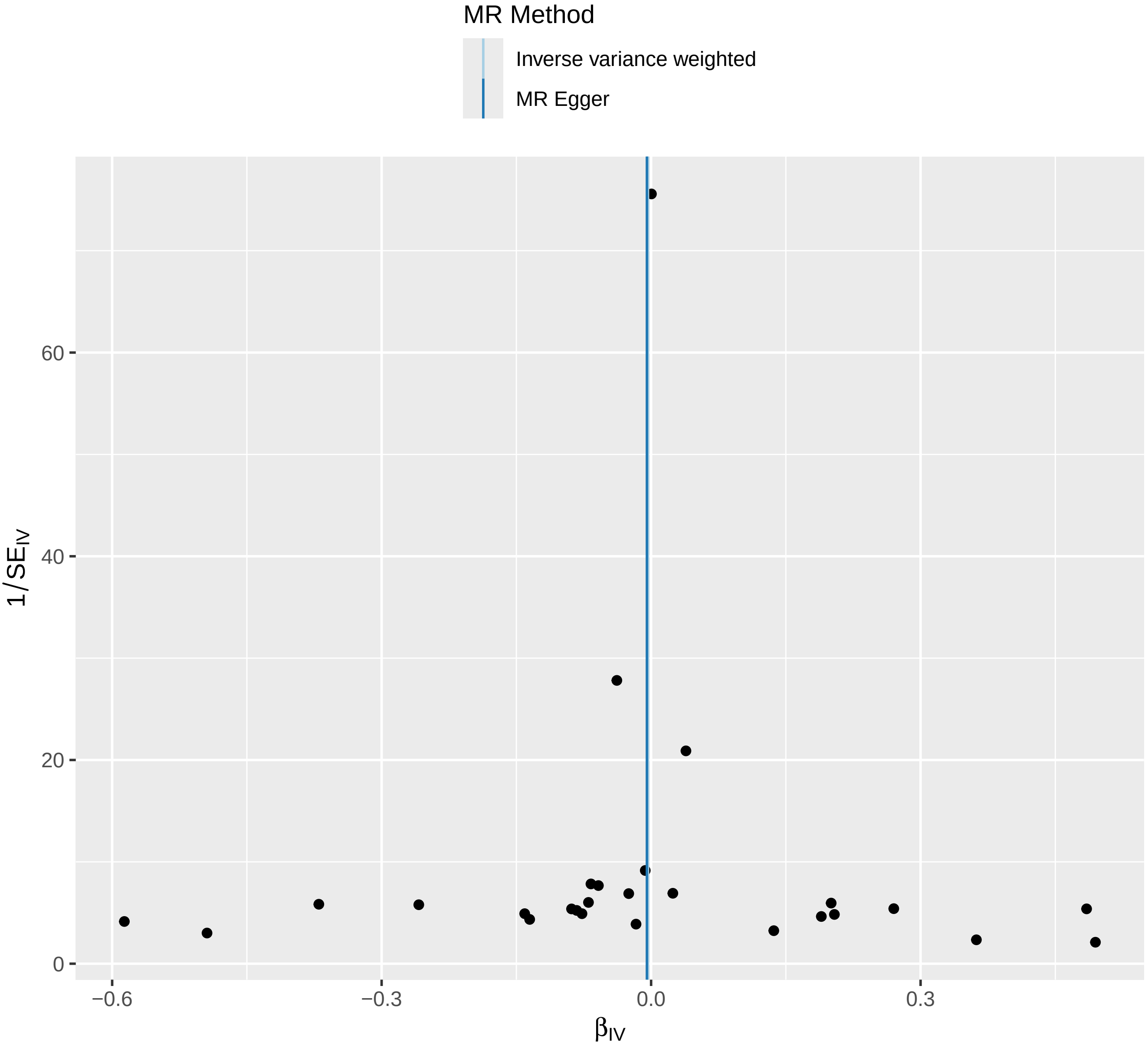

MR Method
Inverse variance weighted
MR Egger
1/SE_IV
60
40
20
0
−0.6
−0.3
0.0
0.3
β_IV

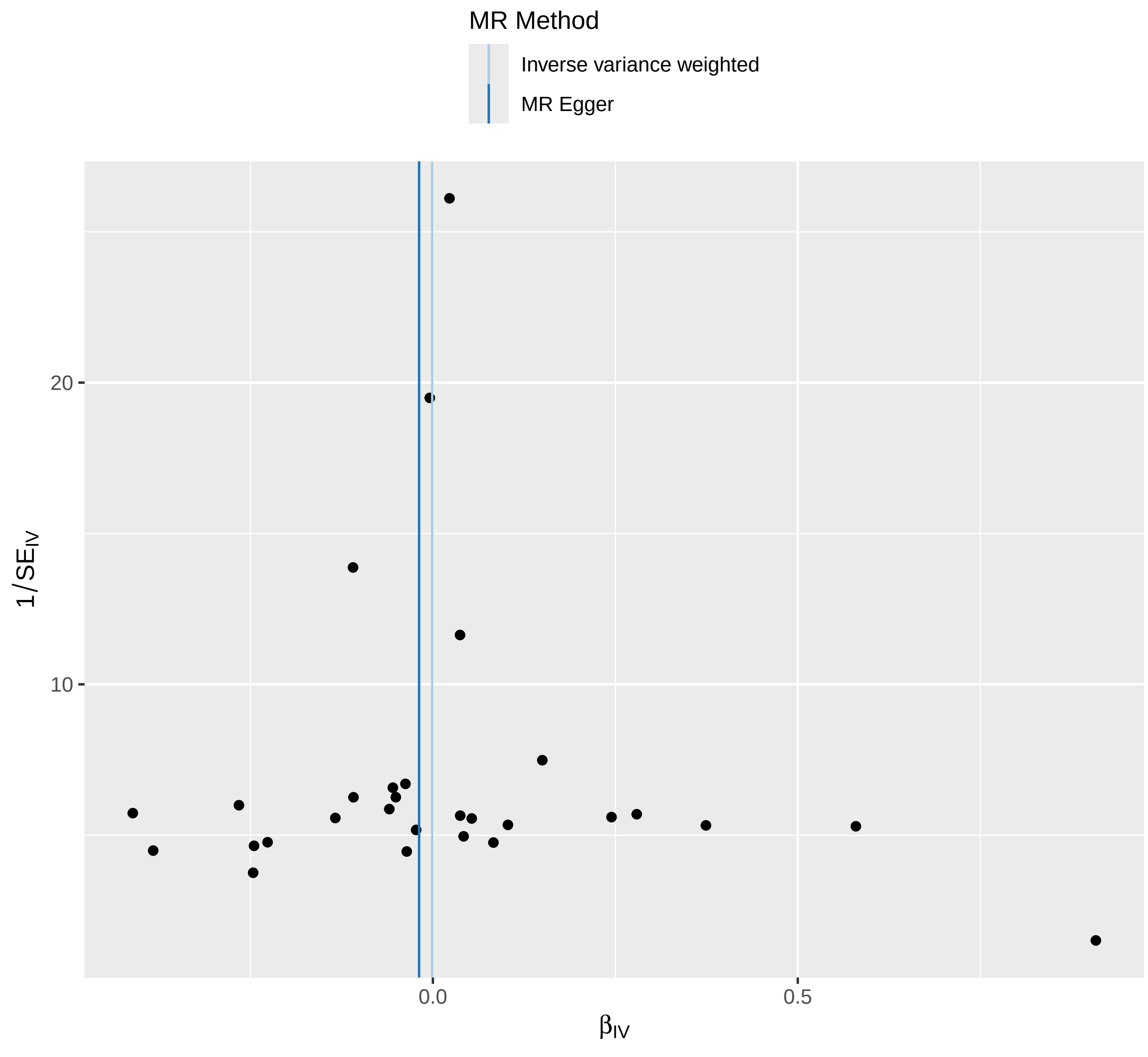

MR Method
Inverse variance weighted
MR Egger
20
10
1/SE$_{IV}$
0.0
0.5
β$_{IV}$

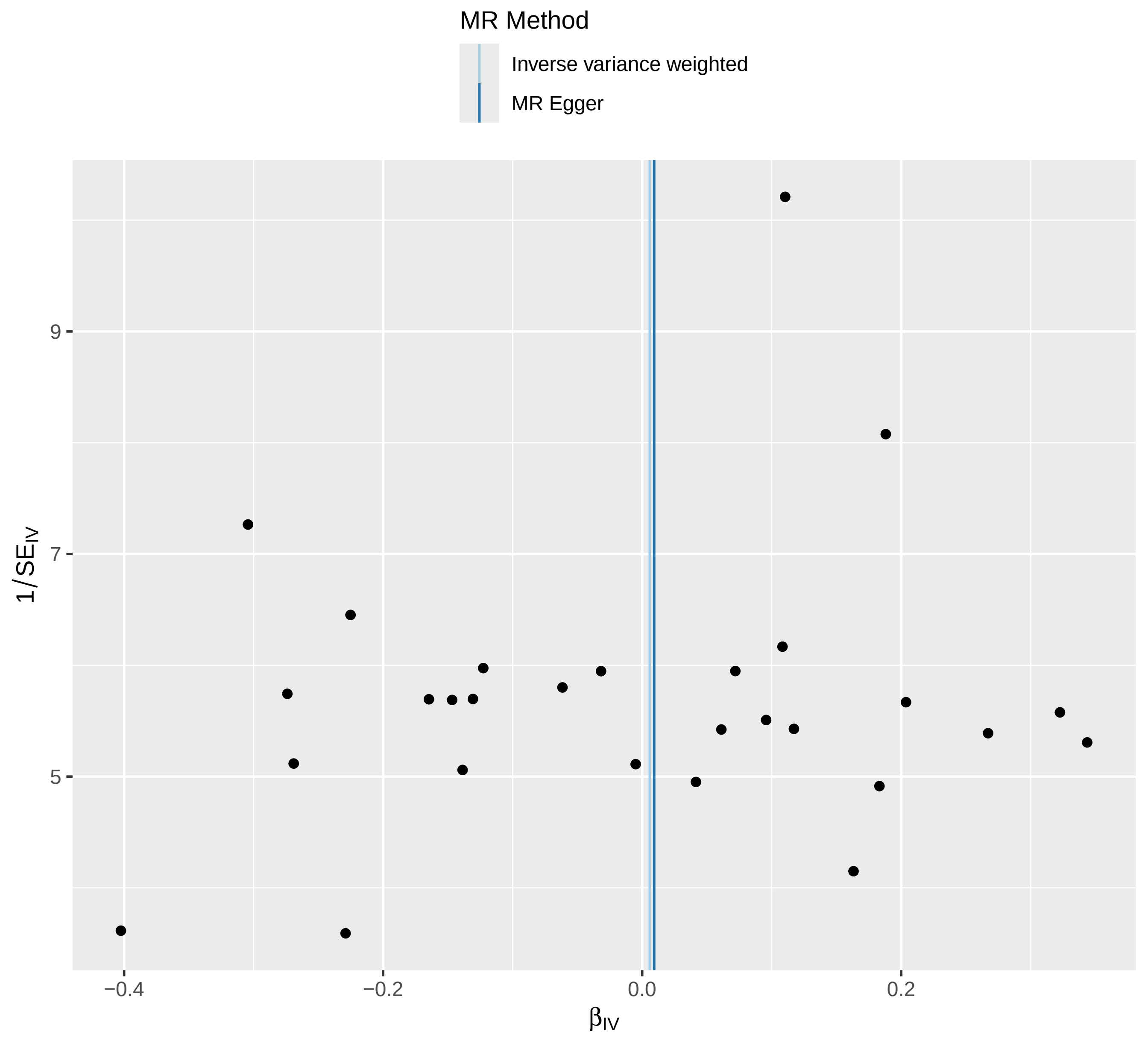
MR Method
Inverse variance weighted
MR Egger
1/SE_{IV}
9
7
5
−0.4
−0.2
0.0
0.2
β_{IV}

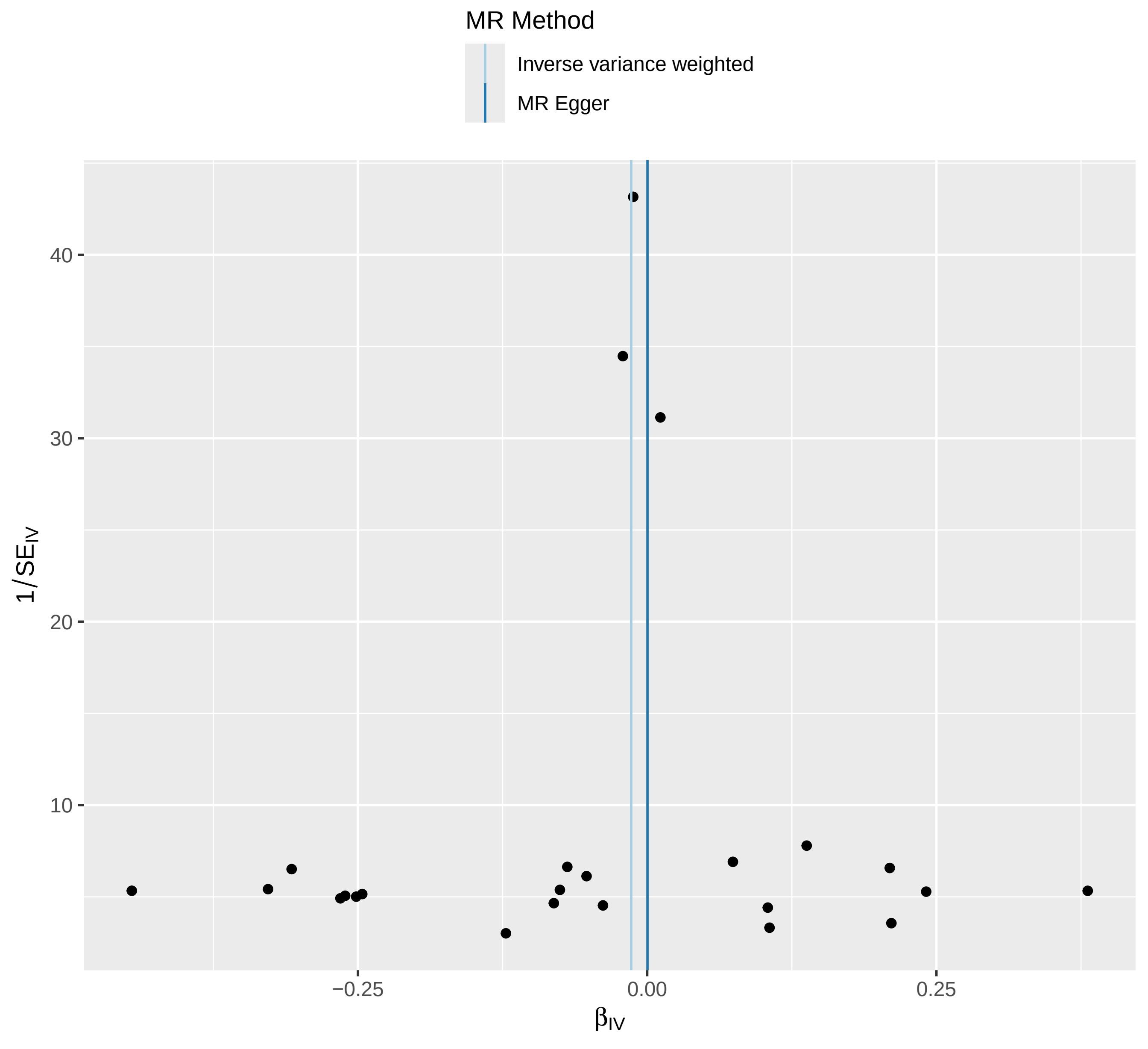

MR Method
Inverse variance weighted
MR Egger
40
30
20
10
1/SE$_{IV}$
−0.25
0.00
0.25
β$_{IV}$

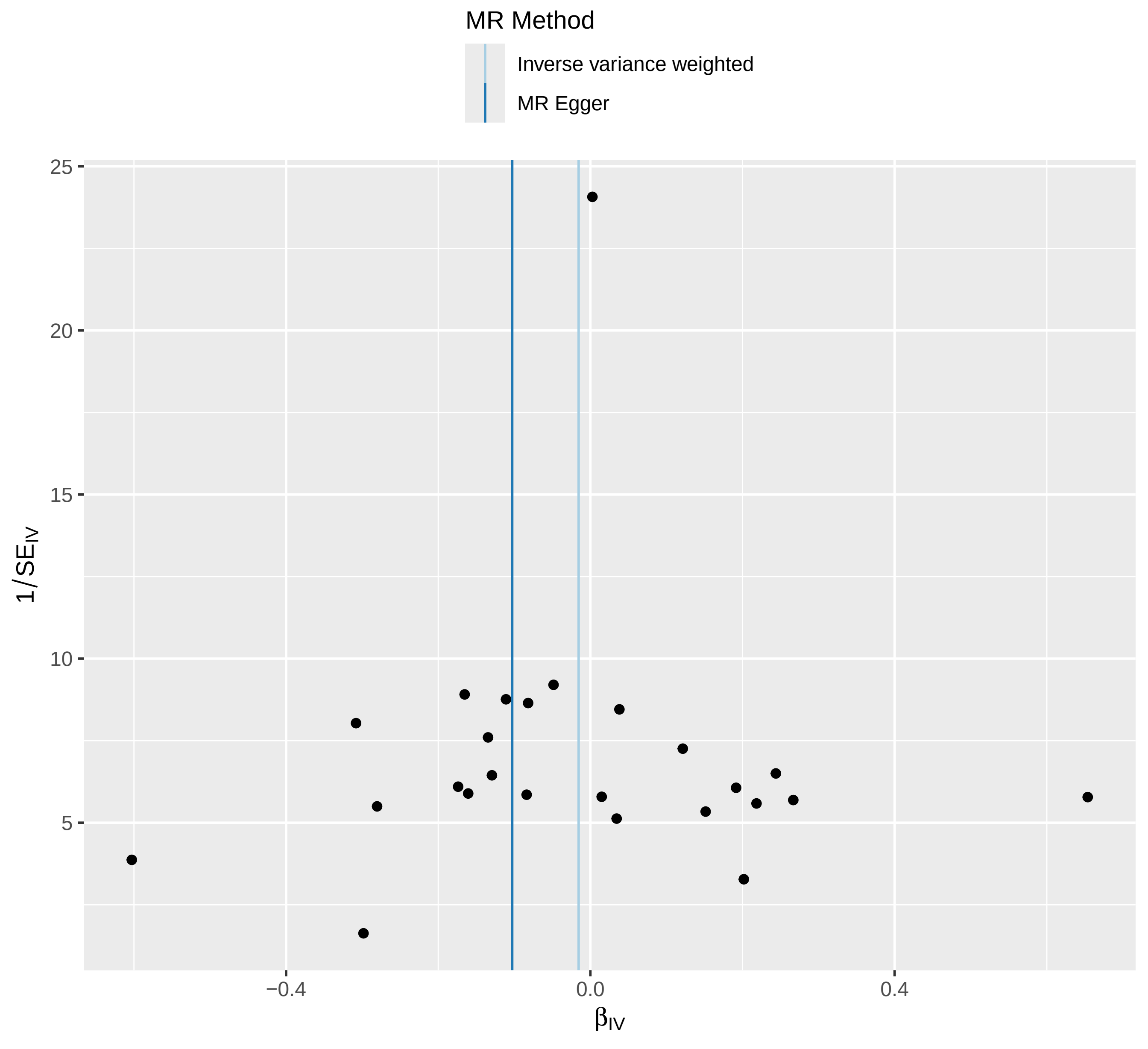

MR Method
Inverse variance weighted
MR Egger
25
20
15
10
5
1/SE_IV
−0.4
0.0
0.4
β_IV

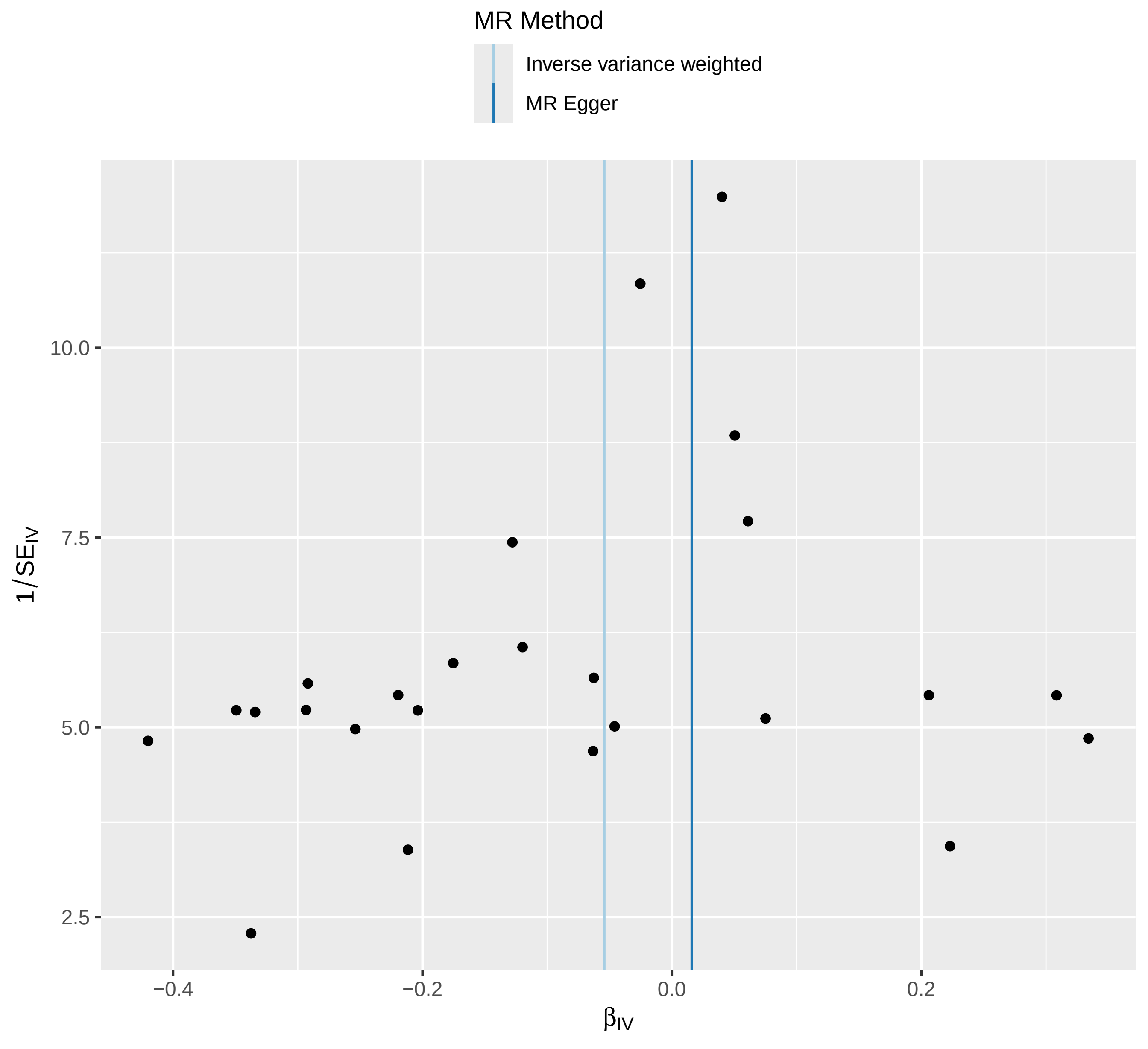

MR Method
Inverse variance weighted
MR Egger
10.0
7.5
5.0
2.5
$1/SE_{IV}$
−0.4
−0.2
0.0
0.2
β_{IV}

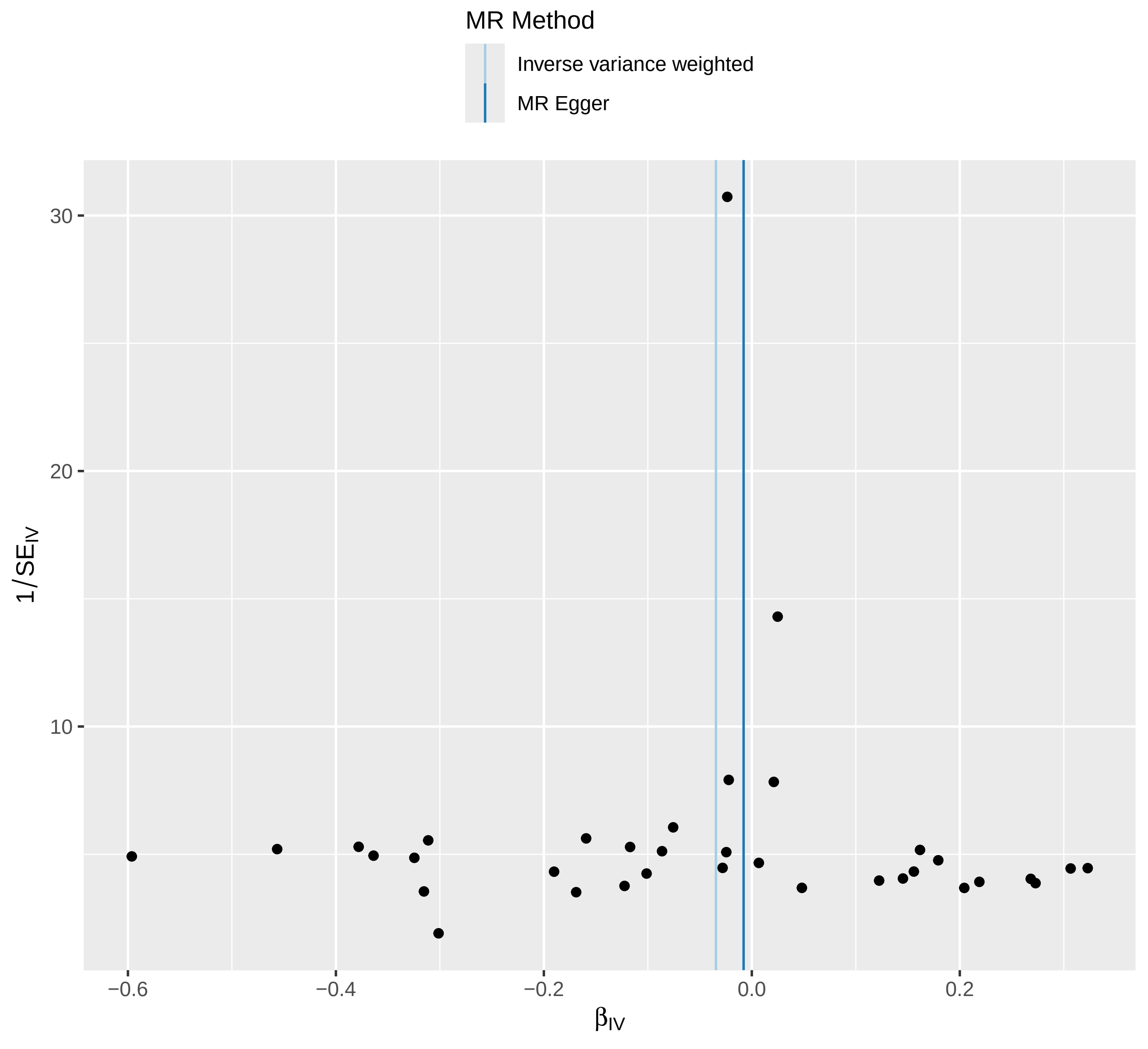
MR Method
Inverse variance weighted
MR Egger
30
20
10
1/SE_IV
−0.6
−0.4
−0.2
0.0
0.2
β_IV

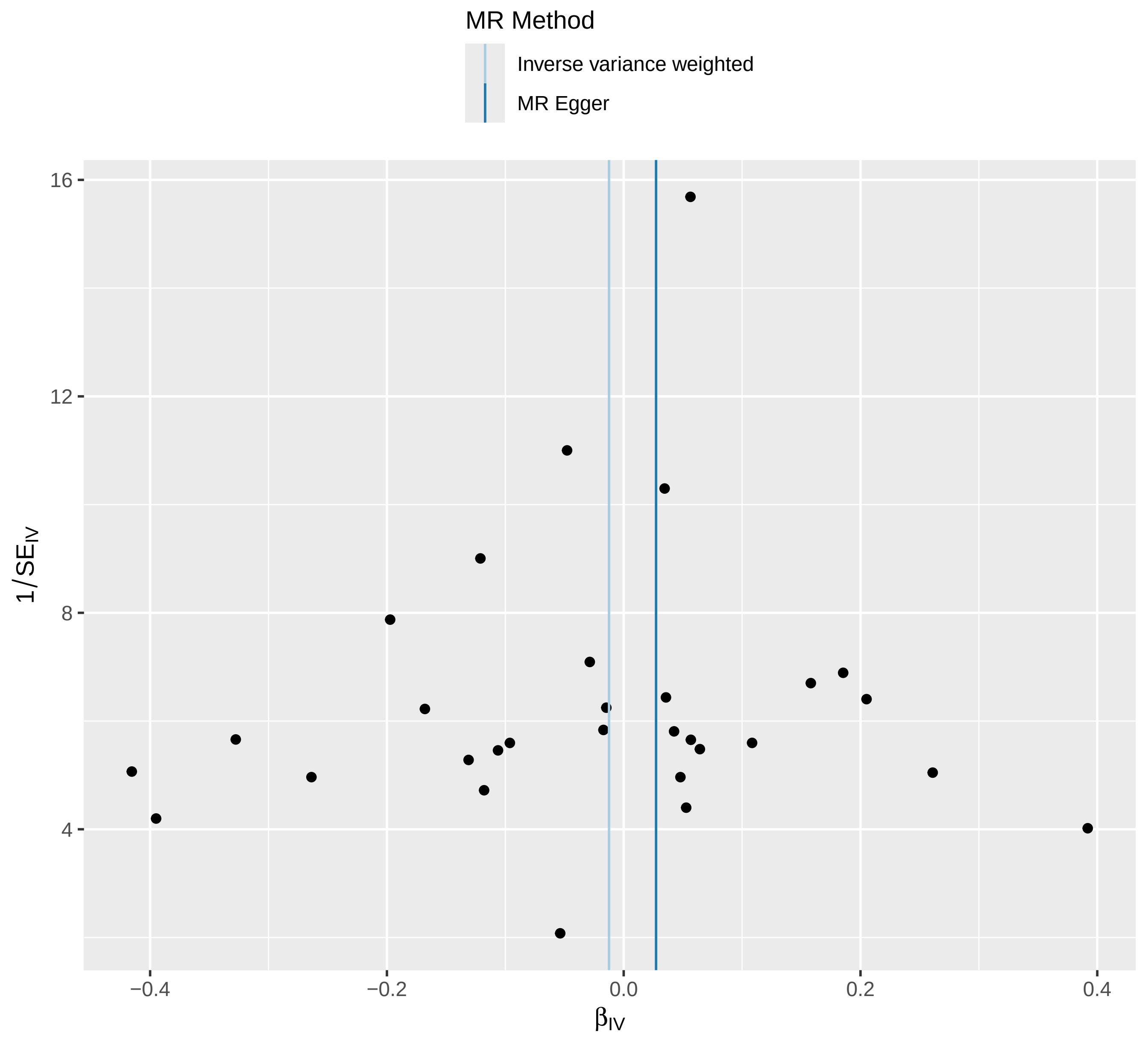
MR Method
Inverse variance weighted
MR Egger
16
12
8
4
1/SE_IV
−0.4
−0.2
0.0
0.2
0.4
β_IV

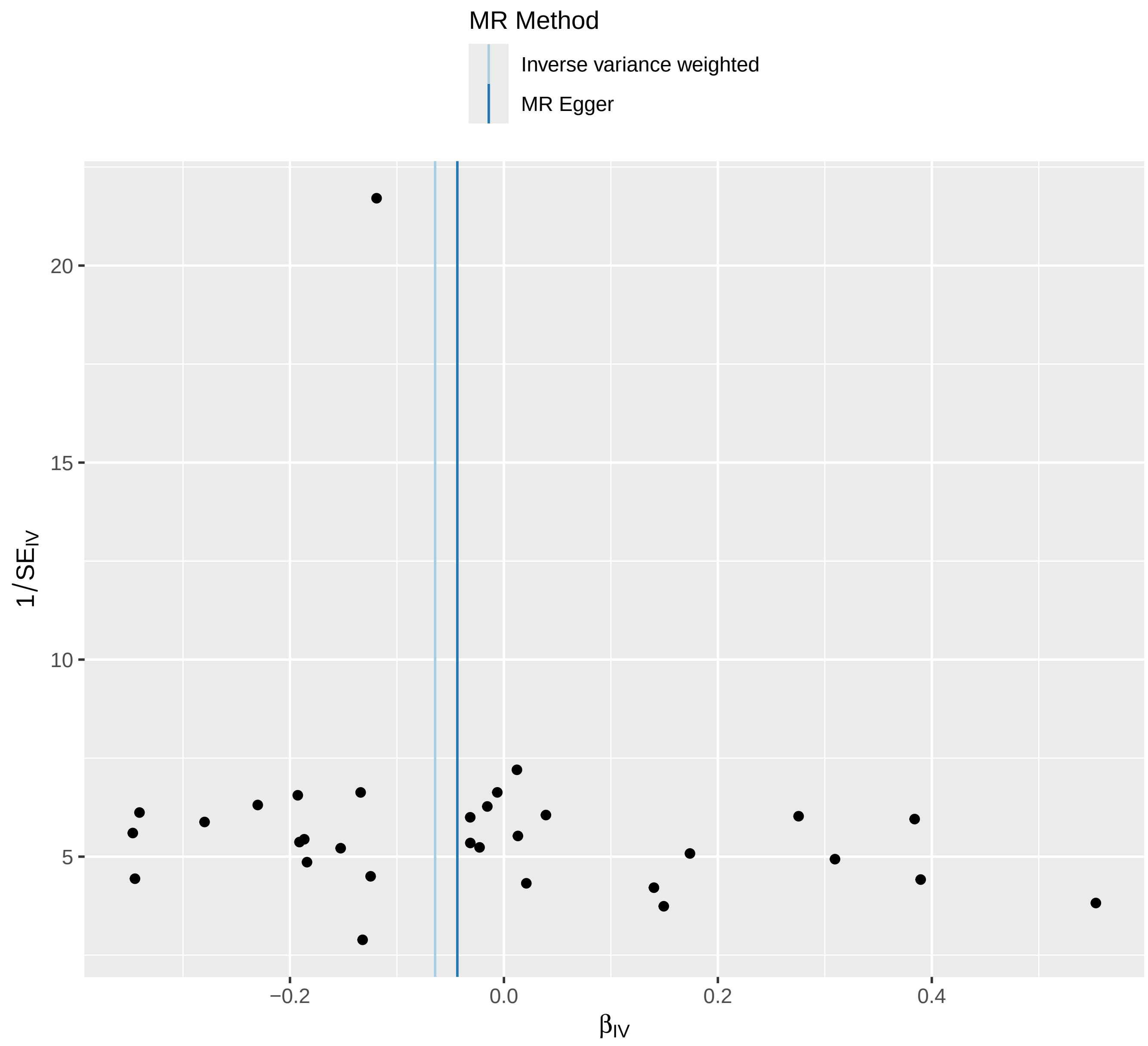
MR Method
Inverse variance weighted
MR Egger
20
15
10
5
1/SE_IV
−0.2
0.0
0.2
0.4
β_IV

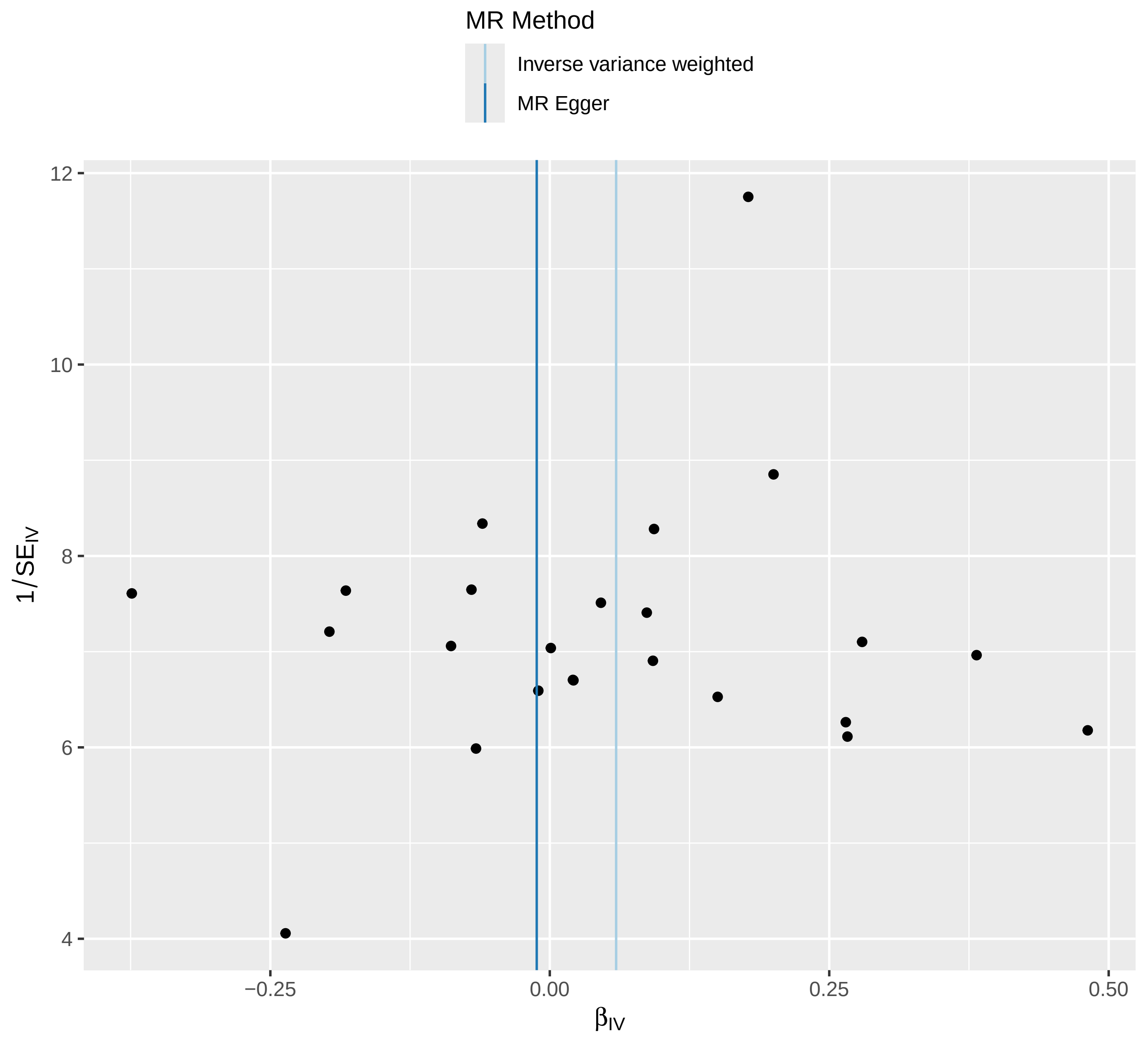
MR Method
Inverse variance weighted
MR Egger
12
10
8
6
4
$1/SE_{IV}$
−0.25
0.00
0.25
0.50
β_{IV}

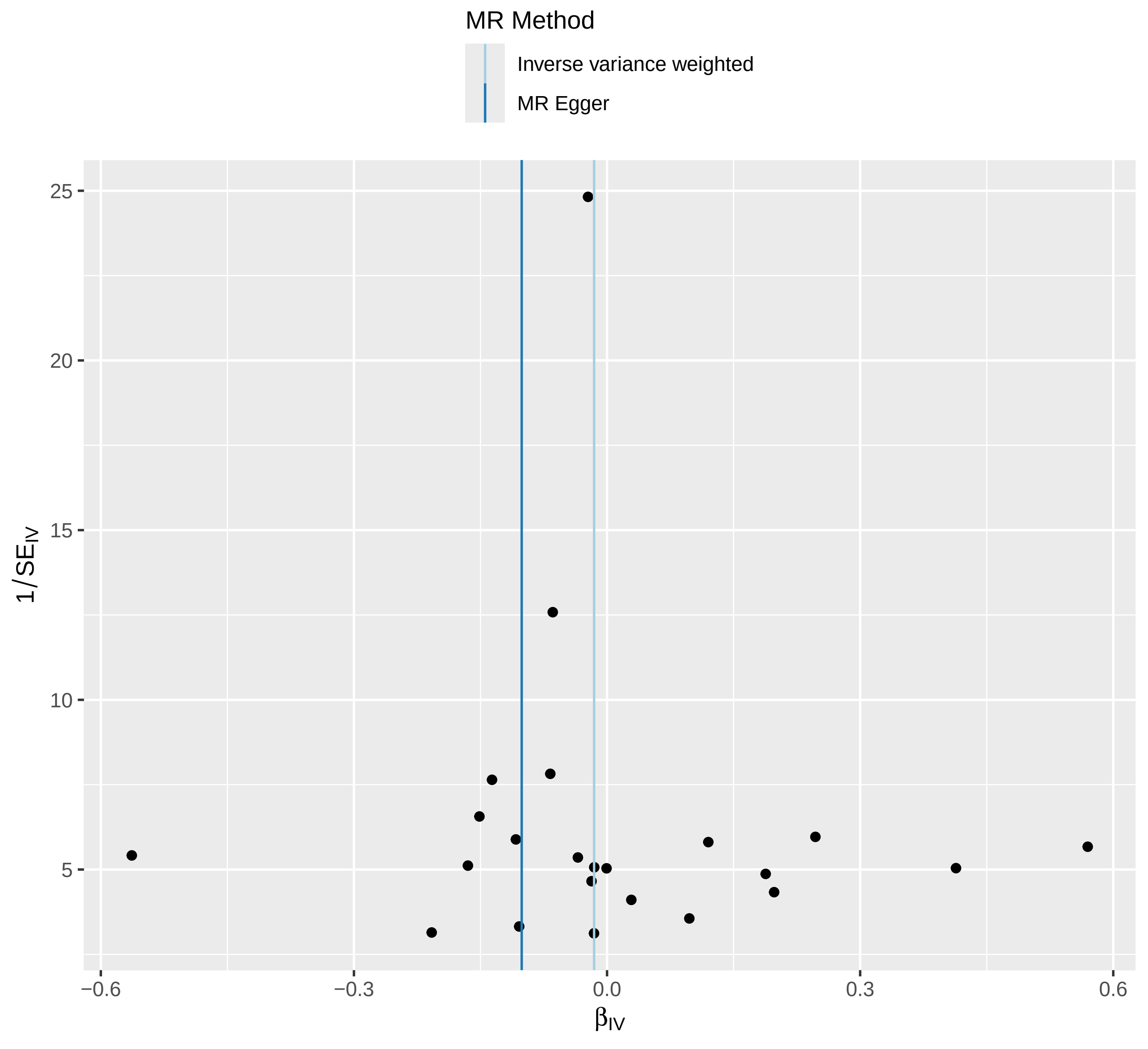

MR Method
Inverse variance weighted
MR Egger
25
20
15
10
5
1/SE_IV
−0.6
−0.3
0.0
0.3
0.6
β_IV

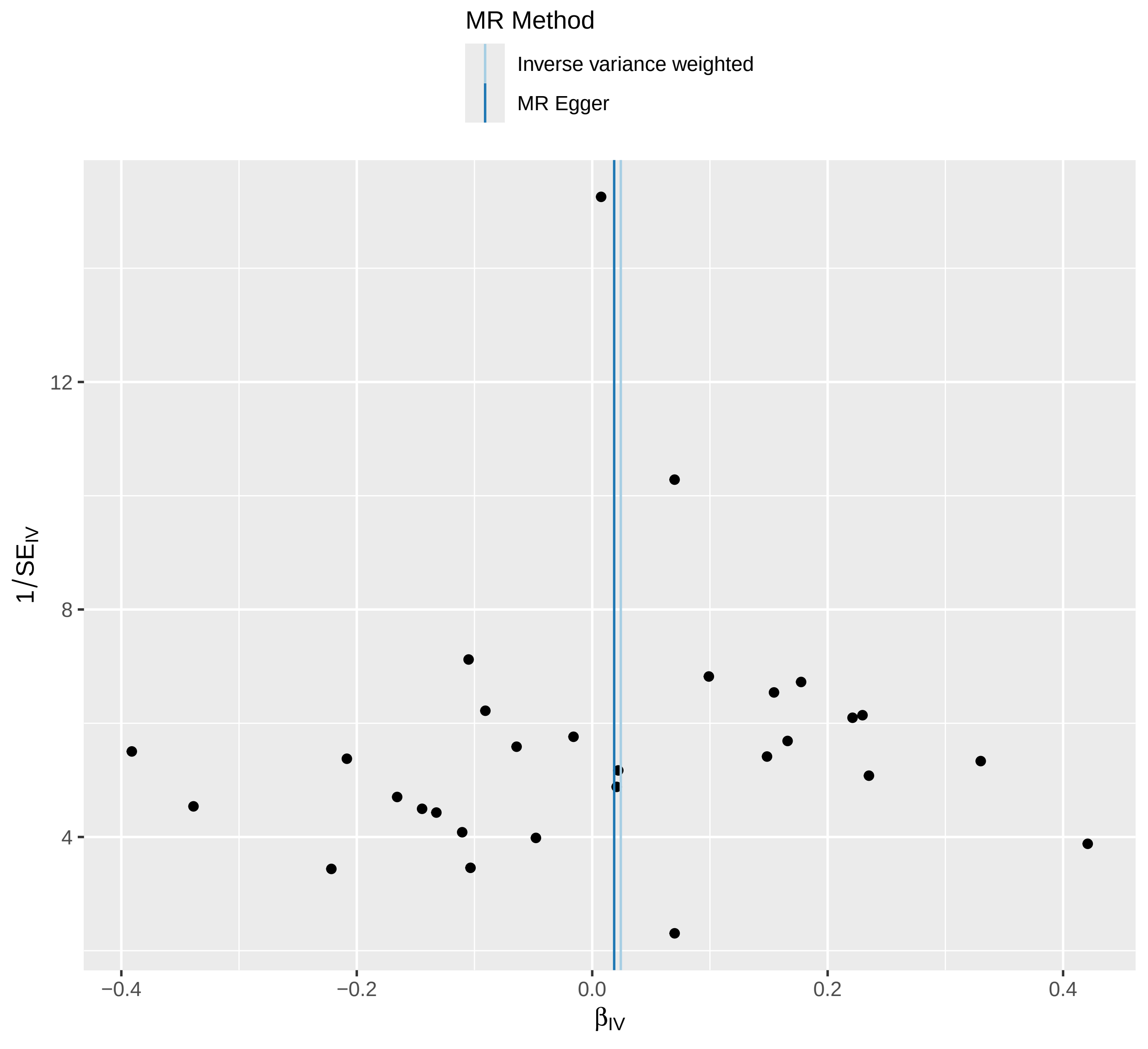
MR Method
Inverse variance weighted
MR Egger
1/SE_IV
12
8
4
−0.4
−0.2
0.0
0.2
0.4
β_IV

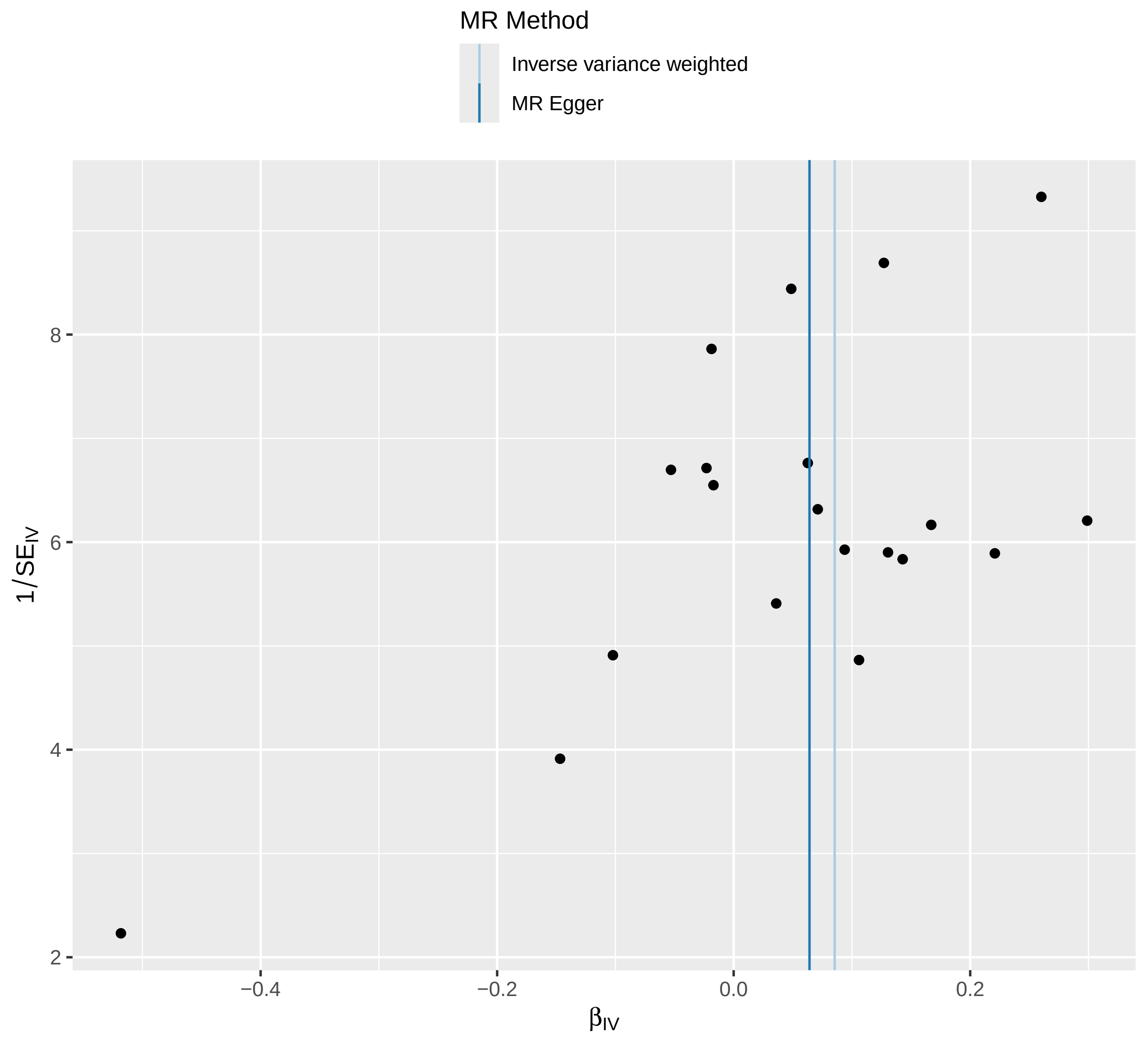
MR Method
Inverse variance weighted
MR Egger
8
6
4
2
$1/SE_{IV}$
−0.4
−0.2
0.0
0.2
β_{IV}

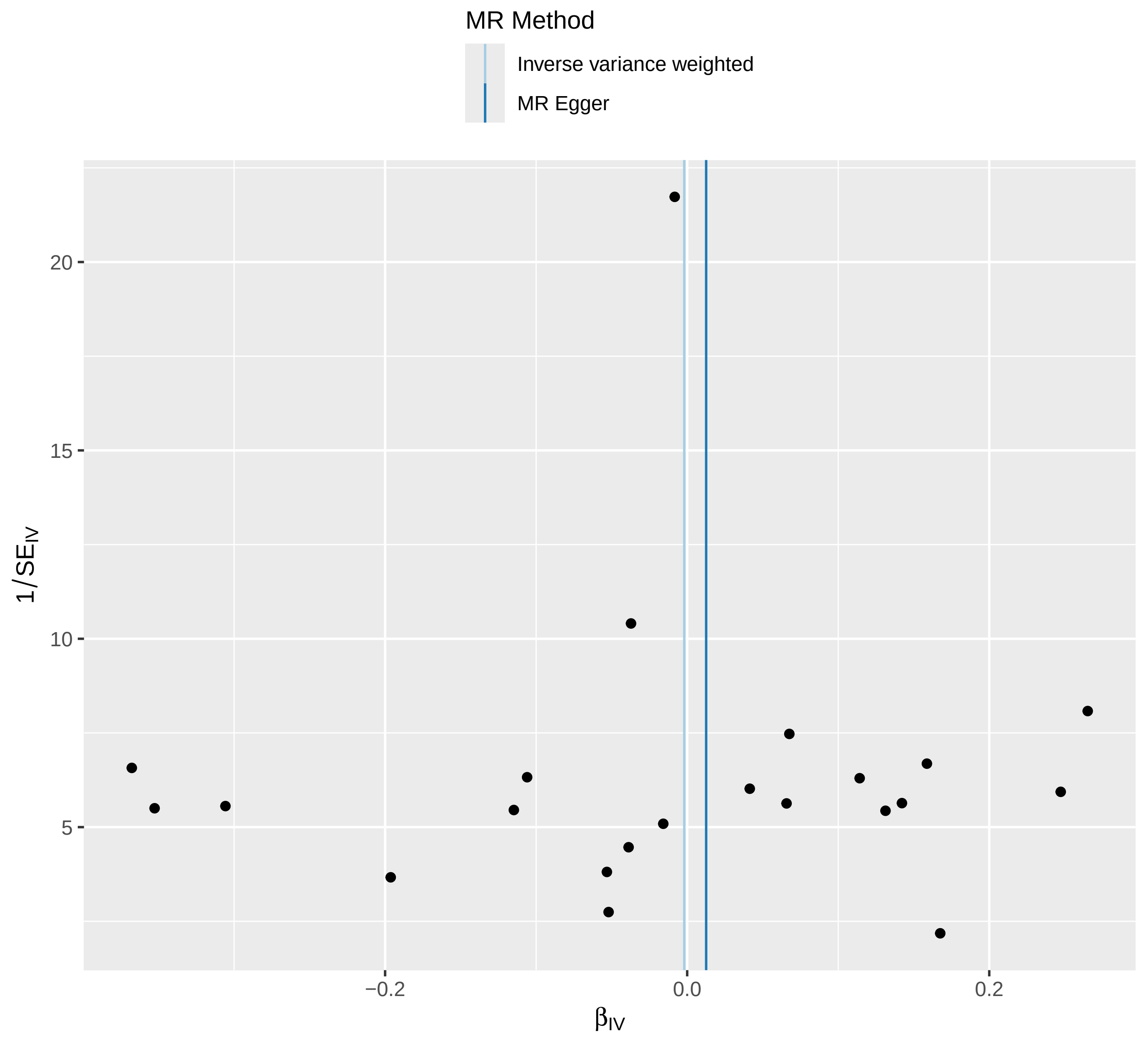

MR Method
Inverse variance weighted
MR Egger
1/SE_IV
β_IV
20
15
10
5
−0.2
0.0
0.2

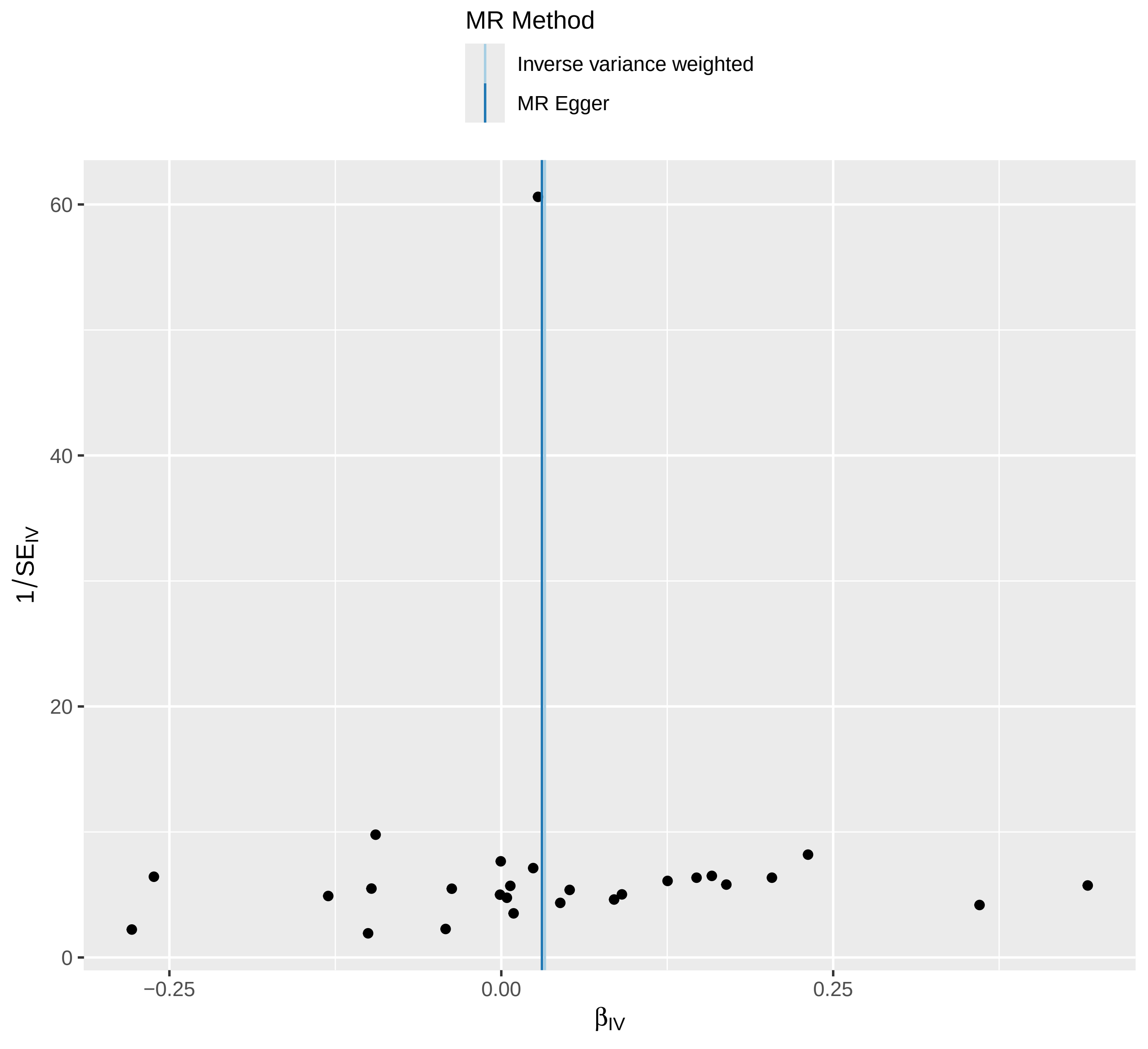

MR Method
Inverse variance weighted
MR Egger
60
40
20
0
1/SE$_{IV}$
−0.25
0.00
0.25
β$_{IV}$

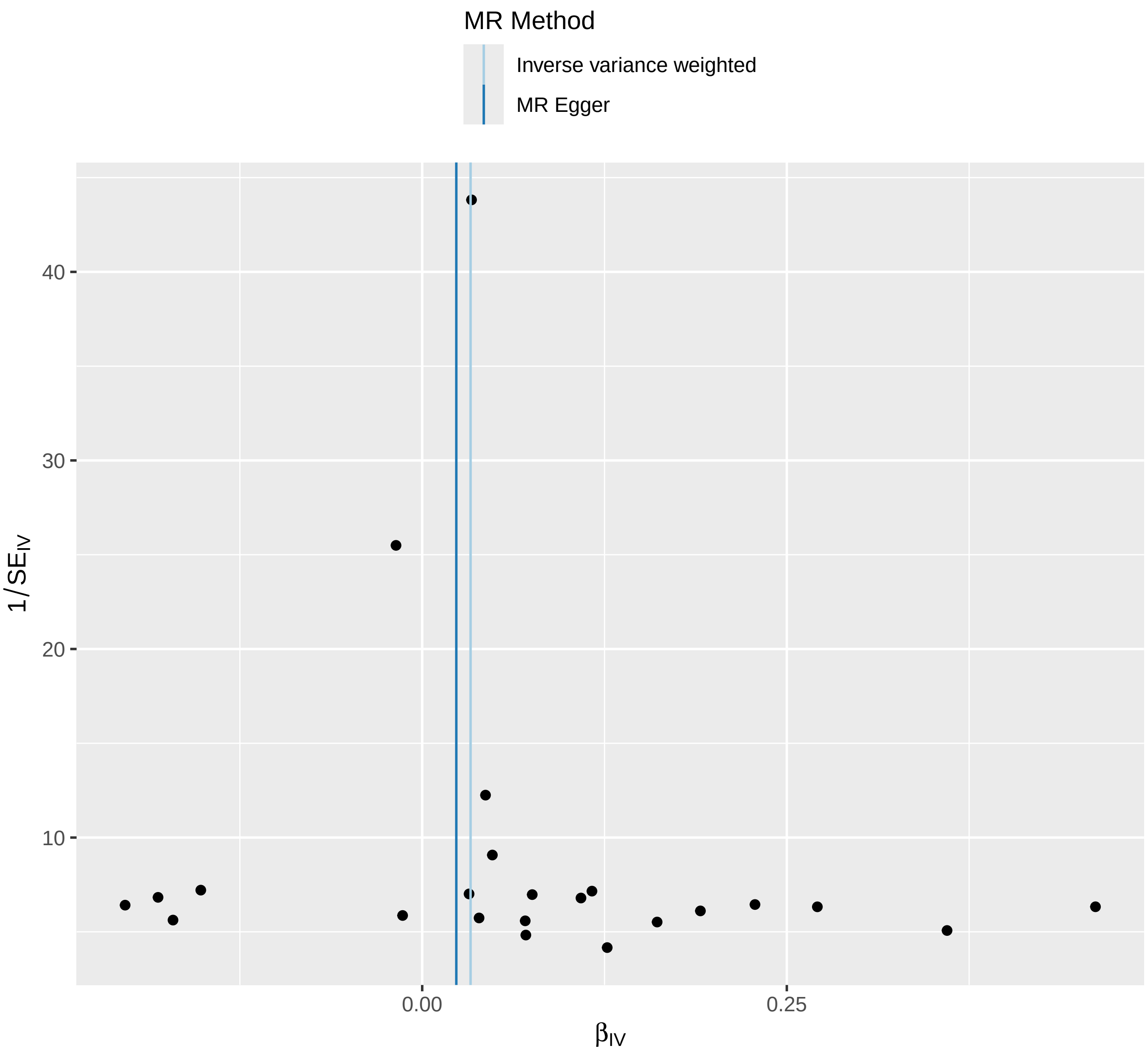
MR Method
Inverse variance weighted
MR Egger
40
30
20
10
1/SE_IV
0.00
0.25
β_IV

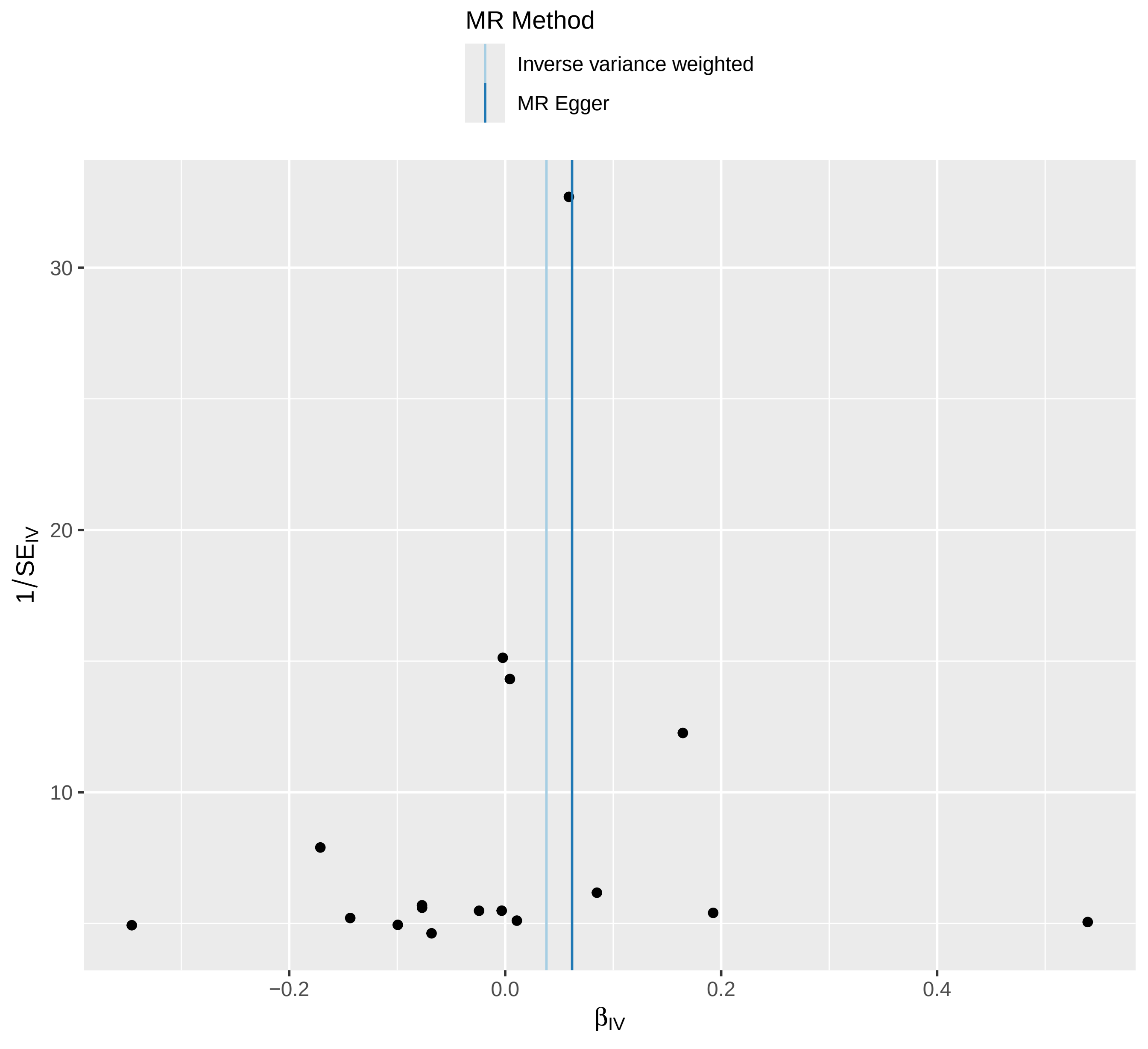
MR Method
Inverse variance weighted
MR Egger
30
20
10
1/SE$_{IV}$
−0.2
0.0
0.2
0.4
β$_{IV}$

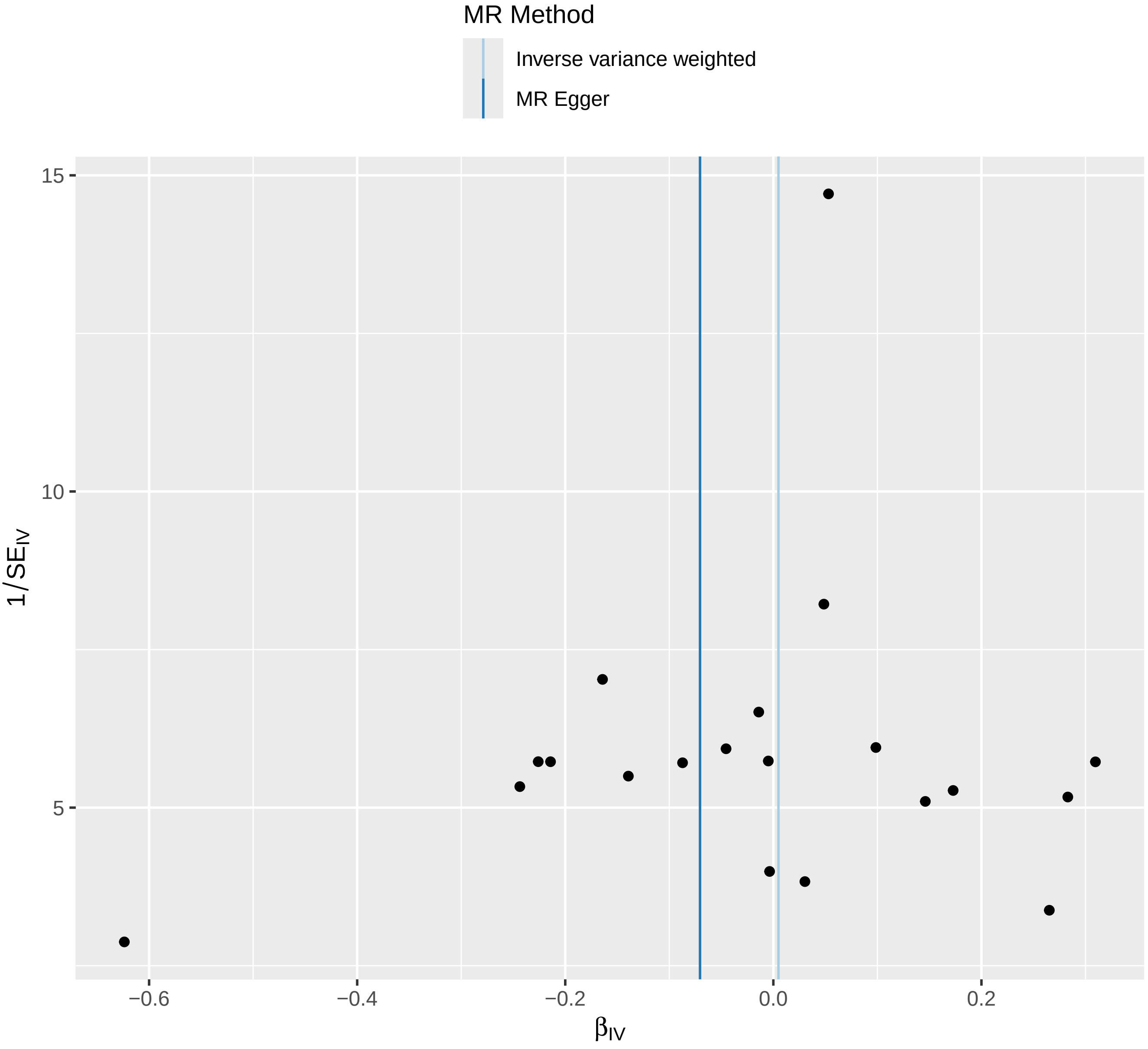
MR Method
Inverse variance weighted
MR Egger
15
10
5
1/SE$_{IV}$
−0.6
−0.4
−0.2
0.0
0.2
β$_{IV}$

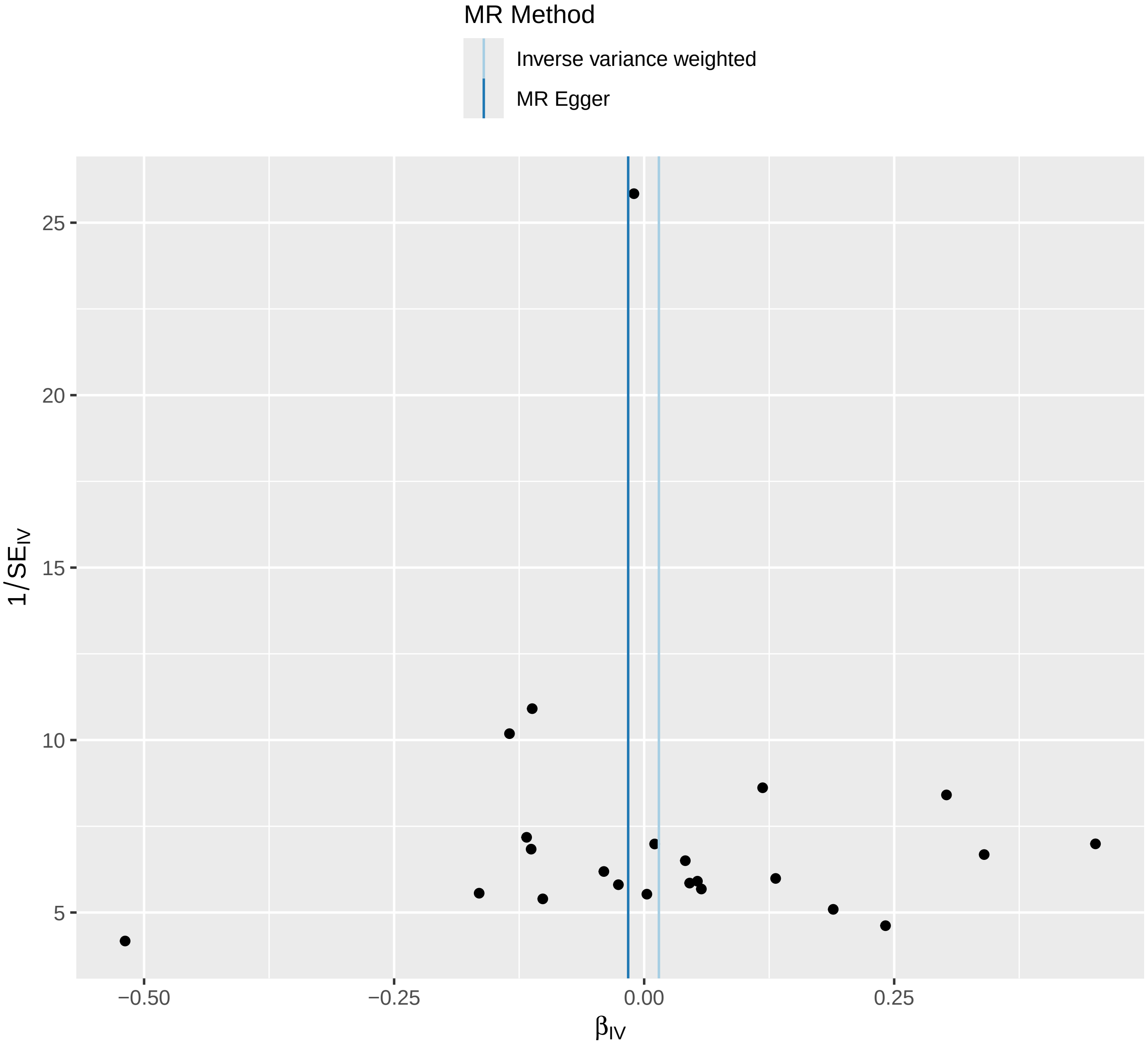
MR Method
Inverse variance weighted
MR Egger
25
20
15
10
5
$1/SE_{IV}$
−0.50
−0.25
0.00
0.25
β_{IV}

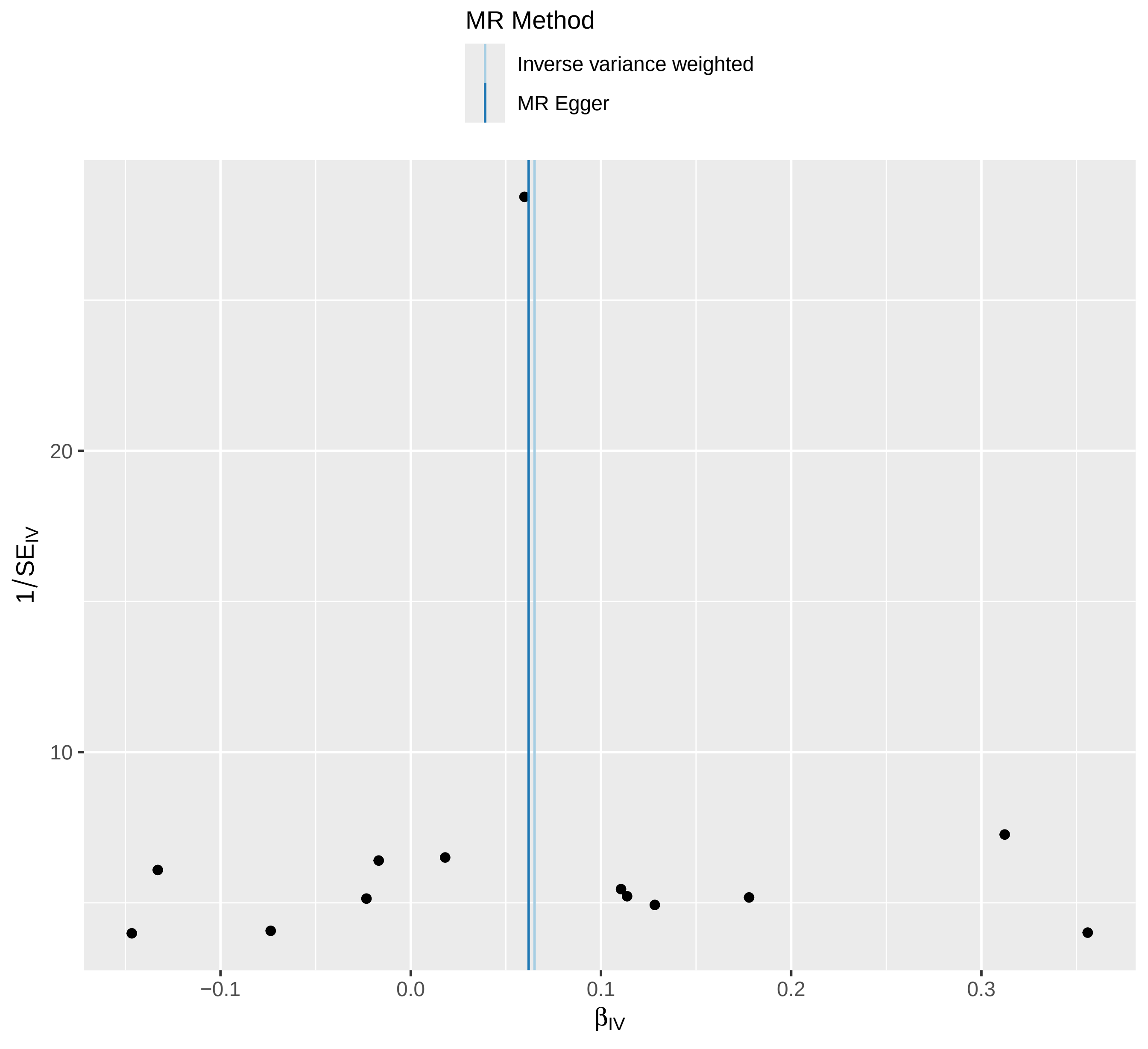

MR Method
Inverse variance weighted
MR Egger
20
10
1/SE_IV
−0.1
0.0
0.1
0.2
0.3
β_IV

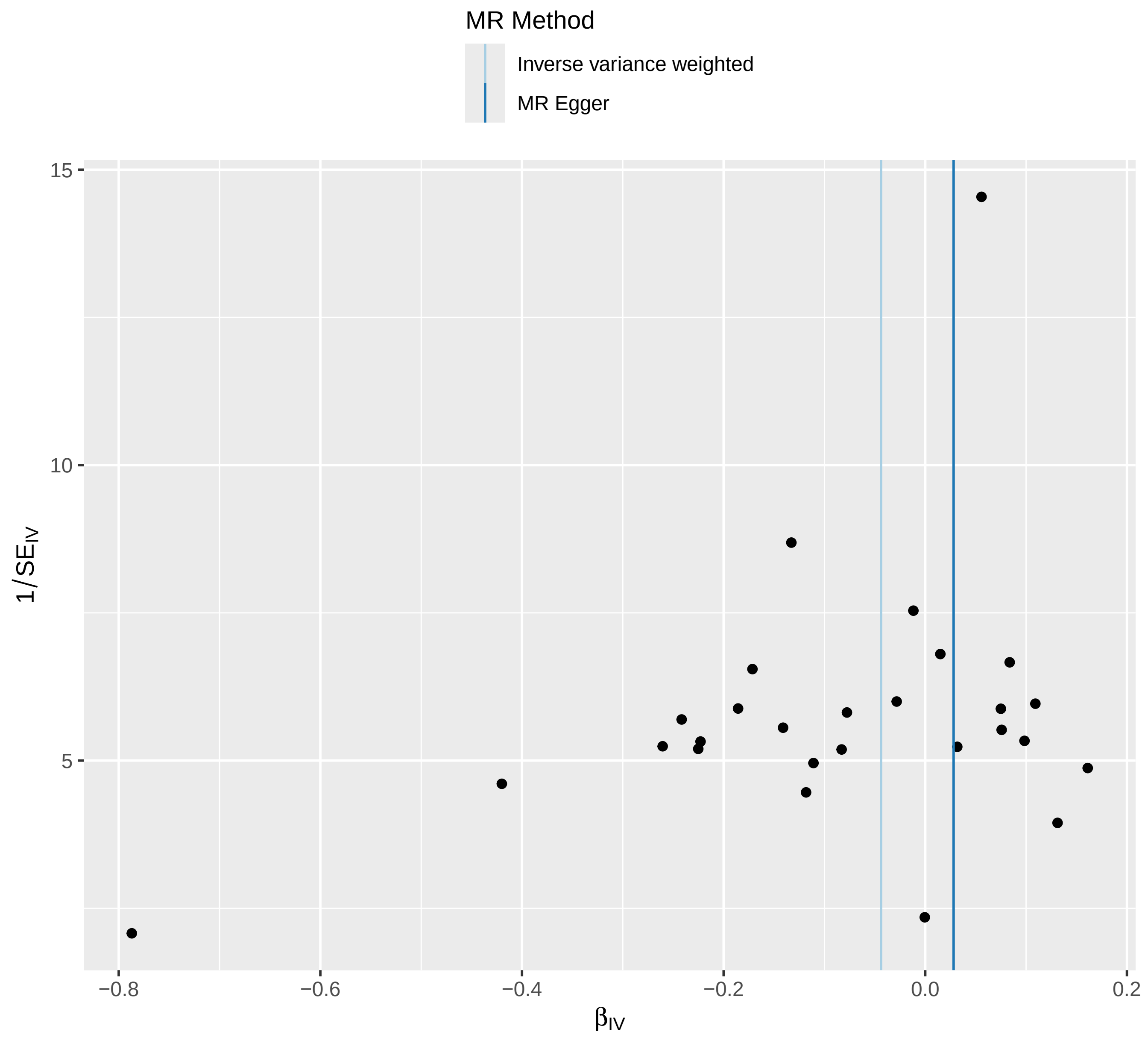
MR Method
Inverse variance weighted
MR Egger
15
10
5
1/SE_IV
−0.8
−0.6
−0.4
−0.2
0.0
0.2
β_IV

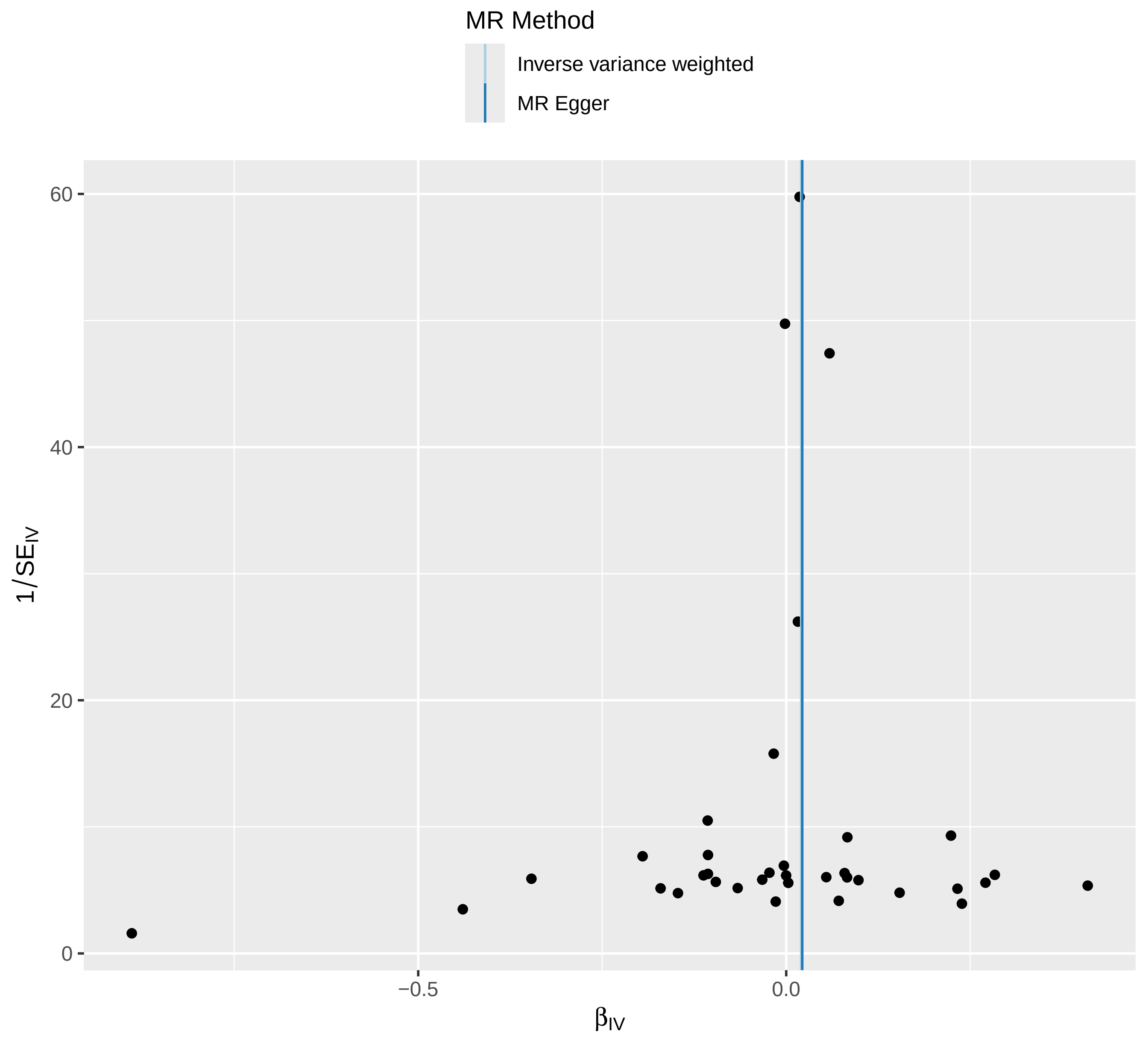

MR Method
Inverse variance weighted
MR Egger
60
40
20
0
−0.5
0.0
$1/SE_{IV}$
β_{IV}

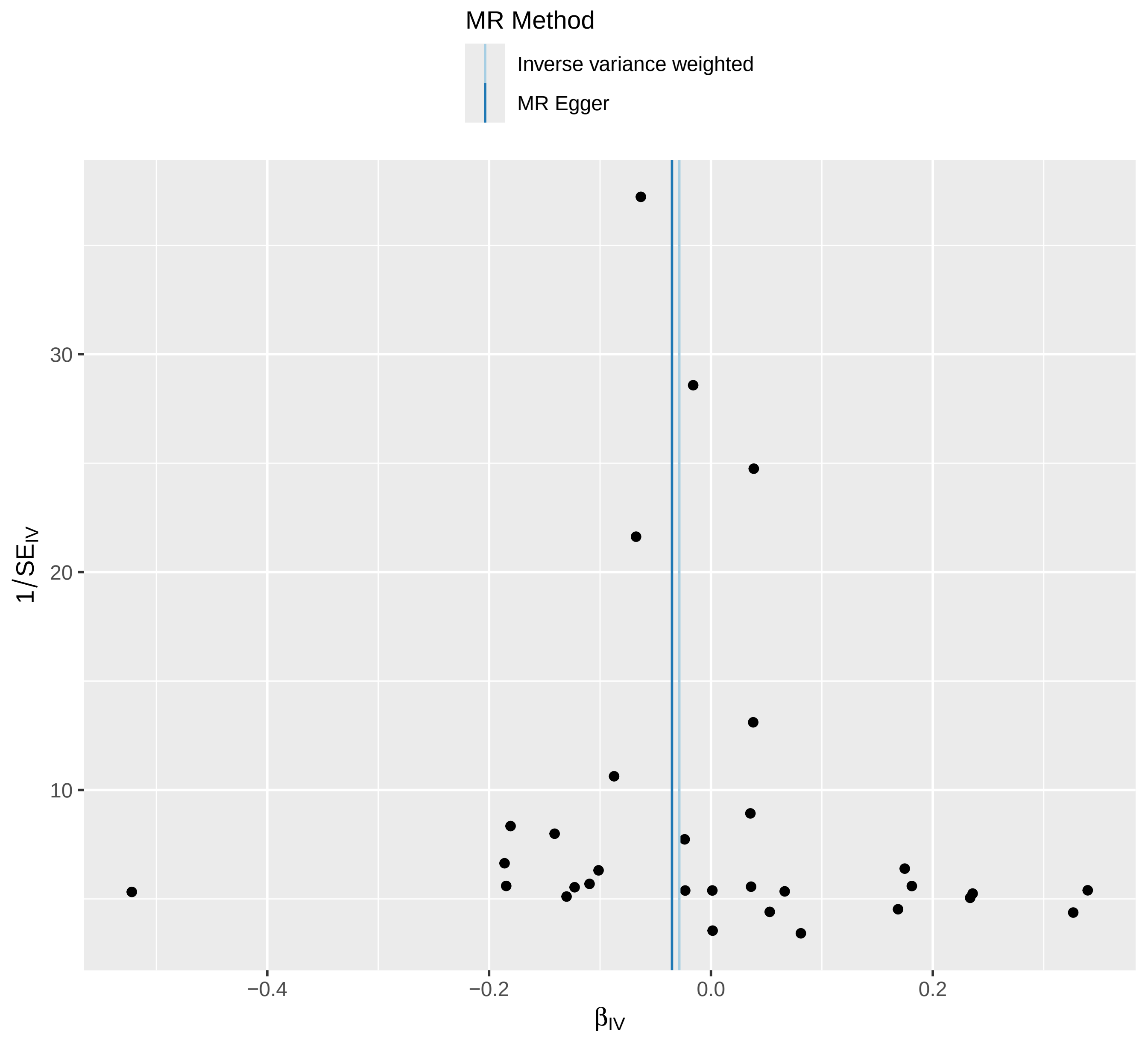
MR Method
Inverse variance weighted
MR Egger
30
20
10
1/SE_{IV}
−0.4
−0.2
0.0
0.2
β_{IV}

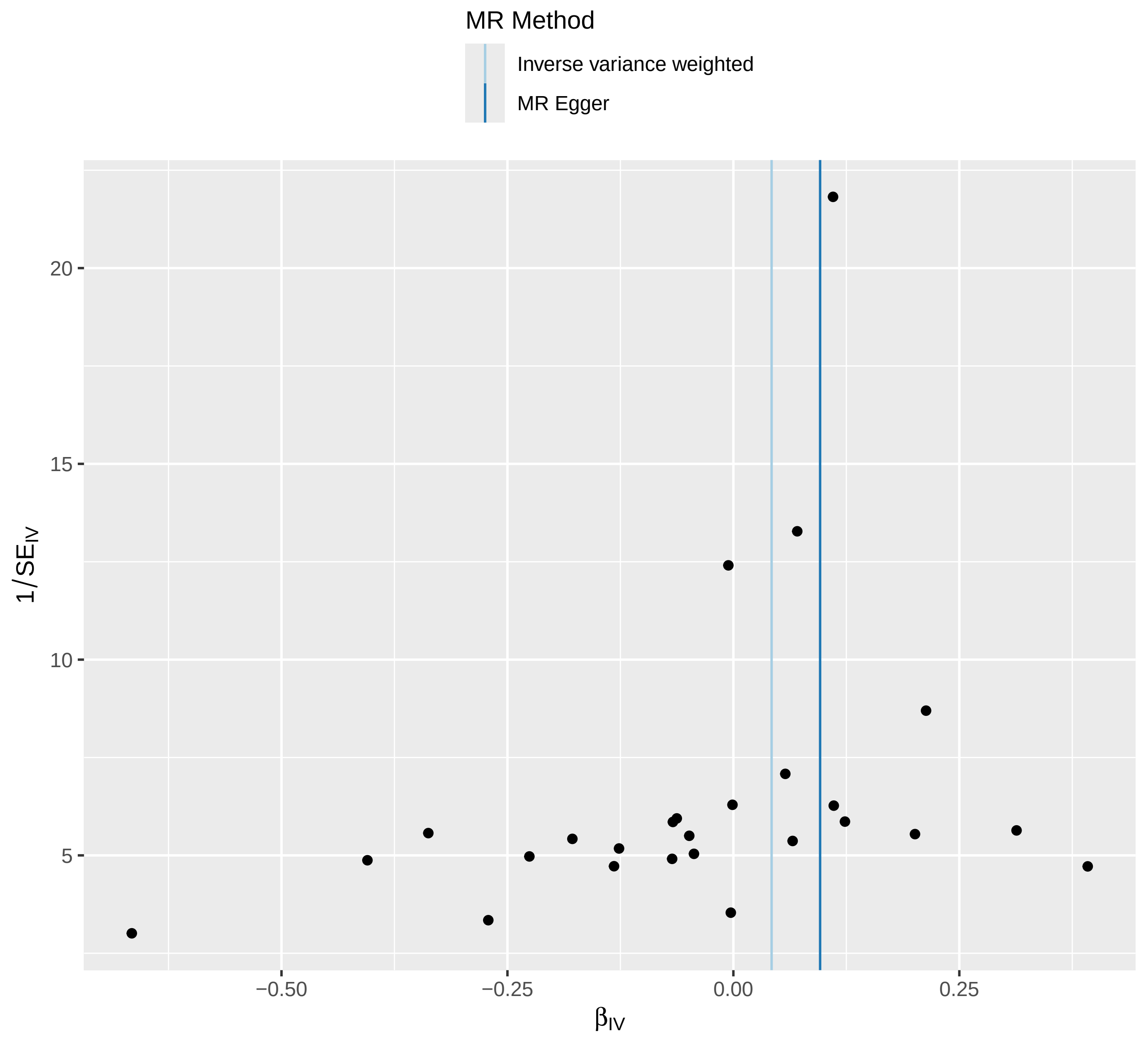

MR Method
Inverse variance weighted
MR Egger
20
15
10
5
1/SE_IV
−0.50
−0.25
0.00
0.25
β_IV

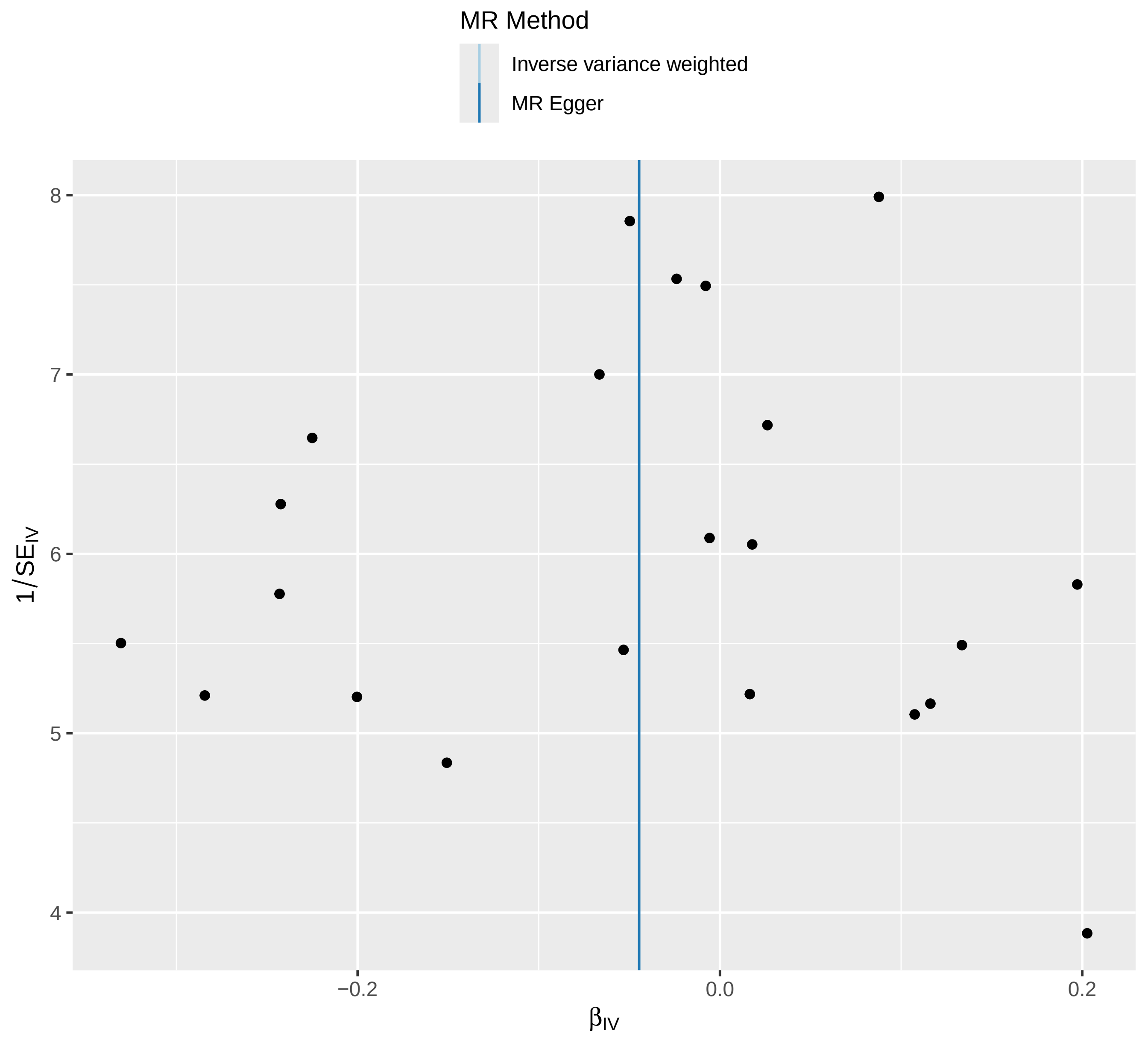
MR Method
Inverse variance weighted
MR Egger
1/SE_IV
8
7
6
5
4
−0.2
0.0
0.2
β_IV

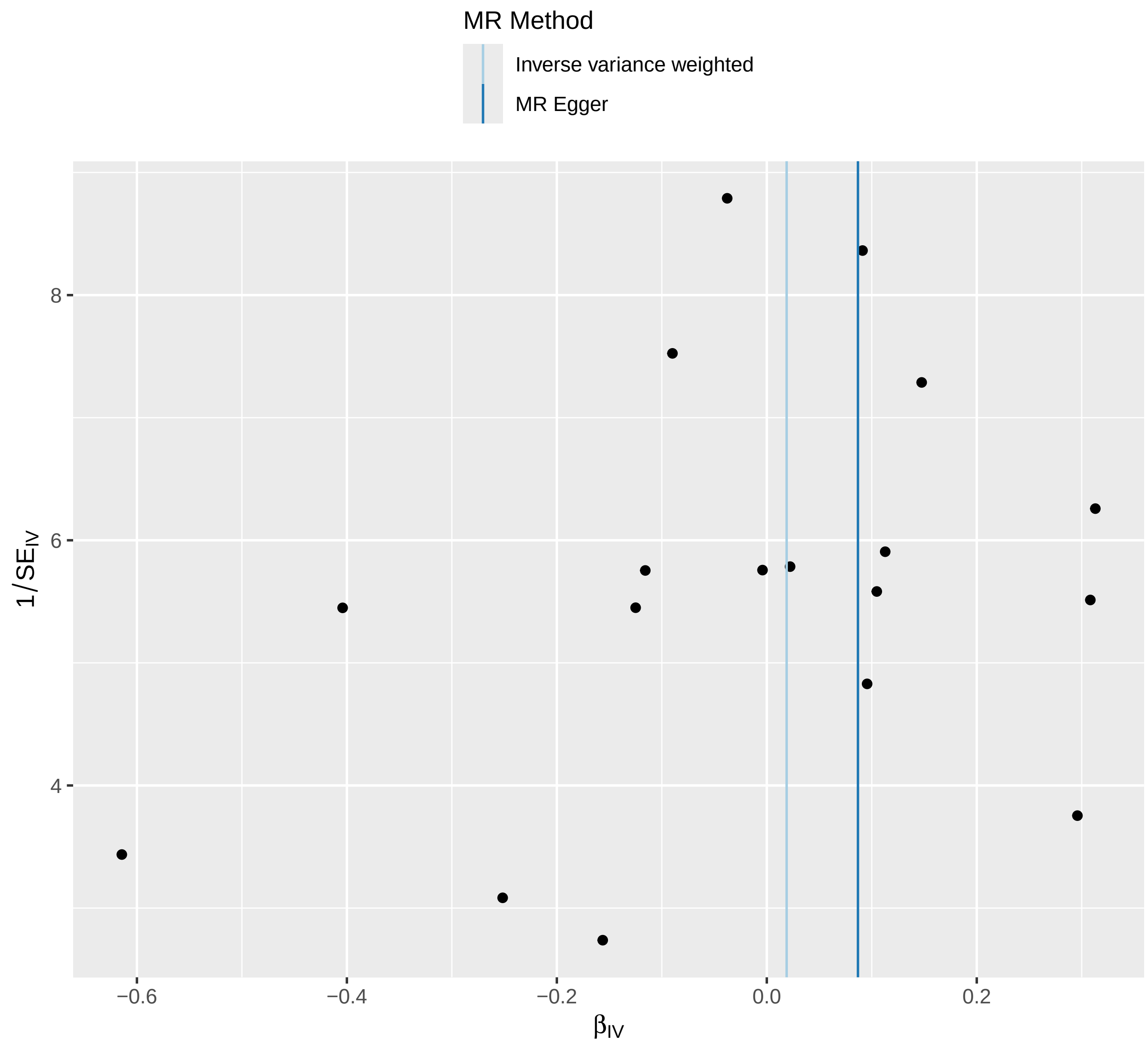
MR Method
Inverse variance weighted
MR Egger
8
6
4
1/SE$_{IV}$
−0.6
−0.4
−0.2
0.0
0.2
β$_{IV}$

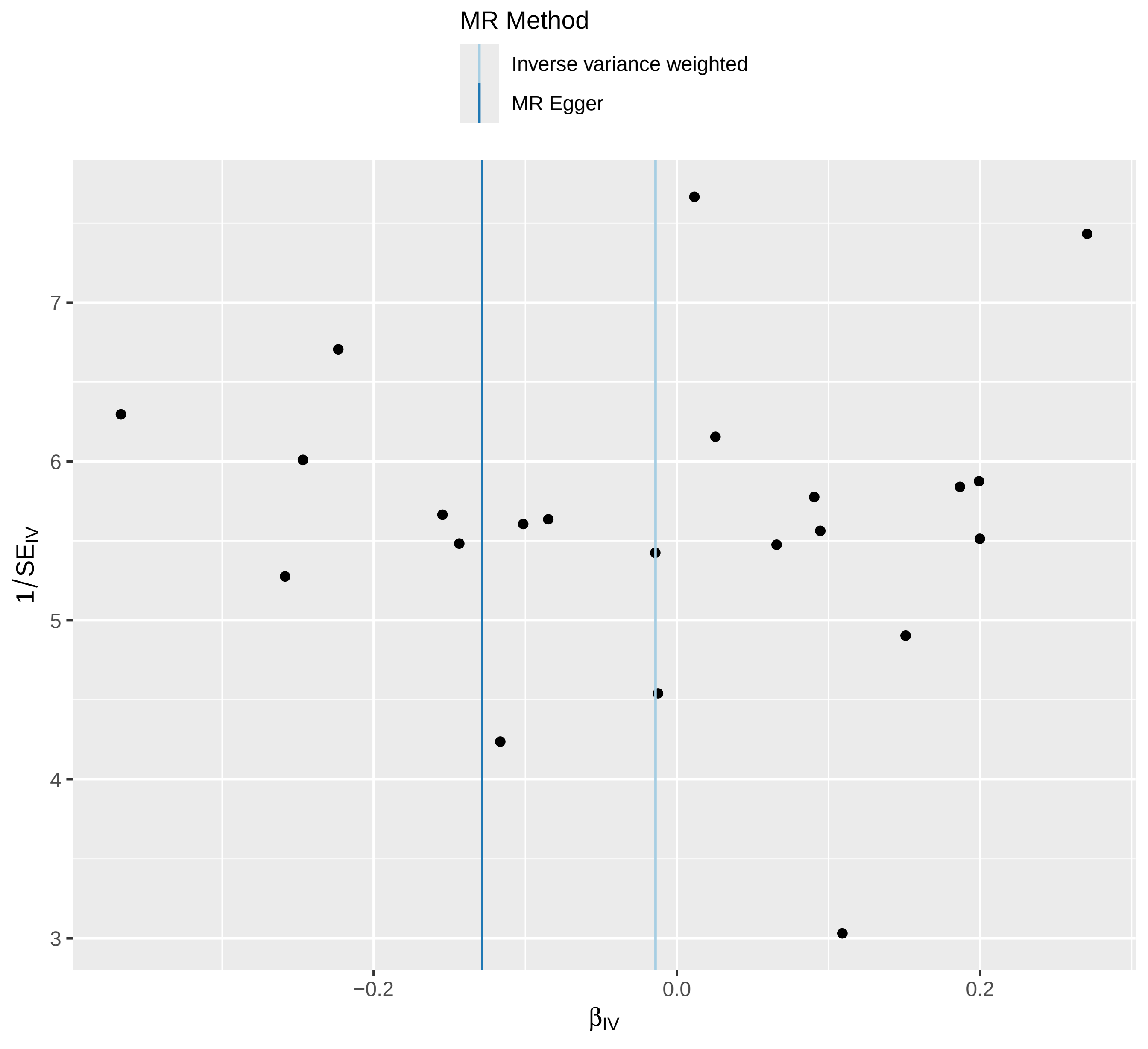
MR Method
Inverse variance weighted
MR Egger
1/SE_IV
7
6
5
4
3
−0.2
0.0
0.2
β_IV

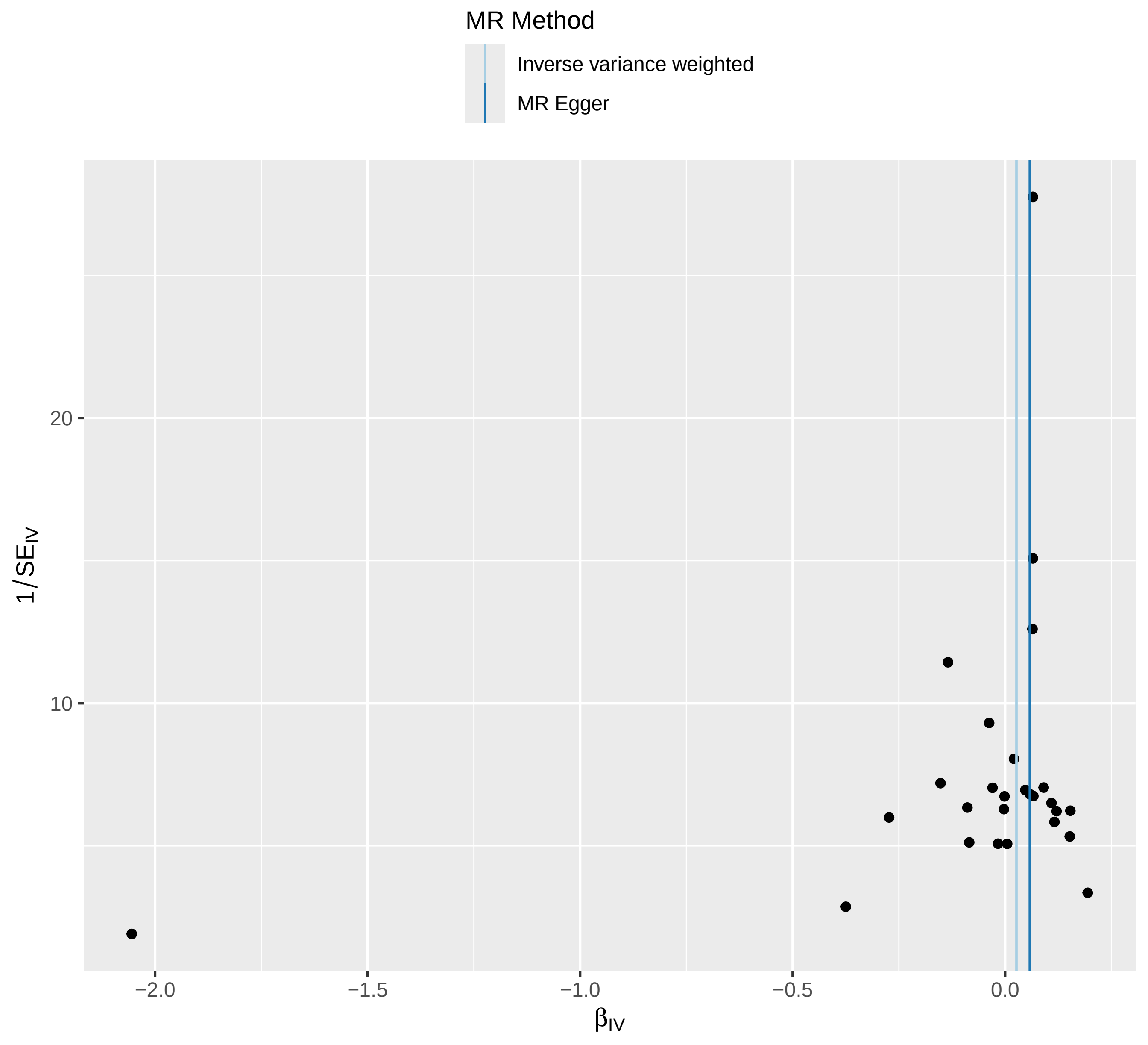

MR Method
Inverse variance weighted
MR Egger
20
10
1/SE_IV
−2.0
−1.5
−1.0
−0.5
0.0
β_IV

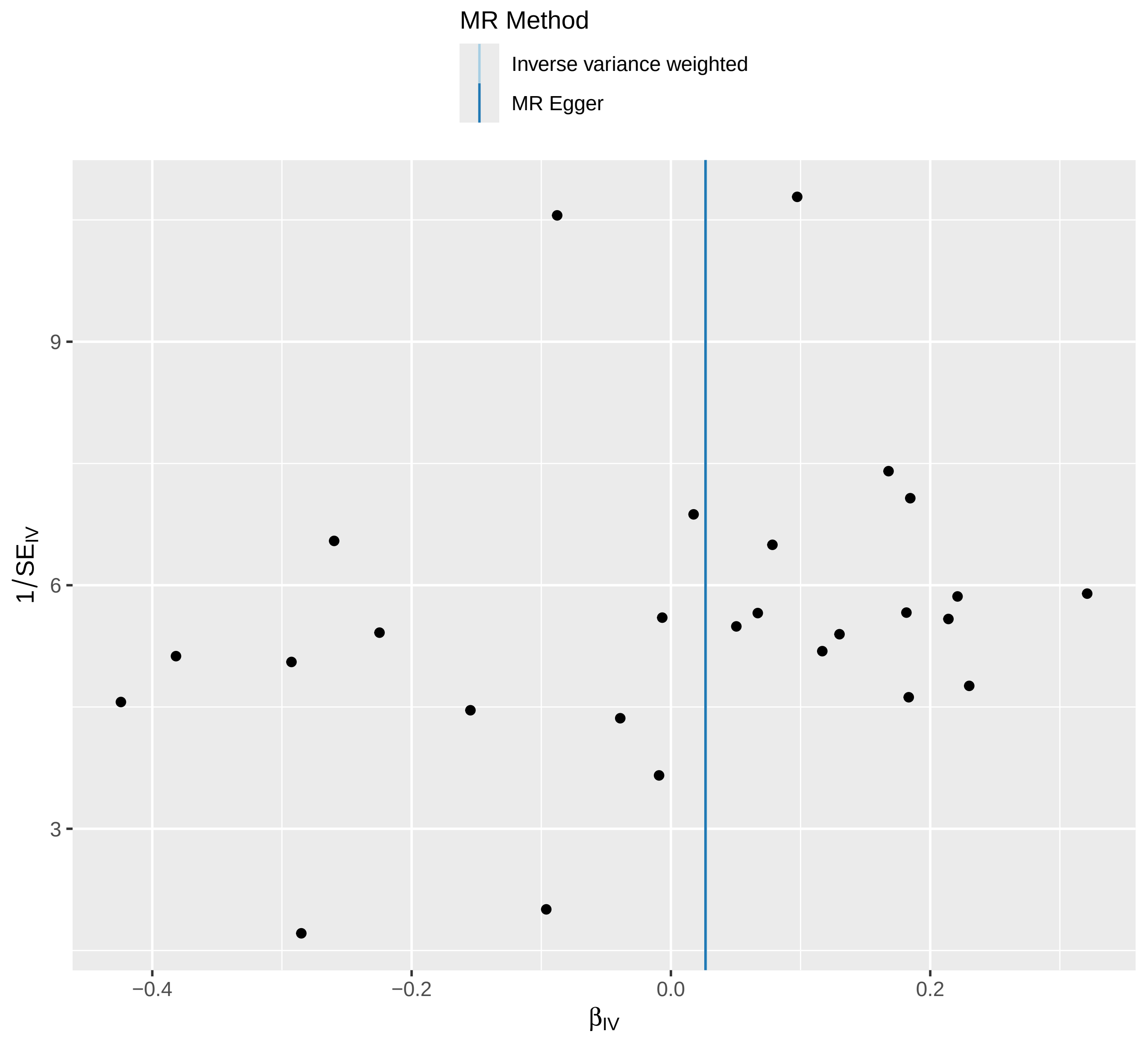

MR Method
Inverse variance weighted
MR Egger
9
6
3
1/SE$_{IV}$
−0.4
−0.2
0.0
0.2
β$_{IV}$

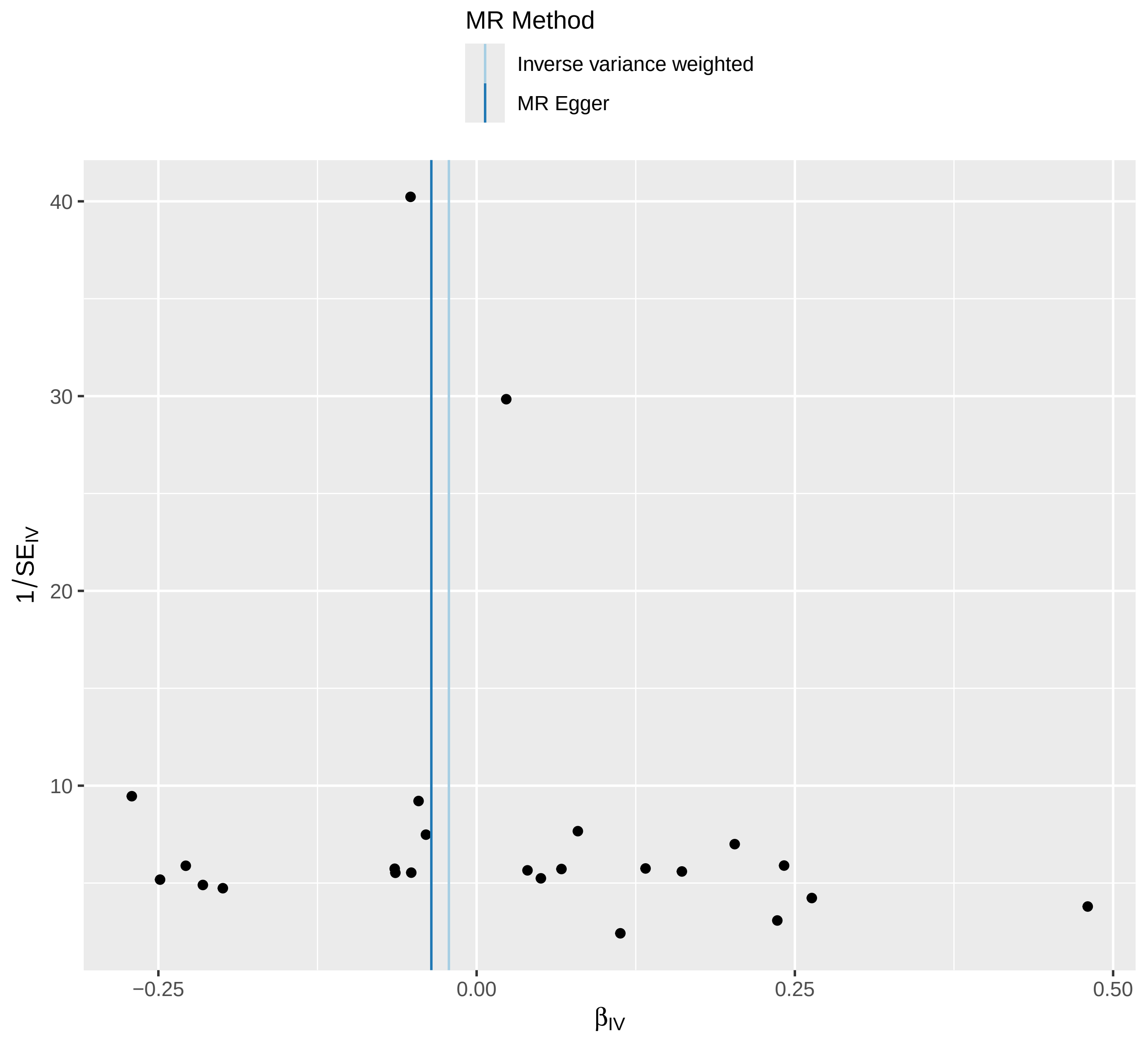

MR Method
Inverse variance weighted
MR Egger
40
30
20
10
1/SE_IV
−0.25
0.00
0.25
0.50
β_IV

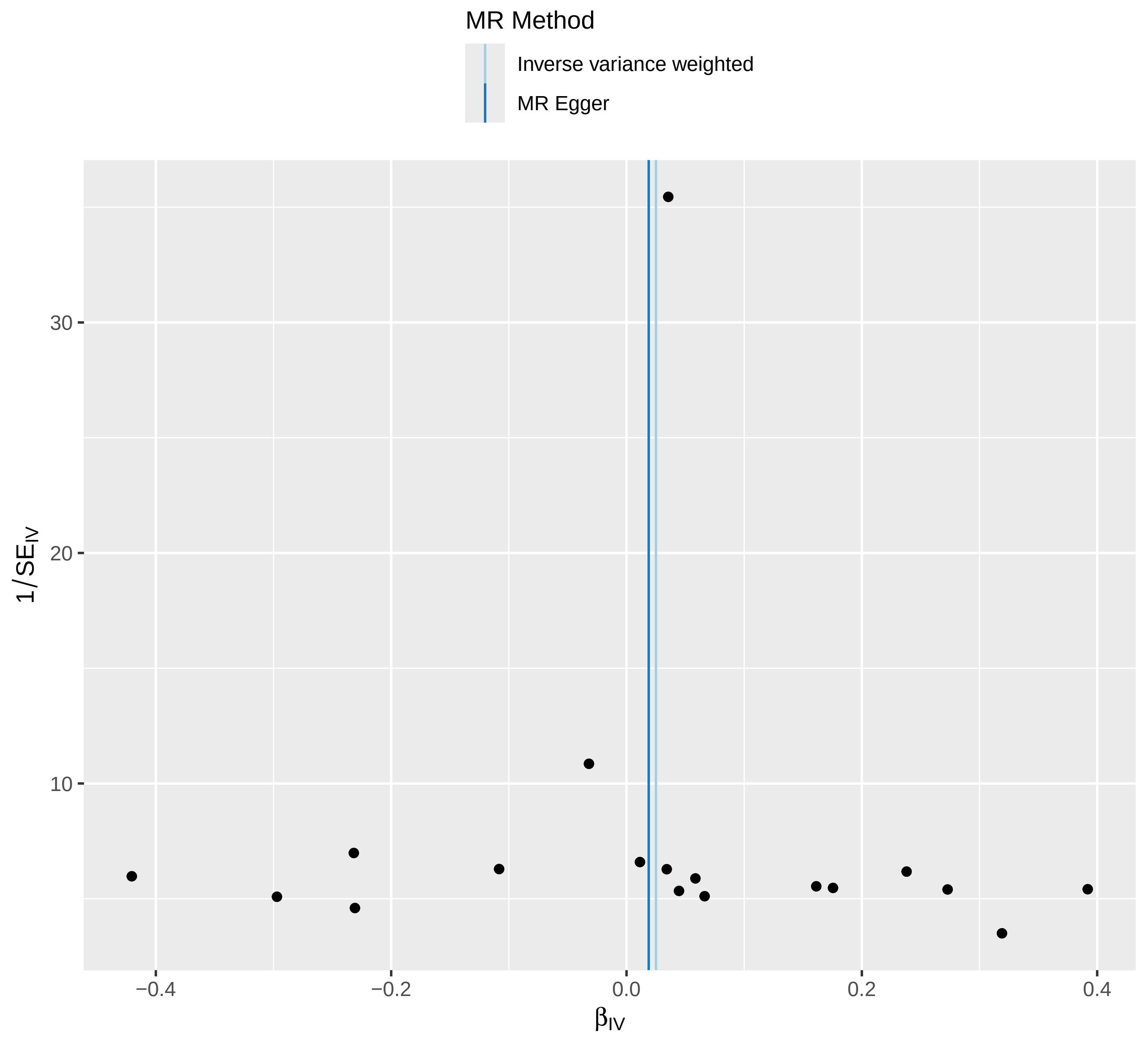
MR Method
Inverse variance weighted
MR Egger
30
20
10
1/SE_IV
−0.4
−0.2
0.0
0.2
0.4
β_IV

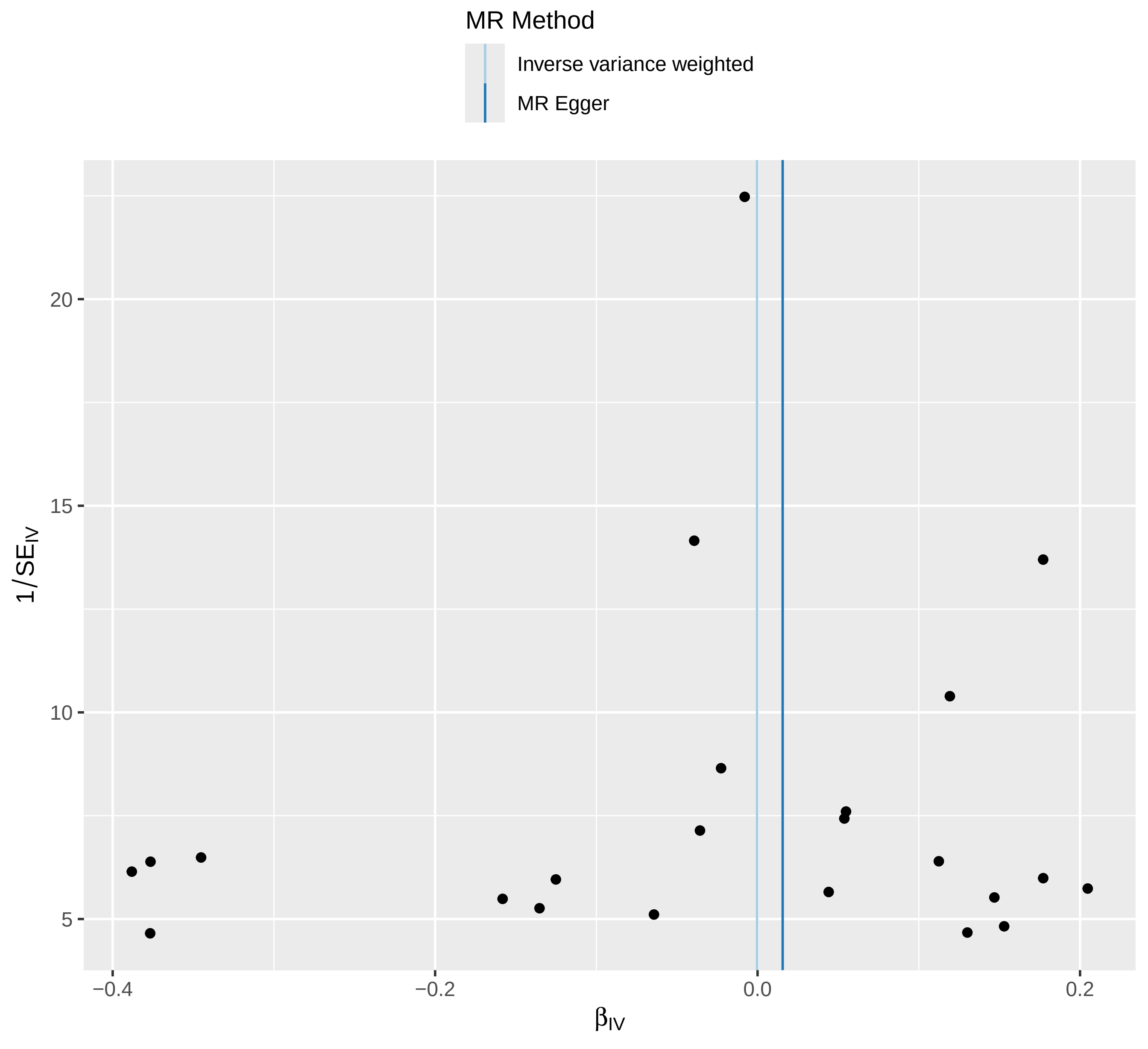
MR Method
Inverse variance weighted
MR Egger
20
15
10
5
1/SE_IV
−0.4
−0.2
0.0
0.2
β_IV

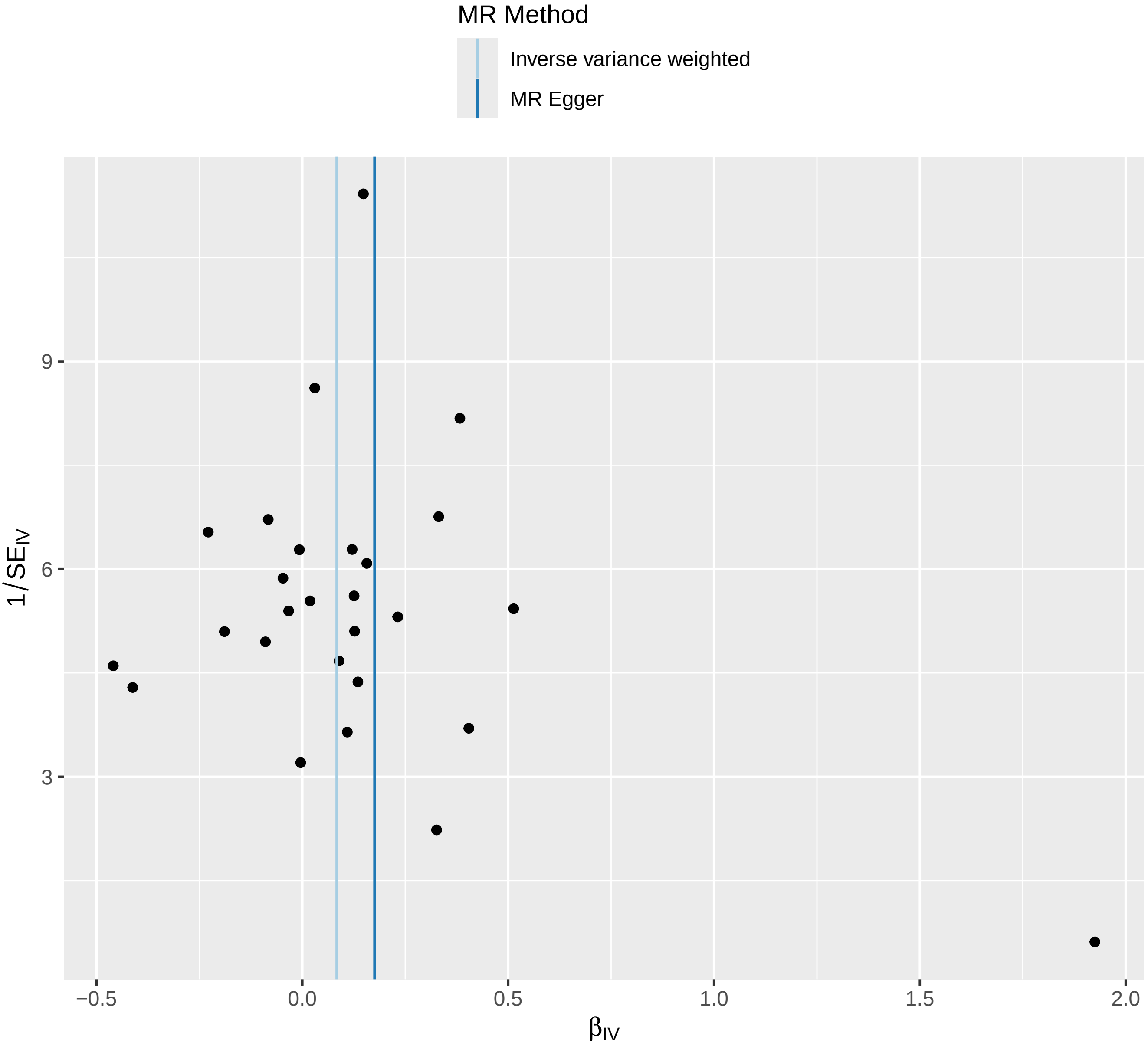
MR Method
Inverse variance weighted
MR Egger
1/SE_IV
9
6
3
β_IV
−0.5
0.0
0.5
1.0
1.5
2.0

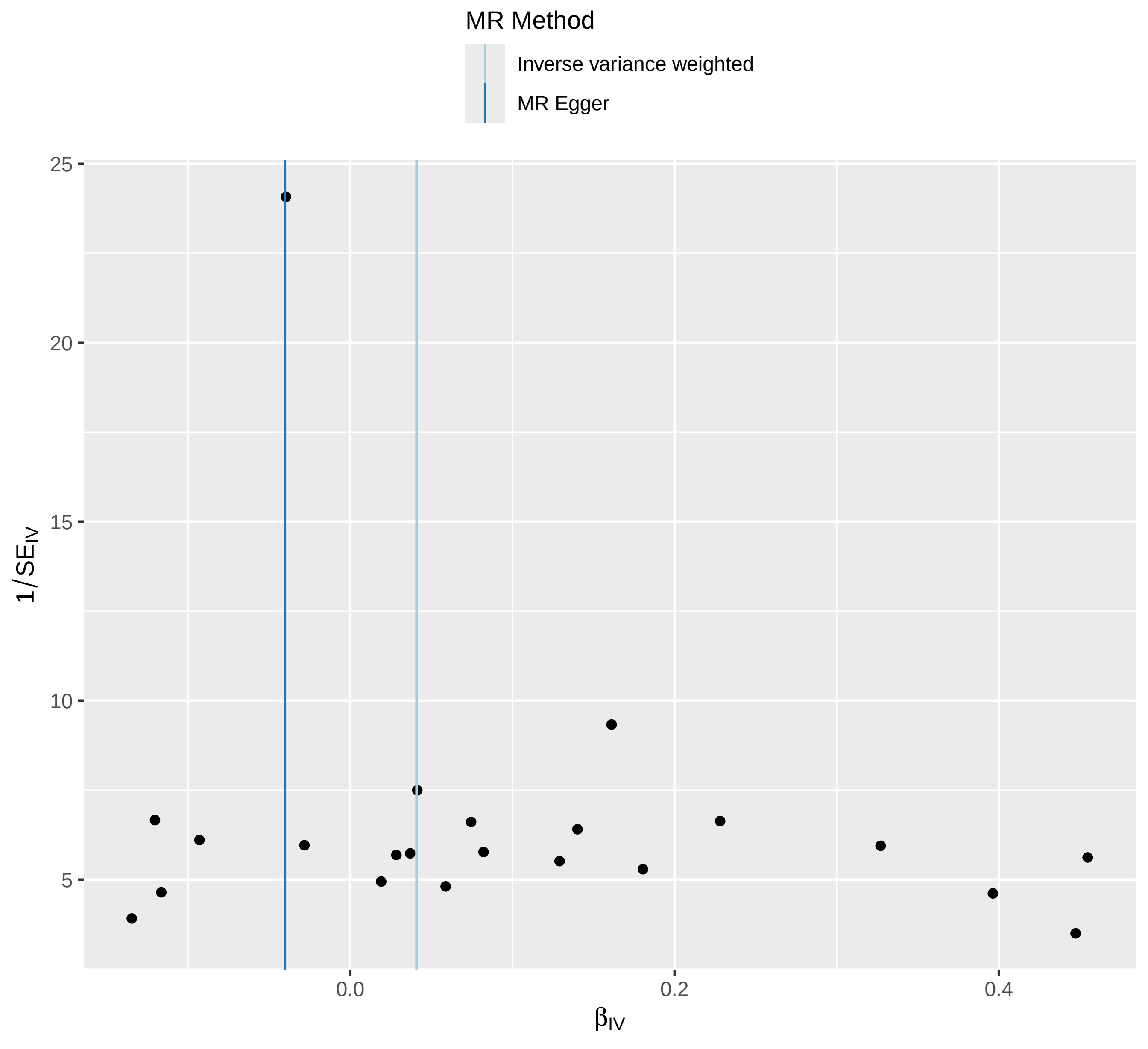

MR Method
Inverse variance weighted
MR Egger
25
20
15
10
5
1/SE_IV
0.0
0.2
0.4
β_IV

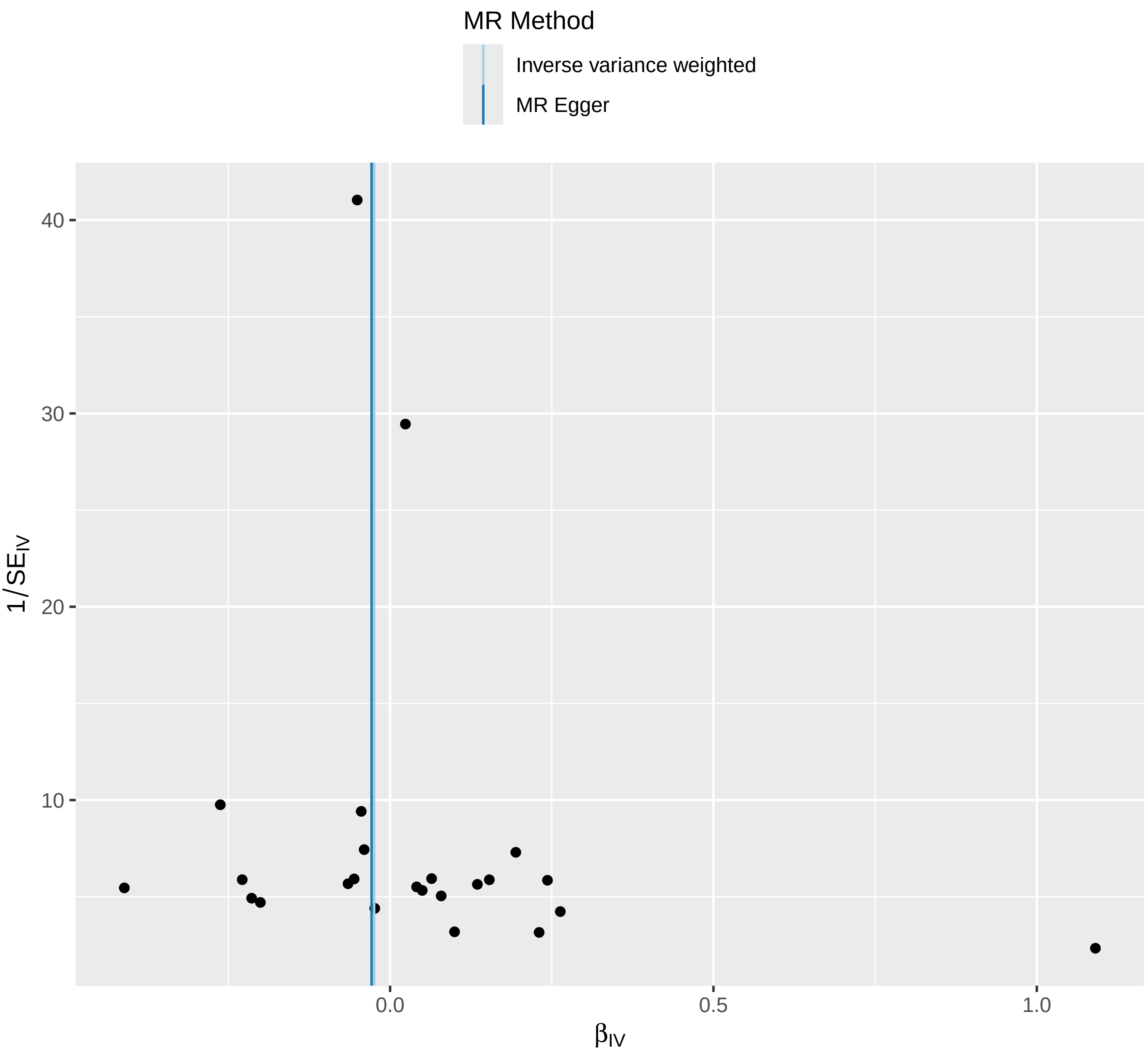
MR Method
Inverse variance weighted
MR Egger
40
30
20
10
1/SE_IV
0.0
0.5
1.0
β_IV

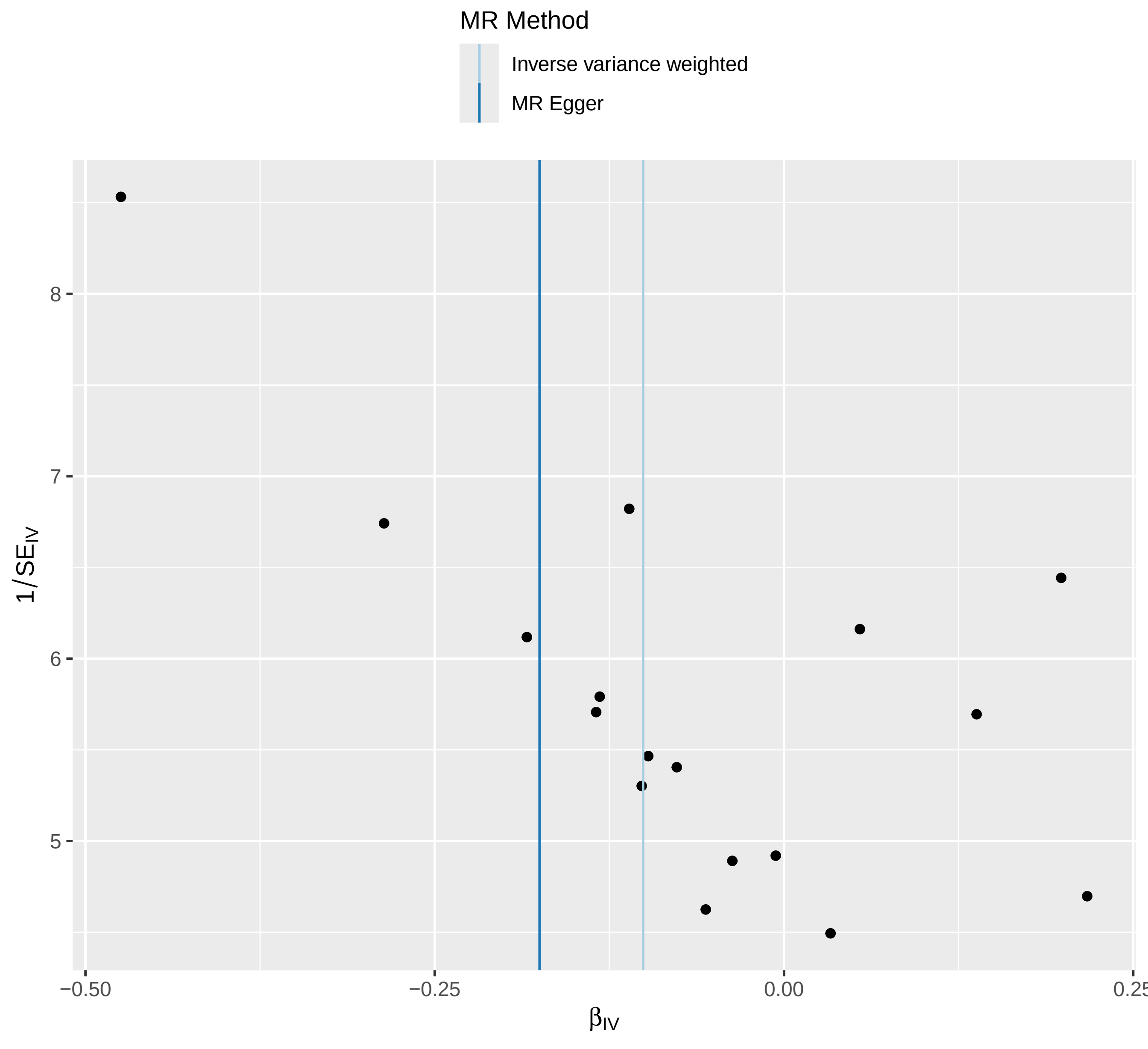
MR Method
Inverse variance weighted
MR Egger
8
7
6
5
1/SE_IV
−0.50
−0.25
0.00
0.25
β_IV

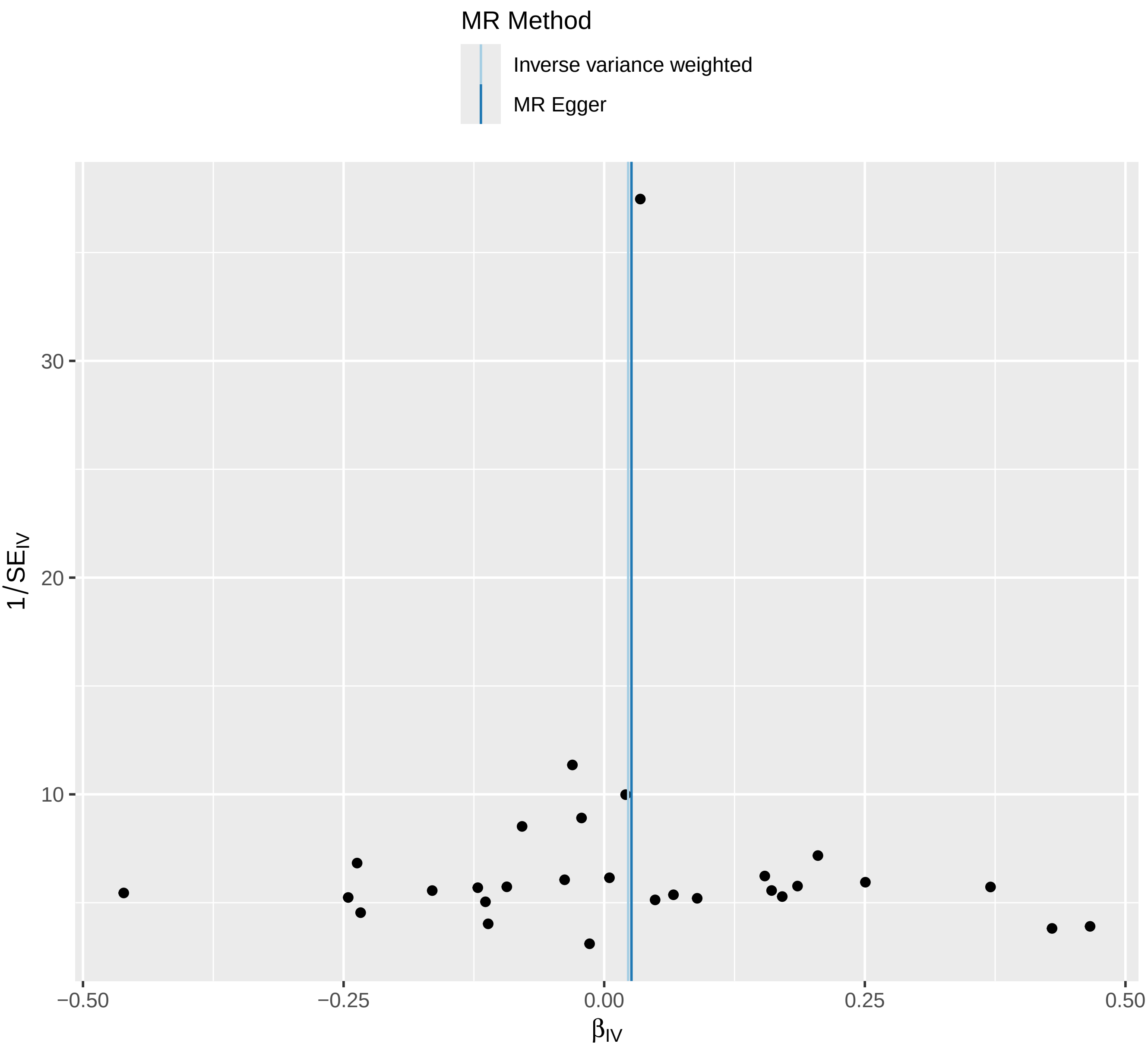

MR Method
Inverse variance weighted
MR Egger
30
20
10
−0.50
−0.25
0.00
0.25
0.50
$1/SE_{IV}$
β_{IV}

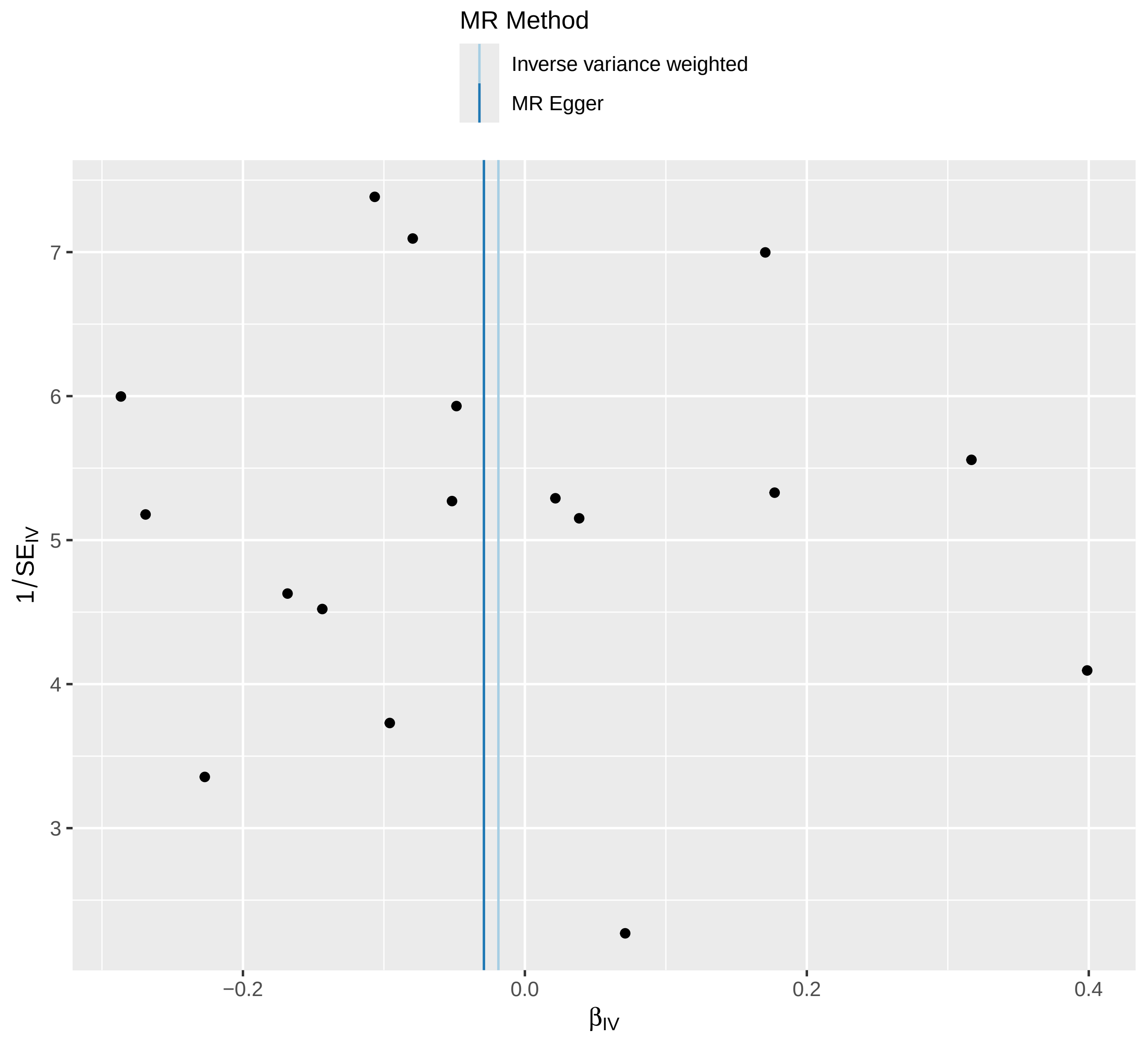

MR Method
Inverse variance weighted
MR Egger
1/SE_IV
7
6
5
4
3
−0.2
0.0
0.2
0.4
β_IV

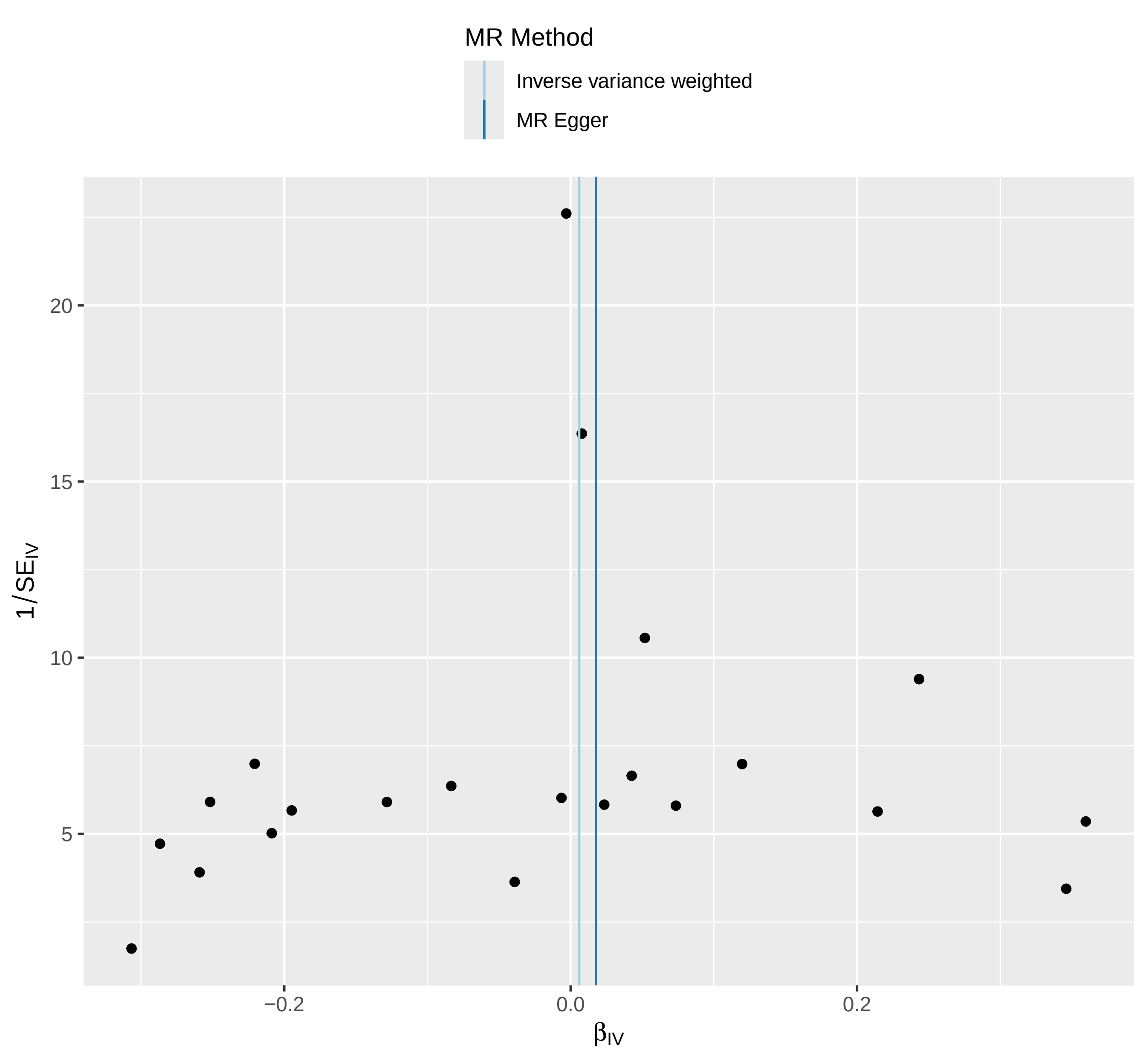
MR Method
Inverse variance weighted
MR Egger
20
15
10
5
1/SE_IV
−0.2
0.0
0.2
β_IV

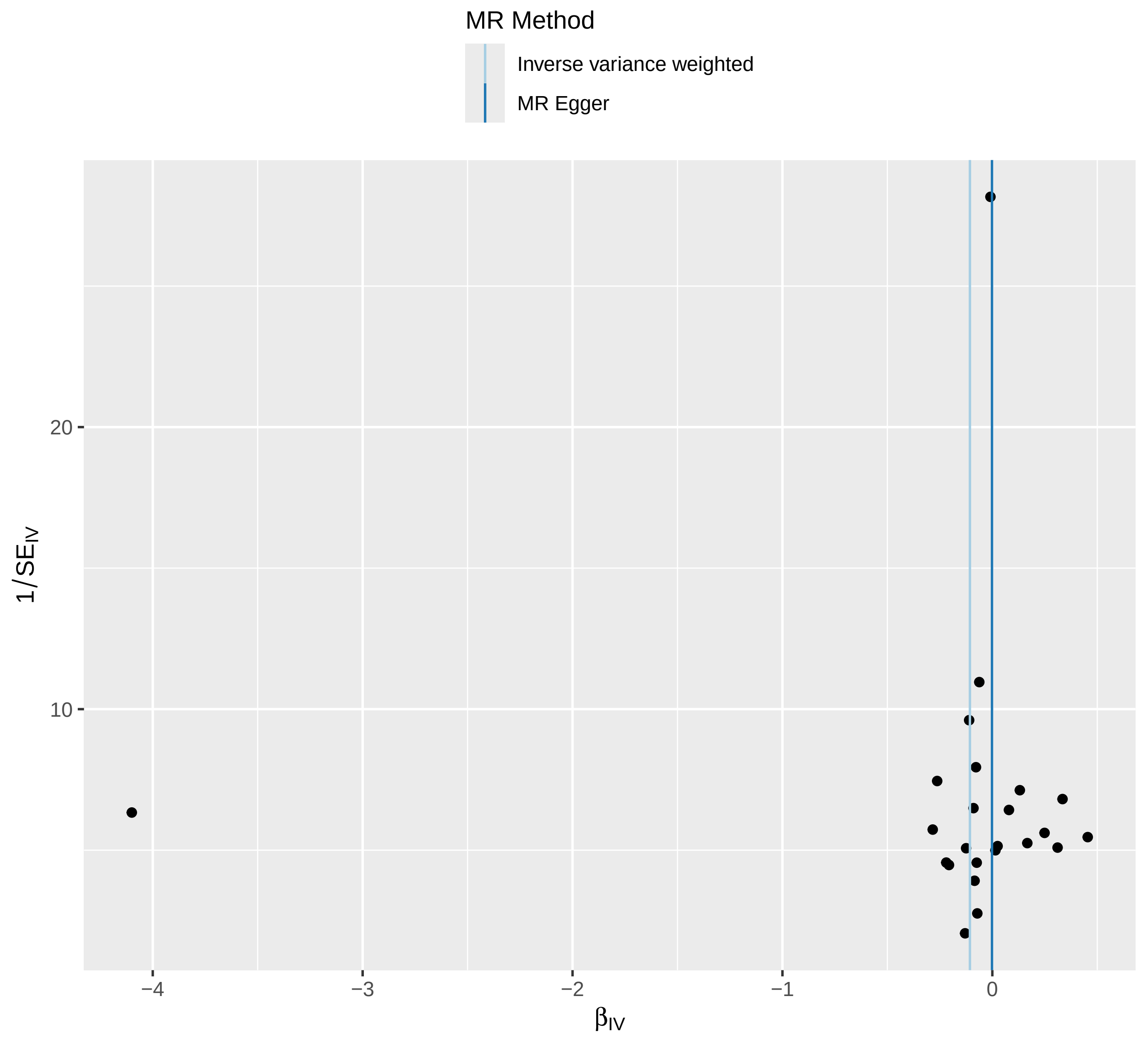
MR Method
Inverse variance weighted
MR Egger
20
10
1/SE_IV
−4
−3
−2
−1
0
β_IV

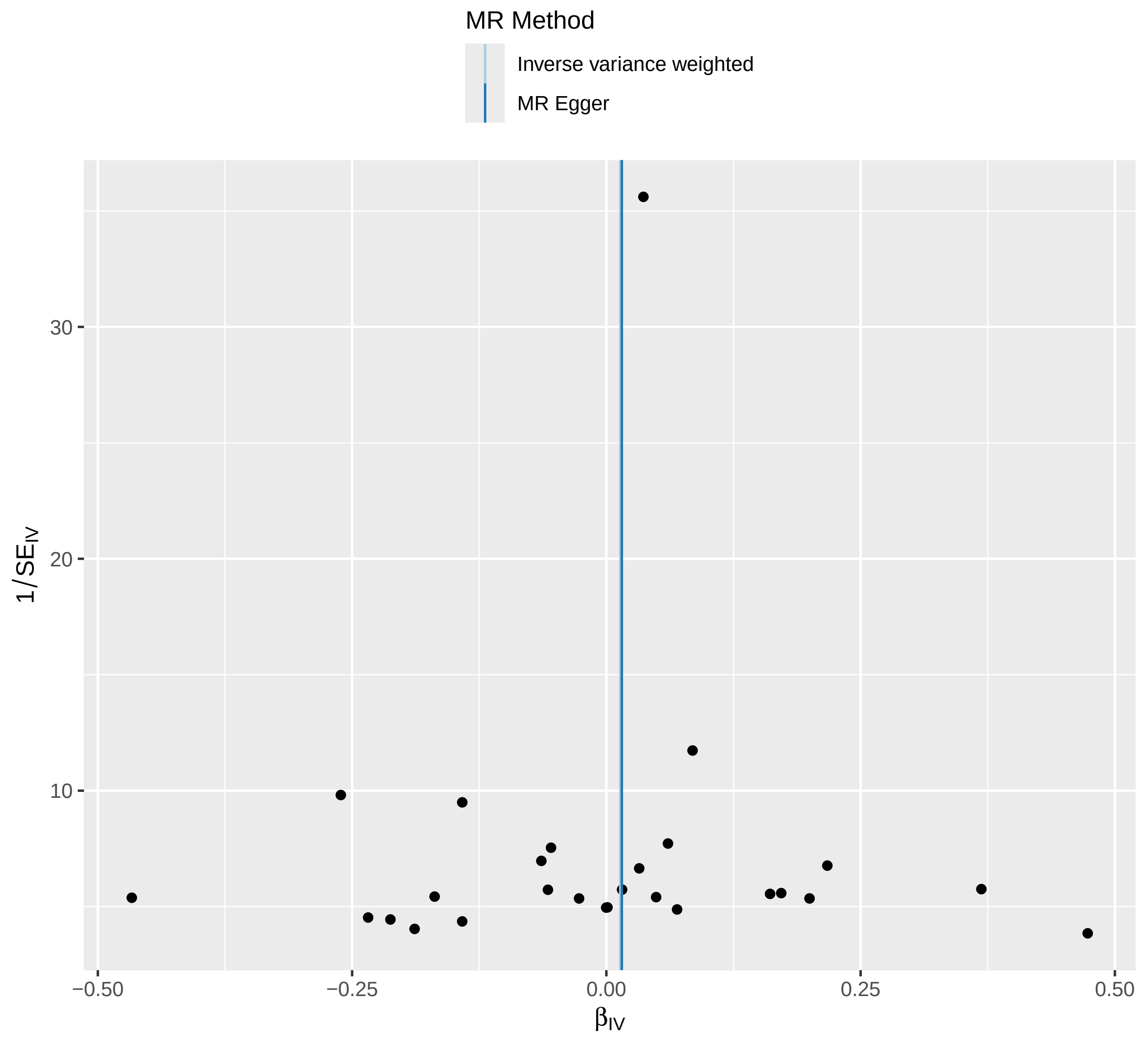
MR Method
Inverse variance weighted
MR Egger
$1/SE_{IV}$
30
20
10
−0.50
−0.25
0.00
0.25
0.50
β_{IV}

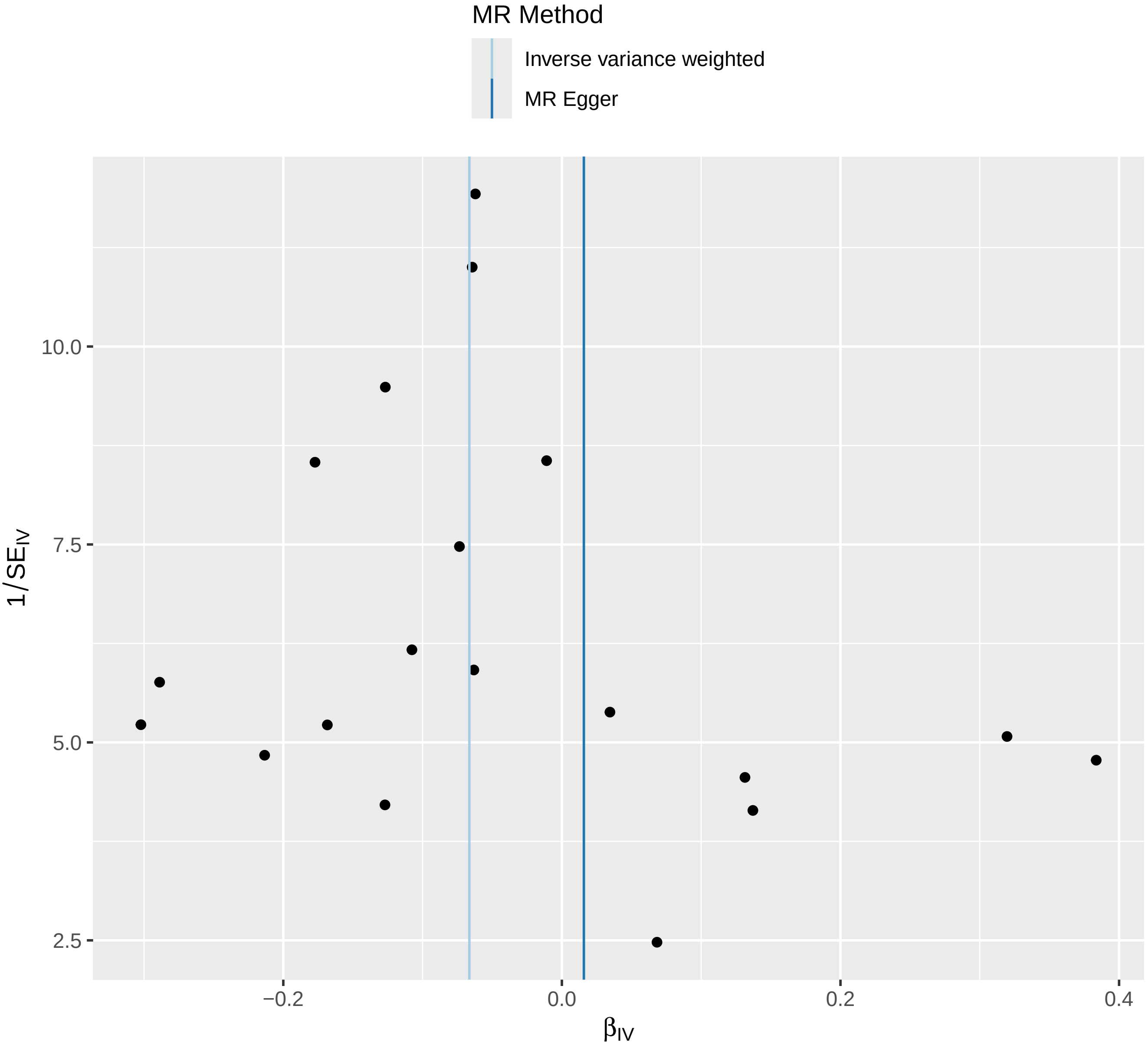
MR Method
Inverse variance weighted
MR Egger
1/SE_IV
10.0
7.5
5.0
2.5
−0.2
0.0
0.2
0.4
β_IV

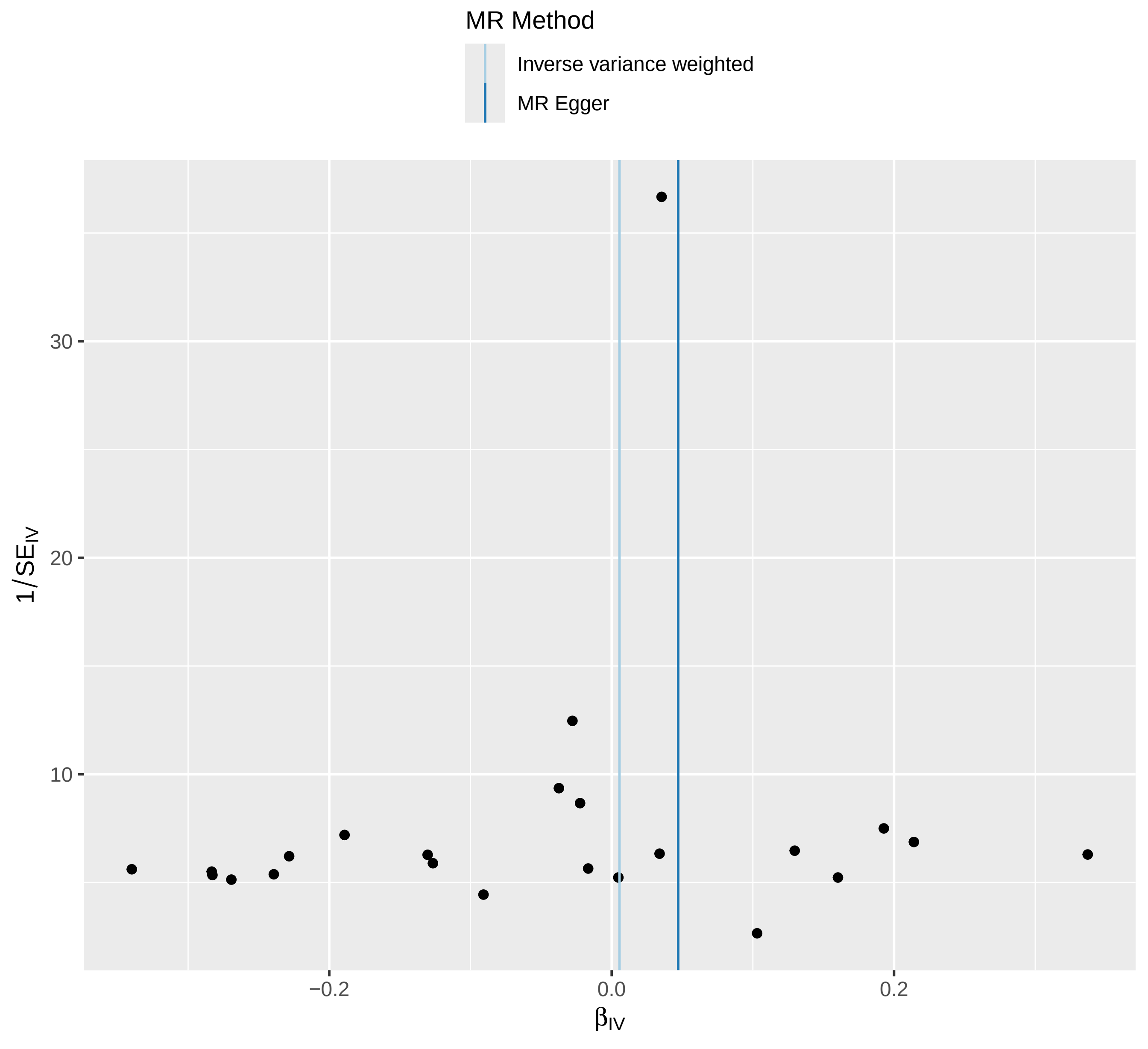
MR Method
Inverse variance weighted
MR Egger
30
20
10
1/SE$_{IV}$
−0.2
0.0
0.2
β_{IV}

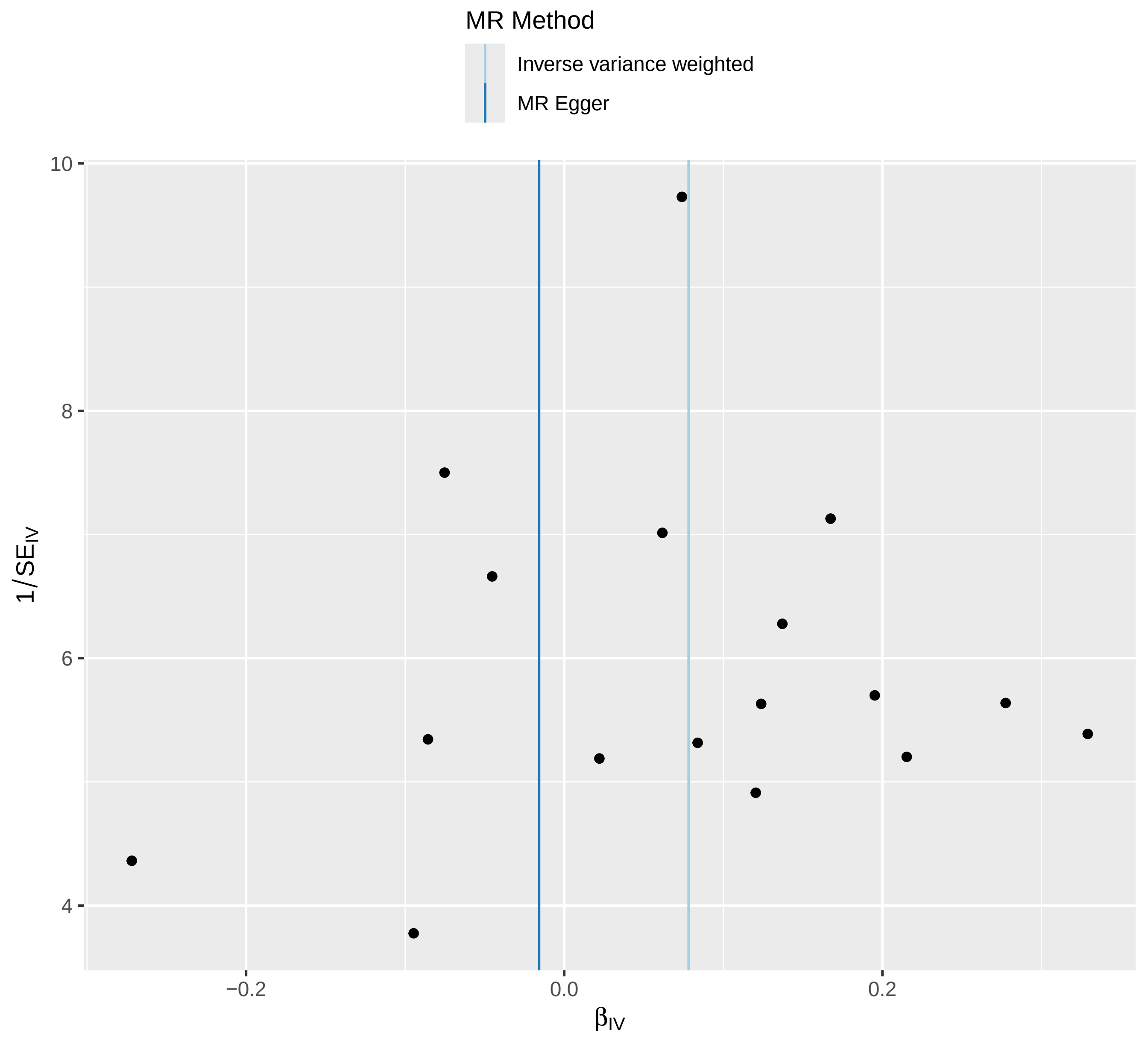

MR Method
Inverse variance weighted
MR Egger
10
8
6
4
1/SE$_{IV}$
−0.2
0.0
0.2
β$_{IV}$

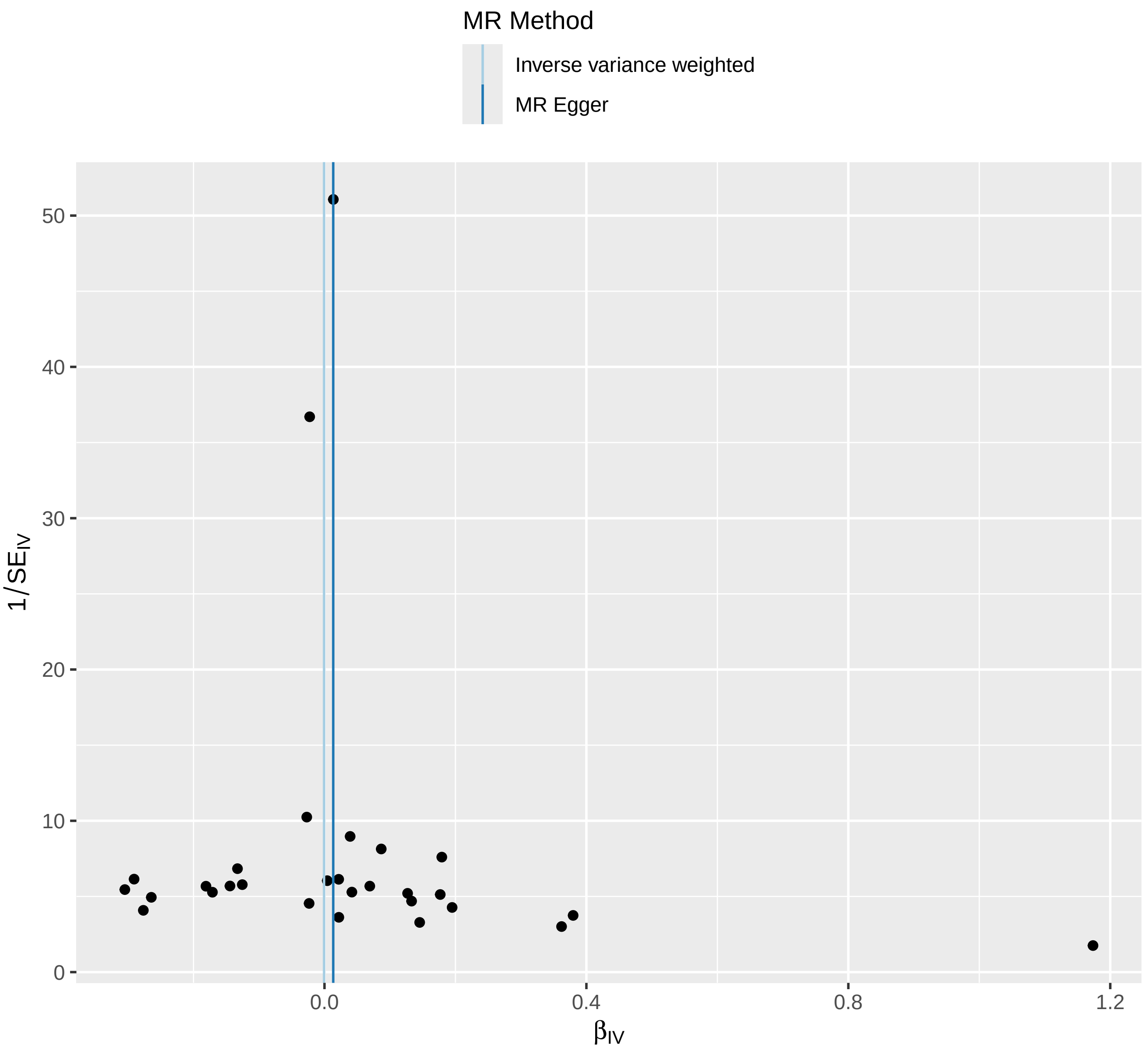
MR Method
Inverse variance weighted
MR Egger
50
40
30
20
10
0
1/SE_IV
0.0
0.4
0.8
1.2
β_IV

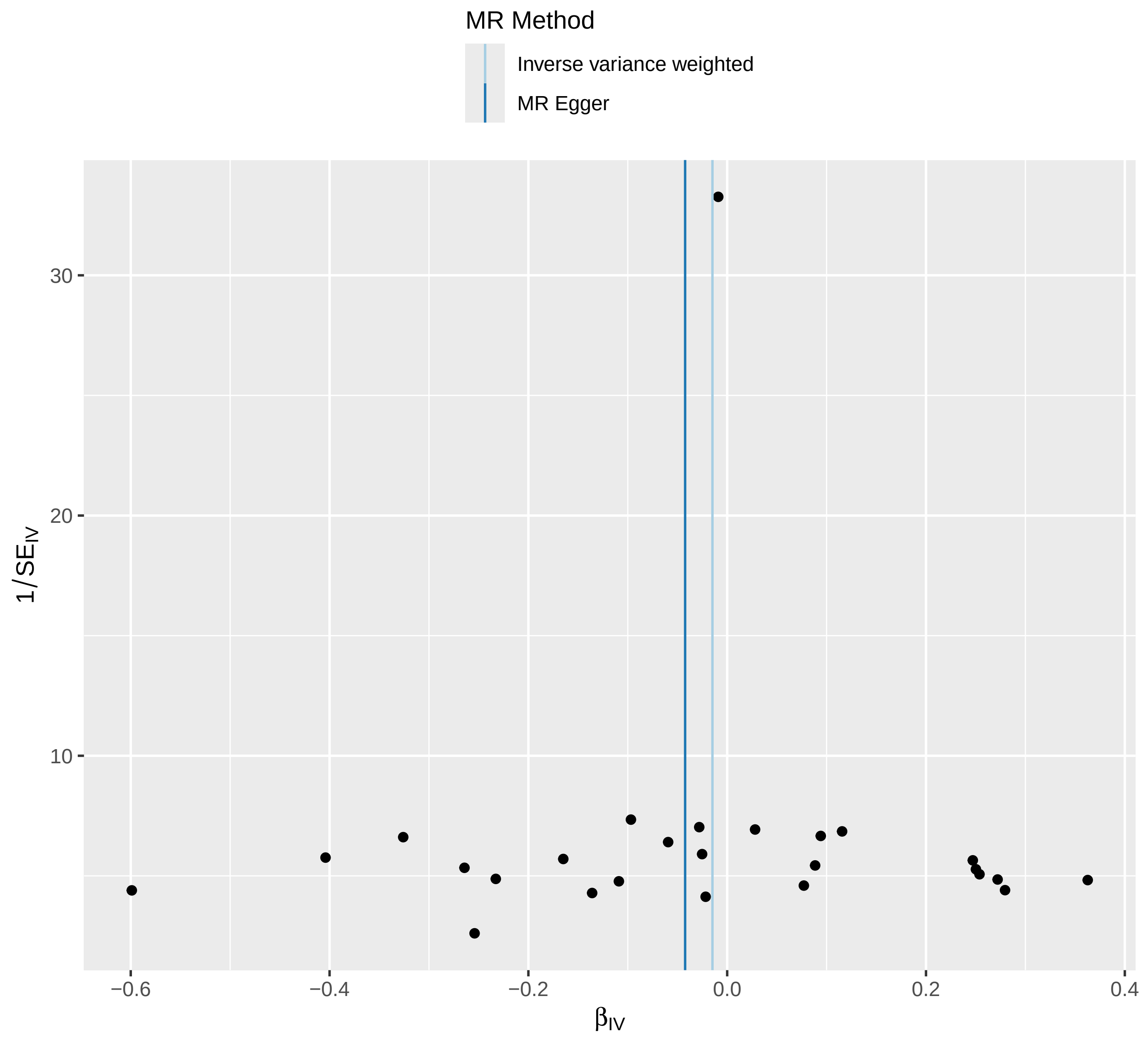
MR Method
Inverse variance weighted
MR Egger
30
20
10
−0.6
−0.4
−0.2
0.0
0.2
0.4
β_{IV}
$1/SE_{IV}$

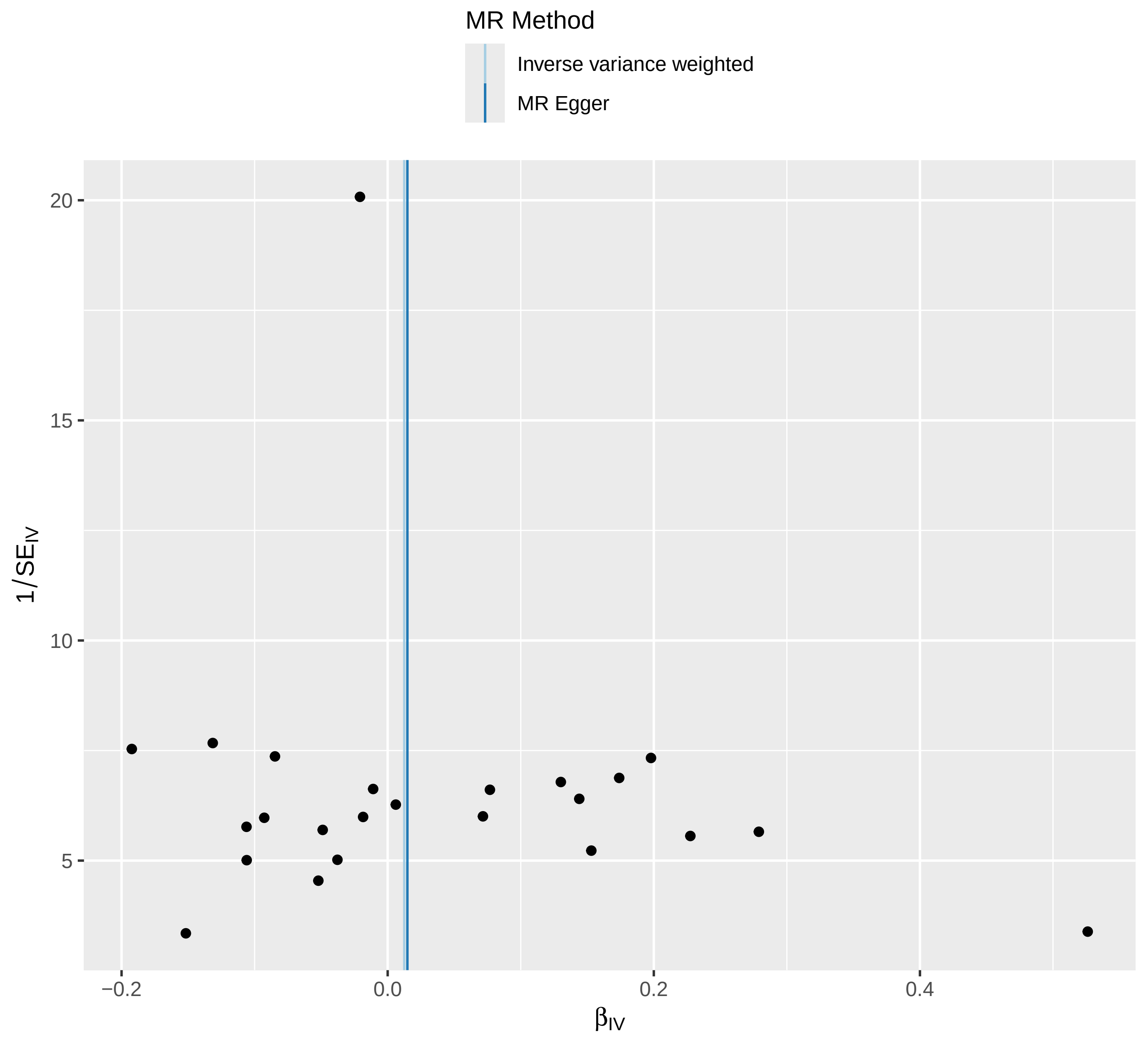

MR Method
Inverse variance weighted
MR Egger
20
15
10
5
1/SE$_{IV}$
−0.2
0.0
0.2
0.4
β$_{IV}$

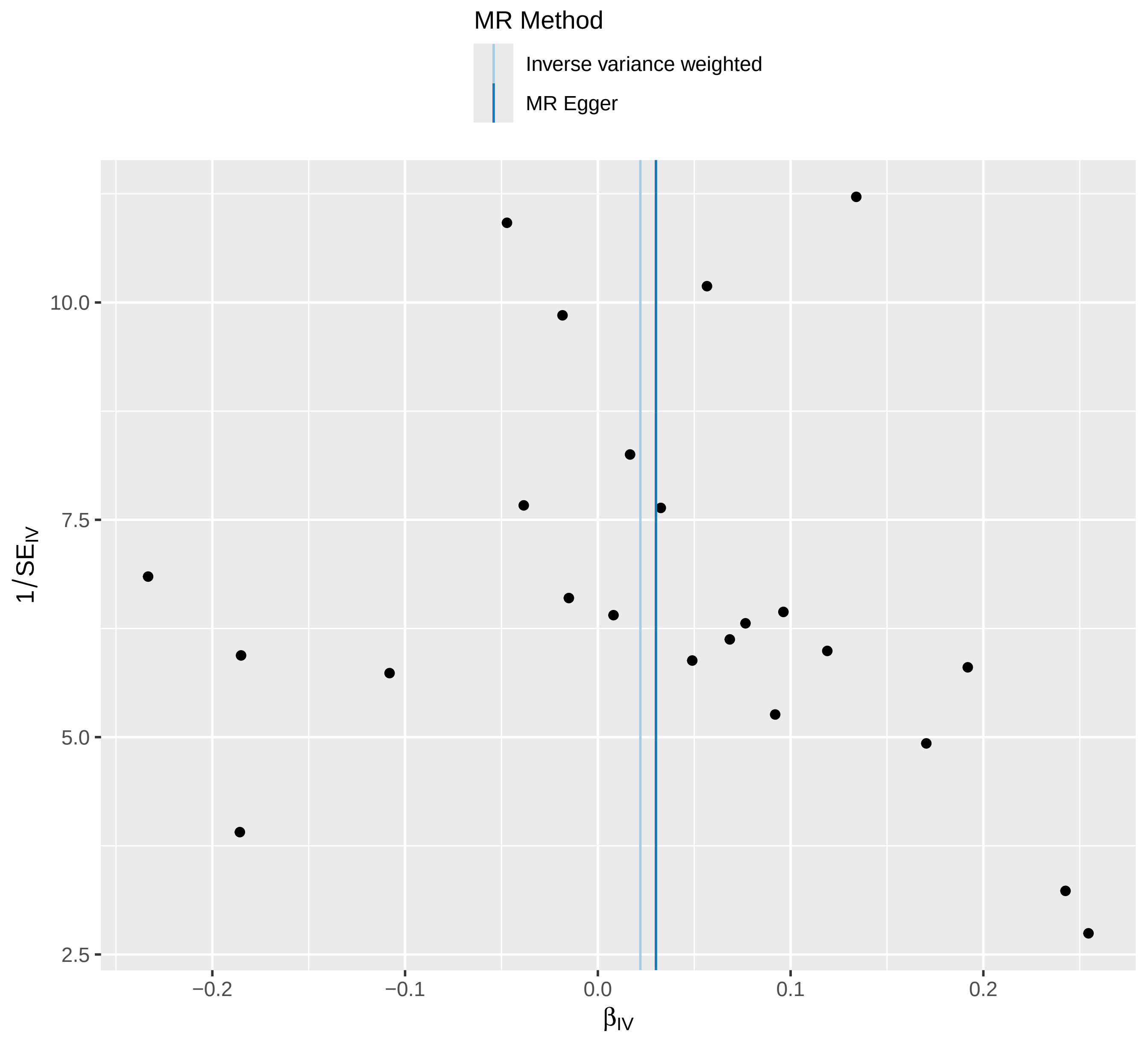
MR Method
Inverse variance weighted
MR Egger
10.0
7.5
5.0
2.5
1/SE_{IV}
−0.2
−0.1
0.0
0.1
0.2
$β_{IV}$

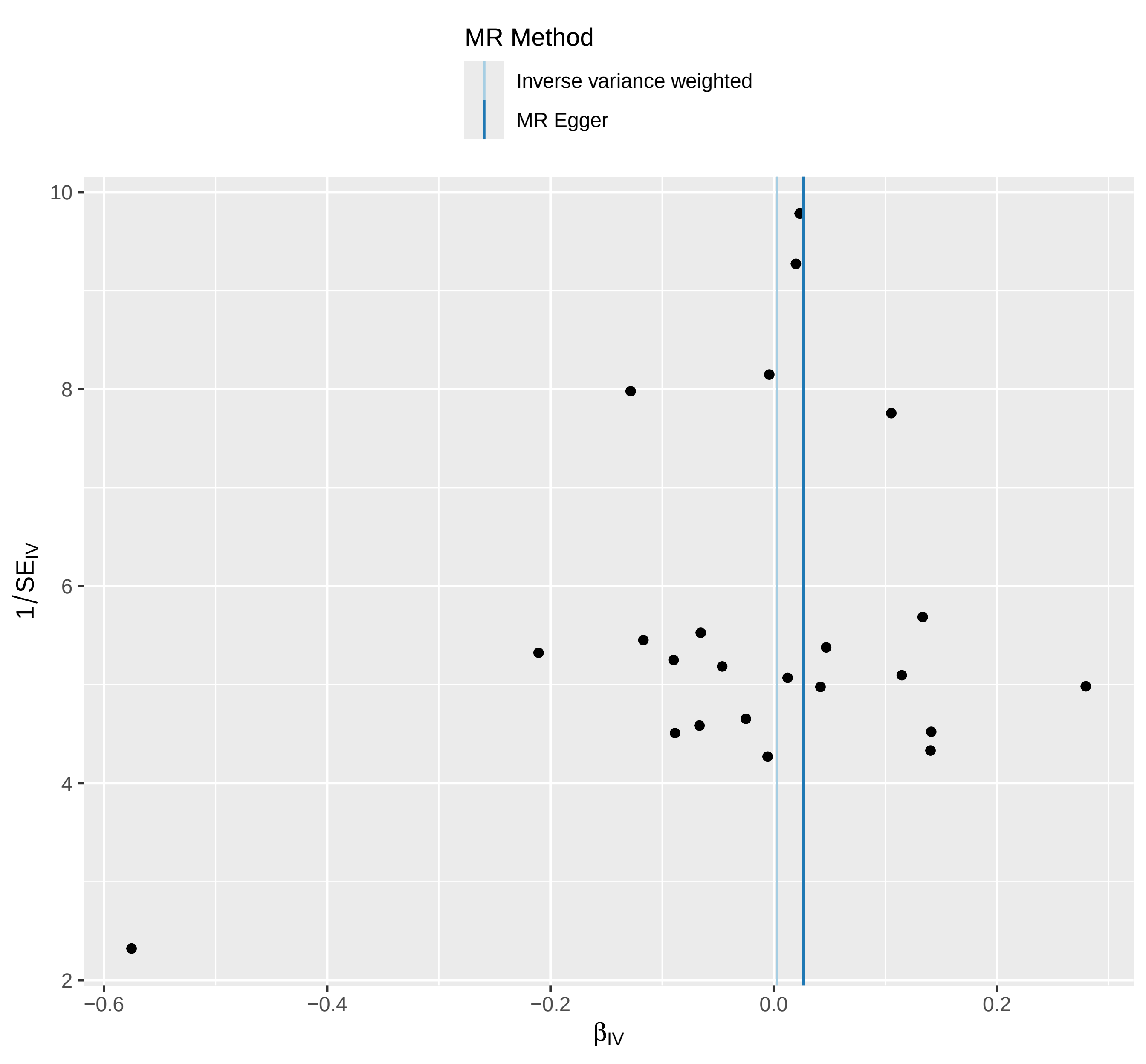

MR Method
Inverse variance weighted
MR Egger
1/SE_IV
10
8
6
4
2
−0.6
−0.4
−0.2
0.0
0.2
β_IV

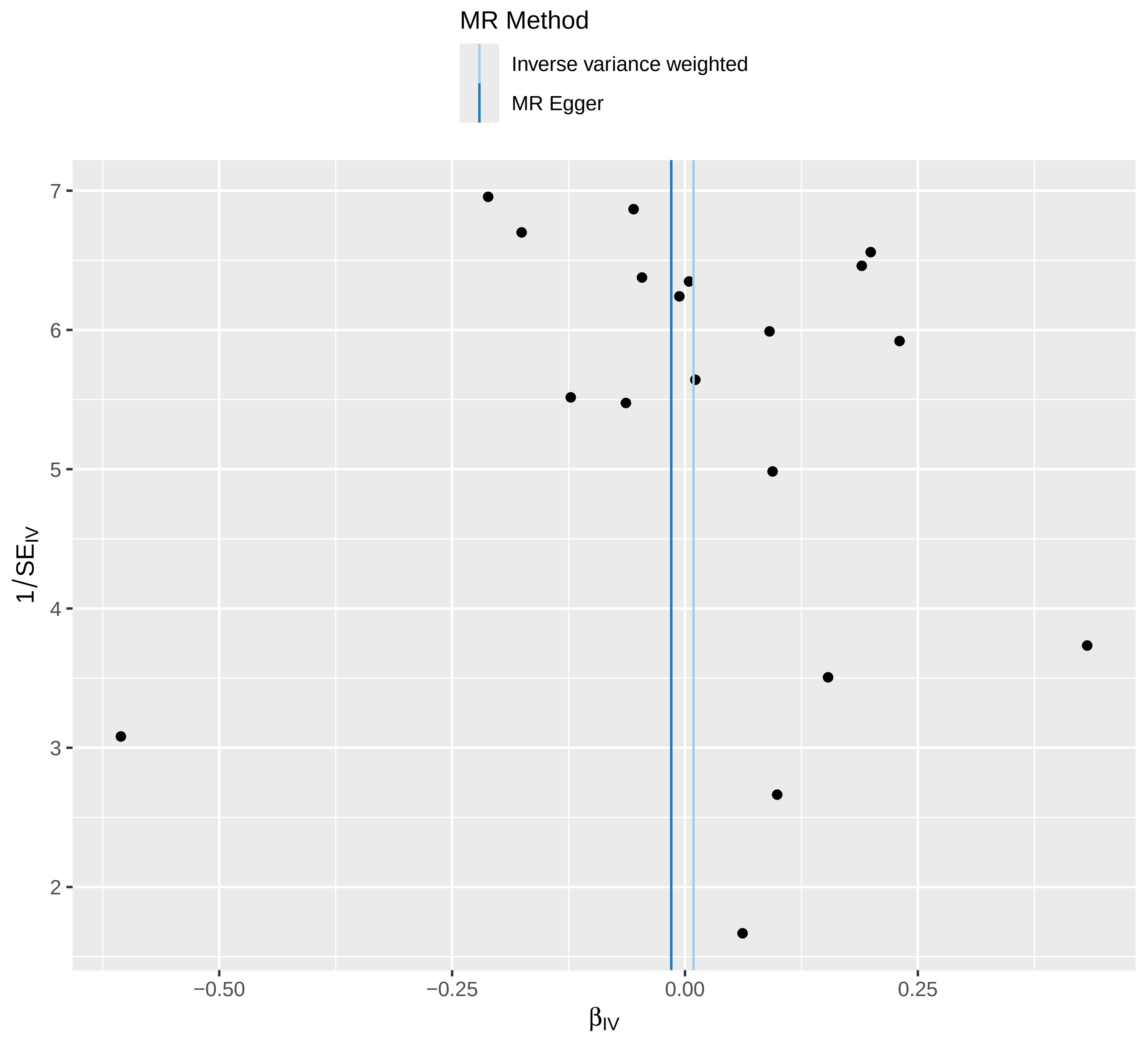
MR Method
Inverse variance weighted
MR Egger
1/SE_IV
7
6
5
4
3
2
−0.50
−0.25
0.00
0.25
β_IV

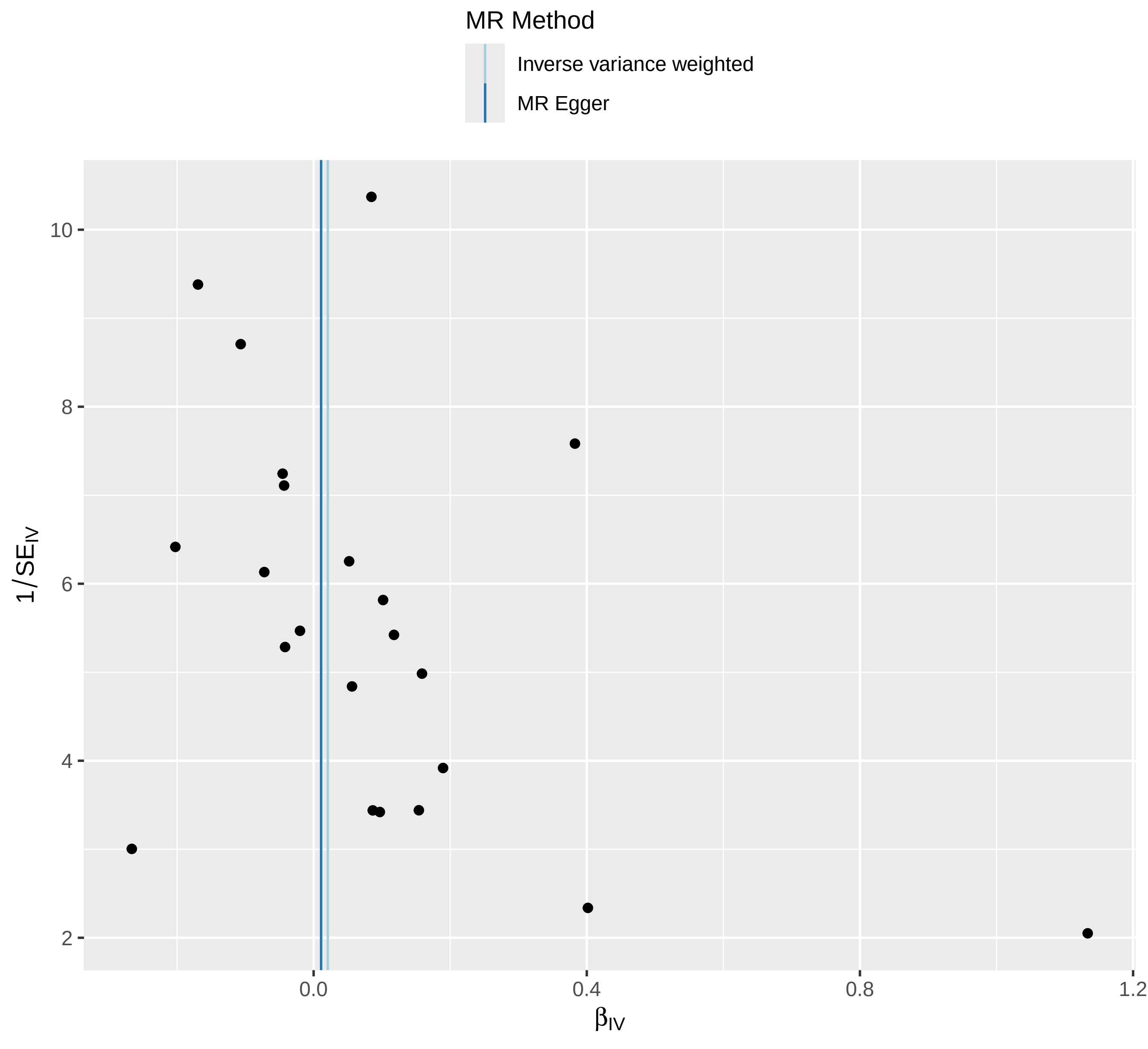
MR Method
Inverse variance weighted
MR Egger
10
8
6
4
2
1/SE_IV
0.0
0.4
0.8
1.2
β_IV

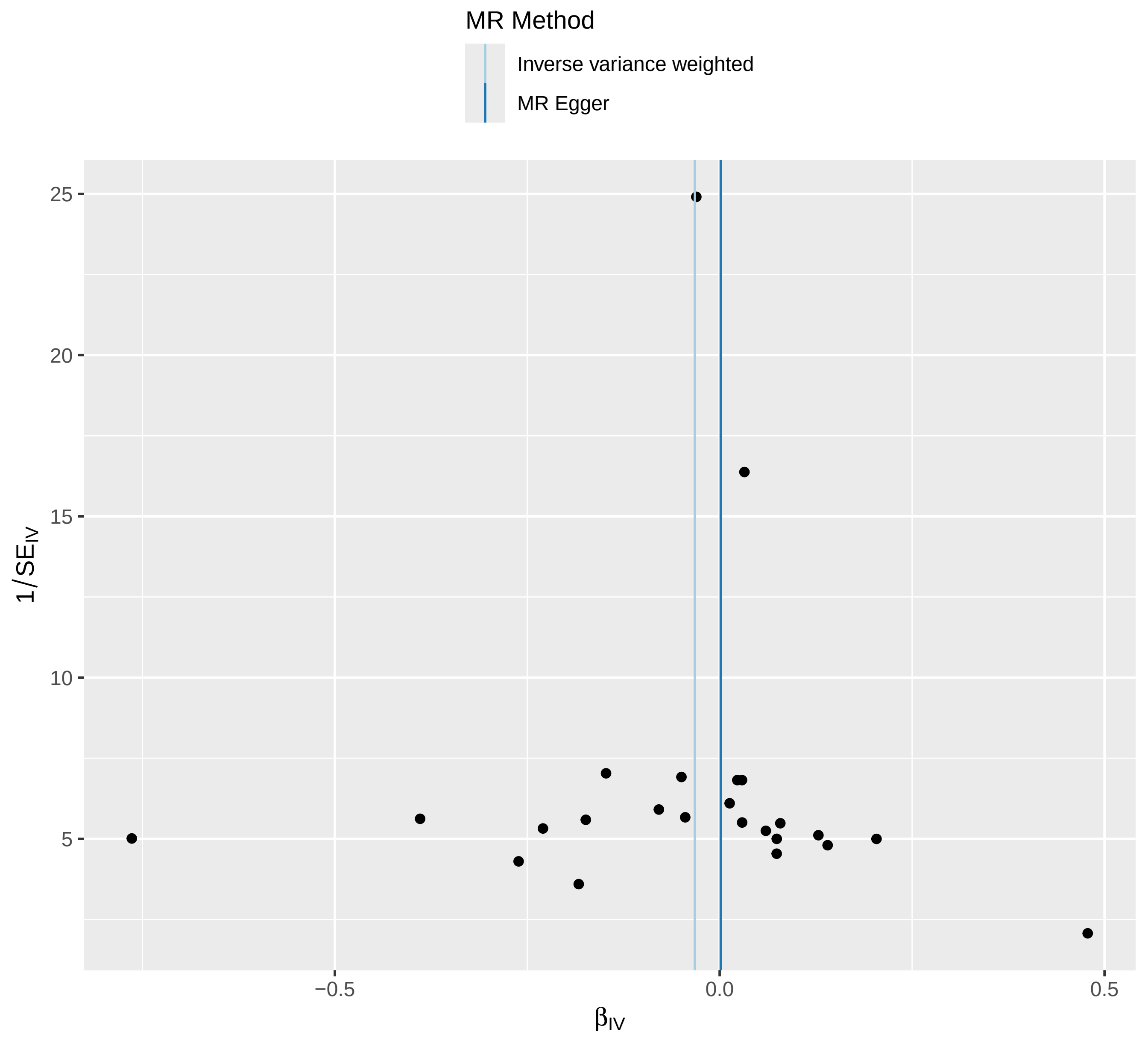

MR Method
Inverse variance weighted
MR Egger
25
20
15
10
5
1/SE_IV
−0.5
0.0
0.5
β_IV

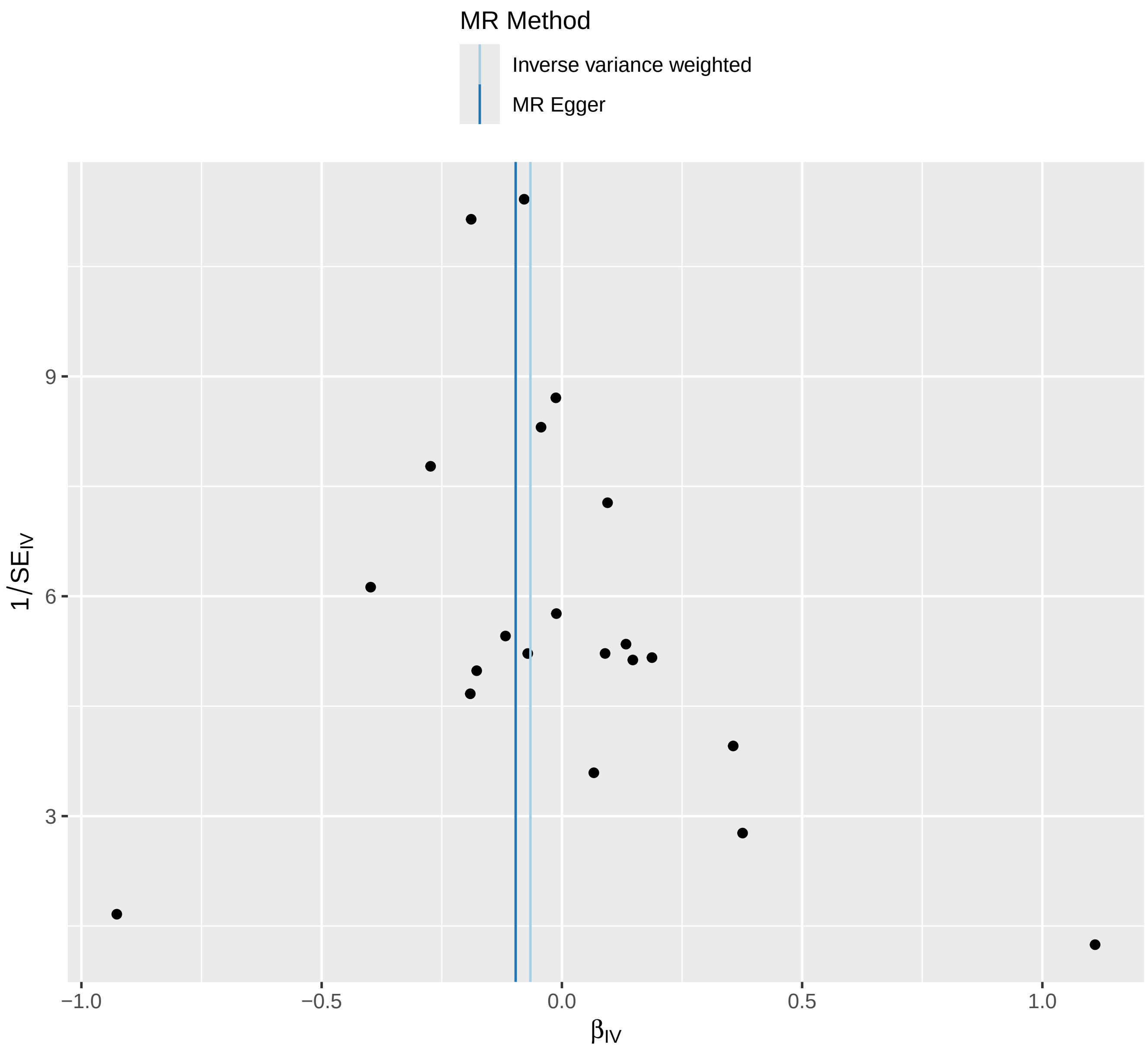

MR Method
Inverse variance weighted
MR Egger
9
6
3
1/SE$_{IV}$
−1.0
−0.5
0.0
0.5
1.0
β$_{IV}$

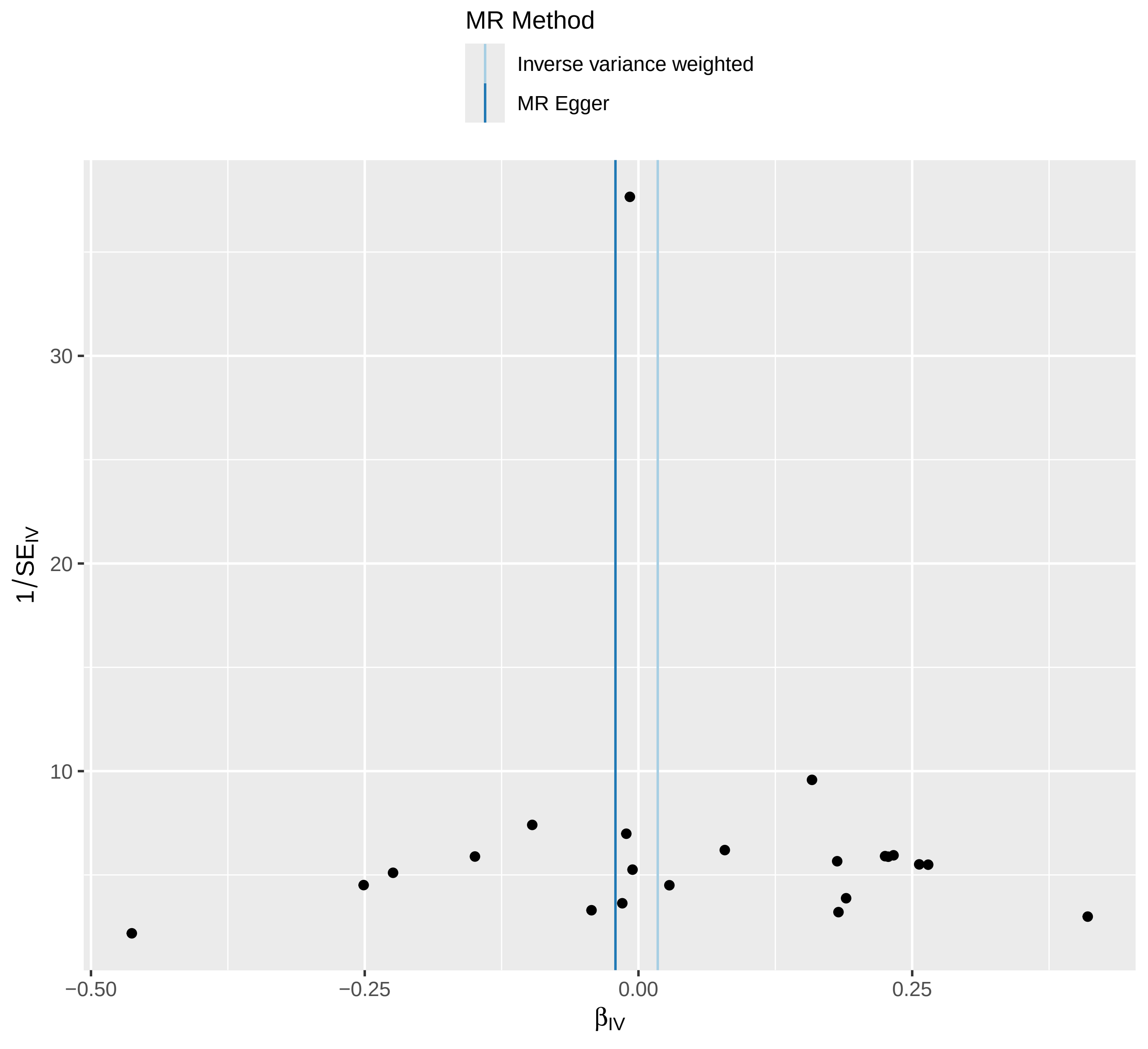
MR Method
Inverse variance weighted
MR Egger
30
20
10
$1/SE_{IV}$
−0.50
−0.25
0.00
0.25
β_{IV}

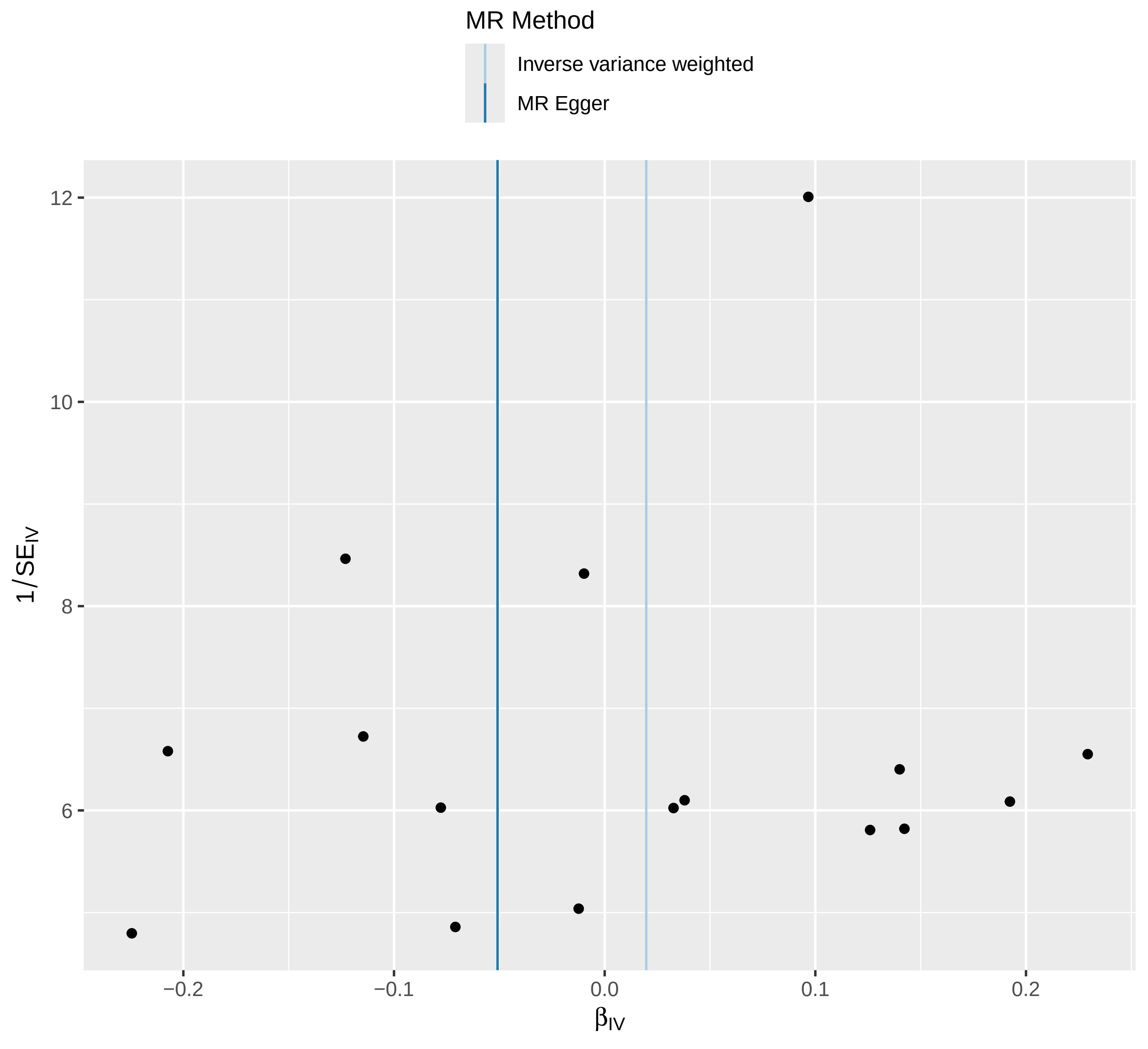

MR Method
Inverse variance weighted
MR Egger
12
10
8
6
1/SE_IV
−0.2
−0.1
0.0
0.1
0.2
β_IV

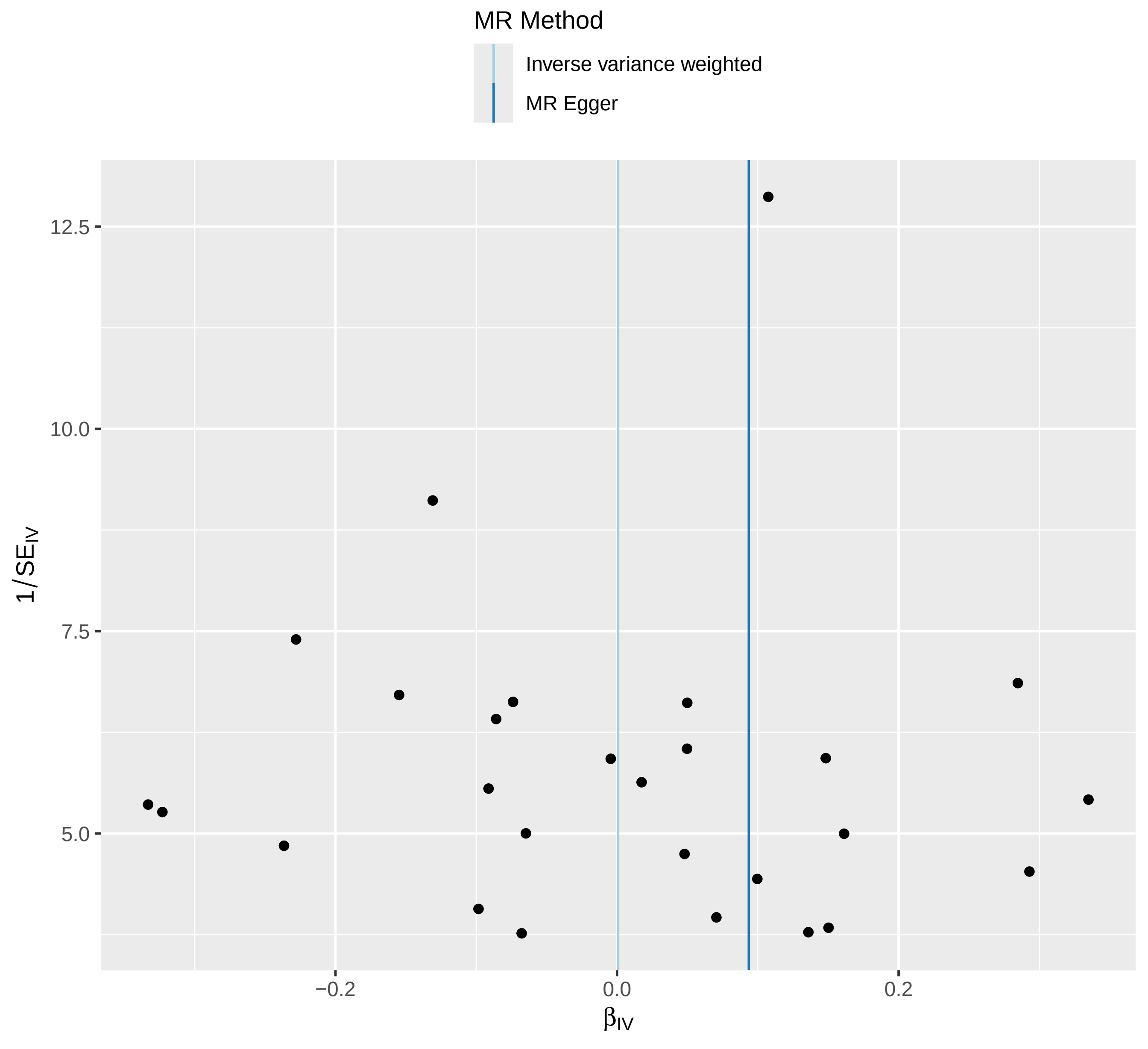

MR Method
Inverse variance weighted
MR Egger
12.5
10.0
7.5
5.0
1/SE_IV
−0.2
0.0
0.2
β_IV

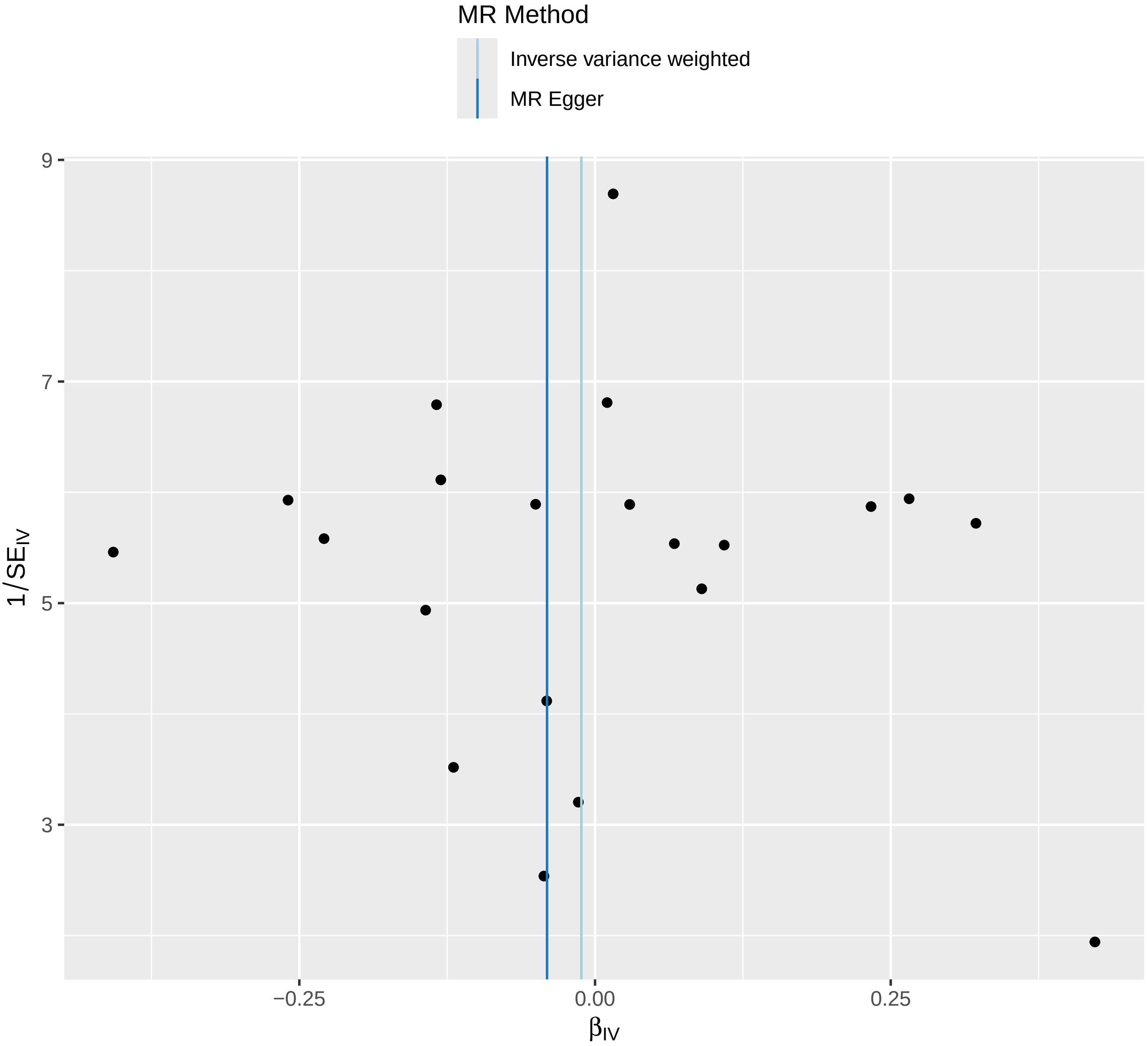

MR Method
Inverse variance weighted
MR Egger
9
7
5
3
$1/SE_{IV}$
−0.25
0.00
0.25
β_{IV}

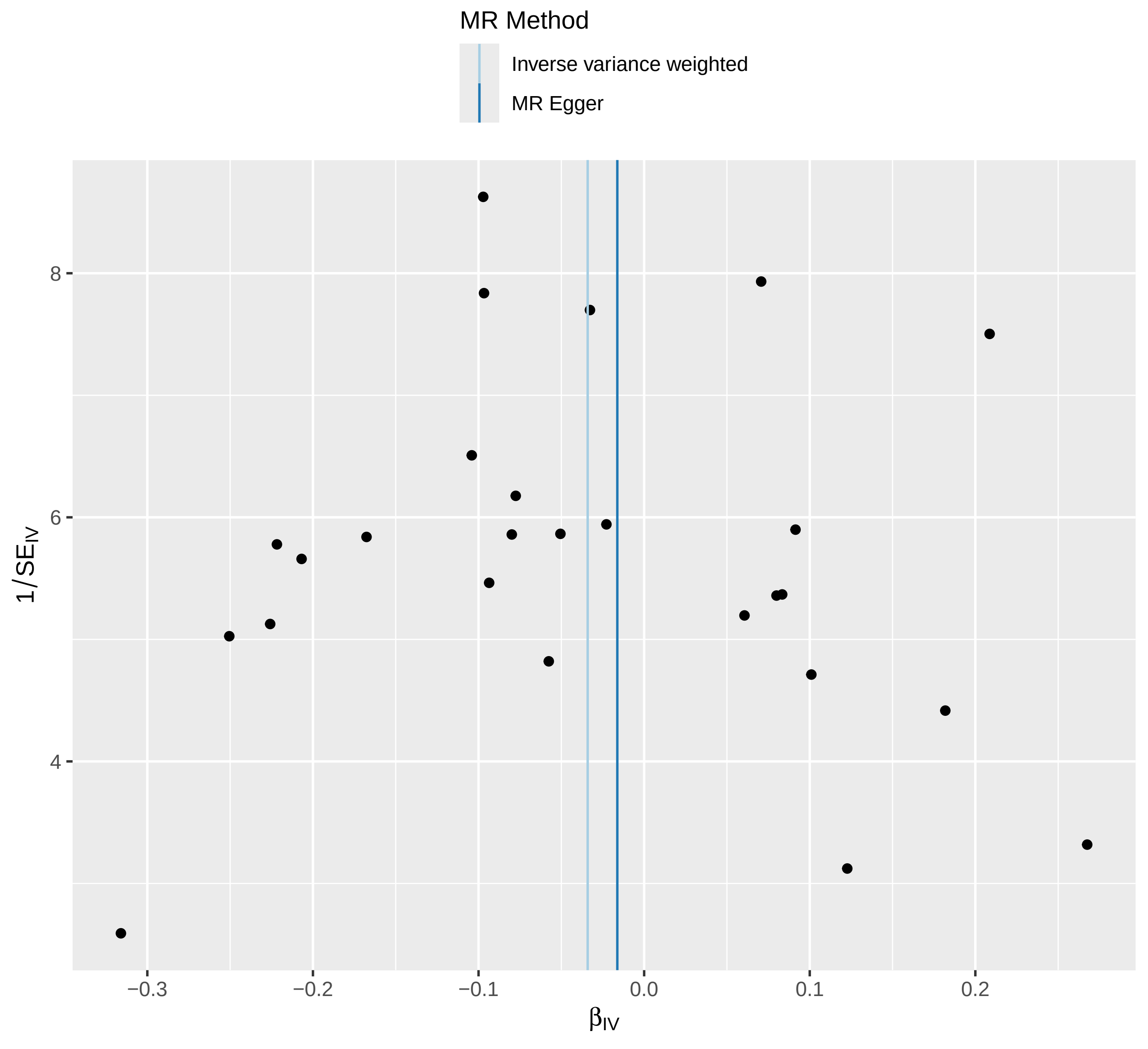
MR Method
Inverse variance weighted
MR Egger
8
6
4
$1/SE_{IV}$
−0.3
−0.2
−0.1
0.0
0.1
0.2
β_{IV}

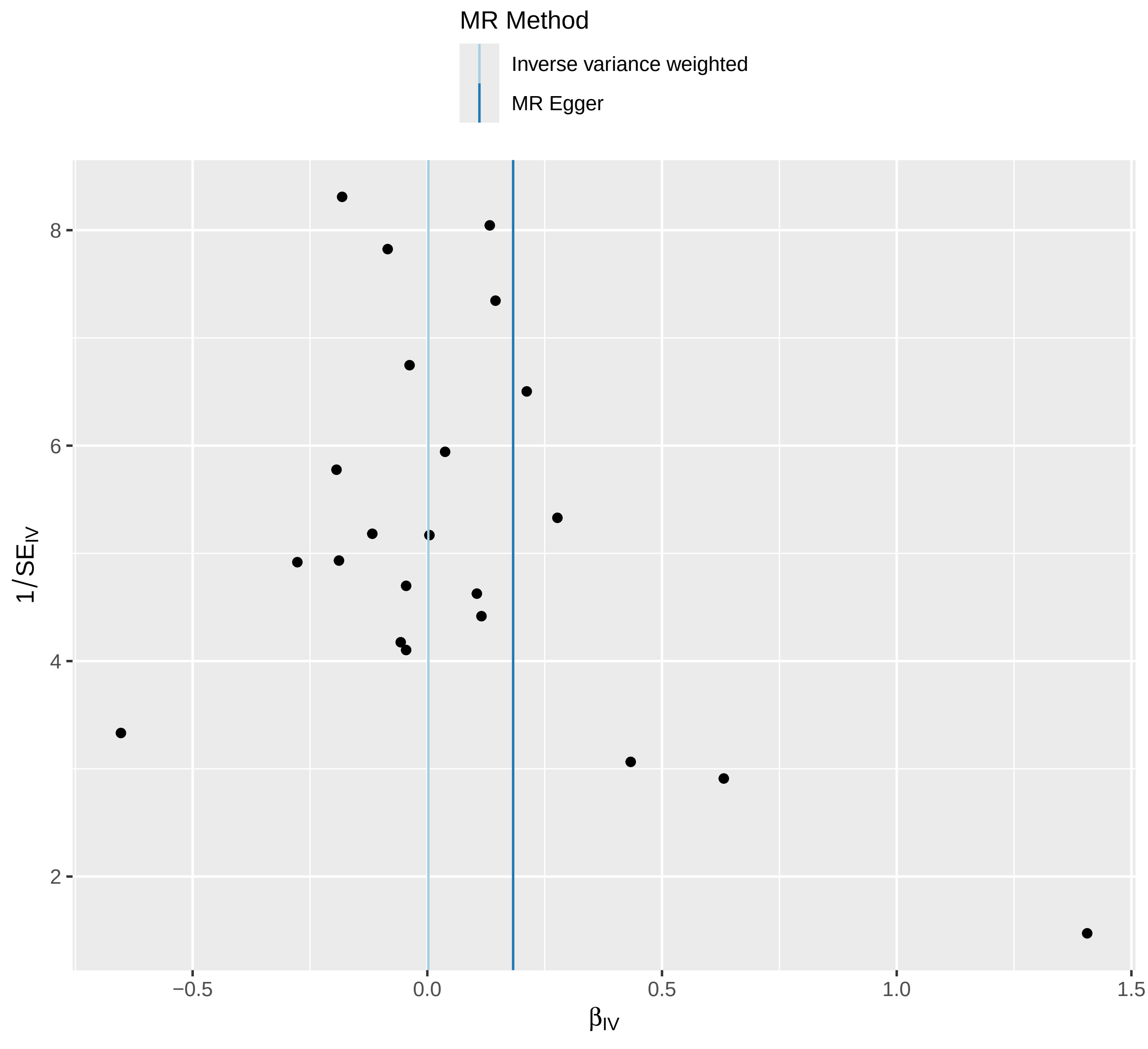

MR Method
Inverse variance weighted
MR Egger
1/SE$_{IV}$
β$_{IV}$
8
6
4
2
−0.5
0.0
0.5
1.0
1.5

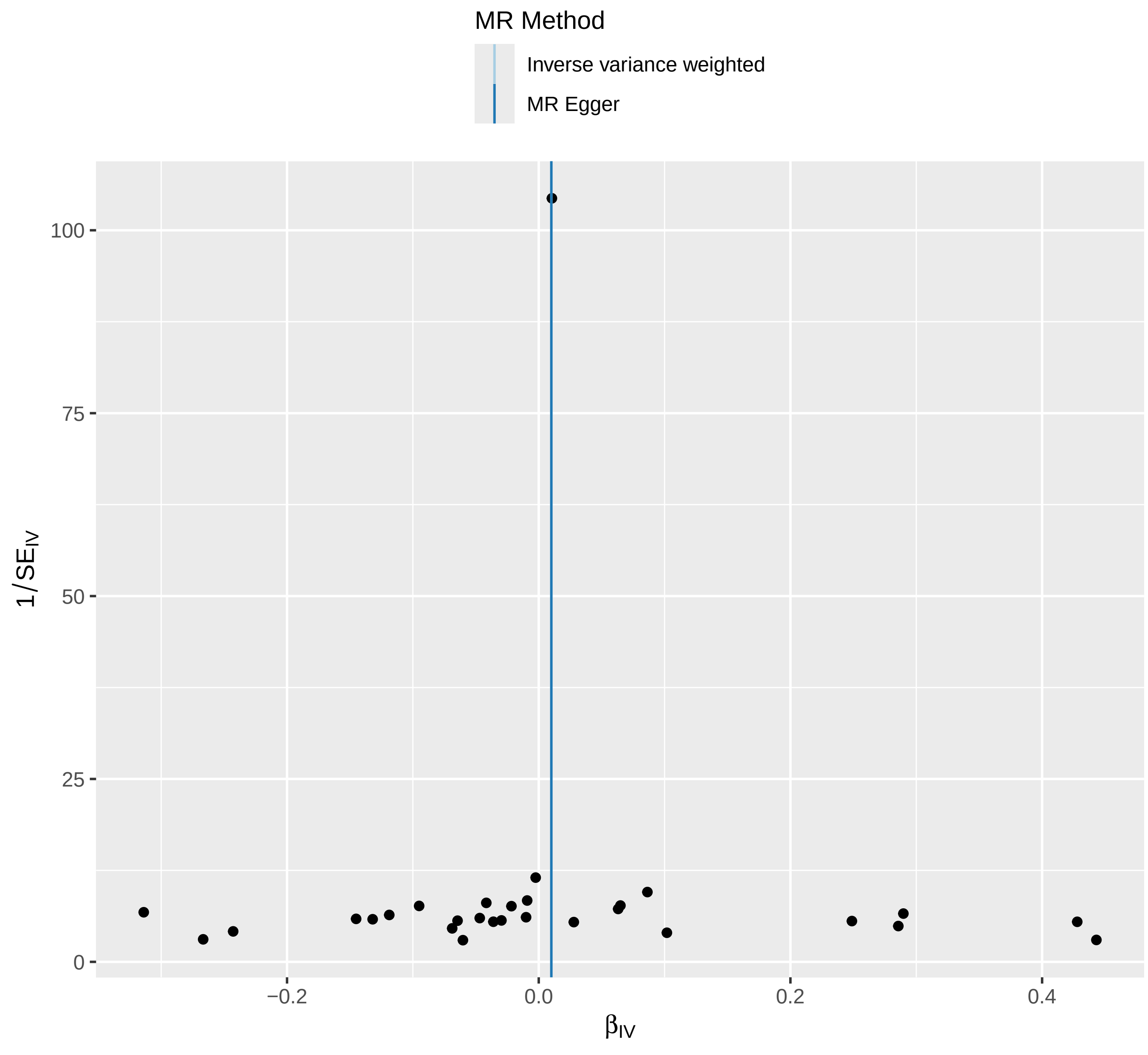
MR Method
Inverse variance weighted
MR Egger
100
75
50
25
0
1/SE_{IV}
−0.2
0.0
0.2
0.4
β_{IV}

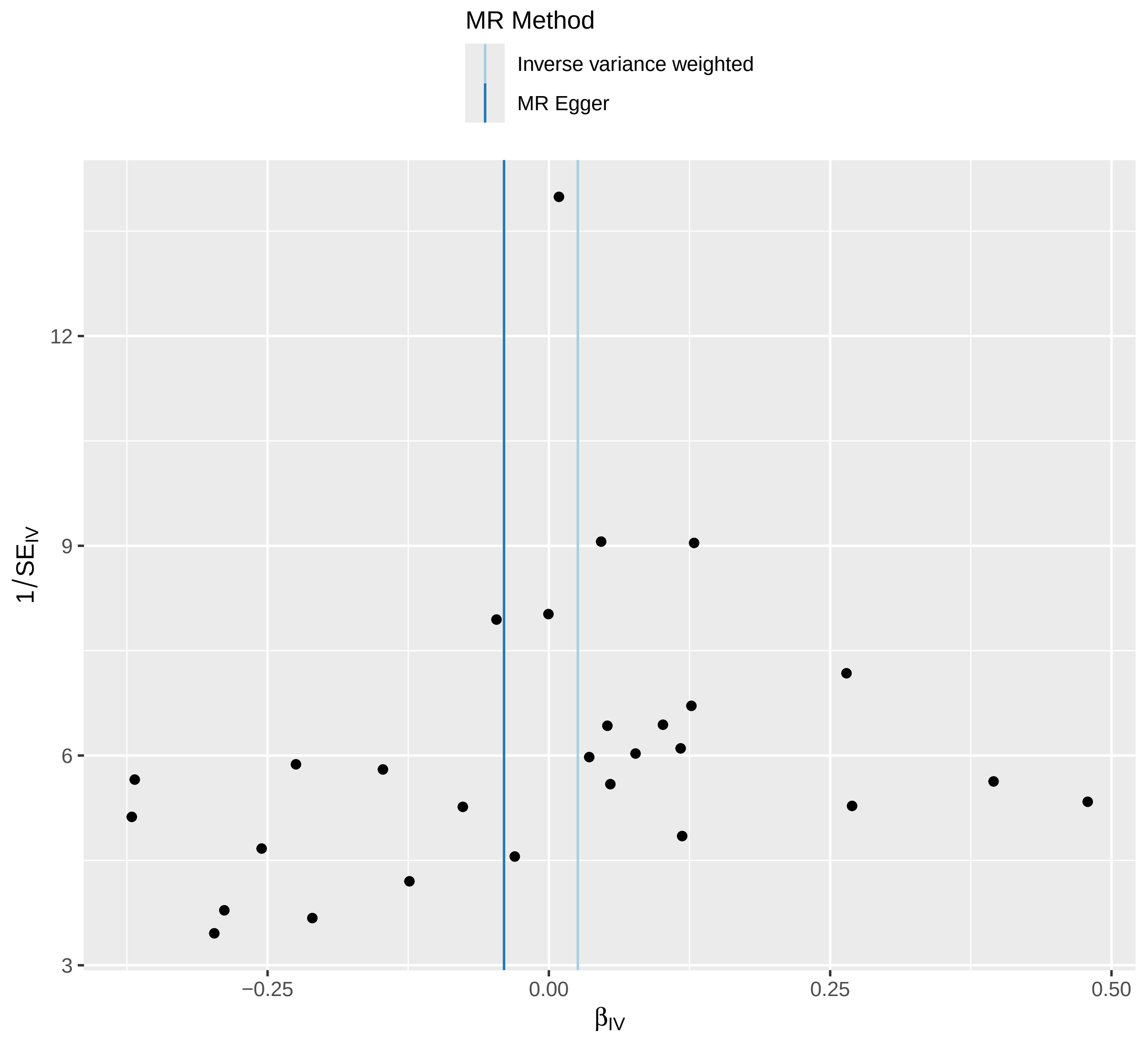

MR Method
Inverse variance weighted
MR Egger
12
9
6
3
1/SE_IV
−0.25
0.00
0.25
0.50
β_IV

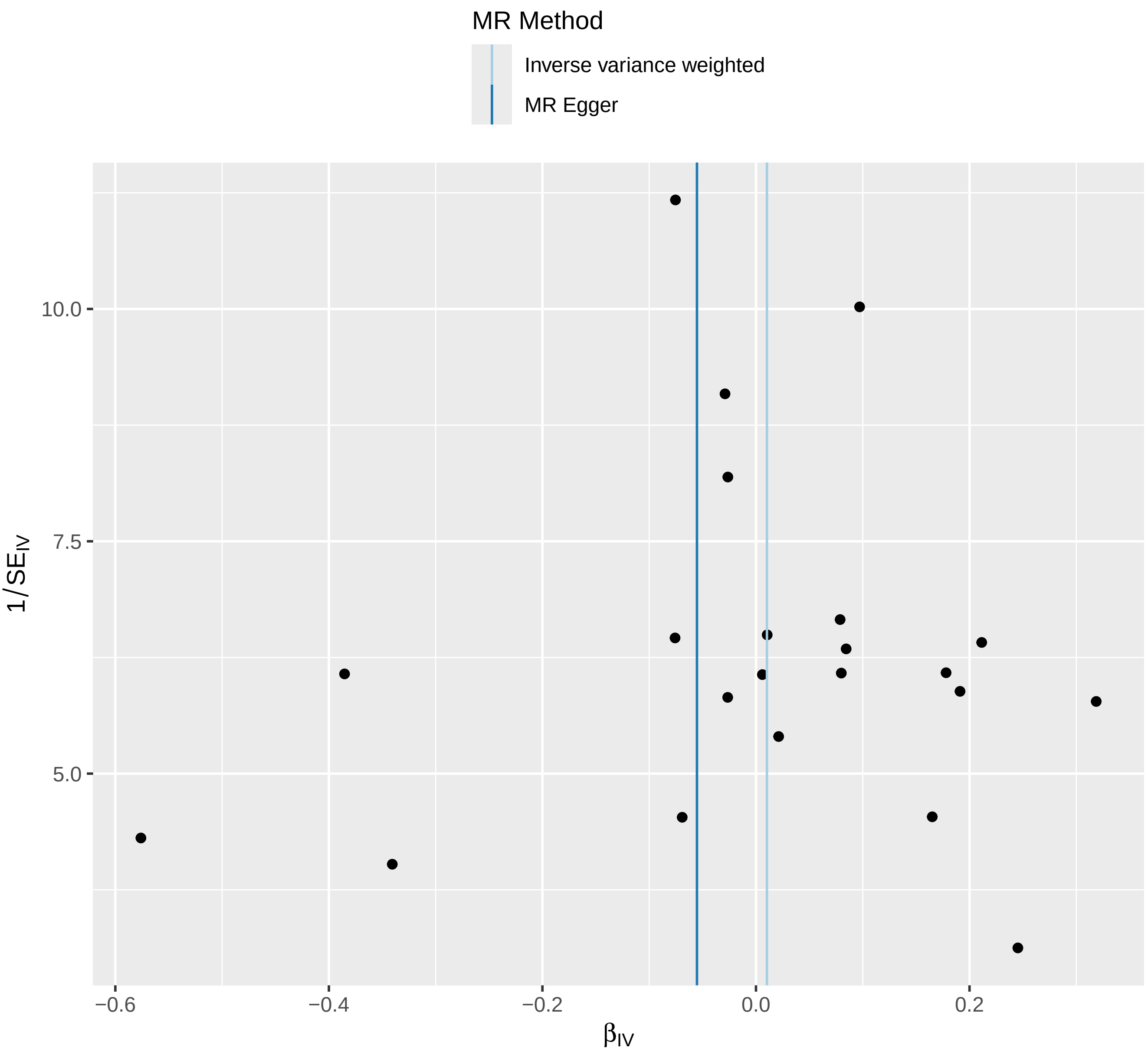
MR Method
Inverse variance weighted
MR Egger
10.0
7.5
5.0
1/SE_IV
−0.6
−0.4
−0.2
0.0
0.2
β_IV

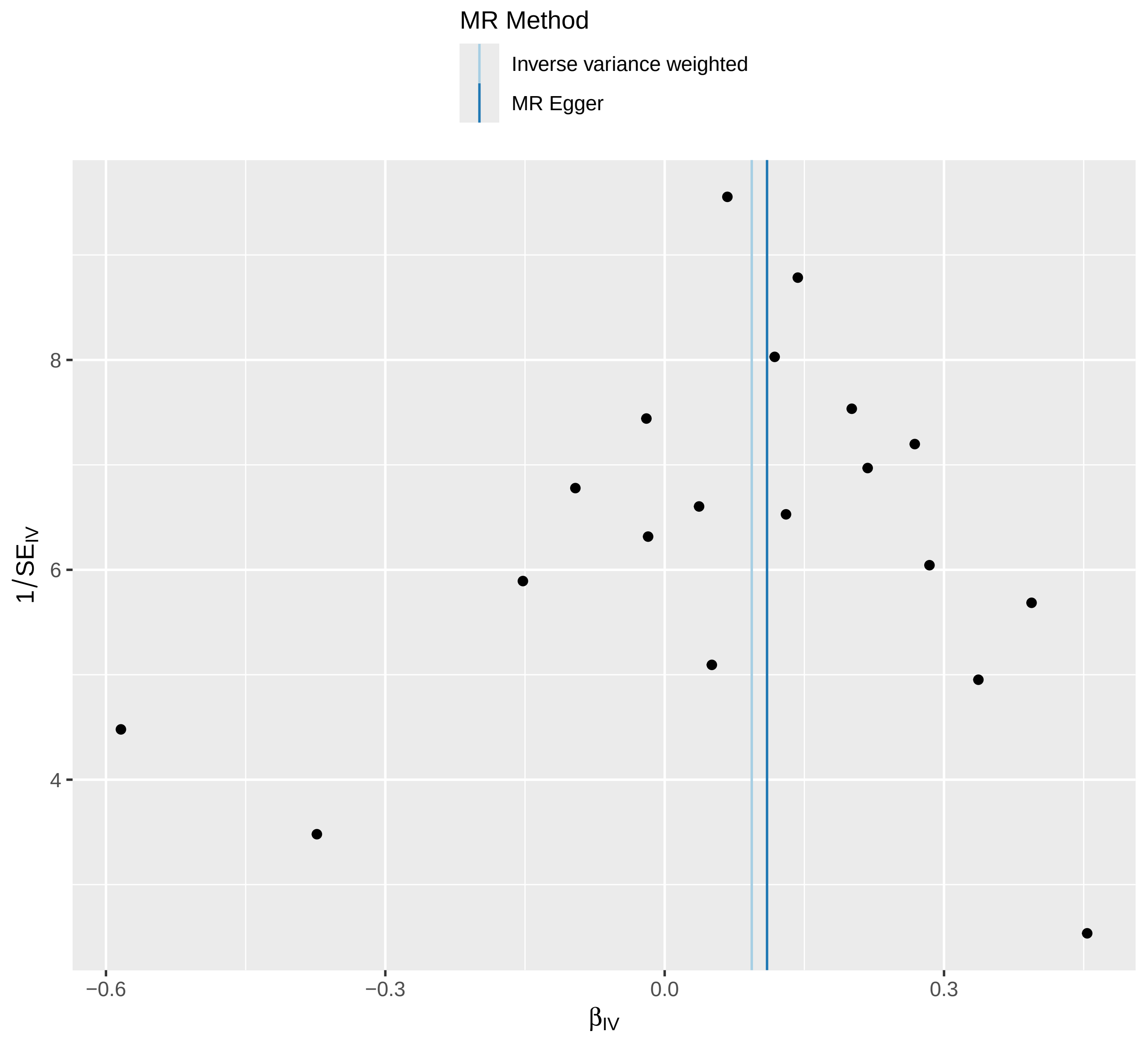

MR Method
Inverse variance weighted
MR Egger
8
6
4
1/SE_IV
−0.6
−0.3
0.0
0.3
β_IV

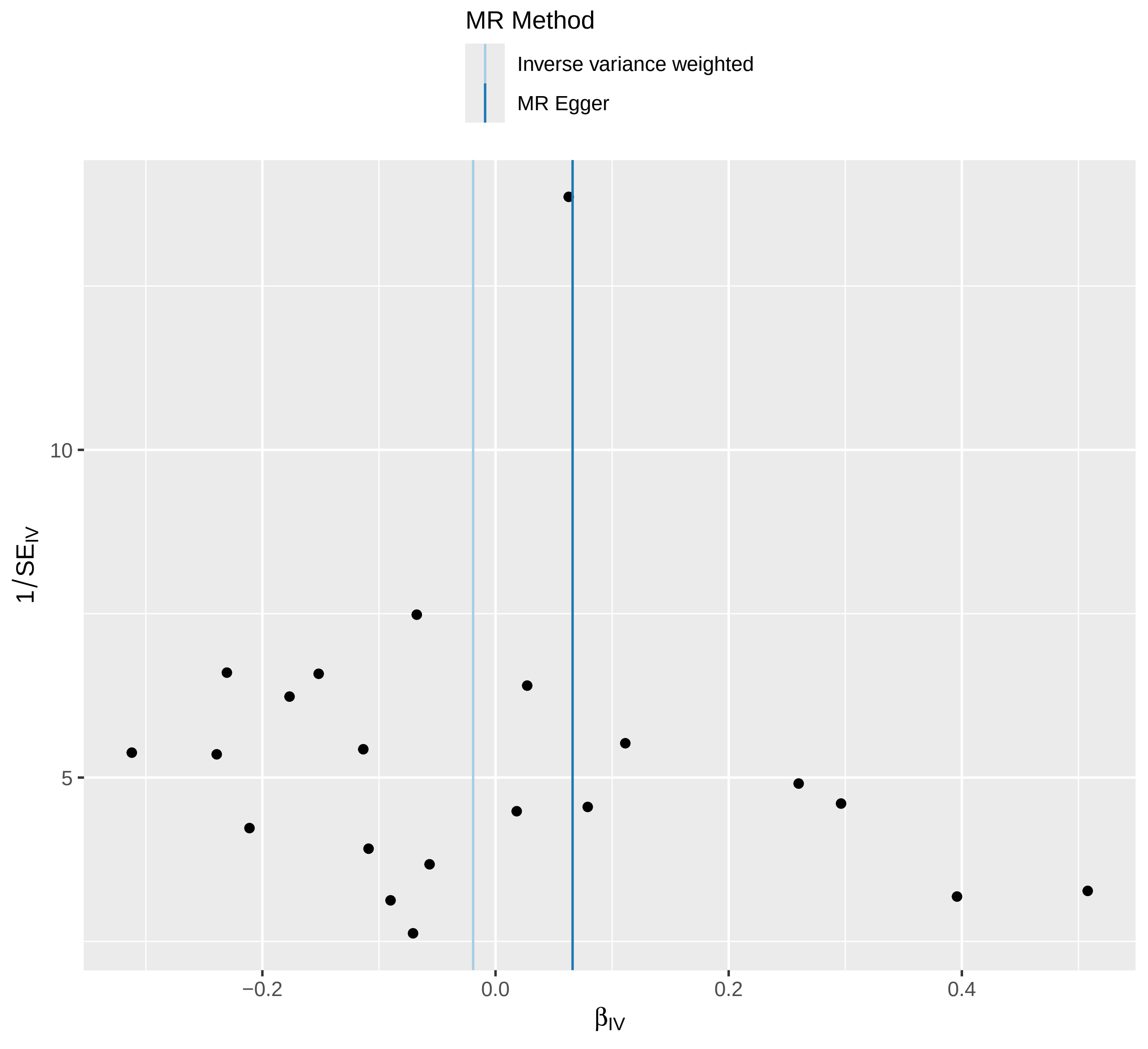

MR Method
Inverse variance weighted
MR Egger
10
5
1/SE_IV
−0.2
0.0
0.2
0.4
β_IV

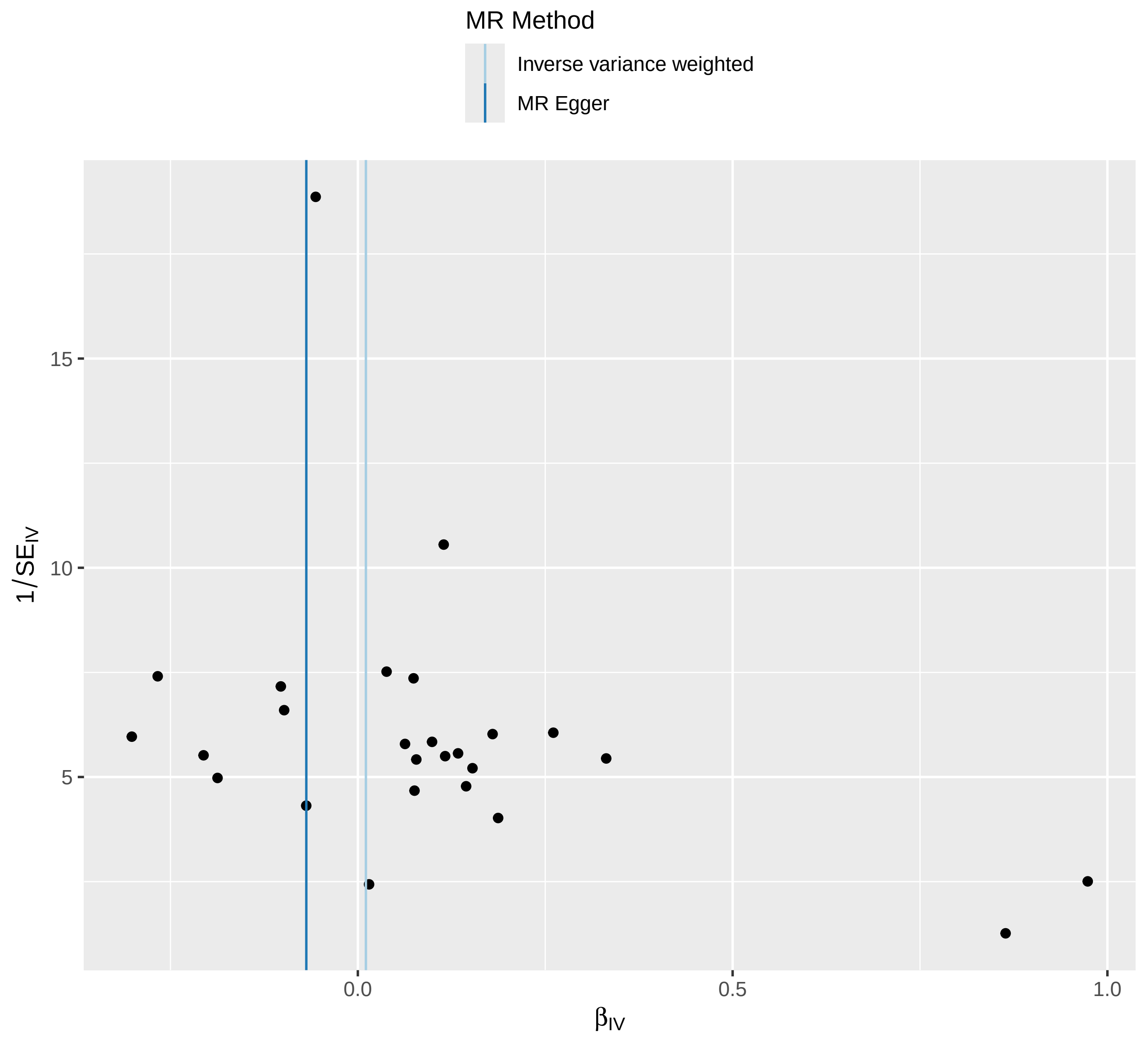
MR Method
Inverse variance weighted
MR Egger
1/SE$_{IV}$
15
10
5
0.0
0.5
1.0
β$_{IV}$

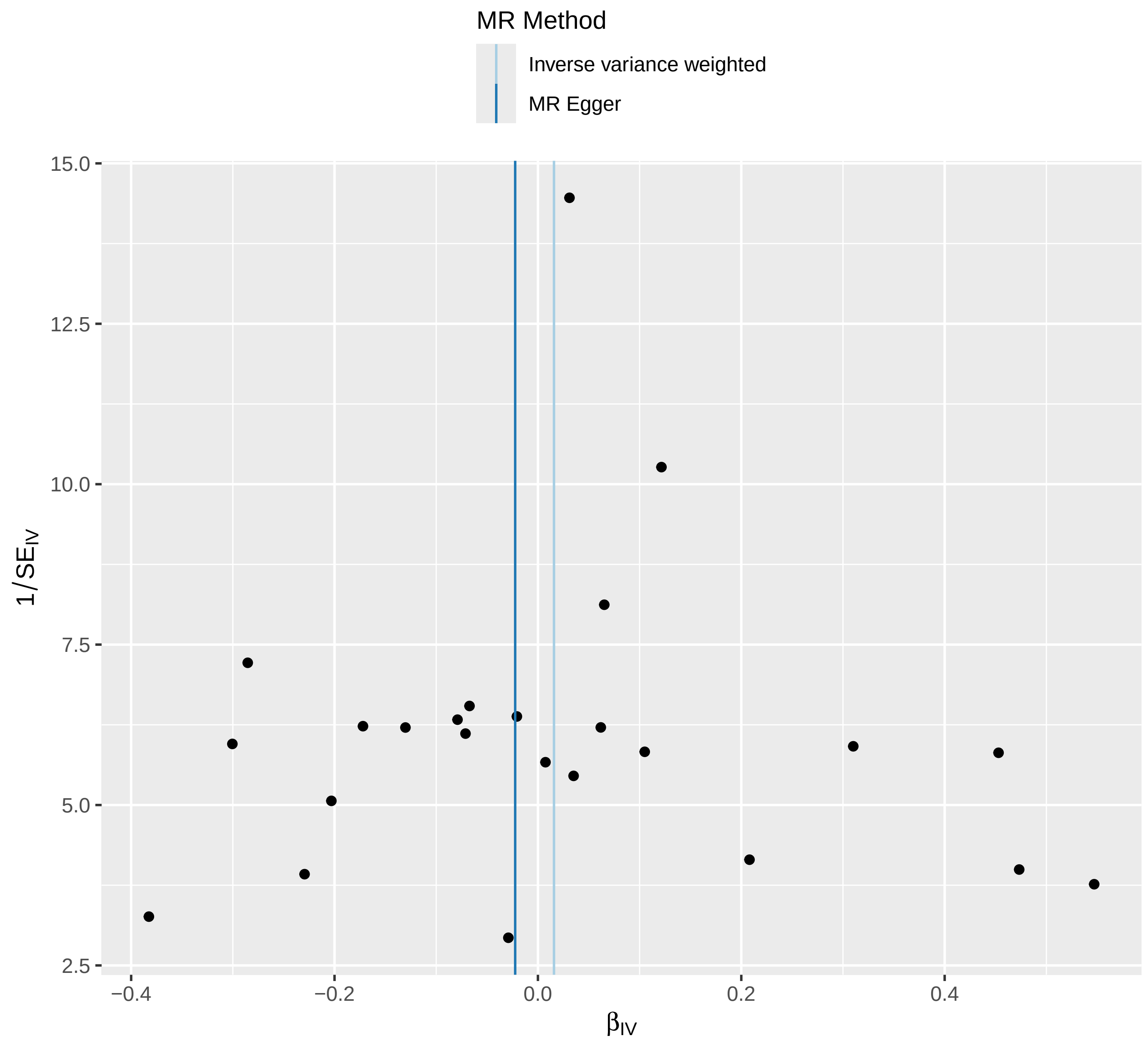
MR Method
Inverse variance weighted
MR Egger
15.0
12.5
10.0
7.5
5.0
2.5
1/SE_IV
−0.4
−0.2
0.0
0.2
0.4
β_IV

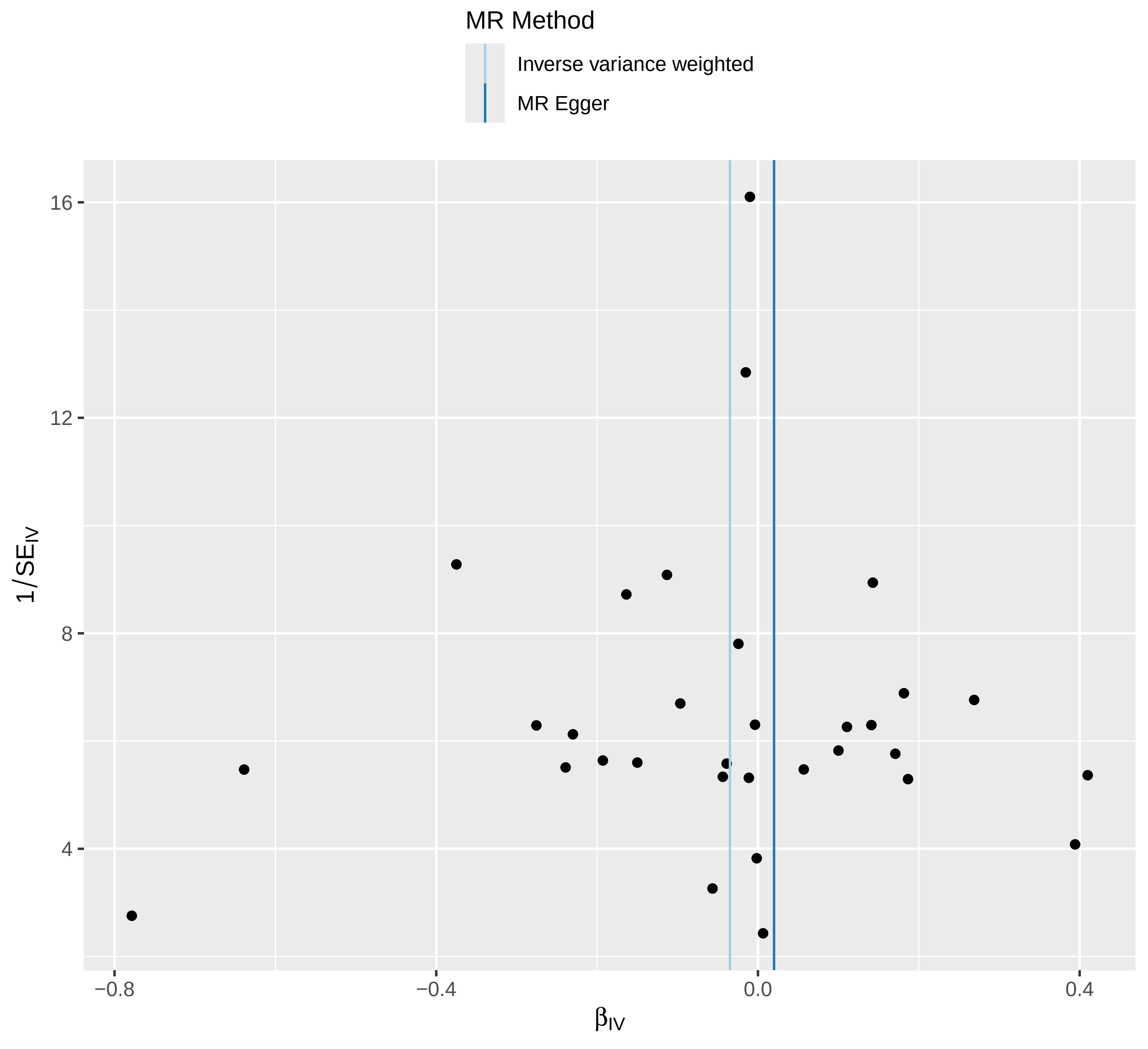
MR Method
Inverse variance weighted
MR Egger
16
12
8
4
$1/SE_{IV}$
−0.8
−0.4
0.0
0.4
β_{IV}

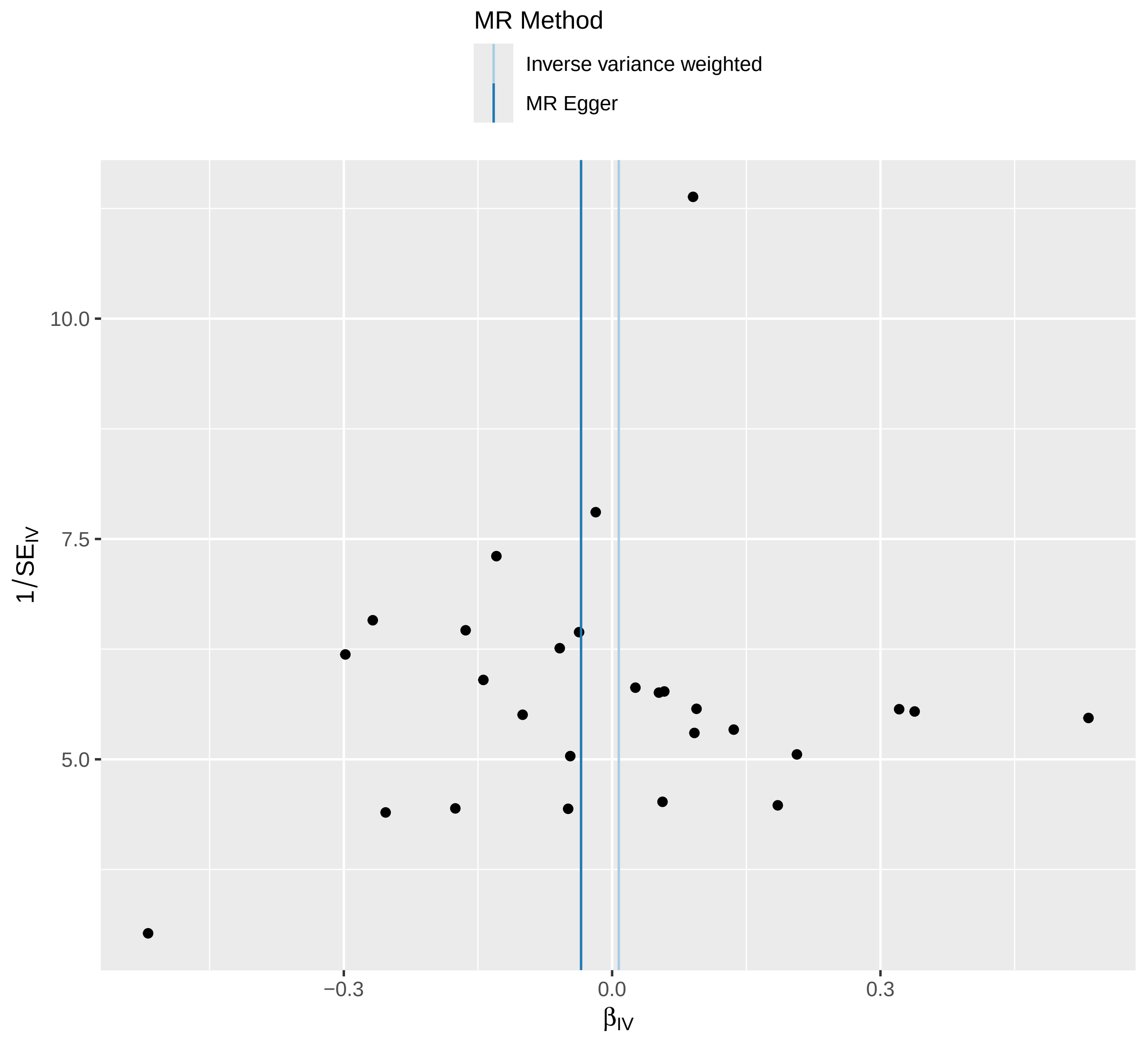
MR Method
Inverse variance weighted
MR Egger
10.0
7.5
5.0
1/SE_IV
−0.3
0.0
0.3
β_IV

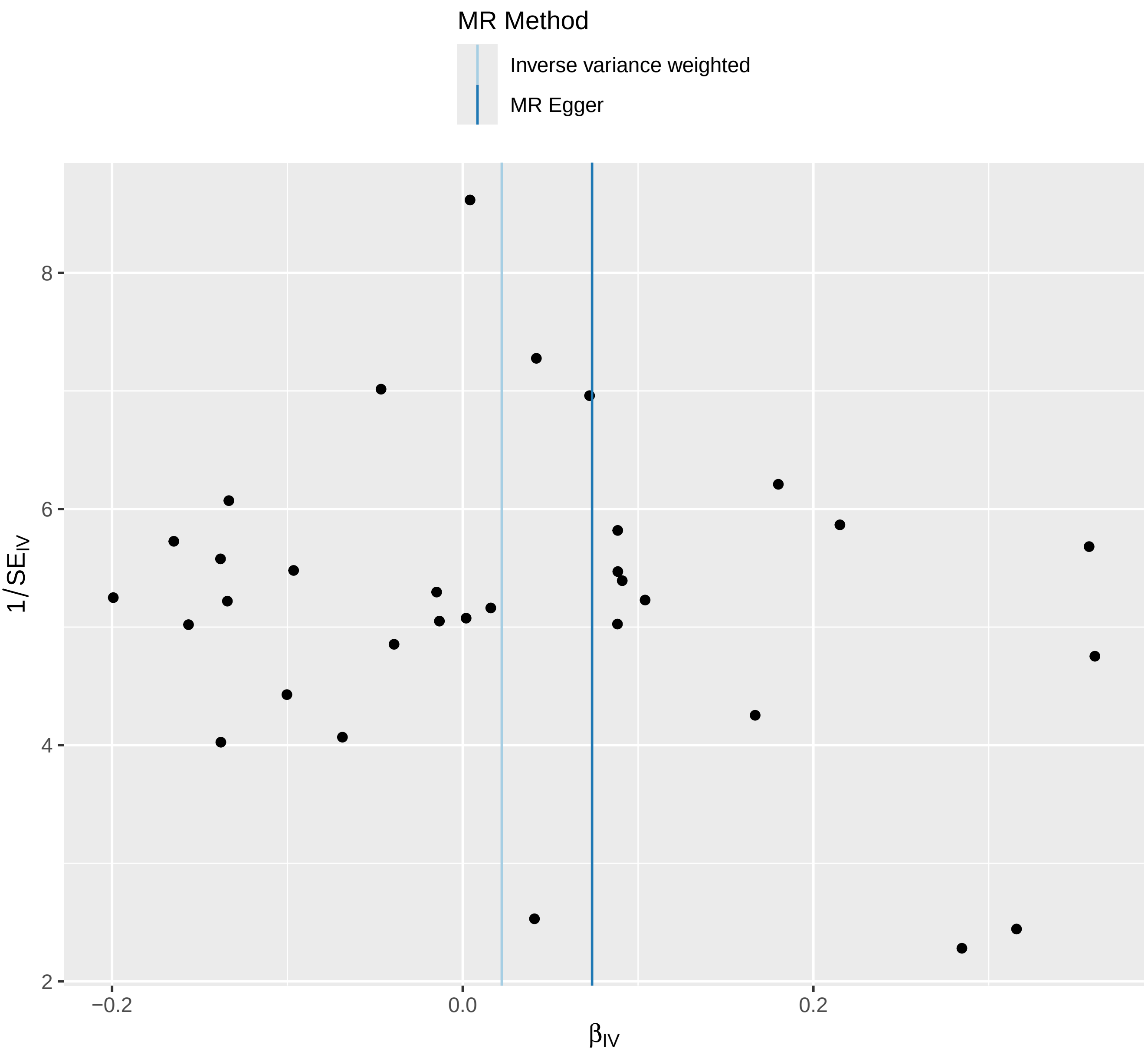

MR Method
Inverse variance weighted
MR Egger
1/SE_IV
8
6
4
2
−0.2
0.0
0.2
β_IV

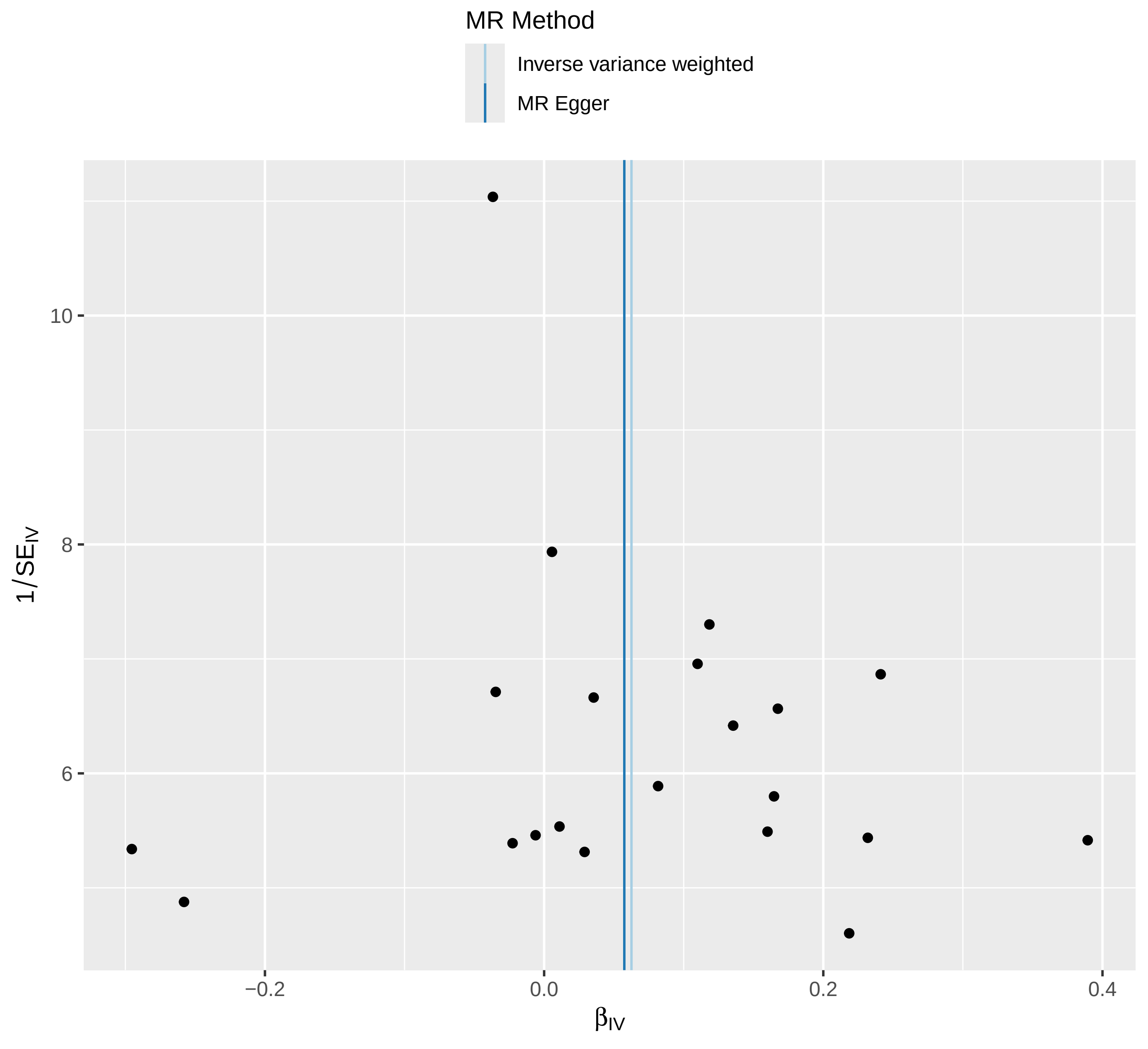
MR Method
Inverse variance weighted
MR Egger
10
8
6
1/SE$_{IV}$
−0.2
0.0
0.2
0.4
β$_{IV}$

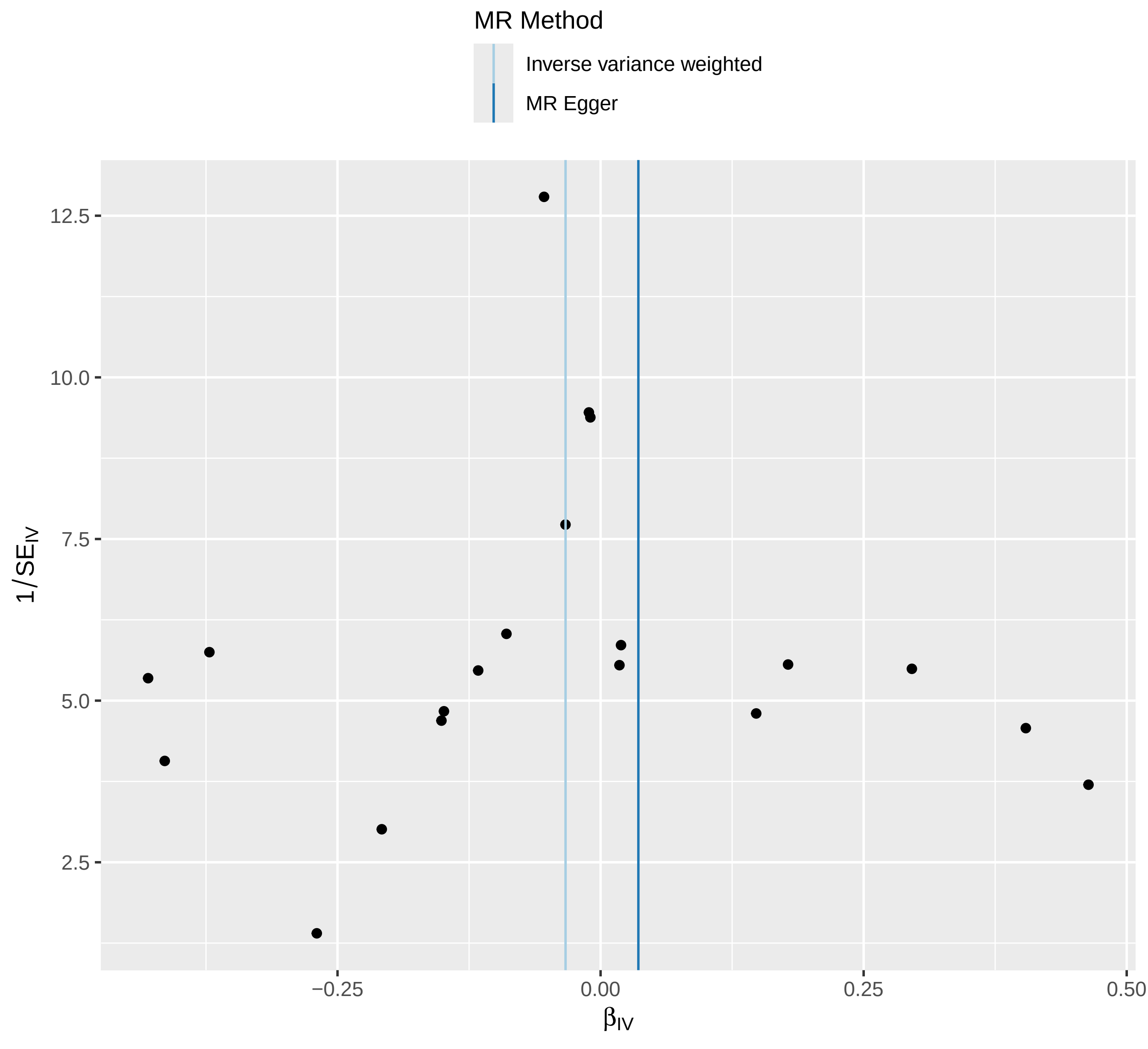
MR Method
Inverse variance weighted
MR Egger
12.5
10.0
7.5
5.0
2.5
$1/SE_{IV}$
−0.25
0.00
0.25
0.50
β_{IV}

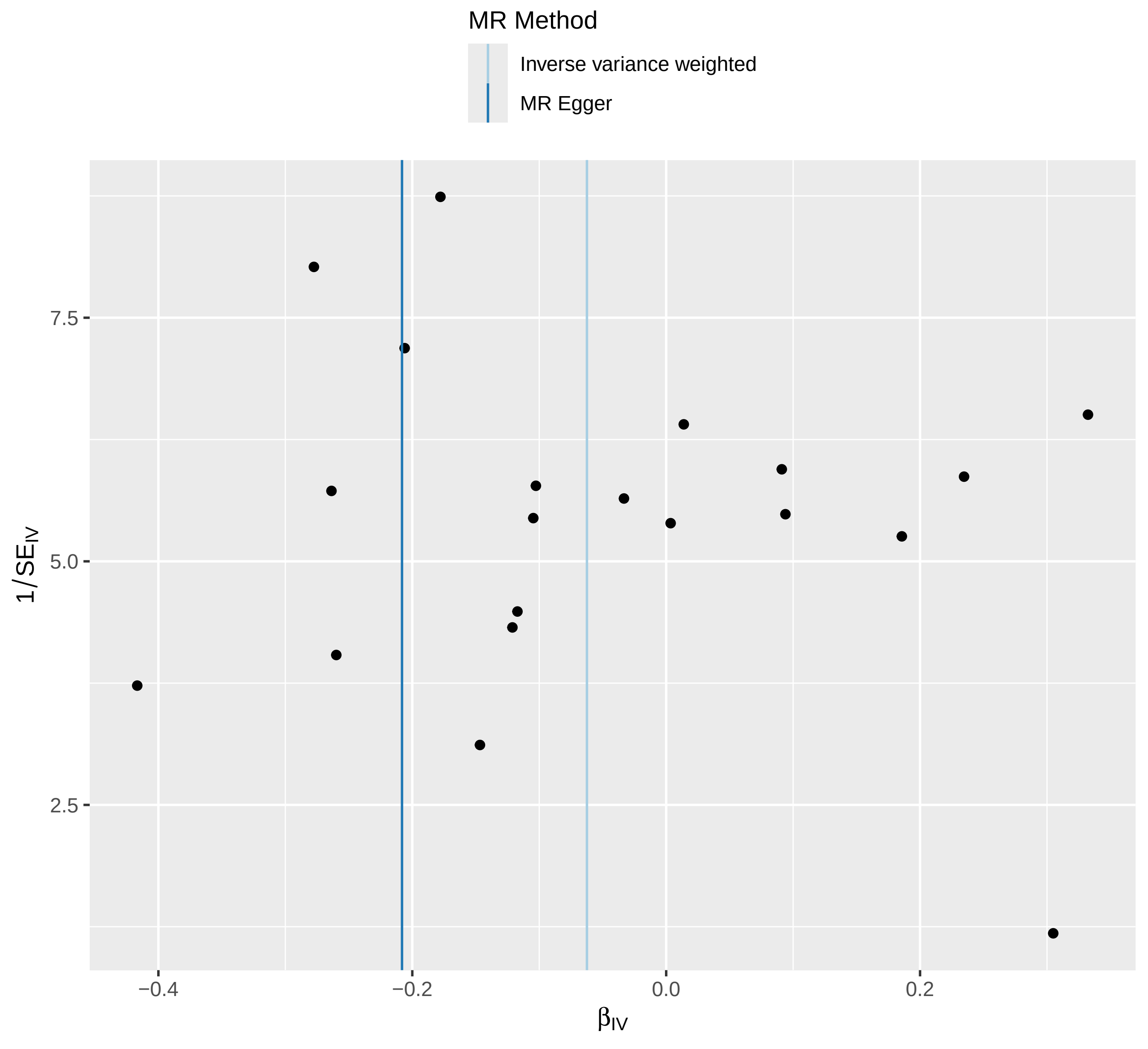

MR Method
Inverse variance weighted
MR Egger
7.5
5.0
2.5
$1/SE_{IV}$
−0.4
−0.2
0.0
0.2
β_{IV}

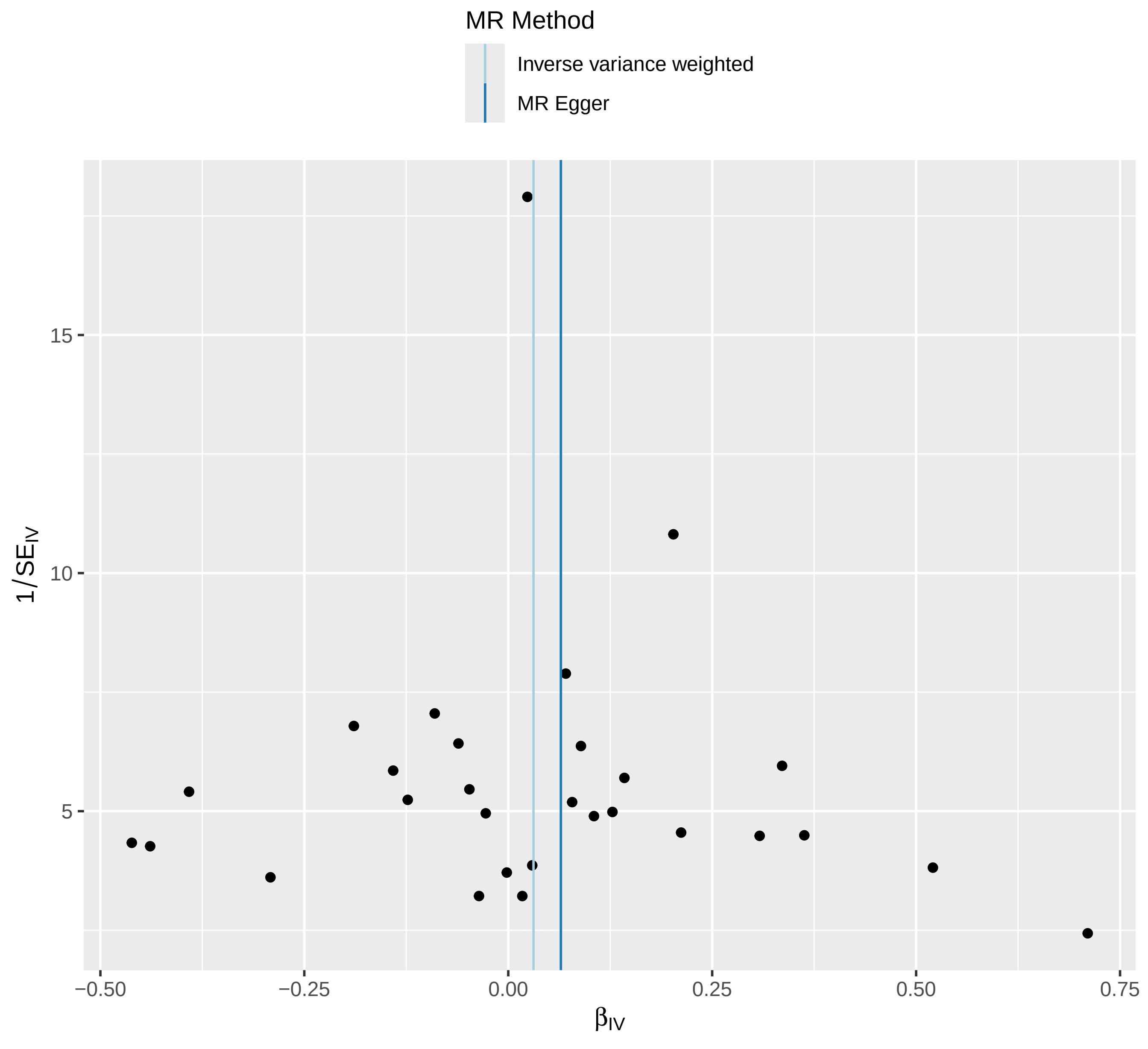
MR Method
Inverse variance weighted
MR Egger
15
10
5
1/SE_IV
−0.50
−0.25
0.00
0.25
0.50
0.75
β_IV

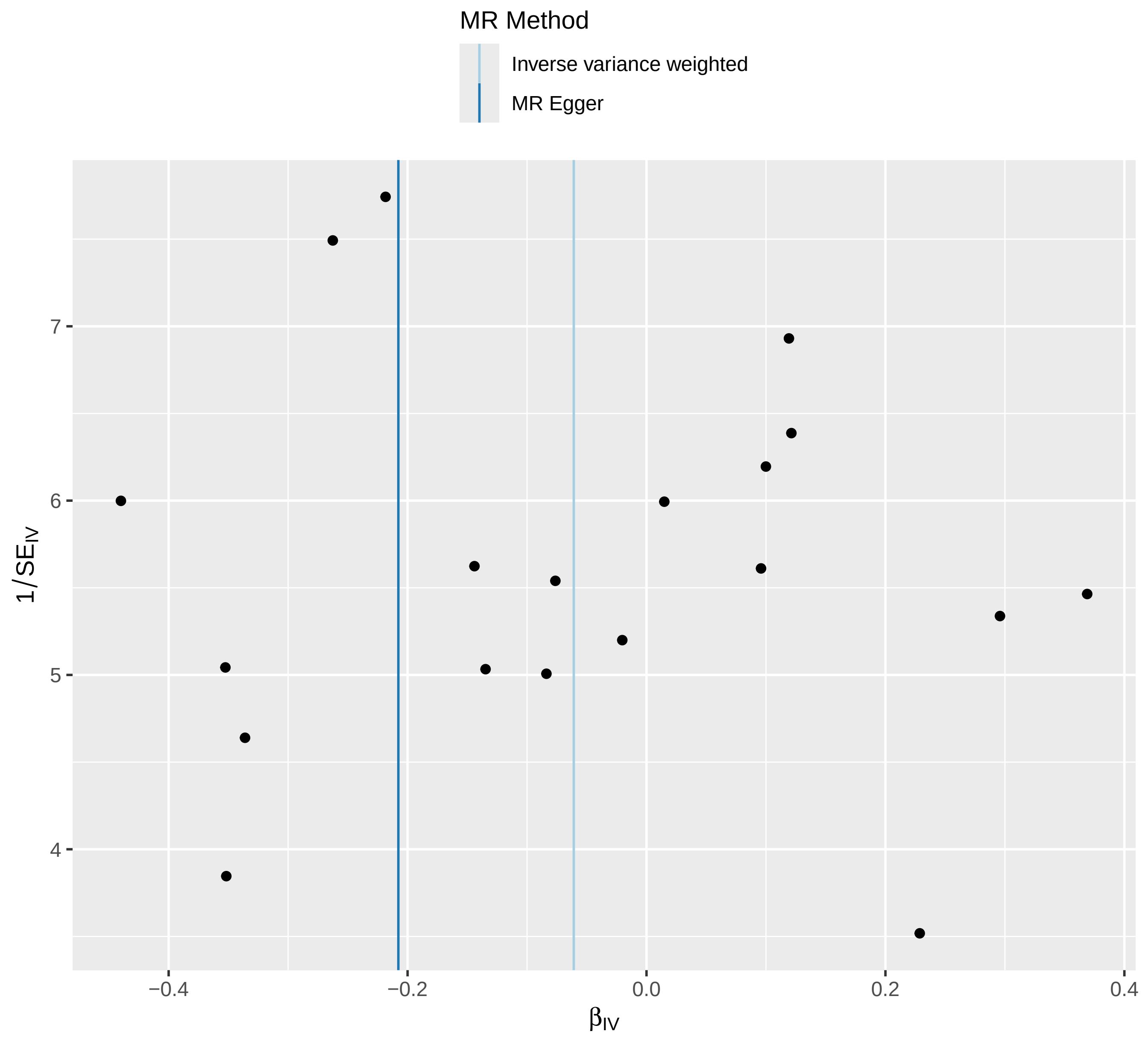
MR Method
Inverse variance weighted
MR Egger
7
6
5
4
1/SE_IV
−0.4
−0.2
0.0
0.2
0.4
β_IV

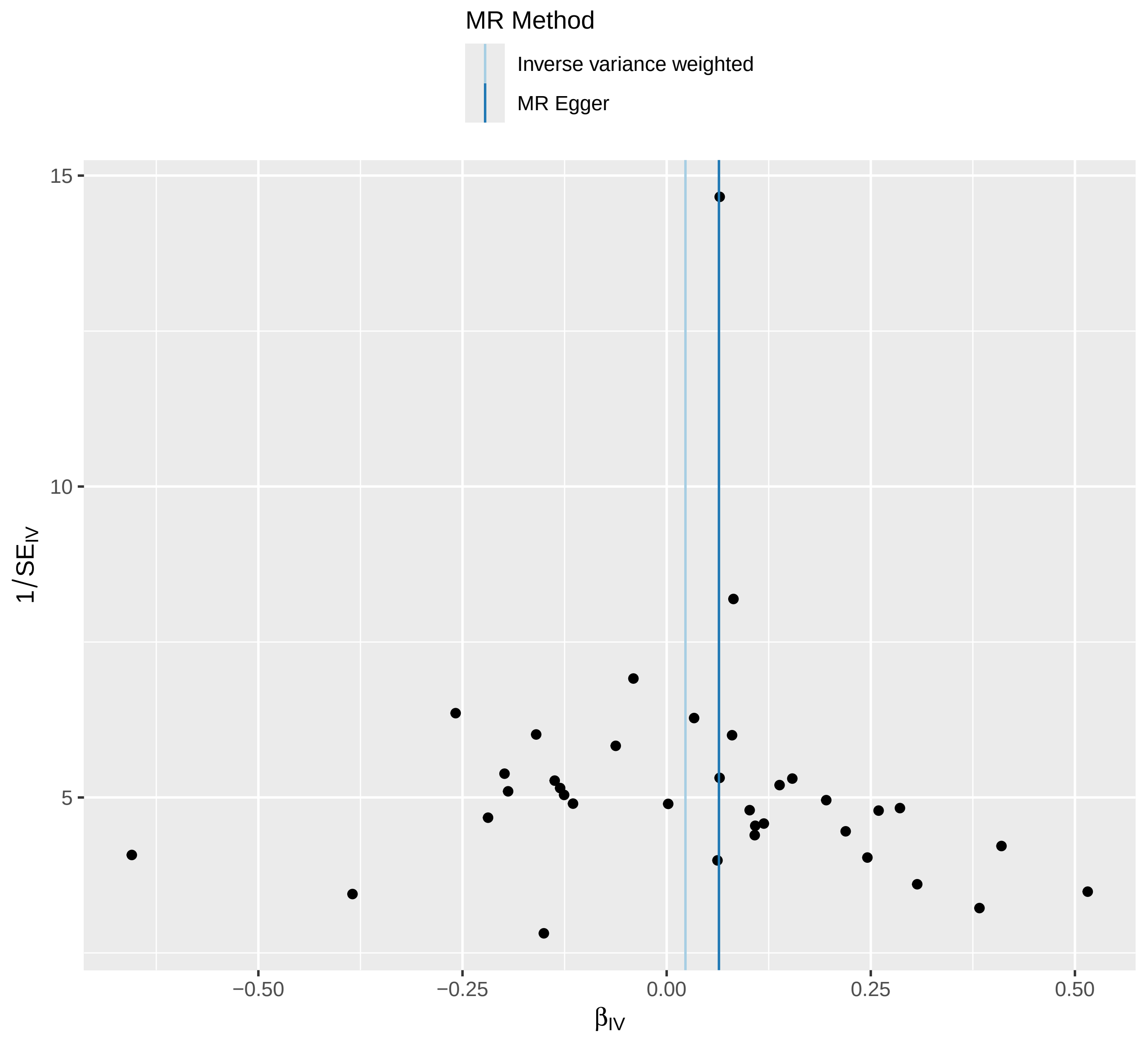
MR Method
Inverse variance weighted
MR Egger
15
10
5
1/SE$_{IV}$
−0.50
−0.25
0.00
0.25
0.50
β$_{IV}$

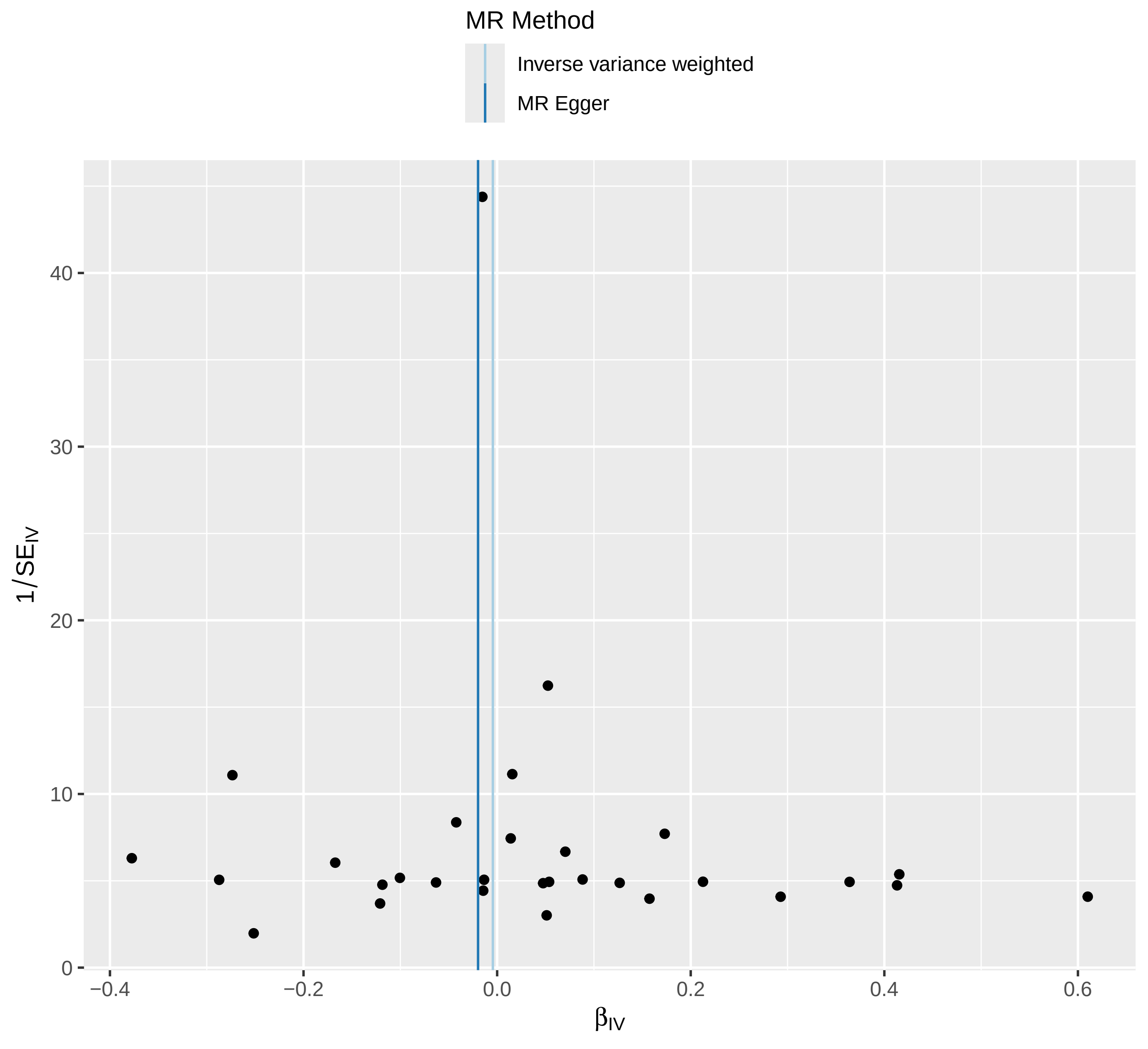
MR Method
Inverse variance weighted
MR Egger
1/SE_IV
β_IV
0
10
20
30
40
−0.4
−0.2
0.0
0.2
0.4
0.6

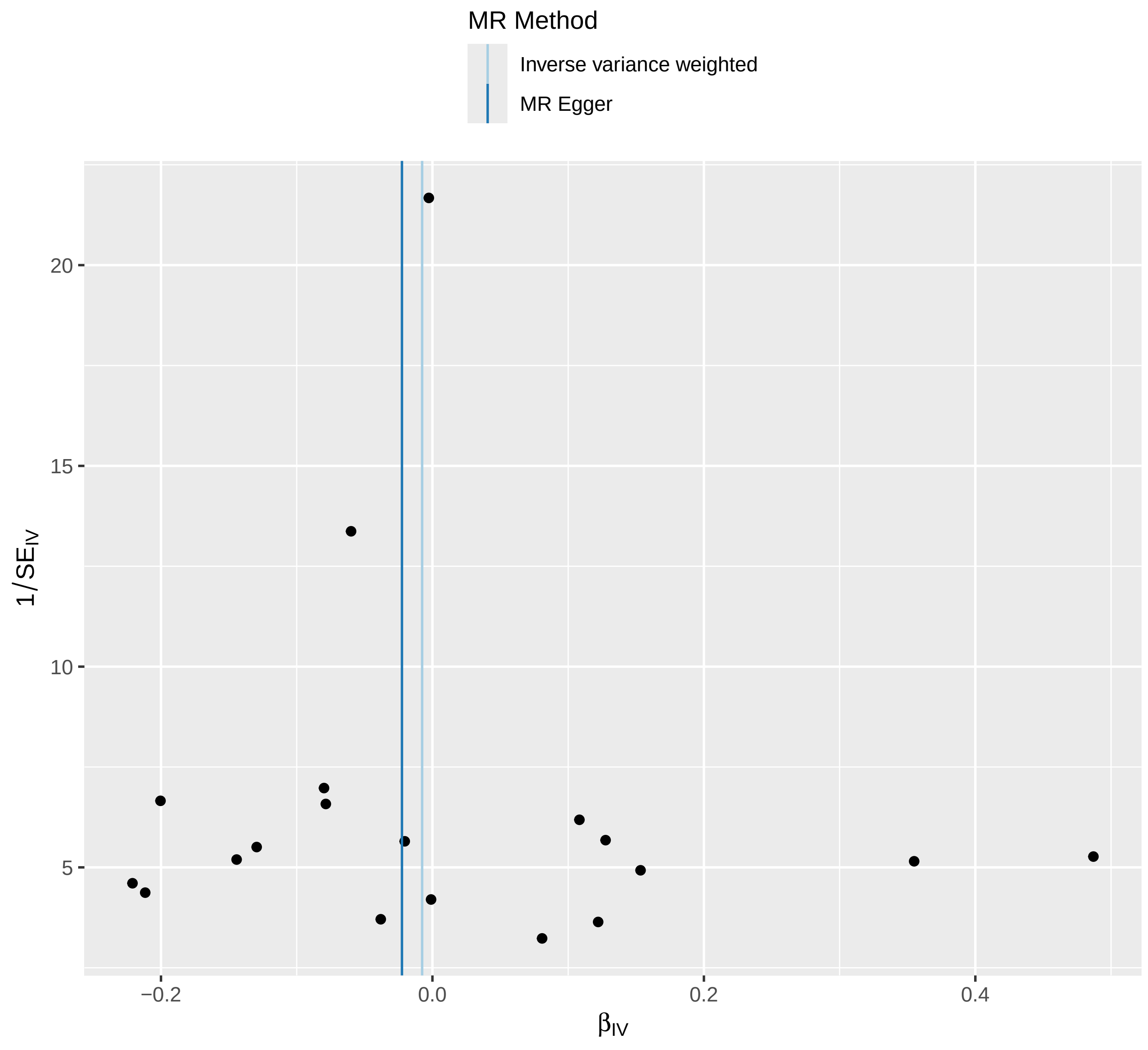
MR Method
Inverse variance weighted
MR Egger
20
15
10
5
1/SE_IV
−0.2
0.0
0.2
0.4
β_IV

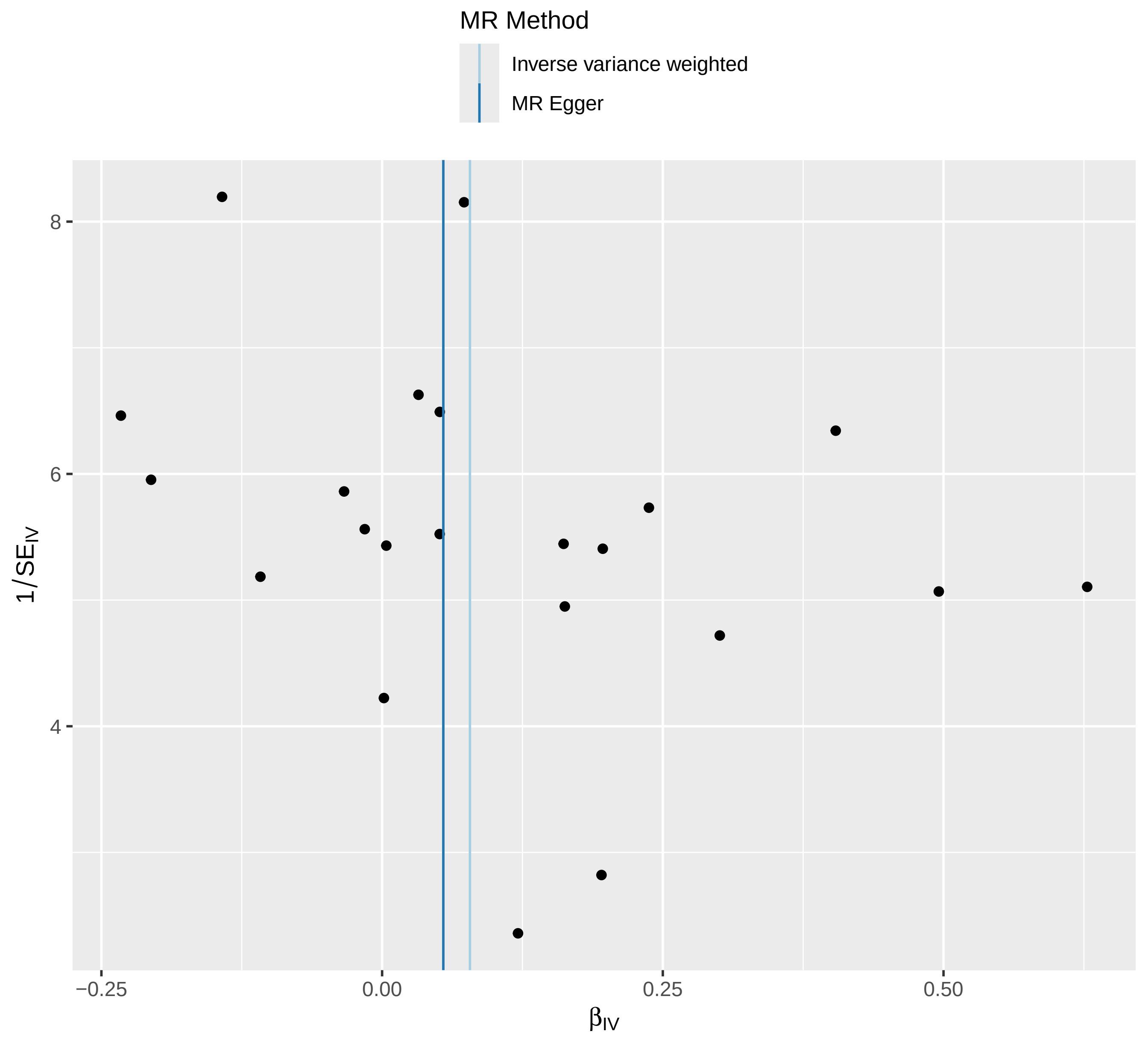

MR Method
Inverse variance weighted
MR Egger
8
6
4
1/SE_IV
−0.25
0.00
0.25
0.50
β_IV

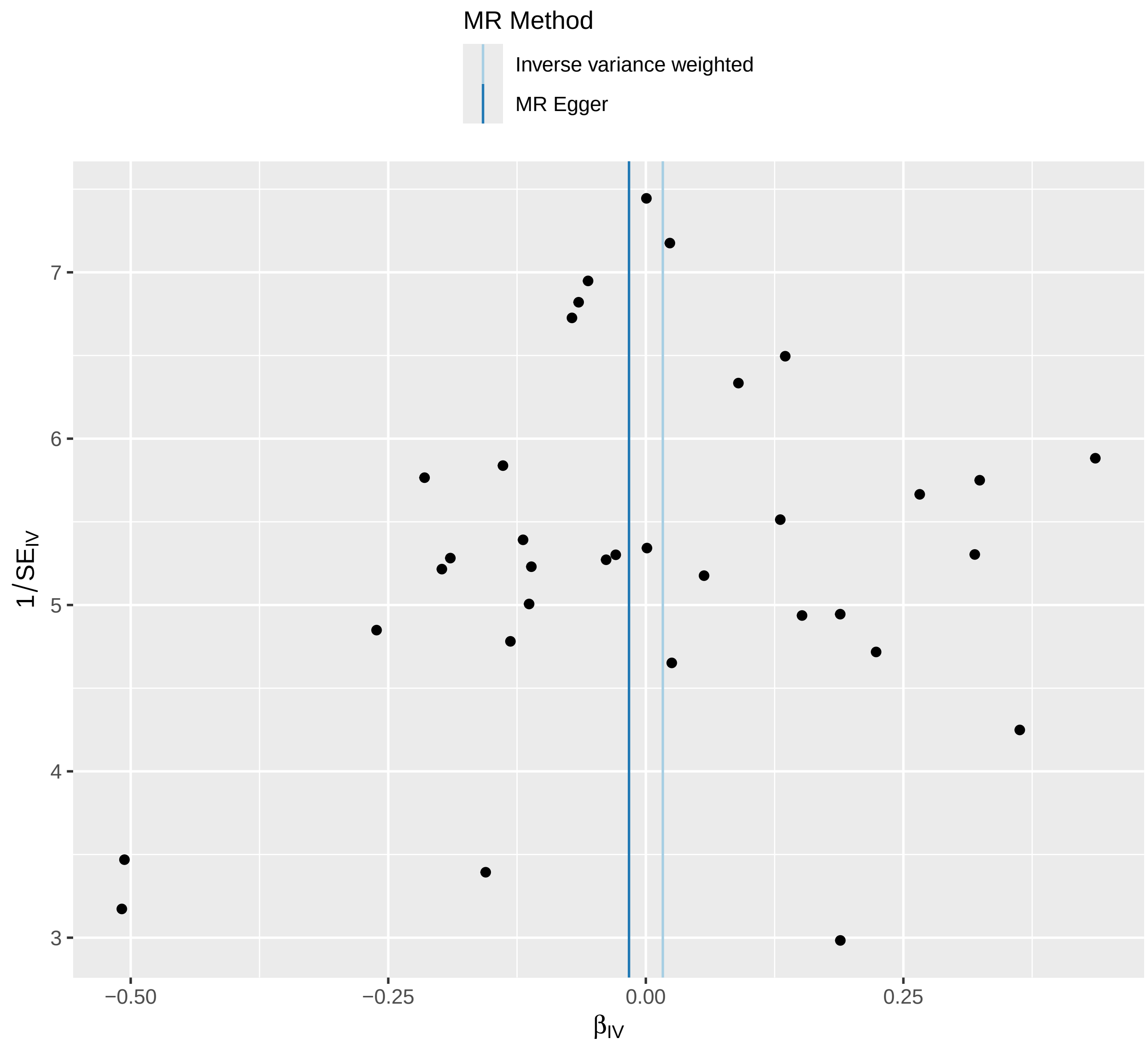

MR Method
Inverse variance weighted
MR Egger
1/SE_IV
7
6
5
4
3
−0.50
−0.25
0.00
0.25
β_IV

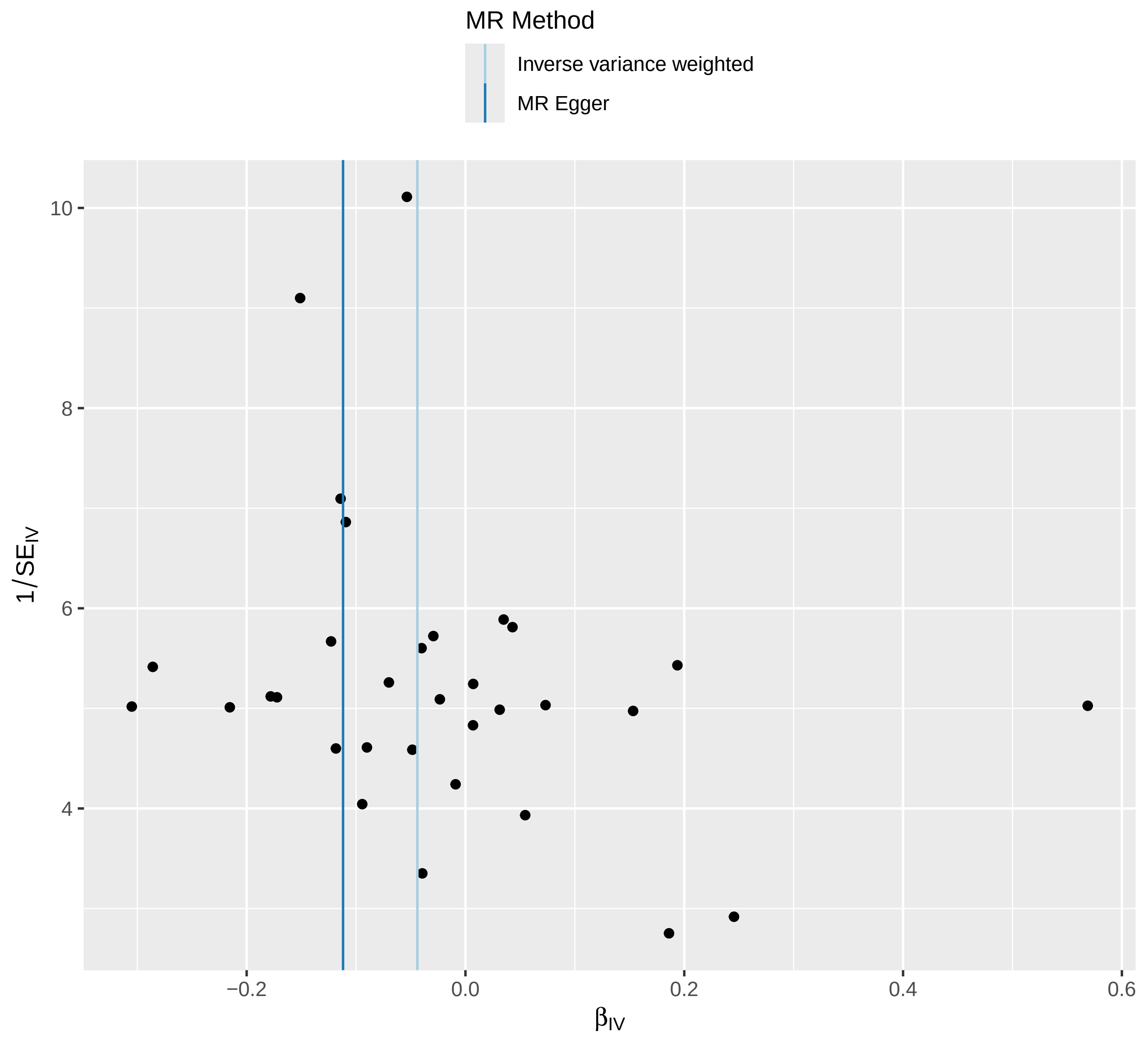
MR Method
Inverse variance weighted
MR Egger
10
8
6
4
−0.2
0.0
0.2
0.4
0.6
$1/SE_{IV}$
β_{IV}

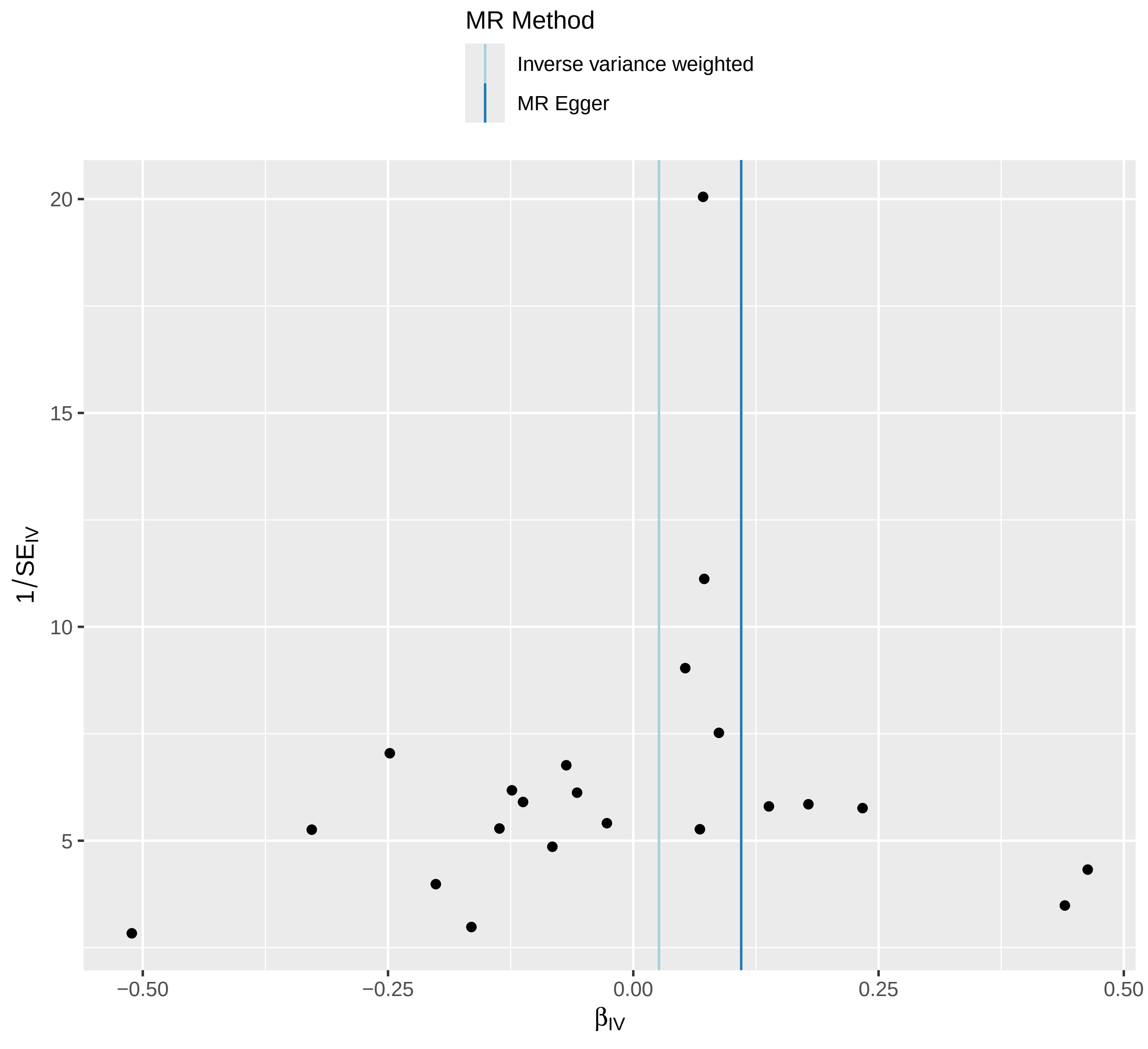

MR Method
Inverse variance weighted
MR Egger
20
15
10
5
1/SE_IV
−0.50
−0.25
0.00
0.25
0.50
β_IV

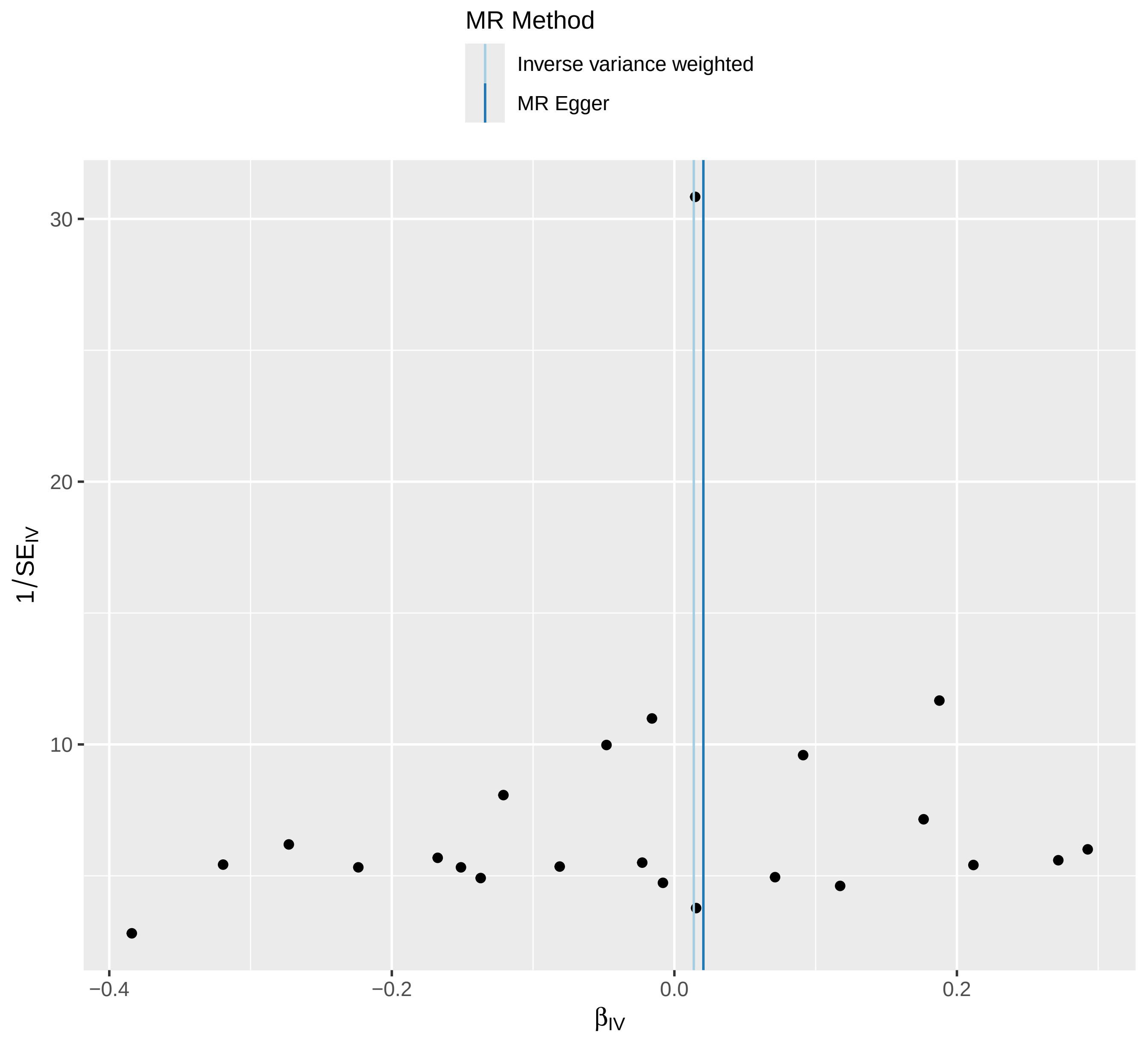
MR Method
Inverse variance weighted
MR Egger
30
20
10
1/SE_IV
−0.4
−0.2
0.0
0.2
β_IV

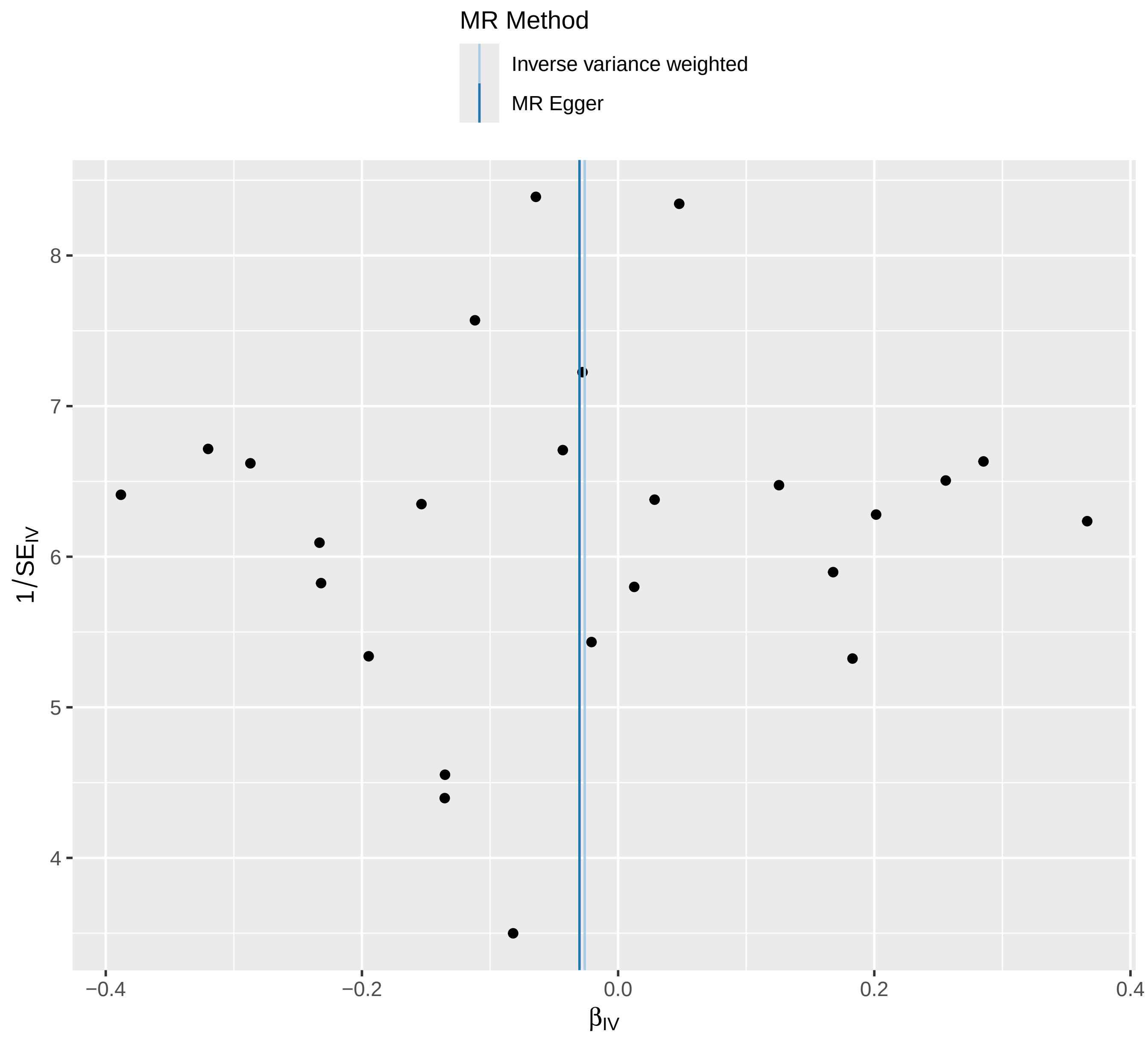

MR Method
Inverse variance weighted
MR Egger
1/SE_IV
β_IV
8
7
6
5
4
−0.4
−0.2
0.0
0.2
0.4

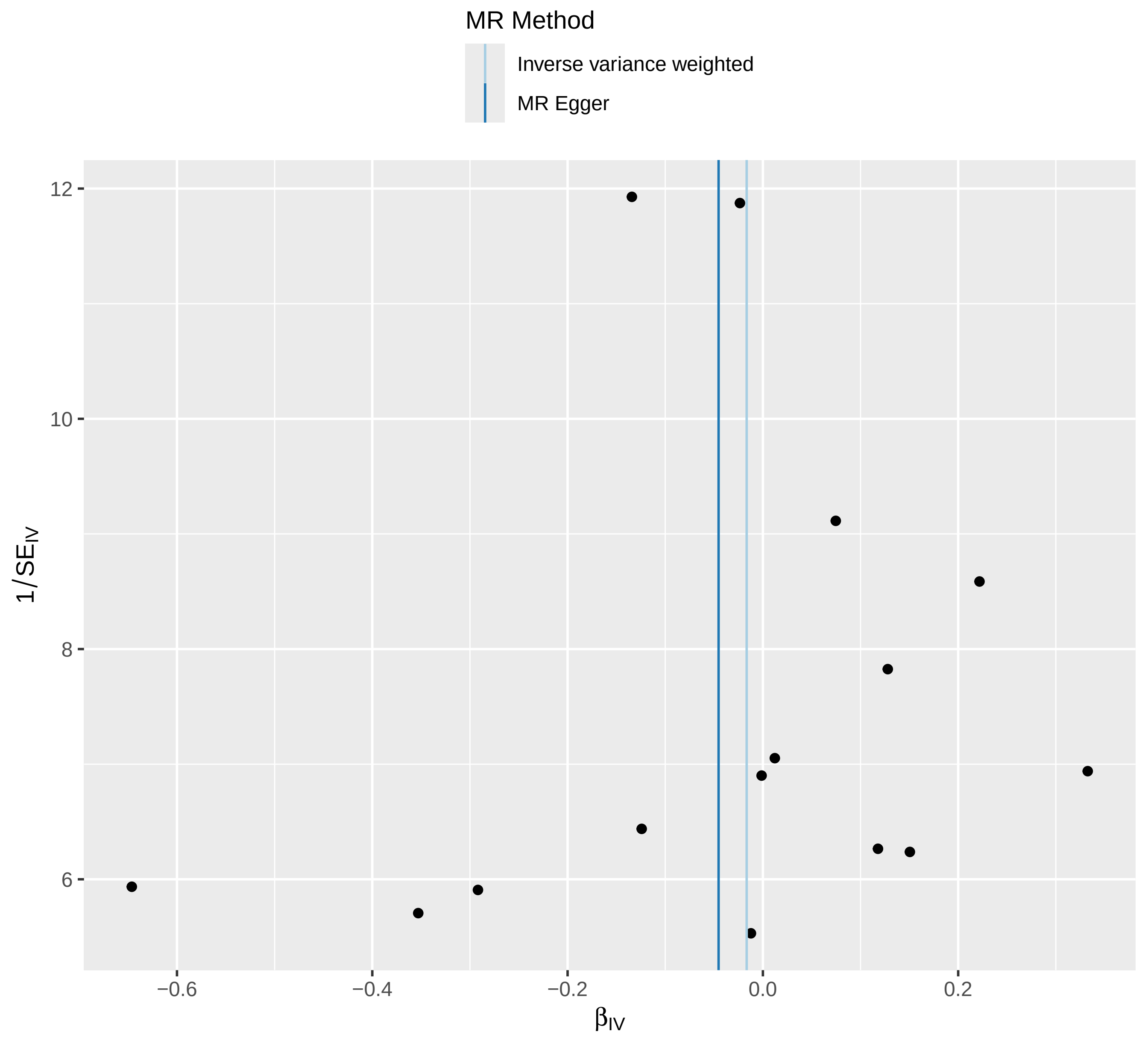

MR Method
Inverse variance weighted
MR Egger
12
10
8
6
1/SE_IV
−0.6
−0.4
−0.2
0.0
0.2
β_IV

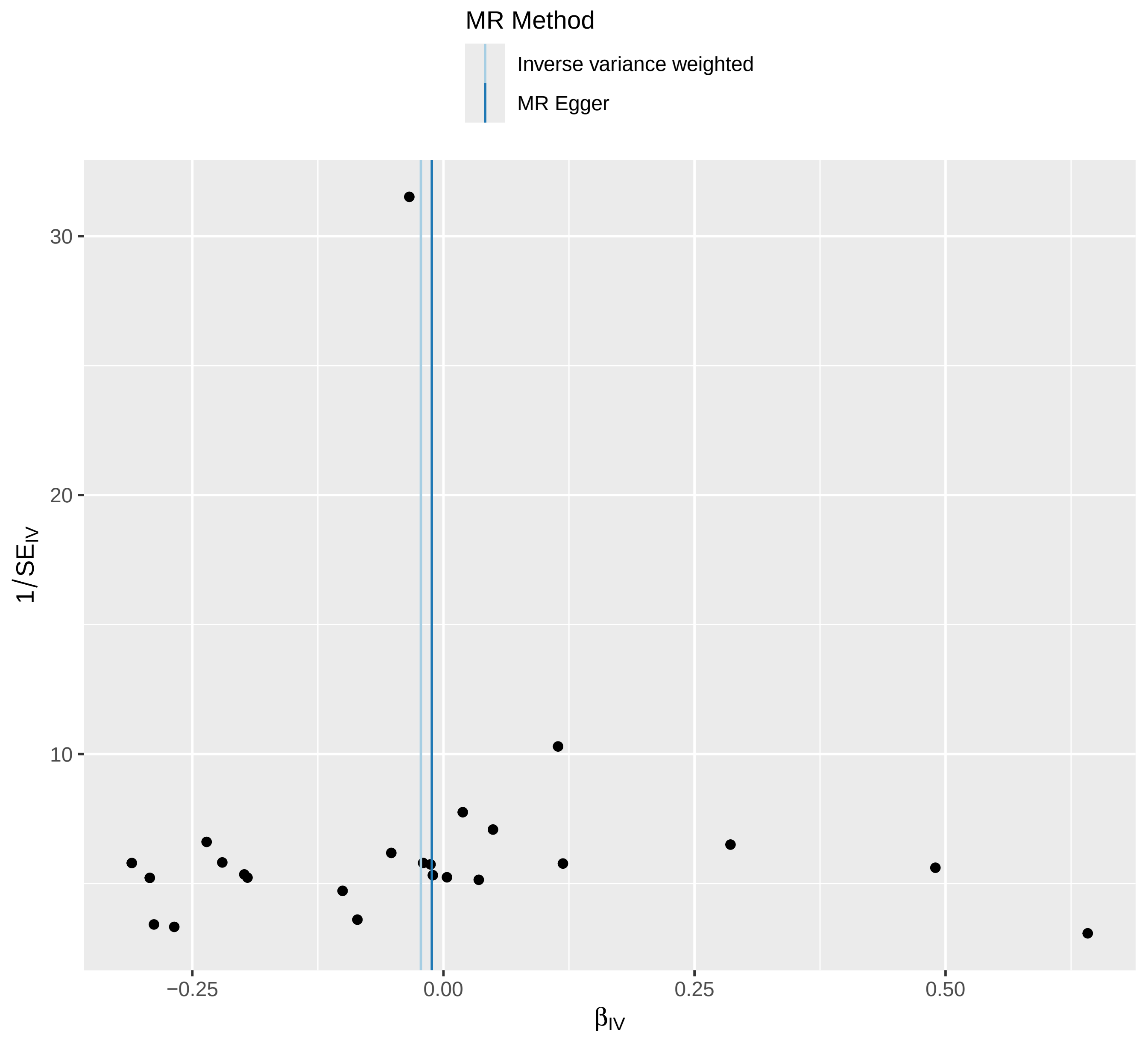
MR Method
Inverse variance weighted
MR Egger
30
20
10
1/SE_{IV}
−0.25
0.00
0.25
0.50
β_{IV}

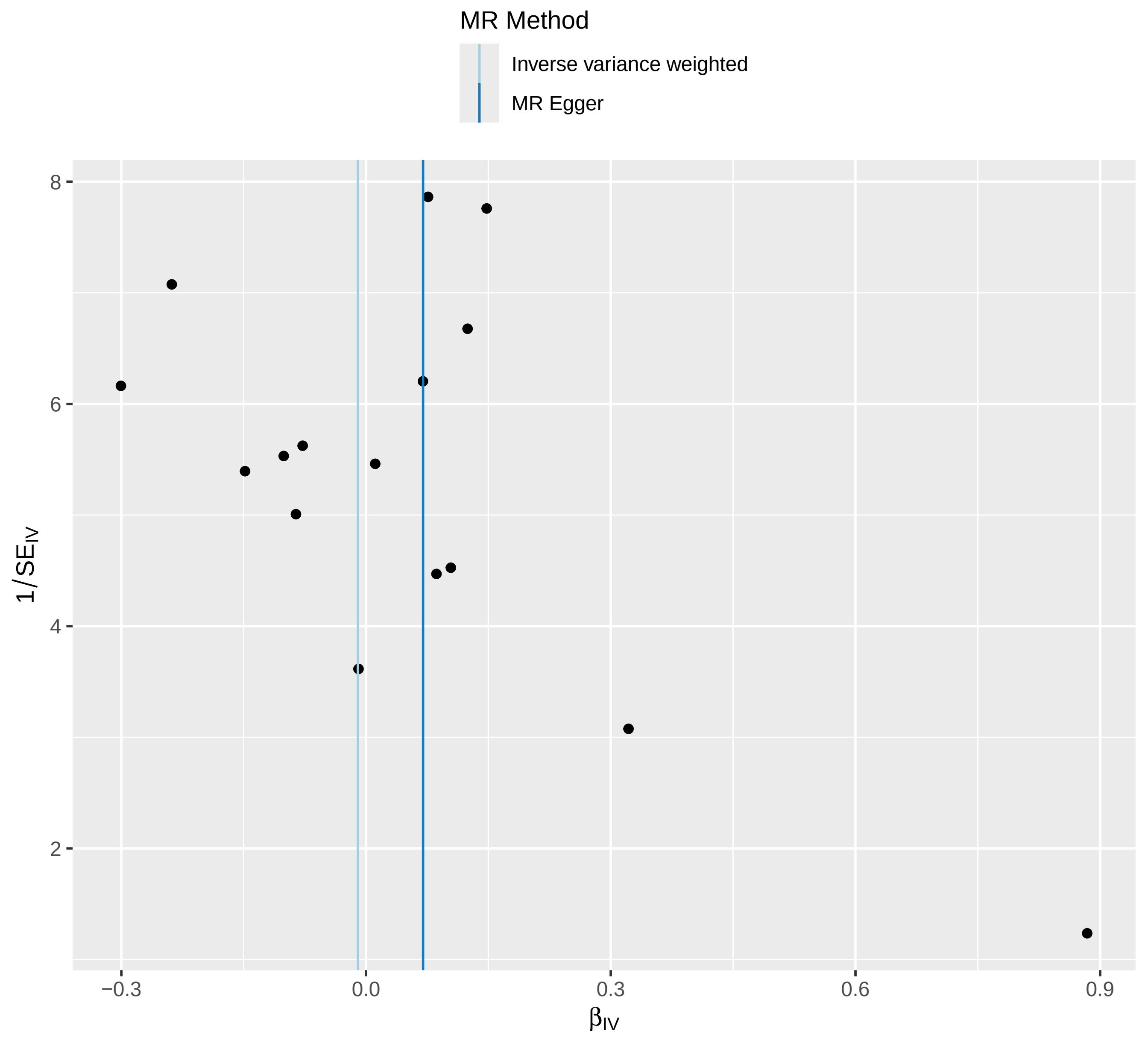

MR Method
Inverse variance weighted
MR Egger
8
6
4
2
1/SE$_{IV}$
−0.3
0.0
0.3
0.6
0.9
β$_{IV}$

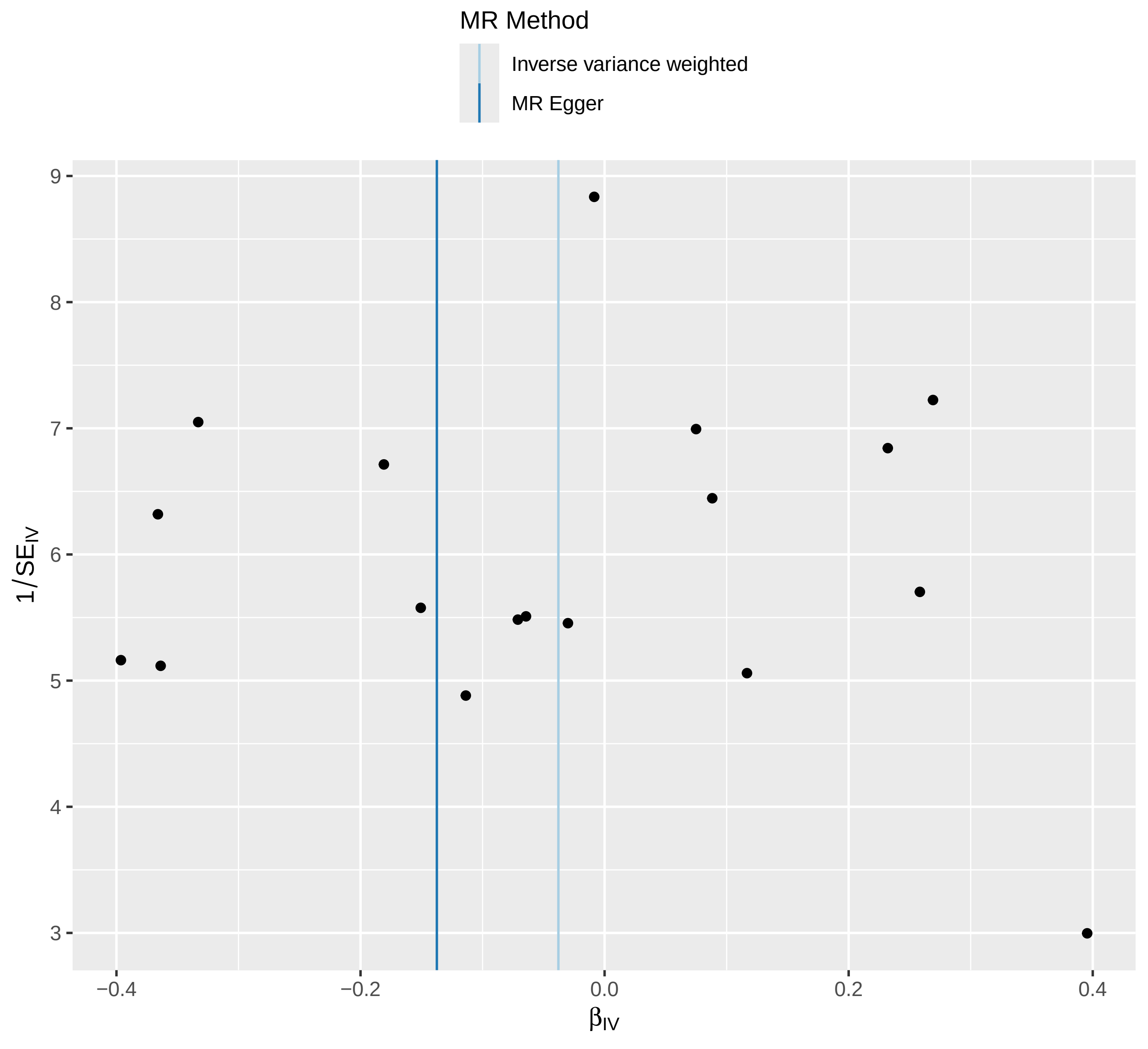

MR Method
Inverse variance weighted
MR Egger
9
8
7
6
5
4
3
1/SE_IV
−0.4
−0.2
0.0
0.2
0.4
β_IV

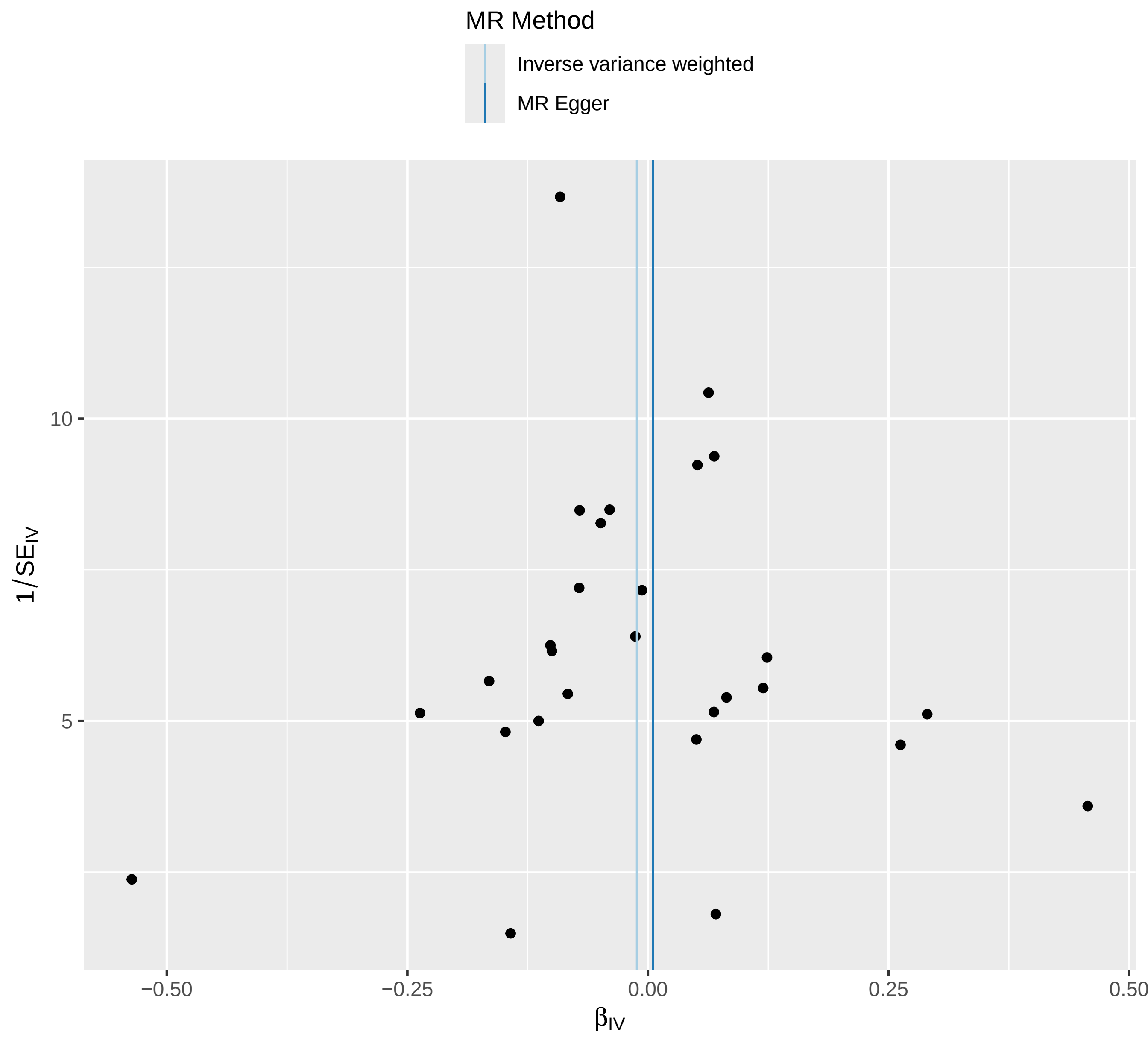

MR Method
Inverse variance weighted
MR Egger
1/SE_IV
10
5
−0.50
−0.25
0.00
0.25
0.50
β_IV

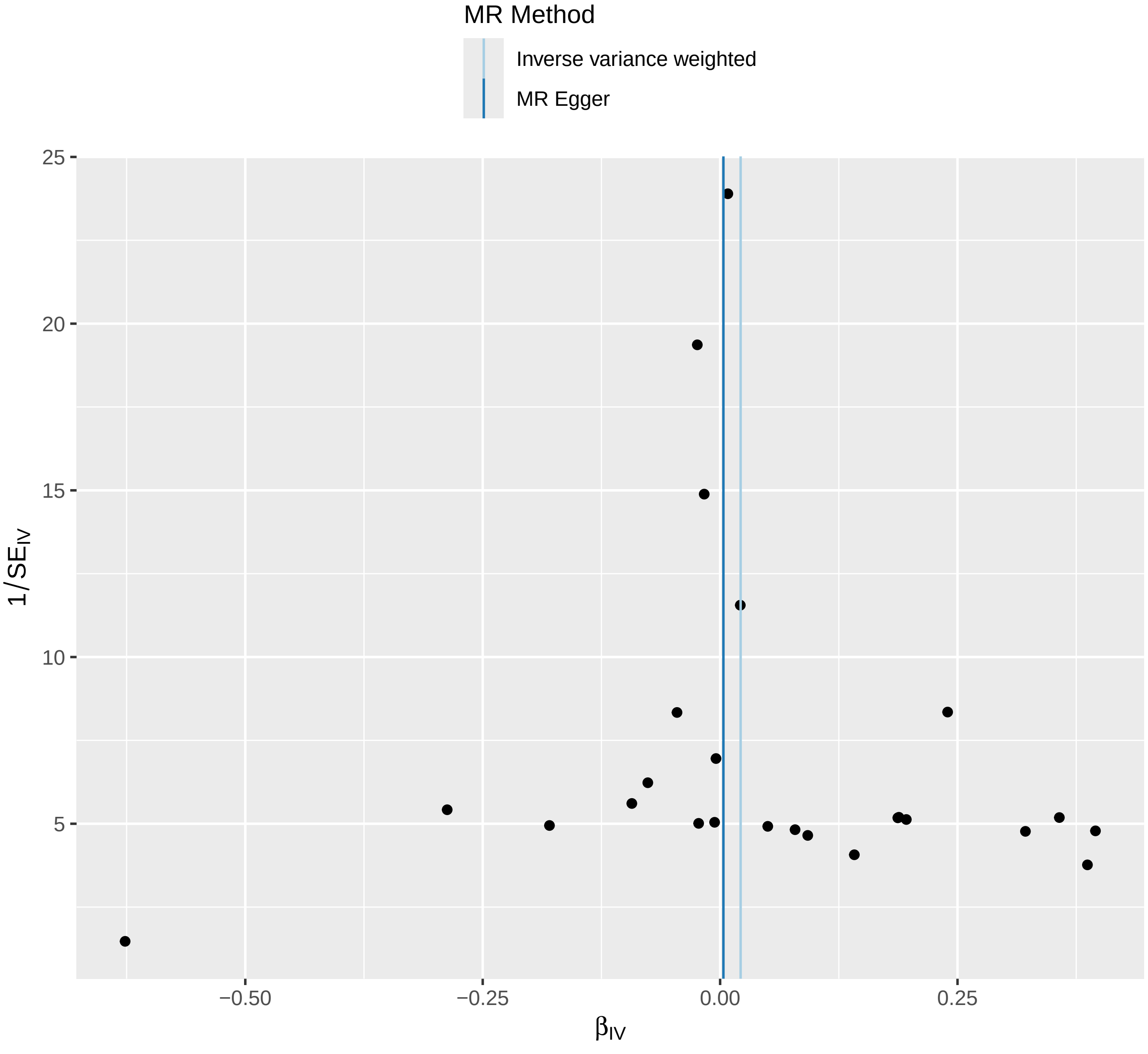
MR Method
Inverse variance weighted
MR Egger
25
20
15
10
5
$1/SE_{IV}$
−0.50
−0.25
0.00
0.25
β_{IV}

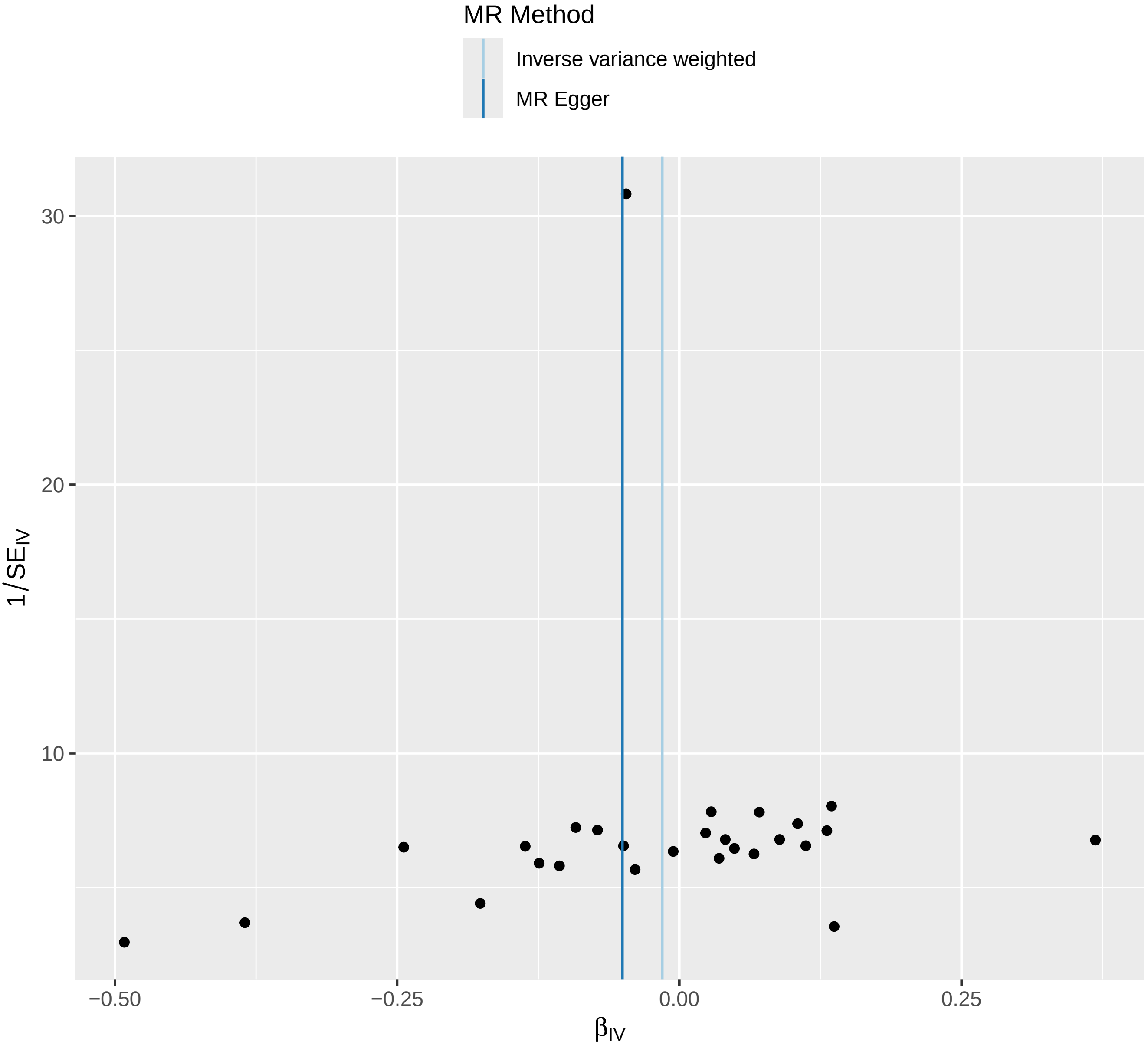

MR Method
Inverse variance weighted
MR Egger
30
20
10
1/SE$_{IV}$
−0.50
−0.25
0.00
0.25
β$_{IV}$

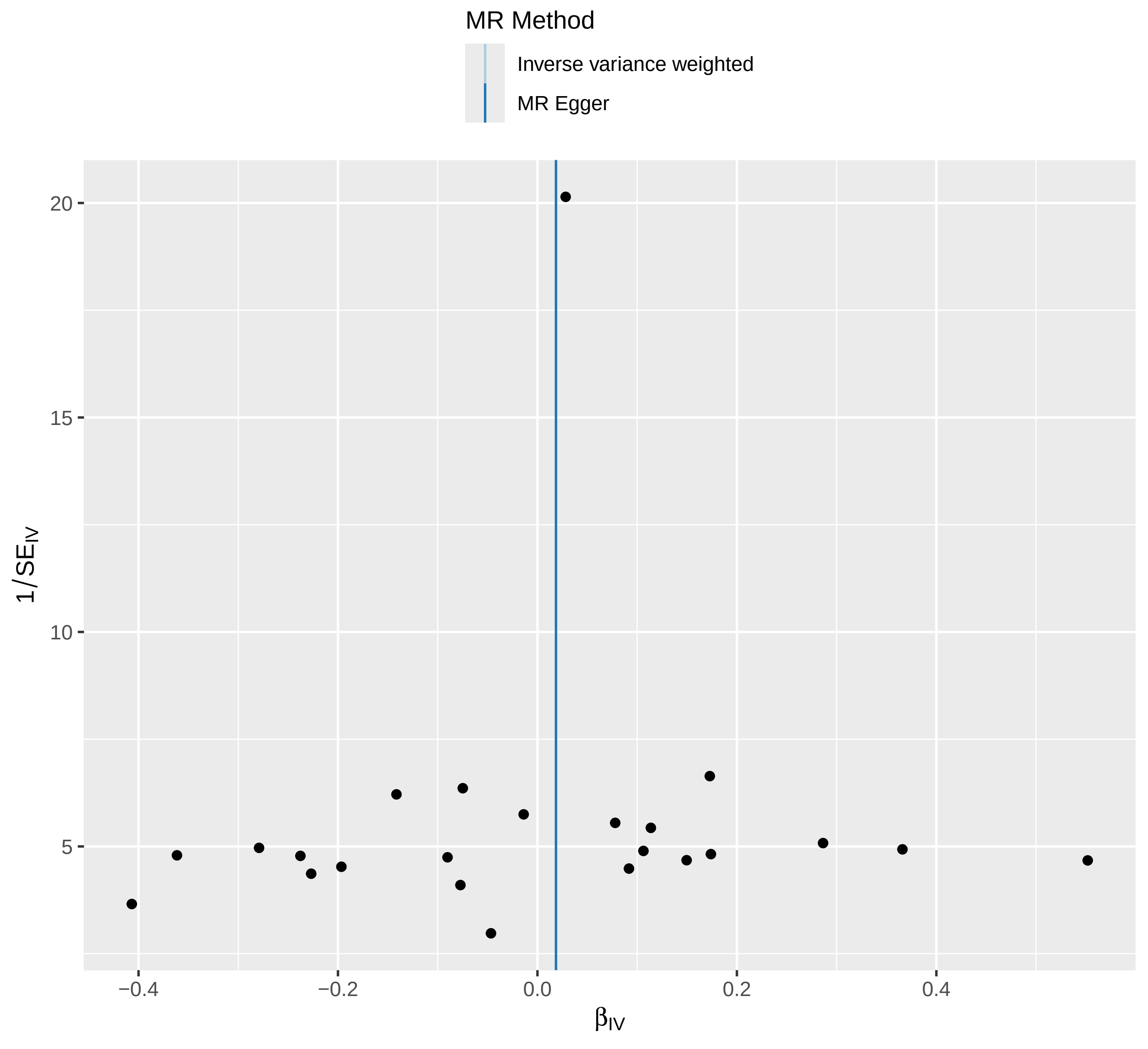
MR Method
Inverse variance weighted
MR Egger
20
15
10
5
1/SE$_{IV}$
−0.4
−0.2
0.0
0.2
0.4
β$_{IV}$

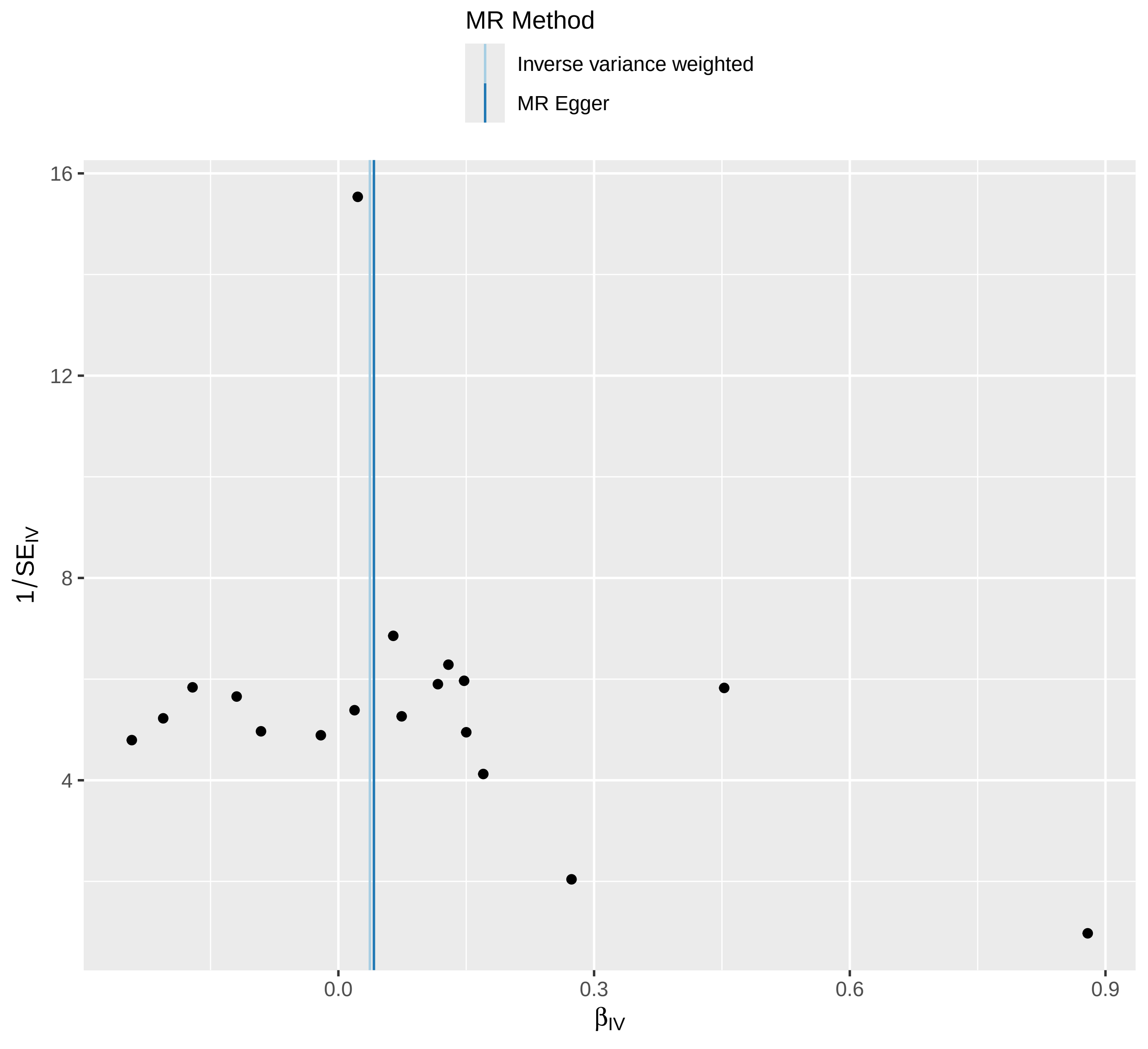

MR Method
Inverse variance weighted
MR Egger
16
12
8
4
1/SE_IV
0.0
0.3
0.6
0.9
β_IV

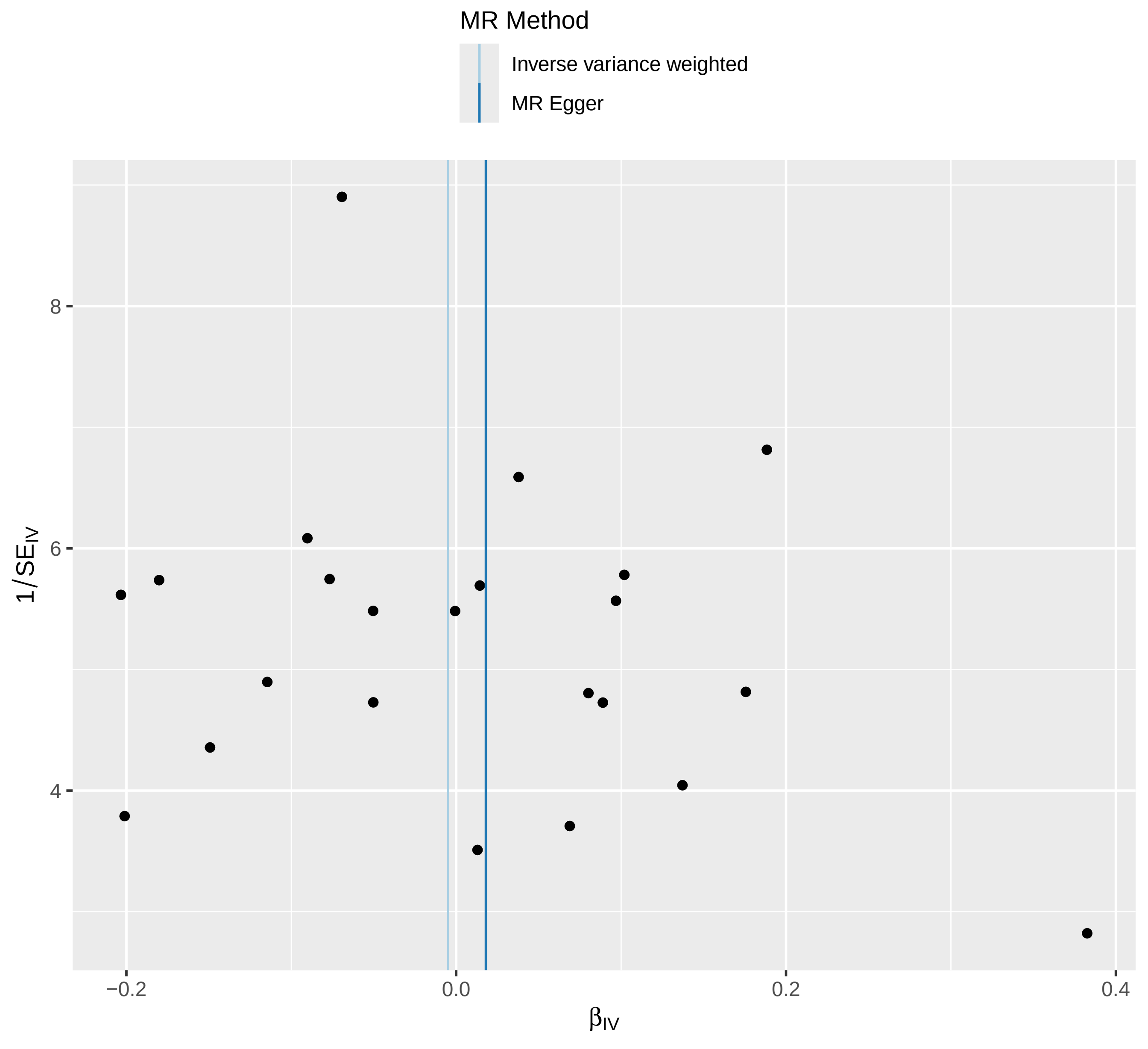

MR Method
Inverse variance weighted
MR Egger
8
6
4
1/SE$_{IV}$
−0.2
0.0
0.2
0.4
β$_{IV}$

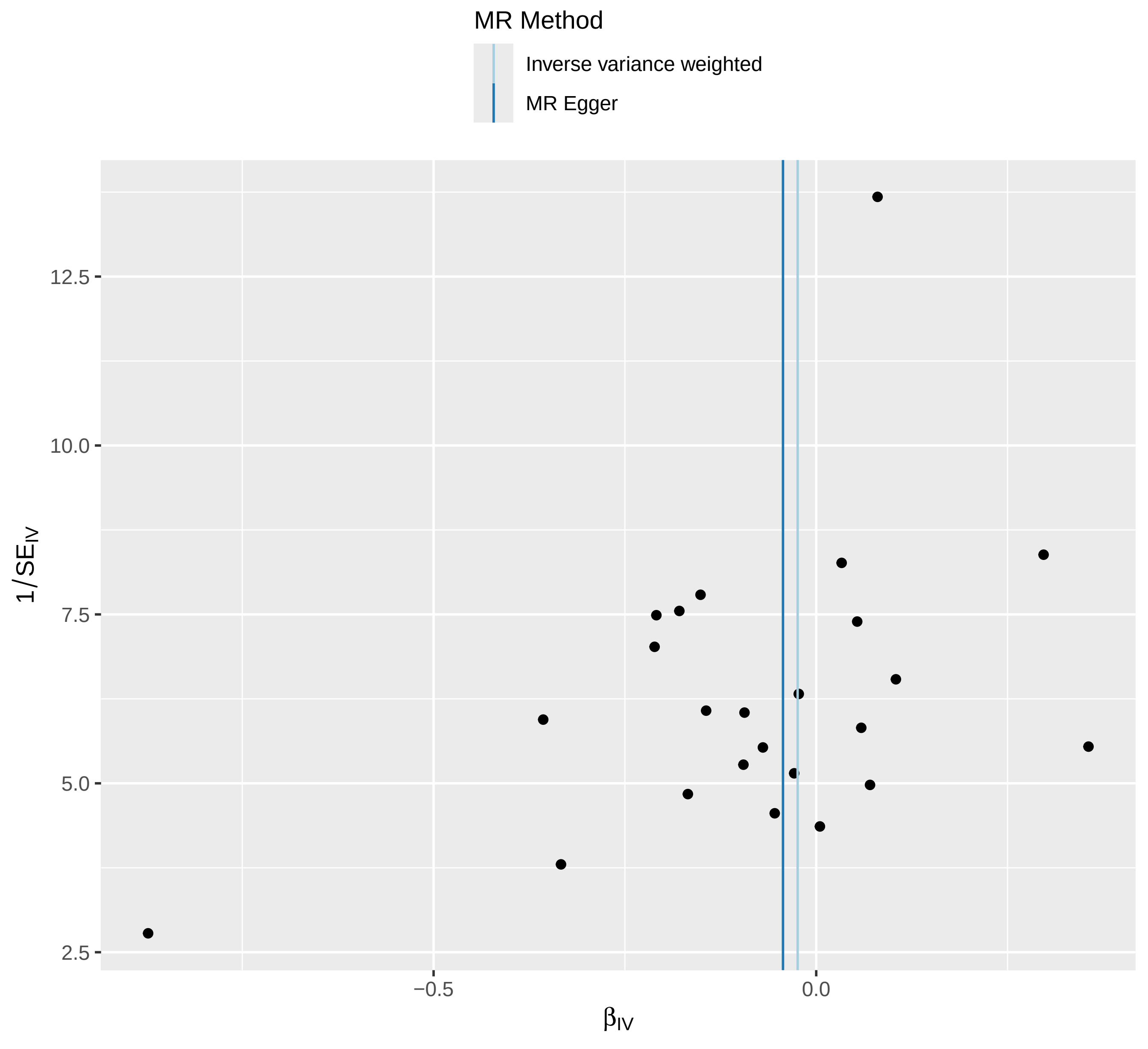

MR Method
Inverse variance weighted
MR Egger
12.5
10.0
7.5
5.0
2.5
1/SE_IV
−0.5
0.0
β_IV

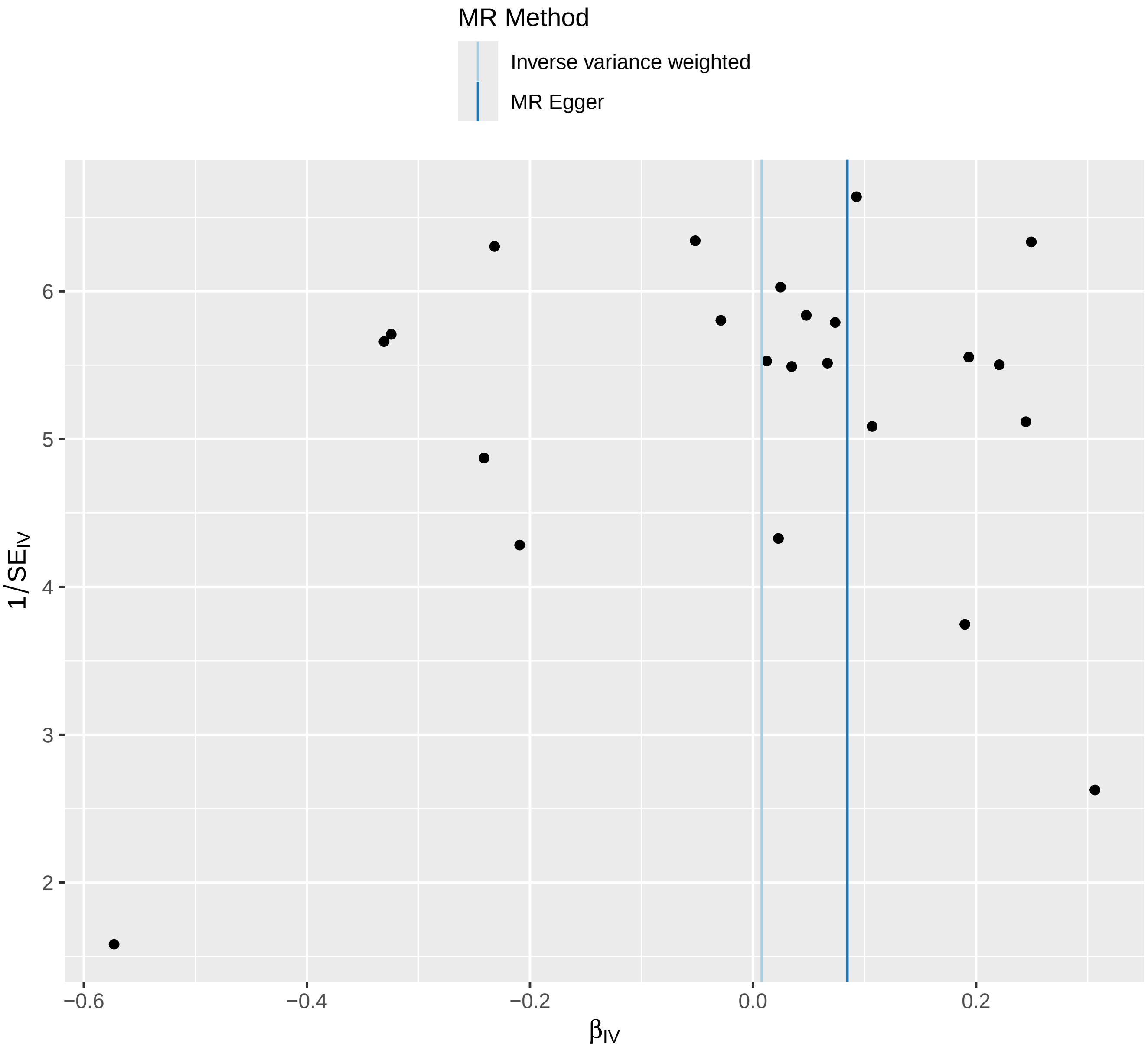

MR Method
Inverse variance weighted
MR Egger
6
5
4
3
2
1/SE_IV
−0.6
−0.4
−0.2
0.0
0.2
β_IV

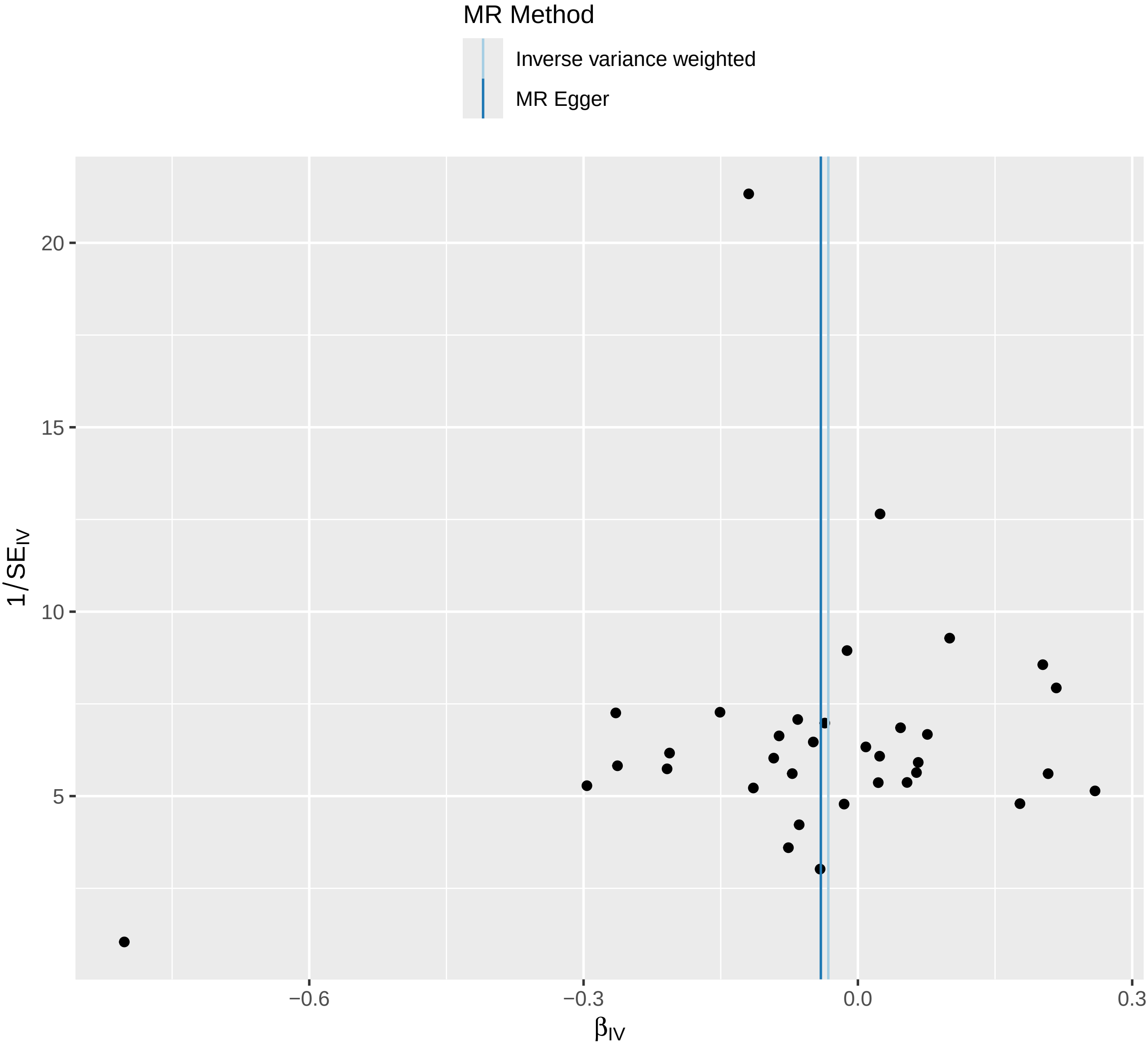
MR Method
Inverse variance weighted
MR Egger
20
15
10
5
1/SE_IV
−0.6
−0.3
0.0
0.3
β_IV

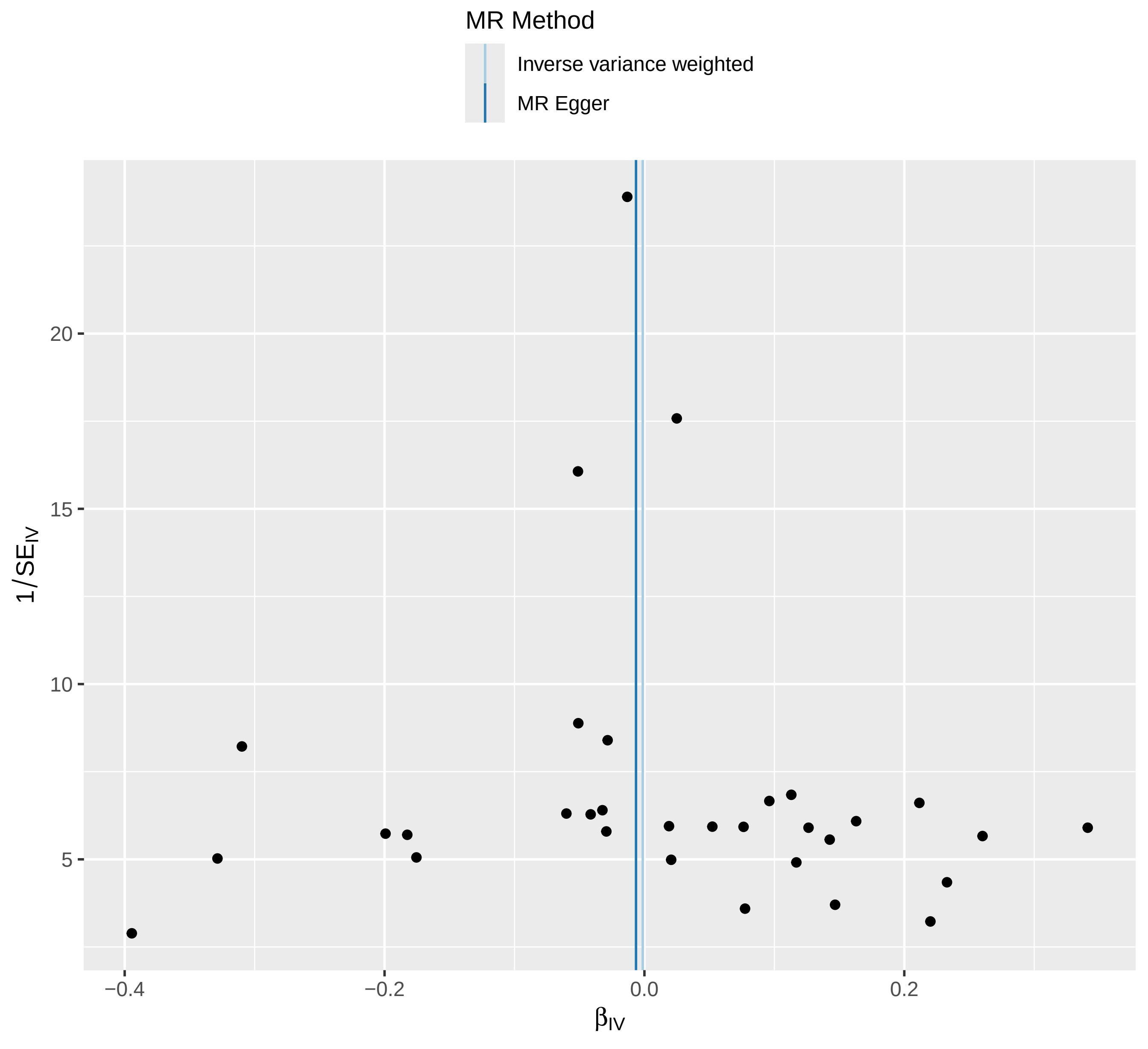
MR Method
Inverse variance weighted
MR Egger
1/SE_IV
20
15
10
5
−0.4
−0.2
0.0
0.2
β_IV

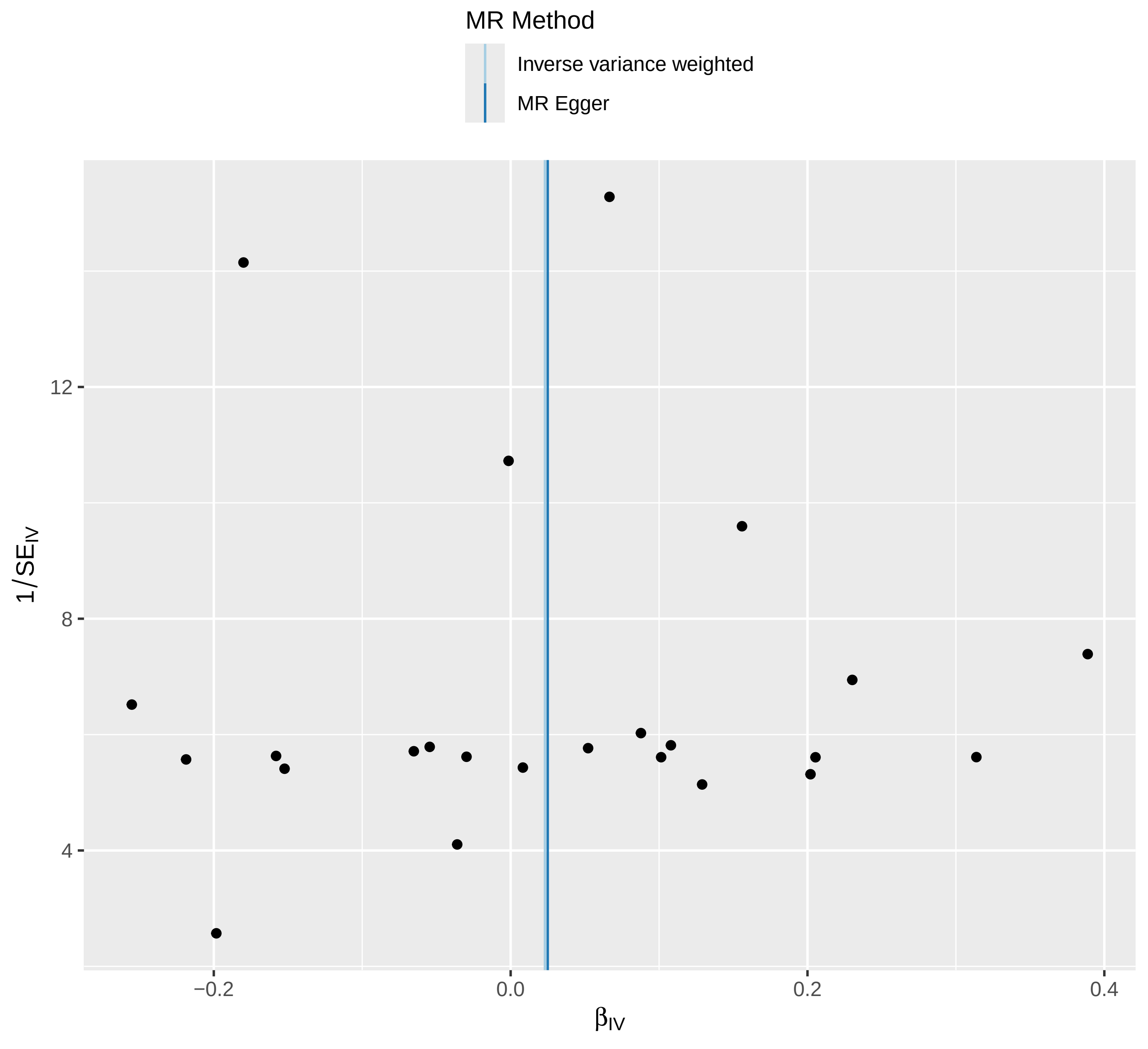

MR Method
Inverse variance weighted
MR Egger
12
8
4
$1/SE_{IV}$
−0.2
0.0
0.2
0.4
β_{IV}

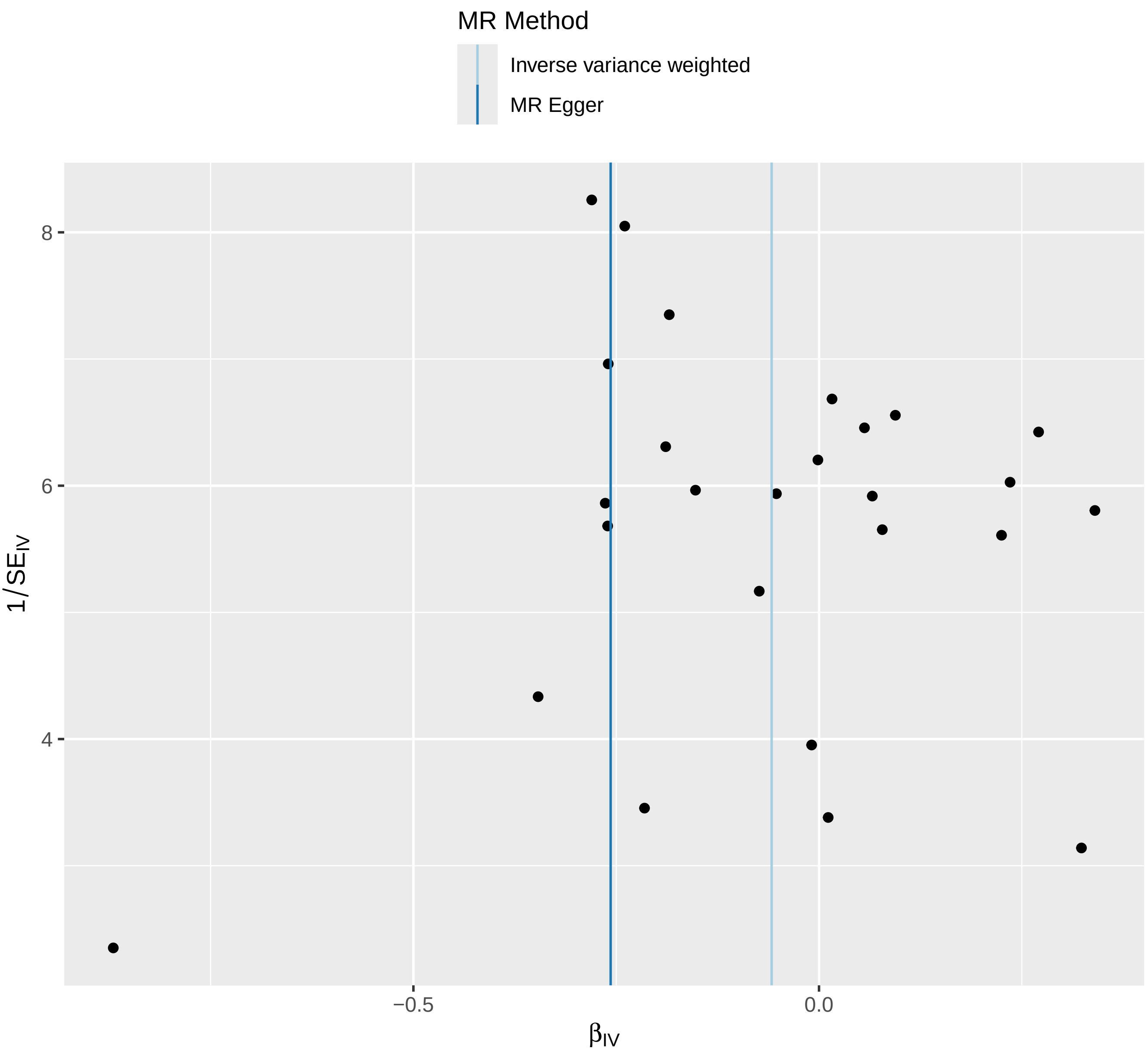

MR Method
Inverse variance weighted
MR Egger
8
6
4
1/SE_IV
−0.5
0.0
β_IV

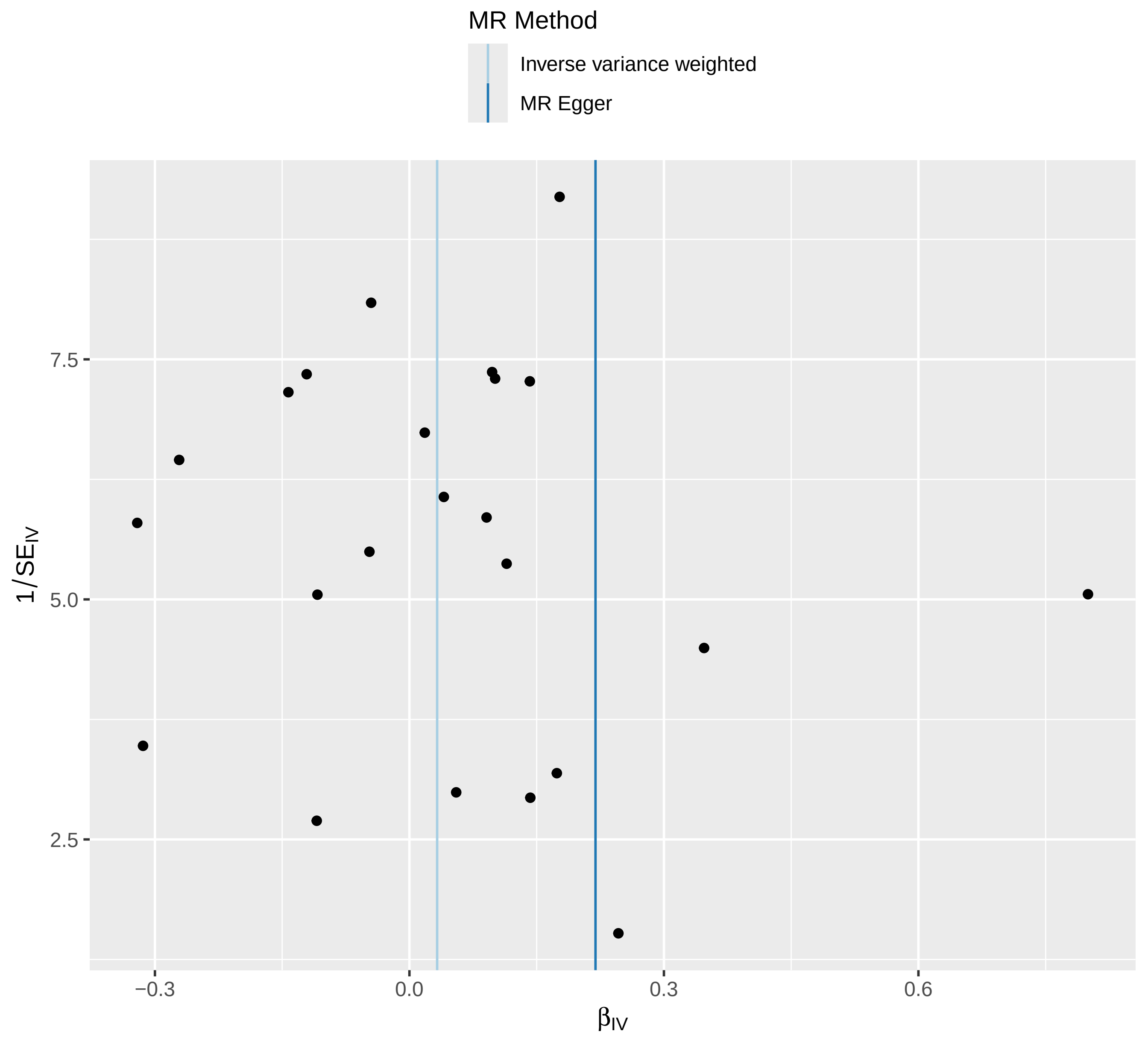

MR Method
Inverse variance weighted
MR Egger
1/SE_IV
7.5
5.0
2.5
−0.3
0.0
0.3
0.6
β_IV

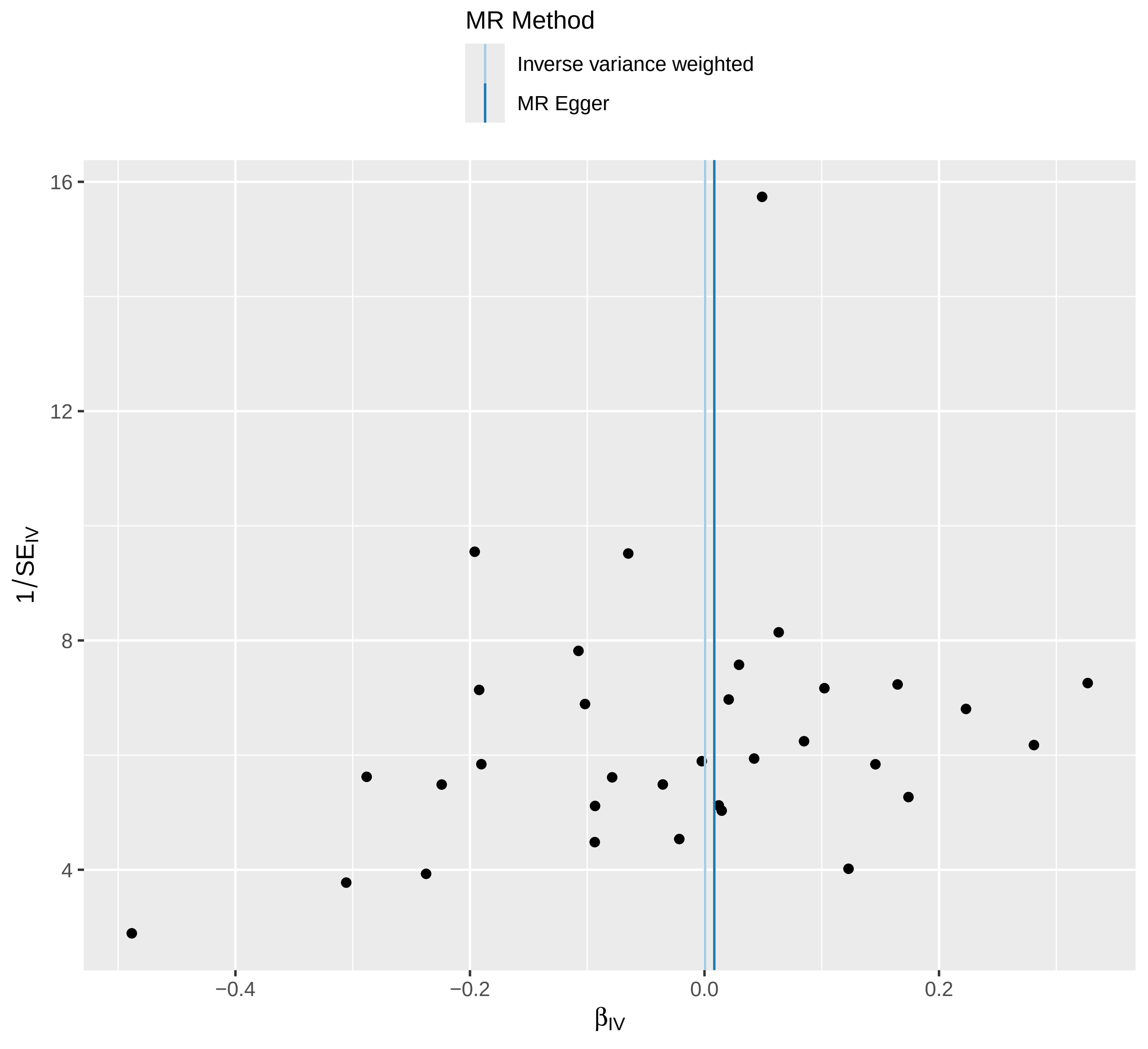

MR Method
Inverse variance weighted
MR Egger
16
12
8
4
1/SE$_{IV}$
−0.4
−0.2
0.0
0.2
β$_{IV}$

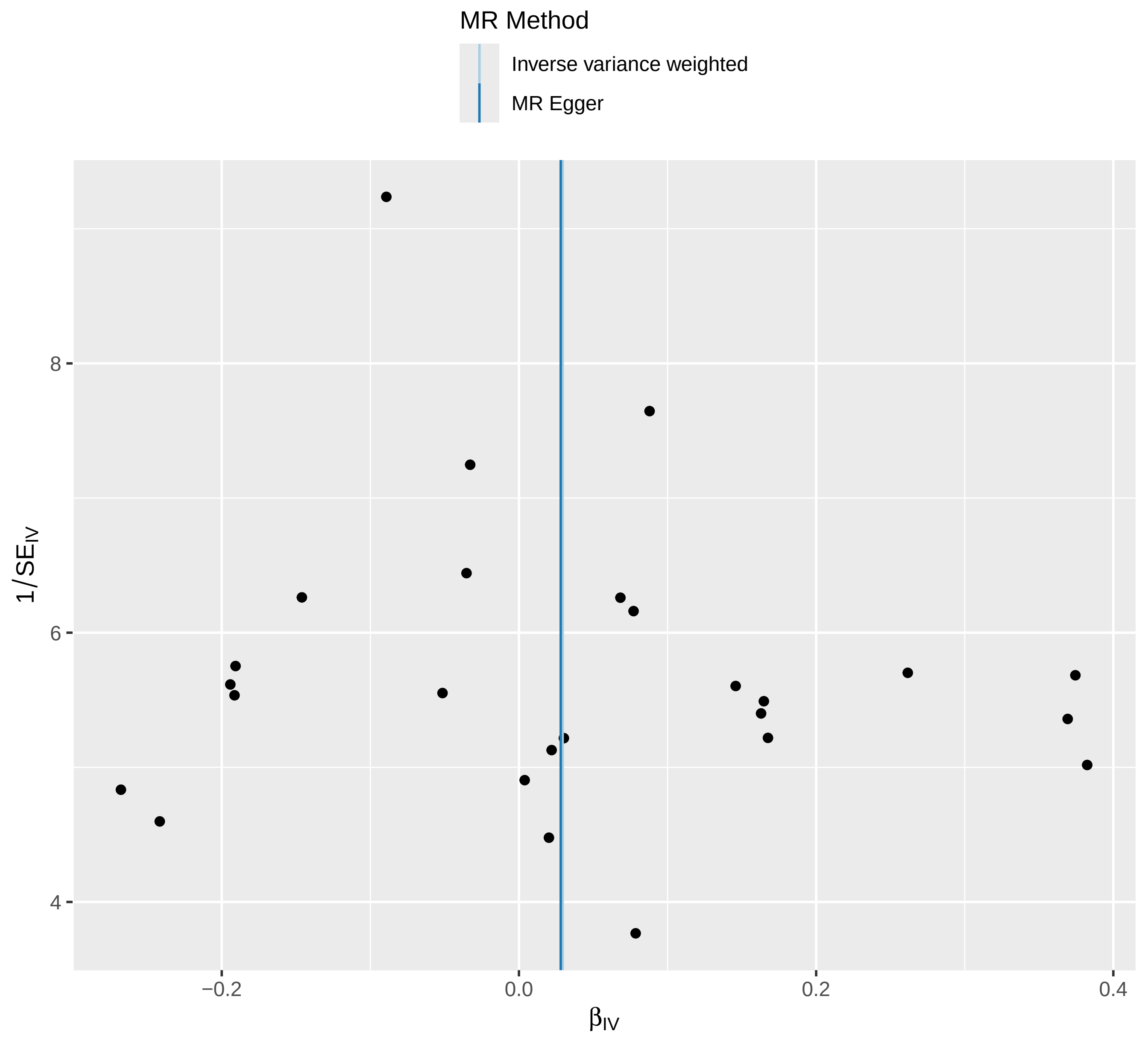
MR Method
Inverse variance weighted
MR Egger
1/SE_IV
8
6
4
β_IV
−0.2
0.0
0.2
0.4

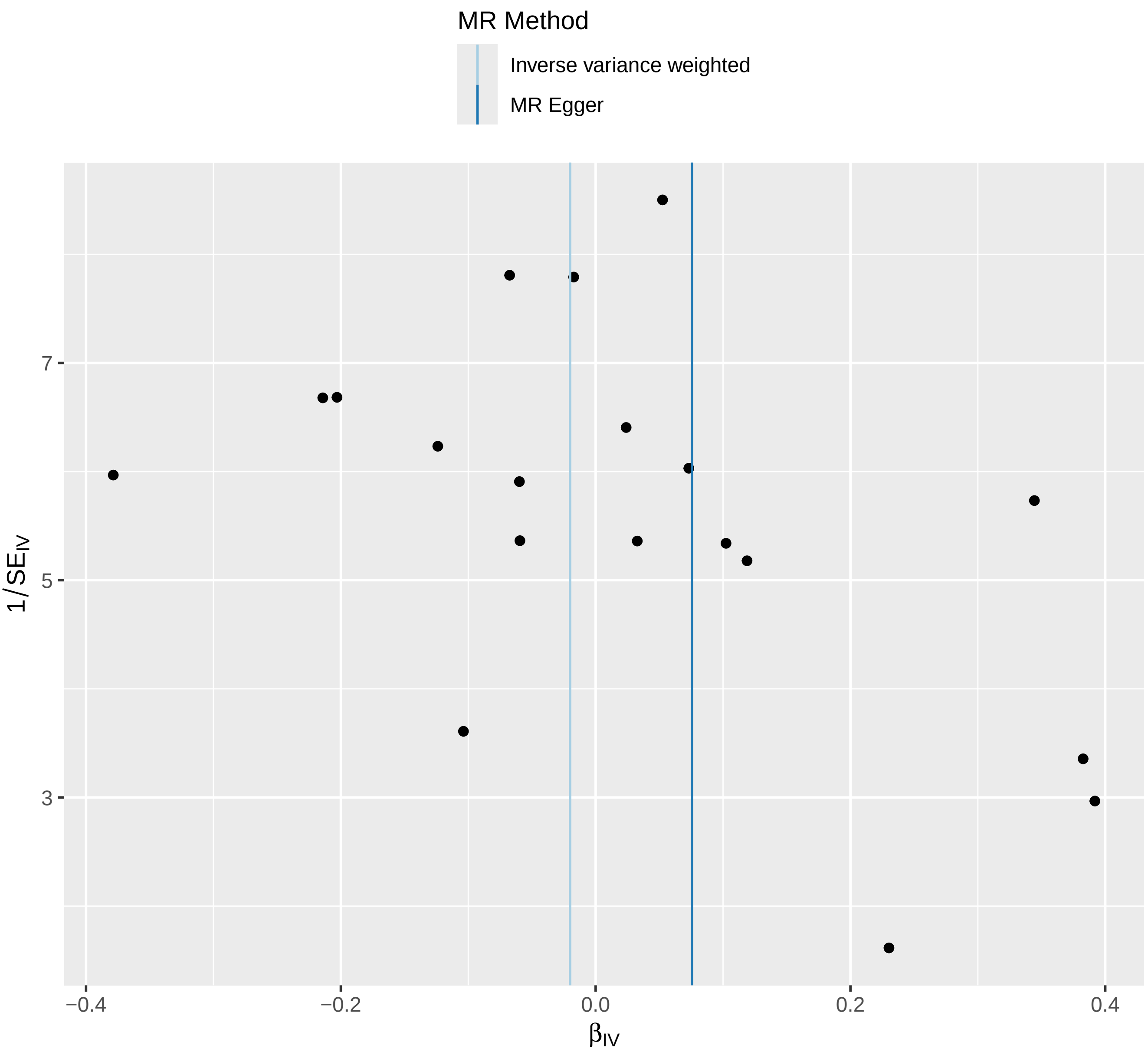
MR Method
Inverse variance weighted
MR Egger
7
5
3
$1/SE_{IV}$
−0.4
−0.2
0.0
0.2
0.4
β_{IV}

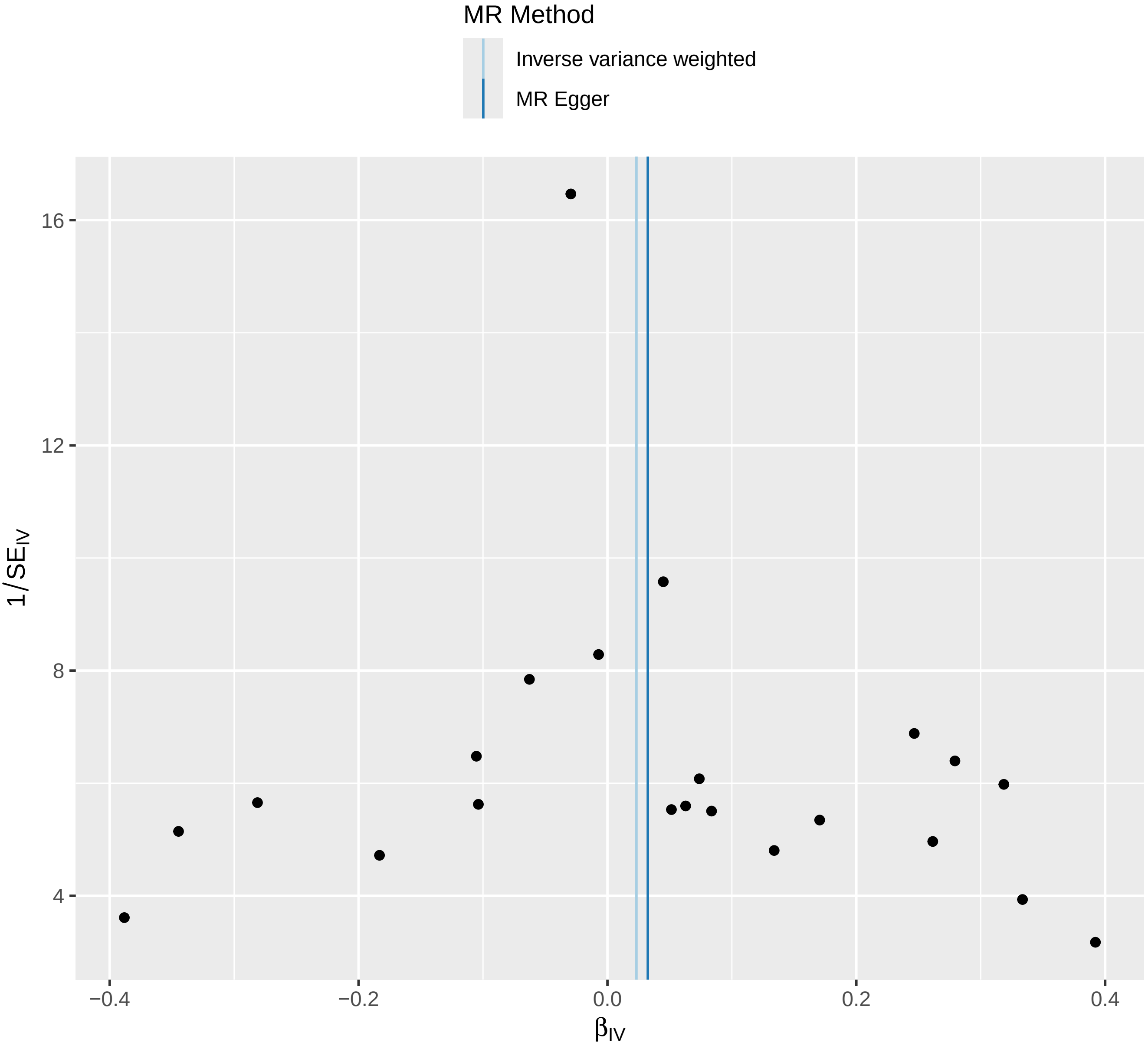

MR Method
Inverse variance weighted
MR Egger
16
12
8
4
1/SE$_{IV}$
−0.4
−0.2
0.0
0.2
0.4
β$_{IV}$

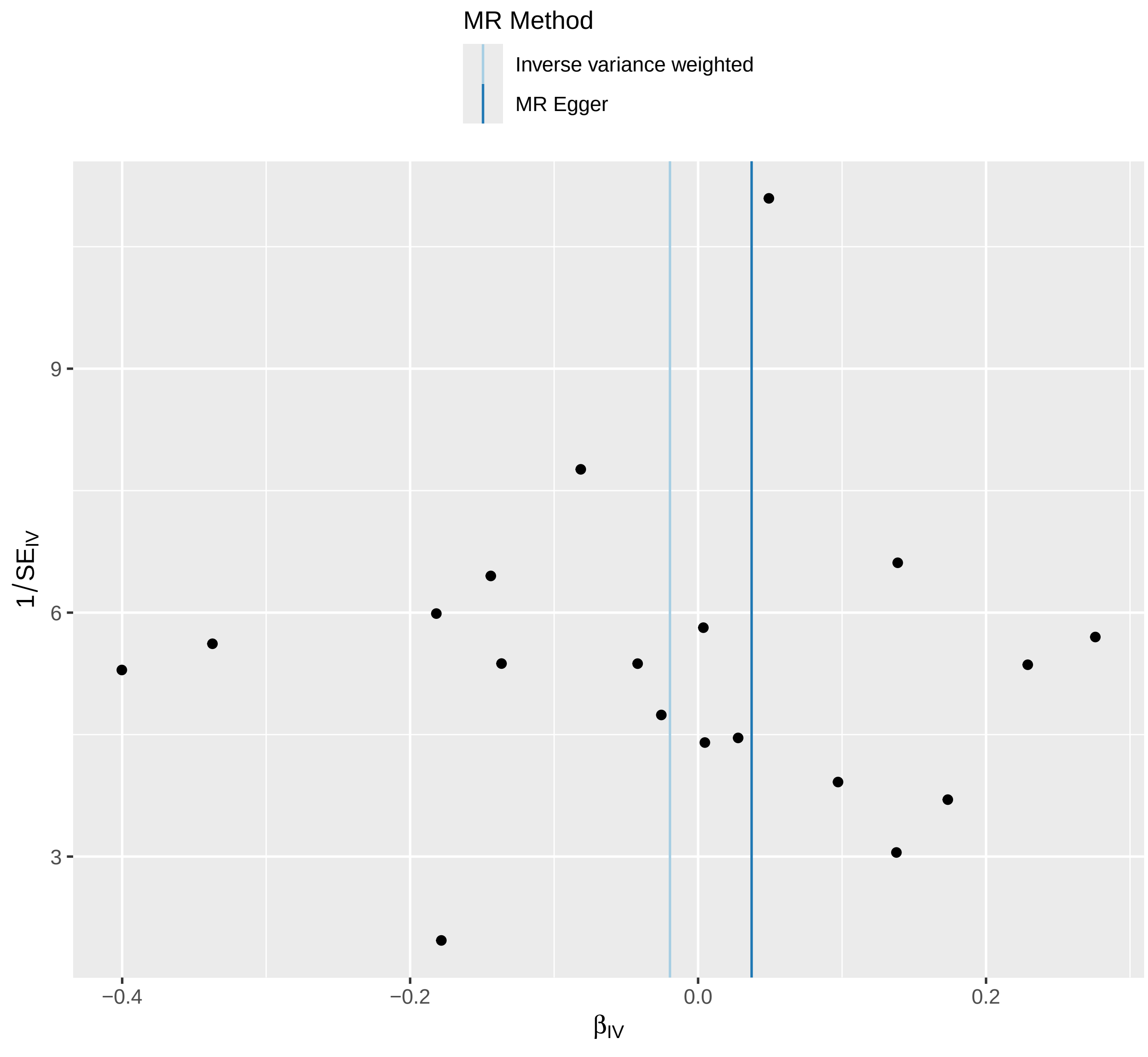

MR Method
Inverse variance weighted
MR Egger
9
6
3
1/SE_IV
−0.4
−0.2
0.0
0.2
β_IV

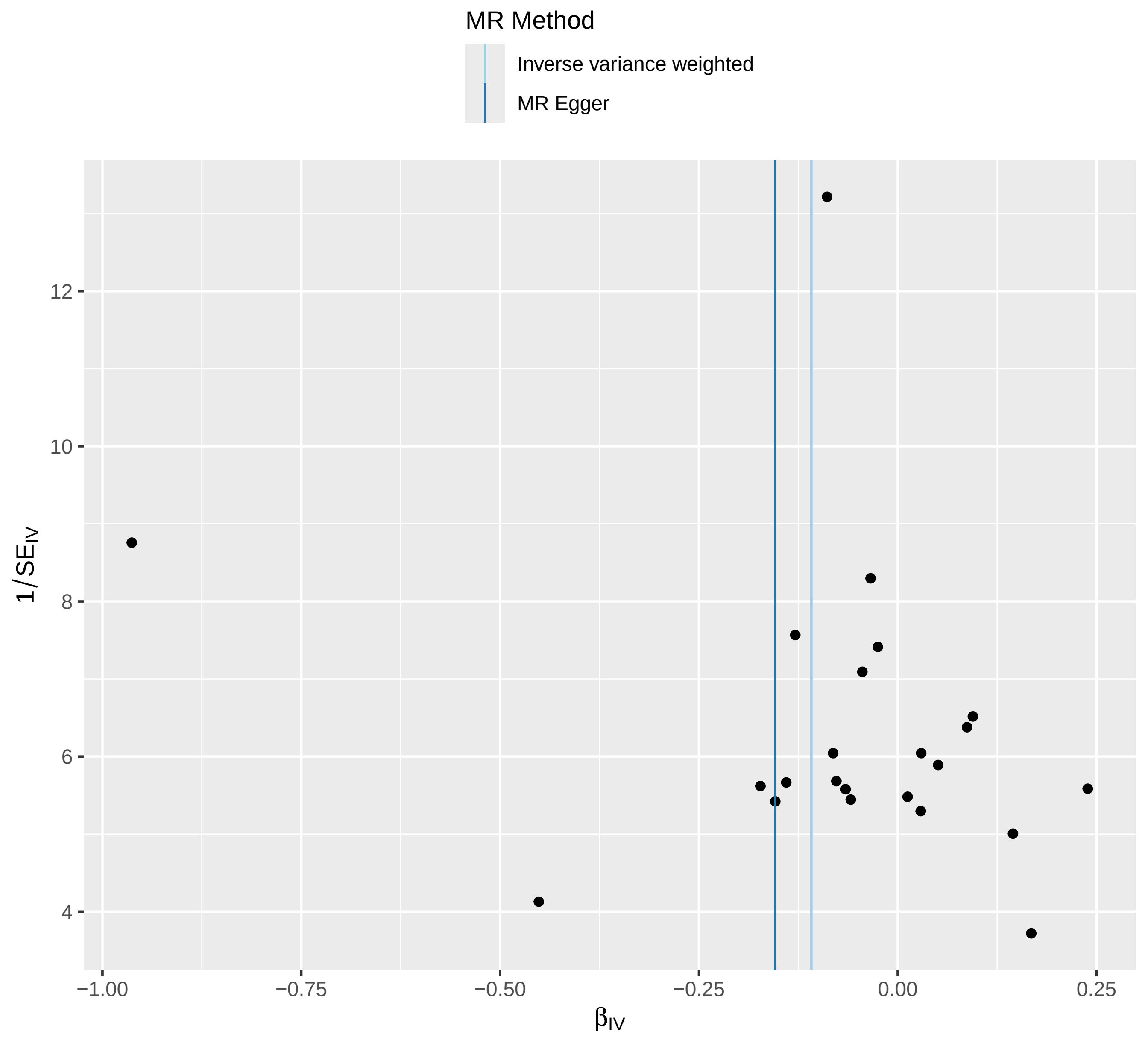

MR Method
Inverse variance weighted
MR Egger
12
10
8
6
4
1/SE$_{IV}$
−1.00
−0.75
−0.50
−0.25
0.00
0.25
β$_{IV}$

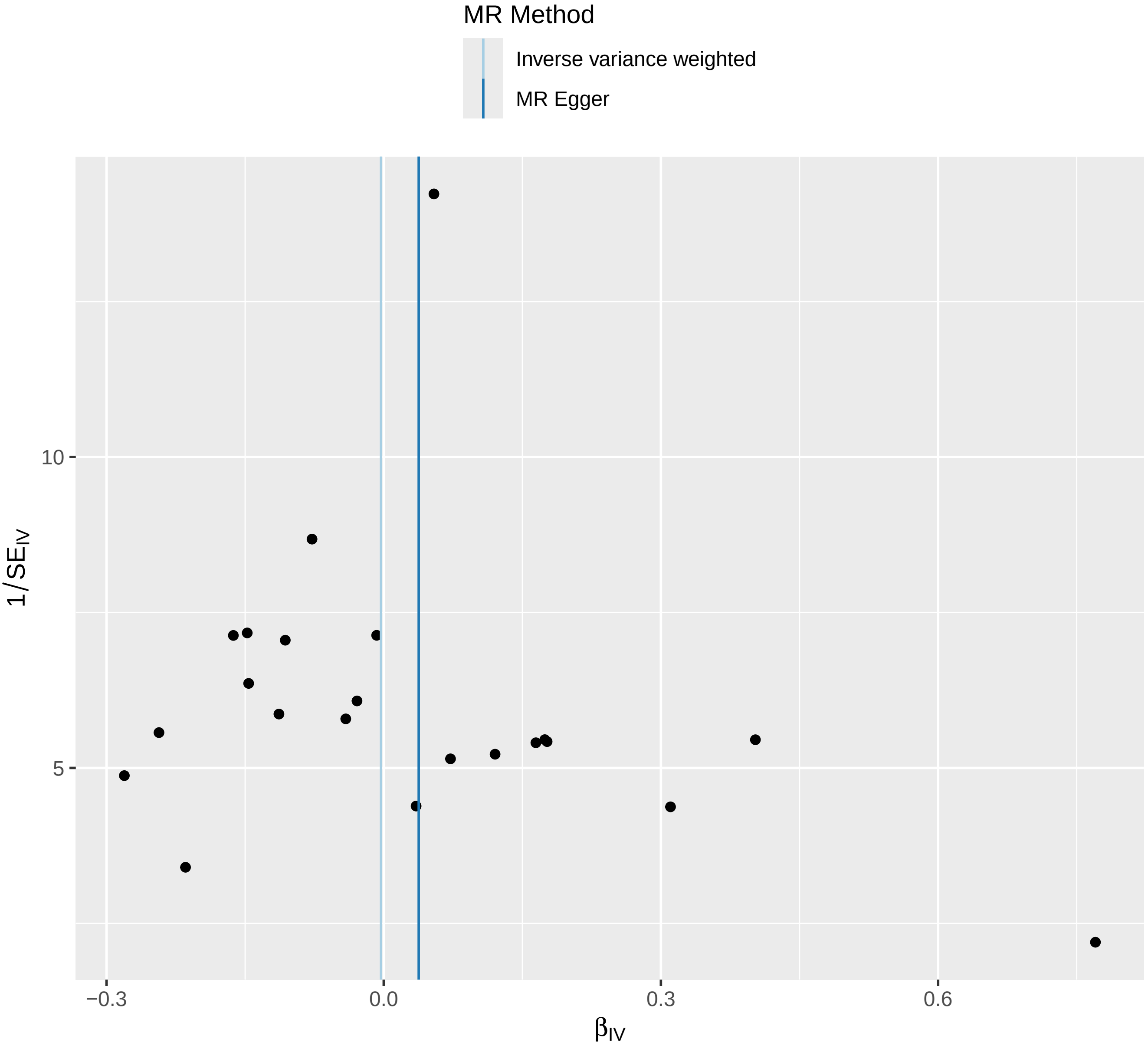

MR Method
Inverse variance weighted
MR Egger
10
5
1/SE_IV
−0.3
0.0
0.3
0.6
β_IV

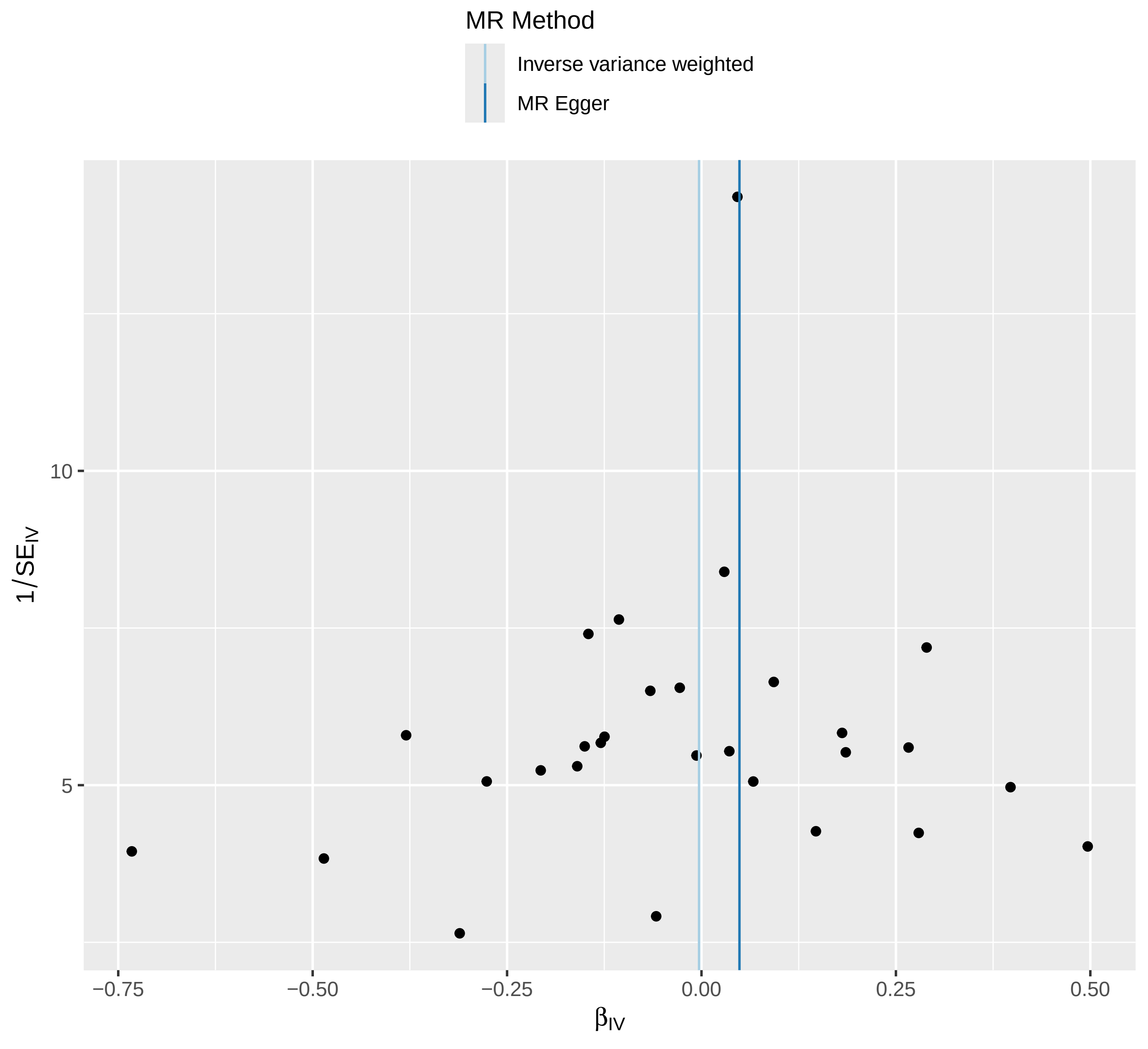
MR Method
Inverse variance weighted
MR Egger
1/SE_IV
10
5
−0.75
−0.50
−0.25
0.00
0.25
0.50
β_IV

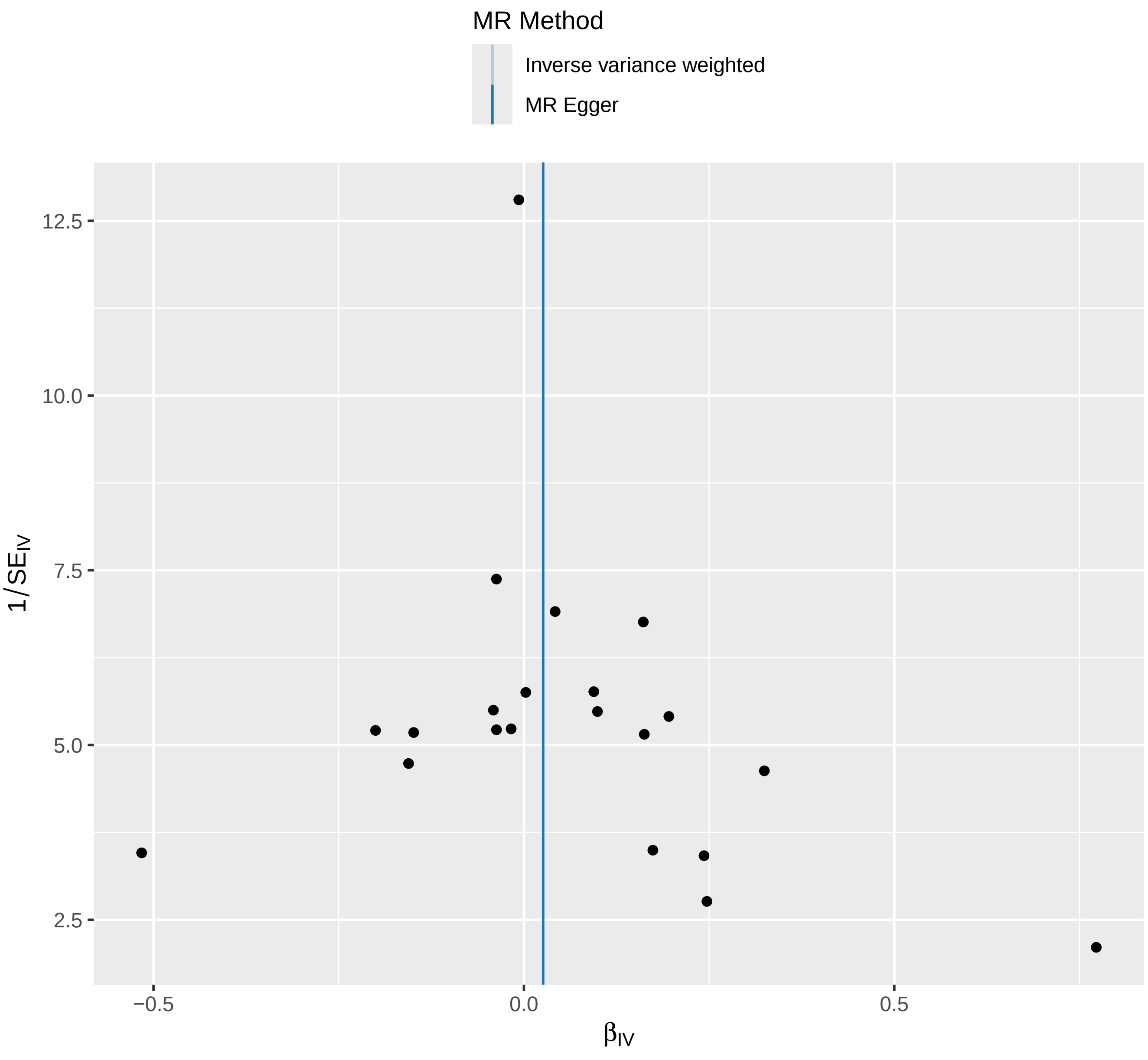

MR Method
Inverse variance weighted
MR Egger
12.5
10.0
7.5
5.0
2.5
1/SE_IV
−0.5
0.0
0.5
β_IV

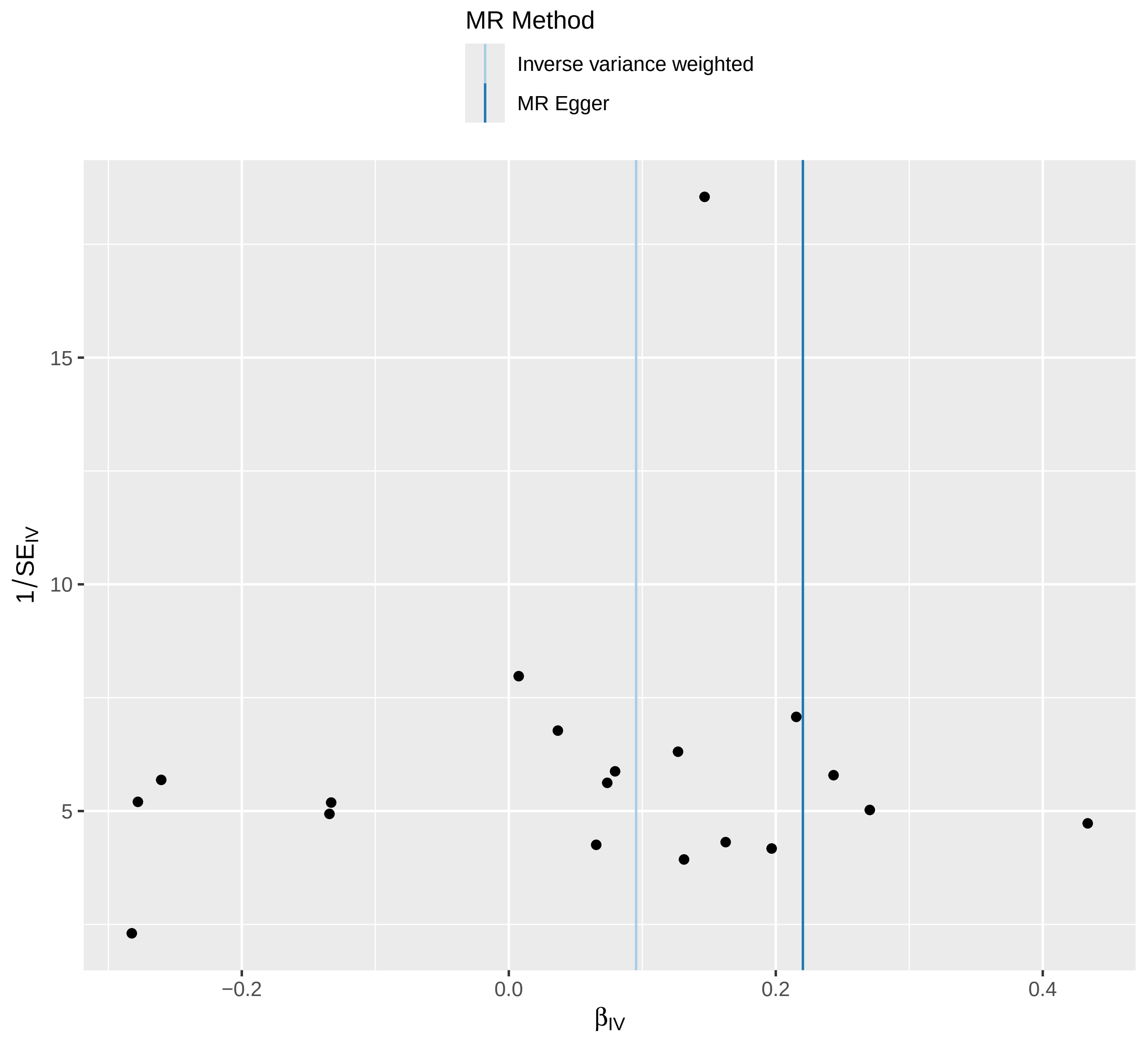
MR Method
Inverse variance weighted
MR Egger
15
10
5
1/SE_IV
−0.2
0.0
0.2
0.4
β_IV

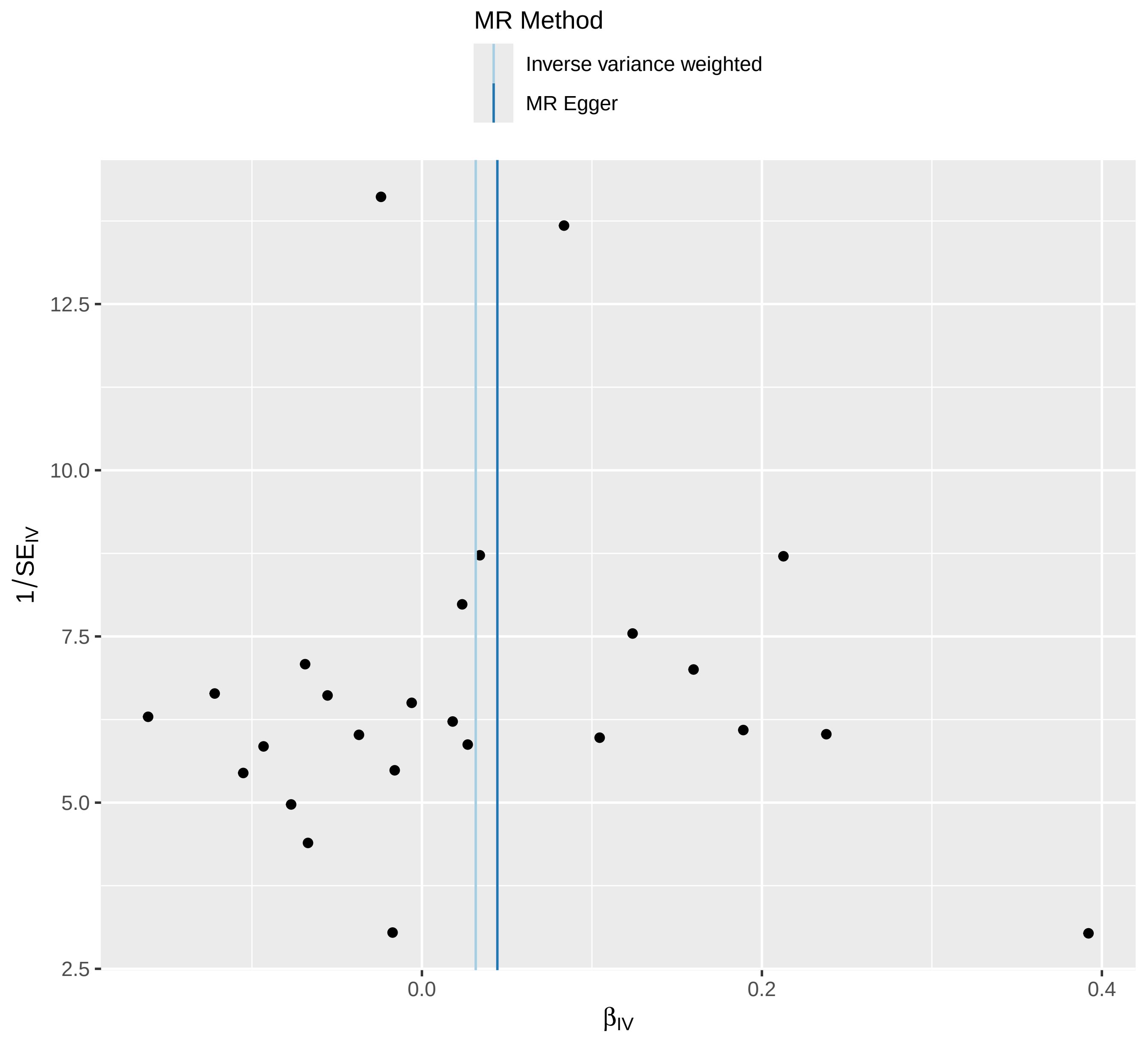

MR Method
Inverse variance weighted
MR Egger
12.5
10.0
7.5
5.0
2.5
1/SE_IV
0.0
0.2
0.4
β_IV

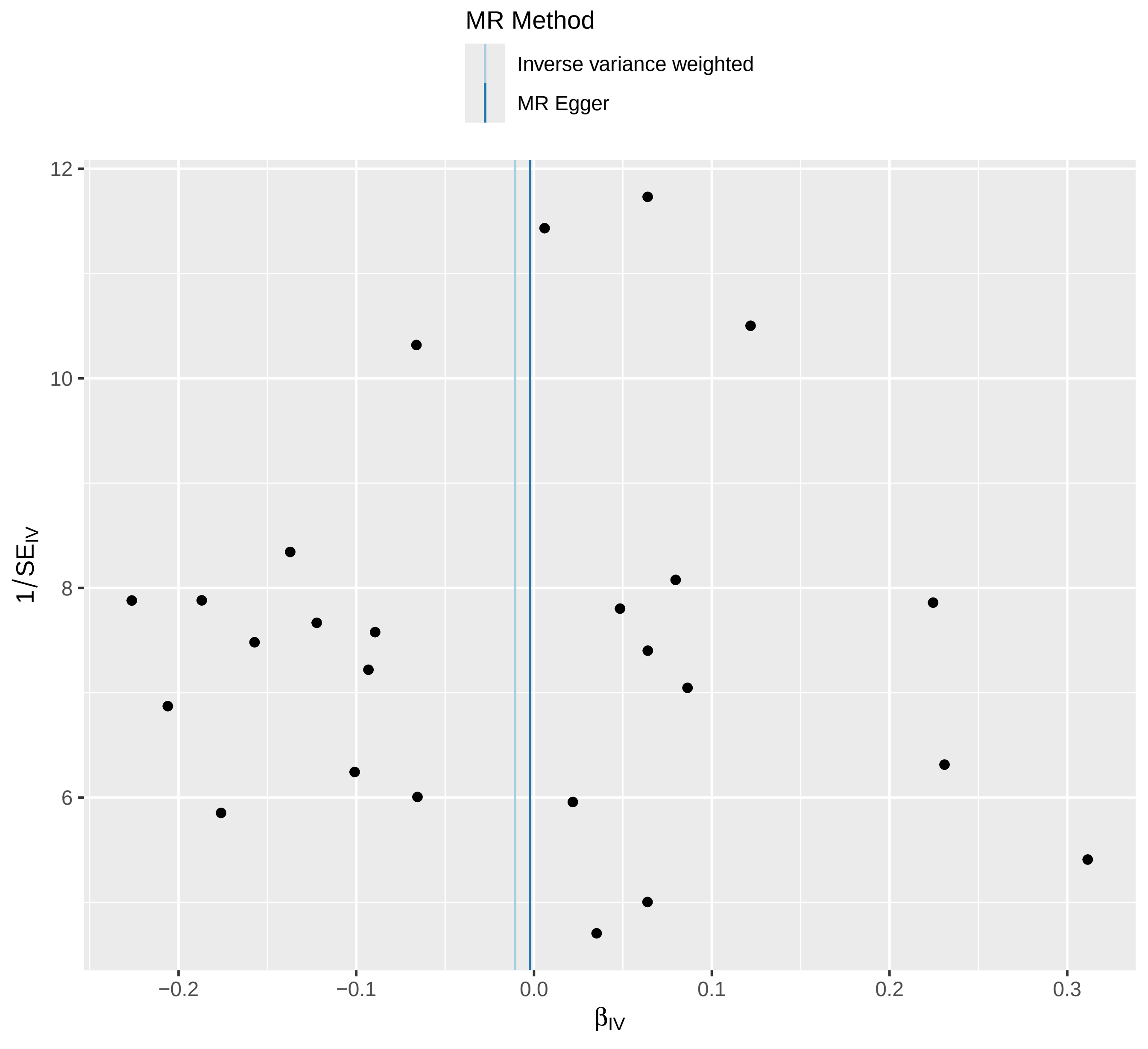
MR Method
Inverse variance weighted
MR Egger
12
10
8
6
−0.2
−0.1
0.0
0.1
0.2
0.3
$1/SE_{IV}$
β_{IV}

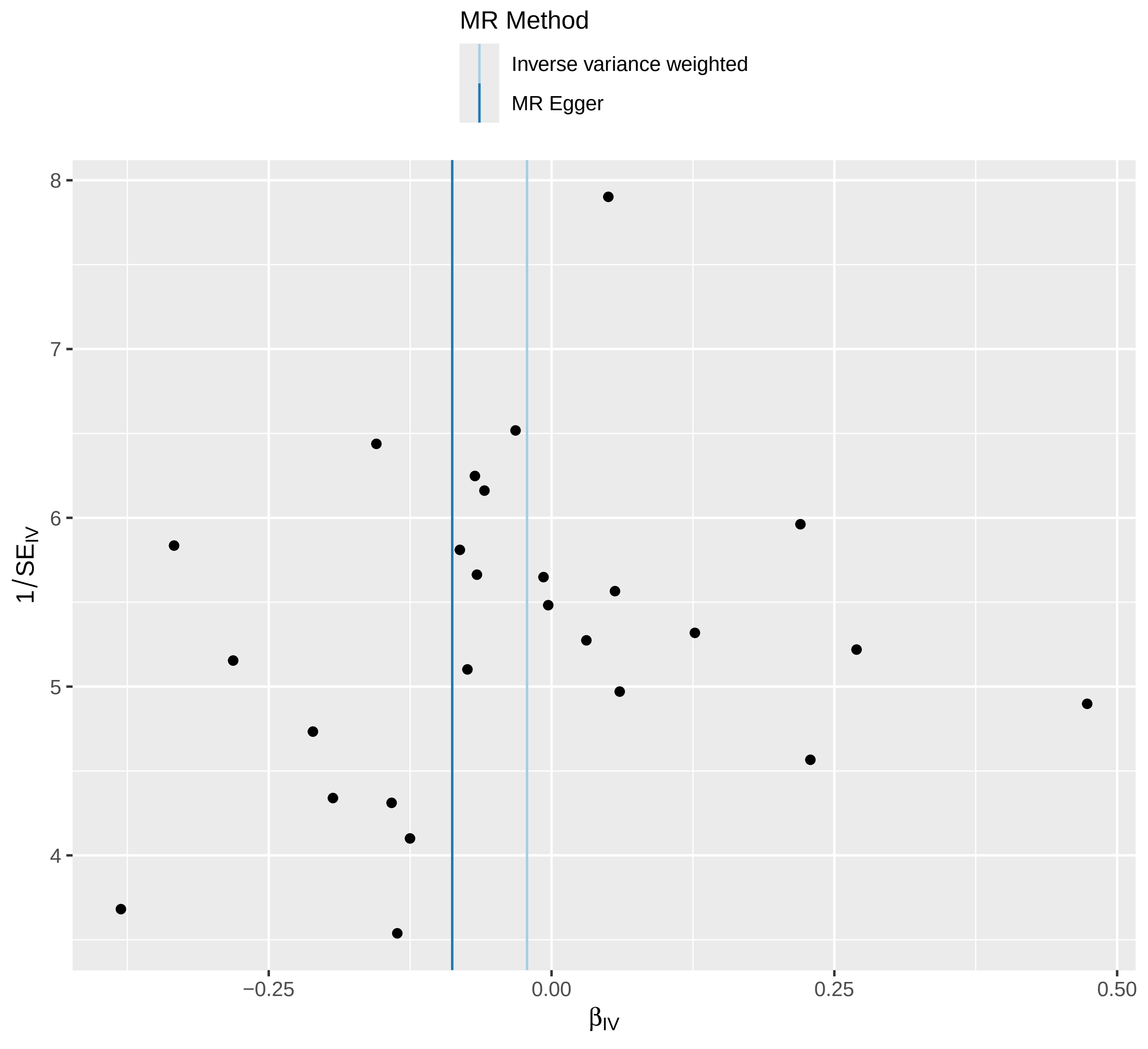
MR Method
Inverse variance weighted
MR Egger
8
7
6
5
4
1/SE_IV
−0.25
0.00
0.25
0.50
β_IV

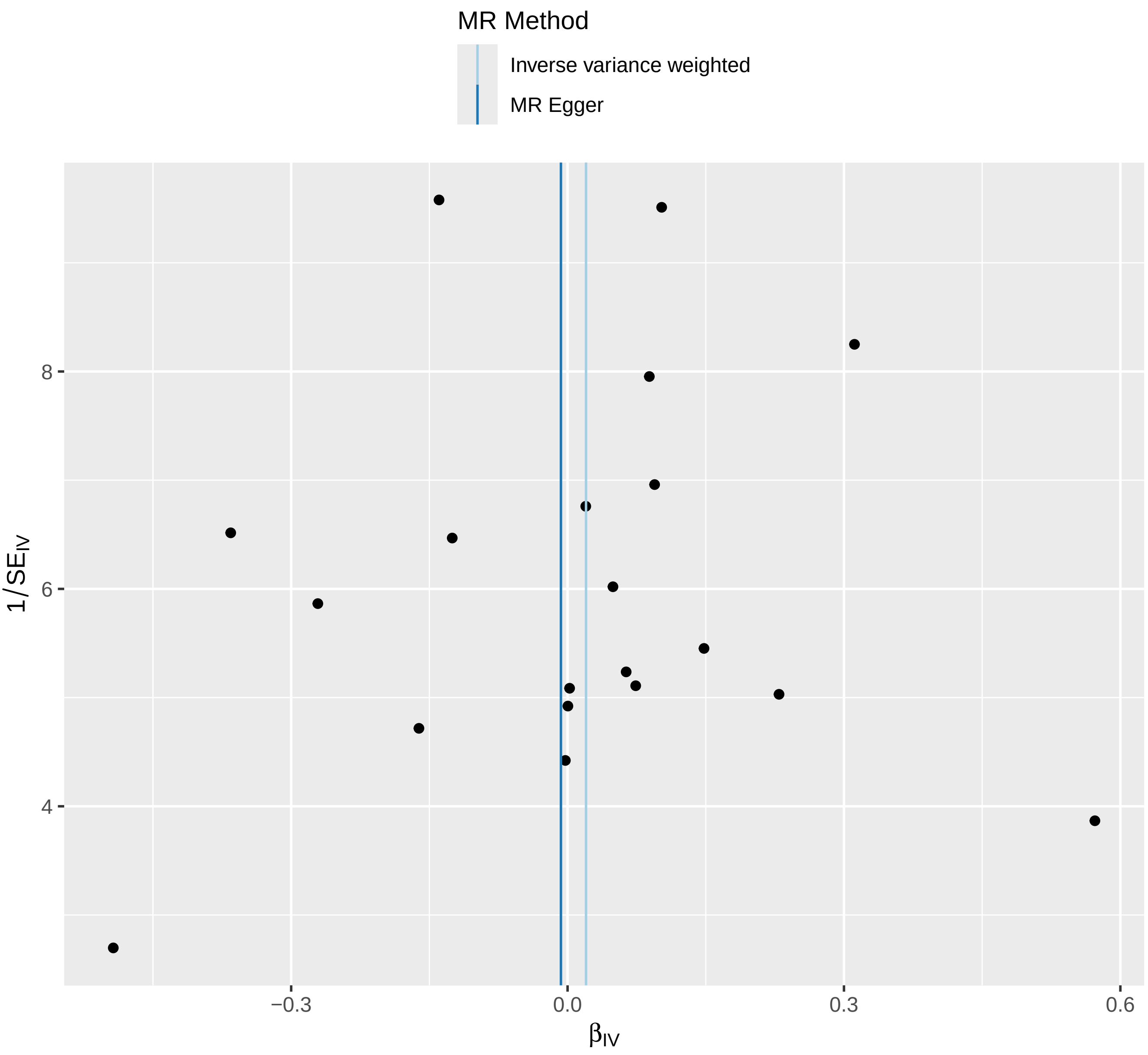

MR Method
Inverse variance weighted
MR Egger
8
6
4
$1/SE_{IV}$
−0.3
0.0
0.3
0.6
β_{IV}

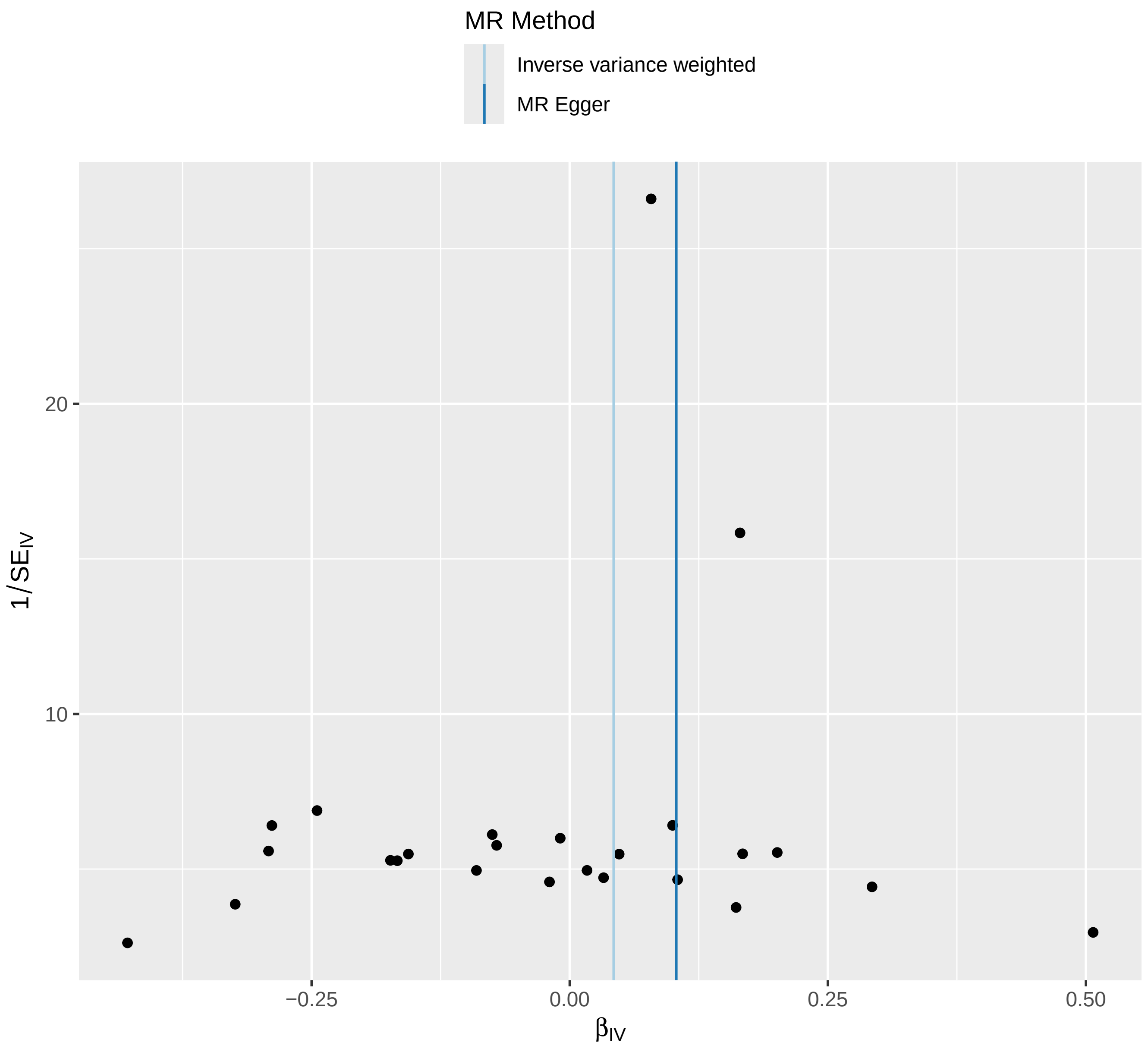

MR Method
Inverse variance weighted
MR Egger
20
10
1/SE_IV
−0.25
0.00
0.25
0.50
β_IV

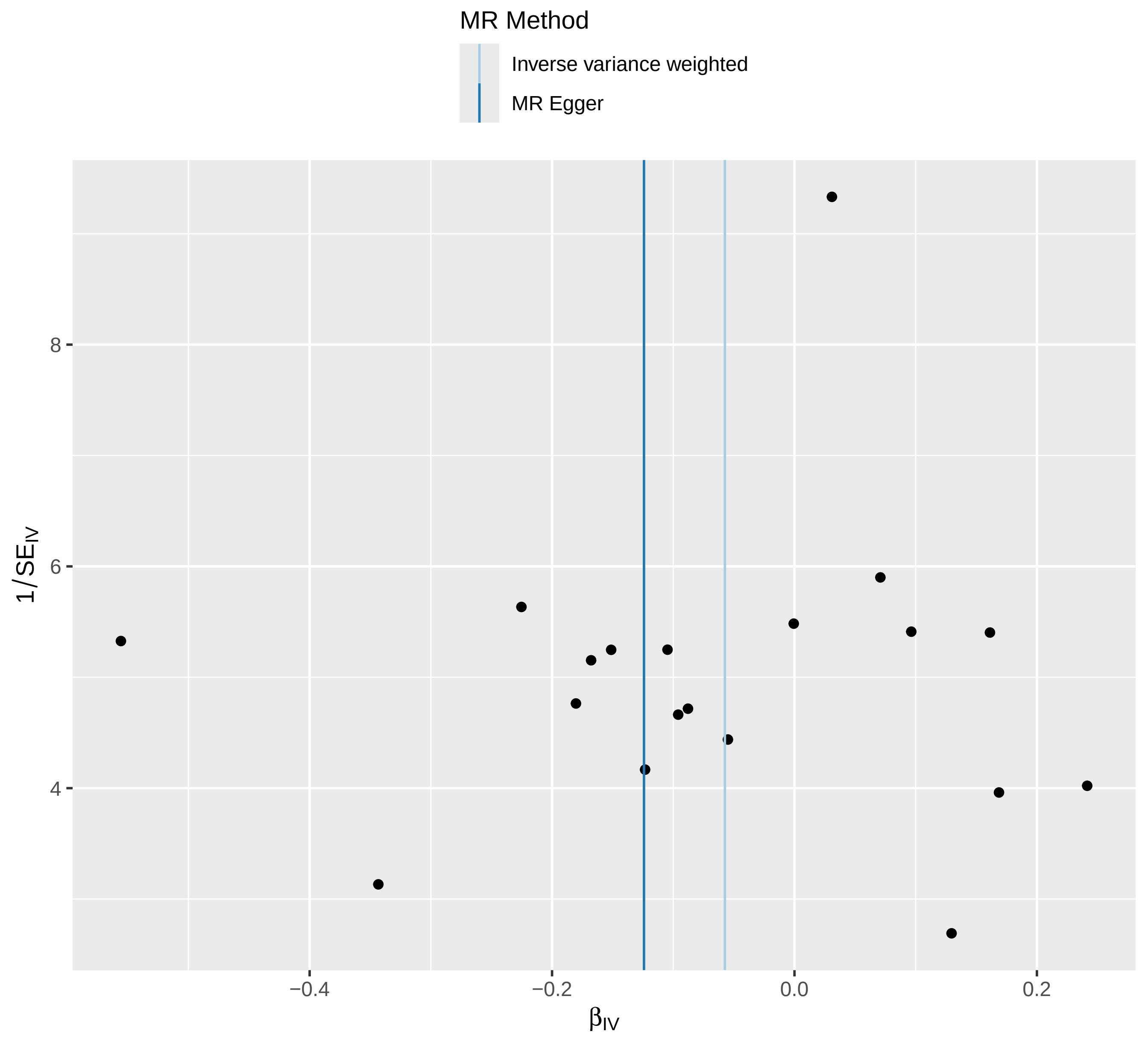
MR Method
Inverse variance weighted
MR Egger
8
6
4
1/SE_IV
−0.4
−0.2
0.0
0.2
β_IV

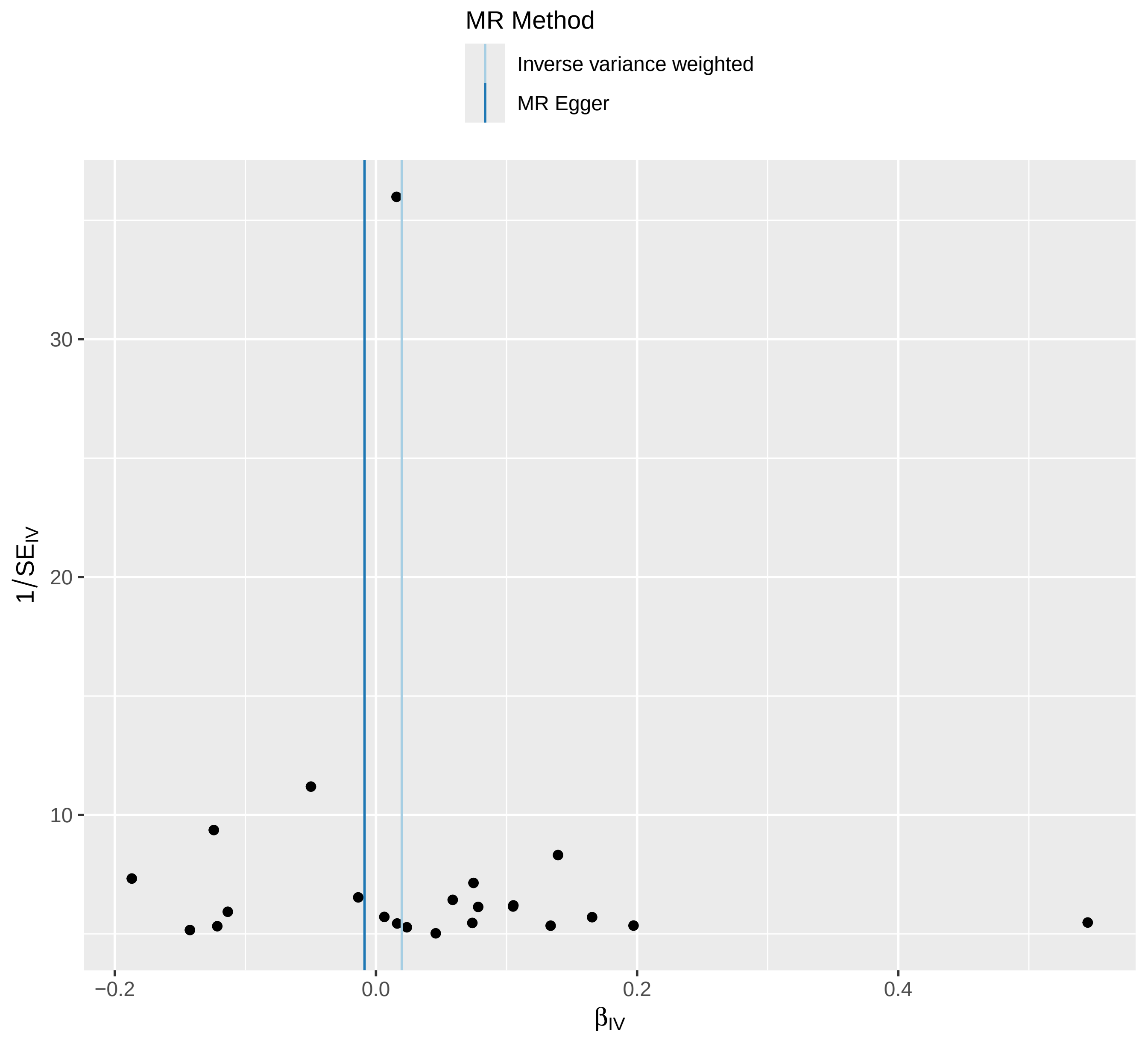
MR Method
Inverse variance weighted
MR Egger
30
20
10
$1/SE_{IV}$
−0.2
0.0
0.2
0.4
β_{IV}

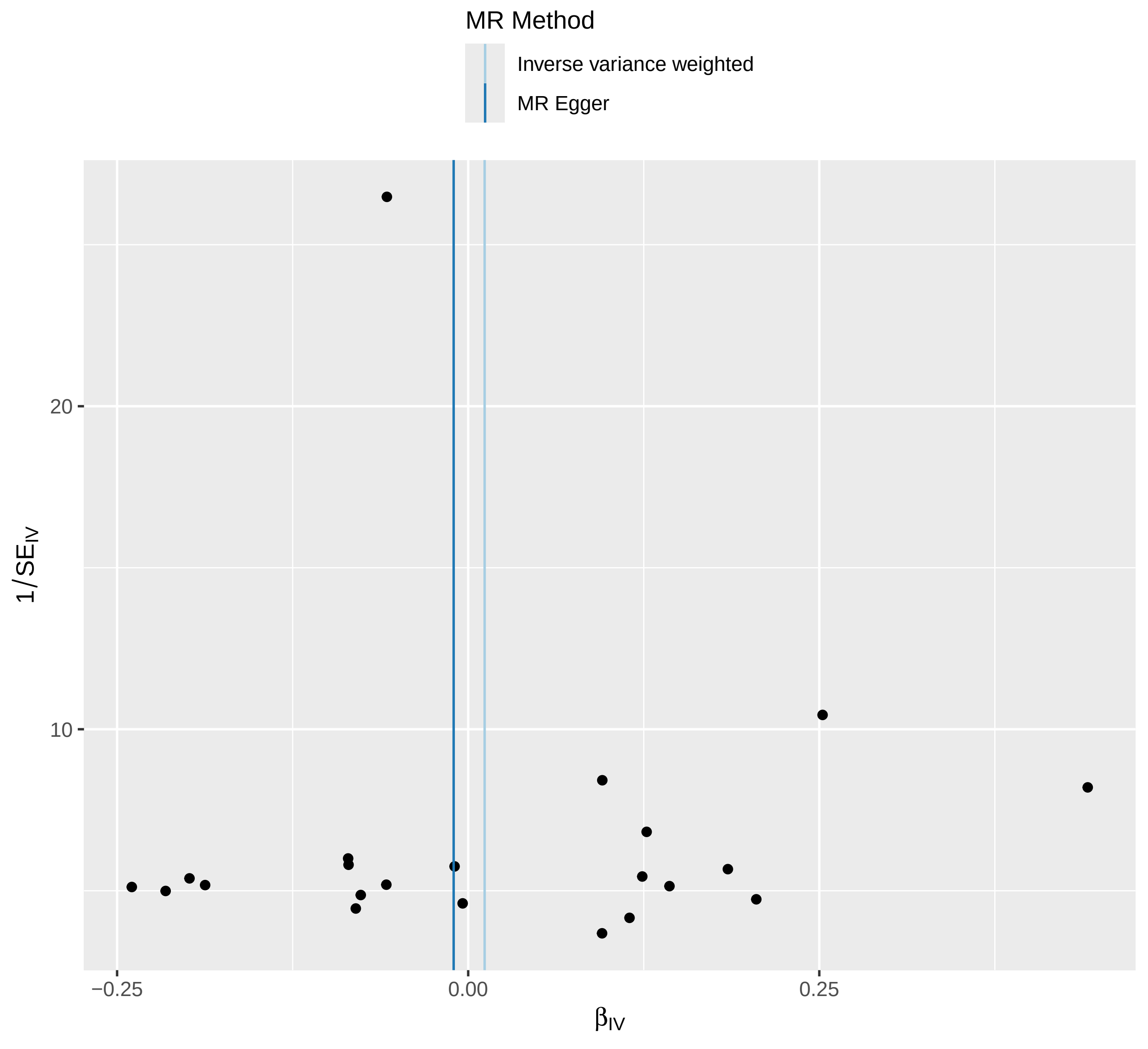

MR Method
Inverse variance weighted
MR Egger
20
10
1/SE_IV
−0.25
0.00
0.25
β_IV

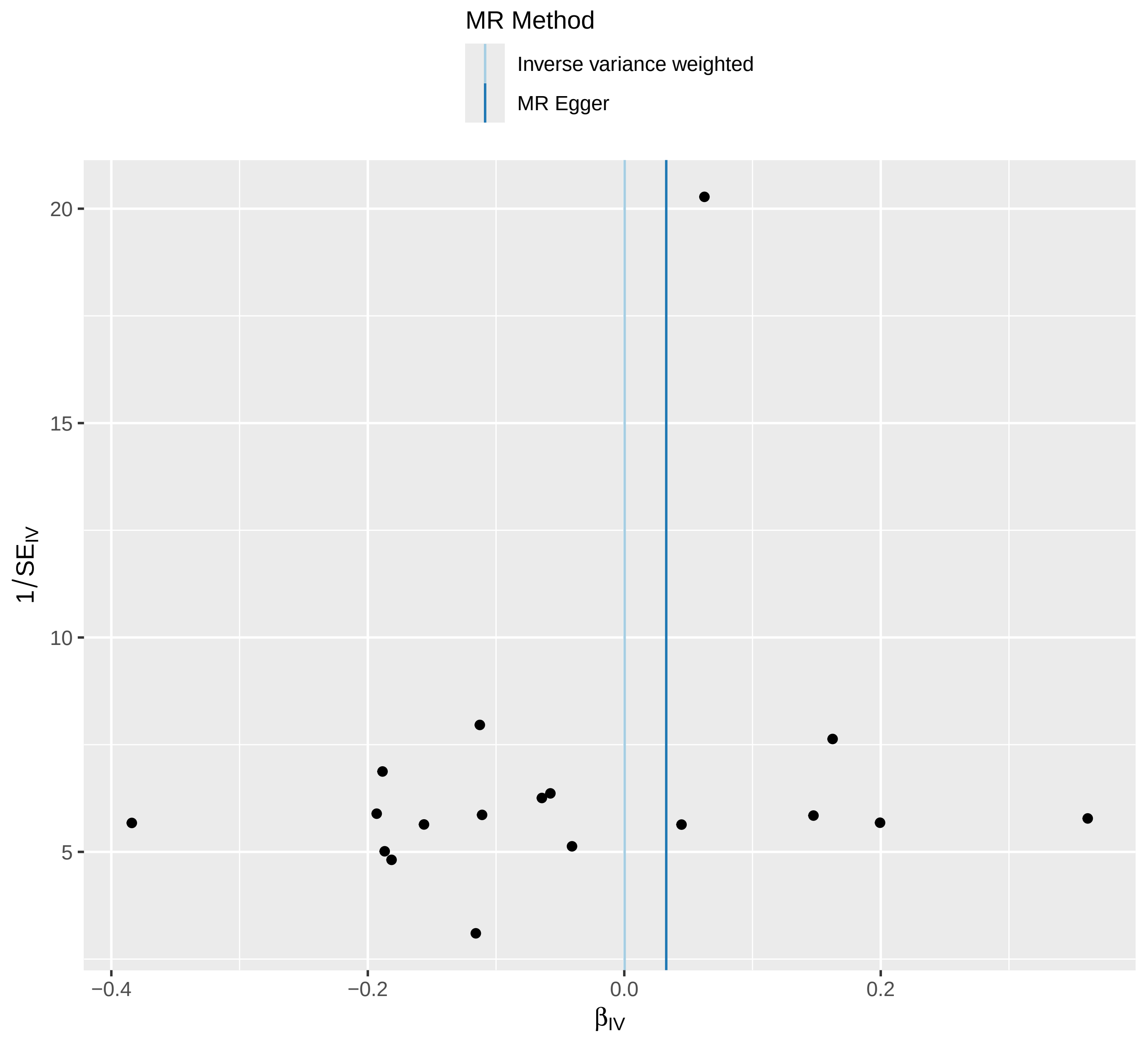
MR Method
Inverse variance weighted
MR Egger
1/SE_IV
20
15
10
5
−0.4
−0.2
0.0
0.2
β_IV

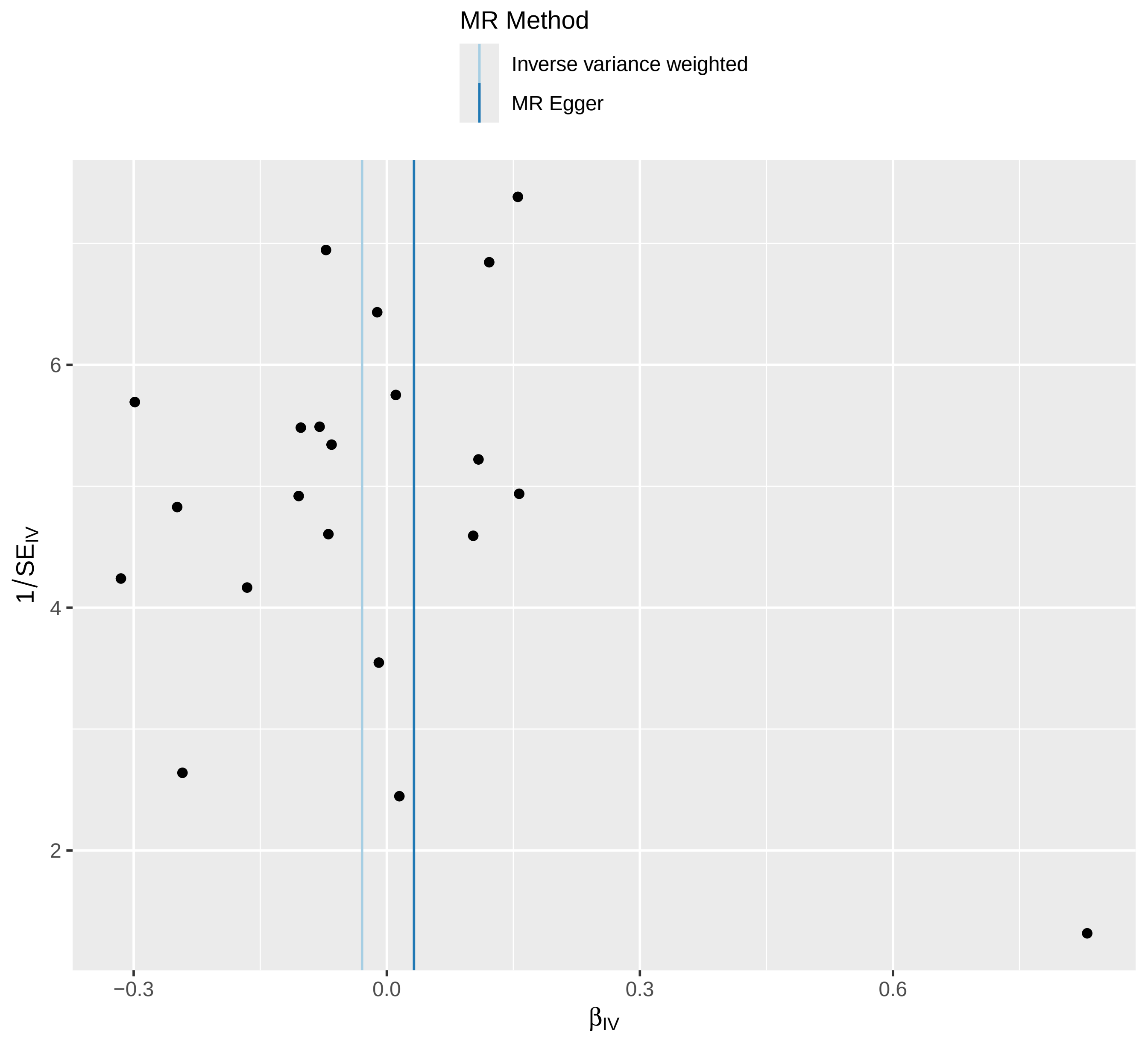
MR Method
Inverse variance weighted
MR Egger
1/SE_IV
β_IV
6
4
2
−0.3
0.0
0.3
0.6

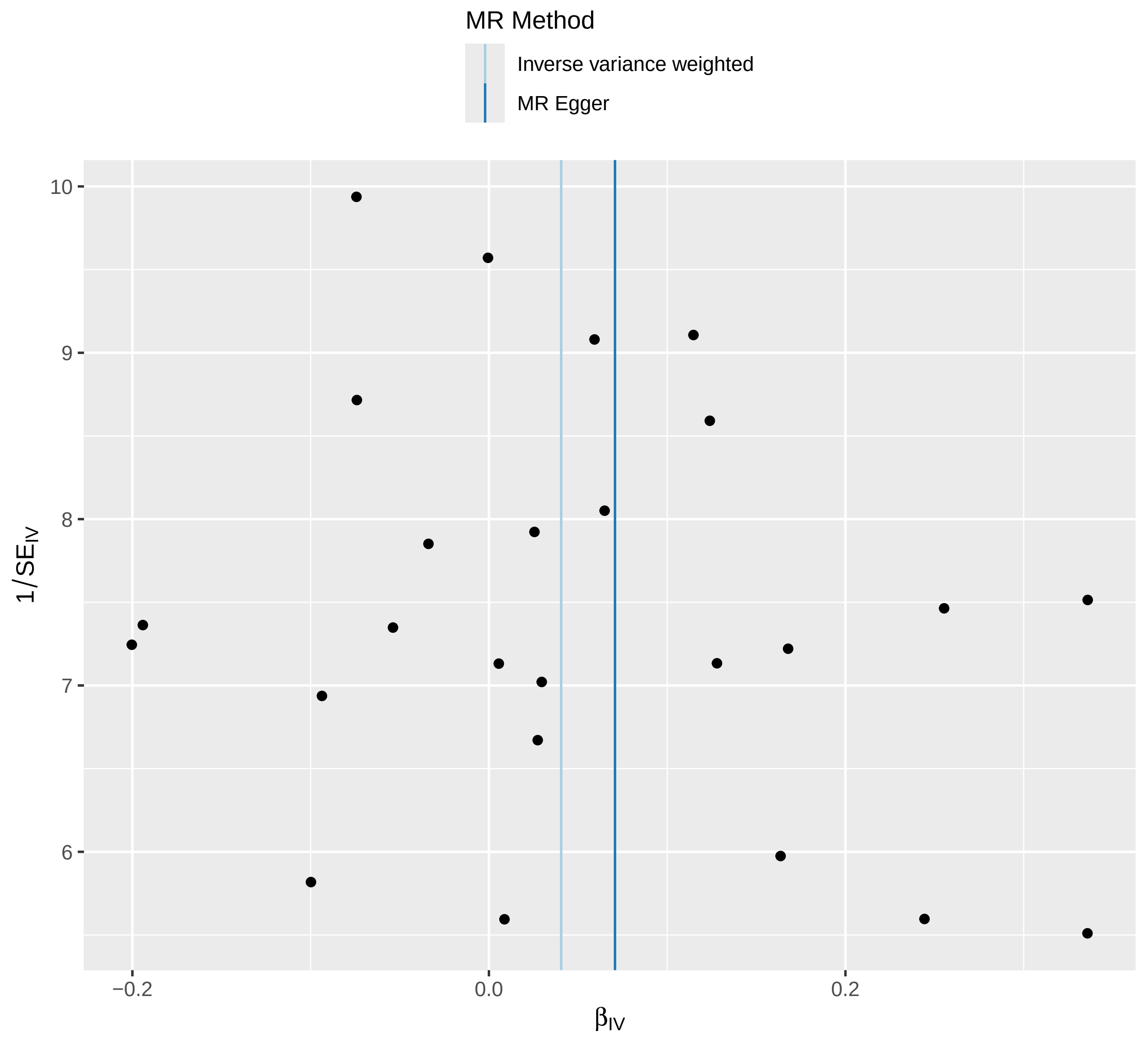
MR Method
Inverse variance weighted
MR Egger
10
9
8
7
6
1/SE_IV
−0.2
0.0
0.2
β_IV

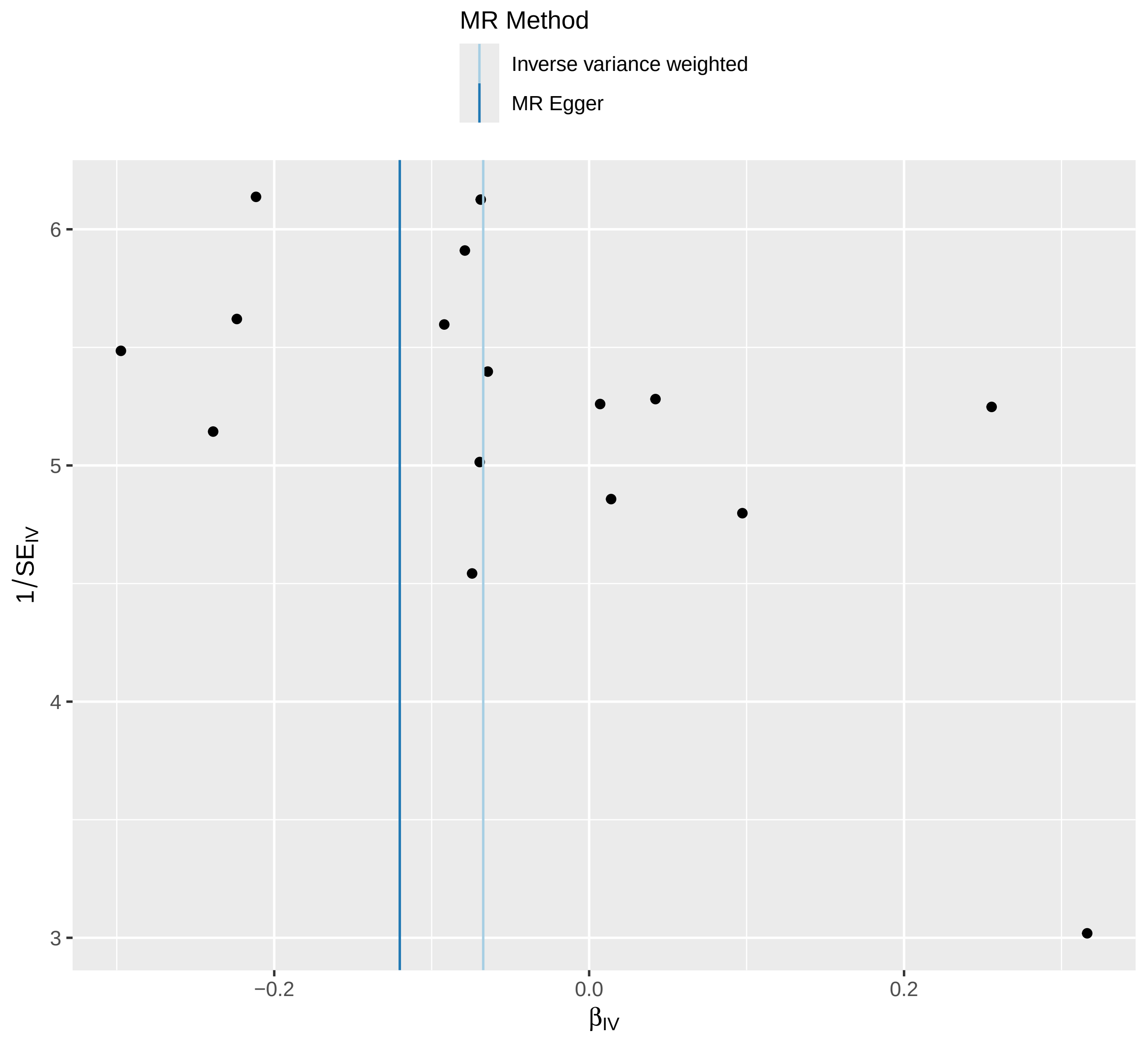
MR Method
Inverse variance weighted
MR Egger
6
5
4
3
1/SE$_{IV}$
−0.2
0.0
0.2
β$_{IV}$

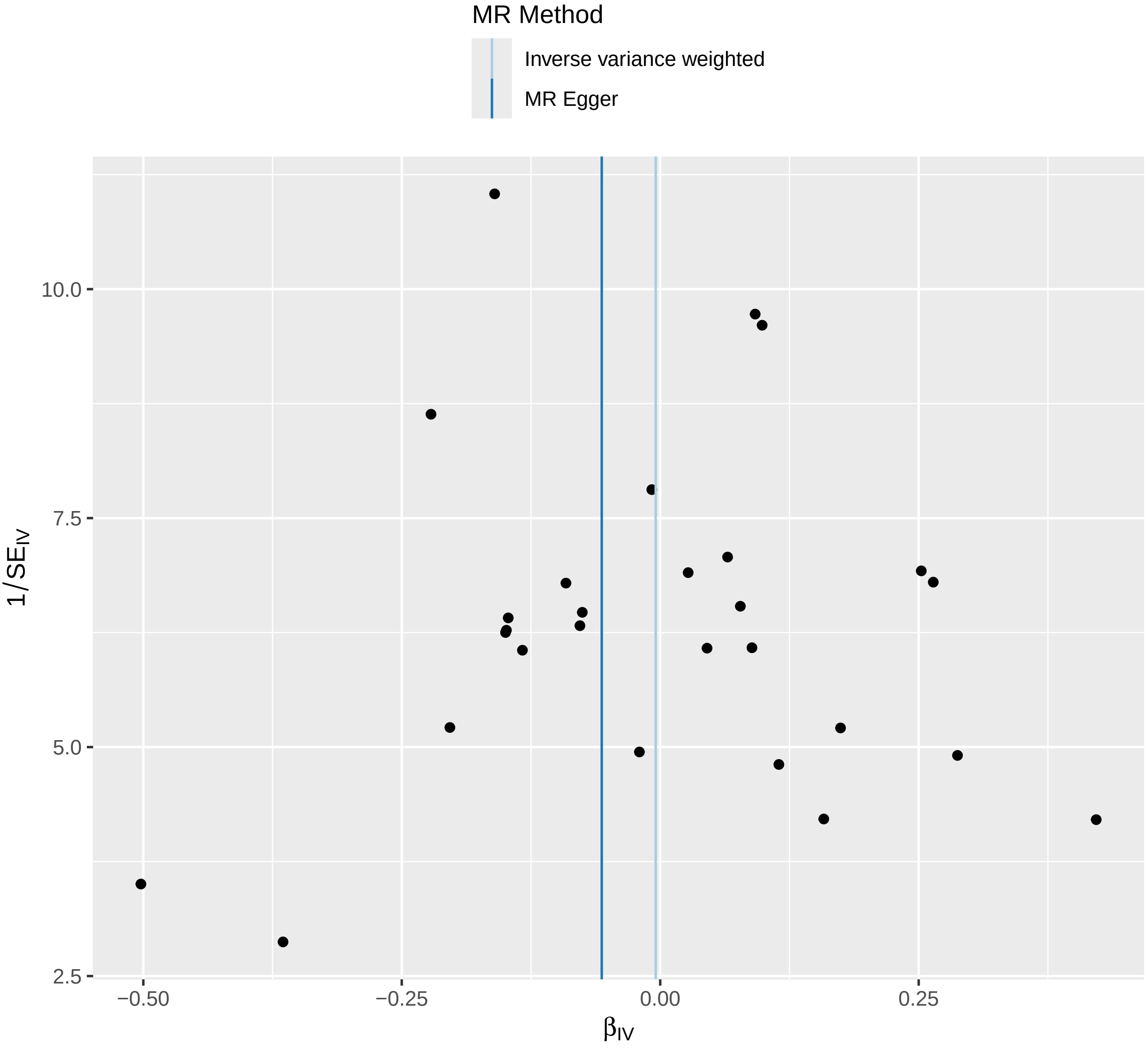
MR Method
Inverse variance weighted
MR Egger
$1/SE_{IV}$
10.0
7.5
5.0
2.5
−0.50
−0.25
0.00
0.25
β_{IV}

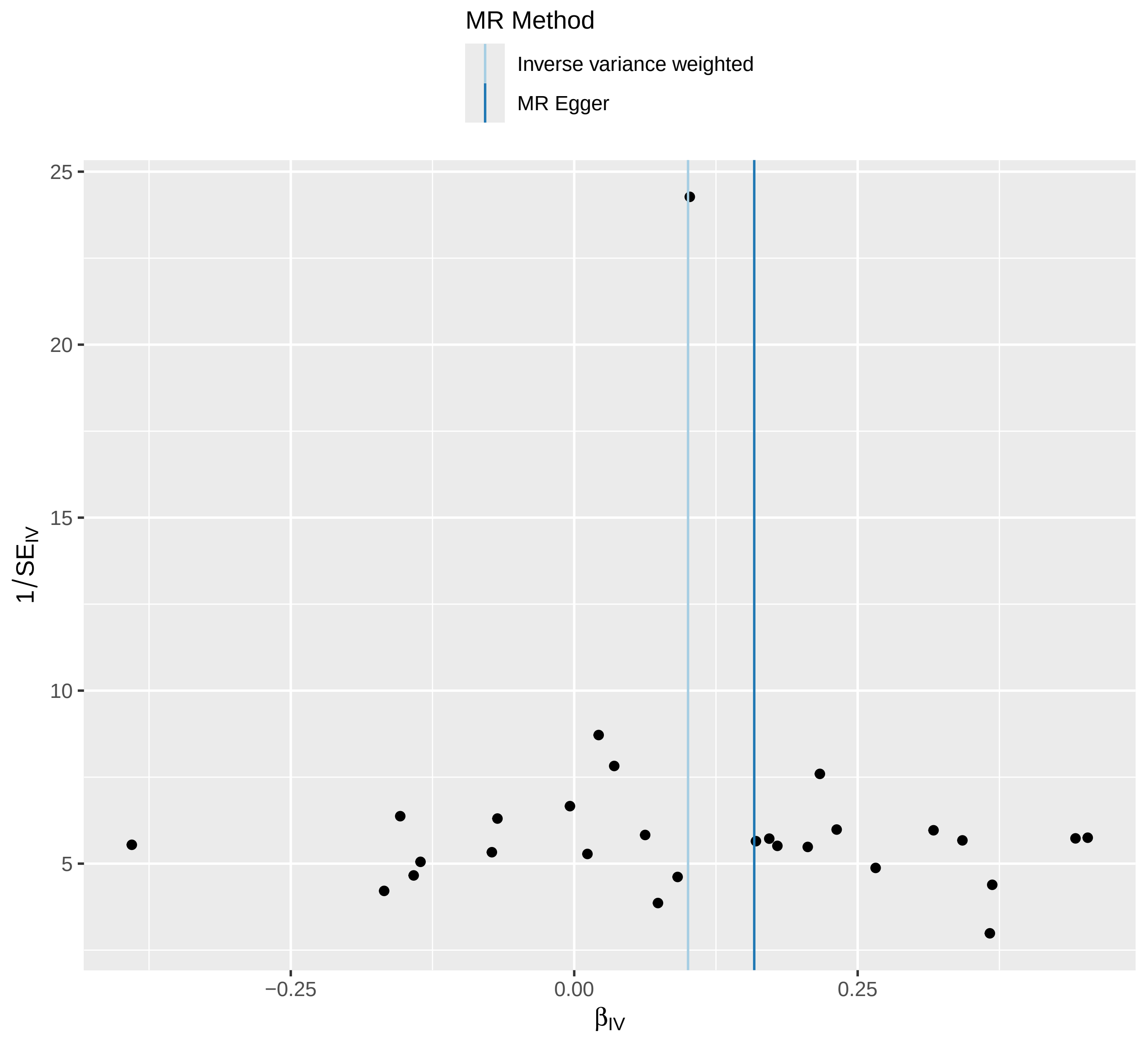
MR Method
Inverse variance weighted
MR Egger
1/SE$_{IV}$
25
20
15
10
5
−0.25
0.00
0.25
β$_{IV}$

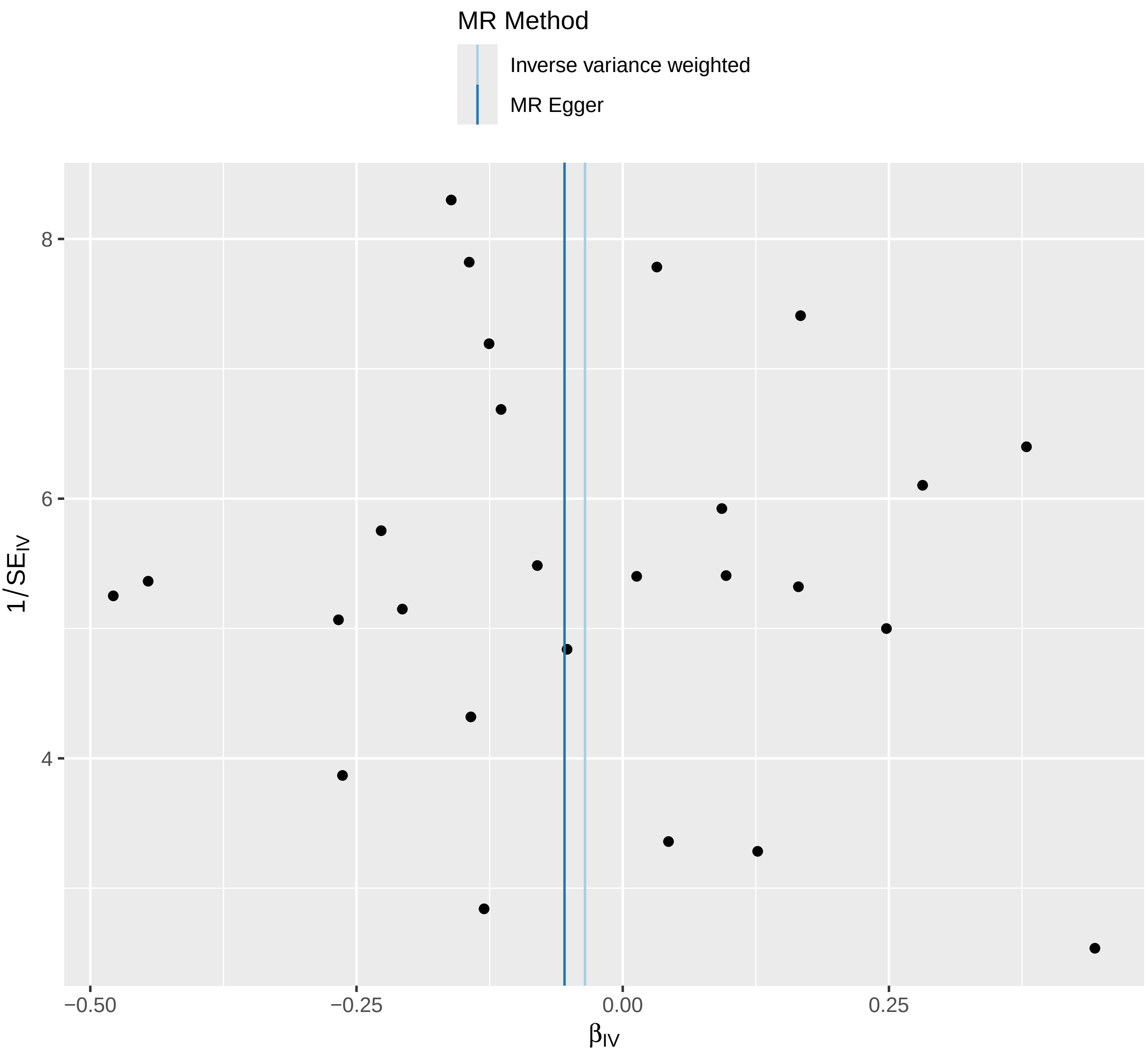
MR Method
Inverse variance weighted
MR Egger
8
6
4
1/SE_IV
−0.50
−0.25
0.00
0.25
β_IV

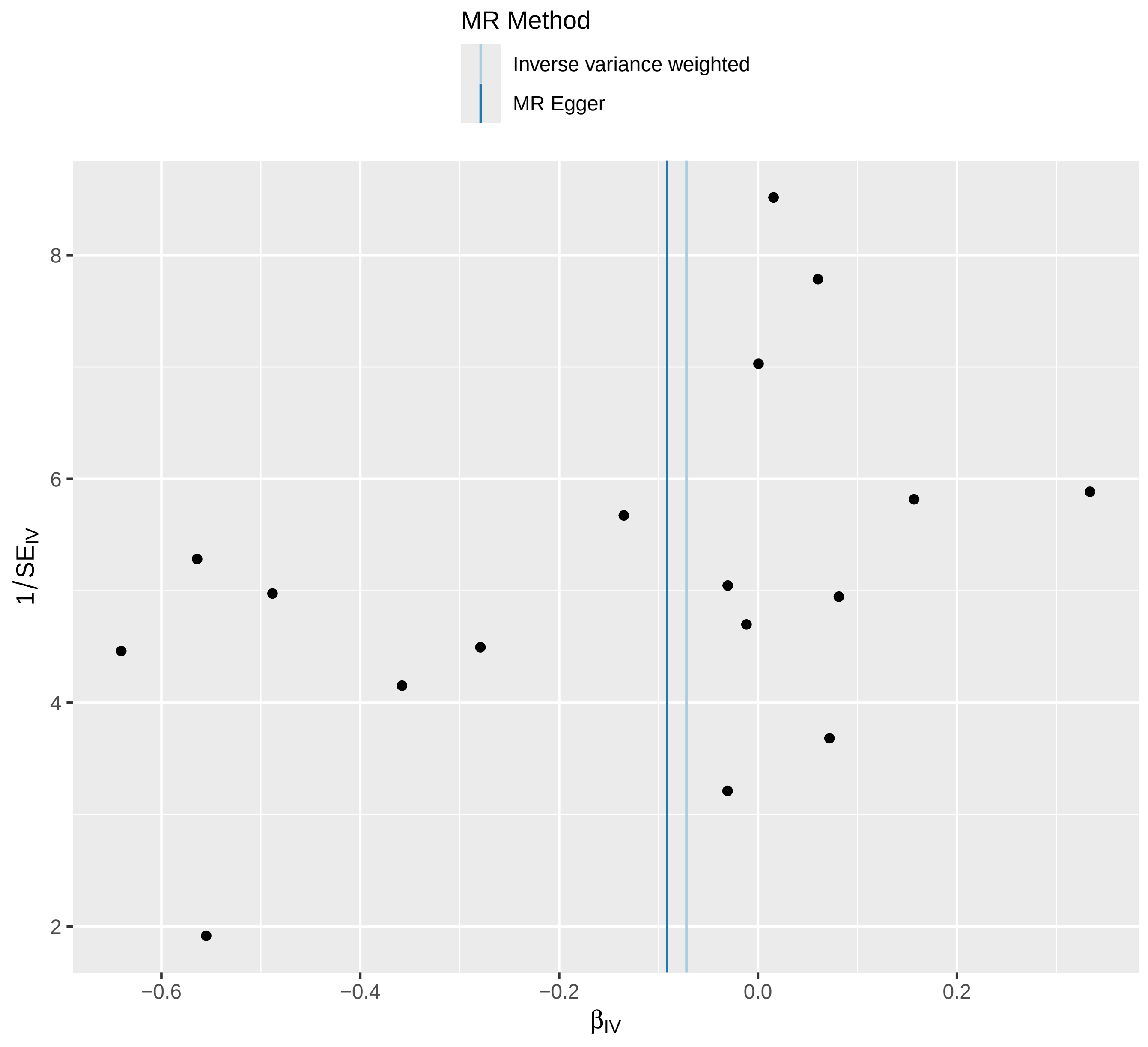

MR Method
Inverse variance weighted
MR Egger
8
6
4
2
1/SE$_{IV}$
−0.6
−0.4
−0.2
0.0
0.2
β$_{IV}$

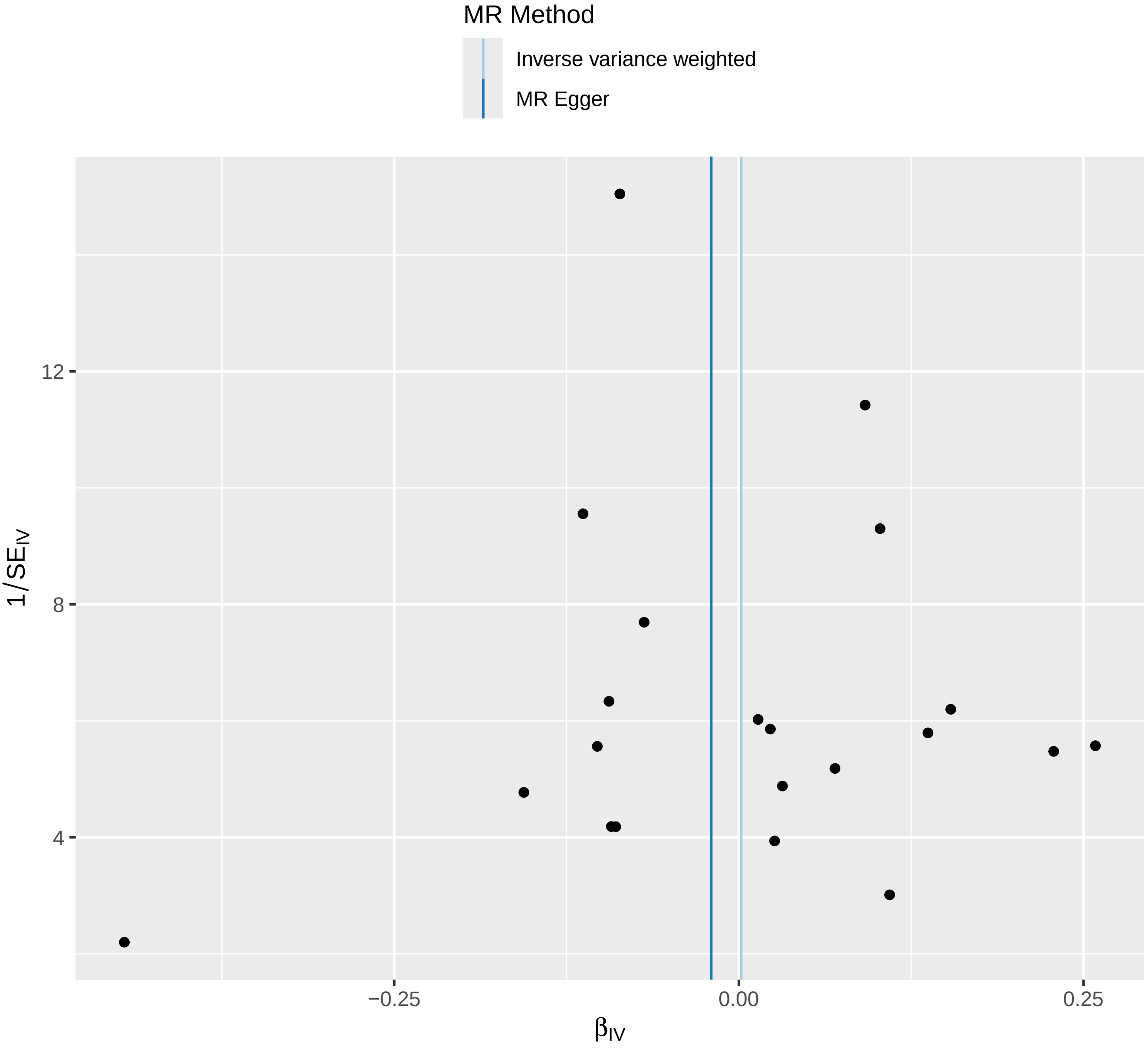
MR Method
Inverse variance weighted
MR Egger
1/SE_IV
12
8
4
−0.25
0.00
0.25
β_IV

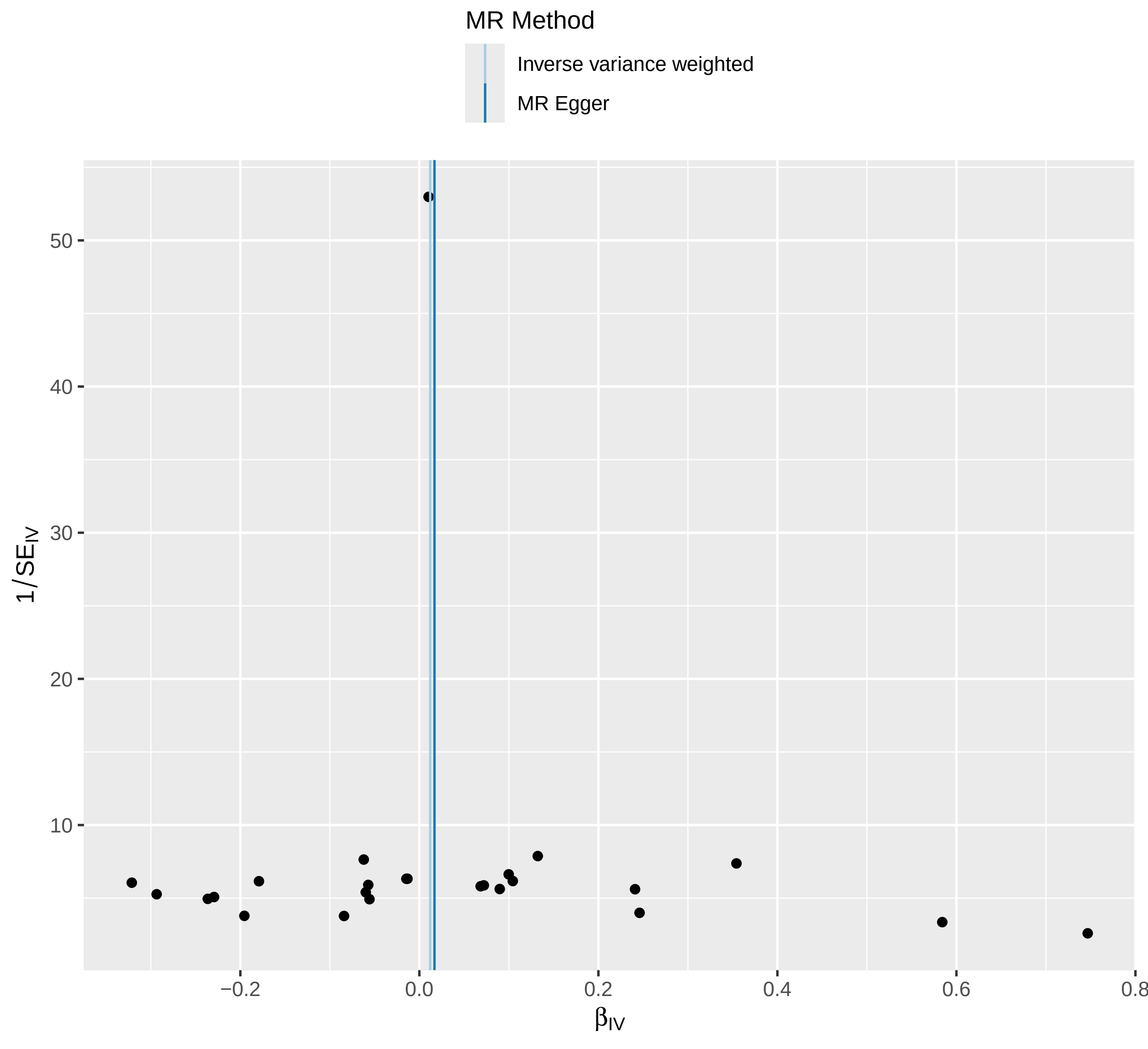

MR Method
Inverse variance weighted
MR Egger
1/SE_IV
β_IV
50
40
30
20
10
−0.2
0.0
0.2
0.4
0.6
0.8

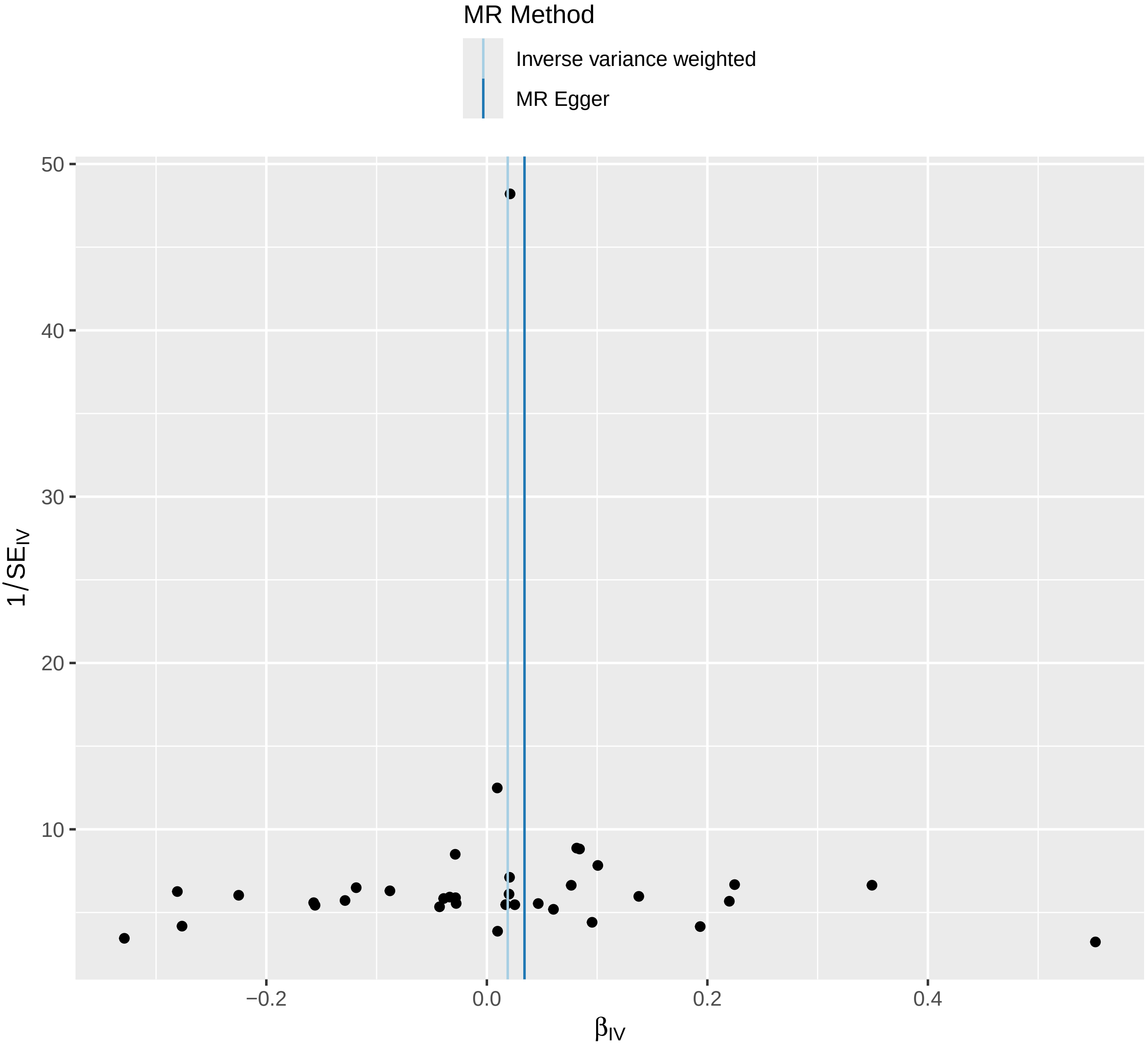
MR Method
Inverse variance weighted
MR Egger
50
40
30
20
10
1/SE_IV
−0.2
0.0
0.2
0.4
β_IV

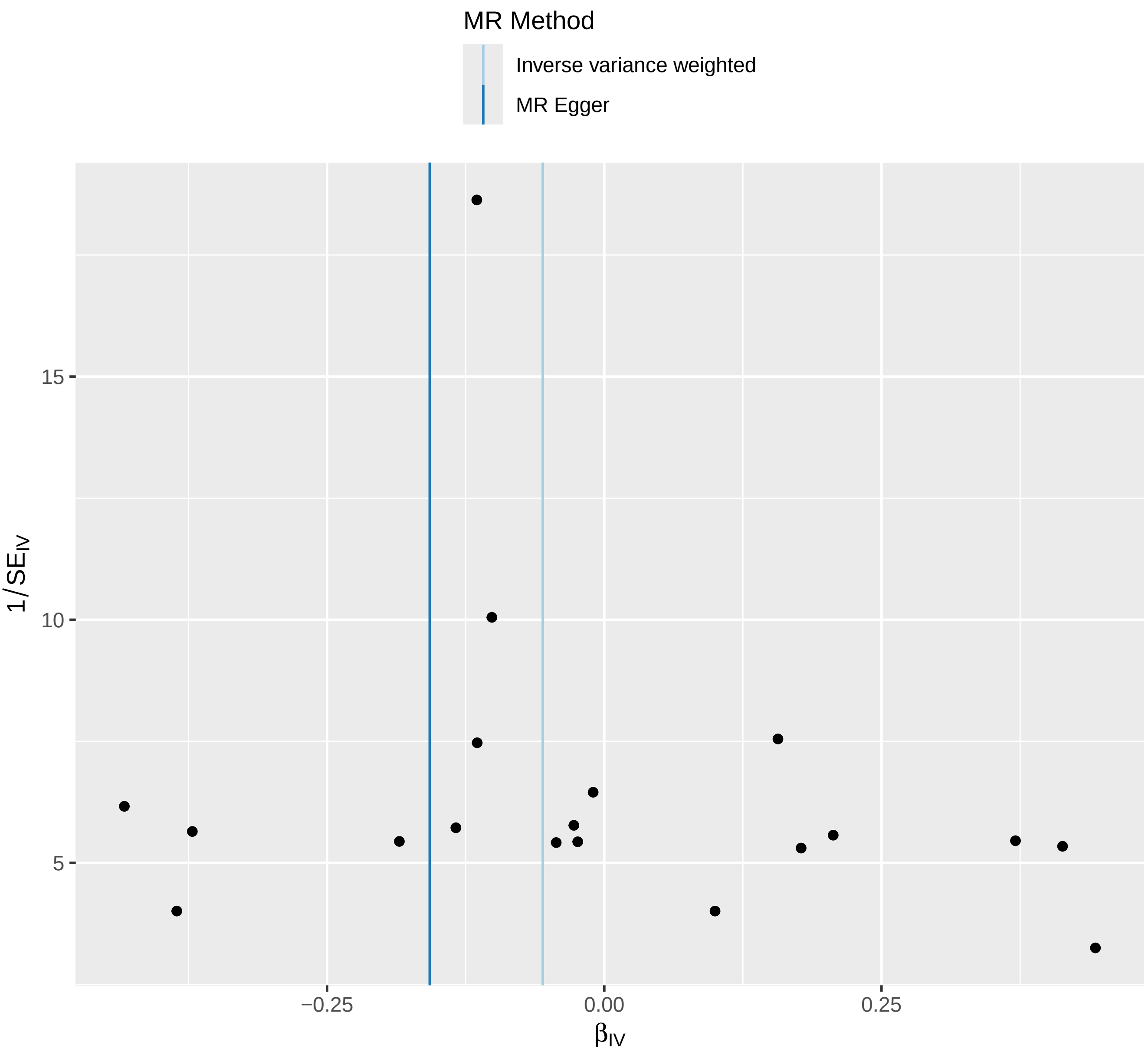

MR Method
Inverse variance weighted
MR Egger
1/SE_IV
15
10
5
−0.25
0.00
0.25
β_IV

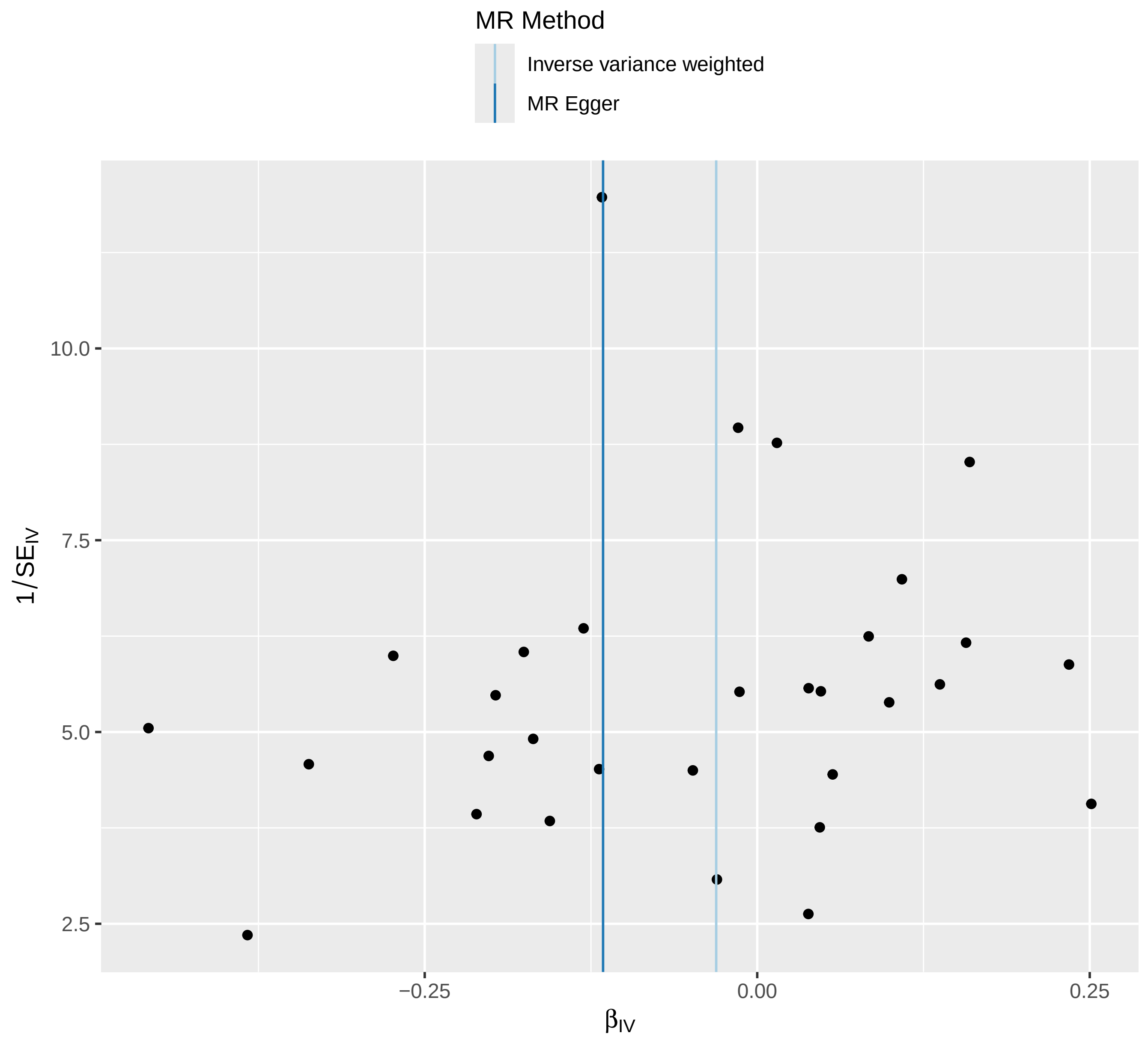
MR Method
Inverse variance weighted
MR Egger
10.0
7.5
5.0
2.5
1/SE_IV
−0.25
0.00
0.25
β_IV

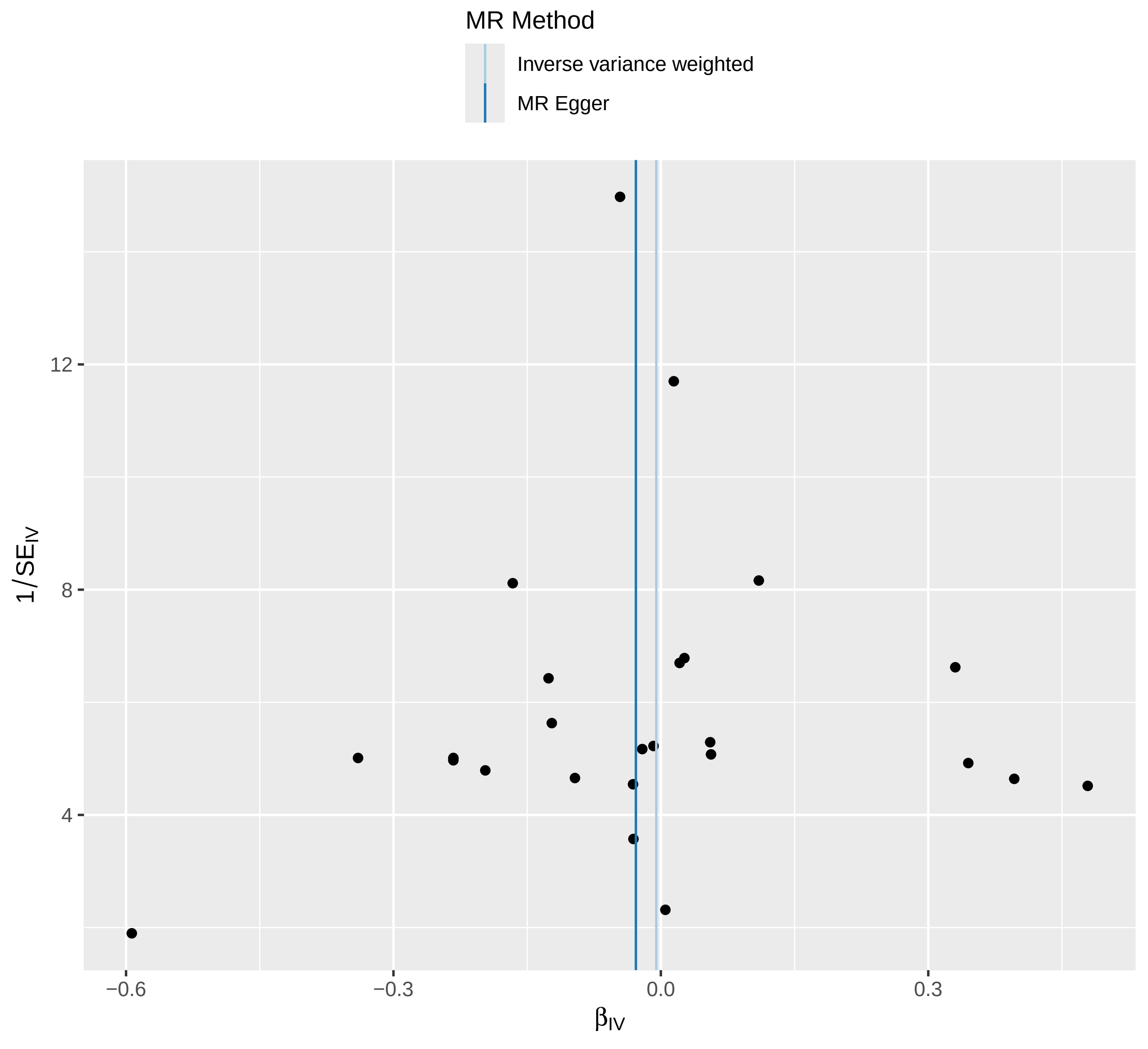

MR Method
Inverse variance weighted
MR Egger
12
8
4
$1/SE_{IV}$
−0.6
−0.3
0.0
0.3
β_{IV}

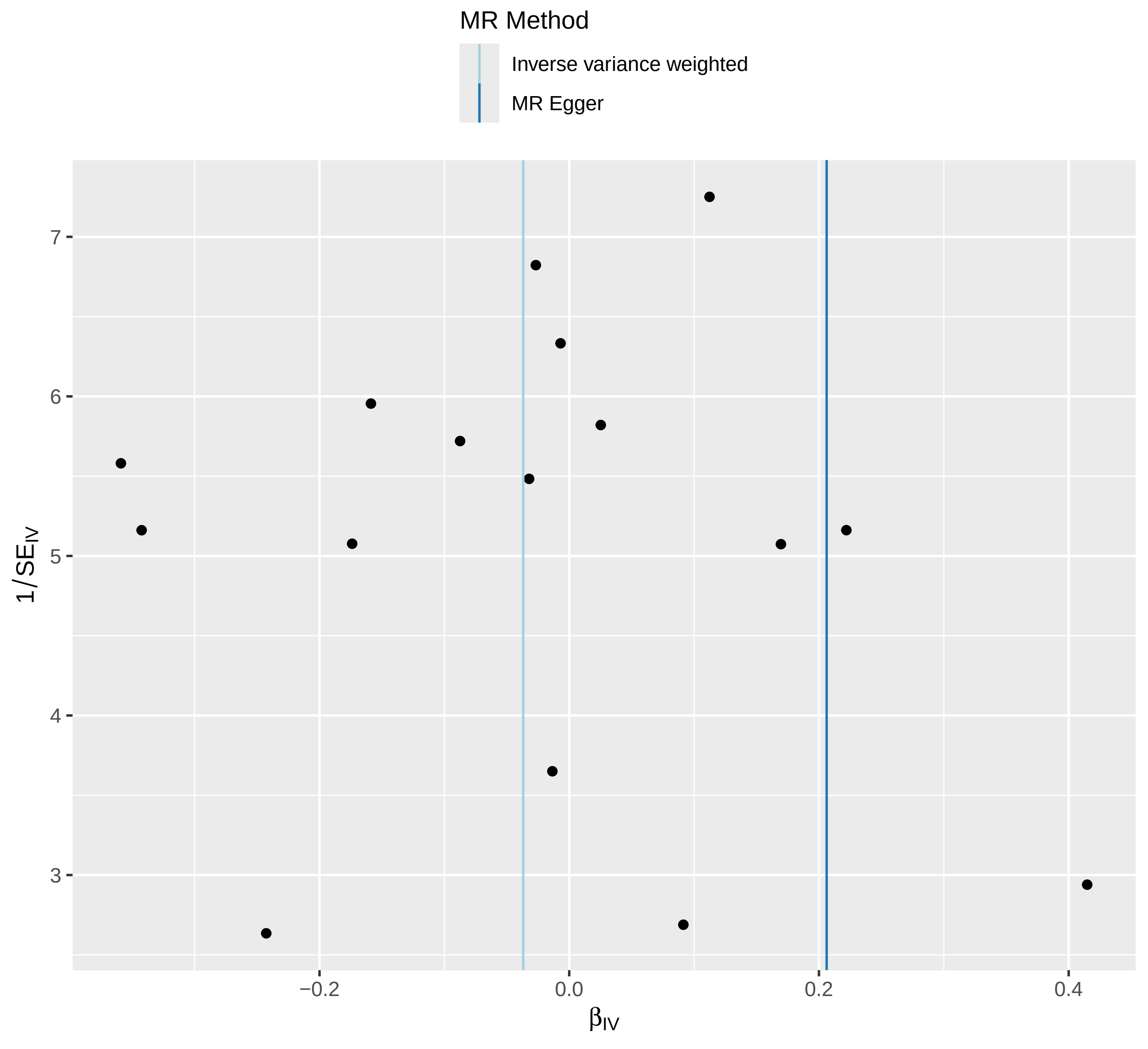

MR Method
Inverse variance weighted
MR Egger
7
6
5
4
3
1/SE_IV
−0.2
0.0
0.2
0.4
β_IV

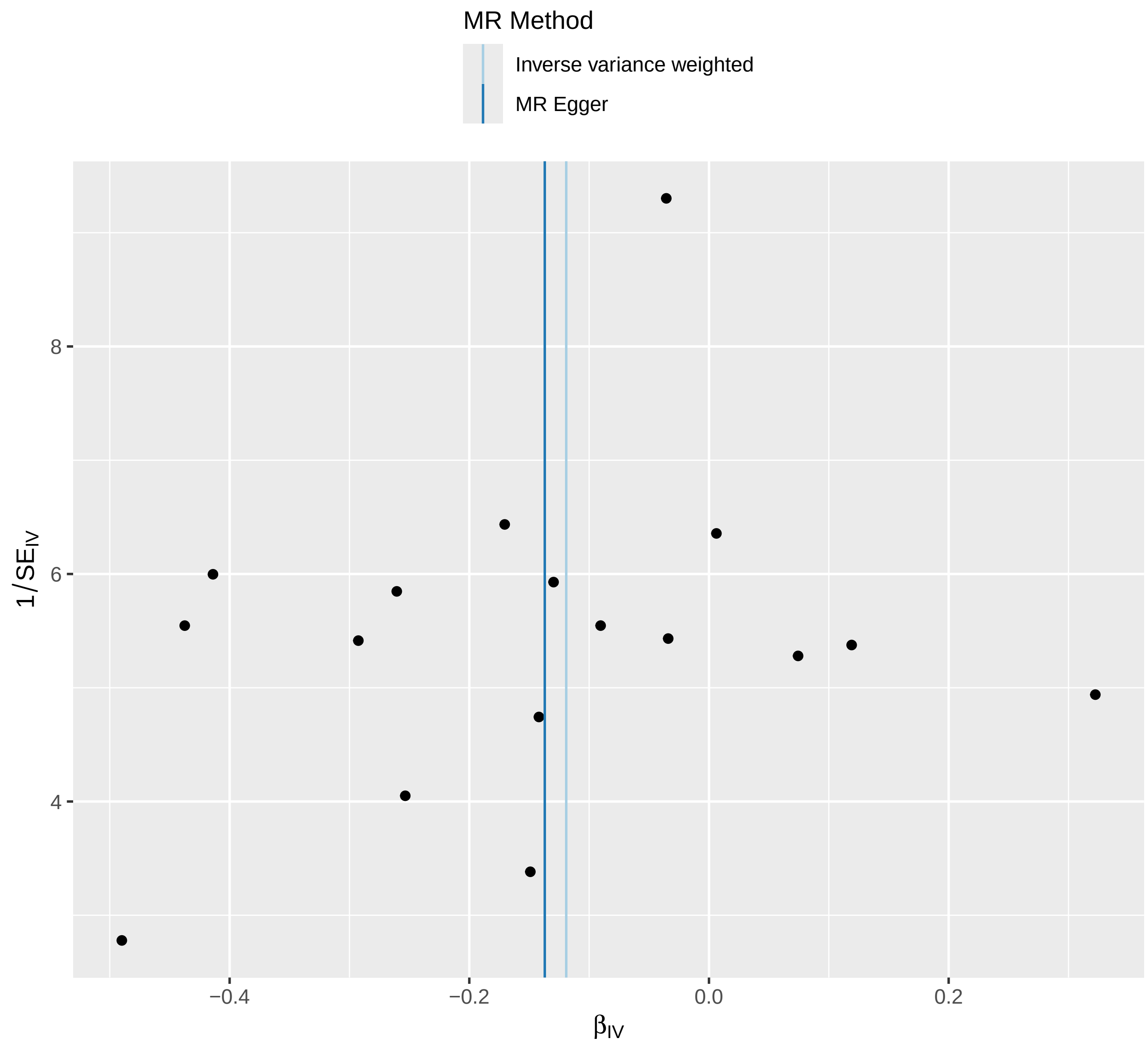
MR Method
Inverse variance weighted
MR Egger
1/SE_IV
8
6
4
β_IV
−0.4
−0.2
0.0
0.2

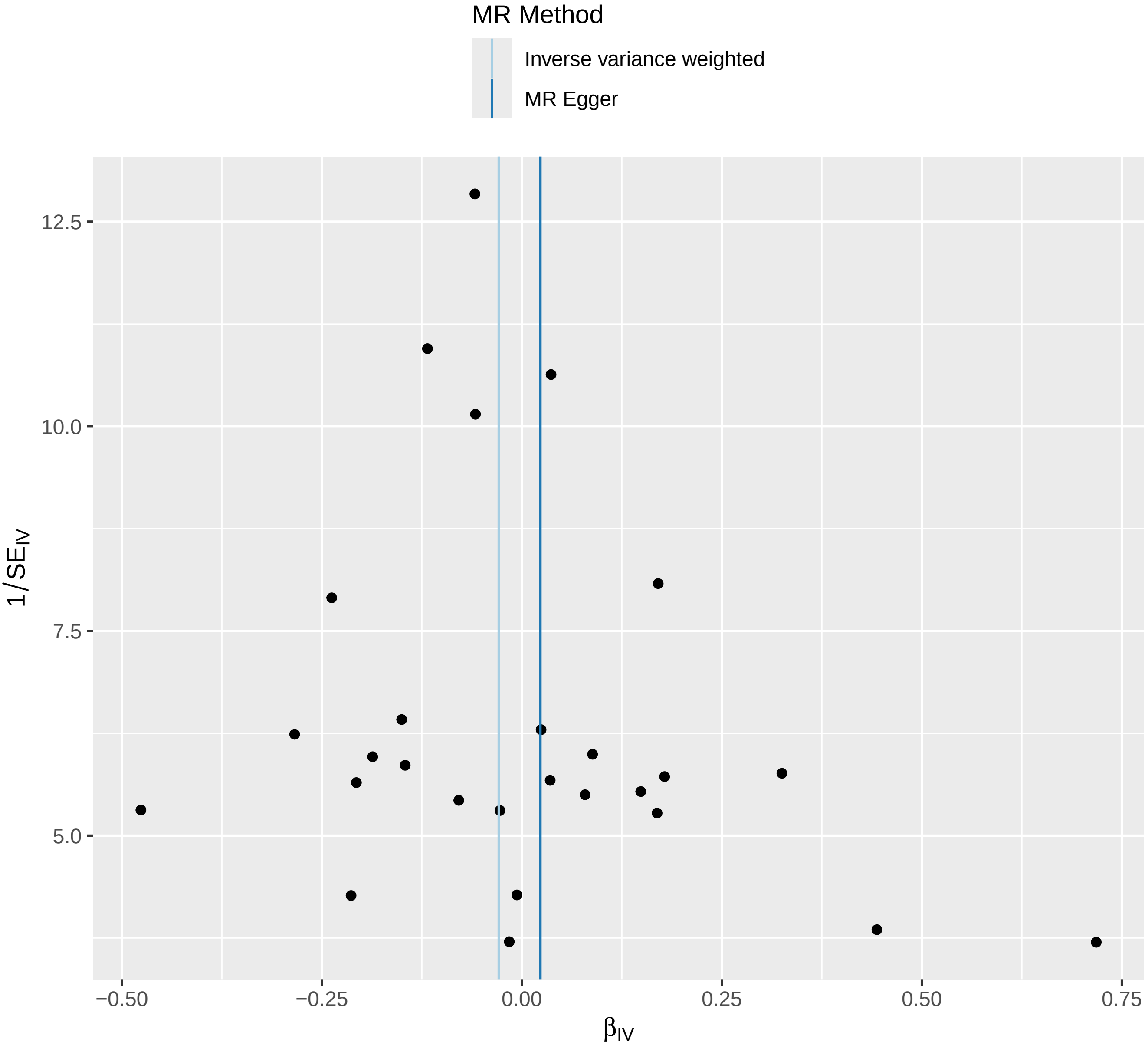

MR Method
Inverse variance weighted
MR Egger
12.5
10.0
7.5
5.0
1/SE_IV
−0.50
−0.25
0.00
0.25
0.50
0.75
β_IV

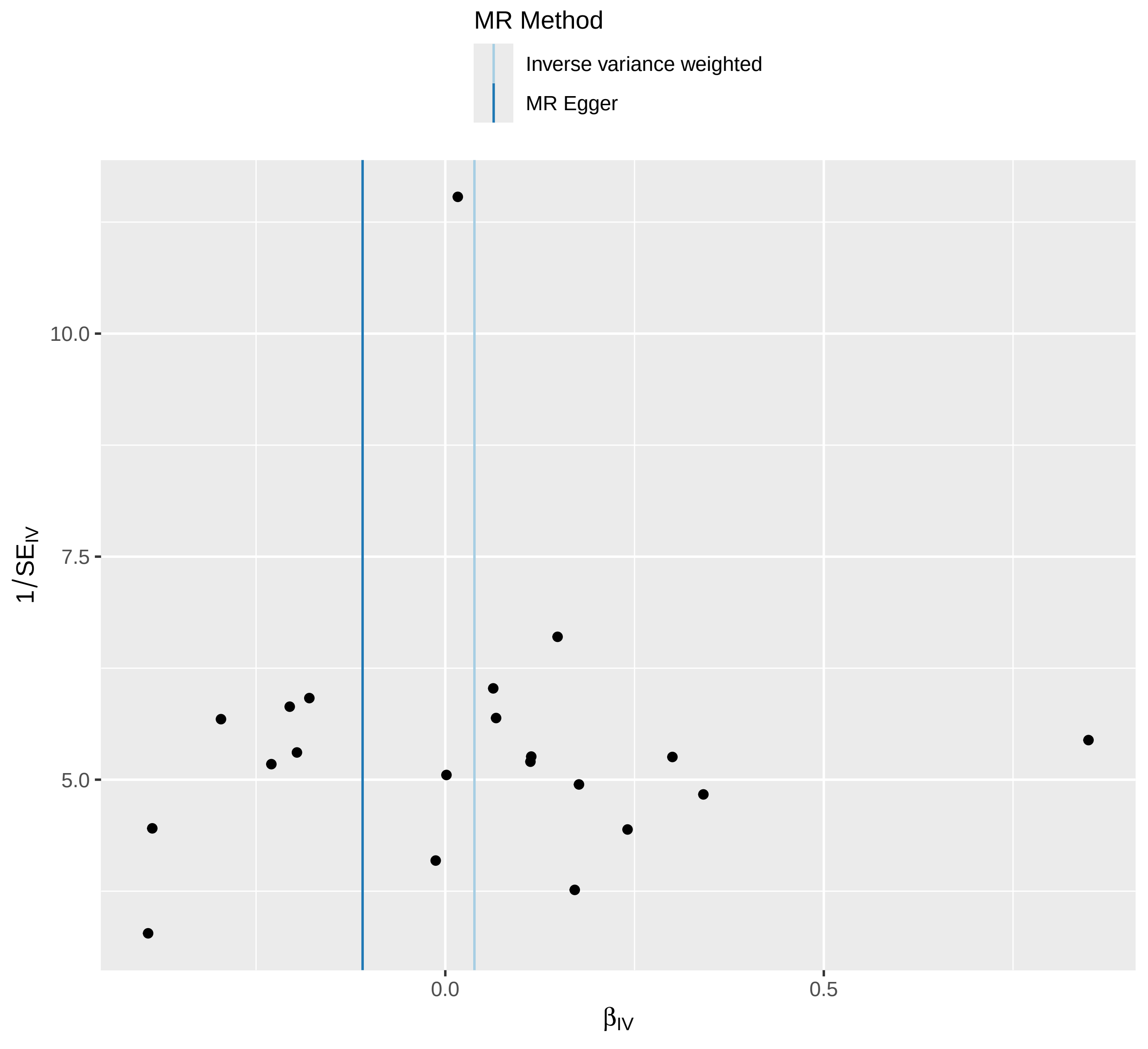
MR Method
Inverse variance weighted
MR Egger
10.0
7.5
5.0
1/SE_IV
0.0
0.5
β_IV

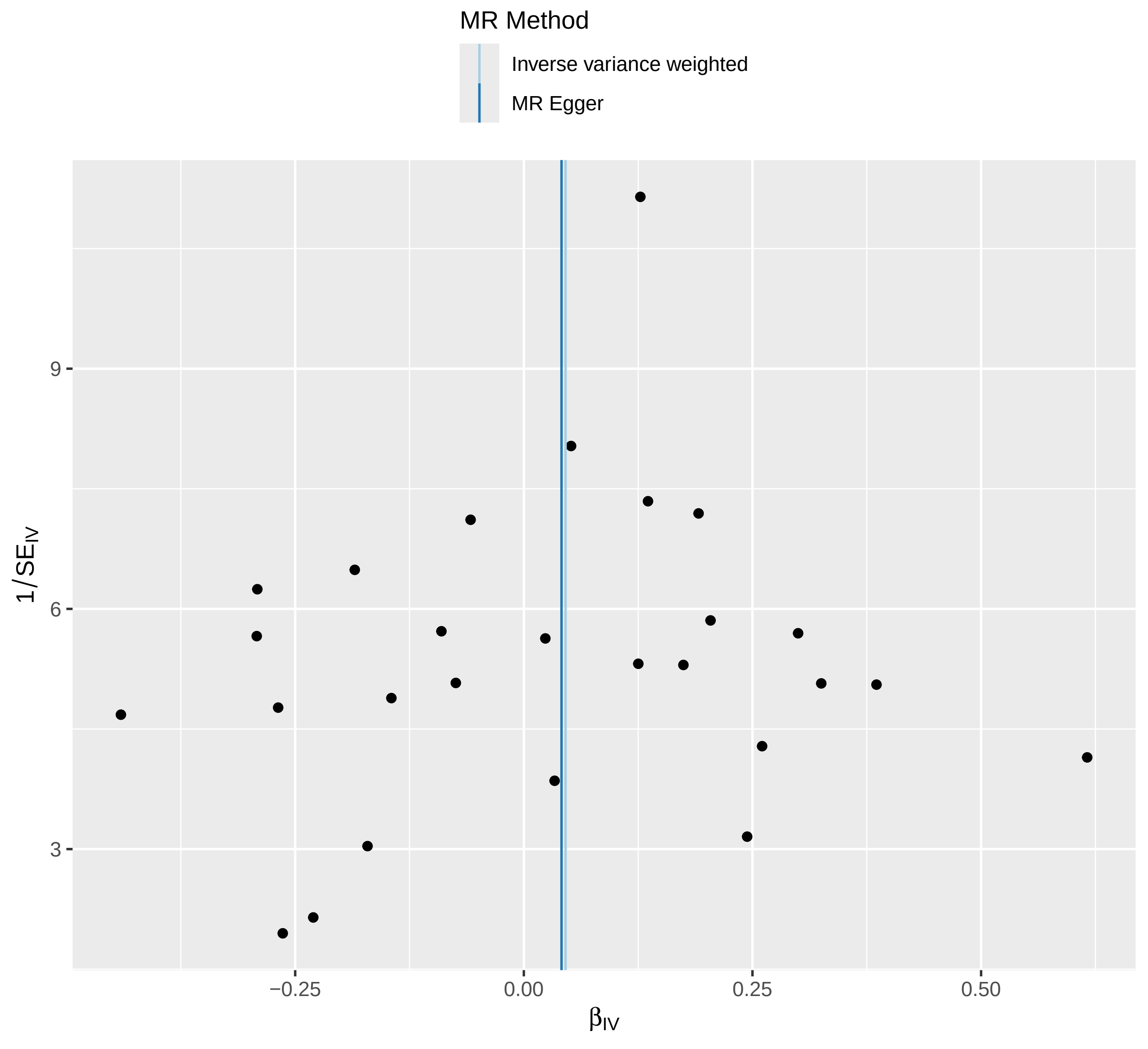
MR Method
Inverse variance weighted
MR Egger
1/SE_IV
9
6
3
−0.25
0.00
0.25
0.50
β_IV

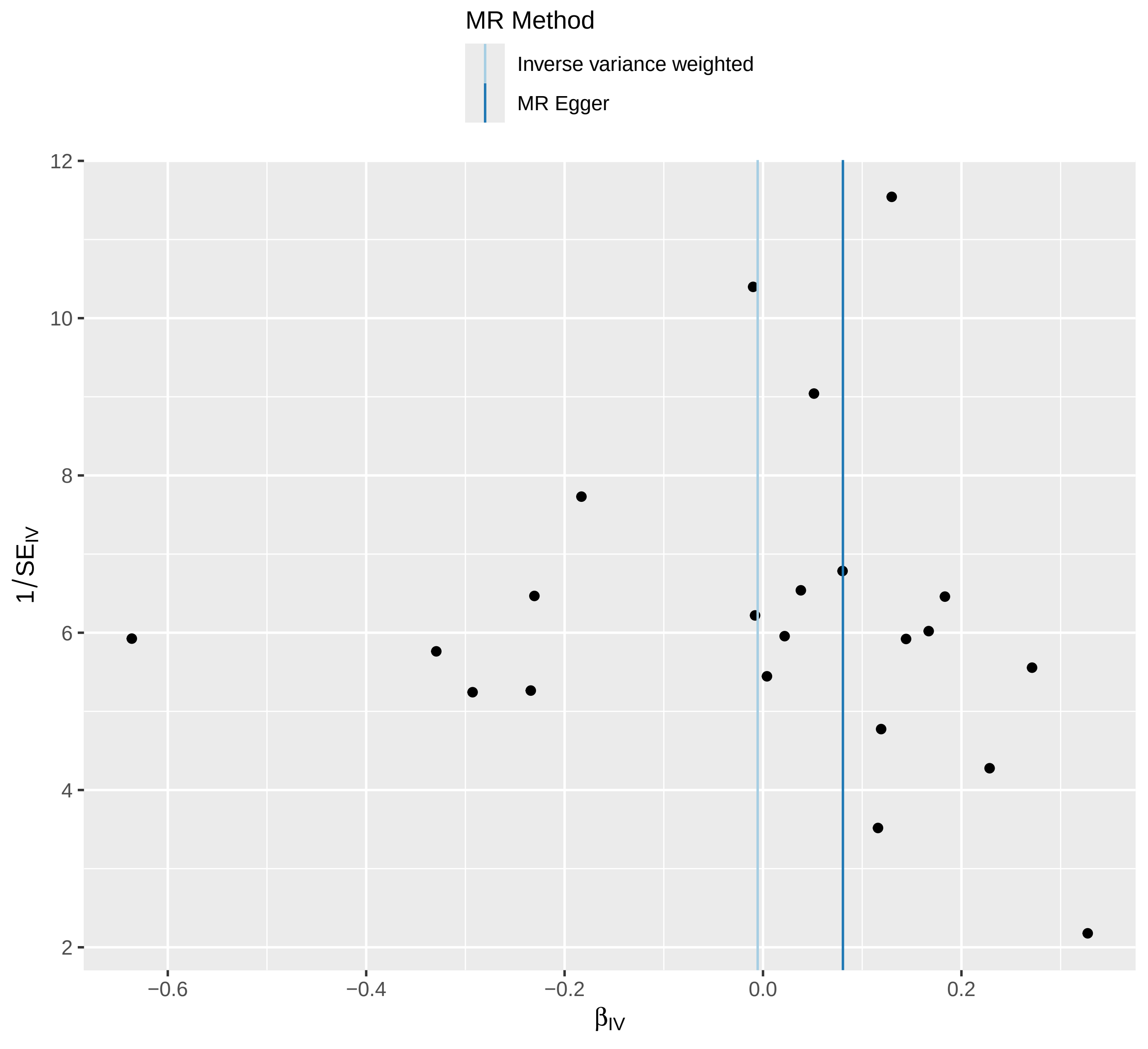
MR Method
Inverse variance weighted
MR Egger
12
10
8
6
4
2
1/SE_IV
−0.6
−0.4
−0.2
0.0
0.2
β_IV

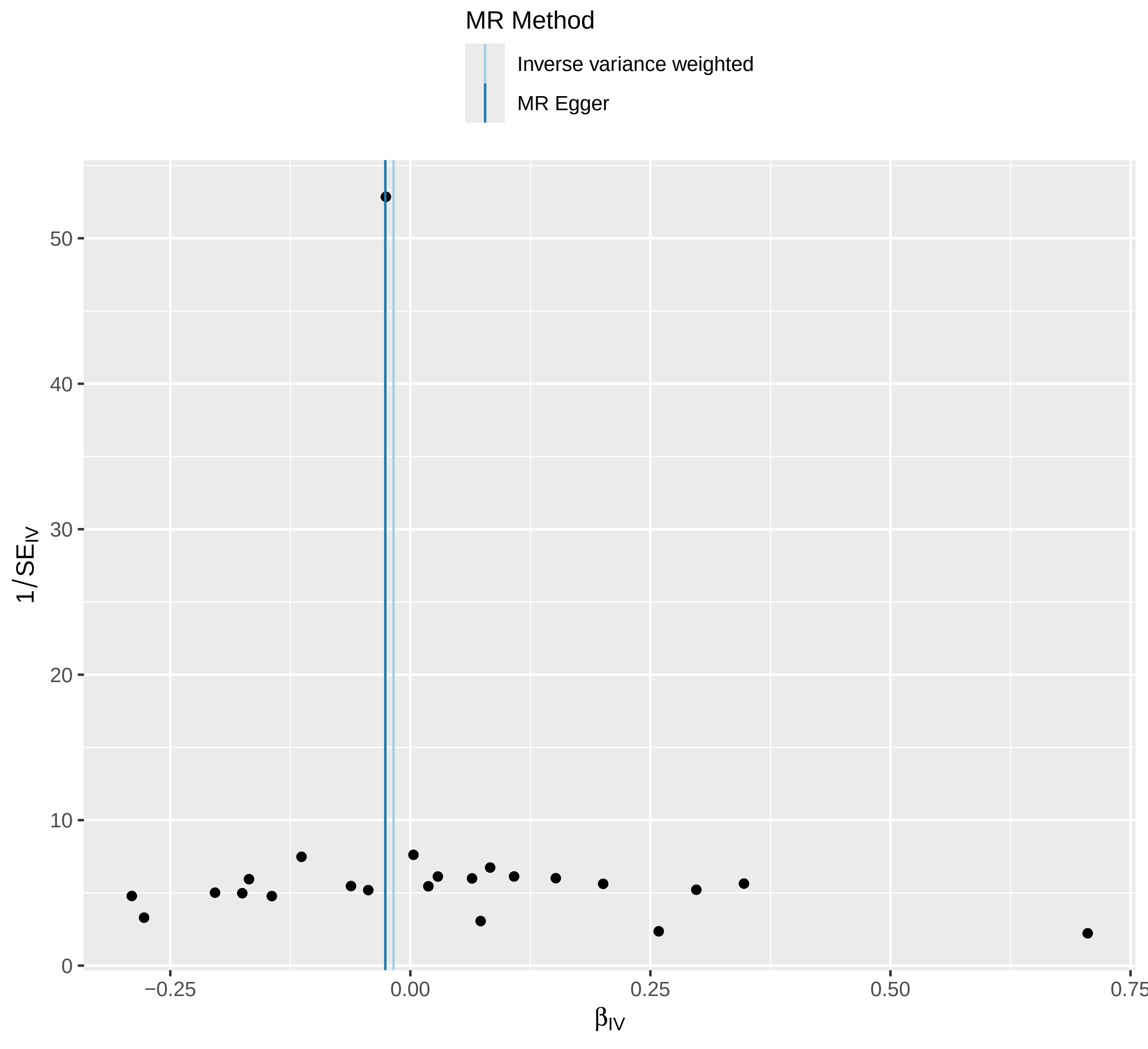

MR Method
Inverse variance weighted
MR Egger
1/SE_IV
β_IV
0
10
20
30
40
50
−0.25
0.00
0.25
0.50
0.75

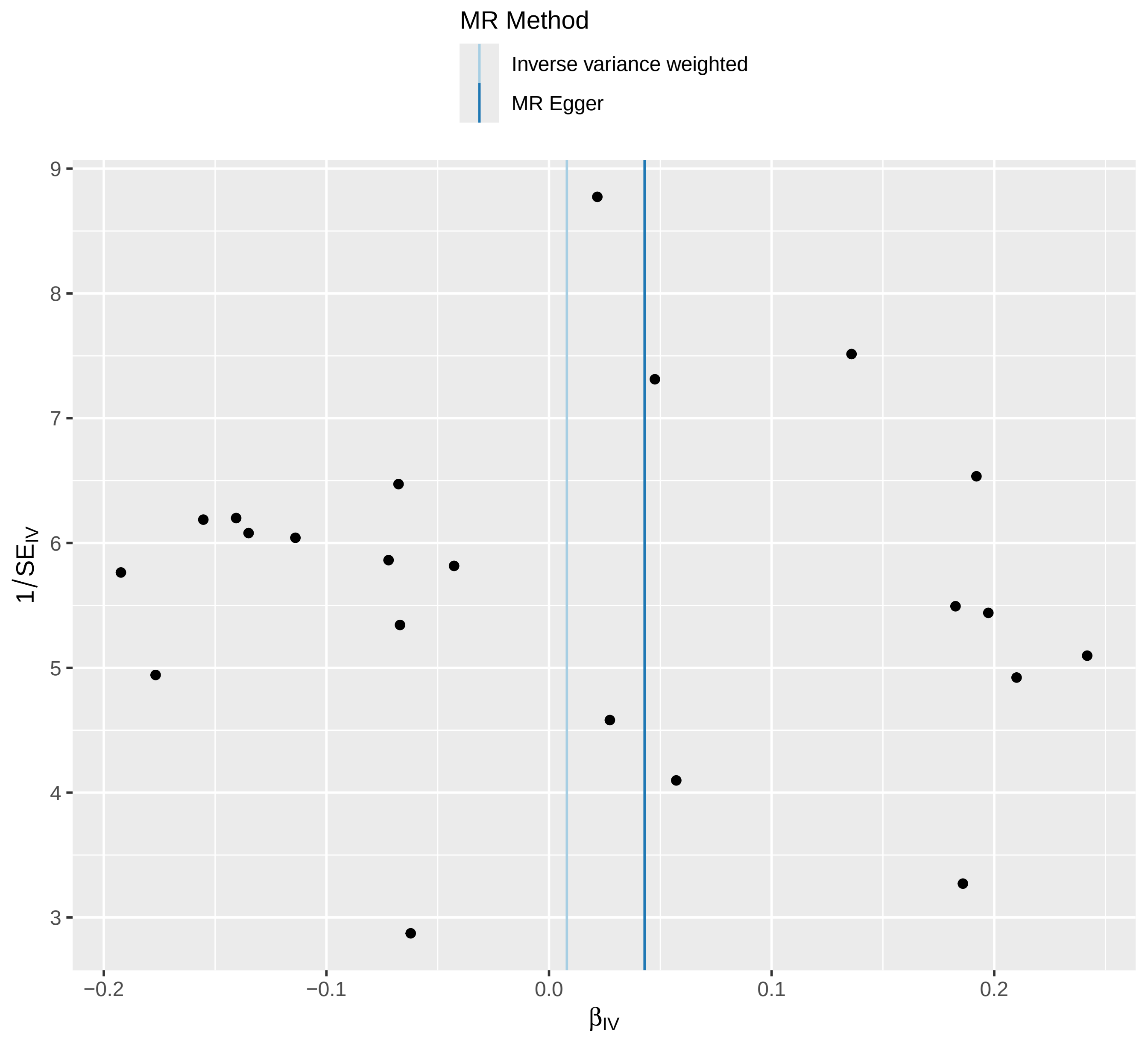

MR Method
Inverse variance weighted
MR Egger
1/SE_{IV}
β_{IV}
9
8
7
6
5
4
3
−0.2
−0.1
0.0
0.1
0.2

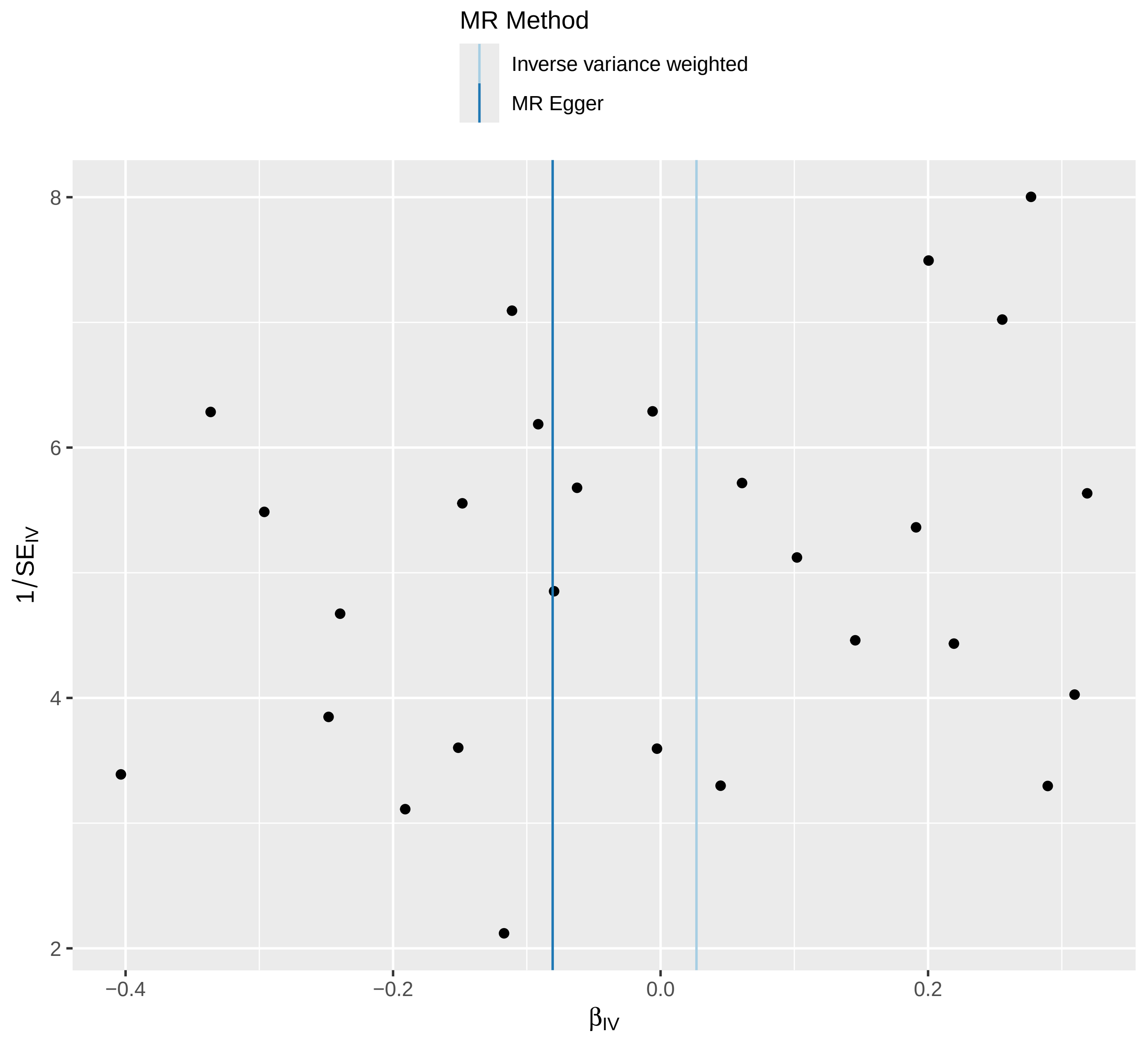
MR Method
Inverse variance weighted
MR Egger
8
6
4
2
1/SE_IV
−0.4
−0.2
0.0
0.2
β_IV

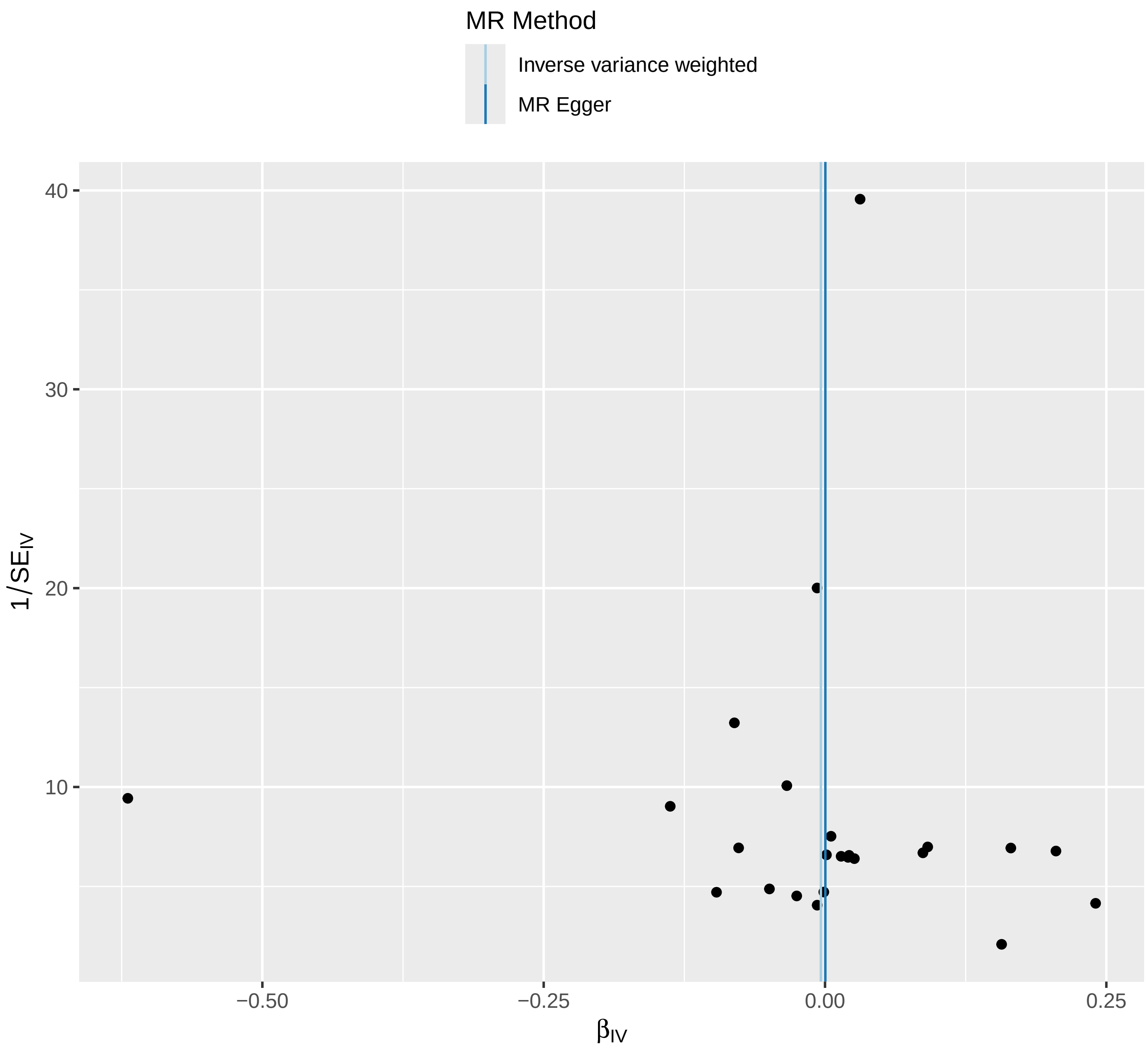
MR Method
Inverse variance weighted
MR Egger
40
30
20
10
1/SE_IV
−0.50
−0.25
0.00
0.25
β_IV

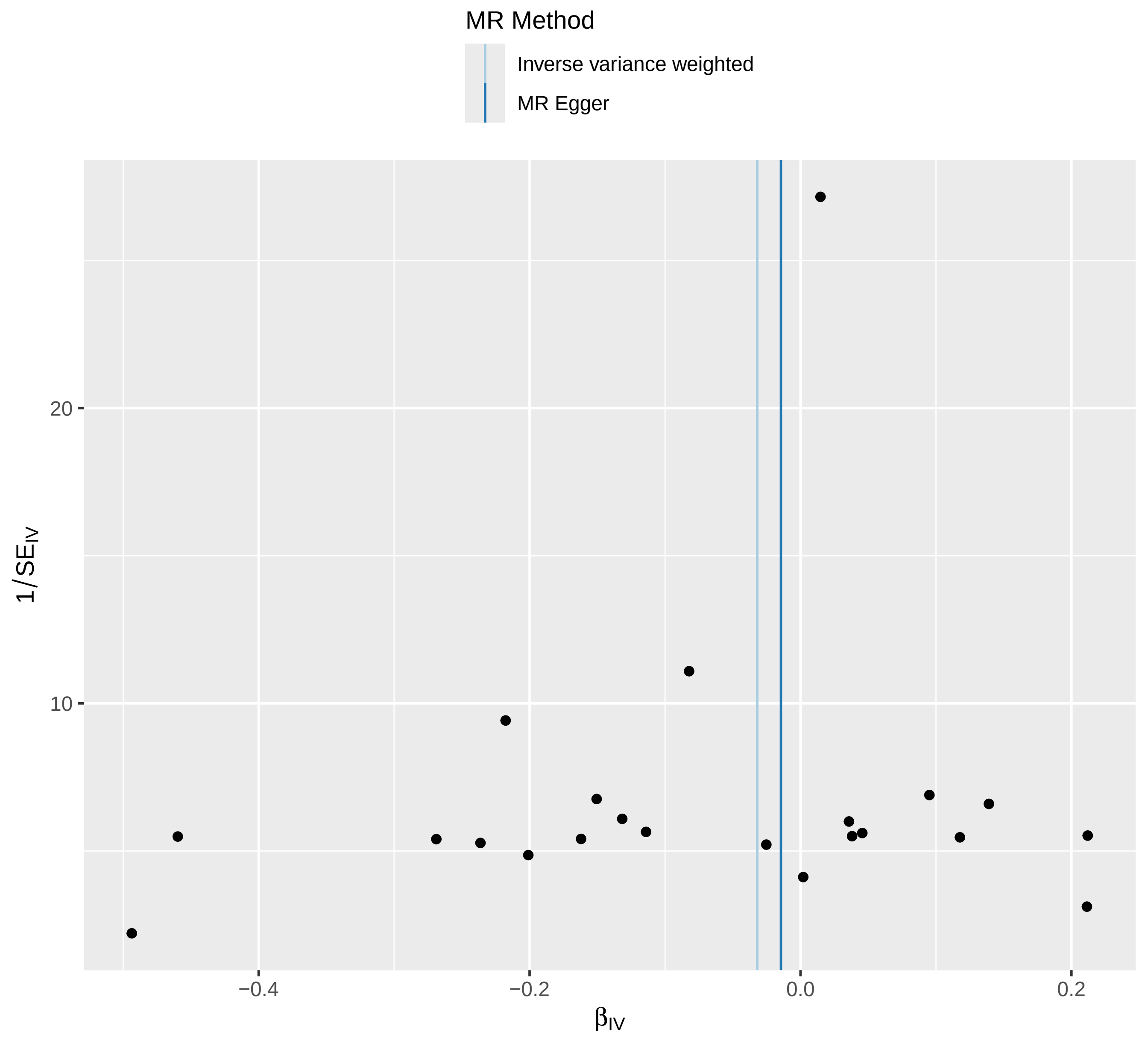

MR Method
Inverse variance weighted
MR Egger
20
10
1/SE_IV
−0.4
−0.2
0.0
0.2
β_IV

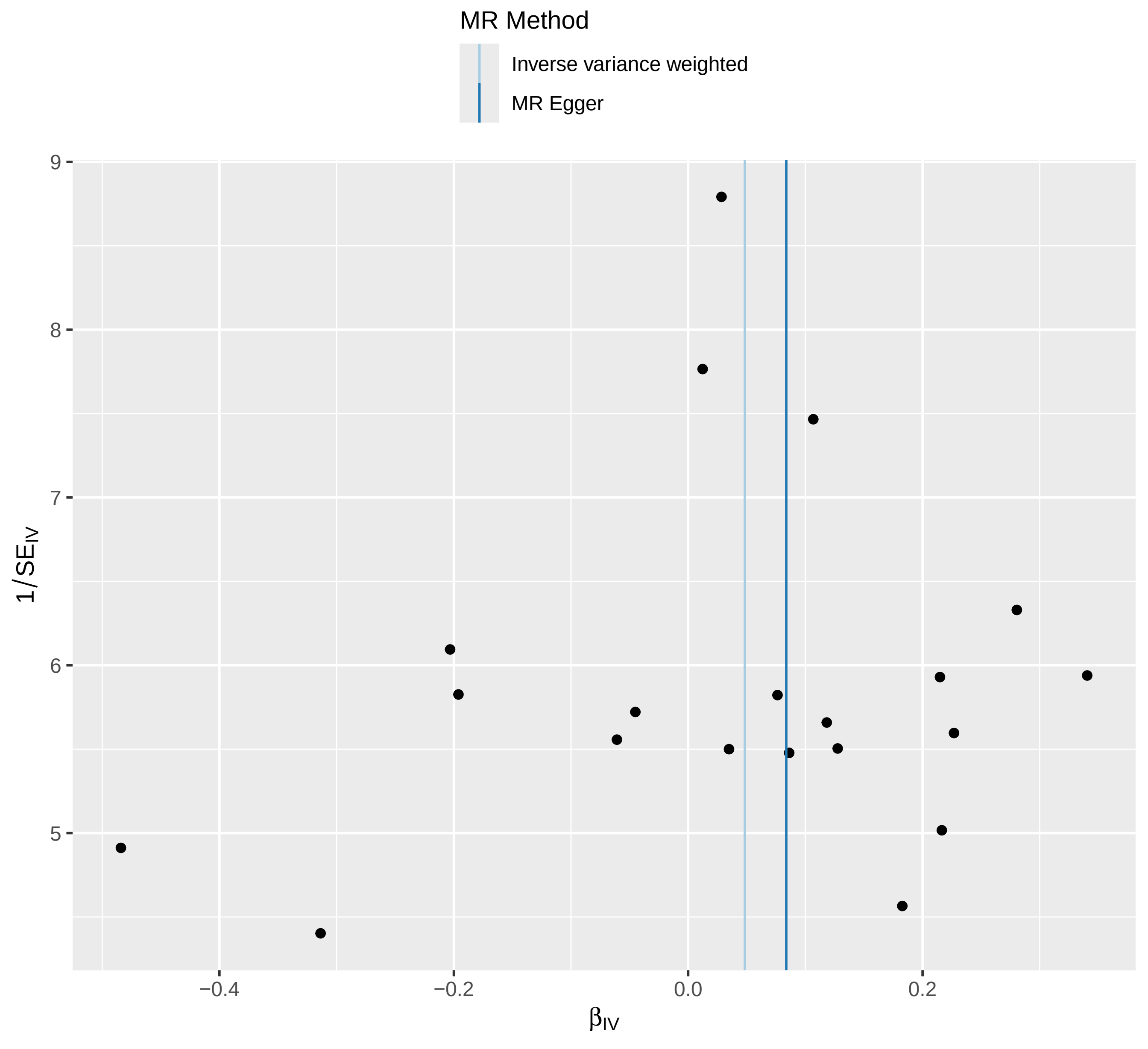
MR Method
Inverse variance weighted
MR Egger
9
8
7
6
5
1/SE_IV
−0.4
−0.2
0.0
0.2
β_IV

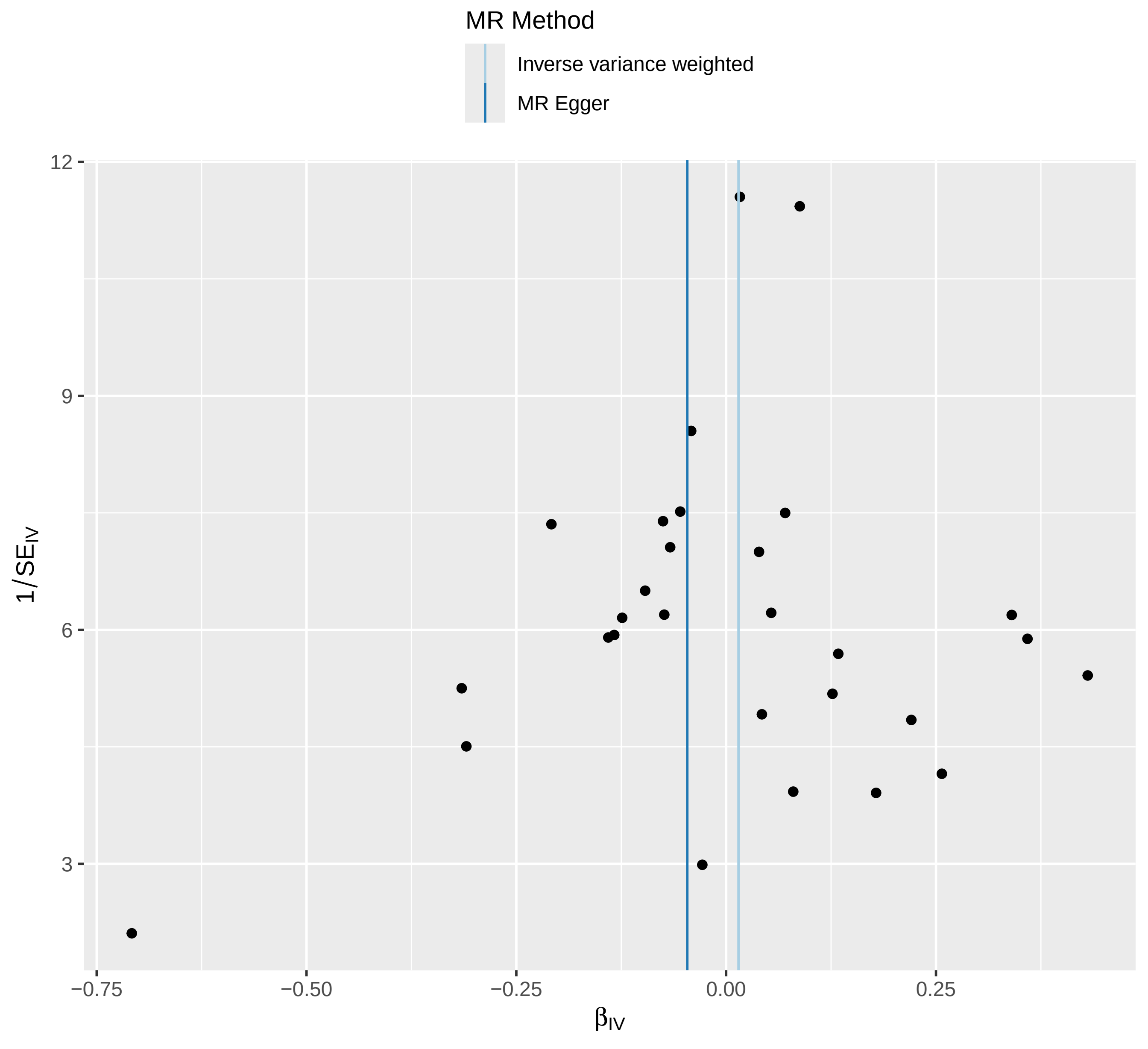
MR Method
Inverse variance weighted
MR Egger
12
9
6
3
1/SE_IV
−0.75
−0.50
−0.25
0.00
0.25
β_IV

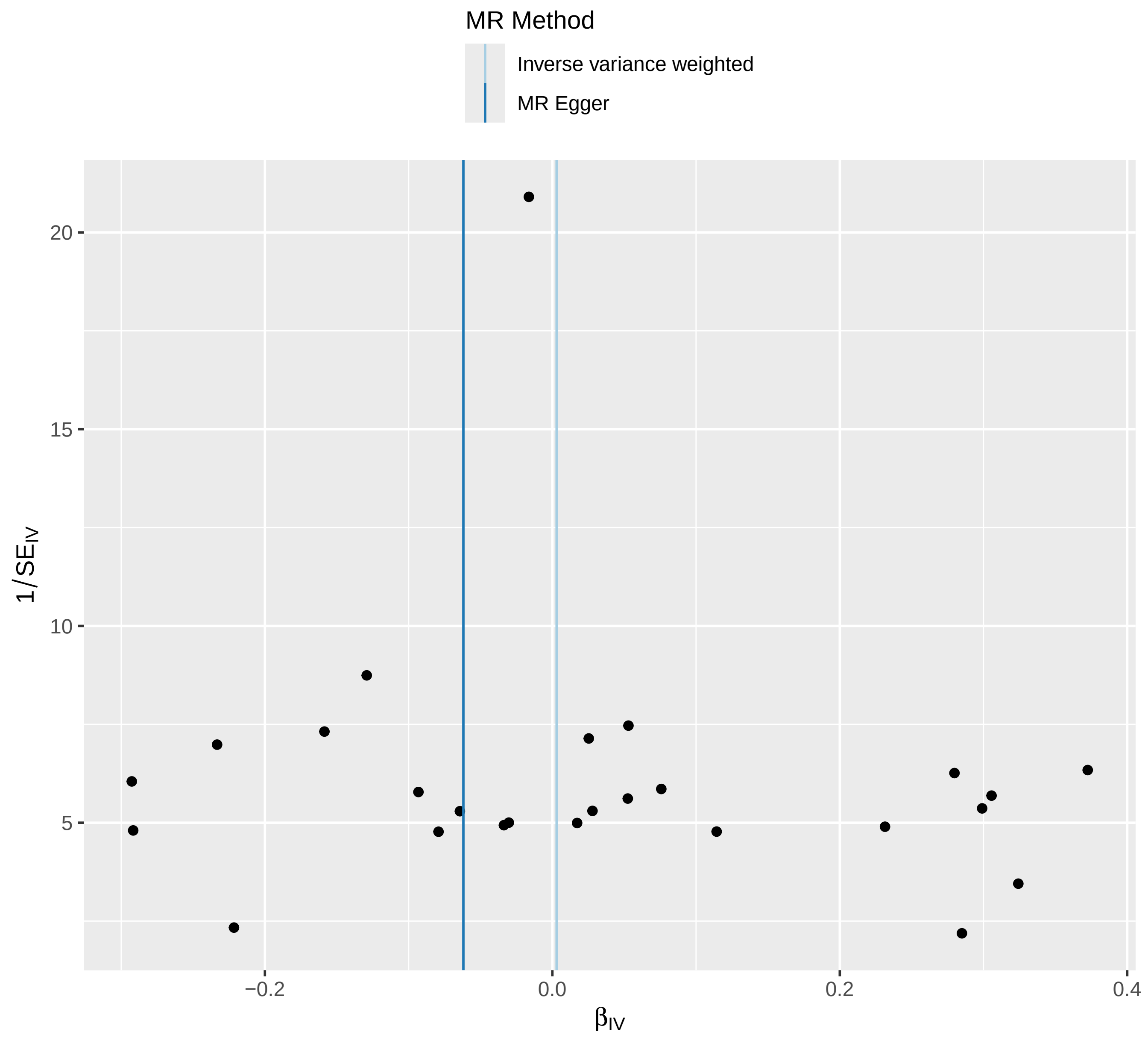
MR Method
Inverse variance weighted
MR Egger
1/SE_IV
20
15
10
5
−0.2
0.0
0.2
0.4
β_IV

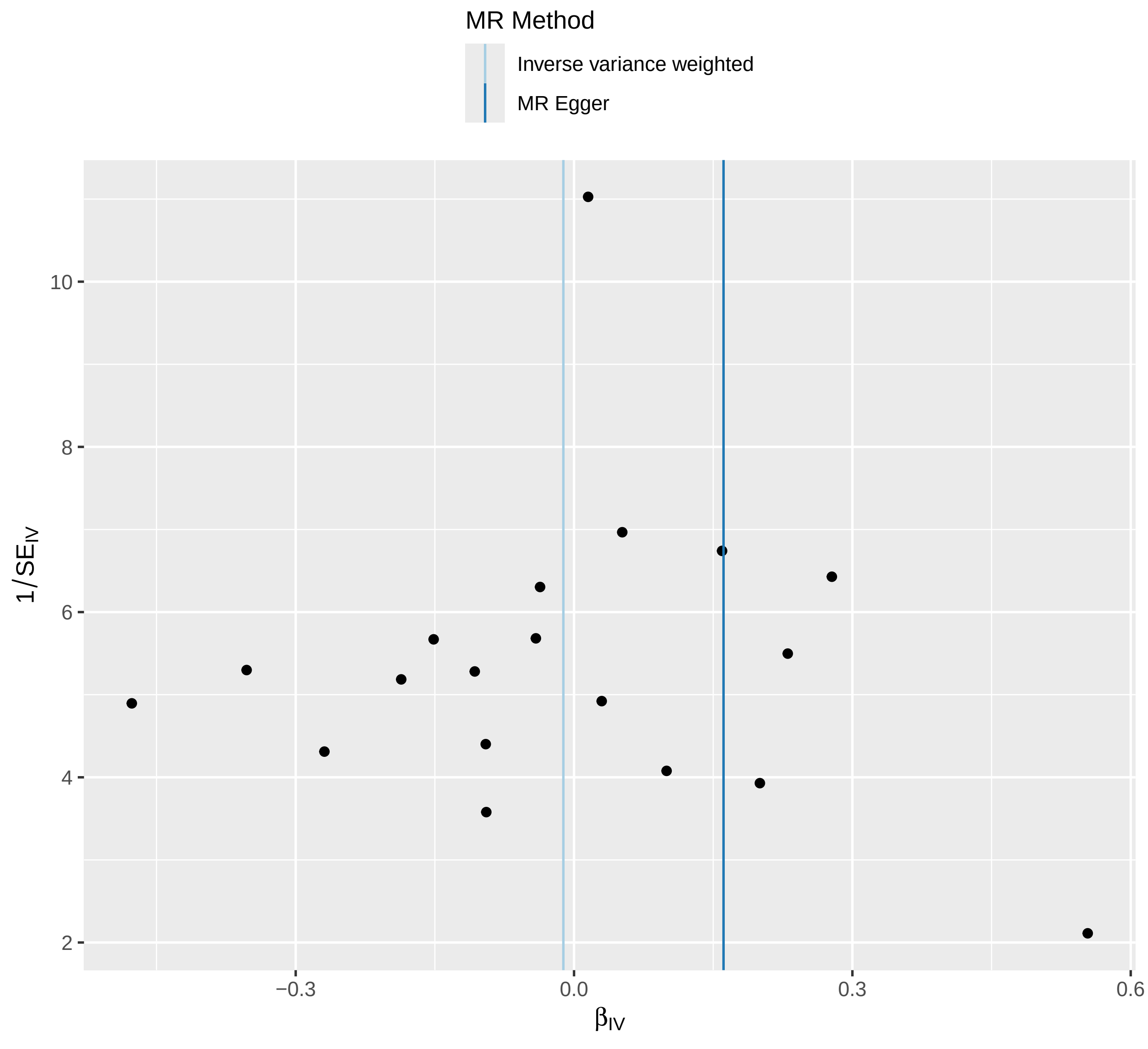

MR Method
Inverse variance weighted
MR Egger
1/SE$_{IV}$
10
8
6
4
2
β_{IV}
−0.3
0.0
0.3
0.6

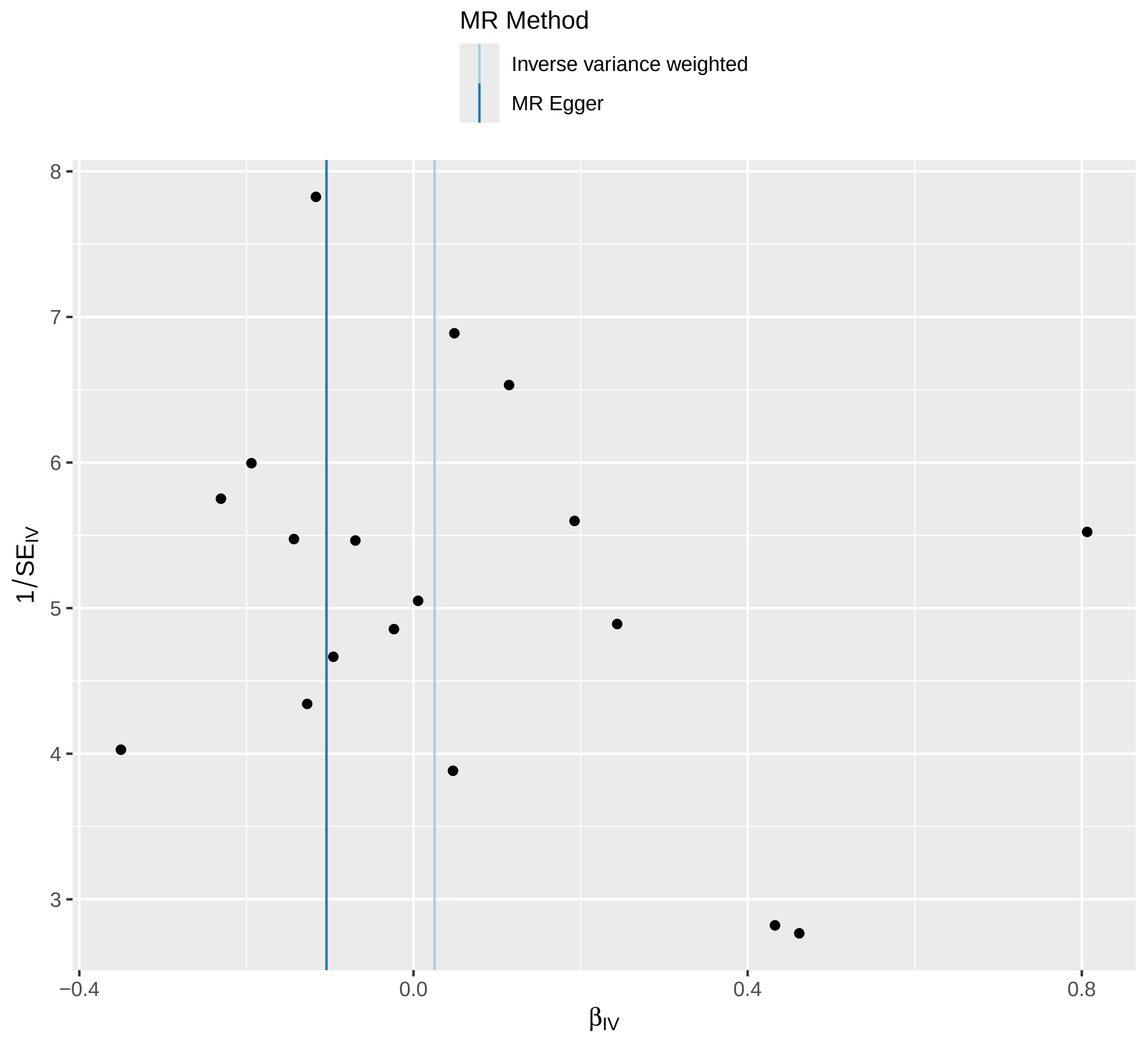

MR Method
Inverse variance weighted
MR Egger
8
7
6
5
4
3
1/SE_IV
−0.4
0.0
0.4
0.8
β_IV

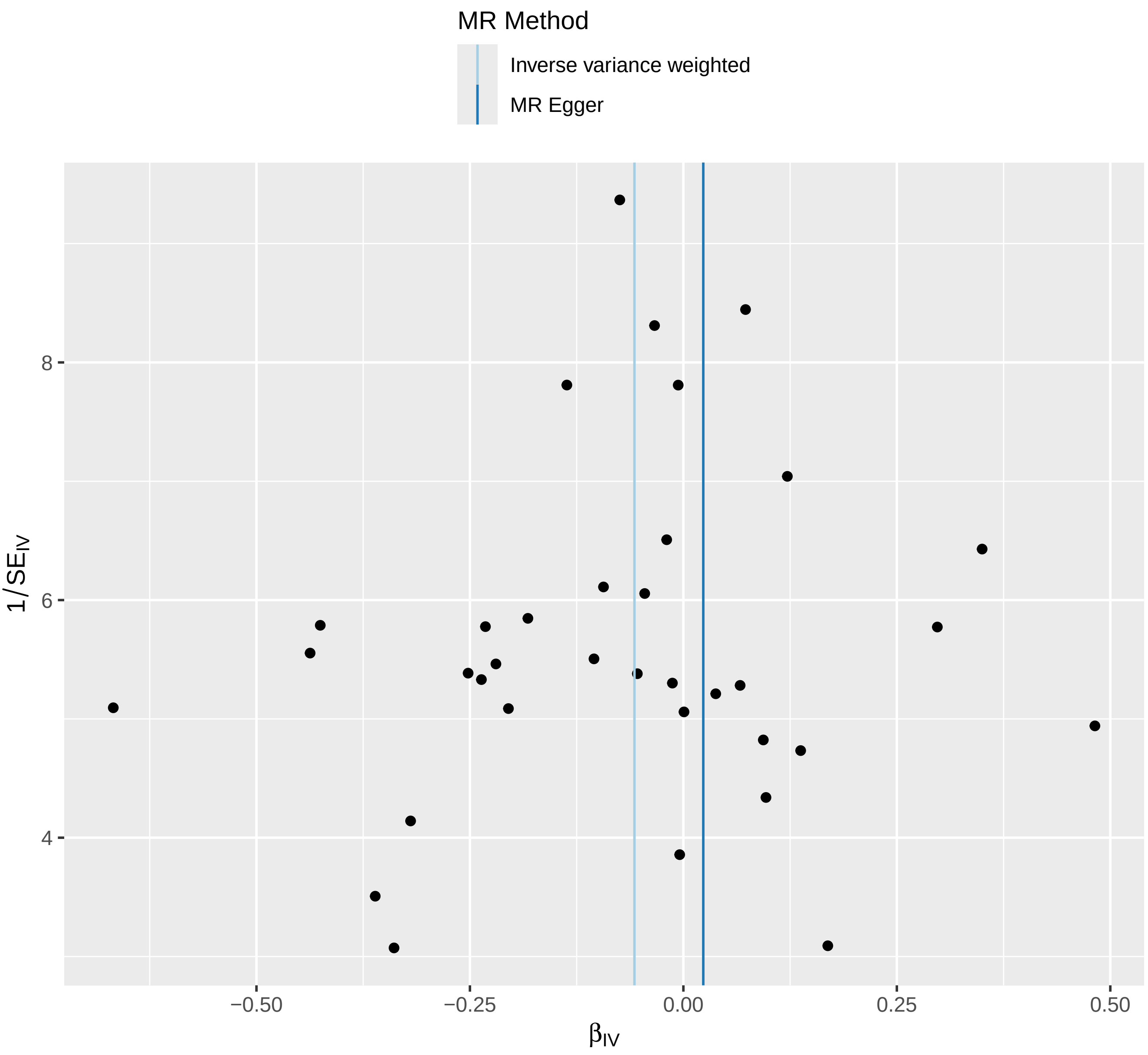

MR Method
Inverse variance weighted
MR Egger
8
6
4
1/SE_IV
−0.50
−0.25
0.00
0.25
0.50
β_IV

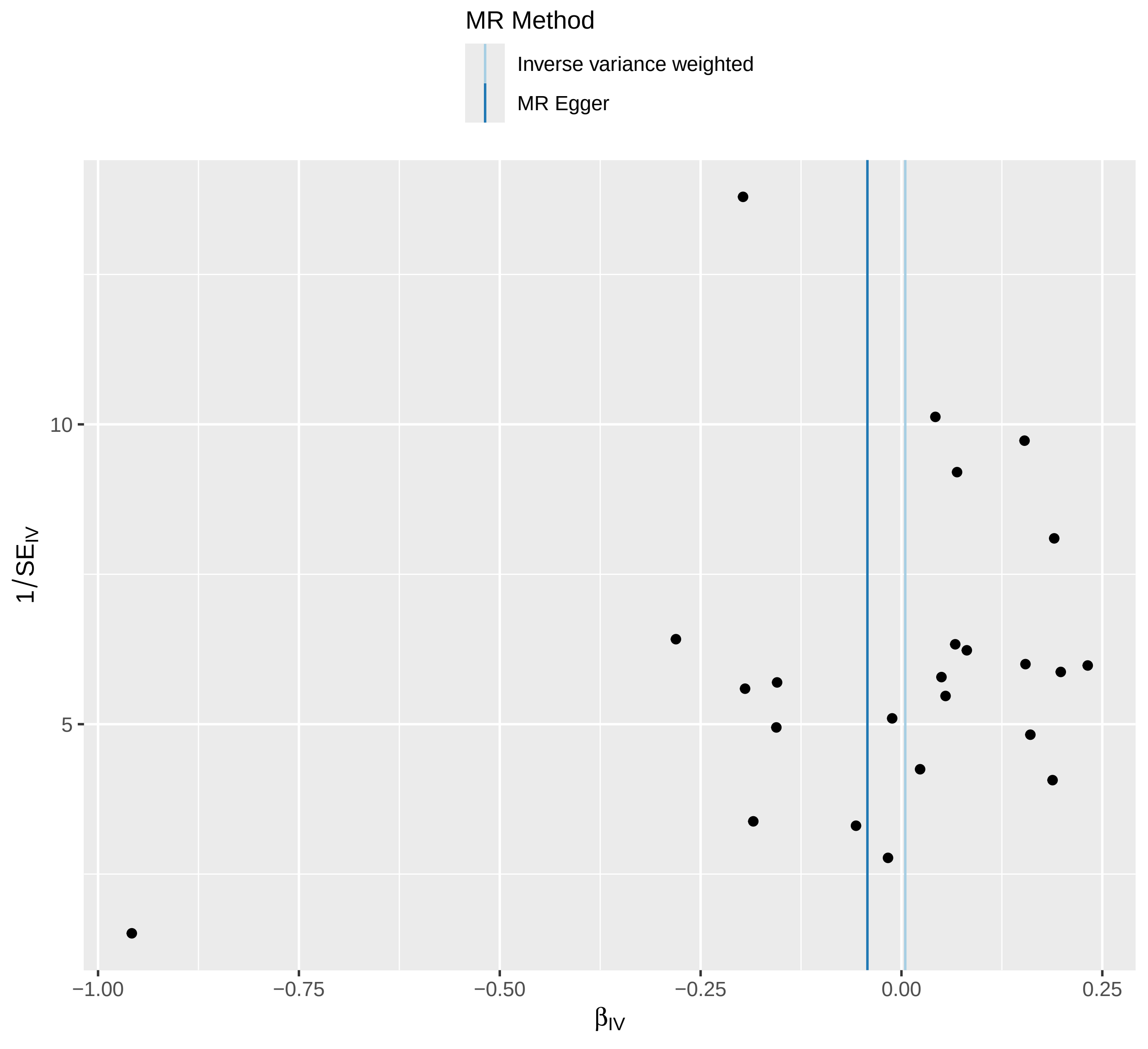

MR Method
Inverse variance weighted
MR Egger
1/SE_IV
10
5
−1.00
−0.75
−0.50
−0.25
0.00
0.25
β_IV

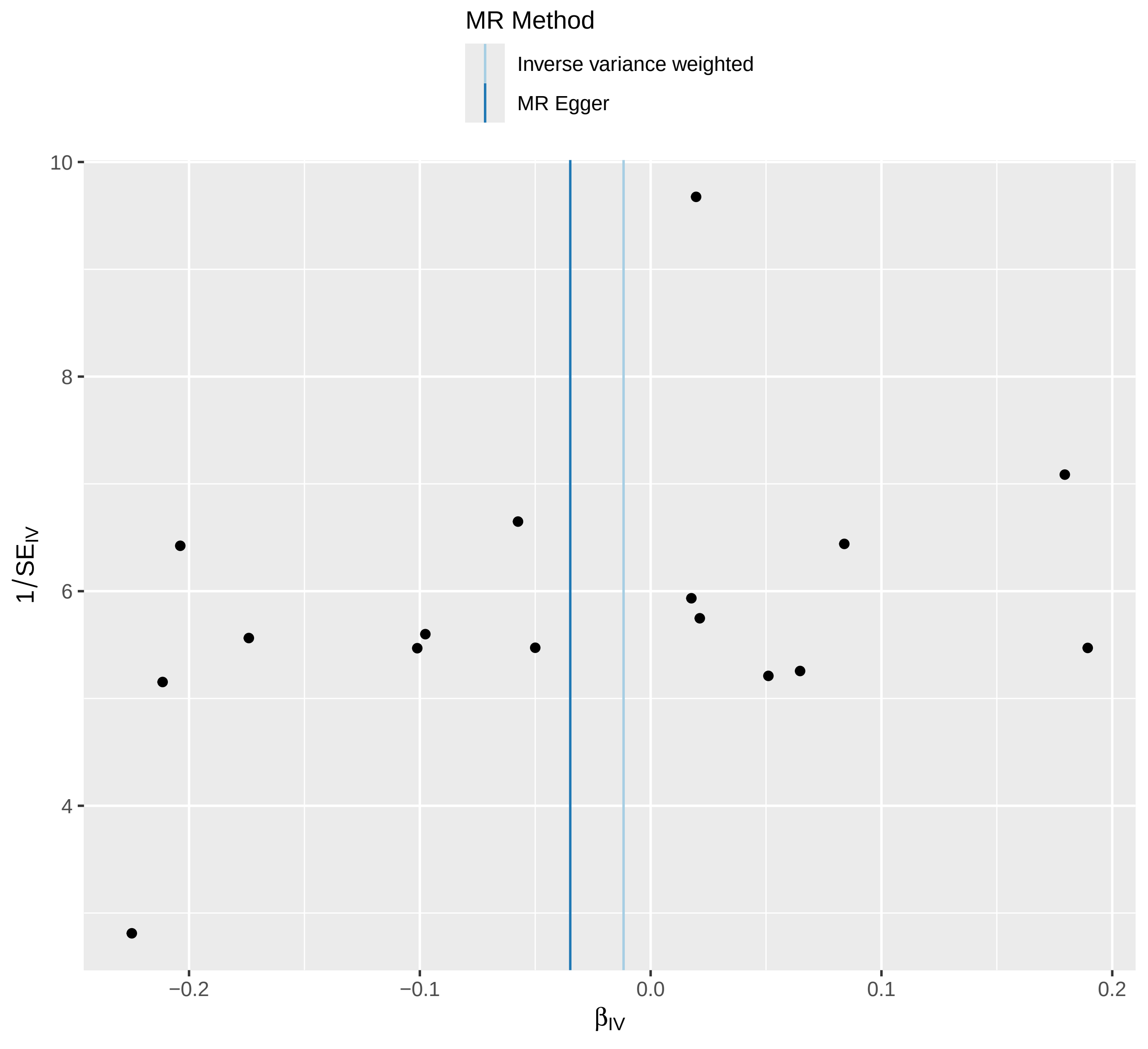
MR Method
Inverse variance weighted
MR Egger
10
8
6
4
1/SE_IV
−0.2
−0.1
0.0
0.1
0.2
β_IV

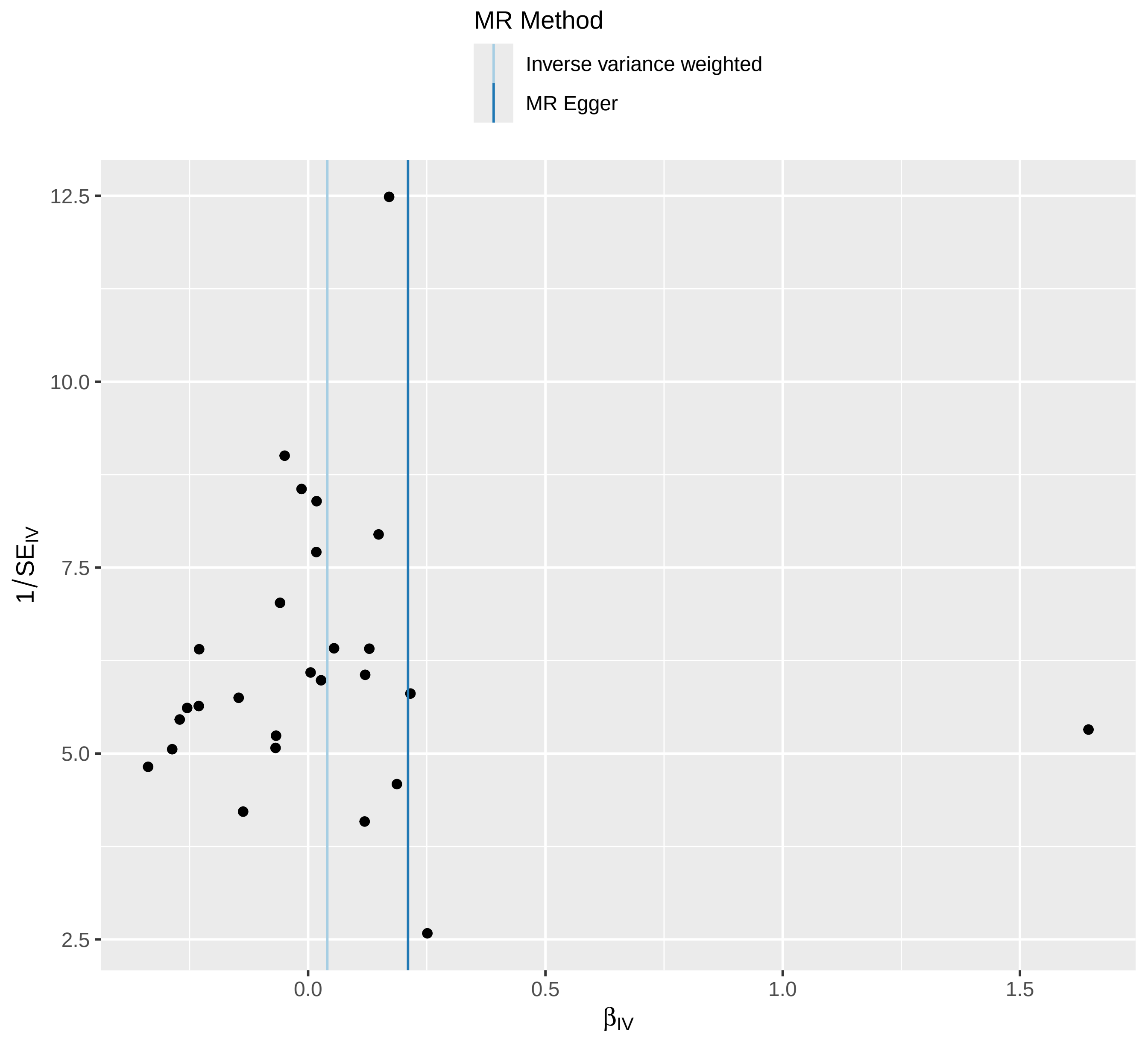
MR Method
Inverse variance weighted
MR Egger
12.5
10.0
7.5
5.0
2.5
0.0
0.5
1.0
1.5
1/SE_IV
β_IV

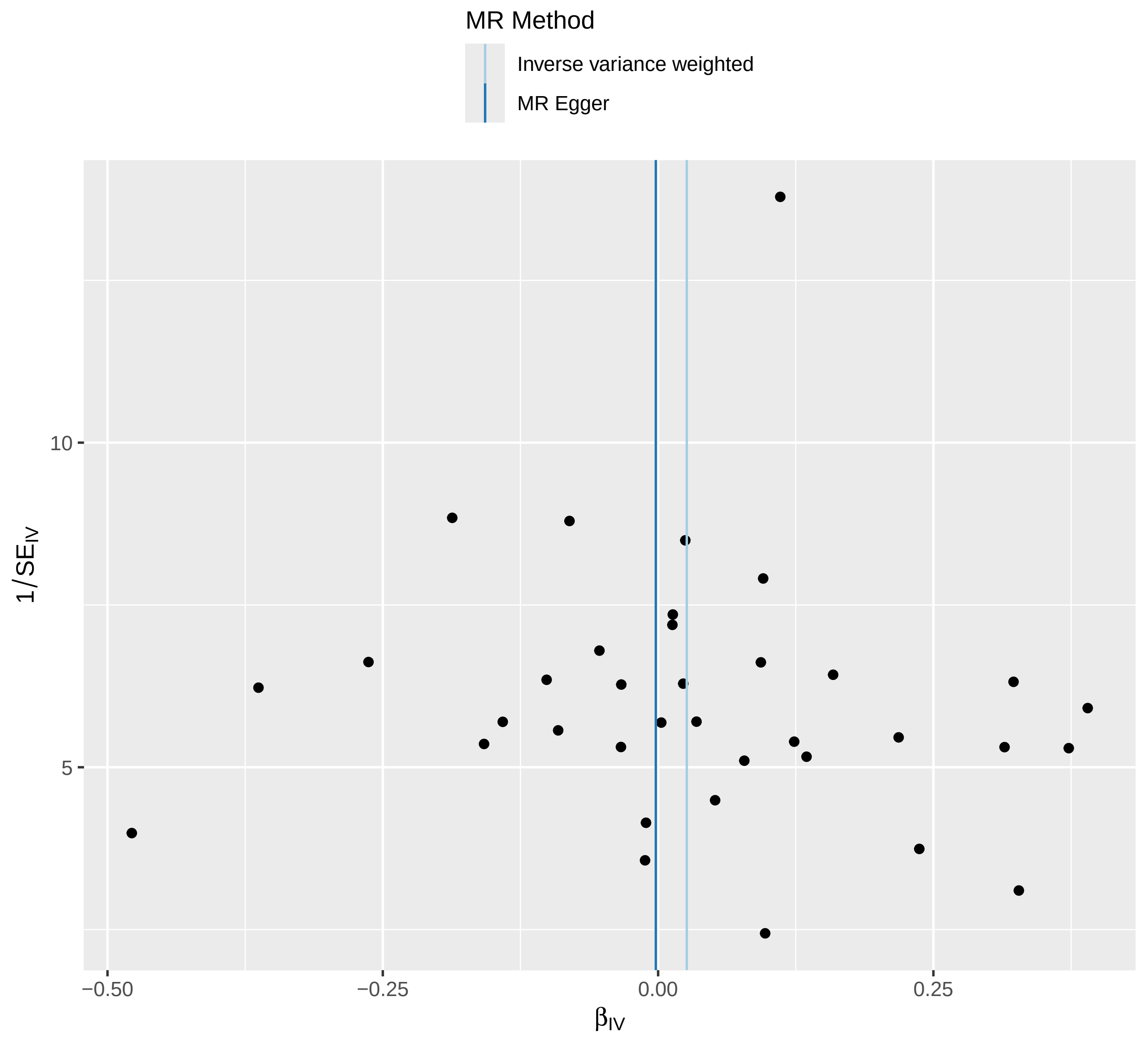

MR Method
Inverse variance weighted
MR Egger
10
5
1/SE$_{IV}$
−0.50
−0.25
0.00
0.25
β$_{IV}$

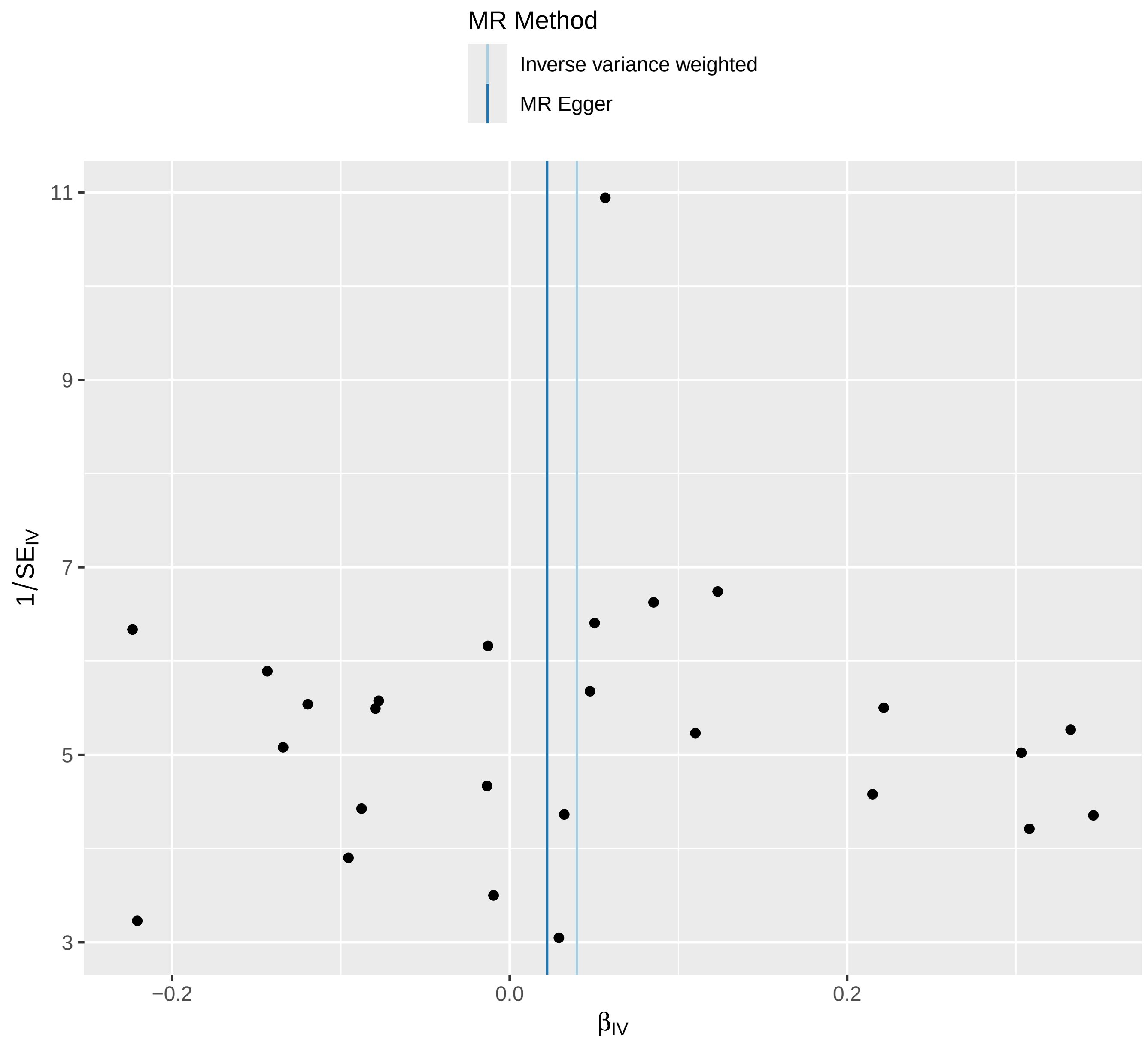

MR Method
Inverse variance weighted
MR Egger
11
9
7
5
3
1/SE_IV
−0.2
0.0
0.2
β_IV

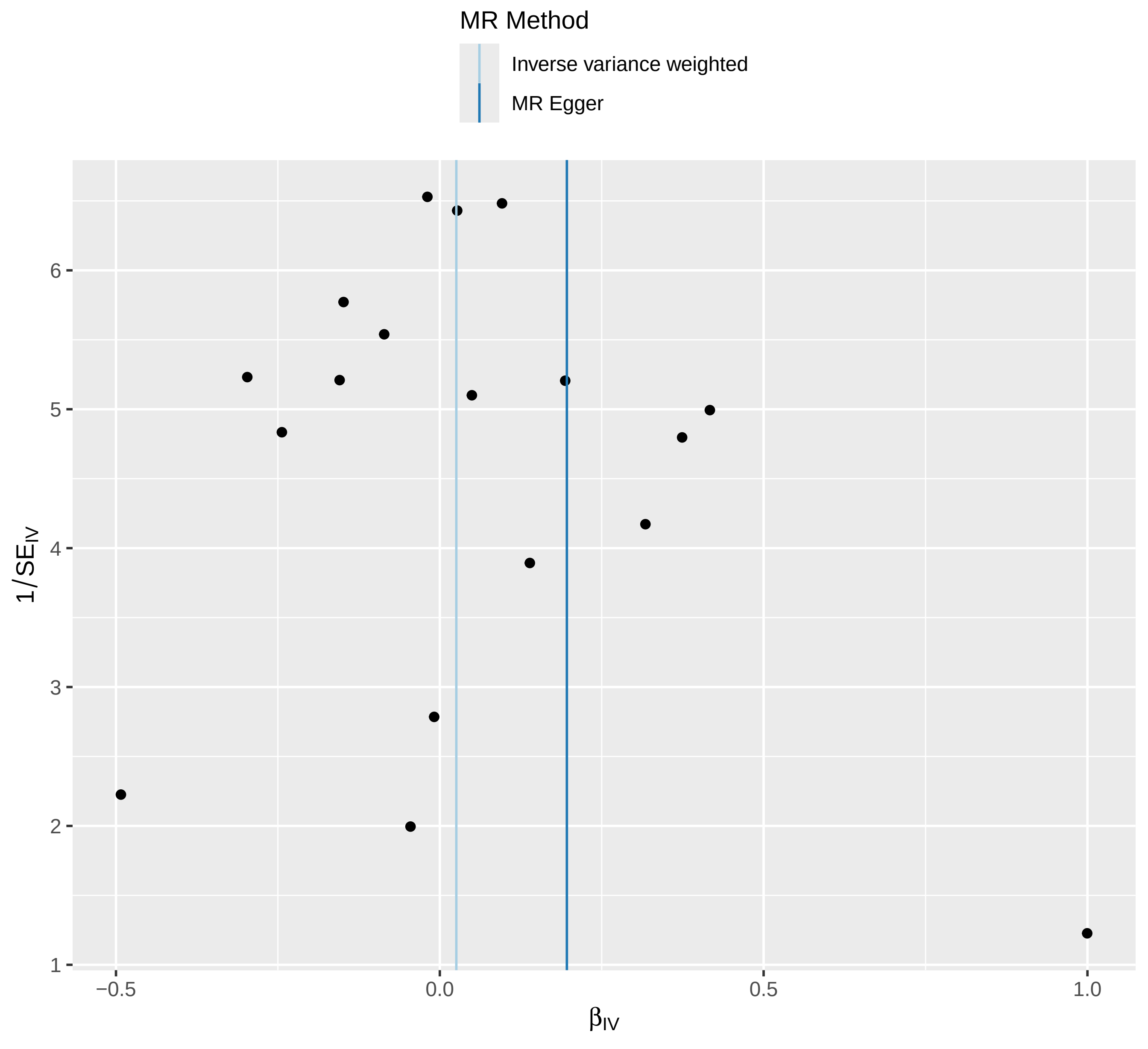

MR Method
Inverse variance weighted
MR Egger
6
5
4
3
2
1
$1/SE_{IV}$
−0.5
0.0
0.5
1.0
β_{IV}

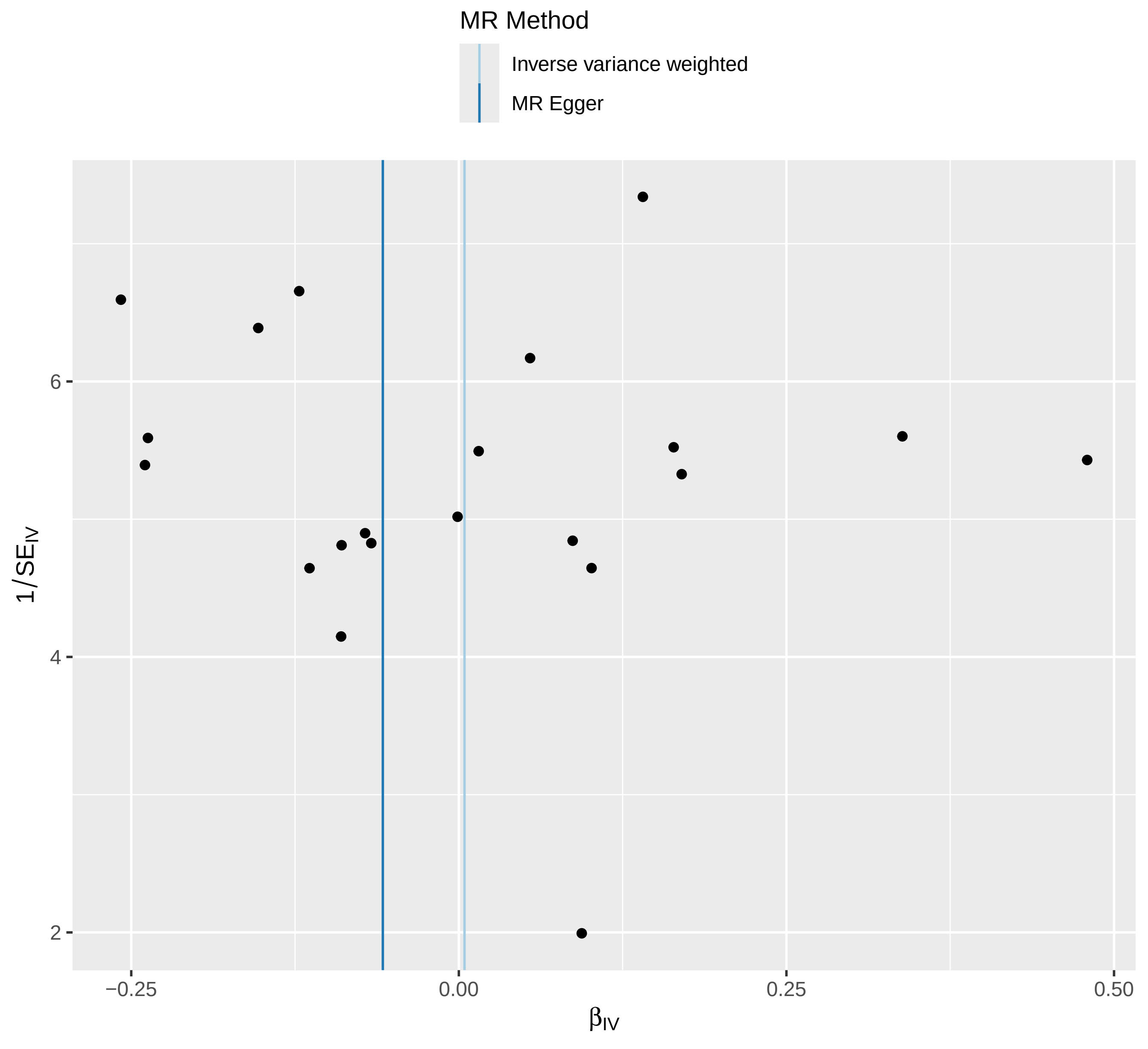

MR Method
Inverse variance weighted
MR Egger
1/SE_IV
6
4
2
−0.25
0.00
0.25
0.50
β_IV

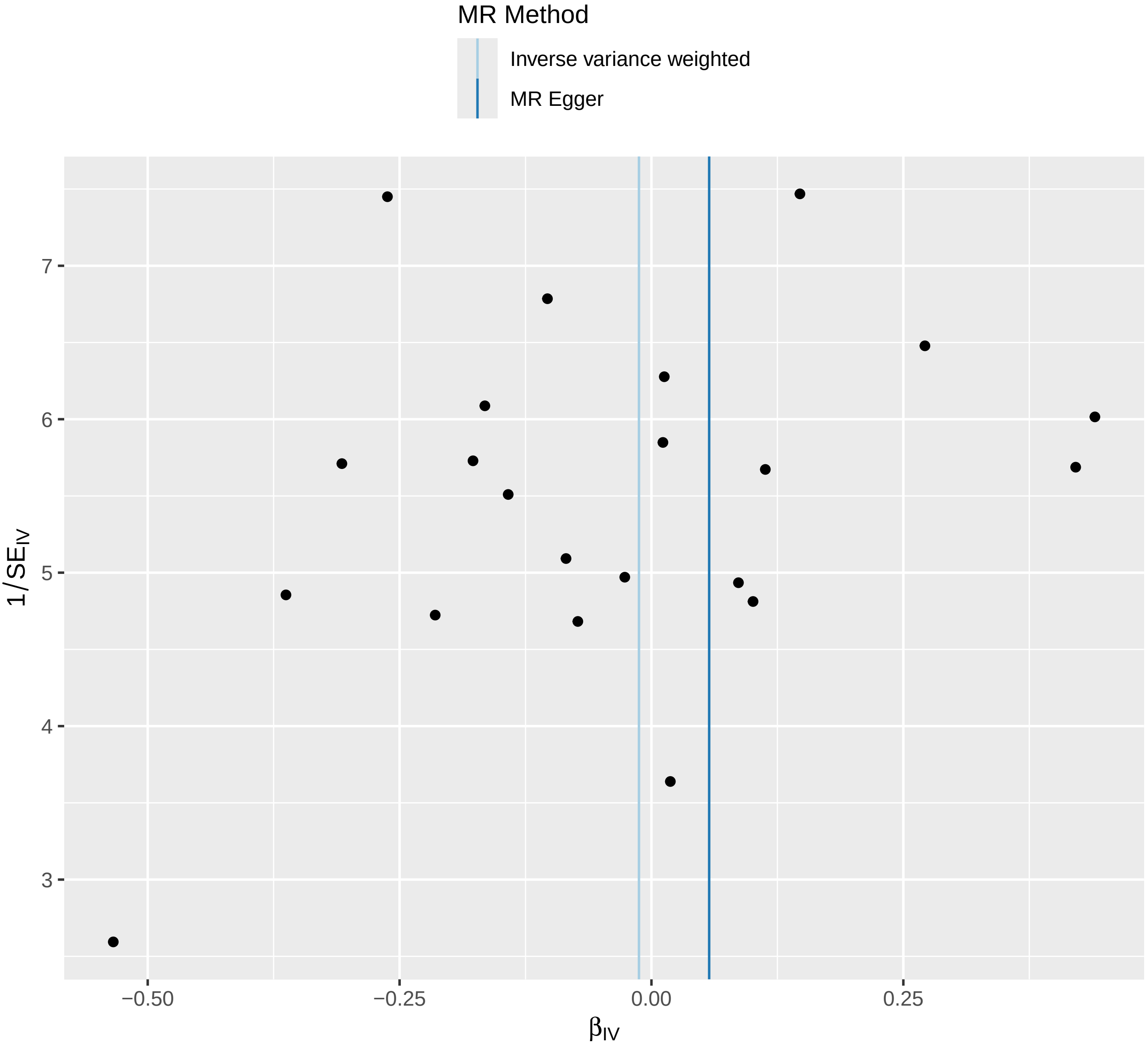
MR Method
Inverse variance weighted
MR Egger
1/SE_IV
7
6
5
4
3
−0.50
−0.25
0.00
0.25
β_IV

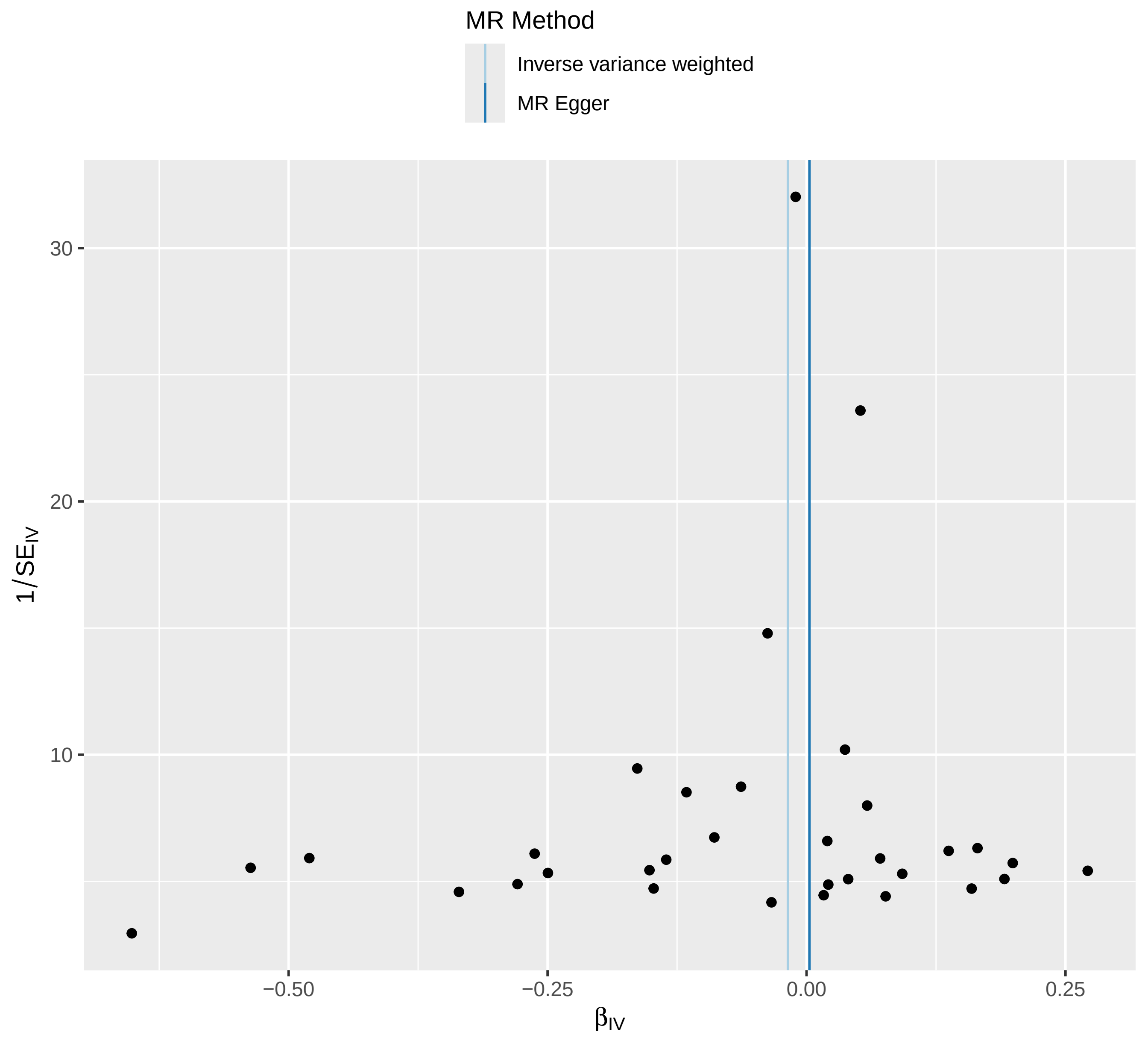

MR Method
Inverse variance weighted
MR Egger
30
20
10
1/SE_IV
−0.50
−0.25
0.00
0.25
β_IV

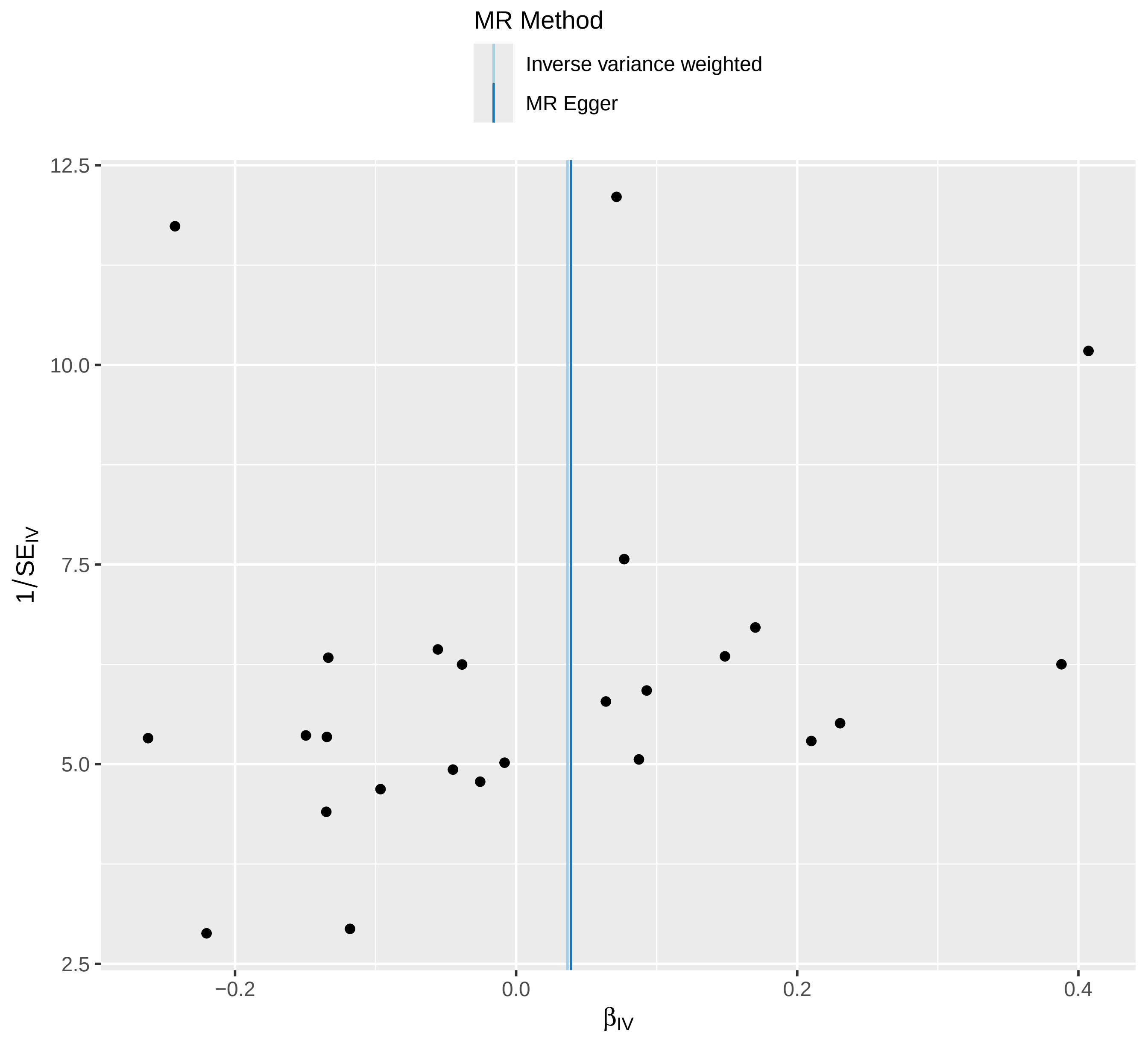
MR Method
Inverse variance weighted
MR Egger
1/SE_IV
β_IV
12.5
10.0
7.5
5.0
2.5
−0.2
0.0
0.2
0.4

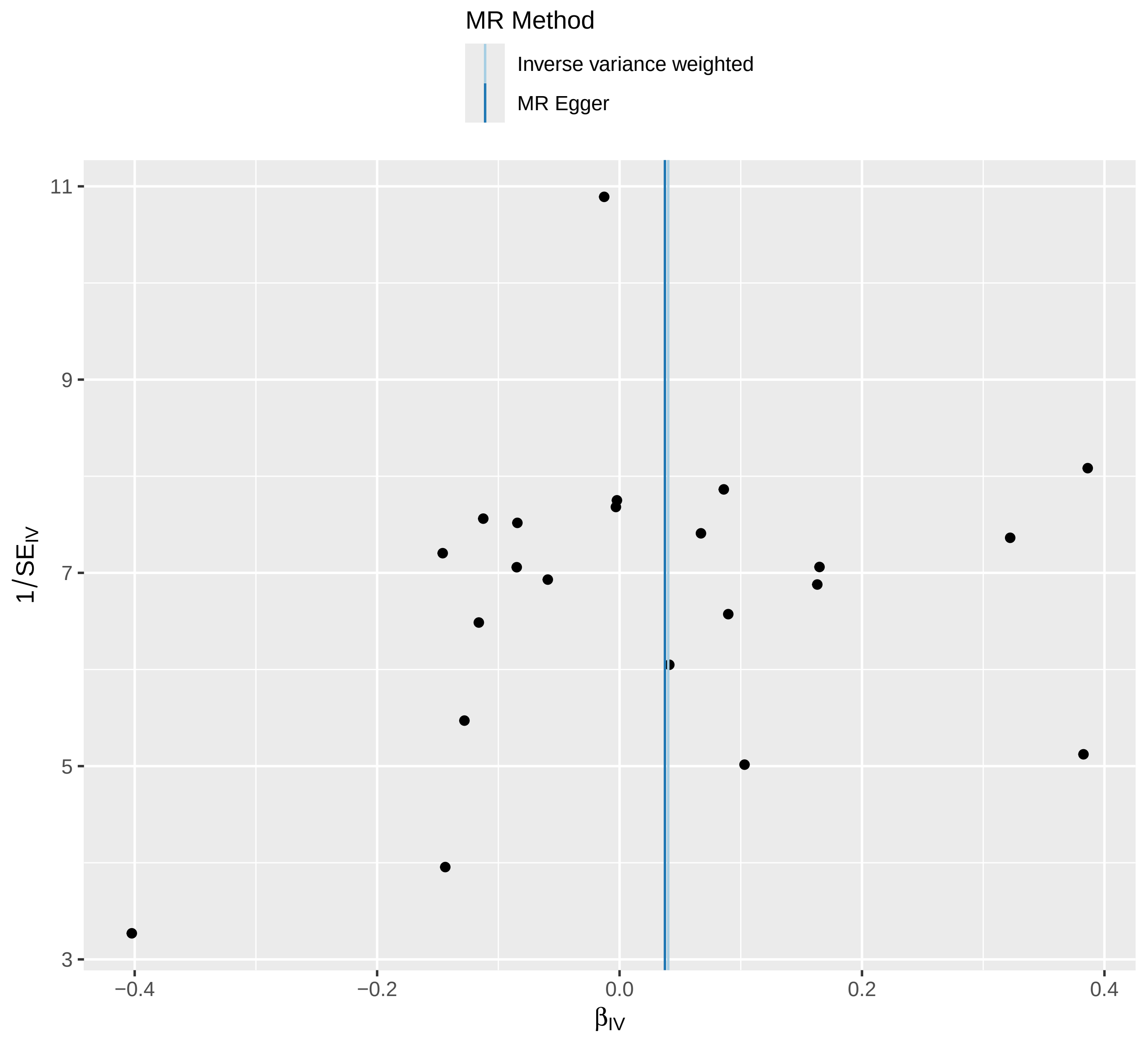
MR Method
Inverse variance weighted
MR Egger
11
9
7
5
3
1/SE_{IV}
−0.4
−0.2
0.0
0.2
0.4
β_{IV}

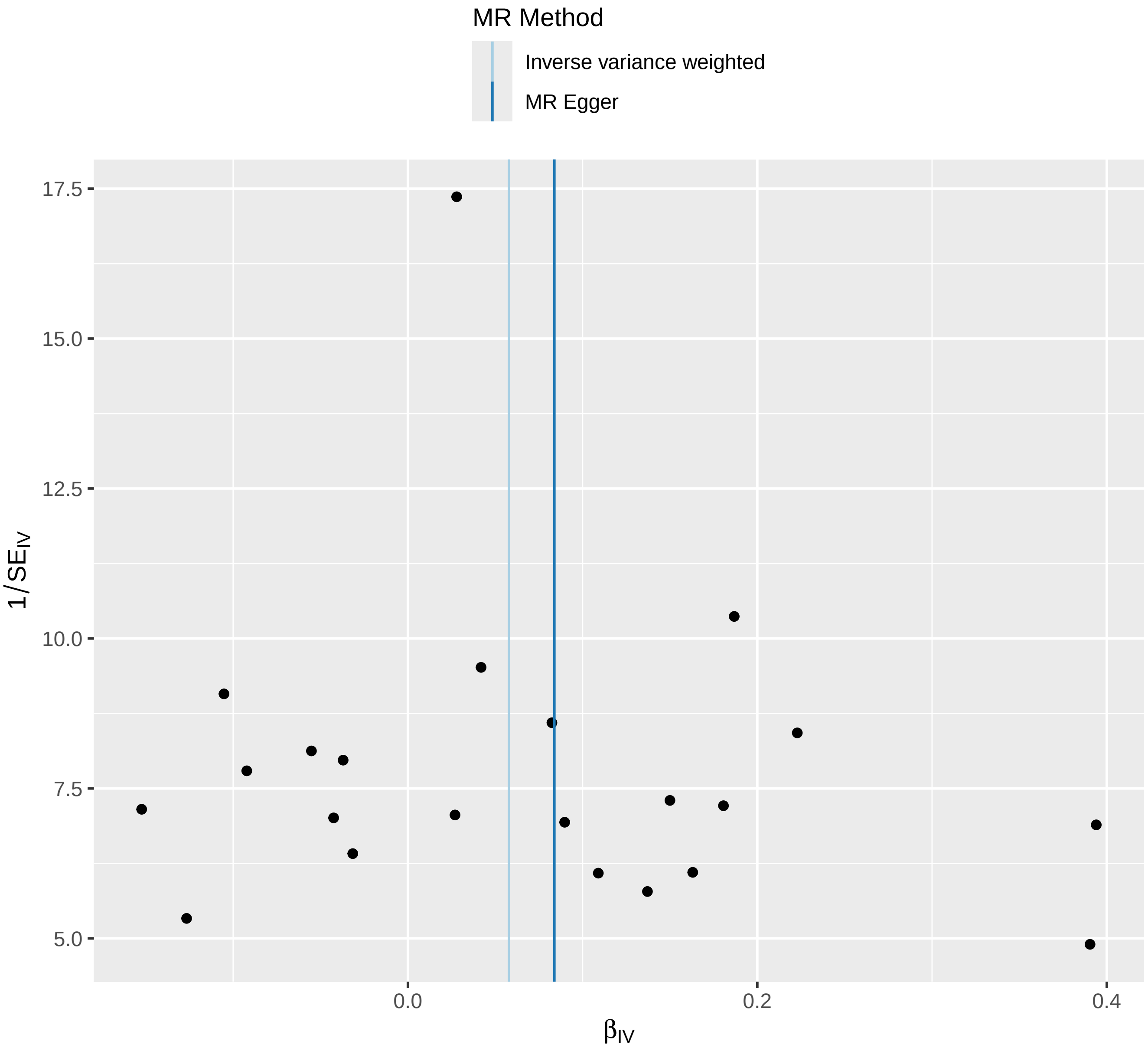
MR Method
Inverse variance weighted
MR Egger
17.5
15.0
12.5
10.0
7.5
5.0
$1/SE_{IV}$
0.0
0.2
0.4
β_{IV}

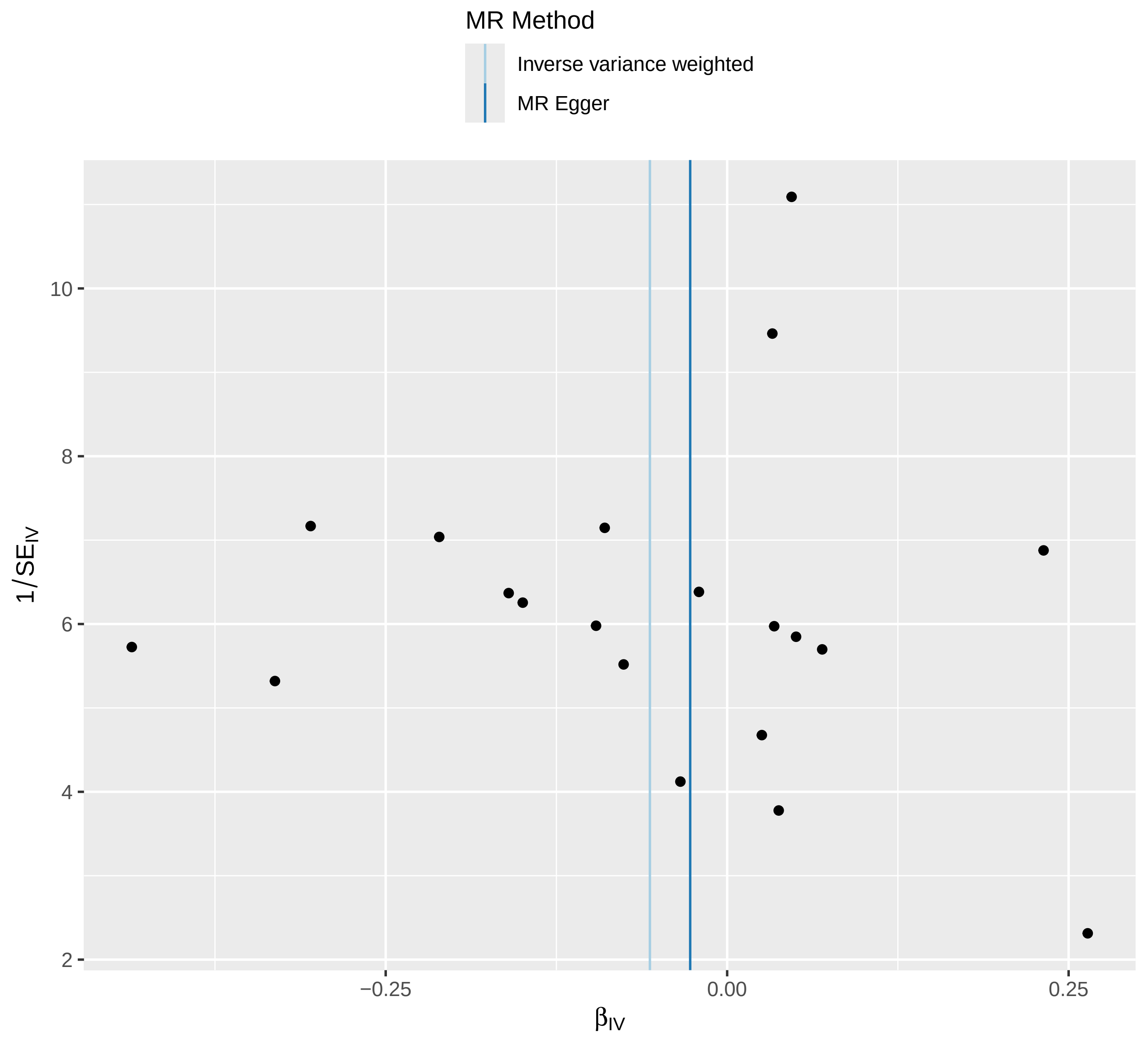

MR Method
Inverse variance weighted
MR Egger
1/SE_IV
10
8
6
4
2
β_IV
−0.25
0.00
0.25

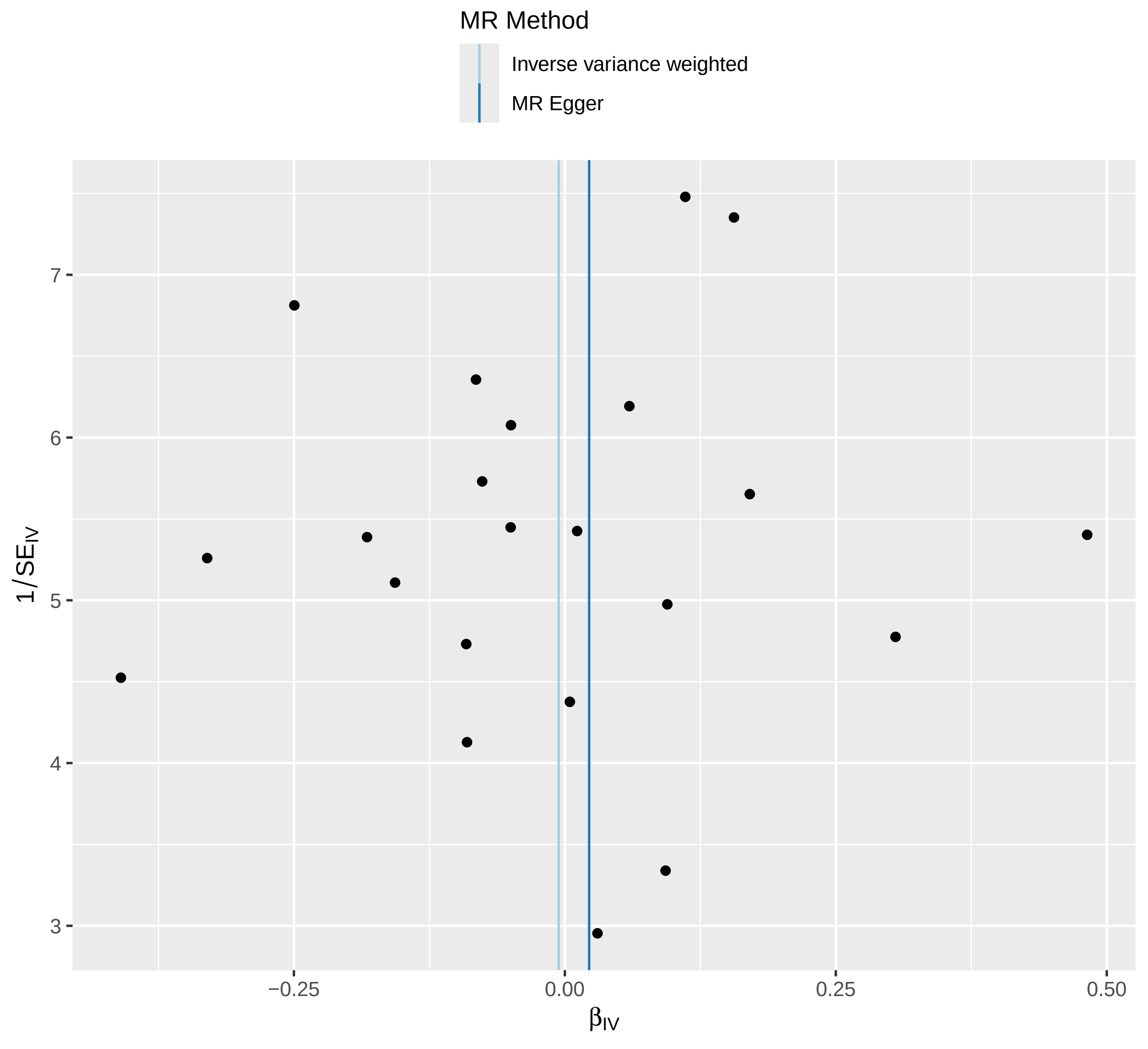
MR Method
Inverse variance weighted
MR Egger
1/SE$_{IV}$
7
6
5
4
3
−0.25
0.00
0.25
0.50
β$_{IV}$

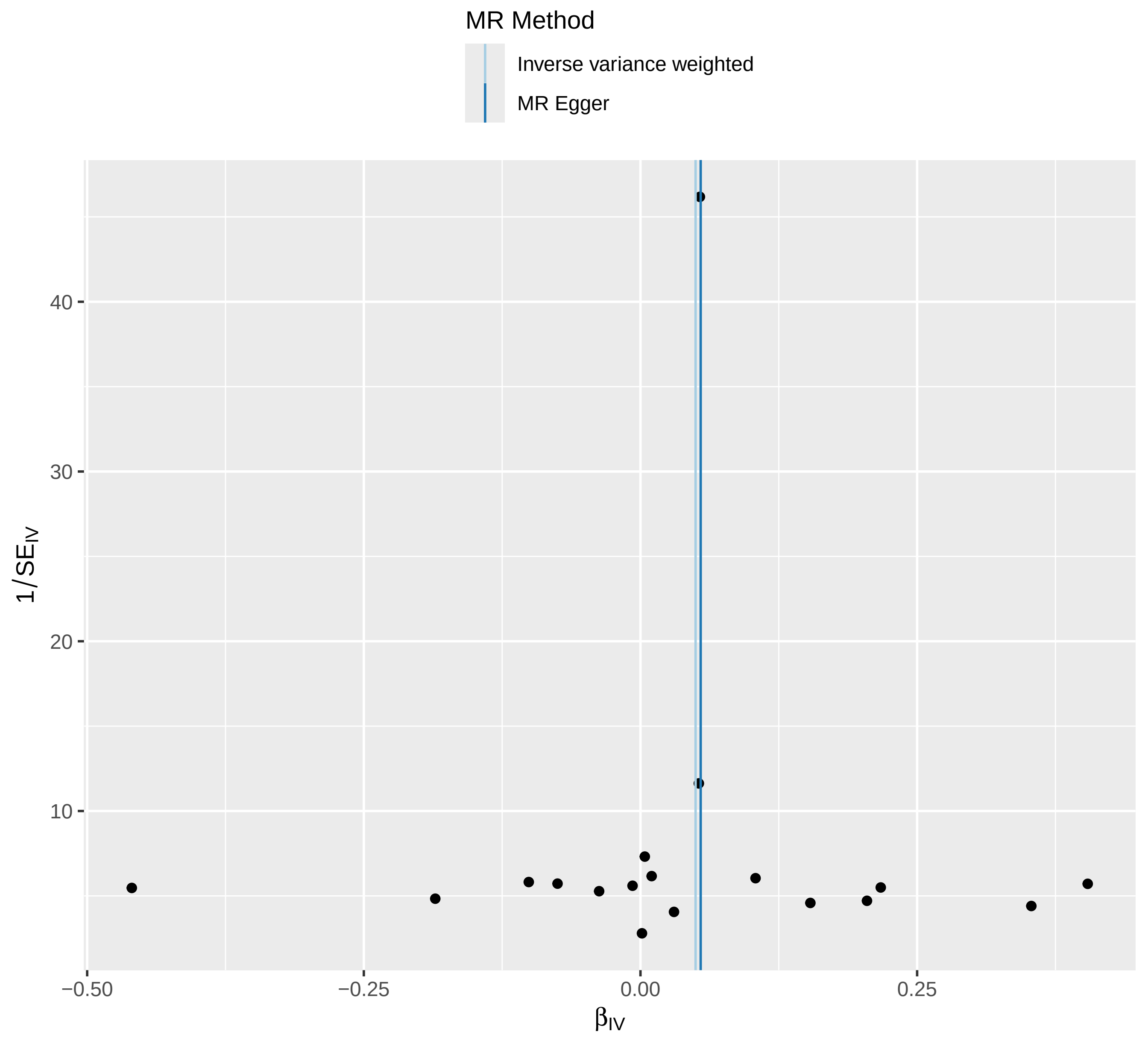
MR Method
Inverse variance weighted
MR Egger
1/SE$_{IV}$
40
30
20
10
−0.50
−0.25
0.00
0.25
β$_{IV}$

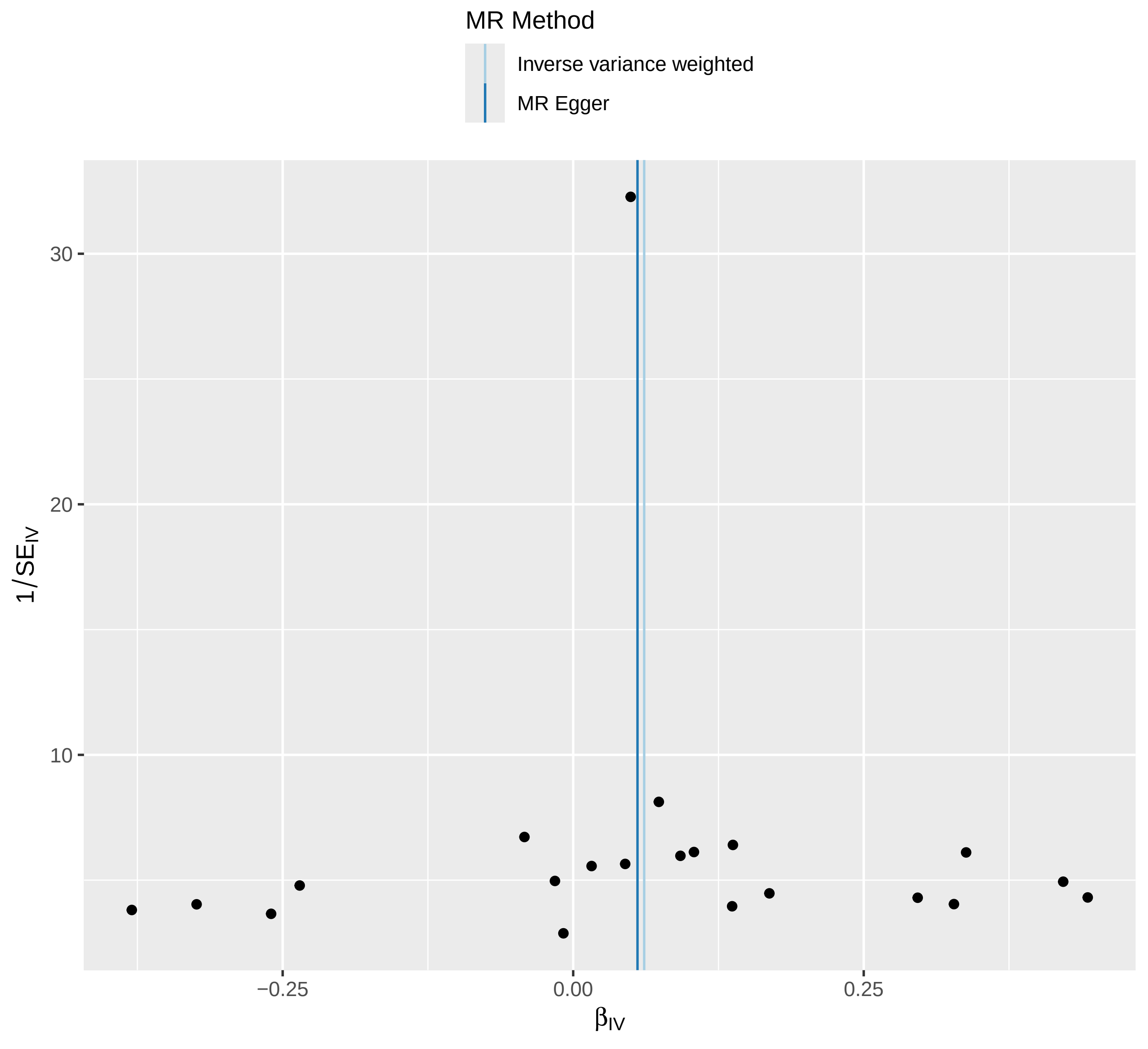
MR Method
Inverse variance weighted
MR Egger
30
20
10
1/SE$_{IV}$
−0.25
0.00
0.25
β$_{IV}$

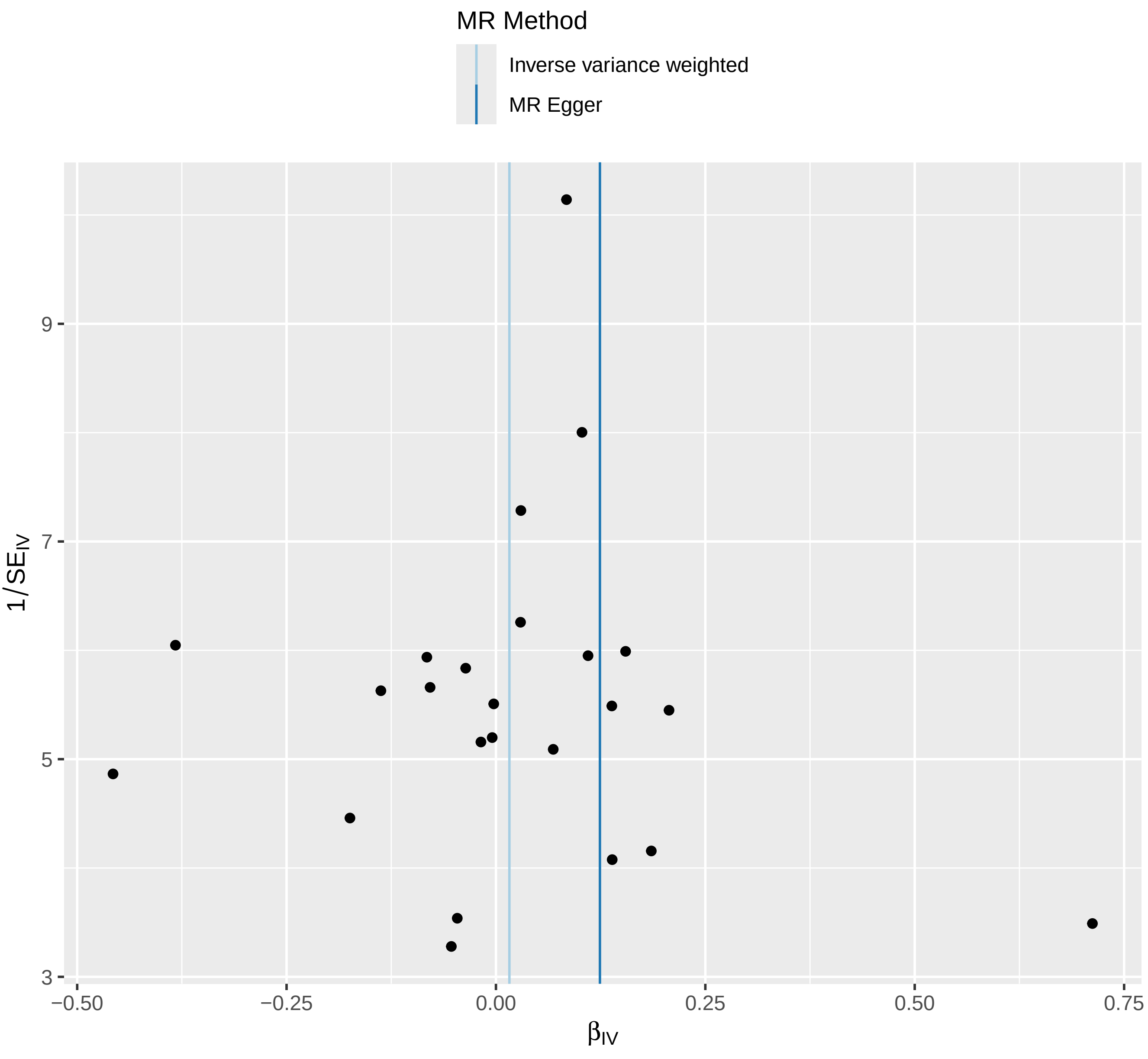
MR Method
Inverse variance weighted
MR Egger
9
7
5
3
−0.50
−0.25
0.00
0.25
0.50
0.75
$1/SE_{IV}$
β_{IV}

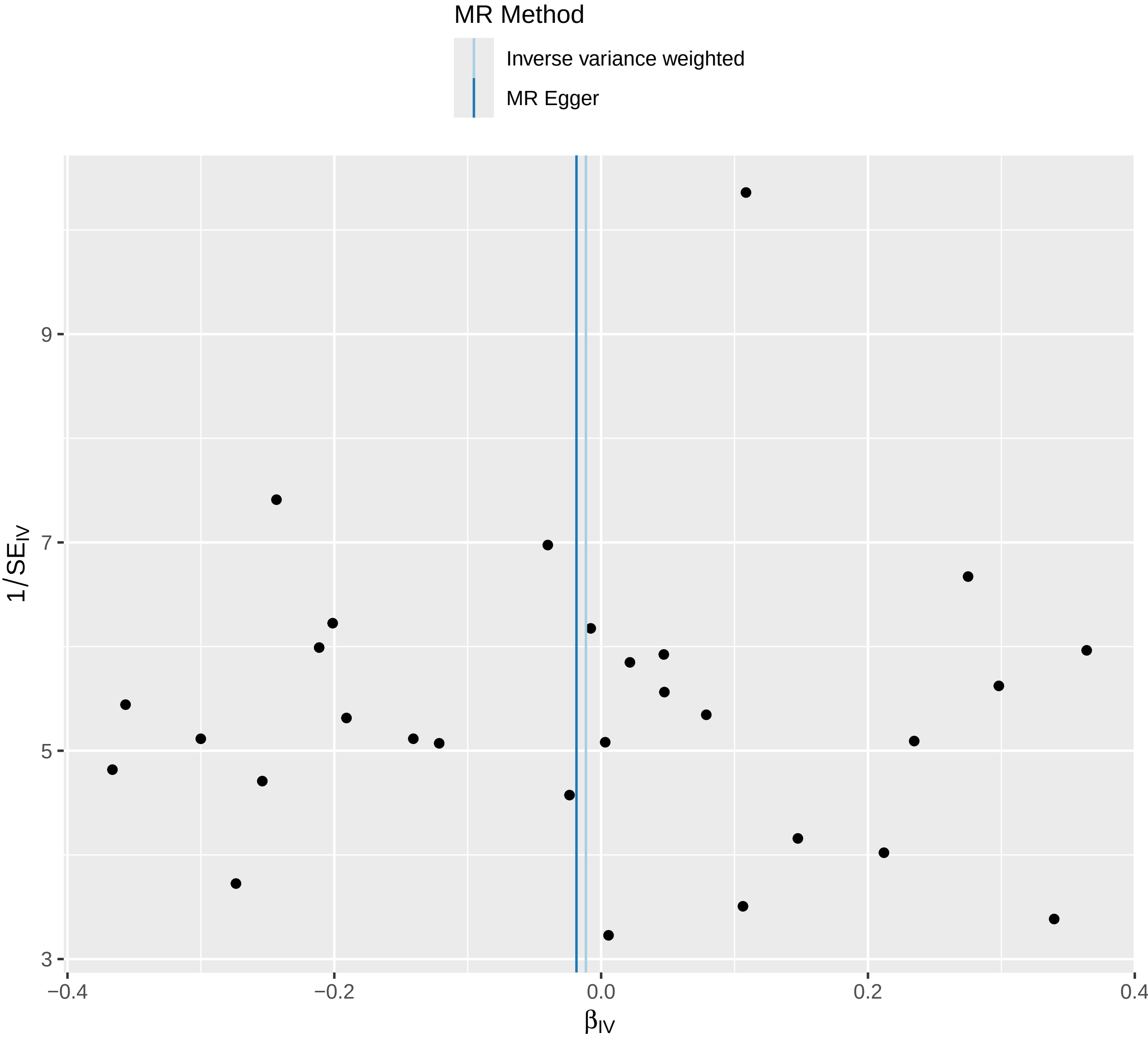
MR Method
Inverse variance weighted
MR Egger
1/SE_IV
9
7
5
3
−0.4
−0.2
0.0
0.2
0.4
β_IV

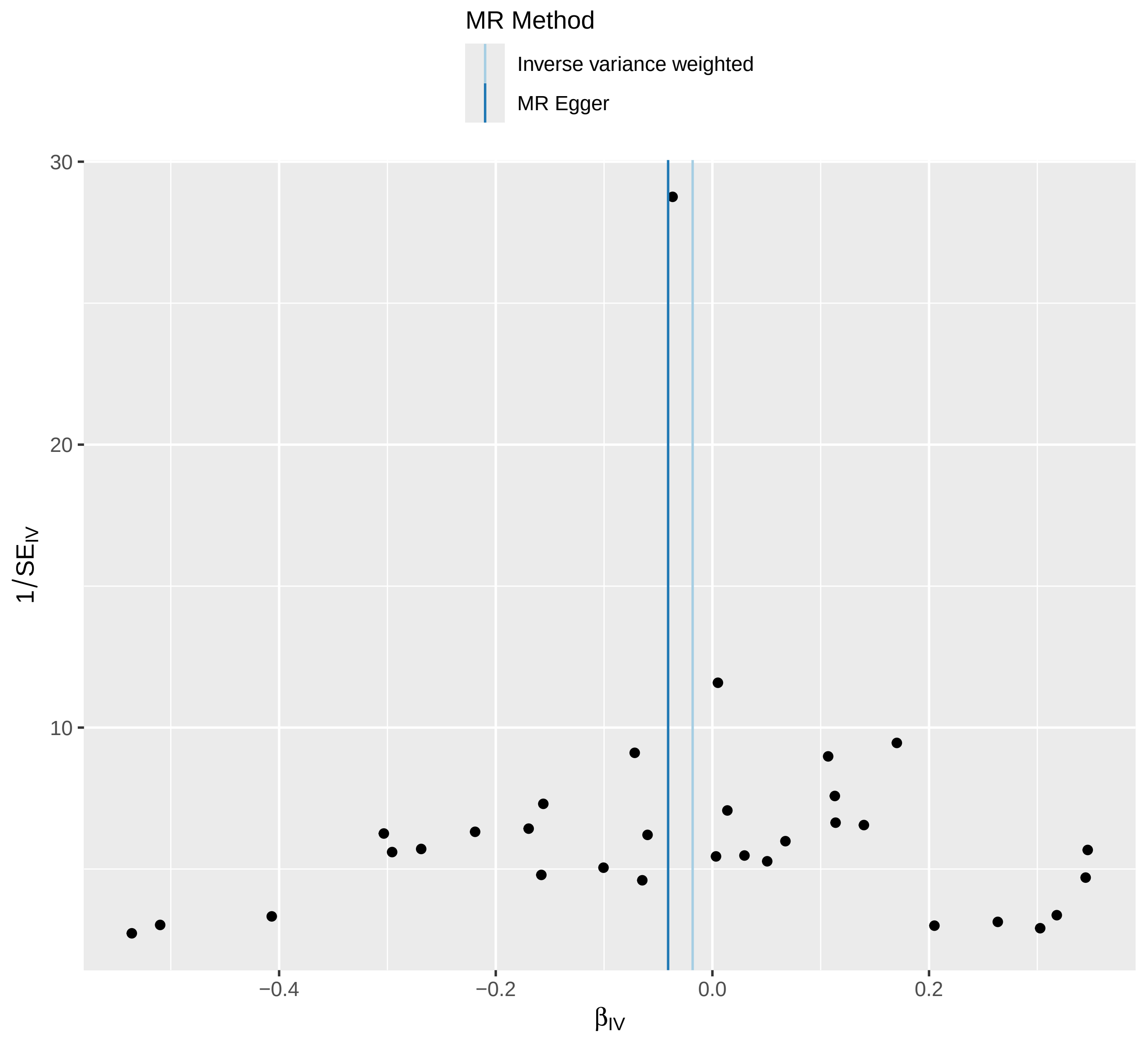
MR Method
Inverse variance weighted
MR Egger
30
20
10
1/SE_IV
−0.4
−0.2
0.0
0.2
β_IV

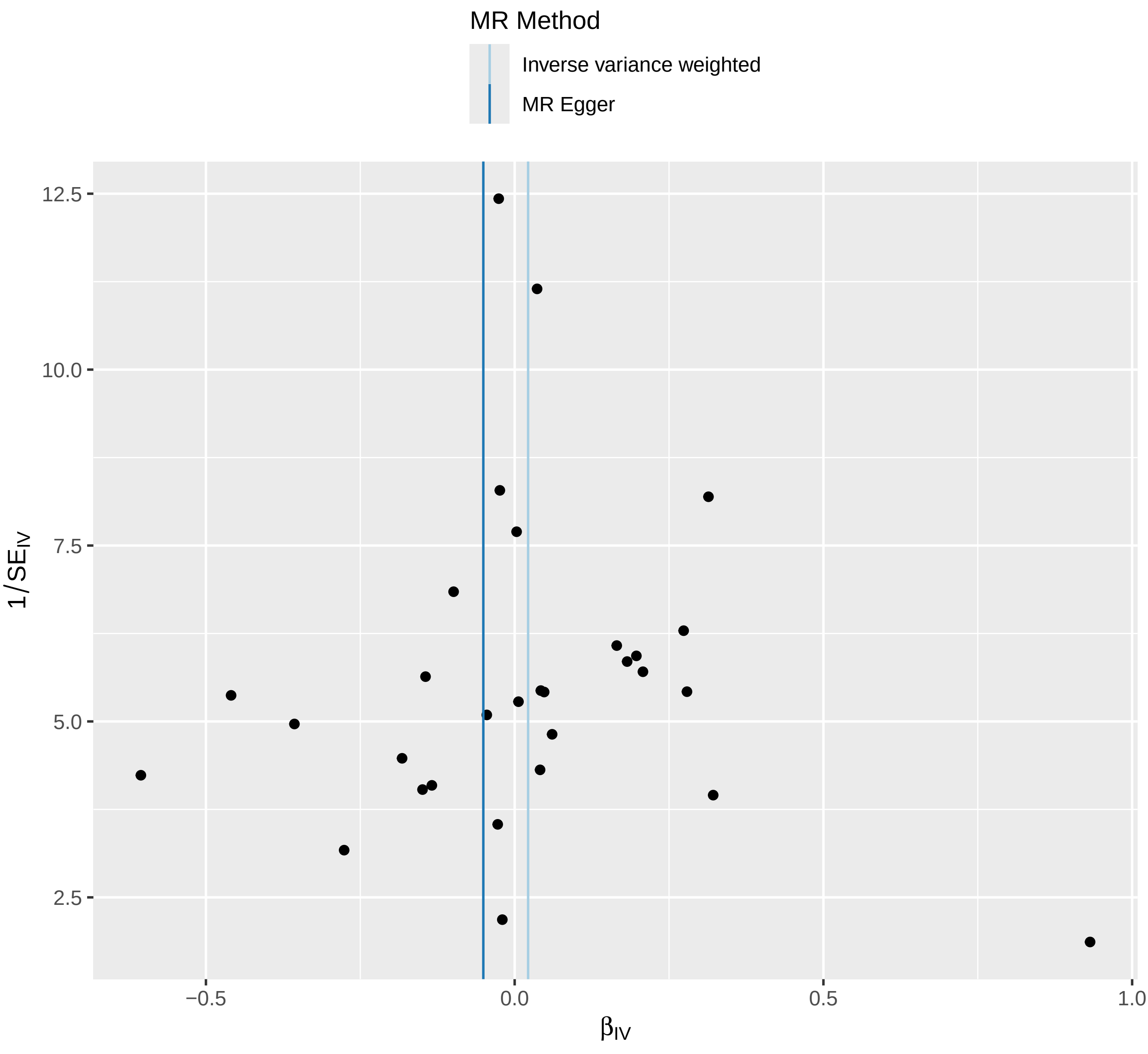

MR Method
Inverse variance weighted
MR Egger
12.5
10.0
7.5
5.0
2.5
1/SE_IV
−0.5
0.0
0.5
1.0
β_IV

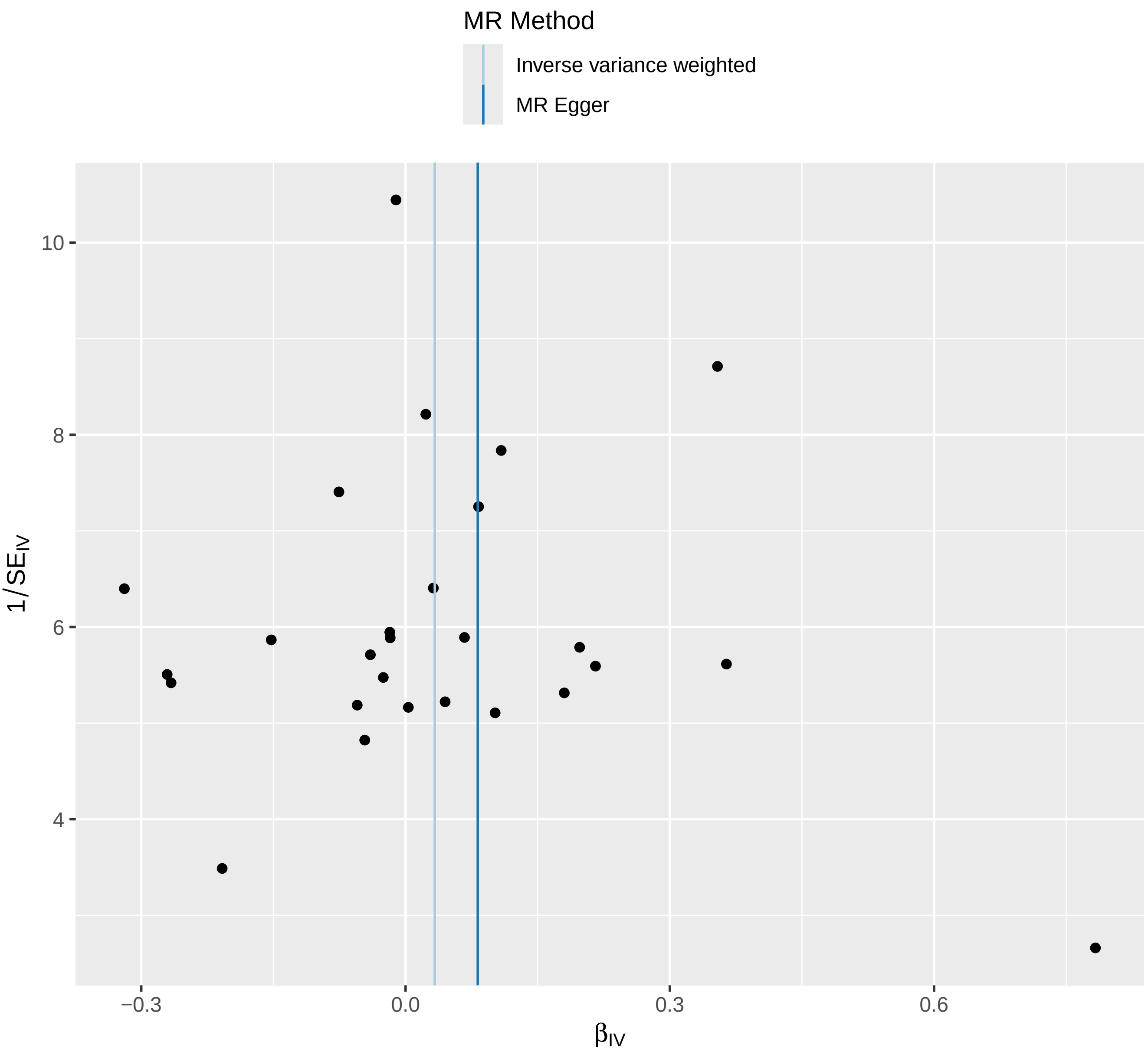
MR Method
Inverse variance weighted
MR Egger
10
8
6
4
$1/SE_{IV}$
−0.3
0.0
0.3
0.6
β_{IV}

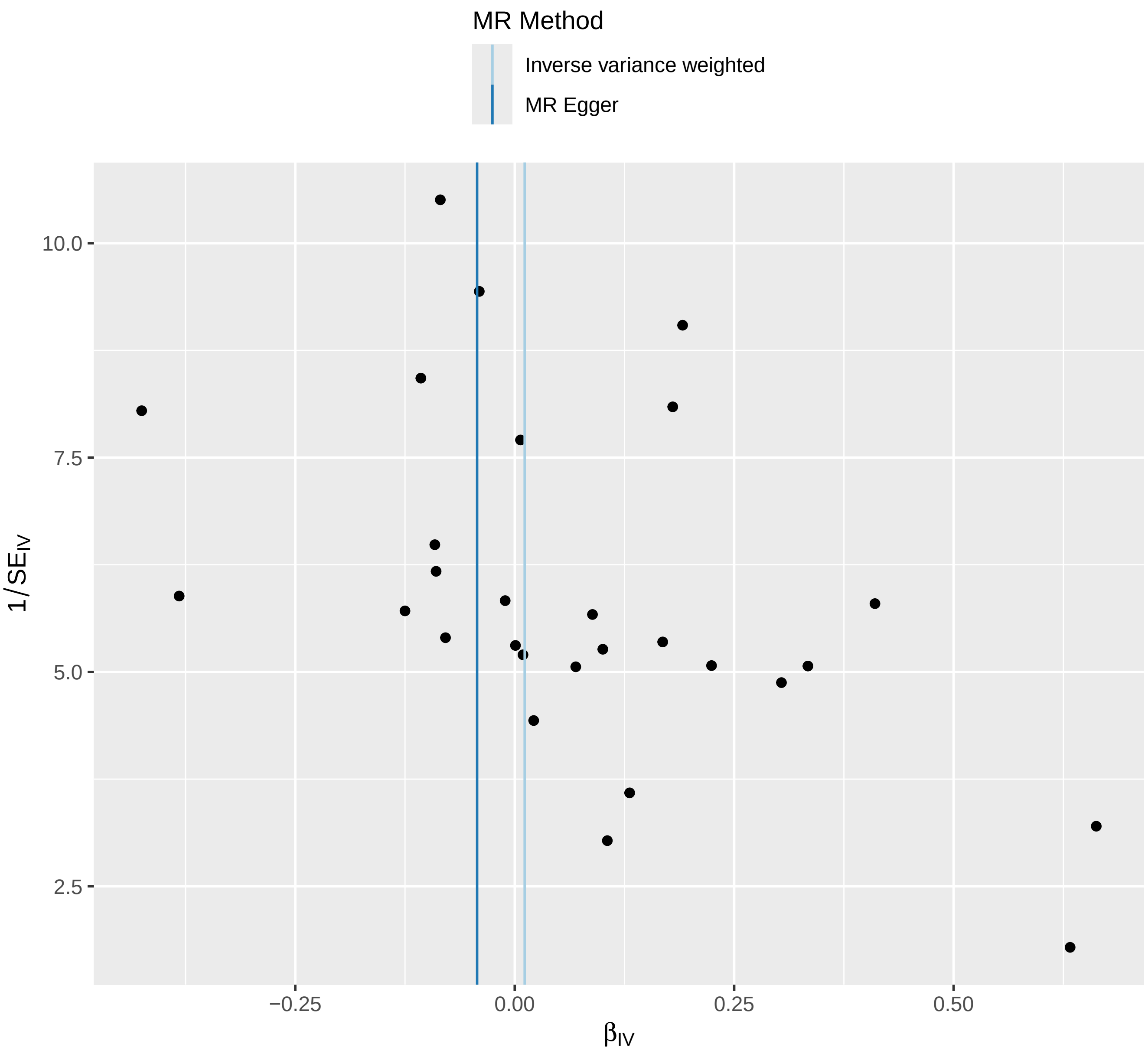
MR Method
Inverse variance weighted
MR Egger
10.0
7.5
5.0
2.5
1/SE$_{IV}$
−0.25
0.00
0.25
0.50
β$_{IV}$

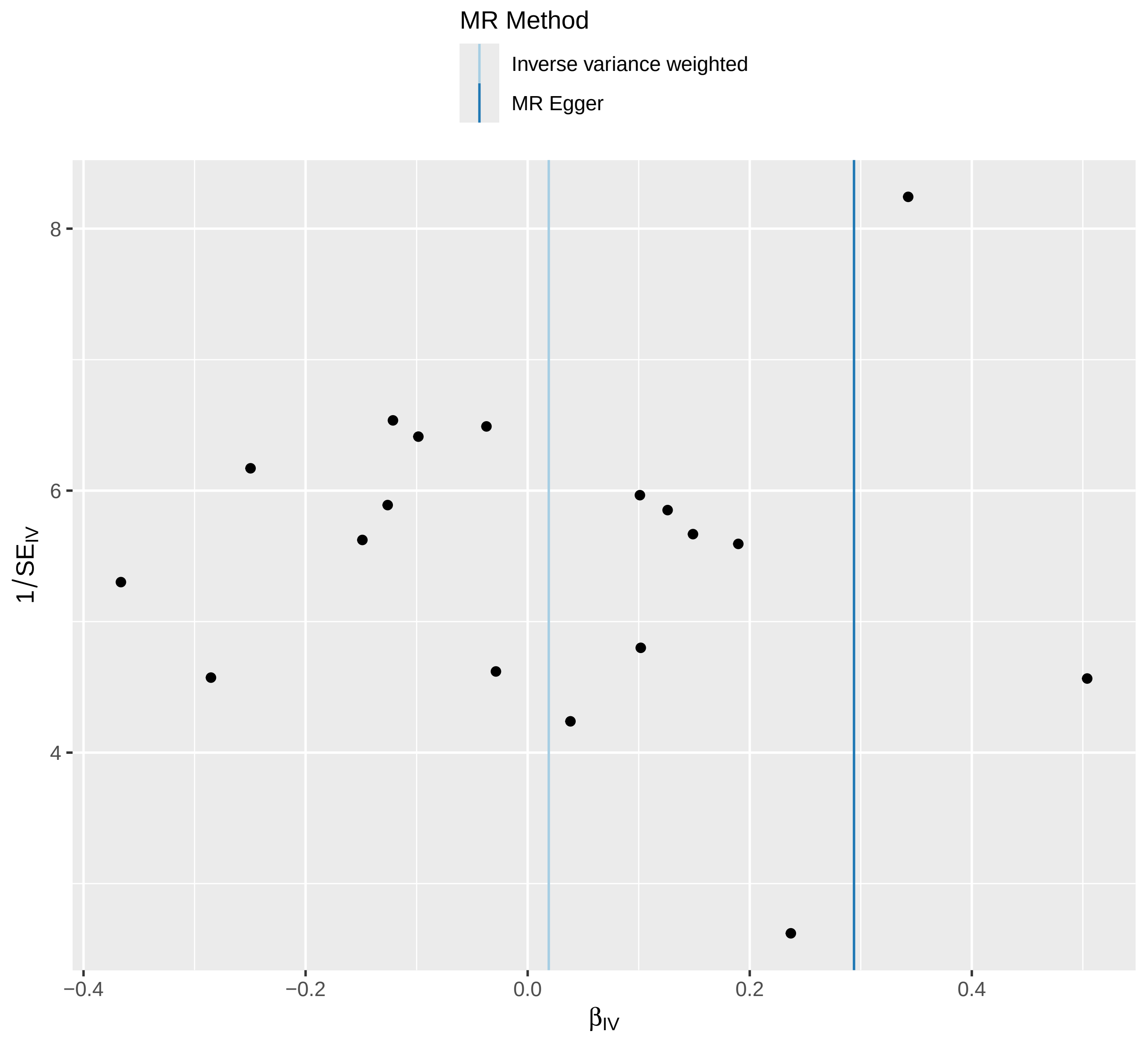
MR Method
Inverse variance weighted
MR Egger
8
6
4
1/SE_IV
−0.4
−0.2
0.0
0.2
0.4
β_IV

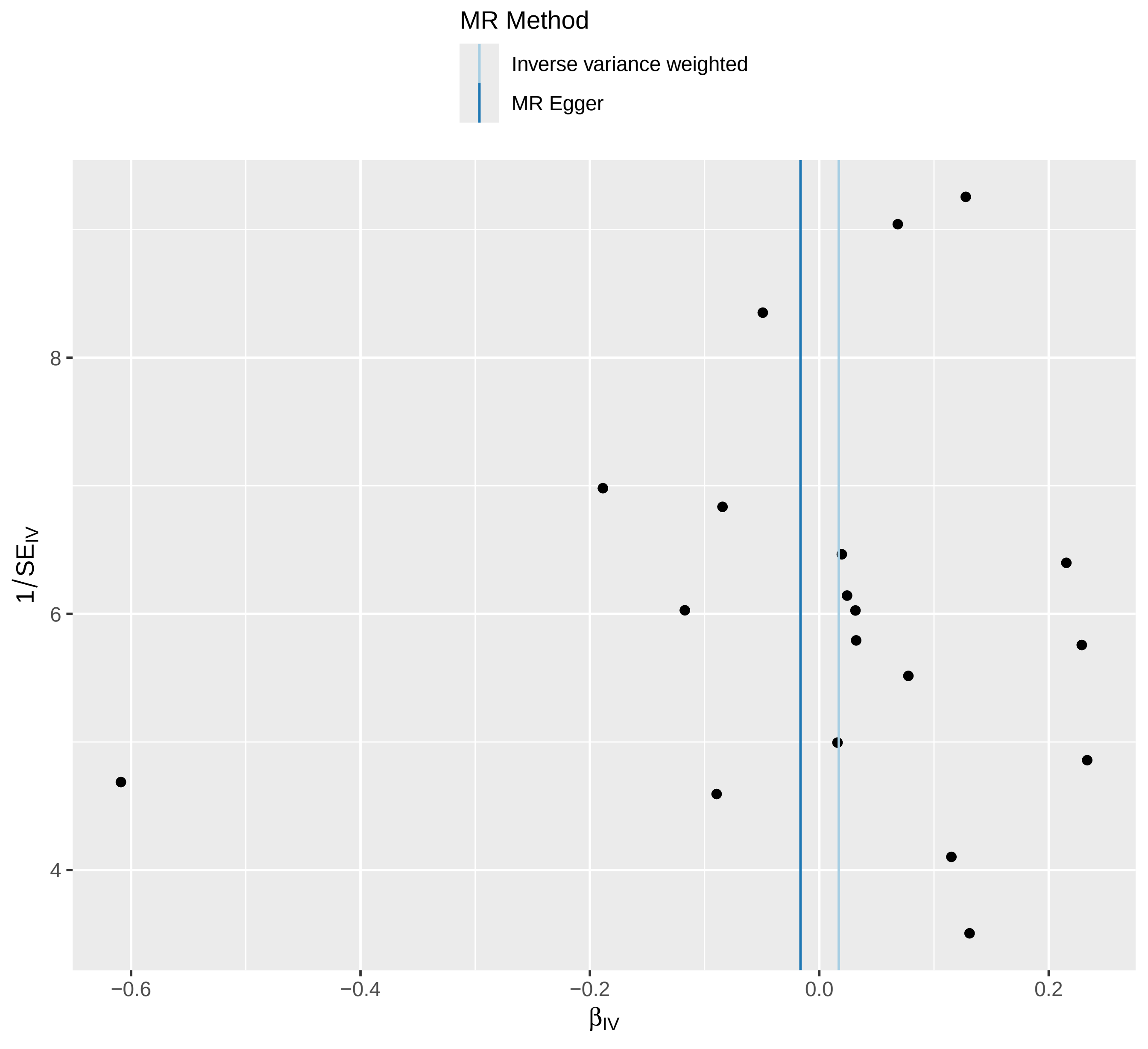

MR Method
Inverse variance weighted
MR Egger
1/SE_IV
8
6
4
−0.6
−0.4
−0.2
0.0
0.2
β_IV

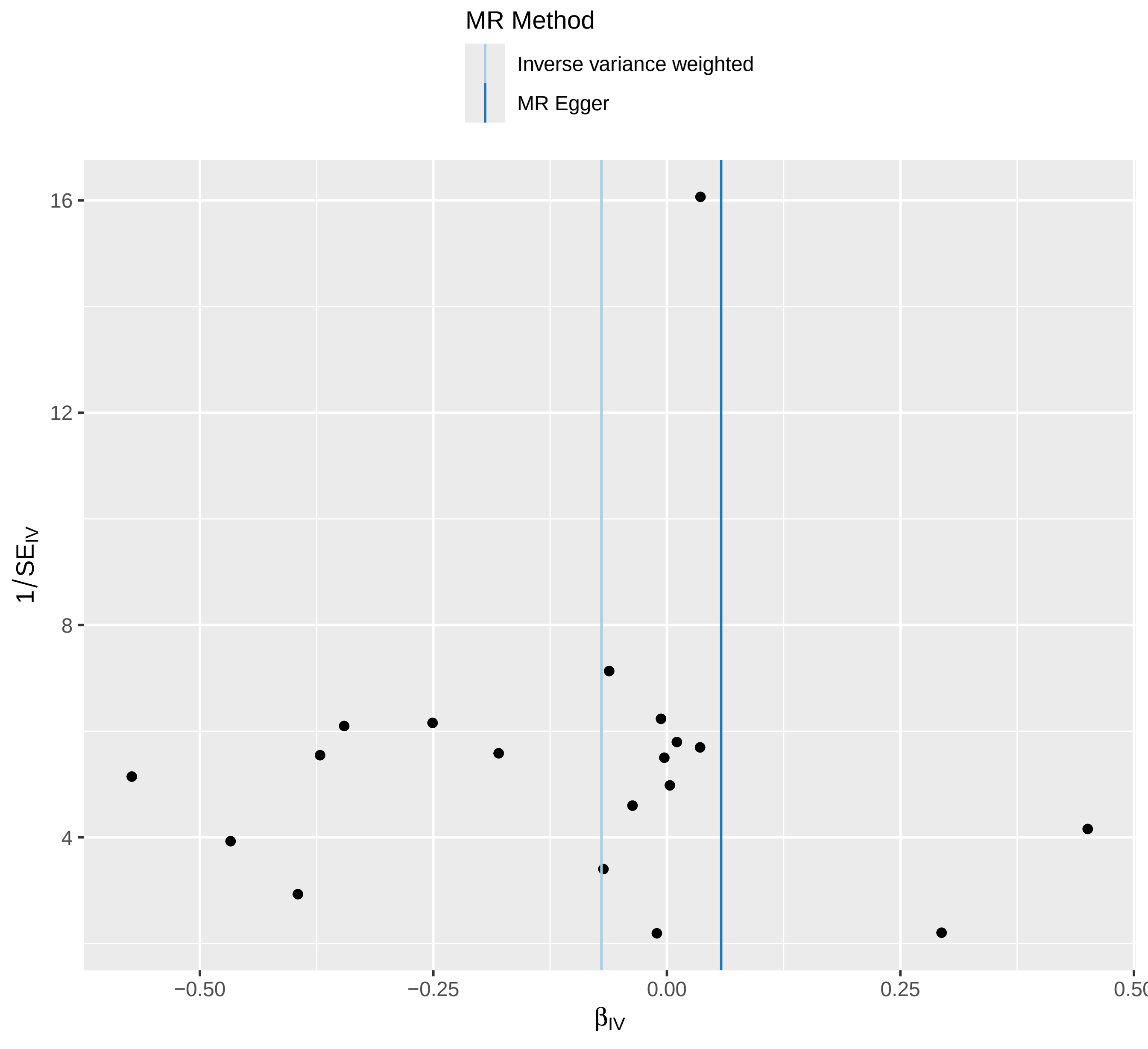
MR Method
Inverse variance weighted
MR Egger
1/SE_IV
16
12
8
4
−0.50
−0.25
0.00
0.25
0.50
β_IV

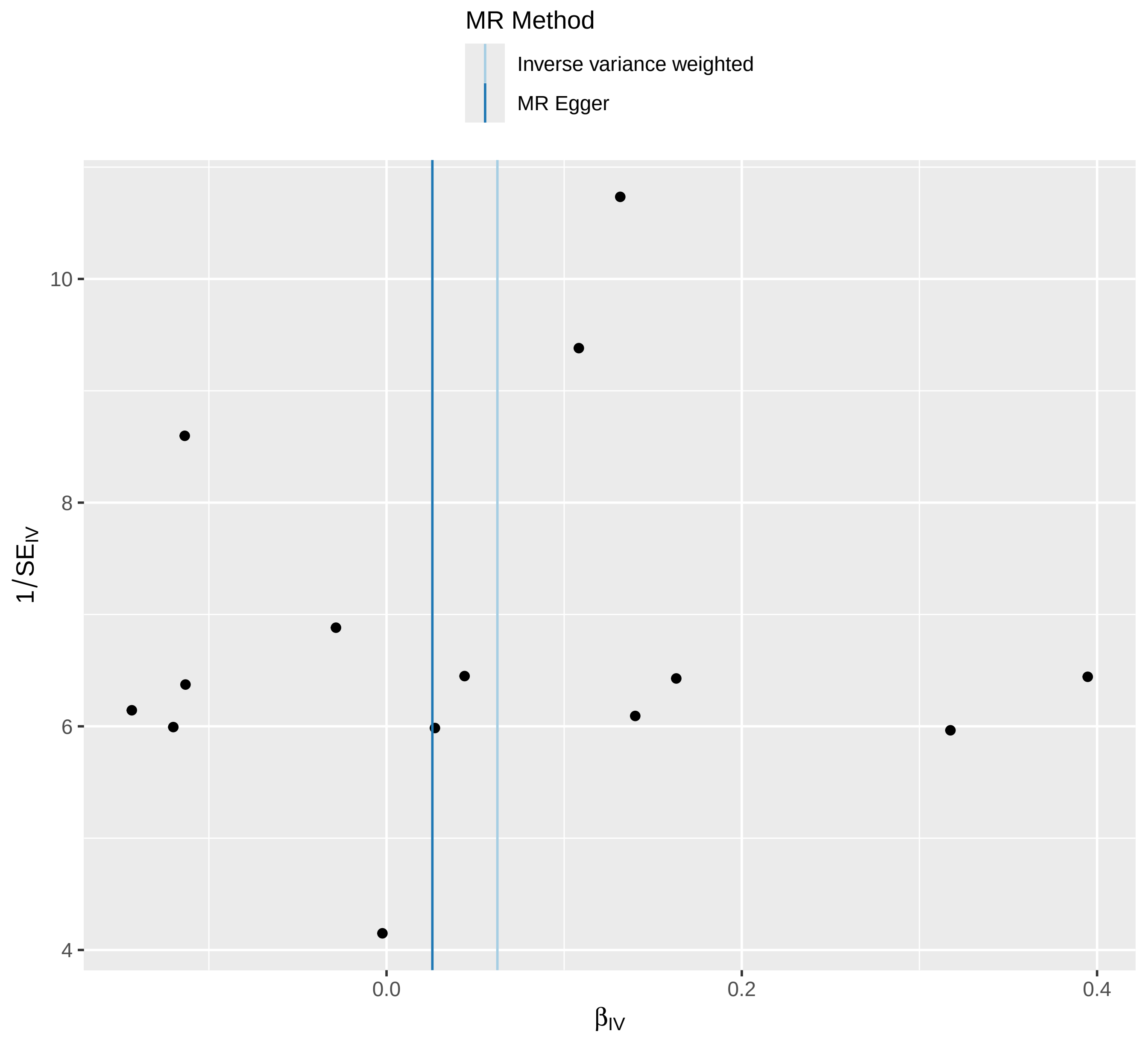
MR Method
Inverse variance weighted
MR Egger
10
8
6
4
0.0
0.2
0.4
$1/SE_{IV}$
β_{IV}

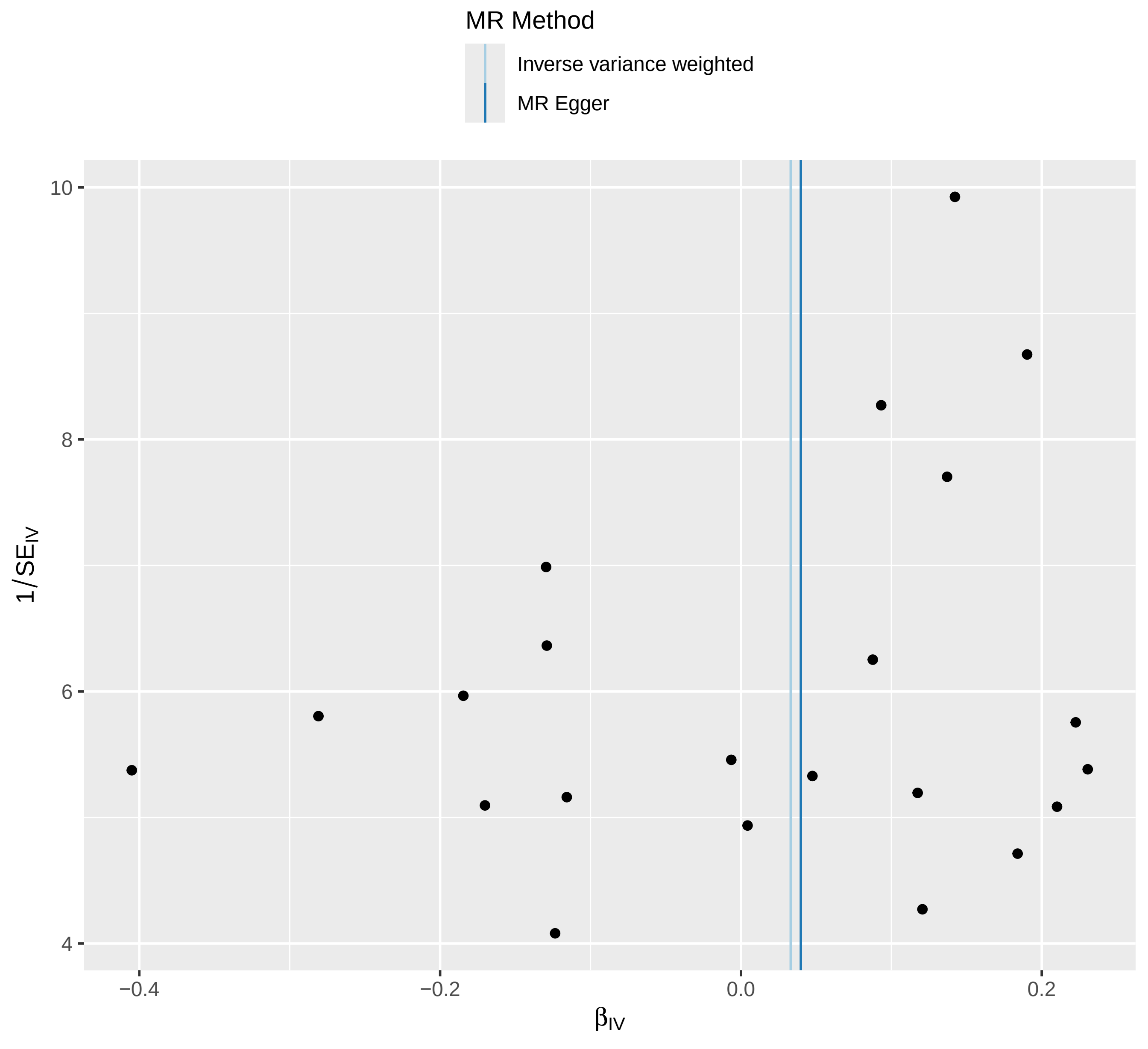

MR Method
Inverse variance weighted
MR Egger
1/SE_IV
10
8
6
4
−0.4
−0.2
0.0
0.2
β_IV

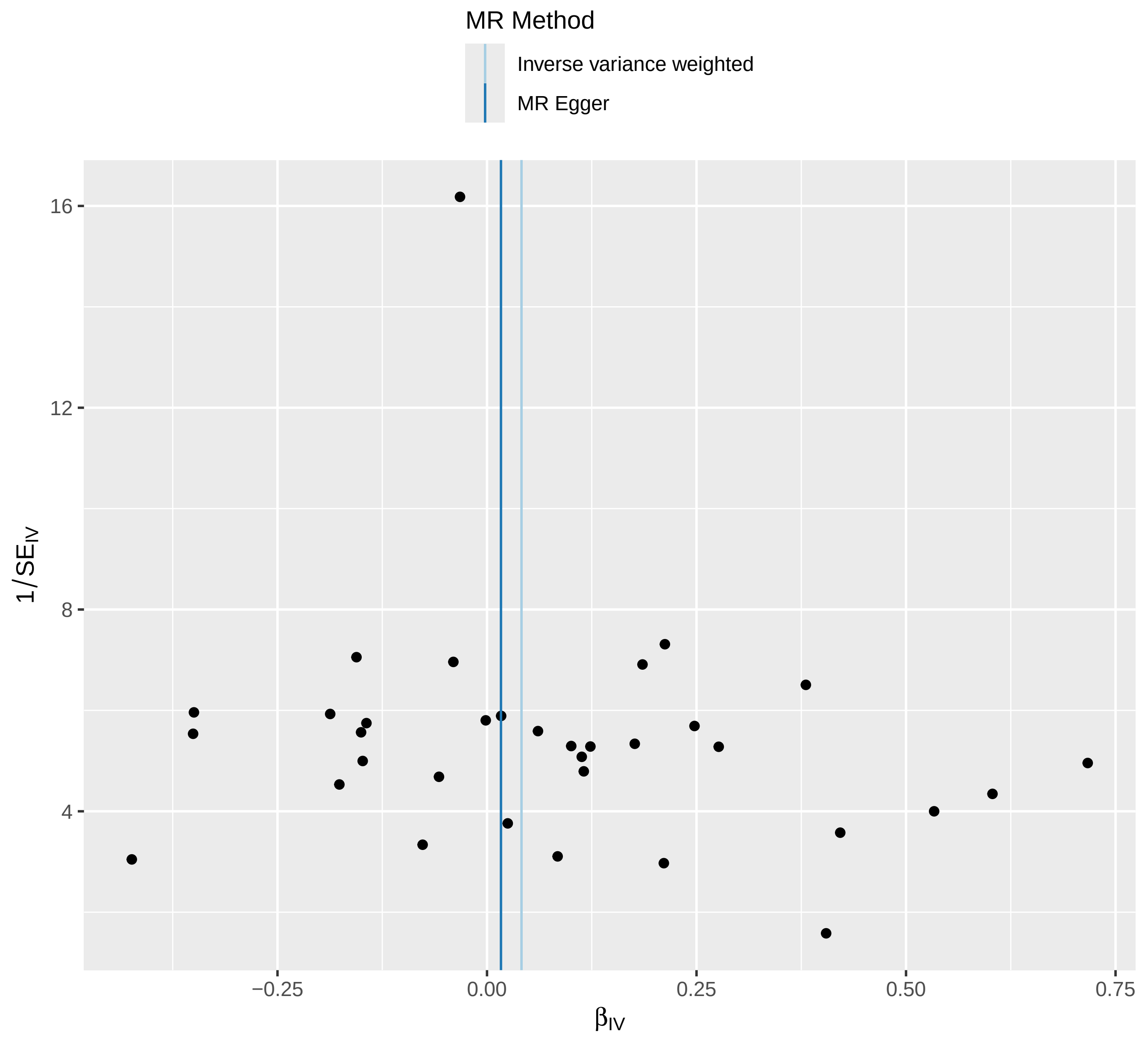
MR Method
Inverse variance weighted
MR Egger
16
12
8
4
1/SE$_{IV}$
−0.25
0.00
0.25
0.50
0.75
β$_{IV}$

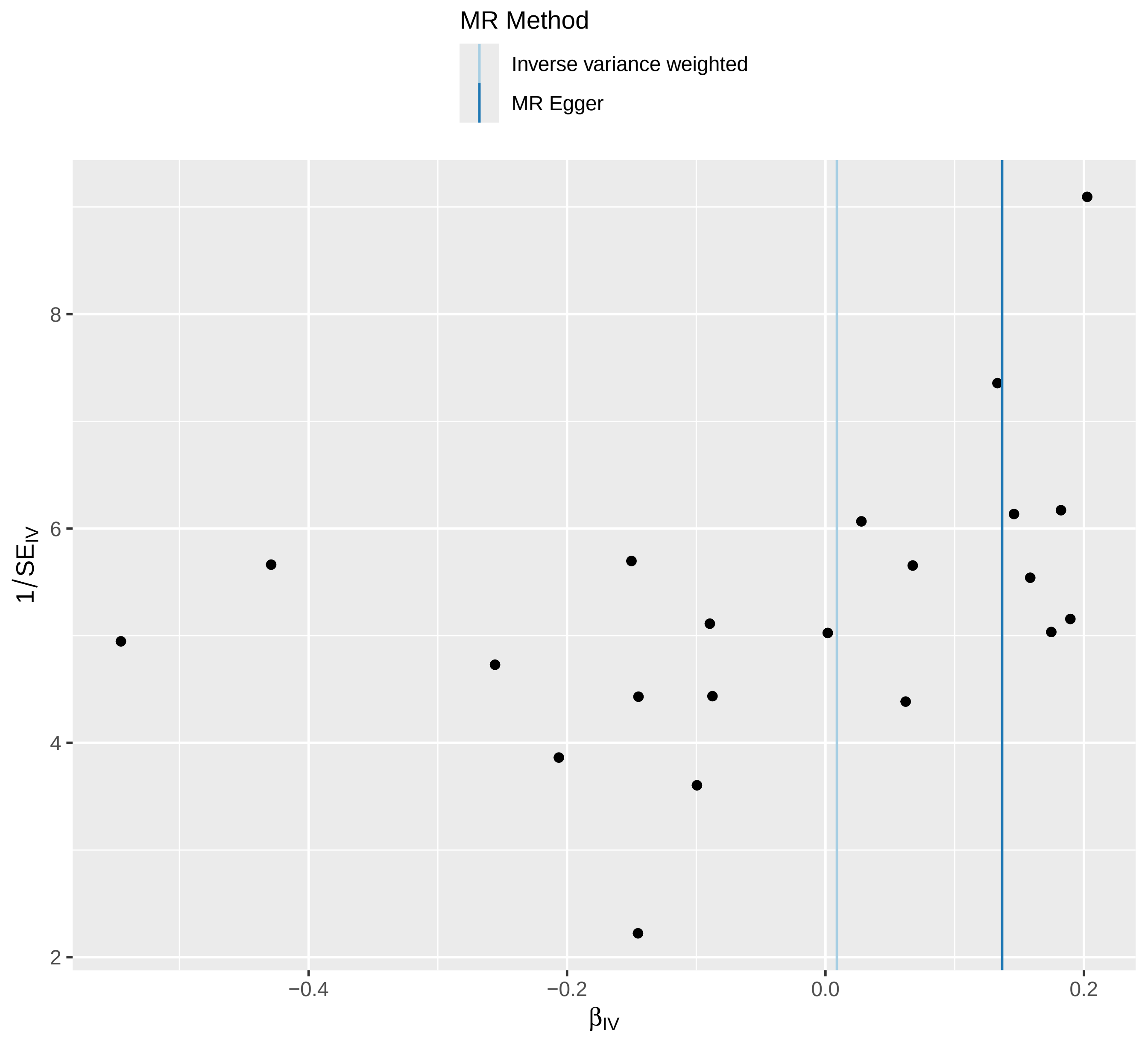

MR Method
Inverse variance weighted
MR Egger
8
6
4
2
1/SE_IV
−0.4
−0.2
0.0
0.2
β_IV

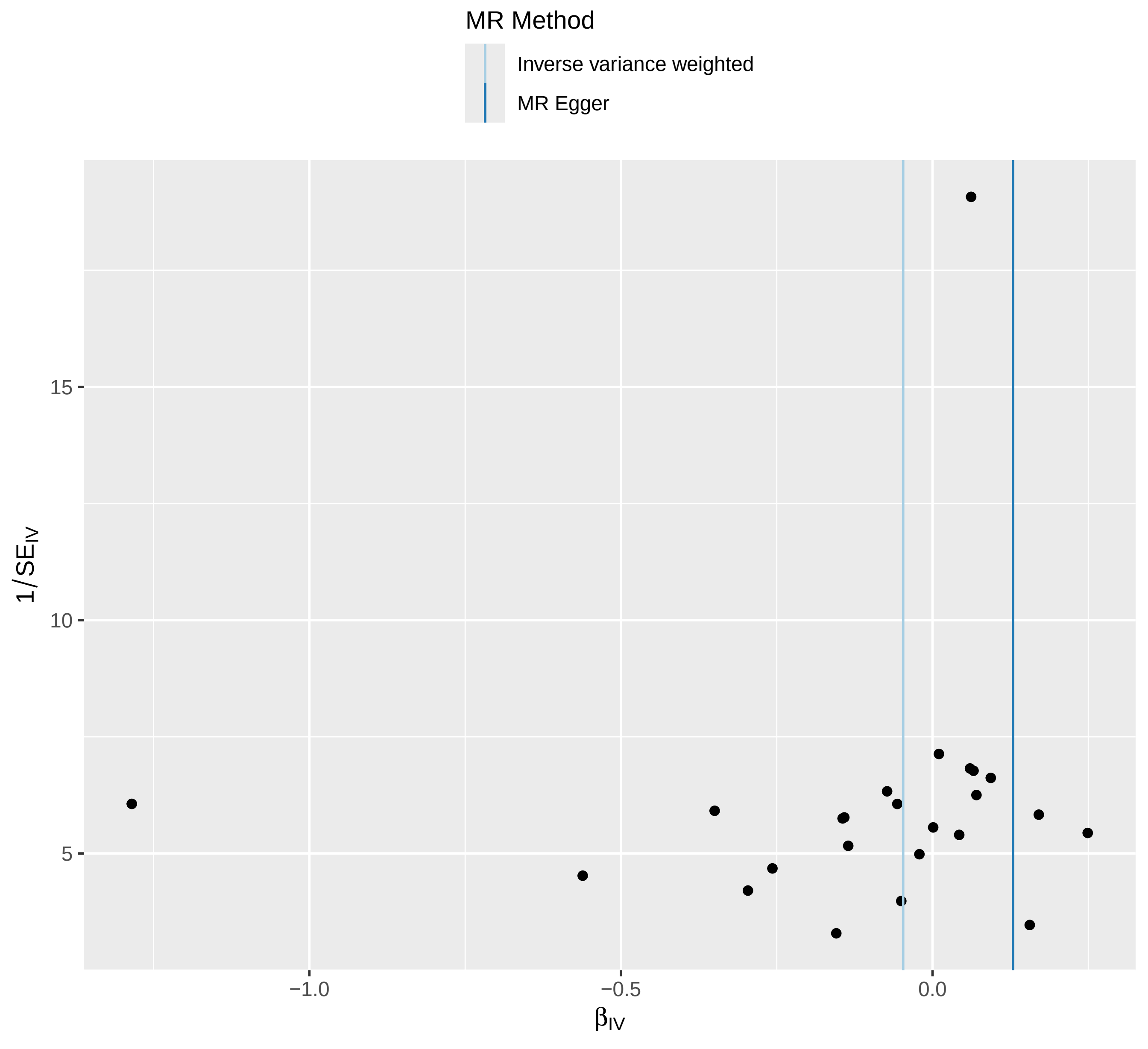

MR Method
Inverse variance weighted
MR Egger
15
10
5
$1/SE_{IV}$
−1.0
−0.5
0.0
β_{IV}

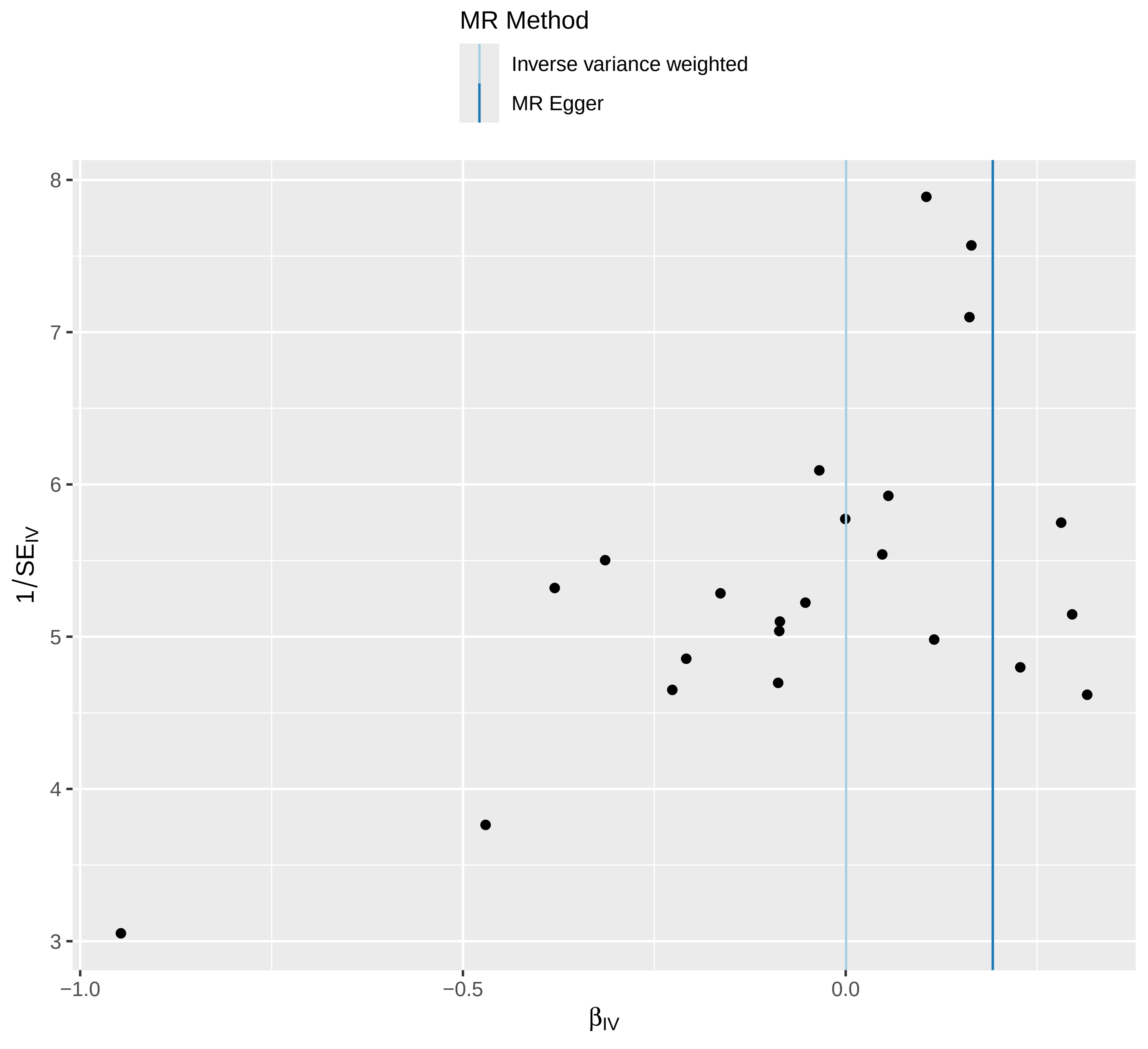

MR Method
Inverse variance weighted
MR Egger
8
7
6
5
4
3
1/SE_IV
−1.0
−0.5
0.0
β_IV

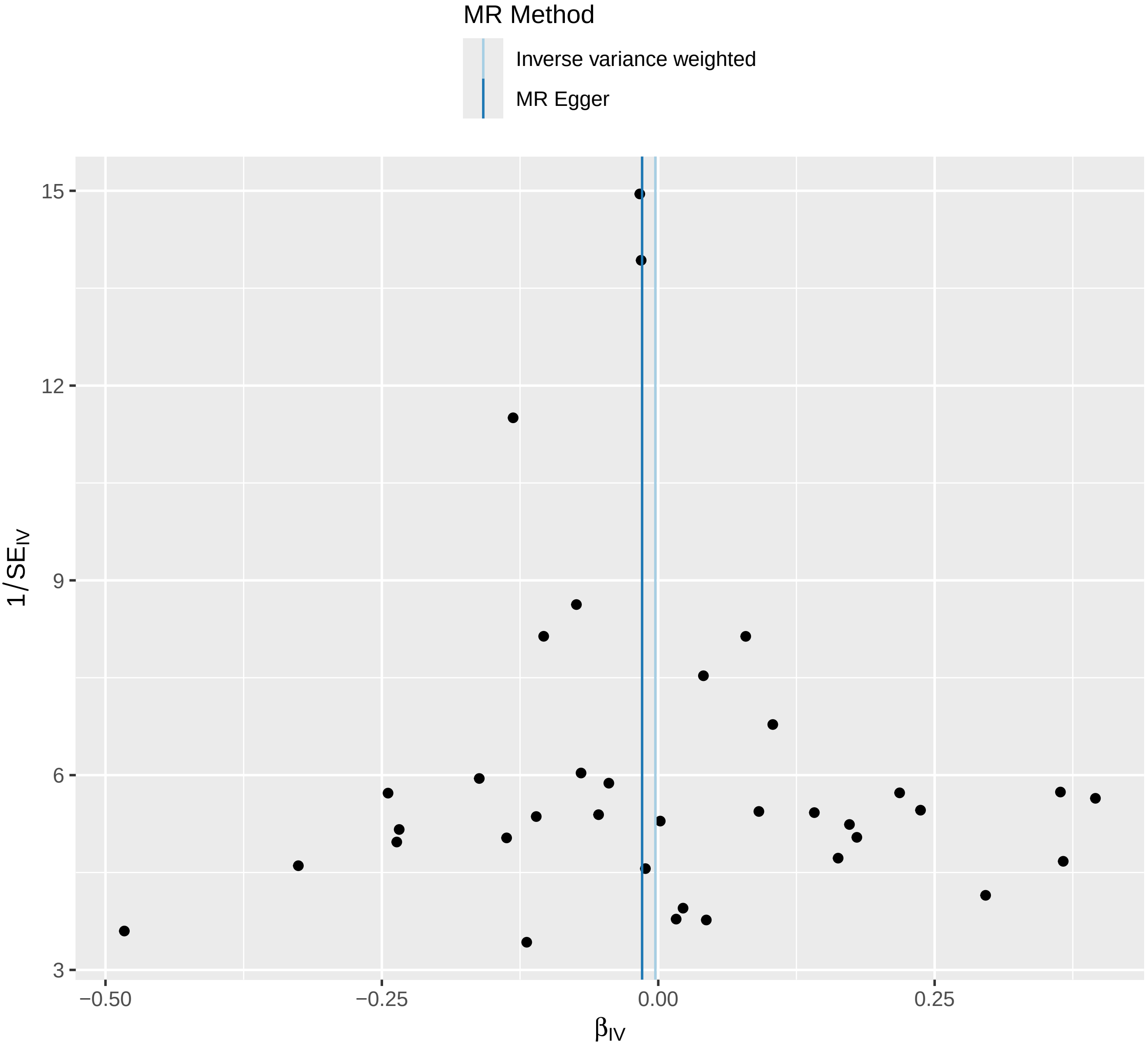
MR Method
Inverse variance weighted
MR Egger
15
12
9
6
3
−0.50
−0.25
0.00
0.25
$1/SE_{IV}$
β_{IV}

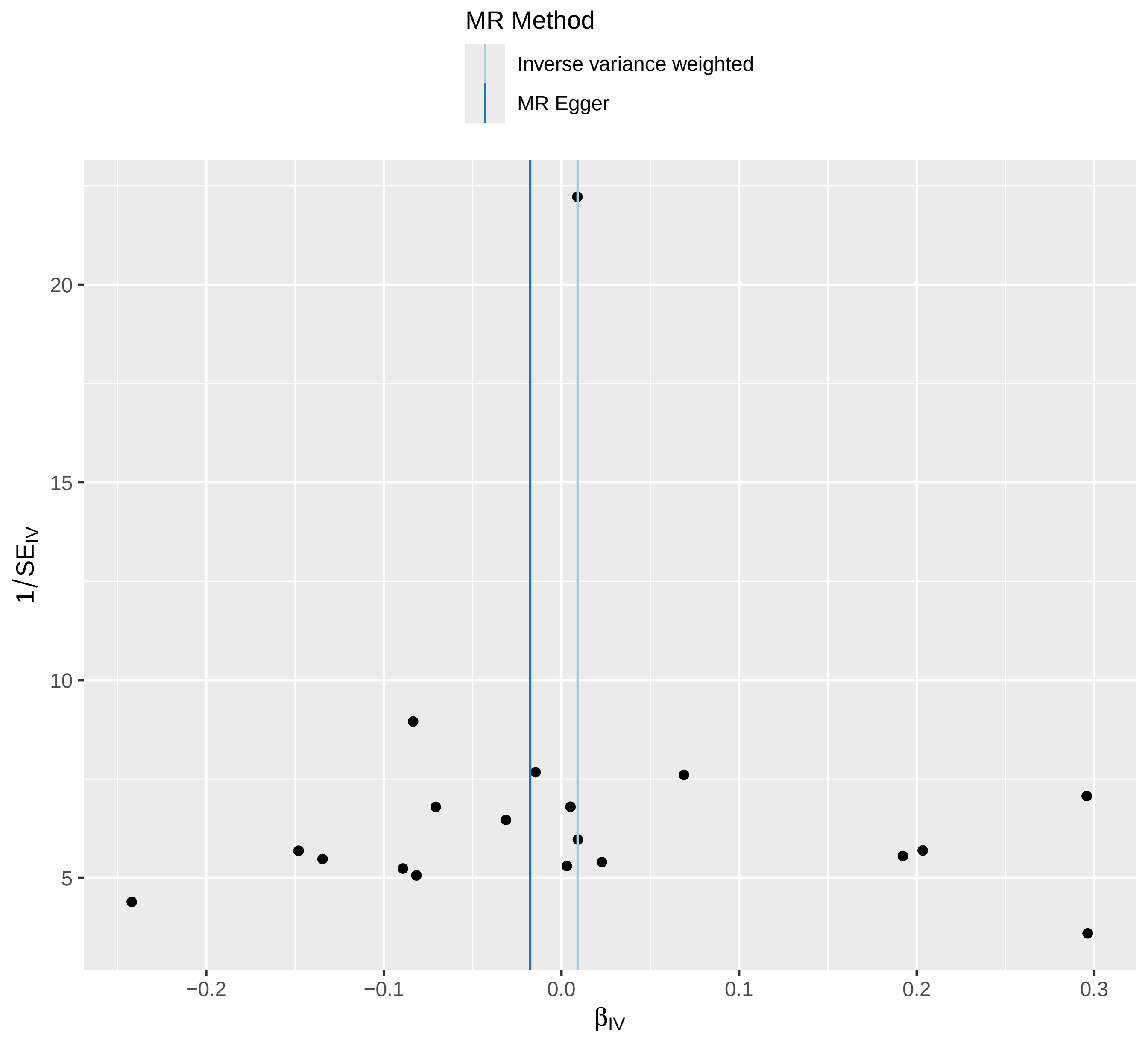
MR Method
Inverse variance weighted
MR Egger
20
15
10
5
1/SE$_{IV}$
−0.2
−0.1
0.0
0.1
0.2
0.3
β$_{IV}$

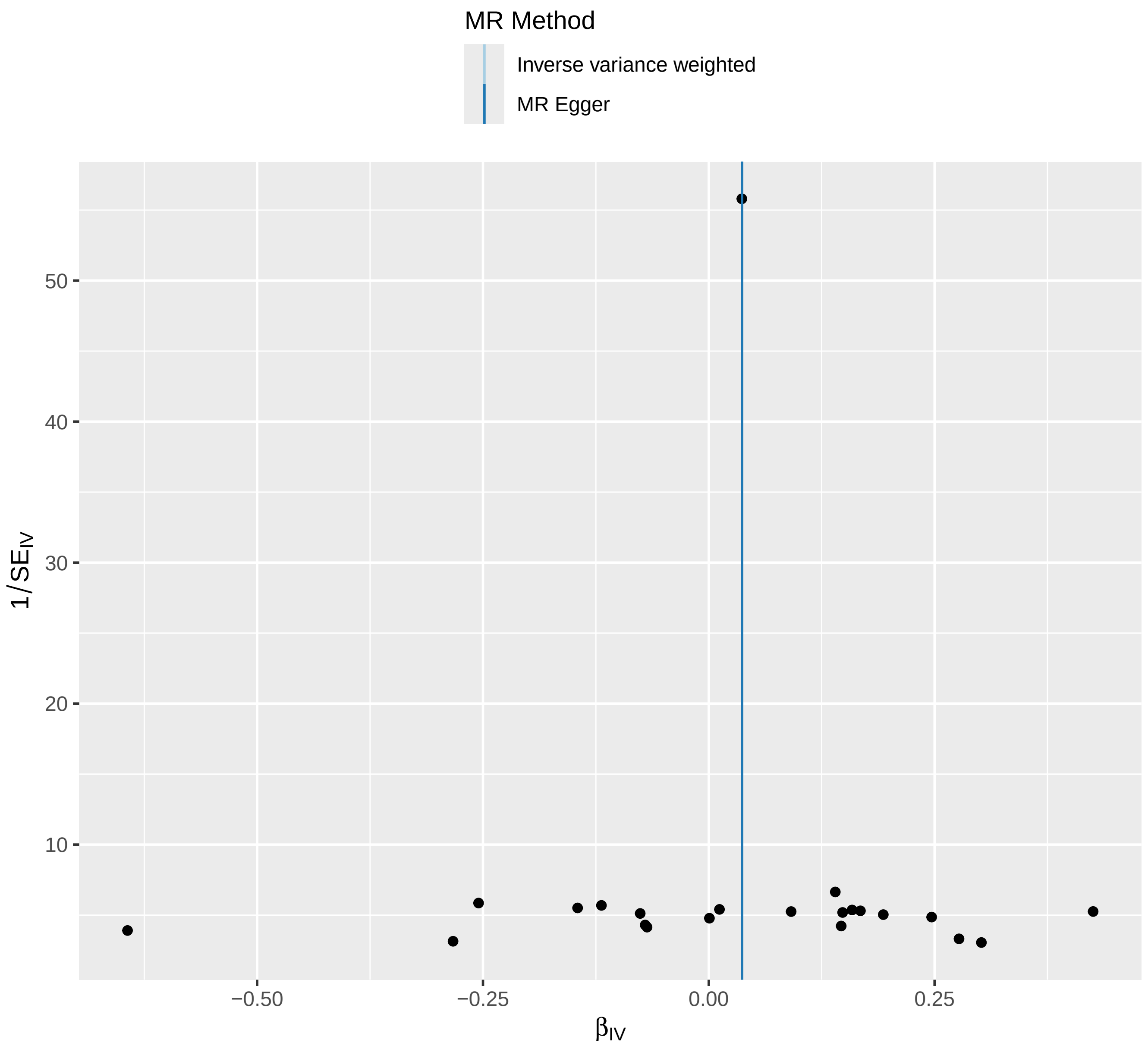
MR Method
Inverse variance weighted
MR Egger
1/SE_{IV}
50
40
30
20
10
−0.50
−0.25
0.00
0.25
β_{IV}

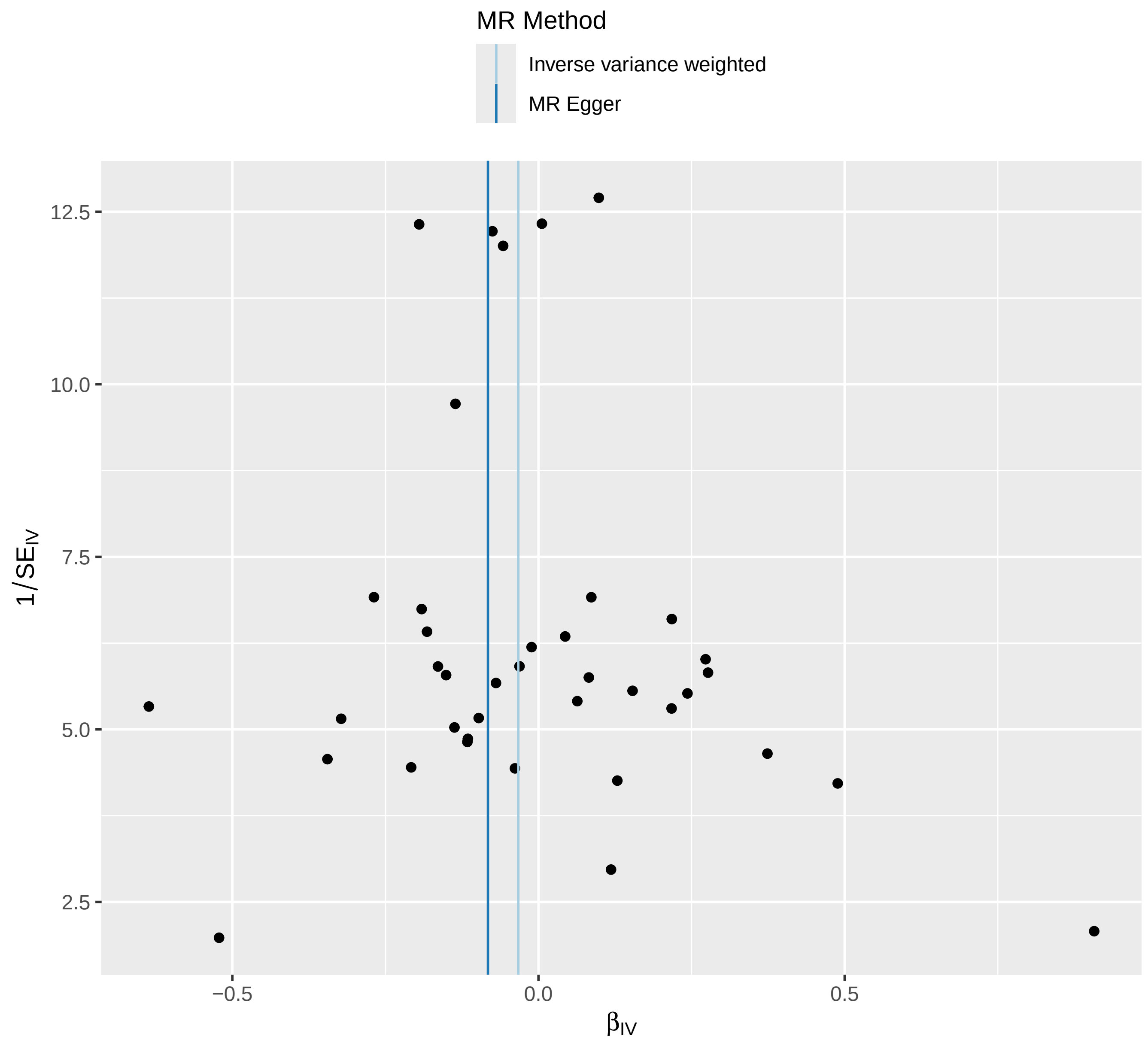
MR Method
Inverse variance weighted
MR Egger
12.5
10.0
7.5
5.0
2.5
1/SE_IV
−0.5
0.0
0.5
β_IV

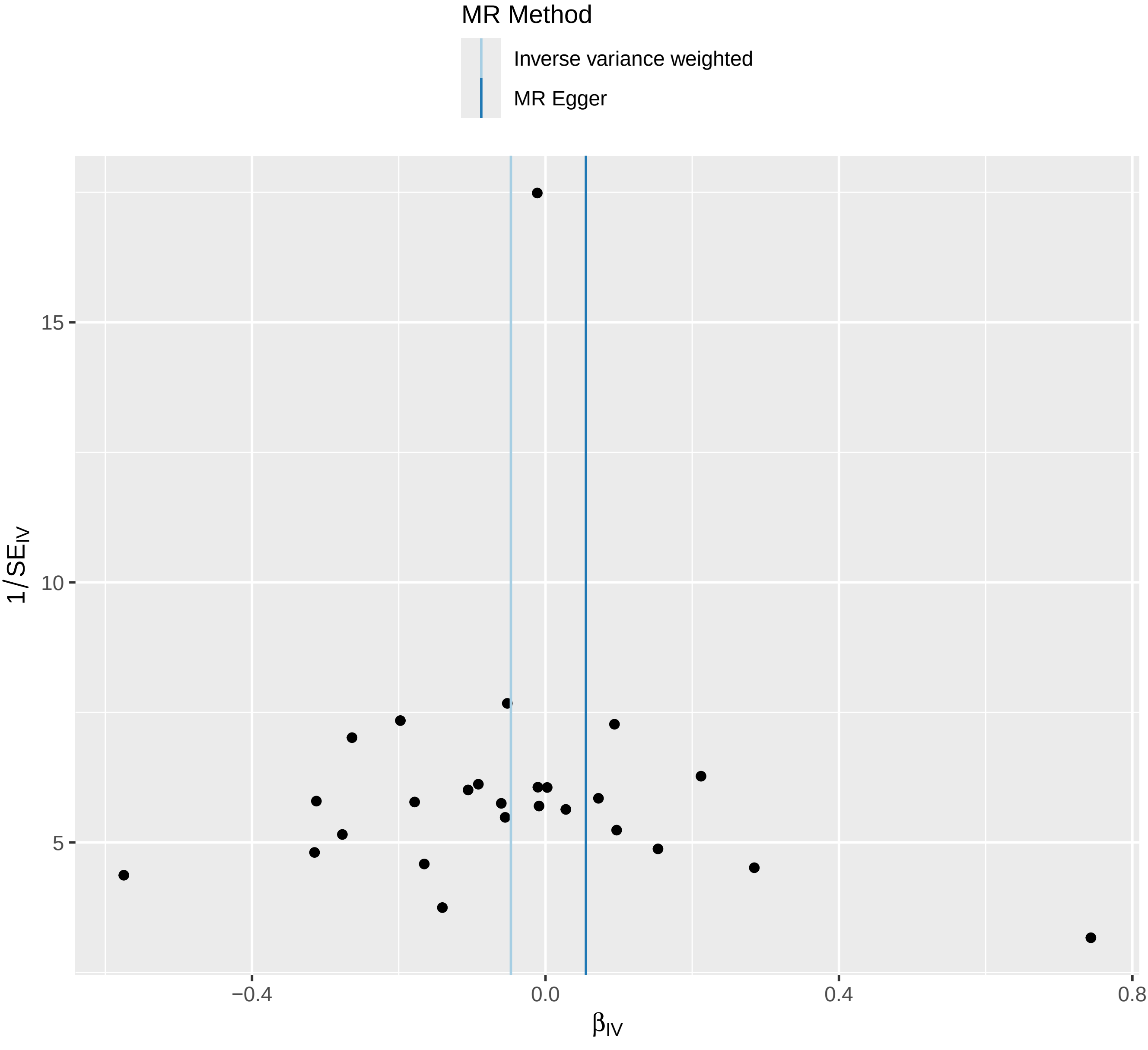

MR Method
Inverse variance weighted
MR Egger
15
10
5
$1/SE_{IV}$
−0.4
0.0
0.4
0.8
β_{IV}

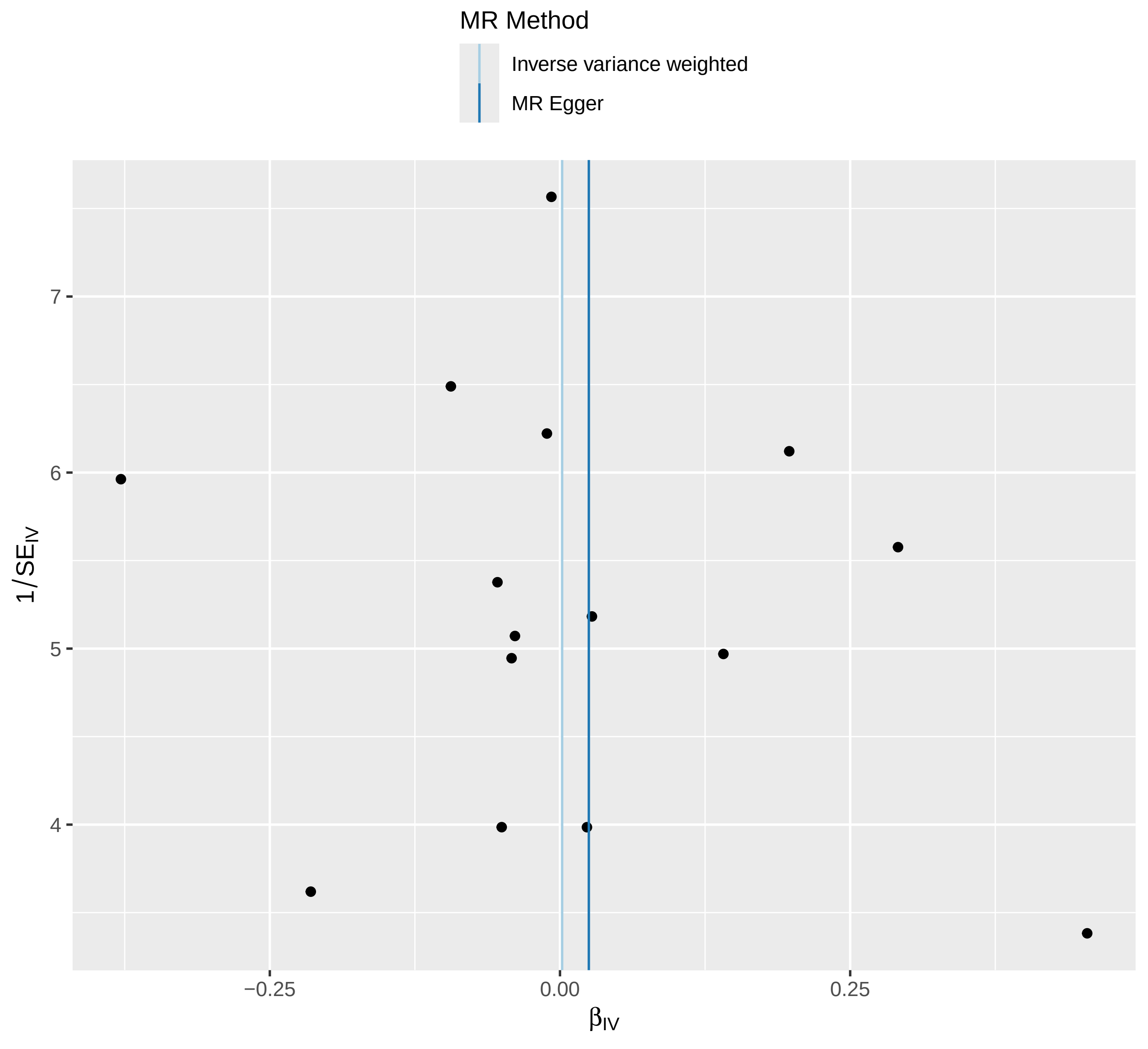
MR Method
Inverse variance weighted
MR Egger
7
6
5
4
1/SE_IV
−0.25
0.00
0.25
β_IV

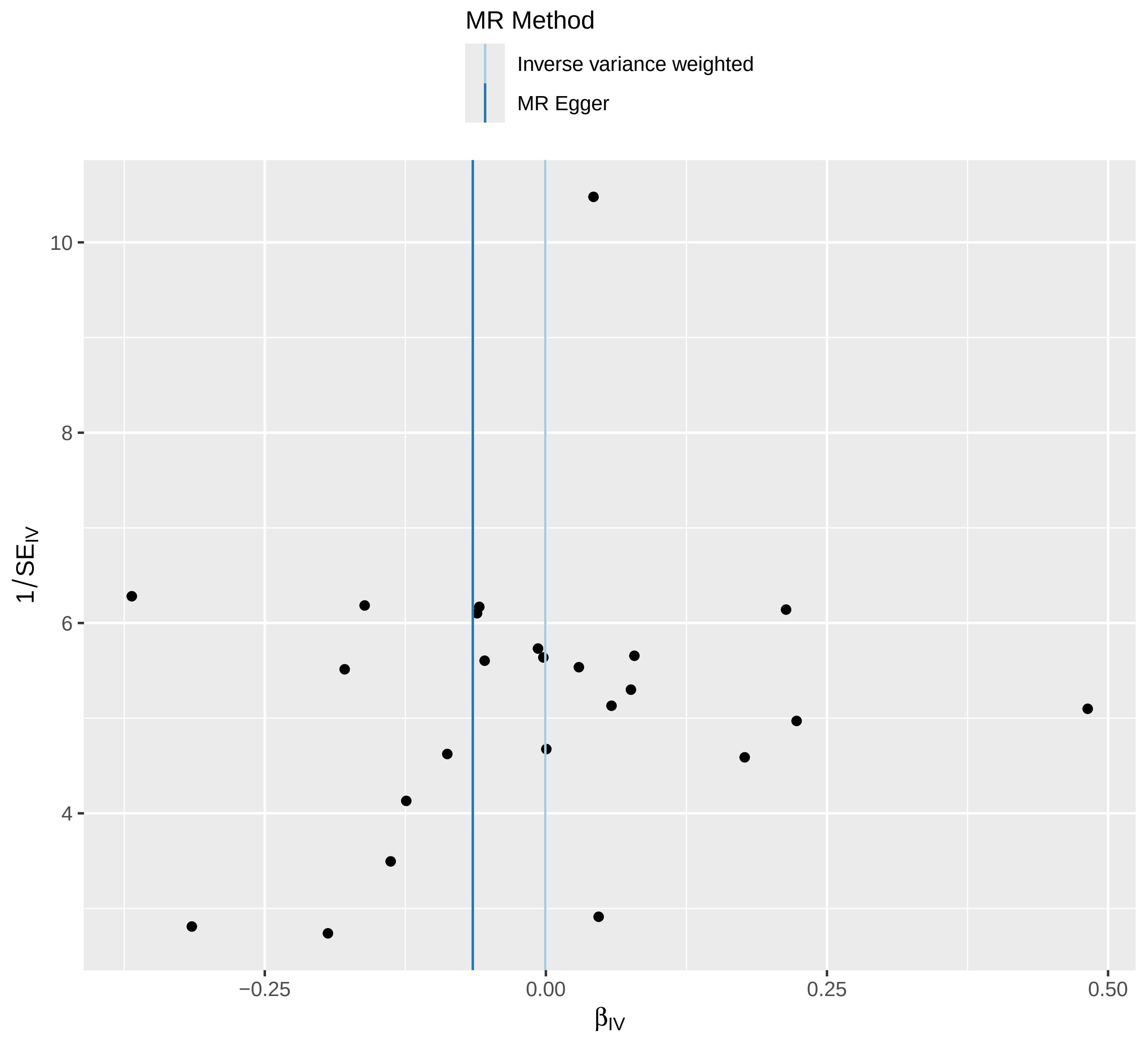
MR Method
Inverse variance weighted
MR Egger
10
8
6
4
−0.25
0.00
0.25
0.50
$1/SE_{IV}$
β_{IV}

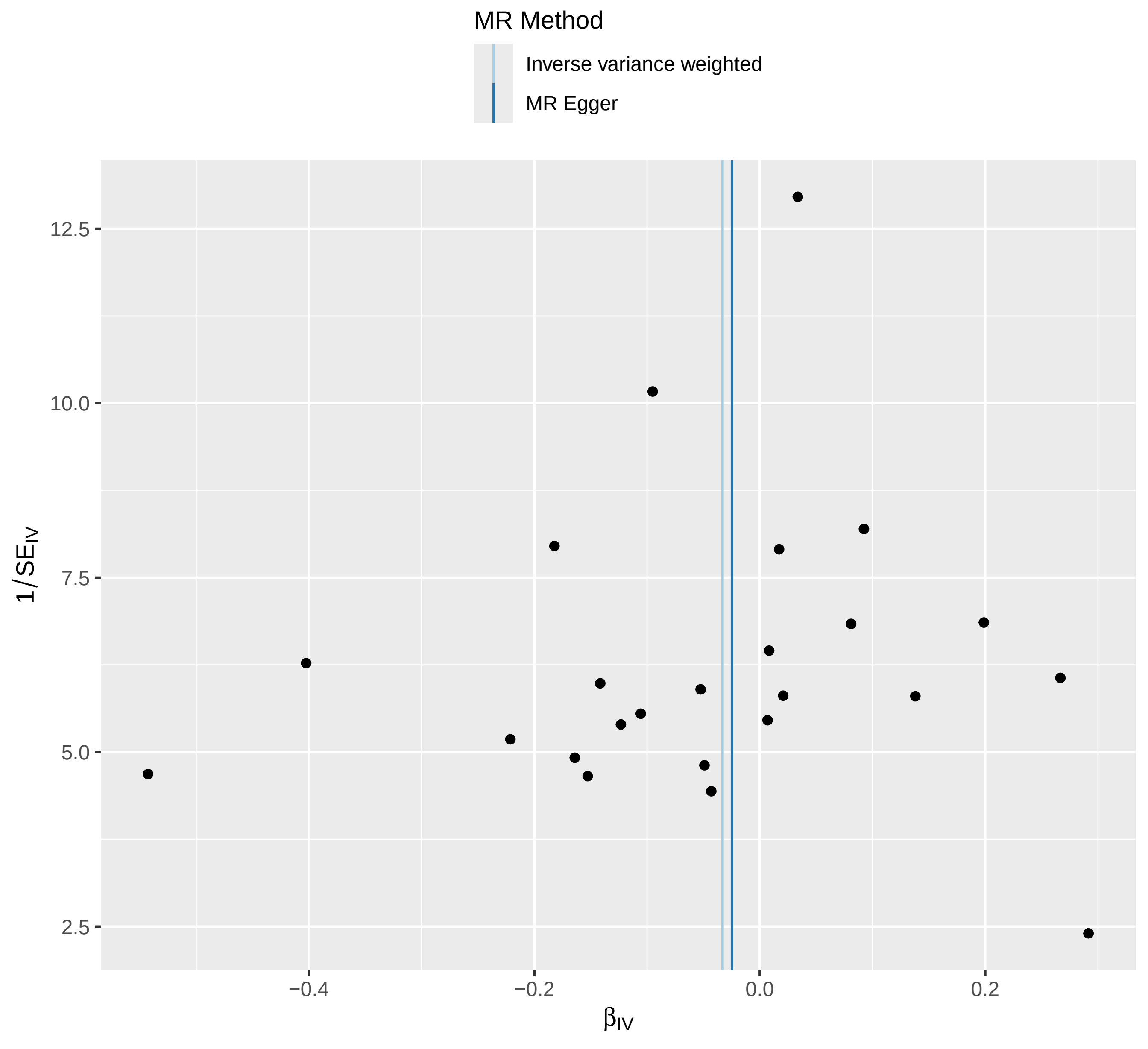
MR Method
Inverse variance weighted
MR Egger
12.5
10.0
7.5
5.0
2.5
1/SE_IV
−0.4
−0.2
0.0
0.2
β_IV

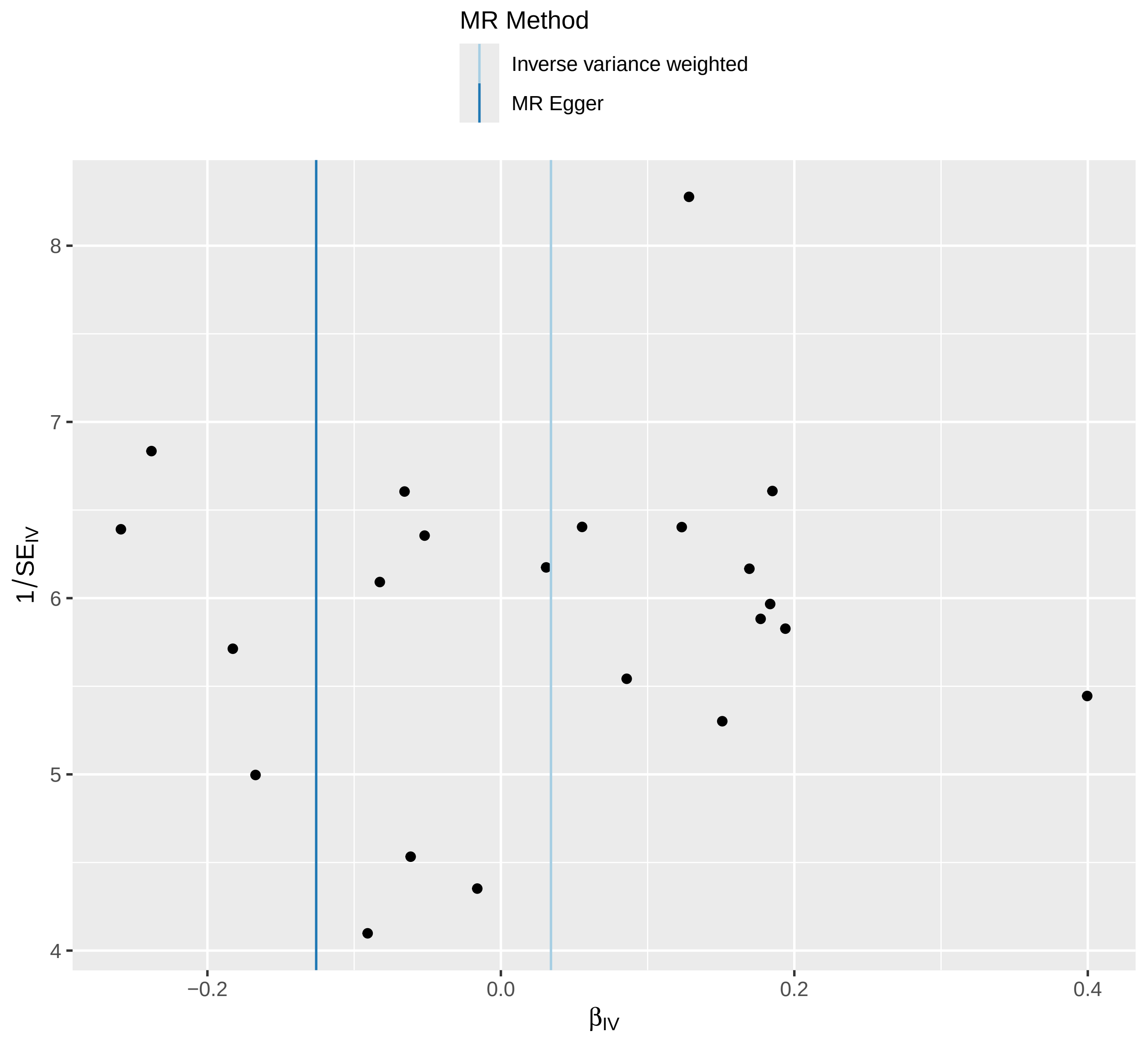
MR Method
Inverse variance weighted
MR Egger
1/SE_IV
β_IV
8
7
6
5
4
−0.2
0.0
0.2
0.4

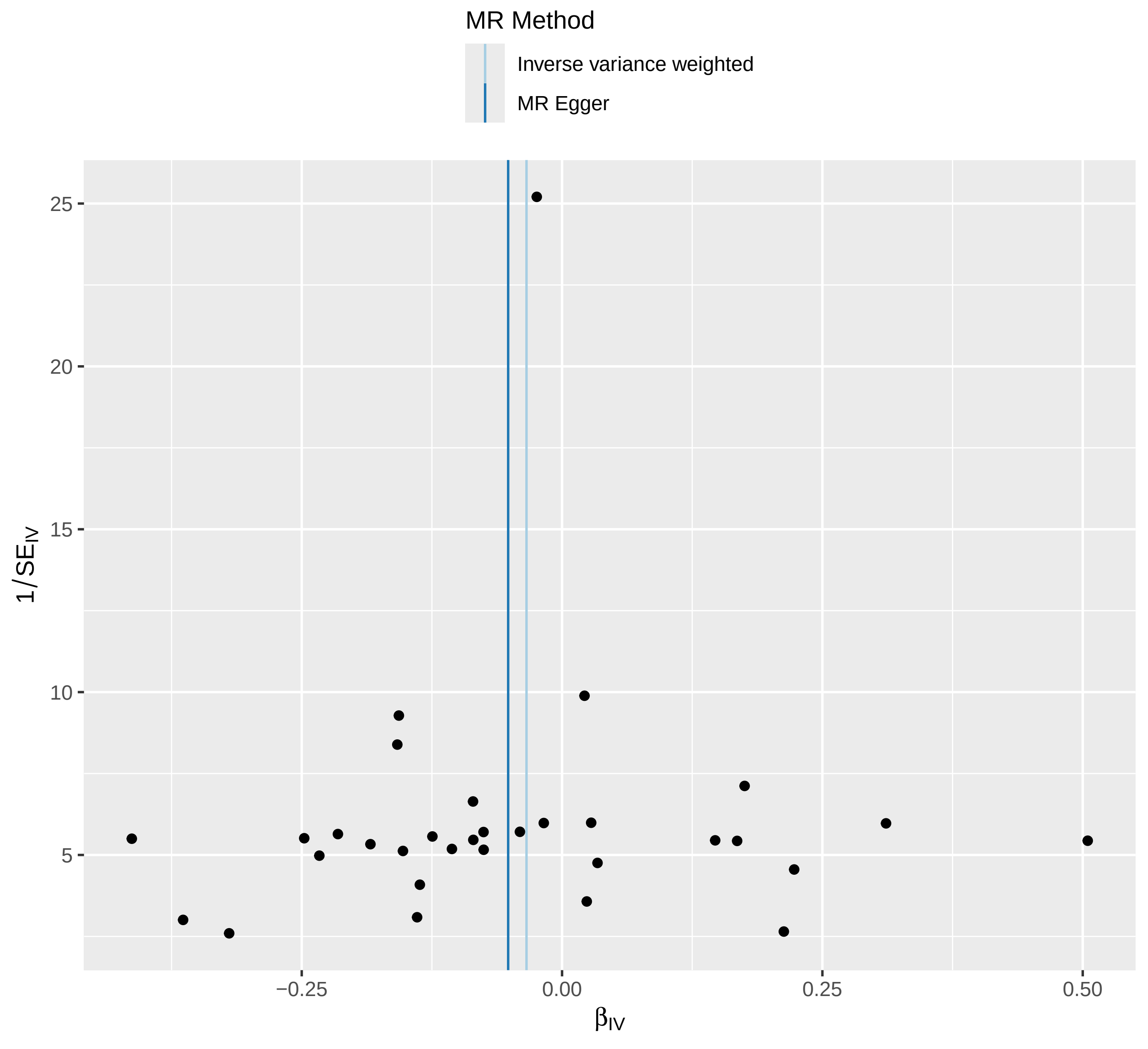
MR Method
Inverse variance weighted
MR Egger
25
20
15
10
5
1/SE_{IV}
−0.25
0.00
0.25
0.50
β_{IV}

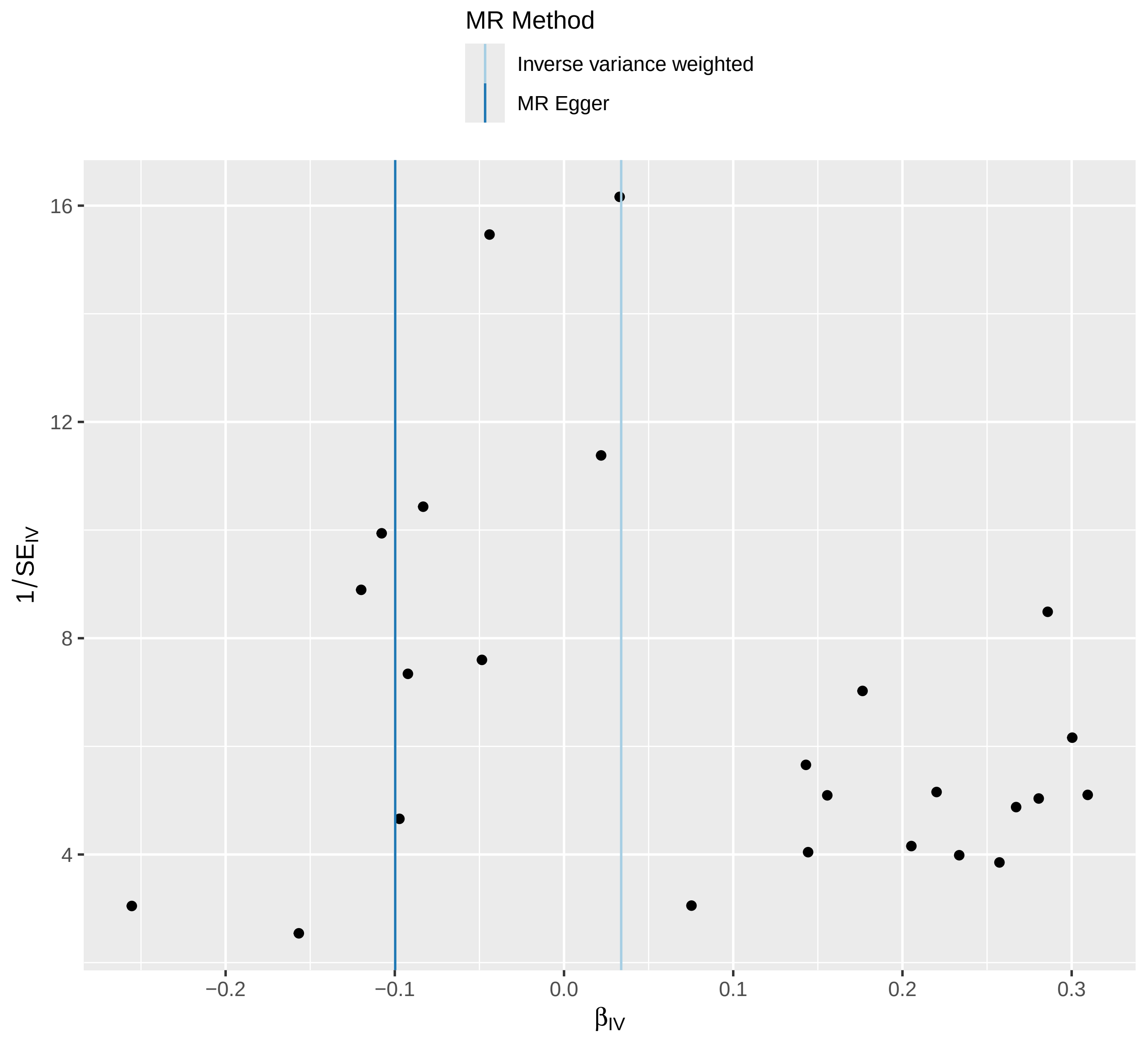
MR Method
Inverse variance weighted
MR Egger
16
12
8
4
1/SE_IV
−0.2
−0.1
0.0
0.1
0.2
0.3
β_IV

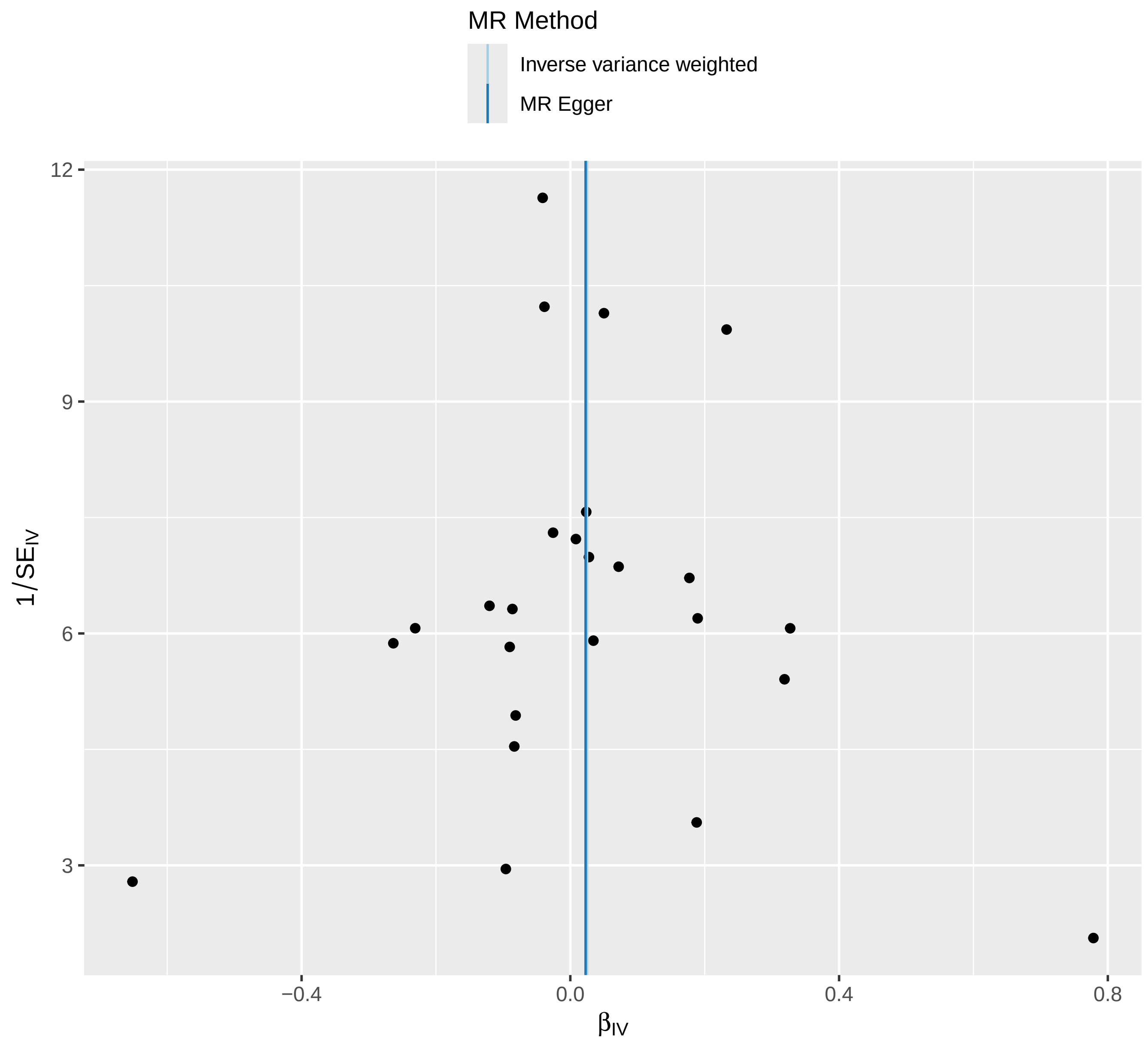
MR Method
Inverse variance weighted
MR Egger
12
9
6
3
$1/SE_{IV}$
−0.4
0.0
0.4
0.8
β_{IV}

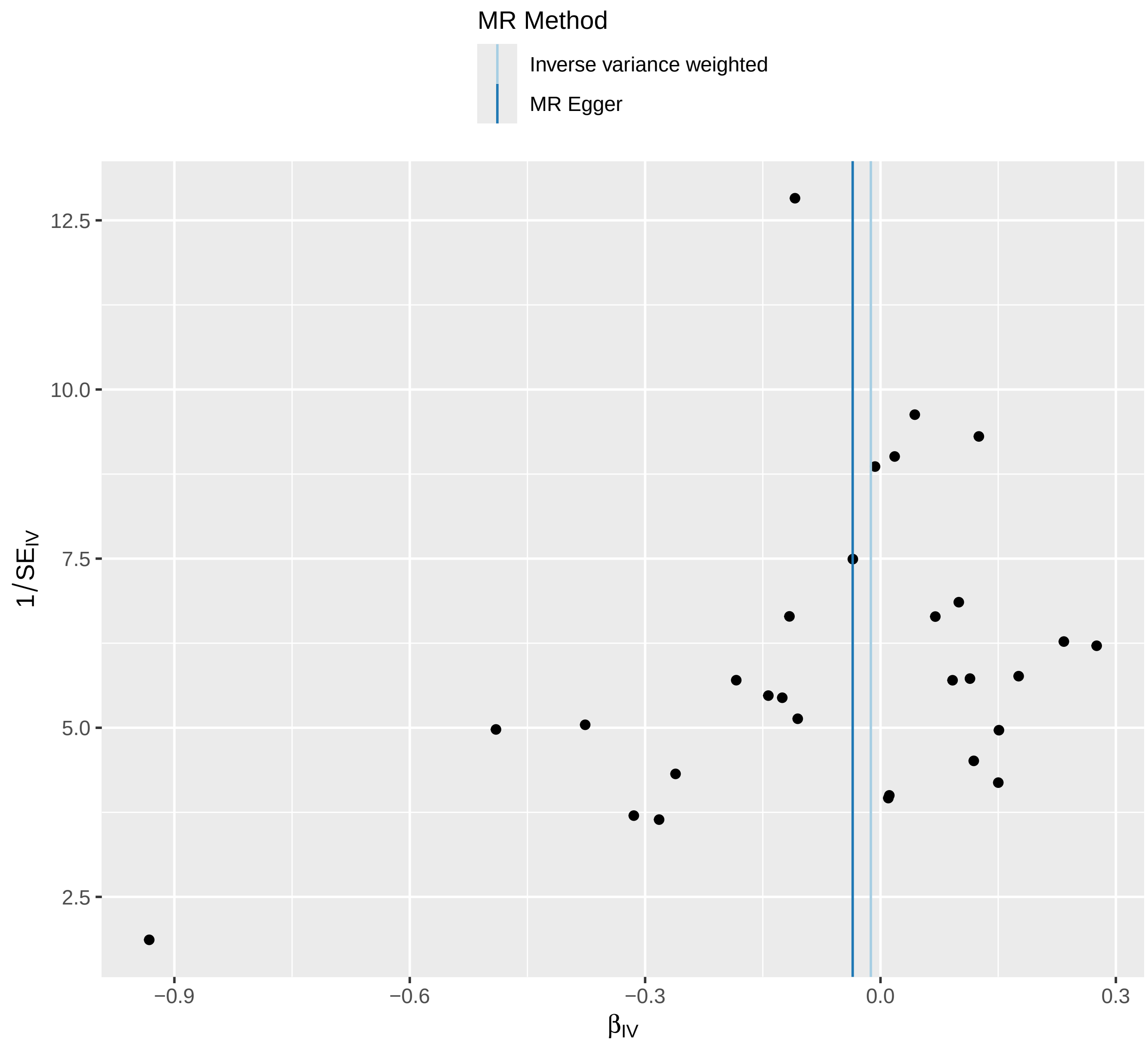
MR Method
Inverse variance weighted
MR Egger
12.5
10.0
7.5
5.0
2.5
$1/SE_{IV}$
−0.9
−0.6
−0.3
0.0
0.3
β_{IV}

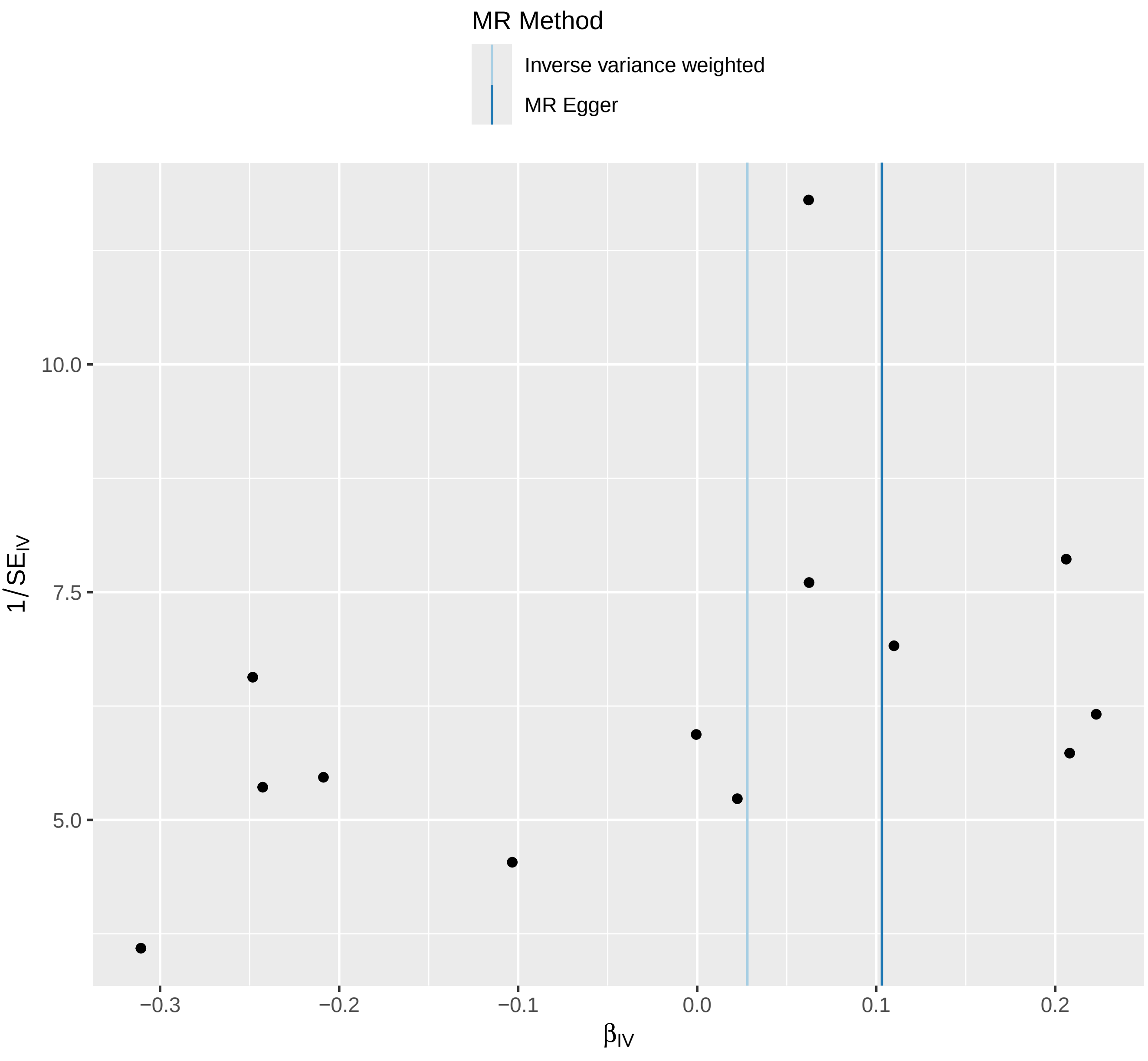

MR Method
Inverse variance weighted
MR Egger
10.0
7.5
5.0
1/SE_IV
−0.3
−0.2
−0.1
0.0
0.1
0.2
β_IV

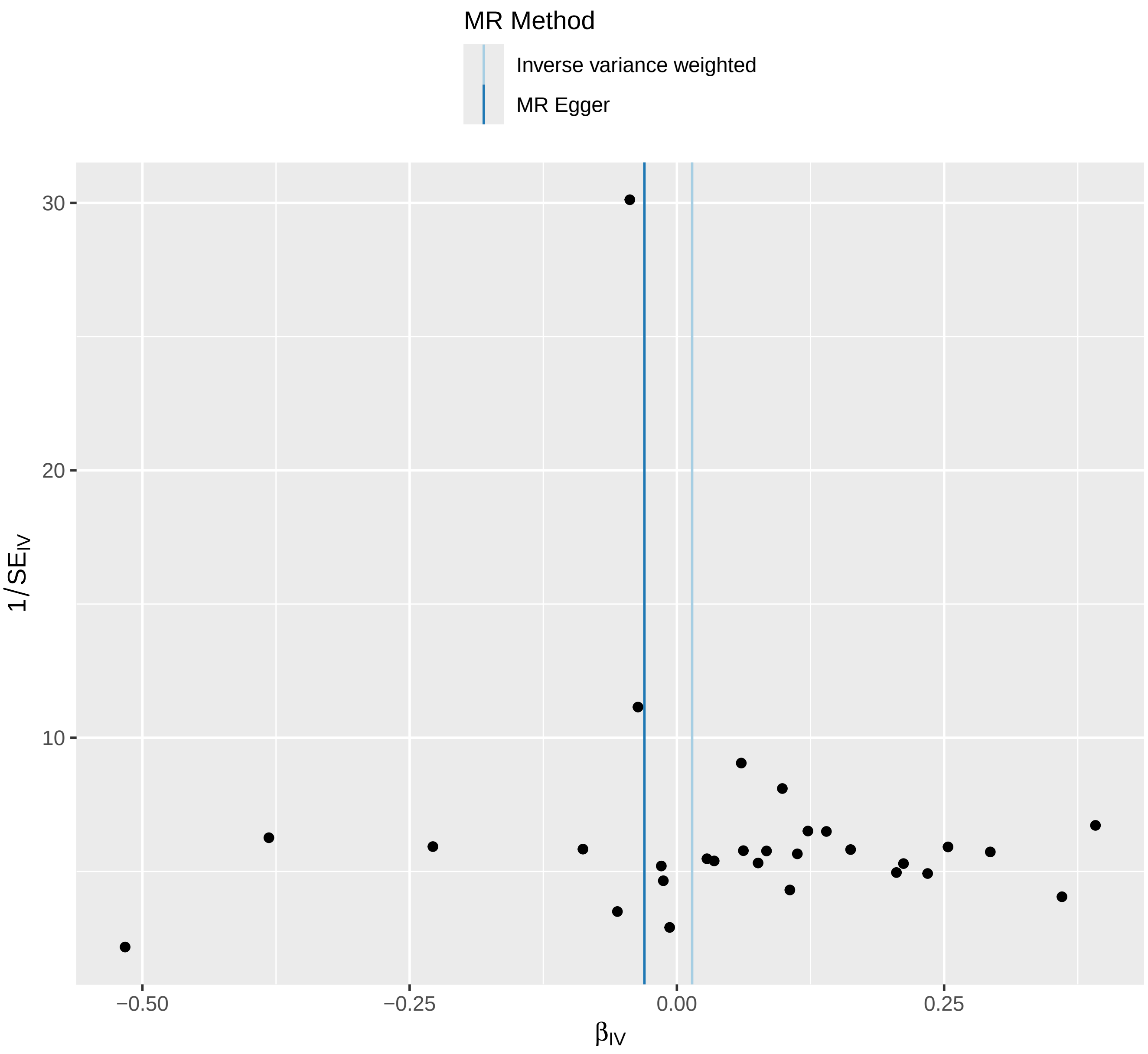
MR Method
Inverse variance weighted
MR Egger
30
20
10
1/SE$_{IV}$
−0.50
−0.25
0.00
0.25
β_{IV}

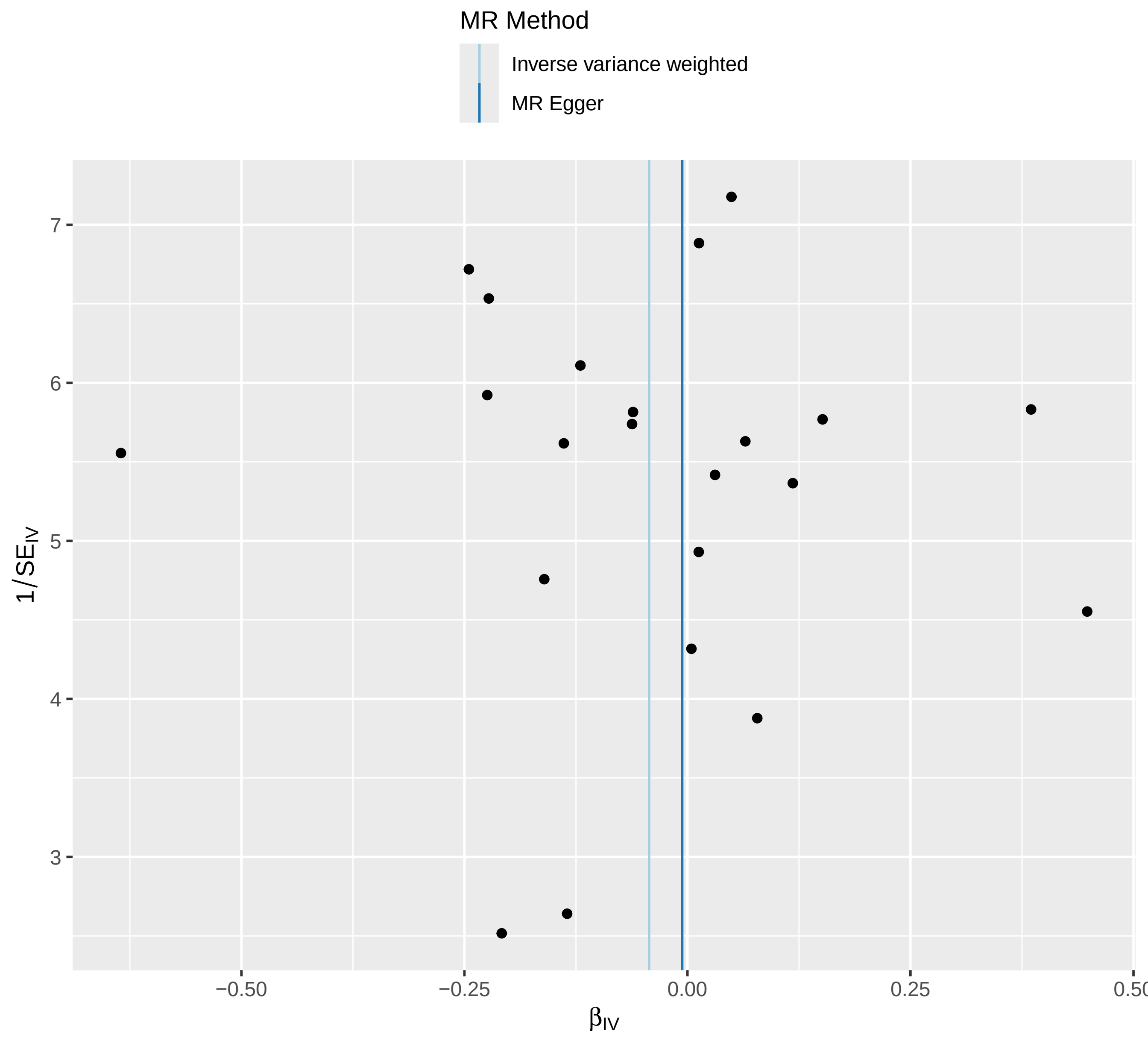

MR Method
Inverse variance weighted
MR Egger
7
6
5
4
3
1/SE$_{IV}$
−0.50
−0.25
0.00
0.25
0.50
β$_{IV}$

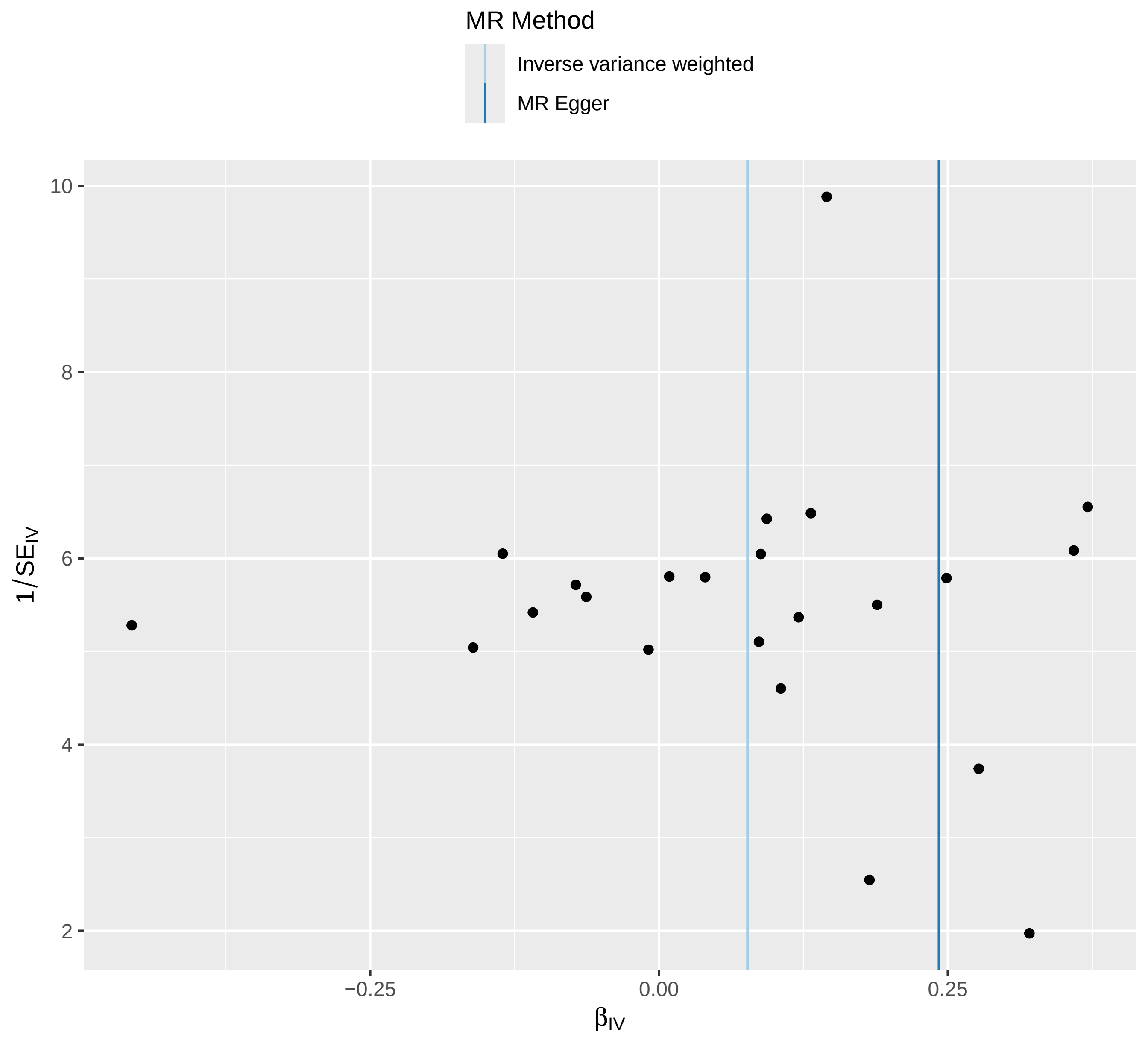
MR Method
Inverse variance weighted
MR Egger
10
8
6
4
2
1/SE_IV
−0.25
0.00
0.25
β_IV

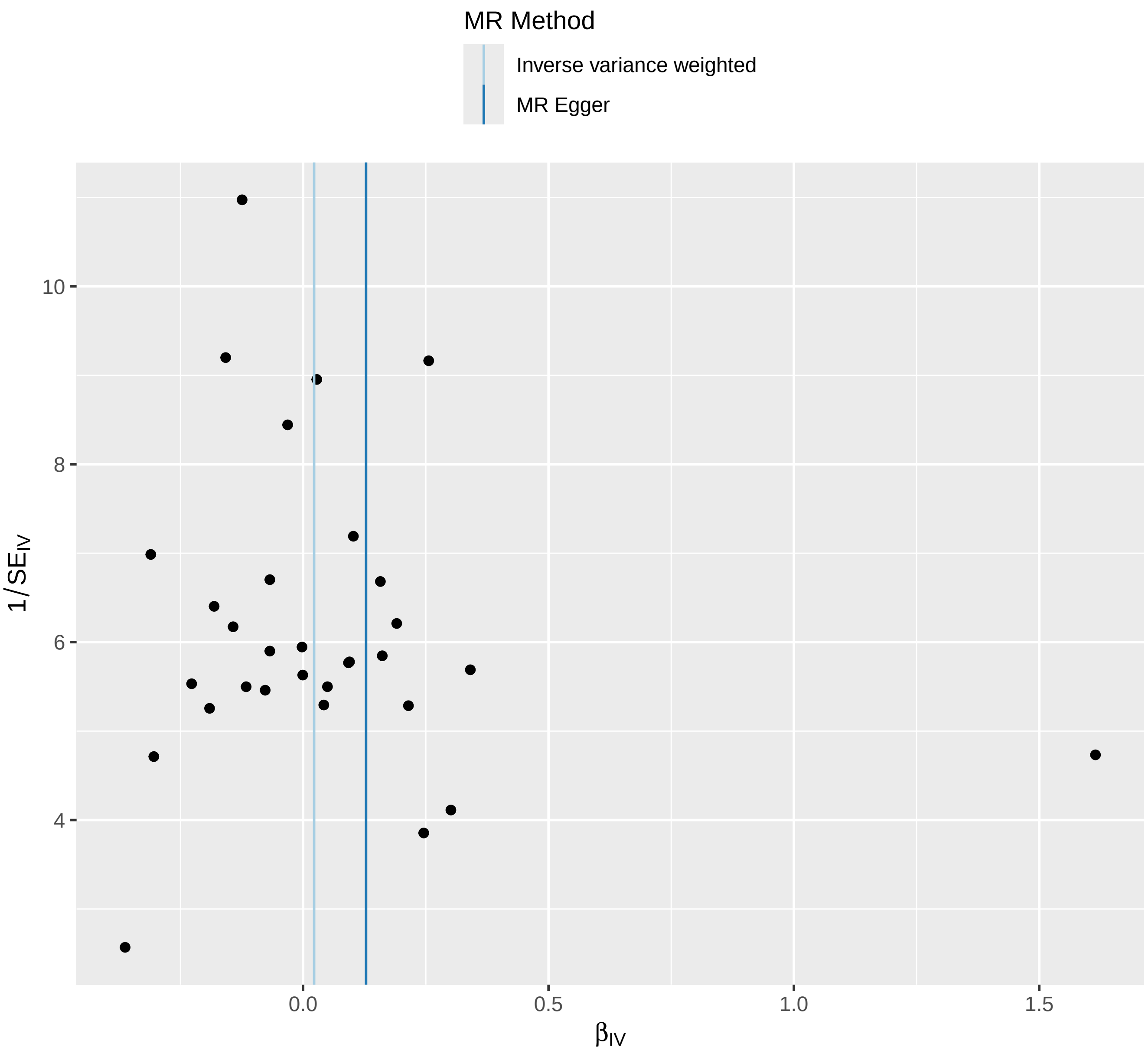

MR Method
Inverse variance weighted
MR Egger
1/SE_IV
10
8
6
4
0.0
0.5
1.0
1.5
β_IV

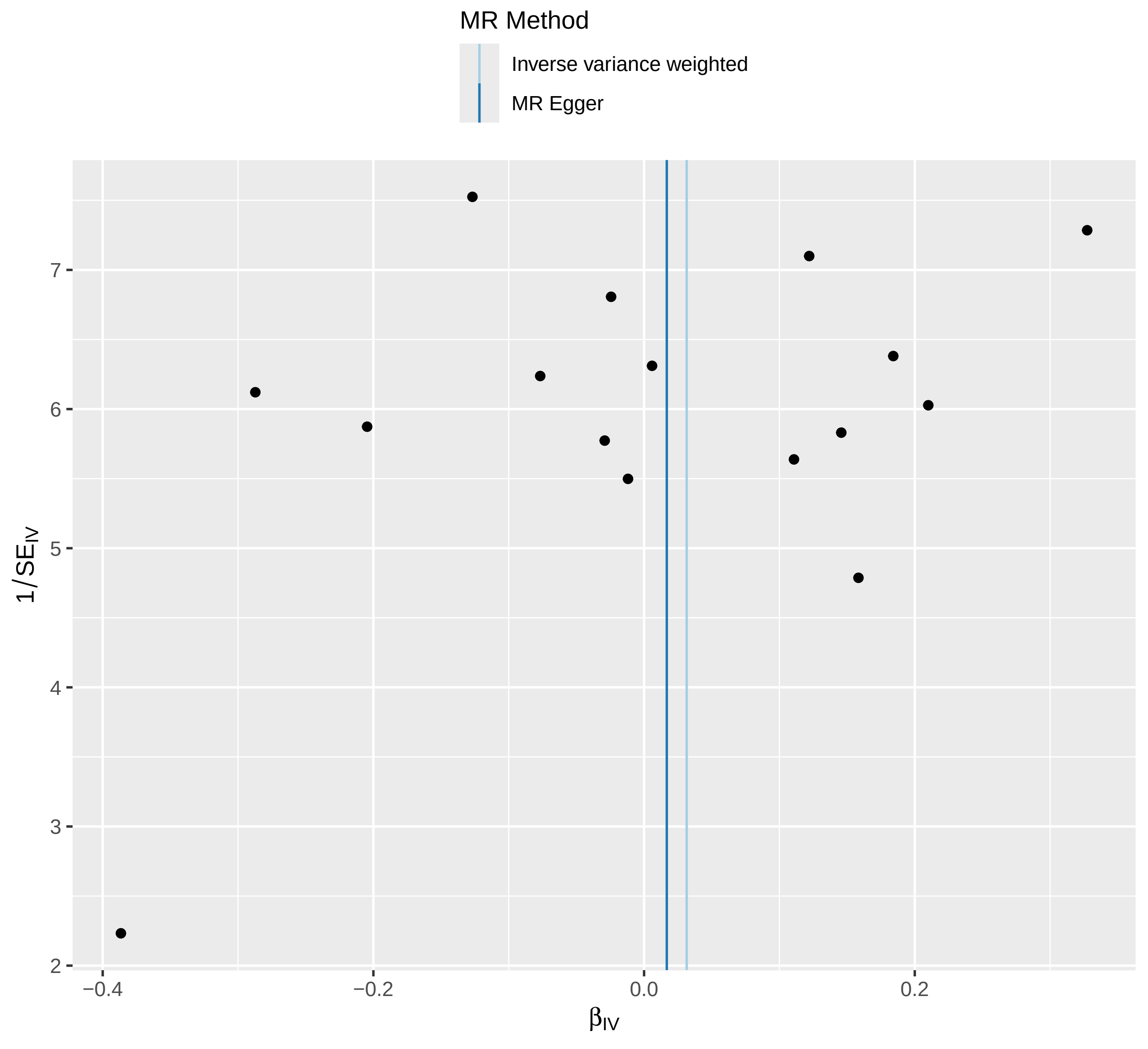
MR Method
Inverse variance weighted
MR Egger
1/SE_IV
β_IV
7
6
5
4
3
2
−0.4
−0.2
0.0
0.2

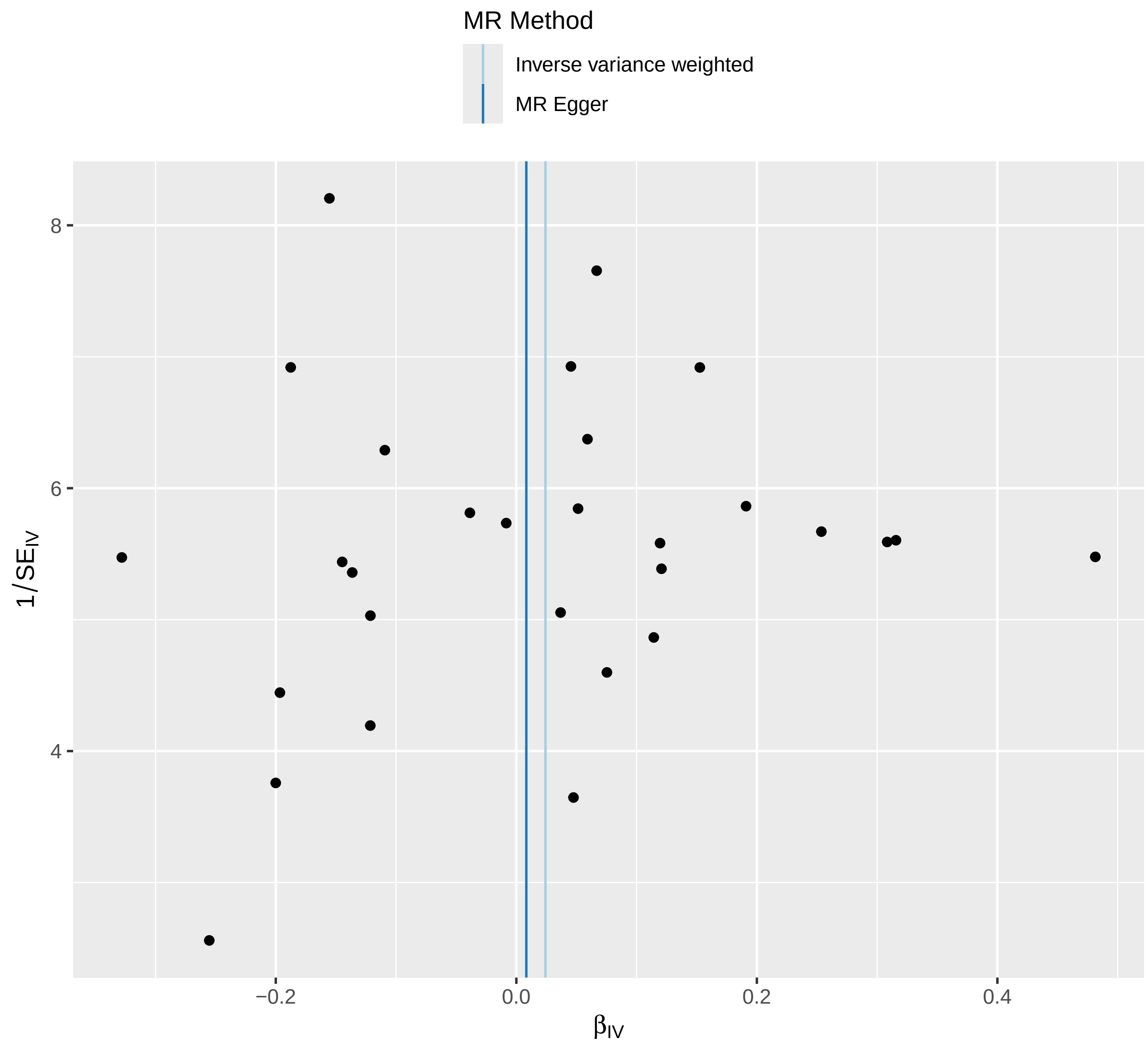

MR Method
Inverse variance weighted
MR Egger
8
6
4
1/SE$_{IV}$
−0.2
0.0
0.2
0.4
β$_{IV}$

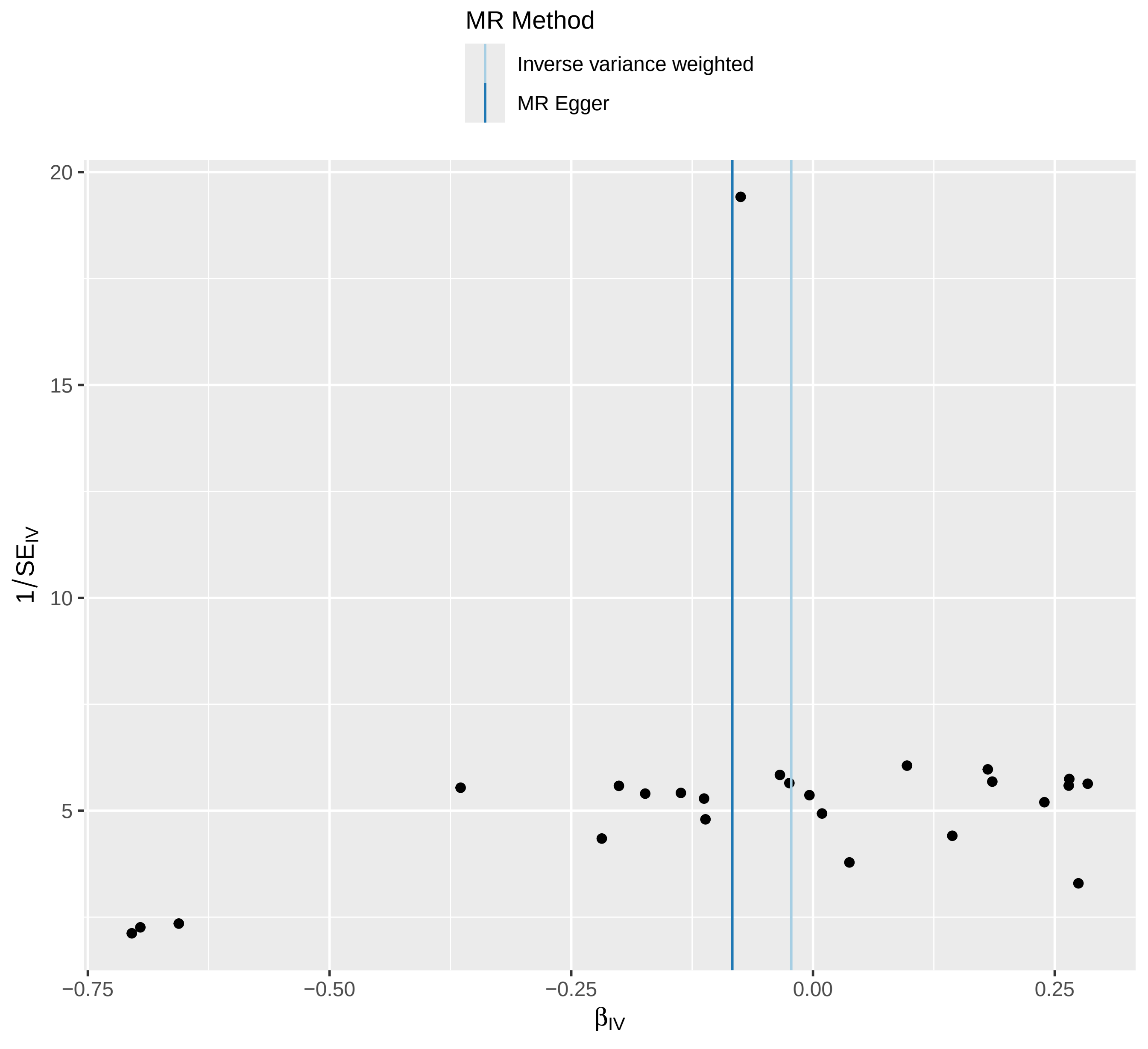
MR Method
Inverse variance weighted
MR Egger
20
15
10
5
1/SE$_{IV}$
−0.75
−0.50
−0.25
0.00
0.25
β$_{IV}$

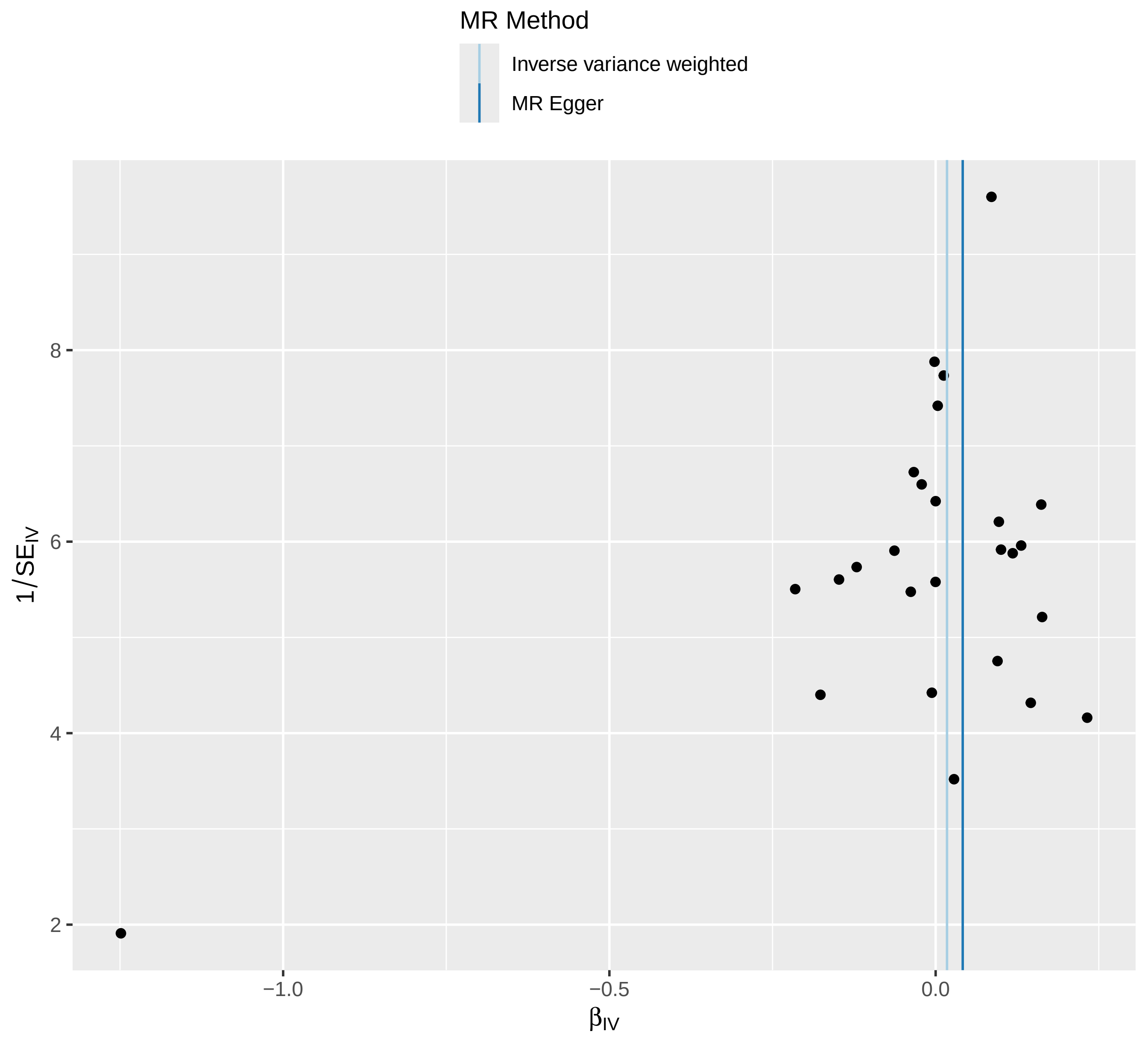

MR Method
Inverse variance weighted
MR Egger
8
6
4
2
1/SE_IV
−1.0
−0.5
0.0
β_IV

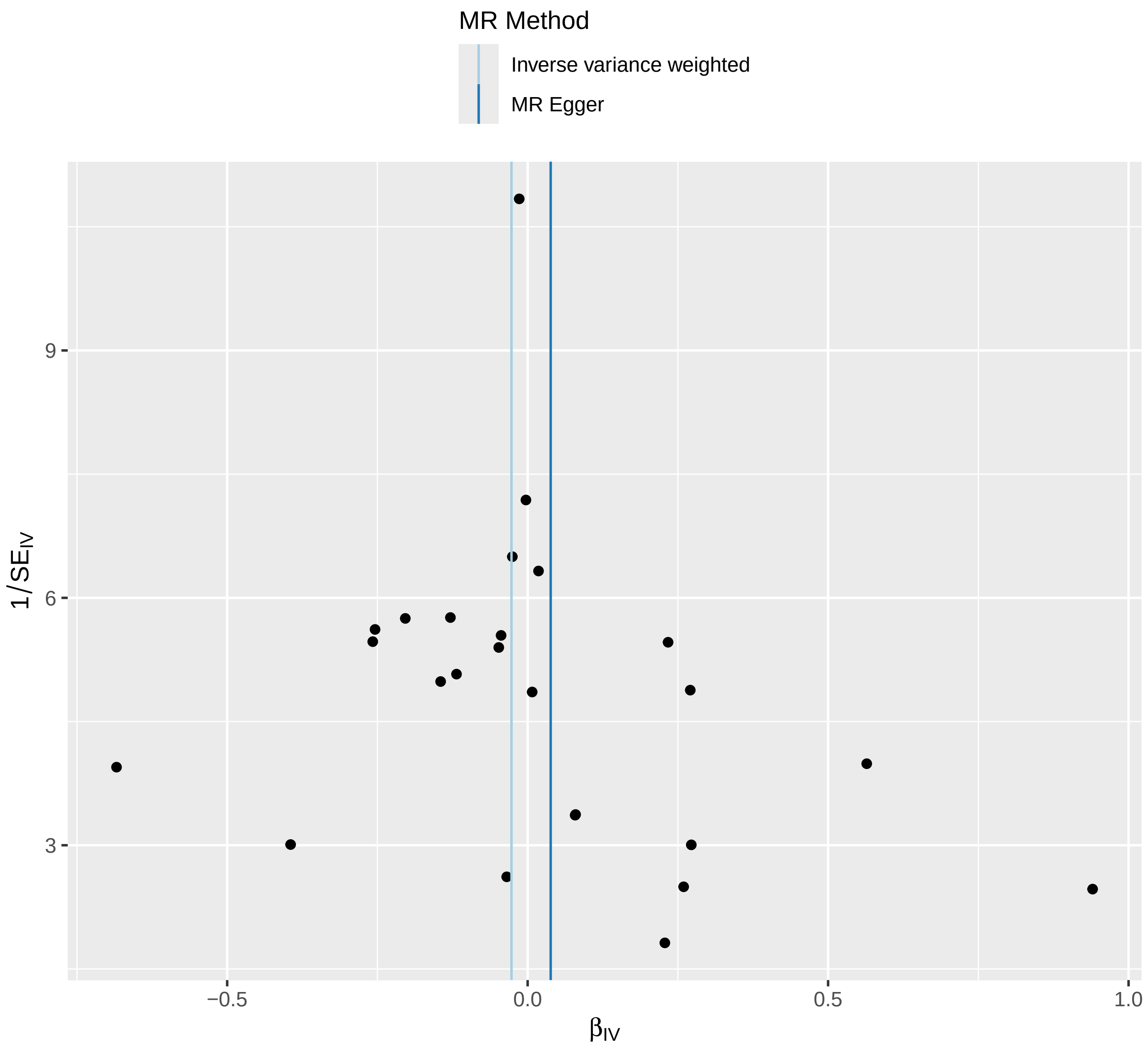
MR Method
Inverse variance weighted
MR Egger
9
6
3
1/SE_IV
−0.5
0.0
0.5
1.0
β_IV

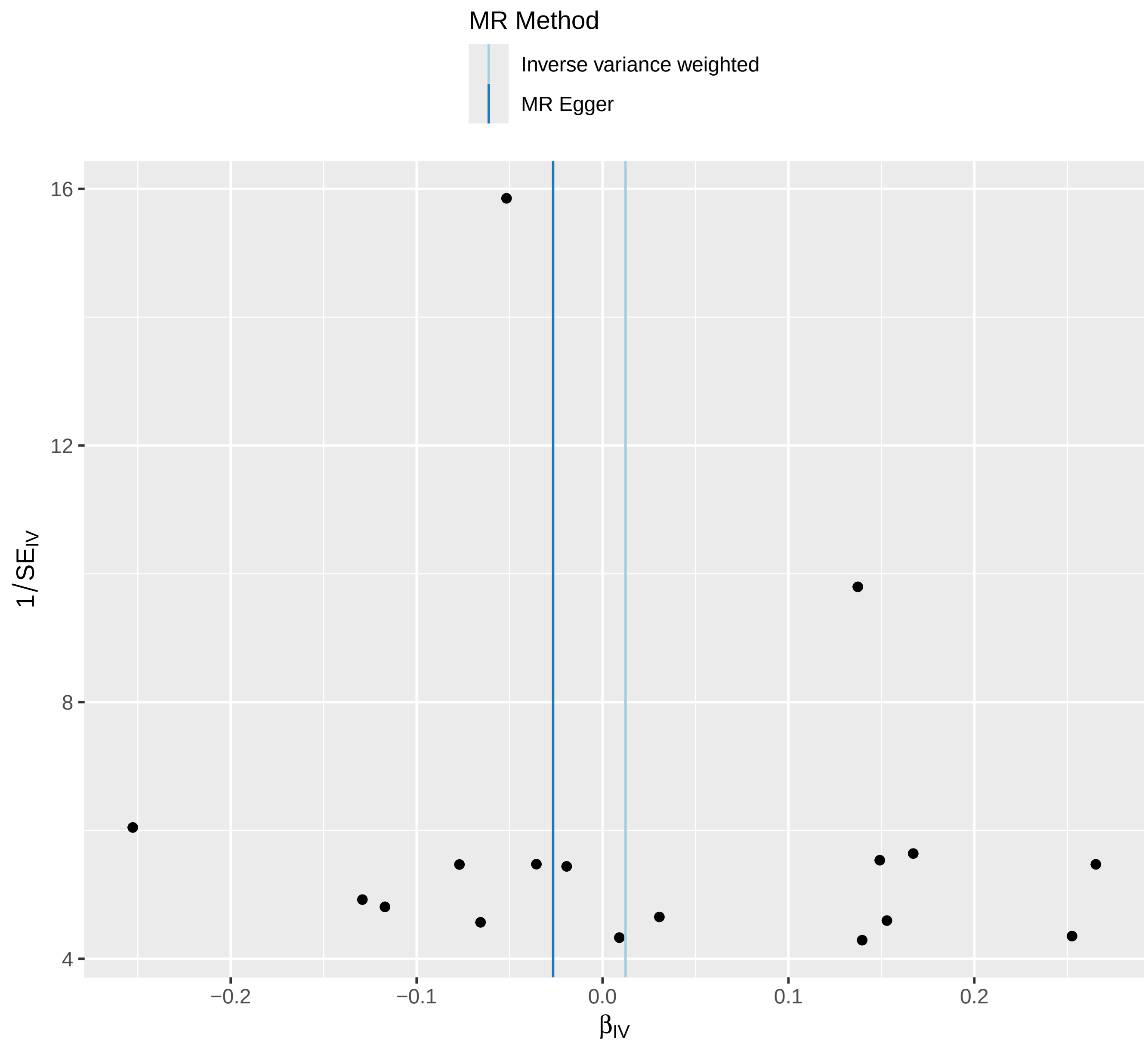
MR Method
Inverse variance weighted
MR Egger
16
12
8
4
1/SE_IV
−0.2
−0.1
0.0
0.1
0.2
β_IV

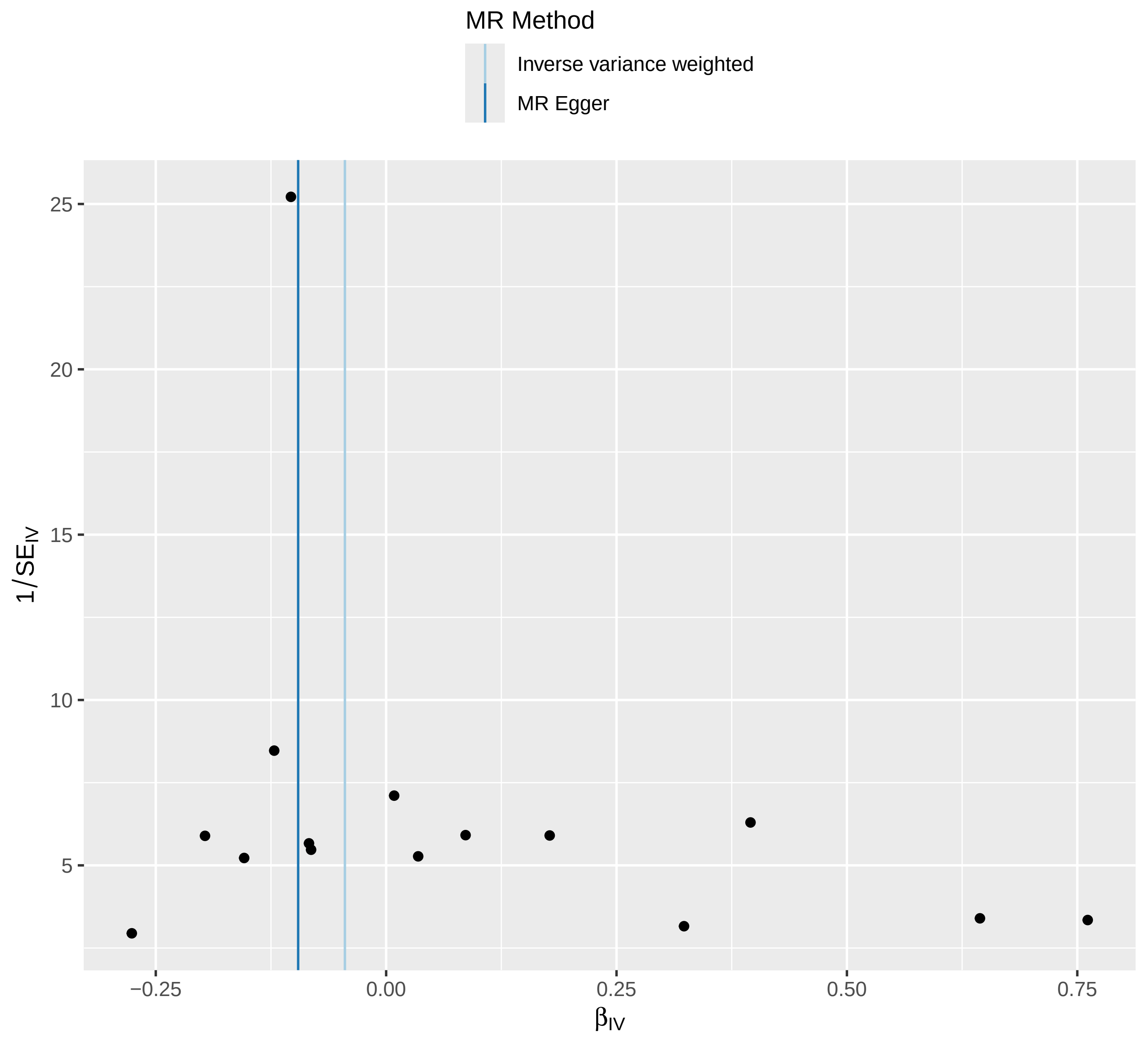

MR Method
Inverse variance weighted
MR Egger
25
20
15
10
5
1/SE$_{IV}$
−0.25
0.00
0.25
0.50
0.75
β$_{IV}$

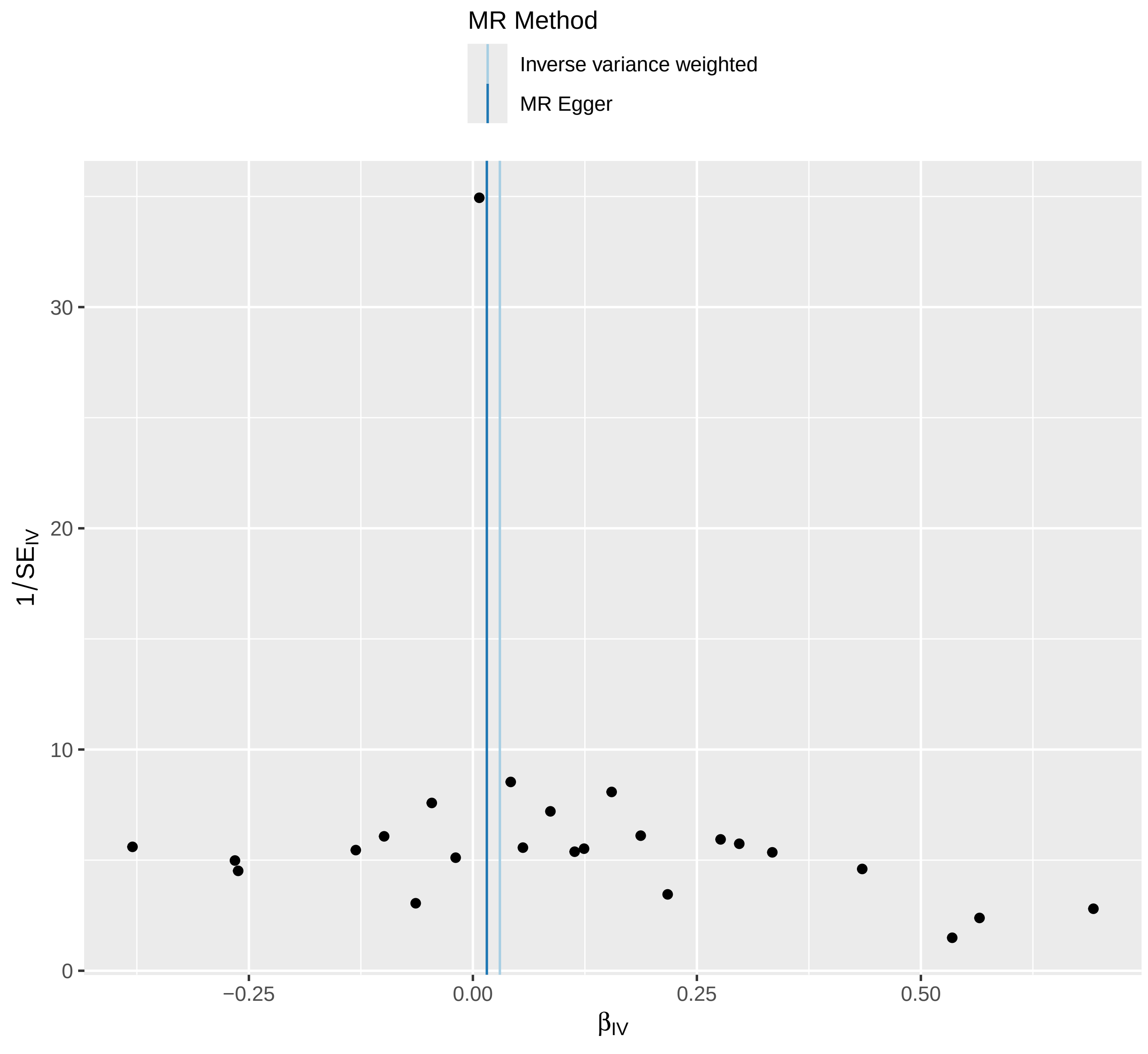

MR Method
Inverse variance weighted
MR Egger
30
20
10
0
1/SE$_{IV}$
−0.25
0.00
0.25
0.50
β$_{IV}$

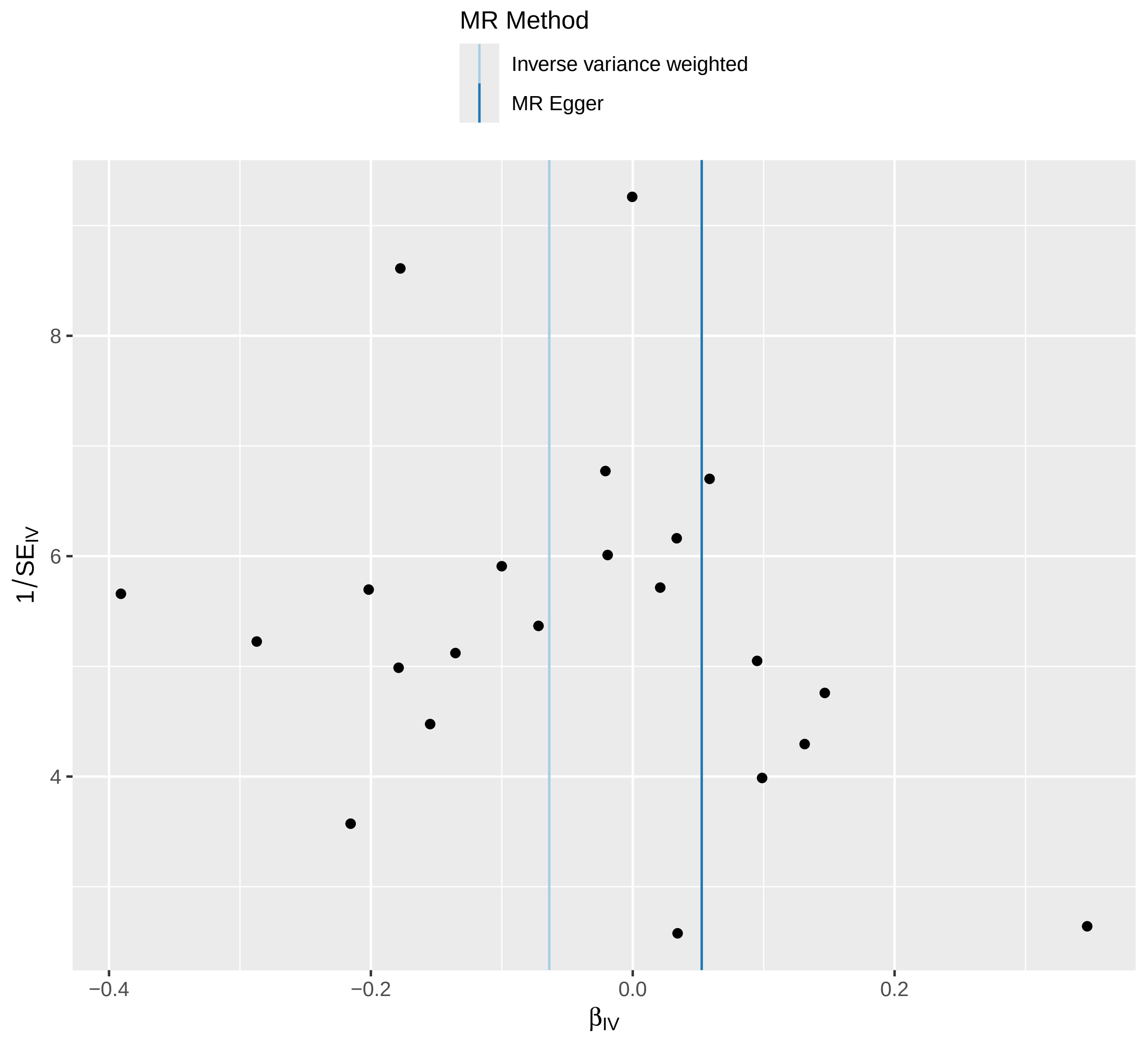
MR Method
Inverse variance weighted
MR Egger
8
6
4
1/SE_IV
−0.4
−0.2
0.0
0.2
β_IV

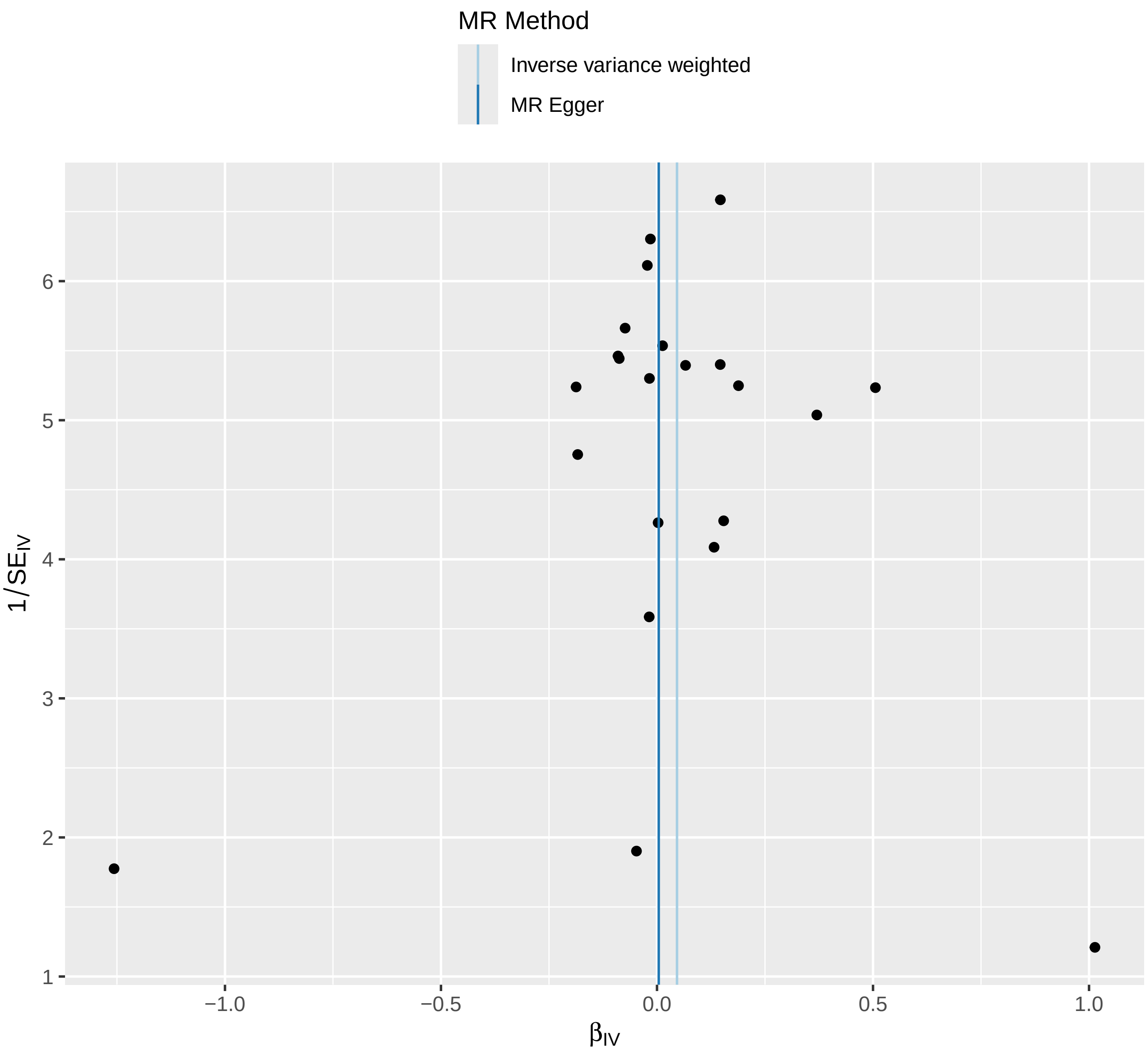
MR Method
Inverse variance weighted
MR Egger
1/SE_IV
6
5
4
3
2
1
−1.0
−0.5
0.0
0.5
1.0
β_IV

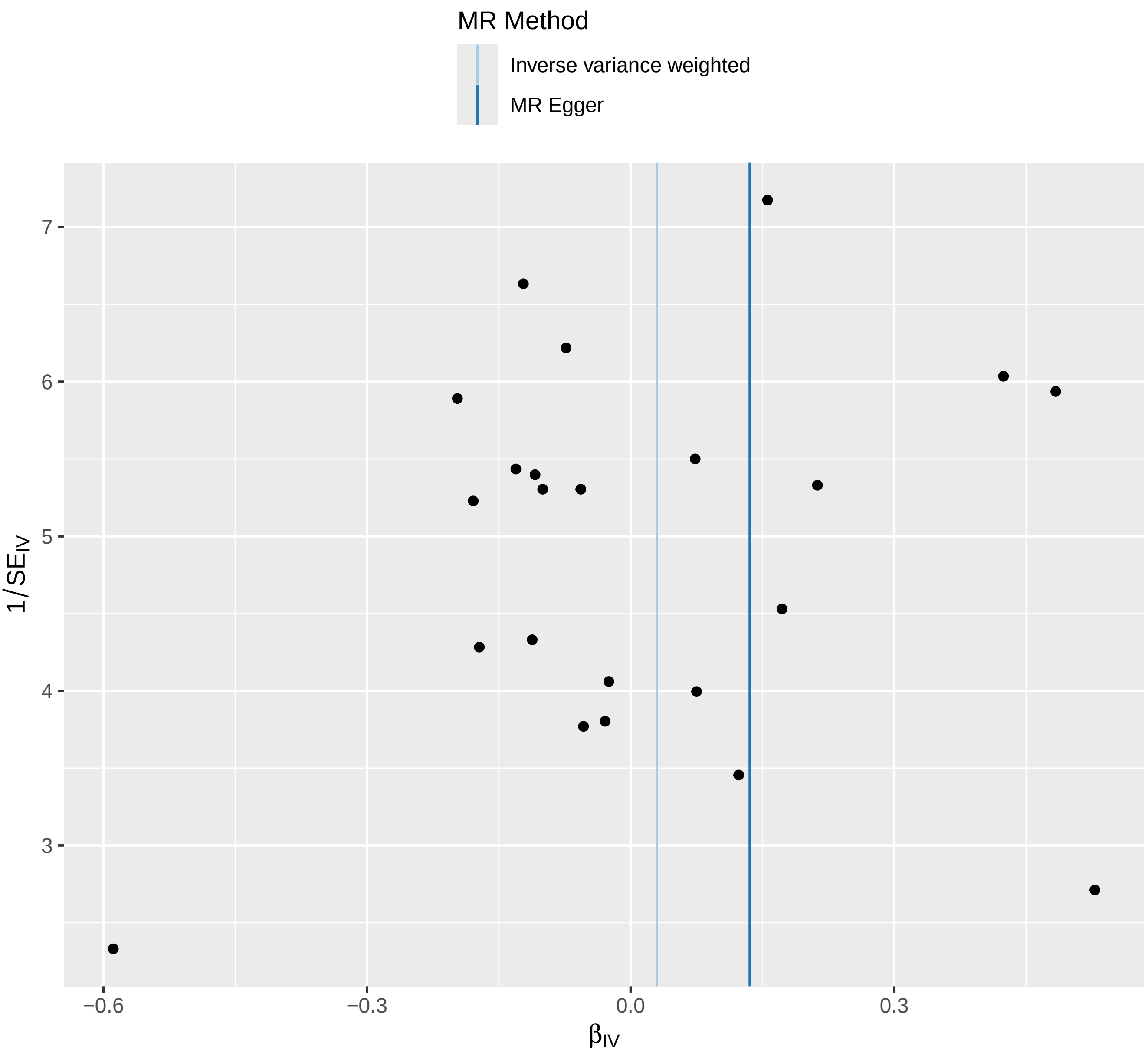
MR Method
Inverse variance weighted
MR Egger
7
6
5
4
3
1/SE_IV
−0.6
−0.3
0.0
0.3
β_IV

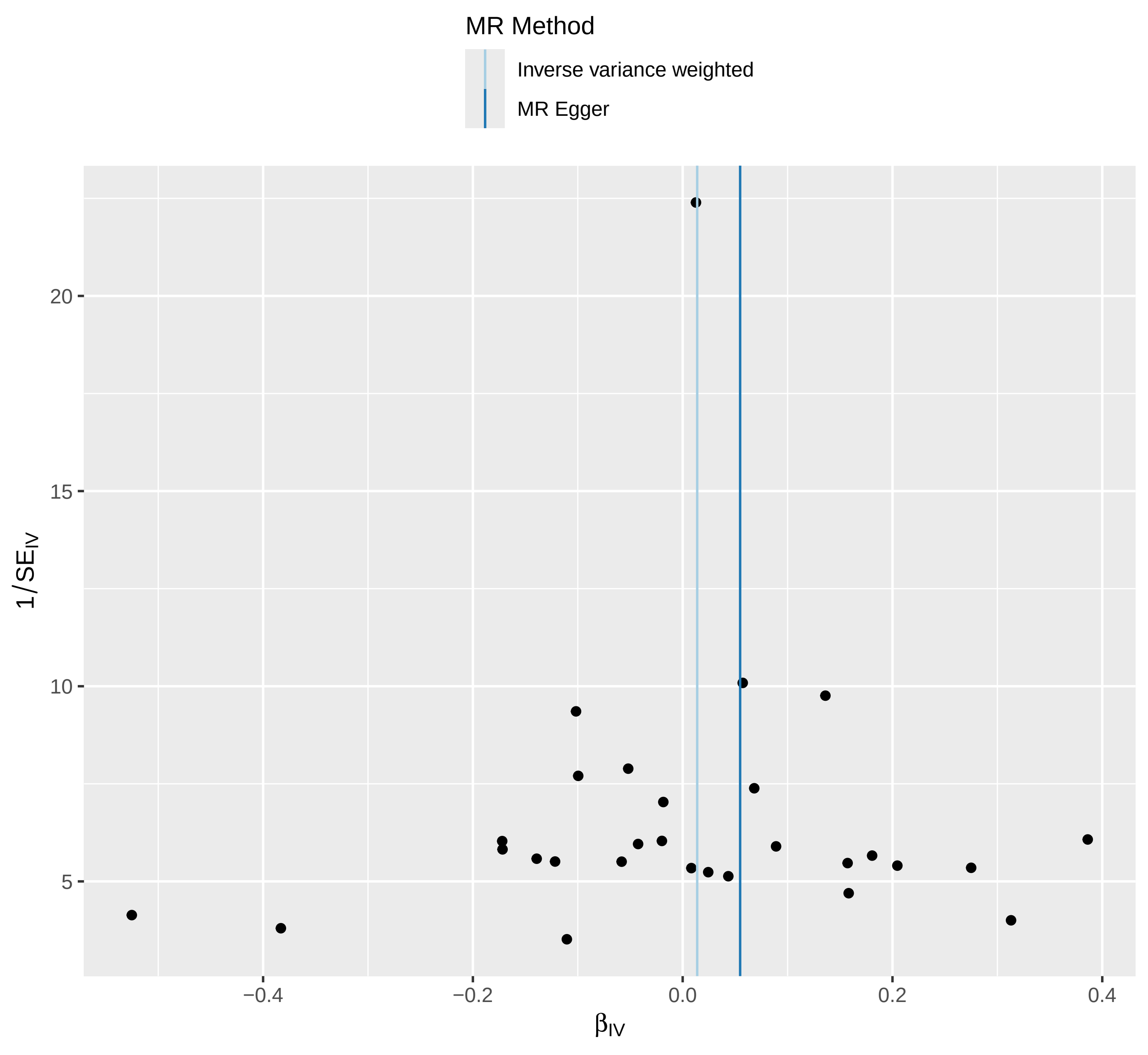
MR Method
Inverse variance weighted
MR Egger
20
15
10
5
−0.4
−0.2
0.0
0.2
0.4
$1/SE_{IV}$
β_{IV}

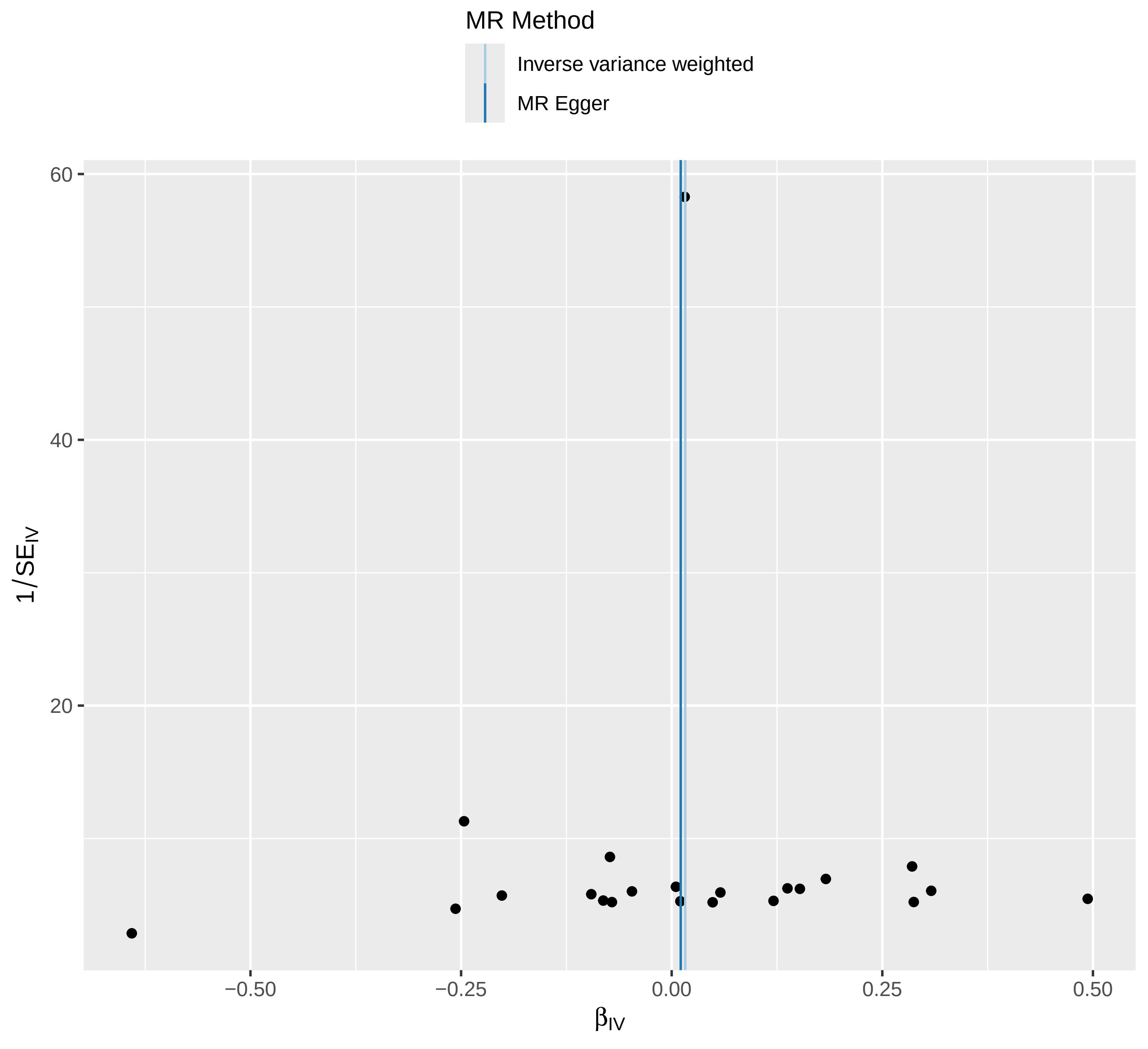
MR Method
Inverse variance weighted
MR Egger
60
40
20
1/SE$_{IV}$
−0.50
−0.25
0.00
0.25
0.50
β$_{IV}$

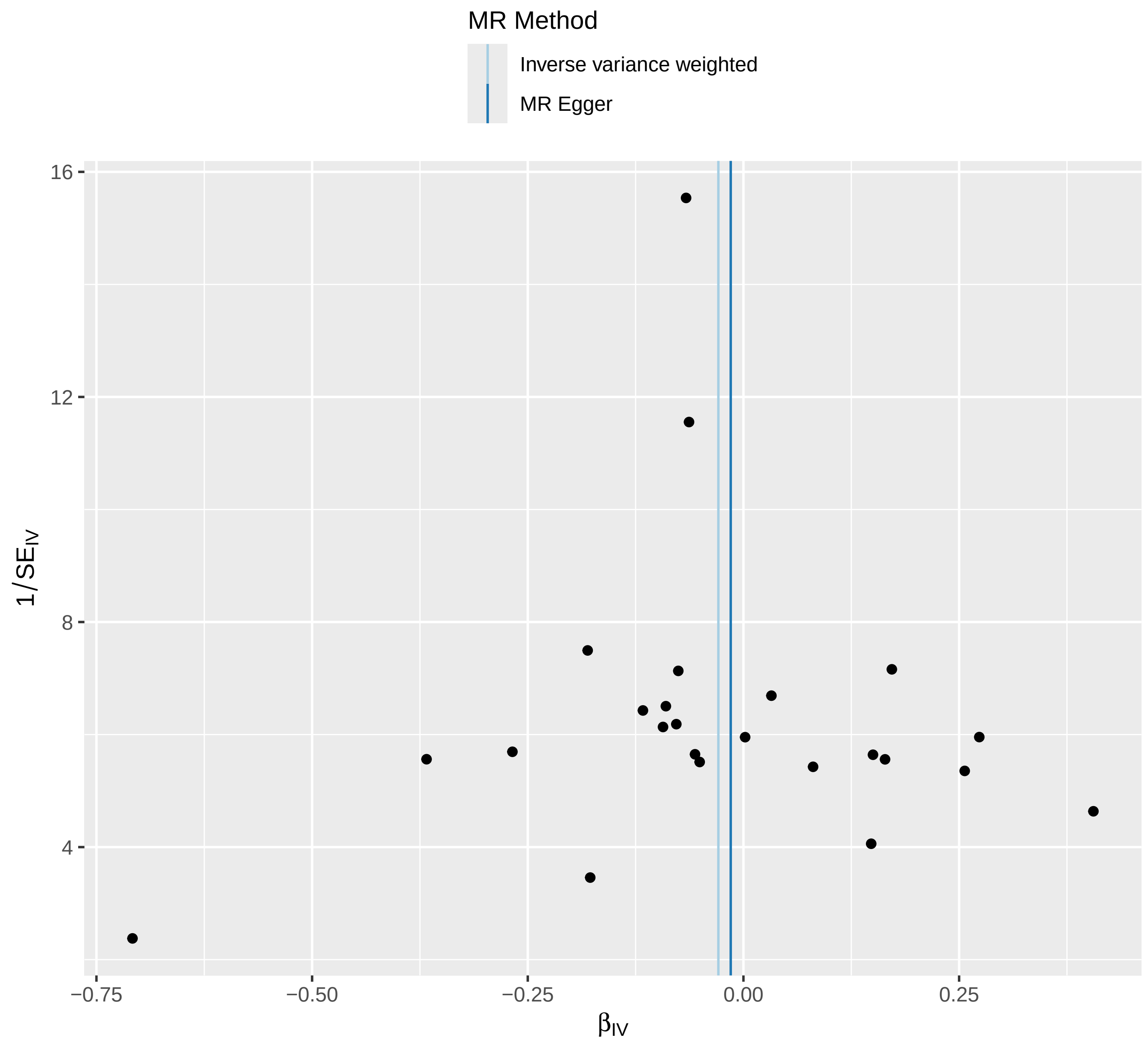

MR Method
Inverse variance weighted
MR Egger
16
12
8
4
1/SE$_{IV}$
−0.75
−0.50
−0.25
0.00
0.25
β$_{IV}$

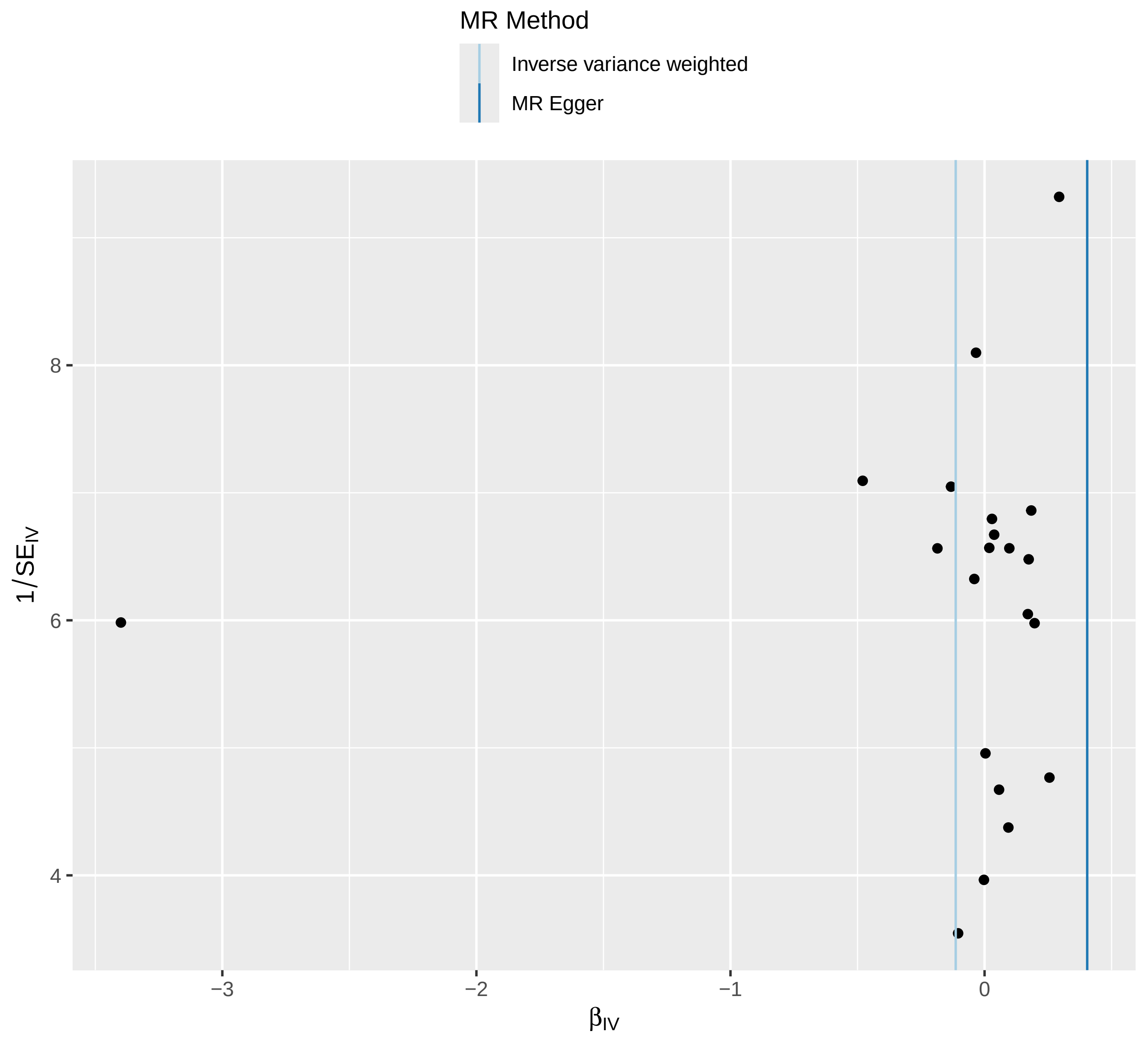

MR Method
Inverse variance weighted
MR Egger
8
6
4
1/SE_IV
−3
−2
−1
0
β_IV

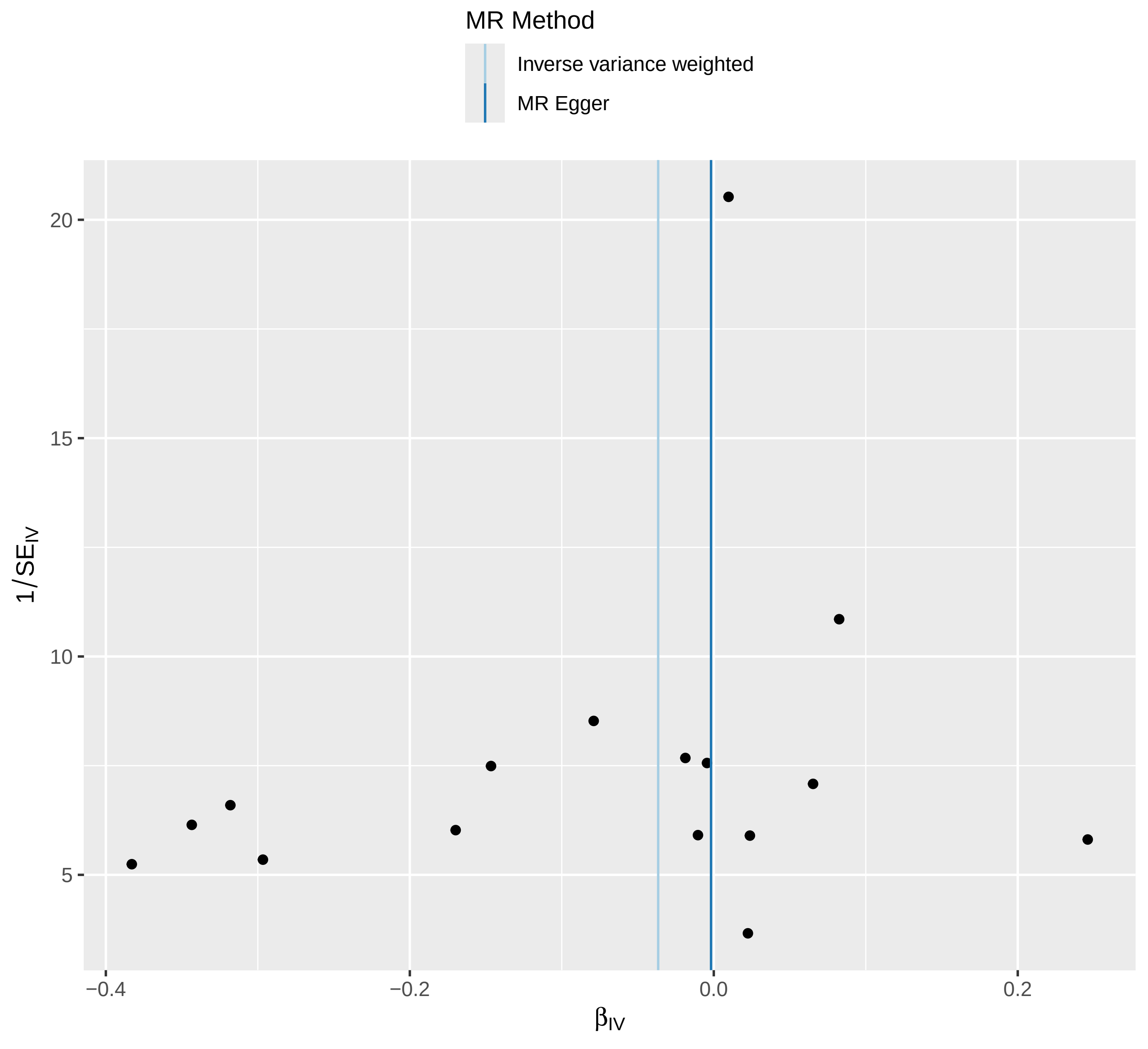

MR Method
Inverse variance weighted
MR Egger
20
15
10
5
1/SE$_{IV}$
−0.4
−0.2
0.0
0.2
β$_{IV}$

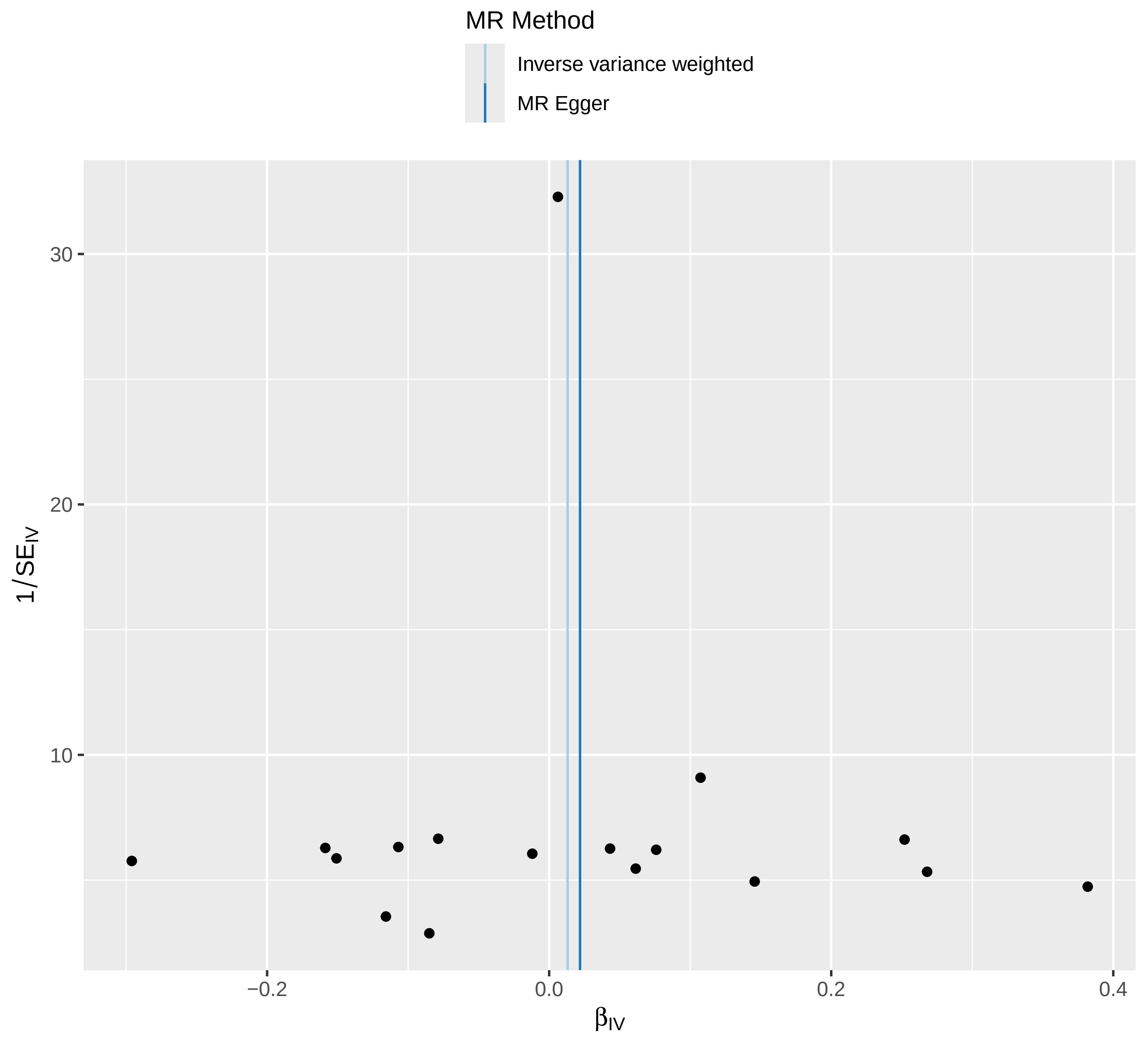

MR Method
Inverse variance weighted
MR Egger
30
20
10
1/SE_IV
−0.2
0.0
0.2
0.4
β_IV

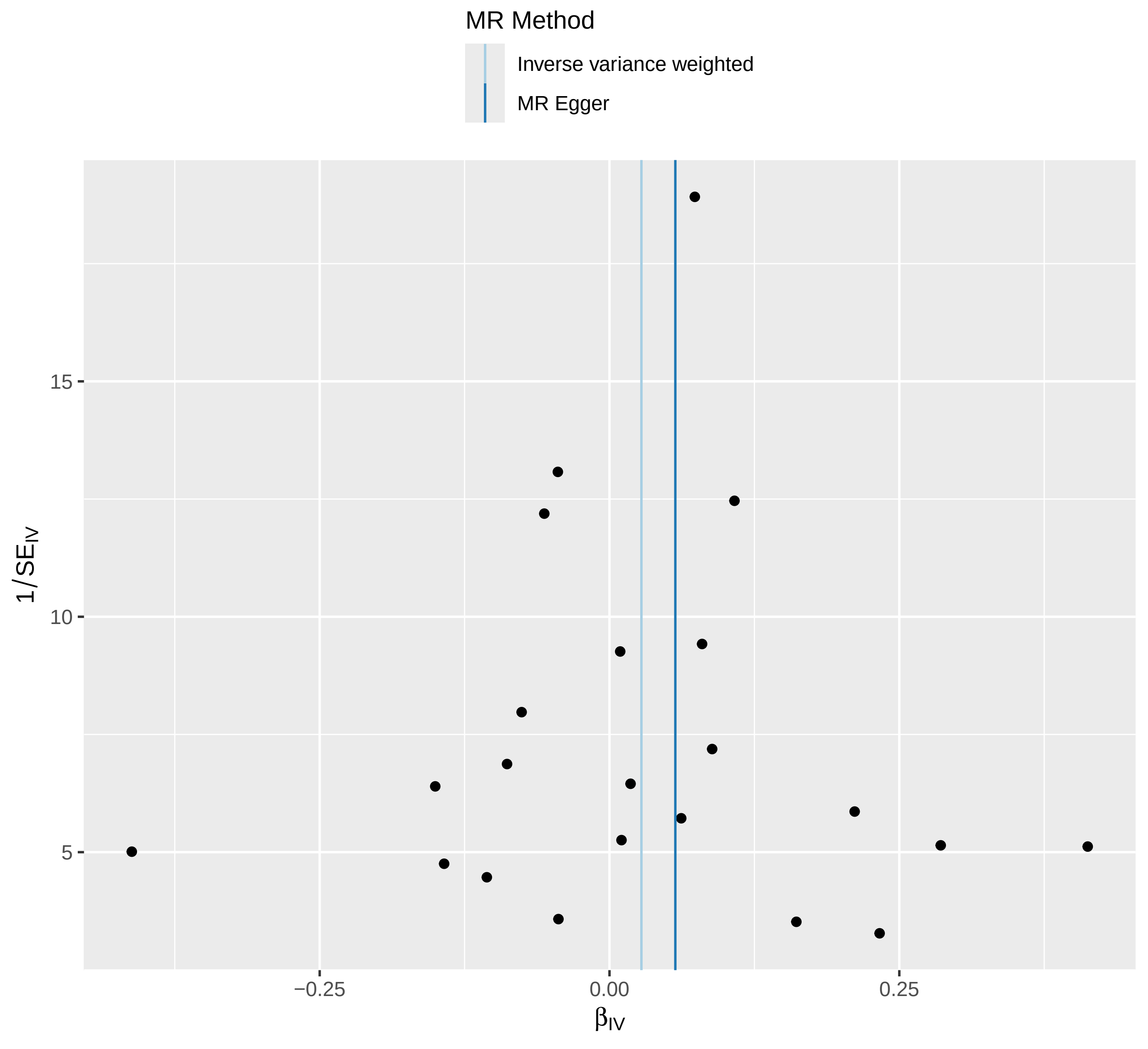
MR Method
Inverse variance weighted
MR Egger
15
10
5
1/SE_IV
−0.25
0.00
0.25
β_IV

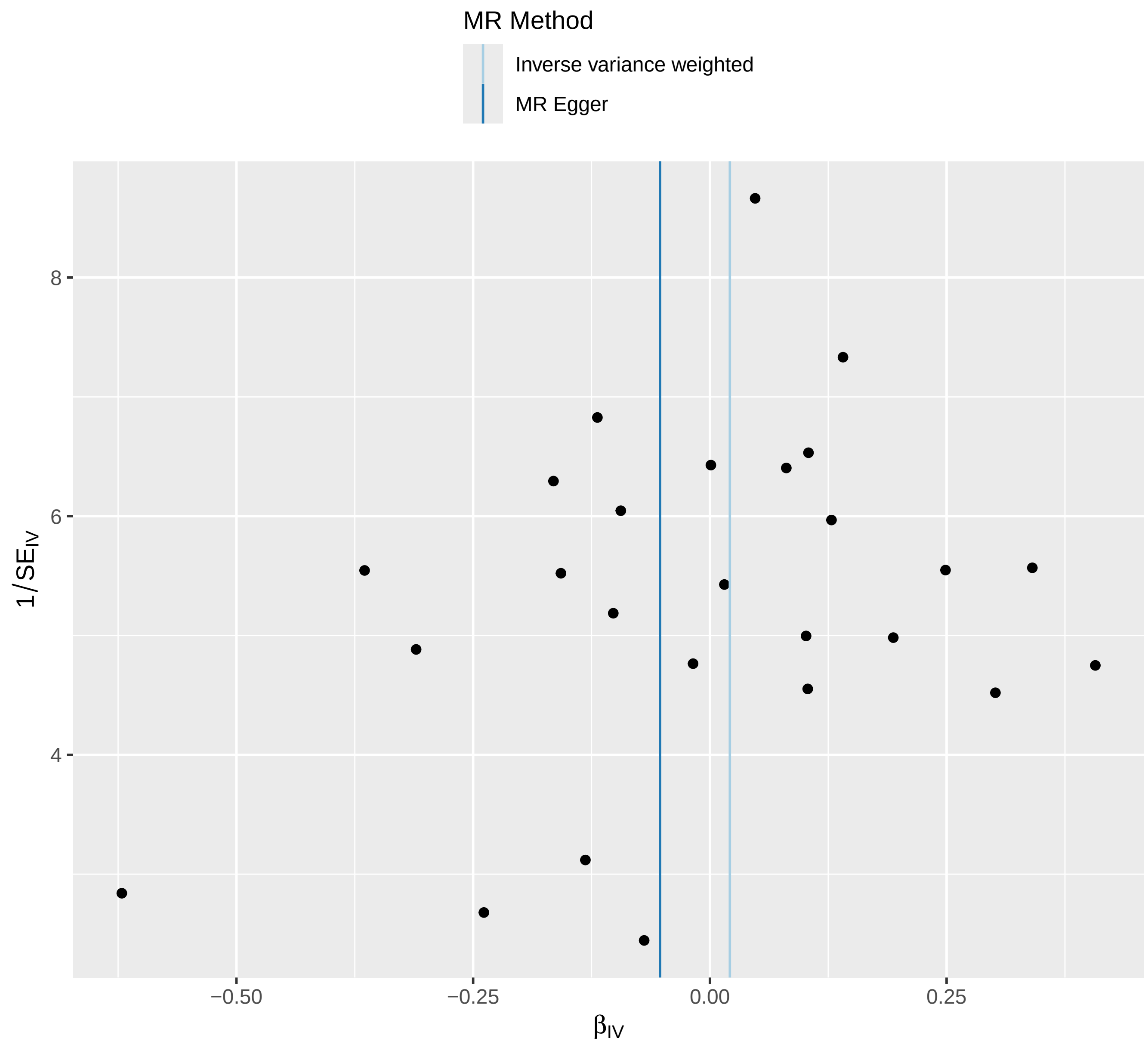

MR Method
Inverse variance weighted
MR Egger
8
6
4
1/SE_IV
−0.50
−0.25
0.00
0.25
β_IV

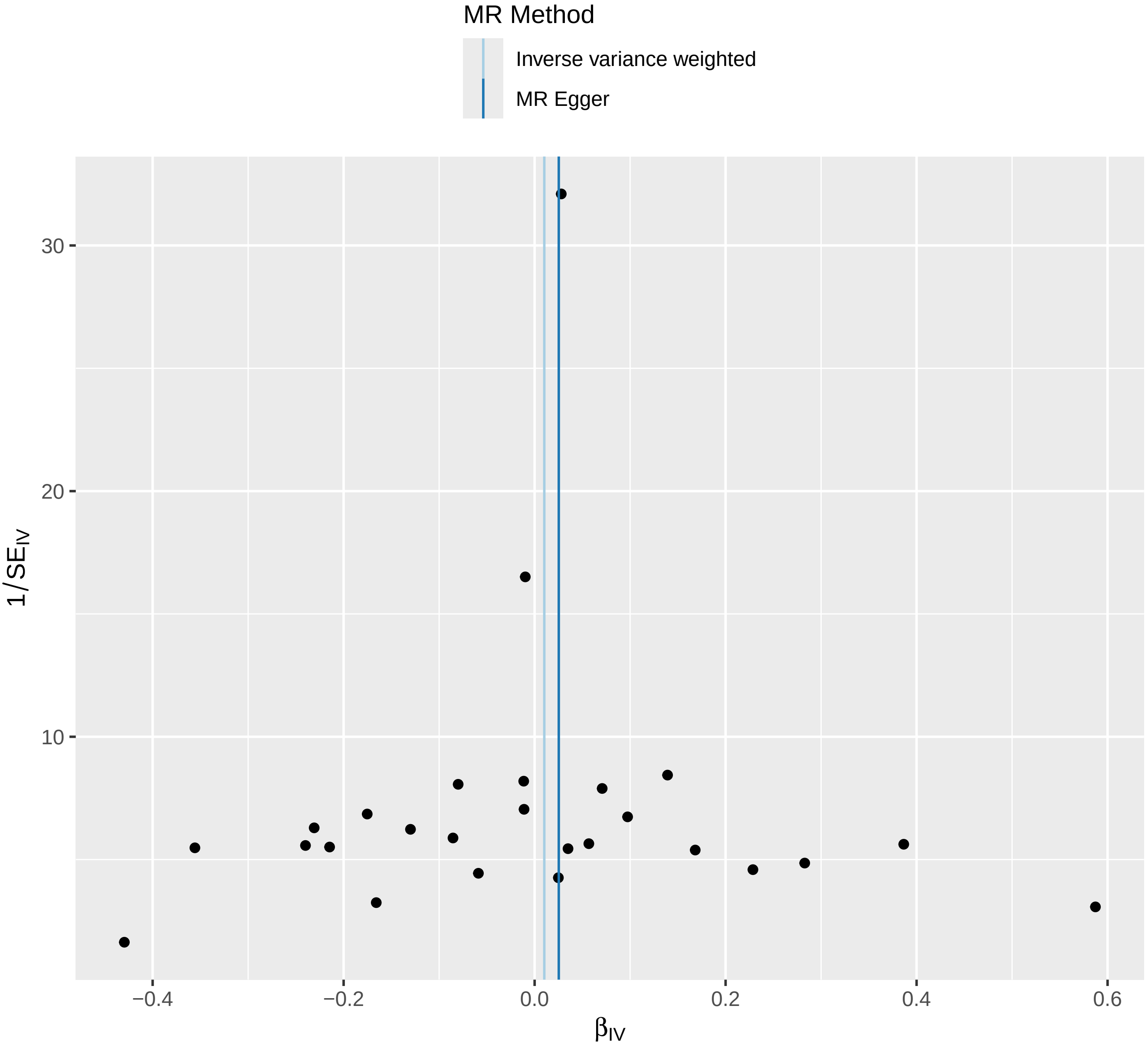
MR Method
Inverse variance weighted
MR Egger
30
20
10
−0.4
−0.2
0.0
0.2
0.4
0.6
$1/SE_{IV}$
β_{IV}

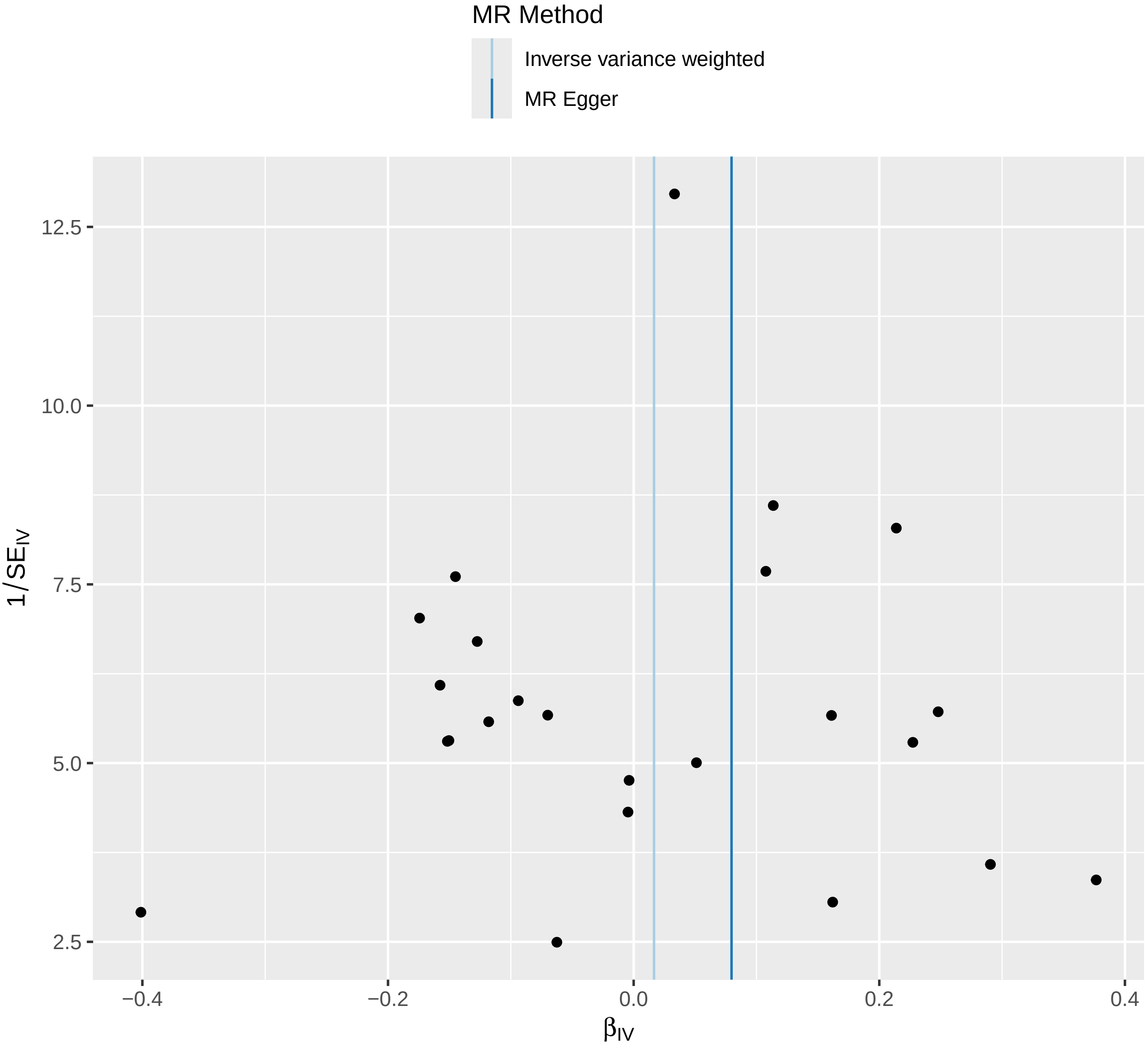
MR Method
Inverse variance weighted
MR Egger
12.5
10.0
7.5
5.0
2.5
$1/SE_{IV}$
−0.4
−0.2
0.0
0.2
0.4
β_{IV}

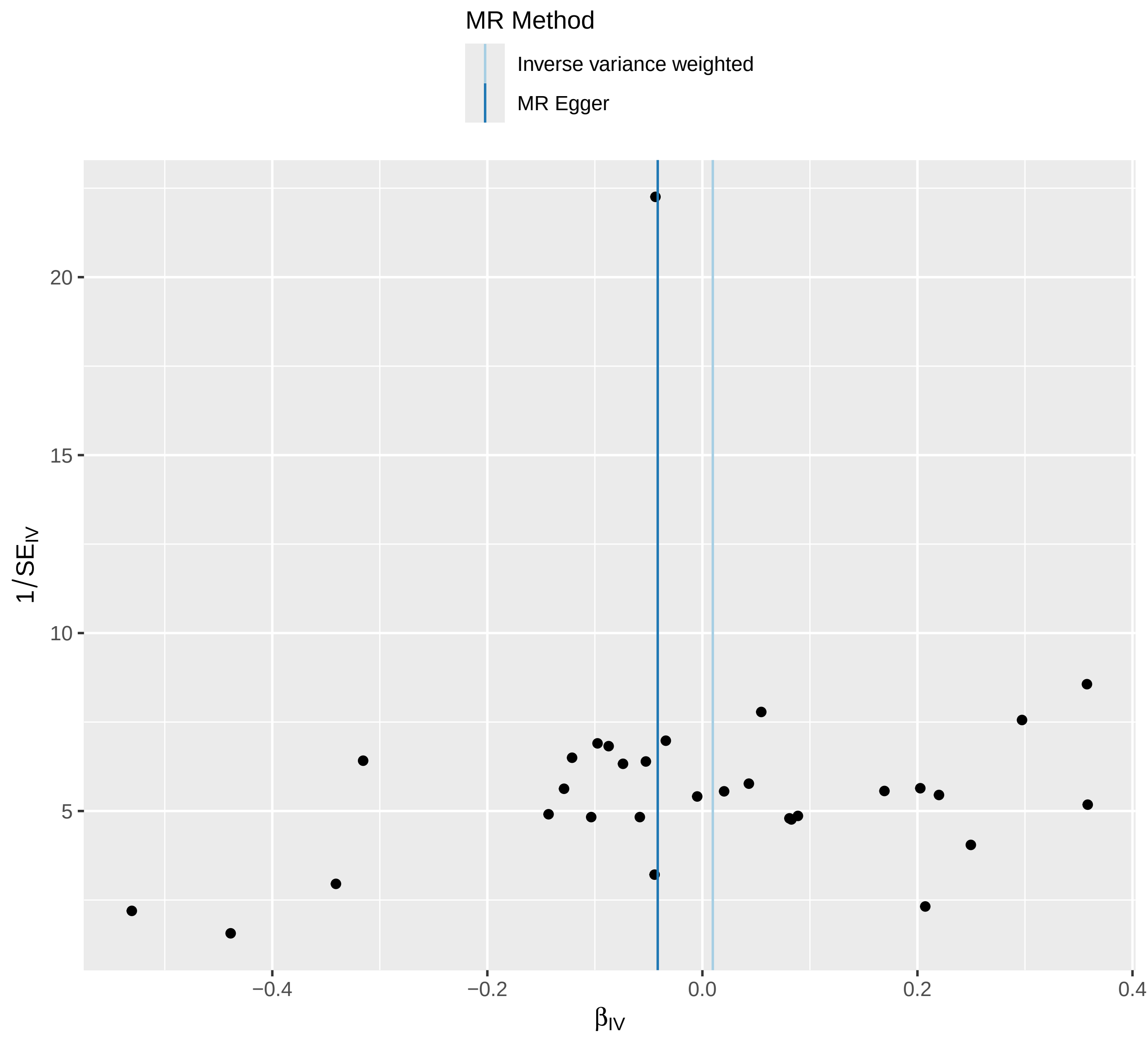

MR Method
Inverse variance weighted
MR Egger
20
15
10
5
1/SE$_{IV}$
−0.4
−0.2
0.0
0.2
0.4
β$_{IV}$

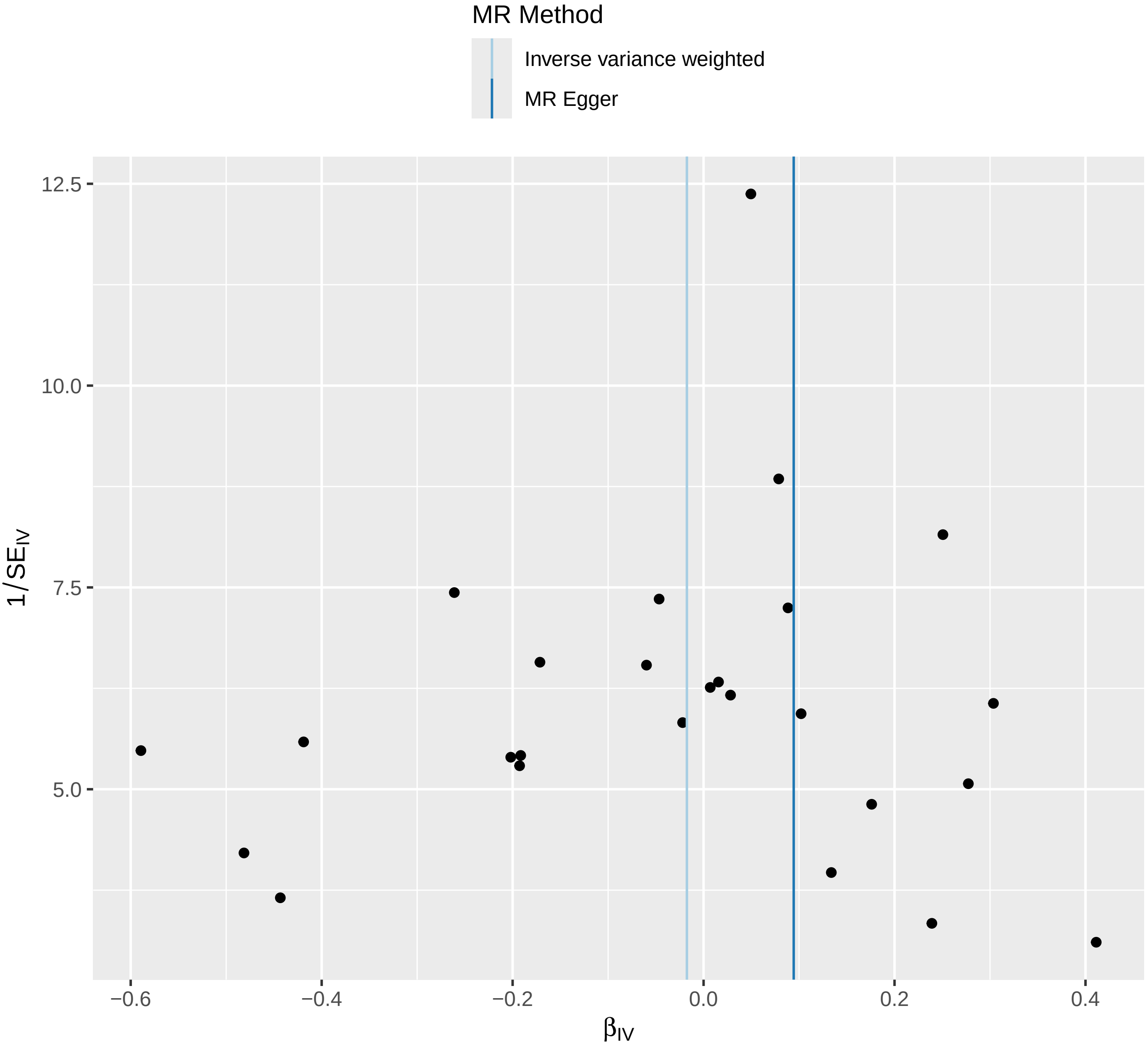

MR Method
Inverse variance weighted
MR Egger
12.5
10.0
7.5
5.0
1/SE_IV
−0.6
−0.4
−0.2
0.0
0.2
0.4
β_IV

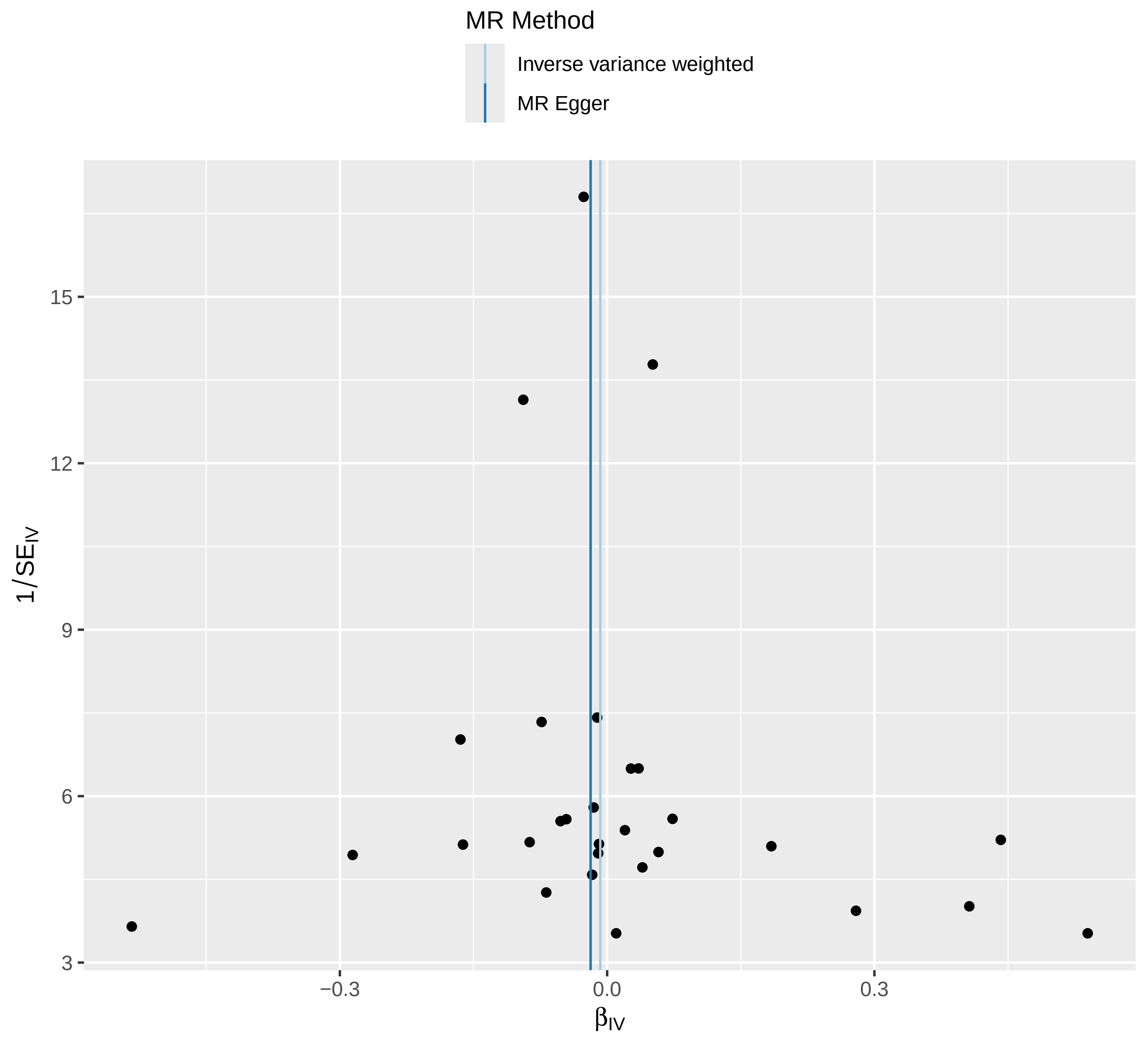
MR Method
Inverse variance weighted
MR Egger
1/SE_IV
15
12
9
6
3
−0.3
0.0
0.3
β_IV

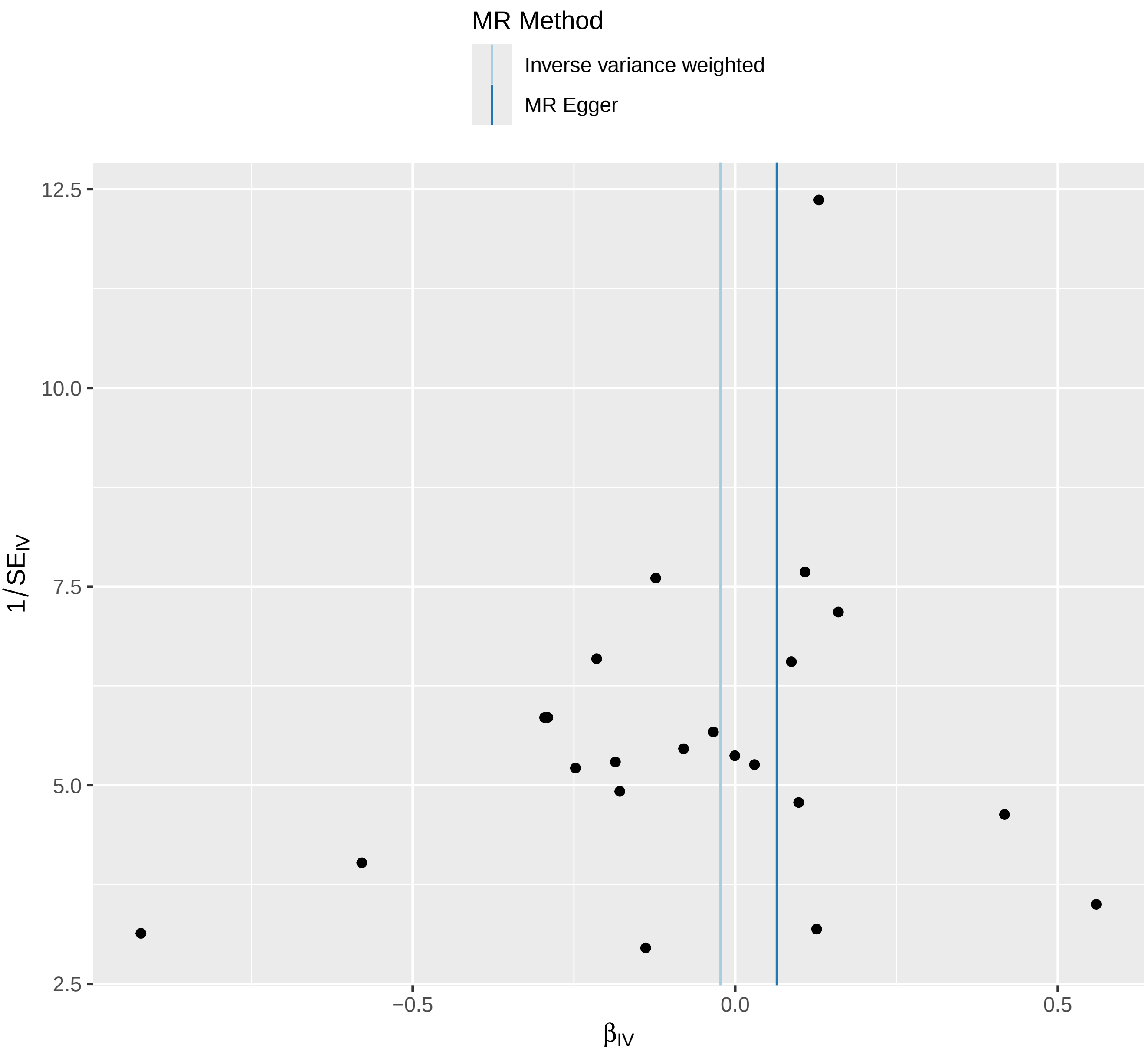
MR Method
Inverse variance weighted
MR Egger
12.5
10.0
7.5
5.0
2.5
1/SE$_{IV}$
−0.5
0.0
0.5
β$_{IV}$

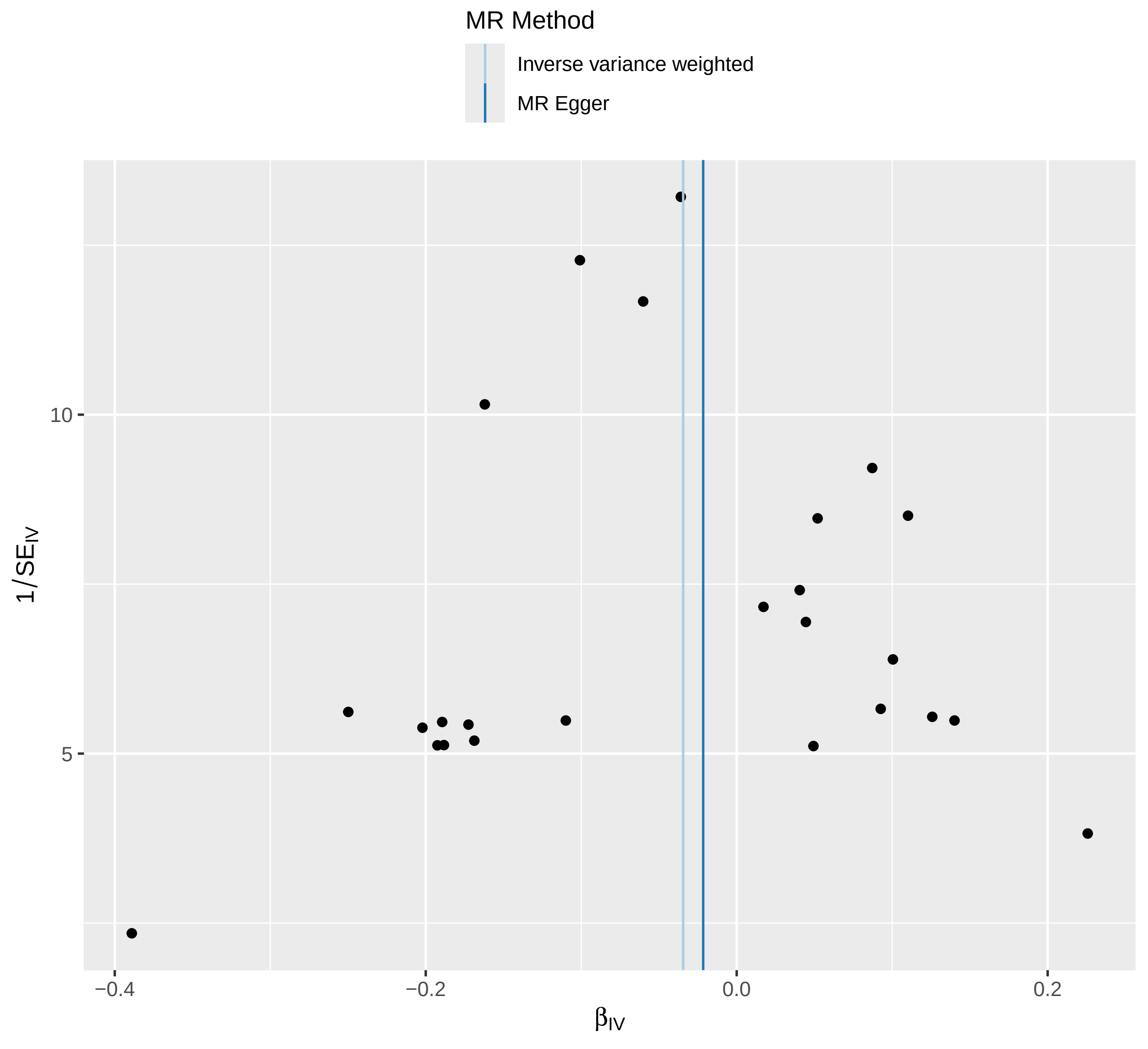
MR Method
Inverse variance weighted
MR Egger
10
5
1/SE_IV
−0.4
−0.2
0.0
0.2
β_IV

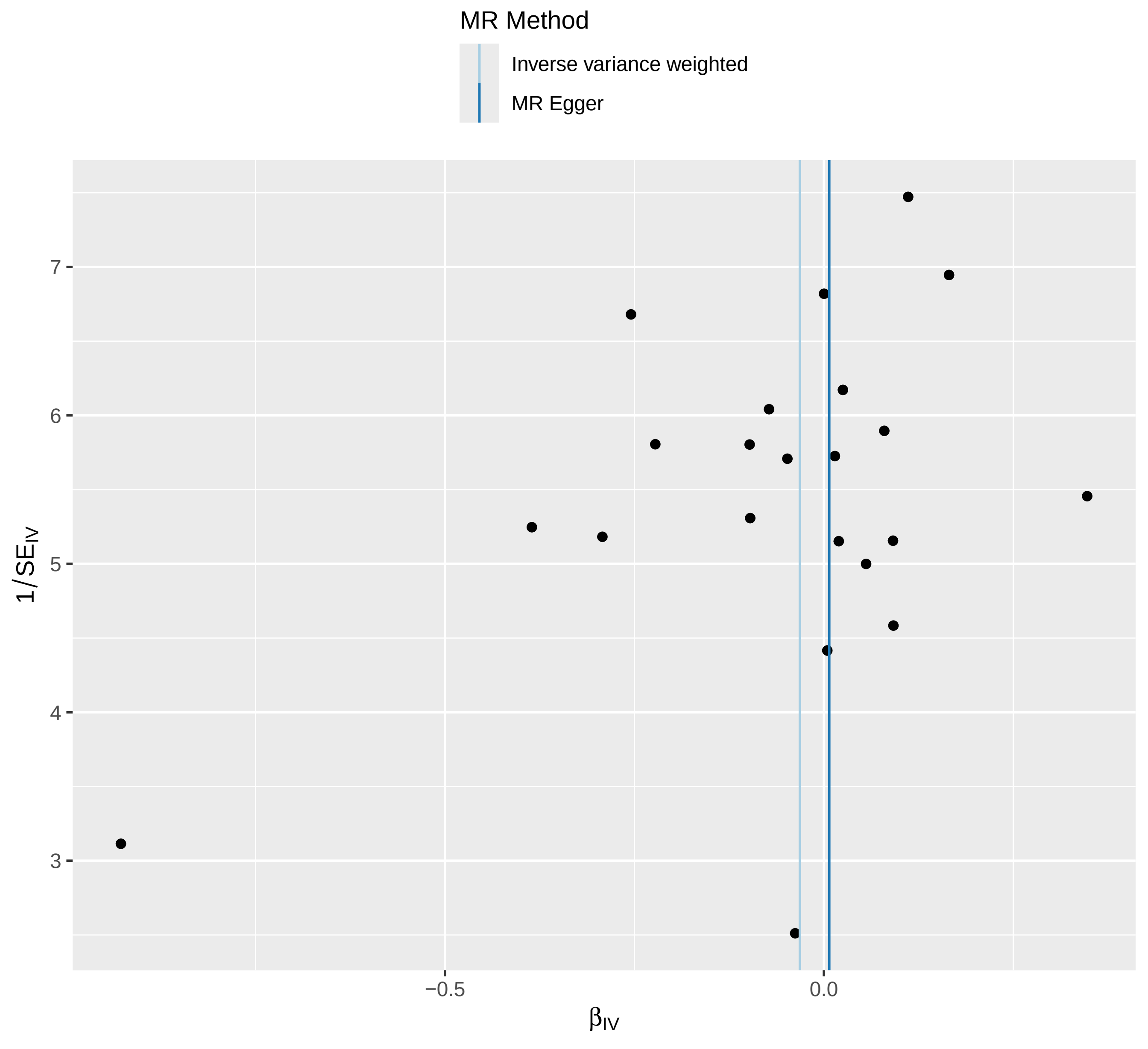

MR Method
Inverse variance weighted
MR Egger
7
6
5
4
3
1/SE_IV
−0.5
0.0
β_IV

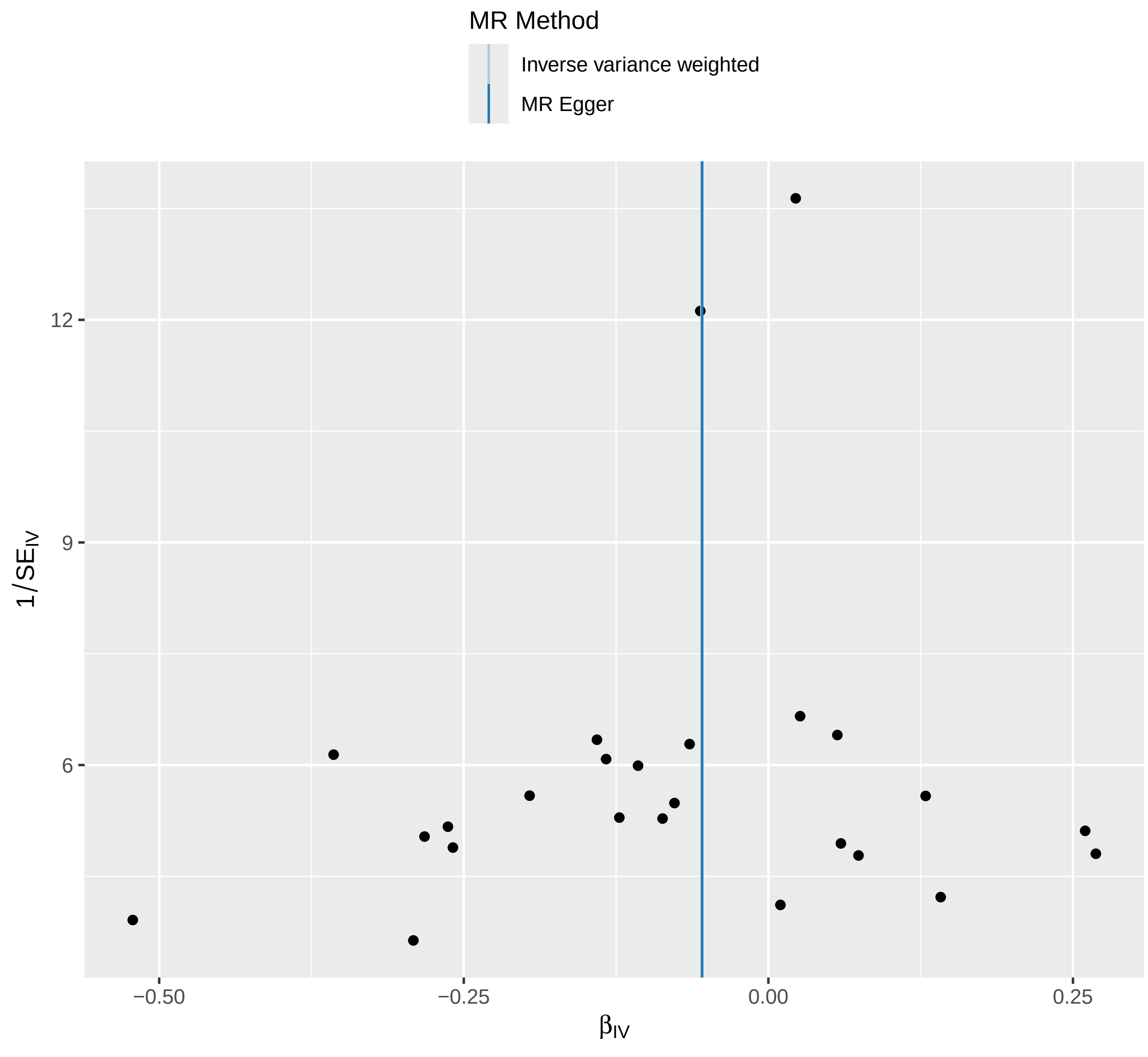
MR Method
Inverse variance weighted
MR Egger
12
9
6
1/SE_IV
−0.50
−0.25
0.00
0.25
β_IV

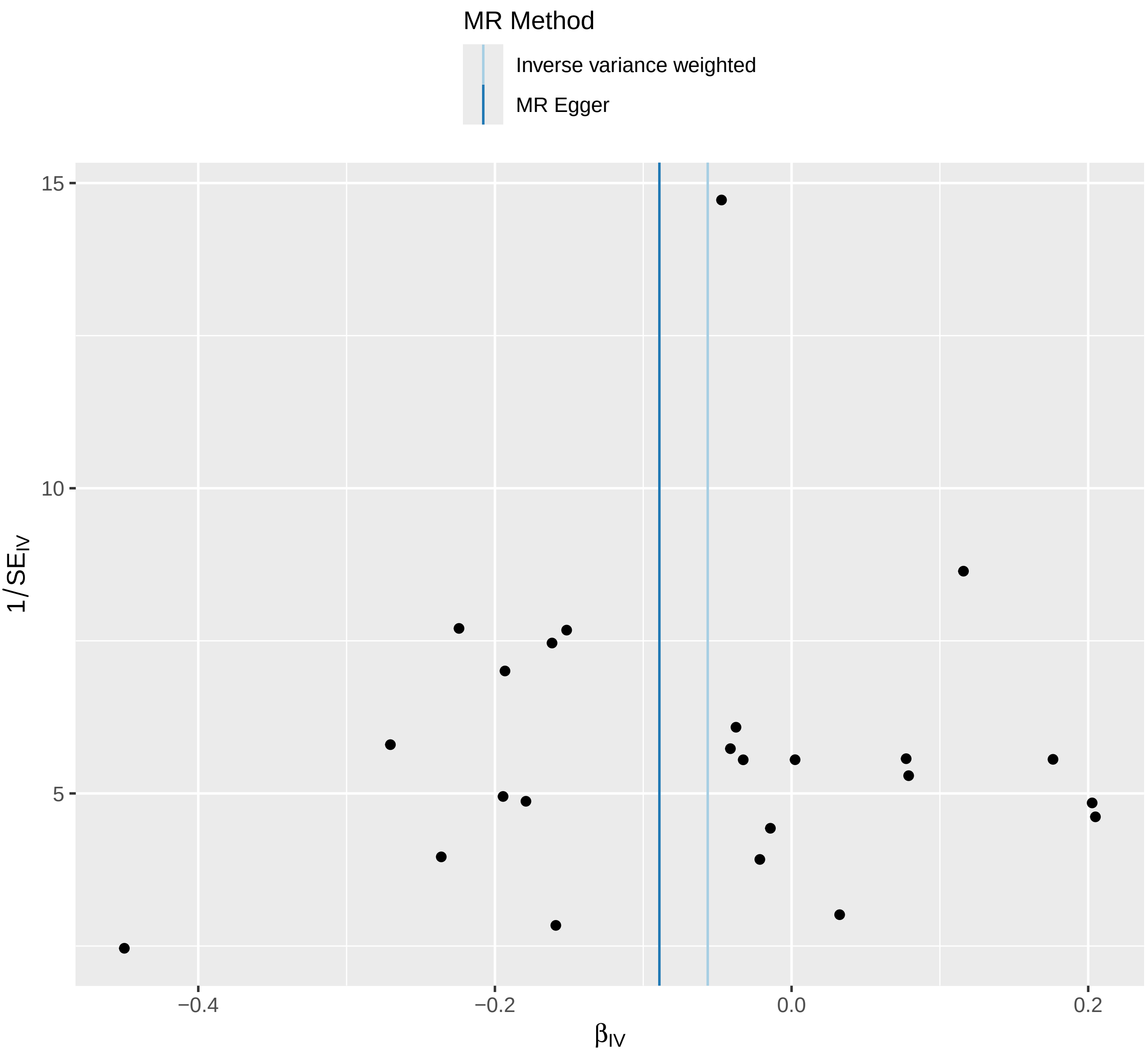

MR Method
Inverse variance weighted
MR Egger
15
10
5
1/SE_IV
−0.4
−0.2
0.0
0.2
β_IV

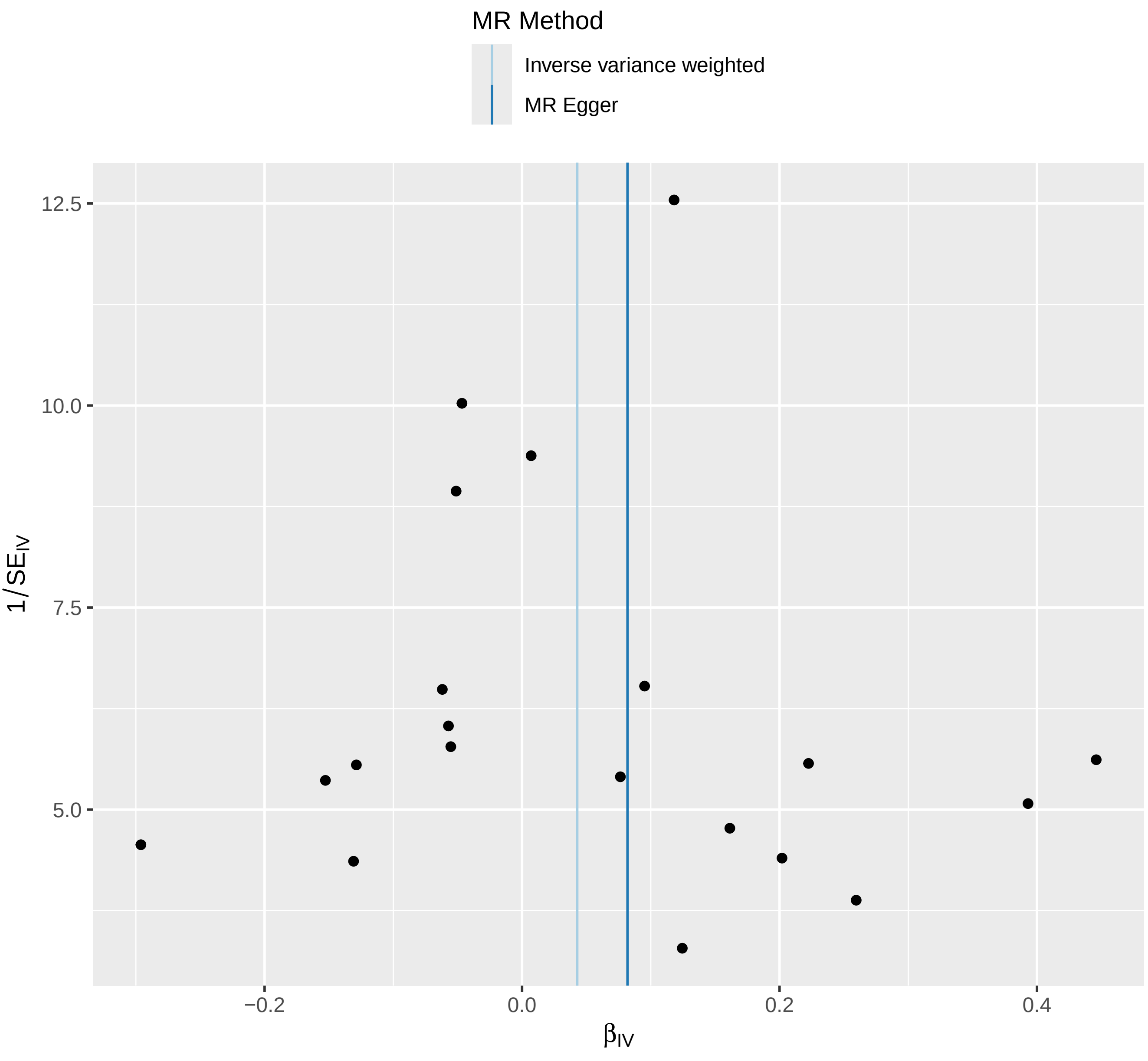

MR Method
Inverse variance weighted
MR Egger
12.5
10.0
7.5
5.0
1/SE_IV
−0.2
0.0
0.2
0.4
β_IV

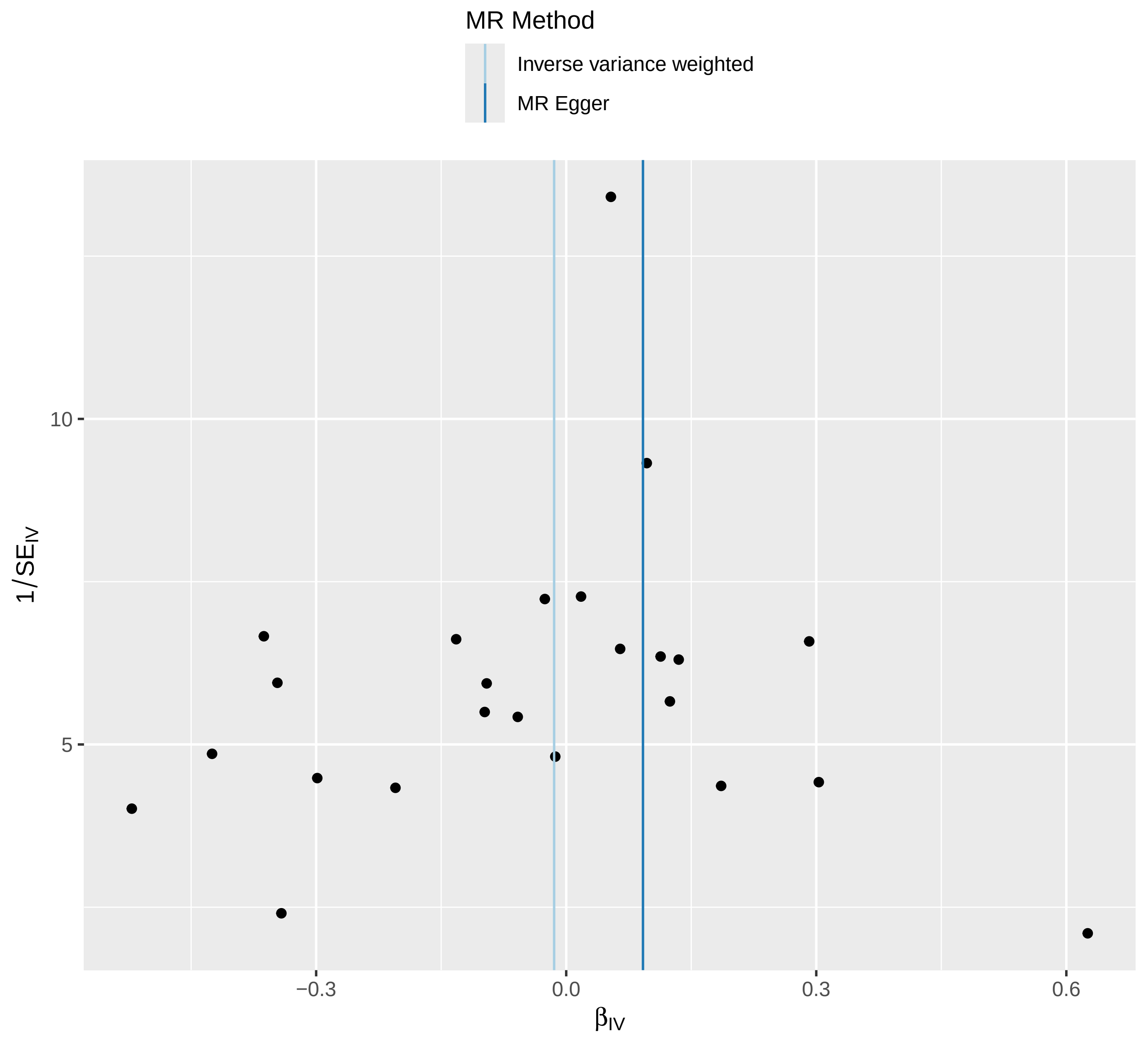
MR Method
Inverse variance weighted
MR Egger
10
5
1/SE$_{IV}$
−0.3
0.0
0.3
0.6
β$_{IV}$

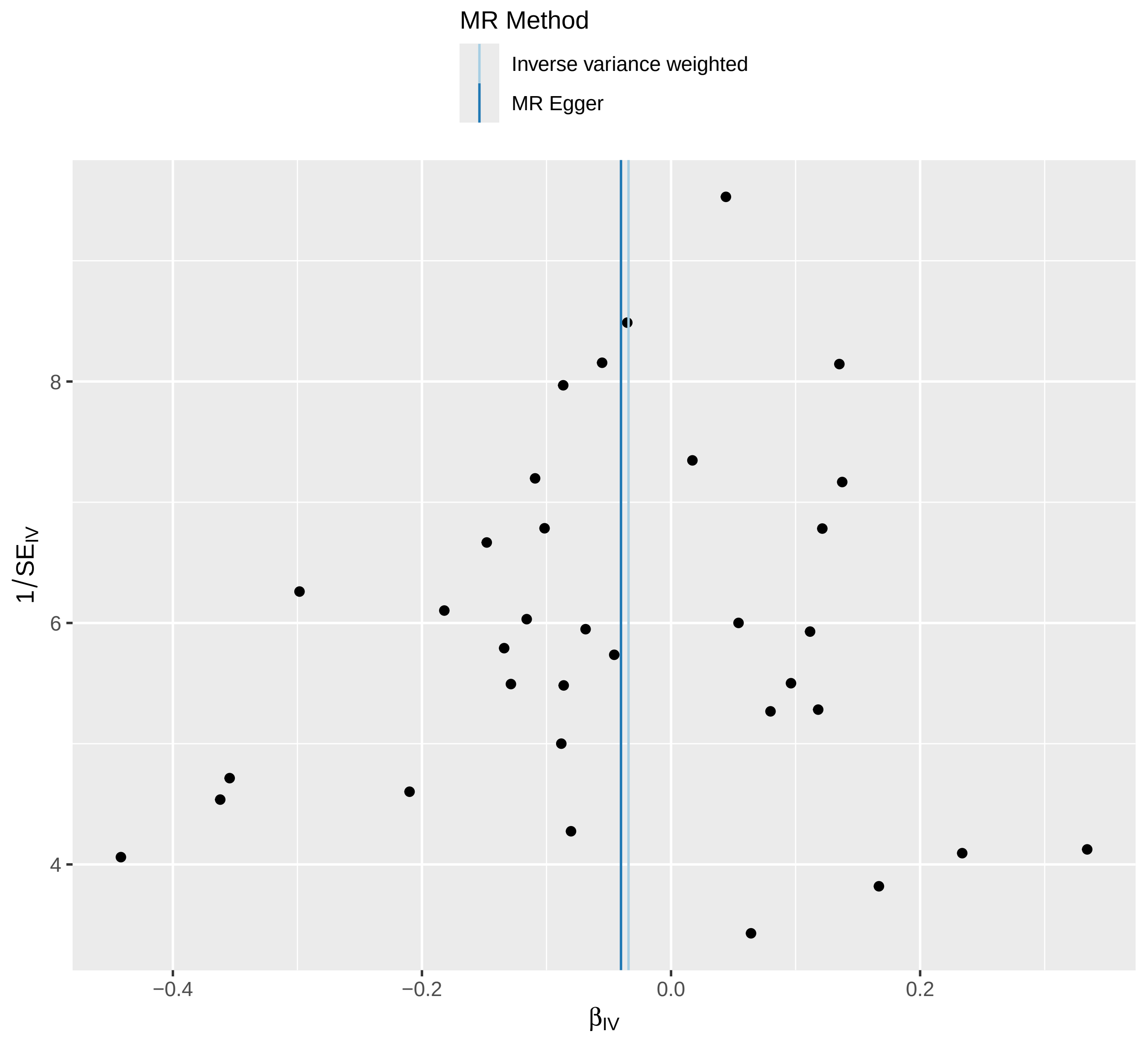

MR Method
Inverse variance weighted
MR Egger
1/SE_IV
8
6
4
−0.4
−0.2
0.0
0.2
β_IV

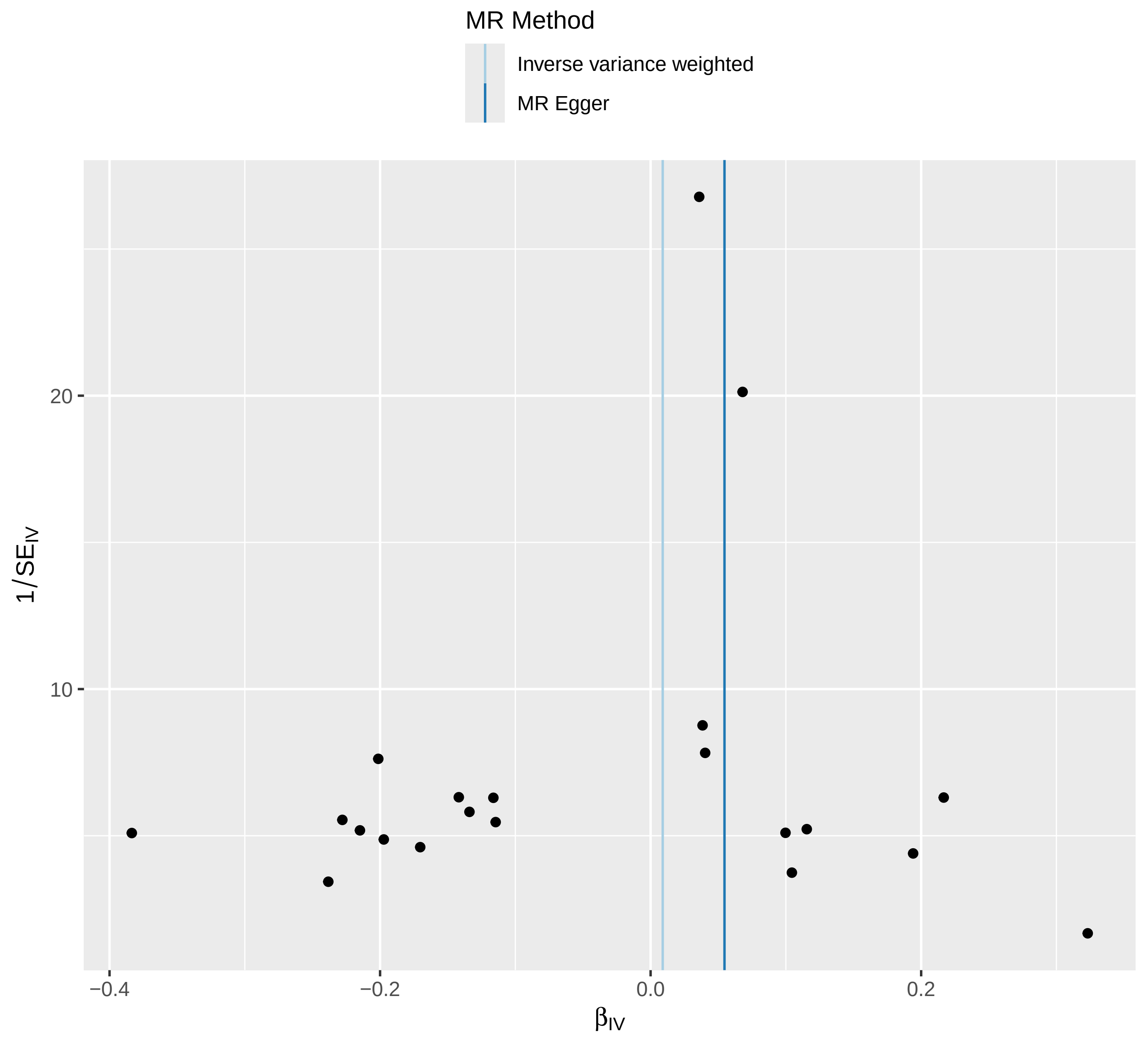
MR Method
Inverse variance weighted
MR Egger
20
10
1/SE_IV
−0.4
−0.2
0.0
0.2
β_IV

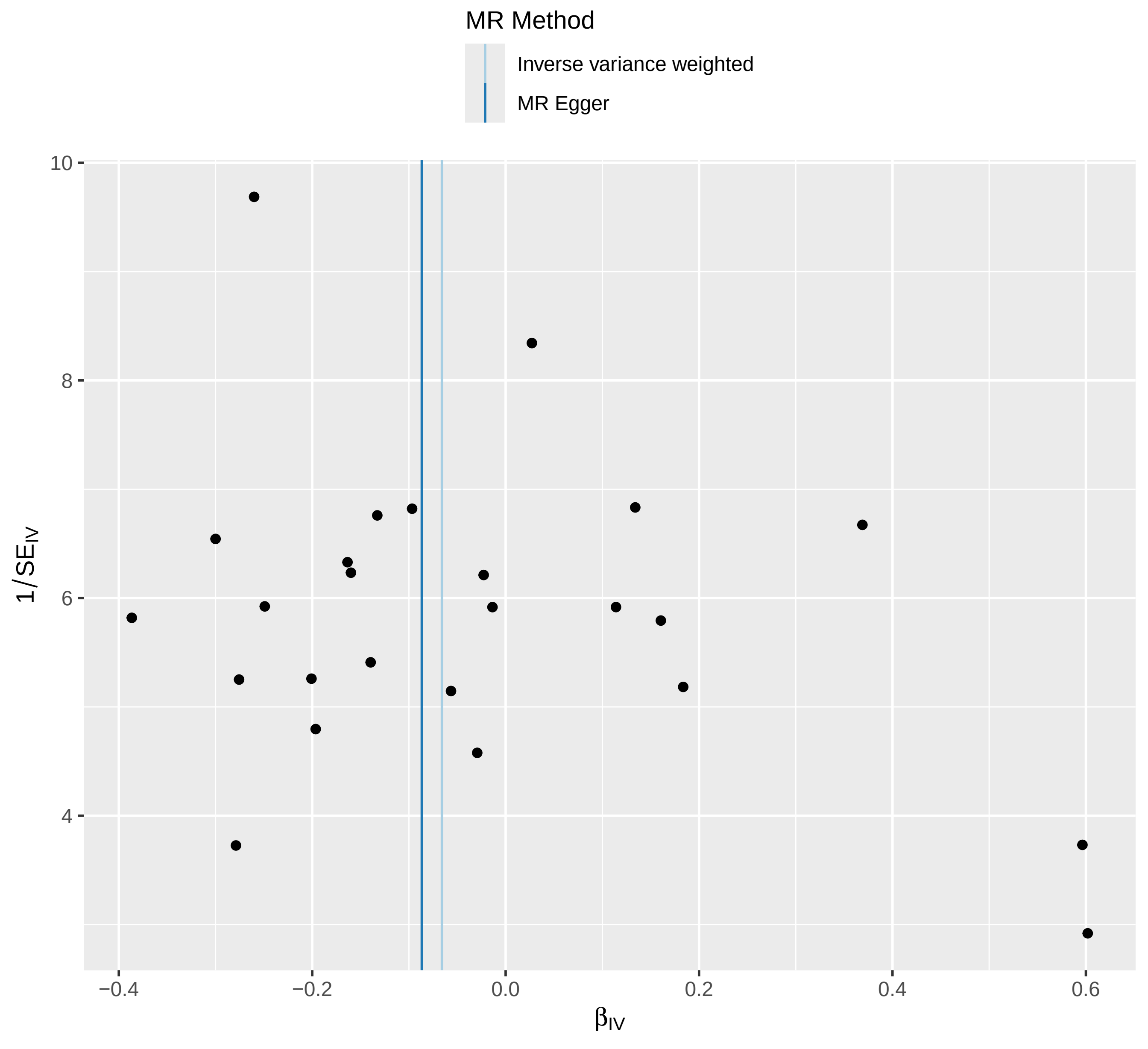
MR Method
Inverse variance weighted
MR Egger
10
8
6
4
$1/SE_{IV}$
−0.4
−0.2
0.0
0.2
0.4
0.6
β_{IV}

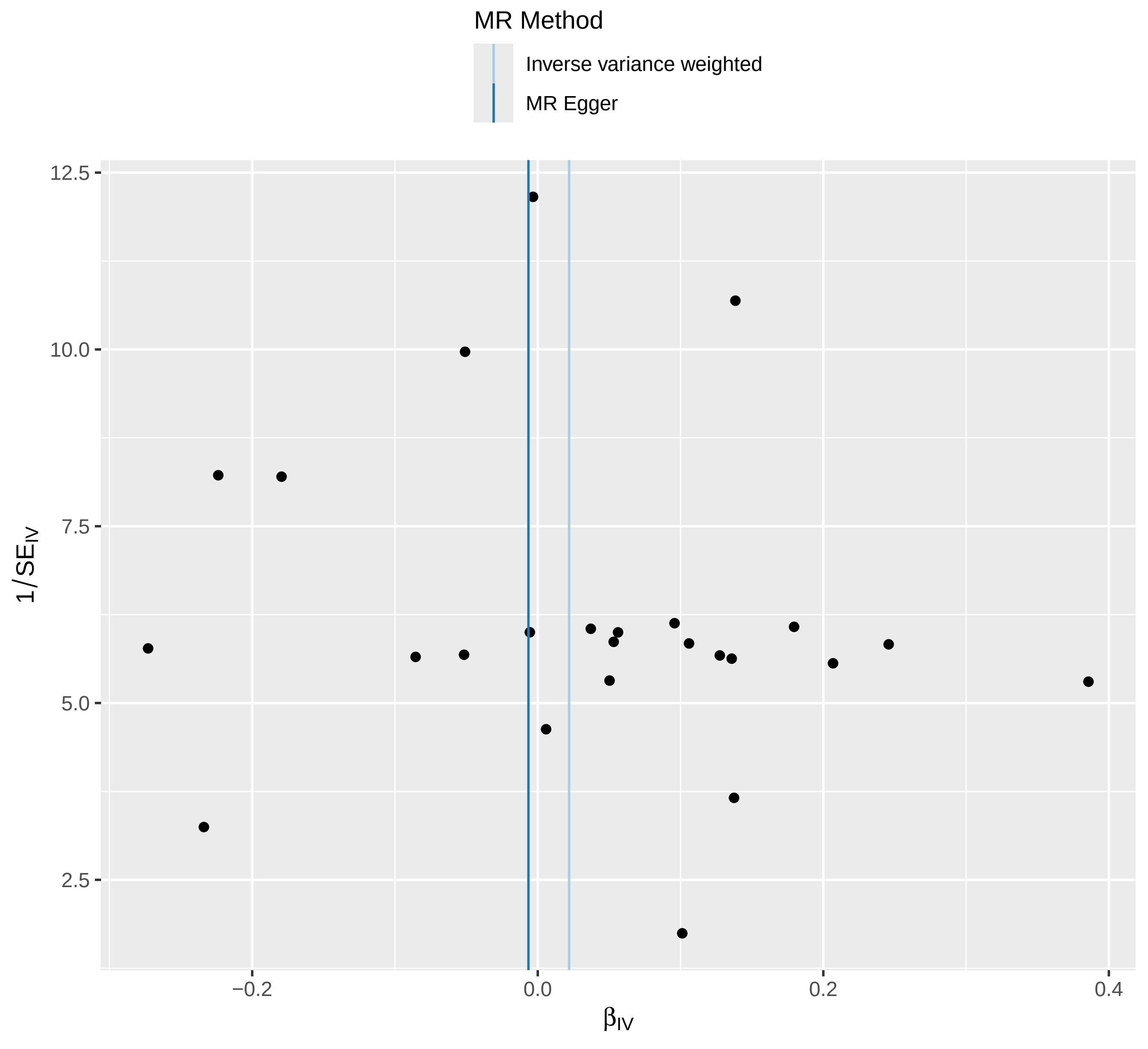
MR Method
Inverse variance weighted
MR Egger
12.5
10.0
7.5
5.0
2.5
1/SE_IV
−0.2
0.0
0.2
0.4
β_IV

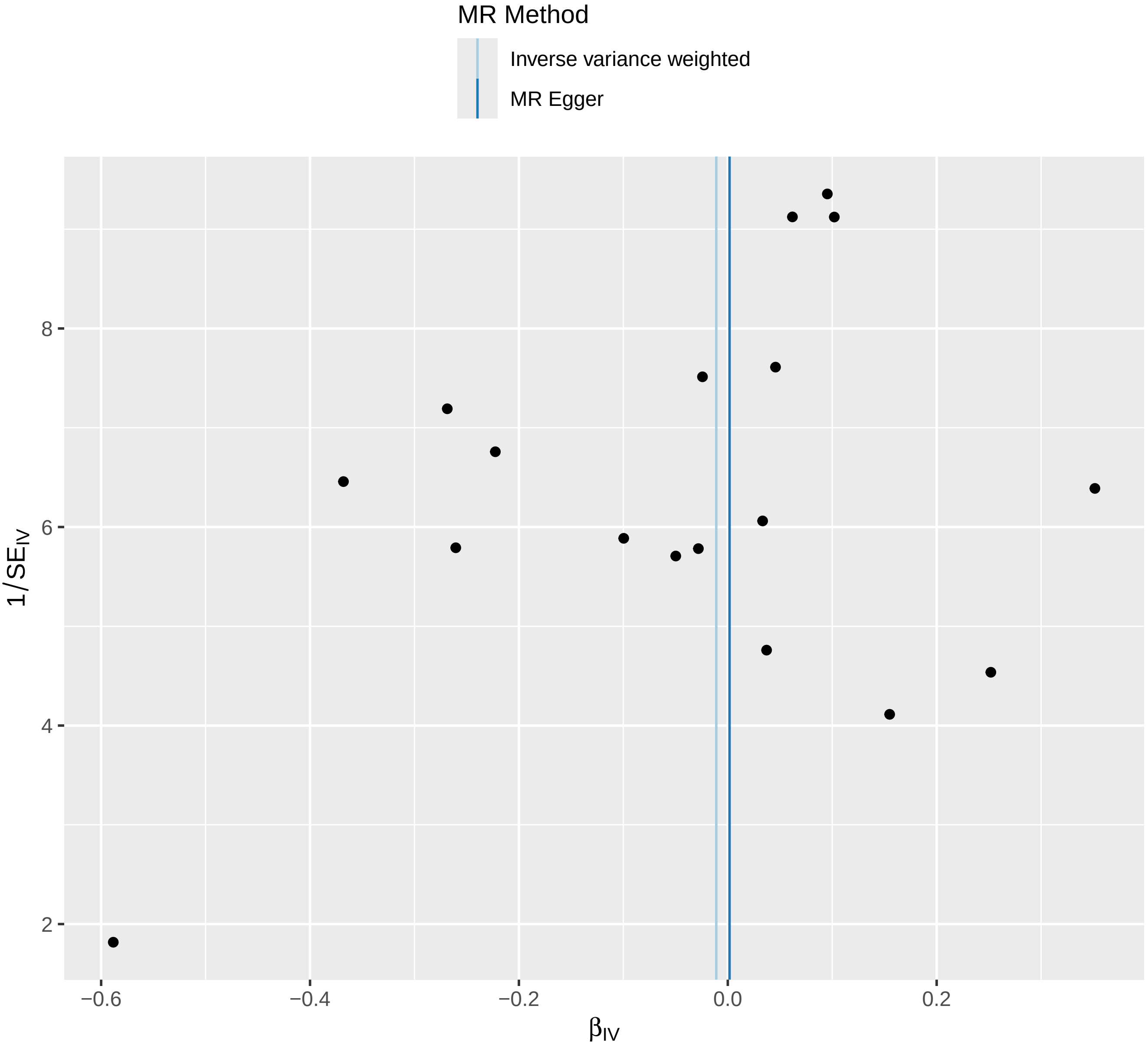
MR Method
Inverse variance weighted
MR Egger
8
6
4
2
−0.6
−0.4
−0.2
0.0
0.2
$1/SE_{IV}$
β_{IV}

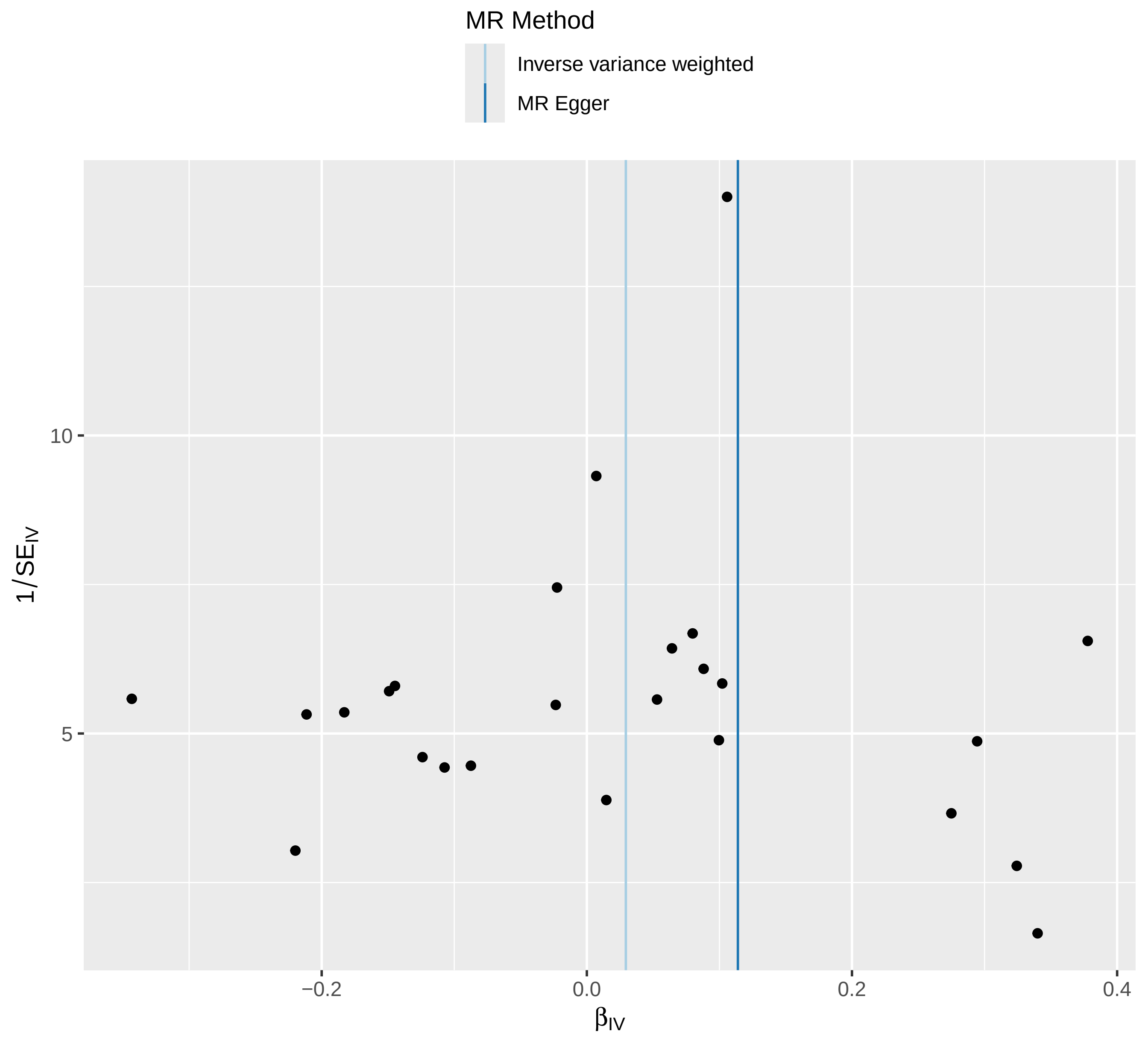

MR Method
Inverse variance weighted
MR Egger
10
5
1/SE_IV
−0.2
0.0
0.2
0.4
β_IV

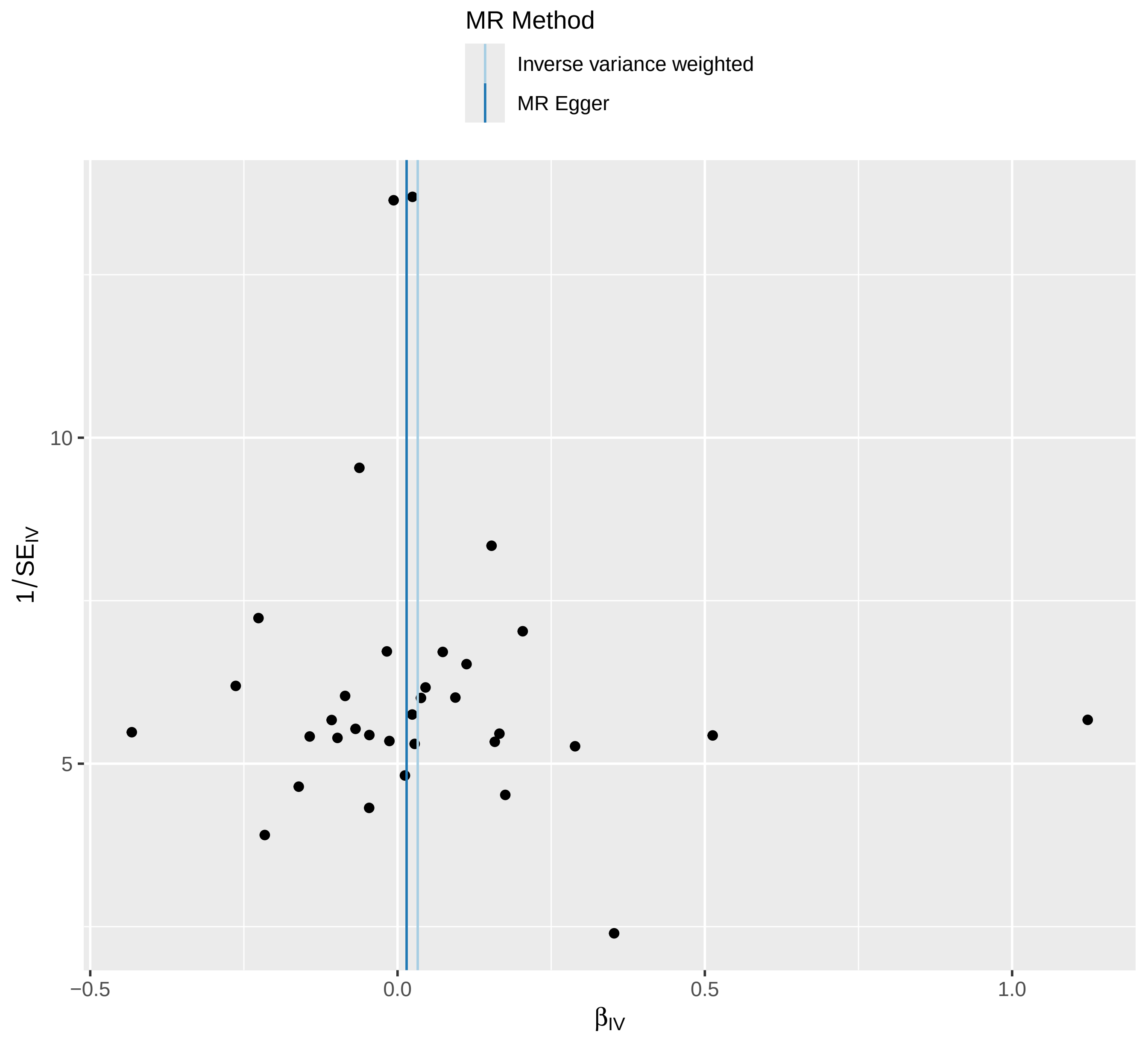
MR Method
Inverse variance weighted
MR Egger
1/SE$_{IV}$
10
5
−0.5
0.0
0.5
1.0
β$_{IV}$

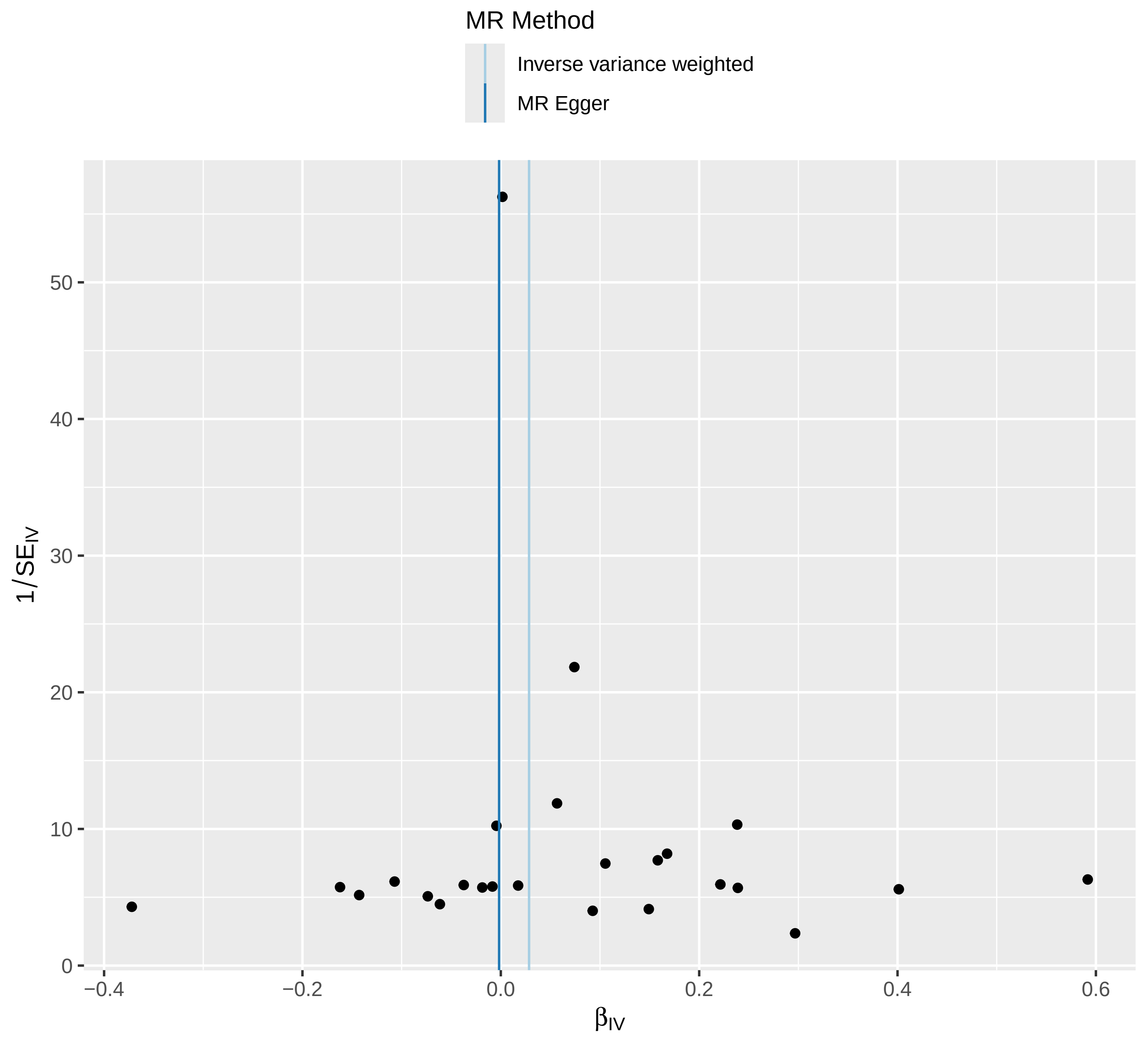
MR Method
Inverse variance weighted
MR Egger
1/SE_IV
β_IV
0
10
20
30
40
50
−0.4
−0.2
0.0
0.2
0.4
0.6

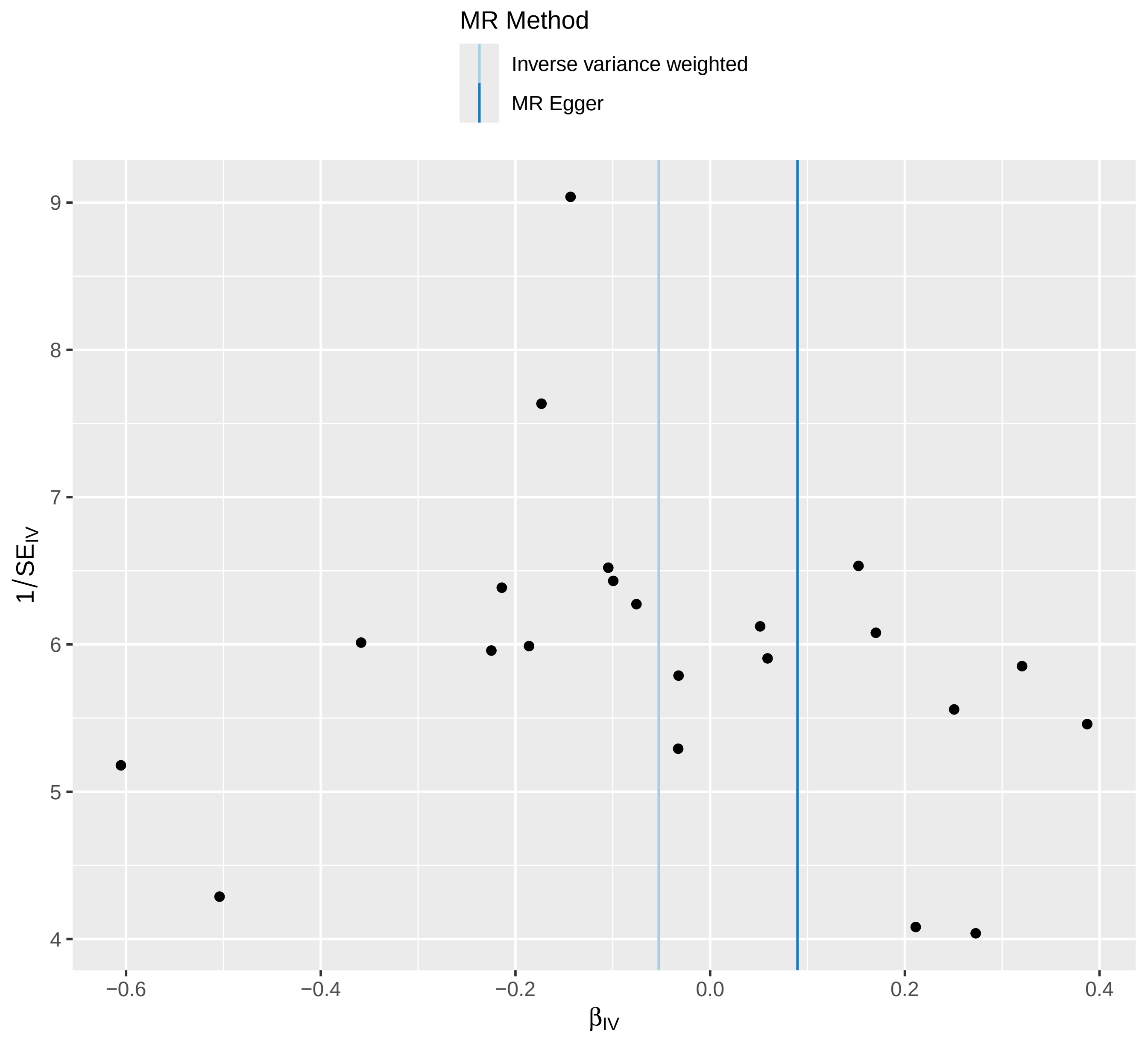

MR Method
Inverse variance weighted
MR Egger
1/SE$_{IV}$
β$_{IV}$
9
8
7
6
5
4
−0.6
−0.4
−0.2
0.0
0.2
0.4

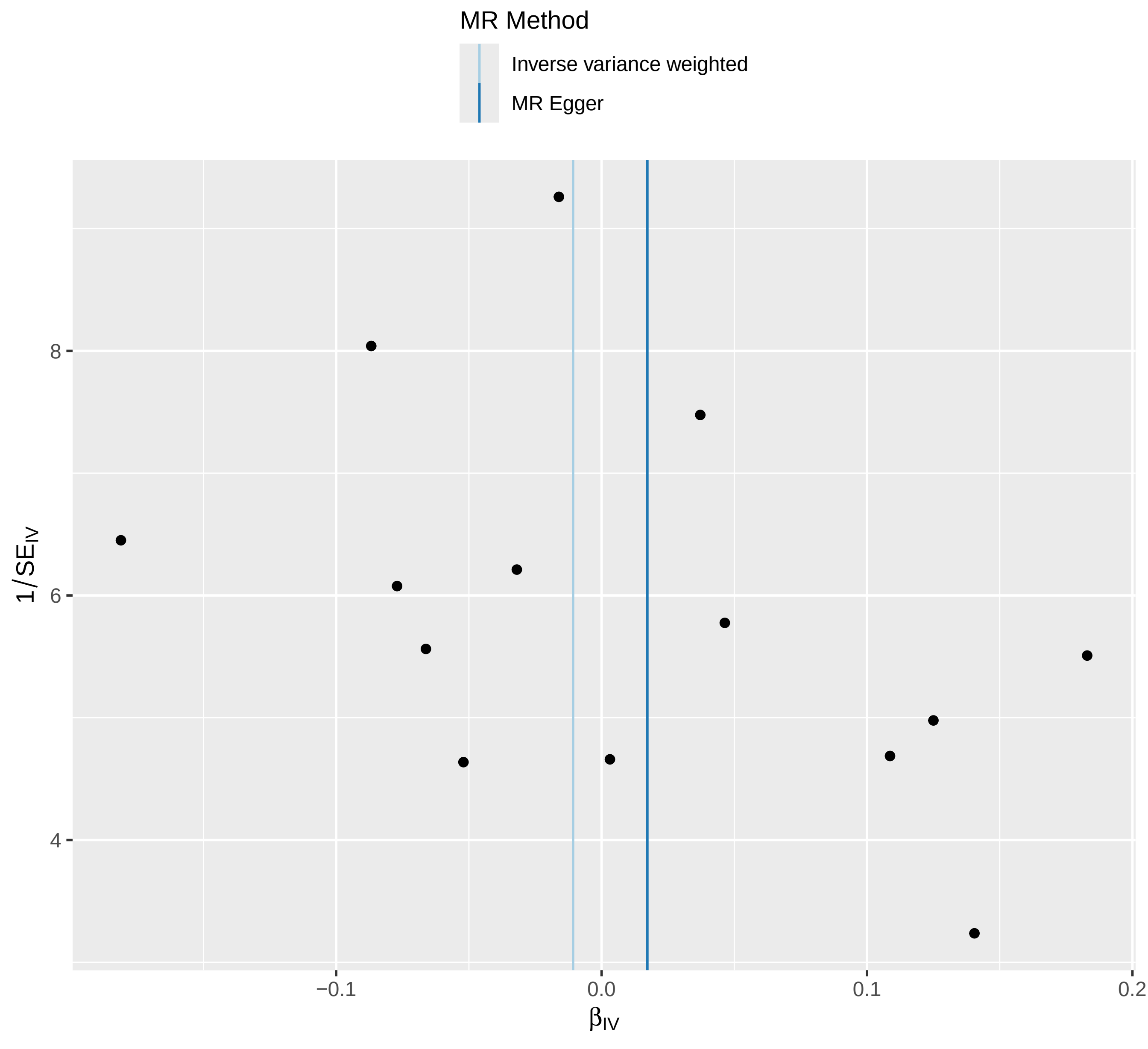

MR Method
Inverse variance weighted
MR Egger
8
6
4
1/SE_IV
−0.1
0.0
0.1
0.2
β_IV

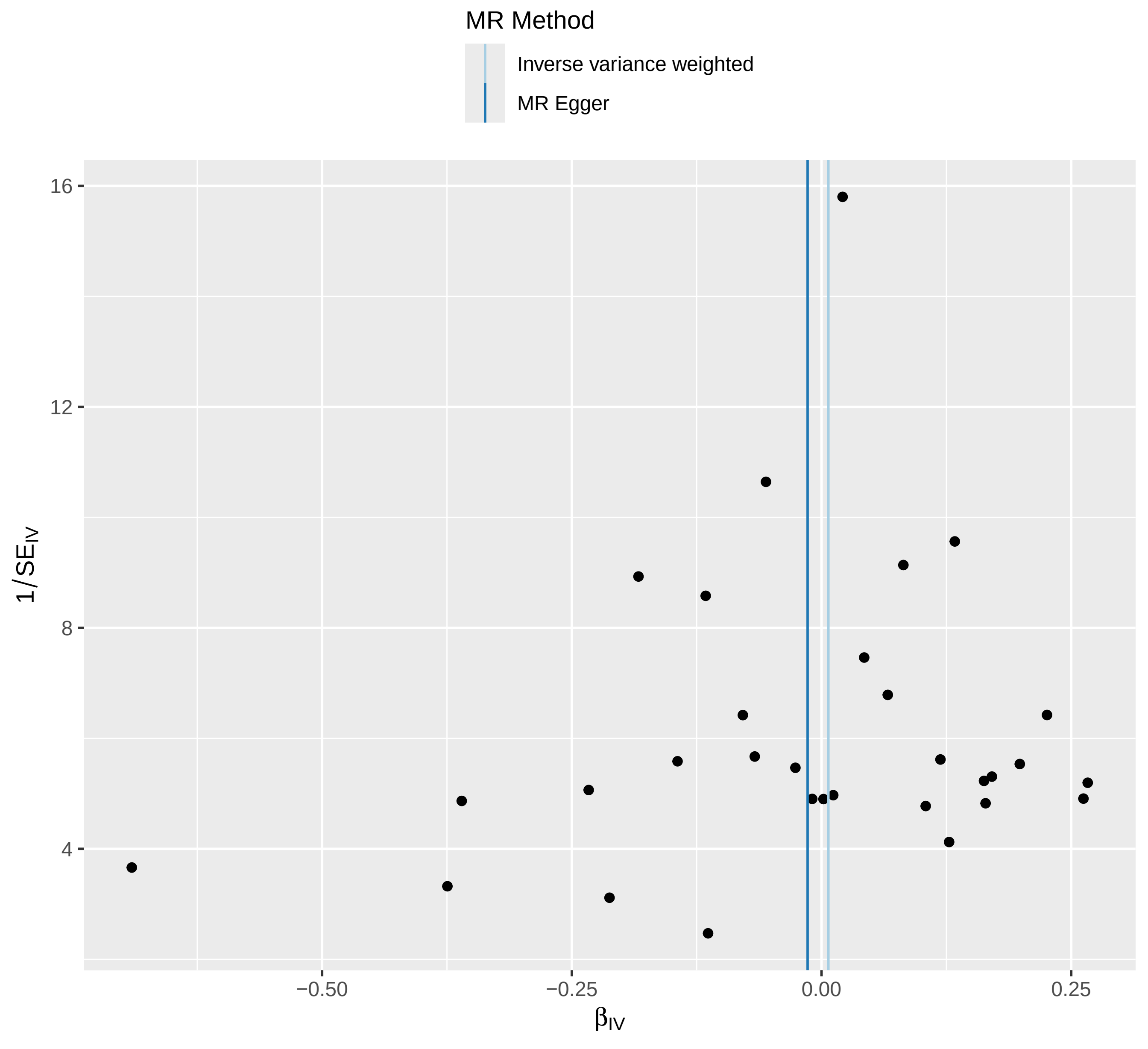

MR Method
Inverse variance weighted
MR Egger
16
12
8
4
1/SE_IV
−0.50
−0.25
0.00
0.25
β_IV

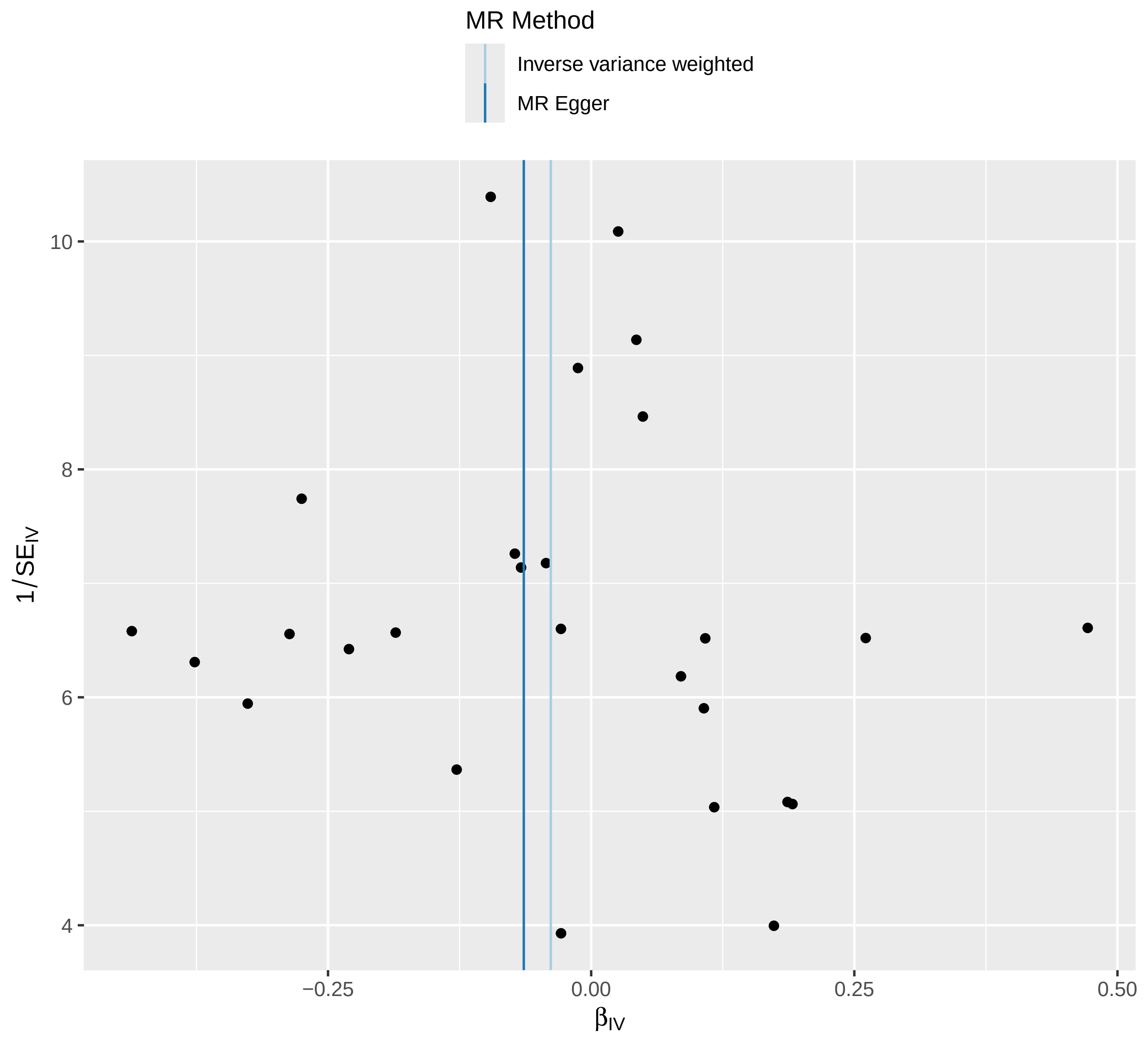

MR Method
Inverse variance weighted
MR Egger
10
8
6
4
1/SE_IV
−0.25
0.00
0.25
0.50
β_IV

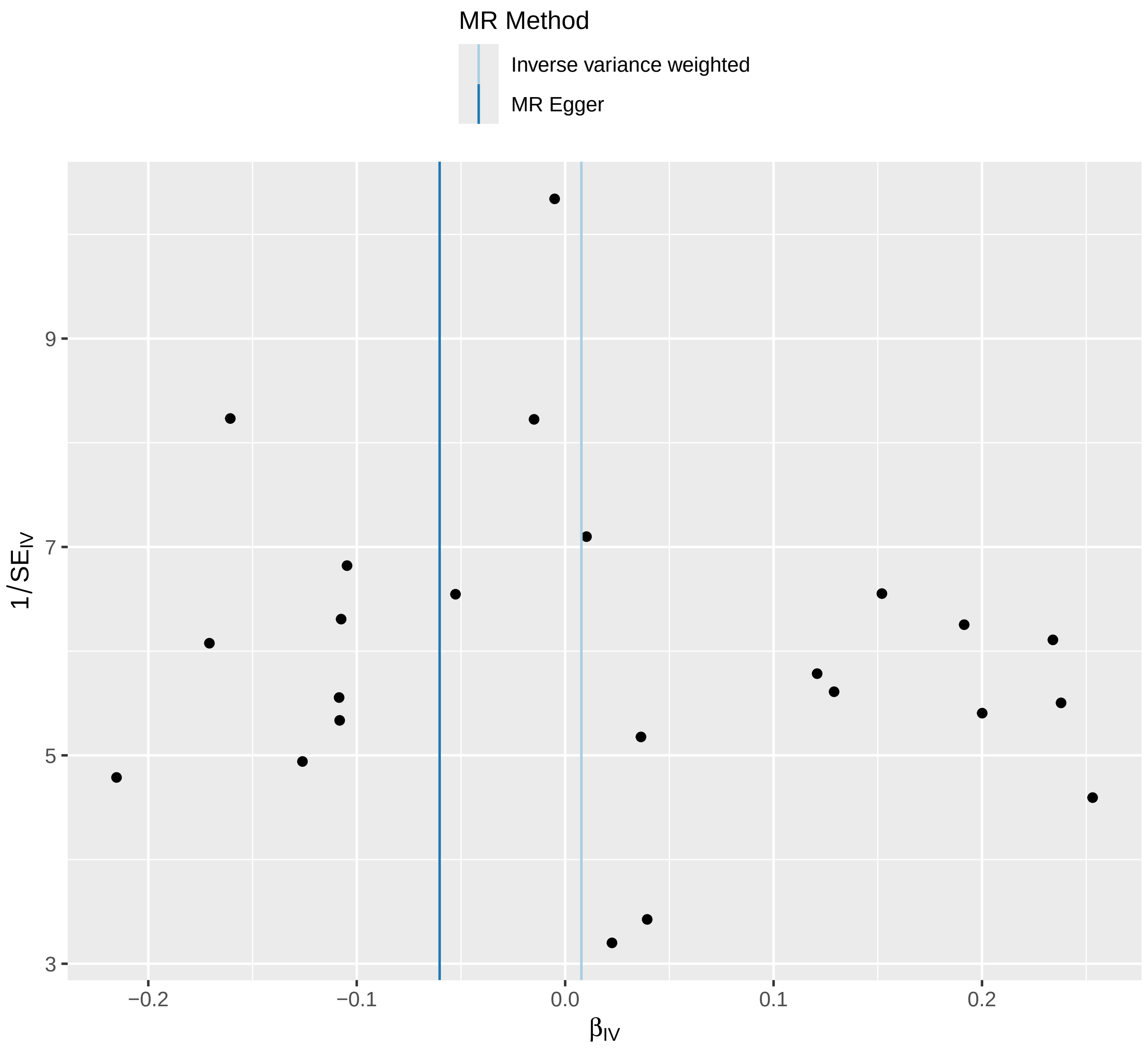
MR Method
Inverse variance weighted
MR Egger
9
7
5
3
1/SE$_{IV}$
−0.2
−0.1
0.0
0.1
0.2
β$_{IV}$

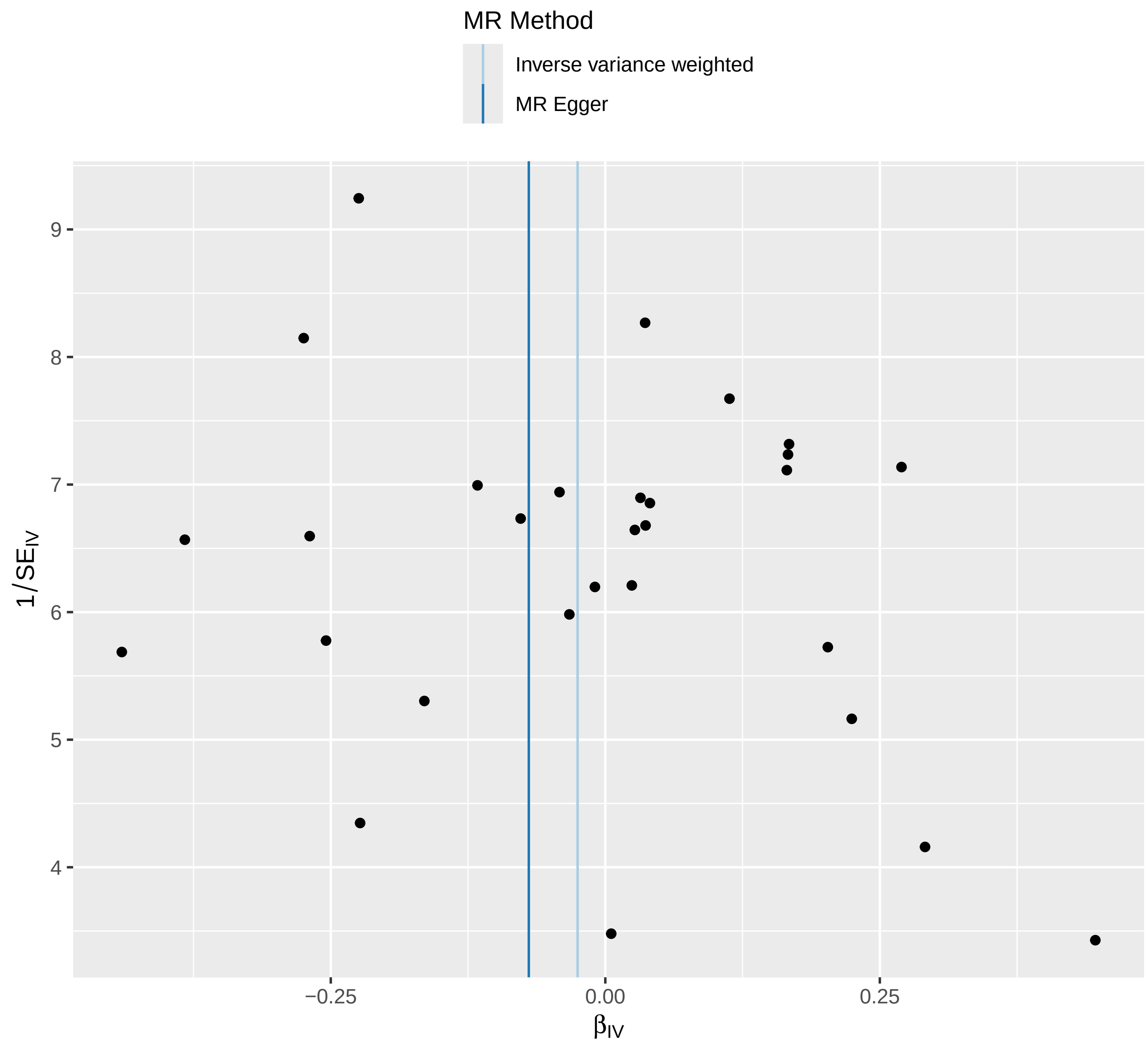
MR Method
Inverse variance weighted
MR Egger
9
8
7
6
5
4
1/SE_IV
−0.25
0.00
0.25
β_IV

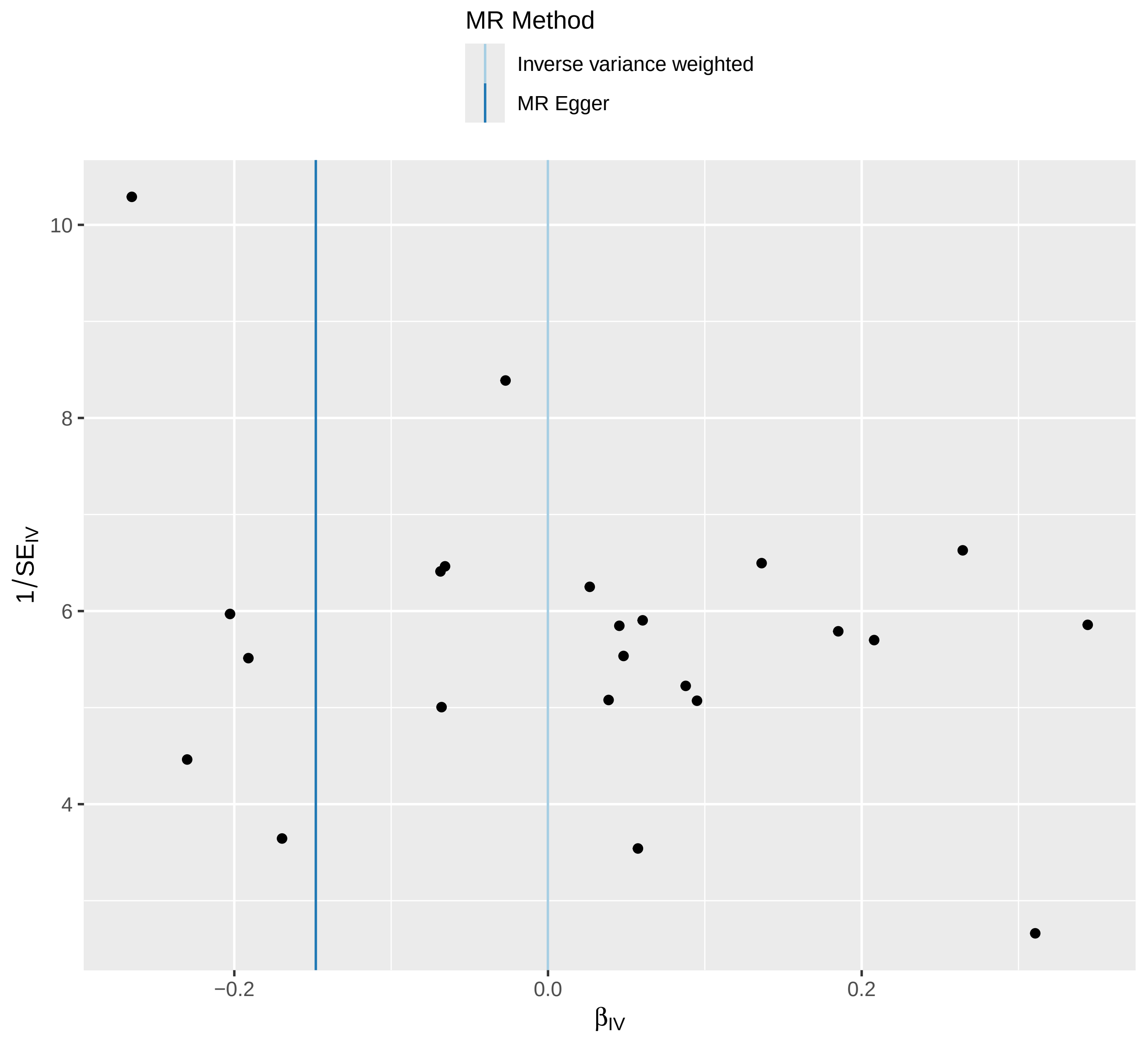
MR Method
Inverse variance weighted
MR Egger
10
8
6
4
1/SE_IV
−0.2
0.0
0.2
β_IV

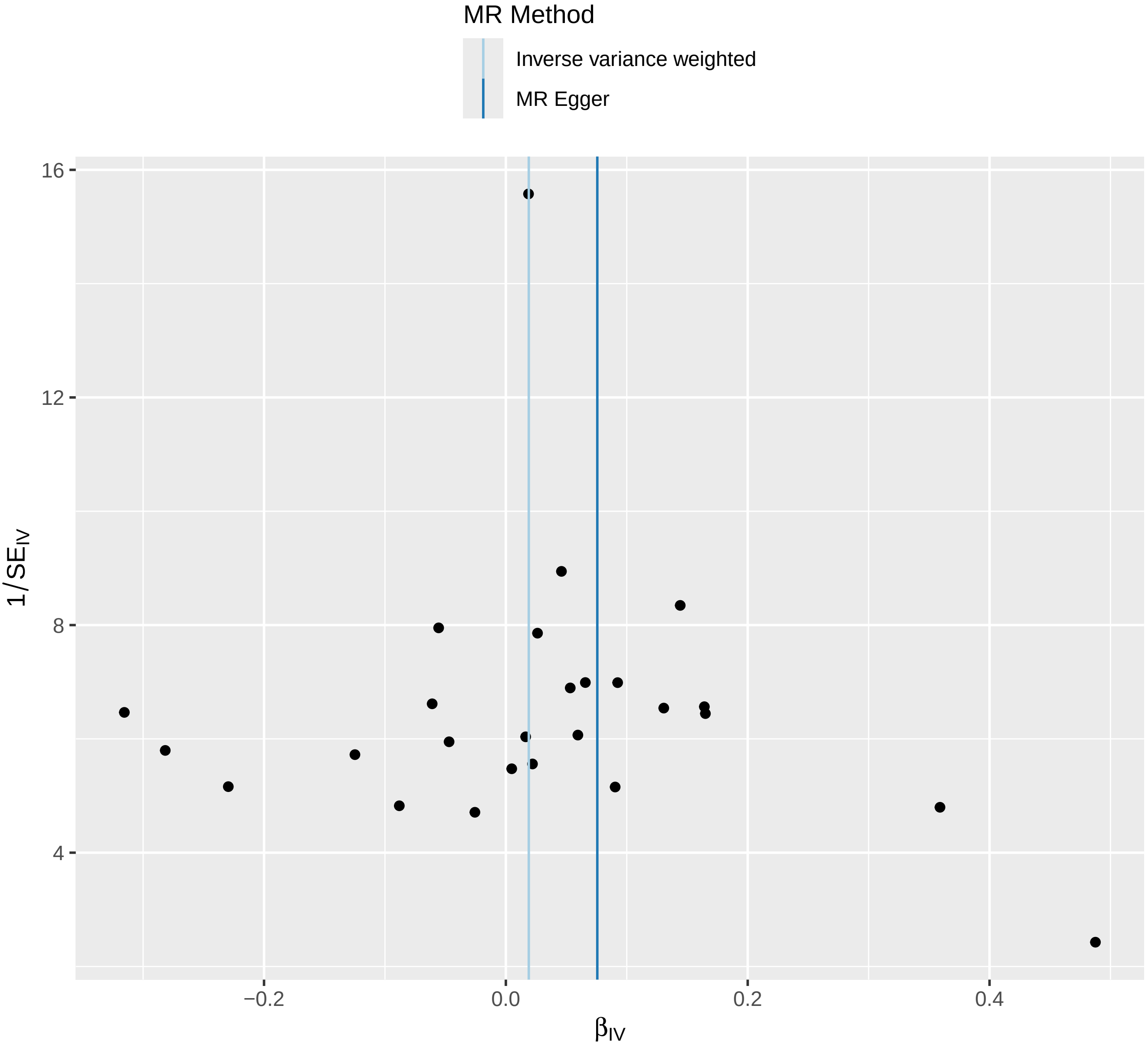
MR Method
Inverse variance weighted
MR Egger
16
12
8
4
1/SE_IV
−0.2
0.0
0.2
0.4
β_IV

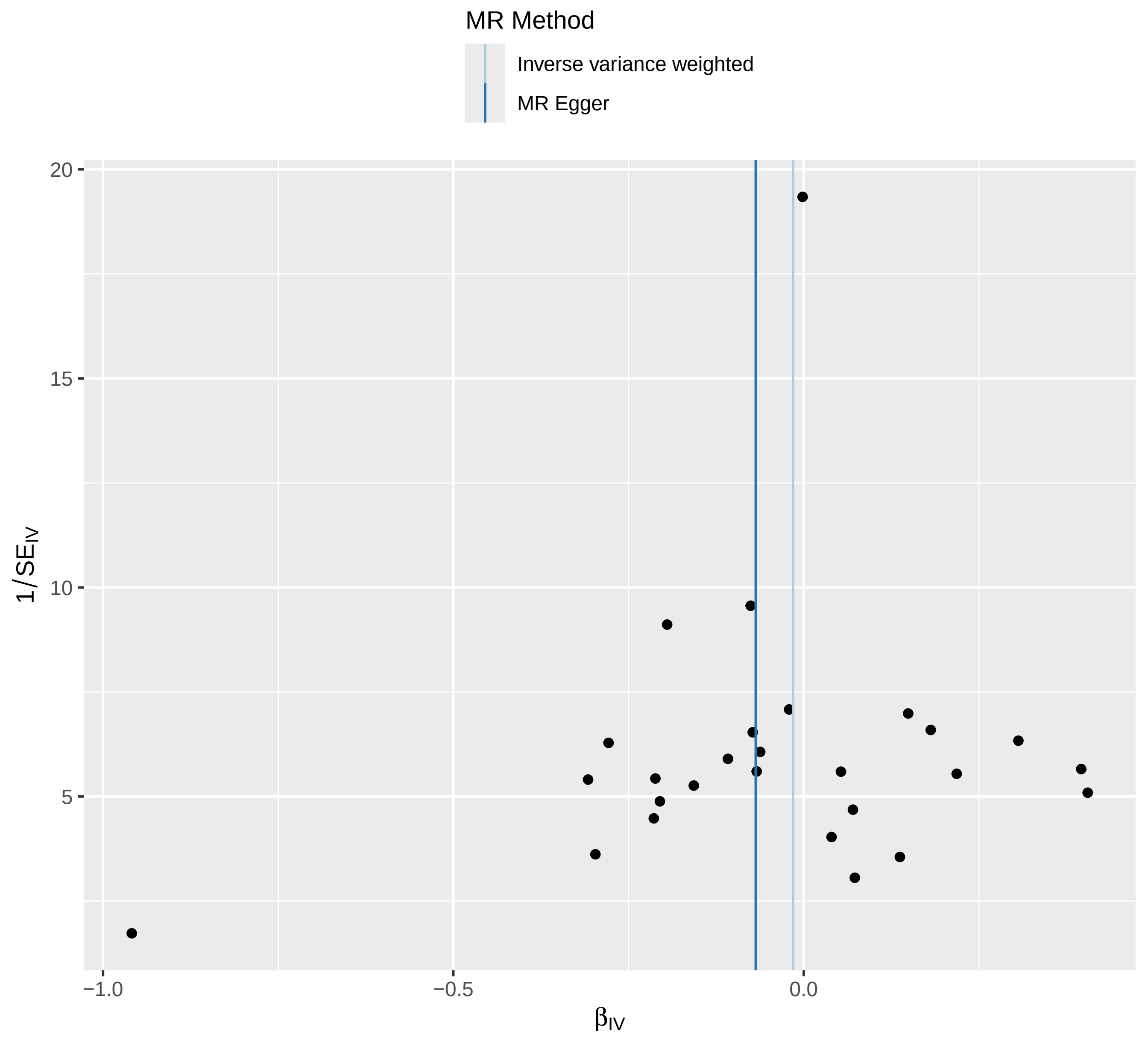
MR Method
Inverse variance weighted
MR Egger
20
15
10
5
1/SE_IV
−1.0
−0.5
0.0
β_IV

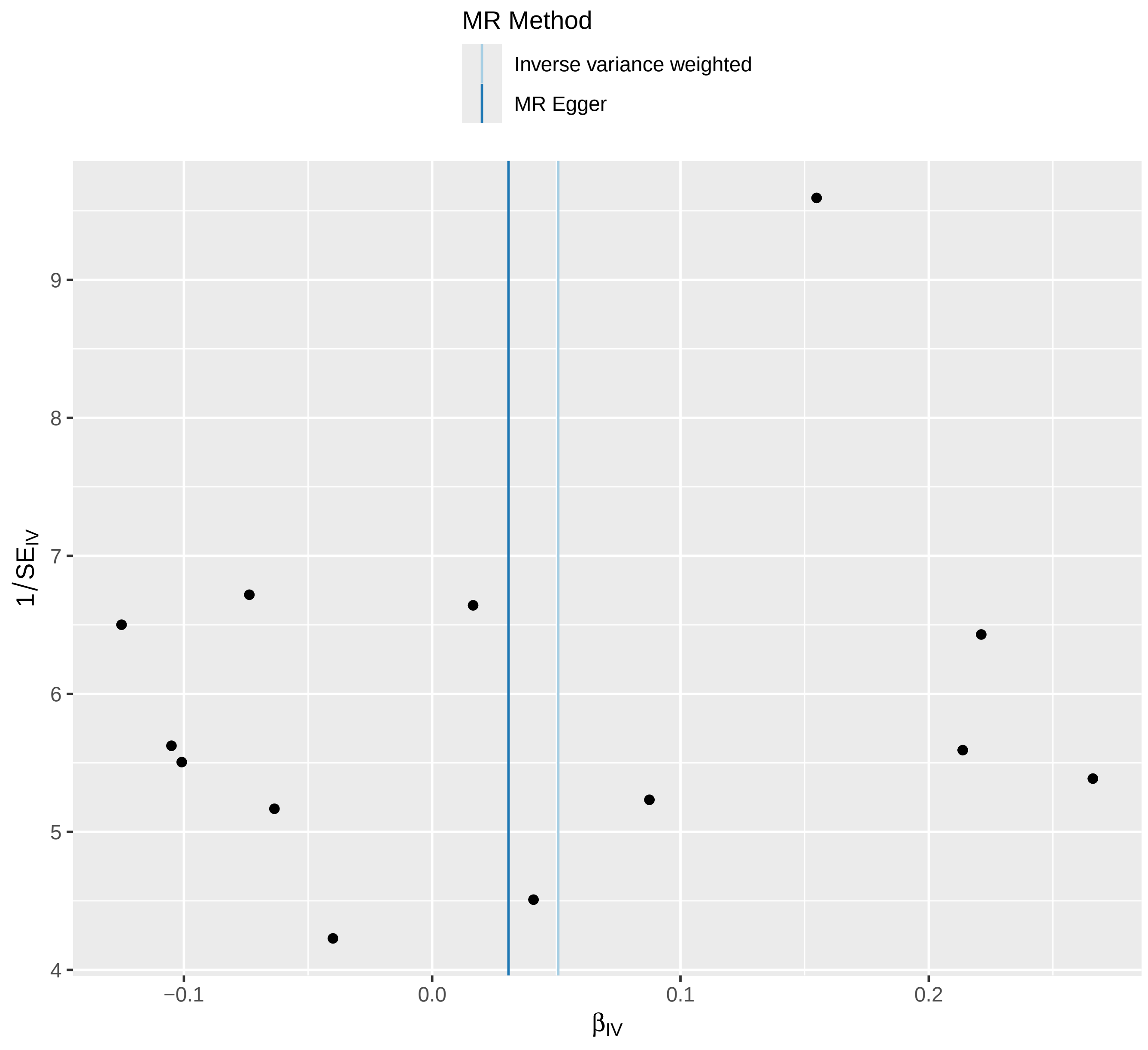
MR Method
Inverse variance weighted
MR Egger
1/SE_IV
β_IV
9
8
7
6
5
4
−0.1
0.0
0.1
0.2

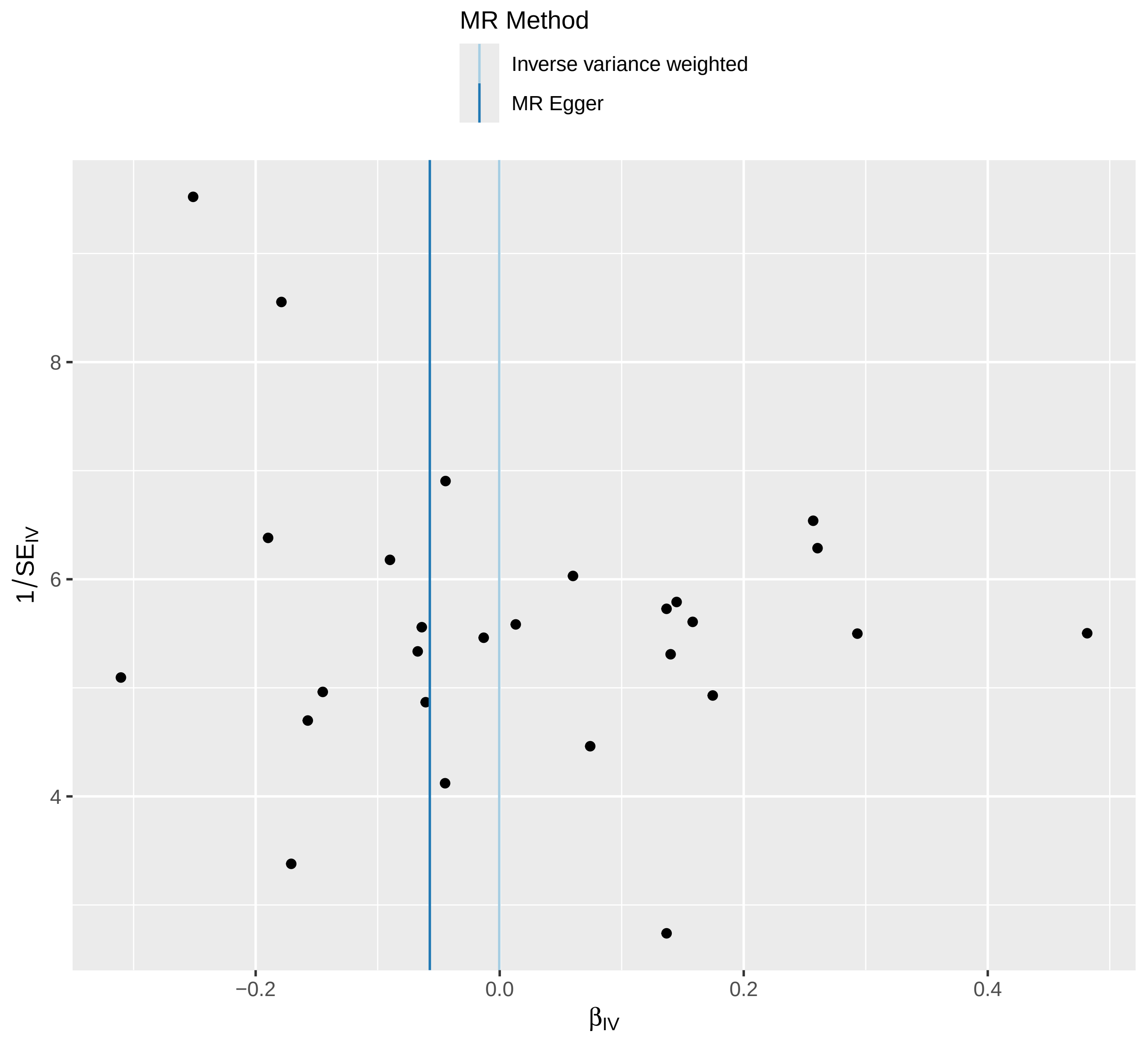
MR Method
Inverse variance weighted
MR Egger
8
6
4
1/SE_IV
−0.2
0.0
0.2
0.4
β_IV

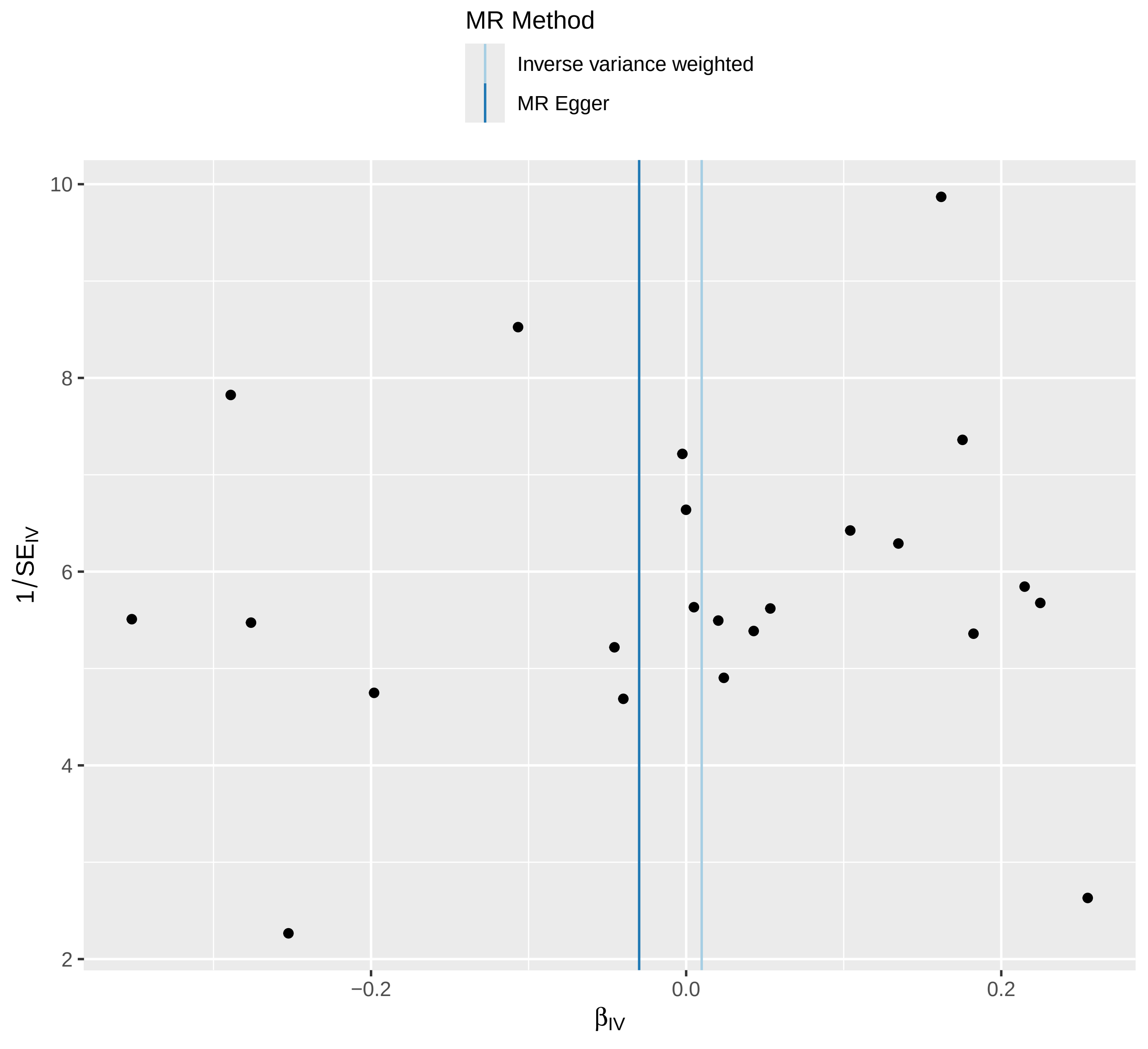
MR Method
Inverse variance weighted
MR Egger
10
8
6
4
2
1/SE$_{IV}$
−0.2
0.0
0.2
β$_{IV}$

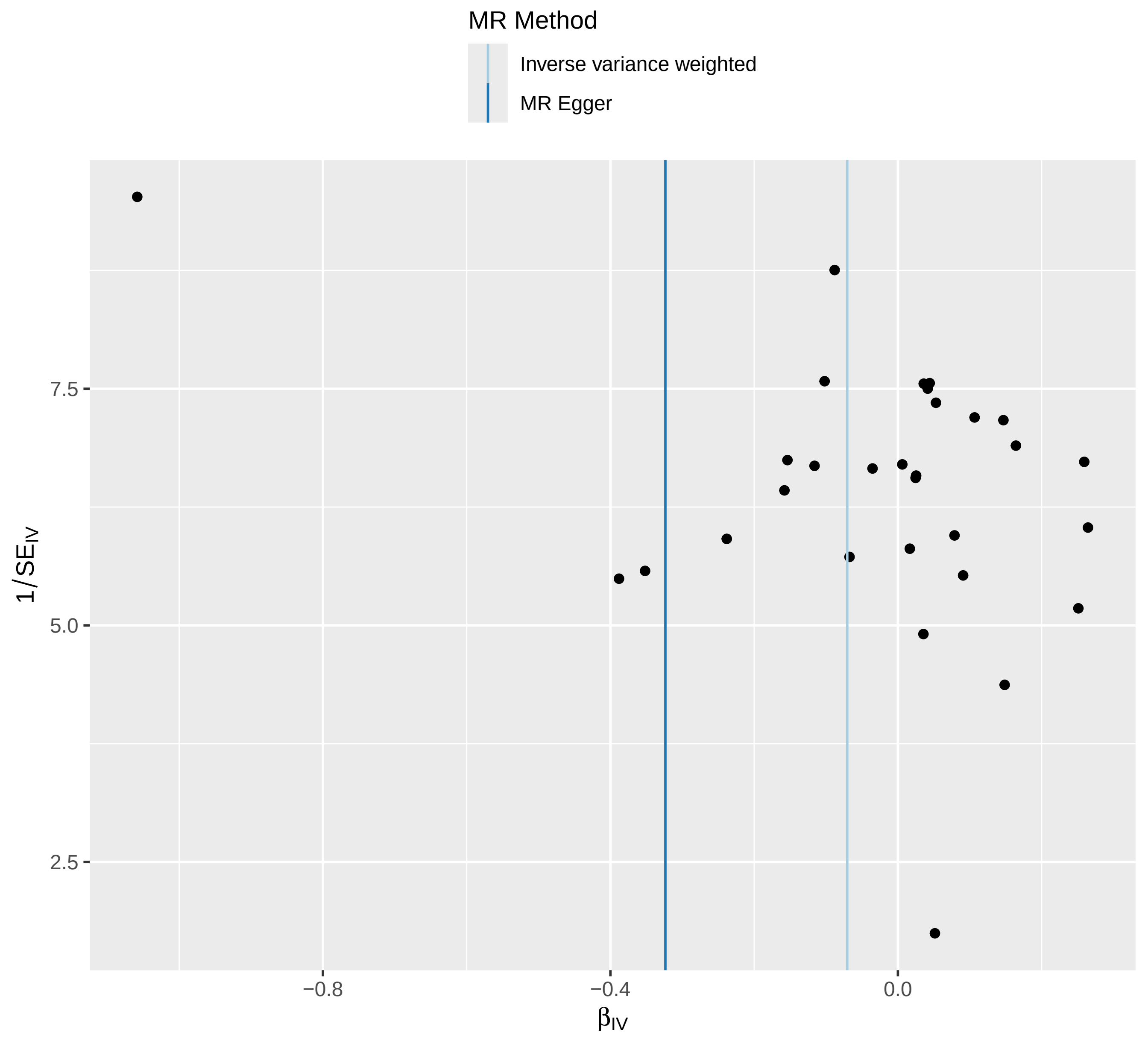
MR Method
Inverse variance weighted
MR Egger
1/SE_IV
7.5
5.0
2.5
−0.8
−0.4
0.0
β_IV

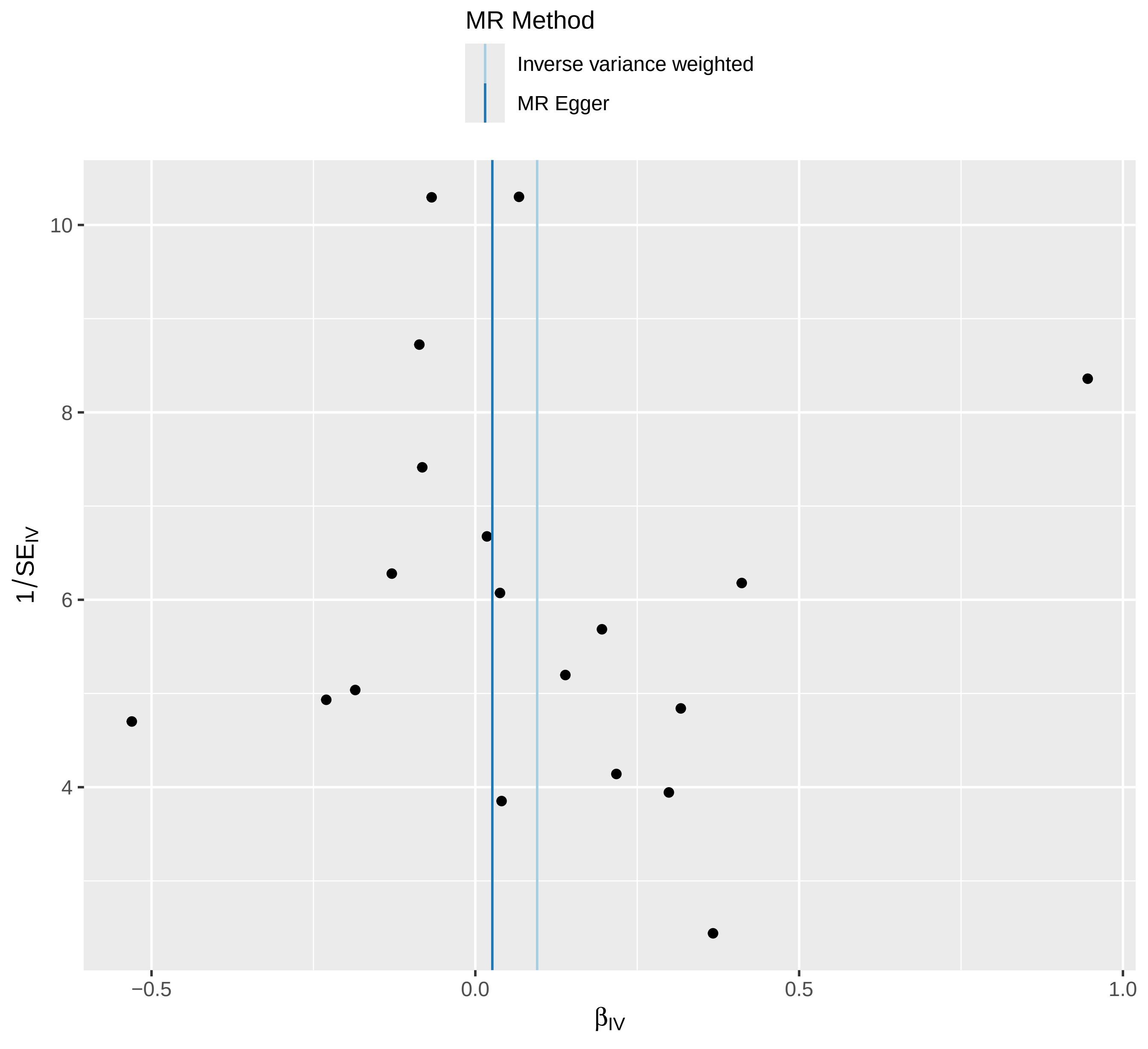
MR Method
Inverse variance weighted
MR Egger
$1/SE_{IV}$
10
8
6
4
−0.5
0.0
0.5
1.0
β_{IV}

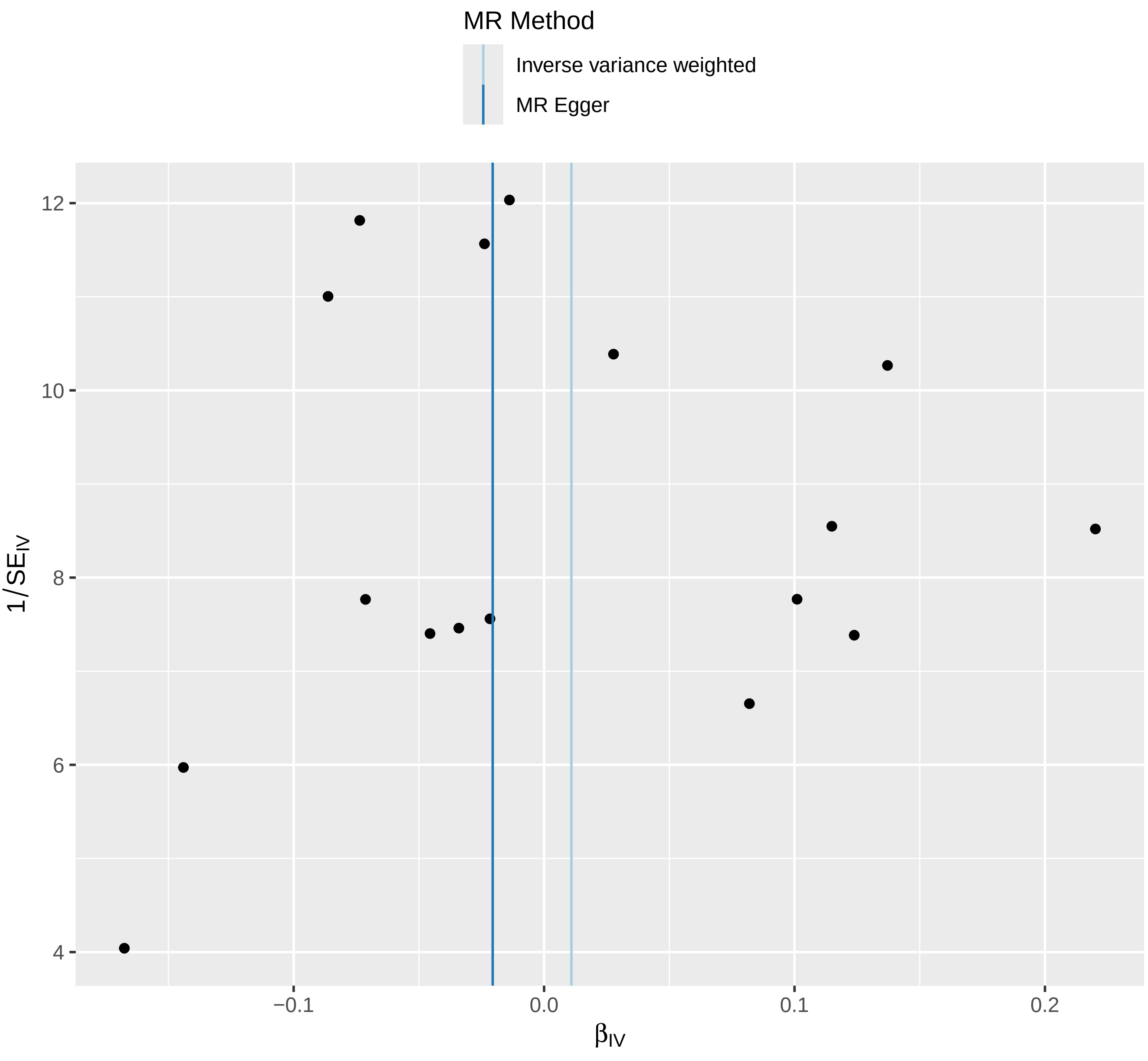
MR Method
Inverse variance weighted
MR Egger
1/SE_IV
β_IV
12
10
8
6
4
−0.1
0.0
0.1
0.2

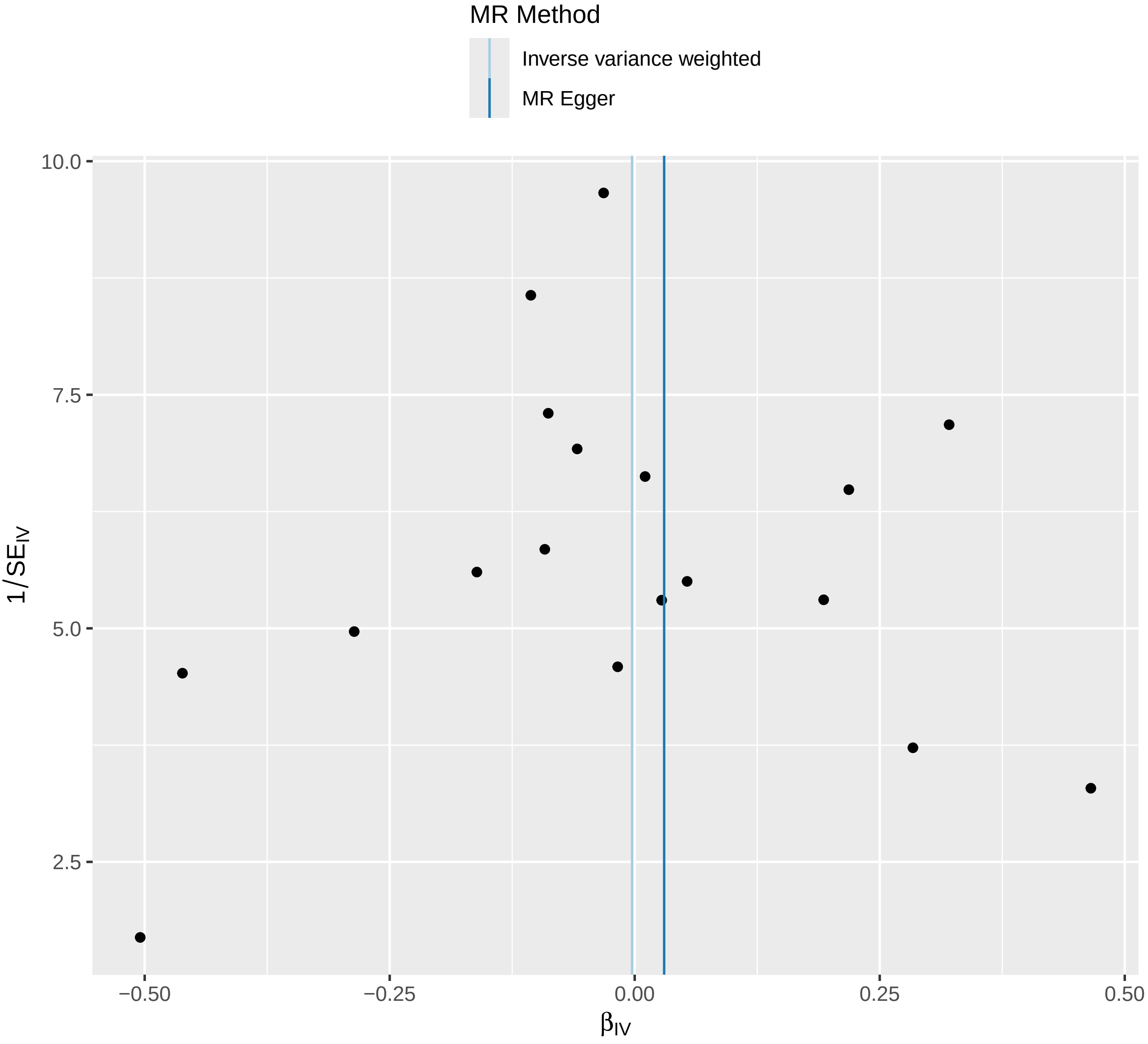

MR Method
Inverse variance weighted
MR Egger
10.0
7.5
5.0
2.5
1/SE_IV
−0.50
−0.25
0.00
0.25
0.50
β_IV

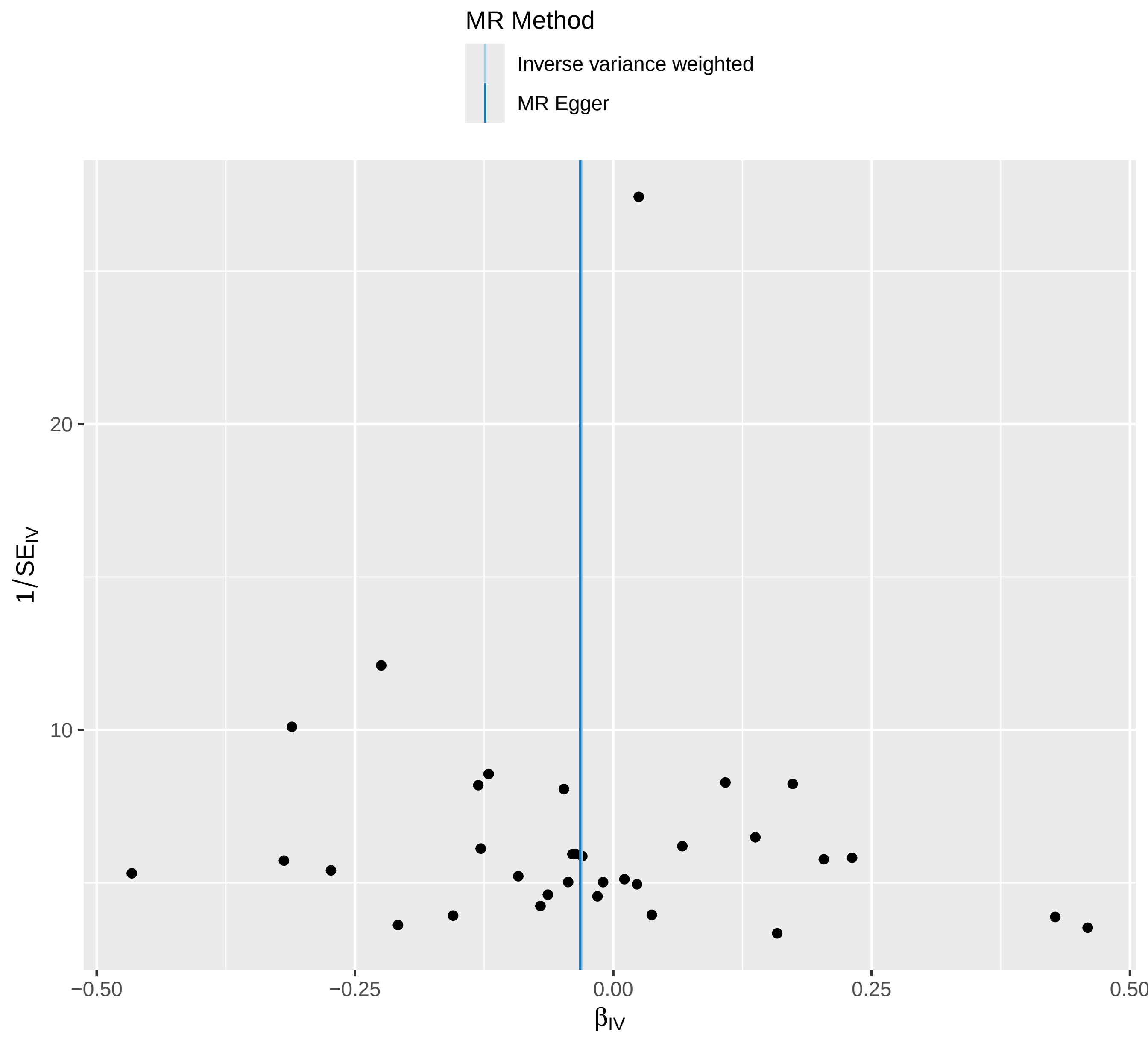

MR Method
Inverse variance weighted
MR Egger
20
10
1/SE_IV
−0.50
−0.25
0.00
0.25
0.50
β_IV

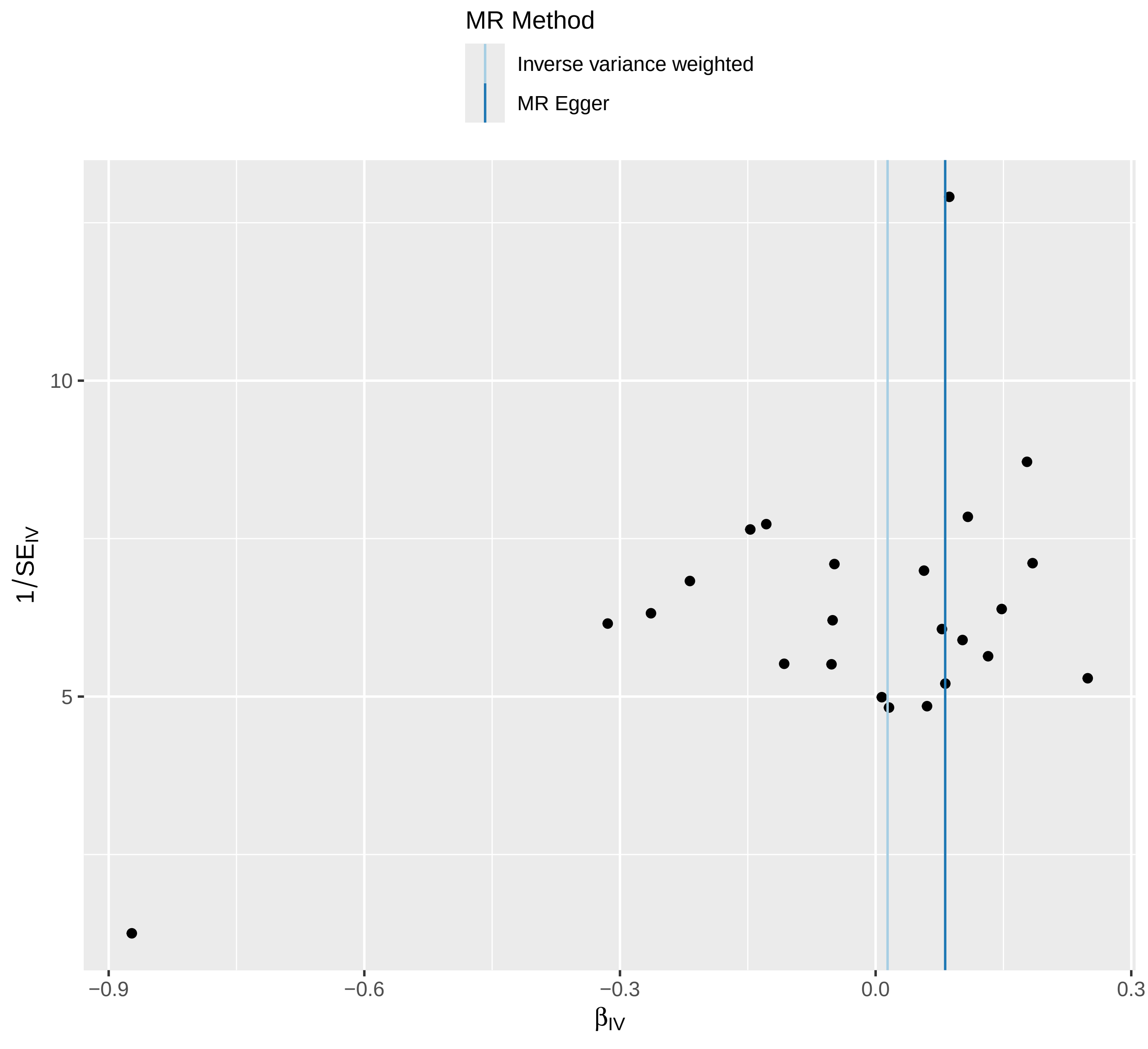

MR Method
Inverse variance weighted
MR Egger
10
5
1/SE_IV
−0.9
−0.6
−0.3
0.0
0.3
β_IV

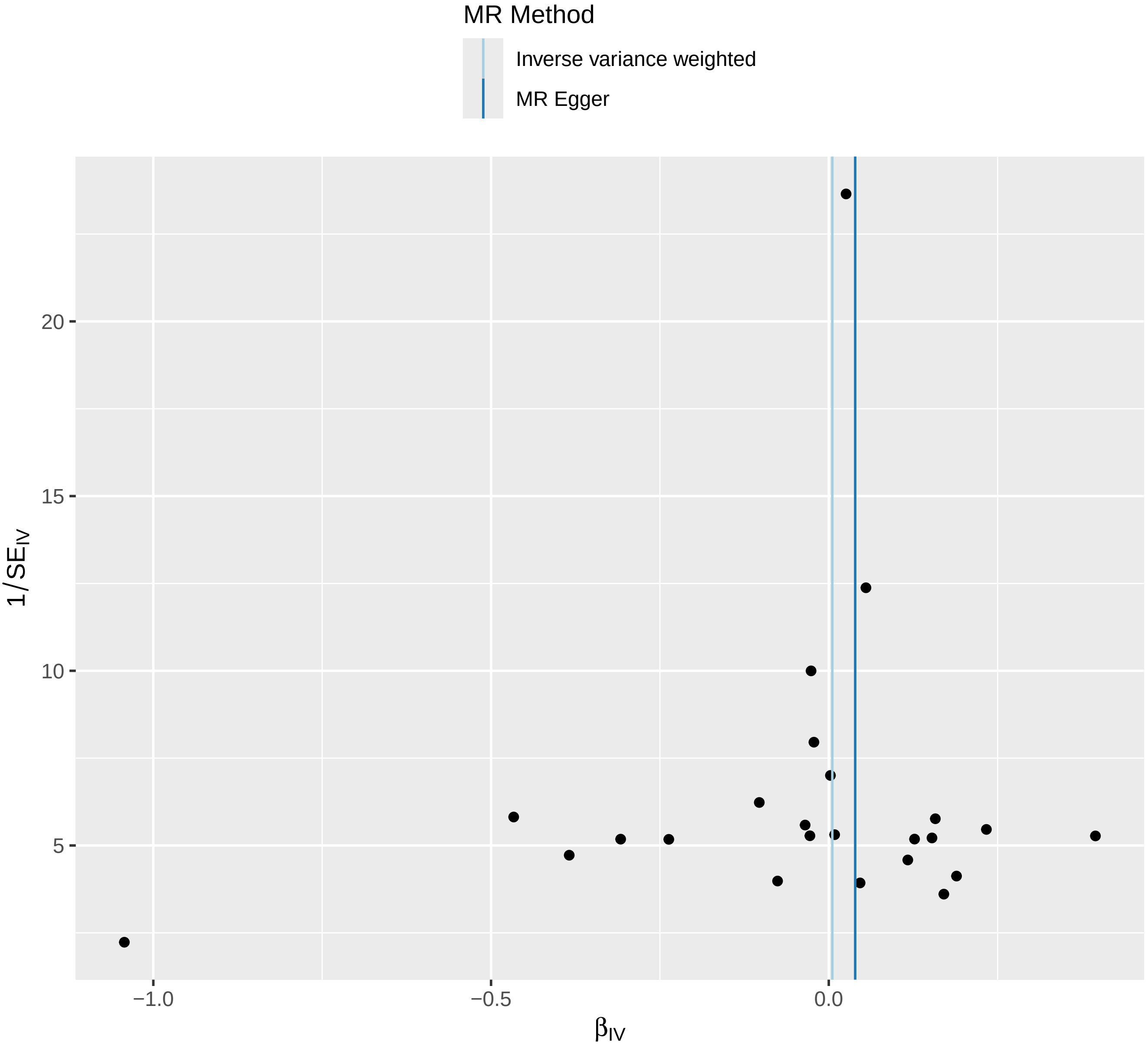
MR Method
Inverse variance weighted
MR Egger
1/SE_IV
20
15
10
5
−1.0
−0.5
0.0
β_IV

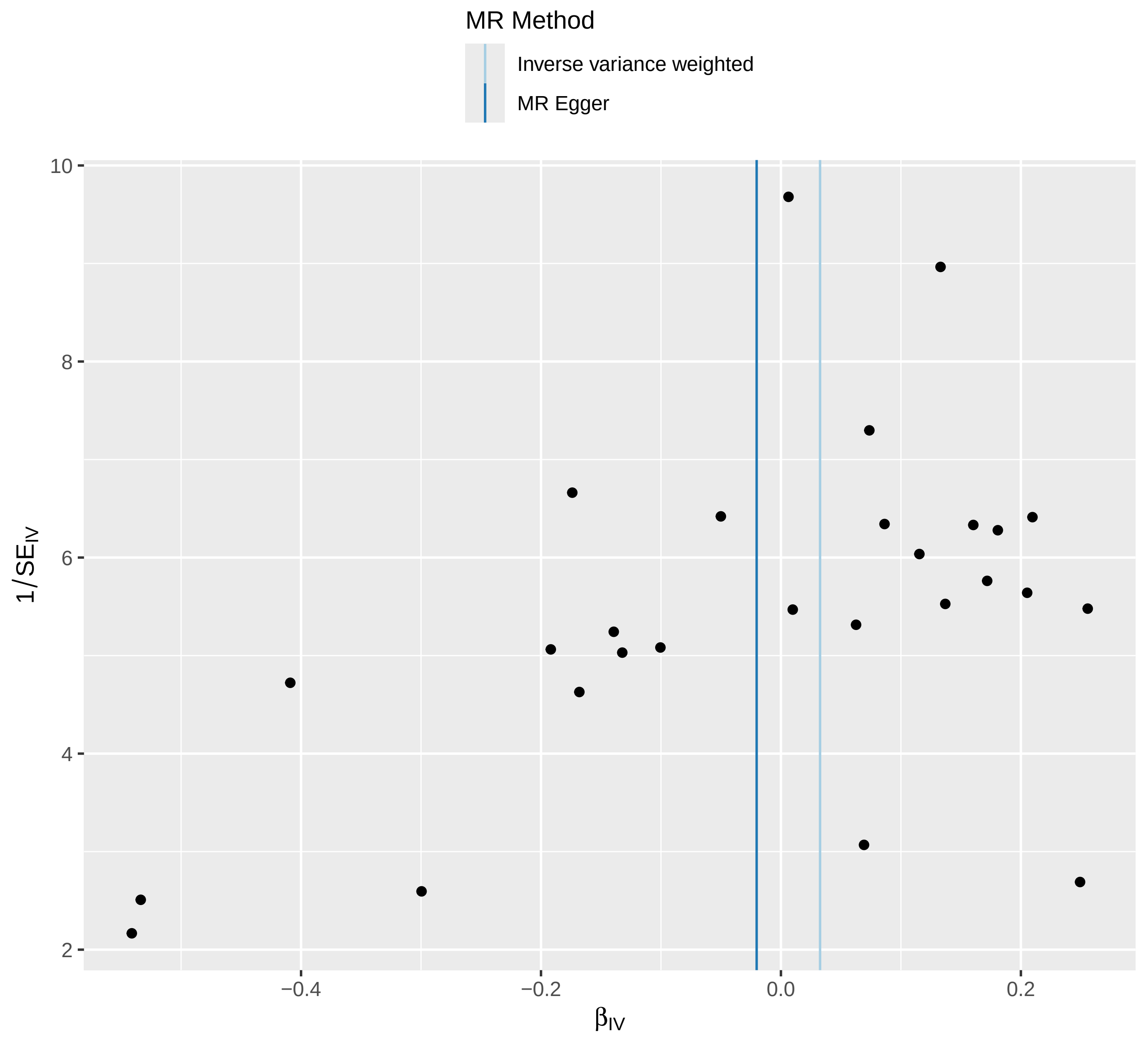
MR Method
Inverse variance weighted
MR Egger
1/SE_{IV}
β_{IV}
10
8
6
4
2
−0.4
−0.2
0.0
0.2

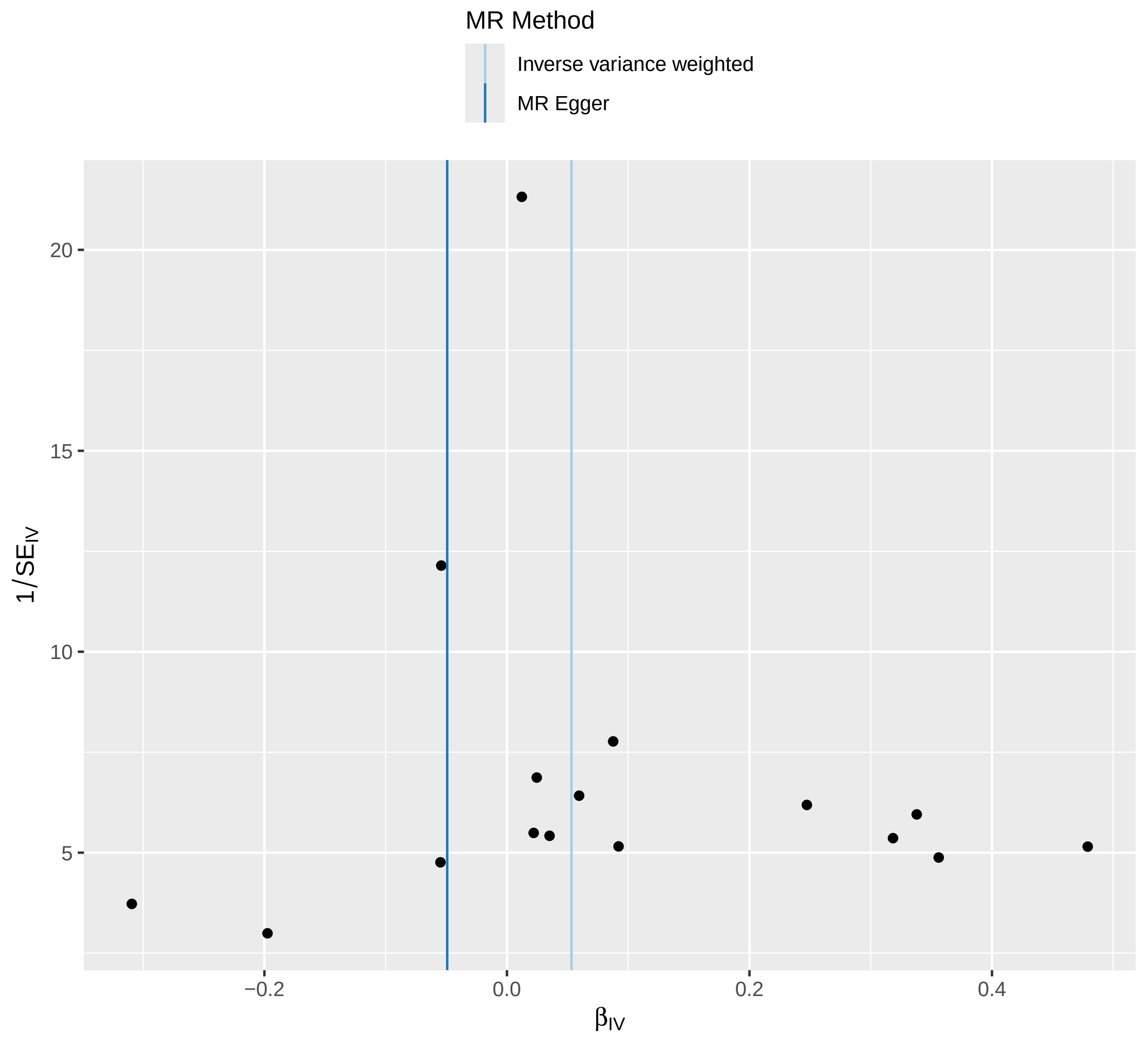

MR Method
Inverse variance weighted
MR Egger
20
15
10
5
1/SE_IV
−0.2
0.0
0.2
0.4
β_IV

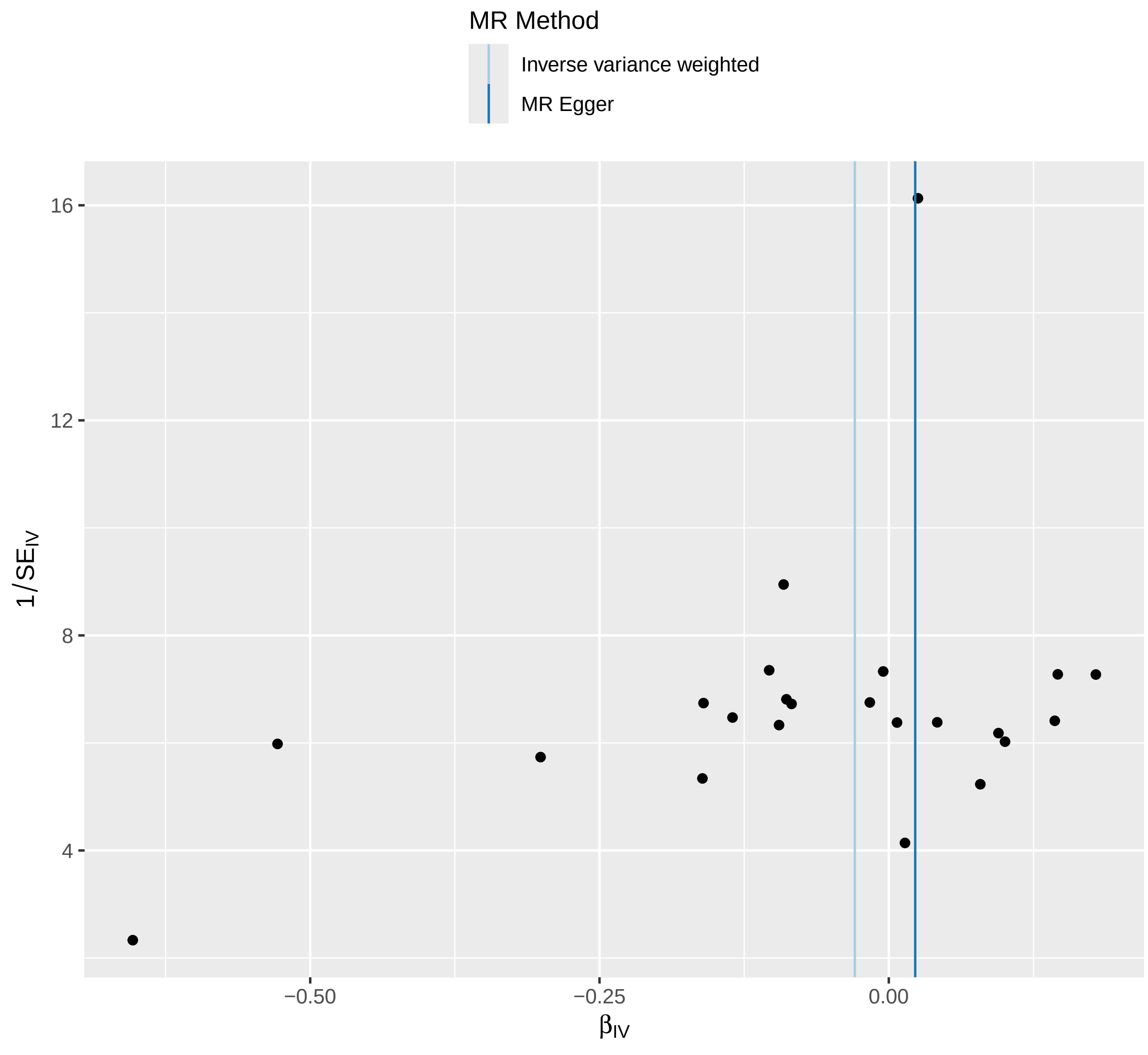
MR Method
Inverse variance weighted
MR Egger
16
12
8
4
1/SE$_{IV}$
−0.50
−0.25
0.00
β$_{IV}$

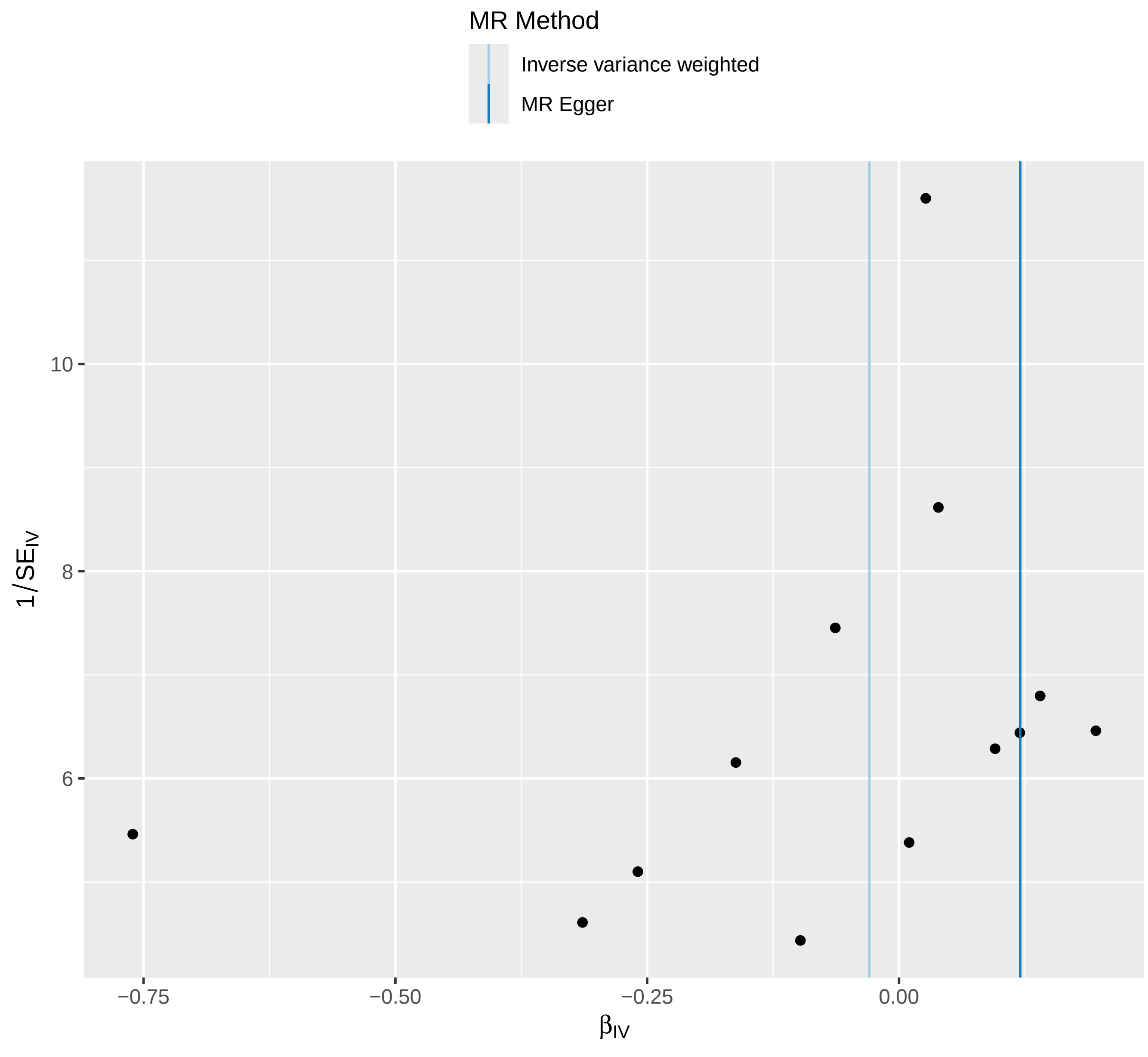
MR Method
Inverse variance weighted
MR Egger
10
8
6
1/SE_IV
−0.75
−0.50
−0.25
0.00
β_IV

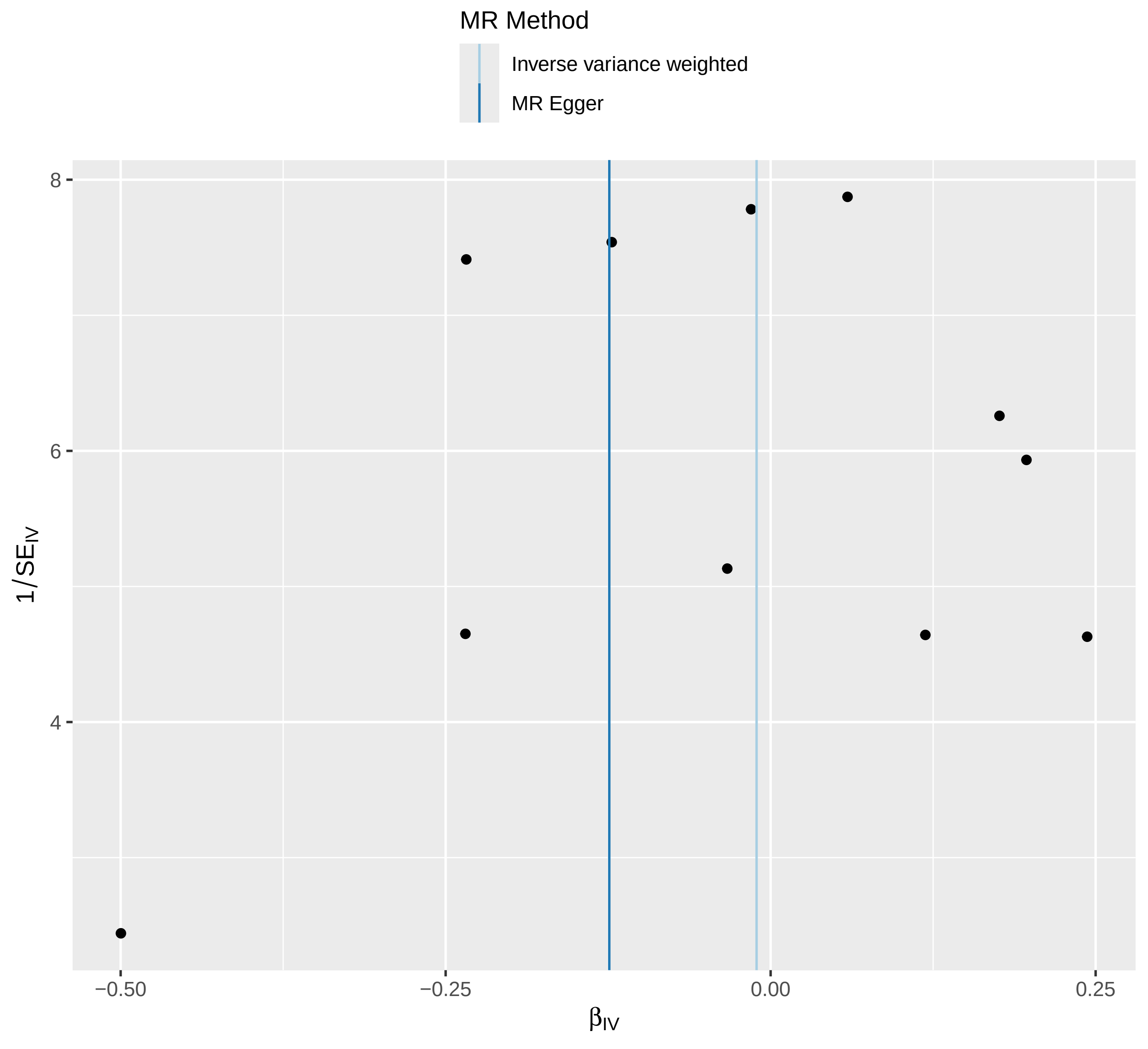
MR Method
Inverse variance weighted
MR Egger
8
6
4
1/SE$_{IV}$
−0.50
−0.25
0.00
0.25
β$_{IV}$

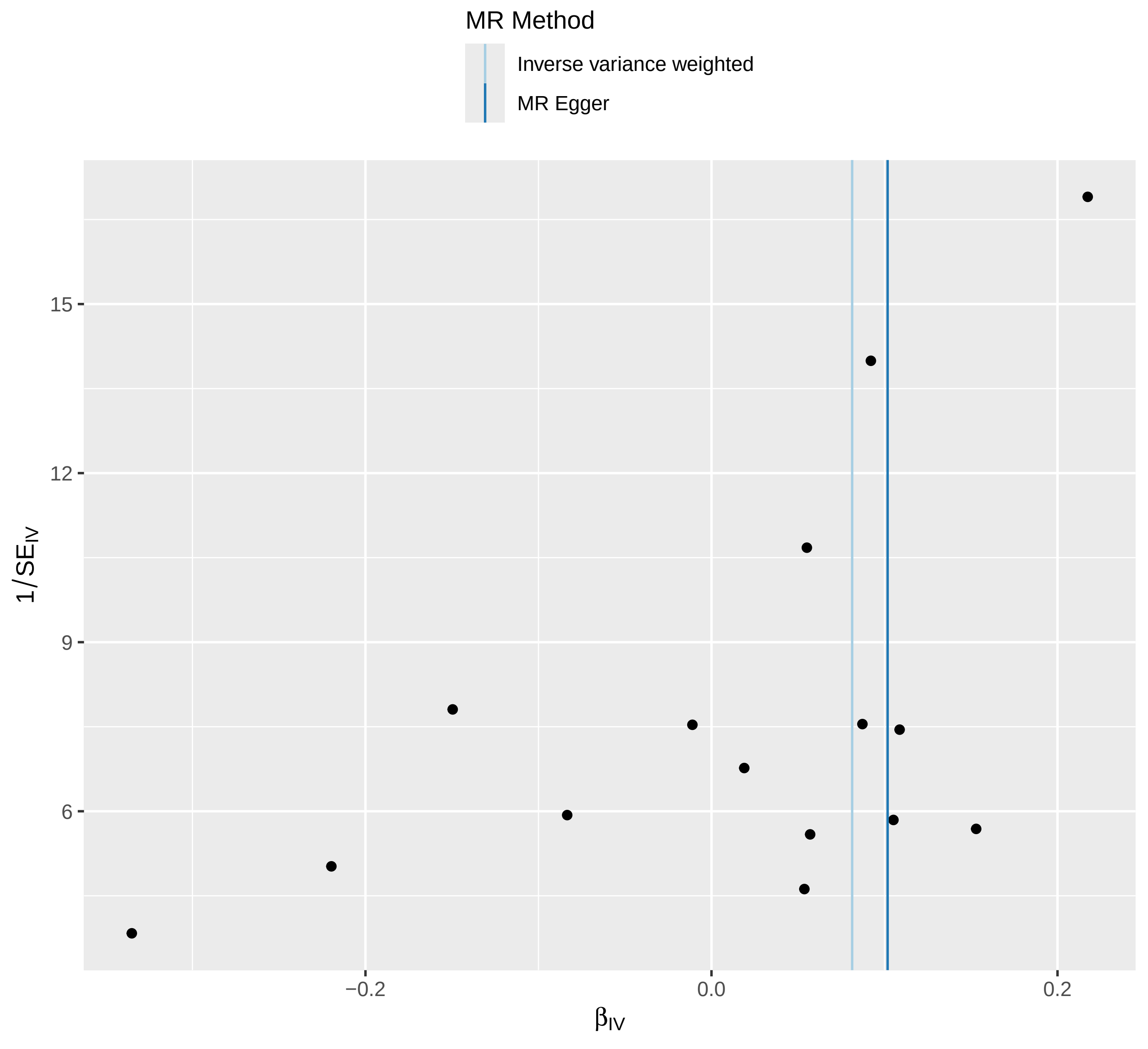

MR Method
Inverse variance weighted
MR Egger
15
12
9
6
1/SE_IV
−0.2
0.0
0.2
β_IV

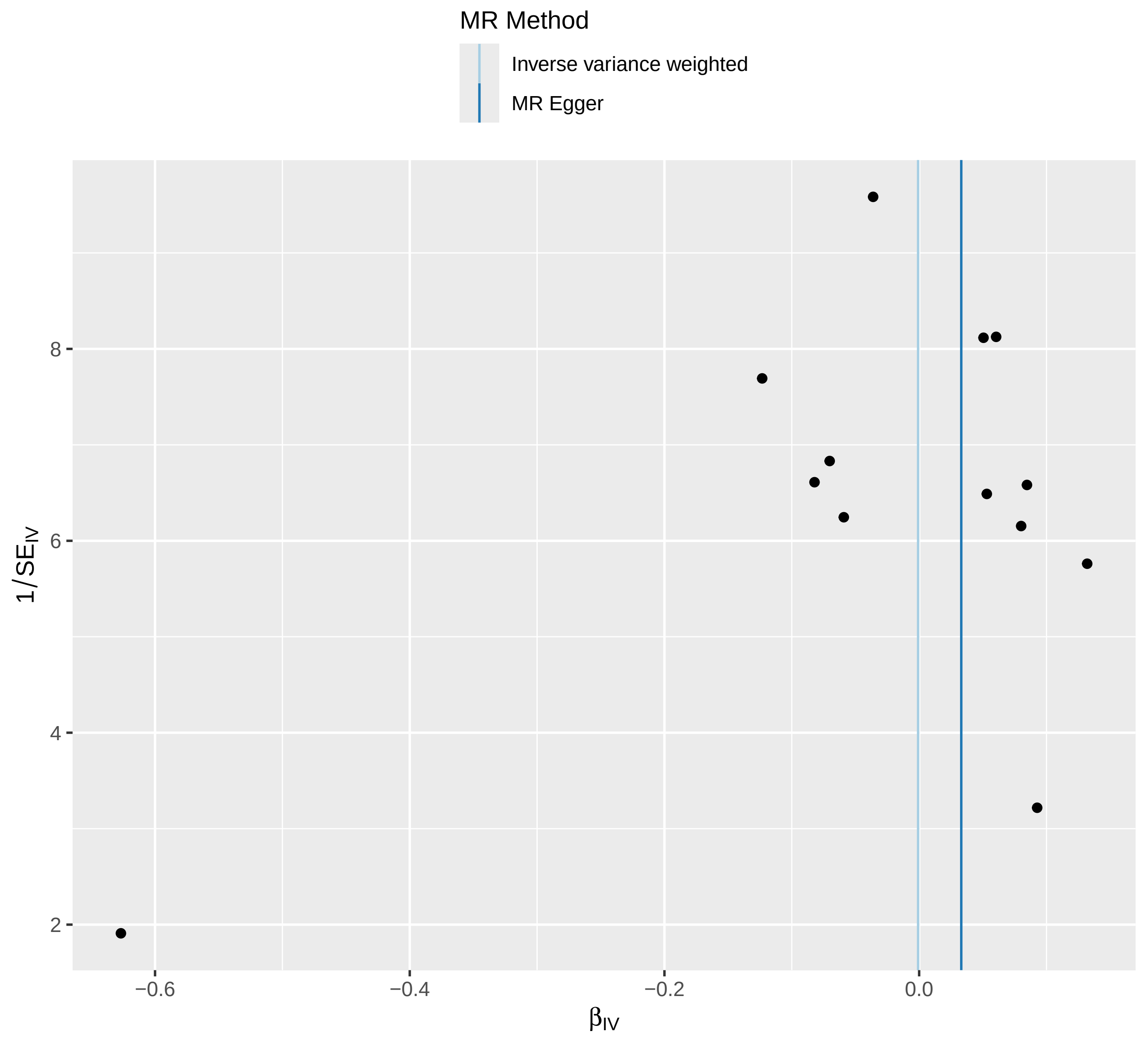
MR Method
Inverse variance weighted
MR Egger
1/SE_IV
8
6
4
2
−0.6
−0.4
−0.2
0.0
β_IV

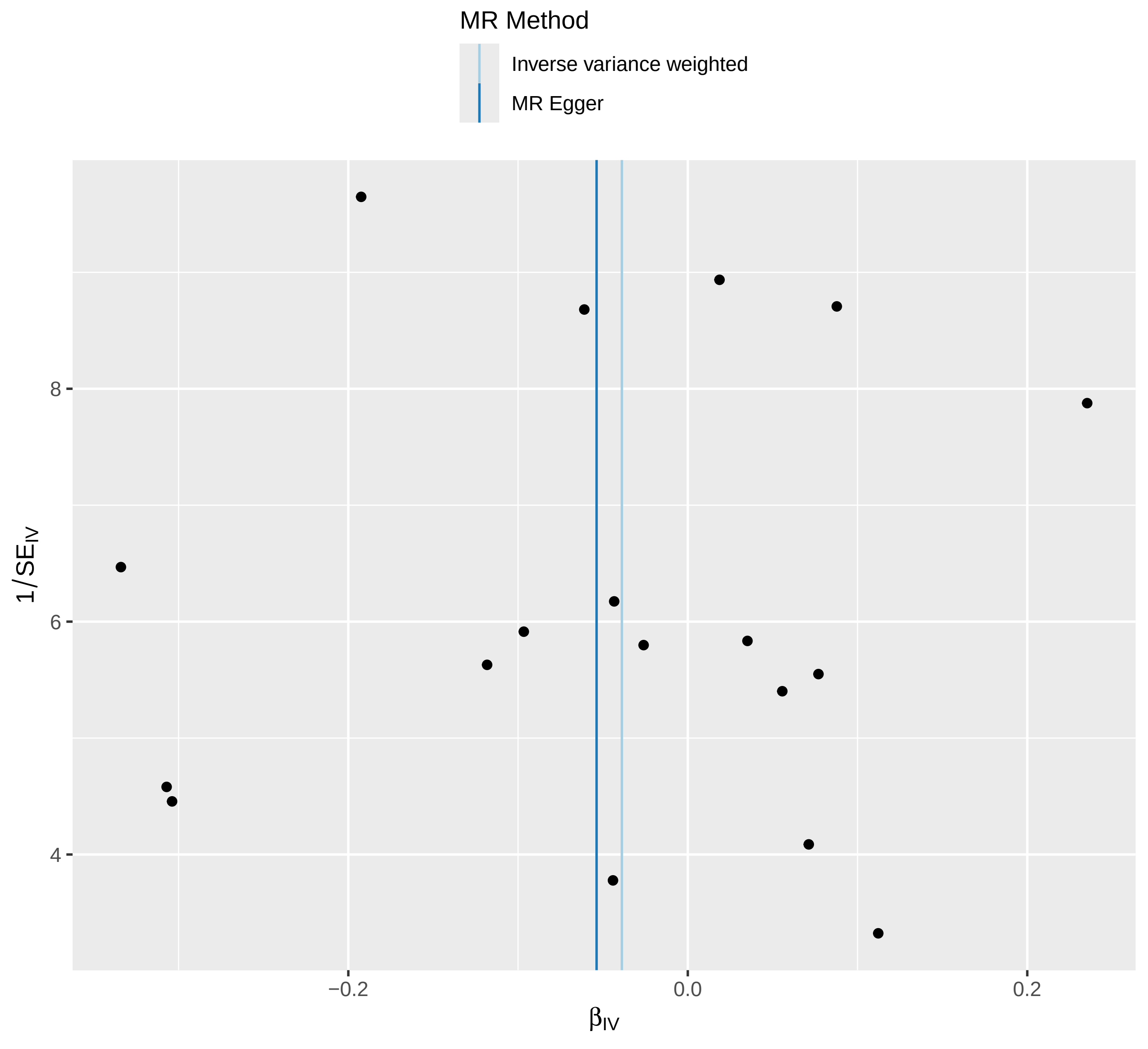

MR Method
Inverse variance weighted
MR Egger
1/SE_IV
8
6
4
−0.2
0.0
0.2
β_IV

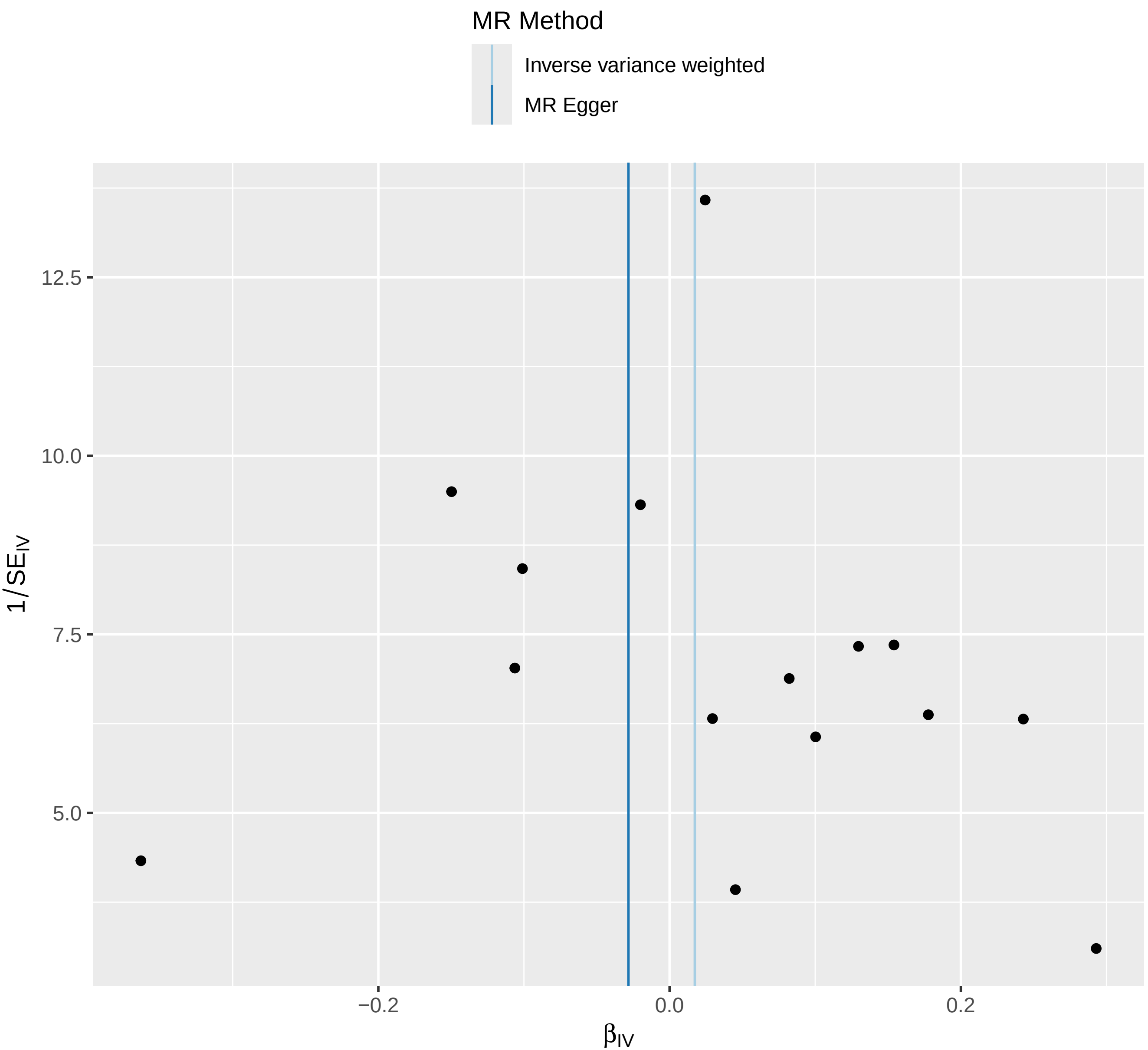
MR Method
Inverse variance weighted
MR Egger
12.5
10.0
7.5
5.0
1/SE$_{IV}$
−0.2
0.0
0.2
β$_{IV}$

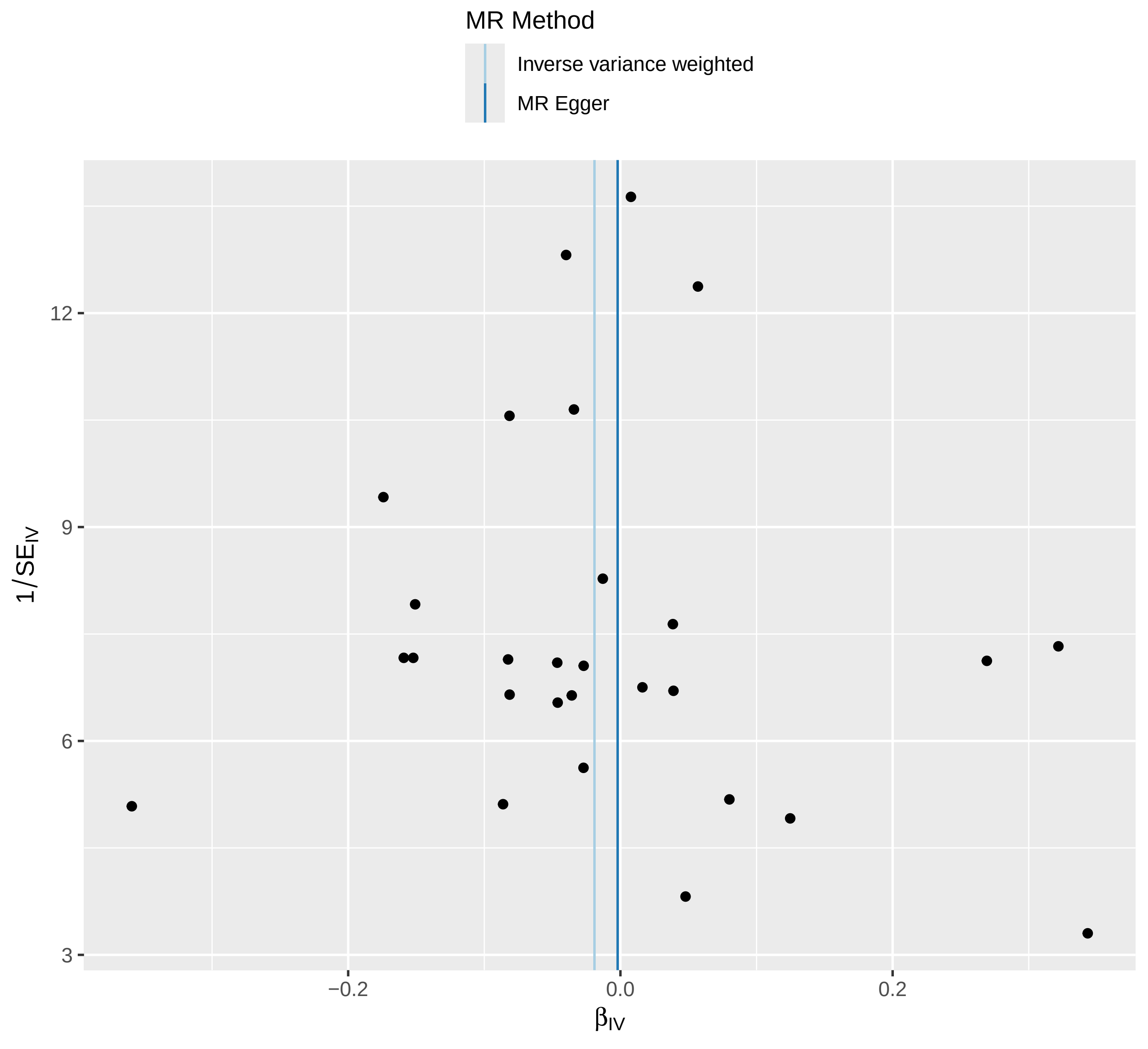

MR Method
Inverse variance weighted
MR Egger
12
9
6
3
1/SE_IV
−0.2
0.0
0.2
β_IV

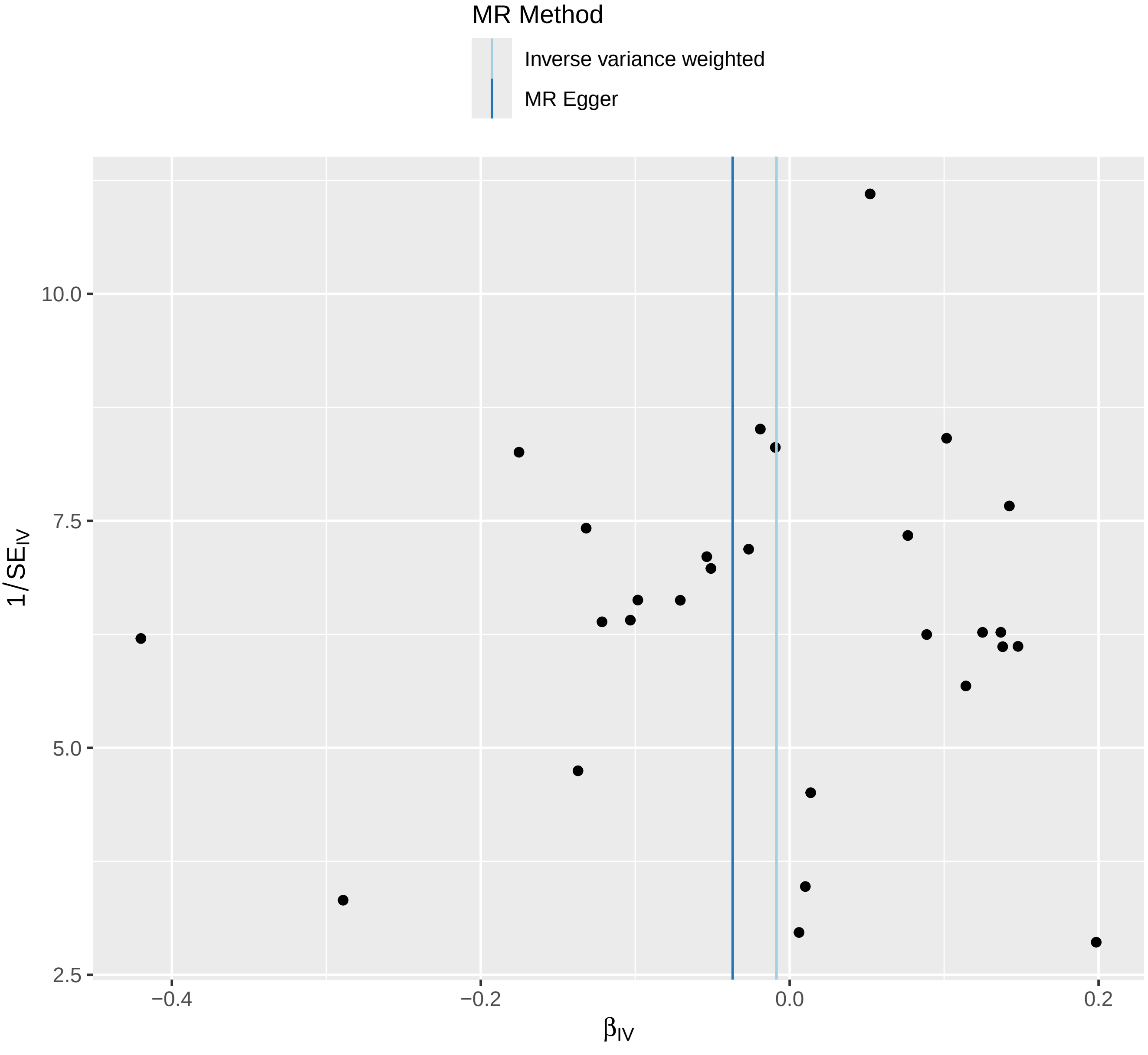
MR Method
Inverse variance weighted
MR Egger
10.0
7.5
5.0
2.5
$1/SE_{IV}$
−0.4
−0.2
0.0
0.2
β_{IV}

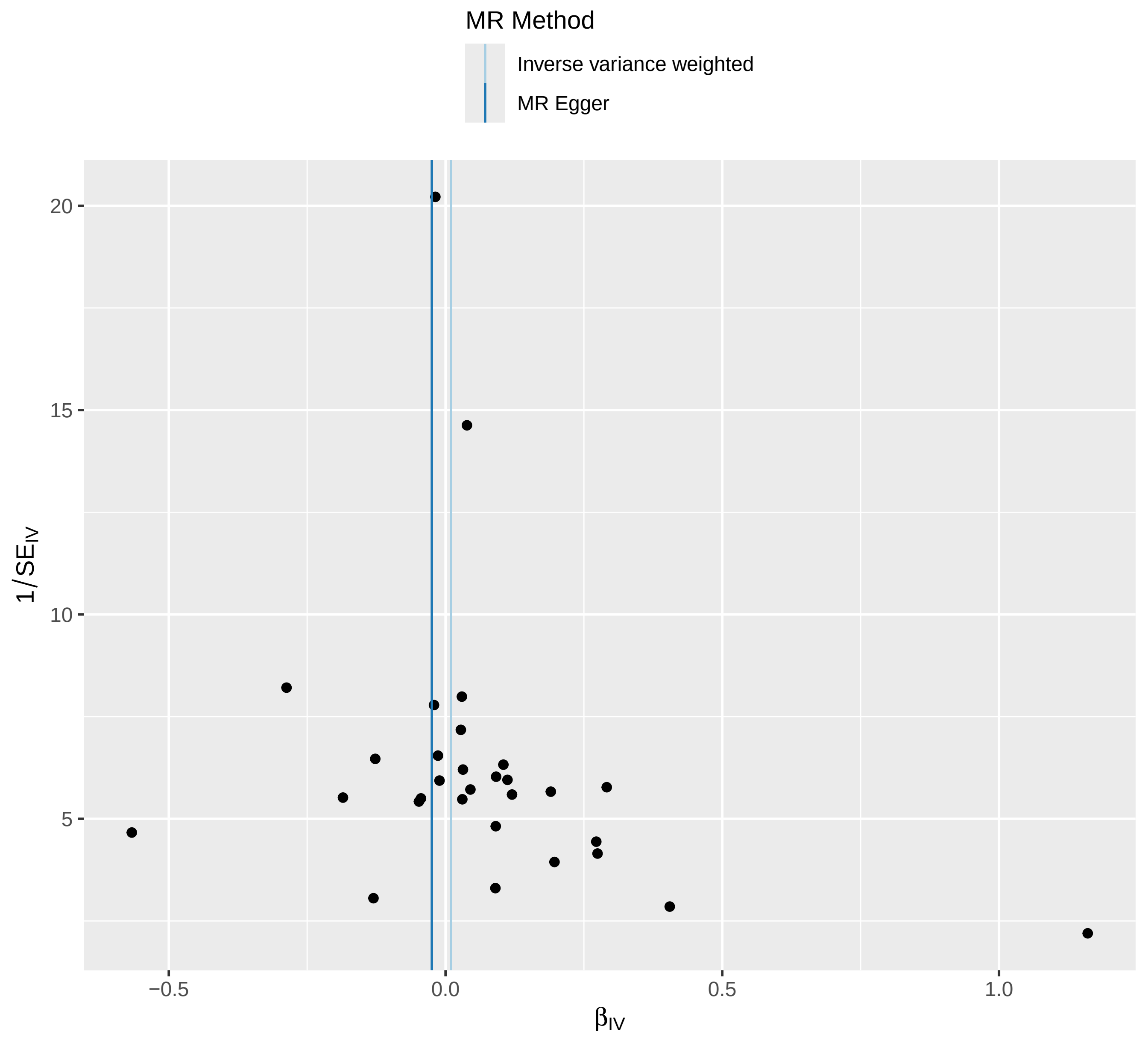

MR Method
Inverse variance weighted
MR Egger
20
15
10
5
$1/SE_{IV}$
−0.5
0.0
0.5
1.0
β_{IV}

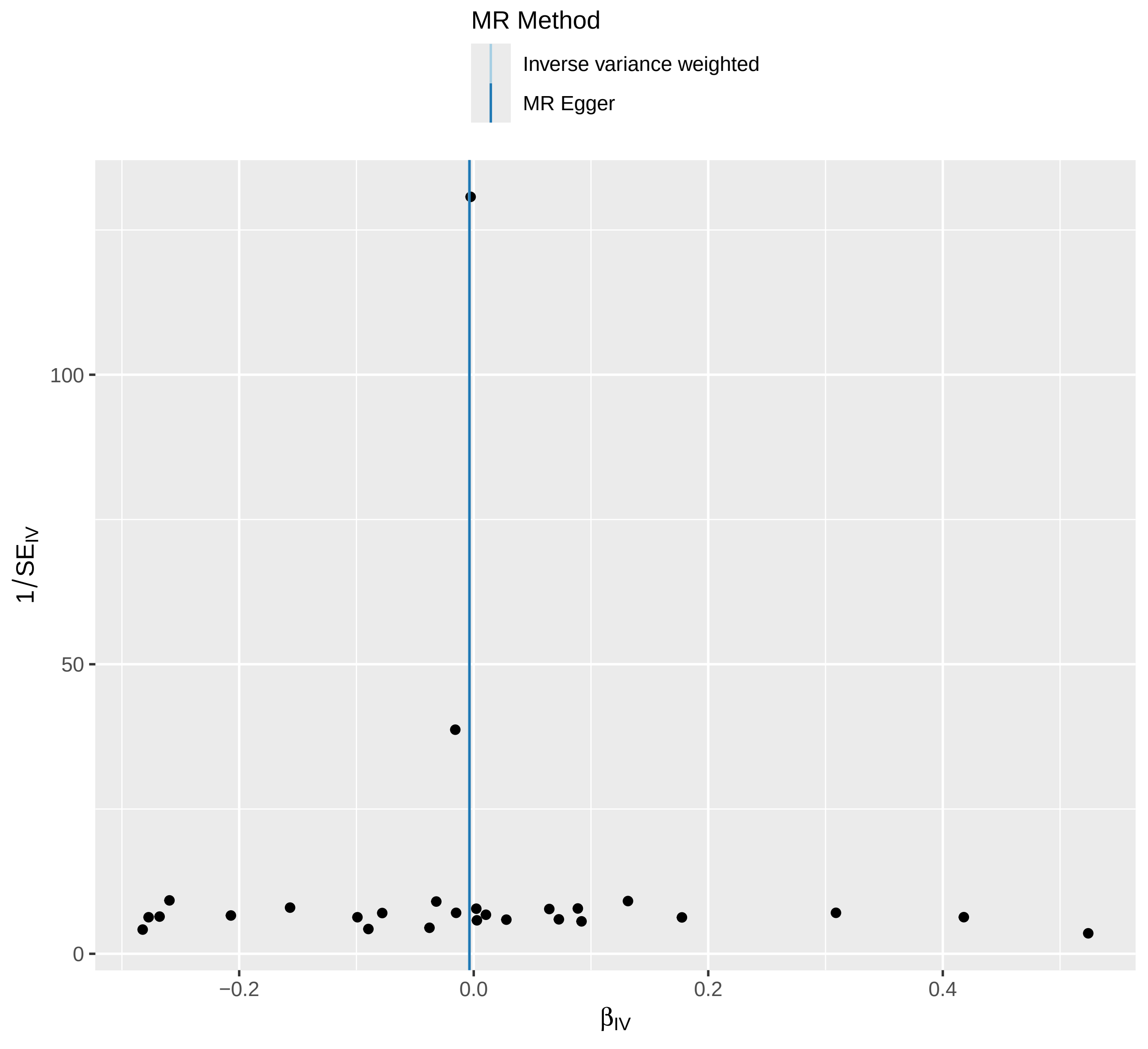

MR Method
Inverse variance weighted
MR Egger
100
50
0
1/SE_IV
−0.2
0.0
0.2
0.4
β_IV

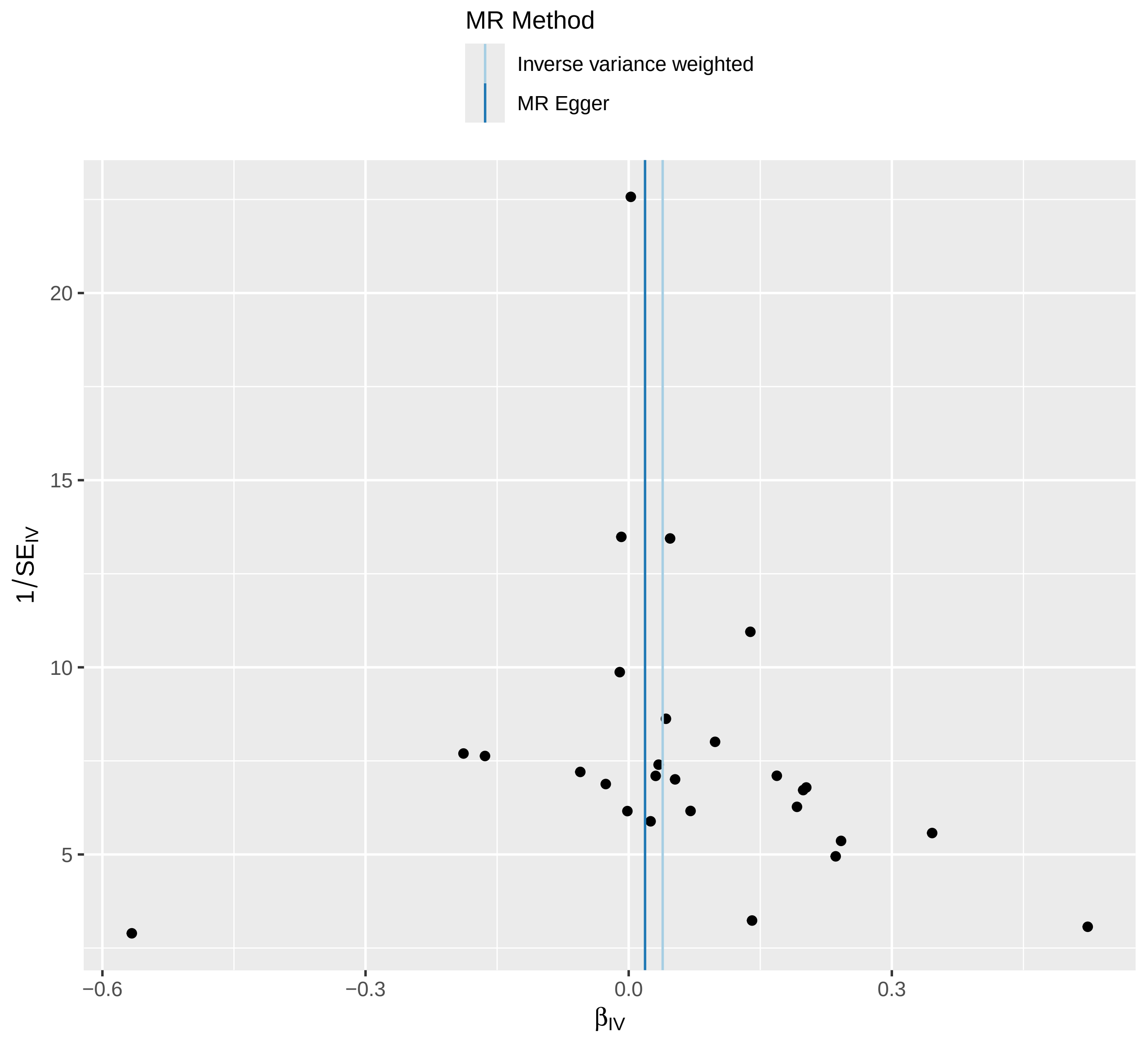

MR Method
Inverse variance weighted
MR Egger
20
15
10
5
1/SE_IV
−0.6
−0.3
0.0
0.3
β_IV

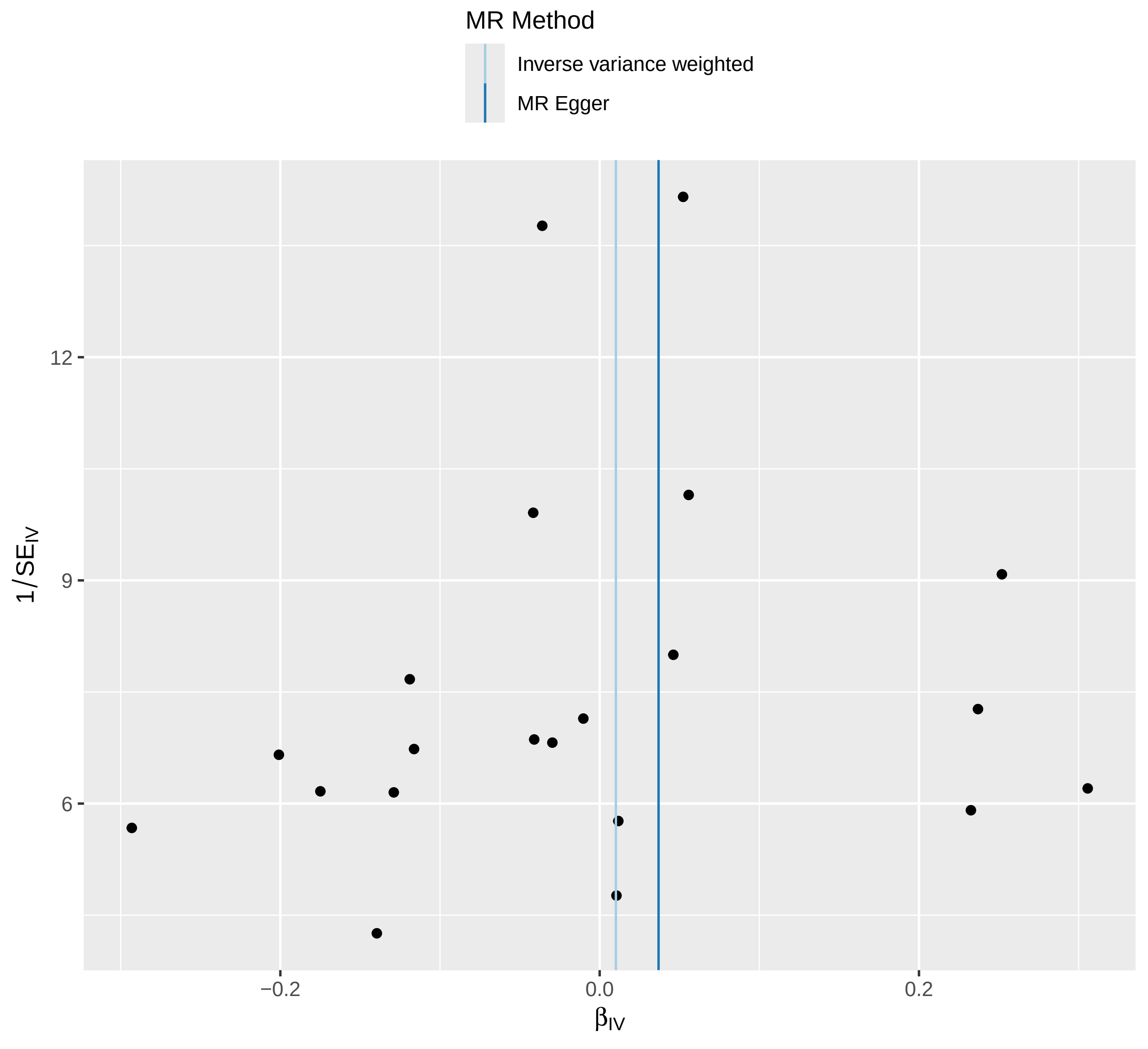

MR Method
Inverse variance weighted
MR Egger
1/SE_IV
12
9
6
−0.2
0.0
0.2
β_IV

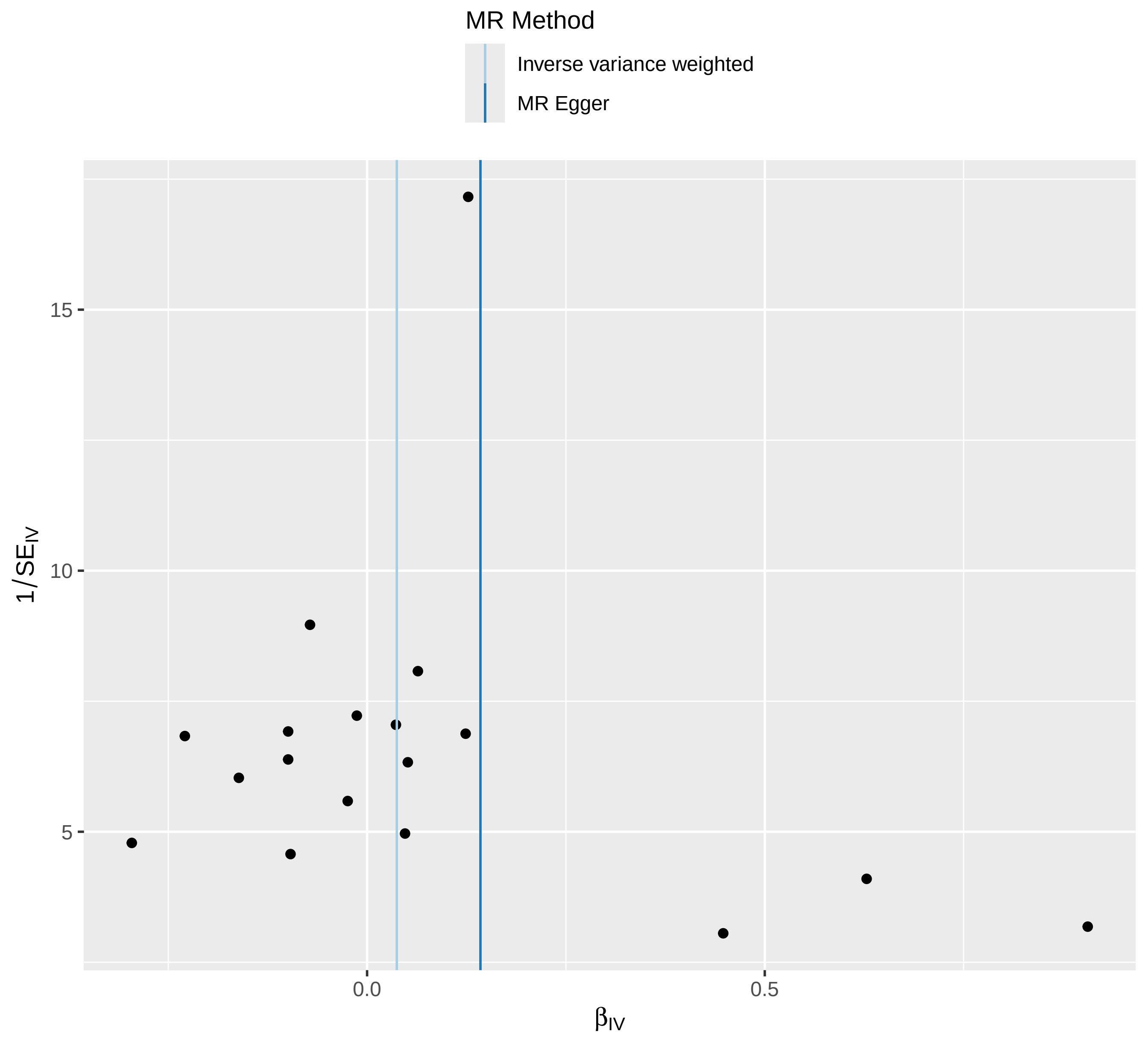

MR Method
Inverse variance weighted
MR Egger
15
10
5
0.0
0.5
$1/SE_{IV}$
β_{IV}

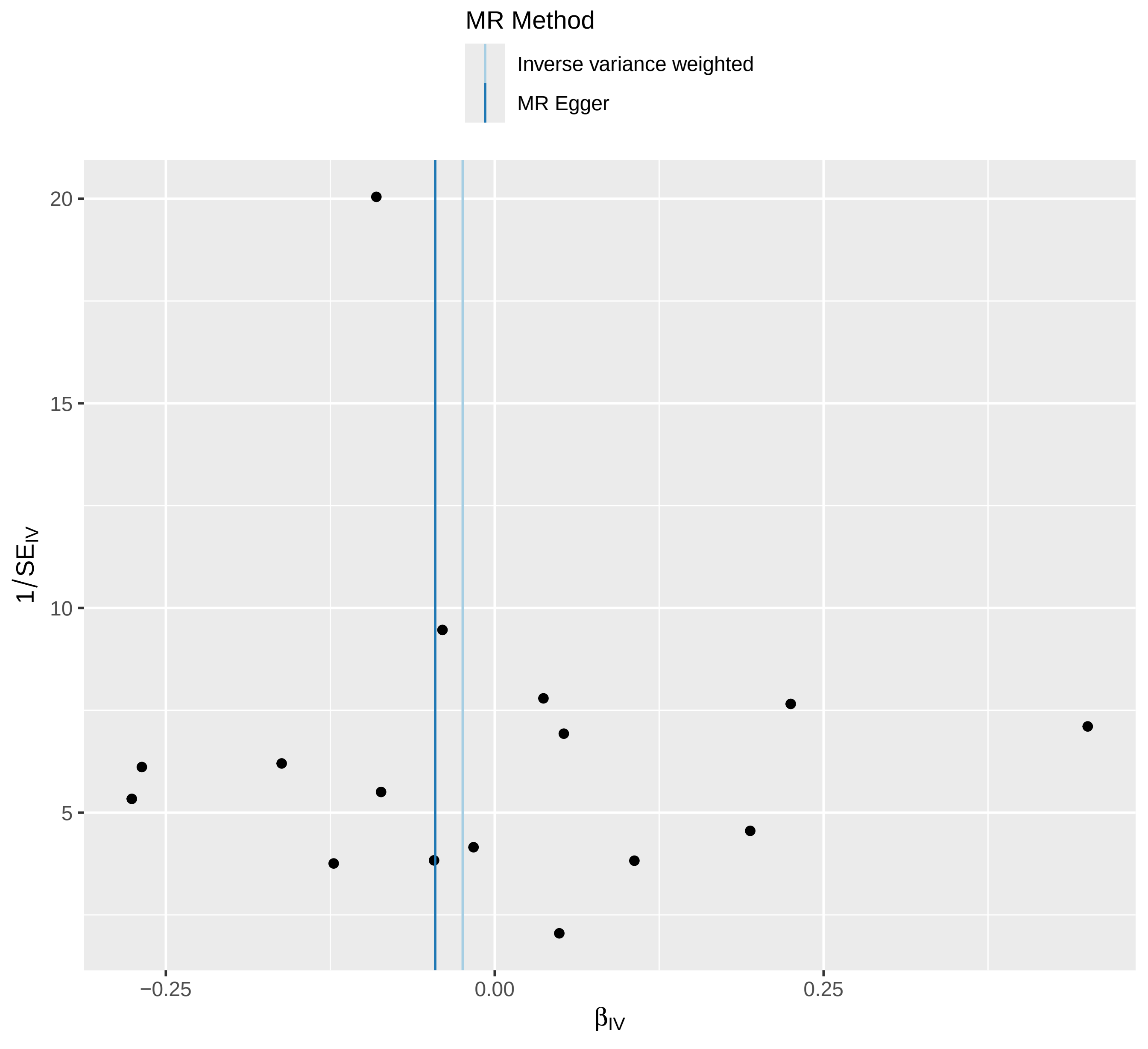
MR Method
Inverse variance weighted
MR Egger
20
15
10
5
1/SE_IV
−0.25
0.00
0.25
β_IV

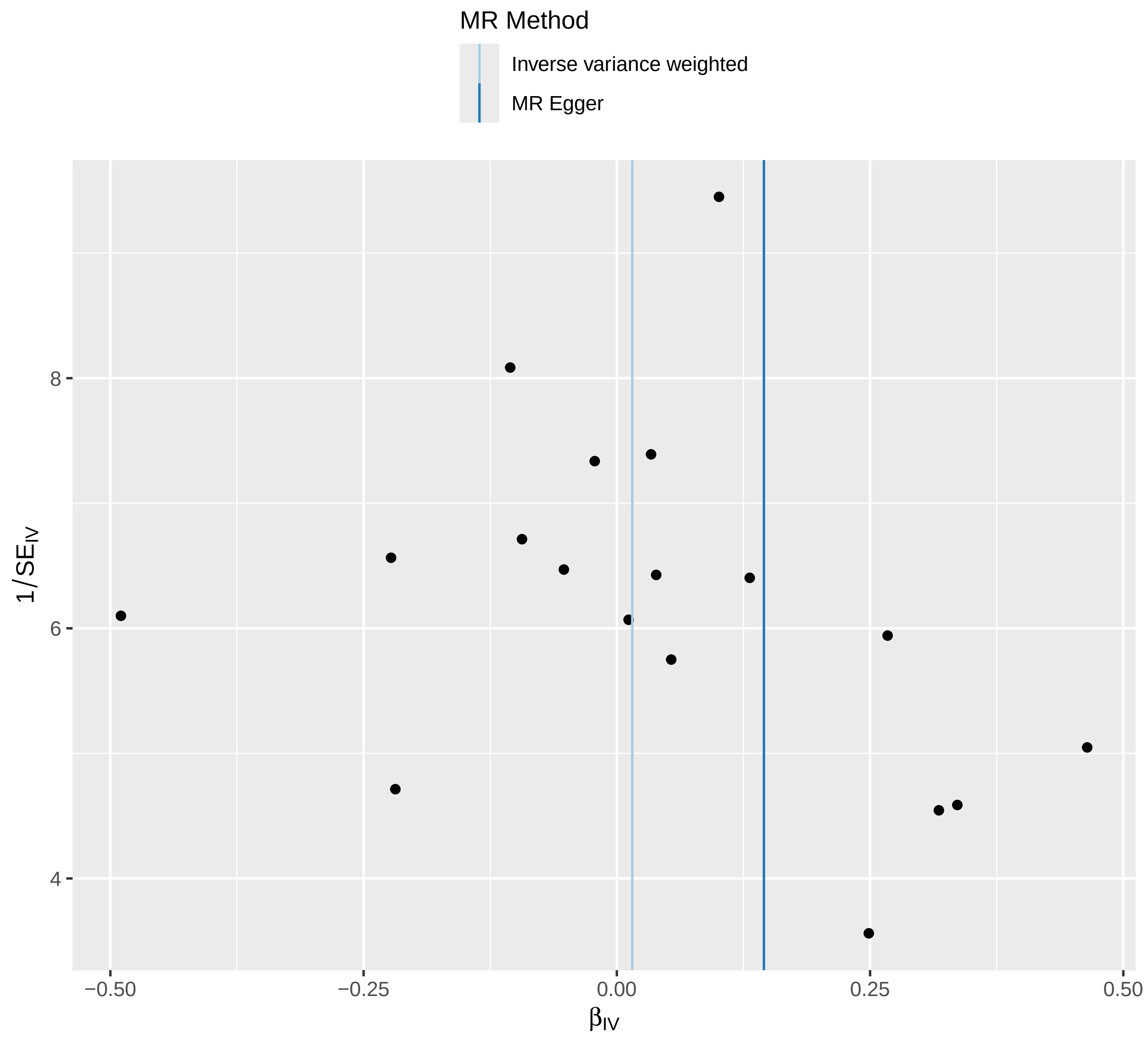

MR Method
Inverse variance weighted
MR Egger
1/SE_IV
8
6
4
−0.50
−0.25
0.00
0.25
0.50
β_IV

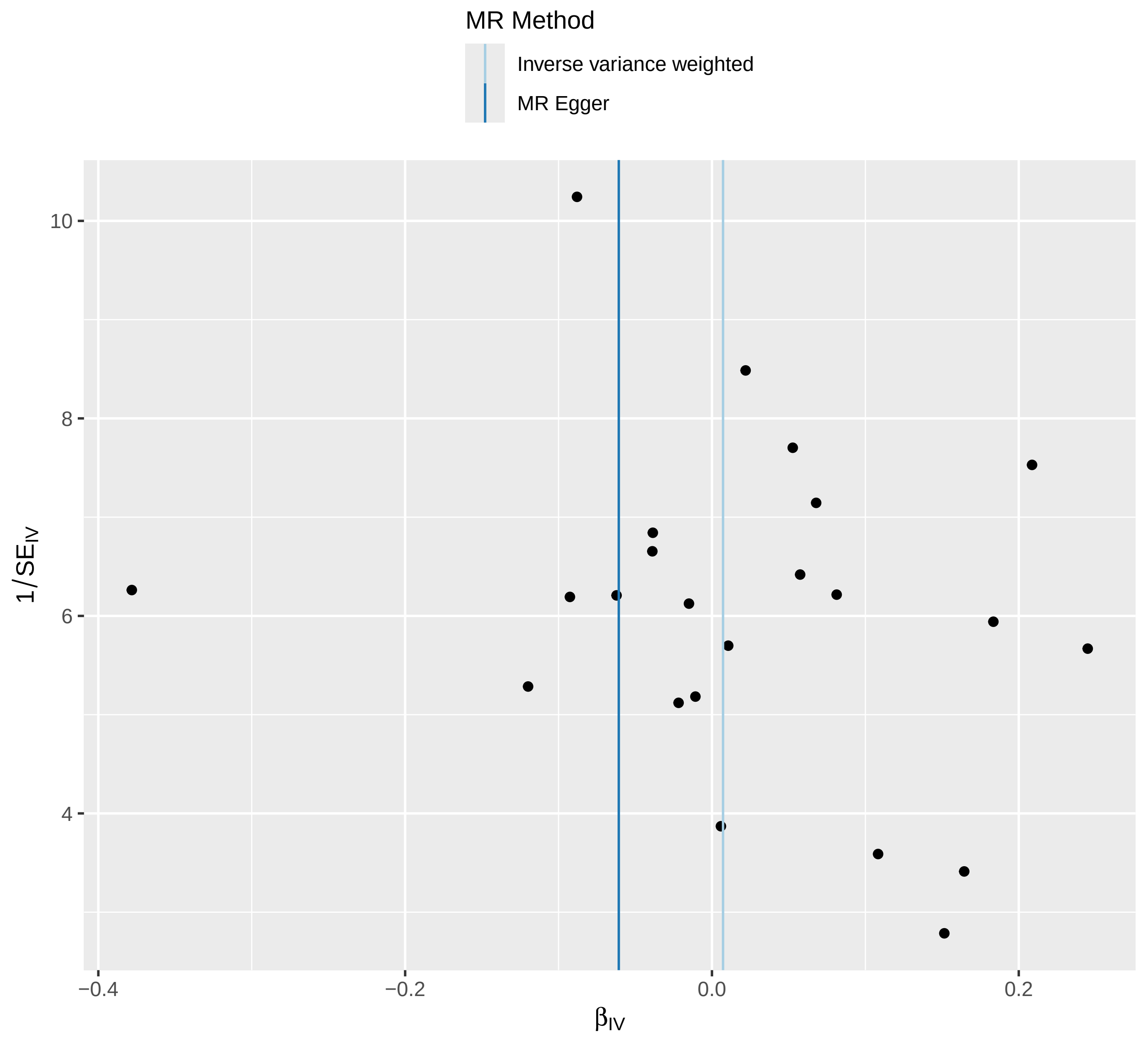
MR Method
Inverse variance weighted
MR Egger
10
8
6
4
1/SE_IV
−0.4
−0.2
0.0
0.2
β_IV

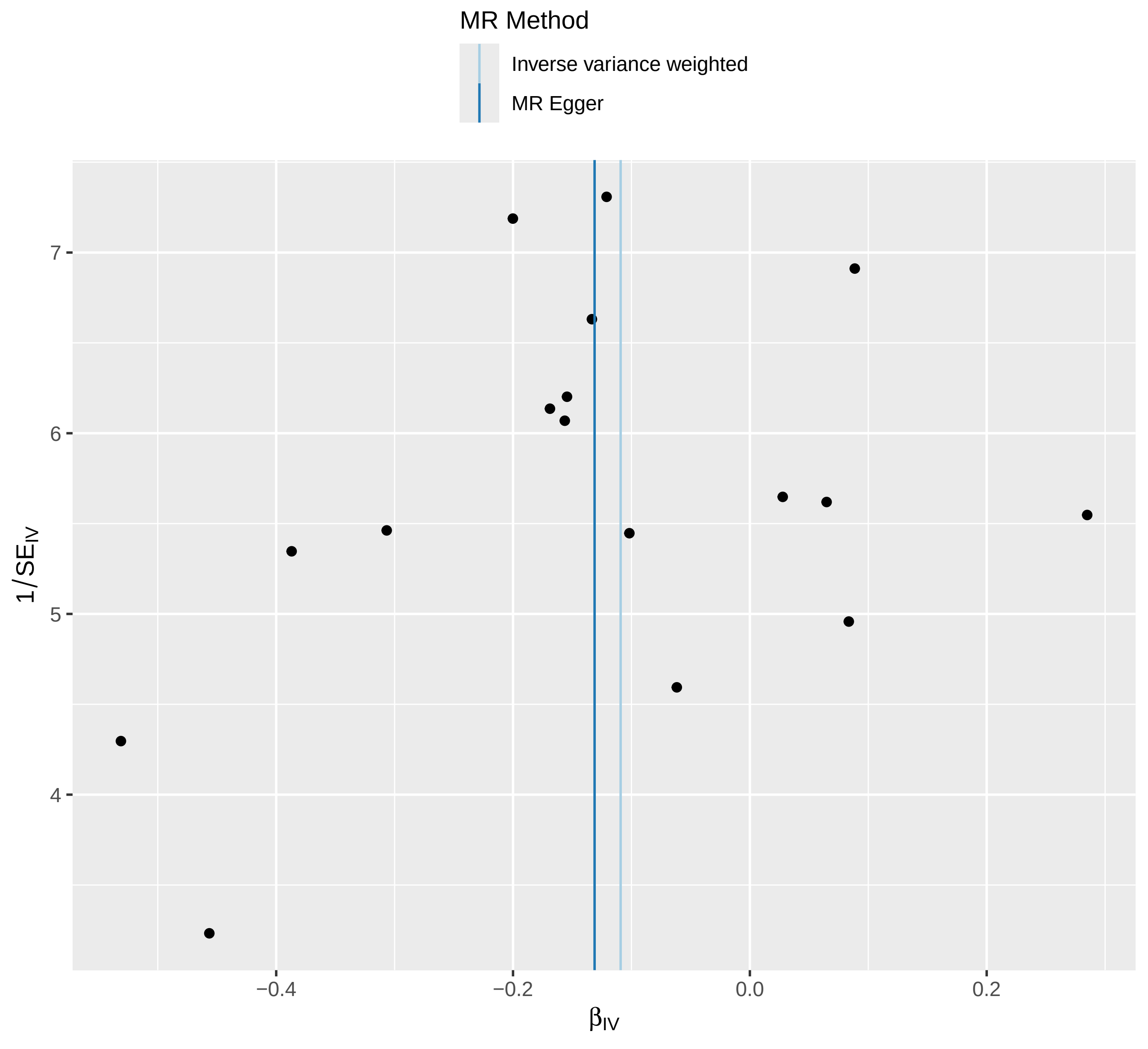

MR Method
Inverse variance weighted
MR Egger
7
6
5
4
1/SE_IV
−0.4
−0.2
0.0
0.2
β_IV

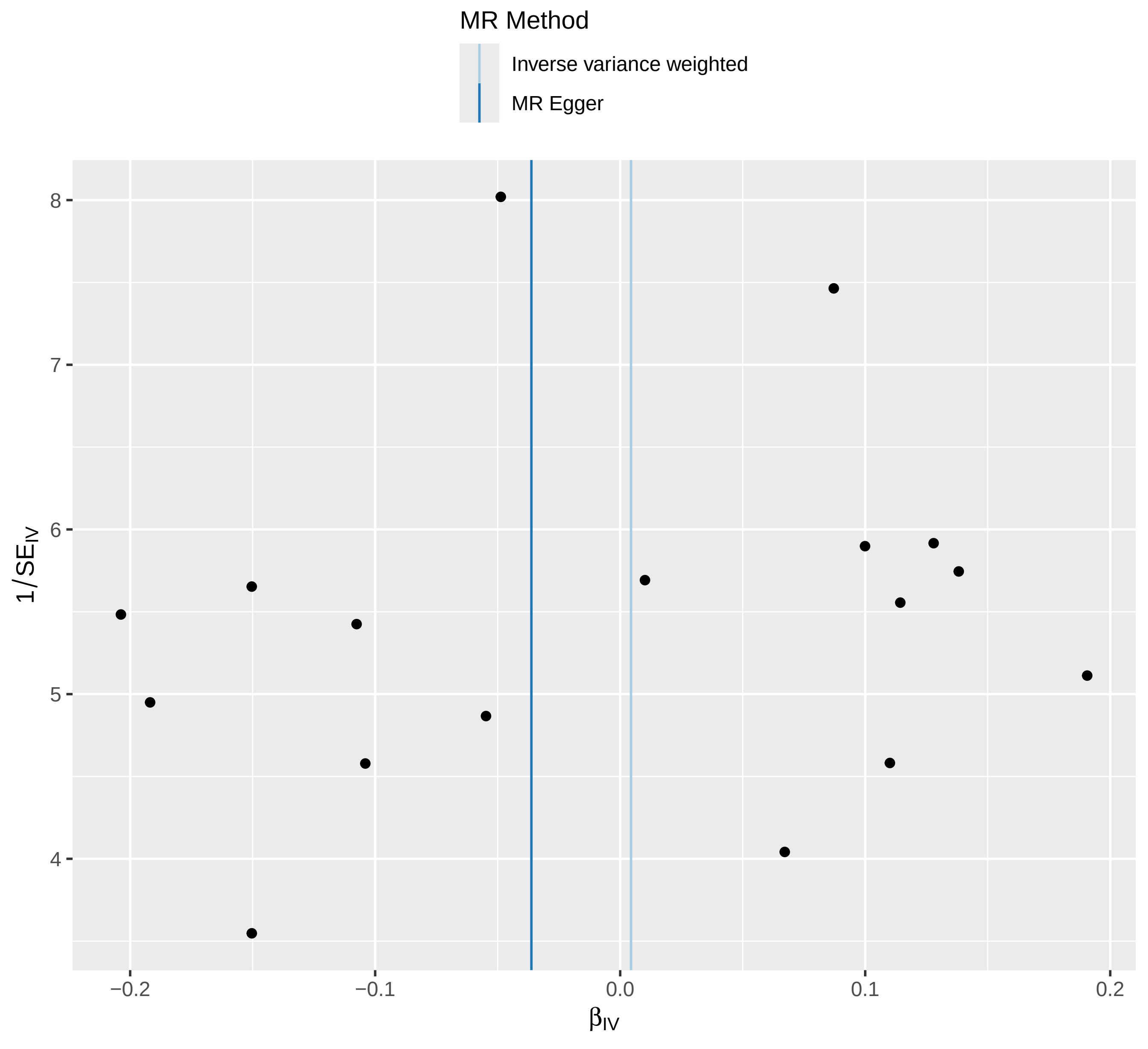

MR Method
Inverse variance weighted
MR Egger
8
7
6
5
4
1/SE_IV
−0.2
−0.1
0.0
0.1
0.2
β_IV

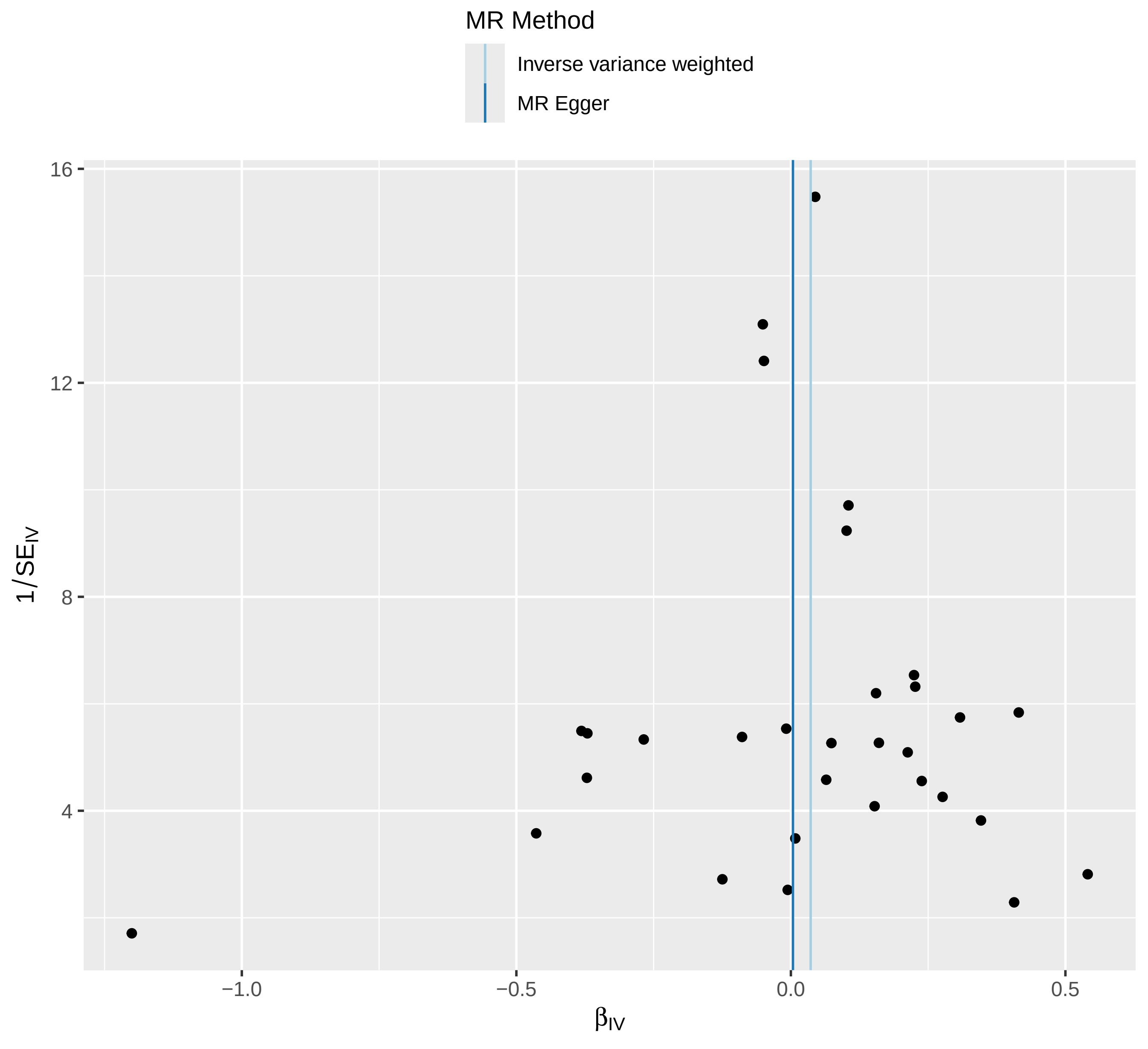

MR Method
Inverse variance weighted
MR Egger
16
12
8
4
1/SE_IV
−1.0
−0.5
0.0
0.5
β_IV

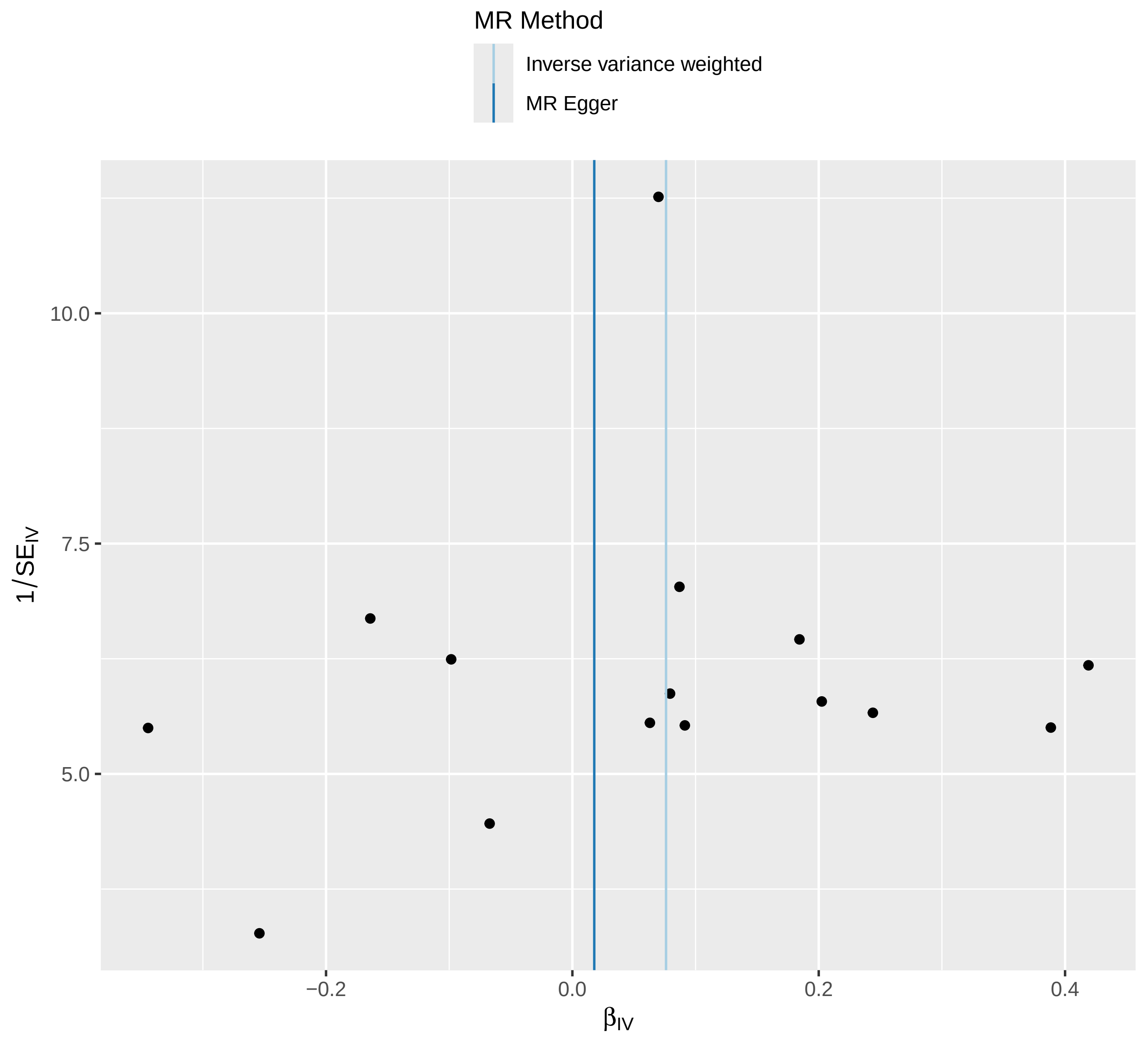
MR Method
Inverse variance weighted
MR Egger
10.0
7.5
5.0
−0.2
0.0
0.2
0.4
$1/SE_{IV}$
β_{IV}

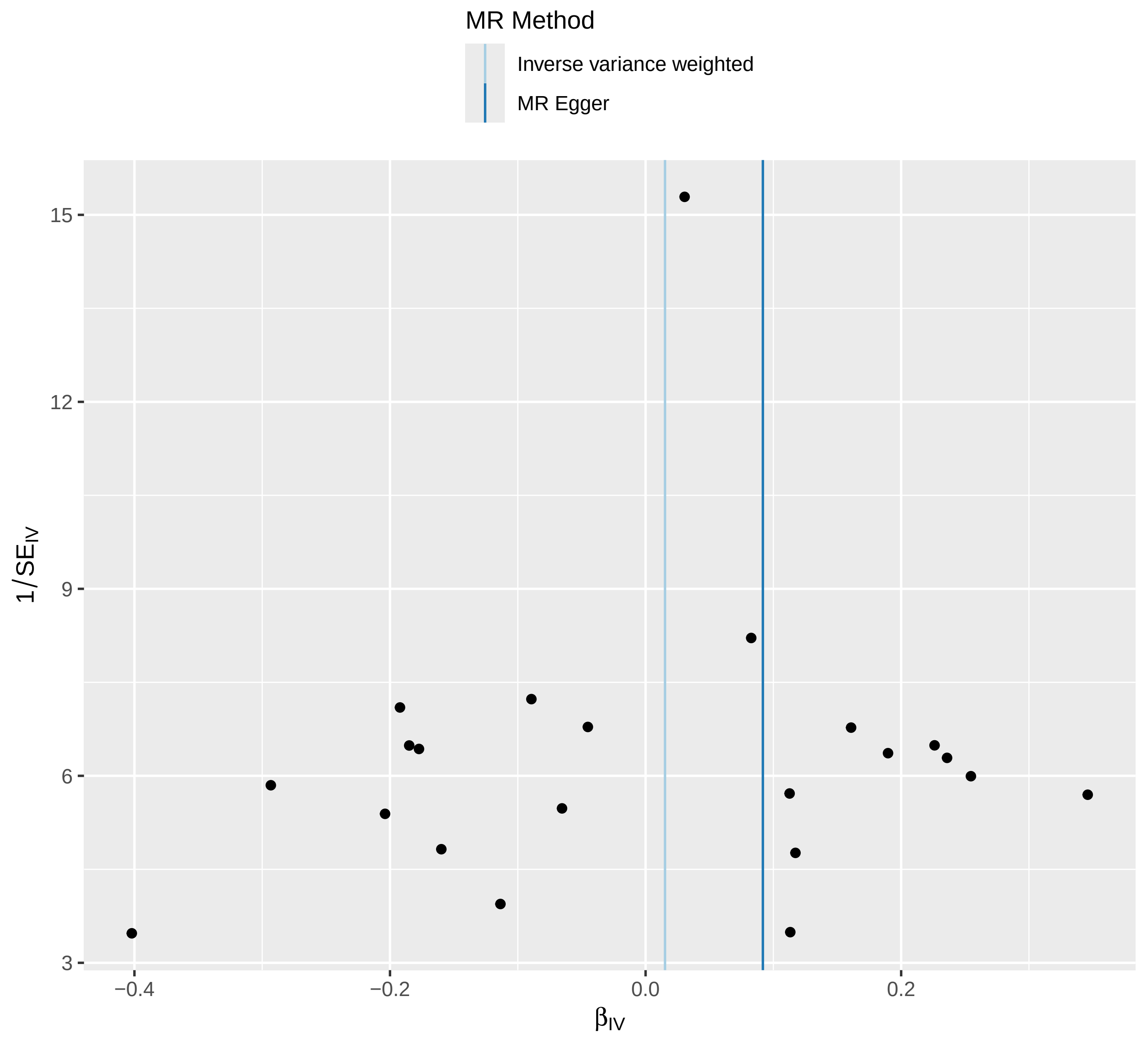
MR Method
Inverse variance weighted
MR Egger
1/SE_IV
β_IV
15
12
9
6
3
−0.4
−0.2
0.0
0.2

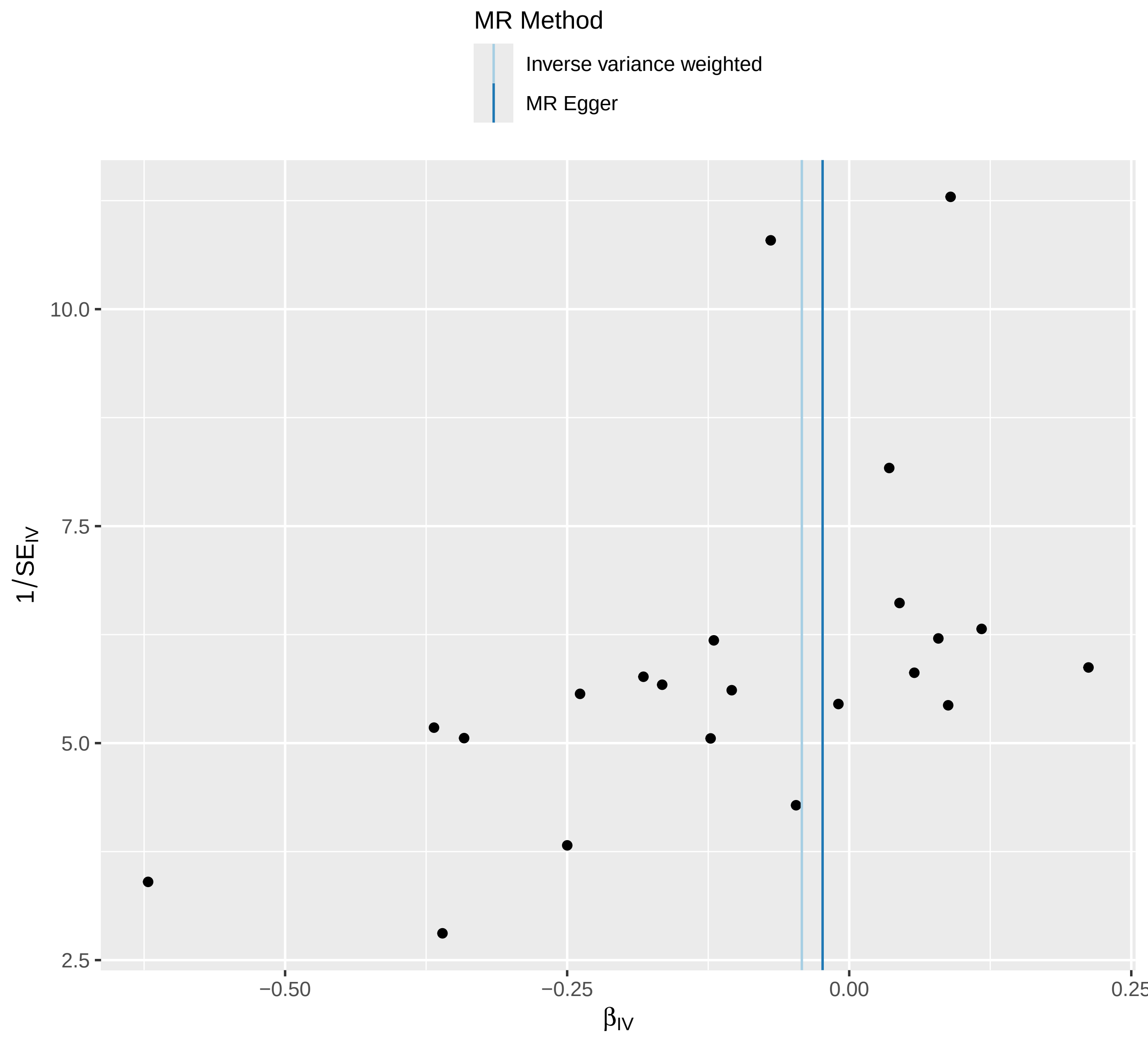

MR Method
Inverse variance weighted
MR Egger
10.0
7.5
5.0
2.5
1/SE_IV
−0.50
−0.25
0.00
0.25
β_IV

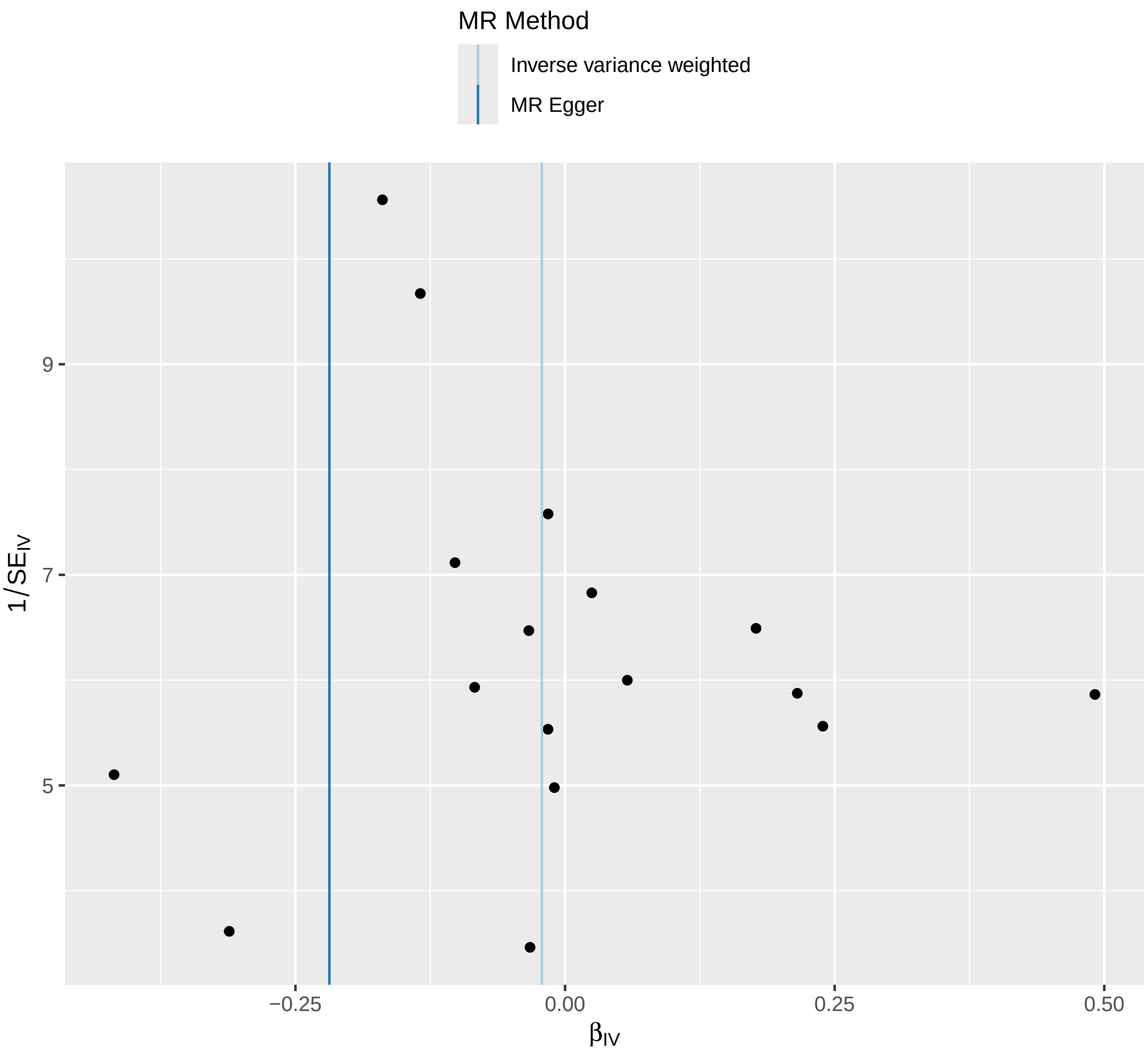

MR Method
Inverse variance weighted
MR Egger
1/SE_IV
9
7
5
−0.25
0.00
0.25
0.50
β_IV

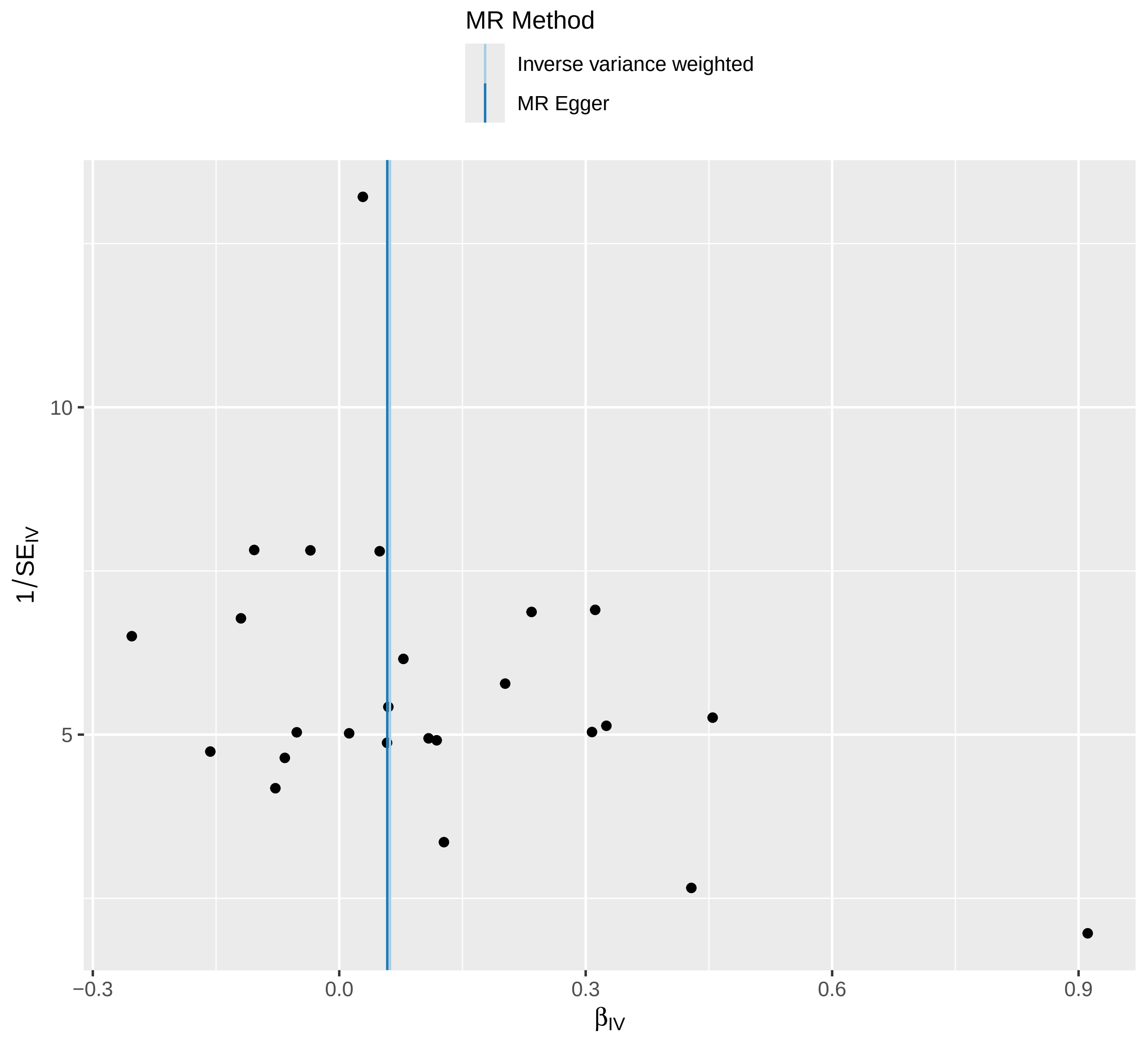
MR Method
Inverse variance weighted
MR Egger
10
5
1/SE$_{IV}$
−0.3
0.0
0.3
0.6
0.9
β$_{IV}$

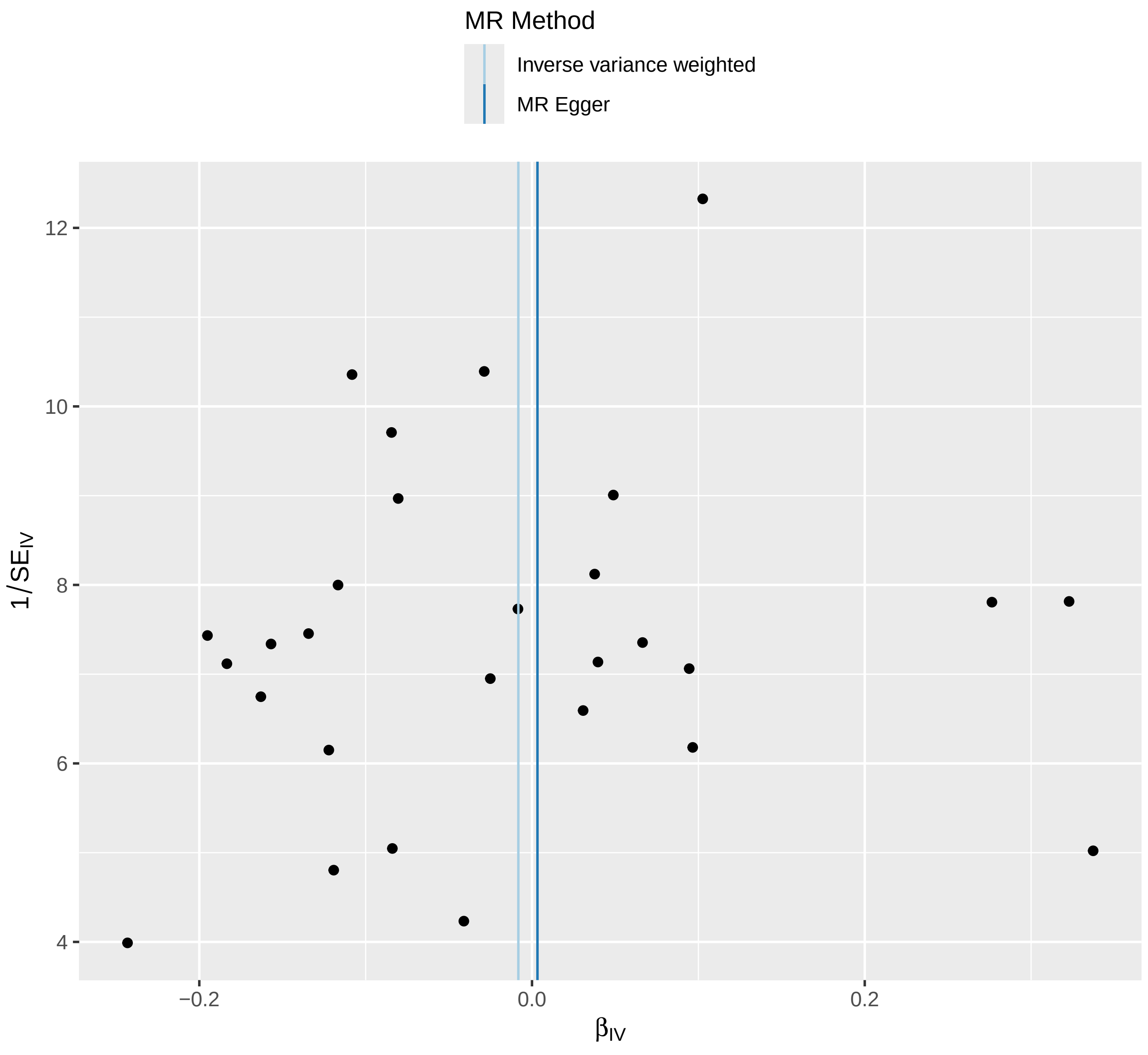

MR Method
Inverse variance weighted
MR Egger
12
10
8
6
4
$1/SE_{IV}$
−0.2
0.0
0.2
β_{IV}

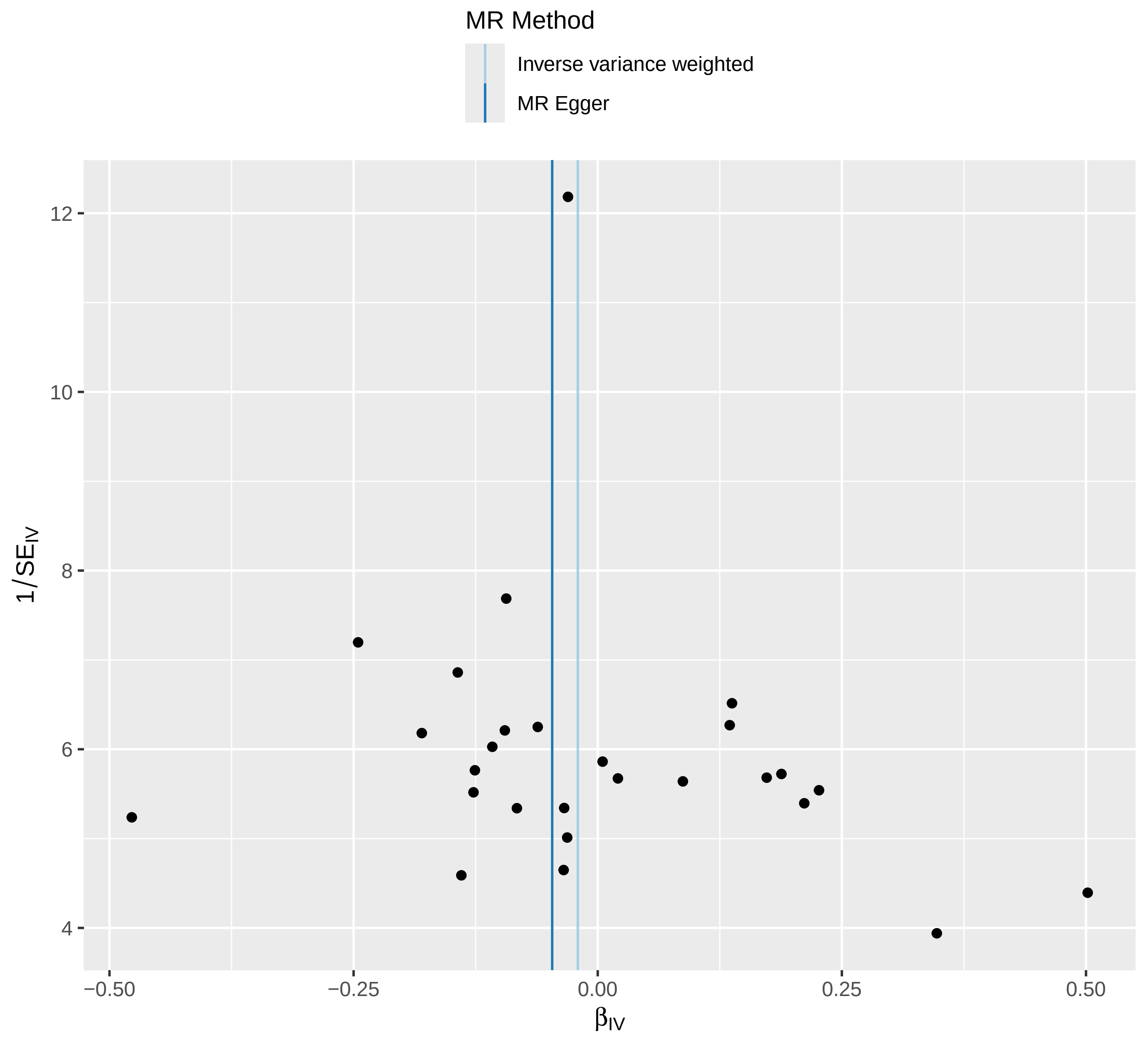
MR Method
Inverse variance weighted
MR Egger
12
10
8
6
4
1/SE$_{IV}$
−0.50
−0.25
0.00
0.25
0.50
β$_{IV}$

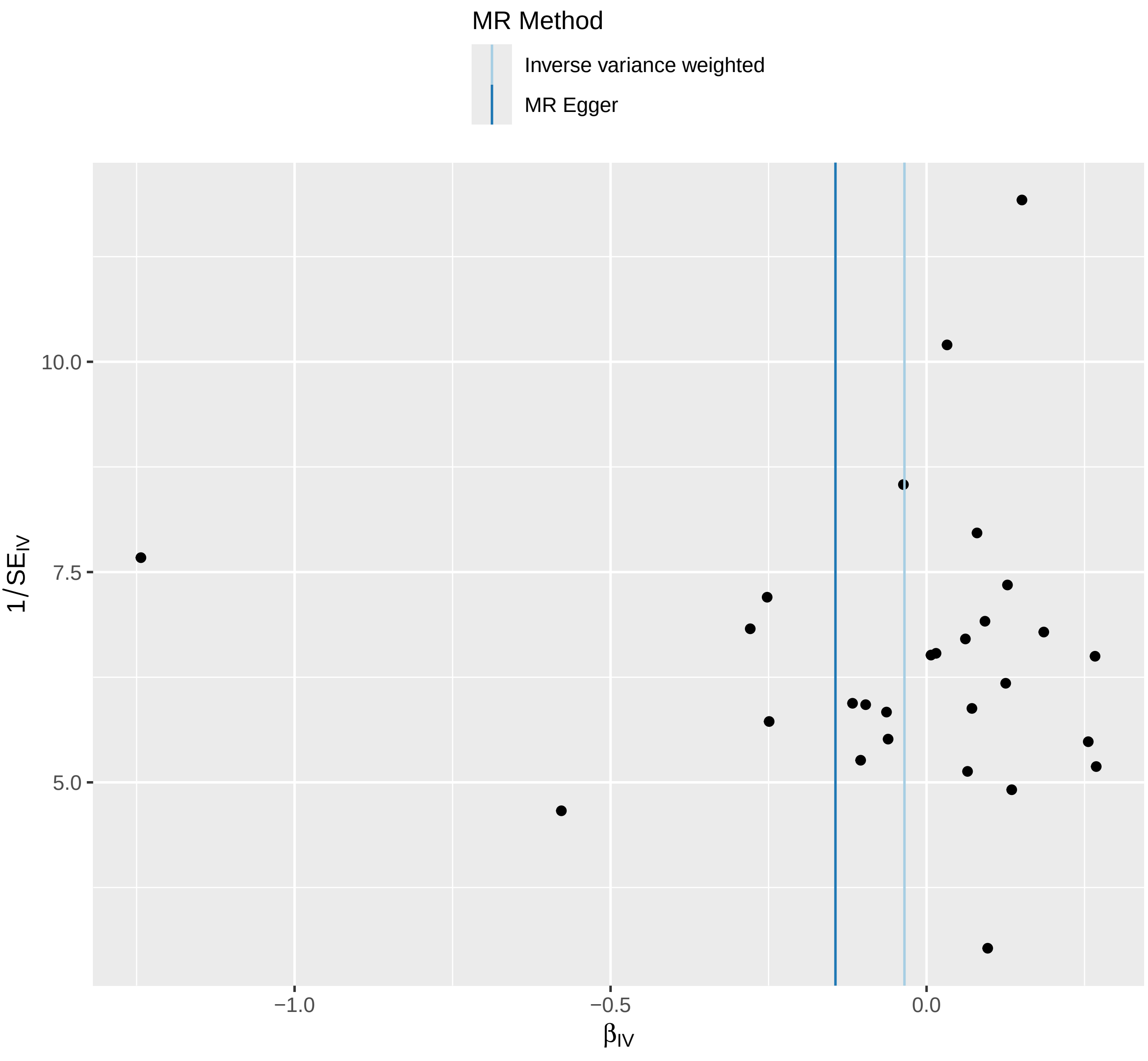

MR Method
Inverse variance weighted
MR Egger
10.0
7.5
5.0
1/SE_IV
−1.0
−0.5
0.0
β_IV

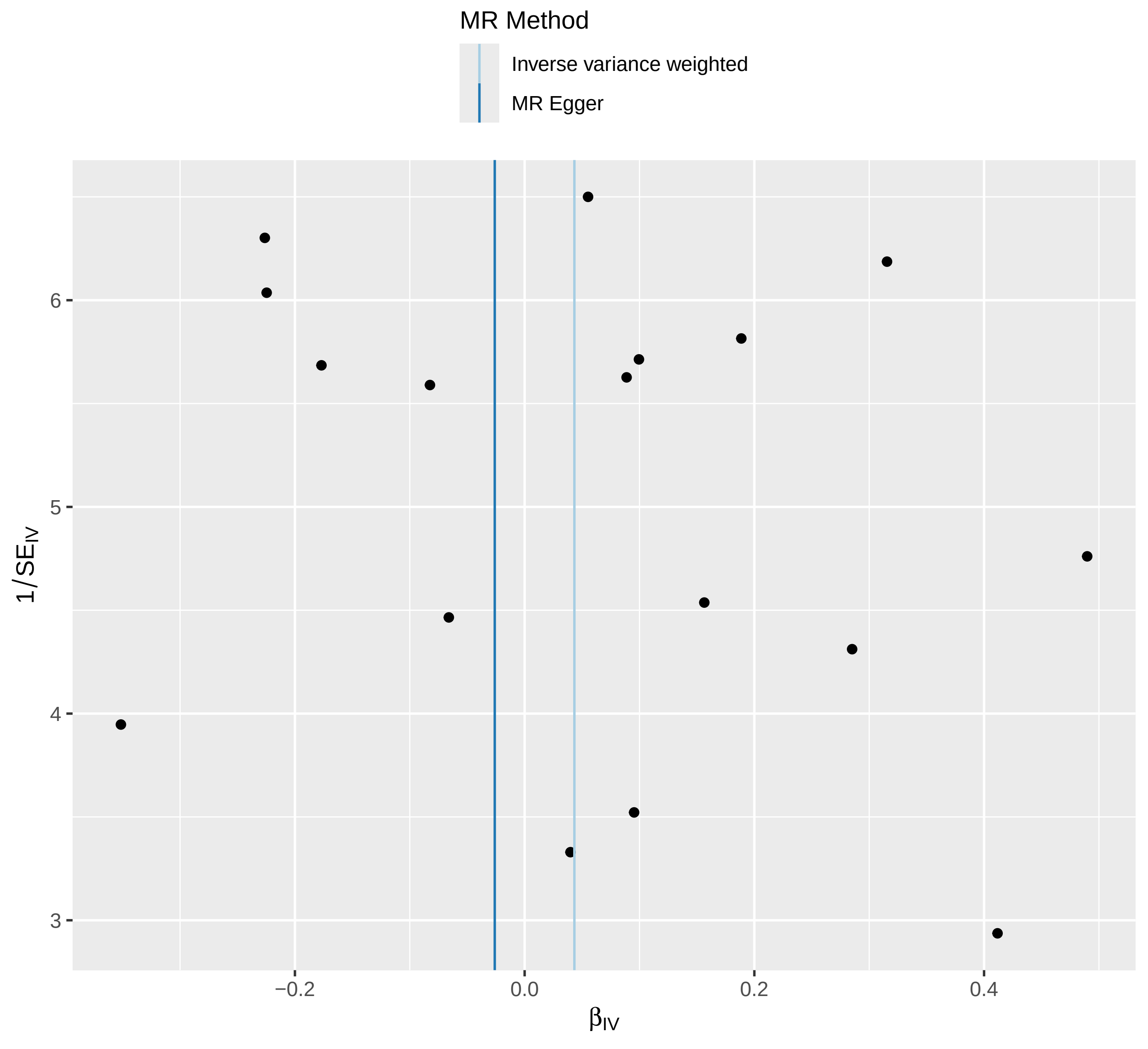

MR Method
Inverse variance weighted
MR Egger
6
5
4
3
1/SE$_{IV}$
−0.2
0.0
0.2
0.4
β_{IV}

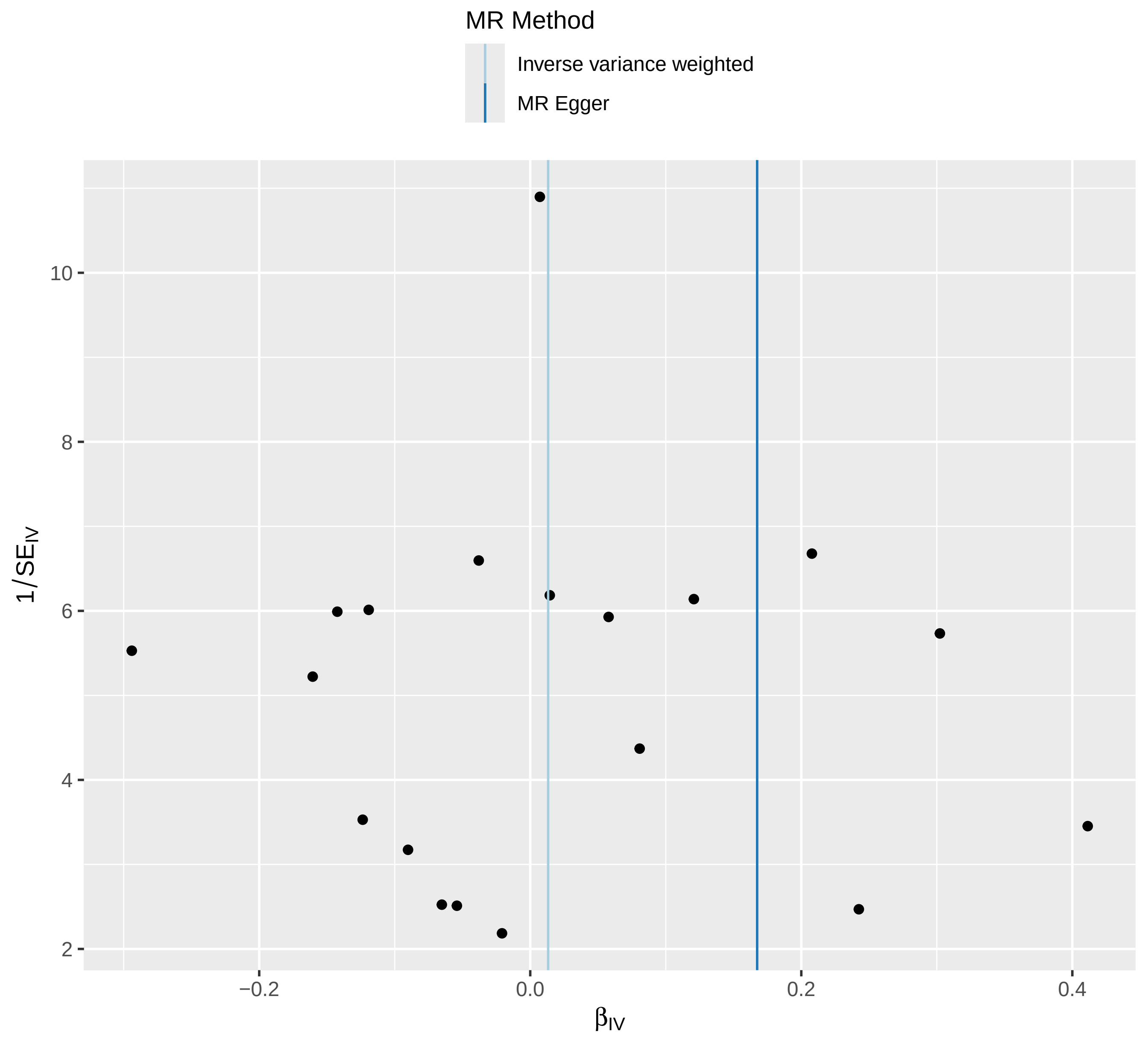
MR Method
Inverse variance weighted
MR Egger
1/SE_IV
10
8
6
4
2
−0.2
0.0
0.2
0.4
β_IV

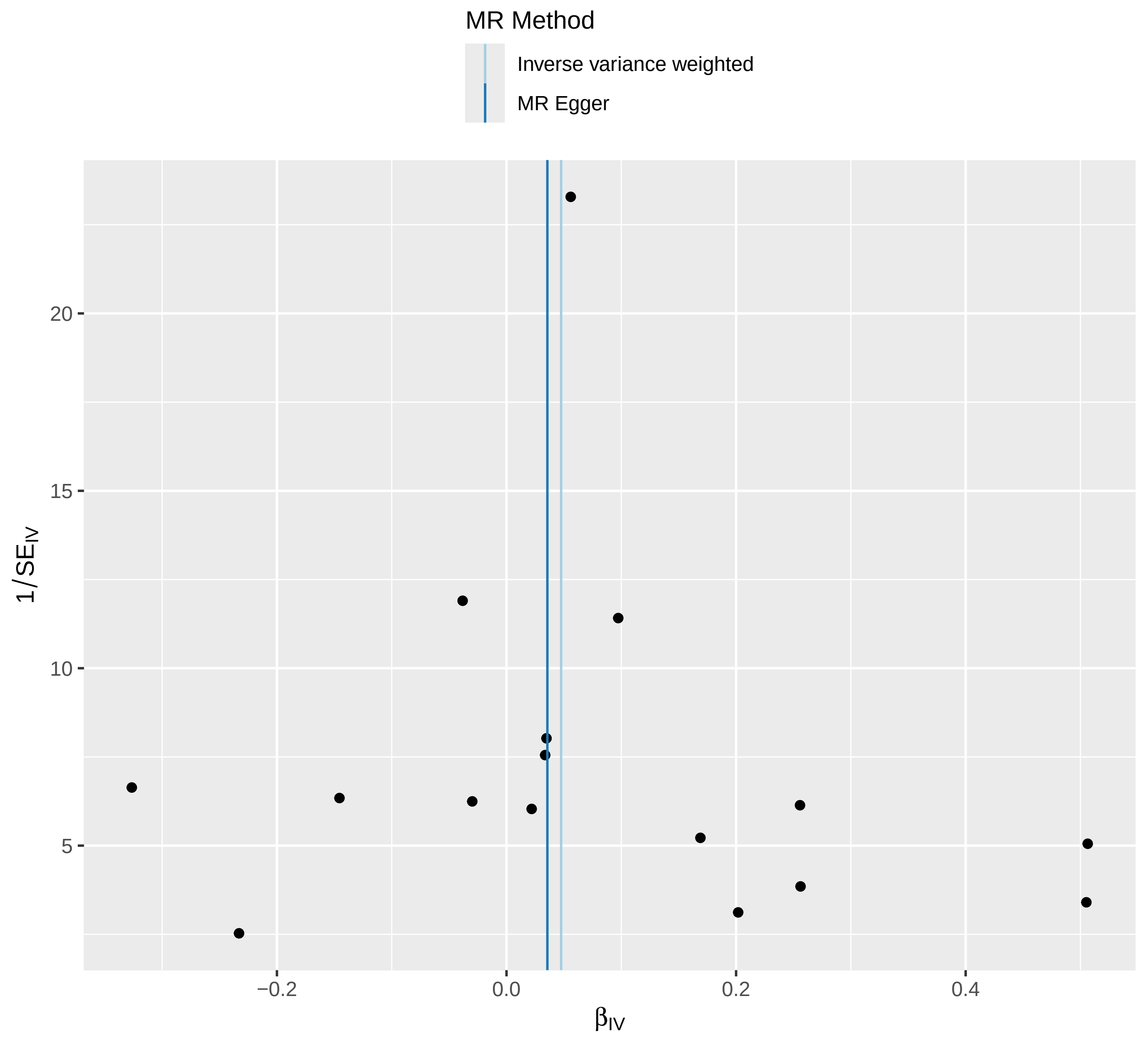
MR Method
Inverse variance weighted
MR Egger
20
15
10
5
1/SE_IV
−0.2
0.0
0.2
0.4
β_IV

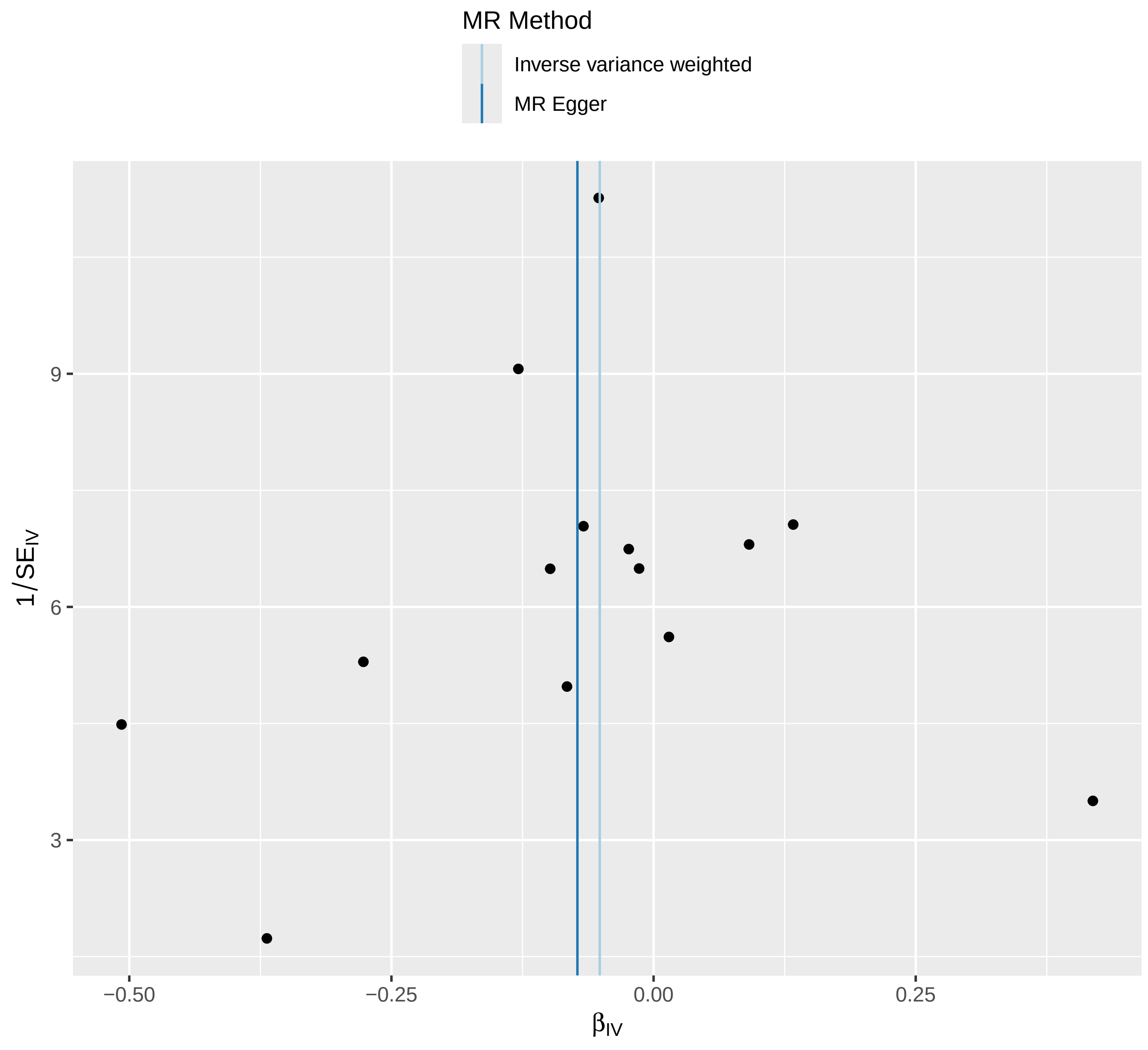

MR Method
Inverse variance weighted
MR Egger
9
6
3
$1/SE_{IV}$
−0.50
−0.25
0.00
0.25
β_{IV}

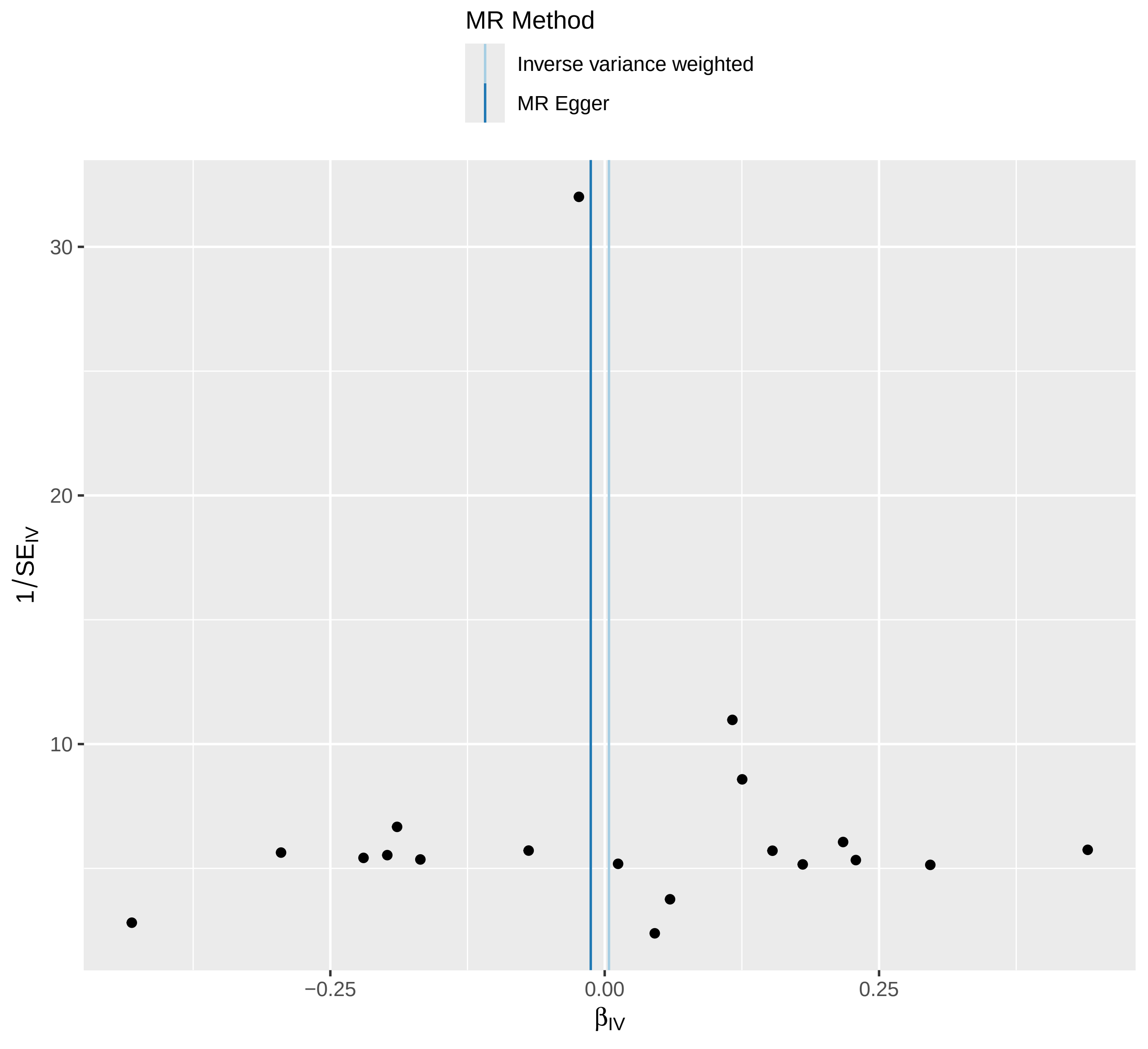
MR Method
Inverse variance weighted
MR Egger
30
20
10
−0.25
0.00
0.25
$1/SE_{IV}$
β_{IV}

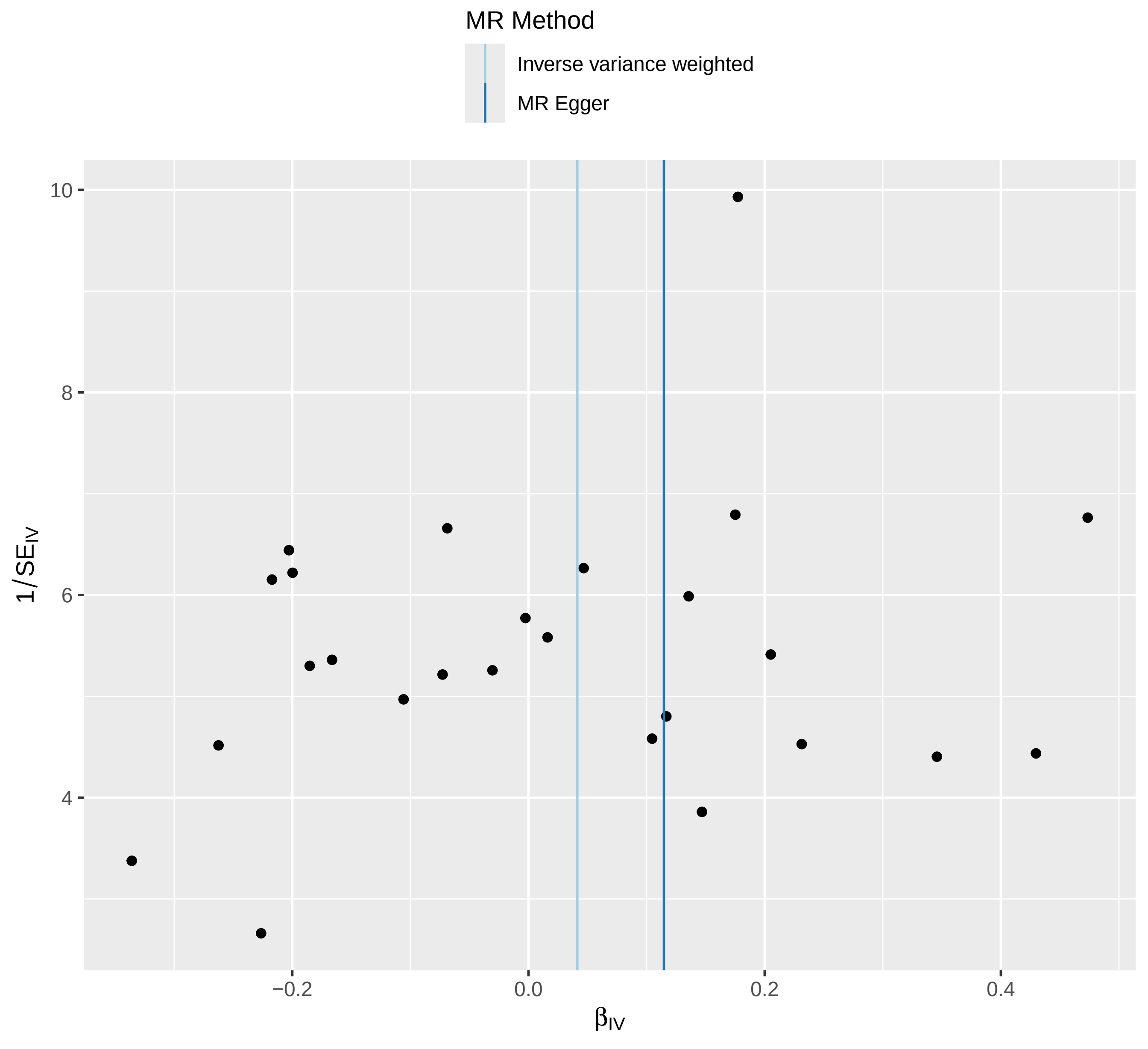
MR Method
Inverse variance weighted
MR Egger
10
8
6
4
1/SE_IV
−0.2
0.0
0.2
0.4
β_IV

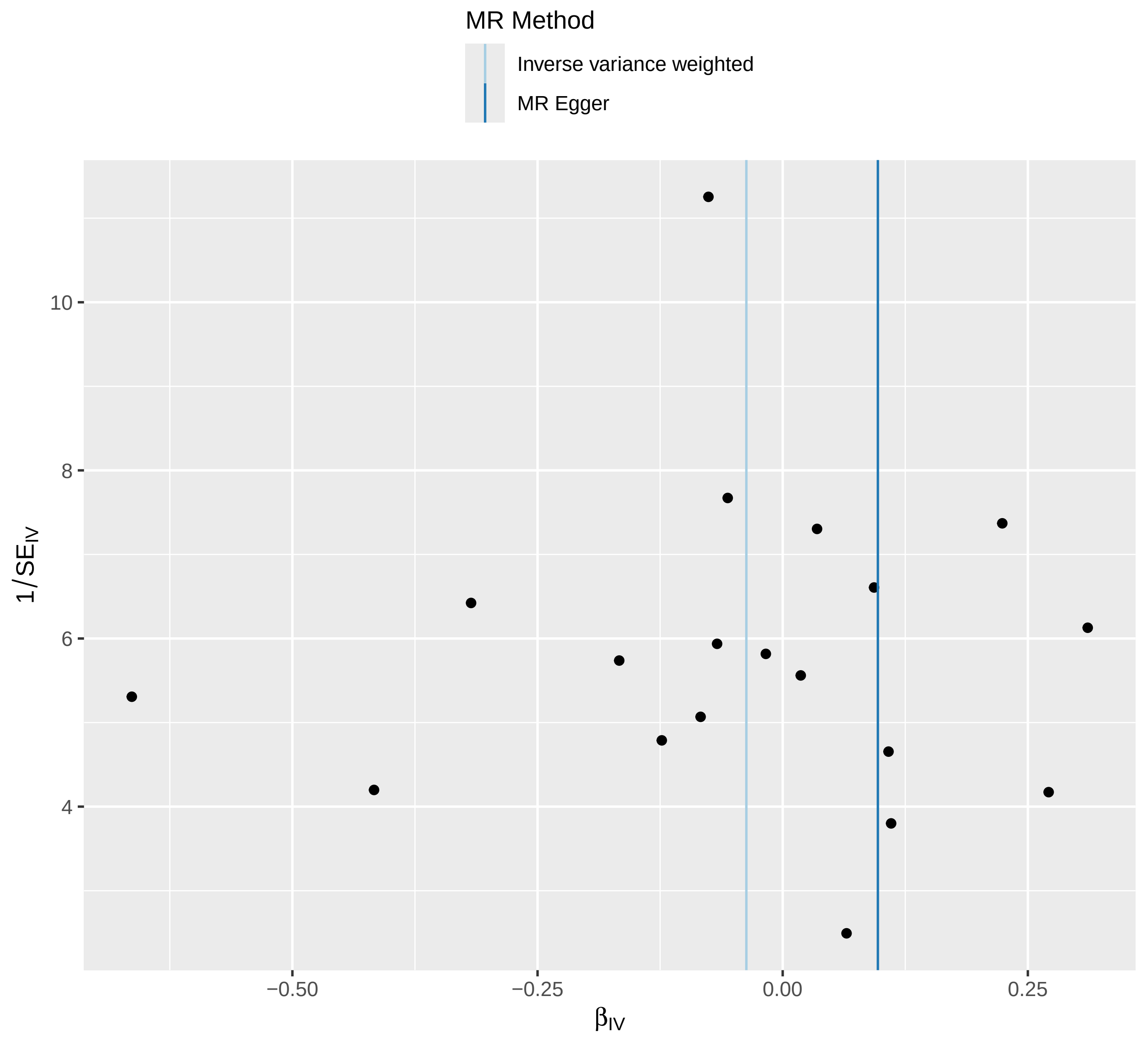
MR Method
Inverse variance weighted
MR Egger
10
8
6
4
1/SE$_{IV}$
−0.50
−0.25
0.00
0.25
β$_{IV}$

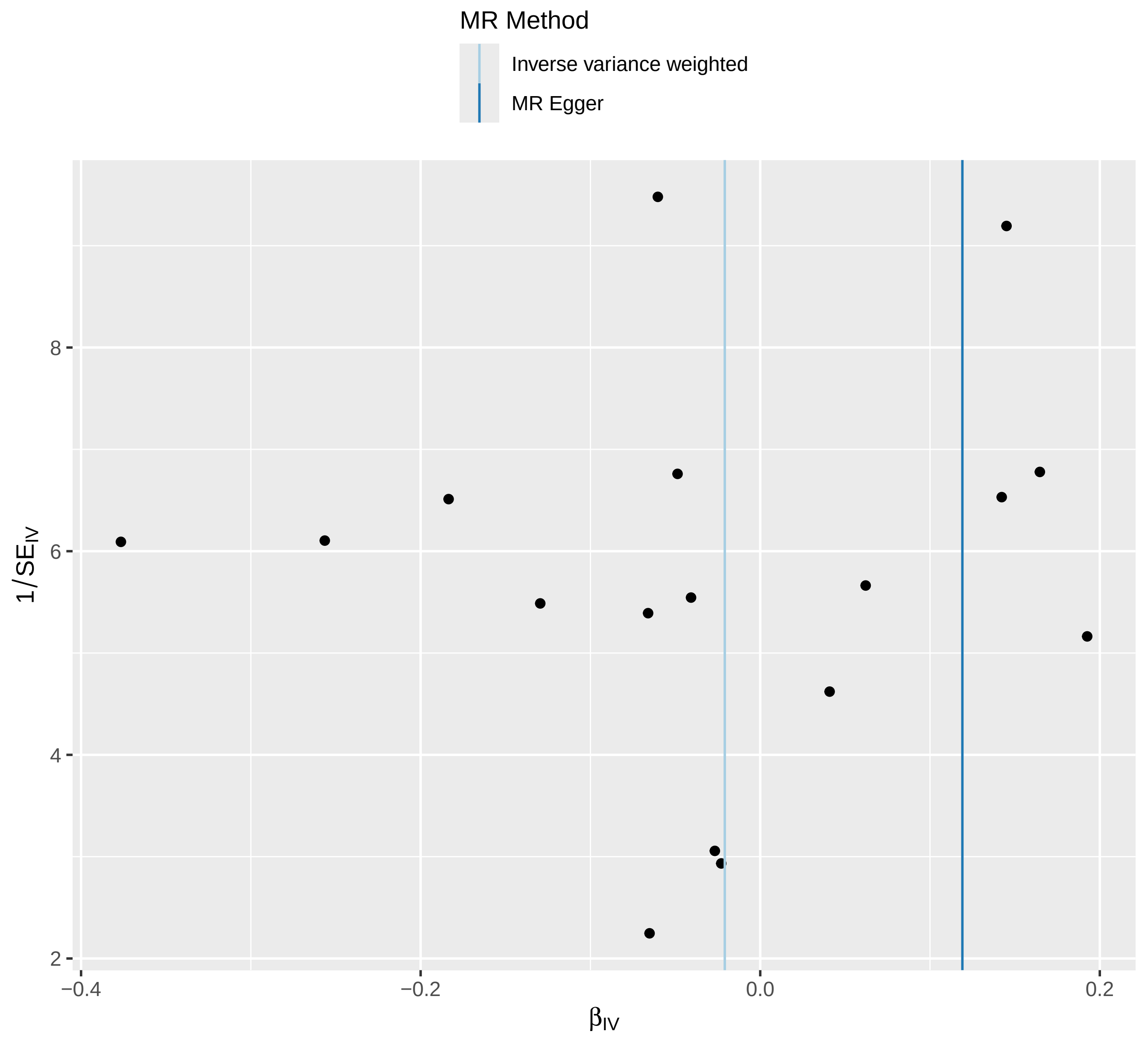
MR Method
Inverse variance weighted
MR Egger
1/SE$_{IV}$
8
6
4
2
−0.4
−0.2
0.0
0.2
β$_{IV}$

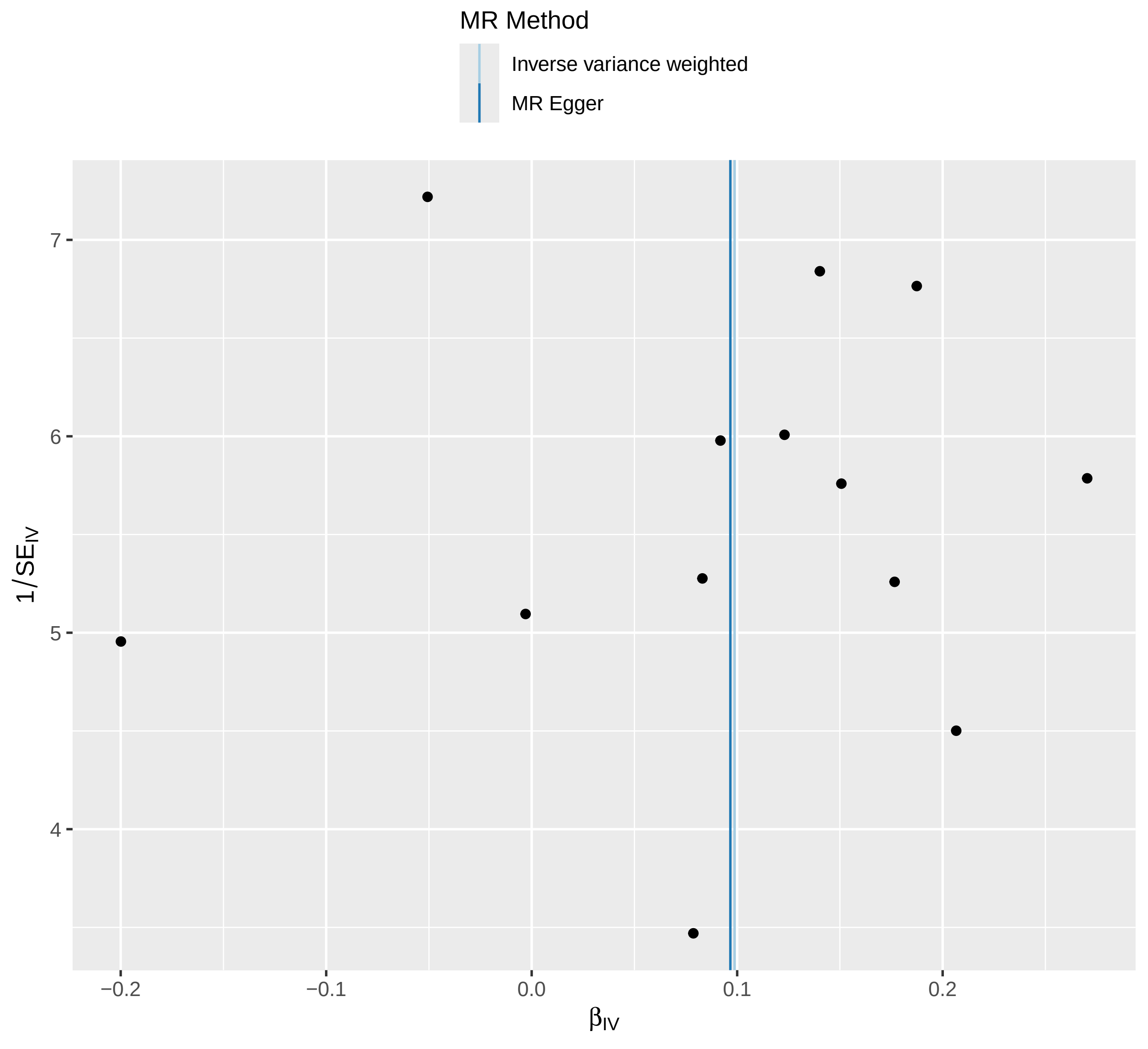

MR Method
Inverse variance weighted
MR Egger
7
6
5
4
1/SE_IV
−0.2
−0.1
0.0
0.1
0.2
β_IV

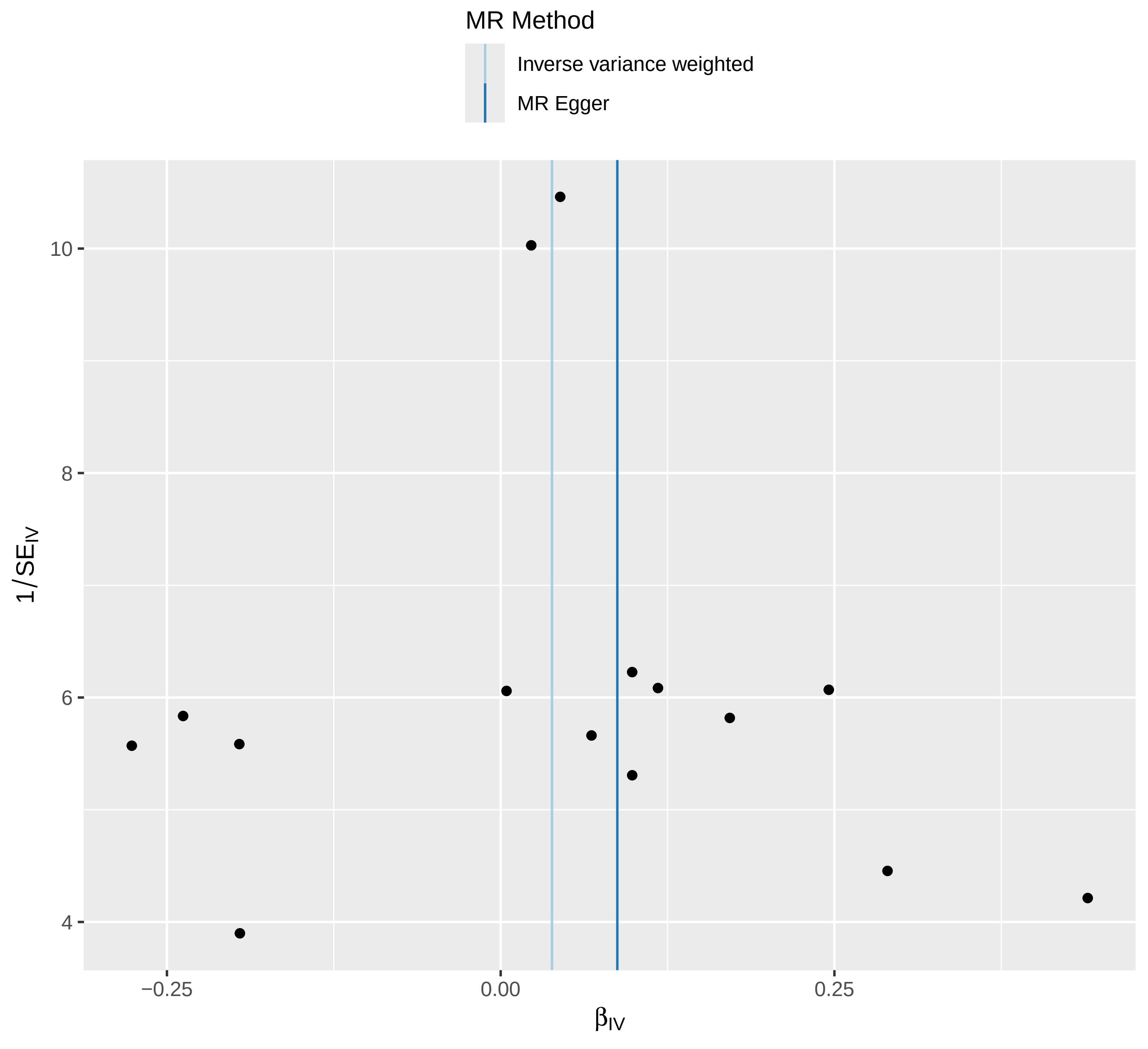
MR Method
Inverse variance weighted
MR Egger
10
8
6
4
1/SE_IV
−0.25
0.00
0.25
β_IV

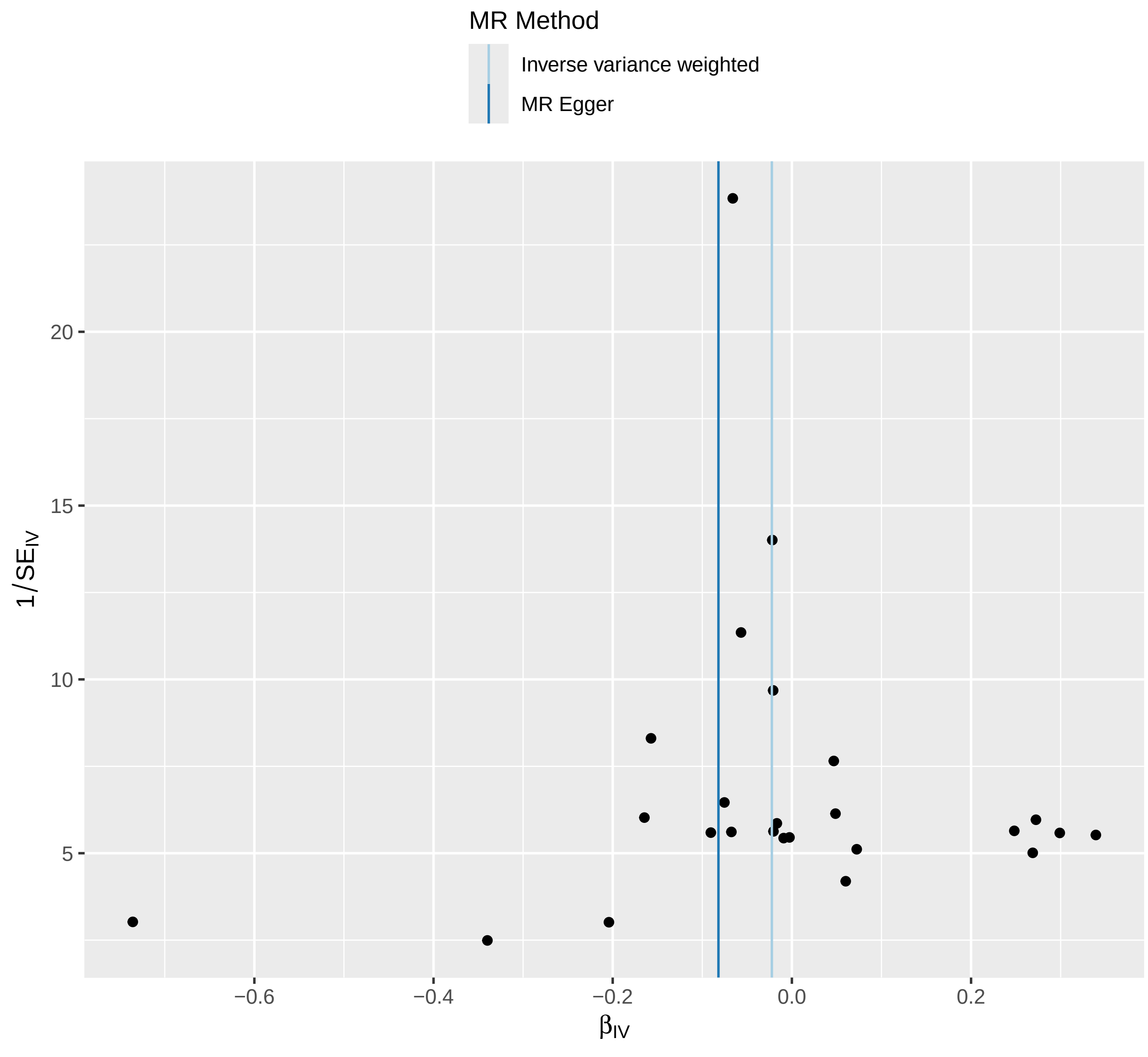
MR Method
Inverse variance weighted
MR Egger
20
15
10
5
1/SE$_{IV}$
−0.6
−0.4
−0.2
0.0
0.2
β$_{IV}$

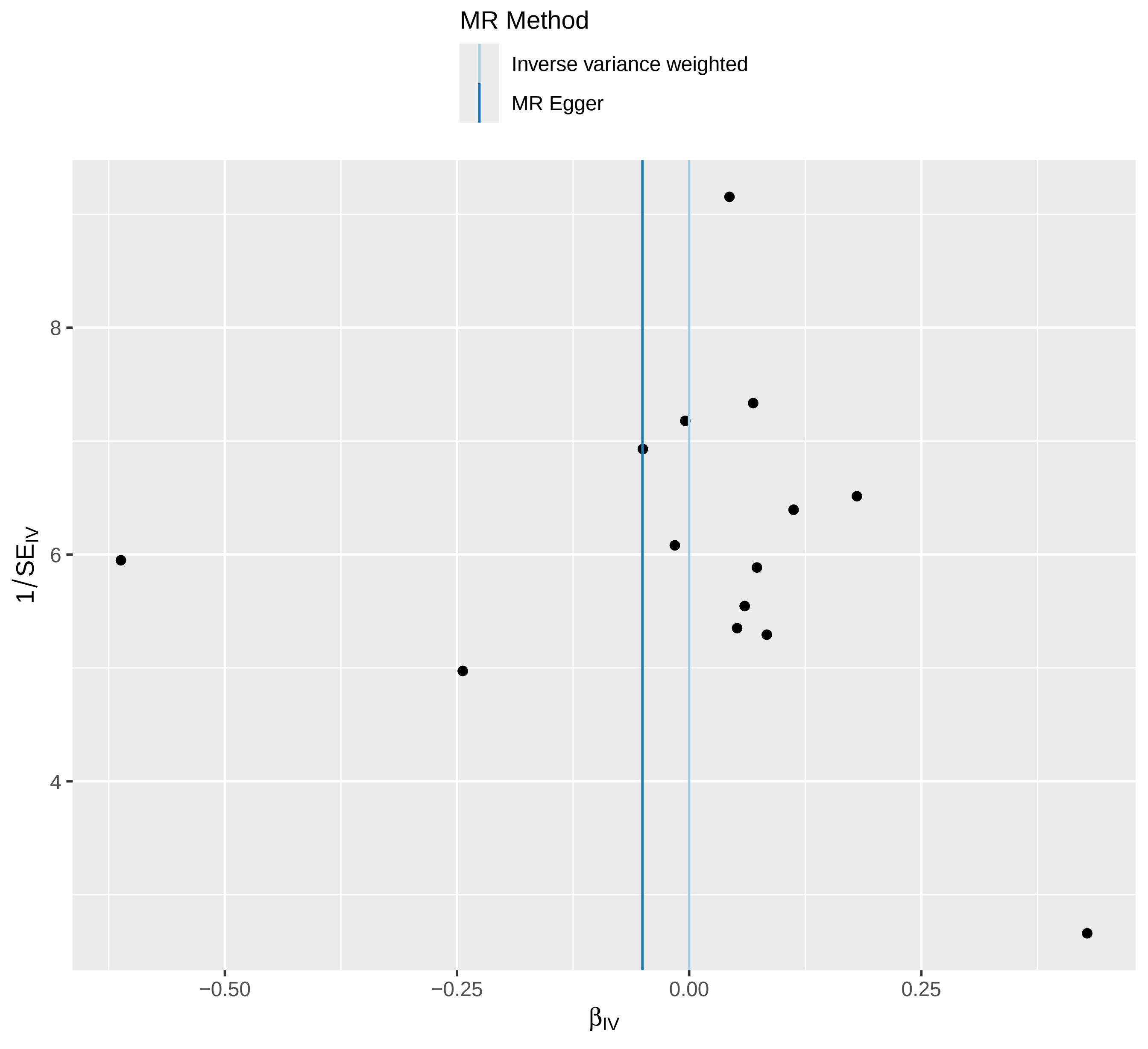

MR Method
Inverse variance weighted
MR Egger
8
6
4
−0.50
−0.25
0.00
0.25
1/SE_IV
β_IV

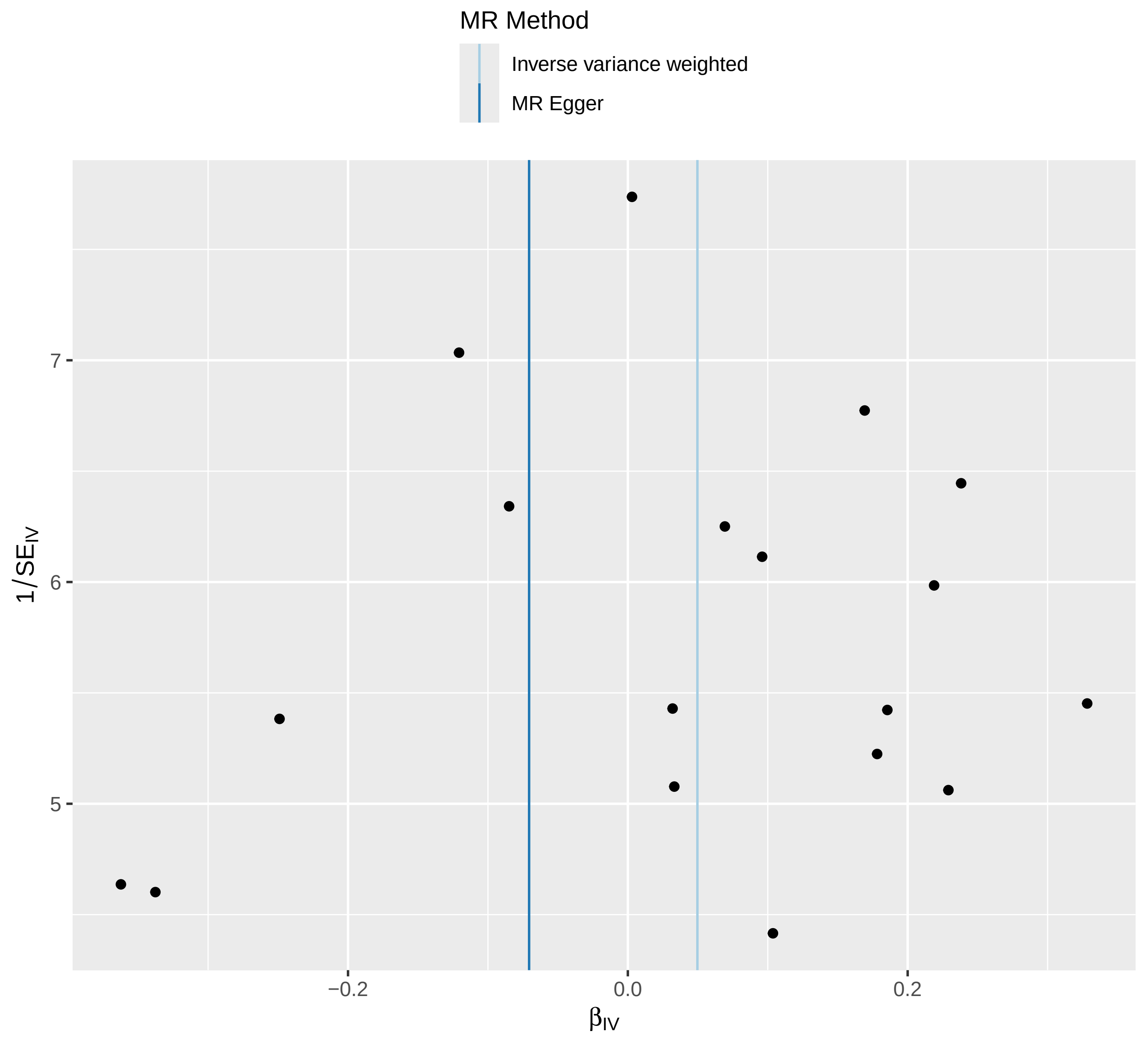

MR Method
Inverse variance weighted
MR Egger
7
6
5
−0.2
0.0
0.2
$1/SE_{IV}$
β_{IV}

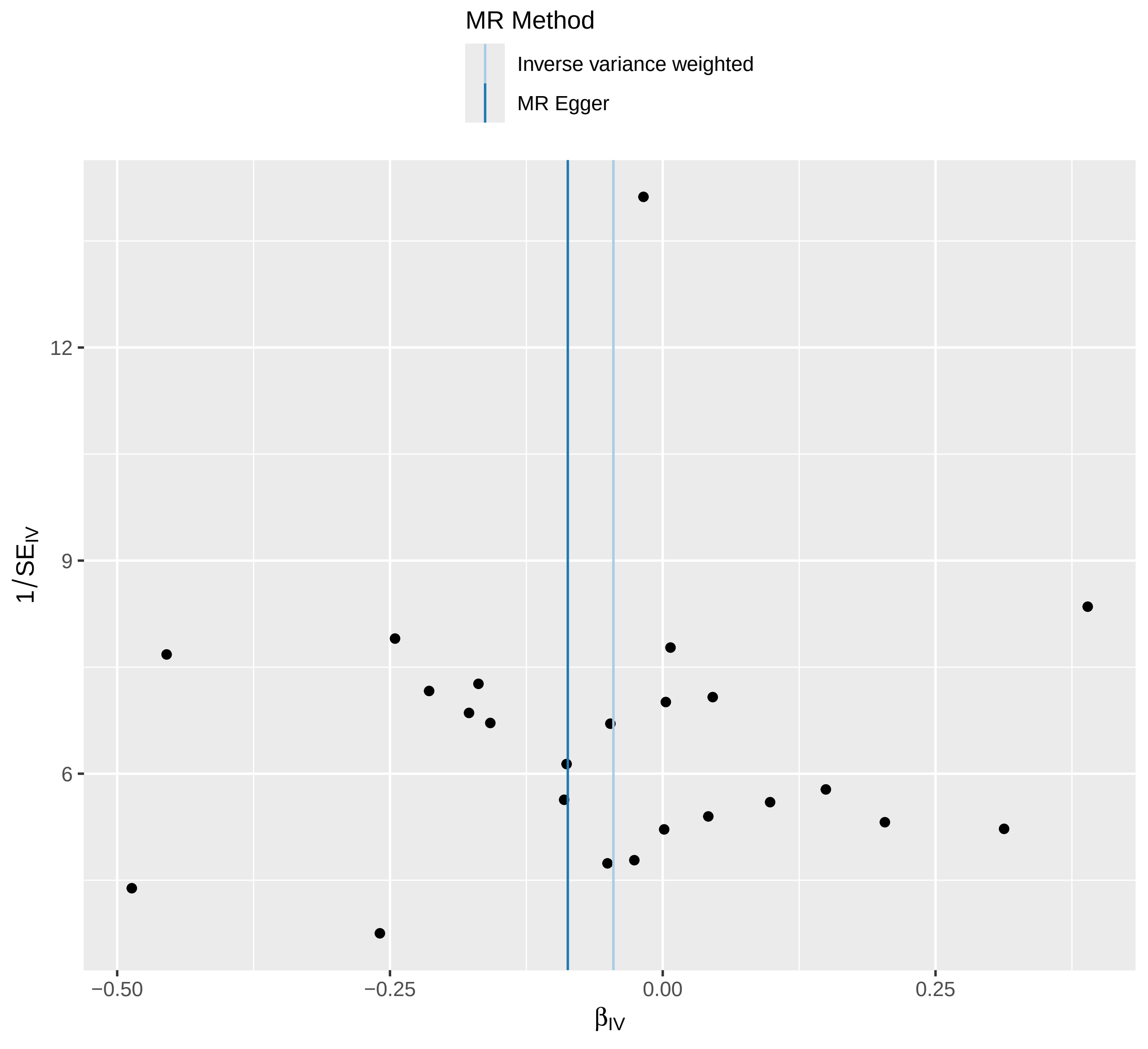
MR Method
Inverse variance weighted
MR Egger
12
9
6
1/SE_IV
−0.50
−0.25
0.00
0.25
β_IV

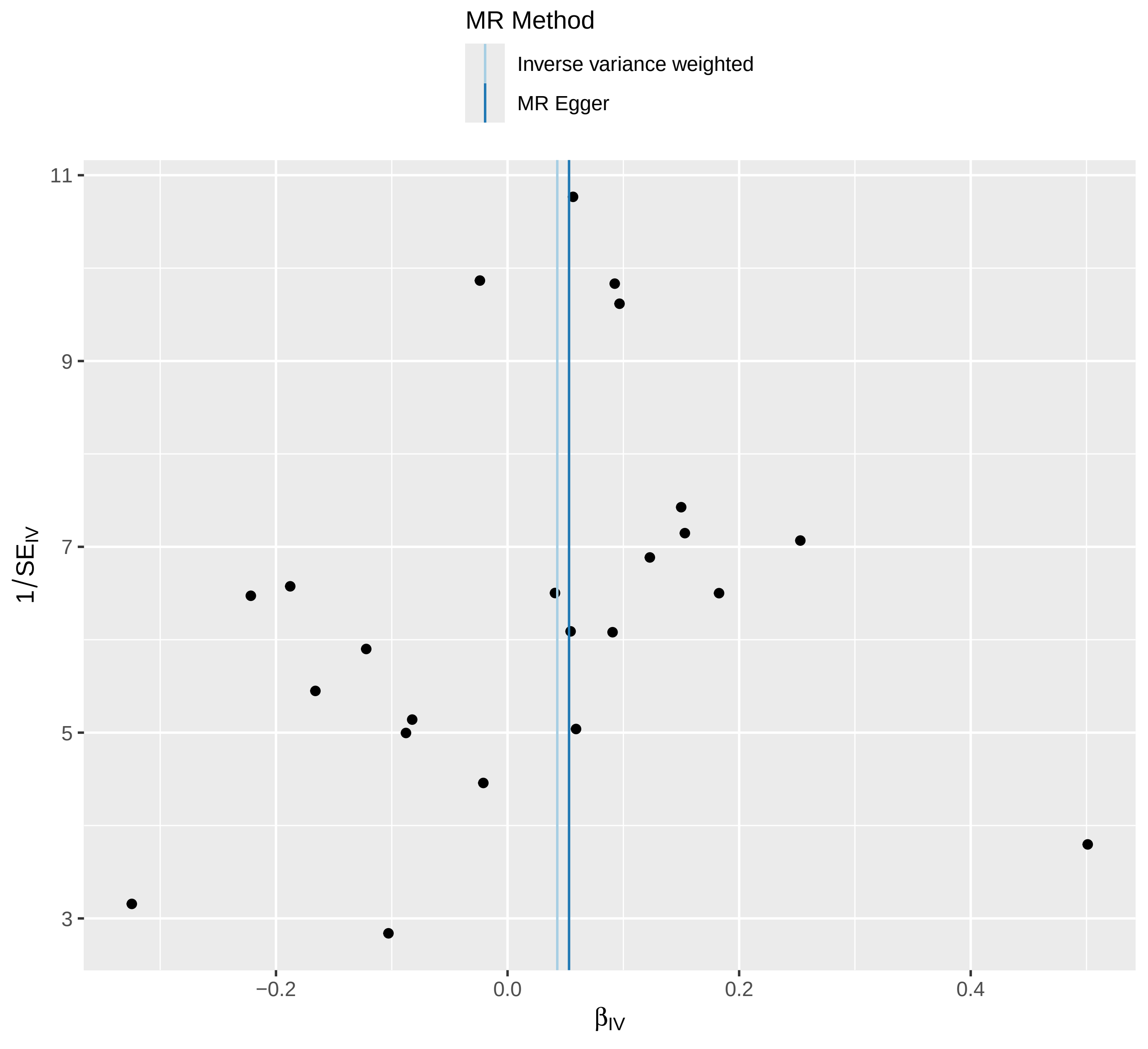
MR Method
Inverse variance weighted
MR Egger
11
9
7
5
3
$1/SE_{IV}$
−0.2
0.0
0.2
0.4
β_{IV}

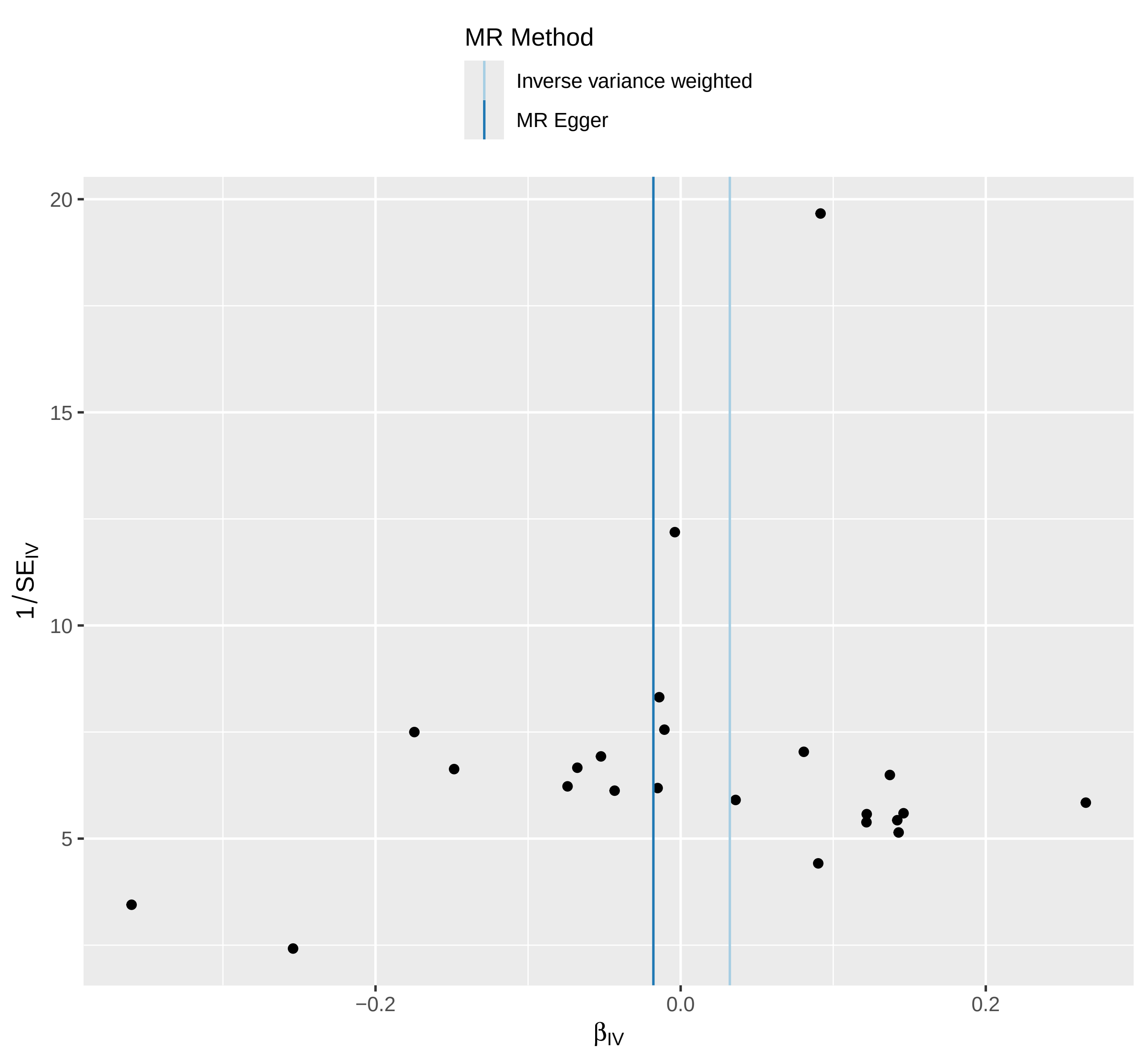
MR Method
Inverse variance weighted
MR Egger
20
15
10
5
1/SE_IV
−0.2
0.0
0.2
β_IV

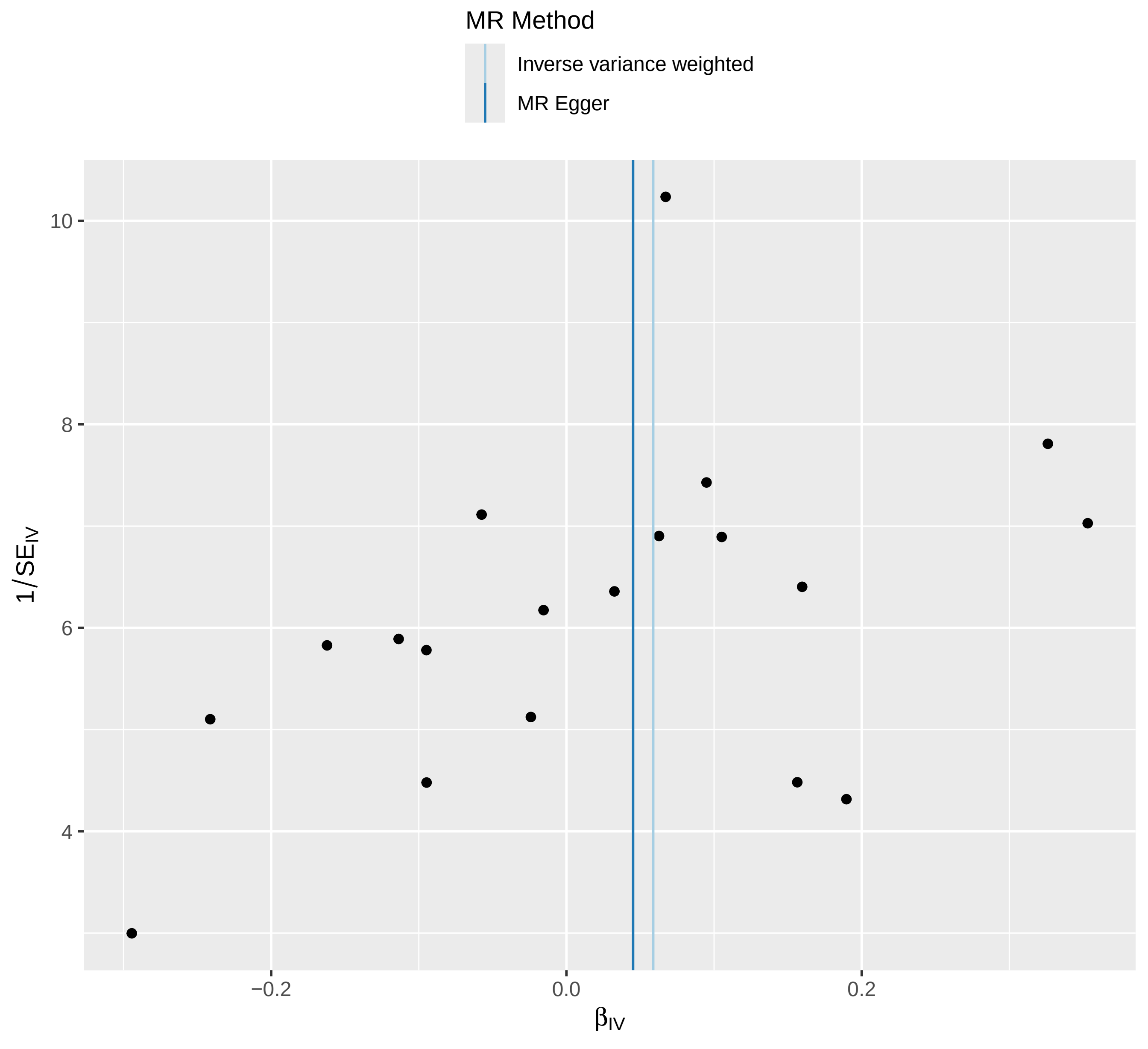
MR Method
Inverse variance weighted
MR Egger
1/SE$_{IV}$
10
8
6
4
−0.2
0.0
0.2
β$_{IV}$

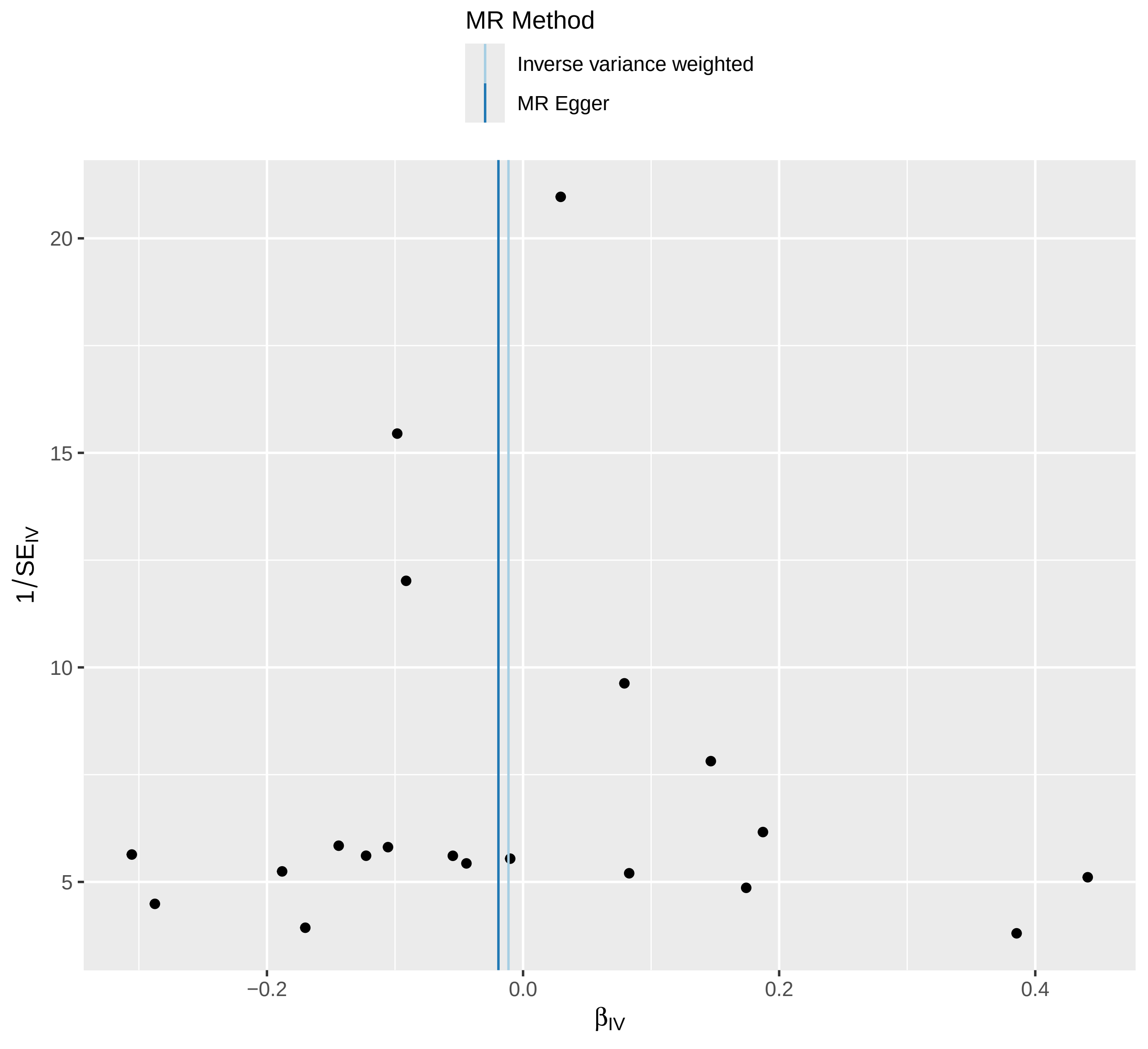
MR Method
Inverse variance weighted
MR Egger
20
15
10
5
1/SE_IV
−0.2
0.0
0.2
0.4
β_IV

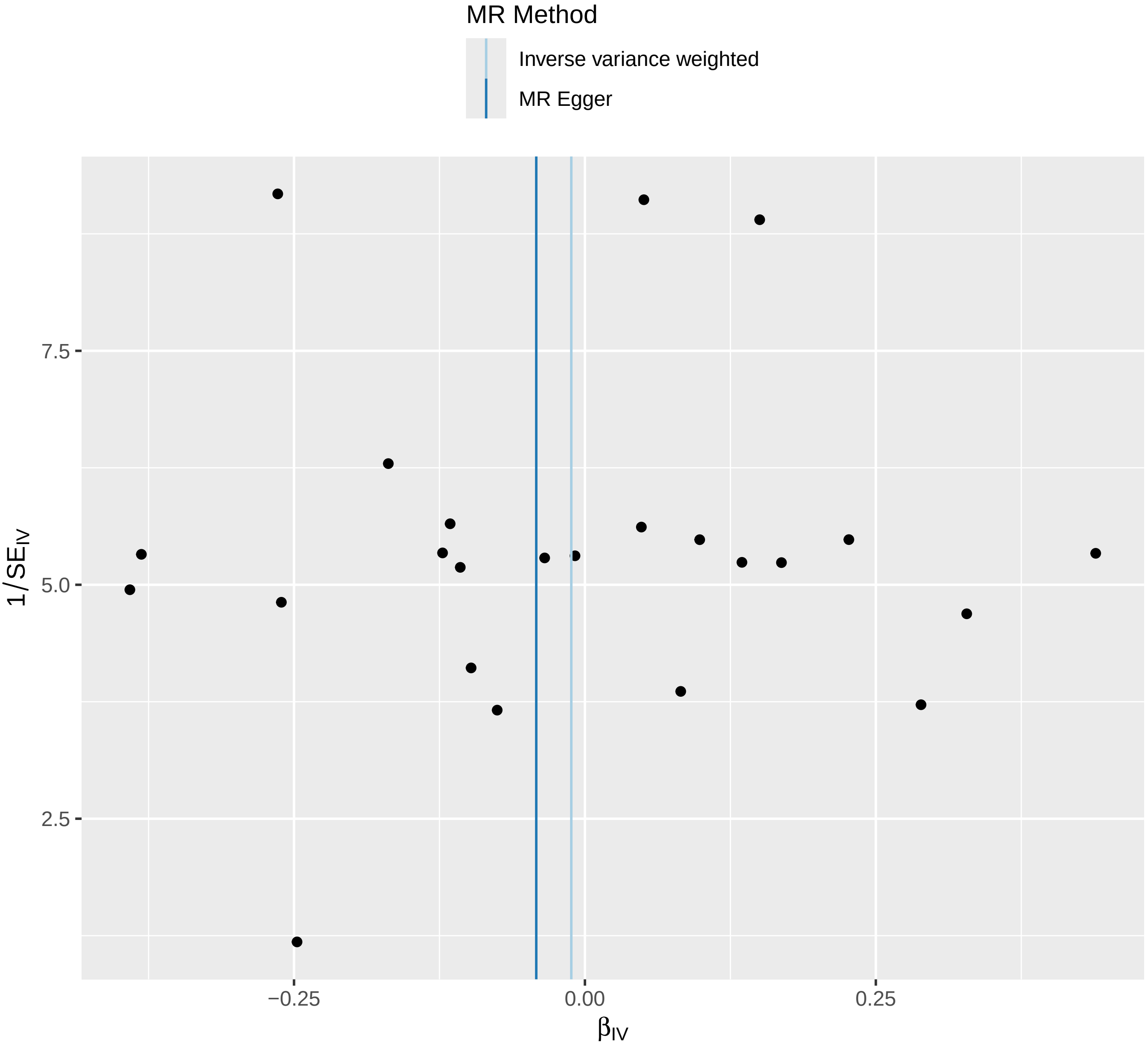
MR Method
Inverse variance weighted
MR Egger
7.5
5.0
2.5
1/SE_IV
−0.25
0.00
0.25
β_IV

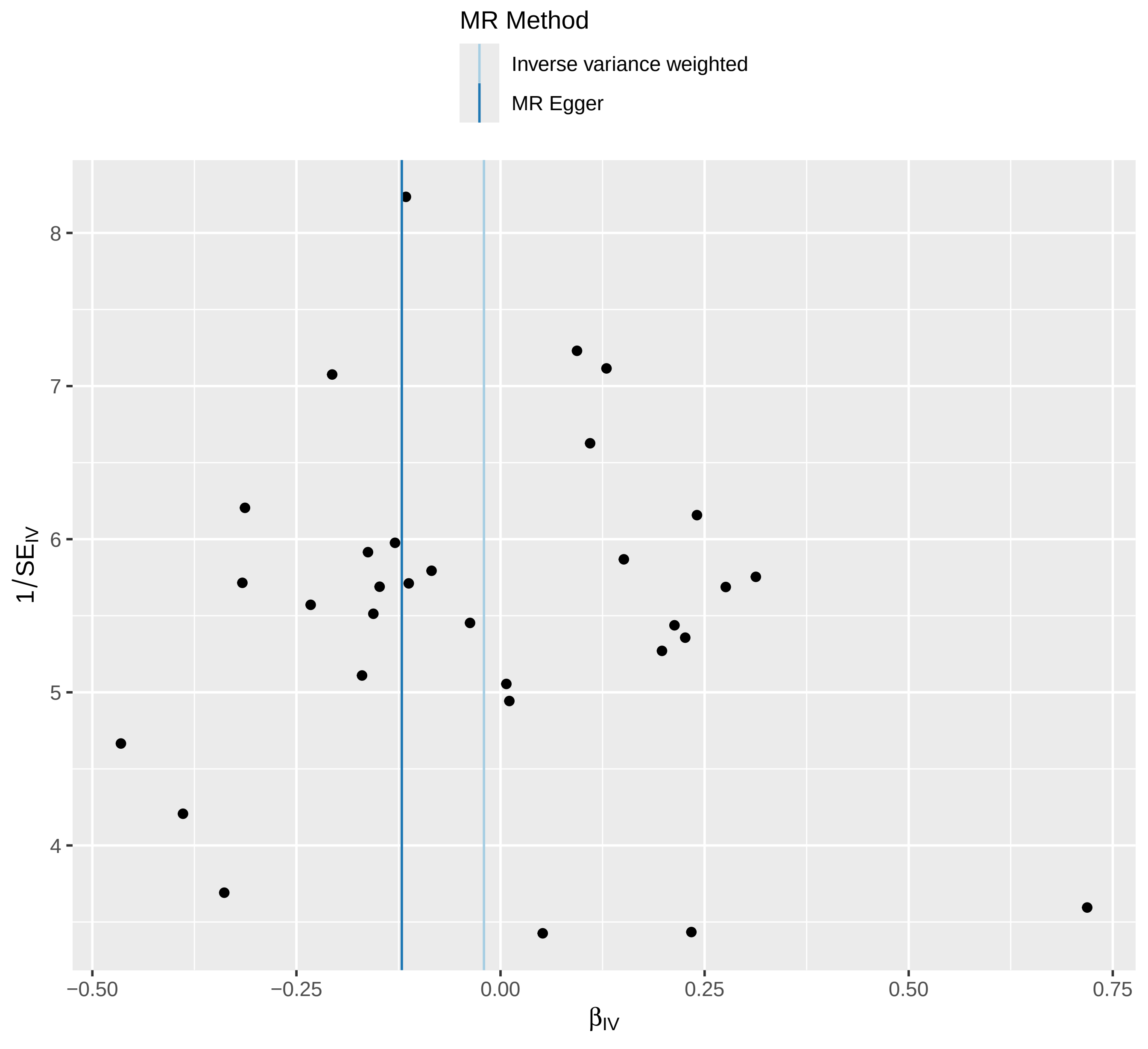

MR Method
Inverse variance weighted
MR Egger
8
7
6
5
4
1/SE$_{IV}$
−0.50
−0.25
0.00
0.25
0.50
0.75
β$_{IV}$

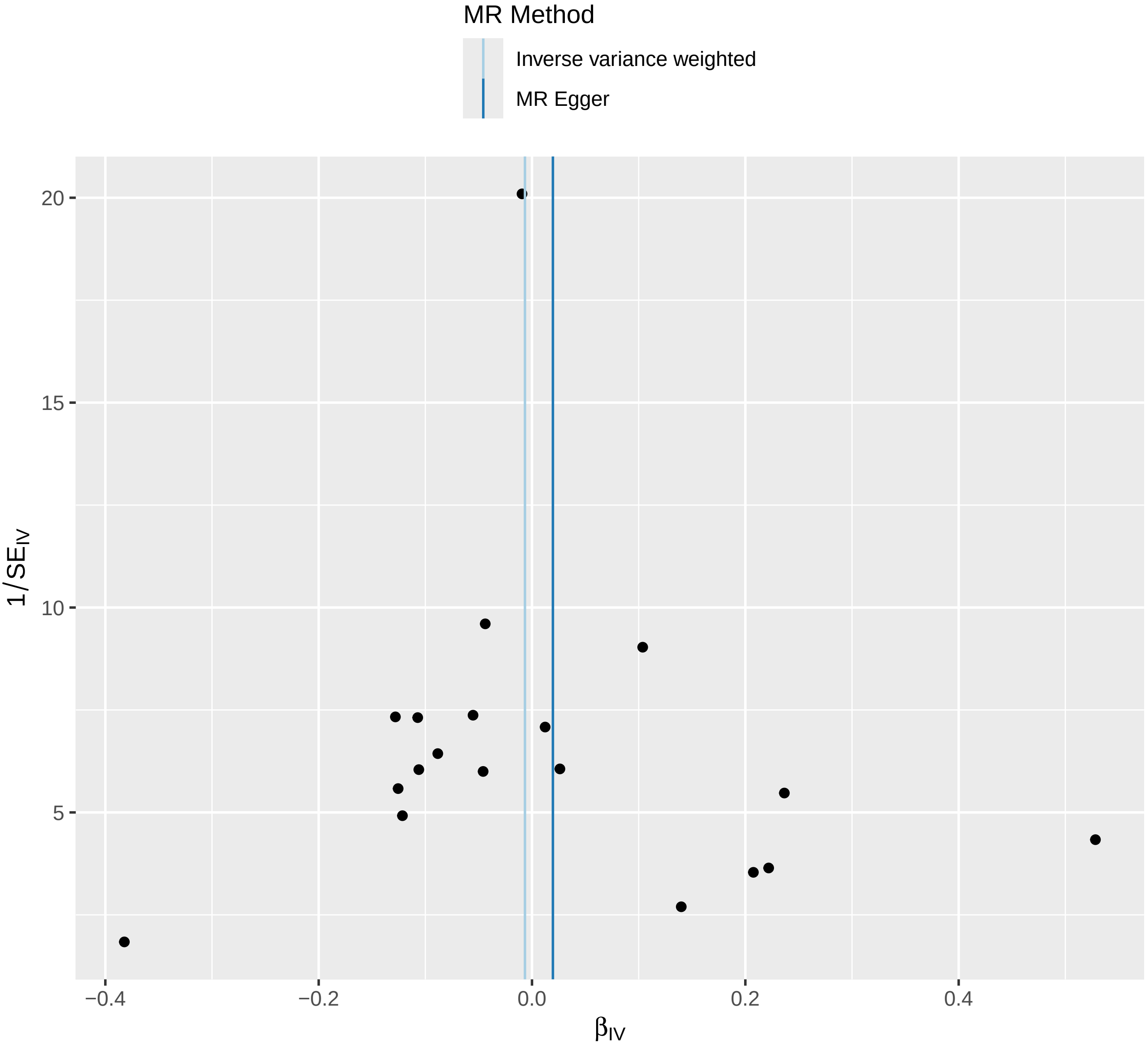
MR Method
Inverse variance weighted
MR Egger
20
15
10
5
1/SE_IV
−0.4
−0.2
0.0
0.2
0.4
β_IV

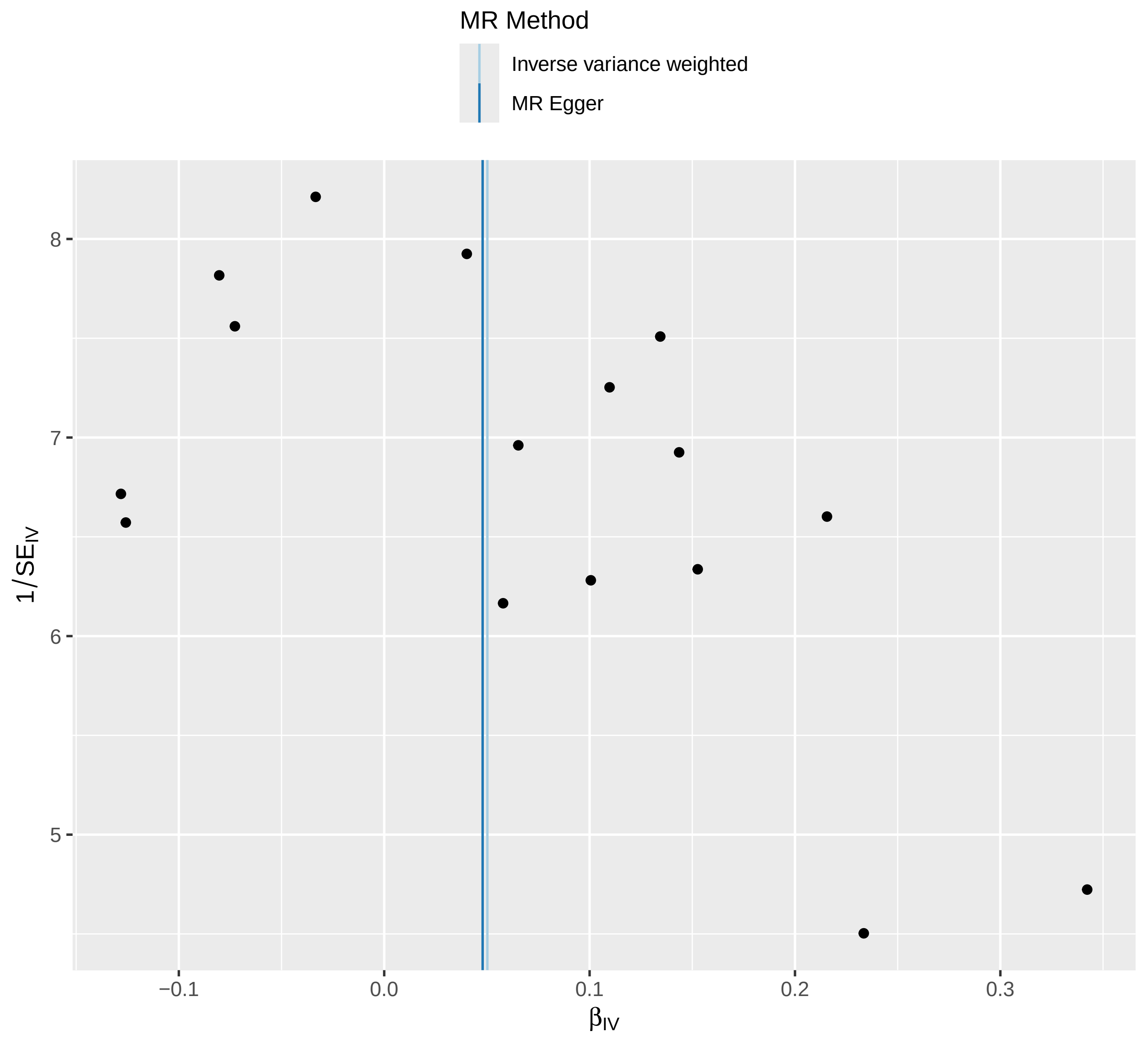

MR Method
Inverse variance weighted
MR Egger
1/SE_IV
8
7
6
5
−0.1
0.0
0.1
0.2
0.3
β_IV

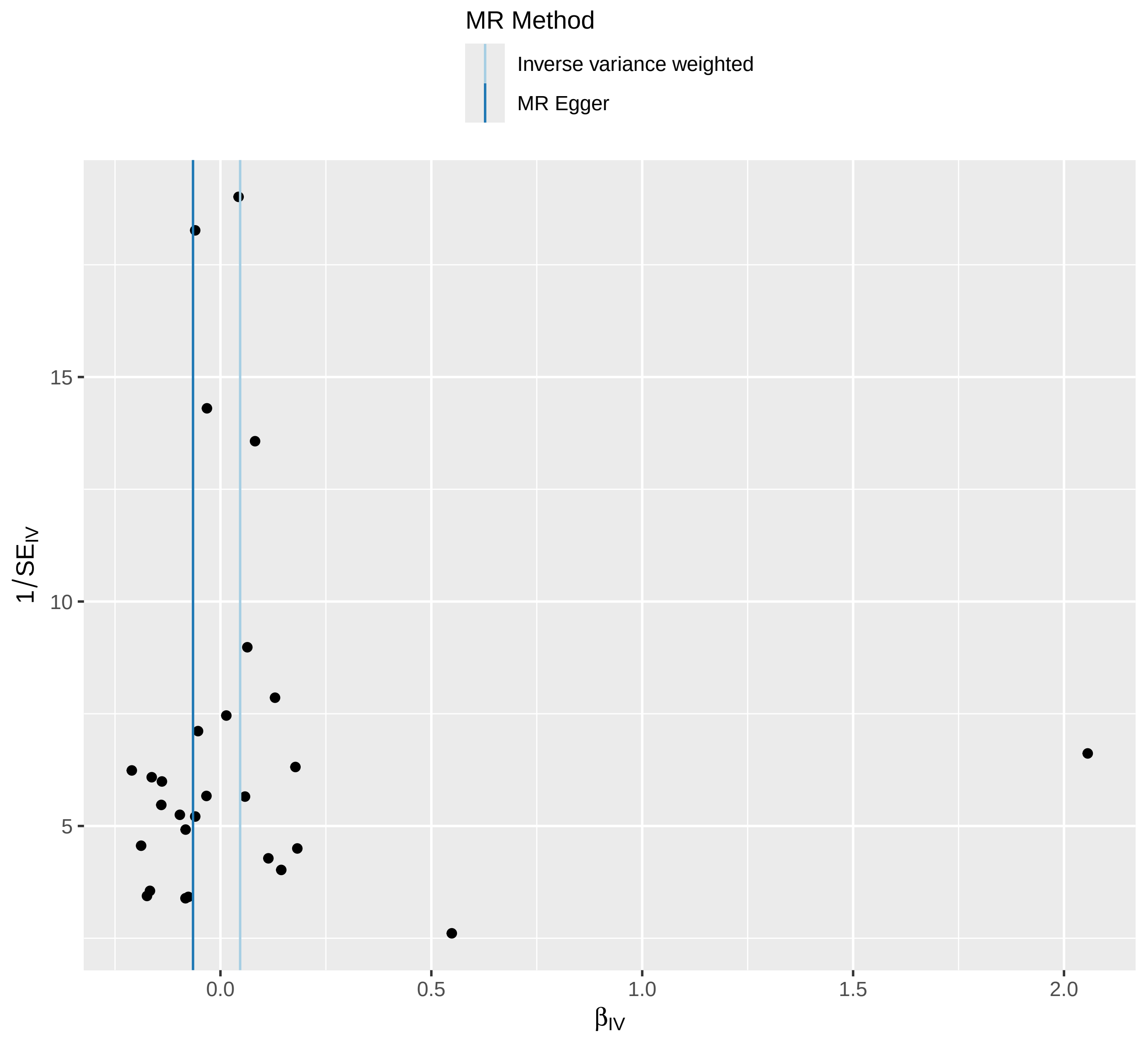
MR Method
Inverse variance weighted
MR Egger
1/SE_IV
15
10
5
0.0
0.5
1.0
1.5
2.0
β_IV

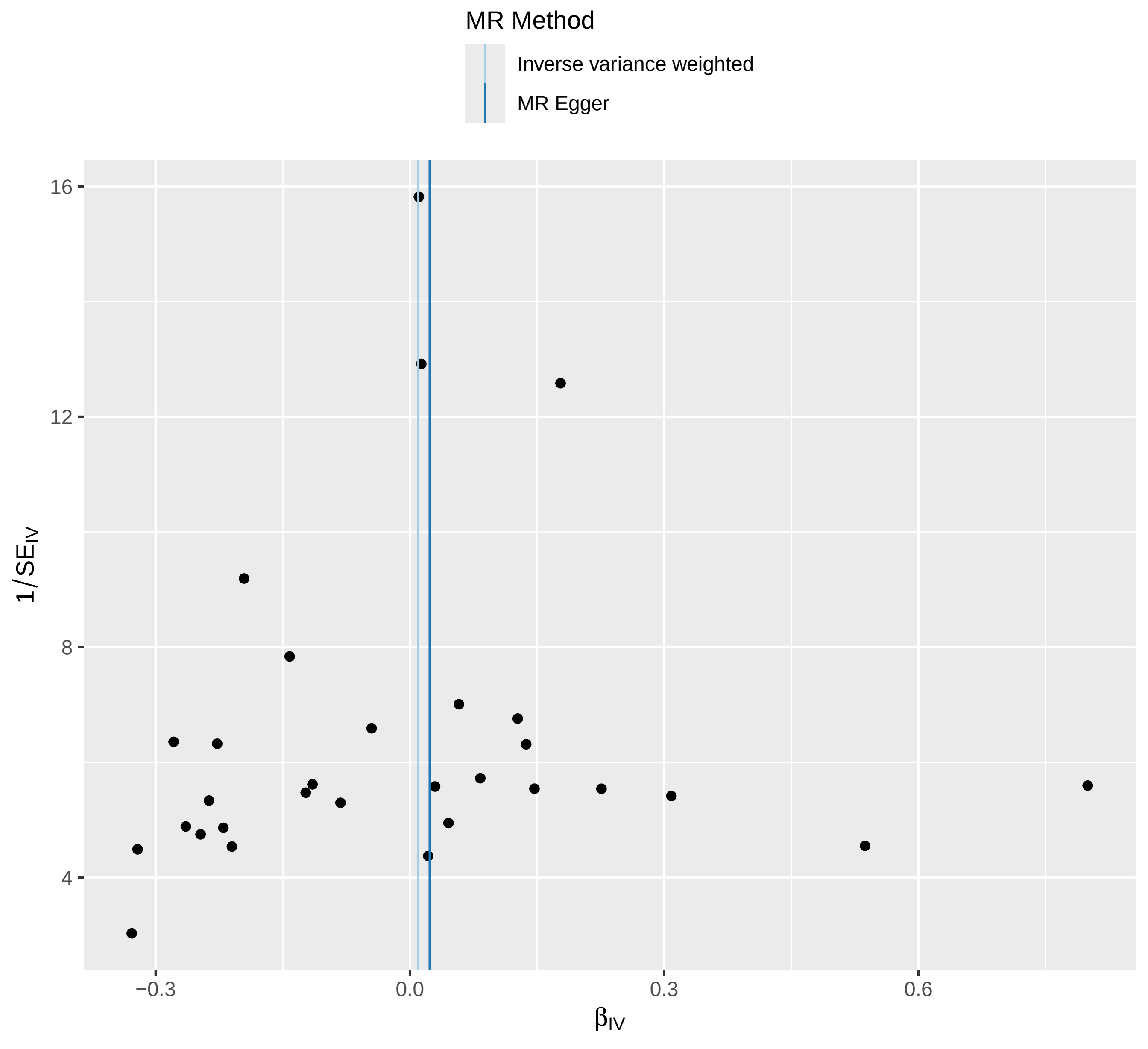

MR Method
Inverse variance weighted
MR Egger
16
12
8
4
1/SE$_{IV}$
−0.3
0.0
0.3
0.6
β$_{IV}$

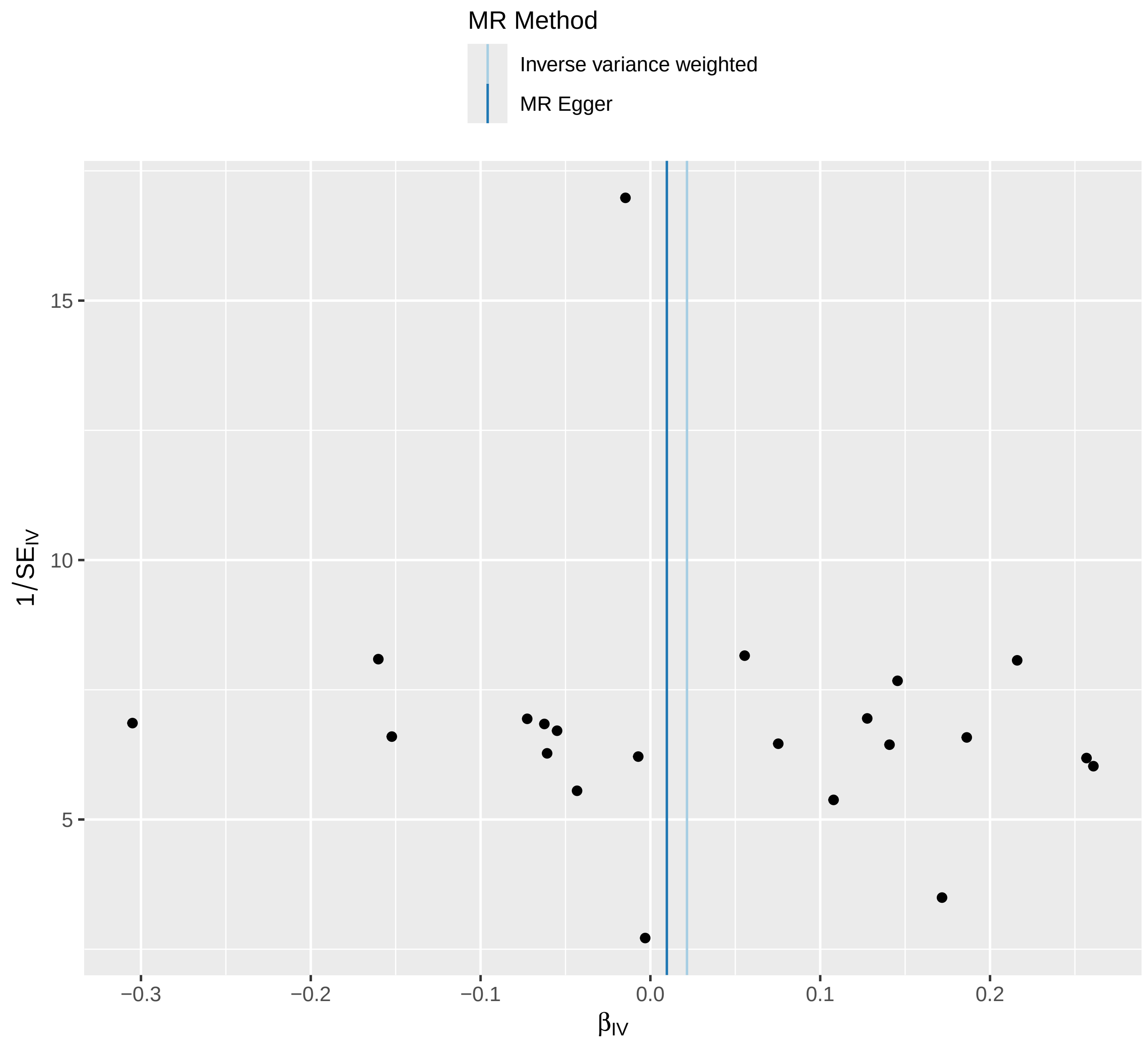

MR Method
Inverse variance weighted
MR Egger
15
10
5
1/SE$_{IV}$
−0.3
−0.2
−0.1
0.0
0.1
0.2
β$_{IV}$

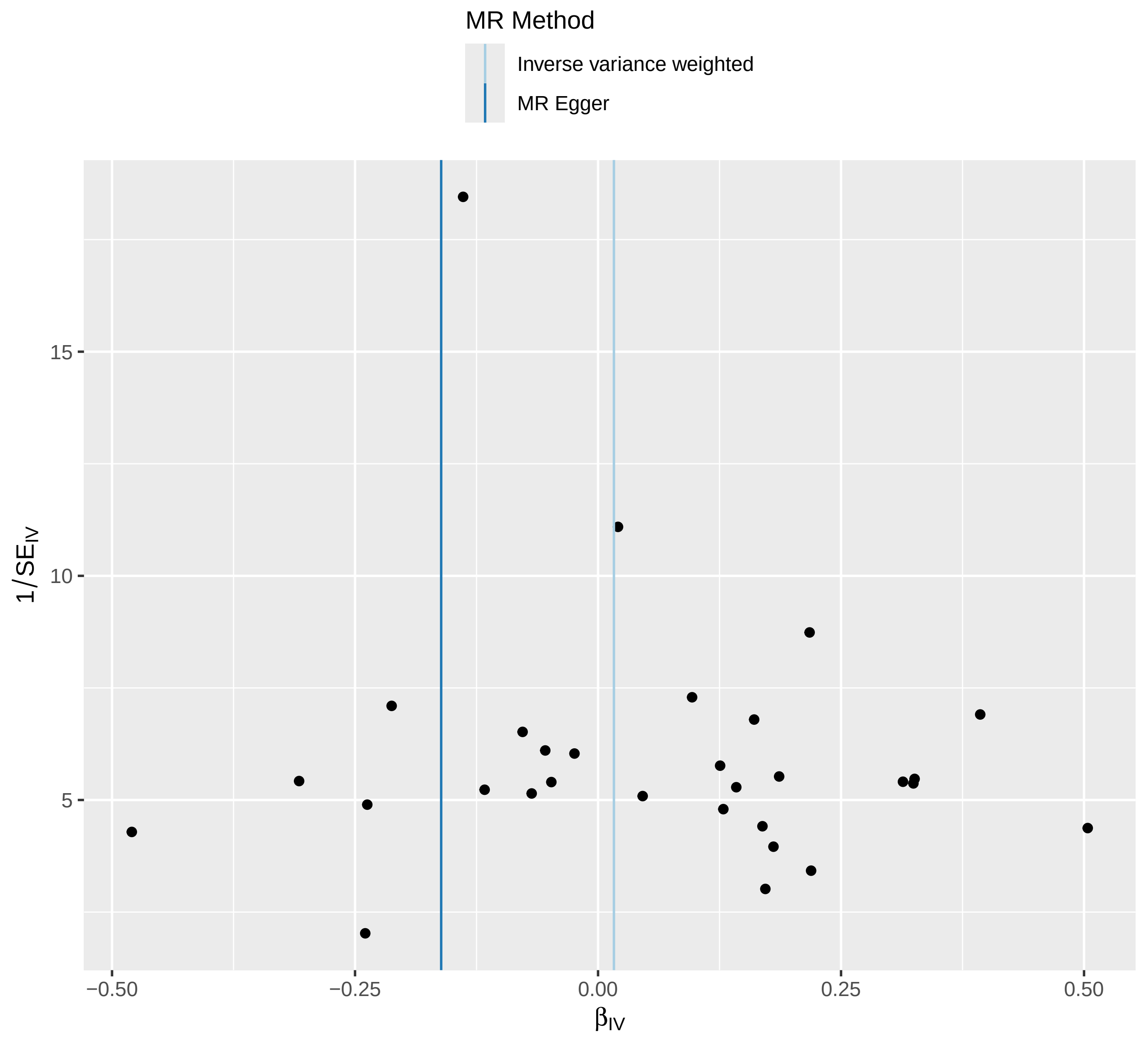

MR Method
Inverse variance weighted
MR Egger
1/SE_IV
15
10
5
−0.50
−0.25
0.00
0.25
0.50
β_IV

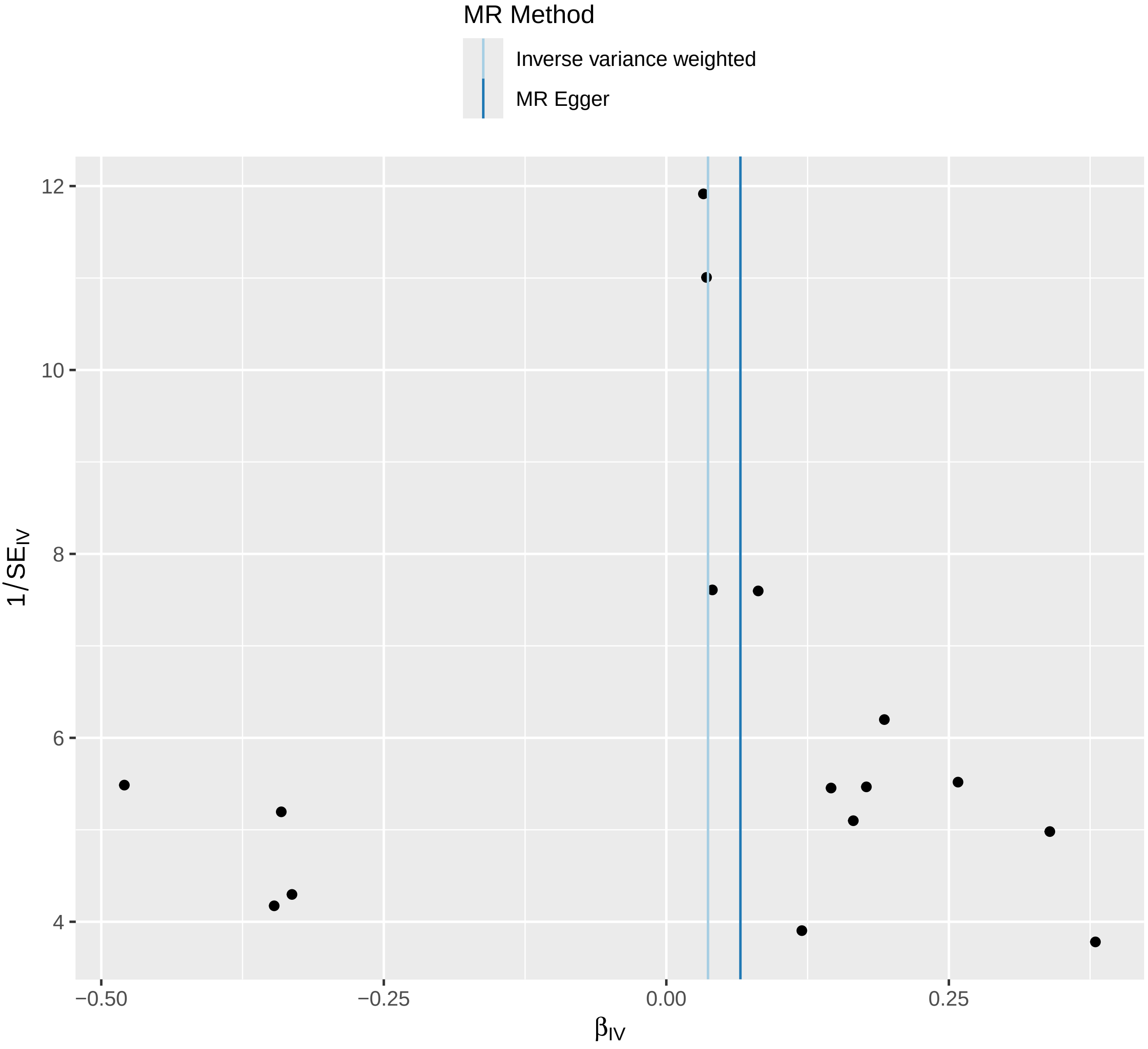
MR Method
Inverse variance weighted
MR Egger
12
10
8
6
4
$1/SE_{IV}$
−0.50
−0.25
0.00
0.25
β_{IV}

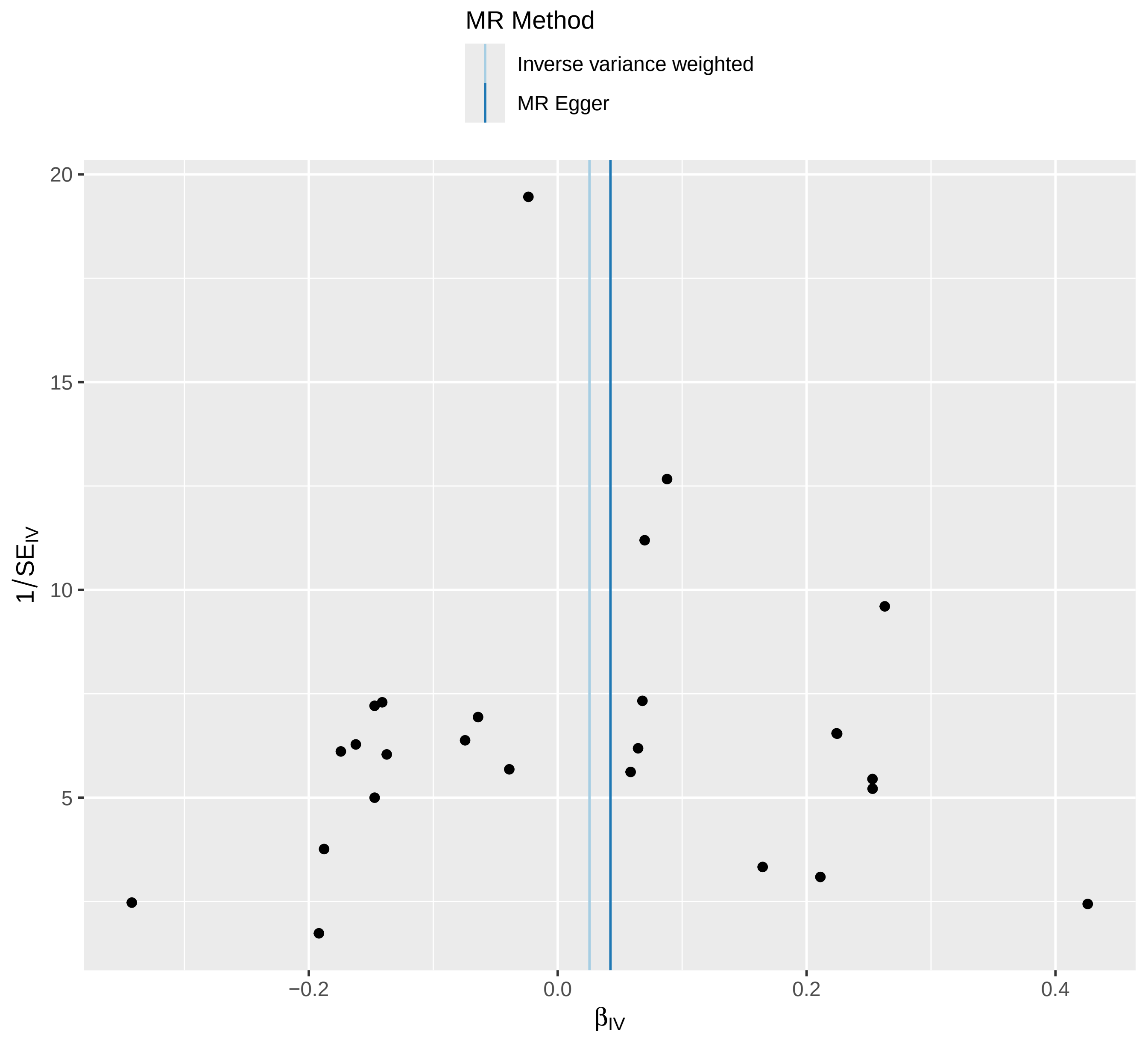
MR Method
Inverse variance weighted
MR Egger
20
15
10
5
1/SE_IV
−0.2
0.0
0.2
0.4
β_IV

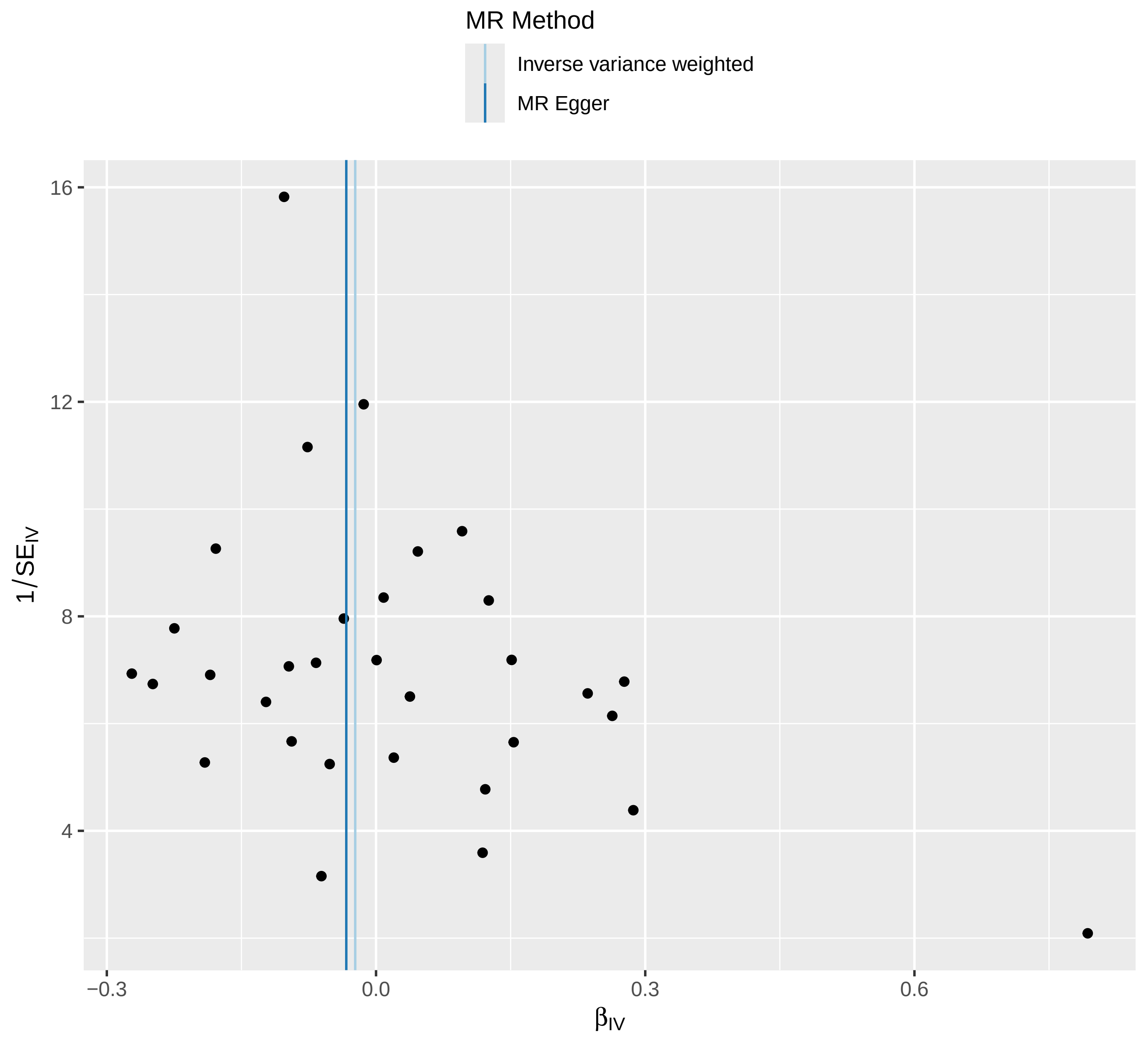

MR Method
Inverse variance weighted
MR Egger
16
12
8
4
1/SE$_{IV}$
−0.3
0.0
0.3
0.6
β$_{IV}$

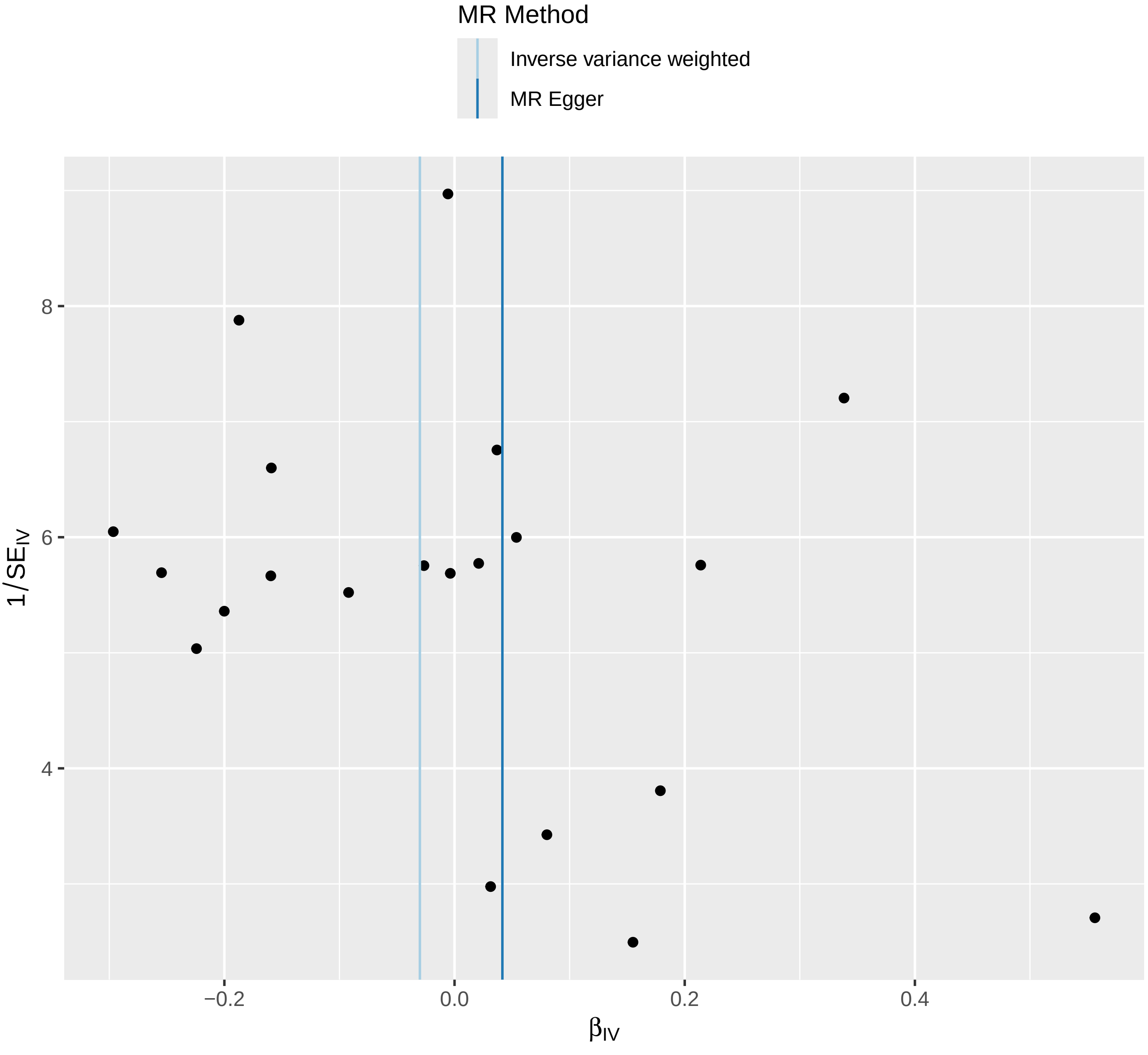
MR Method
Inverse variance weighted
MR Egger
8
6
4
1/SE$_{IV}$
−0.2
0.0
0.2
0.4
β$_{IV}$

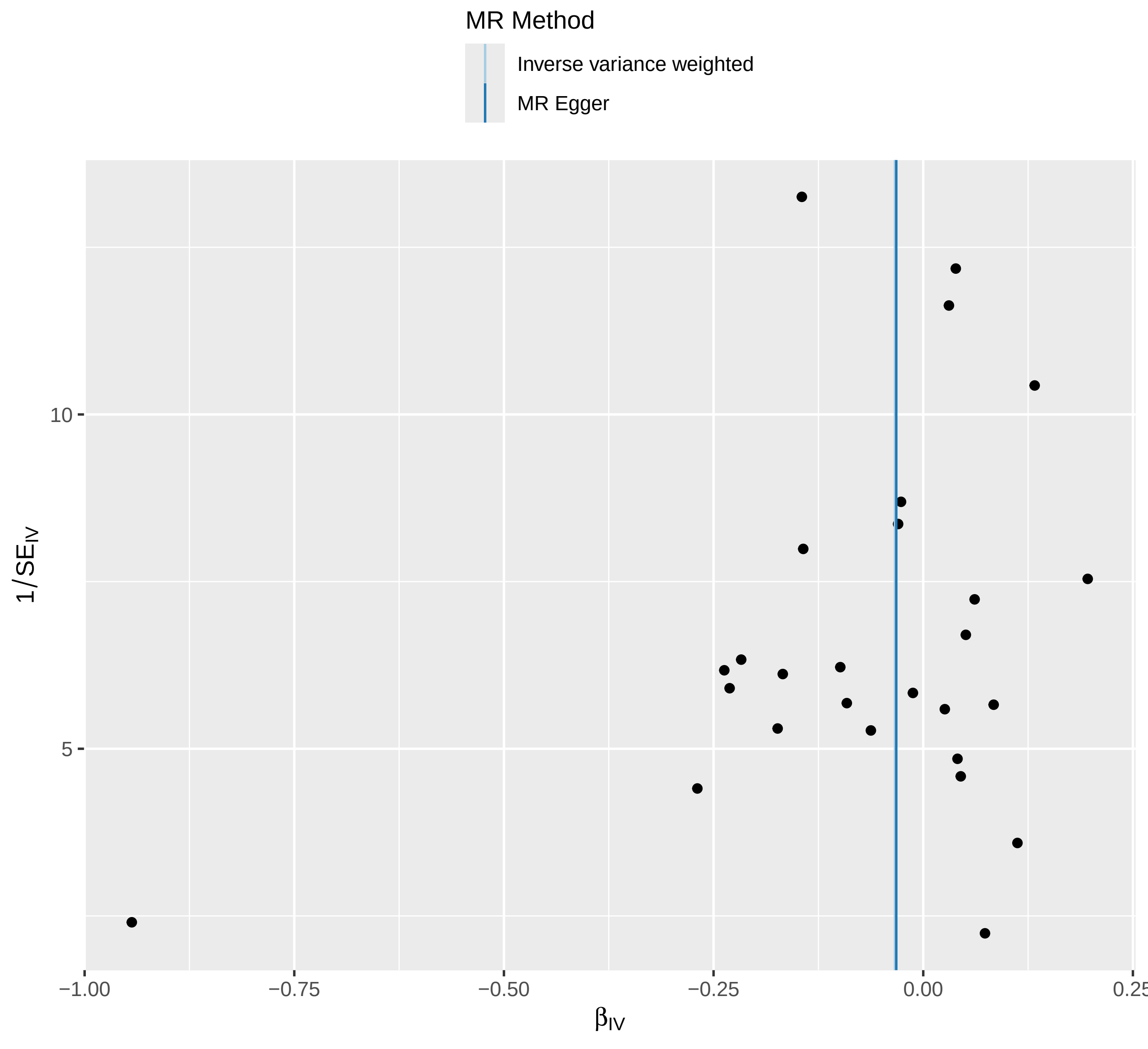
MR Method
Inverse variance weighted
MR Egger
1/SE_IV
10
5
β_IV
−1.00
−0.75
−0.50
−0.25
0.00
0.25

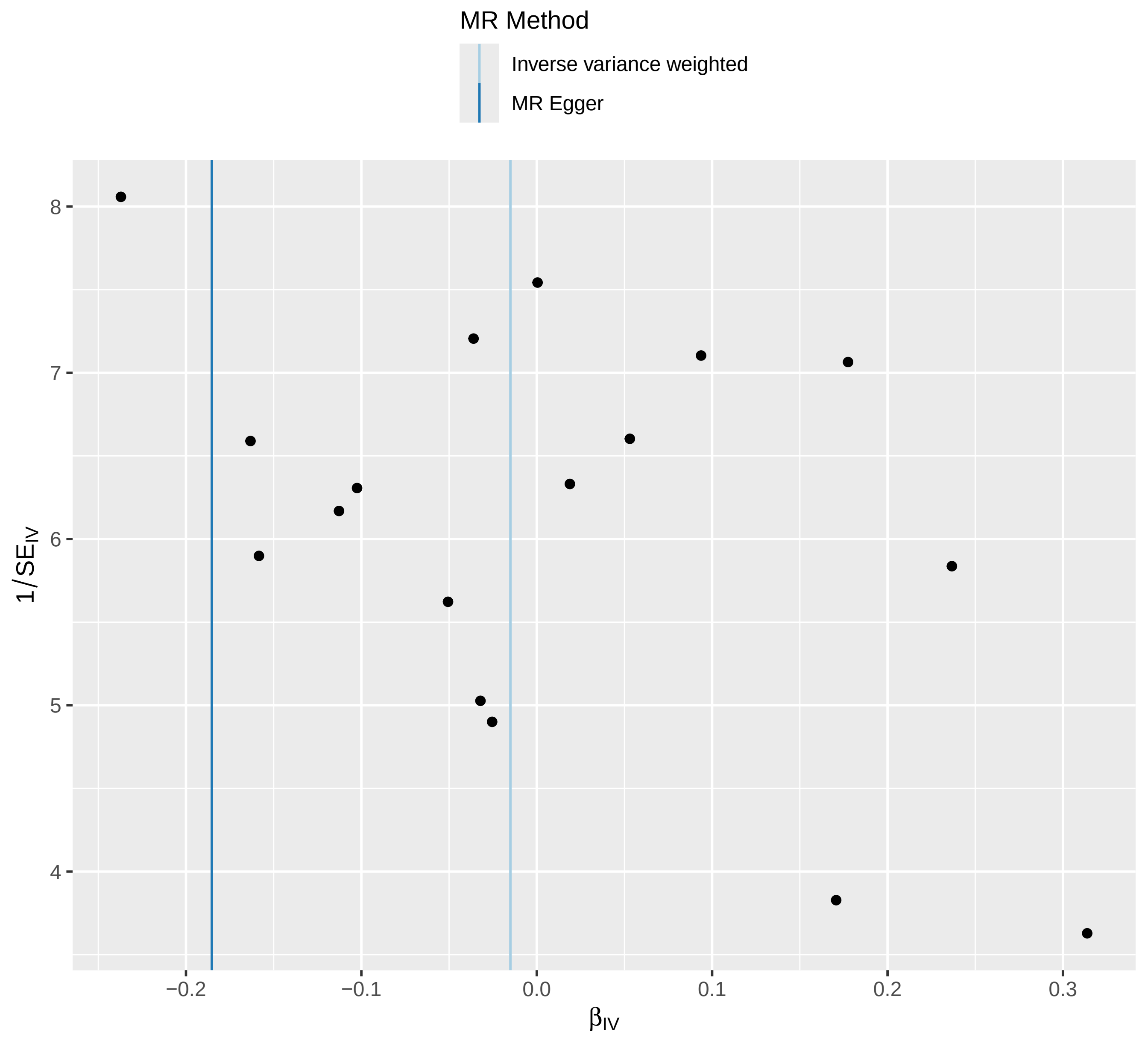
MR Method
Inverse variance weighted
MR Egger
8
7
6
5
4
1/SE_IV
−0.2
−0.1
0.0
0.1
0.2
0.3
β_IV

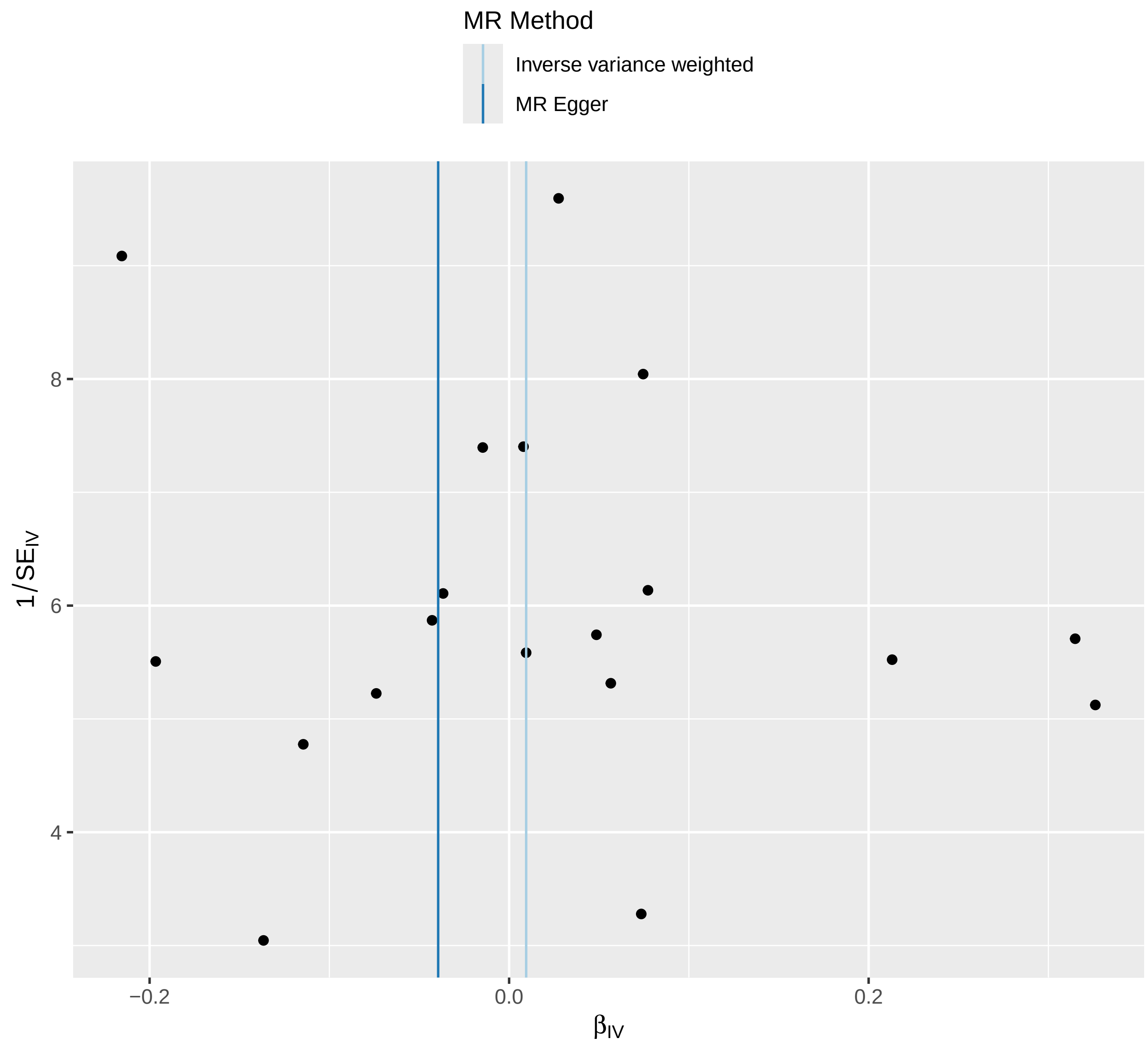
MR Method
Inverse variance weighted
MR Egger
1/SE_IV
8
6
4
−0.2
0.0
0.2
β_IV

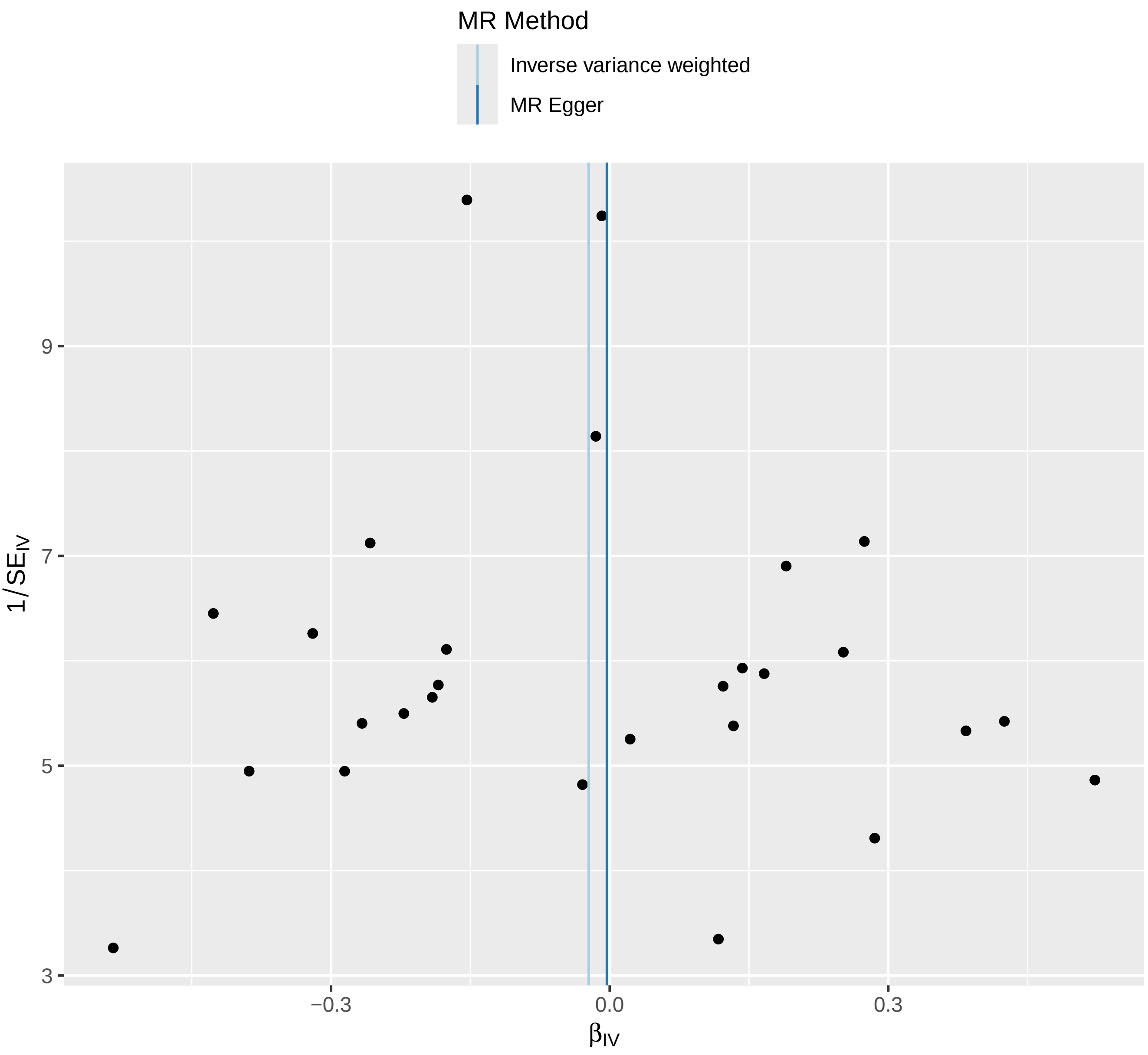
MR Method
Inverse variance weighted
MR Egger
9
7
5
3
−0.3
0.0
0.3
$1/SE_{IV}$
β_{IV}

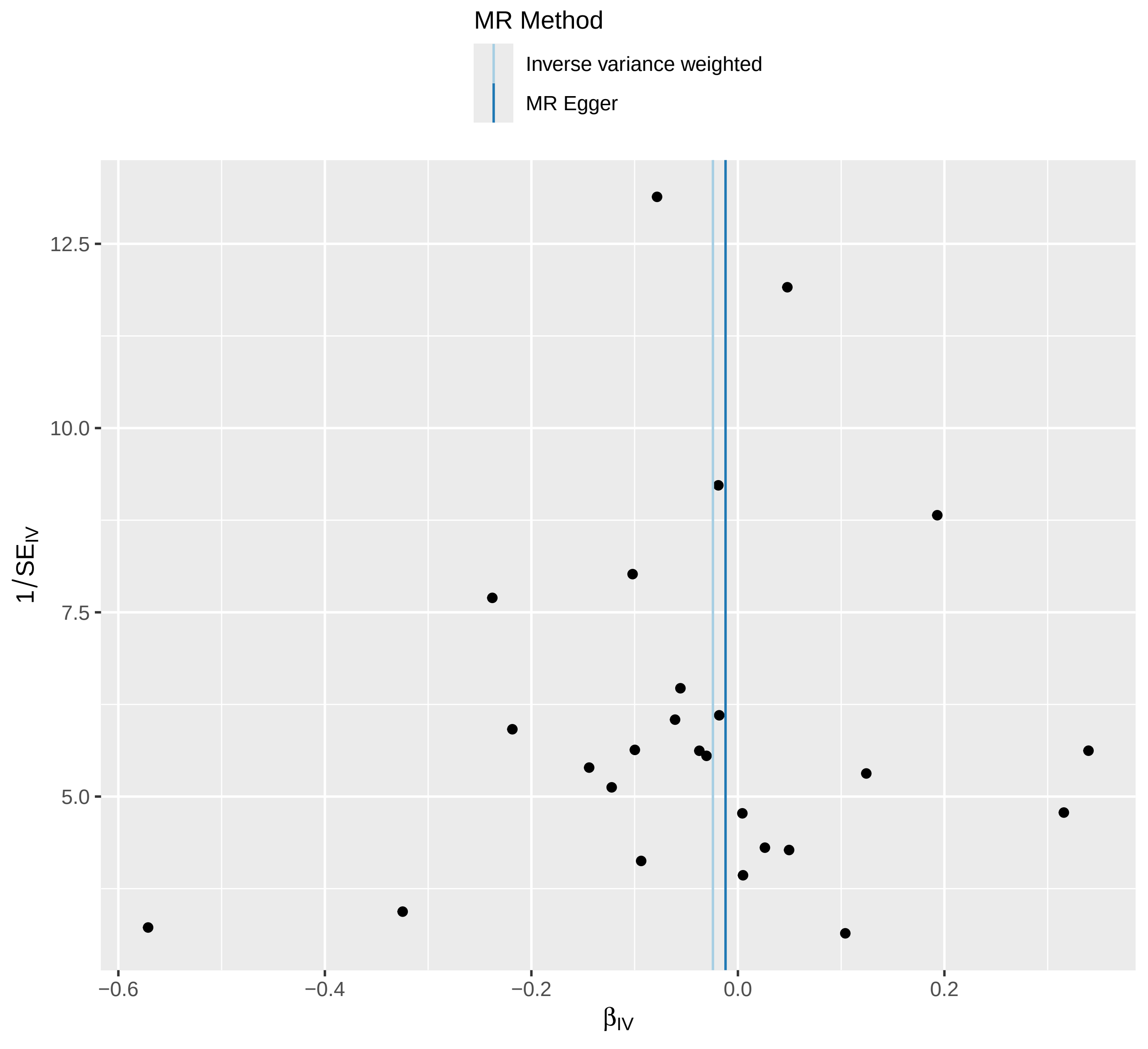
MR Method
Inverse variance weighted
MR Egger
12.5
10.0
7.5
5.0
$1/SE_{IV}$
−0.6
−0.4
−0.2
0.0
0.2
β_{IV}

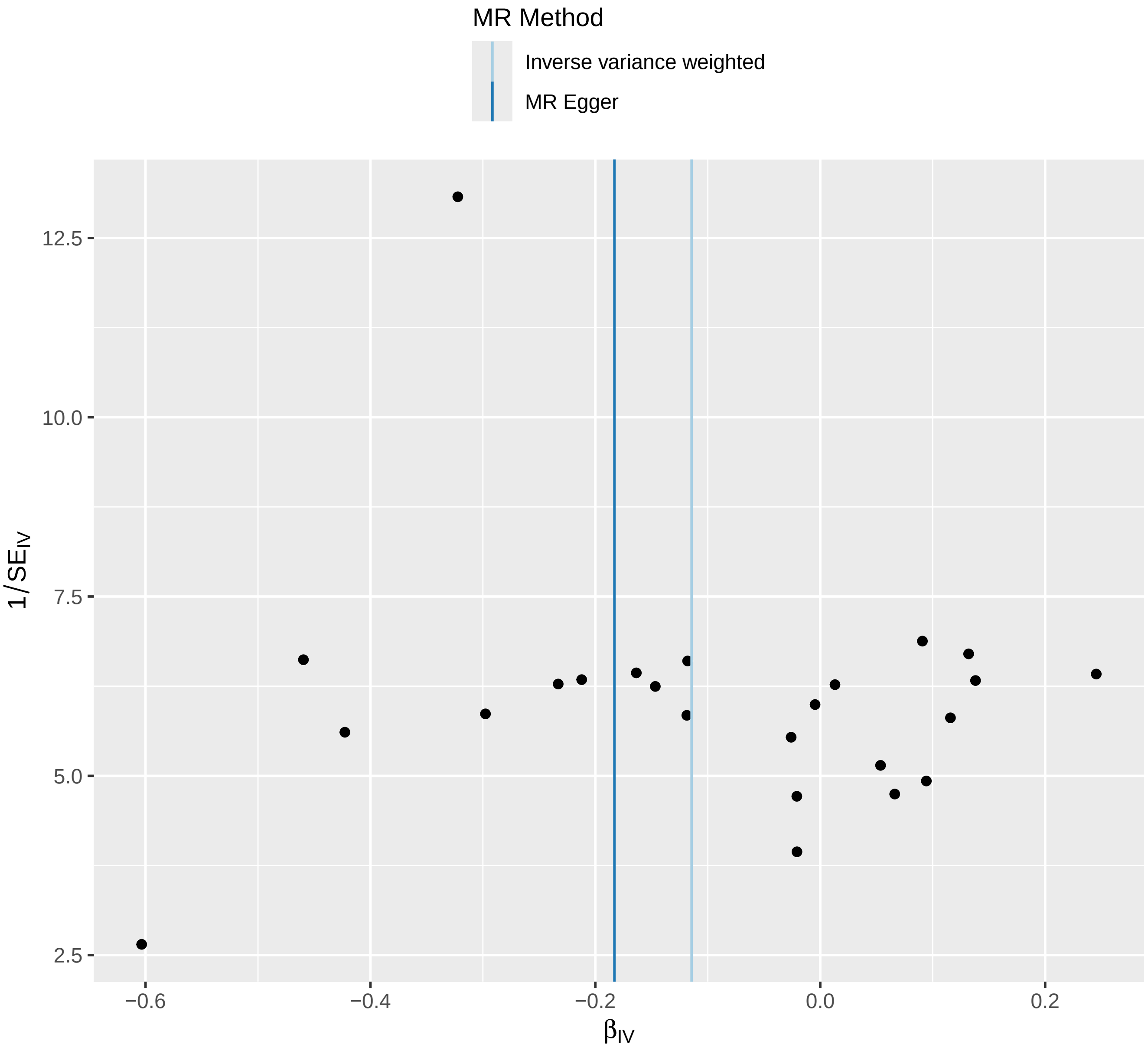

MR Method
Inverse variance weighted
MR Egger
12.5
10.0
7.5
5.0
2.5
1/SE_IV
−0.6
−0.4
−0.2
0.0
0.2
β_IV

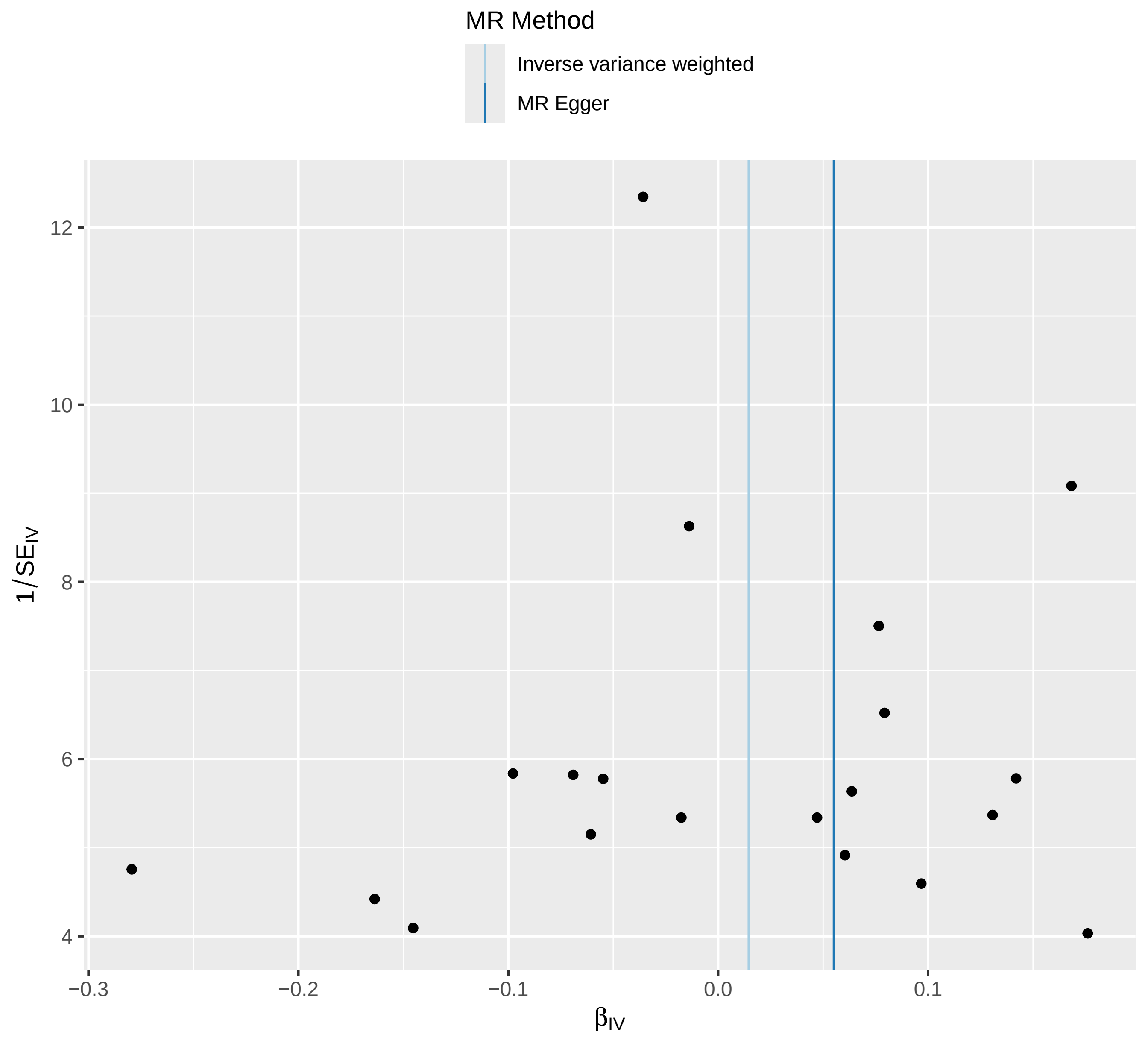

MR Method
Inverse variance weighted
MR Egger
12
10
8
6
4
1/SE_IV
−0.3
−0.2
−0.1
0.0
0.1
β_IV

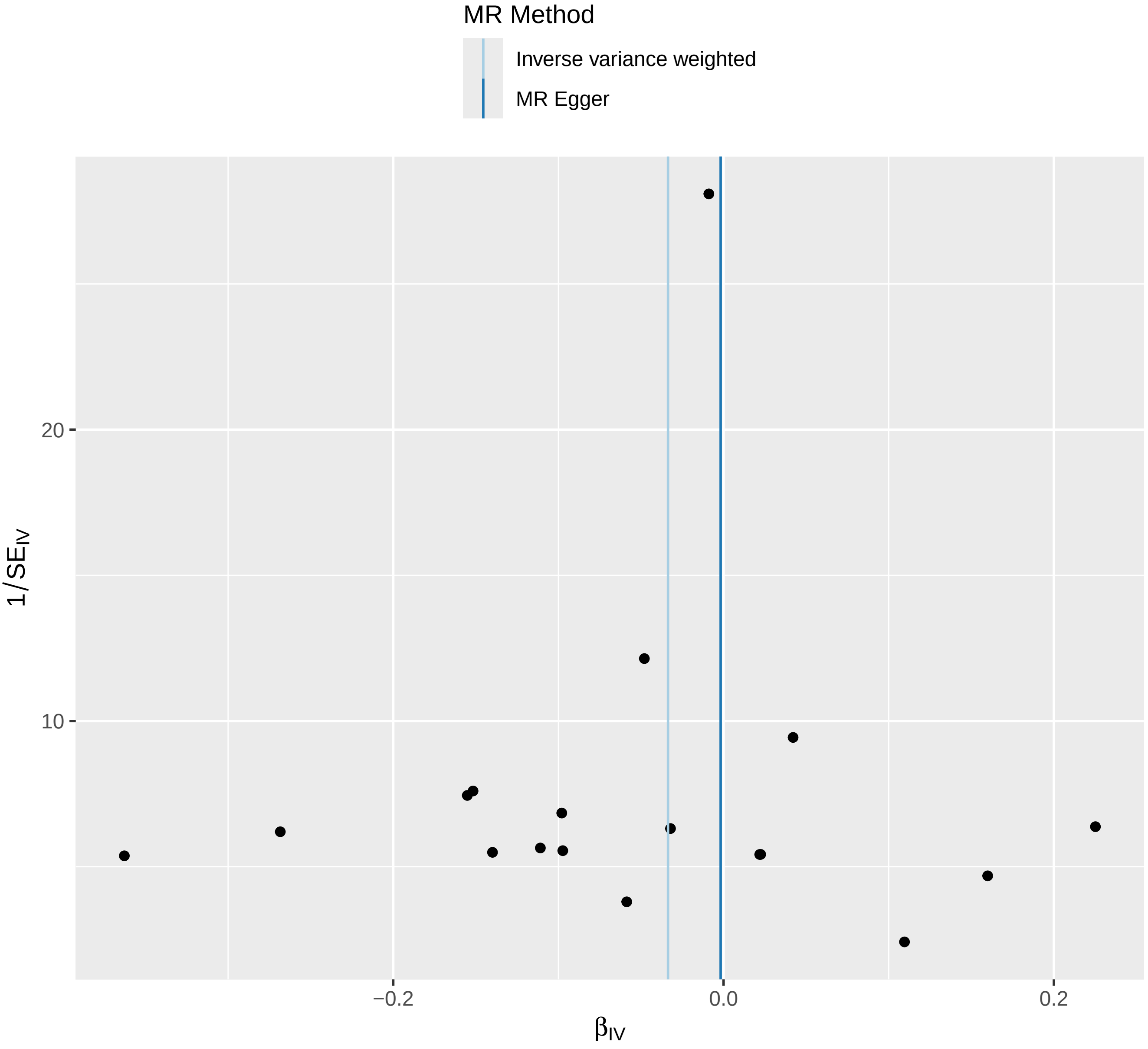
MR Method
Inverse variance weighted
MR Egger
20
10
−0.2
0.0
0.2
βIV
1/SEIV

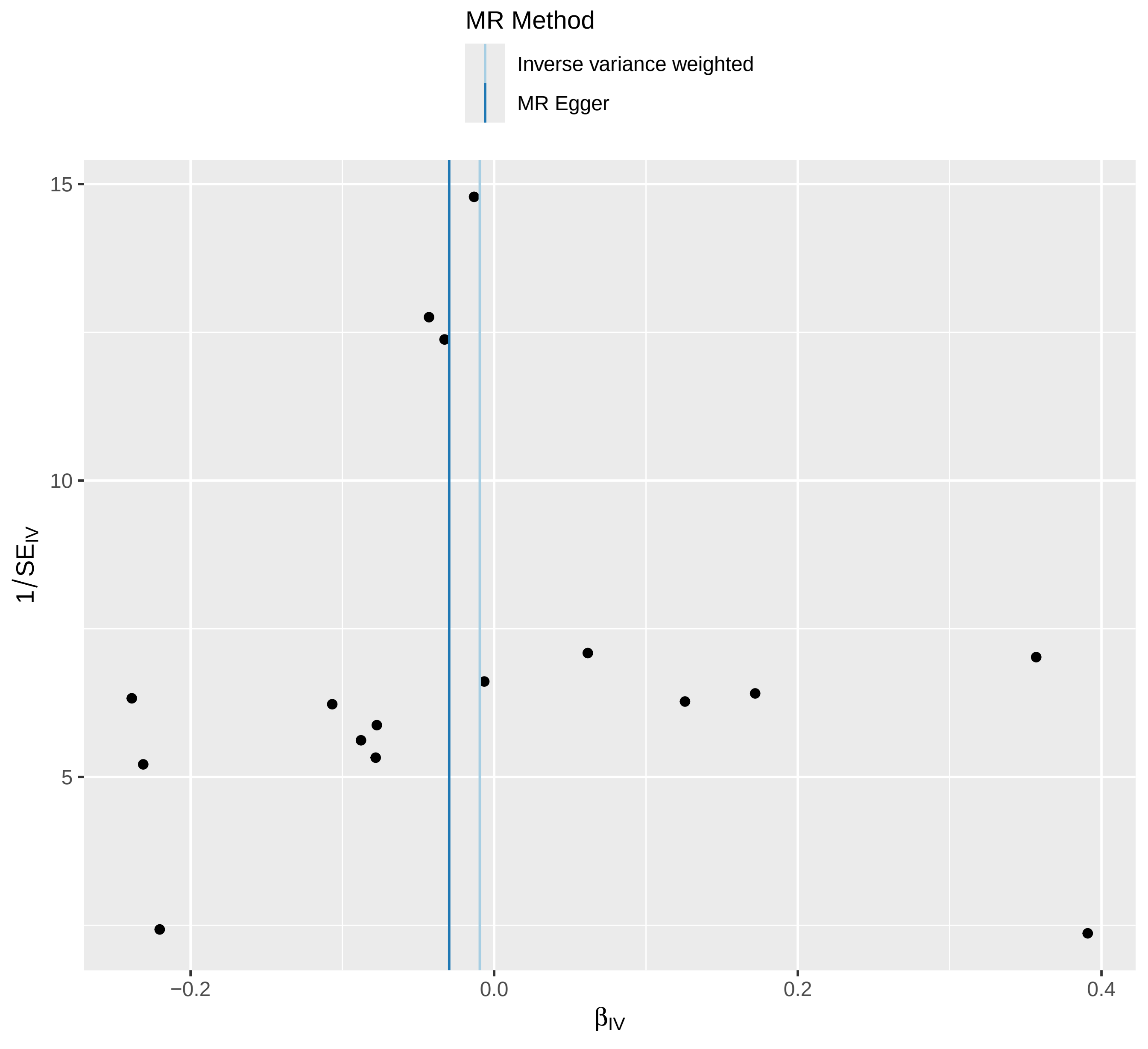

MR Method
Inverse variance weighted
MR Egger
15
10
5
1/SE$_{IV}$
−0.2
0.0
0.2
0.4
β$_{IV}$

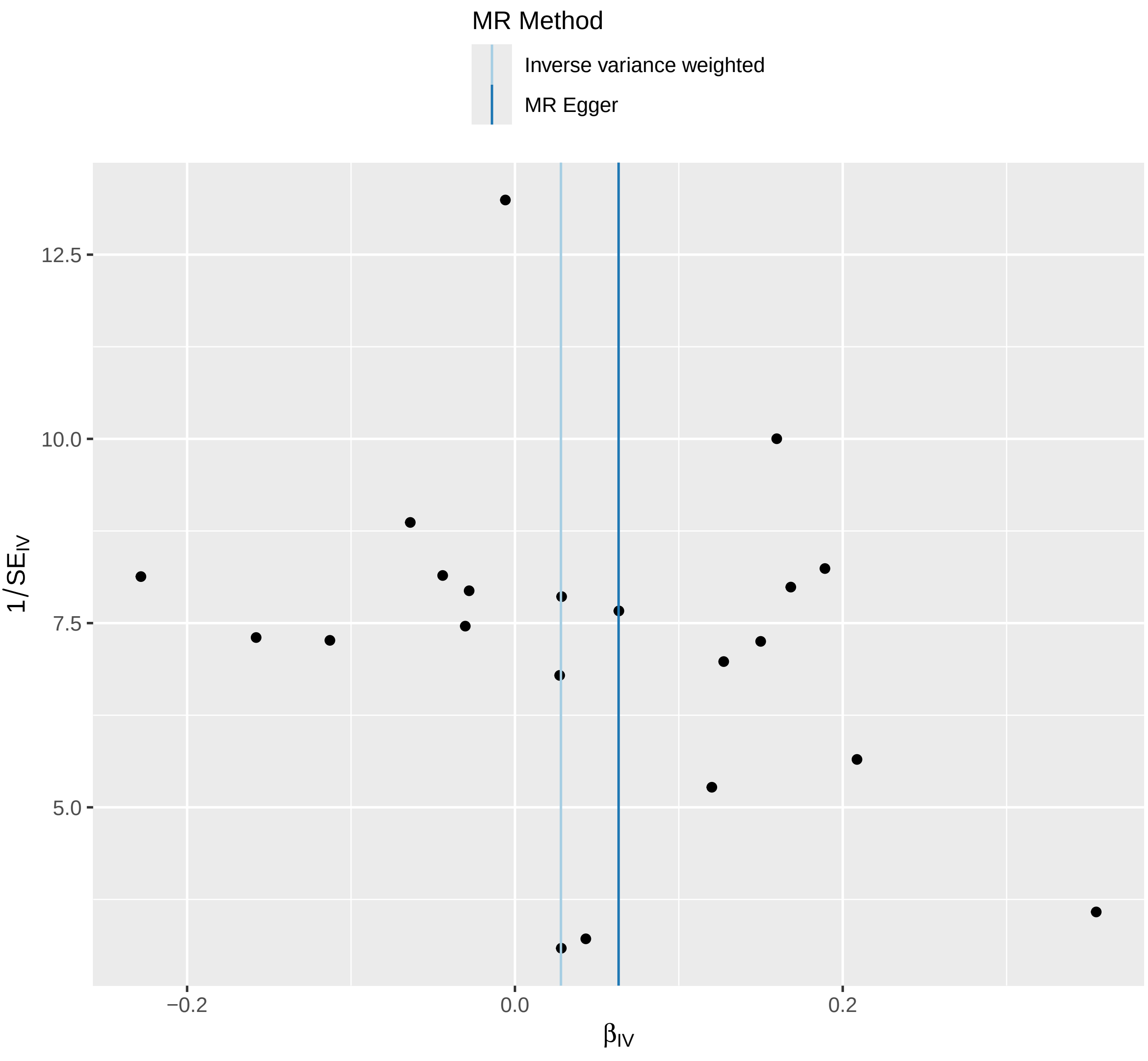
MR Method
Inverse variance weighted
MR Egger
12.5
10.0
7.5
5.0
1/SE_IV
−0.2
0.0
0.2
β_IV

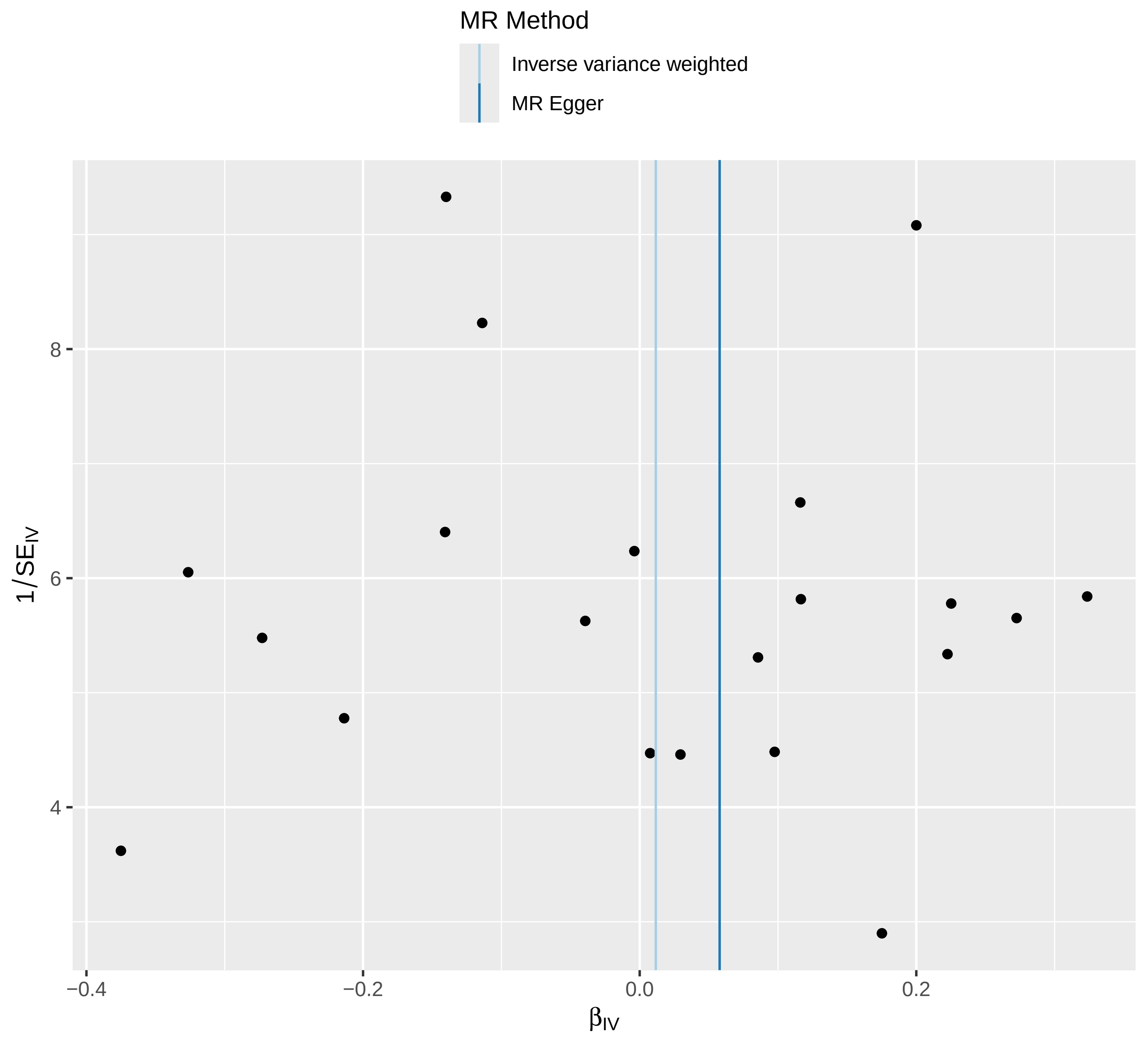

MR Method
Inverse variance weighted
MR Egger
1/SE_IV
8
6
4
−0.4
−0.2
0.0
0.2
β_IV

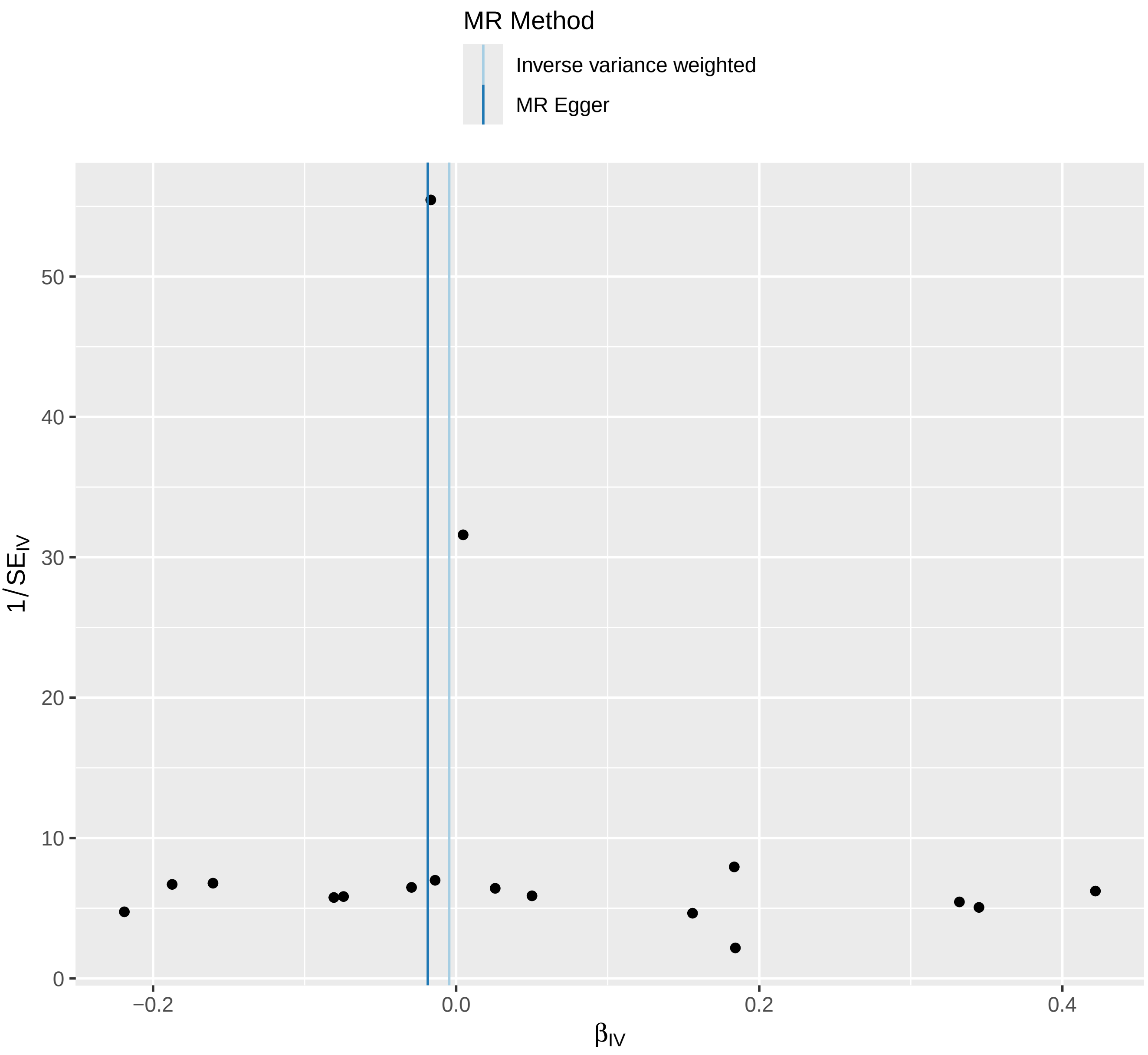
MR Method
Inverse variance weighted
MR Egger
$1/SE_{IV}$
β_{IV}
0
10
20
30
40
50
−0.2
0.0
0.2
0.4

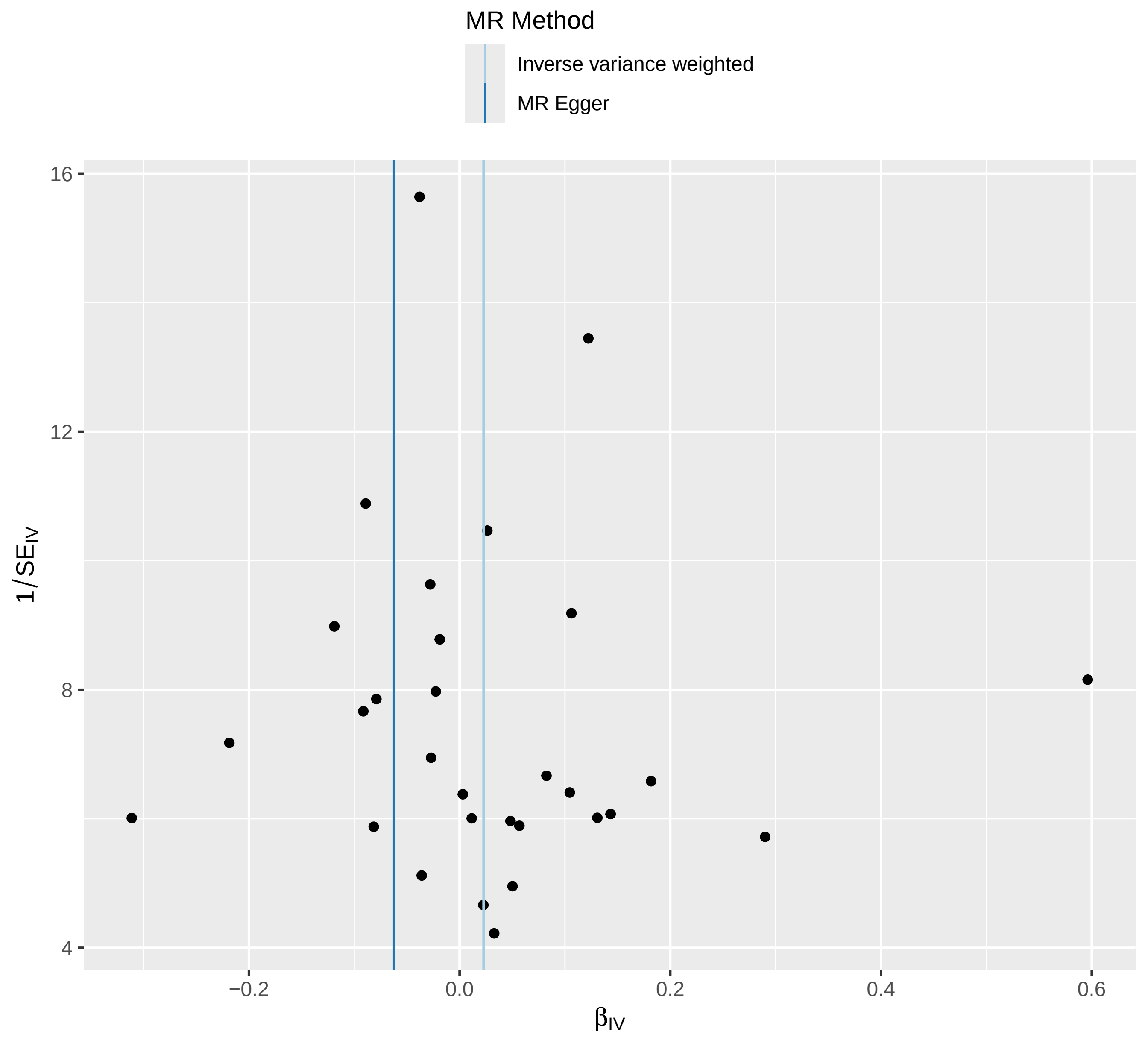
MR Method
Inverse variance weighted
MR Egger
16
12
8
4
$1/SE_{IV}$
−0.2
0.0
0.2
0.4
0.6
β_{IV}

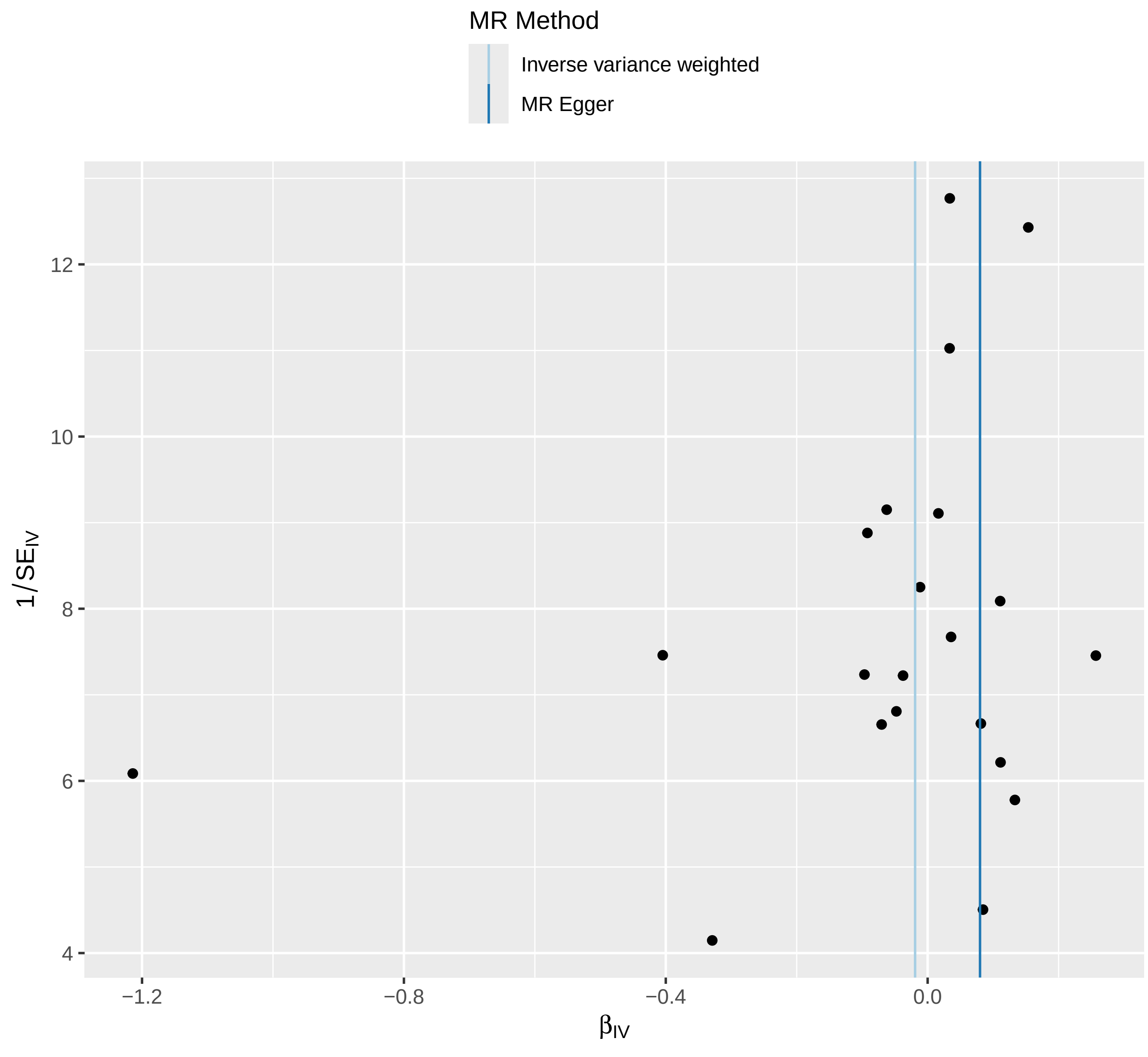
MR Method
Inverse variance weighted
MR Egger
1/SE$_{IV}$
12
10
8
6
4
−1.2
−0.8
−0.4
0.0
β$_{IV}$

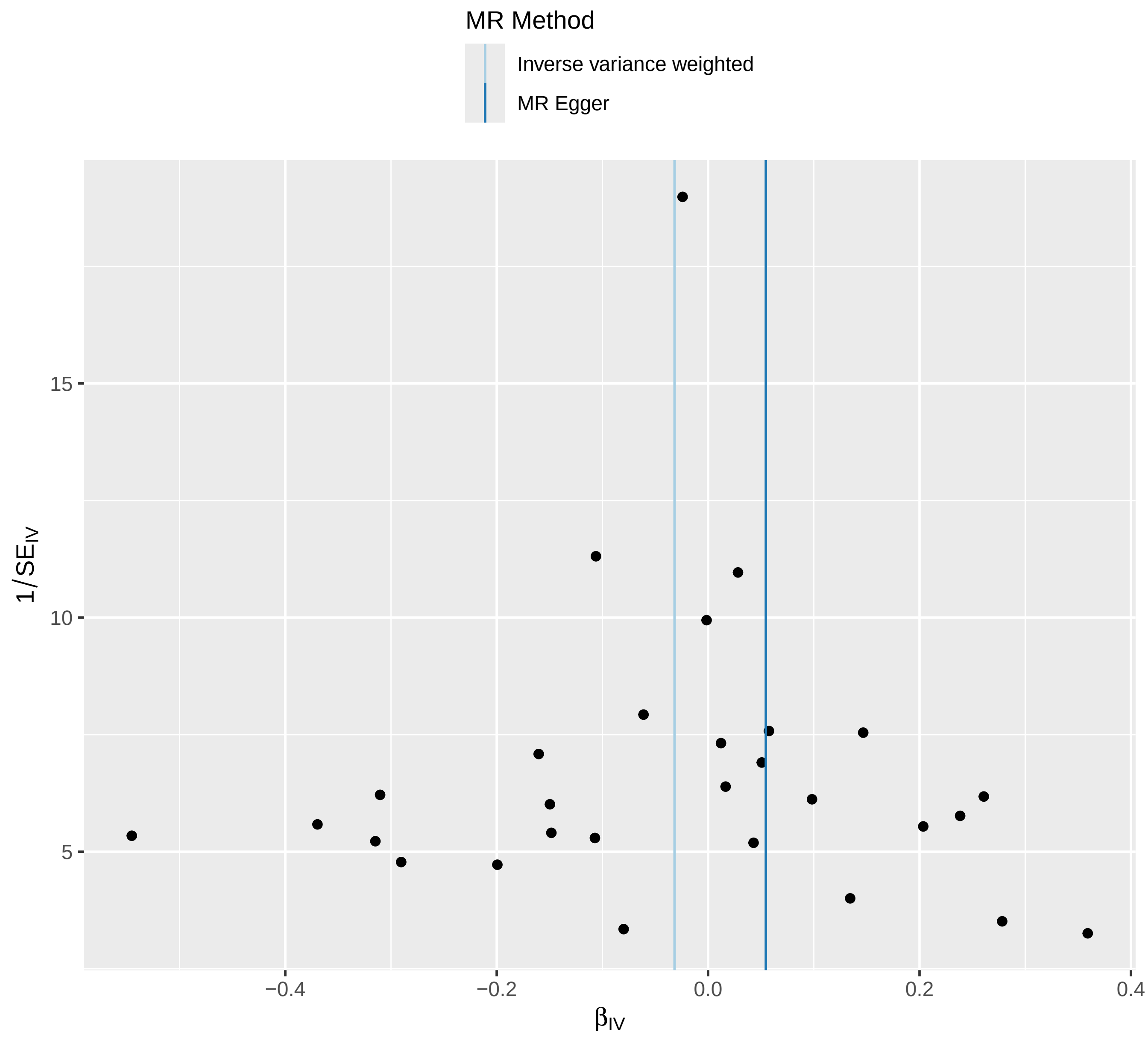
MR Method
Inverse variance weighted
MR Egger
1/SE_IV
15
10
5
−0.4
−0.2
0.0
0.2
0.4
β_IV

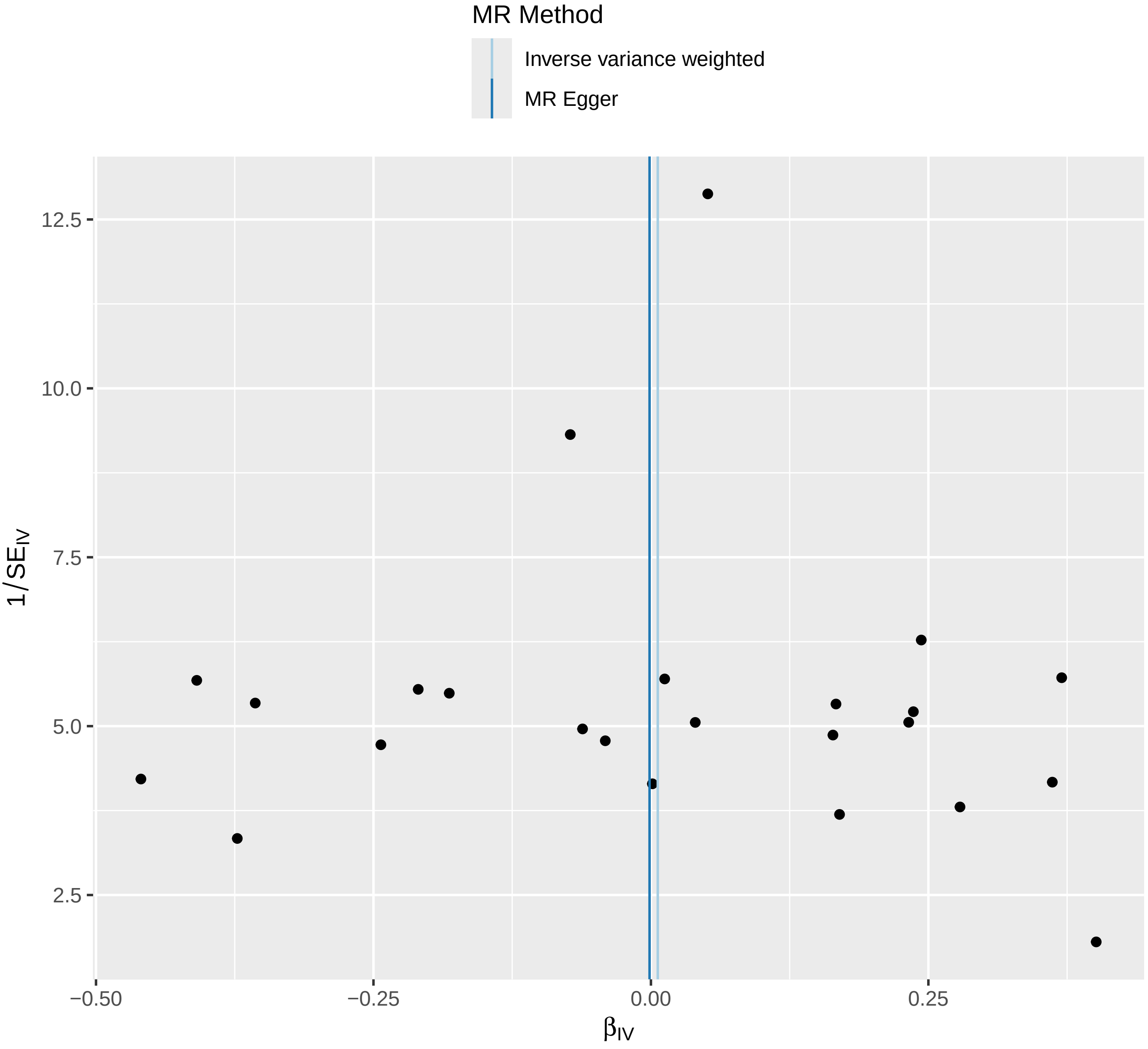

MR Method
Inverse variance weighted
MR Egger
12.5
10.0
7.5
5.0
2.5
1/SE_IV
−0.50
−0.25
0.00
0.25
β_IV

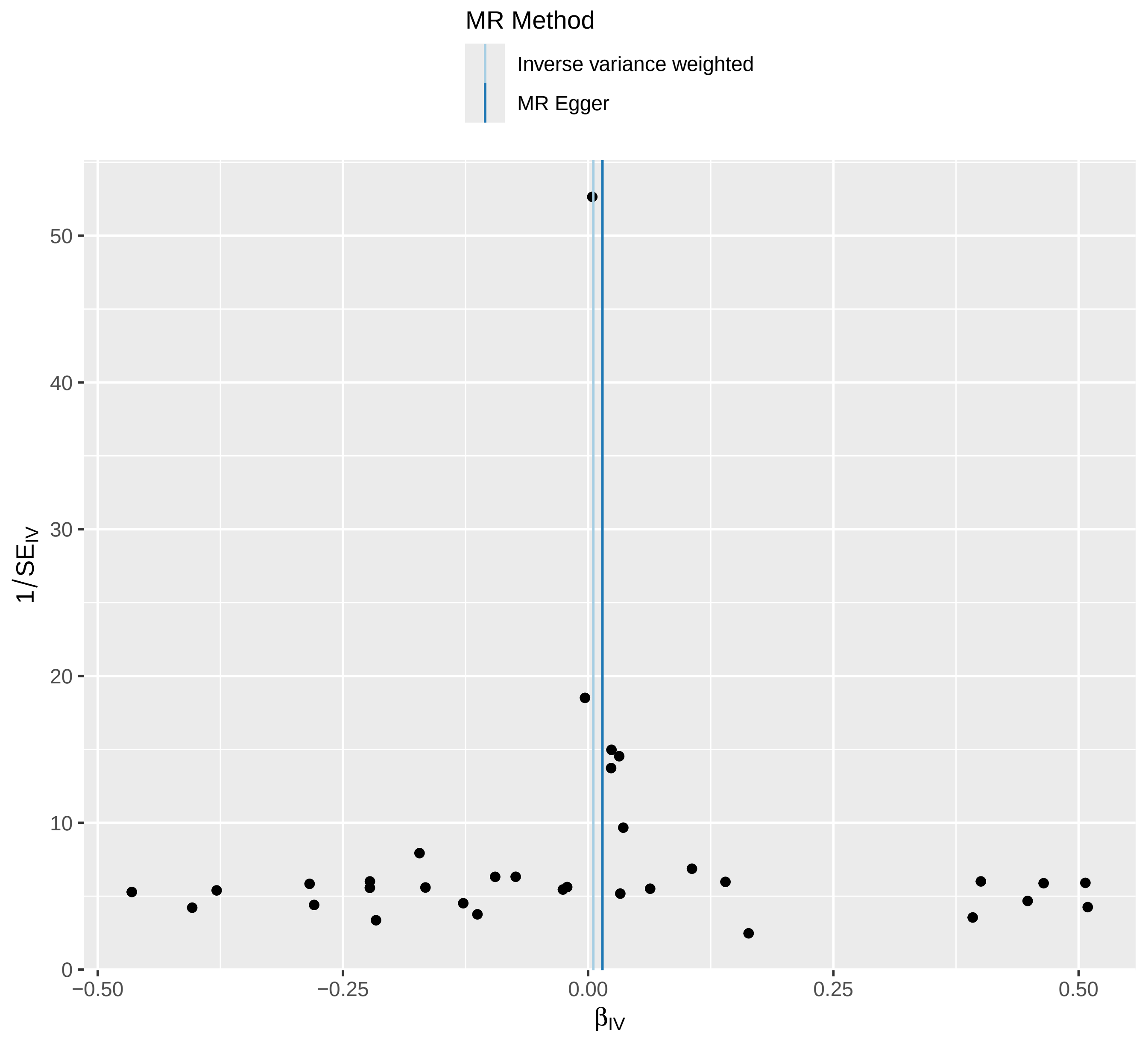
MR Method
Inverse variance weighted
MR Egger
1/SE_IV
β_IV
50
40
30
20
10
0
−0.50
−0.25
0.00
0.25
0.50

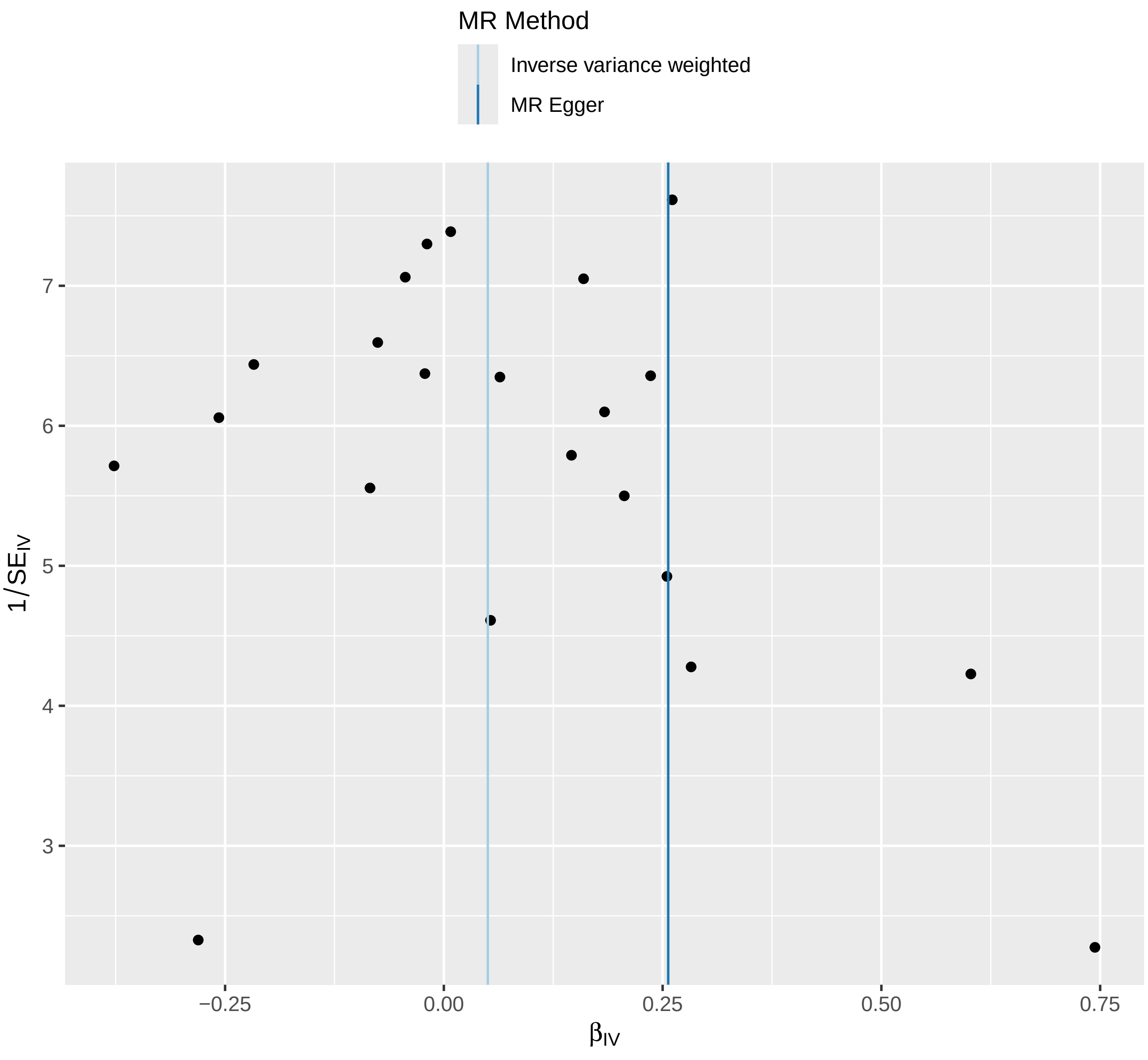
MR Method
Inverse variance weighted
MR Egger
1/SE_IV
β_IV
7
6
5
4
3
−0.25
0.00
0.25
0.50
0.75

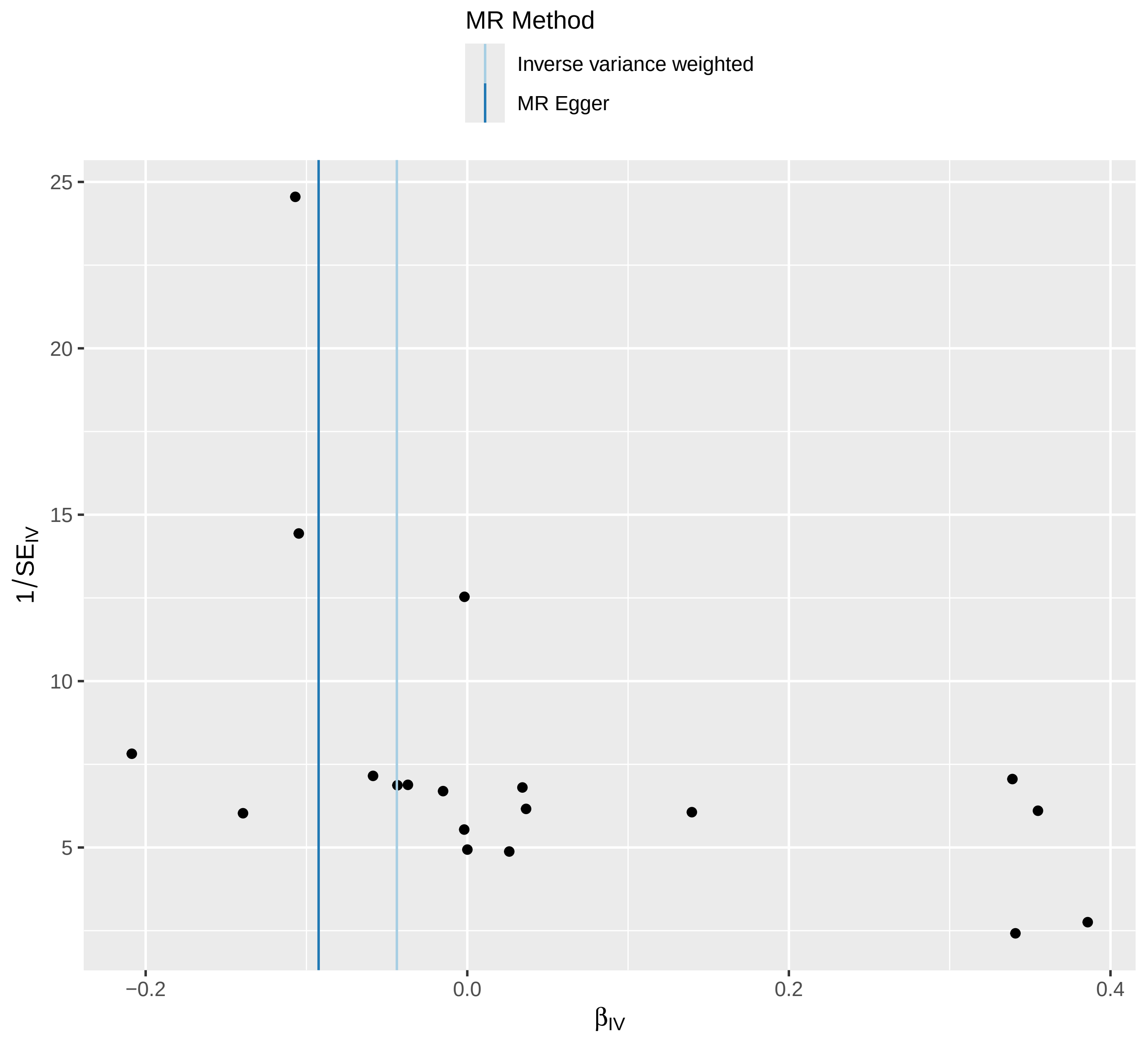
MR Method
Inverse variance weighted
MR Egger
25
20
15
10
5
1/SE_IV
−0.2
0.0
0.2
0.4
β_IV

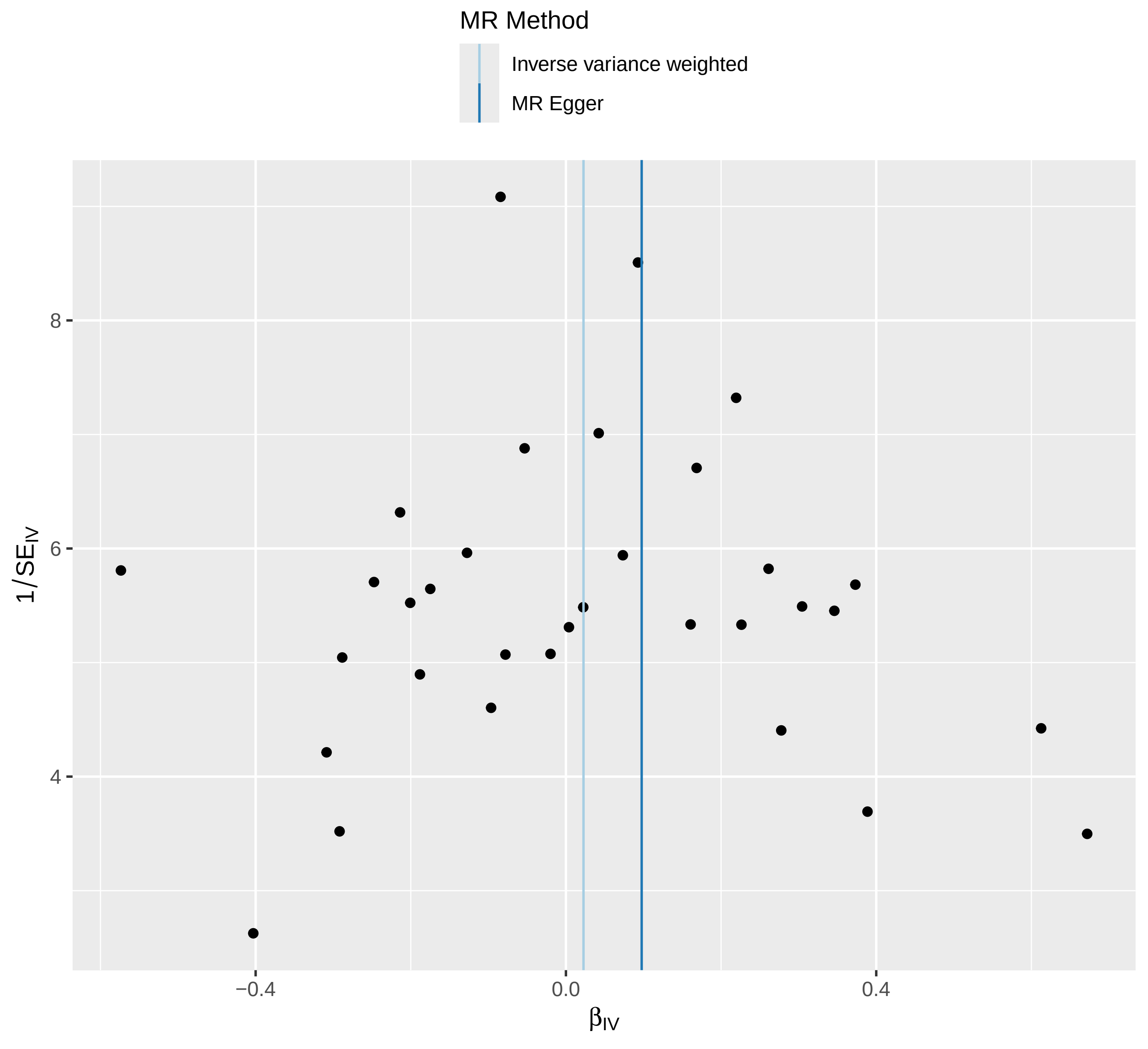
MR Method
Inverse variance weighted
MR Egger
8
6
4
1/SE$_{IV}$
−0.4
0.0
0.4
β$_{IV}$

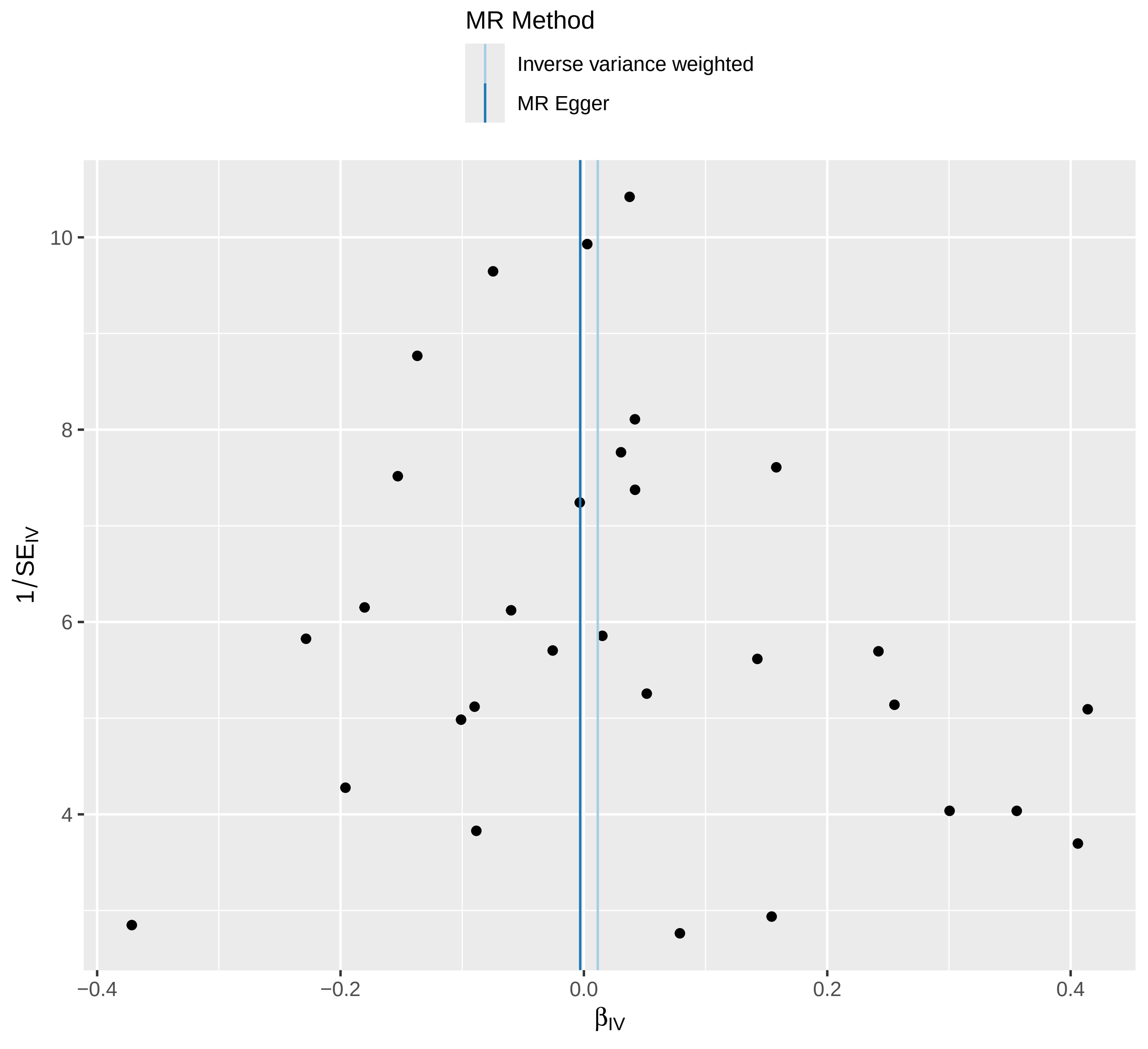
MR Method
Inverse variance weighted
MR Egger
1/SE_IV
β_IV
10
8
6
4
−0.4
−0.2
0.0
0.2
0.4

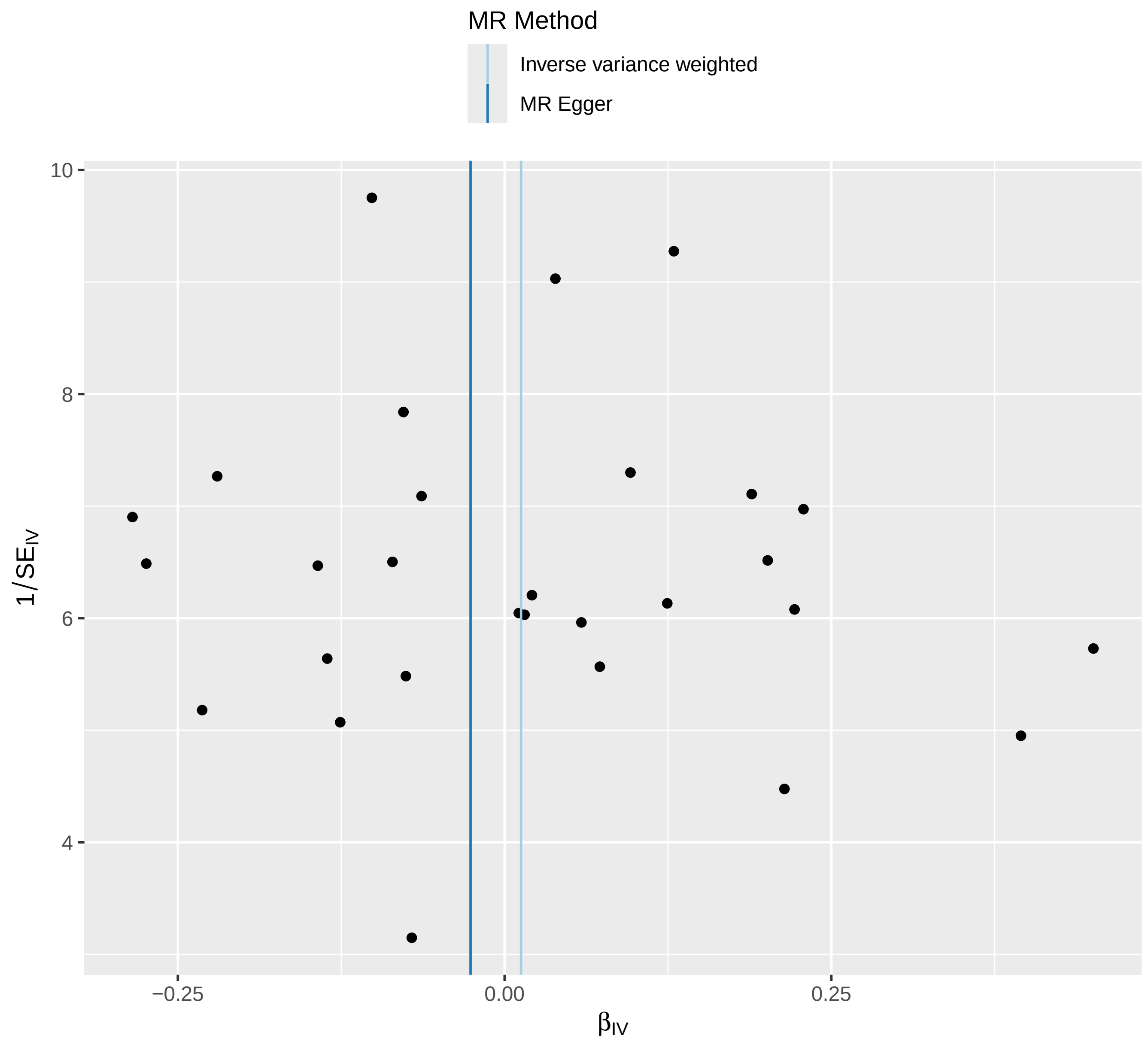
MR Method
Inverse variance weighted
MR Egger
10
8
6
4
1/SE_IV
−0.25
0.00
0.25
β_IV

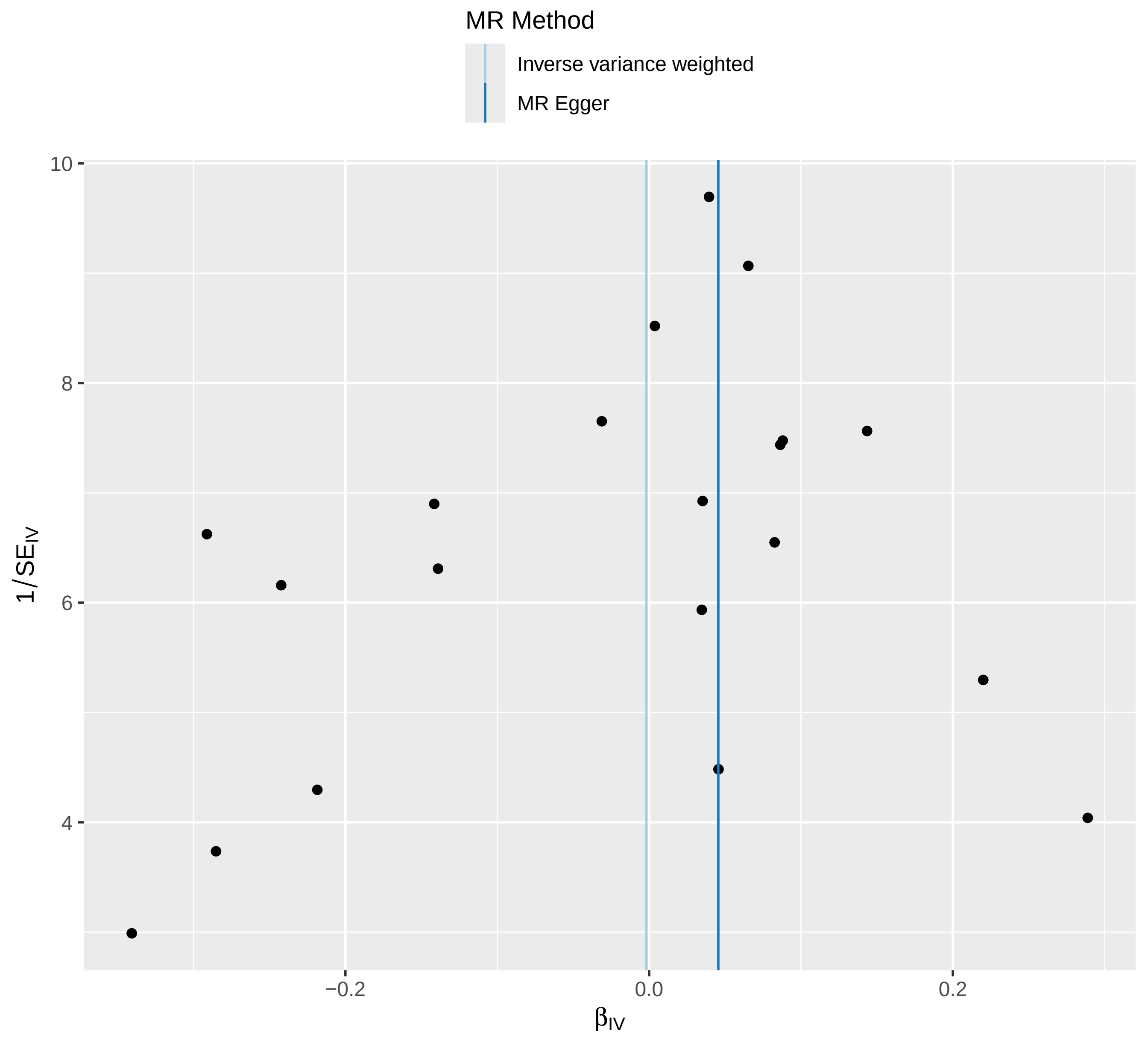

MR Method
Inverse variance weighted
MR Egger
10
8
6
4
1/SE$_{IV}$
−0.2
0.0
0.2
β$_{IV}$

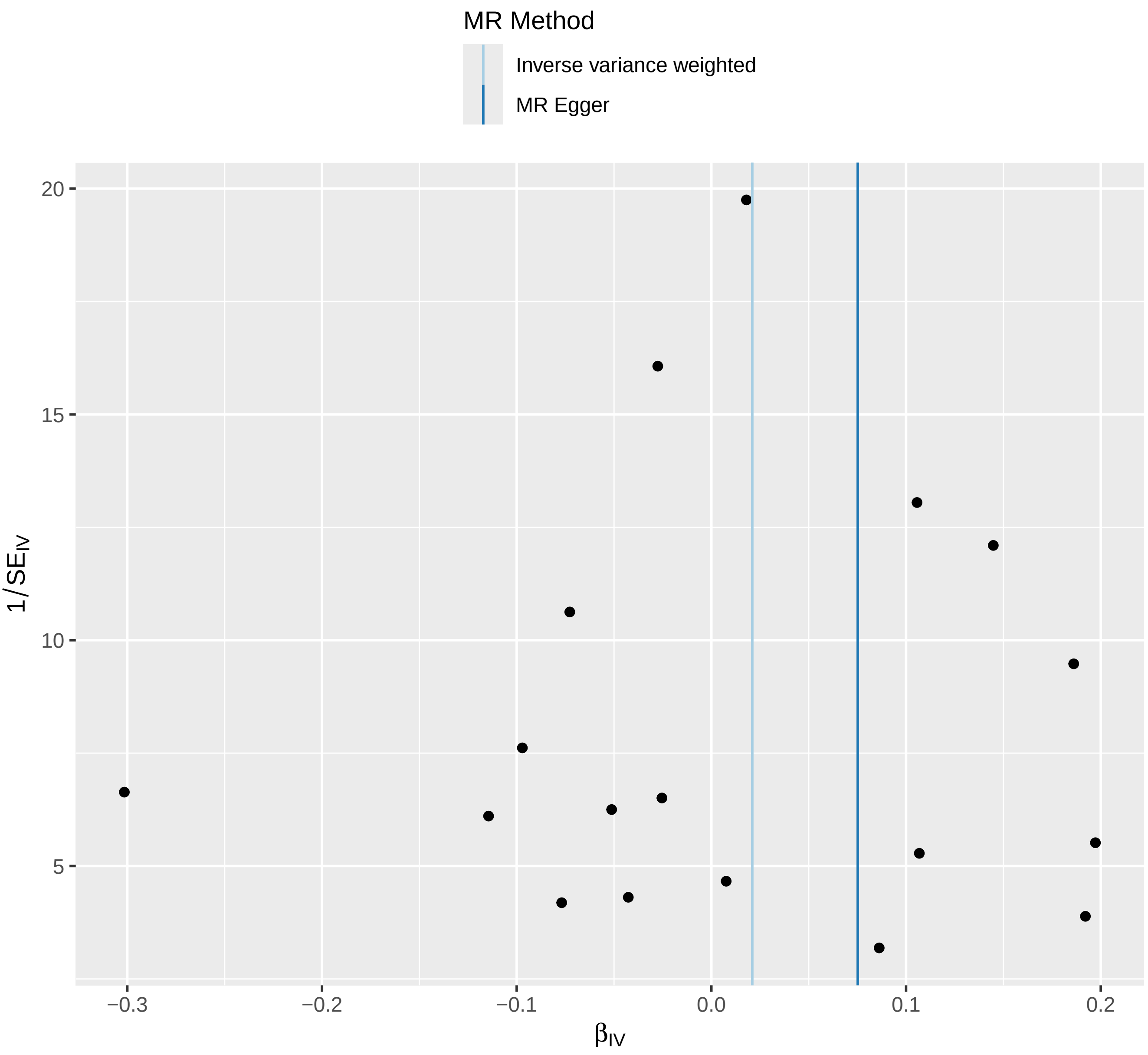
MR Method
Inverse variance weighted
MR Egger
20
15
10
5
1/SE_IV
−0.3
−0.2
−0.1
0.0
0.1
0.2
β_IV

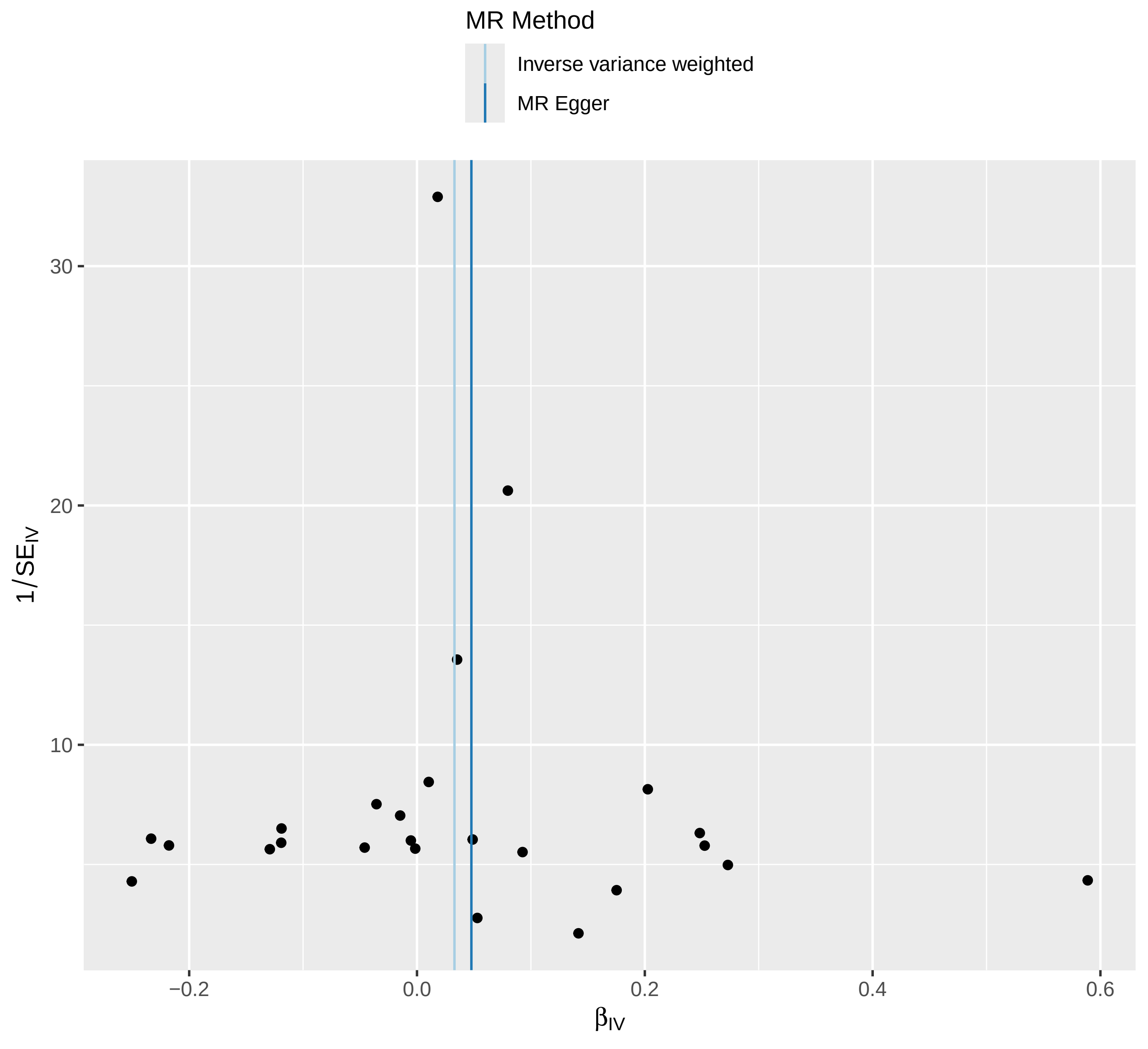
MR Method
Inverse variance weighted
MR Egger
30
20
10
1/SE_IV
−0.2
0.0
0.2
0.4
0.6
β_IV

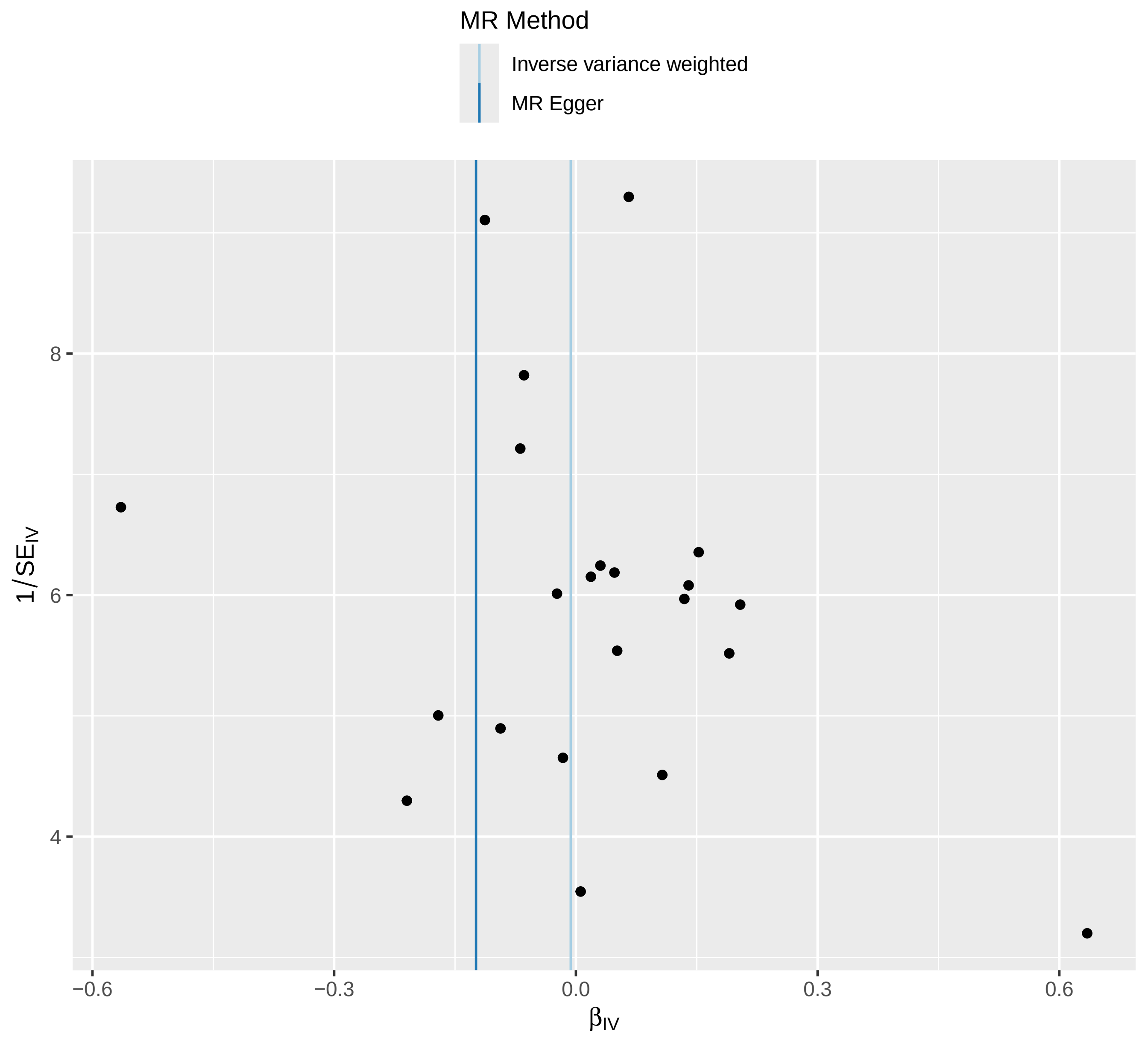
MR Method
Inverse variance weighted
MR Egger
8
6
4
1/SE_IV
−0.6
−0.3
0.0
0.3
0.6
β_IV

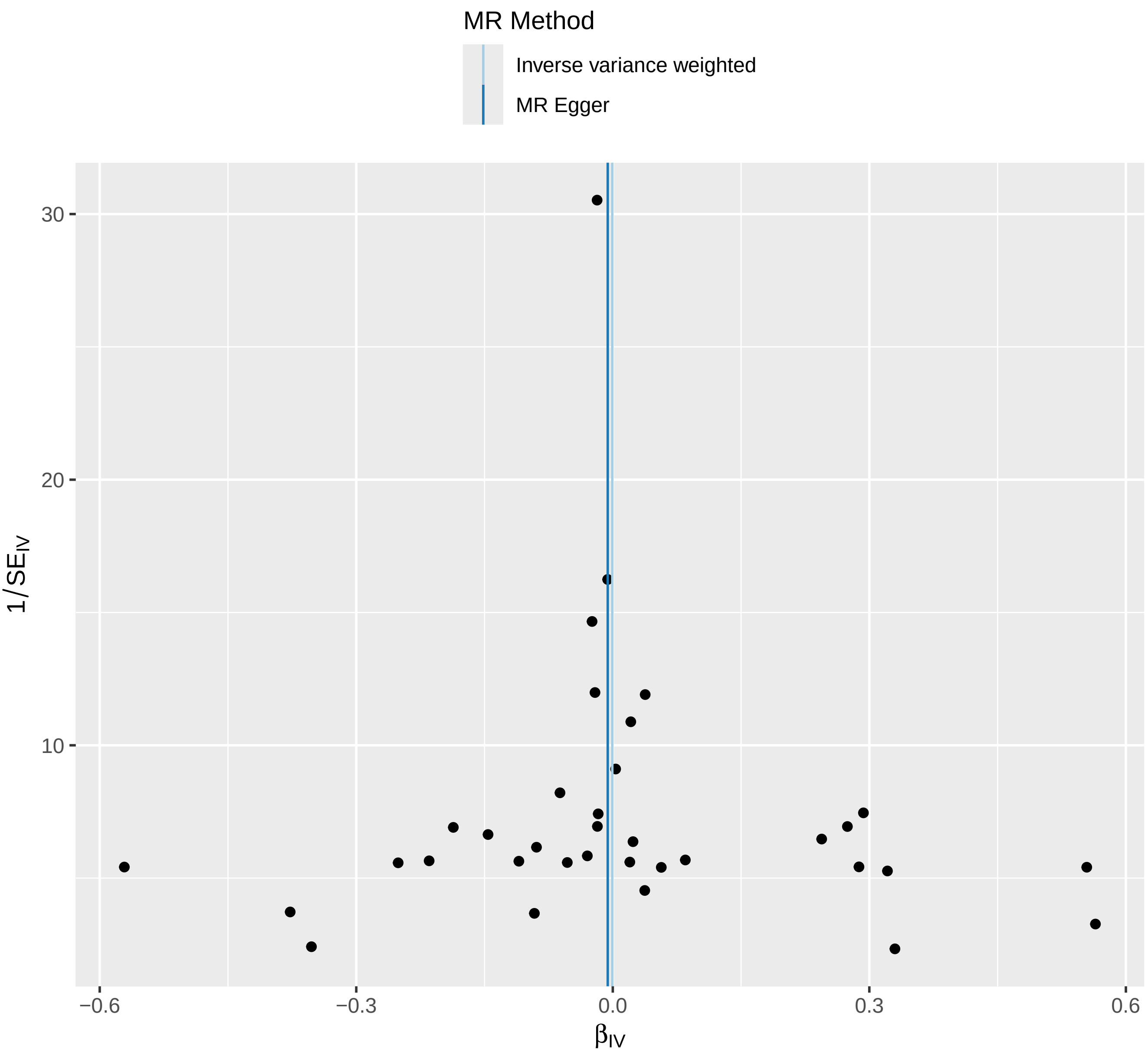
MR Method
Inverse variance weighted
MR Egger
30
20
10
1/SE_{IV}
−0.6
−0.3
0.0
0.3
0.6
β_{IV}

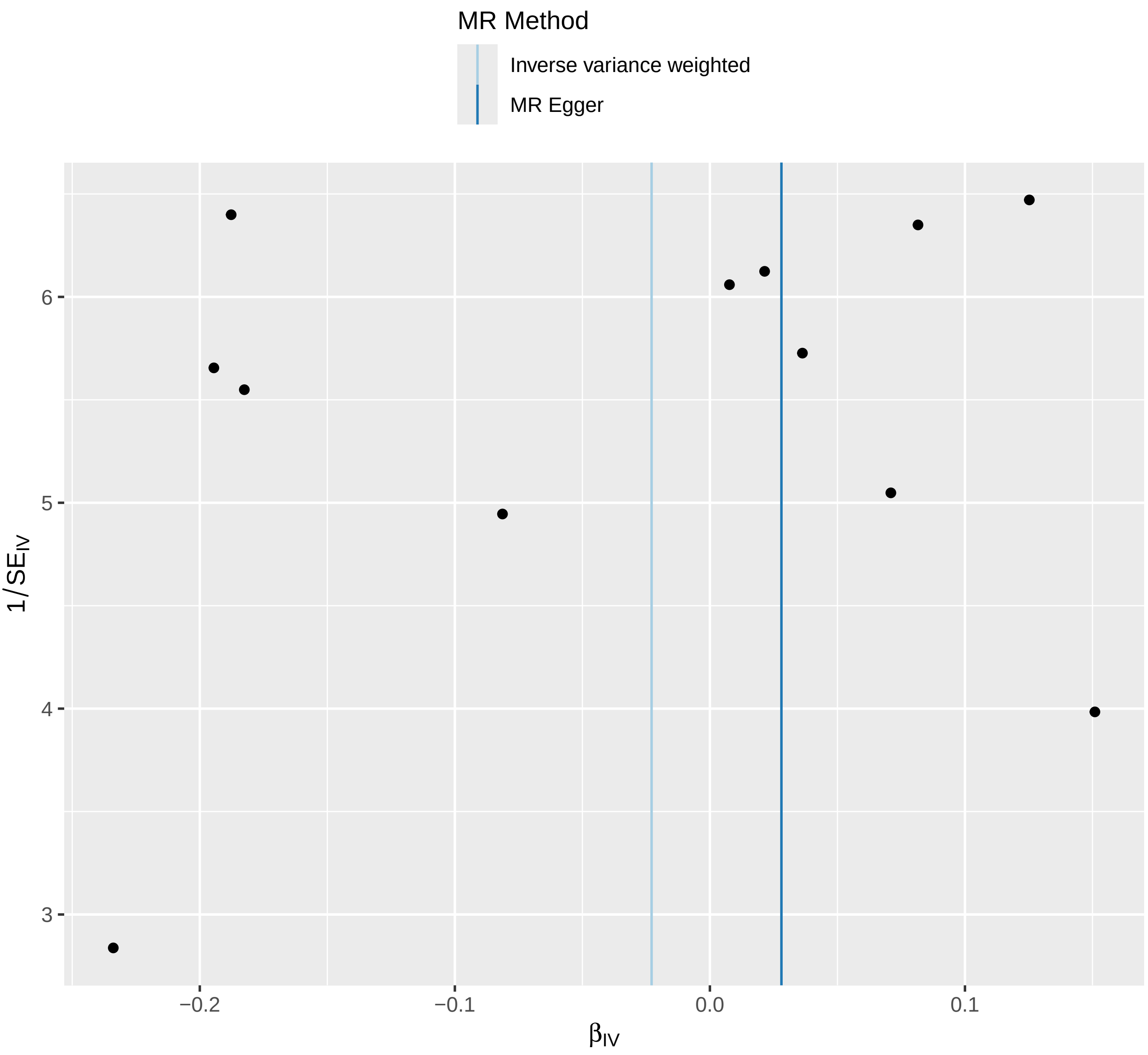

MR Method
Inverse variance weighted
MR Egger
1/SE_IV
6
5
4
3
−0.2
−0.1
0.0
0.1
β_IV

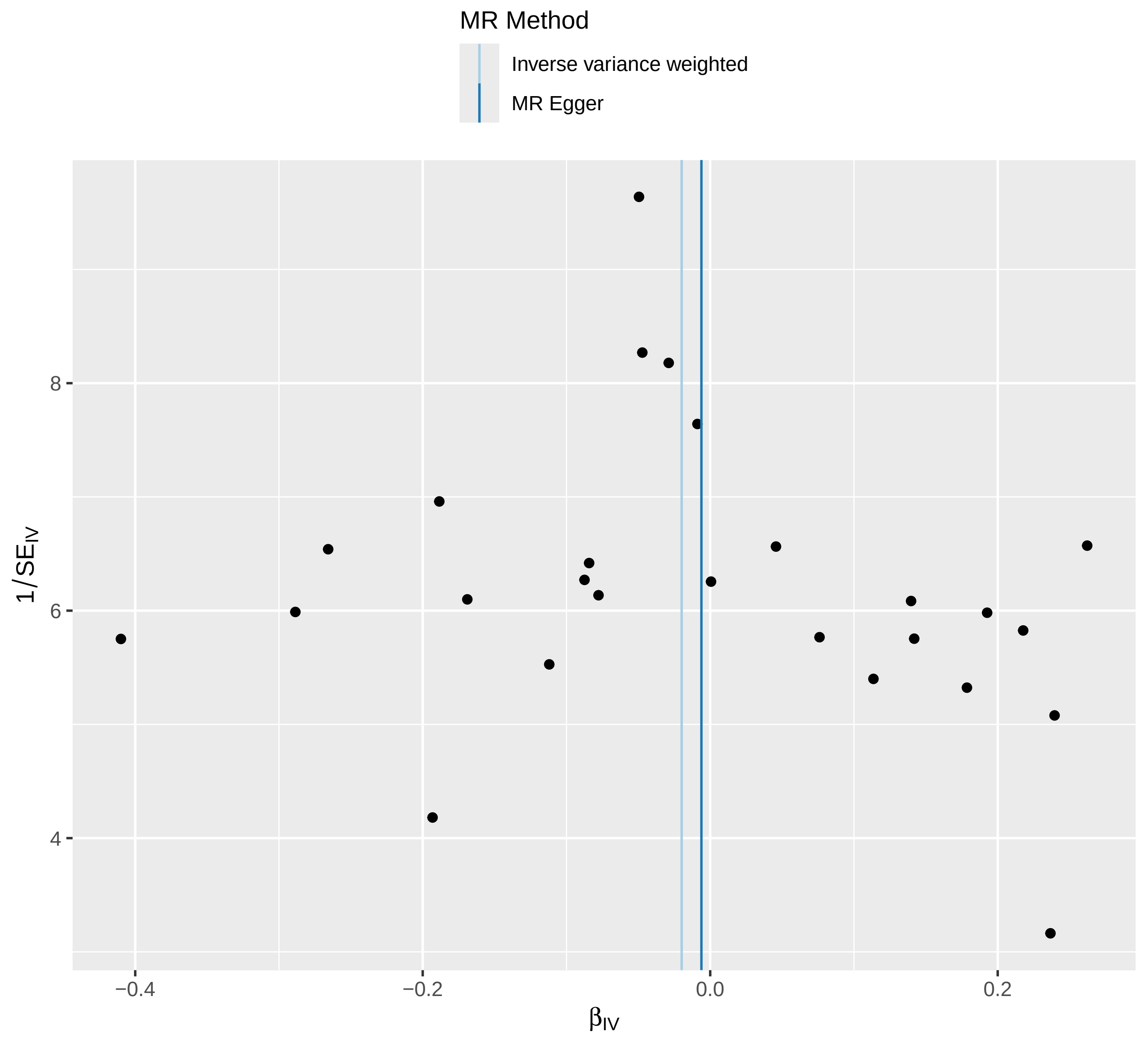
MR Method
Inverse variance weighted
MR Egger
1/SE_IV
8
6
4
−0.4
−0.2
0.0
0.2
β_IV

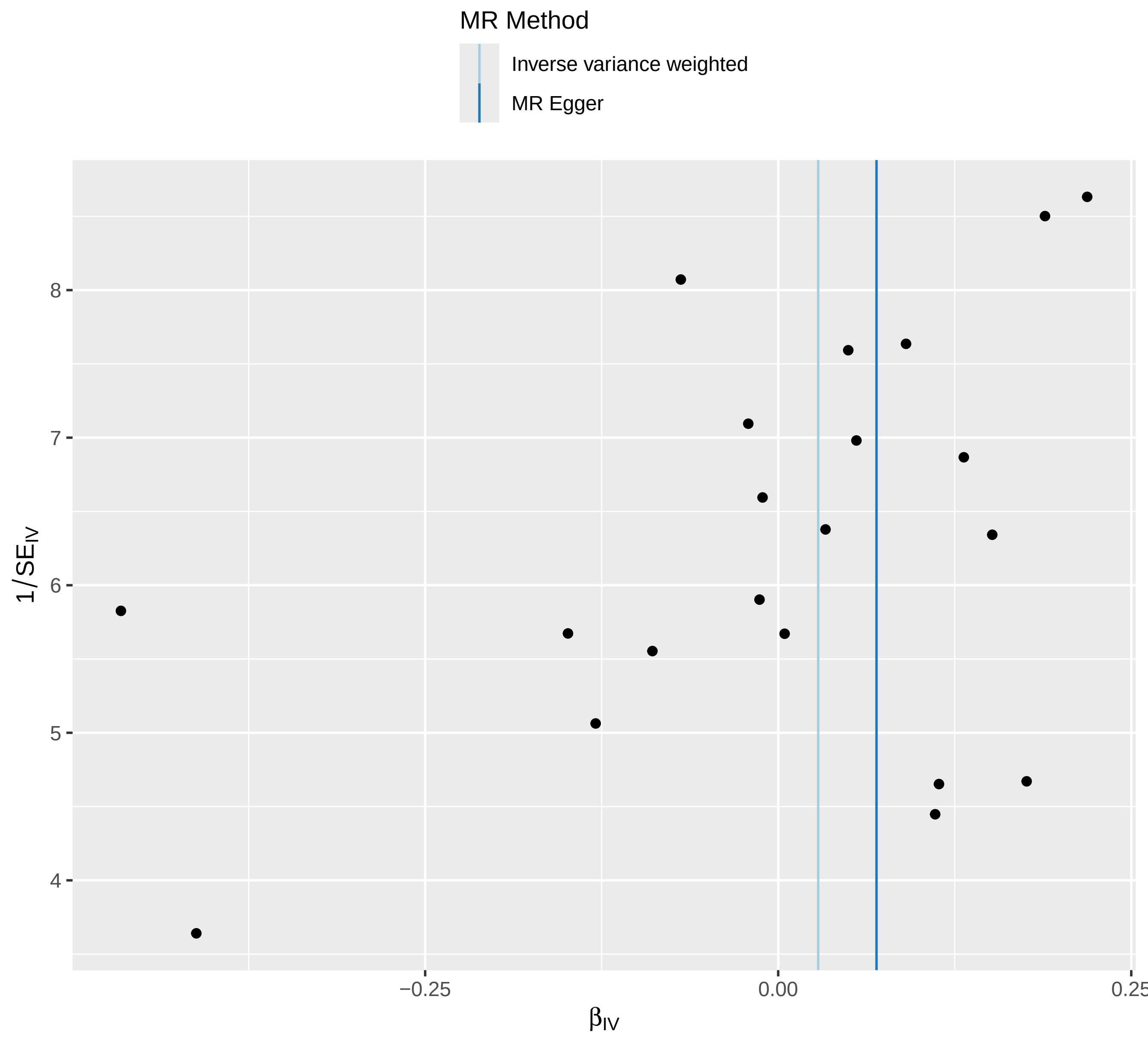

MR Method
Inverse variance weighted
MR Egger
8
7
6
5
4
$1/SE_{IV}$
−0.25
0.00
0.25
β_{IV}

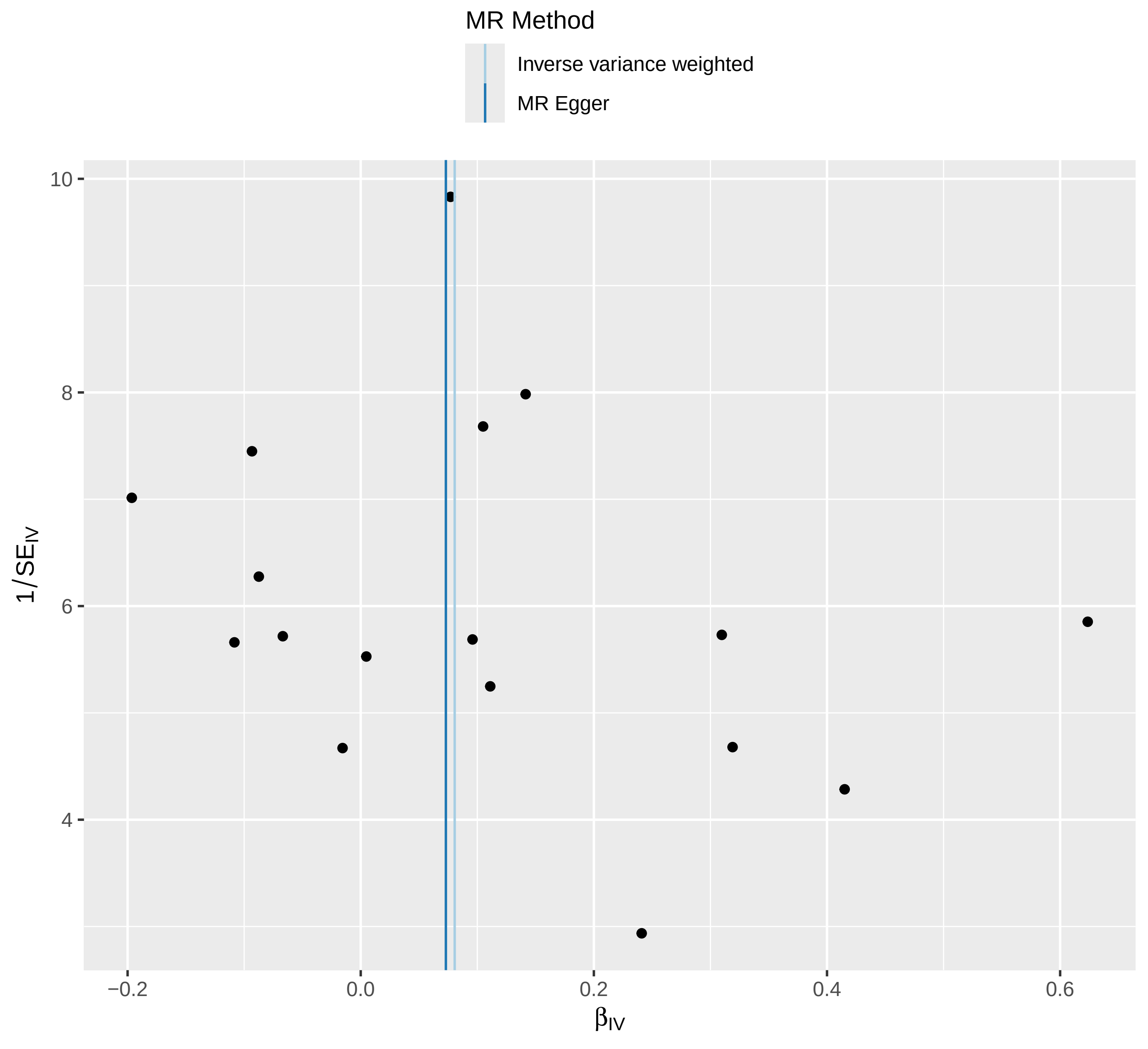
MR Method
Inverse variance weighted
MR Egger
10
8
6
4
1/SE_IV
−0.2
0.0
0.2
0.4
0.6
β_IV

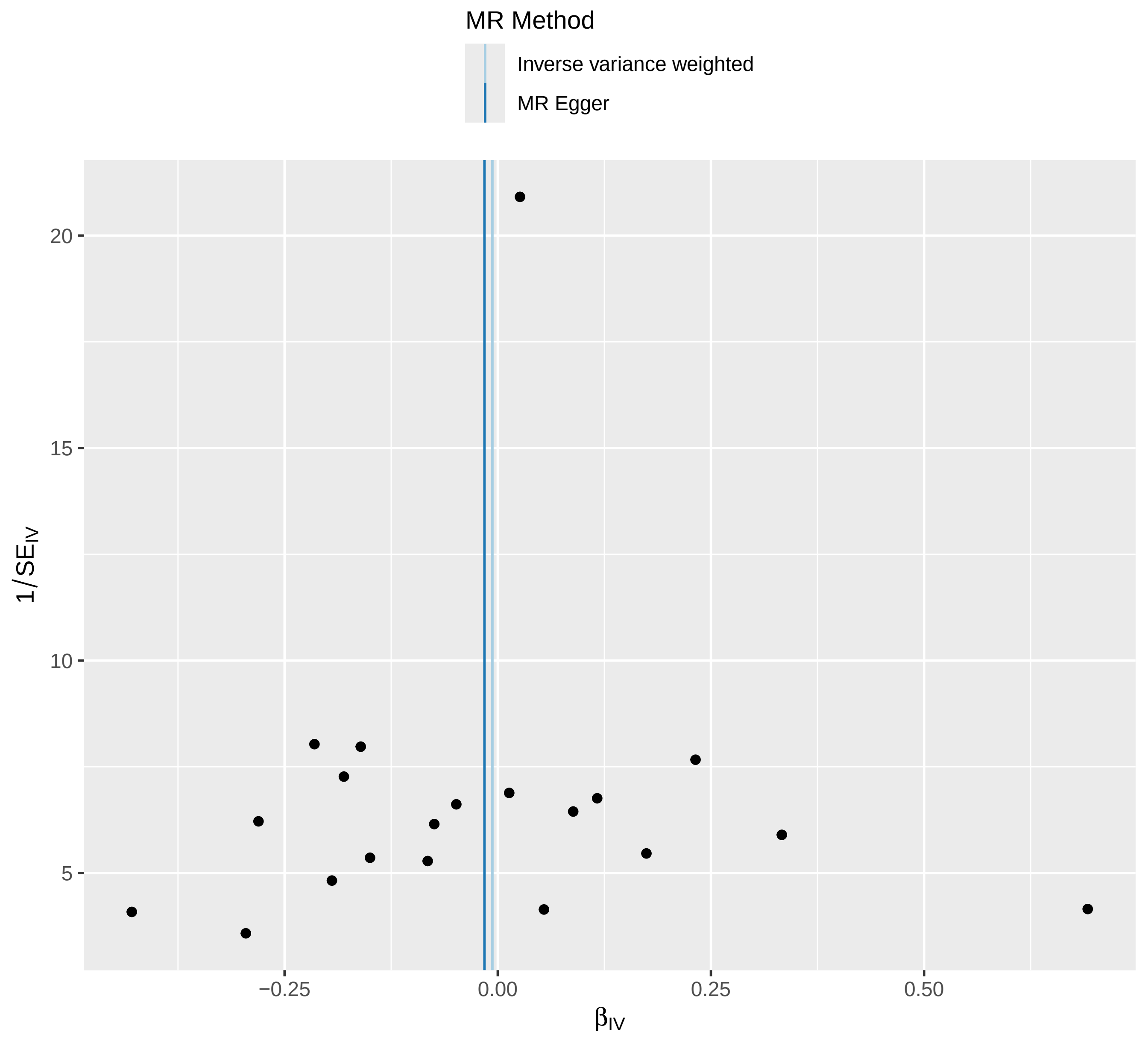

MR Method
Inverse variance weighted
MR Egger
20
15
10
5
1/SE$_{IV}$
−0.25
0.00
0.25
0.50
β$_{IV}$

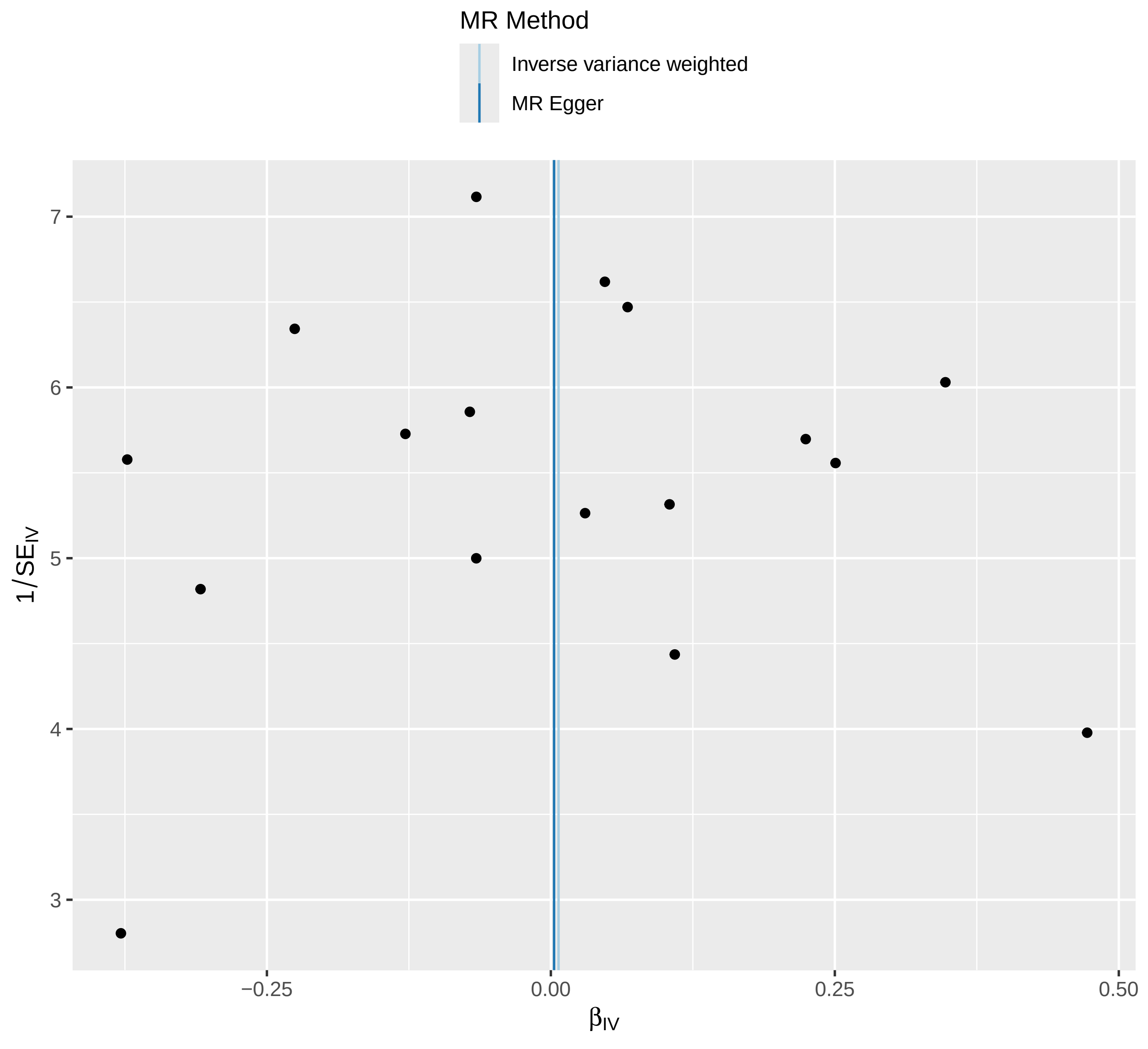
MR Method
Inverse variance weighted
MR Egger
1/SE_IV
β_IV
7
6
5
4
3
−0.25
0.00
0.25
0.50

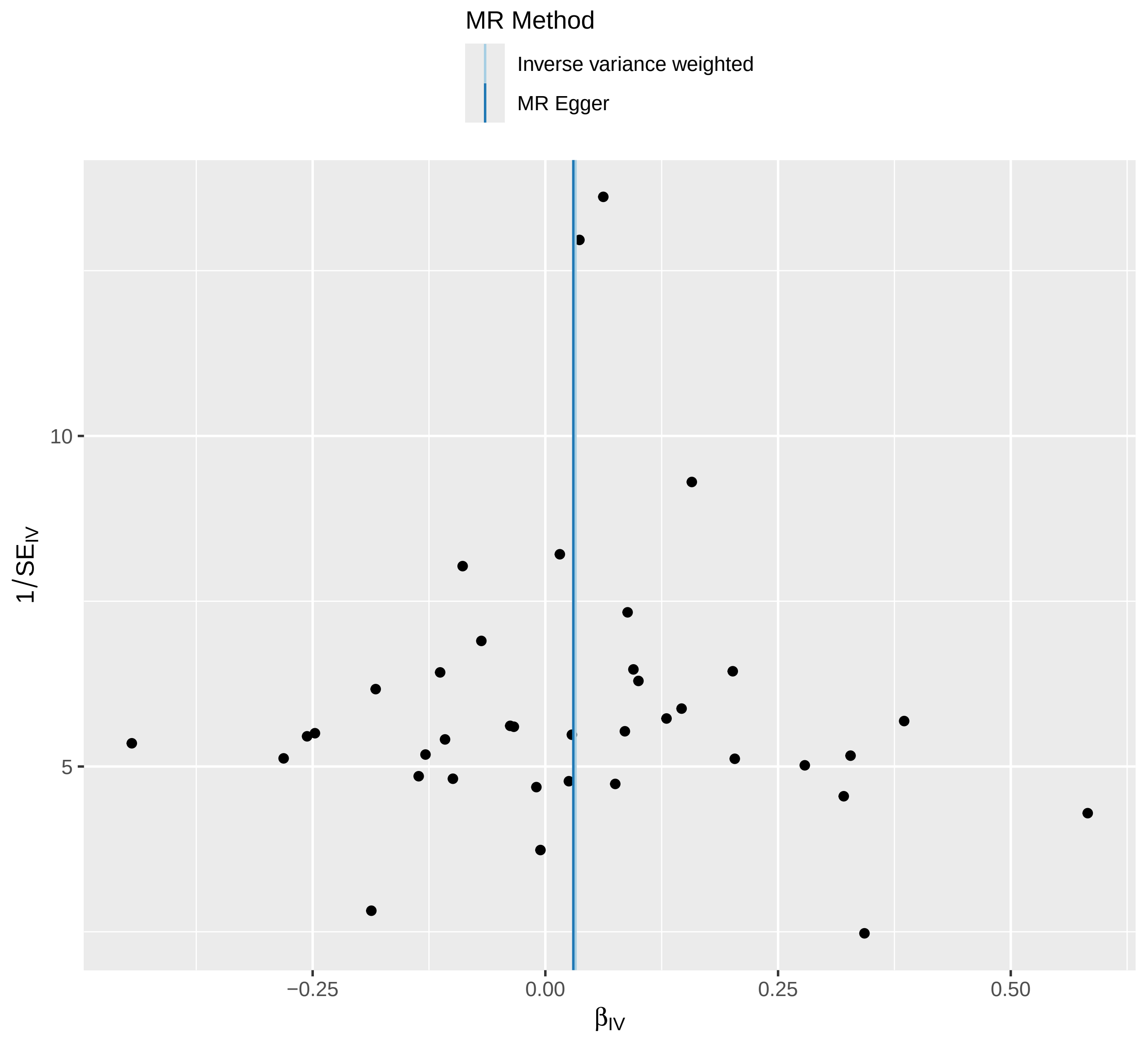
MR Method
Inverse variance weighted
MR Egger
1/SE_IV
10
5
β_IV
−0.25
0.00
0.25
0.50

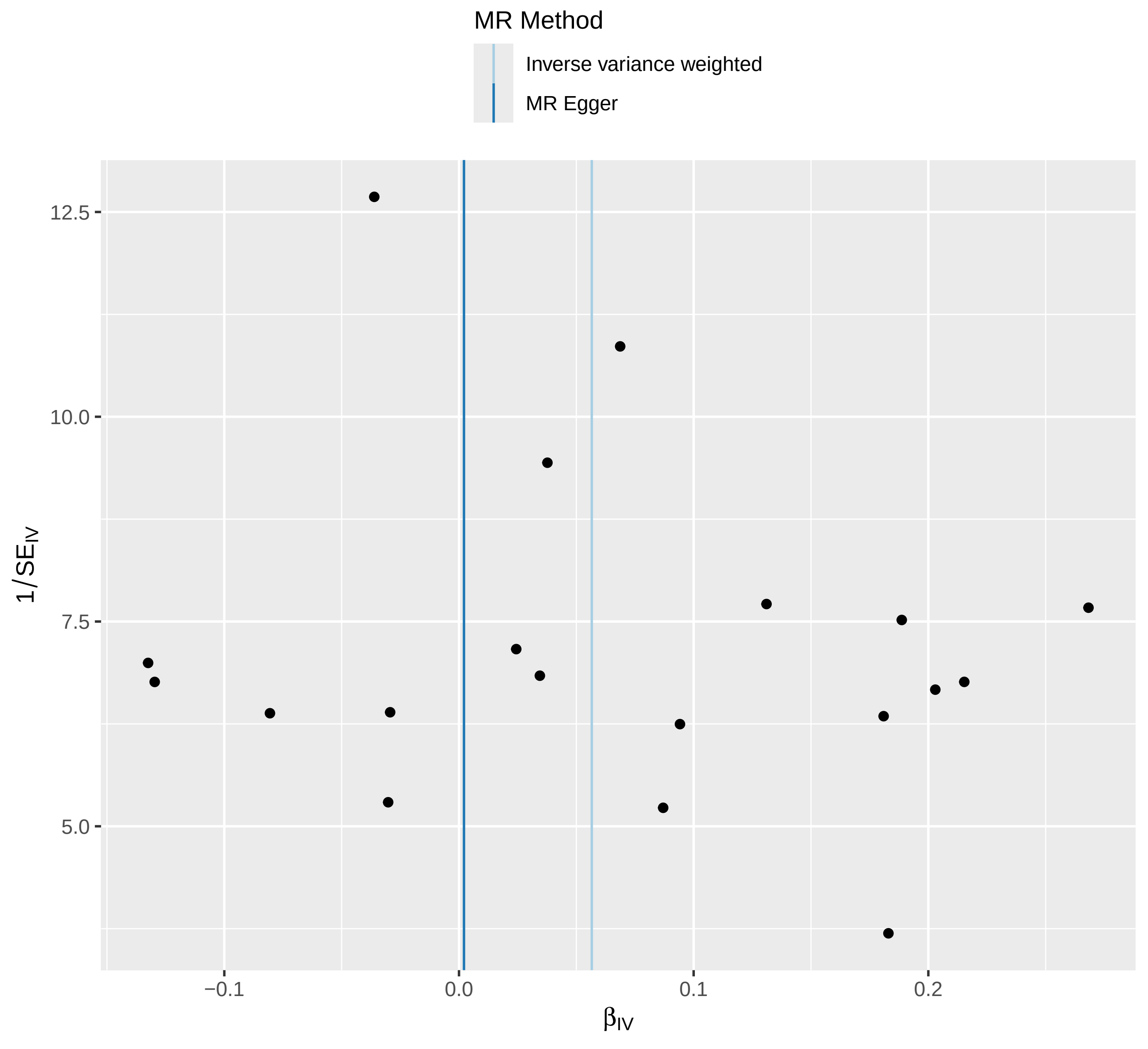

MR Method
Inverse variance weighted
MR Egger
12.5
10.0
7.5
5.0
1/SE_IV
−0.1
0.0
0.1
0.2
β_IV

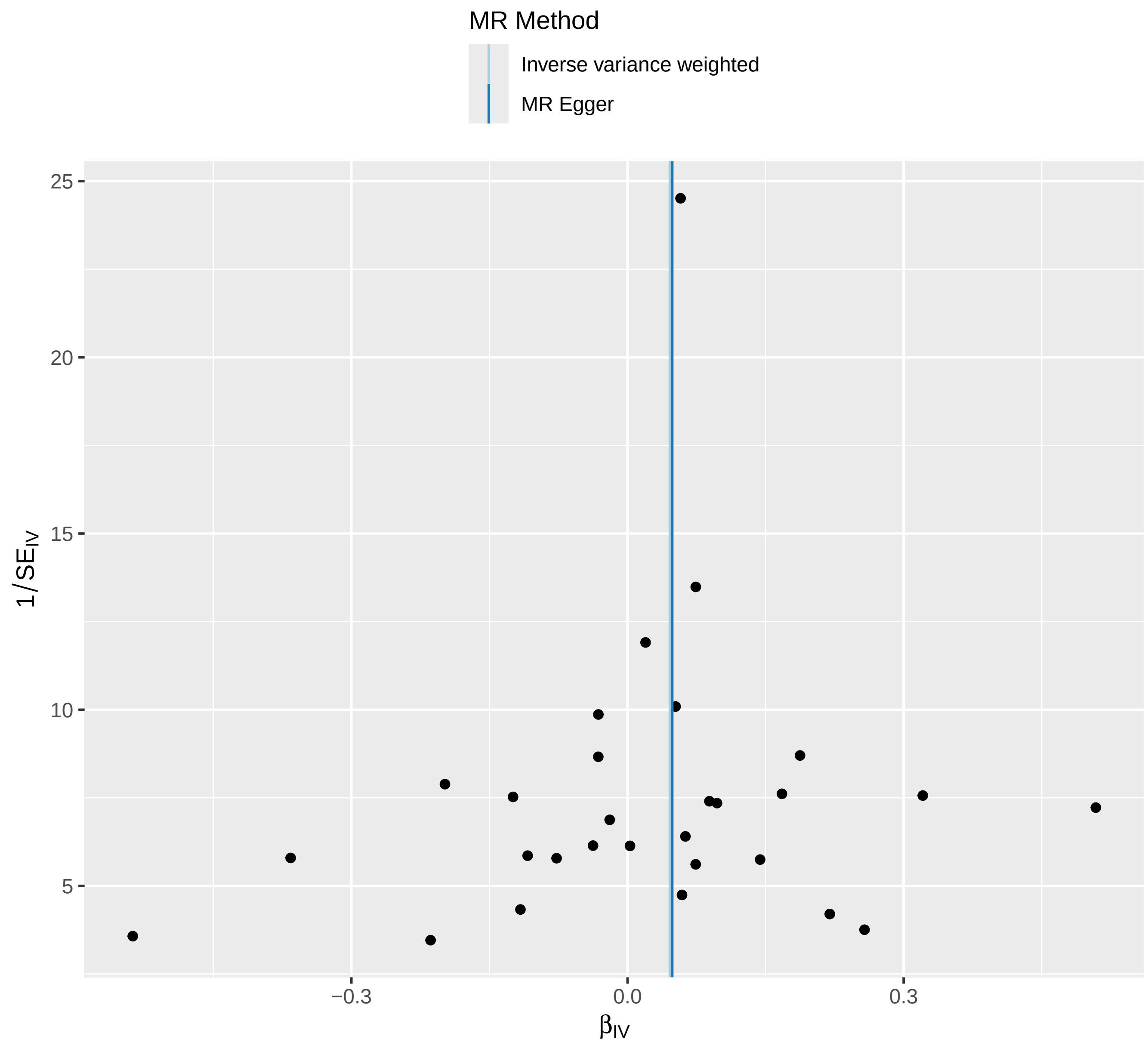

MR Method
Inverse variance weighted
MR Egger
25
20
15
10
5
1/SE_IV
−0.3
0.0
0.3
β_IV

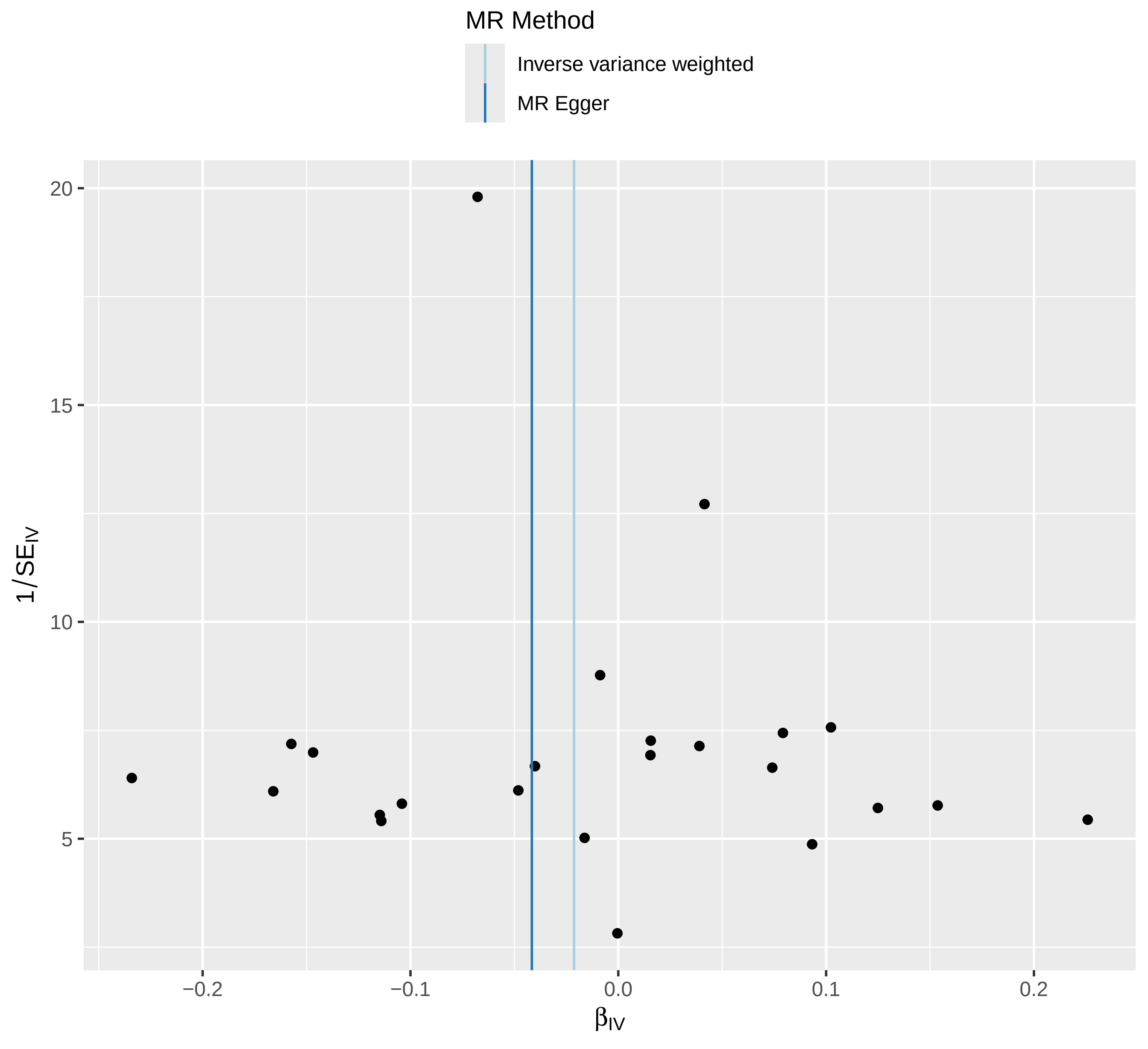

MR Method
Inverse variance weighted
MR Egger
20
15
10
5
1/SE_IV
−0.2
−0.1
0.0
0.1
0.2
β_IV

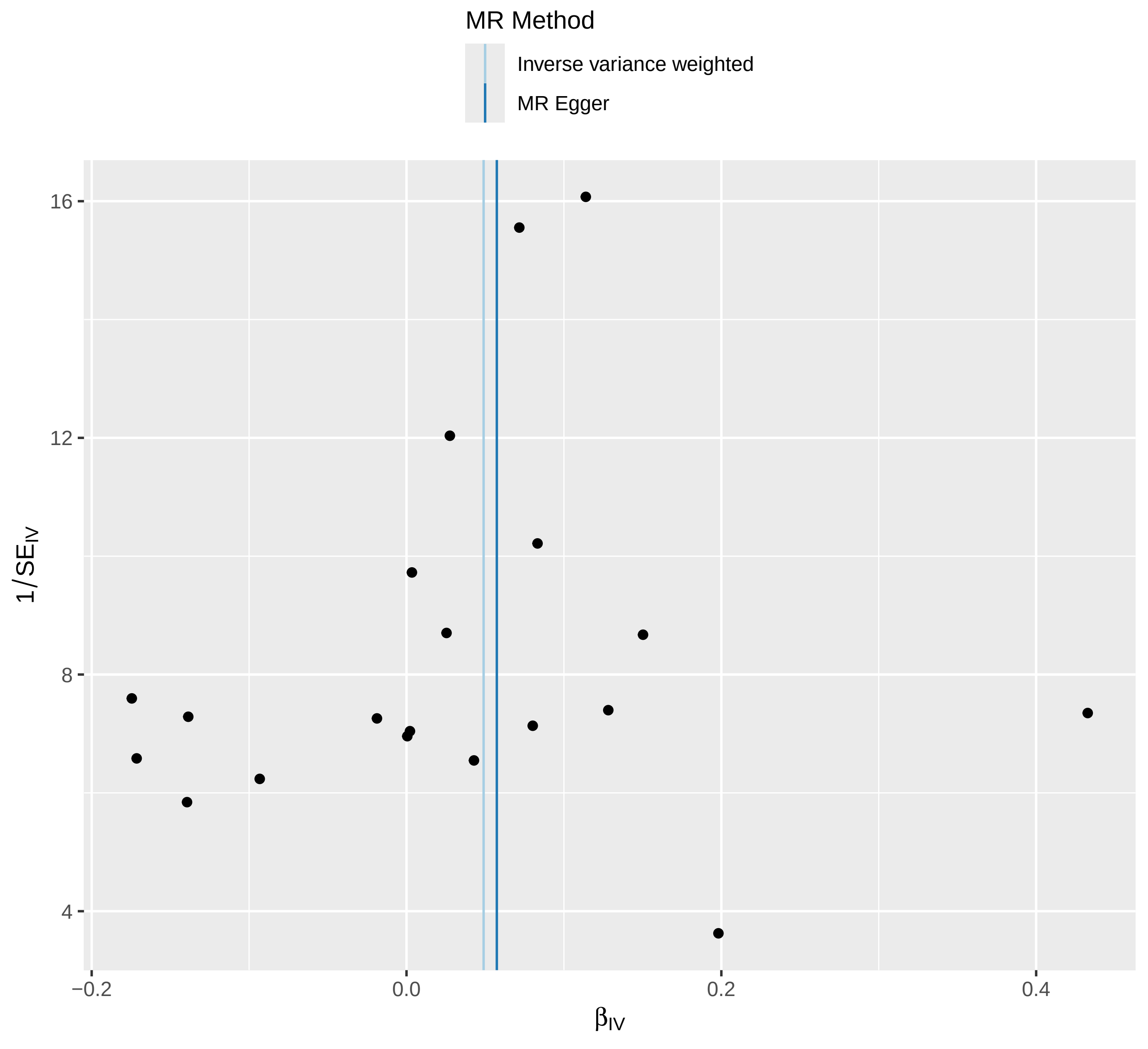

MR Method
Inverse variance weighted
MR Egger
16
12
8
4
1/SE_IV
−0.2
0.0
0.2
0.4
β_IV

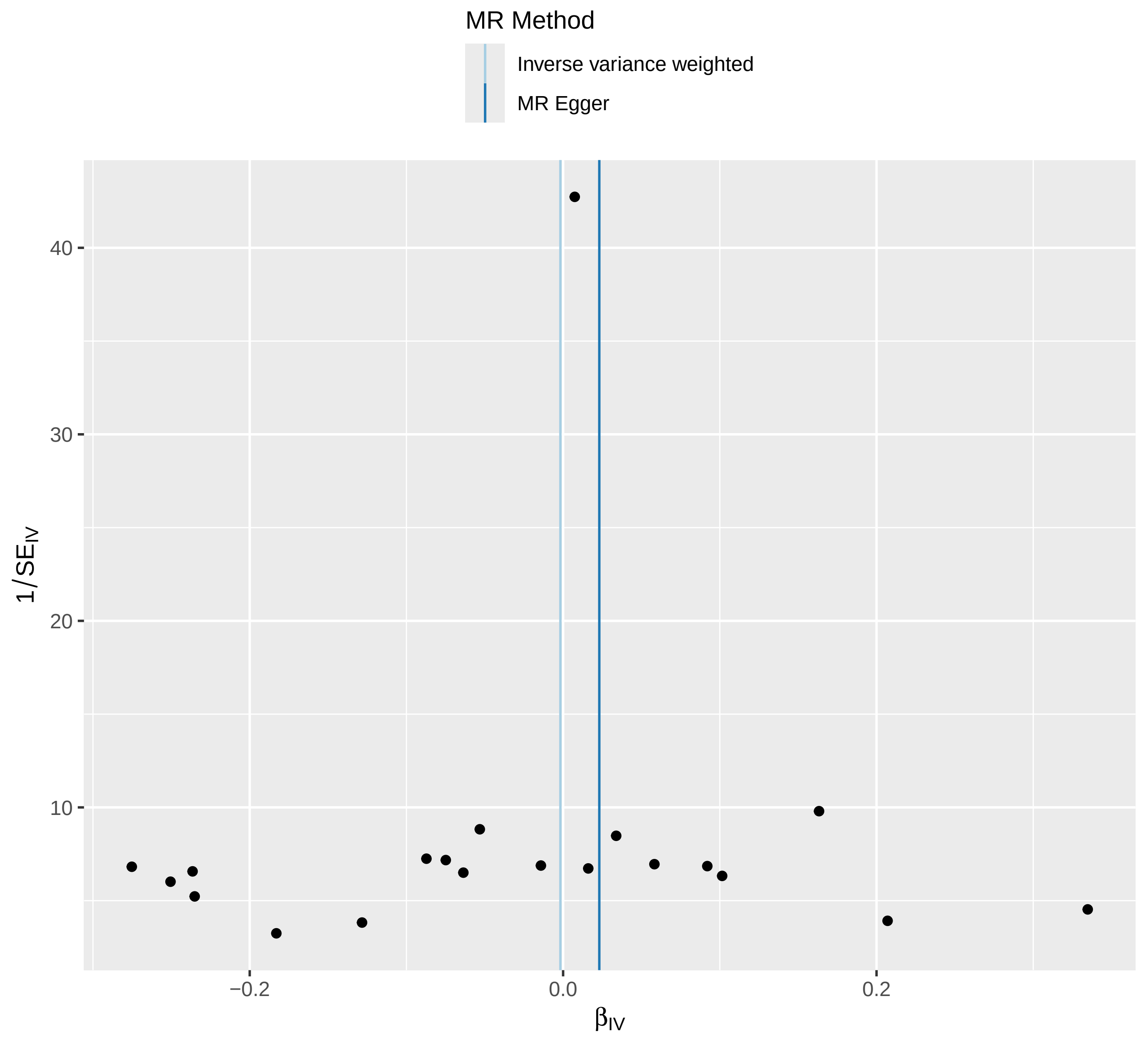
MR Method
Inverse variance weighted
MR Egger
40
30
20
10
1/SE_IV
−0.2
0.0
0.2
β_IV

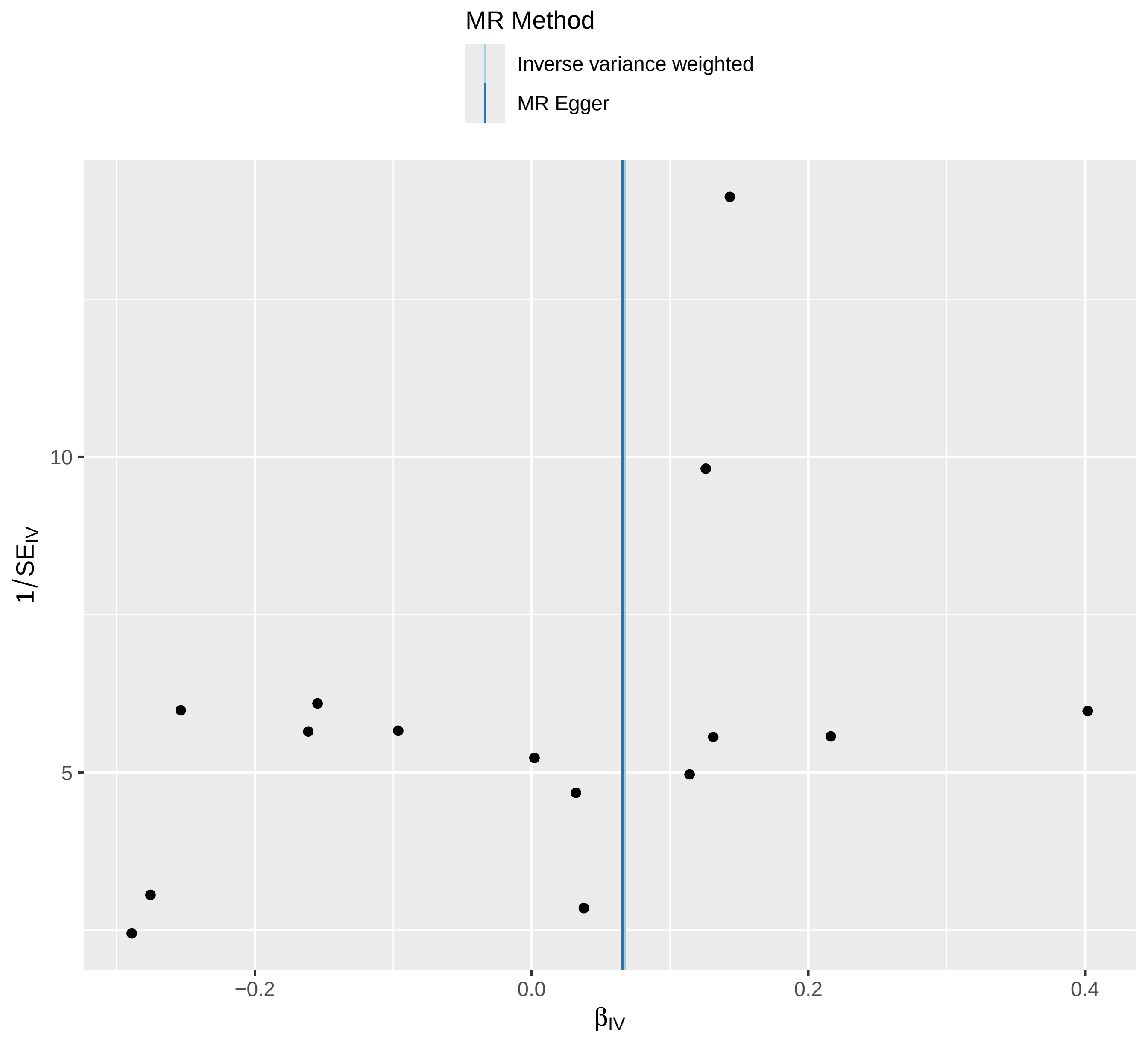
MR Method
Inverse variance weighted
MR Egger
1/SE_IV
10
5
β_IV
−0.2
0.0
0.2
0.4

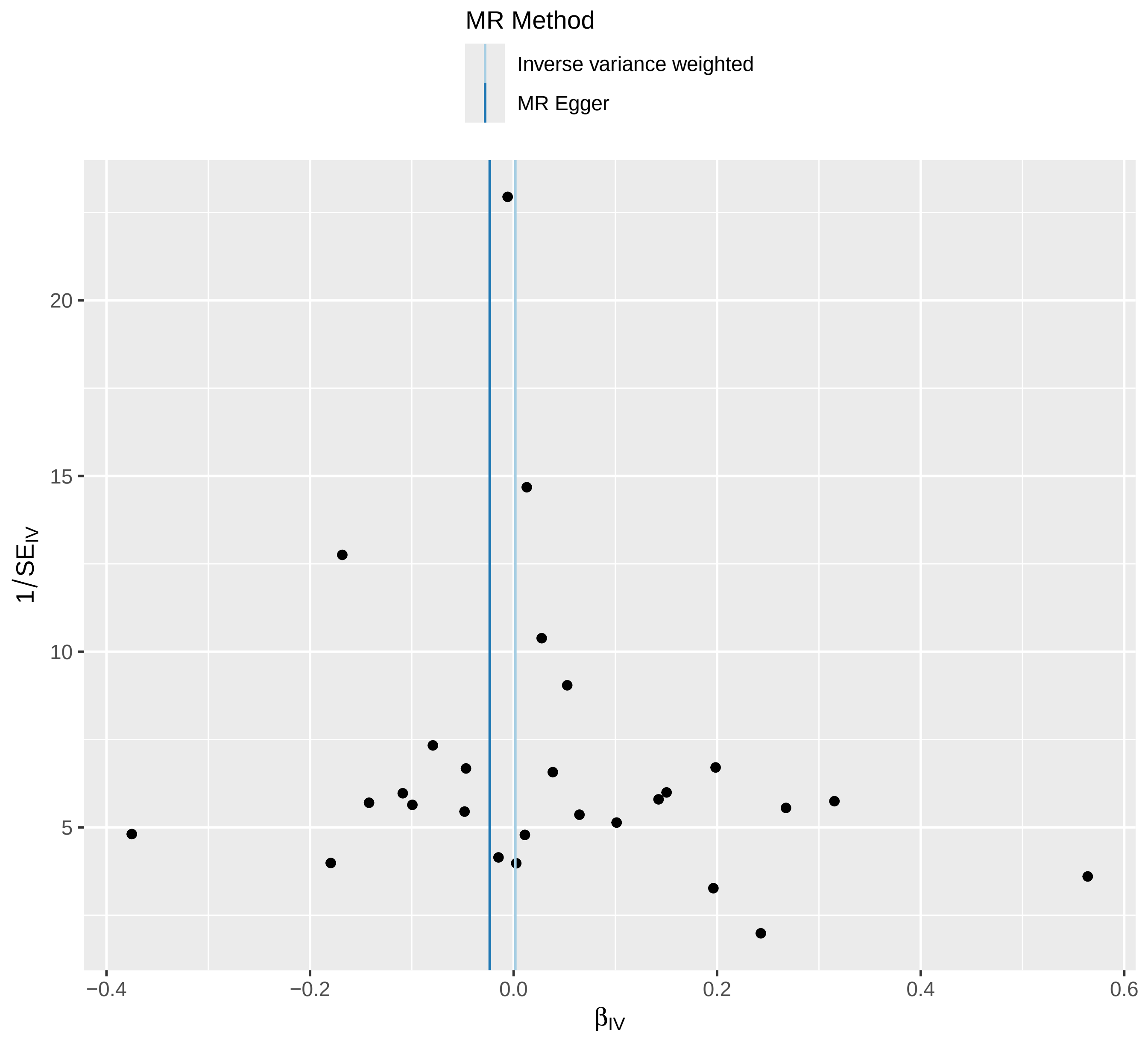

MR Method
Inverse variance weighted
MR Egger
1/SE_IV
20
15
10
5
β_IV
−0.4
−0.2
0.0
0.2
0.4
0.6

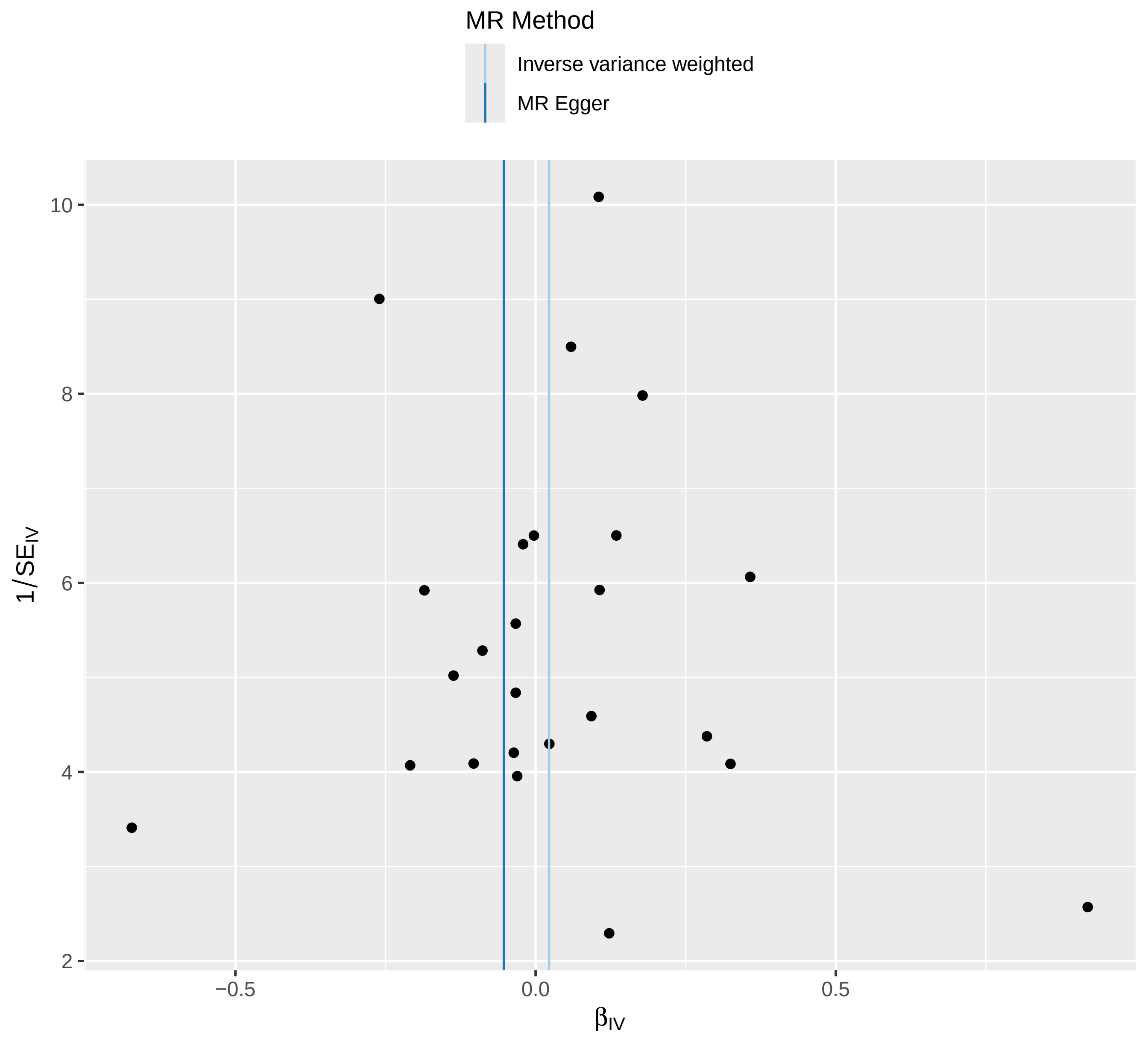
MR Method
Inverse variance weighted
MR Egger
10
8
6
4
2
1/SE$_{IV}$
−0.5
0.0
0.5
β$_{IV}$

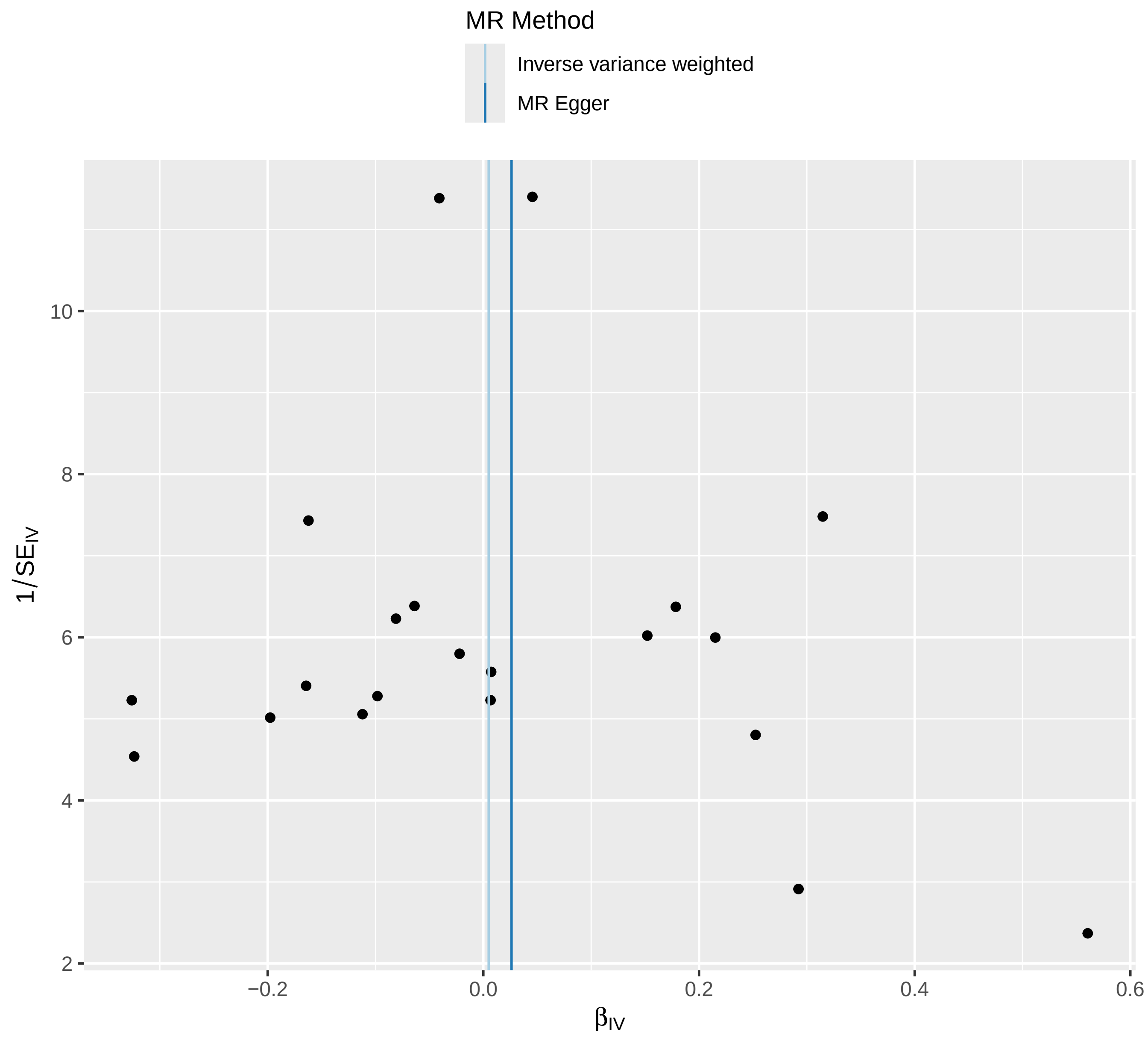
MR Method
Inverse variance weighted
MR Egger
1/SE_IV
β_IV
10
8
6
4
2
−0.2
0.0
0.2
0.4
0.6

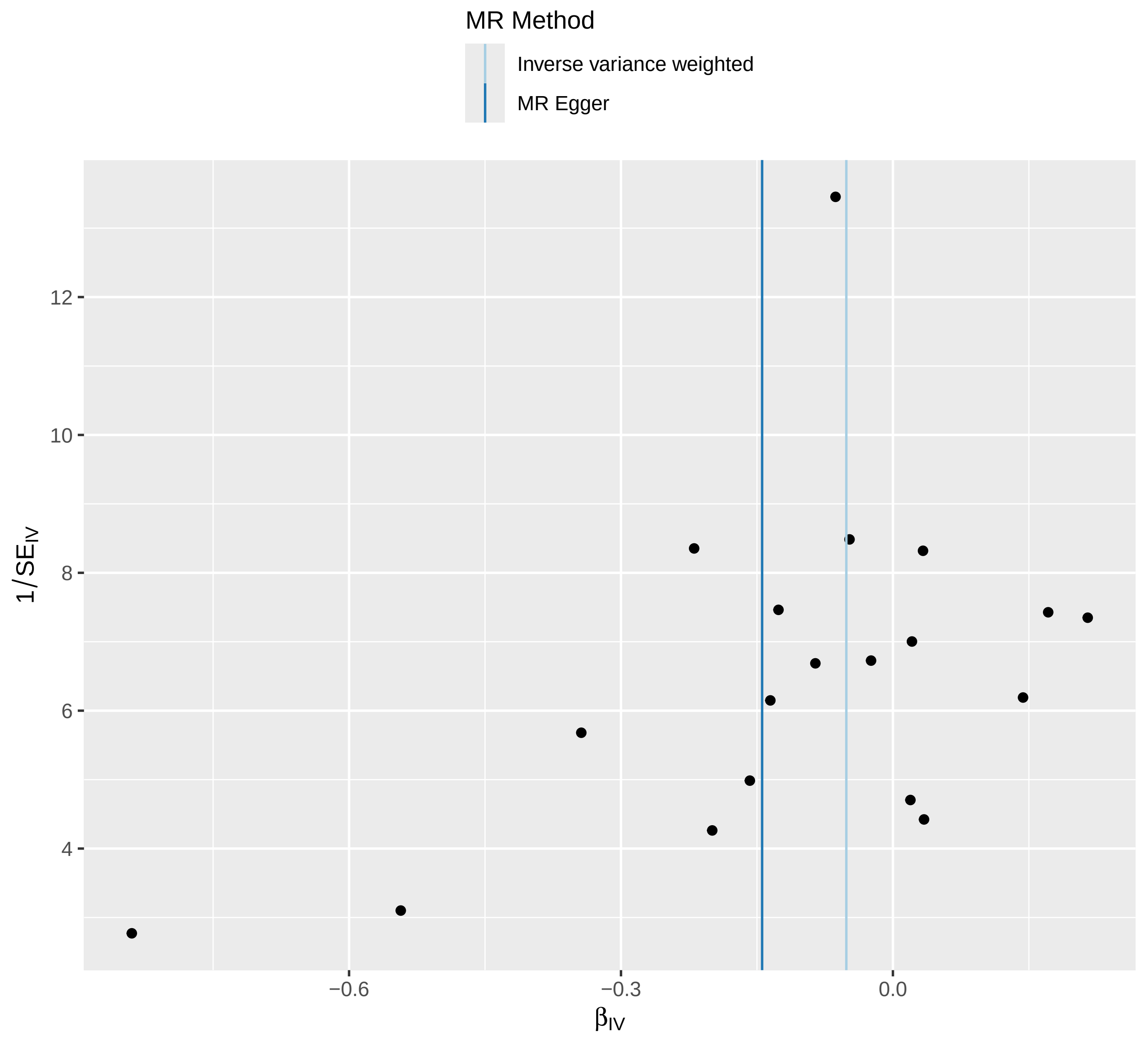
MR Method
Inverse variance weighted
MR Egger
12
10
8
6
4
1/SE$_{IV}$
−0.6
−0.3
0.0
β$_{IV}$

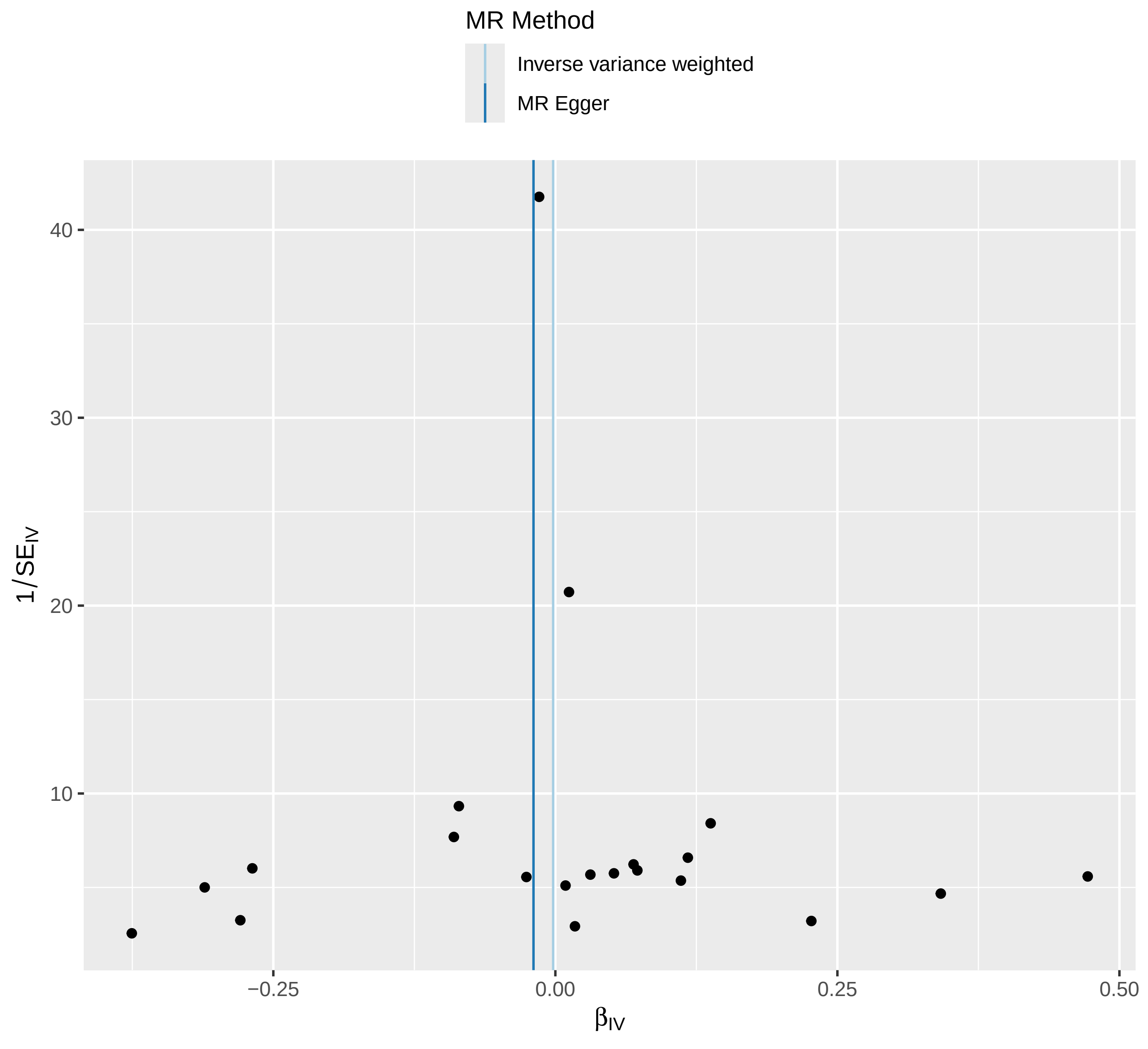

MR Method
Inverse variance weighted
MR Egger
40
30
20
10
1/SE$_{IV}$
−0.25
0.00
0.25
0.50
β$_{IV}$

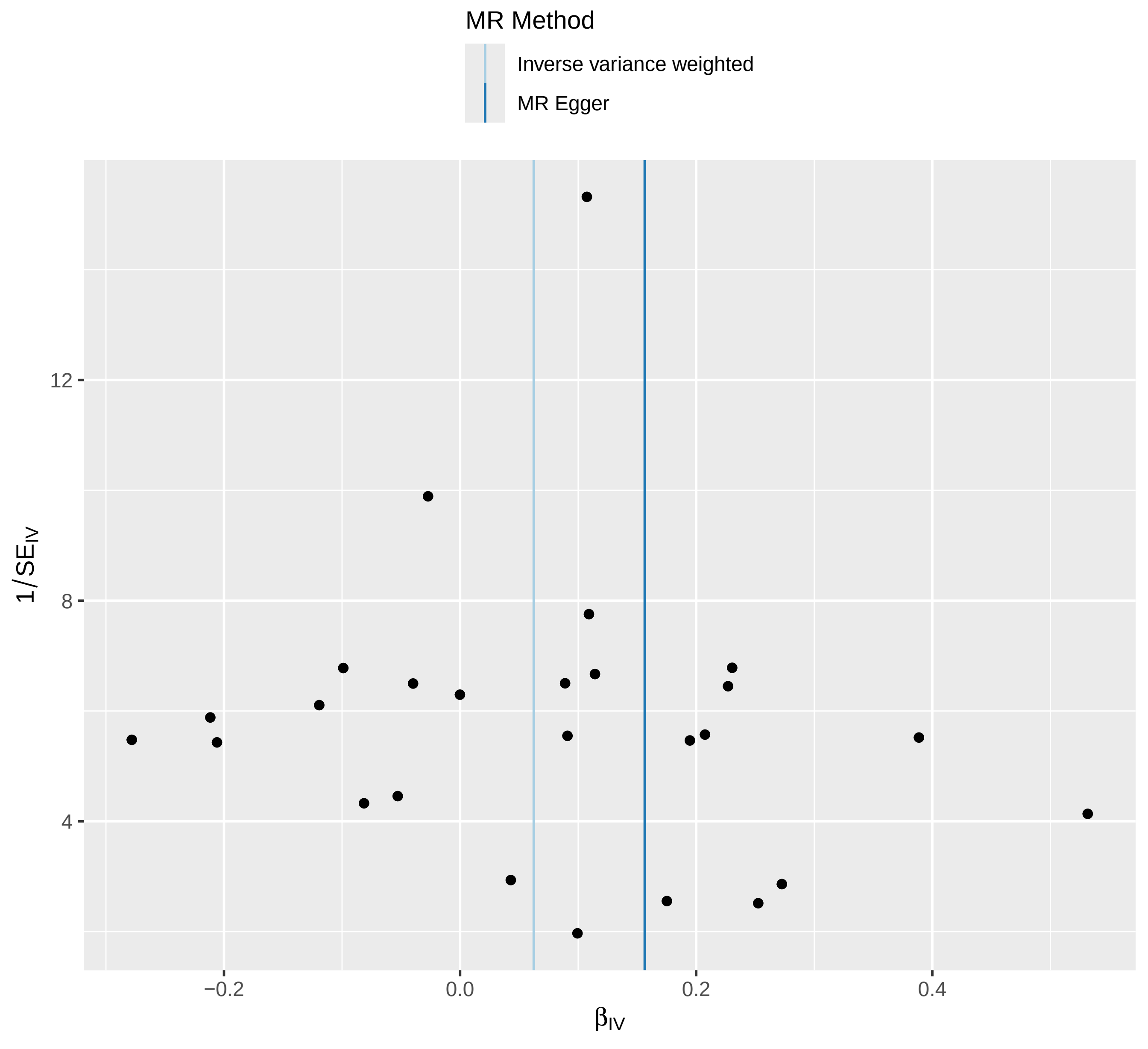
MR Method
Inverse variance weighted
MR Egger
12
8
4
1/SE$_{IV}$
−0.2
0.0
0.2
0.4
β$_{IV}$

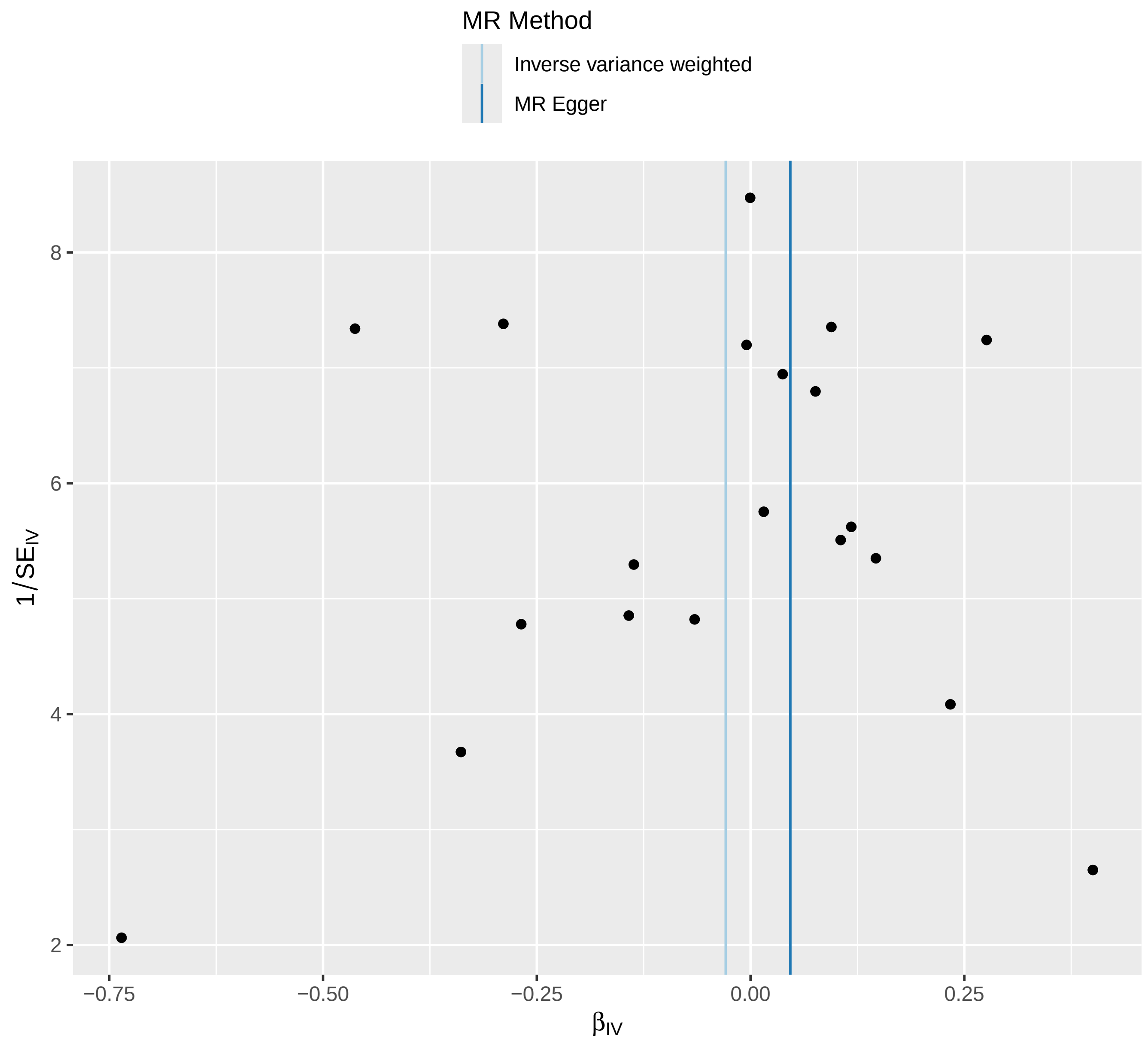
MR Method
Inverse variance weighted
MR Egger
8
6
4
2
1/SE_IV
−0.75
−0.50
−0.25
0.00
0.25
β_IV

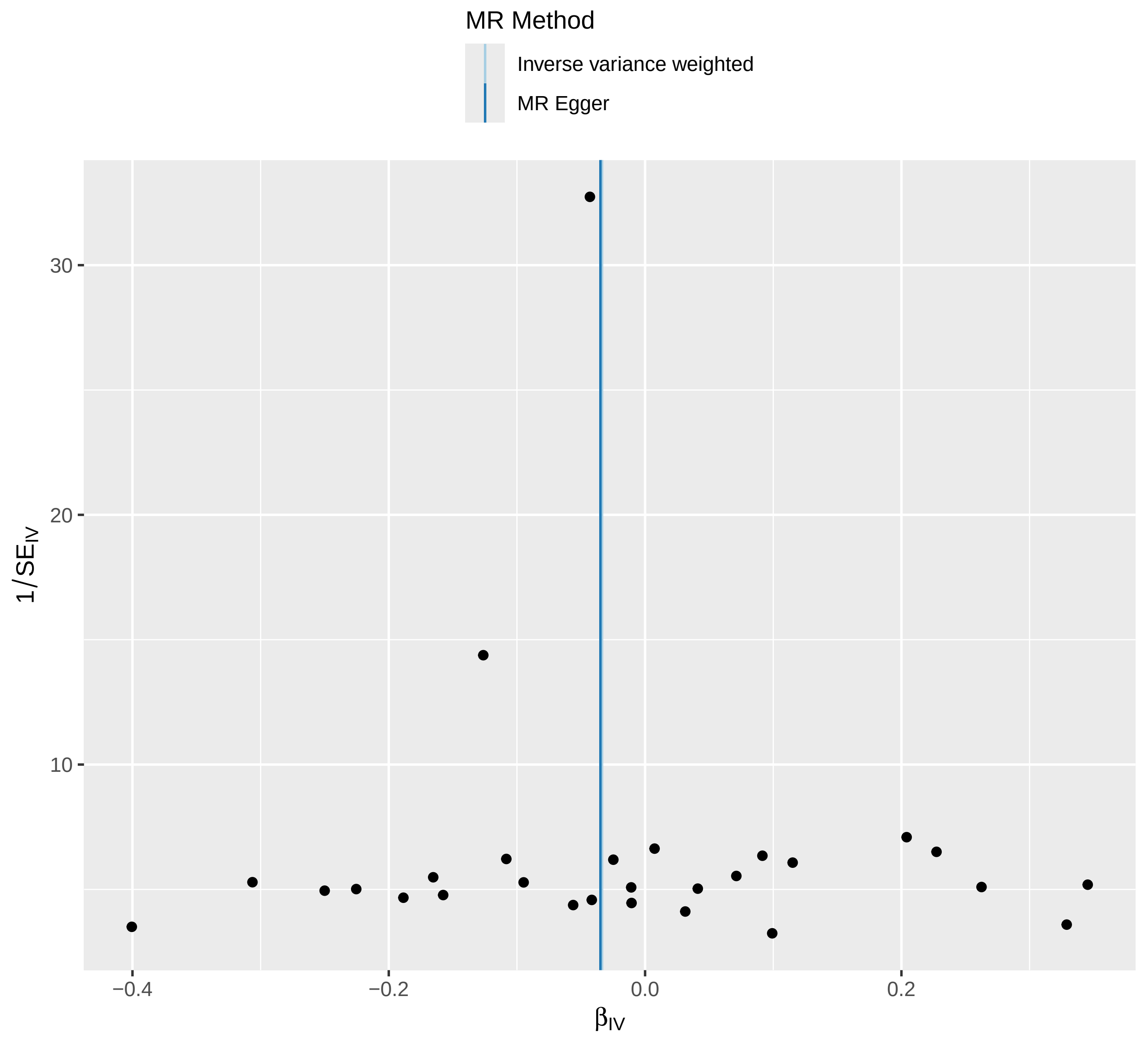
MR Method
Inverse variance weighted
MR Egger
30
20
10
1/SE_IV
−0.4
−0.2
0.0
0.2
β_IV

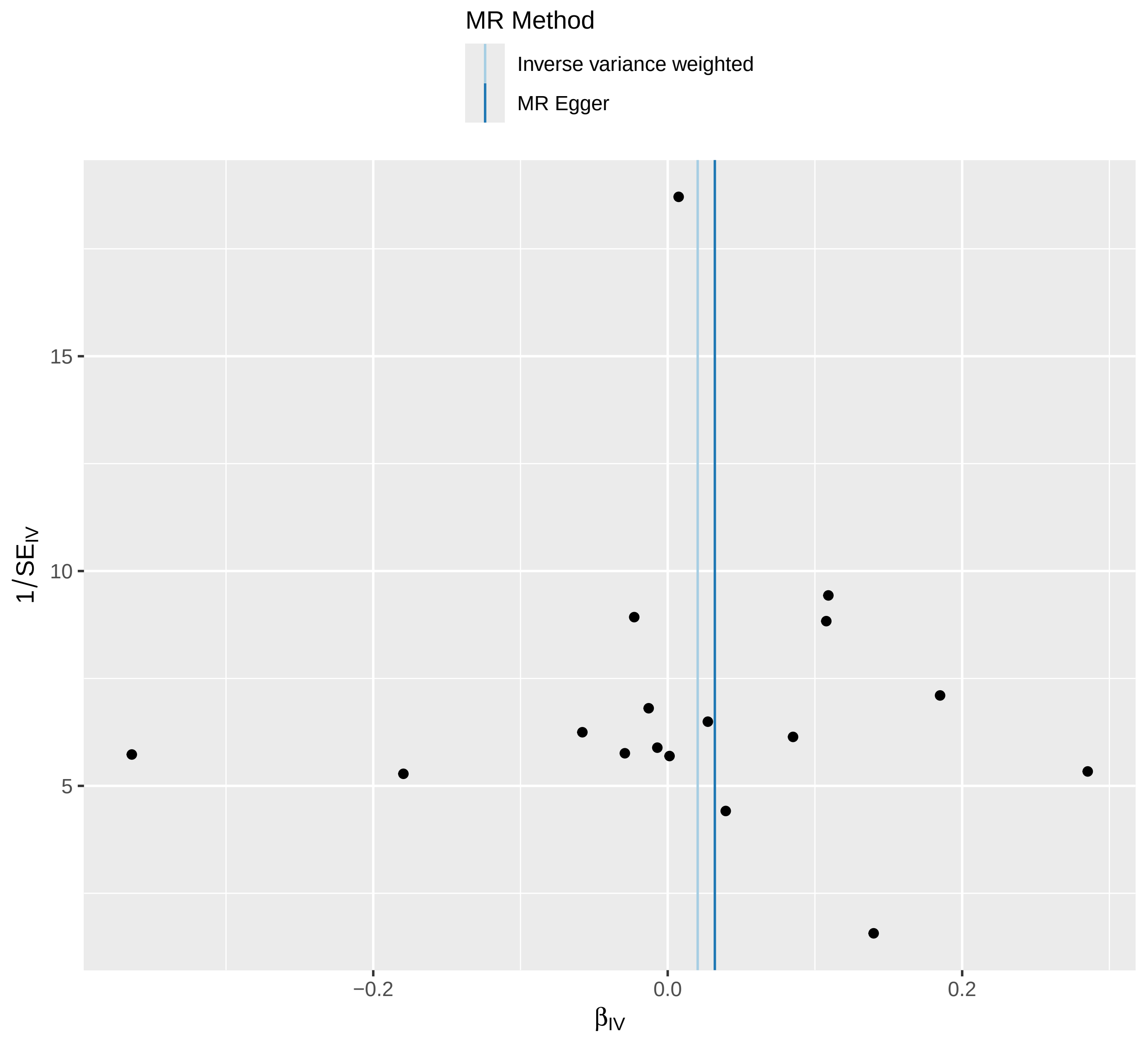

MR Method
Inverse variance weighted
MR Egger
15
10
5
1/SE_IV
−0.2
0.0
0.2
β_IV

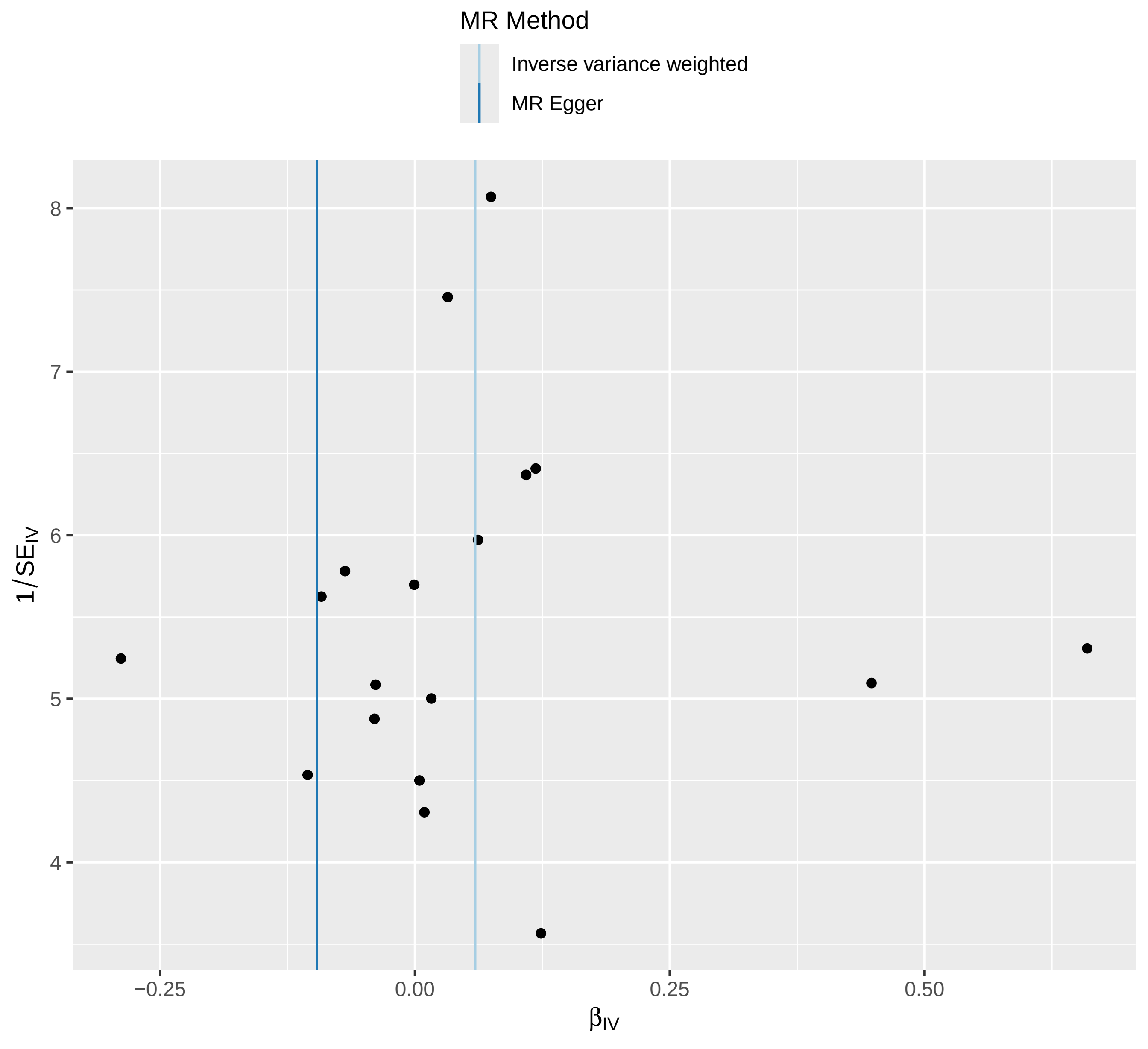
MR Method
Inverse variance weighted
MR Egger
1/SE$_{IV}$
8
7
6
5
4
−0.25
0.00
0.25
0.50
β$_{IV}$

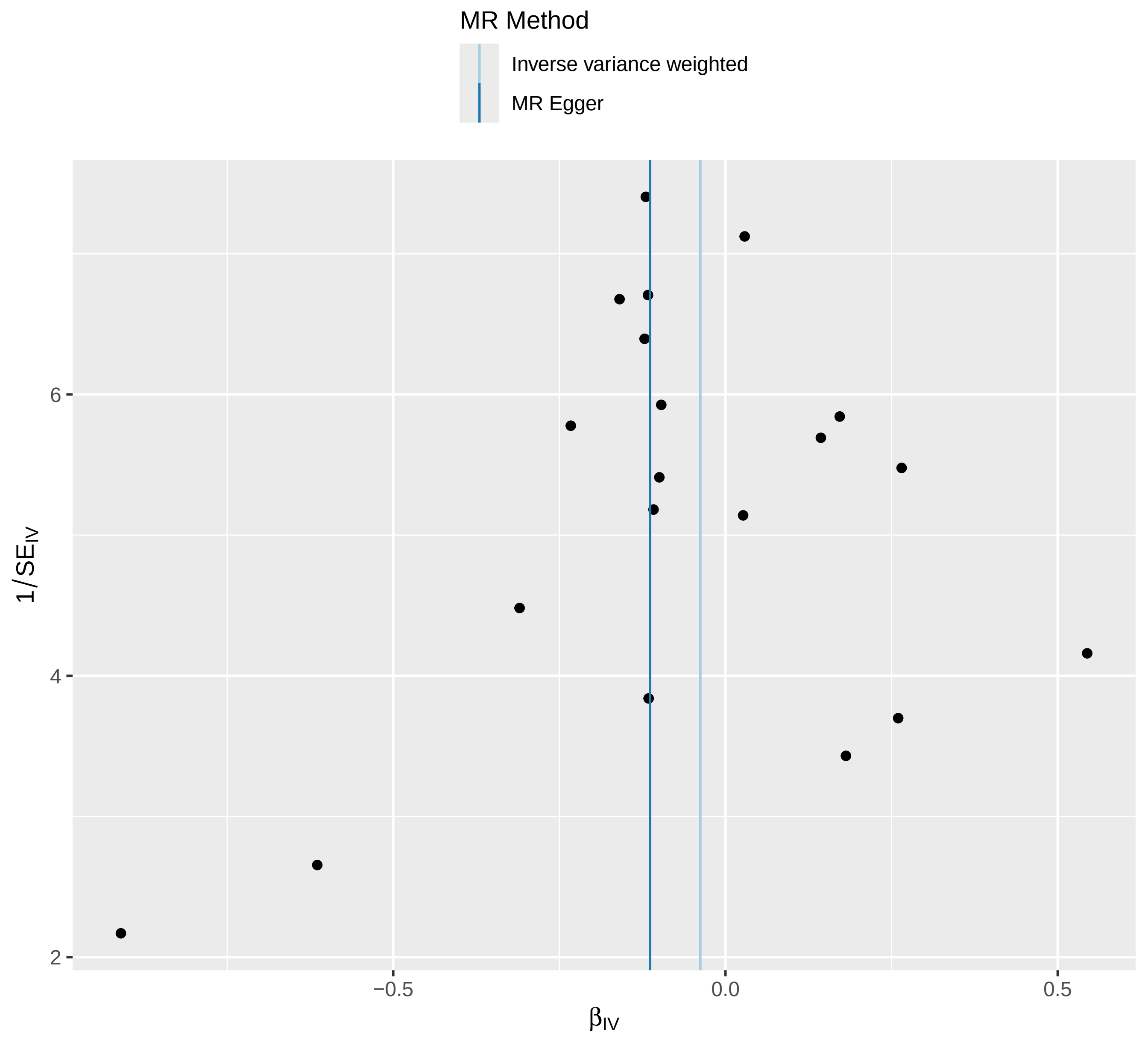
MR Method
Inverse variance weighted
MR Egger
1/SE_IV
6
4
2
−0.5
0.0
0.5
β_IV

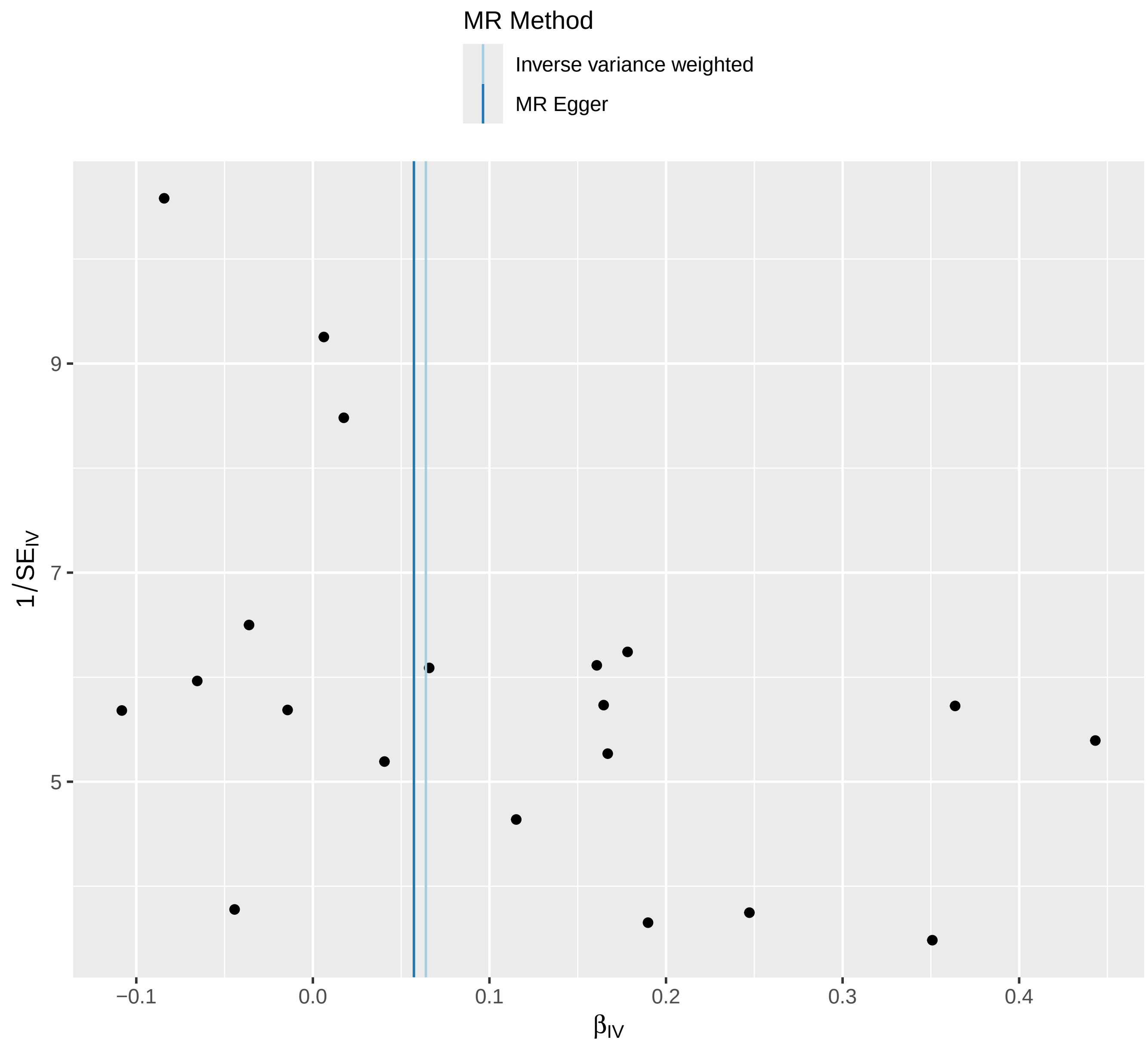
MR Method
Inverse variance weighted
MR Egger
1/SE_IV
9
7
5
−0.1
0.0
0.1
0.2
0.3
0.4
β_IV

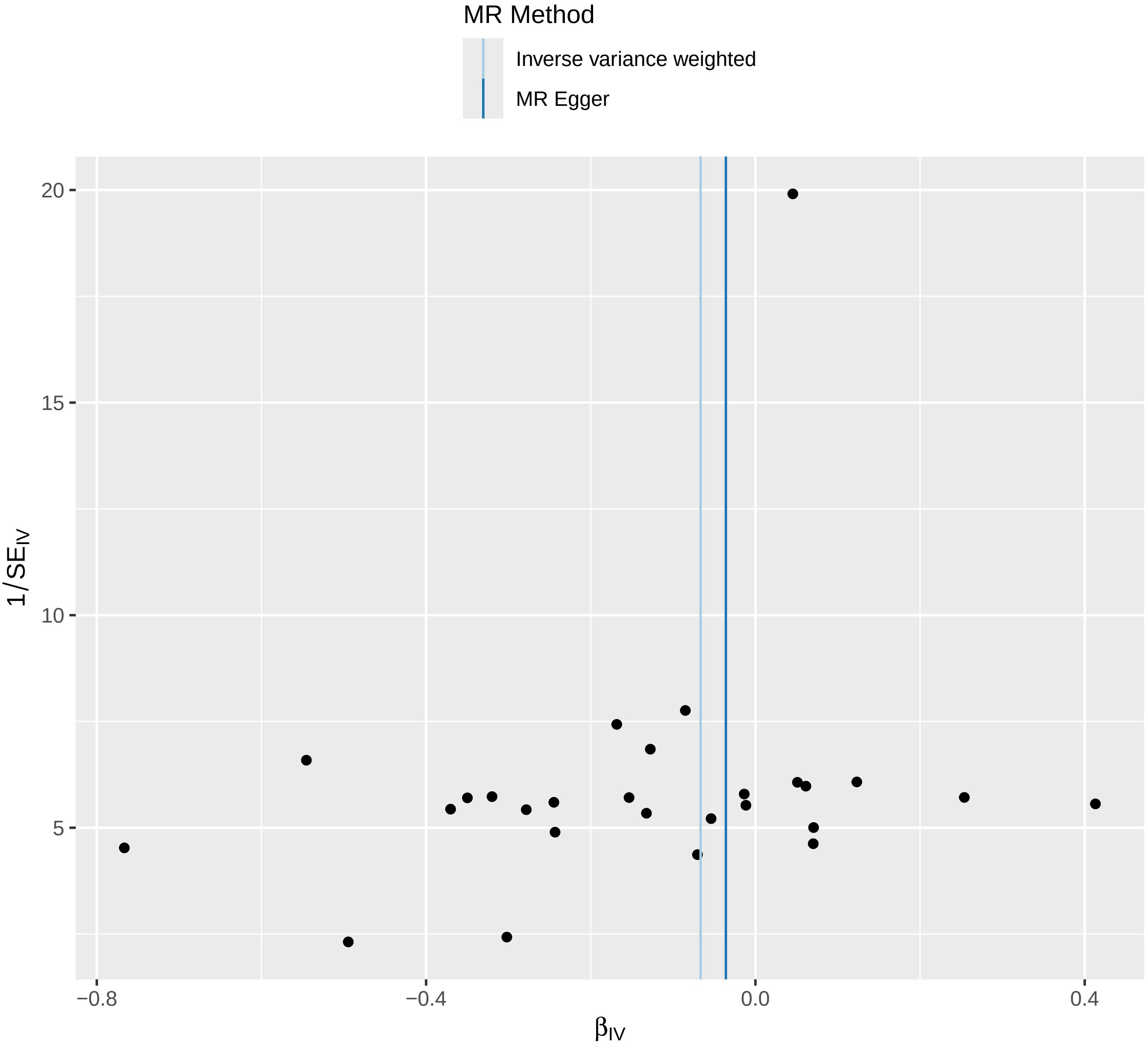
MR Method
Inverse variance weighted
MR Egger
20
15
10
5
1/SE$_{IV}$
−0.8
−0.4
0.0
0.4
β$_{IV}$

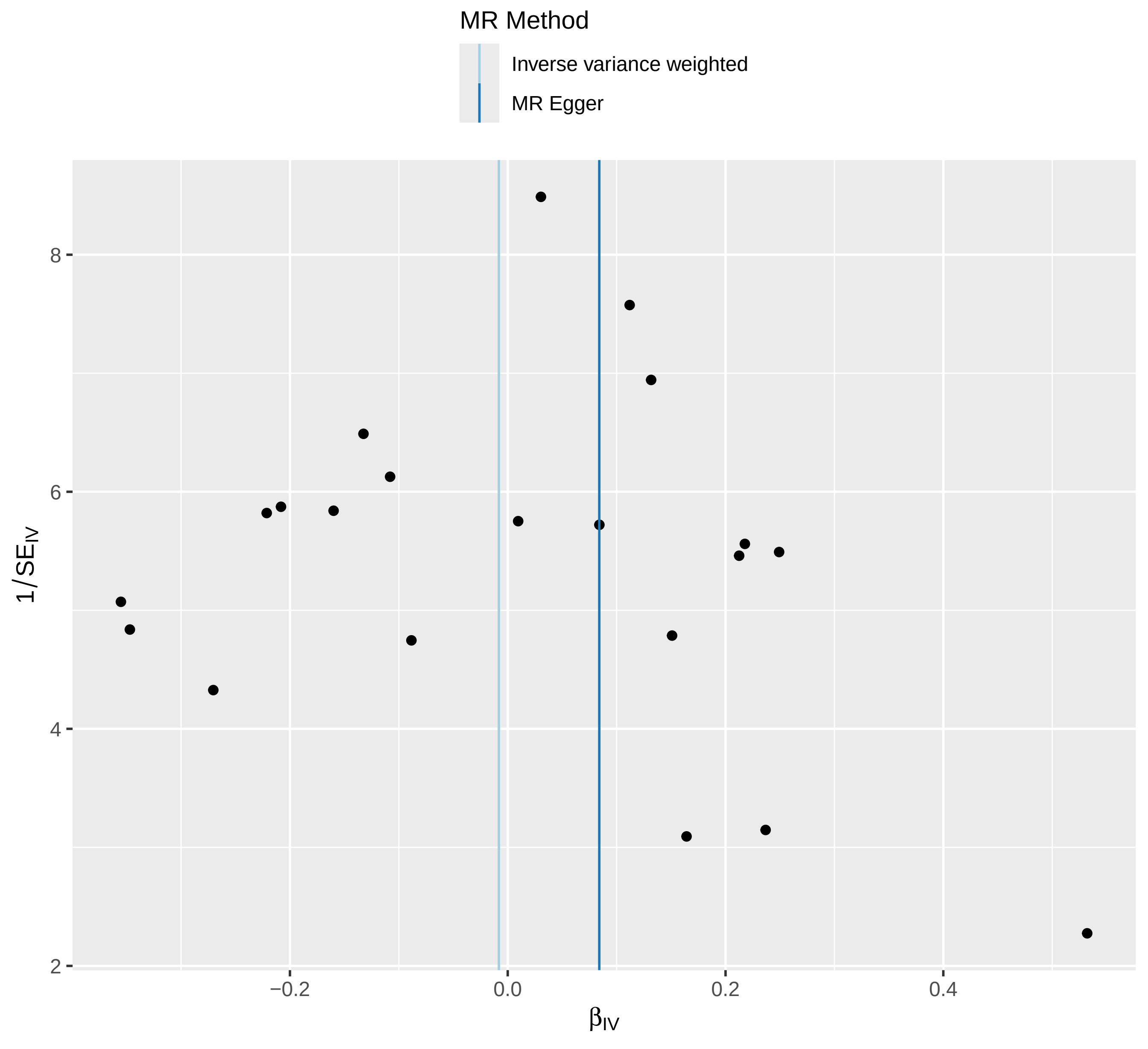

MR Method
Inverse variance weighted
MR Egger
8
6
4
2
1/SE_IV
−0.2
0.0
0.2
0.4
β_IV

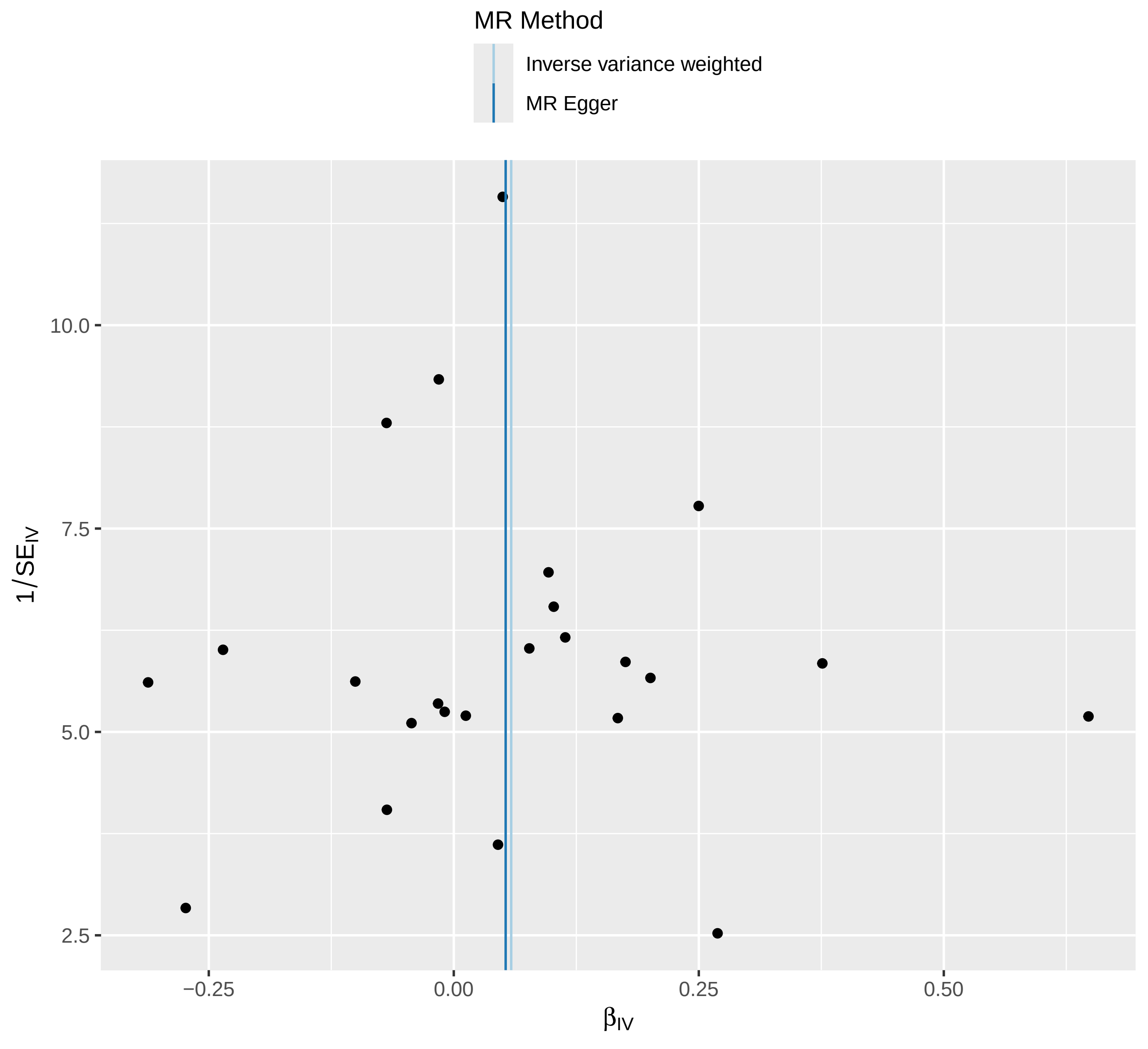

MR Method
Inverse variance weighted
MR Egger
10.0
7.5
5.0
2.5
$1/SE_{IV}$
−0.25
0.00
0.25
0.50
β_{IV}

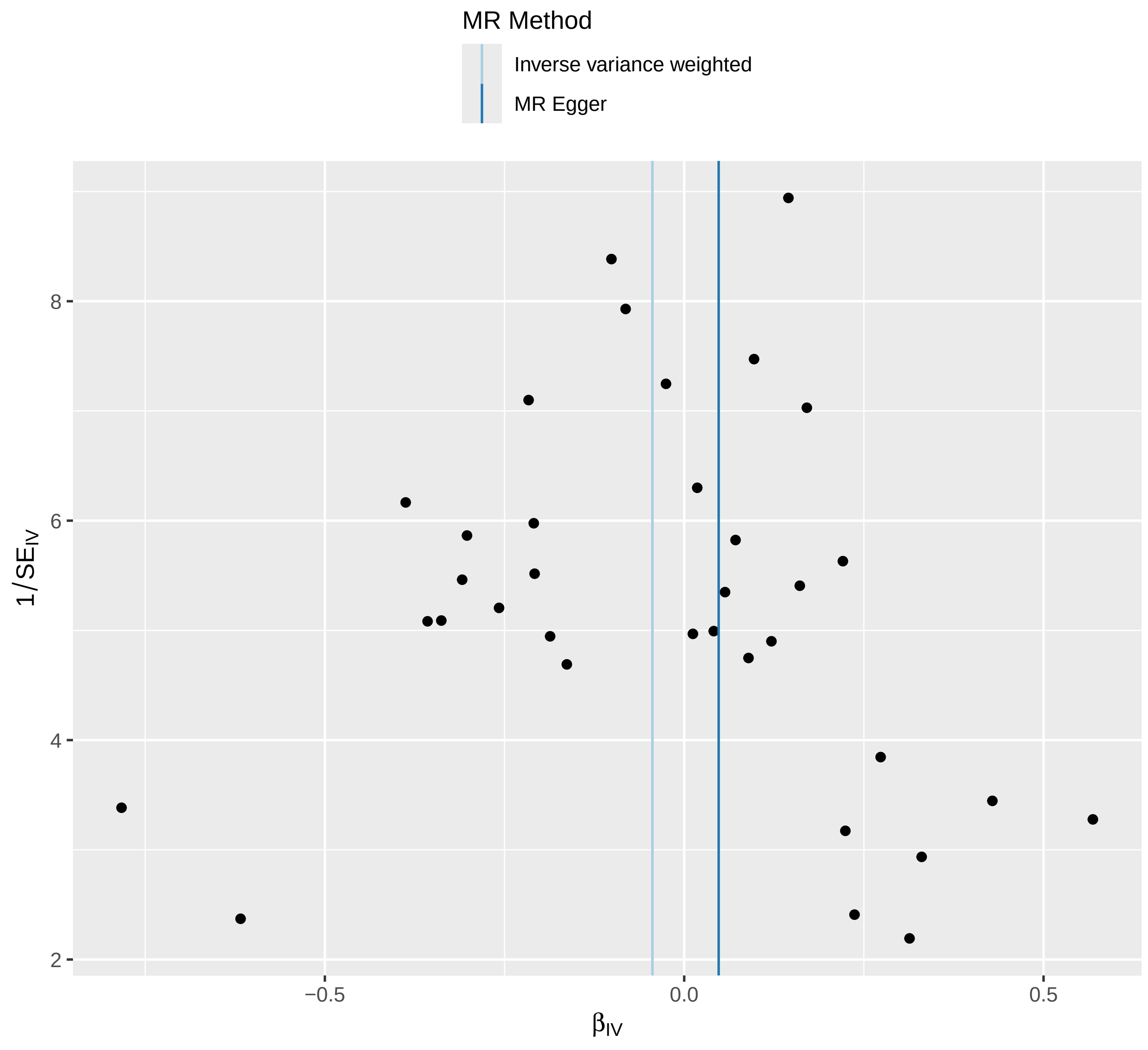
MR Method
Inverse variance weighted
MR Egger
8
6
4
2
1/SE_IV
−0.5
0.0
0.5
β_IV

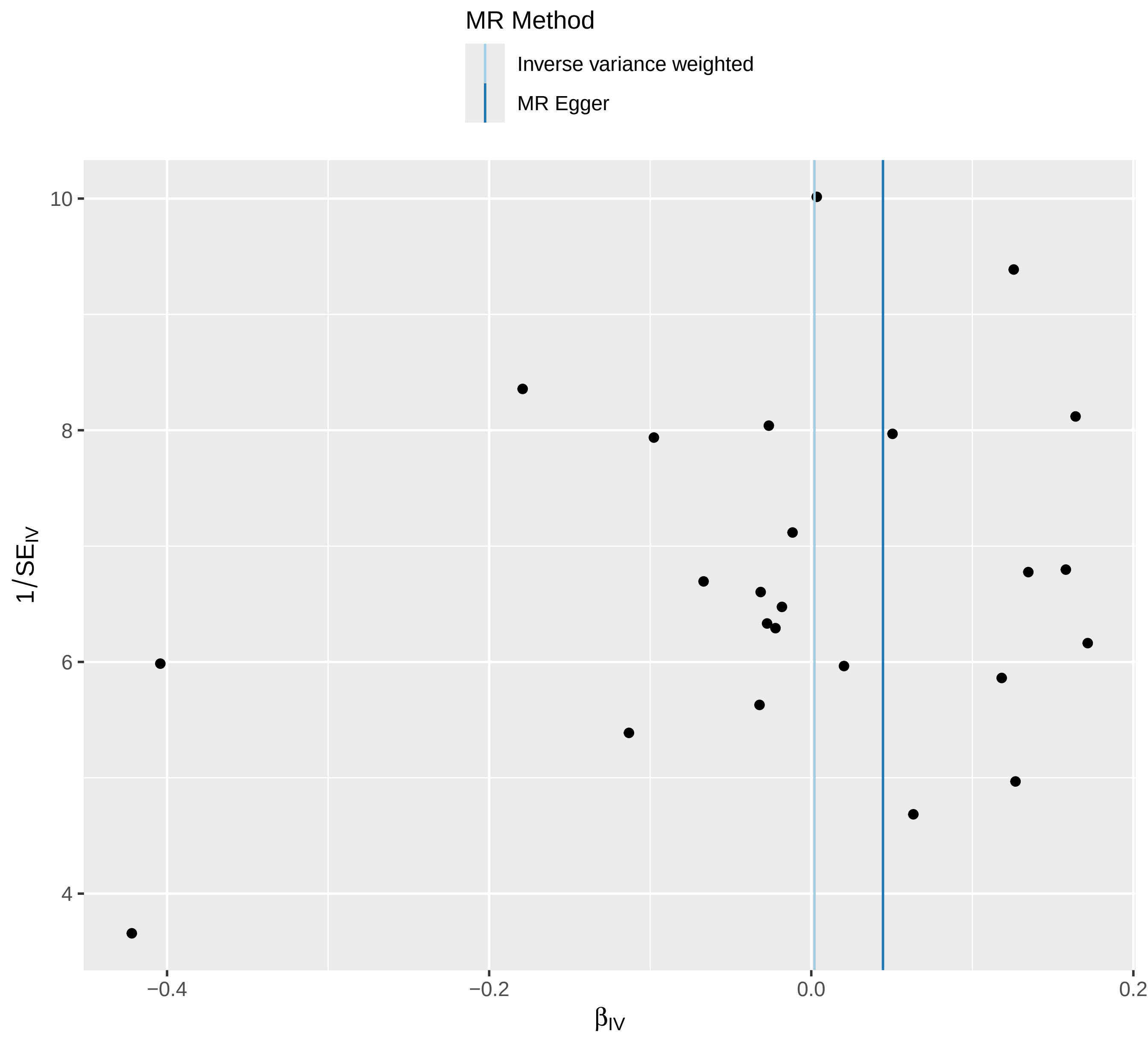
MR Method
Inverse variance weighted
MR Egger
10
8
6
4
1/SE_IV
−0.4
−0.2
0.0
0.2
β_IV

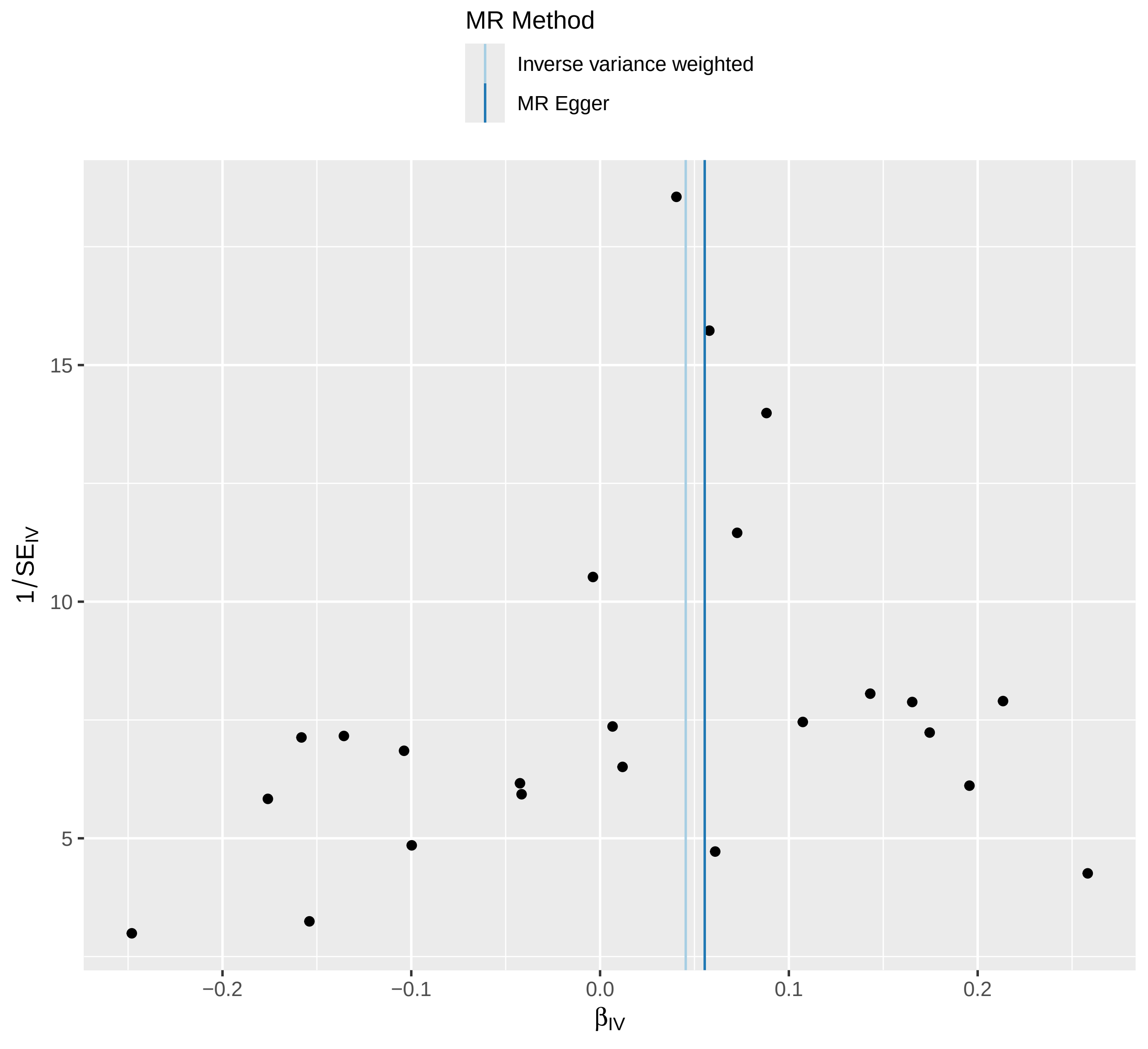

MR Method
Inverse variance weighted
MR Egger
15
10
5
1/SE$_{IV}$
−0.2
−0.1
0.0
0.1
0.2
β$_{IV}$

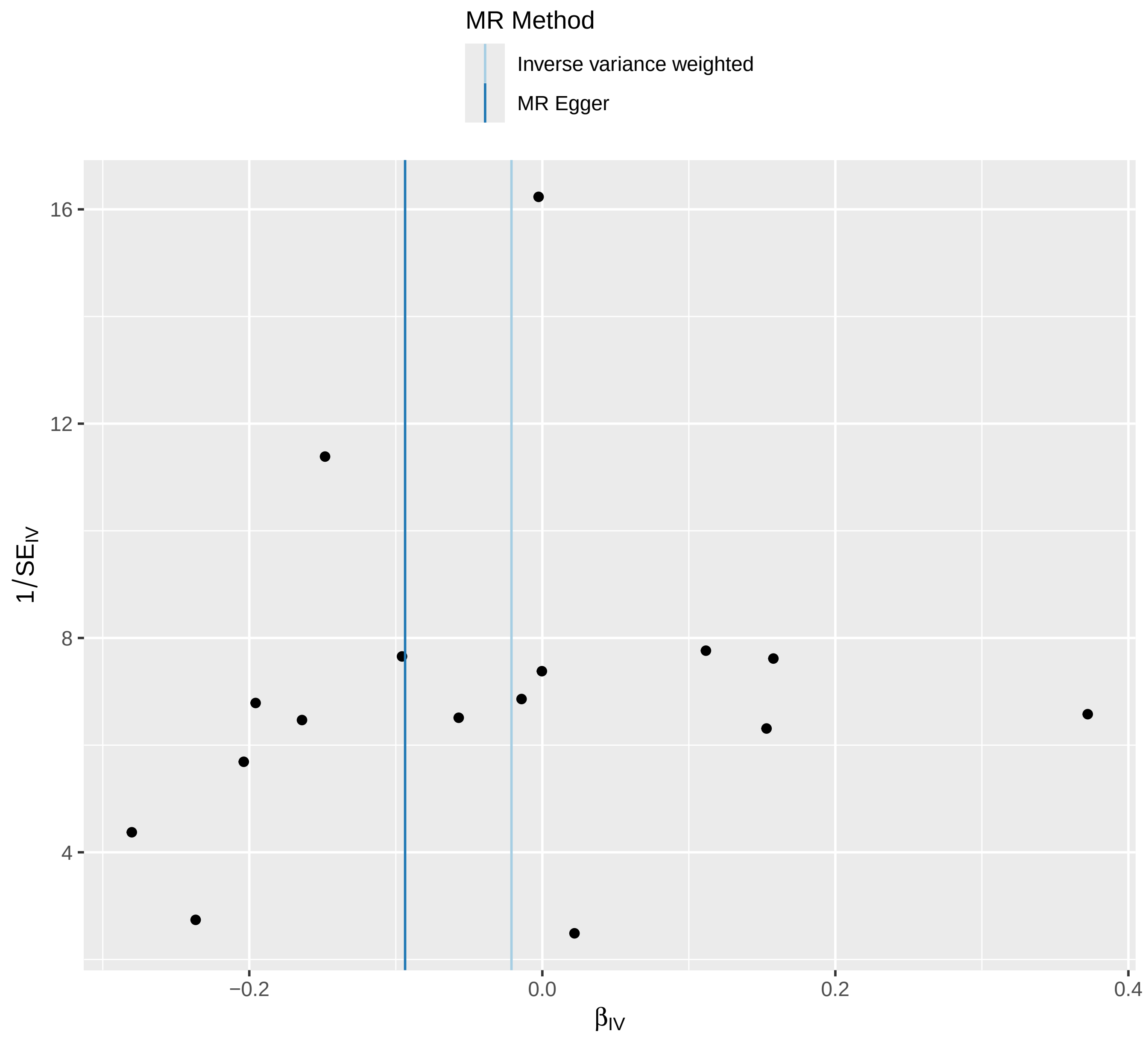

MR Method
Inverse variance weighted
MR Egger
16
12
8
4
−0.2
0.0
0.2
0.4
$1/SE_{IV}$
β_{IV}

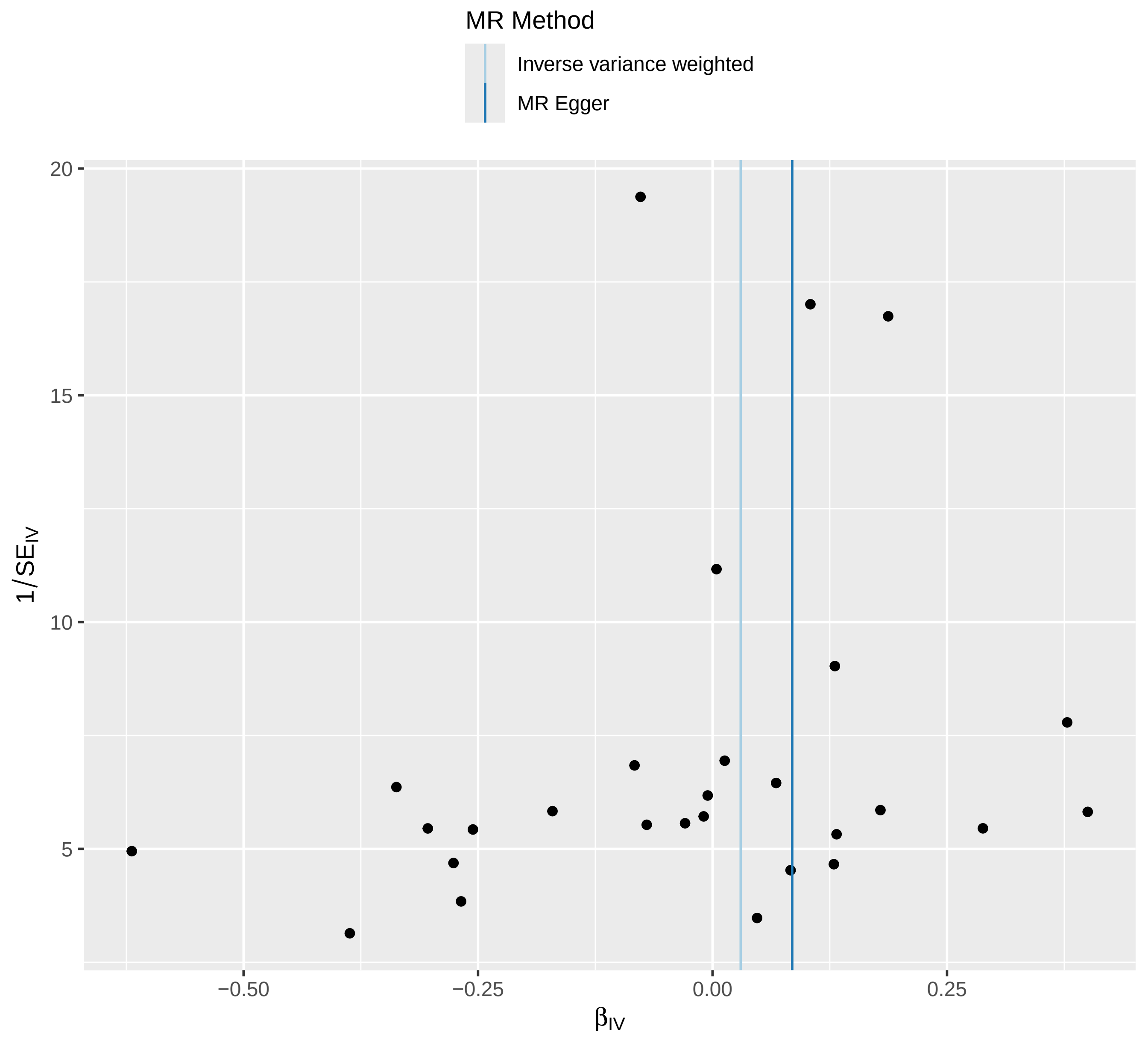

MR Method
Inverse variance weighted
MR Egger
20
15
10
5
1/SE_IV
−0.50
−0.25
0.00
0.25
β_IV

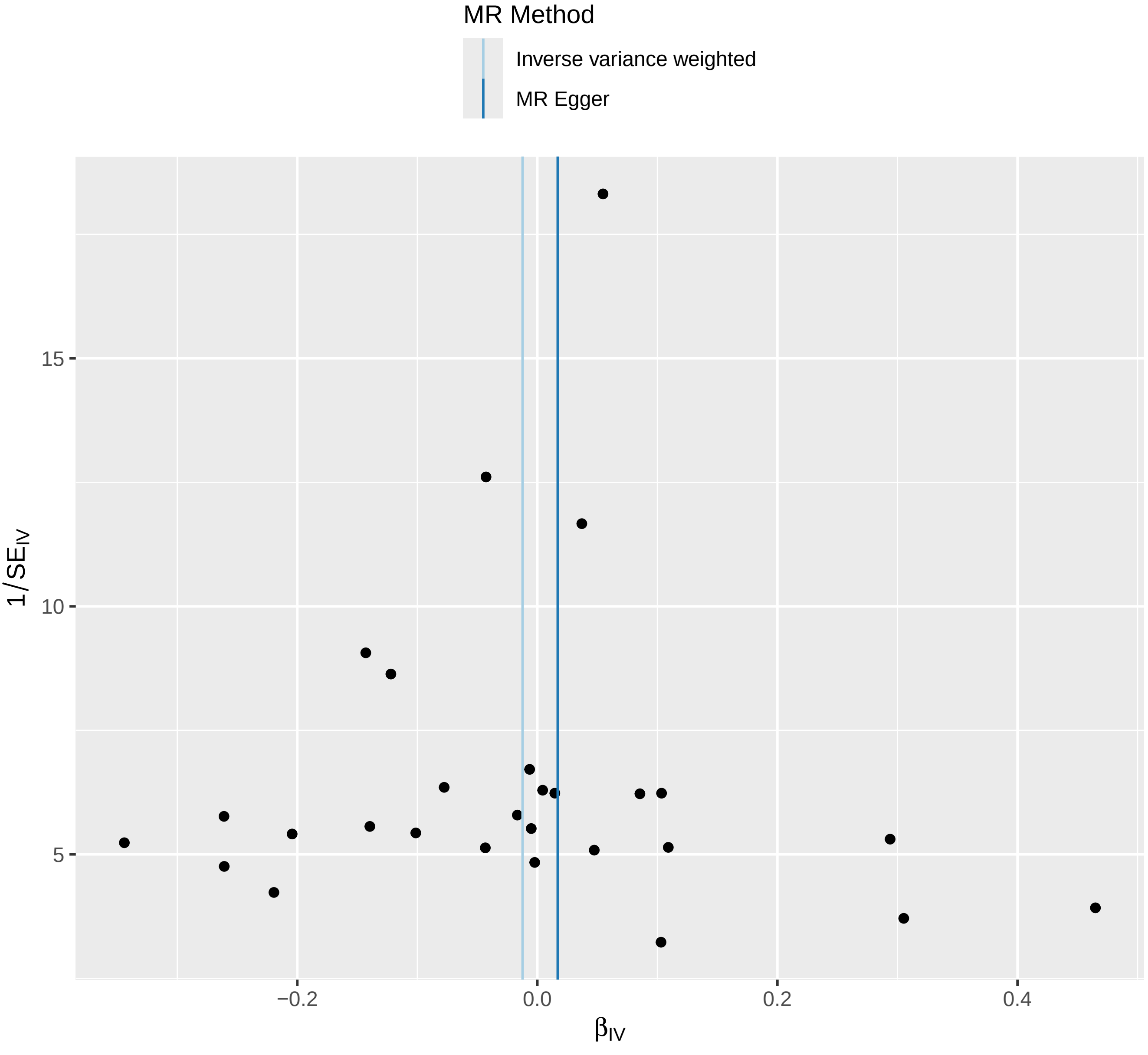
MR Method
Inverse variance weighted
MR Egger
15
10
5
1/SE_IV
−0.2
0.0
0.2
0.4
β_IV

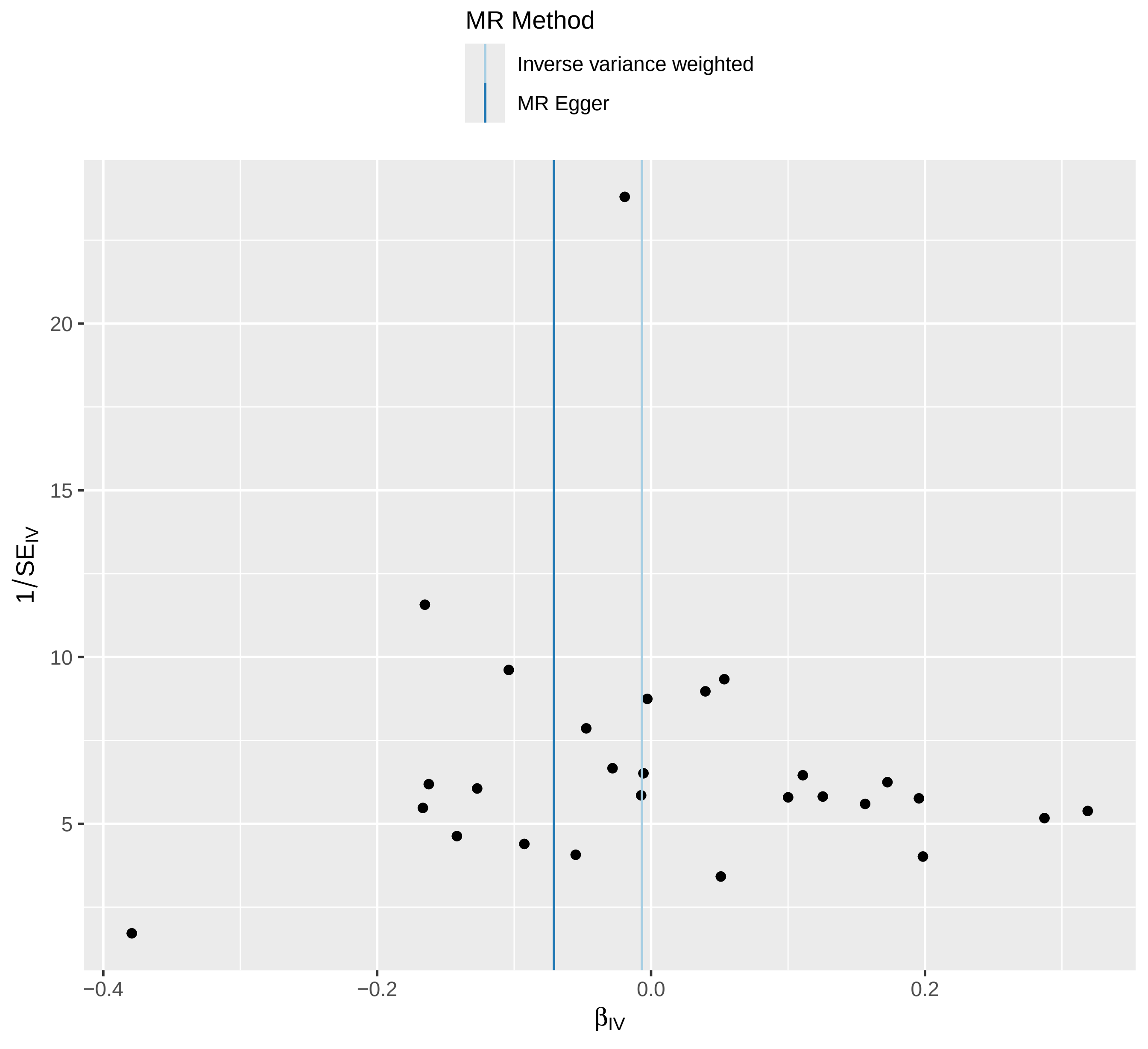

MR Method
Inverse variance weighted
MR Egger
20
15
10
5
1/SE_IV
−0.4
−0.2
0.0
0.2
β_IV

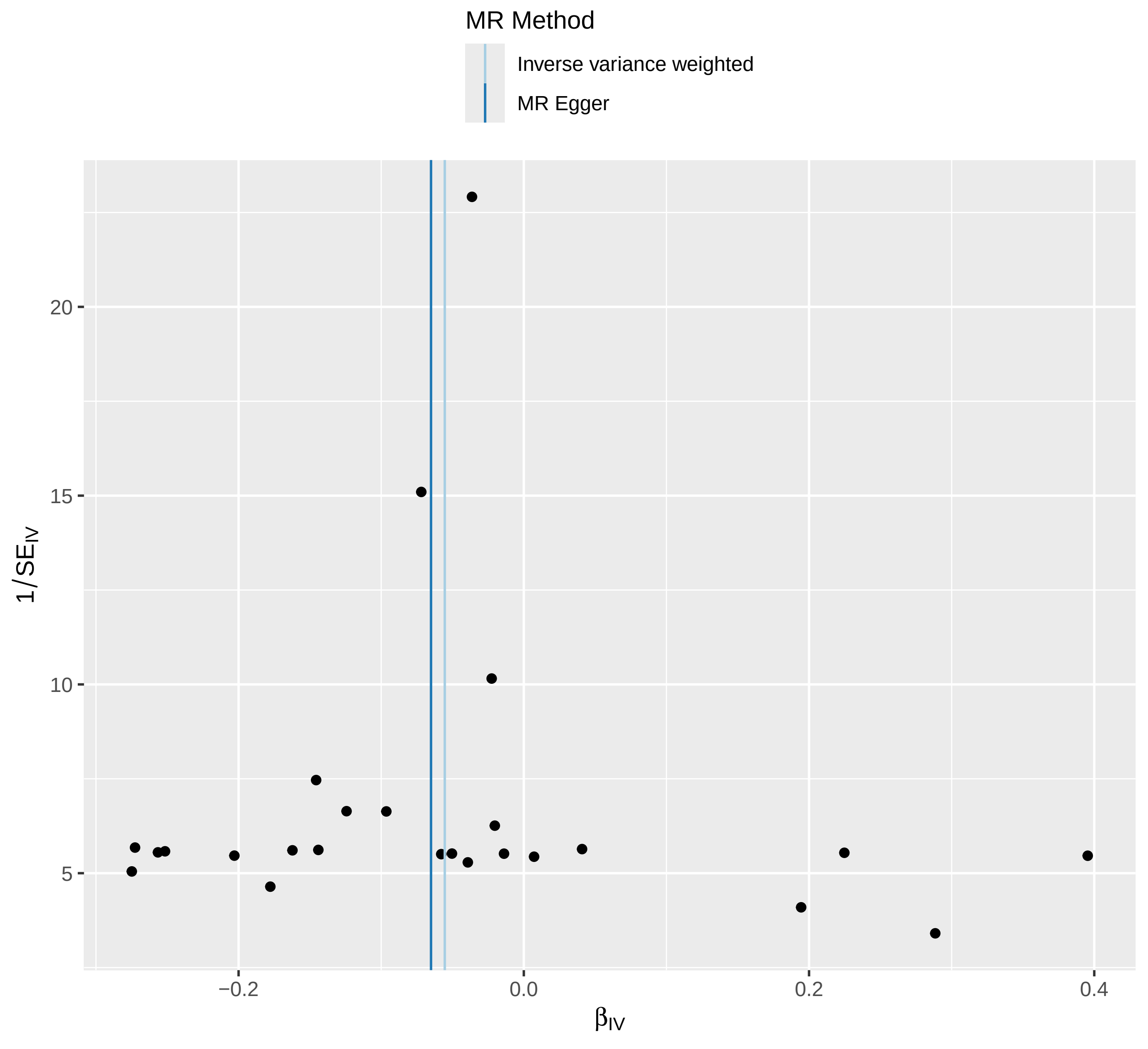
MR Method
Inverse variance weighted
MR Egger
20
15
10
5
1/SE_{IV}
−0.2
0.0
0.2
0.4
β_{IV}

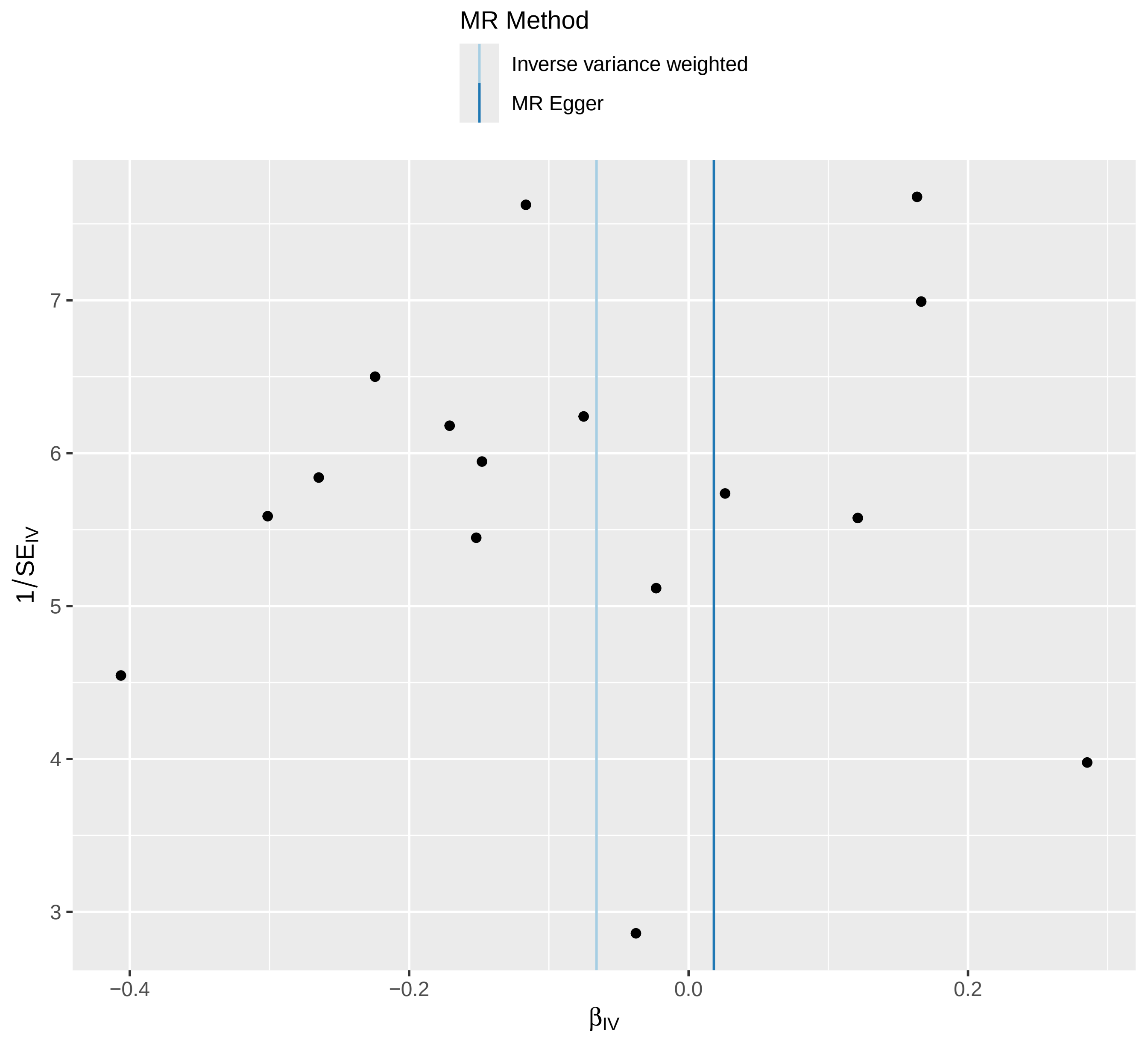
MR Method
Inverse variance weighted
MR Egger
1/SE_IV
7
6
5
4
3
−0.4
−0.2
0.0
0.2
β_IV

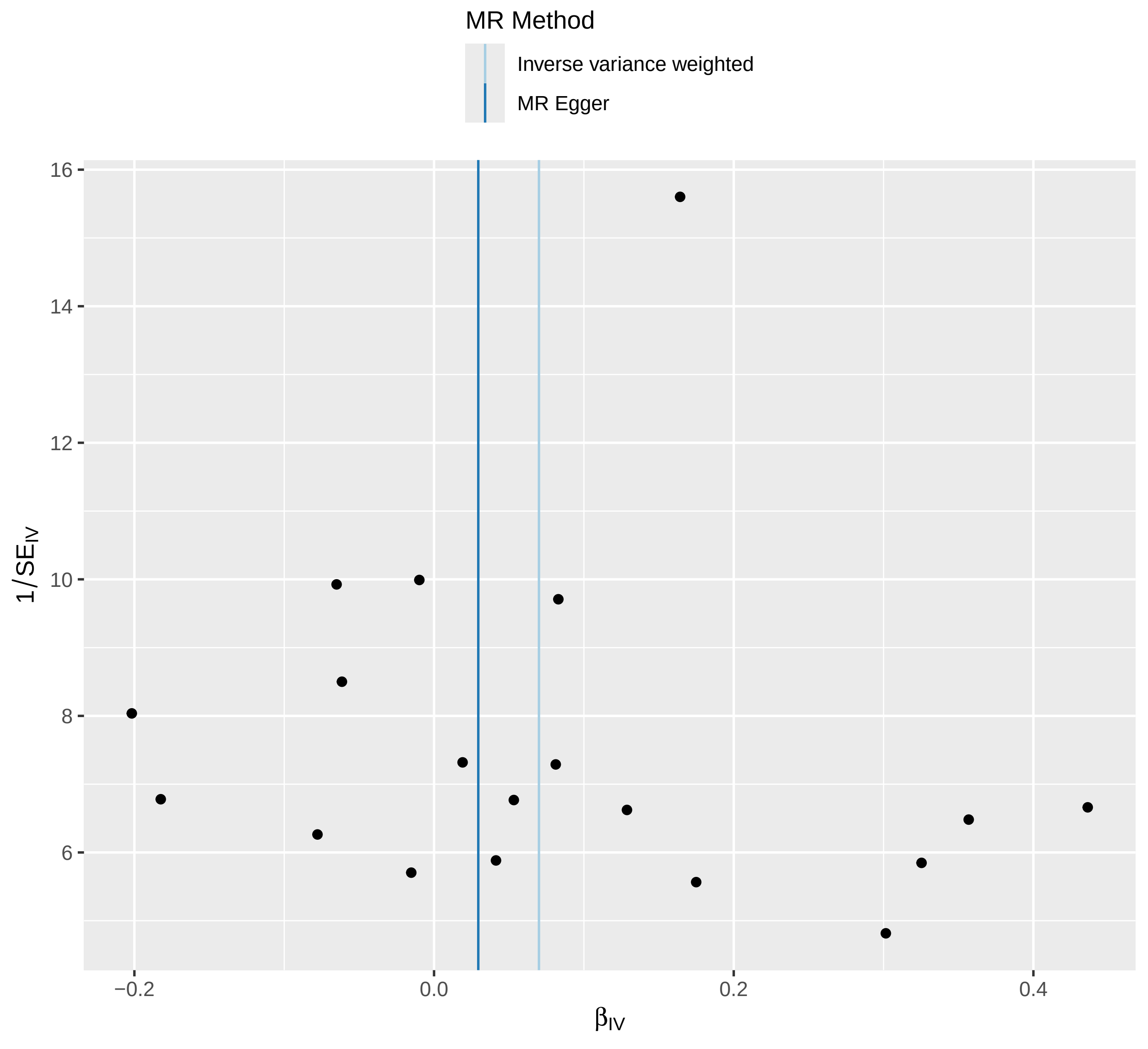

MR Method
Inverse variance weighted
MR Egger
1/SE_IV
β_IV
16
14
12
10
8
6
−0.2
0.0
0.2
0.4

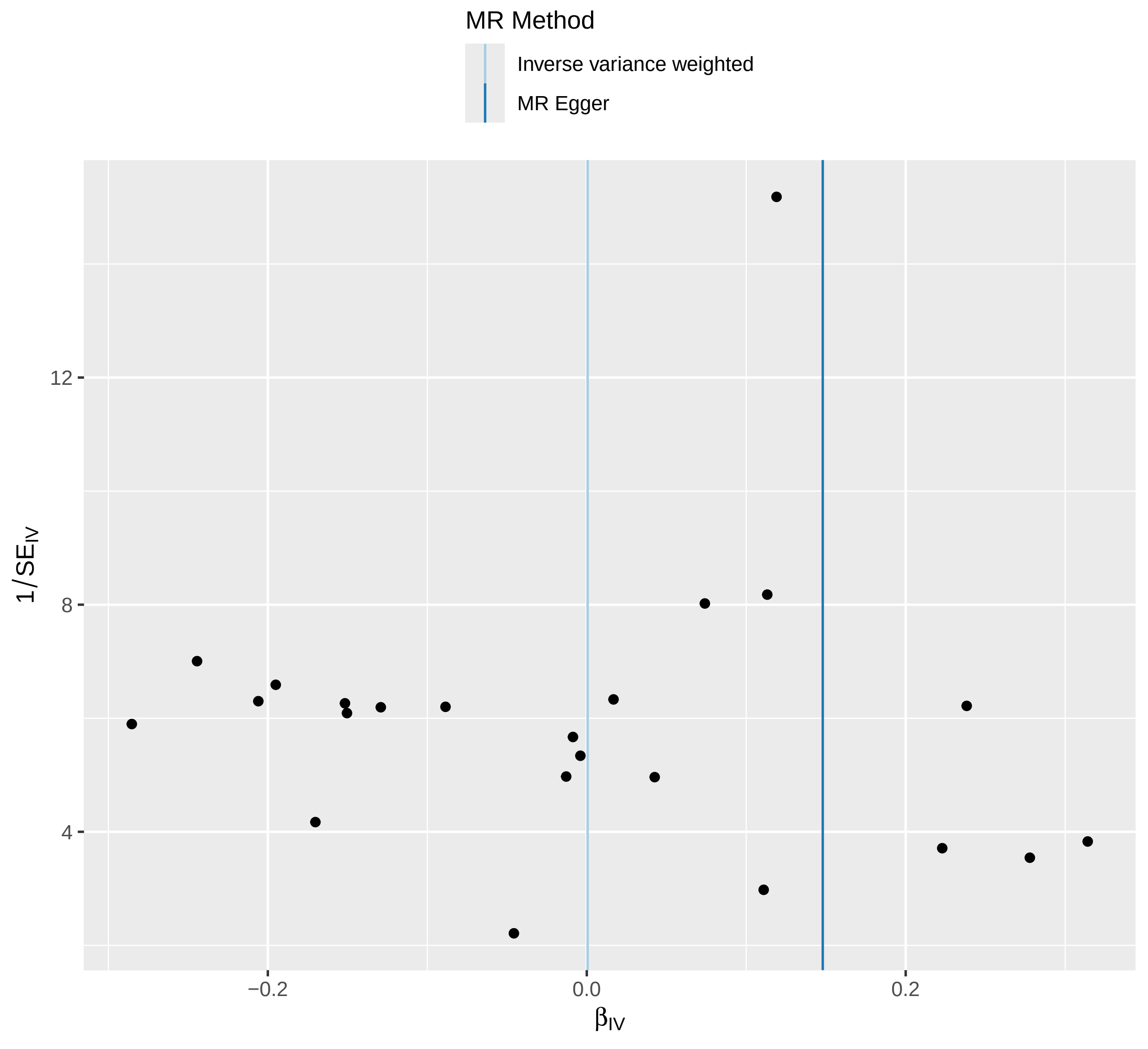
MR Method
Inverse variance weighted
MR Egger
12
8
4
$1/SE_{IV}$
−0.2
0.0
0.2
β_{IV}

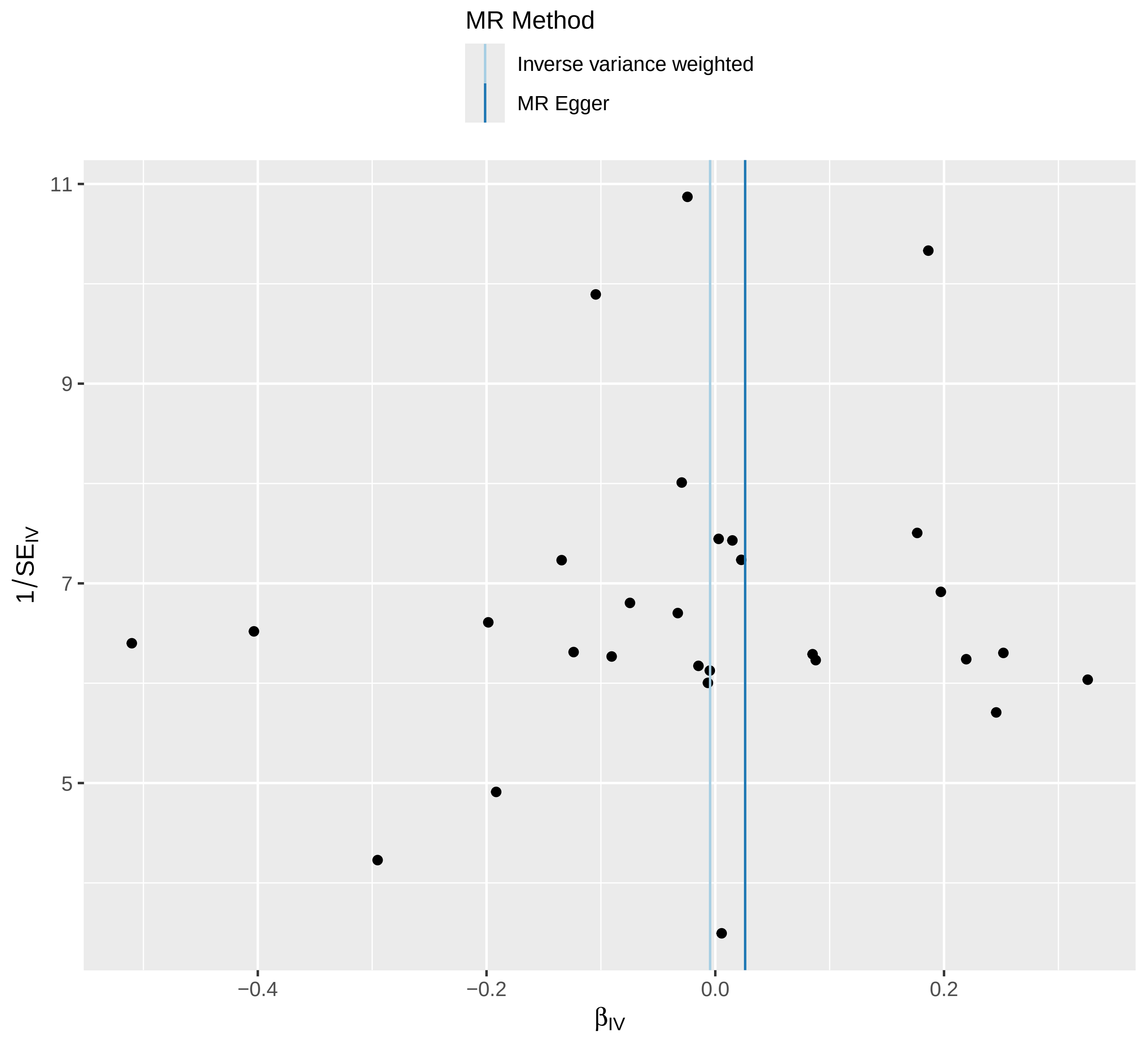

MR Method
Inverse variance weighted
MR Egger
11
9
7
5
1/SE_IV
−0.4
−0.2
0.0
0.2
β_IV

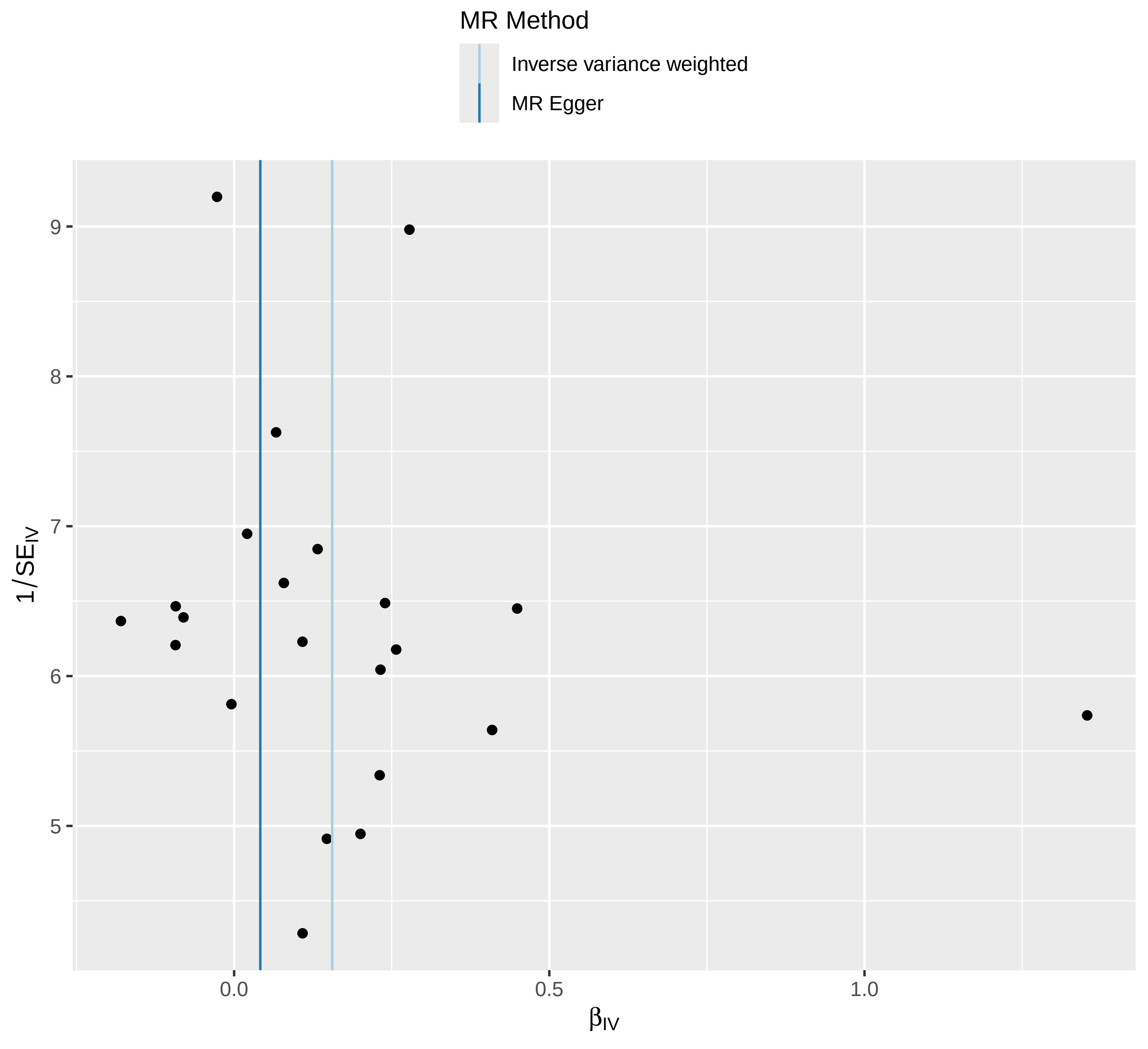
MR Method
Inverse variance weighted
MR Egger
9
8
7
6
5
1/SE_IV
0.0
0.5
1.0
β_IV

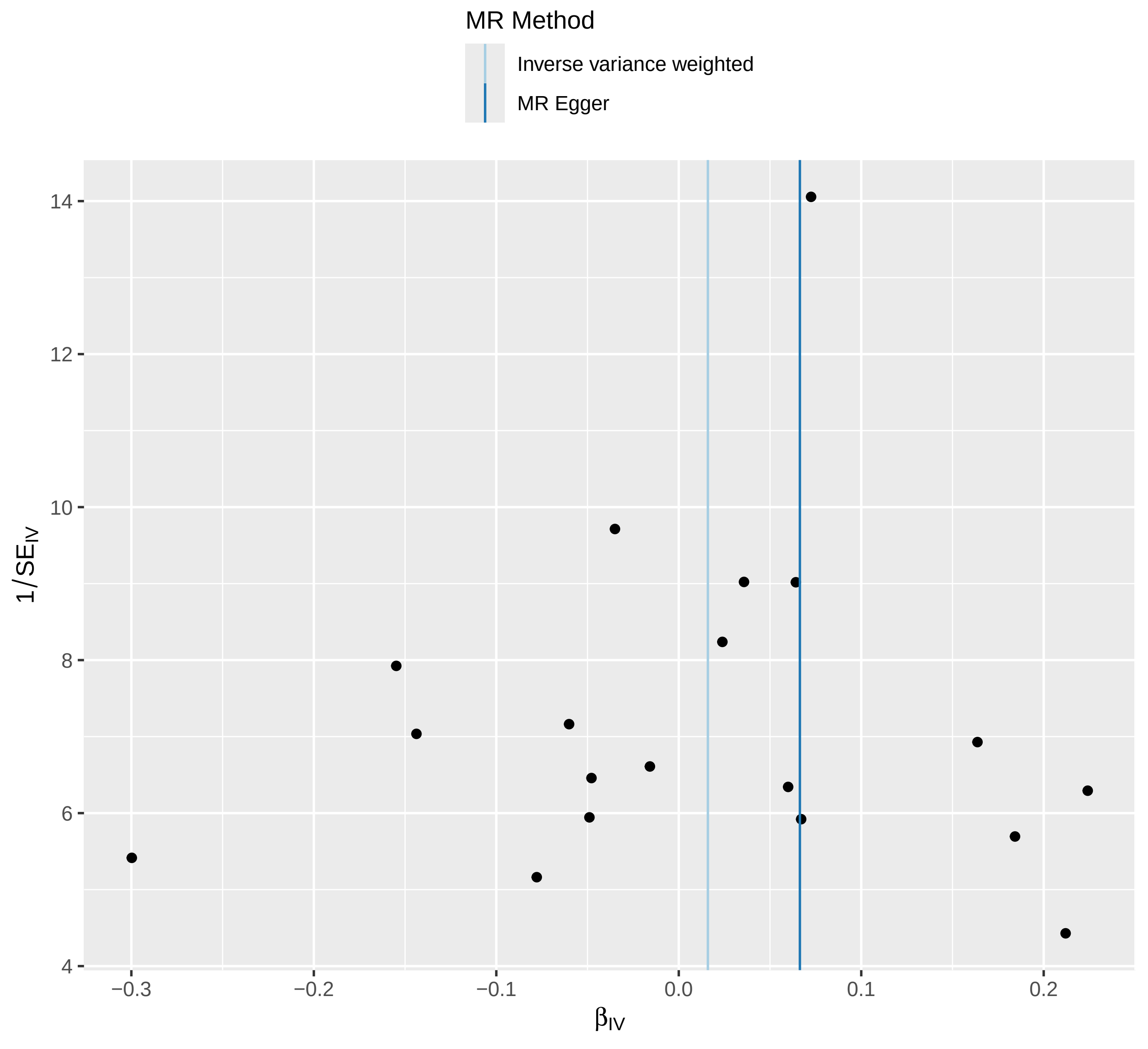

MR Method
Inverse variance weighted
MR Egger
14
12
10
8
6
4
1/SE$_{IV}$
−0.3
−0.2
−0.1
0.0
0.1
0.2
β$_{IV}$

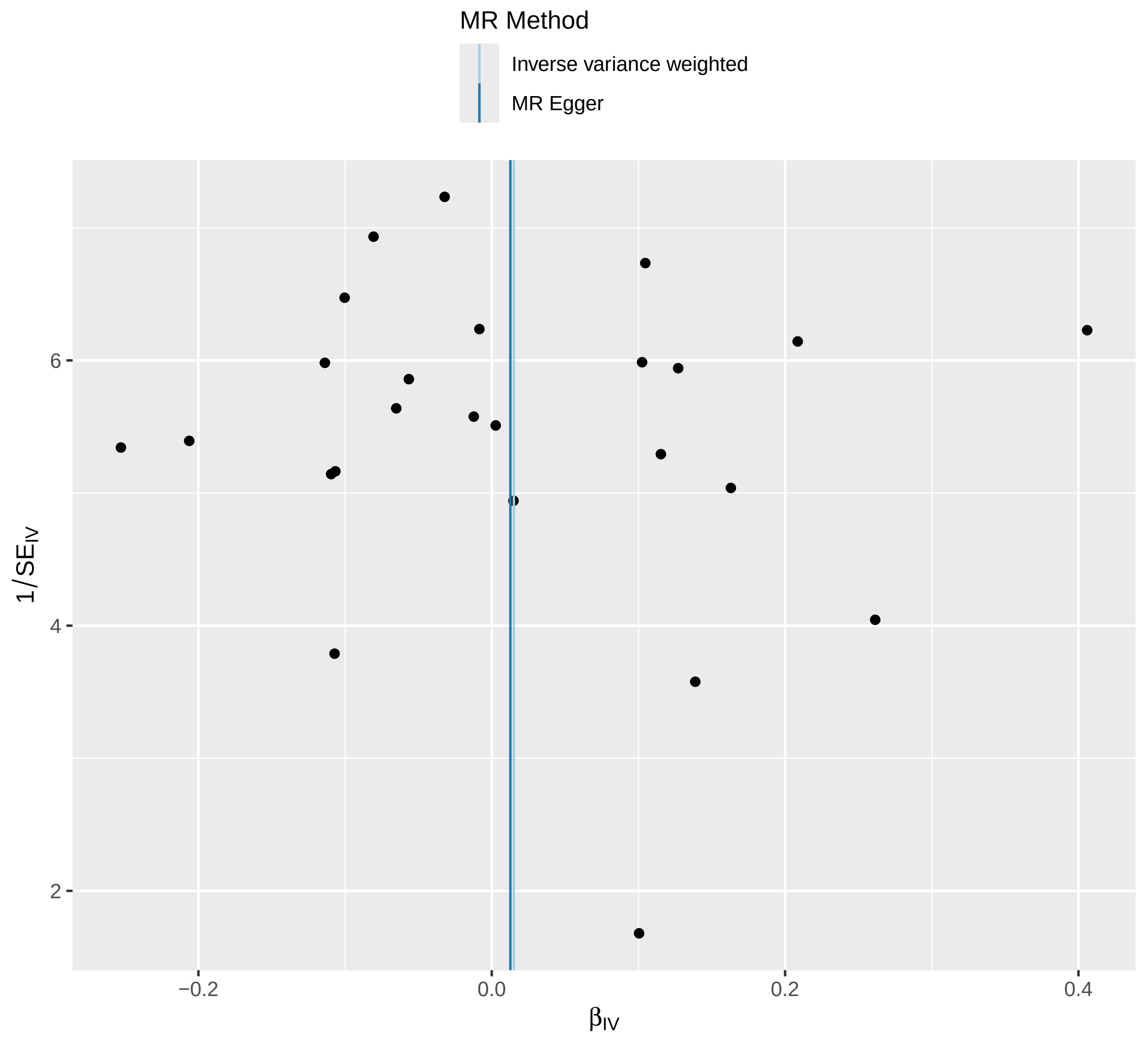
MR Method
Inverse variance weighted
MR Egger
1/SE_IV
β_IV
6
4
2
−0.2
0.0
0.2
0.4

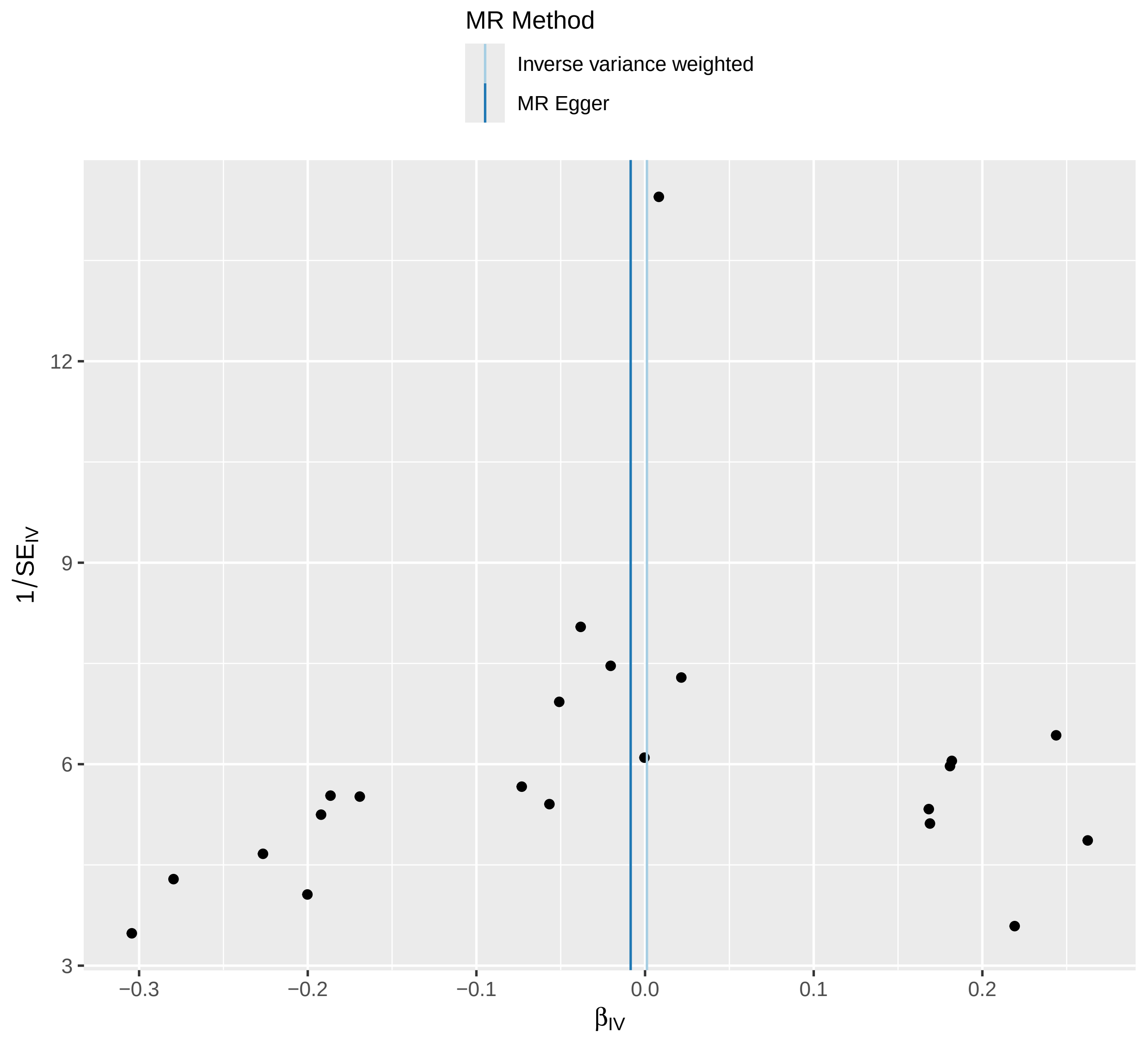
MR Method
Inverse variance weighted
MR Egger
12
9
6
3
−0.3
−0.2
−0.1
0.0
0.1
0.2
$1/SE_{IV}$
β_{IV}

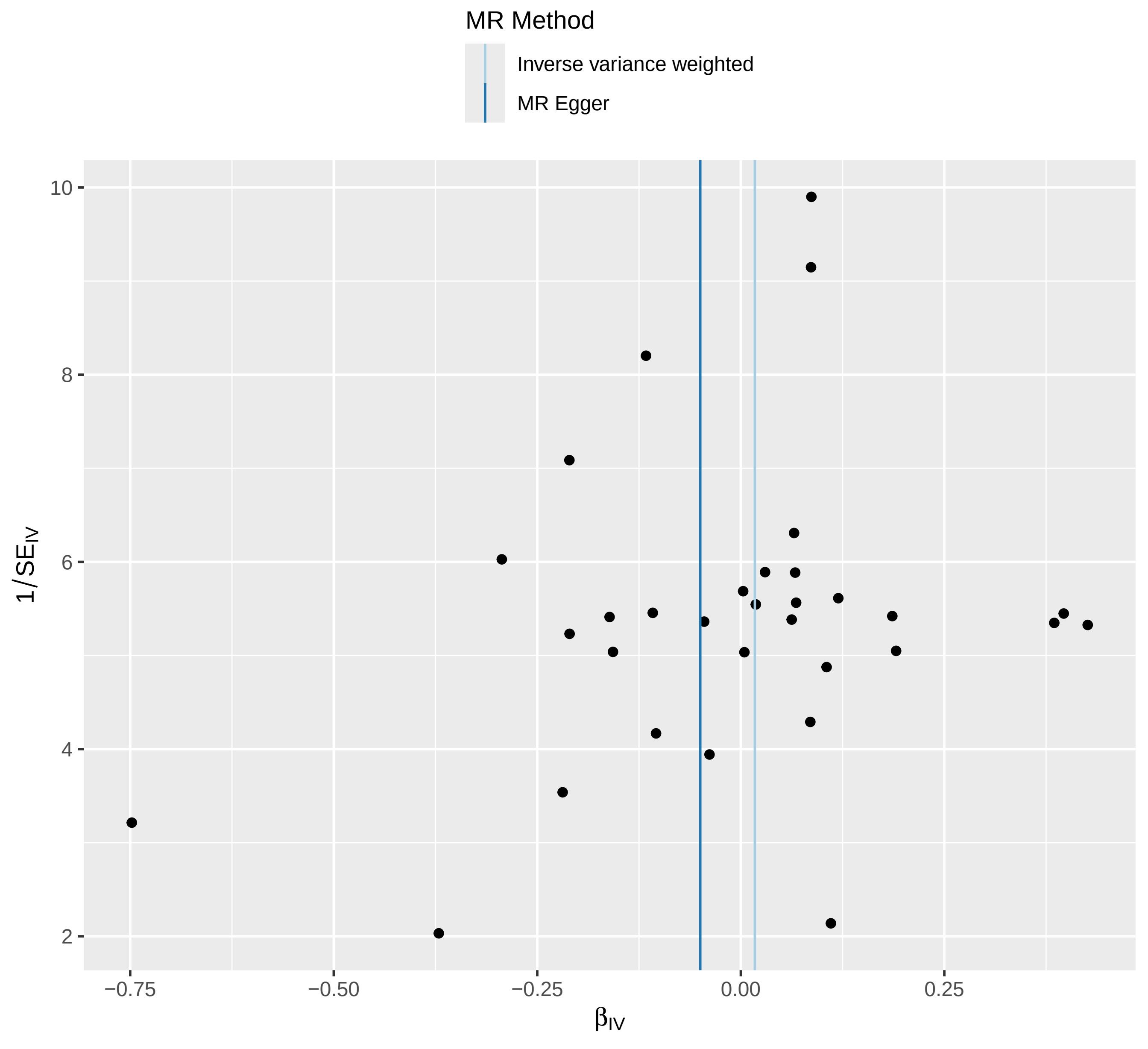

MR Method
Inverse variance weighted
MR Egger
10
8
6
4
2
1/SE_IV
−0.75
−0.50
−0.25
0.00
0.25
β_IV

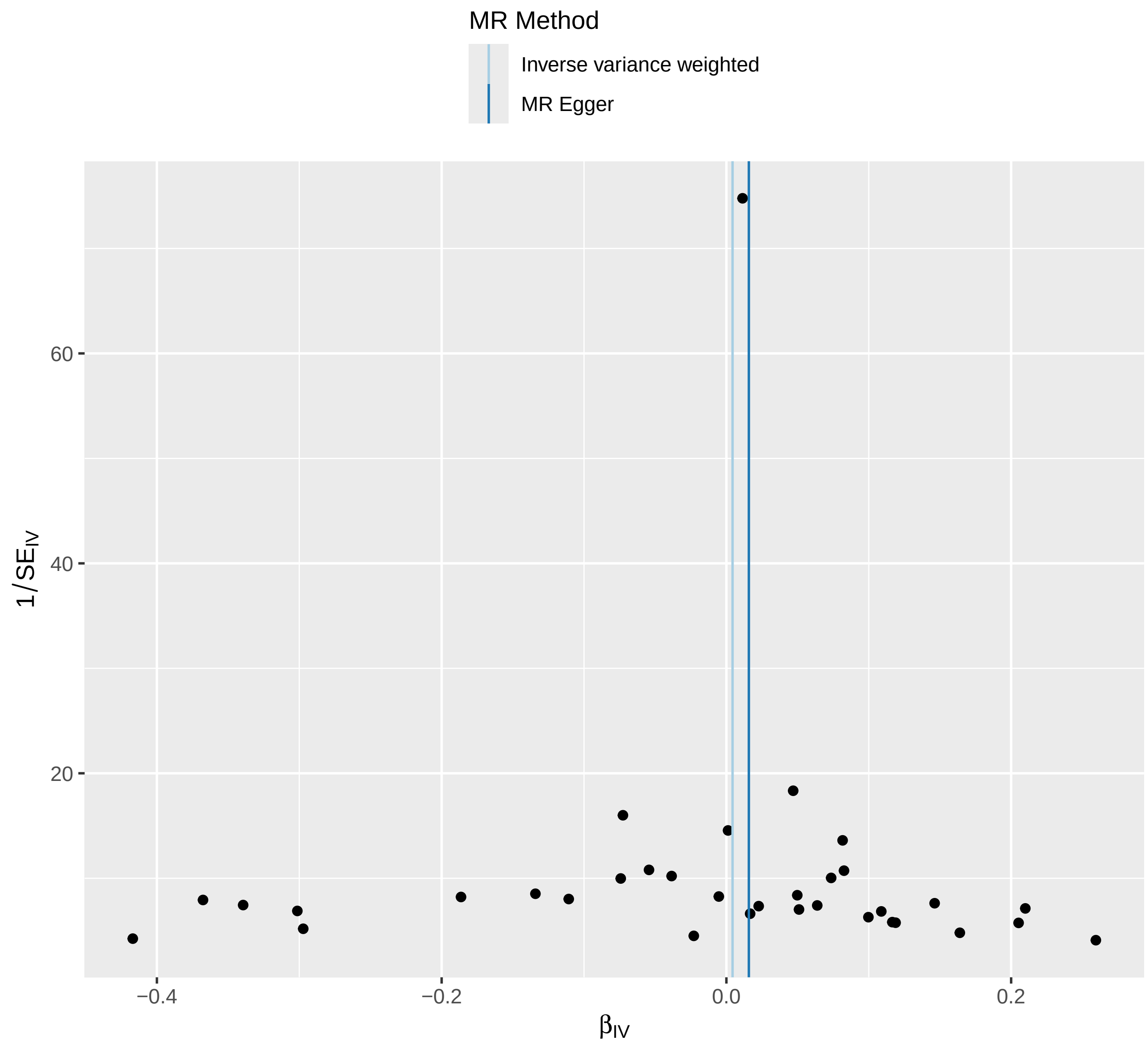
MR Method
Inverse variance weighted
MR Egger
60
40
20
1/SE_IV
−0.4
−0.2
0.0
0.2
β_IV

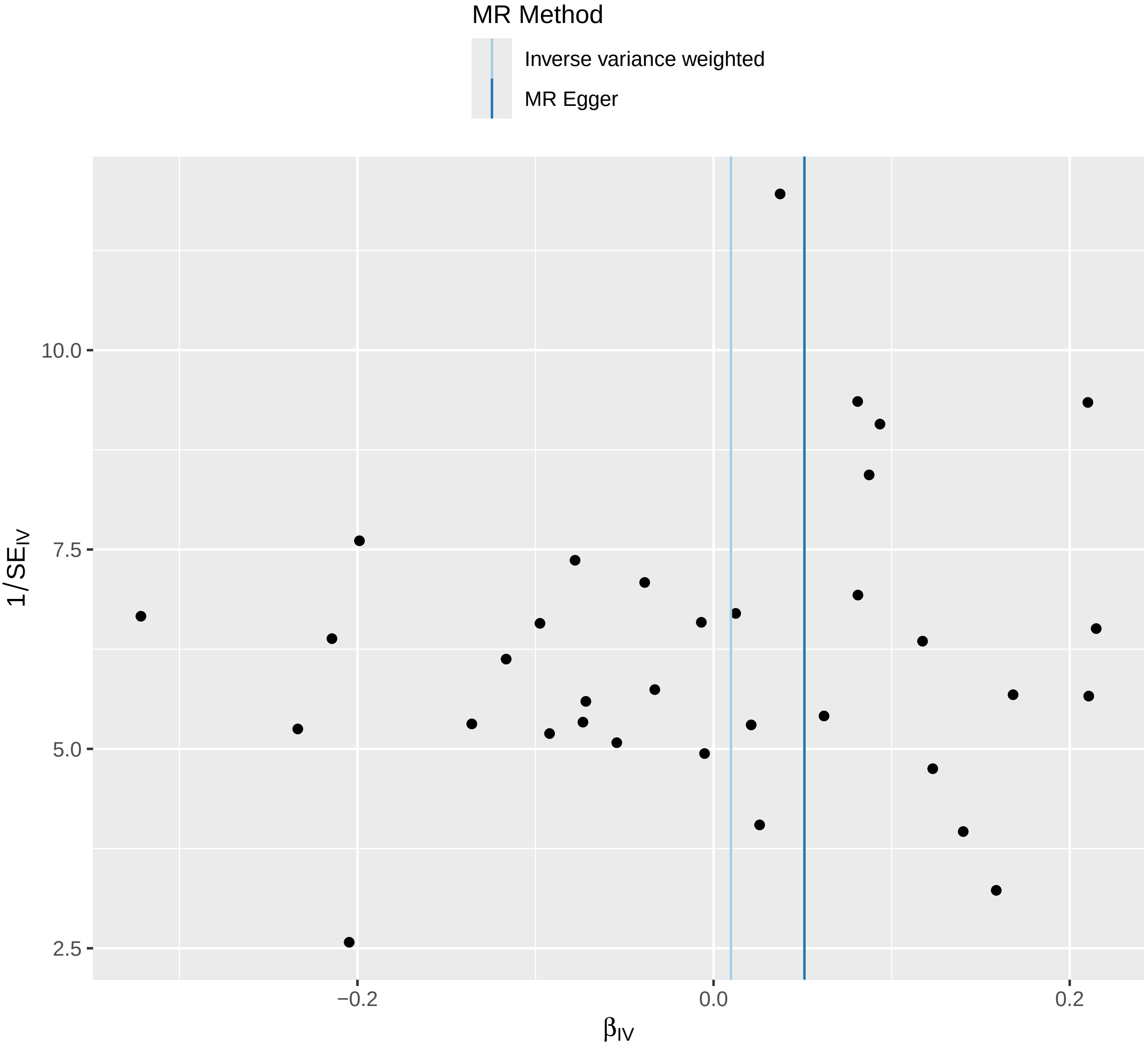
MR Method
Inverse variance weighted
MR Egger
10.0
7.5
5.0
2.5
$1/SE_{IV}$
−0.2
0.0
0.2
β_{IV}

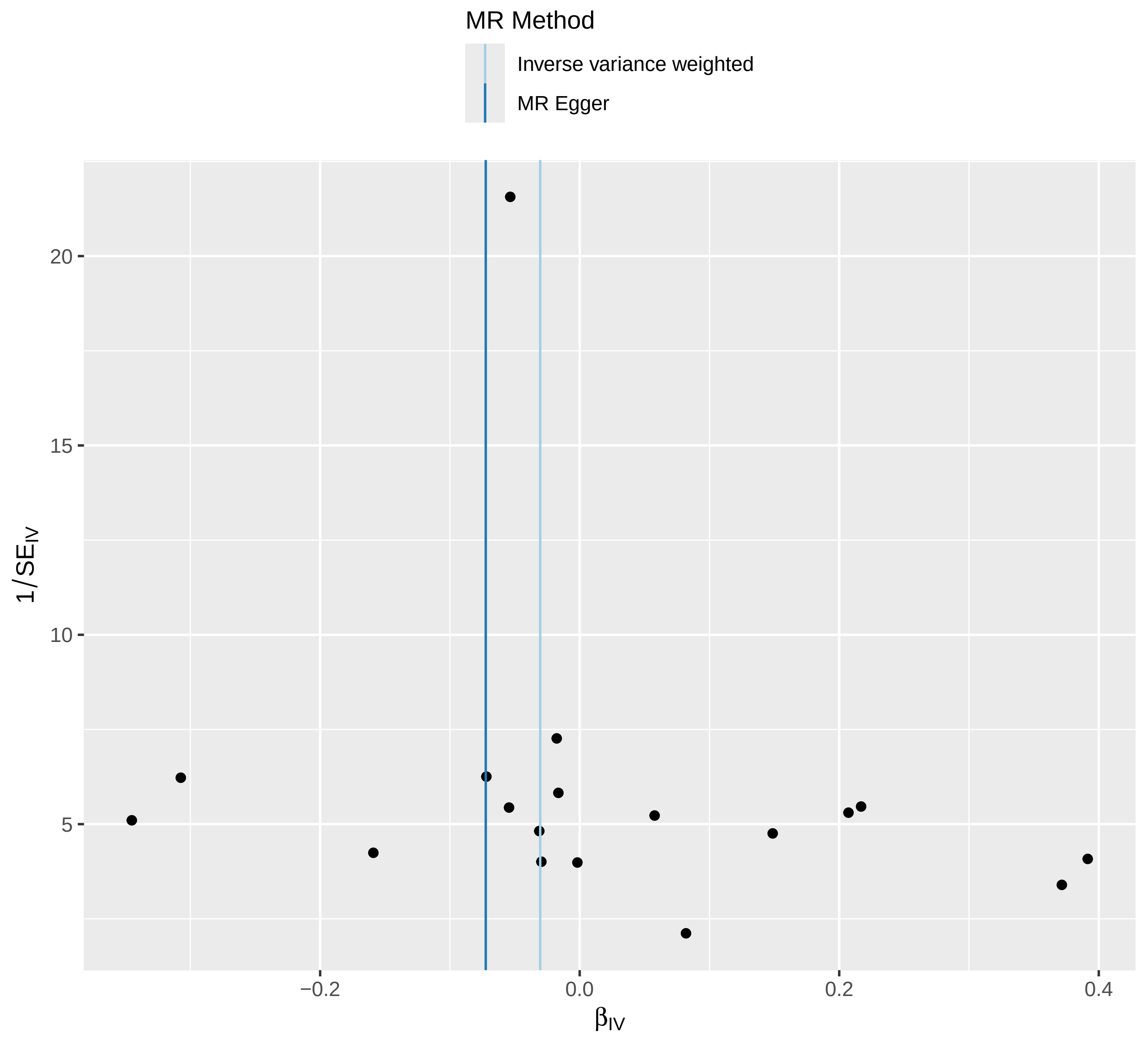

MR Method
Inverse variance weighted
MR Egger
20
15
10
5
1/SE_IV
−0.2
0.0
0.2
0.4
β_IV

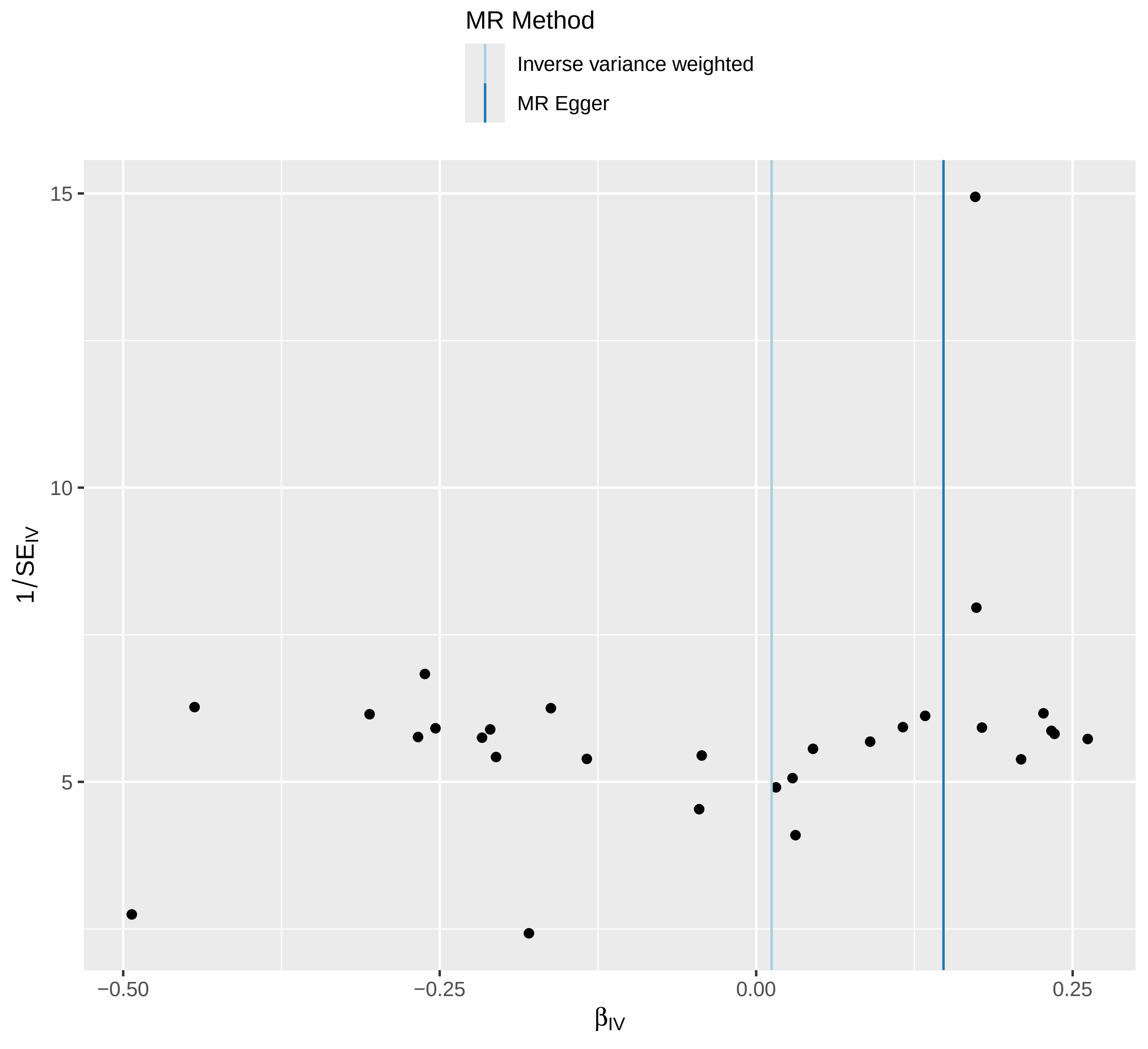
MR Method
Inverse variance weighted
MR Egger
15
10
5
1/SE$_{IV}$
−0.50
−0.25
0.00
0.25
β$_{IV}$

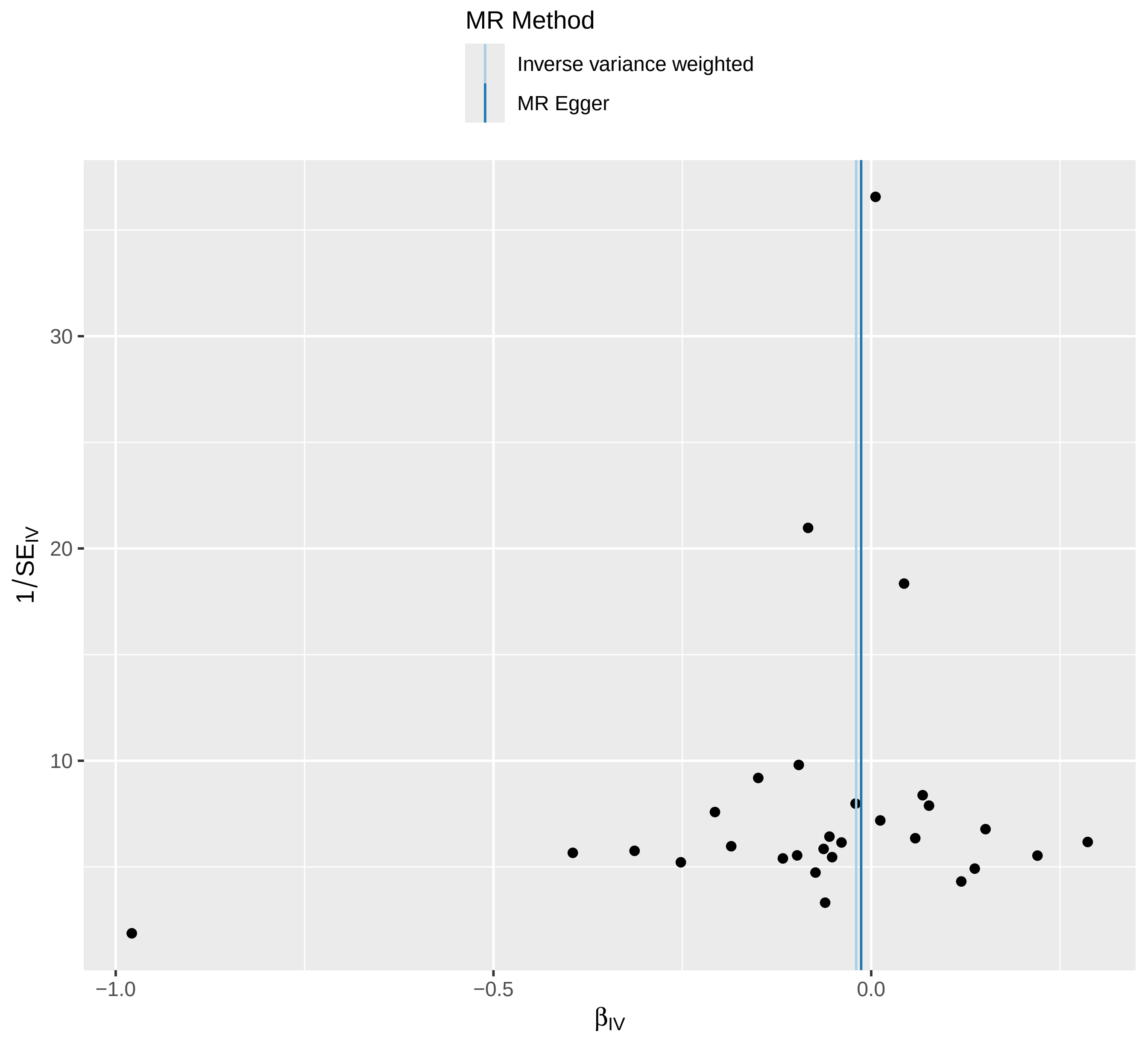
MR Method
Inverse variance weighted
MR Egger
30
20
10
1/SE$_{IV}$
−1.0
−0.5
0.0
β$_{IV}$

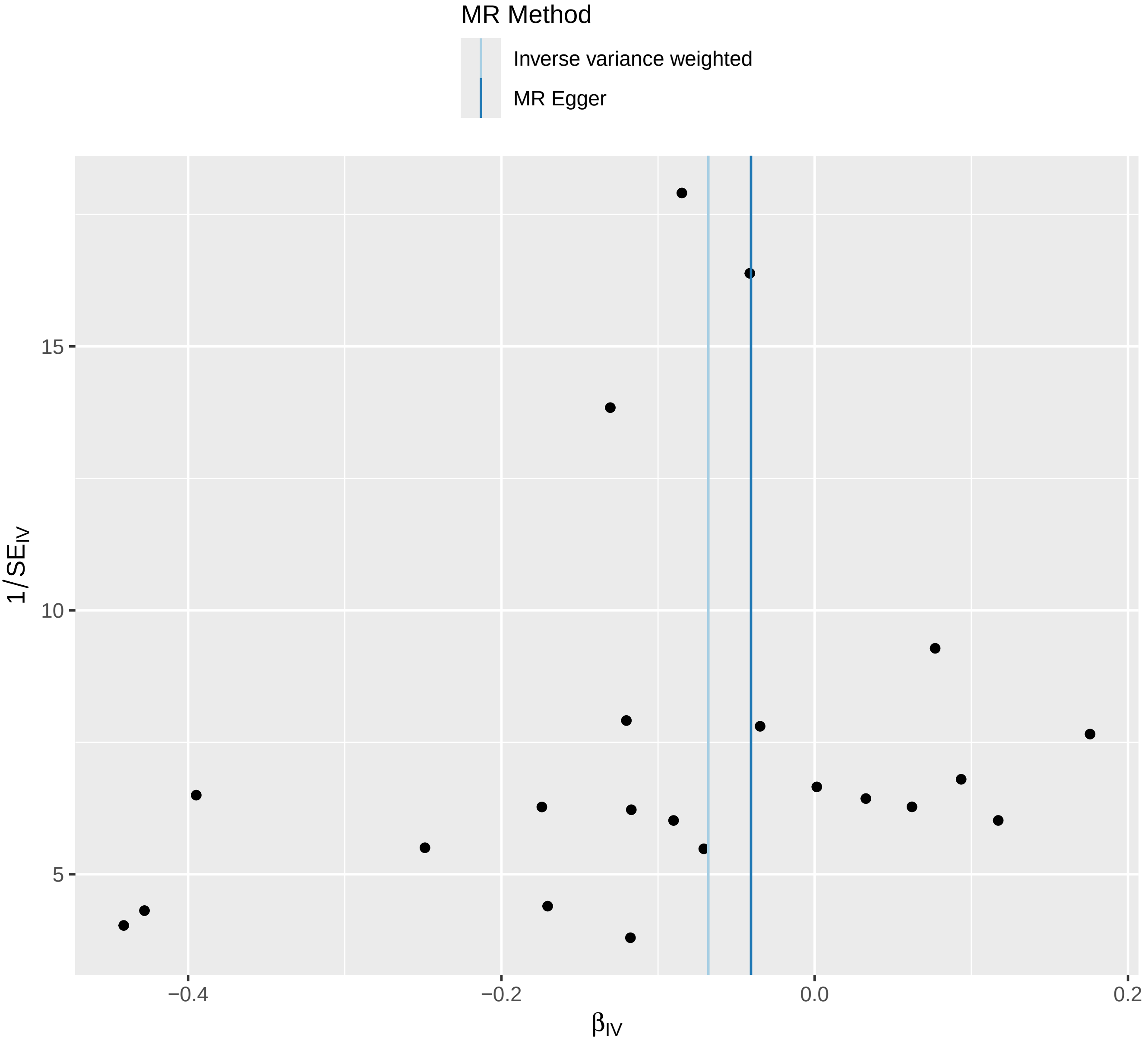
MR Method
Inverse variance weighted
MR Egger
1/SE_IV
15
10
5
−0.4
−0.2
0.0
0.2
β_IV

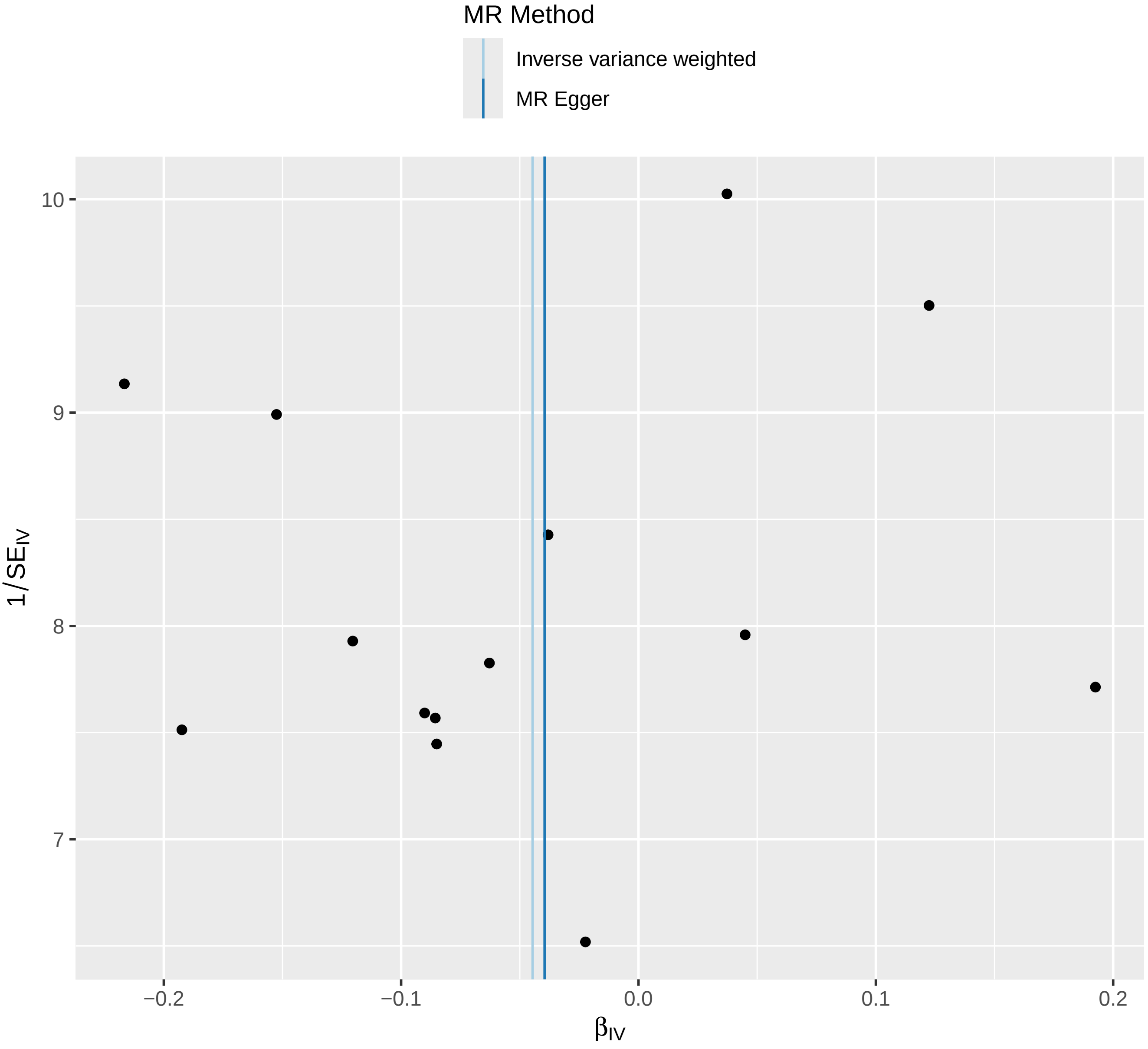
MR Method
Inverse variance weighted
MR Egger
10
9
8
7
1/SE_{IV}
−0.2
−0.1
0.0
0.1
0.2
β_{IV}

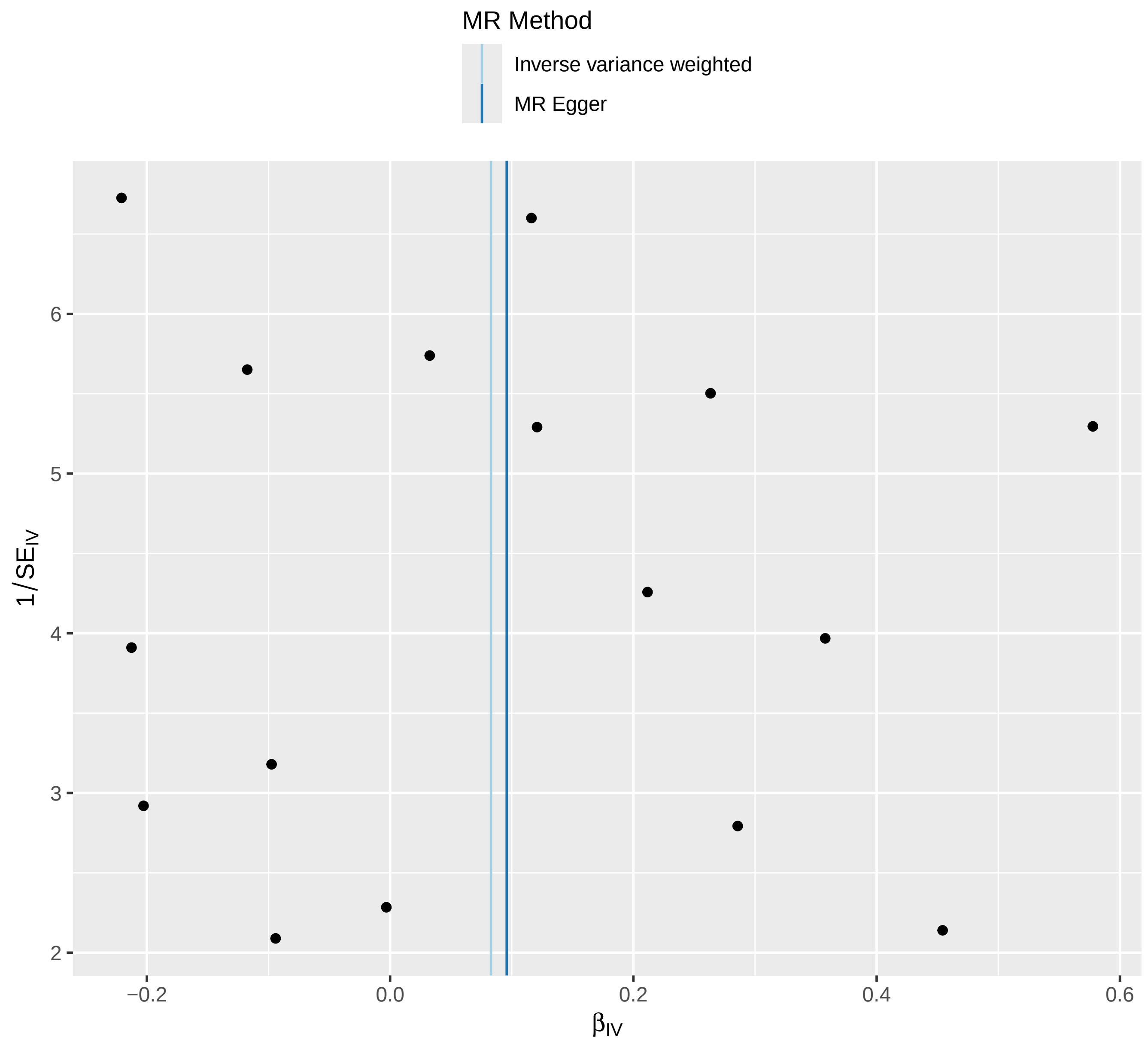

MR Method
Inverse variance weighted
MR Egger
1/SE_IV
β_IV
6
5
4
3
2
−0.2
0.0
0.2
0.4
0.6

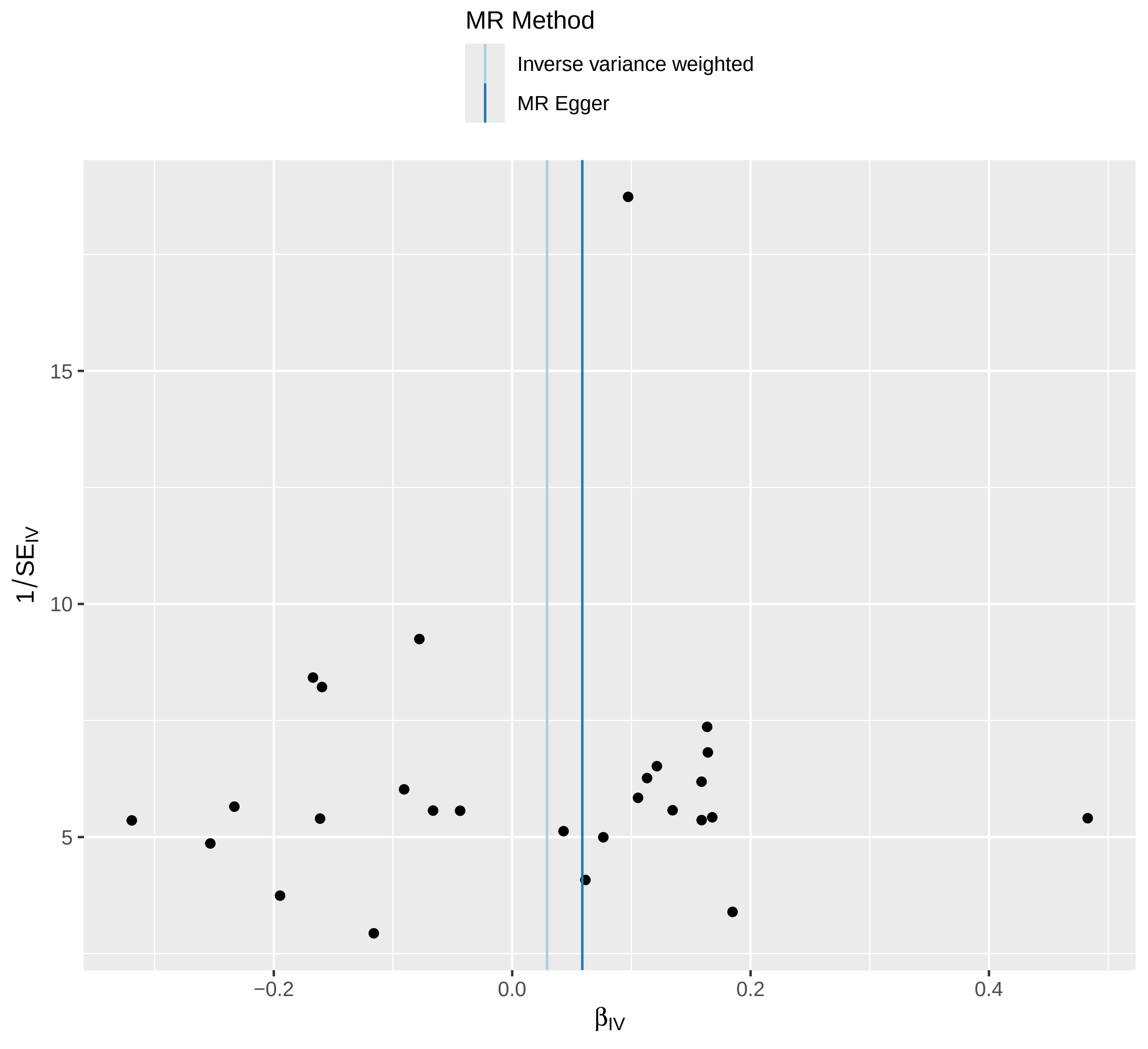

MR Method
Inverse variance weighted
MR Egger
15
10
5
−0.2
0.0
0.2
0.4
$1/SE_{IV}$
β_{IV}

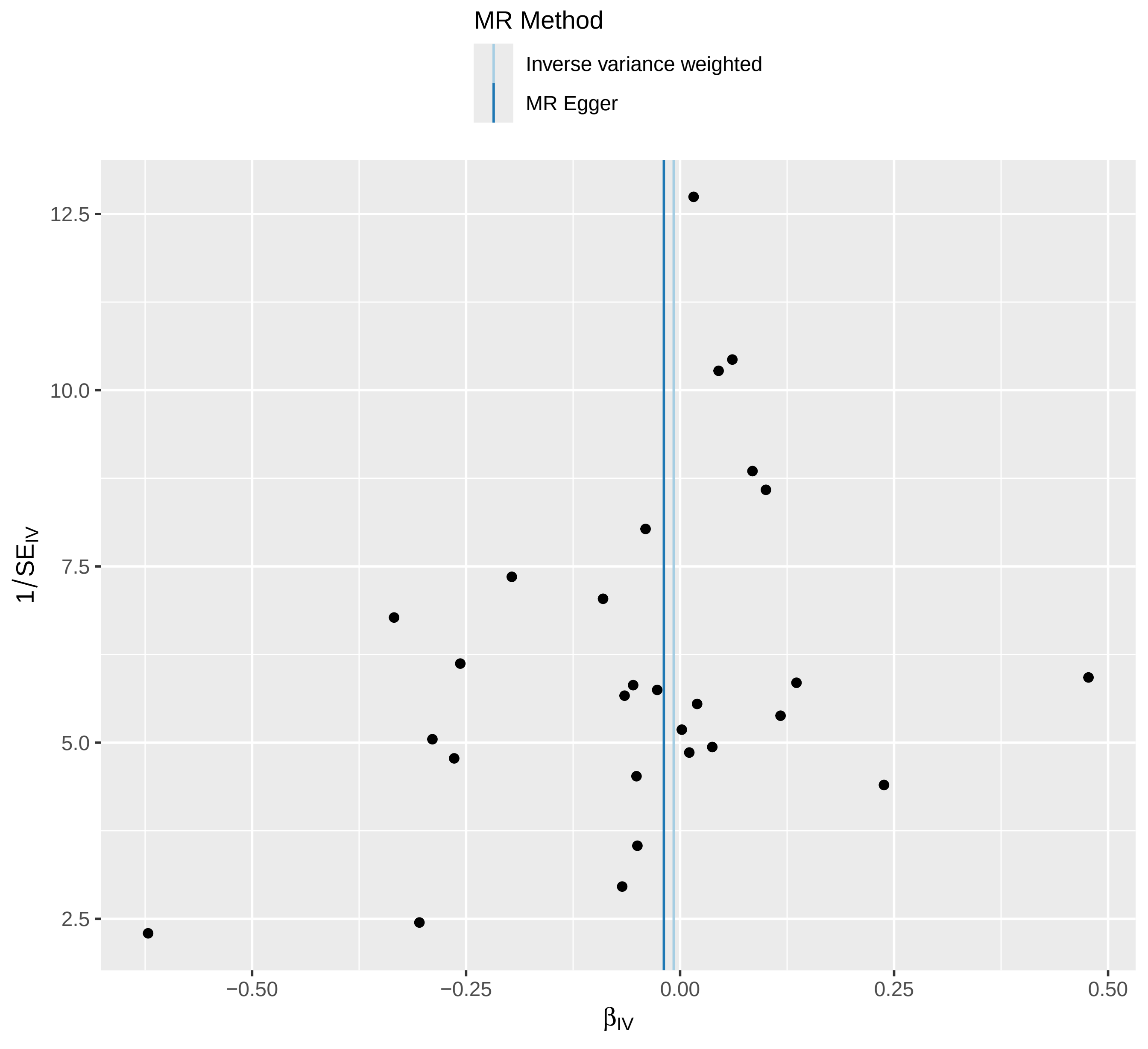
MR Method
Inverse variance weighted
MR Egger
12.5
10.0
7.5
5.0
2.5
$1/SE_{IV}$
−0.50
−0.25
0.00
0.25
0.50
β_{IV}

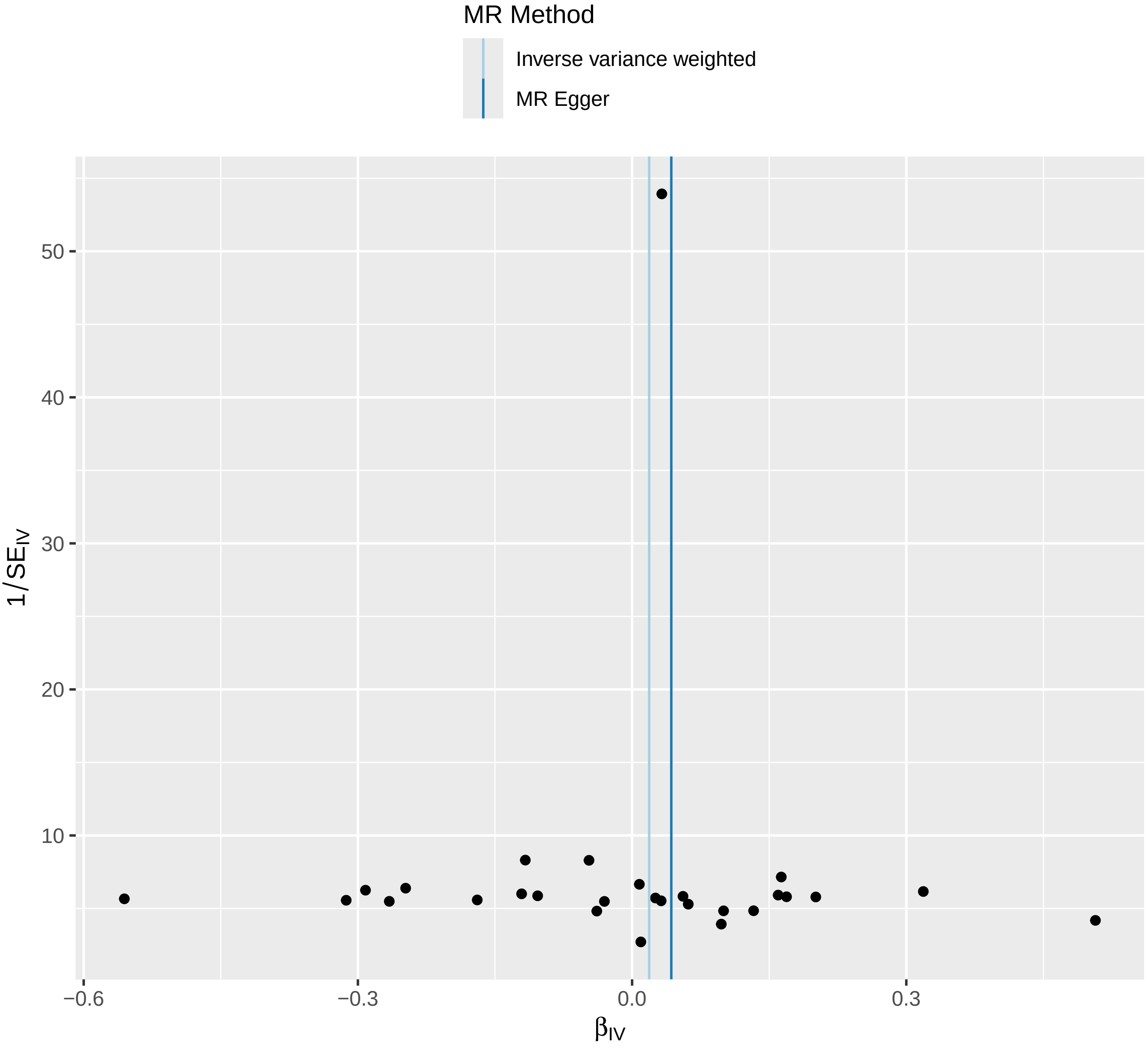
MR Method
Inverse variance weighted
MR Egger
50
40
30
20
10
$1/SE_{IV}$
−0.6
−0.3
0.0
0.3
β_{IV}

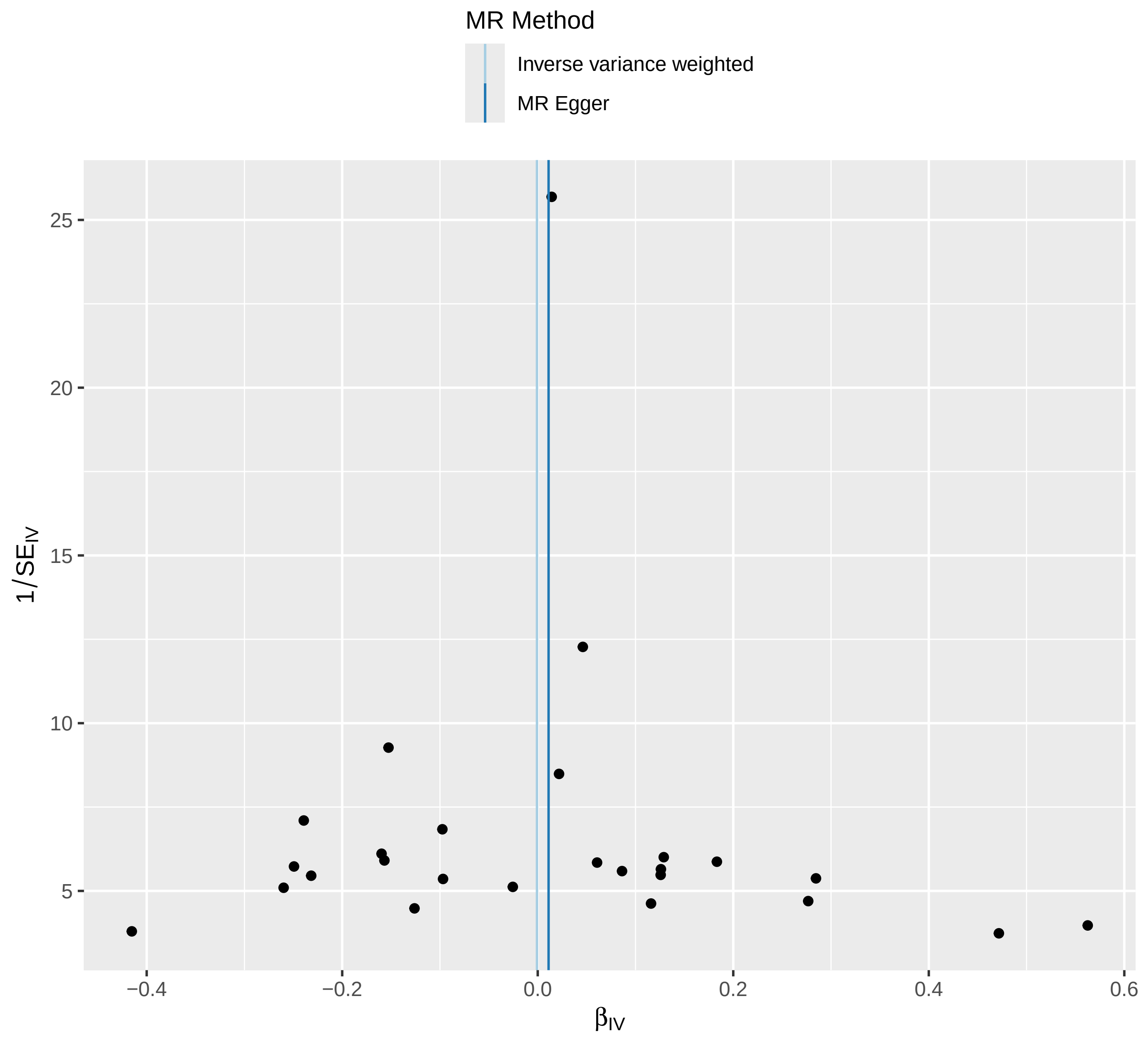
MR Method
Inverse variance weighted
MR Egger
1/SE_IV
25
20
15
10
5
−0.4
−0.2
0.0
0.2
0.4
0.6
β_IV

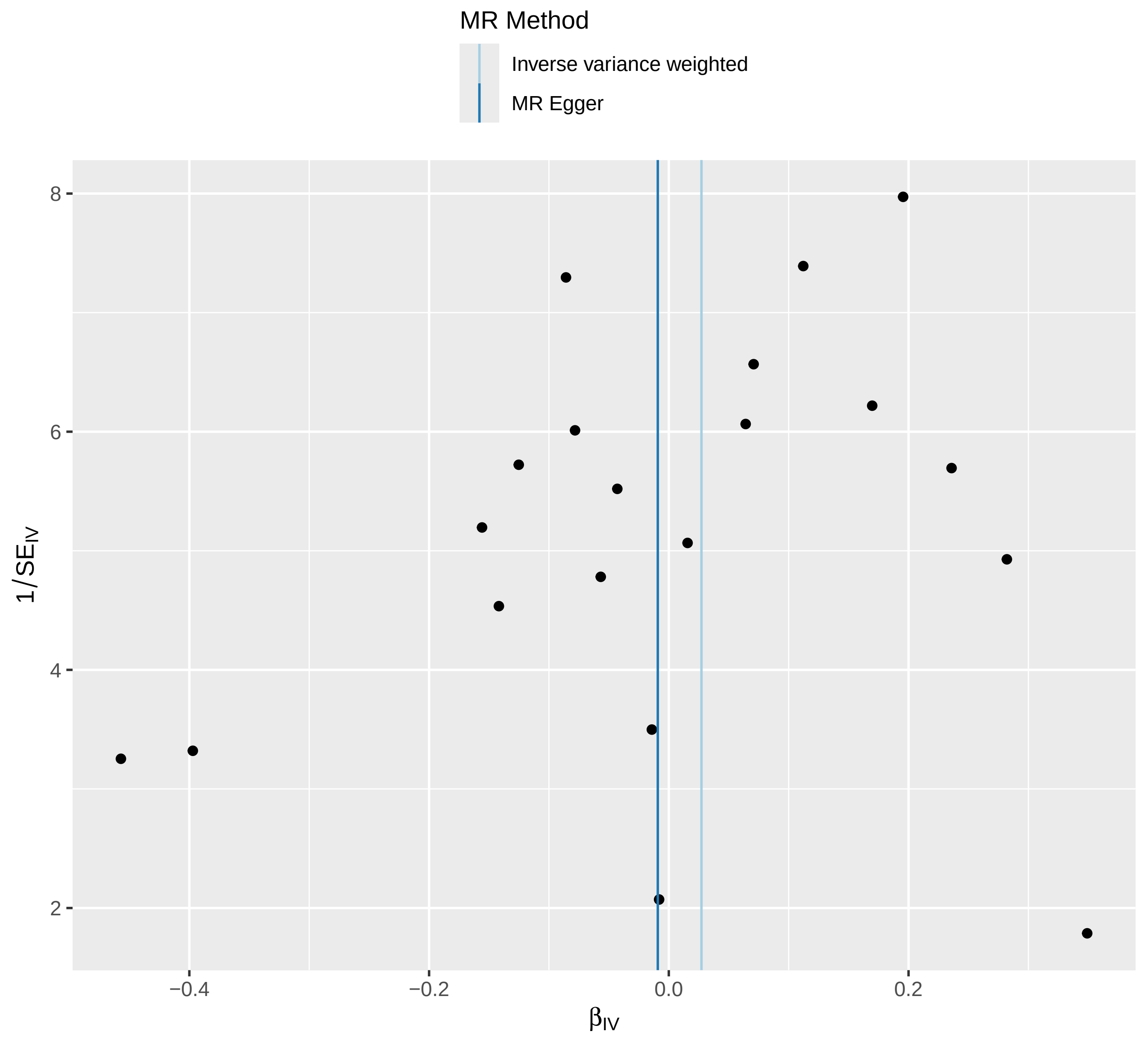
MR Method
Inverse variance weighted
MR Egger
8
6
4
2
−0.4
−0.2
0.0
0.2
$1/SE_{IV}$
β_{IV}

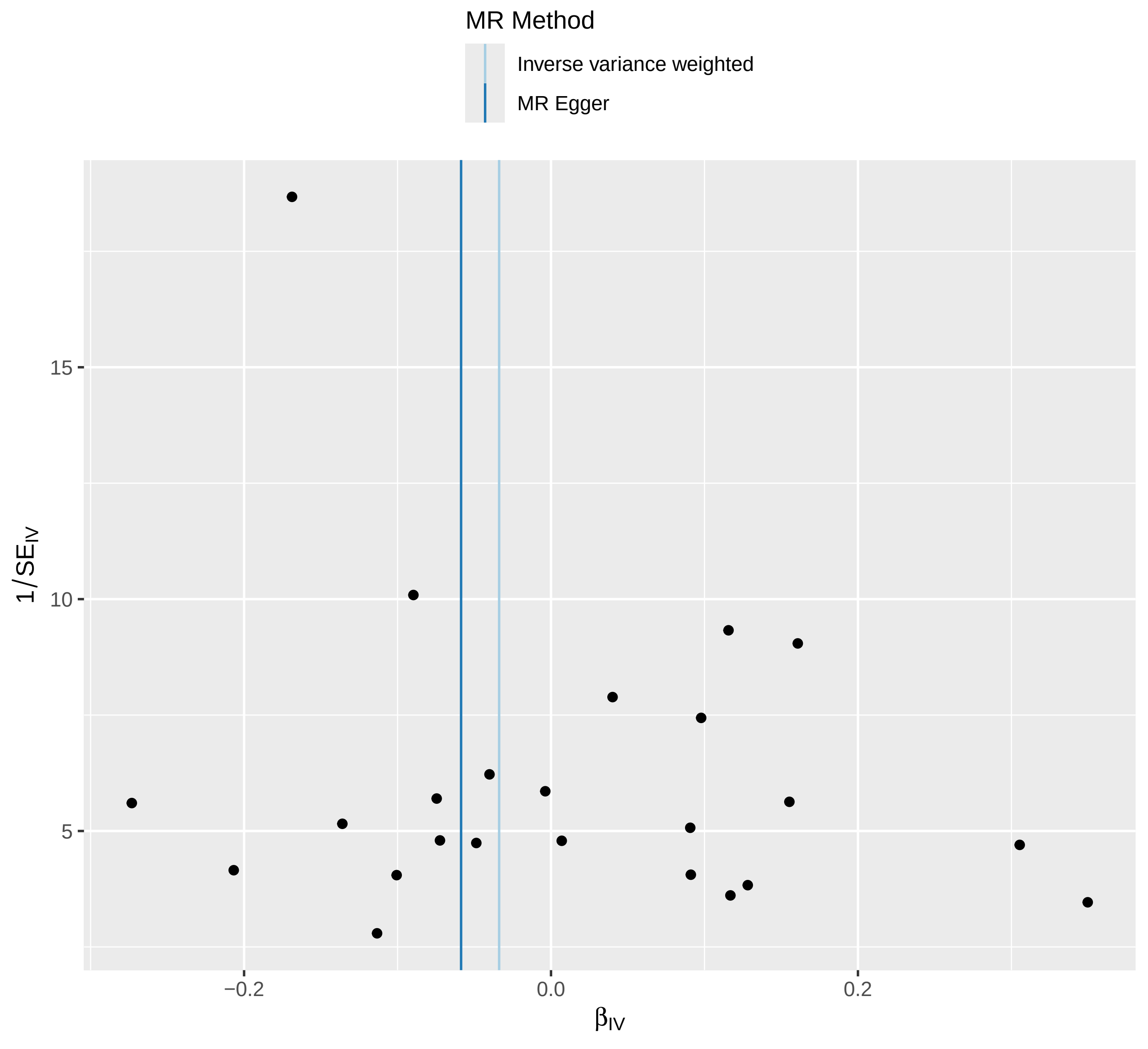
MR Method
Inverse variance weighted
MR Egger
15
10
5
1/SE_IV
−0.2
0.0
0.2
β_IV

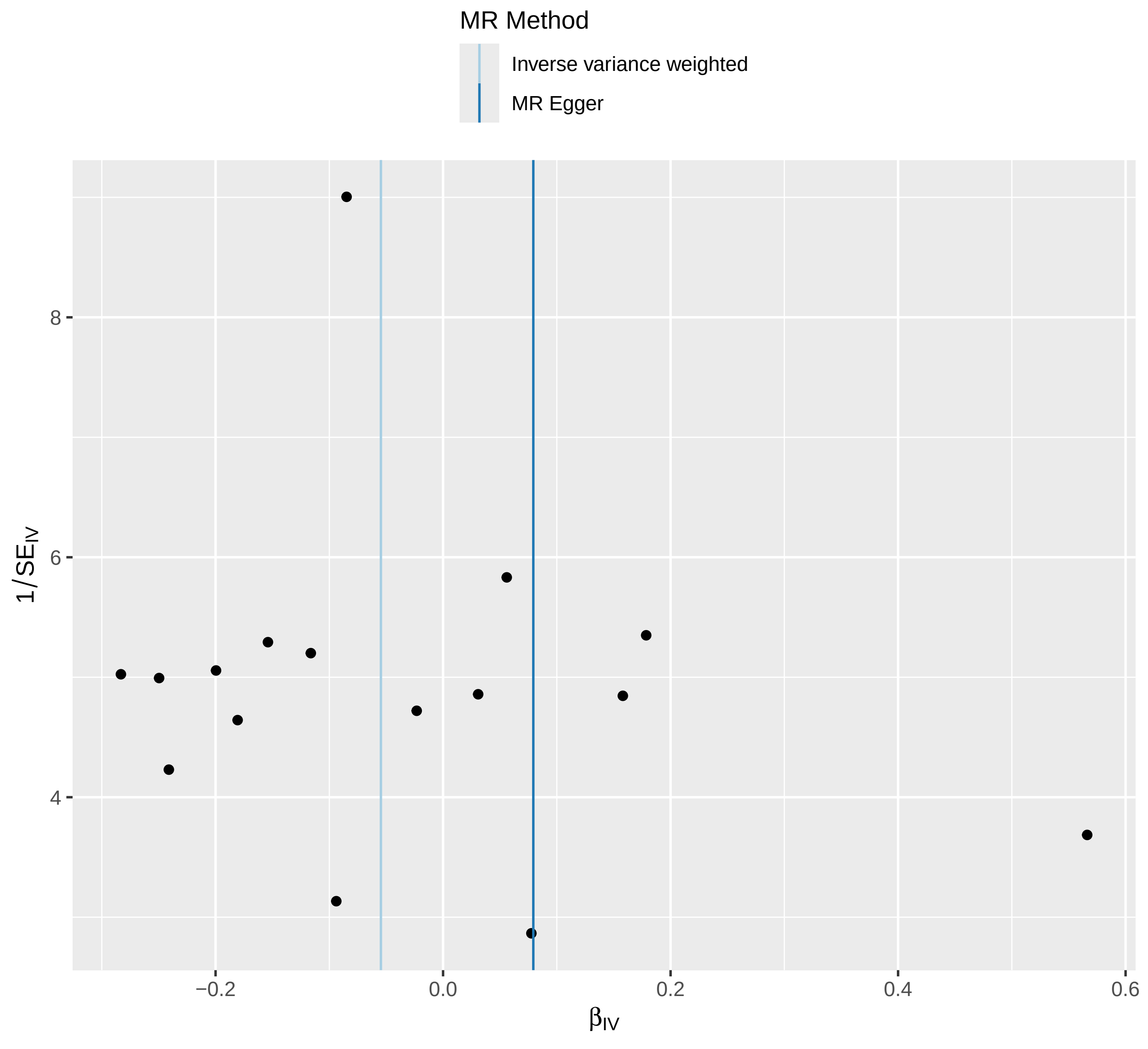
MR Method
Inverse variance weighted
MR Egger
8
6
4
1/SE_IV
−0.2
0.0
0.2
0.4
0.6
β_IV

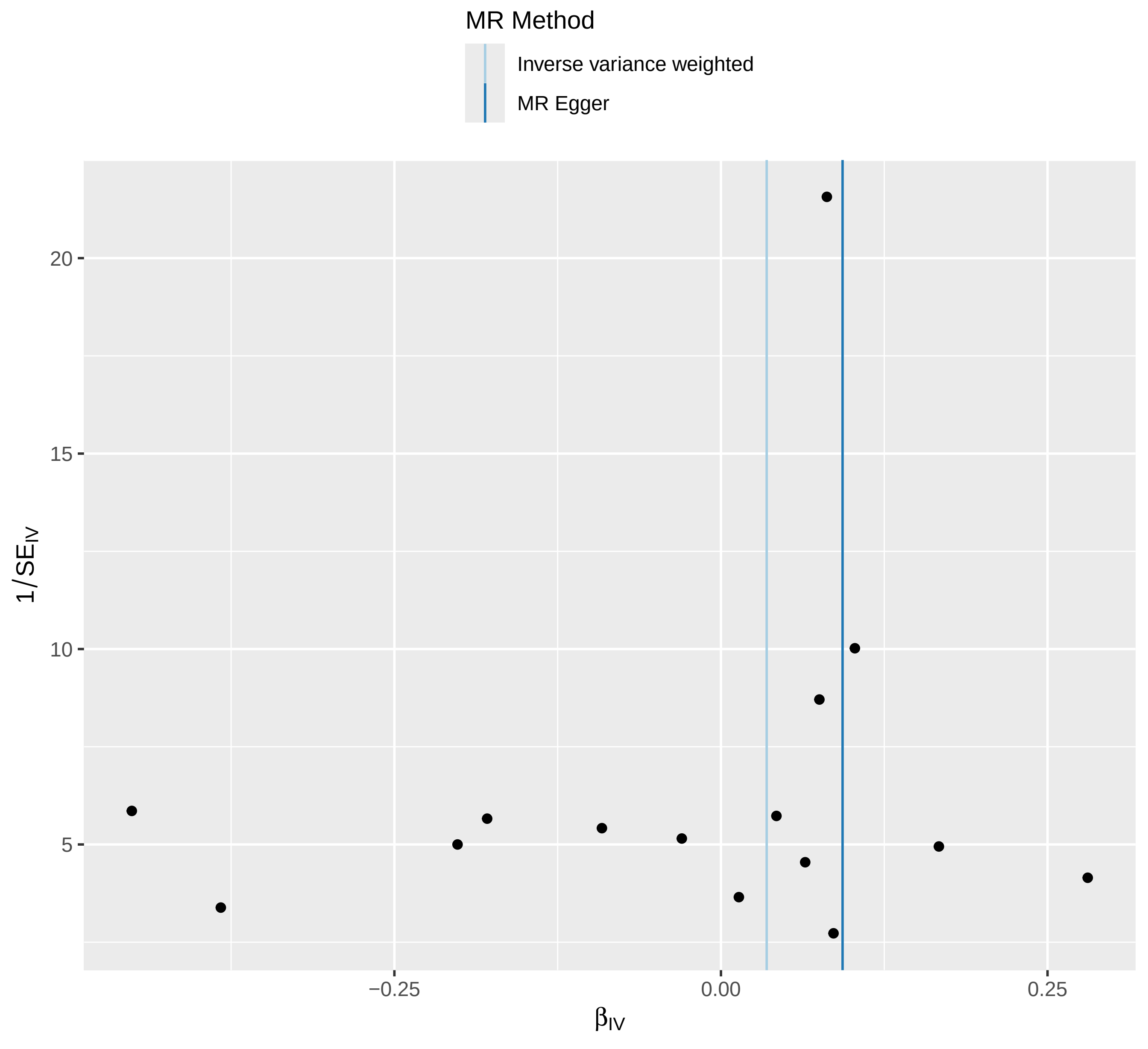

MR Method
Inverse variance weighted
MR Egger
20
15
10
5
1/SE$_{IV}$
−0.25
0.00
0.25
β$_{IV}$

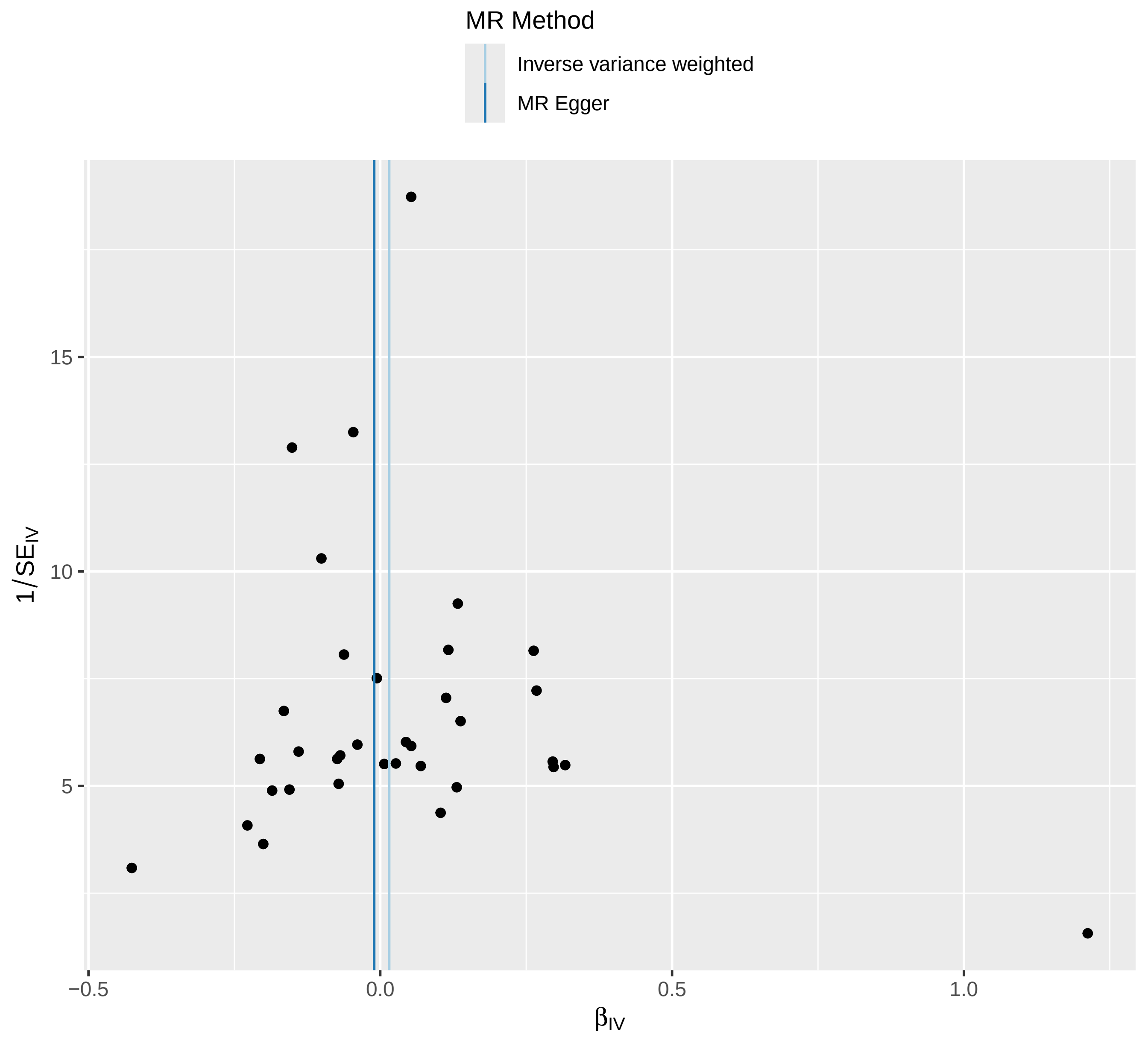

MR Method
Inverse variance weighted
MR Egger
15
10
5
1/SE_IV
−0.5
0.0
0.5
1.0
β_IV

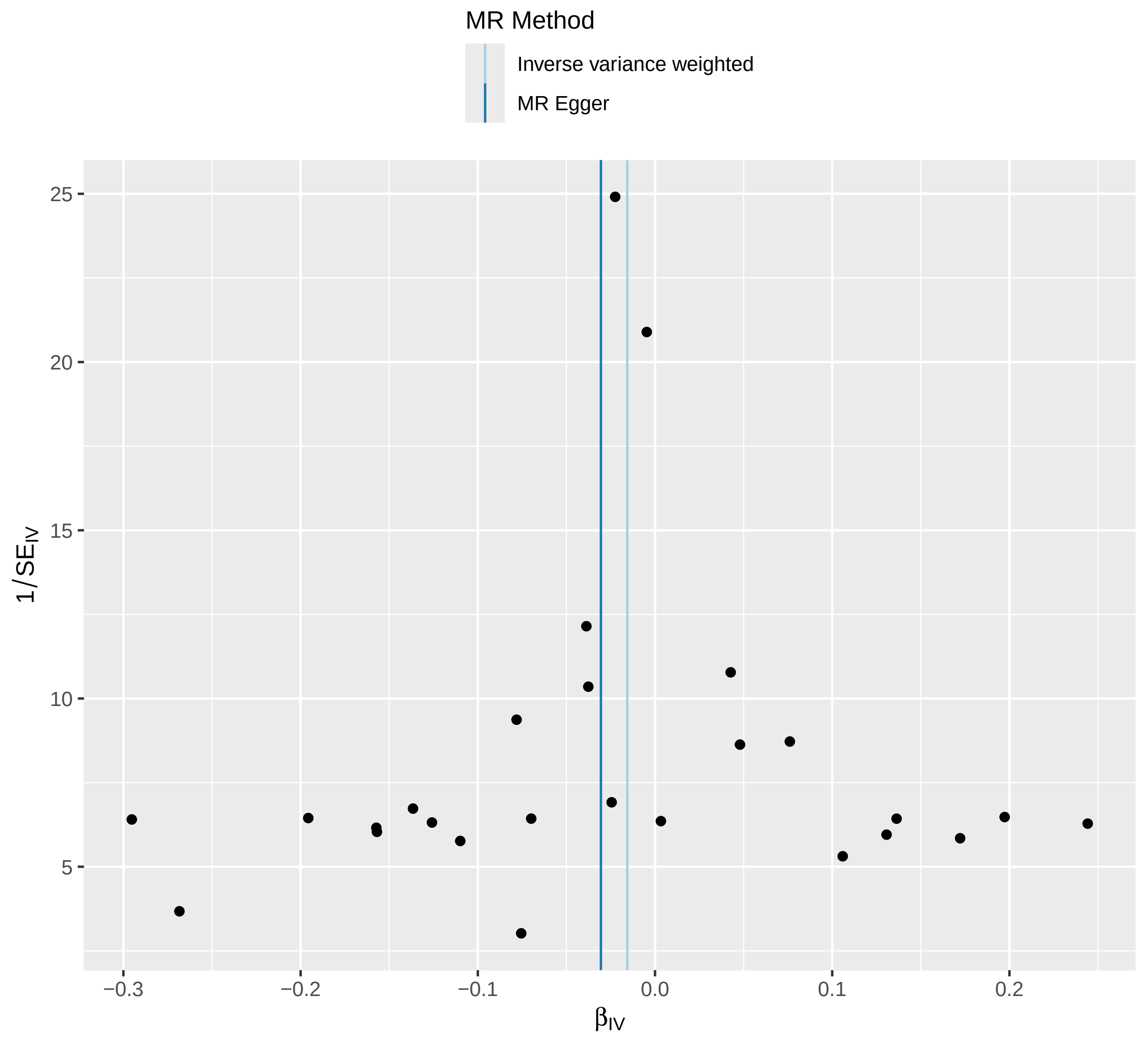
MR Method
Inverse variance weighted
MR Egger
1/SE$_{IV}$
β$_{IV}$
25
20
15
10
5
−0.3
−0.2
−0.1
0.0
0.1
0.2

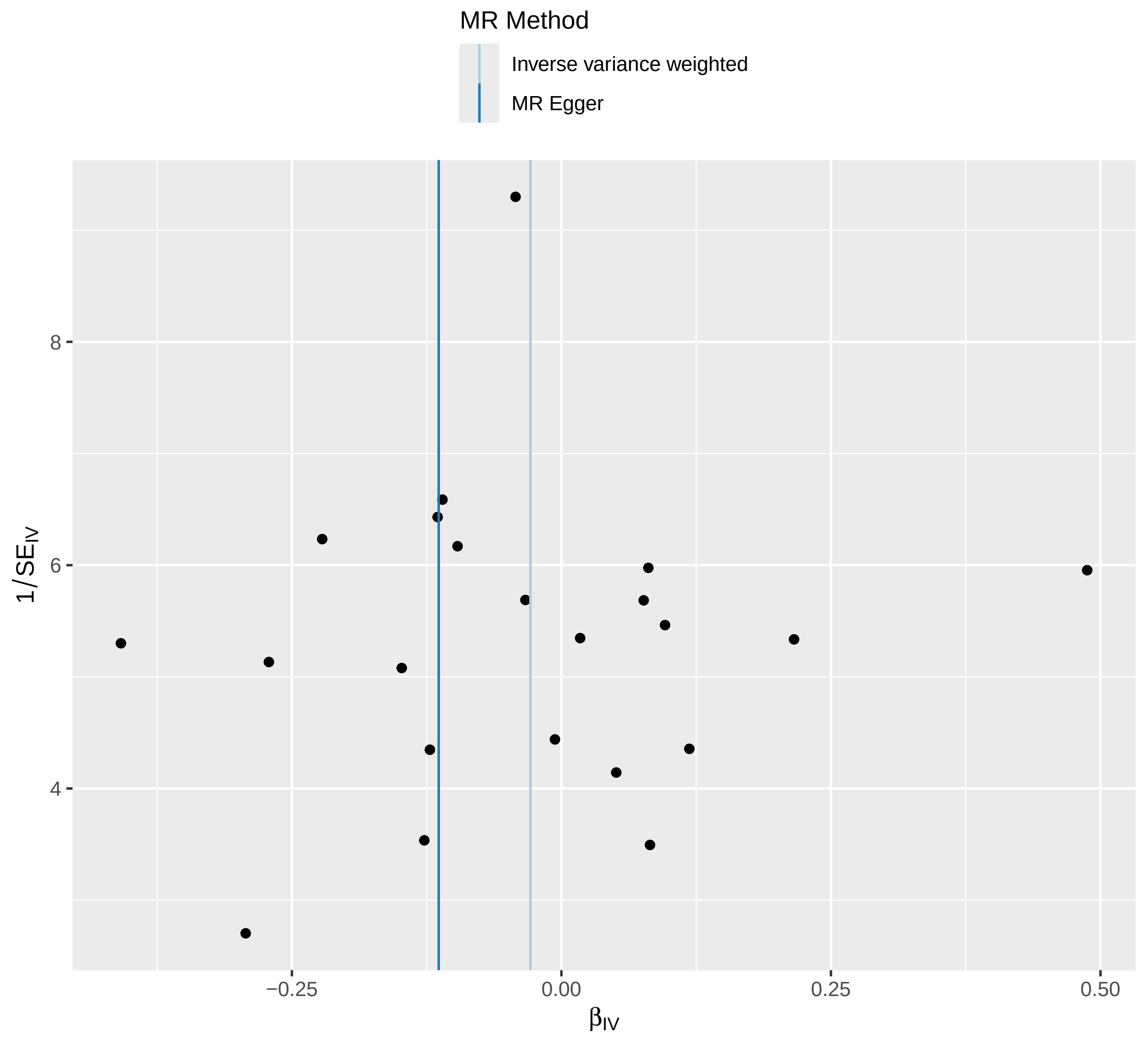
MR Method
Inverse variance weighted
MR Egger
8
6
4
1/SE$_{IV}$
−0.25
0.00
0.25
0.50
β$_{IV}$

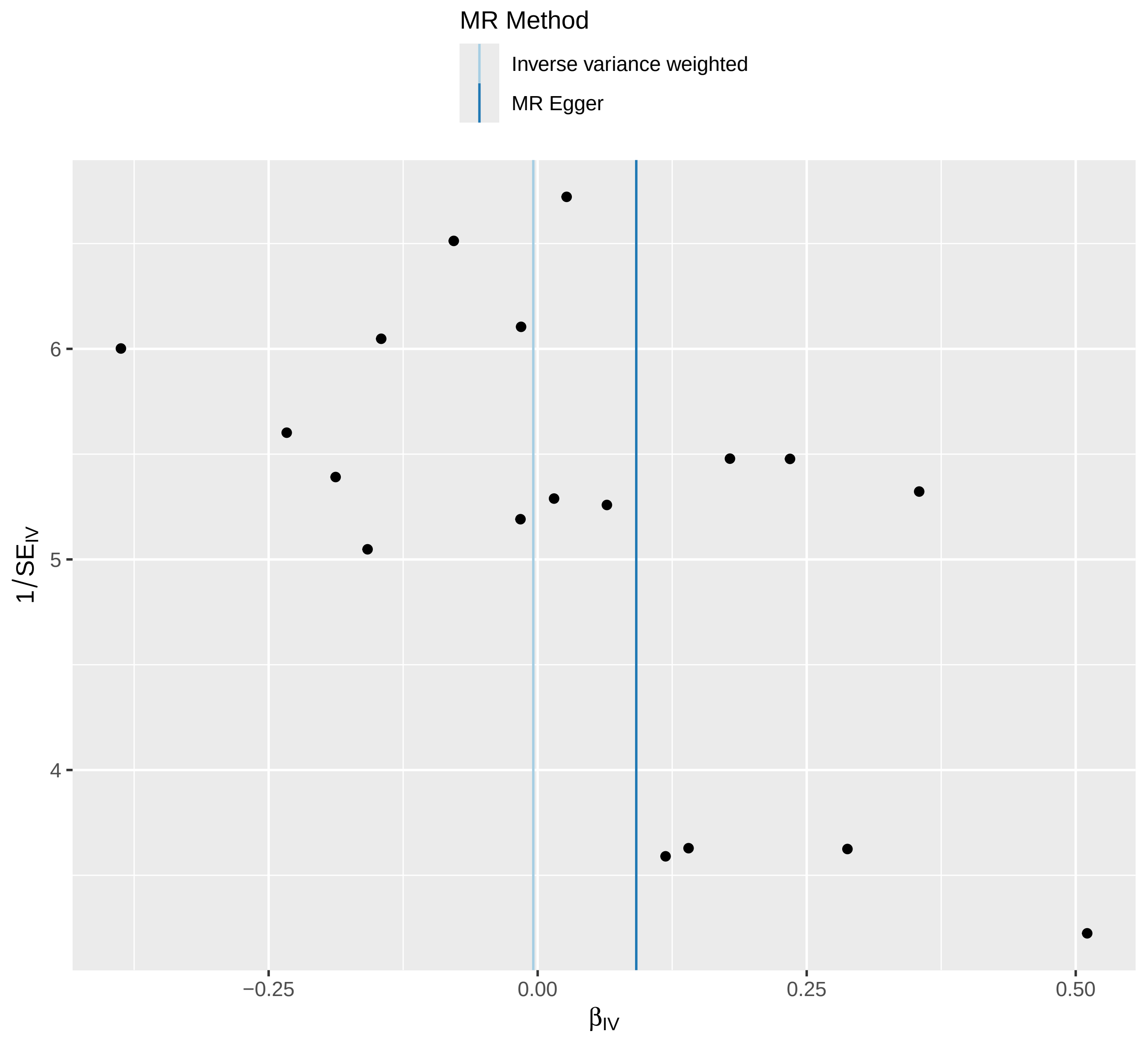
MR Method
Inverse variance weighted
MR Egger
1/SE_IV
6
5
4
−0.25
0.00
0.25
0.50
β_IV

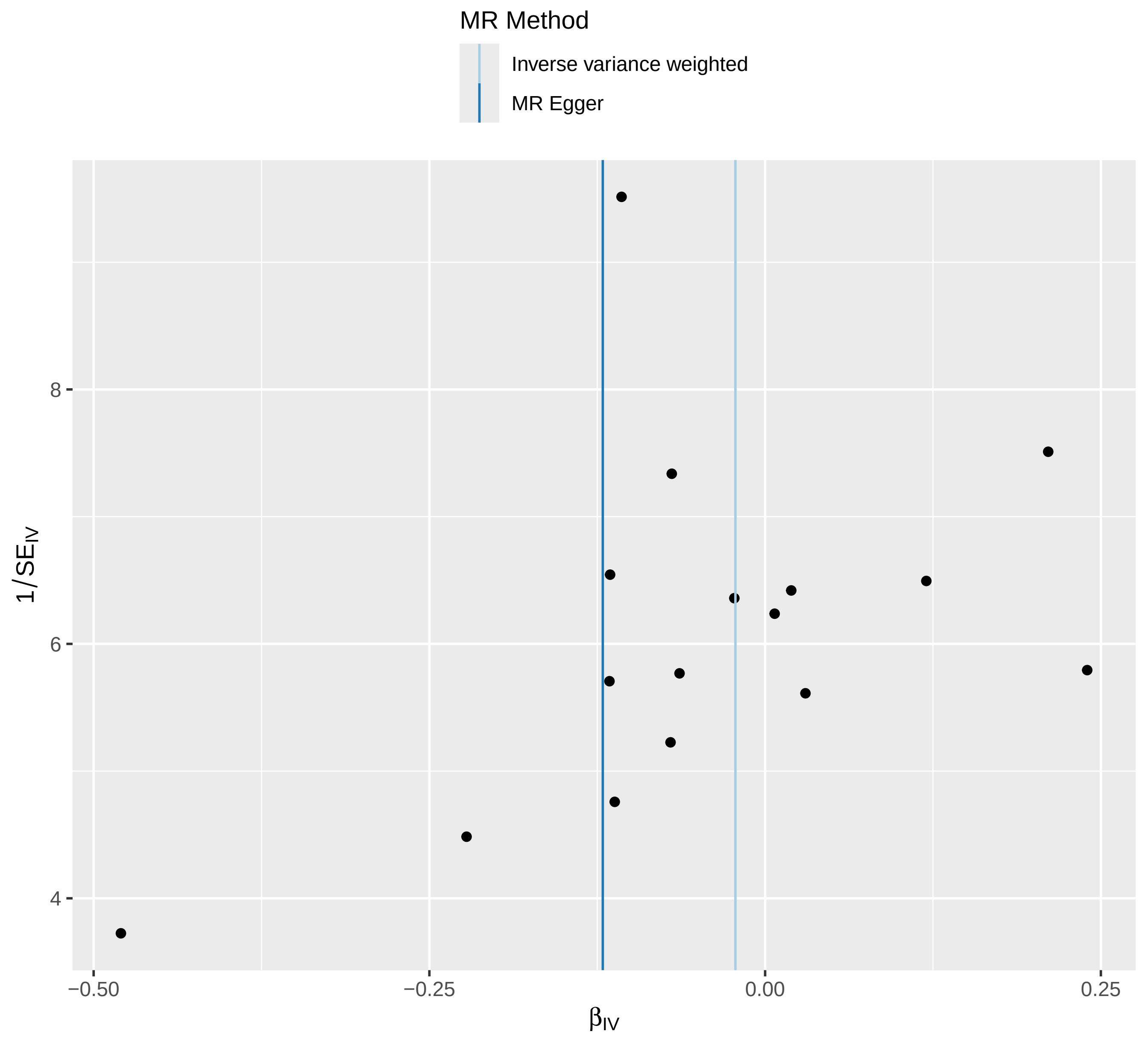

MR Method
Inverse variance weighted
MR Egger
8
6
4
1/SE_IV
−0.50
−0.25
0.00
0.25
β_IV

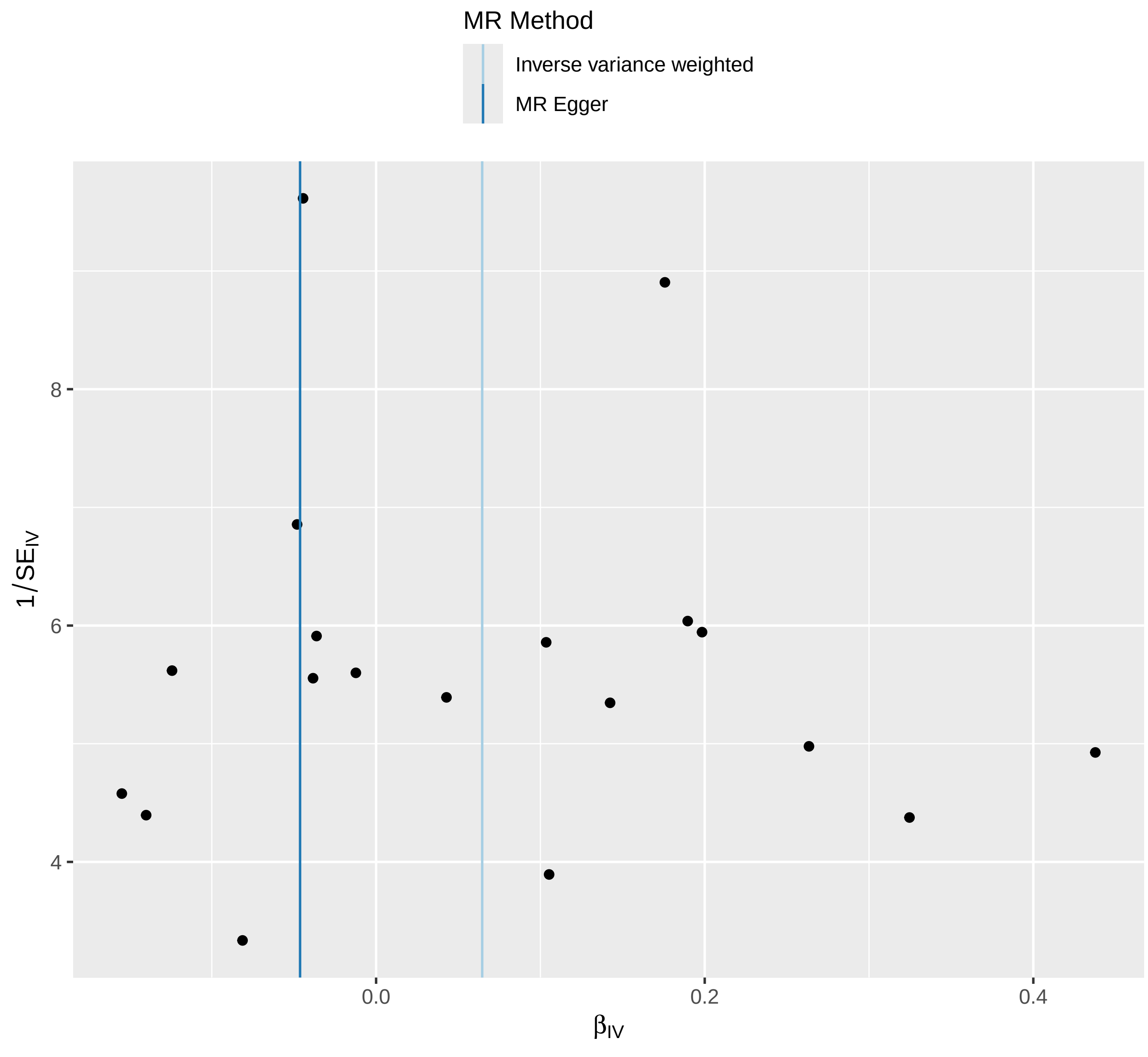
MR Method
Inverse variance weighted
MR Egger
8
6
4
0.0
0.2
0.4
$1/SE_{IV}$
β_{IV}

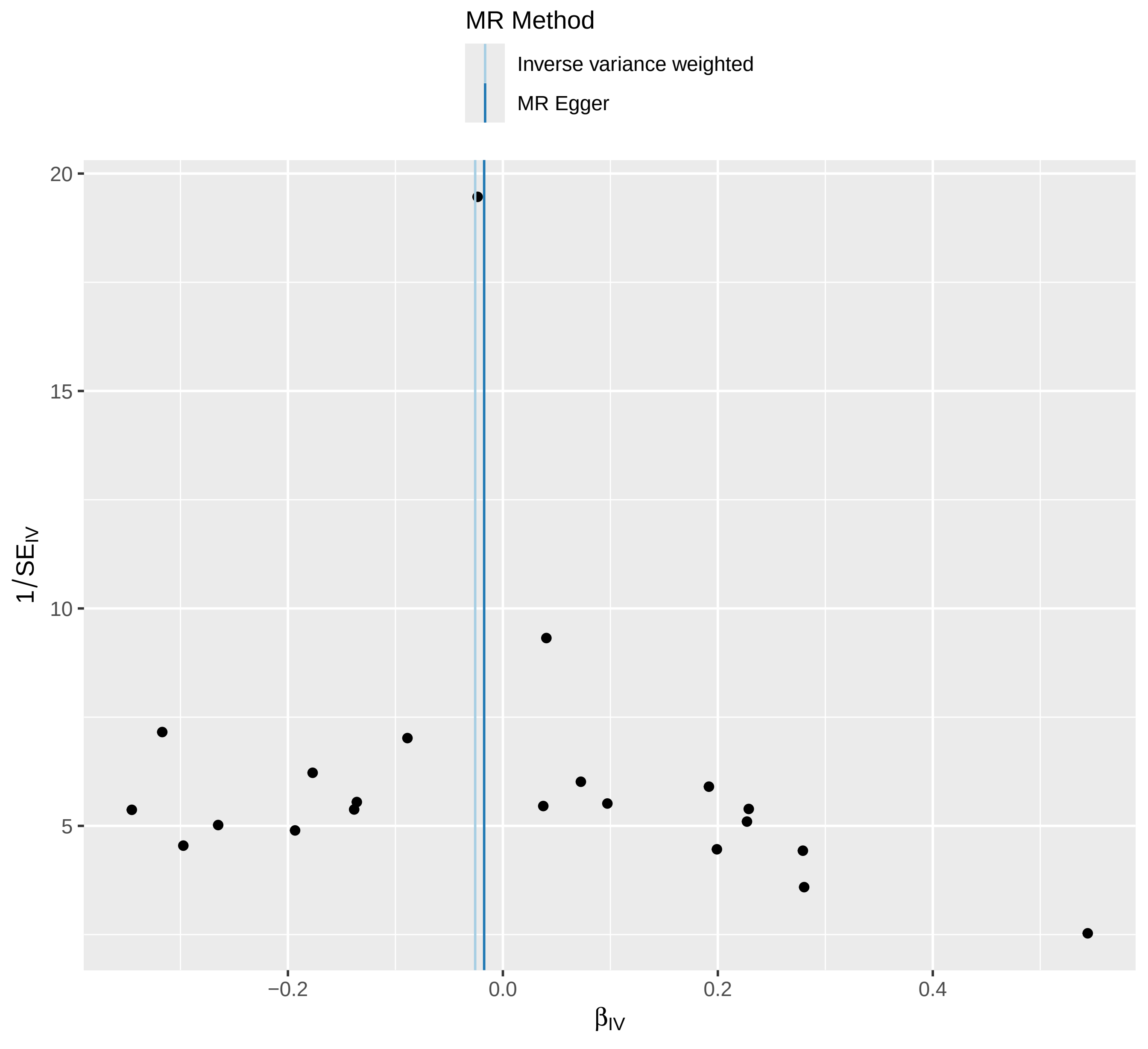

MR Method
Inverse variance weighted
MR Egger
20
15
10
5
1/SE_IV
−0.2
0.0
0.2
0.4
β_IV

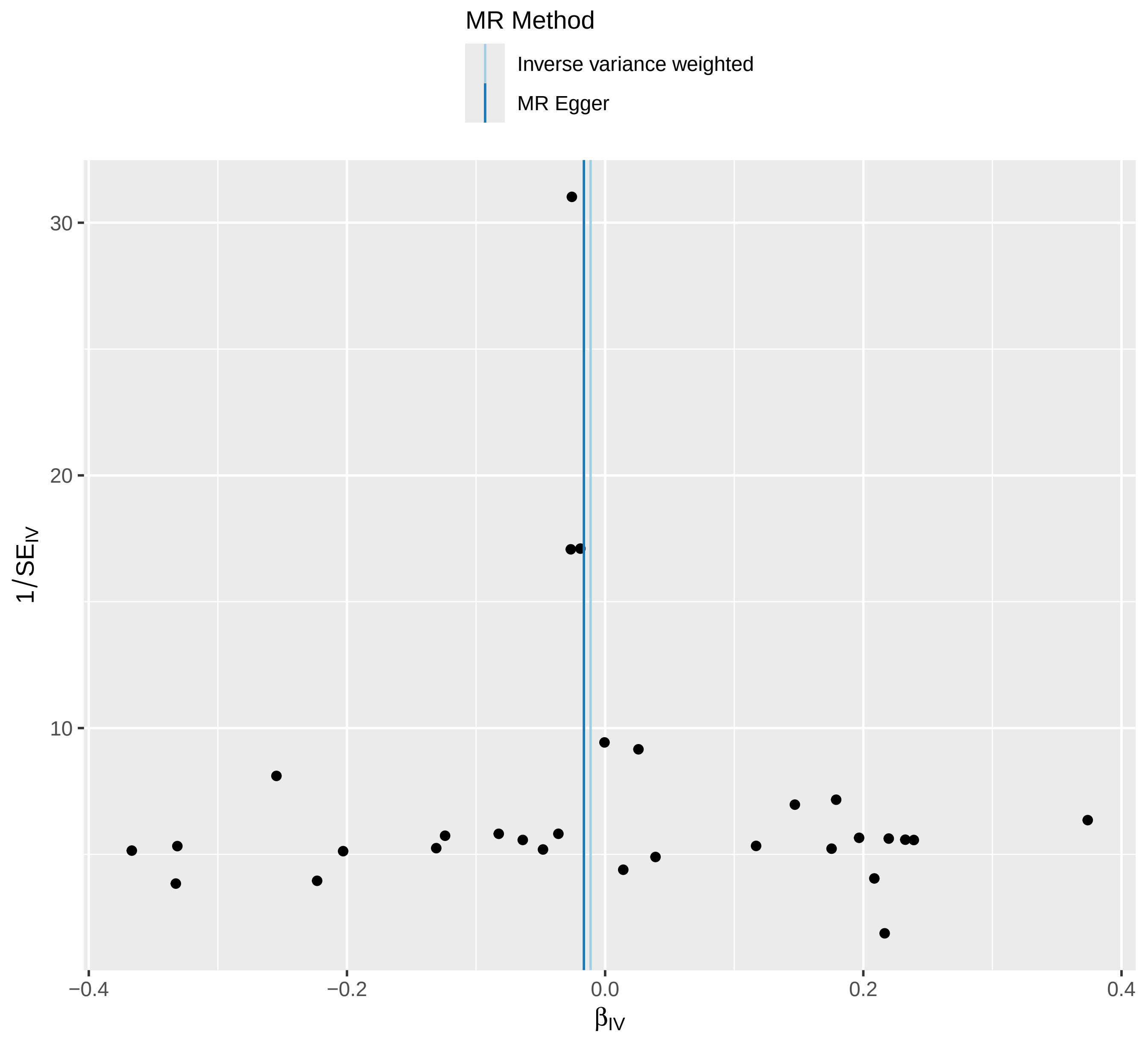

MR Method
Inverse variance weighted
MR Egger
30
20
10
1/SE$_{IV}$
−0.4
−0.2
0.0
0.2
0.4
β$_{IV}$

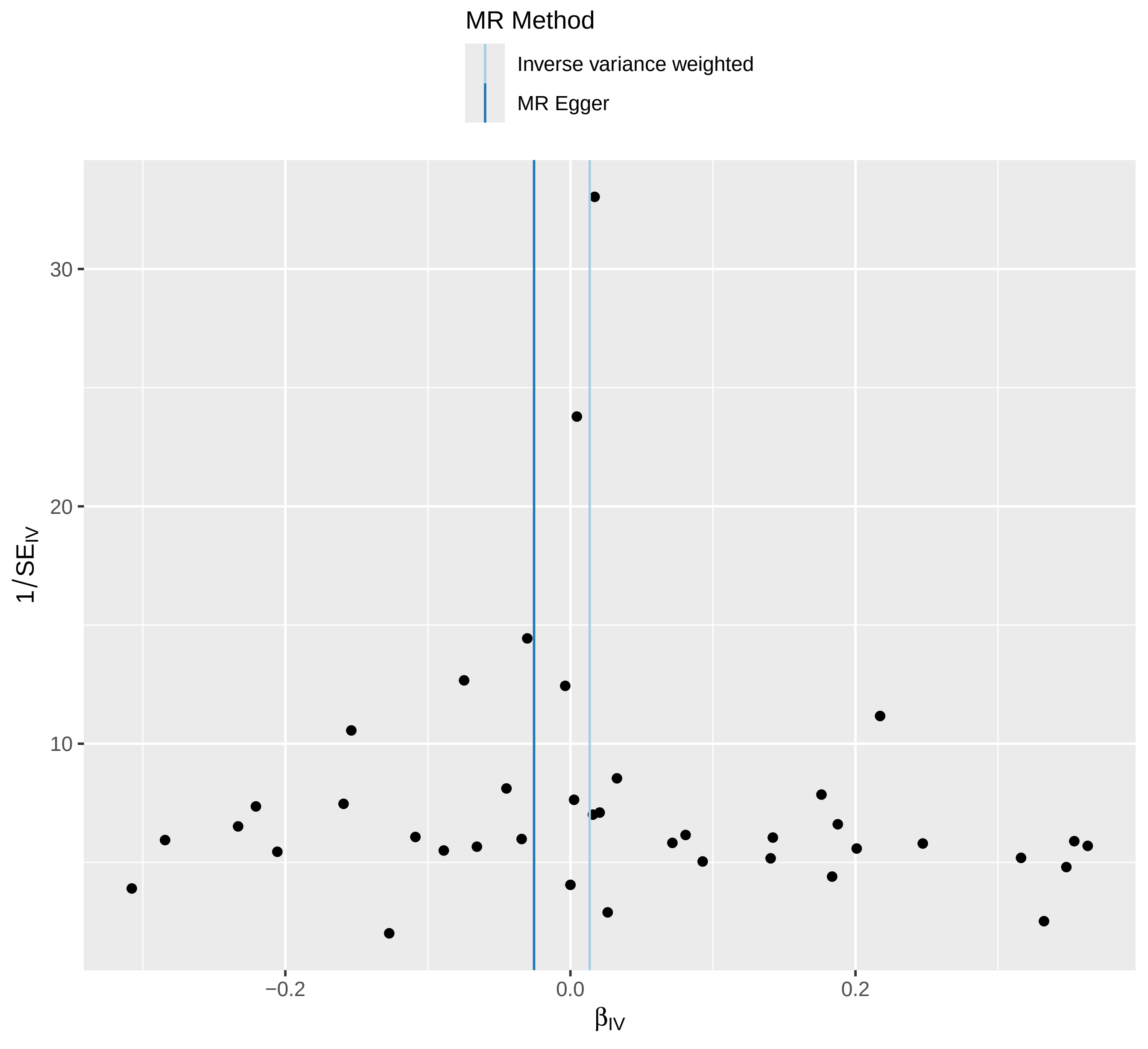
MR Method
Inverse variance weighted
MR Egger
30
20
10
1/SE_IV
−0.2
0.0
0.2
β_IV

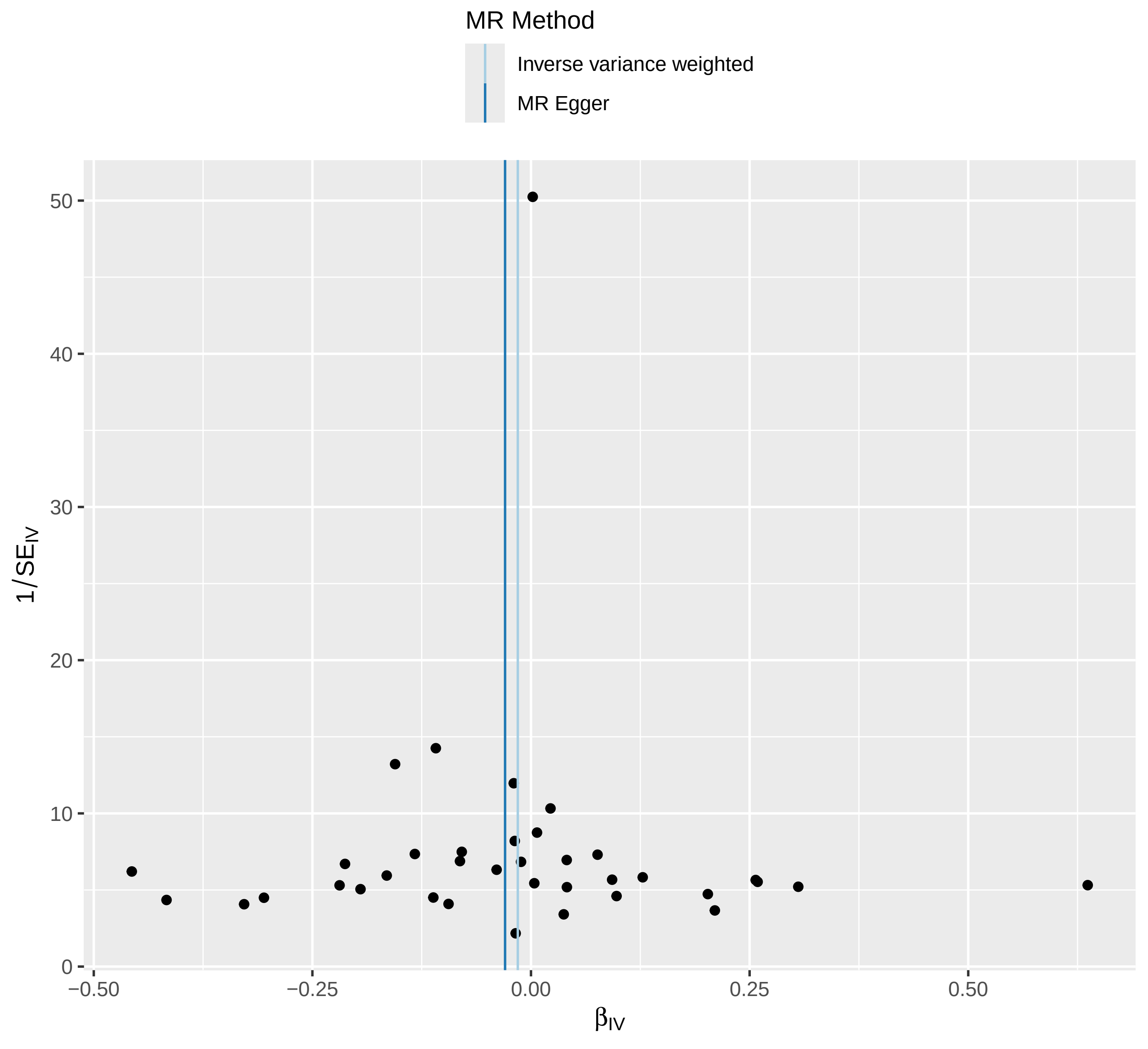
MR Method
Inverse variance weighted
MR Egger
1/SE_IV
β_IV
0
10
20
30
40
50
−0.50
−0.25
0.00
0.25
0.50

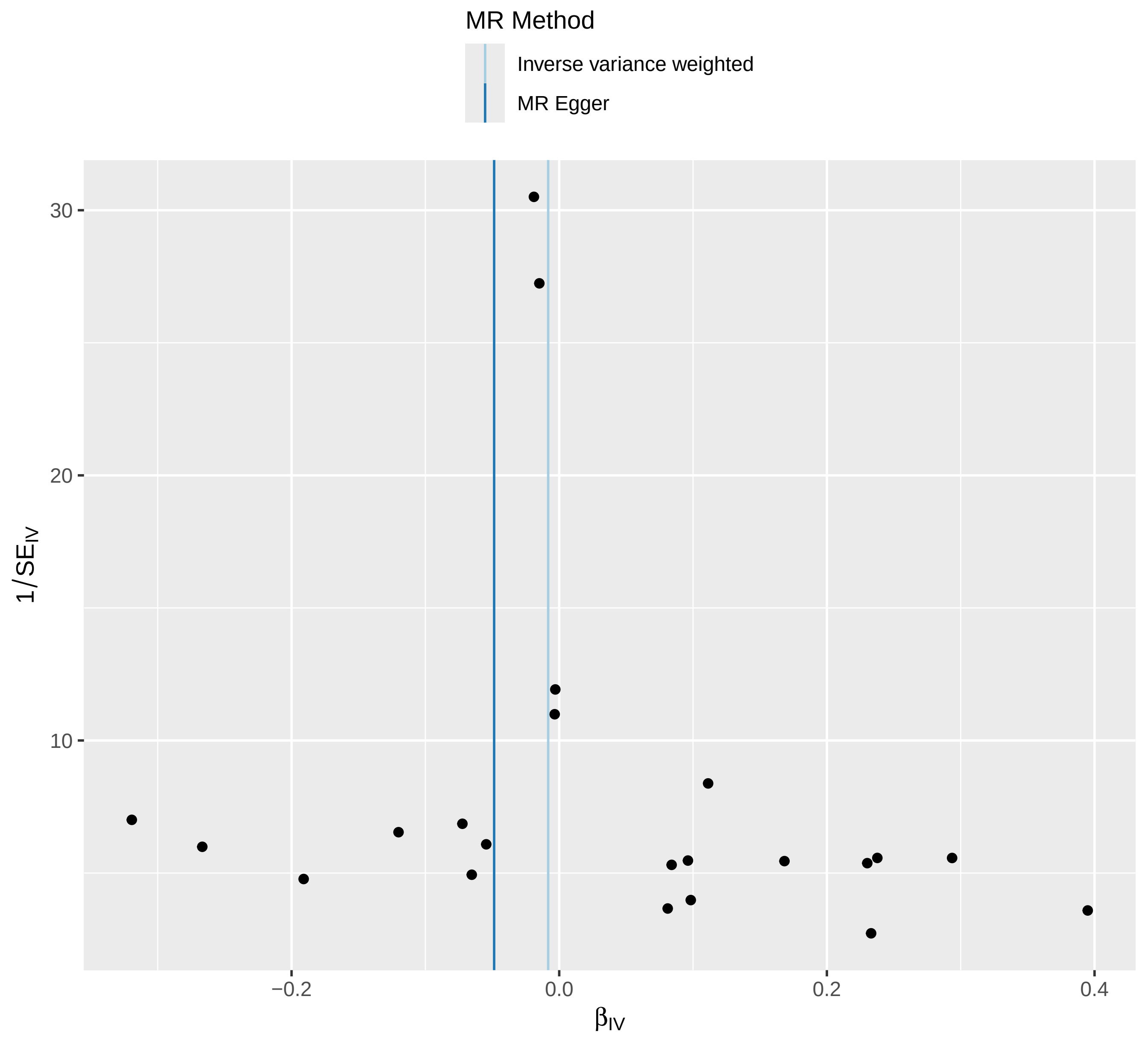
MR Method
Inverse variance weighted
MR Egger
30
20
10
$1/SE_{IV}$
−0.2
0.0
0.2
0.4
β_{IV}

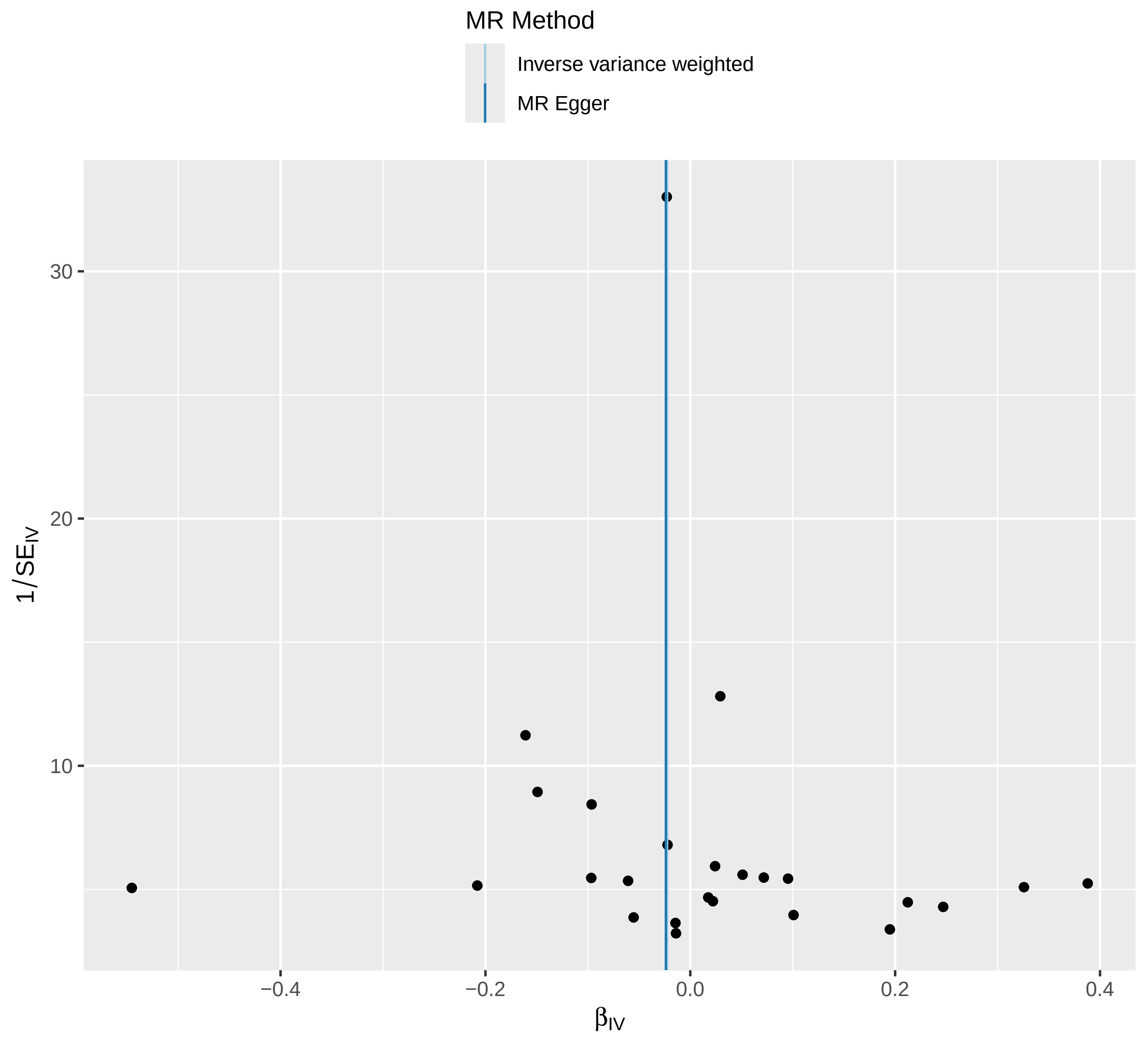
MR Method
Inverse variance weighted
MR Egger
30
20
10
1/SE_IV
−0.4
−0.2
0.0
0.2
0.4
β_IV

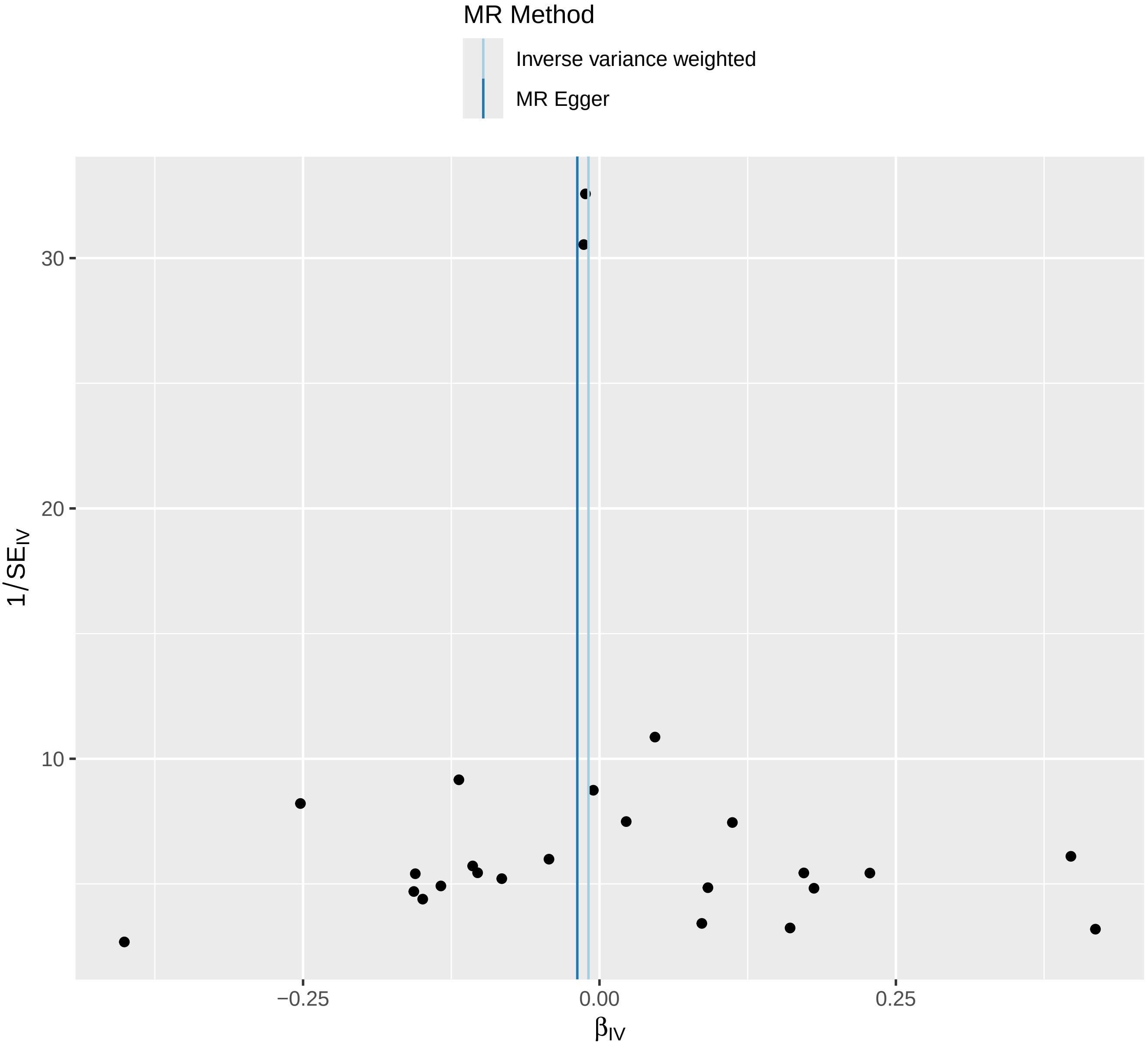

MR Method
Inverse variance weighted
MR Egger
30
20
10
$1/SE_{IV}$
−0.25
0.00
0.25
β_{IV}

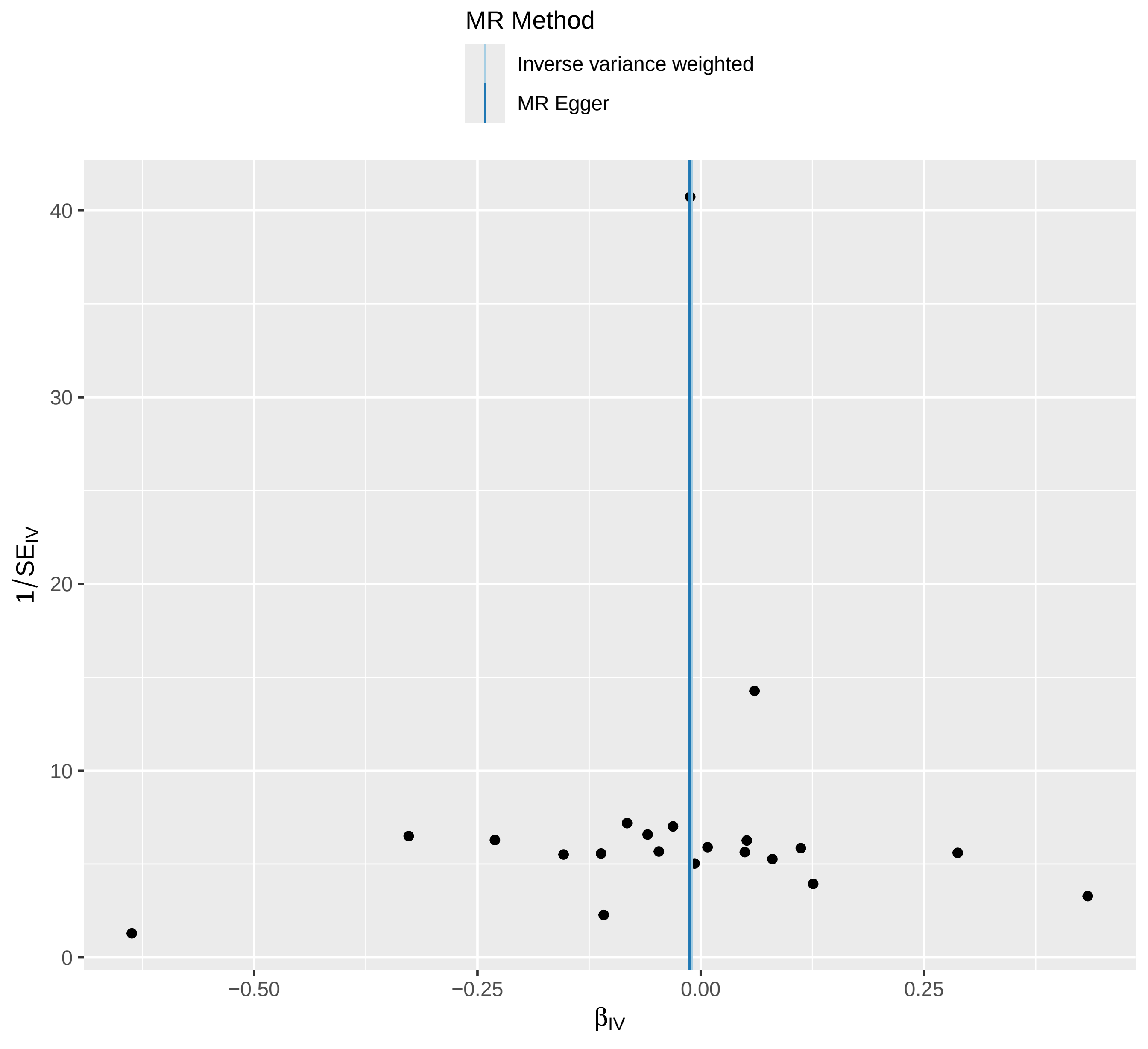

MR Method
Inverse variance weighted
MR Egger
40
30
20
10
0
−0.50
−0.25
0.00
0.25
$1/SE_{IV}$
β_{IV}

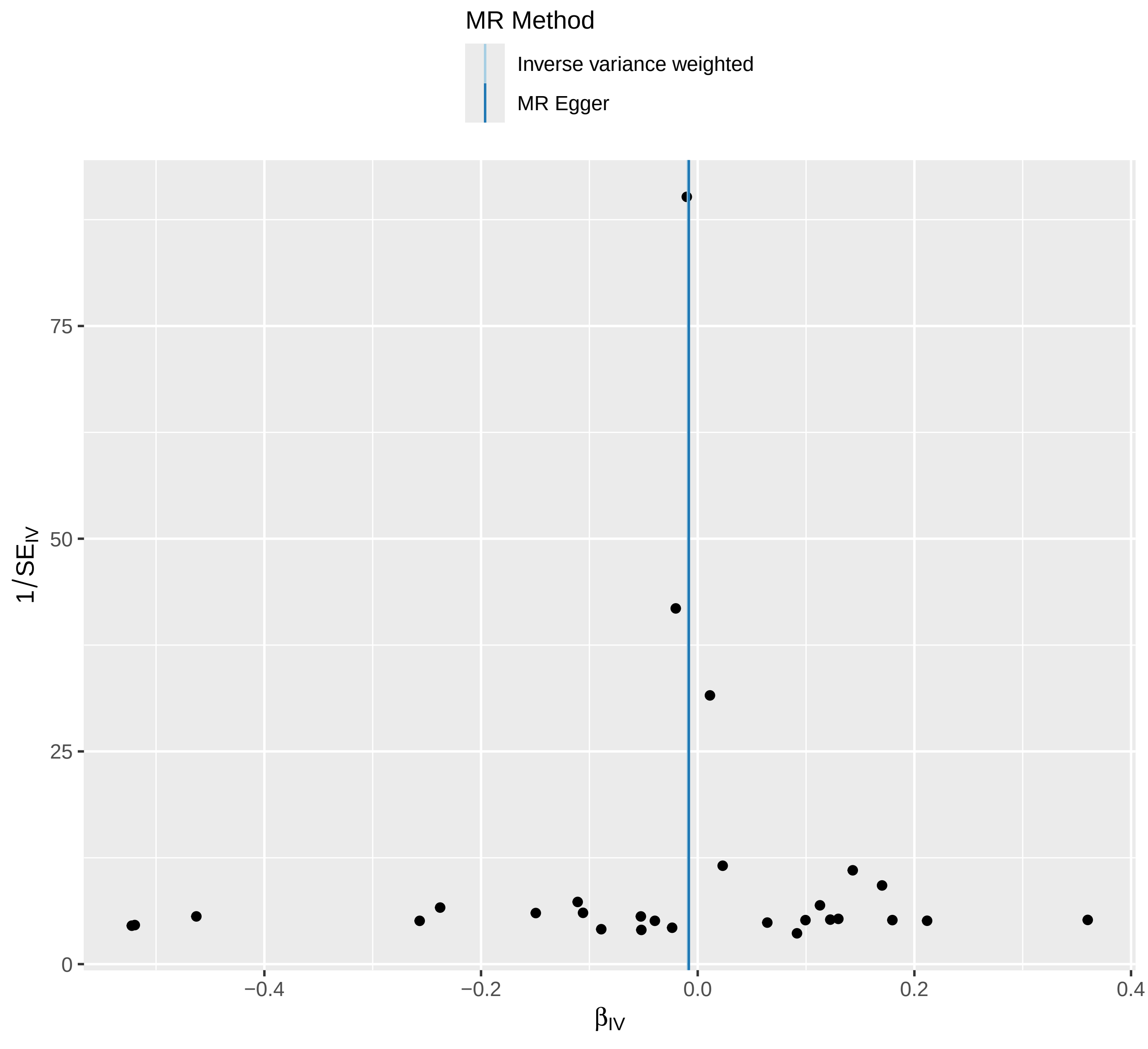

MR Method
Inverse variance weighted
MR Egger
1/SE_IV
75
50
25
0
−0.4
−0.2
0.0
0.2
0.4
β_IV

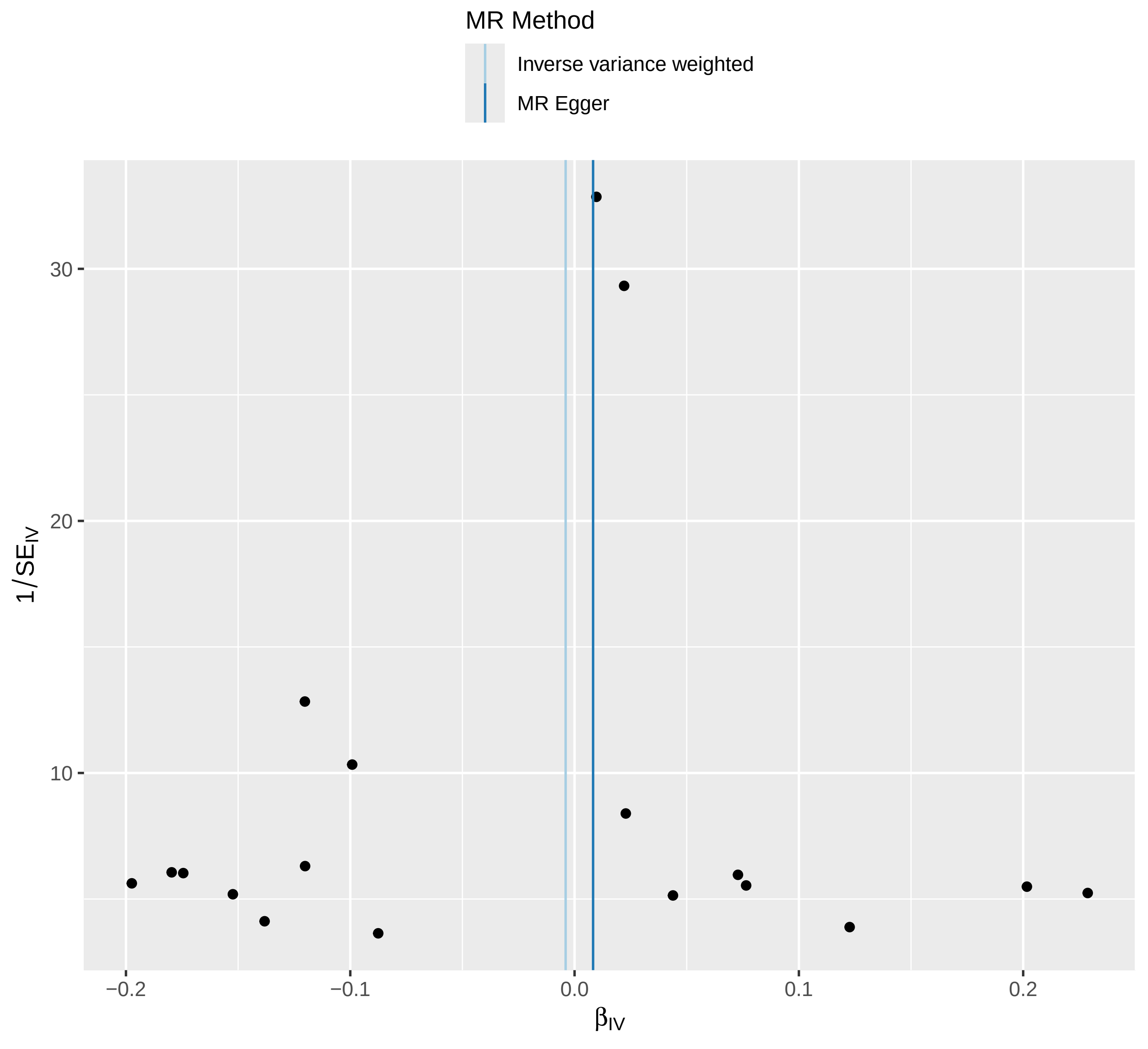
MR Method
Inverse variance weighted
MR Egger
30
20
10
1/SE$_{IV}$
−0.2
−0.1
0.0
0.1
0.2
β$_{IV}$

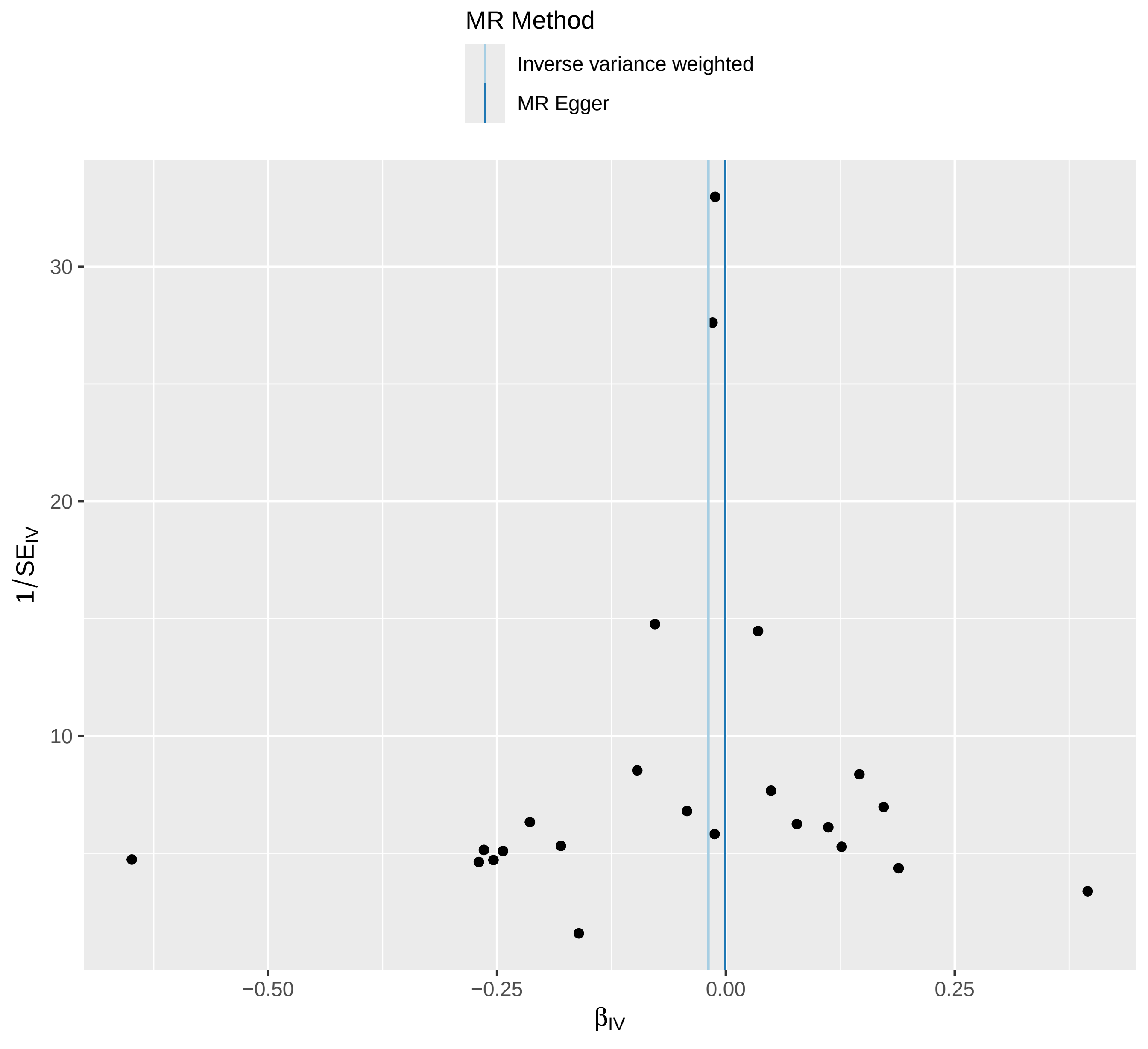
MR Method
Inverse variance weighted
MR Egger
30
20
10
1/SE$_{IV}$
−0.50
−0.25
0.00
0.25
β$_{IV}$

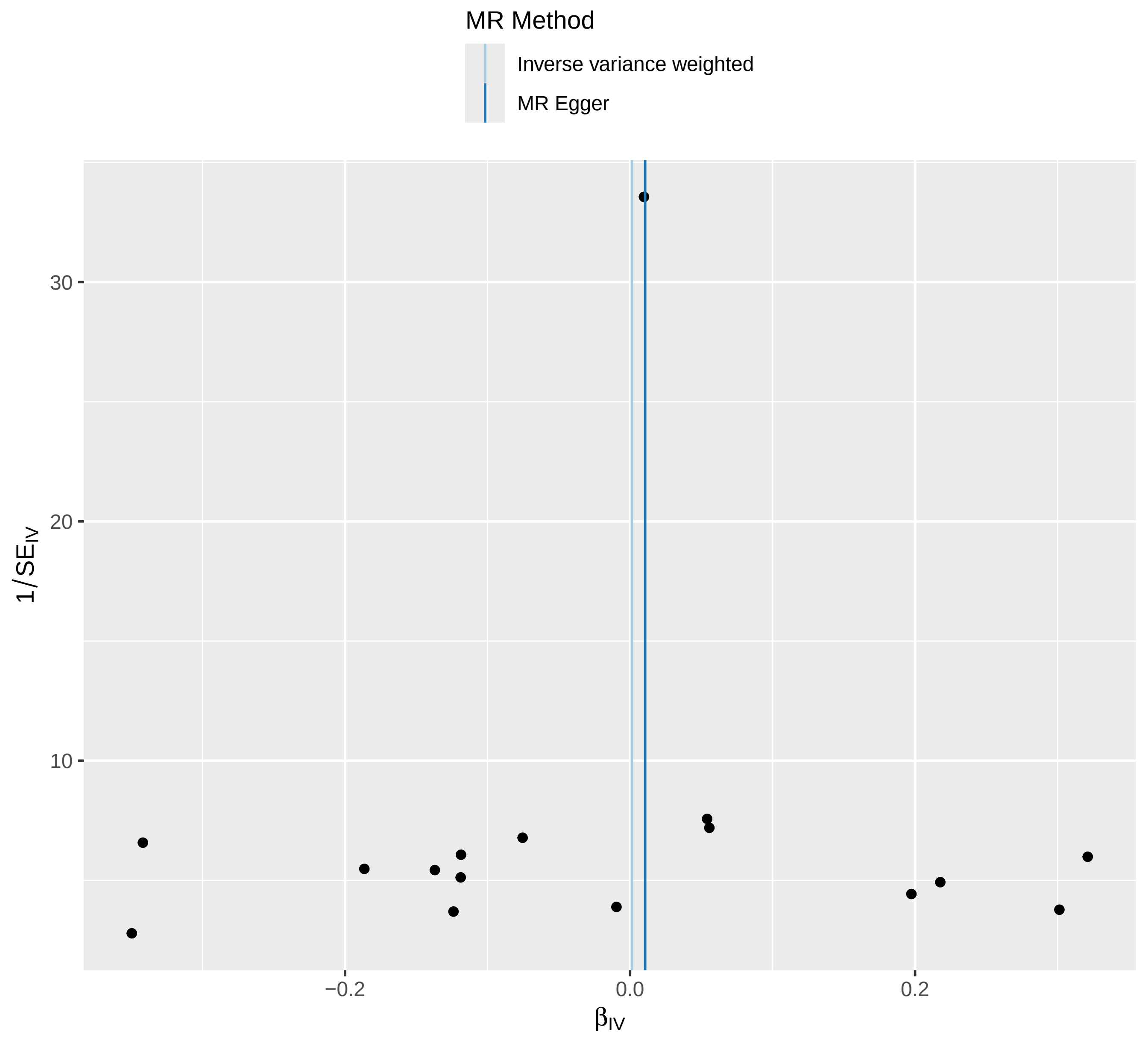

MR Method
Inverse variance weighted
MR Egger
30
20
10
1/SE$_{IV}$
−0.2
0.0
0.2
β$_{IV}$

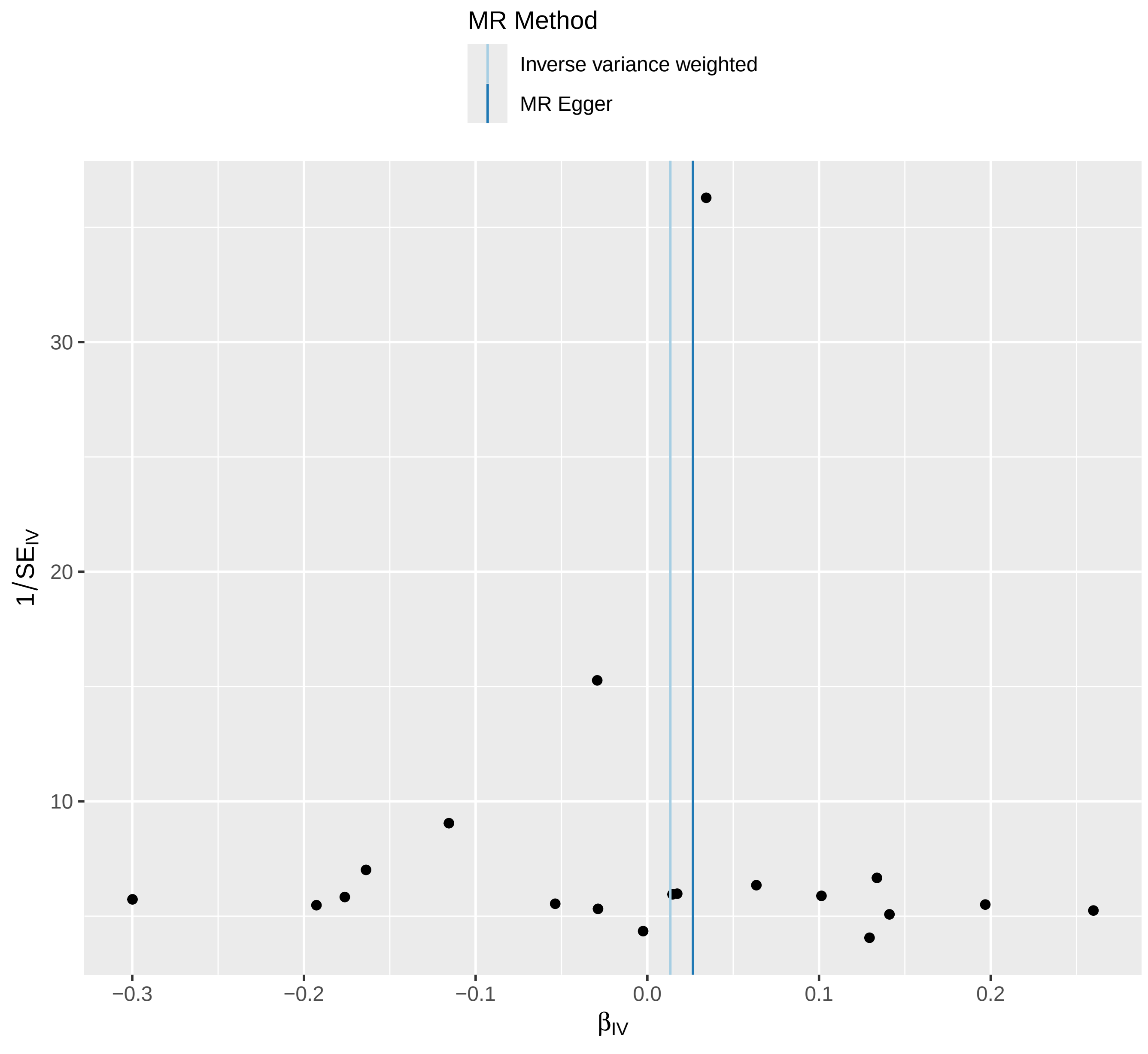
MR Method
Inverse variance weighted
MR Egger
30
20
10
1/SE_IV
−0.3
−0.2
−0.1
0.0
0.1
0.2
β_IV

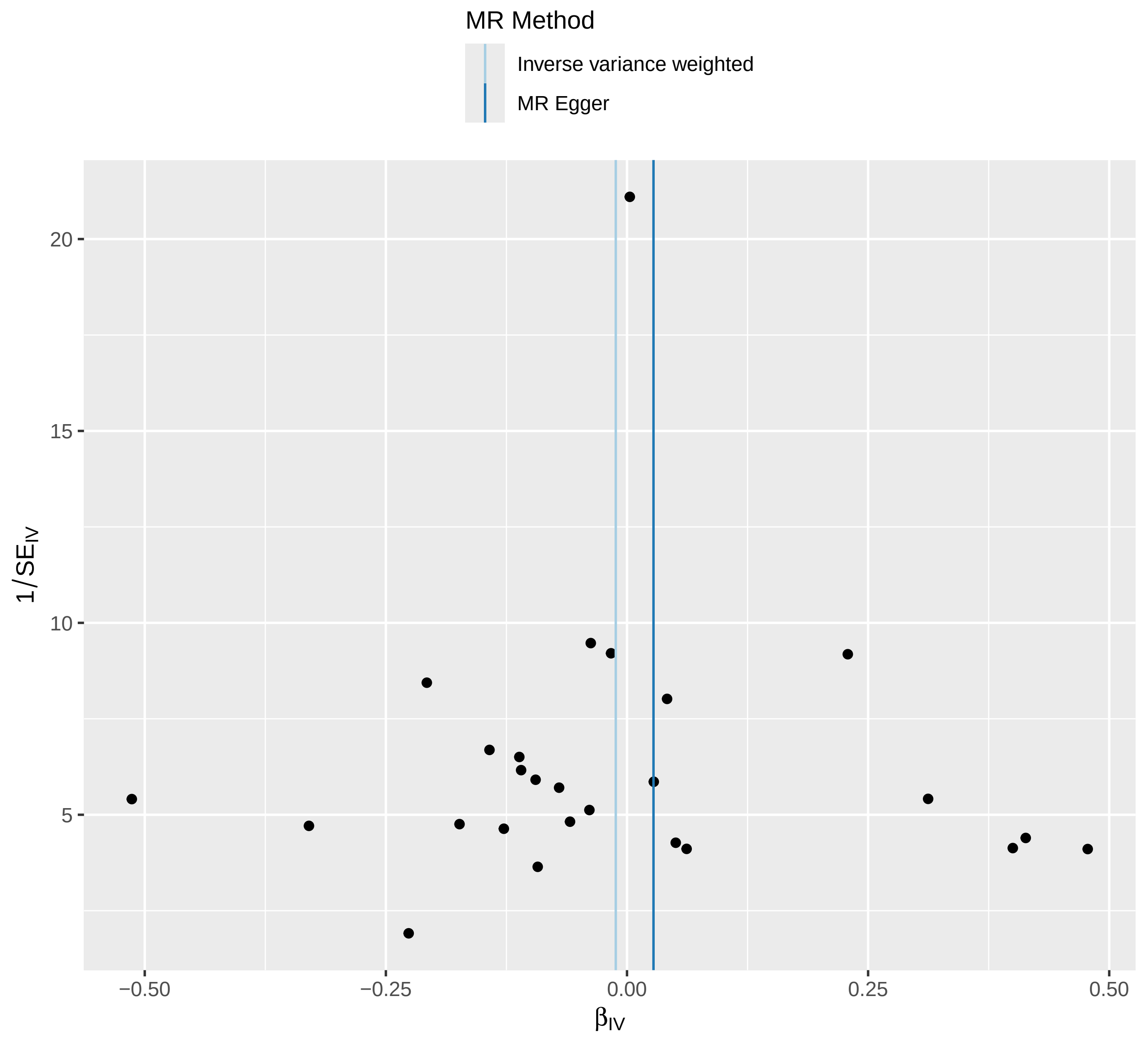

MR Method
Inverse variance weighted
MR Egger
20
15
10
5
1/SE$_{IV}$
−0.50
−0.25
0.00
0.25
0.50
β$_{IV}$

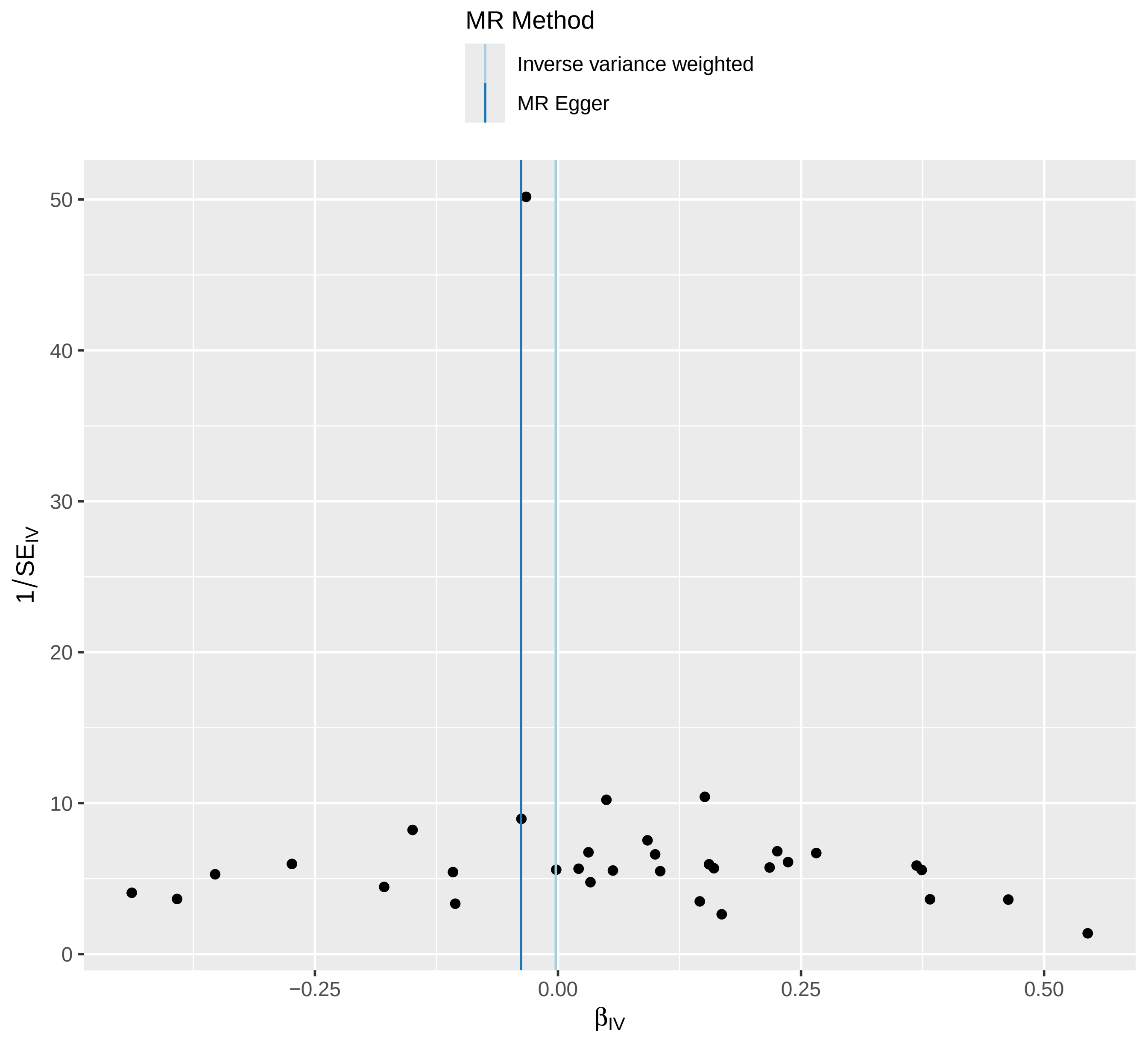
MR Method
Inverse variance weighted
MR Egger
50
40
30
20
10
0
1/SE_IV
−0.25
0.00
0.25
0.50
β_IV

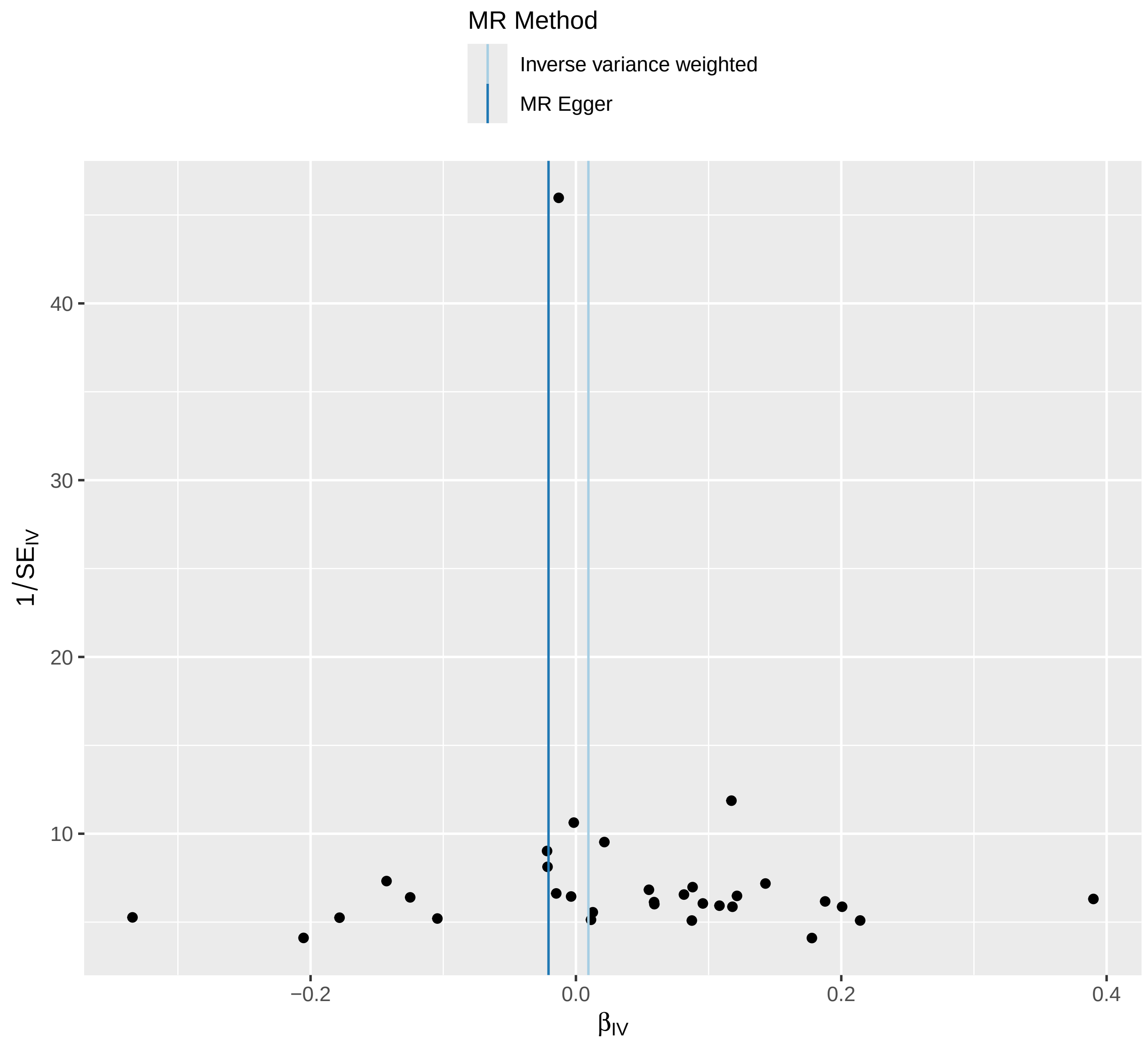
MR Method
Inverse variance weighted
MR Egger
40
30
20
10
1/SE_IV
−0.2
0.0
0.2
0.4
β_IV

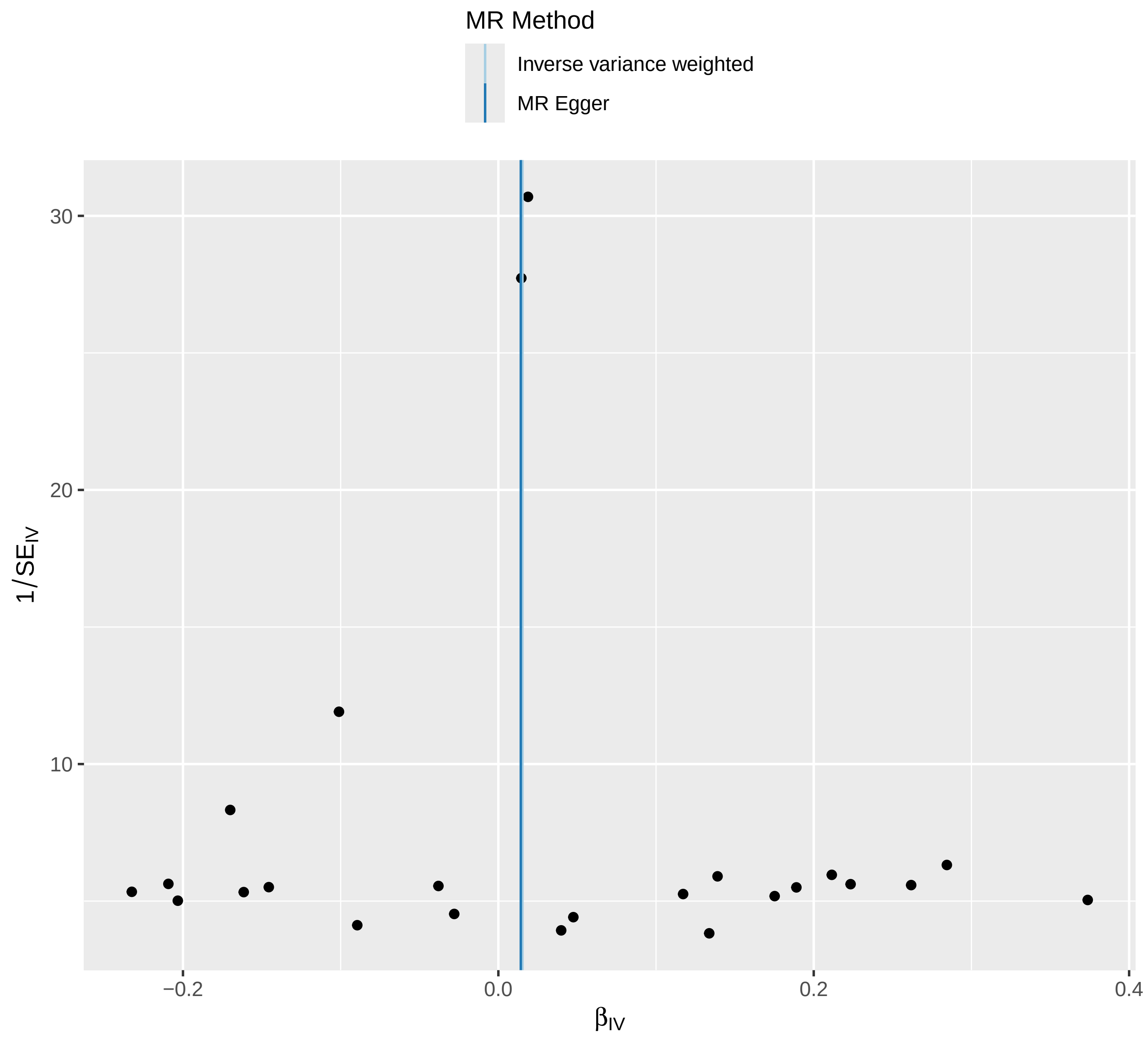
MR Method
Inverse variance weighted
MR Egger
30
20
10
1/SE_IV
−0.2
0.0
0.2
0.4
β_IV

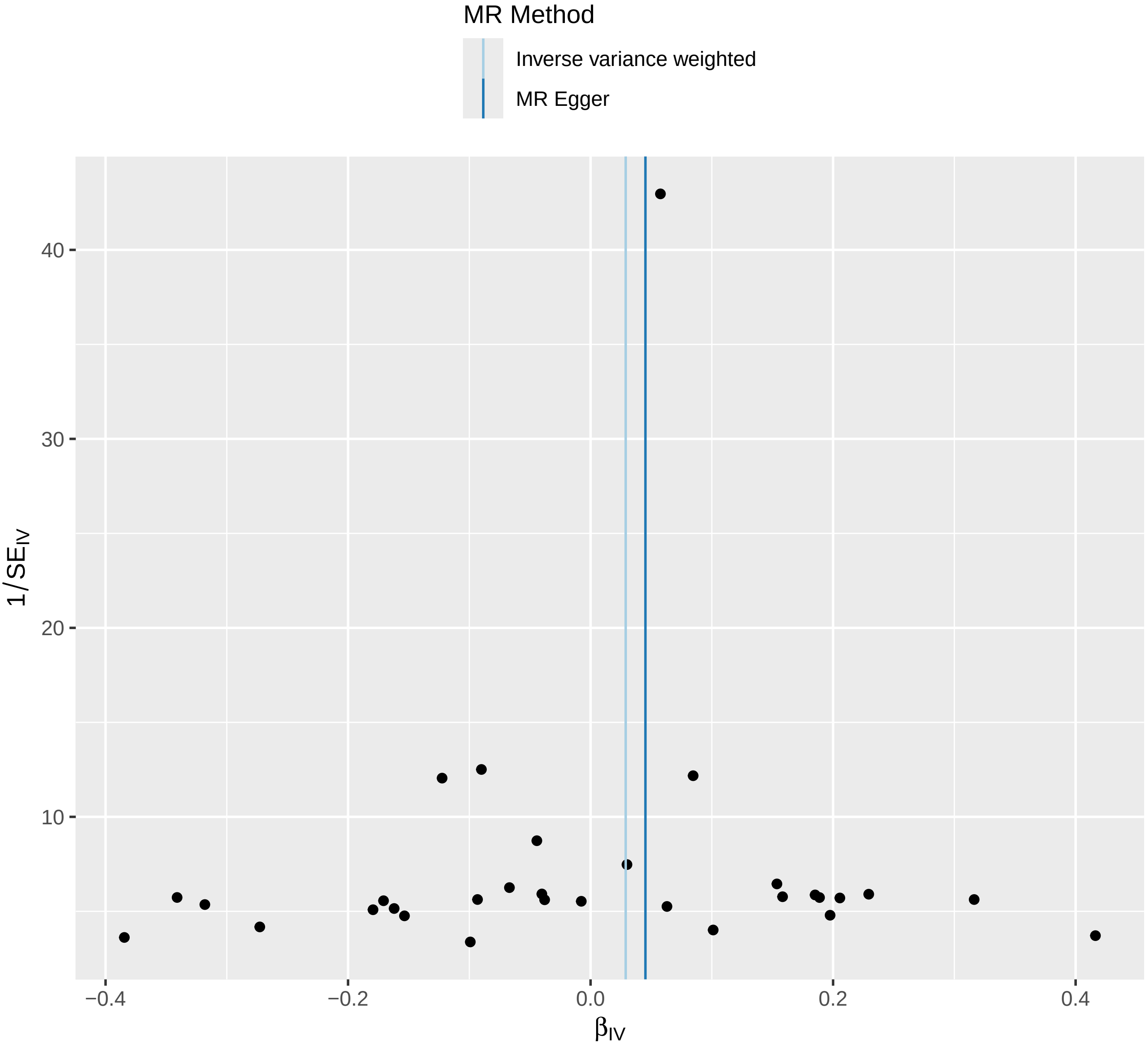
MR Method
Inverse variance weighted
MR Egger
40
30
20
10
1/SE$_{IV}$
−0.4
−0.2
0.0
0.2
0.4
β$_{IV}$

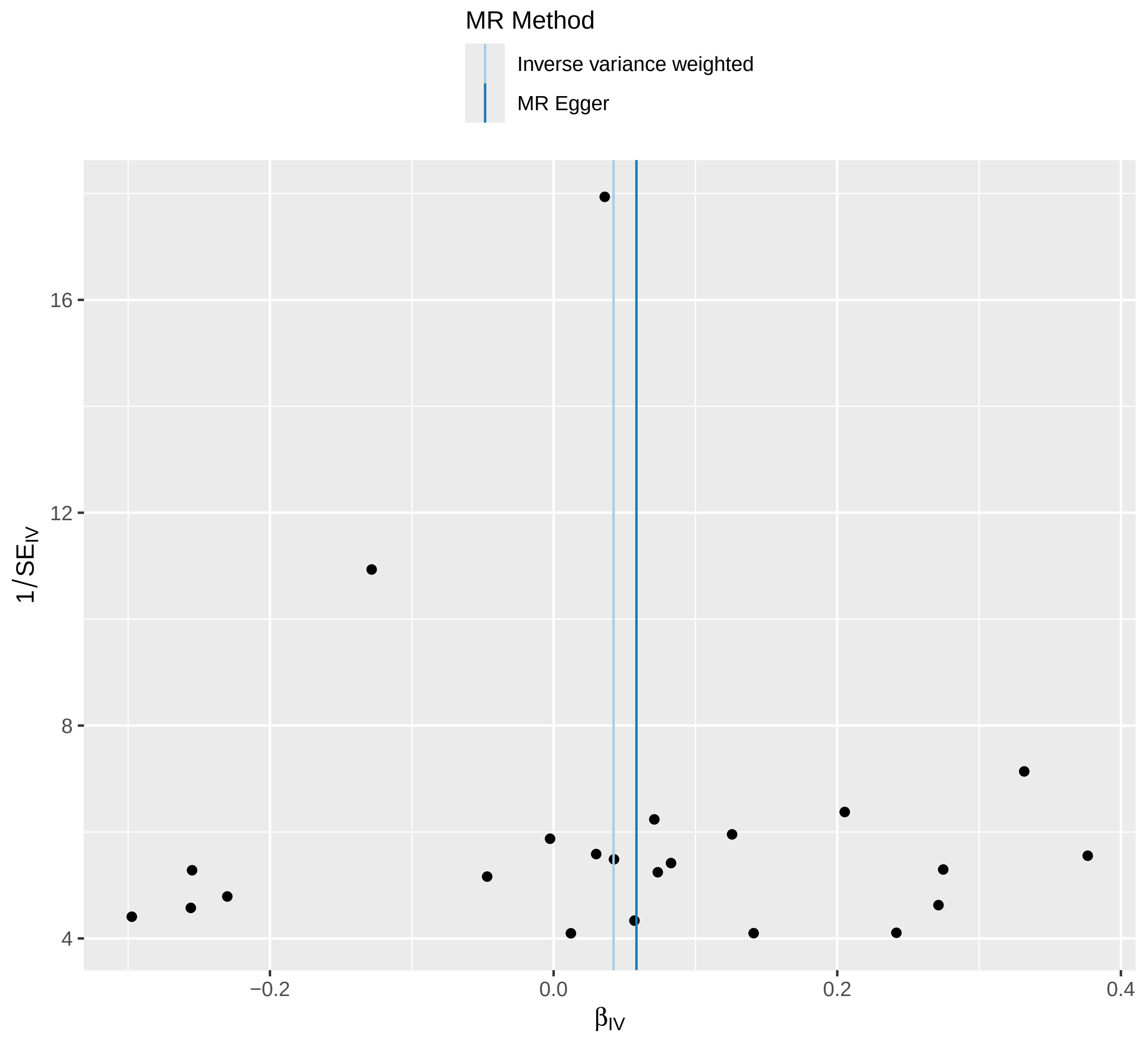
MR Method
Inverse variance weighted
MR Egger
16
12
8
4
1/SE_IV
−0.2
0.0
0.2
0.4
β_IV

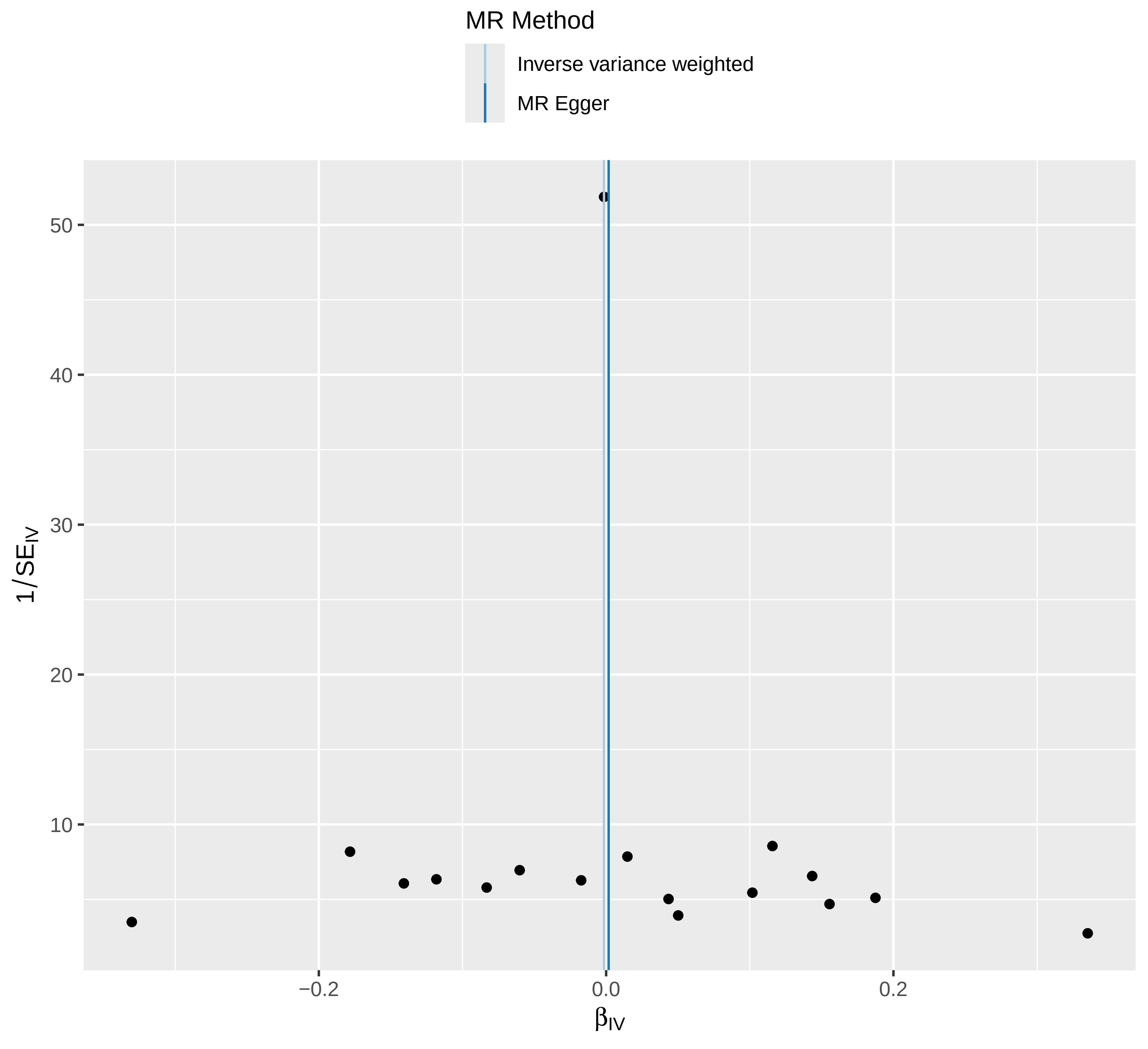

MR Method
Inverse variance weighted
MR Egger
1/SE_IV
50
40
30
20
10
−0.2
0.0
0.2
β_IV

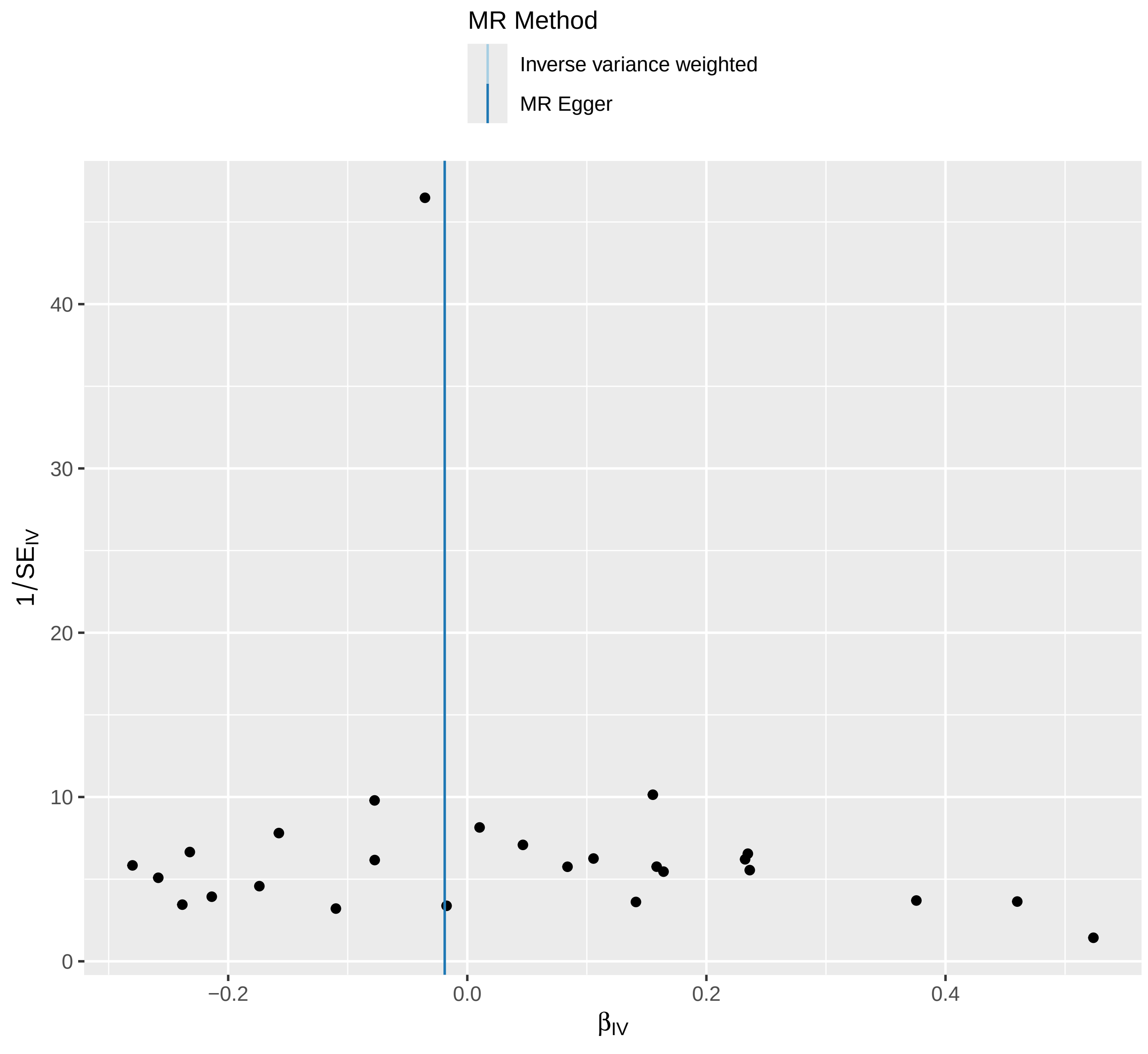

MR Method
Inverse variance weighted
MR Egger
1/SE$_{IV}$
40
30
20
10
0
−0.2
0.0
0.2
0.4
β_{IV}

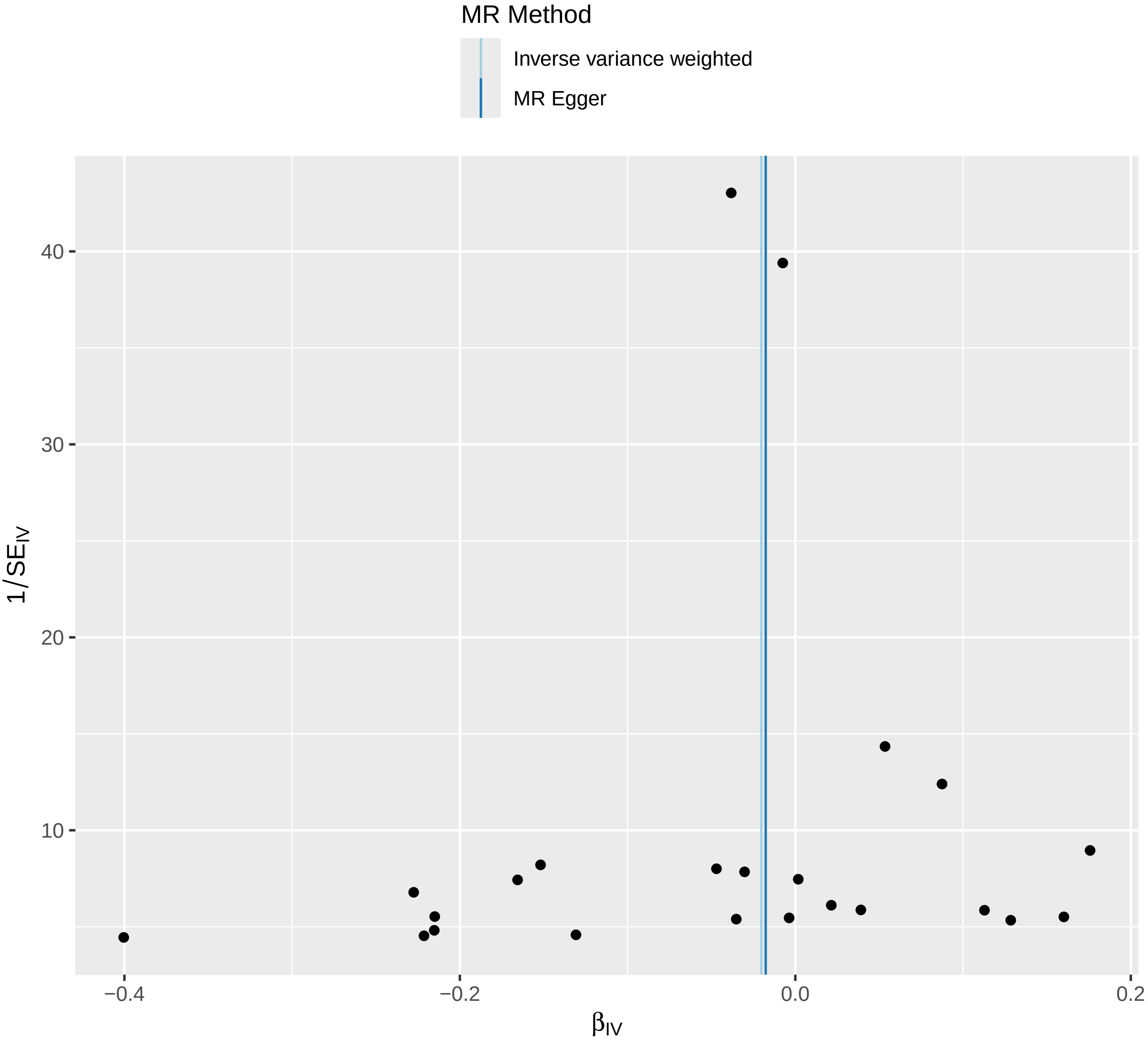
MR Method
Inverse variance weighted
MR Egger
1/SE_IV
40
30
20
10
−0.4
−0.2
0.0
0.2
β_IV

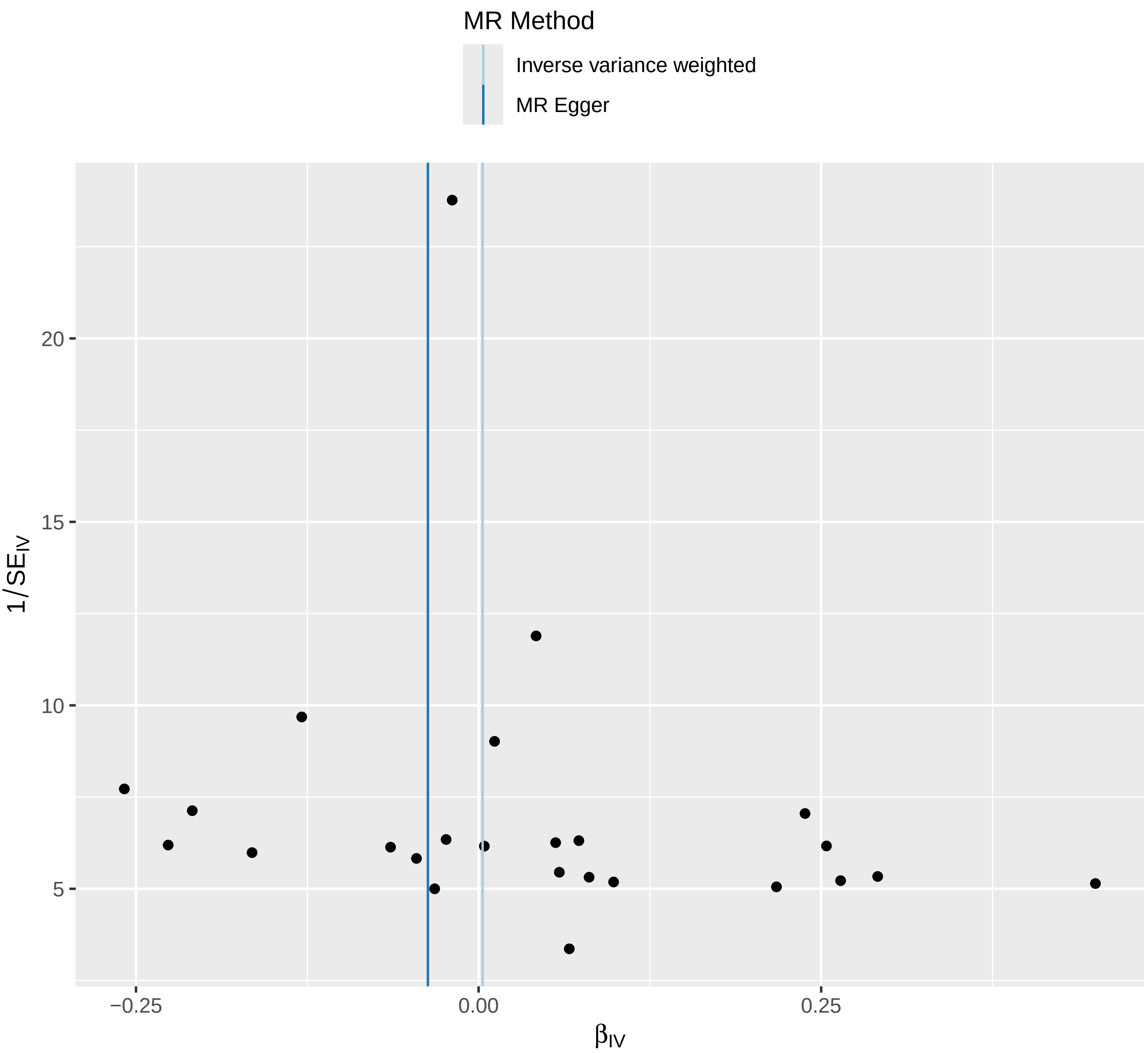
MR Method
Inverse variance weighted
MR Egger
20
15
10
5
1/SE_IV
−0.25
0.00
0.25
β_IV

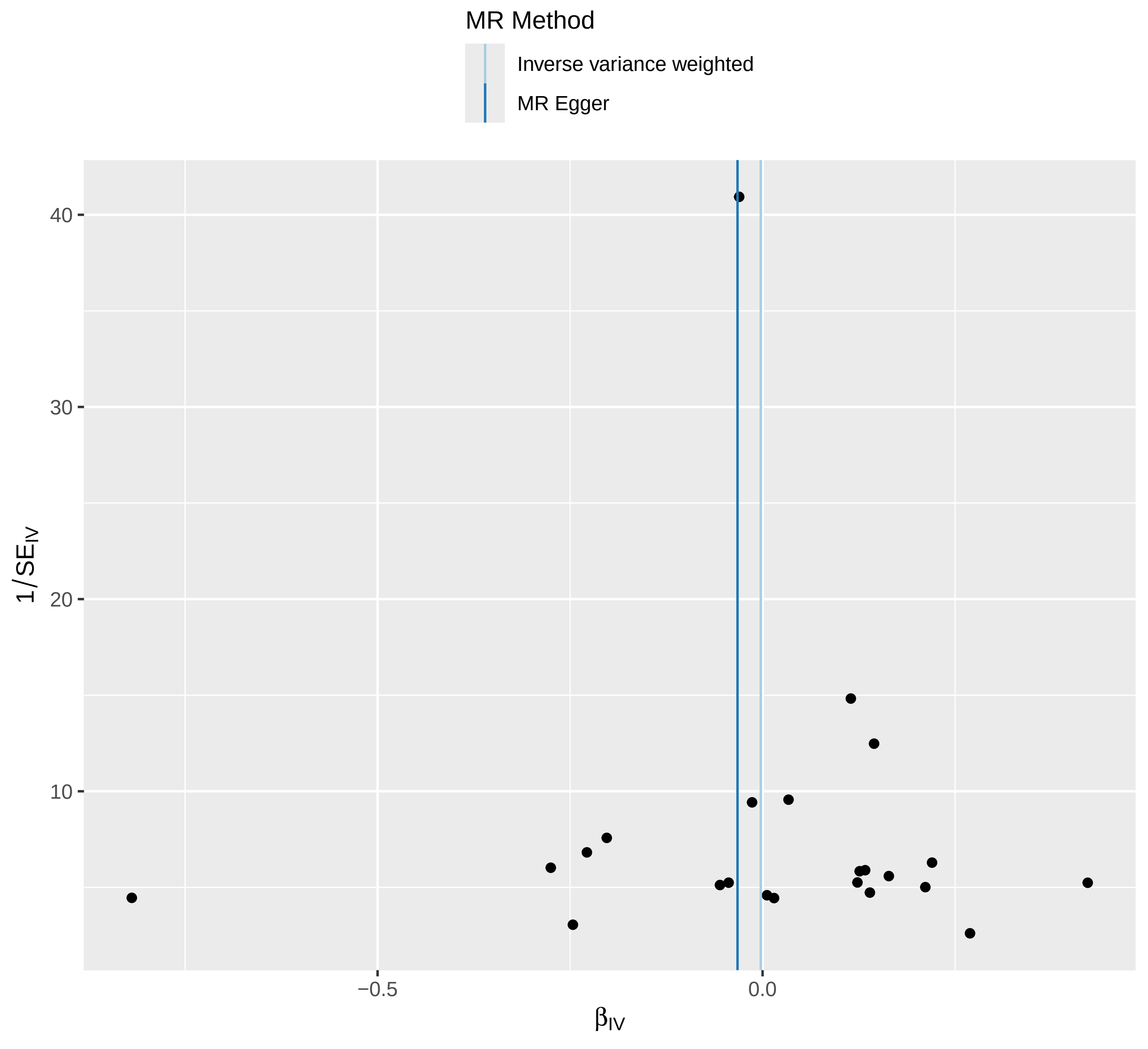
MR Method
Inverse variance weighted
MR Egger
40
30
20
10
1/SE_IV
−0.5
0.0
β_IV

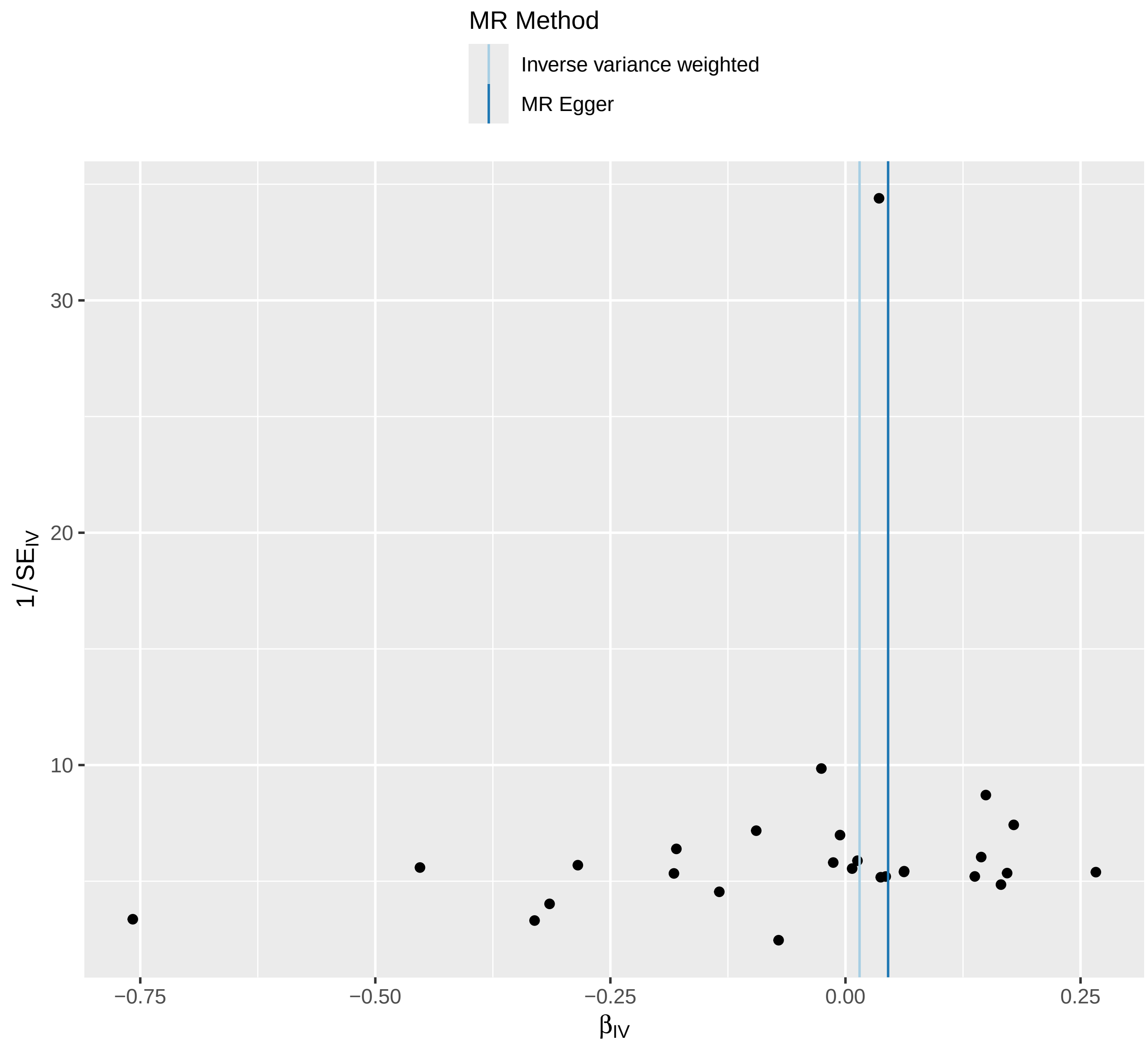
MR Method
Inverse variance weighted
MR Egger
1/SE_IV
30
20
10
−0.75
−0.50
−0.25
0.00
0.25
β_IV

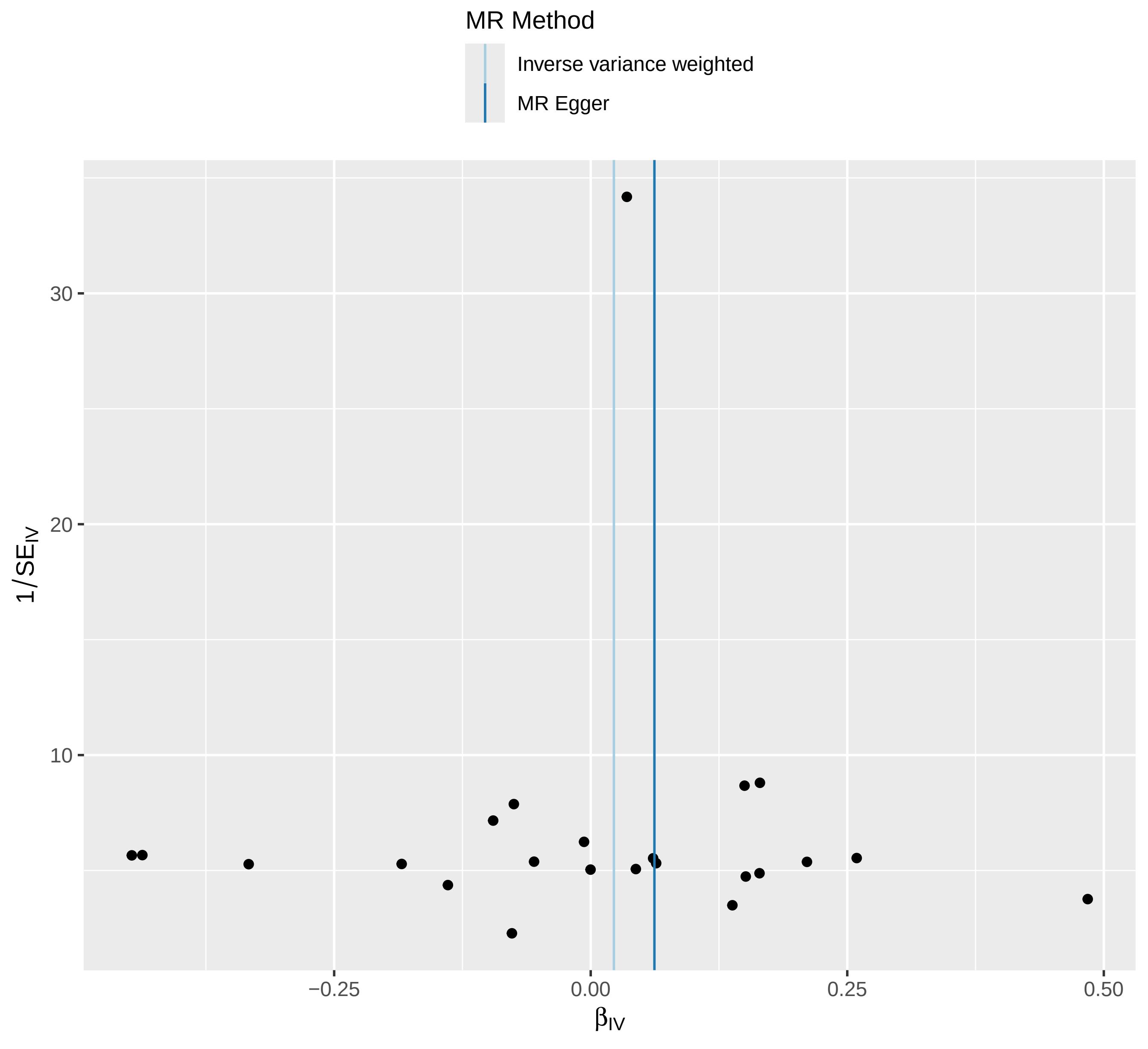
MR Method
Inverse variance weighted
MR Egger
30
20
10
1/SE_IV
−0.25
0.00
0.25
0.50
β_IV

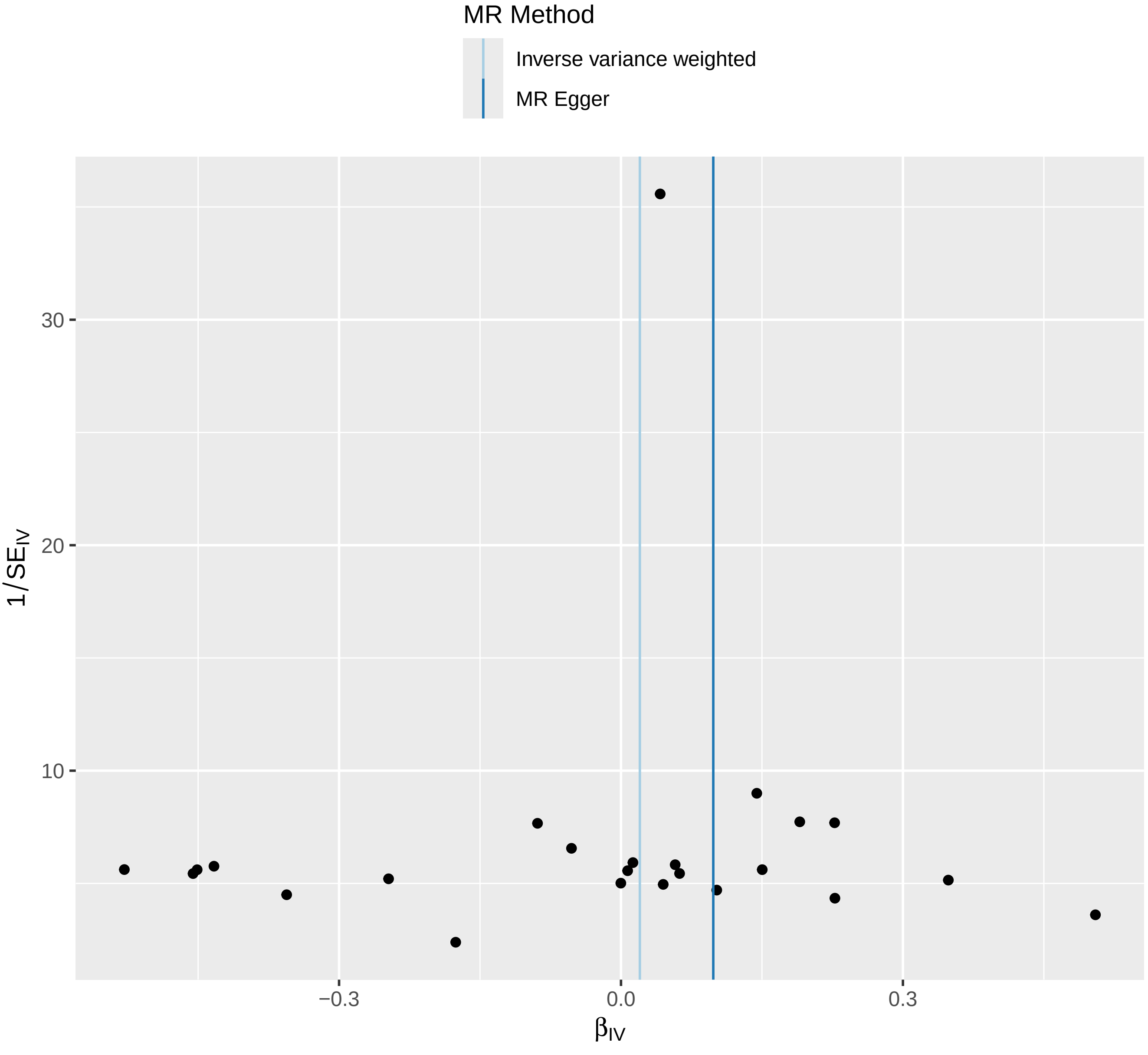
MR Method
Inverse variance weighted
MR Egger
30
20
10
1/SE_IV
−0.3
0.0
0.3
β_IV

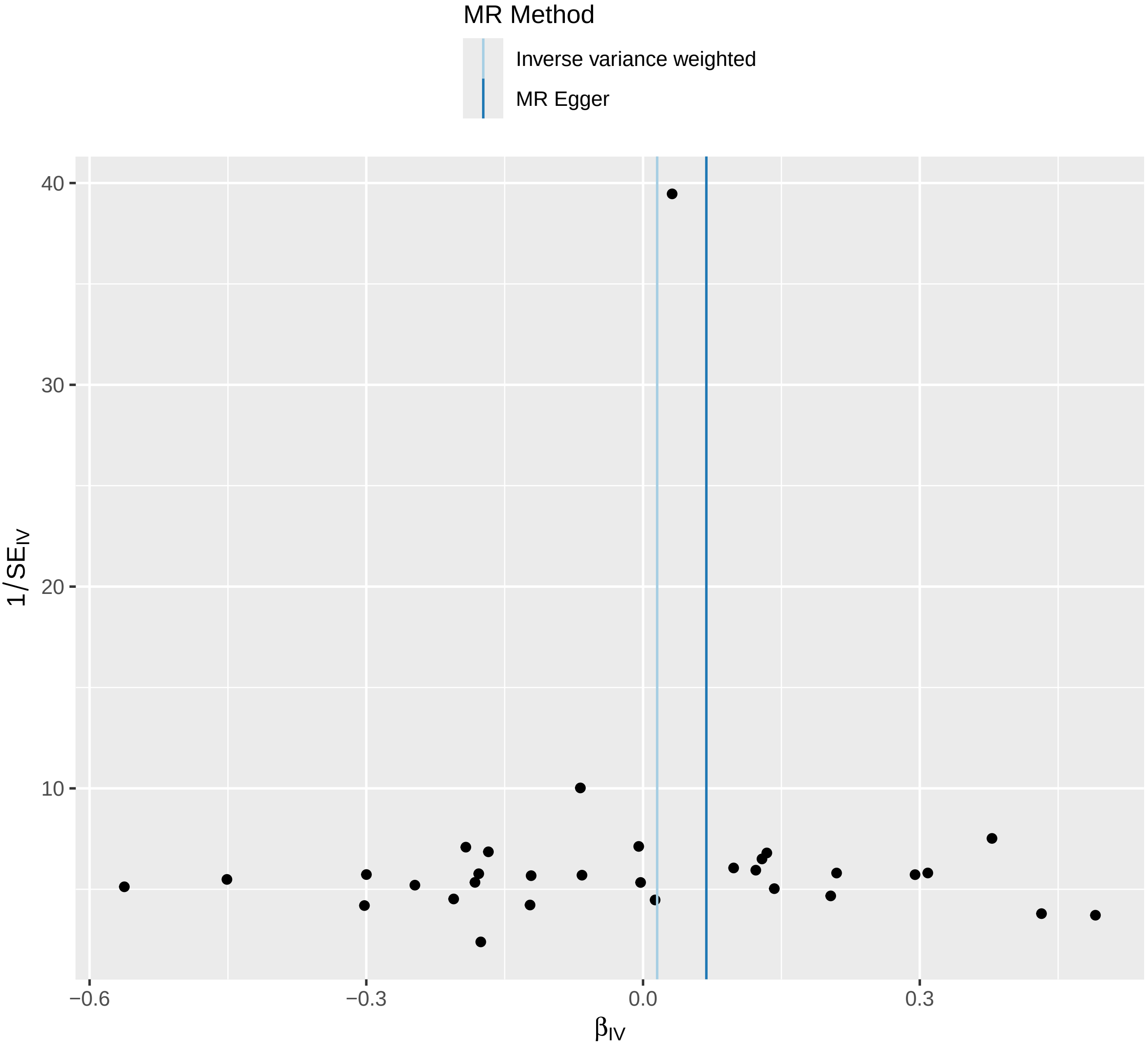
MR Method
Inverse variance weighted
MR Egger
40
30
20
10
1/SE_IV
−0.6
−0.3
0.0
0.3
β_IV

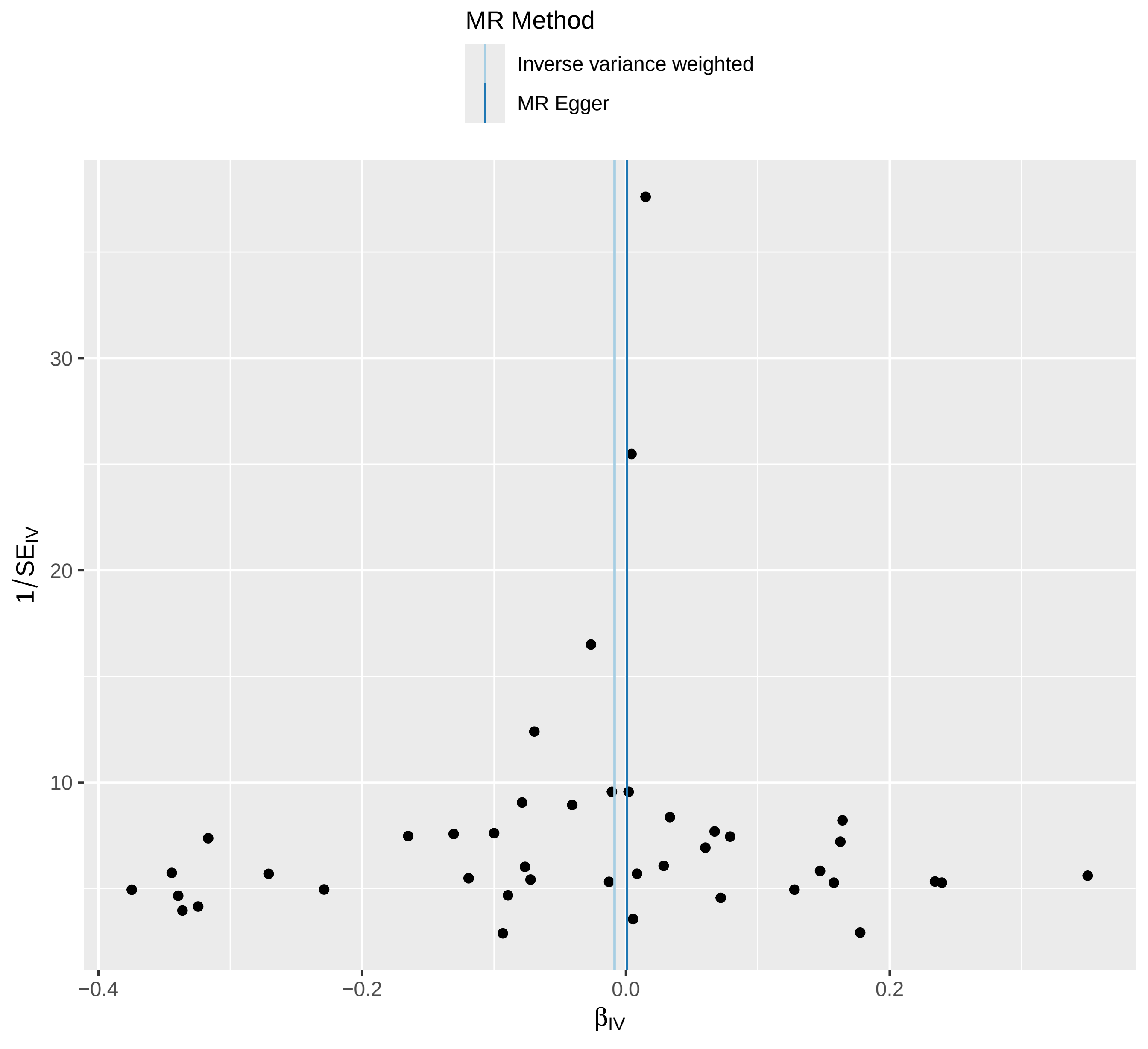
MR Method
Inverse variance weighted
MR Egger
30
20
10
1/SE$_{IV}$
−0.4
−0.2
0.0
0.2
β$_{IV}$

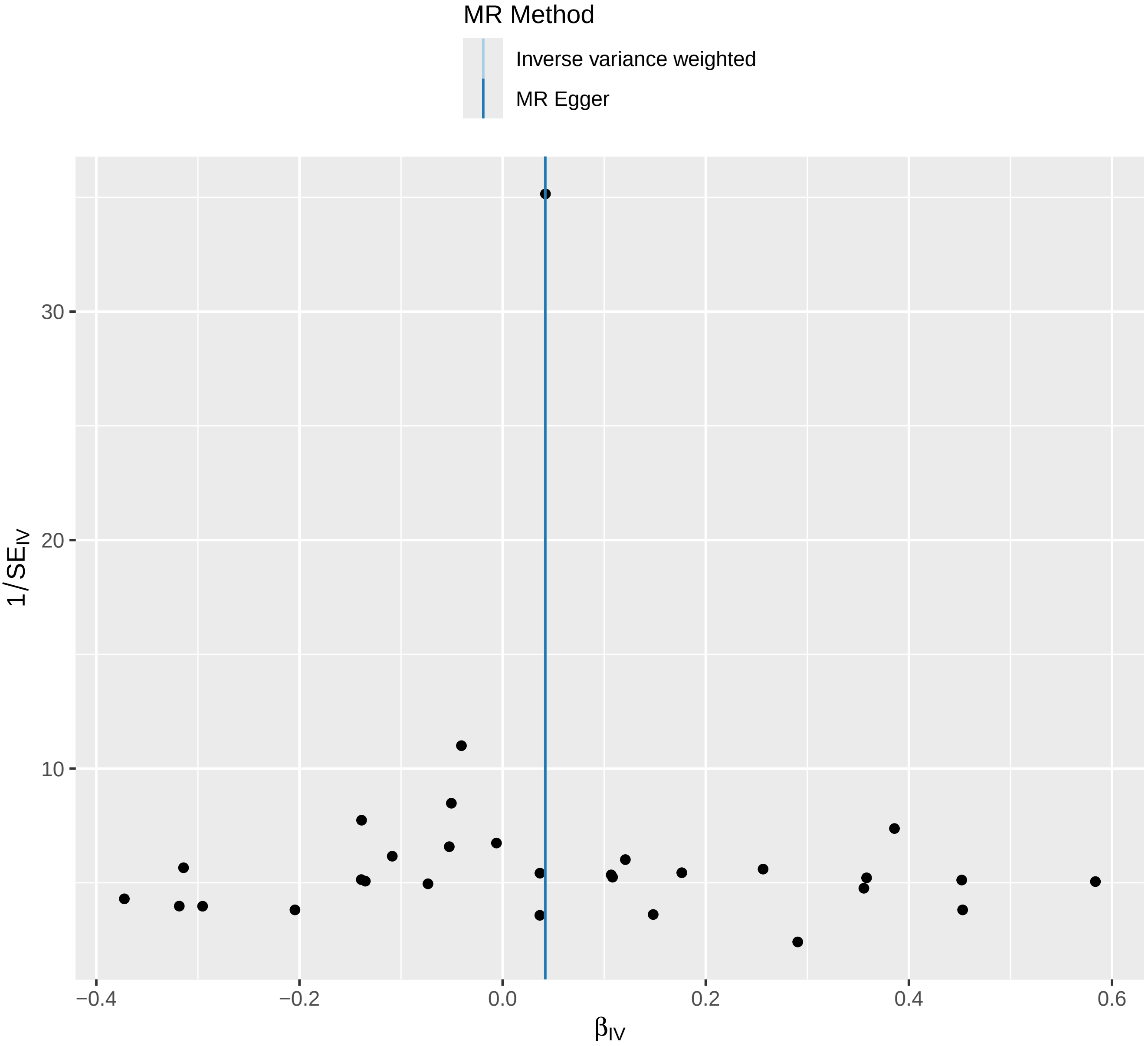
MR Method
Inverse variance weighted
MR Egger
30
20
10
1/SE_IV
−0.4
−0.2
0.0
0.2
0.4
0.6
β_IV

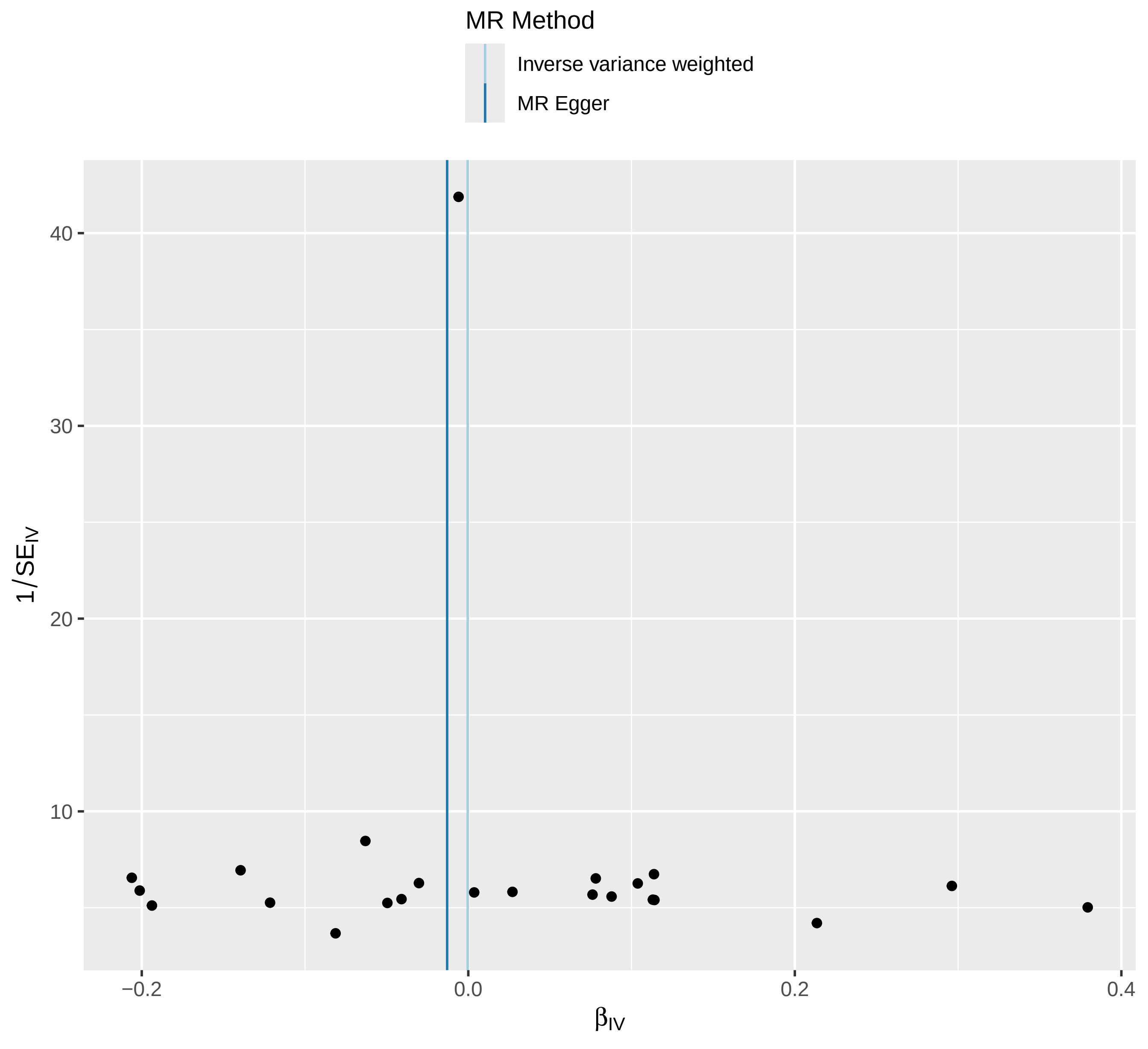
MR Method
Inverse variance weighted
MR Egger
40
30
20
10
1/SE_IV
−0.2
0.0
0.2
0.4
β_IV

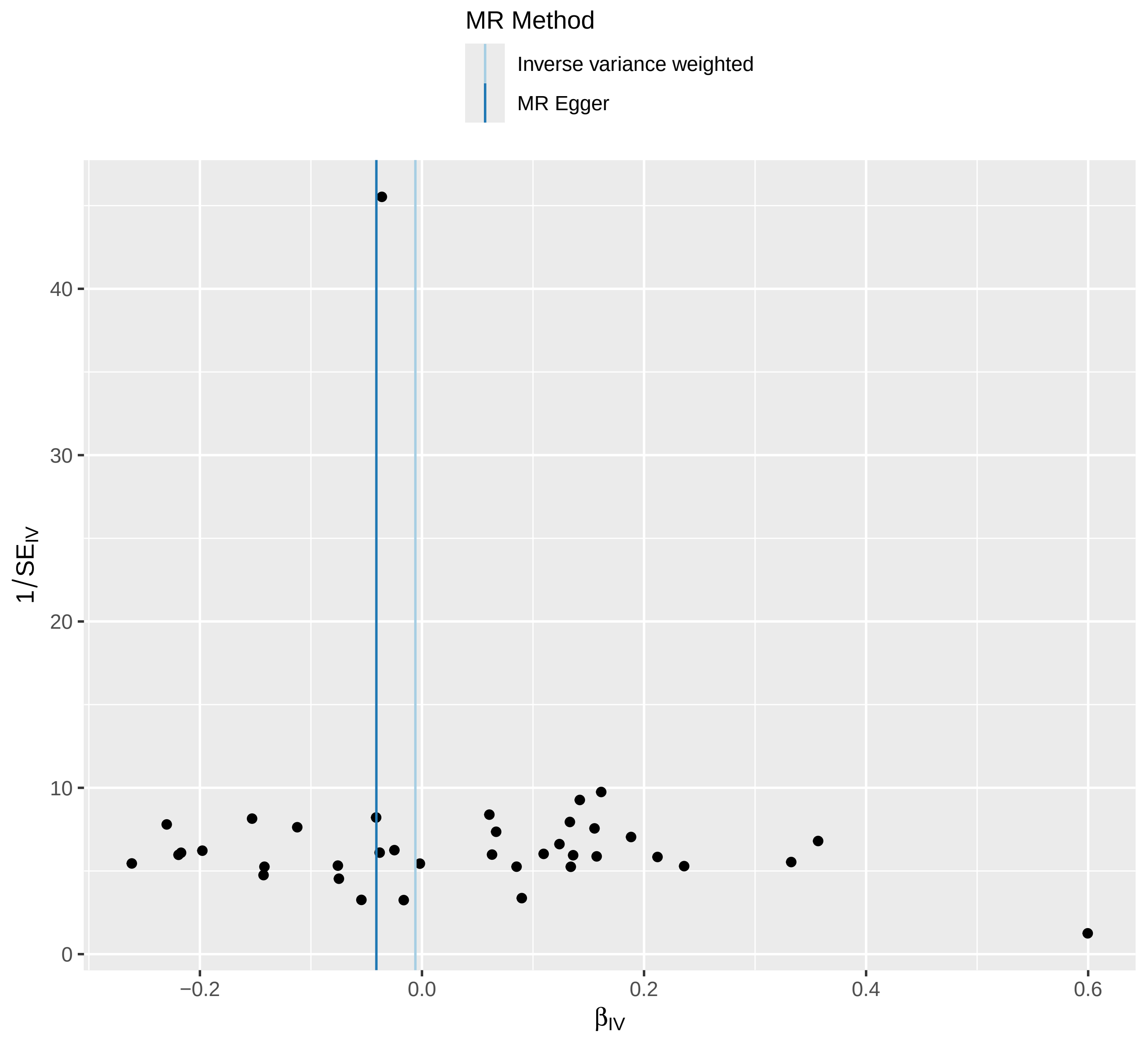
MR Method
Inverse variance weighted
MR Egger
1/SE_IV
0
10
20
30
40
−0.2
0.0
0.2
0.4
0.6
β_IV

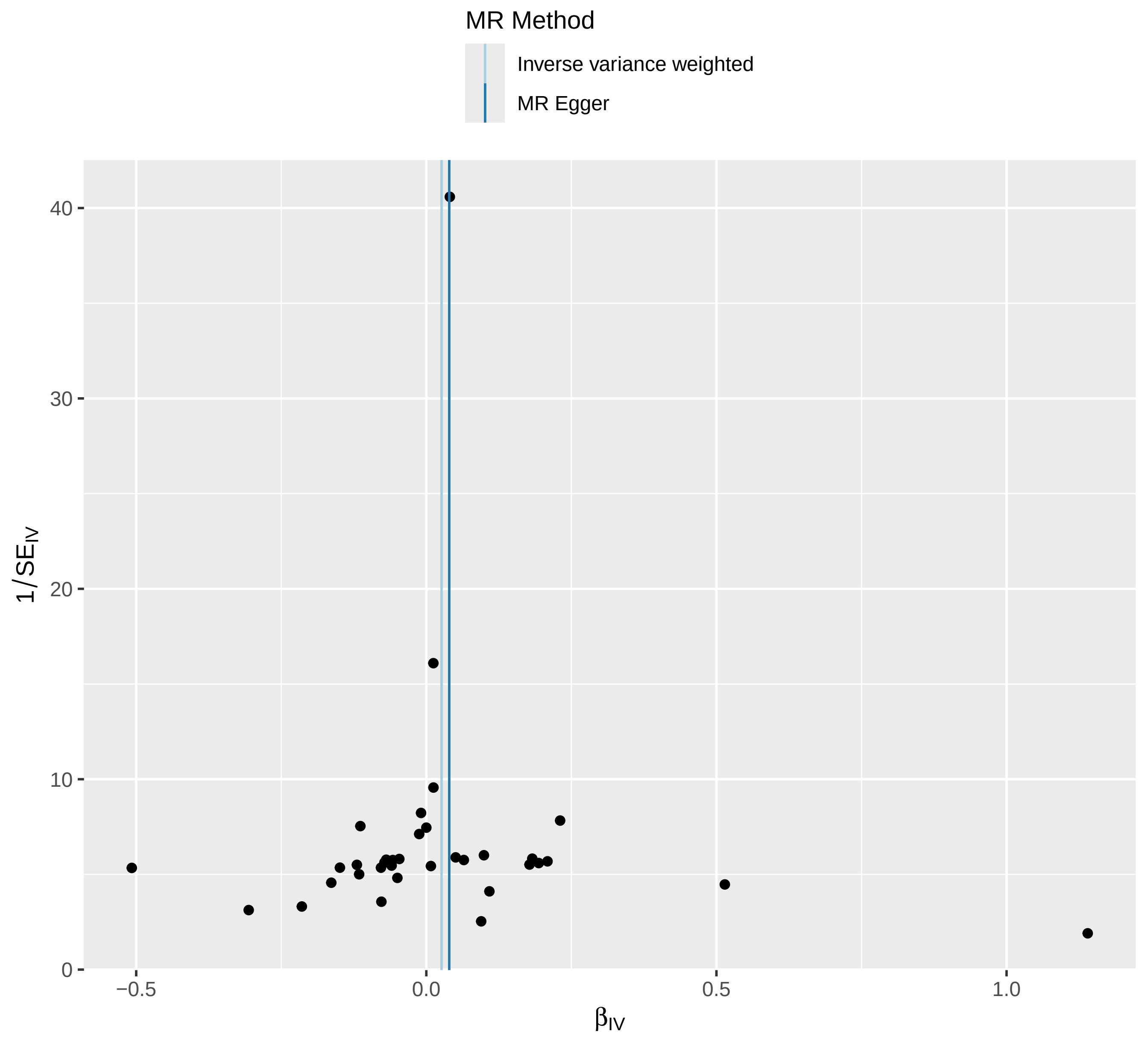

MR Method
Inverse variance weighted
MR Egger
1/SE_IV
40
30
20
10
0
−0.5
0.0
0.5
1.0
β_IV

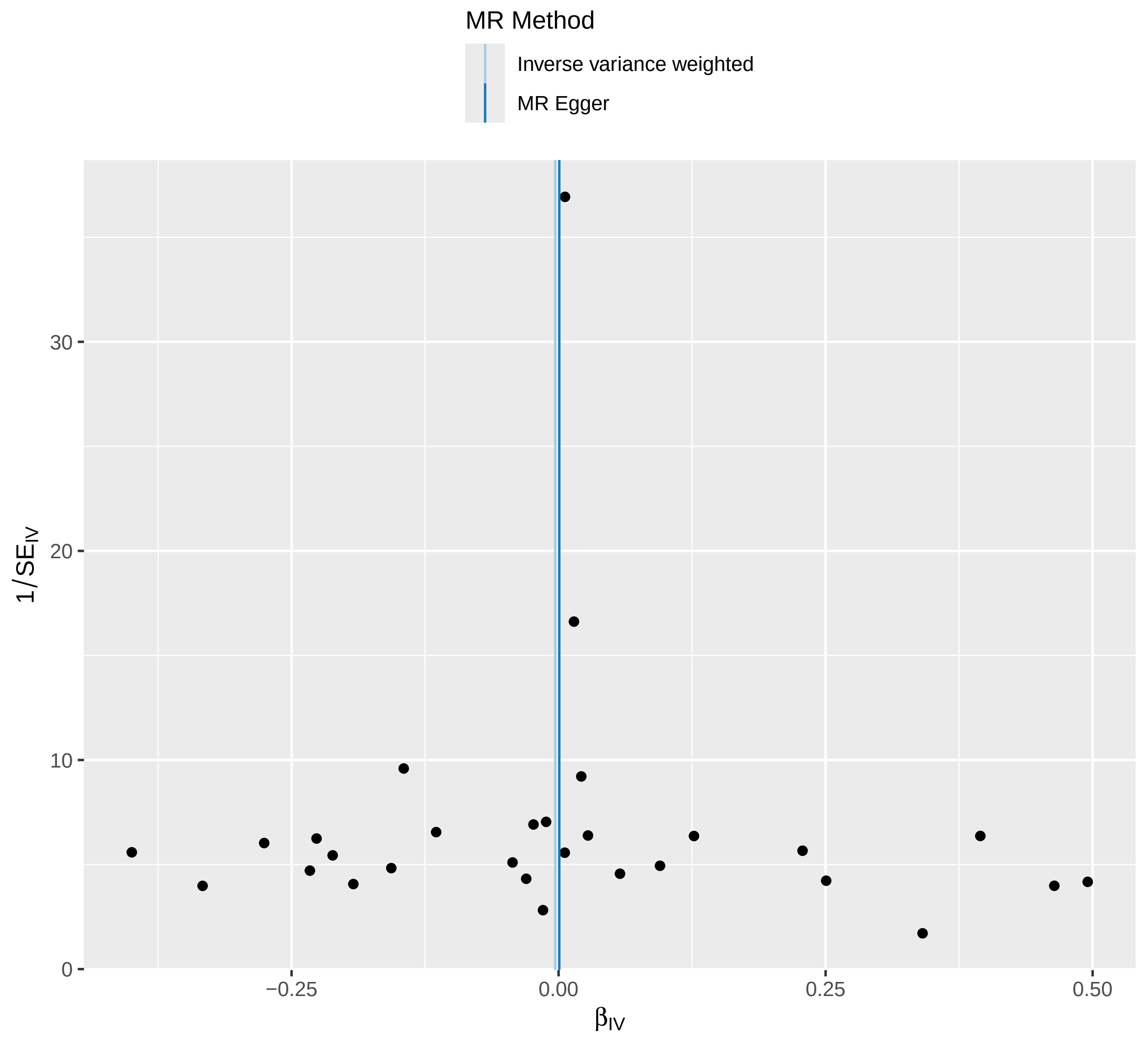

MR Method
Inverse variance weighted
MR Egger
1/SE_IV
30
20
10
0
−0.25
0.00
0.25
0.50
β_IV

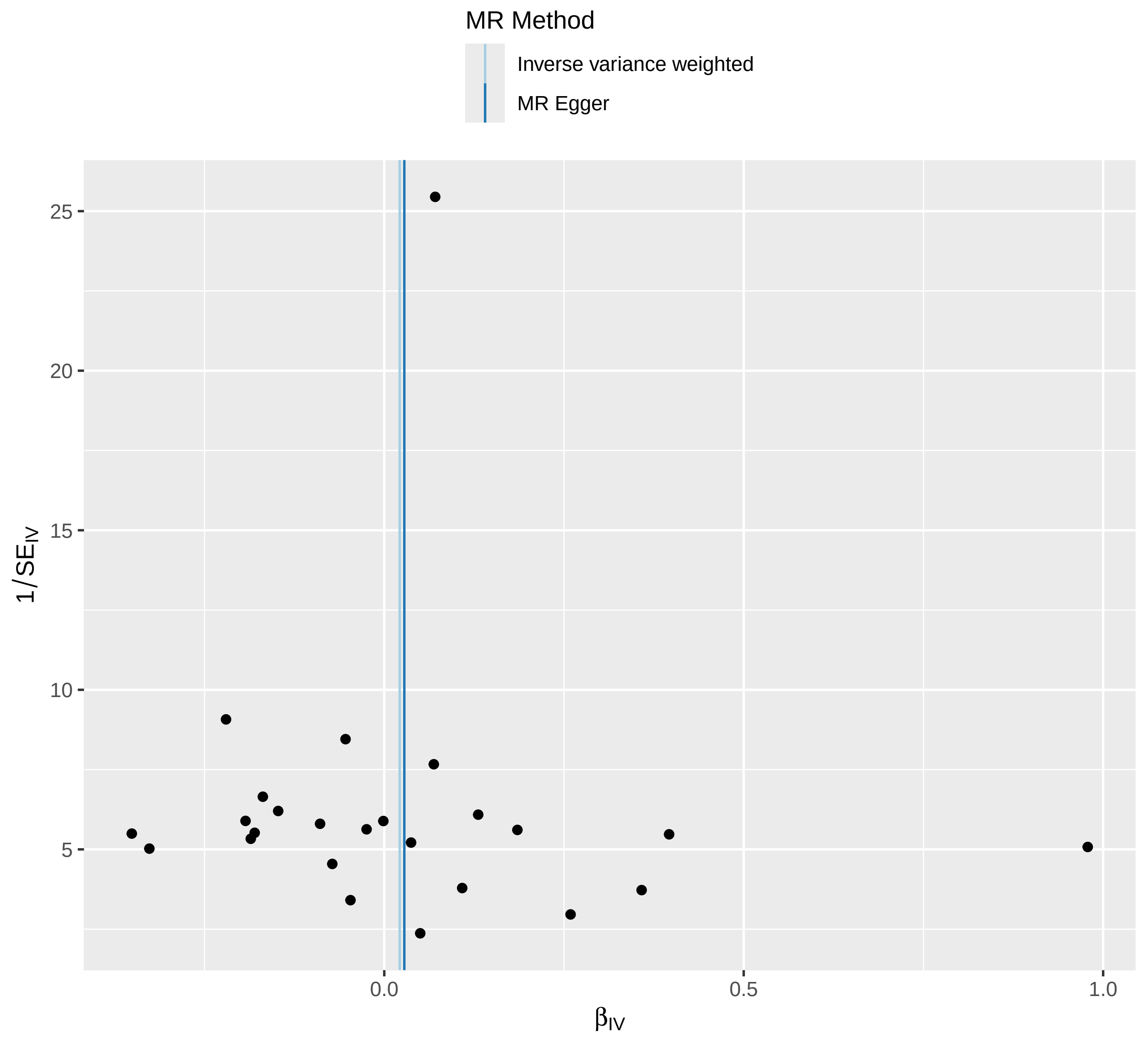

MR Method
Inverse variance weighted
MR Egger
25
20
15
10
5
1/SE_IV
0.0
0.5
1.0
β_IV

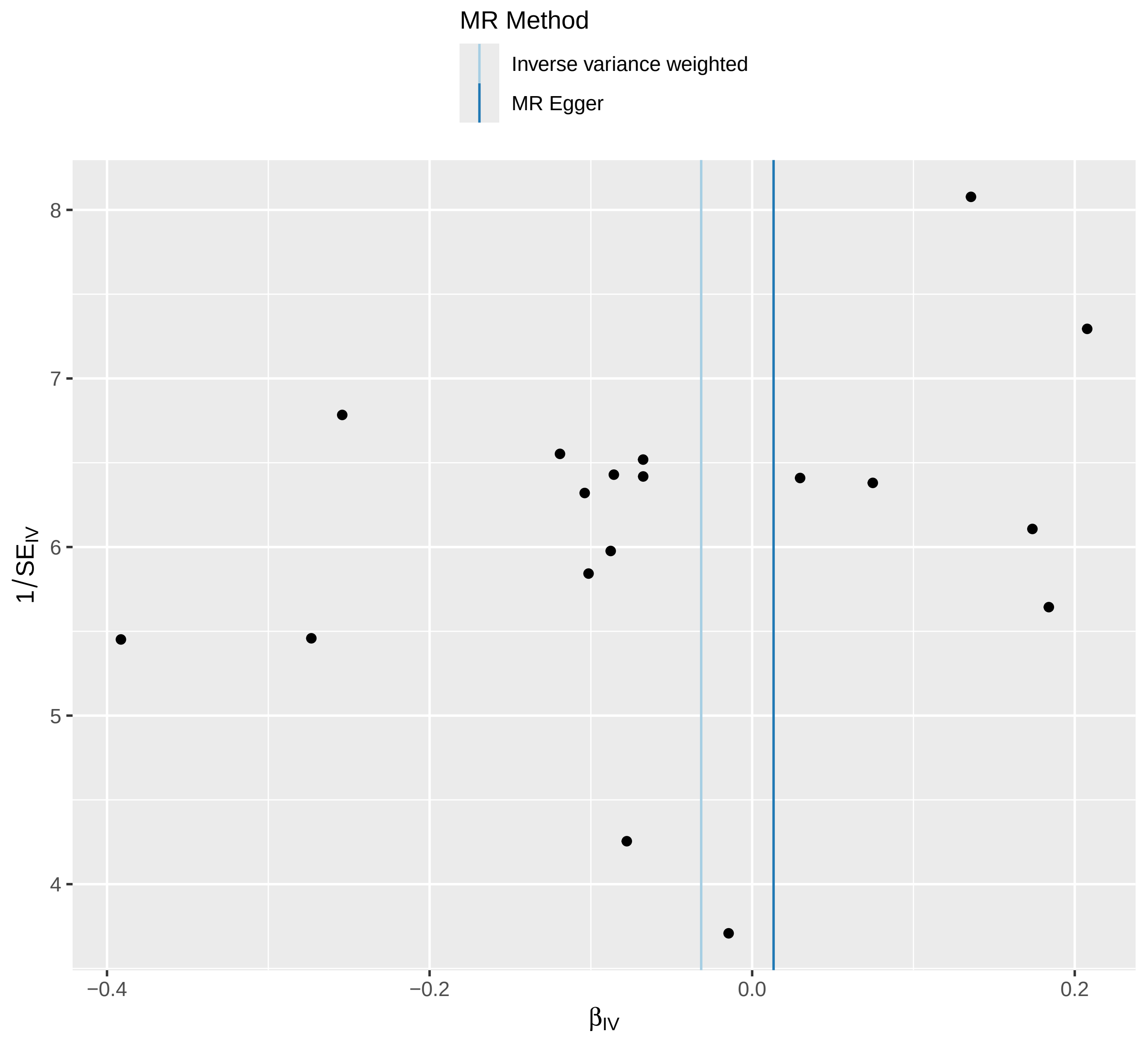

MR Method
Inverse variance weighted
MR Egger
8
7
6
5
4
1/SE_IV
−0.4
−0.2
0.0
0.2
β_IV

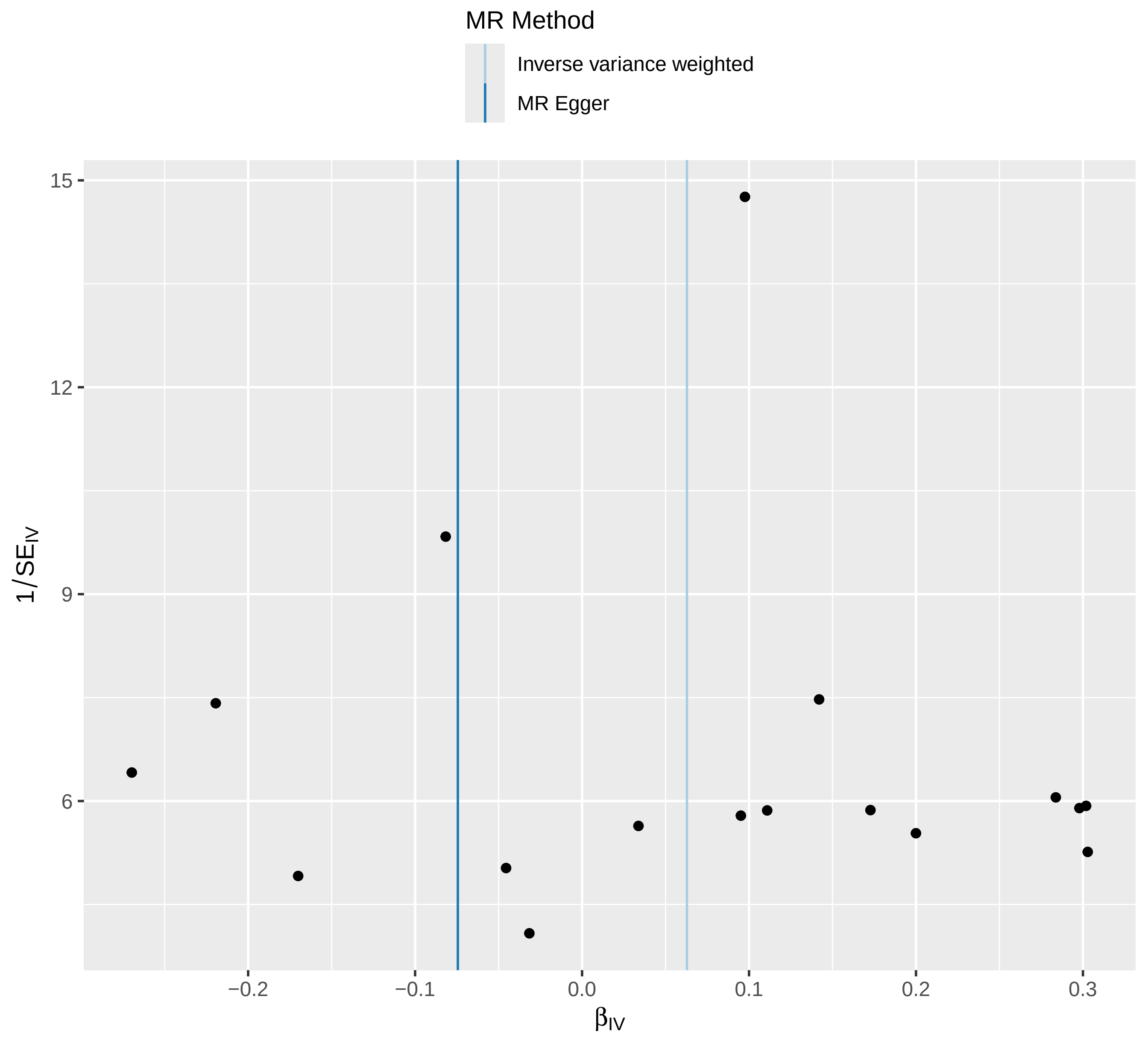

MR Method
Inverse variance weighted
MR Egger
15
12
9
6
$1/SE_{IV}$
−0.2
−0.1
0.0
0.1
0.2
0.3
β_{IV}

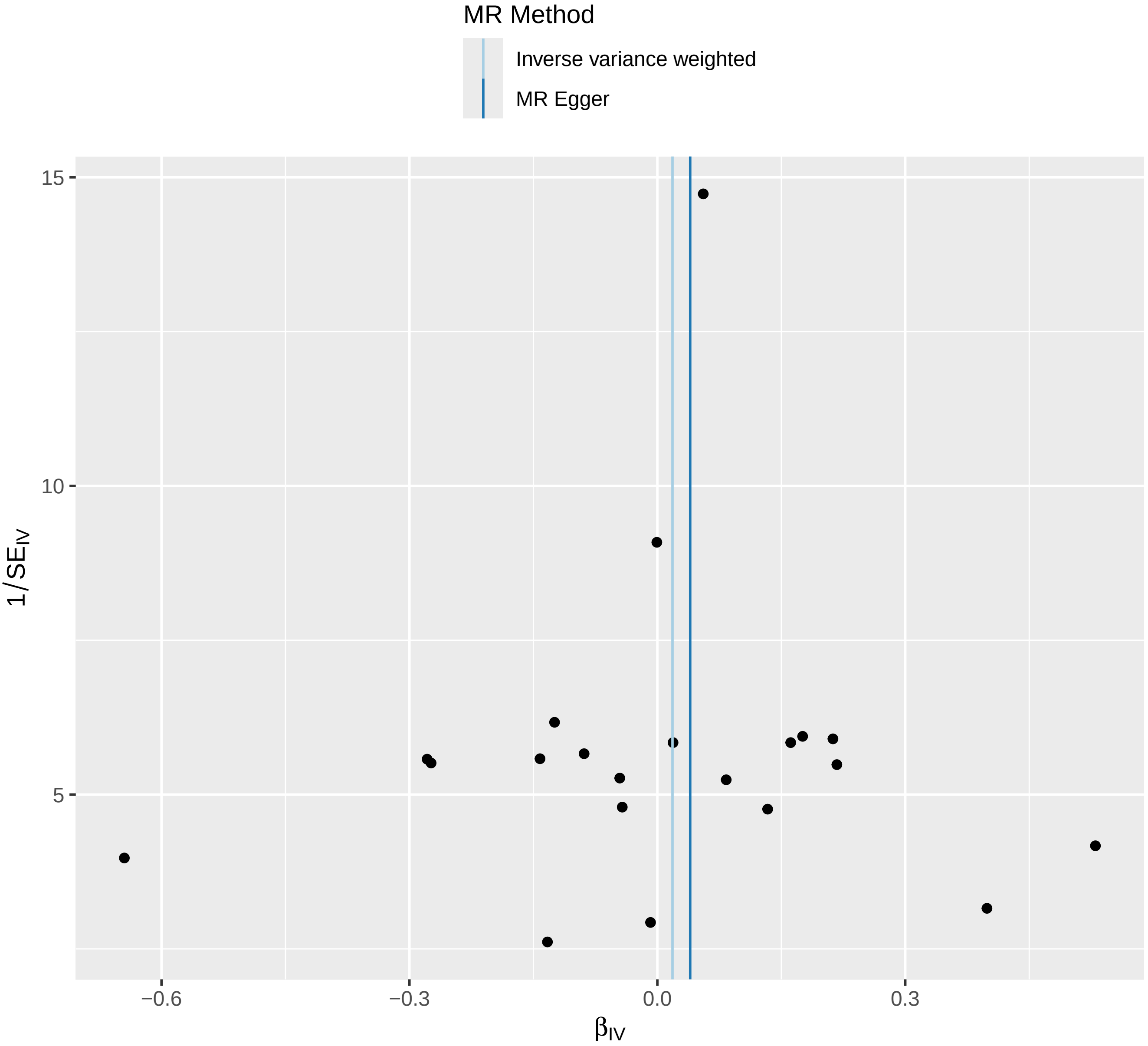

MR Method
Inverse variance weighted
MR Egger
15
10
5
1/SE_IV
−0.6
−0.3
0.0
0.3
β_IV

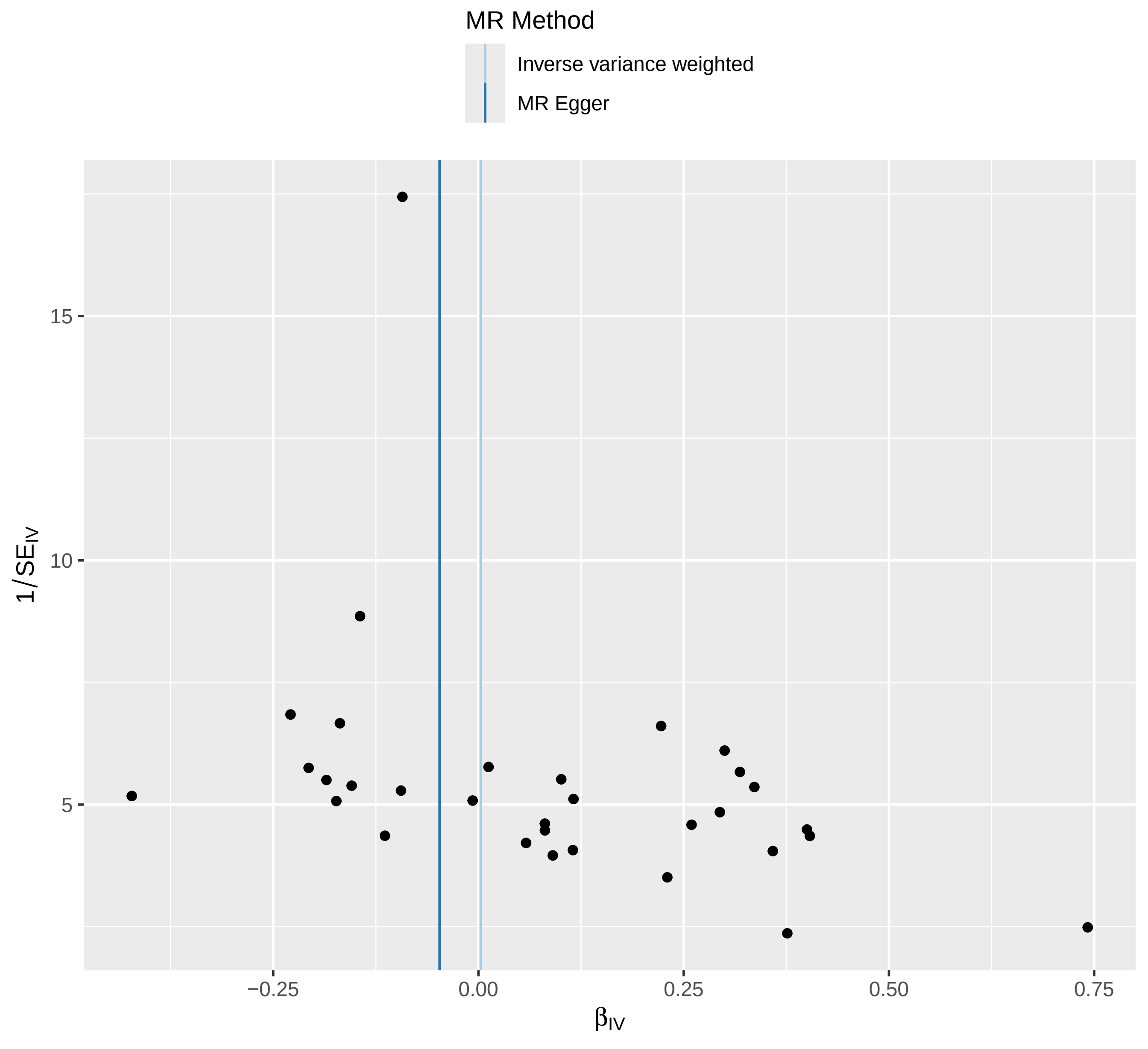
MR Method
Inverse variance weighted
MR Egger
1/SEIV
15
10
5
−0.25
0.00
0.25
0.50
0.75
βIV

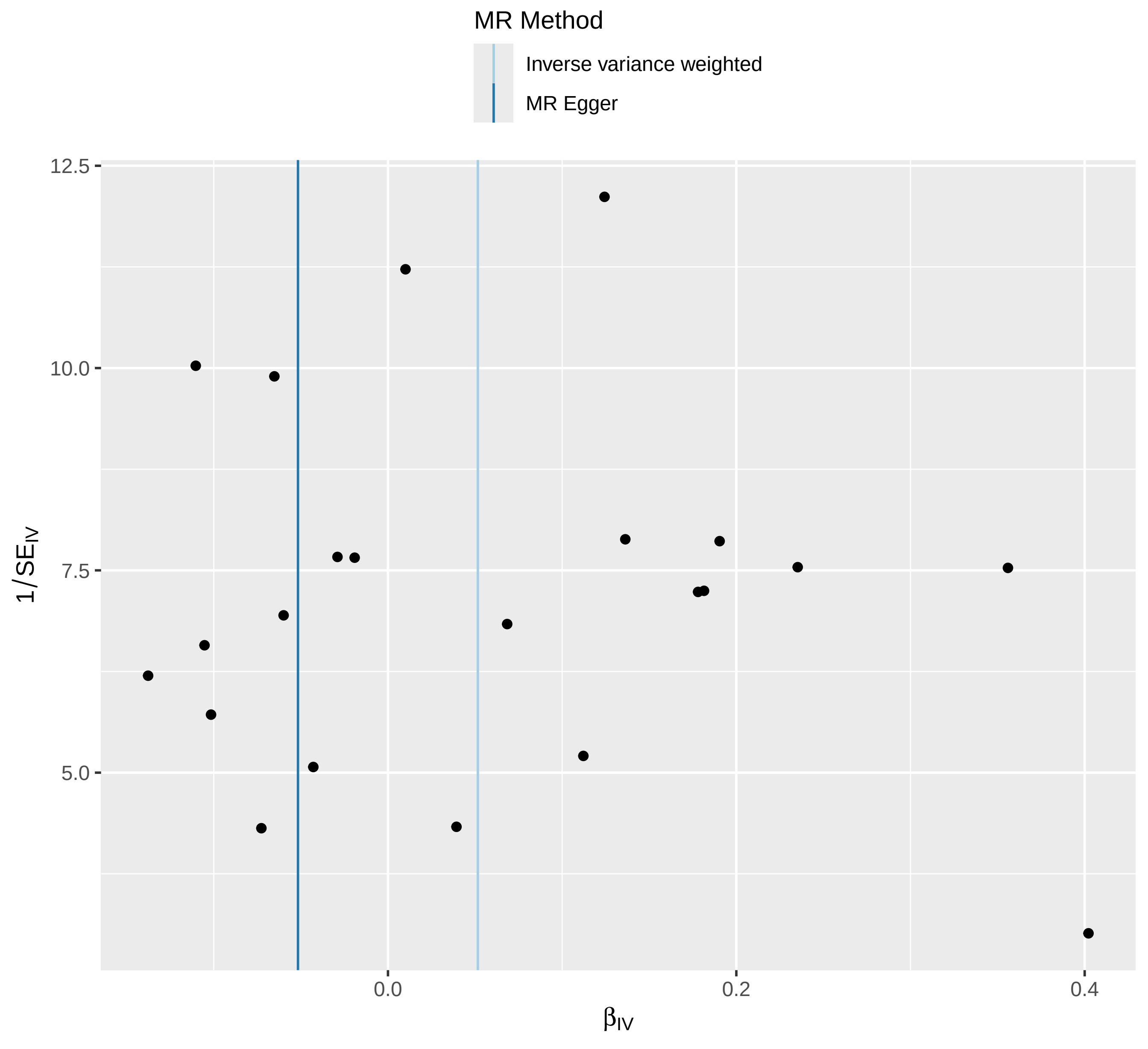
MR Method
Inverse variance weighted
MR Egger
12.5
10.0
7.5
5.0
1/SE_IV
0.0
0.2
0.4
β_IV

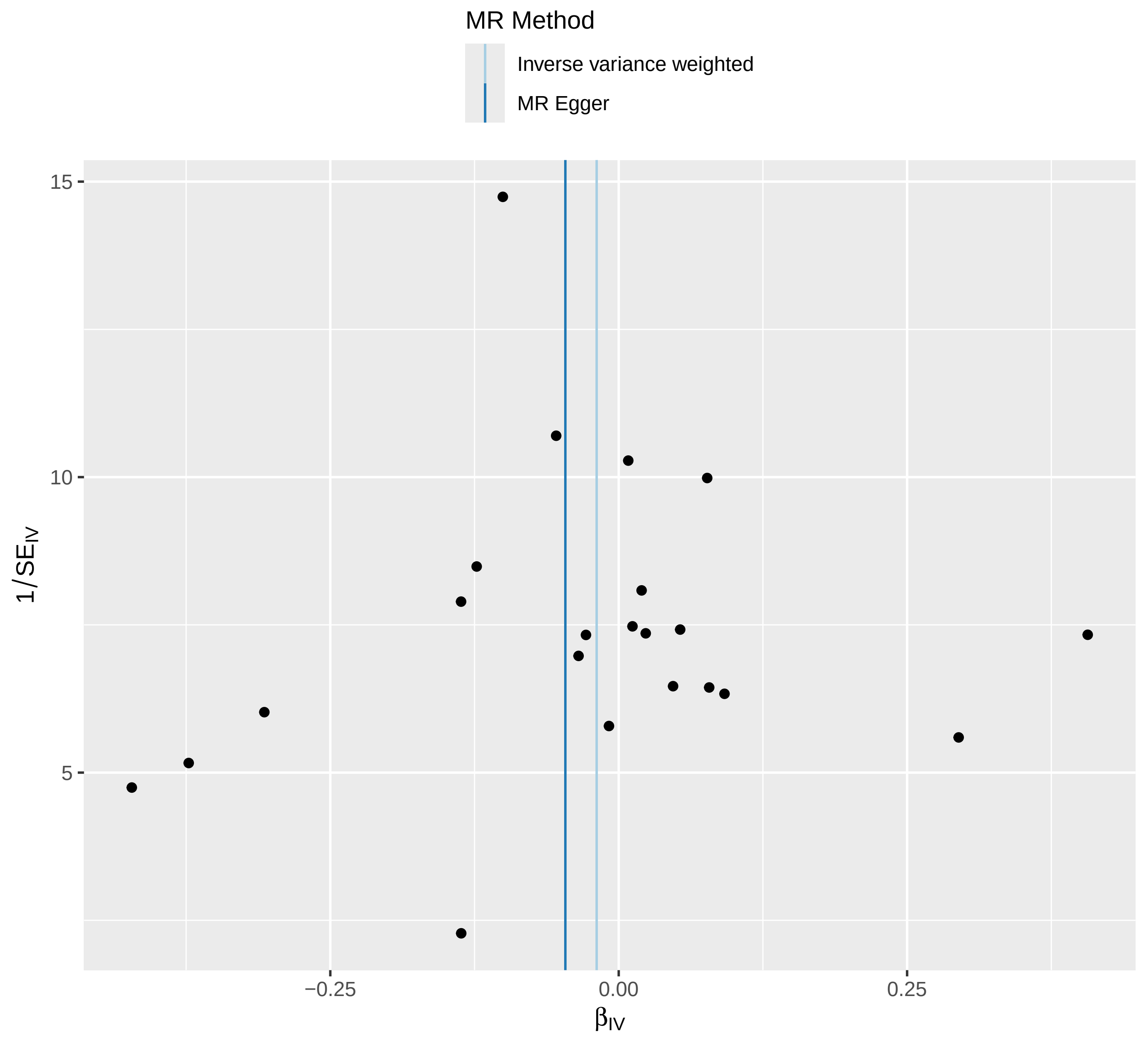
MR Method
Inverse variance weighted
MR Egger
15
10
5
1/SE_IV
−0.25
0.00
0.25
β_IV

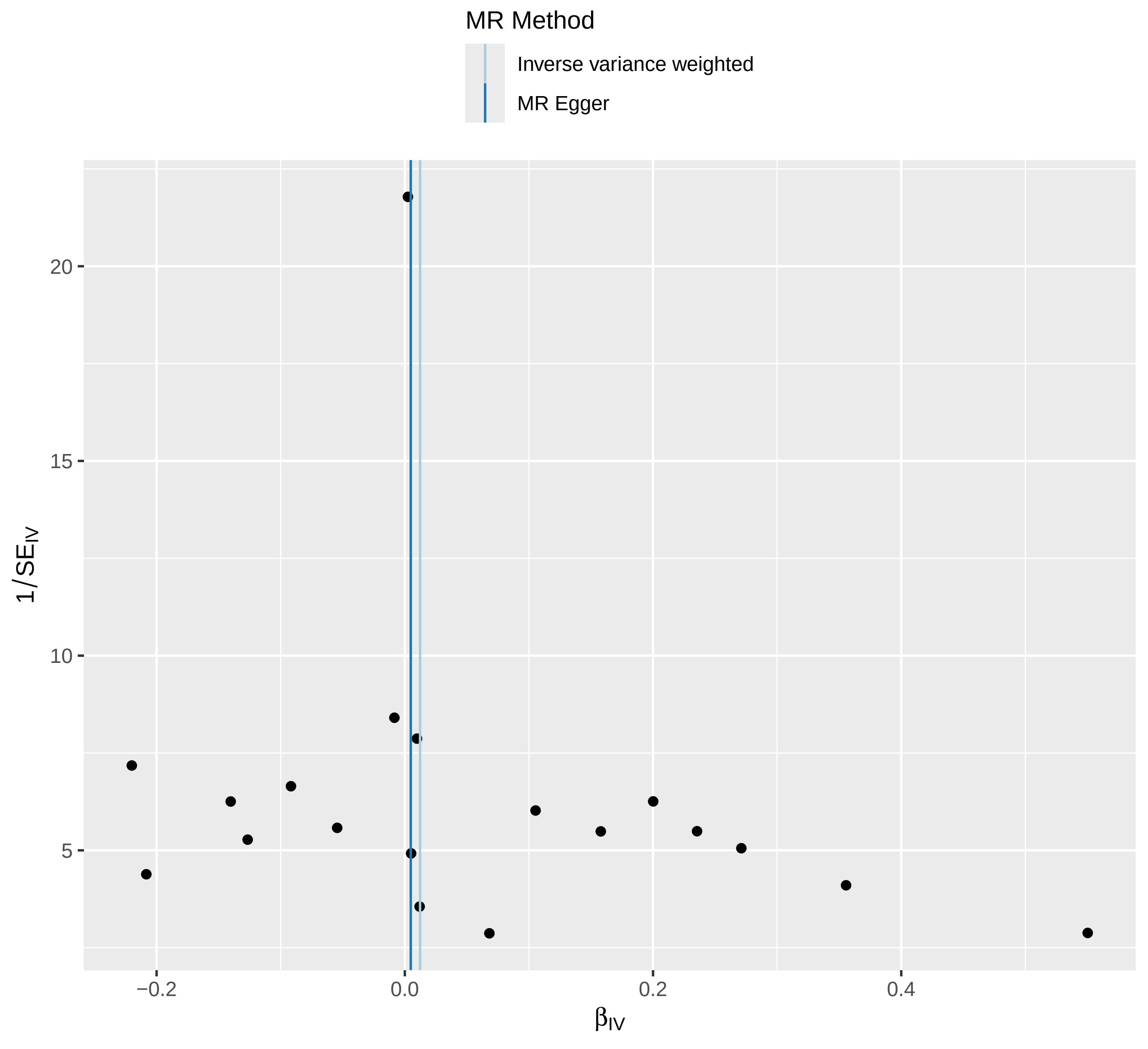
MR Method
Inverse variance weighted
MR Egger
20
15
10
5
1/SE_IV
−0.2
0.0
0.2
0.4
β_IV

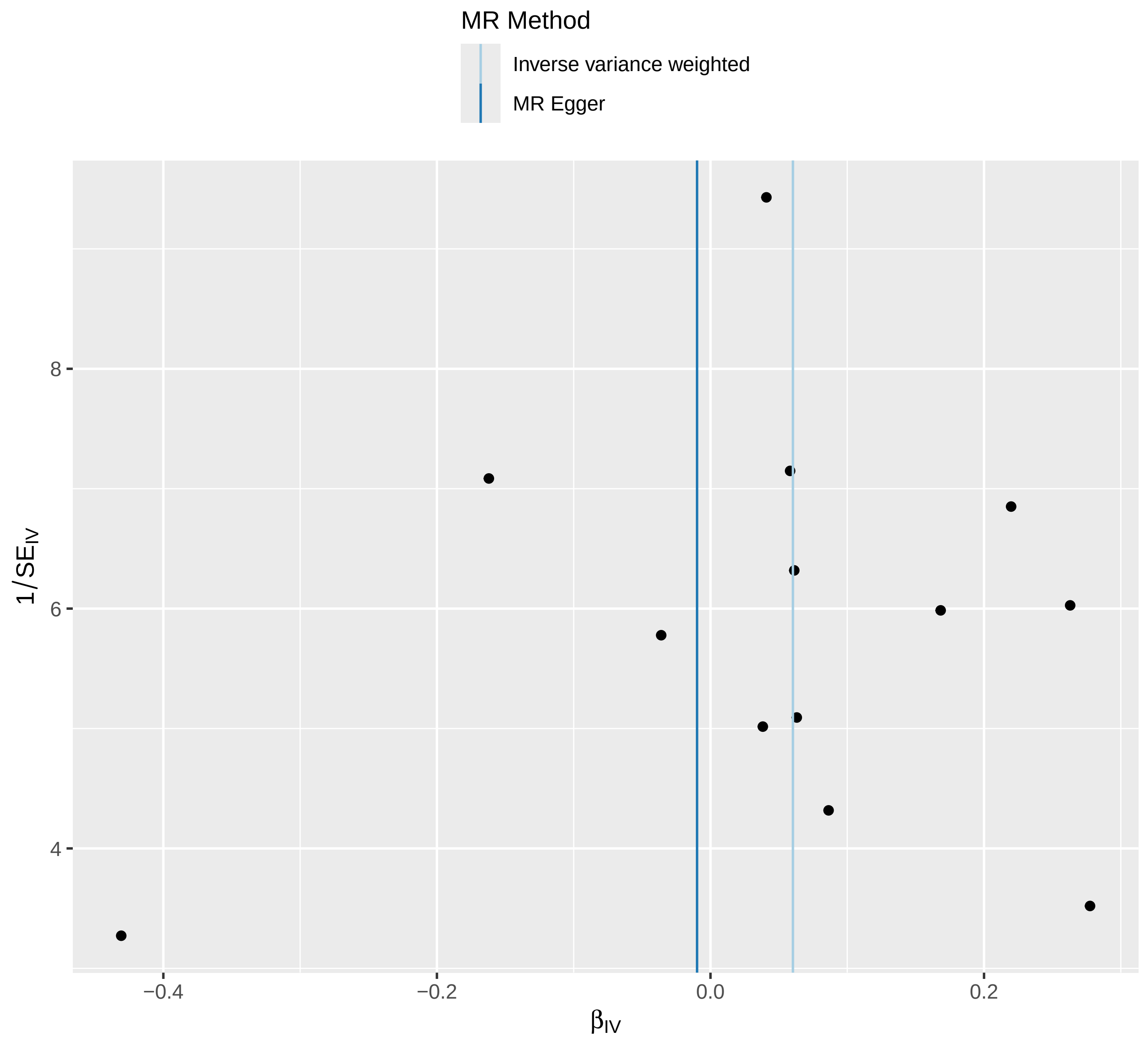
MR Method
Inverse variance weighted
MR Egger
1/SE_IV
8
6
4
−0.4
−0.2
0.0
0.2
β_IV

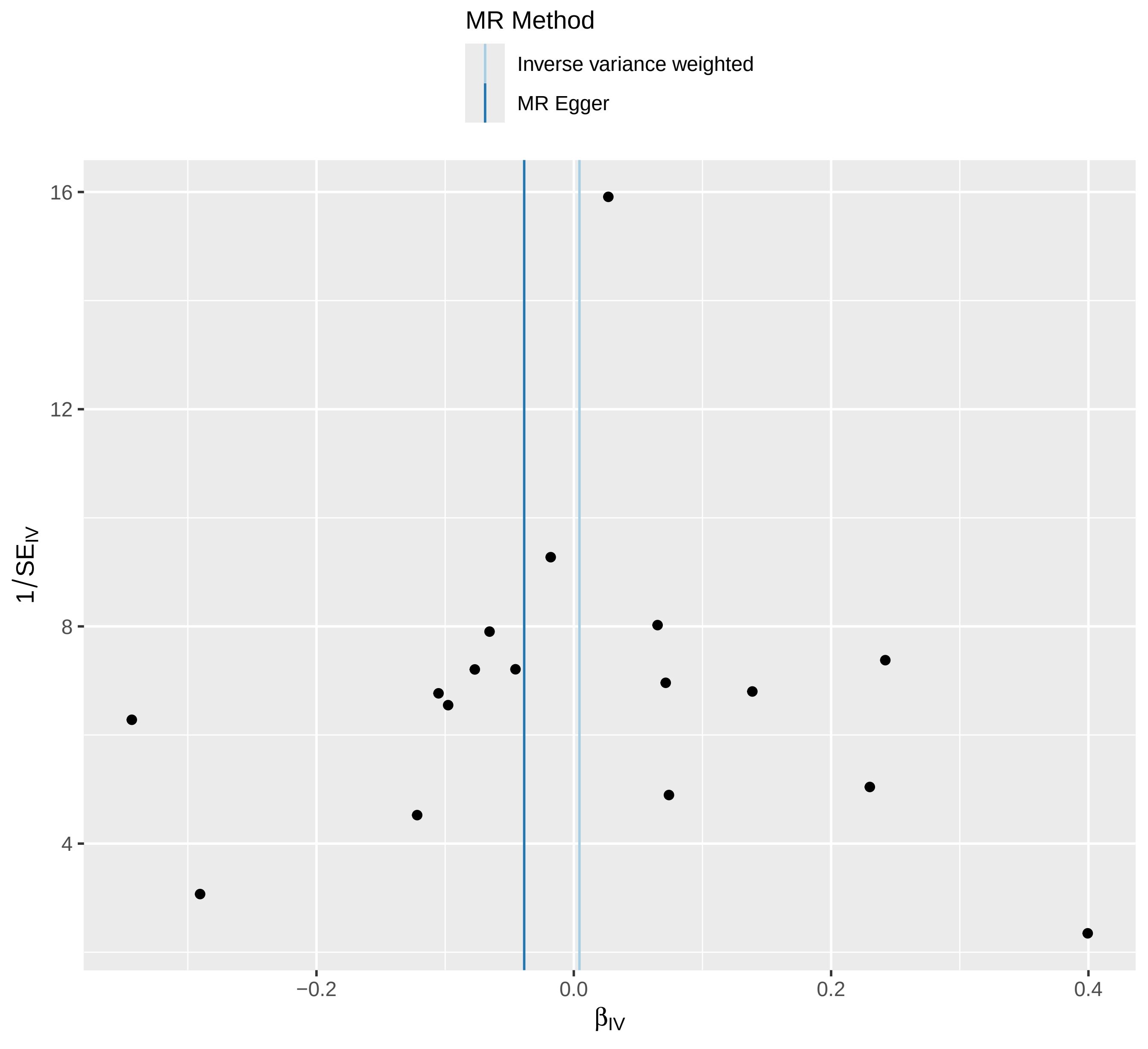
MR Method
Inverse variance weighted
MR Egger
16
12
8
4
1/SE_IV
−0.2
0.0
0.2
0.4
β_IV

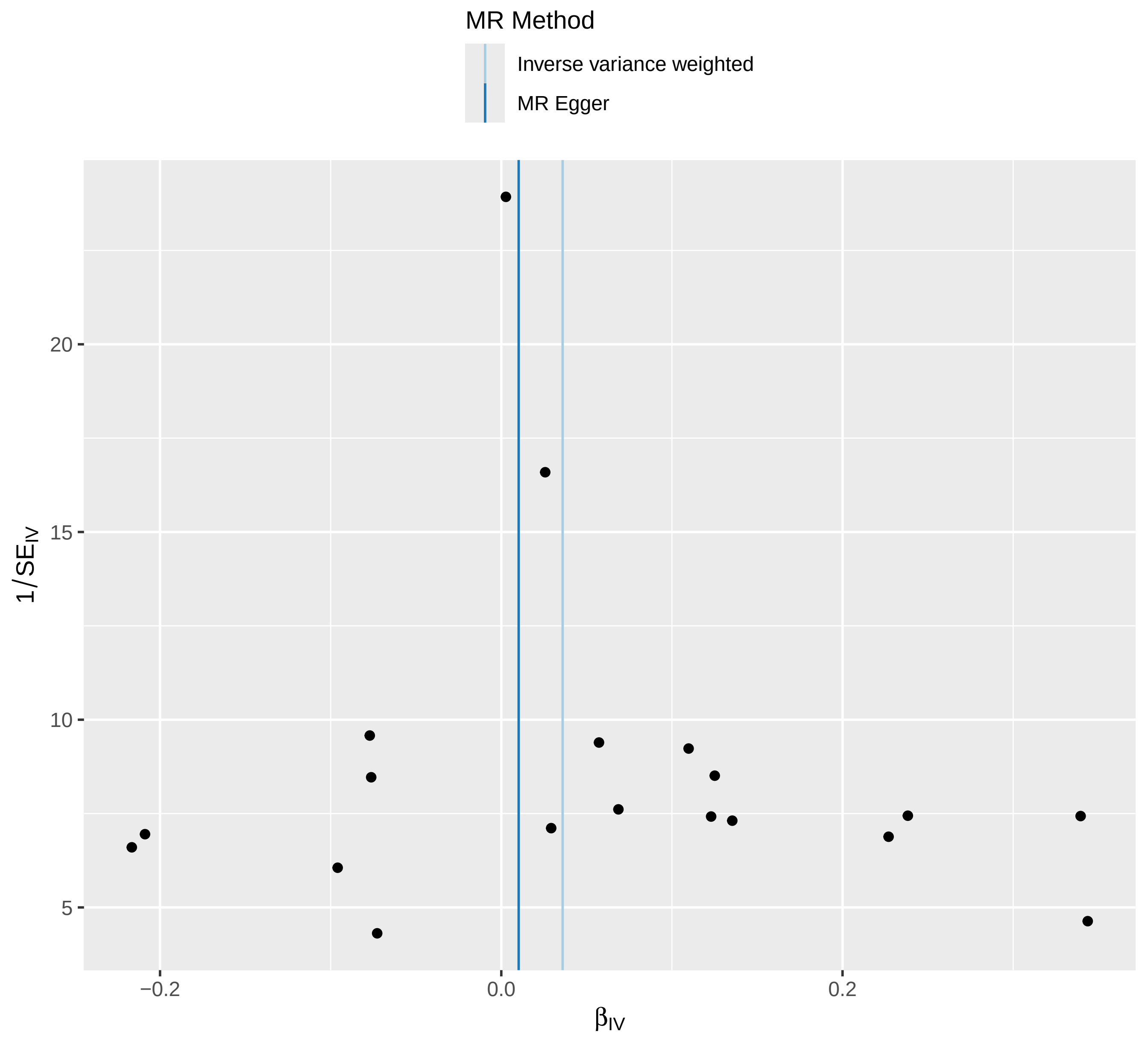
MR Method
Inverse variance weighted
MR Egger
20
15
10
5
1/SE_IV
−0.2
0.0
0.2
β_IV

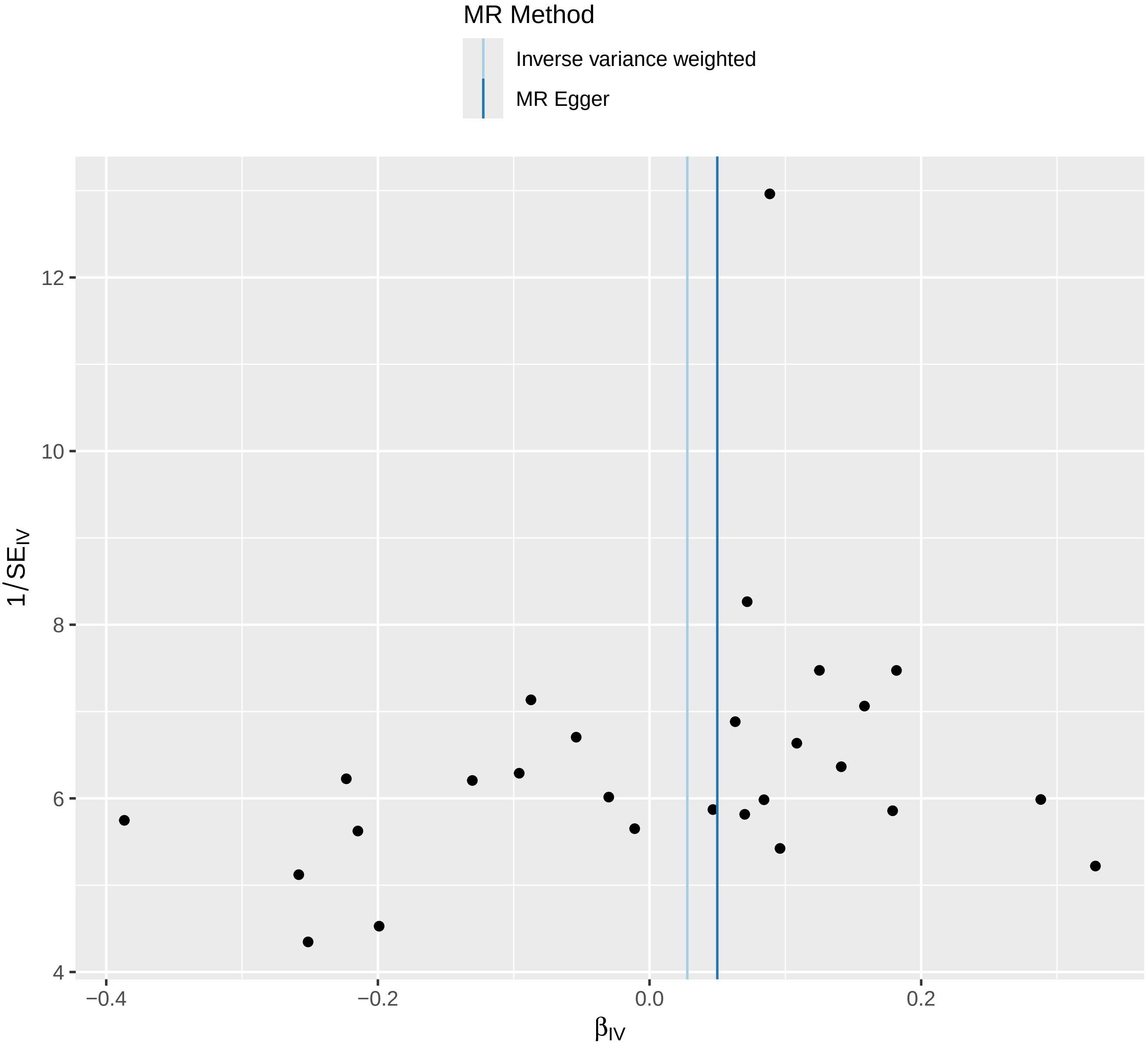

MR Method
Inverse variance weighted
MR Egger
12
10
8
6
4
1/SE$_{IV}$
−0.4
−0.2
0.0
0.2
β$_{IV}$

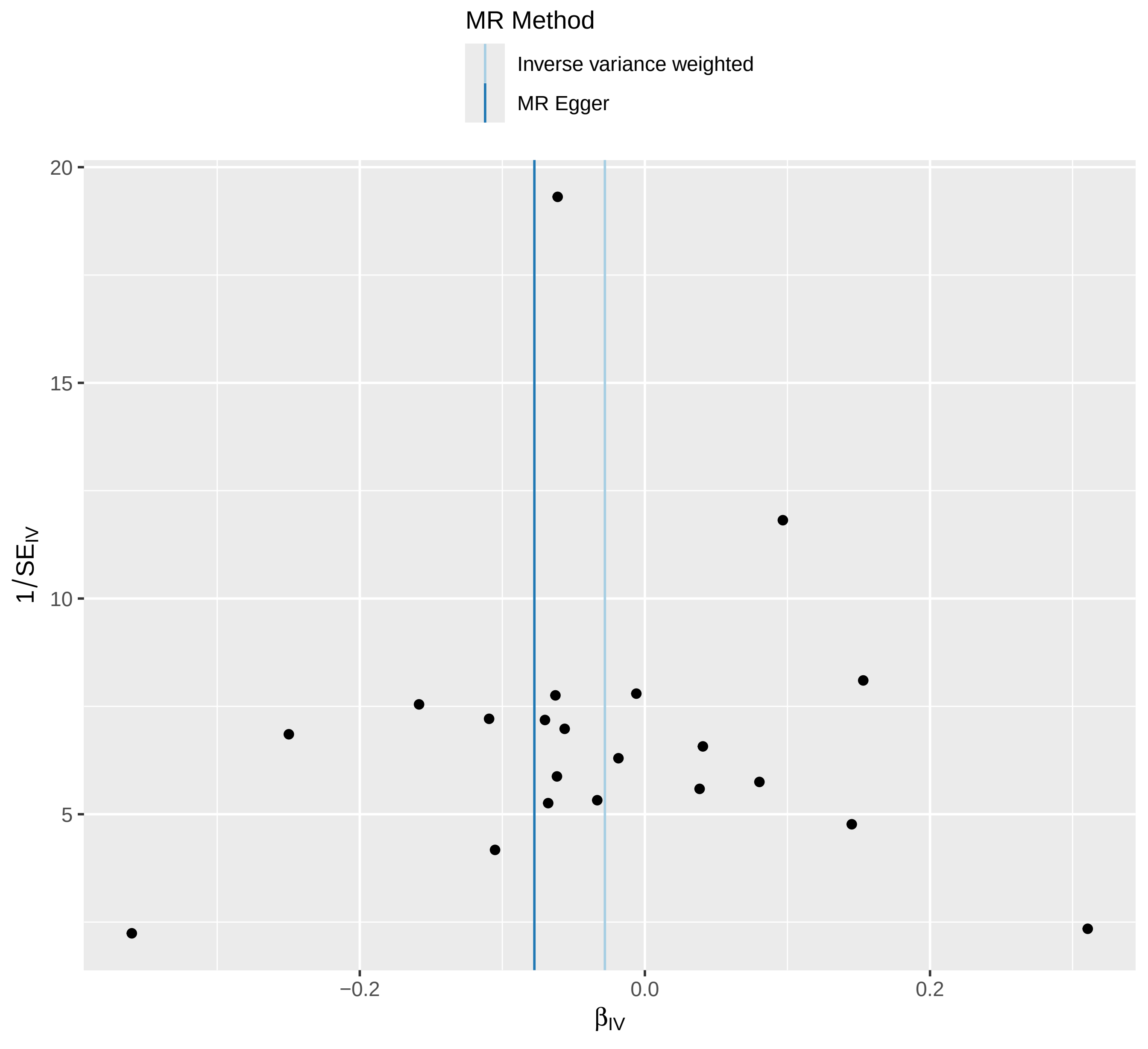
MR Method
Inverse variance weighted
MR Egger
20
15
10
5
1/SE$_{IV}$
−0.2
0.0
0.2
β$_{IV}$

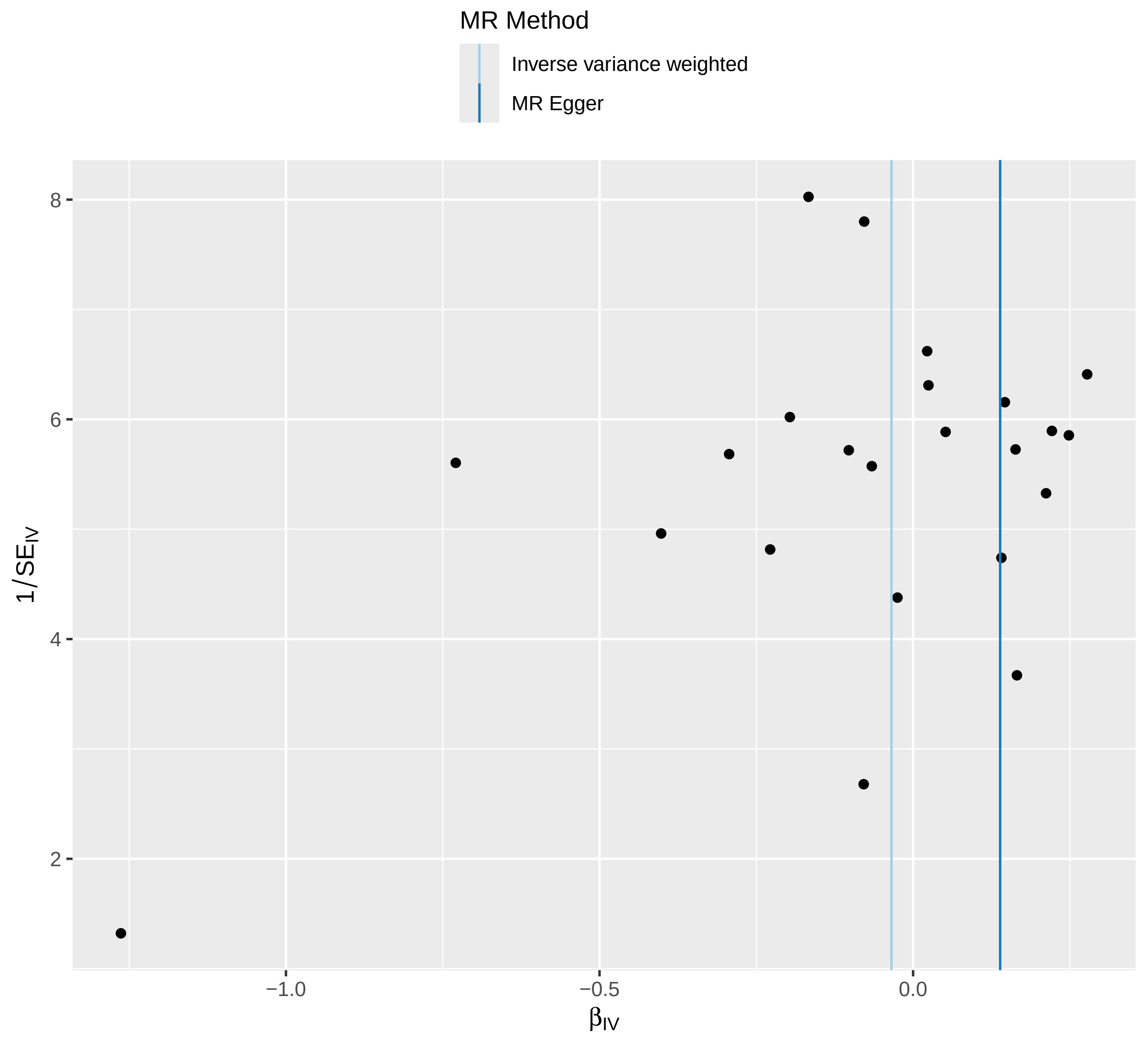
MR Method
Inverse variance weighted
MR Egger
8
6
4
2
1/SE_IV
−1.0
−0.5
0.0
β_IV

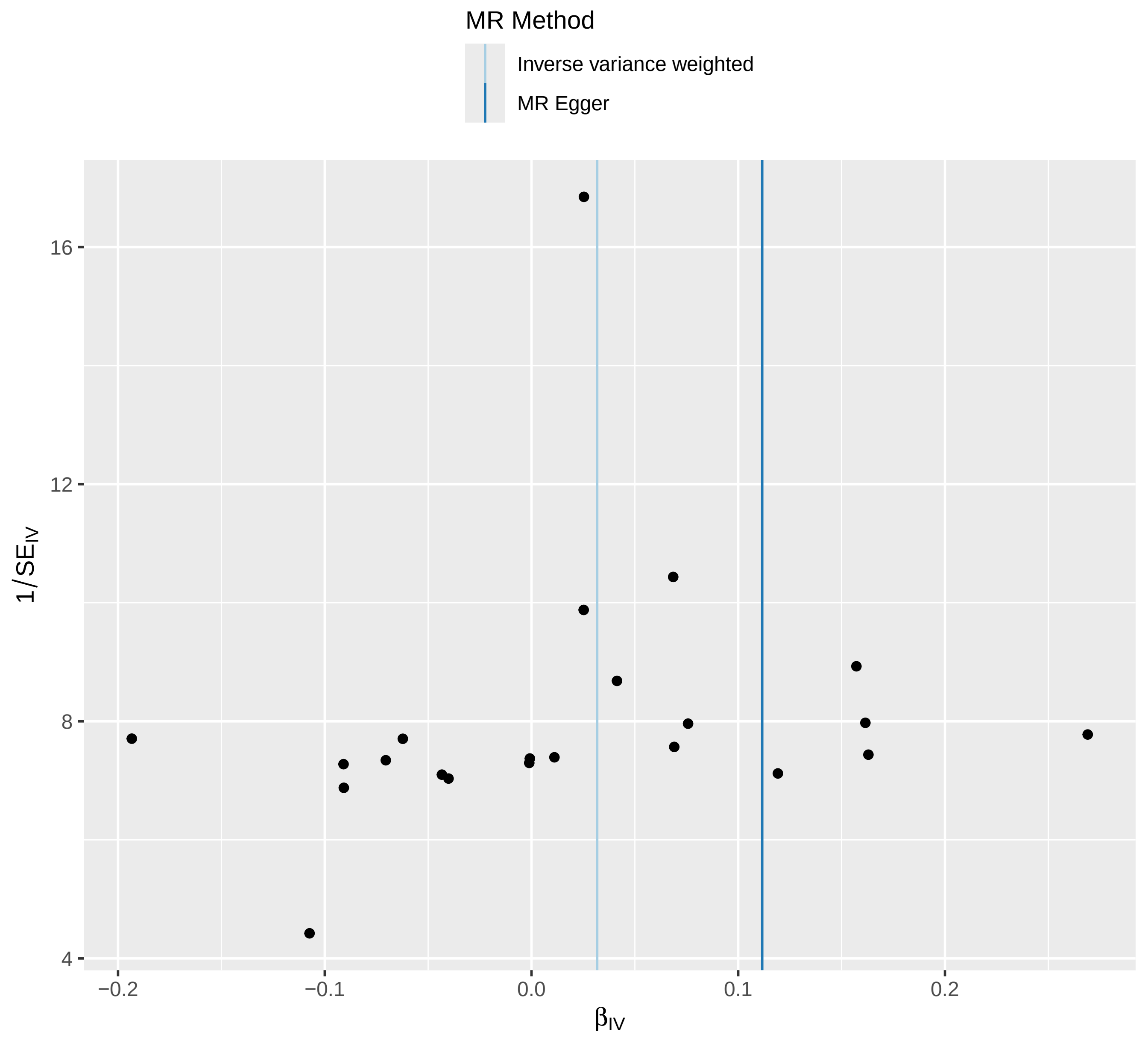

MR Method
Inverse variance weighted
MR Egger
16
12
8
4
1/SE_IV
−0.2
−0.1
0.0
0.1
0.2
β_IV

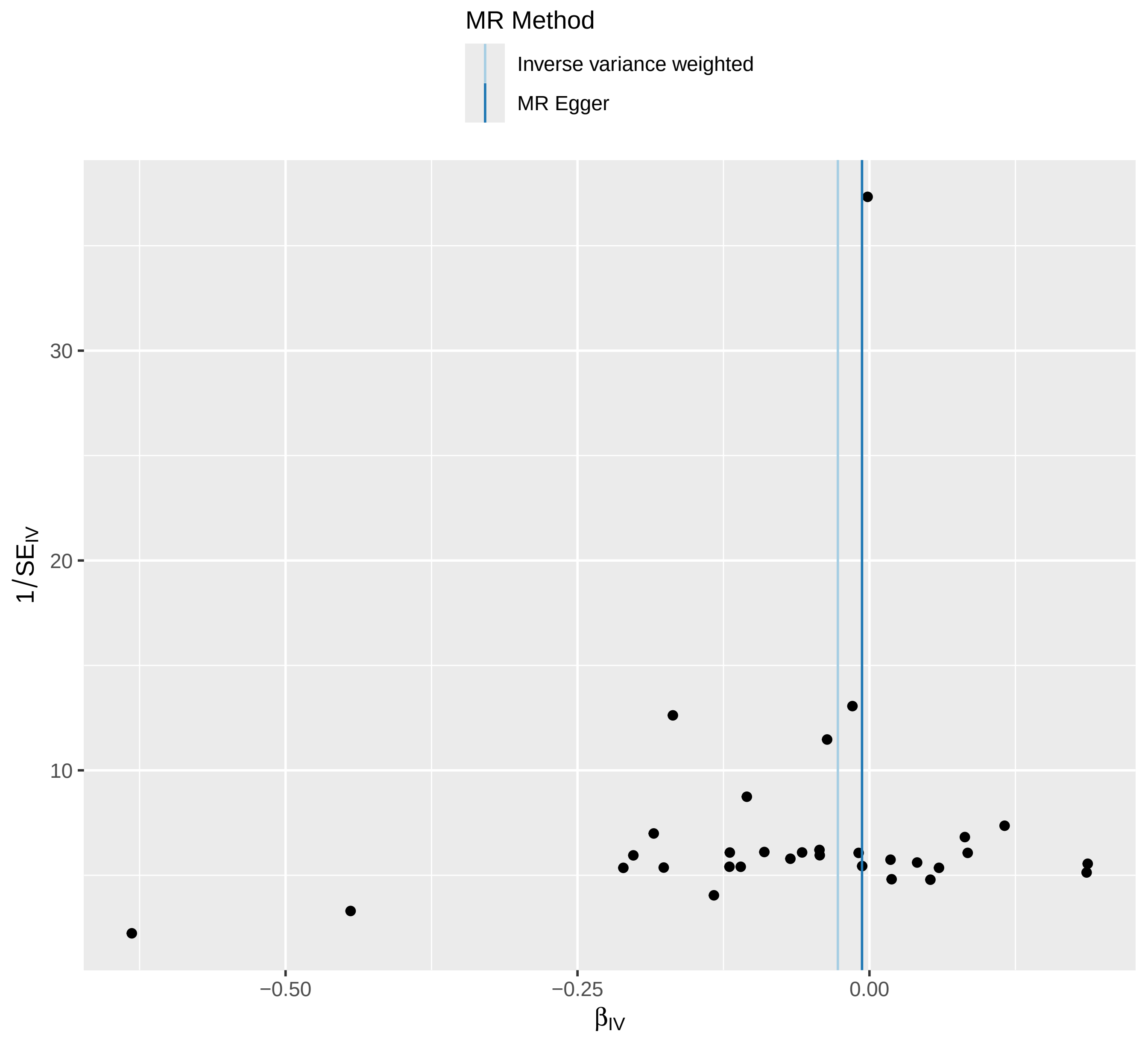

MR Method
Inverse variance weighted
MR Egger
30
20
10
1/SE_IV
−0.50
−0.25
0.00
β_IV

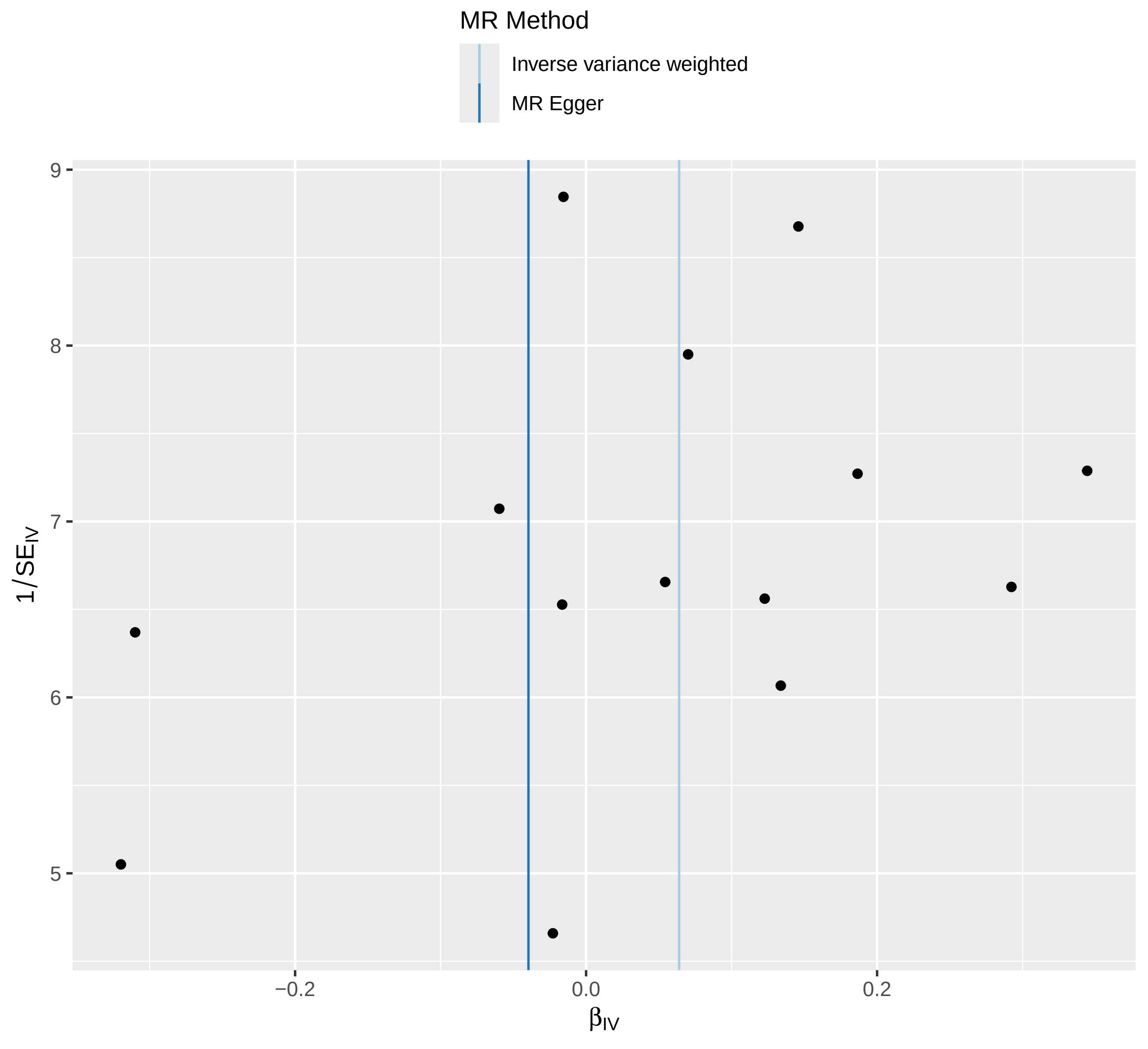
MR Method
Inverse variance weighted
MR Egger
9
8
7
6
5
$1/SE_{IV}$
−0.2
0.0
0.2
β_{IV}

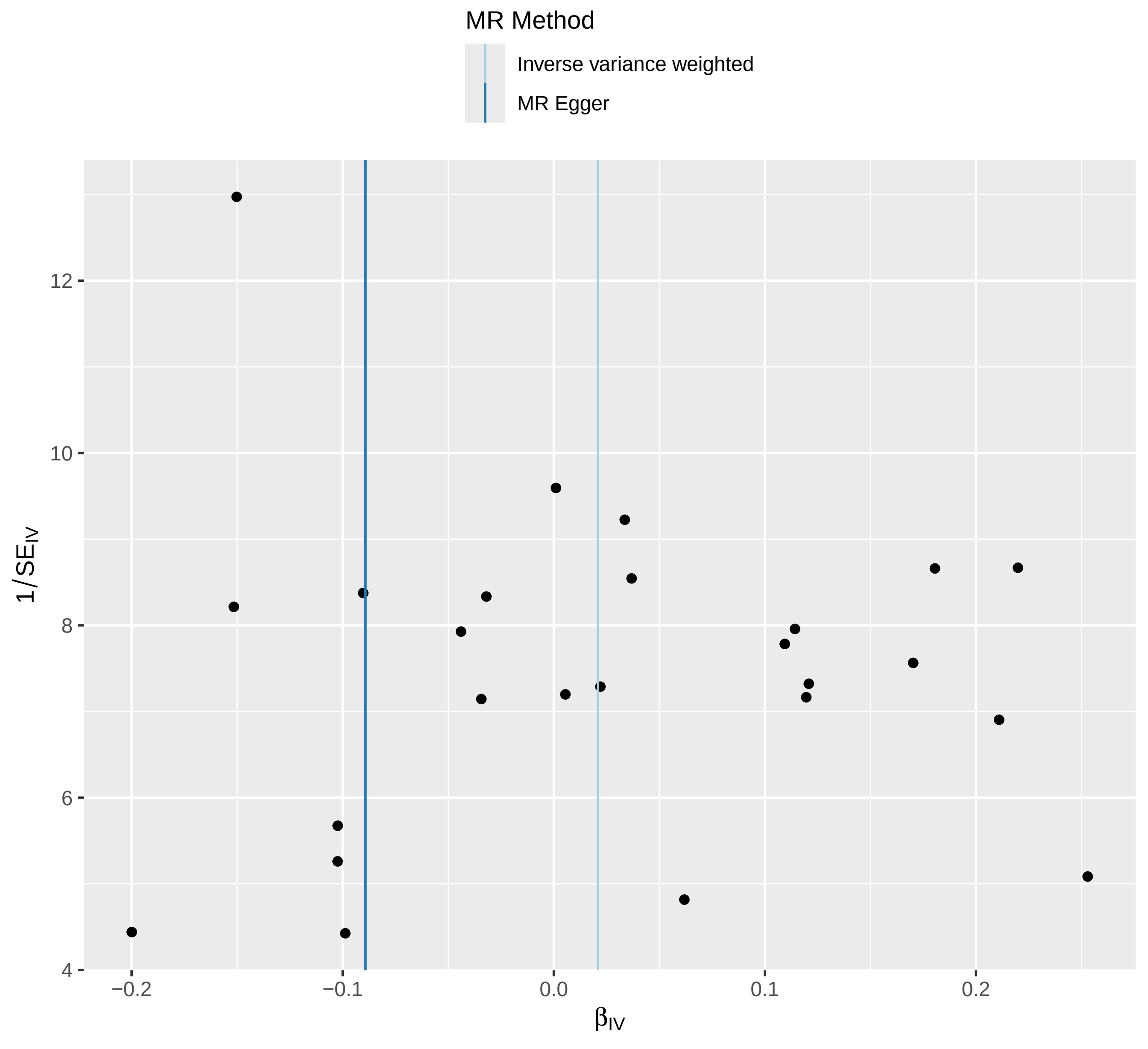
MR Method
Inverse variance weighted
MR Egger
1/SE_IV
β_IV
12
10
8
6
4
−0.2
−0.1
0.0
0.1
0.2

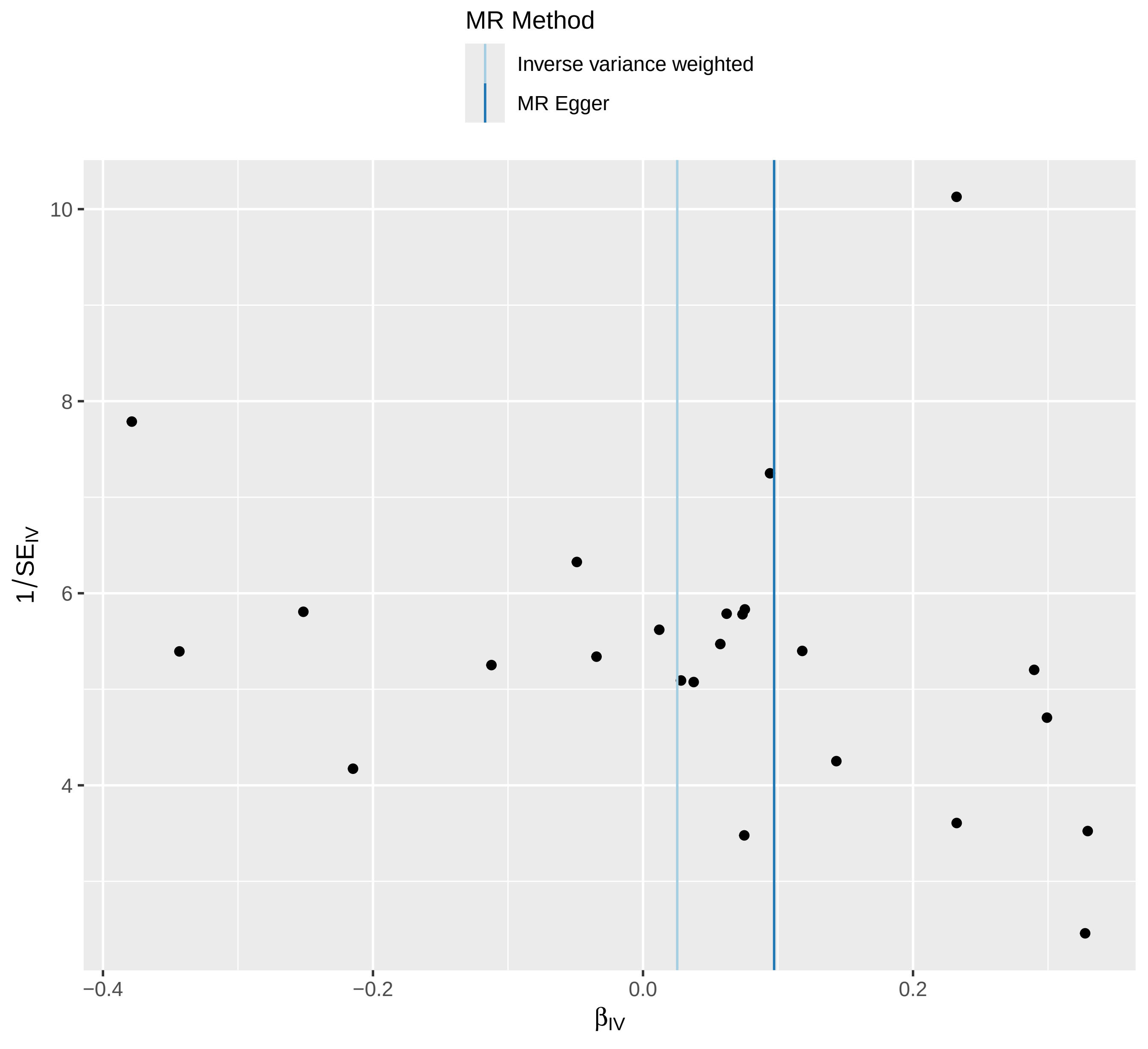
MR Method
Inverse variance weighted
MR Egger
10
8
6
4
1/SE_IV
−0.4
−0.2
0.0
0.2
β_IV

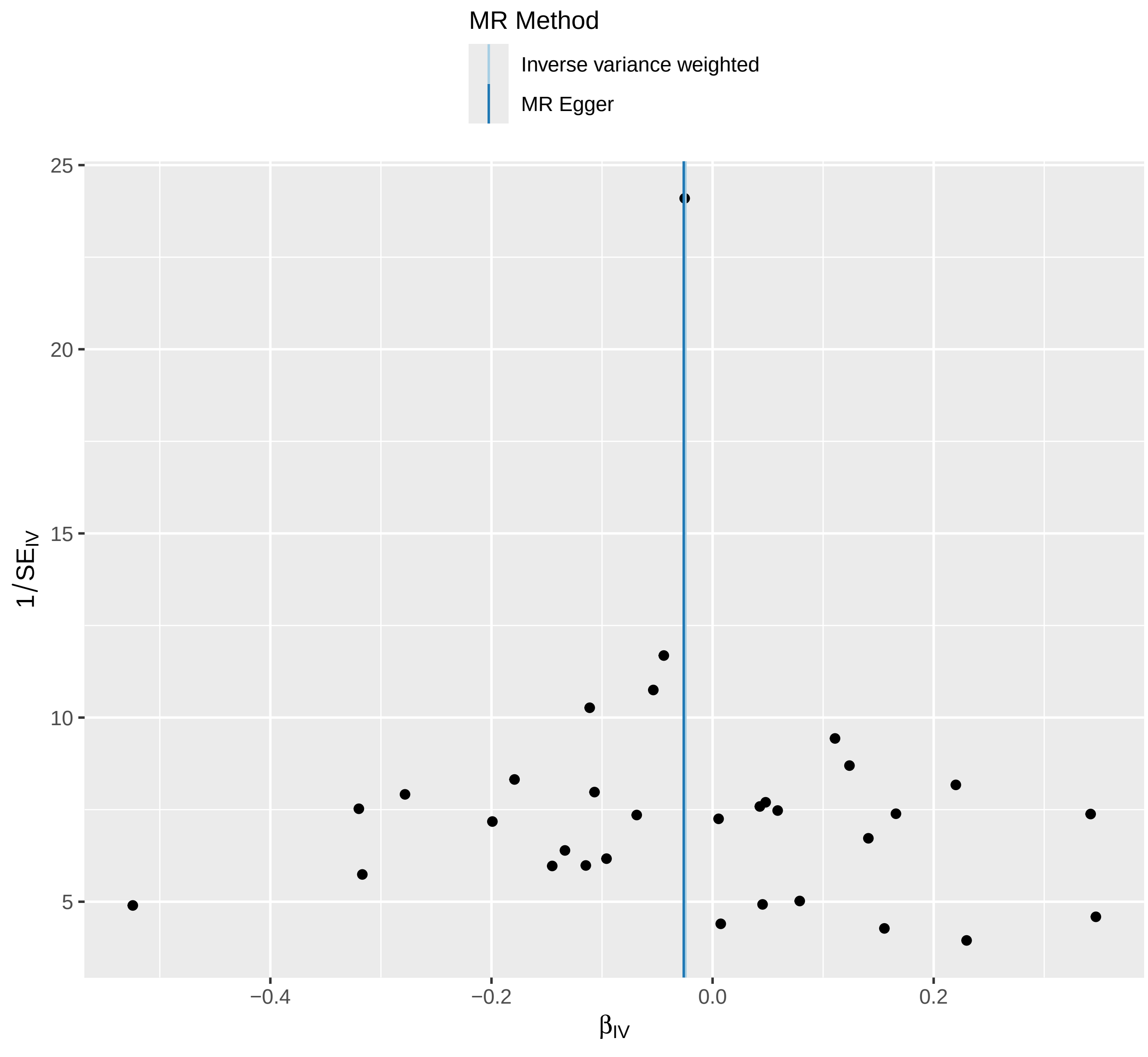
MR Method
Inverse variance weighted
MR Egger
25
20
15
10
5
1/SE_IV
−0.4
−0.2
0.0
0.2
β_IV

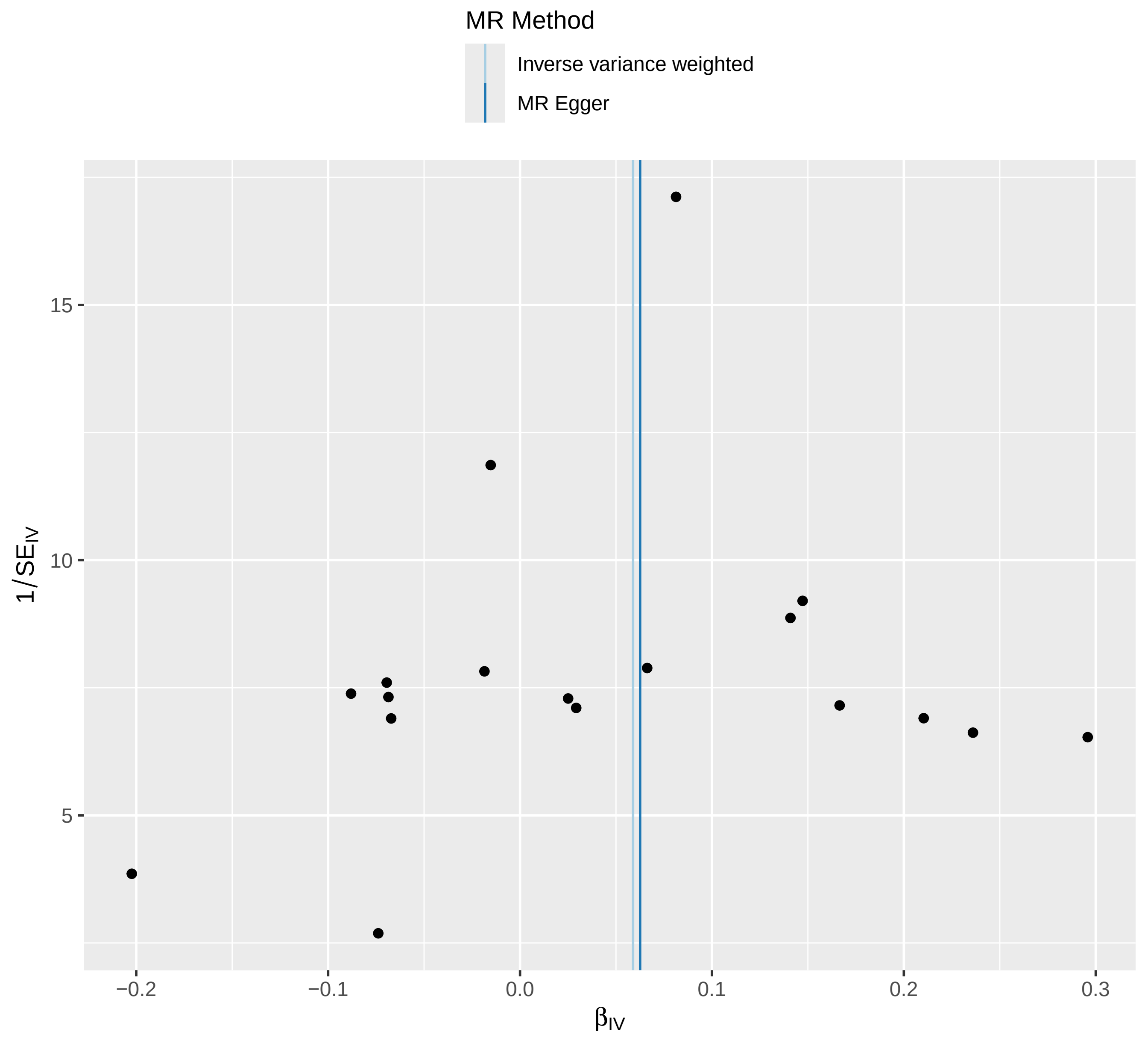
MR Method
Inverse variance weighted
MR Egger
15
10
5
1/SE_IV
−0.2
−0.1
0.0
0.1
0.2
0.3
β_IV

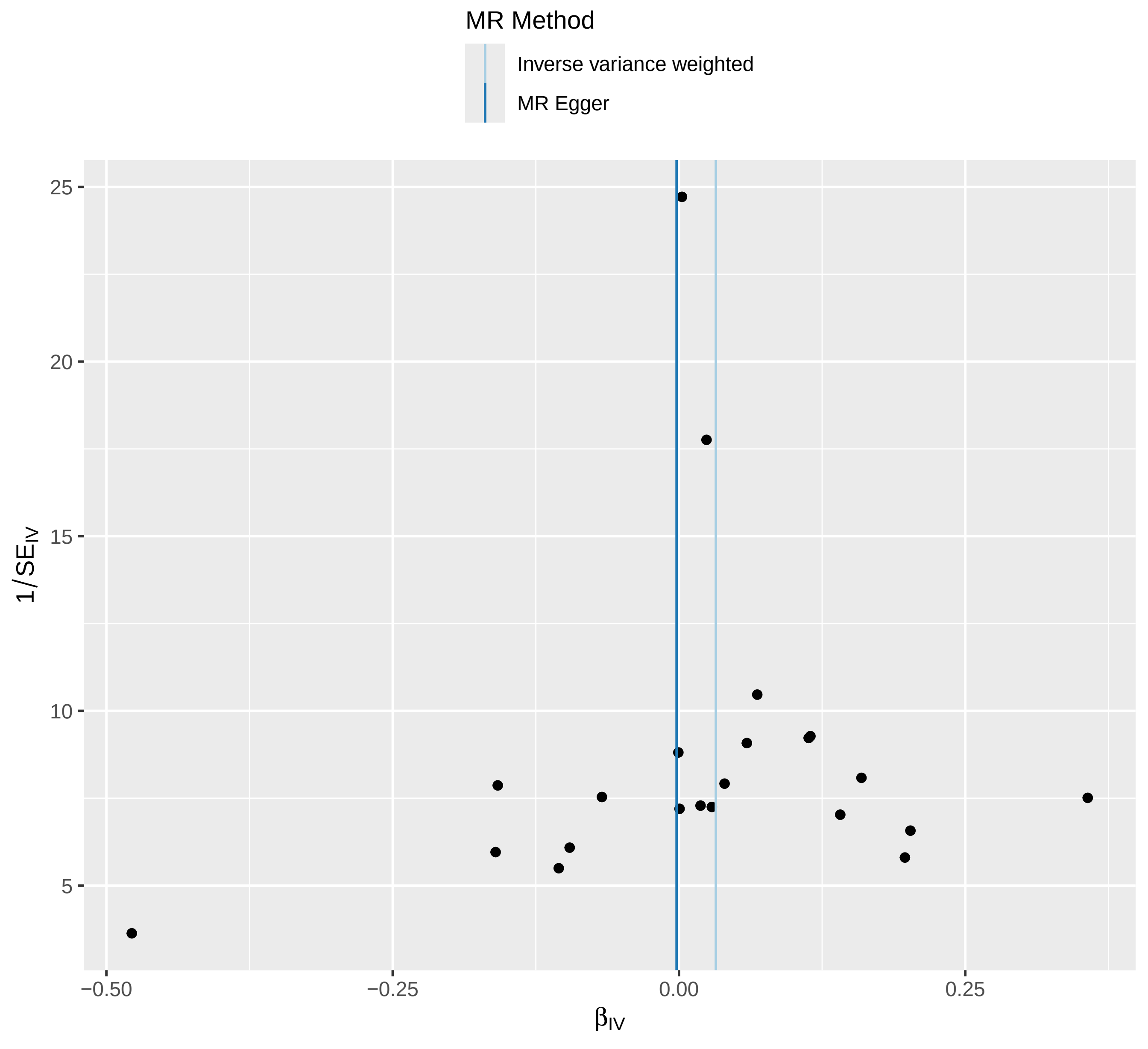
MR Method
Inverse variance weighted
MR Egger
1/SE_IV
25
20
15
10
5
−0.50
−0.25
0.00
0.25
β_IV

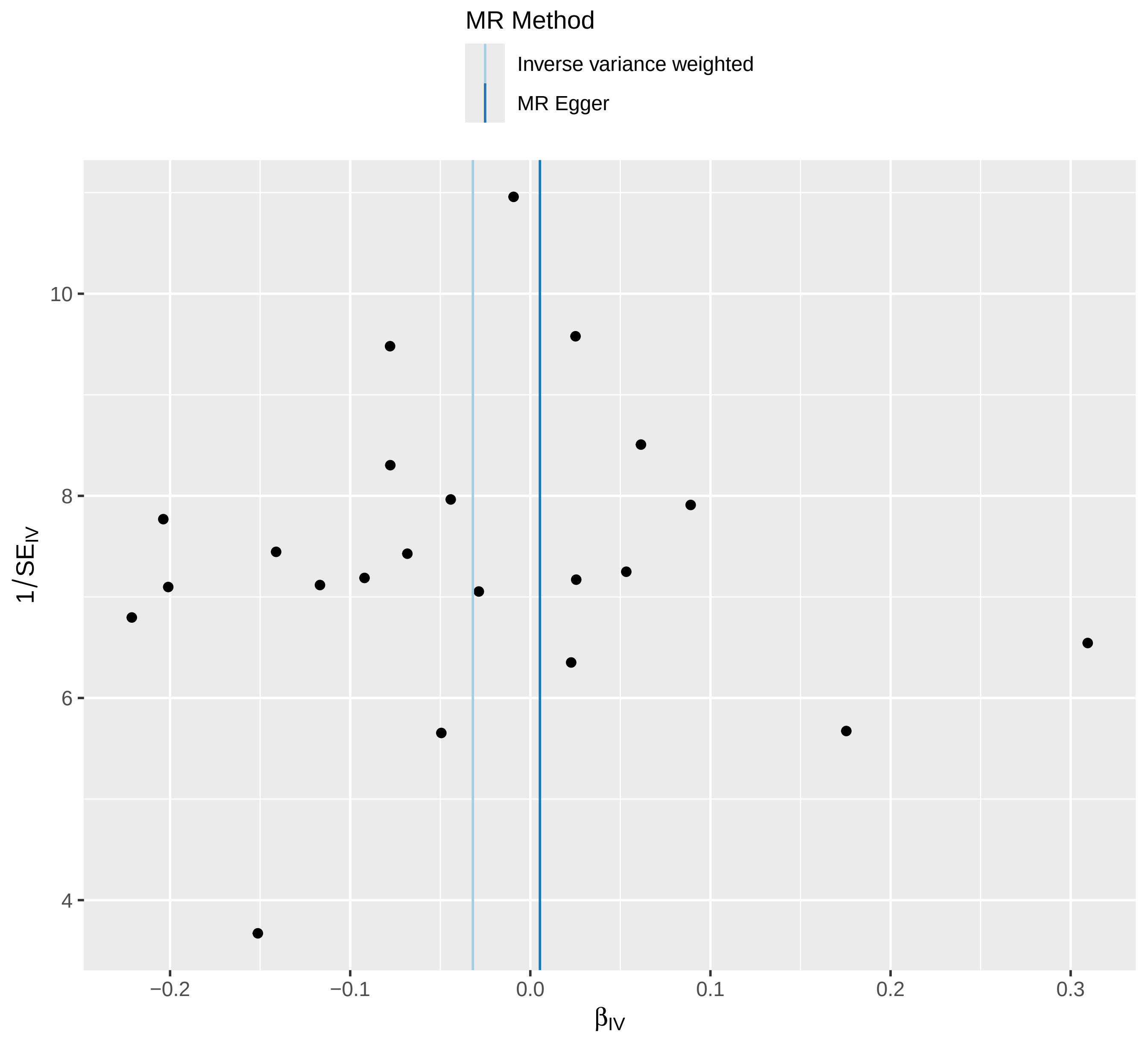
MR Method
Inverse variance weighted
MR Egger
10
8
6
4
1/SE_IV
−0.2
−0.1
0.0
0.1
0.2
0.3
β_IV

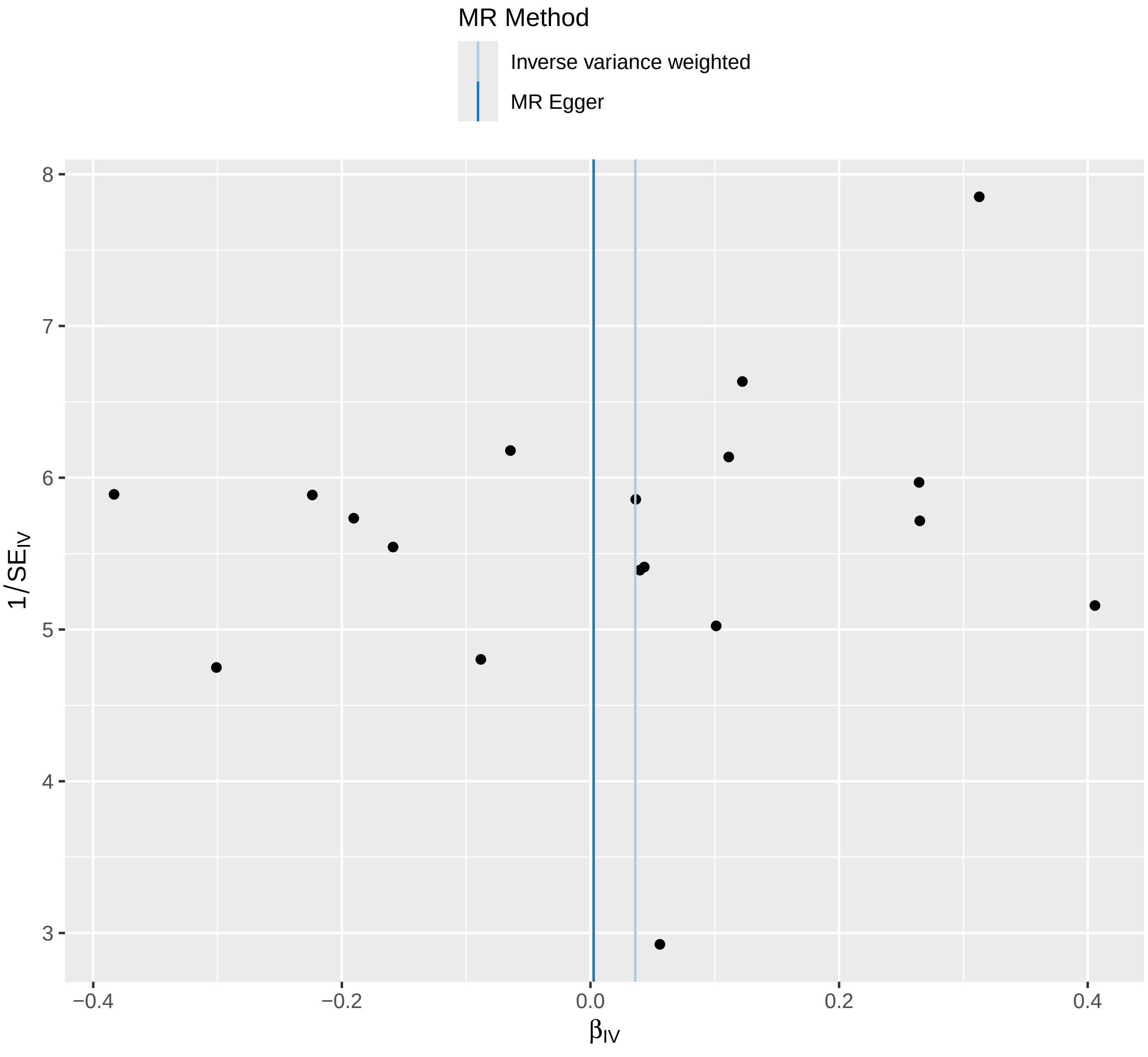

MR Method
Inverse variance weighted
MR Egger
1/SE$_{IV}$
β$_{IV}$
8
7
6
5
4
3
−0.4
−0.2
0.0
0.2
0.4

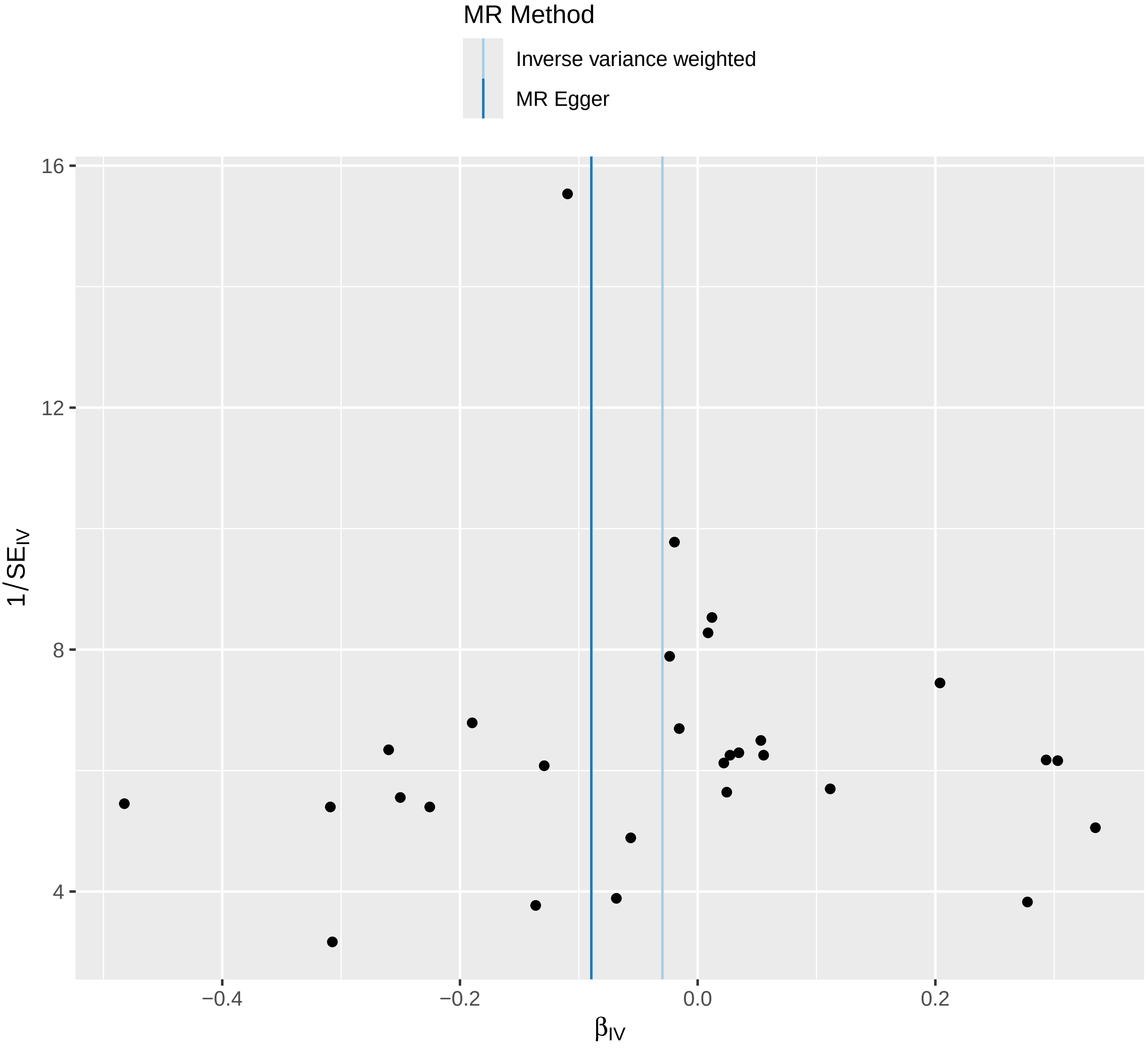
MR Method
Inverse variance weighted
MR Egger
16
12
8
4
1/SE$_{IV}$
−0.4
−0.2
0.0
0.2
β$_{IV}$

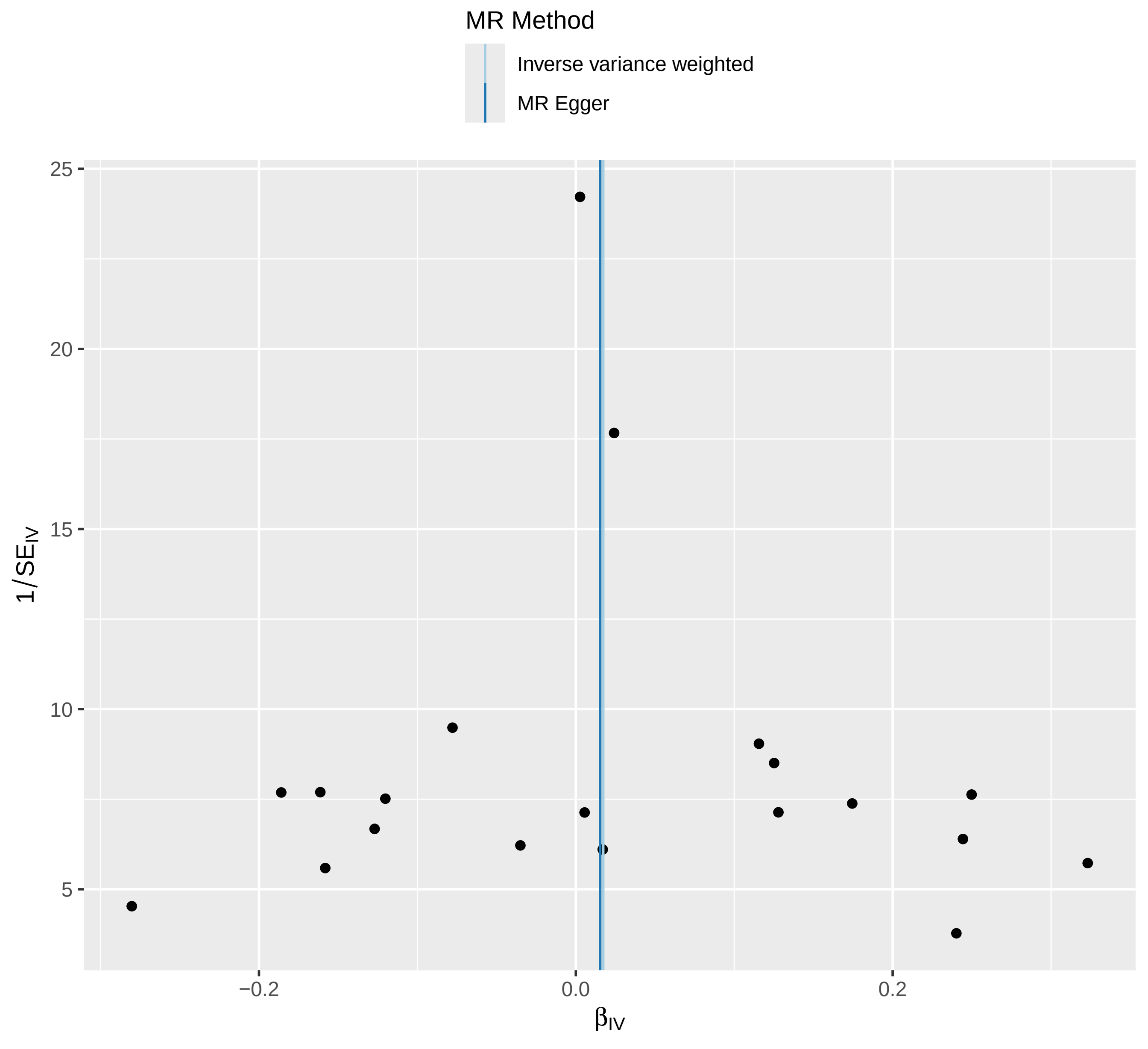

MR Method
Inverse variance weighted
MR Egger
25
20
15
10
5
1/SE$_{IV}$
−0.2
0.0
0.2
β_{IV}

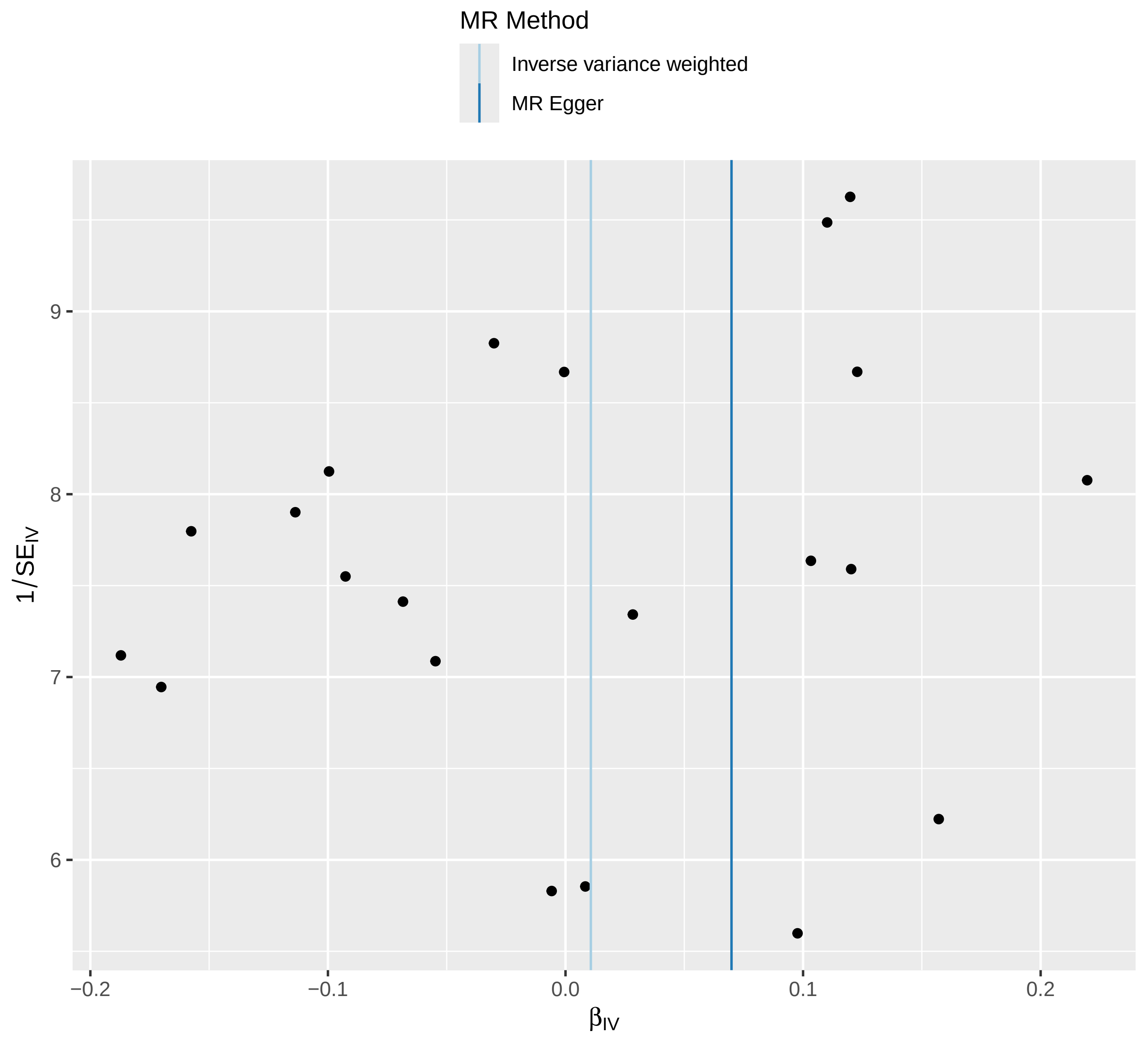
MR Method
Inverse variance weighted
MR Egger
1/SE_IV
β_IV
9
8
7
6
−0.2
−0.1
0.0
0.1
0.2

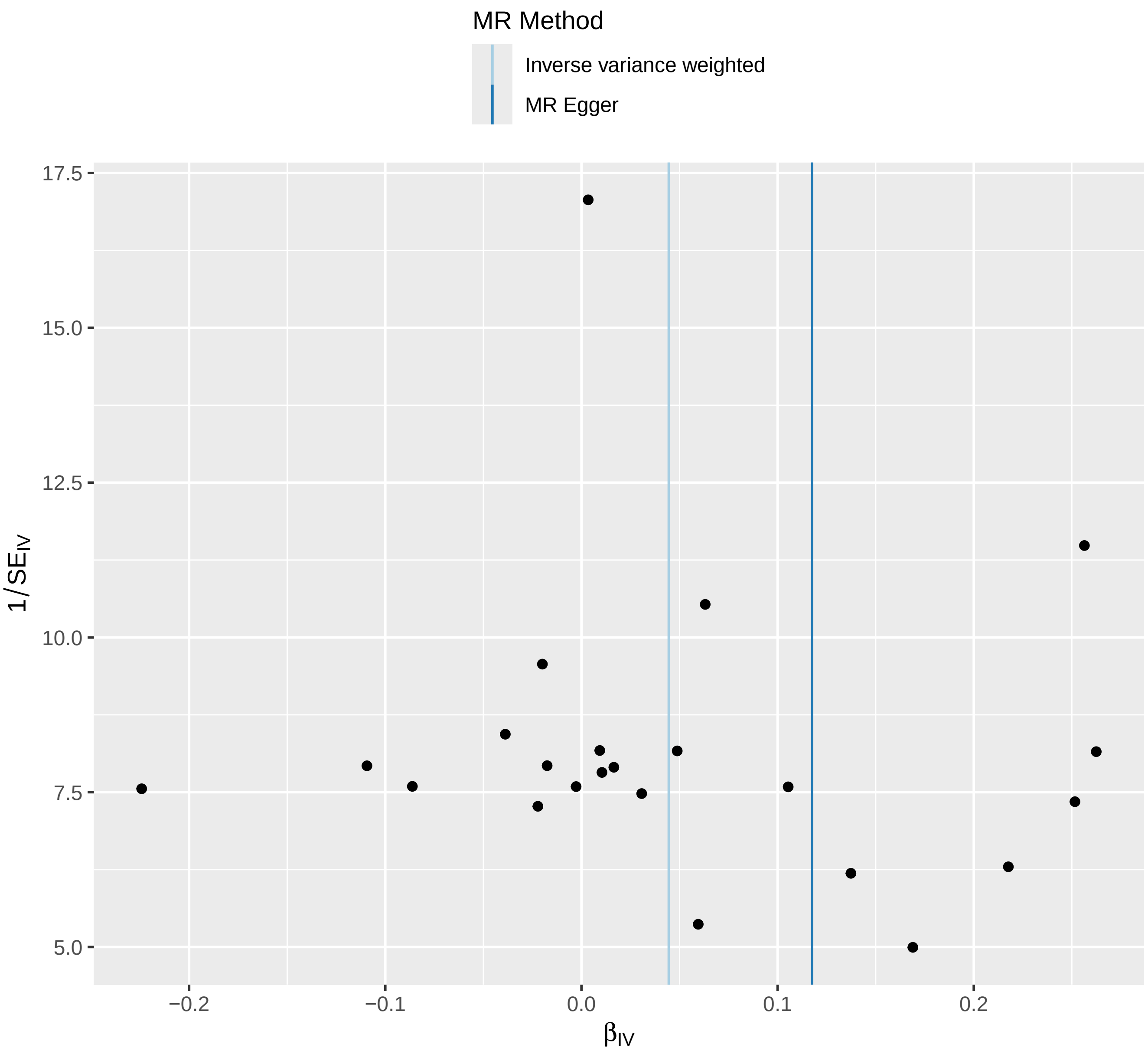

MR Method
Inverse variance weighted
MR Egger
17.5
15.0
12.5
10.0
7.5
5.0
−0.2
−0.1
0.0
0.1
0.2
$1/SE_{IV}$
β_{IV}

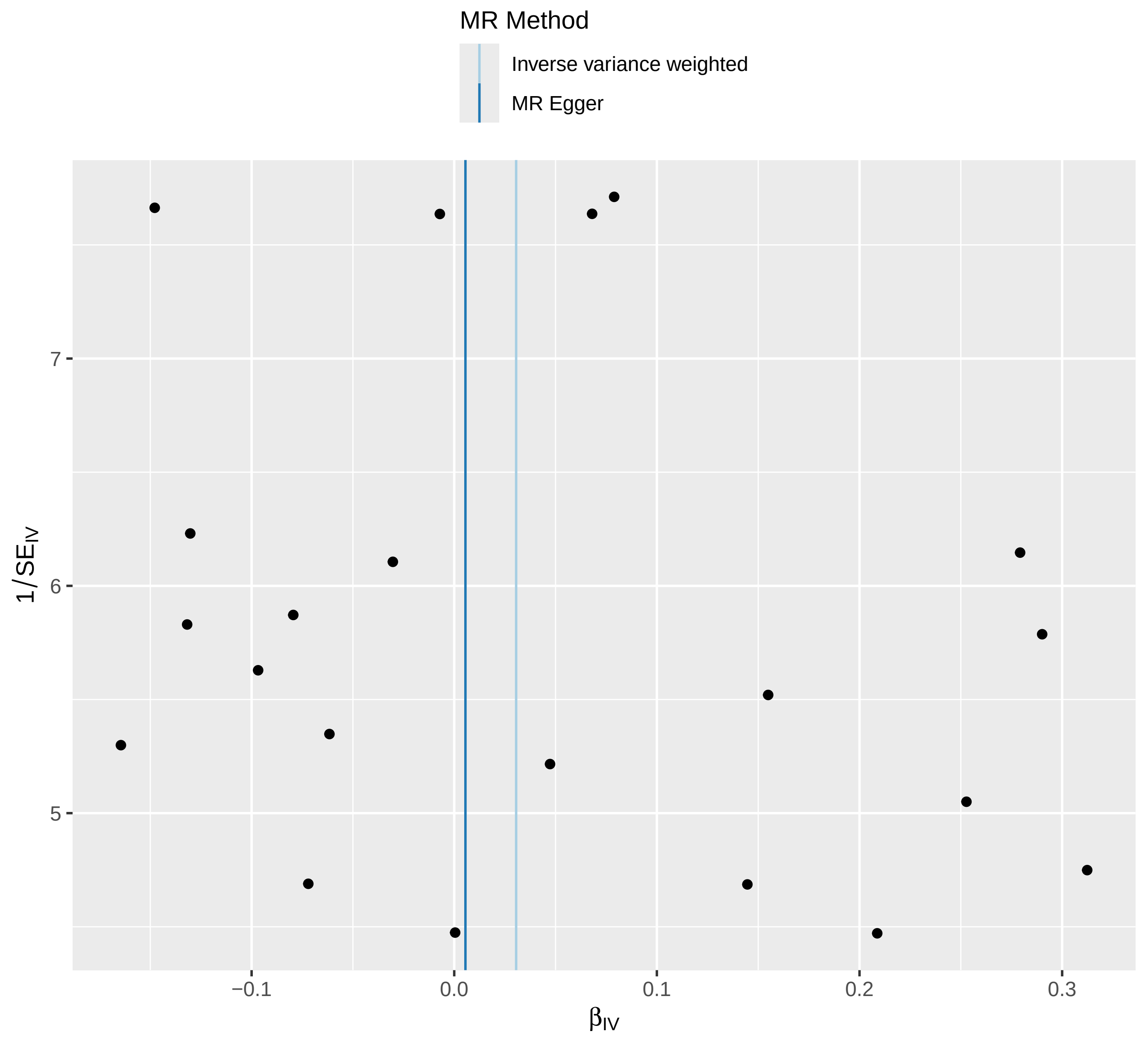

MR Method
Inverse variance weighted
MR Egger
7
6
5
1/SE_IV
−0.1
0.0
0.1
0.2
0.3
β_IV

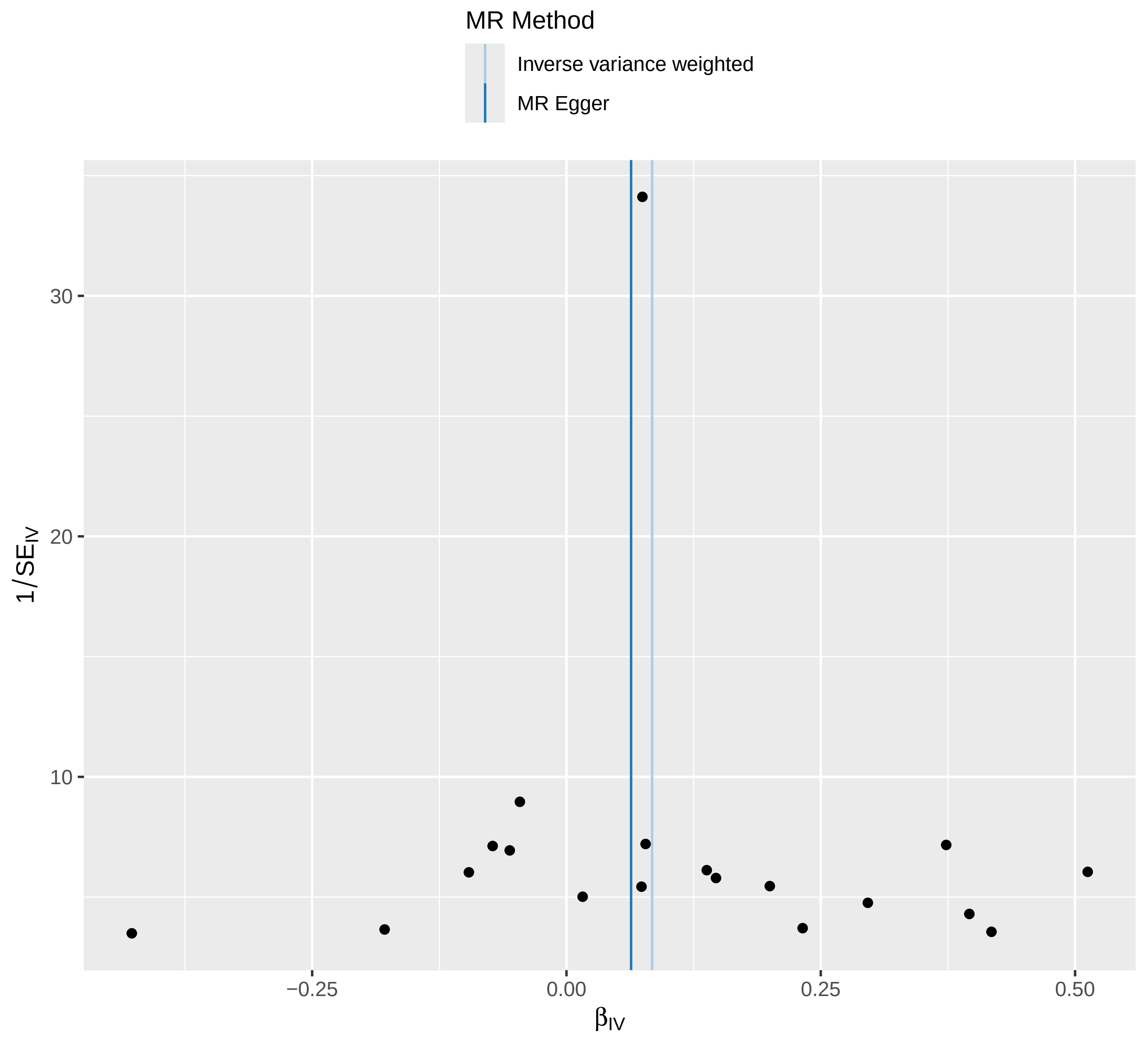
MR Method
Inverse variance weighted
MR Egger
30
20
10
1/SE$_{IV}$
−0.25
0.00
0.25
0.50
β$_{IV}$

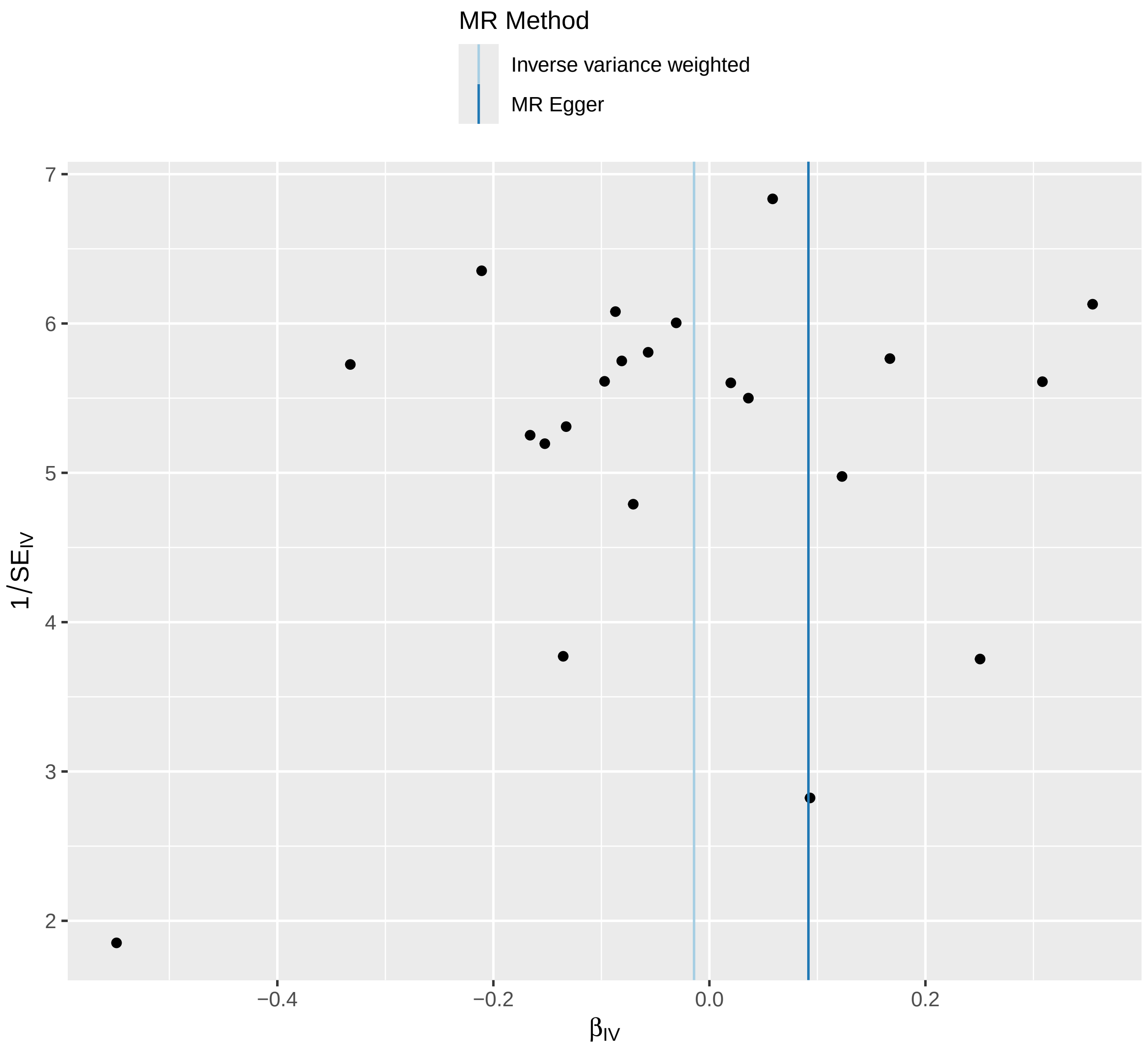
MR Method
Inverse variance weighted
MR Egger
1/SE$_{IV}$
β$_{IV}$
7
6
5
4
3
2
−0.4
−0.2
0.0
0.2

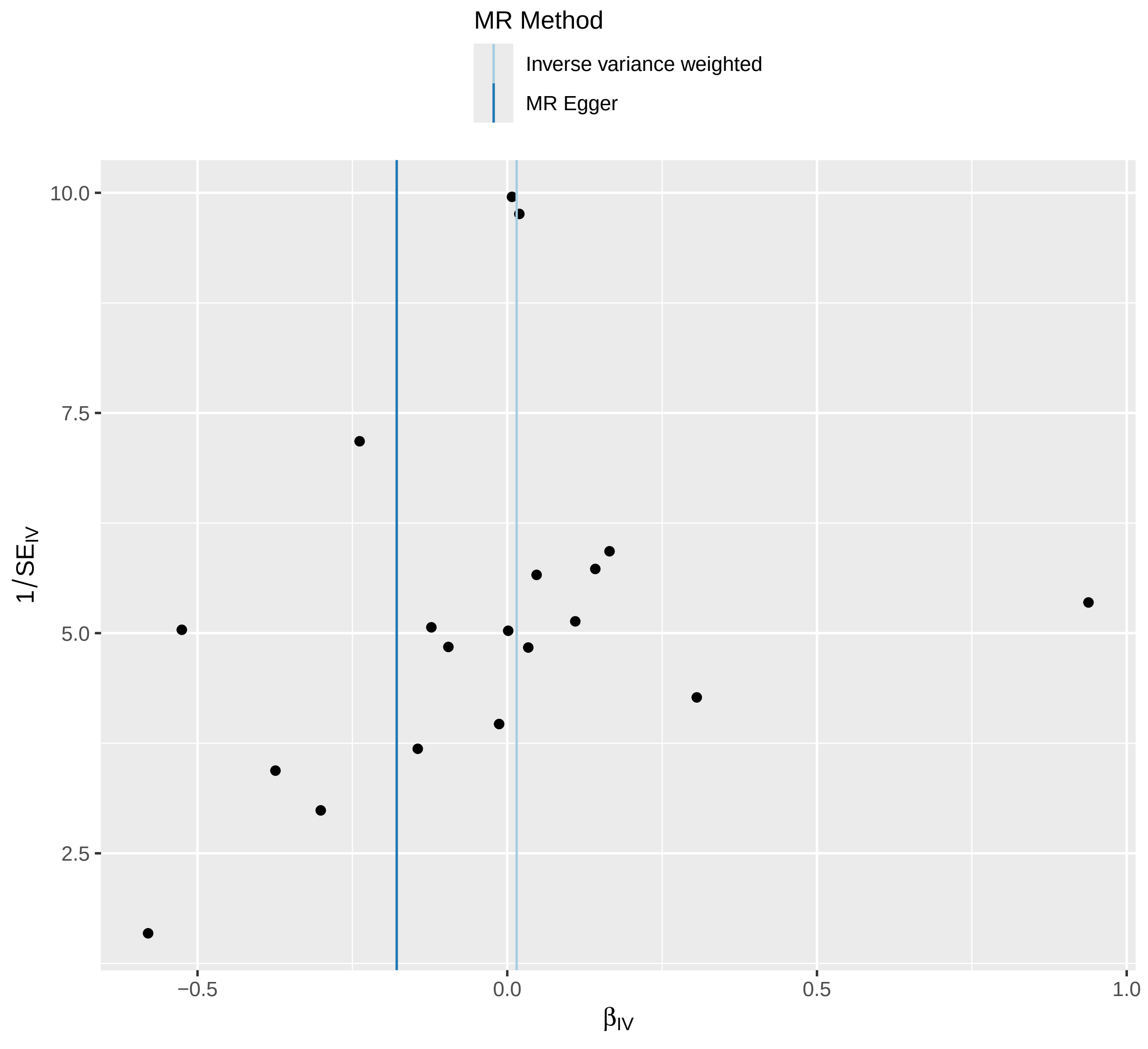

MR Method
Inverse variance weighted
MR Egger
10.0
7.5
5.0
2.5
1/SE$_{IV}$
−0.5
0.0
0.5
1.0
β$_{IV}$

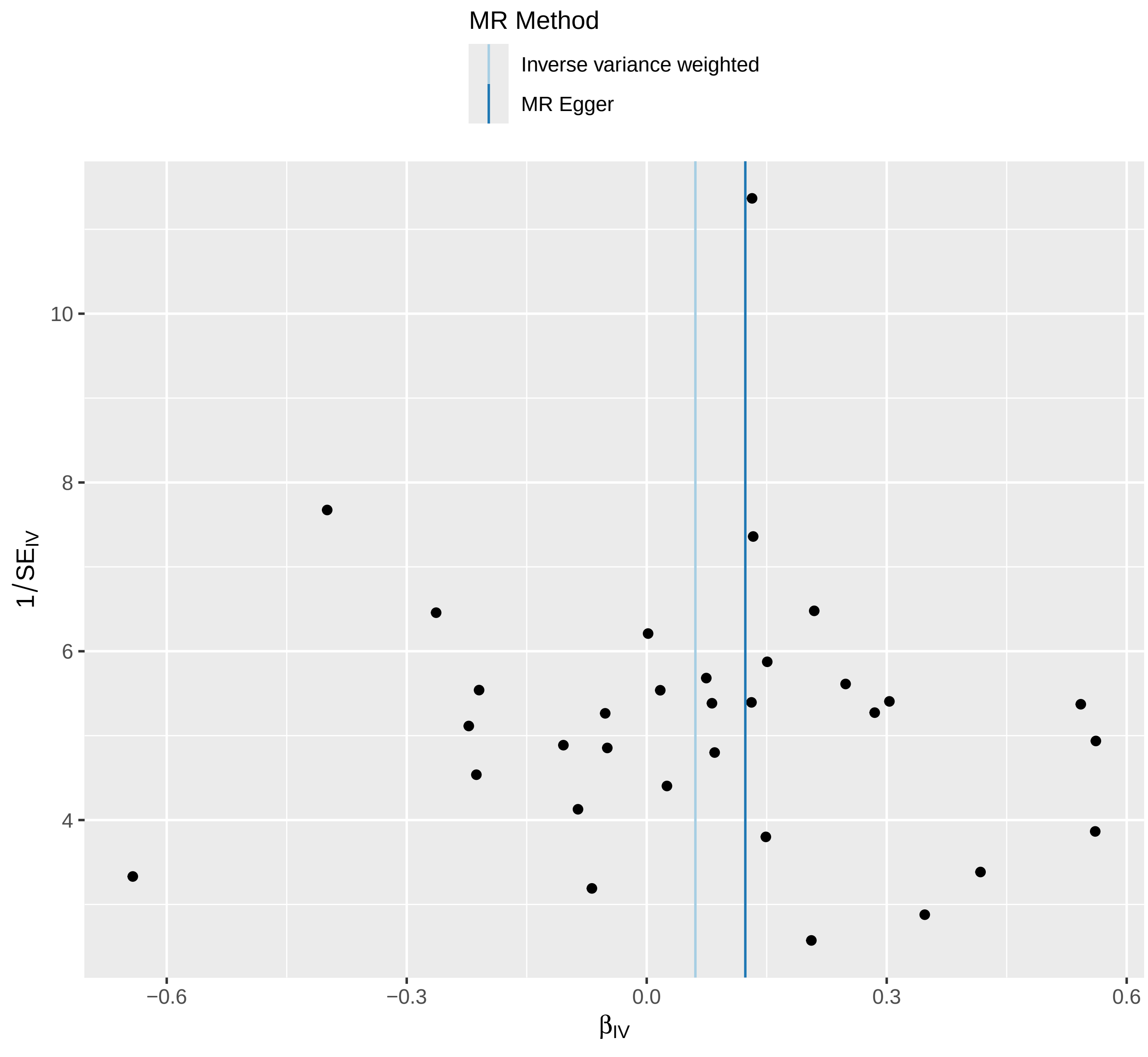
MR Method
Inverse variance weighted
MR Egger
10
8
6
4
1/SE_IV
−0.6
−0.3
0.0
0.3
0.6
β_IV

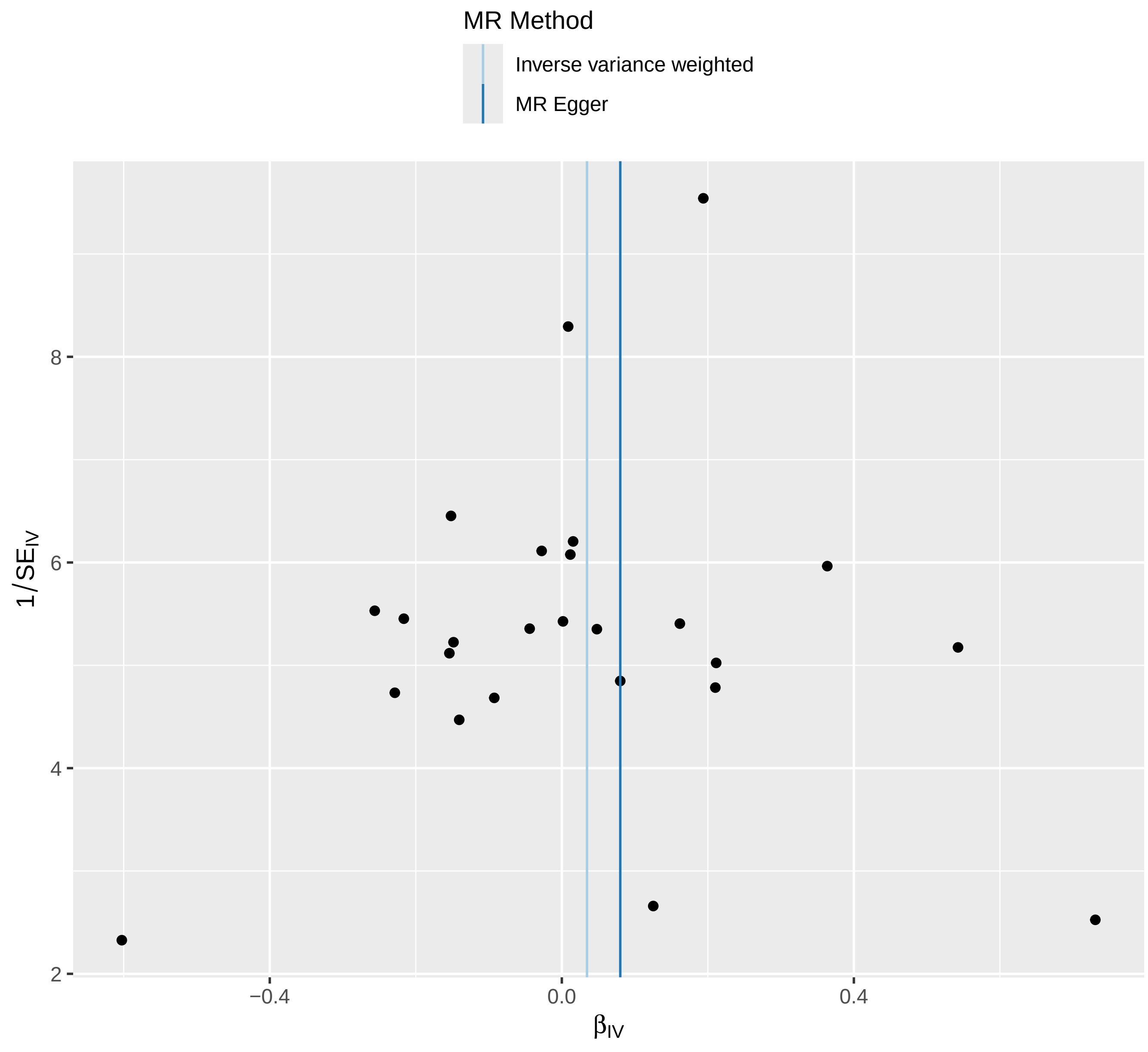
MR Method
Inverse variance weighted
MR Egger
$1/SE_{IV}$
8
6
4
2
−0.4
0.0
0.4
β_{IV}

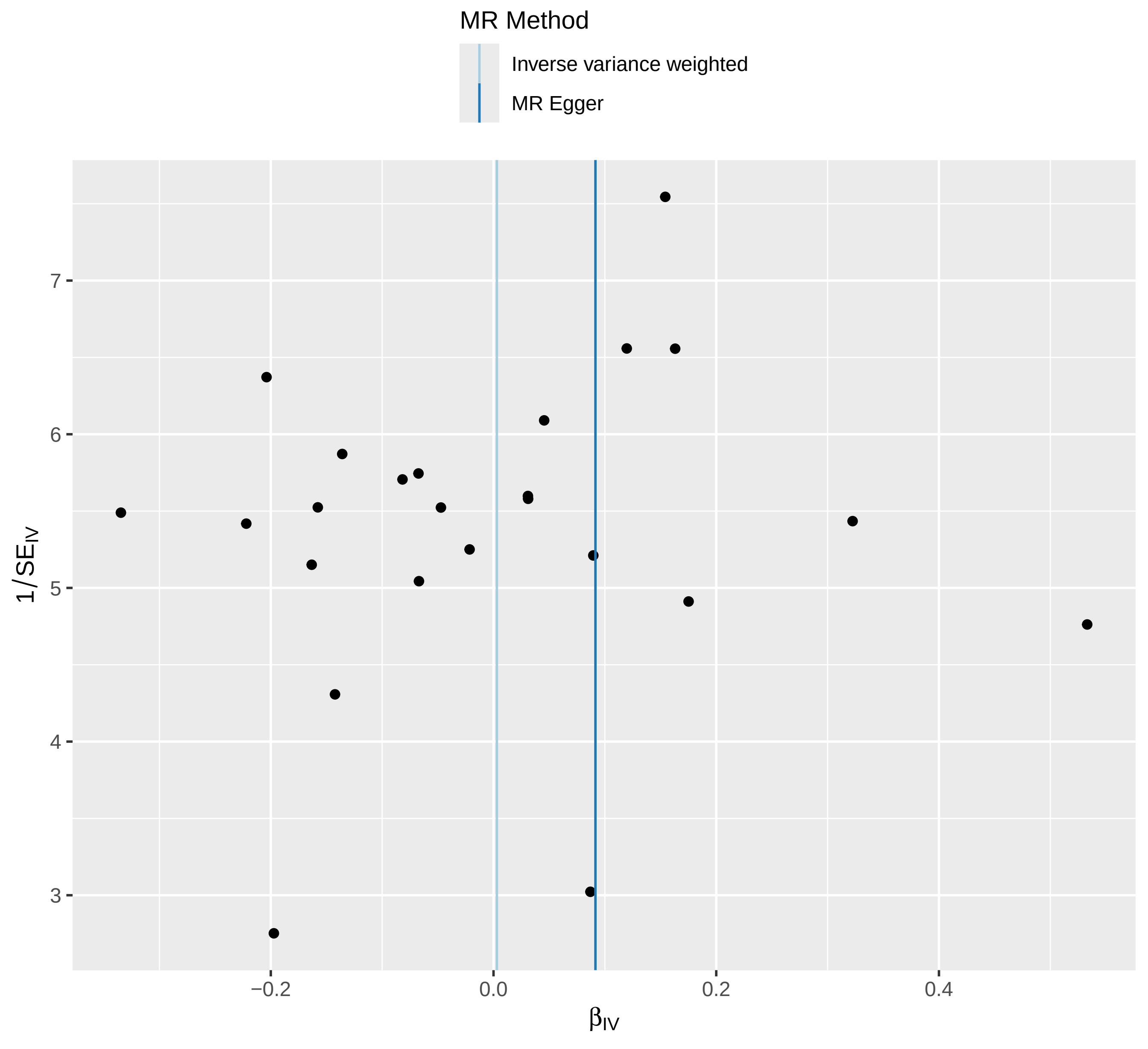

MR Method
Inverse variance weighted
MR Egger
1/SE_IV
7
6
5
4
3
−0.2
0.0
0.2
0.4
β_IV

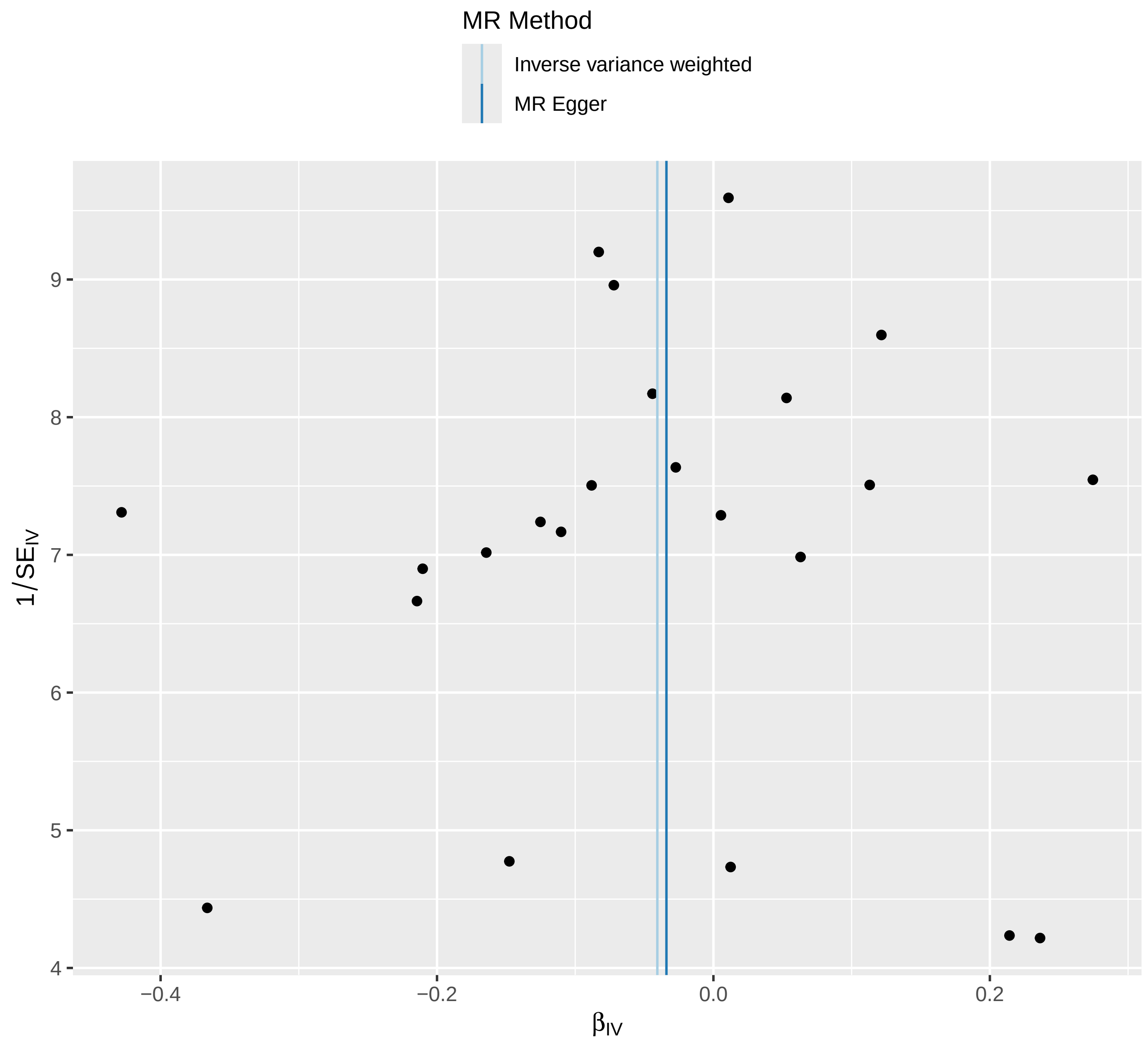
MR Method
Inverse variance weighted
MR Egger
1/SE_IV
β_IV
9
8
7
6
5
4
−0.4
−0.2
0.0
0.2

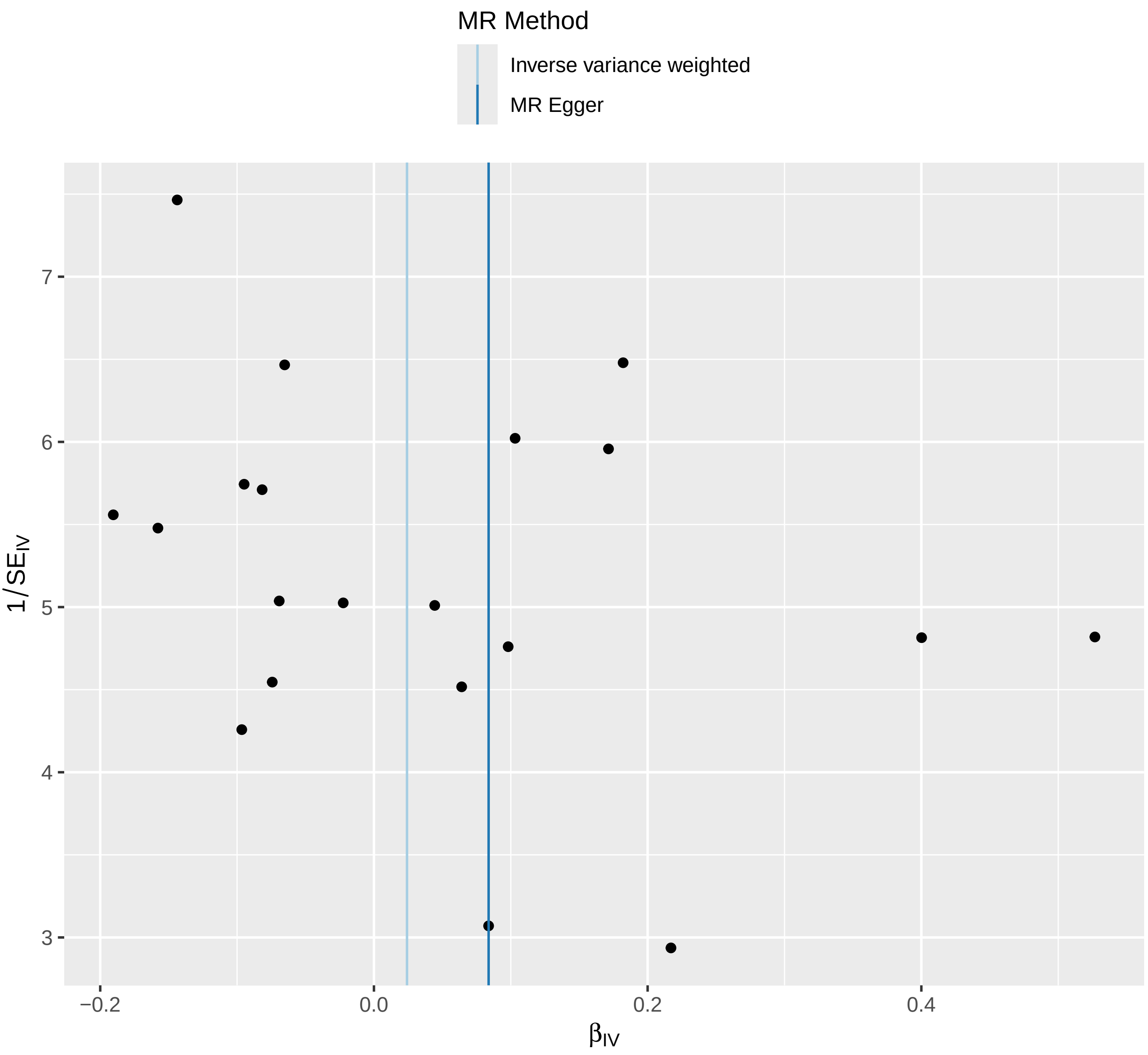

MR Method
Inverse variance weighted
MR Egger
1/SE_IV
β_IV
7
6
5
4
3
−0.2
0.0
0.2
0.4

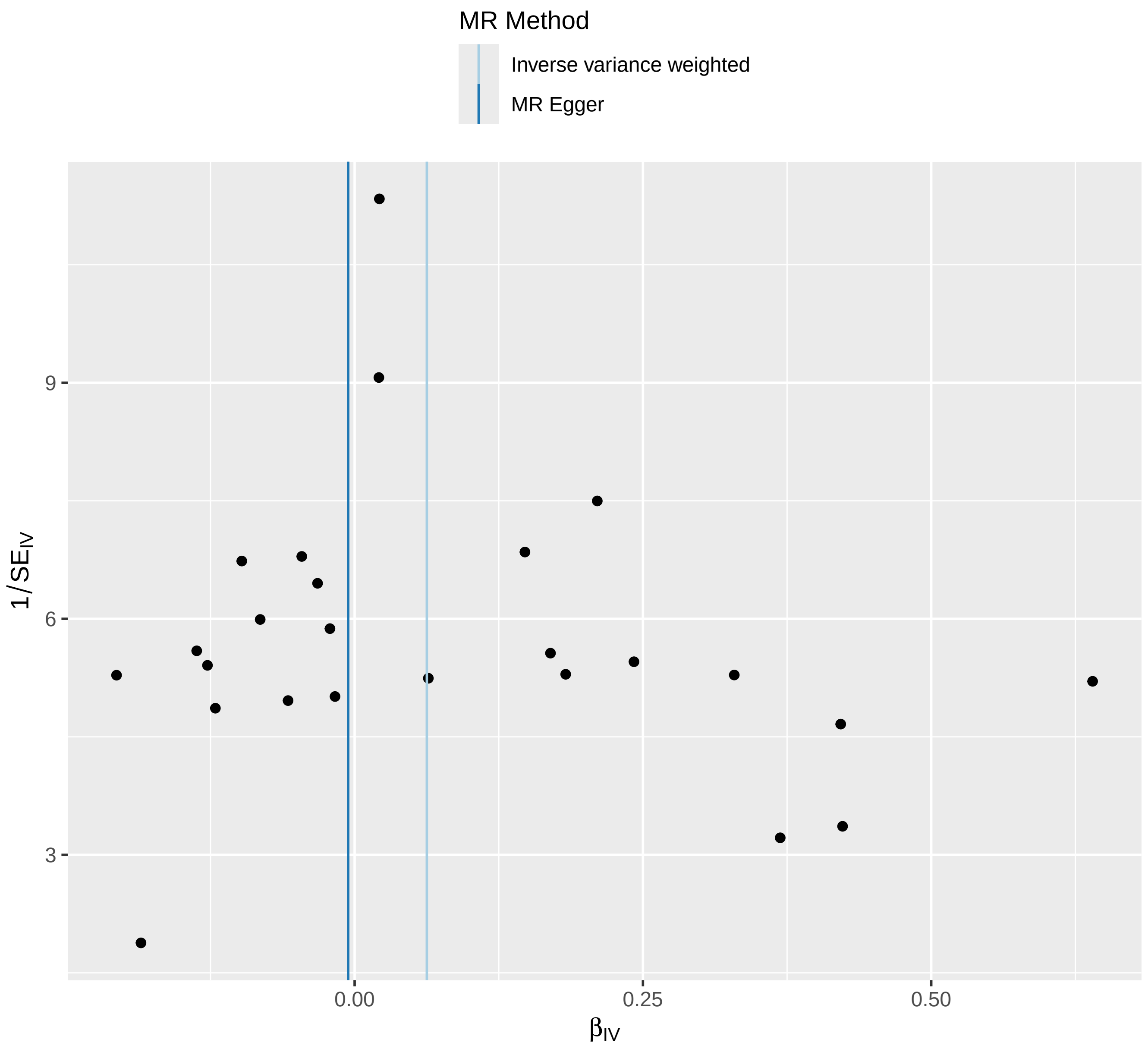
MR Method
Inverse variance weighted
MR Egger
9
6
3
1/SE_IV
0.00
0.25
0.50
β_IV

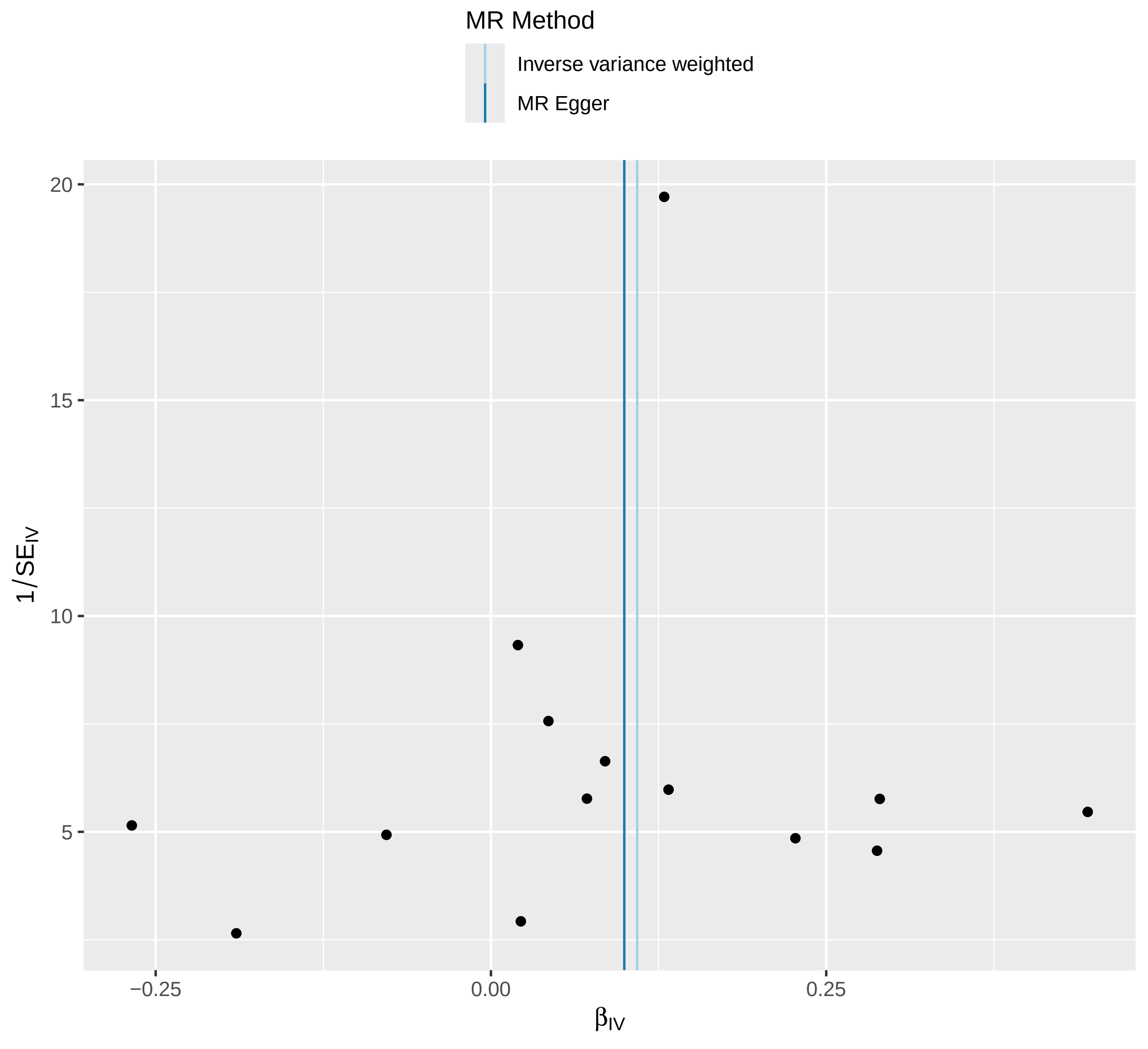
MR Method
Inverse variance weighted
MR Egger
20
15
10
5
1/SE$_{IV}$
−0.25
0.00
0.25
β$_{IV}$

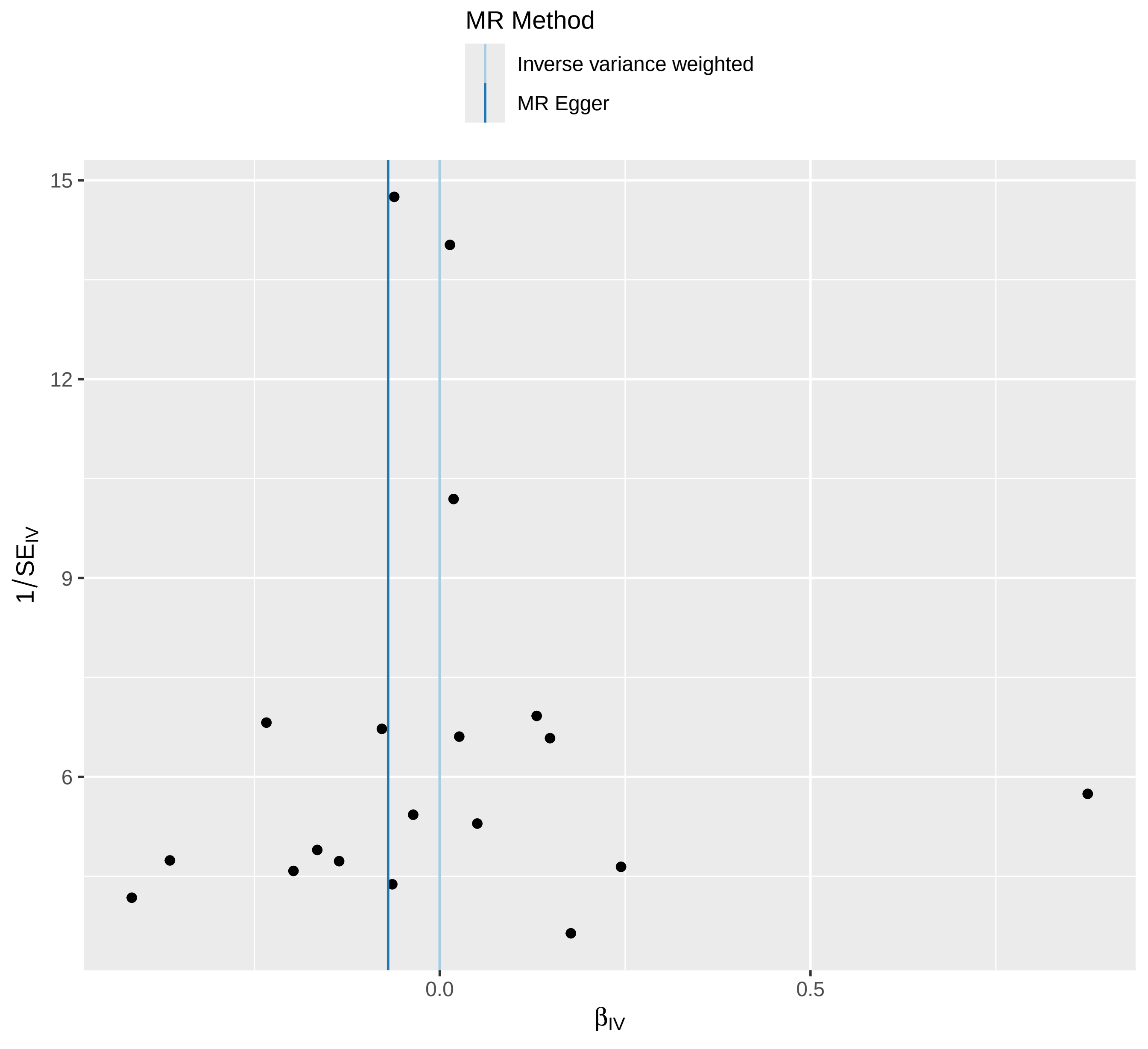
MR Method
Inverse variance weighted
MR Egger
15
12
9
6
1/SE_IV
0.0
0.5
β_IV

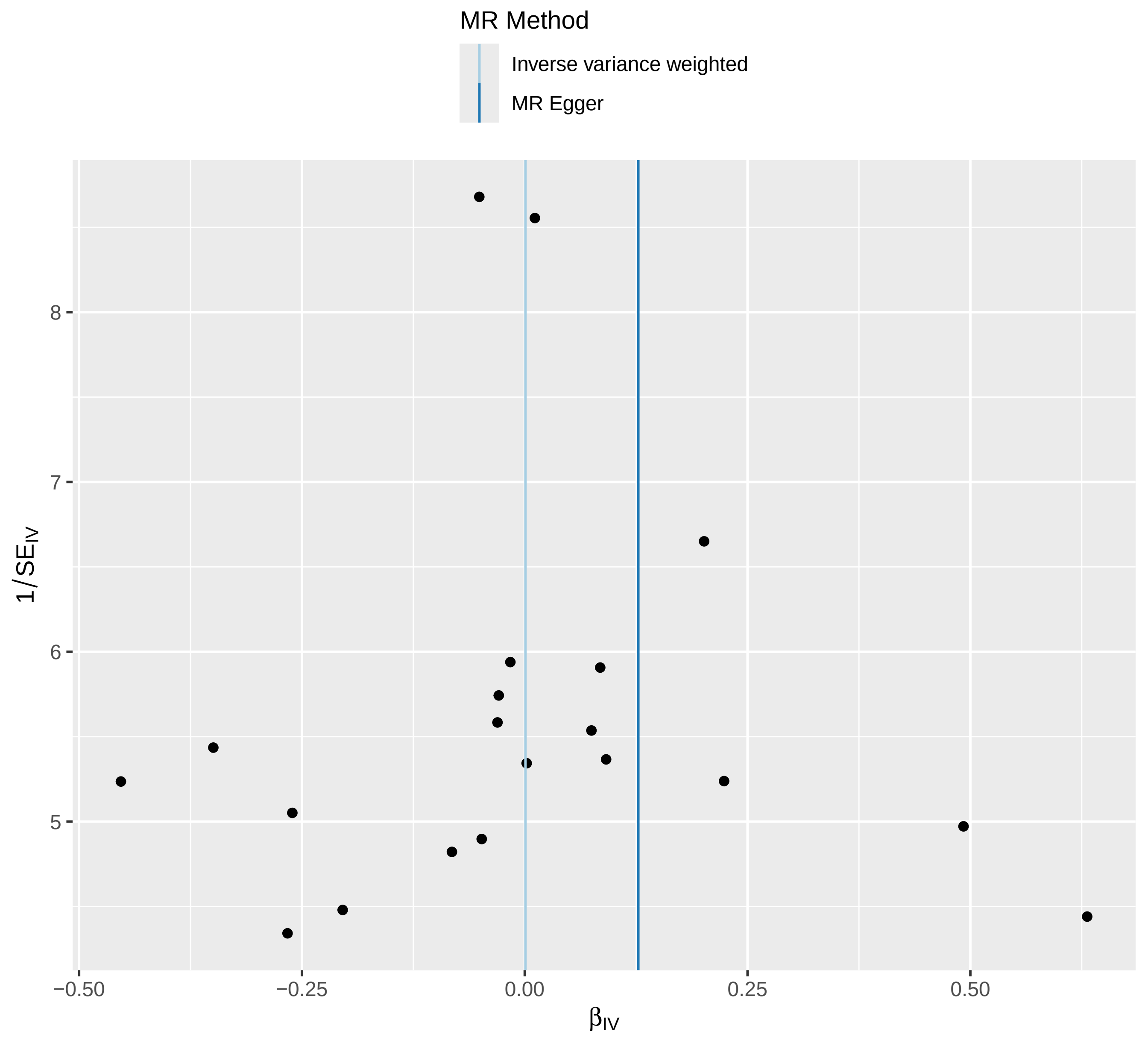
MR Method
Inverse variance weighted
MR Egger
1/SE_IV
8
7
6
5
−0.50
−0.25
0.00
0.25
0.50
β_IV

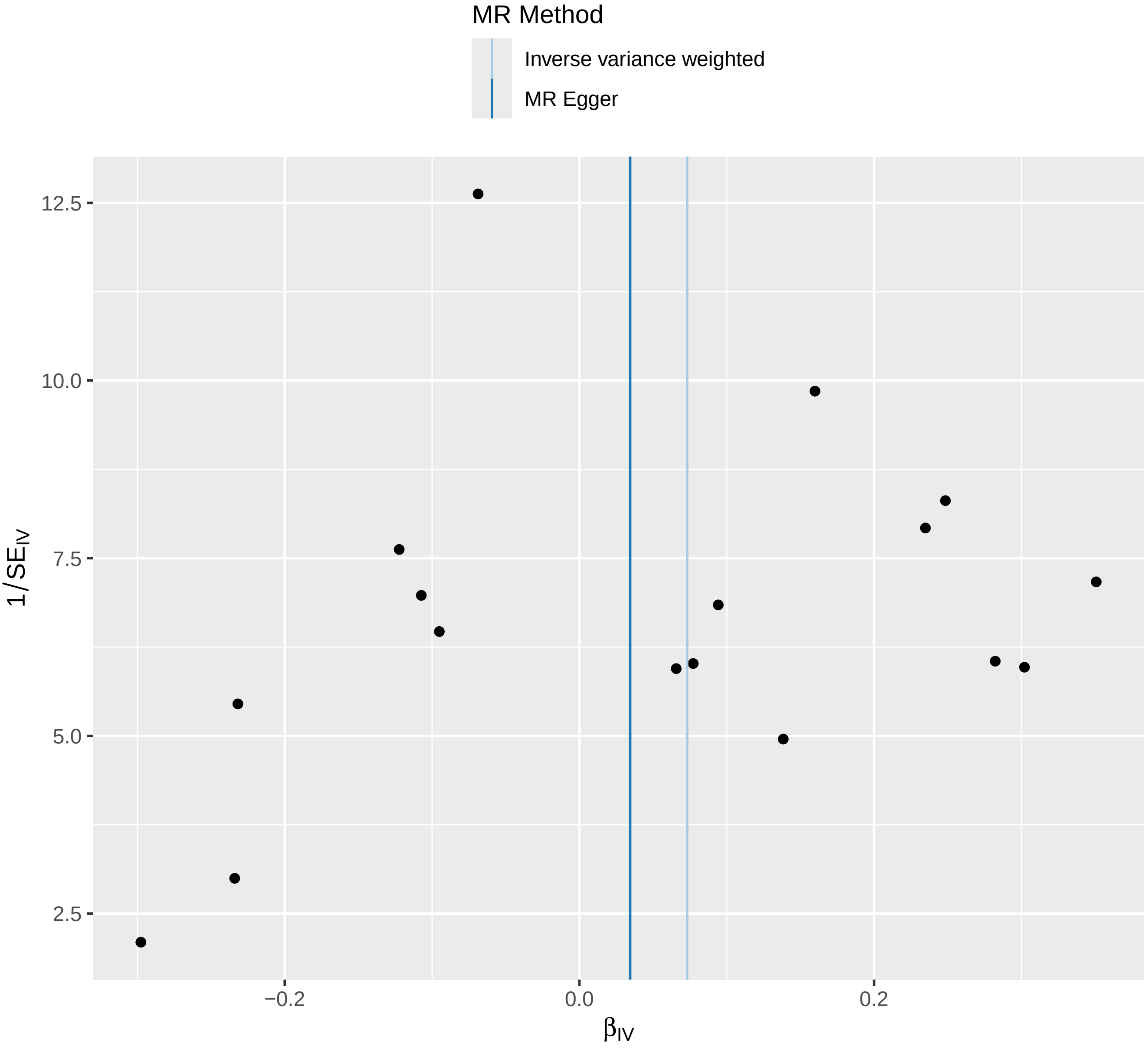
MR Method
Inverse variance weighted
MR Egger
12.5
10.0
7.5
5.0
2.5
1/SE_IV
−0.2
0.0
0.2
β_IV

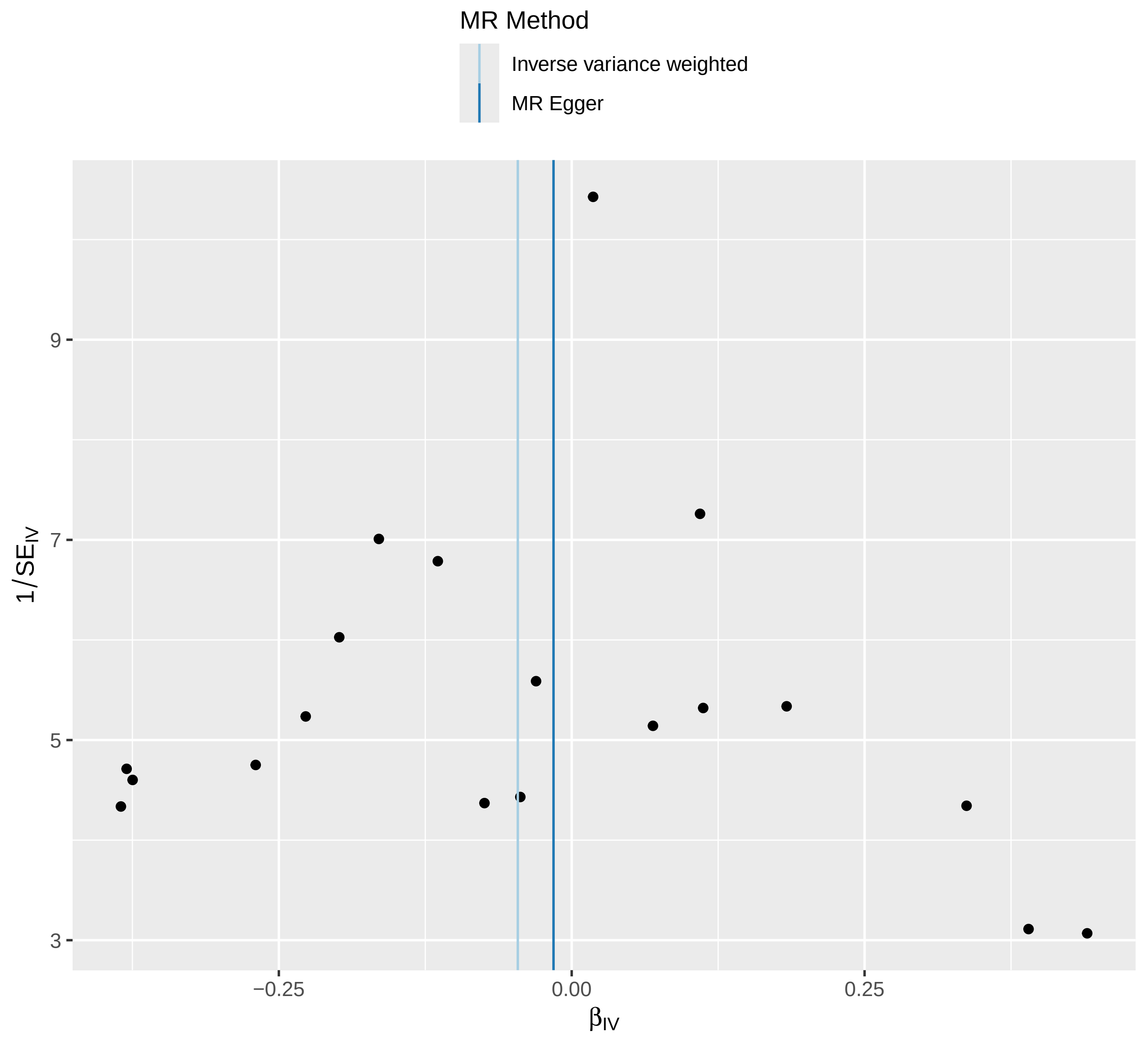
MR Method
Inverse variance weighted
MR Egger
1/SE$_{IV}$
9
7
5
3
−0.25
0.00
0.25
β$_{IV}$

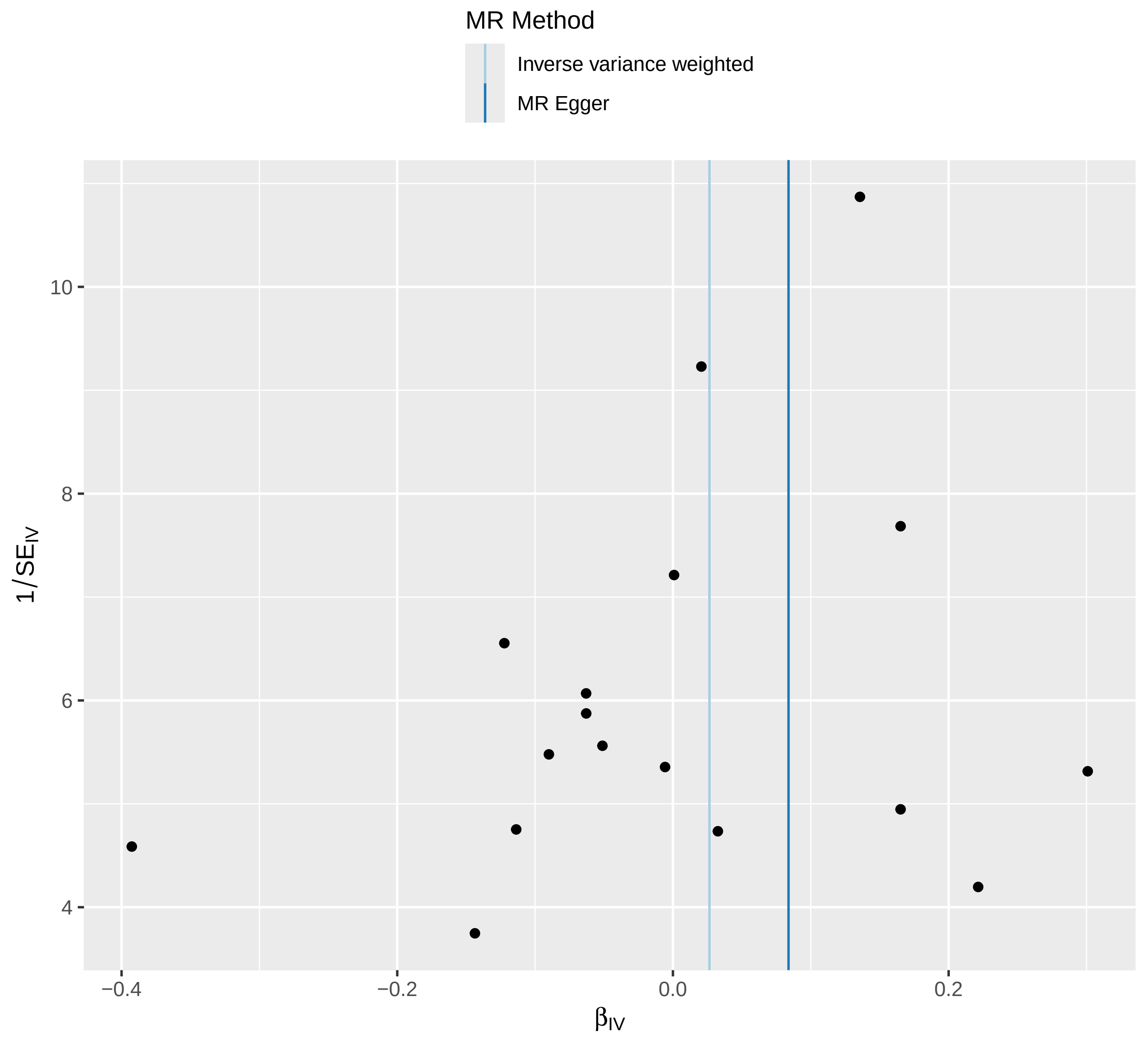
MR Method
Inverse variance weighted
MR Egger
10
8
6
4
1/SE_IV
−0.4
−0.2
0.0
0.2
β_IV

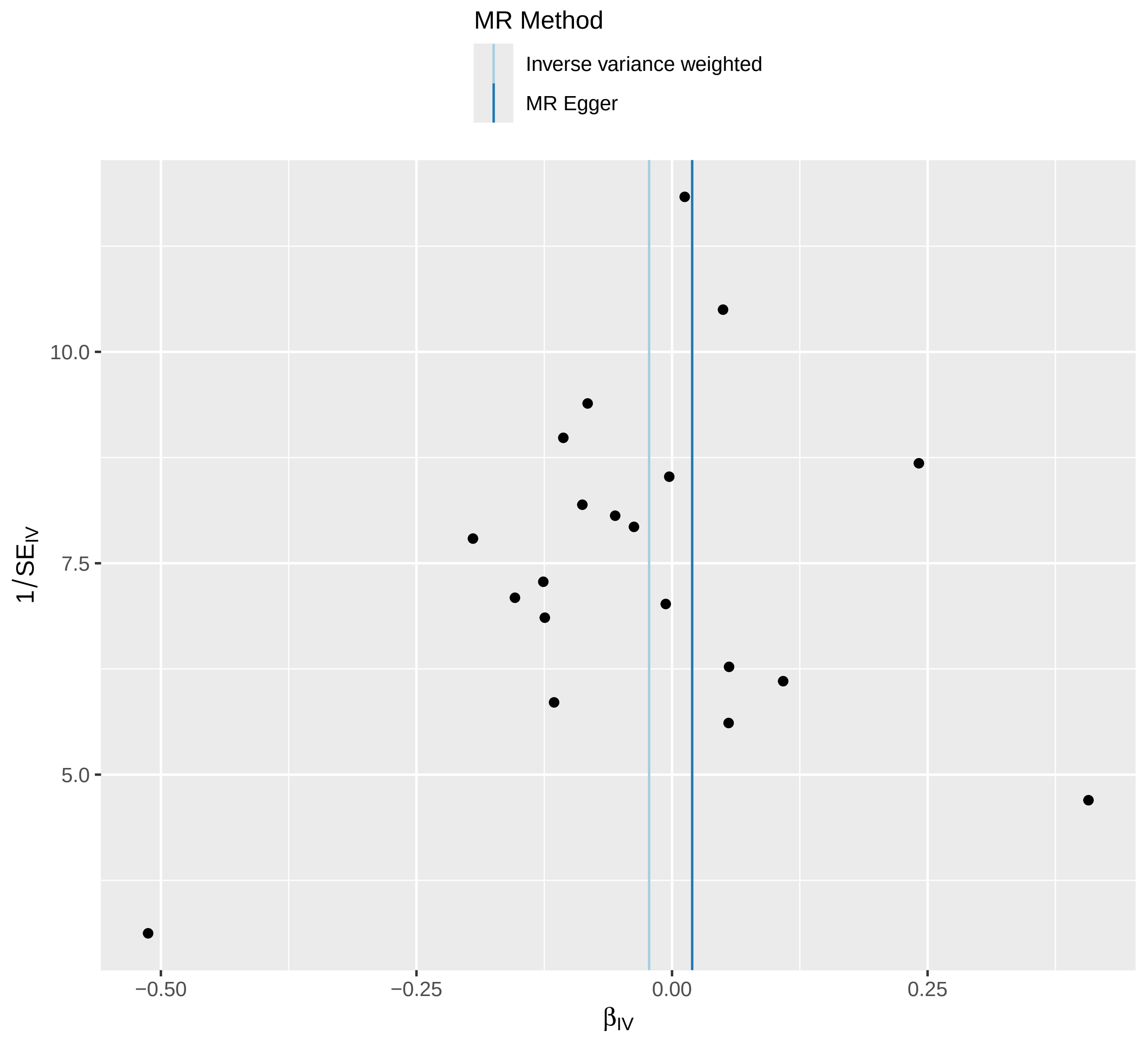

MR Method
Inverse variance weighted
MR Egger
10.0
7.5
5.0
1/SE_IV
−0.50
−0.25
0.00
0.25
β_IV

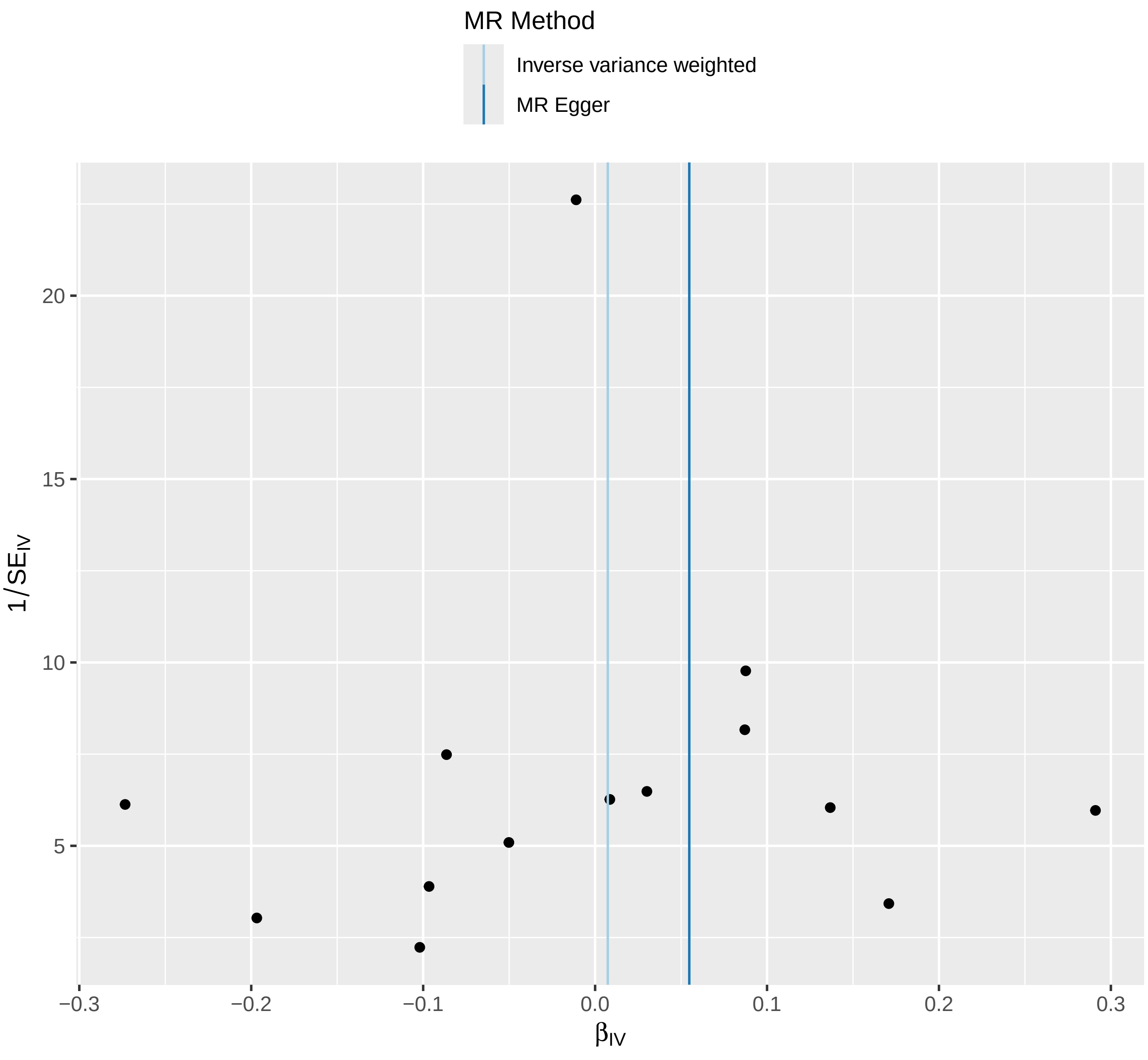
MR Method
Inverse variance weighted
MR Egger
1/SE_IV
β_IV
20
15
10
5
−0.3
−0.2
−0.1
0.0
0.1
0.2
0.3

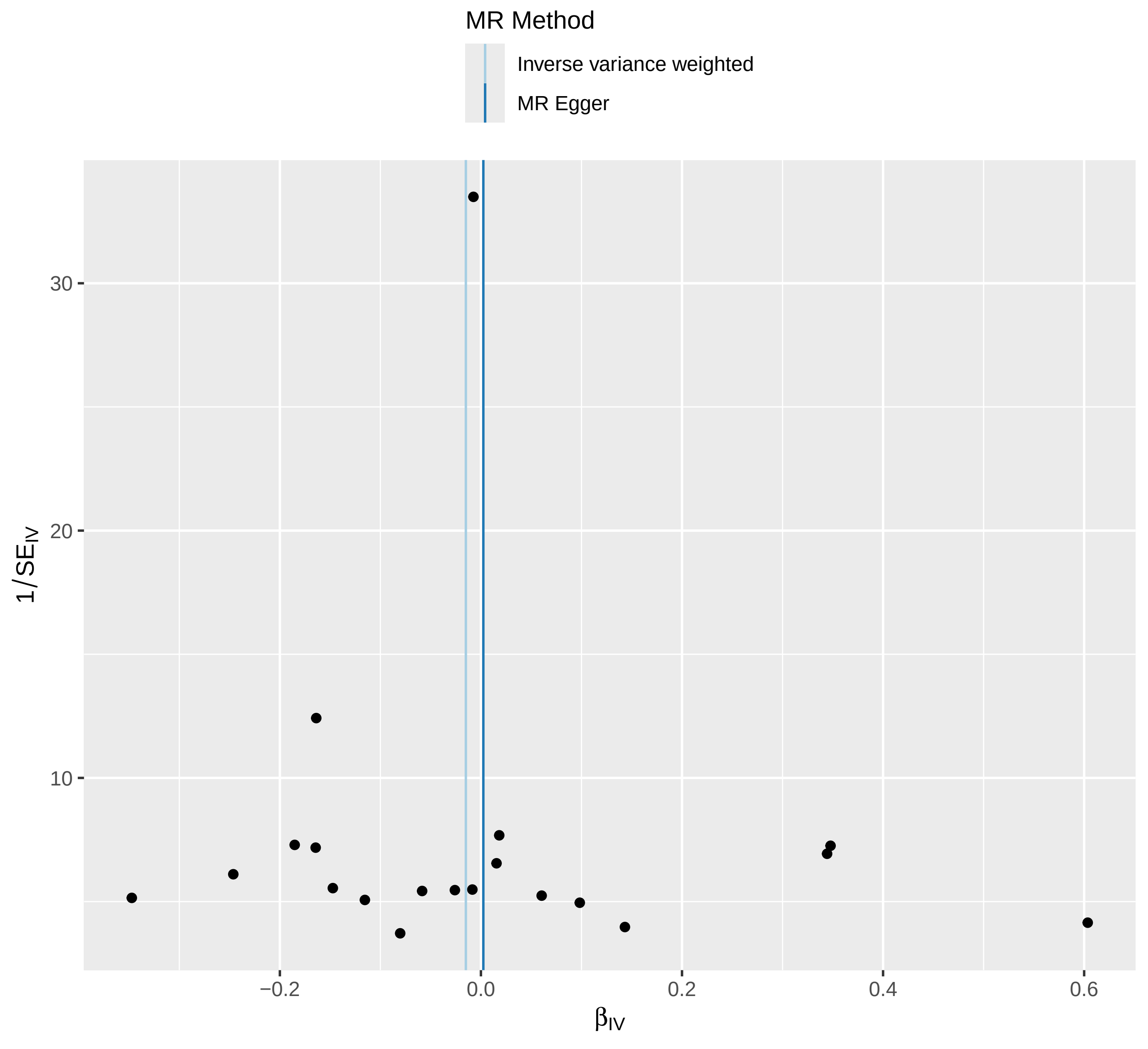
MR Method
Inverse variance weighted
MR Egger
30
20
10
1/SE_IV
−0.2
0.0
0.2
0.4
0.6
β_IV

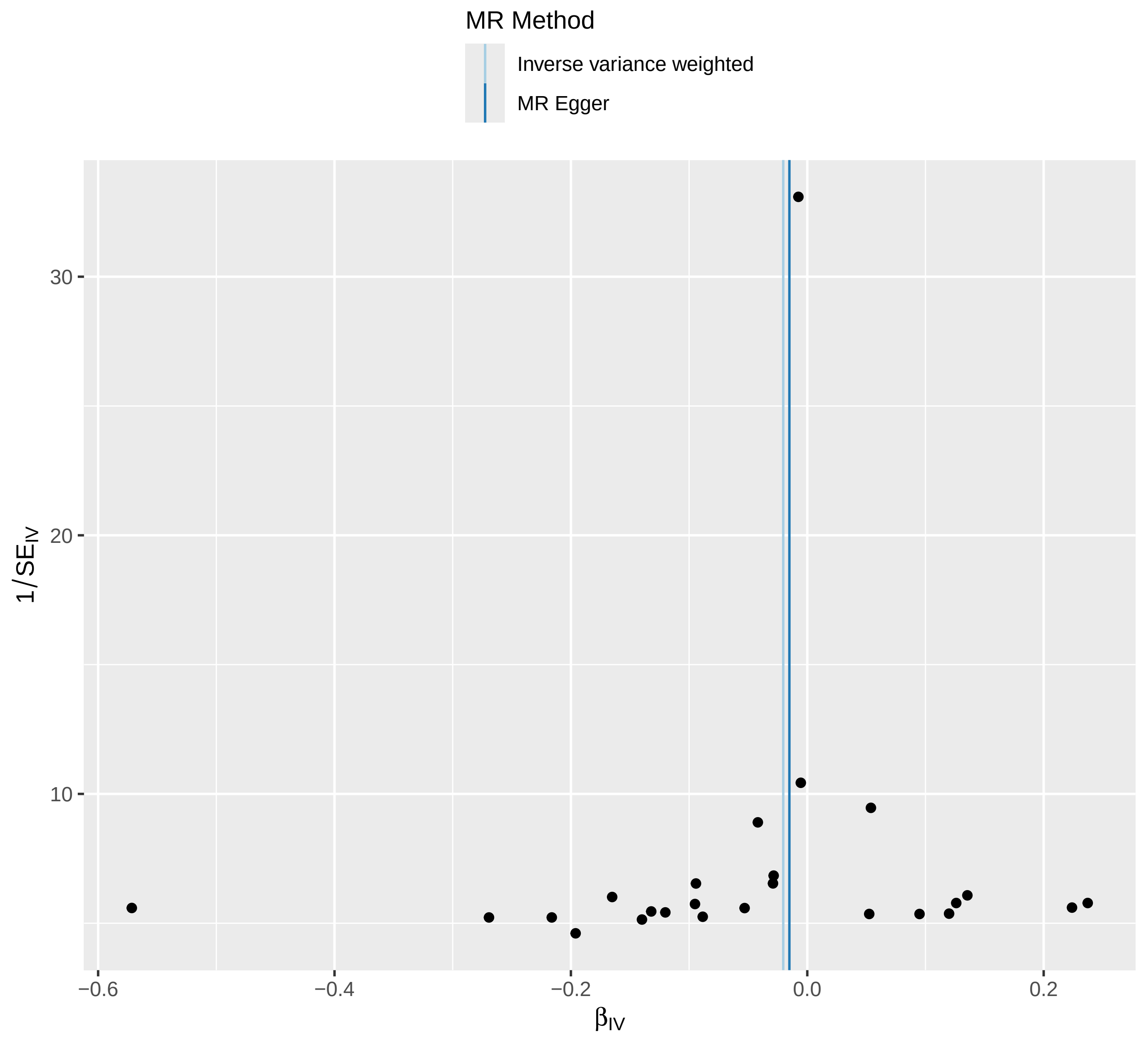
MR Method
Inverse variance weighted
MR Egger
30
20
10
1/SE_IV
−0.6
−0.4
−0.2
0.0
0.2
β_IV

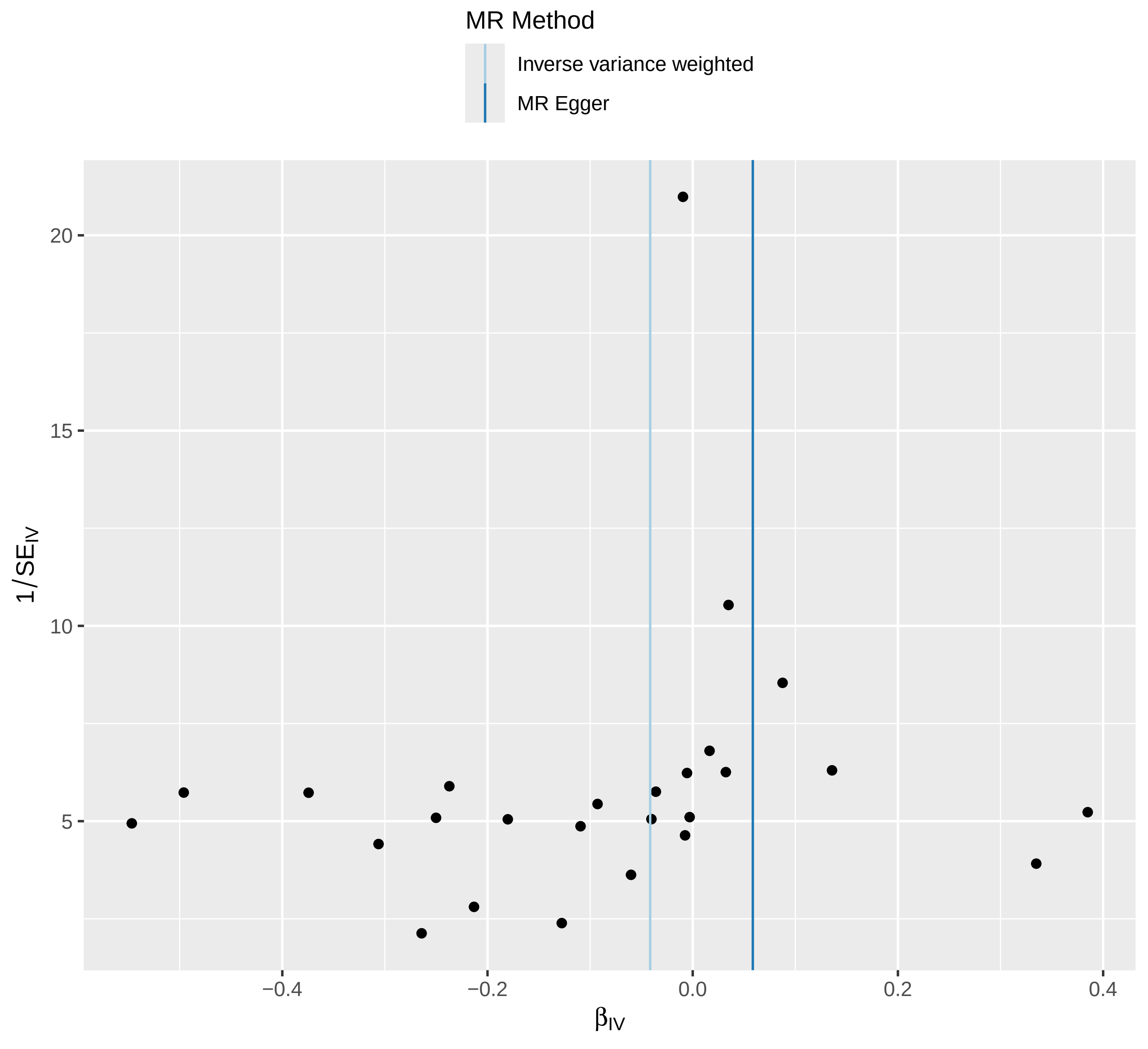
MR Method
Inverse variance weighted
MR Egger
20
15
10
5
1/SE_IV
−0.4
−0.2
0.0
0.2
0.4
β_IV

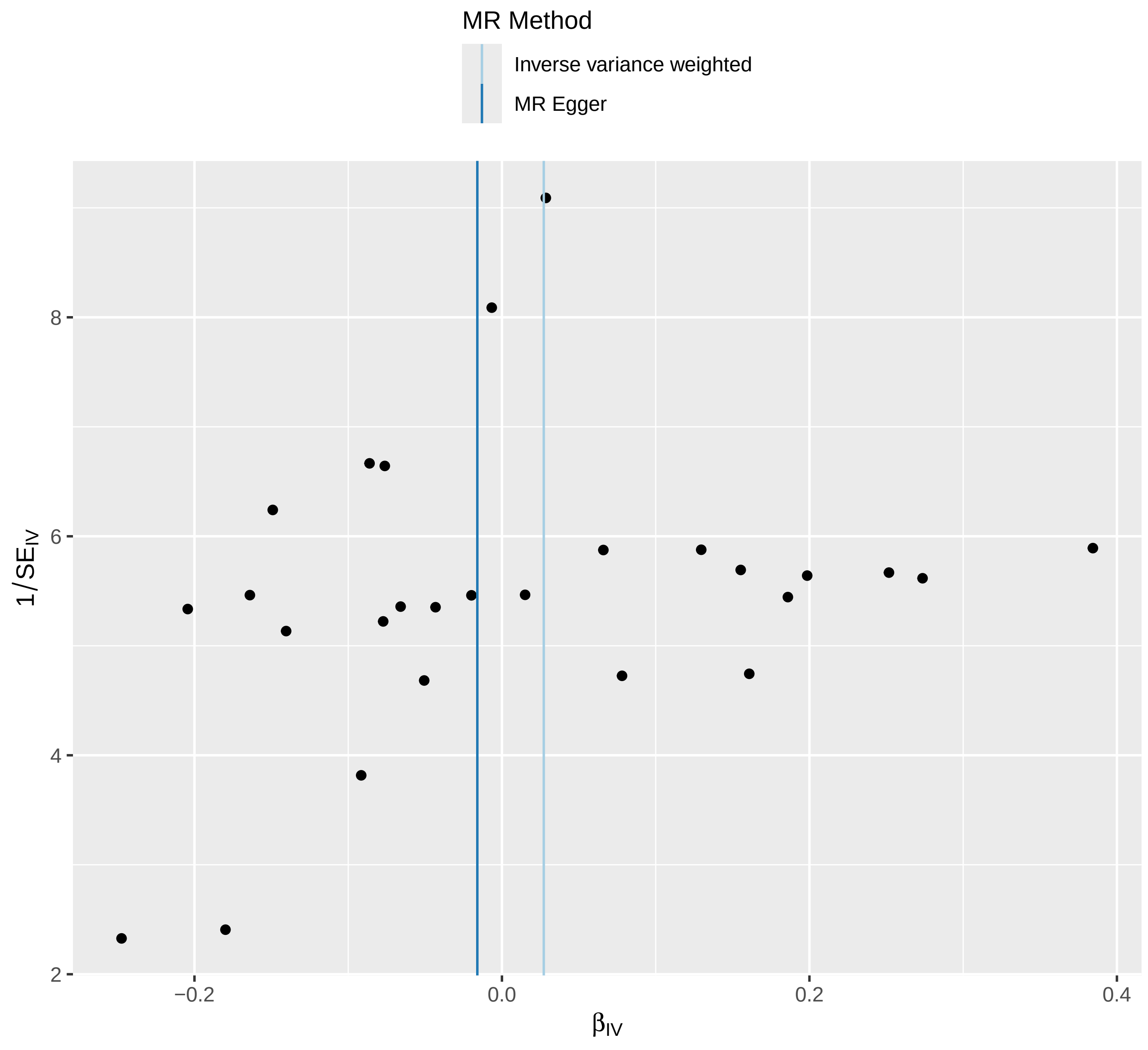
MR Method
Inverse variance weighted
MR Egger
8
6
4
2
1/SE$_{IV}$
−0.2
0.0
0.2
0.4
β$_{IV}$

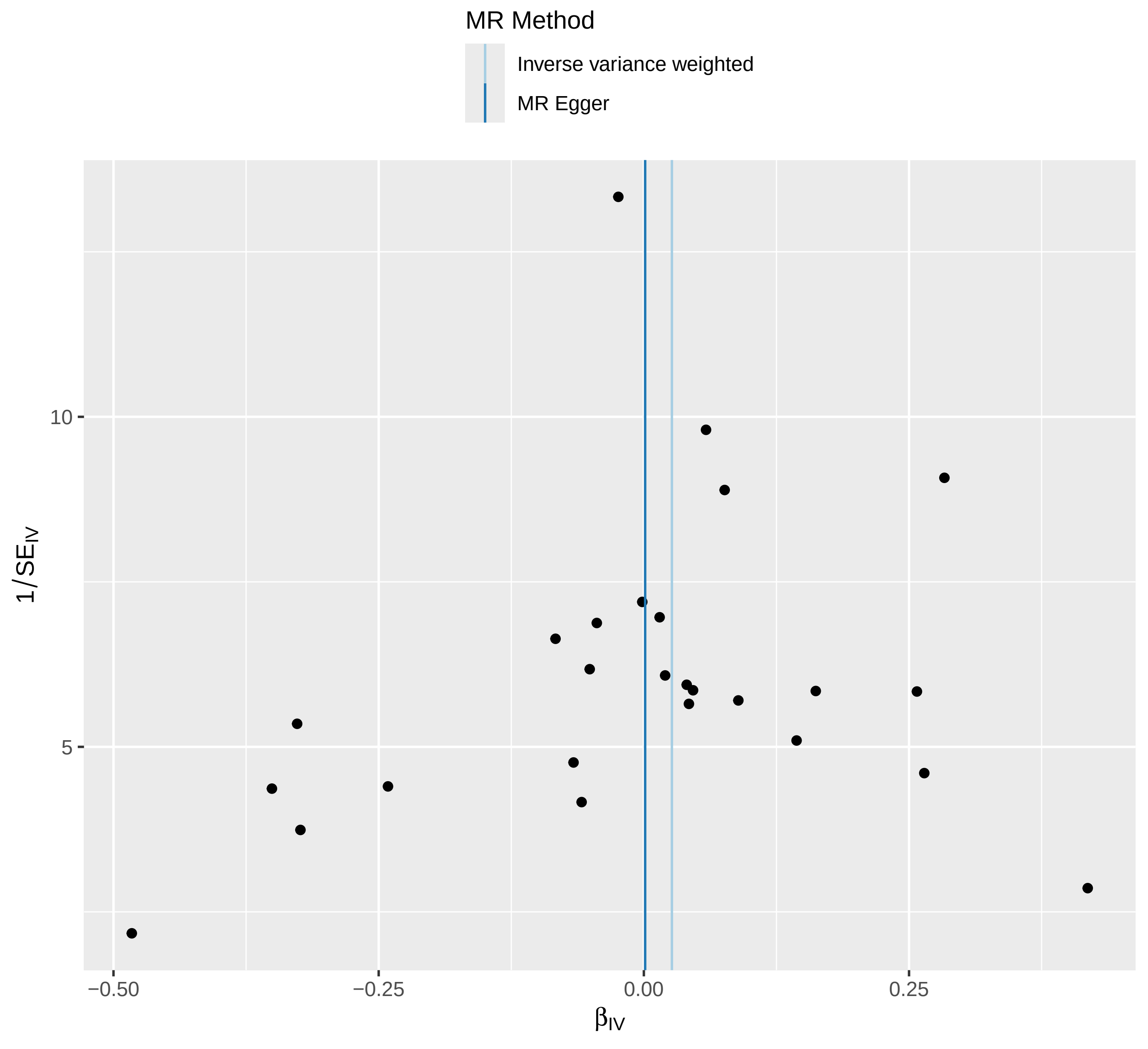
MR Method
Inverse variance weighted
MR Egger
1/SE$_{IV}$
10
5
−0.50
−0.25
0.00
0.25
β$_{IV}$

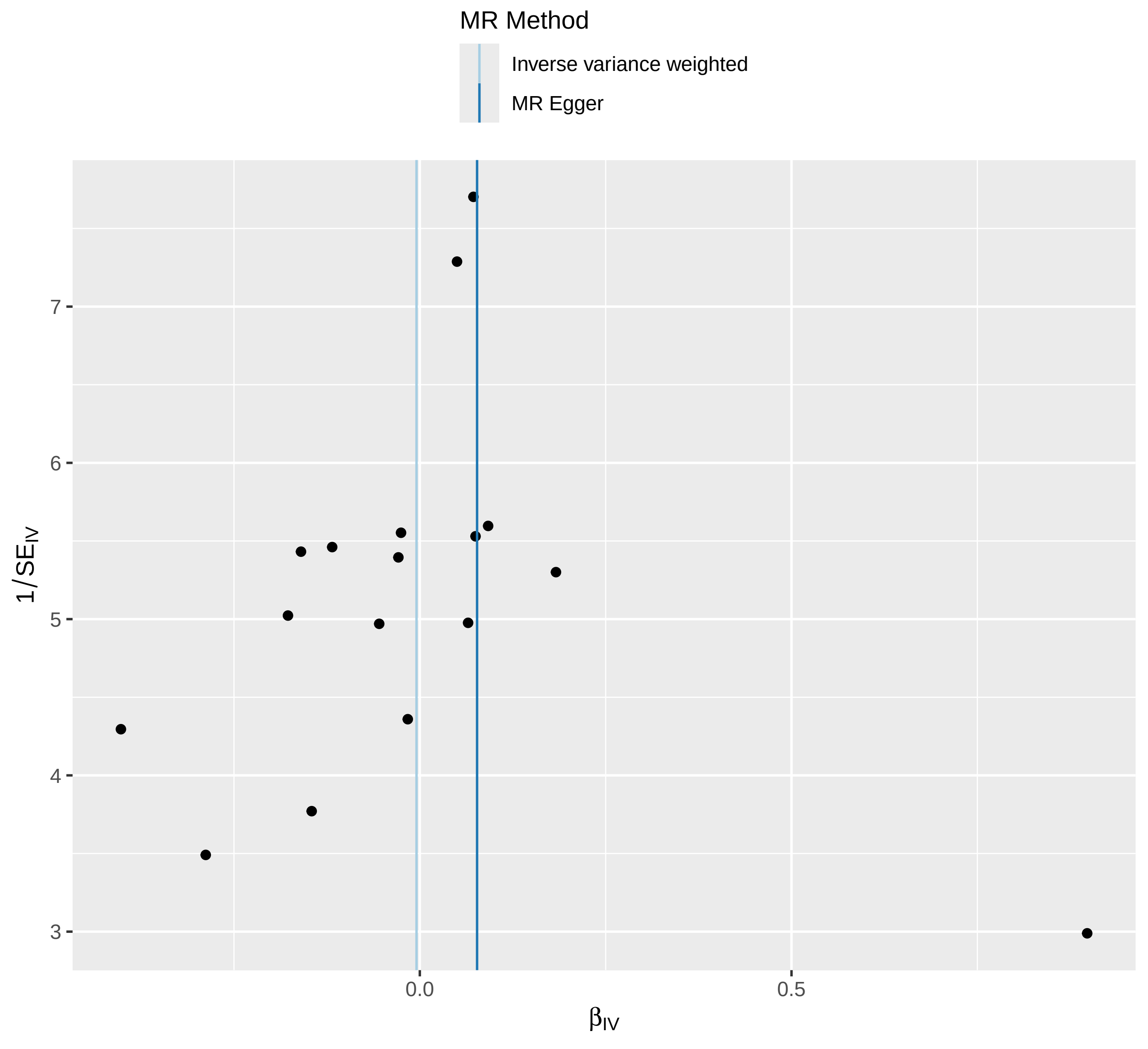

MR Method
Inverse variance weighted
MR Egger
1/SE_IV
7
6
5
4
3
0.0
0.5
β_IV

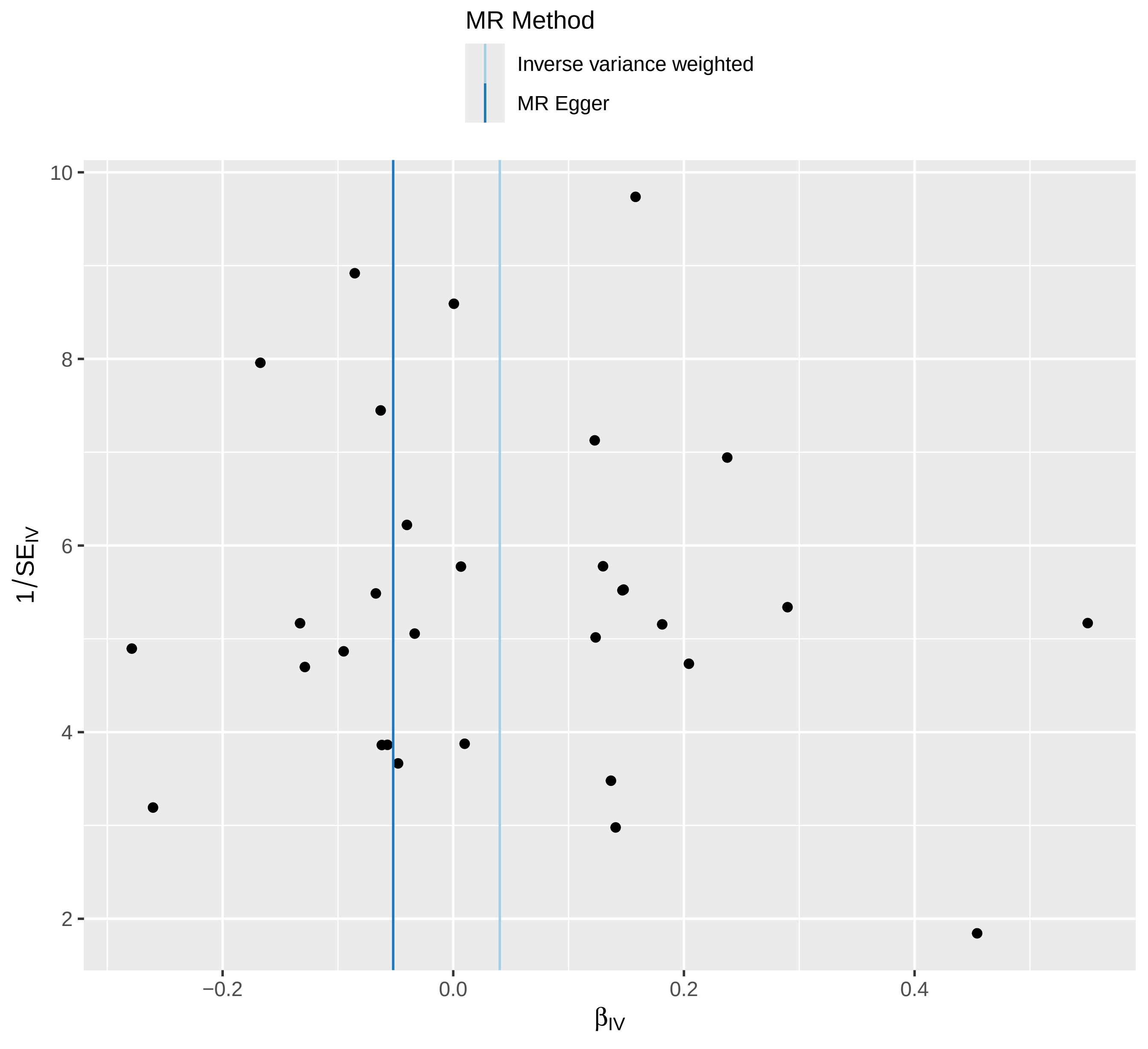
MR Method
Inverse variance weighted
MR Egger
10
8
6
4
2
$1/SE_{IV}$
−0.2
0.0
0.2
0.4
β_{IV}

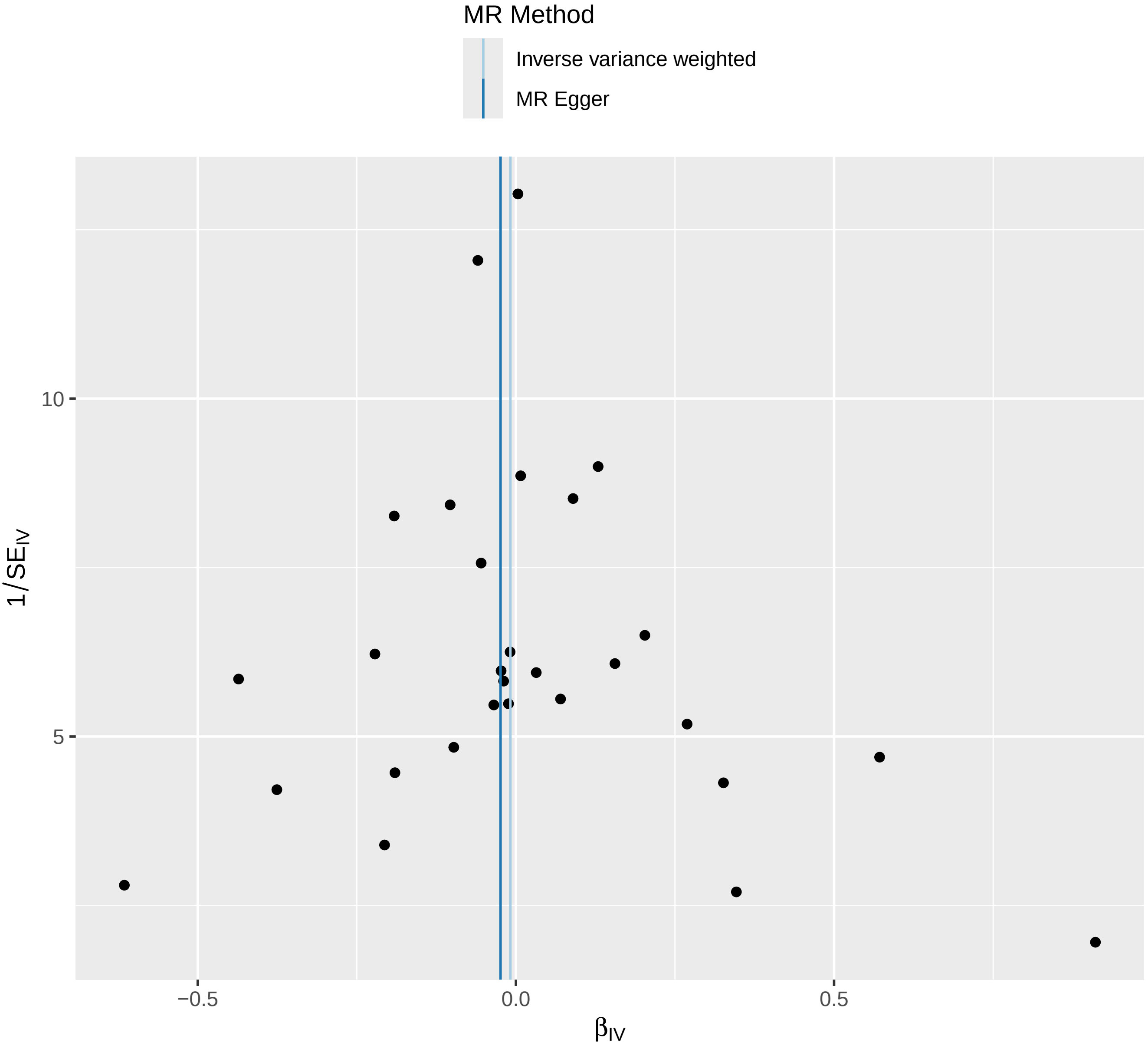

MR Method
Inverse variance weighted
MR Egger
10
5
1/SE_IV
−0.5
0.0
0.5
β_IV

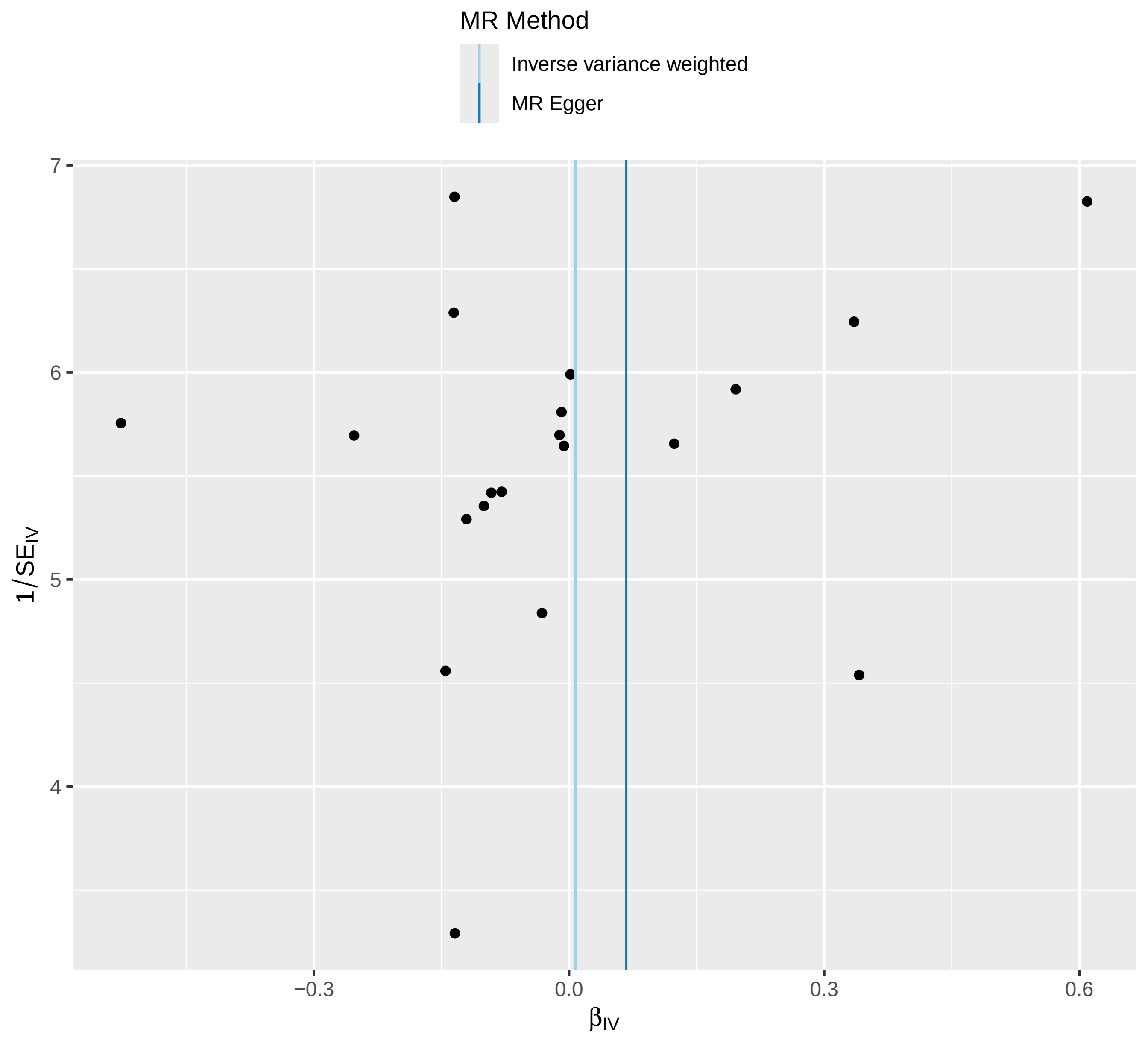

MR Method
Inverse variance weighted
MR Egger
7
6
5
4
1/SE_IV
−0.3
0.0
0.3
0.6
β_IV

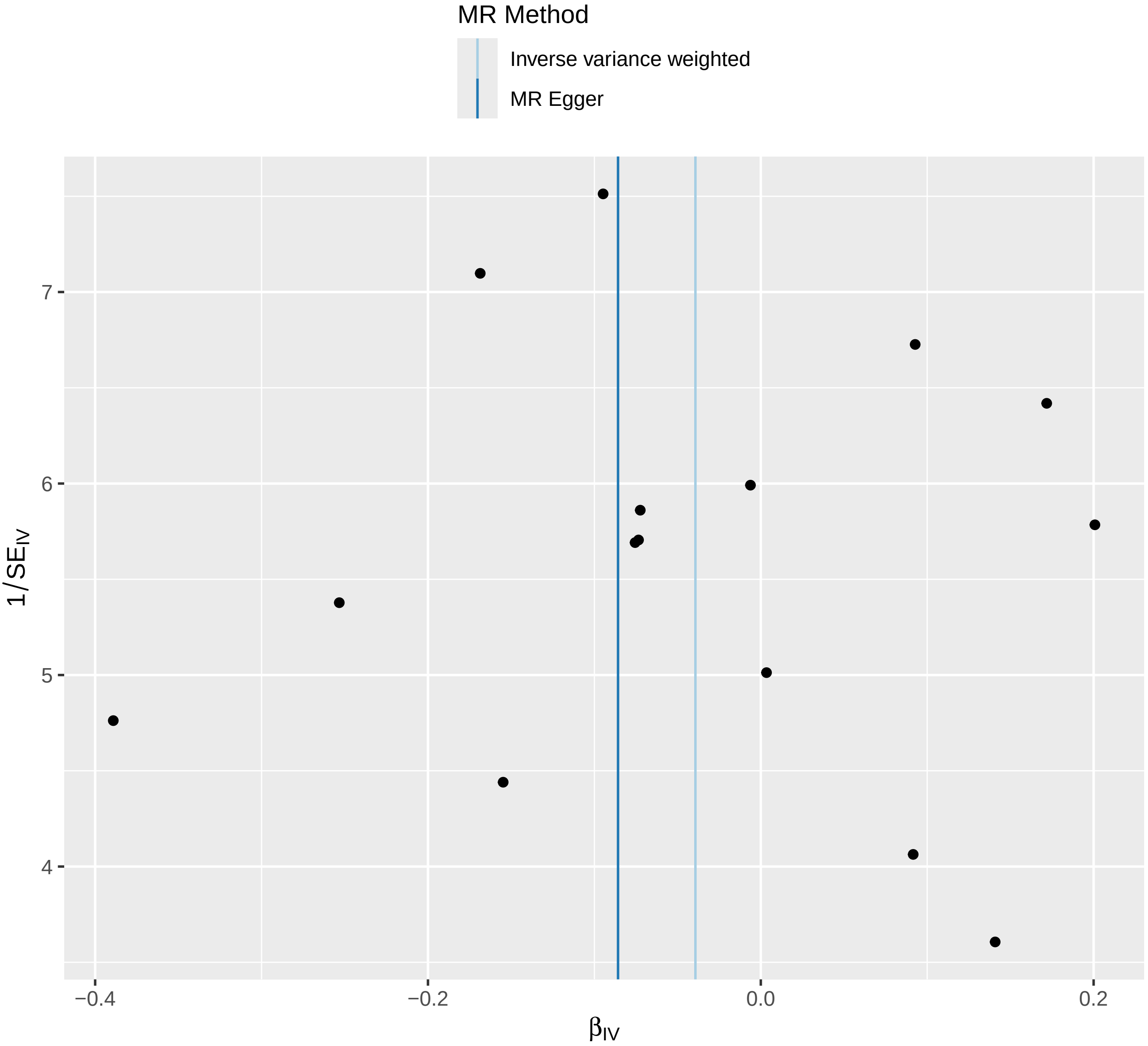
MR Method
Inverse variance weighted
MR Egger
1/SE_IV
β_IV
7
6
5
4
−0.4
−0.2
0.0
0.2

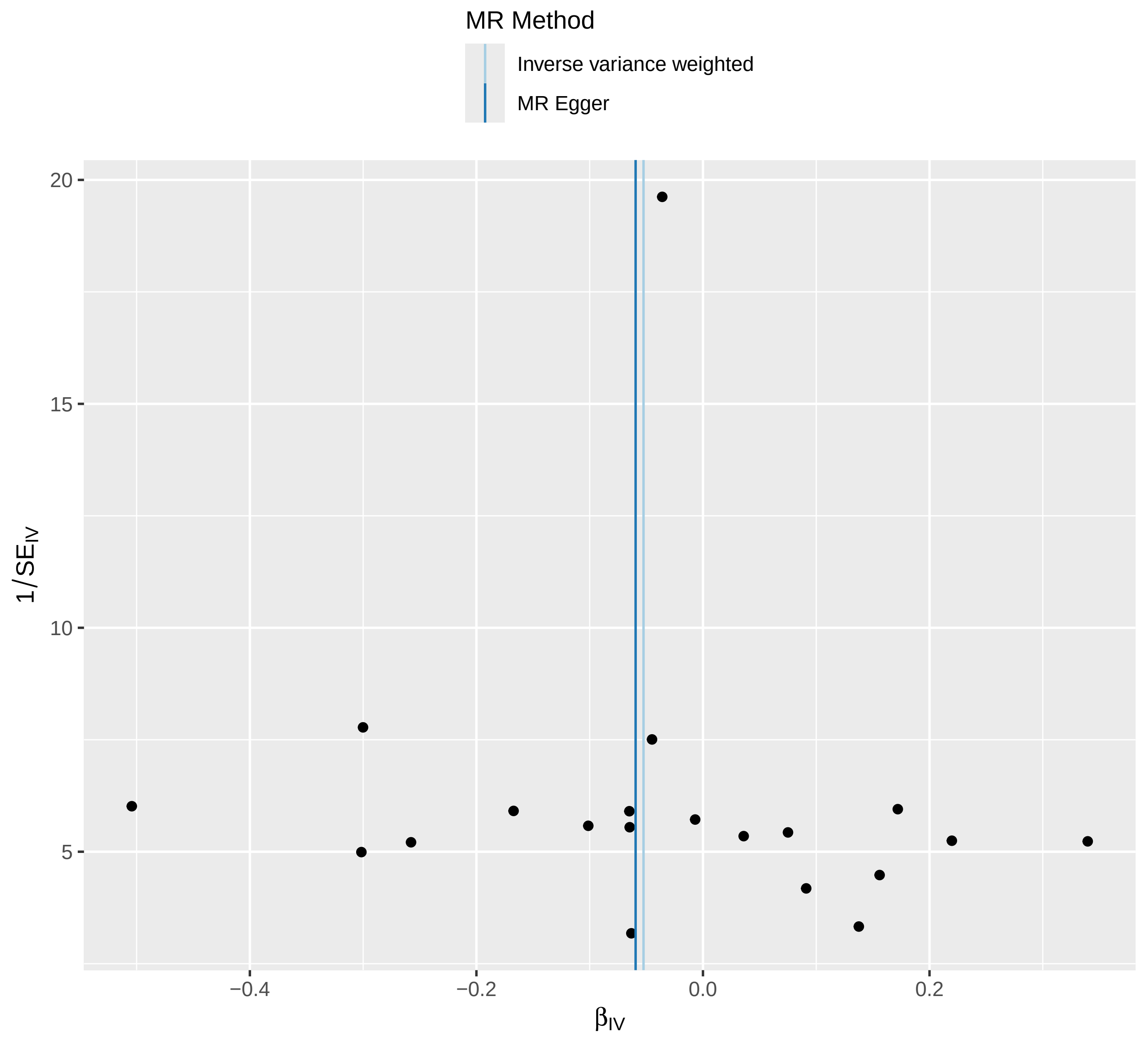
MR Method
Inverse variance weighted
MR Egger
20
15
10
5
1/SE_IV
−0.4
−0.2
0.0
0.2
β_IV

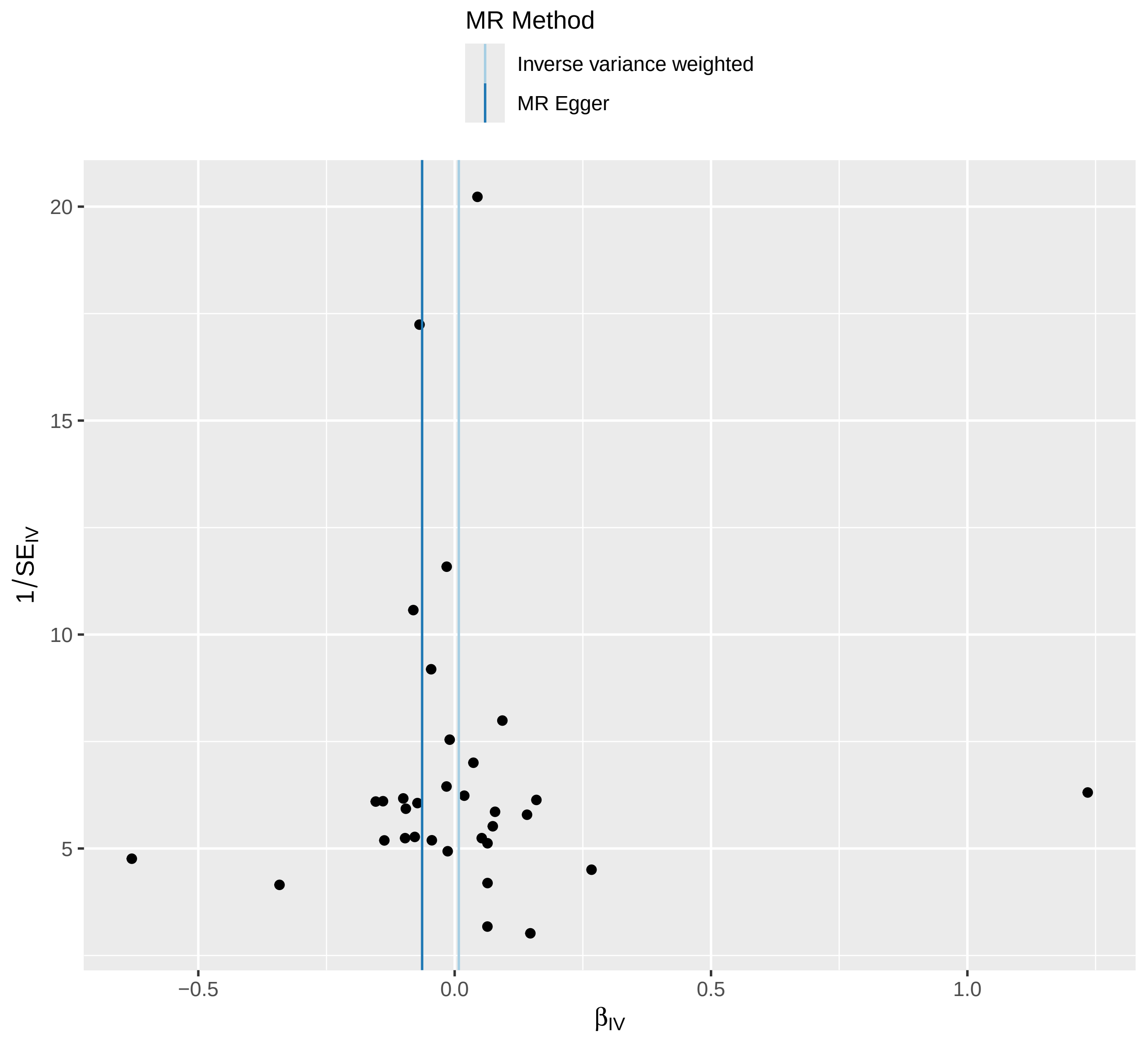

MR Method
Inverse variance weighted
MR Egger
20
15
10
5
1/SE_IV
−0.5
0.0
0.5
1.0
β_IV

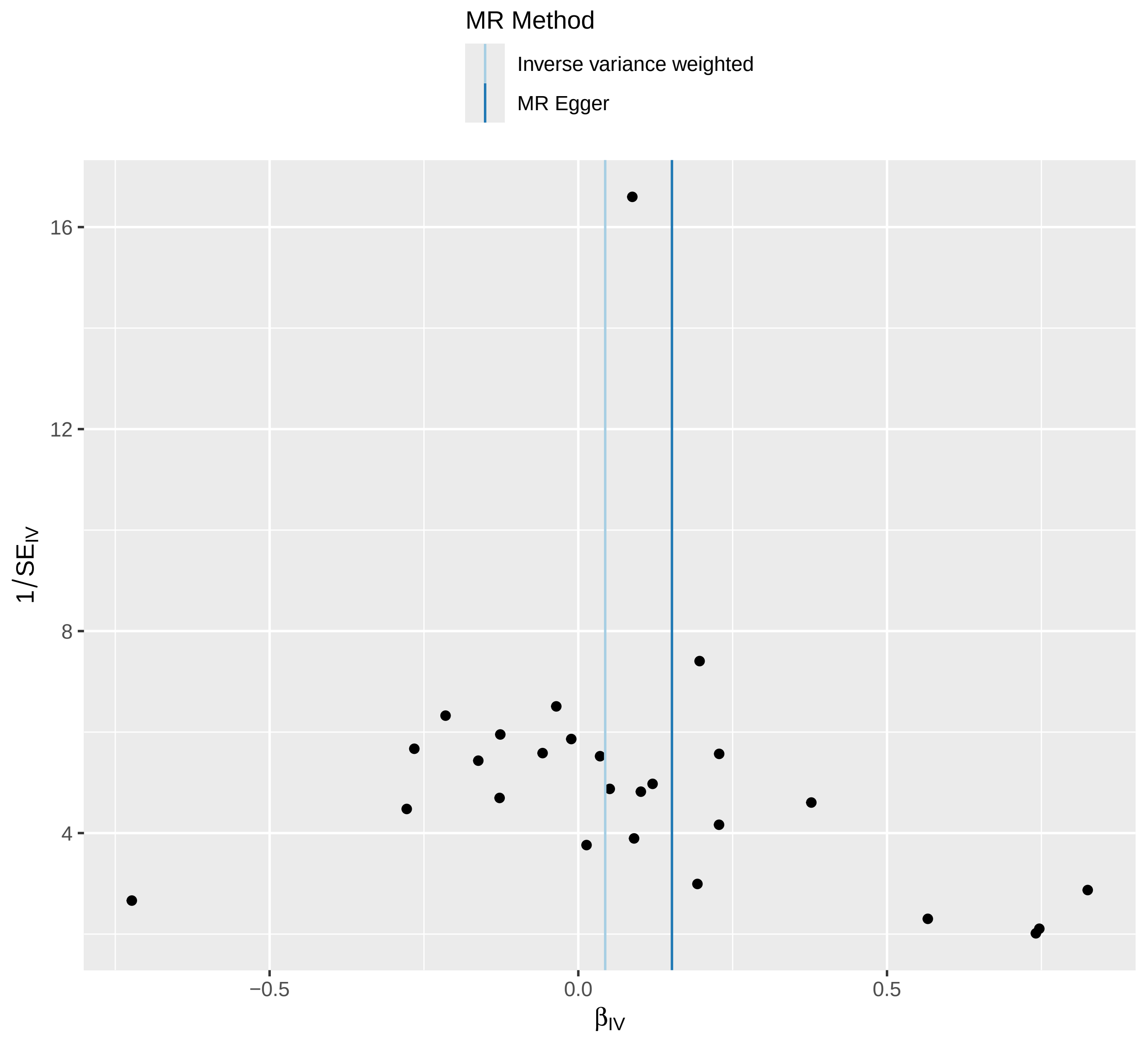

MR Method
Inverse variance weighted
MR Egger
16
12
8
4
1/SE_IV
−0.5
0.0
0.5
β_IV

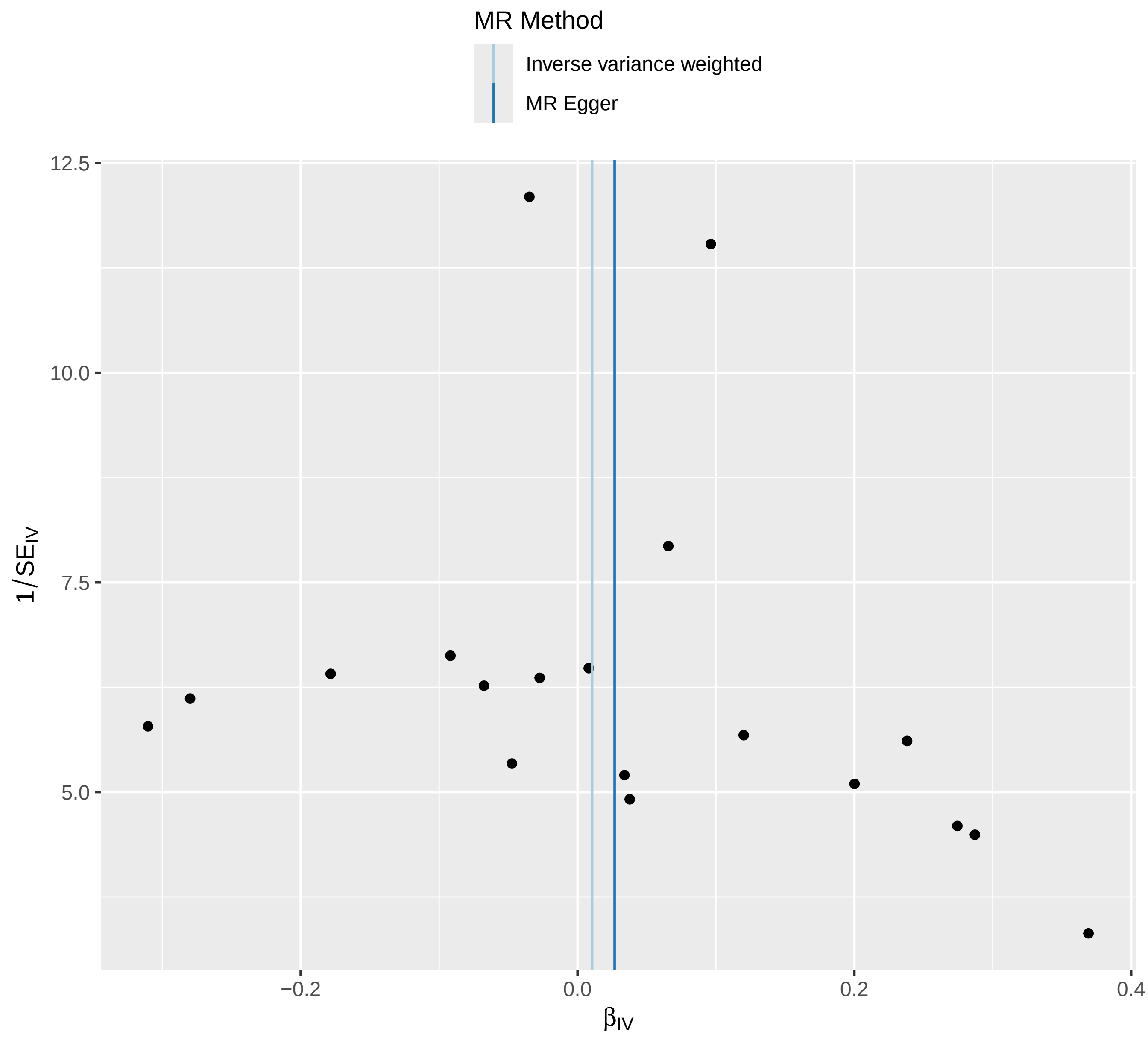
MR Method
Inverse variance weighted
MR Egger
12.5
10.0
7.5
5.0
1/SE$_{IV}$
−0.2
0.0
0.2
0.4
β$_{IV}$

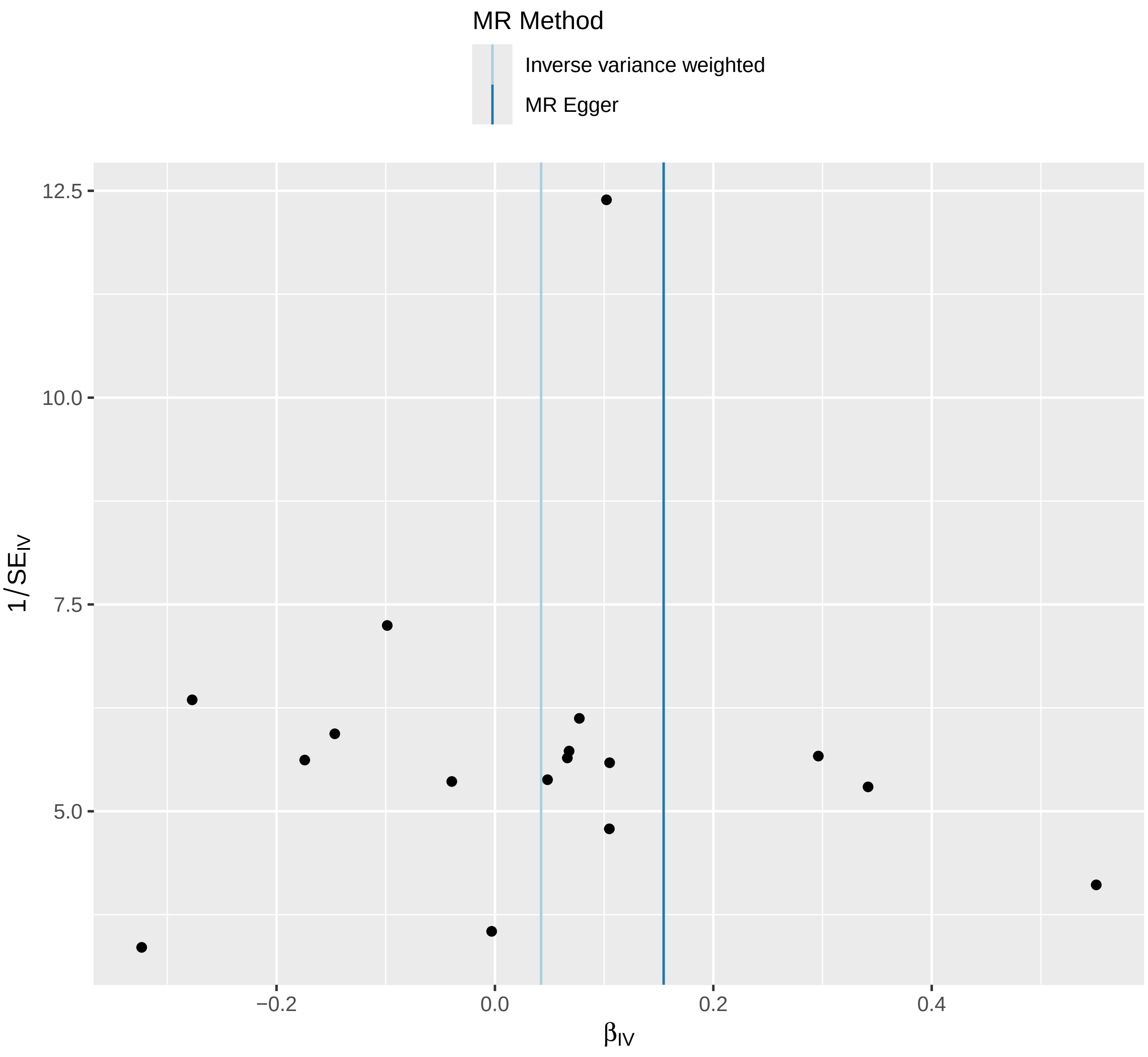
MR Method
Inverse variance weighted
MR Egger
12.5
10.0
7.5
5.0
1/SE_IV
−0.2
0.0
0.2
0.4
β_IV

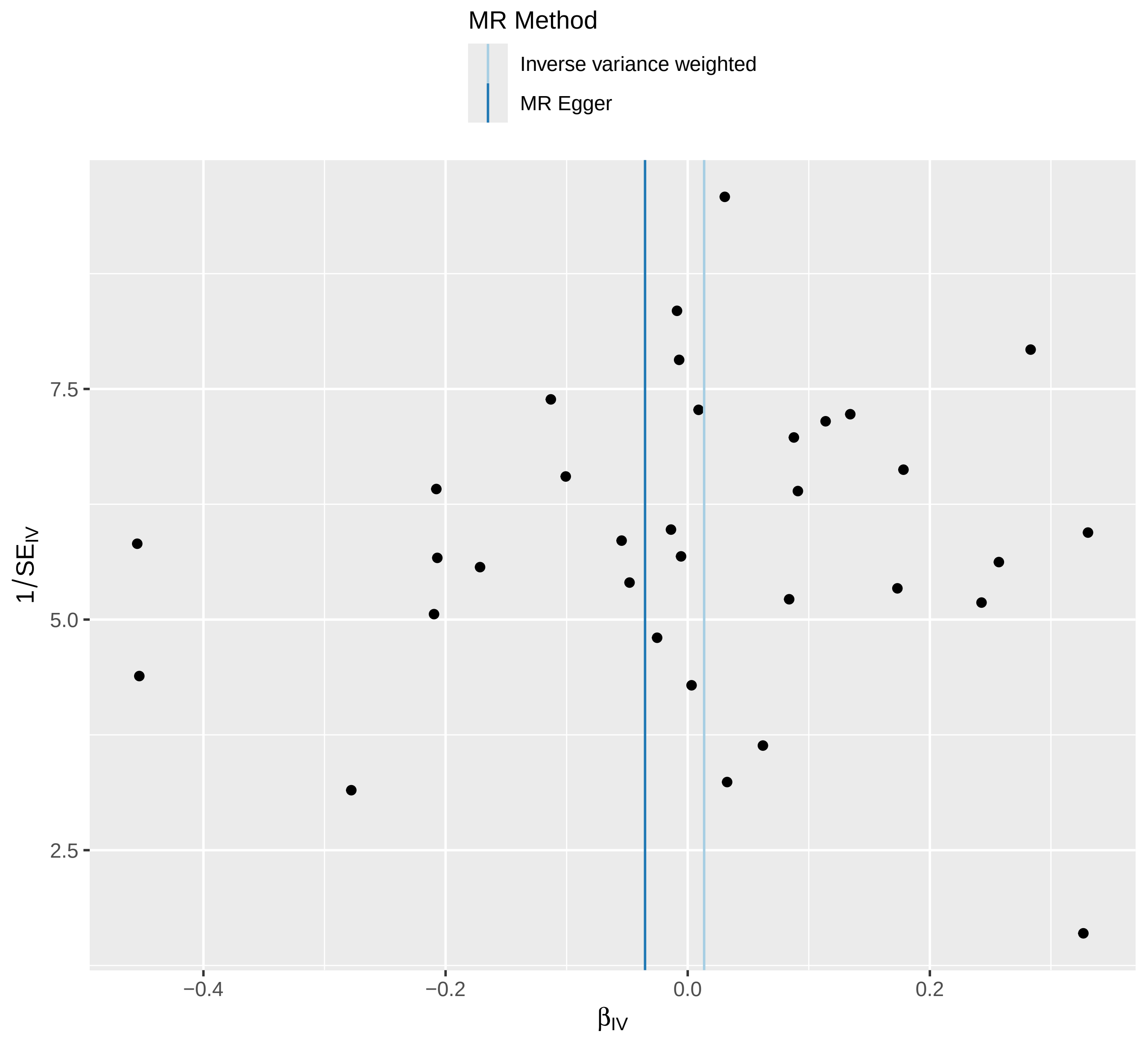

MR Method
Inverse variance weighted
MR Egger
7.5
5.0
2.5
1/SE_IV
−0.4
−0.2
0.0
0.2
β_IV

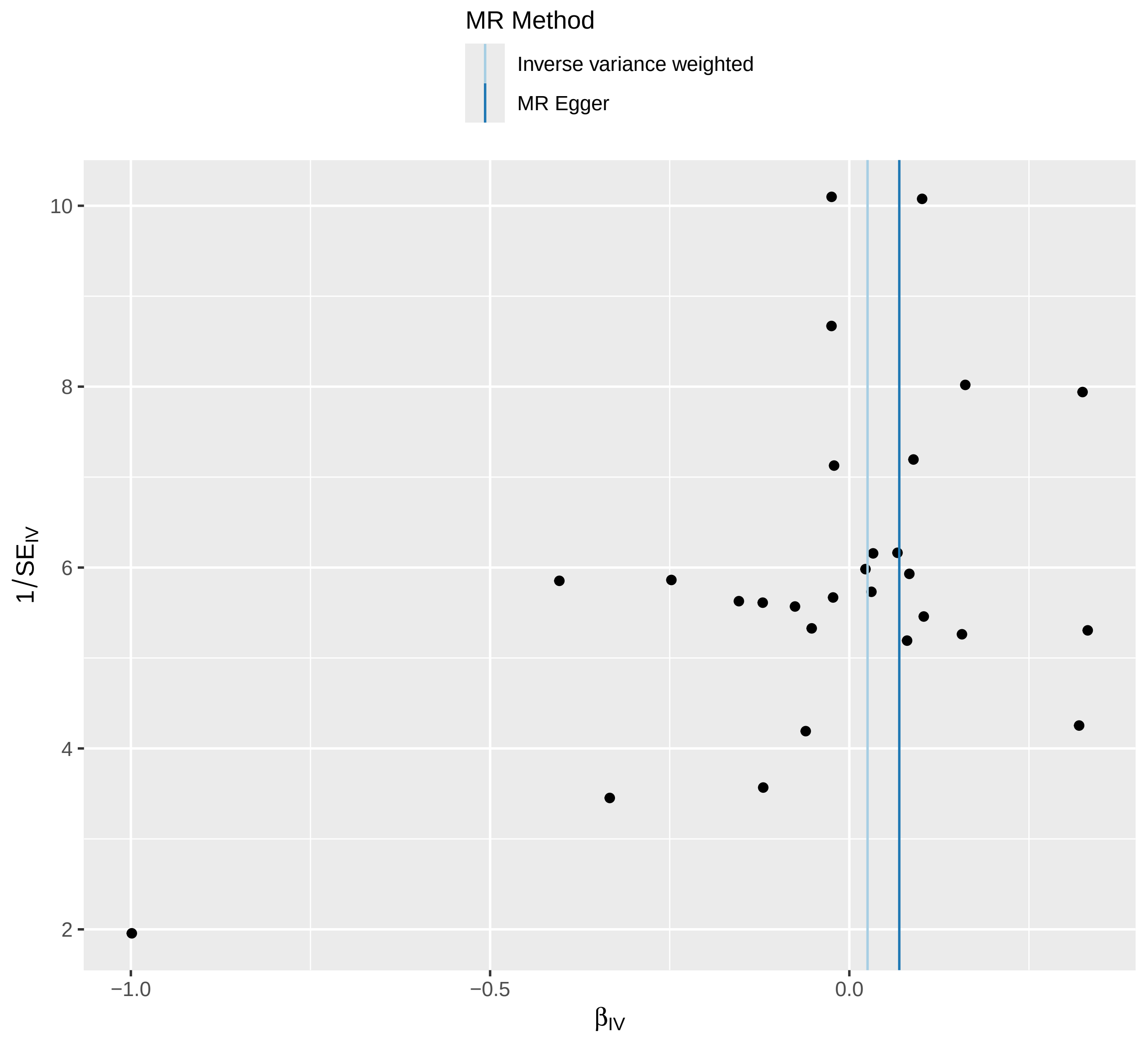

MR Method
Inverse variance weighted
MR Egger
10
8
6
4
2
$1/SE_{IV}$
−1.0
−0.5
0.0
β_{IV}

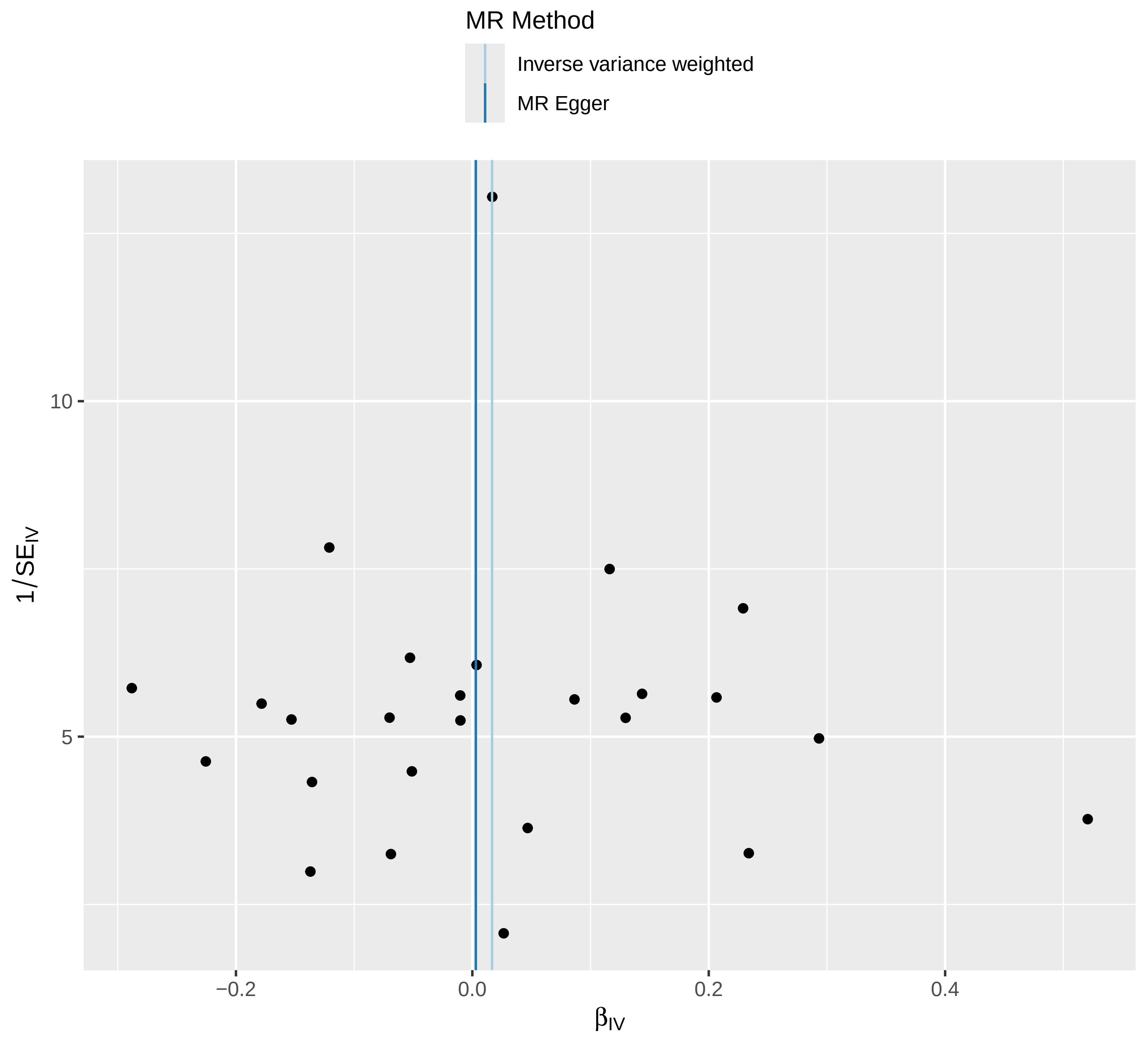

MR Method
Inverse variance weighted
MR Egger
10
5
−0.2
0.0
0.2
0.4
$1/SE_{IV}$
β_{IV}

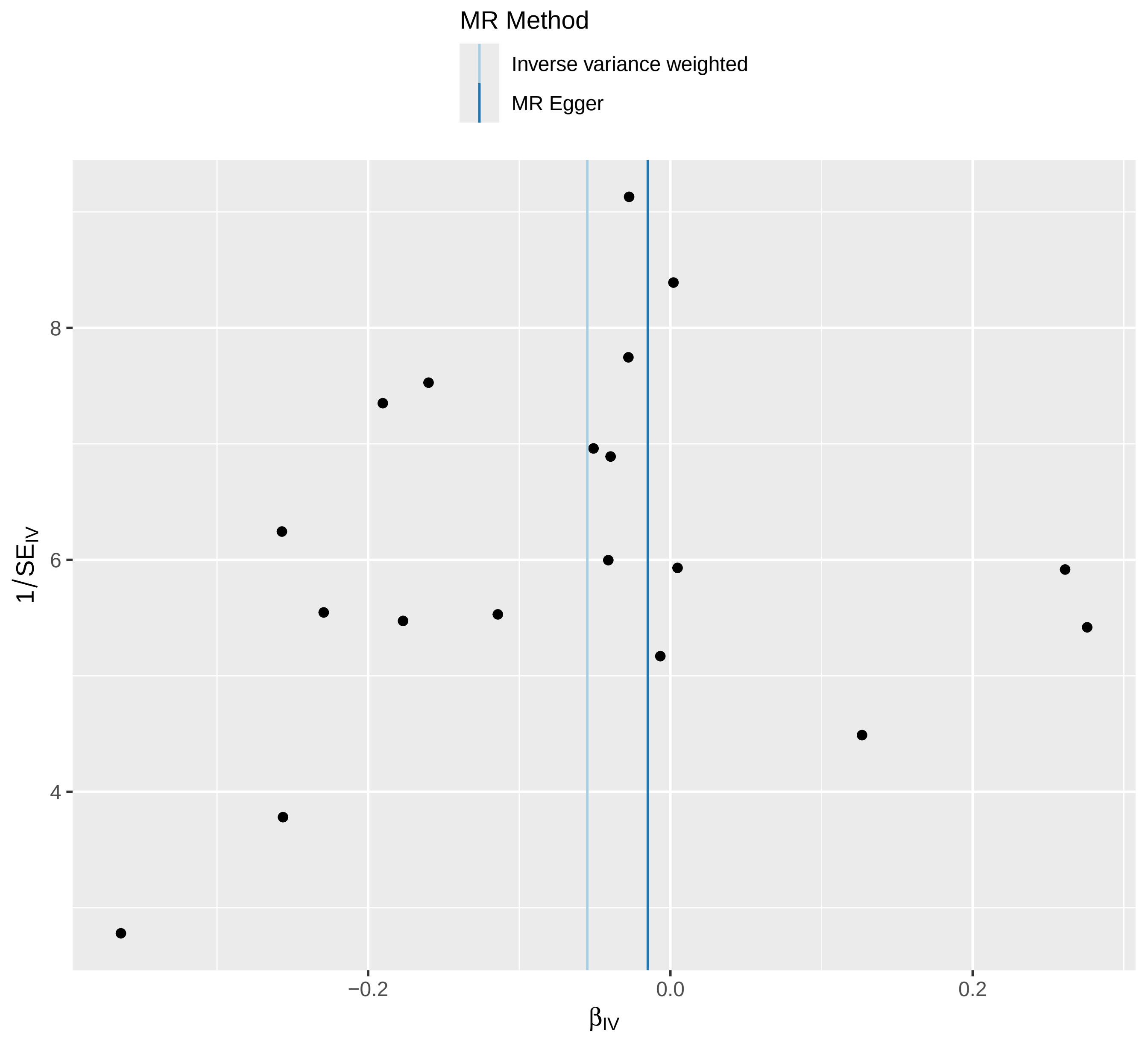

MR Method
Inverse variance weighted
MR Egger
8
6
4
1/SE_IV
−0.2
0.0
0.2
β_IV

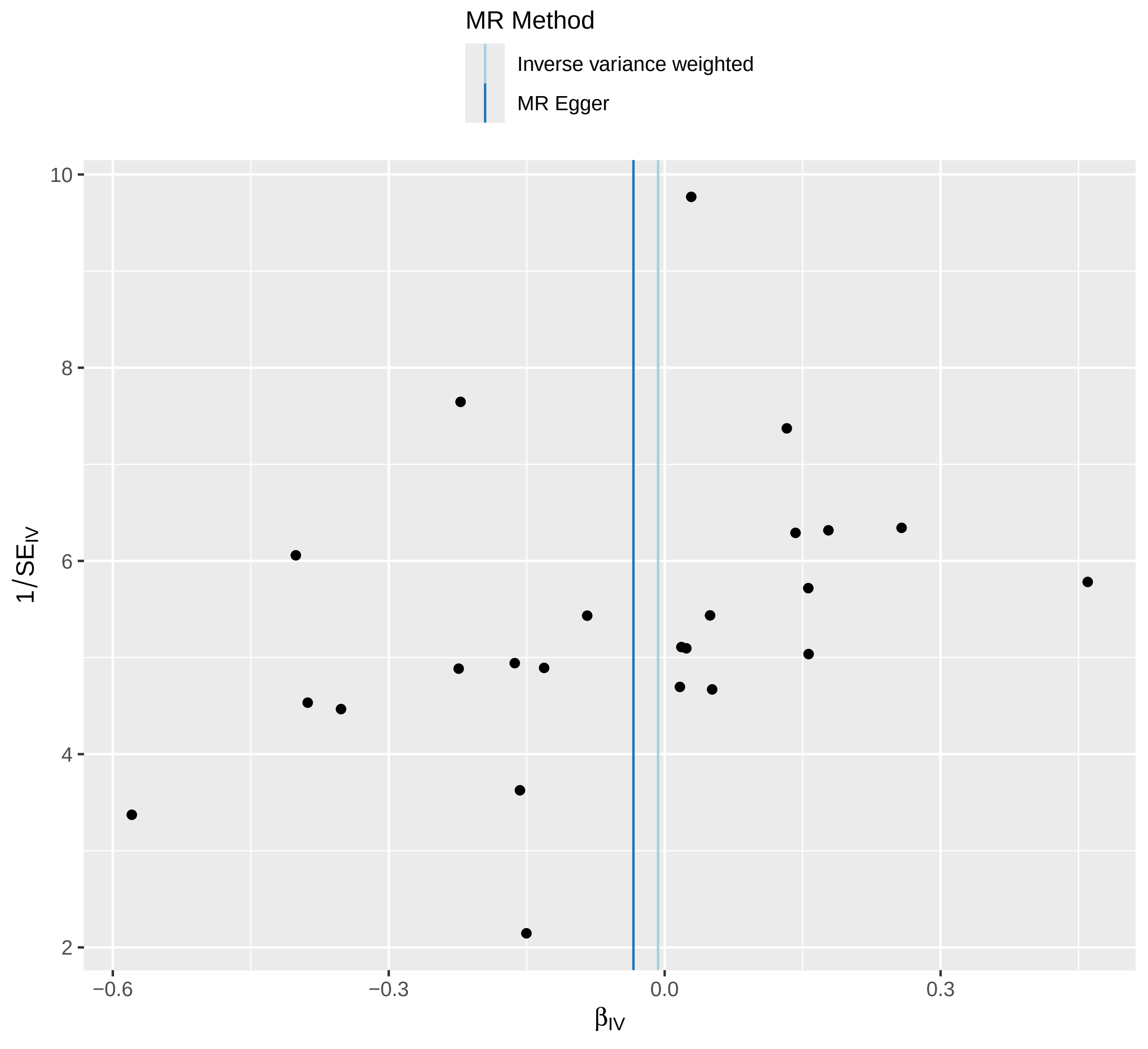

MR Method
Inverse variance weighted
MR Egger
10
8
6
4
2
1/SE_IV
−0.6
−0.3
0.0
0.3
β_IV

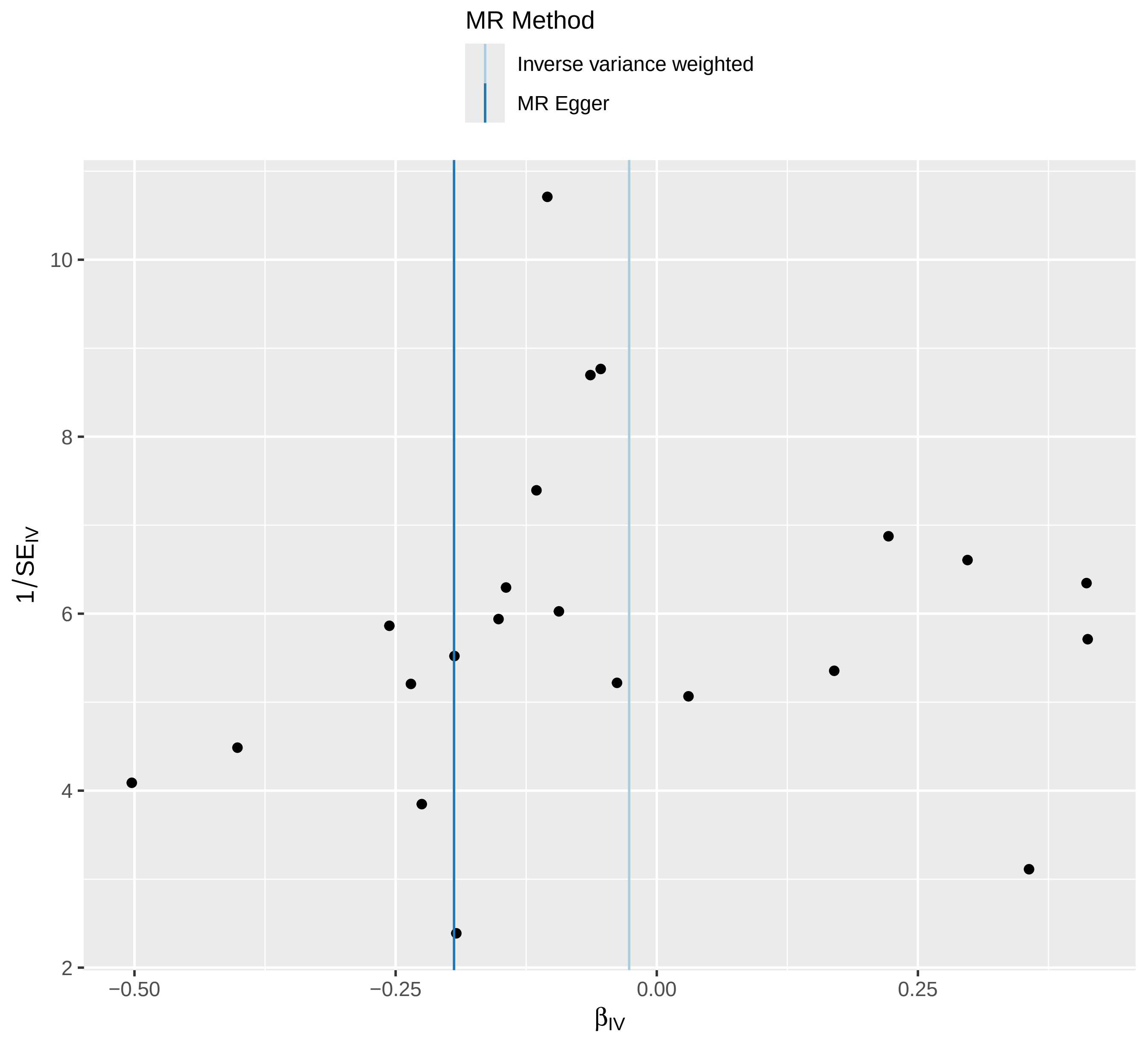
MR Method
Inverse variance weighted
MR Egger
1/SE_IV
10
8
6
4
2
−0.50
−0.25
0.00
0.25
β_IV

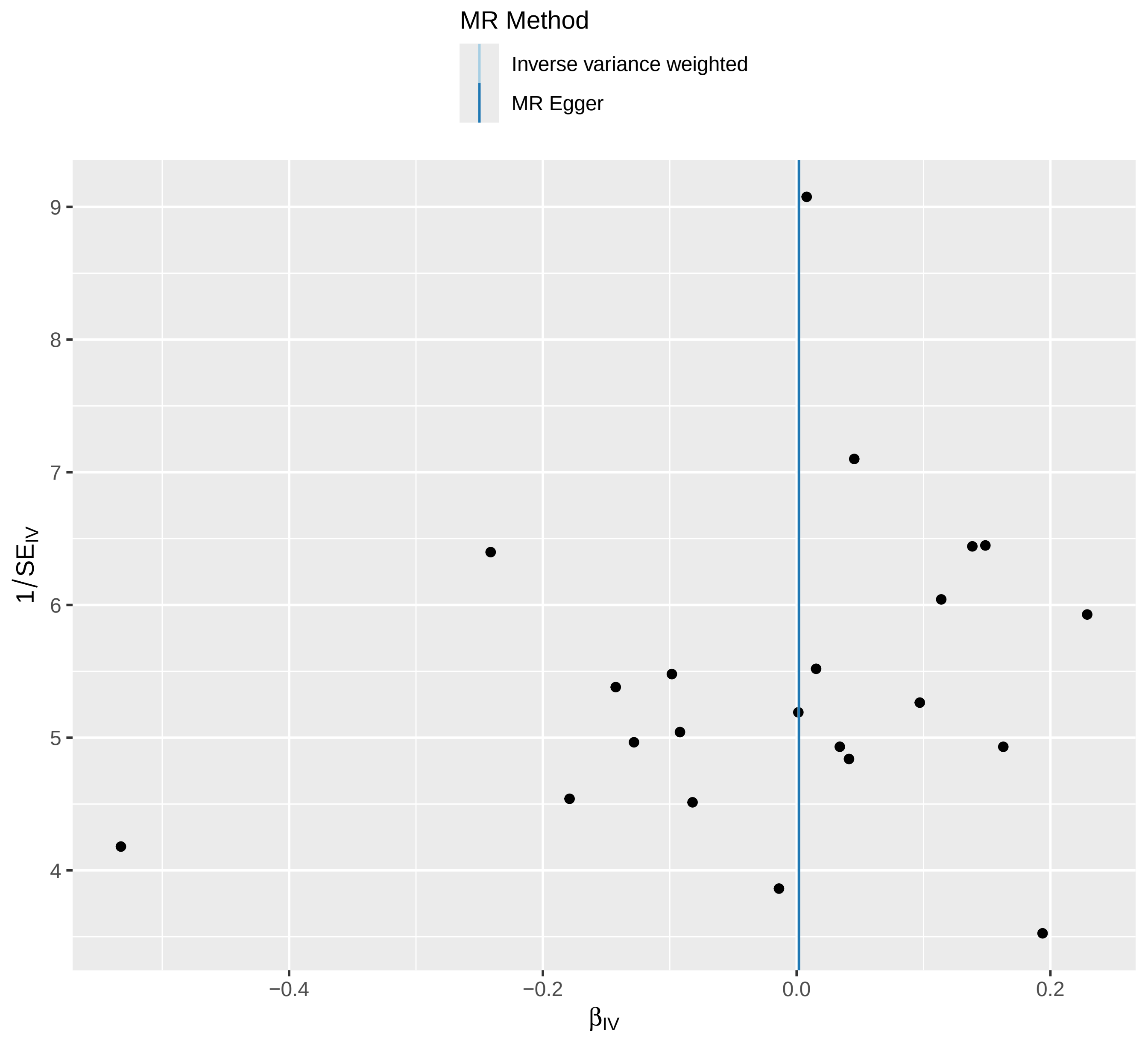

MR Method
Inverse variance weighted
MR Egger
1/SE_{IV}
9
8
7
6
5
4
−0.4
−0.2
0.0
0.2
β_{IV}

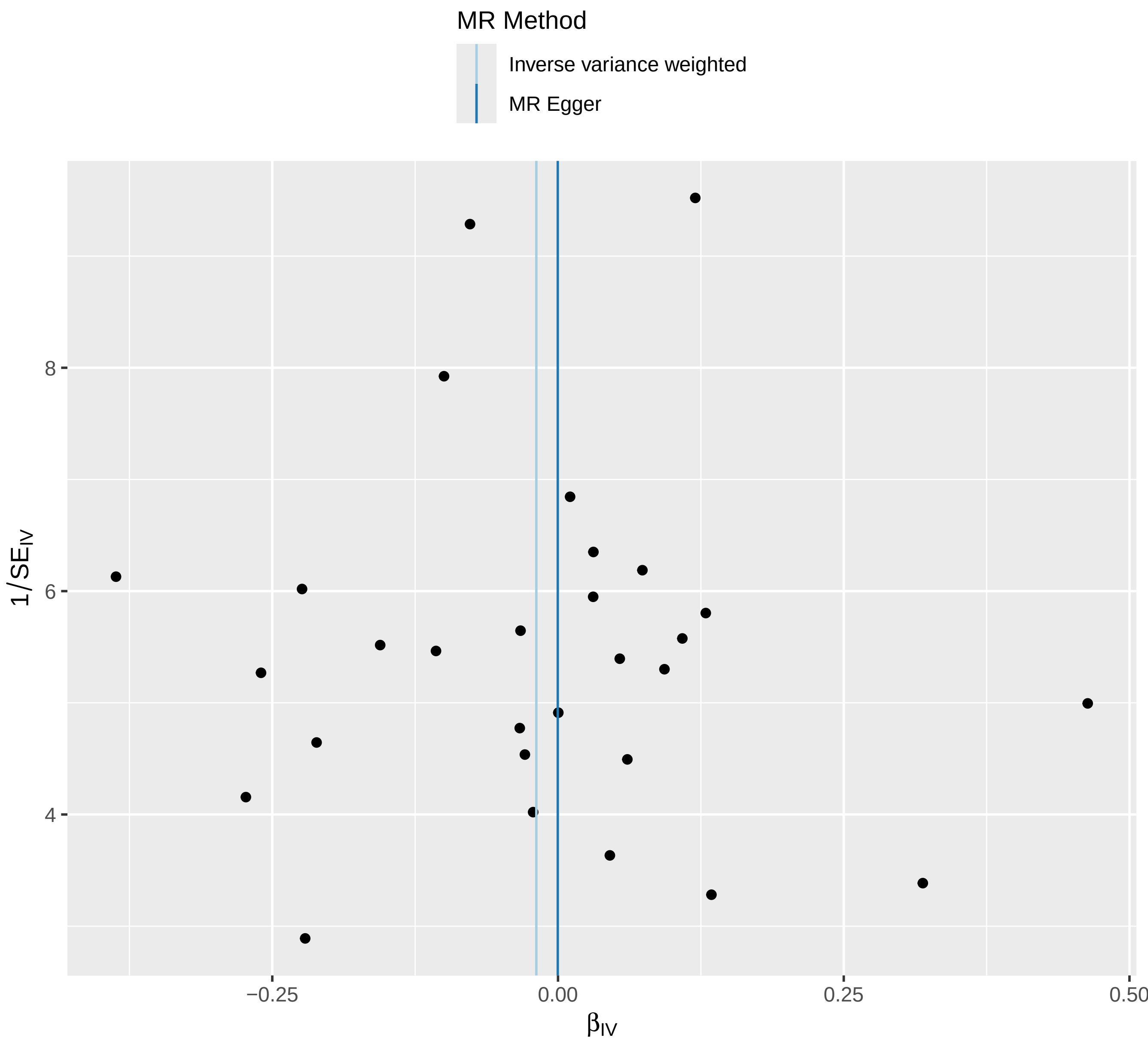
MR Method
Inverse variance weighted
MR Egger
8
6
4
1/SE$_{IV}$
−0.25
0.00
0.25
0.50
β$_{IV}$

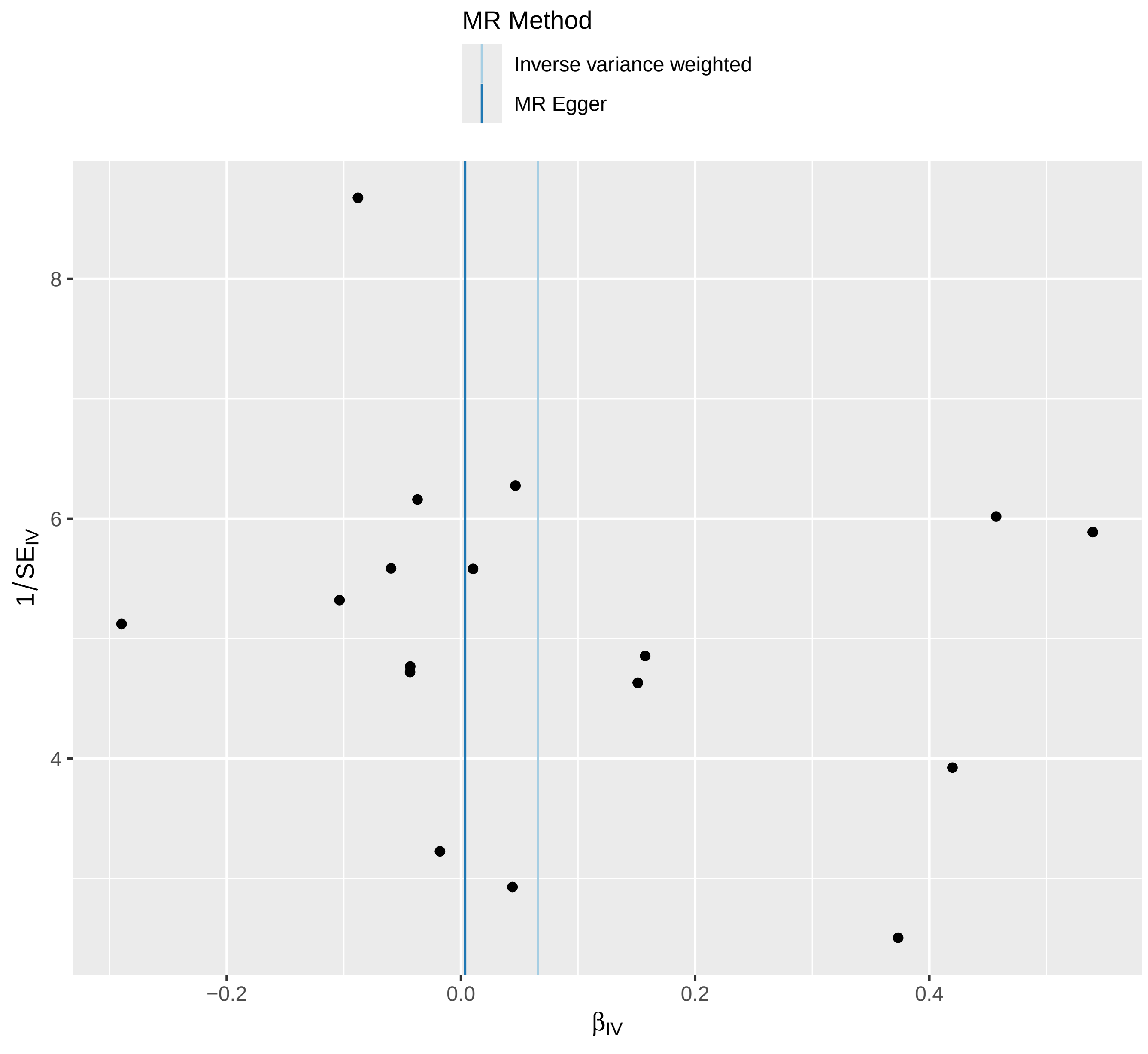

MR Method
Inverse variance weighted
MR Egger
8
6
4
1/SE_IV
−0.2
0.0
0.2
0.4
β_IV

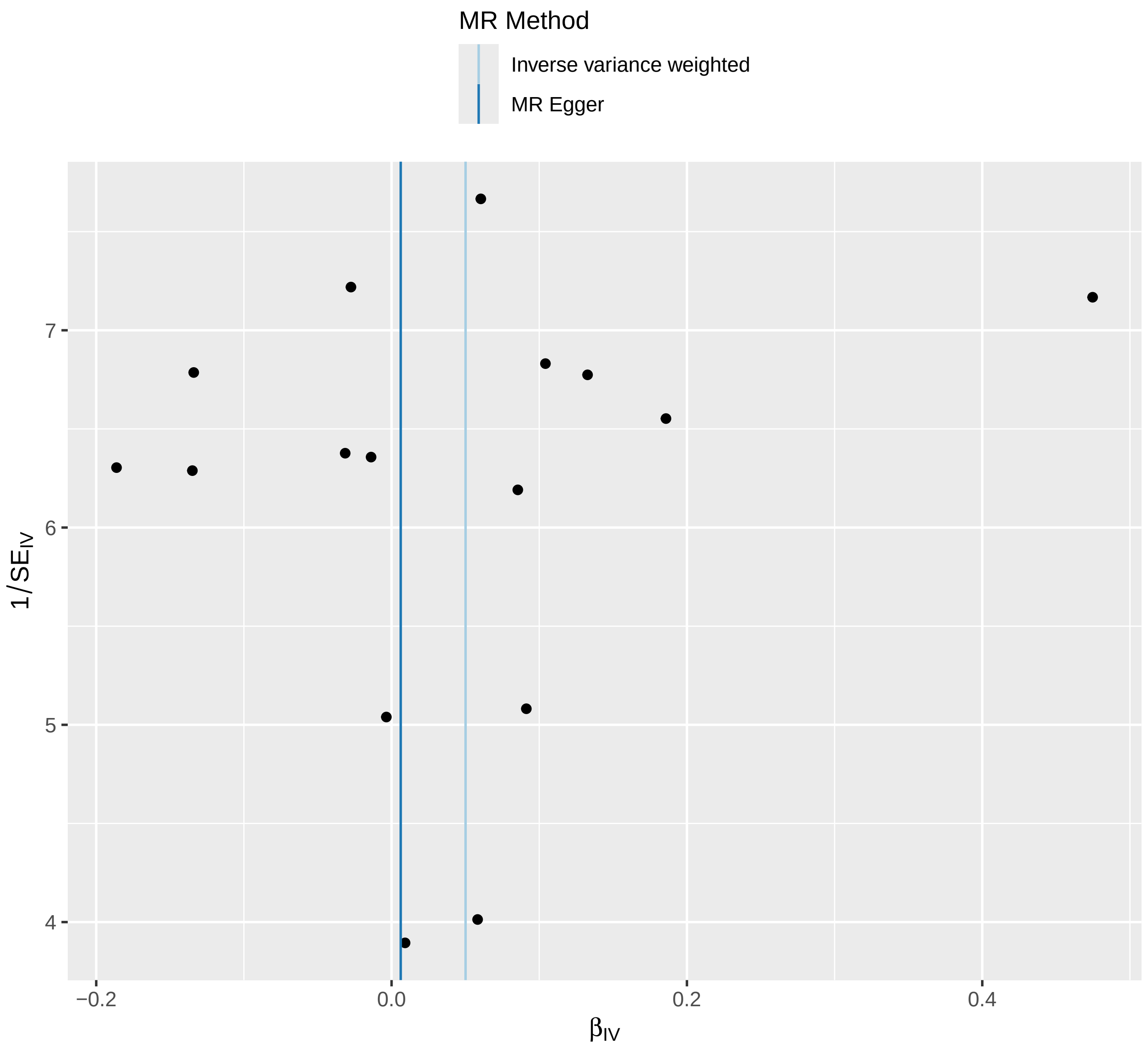

MR Method
Inverse variance weighted
MR Egger
7
6
5
4
1/SE_IV
−0.2
0.0
0.2
0.4
β_IV

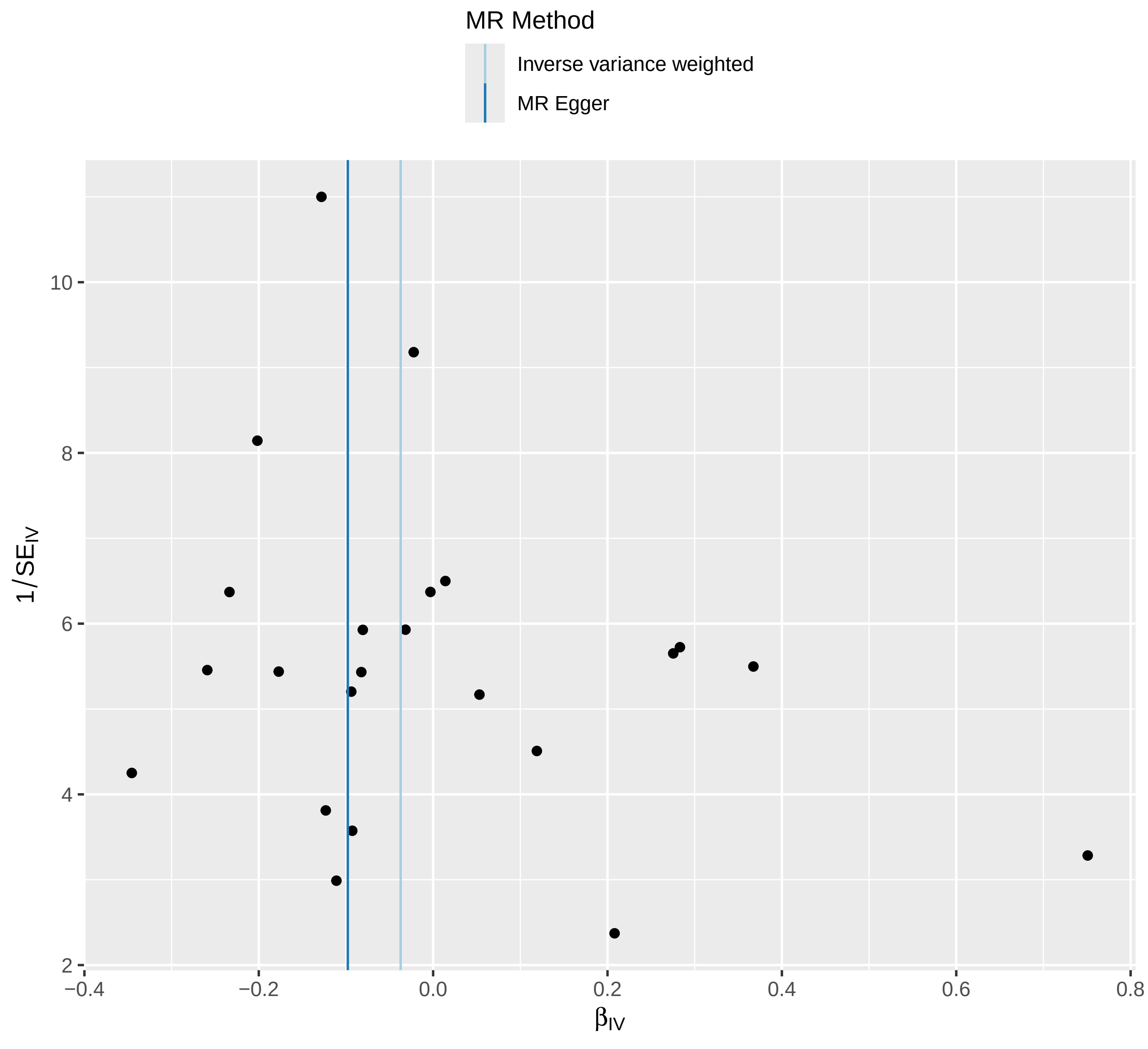
MR Method
Inverse variance weighted
MR Egger
1/SE_{IV}
10
8
6
4
2
−0.4
−0.2
0.0
0.2
0.4
0.6
0.8
β_{IV}

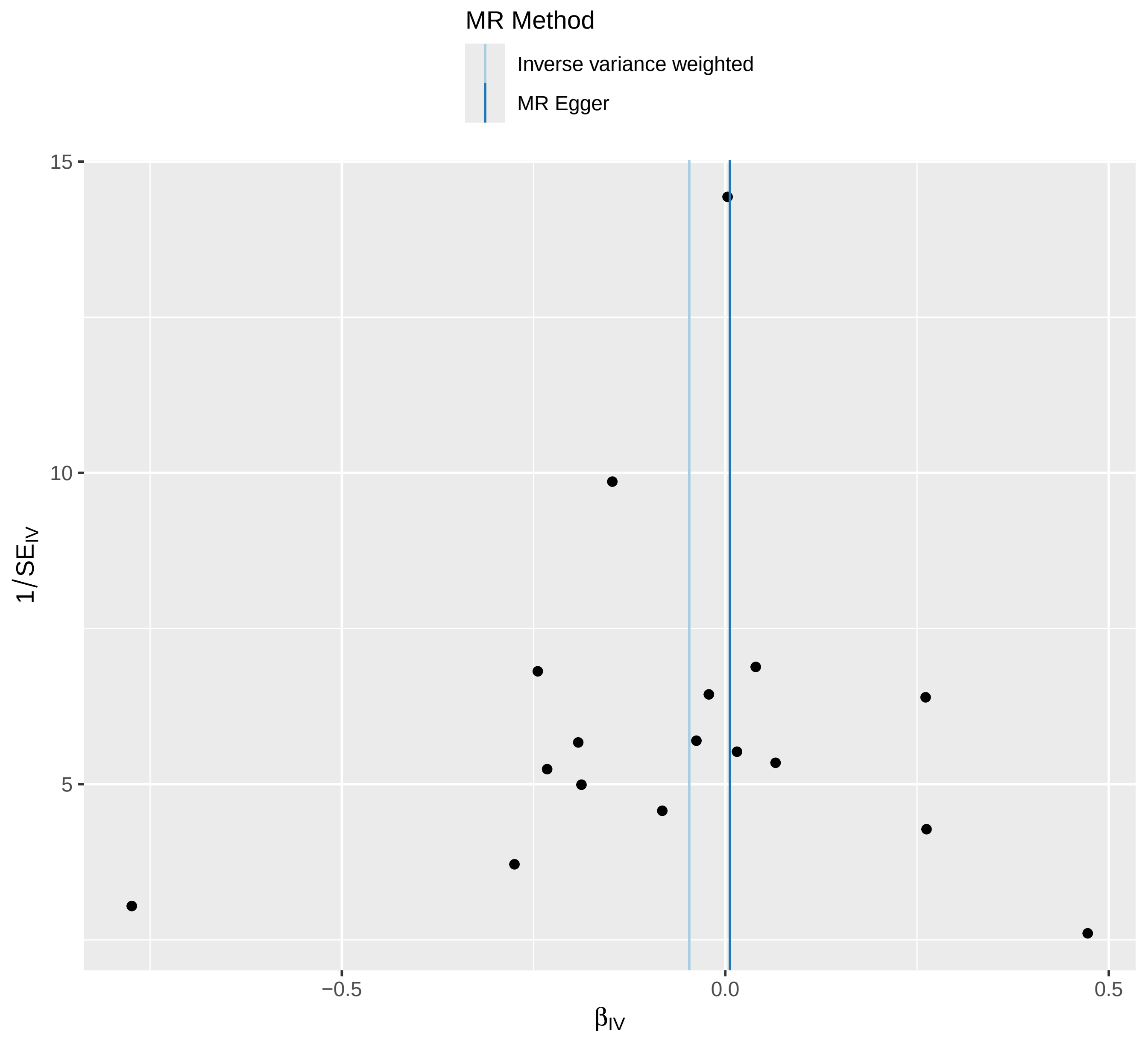
MR Method
Inverse variance weighted
MR Egger
15
10
5
1/SE_IV
−0.5
0.0
0.5
β_IV

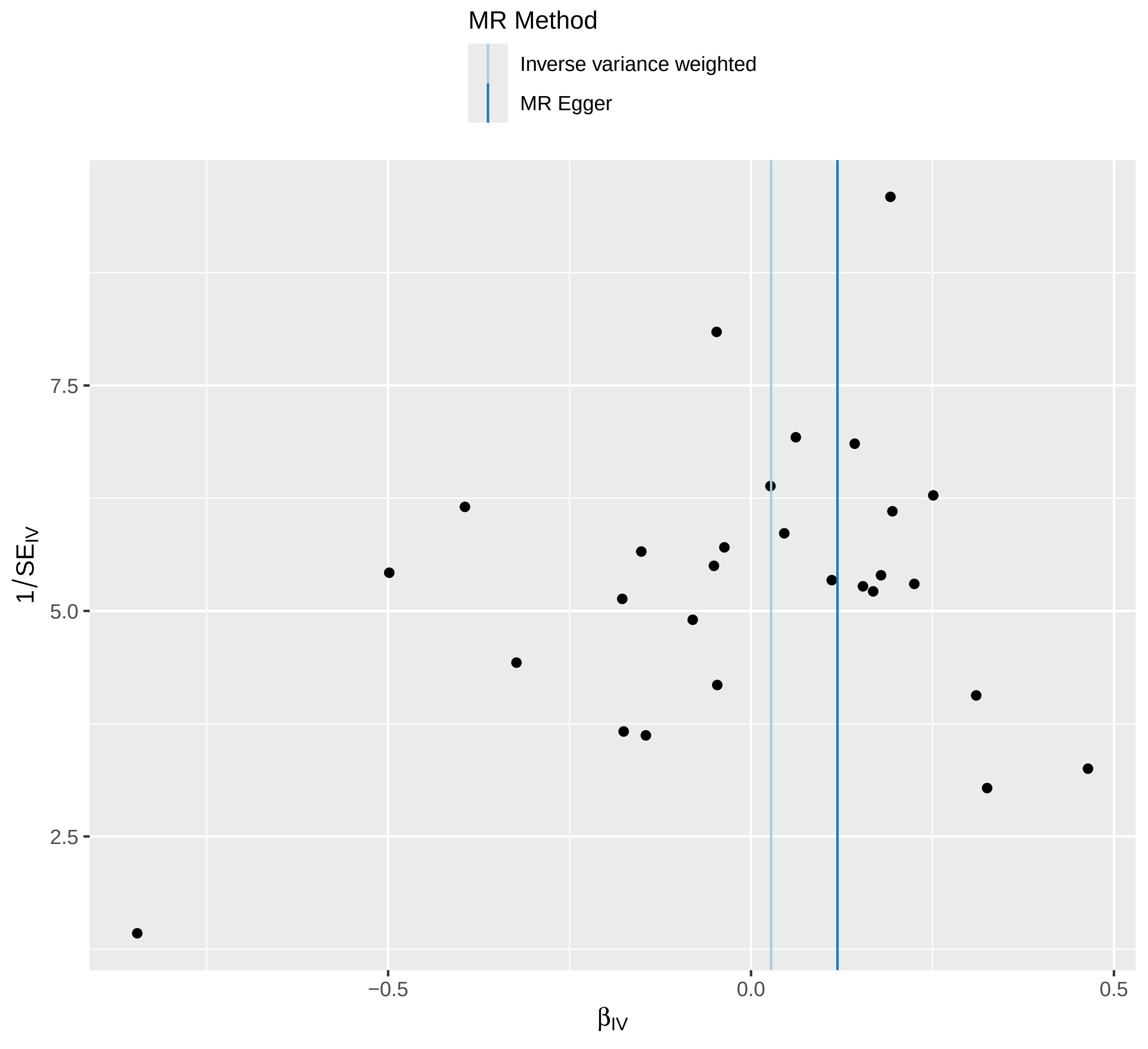
MR Method
Inverse variance weighted
MR Egger
1/SE$_{IV}$
7.5
5.0
2.5
−0.5
0.0
0.5
β$_{IV}$

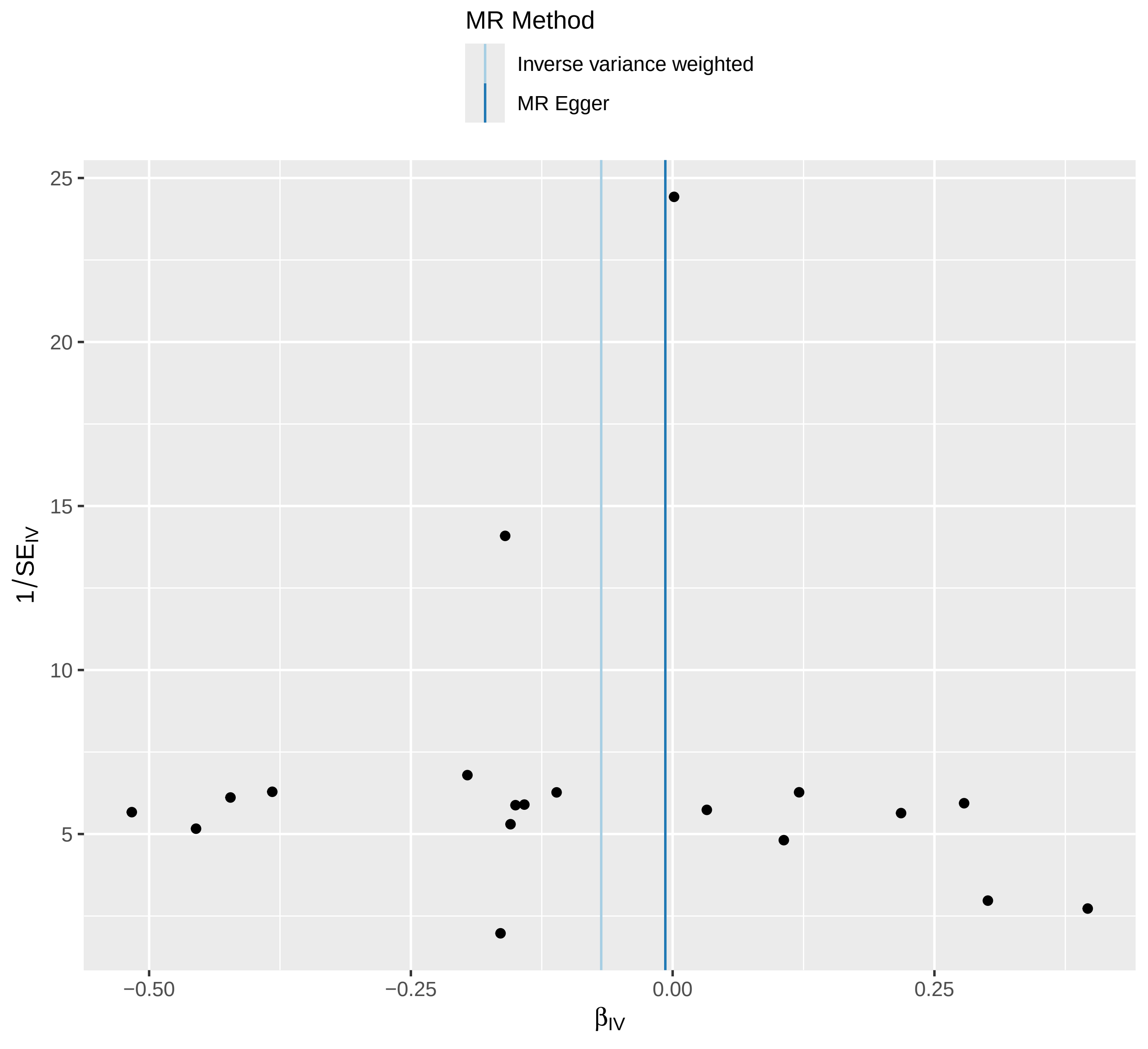
MR Method
Inverse variance weighted
MR Egger
25
20
15
10
5
$1/SE_{IV}$
−0.50
−0.25
0.00
0.25
β_{IV}

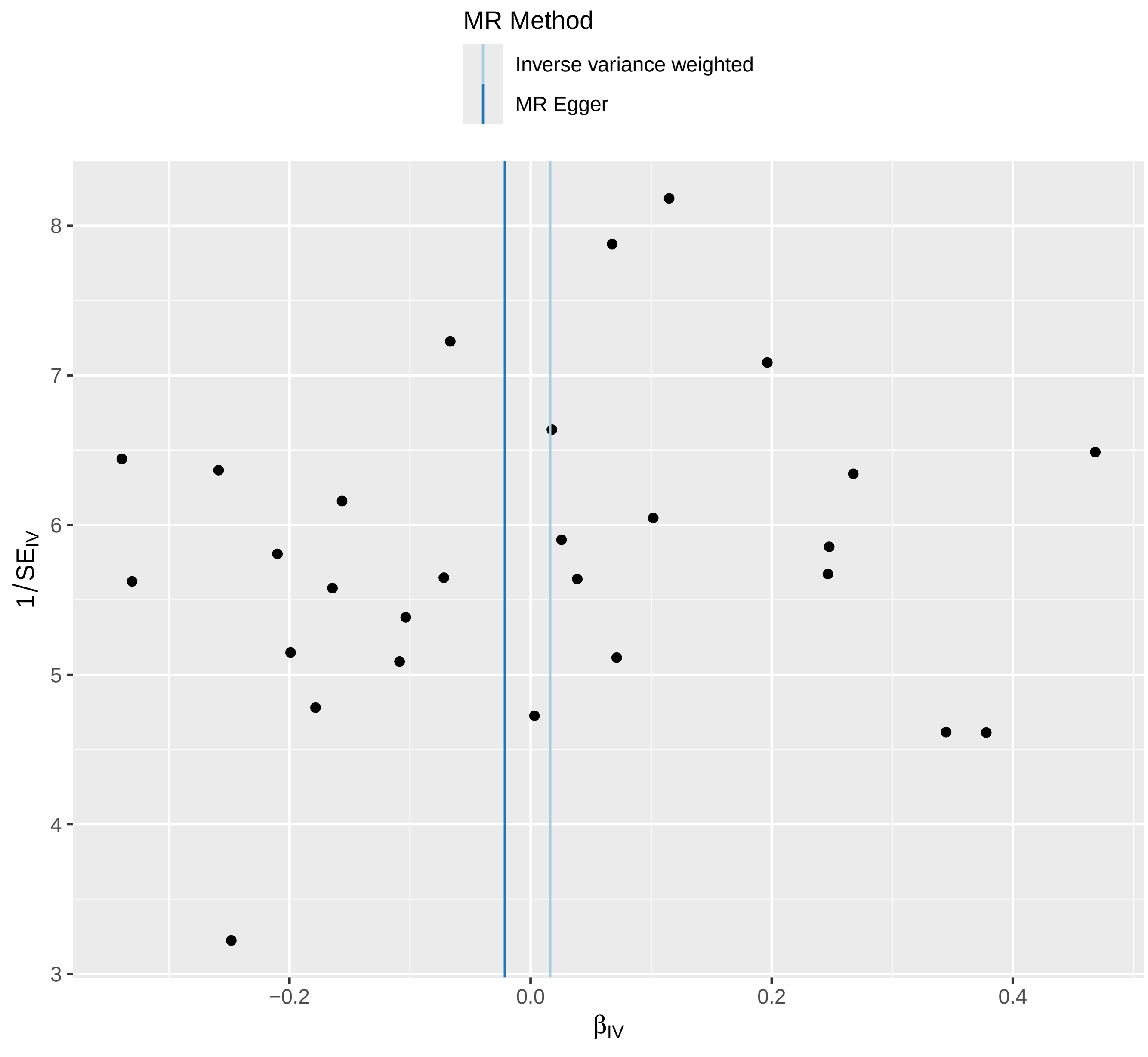

MR Method
Inverse variance weighted
MR Egger
1/SE_IV
8
7
6
5
4
3
−0.2
0.0
0.2
0.4
β_IV

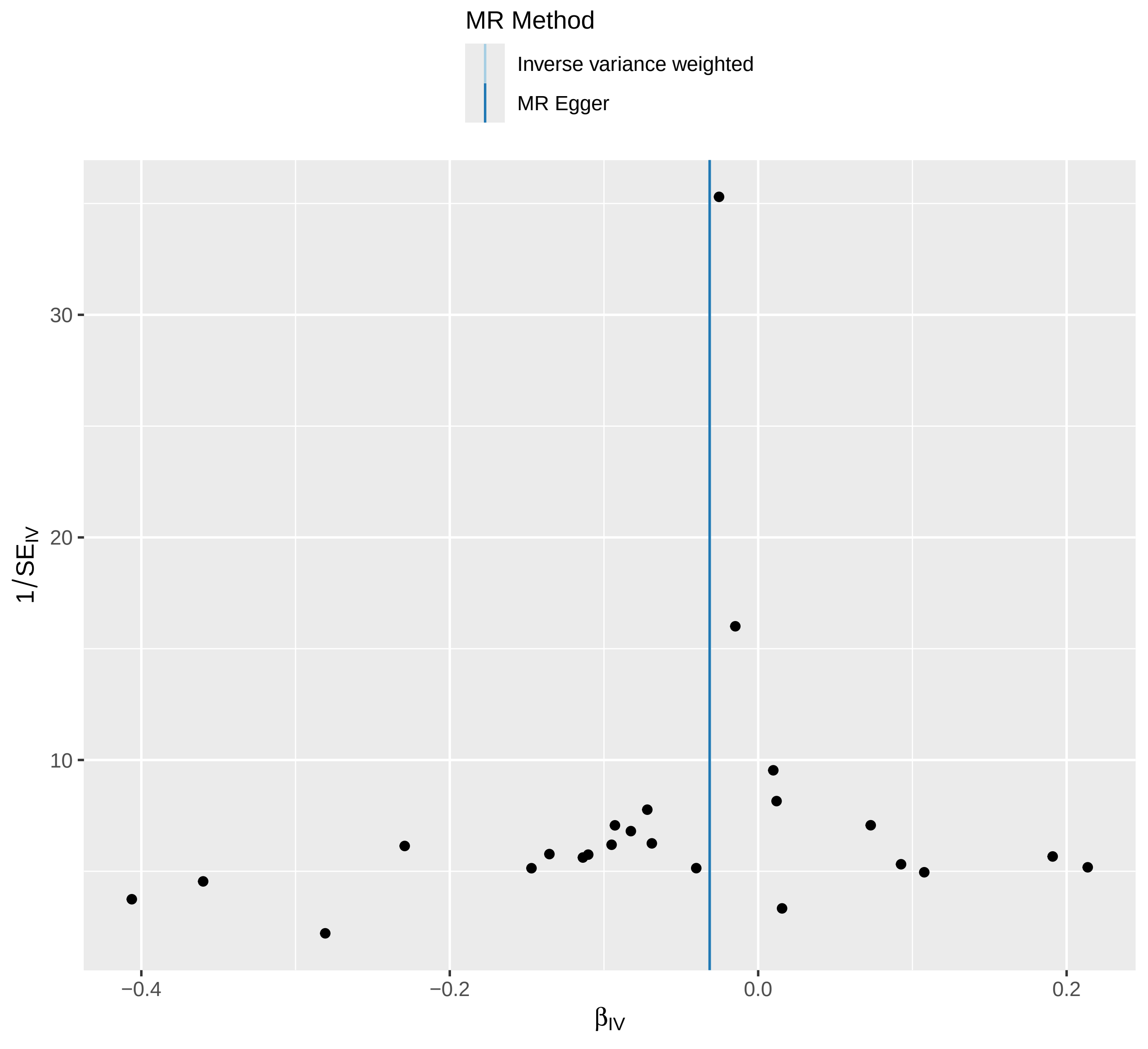
MR Method
Inverse variance weighted
MR Egger
30
20
10
$1/SE_{IV}$
−0.4
−0.2
0.0
0.2
β_{IV}

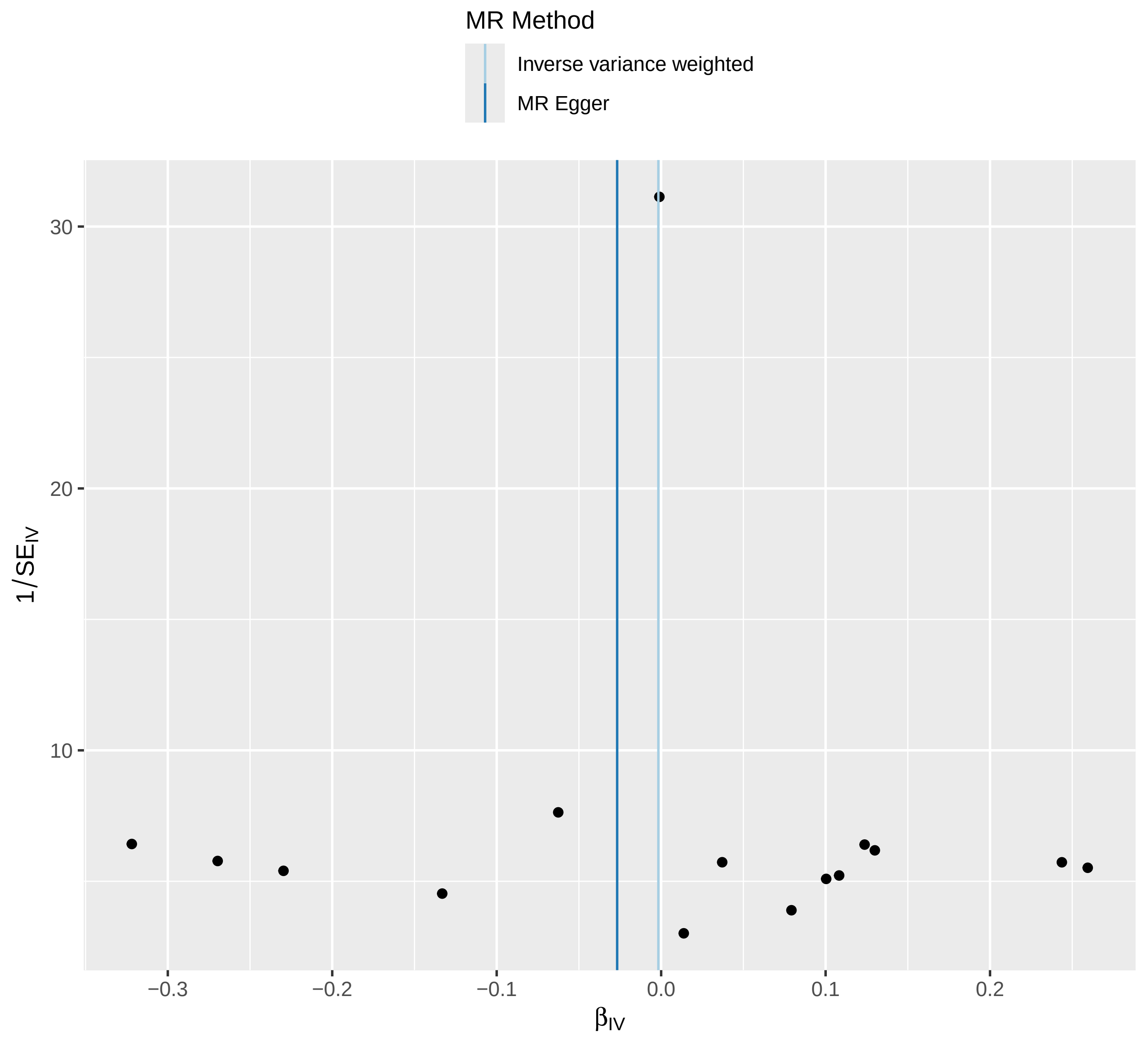
MR Method
Inverse variance weighted
MR Egger
30
20
10
1/SE_IV
−0.3
−0.2
−0.1
0.0
0.1
0.2
β_IV

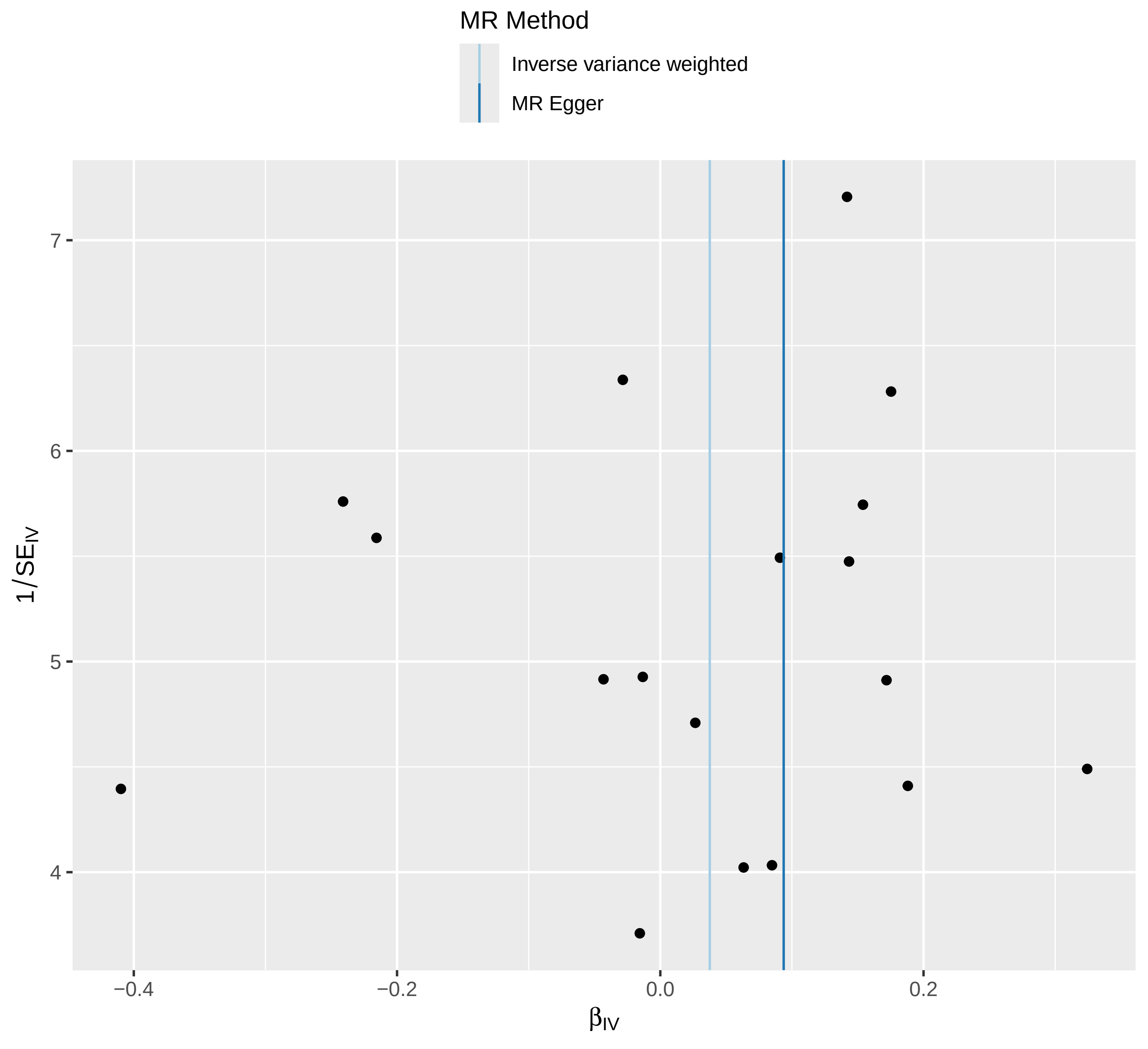
MR Method
Inverse variance weighted
MR Egger
7
6
5
4
1/SE_IV
−0.4
−0.2
0.0
0.2
β_IV

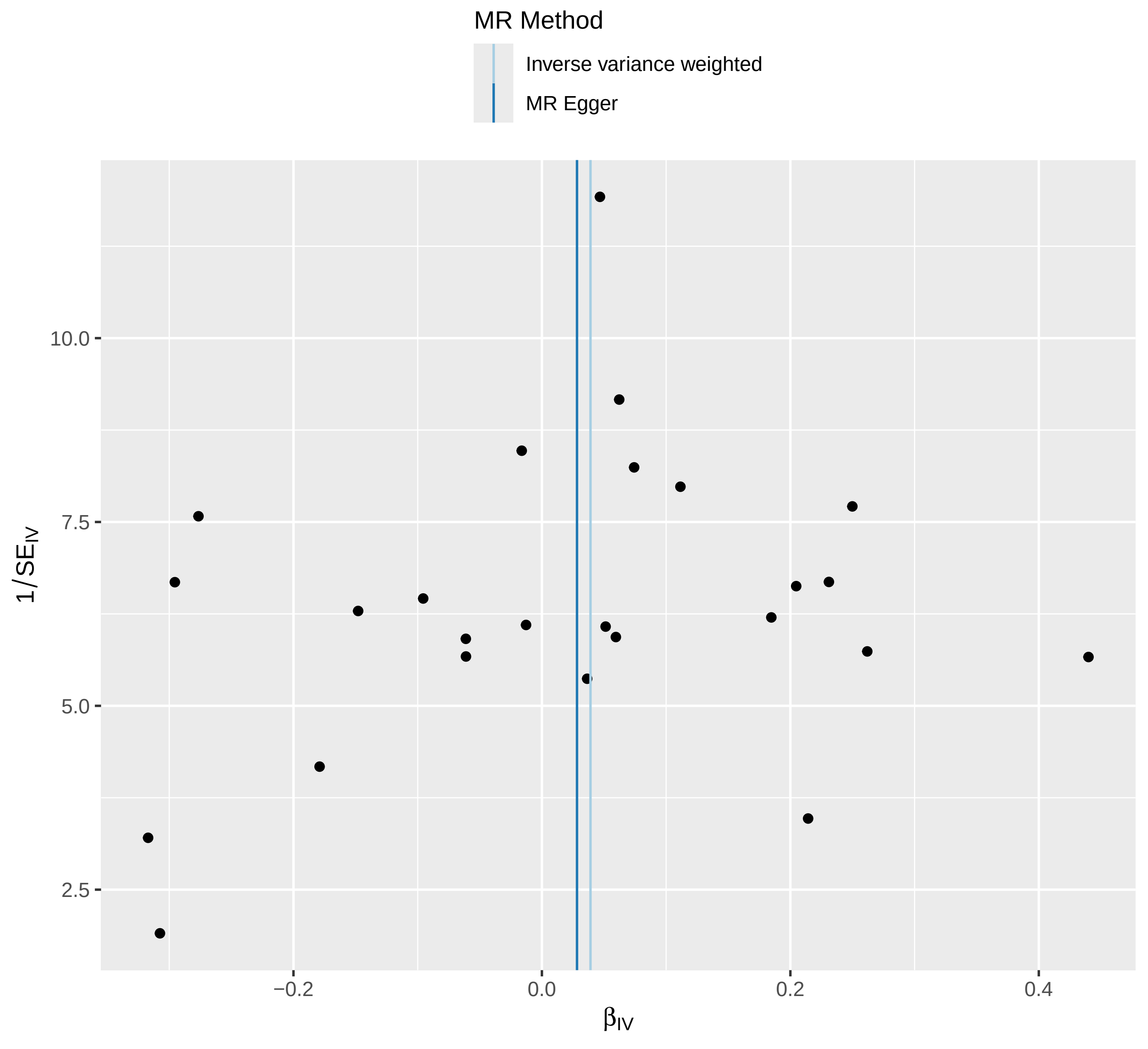
MR Method
Inverse variance weighted
MR Egger
10.0
7.5
5.0
2.5
1/SE_IV
−0.2
0.0
0.2
0.4
β_IV

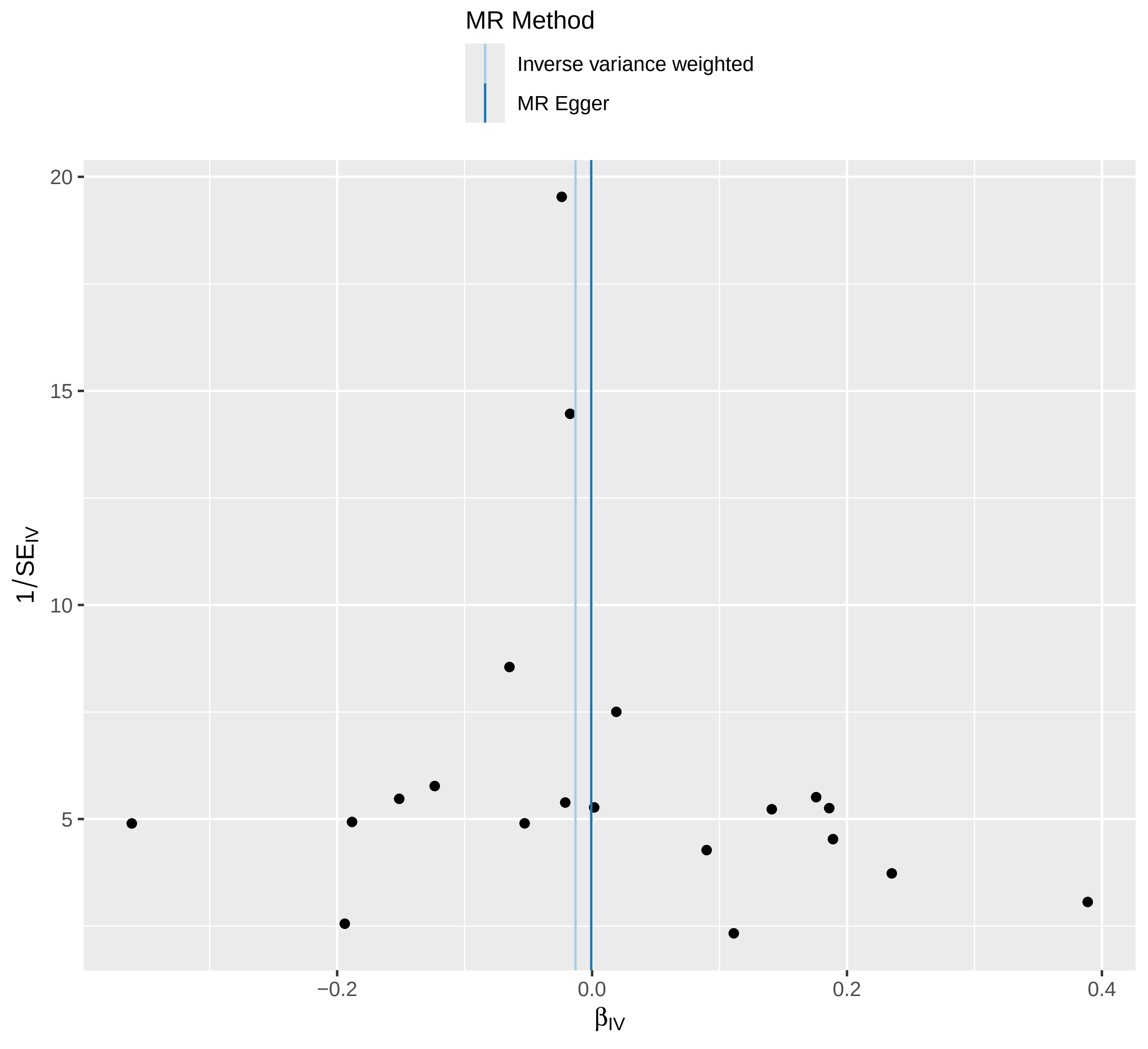
MR Method
Inverse variance weighted
MR Egger
20
15
10
5
1/SE_{IV}
−0.2
0.0
0.2
0.4
β_{IV}

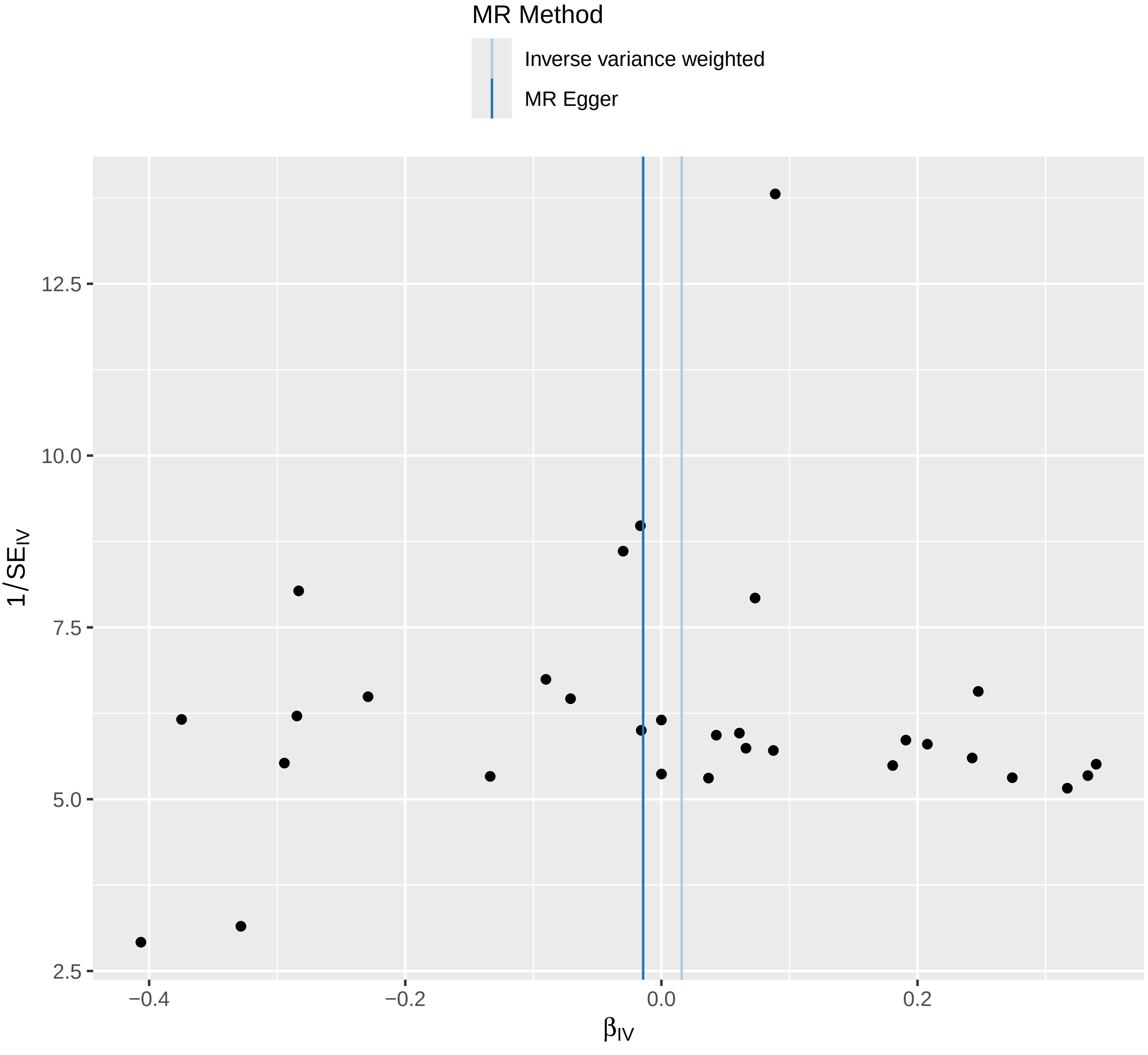
MR Method
Inverse variance weighted
MR Egger
12.5
10.0
7.5
5.0
2.5
$1/SE_{IV}$
−0.4
−0.2
0.0
0.2
β_{IV}

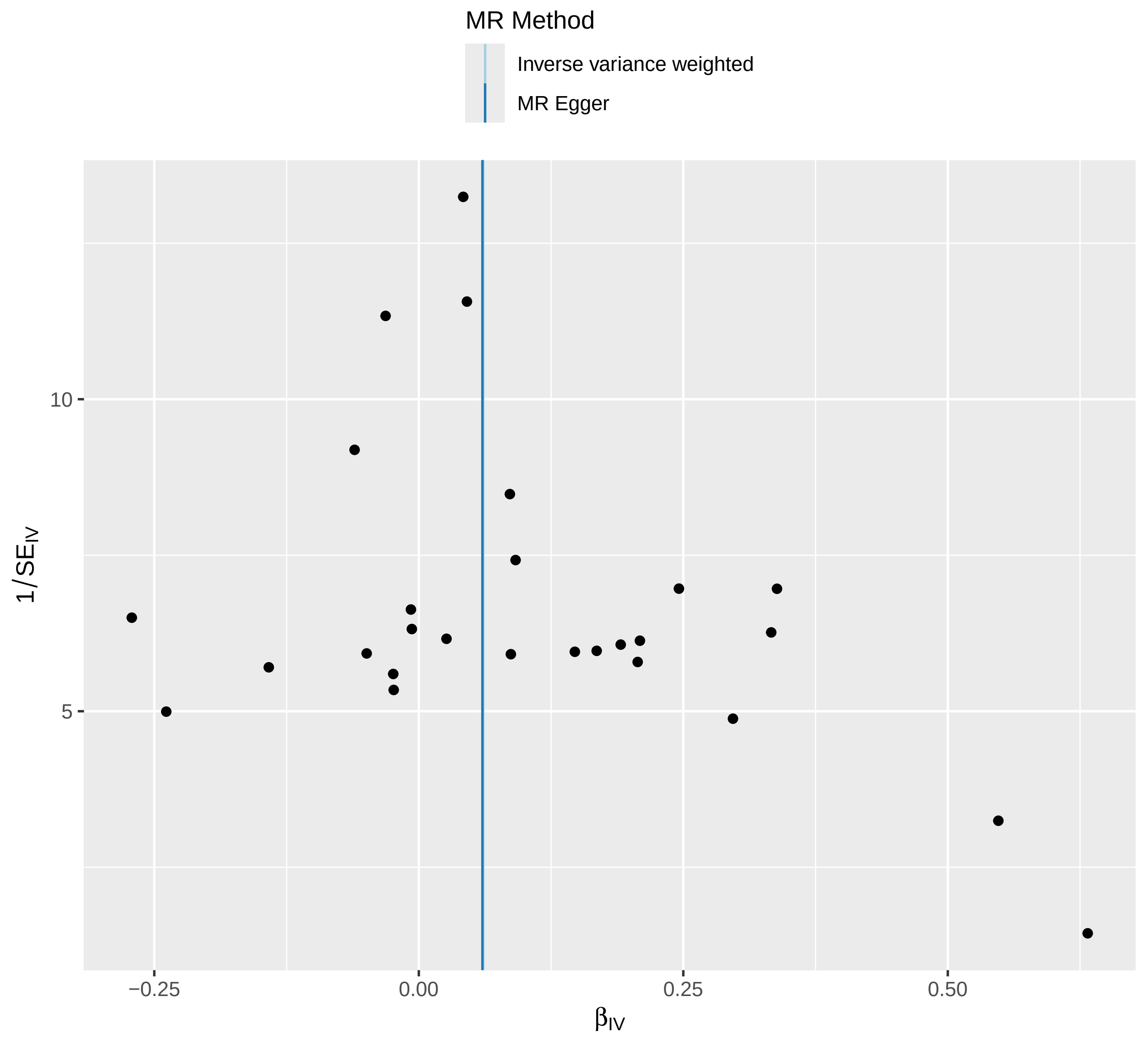
MR Method
Inverse variance weighted
MR Egger
1/SE_IV
10
5
−0.25
0.00
0.25
0.50
β_IV

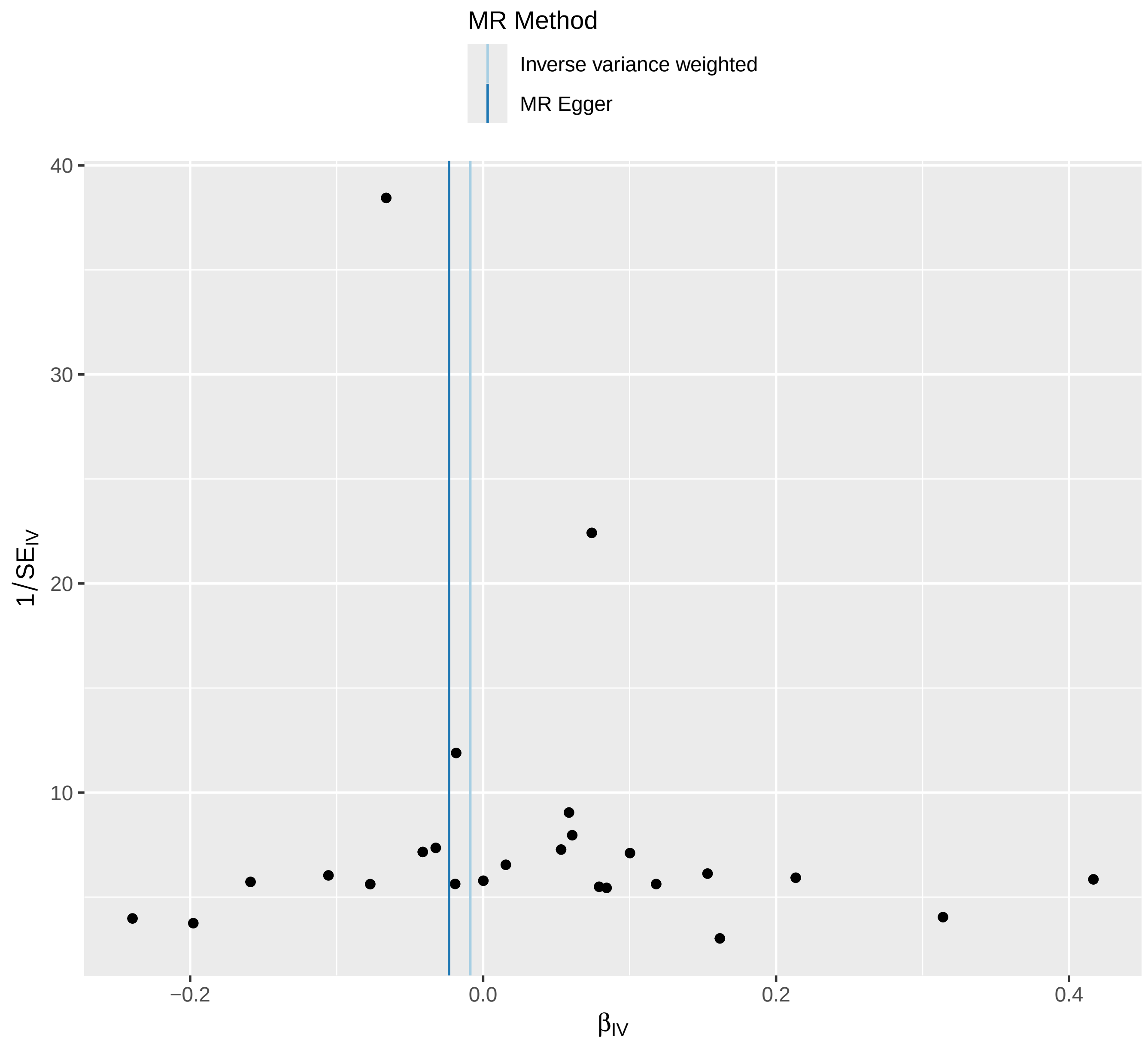
MR Method
Inverse variance weighted
MR Egger
1/SE_IV
β_IV
40
30
20
10
−0.2
0.0
0.2
0.4

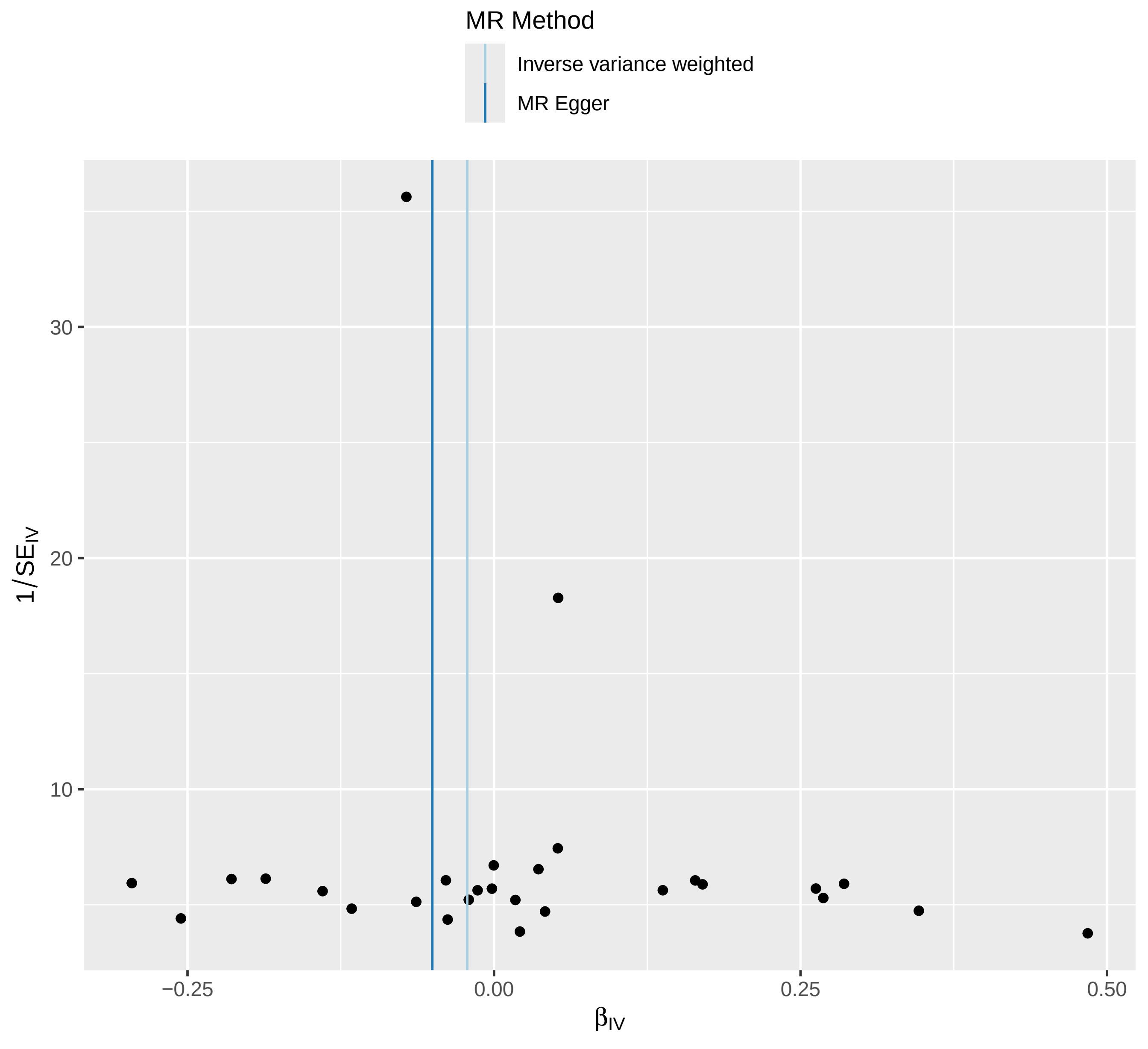

MR Method
Inverse variance weighted
MR Egger
1/SE_IV
30
20
10
−0.25
0.00
0.25
0.50
β_IV

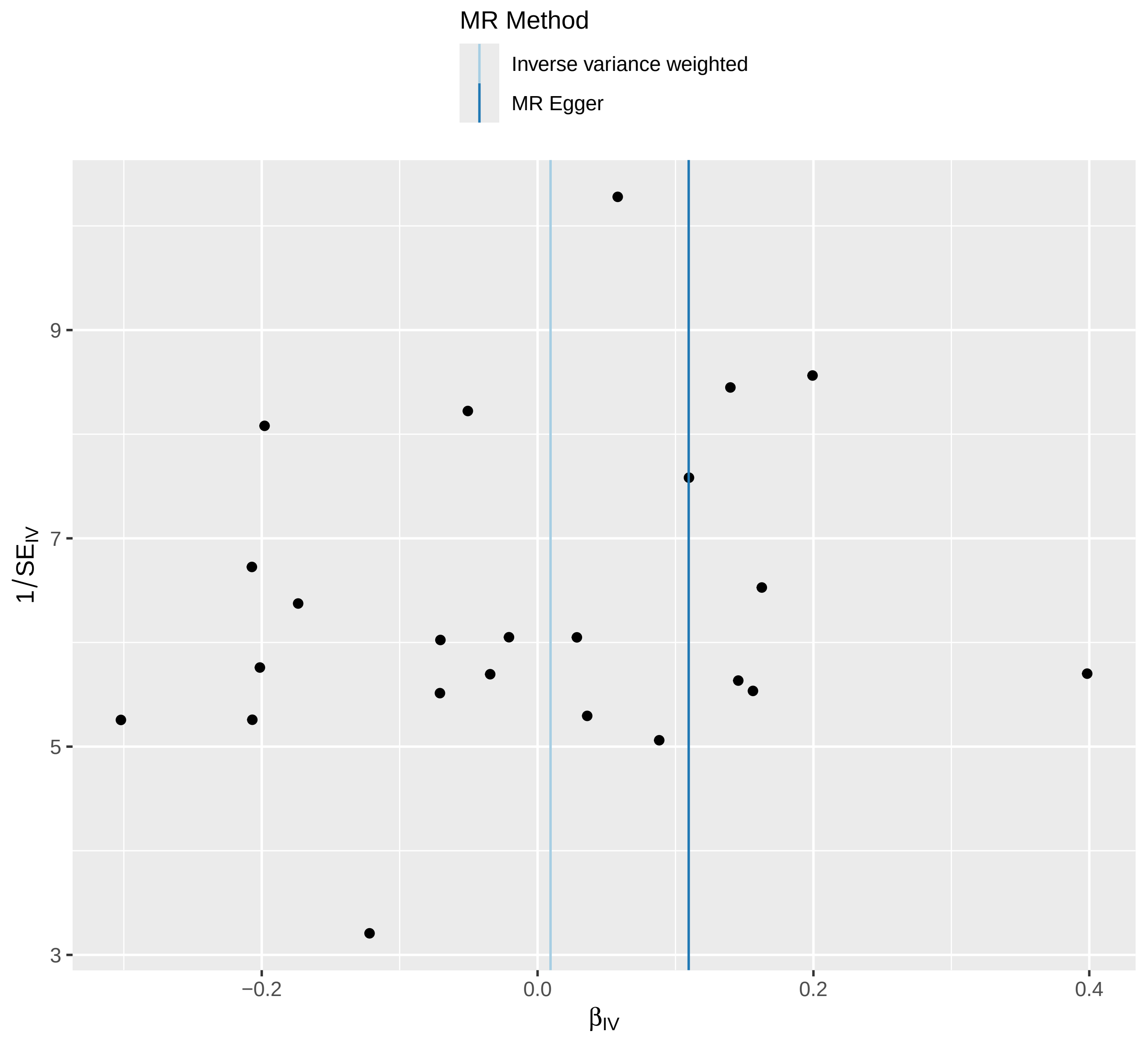

MR Method
Inverse variance weighted
MR Egger
9
7
5
3
1/SE_IV
−0.2
0.0
0.2
0.4
β_IV

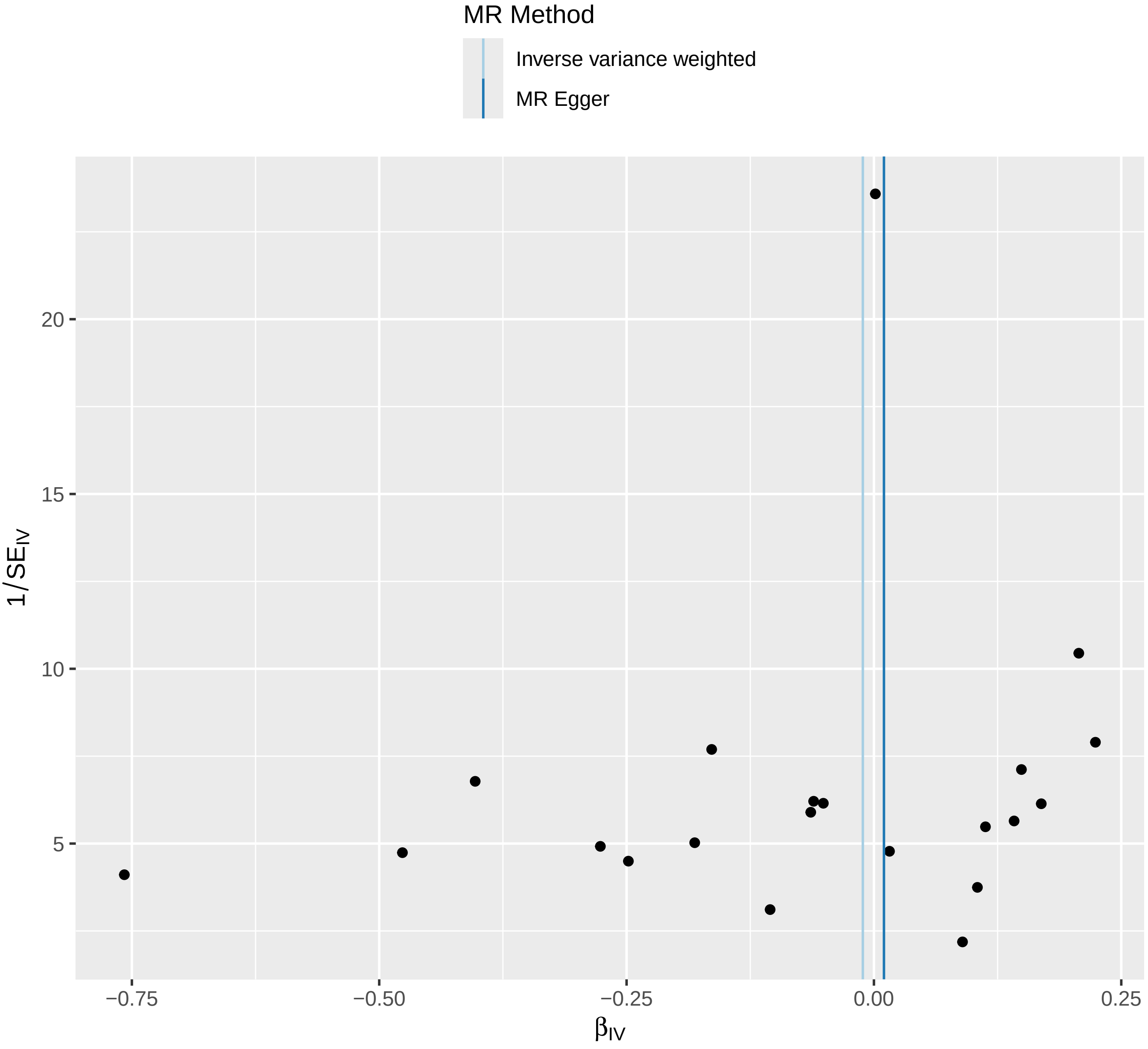

MR Method
Inverse variance weighted
MR Egger
1/SE_IV
20
15
10
5
−0.75
−0.50
−0.25
0.00
0.25
β_IV

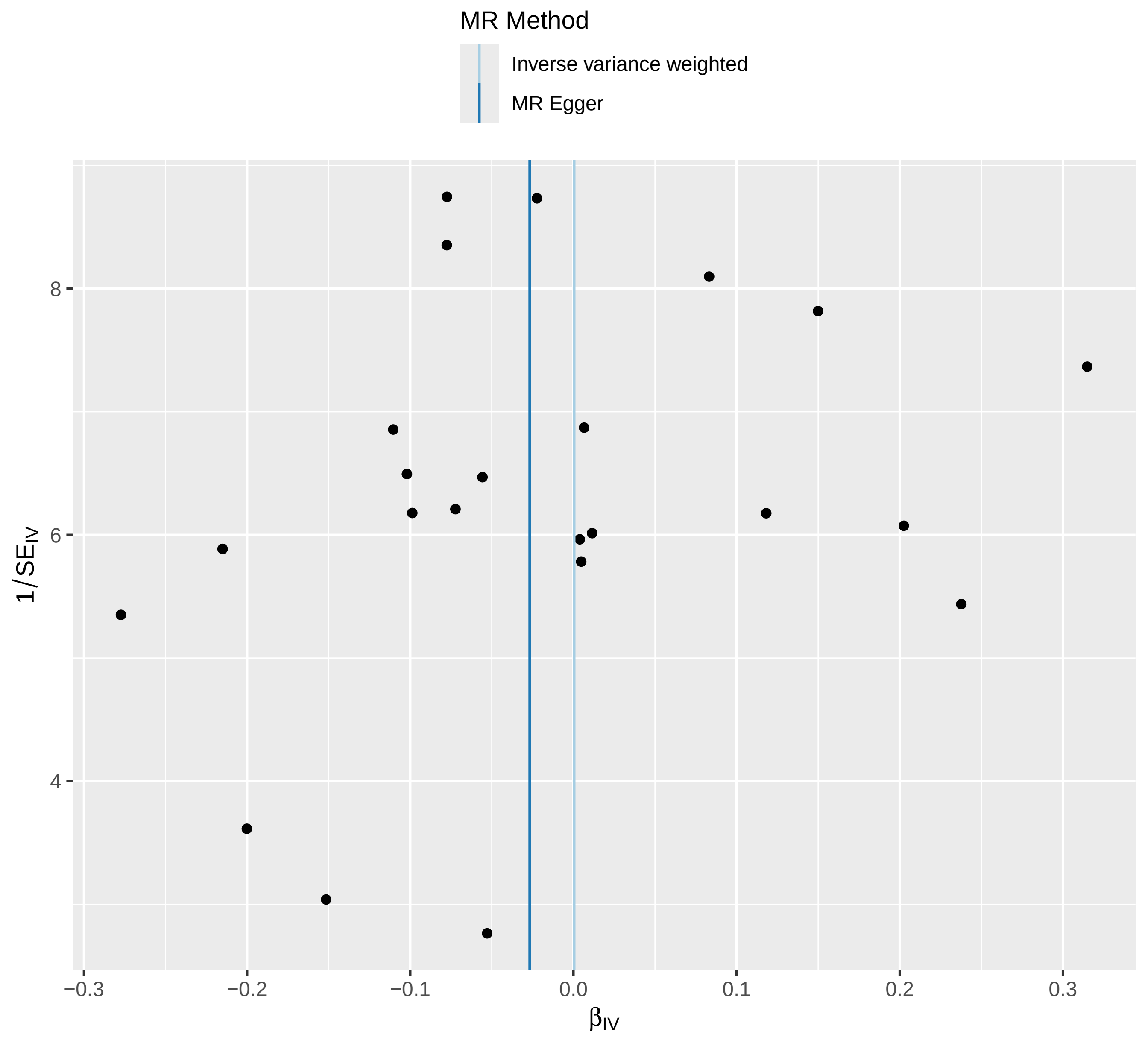
MR Method
Inverse variance weighted
MR Egger
8
6
4
$1/SE_{IV}$
−0.3
−0.2
−0.1
0.0
0.1
0.2
0.3
β_{IV}

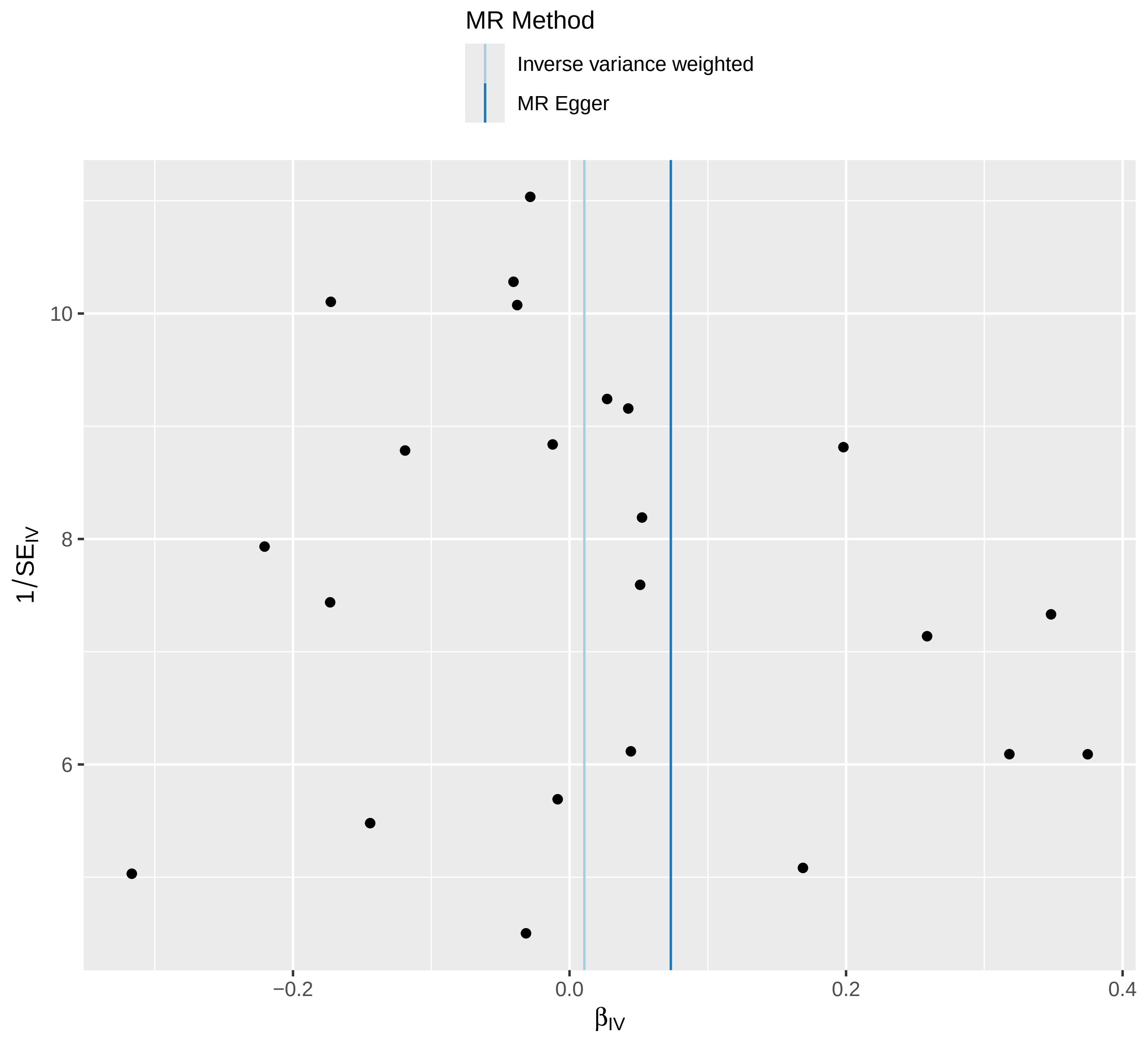
MR Method
Inverse variance weighted
MR Egger
10
8
6
1/SE$_{IV}$
−0.2
0.0
0.2
0.4
β$_{IV}$

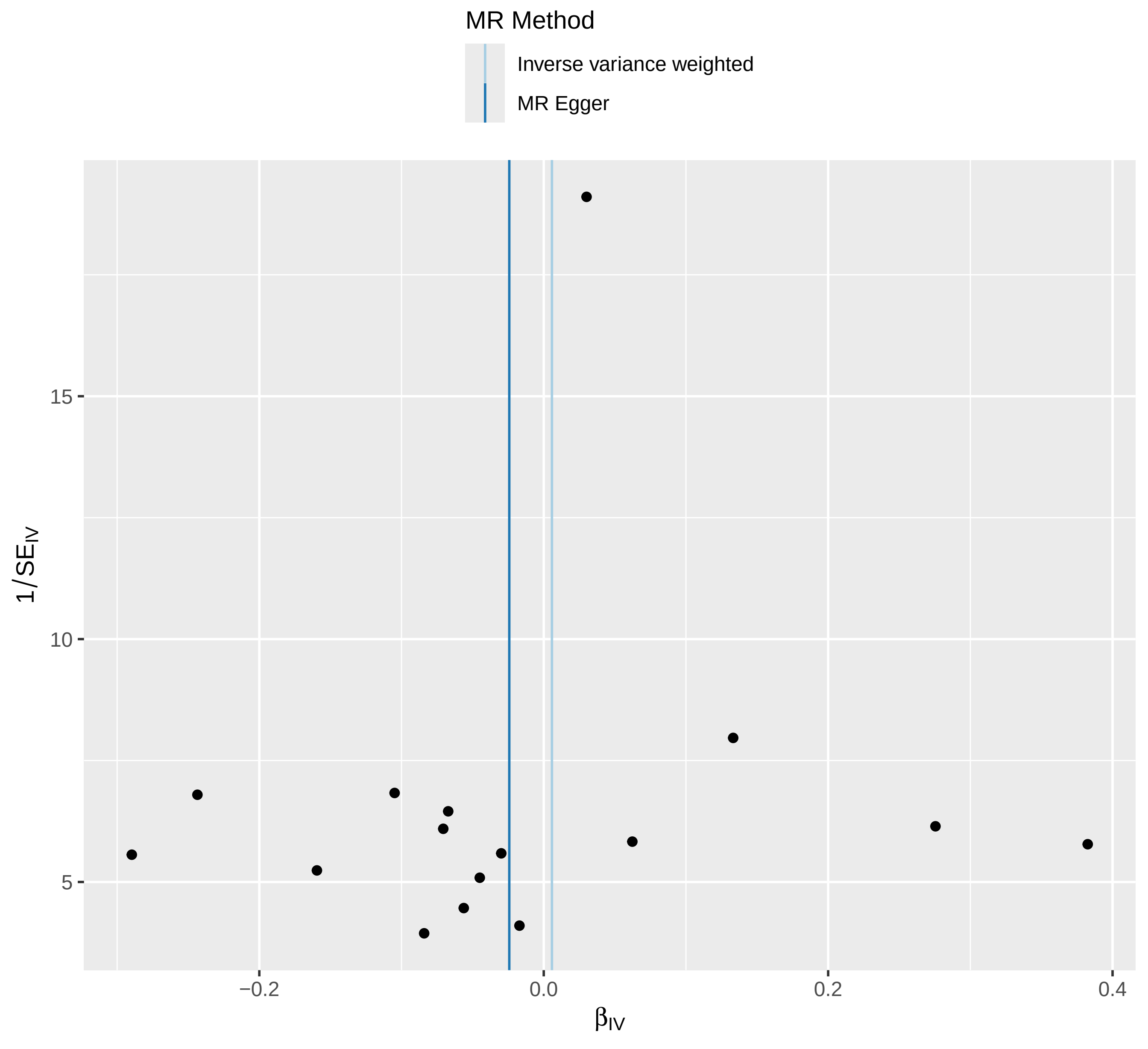

MR Method
Inverse variance weighted
MR Egger
15
10
5
1/SE_IV
−0.2
0.0
0.2
0.4
β_IV

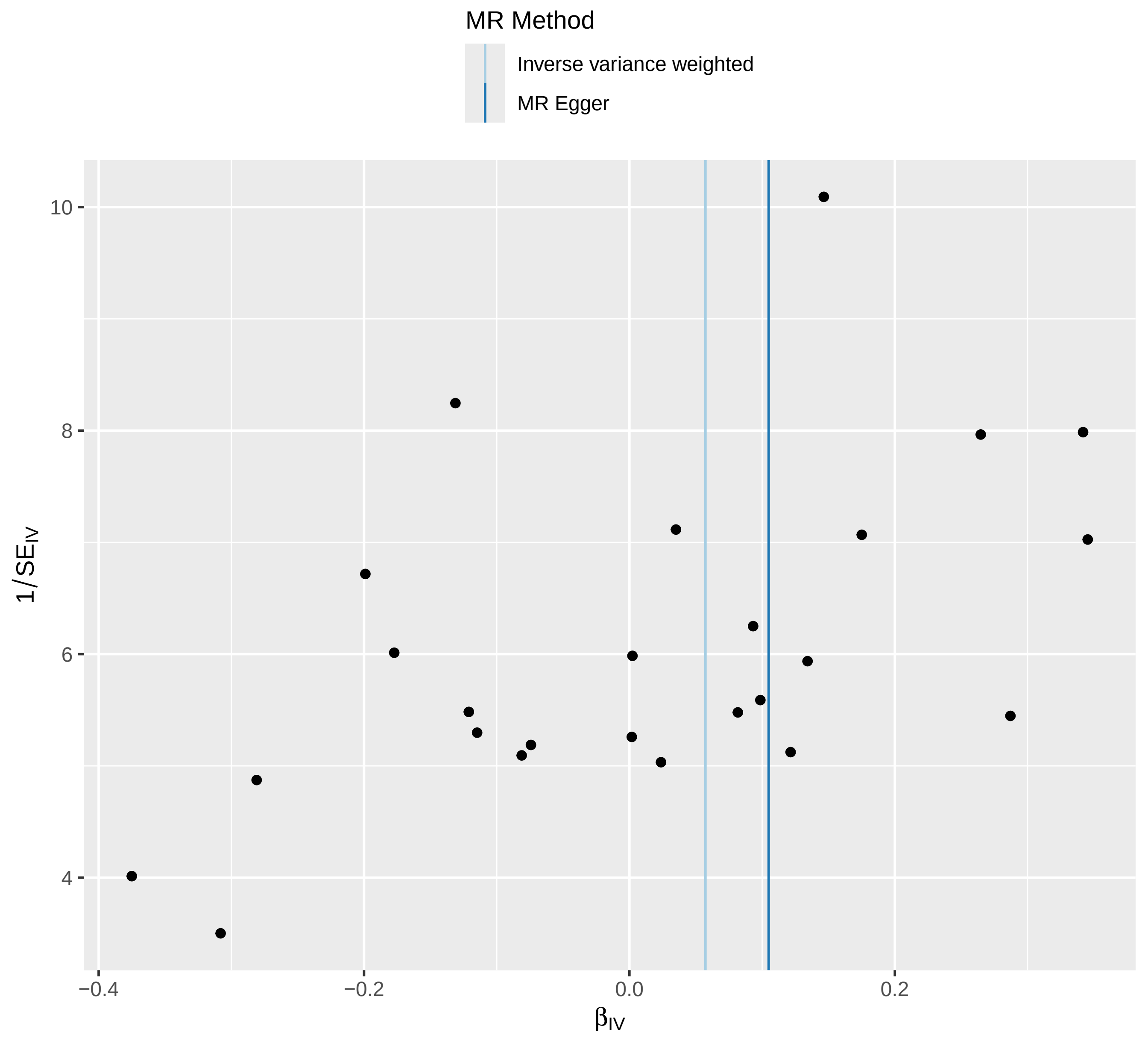
MR Method
Inverse variance weighted
MR Egger
10
8
6
4
1/SE_IV
−0.4
−0.2
0.0
0.2
β_IV

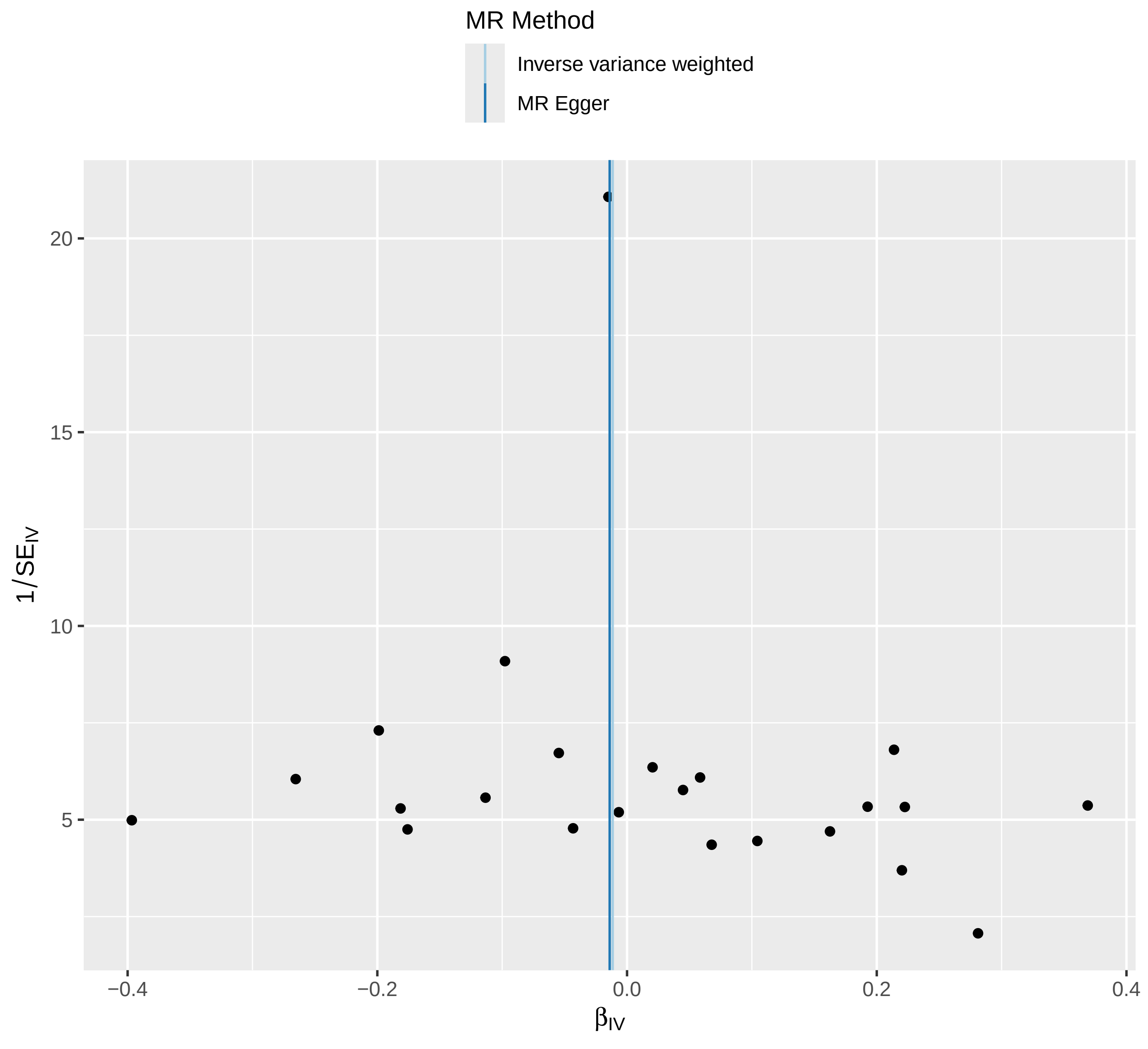

MR Method
Inverse variance weighted
MR Egger
20
15
10
5
1/SE_IV
−0.4
−0.2
0.0
0.2
0.4
β_IV

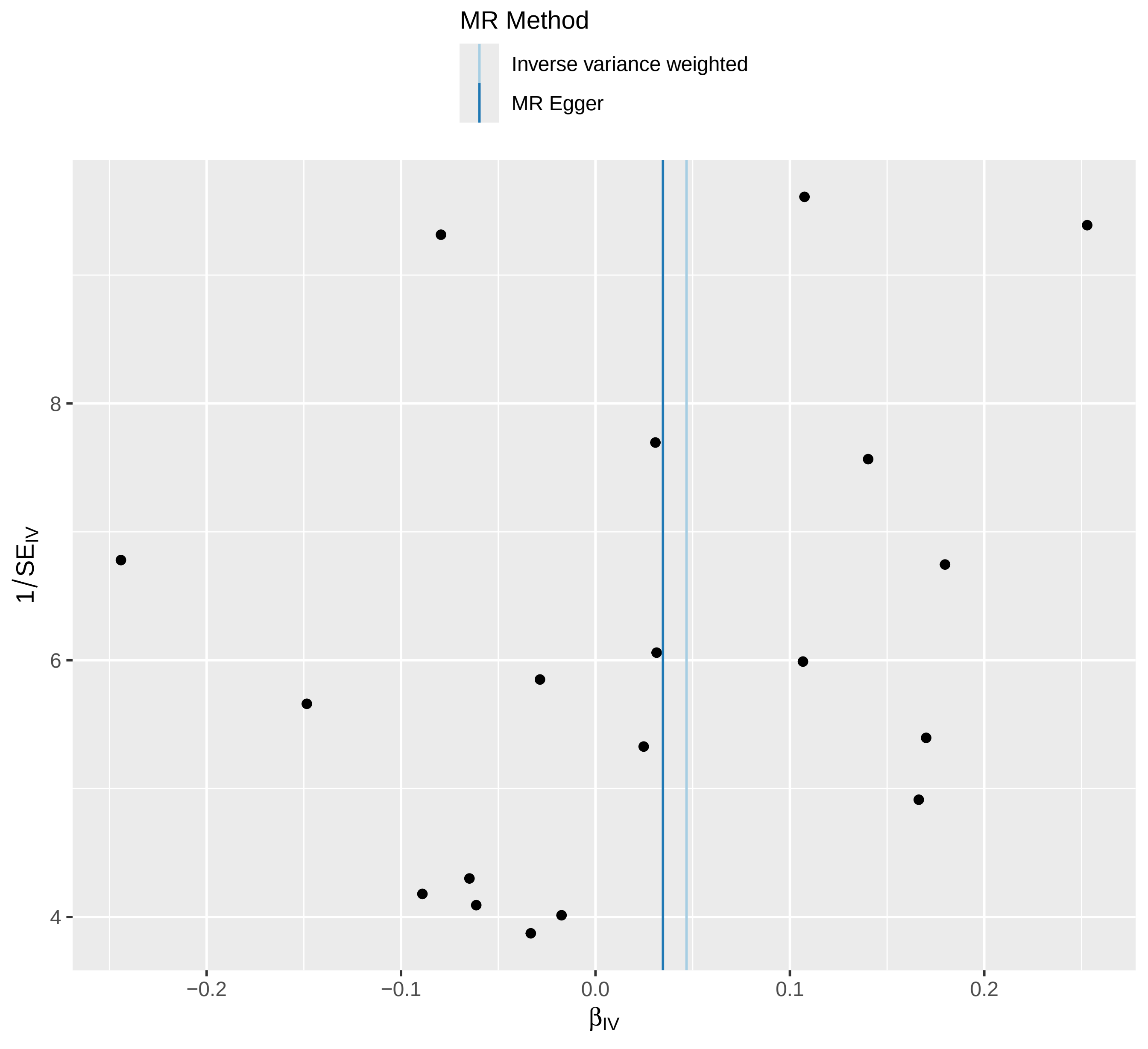
MR Method
Inverse variance weighted
MR Egger
1/SE_IV
8
6
4
−0.2
−0.1
0.0
0.1
0.2
β_IV

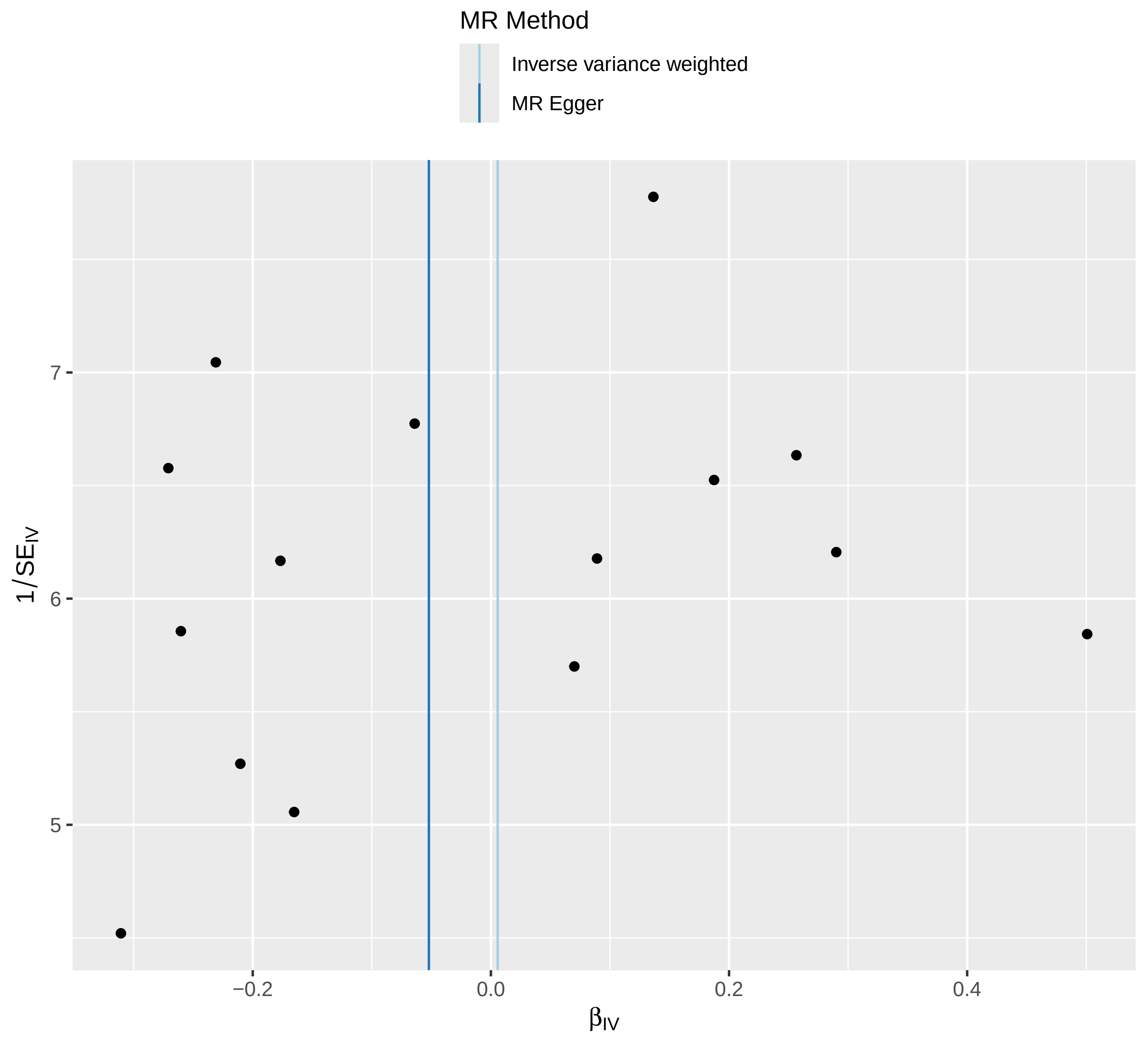

MR Method
Inverse variance weighted
MR Egger
1/SE_IV
7
6
5
−0.2
0.0
0.2
0.4
β_IV

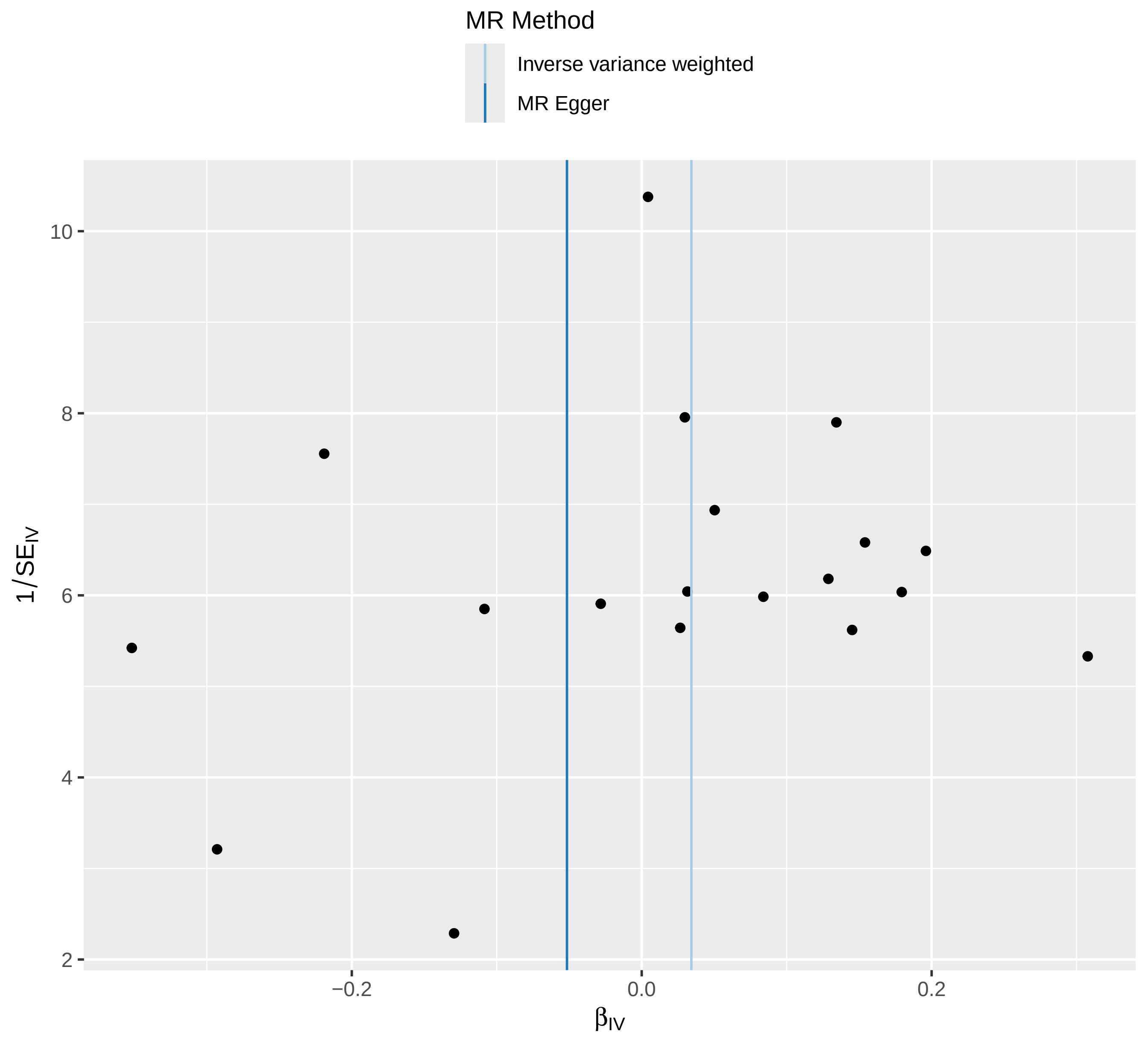
MR Method
Inverse variance weighted
MR Egger
1/SE_IV
10
8
6
4
2
−0.2
0.0
0.2
β_IV

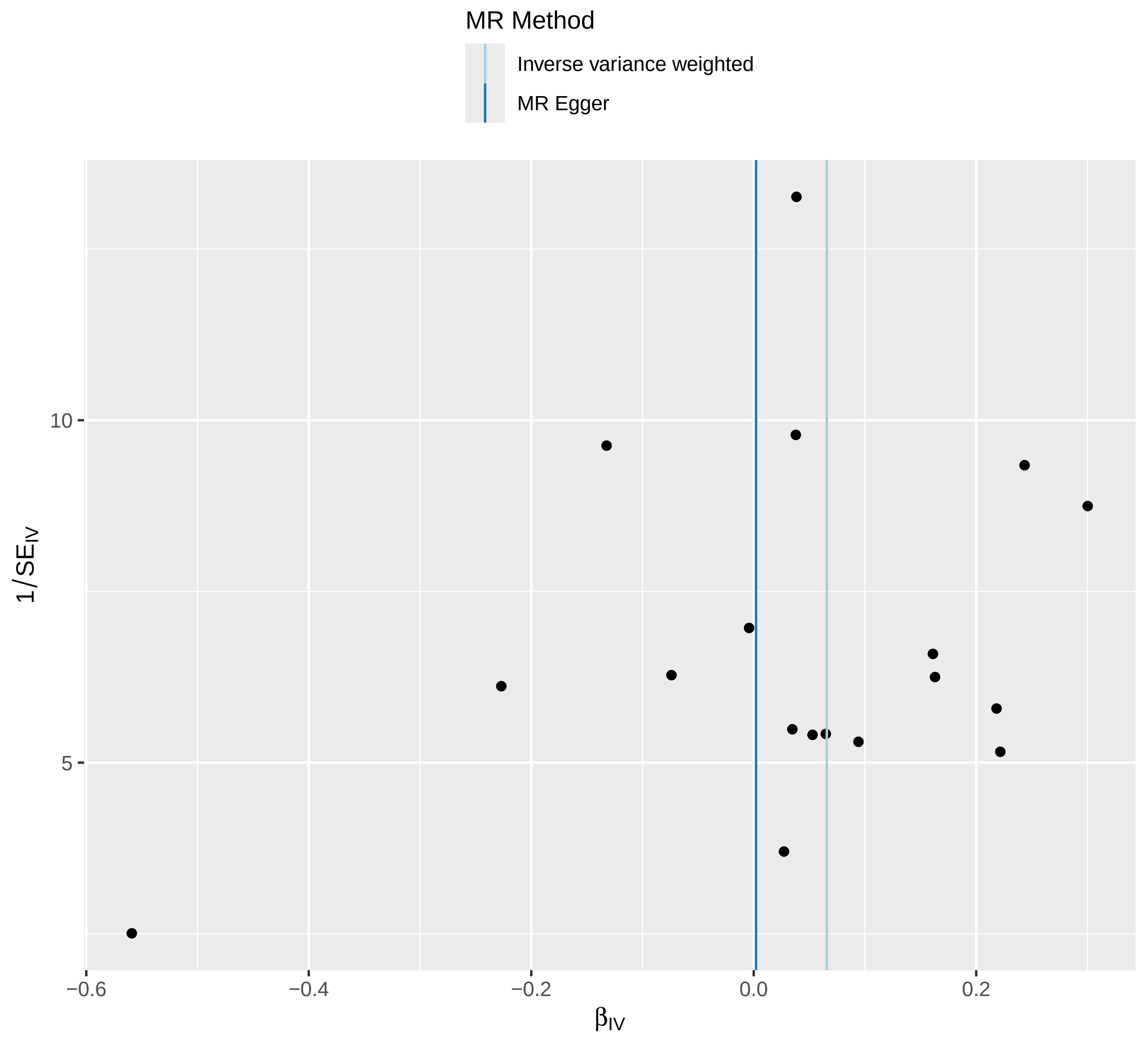
MR Method
Inverse variance weighted
MR Egger
10
5
1/SE_IV
−0.6
−0.4
−0.2
0.0
0.2
β_IV

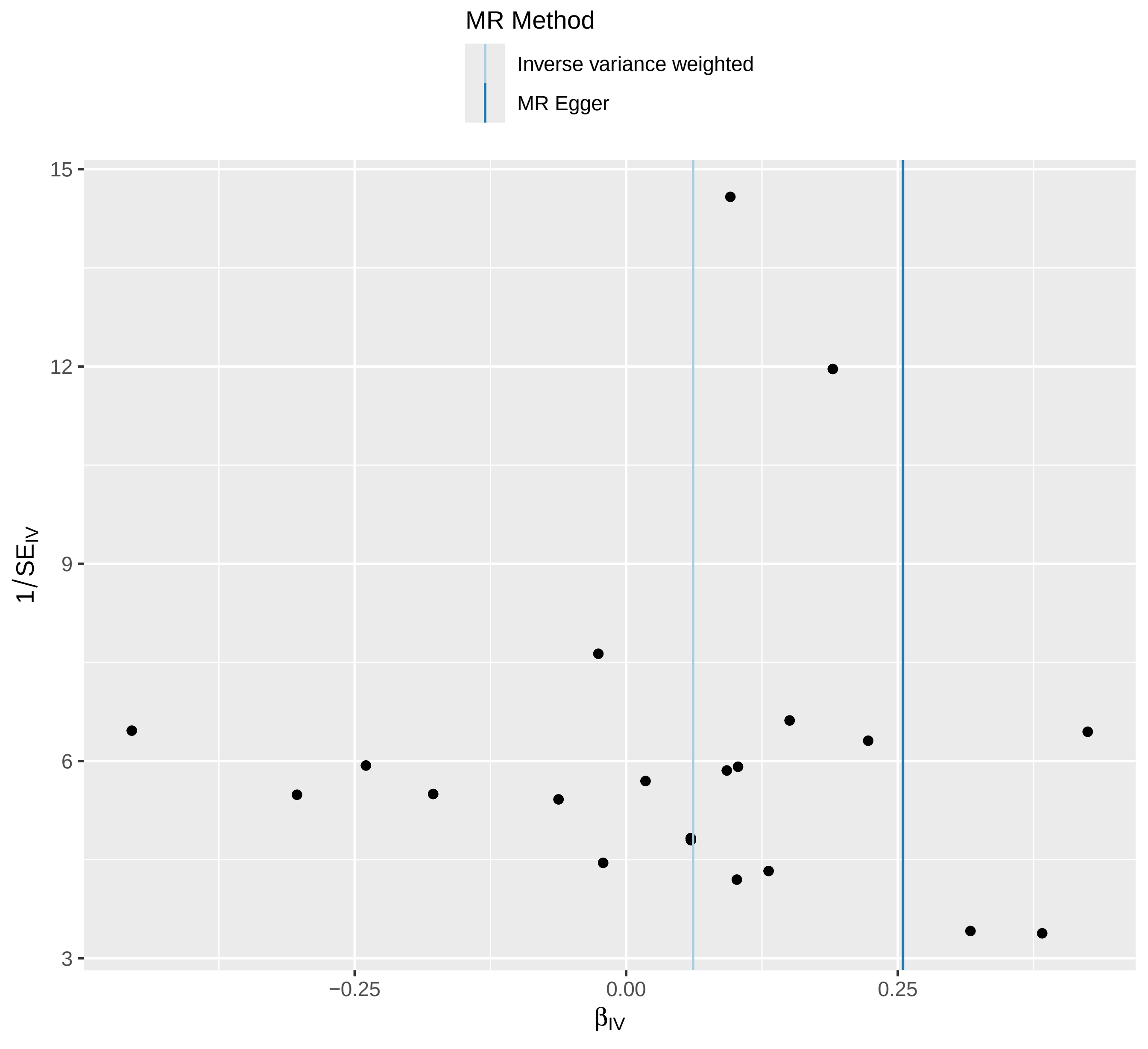
MR Method
Inverse variance weighted
MR Egger
15
12
9
6
3
1/SE_IV
−0.25
0.00
0.25
β_IV

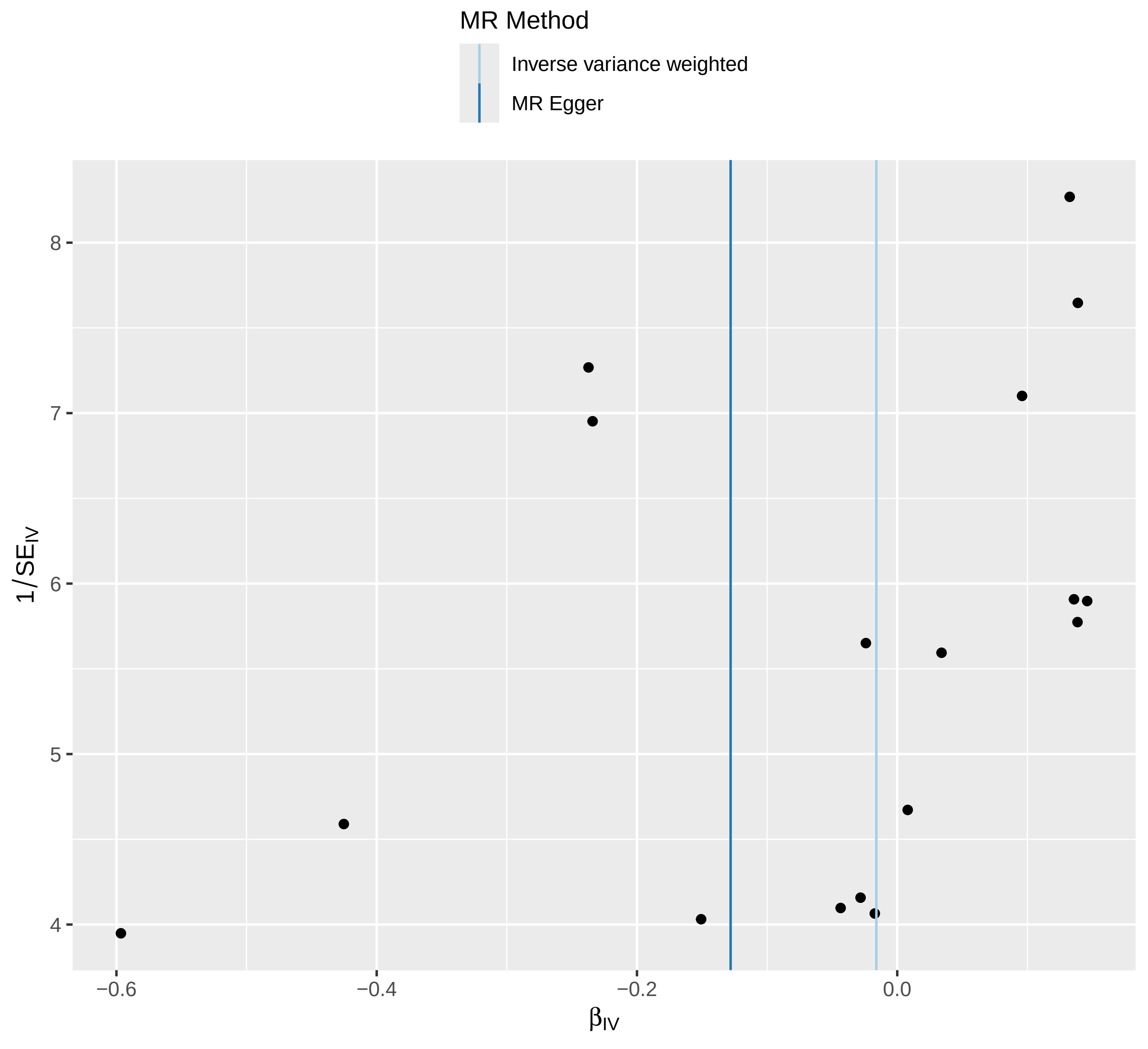

MR Method
Inverse variance weighted
MR Egger
8
7
6
5
4
1/SE_IV
−0.6
−0.4
−0.2
0.0
β_IV

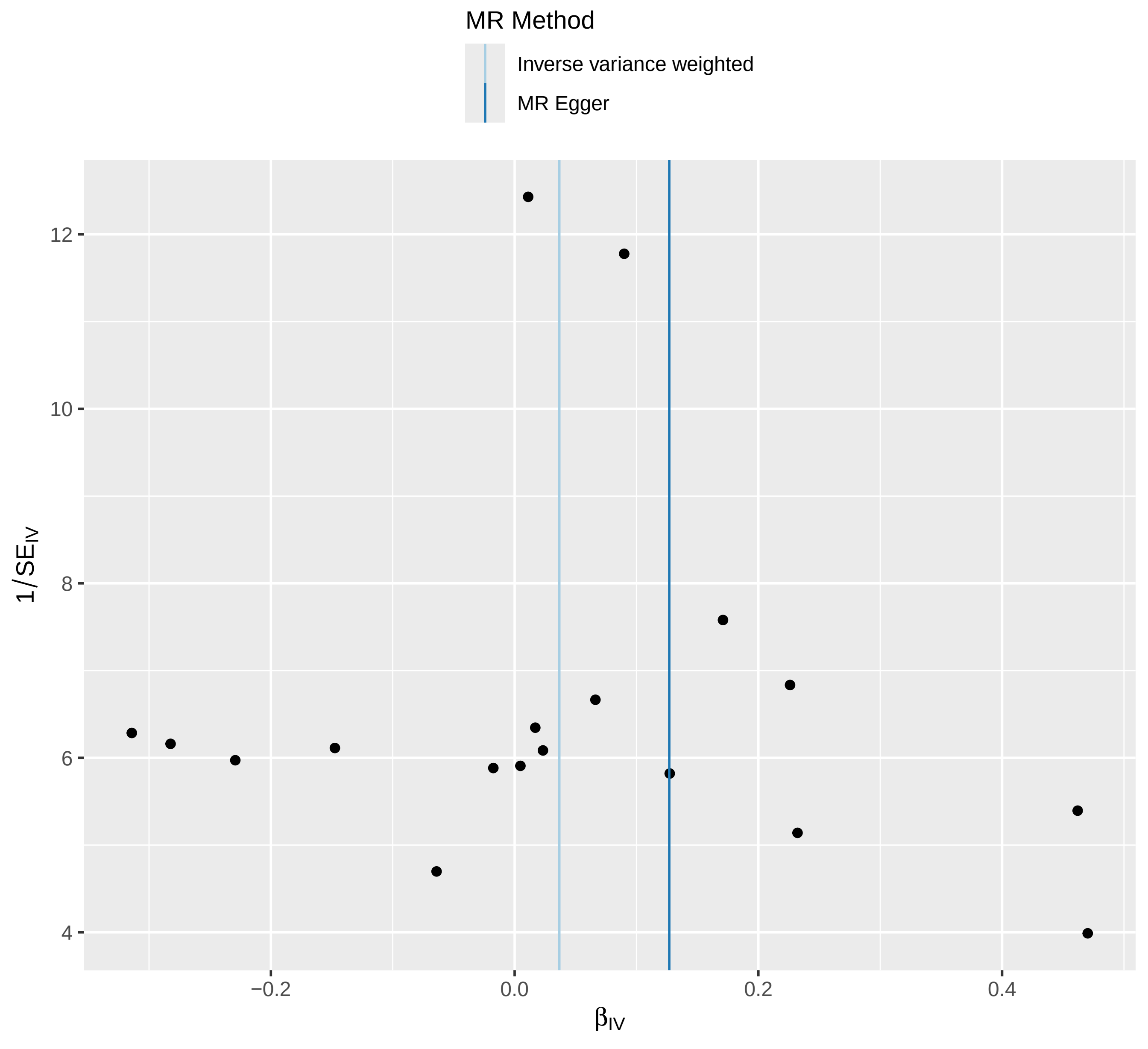

MR Method
Inverse variance weighted
MR Egger
12
10
8
6
4
1/SE_IV
−0.2
0.0
0.2
0.4
β_IV

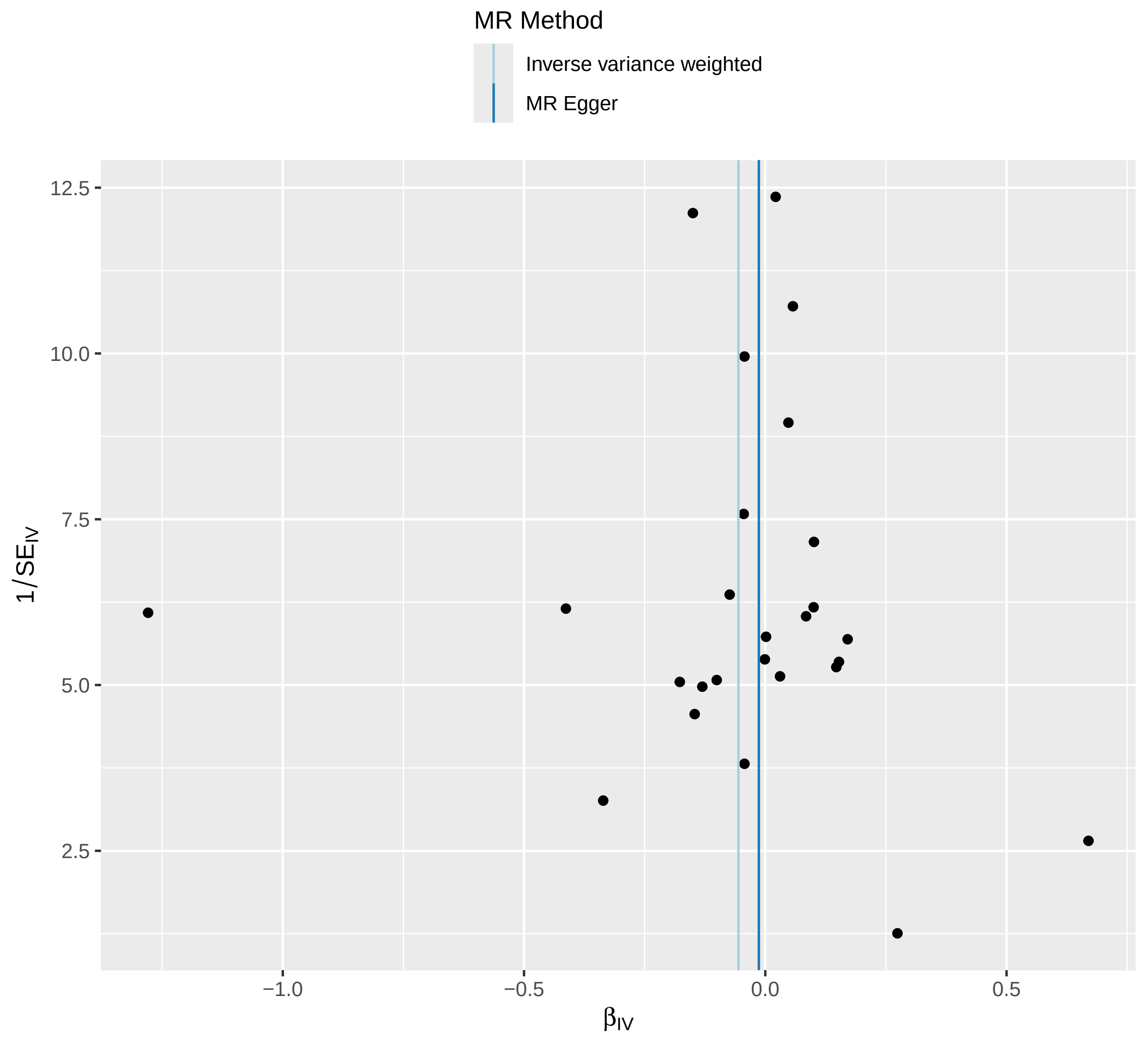

MR Method
Inverse variance weighted
MR Egger
12.5
10.0
7.5
5.0
2.5
1/SE_IV
−1.0
−0.5
0.0
0.5
β_IV

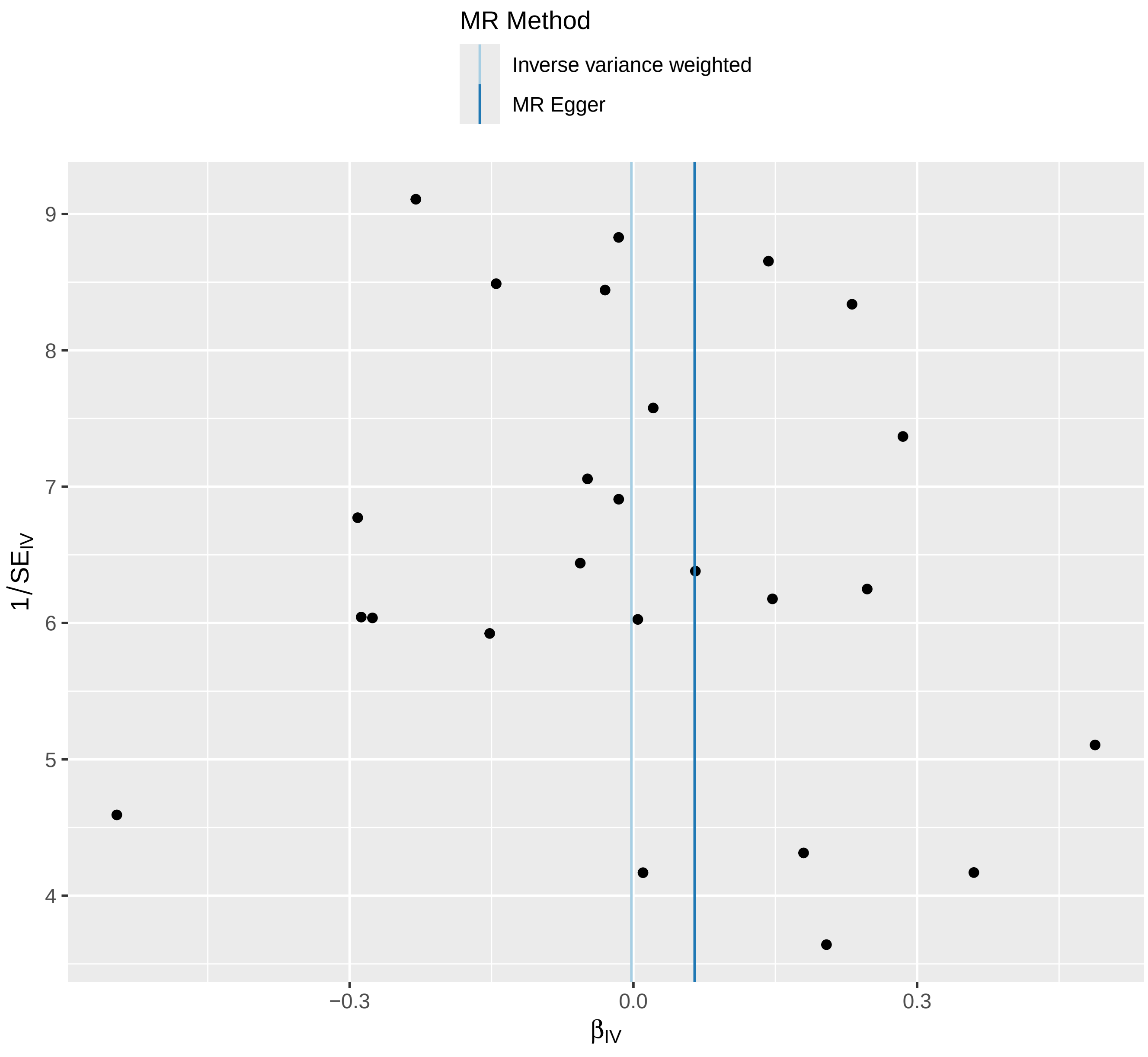
MR Method
Inverse variance weighted
MR Egger
1/SE_IV
9
8
7
6
5
4
−0.3
0.0
0.3
β_IV

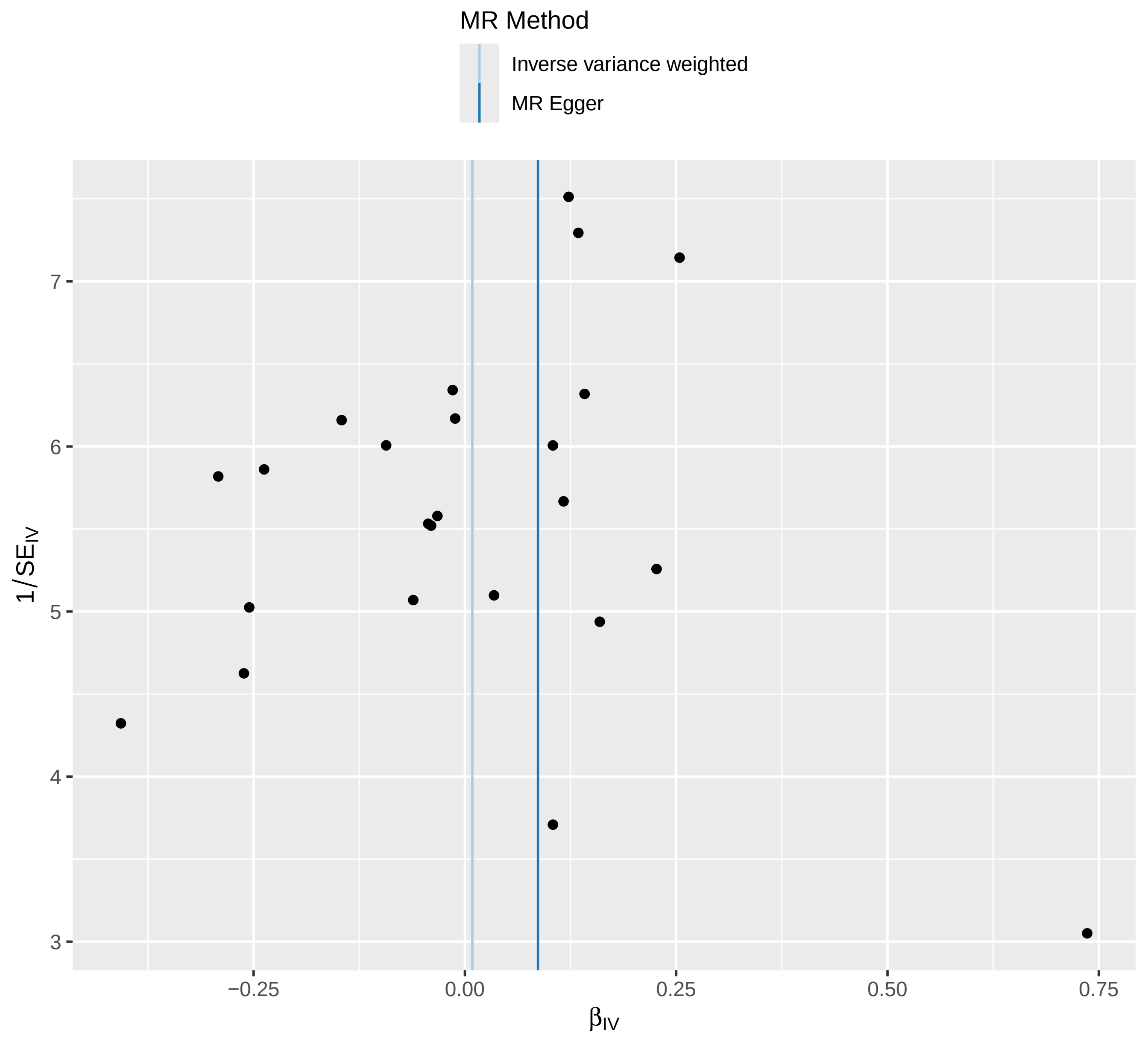
MR Method
Inverse variance weighted
MR Egger
1/SE_IV
7
6
5
4
3
−0.25
0.00
0.25
0.50
0.75
β_IV

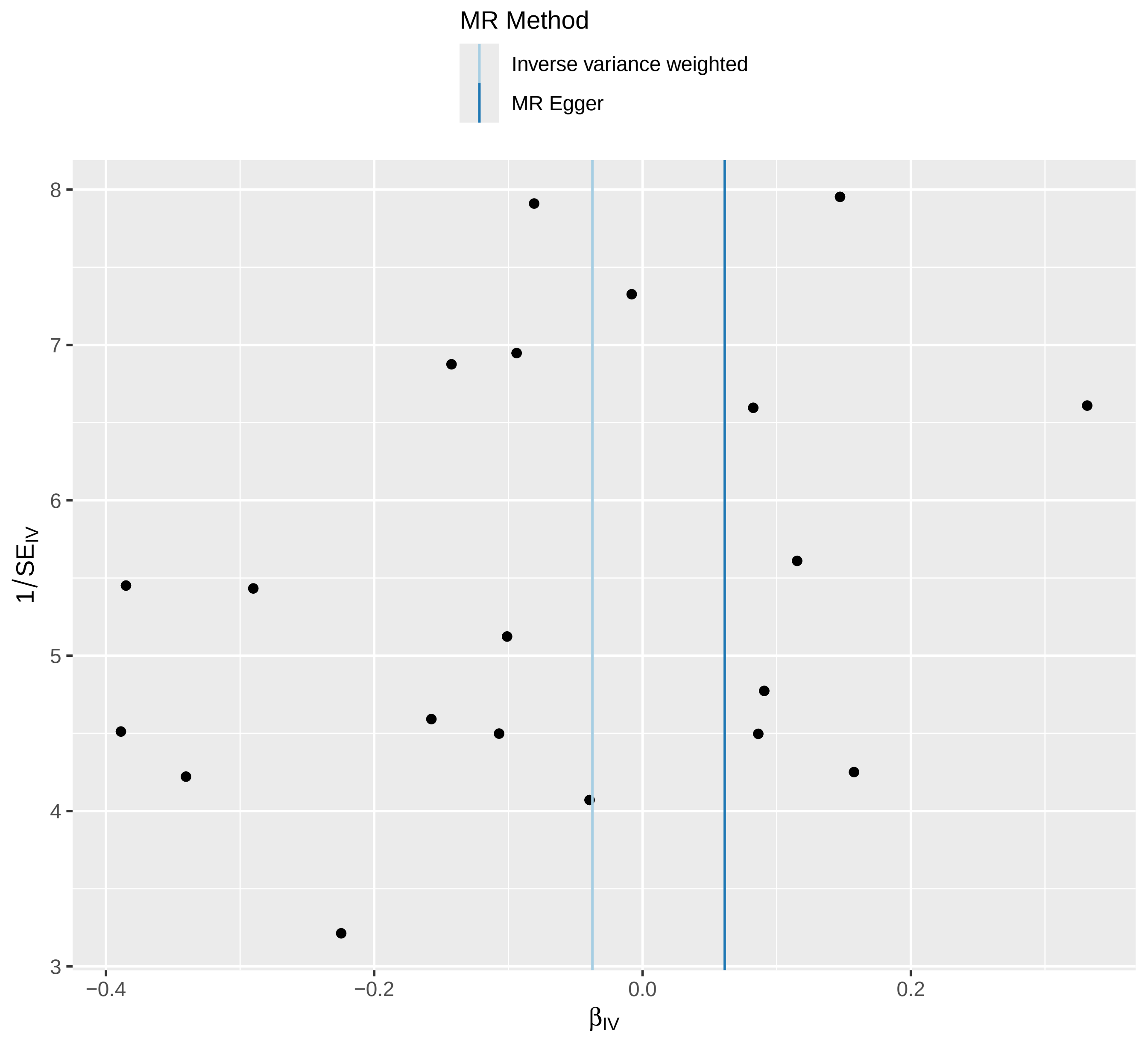
MR Method
Inverse variance weighted
MR Egger
8
7
6
5
4
3
1/SE_IV
−0.4
−0.2
0.0
0.2
β_IV

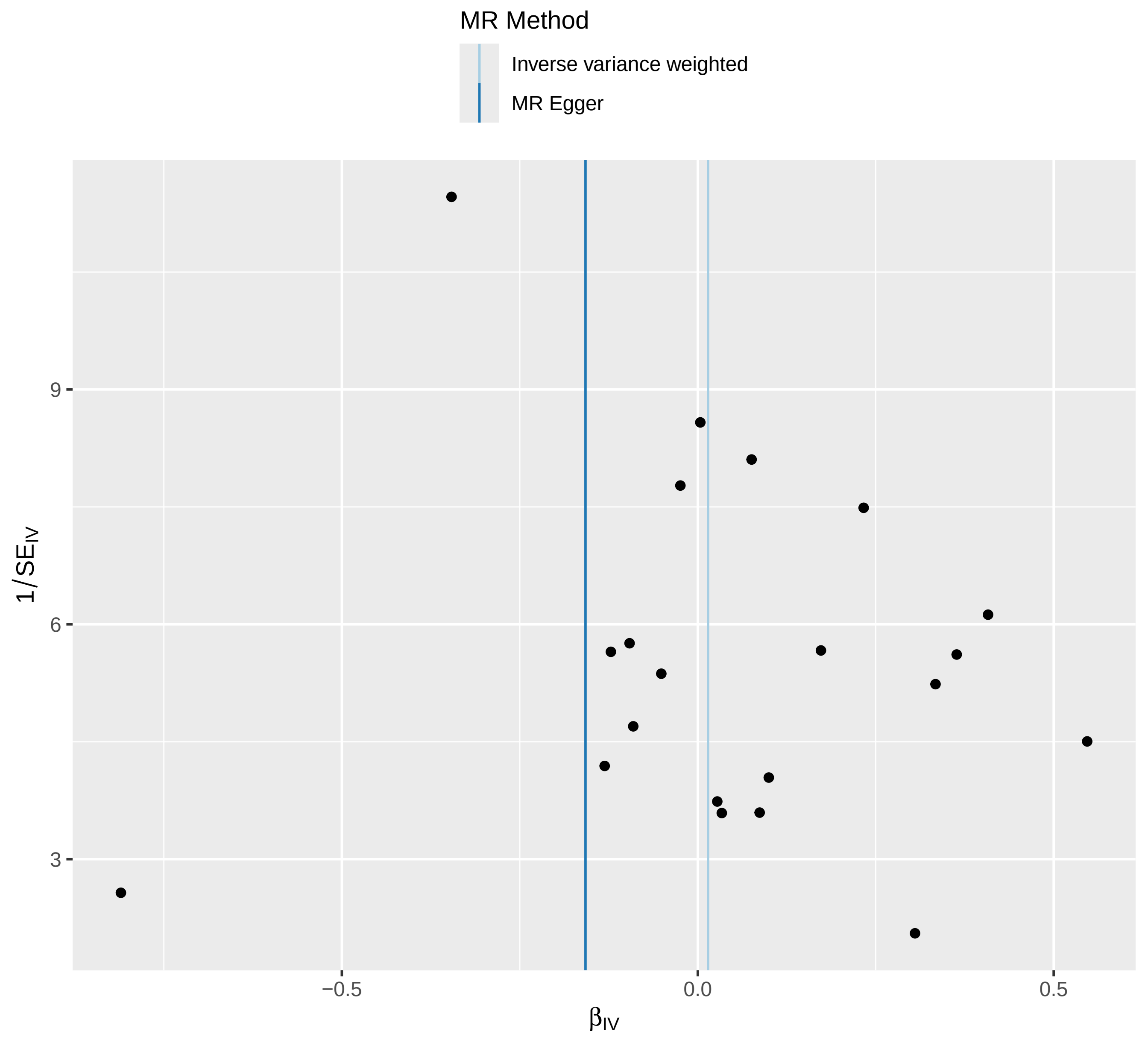
MR Method
Inverse variance weighted
MR Egger
9
6
3
1/SE_IV
−0.5
0.0
0.5
β_IV

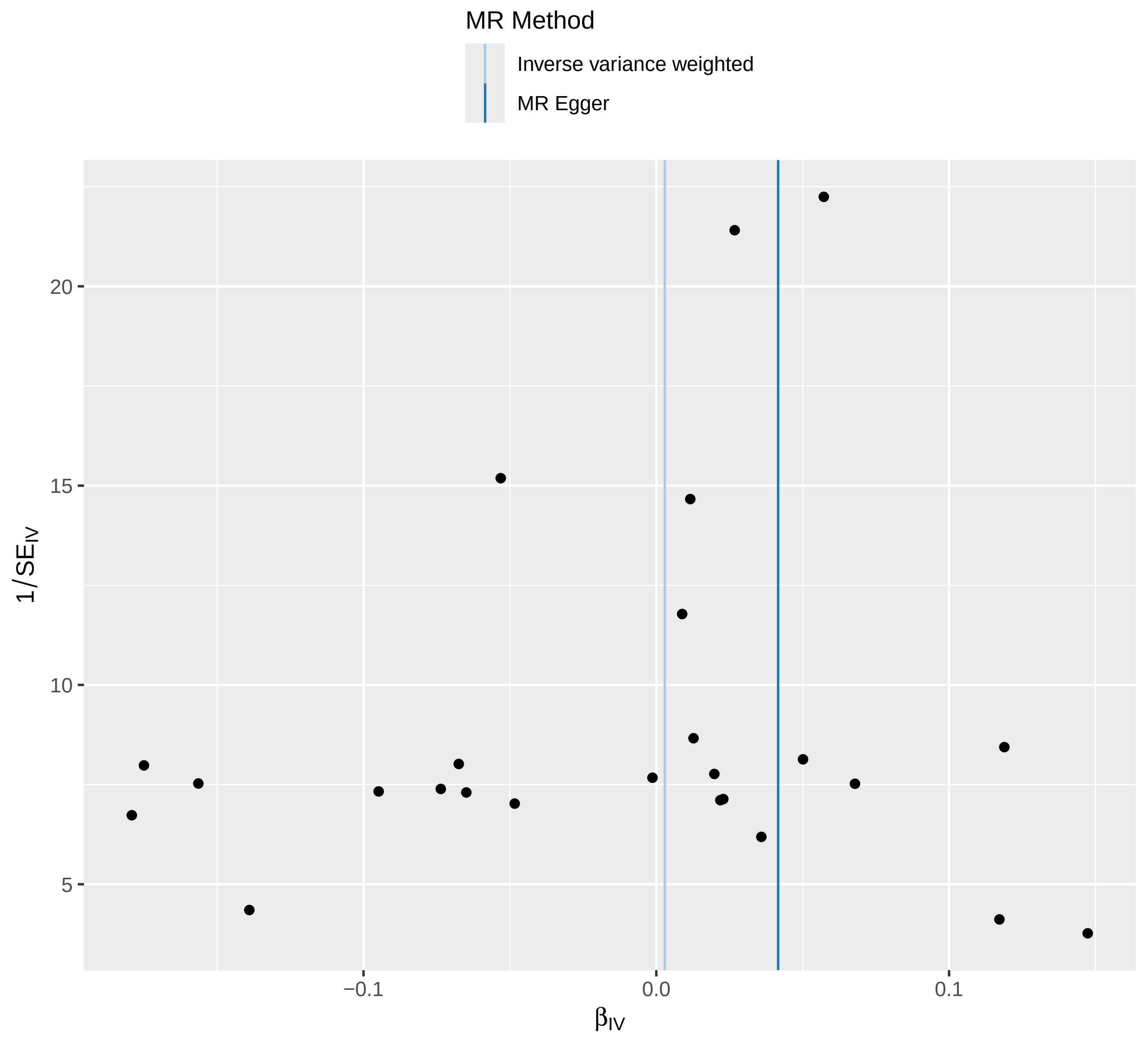

MR Method
Inverse variance weighted
MR Egger
20
15
10
5
1/SE_IV
−0.1
0.0
0.1
β_IV

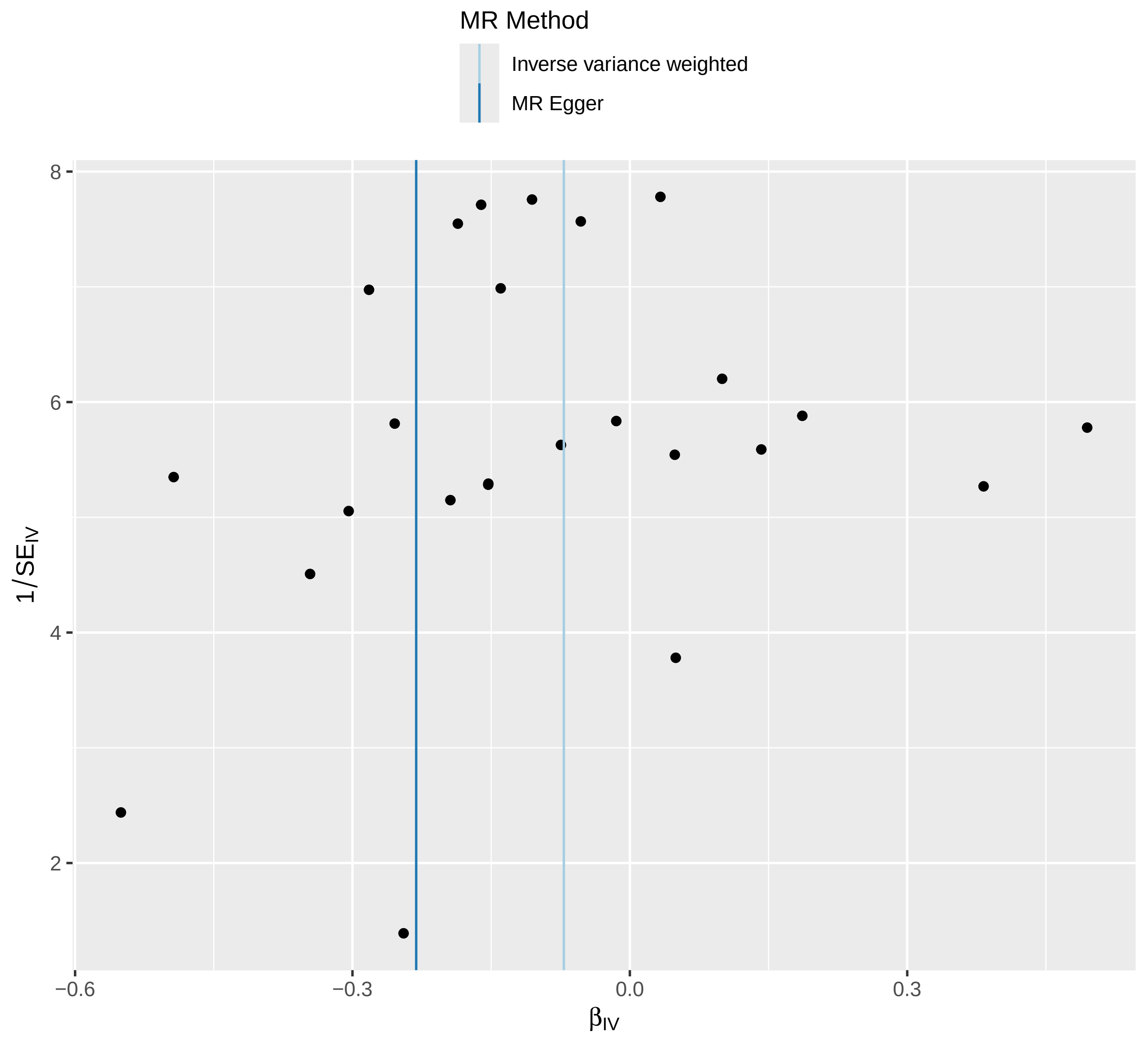
MR Method
Inverse variance weighted
MR Egger
8
6
4
2
$1/SE_{IV}$
−0.6
−0.3
0.0
0.3
β_{IV}

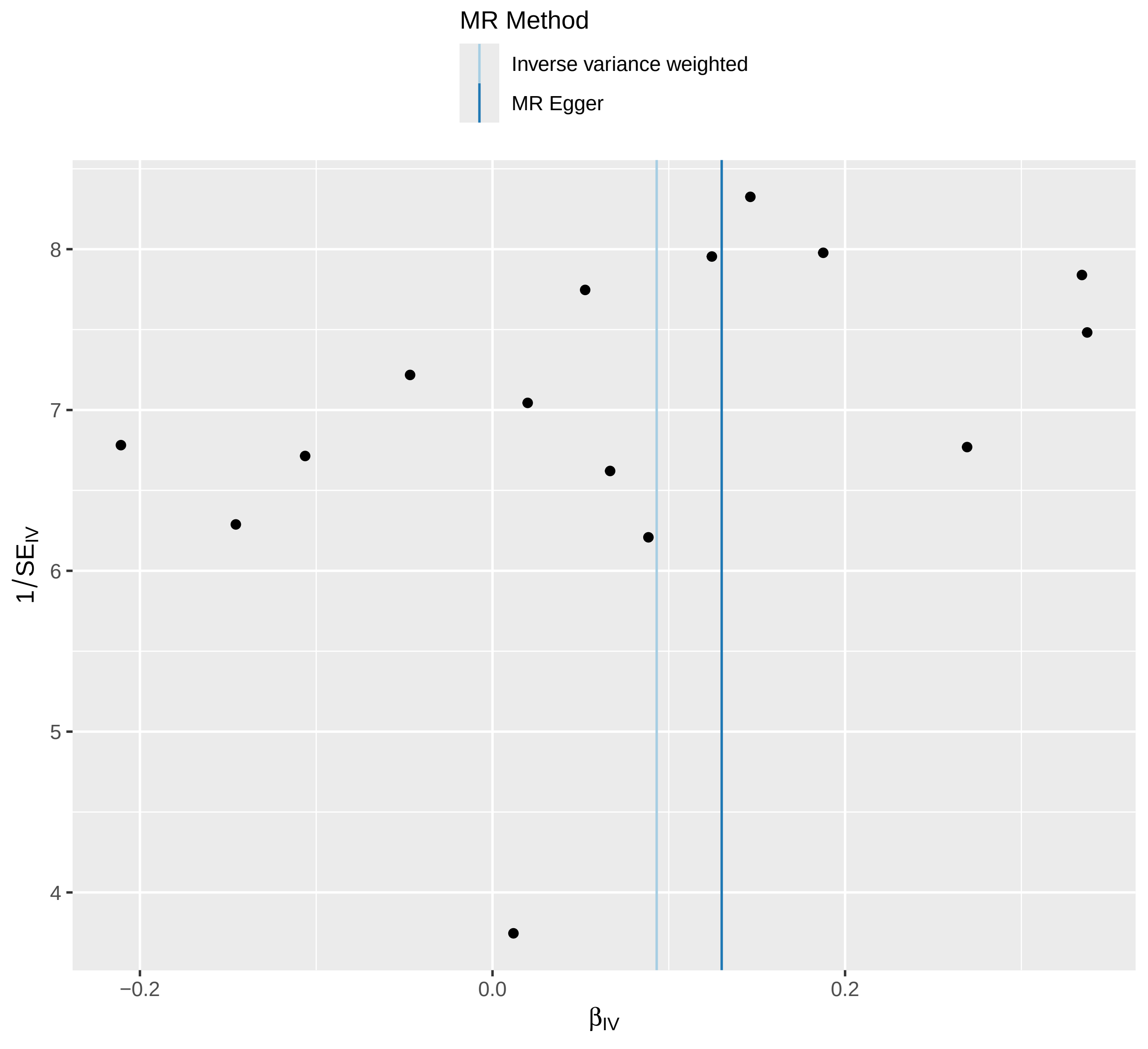

MR Method
Inverse variance weighted
MR Egger
8
7
6
5
4
1/SE_IV
−0.2
0.0
0.2
β_IV

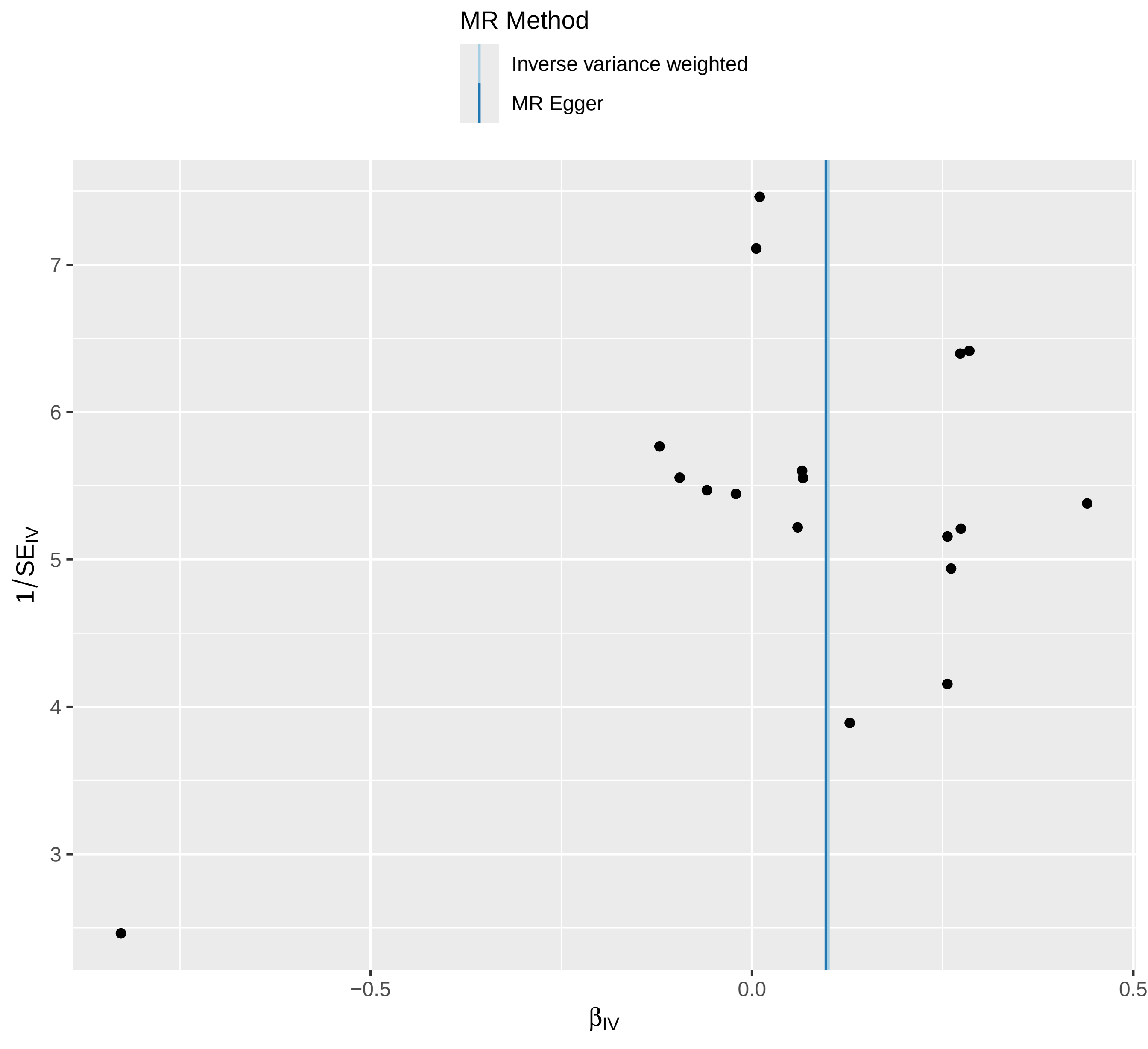

MR Method
Inverse variance weighted
MR Egger
1/SE_IV
7
6
5
4
3
−0.5
0.0
0.5
β_IV

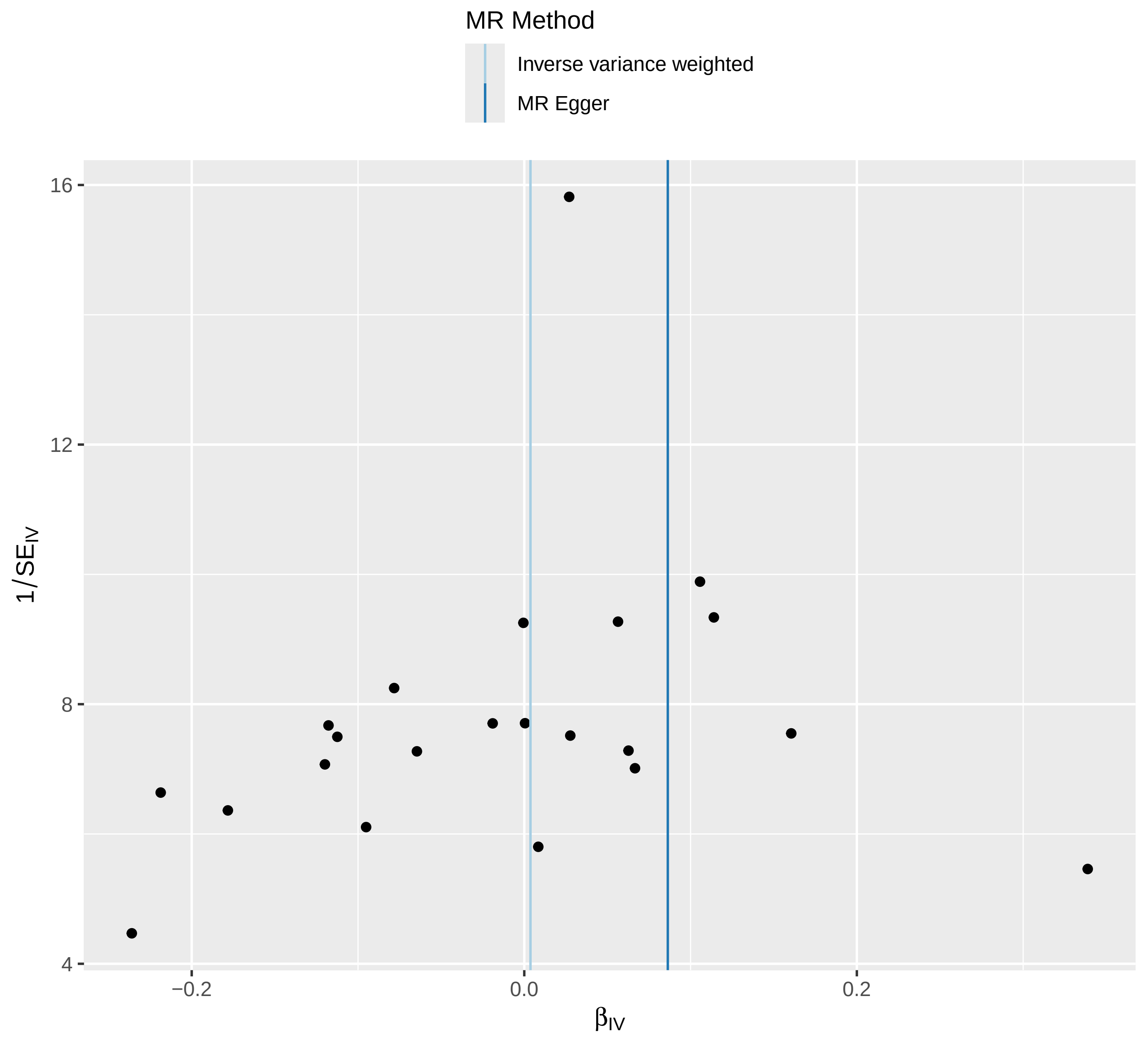

MR Method
Inverse variance weighted
MR Egger
16
12
8
4
1/SE_IV
−0.2
0.0
0.2
β_IV

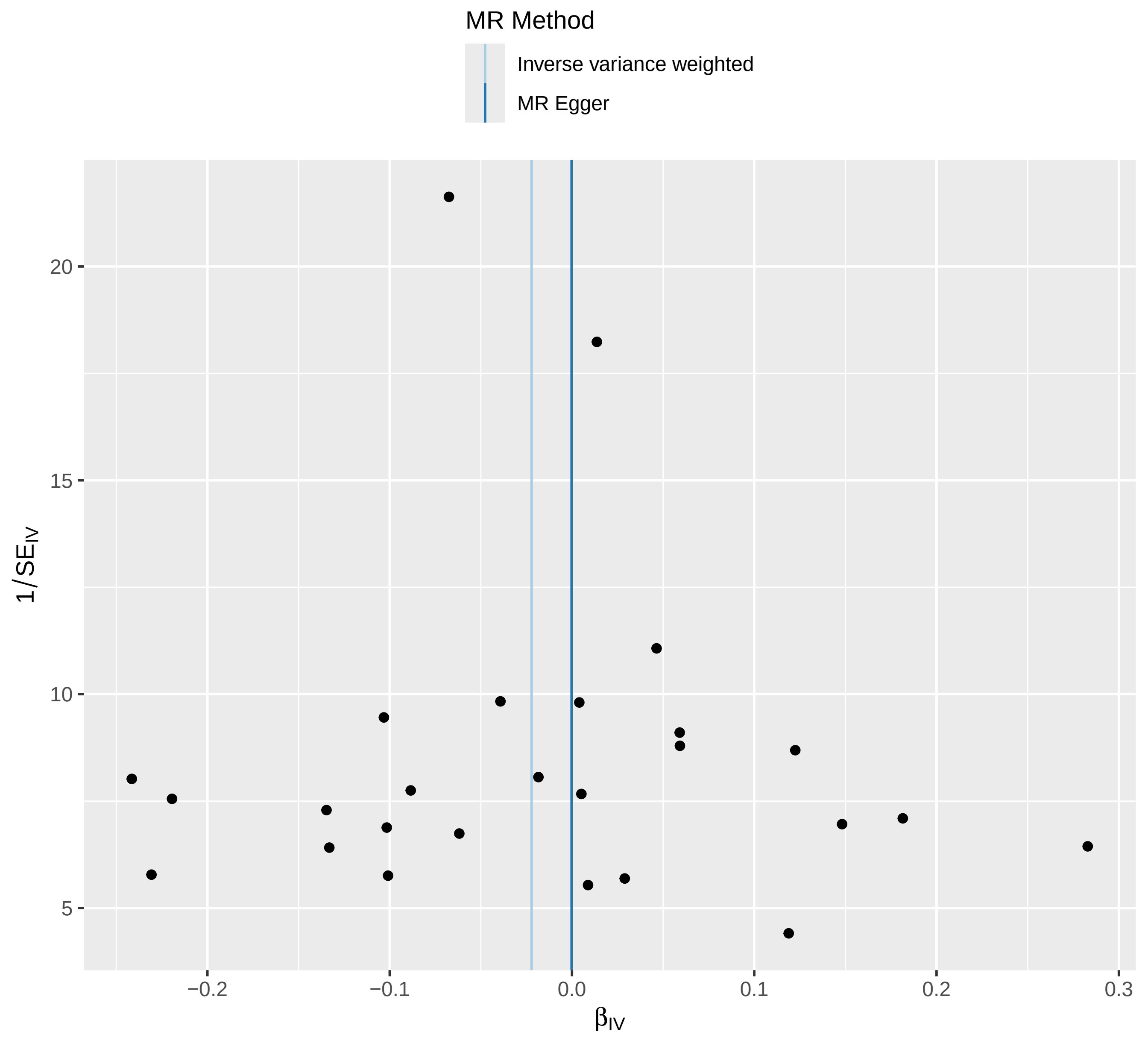

MR Method
Inverse variance weighted
MR Egger
20
15
10
5
1/SE_IV
−0.2
−0.1
0.0
0.1
0.2
0.3
β_IV

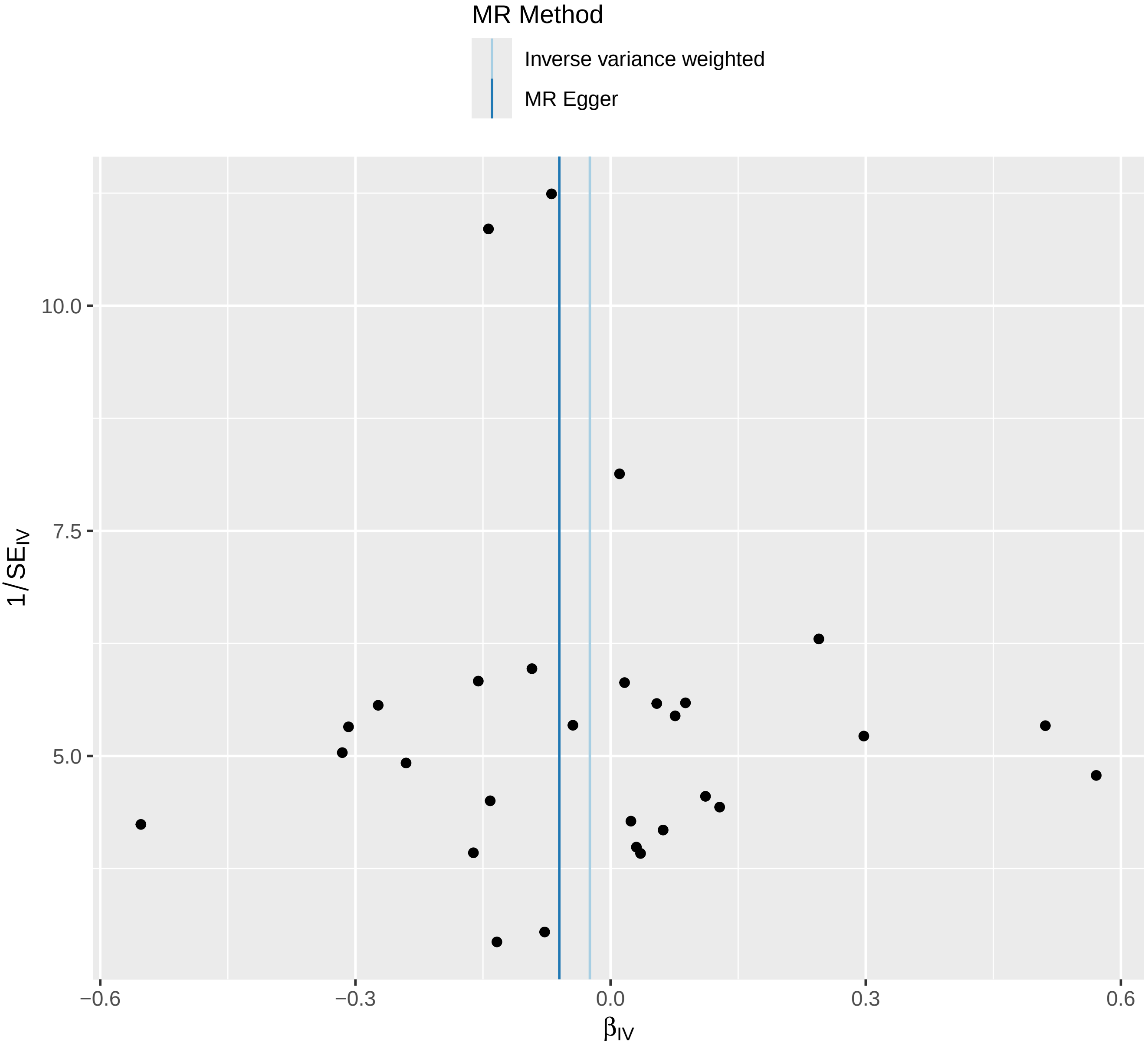
MR Method
Inverse variance weighted
MR Egger
10.0
7.5
5.0
$1/SE_{IV}$
−0.6
−0.3
0.0
0.3
0.6
β_{IV}

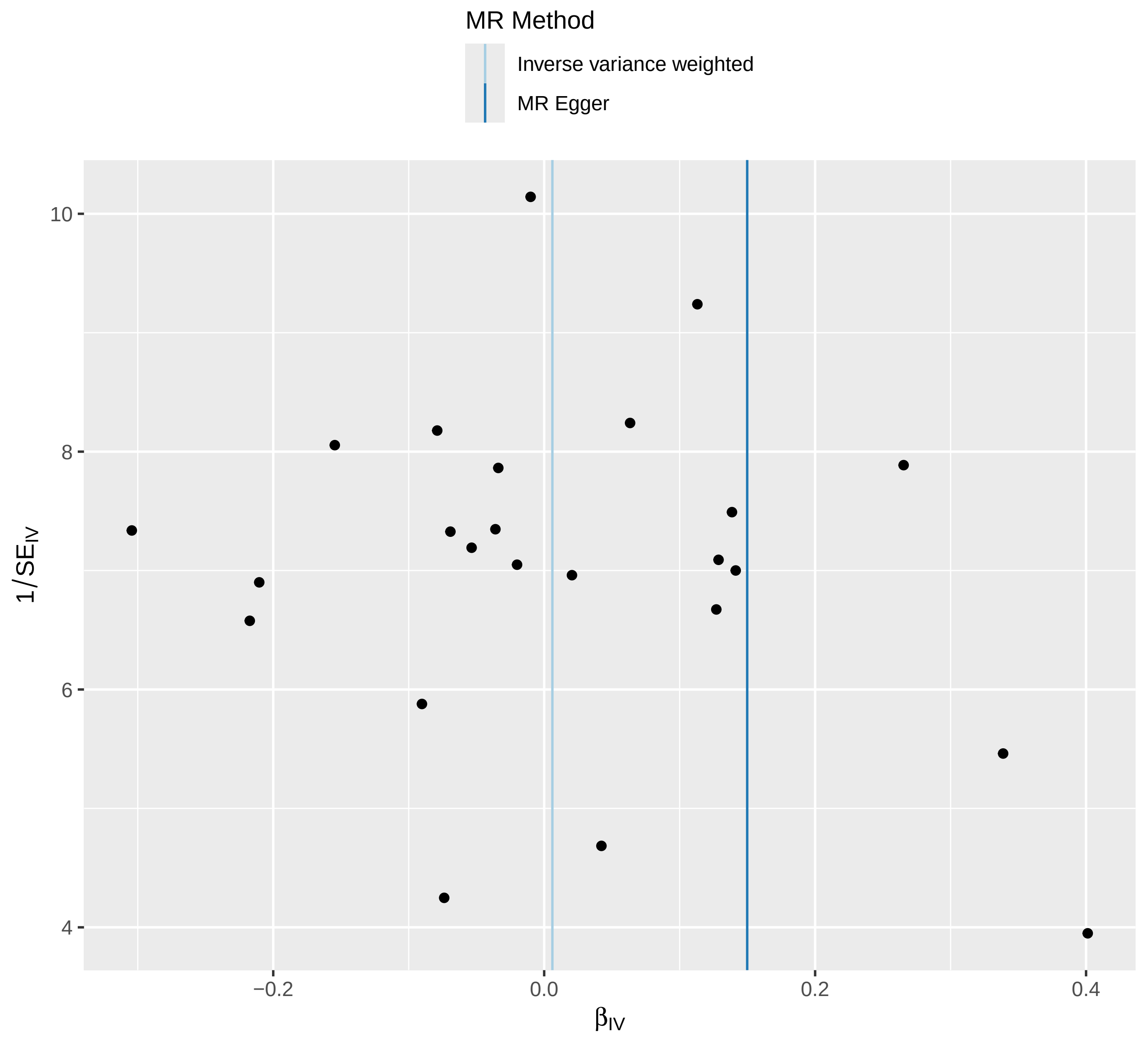
MR Method
Inverse variance weighted
MR Egger
10
8
6
4
−0.2
0.0
0.2
0.4
$1/SE_{IV}$
β_{IV}

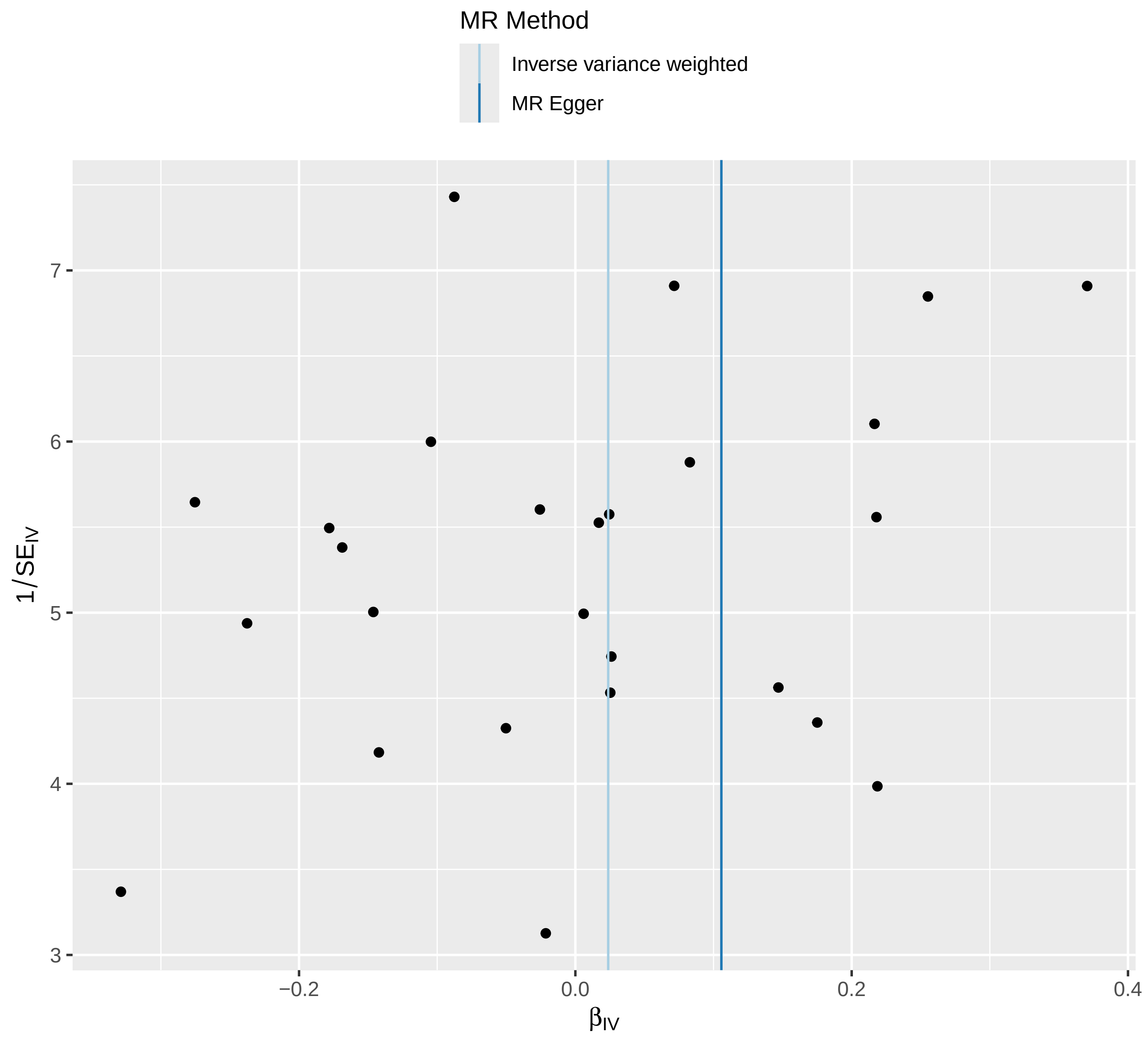
MR Method
Inverse variance weighted
MR Egger
1/SE_IV
7
6
5
4
3
−0.2
0.0
0.2
0.4
β_IV

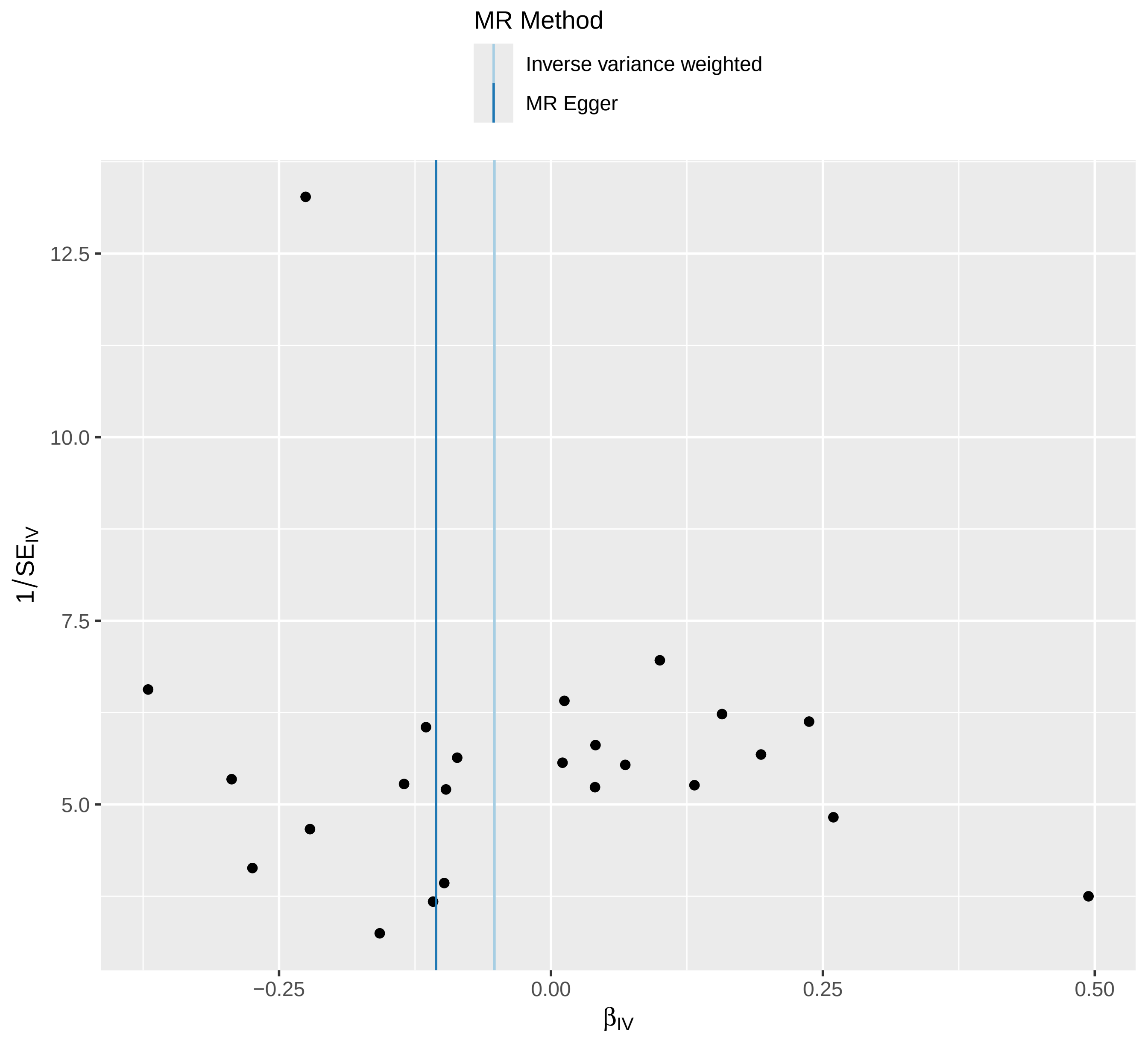

MR Method
Inverse variance weighted
MR Egger
12.5
10.0
7.5
5.0
$1/SE_{IV}$
−0.25
0.00
0.25
0.50
β_{IV}

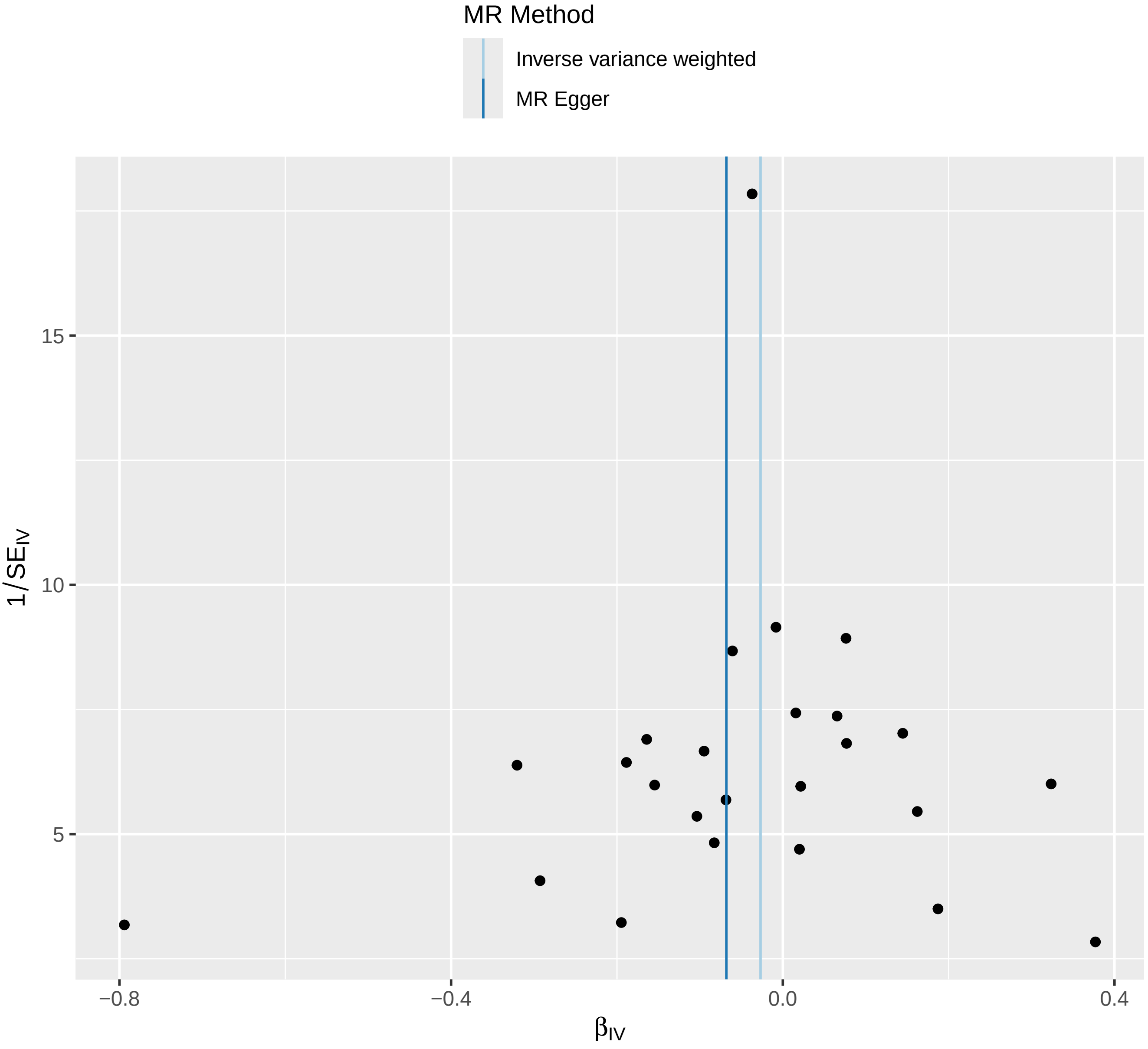
MR Method
Inverse variance weighted
MR Egger
15
10
5
$1/SE_{IV}$
−0.8
−0.4
0.0
0.4
β_{IV}

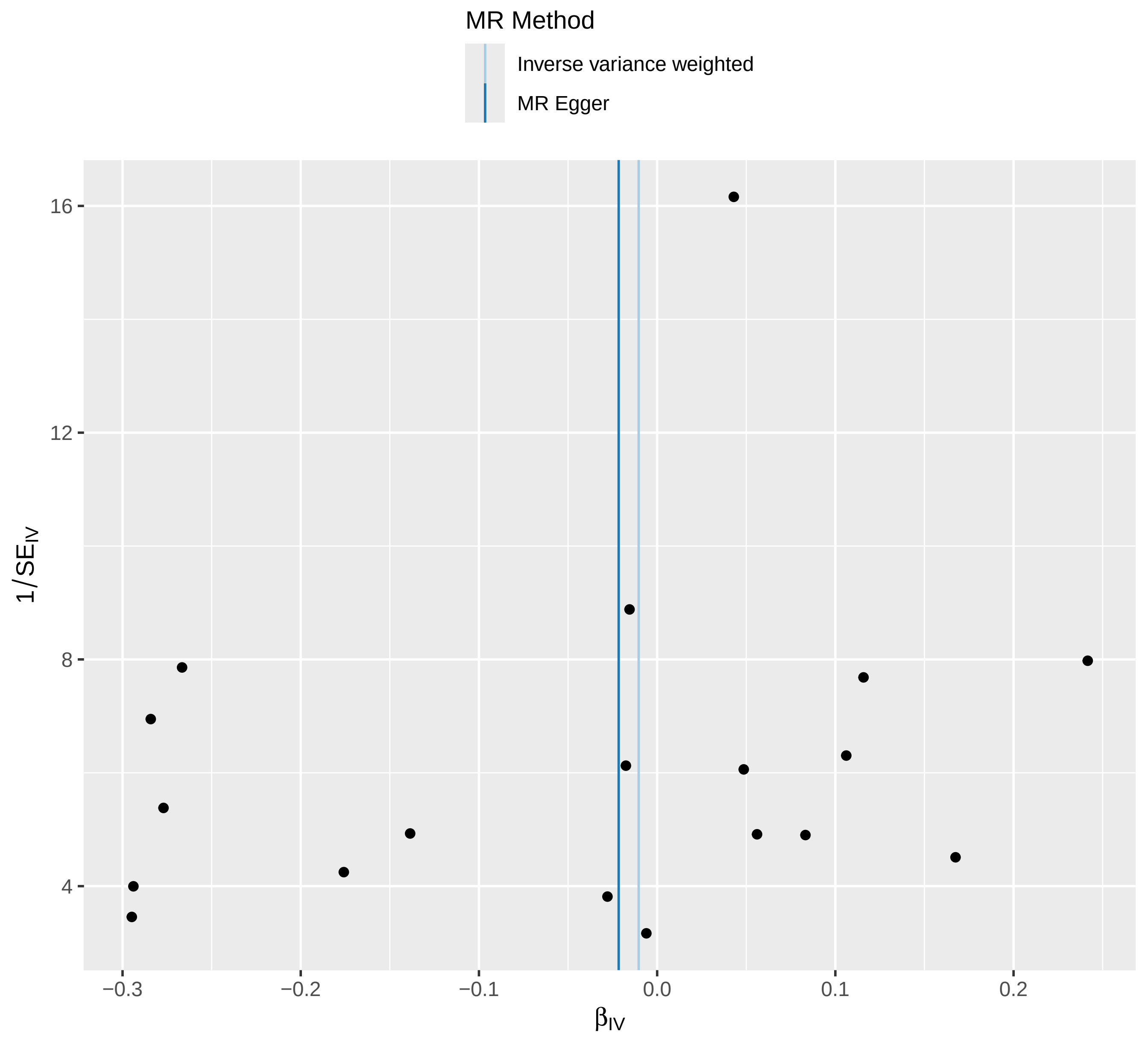
MR Method
Inverse variance weighted
MR Egger
16
12
8
4
1/SE_IV
−0.3
−0.2
−0.1
0.0
0.1
0.2
β_IV

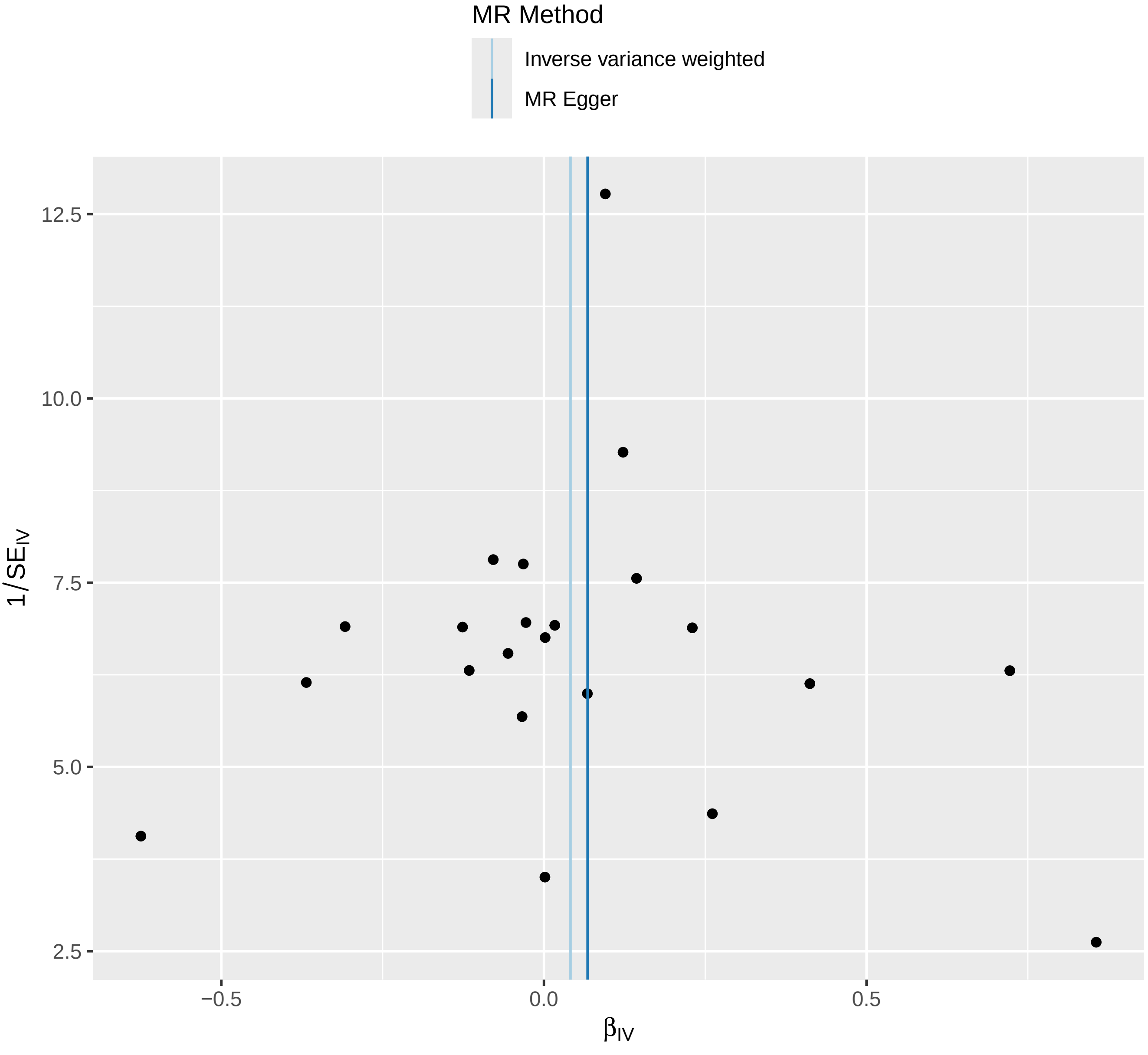

MR Method
Inverse variance weighted
MR Egger
12.5
10.0
7.5
5.0
2.5
−0.5
0.0
0.5
$1/SE_{IV}$
β_{IV}

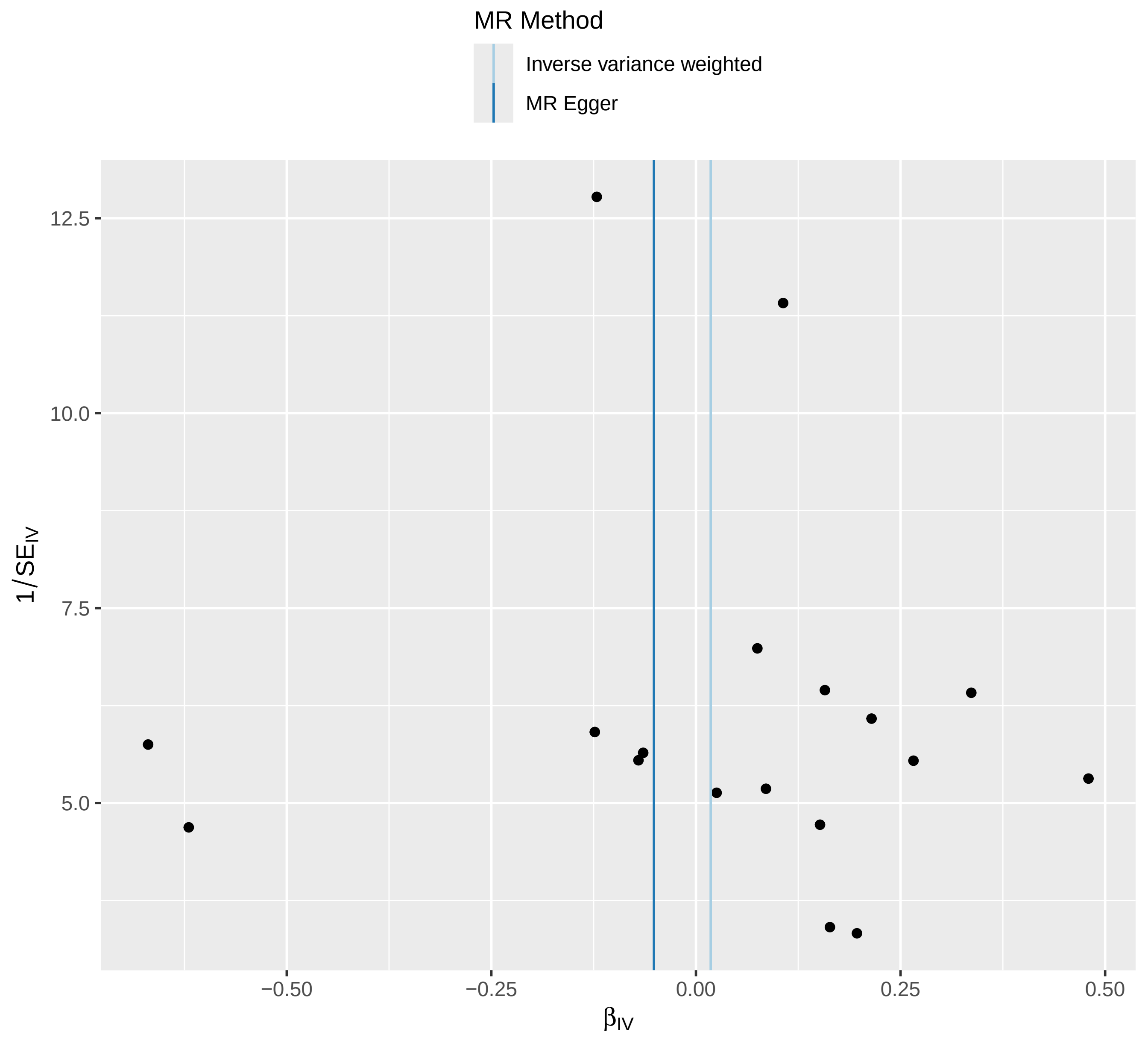

MR Method
Inverse variance weighted
MR Egger
12.5
10.0
7.5
5.0
1/SE_IV
−0.50
−0.25
0.00
0.25
0.50
β_IV

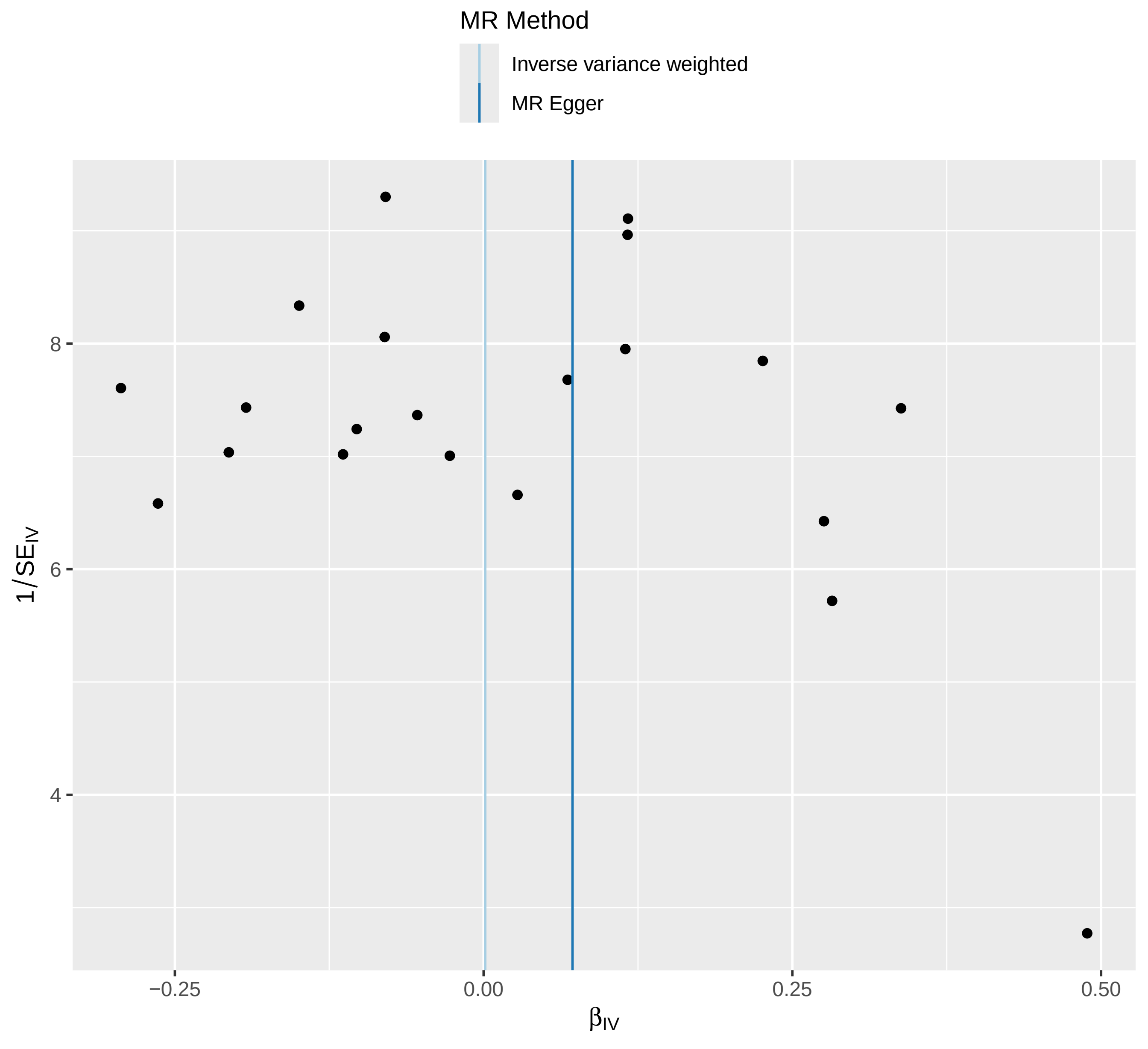

MR Method
Inverse variance weighted
MR Egger
1/SE_IV
8
6
4
−0.25
0.00
0.25
0.50
β_IV

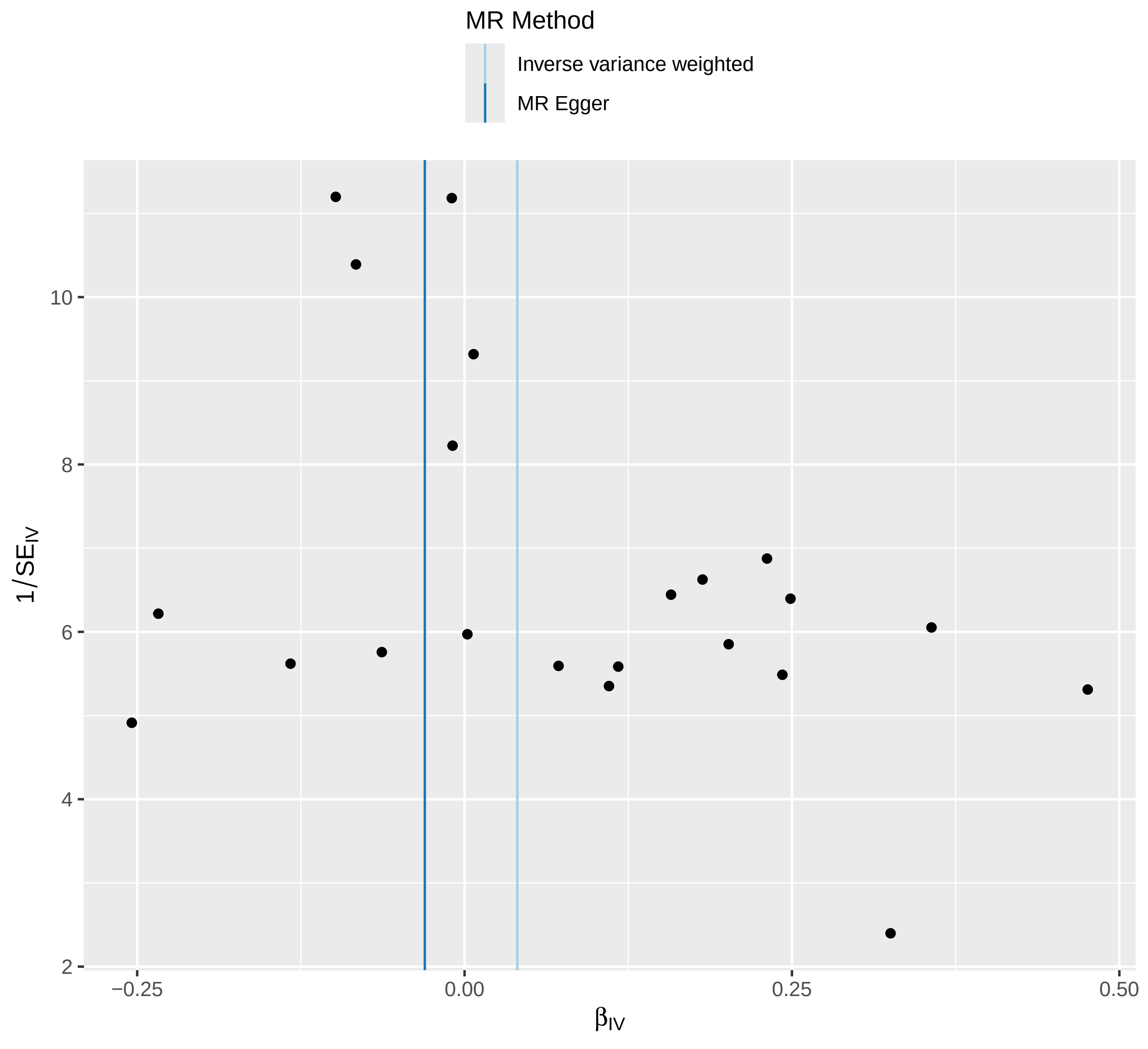

MR Method
Inverse variance weighted
MR Egger
1/SE_IV
β_IV
10
8
6
4
2
−0.25
0.00
0.25
0.50

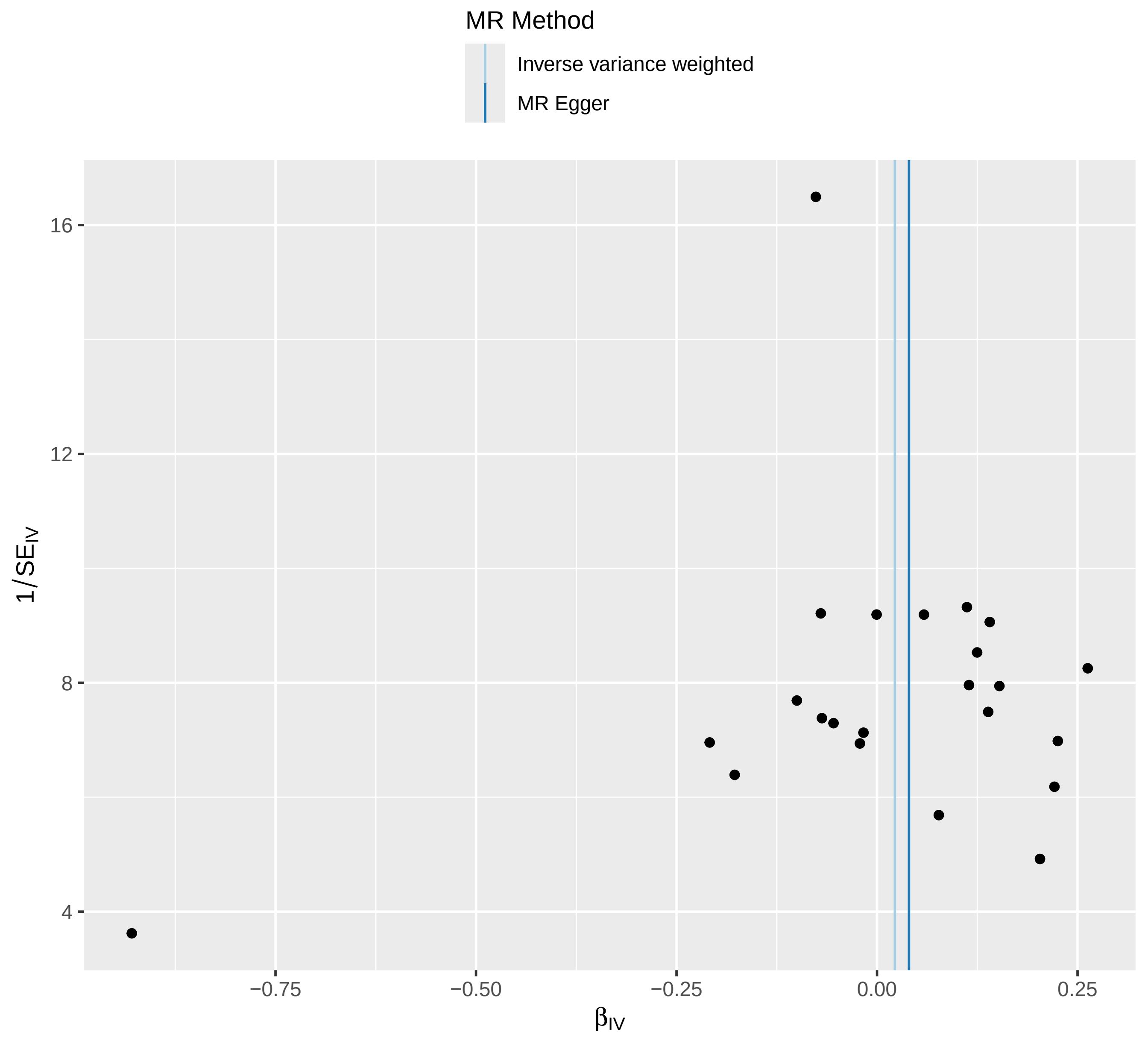

MR Method
Inverse variance weighted
MR Egger
16
12
8
4
1/SE_IV
−0.75
−0.50
−0.25
0.00
0.25
β_IV

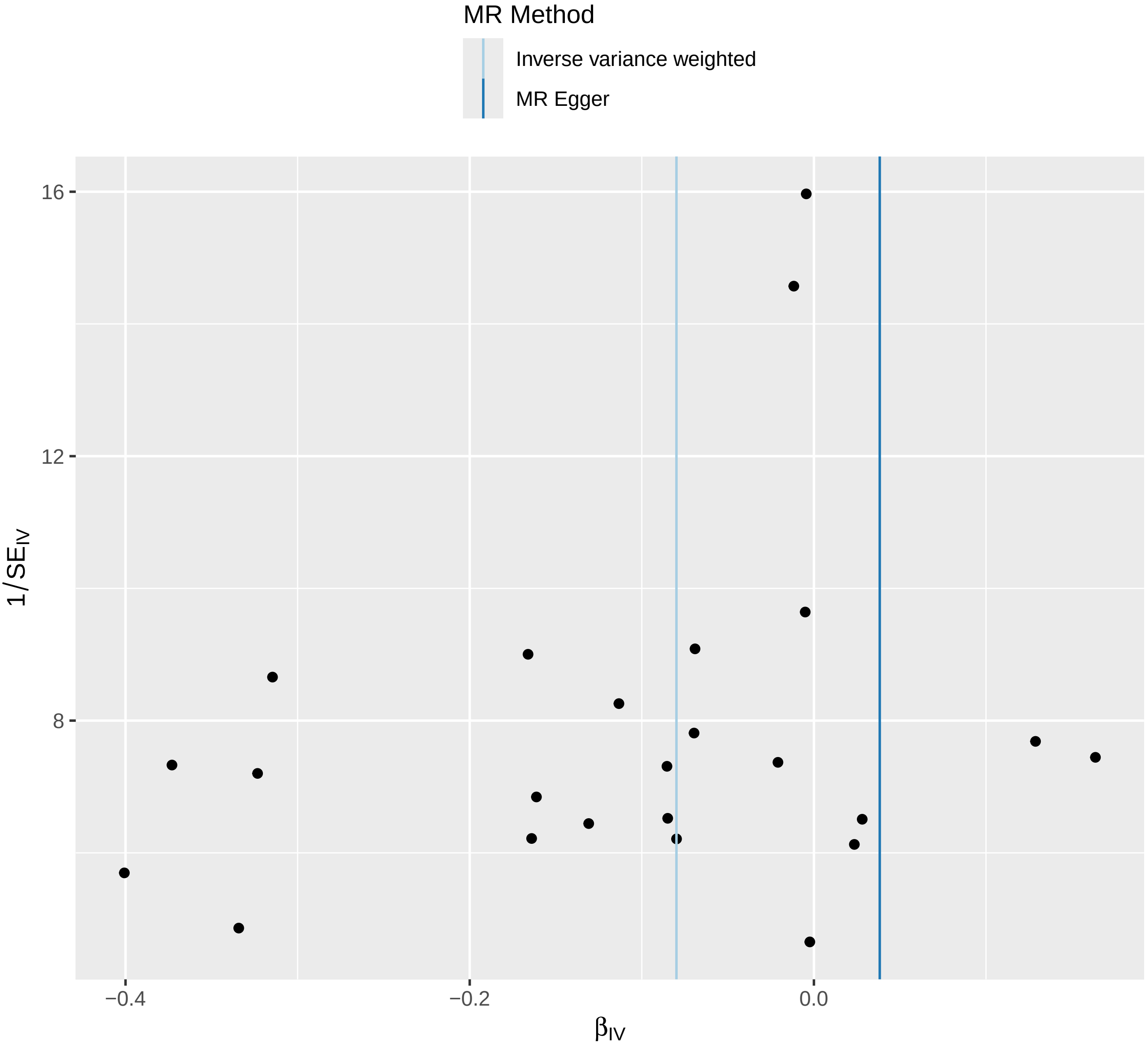
MR Method
Inverse variance weighted
MR Egger
16
12
8
1/SE_IV
−0.4
−0.2
0.0
β_IV

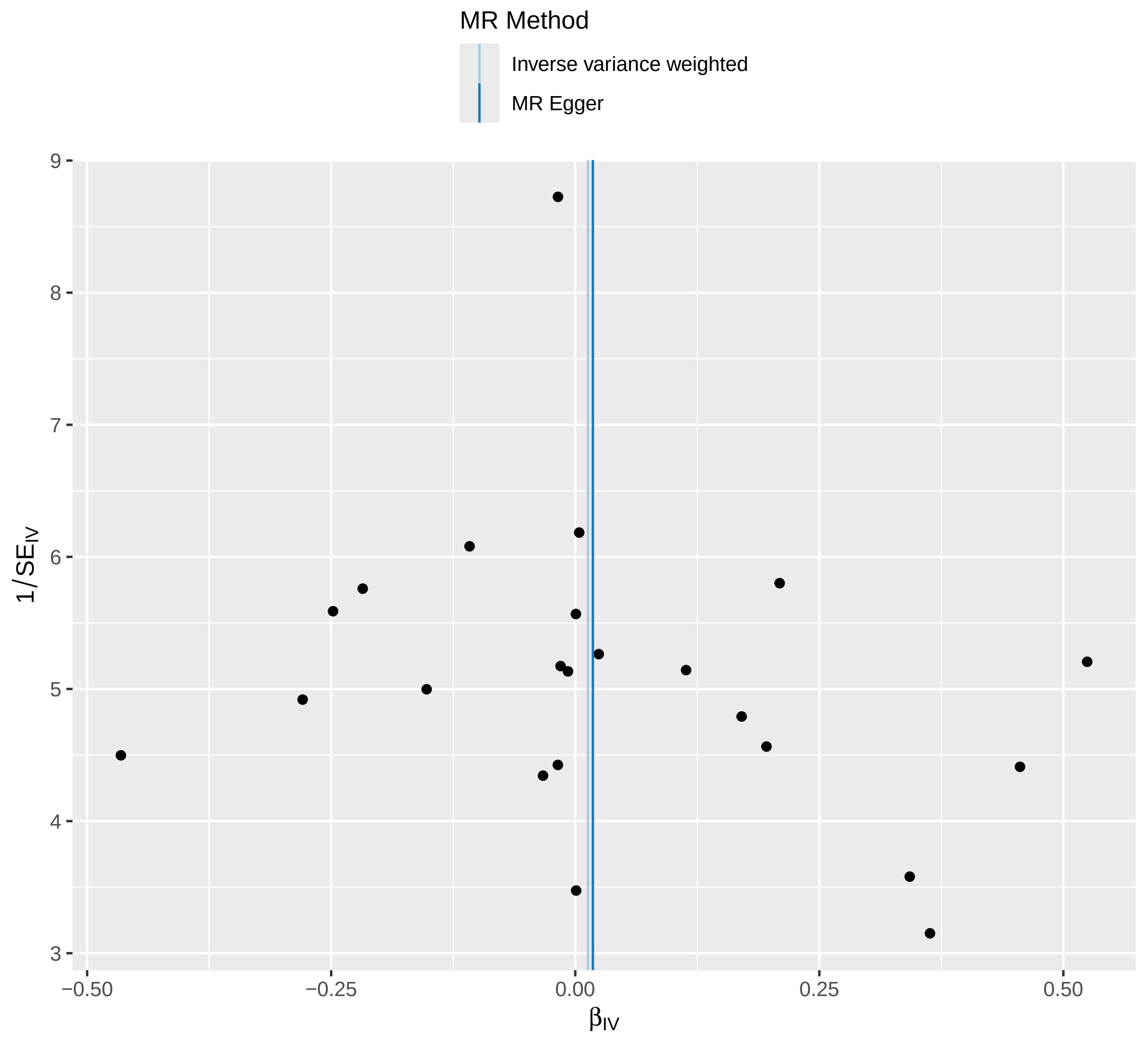

MR Method
Inverse variance weighted
MR Egger
1/SE_IV
β_IV
9
8
7
6
5
4
3
−0.50
−0.25
0.00
0.25
0.50

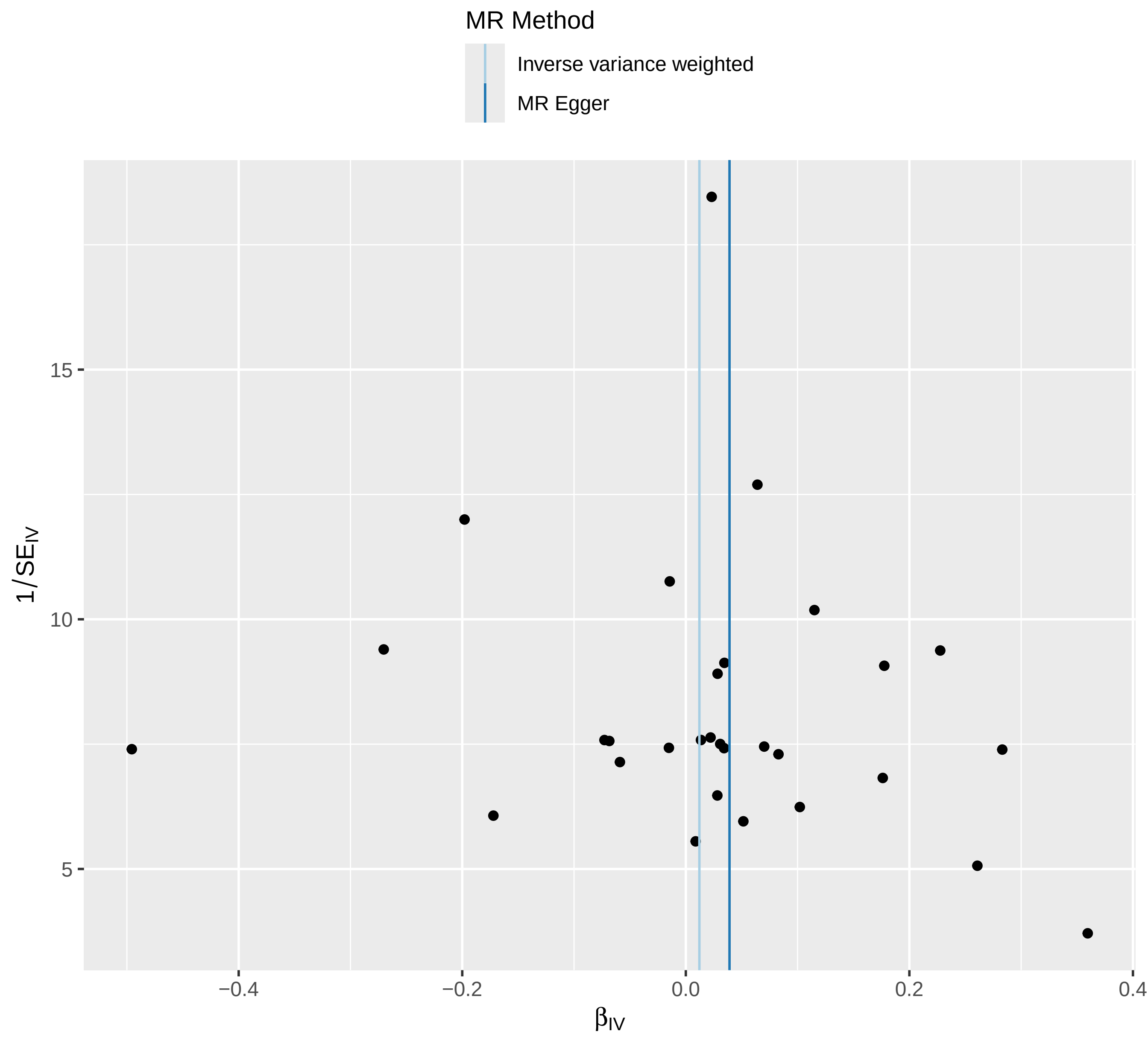
MR Method
Inverse variance weighted
MR Egger
15
10
5
1/SE_IV
−0.4
−0.2
0.0
0.2
0.4
β_IV

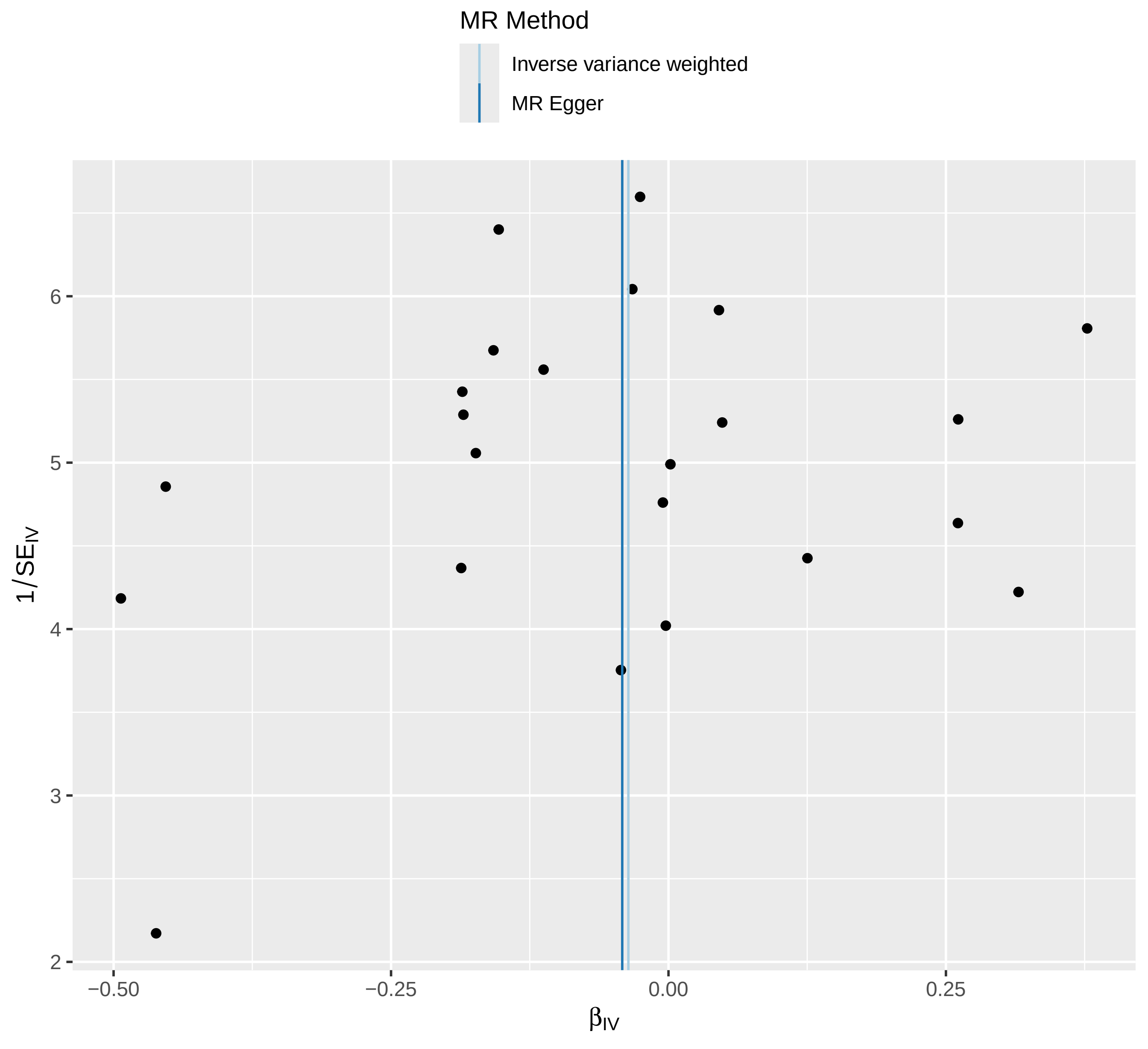

MR Method
Inverse variance weighted
MR Egger
1/SE_IV
6
5
4
3
2
β_IV
−0.50
−0.25
0.00
0.25

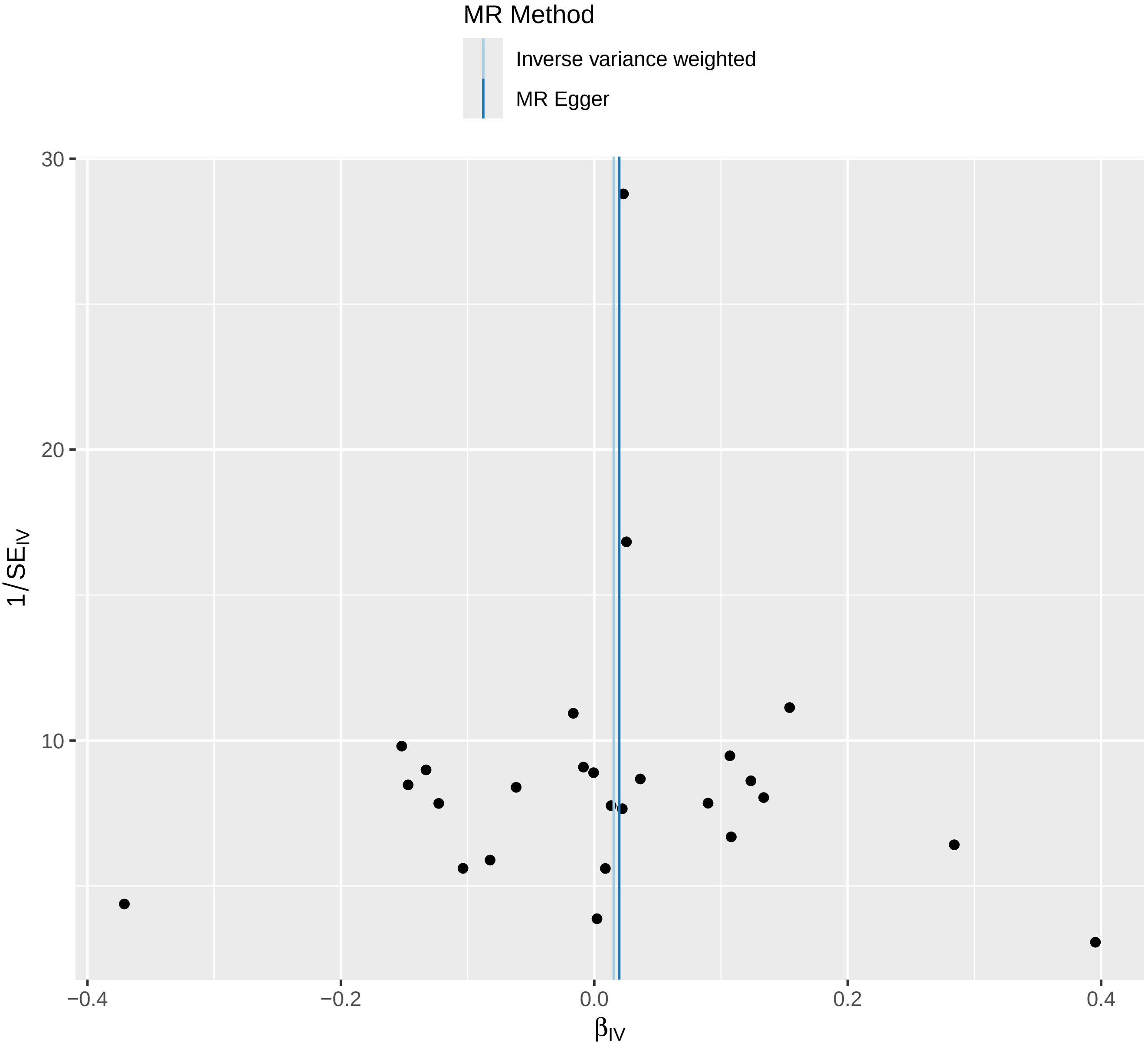
MR Method
Inverse variance weighted
MR Egger
30
20
10
1/SE_IV
−0.4
−0.2
0.0
0.2
0.4
β_IV

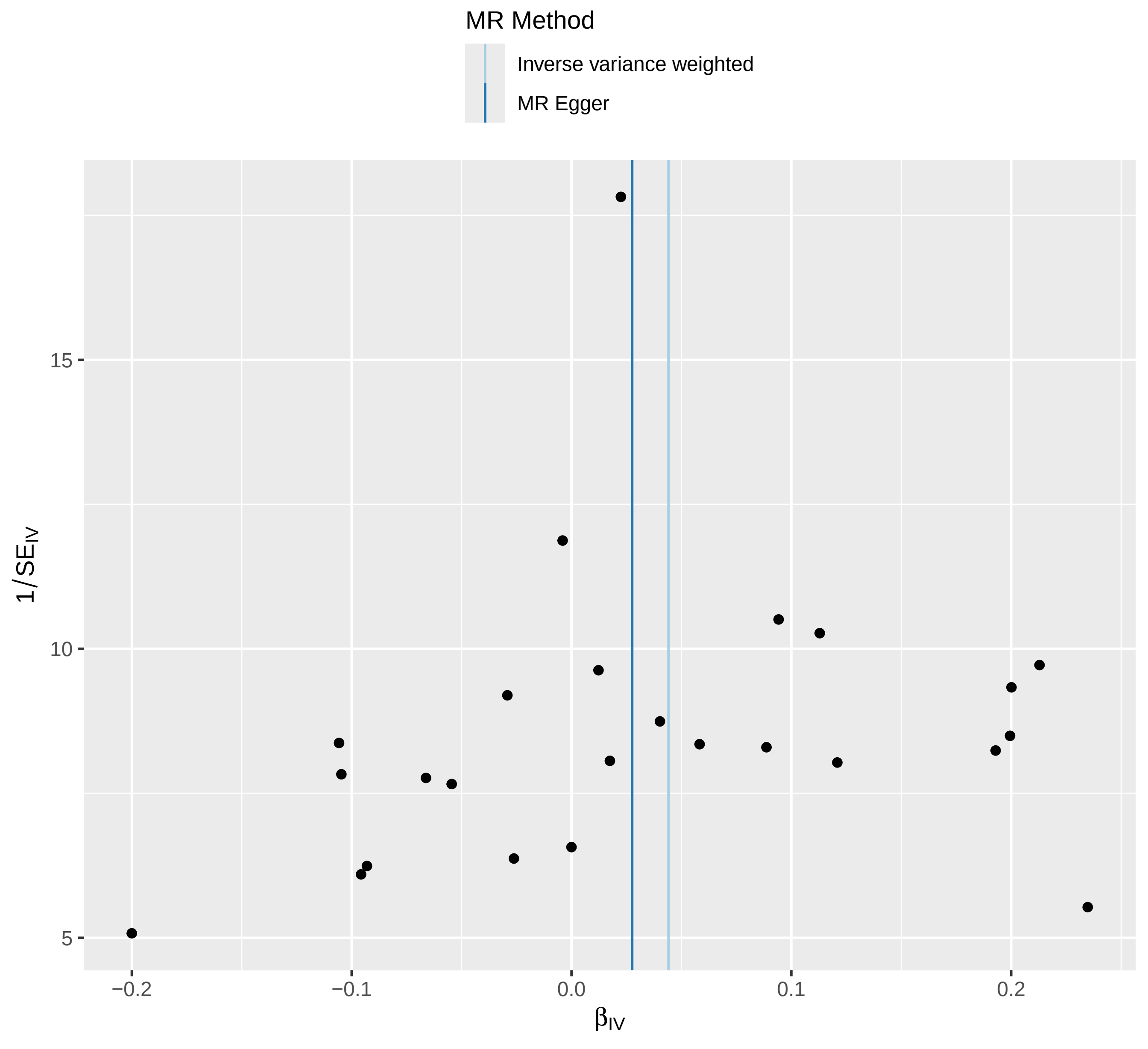
MR Method
Inverse variance weighted
MR Egger
1/SE_IV
15
10
5
−0.2
−0.1
0.0
0.1
0.2
β_IV

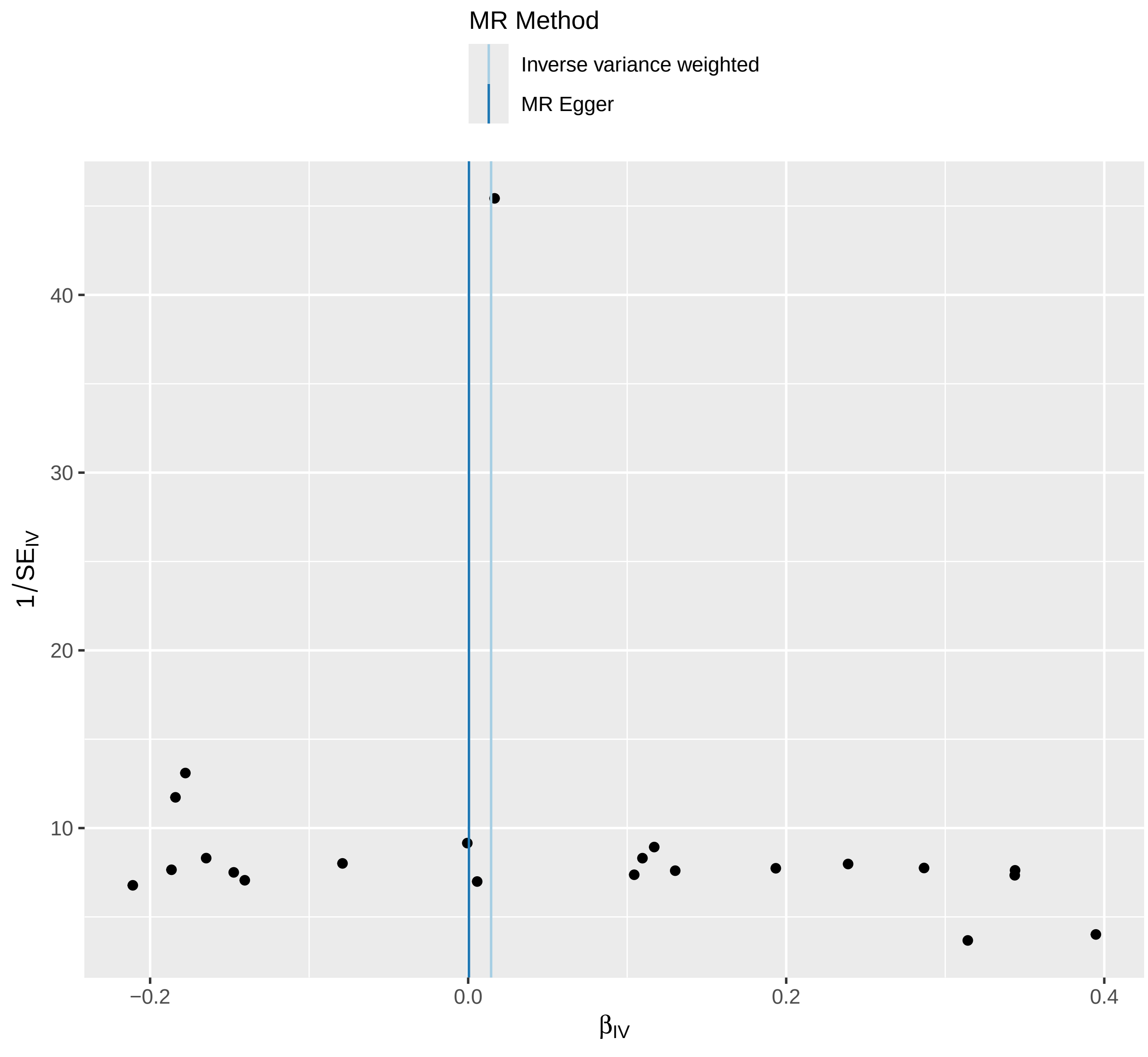

MR Method
Inverse variance weighted
MR Egger
1/SE_IV
40
30
20
10
−0.2
0.0
0.2
0.4
β_IV

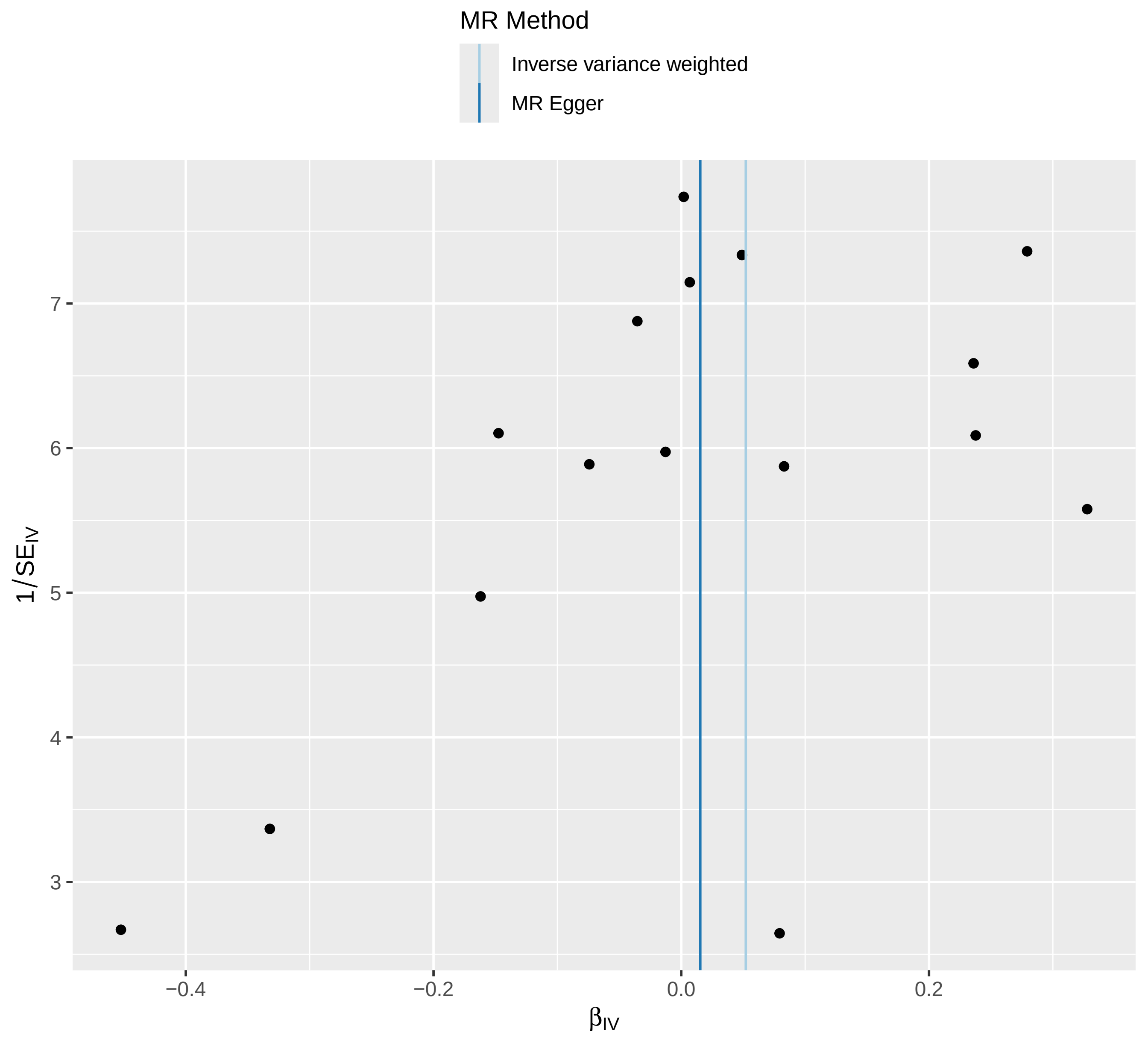

MR Method
Inverse variance weighted
MR Egger
7
6
5
4
3
1/SE_IV
−0.4
−0.2
0.0
0.2
β_IV

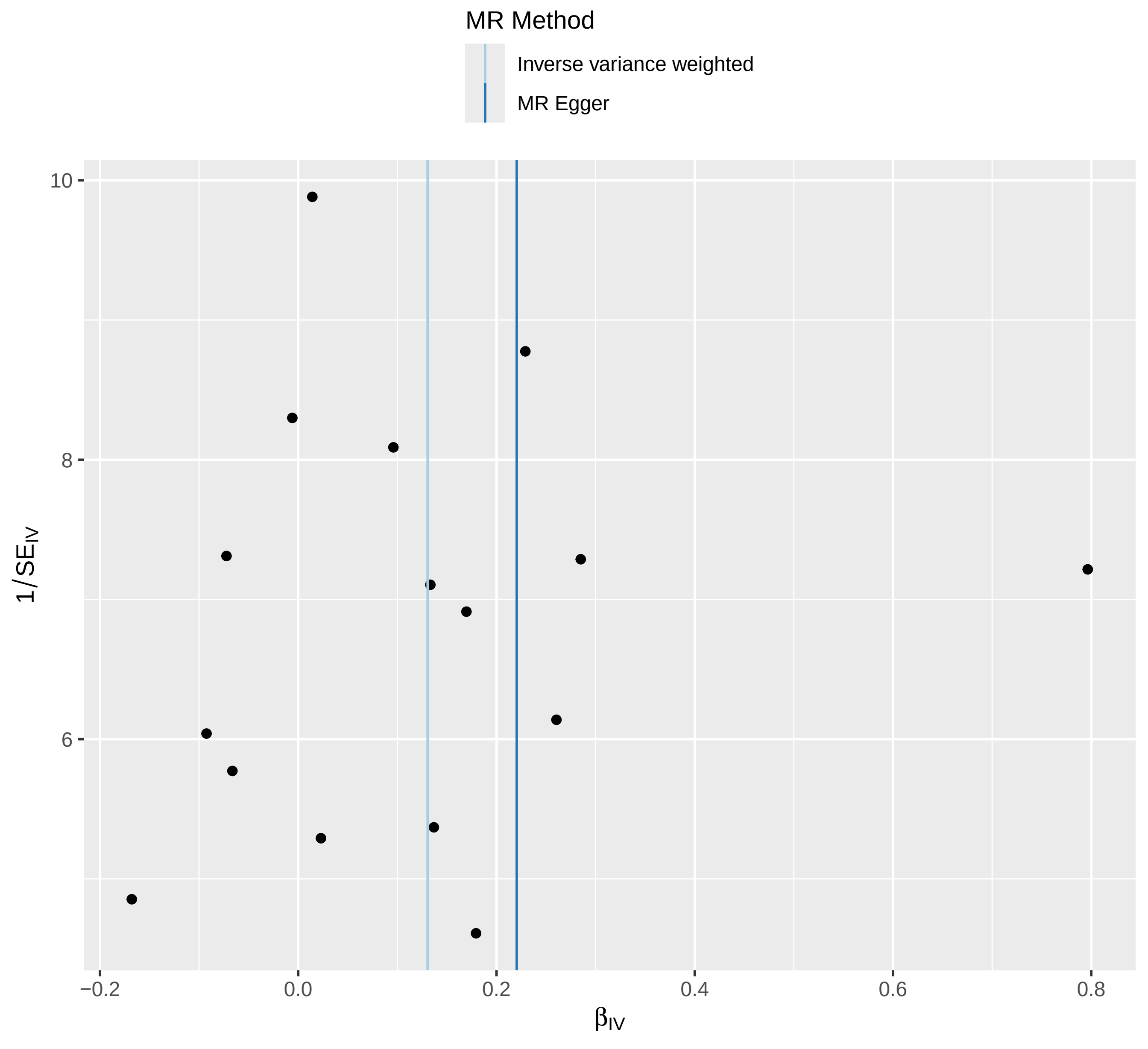
MR Method
Inverse variance weighted
MR Egger
10
8
6
1/SE$_{IV}$
−0.2
0.0
0.2
0.4
0.6
0.8
β$_{IV}$

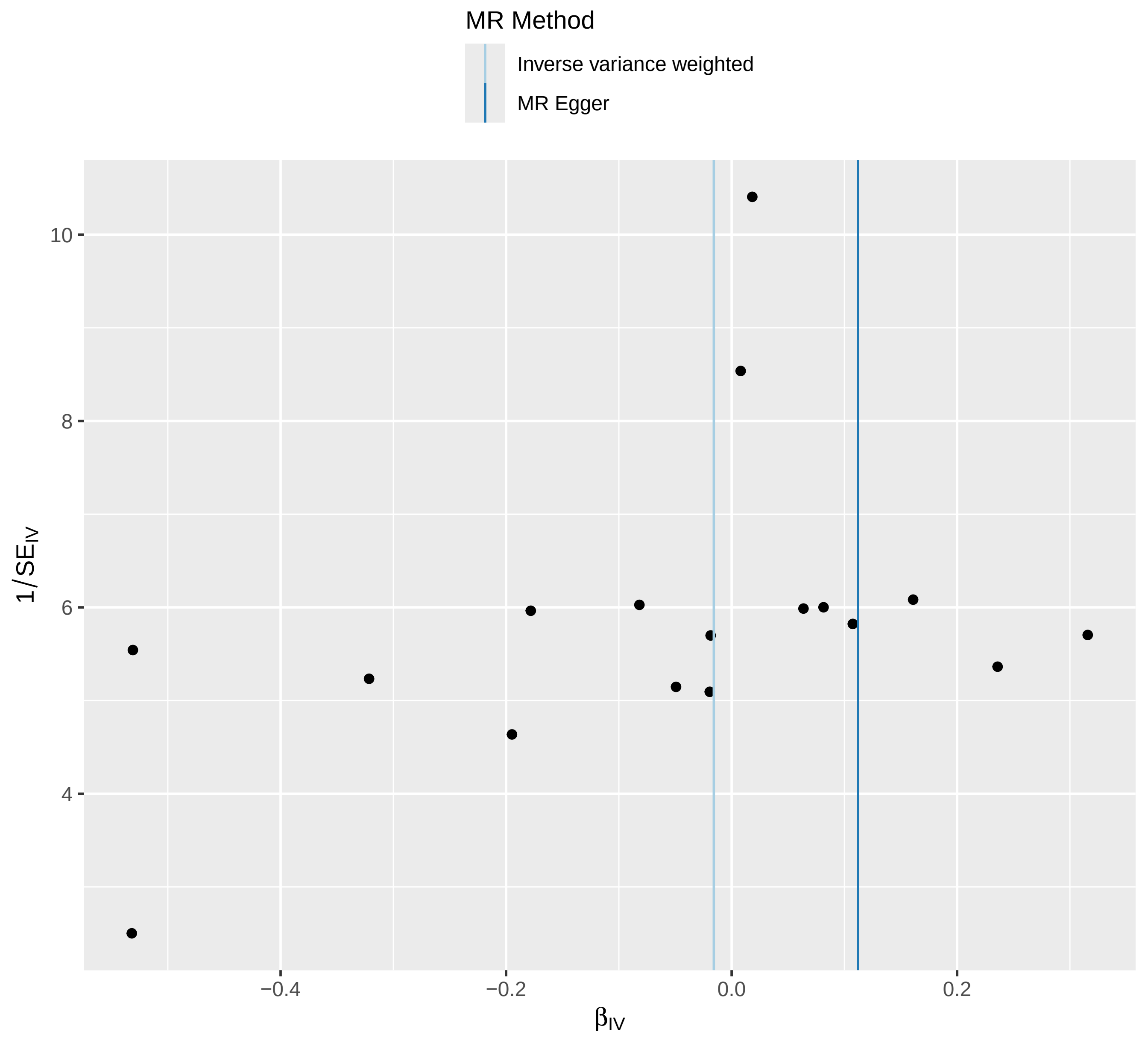
MR Method
Inverse variance weighted
MR Egger
10
8
6
4
1/SE$_{IV}$
−0.4
−0.2
0.0
0.2
β$_{IV}$

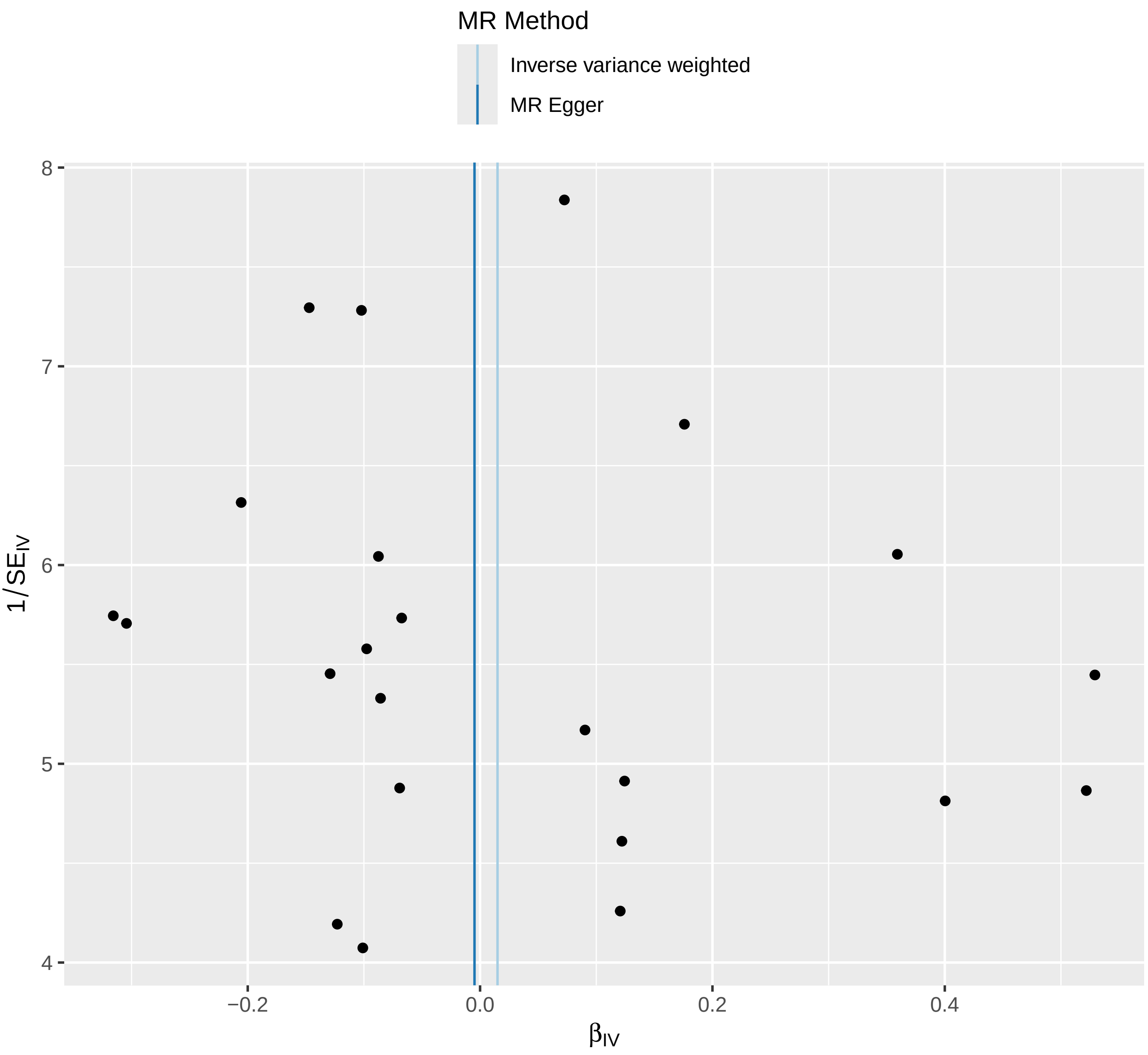

MR Method
Inverse variance weighted
MR Egger
1/SE_IV
8
7
6
5
4
−0.2
0.0
0.2
0.4
β_IV

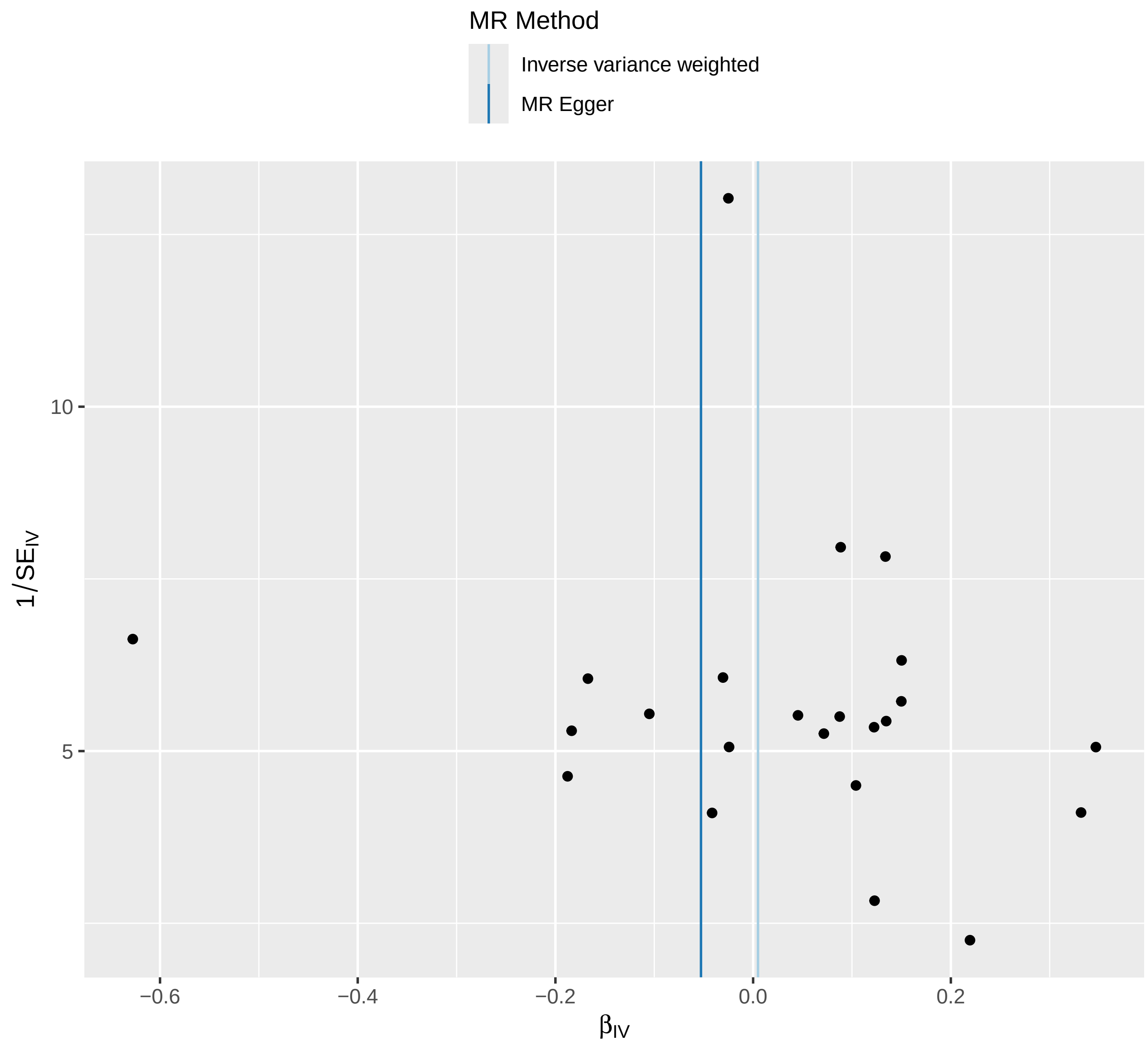

MR Method
Inverse variance weighted
MR Egger
10
5
1/SE_IV
−0.6
−0.4
−0.2
0.0
0.2
β_IV

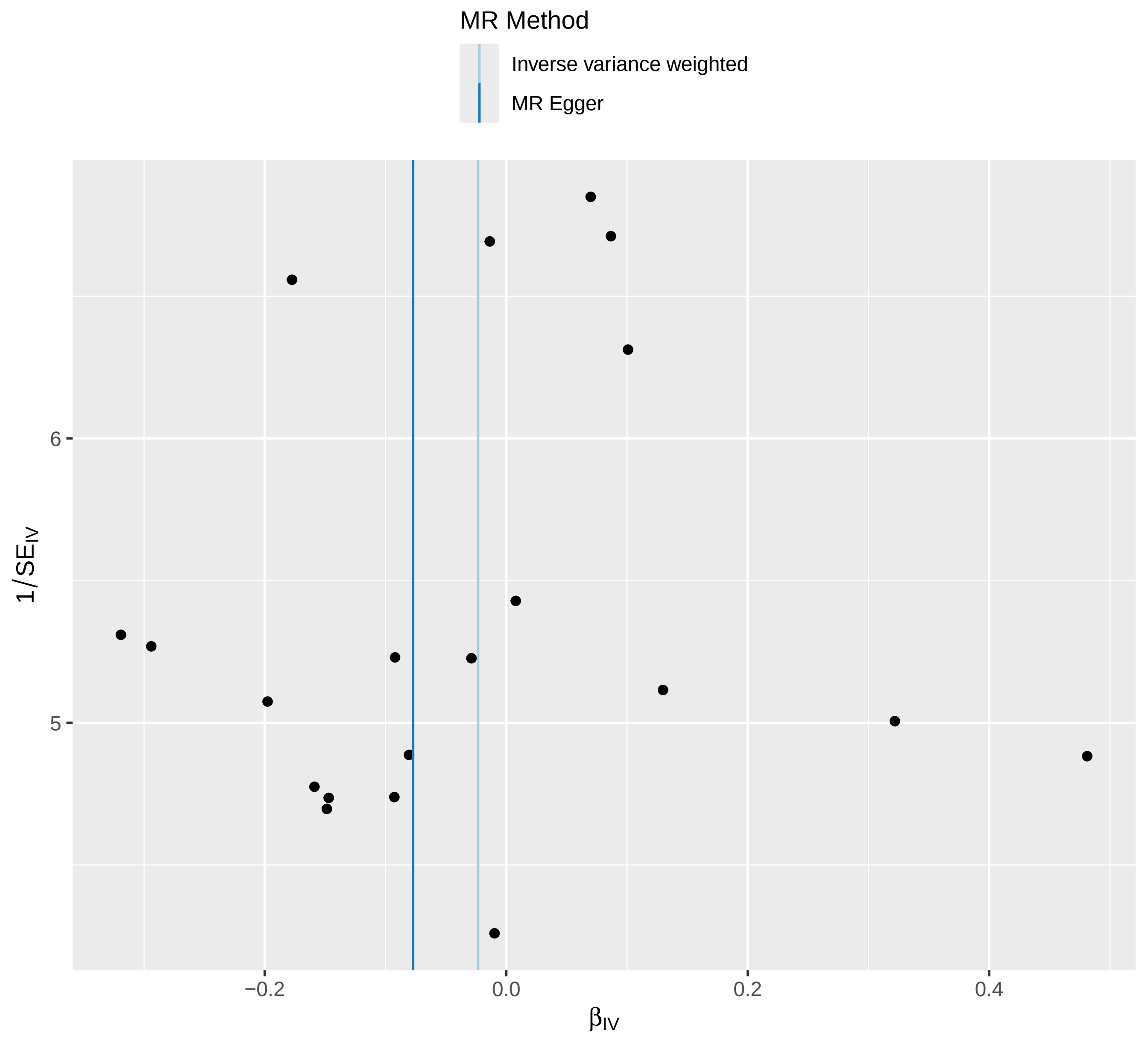

MR Method
Inverse variance weighted
MR Egger
6
5
−0.2
0.0
0.2
0.4
1/SE_IV
β_IV

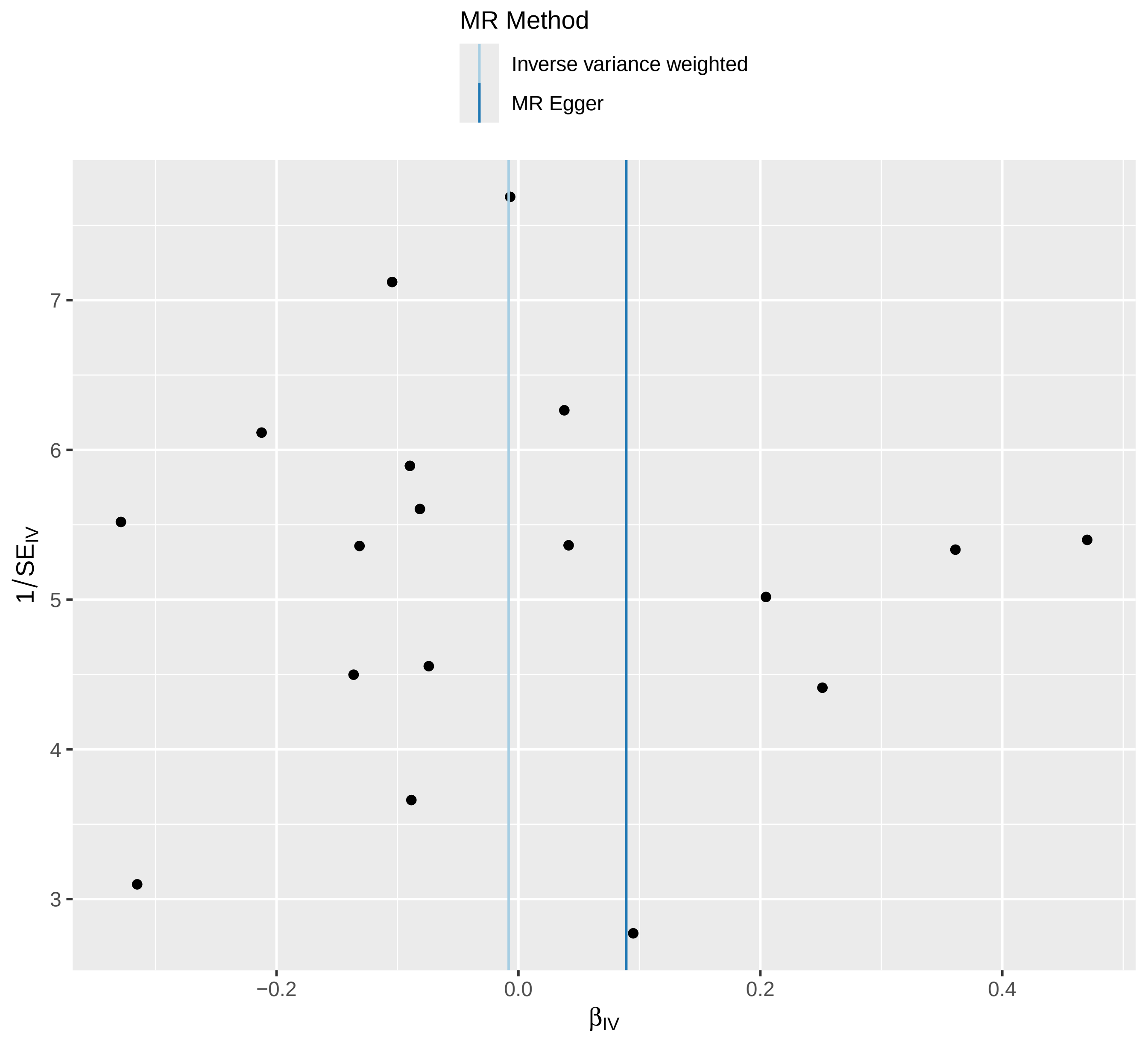

MR Method
Inverse variance weighted
MR Egger
1/SE_IV
β_IV
7
6
5
4
3
−0.2
0.0
0.2
0.4

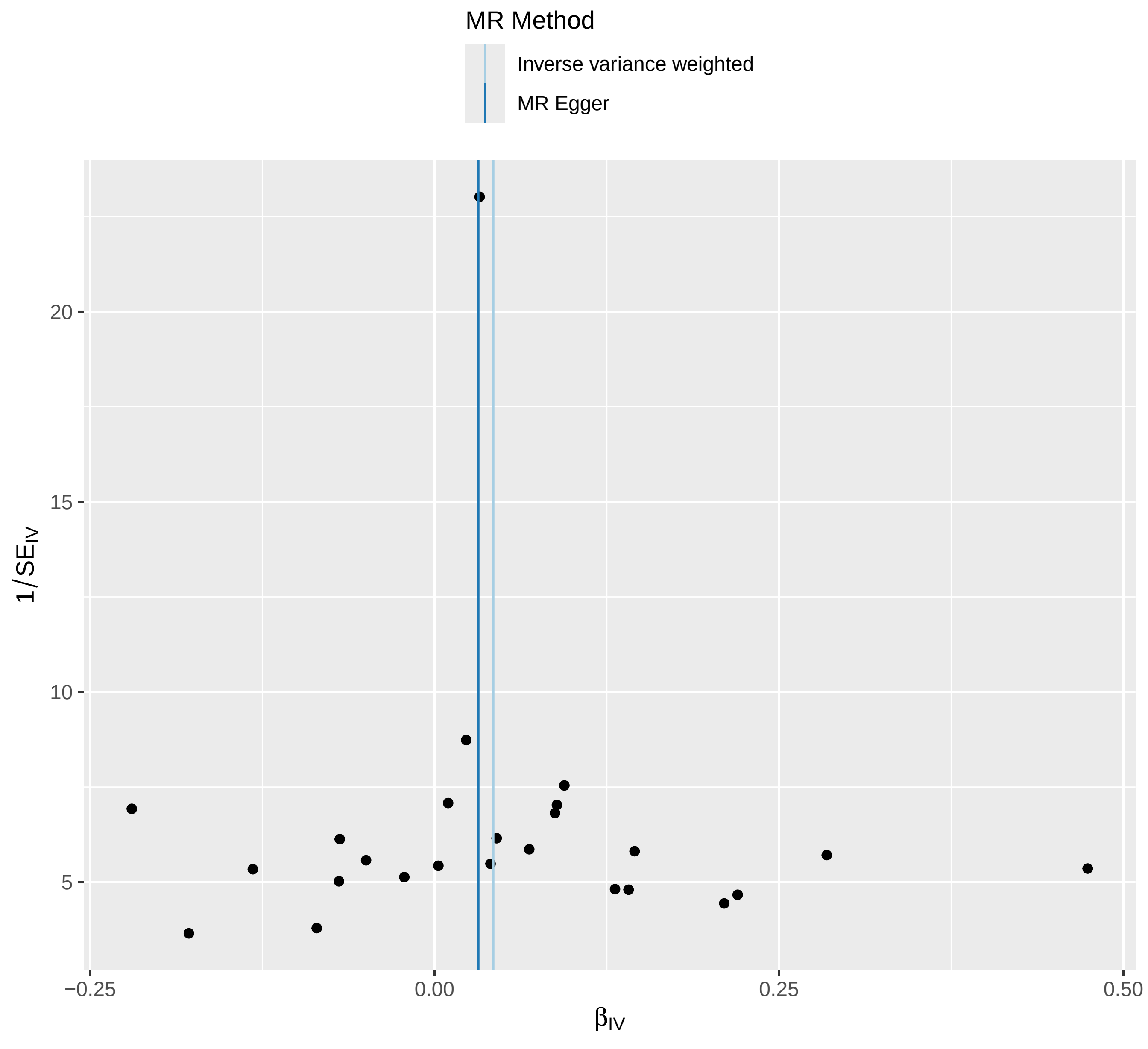
MR Method
Inverse variance weighted
MR Egger
20
15
10
5
1/SE_IV
−0.25
0.00
0.25
0.50
β_IV

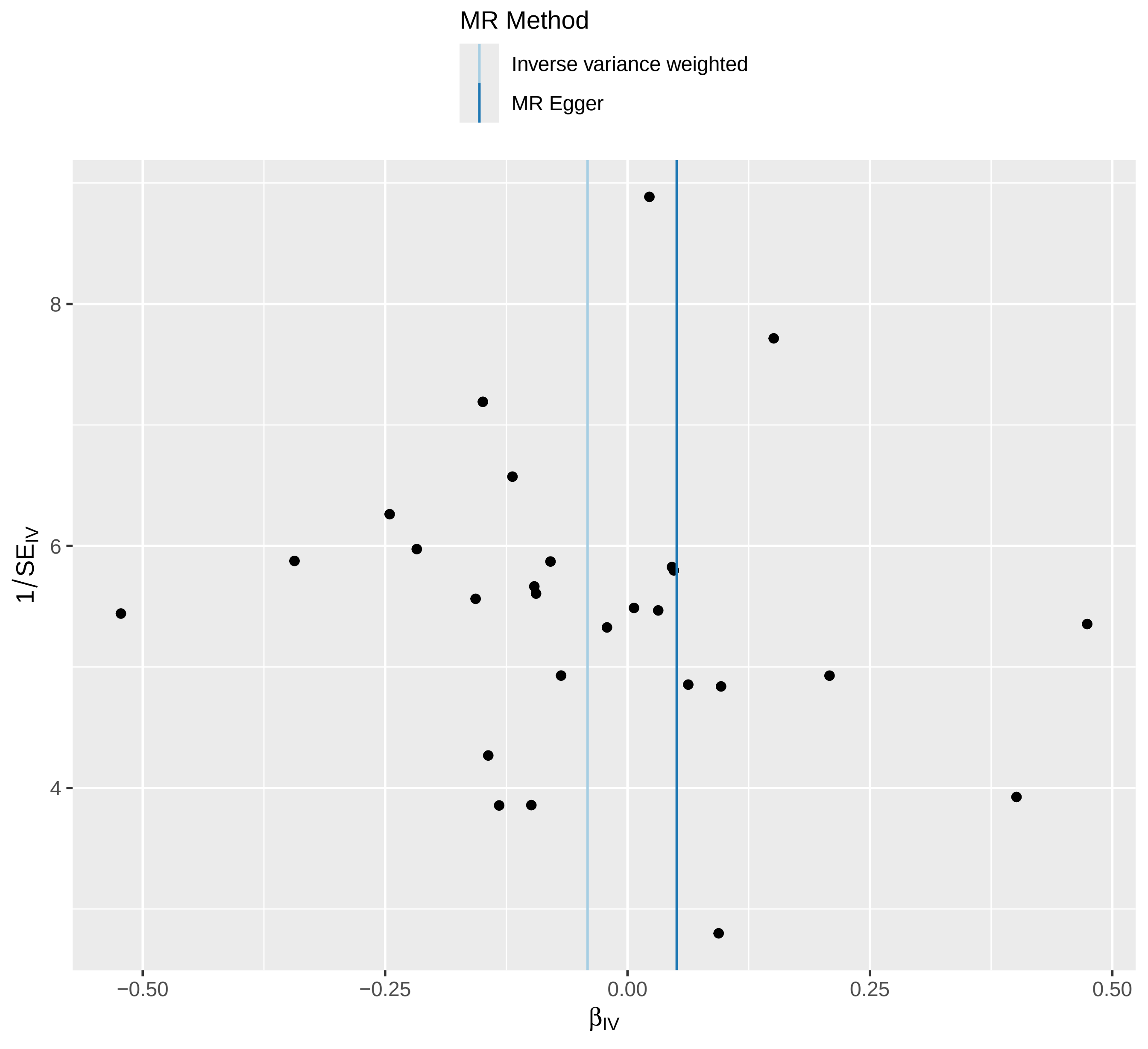

MR Method
Inverse variance weighted
MR Egger
8
6
4
1/SE_IV
−0.50
−0.25
0.00
0.25
0.50
β_IV

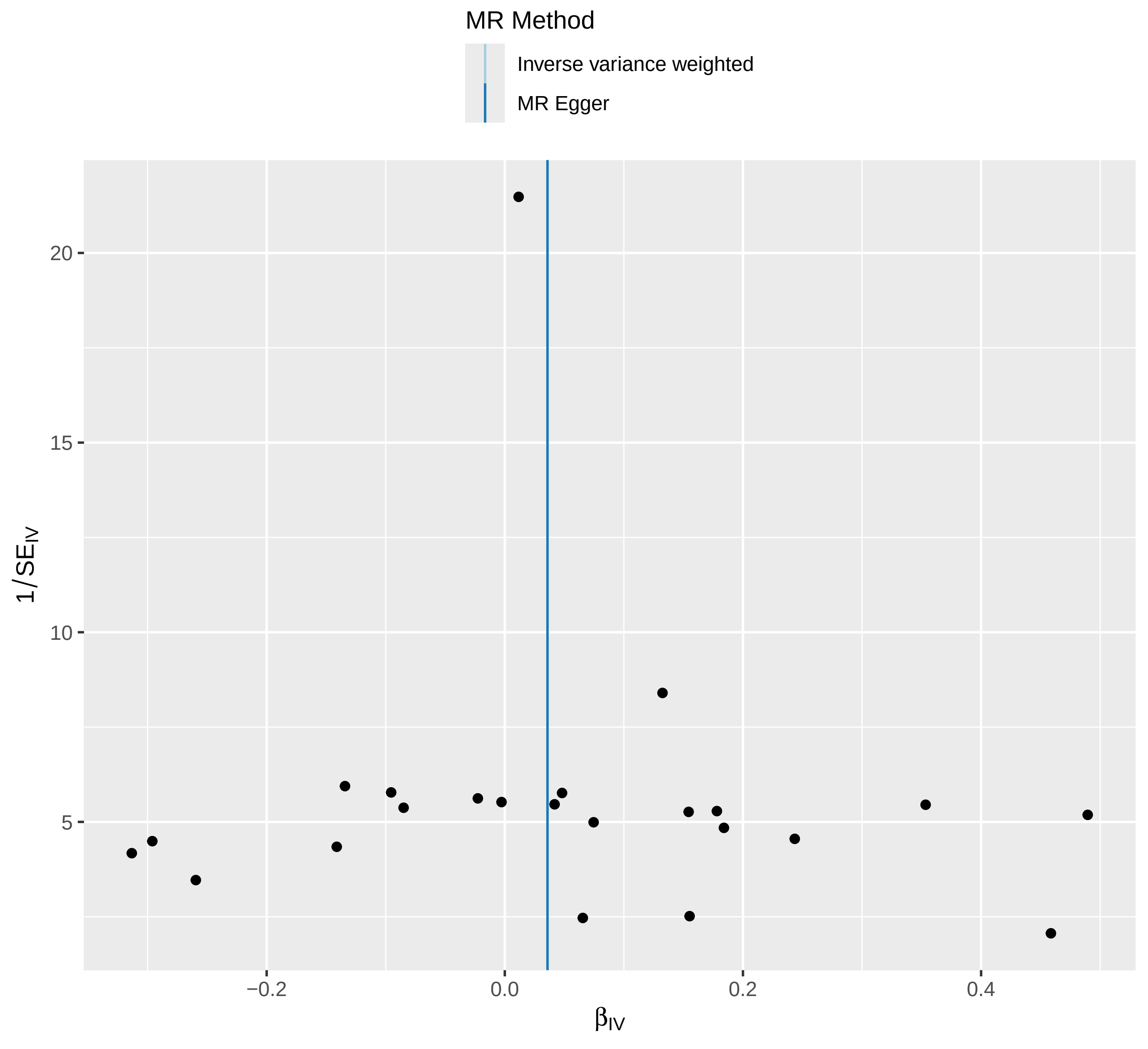
MR Method
Inverse variance weighted
MR Egger
20
15
10
5
1/SE_IV
−0.2
0.0
0.2
0.4
β_IV

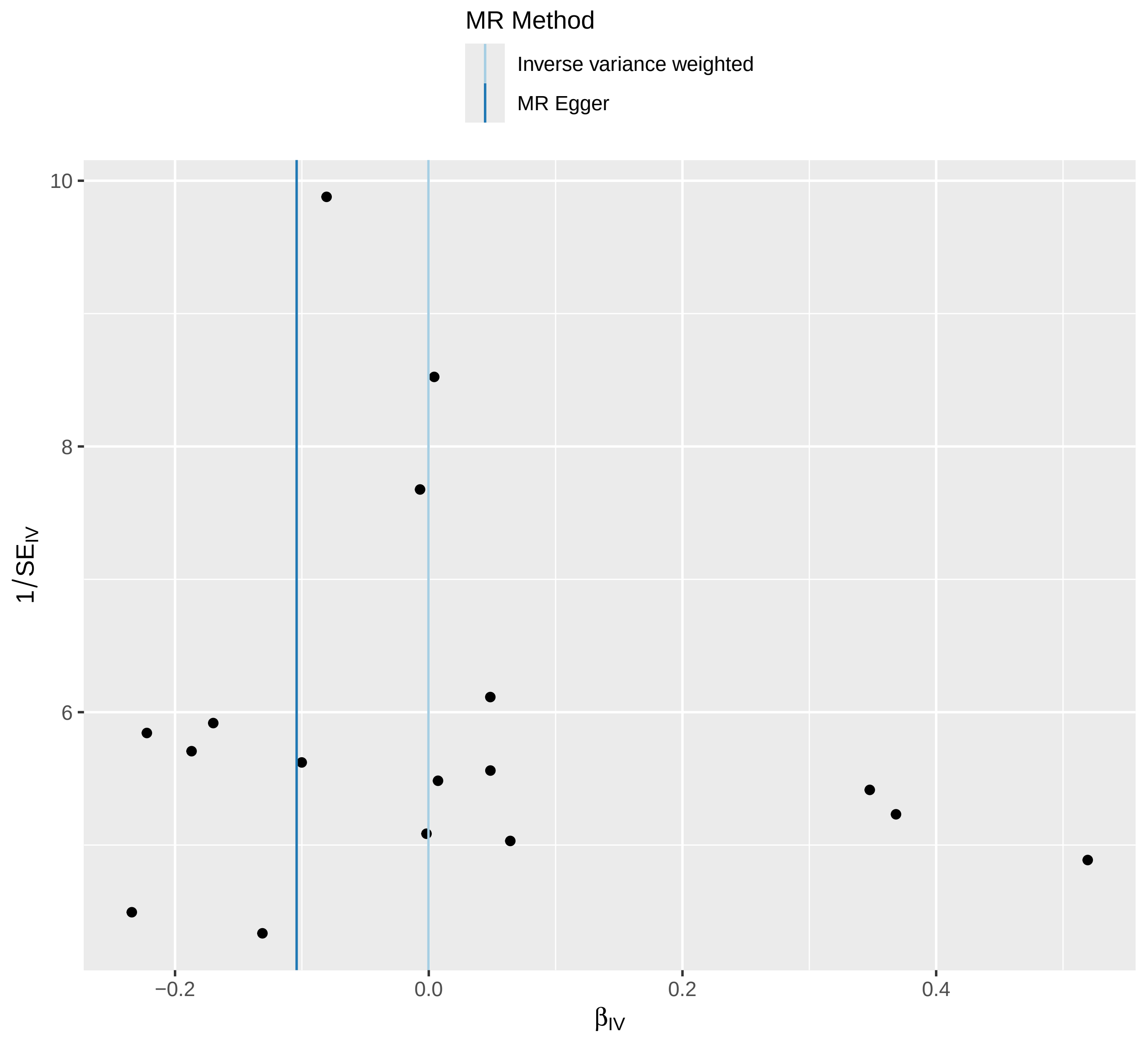
MR Method
Inverse variance weighted
MR Egger
10
8
6
1/SE_IV
−0.2
0.0
0.2
0.4
β_IV

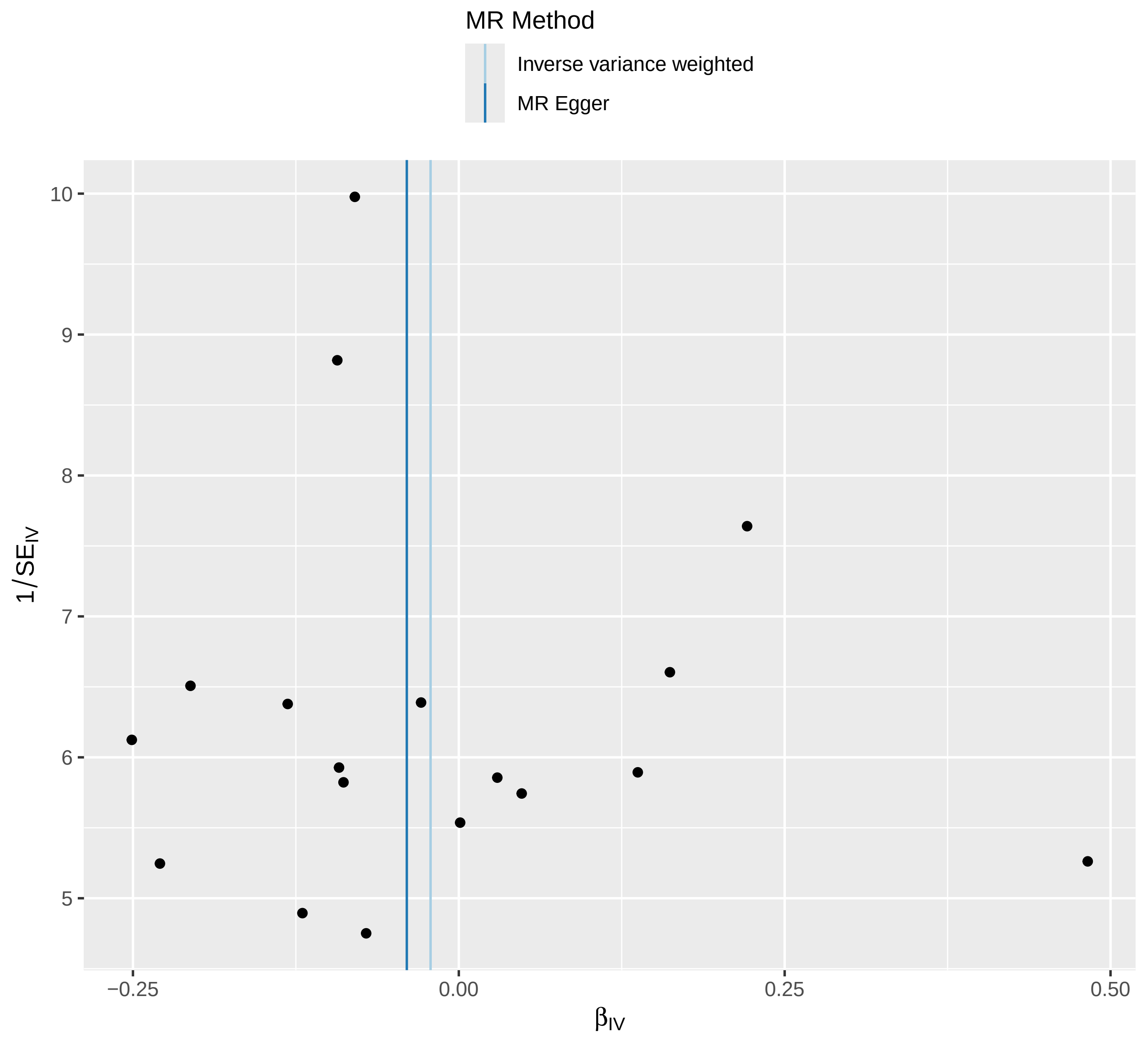
MR Method
Inverse variance weighted
MR Egger
10
9
8
7
6
5
1/SE_IV
−0.25
0.00
0.25
0.50
β_IV

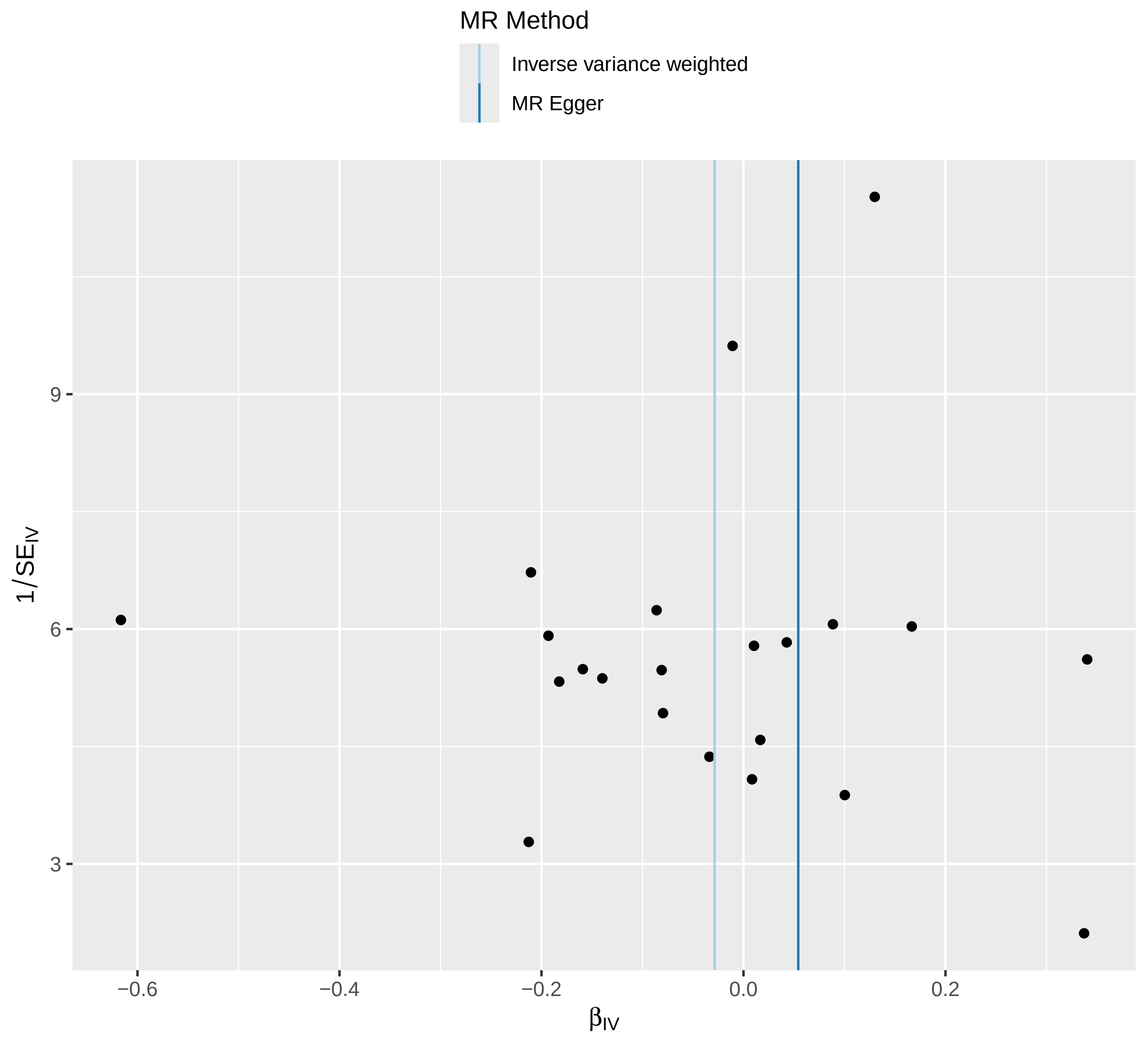
MR Method
Inverse variance weighted
MR Egger
9
6
3
$1/SE_{IV}$
−0.6
−0.4
−0.2
0.0
0.2
β_{IV}

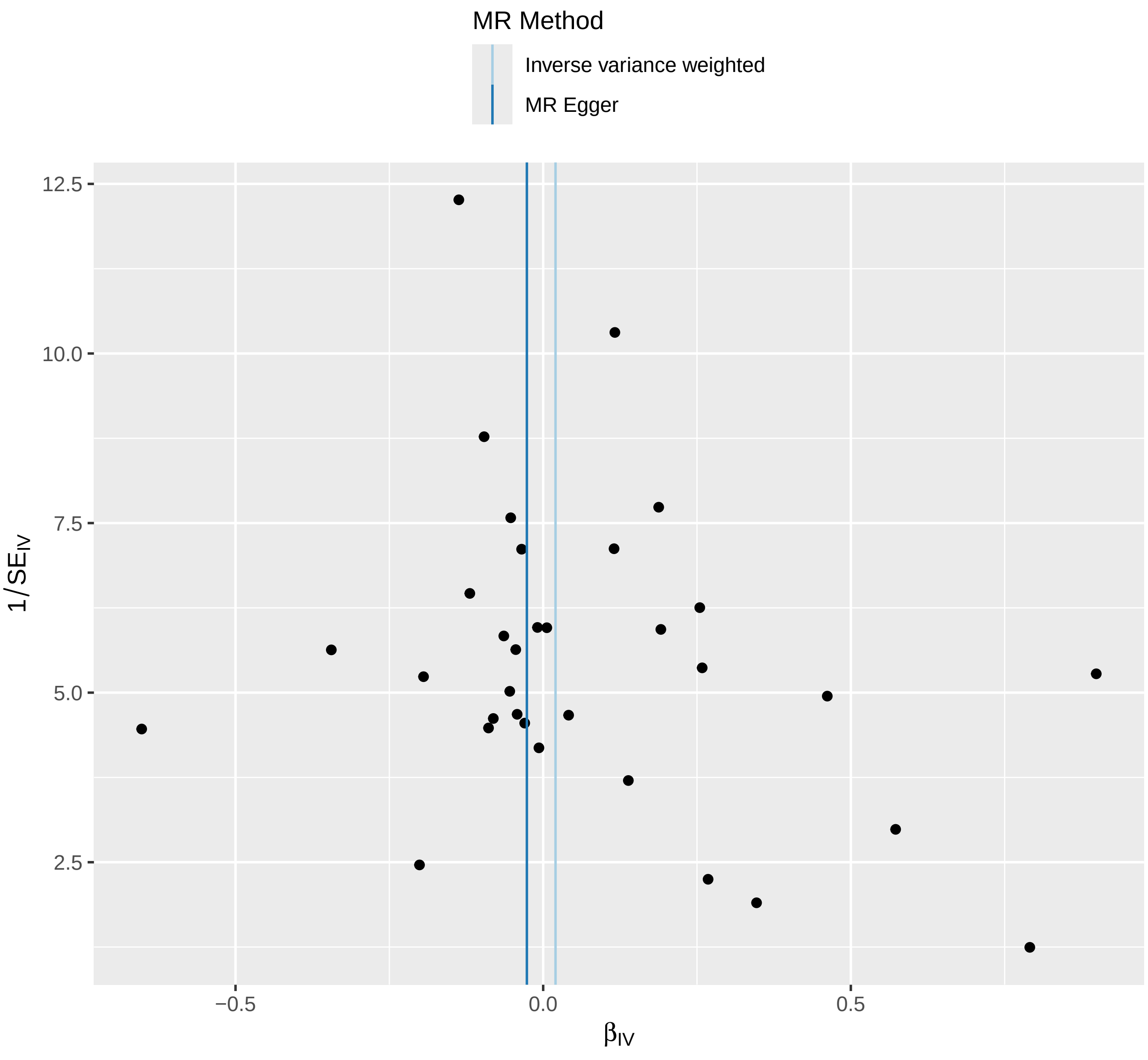

MR Method
Inverse variance weighted
MR Egger
12.5
10.0
7.5
5.0
2.5
1/SE_IV
−0.5
0.0
0.5
β_IV

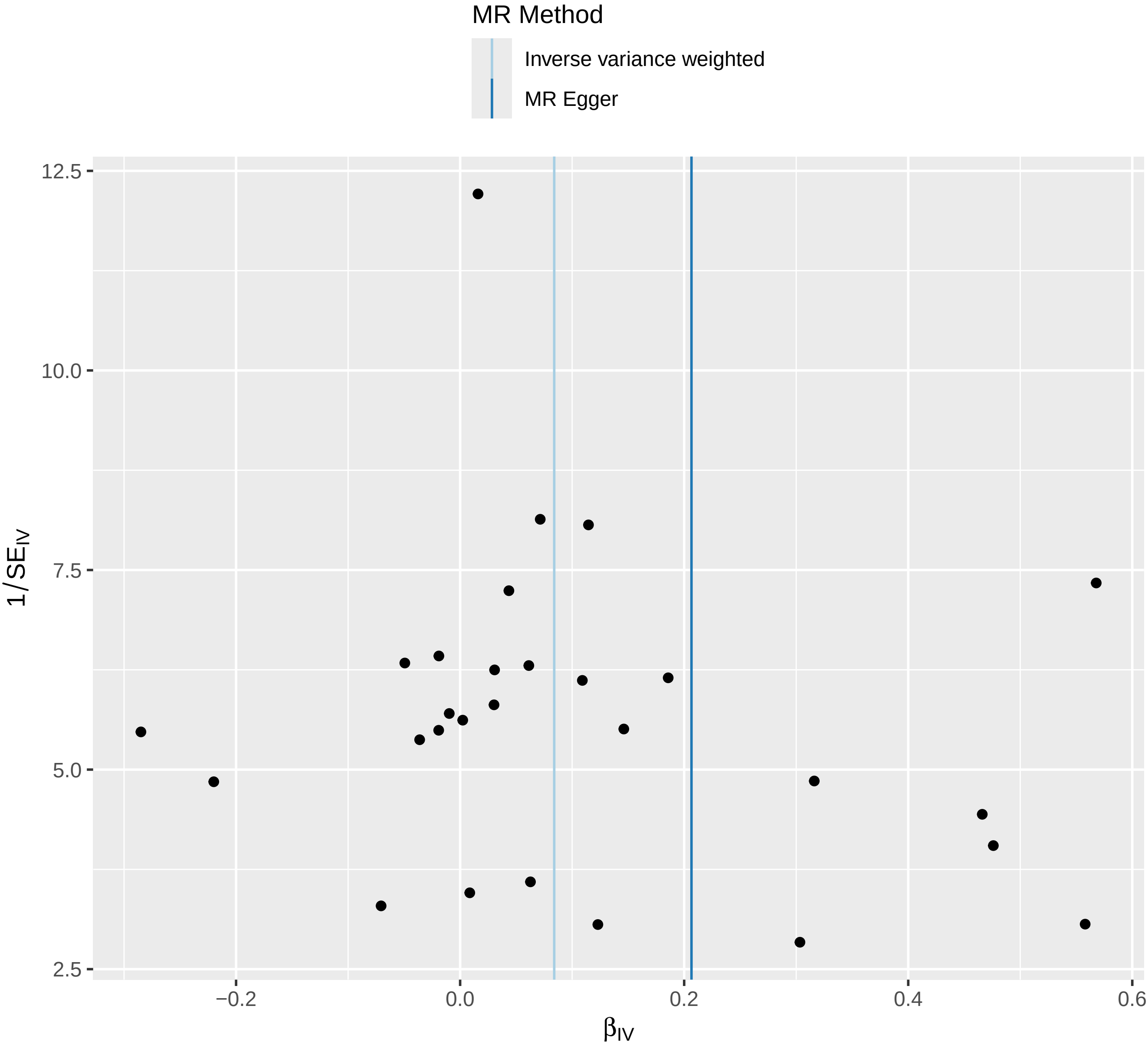

MR Method
Inverse variance weighted
MR Egger
12.5
10.0
7.5
5.0
2.5
−0.2
0.0
0.2
0.4
0.6
$1/SE_{IV}$
β_{IV}

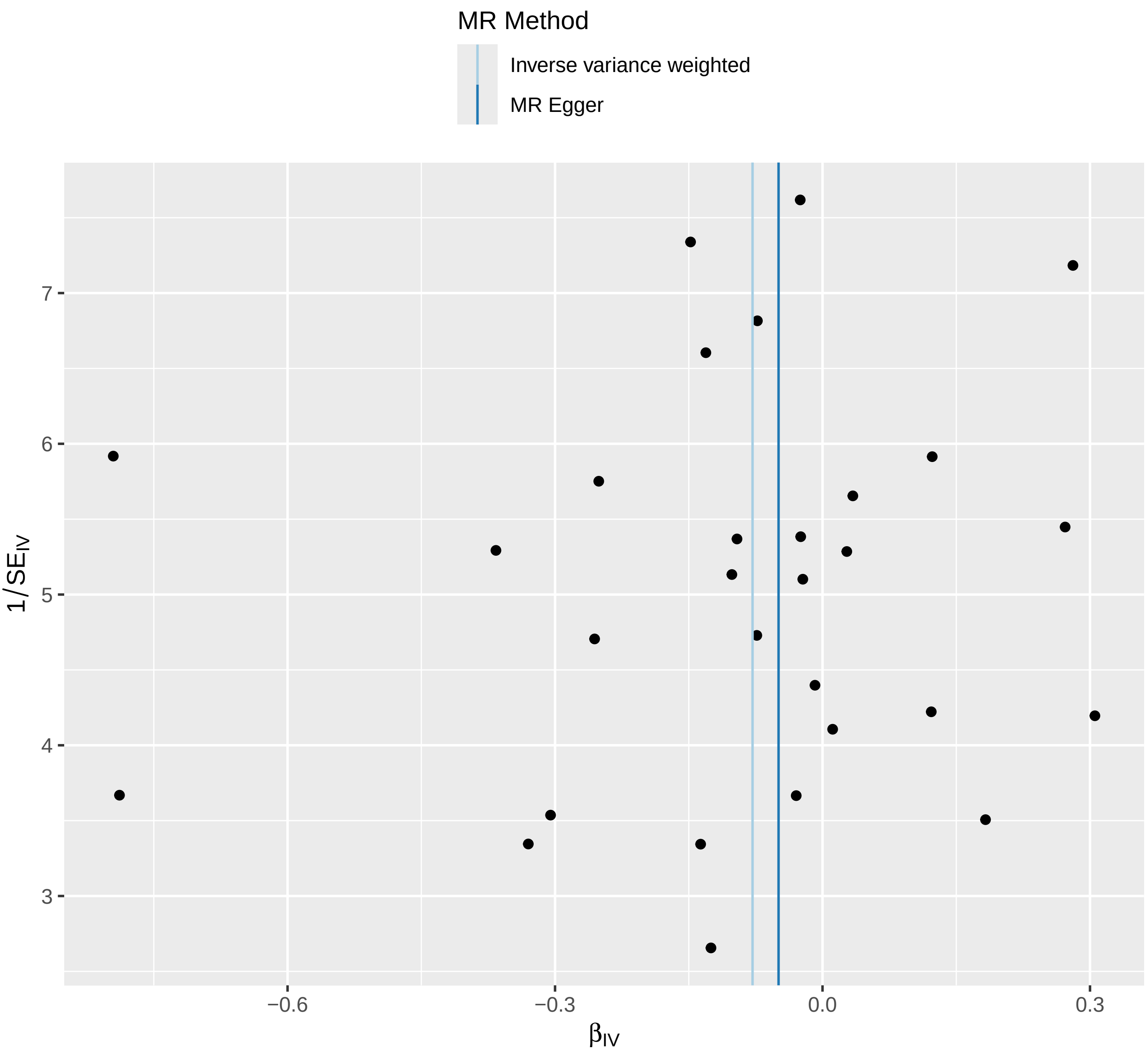
MR Method
Inverse variance weighted
MR Egger
1/SE_IV
β_IV
−0.6
−0.3
0.0
0.3
3
4
5
6
7

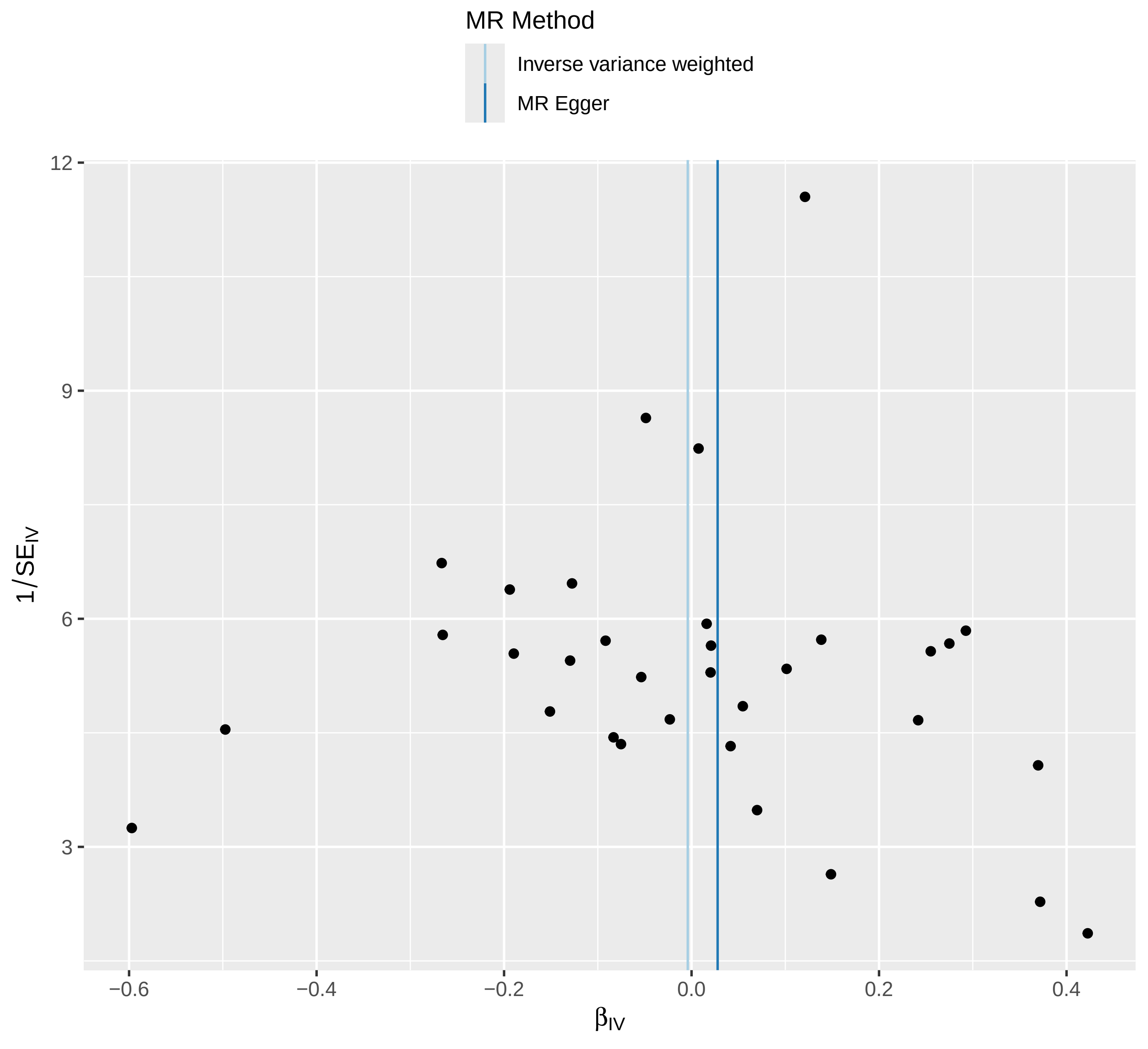

MR Method
Inverse variance weighted
MR Egger
12
9
6
3
1/SE$_{IV}$
−0.6
−0.4
−0.2
0.0
0.2
0.4
β$_{IV}$

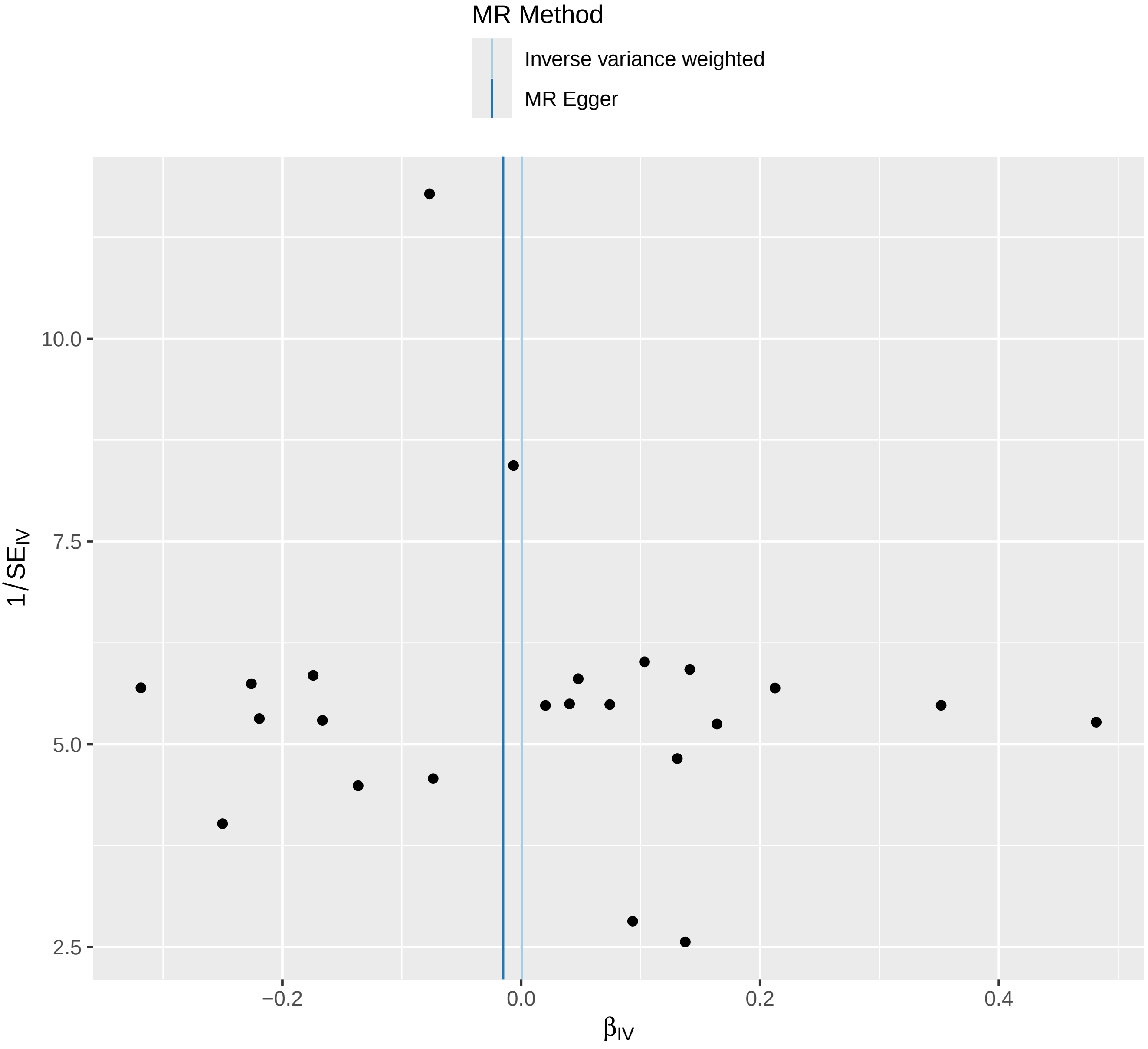
MR Method
Inverse variance weighted
MR Egger
10.0
7.5
5.0
2.5
$1/SE_{IV}$
−0.2
0.0
0.2
0.4
β_{IV}

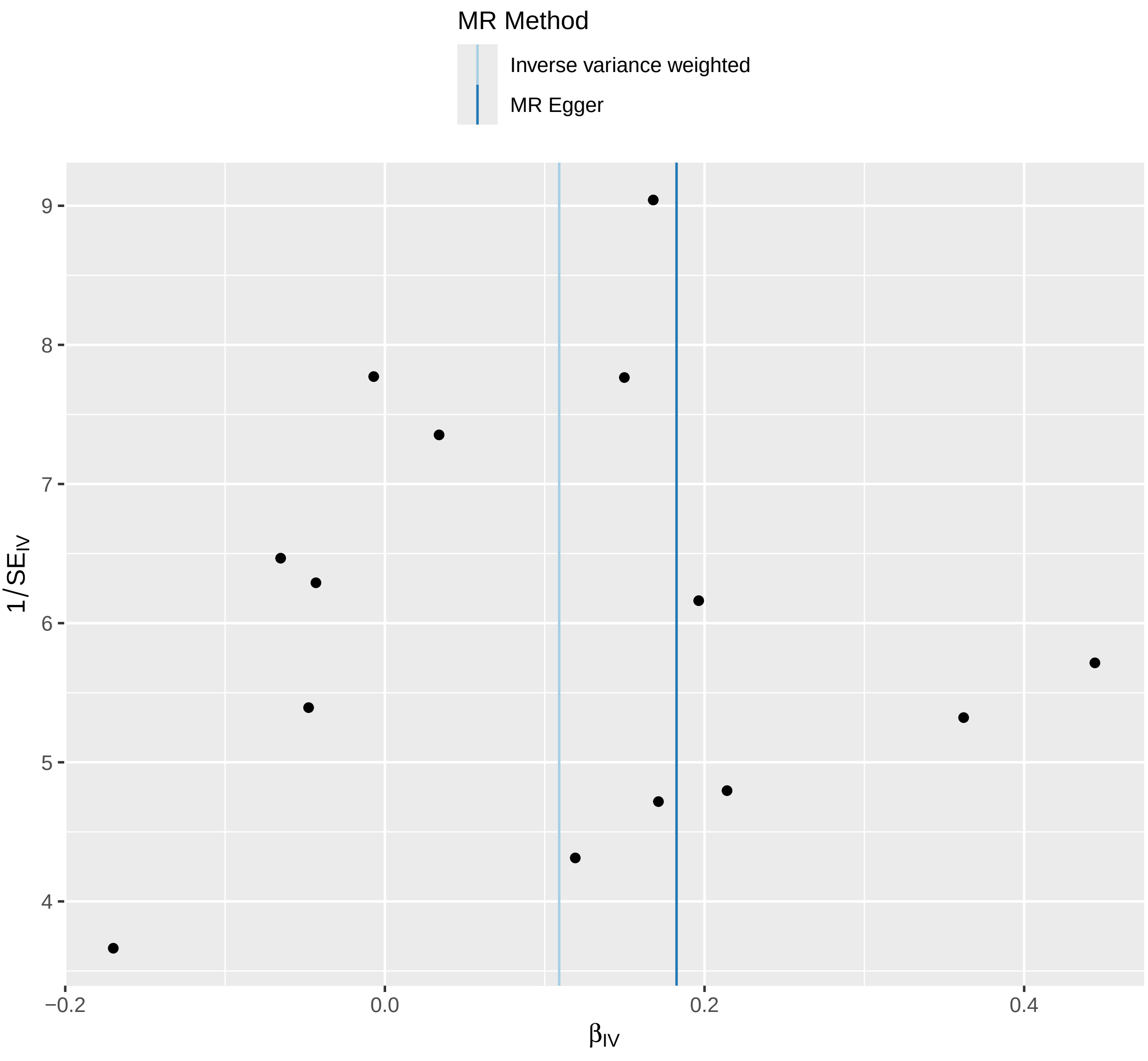
MR Method
Inverse variance weighted
MR Egger
1/SE$_{IV}$
9
8
7
6
5
4
−0.2
0.0
0.2
0.4
β$_{IV}$

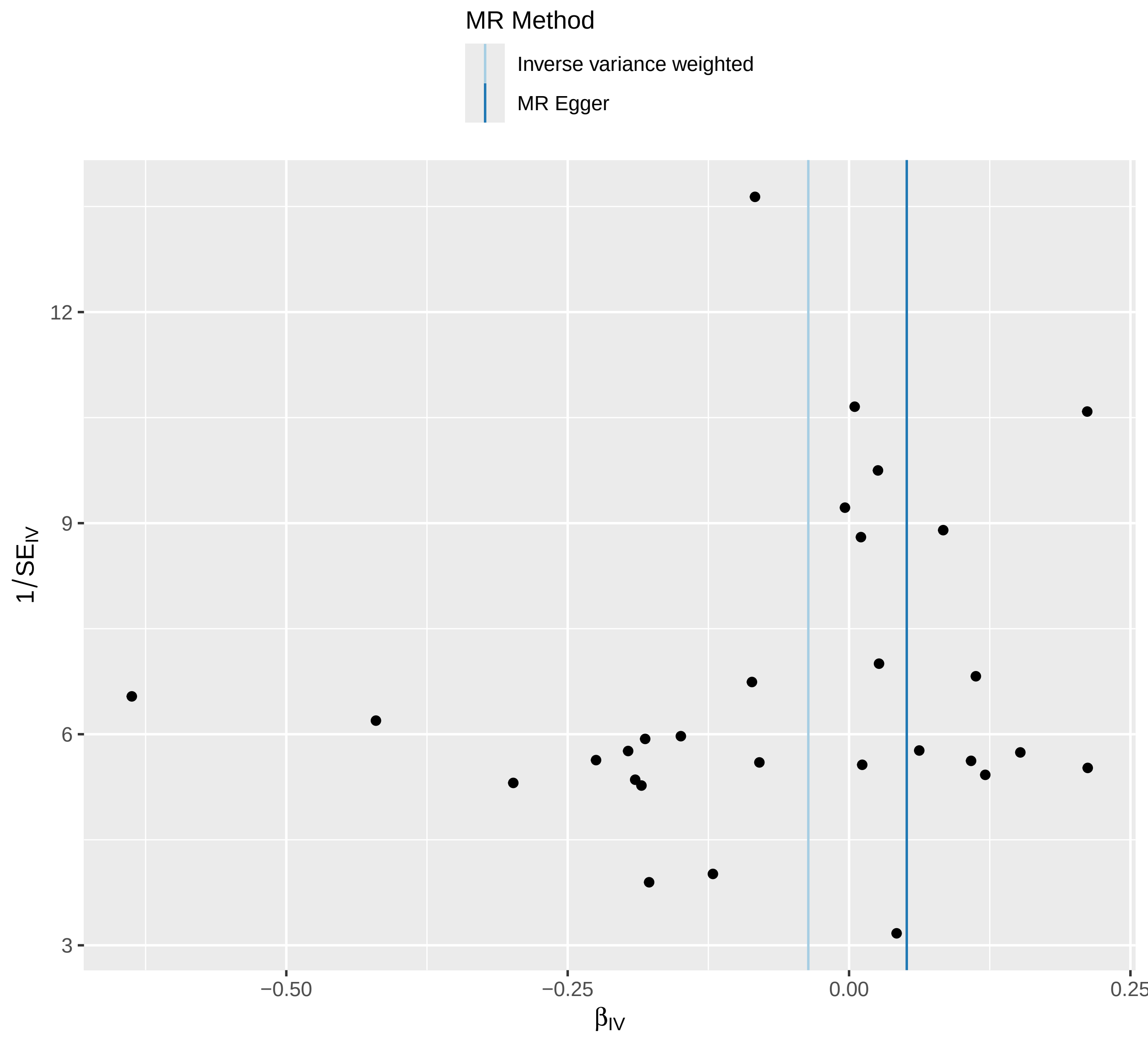

MR Method
Inverse variance weighted
MR Egger
1/SE_IV
β_IV
12
9
6
3
−0.50
−0.25
0.00
0.25

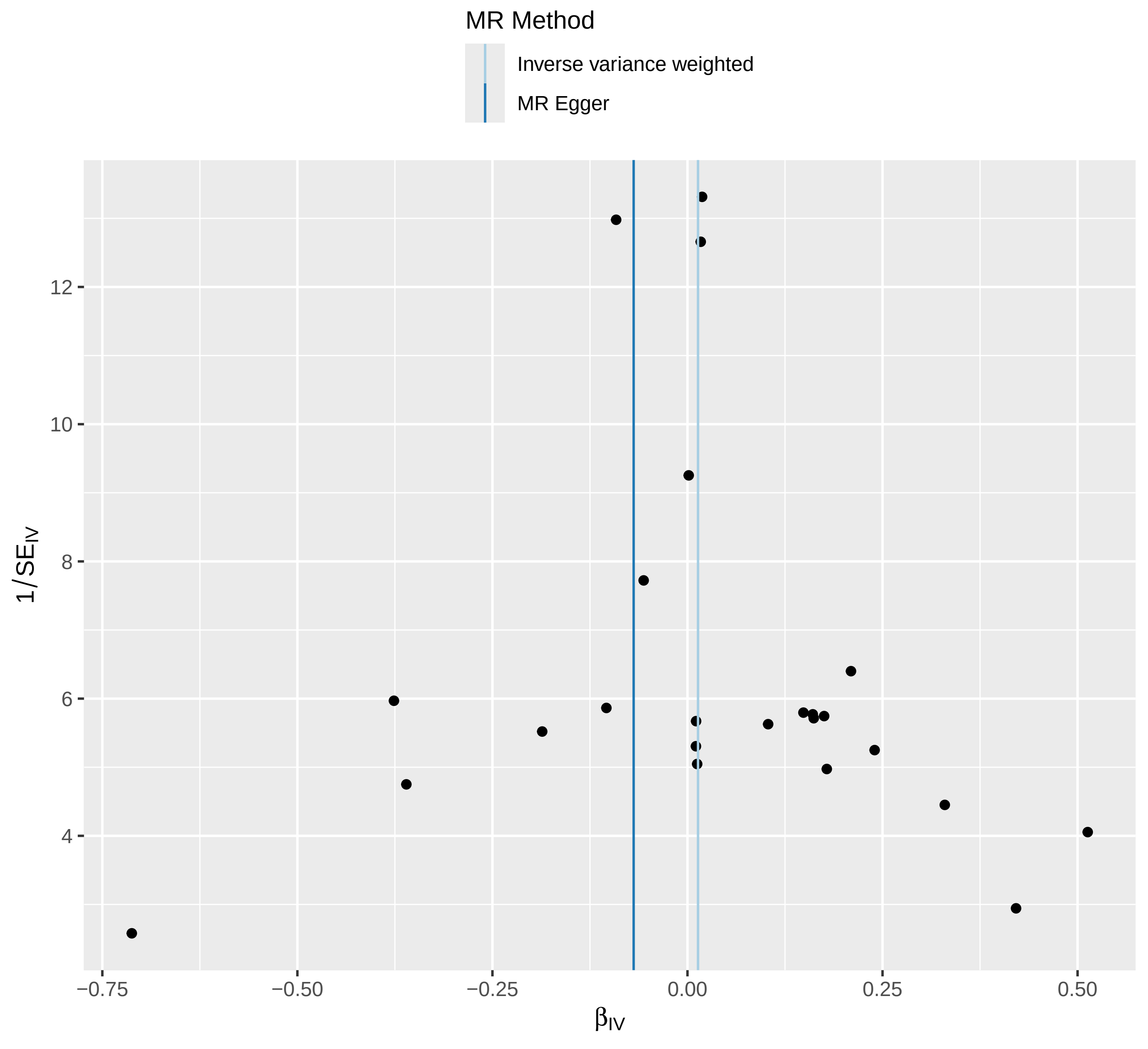
MR Method
Inverse variance weighted
MR Egger
1/SE_IV
β_IV
12
10
8
6
4
−0.75
−0.50
−0.25
0.00
0.25
0.50

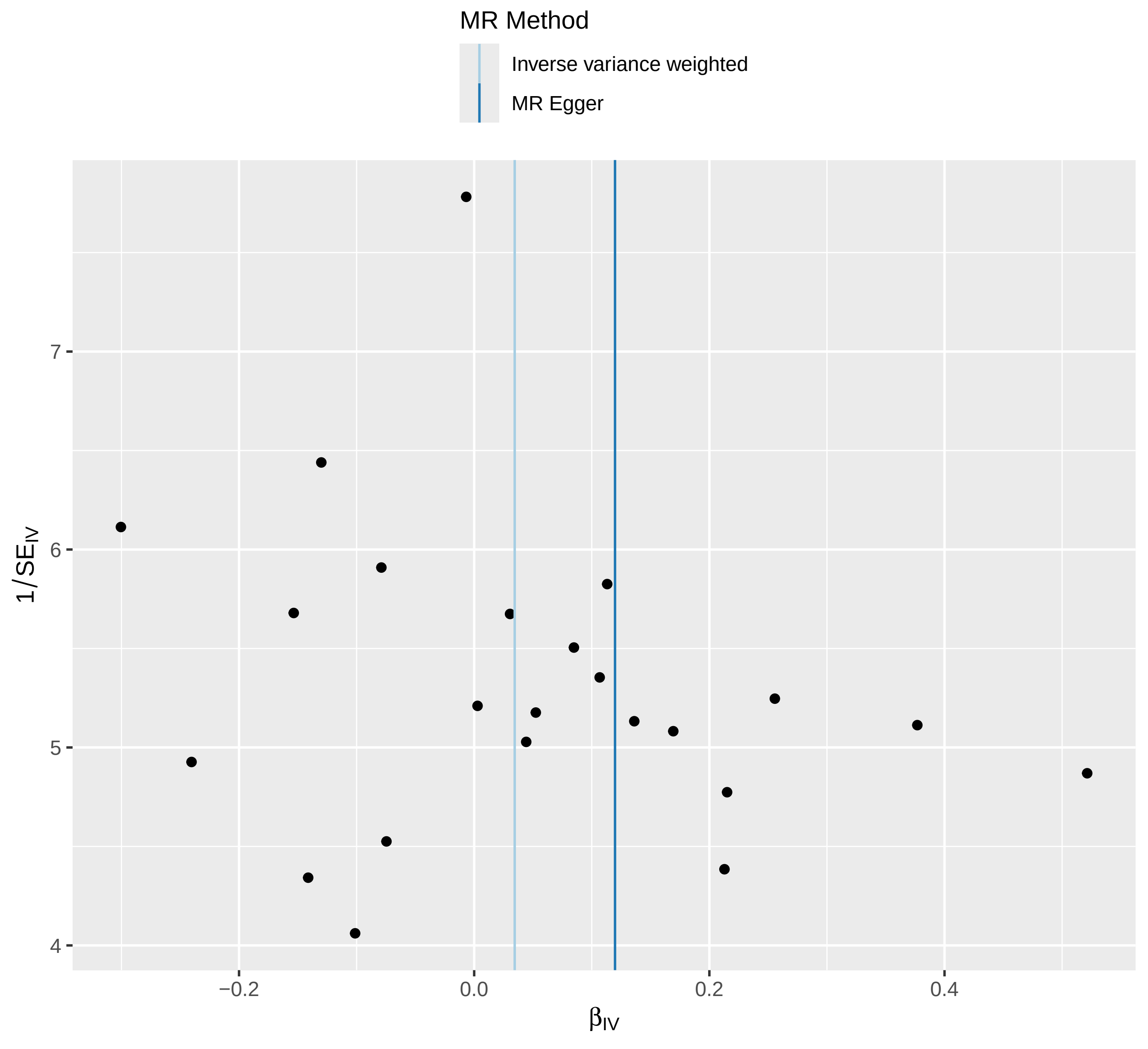
MR Method
Inverse variance weighted
MR Egger
1/SE_IV
β_IV
7
6
5
4
−0.2
0.0
0.2
0.4

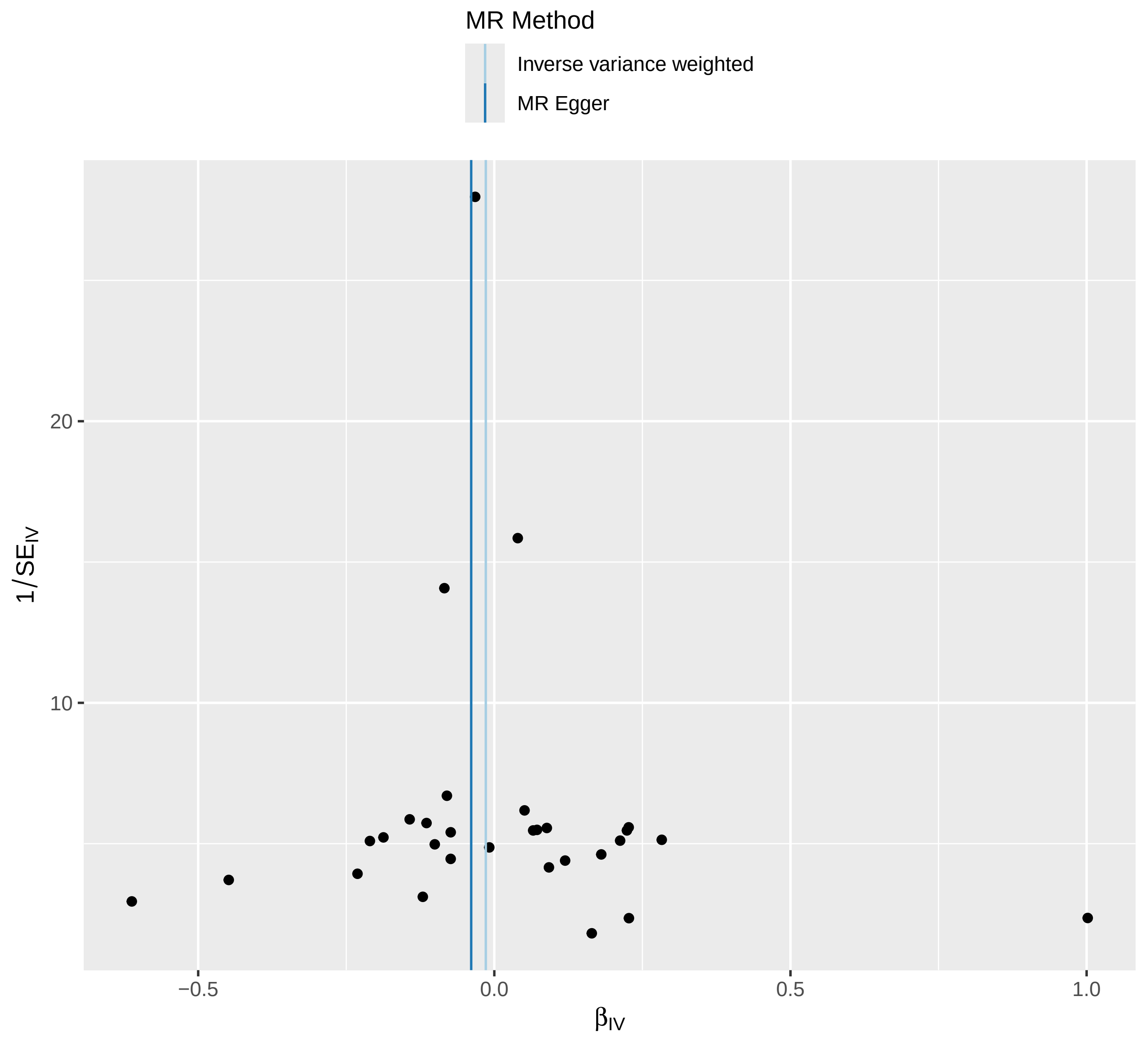
MR Method
Inverse variance weighted
MR Egger
20
10
1/SE_IV
−0.5
0.0
0.5
1.0
β_IV

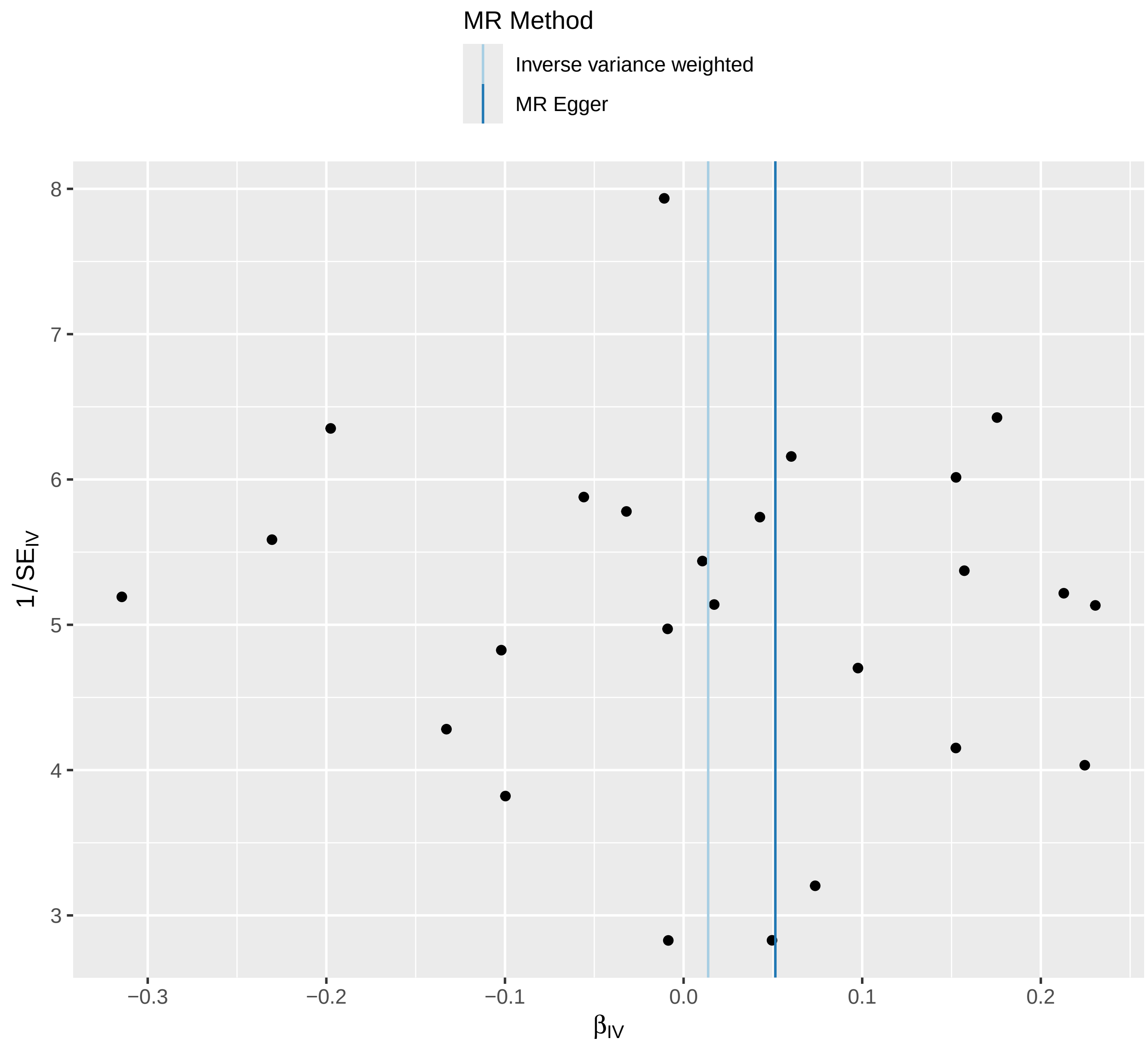
MR Method
Inverse variance weighted
MR Egger
1/SE_IV
β_IV
8
7
6
5
4
3
−0.3
−0.2
−0.1
0.0
0.1
0.2

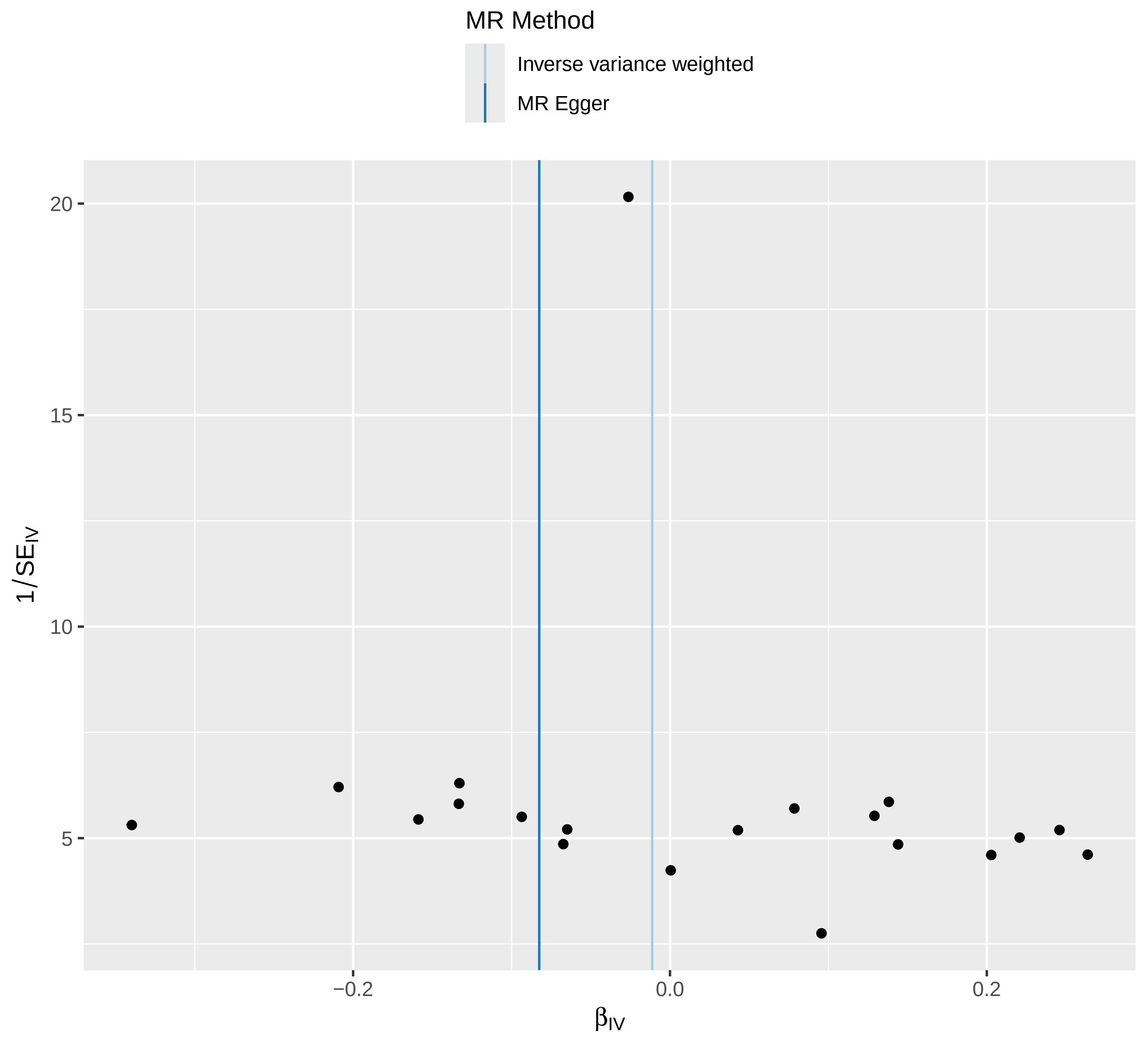

MR Method
Inverse variance weighted
MR Egger
20
15
10
5
1/SE_IV
−0.2
0.0
0.2
β_IV

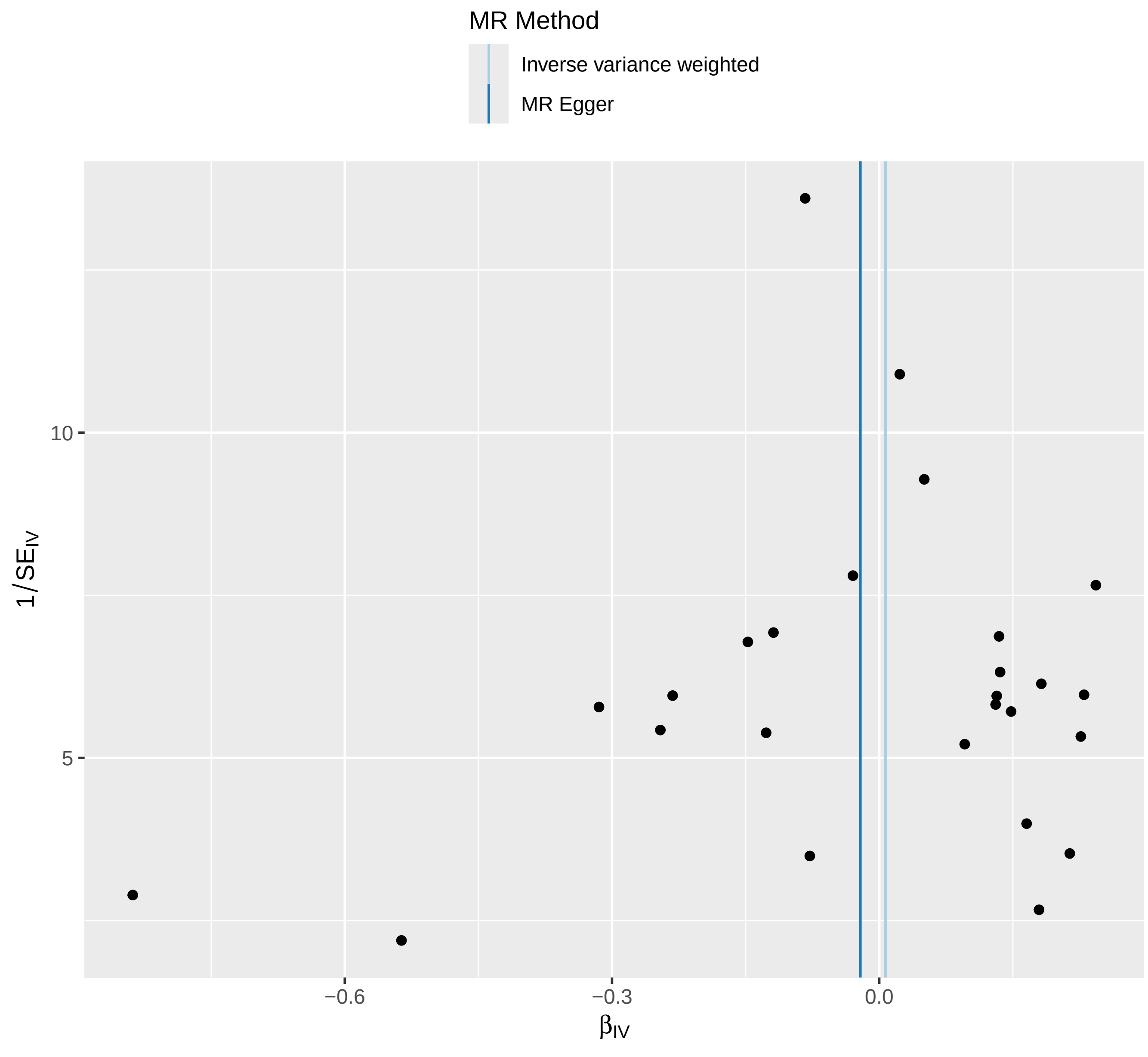
MR Method
Inverse variance weighted
MR Egger
10
5
$1/SE_{IV}$
−0.6
−0.3
0.0
β_{IV}

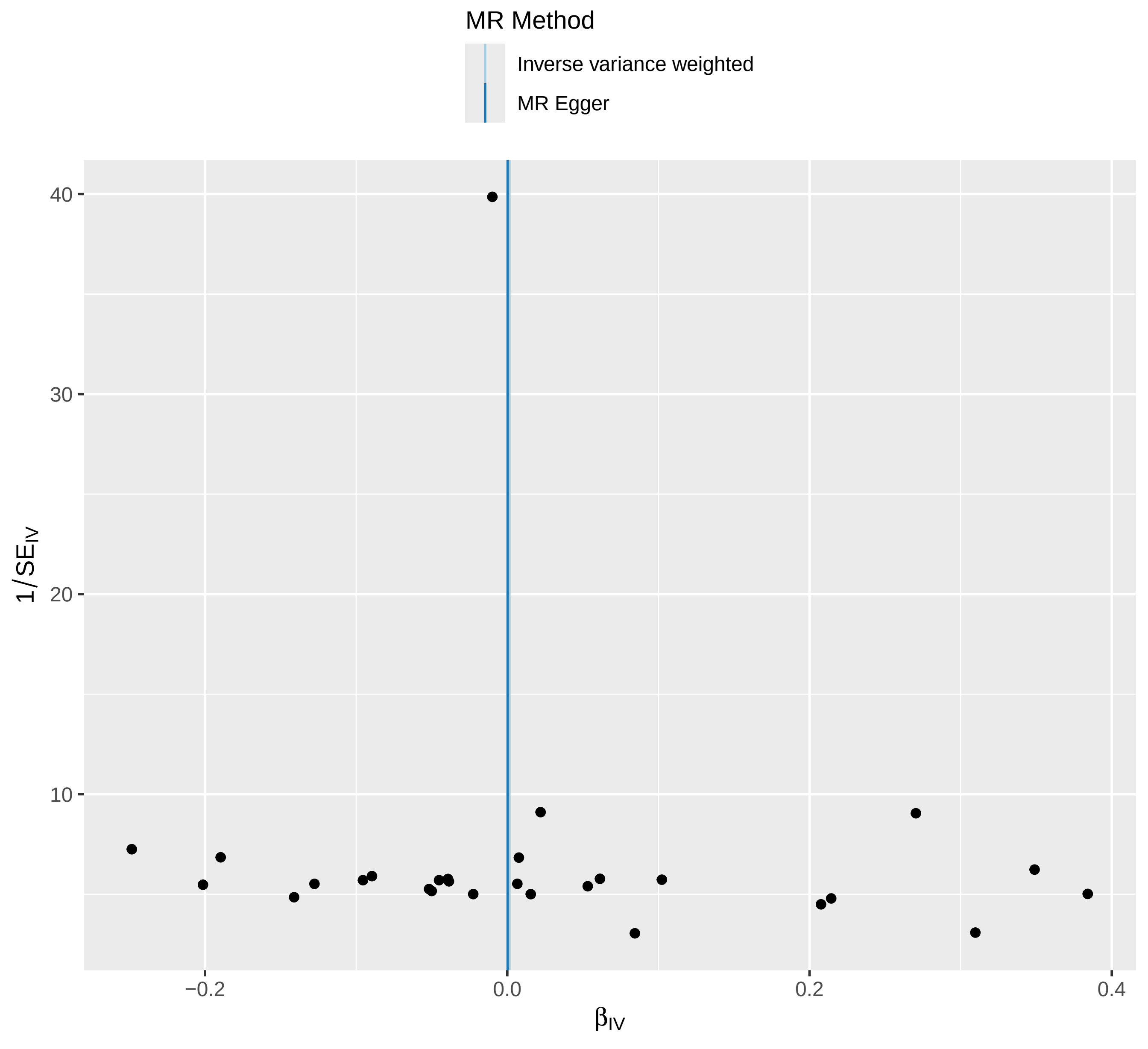
MR Method
Inverse variance weighted
MR Egger
40
30
20
10
$1/SE_{IV}$
−0.2
0.0
0.2
0.4
β_{IV}

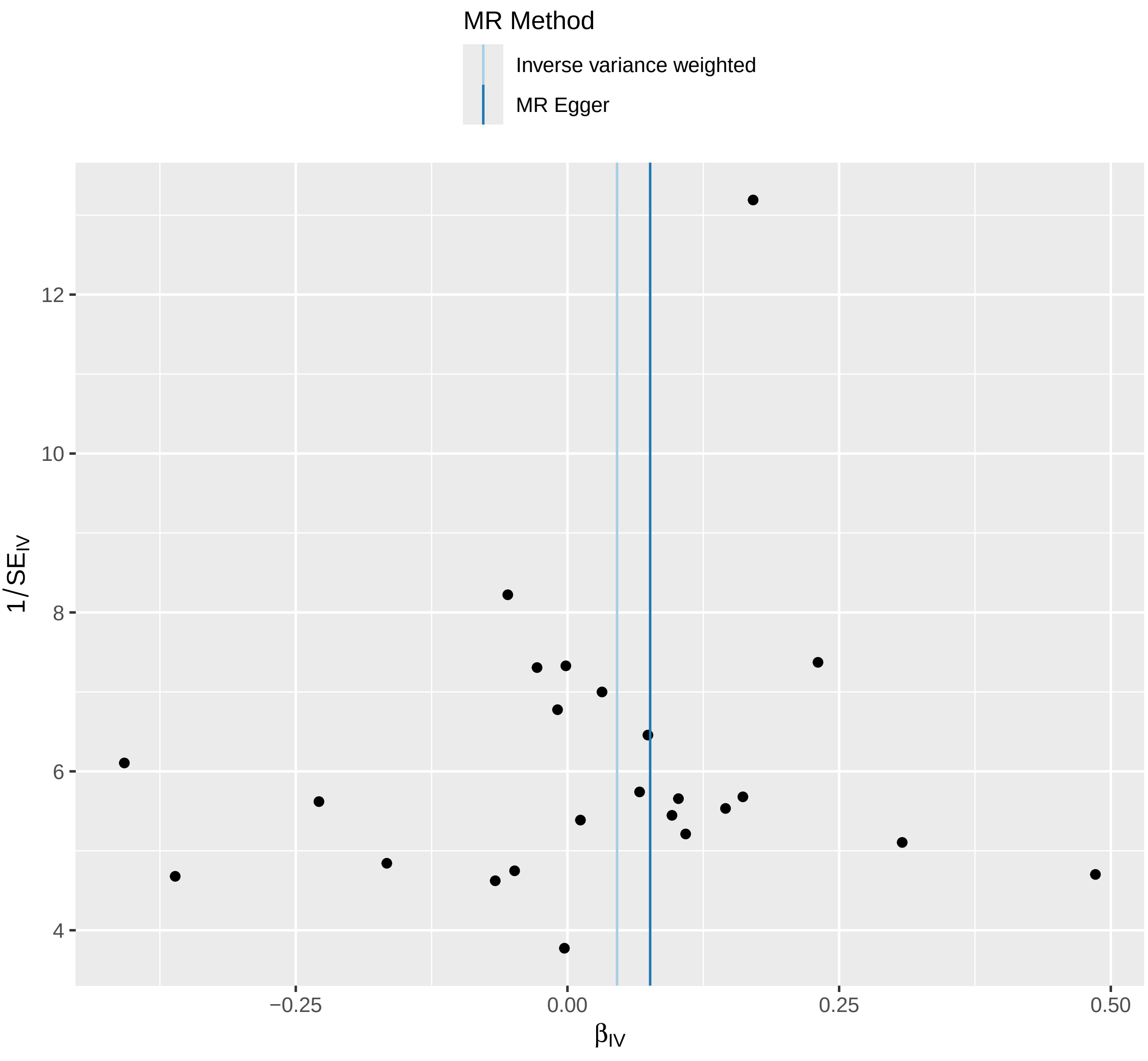
MR Method
Inverse variance weighted
MR Egger
1/SE$_{IV}$
12
10
8
6
4
−0.25
0.00
0.25
0.50
β$_{IV}$

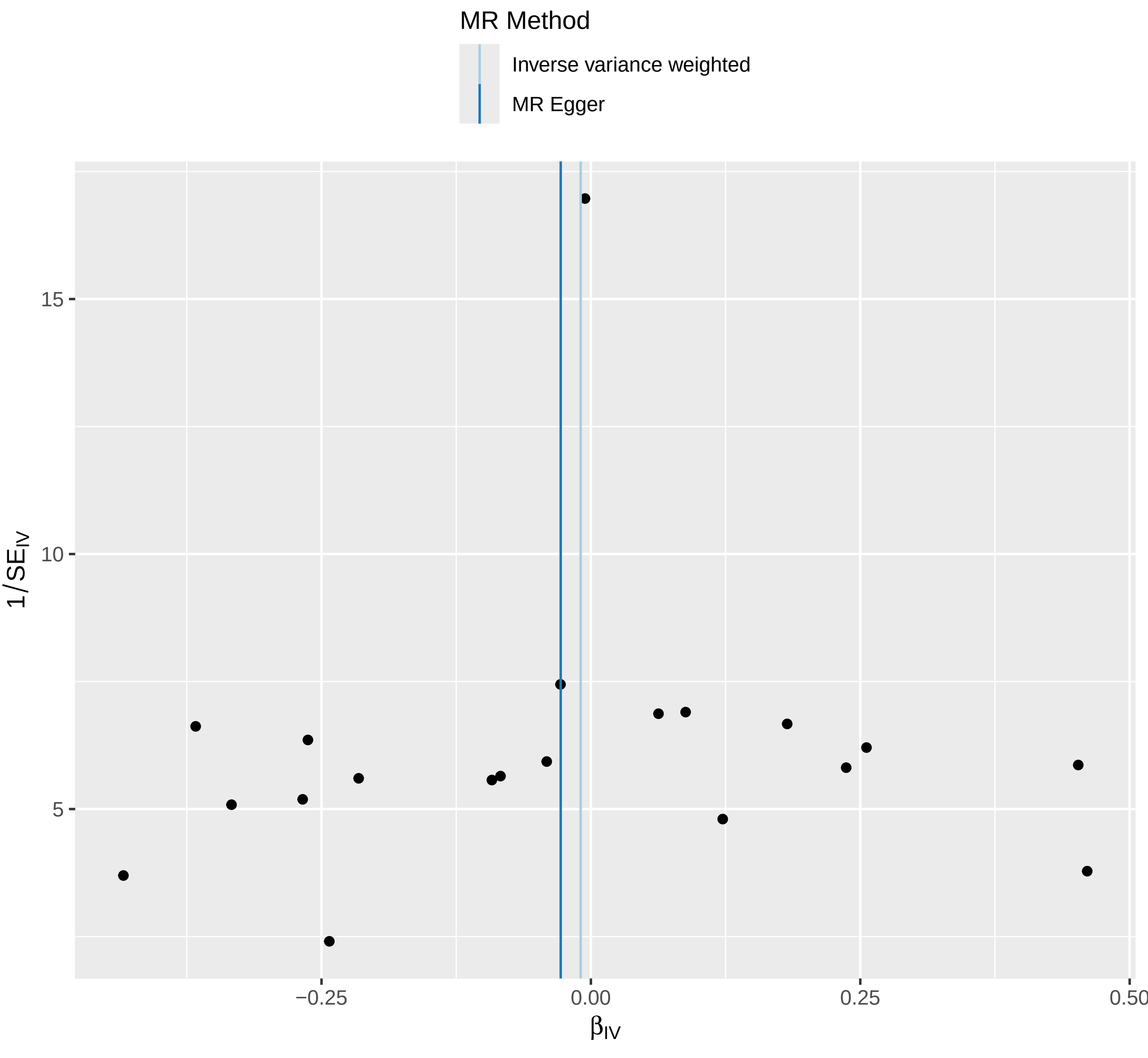

MR Method
Inverse variance weighted
MR Egger
15
10
5
1/SE$_{IV}$
−0.25
0.00
0.25
0.50
β$_{IV}$

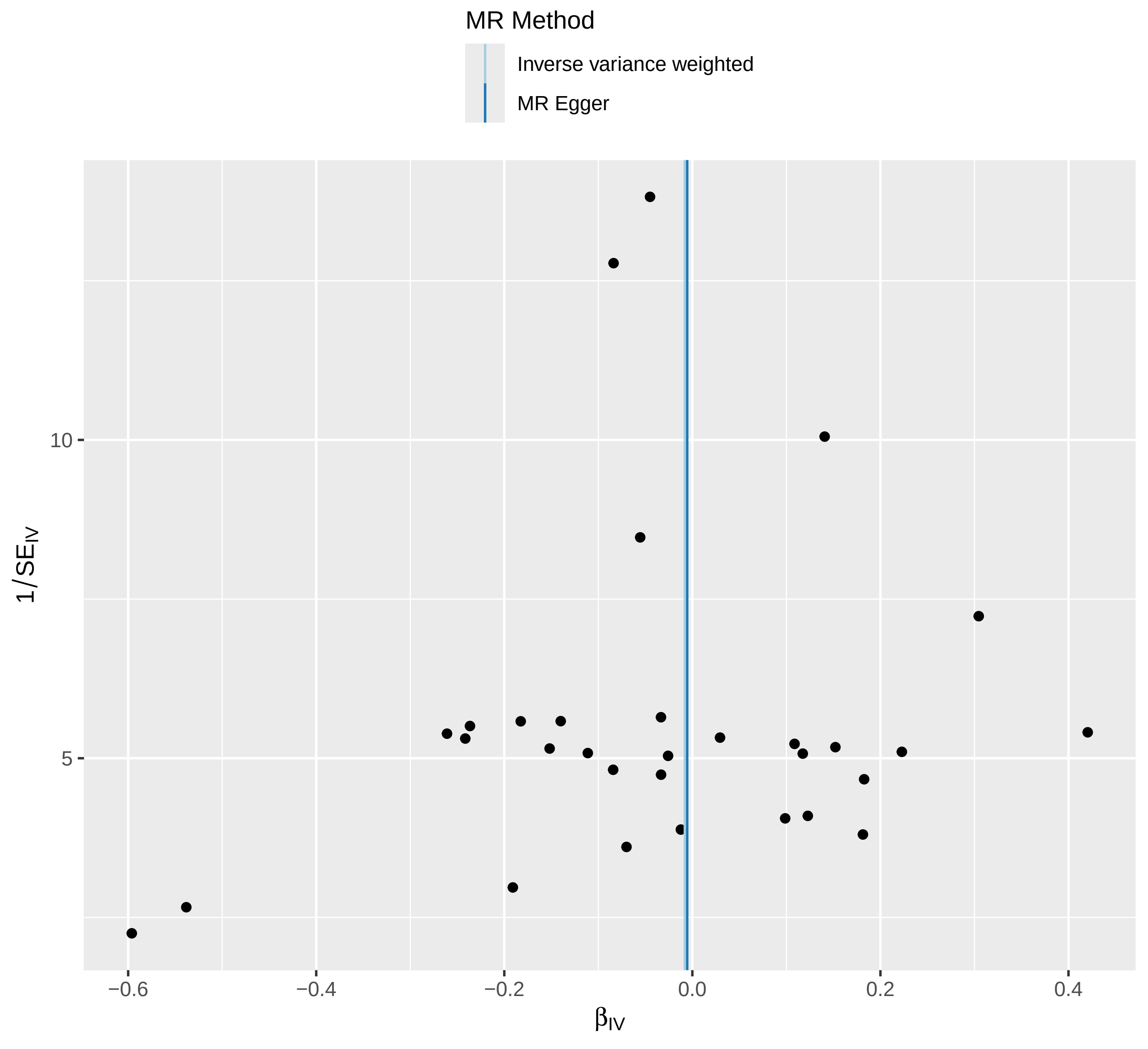
MR Method
Inverse variance weighted
MR Egger
1/SE_IV
10
5
−0.6
−0.4
−0.2
0.0
0.2
0.4
β_IV

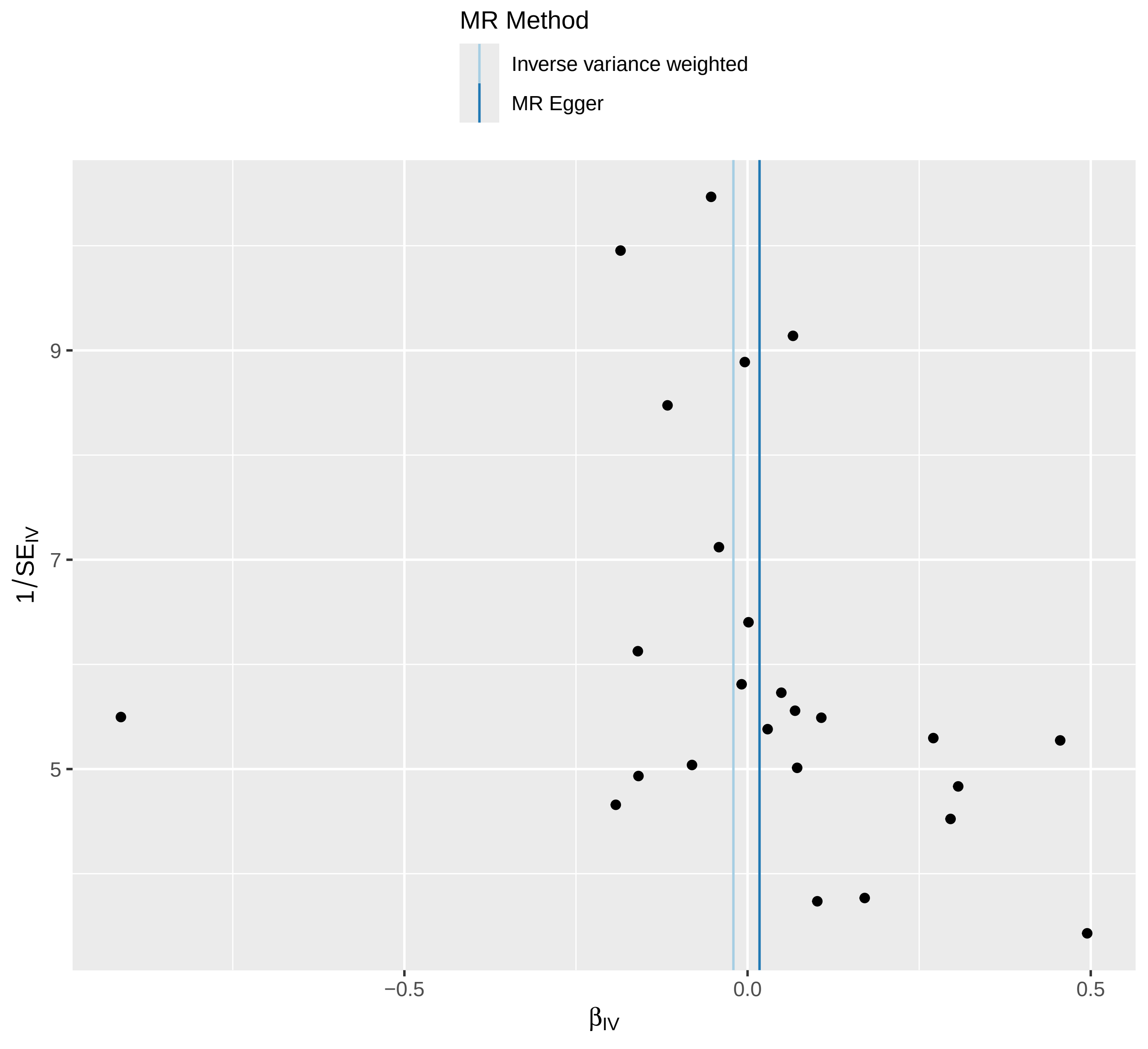

MR Method
Inverse variance weighted
MR Egger
1/SE_IV
9
7
5
−0.5
0.0
0.5
β_IV

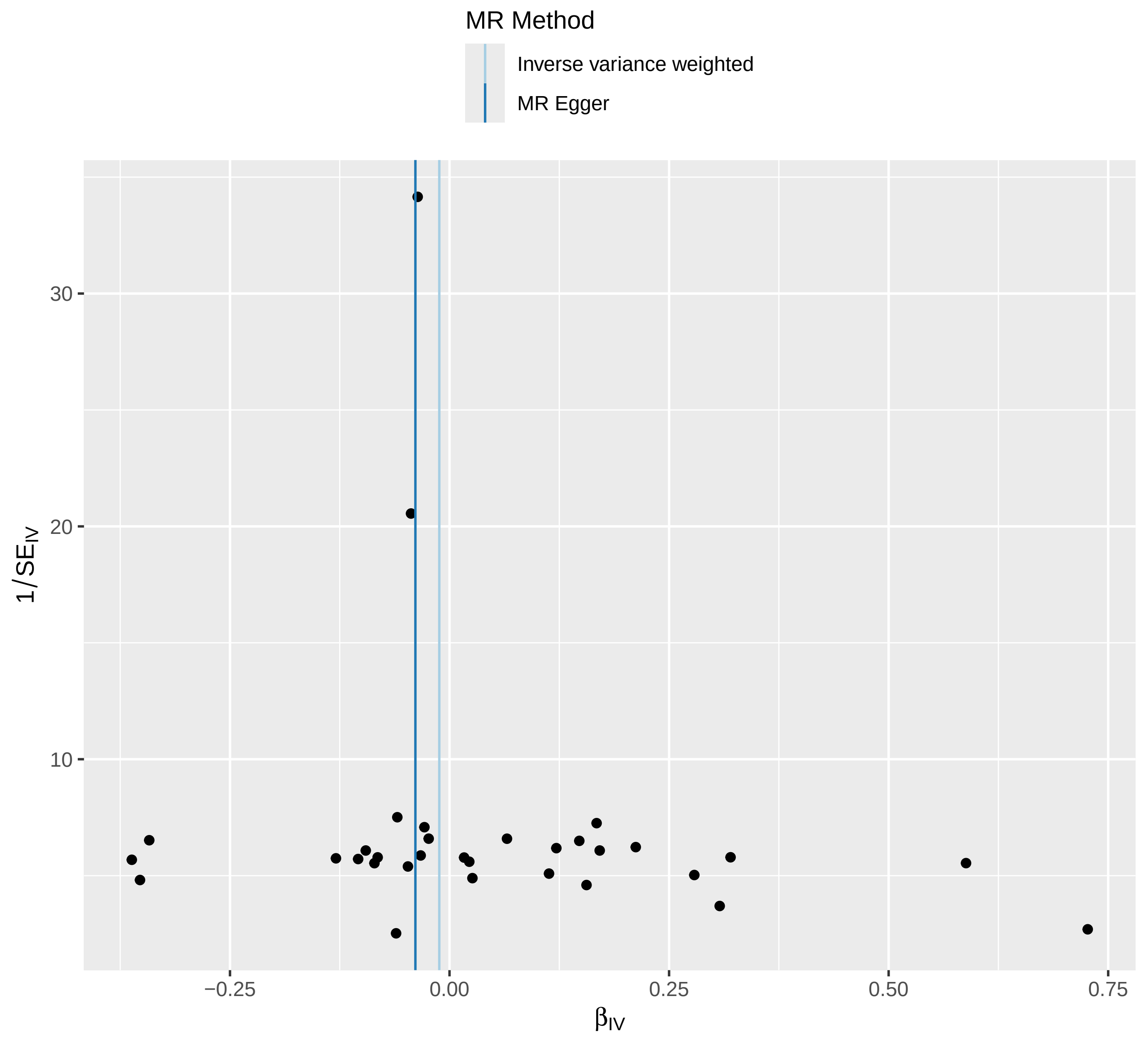
MR Method
Inverse variance weighted
MR Egger
30
20
10
1/SE_IV
−0.25
0.00
0.25
0.50
0.75
β_IV

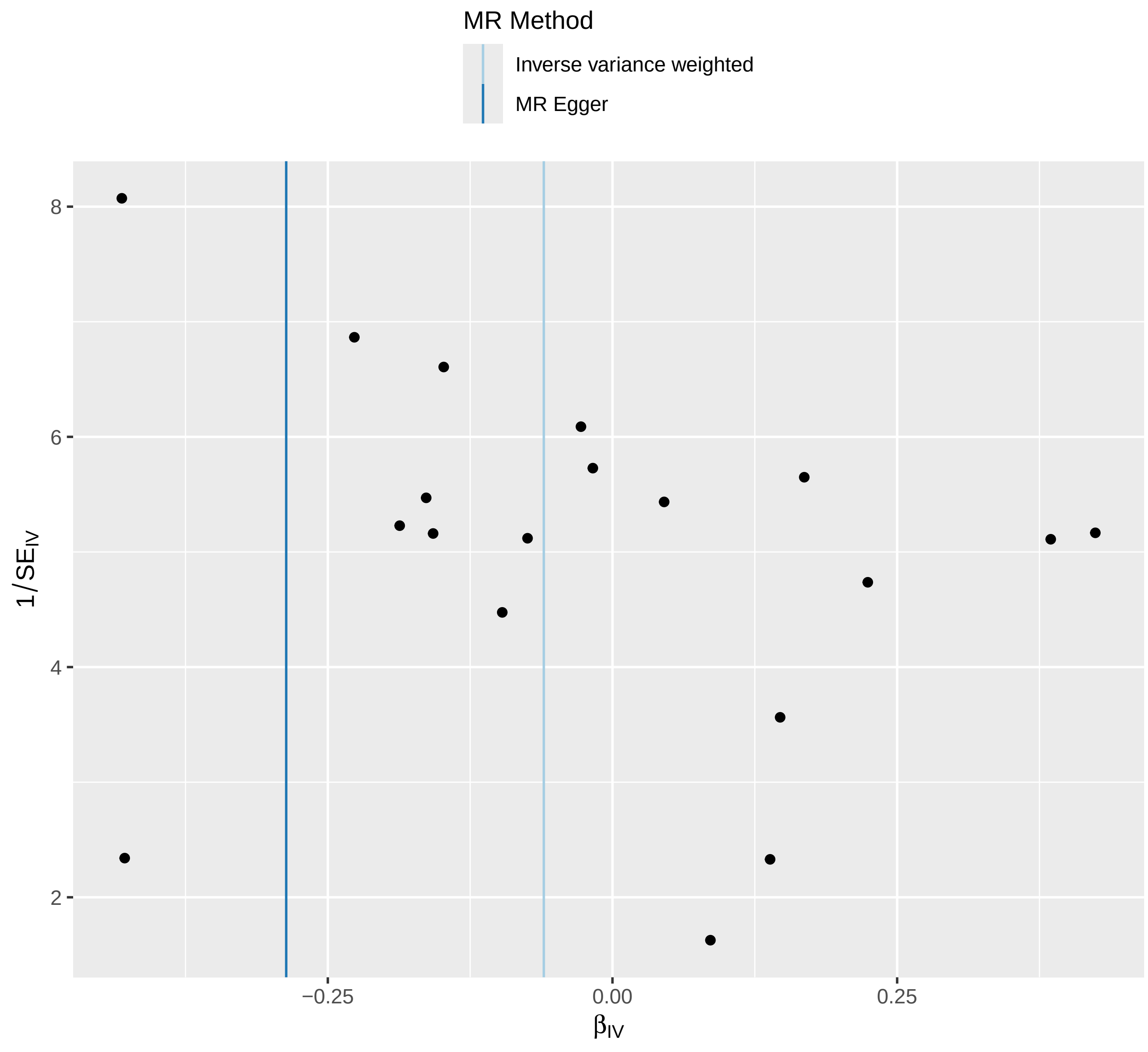

MR Method
Inverse variance weighted
MR Egger
8
6
4
2
1/SE$_{IV}$
−0.25
0.00
0.25
β$_{IV}$

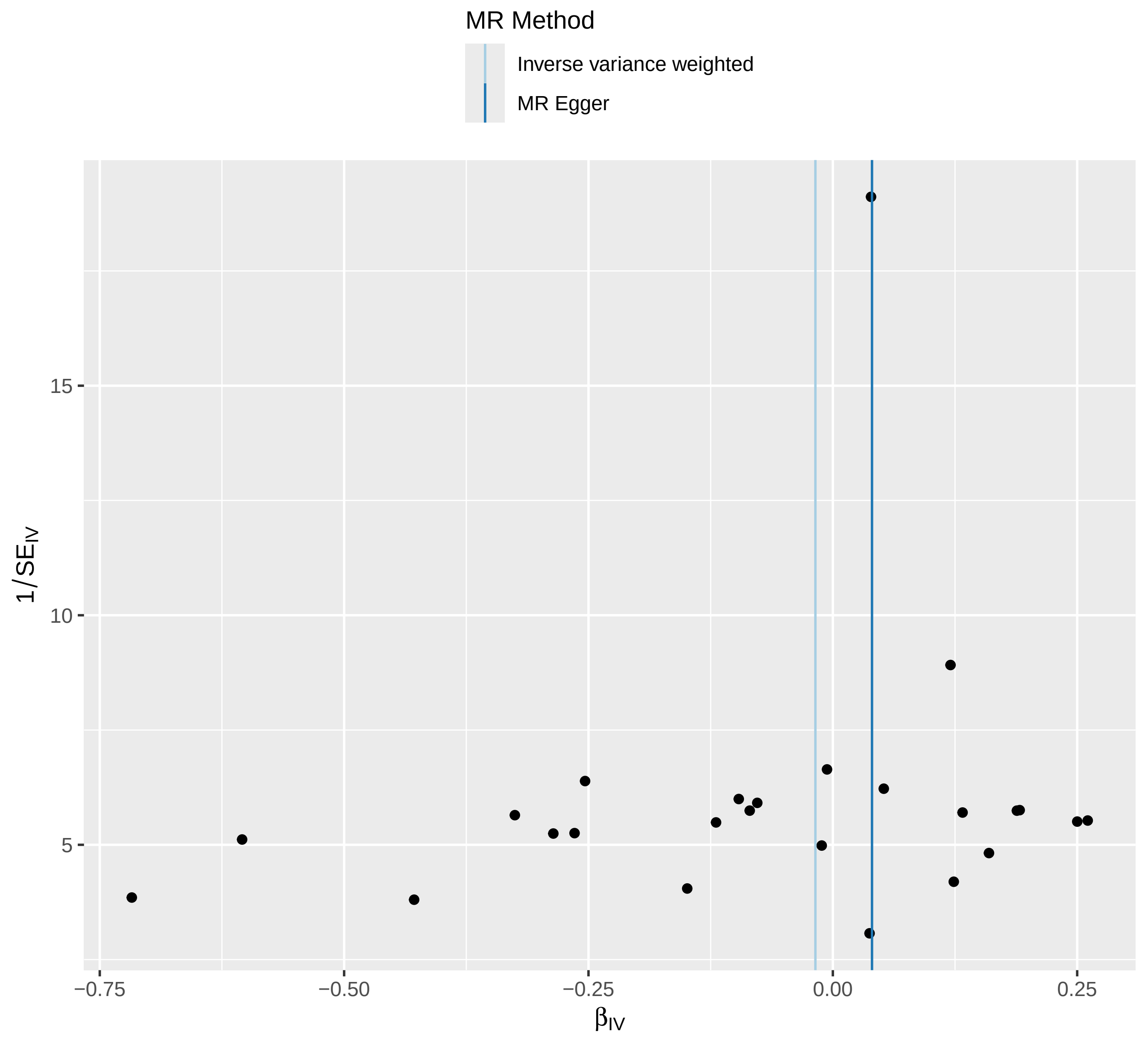
MR Method
Inverse variance weighted
MR Egger
1/SE_IV
β_IV
15
10
5
−0.75
−0.50
−0.25
0.00
0.25

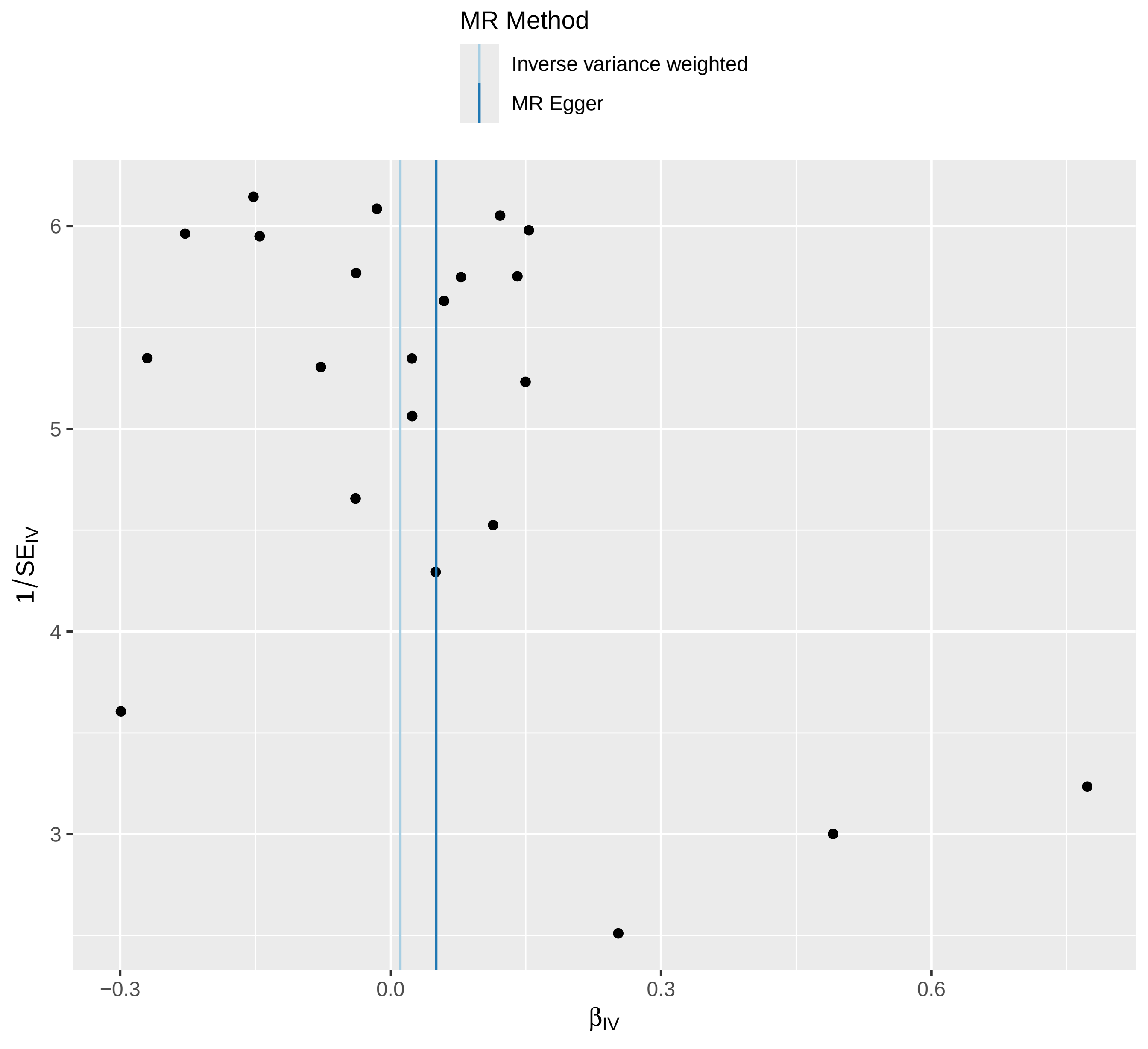

MR Method
Inverse variance weighted
MR Egger
6
5
4
3
1/SE_IV
−0.3
0.0
0.3
0.6
β_IV

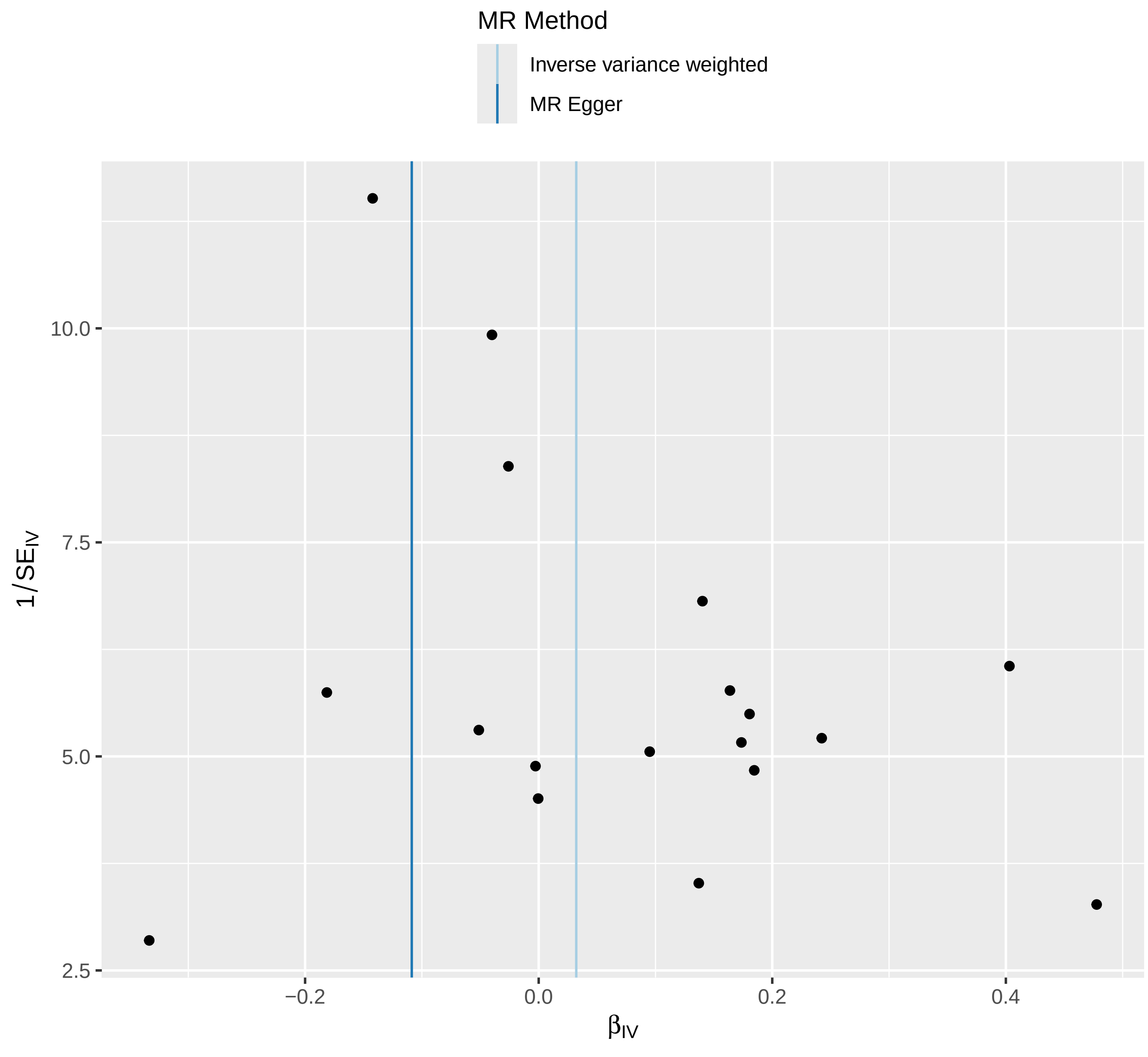
MR Method
Inverse variance weighted
MR Egger
10.0
7.5
5.0
2.5
1/SE$_{IV}$
−0.2
0.0
0.2
0.4
β$_{IV}$

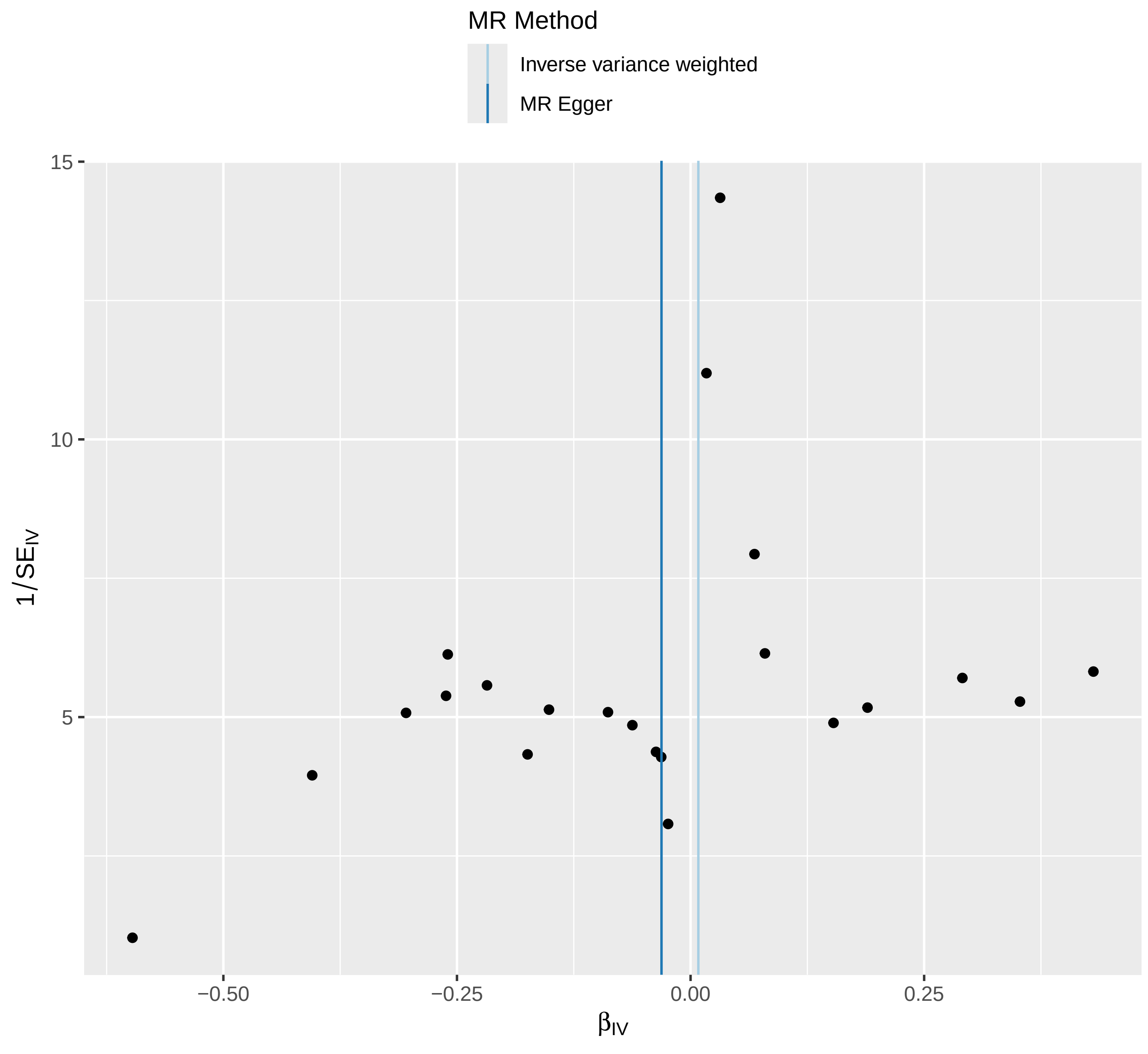
MR Method
Inverse variance weighted
MR Egger
15
10
5
1/SE_IV
−0.50
−0.25
0.00
0.25
β_IV

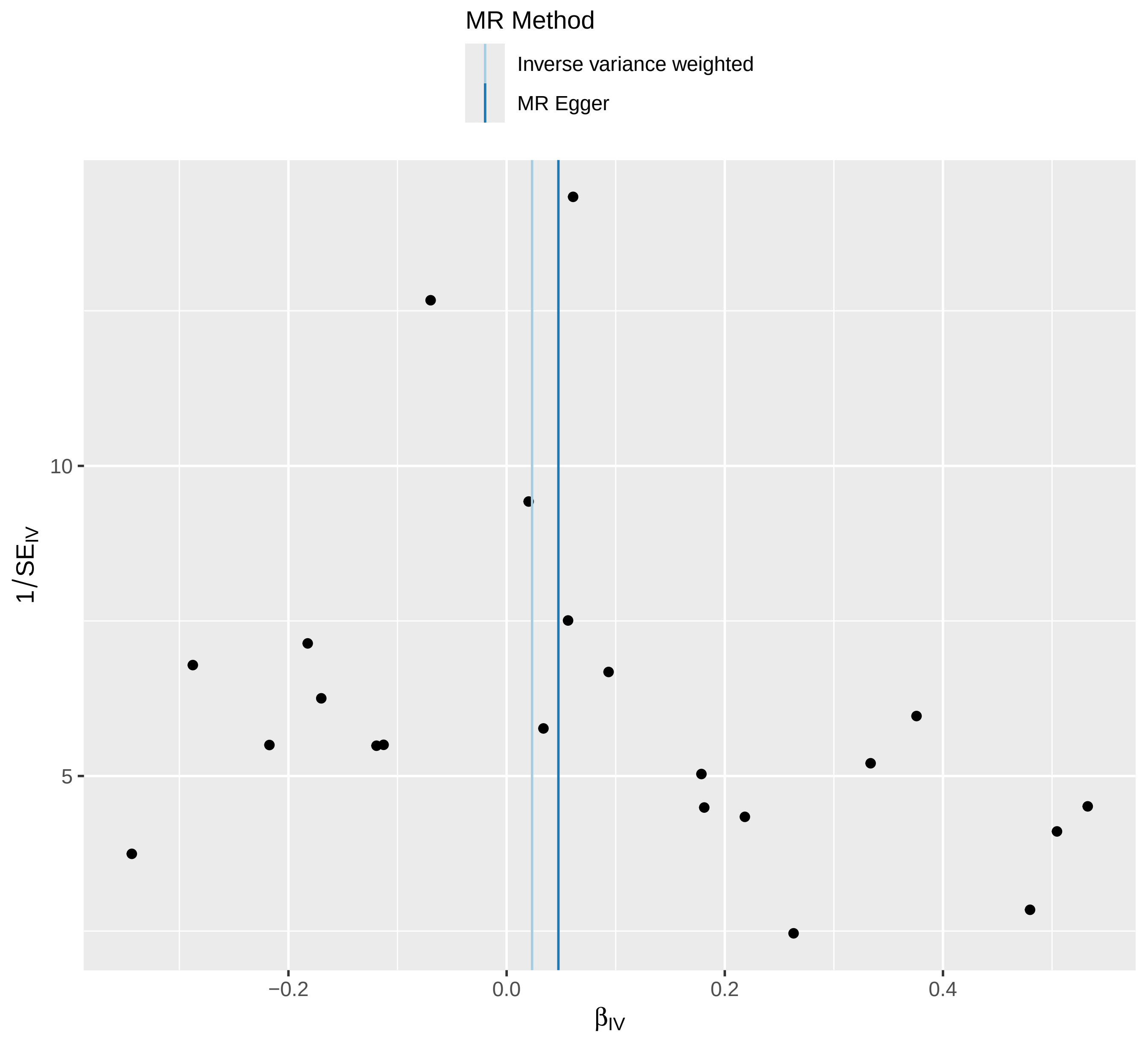
MR Method
Inverse variance weighted
MR Egger
10
5
1/SE_IV
−0.2
0.0
0.2
0.4
β_IV

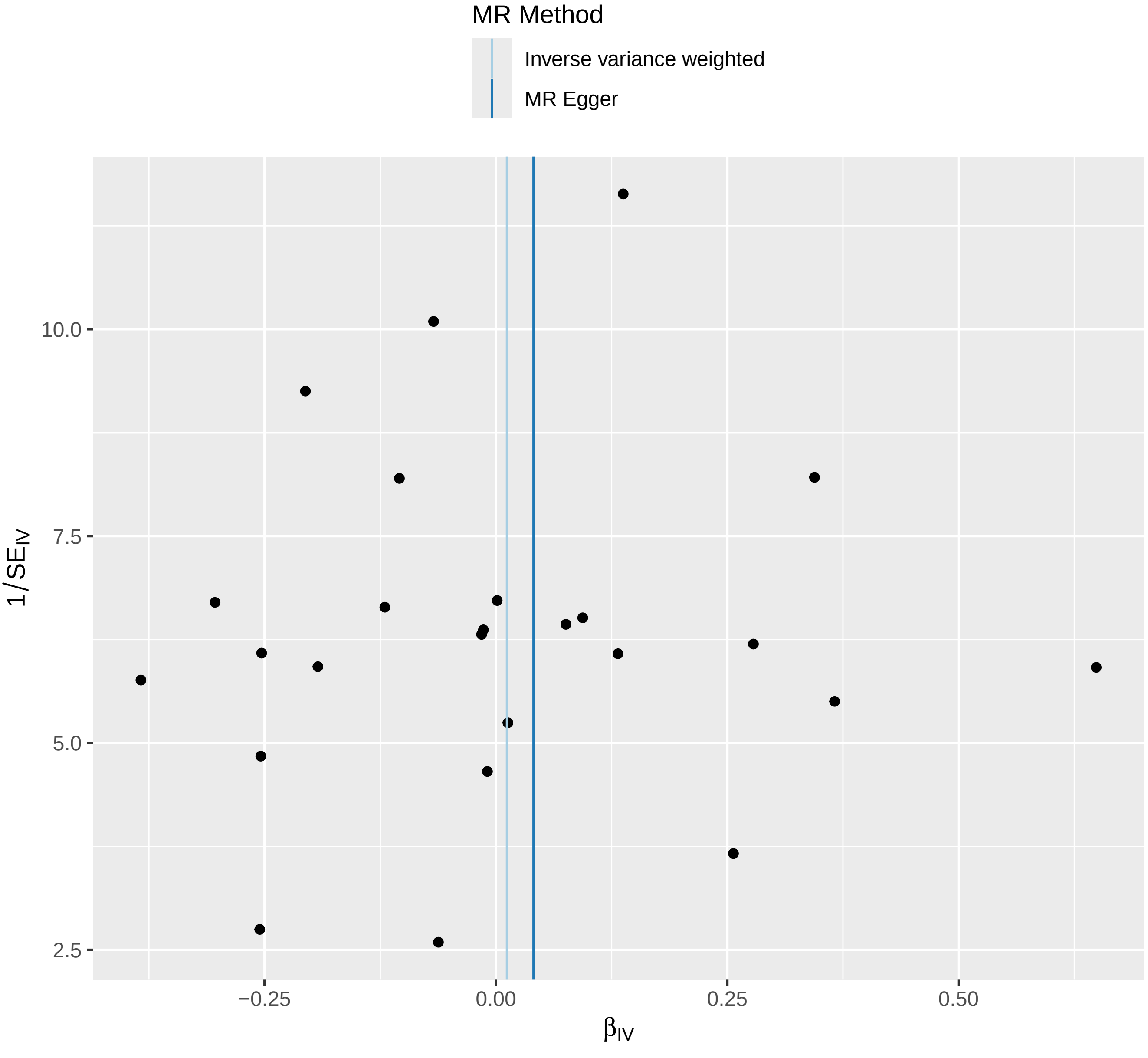
MR Method
Inverse variance weighted
MR Egger
10.0
7.5
5.0
2.5
$1/SE_{IV}$
−0.25
0.00
0.25
0.50
β_{IV}

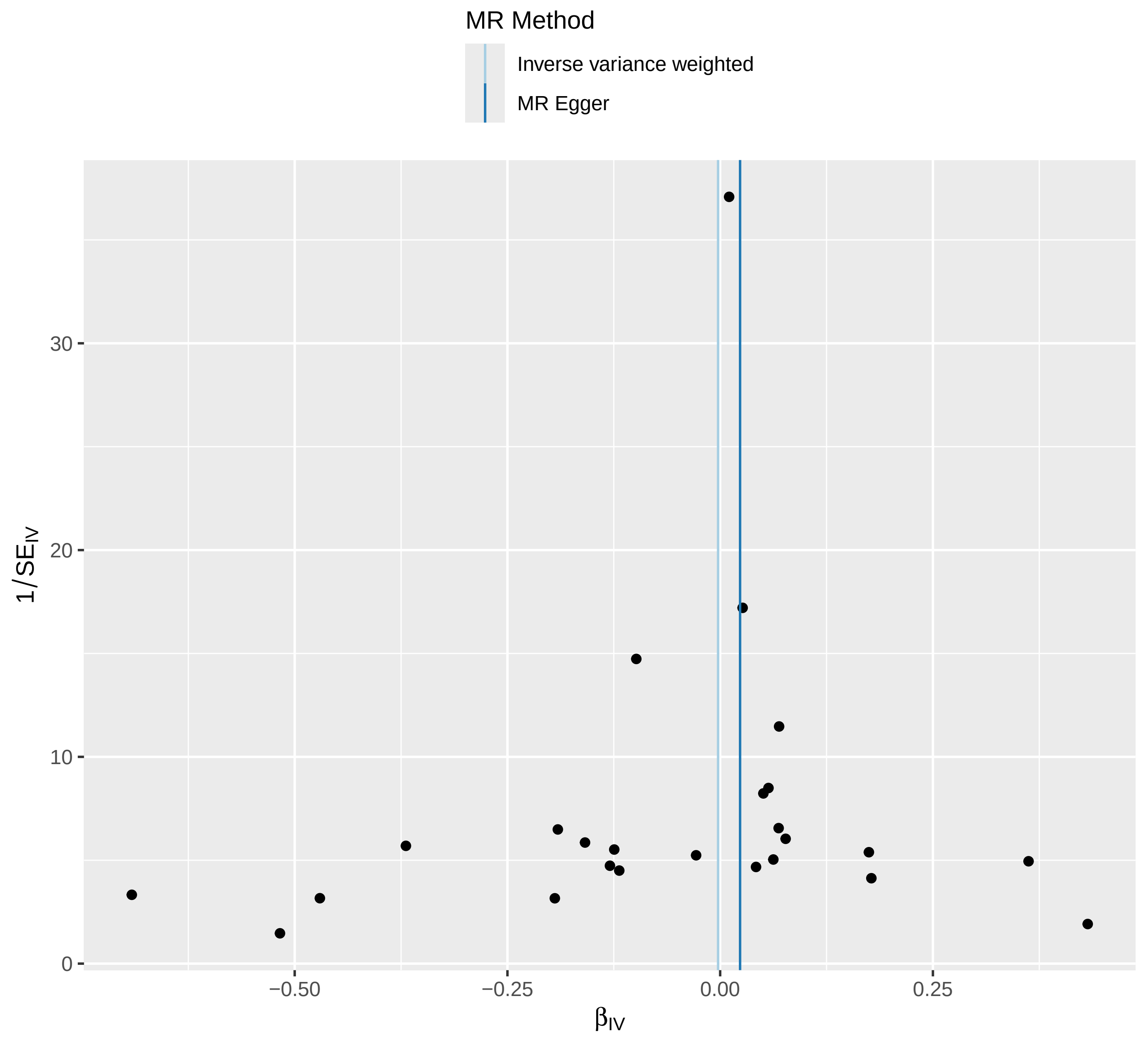
MR Method
Inverse variance weighted
MR Egger
1/SE$_{IV}$
30
20
10
0
−0.50
−0.25
0.00
0.25
β$_{IV}$

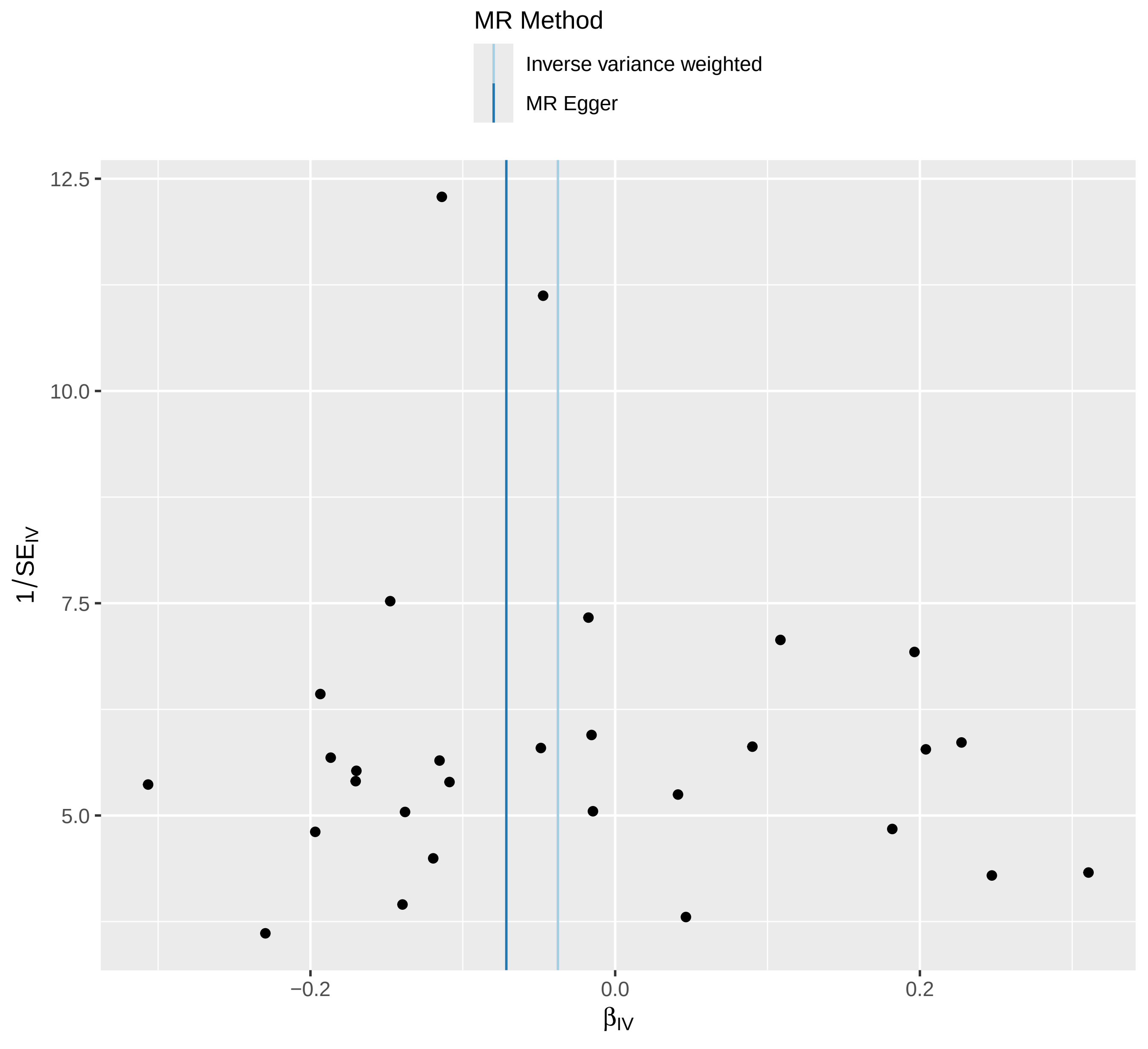
MR Method
Inverse variance weighted
MR Egger
12.5
10.0
7.5
5.0
$1/SE_{IV}$
−0.2
0.0
0.2
β_{IV}

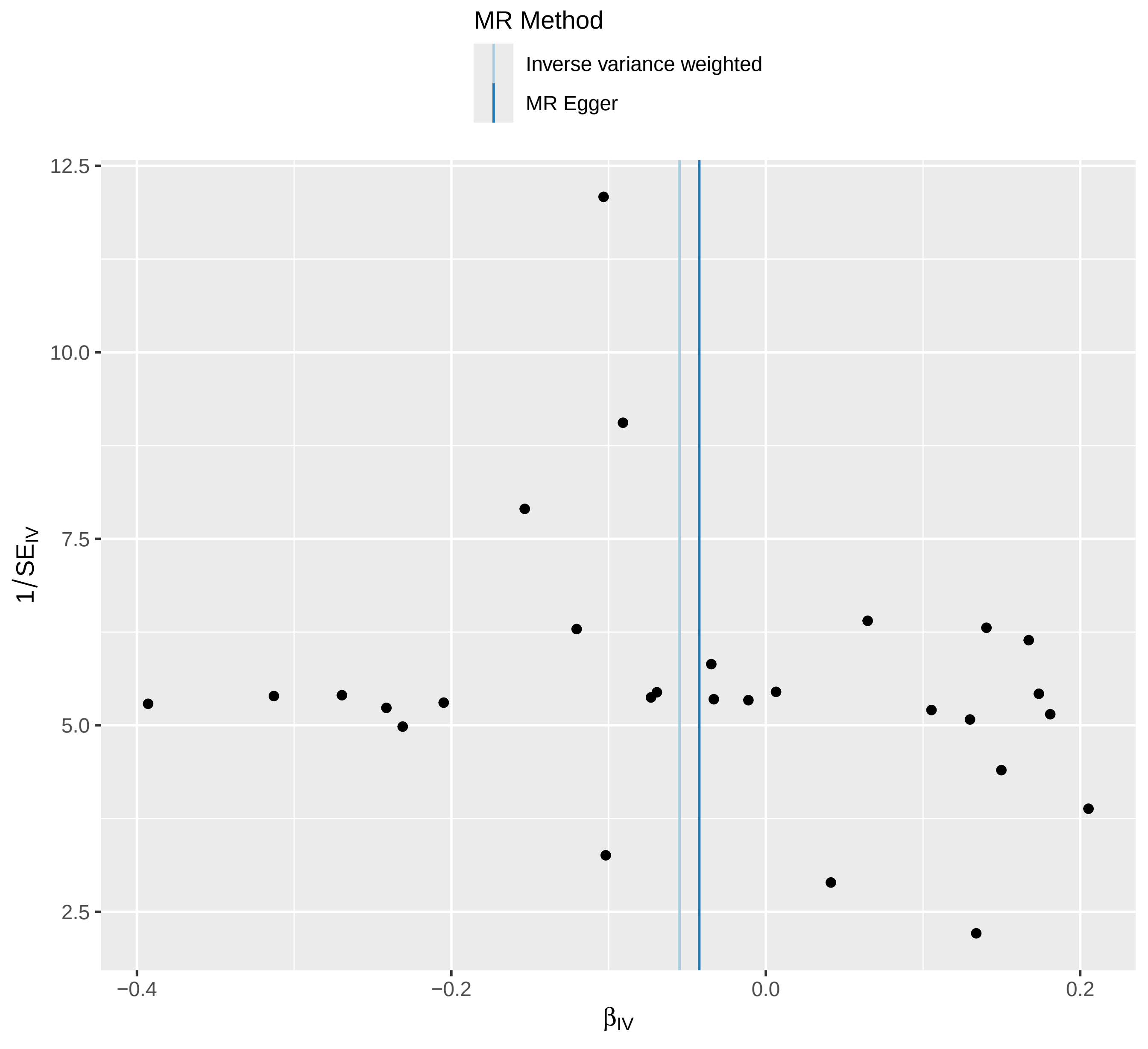
MR Method
Inverse variance weighted
MR Egger
12.5
10.0
7.5
5.0
2.5
1/SE_IV
−0.4
−0.2
0.0
0.2
β_IV

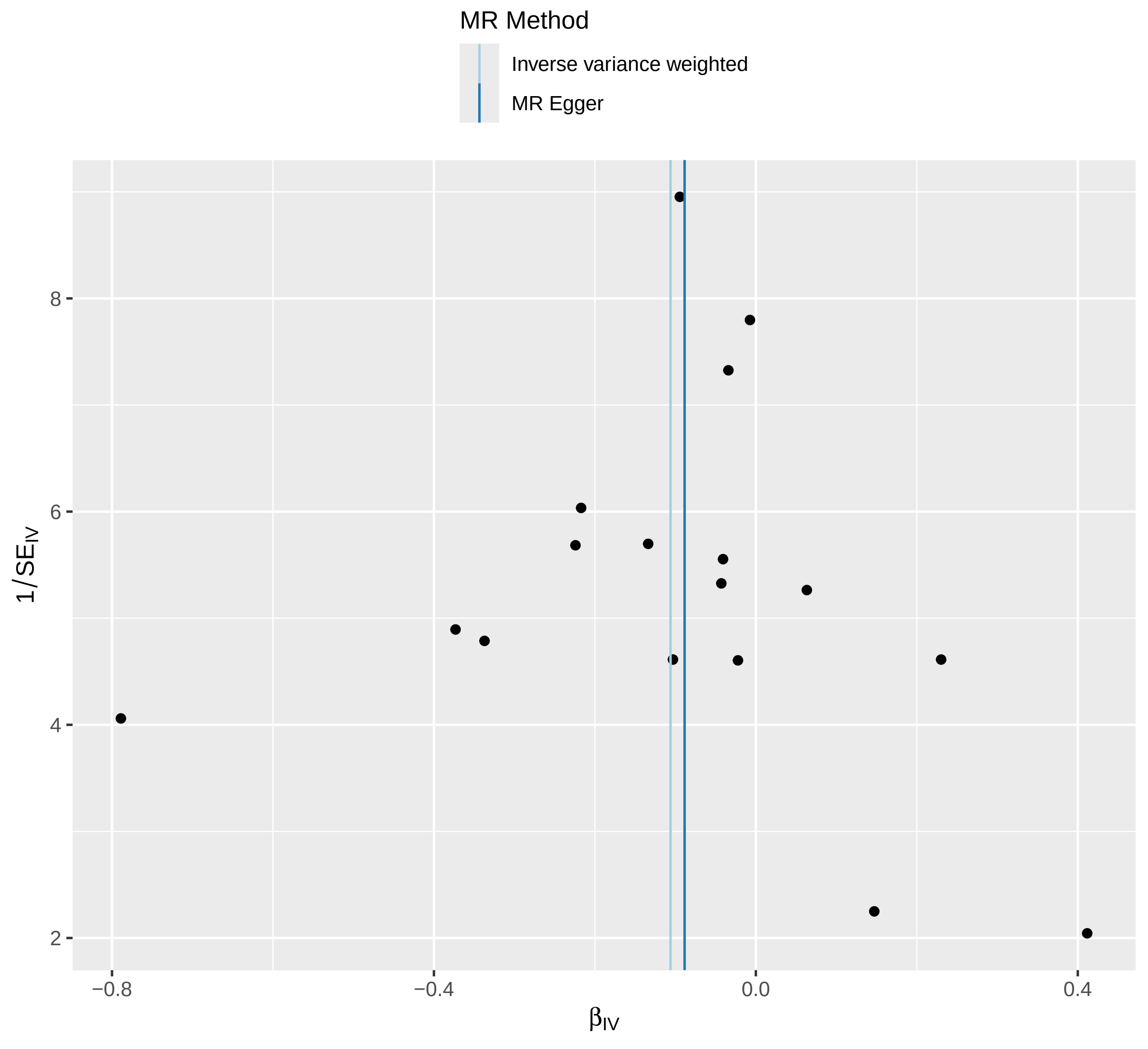

MR Method
Inverse variance weighted
MR Egger
8
6
4
2
1/SE_{IV}
−0.8
−0.4
0.0
0.4
β_{IV}

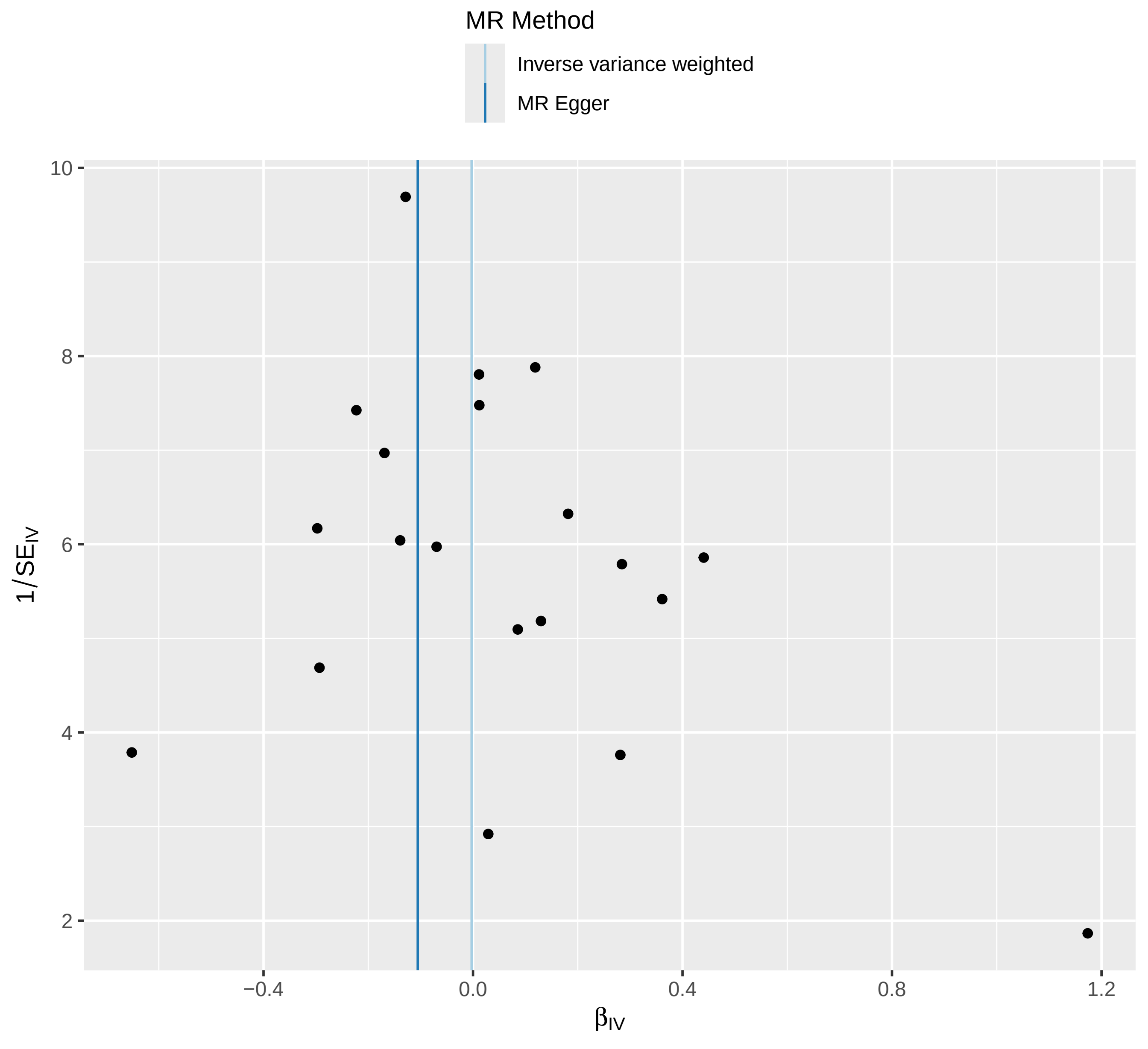

MR Method
Inverse variance weighted
MR Egger
10
8
6
4
2
1/SE$_{IV}$
−0.4
0.0
0.4
0.8
1.2
β$_{IV}$

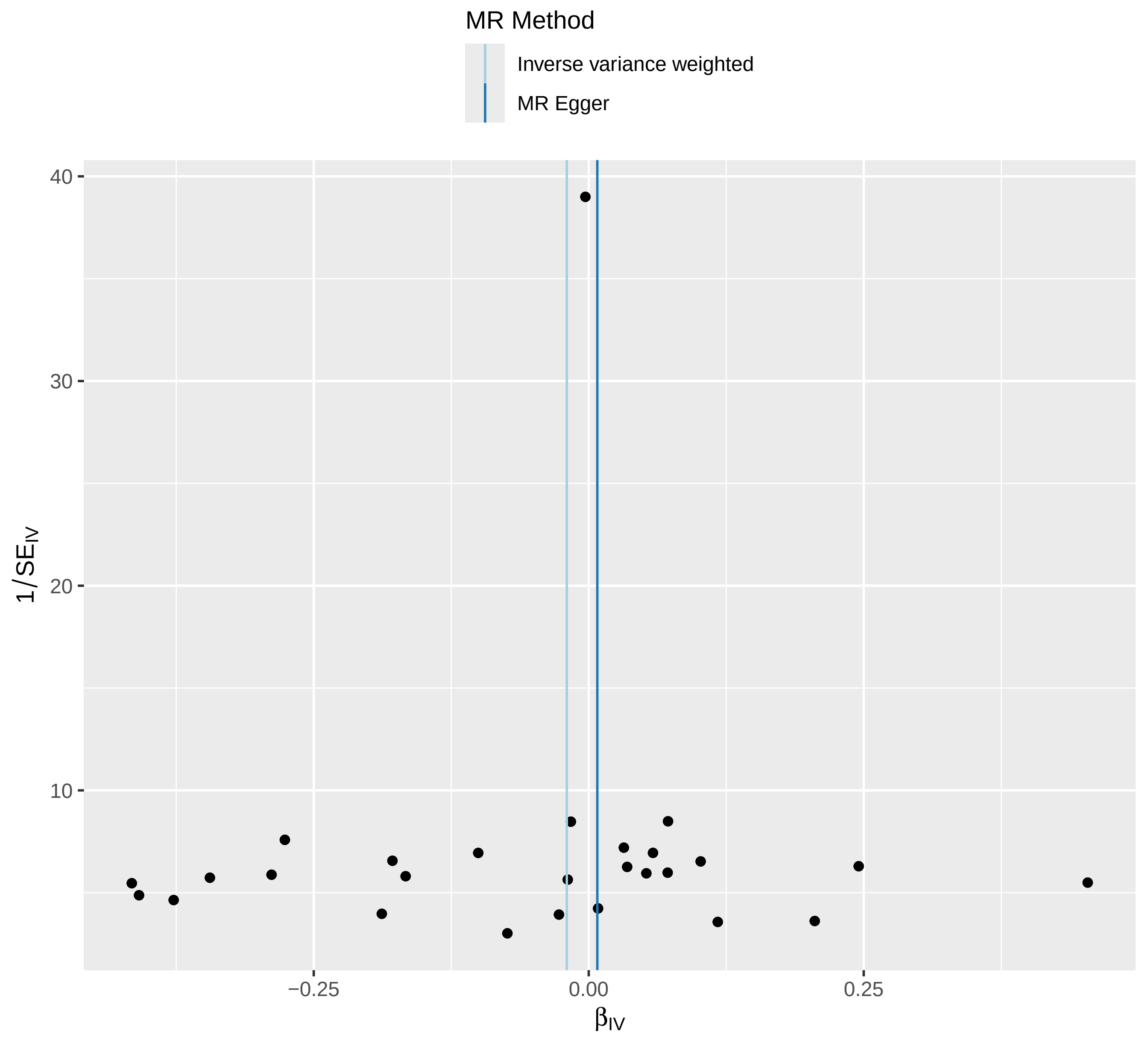
MR Method
Inverse variance weighted
MR Egger
40
30
20
10
1/SE$_{IV}$
−0.25
0.00
0.25
β$_{IV}$

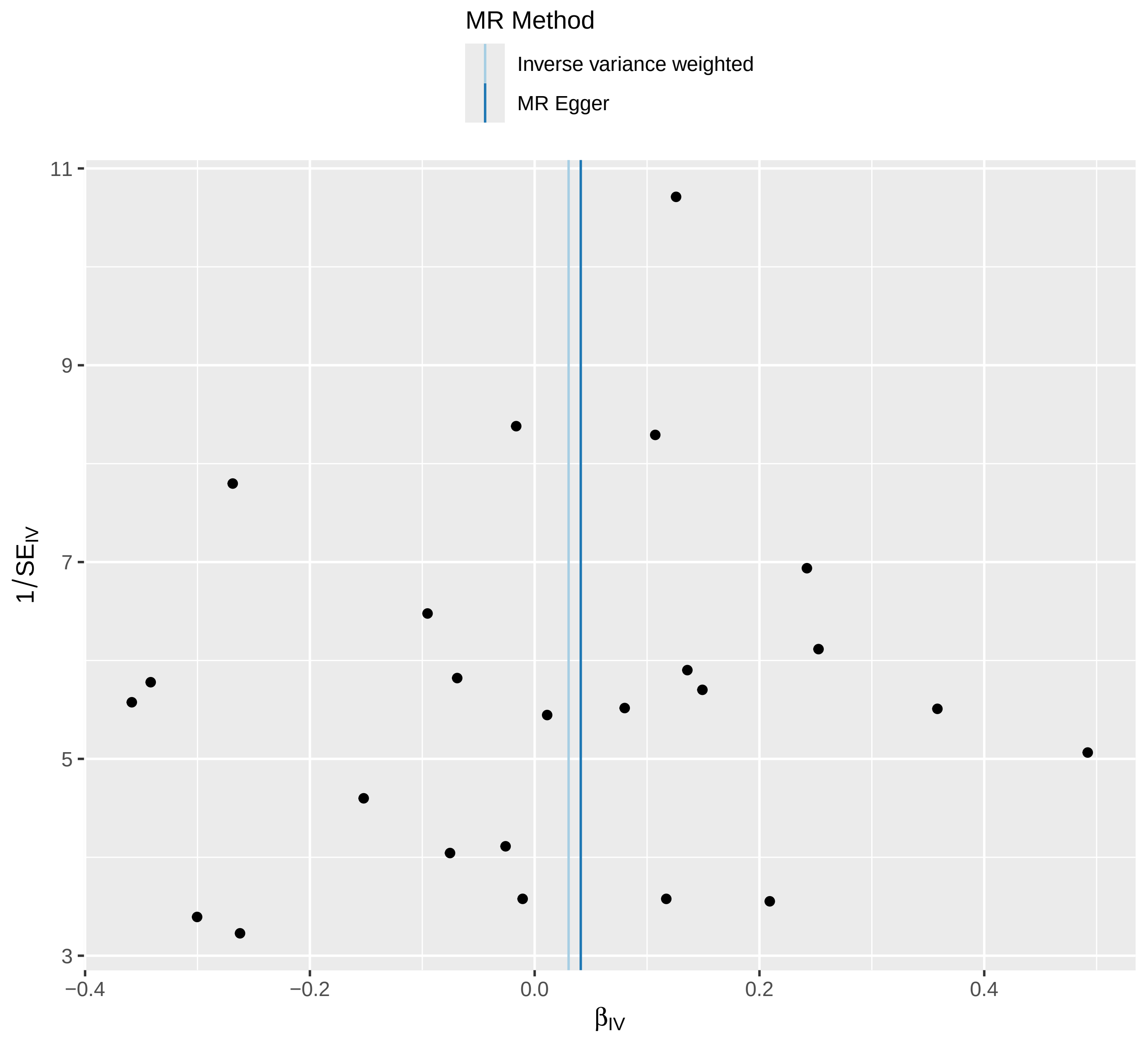
MR Method
Inverse variance weighted
MR Egger
11
9
7
5
3
1/SE_IV
−0.4
−0.2
0.0
0.2
0.4
β_IV

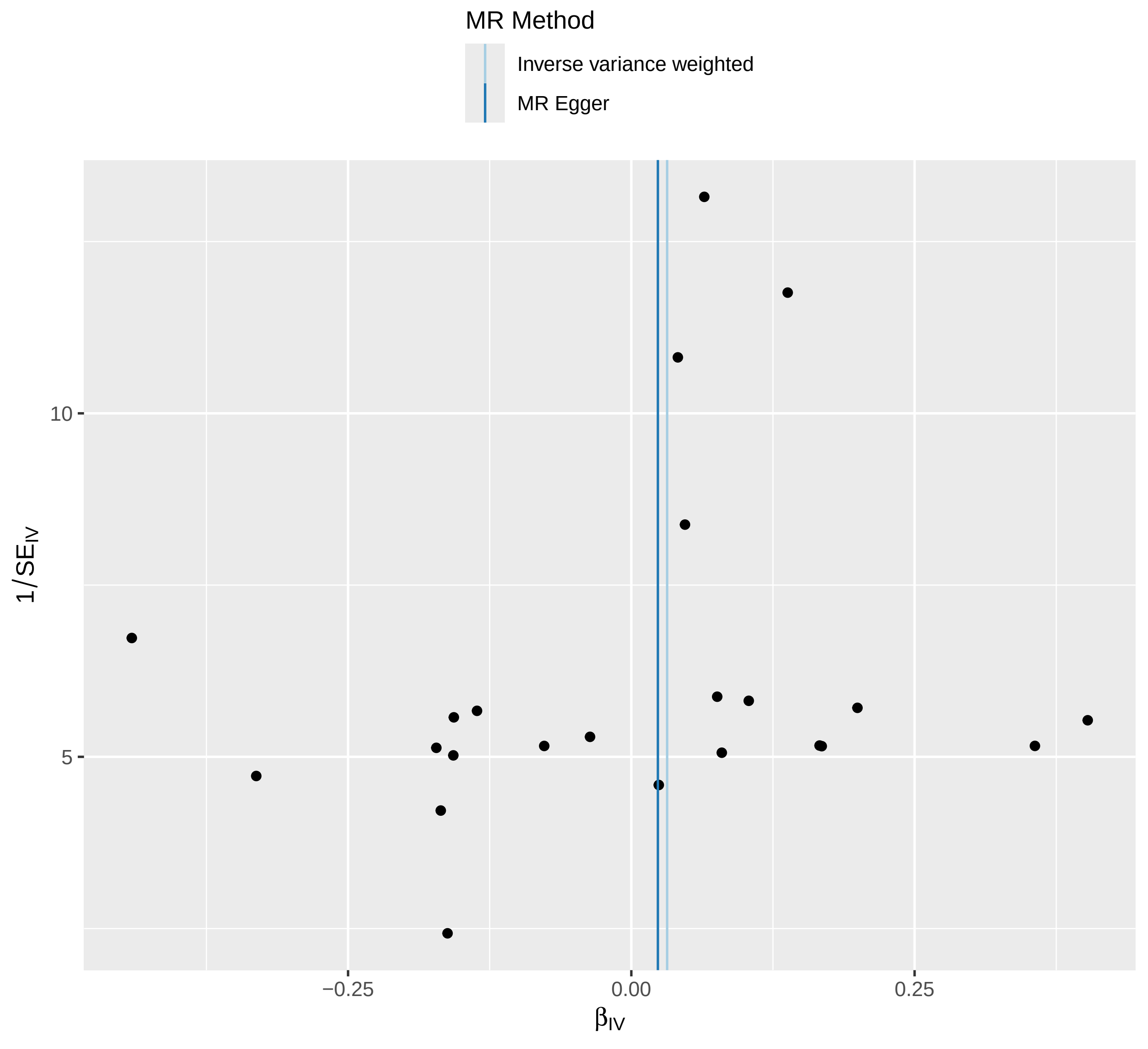
MR Method
Inverse variance weighted
MR Egger
10
5
1/SE$_{IV}$
−0.25
0.00
0.25
β$_{IV}$

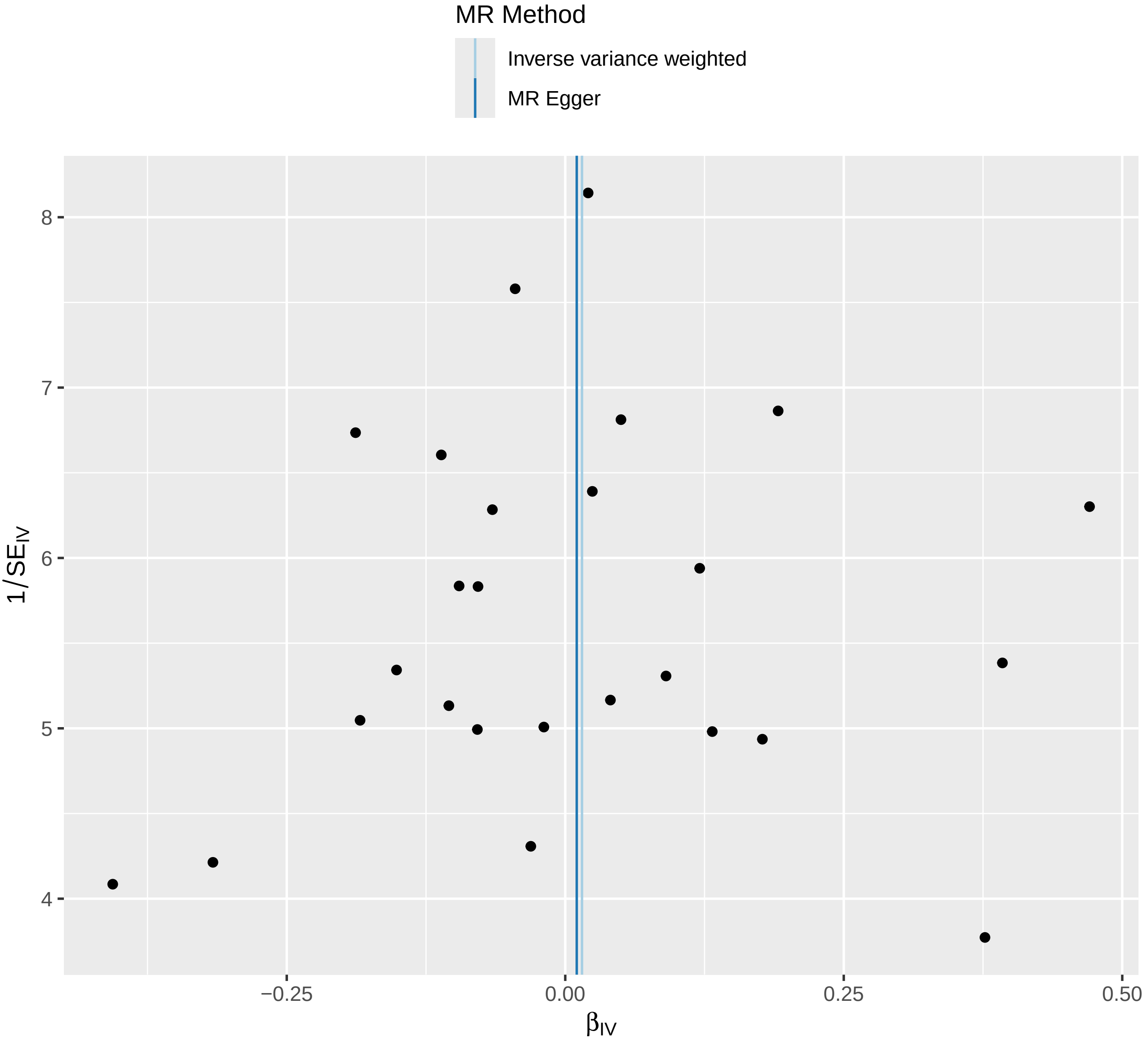

MR Method
Inverse variance weighted
MR Egger
8
7
6
5
4
1/SE$_{IV}$
−0.25
0.00
0.25
0.50
β$_{IV}$

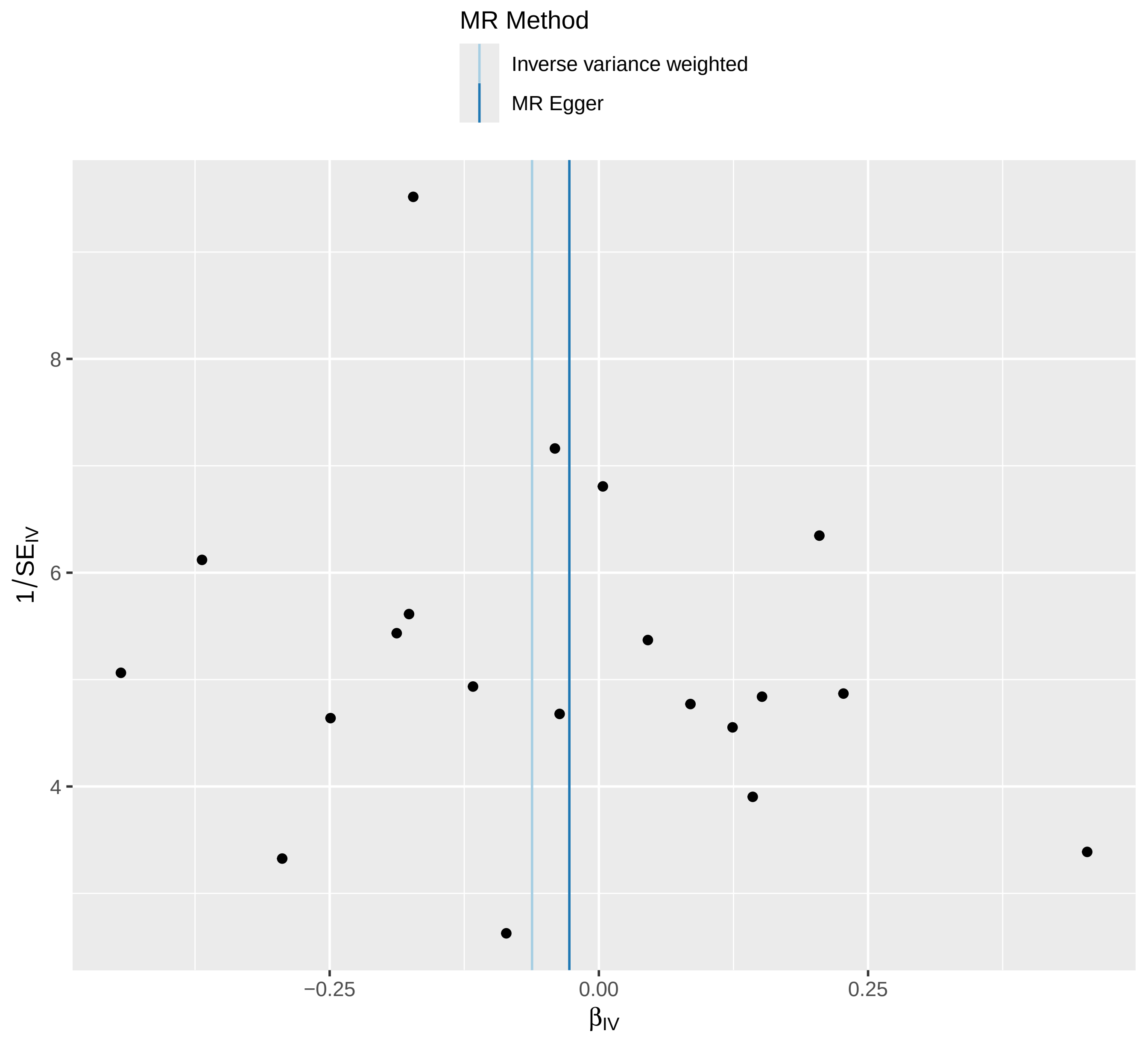

MR Method
Inverse variance weighted
MR Egger
8
6
4
1/SE$_{IV}$
−0.25
0.00
0.25
β_{IV}

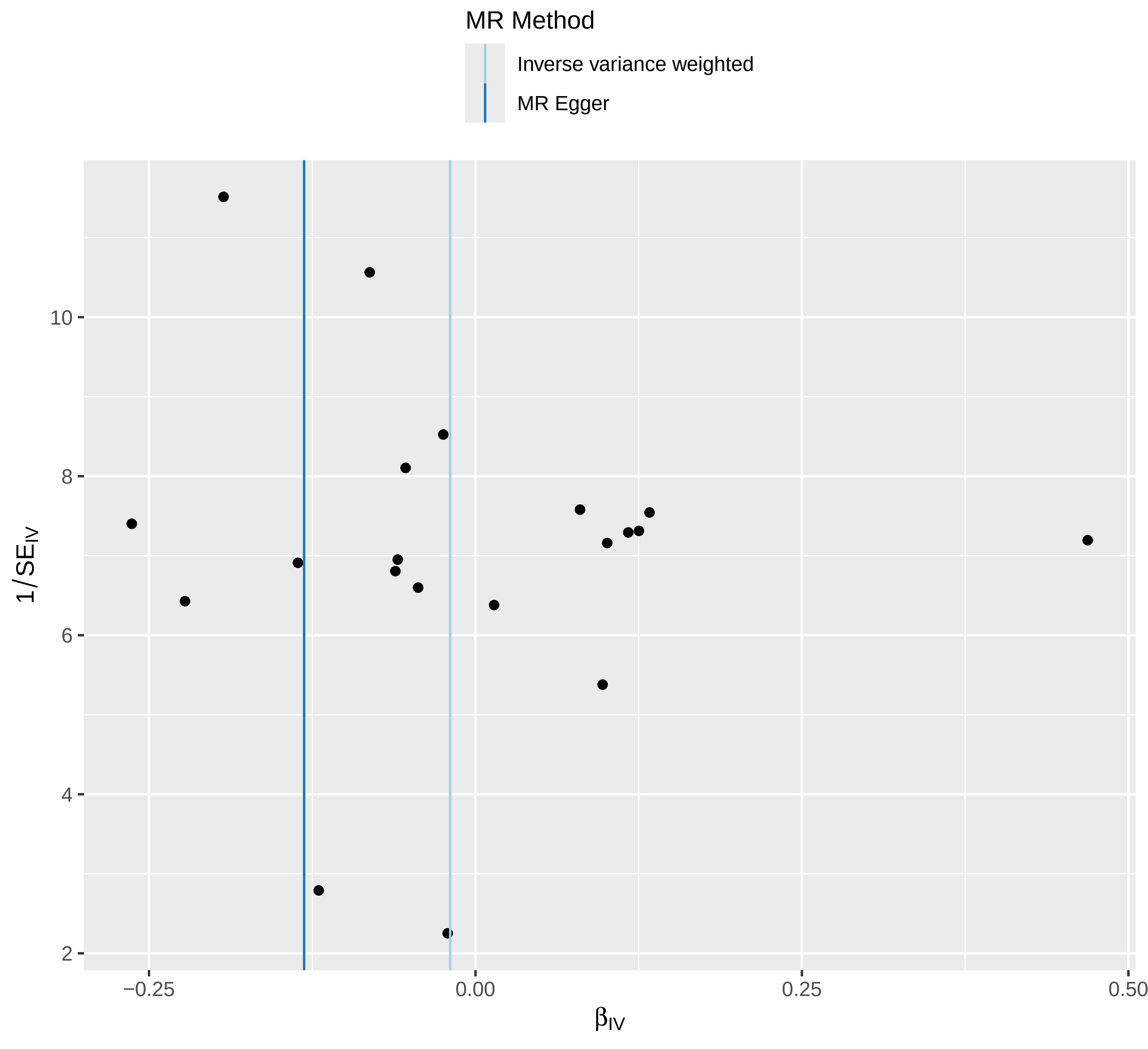

MR Method
Inverse variance weighted
MR Egger
1/SE_IV
β_IV
10
8
6
4
2
−0.25
0.00
0.25
0.50

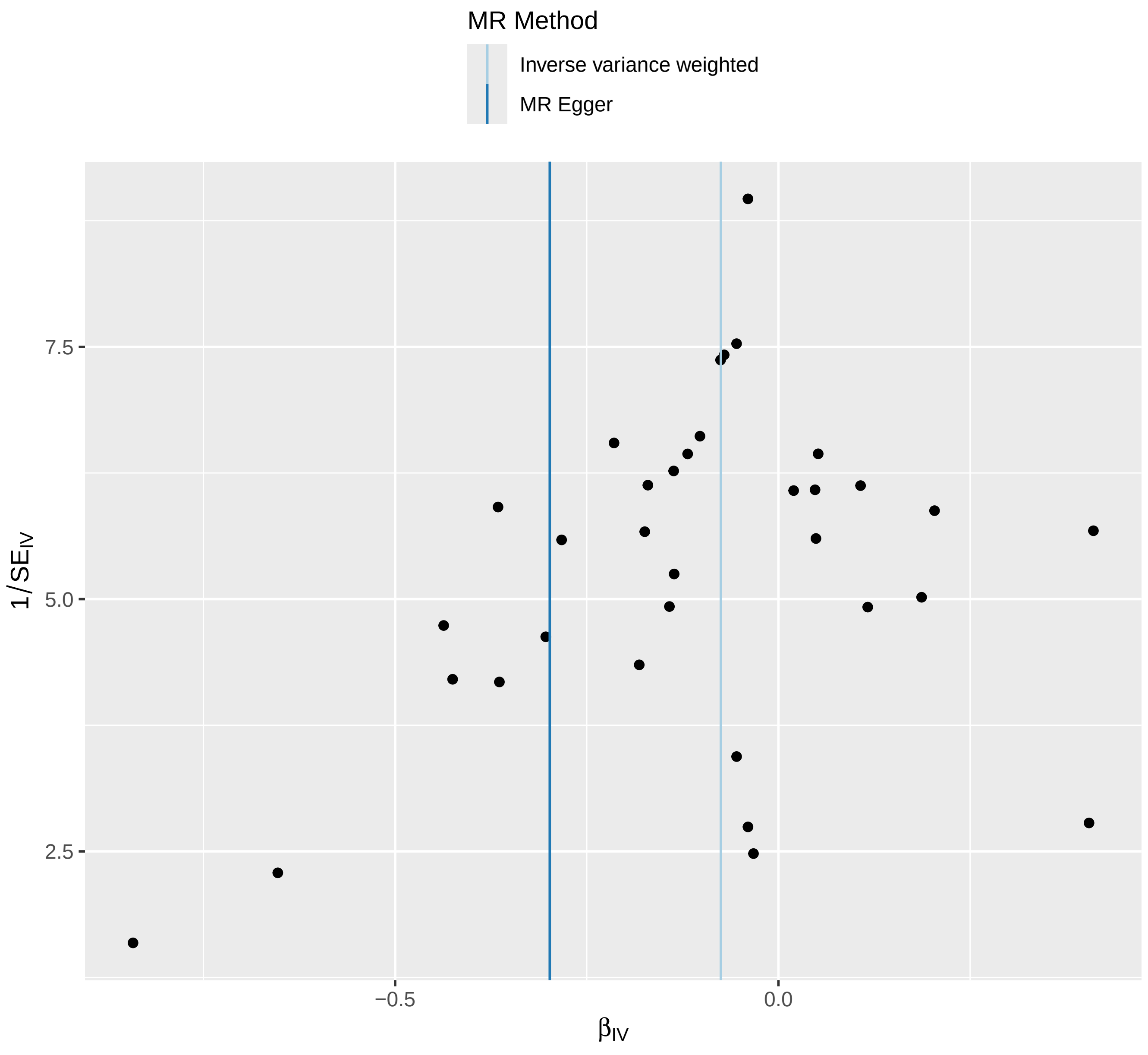

MR Method
Inverse variance weighted
MR Egger
7.5
5.0
2.5
1/SE$_{IV}$
−0.5
0.0
β$_{IV}$

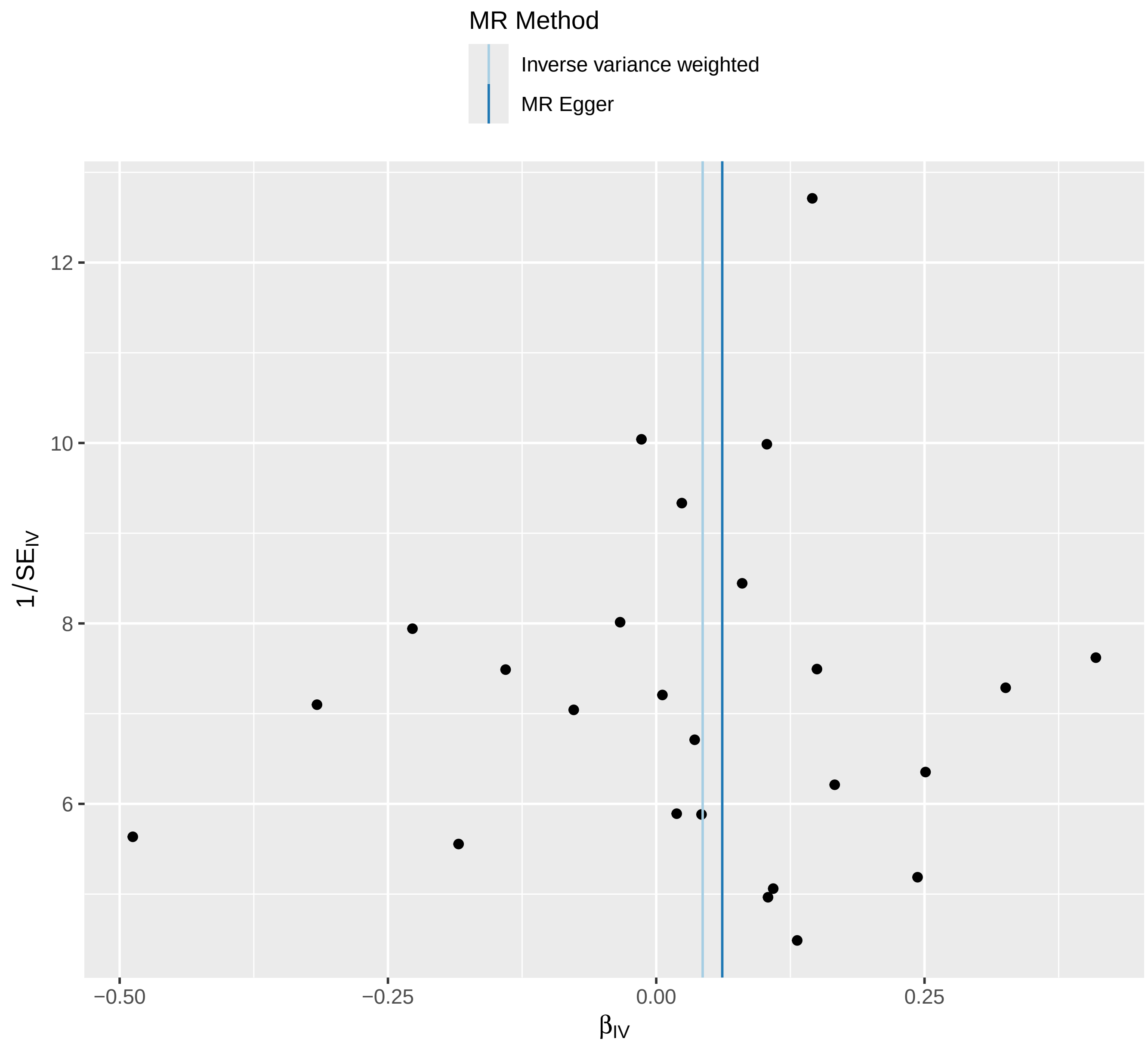

MR Method
Inverse variance weighted
MR Egger
12
10
8
6
1/SE_IV
−0.50
−0.25
0.00
0.25
β_IV

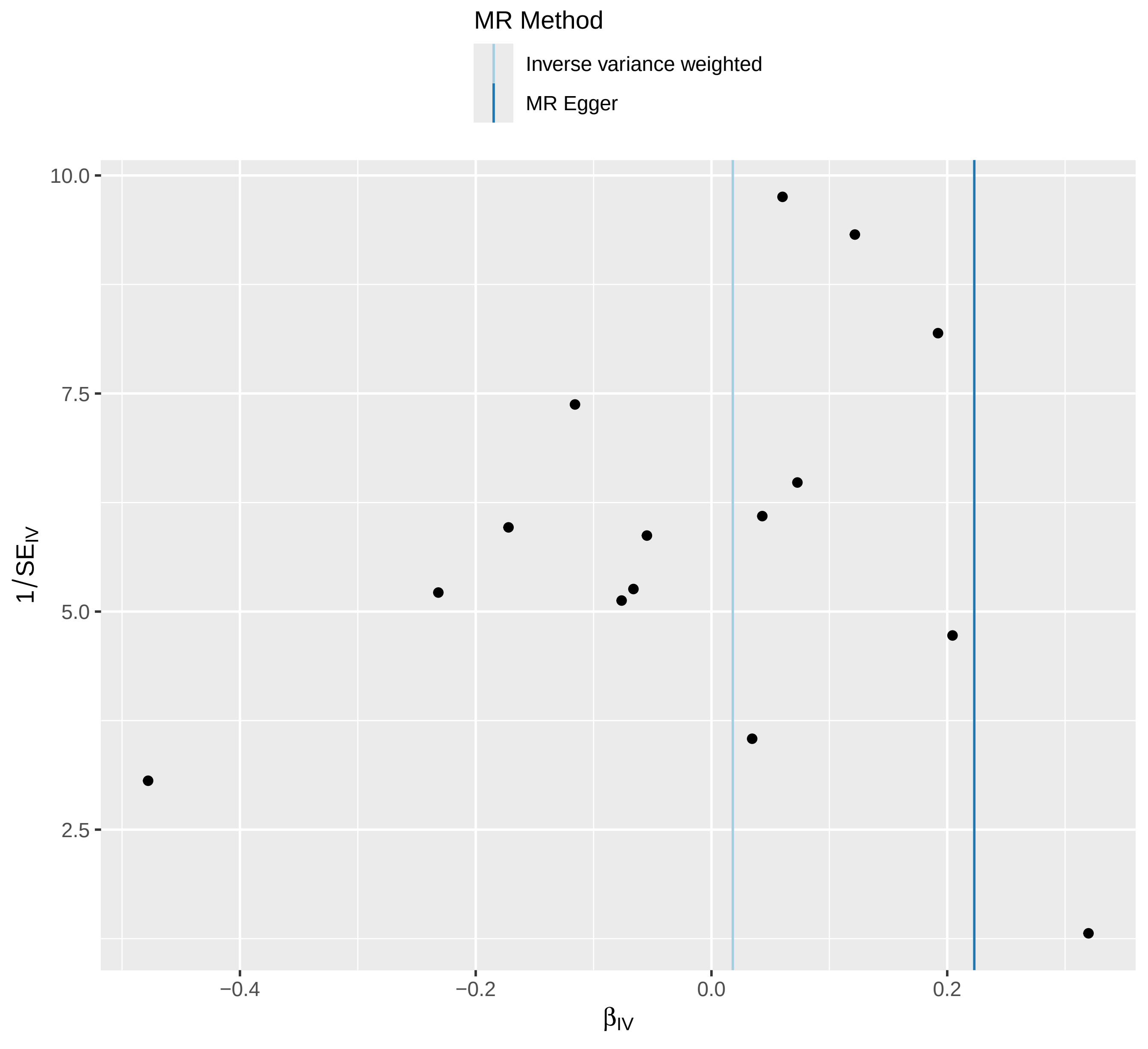

MR Method
Inverse variance weighted
MR Egger
10.0
7.5
5.0
2.5
1/SE$_{IV}$
−0.4
−0.2
0.0
0.2
β$_{IV}$

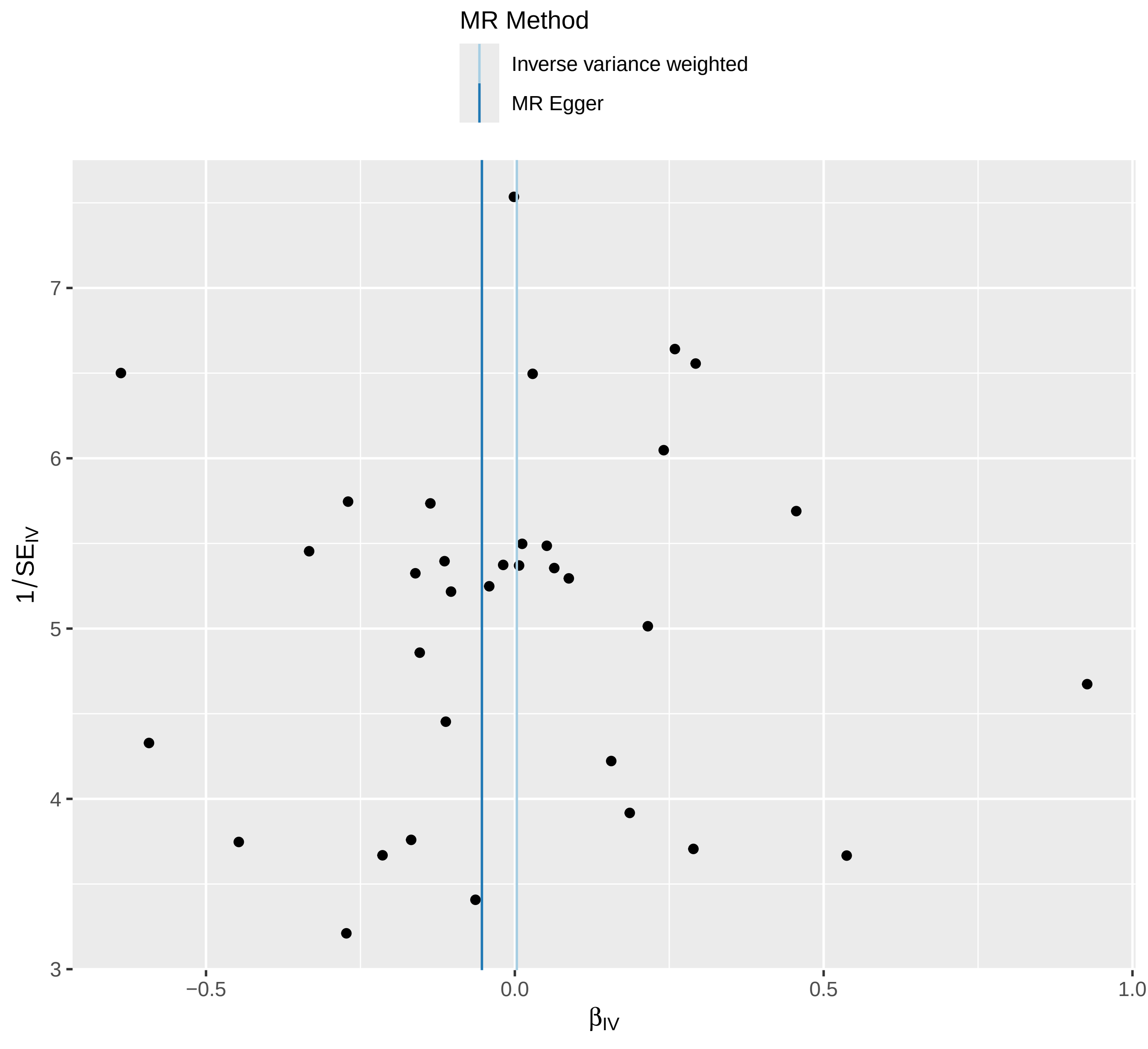
MR Method
Inverse variance weighted
MR Egger
1/SE_IV
7
6
5
4
3
−0.5
0.0
0.5
1.0
β_IV

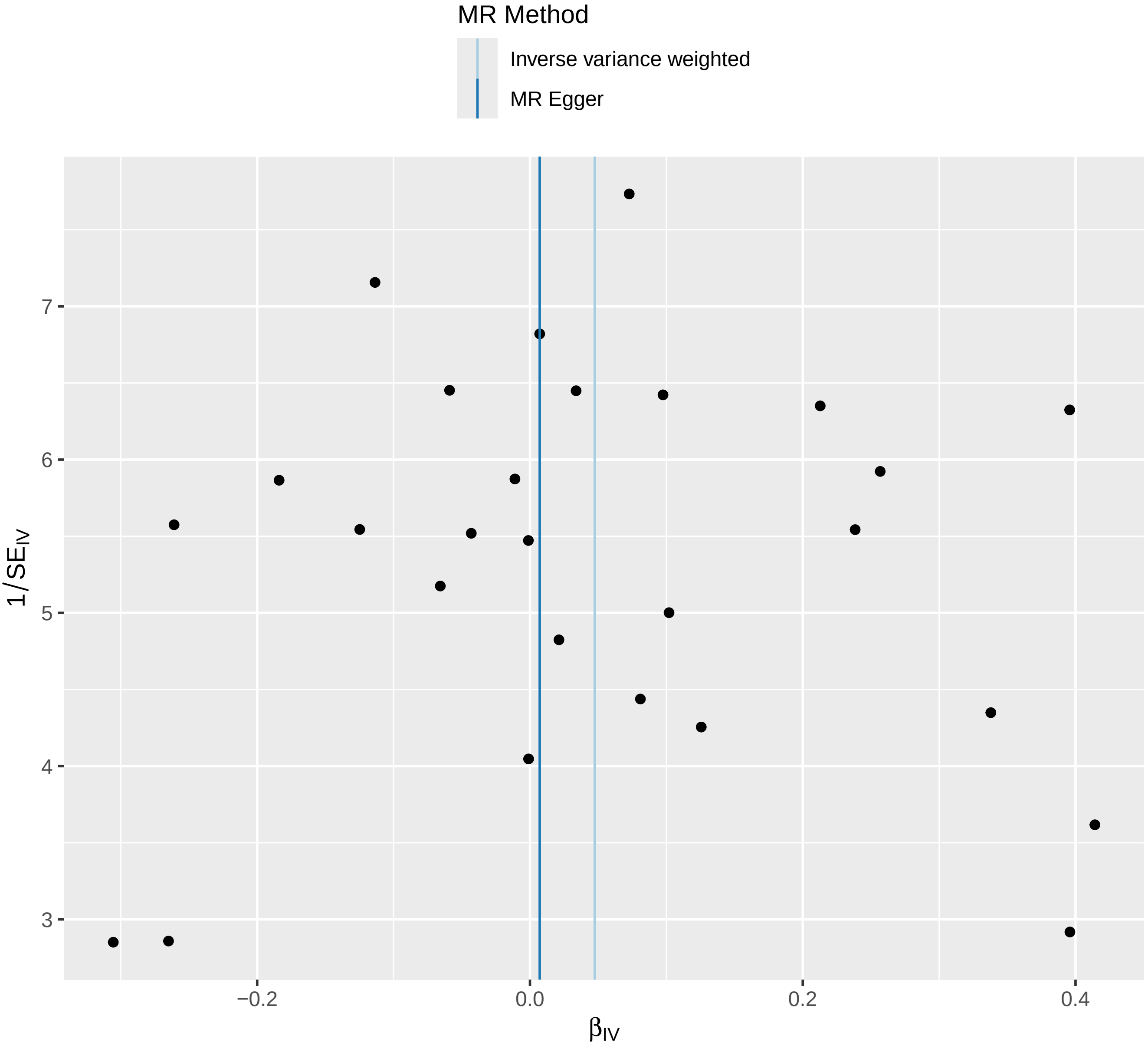

MR Method
Inverse variance weighted
MR Egger
1/SE_{IV}
β_{IV}
7
6
5
4
3
−0.2
0.0
0.2
0.4

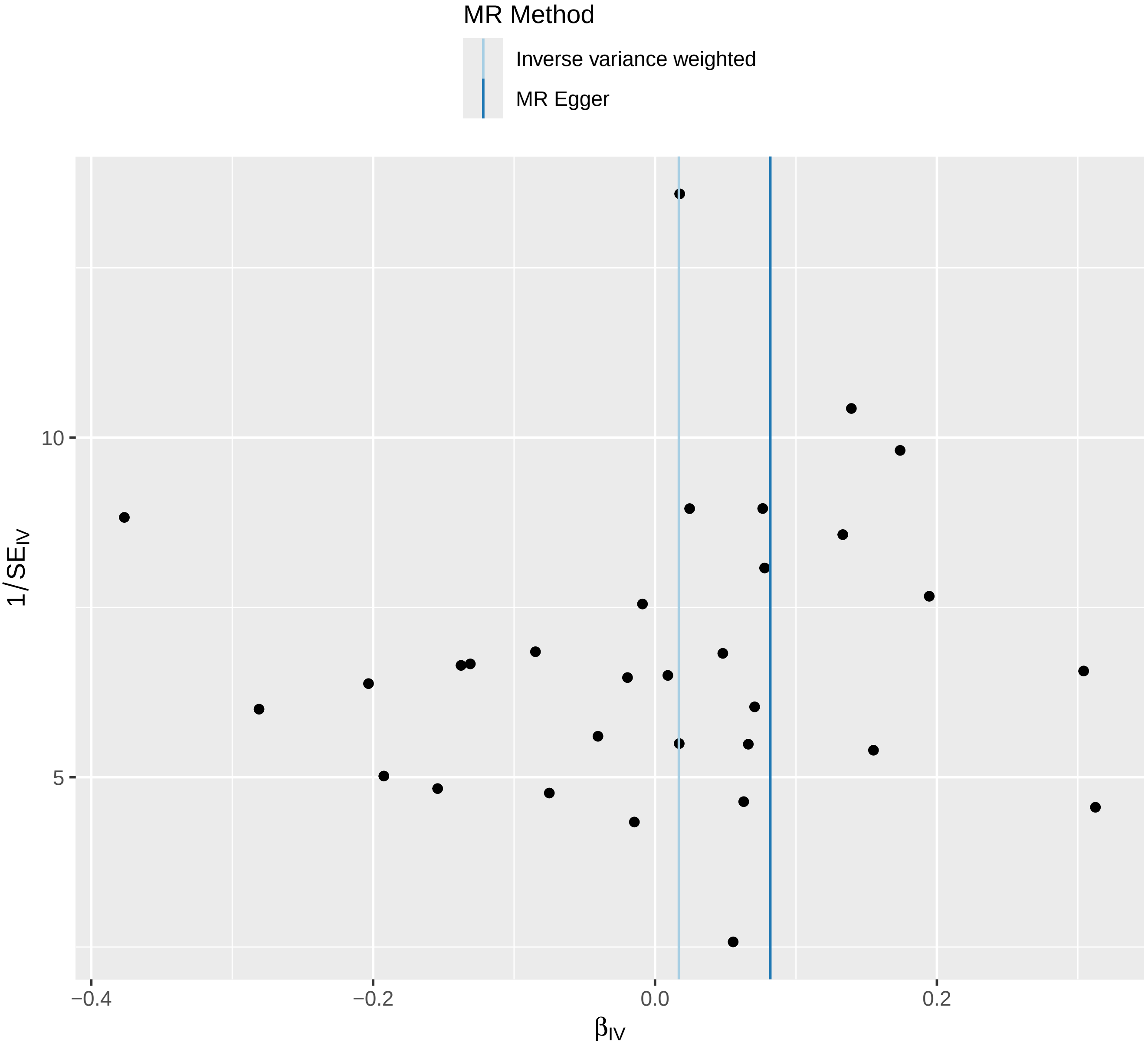

MR Method
Inverse variance weighted
MR Egger
10
5
1/SE_IV
−0.4
−0.2
0.0
0.2
β_IV

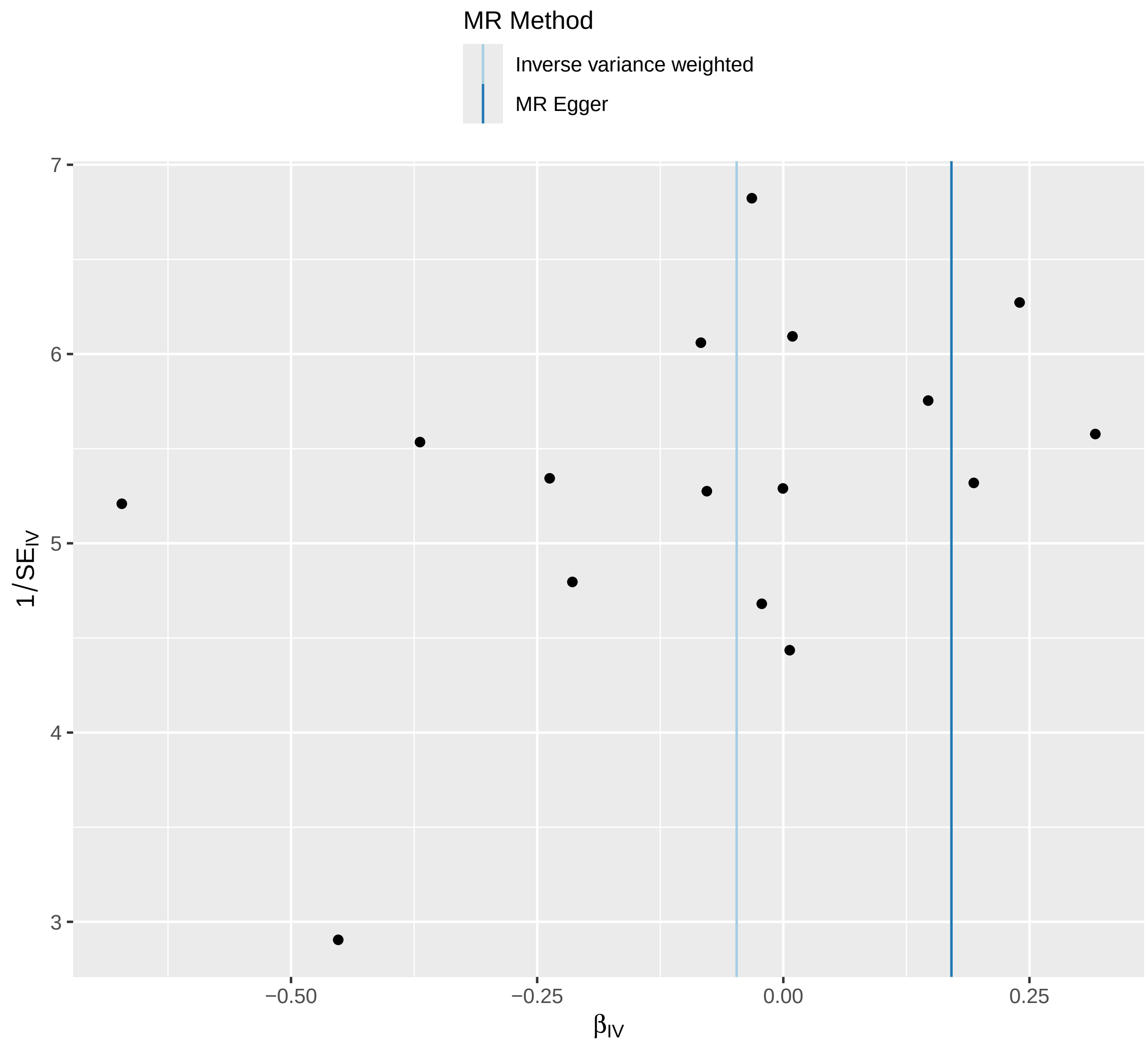
MR Method
Inverse variance weighted
MR Egger
7
6
5
4
3
1/SE_IV
−0.50
−0.25
0.00
0.25
β_IV

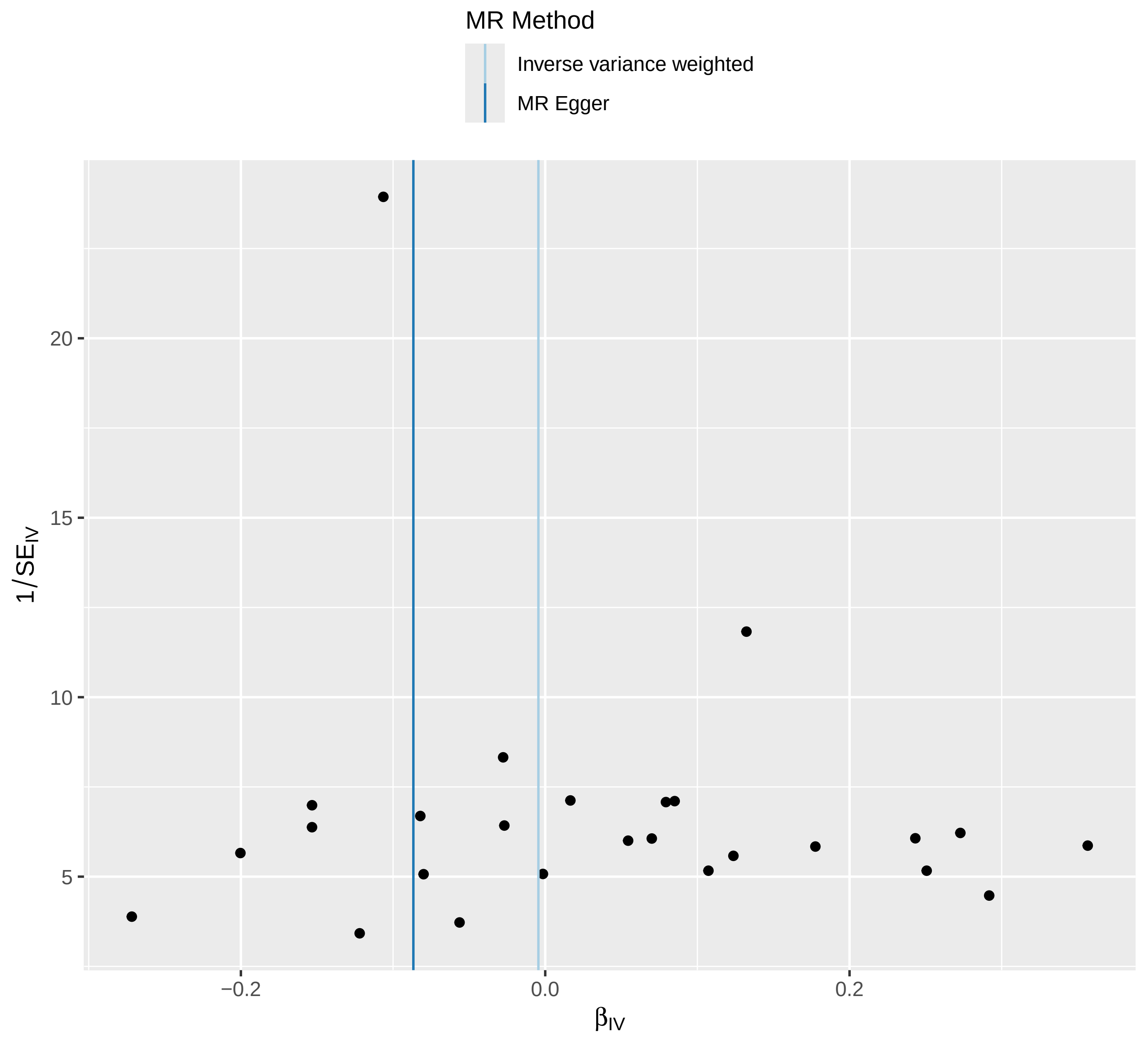
MR Method
Inverse variance weighted
MR Egger
20
15
10
5
−0.2
0.0
0.2
$1/SE_{IV}$
β_{IV}

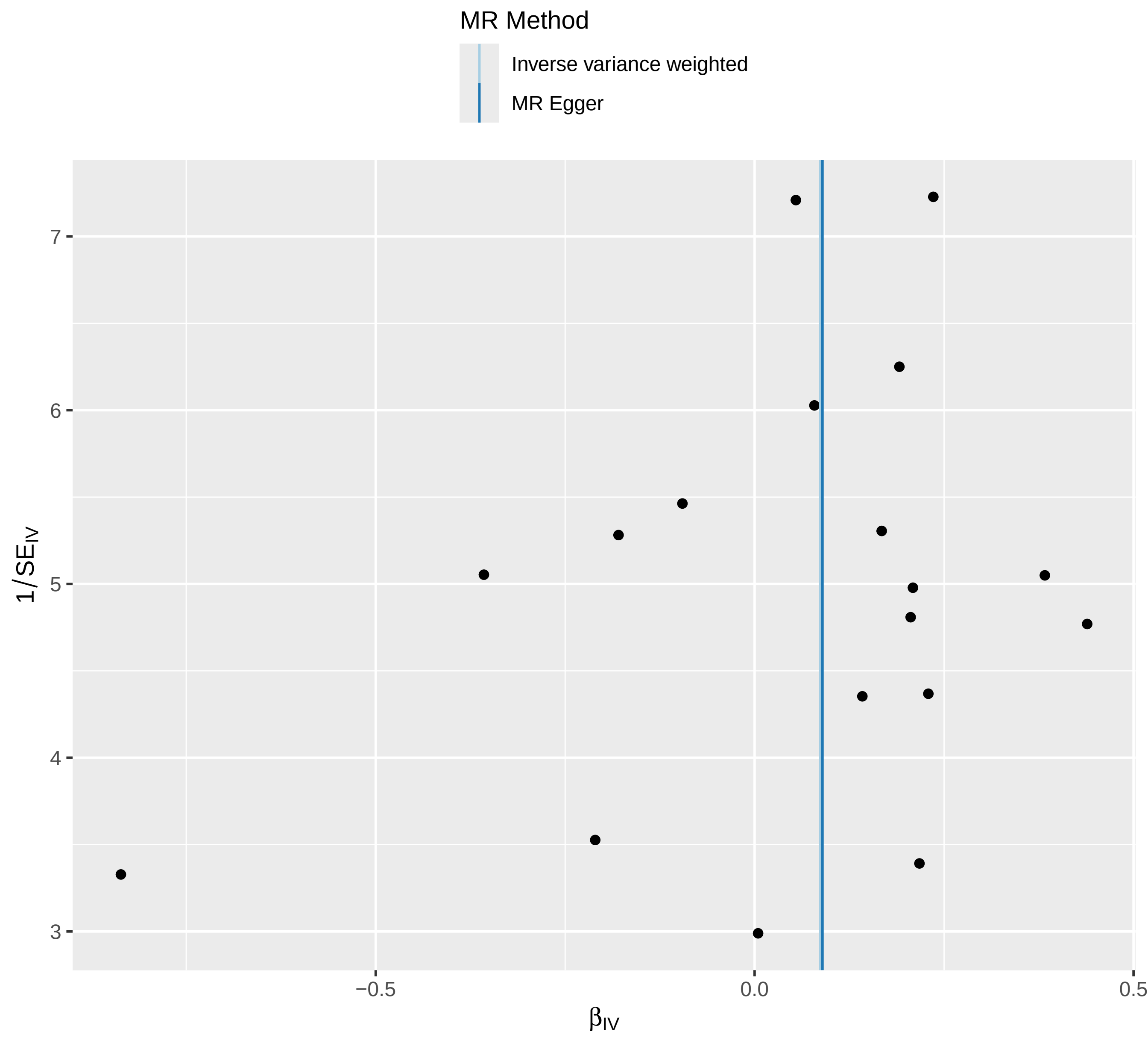

MR Method
Inverse variance weighted
MR Egger
7
6
5
4
3
1/SE_IV
−0.5
0.0
0.5
β_IV

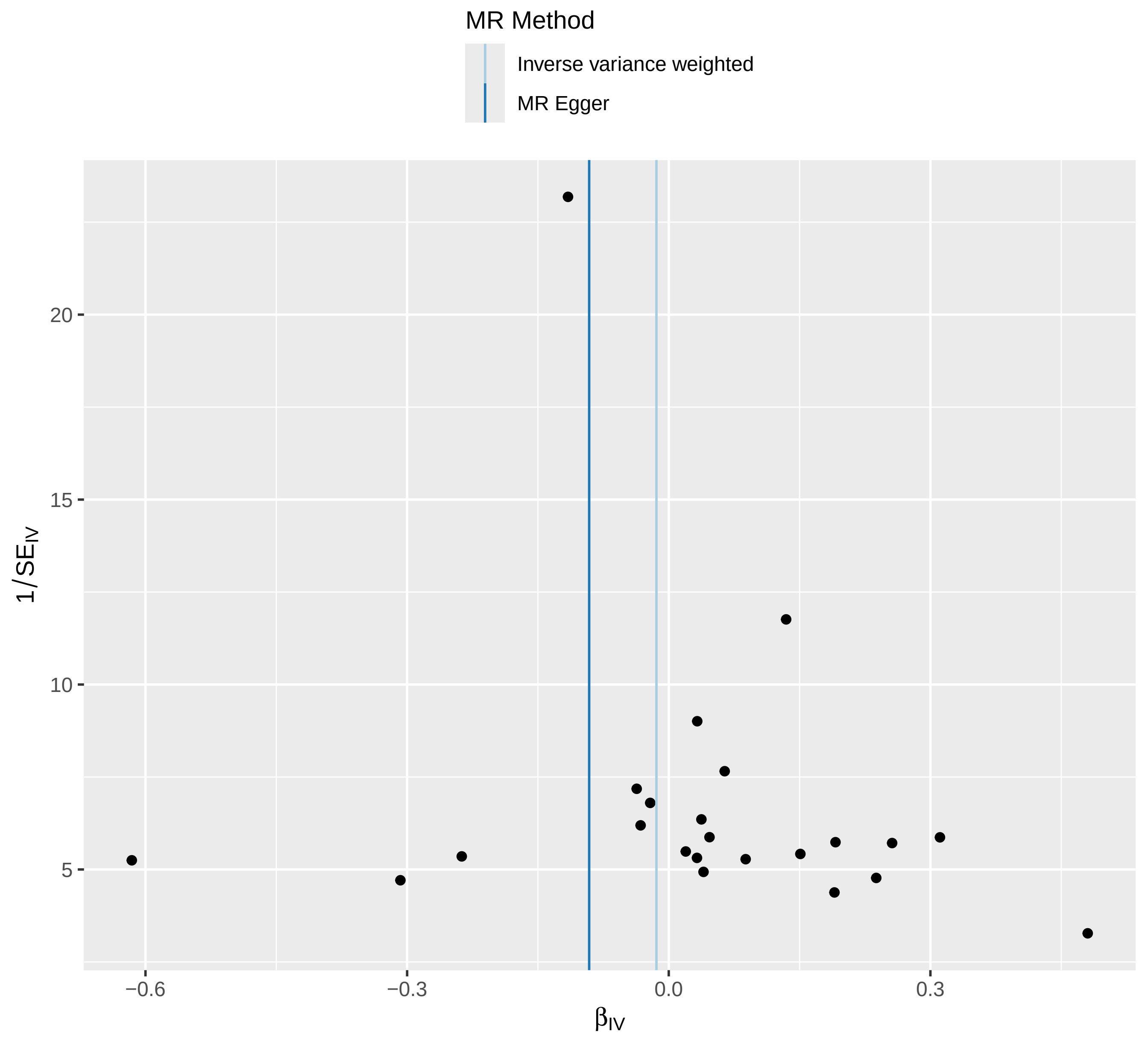
MR Method
Inverse variance weighted
MR Egger
20
15
10
5
1/SE_IV
−0.6
−0.3
0.0
0.3
β_IV

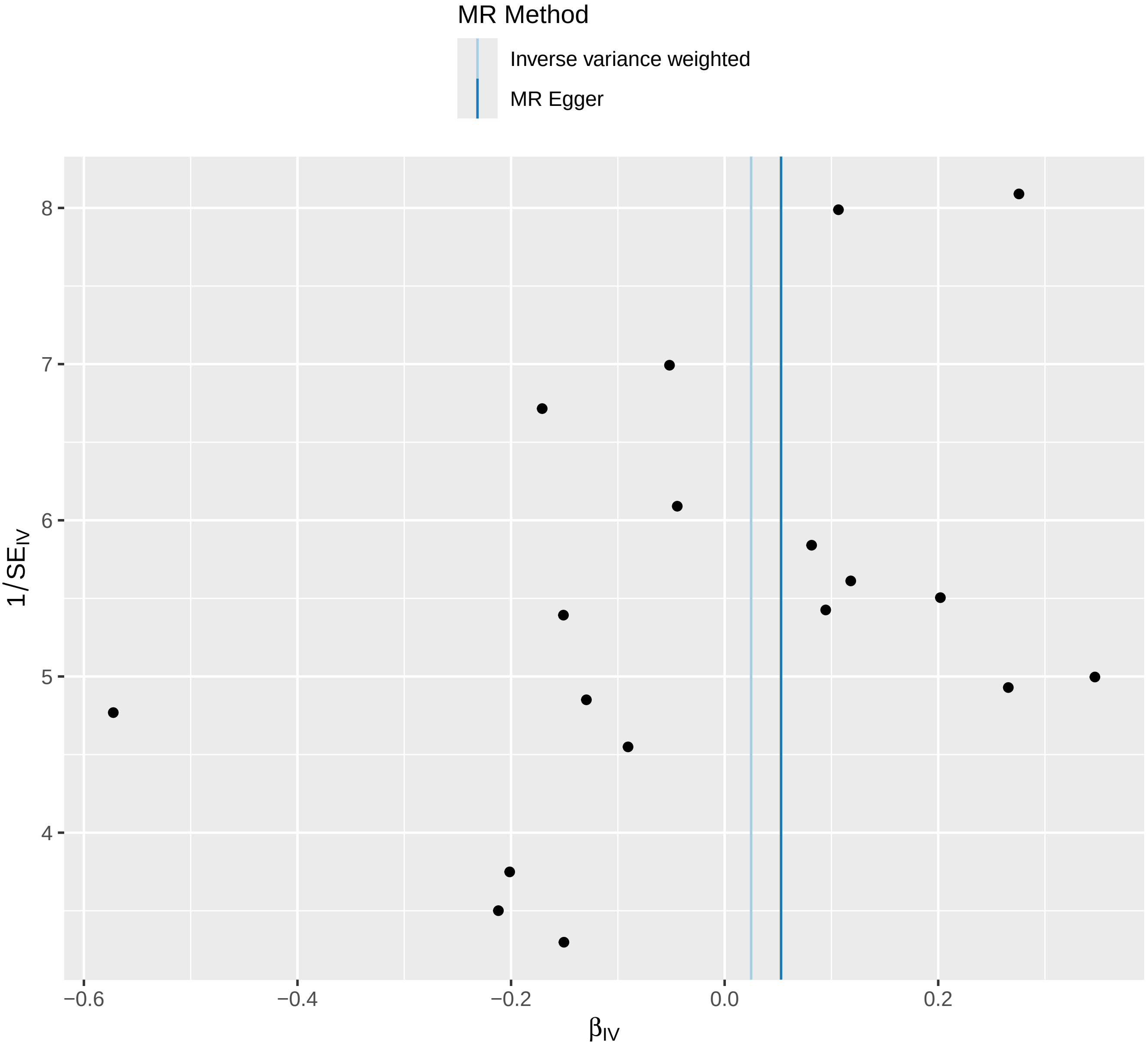

MR Method
Inverse variance weighted
MR Egger
1/SE_IV
β_IV
−0.6
−0.4
−0.2
0.0
0.2
4
5
6
7
8

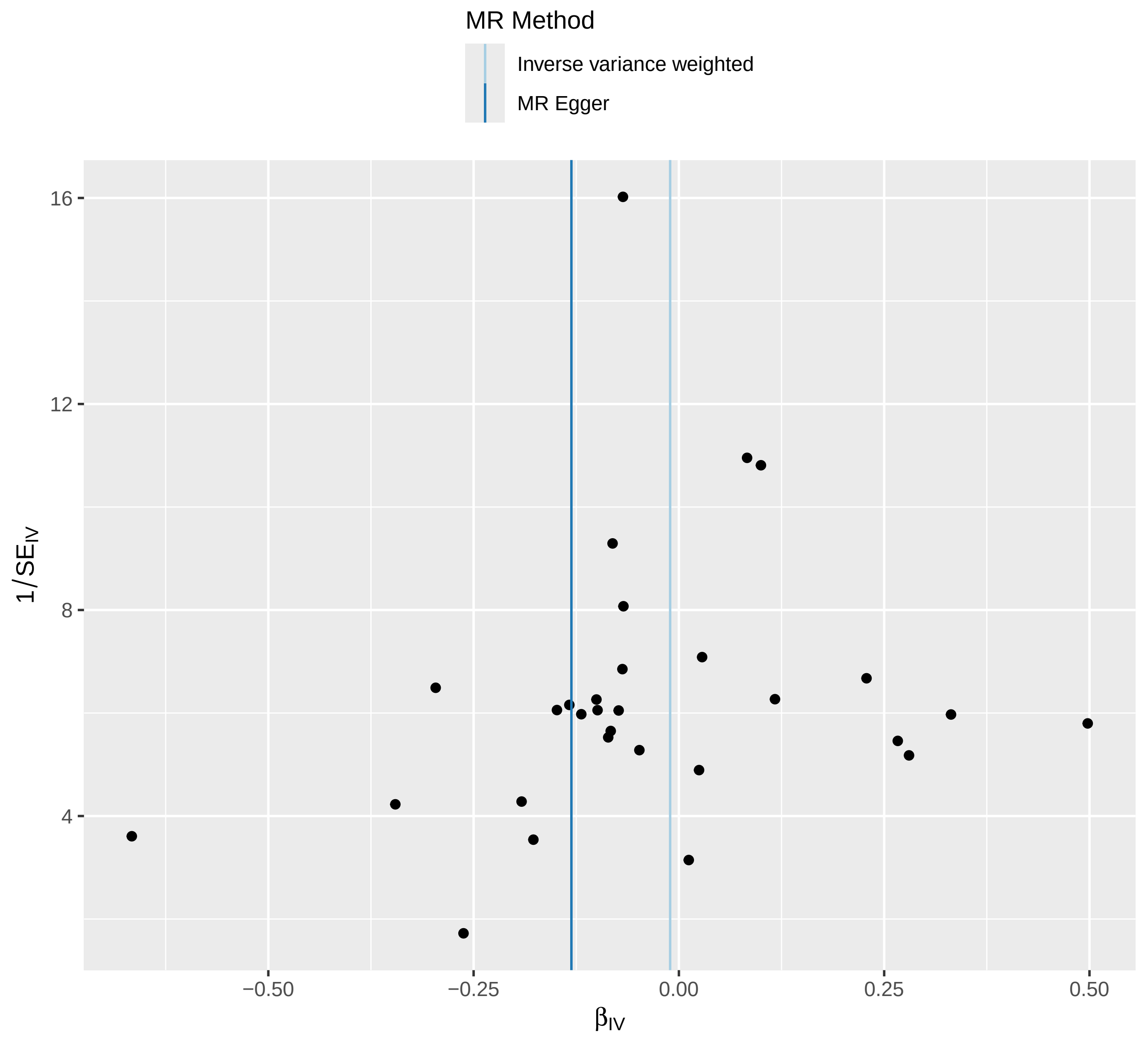

MR Method
Inverse variance weighted
MR Egger
16
12
8
4
$1/SE_{IV}$
−0.50
−0.25
0.00
0.25
0.50
β_{IV}

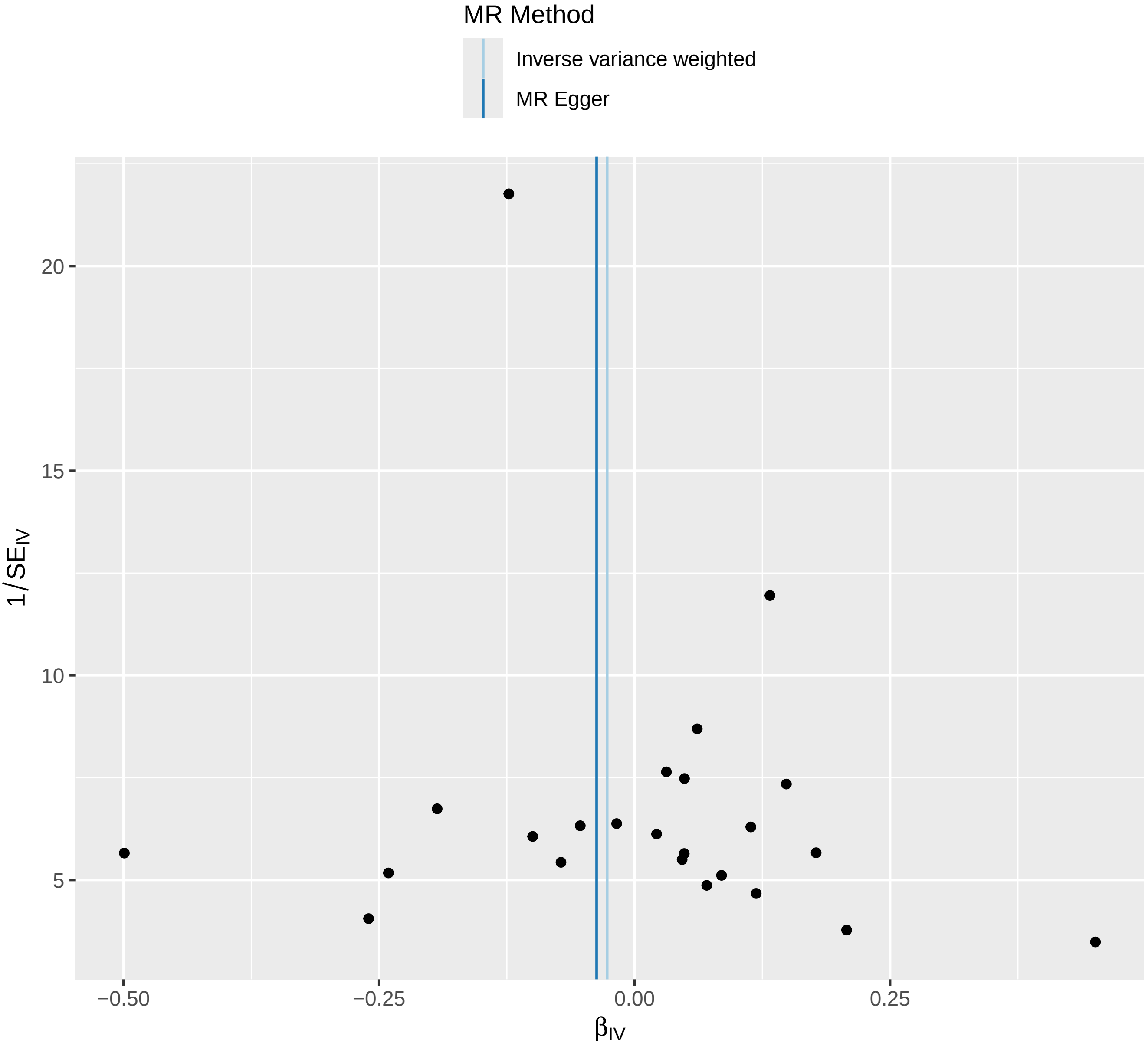
MR Method
Inverse variance weighted
MR Egger
20
15
10
5
1/SE_{IV}
−0.50
−0.25
0.00
0.25
β_{IV}

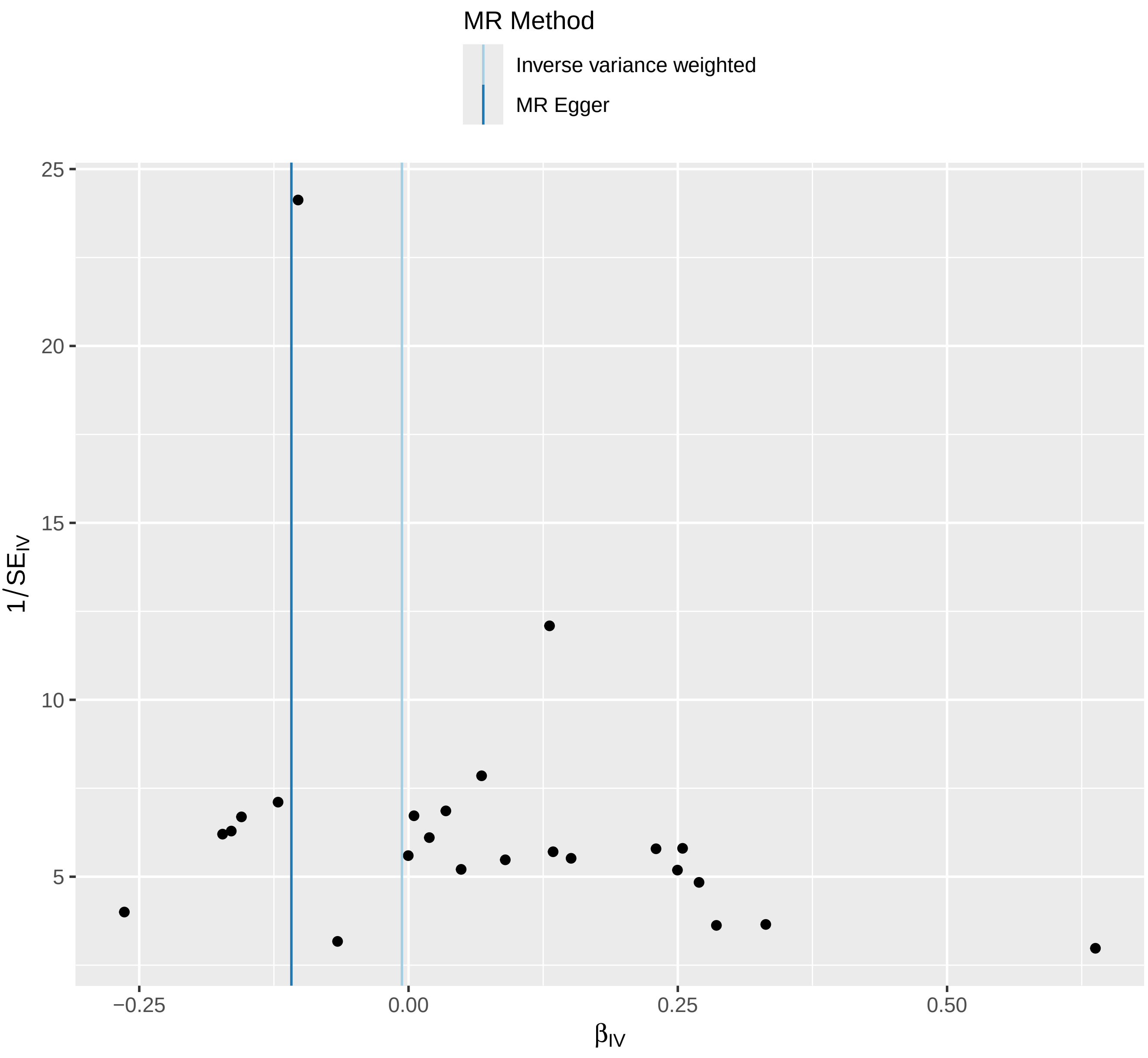

MR Method
Inverse variance weighted
MR Egger
25
20
15
10
5
$1/SE_{IV}$
−0.25
0.00
0.25
0.50
β_{IV}

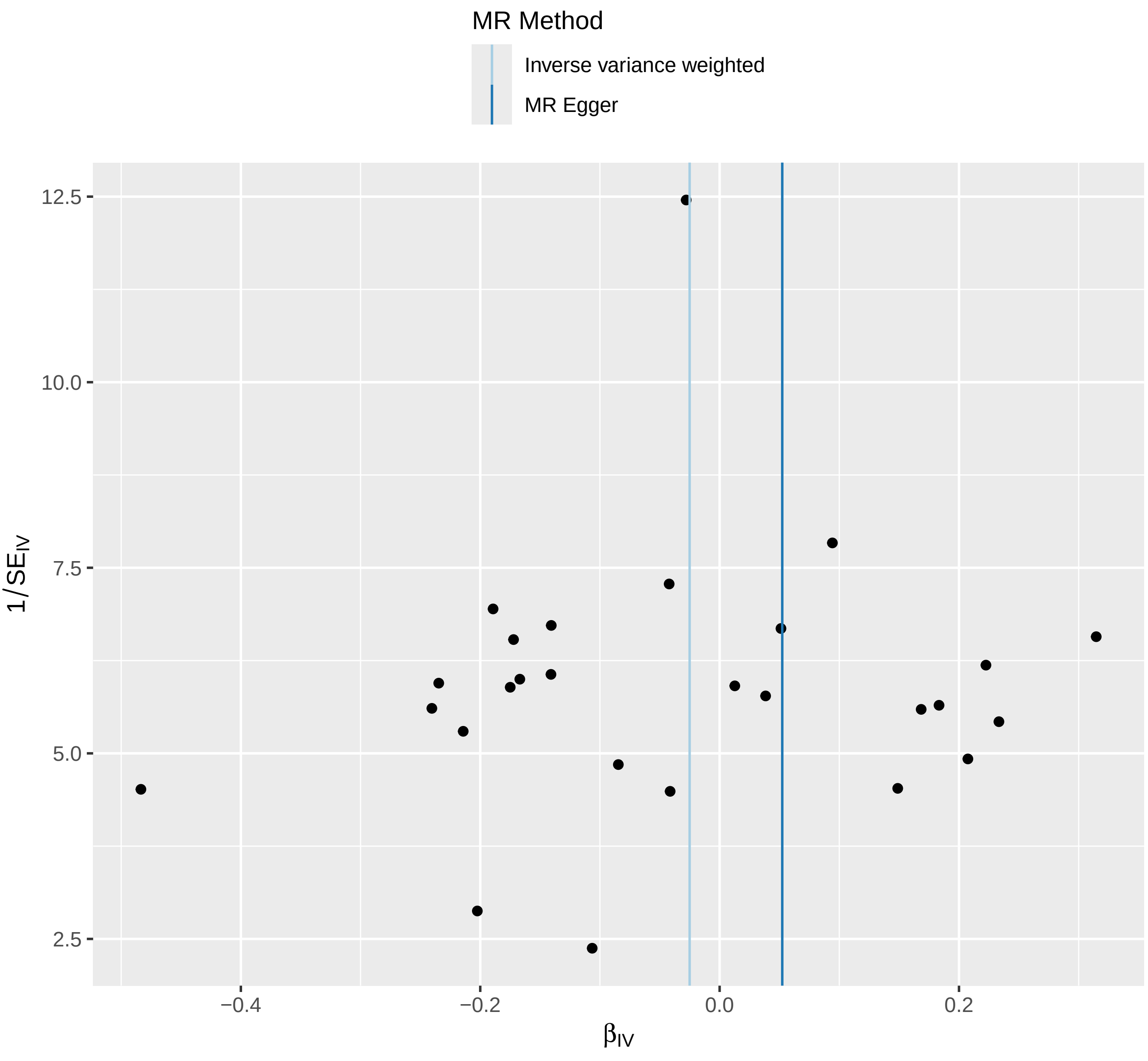
MR Method
Inverse variance weighted
MR Egger
12.5
10.0
7.5
5.0
2.5
$1/SE_{IV}$
−0.4
−0.2
0.0
0.2
β_{IV}

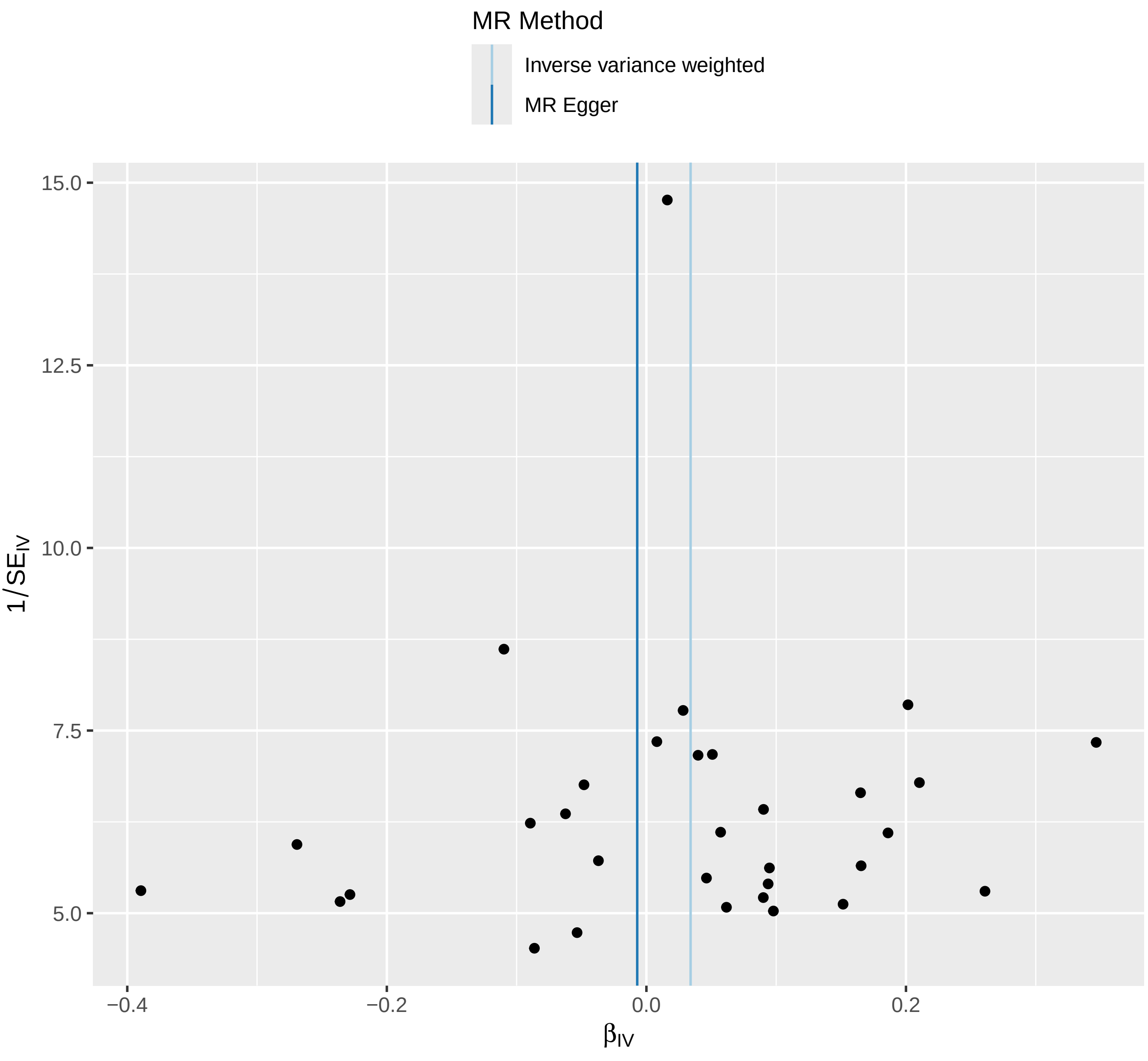
MR Method
Inverse variance weighted
MR Egger
15.0
12.5
10.0
7.5
5.0
$1/SE_{IV}$
−0.4
−0.2
0.0
0.2
β_{IV}

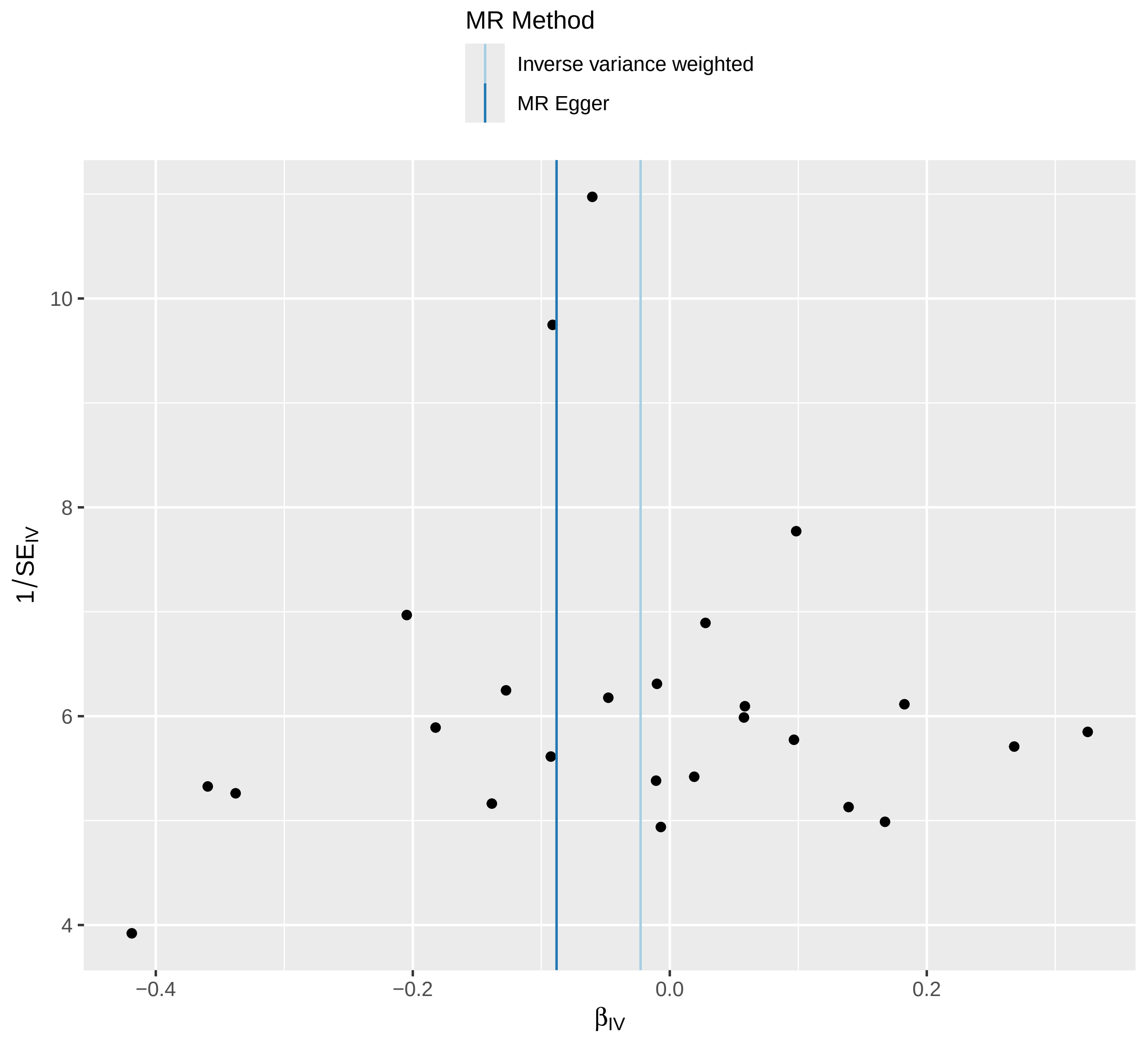
MR Method
Inverse variance weighted
MR Egger
10
8
6
4
1/SE_IV
−0.4
−0.2
0.0
0.2
β_IV

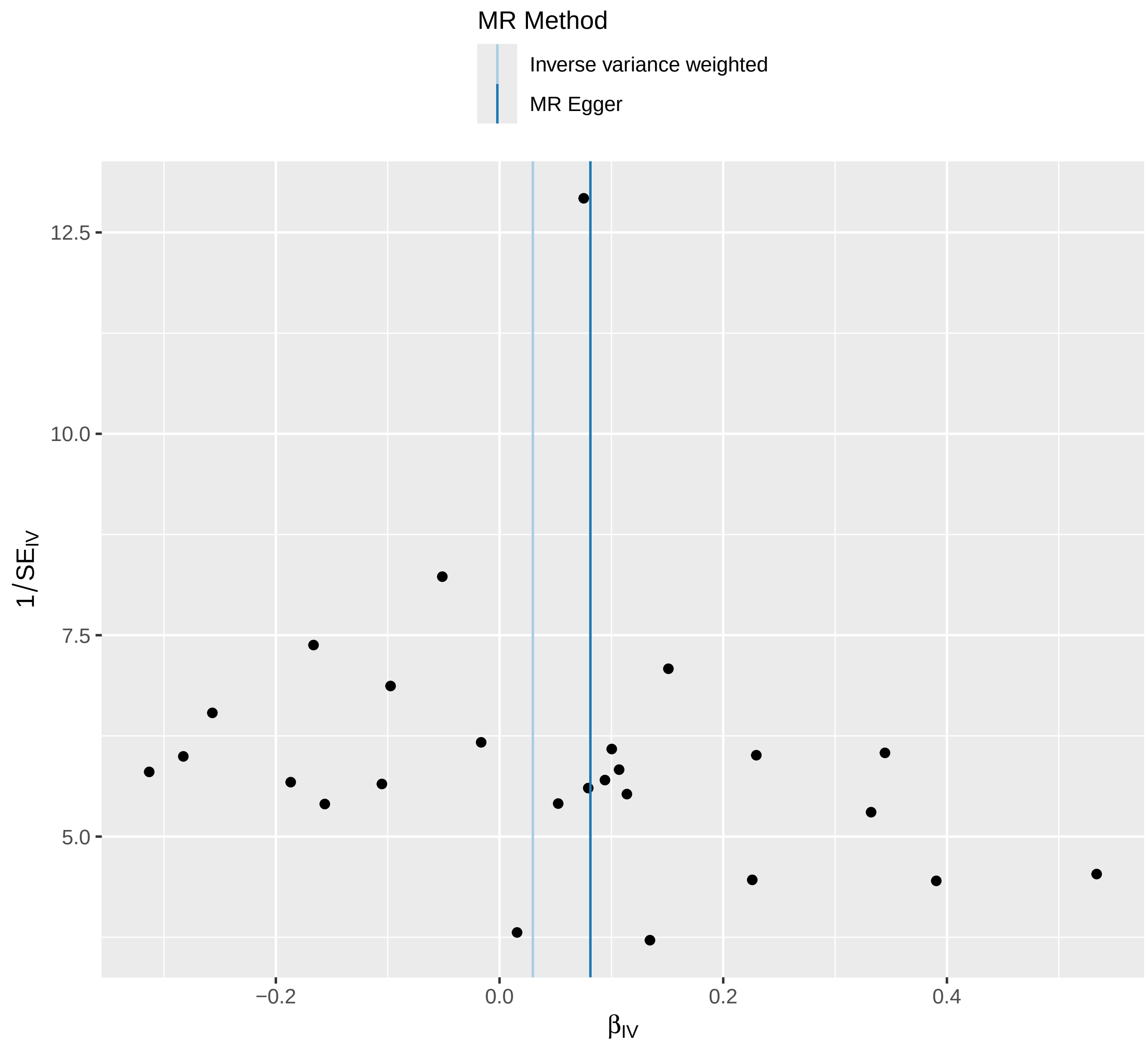

MR Method
Inverse variance weighted
MR Egger
12.5
10.0
7.5
5.0
−0.2
0.0
0.2
0.4
$1/SE_{IV}$
β_{IV}

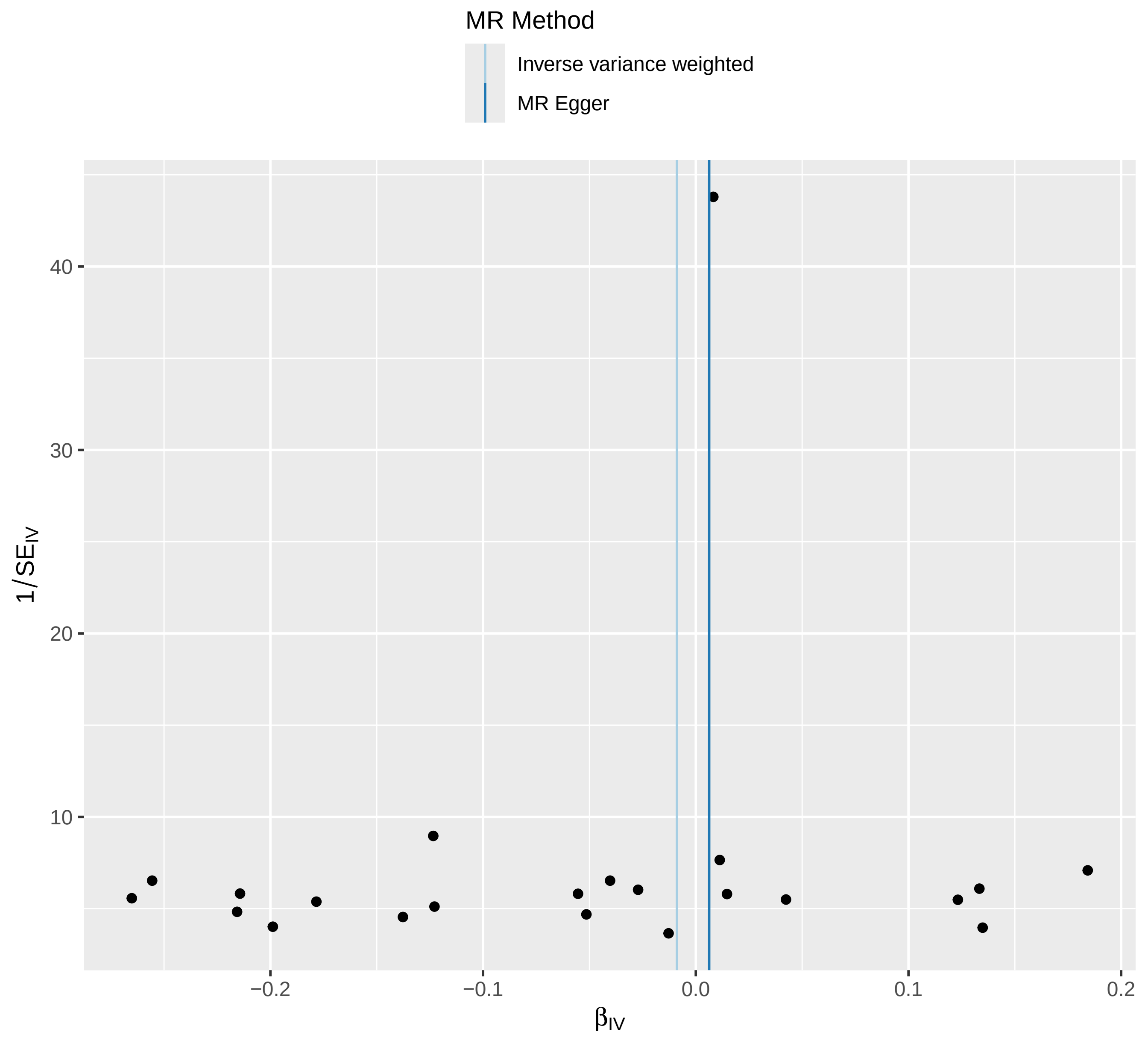

MR Method
Inverse variance weighted
MR Egger
40
30
20
10
1/SE_IV
−0.2
−0.1
0.0
0.1
0.2
β_IV

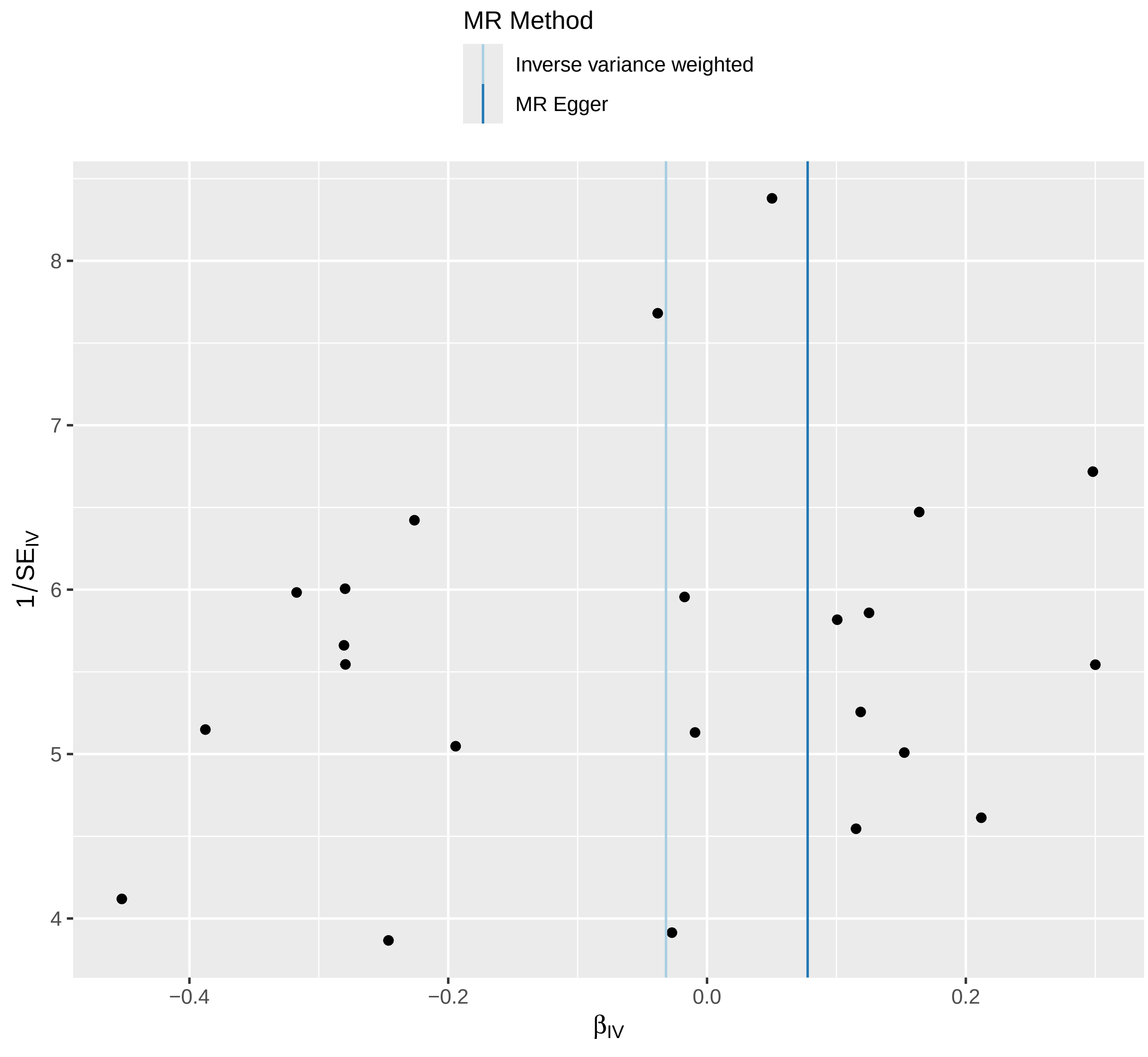
MR Method
Inverse variance weighted
MR Egger
1/SE_IV
β_IV
8
7
6
5
4
−0.4
−0.2
0.0
0.2

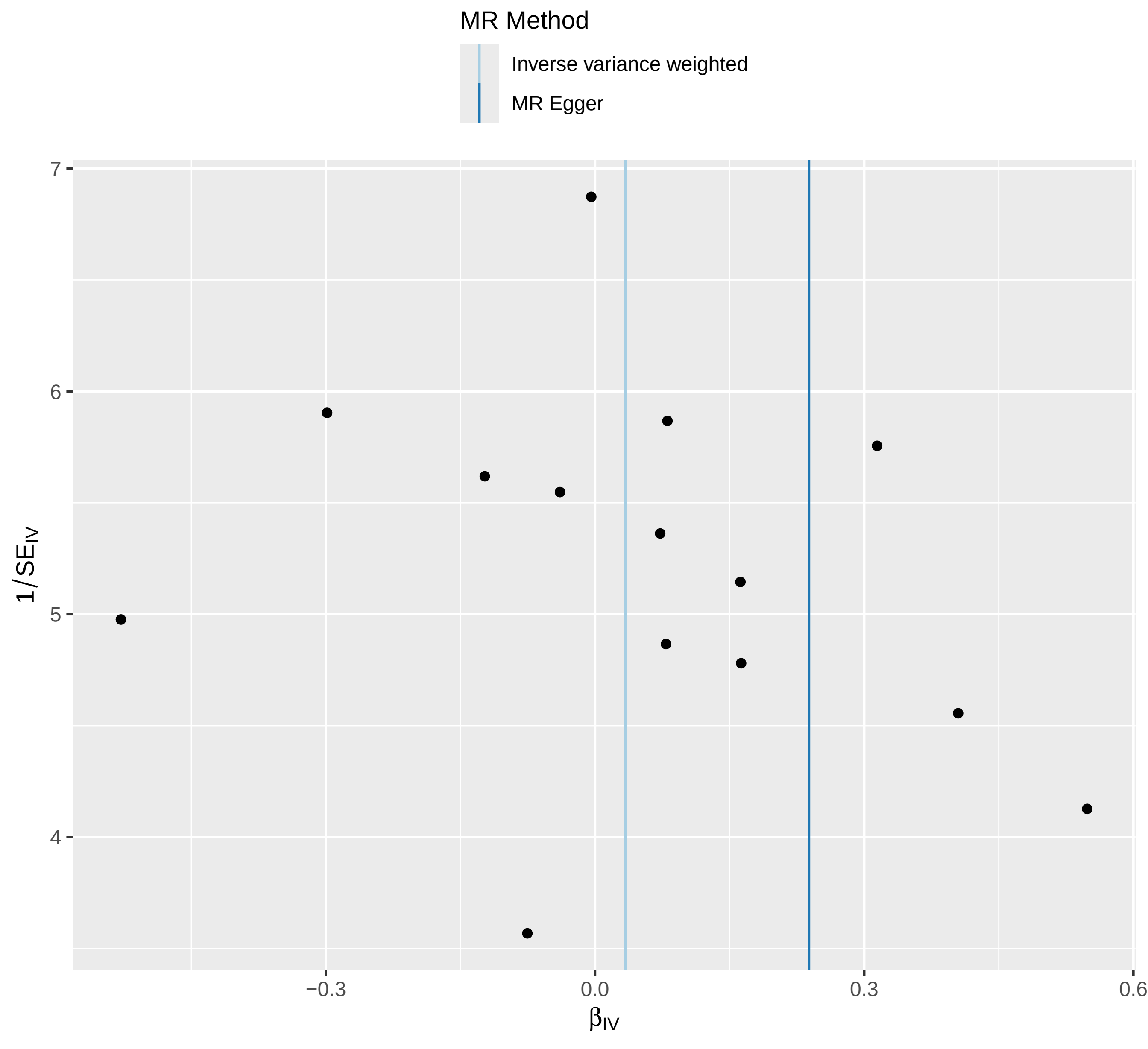
MR Method
Inverse variance weighted
MR Egger
7
6
5
4
1/SE_IV
−0.3
0.0
0.3
0.6
β_IV

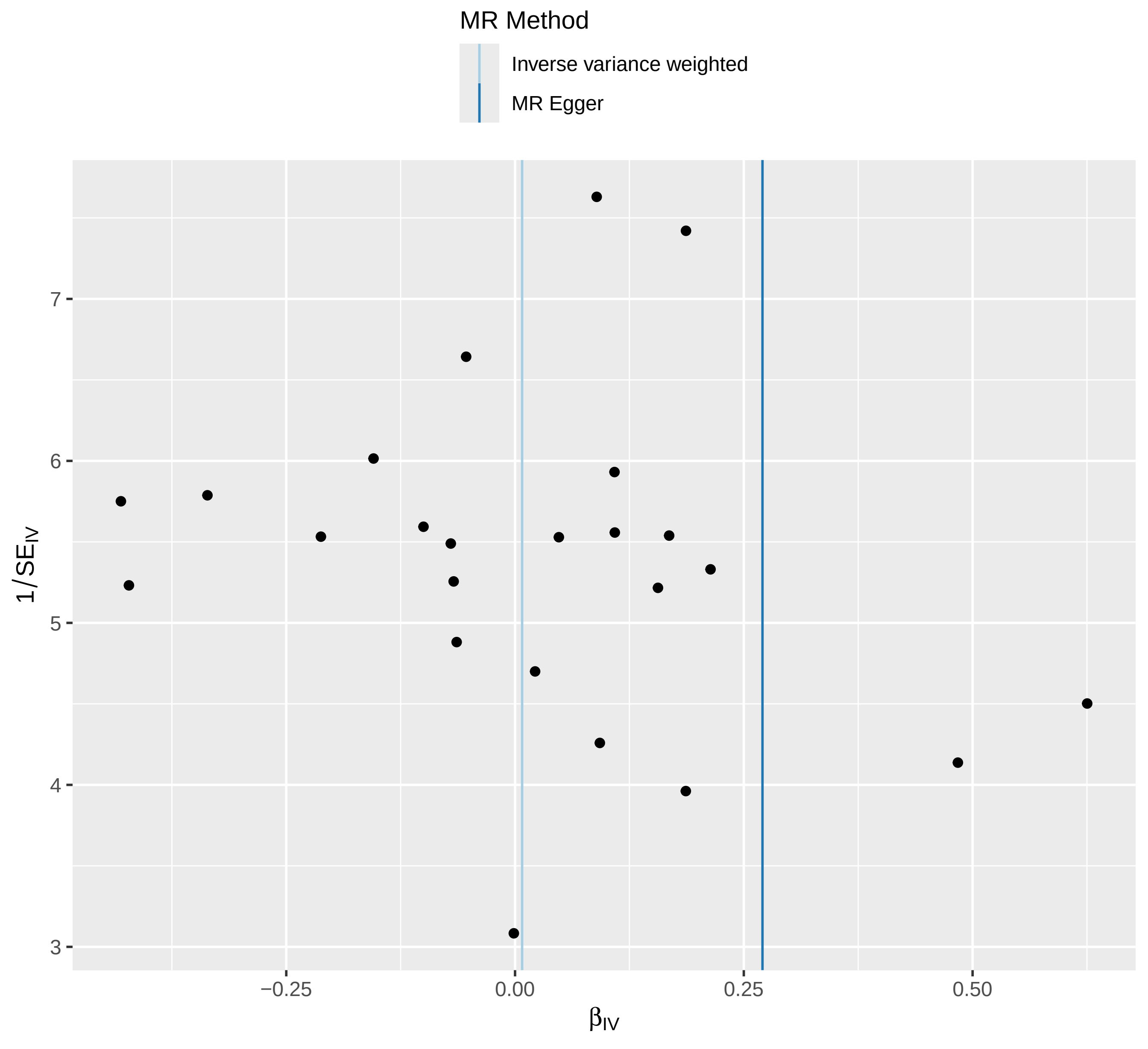
MR Method
Inverse variance weighted
MR Egger
1/SE_IV
7
6
5
4
3
β_IV
−0.25
0.00
0.25
0.50

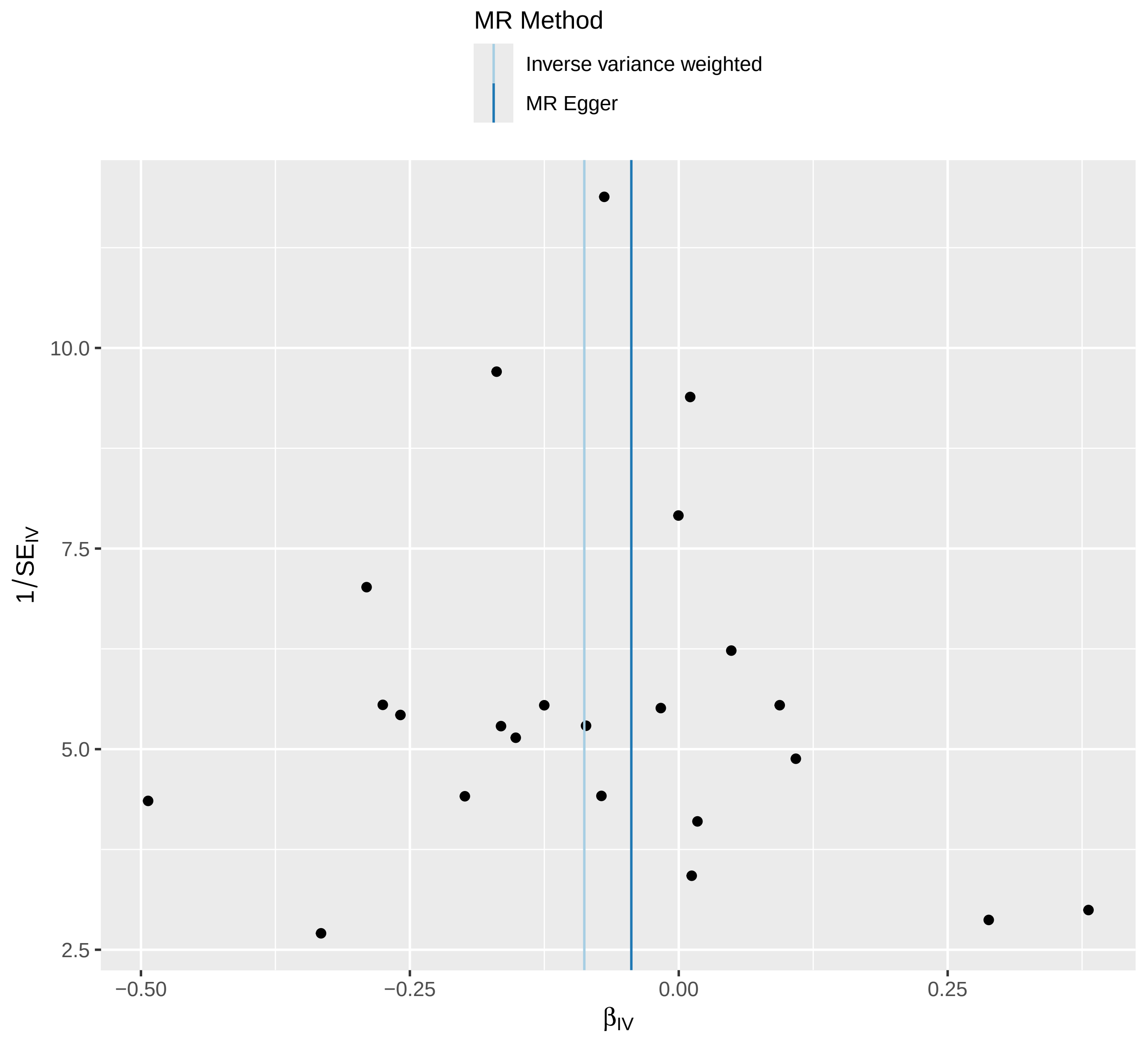
MR Method
Inverse variance weighted
MR Egger
10.0
7.5
5.0
2.5
$1/SE_{IV}$
−0.50
−0.25
0.00
0.25
β_{IV}

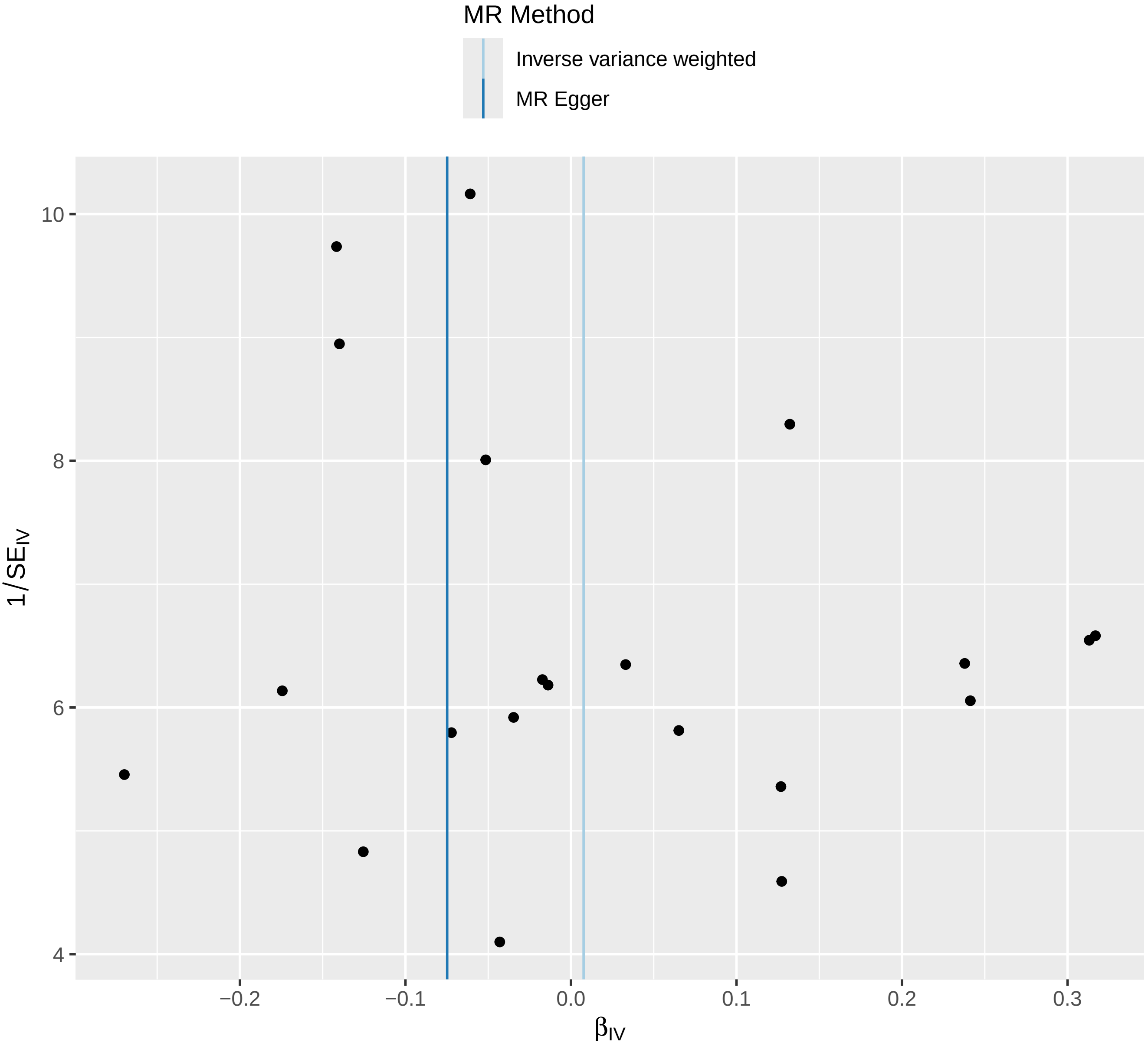
MR Method
Inverse variance weighted
MR Egger
1/SE_IV
10
8
6
4
−0.2
−0.1
0.0
0.1
0.2
0.3
β_IV

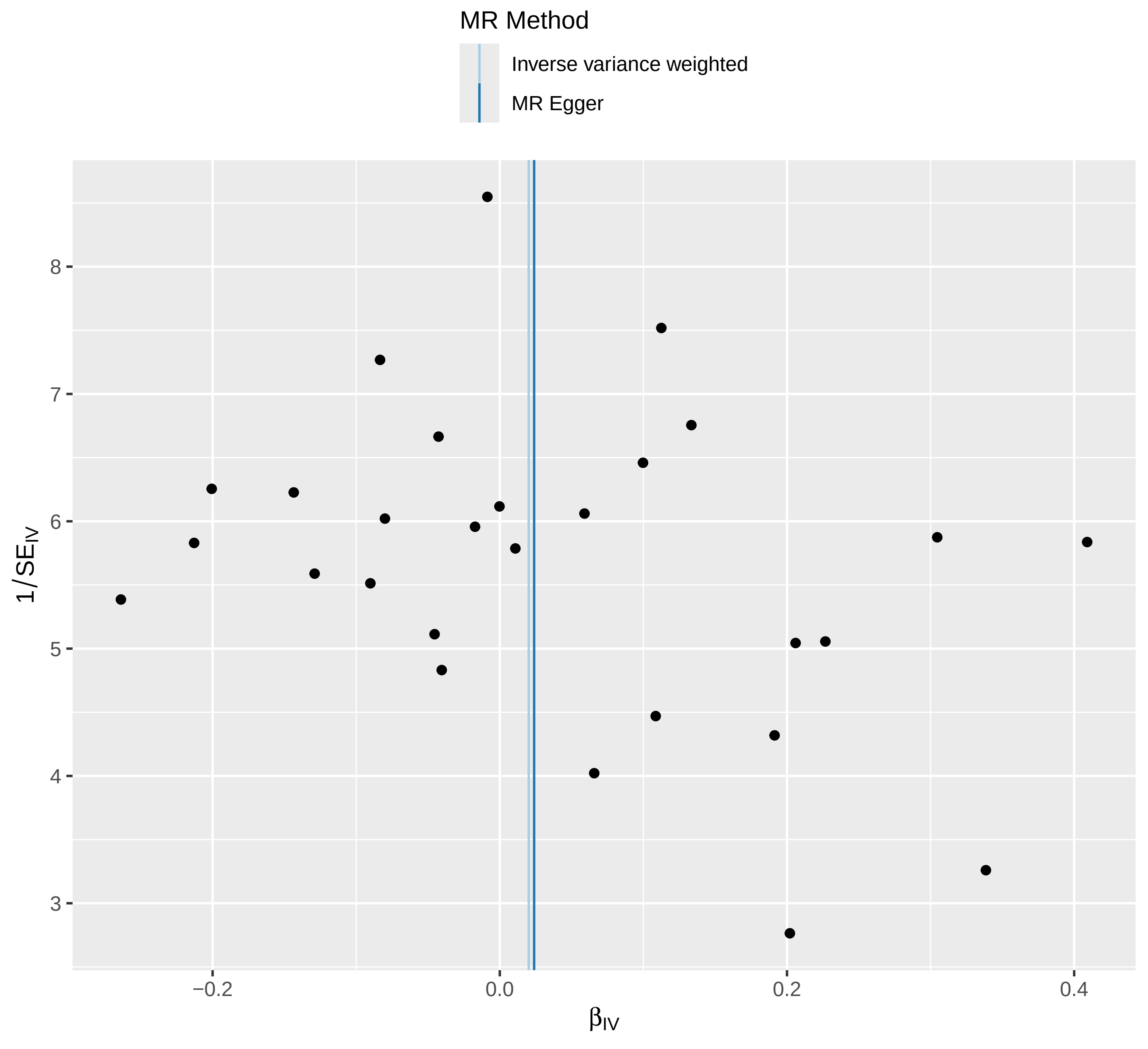
MR Method
Inverse variance weighted
MR Egger
1/SE_IV
β_IV
8
7
6
5
4
3
−0.2
0.0
0.2
0.4

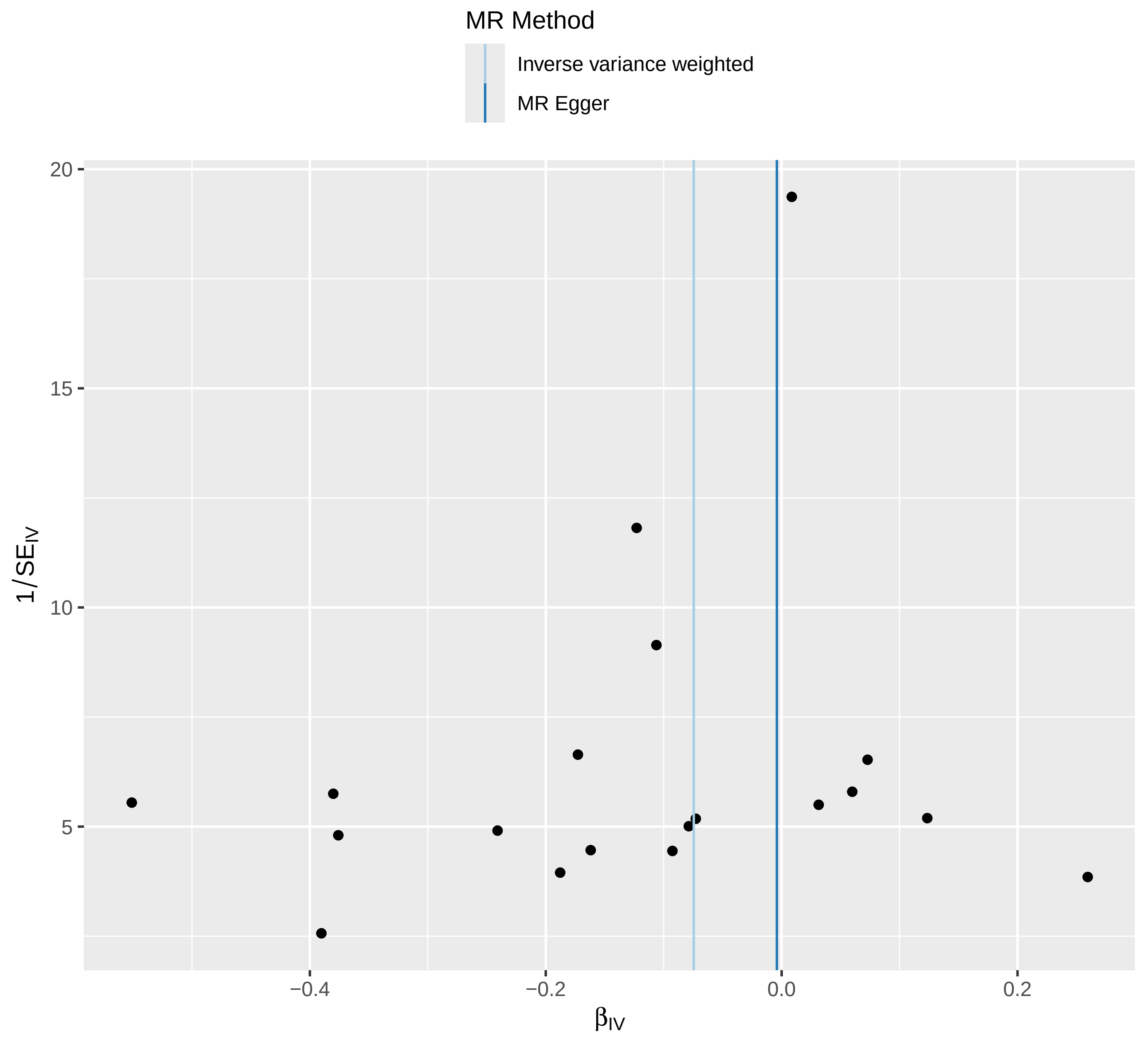
MR Method
Inverse variance weighted
MR Egger
20
15
10
5
1/SE$_{IV}$
−0.4
−0.2
0.0
0.2
β$_{IV}$

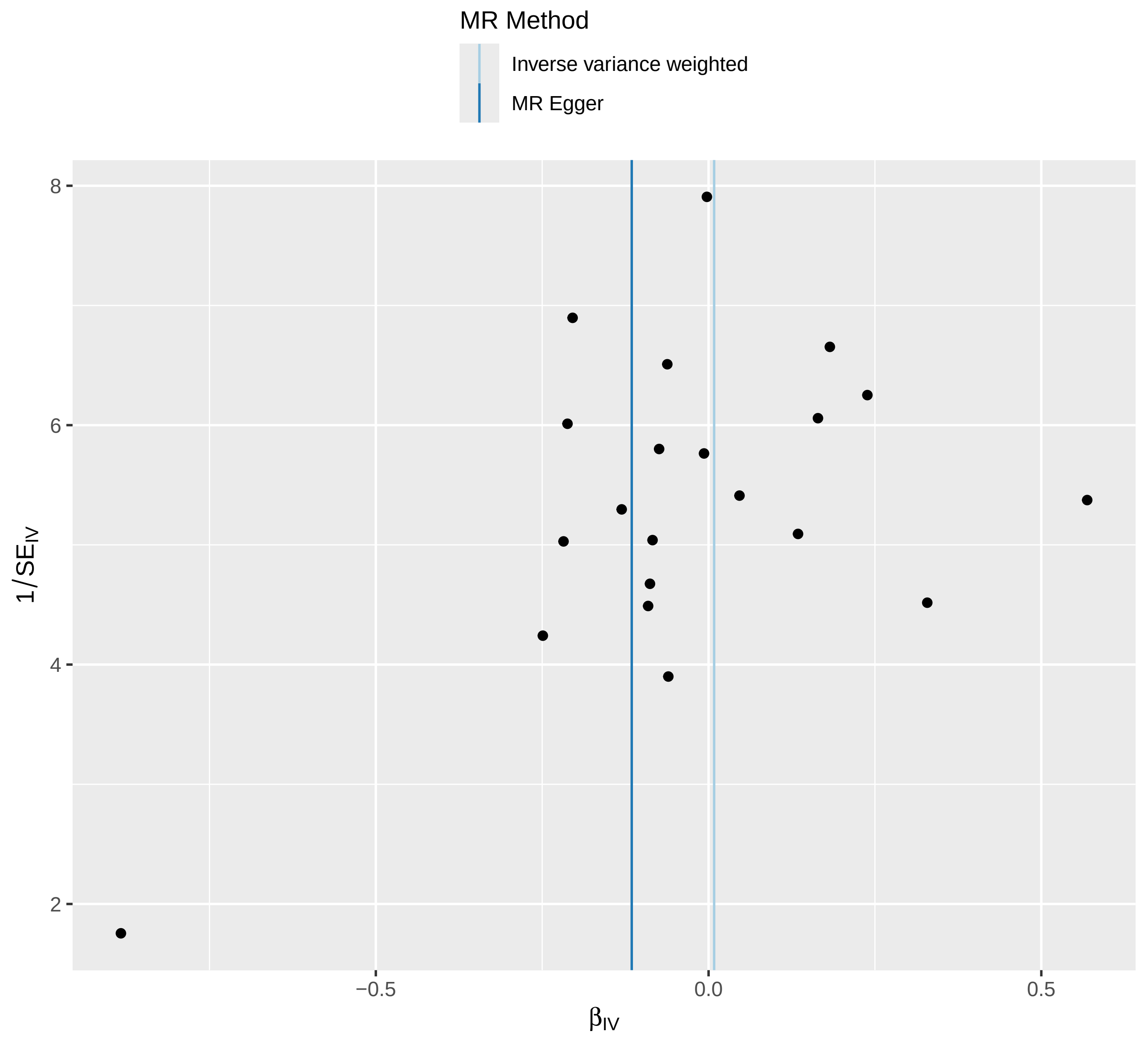

MR Method
Inverse variance weighted
MR Egger
8
6
4
2
1/SE_IV
−0.5
0.0
0.5
β_IV

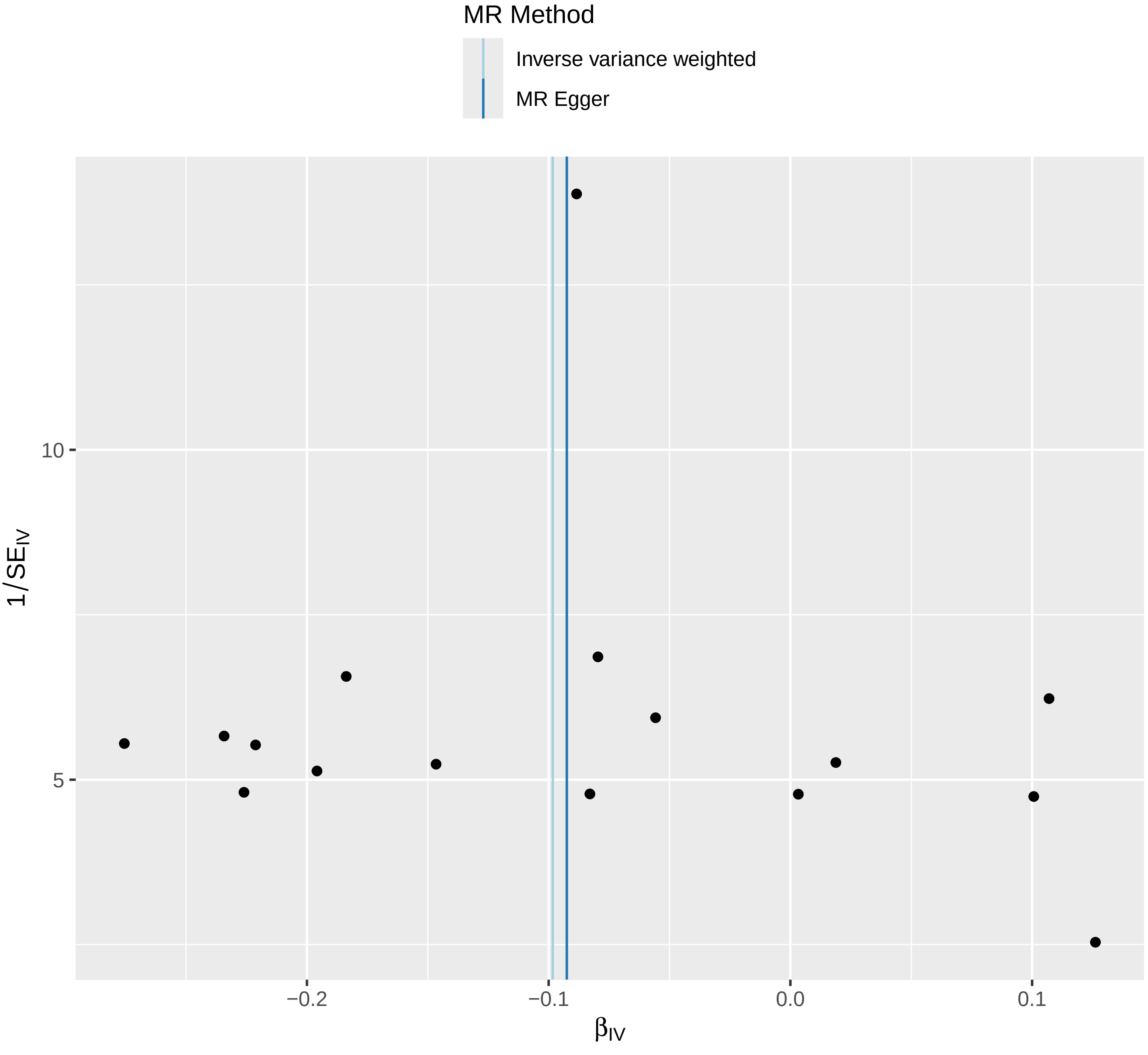
MR Method
Inverse variance weighted
MR Egger
10
5
1/SE_IV
−0.2
−0.1
0.0
0.1
β_IV

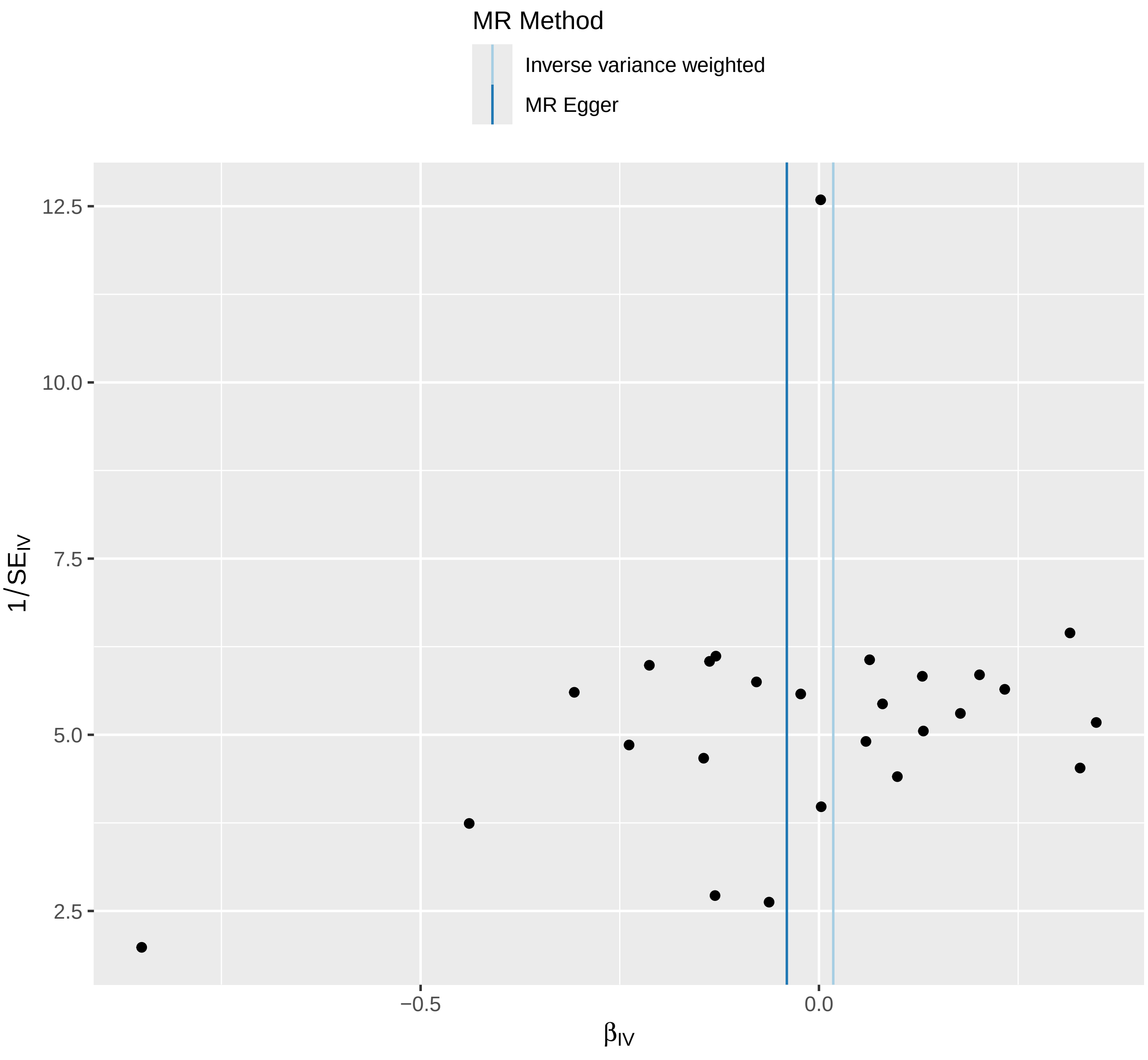
MR Method
Inverse variance weighted
MR Egger
12.5
10.0
7.5
5.0
2.5
1/SE_IV
−0.5
0.0
β_IV

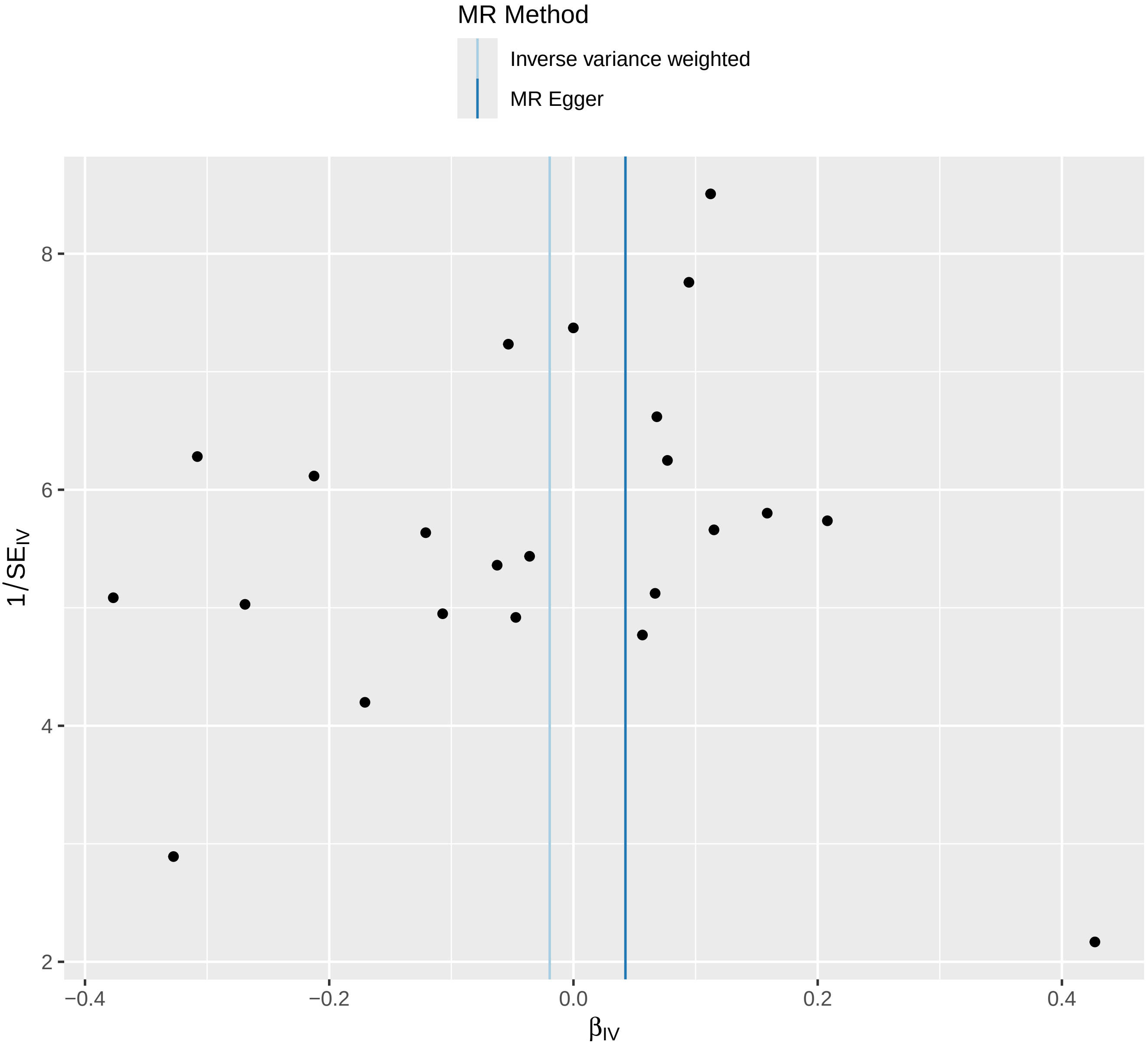
MR Method
Inverse variance weighted
MR Egger
8
6
4
2
1/SE$_{IV}$
−0.4
−0.2
0.0
0.2
0.4
β$_{IV}$

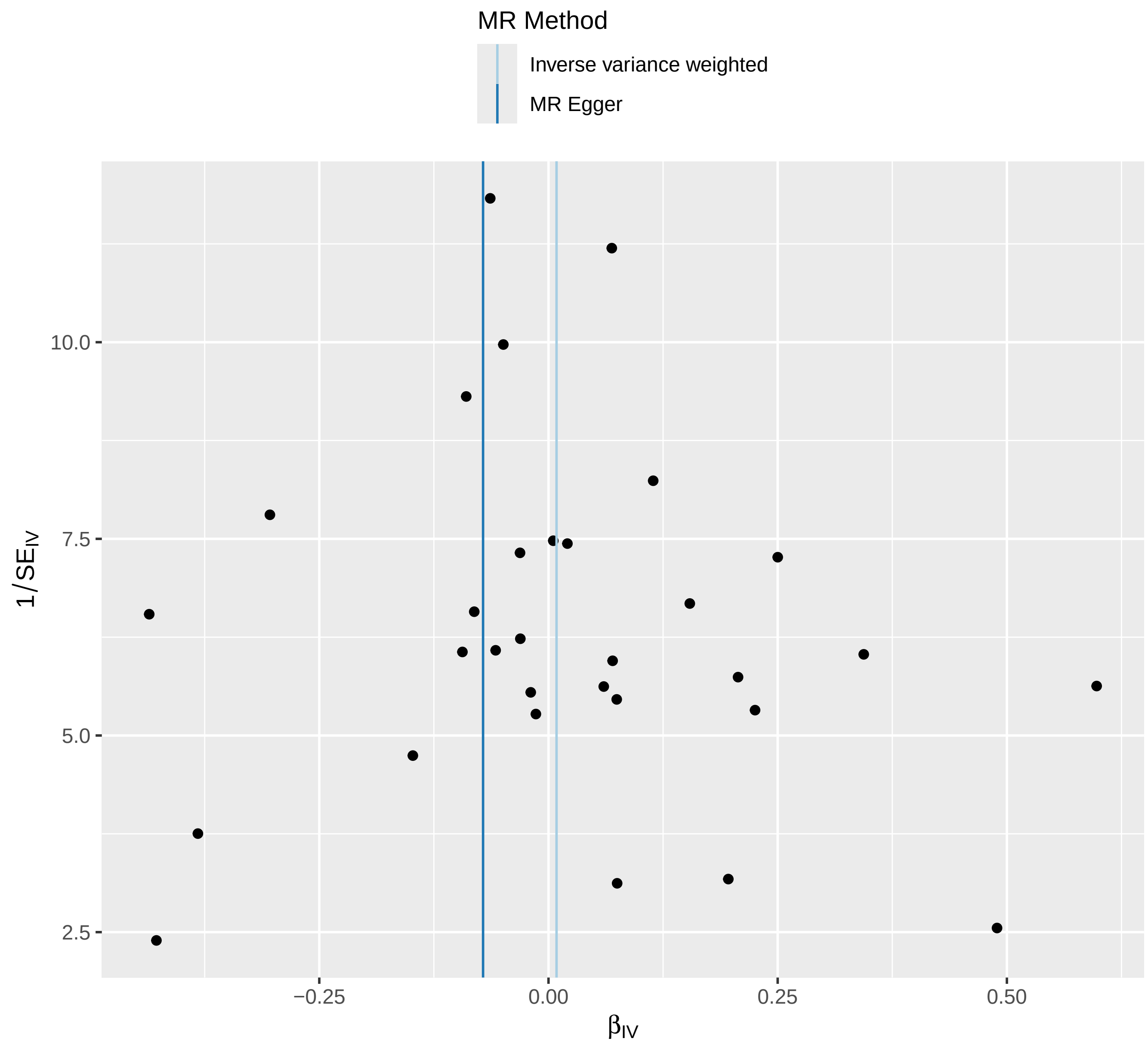
MR Method
Inverse variance weighted
MR Egger
1/SE$_{IV}$
10.0
7.5
5.0
2.5
−0.25
0.00
0.25
0.50
β$_{IV}$

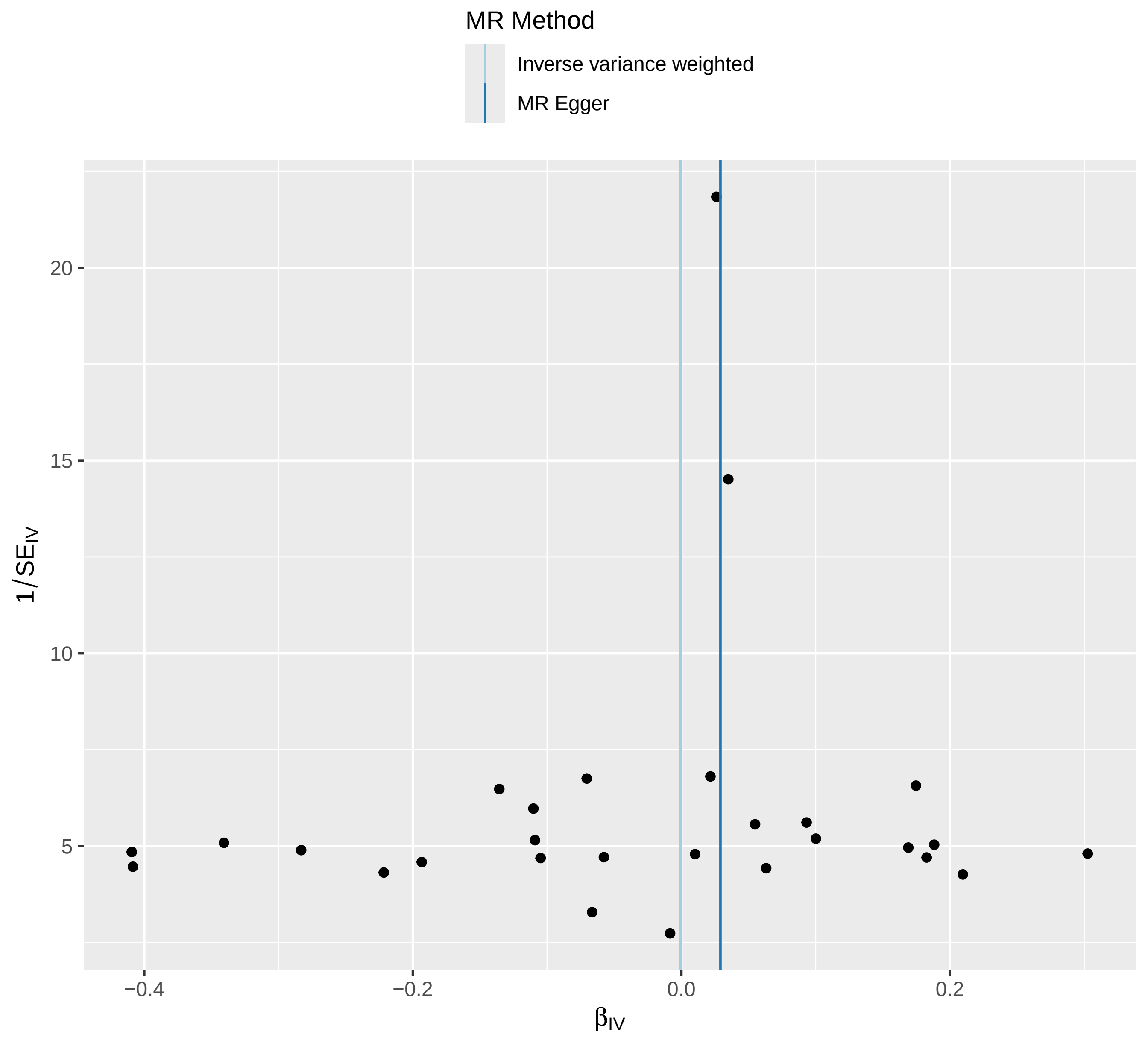

MR Method
Inverse variance weighted
MR Egger
20
15
10
5
1/SE_IV
−0.4
−0.2
0.0
0.2
β_IV

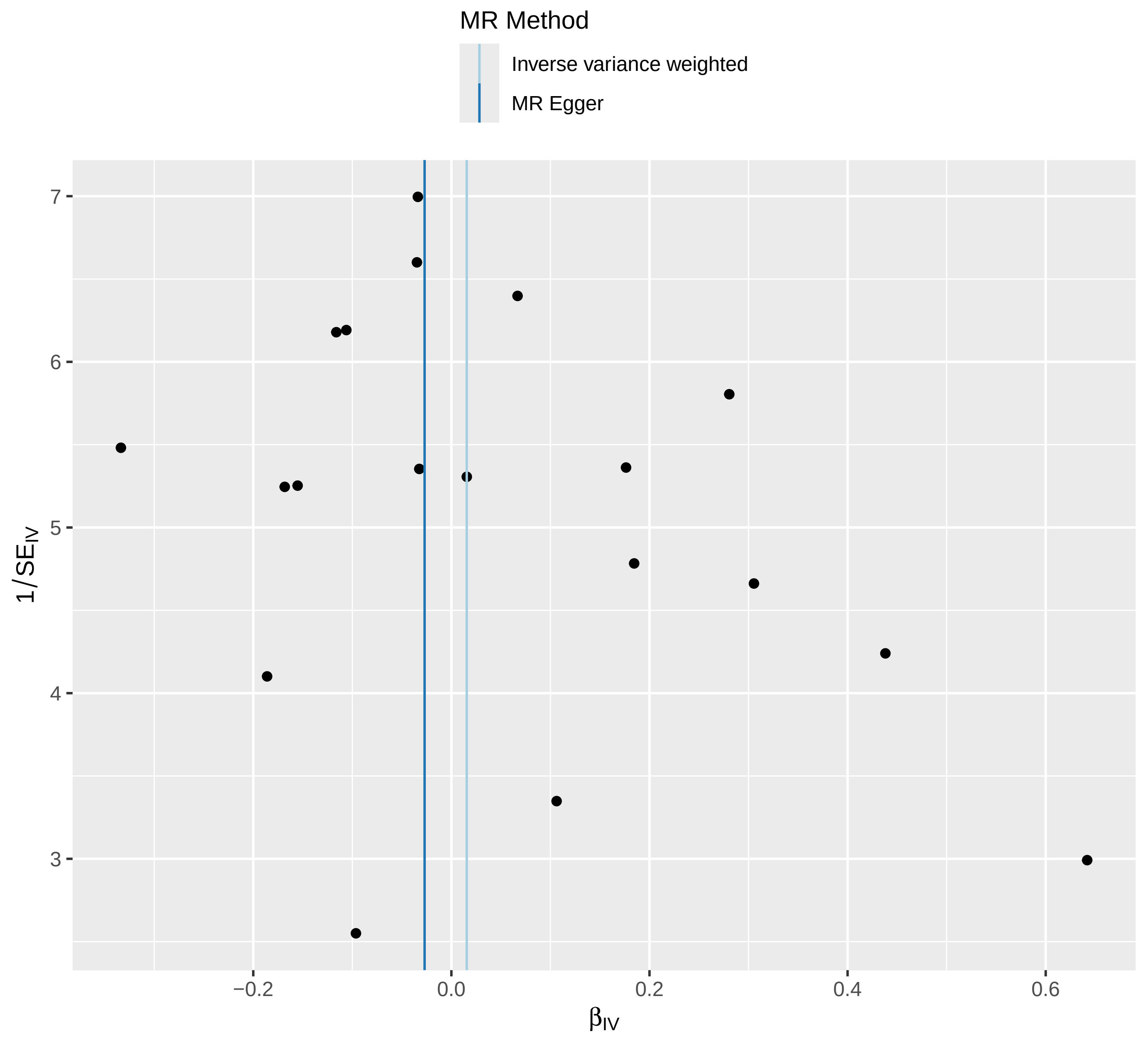
MR Method
Inverse variance weighted
MR Egger
7
6
5
4
3
1/SE_IV
−0.2
0.0
0.2
0.4
0.6
β_IV

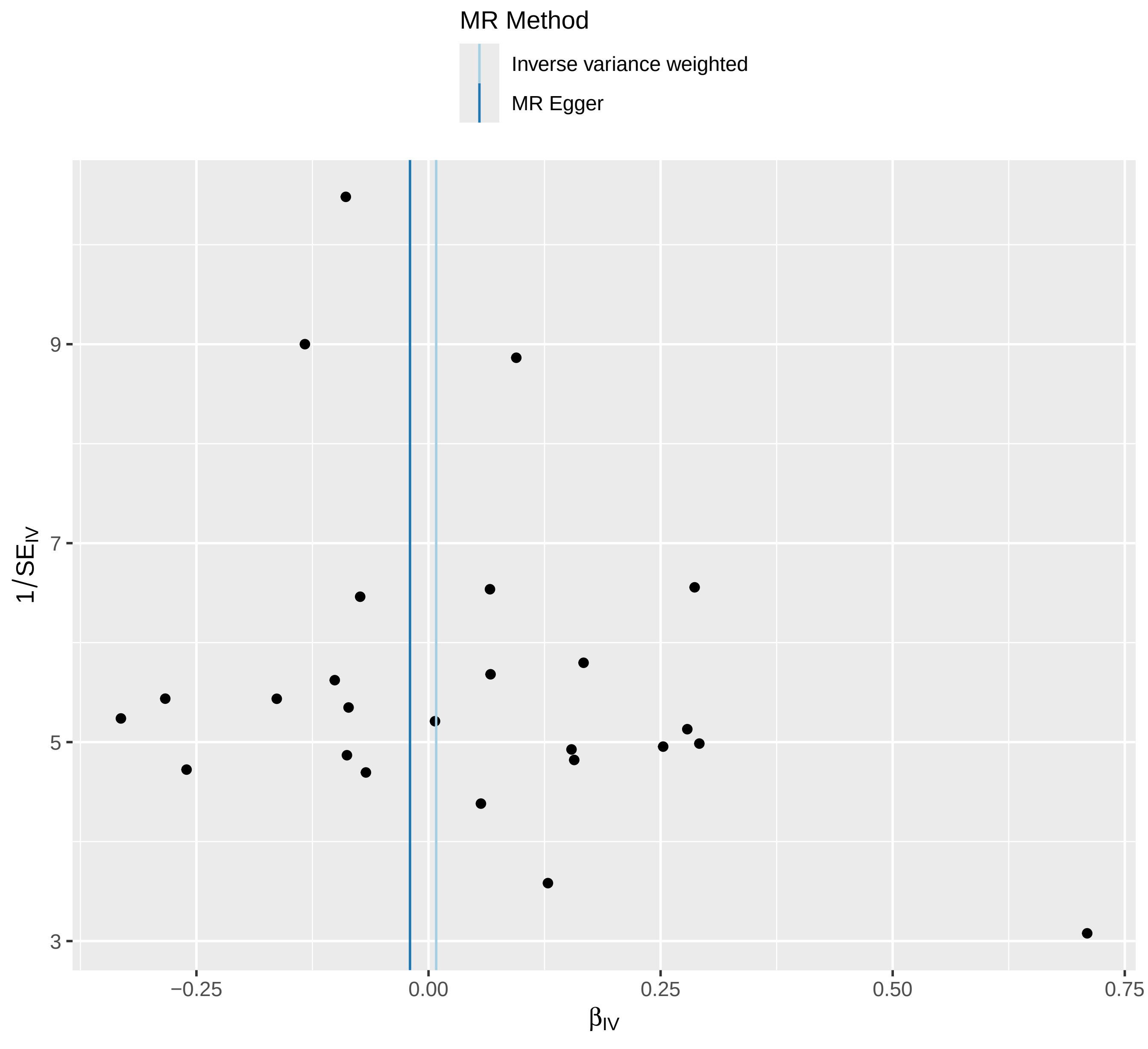
MR Method
Inverse variance weighted
MR Egger
1/SE$_{IV}$
9
7
5
3
−0.25
0.00
0.25
0.50
0.75
β$_{IV}$

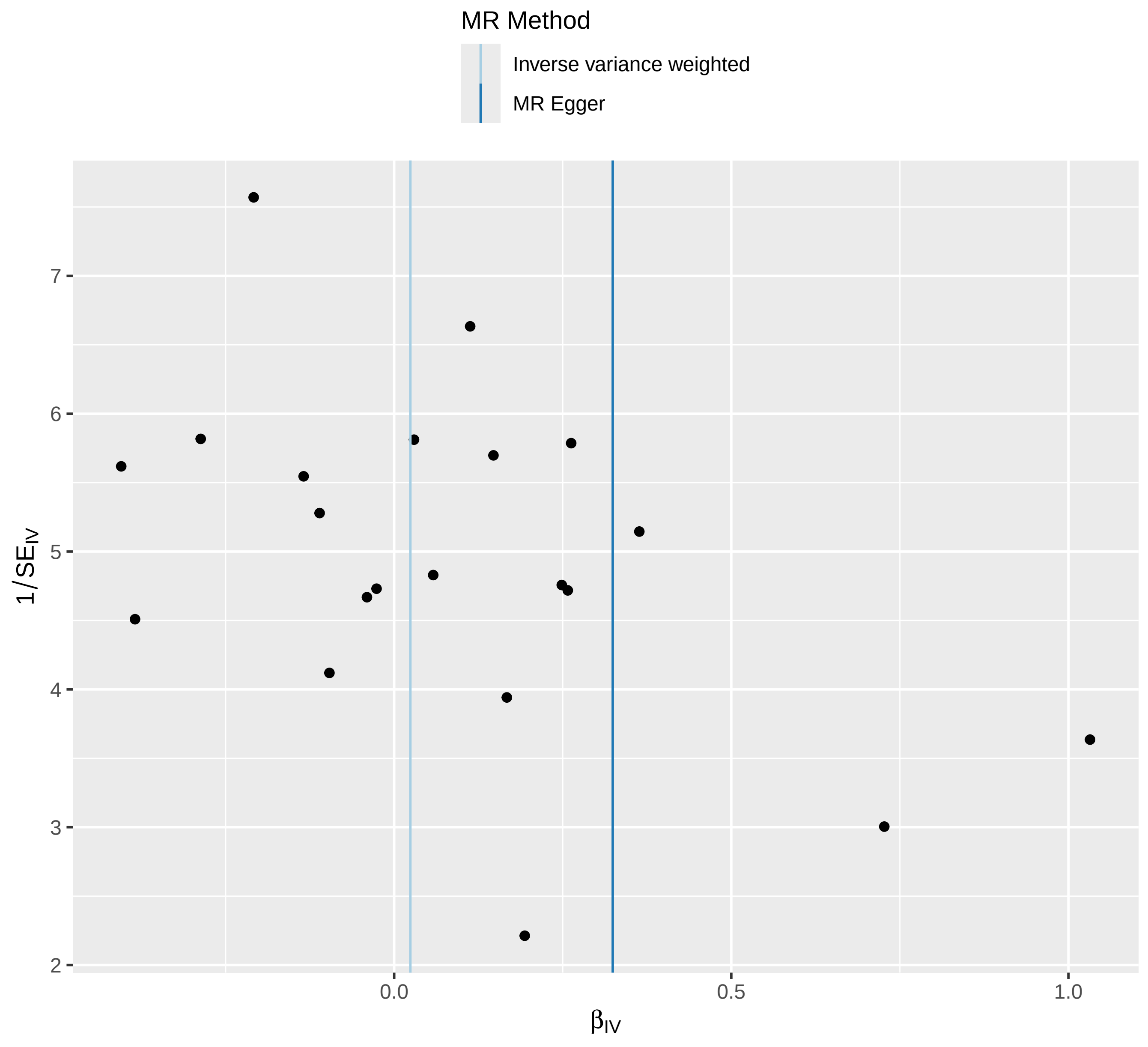
MR Method
Inverse variance weighted
MR Egger
1/SE_IV
7
6
5
4
3
2
0.0
0.5
1.0
β_IV

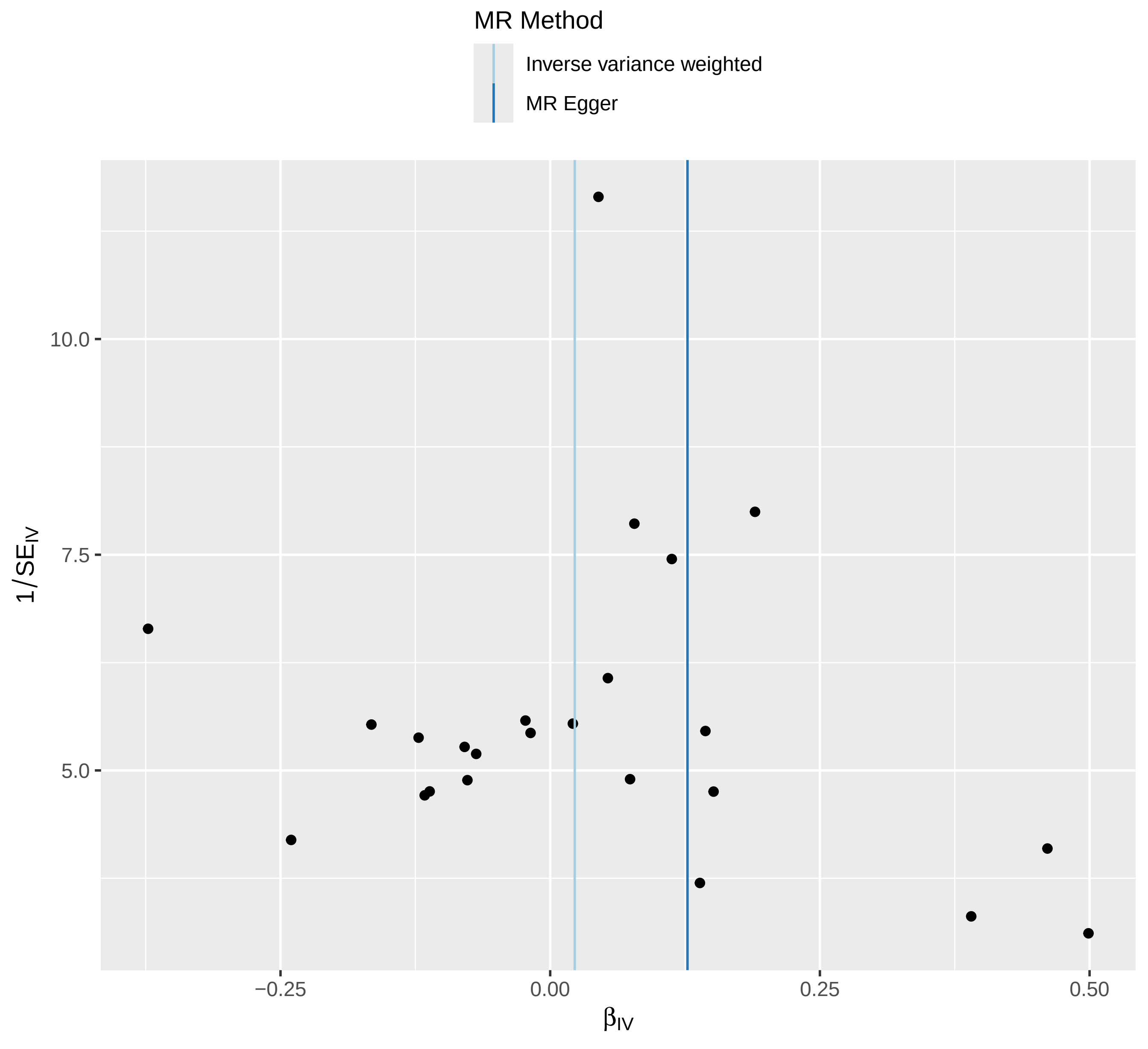

MR Method
Inverse variance weighted
MR Egger
10.0
7.5
5.0
1/SE_IV
−0.25
0.00
0.25
0.50
β_IV

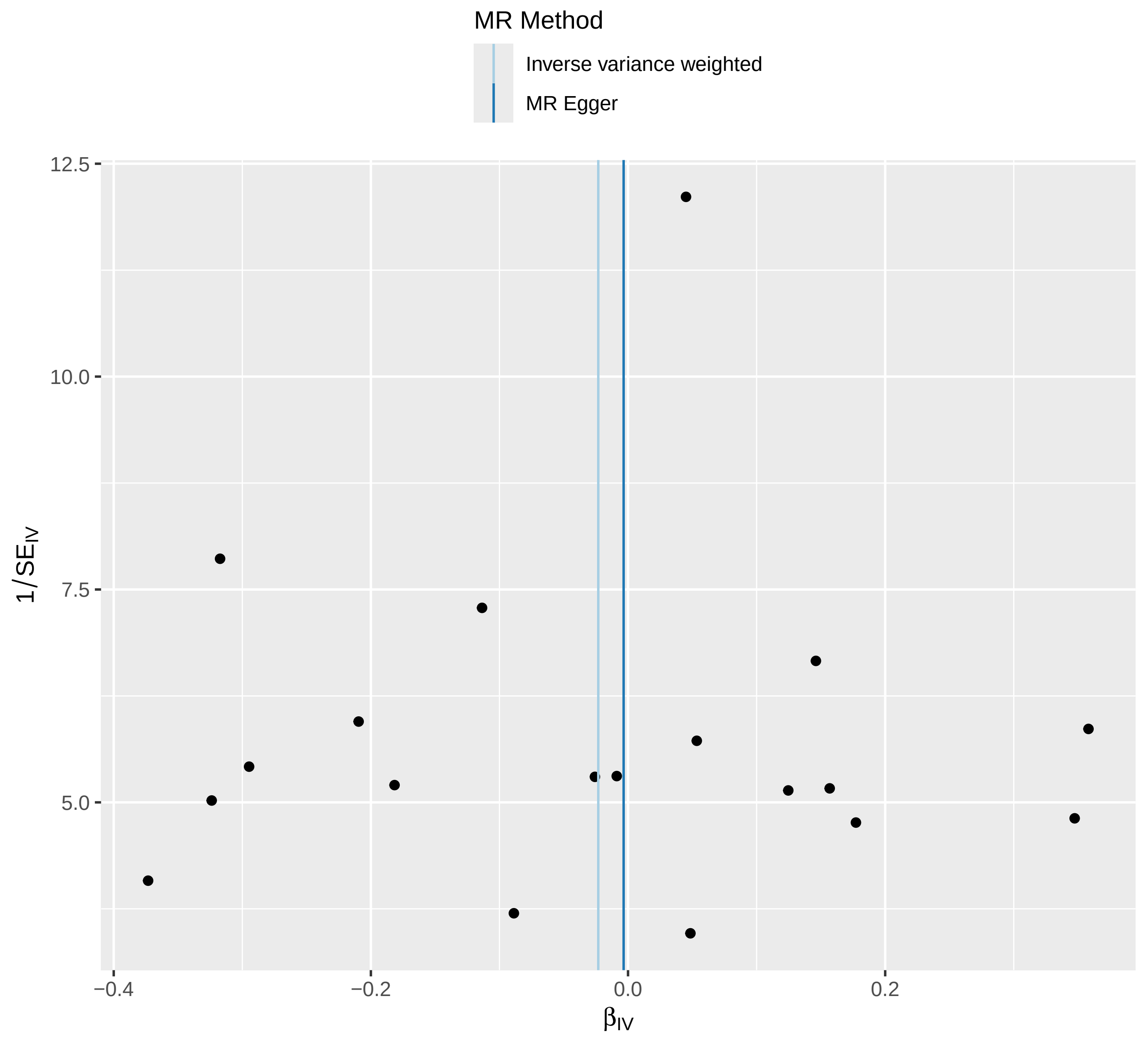

MR Method
Inverse variance weighted
MR Egger
12.5
10.0
7.5
5.0
−0.4
−0.2
0.0
0.2
$1/SE_{IV}$
β_{IV}

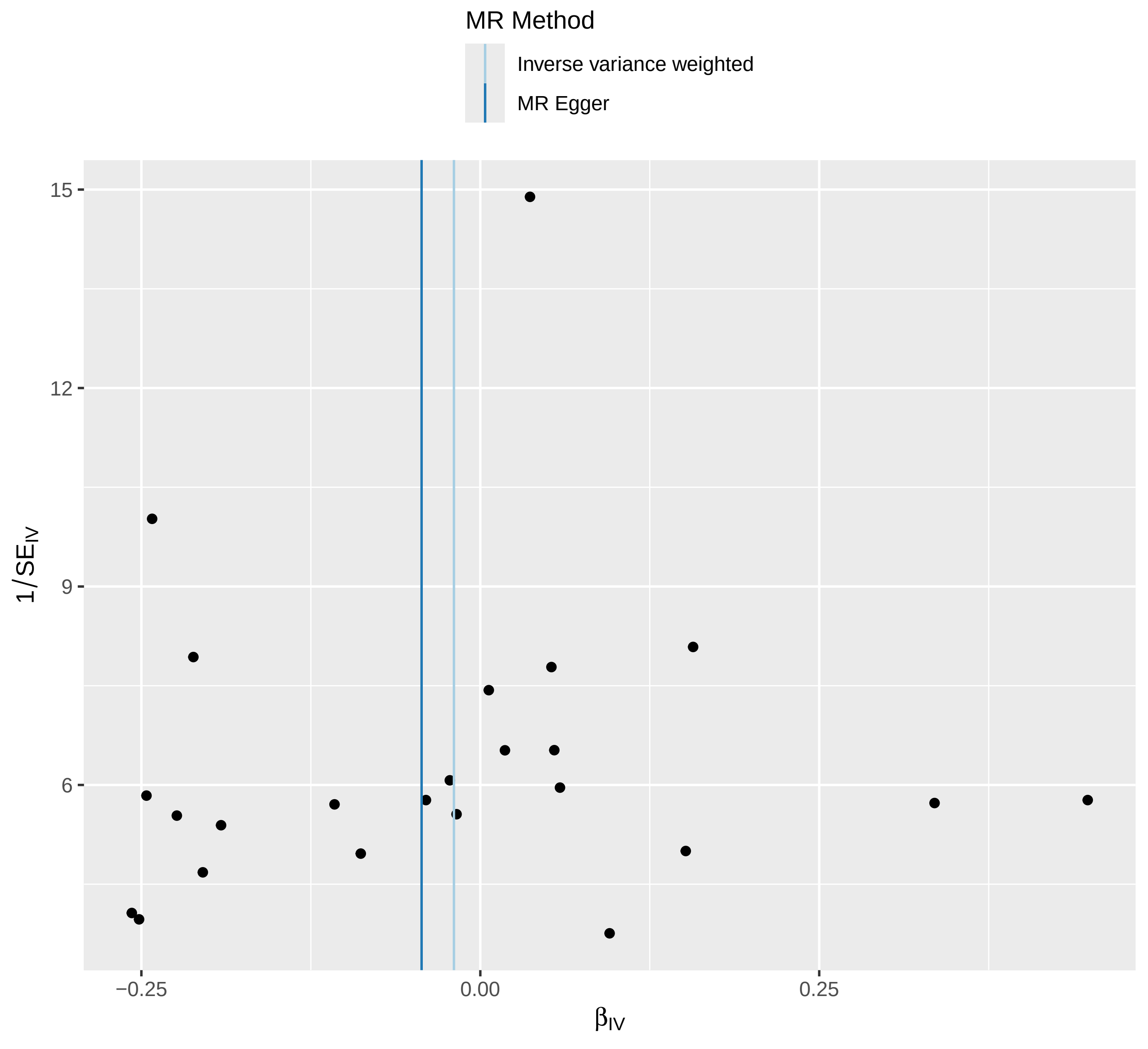
MR Method
Inverse variance weighted
MR Egger
15
12
9
6
1/SE_IV
−0.25
0.00
0.25
β_IV

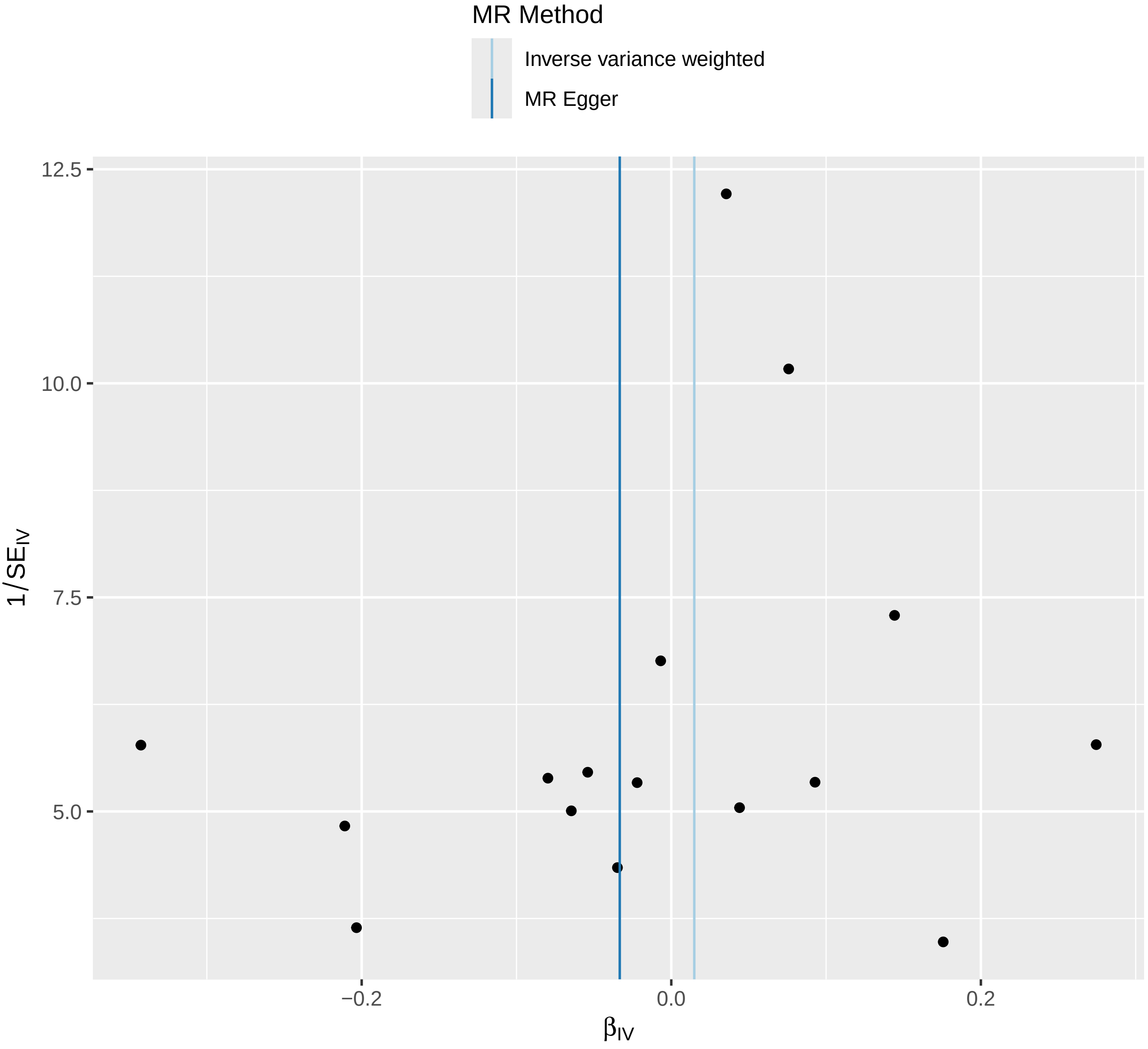
MR Method
Inverse variance weighted
MR Egger
12.5
10.0
7.5
5.0
1/SE_IV
−0.2
0.0
0.2
β_IV

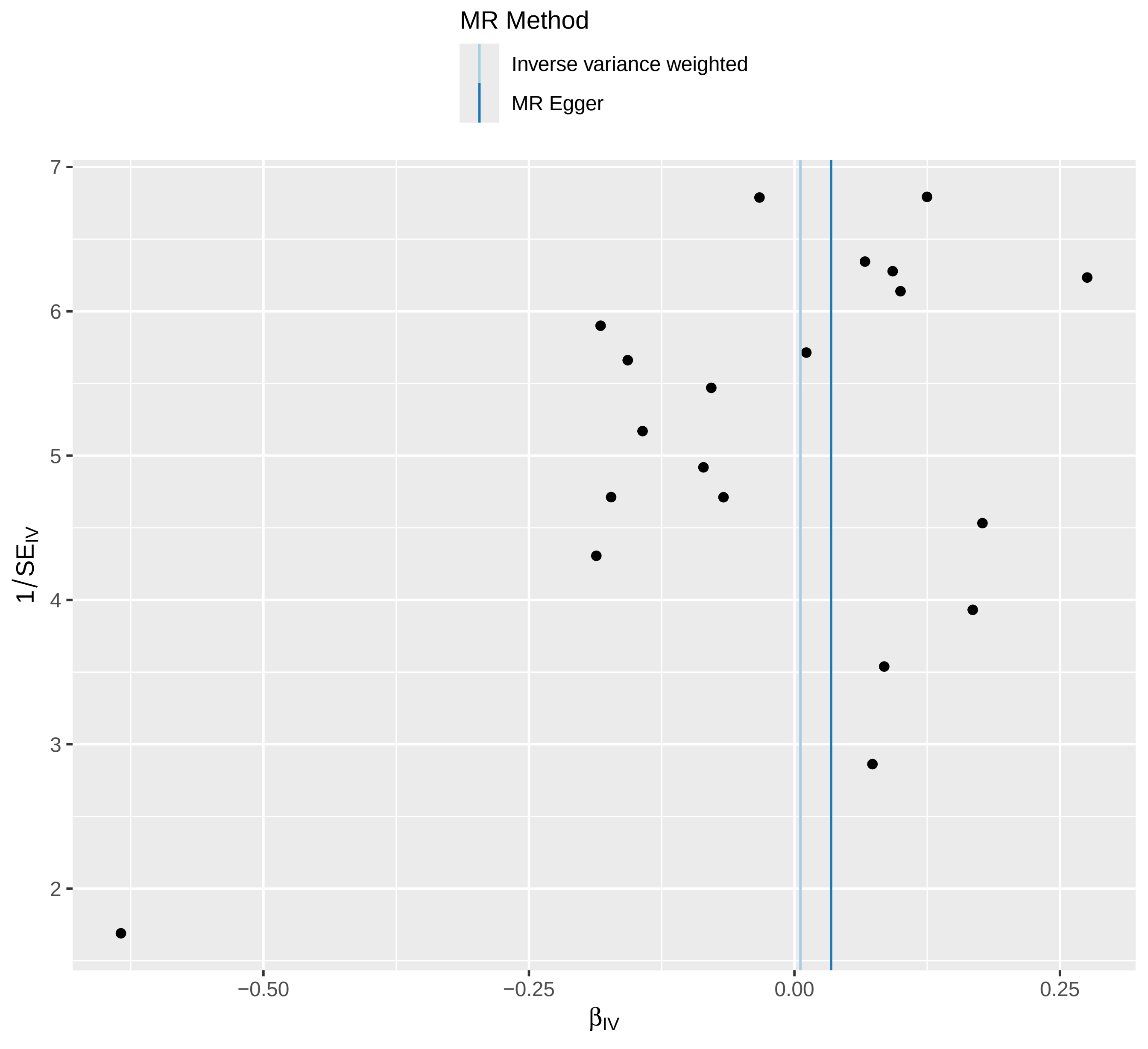
MR Method
Inverse variance weighted
MR Egger
7
6
5
4
3
2
1/SE_IV
−0.50
−0.25
0.00
0.25
β_IV

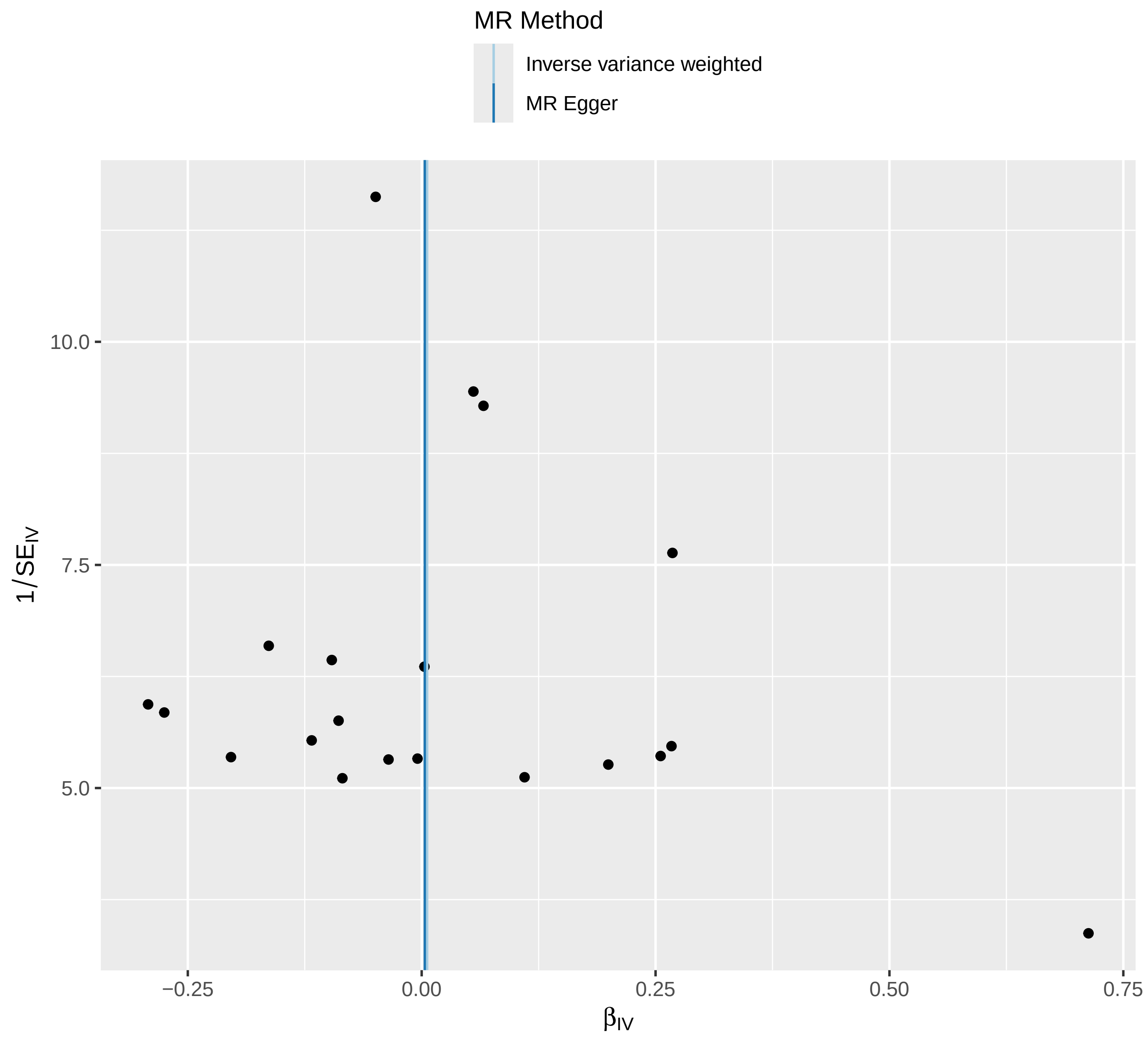
MR Method
Inverse variance weighted
MR Egger
10.0
7.5
5.0
1/SE_IV
−0.25
0.00
0.25
0.50
0.75
β_IV

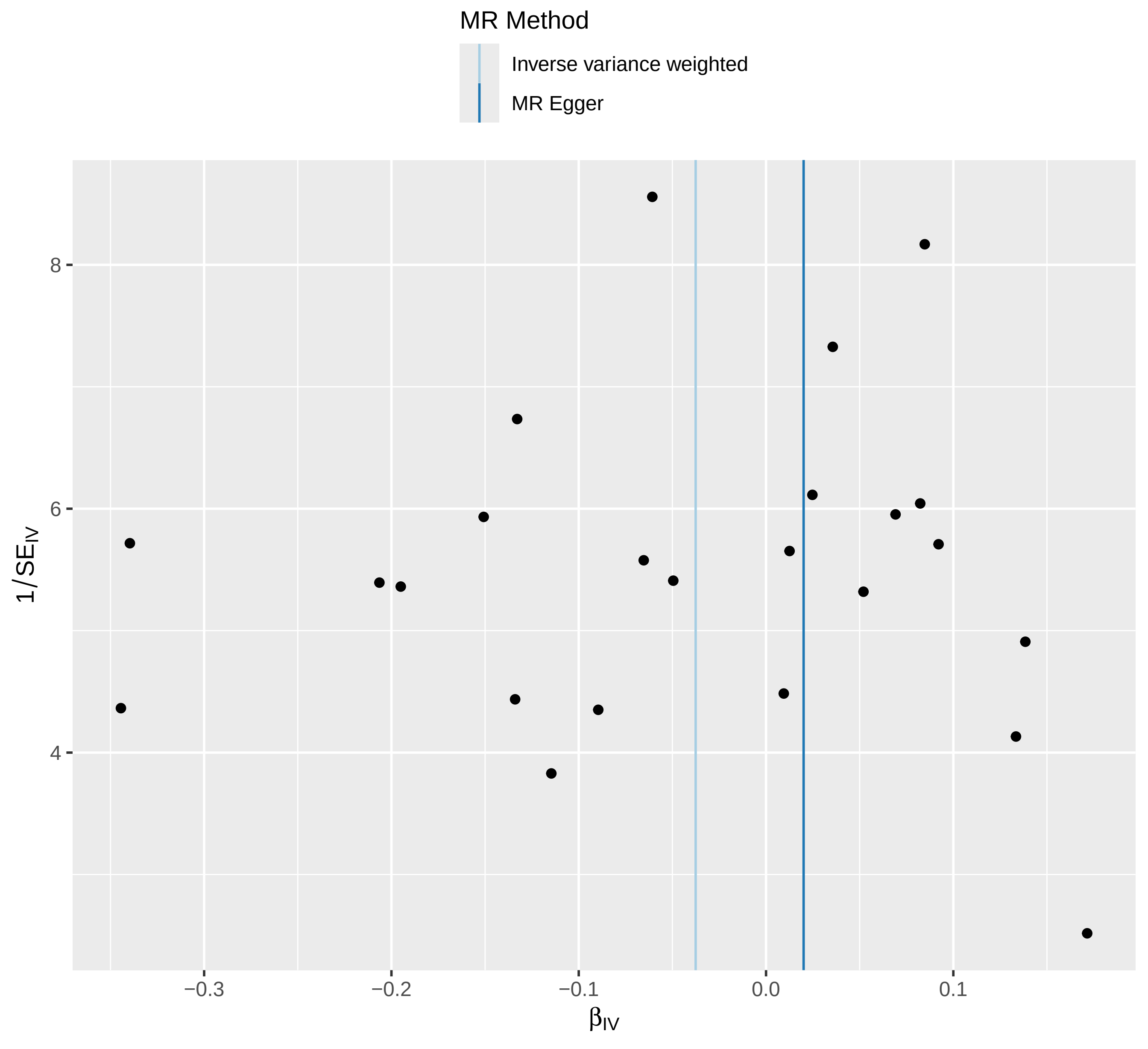
MR Method
Inverse variance weighted
MR Egger
8
6
4
1/SE_IV
−0.3
−0.2
−0.1
0.0
0.1
β_IV

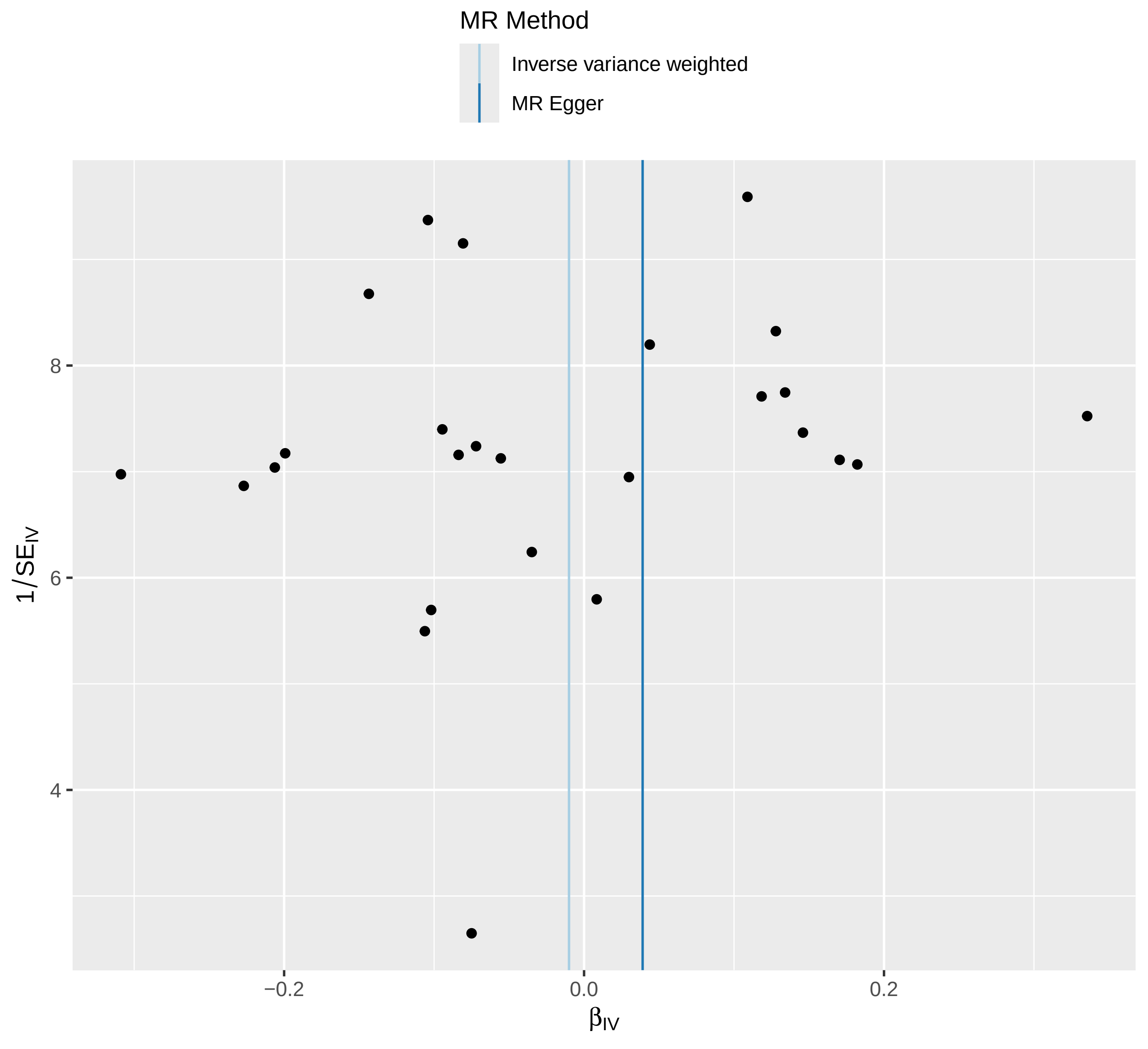
MR Method
Inverse variance weighted
MR Egger
8
6
4
1/SE$_{IV}$
−0.2
0.0
0.2
β$_{IV}$

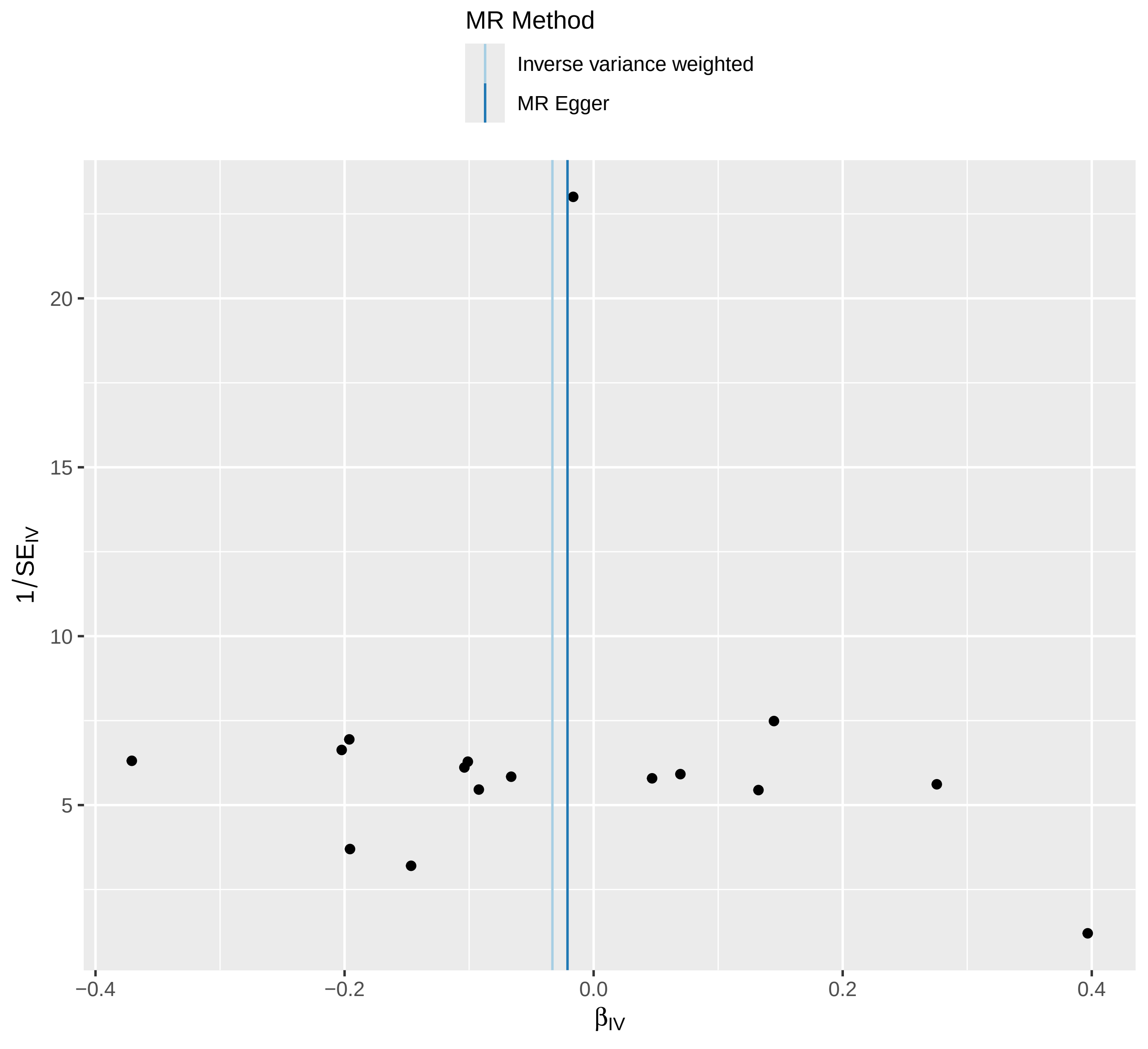
MR Method
Inverse variance weighted
MR Egger
20
15
10
5
$1/SE_{IV}$
−0.4
−0.2
0.0
0.2
0.4
β_{IV}

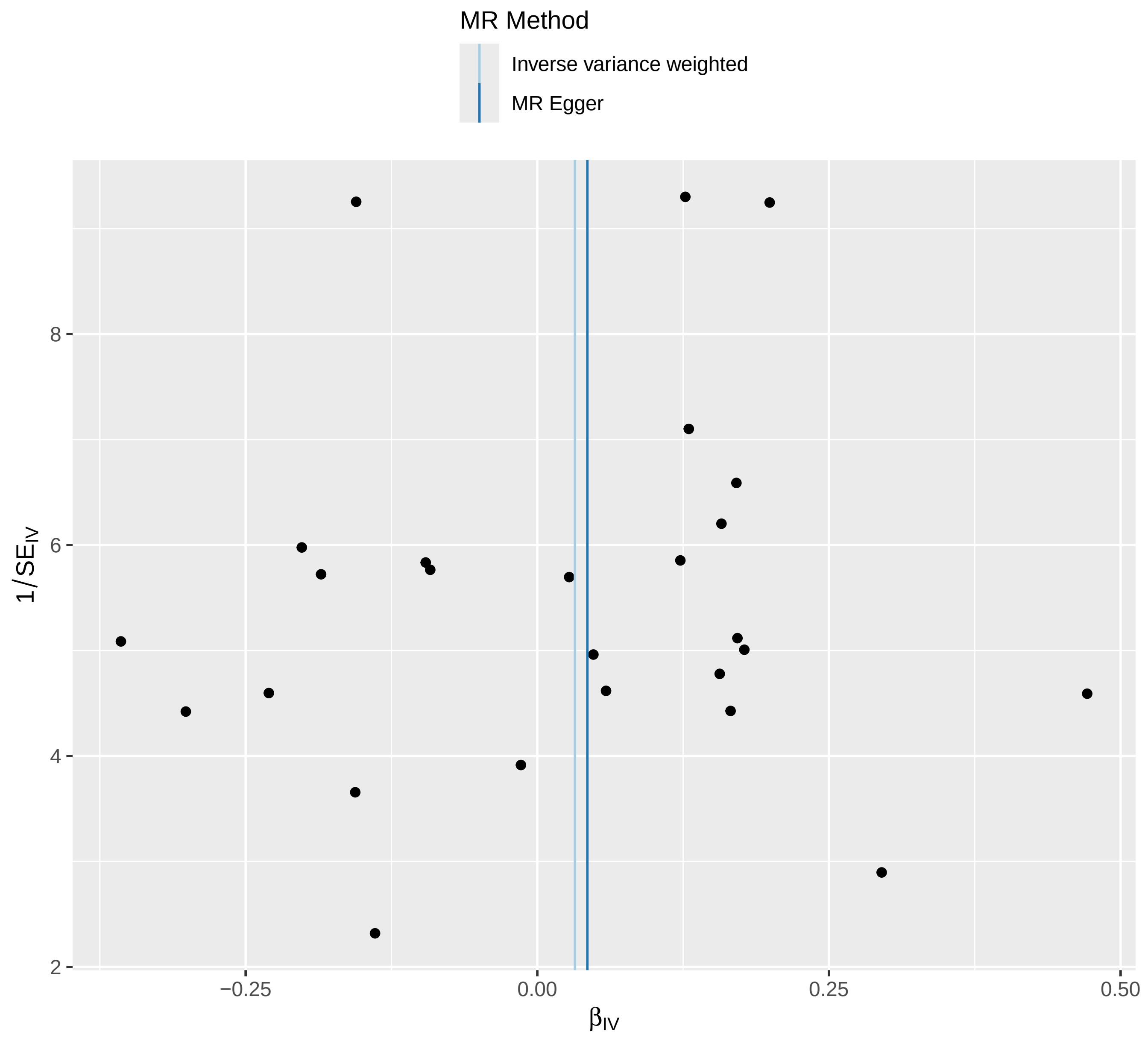
MR Method
Inverse variance weighted
MR Egger
8
6
4
2
1/SE_IV
−0.25
0.00
0.25
0.50
β_IV

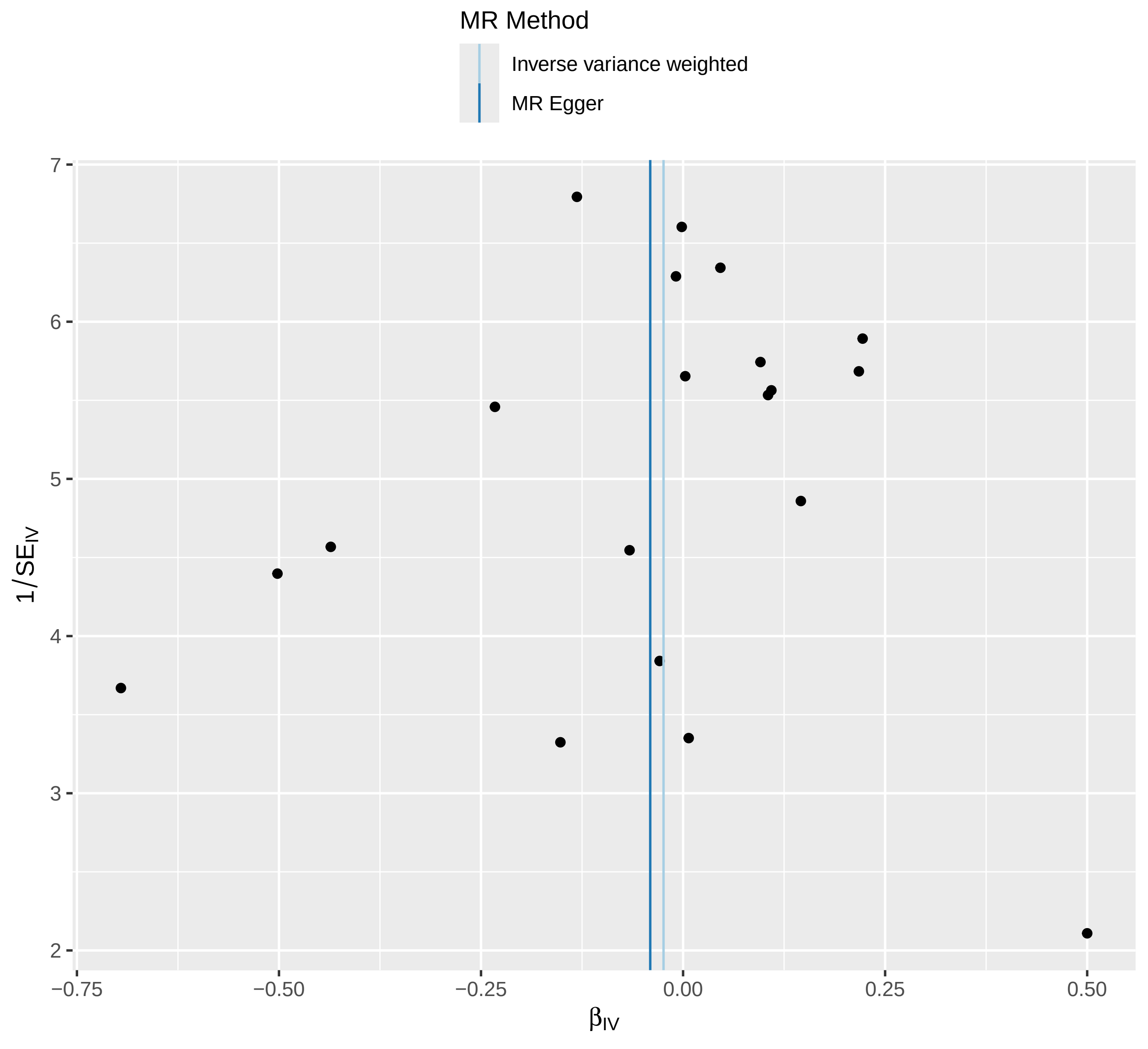
MR Method
Inverse variance weighted
MR Egger
7
6
5
4
3
2
1/SE$_{IV}$
−0.75
−0.50
−0.25
0.00
0.25
0.50
β$_{IV}$

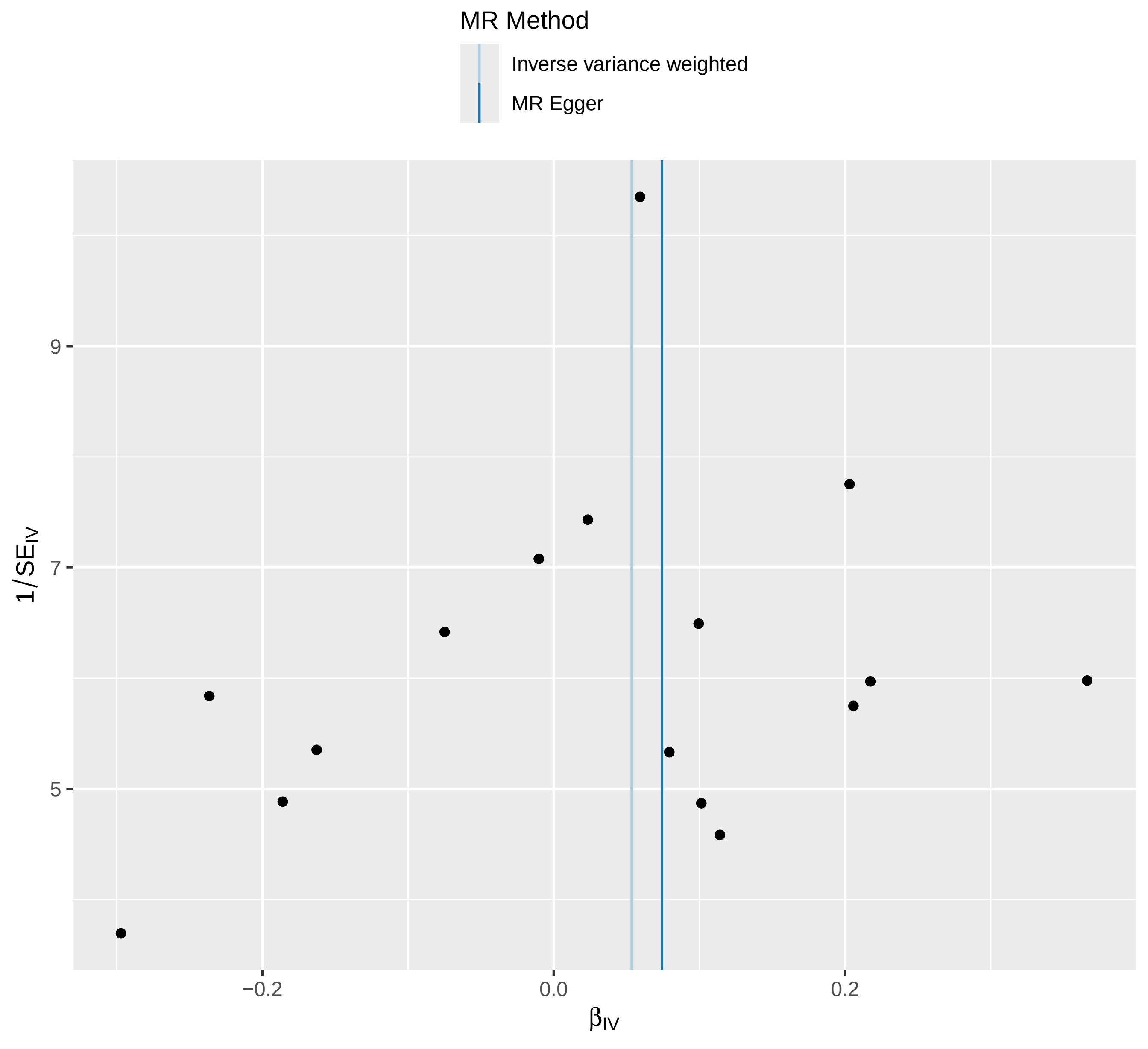
MR Method
Inverse variance weighted
MR Egger
9
7
5
$1/SE_{IV}$
−0.2
0.0
0.2
β_{IV}

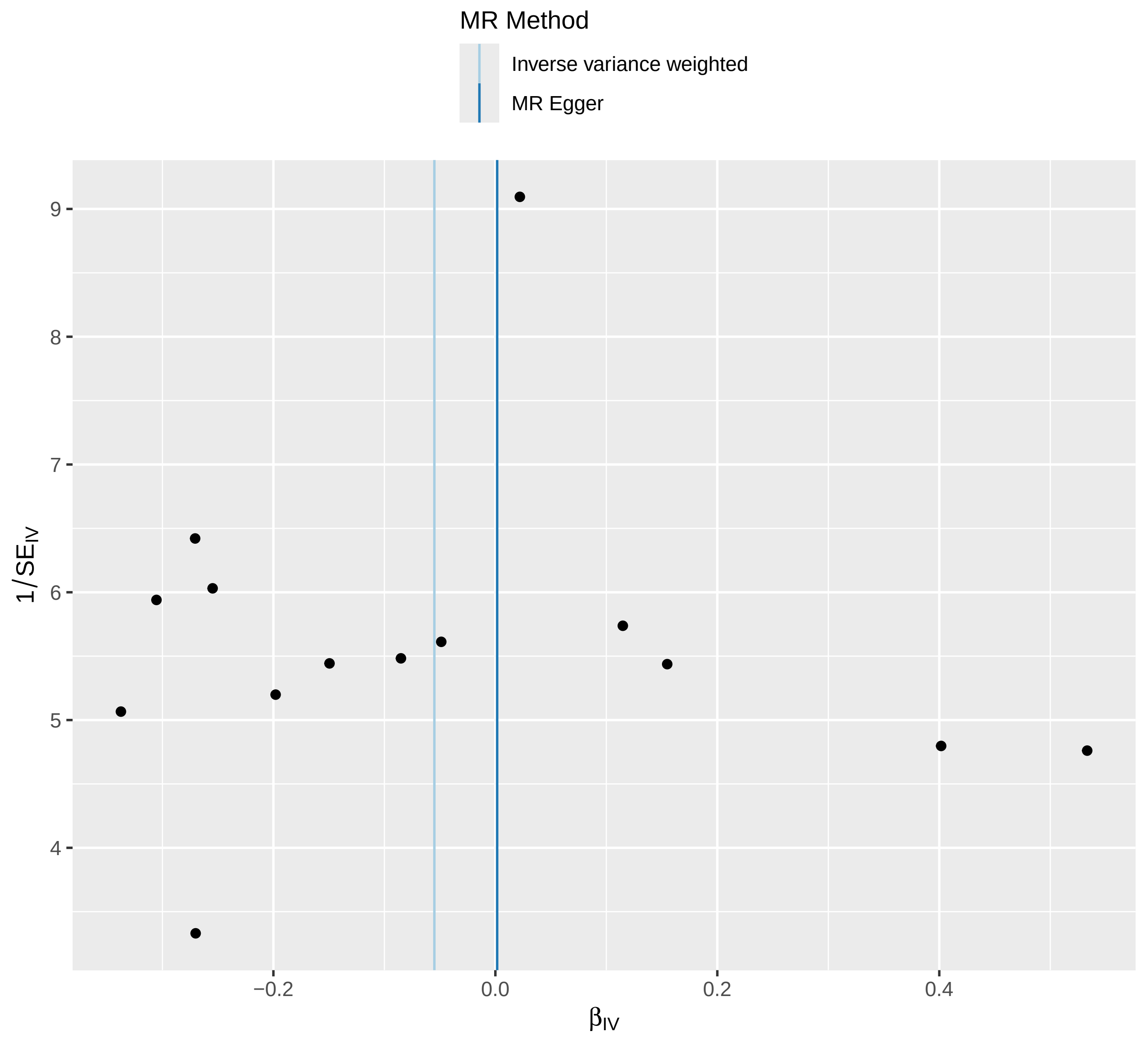
MR Method
Inverse variance weighted
MR Egger
9
8
7
6
5
4
1/SE_IV
−0.2
0.0
0.2
0.4
β_IV

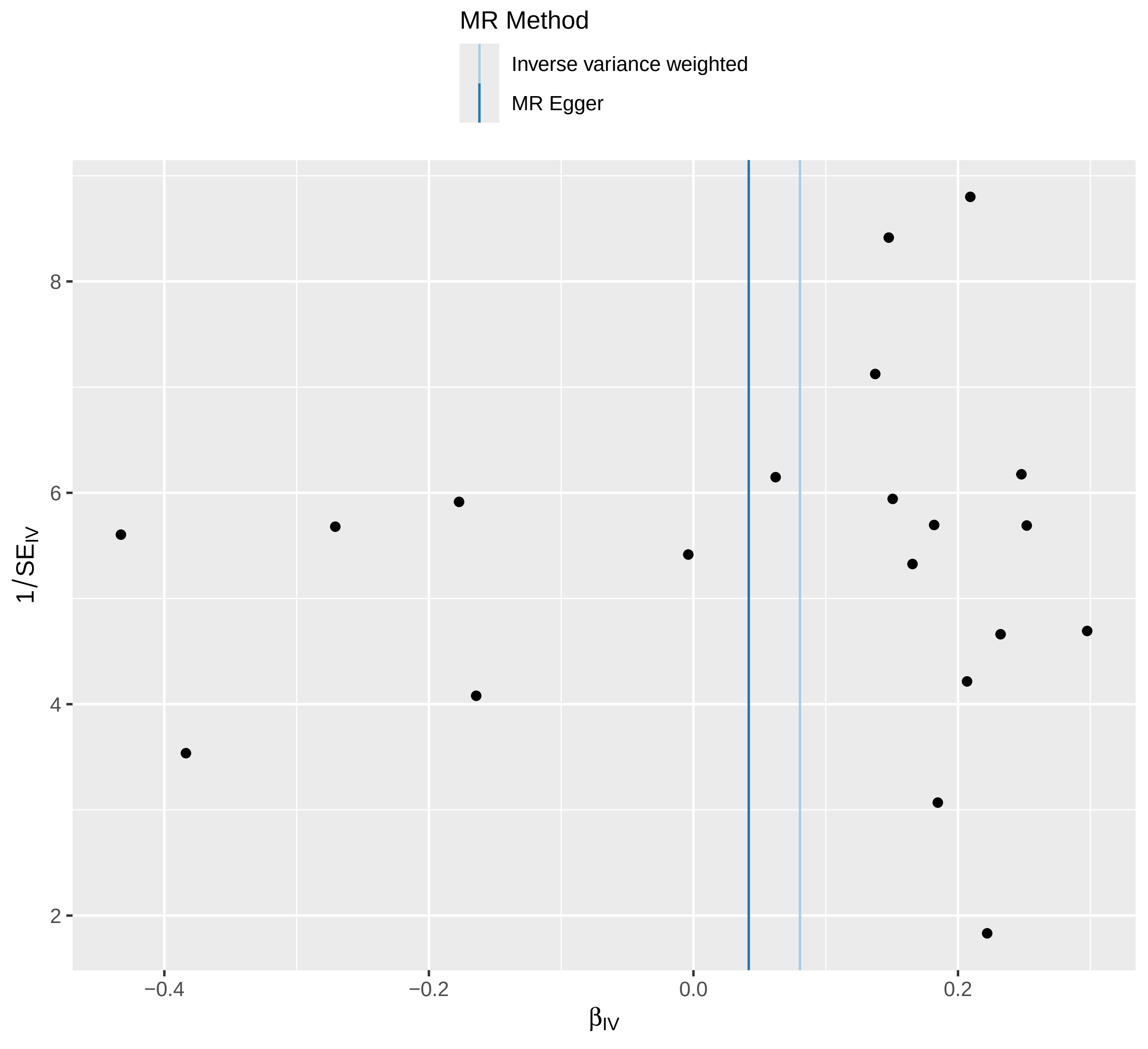
MR Method
Inverse variance weighted
MR Egger
1/SE_IV
8
6
4
2
β_IV
−0.4
−0.2
0.0
0.2

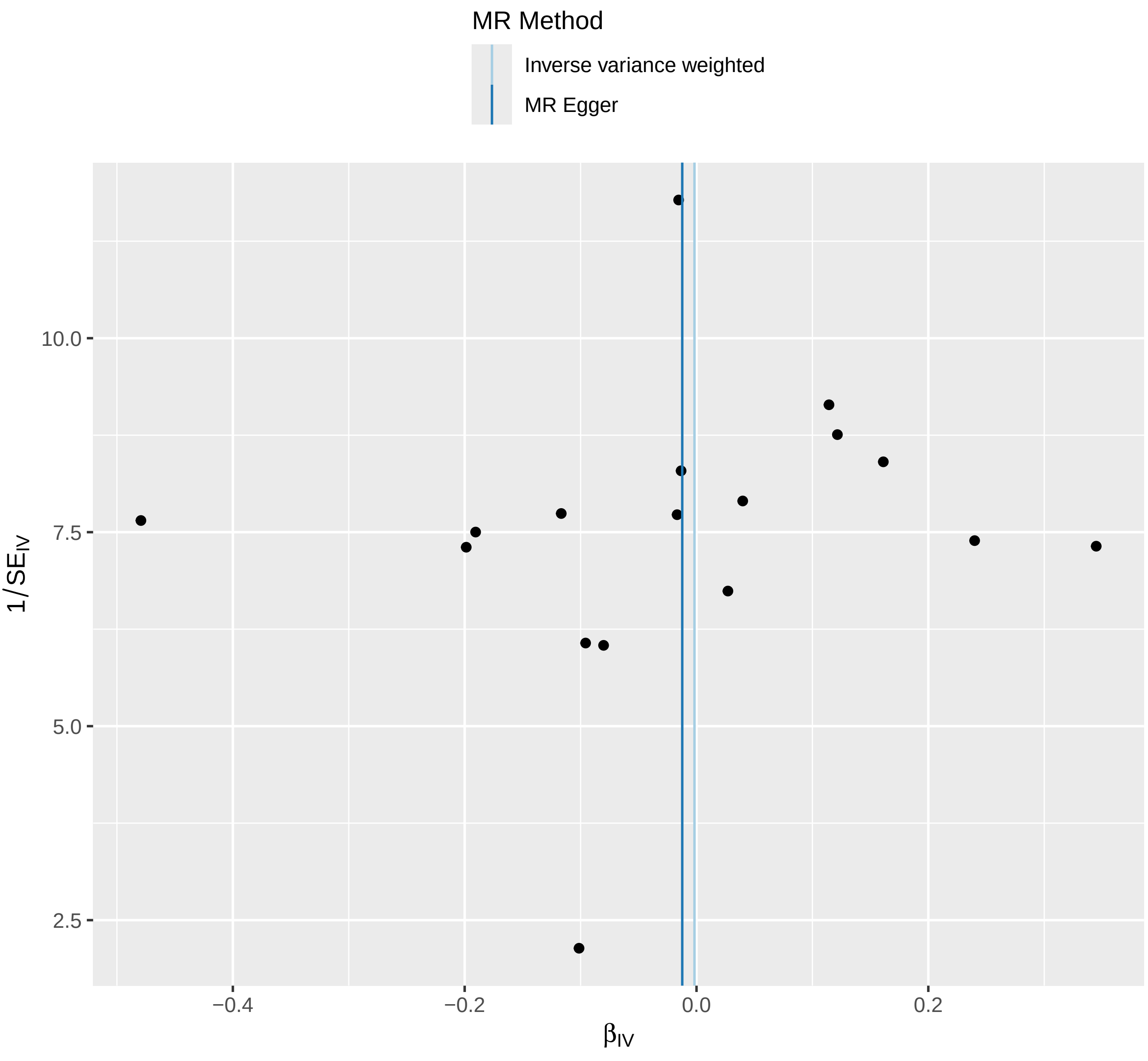
MR Method
Inverse variance weighted
MR Egger
10.0
7.5
5.0
2.5
1/SE_IV
−0.4
−0.2
0.0
0.2
β_IV

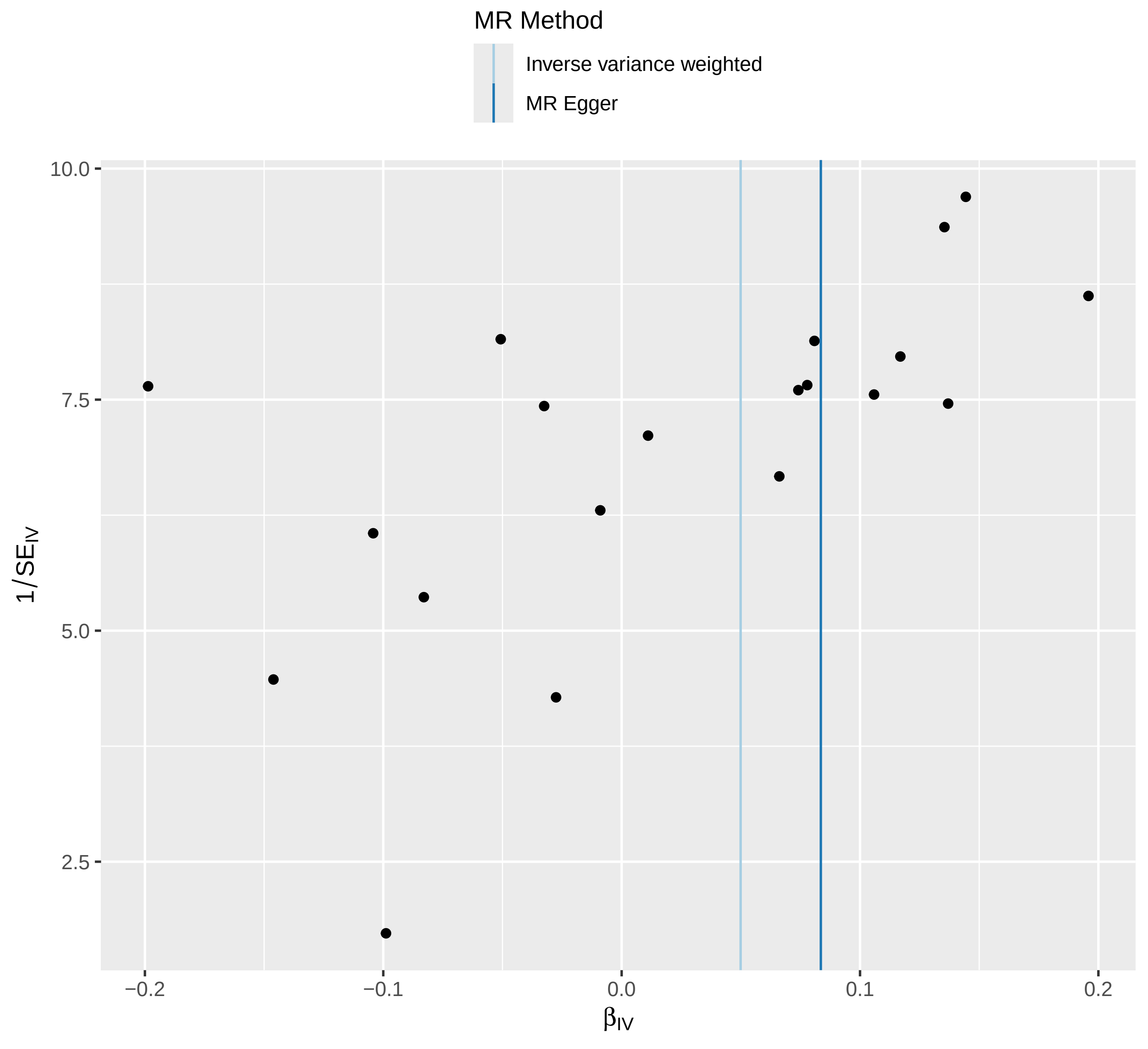
MR Method
Inverse variance weighted
MR Egger
10.0
7.5
5.0
2.5
1/SE_IV
−0.2
−0.1
0.0
0.1
0.2
β_IV

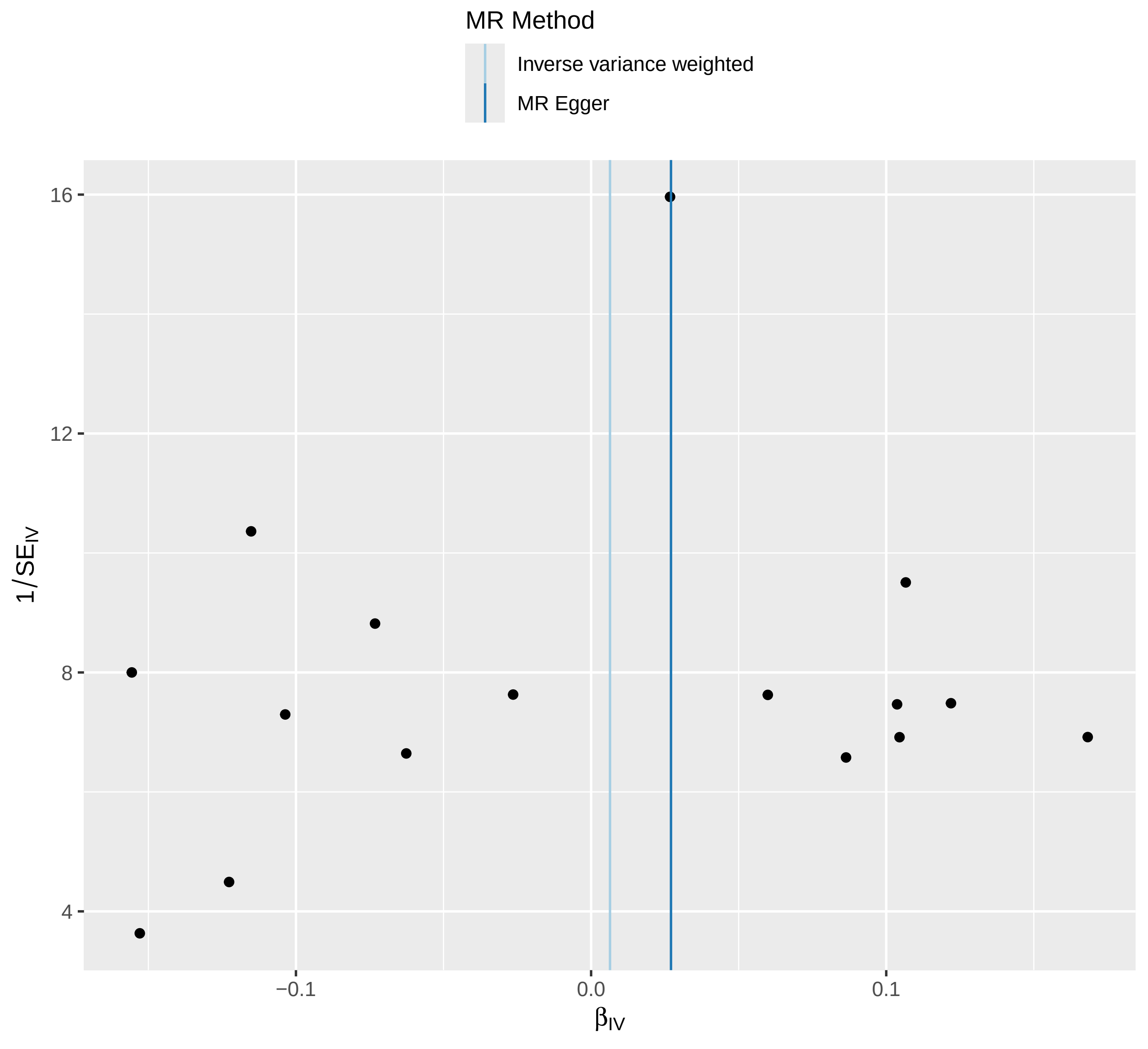

MR Method
Inverse variance weighted
MR Egger
16
12
8
4
1/SE$_{IV}$
−0.1
0.0
0.1
β$_{IV}$

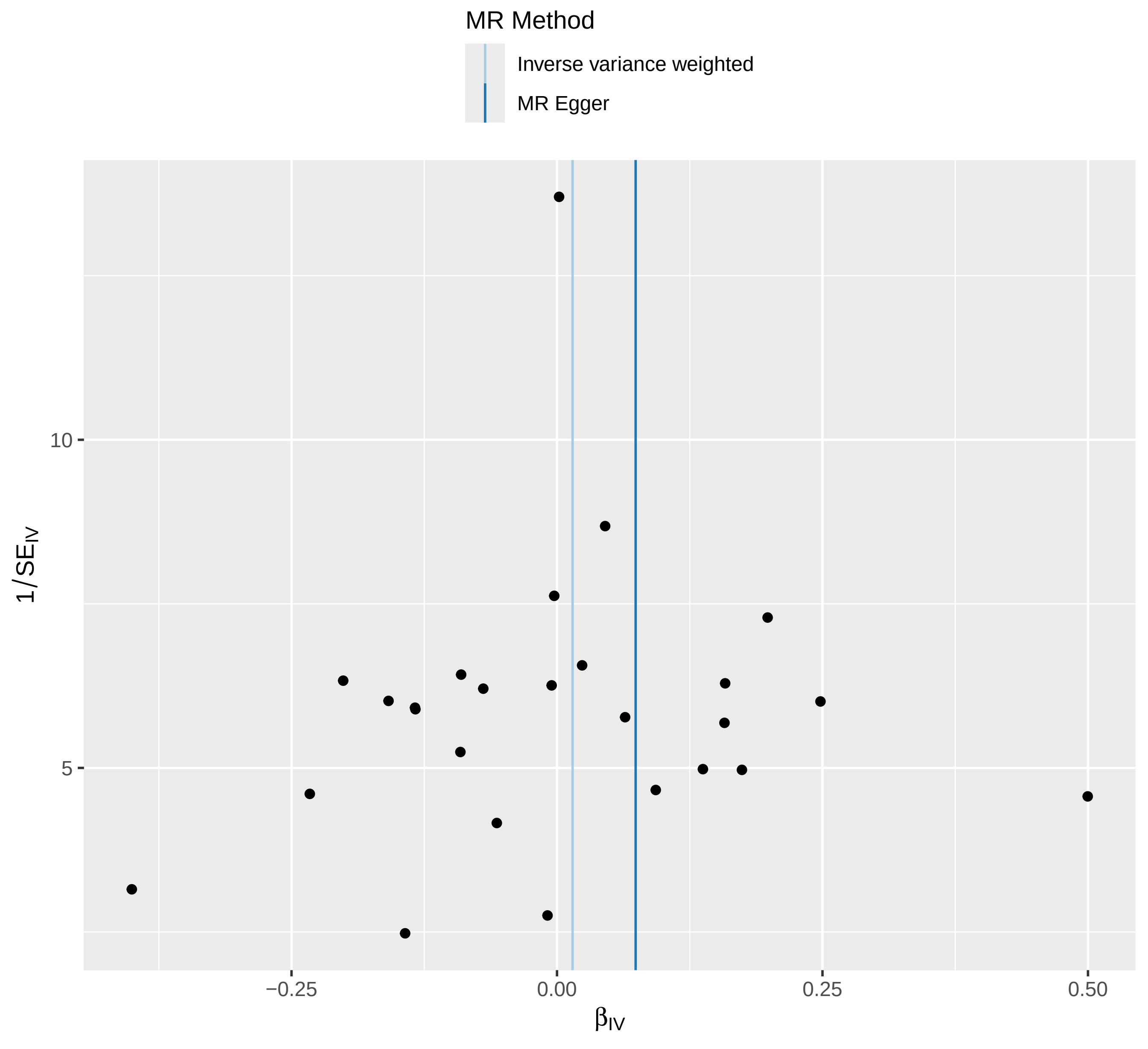

MR Method
Inverse variance weighted
MR Egger
1/SE_IV
10
5
−0.25
0.00
0.25
0.50
β_IV

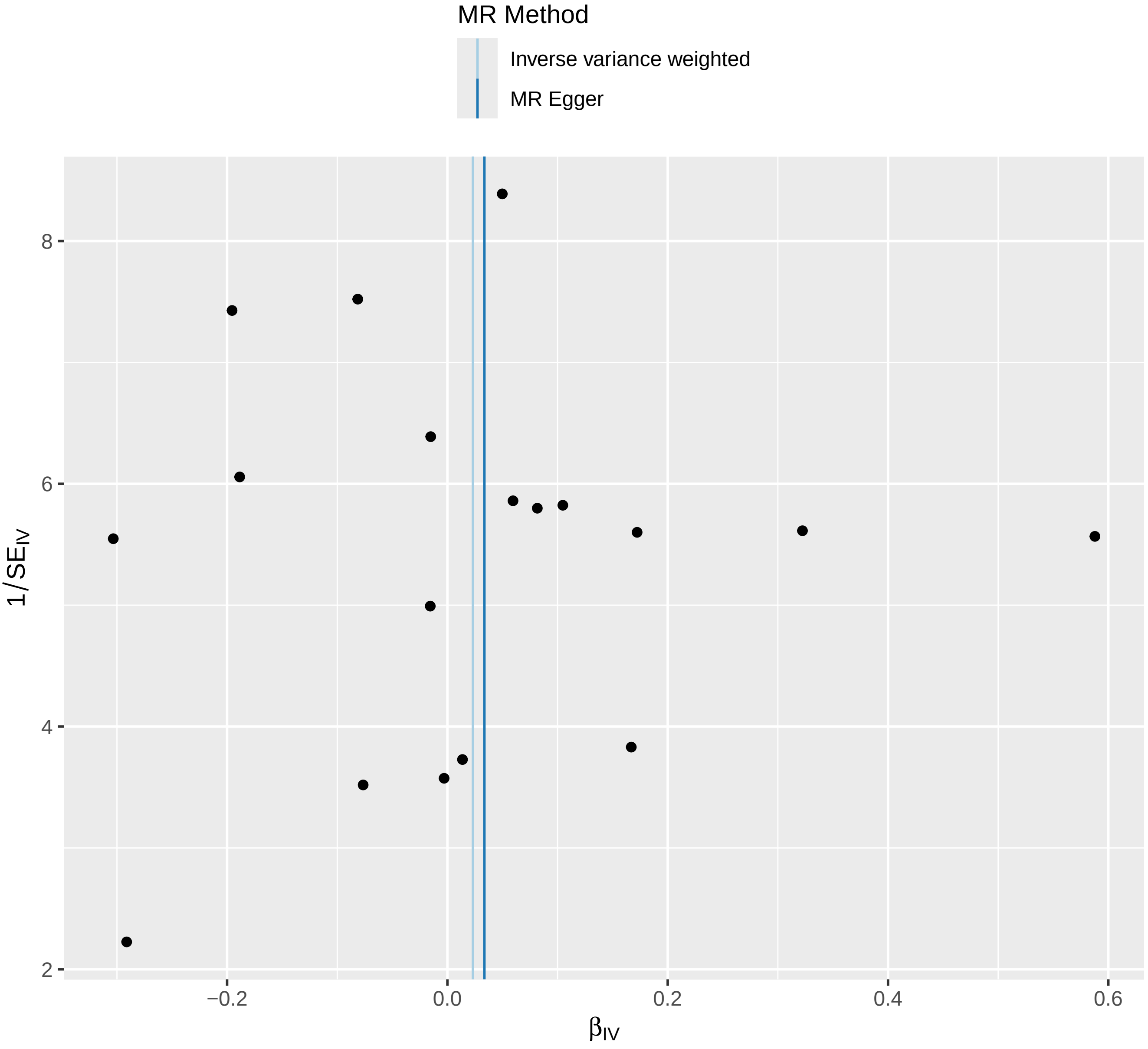
MR Method
Inverse variance weighted
MR Egger
1/SE_IV
β_IV
8
6
4
2
−0.2
0.0
0.2
0.4
0.6

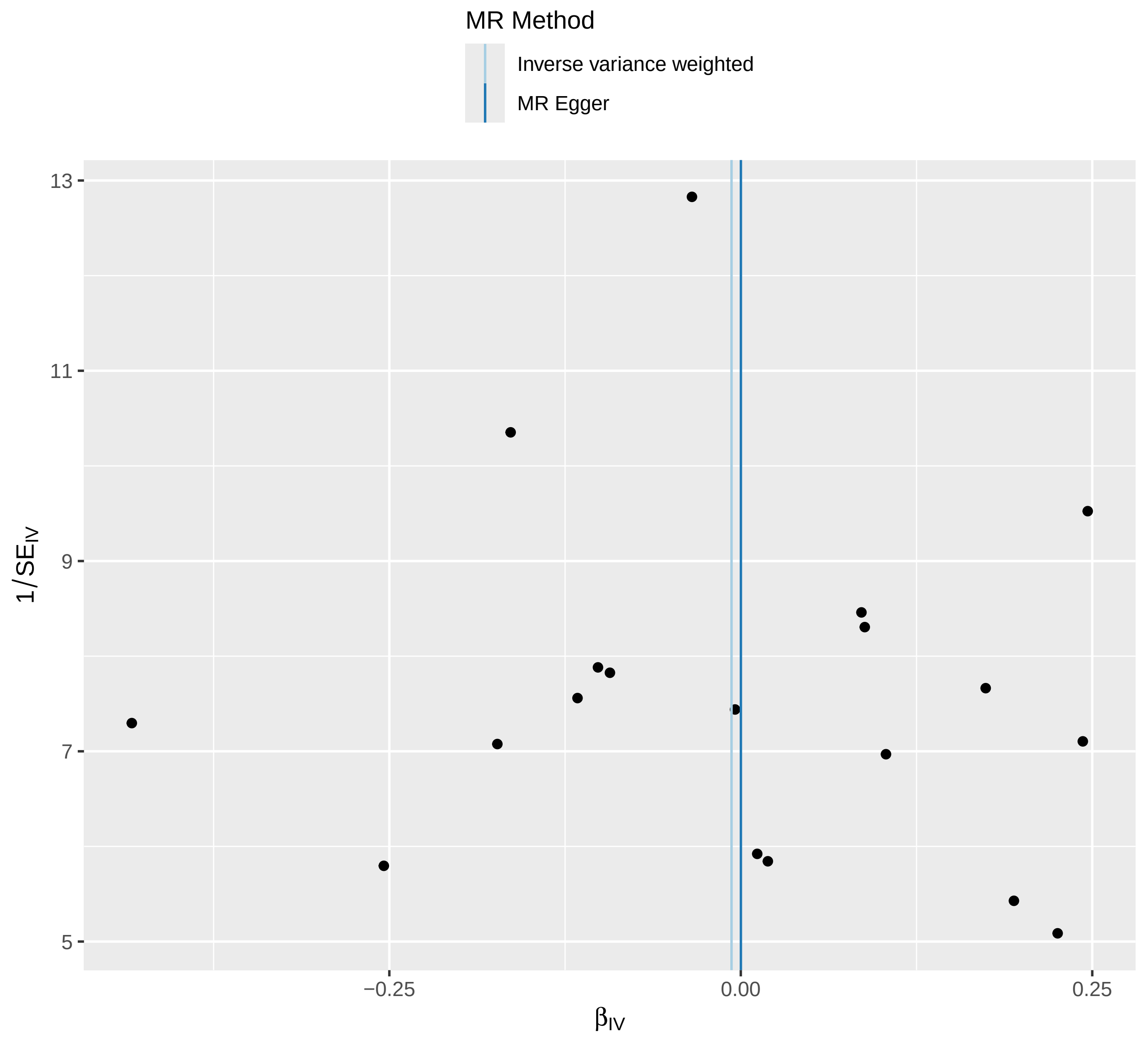
MR Method
Inverse variance weighted
MR Egger
13
11
9
7
5
1/SE_IV
−0.25
0.00
0.25
β_IV

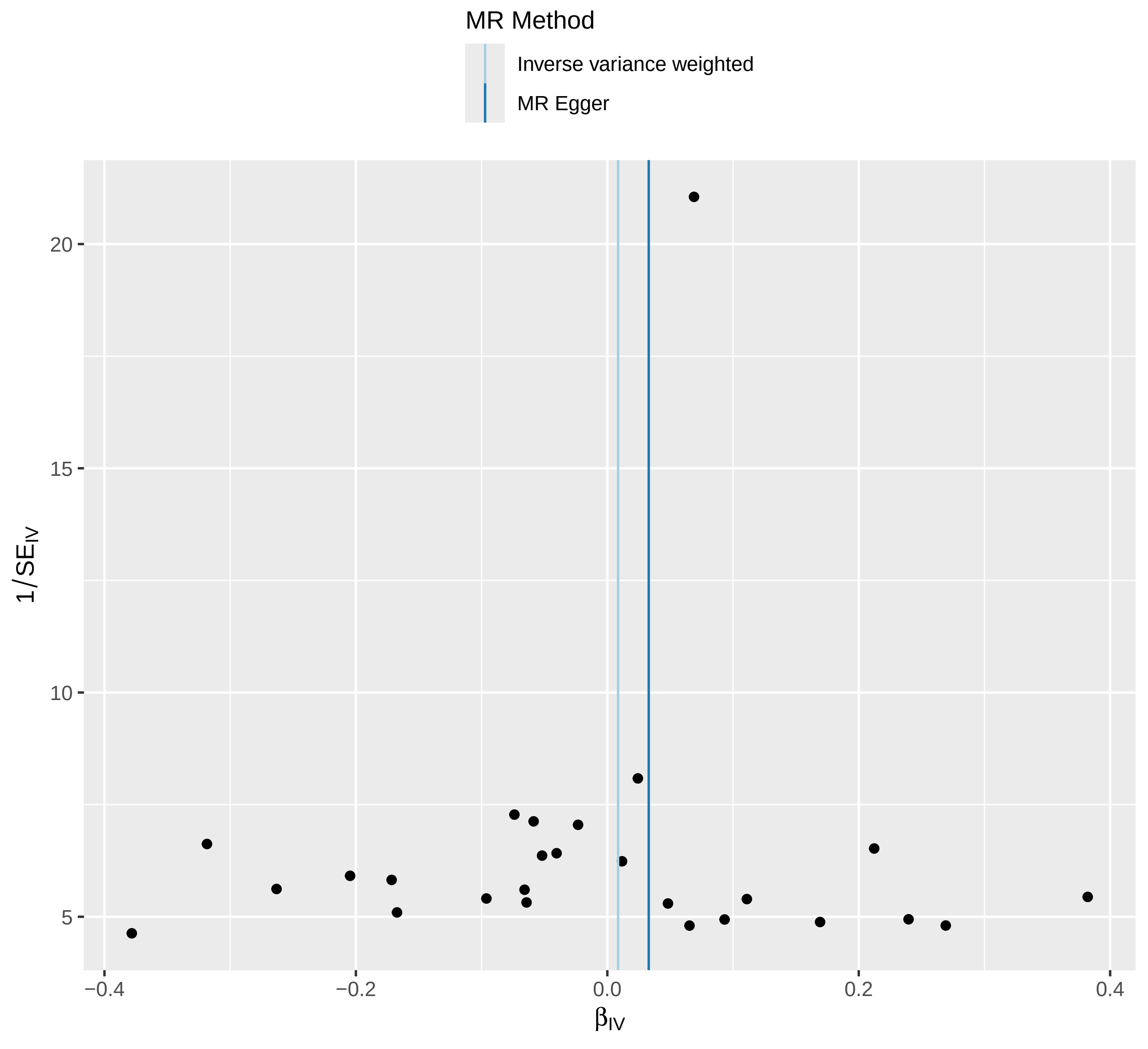
MR Method
Inverse variance weighted
MR Egger
20
15
10
5
1/SE_IV
−0.4
−0.2
0.0
0.2
0.4
β_IV

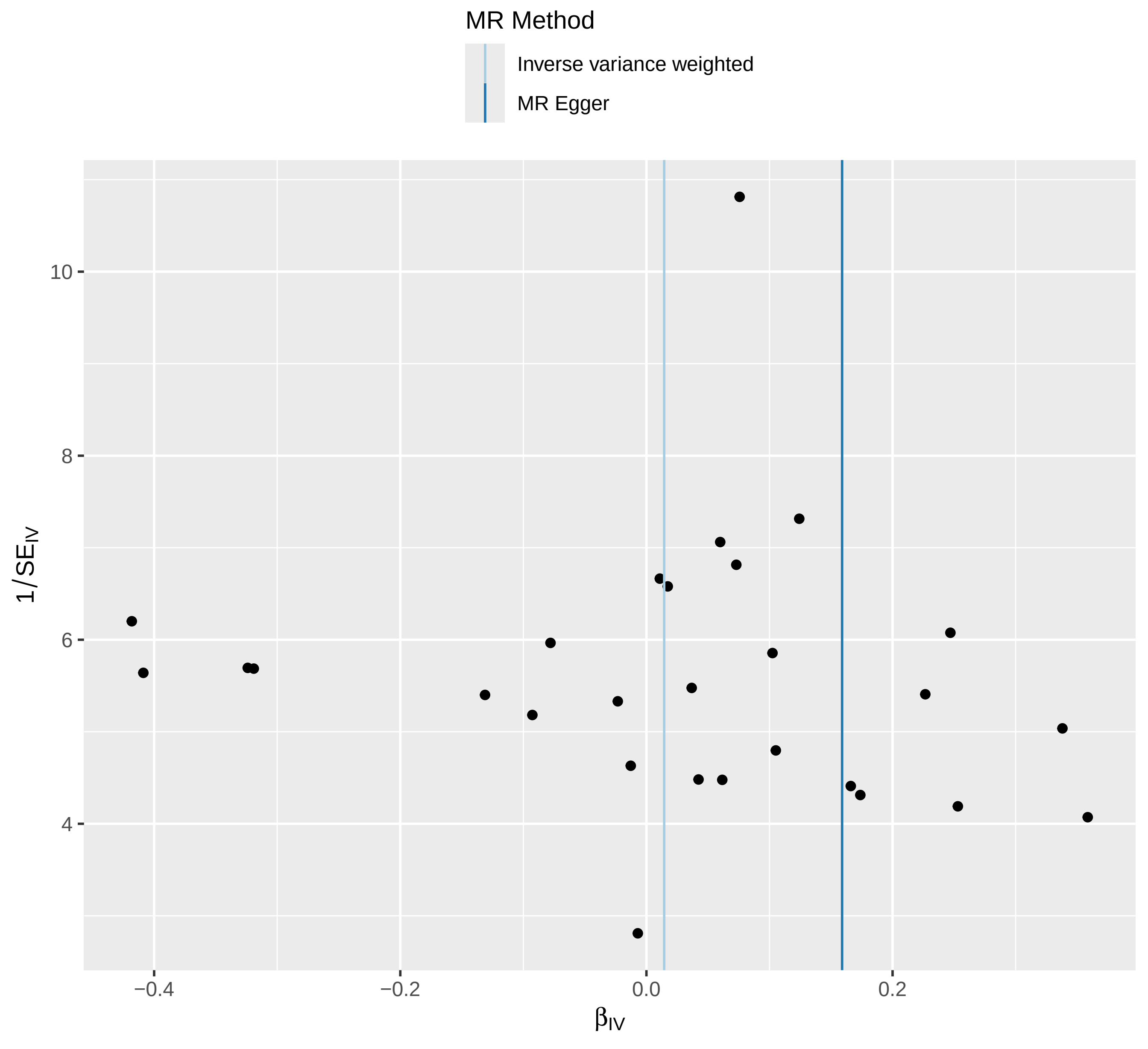
MR Method
Inverse variance weighted
MR Egger
10
8
6
4
−0.4
−0.2
0.0
0.2
$1/SE_{IV}$
$β_{IV}$

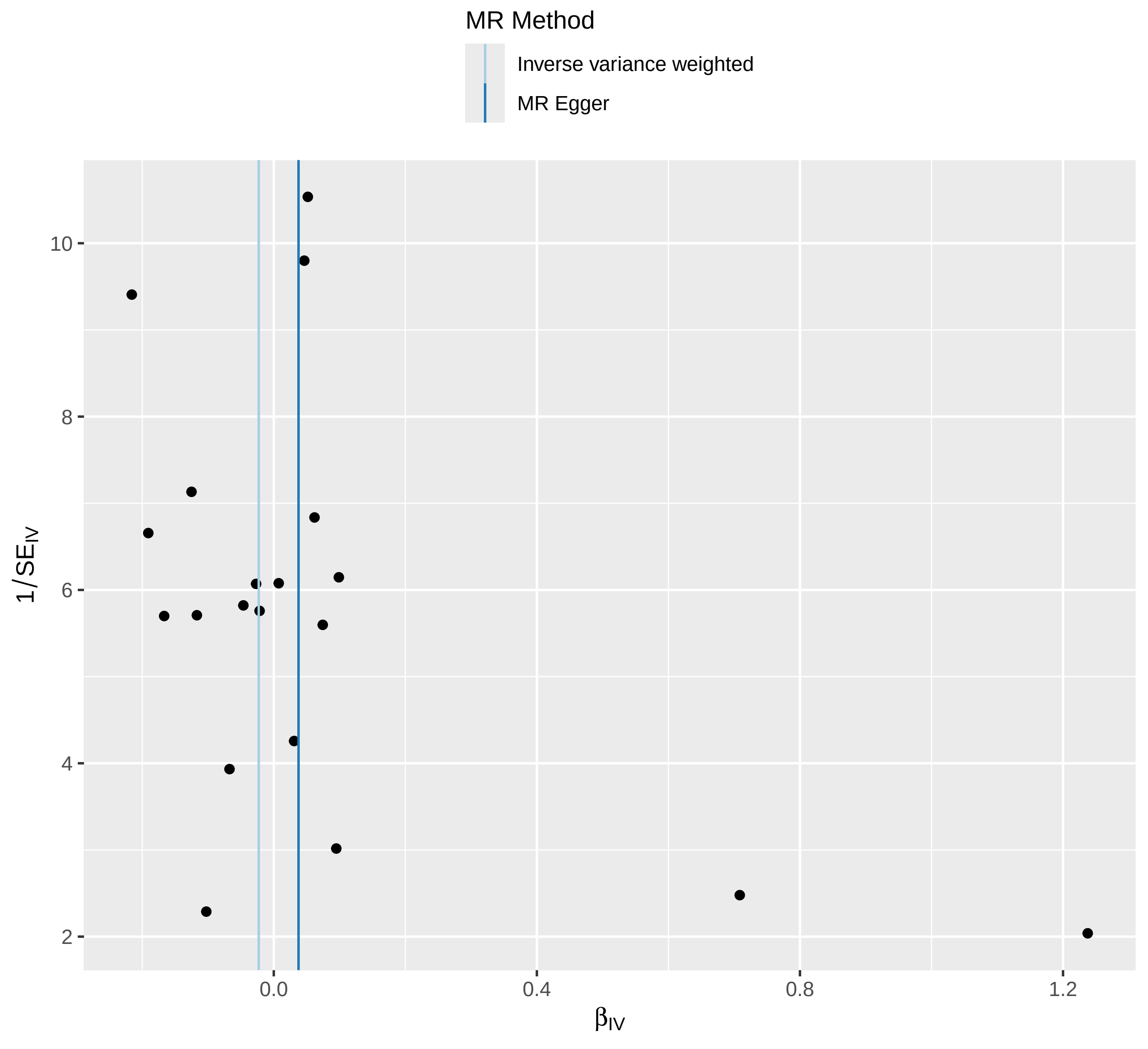
MR Method
Inverse variance weighted
MR Egger
1/SE_IV
β_IV
10
8
6
4
2
0.0
0.4
0.8
1.2

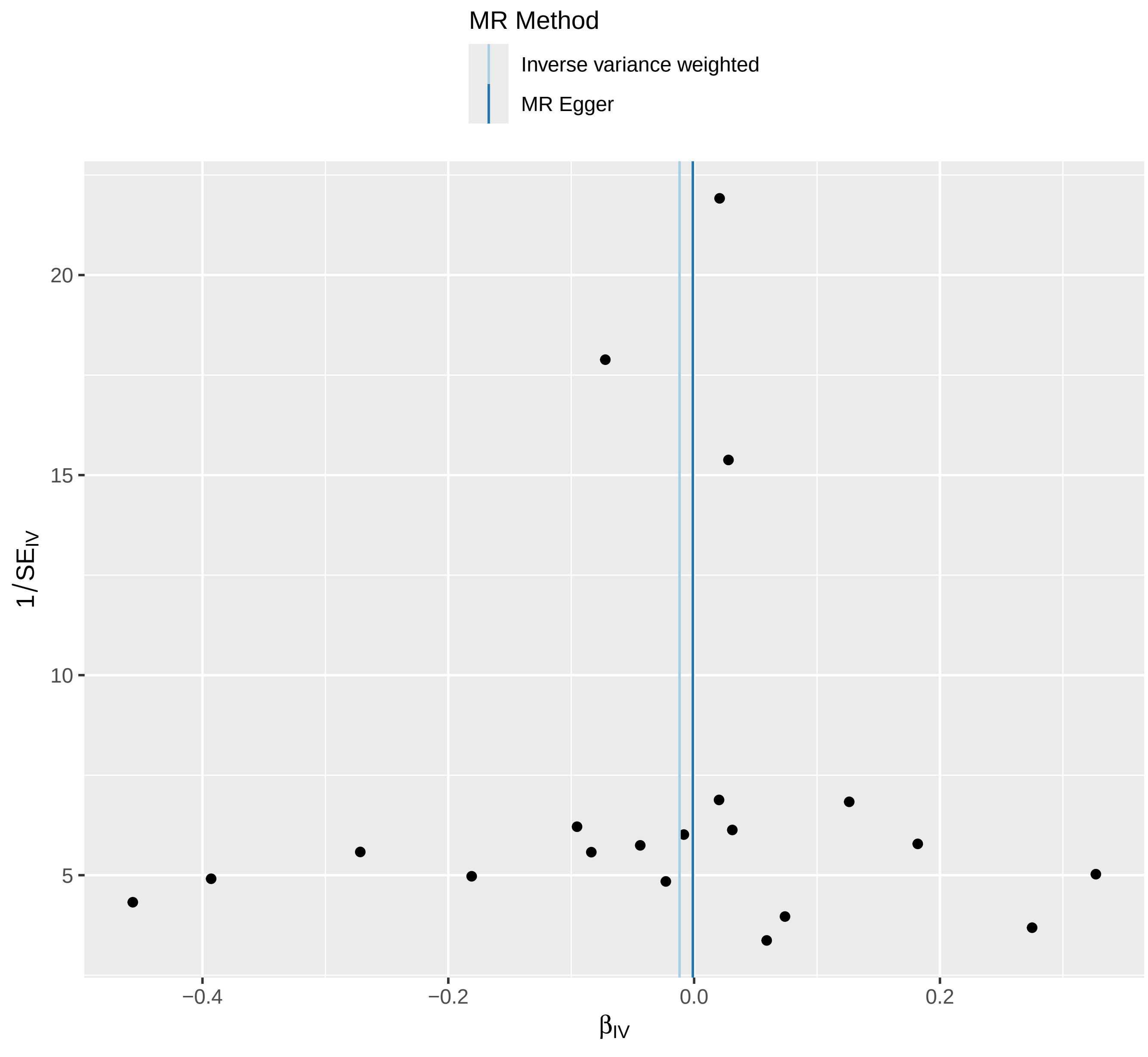

MR Method
Inverse variance weighted
MR Egger
20
15
10
5
1/SE_IV
−0.4
−0.2
0.0
0.2
β_IV

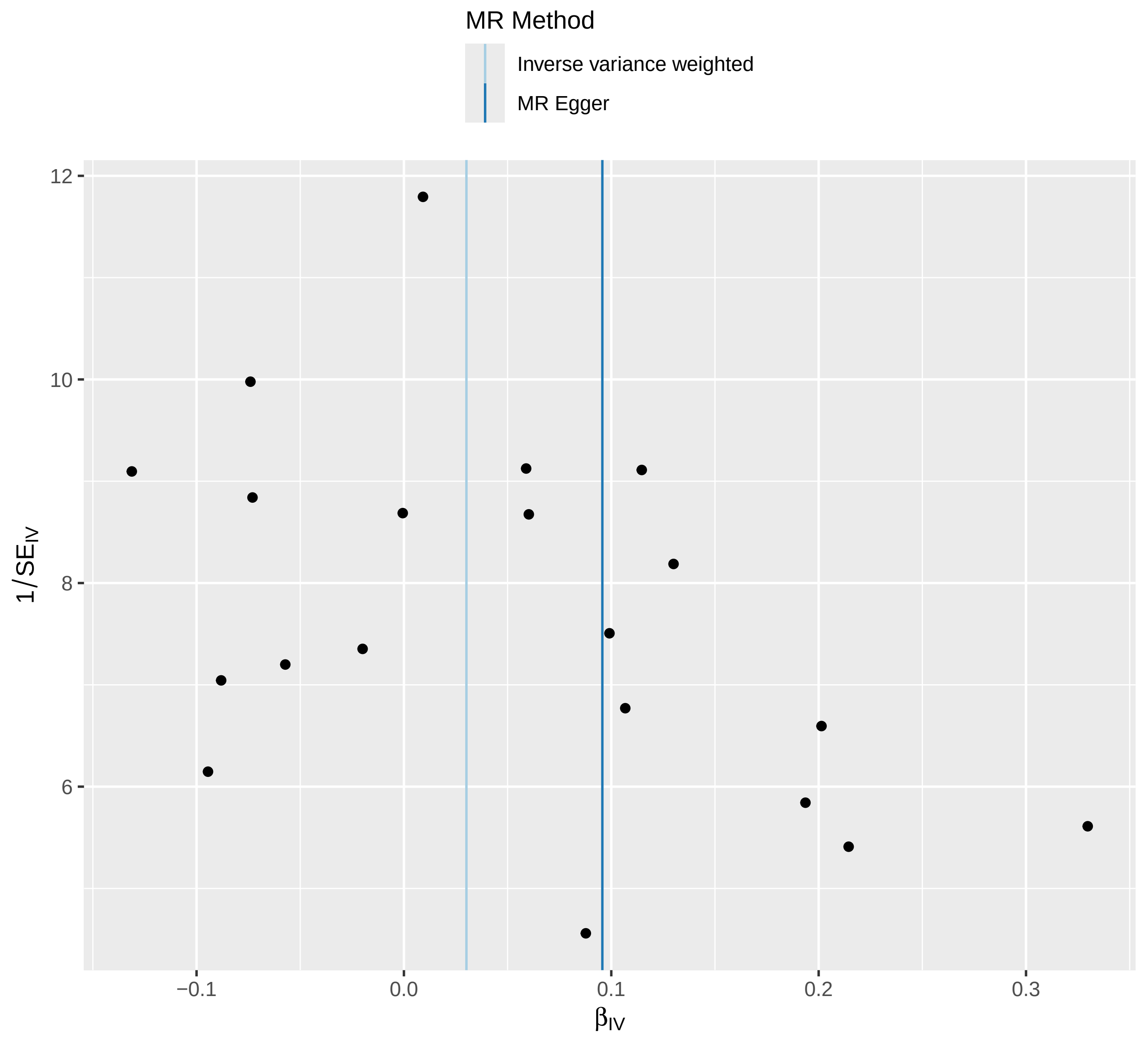
MR Method
Inverse variance weighted
MR Egger
12
10
8
6
1/SE$_{IV}$
−0.1
0.0
0.1
0.2
0.3
β$_{IV}$

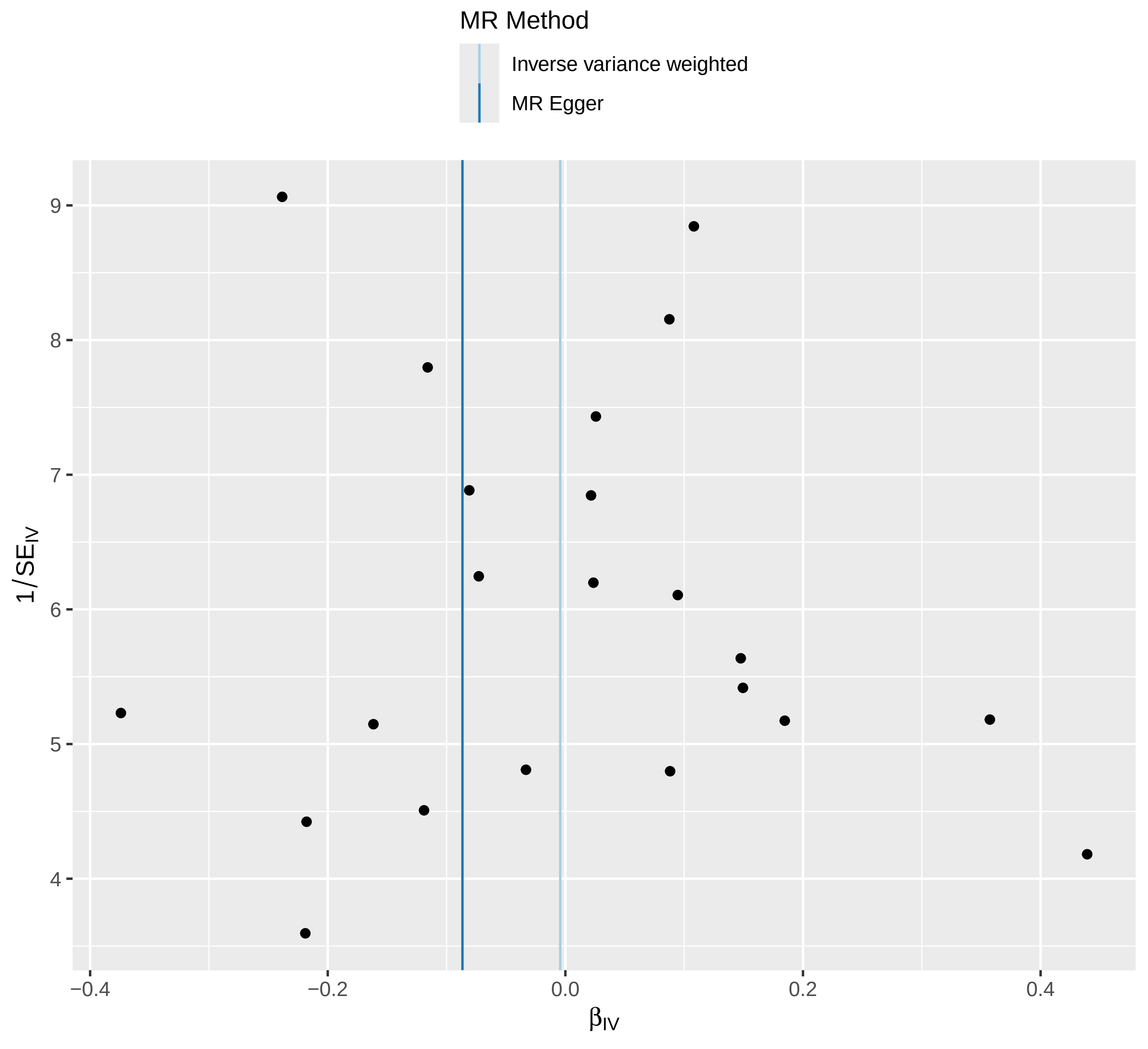
MR Method
Inverse variance weighted
MR Egger
1/SE_IV
9
8
7
6
5
4
−0.4
−0.2
0.0
0.2
0.4
β_IV

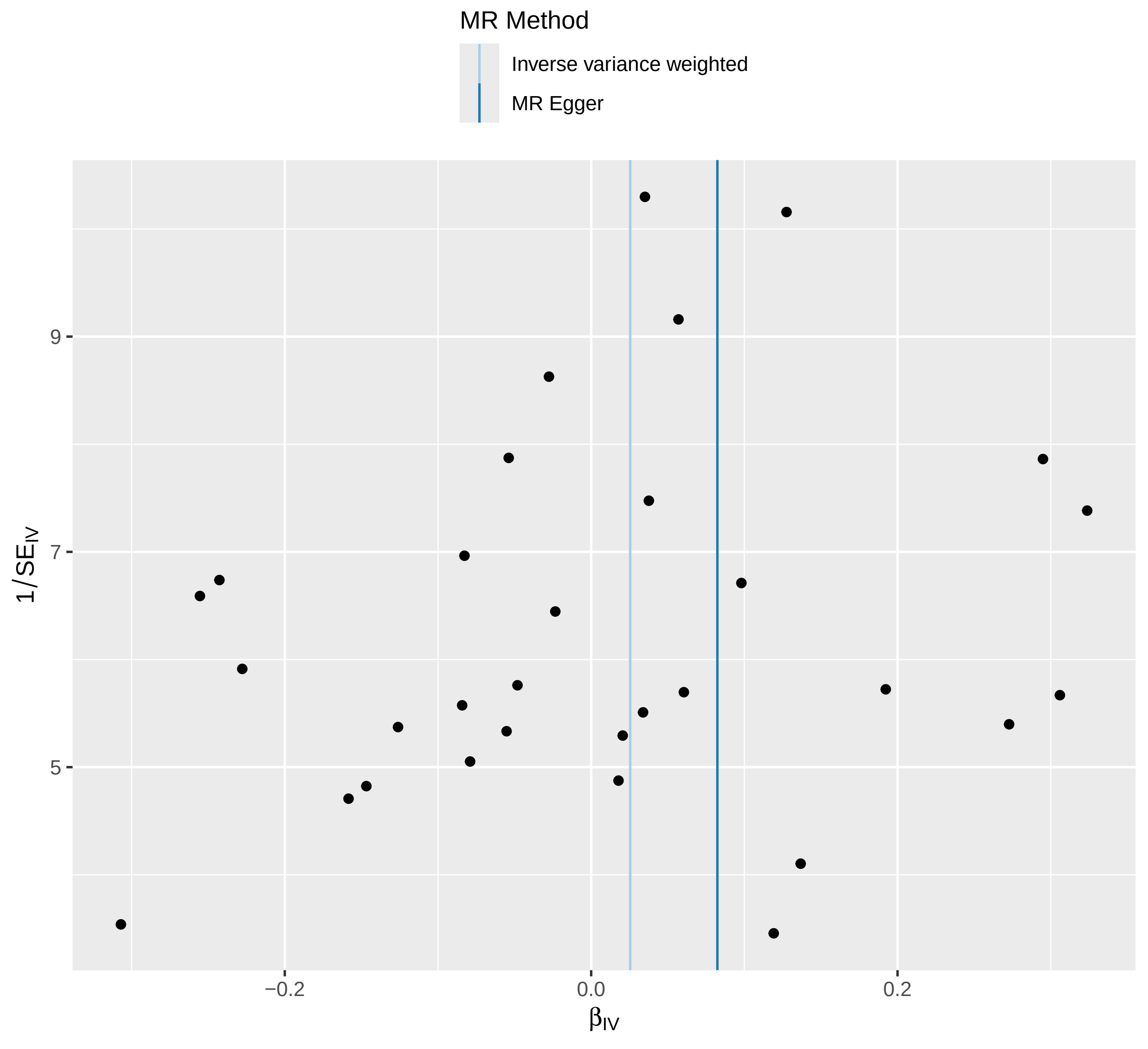
MR Method
Inverse variance weighted
MR Egger
9
7
5
1/SE_IV
−0.2
0.0
0.2
β_IV

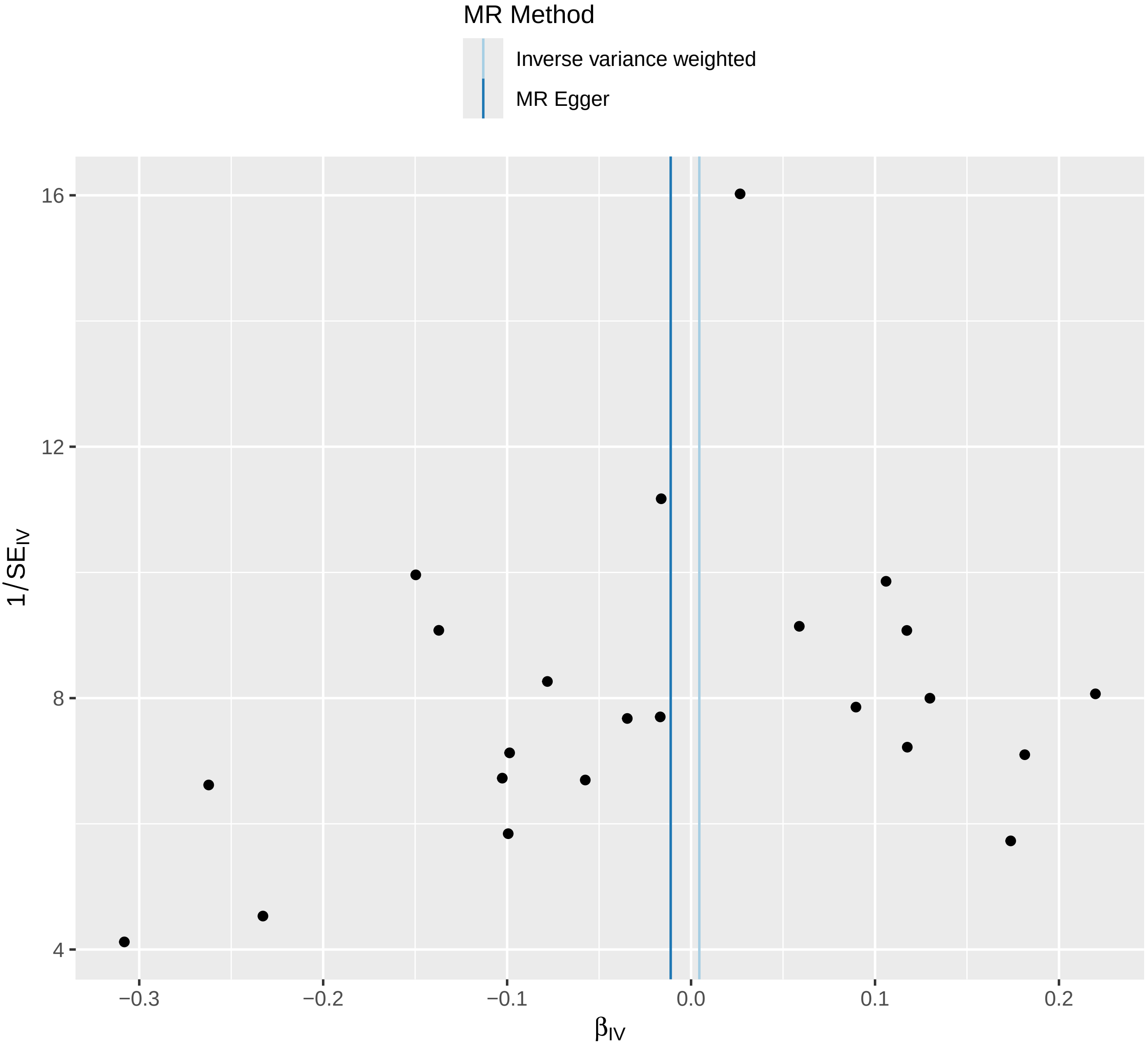

MR Method
Inverse variance weighted
MR Egger
16
12
8
4
1/SE_IV
−0.3
−0.2
−0.1
0.0
0.1
0.2
β_IV

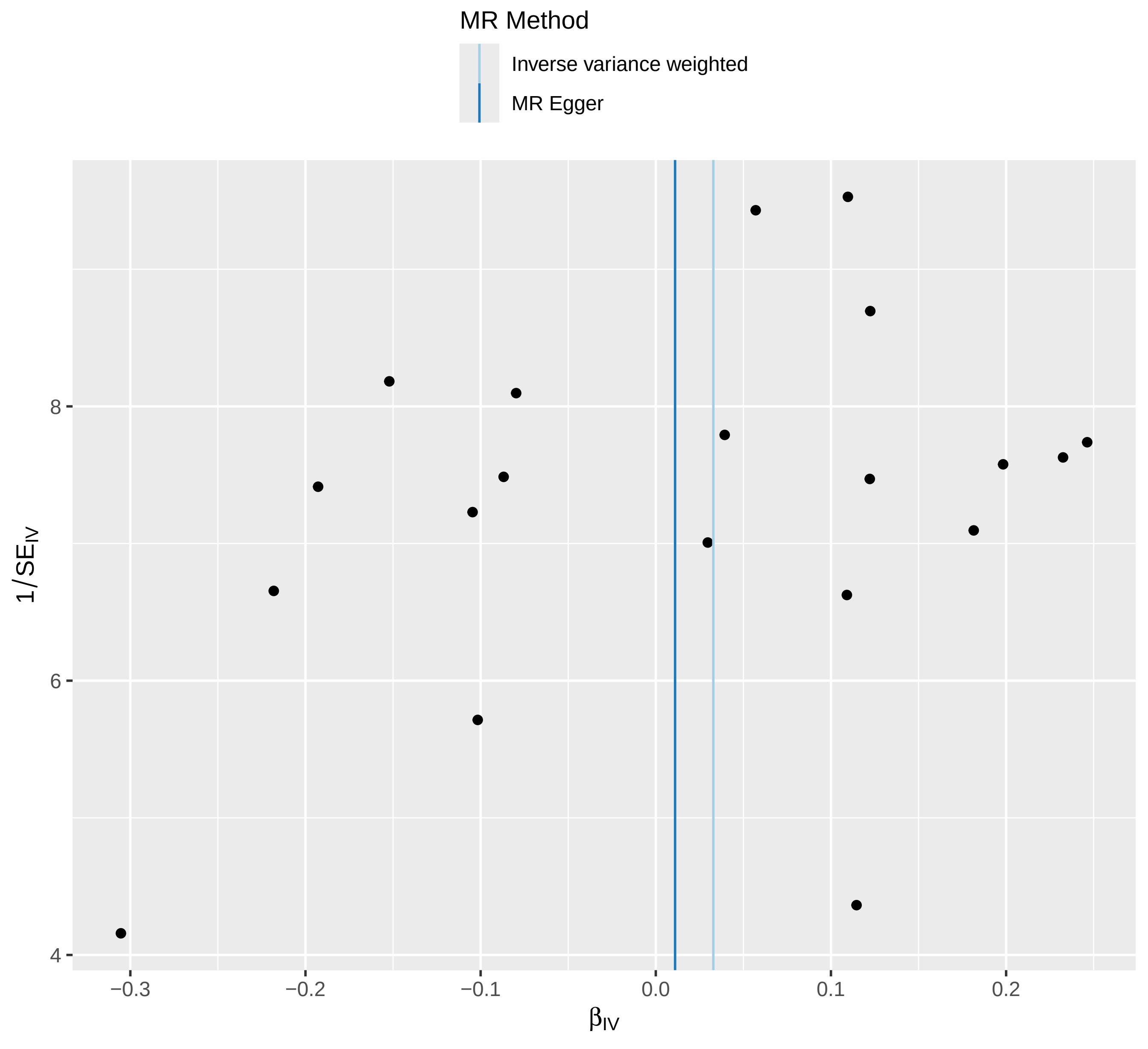
MR Method
Inverse variance weighted
MR Egger
1/SE_{IV}
8
6
4
−0.3
−0.2
−0.1
0.0
0.1
0.2
β_{IV}

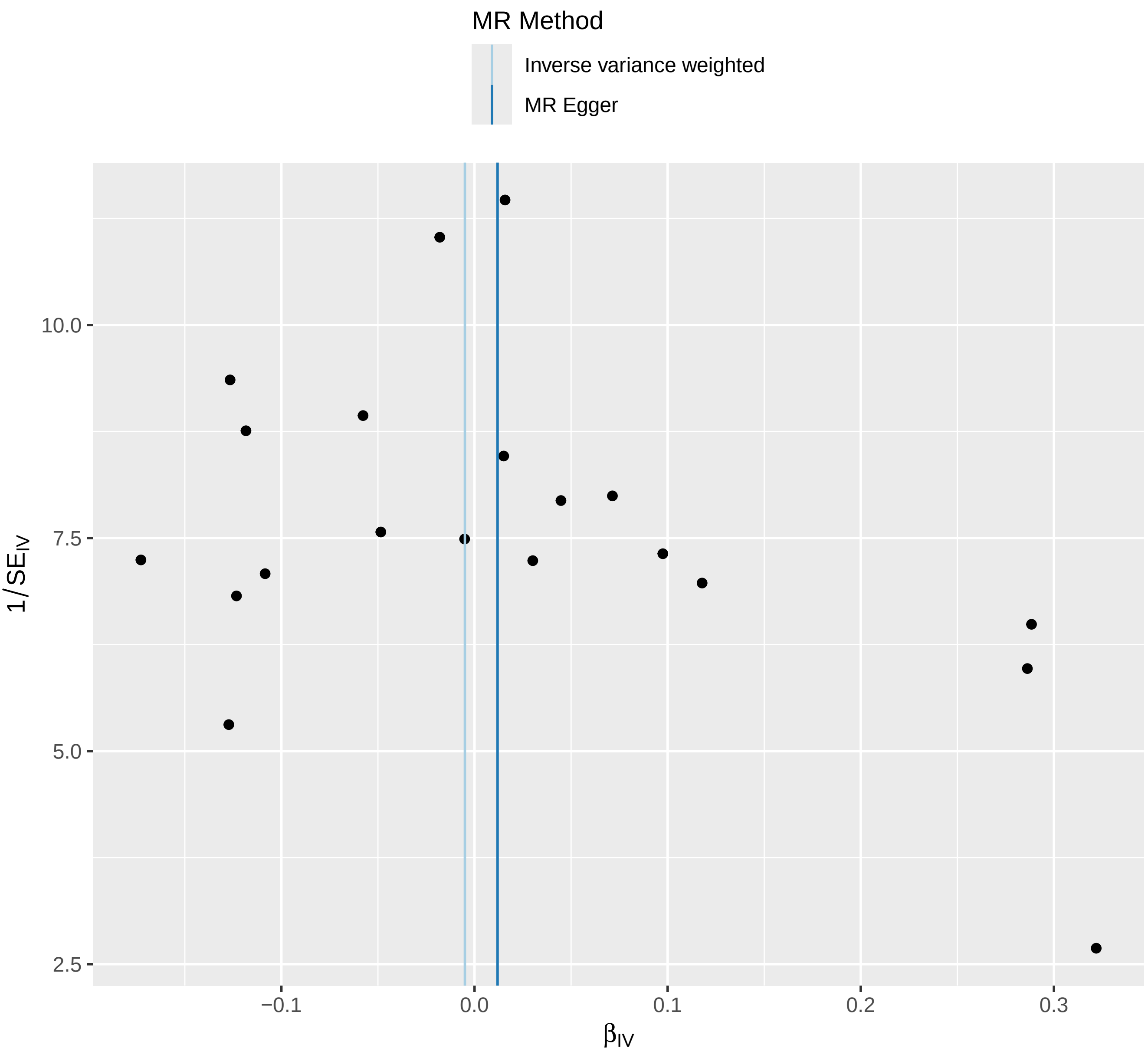
MR Method
Inverse variance weighted
MR Egger
10.0
7.5
5.0
2.5
1/SE$_{IV}$
−0.1
0.0
0.1
0.2
0.3
β$_{IV}$

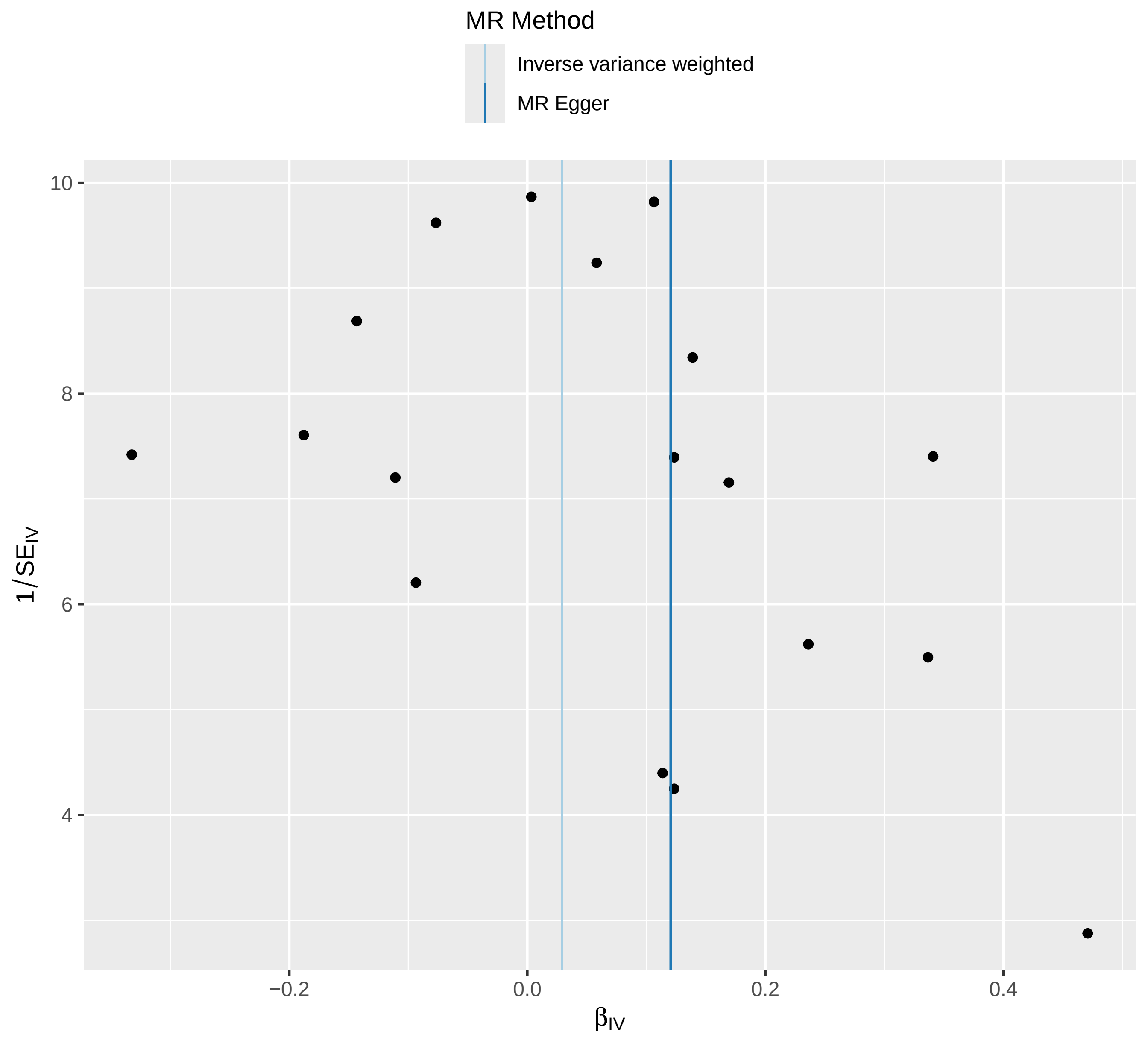
MR Method
Inverse variance weighted
MR Egger
10
8
6
4
1/SE$_{IV}$
−0.2
0.0
0.2
0.4
β$_{IV}$

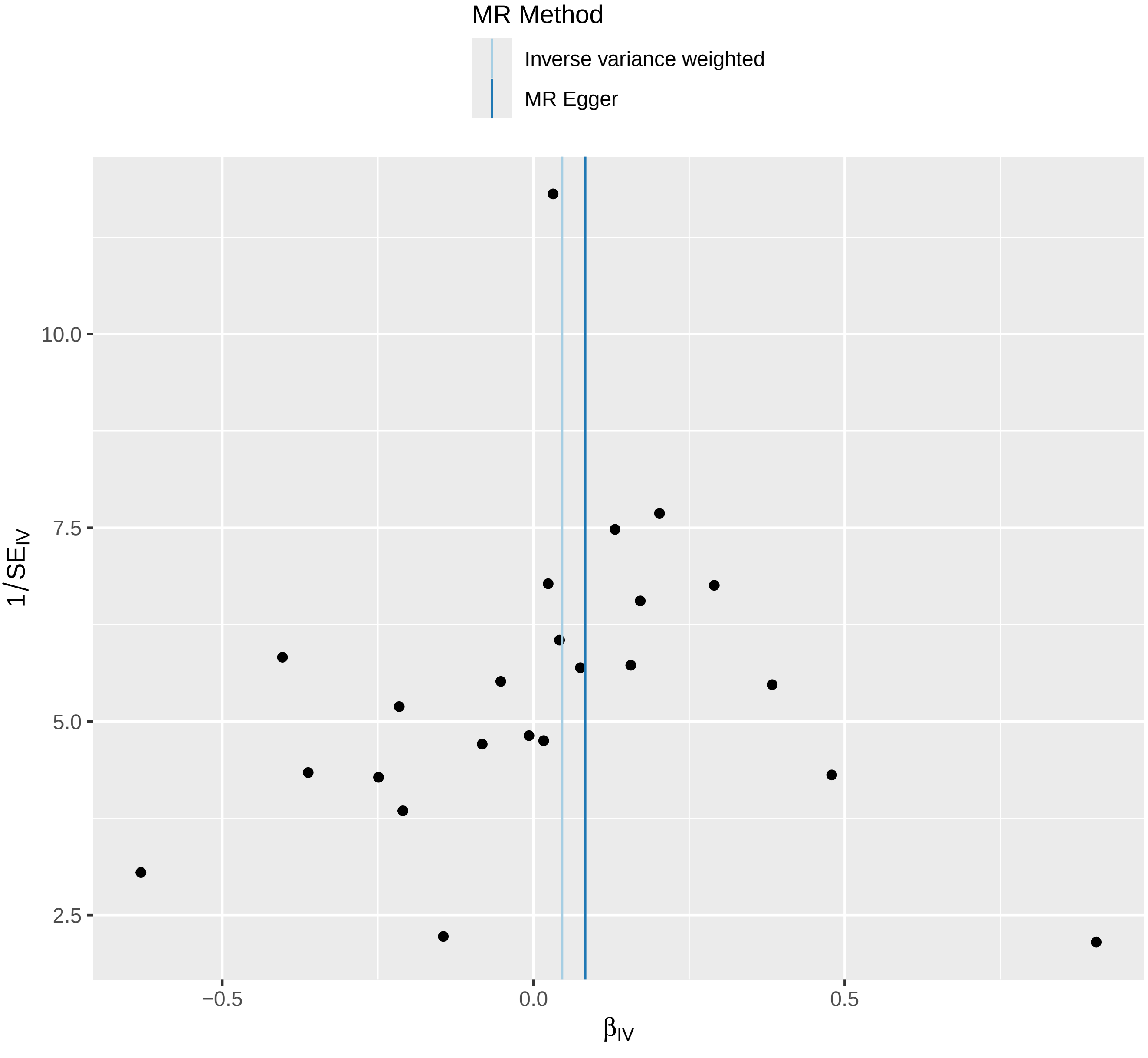
MR Method
Inverse variance weighted
MR Egger
10.0
7.5
5.0
2.5
1/SE_IV
−0.5
0.0
0.5
β_IV

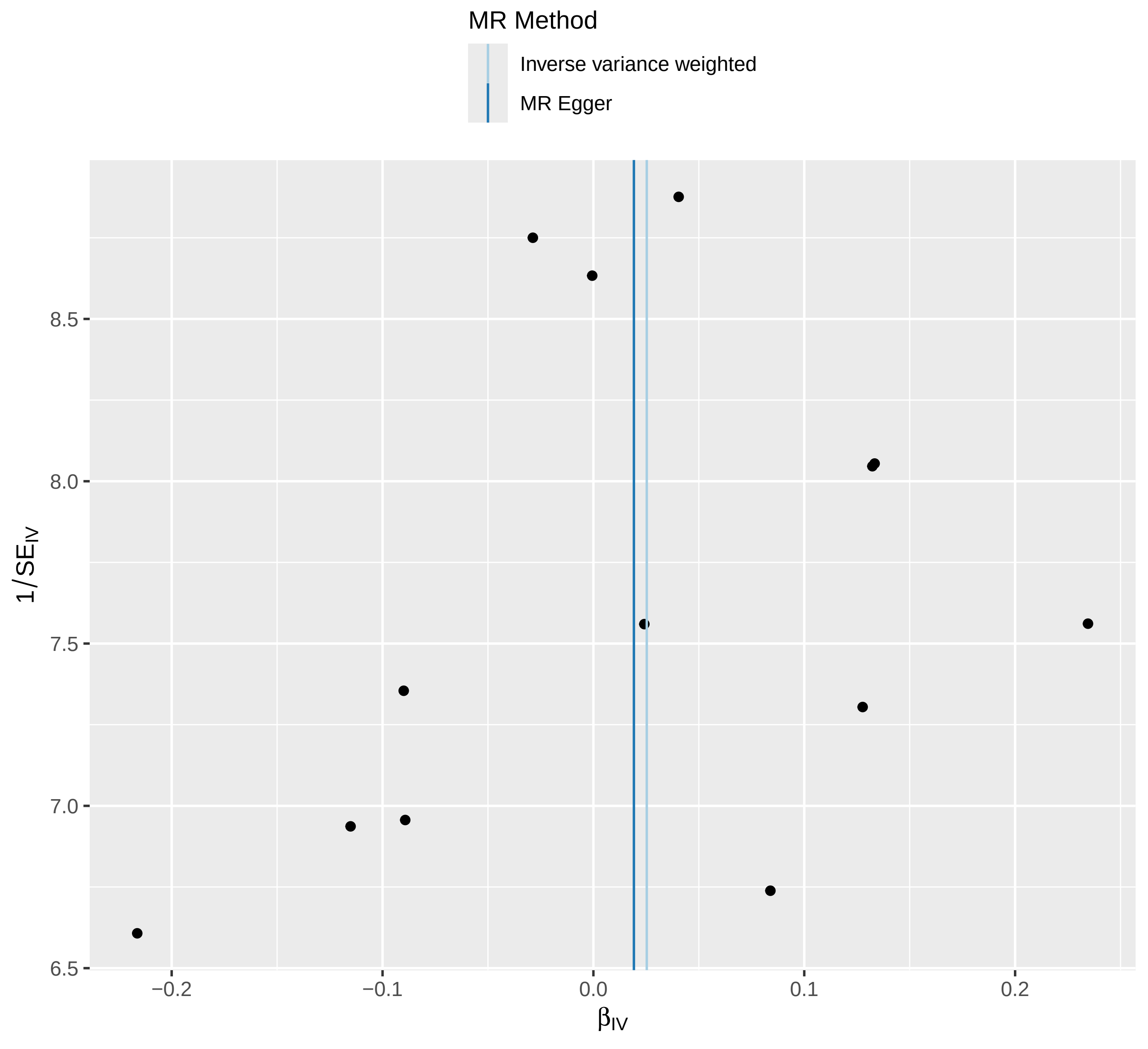
MR Method
Inverse variance weighted
MR Egger
1/SE_IV
β_IV
8.5
8.0
7.5
7.0
6.5
−0.2
−0.1
0.0
0.1
0.2

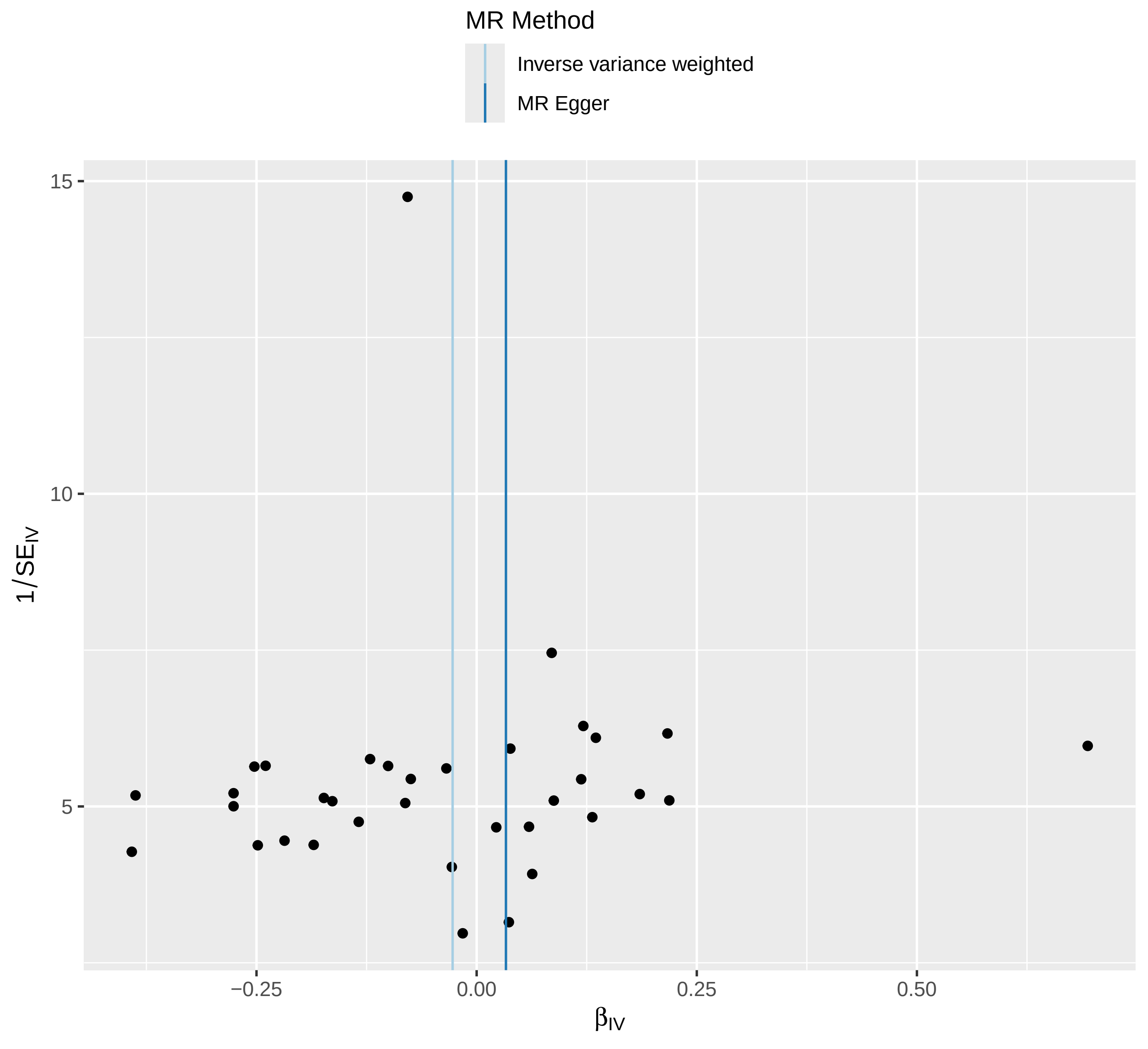

MR Method
Inverse variance weighted
MR Egger
15
10
5
1/SE_IV
−0.25
0.00
0.25
0.50
β_IV

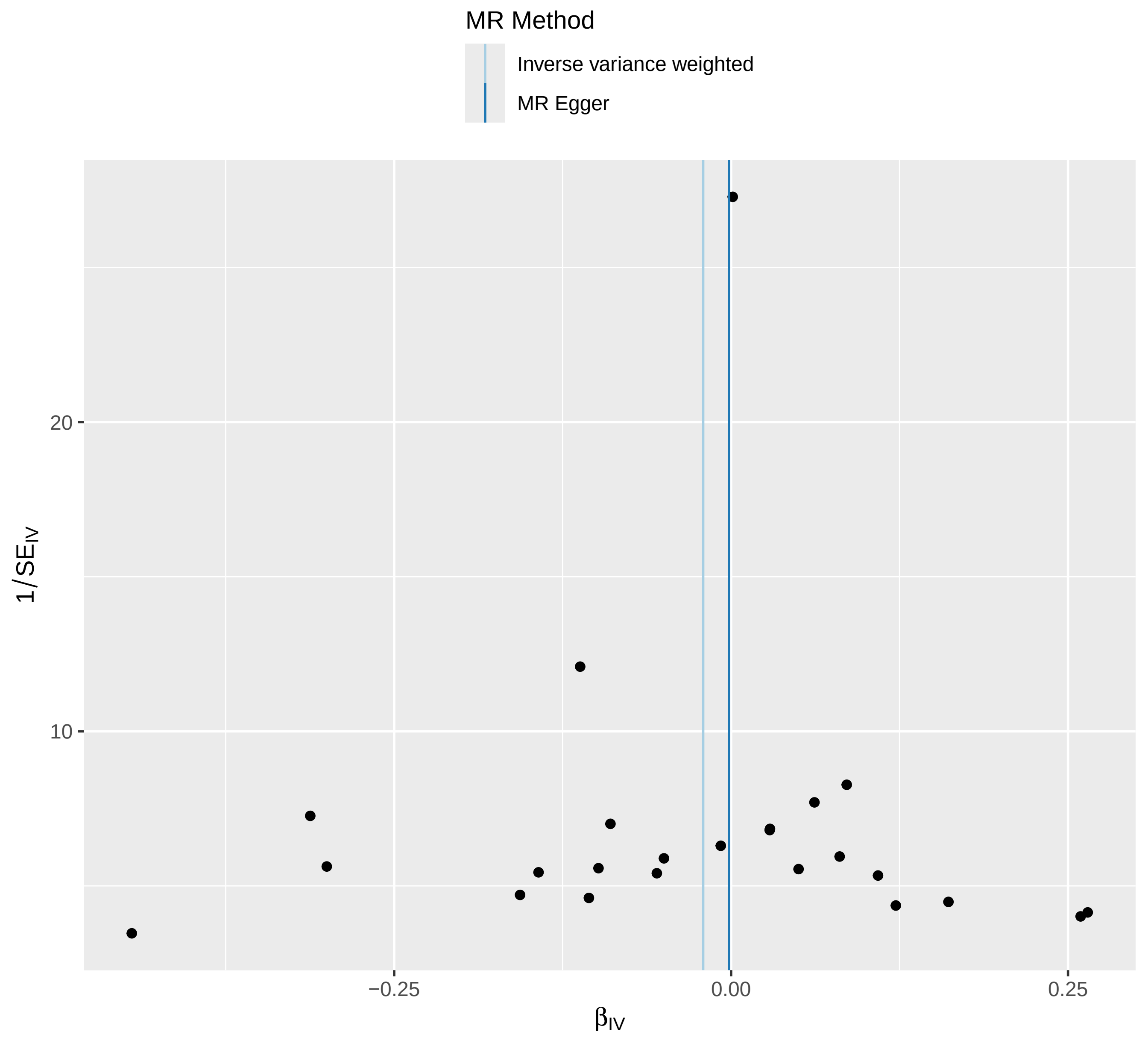
MR Method
Inverse variance weighted
MR Egger
20
10
1/SE$_{IV}$
−0.25
0.00
0.25
β$_{IV}$

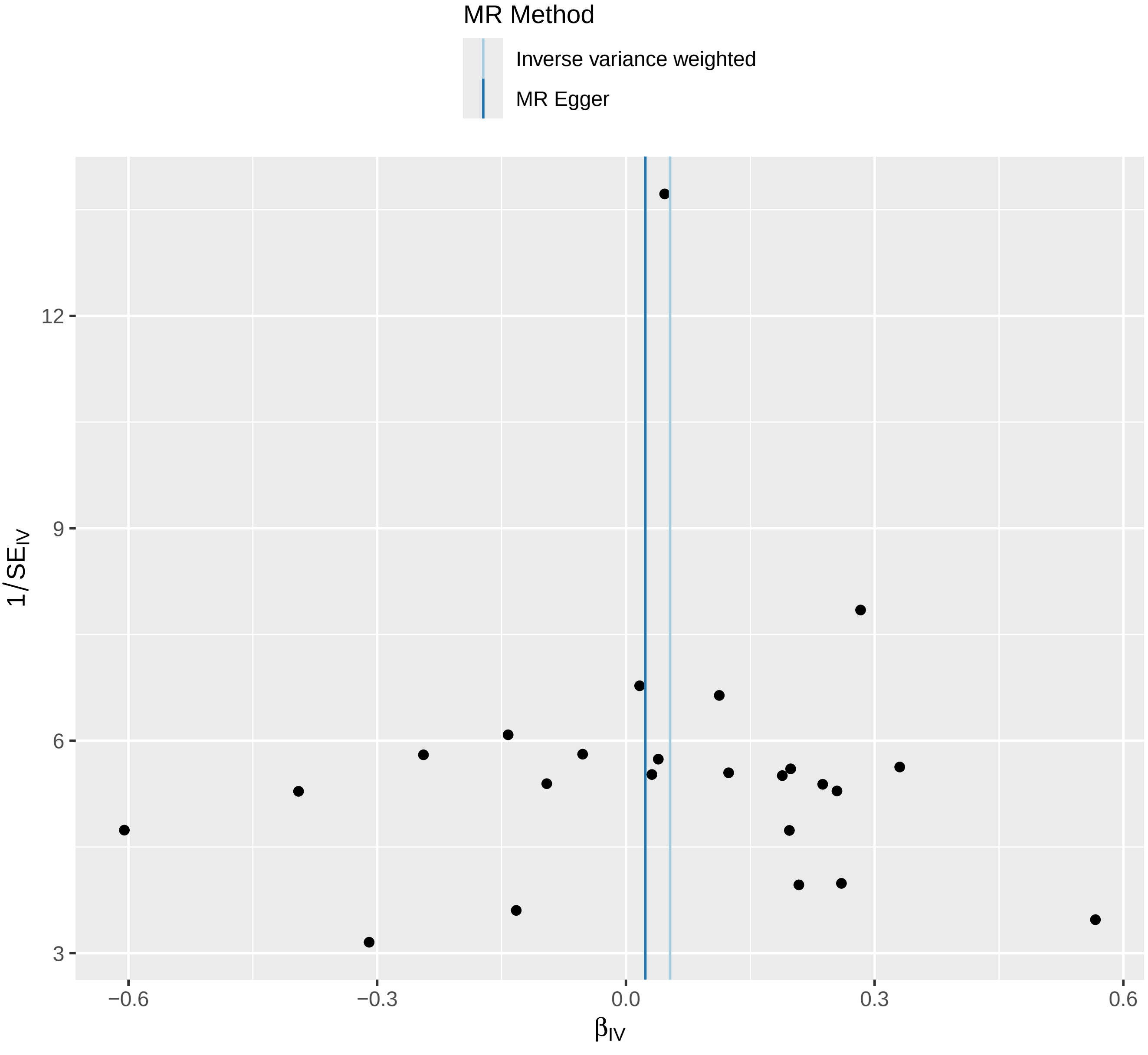
MR Method
Inverse variance weighted
MR Egger
1/SE_IV
12
9
6
3
−0.6
−0.3
0.0
0.3
0.6
β_IV

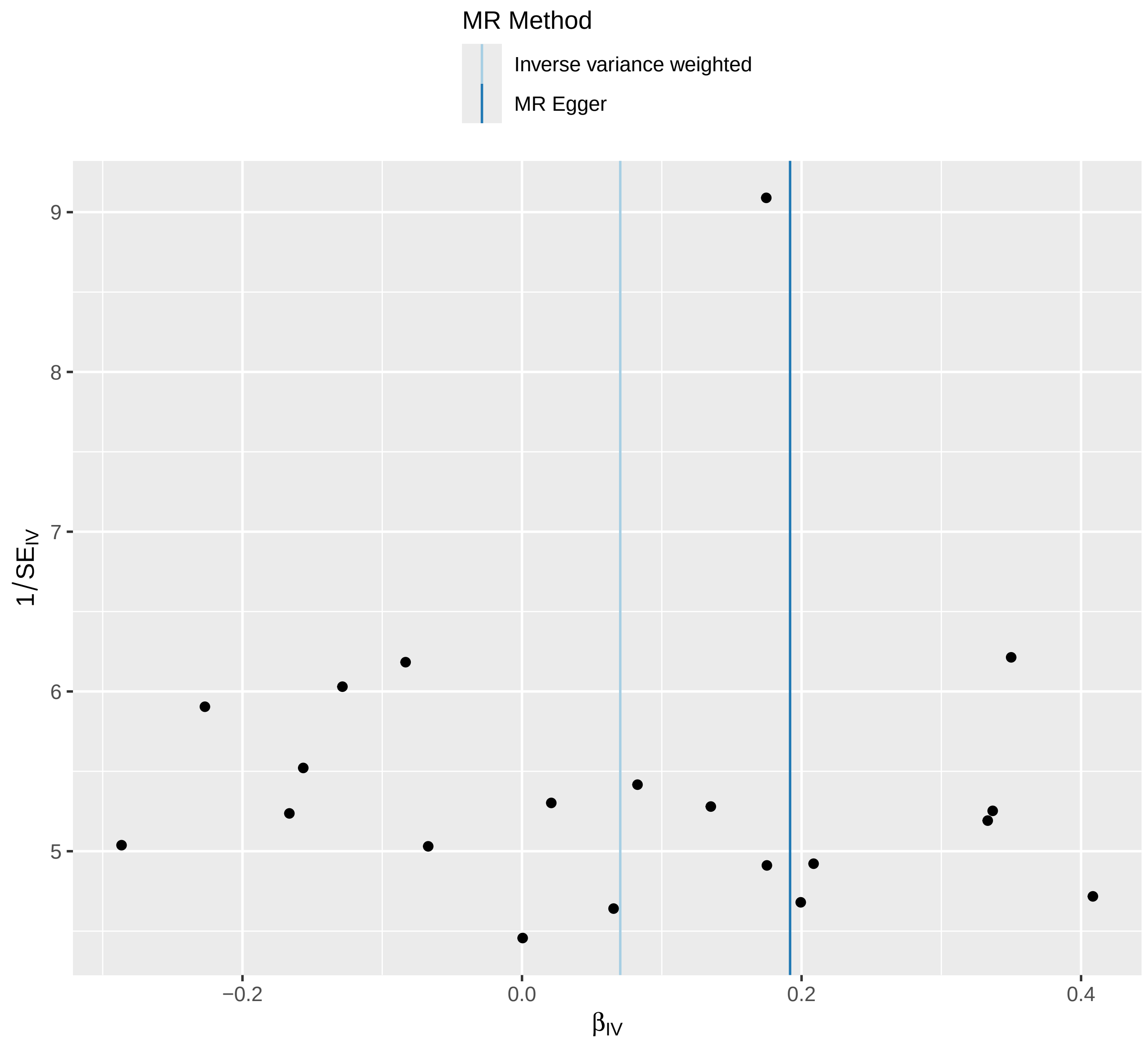
MR Method
Inverse variance weighted
MR Egger
1/SE_IV
9
8
7
6
5
−0.2
0.0
0.2
0.4
β_IV

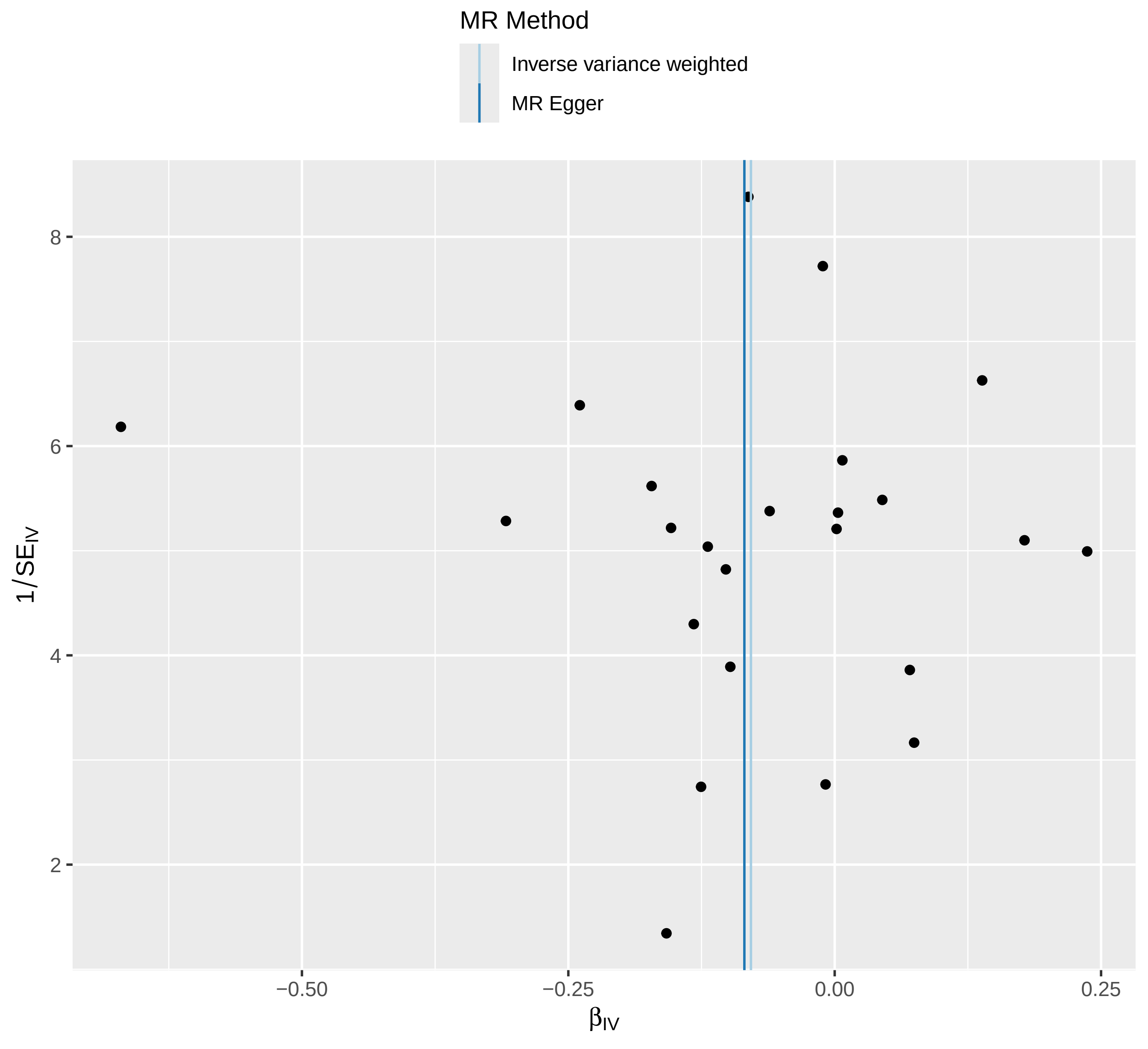
MR Method
Inverse variance weighted
MR Egger
8
6
4
2
1/SE_IV
−0.50
−0.25
0.00
0.25
β_IV

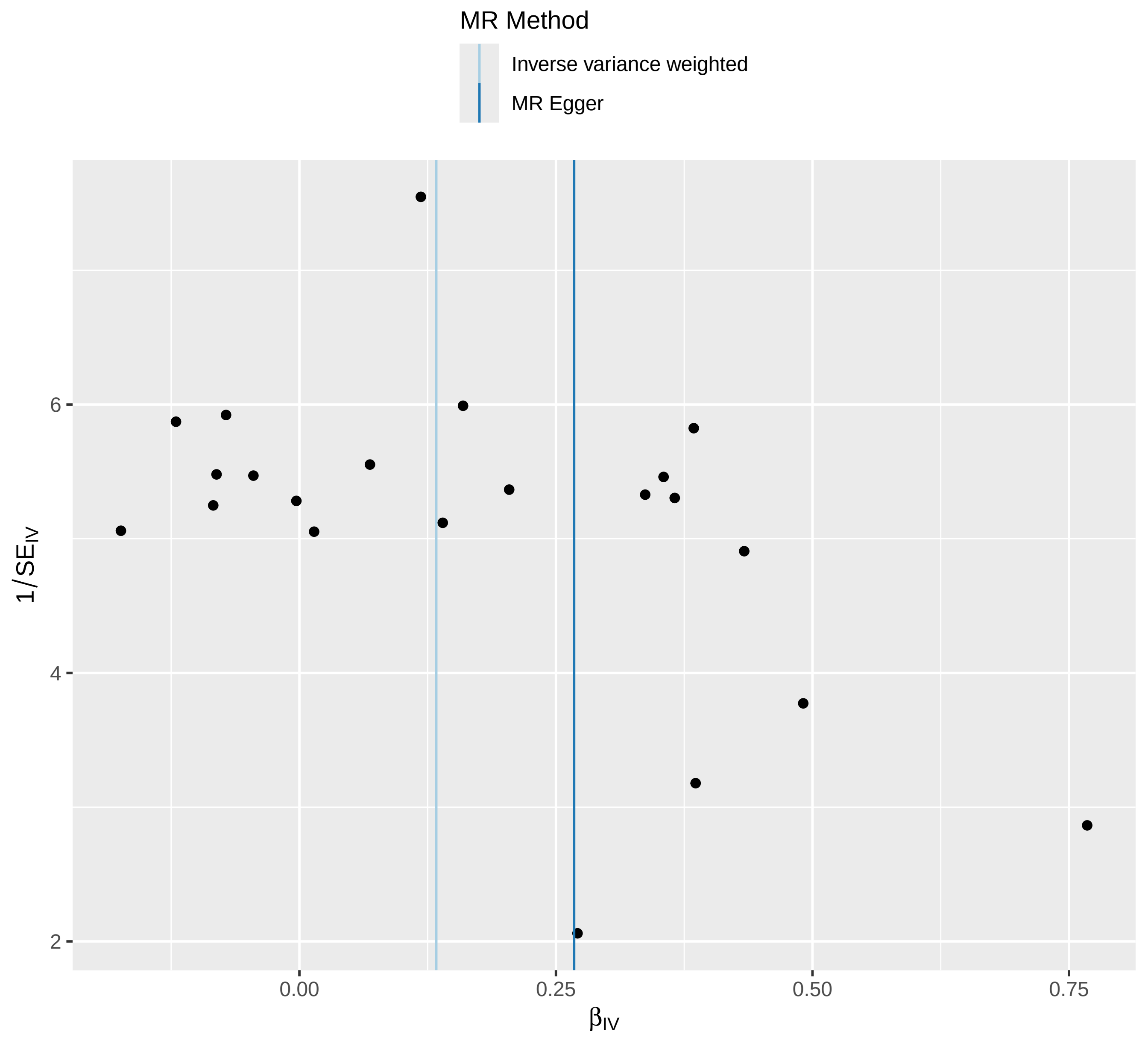

MR Method
Inverse variance weighted
MR Egger
6
4
2
0.00
0.25
0.50
0.75
$1/SE_{IV}$
β_{IV}

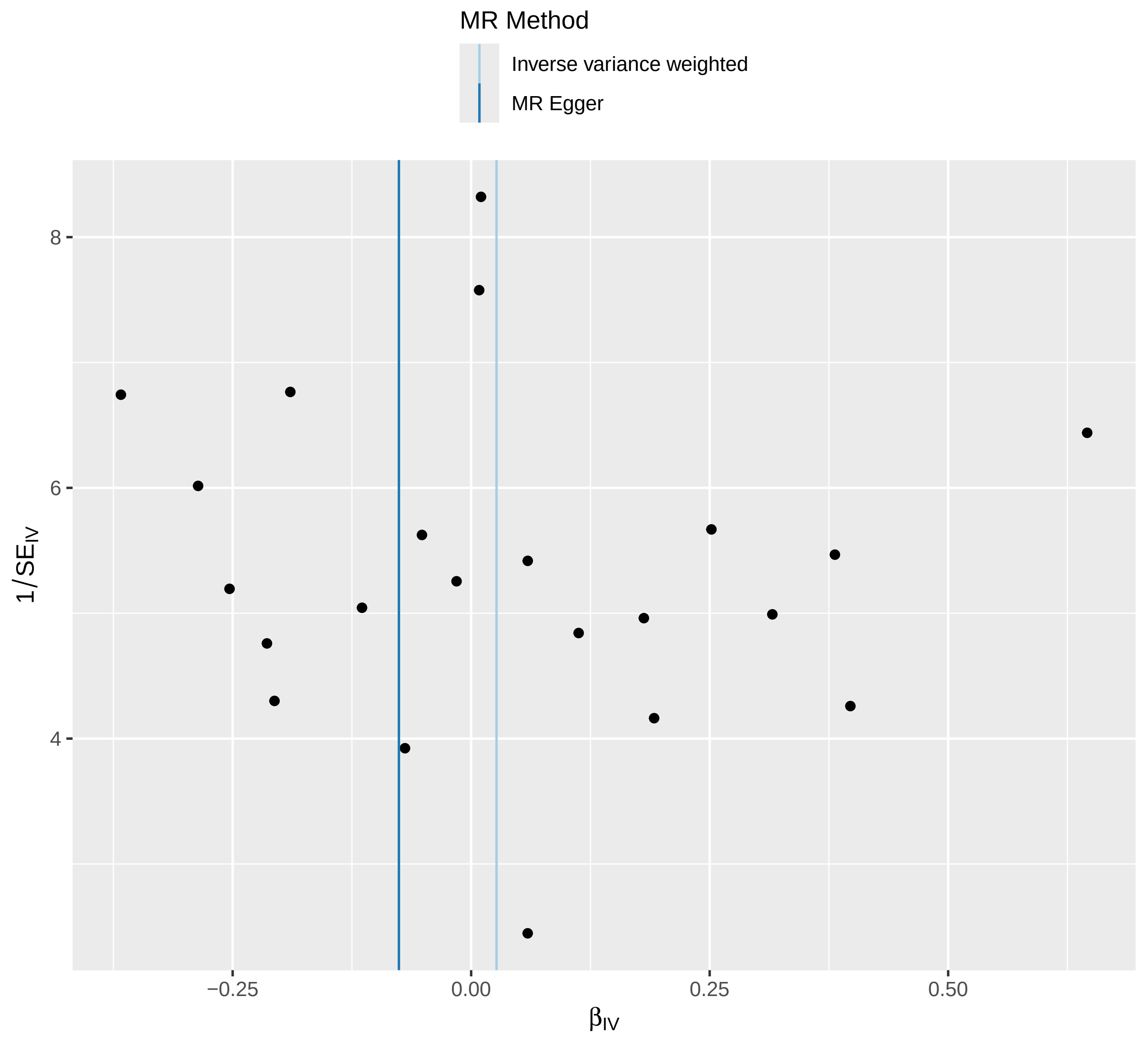
MR Method
Inverse variance weighted
MR Egger
8
6
4
1/SE_IV
−0.25
0.00
0.25
0.50
β_IV

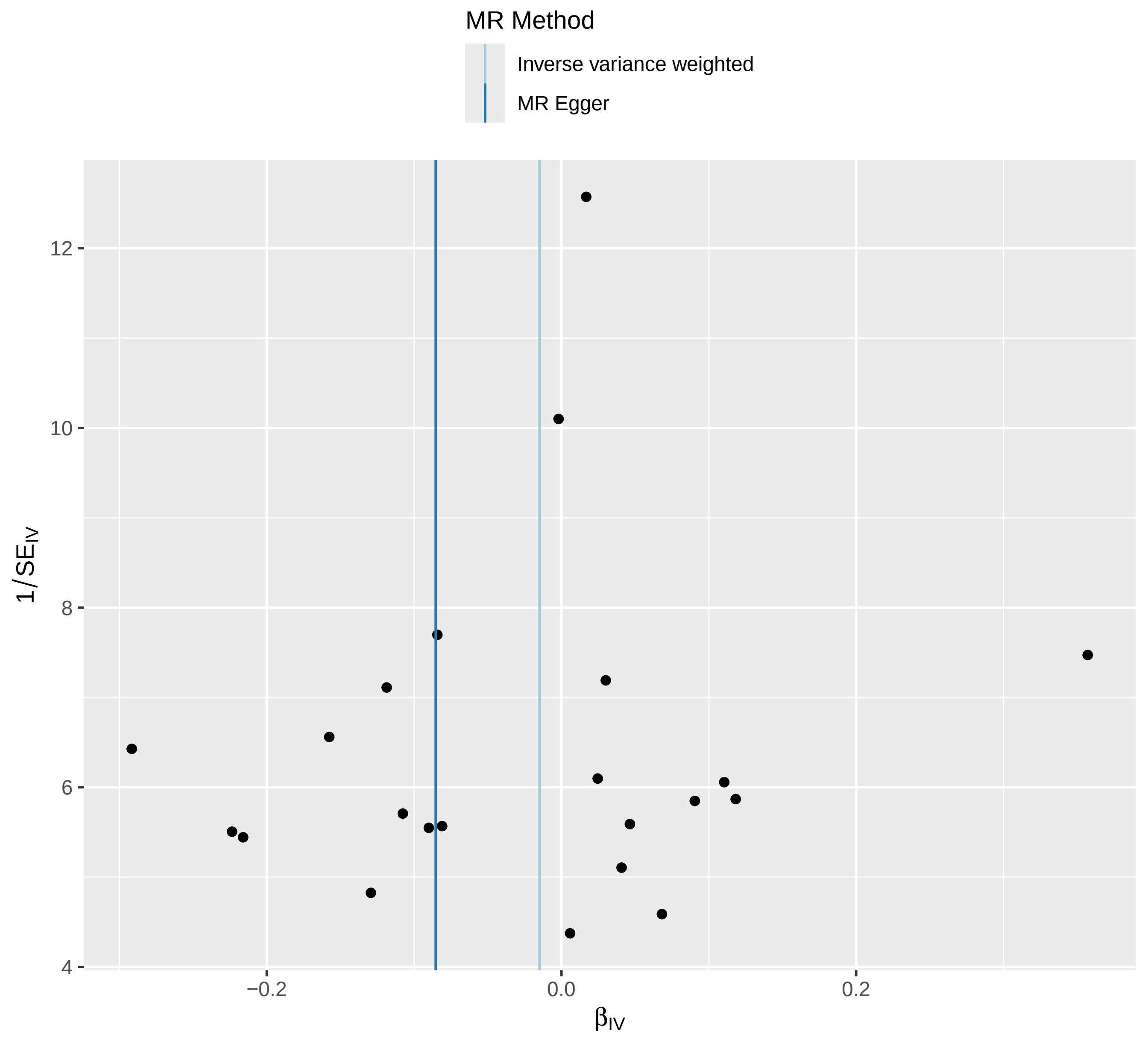

MR Method
Inverse variance weighted
MR Egger
1/SE_IV
12
10
8
6
4
−0.2
0.0
0.2
β_IV

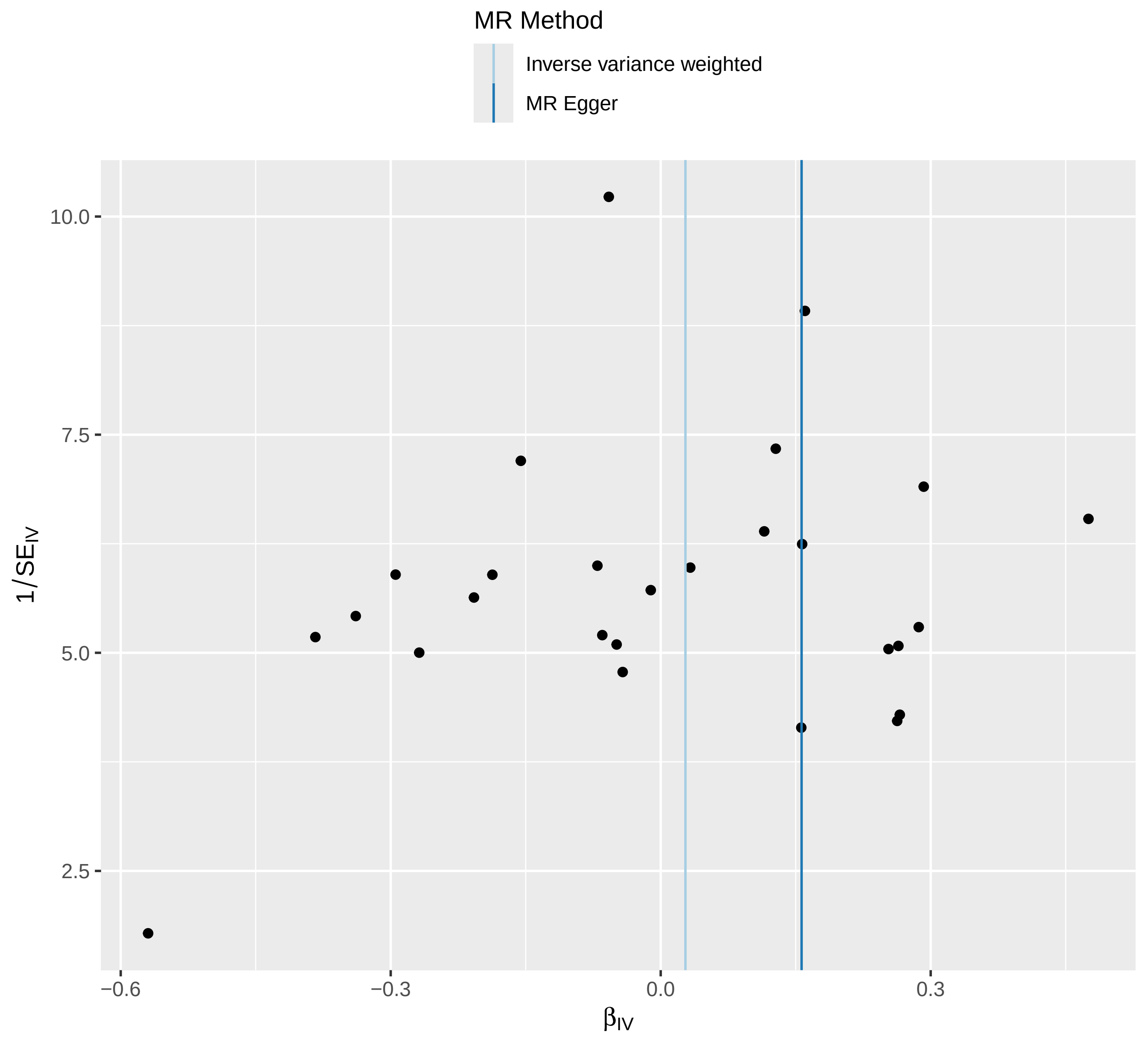
MR Method
Inverse variance weighted
MR Egger
10.0
7.5
5.0
2.5
$1/SE_{IV}$
−0.6
−0.3
0.0
0.3
β_{IV}

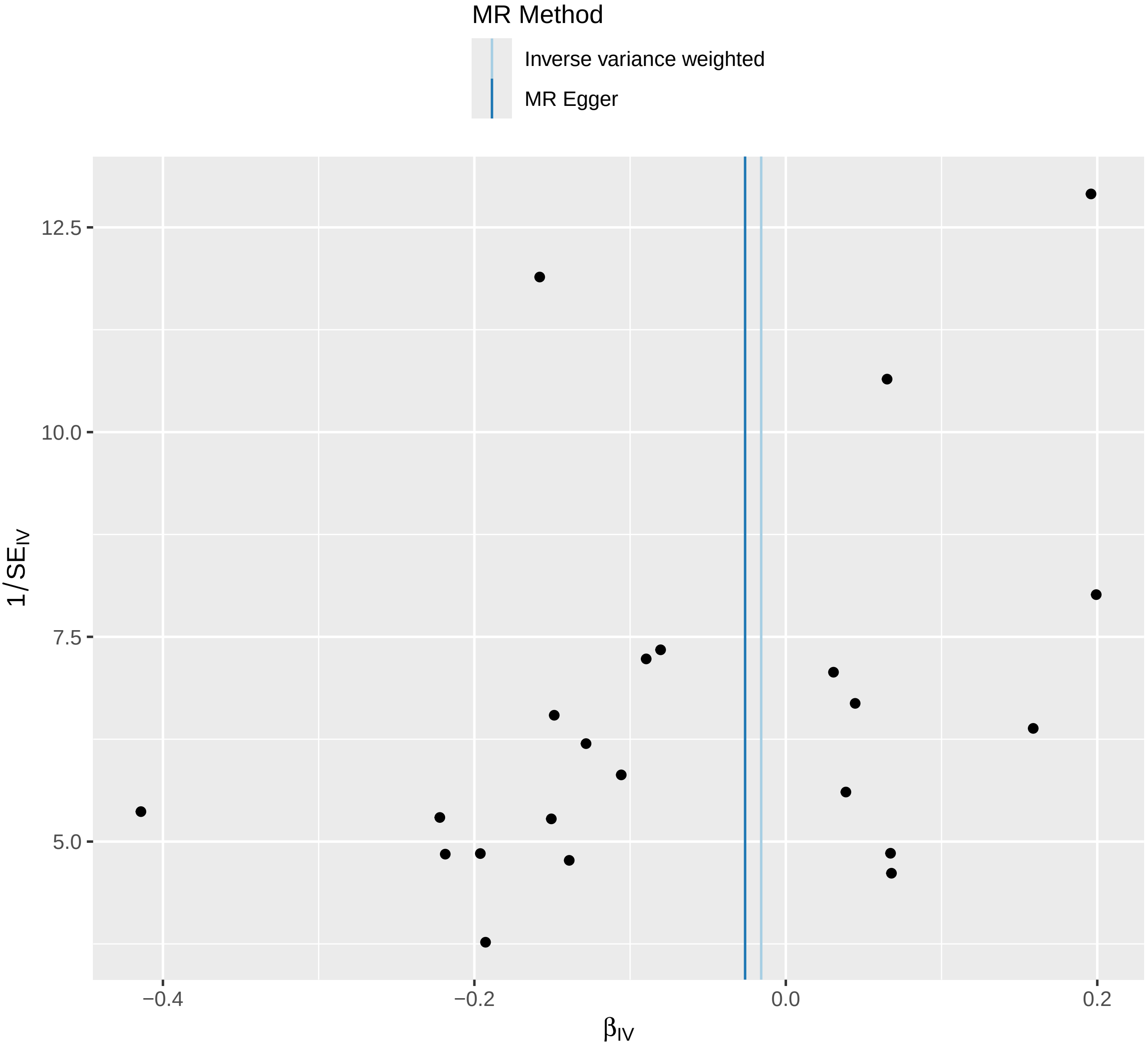
MR Method
Inverse variance weighted
MR Egger
12.5
10.0
7.5
5.0
1/SE_{IV}
−0.4
−0.2
0.0
0.2
$β_{IV}$

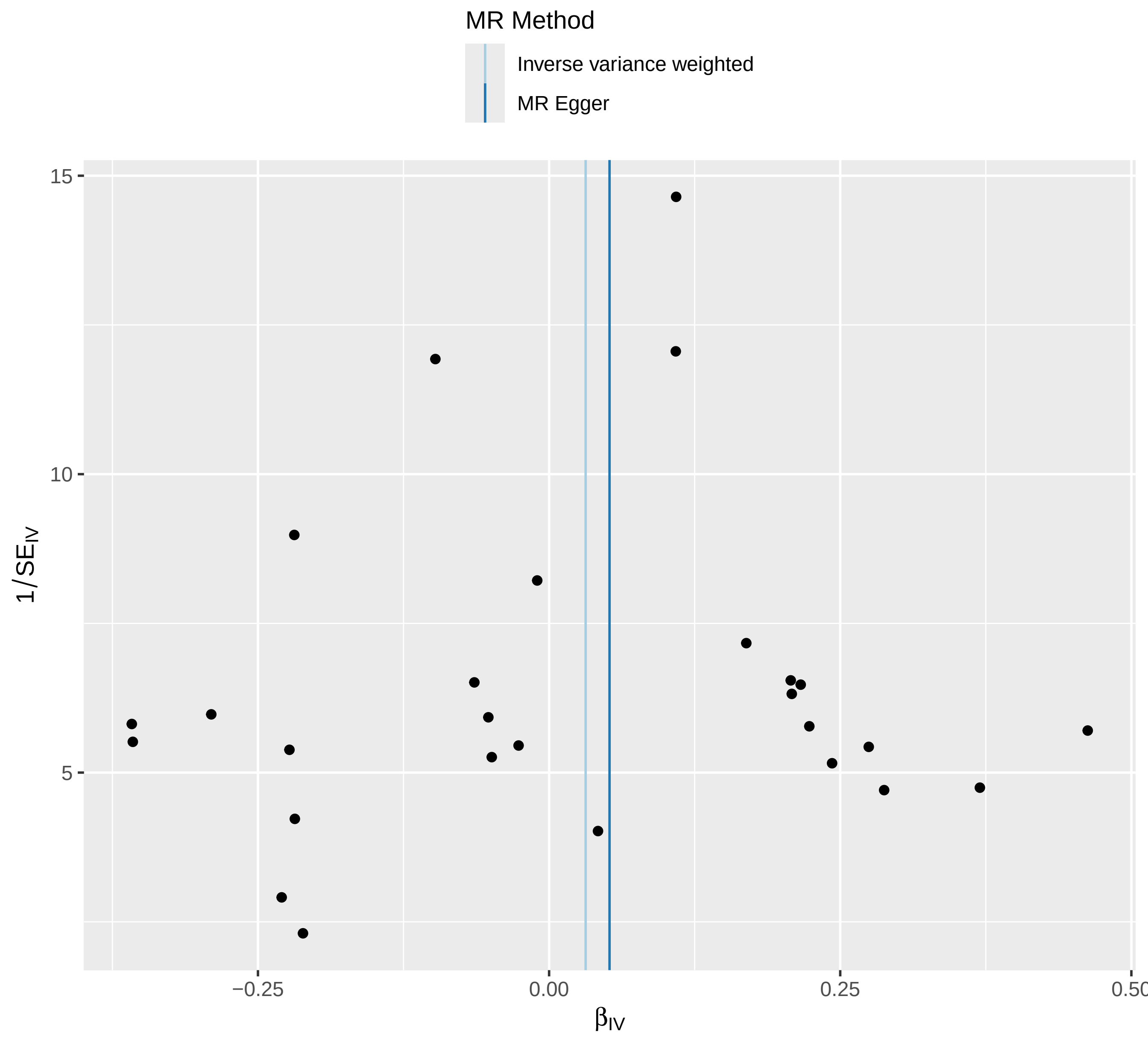
MR Method
Inverse variance weighted
MR Egger
15
10
5
1/SE$_{IV}$
−0.25
0.00
0.25
0.50
β$_{IV}$

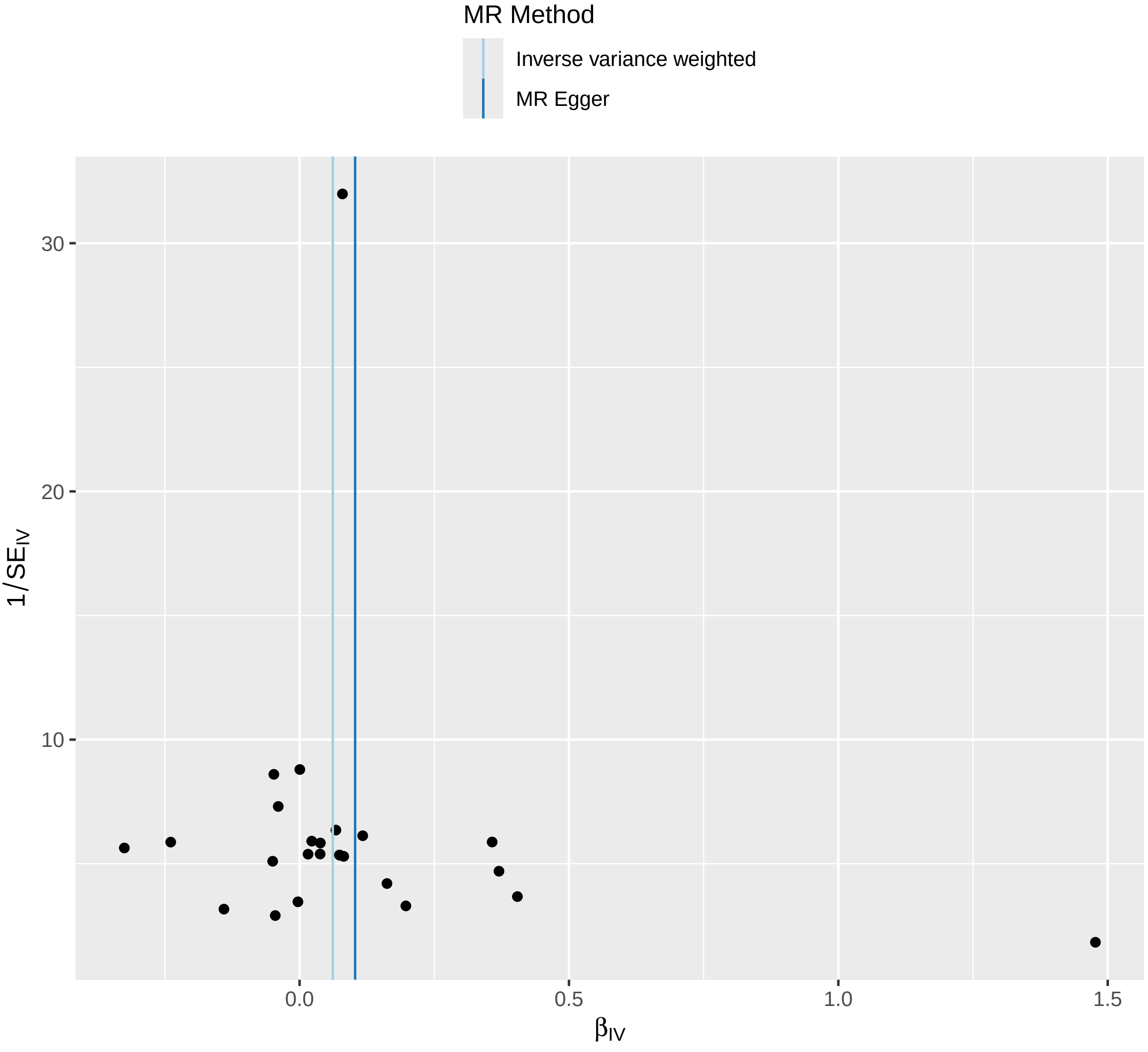
MR Method
Inverse variance weighted
MR Egger
30
20
10
1/SE_{IV}
0.0
0.5
1.0
1.5
$β_{IV}$

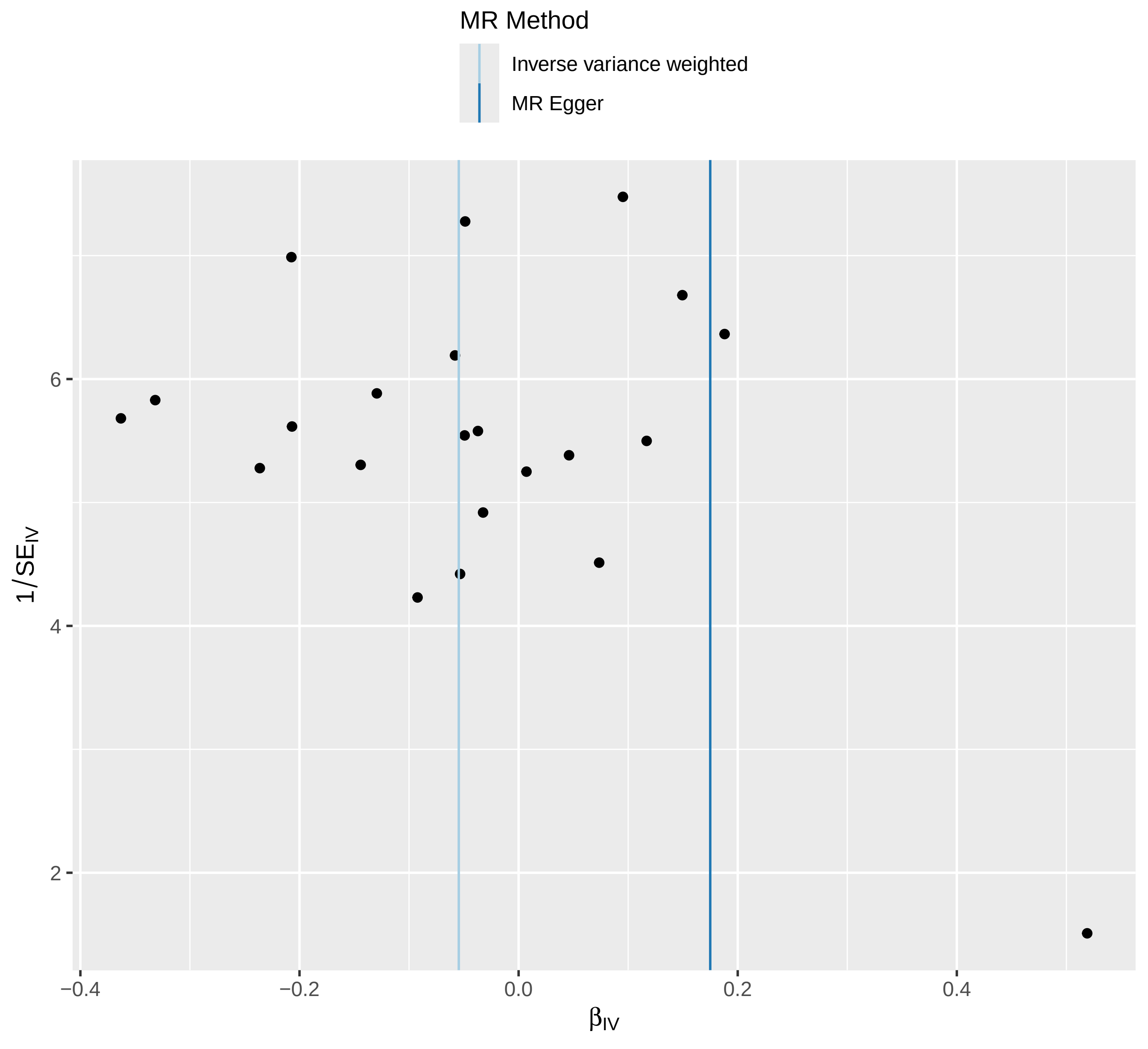

MR Method
Inverse variance weighted
MR Egger
6
4
2
1/SE_IV
−0.4
−0.2
0.0
0.2
0.4
β_IV

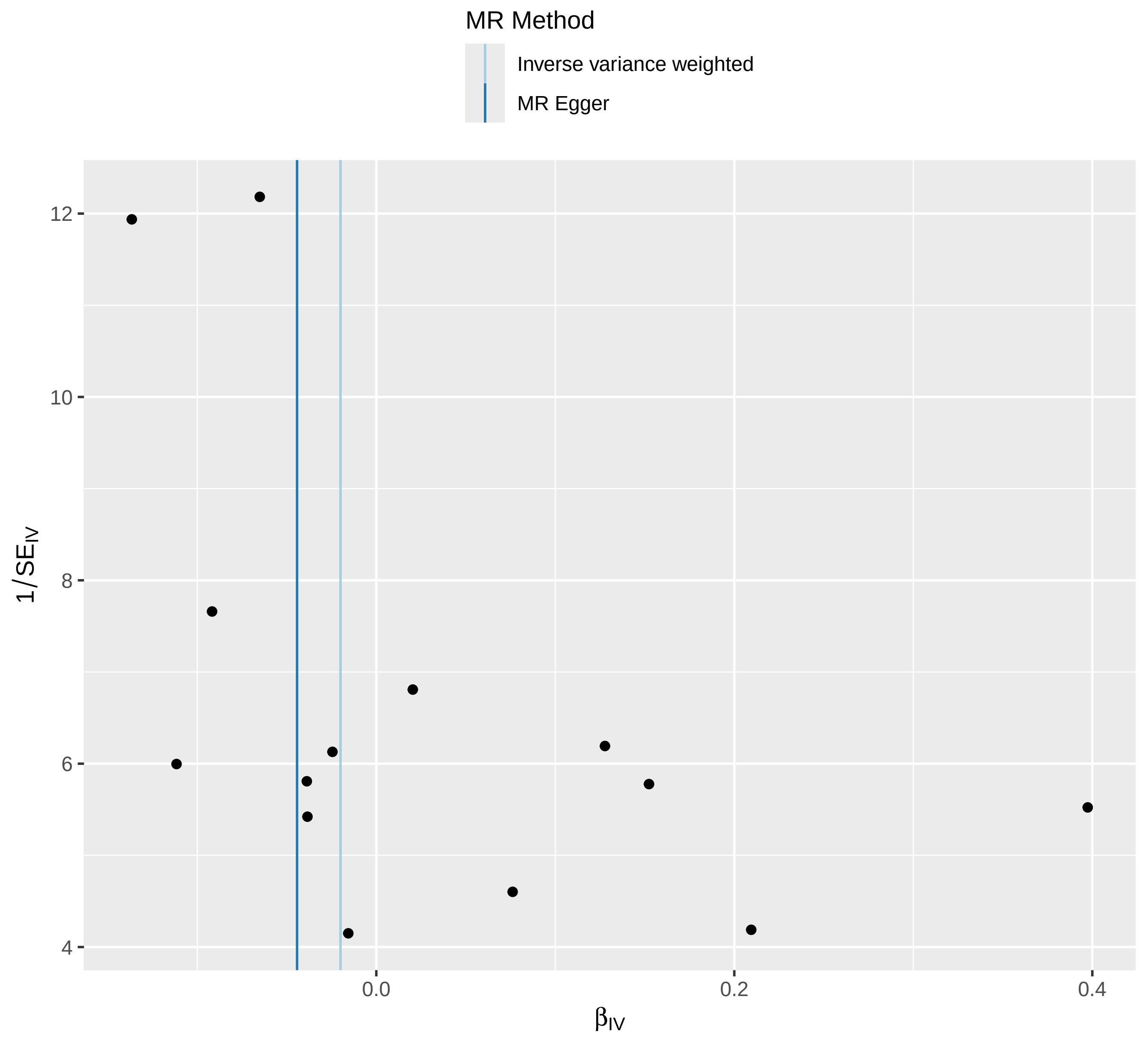
MR Method
Inverse variance weighted
MR Egger
1/SE_IV
12
10
8
6
4
β_IV
0.0
0.2
0.4

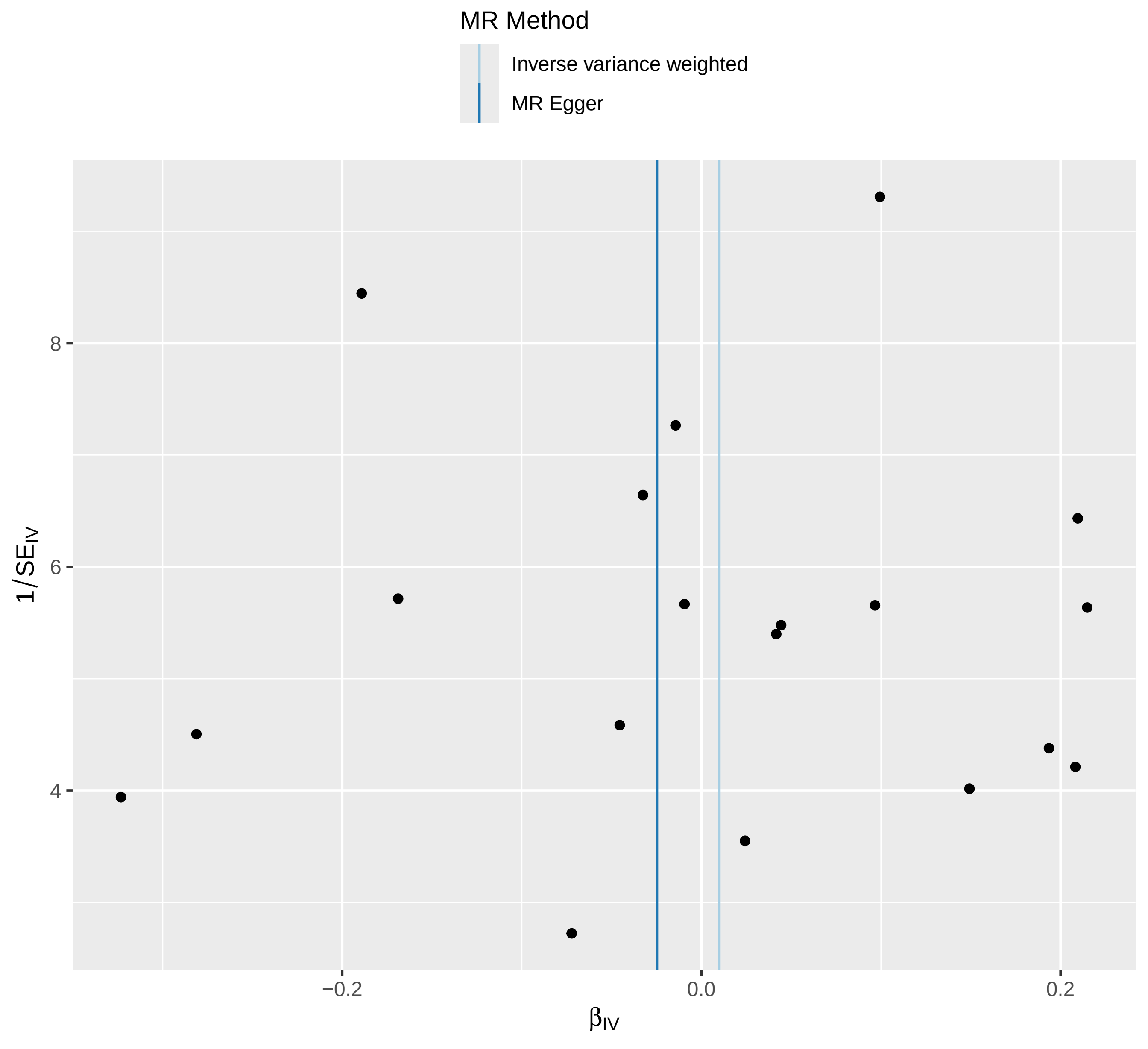

MR Method
Inverse variance weighted
MR Egger
8
6
4
$1/SE_{IV}$
−0.2
0.0
0.2
β_{IV}

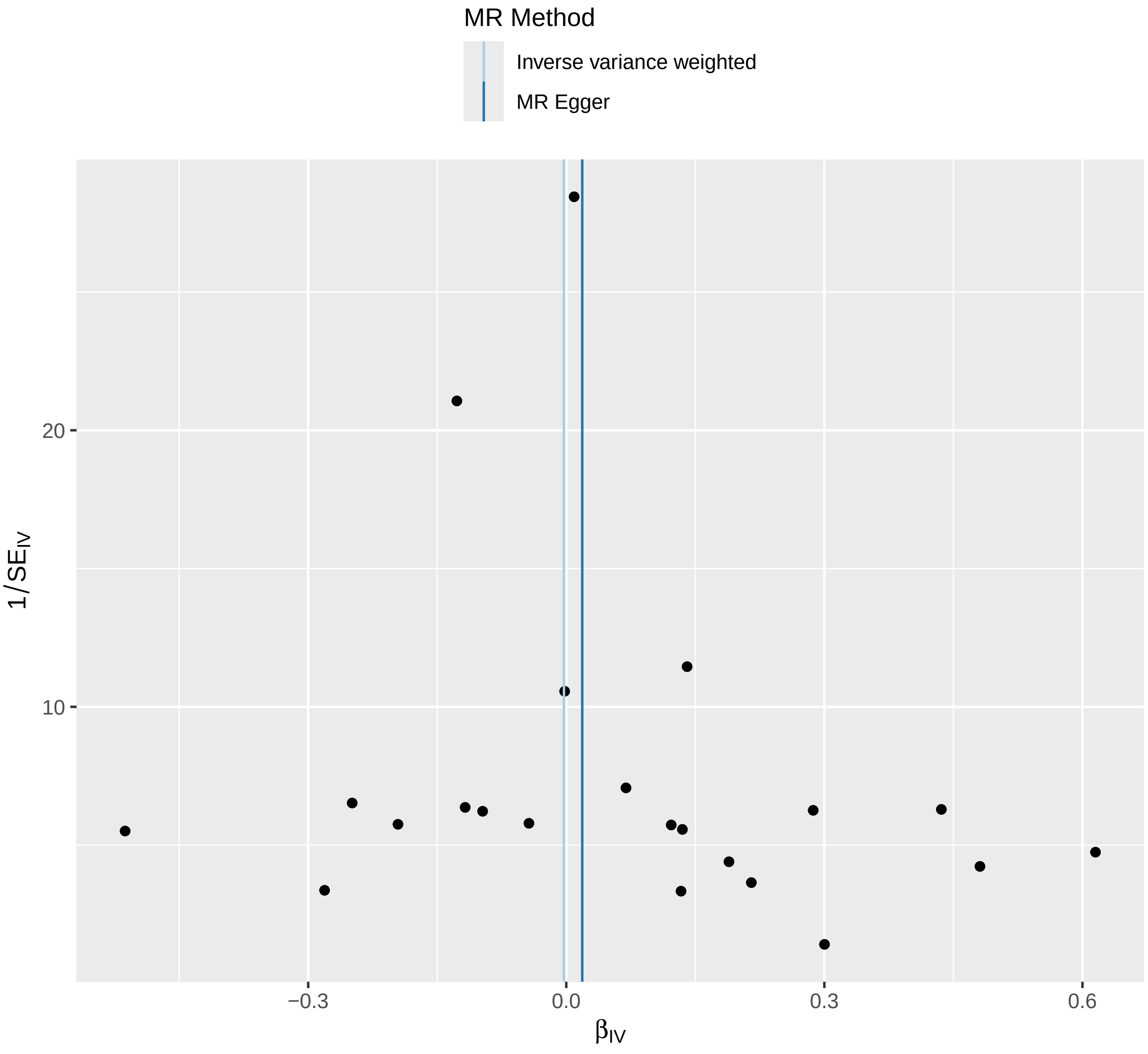

MR Method
Inverse variance weighted
MR Egger
20
10
1/SE$_{IV}$
−0.3
0.0
0.3
0.6
β$_{IV}$

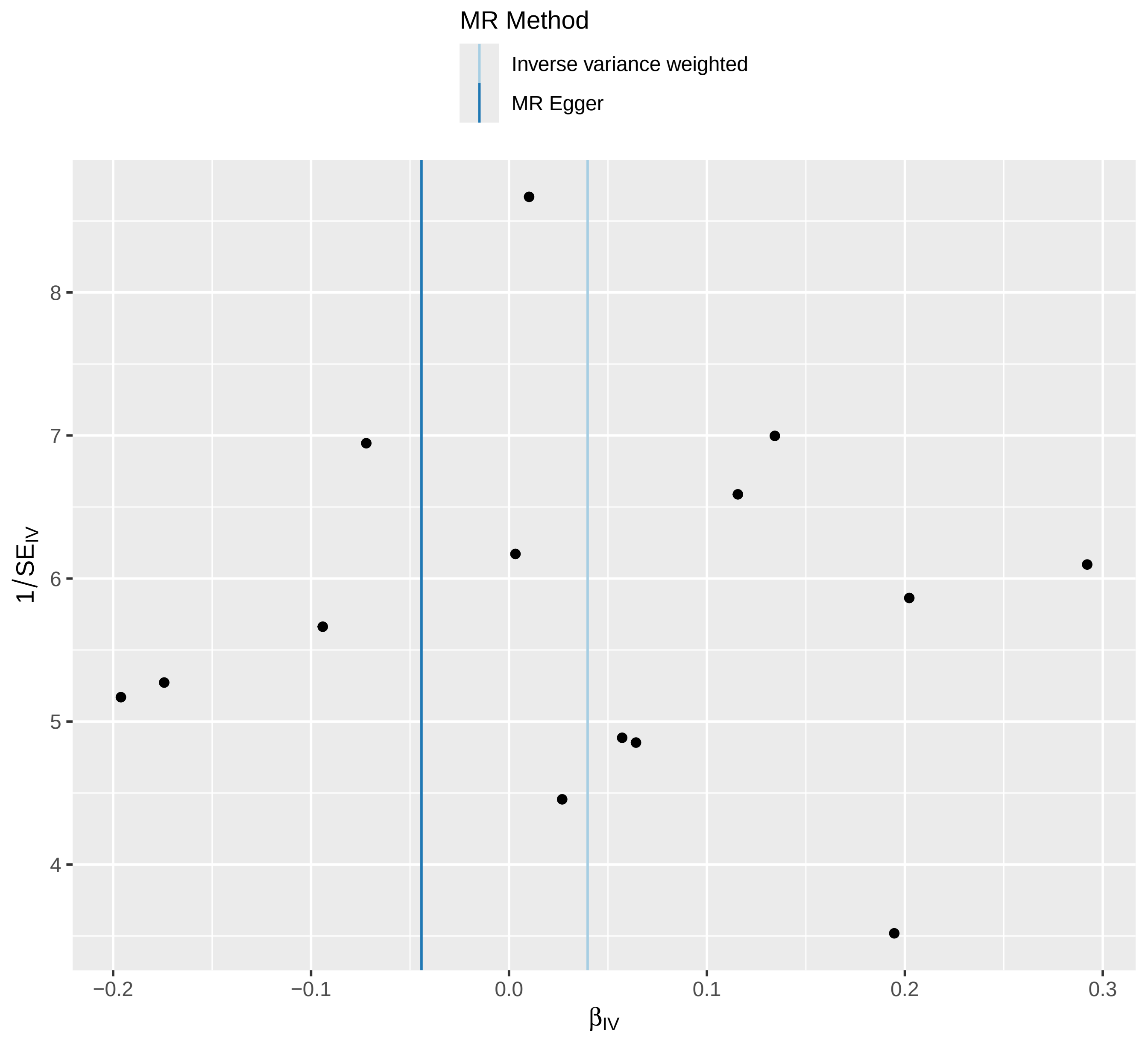
MR Method
Inverse variance weighted
MR Egger
1/SE_IV
8
7
6
5
4
−0.2
−0.1
0.0
0.1
0.2
0.3
β_IV

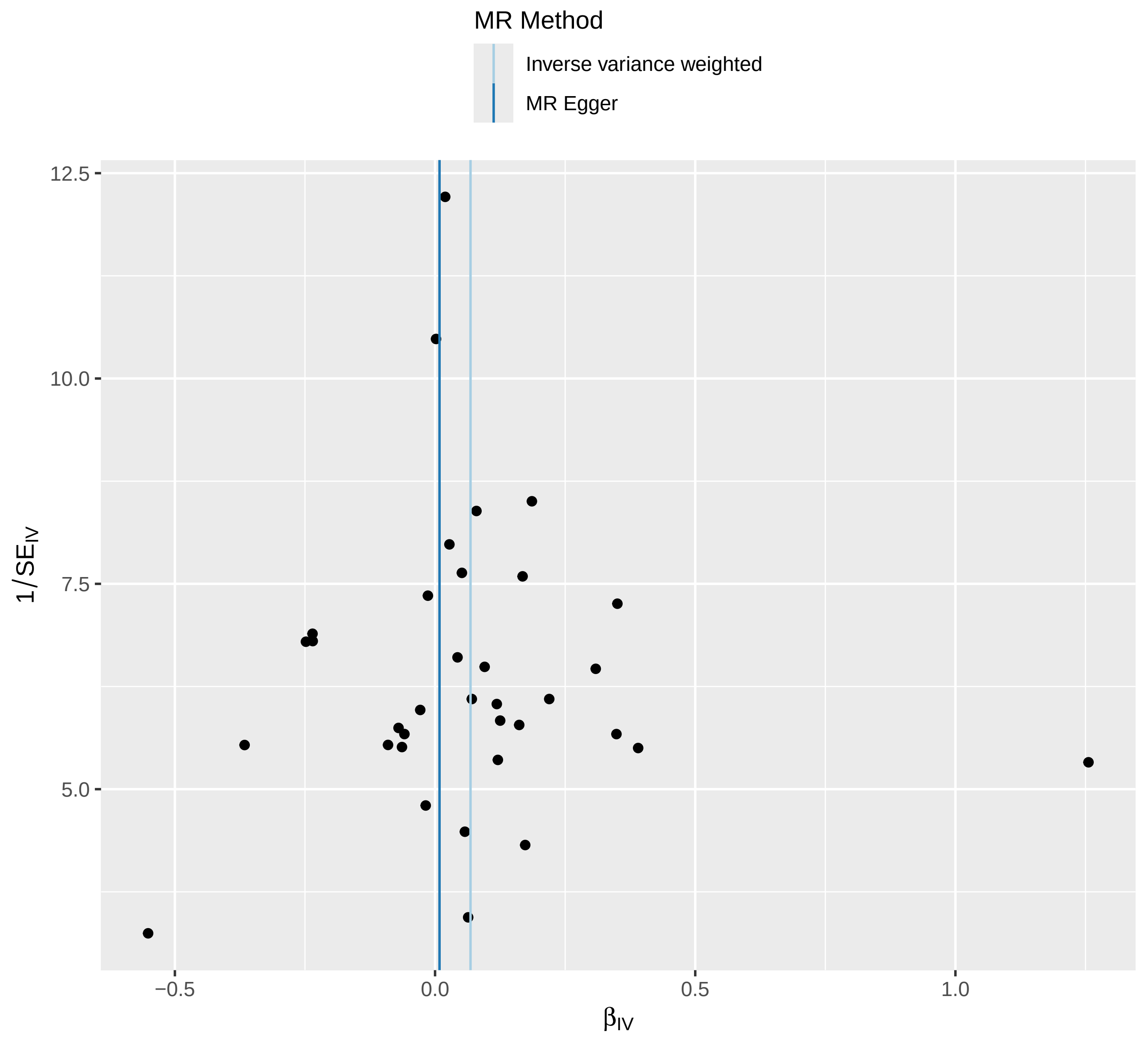
MR Method
Inverse variance weighted
MR Egger
12.5
10.0
7.5
5.0
1/SE$_{IV}$
−0.5
0.0
0.5
1.0
β$_{IV}$

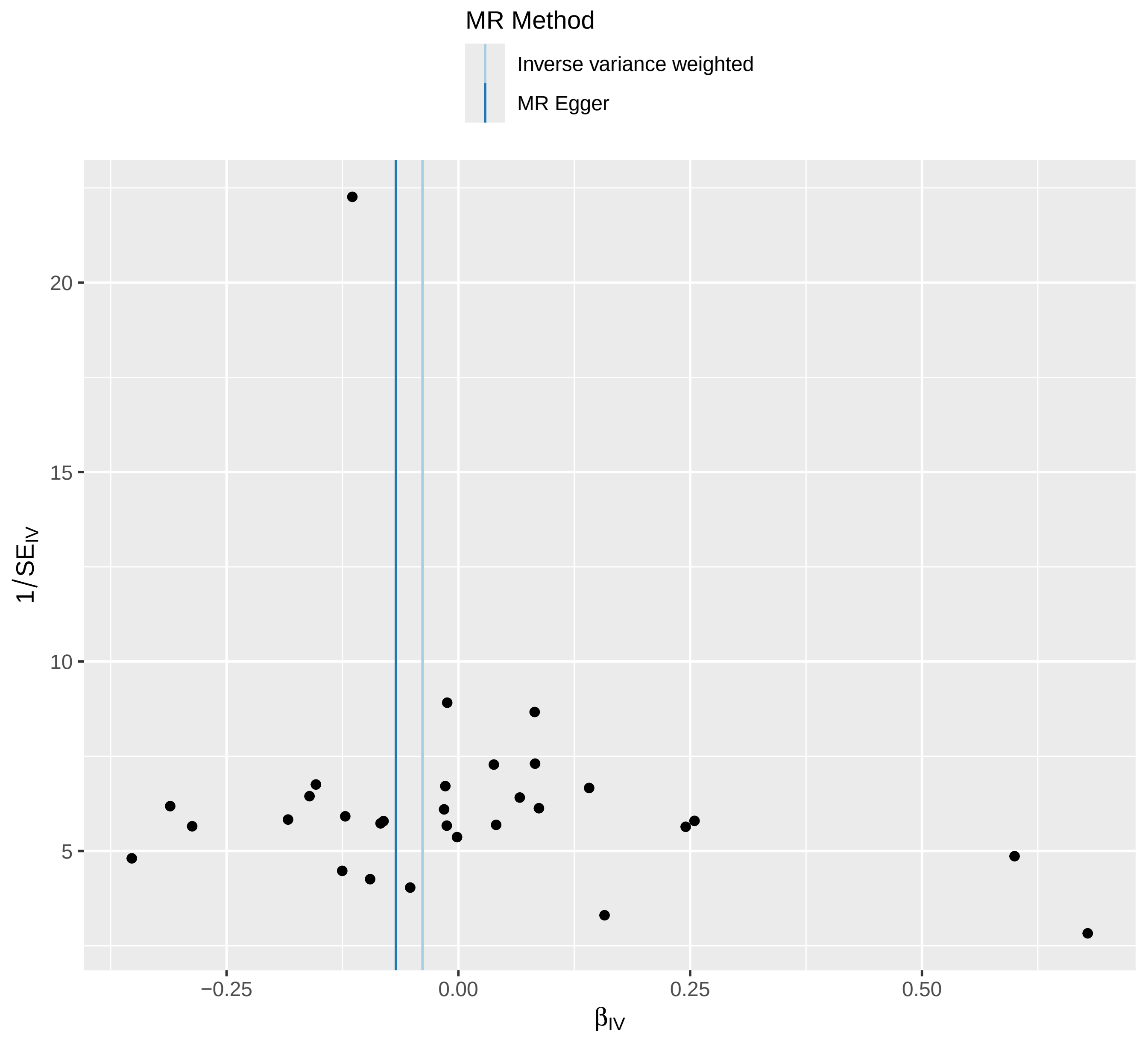
MR Method
Inverse variance weighted
MR Egger
1/SE_IV
β_IV
20
15
10
5
−0.25
0.00
0.25
0.50

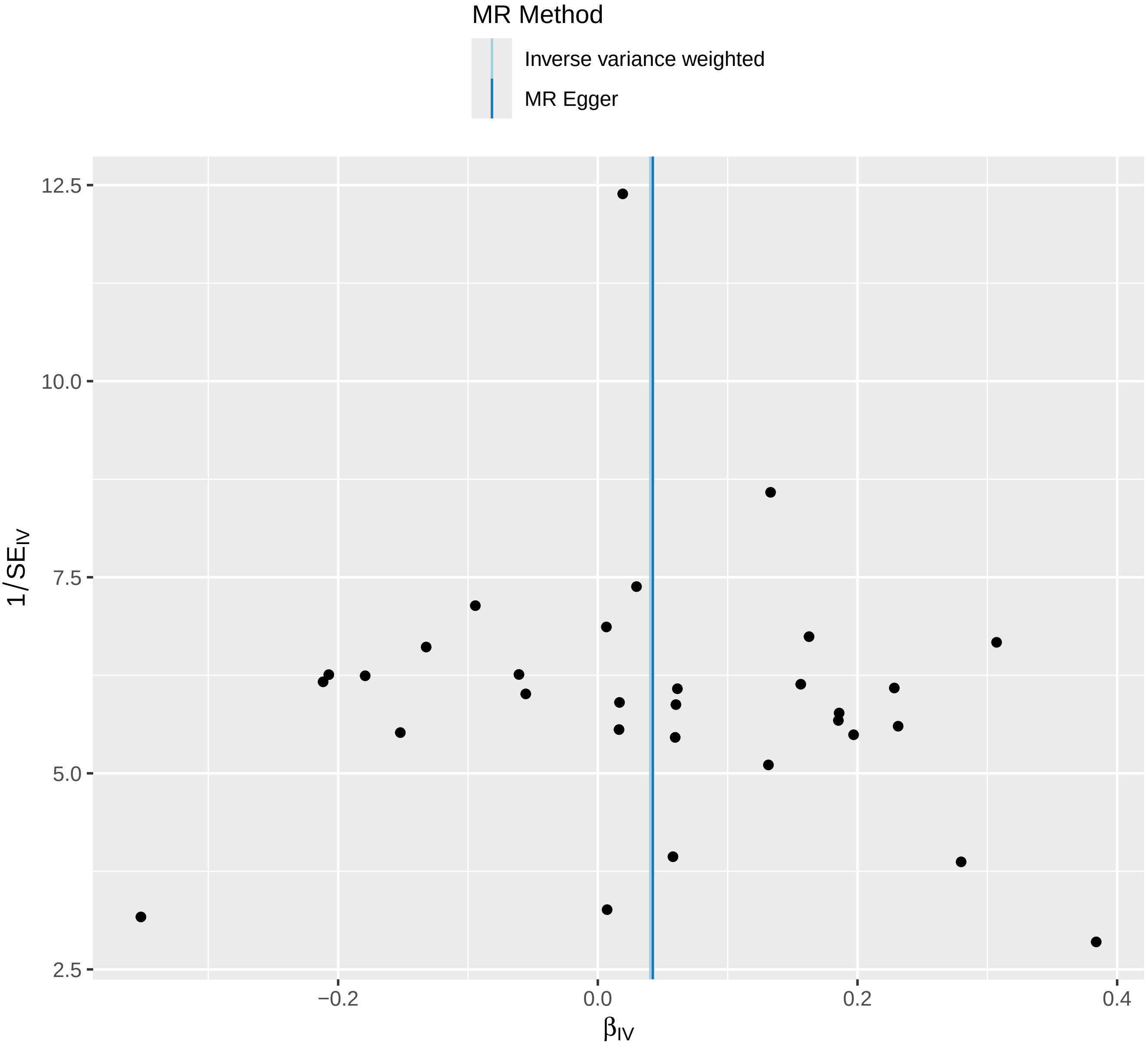
MR Method
Inverse variance weighted
MR Egger
12.5
10.0
7.5
5.0
2.5
1/SE_IV
−0.2
0.0
0.2
0.4
β_IV

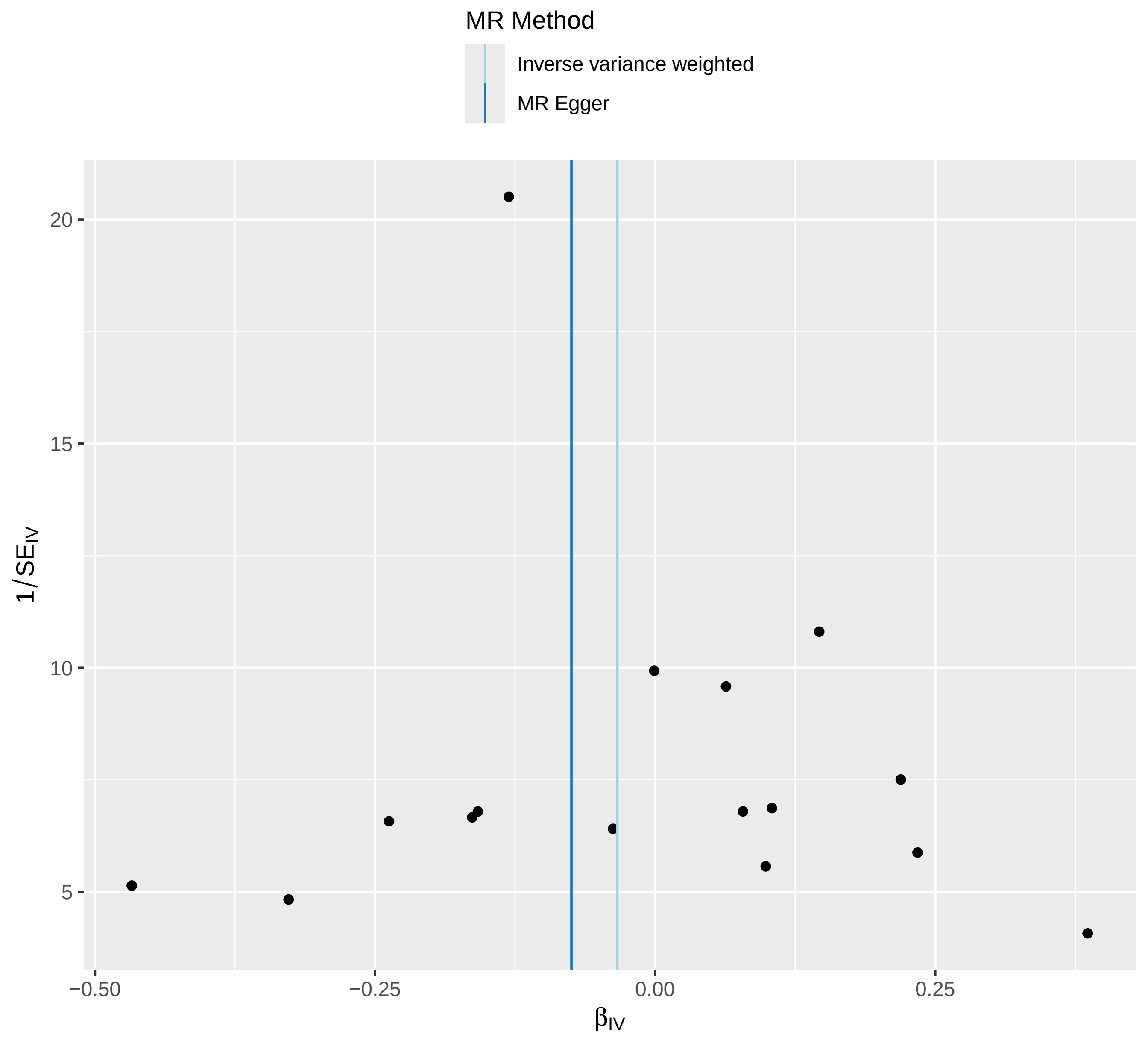

MR Method
Inverse variance weighted
MR Egger
20
15
10
5
1/SE_IV
−0.50
−0.25
0.00
0.25
β_IV

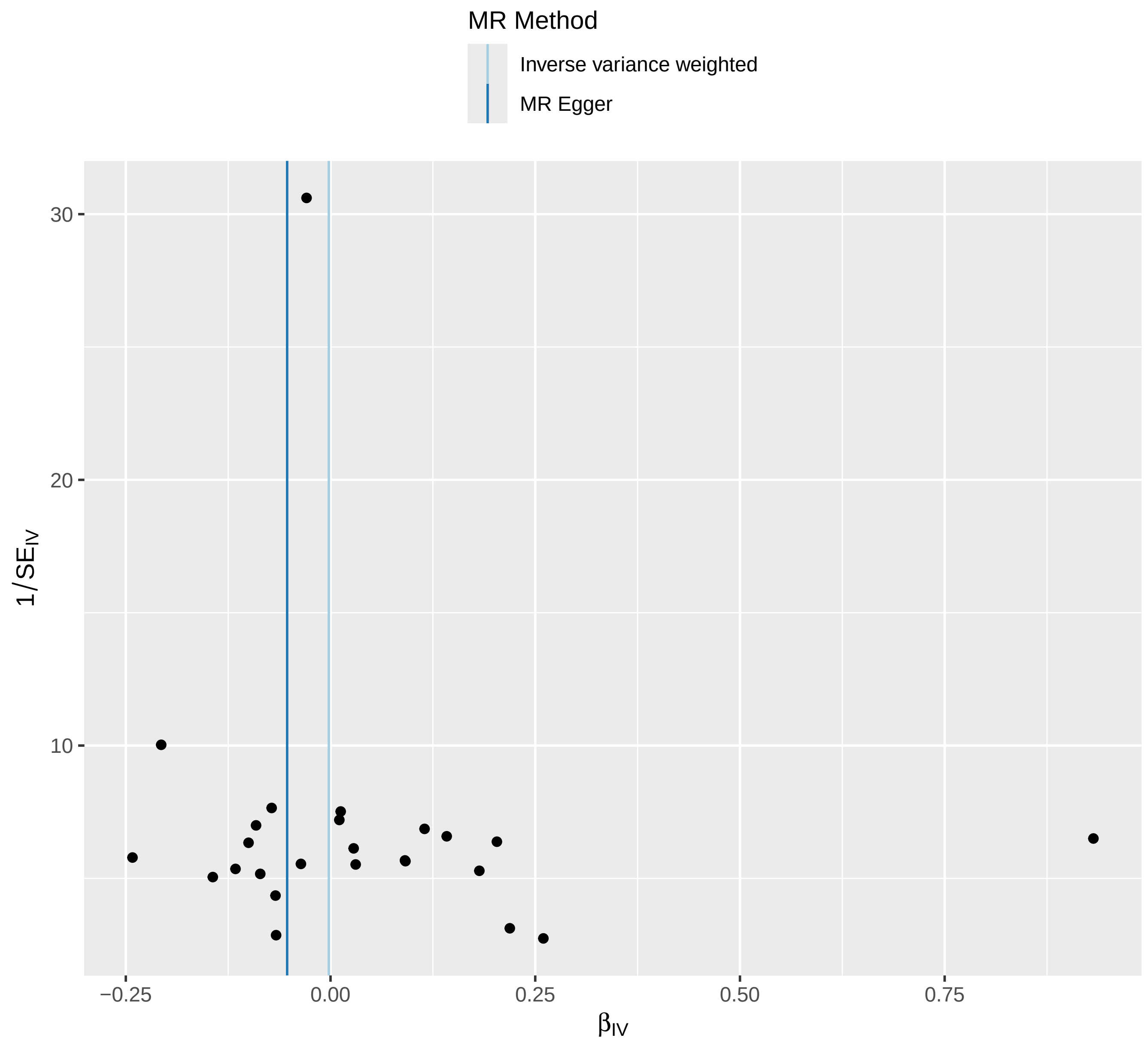
MR Method
Inverse variance weighted
MR Egger
30
20
10
1/SE_IV
−0.25
0.00
0.25
0.50
0.75
β_IV

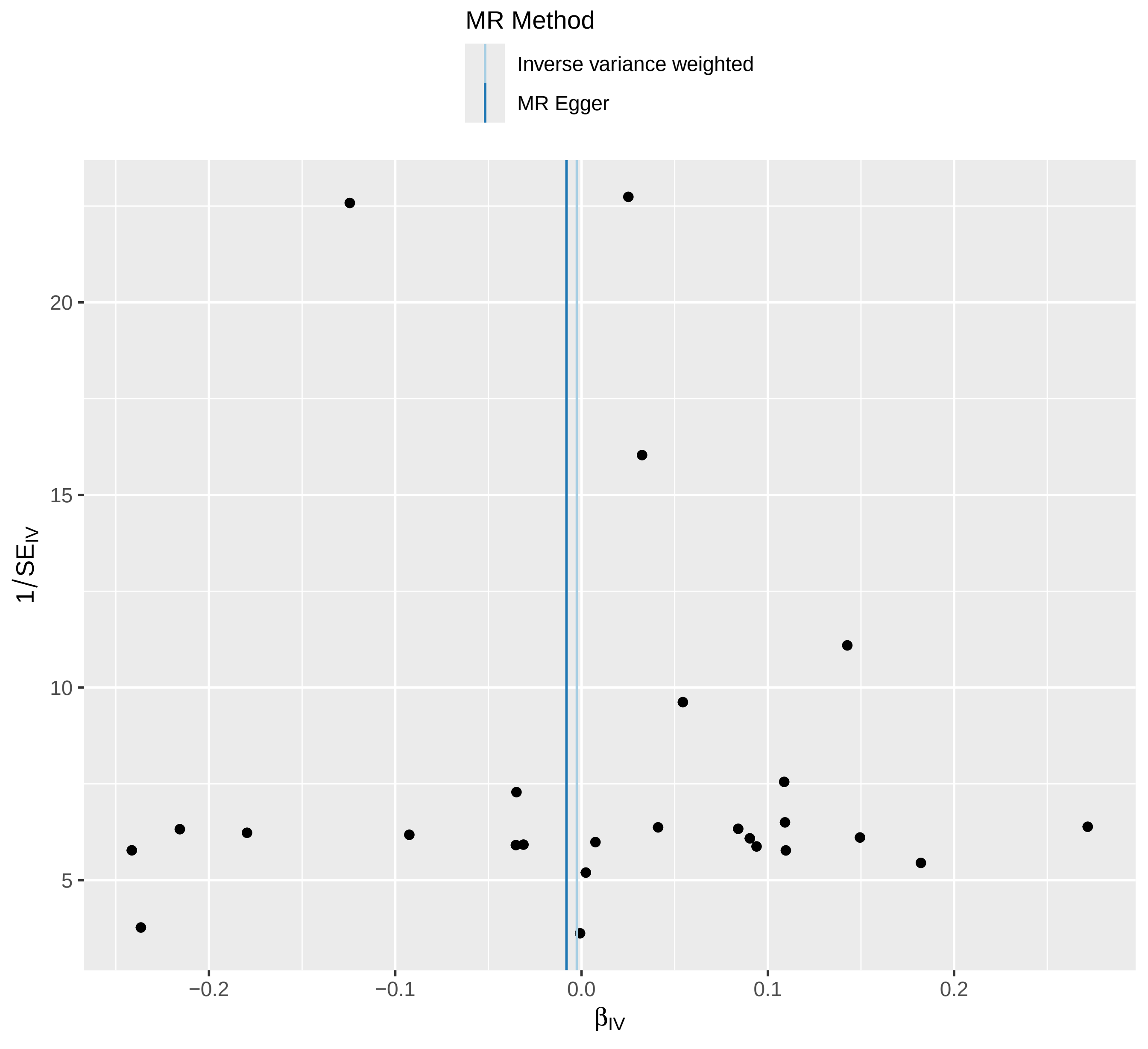

MR Method
Inverse variance weighted
MR Egger
20
15
10
5
1/SE$_{IV}$
−0.2
−0.1
0.0
0.1
0.2
β$_{IV}$

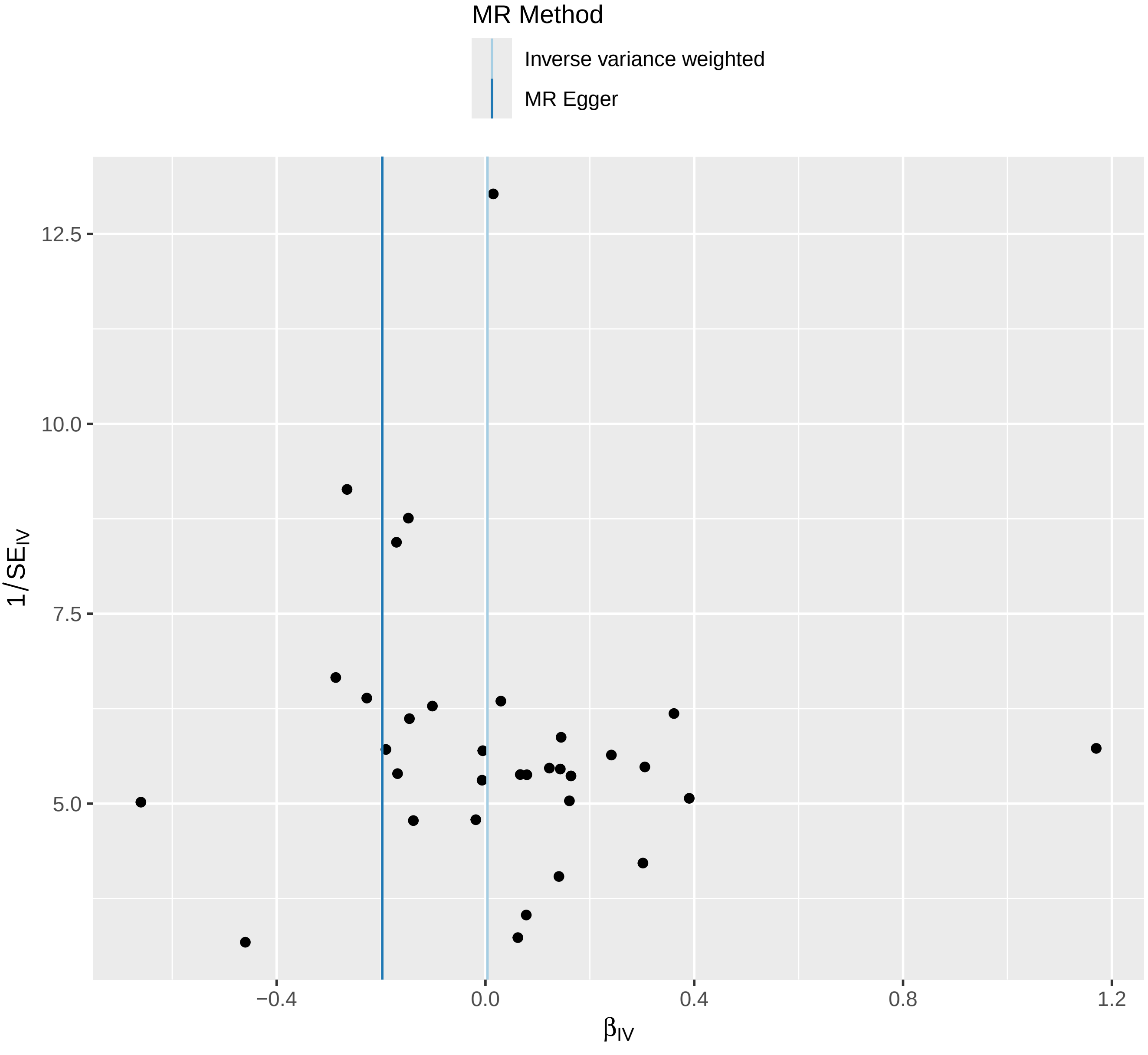

MR Method
Inverse variance weighted
MR Egger
12.5
10.0
7.5
5.0
1/SE_IV
−0.4
0.0
0.4
0.8
1.2
β_IV

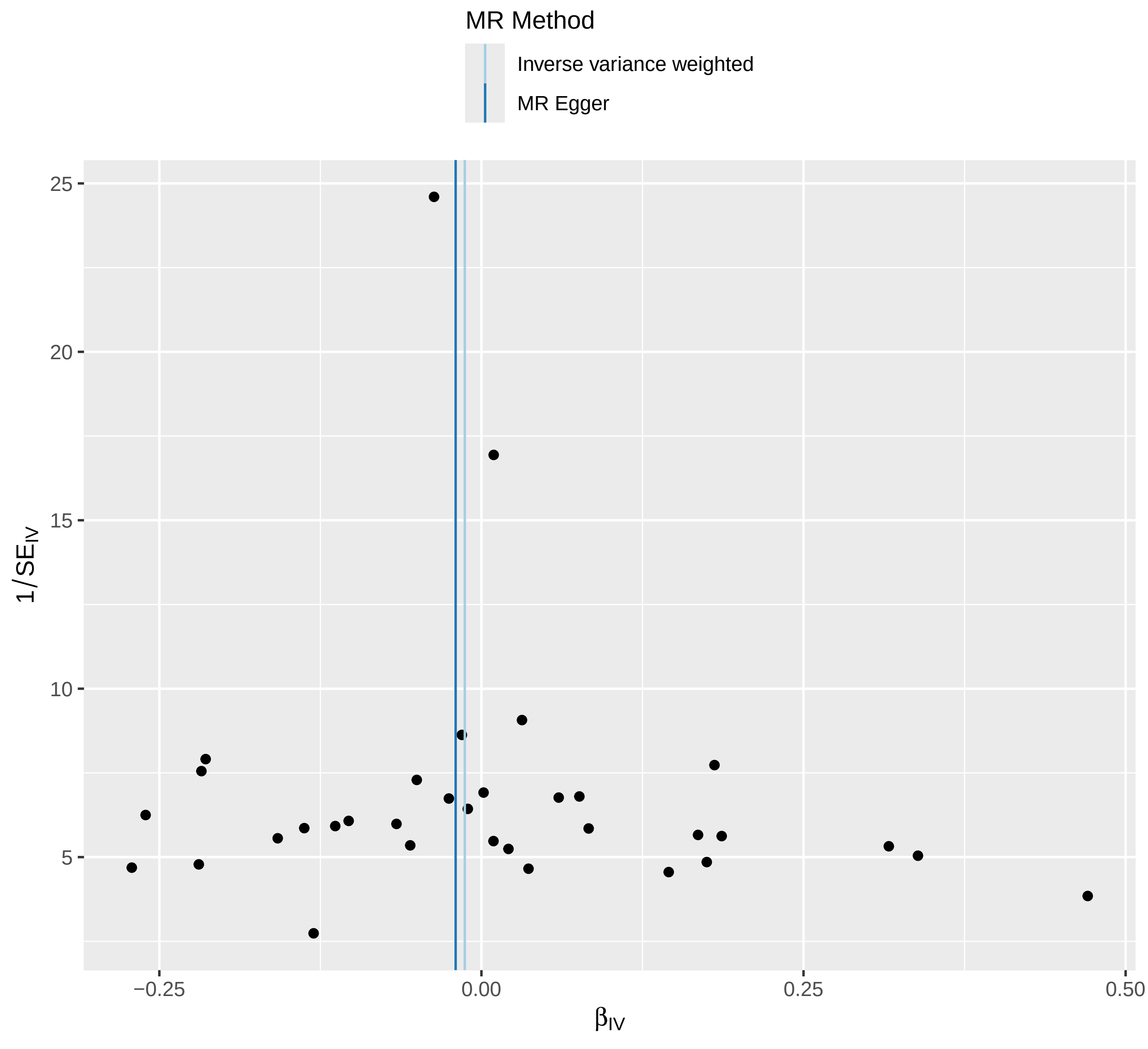

MR Method
Inverse variance weighted
MR Egger
25
20
15
10
5
1/SE$_{IV}$
−0.25
0.00
0.25
0.50
β$_{IV}$

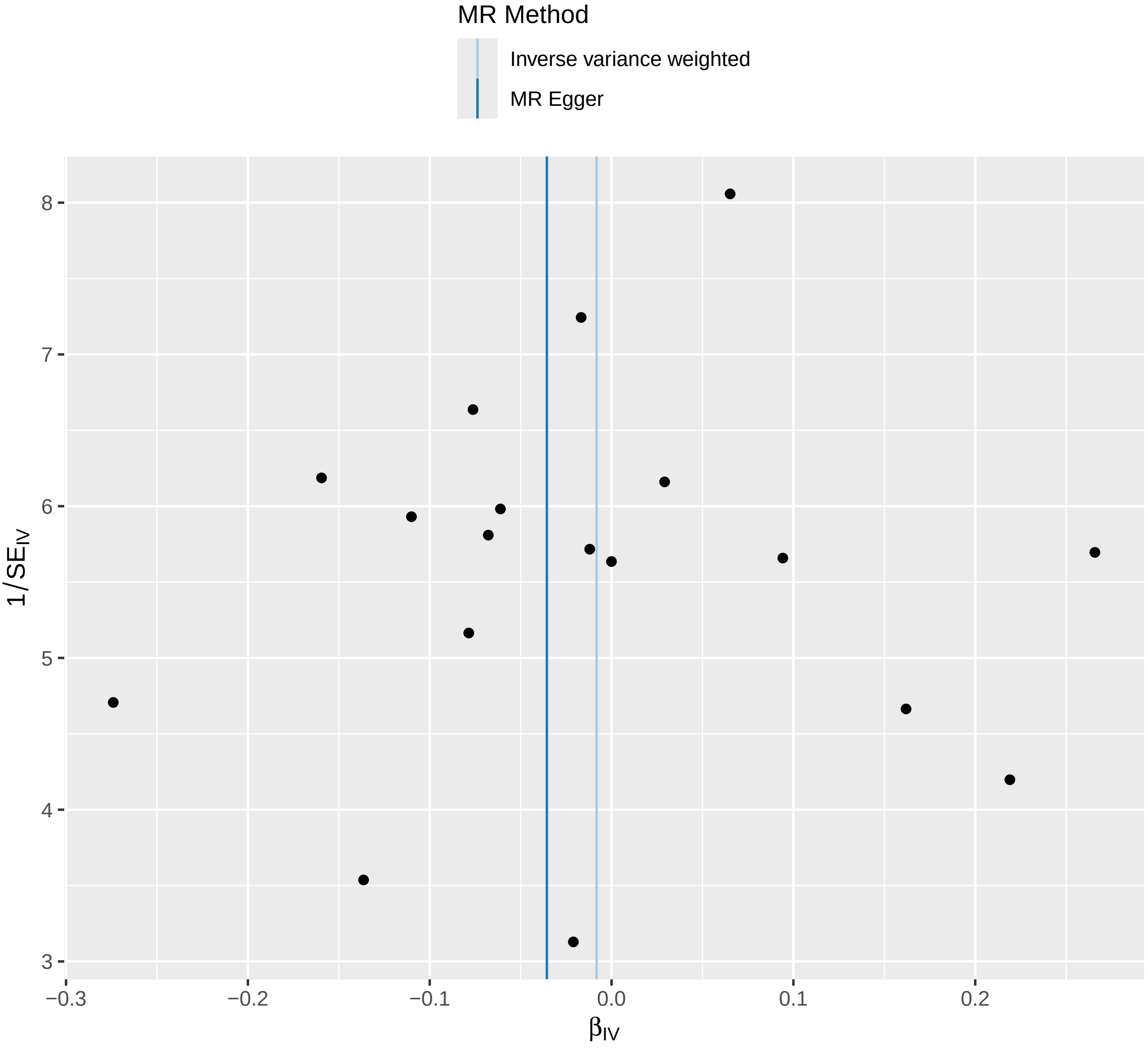
MR Method
Inverse variance weighted
MR Egger
1/SE_IV
β_IV
8
7
6
5
4
3
−0.3
−0.2
−0.1
0.0
0.1
0.2

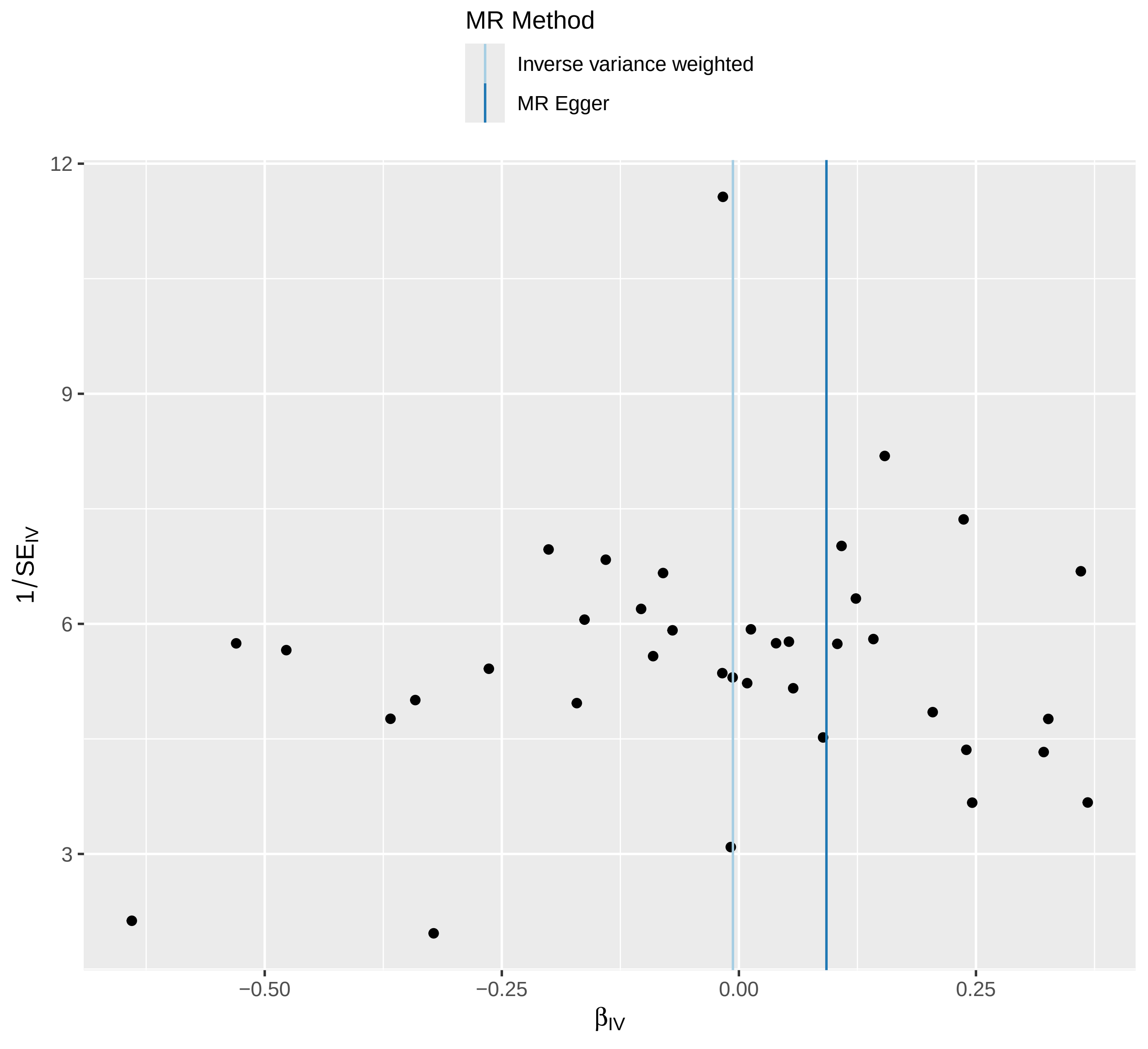
MR Method
Inverse variance weighted
MR Egger
12
9
6
3
1/SE_IV
−0.50
−0.25
0.00
0.25
β_IV

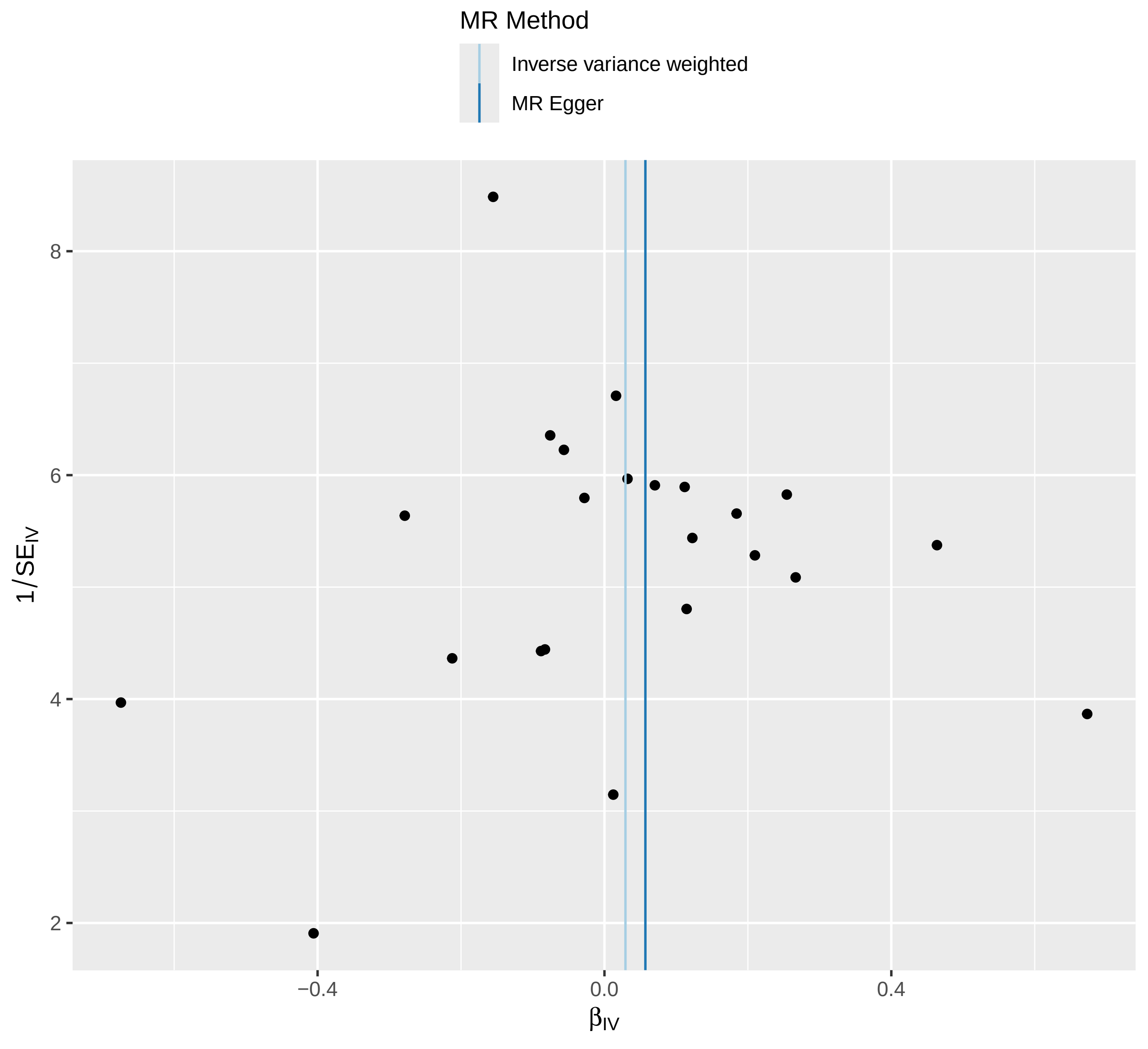
MR Method
Inverse variance weighted
MR Egger
1/SE$_{IV}$
8
6
4
2
−0.4
0.0
0.4
β$_{IV}$

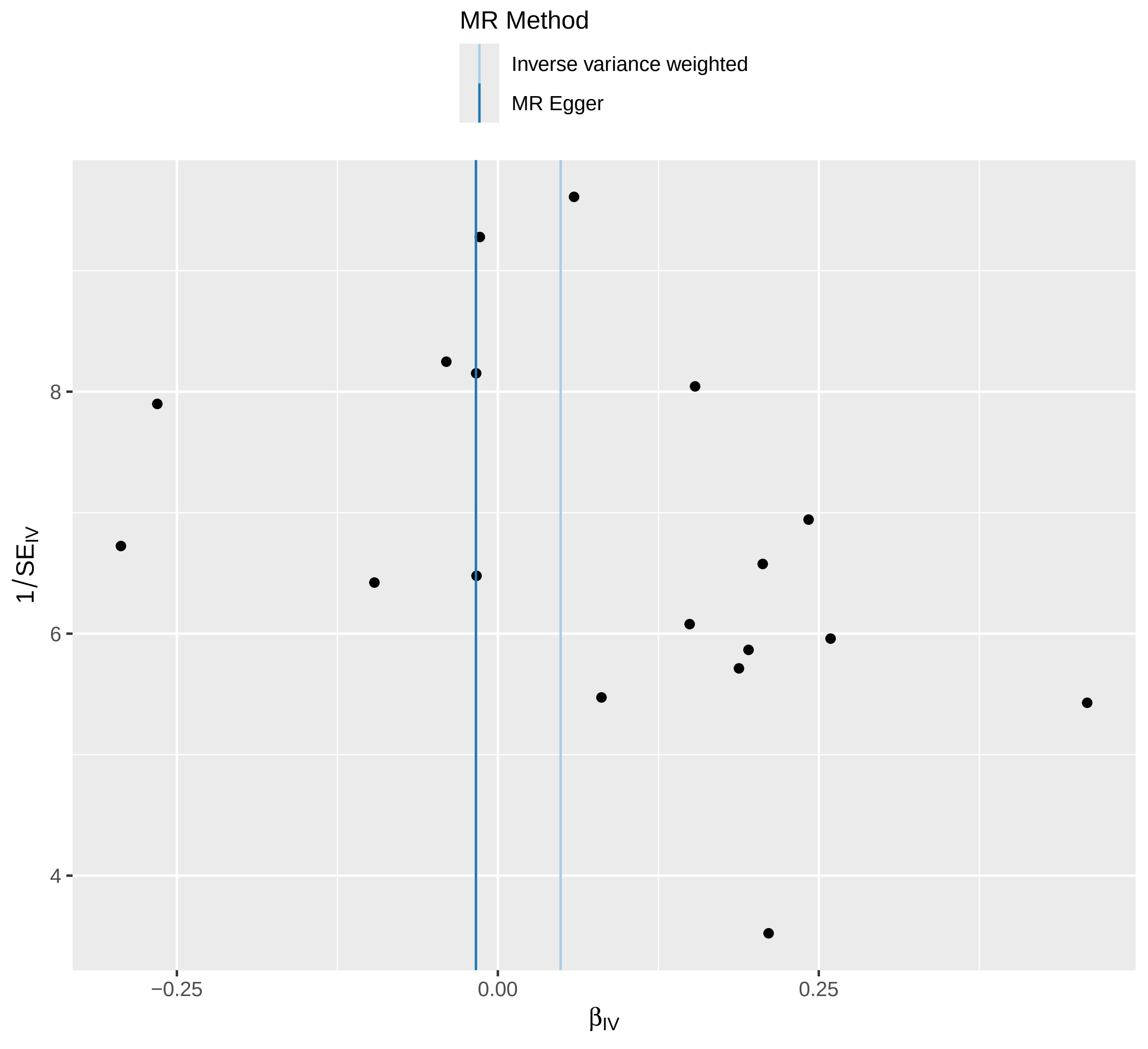

MR Method
Inverse variance weighted
MR Egger
1/SE_IV
8
6
4
−0.25
0.00
0.25
β_IV

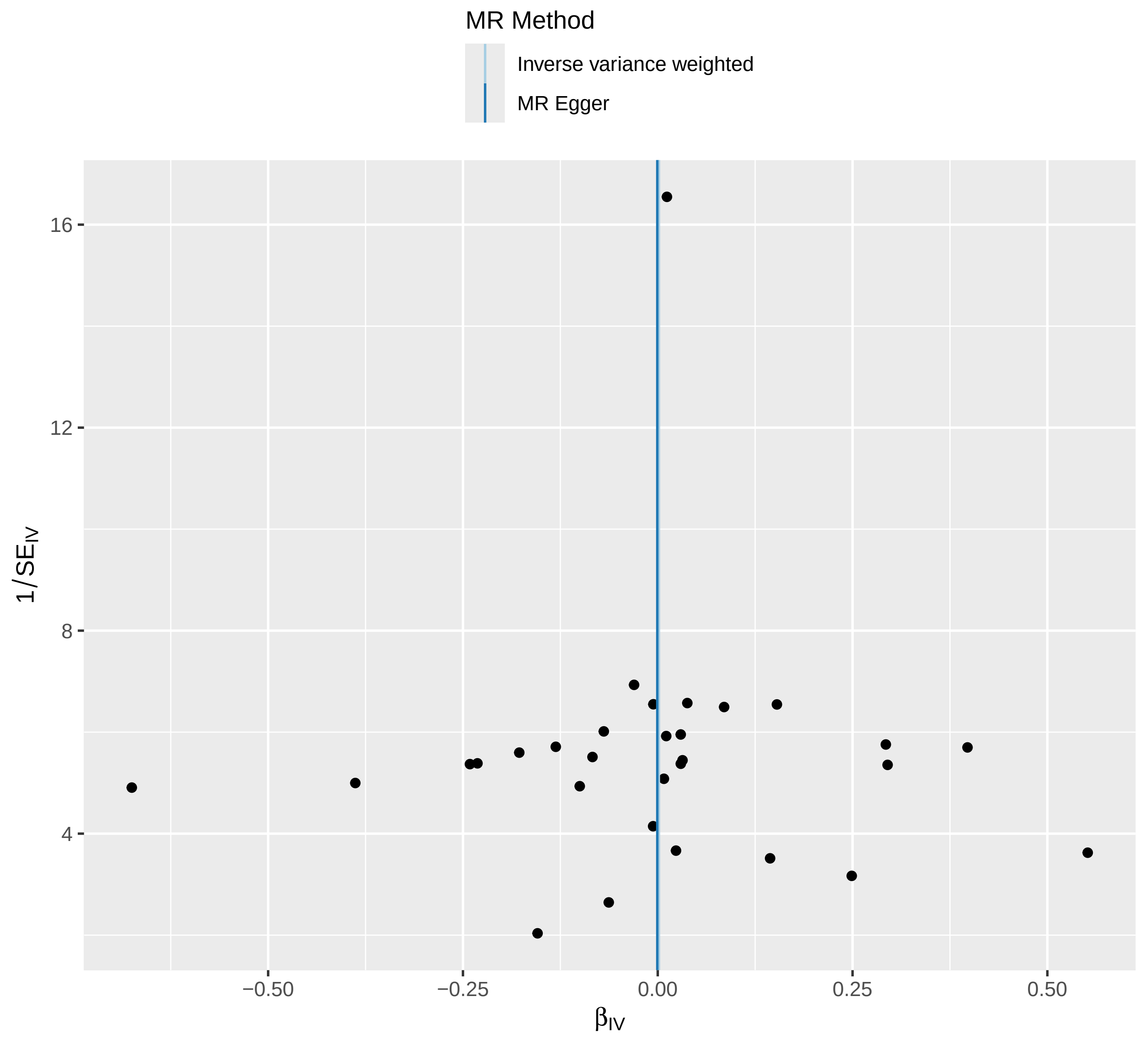

MR Method
Inverse variance weighted
MR Egger
16
12
8
4
1/SE_IV
−0.50
−0.25
0.00
0.25
0.50
β_IV

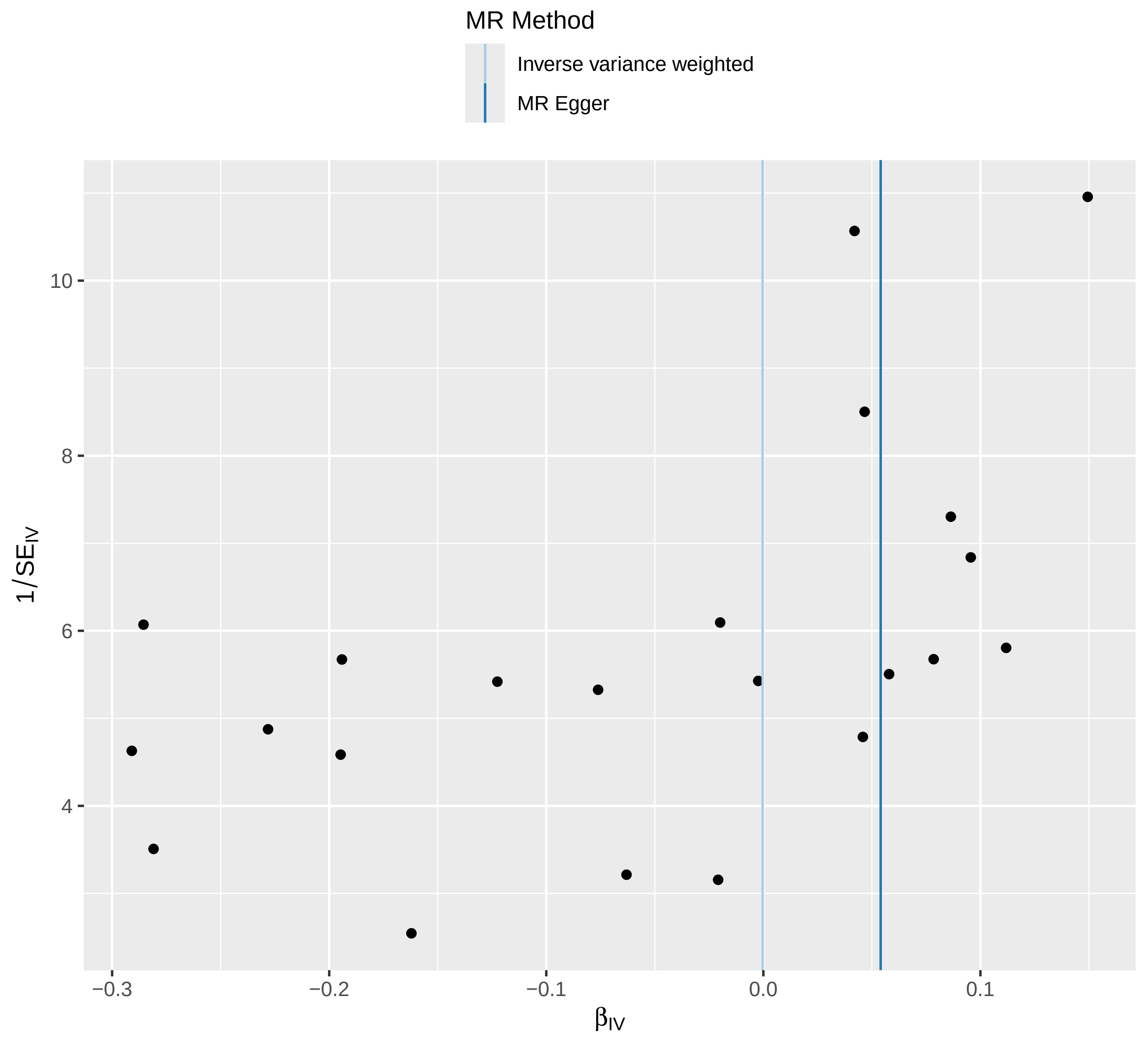
MR Method
Inverse variance weighted
MR Egger
10
8
6
4
1/SE_IV
−0.3
−0.2
−0.1
0.0
0.1
β_IV

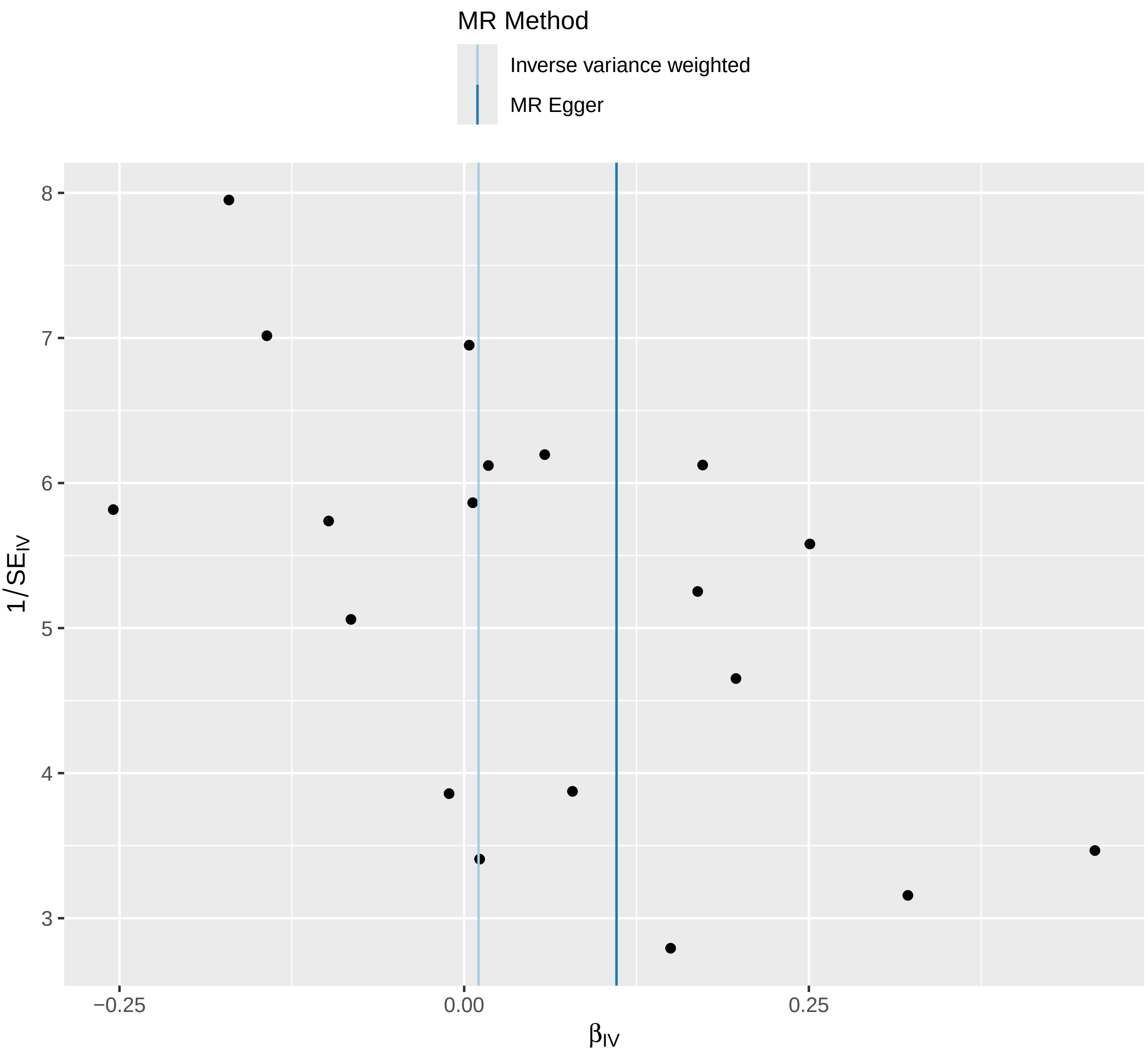

MR Method
Inverse variance weighted
MR Egger
1/SE_IV
β_IV
8
7
6
5
4
3
−0.25
0.00
0.25

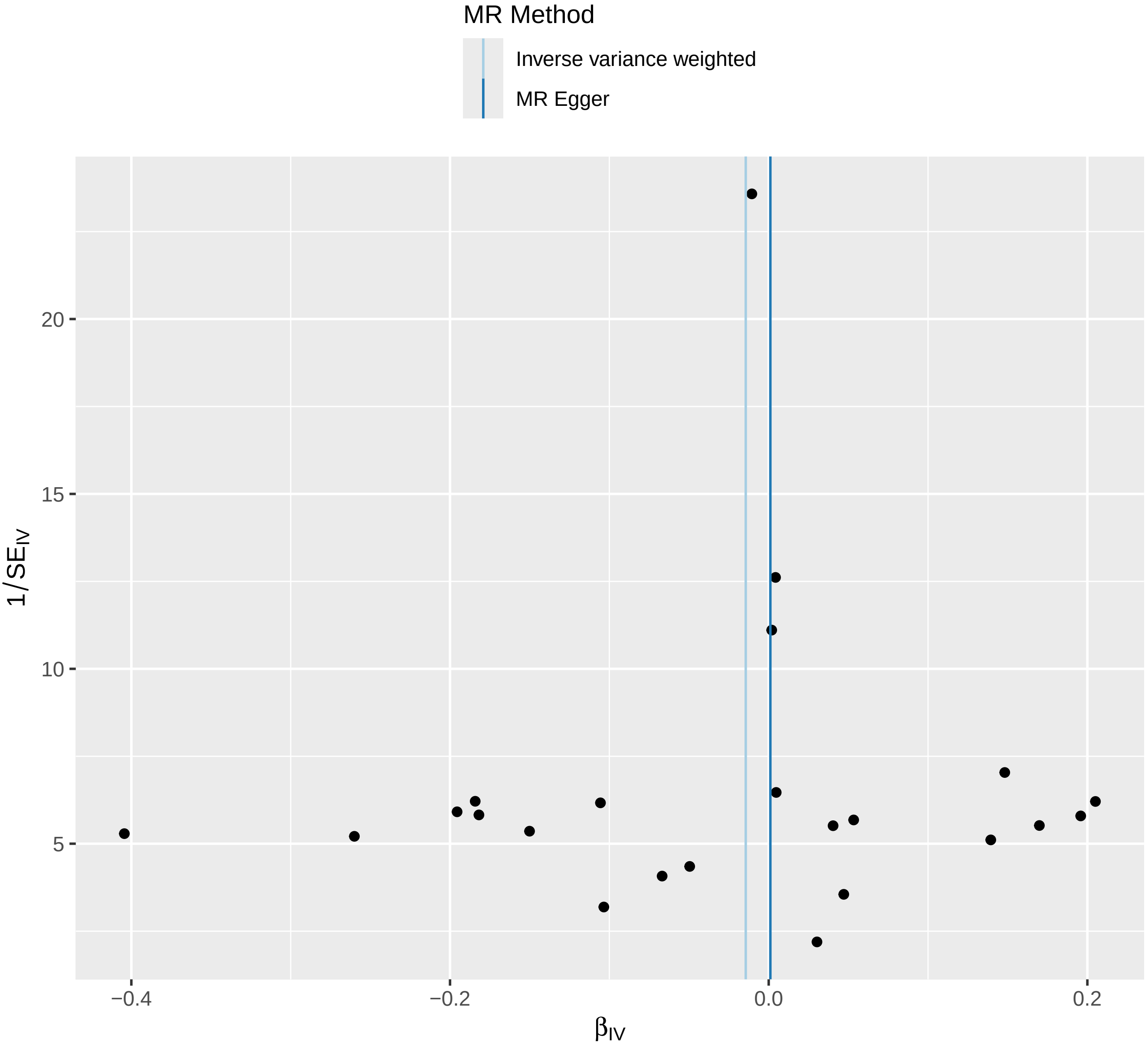
MR Method
Inverse variance weighted
MR Egger
20
15
10
5
−0.4
−0.2
0.0
0.2
$1/SE_{IV}$
β_{IV}

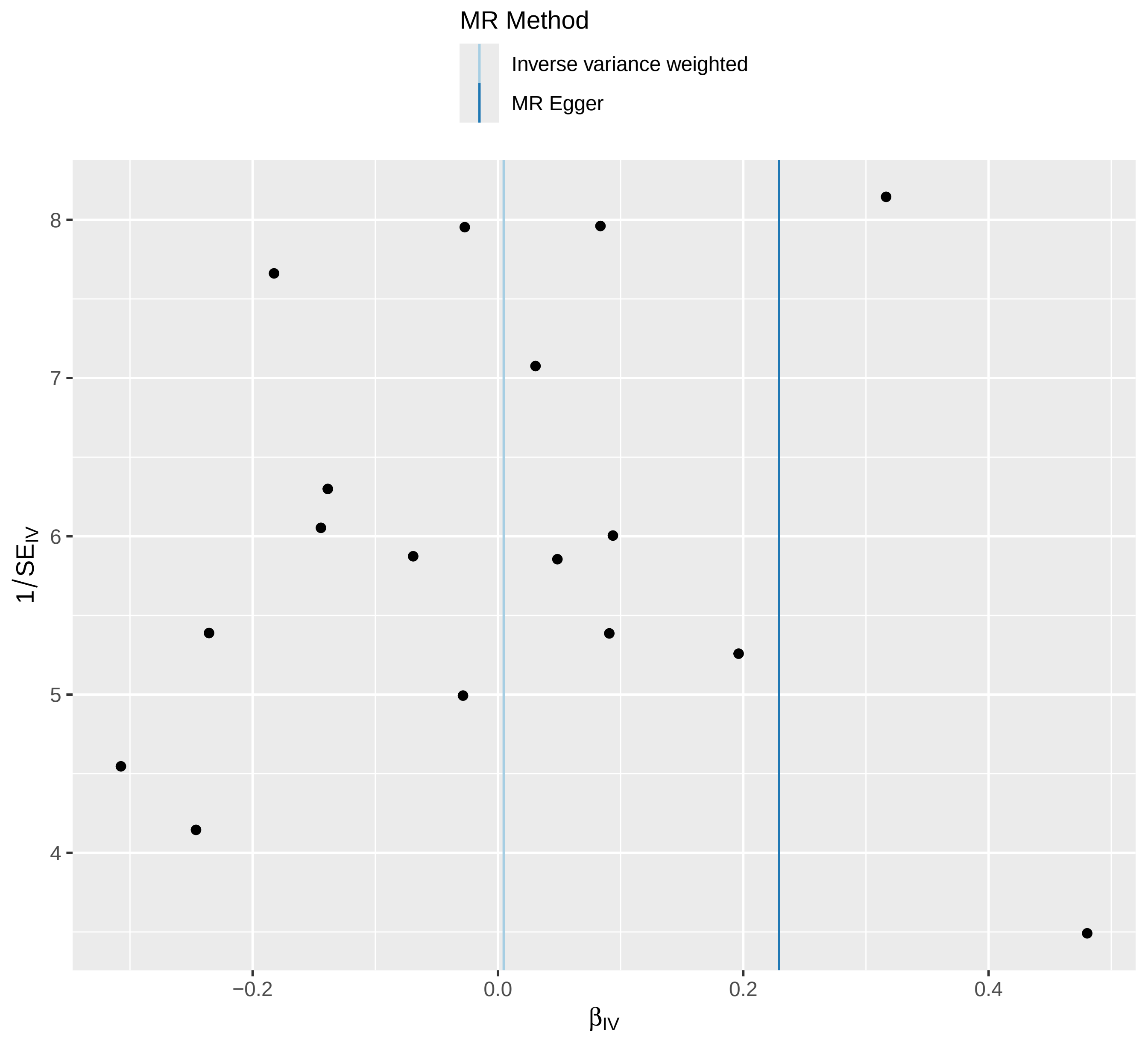
MR Method
Inverse variance weighted
MR Egger
8
7
6
5
4
1/SE_IV
−0.2
0.0
0.2
0.4
β_IV

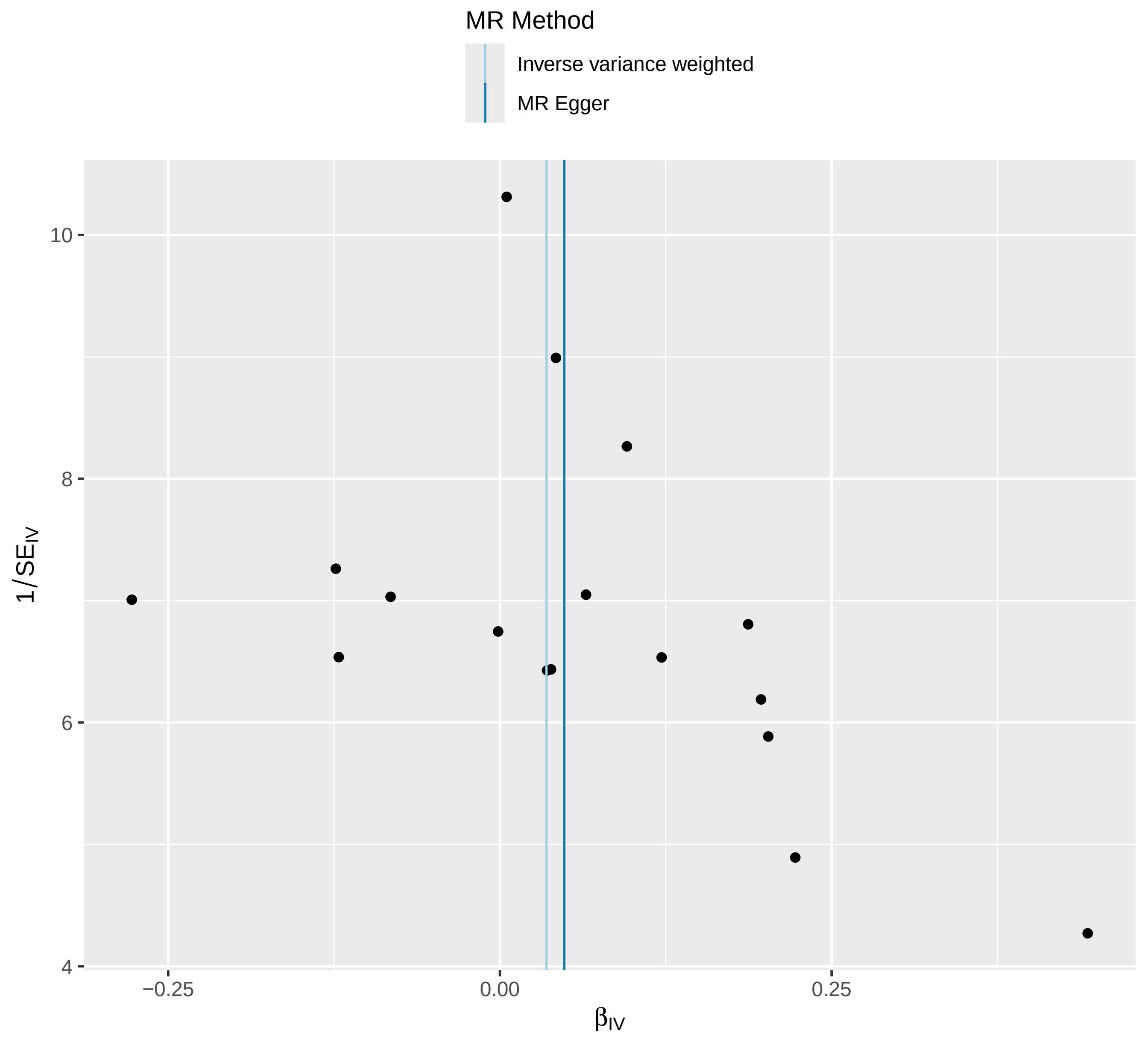

MR Method
Inverse variance weighted
MR Egger
10
8
6
4
1/SE_IV
−0.25
0.00
0.25
β_IV

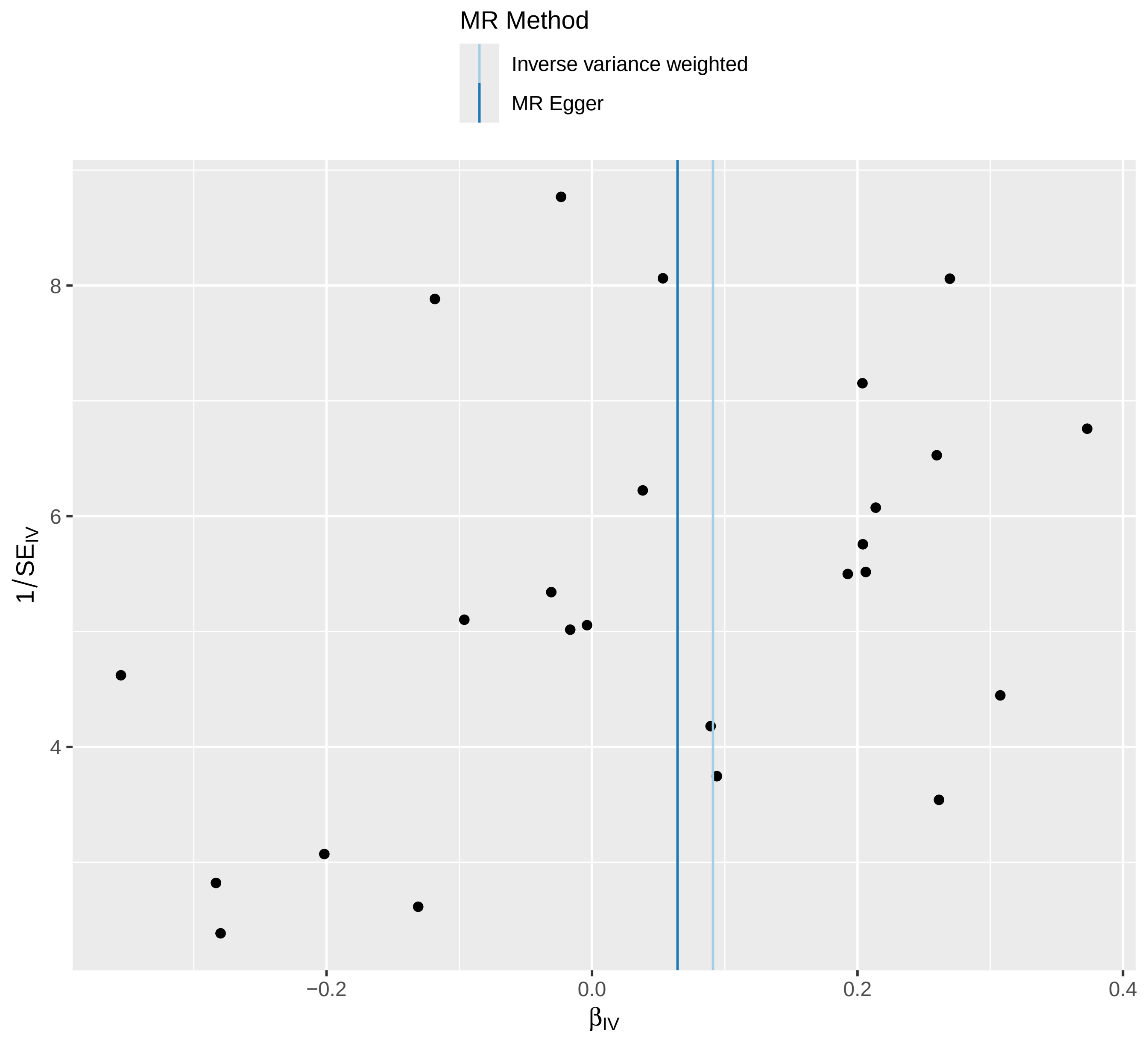

MR Method
Inverse variance weighted
MR Egger
8
6
4
$1/SE_{IV}$
−0.2
0.0
0.2
0.4
β_{IV}

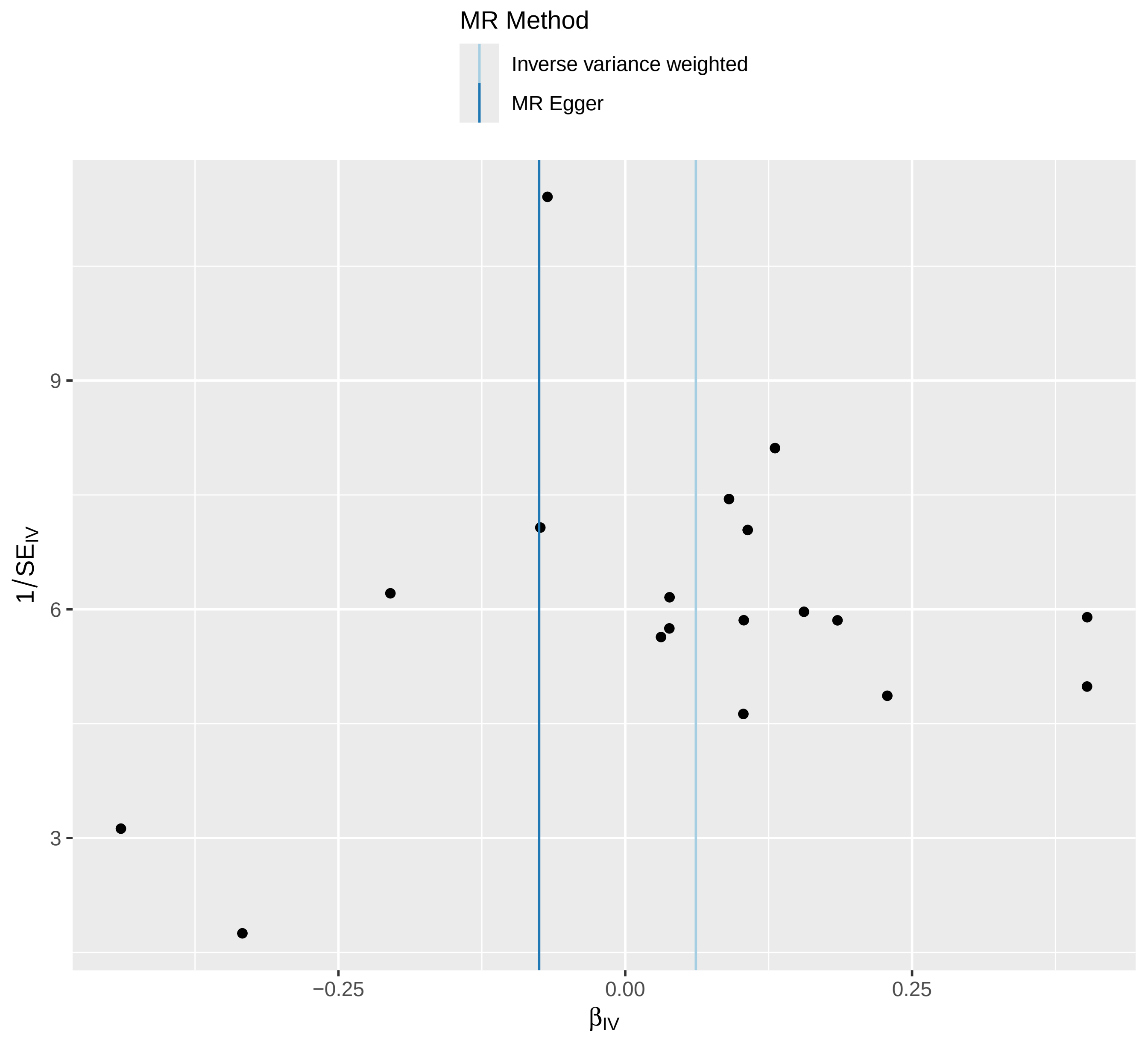

MR Method
Inverse variance weighted
MR Egger
9
6
3
1/SE_IV
−0.25
0.00
0.25
β_IV

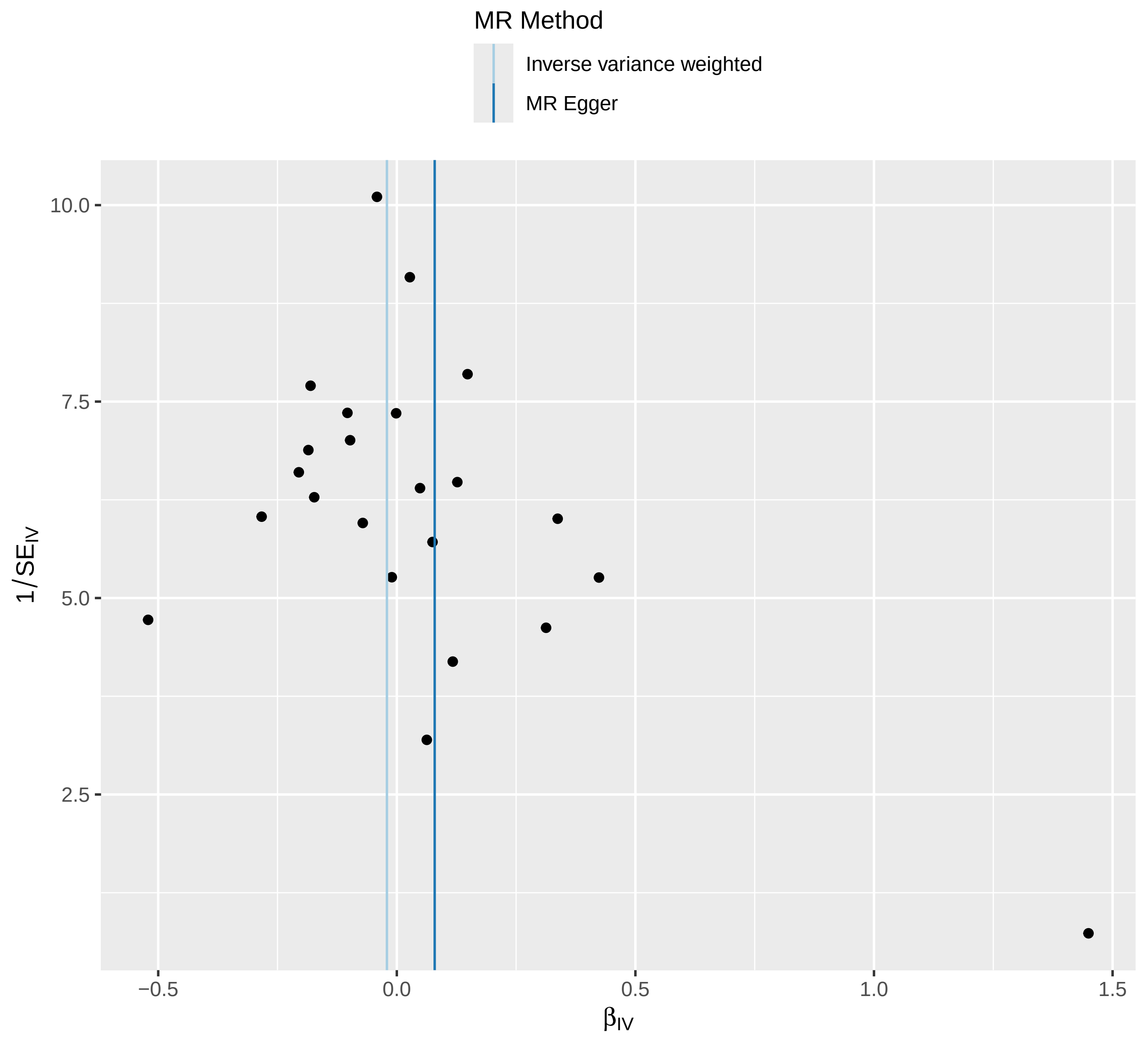

MR Method
Inverse variance weighted
MR Egger
10.0
7.5
5.0
2.5
1/SE_IV
−0.5
0.0
0.5
1.0
1.5
β_IV

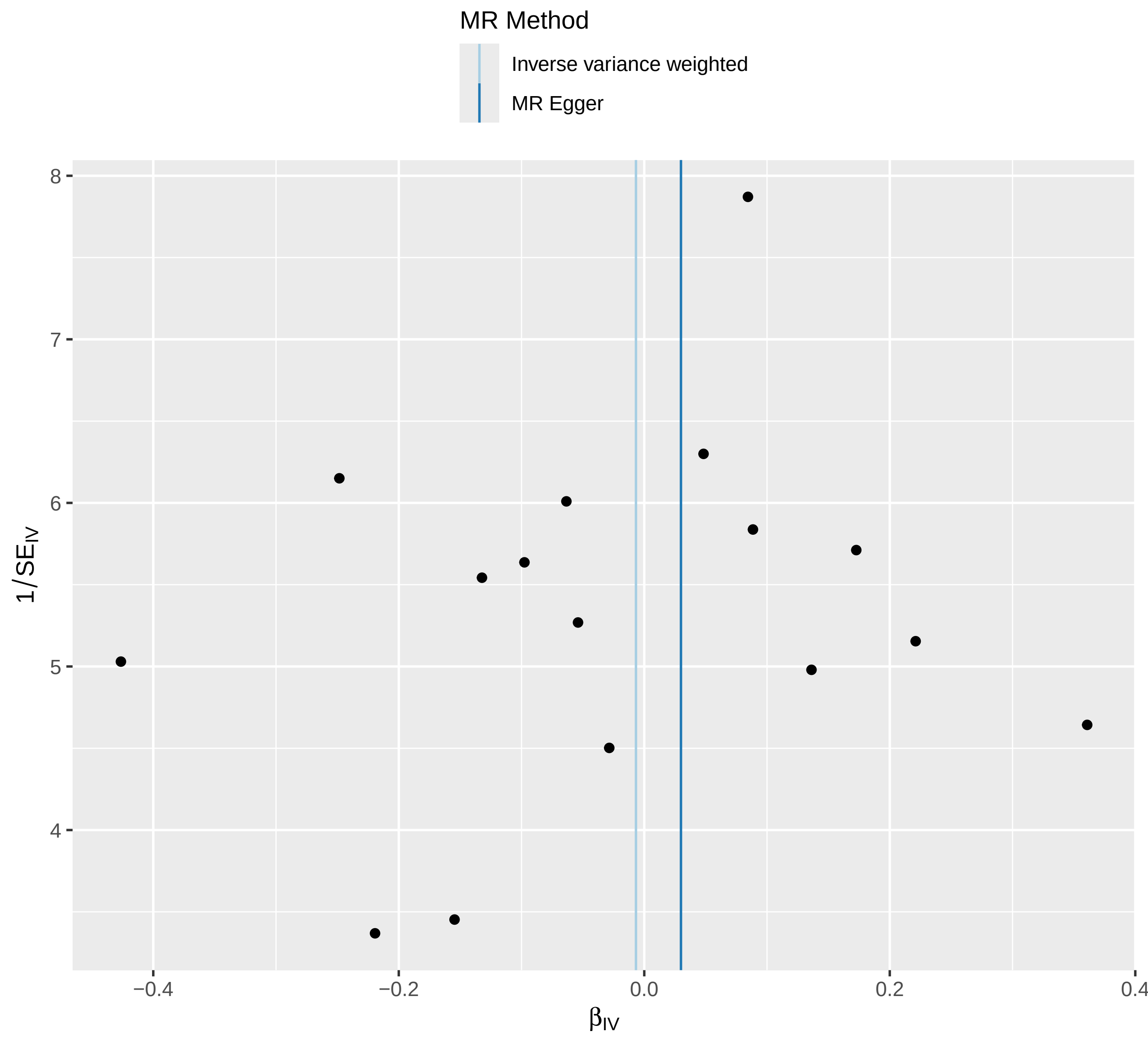

MR Method
Inverse variance weighted
MR Egger
8
7
6
5
4
1/SE$_{IV}$
−0.4
−0.2
0.0
0.2
0.4
β$_{IV}$

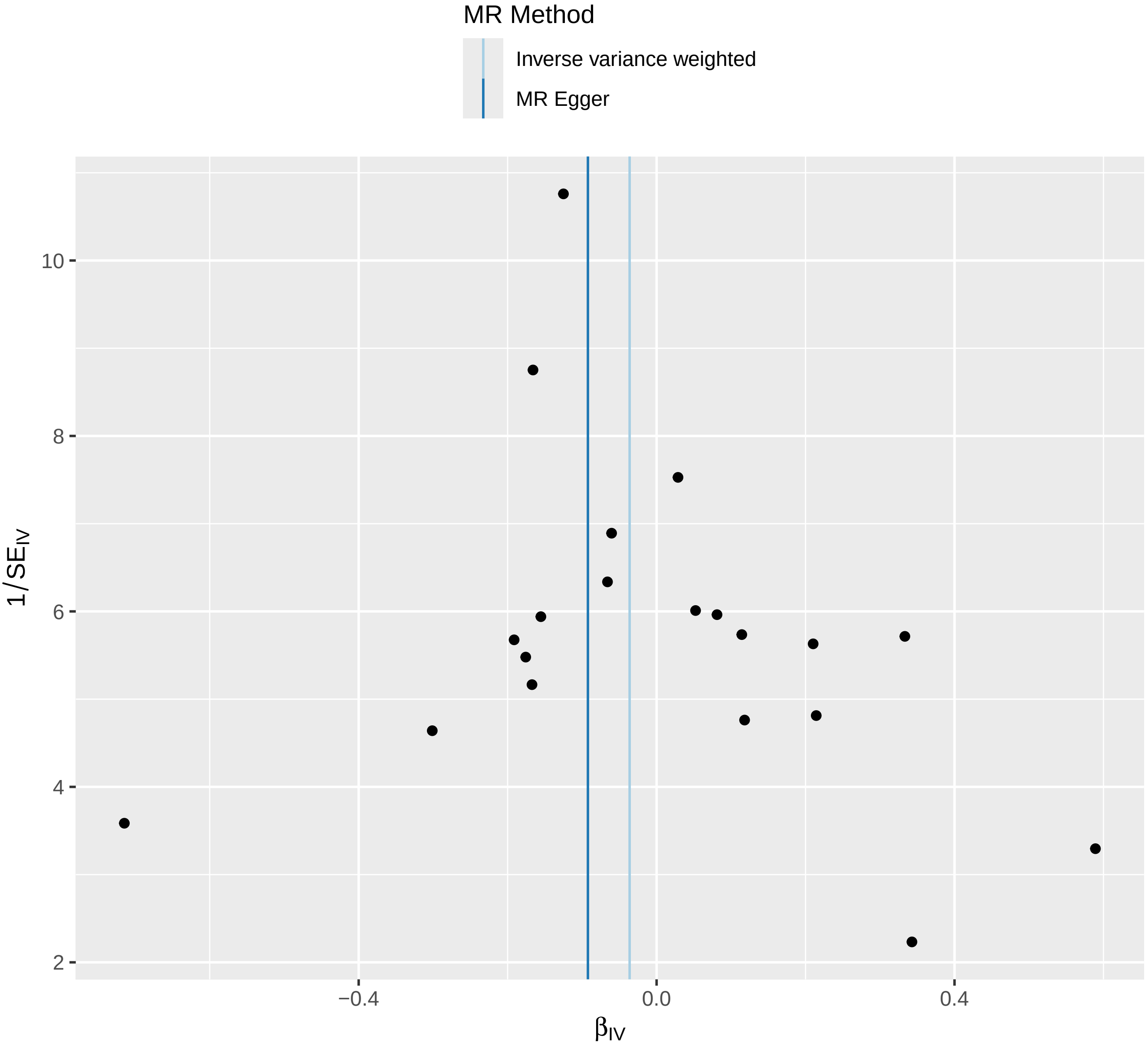

MR Method
Inverse variance weighted
MR Egger
10
8
6
4
2
1/SE_IV
−0.4
0.0
0.4
β_IV

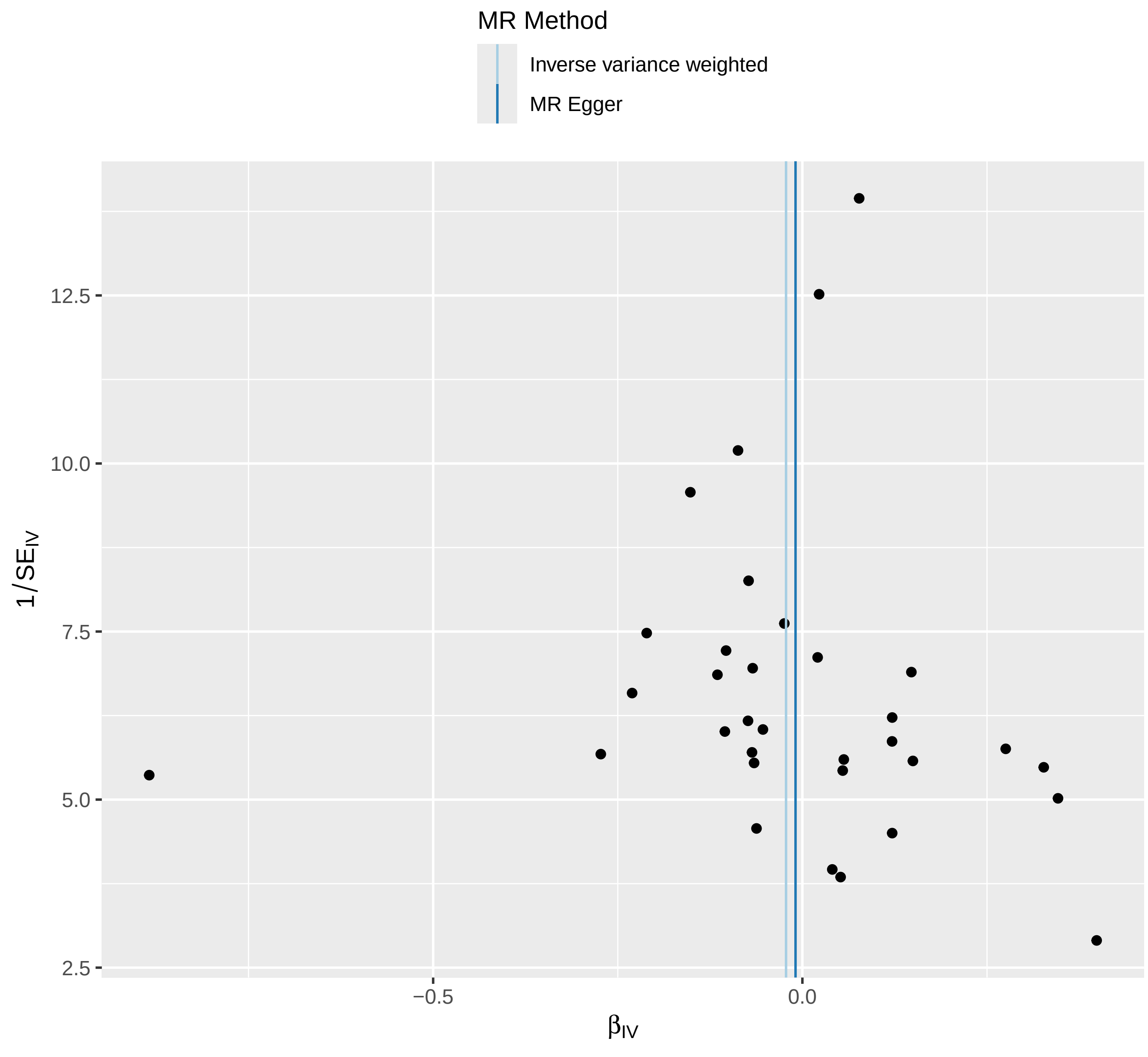

MR Method
Inverse variance weighted
MR Egger
12.5
10.0
7.5
5.0
2.5
1/SE$_{IV}$
−0.5
0.0
β$_{IV}$

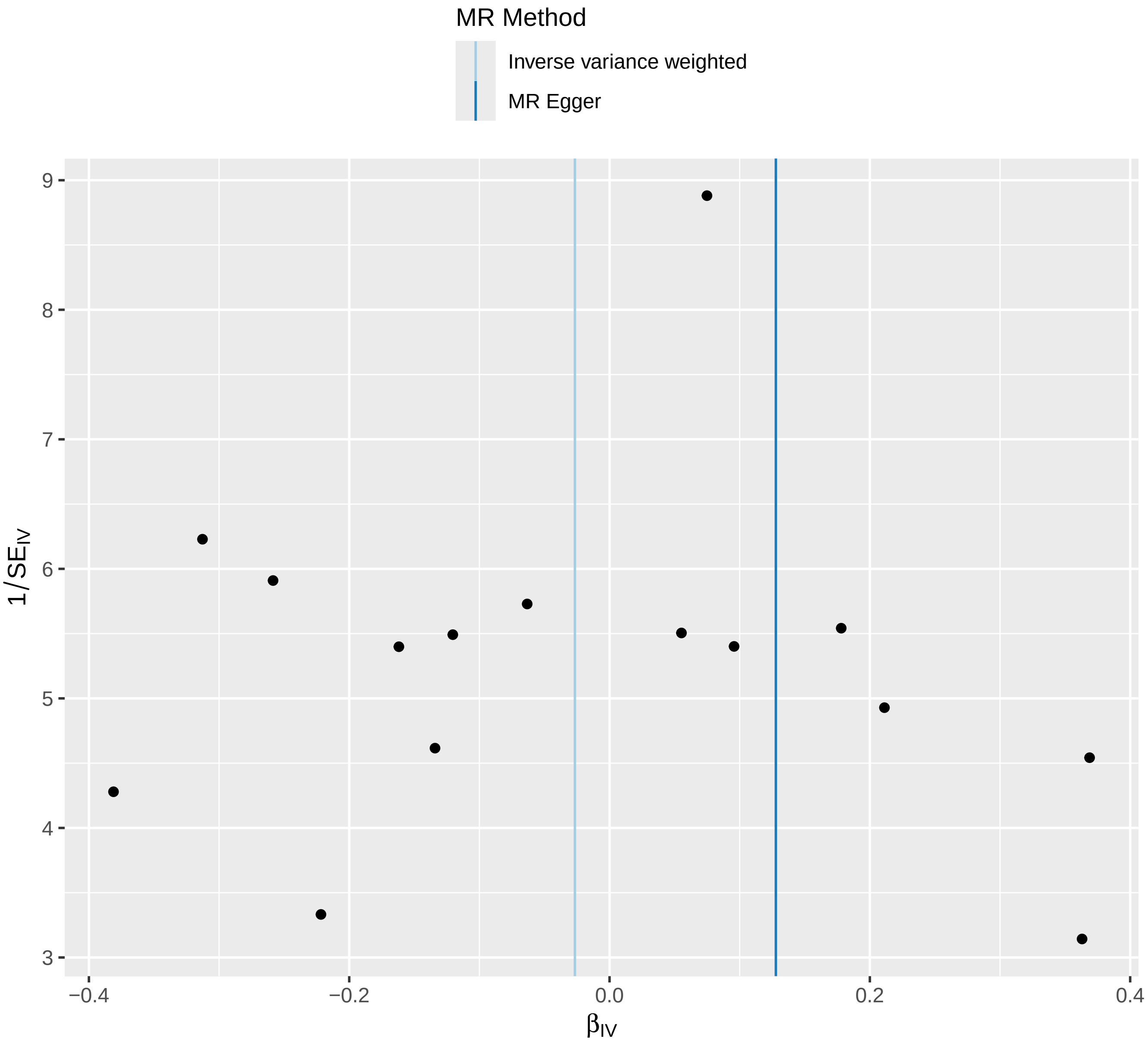

MR Method
Inverse variance weighted
MR Egger
9
8
7
6
5
4
3
1/SE_IV
−0.4
−0.2
0.0
0.2
0.4
β_IV

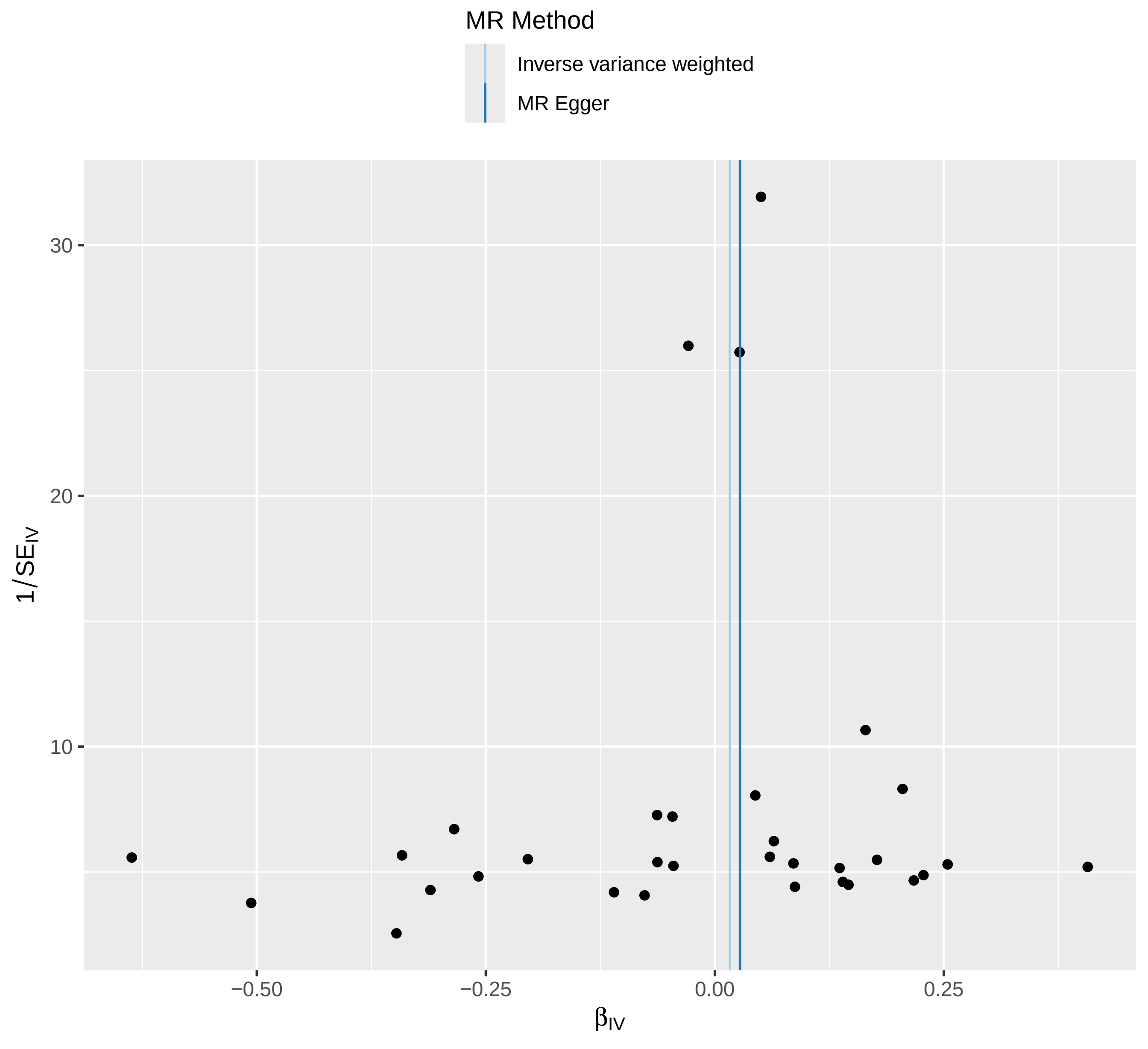

MR Method
Inverse variance weighted
MR Egger
30
20
10
1/SE$_{IV}$
−0.50
−0.25
0.00
0.25
β$_{IV}$

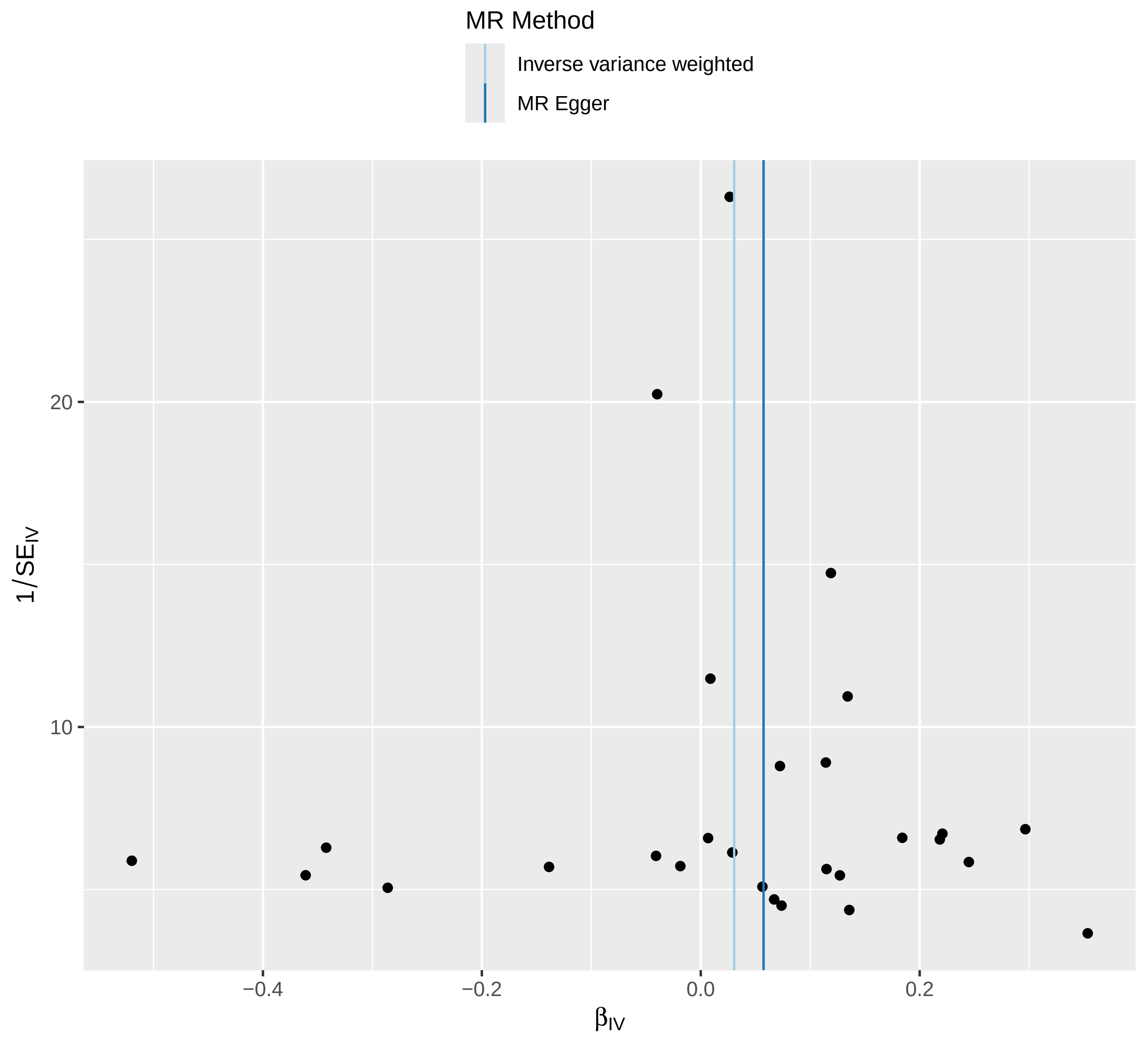
MR Method
Inverse variance weighted
MR Egger
20
10
1/SE$_{IV}$
−0.4
−0.2
0.0
0.2
β$_{IV}$

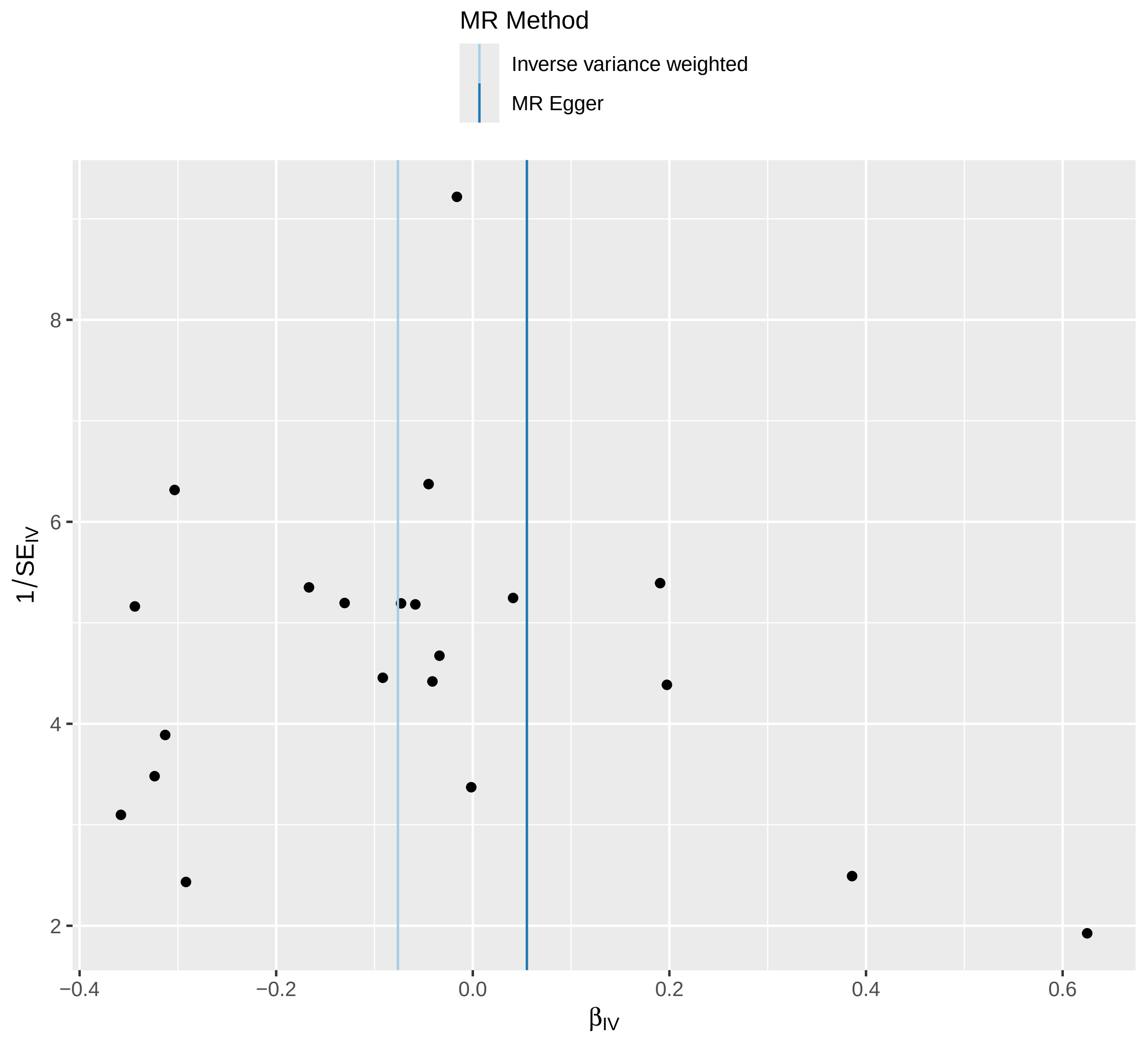

MR Method
Inverse variance weighted
MR Egger
8
6
4
2
$1/SE_{IV}$
−0.4
−0.2
0.0
0.2
0.4
0.6
β_{IV}

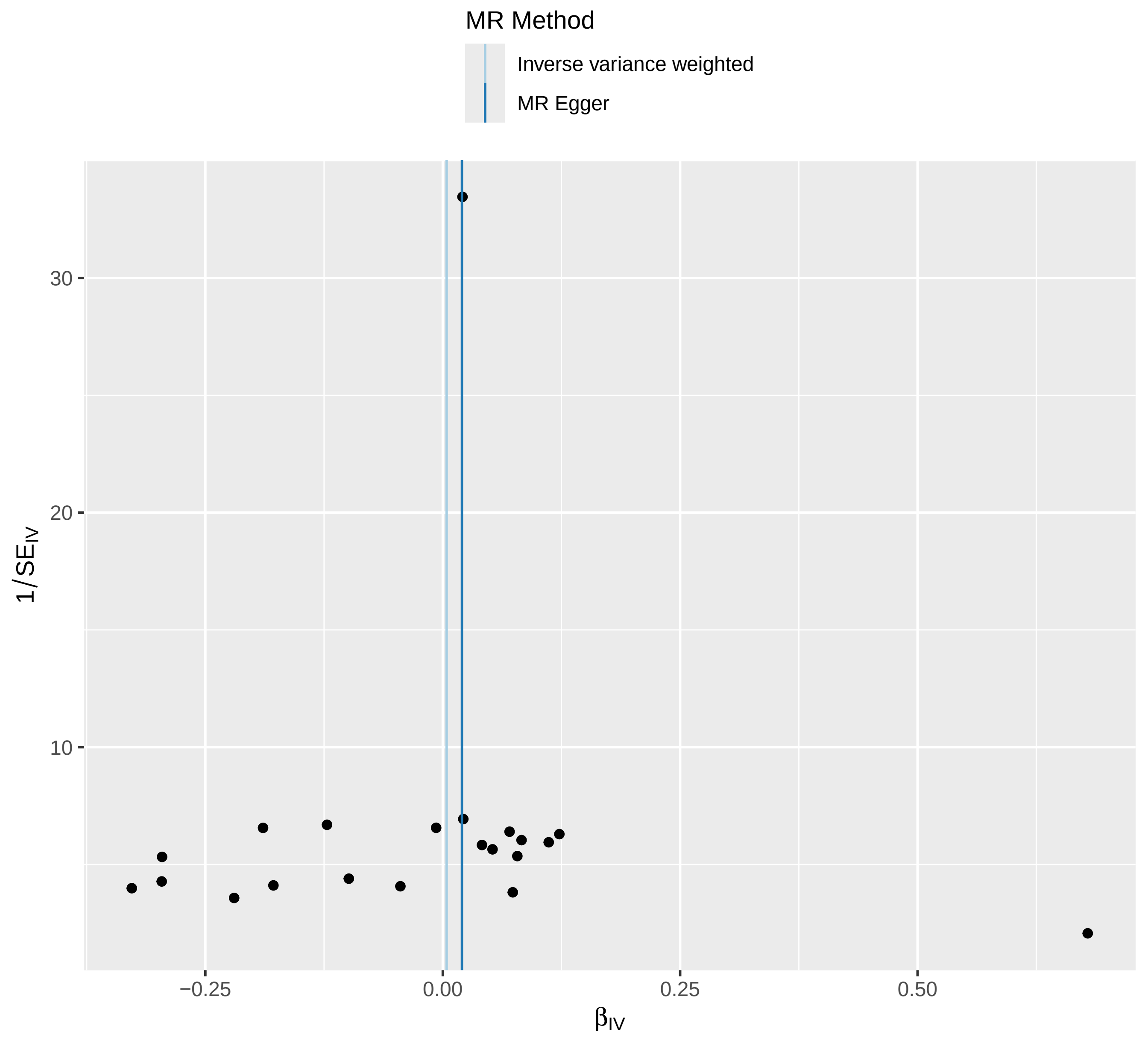
MR Method
Inverse variance weighted
MR Egger
30
20
10
1/SE_IV
−0.25
0.00
0.25
0.50
β_IV

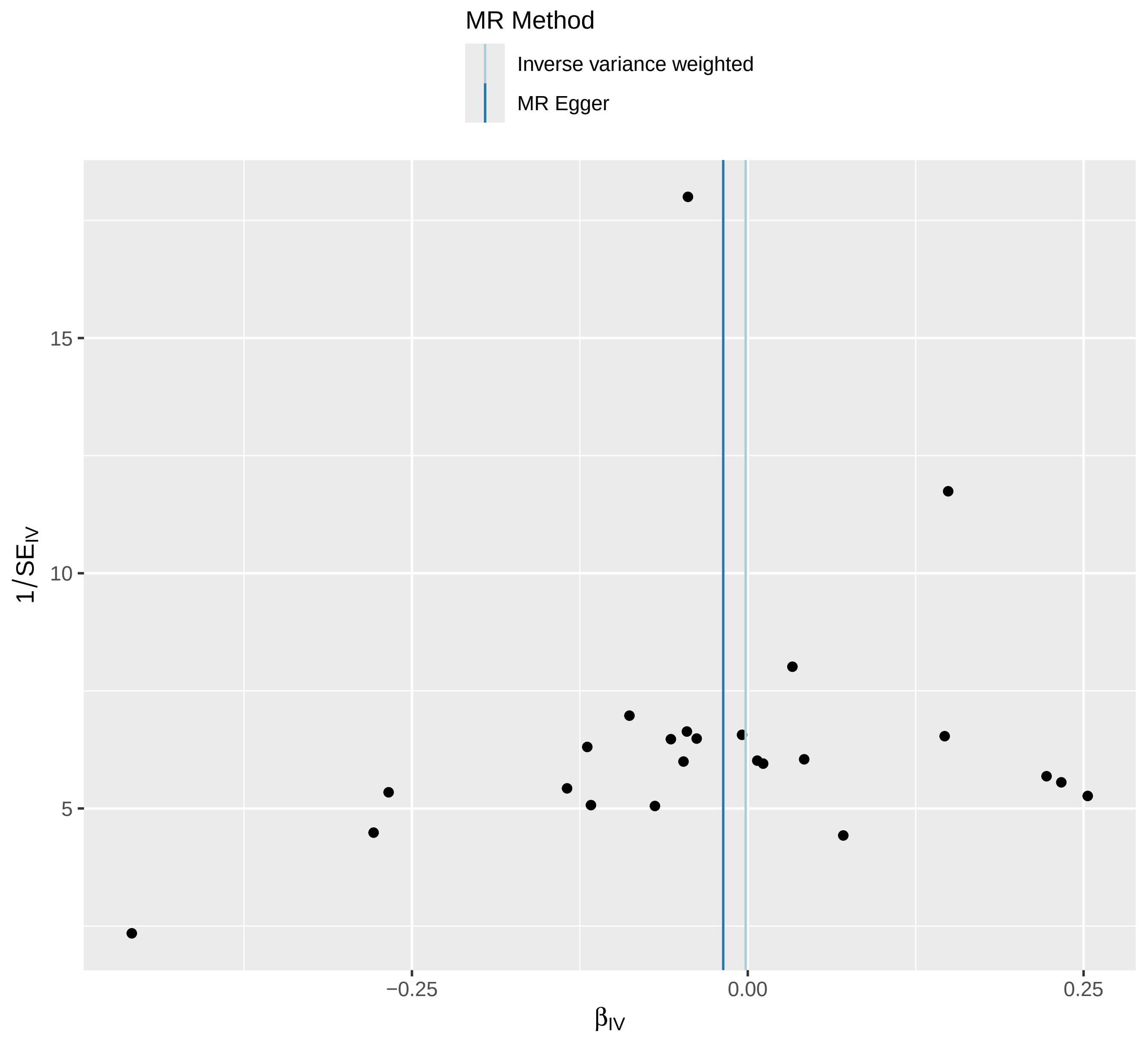
MR Method
Inverse variance weighted
MR Egger
15
10
5
1/SE$_{IV}$
−0.25
0.00
0.25
β$_{IV}$

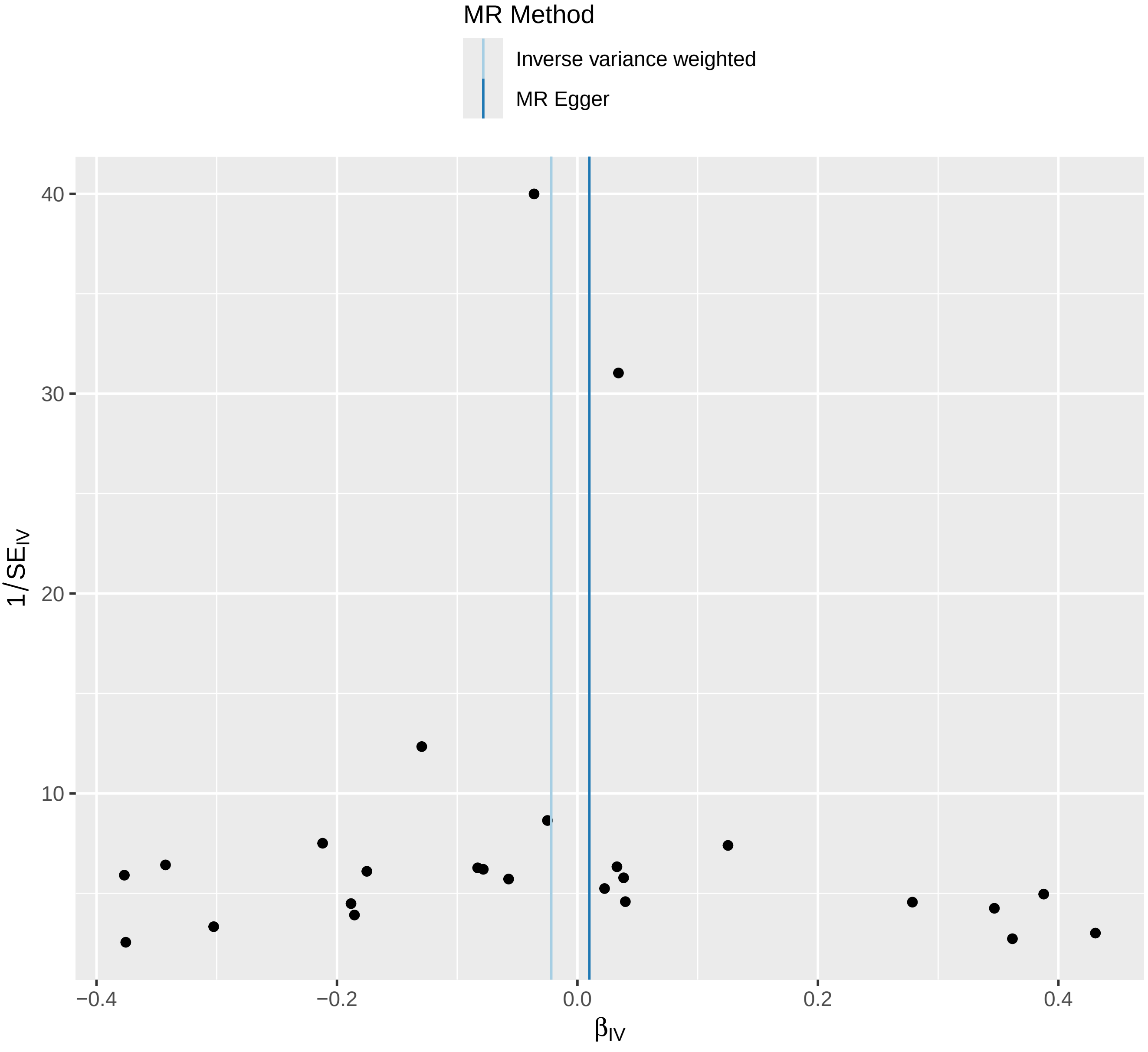
MR Method
Inverse variance weighted
MR Egger
40
30
20
10
1/SE_IV
−0.4
−0.2
0.0
0.2
0.4
β_IV

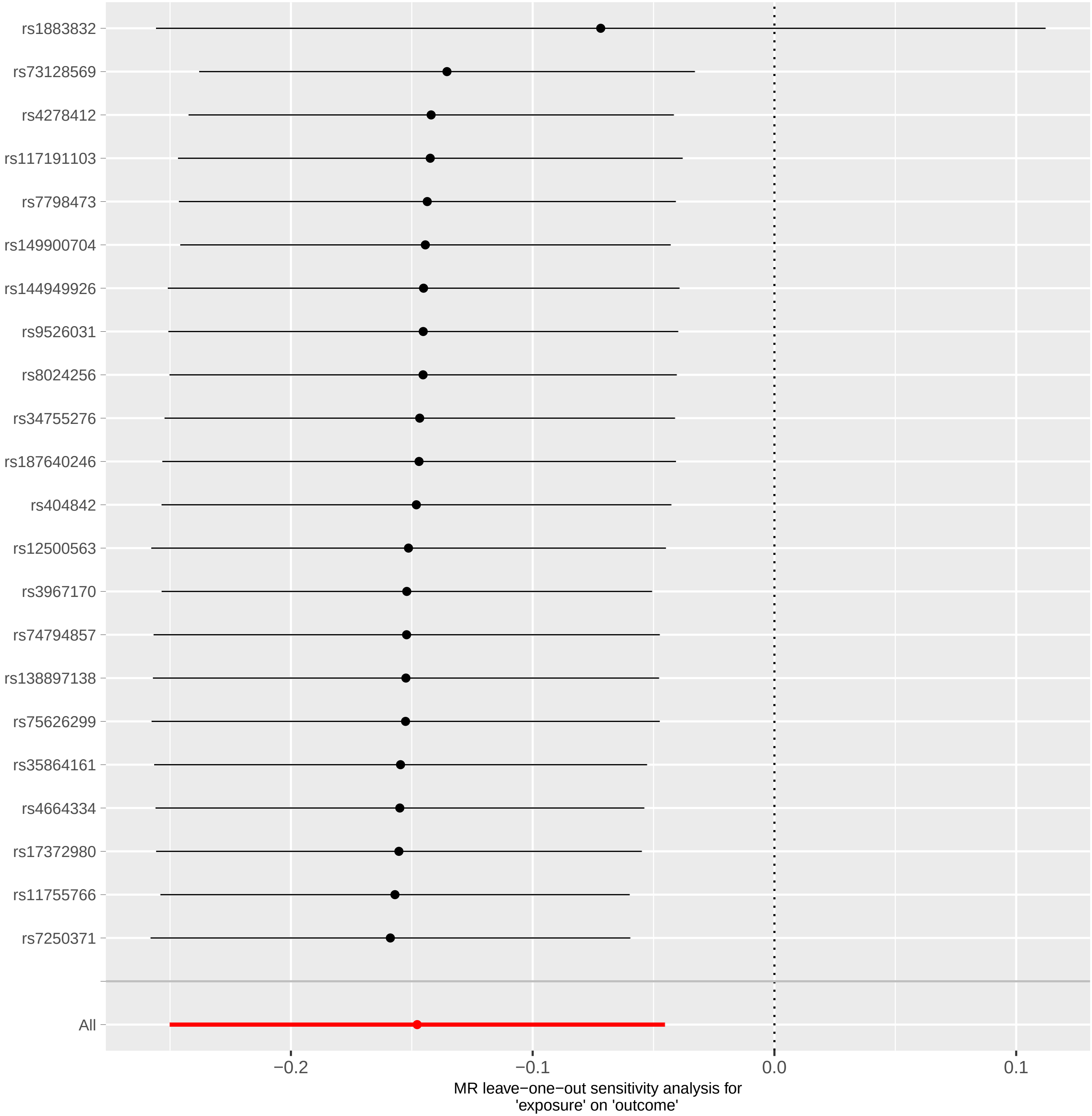
rs1883832
rs73128569
rs4278412
rs117191103
rs7798473
rs149900704
rs144949926
rs9526031
rs8024256
rs34755276
rs187640246
rs404842
rs12500563
rs3967170
rs74794857
rs138897138
rs75626299
rs35864161
rs4664334
rs17372980
rs11755766
rs7250371
All
−0.2
−0.1
0.0
0.1
MR leave−one−out sensitivity analysis for
'exposure' on 'outcome'

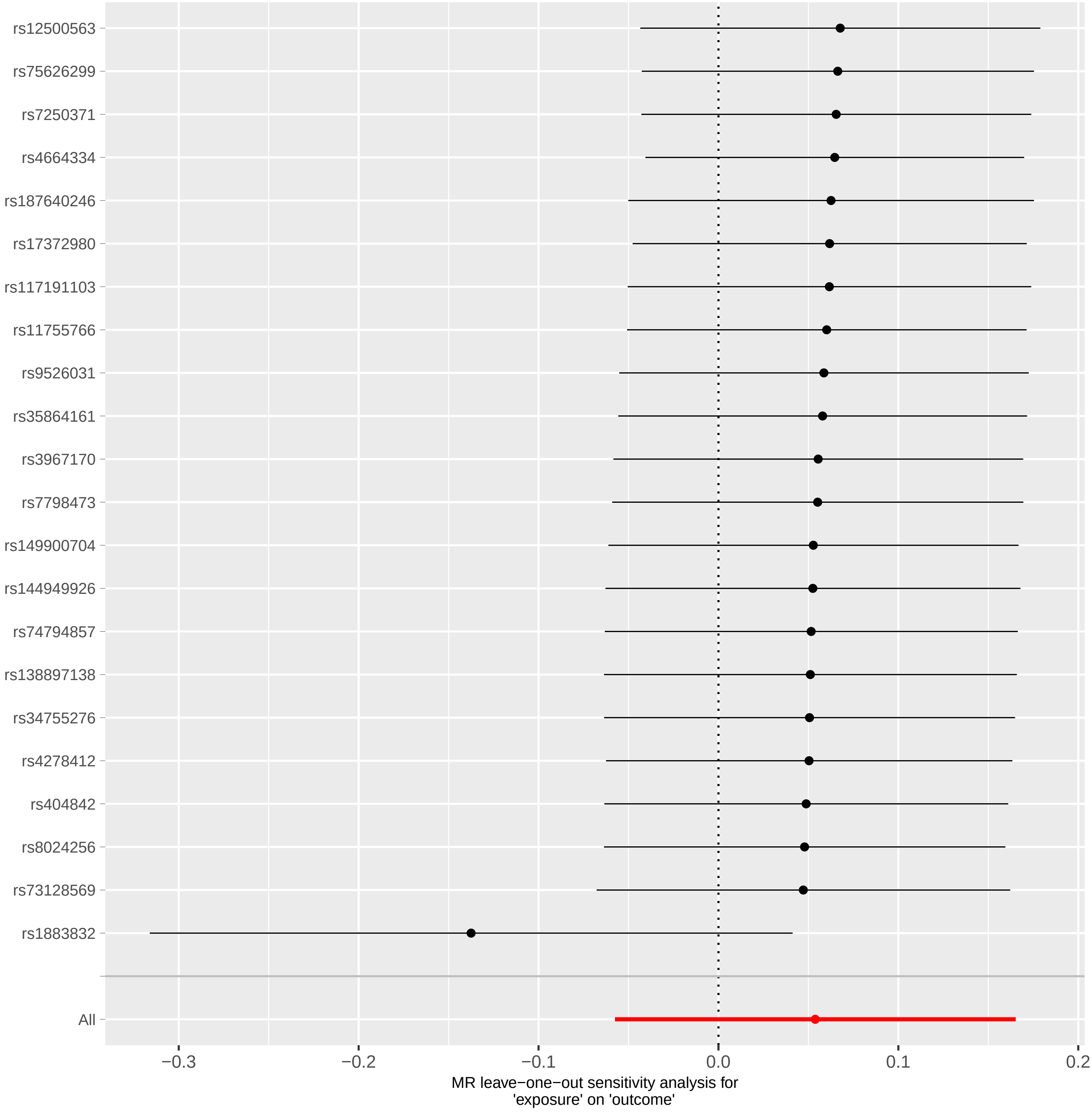

rs12500563
rs75626299
rs7250371
rs4664334
rs187640246
rs17372980
rs117191103
rs11755766
rs9526031
rs35864161
rs3967170
rs7798473
rs149900704
rs144949926
rs74794857
rs138897138
rs34755276
rs4278412
rs404842
rs8024256
rs73128569
rs1883832
All
−0.3
−0.2
−0.1
0.0
0.1
0.2
MR leave−one−out sensitivity analysis for
'exposure' on 'outcome'

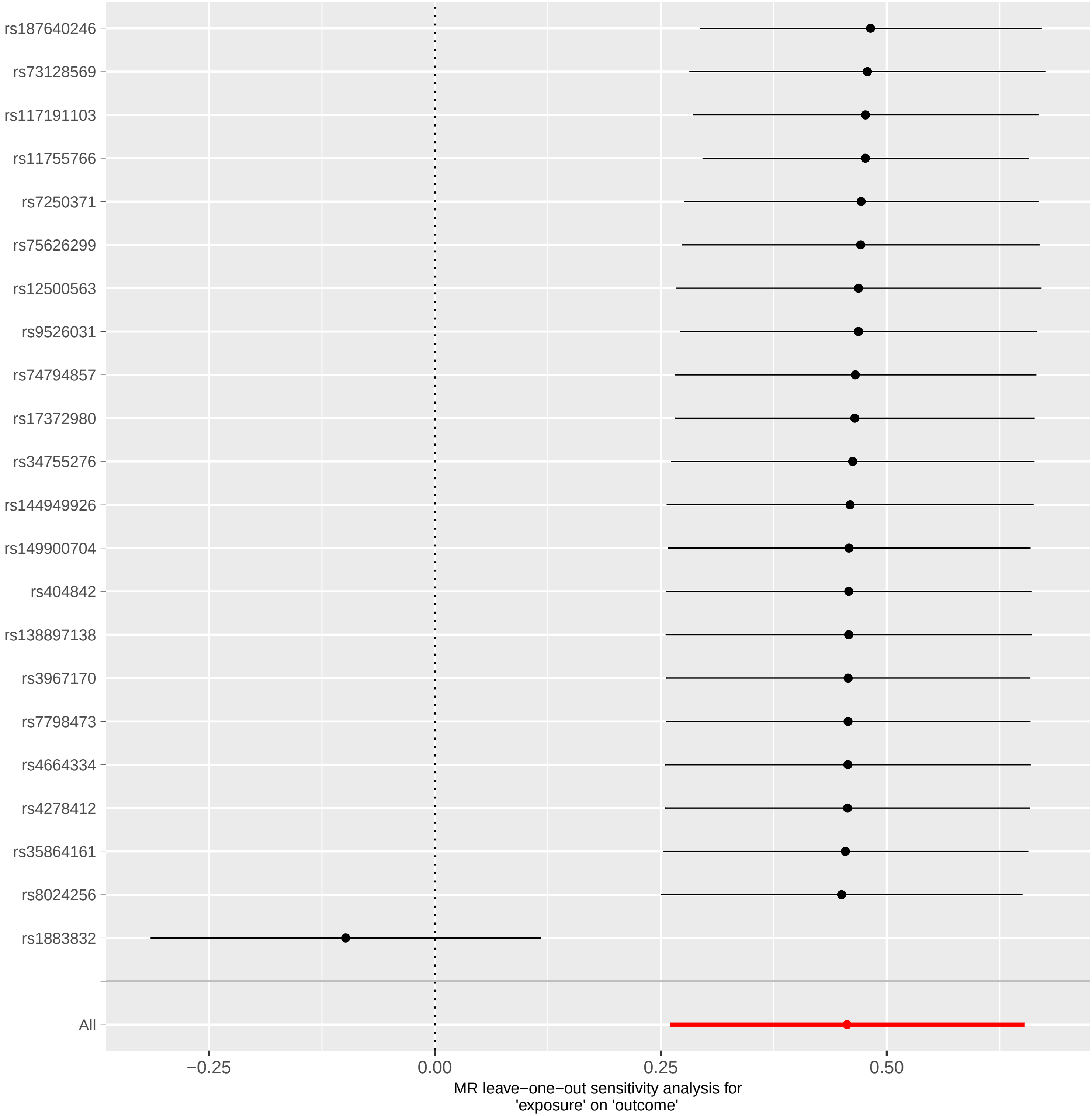
rs187640246
rs73128569
rs117191103
rs11755766
rs7250371
rs75626299
rs12500563
rs9526031
rs74794857
rs17372980
rs34755276
rs144949926
rs149900704
rs404842
rs138897138
rs3967170
rs7798473
rs4664334
rs4278412
rs35864161
rs8024256
rs1883832
All
−0.25
0.00
0.25
0.50
MR leave−one−out sensitivity analysis for
'exposure' on 'outcome'

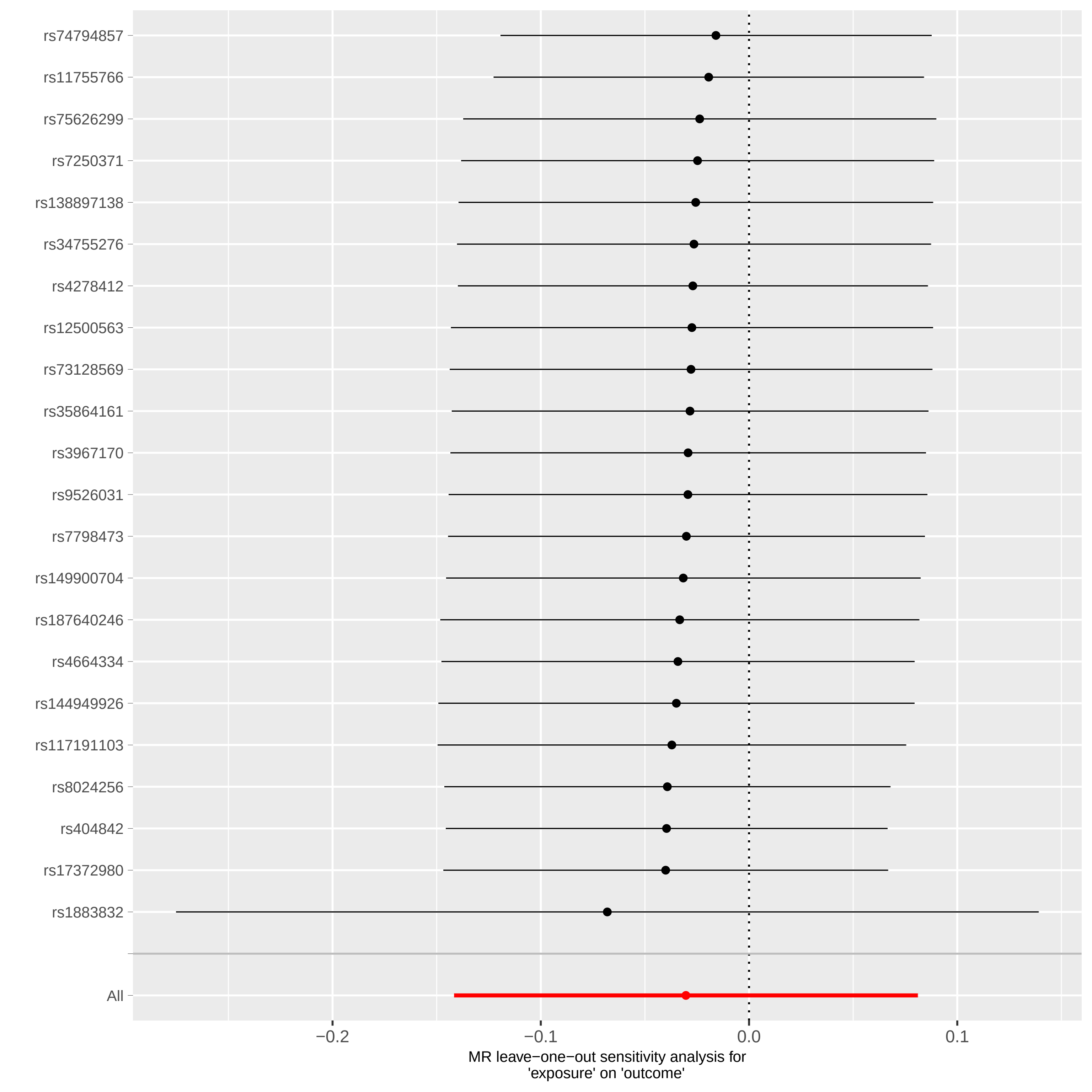

rs74794857
rs11755766
rs75626299
rs7250371
rs138897138
rs34755276
rs4278412
rs12500563
rs73128569
rs35864161
rs3967170
rs9526031
rs7798473
rs149900704
rs187640246
rs4664334
rs144949926
rs117191103
rs8024256
rs404842
rs17372980
rs1883832
All
−0.2
−0.1
0.0
0.1
MR leave−one−out sensitivity analysis for
'exposure' on 'outcome'

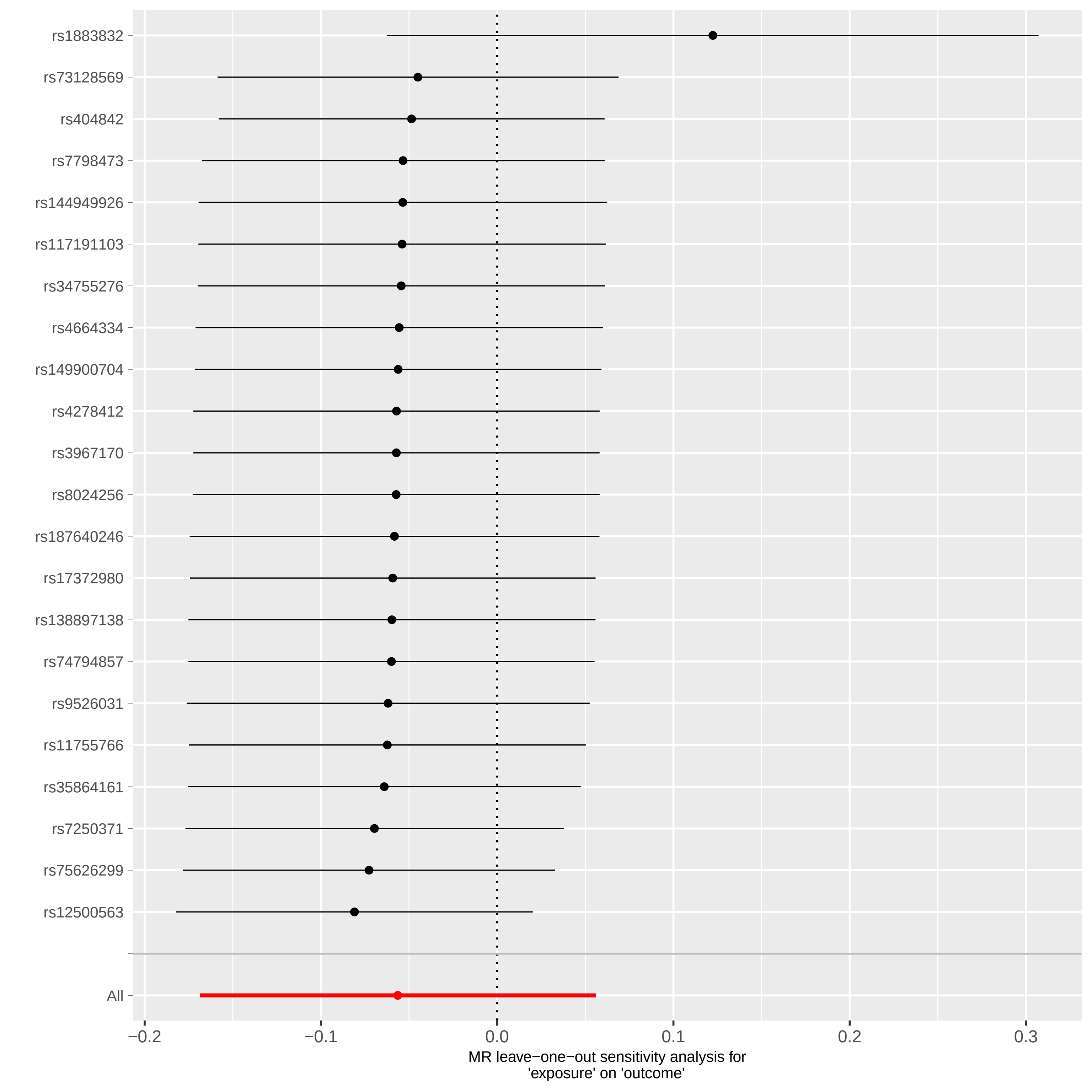

rs1883832
rs73128569
rs404842
rs7798473
rs144949926
rs117191103
rs34755276
rs4664334
rs149900704
rs4278412
rs3967170
rs8024256
rs187640246
rs17372980
rs138897138
rs74794857
rs9526031
rs11755766
rs35864161
rs7250371
rs75626299
rs12500563
All
−0.2
−0.1
0.0
0.1
0.2
0.3
MR leave−one−out sensitivity analysis for
'exposure' on 'outcome'

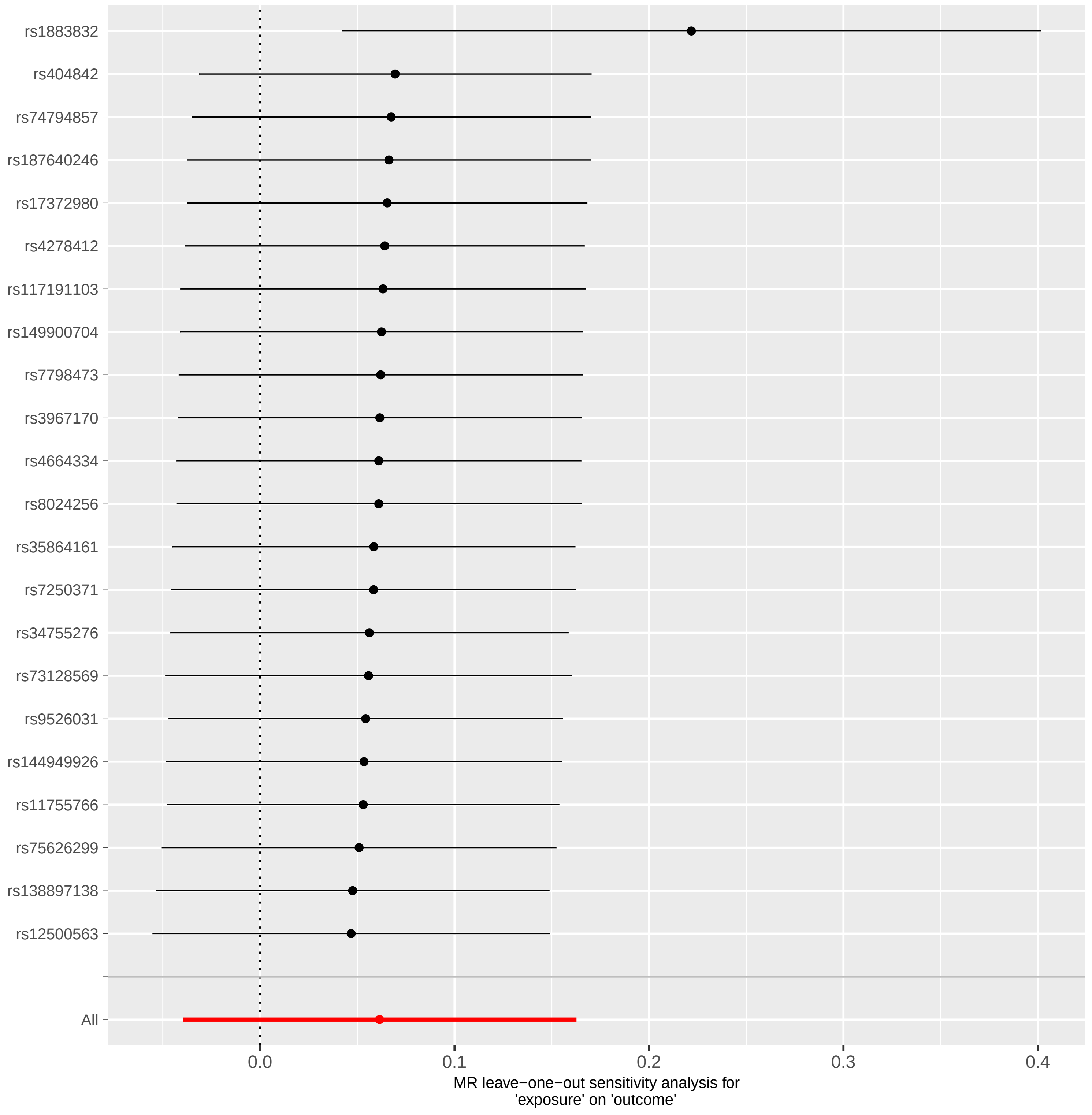

rs1883832
rs404842
rs74794857
rs187640246
rs17372980
rs4278412
rs117191103
rs149900704
rs7798473
rs3967170
rs4664334
rs8024256
rs35864161
rs7250371
rs34755276
rs73128569
rs9526031
rs144949926
rs11755766
rs75626299
rs138897138
rs12500563
All
0.0
0.1
0.2
0.3
0.4
MR leave−one−out sensitivity analysis for
'exposure' on 'outcome'

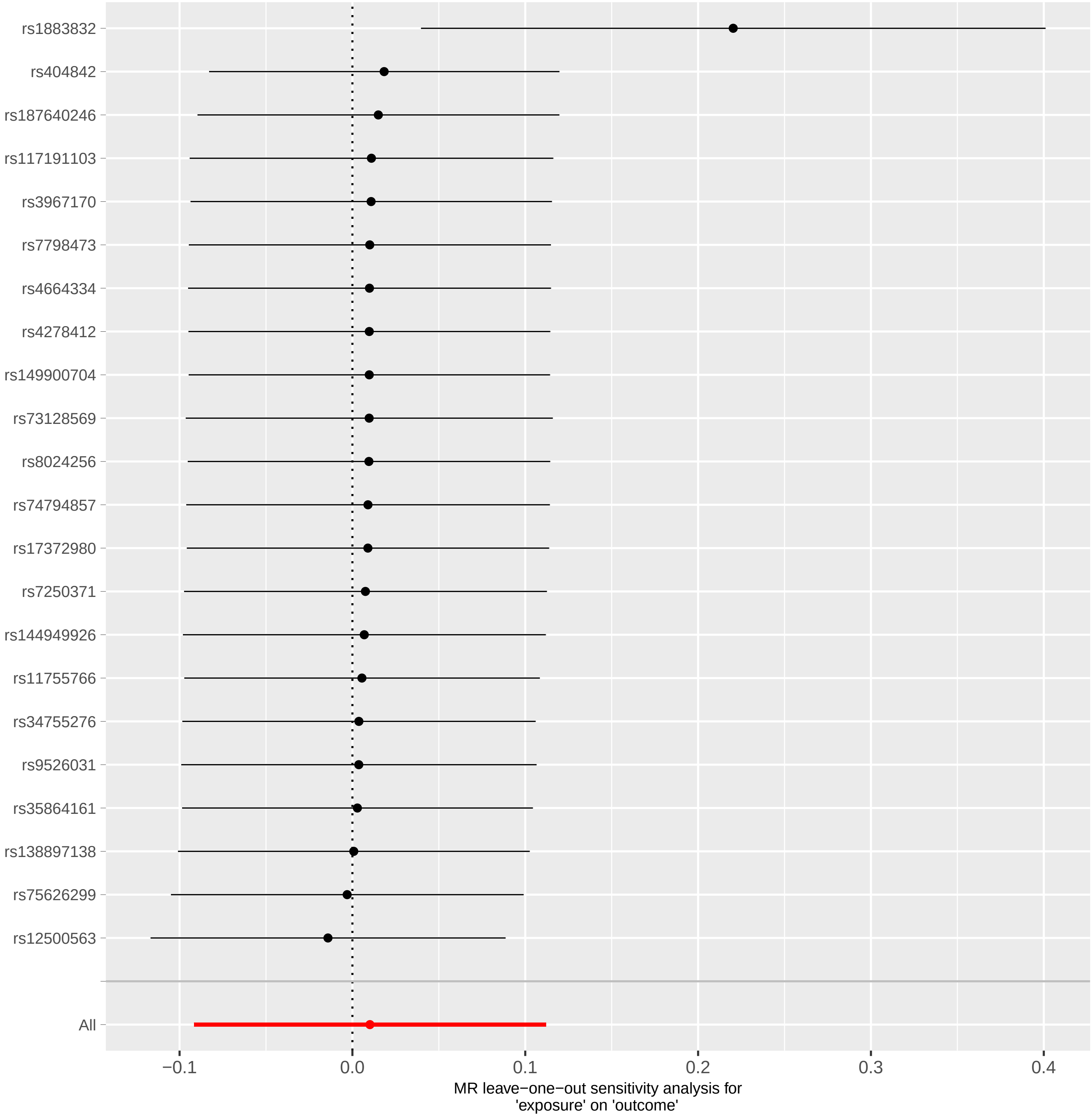
rs1883832
rs404842
rs187640246
rs117191103
rs3967170
rs7798473
rs4664334
rs4278412
rs149900704
rs73128569
rs8024256
rs74794857
rs17372980
rs7250371
rs144949926
rs11755766
rs34755276
rs9526031
rs35864161
rs138897138
rs75626299
rs12500563
All
−0.1
0.0
0.1
0.2
0.3
0.4
MR leave−one−out sensitivity analysis for
'exposure' on 'outcome'

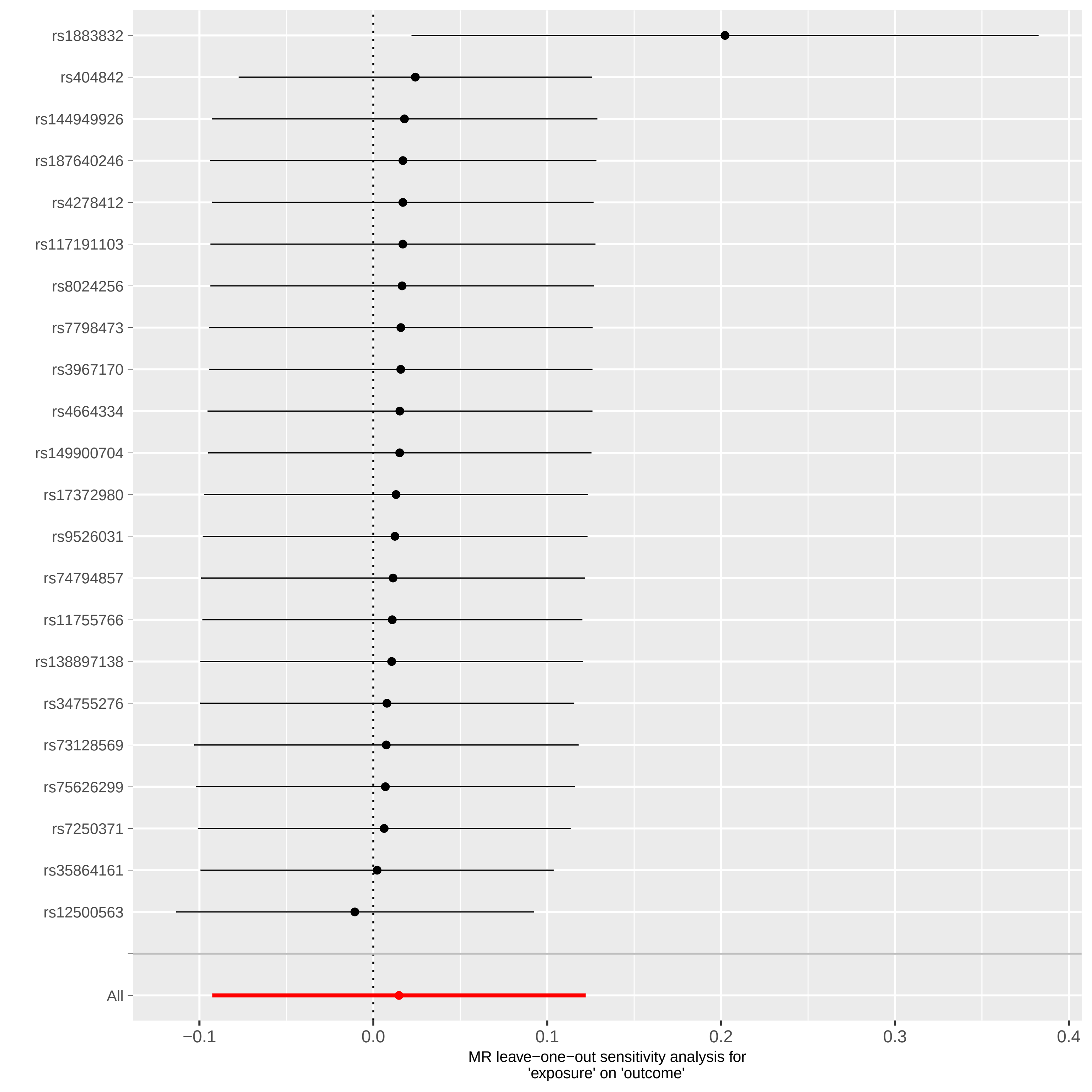
rs1883832
rs404842
rs144949926
rs187640246
rs4278412
rs117191103
rs8024256
rs7798473
rs3967170
rs4664334
rs149900704
rs17372980
rs9526031
rs74794857
rs11755766
rs138897138
rs34755276
rs73128569
rs75626299
rs7250371
rs35864161
rs12500563
All
−0.1
0.0
0.1
0.2
0.3
0.4
MR leave−one−out sensitivity analysis for
'exposure' on 'outcome'

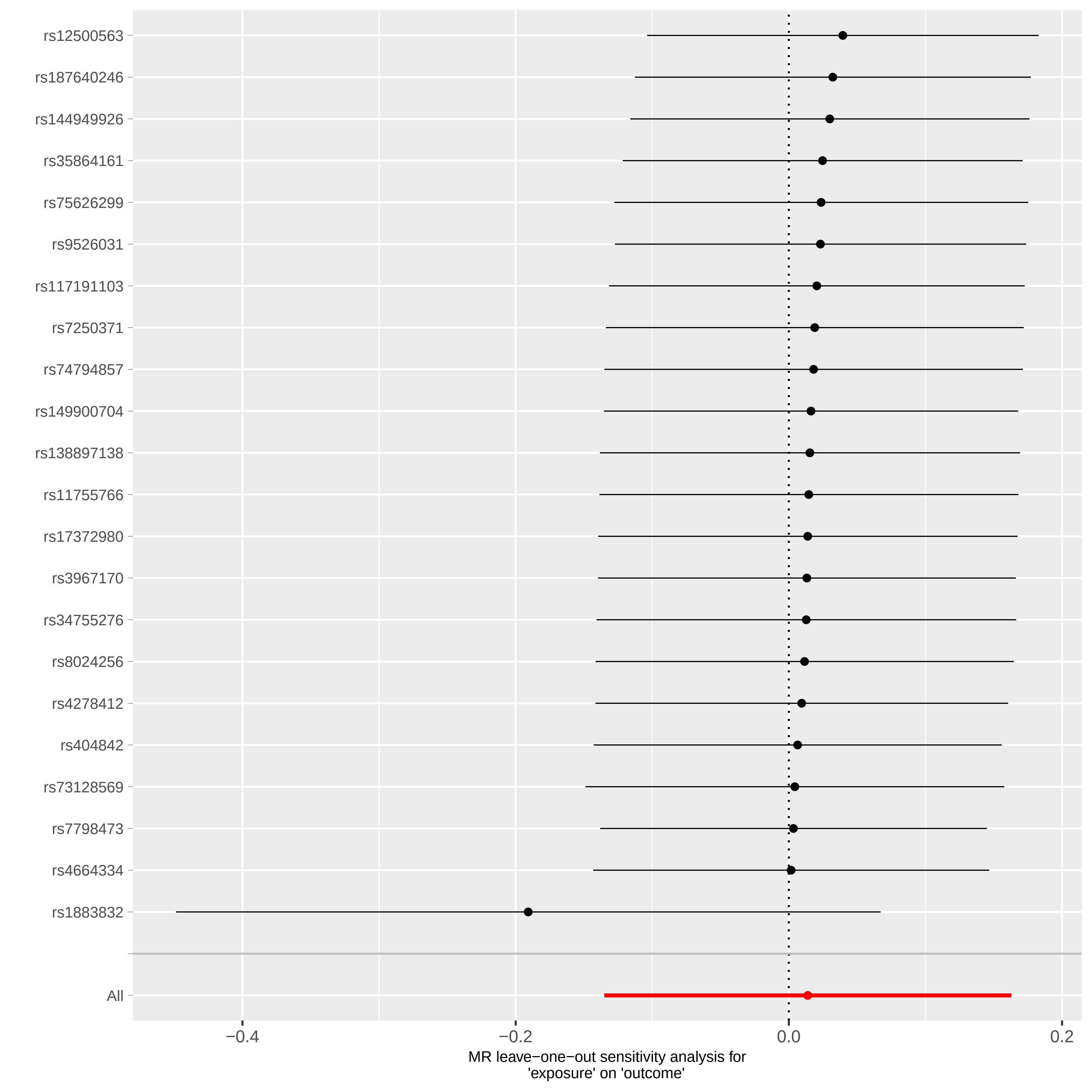

rs12500563
rs187640246
rs144949926
rs35864161
rs75626299
rs9526031
rs117191103
rs7250371
rs74794857
rs149900704
rs138897138
rs11755766
rs17372980
rs3967170
rs34755276
rs8024256
rs4278412
rs404842
rs73128569
rs7798473
rs4664334
rs1883832
All
−0.4
−0.2
0.0
0.2
MR leave−one−out sensitivity analysis for
'exposure' on 'outcome'

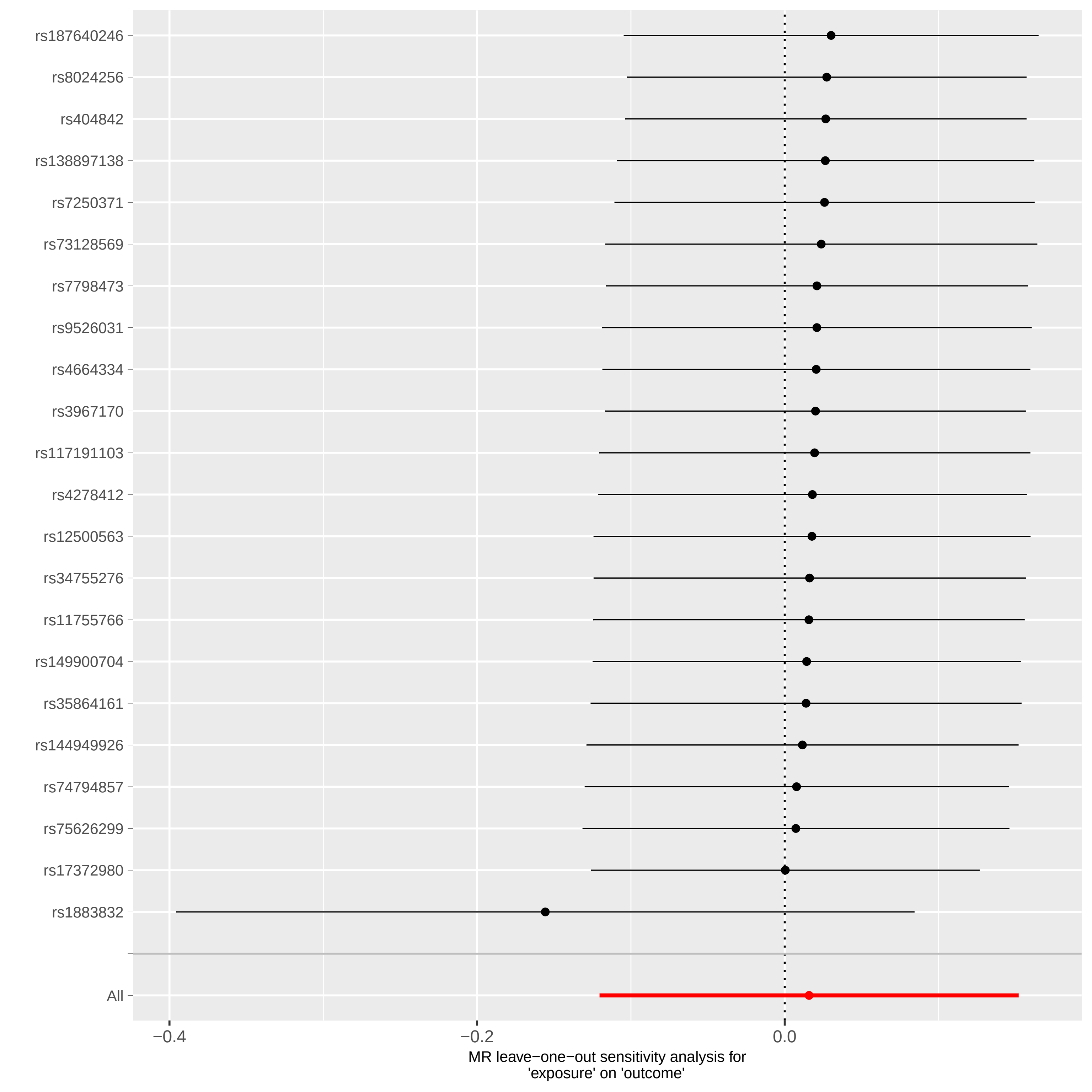
rs187640246
rs8024256
rs404842
rs138897138
rs7250371
rs73128569
rs7798473
rs9526031
rs4664334
rs3967170
rs117191103
rs4278412
rs12500563
rs34755276
rs11755766
rs149900704
rs35864161
rs144949926
rs74794857
rs75626299
rs17372980
rs1883832
All
−0.4
−0.2
0.0
MR leave−one−out sensitivity analysis for
'exposure' on 'outcome'

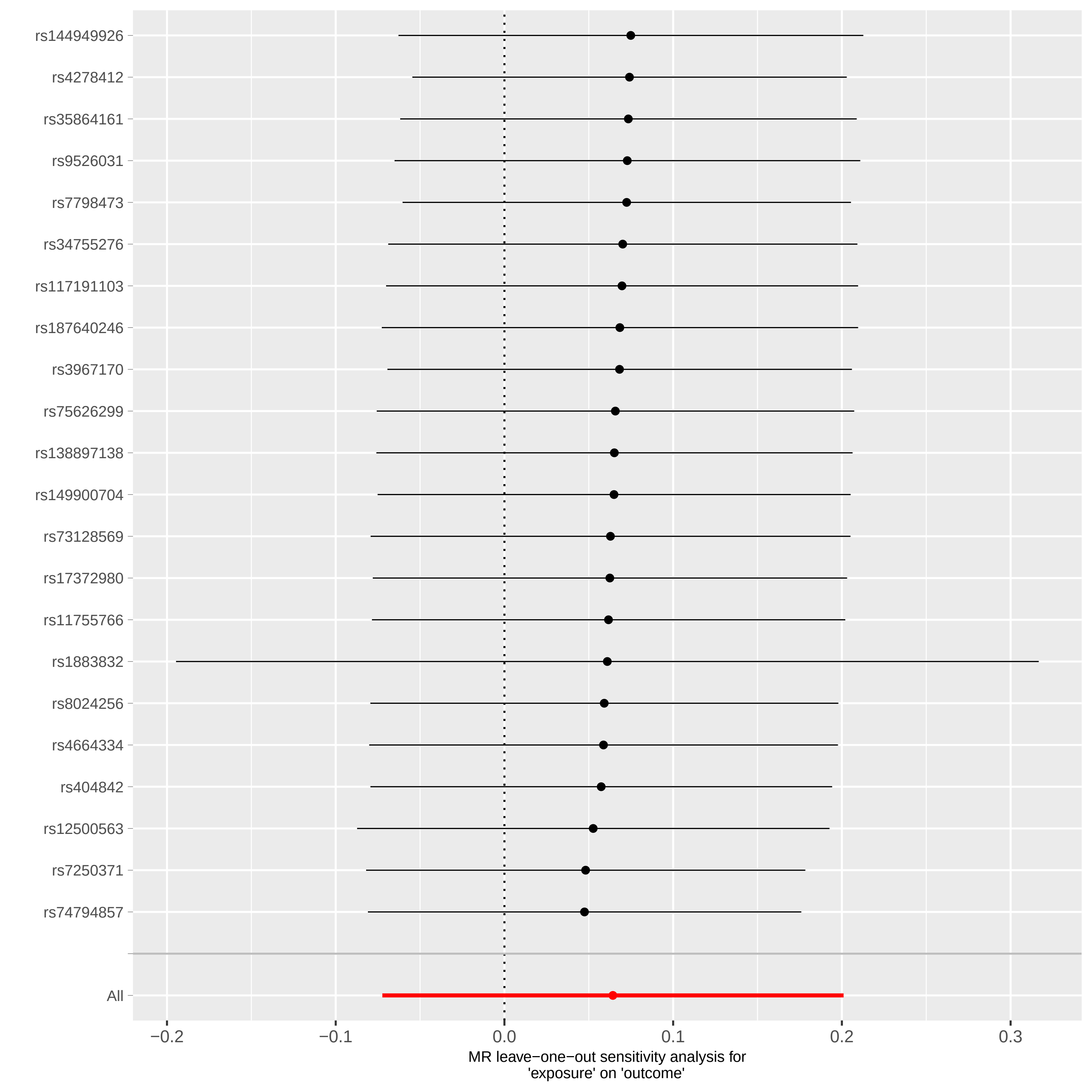

rs144949926
rs4278412
rs35864161
rs9526031
rs7798473
rs34755276
rs117191103
rs187640246
rs3967170
rs75626299
rs138897138
rs149900704
rs73128569
rs17372980
rs11755766
rs1883832
rs8024256
rs4664334
rs404842
rs12500563
rs7250371
rs74794857
All
−0.2
−0.1
0.0
0.1
0.2
0.3
MR leave−one−out sensitivity analysis for
'exposure' on 'outcome'

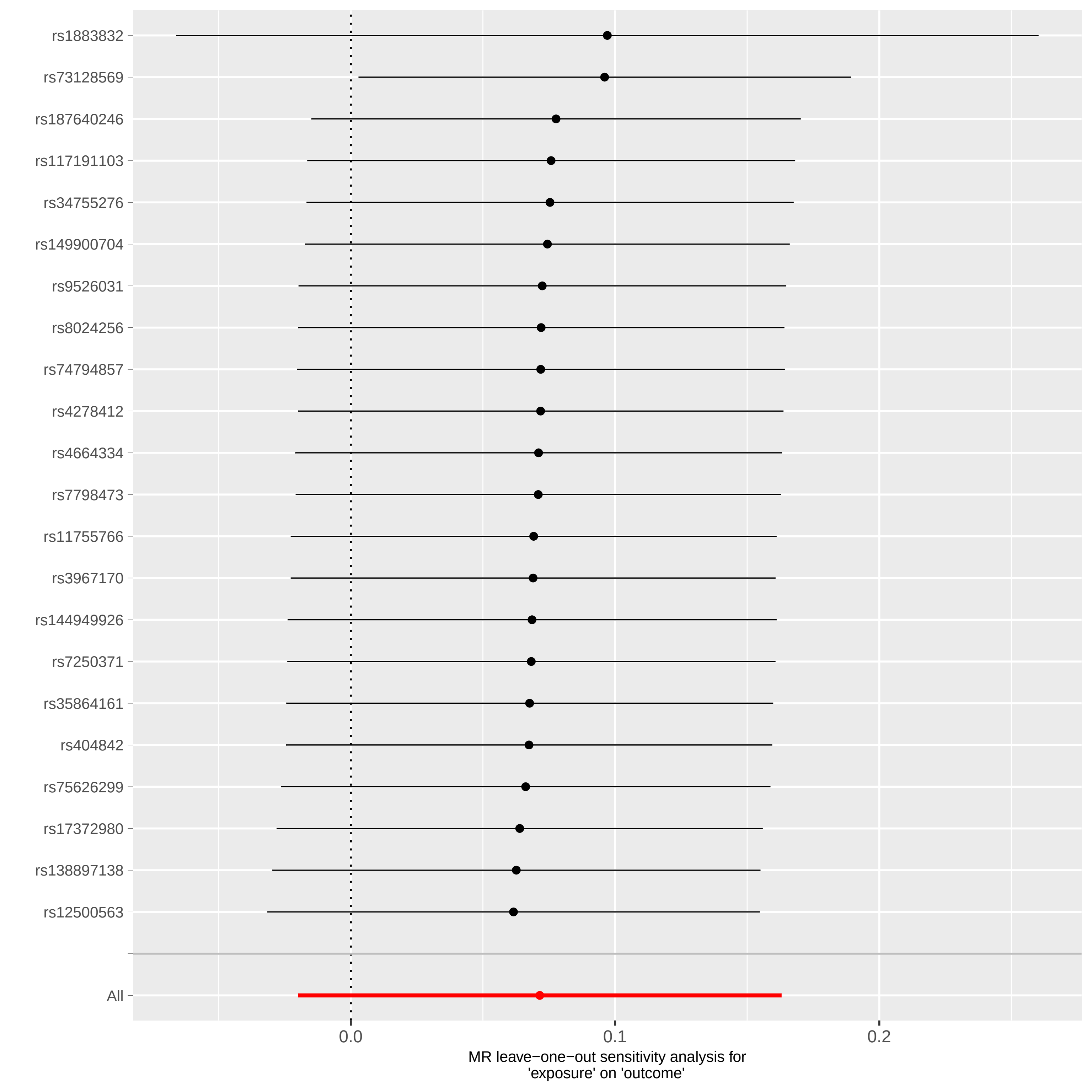
rs1883832
rs73128569
rs187640246
rs117191103
rs34755276
rs149900704
rs9526031
rs8024256
rs74794857
rs4278412
rs4664334
rs7798473
rs11755766
rs3967170
rs144949926
rs7250371
rs35864161
rs404842
rs75626299
rs17372980
rs138897138
rs12500563
All
0.0
0.1
0.2
MR leave−one−out sensitivity analysis for
'exposure' on 'outcome'

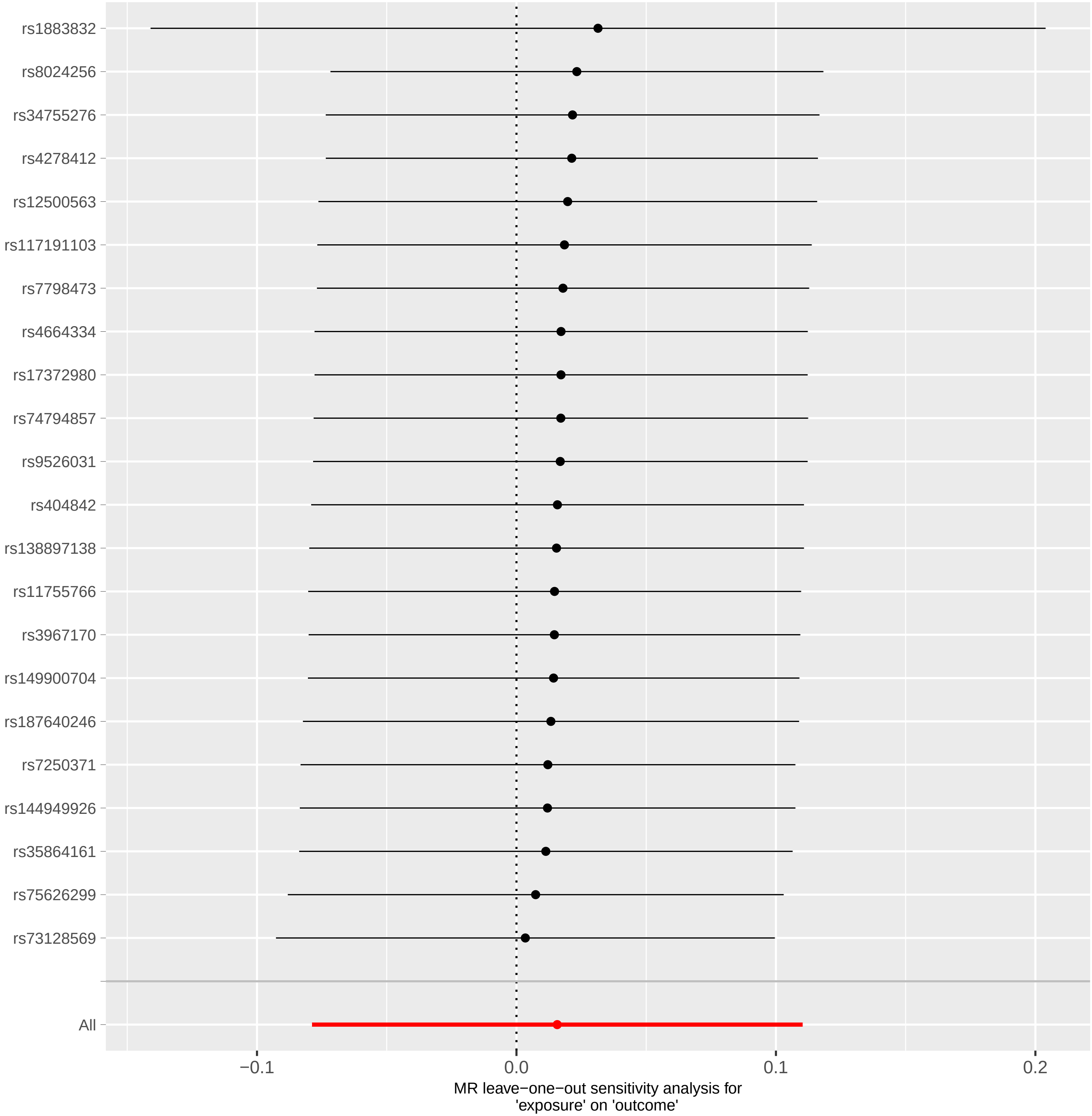
rs1883832
rs8024256
rs34755276
rs4278412
rs12500563
rs117191103
rs7798473
rs4664334
rs17372980
rs74794857
rs9526031
rs404842
rs138897138
rs11755766
rs3967170
rs149900704
rs187640246
rs7250371
rs144949926
rs35864161
rs75626299
rs73128569
All
−0.1
0.0
0.1
0.2
MR leave−one−out sensitivity analysis for
'exposure' on 'outcome'

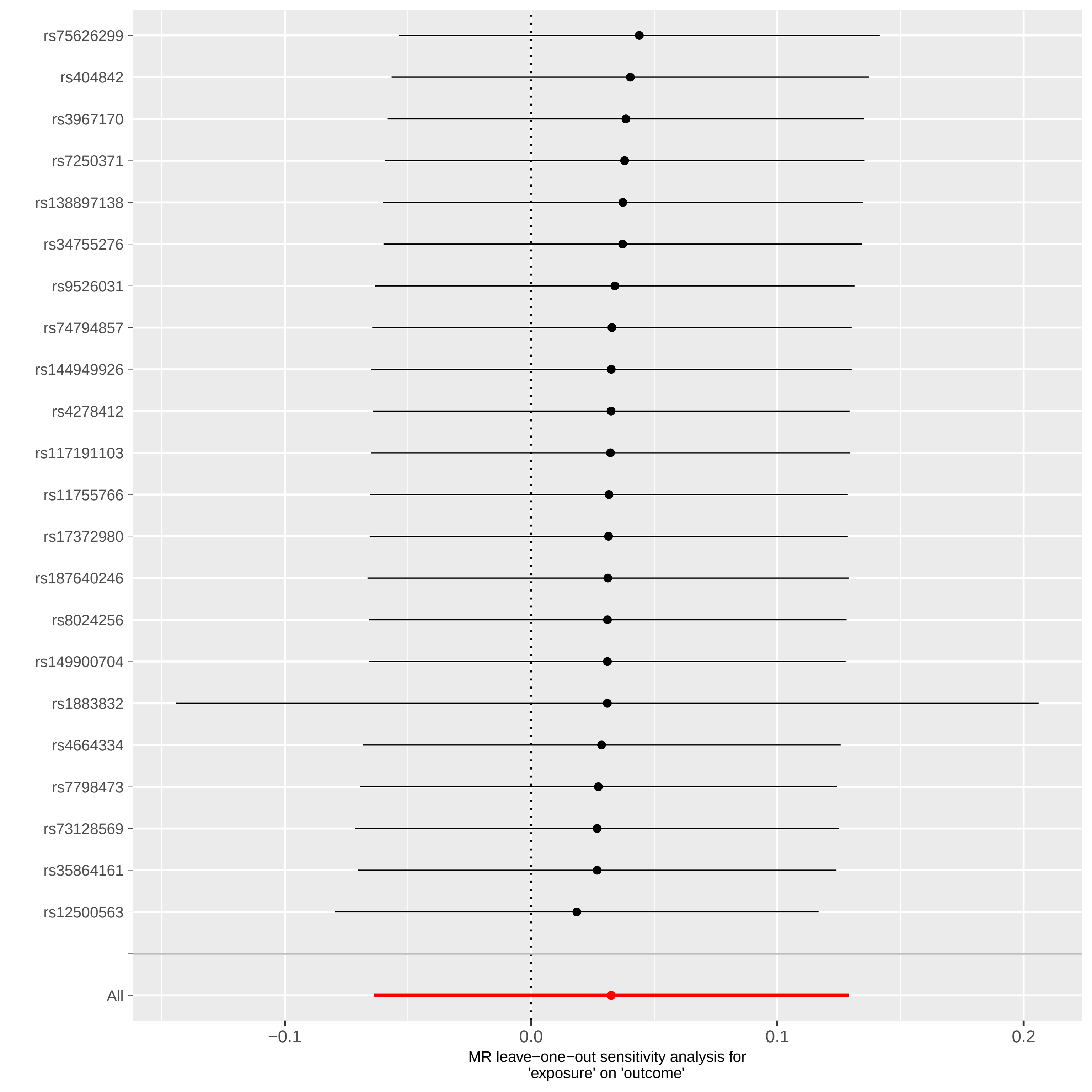
rs75626299
rs404842
rs3967170
rs7250371
rs138897138
rs34755276
rs9526031
rs74794857
rs144949926
rs4278412
rs117191103
rs11755766
rs17372980
rs187640246
rs8024256
rs149900704
rs1883832
rs4664334
rs7798473
rs73128569
rs35864161
rs12500563
All
−0.1
0.0
0.1
0.2
MR leave−one−out sensitivity analysis for
'exposure' on 'outcome'

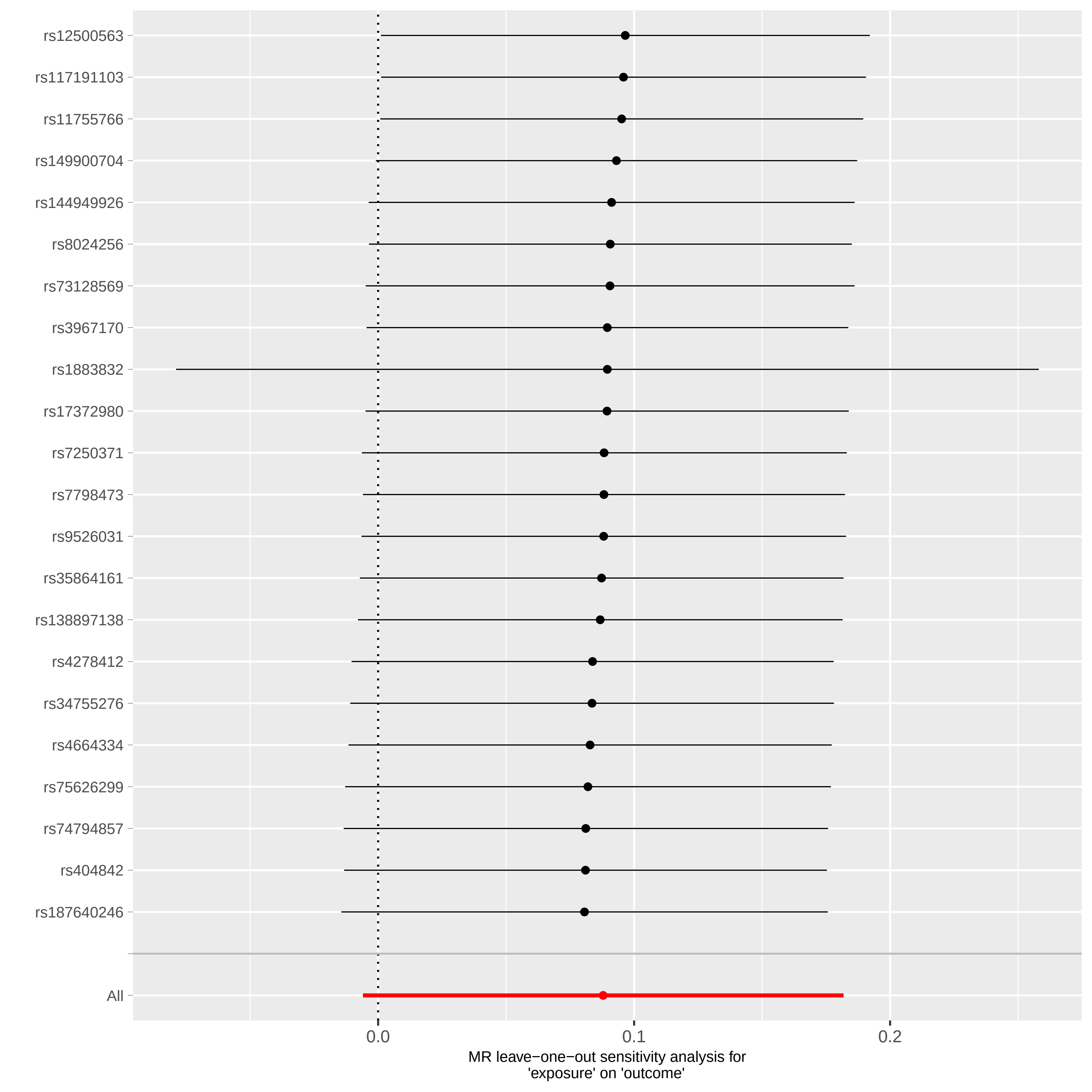

rs12500563
rs117191103
rs11755766
rs149900704
rs144949926
rs8024256
rs73128569
rs3967170
rs1883832
rs17372980
rs7250371
rs7798473
rs9526031
rs35864161
rs138897138
rs4278412
rs34755276
rs4664334
rs75626299
rs74794857
rs404842
rs187640246
All
0.0
0.1
0.2
MR leave−one−out sensitivity analysis for
'exposure' on 'outcome'

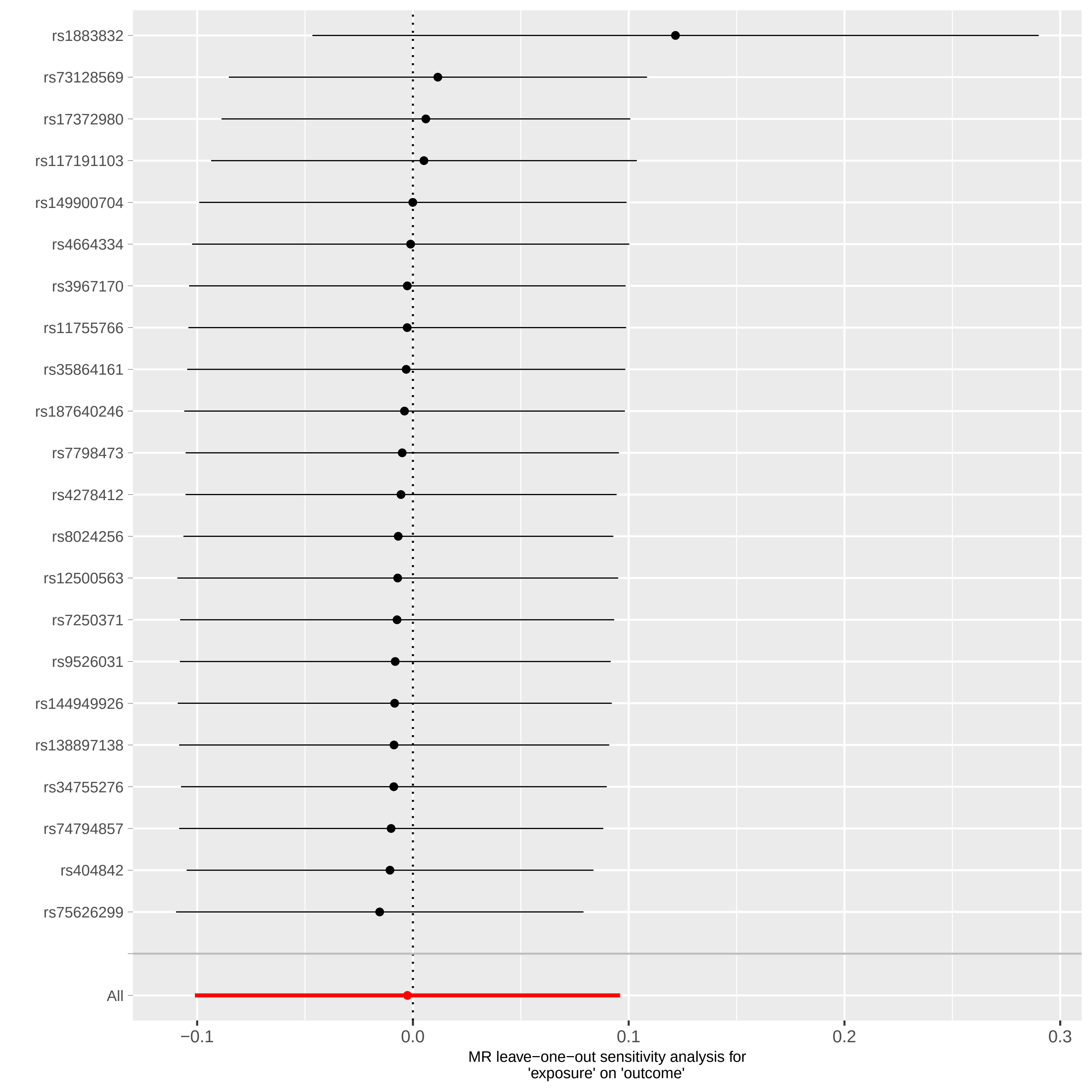

rs1883832
rs73128569
rs17372980
rs117191103
rs149900704
rs4664334
rs3967170
rs11755766
rs35864161
rs187640246
rs7798473
rs4278412
rs8024256
rs12500563
rs7250371
rs9526031
rs144949926
rs138897138
rs34755276
rs74794857
rs404842
rs75626299
All
−0.1
0.0
0.1
0.2
0.3
MR leave−one−out sensitivity analysis for
'exposure' on 'outcome'

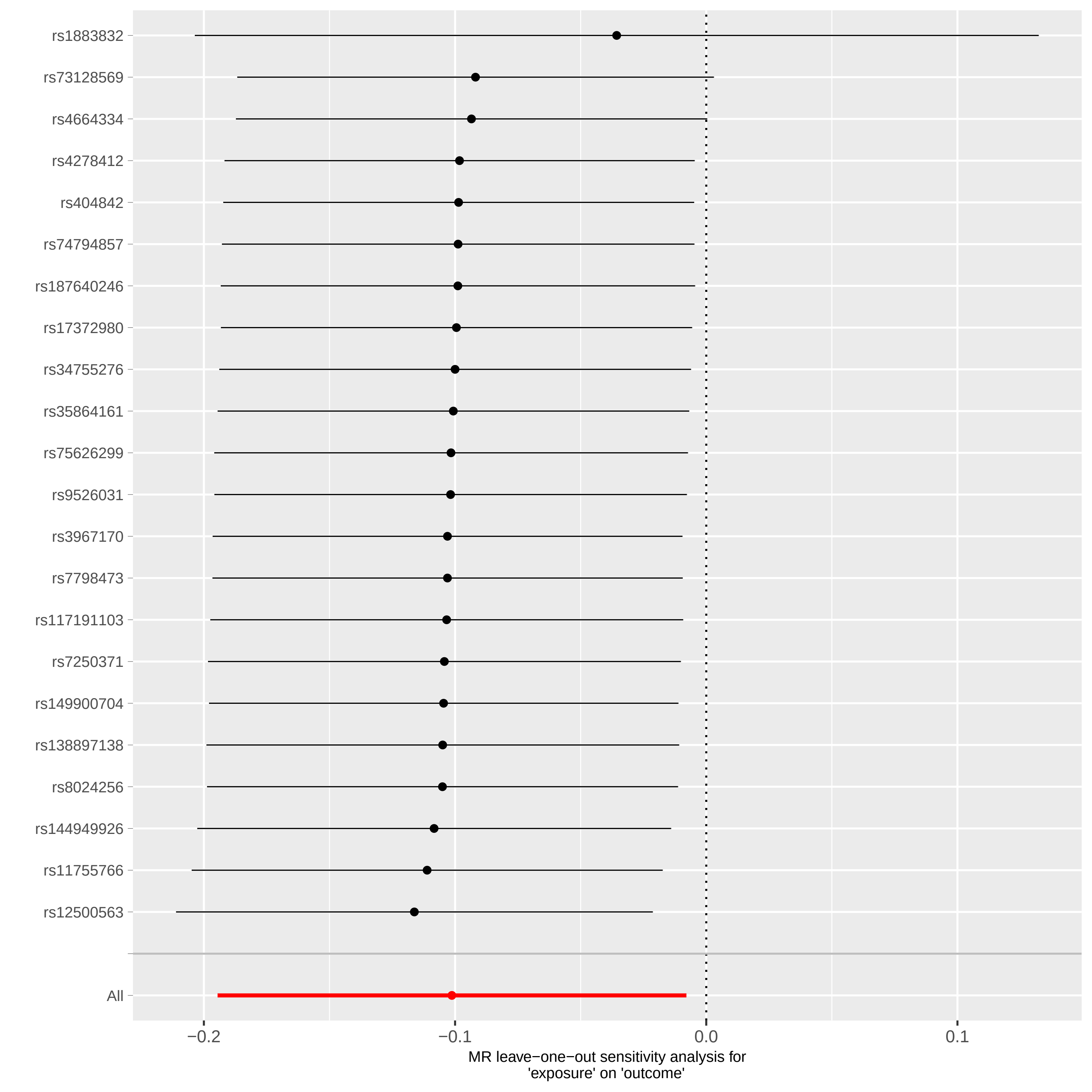
rs1883832
rs73128569
rs4664334
rs4278412
rs404842
rs74794857
rs187640246
rs17372980
rs34755276
rs35864161
rs75626299
rs9526031
rs3967170
rs7798473
rs117191103
rs7250371
rs149900704
rs138897138
rs8024256
rs144949926
rs11755766
rs12500563
All
−0.2
−0.1
0.0
0.1
MR leave−one−out sensitivity analysis for
'exposure' on 'outcome'

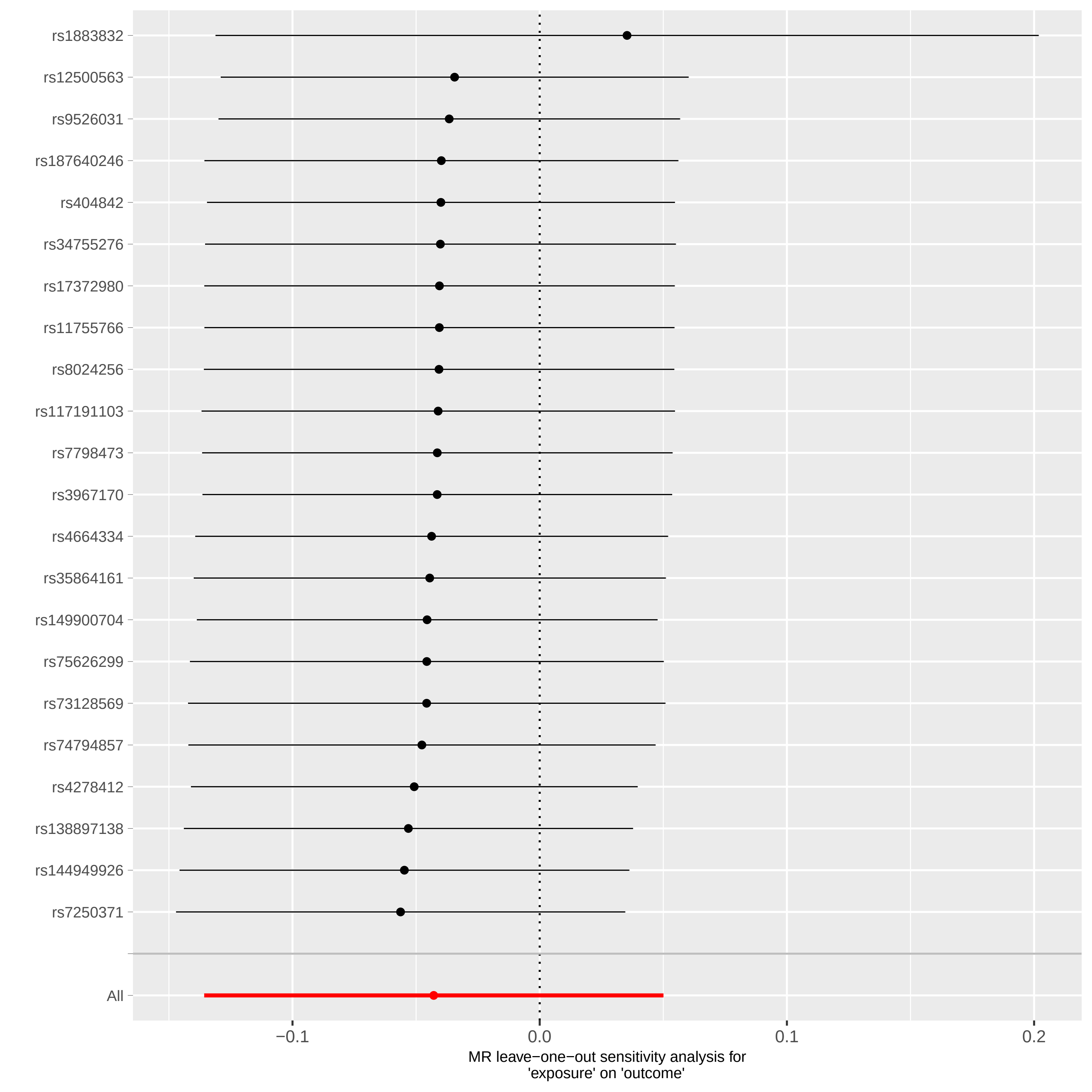

rs1883832
rs12500563
rs9526031
rs187640246
rs404842
rs34755276
rs17372980
rs11755766
rs8024256
rs117191103
rs7798473
rs3967170
rs4664334
rs35864161
rs149900704
rs75626299
rs73128569
rs74794857
rs4278412
rs138897138
rs144949926
rs7250371
All
−0.1
0.0
0.1
0.2
MR leave−one−out sensitivity analysis for
'exposure' on 'outcome'

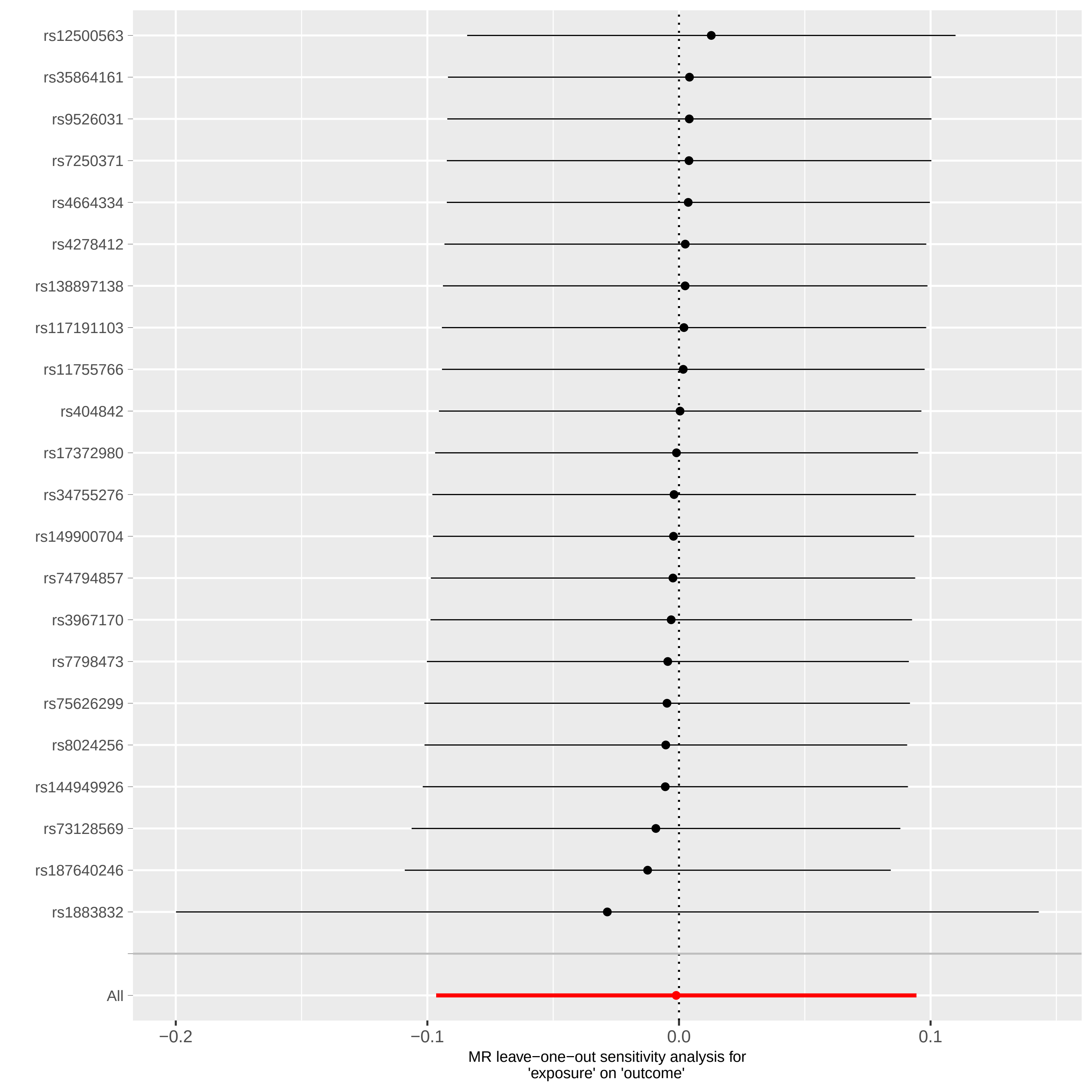

rs12500563
rs35864161
rs9526031
rs7250371
rs4664334
rs4278412
rs138897138
rs117191103
rs11755766
rs404842
rs17372980
rs34755276
rs149900704
rs74794857
rs3967170
rs7798473
rs75626299
rs8024256
rs144949926
rs73128569
rs187640246
rs1883832
All
−0.2
−0.1
0.0
0.1
MR leave−one−out sensitivity analysis for
'exposure' on 'outcome'

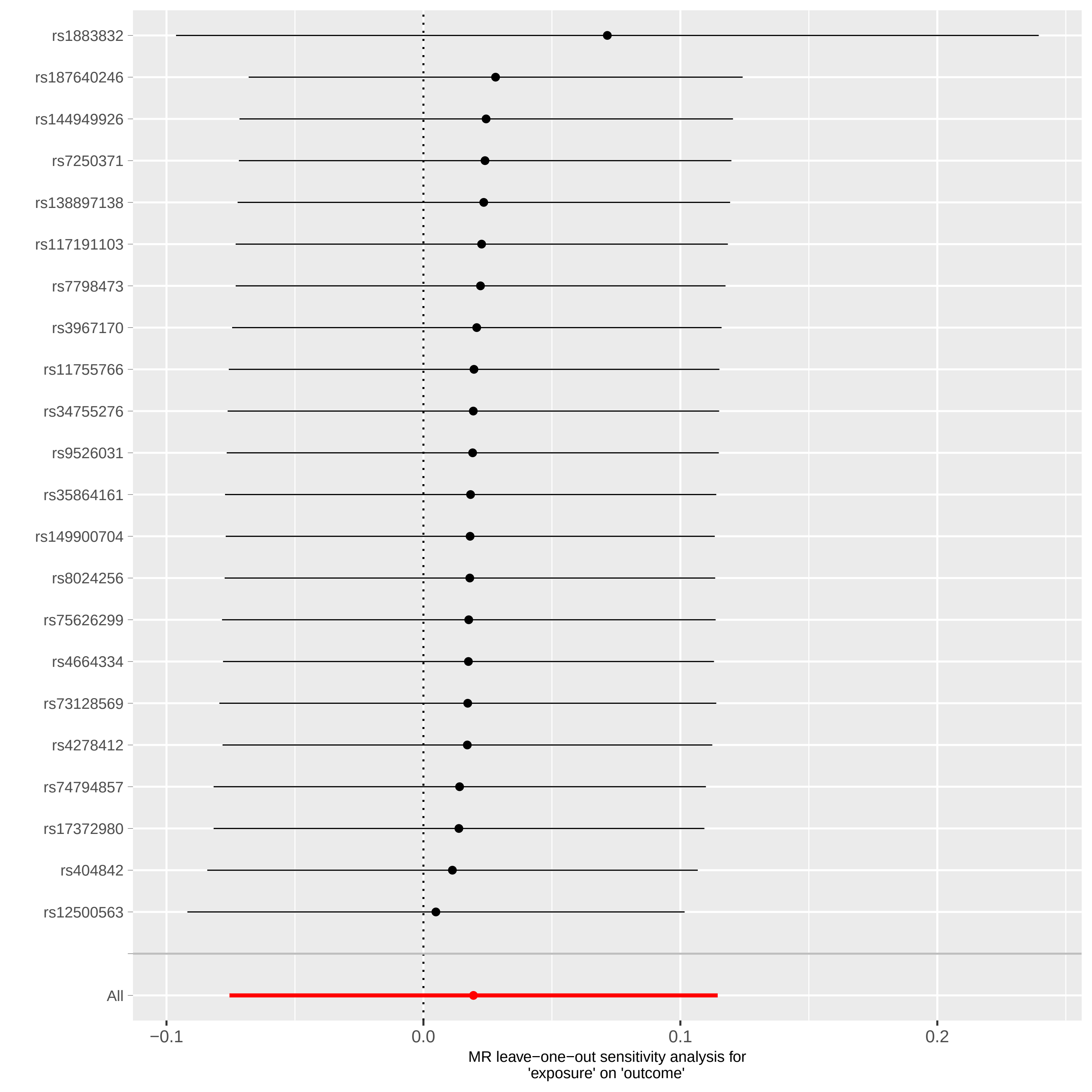

rs1883832
rs187640246
rs144949926
rs7250371
rs138897138
rs117191103
rs7798473
rs3967170
rs11755766
rs34755276
rs9526031
rs35864161
rs149900704
rs8024256
rs75626299
rs4664334
rs73128569
rs4278412
rs74794857
rs17372980
rs404842
rs12500563
All
−0.1
0.0
0.1
0.2
MR leave−one−out sensitivity analysis for
'exposure' on 'outcome'

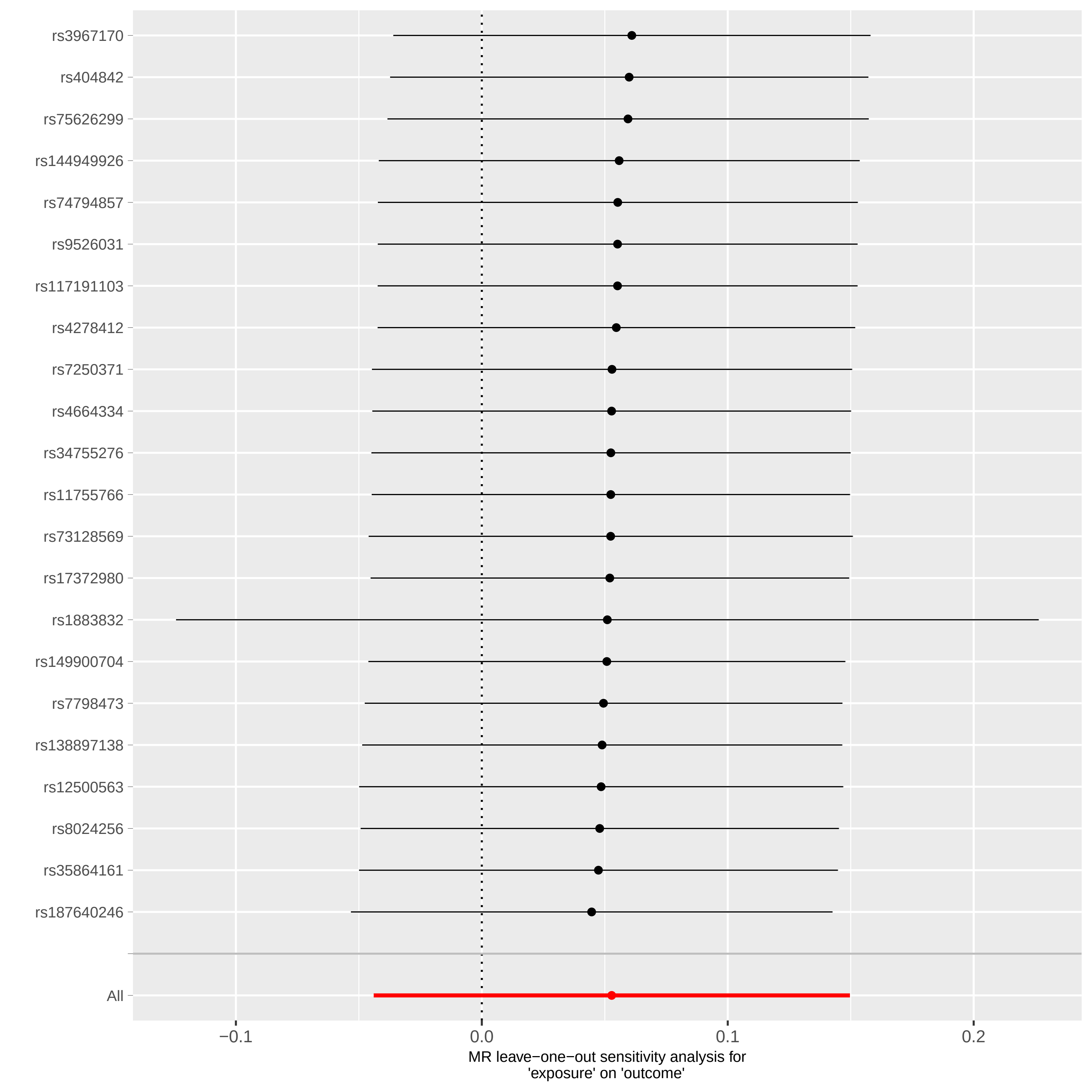
rs3967170
rs404842
rs75626299
rs144949926
rs74794857
rs9526031
rs117191103
rs4278412
rs7250371
rs4664334
rs34755276
rs11755766
rs73128569
rs17372980
rs1883832
rs149900704
rs7798473
rs138897138
rs12500563
rs8024256
rs35864161
rs187640246
All
−0.1
0.0
0.1
0.2
MR leave−one−out sensitivity analysis for
'exposure' on 'outcome'

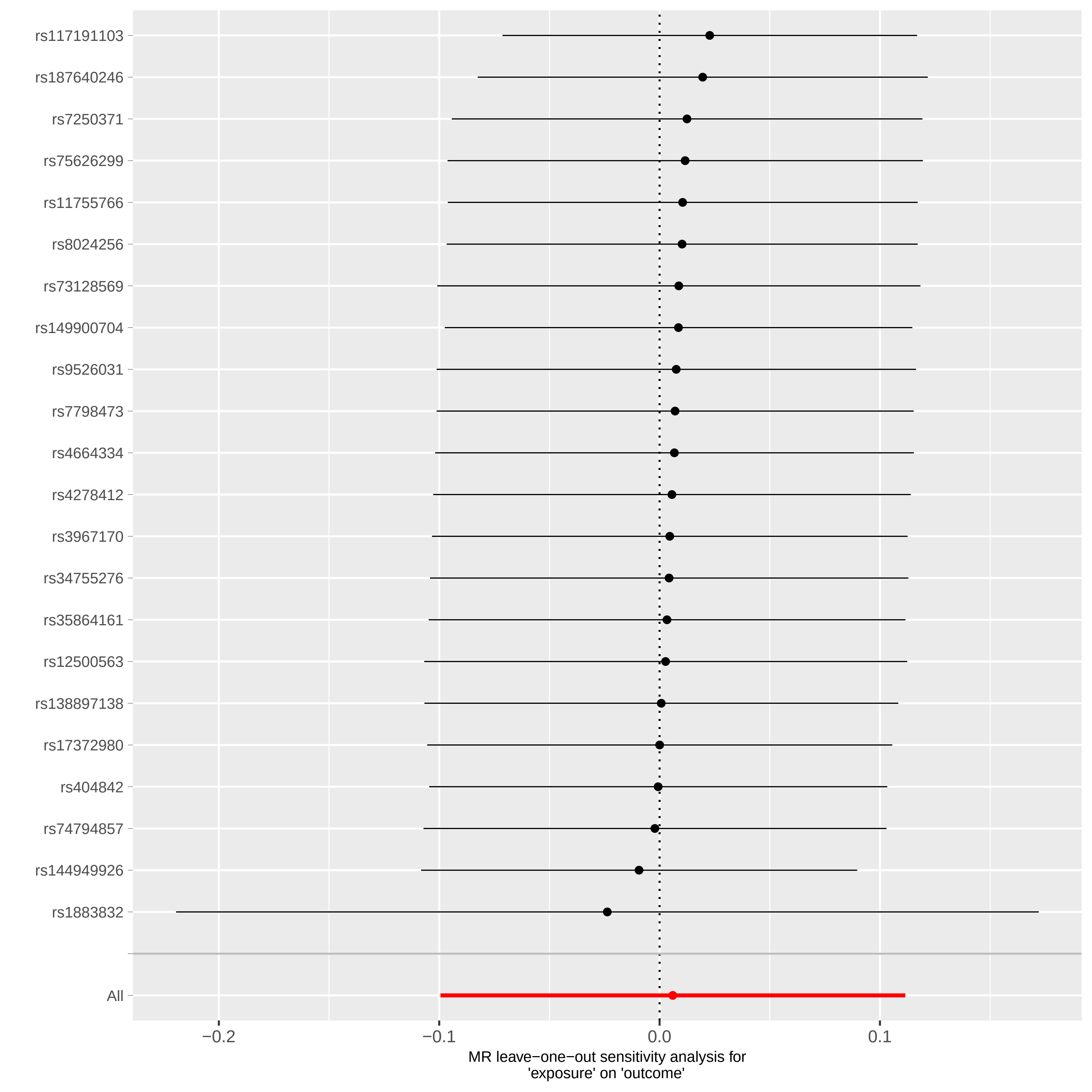
rs117191103
rs187640246
rs7250371
rs75626299
rs11755766
rs8024256
rs73128569
rs149900704
rs9526031
rs7798473
rs4664334
rs4278412
rs3967170
rs34755276
rs35864161
rs12500563
rs138897138
rs17372980
rs404842
rs74794857
rs144949926
rs1883832
All
−0.2
−0.1
0.0
0.1
MR leave−one−out sensitivity analysis for
'exposure' on 'outcome'

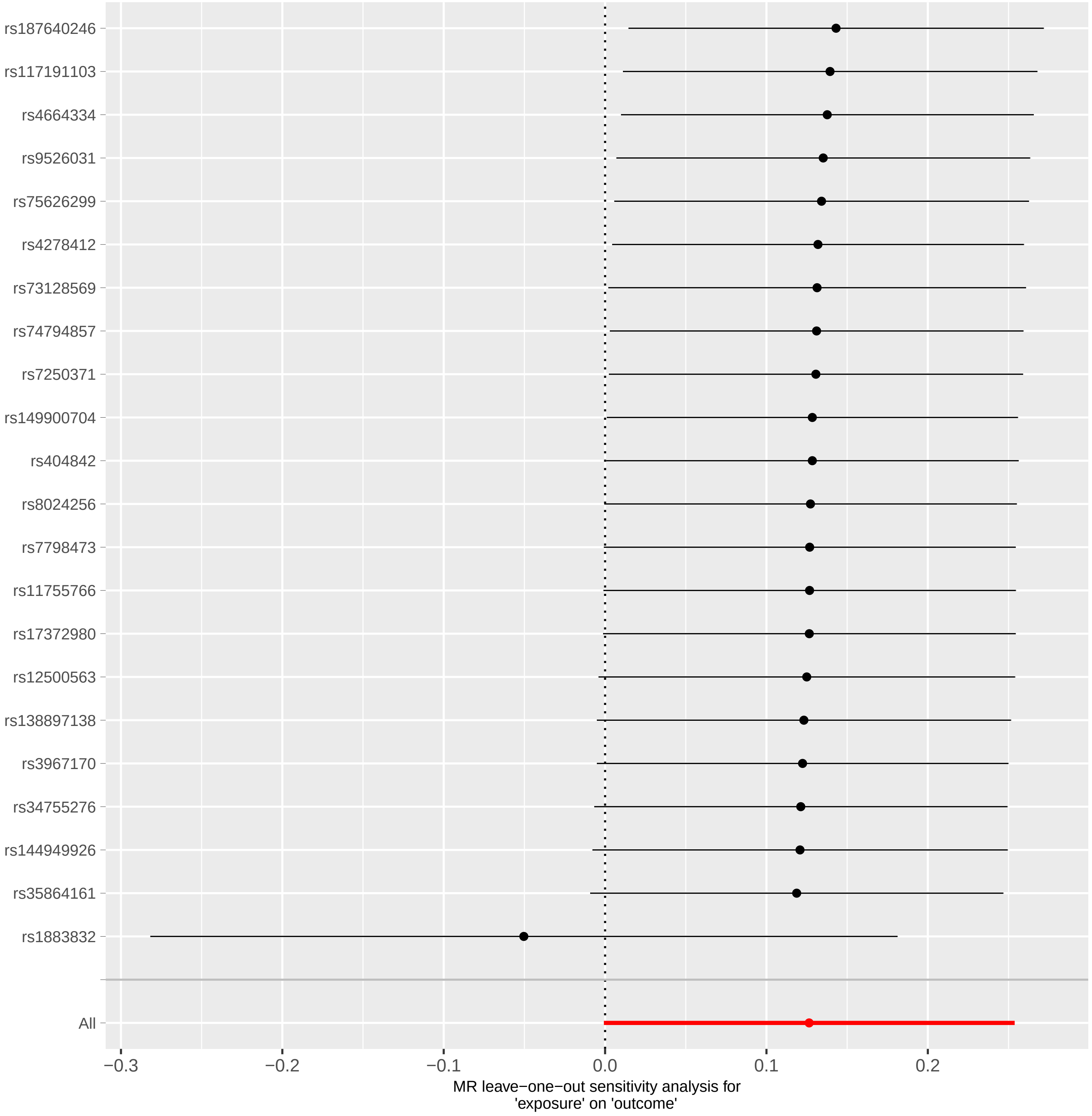
rs187640246
rs117191103
rs4664334
rs9526031
rs75626299
rs4278412
rs73128569
rs74794857
rs7250371
rs149900704
rs404842
rs8024256
rs7798473
rs11755766
rs17372980
rs12500563
rs138897138
rs3967170
rs34755276
rs144949926
rs35864161
rs1883832
All
−0.3
−0.2
−0.1
0.0
0.1
0.2
MR leave−one−out sensitivity analysis for
'exposure' on 'outcome'

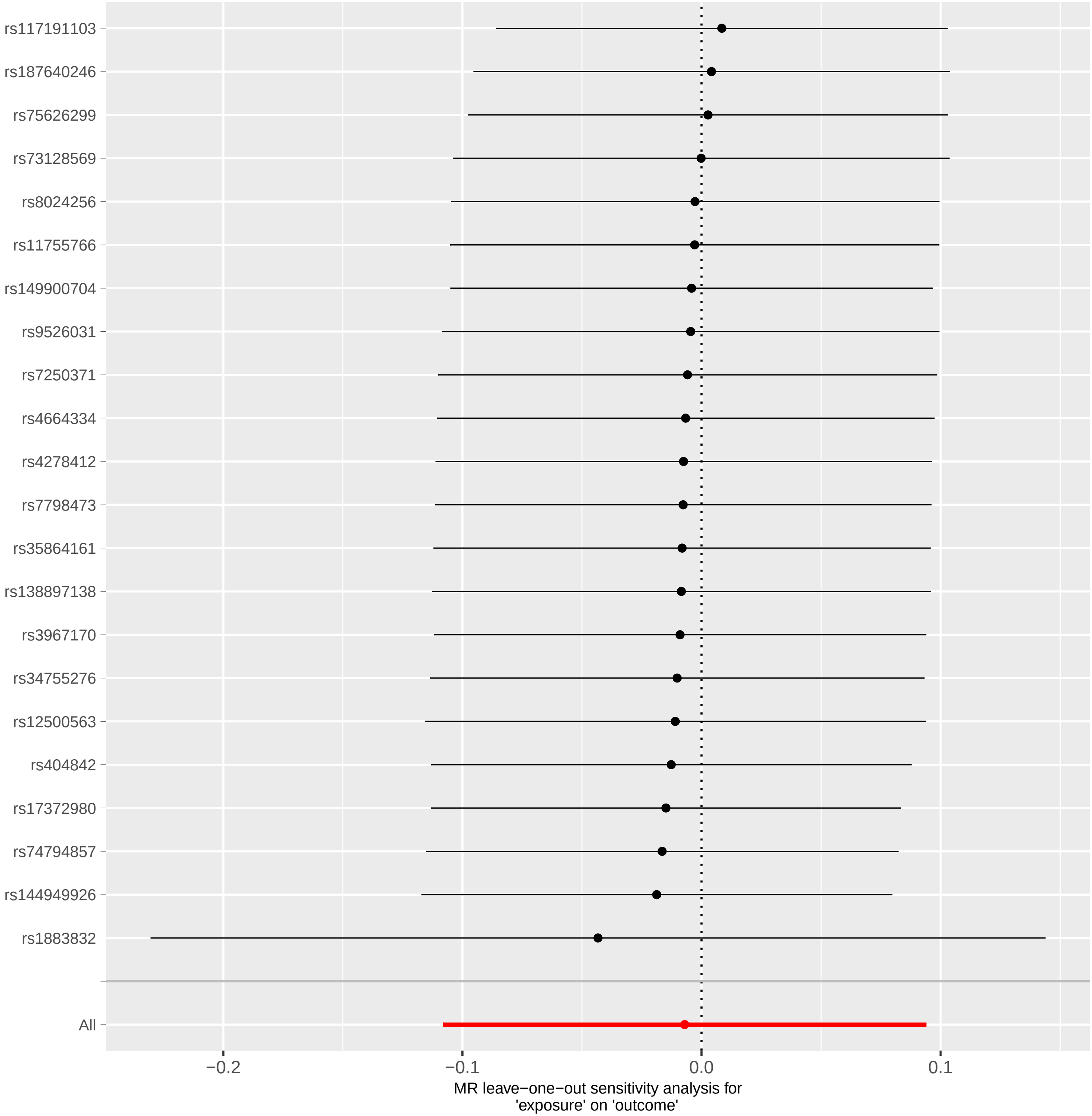
rs117191103
rs187640246
rs75626299
rs73128569
rs8024256
rs11755766
rs149900704
rs9526031
rs7250371
rs4664334
rs4278412
rs7798473
rs35864161
rs138897138
rs3967170
rs34755276
rs12500563
rs404842
rs17372980
rs74794857
rs144949926
rs1883832
All
−0.2
−0.1
0.0
0.1
MR leave−one−out sensitivity analysis for
'exposure' on 'outcome'

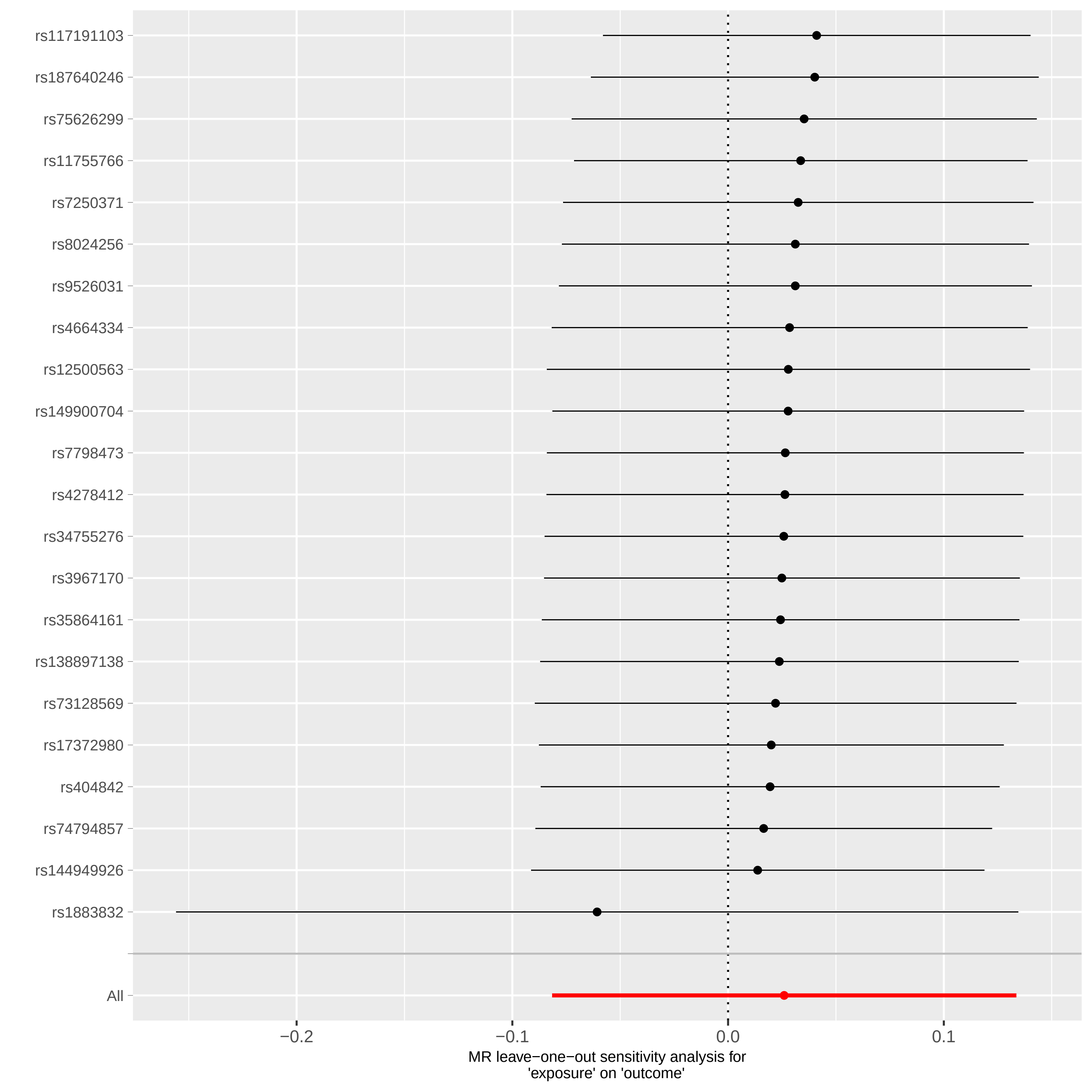

rs117191103
rs187640246
rs75626299
rs11755766
rs7250371
rs8024256
rs9526031
rs4664334
rs12500563
rs149900704
rs7798473
rs4278412
rs34755276
rs3967170
rs35864161
rs138897138
rs73128569
rs17372980
rs404842
rs74794857
rs144949926
rs1883832
All
−0.2
−0.1
0.0
0.1
MR leave−one−out sensitivity analysis for
'exposure' on 'outcome'

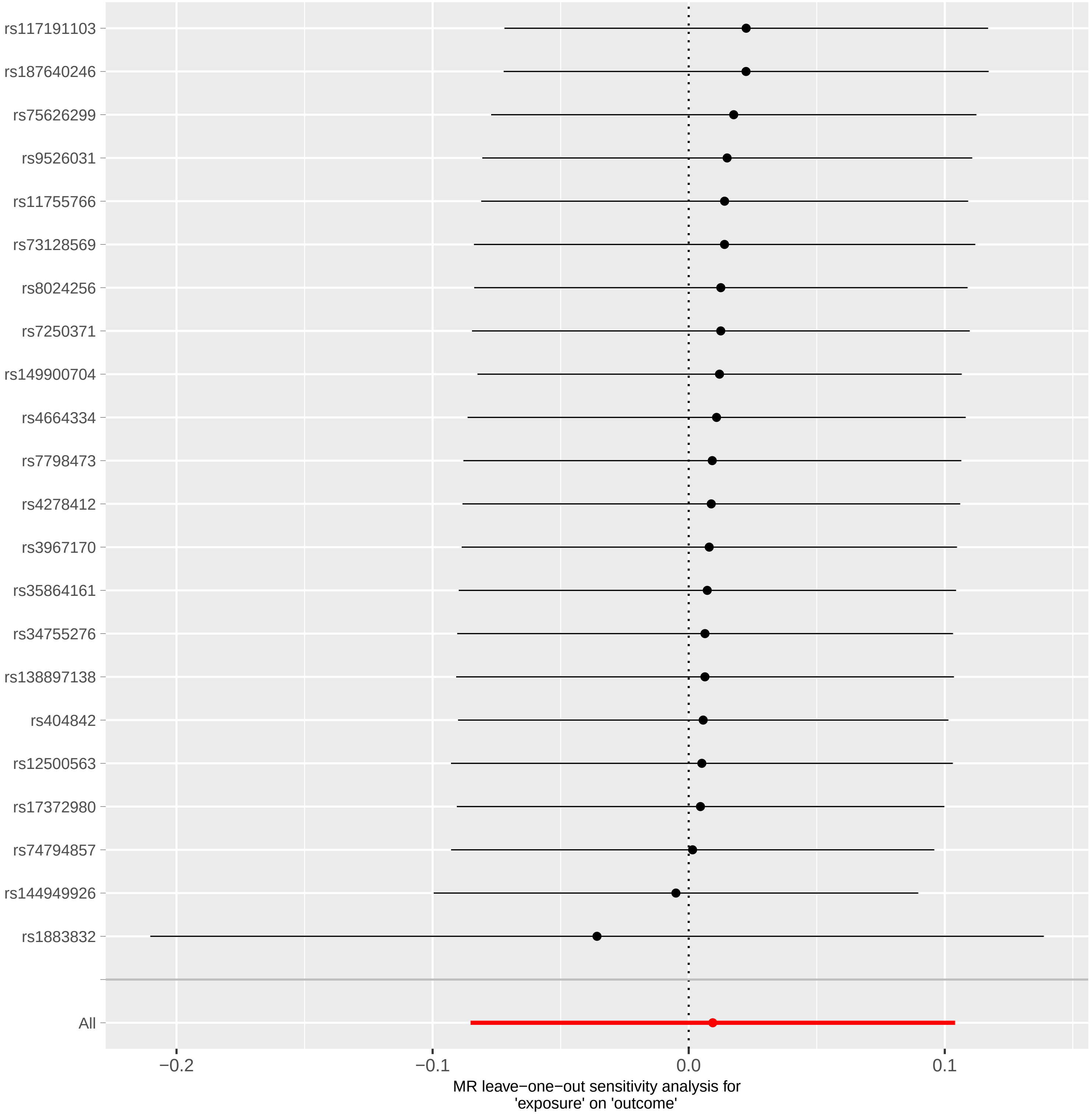

rs117191103
rs187640246
rs75626299
rs9526031
rs11755766
rs73128569
rs8024256
rs7250371
rs149900704
rs4664334
rs7798473
rs4278412
rs3967170
rs35864161
rs34755276
rs138897138
rs404842
rs12500563
rs17372980
rs74794857
rs144949926
rs1883832
All
−0.2
−0.1
0.0
0.1
MR leave−one−out sensitivity analysis for
'exposure' on 'outcome'

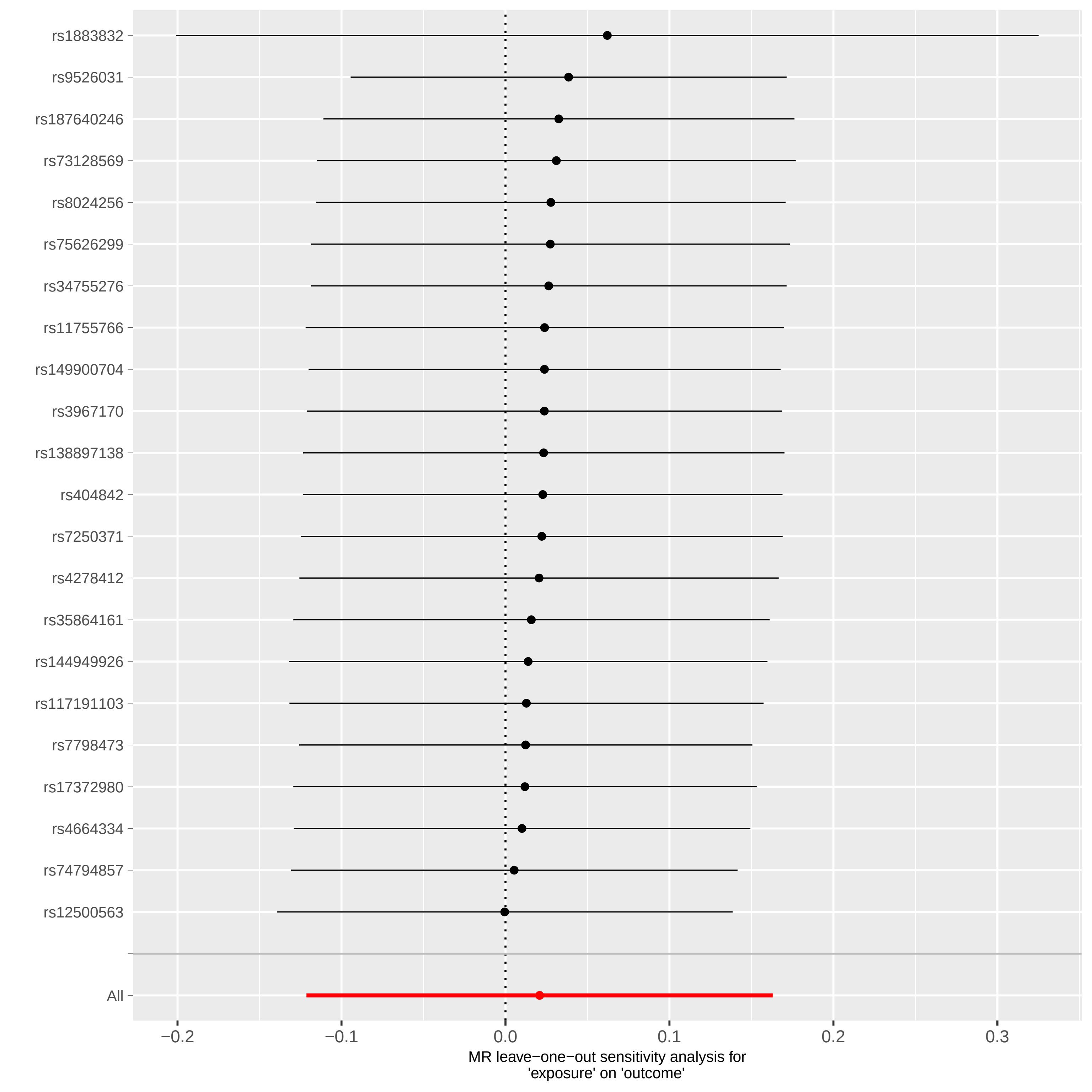
rs1883832
rs9526031
rs187640246
rs73128569
rs8024256
rs75626299
rs34755276
rs11755766
rs149900704
rs3967170
rs138897138
rs404842
rs7250371
rs4278412
rs35864161
rs144949926
rs117191103
rs7798473
rs17372980
rs4664334
rs74794857
rs12500563
All
−0.2
−0.1
0.0
0.1
0.2
0.3
MR leave−one−out sensitivity analysis for
'exposure' on 'outcome'

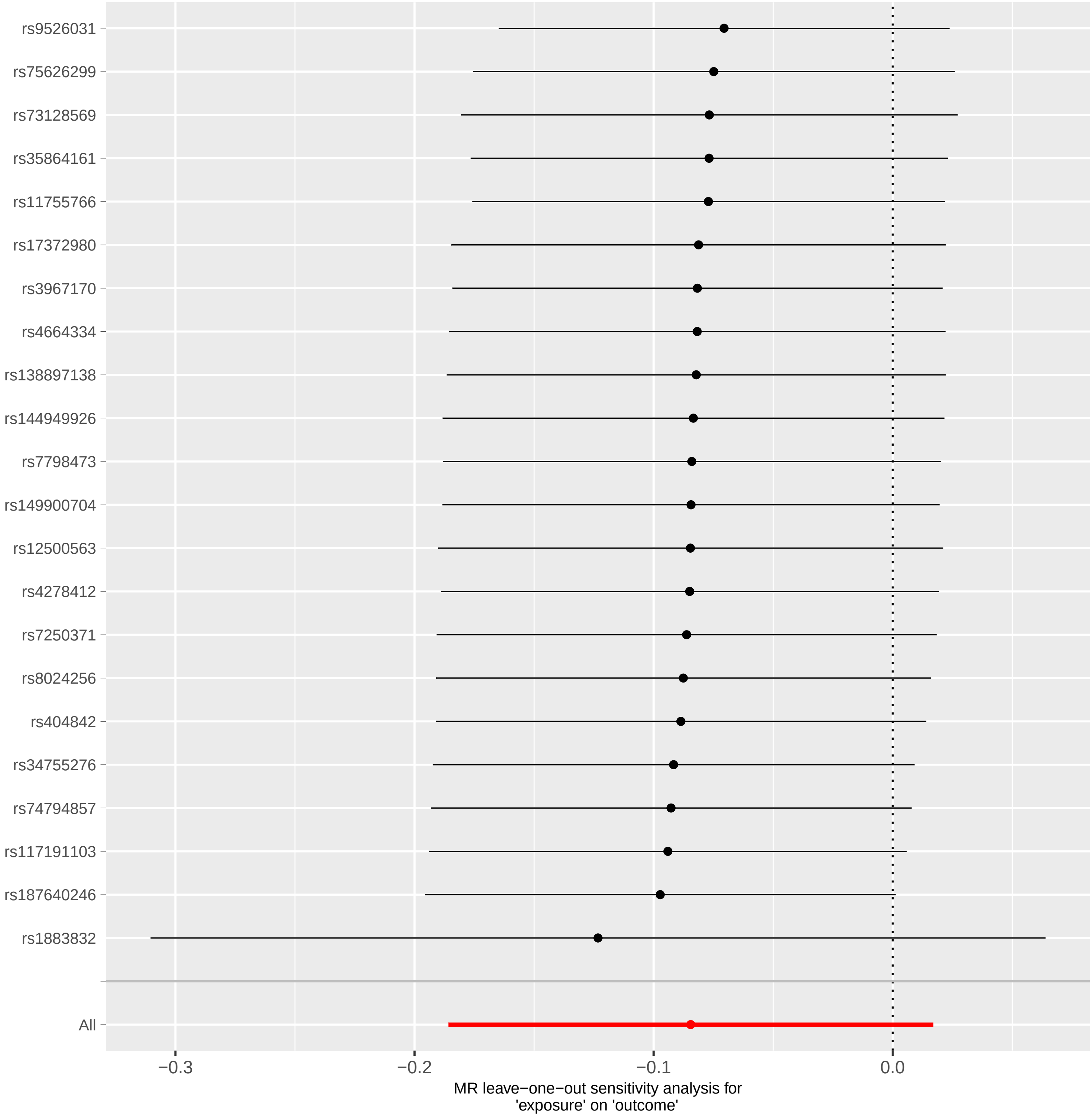

rs9526031
rs75626299
rs73128569
rs35864161
rs11755766
rs17372980
rs3967170
rs4664334
rs138897138
rs144949926
rs7798473
rs149900704
rs12500563
rs4278412
rs7250371
rs8024256
rs404842
rs34755276
rs74794857
rs117191103
rs187640246
rs1883832
All
−0.3
−0.2
−0.1
0.0
MR leave−one−out sensitivity analysis for
'exposure' on 'outcome'

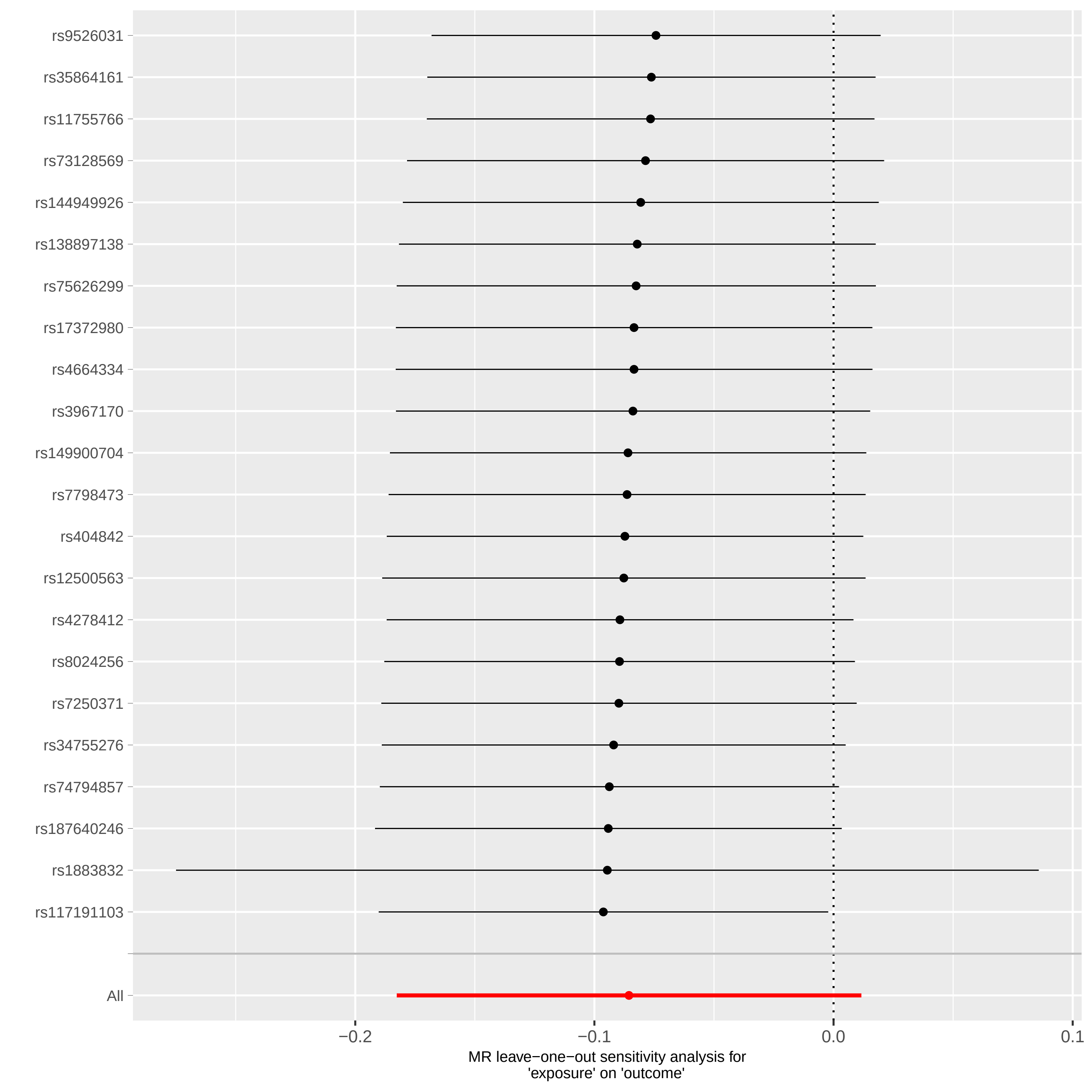
rs9526031
rs35864161
rs11755766
rs73128569
rs144949926
rs138897138
rs75626299
rs17372980
rs4664334
rs3967170
rs149900704
rs7798473
rs404842
rs12500563
rs4278412
rs8024256
rs7250371
rs34755276
rs74794857
rs187640246
rs1883832
rs117191103
All
−0.2
−0.1
0.0
0.1
MR leave−one−out sensitivity analysis for
'exposure' on 'outcome'

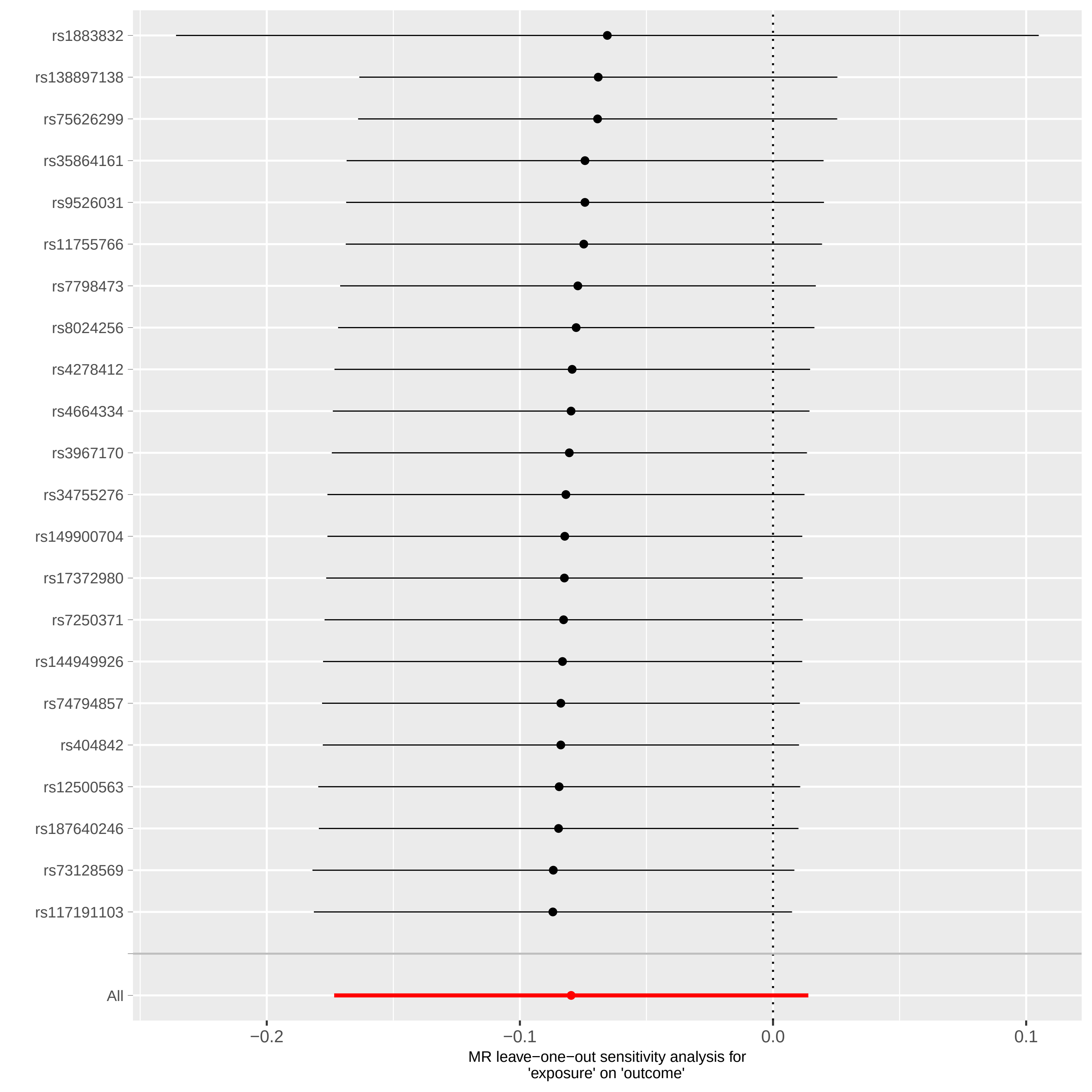
rs1883832
rs138897138
rs75626299
rs35864161
rs9526031
rs11755766
rs7798473
rs8024256
rs4278412
rs4664334
rs3967170
rs34755276
rs149900704
rs17372980
rs7250371
rs144949926
rs74794857
rs404842
rs12500563
rs187640246
rs73128569
rs117191103
All
−0.2
−0.1
0.0
0.1
MR leave−one−out sensitivity analysis for
'exposure' on 'outcome'

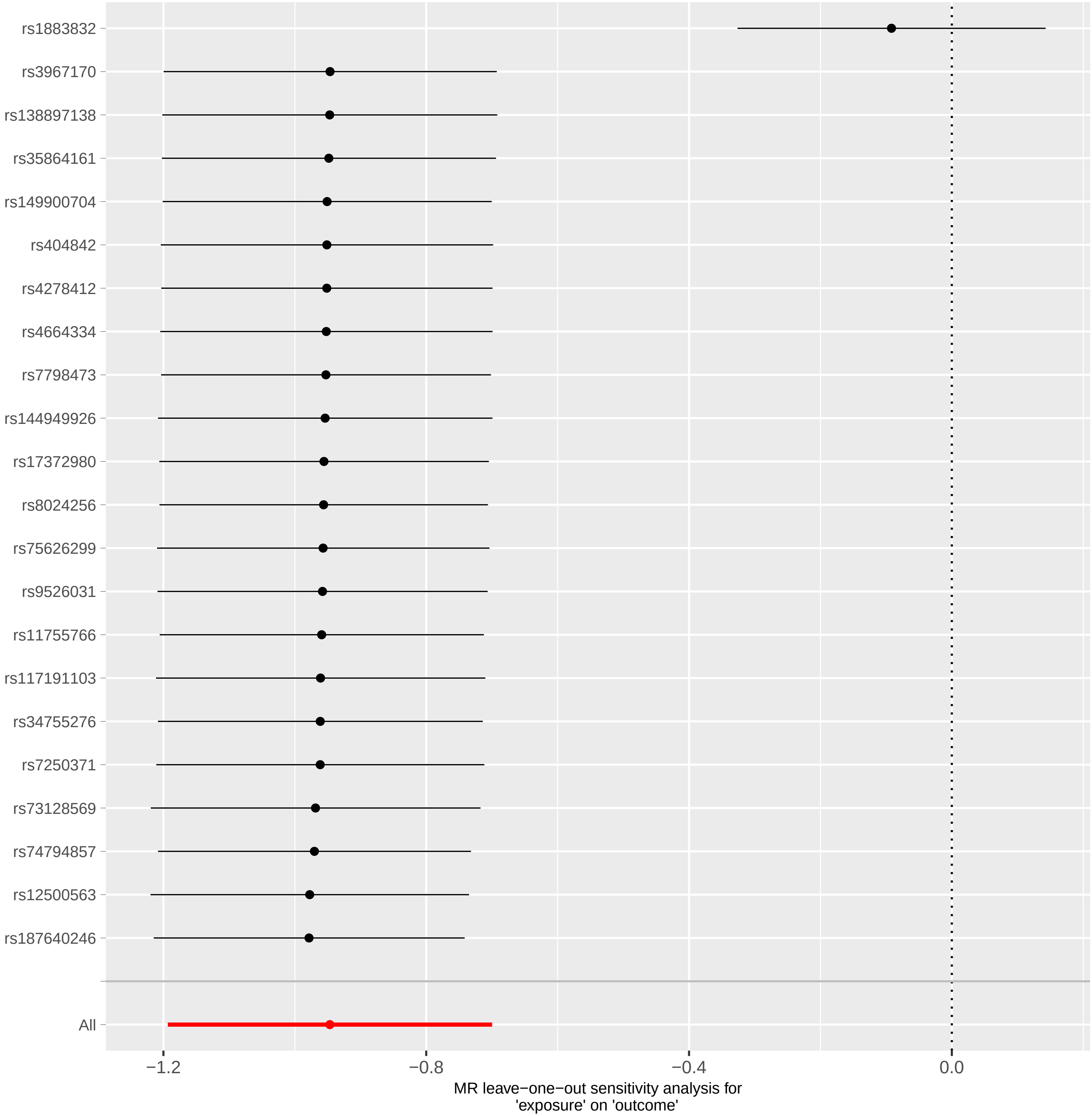

rs1883832
rs3967170
rs138897138
rs35864161
rs149900704
rs404842
rs4278412
rs4664334
rs7798473
rs144949926
rs17372980
rs8024256
rs75626299
rs9526031
rs11755766
rs117191103
rs34755276
rs7250371
rs73128569
rs74794857
rs12500563
rs187640246
All
−1.2
−0.8
−0.4
0.0
MR leave−one−out sensitivity analysis for
'exposure' on 'outcome'

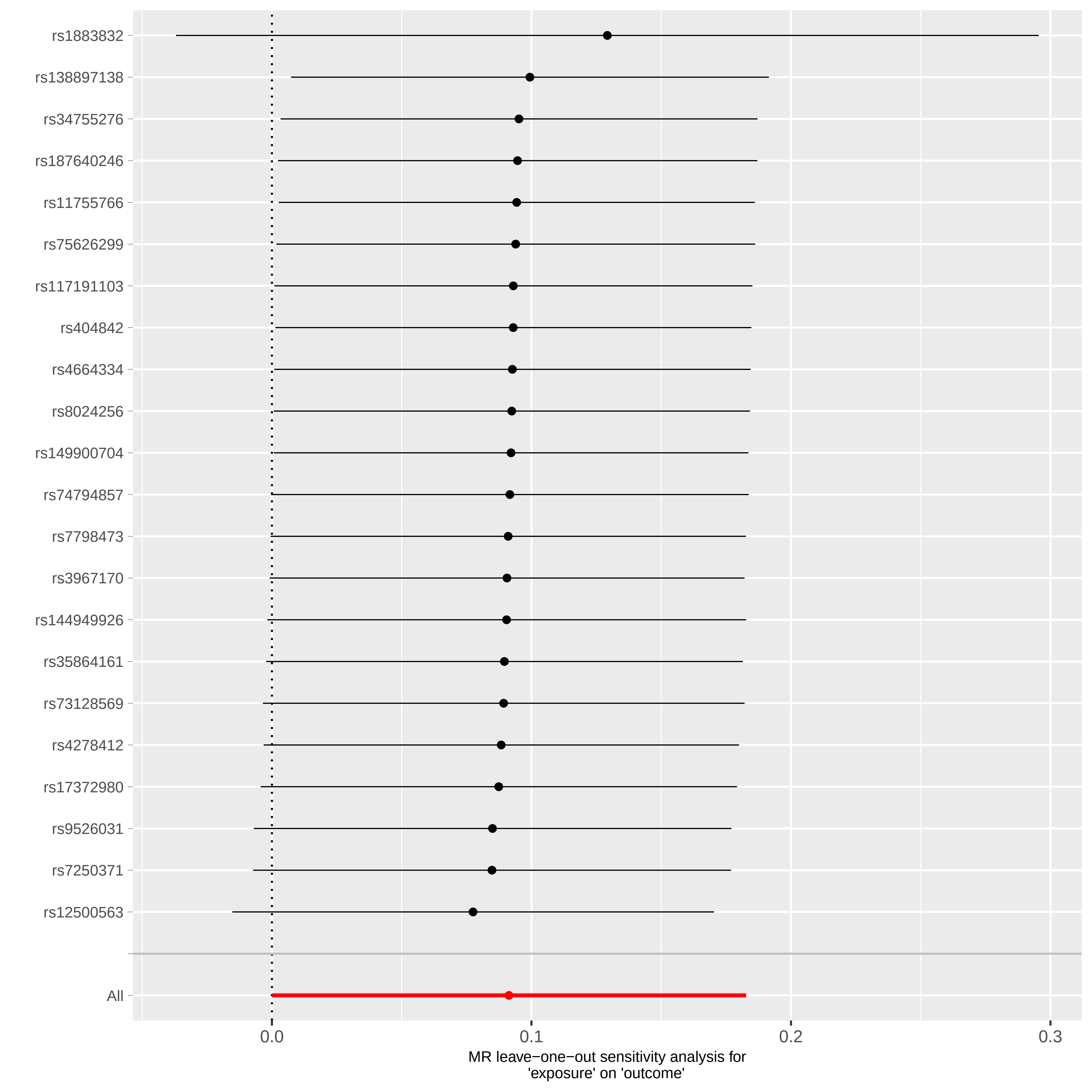
rs1883832
rs138897138
rs34755276
rs187640246
rs11755766
rs75626299
rs117191103
rs404842
rs4664334
rs8024256
rs149900704
rs74794857
rs7798473
rs3967170
rs144949926
rs35864161
rs73128569
rs4278412
rs17372980
rs9526031
rs7250371
rs12500563
All
0.0
0.1
0.2
0.3
MR leave−one−out sensitivity analysis for
'exposure' on 'outcome'

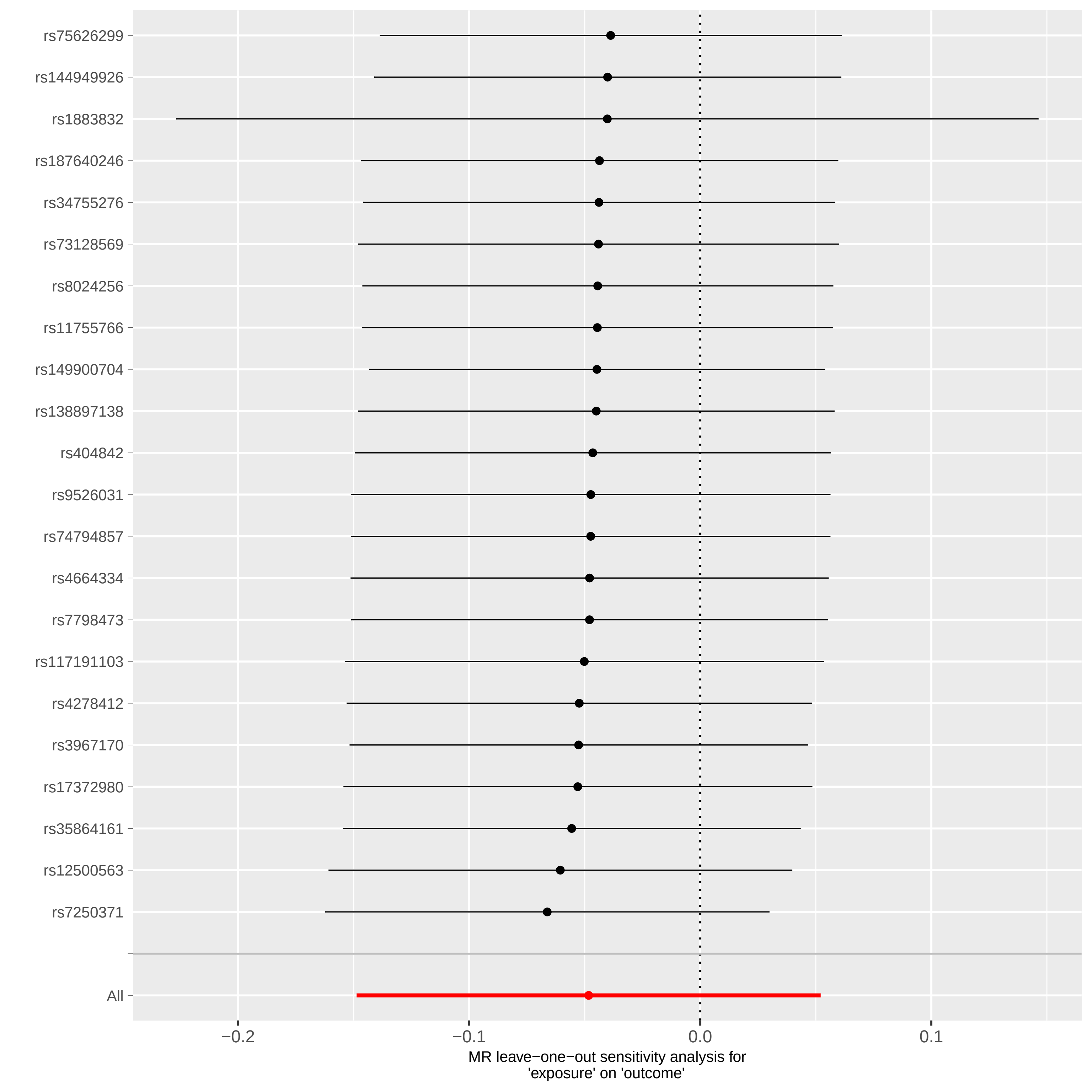
rs75626299
rs144949926
rs1883832
rs187640246
rs34755276
rs73128569
rs8024256
rs11755766
rs149900704
rs138897138
rs404842
rs9526031
rs74794857
rs4664334
rs7798473
rs117191103
rs4278412
rs3967170
rs17372980
rs35864161
rs12500563
rs7250371
All
−0.2
−0.1
0.0
0.1
MR leave−one−out sensitivity analysis for
'exposure' on 'outcome'

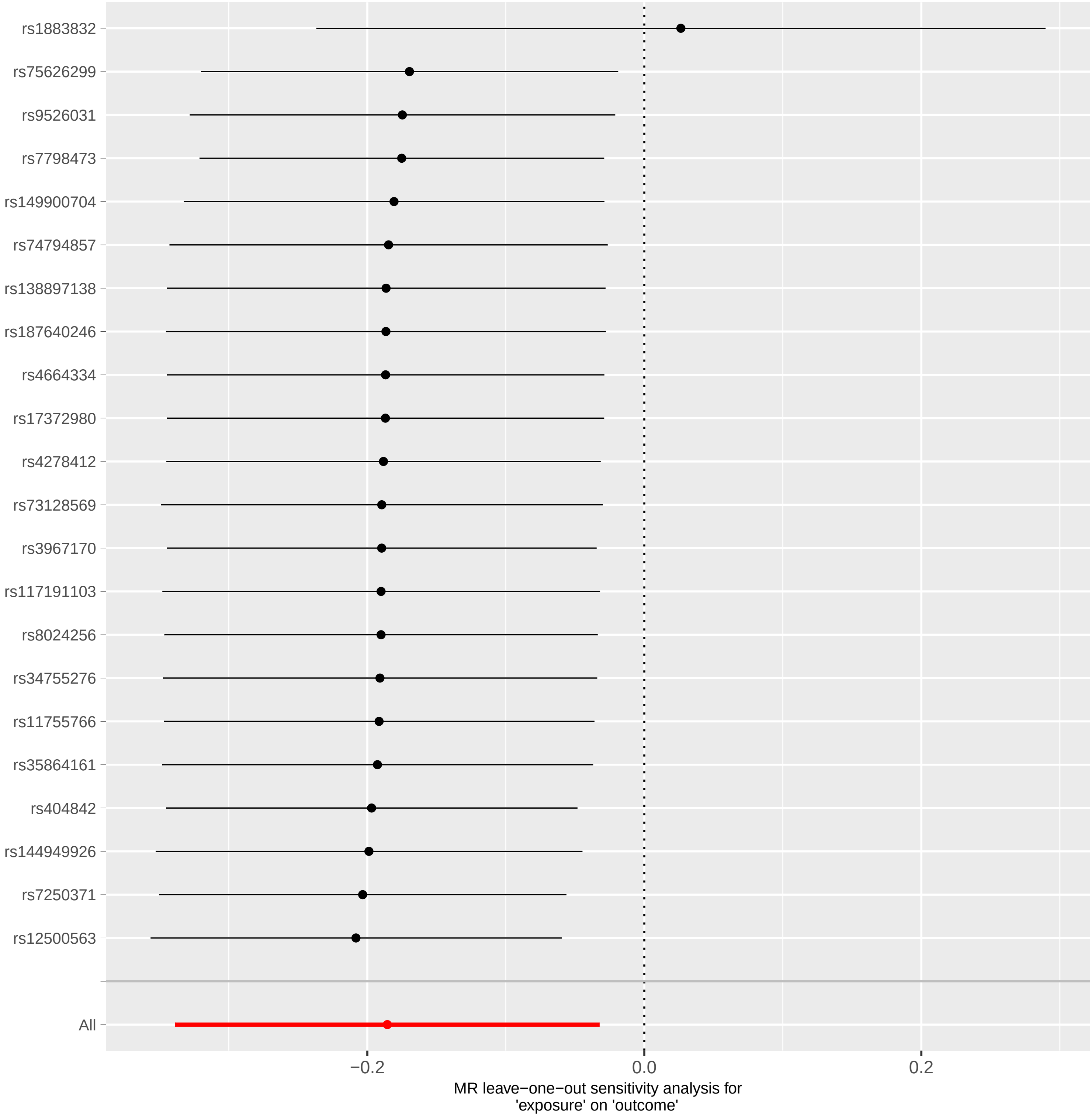
rs1883832
rs75626299
rs9526031
rs7798473
rs149900704
rs74794857
rs138897138
rs187640246
rs4664334
rs17372980
rs4278412
rs73128569
rs3967170
rs117191103
rs8024256
rs34755276
rs11755766
rs35864161
rs404842
rs144949926
rs7250371
rs12500563
All
−0.2
0.0
0.2
MR leave−one−out sensitivity analysis for
'exposure' on 'outcome'

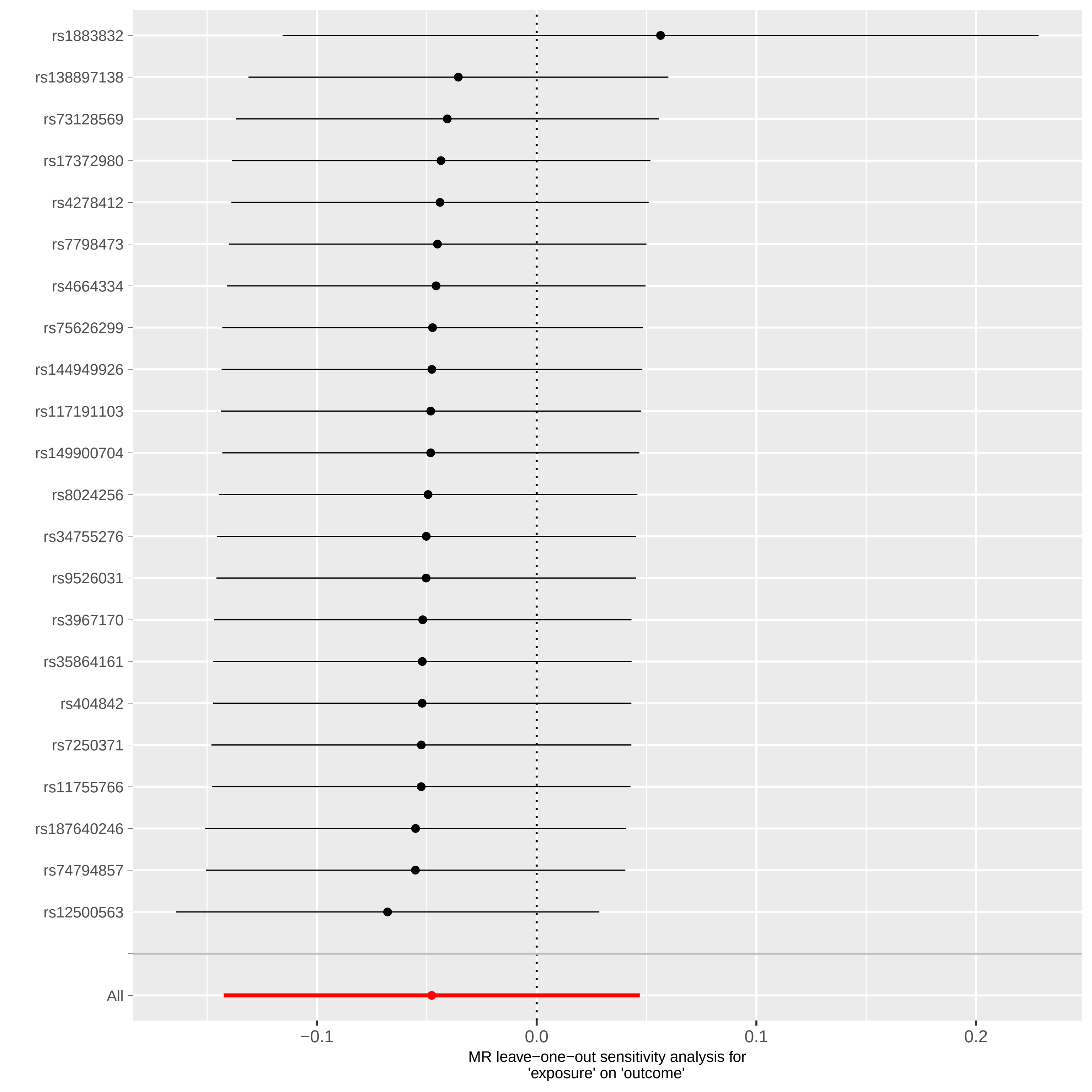
rs1883832
rs138897138
rs73128569
rs17372980
rs4278412
rs7798473
rs4664334
rs75626299
rs144949926
rs117191103
rs149900704
rs8024256
rs34755276
rs9526031
rs3967170
rs35864161
rs404842
rs7250371
rs11755766
rs187640246
rs74794857
rs12500563
All
−0.1
0.0
0.1
0.2
MR leave−one−out sensitivity analysis for
'exposure' on 'outcome'

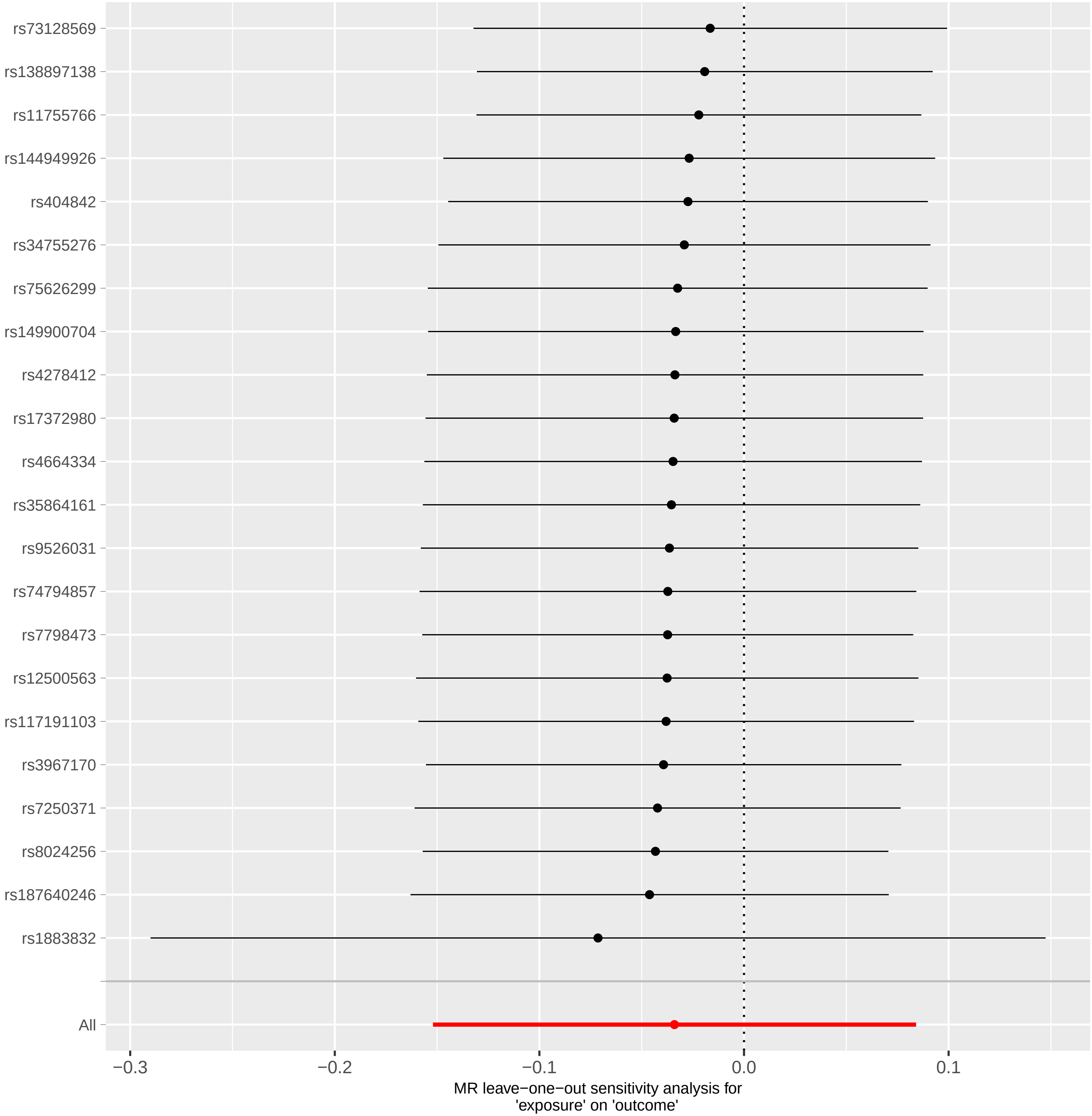

rs73128569
rs138897138
rs11755766
rs144949926
rs404842
rs34755276
rs75626299
rs149900704
rs4278412
rs17372980
rs4664334
rs35864161
rs9526031
rs74794857
rs7798473
rs12500563
rs117191103
rs3967170
rs7250371
rs8024256
rs187640246
rs1883832
All
−0.3
−0.2
−0.1
0.0
0.1
MR leave−one−out sensitivity analysis for
'exposure' on 'outcome'

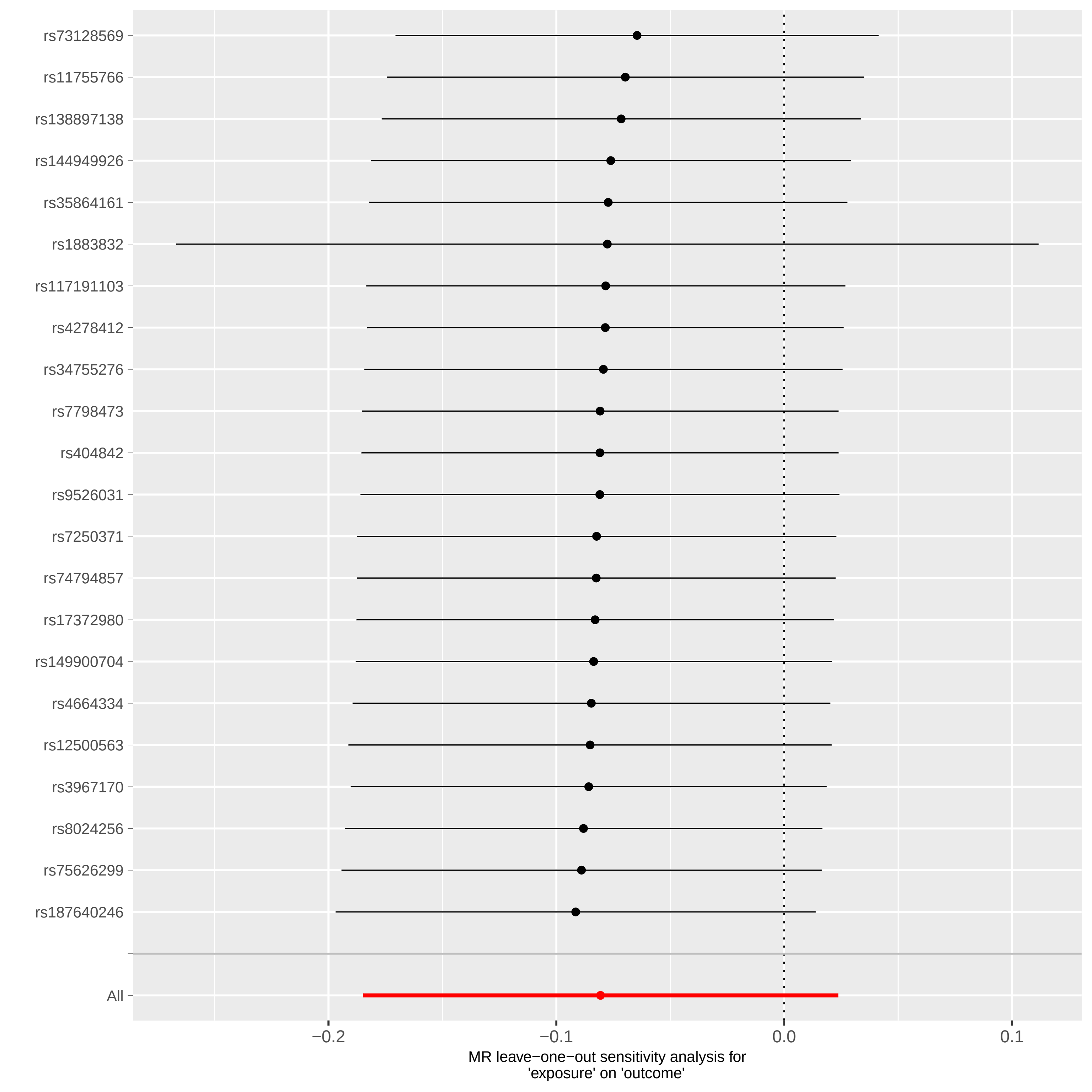
rs73128569
rs11755766
rs138897138
rs144949926
rs35864161
rs1883832
rs117191103
rs4278412
rs34755276
rs7798473
rs404842
rs9526031
rs7250371
rs74794857
rs17372980
rs149900704
rs4664334
rs12500563
rs3967170
rs8024256
rs75626299
rs187640246
All
−0.2
−0.1
0.0
0.1
MR leave−one−out sensitivity analysis for
'exposure' on 'outcome'

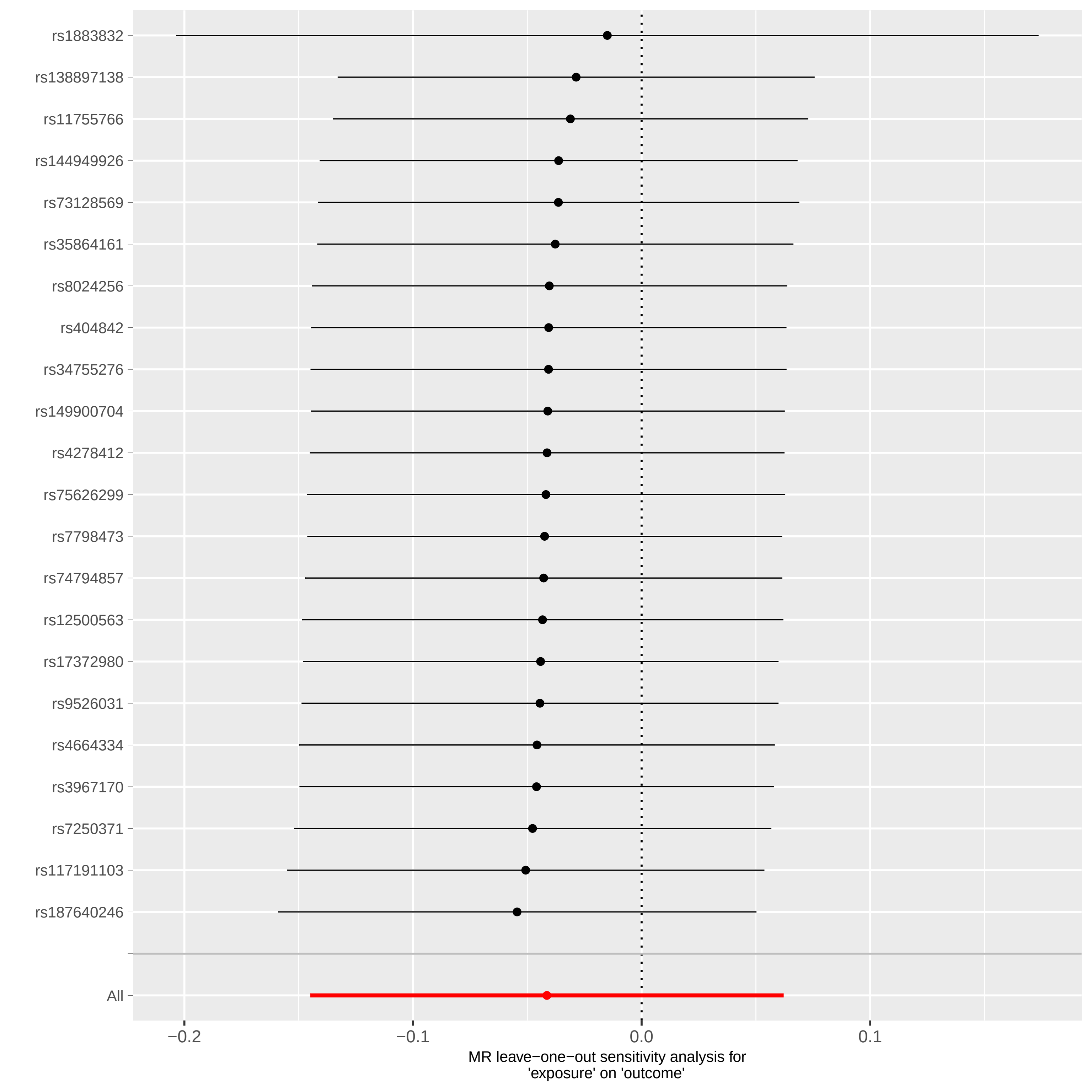
rs1883832
rs138897138
rs11755766
rs144949926
rs73128569
rs35864161
rs8024256
rs404842
rs34755276
rs149900704
rs4278412
rs75626299
rs7798473
rs74794857
rs12500563
rs17372980
rs9526031
rs4664334
rs3967170
rs7250371
rs117191103
rs187640246
All
−0.2
−0.1
0.0
0.1
MR leave−one−out sensitivity analysis for
'exposure' on 'outcome'

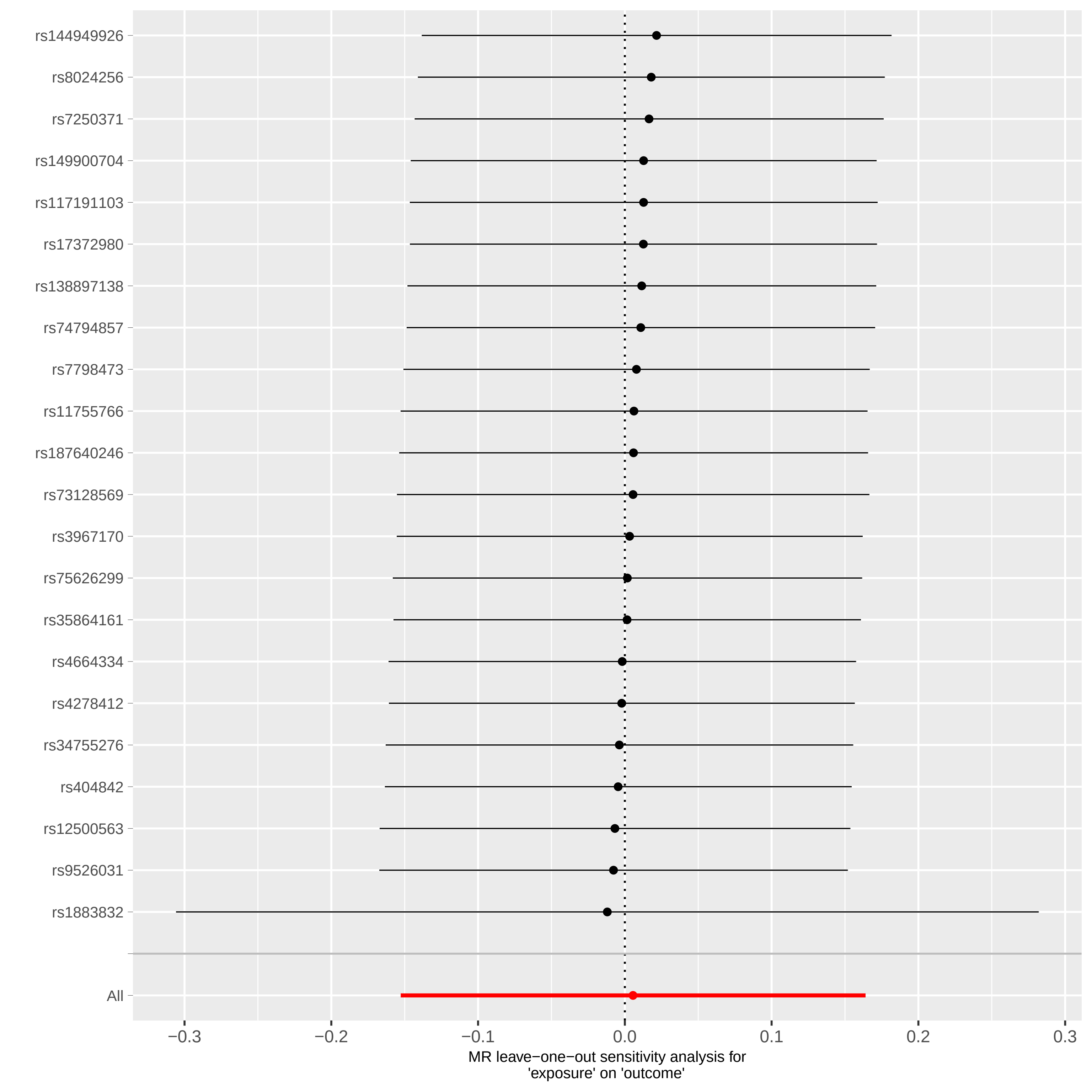
rs144949926
rs8024256
rs7250371
rs149900704
rs117191103
rs17372980
rs138897138
rs74794857
rs7798473
rs11755766
rs187640246
rs73128569
rs3967170
rs75626299
rs35864161
rs4664334
rs4278412
rs34755276
rs404842
rs12500563
rs9526031
rs1883832
All
−0.3
−0.2
−0.1
0.0
0.1
0.2
0.3
MR leave−one−out sensitivity analysis for
'exposure' on 'outcome'

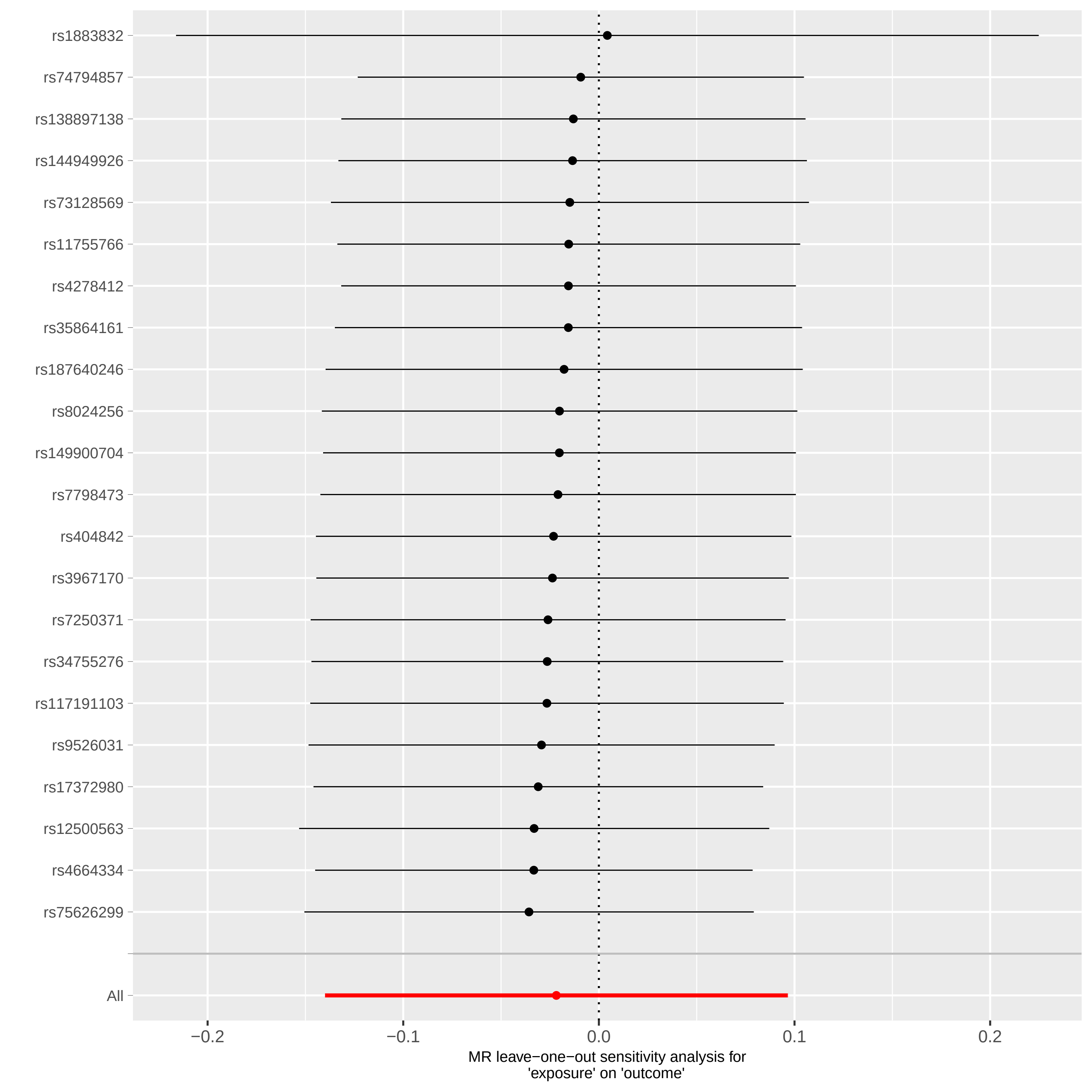
rs1883832
rs74794857
rs138897138
rs144949926
rs73128569
rs11755766
rs4278412
rs35864161
rs187640246
rs8024256
rs149900704
rs7798473
rs404842
rs3967170
rs7250371
rs34755276
rs117191103
rs9526031
rs17372980
rs12500563
rs4664334
rs75626299
All
−0.2
−0.1
0.0
0.1
0.2
MR leave−one−out sensitivity analysis for
'exposure' on 'outcome'

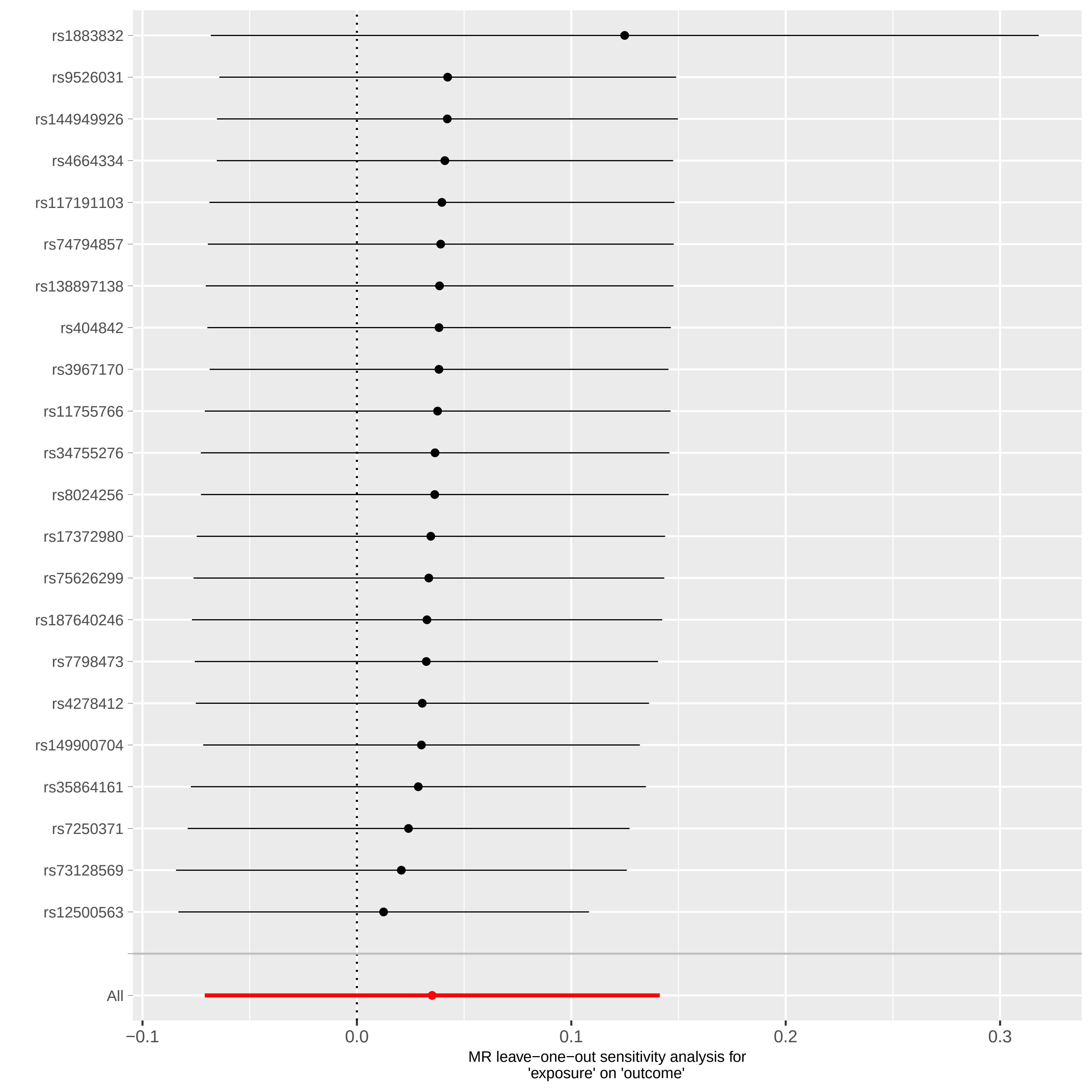

rs1883832
rs9526031
rs144949926
rs4664334
rs117191103
rs74794857
rs138897138
rs404842
rs3967170
rs11755766
rs34755276
rs8024256
rs17372980
rs75626299
rs187640246
rs7798473
rs4278412
rs149900704
rs35864161
rs7250371
rs73128569
rs12500563
All
−0.1
0.0
0.1
0.2
0.3
MR leave−one−out sensitivity analysis for
'exposure' on 'outcome'

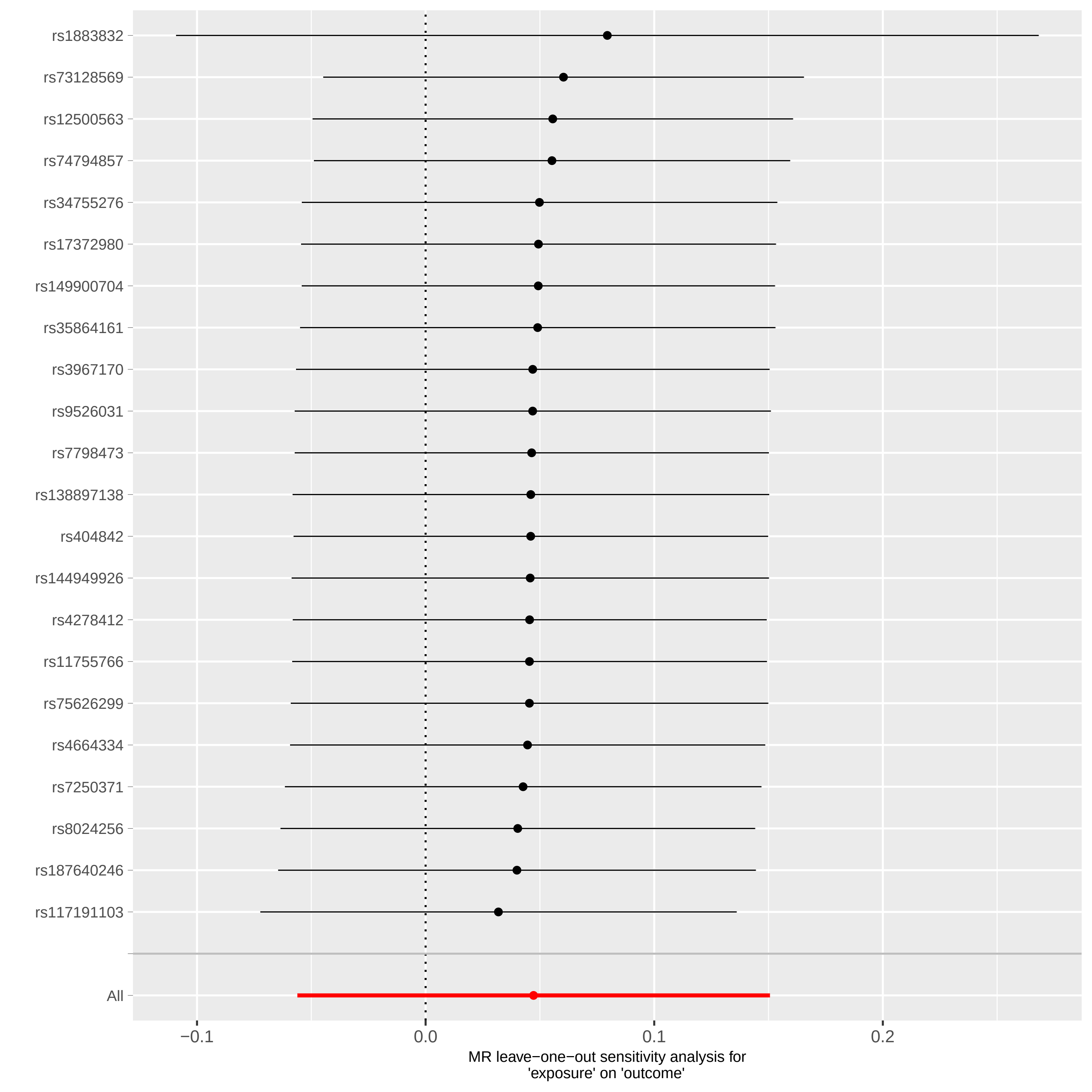
rs1883832
rs73128569
rs12500563
rs74794857
rs34755276
rs17372980
rs149900704
rs35864161
rs3967170
rs9526031
rs7798473
rs138897138
rs404842
rs144949926
rs4278412
rs11755766
rs75626299
rs4664334
rs7250371
rs8024256
rs187640246
rs117191103
All
−0.1
0.0
0.1
0.2
MR leave−one−out sensitivity analysis for
'exposure' on 'outcome'

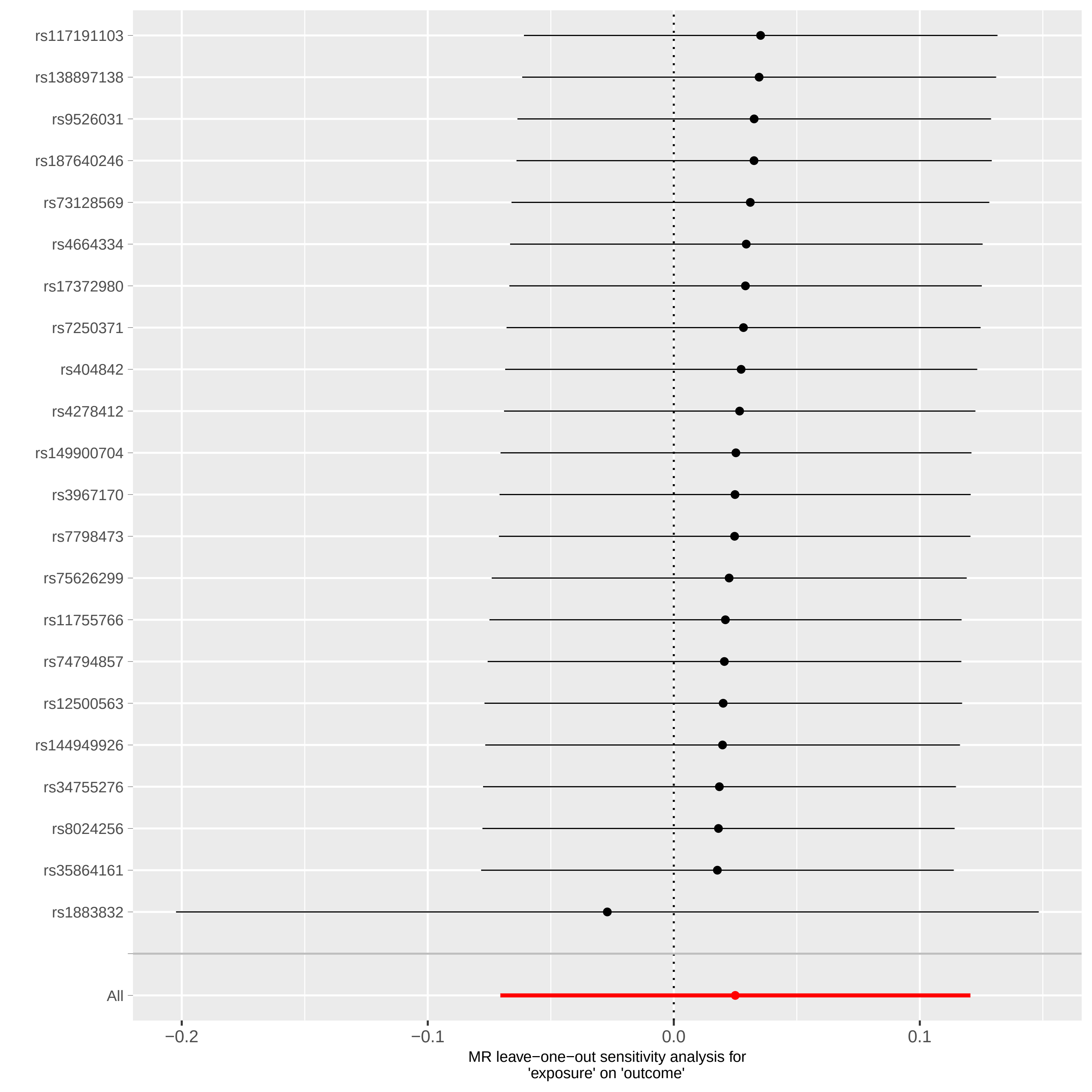
rs117191103
rs138897138
rs9526031
rs187640246
rs73128569
rs4664334
rs17372980
rs7250371
rs404842
rs4278412
rs149900704
rs3967170
rs7798473
rs75626299
rs11755766
rs74794857
rs12500563
rs144949926
rs34755276
rs8024256
rs35864161
rs1883832
All
−0.2
−0.1
0.0
0.1
MR leave−one−out sensitivity analysis for
'exposure' on 'outcome'

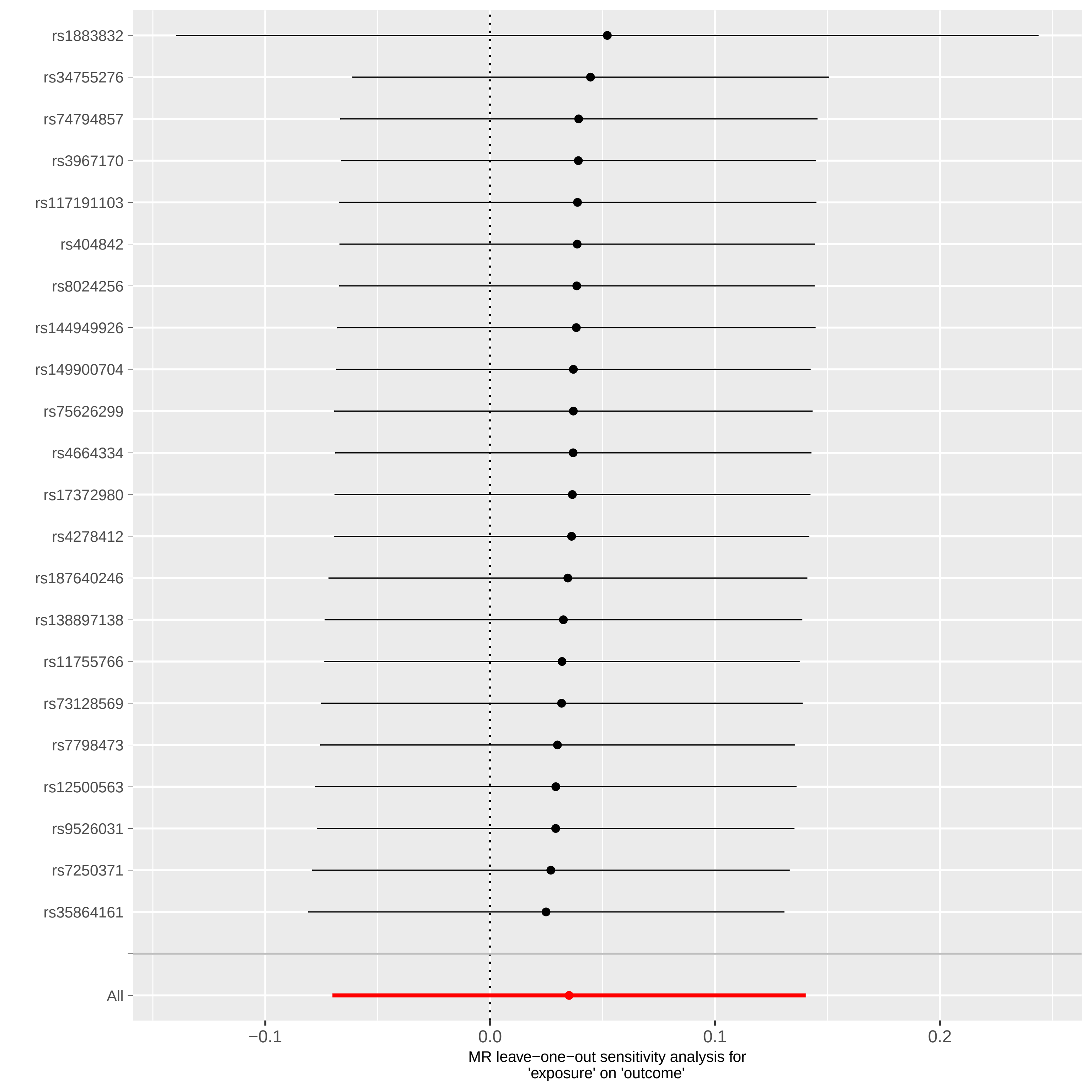
rs1883832
rs34755276
rs74794857
rs3967170
rs117191103
rs404842
rs8024256
rs144949926
rs149900704
rs75626299
rs4664334
rs17372980
rs4278412
rs187640246
rs138897138
rs11755766
rs73128569
rs7798473
rs12500563
rs9526031
rs7250371
rs35864161
All
−0.1
0.0
0.1
0.2
MR leave−one−out sensitivity analysis for
'exposure' on 'outcome'

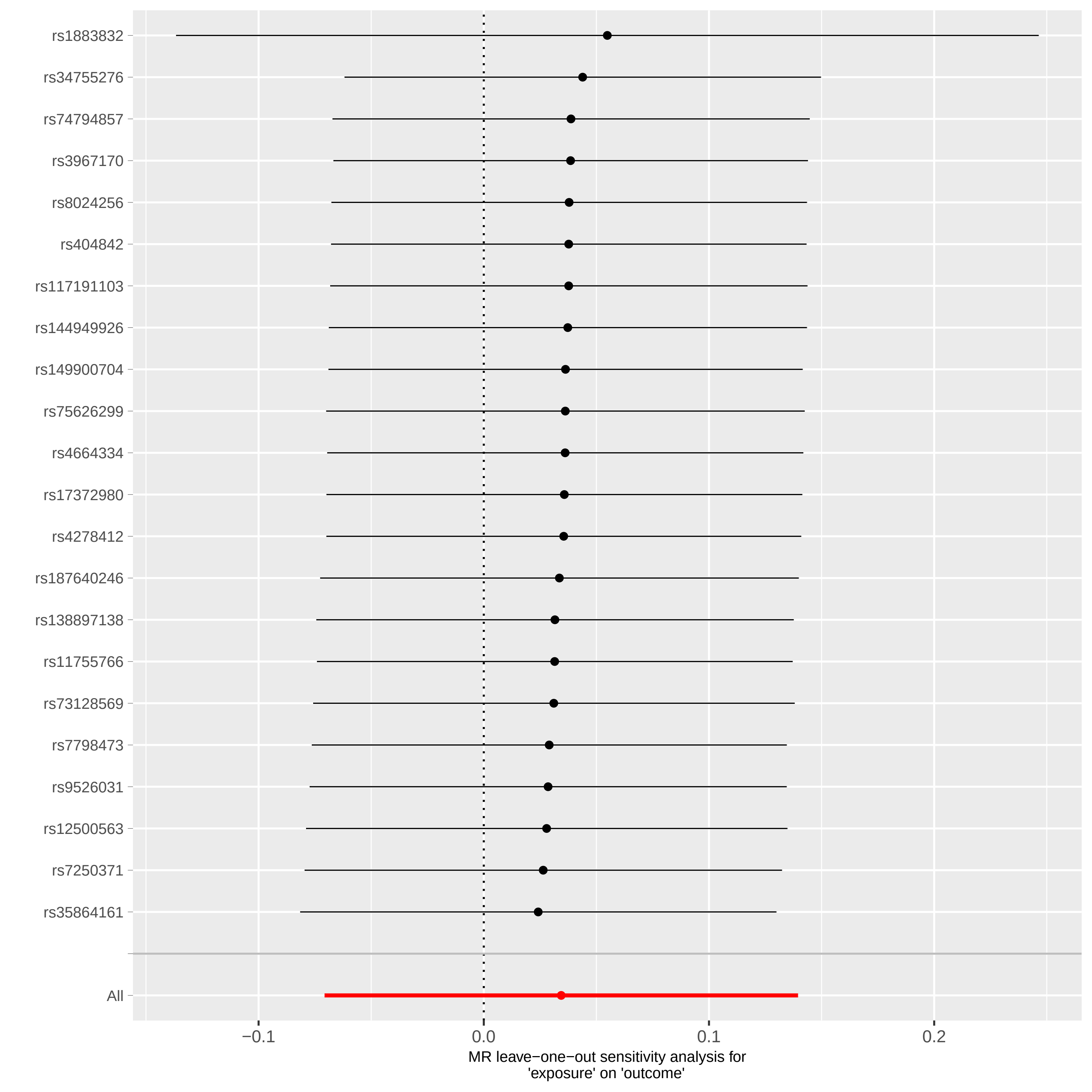
rs1883832
rs34755276
rs74794857
rs3967170
rs8024256
rs404842
rs117191103
rs144949926
rs149900704
rs75626299
rs4664334
rs17372980
rs4278412
rs187640246
rs138897138
rs11755766
rs73128569
rs7798473
rs9526031
rs12500563
rs7250371
rs35864161
All
−0.1
0.0
0.1
0.2
MR leave−one−out sensitivity analysis for
'exposure' on 'outcome'

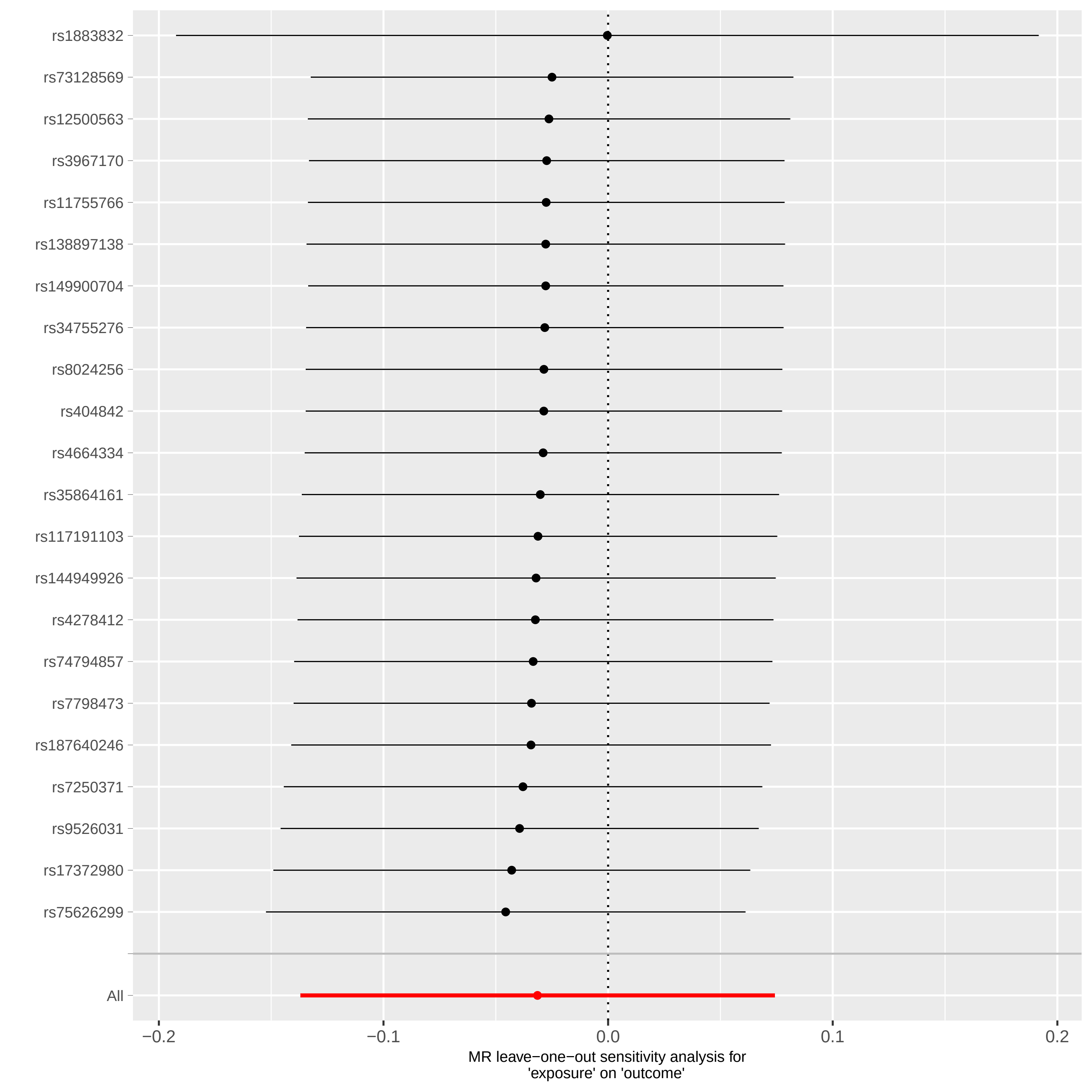
rs1883832
rs73128569
rs12500563
rs3967170
rs11755766
rs138897138
rs149900704
rs34755276
rs8024256
rs404842
rs4664334
rs35864161
rs117191103
rs144949926
rs4278412
rs74794857
rs7798473
rs187640246
rs7250371
rs9526031
rs17372980
rs75626299
All
−0.2
−0.1
0.0
0.1
0.2
MR leave−one−out sensitivity analysis for
'exposure' on 'outcome'

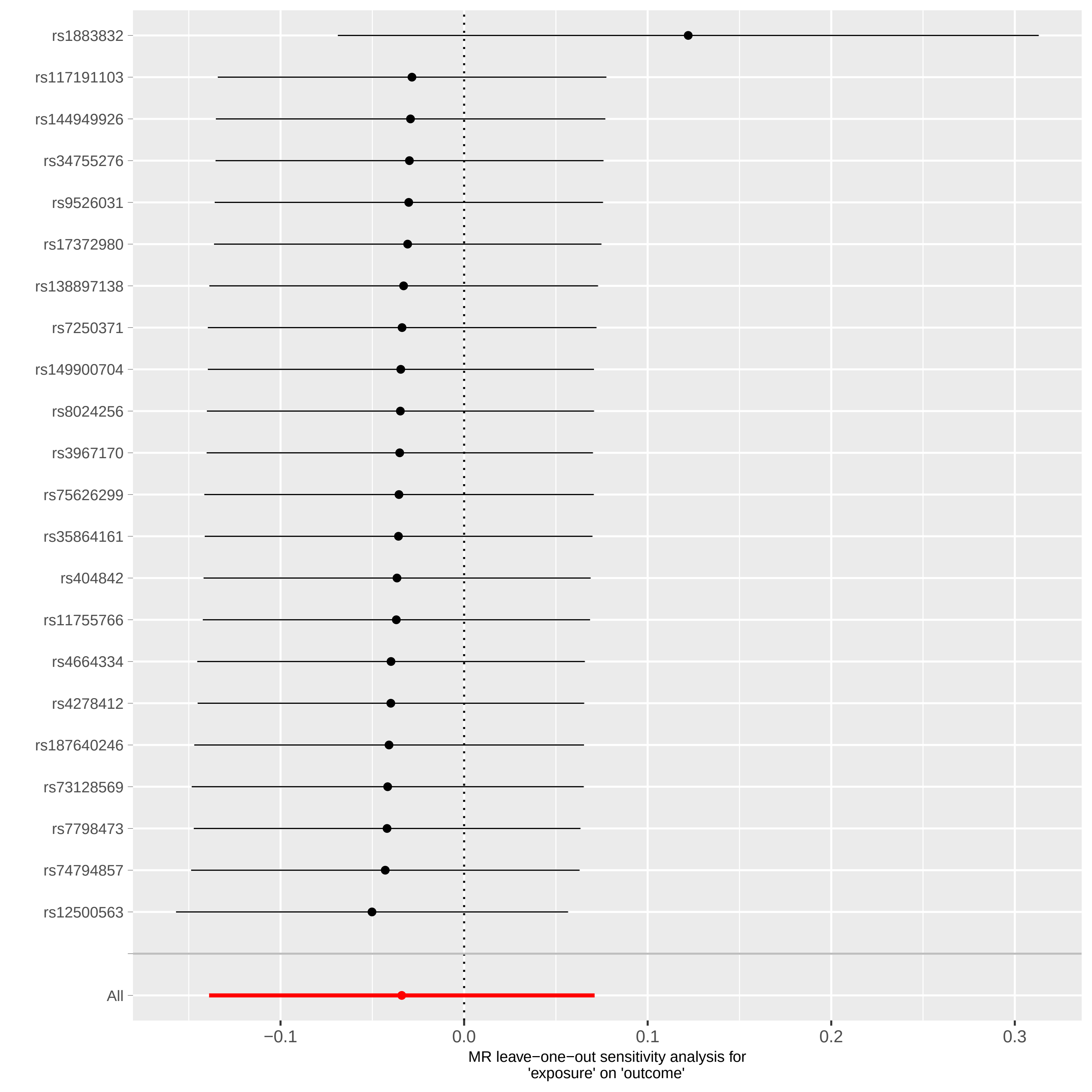

rs1883832
rs117191103
rs144949926
rs34755276
rs9526031
rs17372980
rs138897138
rs7250371
rs149900704
rs8024256
rs3967170
rs75626299
rs35864161
rs404842
rs11755766
rs4664334
rs4278412
rs187640246
rs73128569
rs7798473
rs74794857
rs12500563
All
−0.1
0.0
0.1
0.2
0.3
MR leave−one−out sensitivity analysis for
'exposure' on 'outcome'

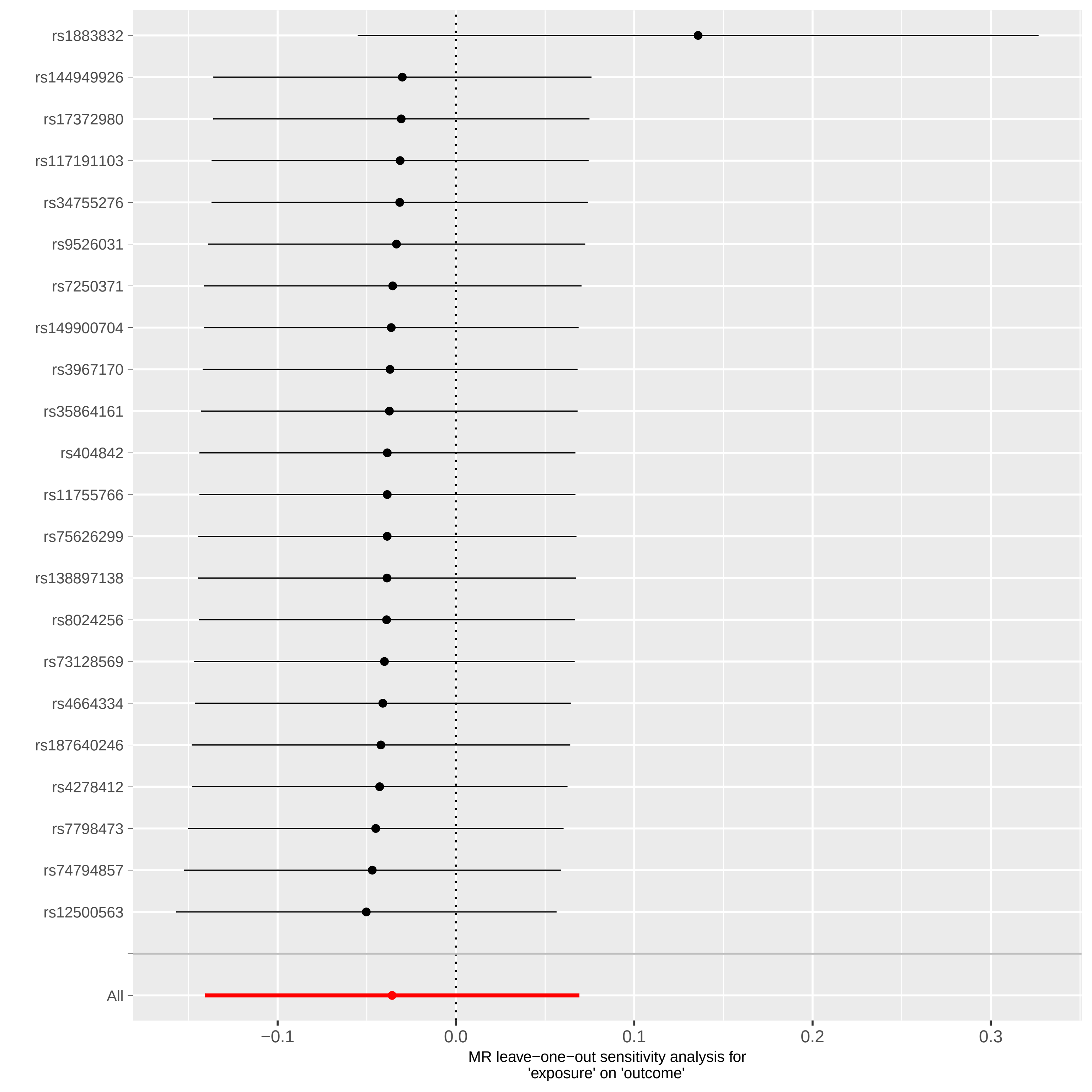
rs1883832
rs144949926
rs17372980
rs117191103
rs34755276
rs9526031
rs7250371
rs149900704
rs3967170
rs35864161
rs404842
rs11755766
rs75626299
rs138897138
rs8024256
rs73128569
rs4664334
rs187640246
rs4278412
rs7798473
rs74794857
rs12500563
All
−0.1
0.0
0.1
0.2
0.3
MR leave−one−out sensitivity analysis for
'exposure' on 'outcome'

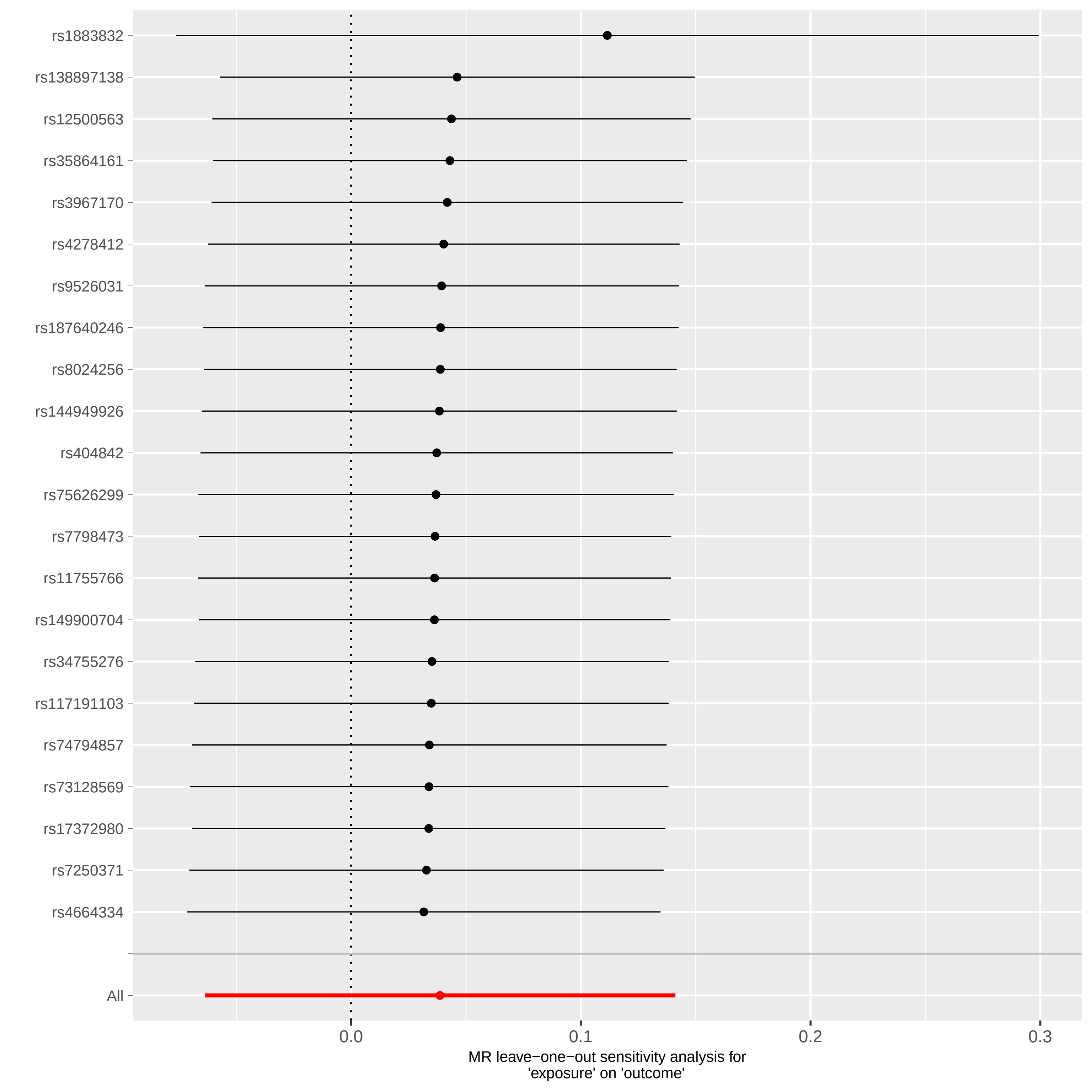

rs1883832
rs138897138
rs12500563
rs35864161
rs3967170
rs4278412
rs9526031
rs187640246
rs8024256
rs144949926
rs404842
rs75626299
rs7798473
rs11755766
rs149900704
rs34755276
rs117191103
rs74794857
rs73128569
rs17372980
rs7250371
rs4664334
All
0.0
0.1
0.2
0.3
MR leave−one−out sensitivity analysis for
'exposure' on 'outcome'

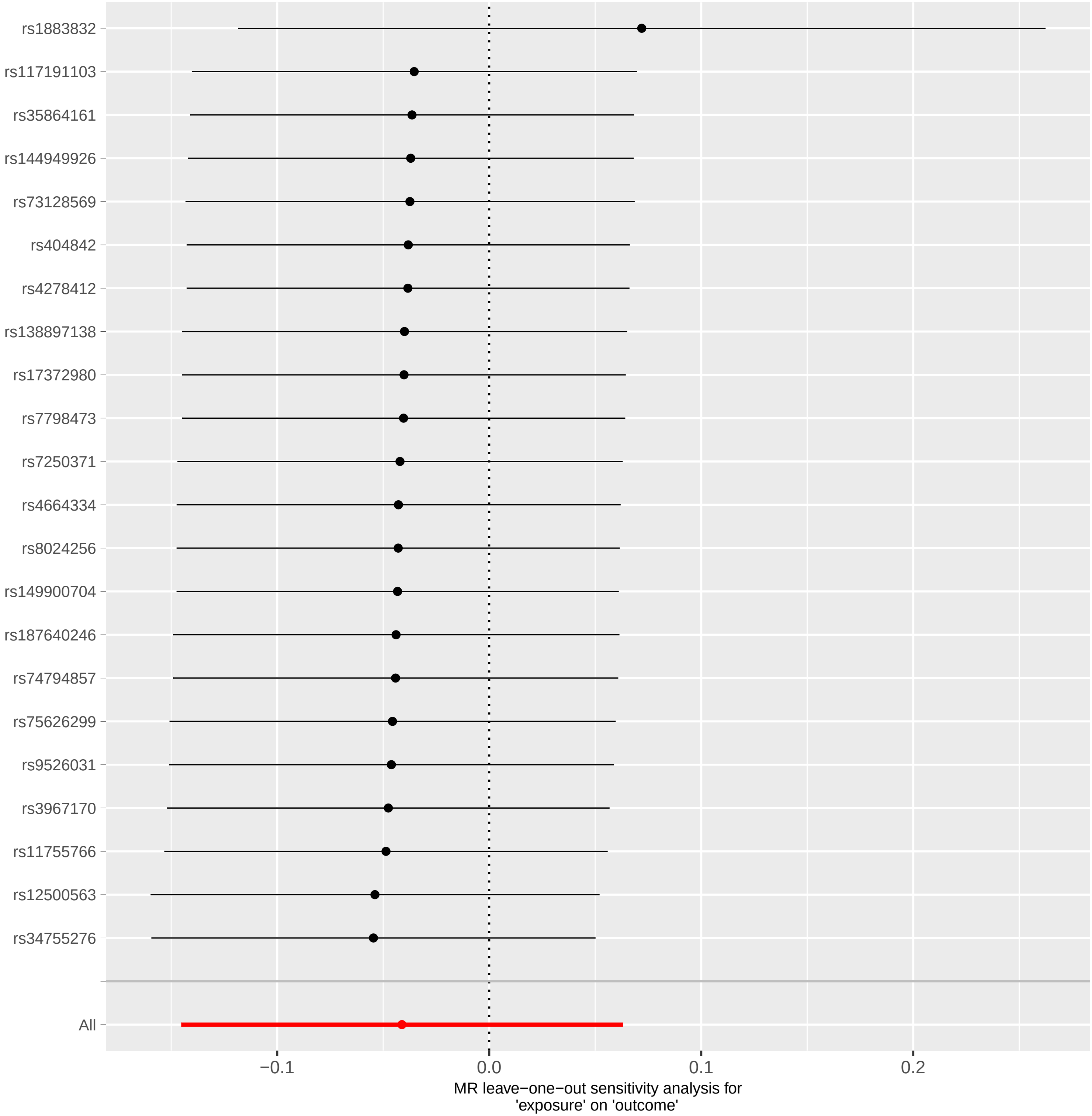
rs1883832
rs117191103
rs35864161
rs144949926
rs73128569
rs404842
rs4278412
rs138897138
rs17372980
rs7798473
rs7250371
rs4664334
rs8024256
rs149900704
rs187640246
rs74794857
rs75626299
rs9526031
rs3967170
rs11755766
rs12500563
rs34755276
All
−0.1
0.0
0.1
0.2
MR leave−one−out sensitivity analysis for
'exposure' on 'outcome'

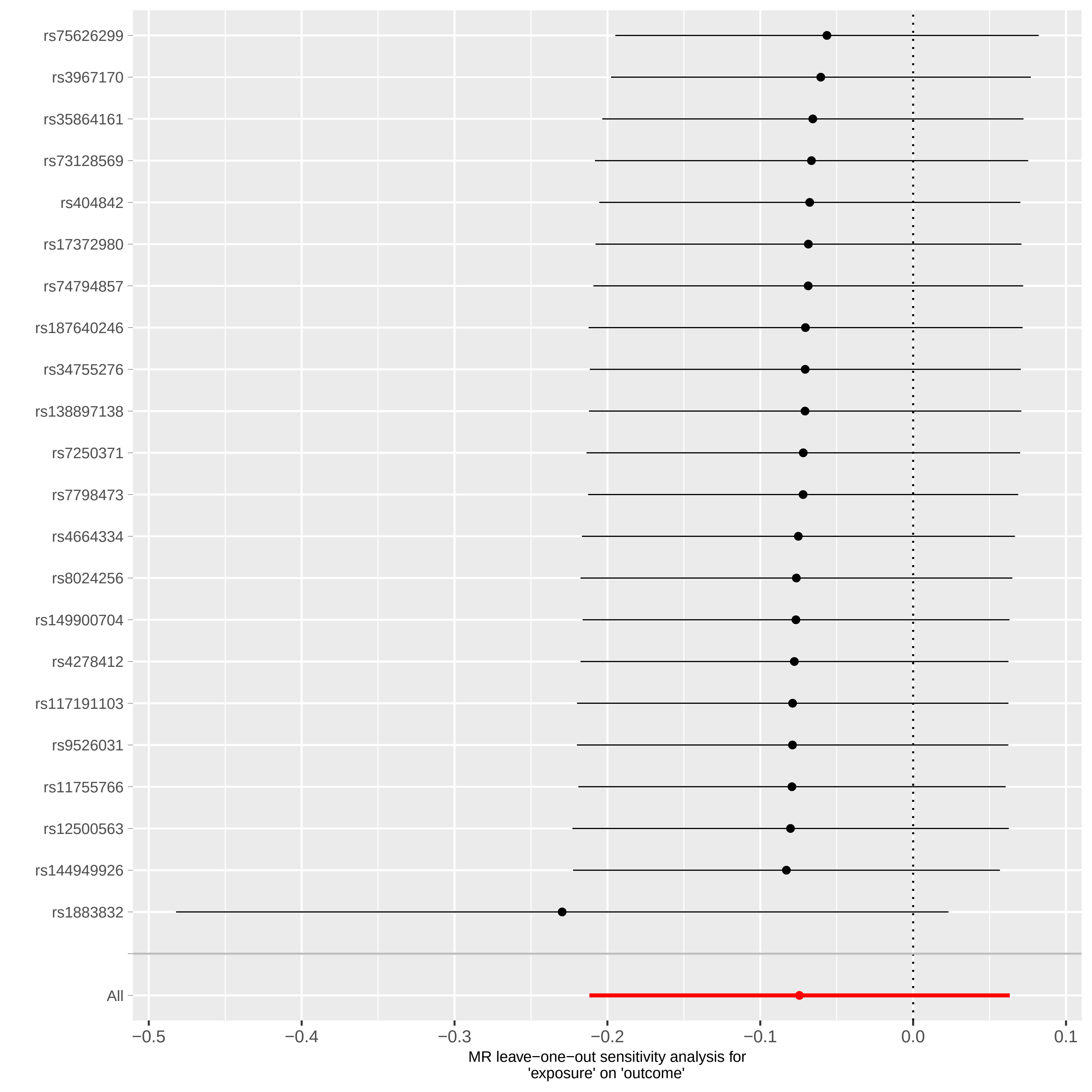

rs75626299
rs3967170
rs35864161
rs73128569
rs404842
rs17372980
rs74794857
rs187640246
rs34755276
rs138897138
rs7250371
rs7798473
rs4664334
rs8024256
rs149900704
rs4278412
rs117191103
rs9526031
rs11755766
rs12500563
rs144949926
rs1883832
All
−0.5
−0.4
−0.3
−0.2
−0.1
0.0
0.1
MR leave−one−out sensitivity analysis for
'exposure' on 'outcome'

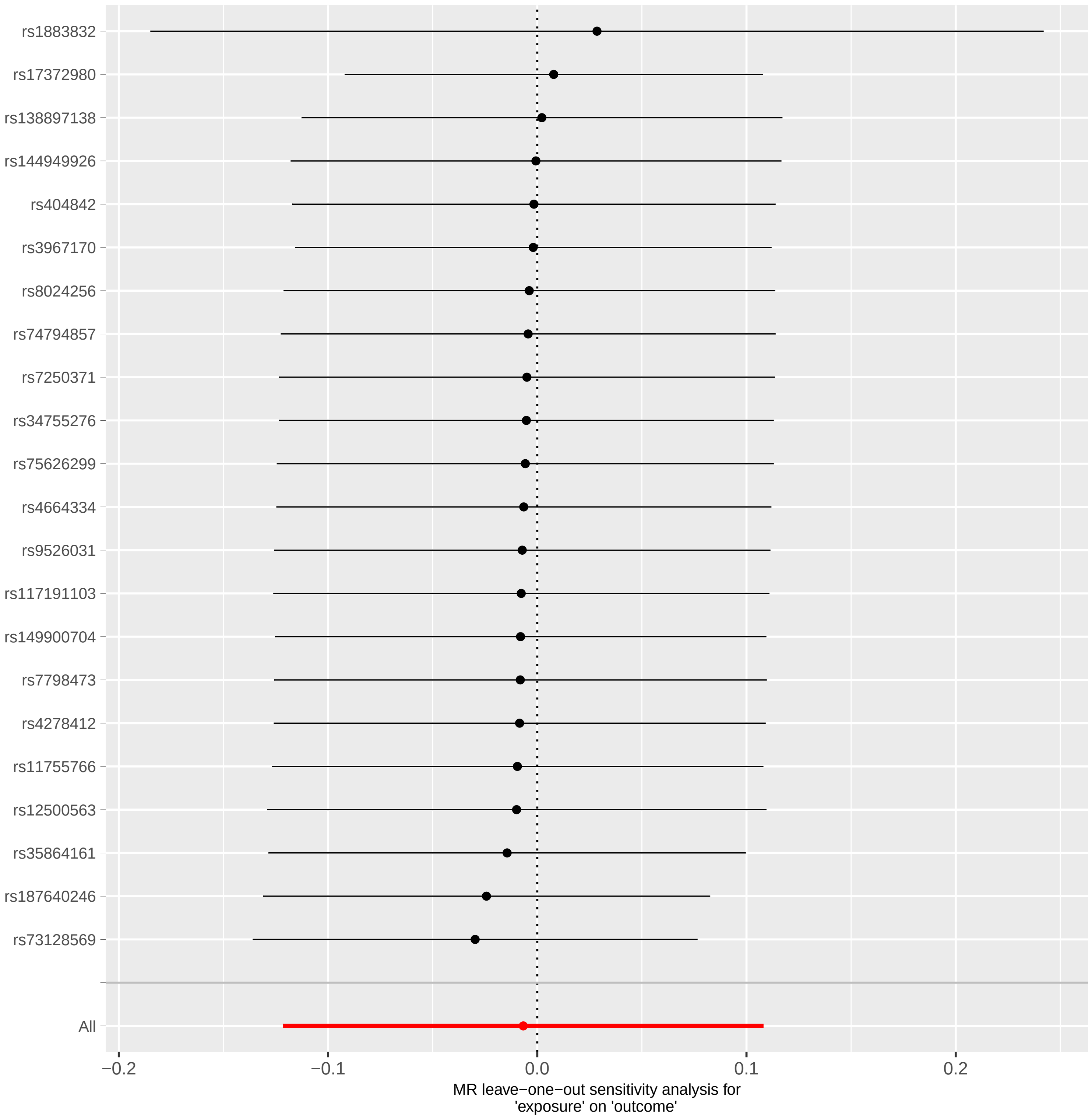
rs1883832
rs17372980
rs138897138
rs144949926
rs404842
rs3967170
rs8024256
rs74794857
rs7250371
rs34755276
rs75626299
rs4664334
rs9526031
rs117191103
rs149900704
rs7798473
rs4278412
rs11755766
rs12500563
rs35864161
rs187640246
rs73128569
All
−0.2
−0.1
0.0
0.1
0.2
MR leave−one−out sensitivity analysis for
'exposure' on 'outcome'

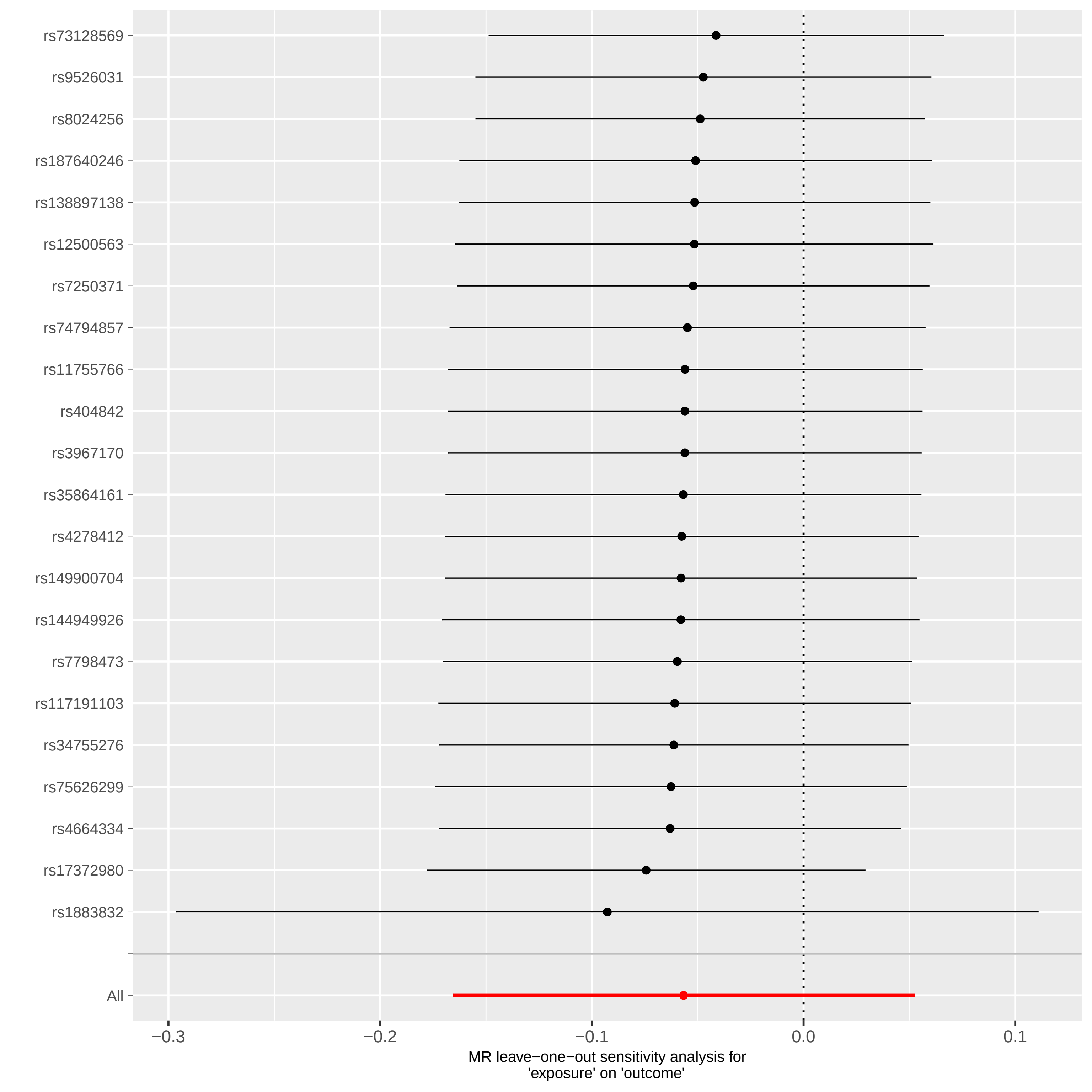

rs73128569
rs9526031
rs8024256
rs187640246
rs138897138
rs12500563
rs7250371
rs74794857
rs11755766
rs404842
rs3967170
rs35864161
rs4278412
rs149900704
rs144949926
rs7798473
rs117191103
rs34755276
rs75626299
rs4664334
rs17372980
rs1883832
All
−0.3
−0.2
−0.1
0.0
0.1
MR leave−one−out sensitivity analysis for
'exposure' on 'outcome'

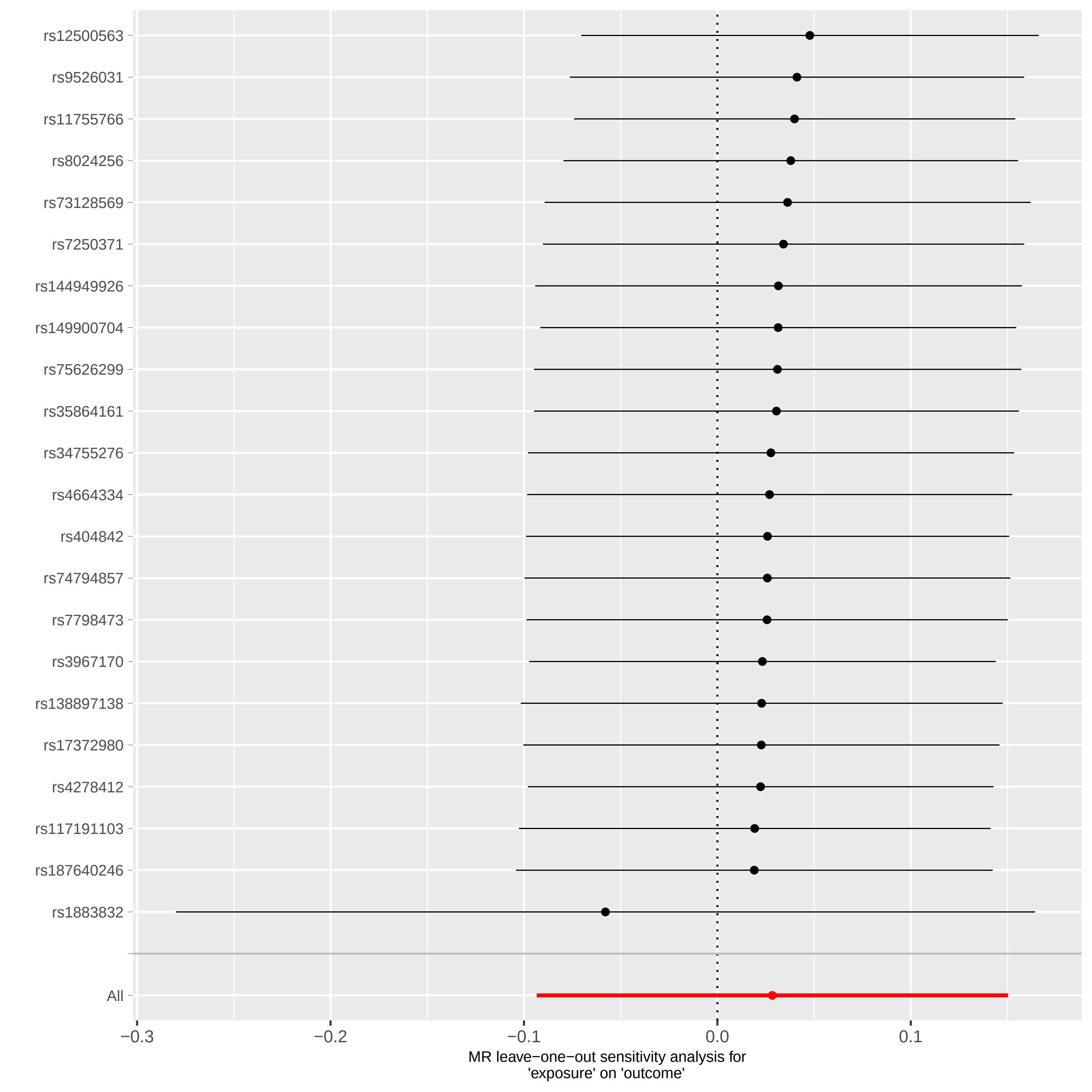
rs12500563
rs9526031
rs11755766
rs8024256
rs73128569
rs7250371
rs144949926
rs149900704
rs75626299
rs35864161
rs34755276
rs4664334
rs404842
rs74794857
rs7798473
rs3967170
rs138897138
rs17372980
rs4278412
rs117191103
rs187640246
rs1883832
All
−0.3
−0.2
−0.1
0.0
0.1
MR leave−one−out sensitivity analysis for
'exposure' on 'outcome'

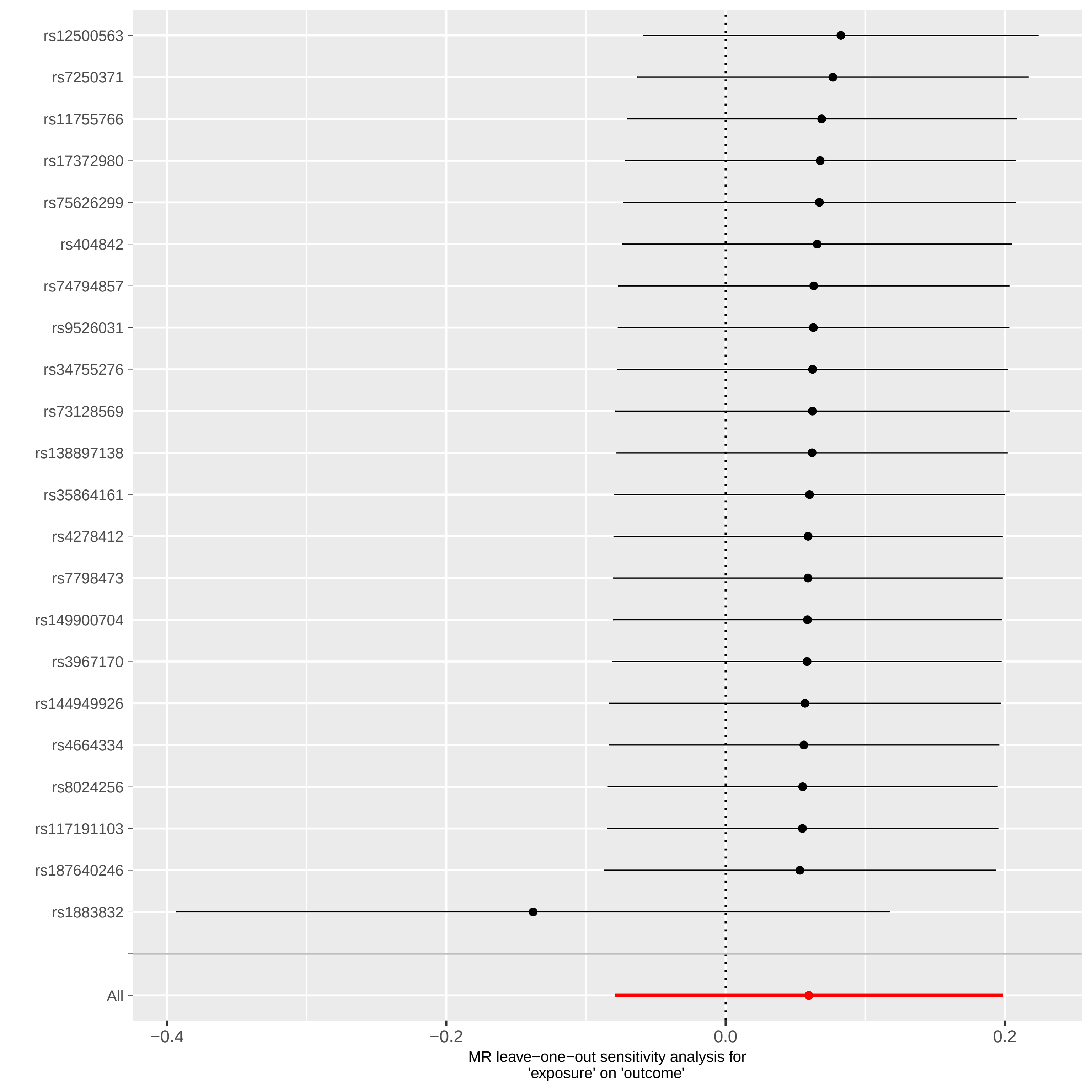
rs12500563
rs7250371
rs11755766
rs17372980
rs75626299
rs404842
rs74794857
rs9526031
rs34755276
rs73128569
rs138897138
rs35864161
rs4278412
rs7798473
rs149900704
rs3967170
rs144949926
rs4664334
rs8024256
rs117191103
rs187640246
rs1883832
All
−0.4
−0.2
0.0
0.2
MR leave−one−out sensitivity analysis for
'exposure' on 'outcome'

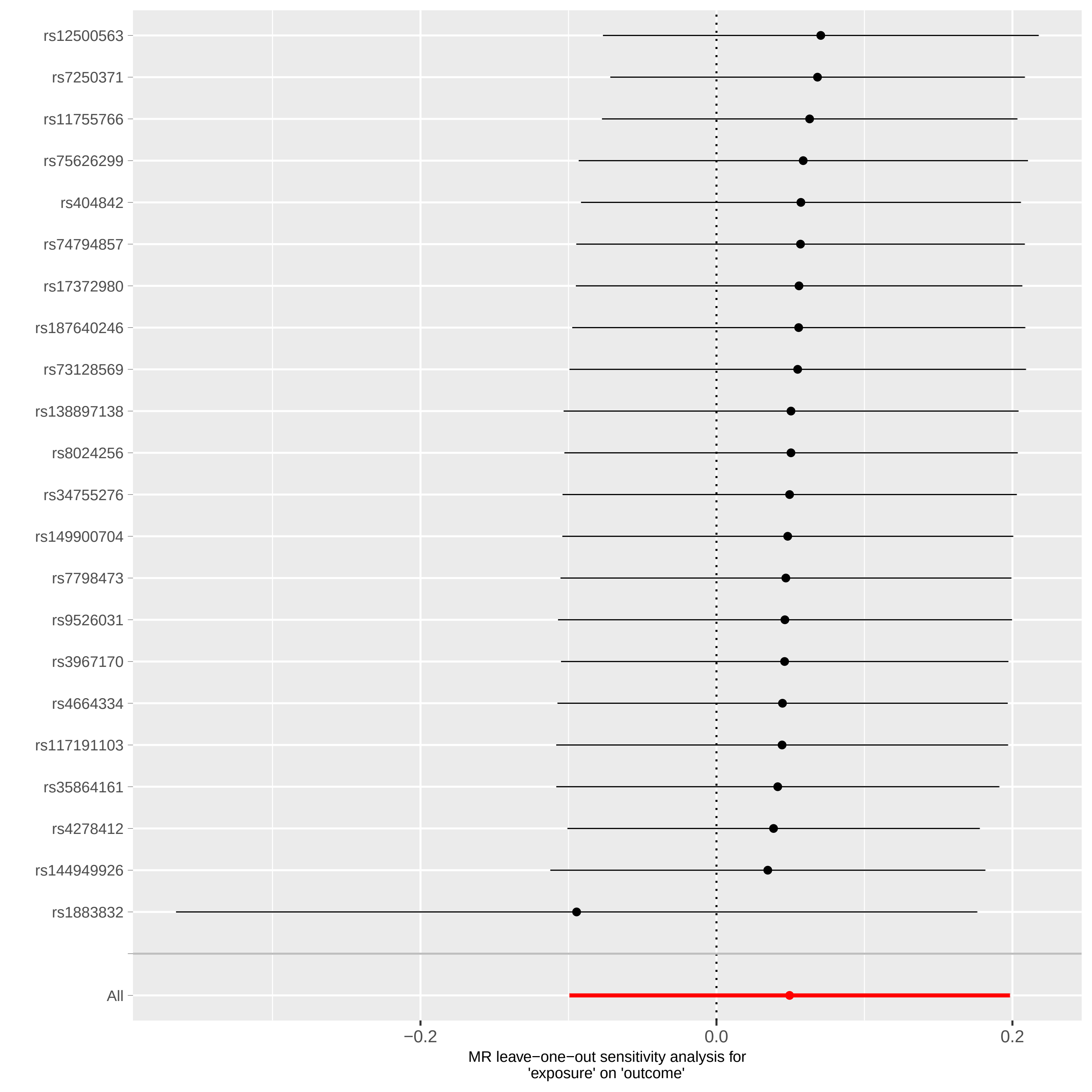
rs12500563
rs7250371
rs11755766
rs75626299
rs404842
rs74794857
rs17372980
rs187640246
rs73128569
rs138897138
rs8024256
rs34755276
rs149900704
rs7798473
rs9526031
rs3967170
rs4664334
rs117191103
rs35864161
rs4278412
rs144949926
rs1883832
All
−0.2
0.0
0.2
MR leave−one−out sensitivity analysis for
'exposure' on 'outcome'

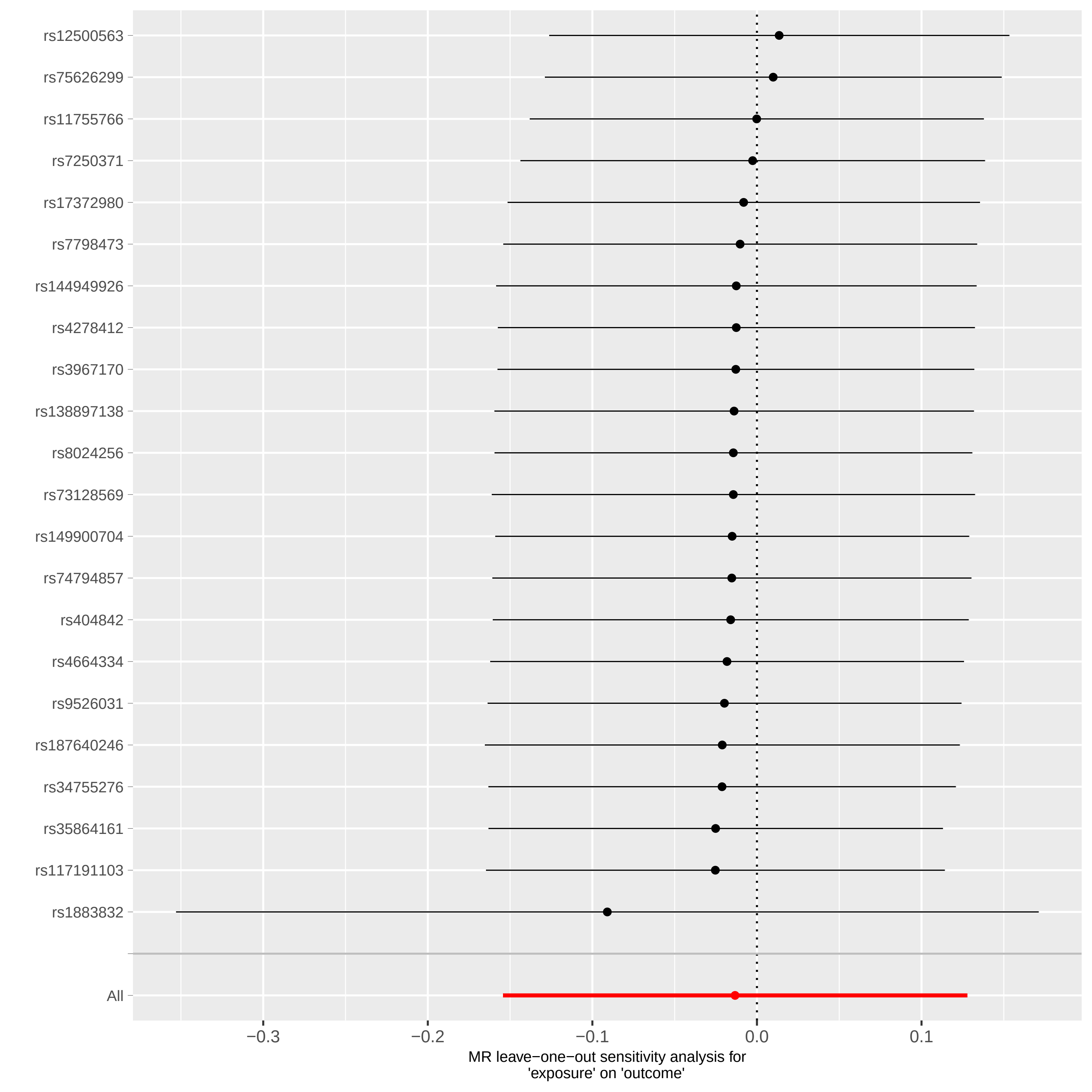
rs12500563
rs75626299
rs11755766
rs7250371
rs17372980
rs7798473
rs144949926
rs4278412
rs3967170
rs138897138
rs8024256
rs73128569
rs149900704
rs74794857
rs404842
rs4664334
rs9526031
rs187640246
rs34755276
rs35864161
rs117191103
rs1883832
All
−0.3
−0.2
−0.1
0.0
0.1
MR leave−one−out sensitivity analysis for
'exposure' on 'outcome'

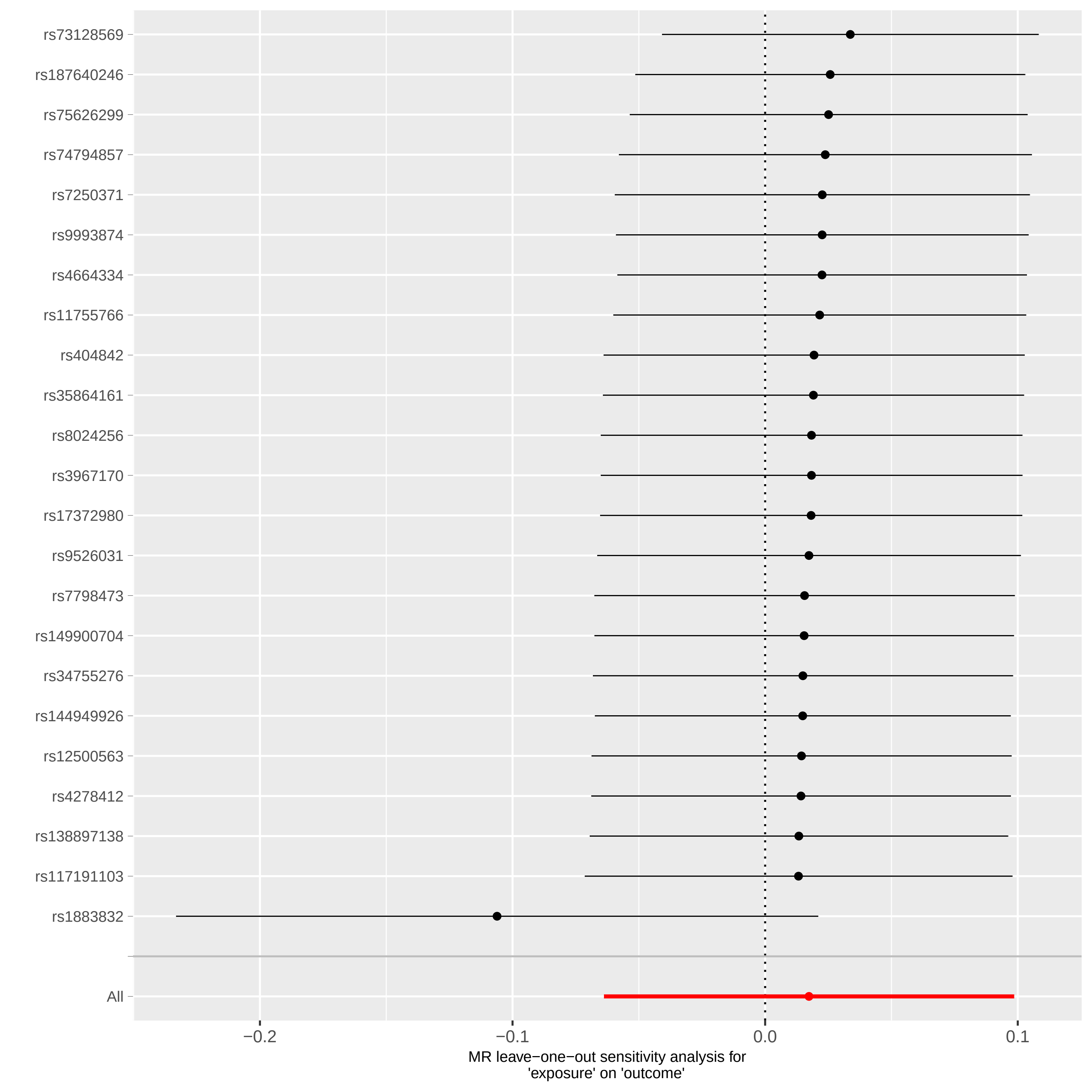
rs73128569
rs187640246
rs75626299
rs74794857
rs7250371
rs9993874
rs4664334
rs11755766
rs404842
rs35864161
rs8024256
rs3967170
rs17372980
rs9526031
rs7798473
rs149900704
rs34755276
rs144949926
rs12500563
rs4278412
rs138897138
rs117191103
rs1883832
All
−0.2
−0.1
0.0
0.1
MR leave−one−out sensitivity analysis for
'exposure' on 'outcome'

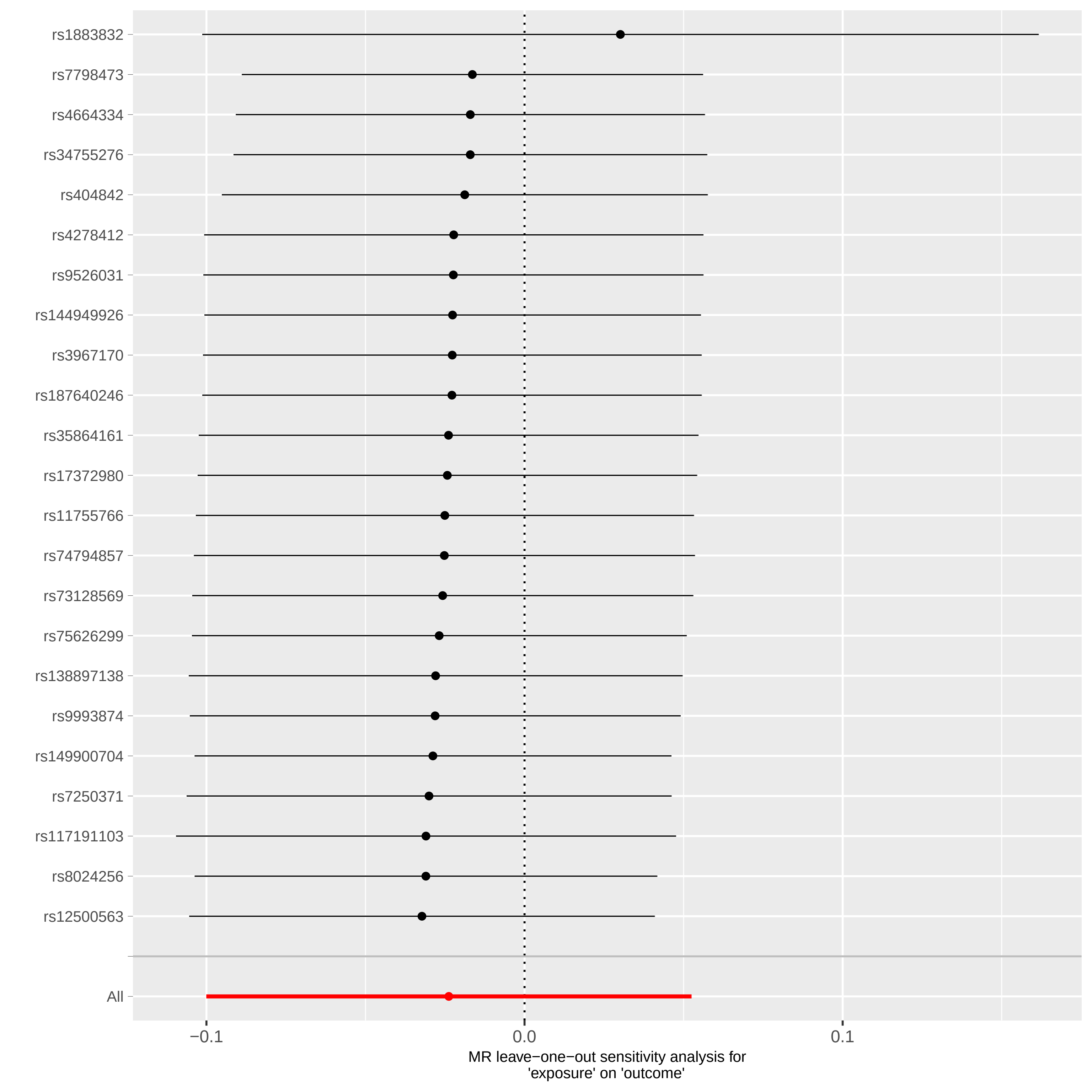
rs1883832
rs7798473
rs4664334
rs34755276
rs404842
rs4278412
rs9526031
rs144949926
rs3967170
rs187640246
rs35864161
rs17372980
rs11755766
rs74794857
rs73128569
rs75626299
rs138897138
rs9993874
rs149900704
rs7250371
rs117191103
rs8024256
rs12500563
All
−0.1
0.0
0.1
MR leave−one−out sensitivity analysis for
'exposure' on 'outcome'

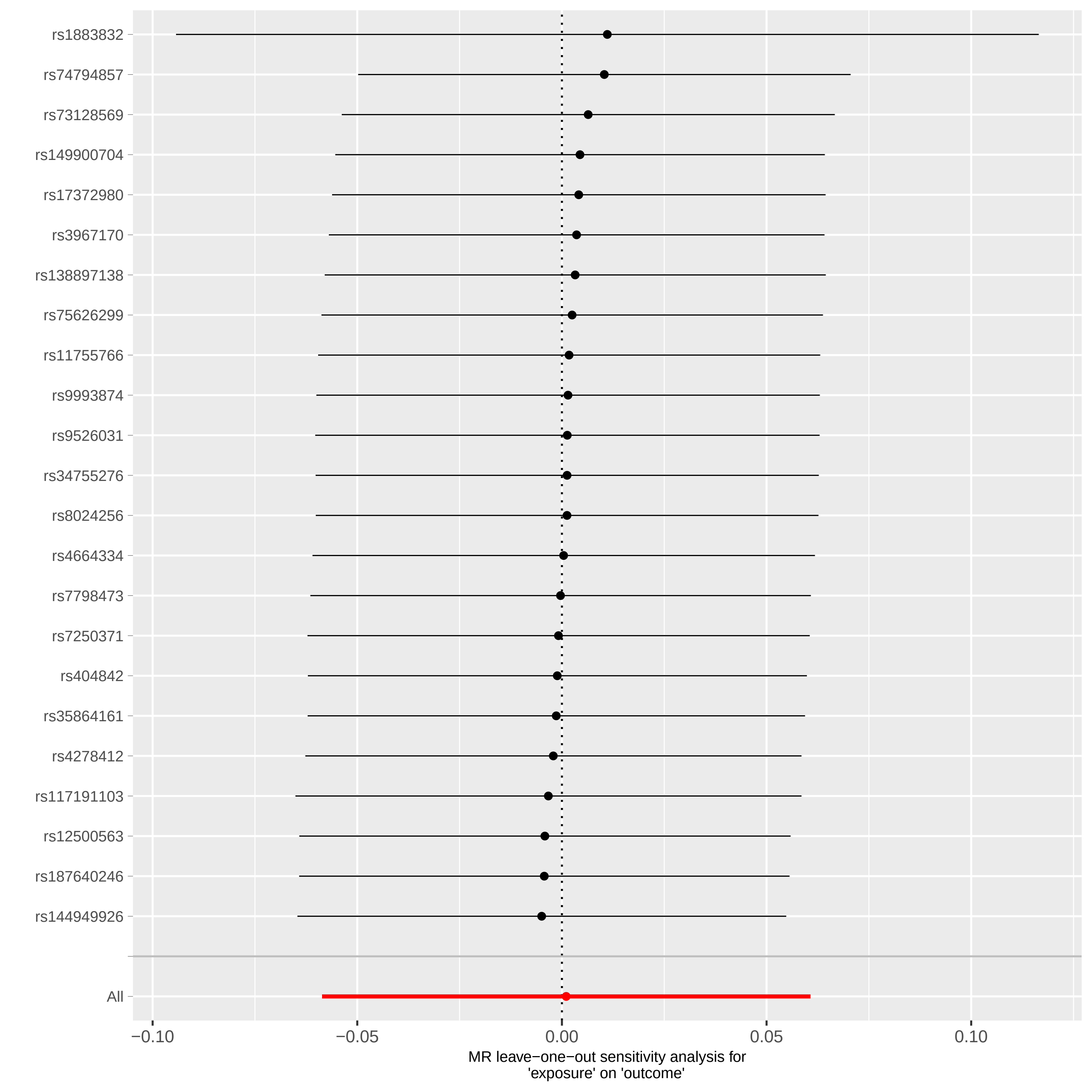

rs1883832
rs74794857
rs73128569
rs149900704
rs17372980
rs3967170
rs138897138
rs75626299
rs11755766
rs9993874
rs9526031
rs34755276
rs8024256
rs4664334
rs7798473
rs7250371
rs404842
rs35864161
rs4278412
rs117191103
rs12500563
rs187640246
rs144949926
All
−0.10
−0.05
0.00
0.05
0.10
MR leave−one−out sensitivity analysis for
'exposure' on 'outcome'

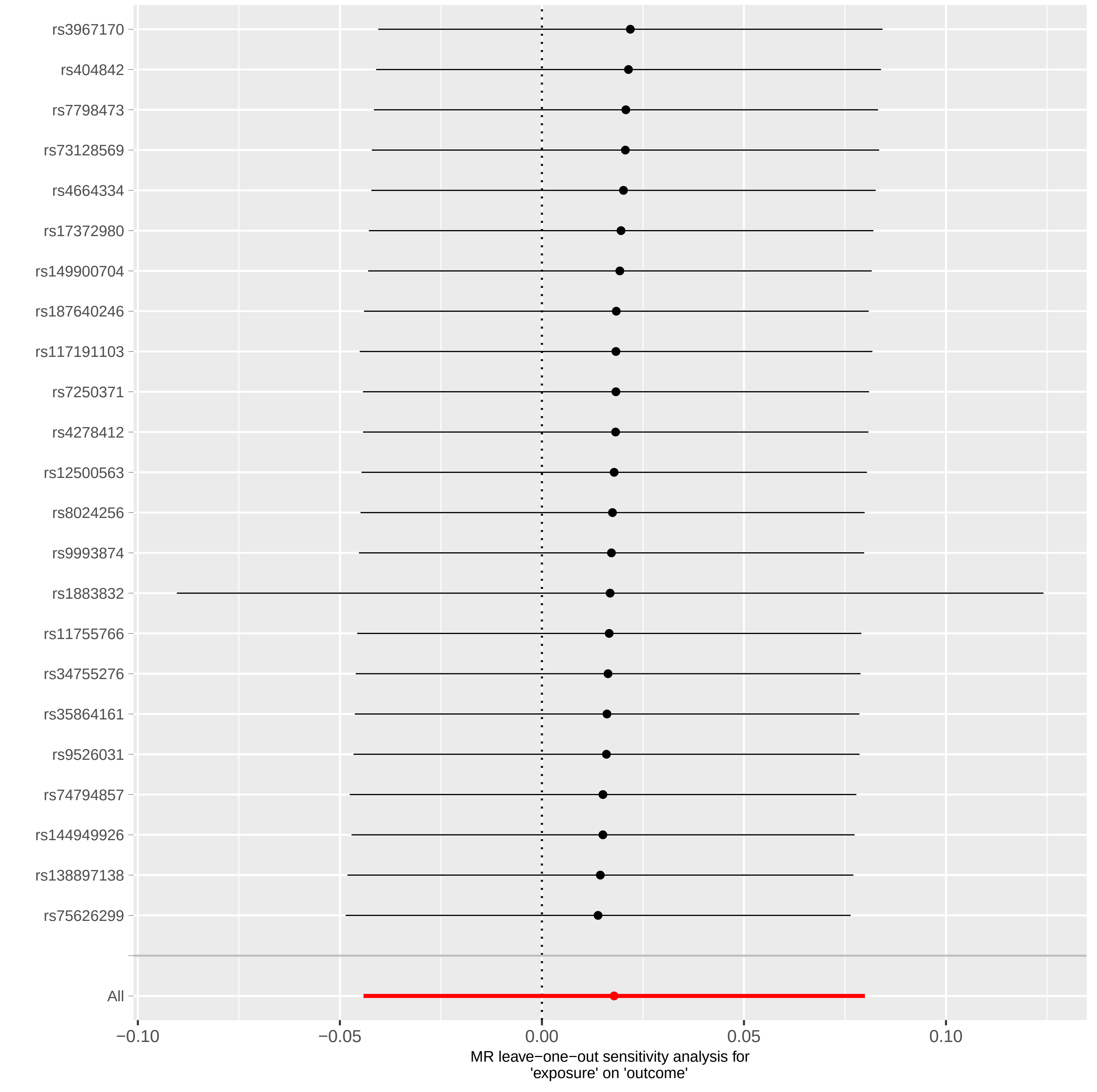

rs3967170
rs404842
rs7798473
rs73128569
rs4664334
rs17372980
rs149900704
rs187640246
rs117191103
rs7250371
rs4278412
rs12500563
rs8024256
rs9993874
rs1883832
rs11755766
rs34755276
rs35864161
rs9526031
rs74794857
rs144949926
rs138897138
rs75626299
All
−0.10
−0.05
0.00
0.05
0.10
MR leave−one−out sensitivity analysis for
'exposure' on 'outcome'

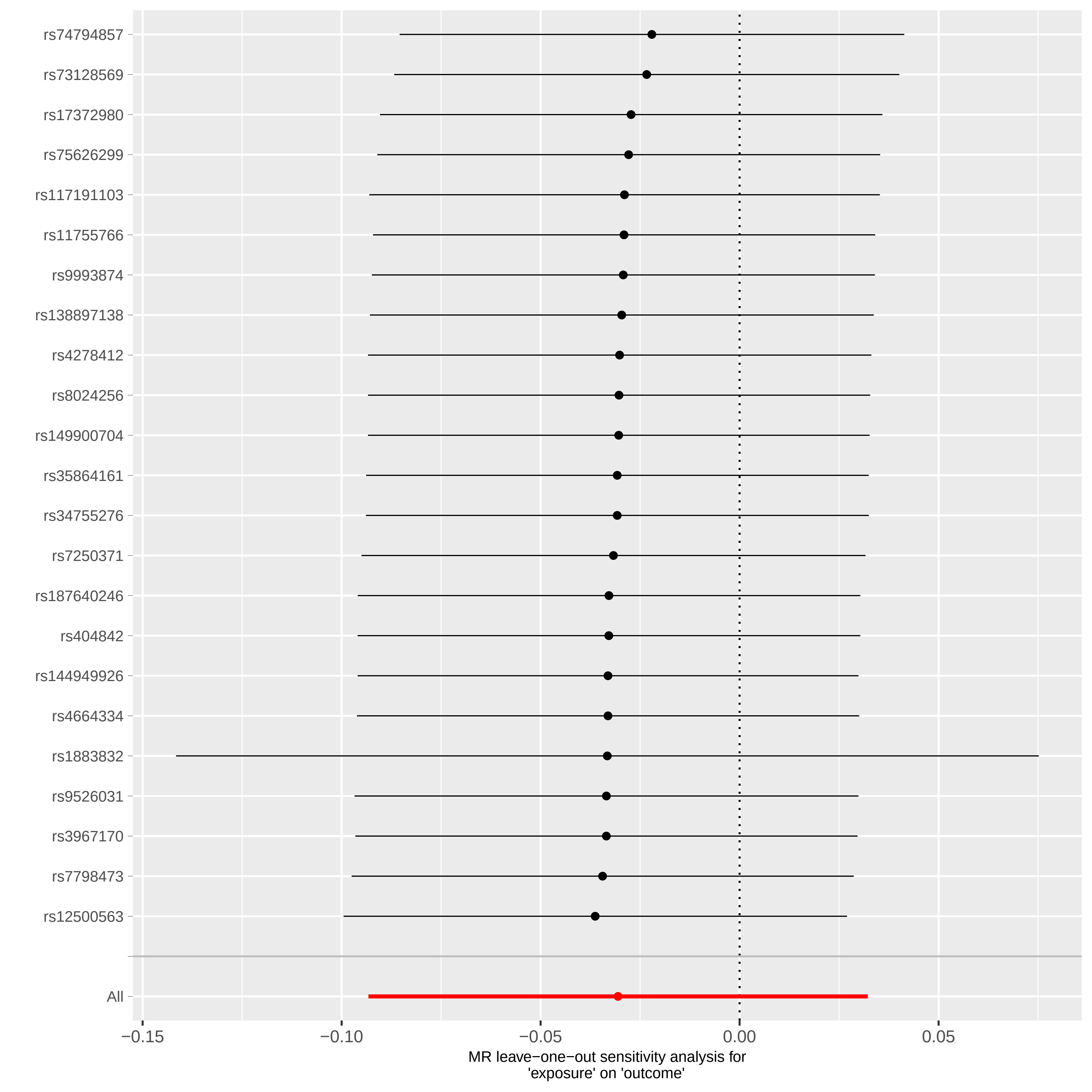

rs74794857
rs73128569
rs17372980
rs75626299
rs117191103
rs11755766
rs9993874
rs138897138
rs4278412
rs8024256
rs149900704
rs35864161
rs34755276
rs7250371
rs187640246
rs404842
rs144949926
rs4664334
rs1883832
rs9526031
rs3967170
rs7798473
rs12500563
All
−0.15
−0.10
−0.05
0.00
0.05
MR leave−one−out sensitivity analysis for
'exposure' on 'outcome'

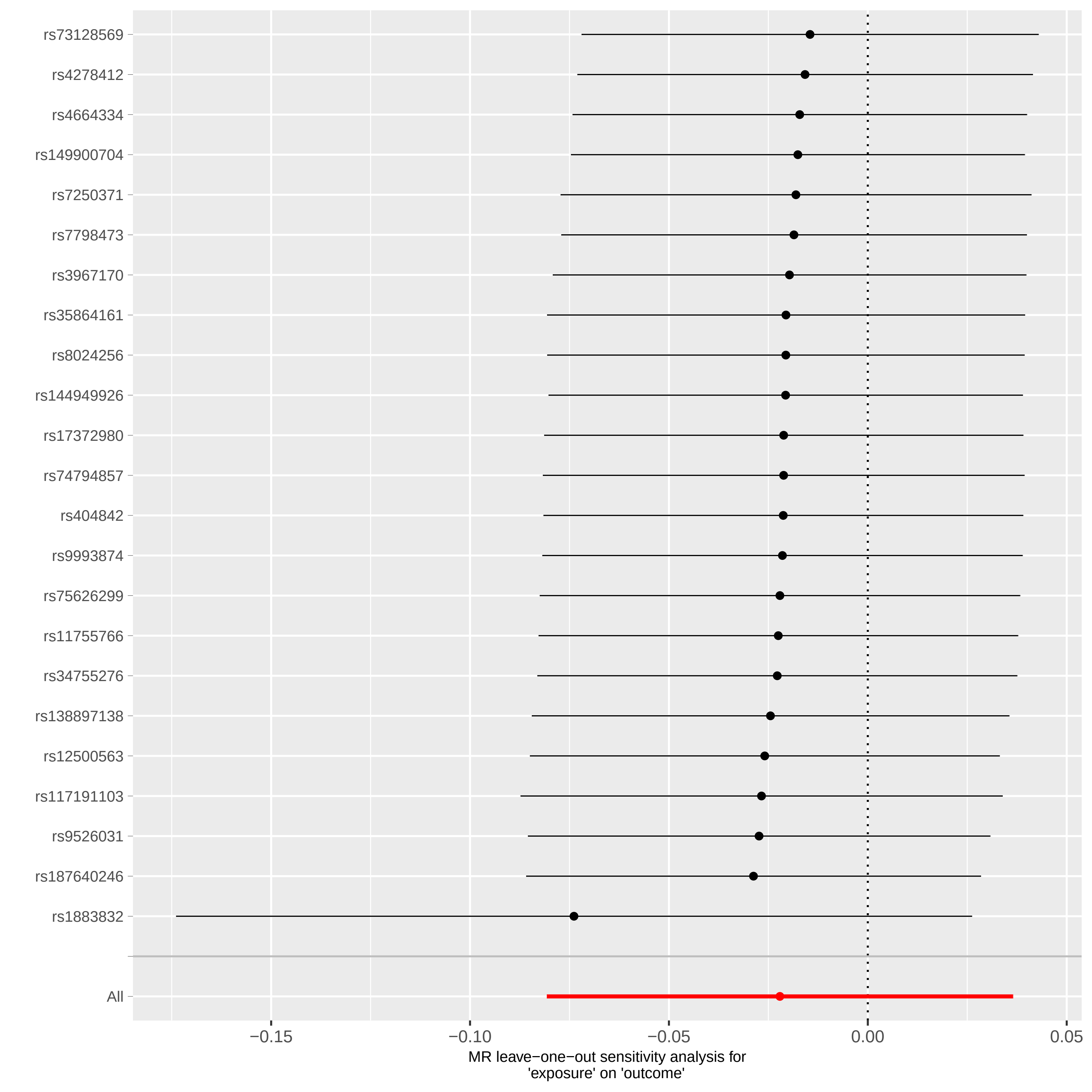
rs73128569
rs4278412
rs4664334
rs149900704
rs7250371
rs7798473
rs3967170
rs35864161
rs8024256
rs144949926
rs17372980
rs74794857
rs404842
rs9993874
rs75626299
rs11755766
rs34755276
rs138897138
rs12500563
rs117191103
rs9526031
rs187640246
rs1883832
All
−0.15
−0.10
−0.05
0.00
0.05
MR leave−one−out sensitivity analysis for
'exposure' on 'outcome'

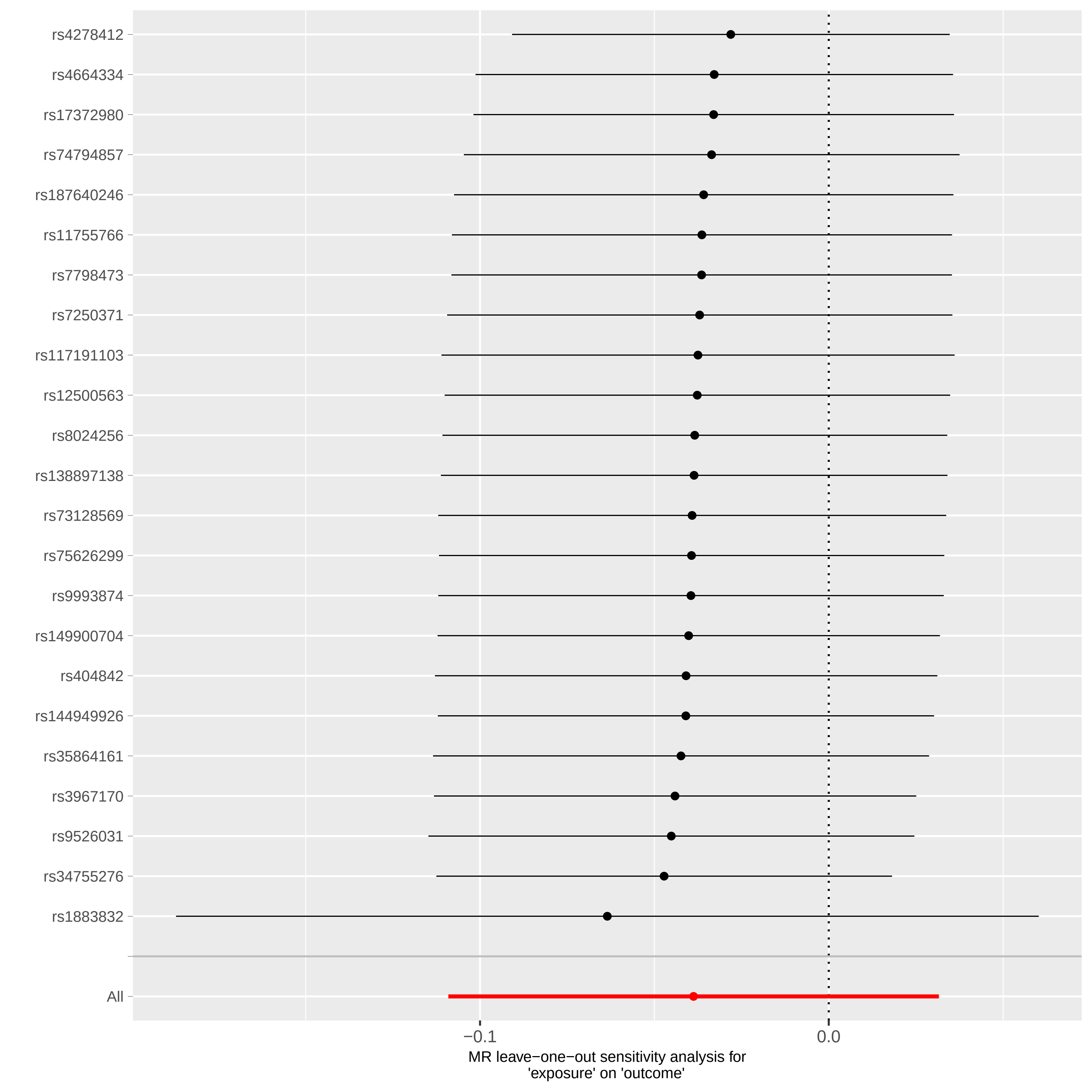
rs4278412
rs4664334
rs17372980
rs74794857
rs187640246
rs11755766
rs7798473
rs7250371
rs117191103
rs12500563
rs8024256
rs138897138
rs73128569
rs75626299
rs9993874
rs149900704
rs404842
rs144949926
rs35864161
rs3967170
rs9526031
rs34755276
rs1883832
All
−0.1
0.0
MR leave−one−out sensitivity analysis for
'exposure' on 'outcome'

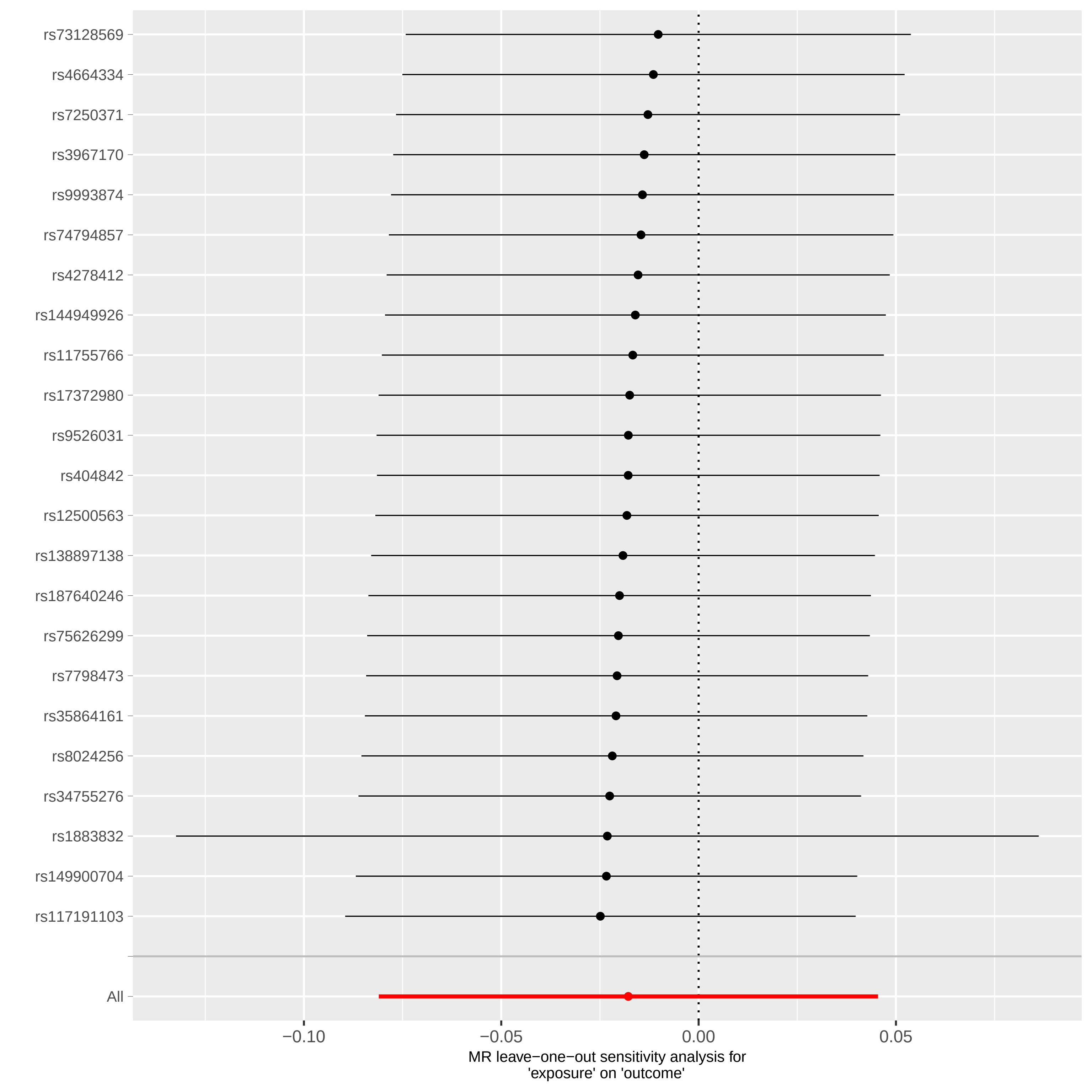
rs73128569
rs4664334
rs7250371
rs3967170
rs9993874
rs74794857
rs4278412
rs144949926
rs11755766
rs17372980
rs9526031
rs404842
rs12500563
rs138897138
rs187640246
rs75626299
rs7798473
rs35864161
rs8024256
rs34755276
rs1883832
rs149900704
rs117191103
All
−0.10
−0.05
0.00
0.05
MR leave−one−out sensitivity analysis for
'exposure' on 'outcome'

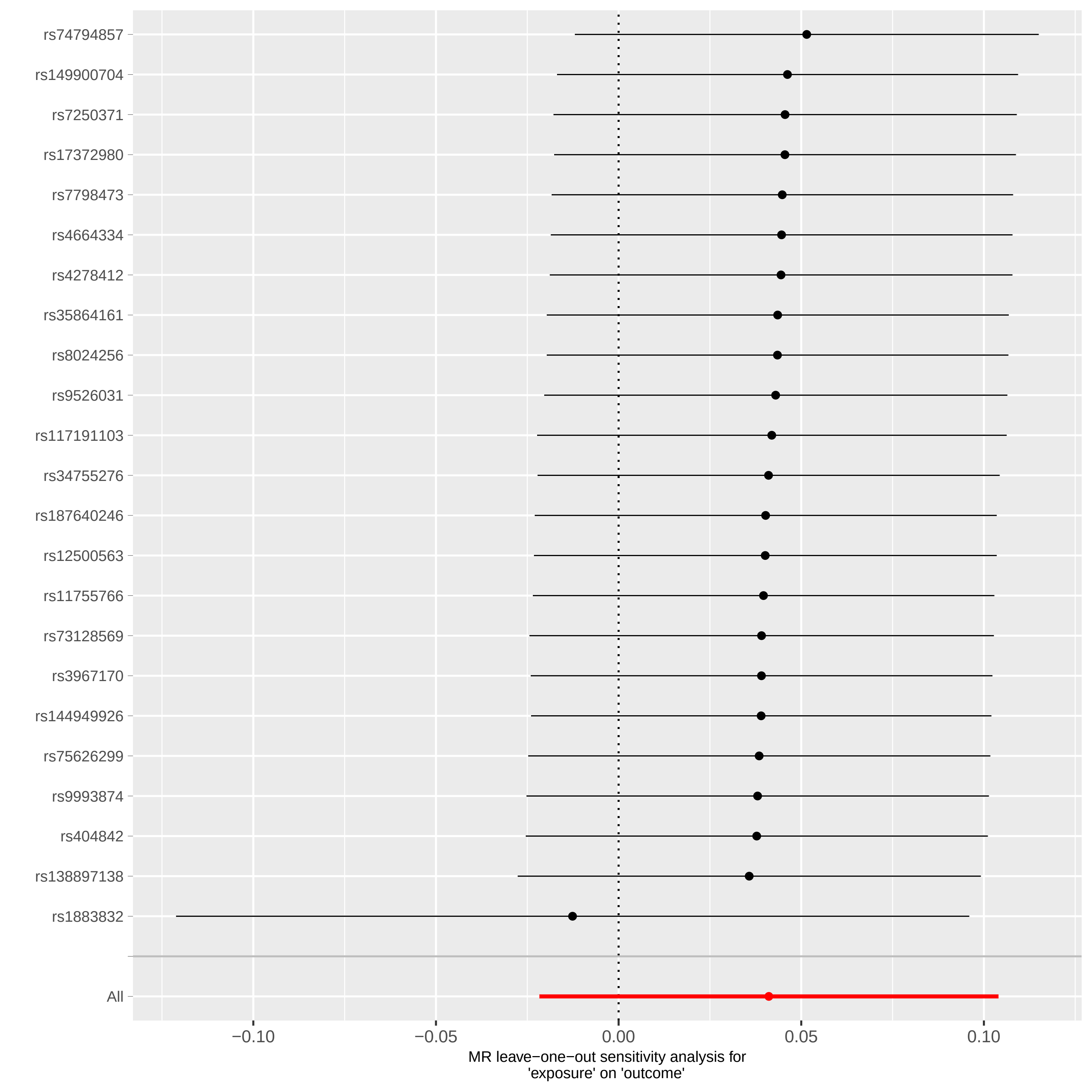
rs74794857
rs149900704
rs7250371
rs17372980
rs7798473
rs4664334
rs4278412
rs35864161
rs8024256
rs9526031
rs117191103
rs34755276
rs187640246
rs12500563
rs11755766
rs73128569
rs3967170
rs144949926
rs75626299
rs9993874
rs404842
rs138897138
rs1883832
All
−0.10
−0.05
0.00
0.05
0.10
MR leave−one−out sensitivity analysis for
'exposure' on 'outcome'

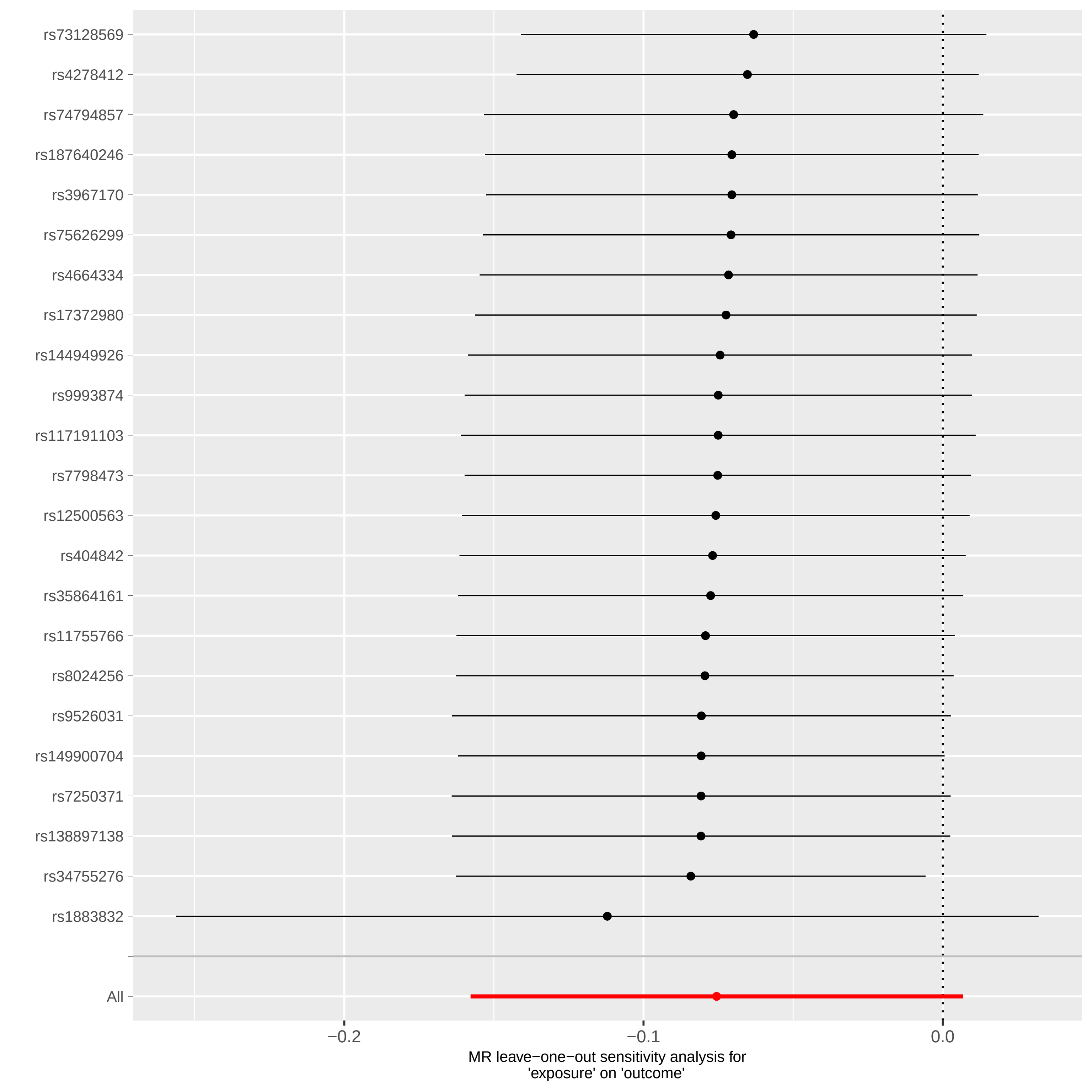
rs73128569
rs4278412
rs74794857
rs187640246
rs3967170
rs75626299
rs4664334
rs17372980
rs144949926
rs9993874
rs117191103
rs7798473
rs12500563
rs404842
rs35864161
rs11755766
rs8024256
rs9526031
rs149900704
rs7250371
rs138897138
rs34755276
rs1883832
All
−0.2
−0.1
0.0
MR leave−one−out sensitivity analysis for
'exposure' on 'outcome'

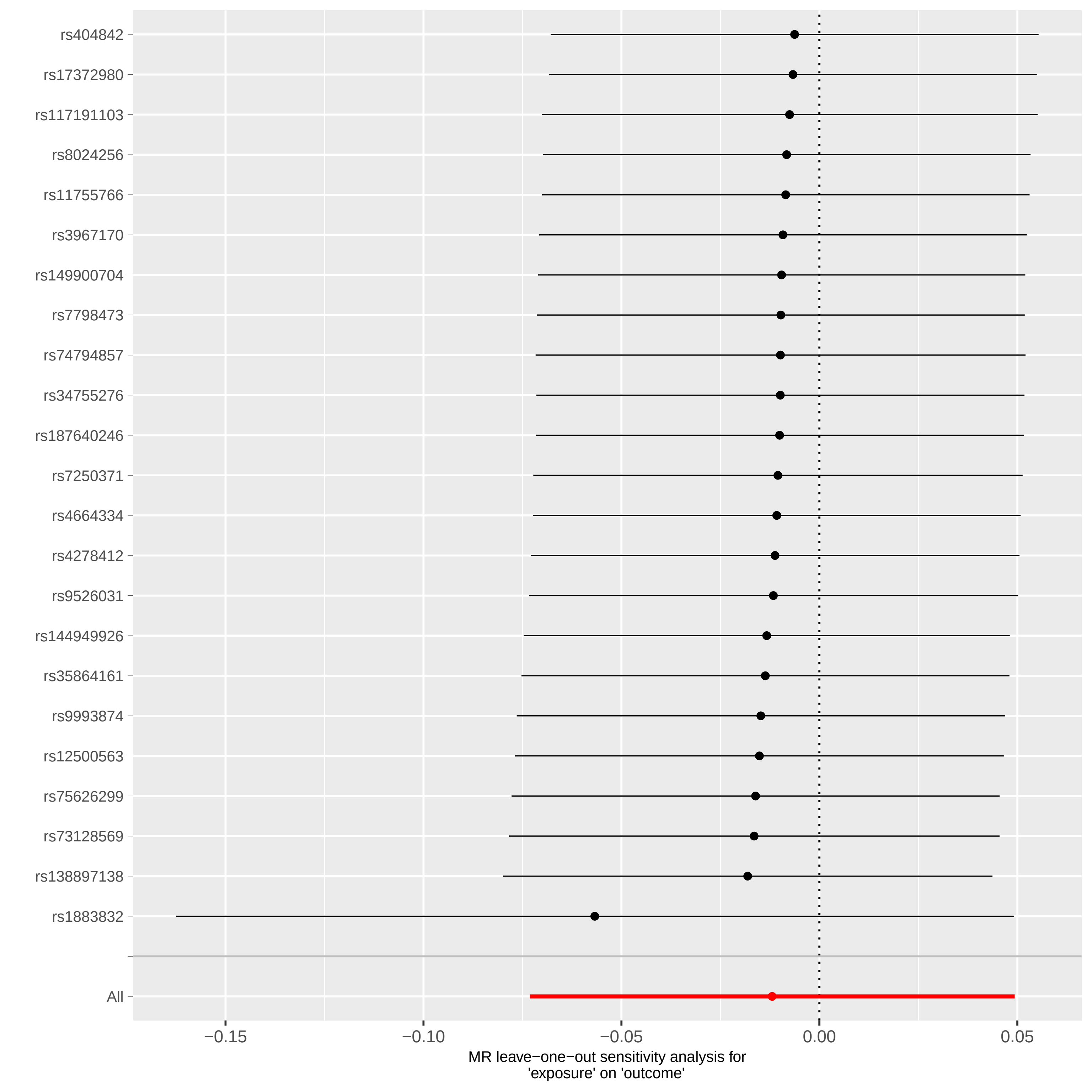

rs404842
rs17372980
rs117191103
rs8024256
rs11755766
rs3967170
rs149900704
rs7798473
rs74794857
rs34755276
rs187640246
rs7250371
rs4664334
rs4278412
rs9526031
rs144949926
rs35864161
rs9993874
rs12500563
rs75626299
rs73128569
rs138897138
rs1883832
All
−0.15
−0.10
−0.05
0.00
0.05
MR leave−one−out sensitivity analysis for
'exposure' on 'outcome'

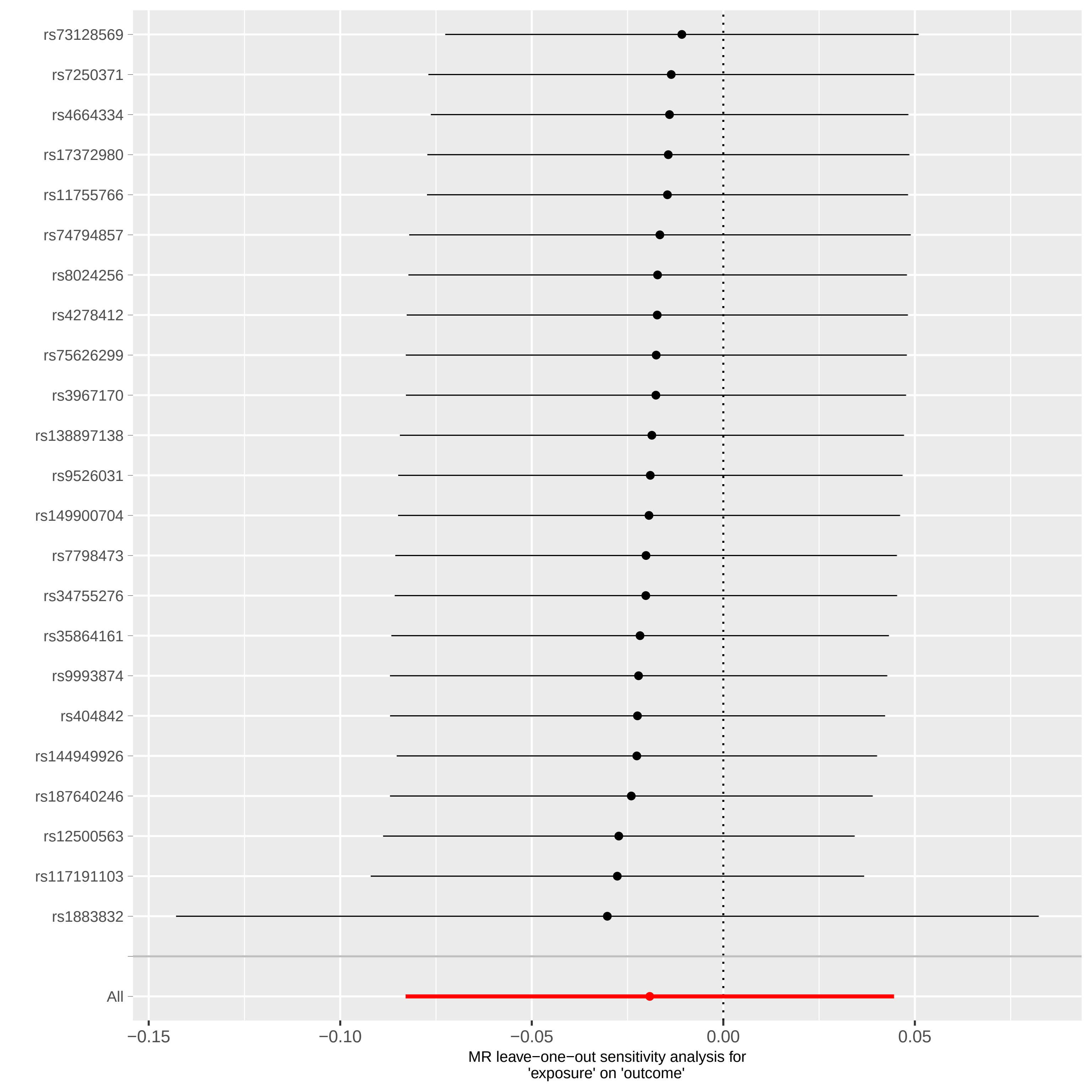

rs73128569
rs7250371
rs4664334
rs17372980
rs11755766
rs74794857
rs8024256
rs4278412
rs75626299
rs3967170
rs138897138
rs9526031
rs149900704
rs7798473
rs34755276
rs35864161
rs9993874
rs404842
rs144949926
rs187640246
rs12500563
rs117191103
rs1883832
All
−0.15
−0.10
−0.05
0.00
0.05
MR leave−one−out sensitivity analysis for
'exposure' on 'outcome'

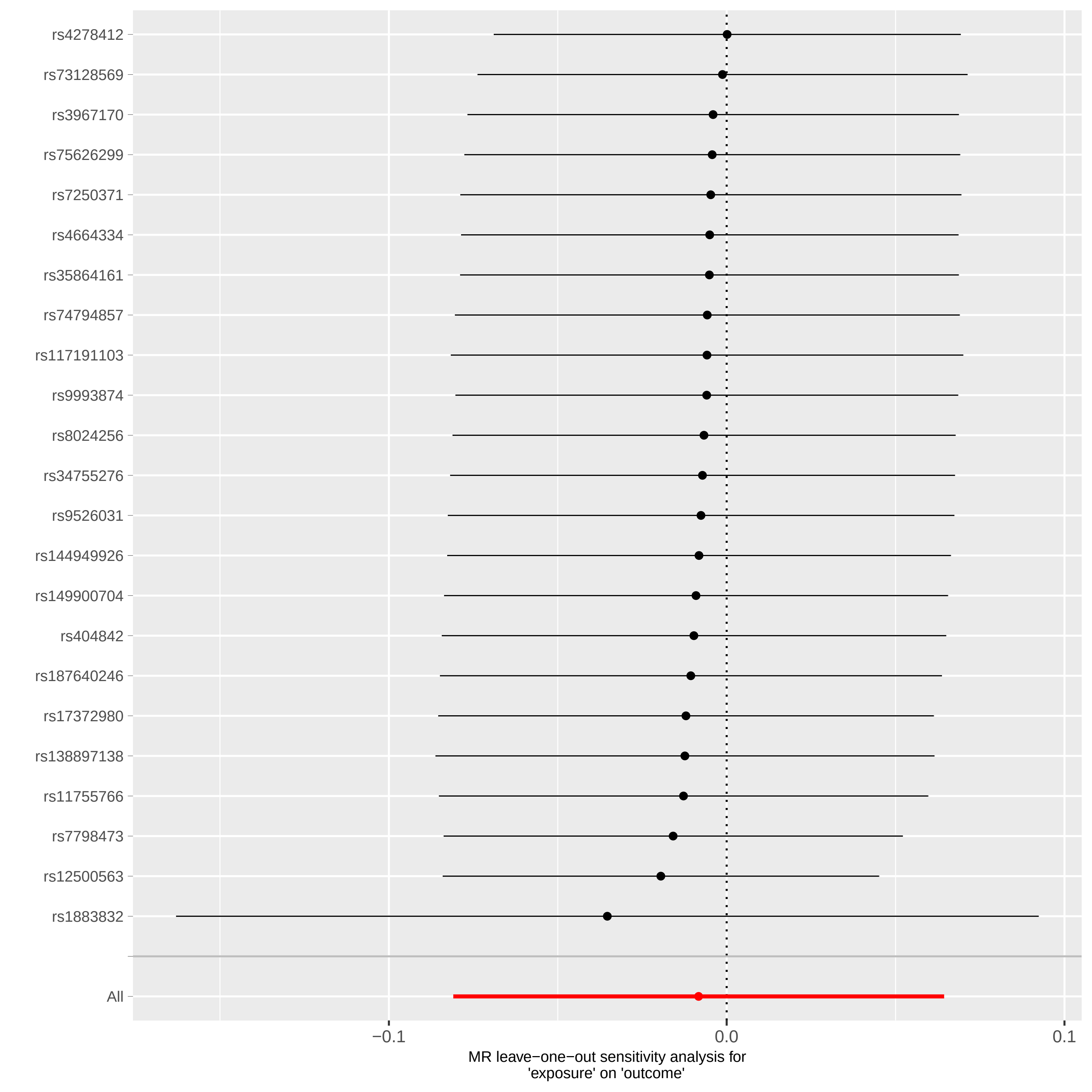

rs4278412
rs73128569
rs3967170
rs75626299
rs7250371
rs4664334
rs35864161
rs74794857
rs117191103
rs9993874
rs8024256
rs34755276
rs9526031
rs144949926
rs149900704
rs404842
rs187640246
rs17372980
rs138897138
rs11755766
rs7798473
rs12500563
rs1883832
All
−0.1
0.0
0.1
MR leave−one−out sensitivity analysis for
'exposure' on 'outcome'

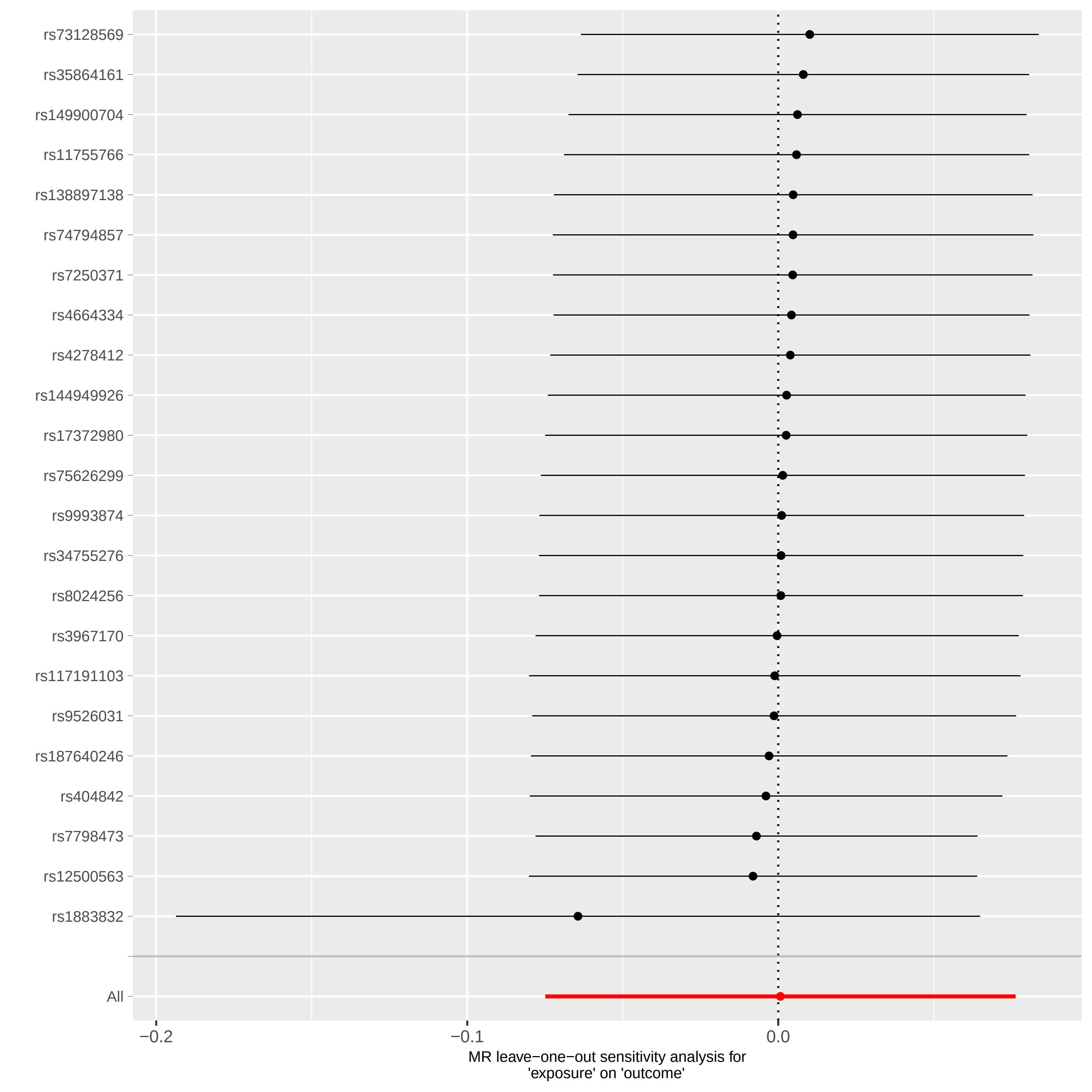
rs73128569
rs35864161
rs149900704
rs11755766
rs138897138
rs74794857
rs7250371
rs4664334
rs4278412
rs144949926
rs17372980
rs75626299
rs9993874
rs34755276
rs8024256
rs3967170
rs117191103
rs9526031
rs187640246
rs404842
rs7798473
rs12500563
rs1883832
All
−0.2
−0.1
0.0
MR leave−one−out sensitivity analysis for
'exposure' on 'outcome'

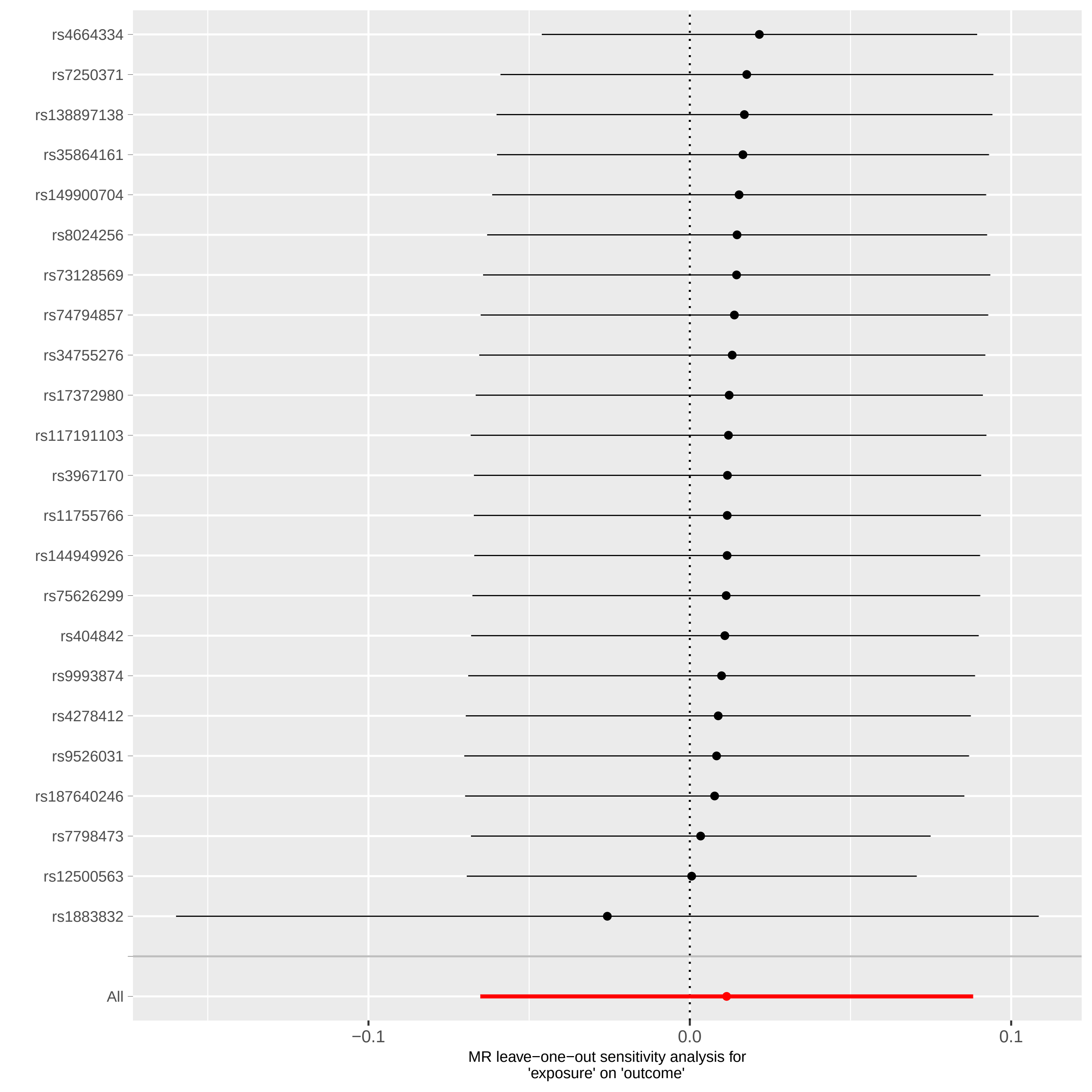
rs4664334
rs7250371
rs138897138
rs35864161
rs149900704
rs8024256
rs73128569
rs74794857
rs34755276
rs17372980
rs117191103
rs3967170
rs11755766
rs144949926
rs75626299
rs404842
rs9993874
rs4278412
rs9526031
rs187640246
rs7798473
rs12500563
rs1883832
All
−0.1
0.0
0.1
MR leave−one−out sensitivity analysis for
'exposure' on 'outcome'

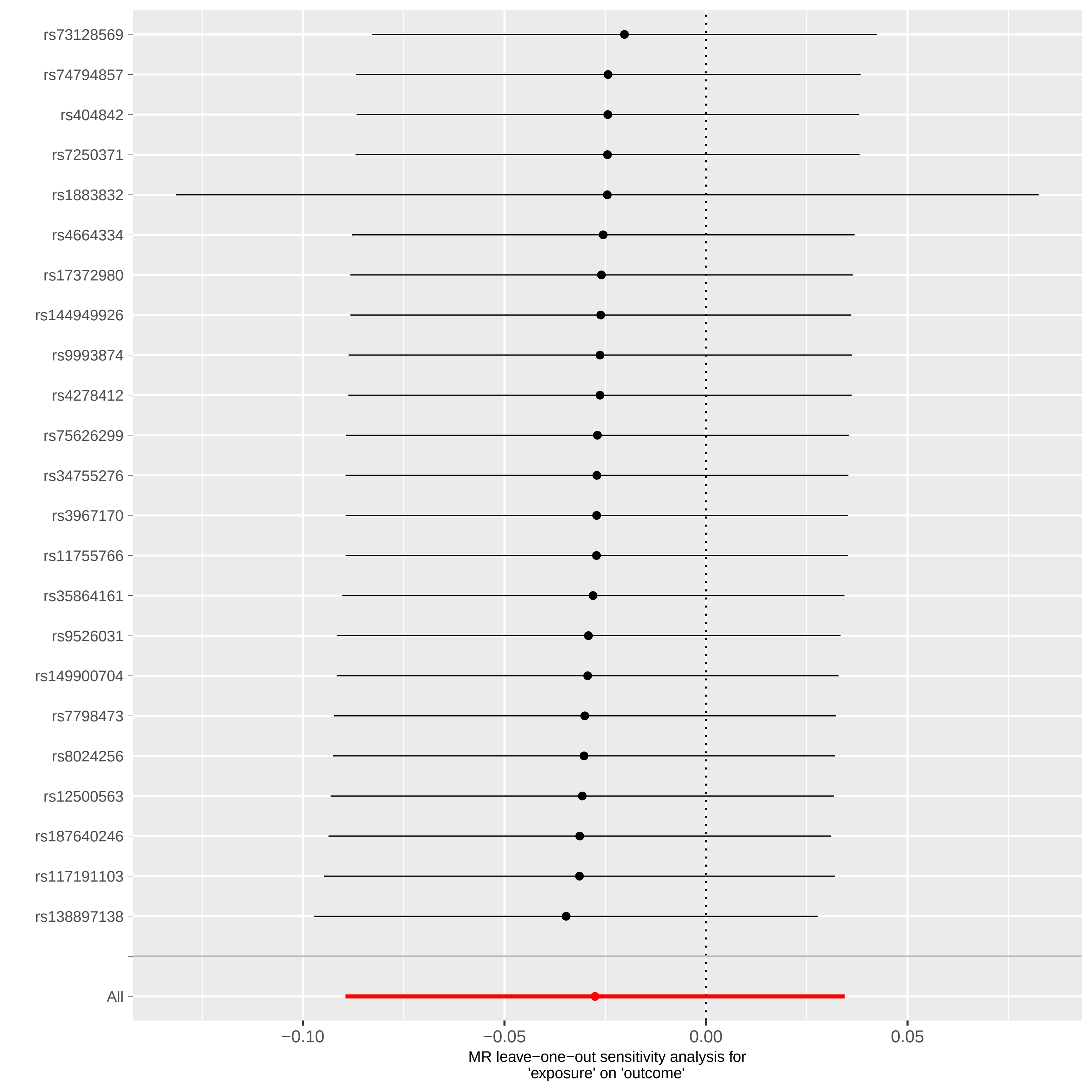
rs73128569
rs74794857
rs404842
rs7250371
rs1883832
rs4664334
rs17372980
rs144949926
rs9993874
rs4278412
rs75626299
rs34755276
rs3967170
rs11755766
rs35864161
rs9526031
rs149900704
rs7798473
rs8024256
rs12500563
rs187640246
rs117191103
rs138897138
All
−0.10
−0.05
0.00
0.05
MR leave−one−out sensitivity analysis for
'exposure' on 'outcome'

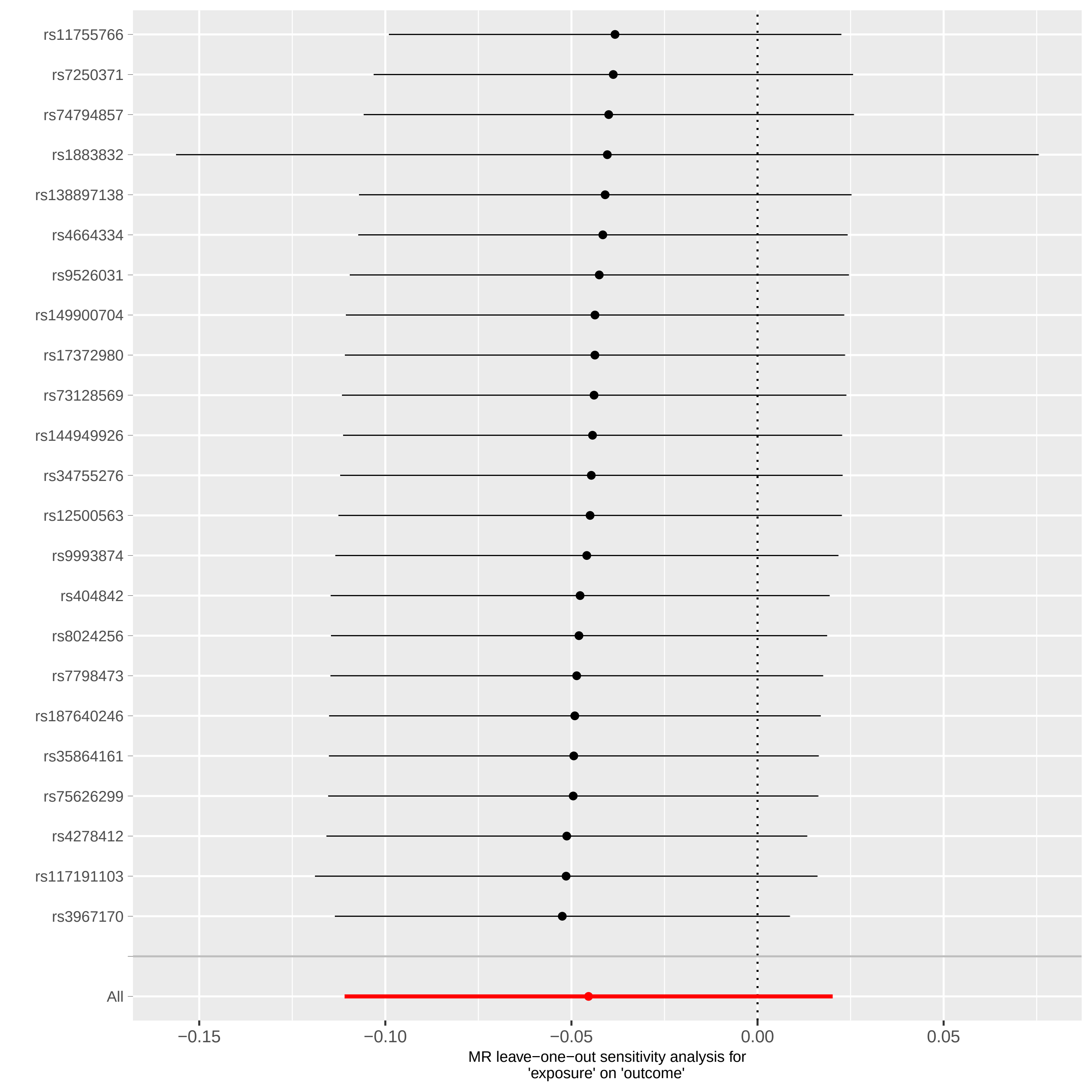

rs11755766
rs7250371
rs74794857
rs1883832
rs138897138
rs4664334
rs9526031
rs149900704
rs17372980
rs73128569
rs144949926
rs34755276
rs12500563
rs9993874
rs404842
rs8024256
rs7798473
rs187640246
rs35864161
rs75626299
rs4278412
rs117191103
rs3967170
All
−0.15
−0.10
−0.05
0.00
0.05
MR leave−one−out sensitivity analysis for
'exposure' on 'outcome'

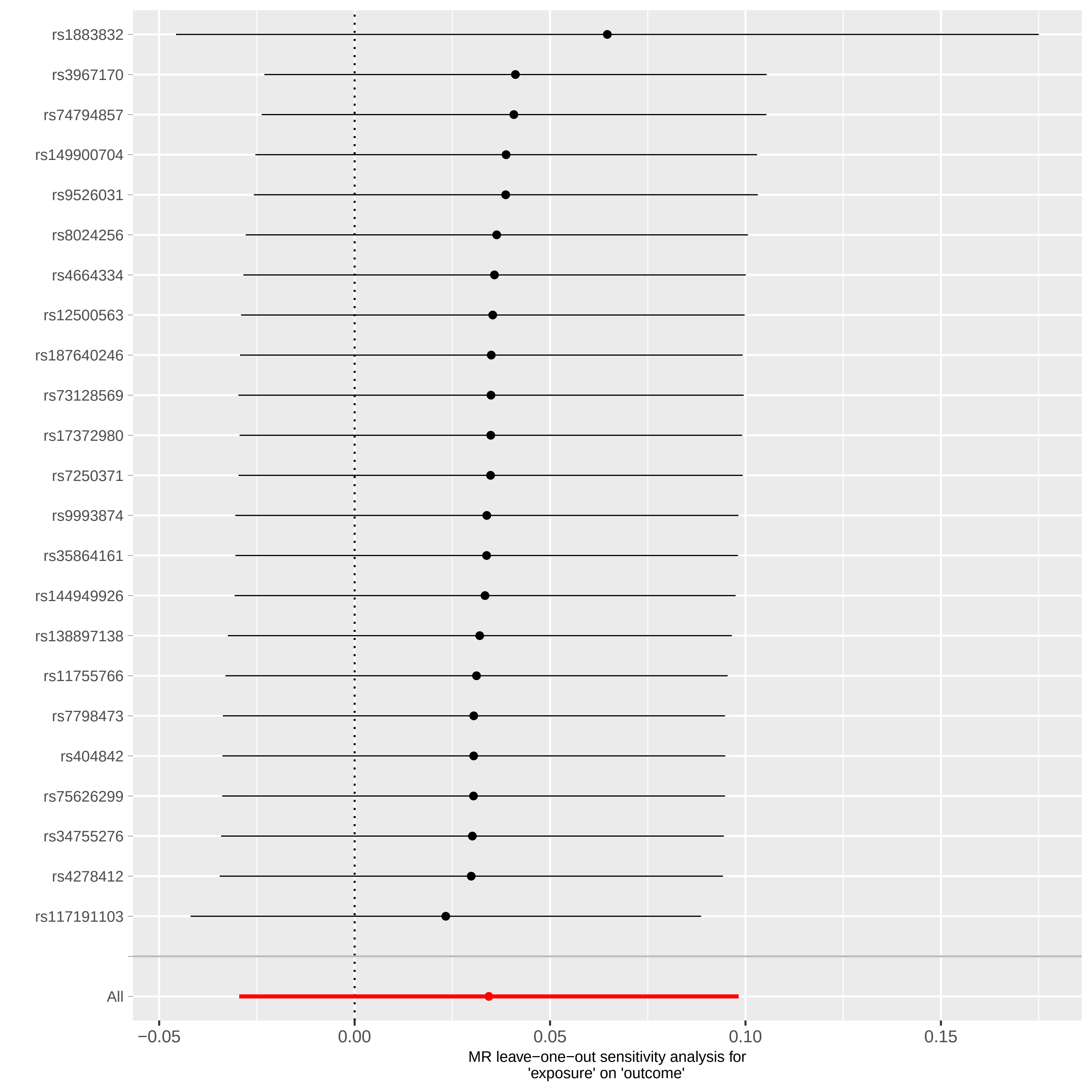

rs1883832
rs3967170
rs74794857
rs149900704
rs9526031
rs8024256
rs4664334
rs12500563
rs187640246
rs73128569
rs17372980
rs7250371
rs9993874
rs35864161
rs144949926
rs138897138
rs11755766
rs7798473
rs404842
rs75626299
rs34755276
rs4278412
rs117191103
All
−0.05
0.00
0.05
0.10
0.15
MR leave−one−out sensitivity analysis for
'exposure' on 'outcome'

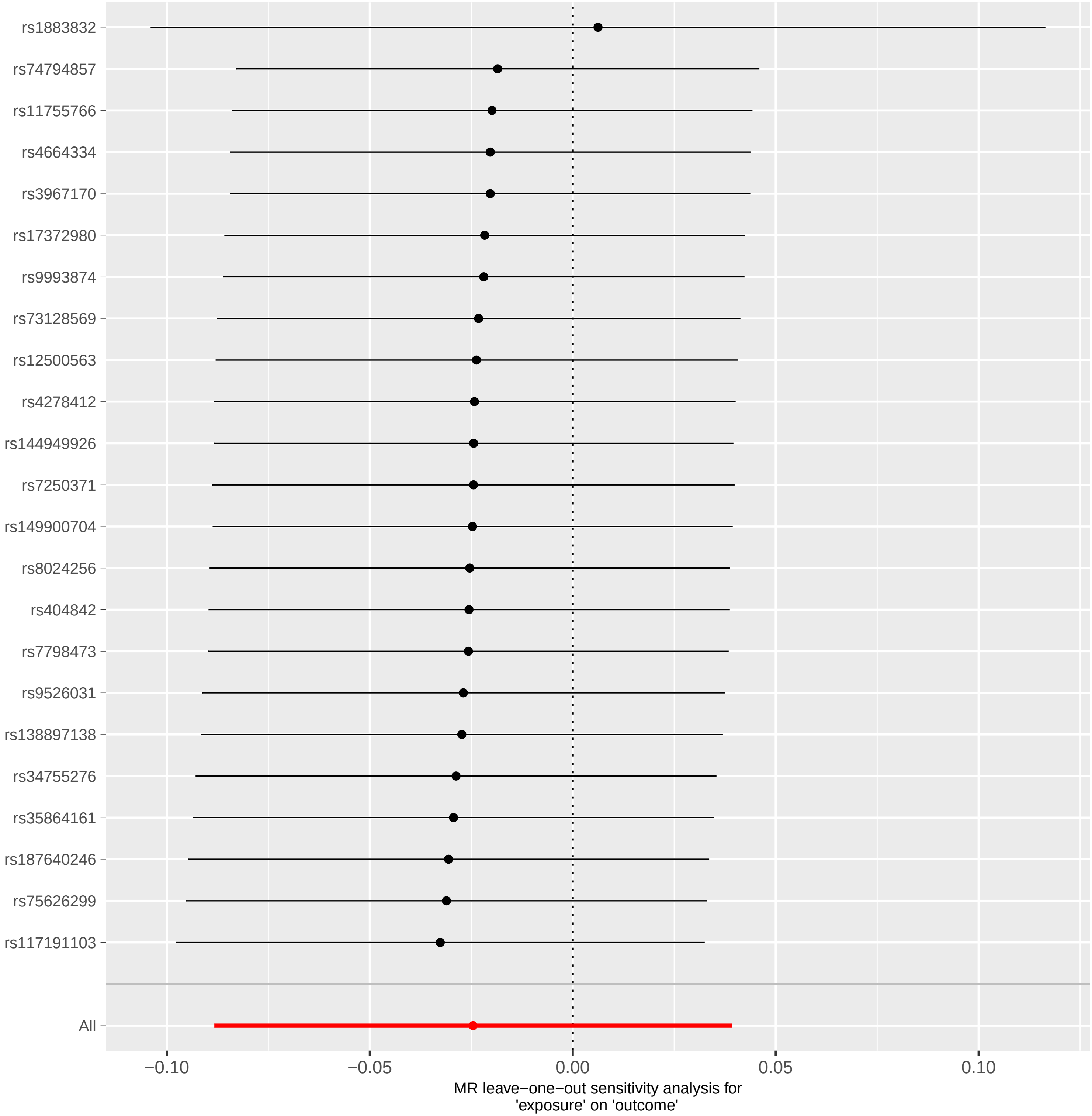

rs1883832
rs74794857
rs11755766
rs4664334
rs3967170
rs17372980
rs9993874
rs73128569
rs12500563
rs4278412
rs144949926
rs7250371
rs149900704
rs8024256
rs404842
rs7798473
rs9526031
rs138897138
rs34755276
rs35864161
rs187640246
rs75626299
rs117191103
All
−0.10
−0.05
0.00
0.05
0.10
MR leave−one−out sensitivity analysis for
'exposure' on 'outcome'

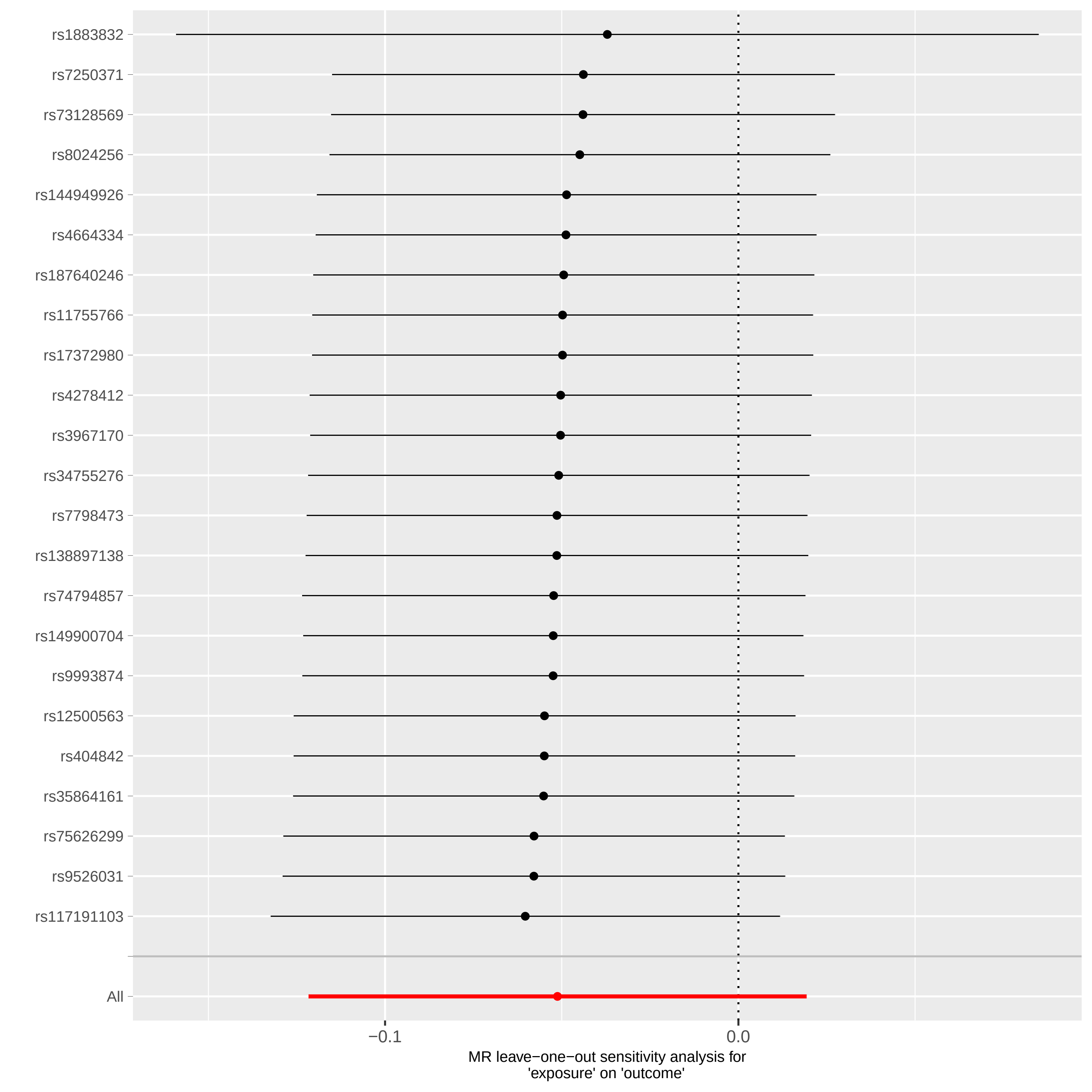

rs1883832
rs7250371
rs73128569
rs8024256
rs144949926
rs4664334
rs187640246
rs11755766
rs17372980
rs4278412
rs3967170
rs34755276
rs7798473
rs138897138
rs74794857
rs149900704
rs9993874
rs12500563
rs404842
rs35864161
rs75626299
rs9526031
rs117191103
All
−0.1
0.0
MR leave−one−out sensitivity analysis for
'exposure' on 'outcome'

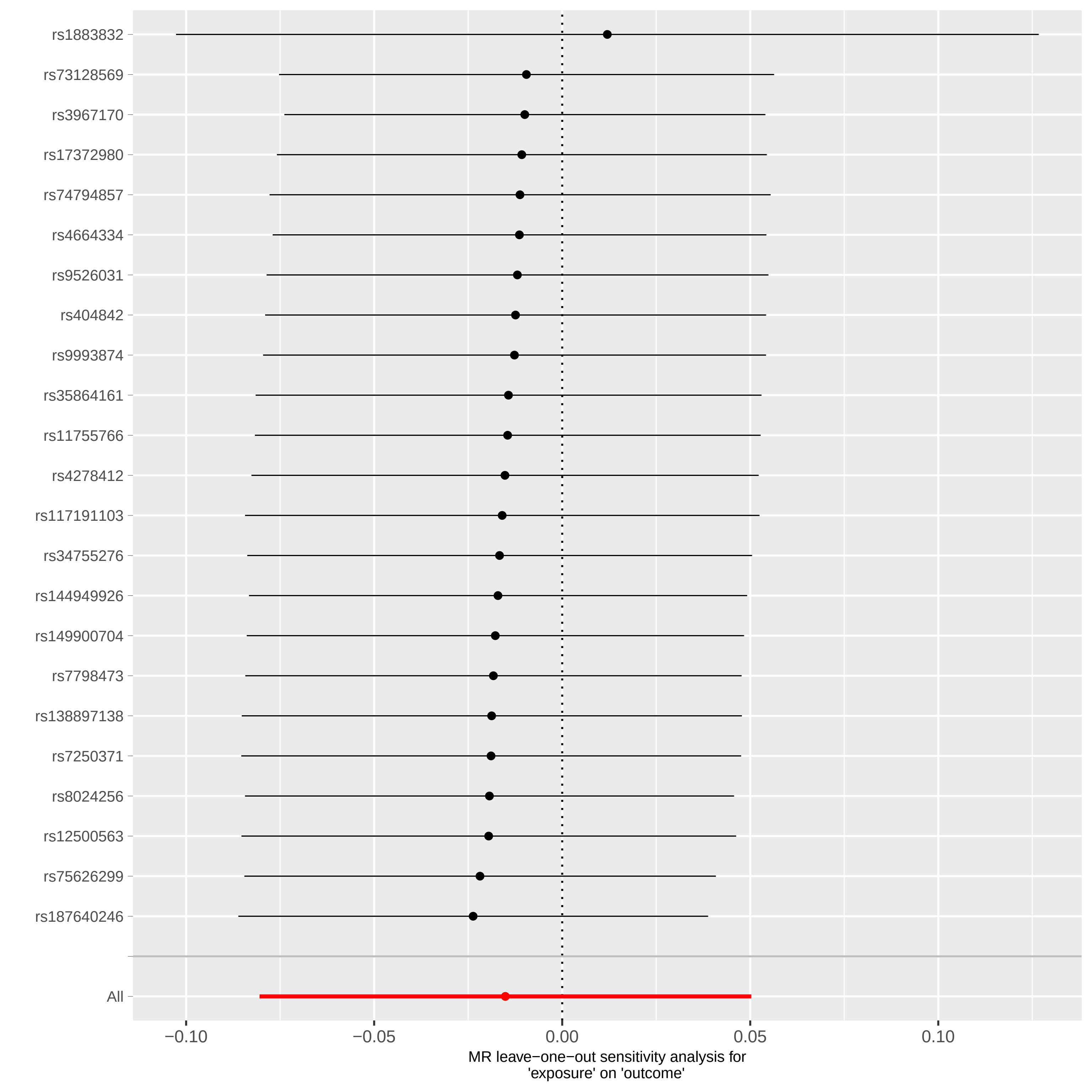
rs1883832
rs73128569
rs3967170
rs17372980
rs74794857
rs4664334
rs9526031
rs404842
rs9993874
rs35864161
rs11755766
rs4278412
rs117191103
rs34755276
rs144949926
rs149900704
rs7798473
rs138897138
rs7250371
rs8024256
rs12500563
rs75626299
rs187640246
All
−0.10
−0.05
0.00
0.05
0.10
MR leave−one−out sensitivity analysis for
'exposure' on 'outcome'

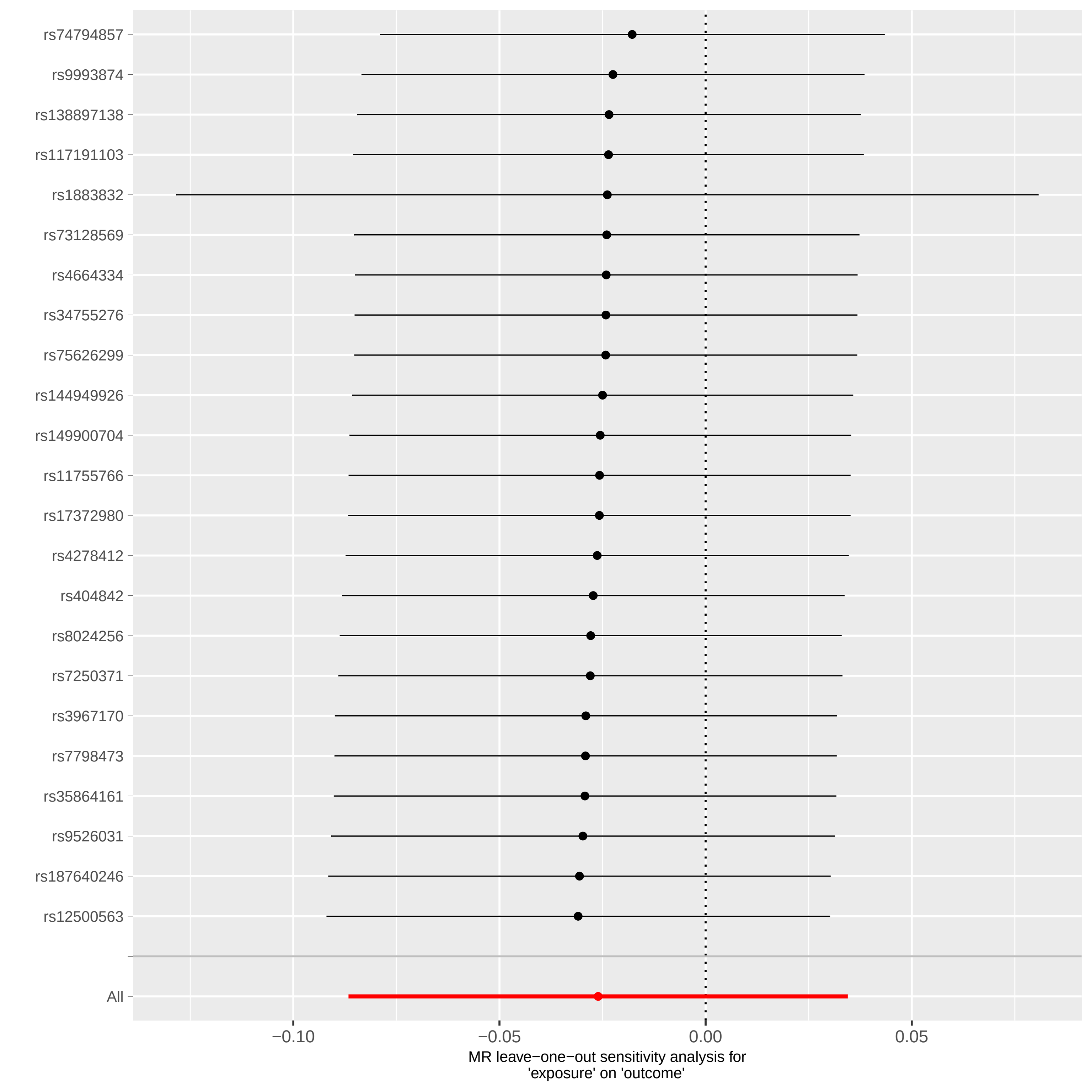

rs74794857
rs9993874
rs138897138
rs117191103
rs1883832
rs73128569
rs4664334
rs34755276
rs75626299
rs144949926
rs149900704
rs11755766
rs17372980
rs4278412
rs404842
rs8024256
rs7250371
rs3967170
rs7798473
rs35864161
rs9526031
rs187640246
rs12500563
All
−0.10
−0.05
0.00
0.05
MR leave−one−out sensitivity analysis for
'exposure' on 'outcome'

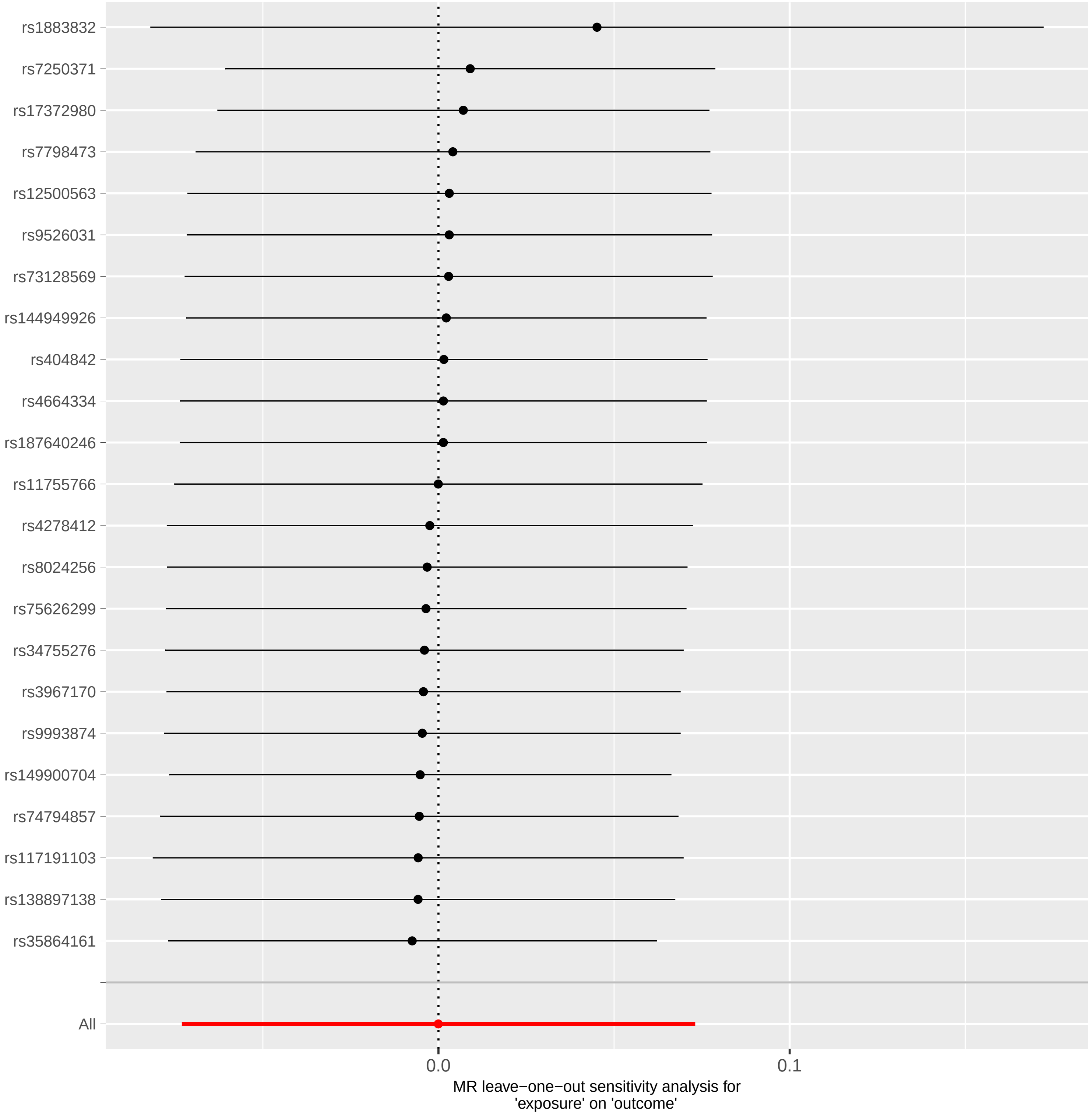
rs1883832
rs7250371
rs17372980
rs7798473
rs12500563
rs9526031
rs73128569
rs144949926
rs404842
rs4664334
rs187640246
rs11755766
rs4278412
rs8024256
rs75626299
rs34755276
rs3967170
rs9993874
rs149900704
rs74794857
rs117191103
rs138897138
rs35864161
All
0.0
0.1
MR leave−one−out sensitivity analysis for
'exposure' on 'outcome'

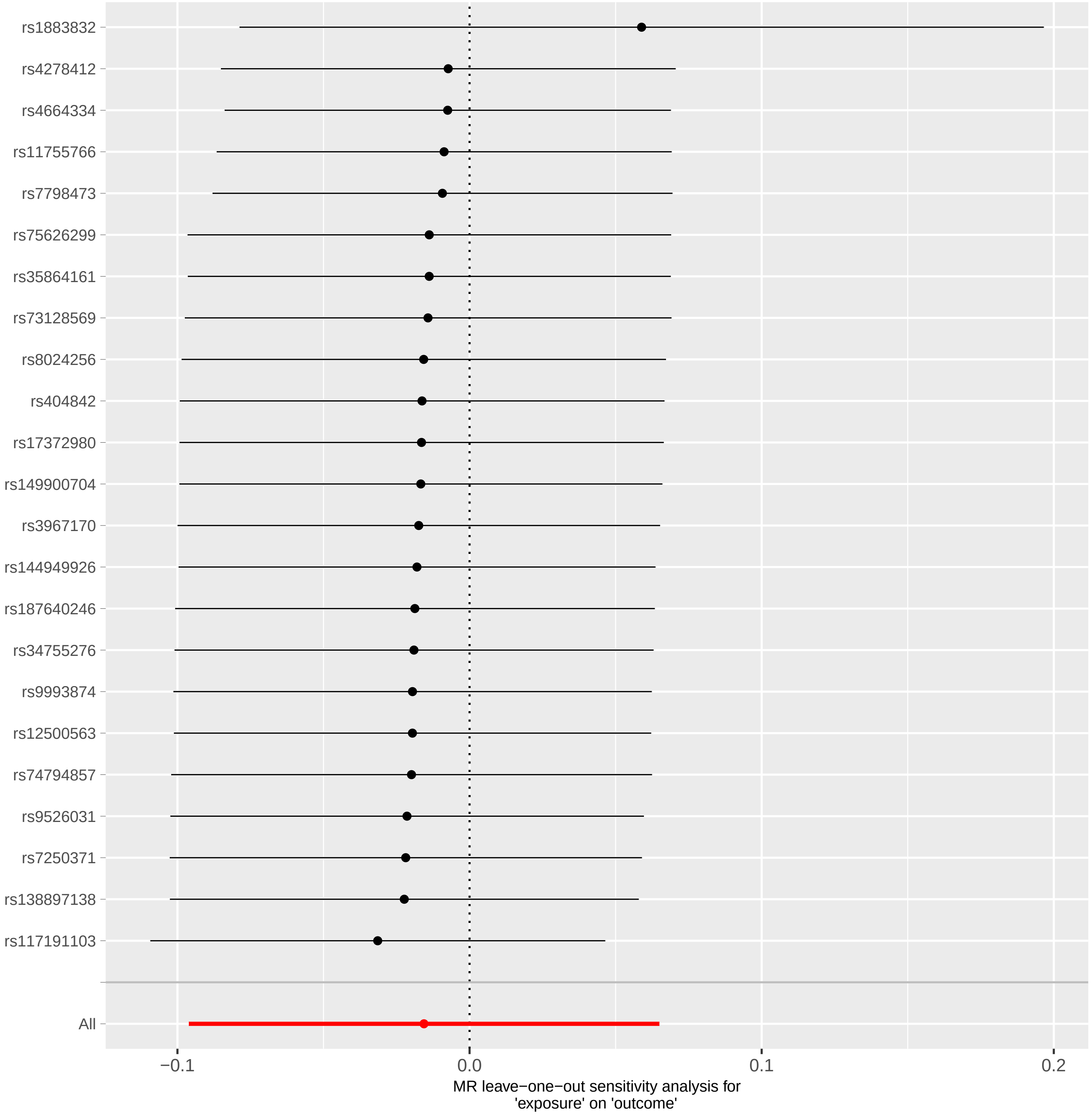
rs1883832
rs4278412
rs4664334
rs11755766
rs7798473
rs75626299
rs35864161
rs73128569
rs8024256
rs404842
rs17372980
rs149900704
rs3967170
rs144949926
rs187640246
rs34755276
rs9993874
rs12500563
rs74794857
rs9526031
rs7250371
rs138897138
rs117191103
All
−0.1
0.0
0.1
0.2
MR leave−one−out sensitivity analysis for
'exposure' on 'outcome'

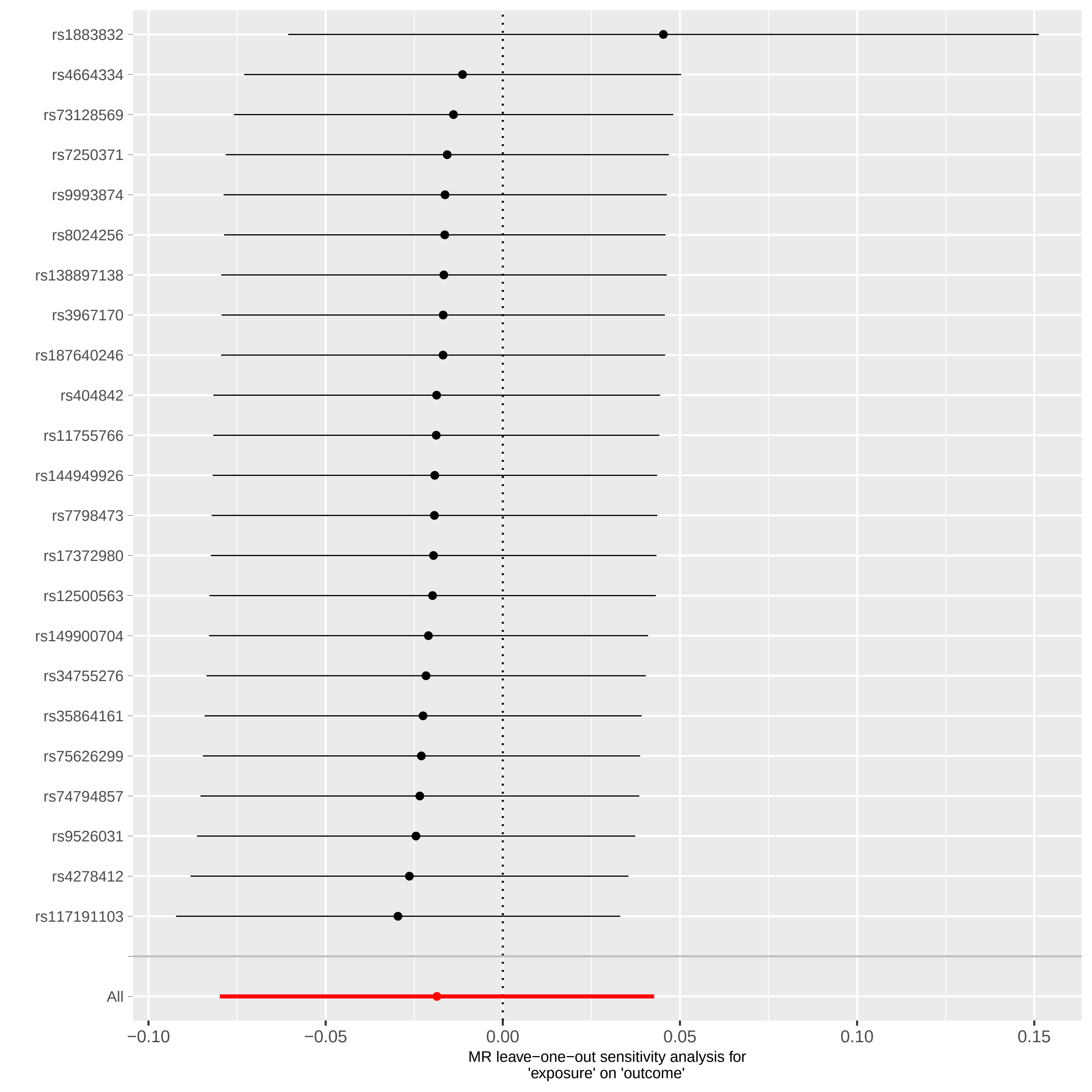
rs1883832
rs4664334
rs73128569
rs7250371
rs9993874
rs8024256
rs138897138
rs3967170
rs187640246
rs404842
rs11755766
rs144949926
rs7798473
rs17372980
rs12500563
rs149900704
rs34755276
rs35864161
rs75626299
rs74794857
rs9526031
rs4278412
rs117191103
All
−0.10
−0.05
0.00
0.05
0.10
0.15
MR leave−one−out sensitivity analysis for
'exposure' on 'outcome'

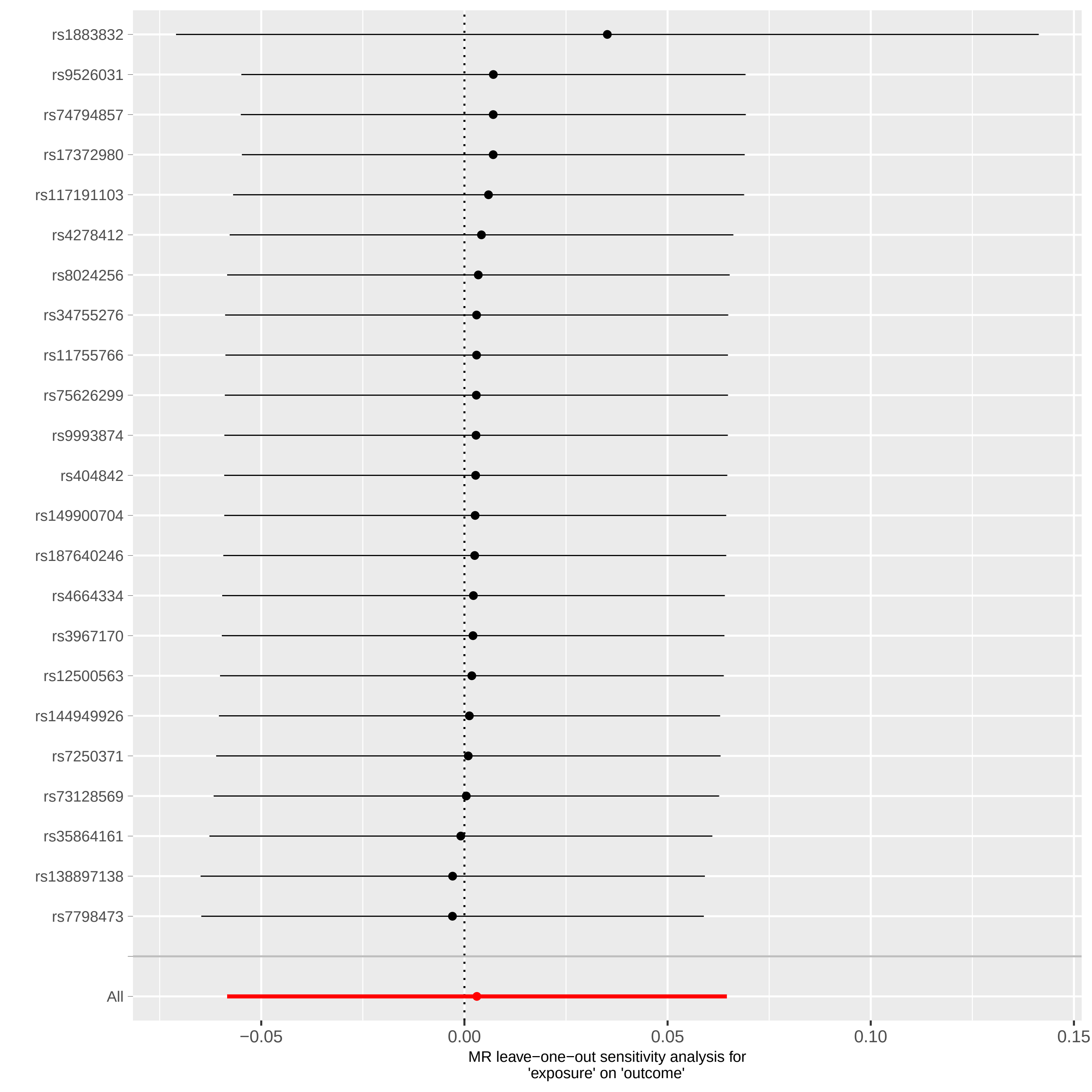

rs1883832
rs9526031
rs74794857
rs17372980
rs117191103
rs4278412
rs8024256
rs34755276
rs11755766
rs75626299
rs9993874
rs404842
rs149900704
rs187640246
rs4664334
rs3967170
rs12500563
rs144949926
rs7250371
rs73128569
rs35864161
rs138897138
rs7798473
All
−0.05
0.00
0.05
0.10
0.15
MR leave−one−out sensitivity analysis for
'exposure' on 'outcome'

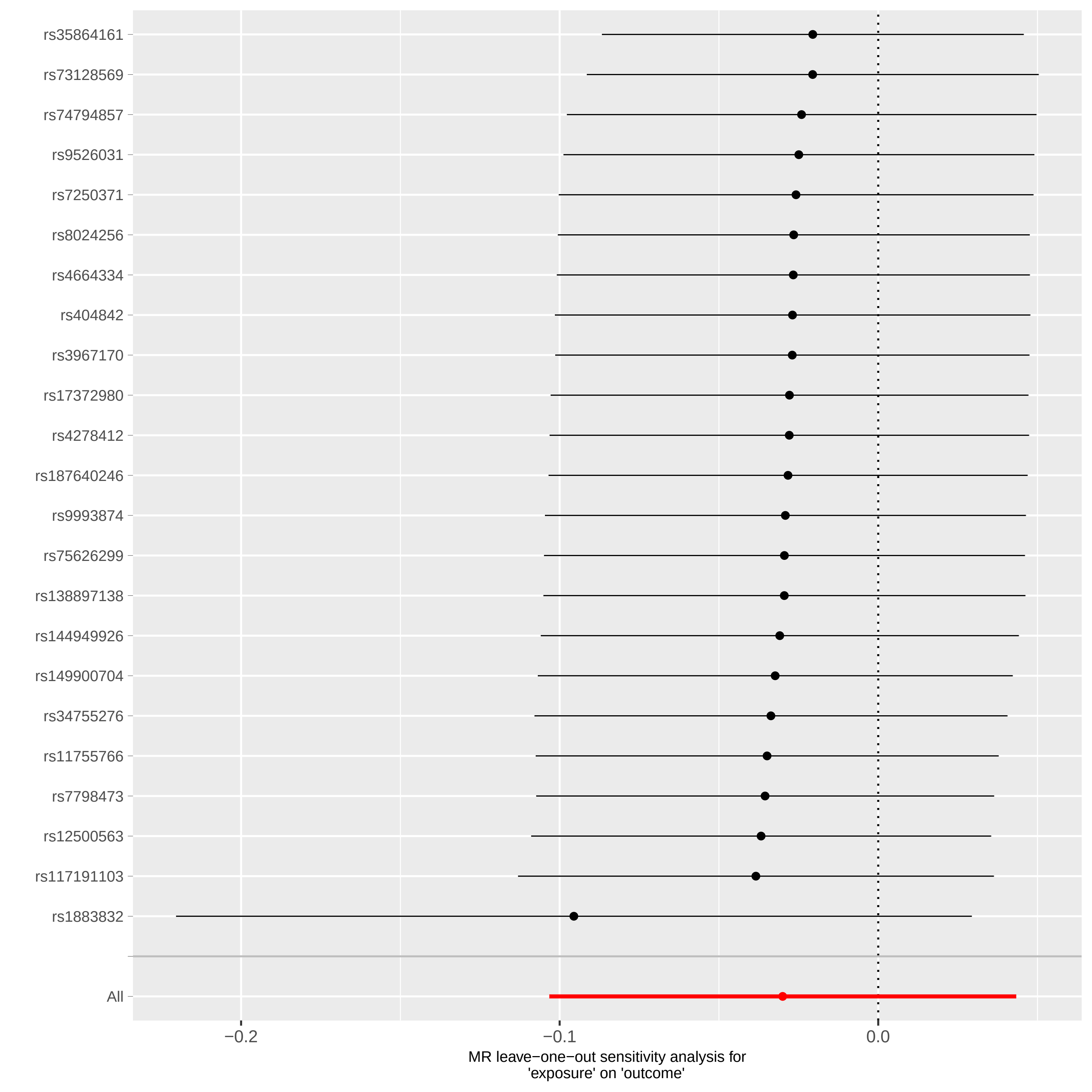
rs35864161
rs73128569
rs74794857
rs9526031
rs7250371
rs8024256
rs4664334
rs404842
rs3967170
rs17372980
rs4278412
rs187640246
rs9993874
rs75626299
rs138897138
rs144949926
rs149900704
rs34755276
rs11755766
rs7798473
rs12500563
rs117191103
rs1883832
All
−0.2
−0.1
0.0
MR leave−one−out sensitivity analysis for
'exposure' on 'outcome'

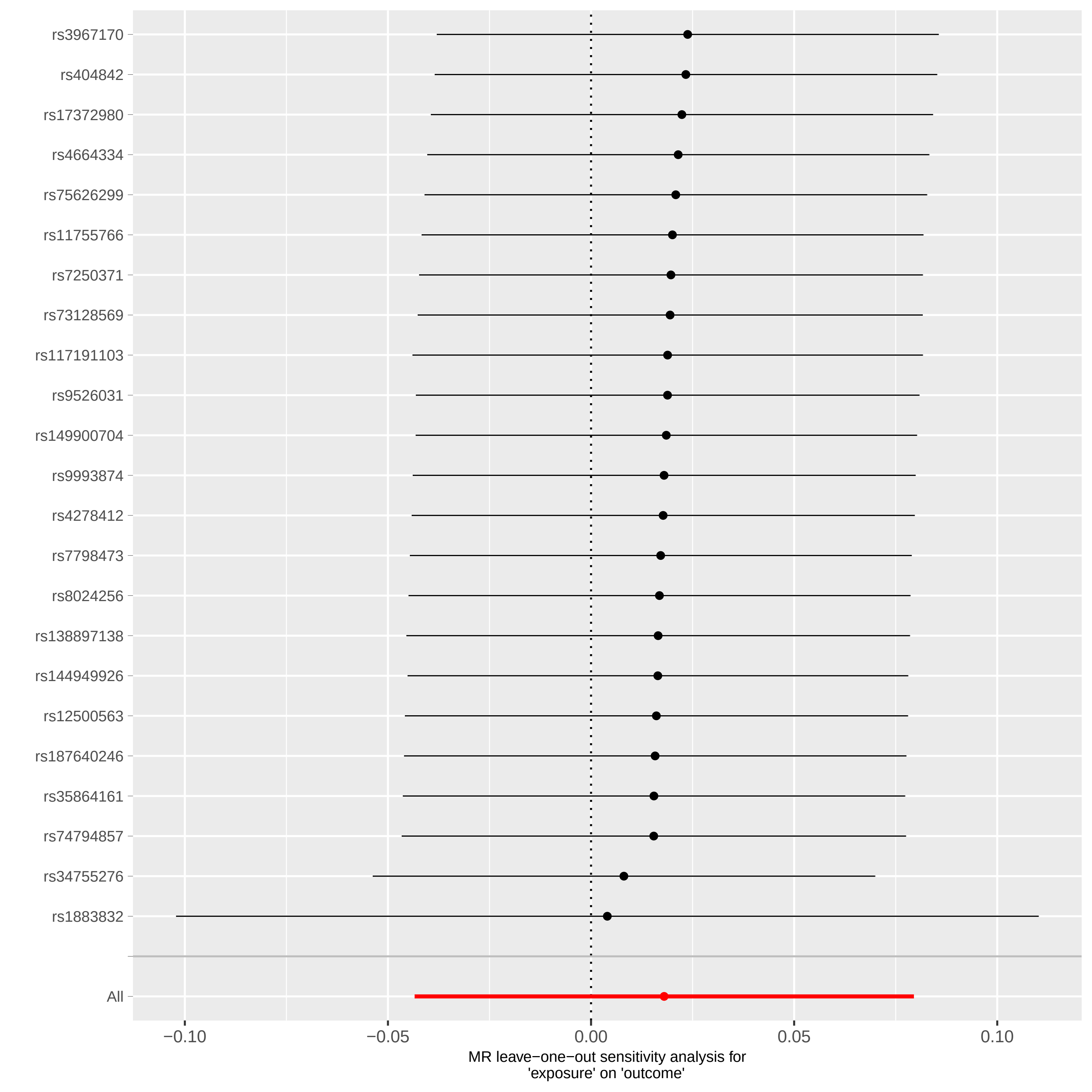

rs3967170
rs404842
rs17372980
rs4664334
rs75626299
rs11755766
rs7250371
rs73128569
rs117191103
rs9526031
rs149900704
rs9993874
rs4278412
rs7798473
rs8024256
rs138897138
rs144949926
rs12500563
rs187640246
rs35864161
rs74794857
rs34755276
rs1883832
All
−0.10
−0.05
0.00
0.05
0.10
MR leave−one−out sensitivity analysis for
'exposure' on 'outcome'

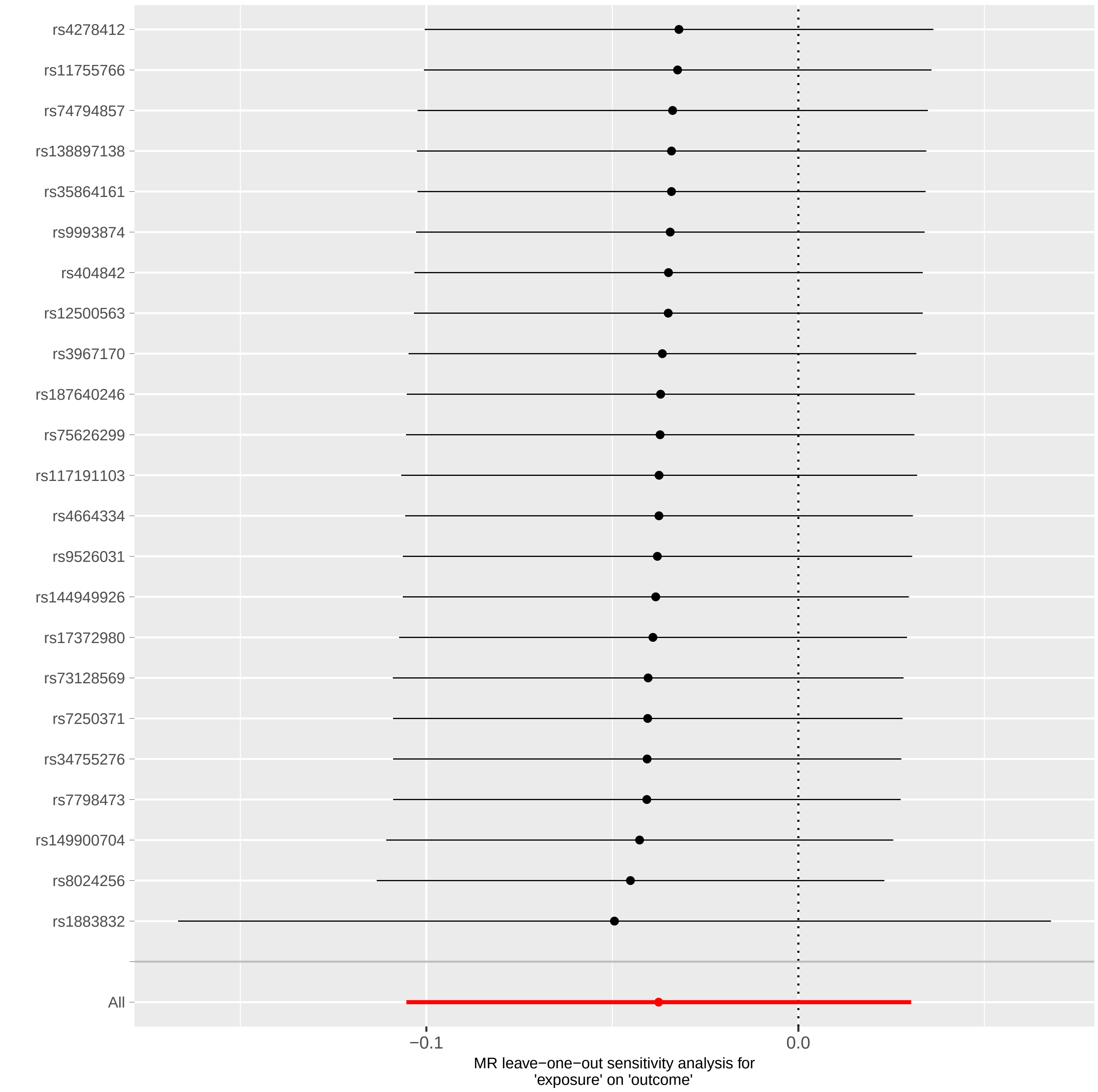

rs4278412
rs11755766
rs74794857
rs138897138
rs35864161
rs9993874
rs404842
rs12500563
rs3967170
rs187640246
rs75626299
rs117191103
rs4664334
rs9526031
rs144949926
rs17372980
rs73128569
rs7250371
rs34755276
rs7798473
rs149900704
rs8024256
rs1883832
All
−0.1
0.0
MR leave−one−out sensitivity analysis for
'exposure' on 'outcome'

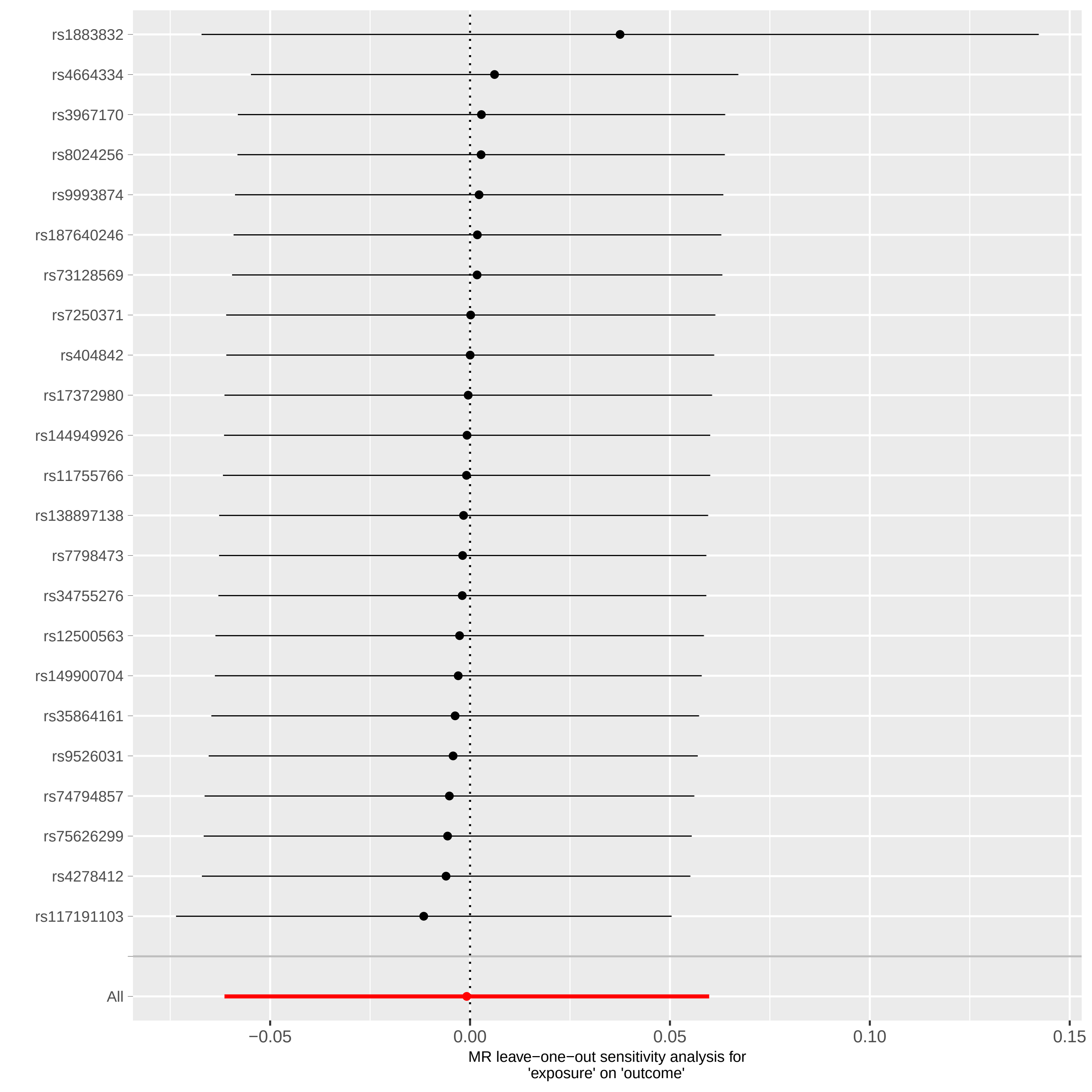

rs1883832
rs4664334
rs3967170
rs8024256
rs9993874
rs187640246
rs73128569
rs7250371
rs404842
rs17372980
rs144949926
rs11755766
rs138897138
rs7798473
rs34755276
rs12500563
rs149900704
rs35864161
rs9526031
rs74794857
rs75626299
rs4278412
rs117191103
All
−0.05
0.00
0.05
0.10
0.15
MR leave−one−out sensitivity analysis for
'exposure' on 'outcome'

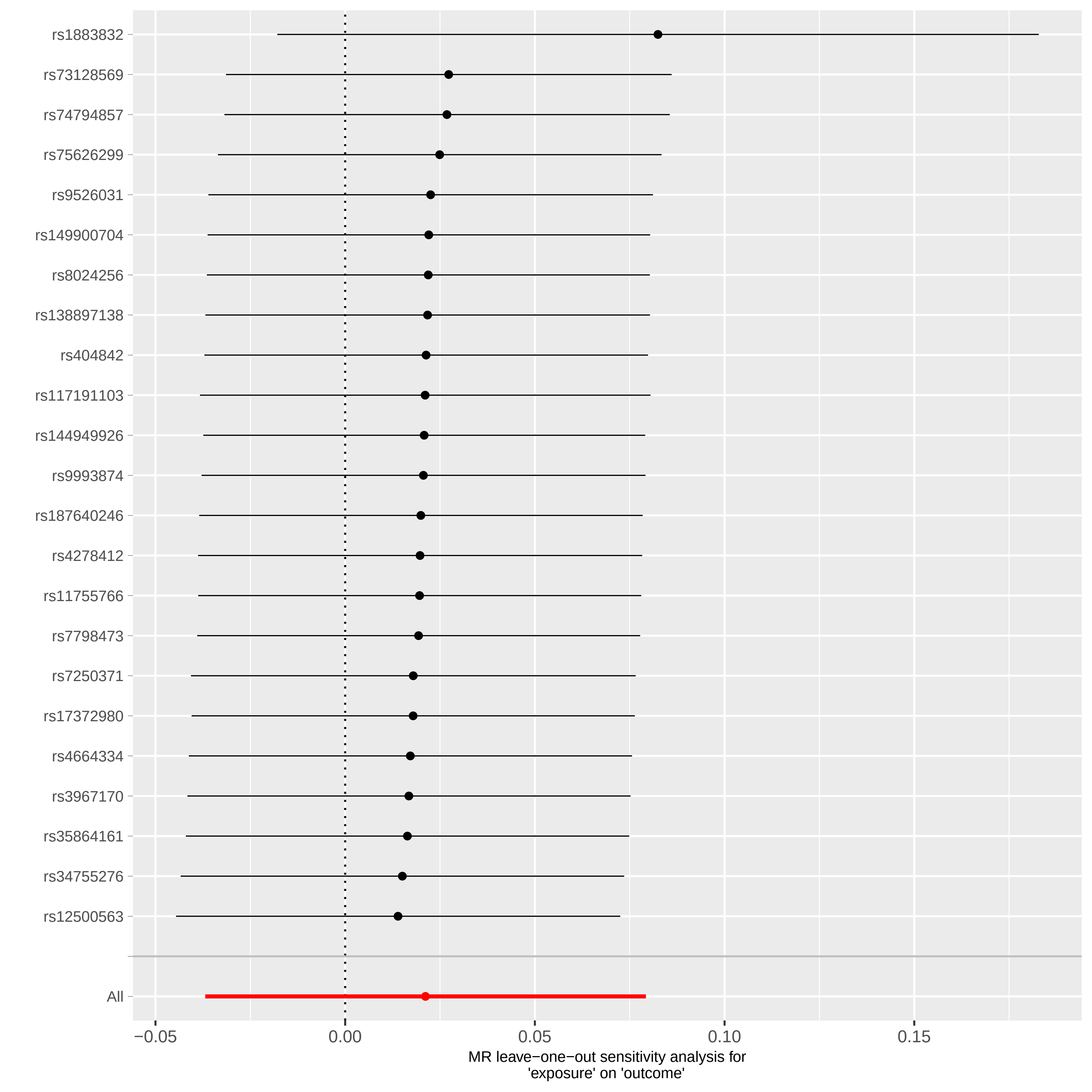
rs1883832
rs73128569
rs74794857
rs75626299
rs9526031
rs149900704
rs8024256
rs138897138
rs404842
rs117191103
rs144949926
rs9993874
rs187640246
rs4278412
rs11755766
rs7798473
rs7250371
rs17372980
rs4664334
rs3967170
rs35864161
rs34755276
rs12500563
All
−0.05
0.00
0.05
0.10
0.15
MR leave−one−out sensitivity analysis for
'exposure' on 'outcome'

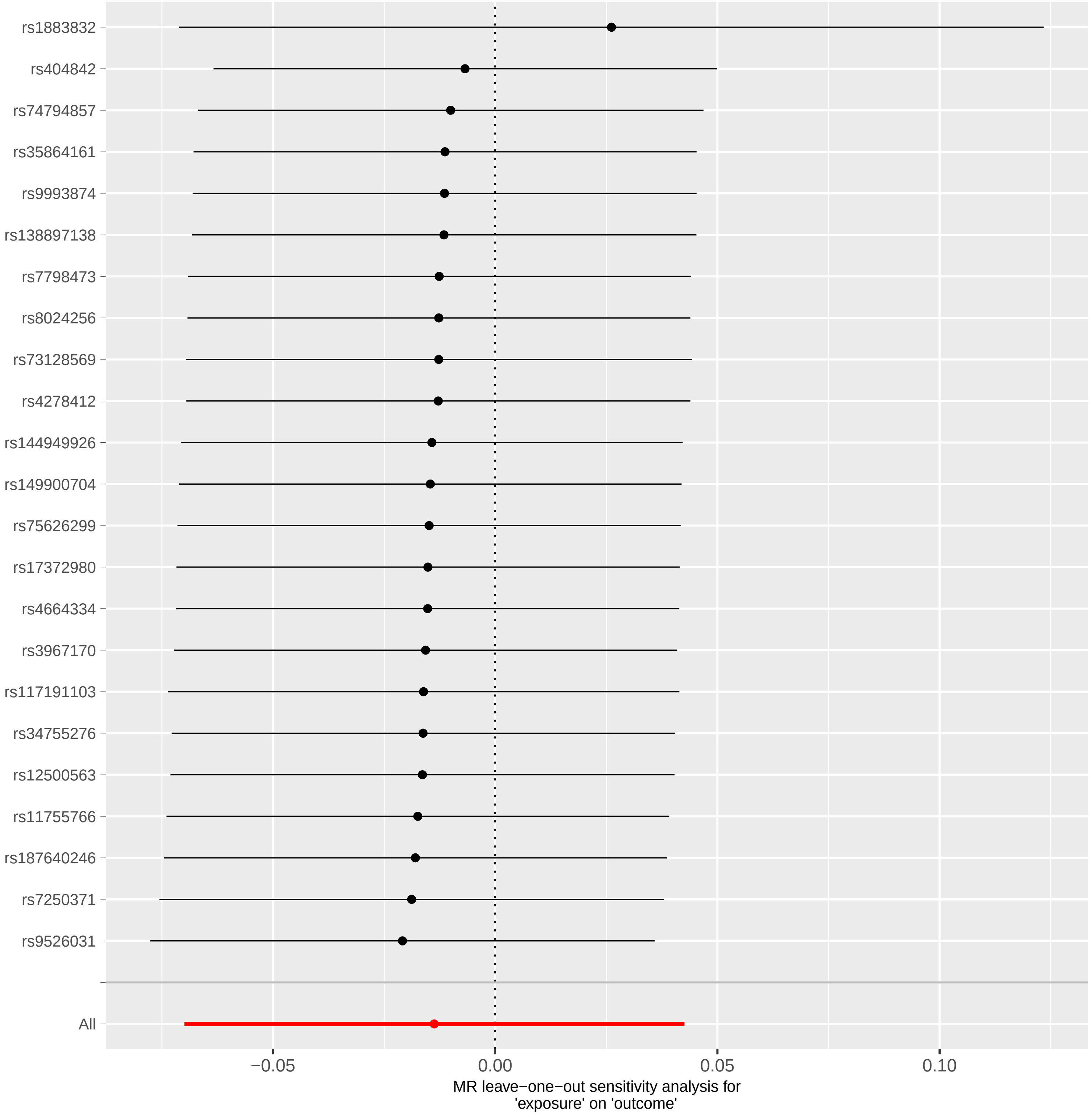
rs1883832
rs404842
rs74794857
rs35864161
rs9993874
rs138897138
rs7798473
rs8024256
rs73128569
rs4278412
rs144949926
rs149900704
rs75626299
rs17372980
rs4664334
rs3967170
rs117191103
rs34755276
rs12500563
rs11755766
rs187640246
rs7250371
rs9526031
All
−0.05
0.00
0.05
0.10
MR leave−one−out sensitivity analysis for
'exposure' on 'outcome'

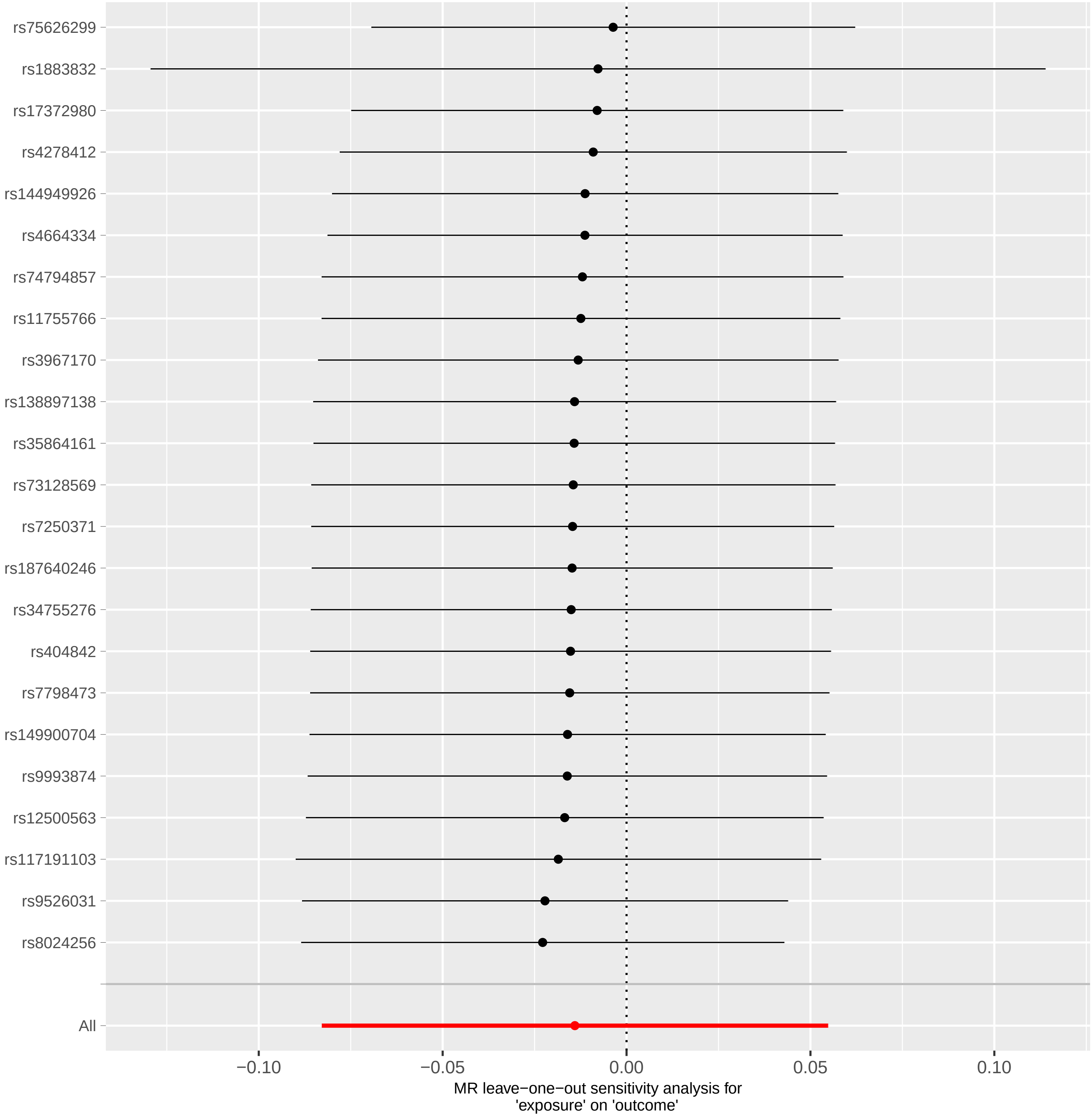

rs75626299
rs1883832
rs17372980
rs4278412
rs144949926
rs4664334
rs74794857
rs11755766
rs3967170
rs138897138
rs35864161
rs73128569
rs7250371
rs187640246
rs34755276
rs404842
rs7798473
rs149900704
rs9993874
rs12500563
rs117191103
rs9526031
rs8024256
All
−0.10
−0.05
0.00
0.05
0.10
MR leave−one−out sensitivity analysis for
'exposure' on 'outcome'

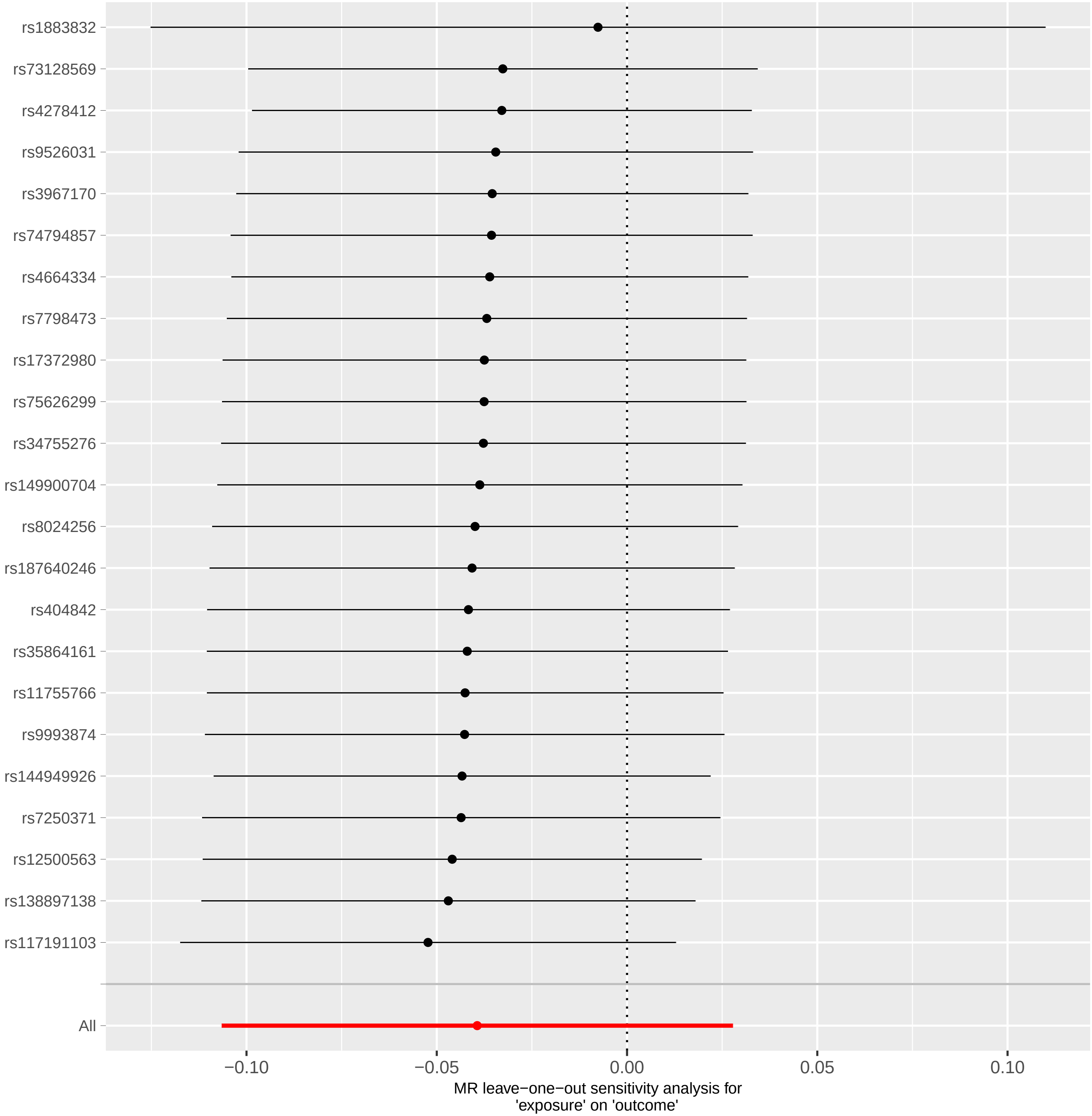
rs1883832
rs73128569
rs4278412
rs9526031
rs3967170
rs74794857
rs4664334
rs7798473
rs17372980
rs75626299
rs34755276
rs149900704
rs8024256
rs187640246
rs404842
rs35864161
rs11755766
rs9993874
rs144949926
rs7250371
rs12500563
rs138897138
rs117191103
All
−0.10
−0.05
0.00
0.05
0.10
MR leave−one−out sensitivity analysis for
'exposure' on 'outcome'

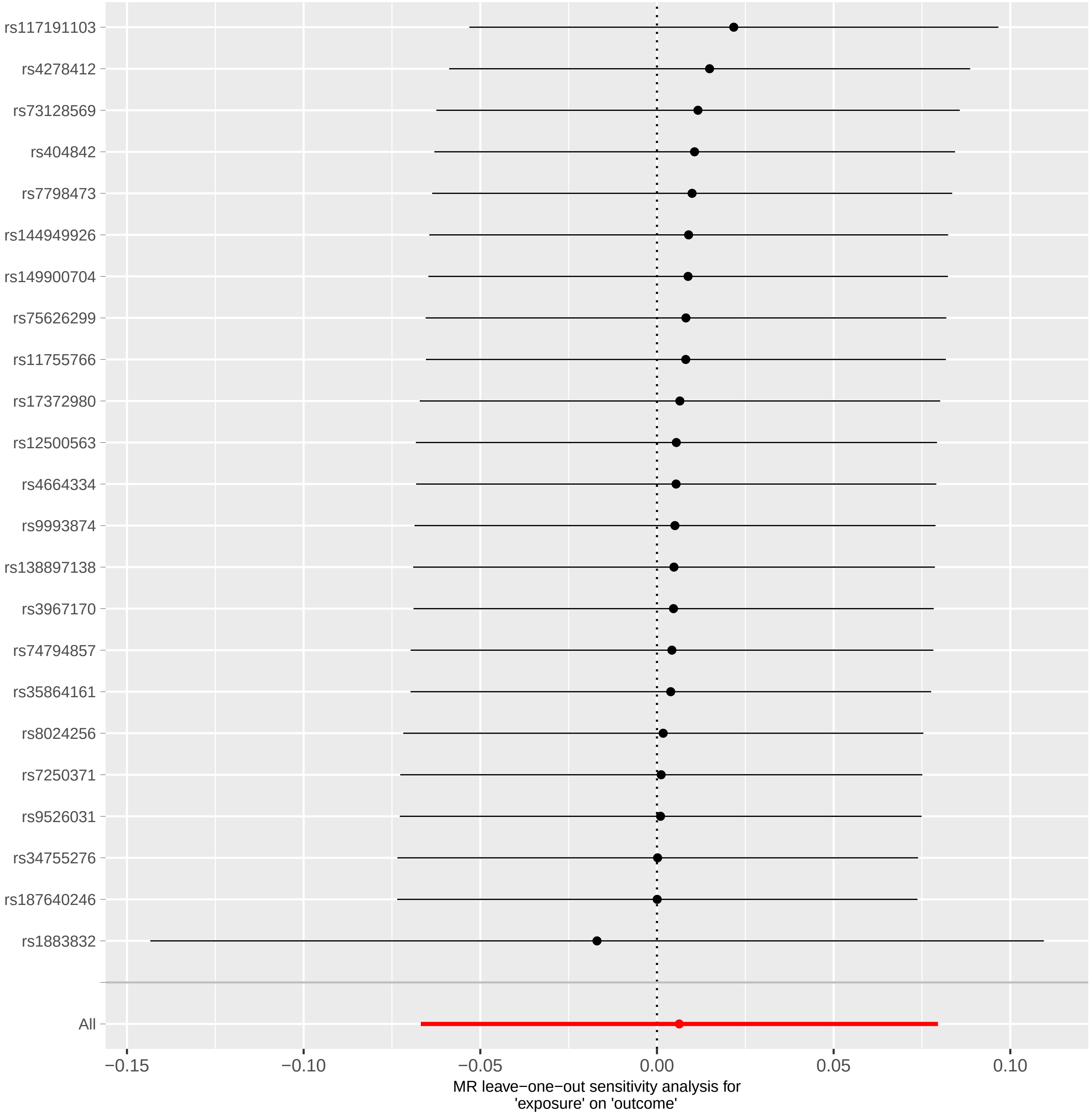
rs117191103
rs4278412
rs73128569
rs404842
rs7798473
rs144949926
rs149900704
rs75626299
rs11755766
rs17372980
rs12500563
rs4664334
rs9993874
rs138897138
rs3967170
rs74794857
rs35864161
rs8024256
rs7250371
rs9526031
rs34755276
rs187640246
rs1883832
All
−0.15
−0.10
−0.05
0.00
0.05
0.10
MR leave−one−out sensitivity analysis for
'exposure' on 'outcome'

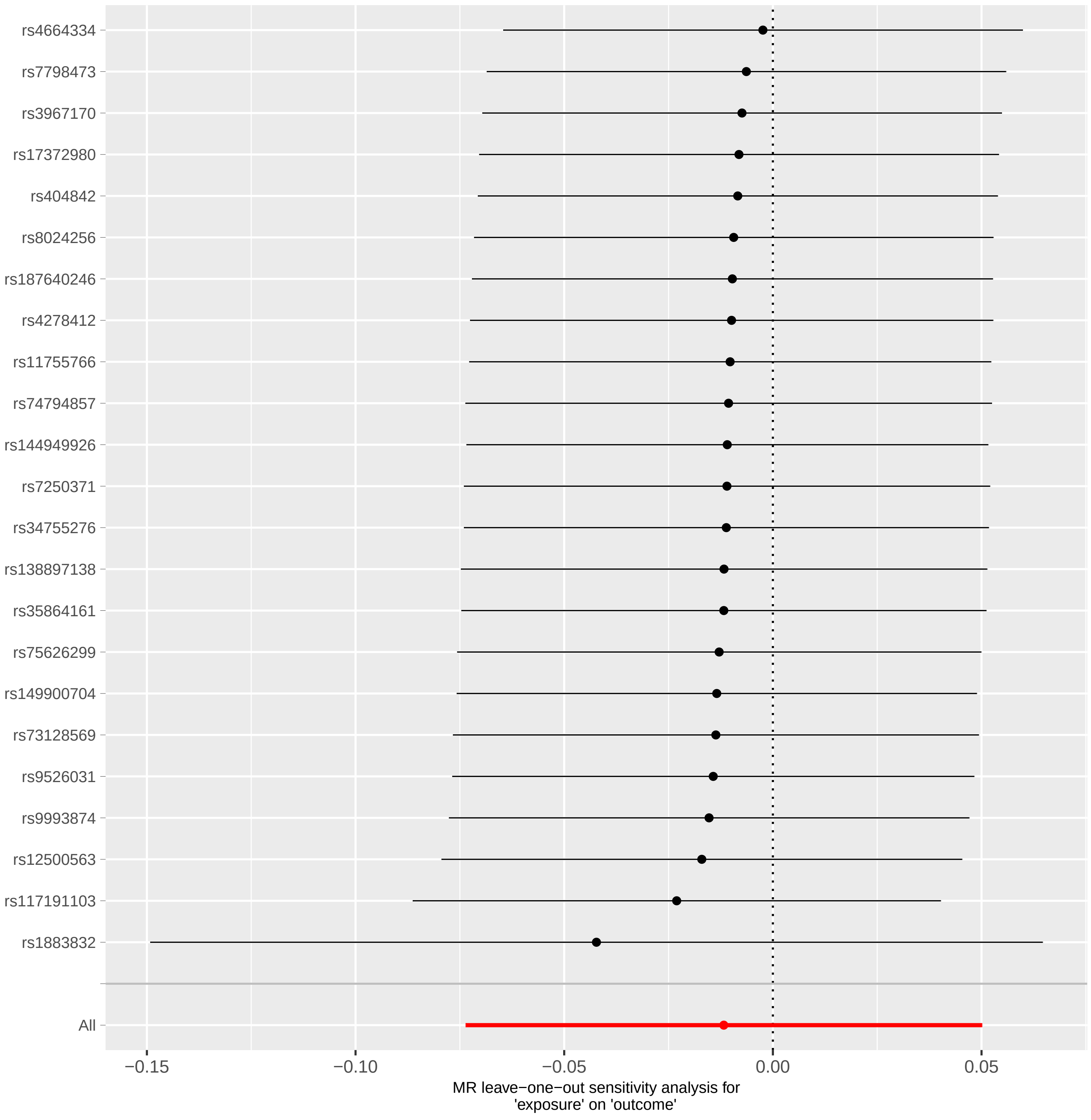
rs4664334
rs7798473
rs3967170
rs17372980
rs404842
rs8024256
rs187640246
rs4278412
rs11755766
rs74794857
rs144949926
rs7250371
rs34755276
rs138897138
rs35864161
rs75626299
rs149900704
rs73128569
rs9526031
rs9993874
rs12500563
rs117191103
rs1883832
All
−0.15
−0.10
−0.05
0.00
0.05
MR leave−one−out sensitivity analysis for
'exposure' on 'outcome'

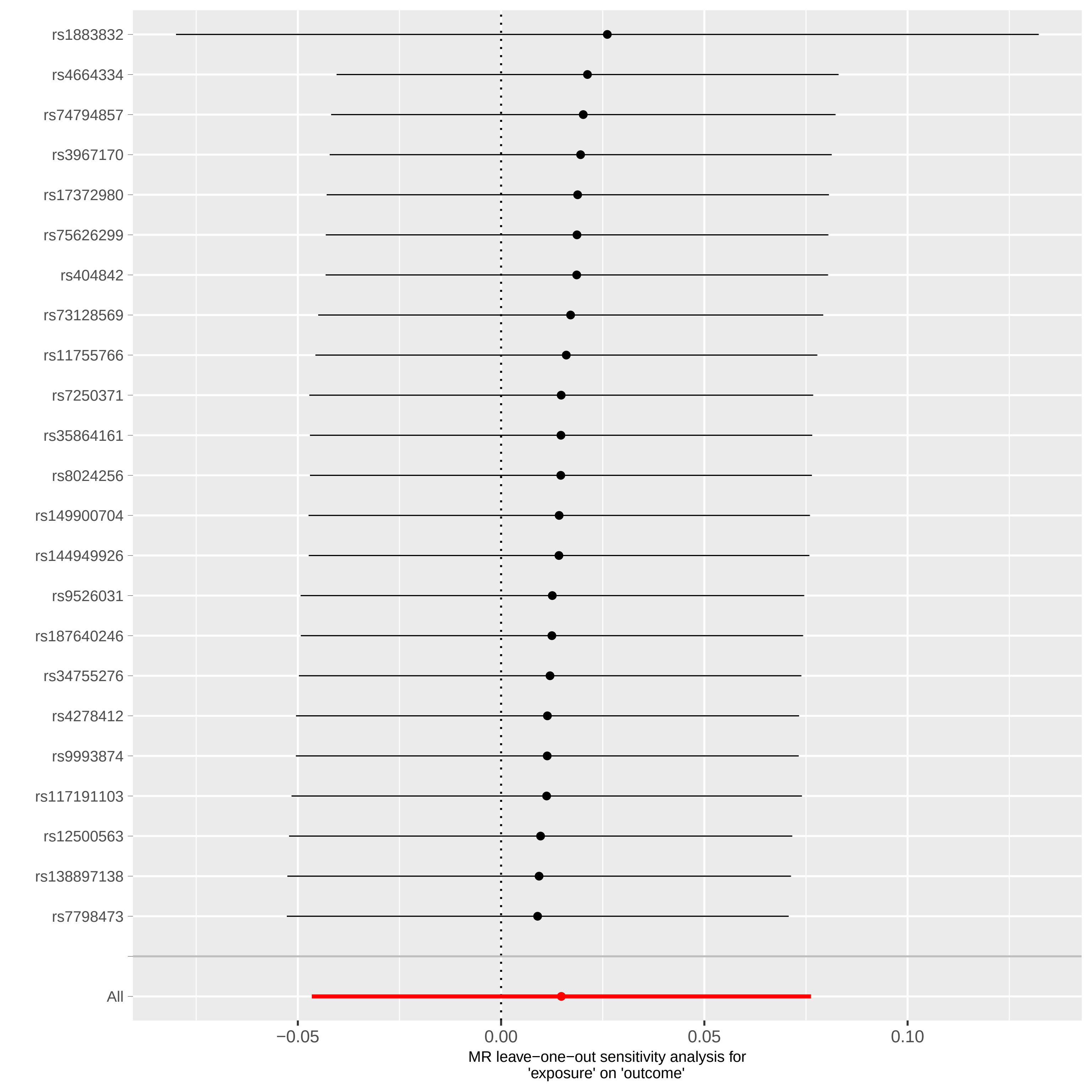

rs1883832
rs4664334
rs74794857
rs3967170
rs17372980
rs75626299
rs404842
rs73128569
rs11755766
rs7250371
rs35864161
rs8024256
rs149900704
rs144949926
rs9526031
rs187640246
rs34755276
rs4278412
rs9993874
rs117191103
rs12500563
rs138897138
rs7798473
All
−0.05
0.00
0.05
0.10
MR leave−one−out sensitivity analysis for
'exposure' on 'outcome'

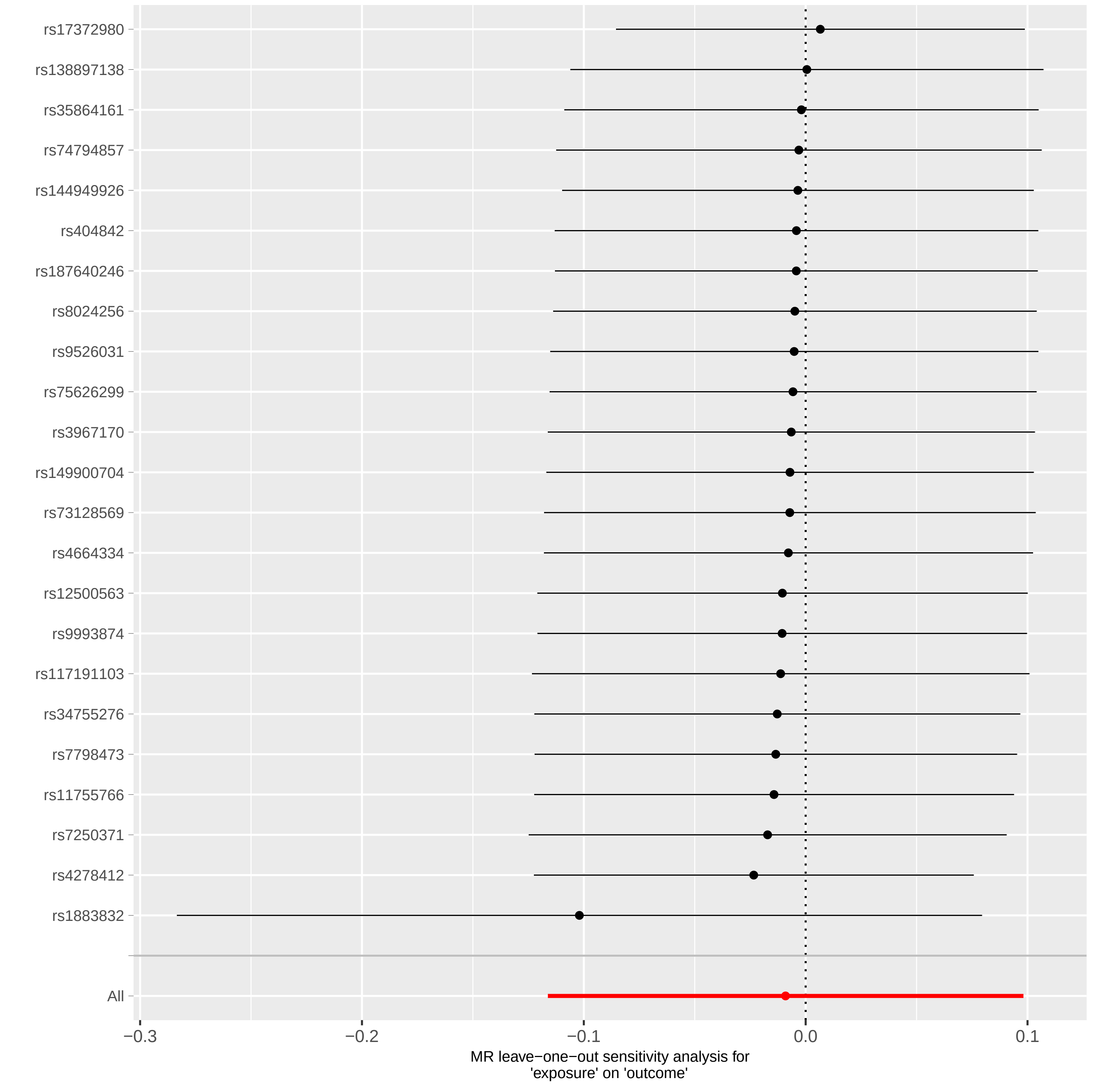

rs17372980
rs138897138
rs35864161
rs74794857
rs144949926
rs404842
rs187640246
rs8024256
rs9526031
rs75626299
rs3967170
rs149900704
rs73128569
rs4664334
rs12500563
rs9993874
rs117191103
rs34755276
rs7798473
rs11755766
rs7250371
rs4278412
rs1883832
All
−0.3
−0.2
−0.1
0.0
0.1
MR leave−one−out sensitivity analysis for
'exposure' on 'outcome'

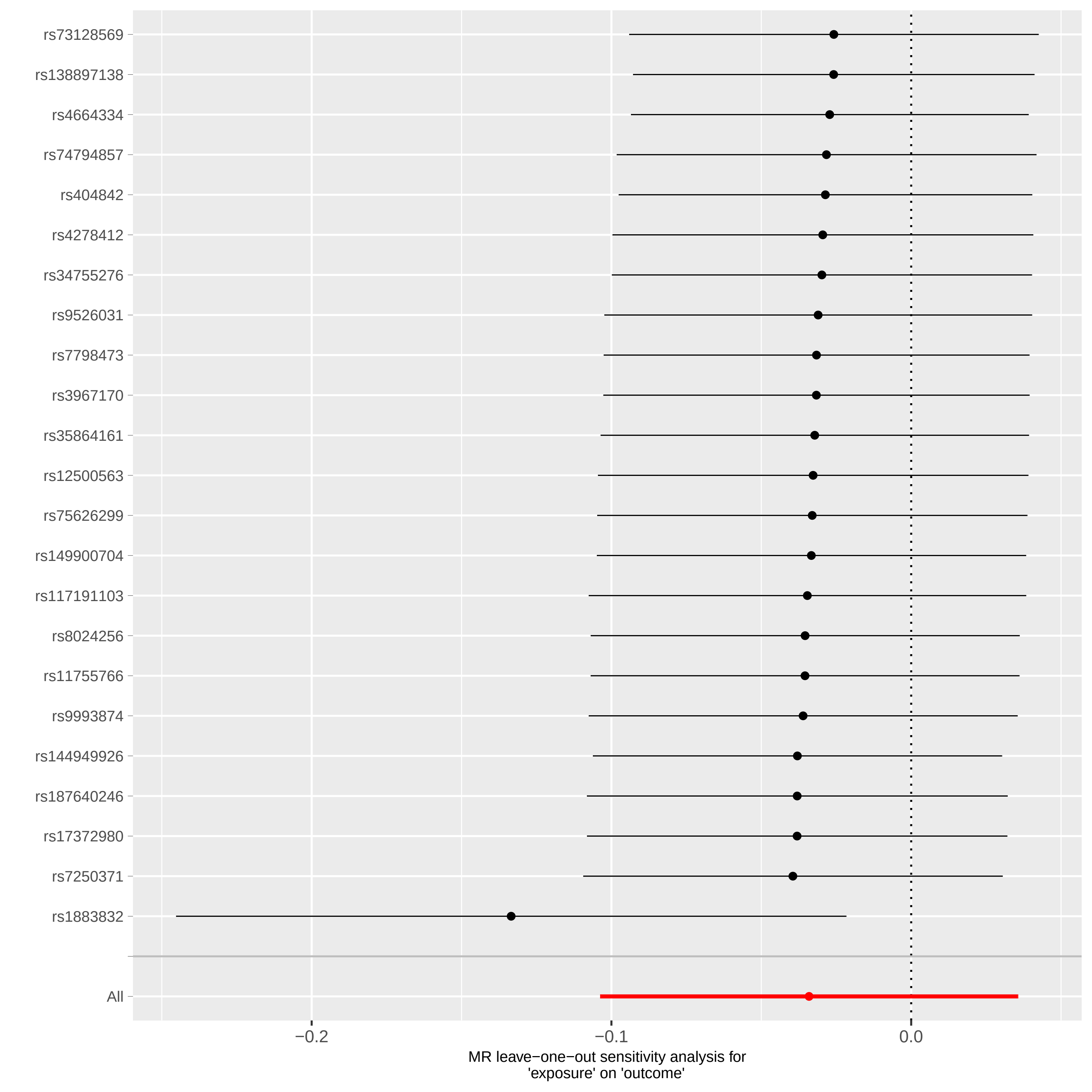
rs73128569
rs138897138
rs4664334
rs74794857
rs404842
rs4278412
rs34755276
rs9526031
rs7798473
rs3967170
rs35864161
rs12500563
rs75626299
rs149900704
rs117191103
rs8024256
rs11755766
rs9993874
rs144949926
rs187640246
rs17372980
rs7250371
rs1883832
All
−0.2
−0.1
0.0
MR leave−one−out sensitivity analysis for
'exposure' on 'outcome'

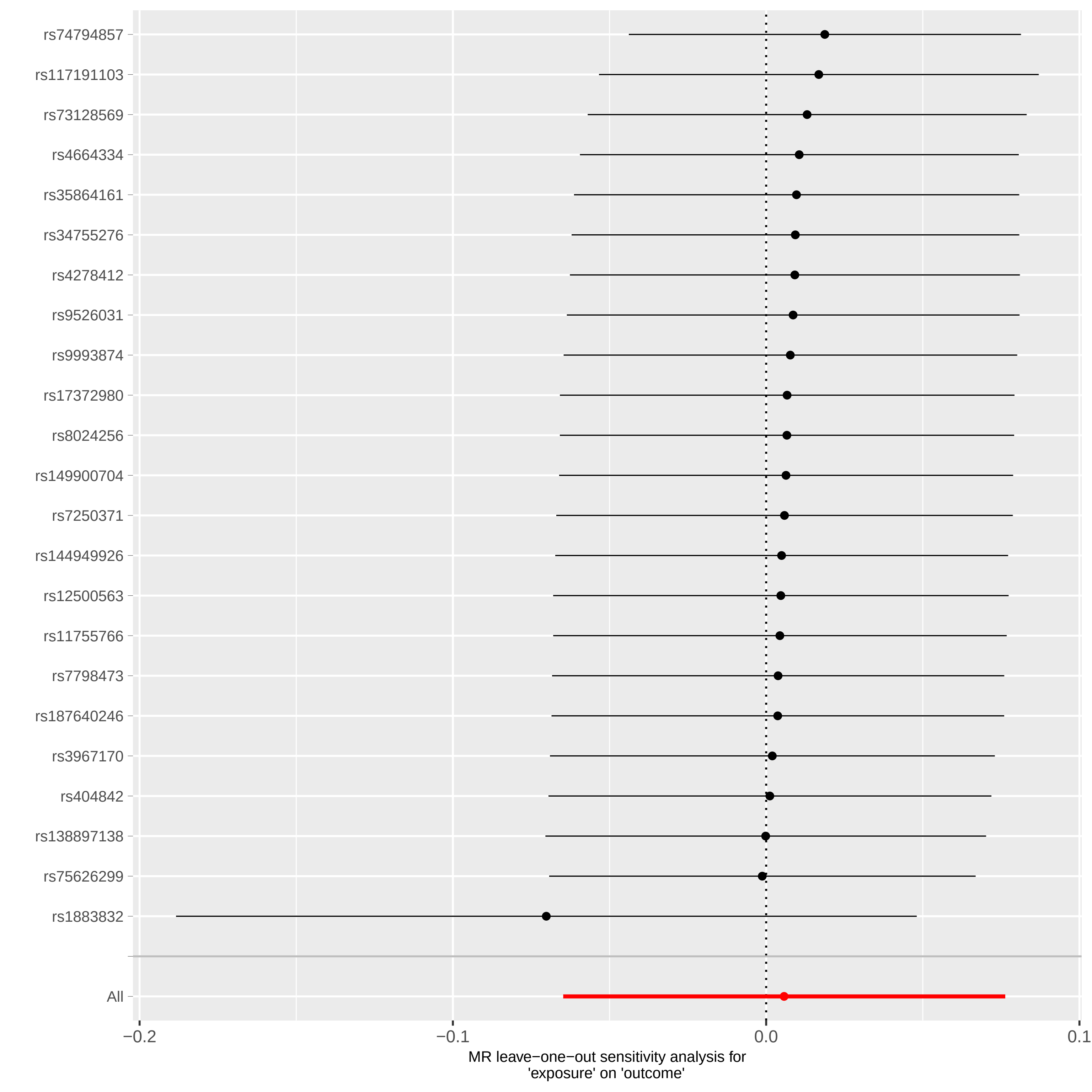

rs74794857
rs117191103
rs73128569
rs4664334
rs35864161
rs34755276
rs4278412
rs9526031
rs9993874
rs17372980
rs8024256
rs149900704
rs7250371
rs144949926
rs12500563
rs11755766
rs7798473
rs187640246
rs3967170
rs404842
rs138897138
rs75626299
rs1883832
All
−0.2
−0.1
0.0
0.1
MR leave−one−out sensitivity analysis for
'exposure' on 'outcome'

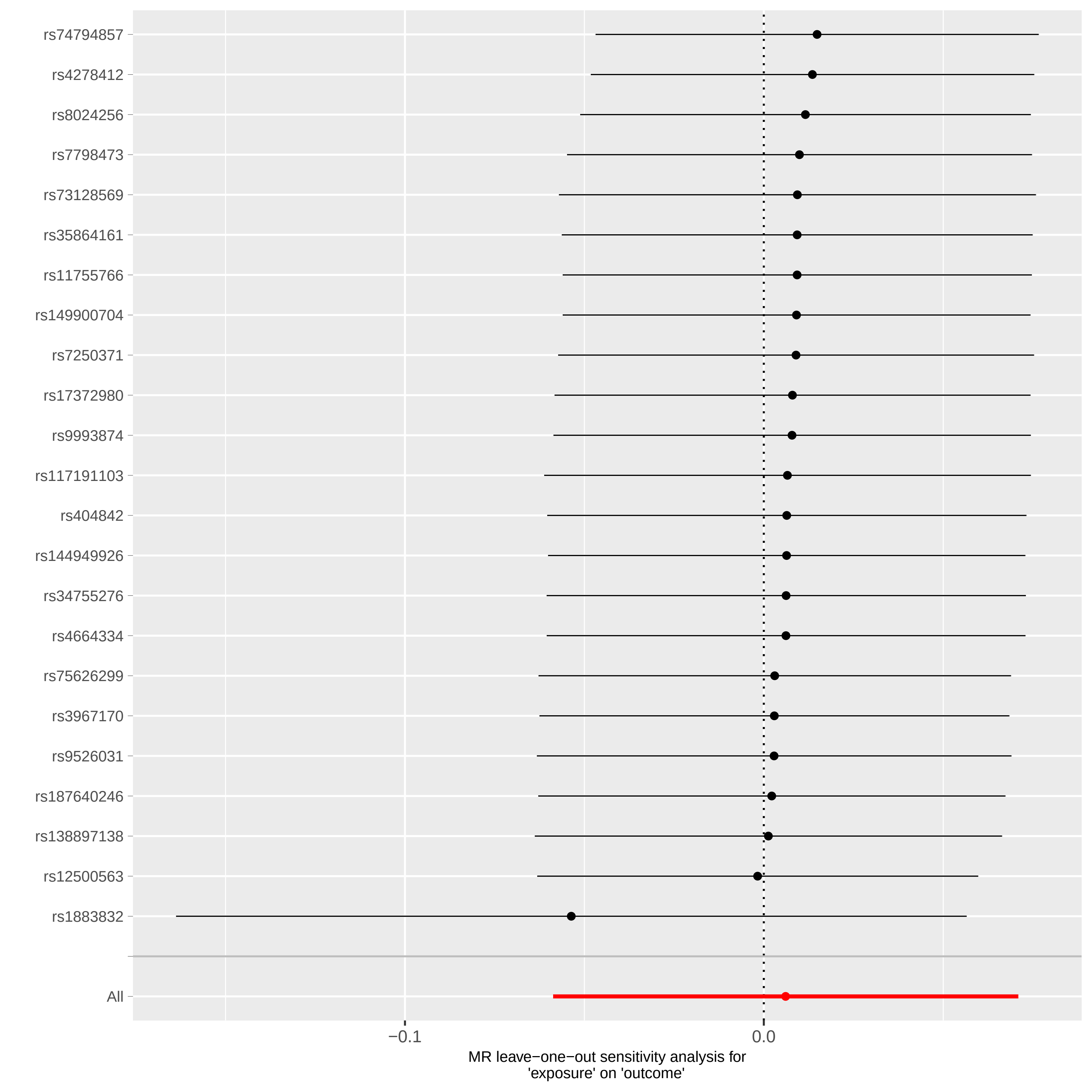

rs74794857
rs4278412
rs8024256
rs7798473
rs73128569
rs35864161
rs11755766
rs149900704
rs7250371
rs17372980
rs9993874
rs117191103
rs404842
rs144949926
rs34755276
rs4664334
rs75626299
rs3967170
rs9526031
rs187640246
rs138897138
rs12500563
rs1883832
All
−0.1
0.0
MR leave−one−out sensitivity analysis for
'exposure' on 'outcome'

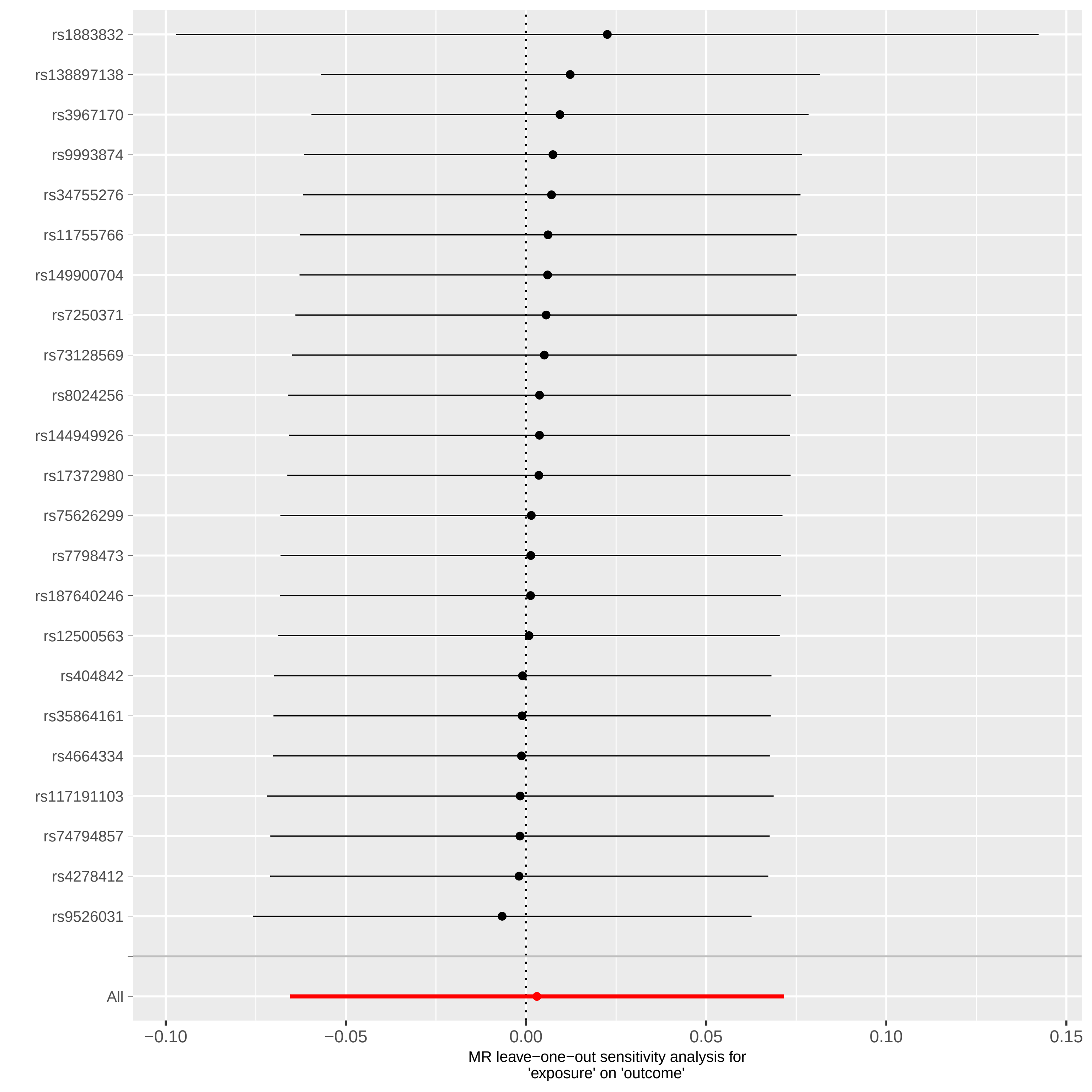
rs1883832
rs138897138
rs3967170
rs9993874
rs34755276
rs11755766
rs149900704
rs7250371
rs73128569
rs8024256
rs144949926
rs17372980
rs75626299
rs7798473
rs187640246
rs12500563
rs404842
rs35864161
rs4664334
rs117191103
rs74794857
rs4278412
rs9526031
All
−0.10
−0.05
0.00
0.05
0.10
0.15
MR leave−one−out sensitivity analysis for
'exposure' on 'outcome'

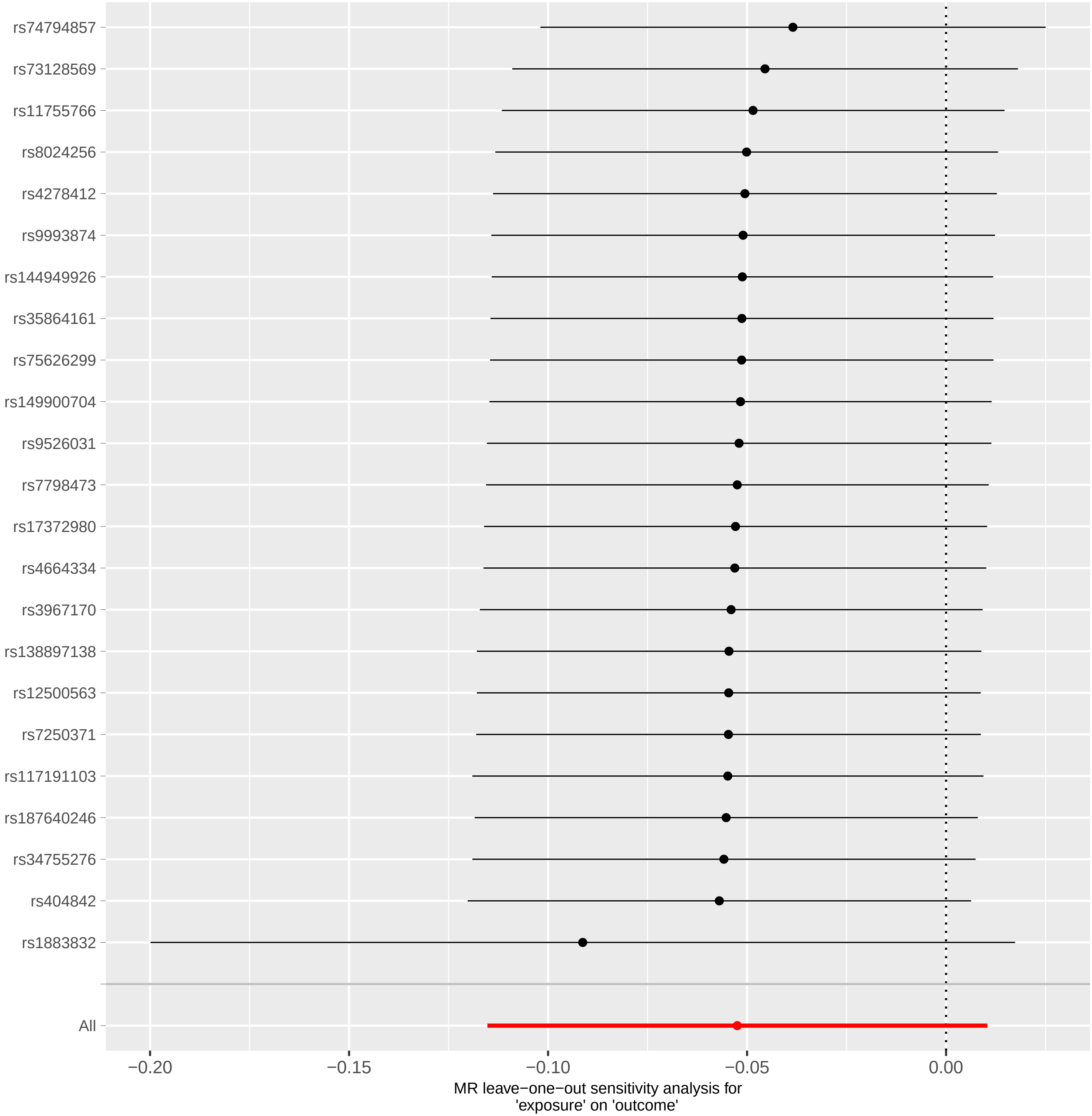
rs74794857
rs73128569
rs11755766
rs8024256
rs4278412
rs9993874
rs144949926
rs35864161
rs75626299
rs149900704
rs9526031
rs7798473
rs17372980
rs4664334
rs3967170
rs138897138
rs12500563
rs7250371
rs117191103
rs187640246
rs34755276
rs404842
rs1883832
All
−0.20
−0.15
−0.10
−0.05
0.00
MR leave−one−out sensitivity analysis for
'exposure' on 'outcome'

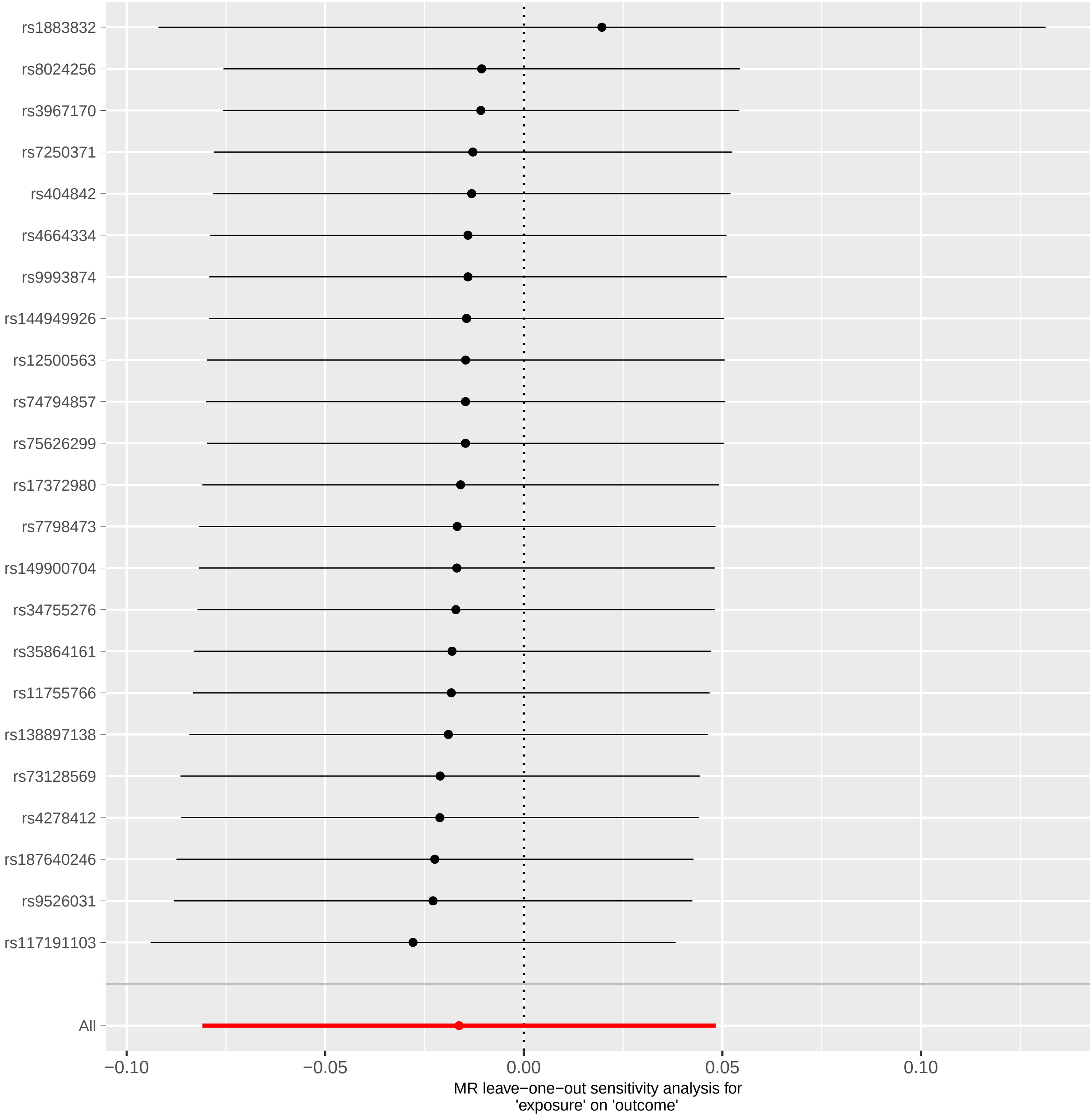
rs1883832
rs8024256
rs3967170
rs7250371
rs404842
rs4664334
rs9993874
rs144949926
rs12500563
rs74794857
rs75626299
rs17372980
rs7798473
rs149900704
rs34755276
rs35864161
rs11755766
rs138897138
rs73128569
rs4278412
rs187640246
rs9526031
rs117191103
All
−0.10
−0.05
0.00
0.05
0.10
MR leave−one−out sensitivity analysis for
'exposure' on 'outcome'

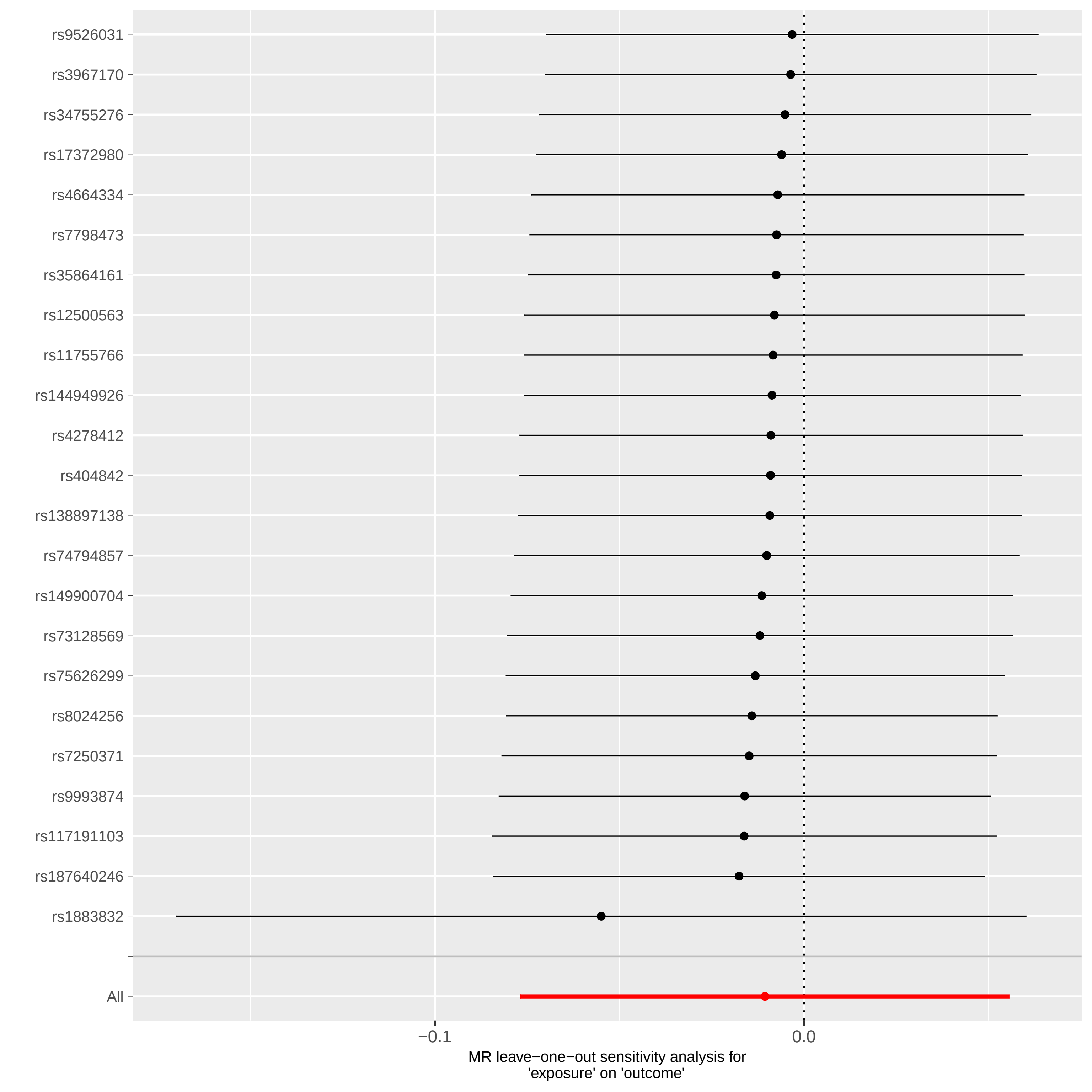
rs9526031
rs3967170
rs34755276
rs17372980
rs4664334
rs7798473
rs35864161
rs12500563
rs11755766
rs144949926
rs4278412
rs404842
rs138897138
rs74794857
rs149900704
rs73128569
rs75626299
rs8024256
rs7250371
rs9993874
rs117191103
rs187640246
rs1883832
All
−0.1
0.0
MR leave−one−out sensitivity analysis for
'exposure' on 'outcome'

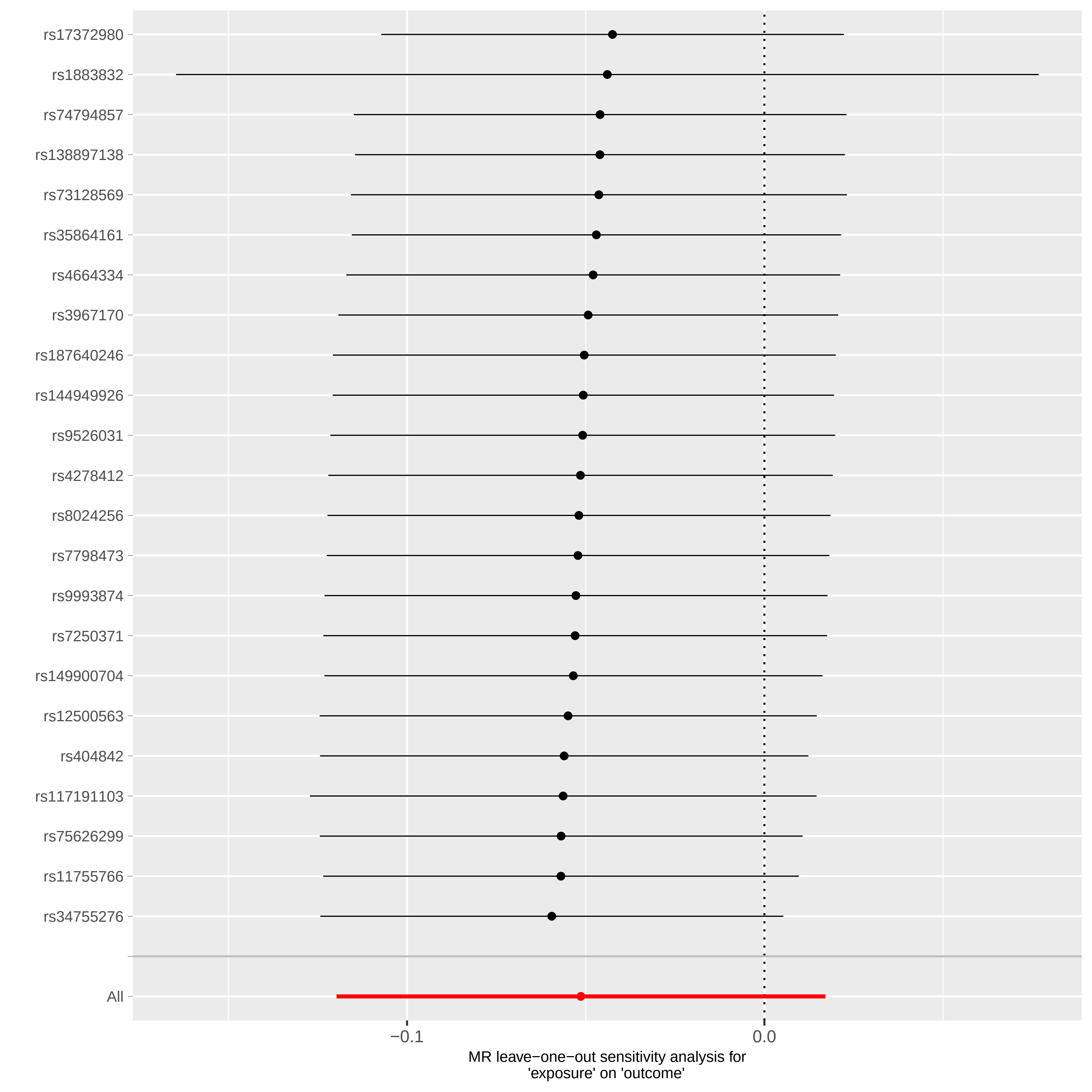

rs17372980
rs1883832
rs74794857
rs138897138
rs73128569
rs35864161
rs4664334
rs3967170
rs187640246
rs144949926
rs9526031
rs4278412
rs8024256
rs7798473
rs9993874
rs7250371
rs149900704
rs12500563
rs404842
rs117191103
rs75626299
rs11755766
rs34755276
All
−0.1
0.0
MR leave−one−out sensitivity analysis for
'exposure' on 'outcome'

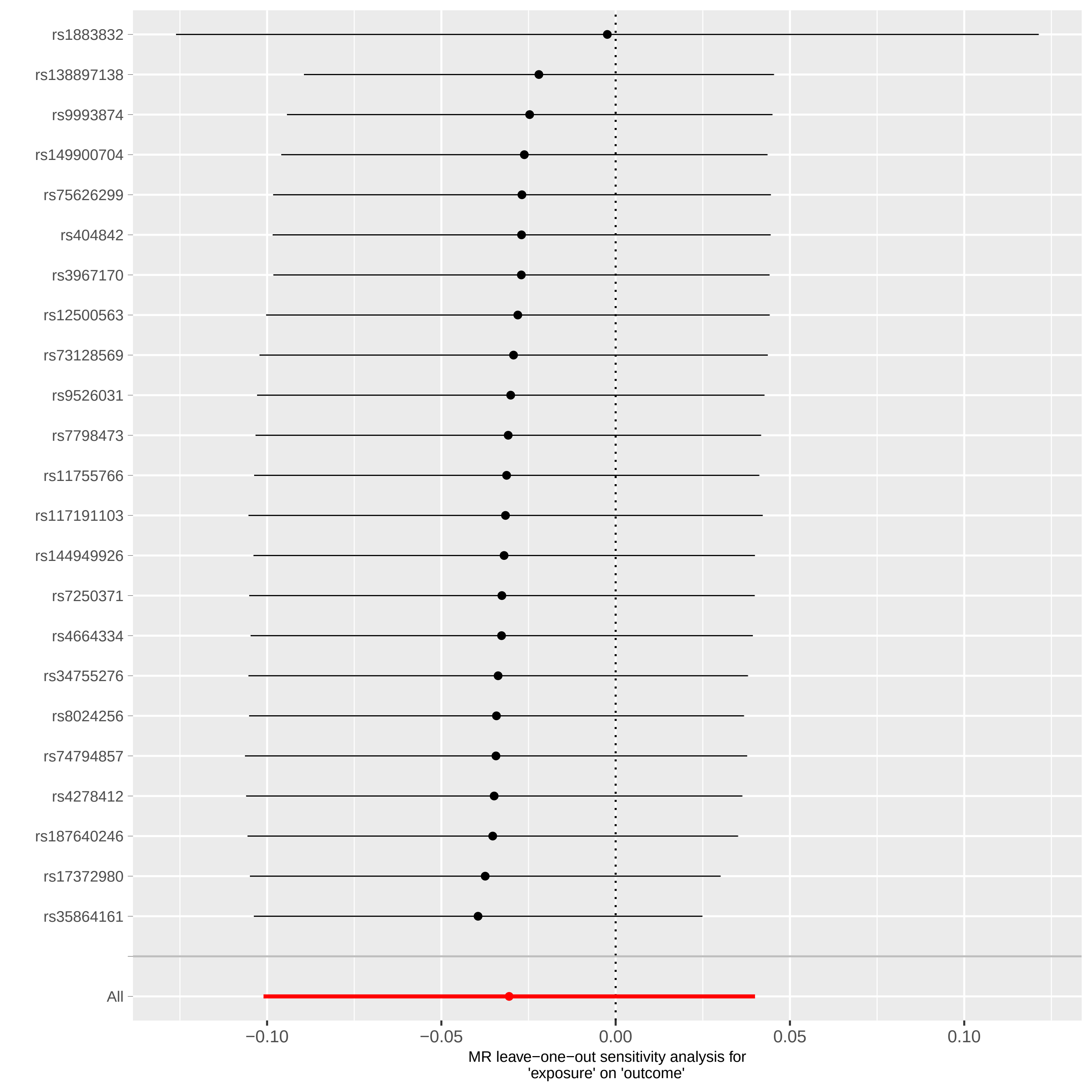
rs1883832
rs138897138
rs9993874
rs149900704
rs75626299
rs404842
rs3967170
rs12500563
rs73128569
rs9526031
rs7798473
rs11755766
rs117191103
rs144949926
rs7250371
rs4664334
rs34755276
rs8024256
rs74794857
rs4278412
rs187640246
rs17372980
rs35864161
All
−0.10
−0.05
0.00
0.05
0.10
MR leave−one−out sensitivity analysis for
'exposure' on 'outcome'

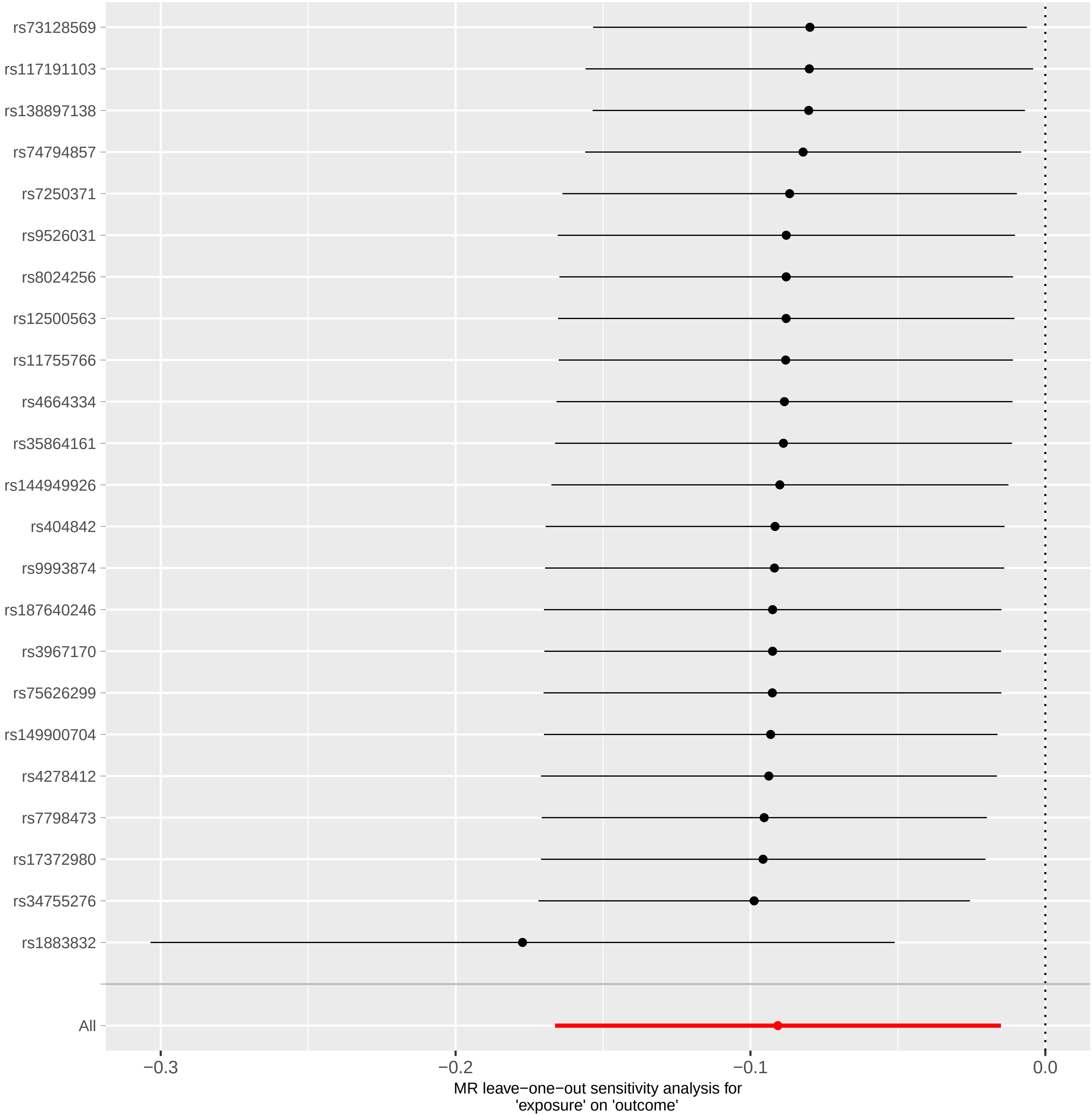

rs73128569
rs117191103
rs138897138
rs74794857
rs7250371
rs9526031
rs8024256
rs12500563
rs11755766
rs4664334
rs35864161
rs144949926
rs404842
rs9993874
rs187640246
rs3967170
rs75626299
rs149900704
rs4278412
rs7798473
rs17372980
rs34755276
rs1883832
All
−0.3
−0.2
−0.1
0.0
MR leave−one−out sensitivity analysis for
'exposure' on 'outcome'

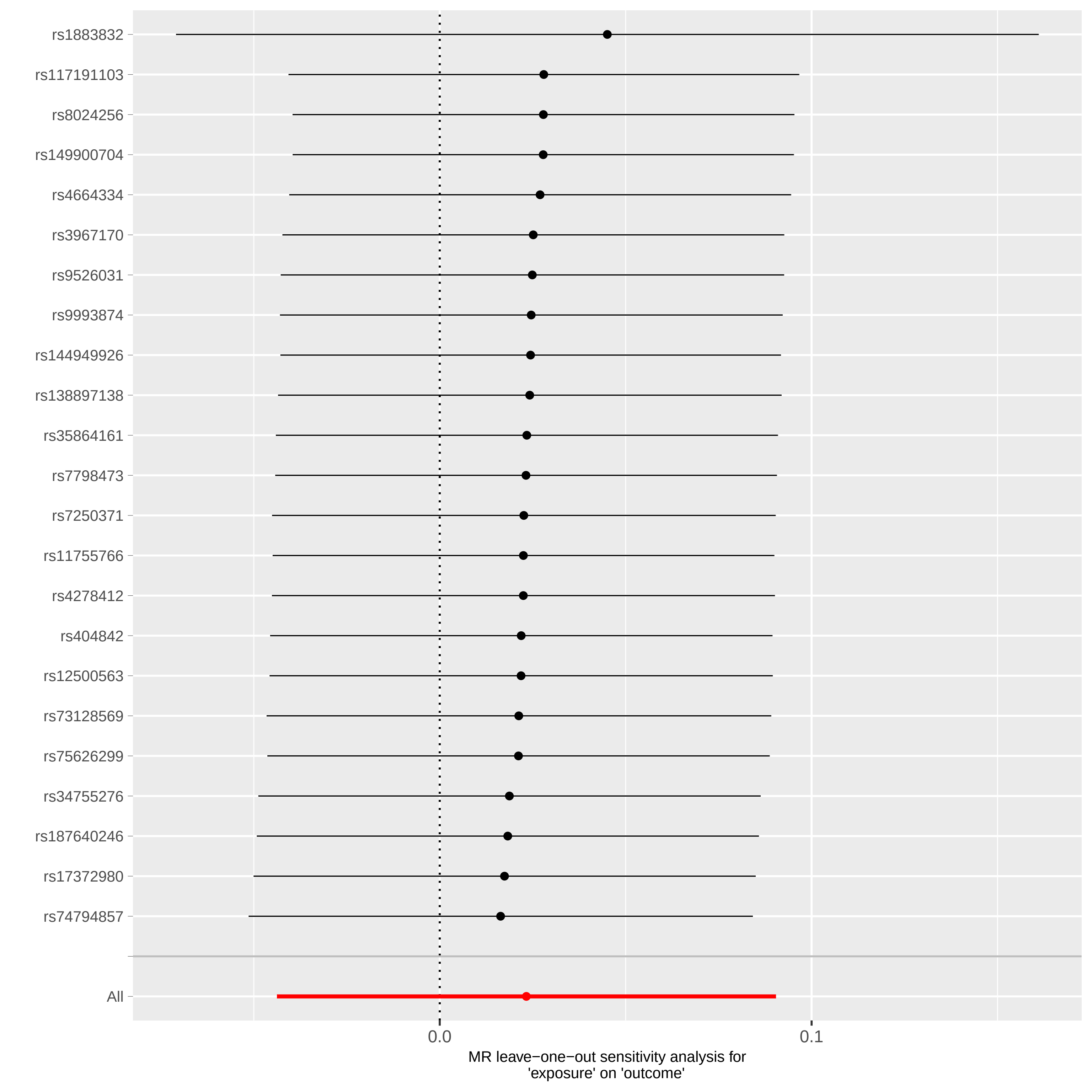
rs1883832
rs117191103
rs8024256
rs149900704
rs4664334
rs3967170
rs9526031
rs9993874
rs144949926
rs138897138
rs35864161
rs7798473
rs7250371
rs11755766
rs4278412
rs404842
rs12500563
rs73128569
rs75626299
rs34755276
rs187640246
rs17372980
rs74794857
All
0.0
0.1
MR leave−one−out sensitivity analysis for
'exposure' on 'outcome'

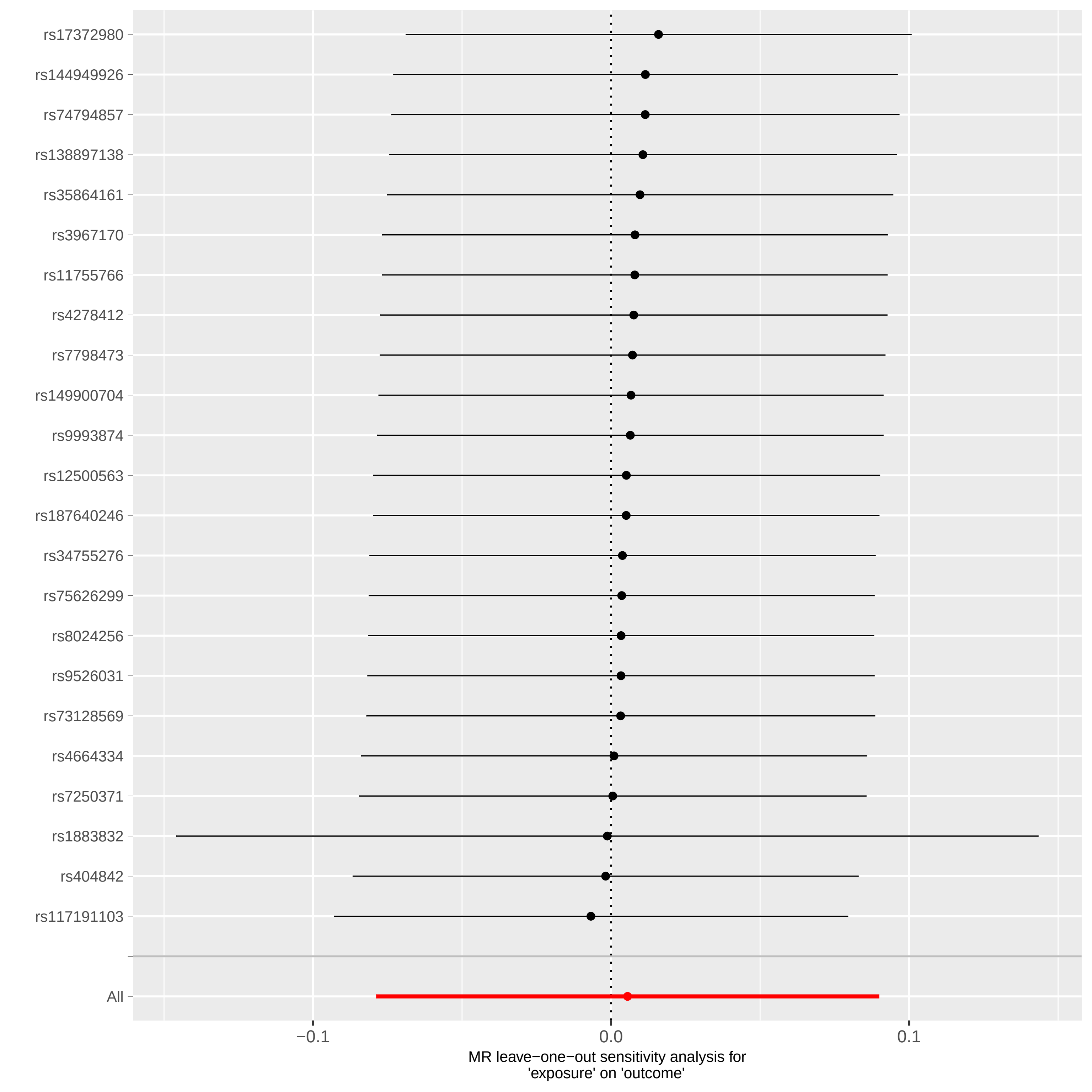
rs17372980
rs144949926
rs74794857
rs138897138
rs35864161
rs3967170
rs11755766
rs4278412
rs7798473
rs149900704
rs9993874
rs12500563
rs187640246
rs34755276
rs75626299
rs8024256
rs9526031
rs73128569
rs4664334
rs7250371
rs1883832
rs404842
rs117191103
All
−0.1
0.0
0.1
MR leave−one−out sensitivity analysis for
'exposure' on 'outcome'

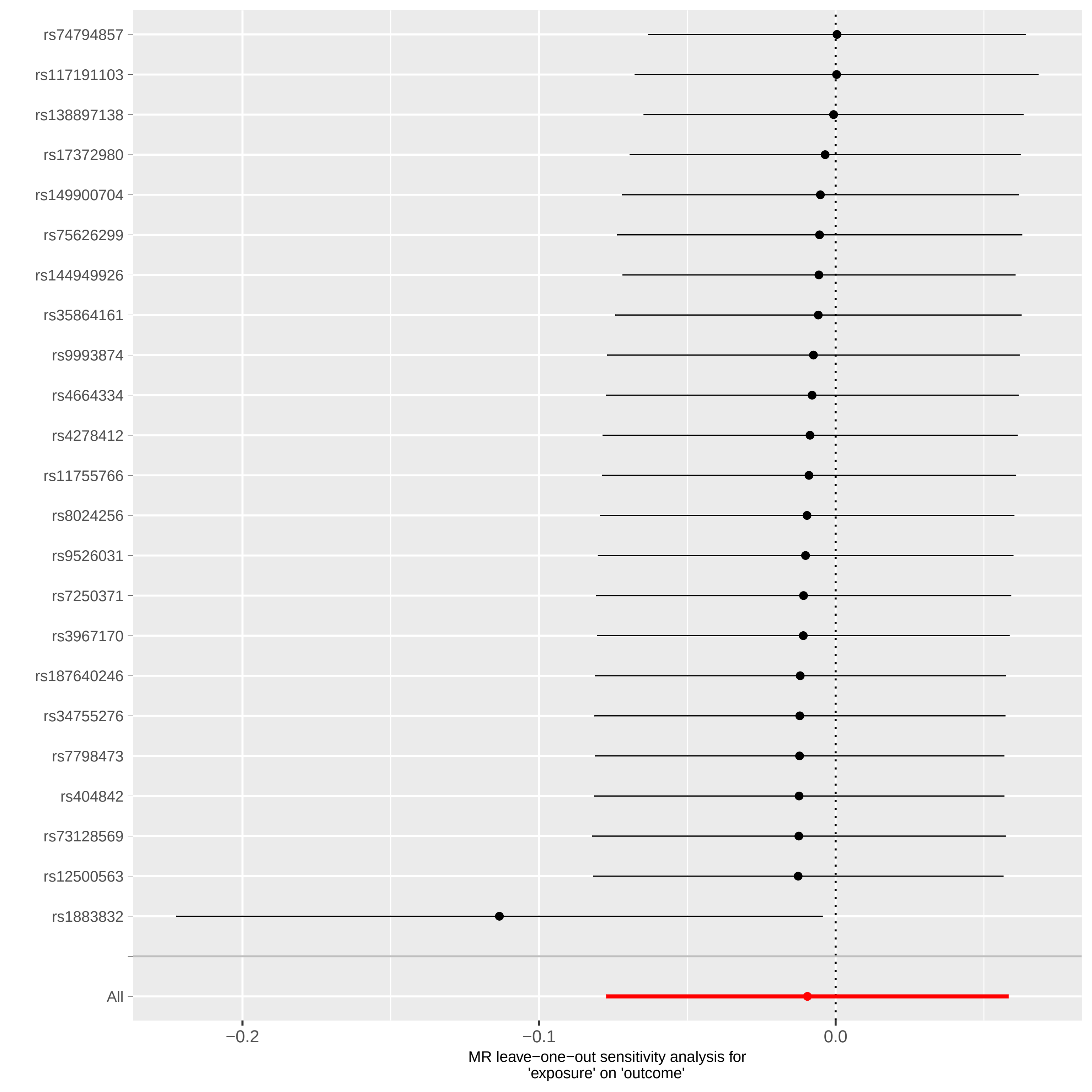

rs74794857
rs117191103
rs138897138
rs17372980
rs149900704
rs75626299
rs144949926
rs35864161
rs9993874
rs4664334
rs4278412
rs11755766
rs8024256
rs9526031
rs7250371
rs3967170
rs187640246
rs34755276
rs7798473
rs404842
rs73128569
rs12500563
rs1883832
All
−0.2
−0.1
0.0
MR leave−one−out sensitivity analysis for
'exposure' on 'outcome'

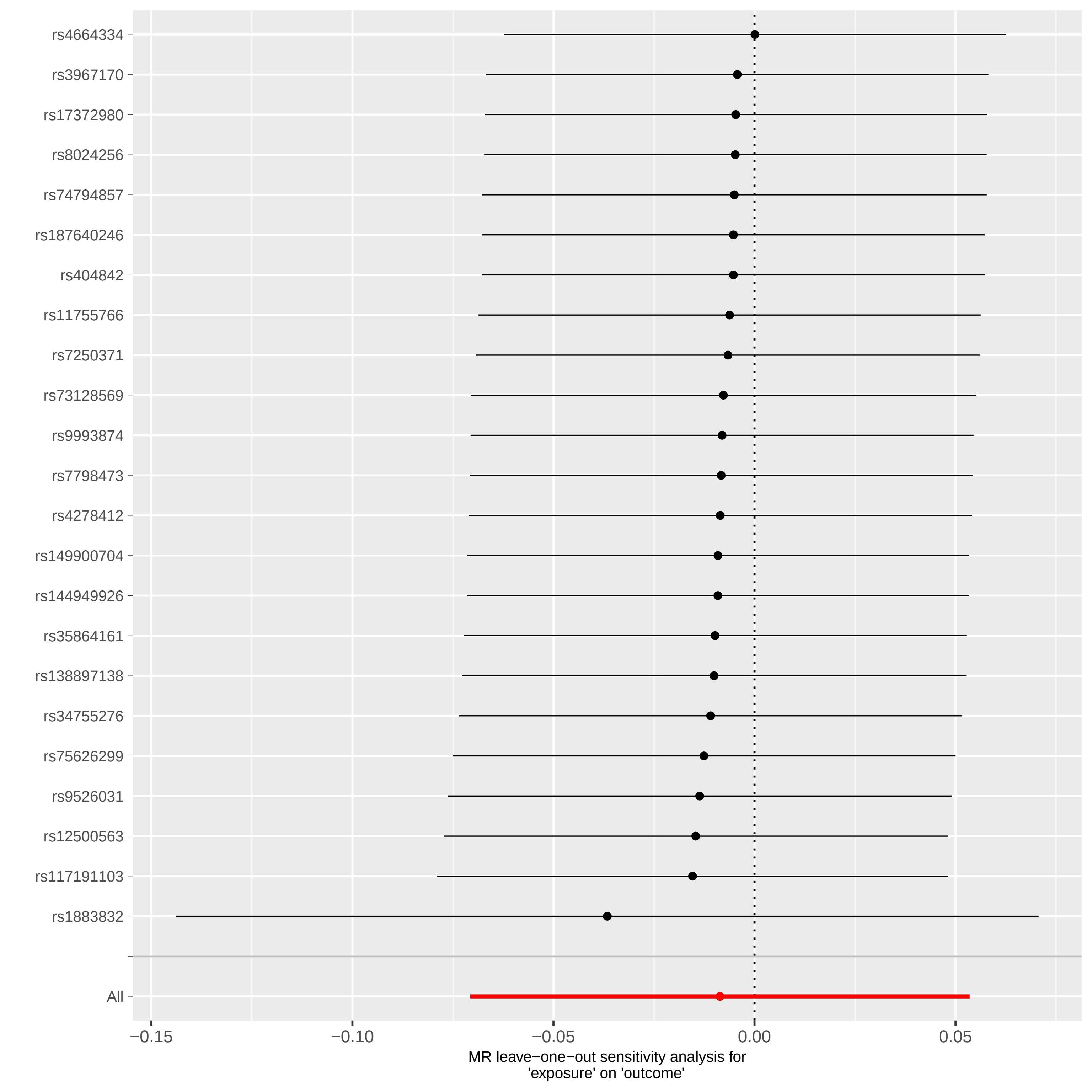

rs4664334
rs3967170
rs17372980
rs8024256
rs74794857
rs187640246
rs404842
rs11755766
rs7250371
rs73128569
rs9993874
rs7798473
rs4278412
rs149900704
rs144949926
rs35864161
rs138897138
rs34755276
rs75626299
rs9526031
rs12500563
rs117191103
rs1883832
All
−0.15
−0.10
−0.05
0.00
0.05
MR leave−one−out sensitivity analysis for
'exposure' on 'outcome'

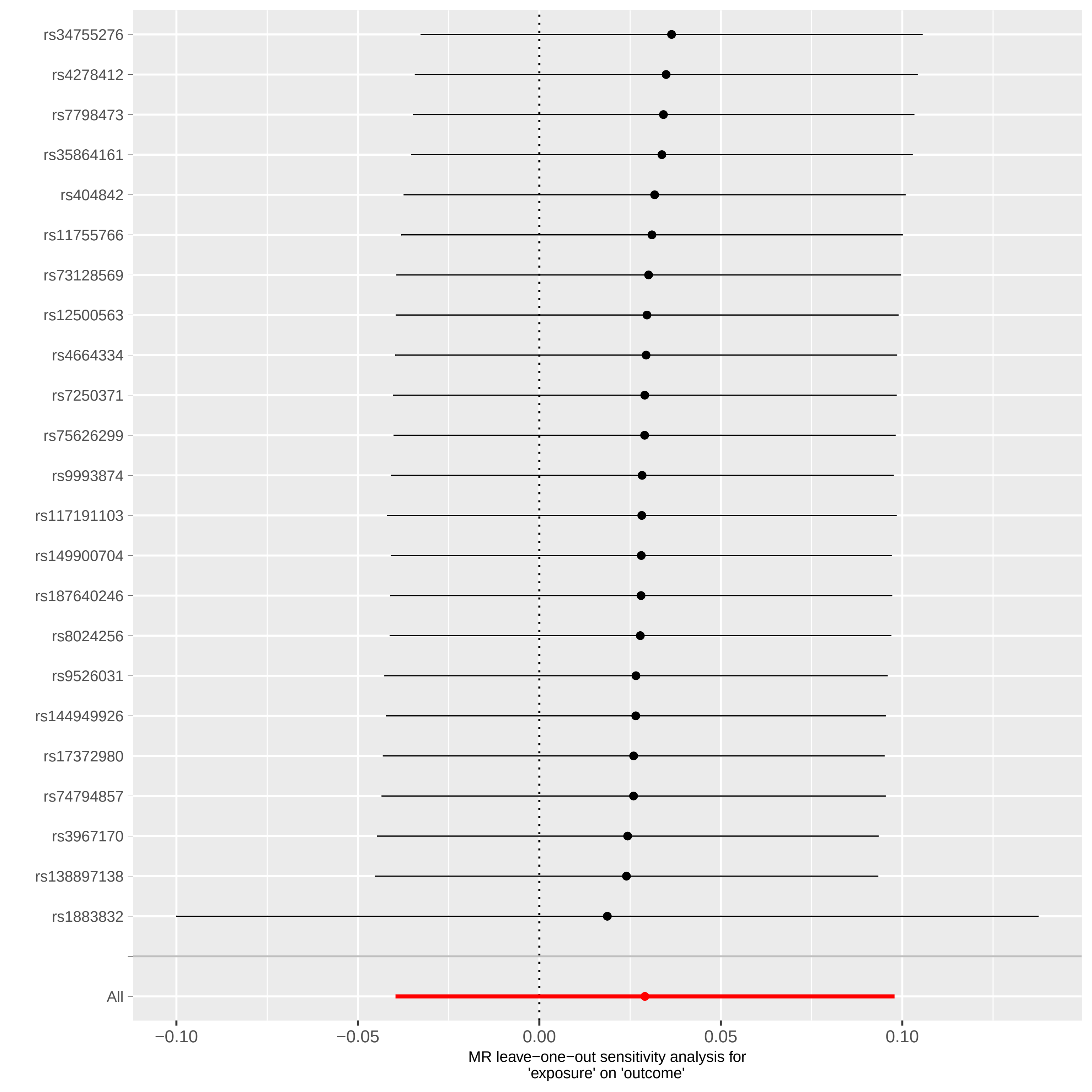

rs34755276
rs4278412
rs7798473
rs35864161
rs404842
rs11755766
rs73128569
rs12500563
rs4664334
rs7250371
rs75626299
rs9993874
rs117191103
rs149900704
rs187640246
rs8024256
rs9526031
rs144949926
rs17372980
rs74794857
rs3967170
rs138897138
rs1883832
All
−0.10
−0.05
0.00
0.05
0.10
MR leave−one−out sensitivity analysis for
'exposure' on 'outcome'

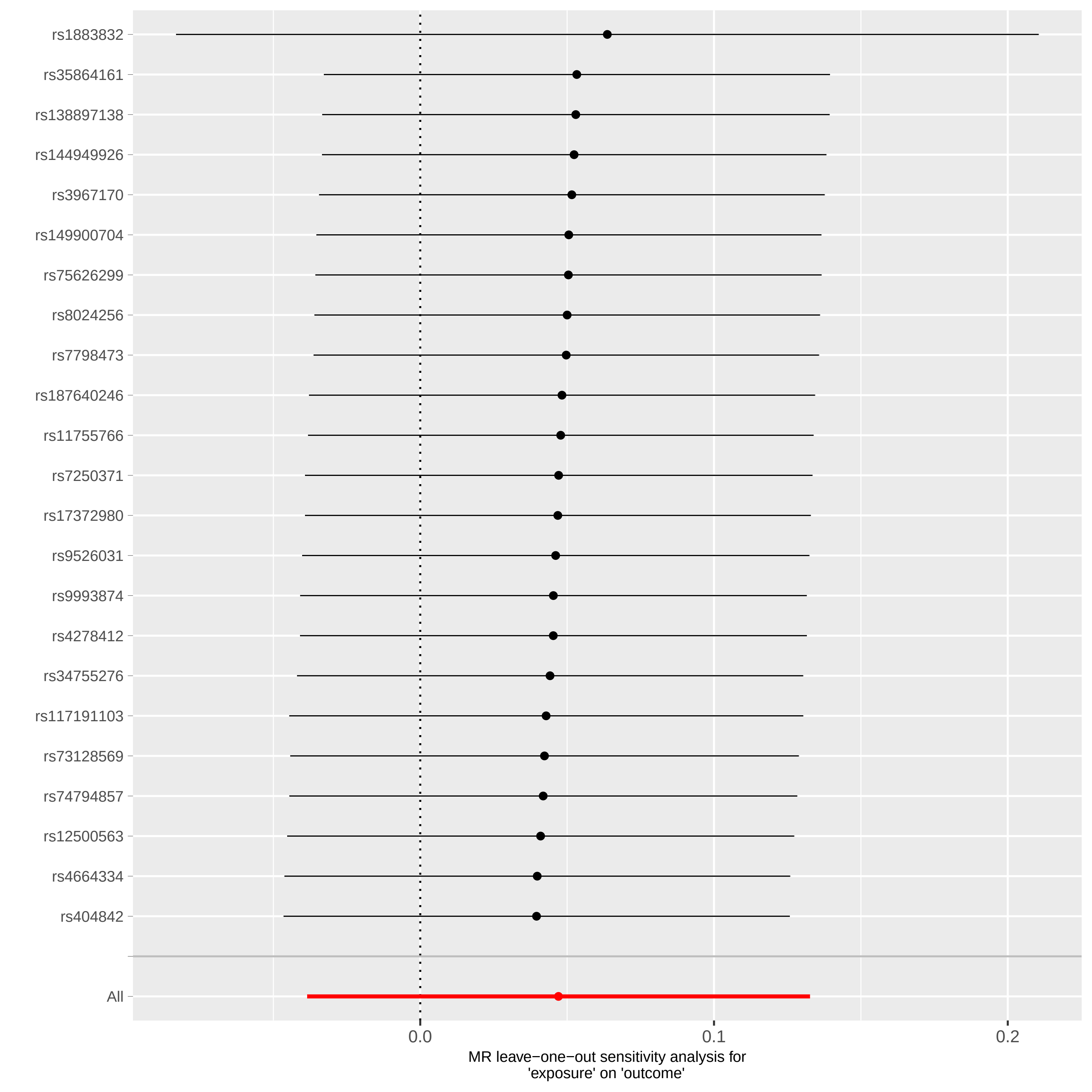

rs1883832
rs35864161
rs138897138
rs144949926
rs3967170
rs149900704
rs75626299
rs8024256
rs7798473
rs187640246
rs11755766
rs7250371
rs17372980
rs9526031
rs9993874
rs4278412
rs34755276
rs117191103
rs73128569
rs74794857
rs12500563
rs4664334
rs404842
All
0.0
0.1
0.2
MR leave−one−out sensitivity analysis for
'exposure' on 'outcome'

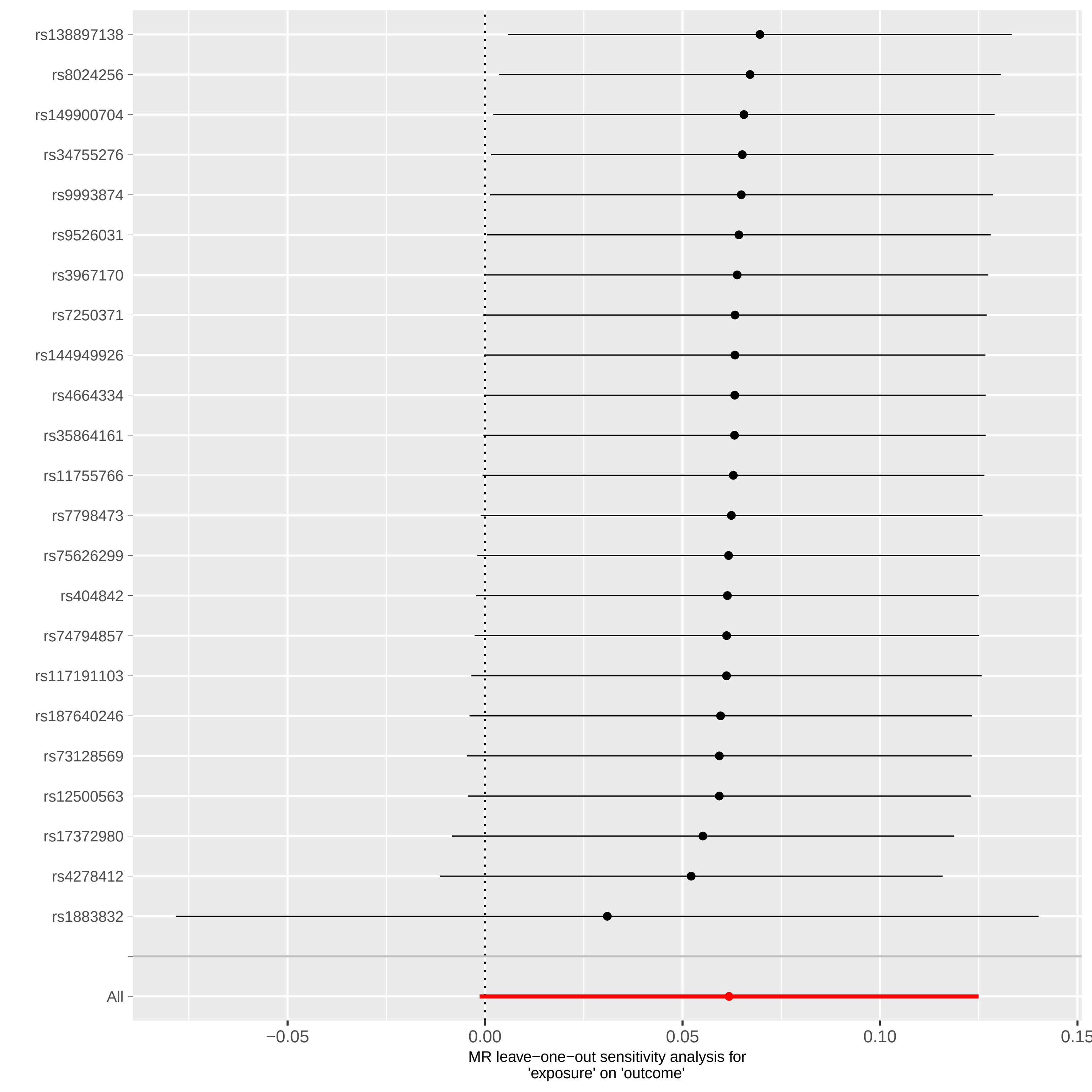
rs138897138
rs8024256
rs149900704
rs34755276
rs9993874
rs9526031
rs3967170
rs7250371
rs144949926
rs4664334
rs35864161
rs11755766
rs7798473
rs75626299
rs404842
rs74794857
rs117191103
rs187640246
rs73128569
rs12500563
rs17372980
rs4278412
rs1883832
All
−0.05
0.00
0.05
0.10
0.15
MR leave−one−out sensitivity analysis for
'exposure' on 'outcome'

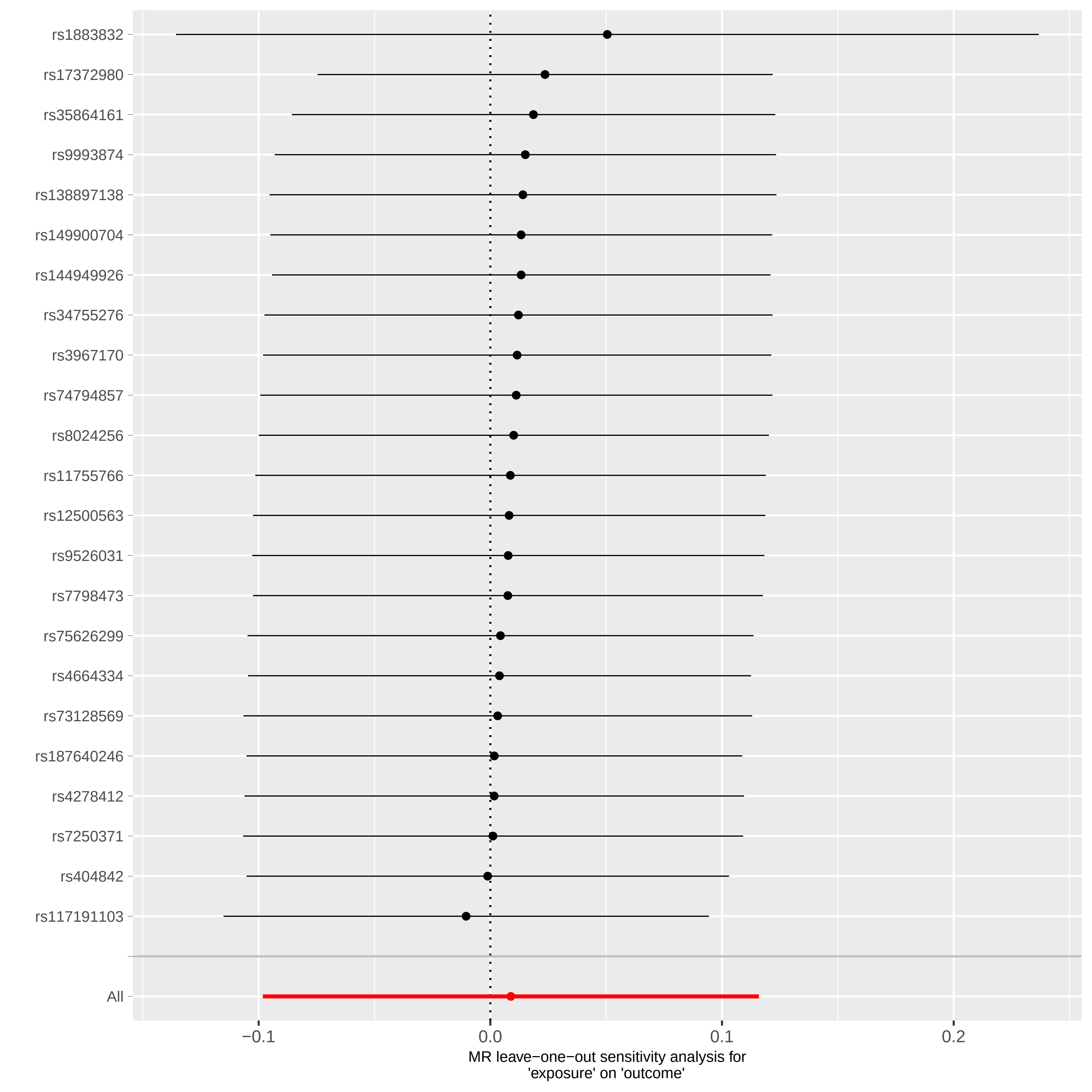
rs1883832
rs17372980
rs35864161
rs9993874
rs138897138
rs149900704
rs144949926
rs34755276
rs3967170
rs74794857
rs8024256
rs11755766
rs12500563
rs9526031
rs7798473
rs75626299
rs4664334
rs73128569
rs187640246
rs4278412
rs7250371
rs404842
rs117191103
All
−0.1
0.0
0.1
0.2
MR leave−one−out sensitivity analysis for
'exposure' on 'outcome'

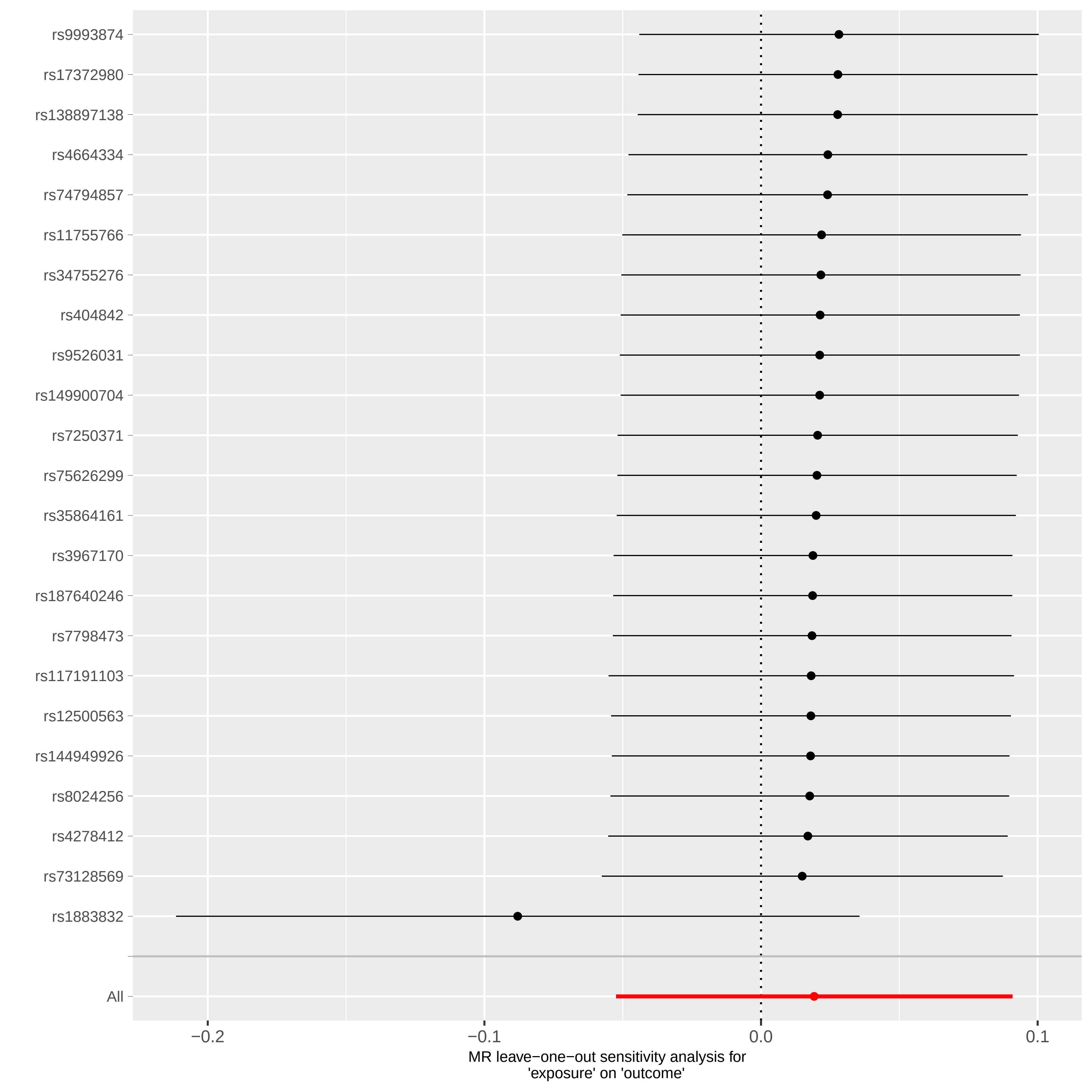
rs9993874
rs17372980
rs138897138
rs4664334
rs74794857
rs11755766
rs34755276
rs404842
rs9526031
rs149900704
rs7250371
rs75626299
rs35864161
rs3967170
rs187640246
rs7798473
rs117191103
rs12500563
rs144949926
rs8024256
rs4278412
rs73128569
rs1883832
All
−0.2
−0.1
0.0
0.1
MR leave−one−out sensitivity analysis for
'exposure' on 'outcome'

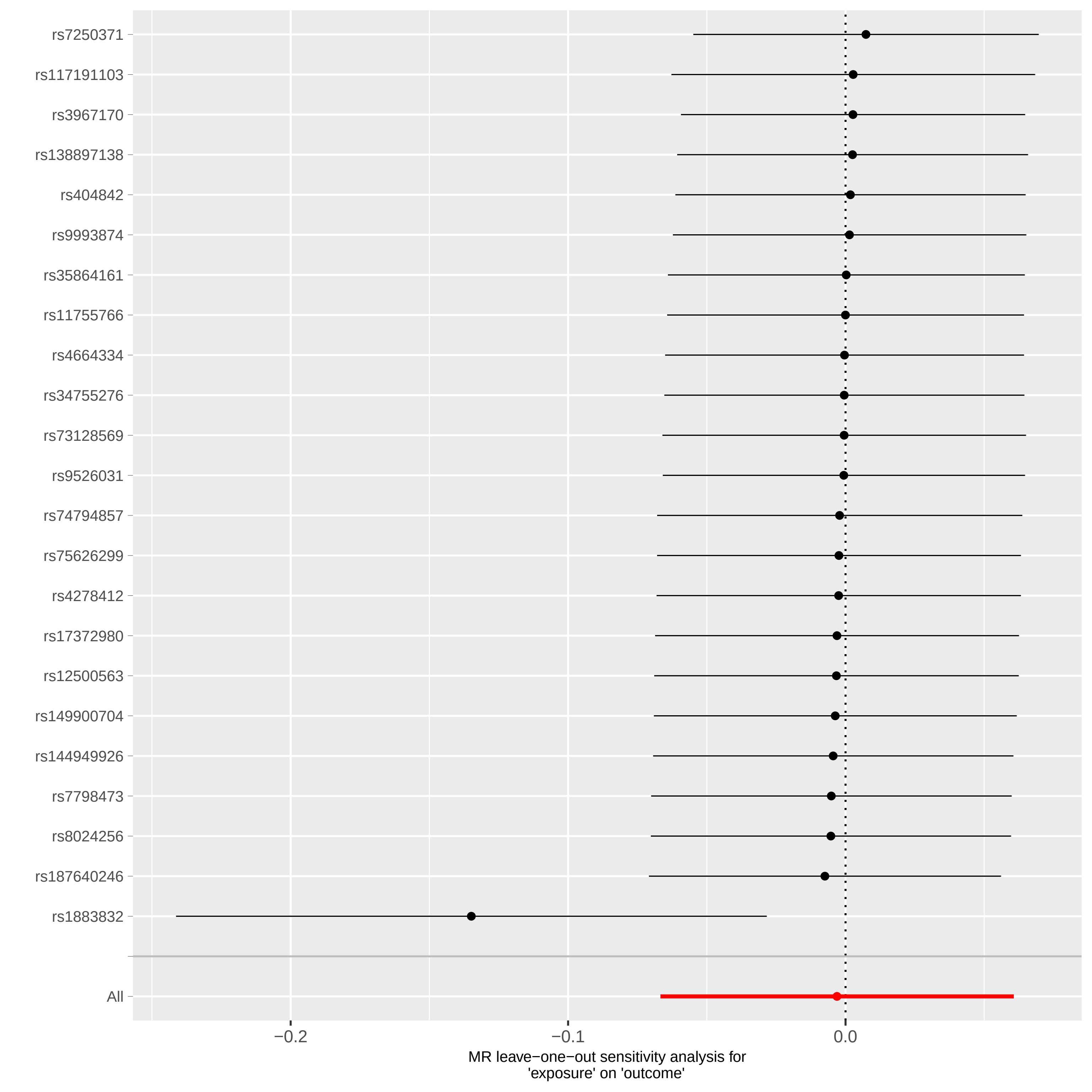
rs7250371
rs117191103
rs3967170
rs138897138
rs404842
rs9993874
rs35864161
rs11755766
rs4664334
rs34755276
rs73128569
rs9526031
rs74794857
rs75626299
rs4278412
rs17372980
rs12500563
rs149900704
rs144949926
rs7798473
rs8024256
rs187640246
rs1883832
All
−0.2
−0.1
0.0
MR leave−one−out sensitivity analysis for
'exposure' on 'outcome'

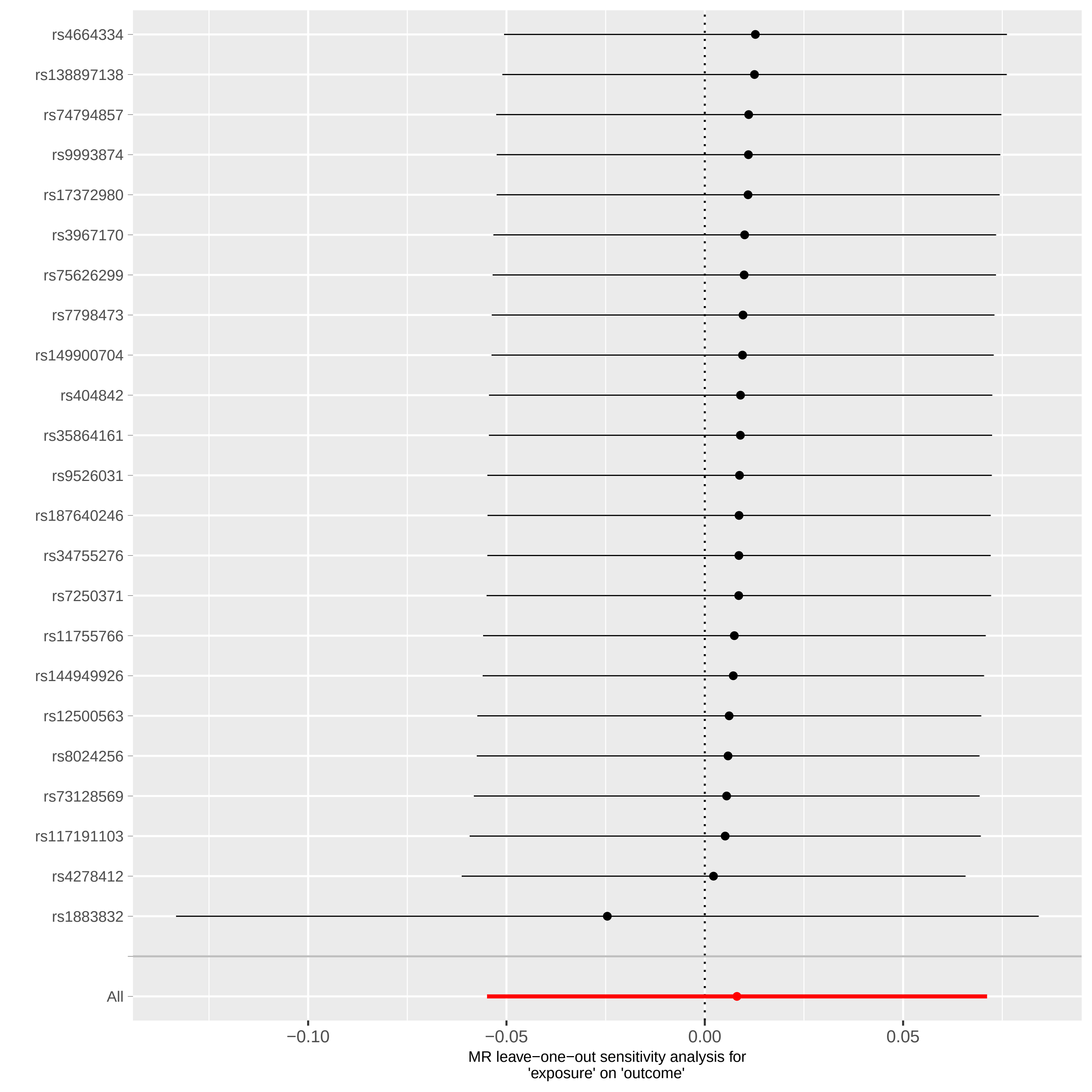
rs4664334
rs138897138
rs74794857
rs9993874
rs17372980
rs3967170
rs75626299
rs7798473
rs149900704
rs404842
rs35864161
rs9526031
rs187640246
rs34755276
rs7250371
rs11755766
rs144949926
rs12500563
rs8024256
rs73128569
rs117191103
rs4278412
rs1883832
All
−0.10
−0.05
0.00
0.05
MR leave−one−out sensitivity analysis for
'exposure' on 'outcome'

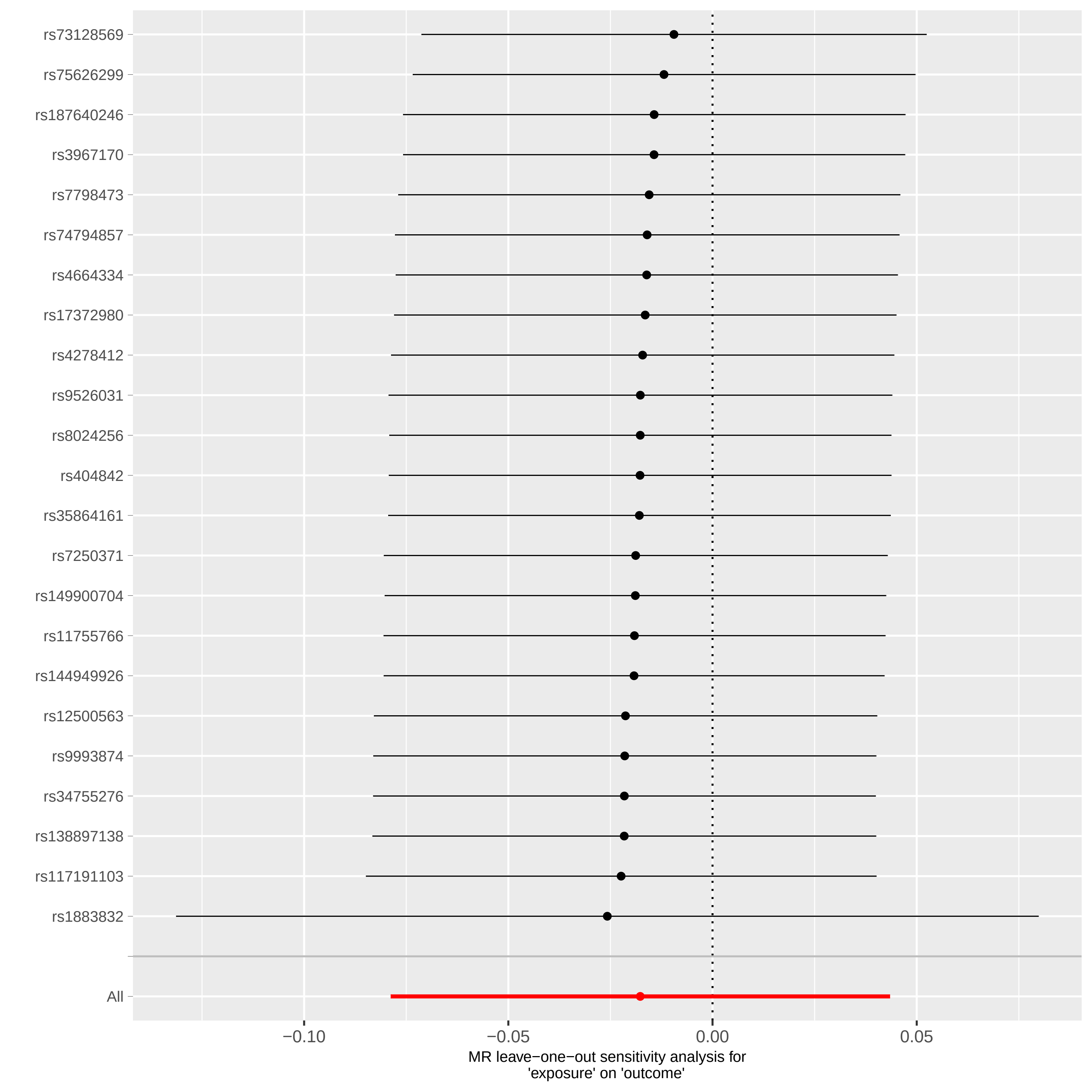
rs73128569
rs75626299
rs187640246
rs3967170
rs7798473
rs74794857
rs4664334
rs17372980
rs4278412
rs9526031
rs8024256
rs404842
rs35864161
rs7250371
rs149900704
rs11755766
rs144949926
rs12500563
rs9993874
rs34755276
rs138897138
rs117191103
rs1883832
All
−0.10
−0.05
0.00
0.05
MR leave−one−out sensitivity analysis for
'exposure' on 'outcome'

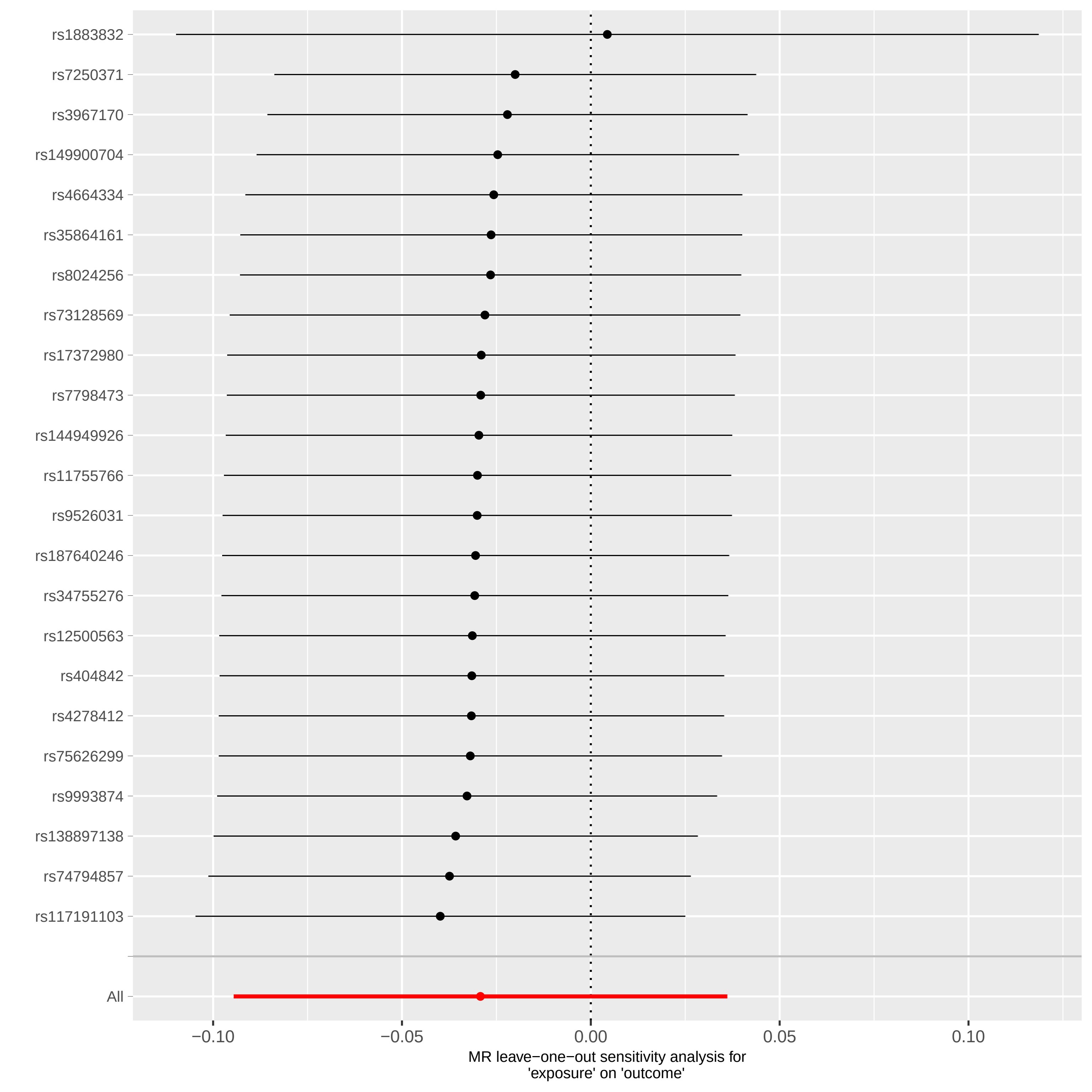

rs1883832
rs7250371
rs3967170
rs149900704
rs4664334
rs35864161
rs8024256
rs73128569
rs17372980
rs7798473
rs144949926
rs11755766
rs9526031
rs187640246
rs34755276
rs12500563
rs404842
rs4278412
rs75626299
rs9993874
rs138897138
rs74794857
rs117191103
All
−0.10
−0.05
0.00
0.05
0.10
MR leave−one−out sensitivity analysis for
'exposure' on 'outcome'

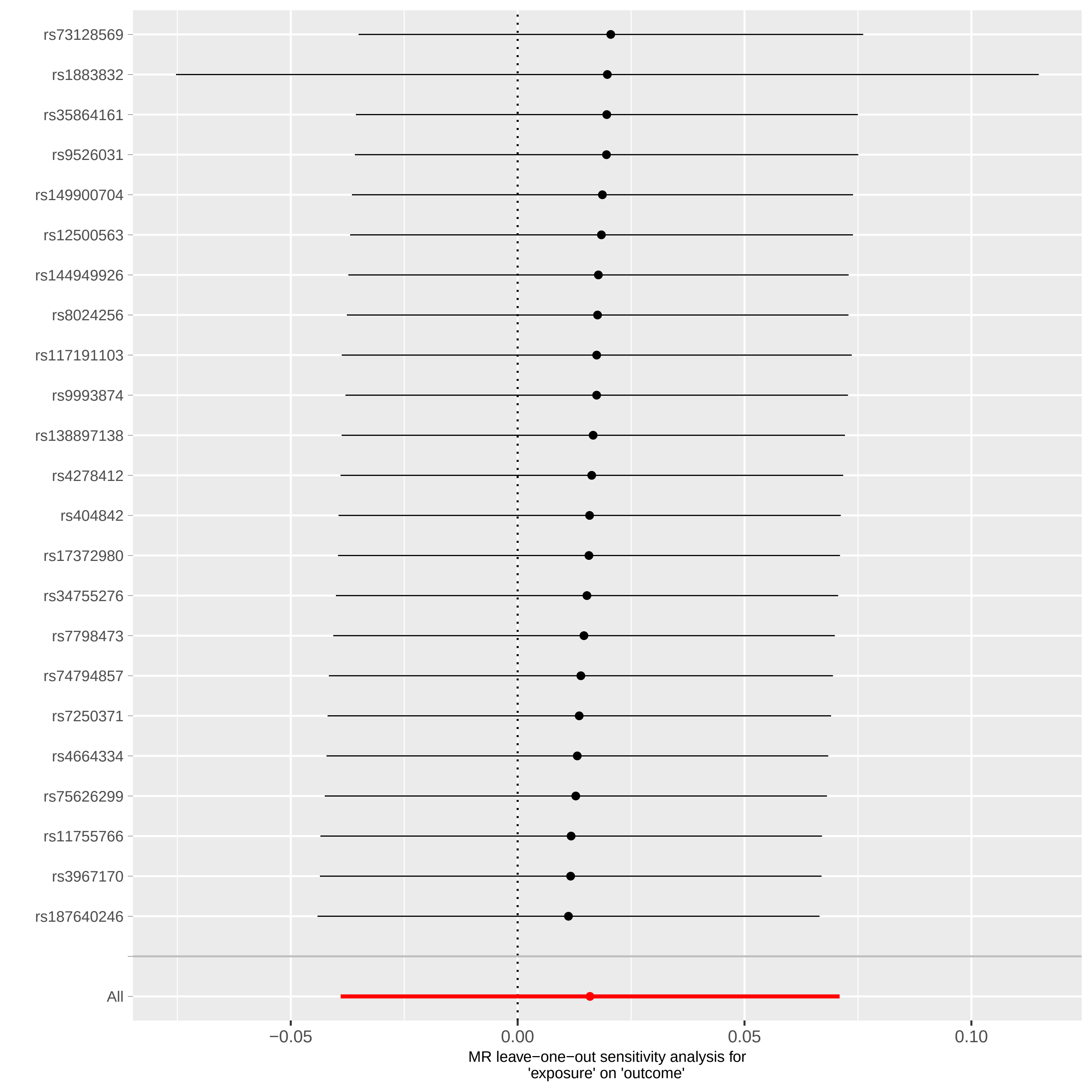
rs73128569
rs1883832
rs35864161
rs9526031
rs149900704
rs12500563
rs144949926
rs8024256
rs117191103
rs9993874
rs138897138
rs4278412
rs404842
rs17372980
rs34755276
rs7798473
rs74794857
rs7250371
rs4664334
rs75626299
rs11755766
rs3967170
rs187640246
All
−0.05
0.00
0.05
0.10
MR leave−one−out sensitivity analysis for
'exposure' on 'outcome'

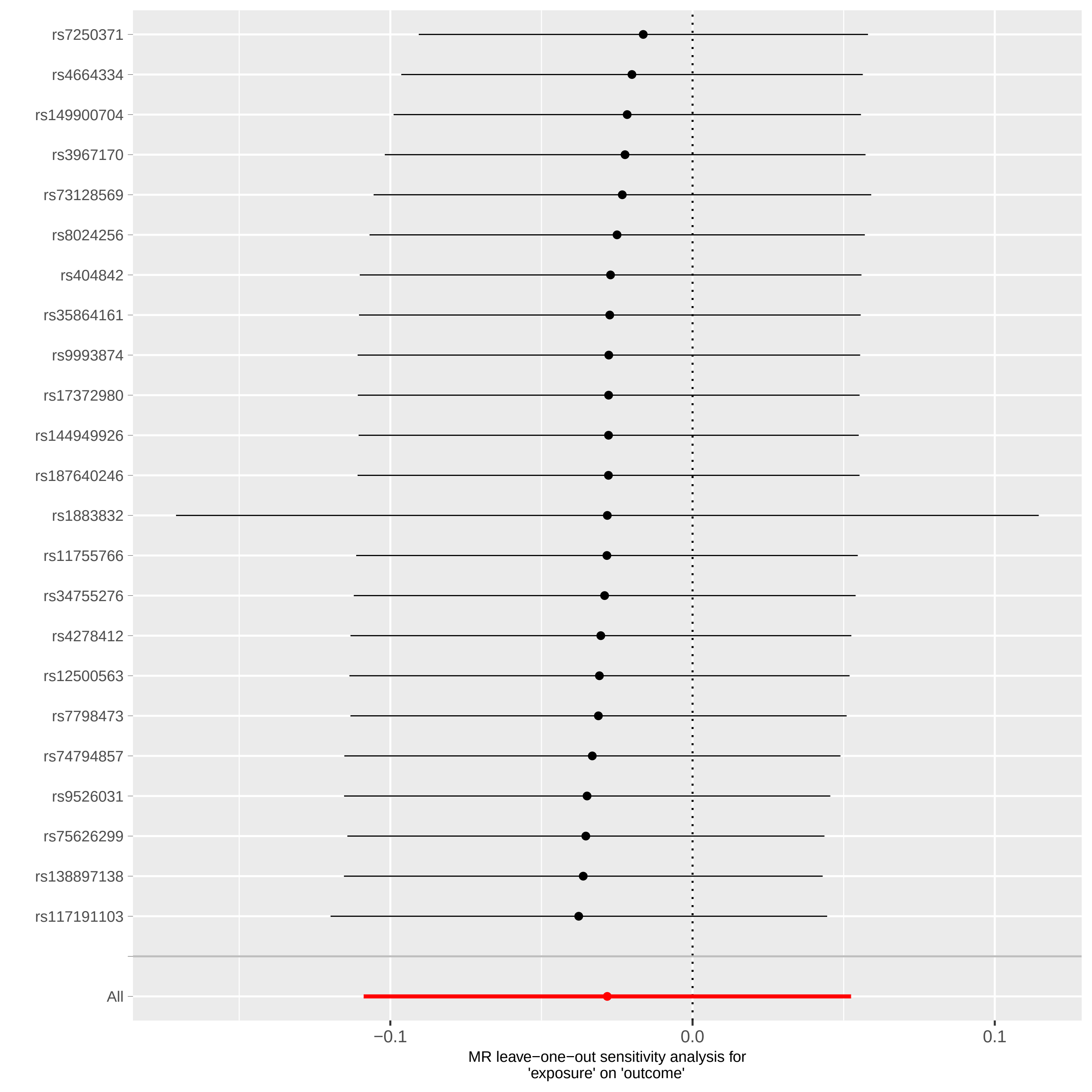

rs7250371
rs4664334
rs149900704
rs3967170
rs73128569
rs8024256
rs404842
rs35864161
rs9993874
rs17372980
rs144949926
rs187640246
rs1883832
rs11755766
rs34755276
rs4278412
rs12500563
rs7798473
rs74794857
rs9526031
rs75626299
rs138897138
rs117191103
All
−0.1
0.0
0.1
MR leave−one−out sensitivity analysis for
'exposure' on 'outcome'

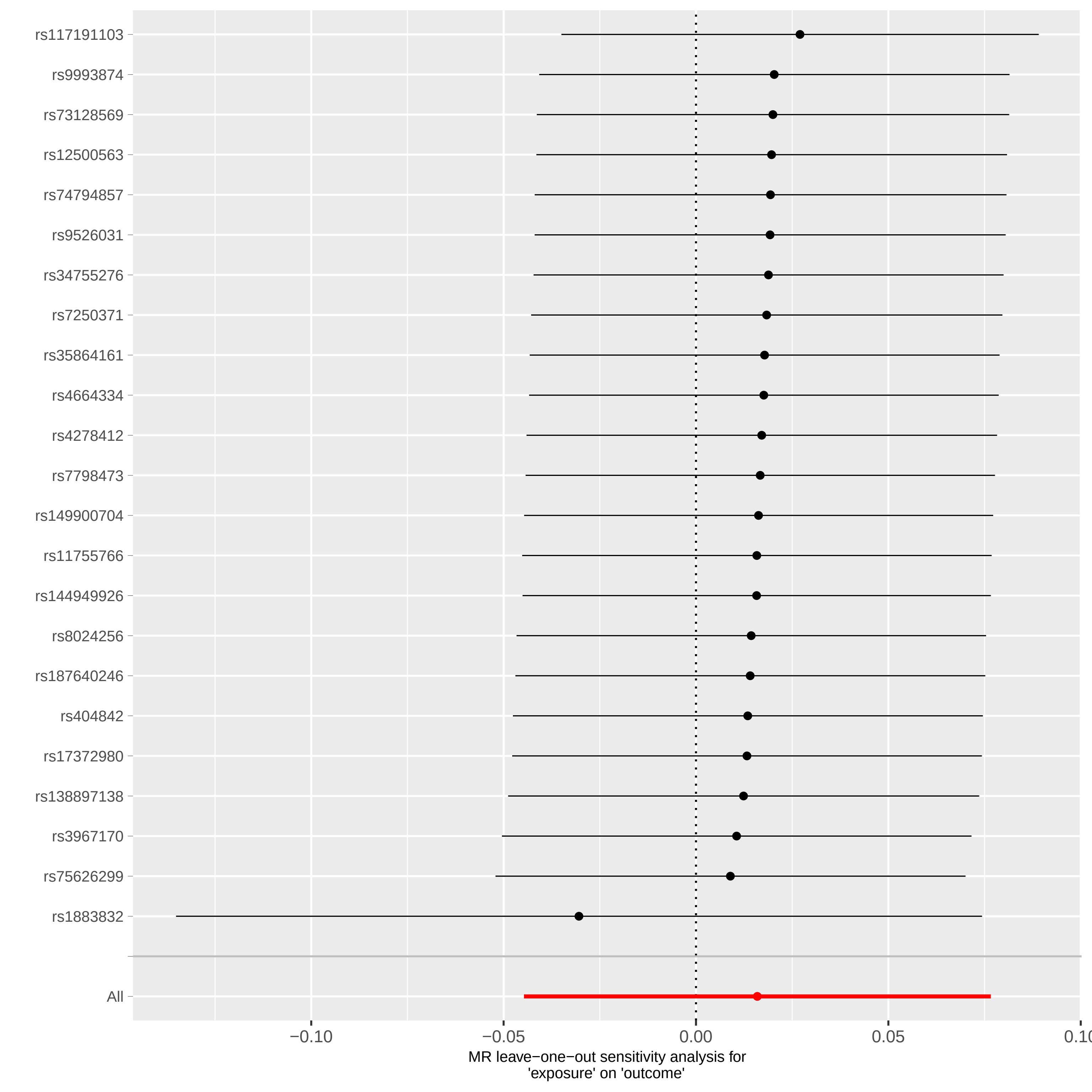

rs117191103
rs9993874
rs73128569
rs12500563
rs74794857
rs9526031
rs34755276
rs7250371
rs35864161
rs4664334
rs4278412
rs7798473
rs149900704
rs11755766
rs144949926
rs8024256
rs187640246
rs404842
rs17372980
rs138897138
rs3967170
rs75626299
rs1883832
All
−0.10
−0.05
0.00
0.05
MR leave−one−out sensitivity analysis for
'exposure' on 'outcome'

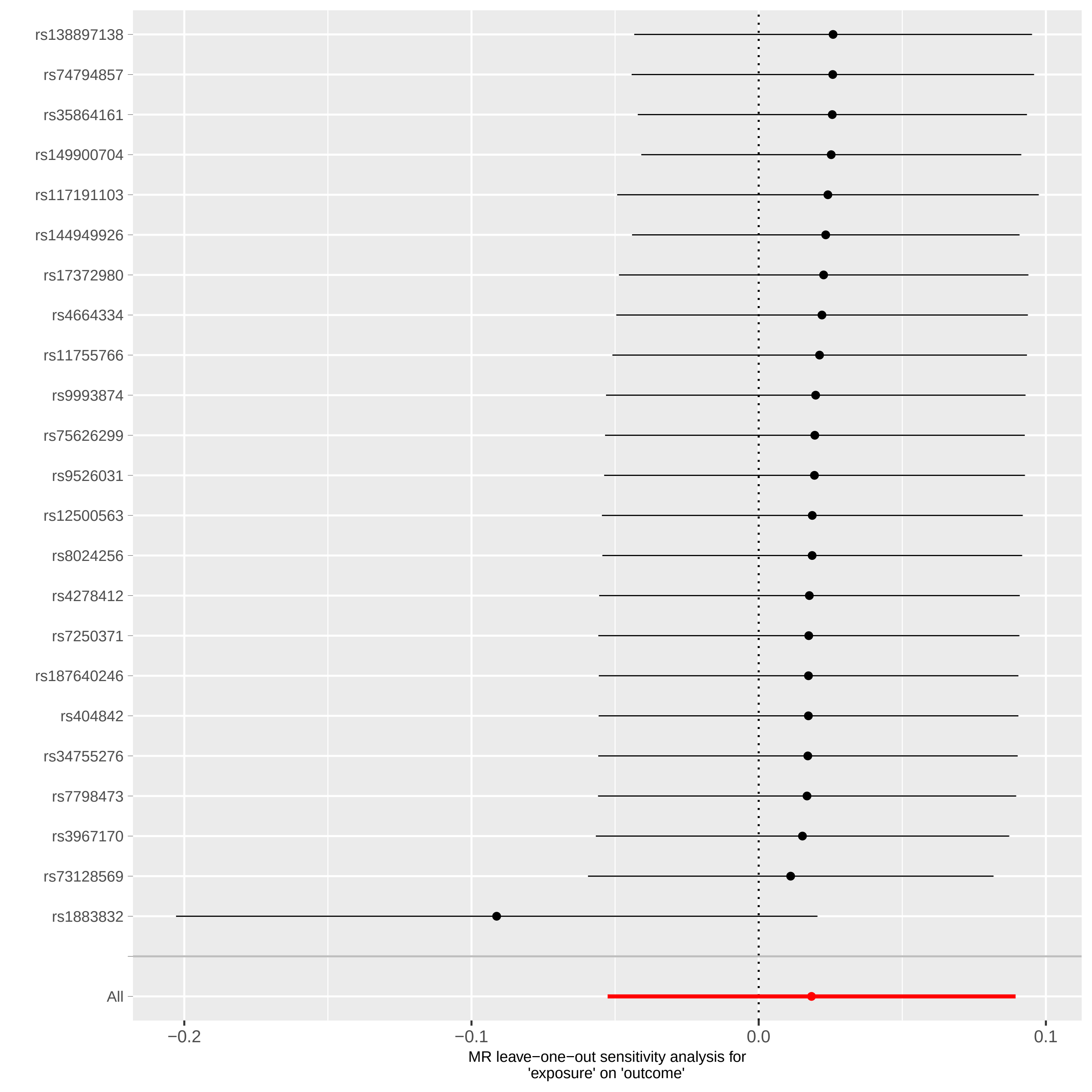
rs138897138
rs74794857
rs35864161
rs149900704
rs117191103
rs144949926
rs17372980
rs4664334
rs11755766
rs9993874
rs75626299
rs9526031
rs12500563
rs8024256
rs4278412
rs7250371
rs187640246
rs404842
rs34755276
rs7798473
rs3967170
rs73128569
rs1883832
All
−0.2
−0.1
0.0
0.1
MR leave−one−out sensitivity analysis for
'exposure' on 'outcome'

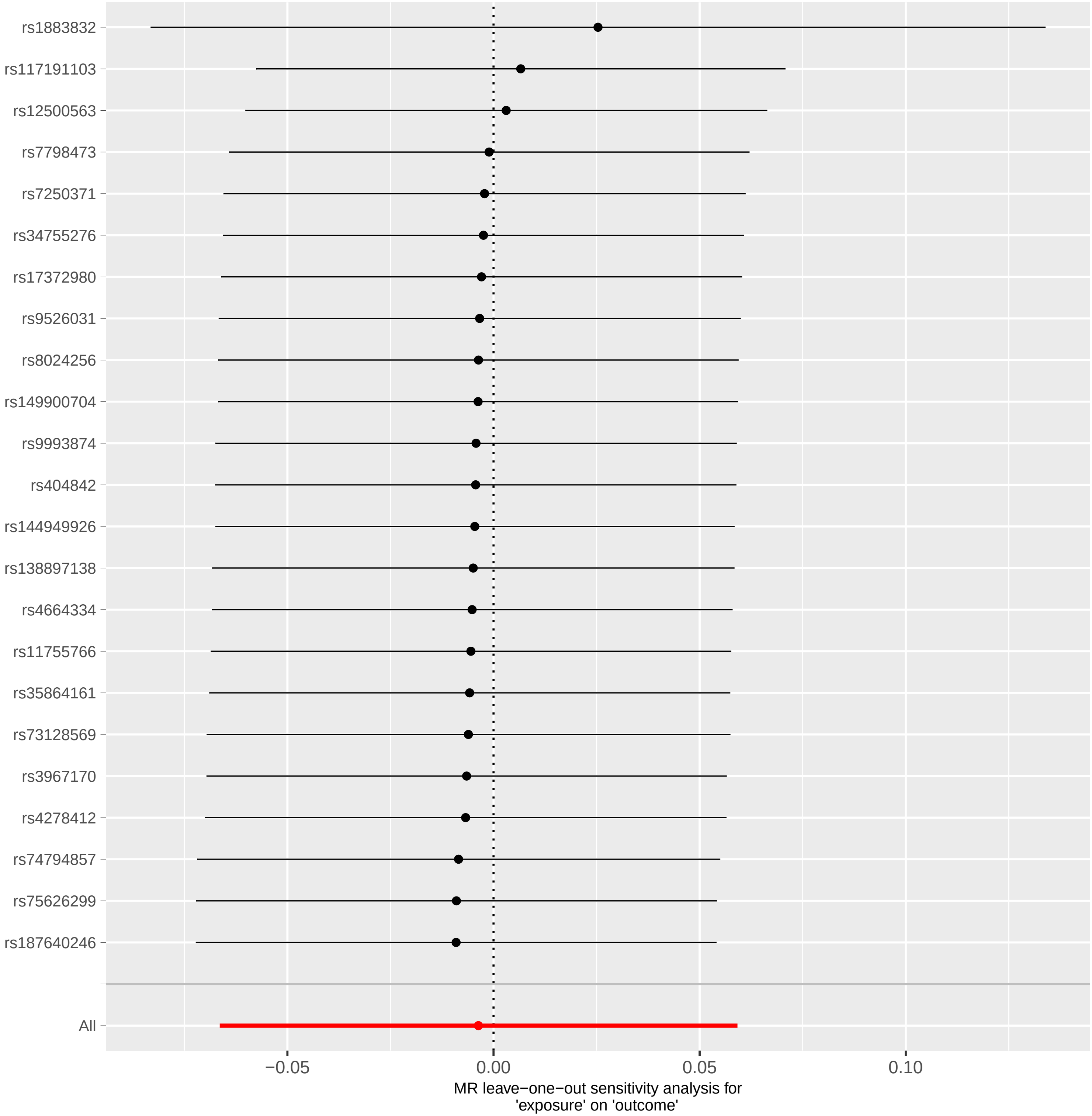
rs1883832
rs117191103
rs12500563
rs7798473
rs7250371
rs34755276
rs17372980
rs9526031
rs8024256
rs149900704
rs9993874
rs404842
rs144949926
rs138897138
rs4664334
rs11755766
rs35864161
rs73128569
rs3967170
rs4278412
rs74794857
rs75626299
rs187640246
All
−0.05
0.00
0.05
0.10
MR leave−one−out sensitivity analysis for
'exposure' on 'outcome'

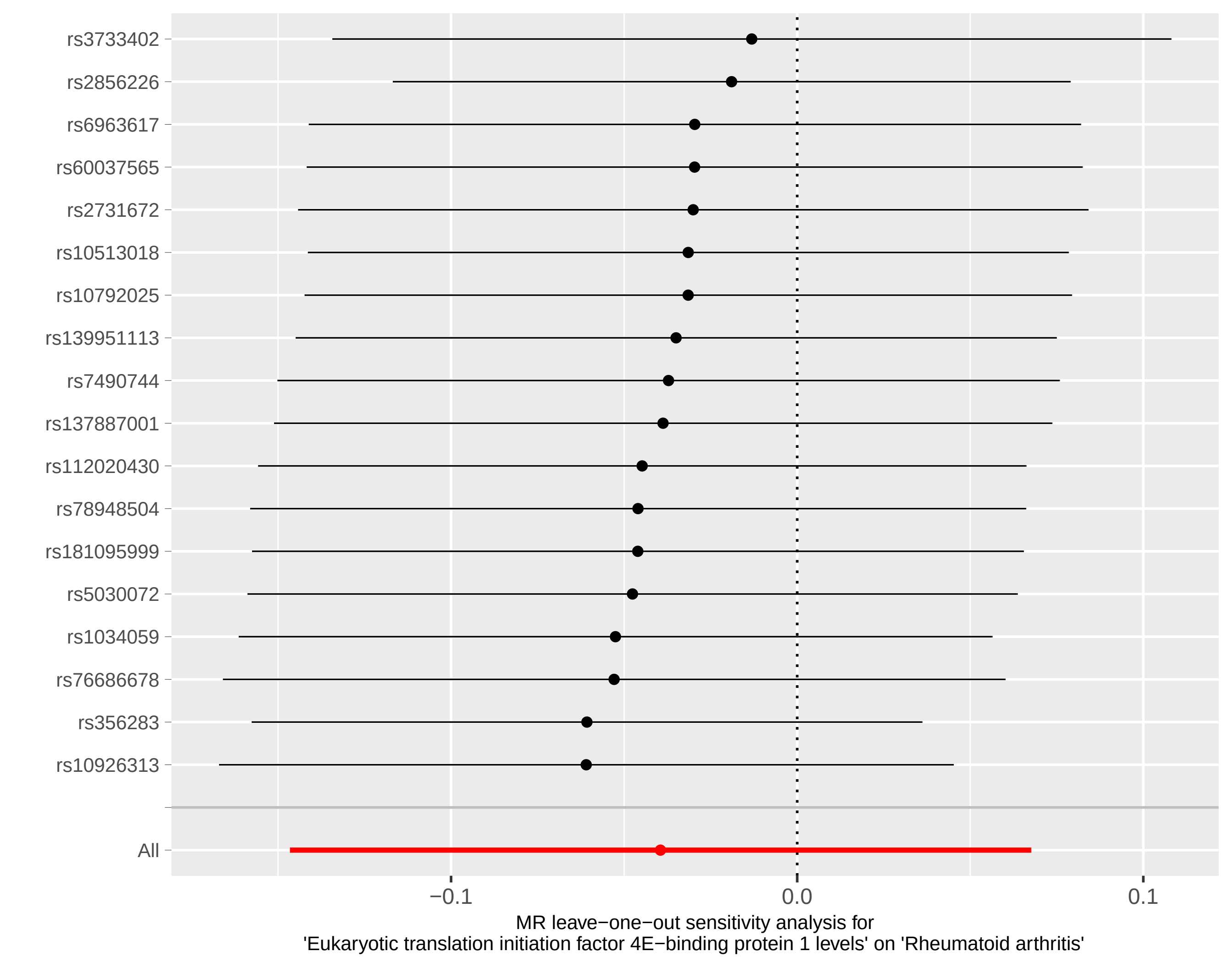
rs3733402
rs2856226
rs6963617
rs60037565
rs2731672
rs10513018
rs10792025
rs139951113
rs7490744
rs137887001
rs112020430
rs78948504
rs181095999
rs5030072
rs1034059
rs76686678
rs356283
rs10926313
All
−0.1
0.0
0.1
MR leave−one−out sensitivity analysis for
'Eukaryotic translation initiation factor 4E−binding protein 1 levels' on 'Rheumatoid arthritis'

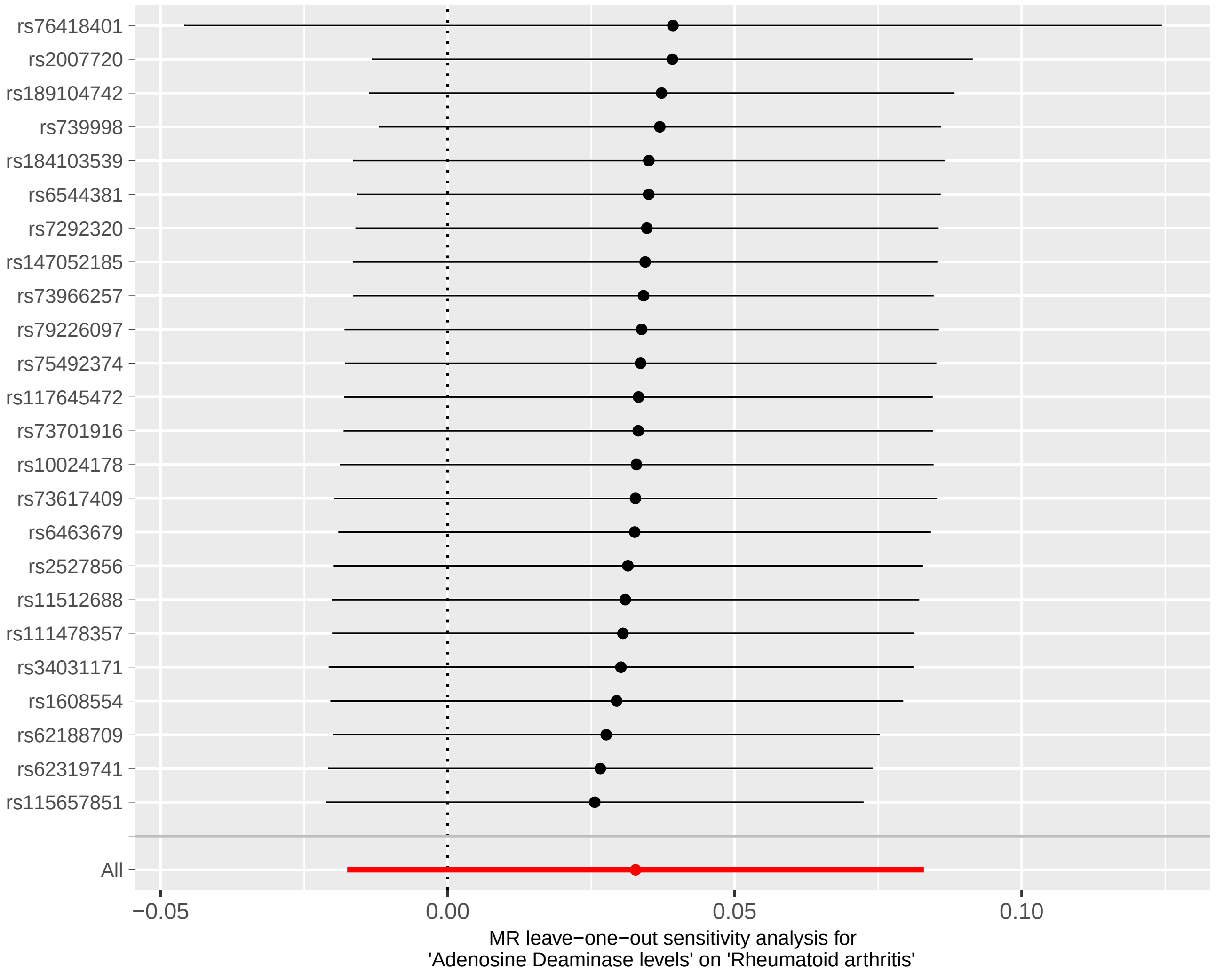
rs76418401
rs2007720
rs189104742
rs739998
rs184103539
rs6544381
rs7292320
rs147052185
rs73966257
rs79226097
rs75492374
rs117645472
rs73701916
rs10024178
rs73617409
rs6463679
rs2527856
rs11512688
rs111478357
rs34031171
rs1608554
rs62188709
rs62319741
rs115657851
All
−0.05
0.00
0.05
0.10
MR leave−one−out sensitivity analysis for
'Adenosine Deaminase levels' on 'Rheumatoid arthritis'

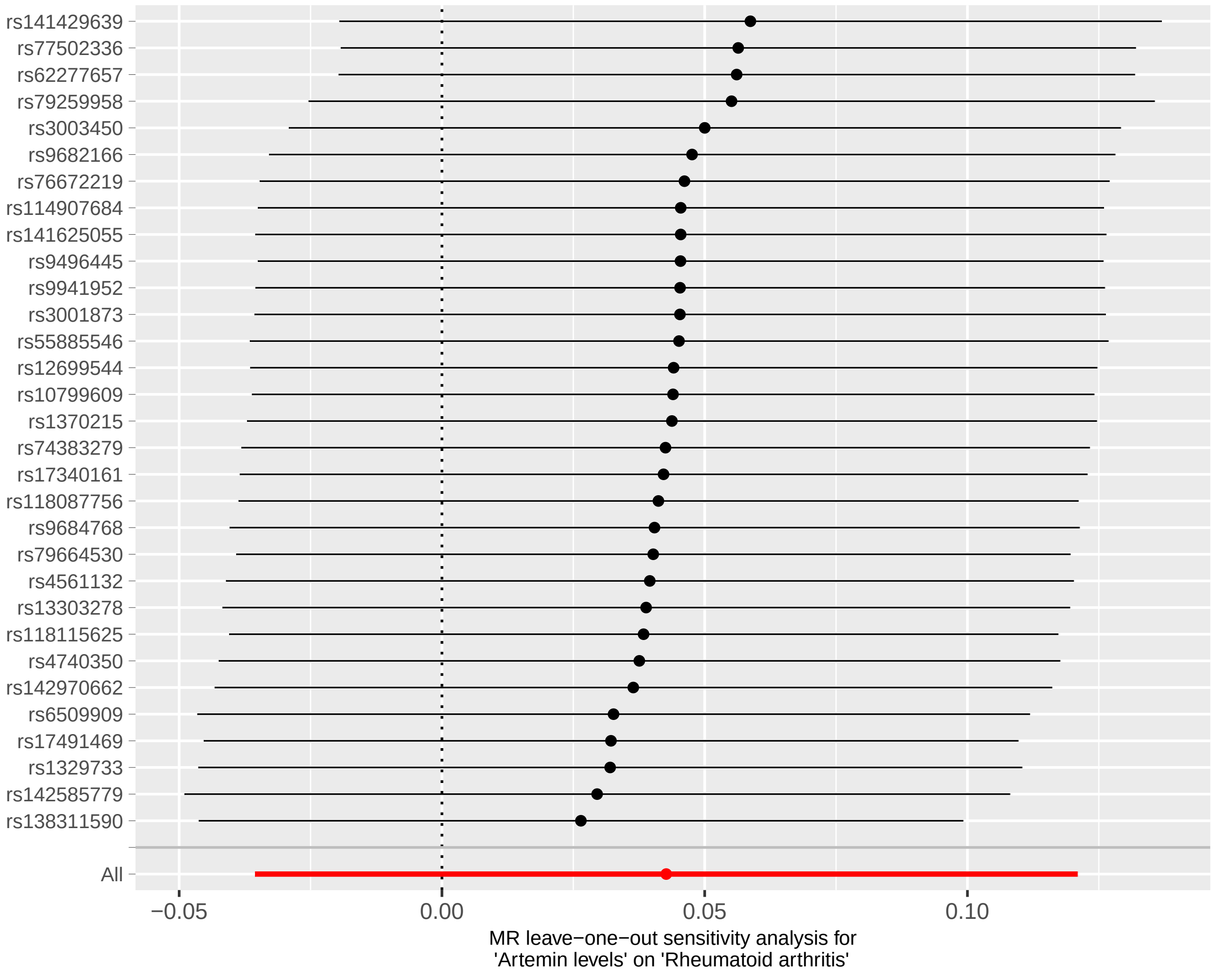

rs141429639
rs77502336
rs62277657
rs79259958
rs3003450
rs9682166
rs76672219
rs114907684
rs141625055
rs9496445
rs9941952
rs3001873
rs55885546
rs12699544
rs10799609
rs1370215
rs74383279
rs17340161
rs118087756
rs9684768
rs79664530
rs4561132
rs13303278
rs118115625
rs4740350
rs142970662
rs6509909
rs17491469
rs1329733
rs142585779
rs138311590
All
−0.05
0.00
0.05
0.10
MR leave−one−out sensitivity analysis for
'Artemin levels' on 'Rheumatoid arthritis'

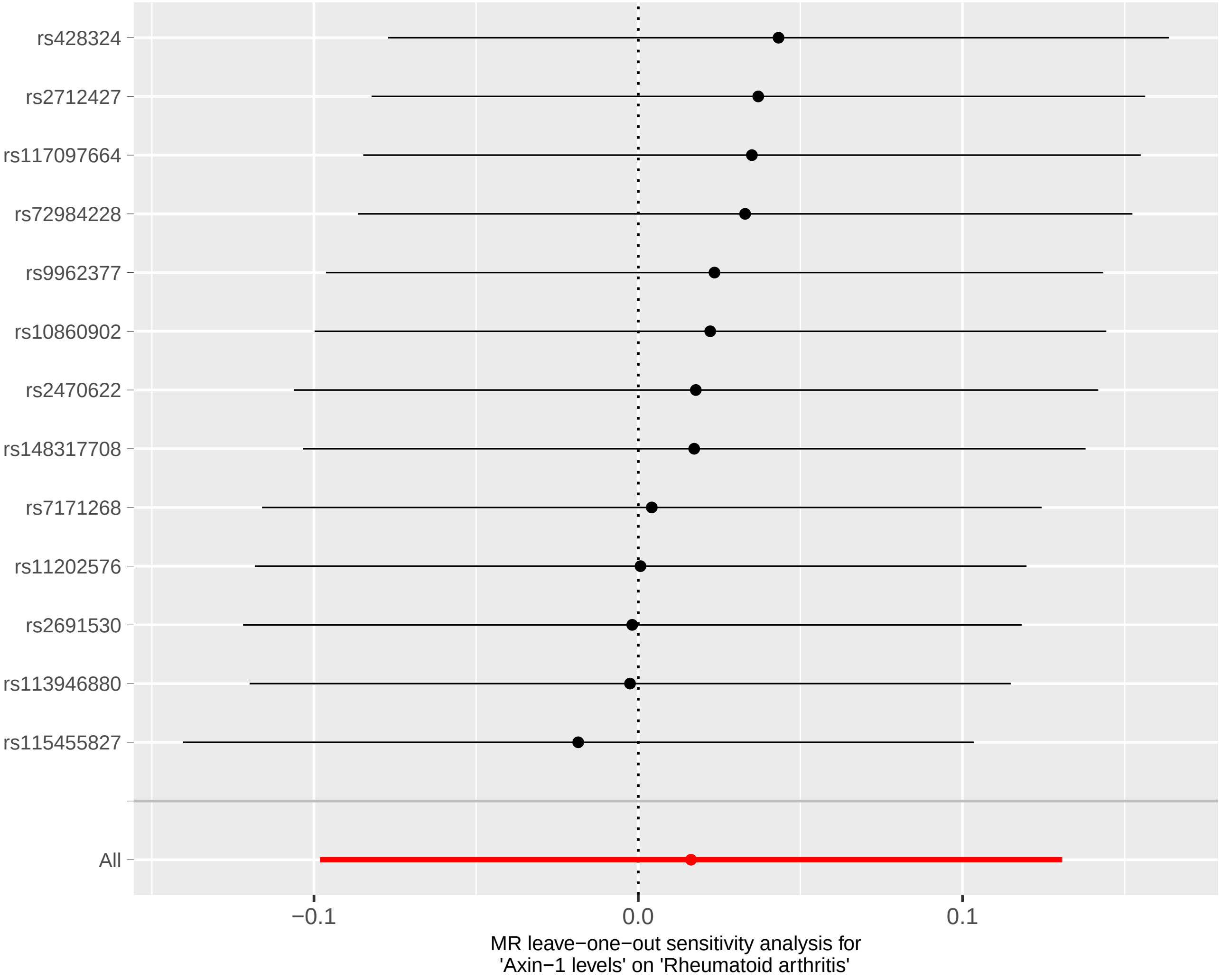
rs428324
rs2712427
rs117097664
rs72984228
rs9962377
rs10860902
rs2470622
rs148317708
rs7171268
rs11202576
rs2691530
rs113946880
rs115455827
All
−0.1
0.0
0.1
MR leave−one−out sensitivity analysis for
'Axin−1 levels' on 'Rheumatoid arthritis'

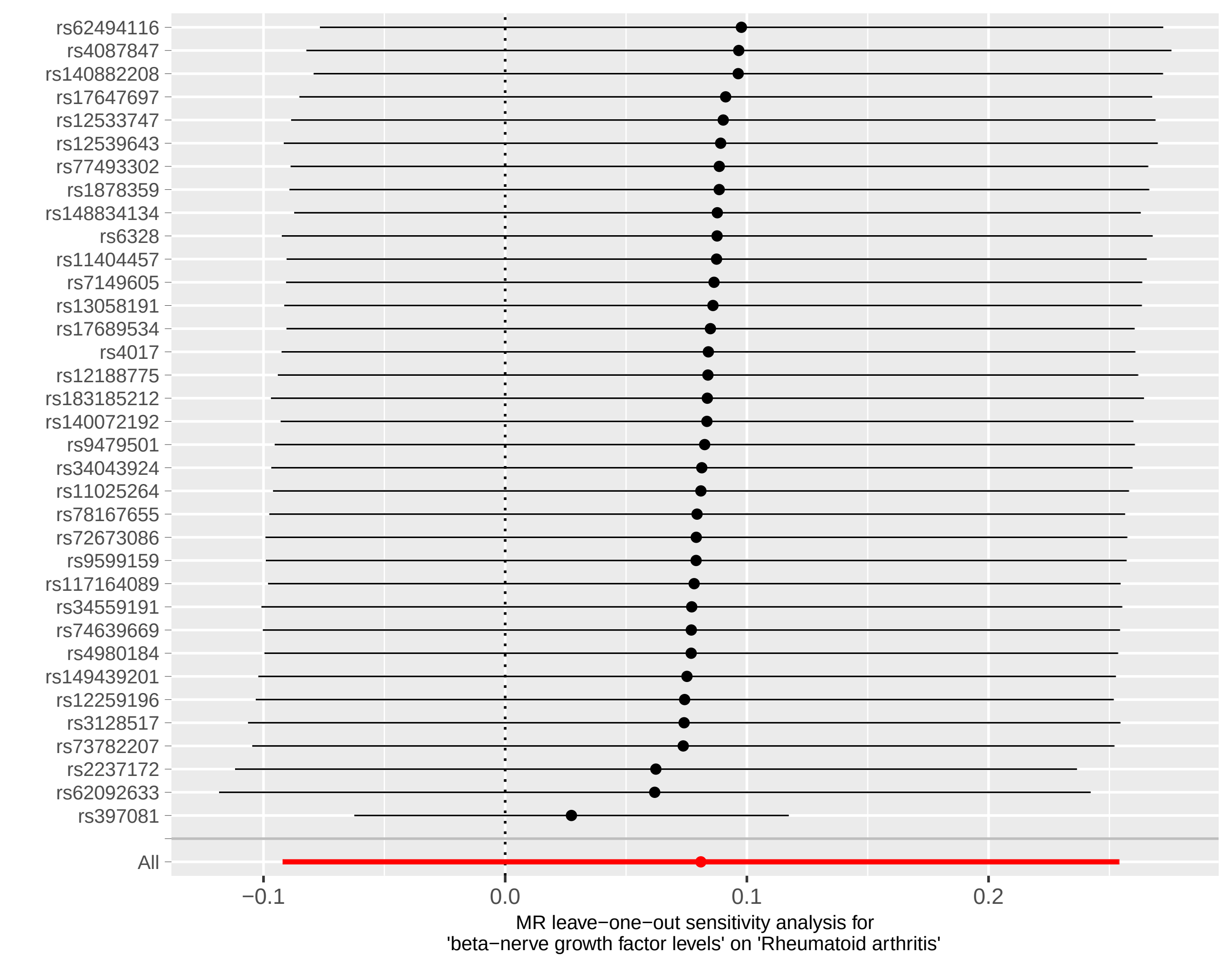
rs62494116
rs4087847
rs140882208
rs17647697
rs12533747
rs12539643
rs77493302
rs1878359
rs148834134
rs6328
rs11404457
rs7149605
rs13058191
rs17689534
rs4017
rs12188775
rs183185212
rs140072192
rs9479501
rs34043924
rs11025264
rs78167655
rs72673086
rs9599159
rs117164089
rs34559191
rs74639669
rs4980184
rs149439201
rs12259196
rs3128517
rs73782207
rs2237172
rs62092633
rs397081
All
−0.1
0.0
0.1
0.2
MR leave−one−out sensitivity analysis for
'beta−nerve growth factor levels' on 'Rheumatoid arthritis'

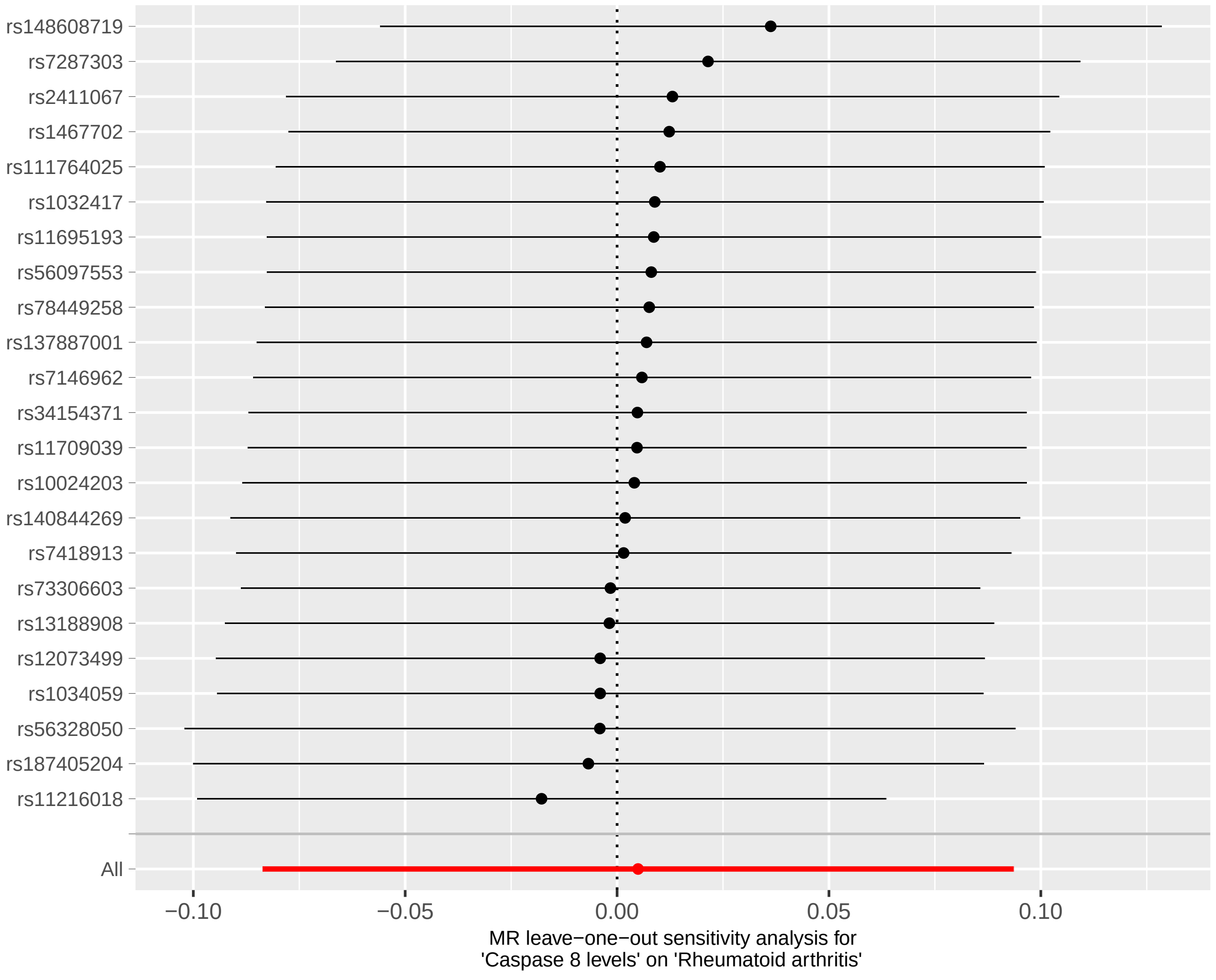
rs148608719
rs7287303
rs2411067
rs1467702
rs111764025
rs1032417
rs11695193
rs56097553
rs78449258
rs137887001
rs7146962
rs34154371
rs11709039
rs10024203
rs140844269
rs7418913
rs73306603
rs13188908
rs12073499
rs1034059
rs56328050
rs187405204
rs11216018
All
−0.10
−0.05
0.00
0.05
0.10
MR leave−one−out sensitivity analysis for
'Caspase 8 levels' on 'Rheumatoid arthritis'

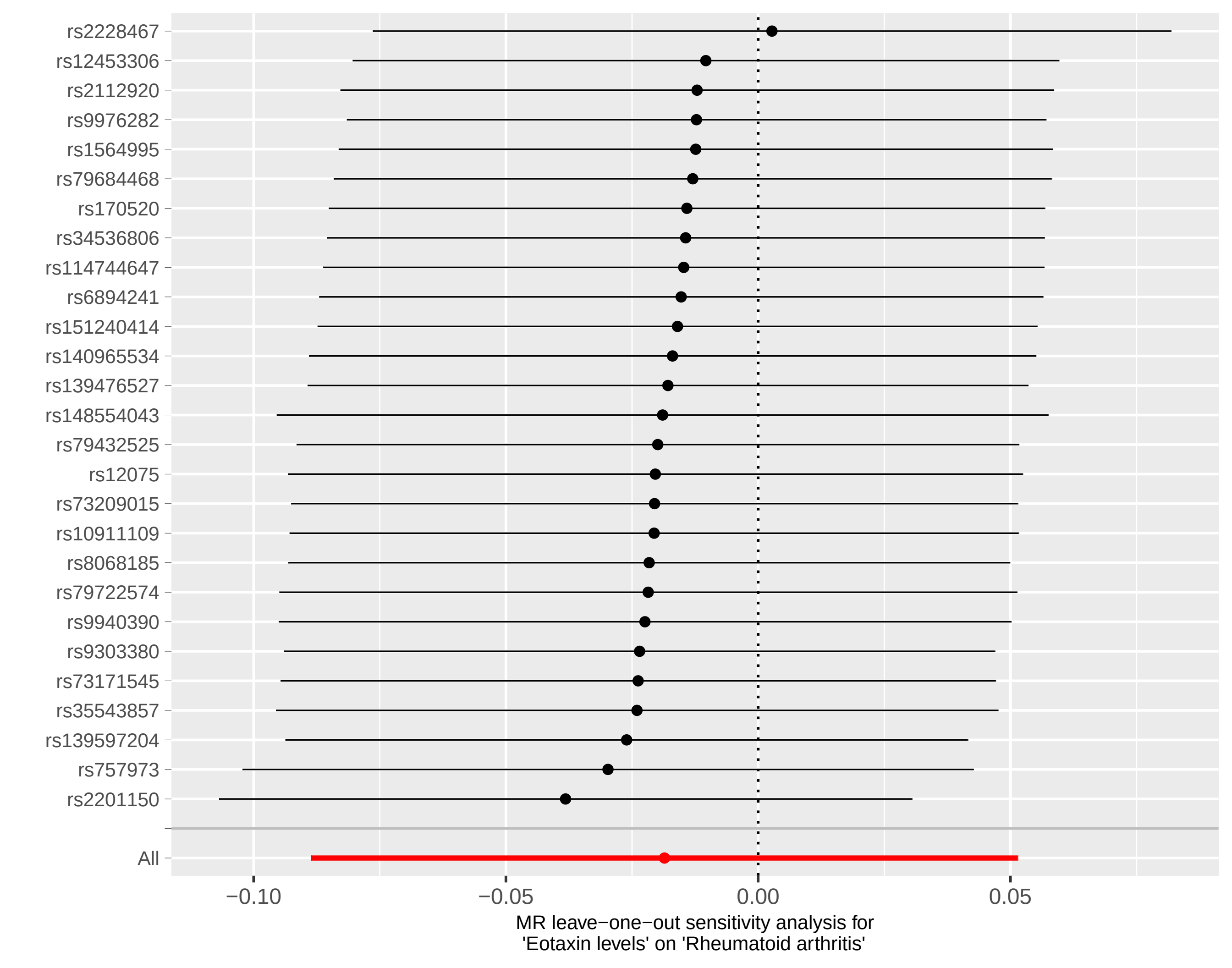
rs2228467
rs12453306
rs2112920
rs9976282
rs1564995
rs79684468
rs170520
rs34536806
rs114744647
rs6894241
rs151240414
rs140965534
rs139476527
rs148554043
rs79432525
rs12075
rs73209015
rs10911109
rs8068185
rs79722574
rs9940390
rs9303380
rs73171545
rs35543857
rs139597204
rs757973
rs2201150
All
−0.10
−0.05
0.00
0.05
MR leave−one−out sensitivity analysis for
'Eotaxin levels' on 'Rheumatoid arthritis'

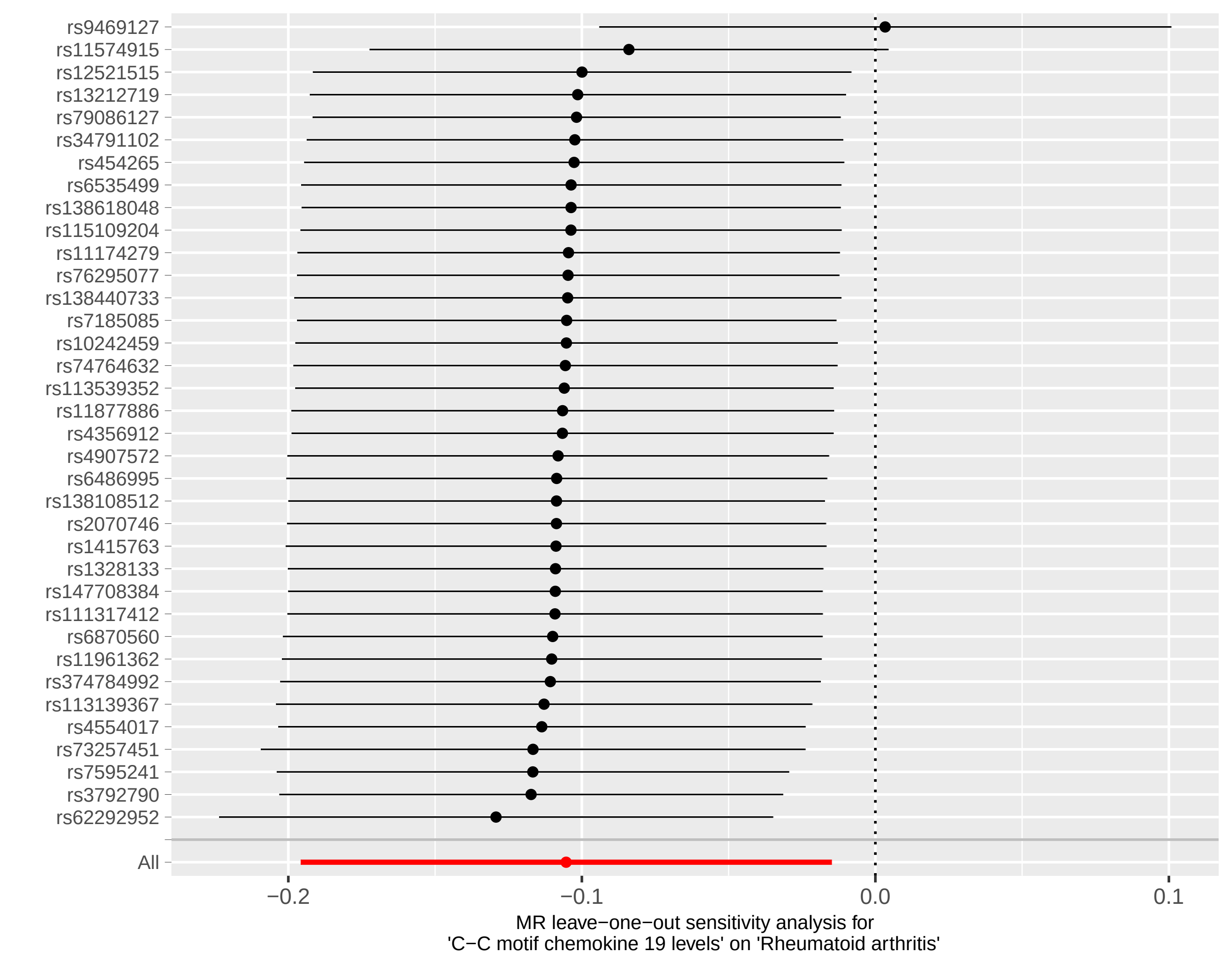

rs9469127
rs11574915
rs12521515
rs13212719
rs79086127
rs34791102
rs454265
rs6535499
rs138618048
rs115109204
rs11174279
rs76295077
rs138440733
rs7185085
rs10242459
rs74764632
rs113539352
rs11877886
rs4356912
rs4907572
rs6486995
rs138108512
rs2070746
rs1415763
rs1328133
rs147708384
rs111317412
rs6870560
rs11961362
rs374784992
rs113139367
rs4554017
rs73257451
rs7595241
rs3792790
rs62292952
All
−0.2
−0.1
0.0
0.1
MR leave−one−out sensitivity analysis for
'C−C motif chemokine 19 levels' on 'Rheumatoid arthritis'

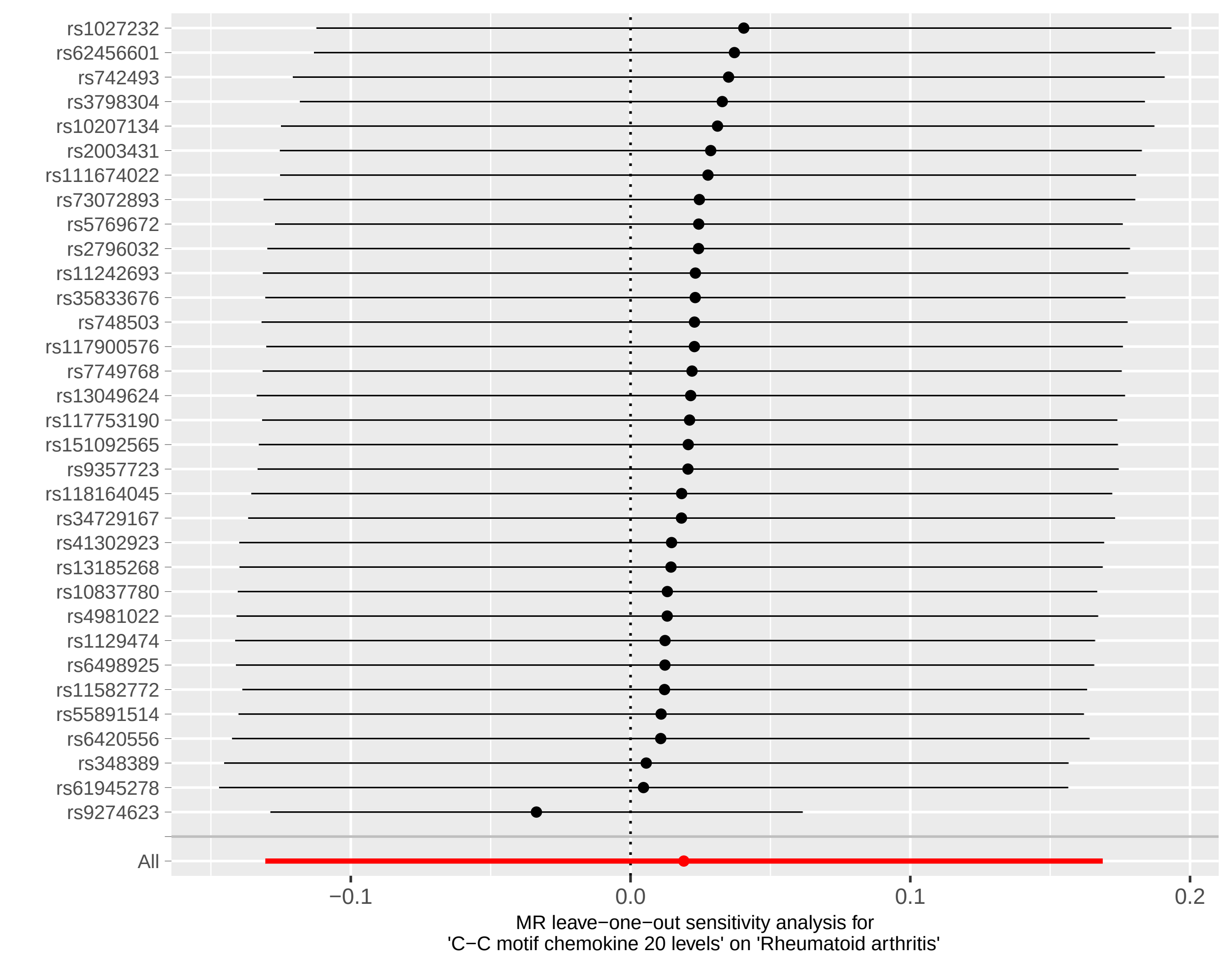
rs1027232
rs62456601
rs742493
rs3798304
rs10207134
rs2003431
rs111674022
rs73072893
rs5769672
rs2796032
rs11242693
rs35833676
rs748503
rs117900576
rs7749768
rs13049624
rs117753190
rs151092565
rs9357723
rs118164045
rs34729167
rs41302923
rs13185268
rs10837780
rs4981022
rs1129474
rs6498925
rs11582772
rs55891514
rs6420556
rs348389
rs61945278
rs9274623
All
−0.1
0.0
0.1
0.2
MR leave−one−out sensitivity analysis for
'C−C motif chemokine 20 levels' on 'Rheumatoid arthritis'

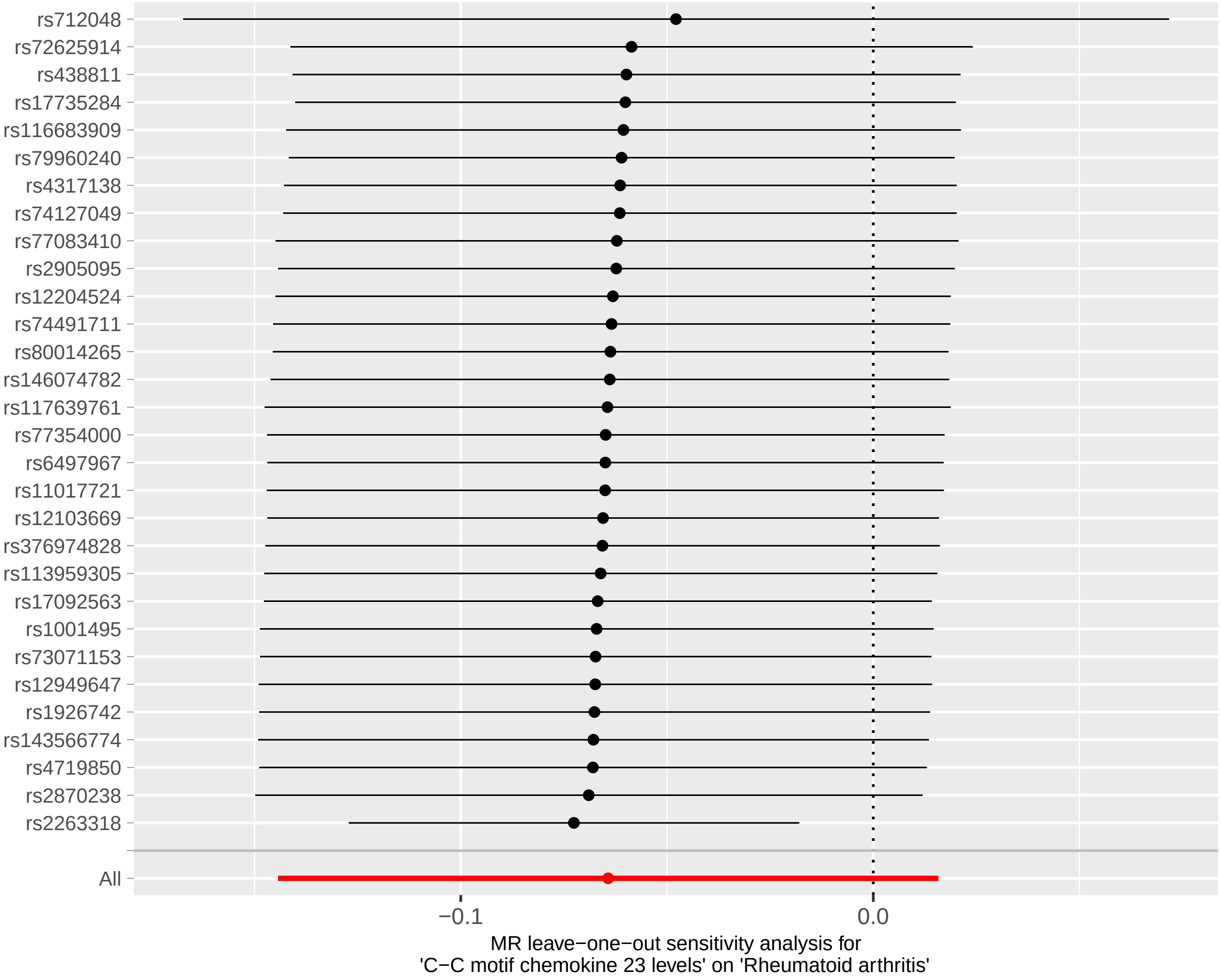
rs712048
rs72625914
rs438811
rs17735284
rs116683909
rs79960240
rs4317138
rs74127049
rs77083410
rs2905095
rs12204524
rs74491711
rs80014265
rs146074782
rs117639761
rs77354000
rs6497967
rs11017721
rs12103669
rs376974828
rs113959305
rs17092563
rs1001495
rs73071153
rs12949647
rs1926742
rs143566774
rs4719850
rs2870238
rs2263318
All
−0.1
0.0
MR leave−one−out sensitivity analysis for
'C−C motif chemokine 23 levels' on 'Rheumatoid arthritis'

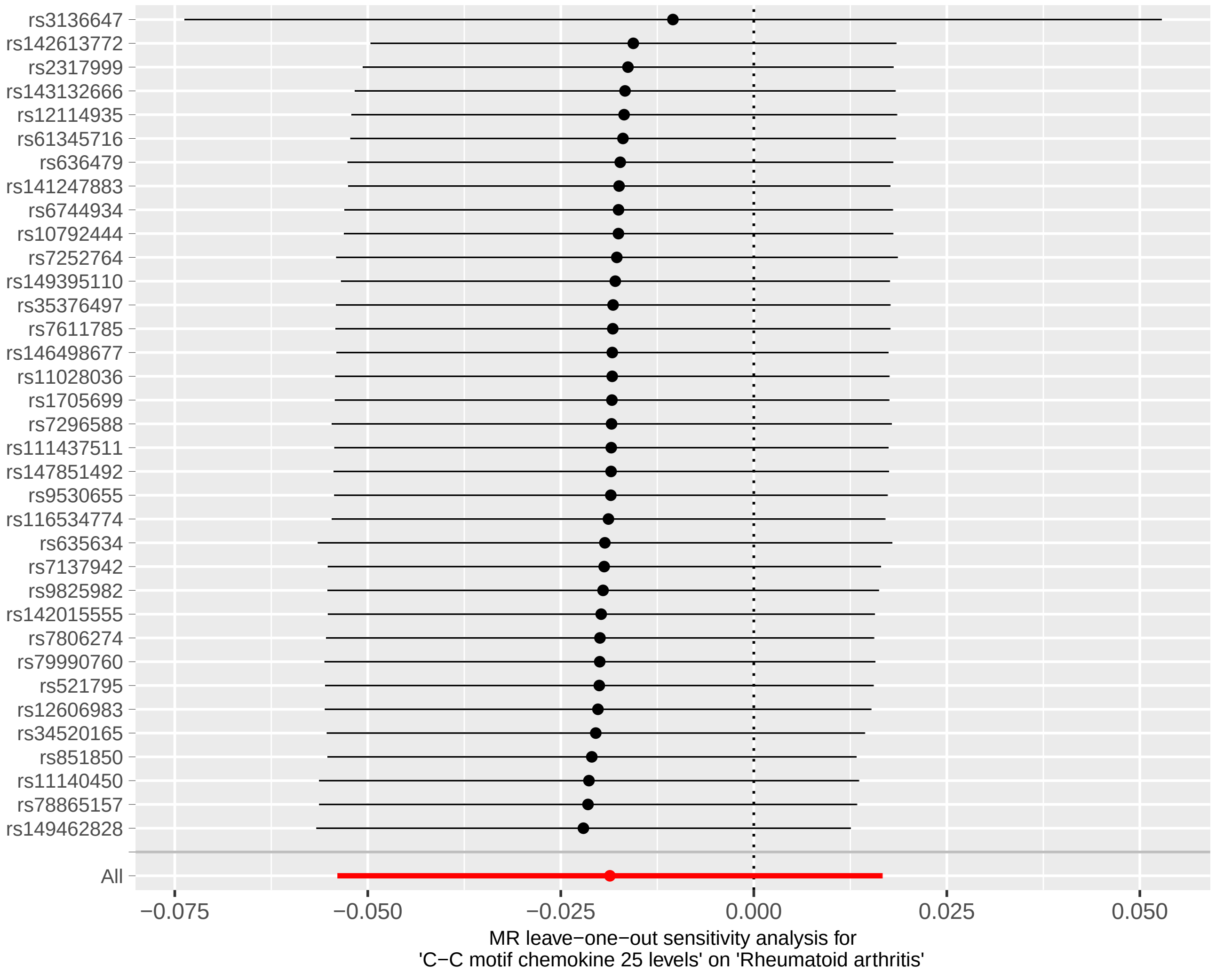
rs3136647
rs142613772
rs2317999
rs143132666
rs12114935
rs61345716
rs636479
rs141247883
rs6744934
rs10792444
rs7252764
rs149395110
rs35376497
rs7611785
rs146498677
rs11028036
rs1705699
rs7296588
rs111437511
rs147851492
rs9530655
rs116534774
rs635634
rs7137942
rs9825982
rs142015555
rs7806274
rs79990760
rs521795
rs12606983
rs34520165
rs851850
rs11140450
rs78865157
rs149462828
All
−0.075
−0.050
−0.025
0.000
0.025
0.050
MR leave−one−out sensitivity analysis for
'C−C motif chemokine 25 levels' on 'Rheumatoid arthritis'

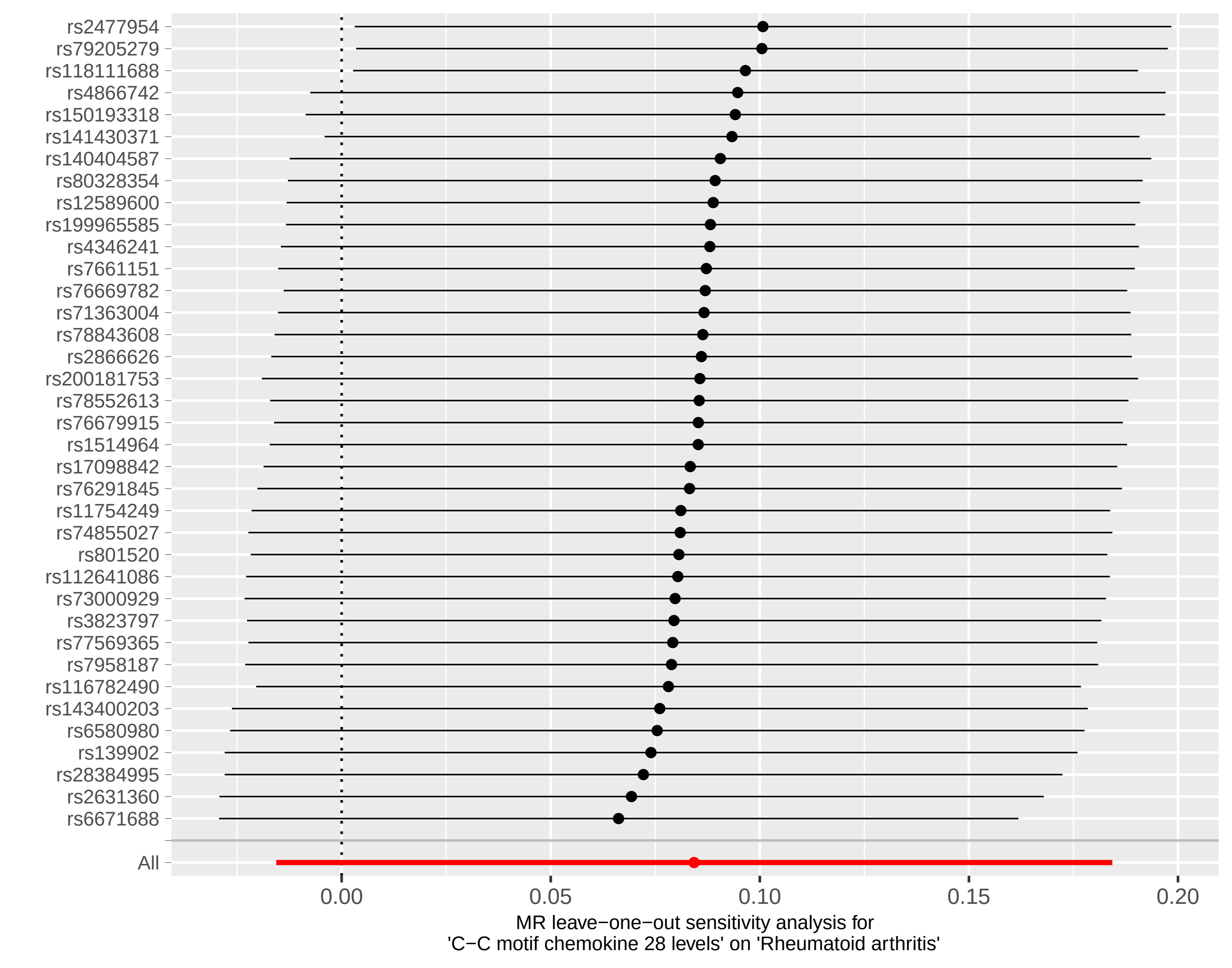
rs2477954
rs79205279
rs118111688
rs4866742
rs150193318
rs141430371
rs140404587
rs80328354
rs12589600
rs199965585
rs4346241
rs7661151
rs76669782
rs71363004
rs78843608
rs2866626
rs200181753
rs78552613
rs76679915
rs1514964
rs17098842
rs76291845
rs11754249
rs74855027
rs801520
rs112641086
rs73000929
rs3823797
rs77569365
rs7958187
rs116782490
rs143400203
rs6580980
rs139902
rs28384995
rs2631360
rs6671688
All
0.00
0.05
0.10
0.15
0.20
MR leave−one−out sensitivity analysis for
'C−C motif chemokine 28 levels' on 'Rheumatoid arthritis'

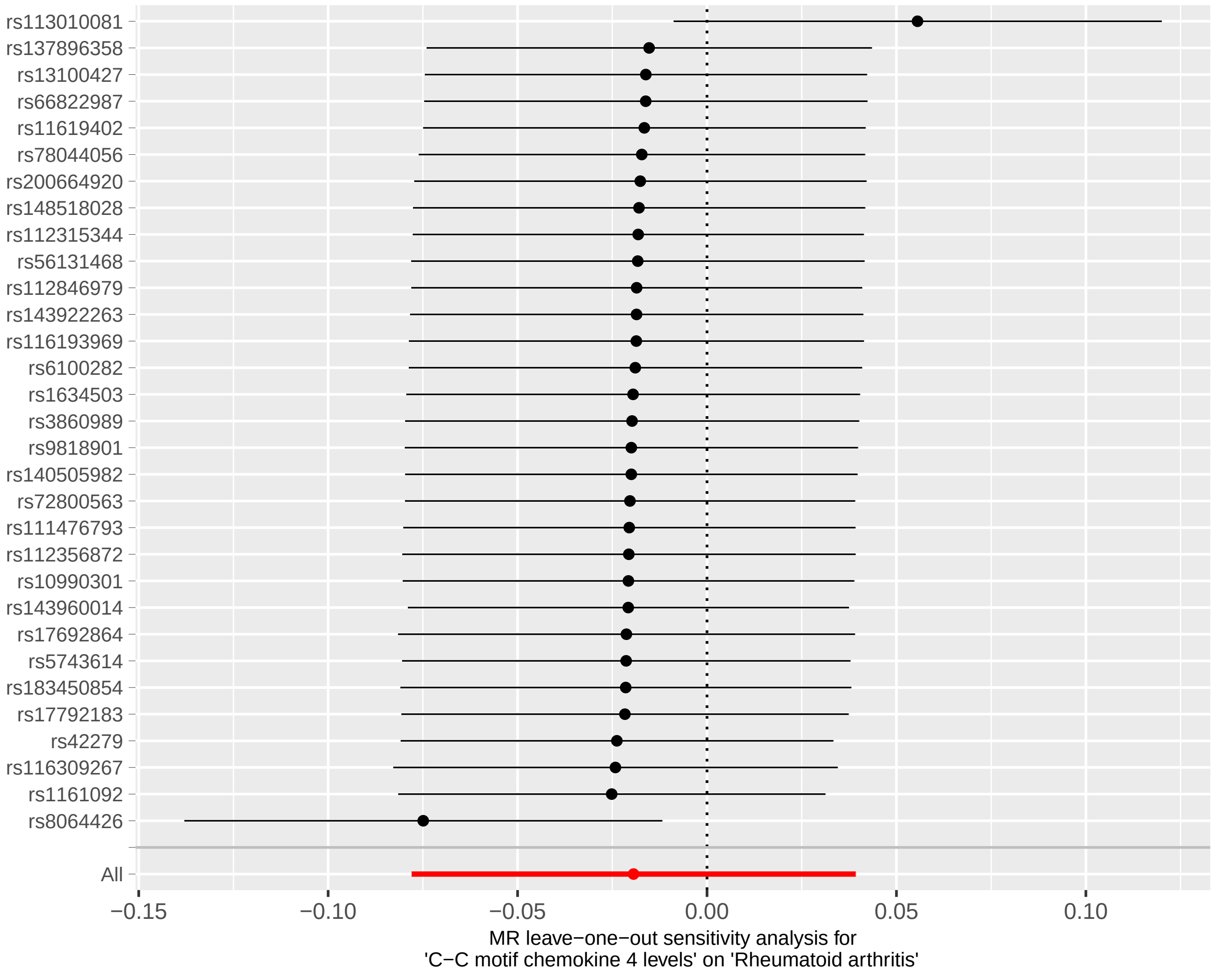
rs113010081
rs137896358
rs13100427
rs66822987
rs11619402
rs78044056
rs200664920
rs148518028
rs112315344
rs56131468
rs112846979
rs143922263
rs116193969
rs6100282
rs1634503
rs3860989
rs9818901
rs140505982
rs72800563
rs111476793
rs112356872
rs10990301
rs143960014
rs17692864
rs5743614
rs183450854
rs17792183
rs42279
rs116309267
rs1161092
rs8064426
All
−0.15
−0.10
−0.05
0.00
0.05
0.10
MR leave−one−out sensitivity analysis for
'C−C motif chemokine 4 levels' on 'Rheumatoid arthritis'

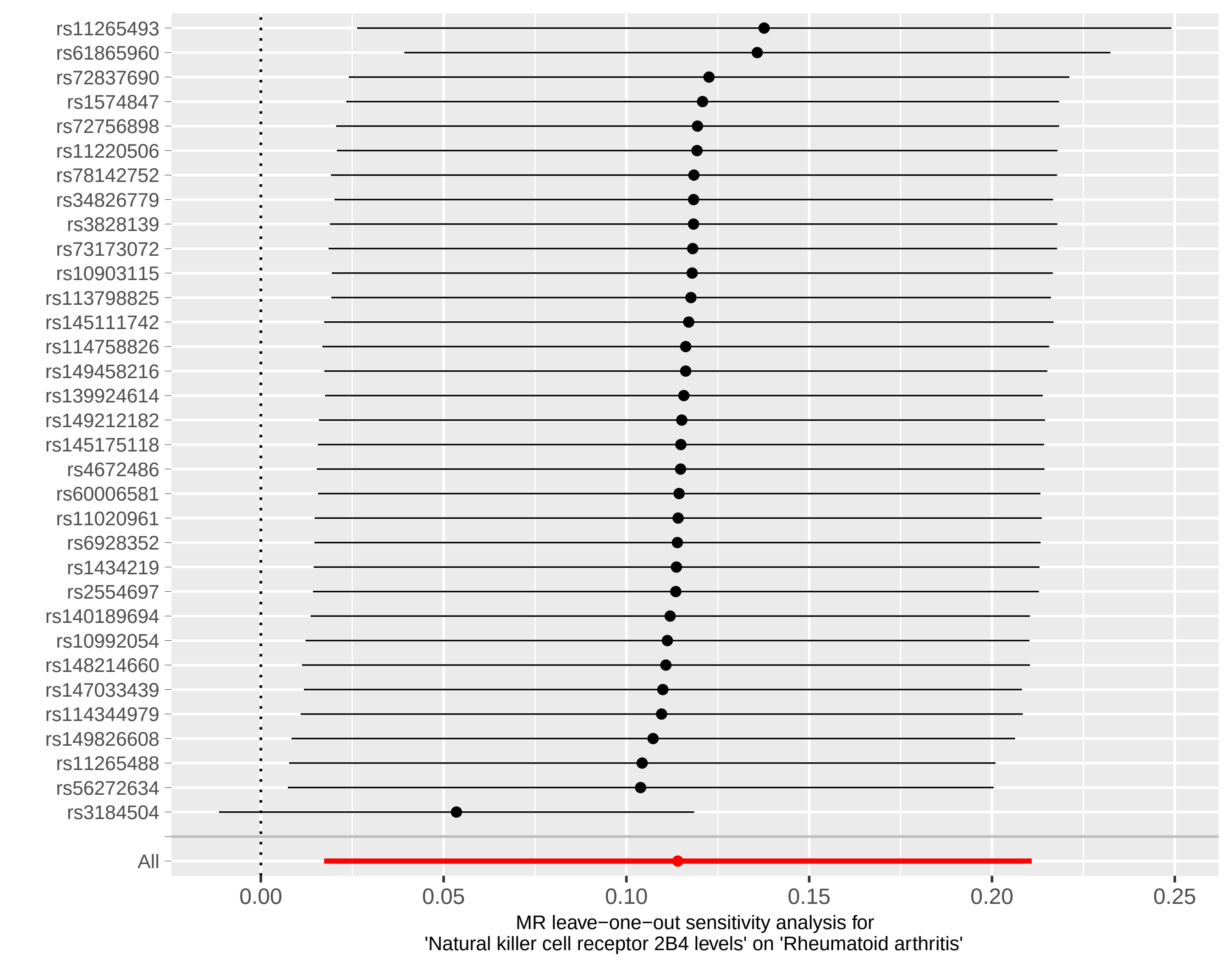
rs11265493
rs61865960
rs72837690
rs1574847
rs72756898
rs11220506
rs78142752
rs34826779
rs3828139
rs73173072
rs10903115
rs113798825
rs145111742
rs114758826
rs149458216
rs139924614
rs149212182
rs145175118
rs4672486
rs60006581
rs11020961
rs6928352
rs1434219
rs2554697
rs140189694
rs10992054
rs148214660
rs147033439
rs114344979
rs149826608
rs11265488
rs56272634
rs3184504
All
0.00
0.05
0.10
0.15
0.20
0.25
MR leave−one−out sensitivity analysis for
'Natural killer cell receptor 2B4 levels' on 'Rheumatoid arthritis'

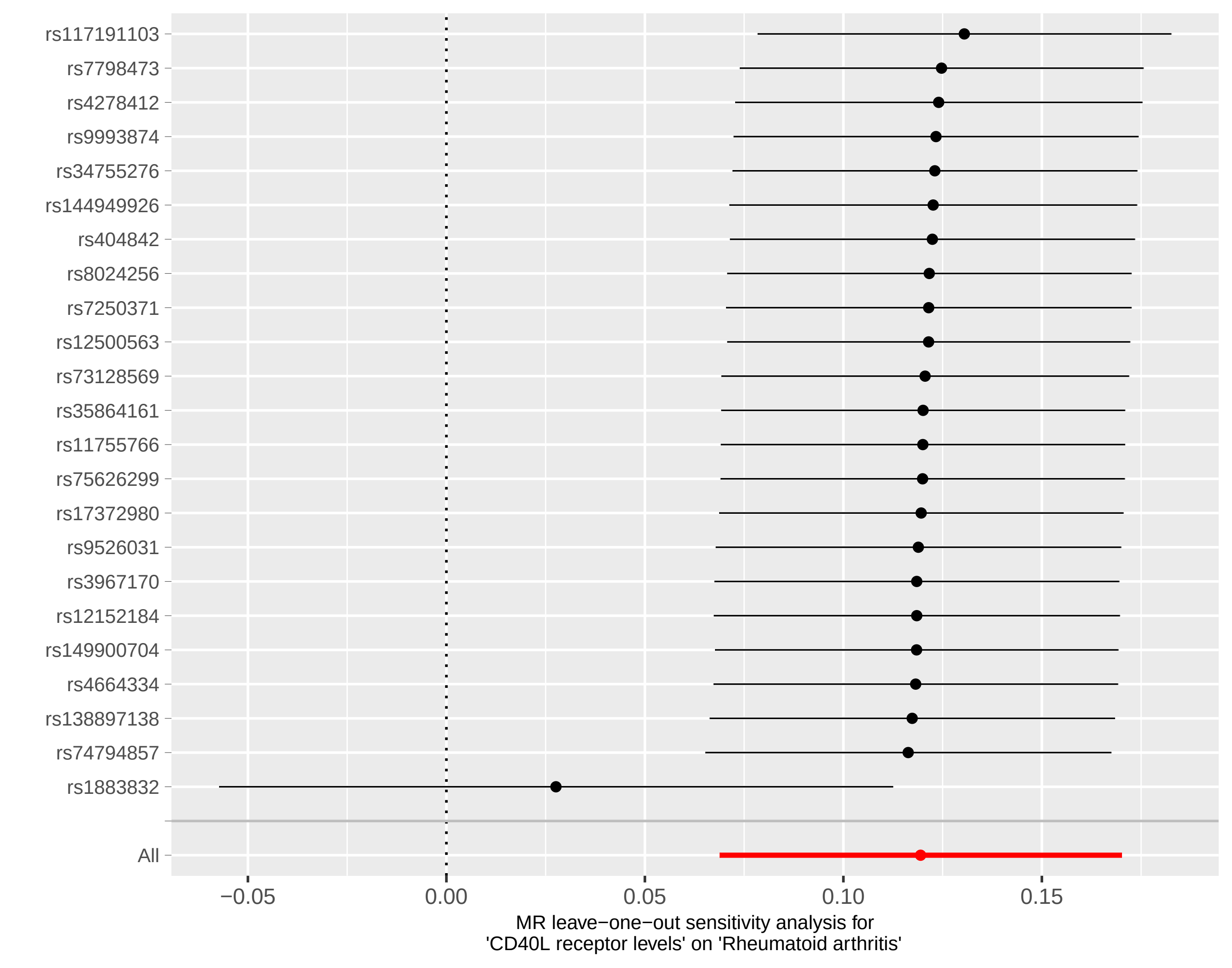

rs117191103
rs7798473
rs4278412
rs9993874
rs34755276
rs144949926
rs404842
rs8024256
rs7250371
rs12500563
rs73128569
rs35864161
rs11755766
rs75626299
rs17372980
rs9526031
rs3967170
rs12152184
rs149900704
rs4664334
rs138897138
rs74794857
rs1883832
All
−0.05
0.00
0.05
0.10
0.15
MR leave−one−out sensitivity analysis for
'CD40L receptor levels' on 'Rheumatoid arthritis'

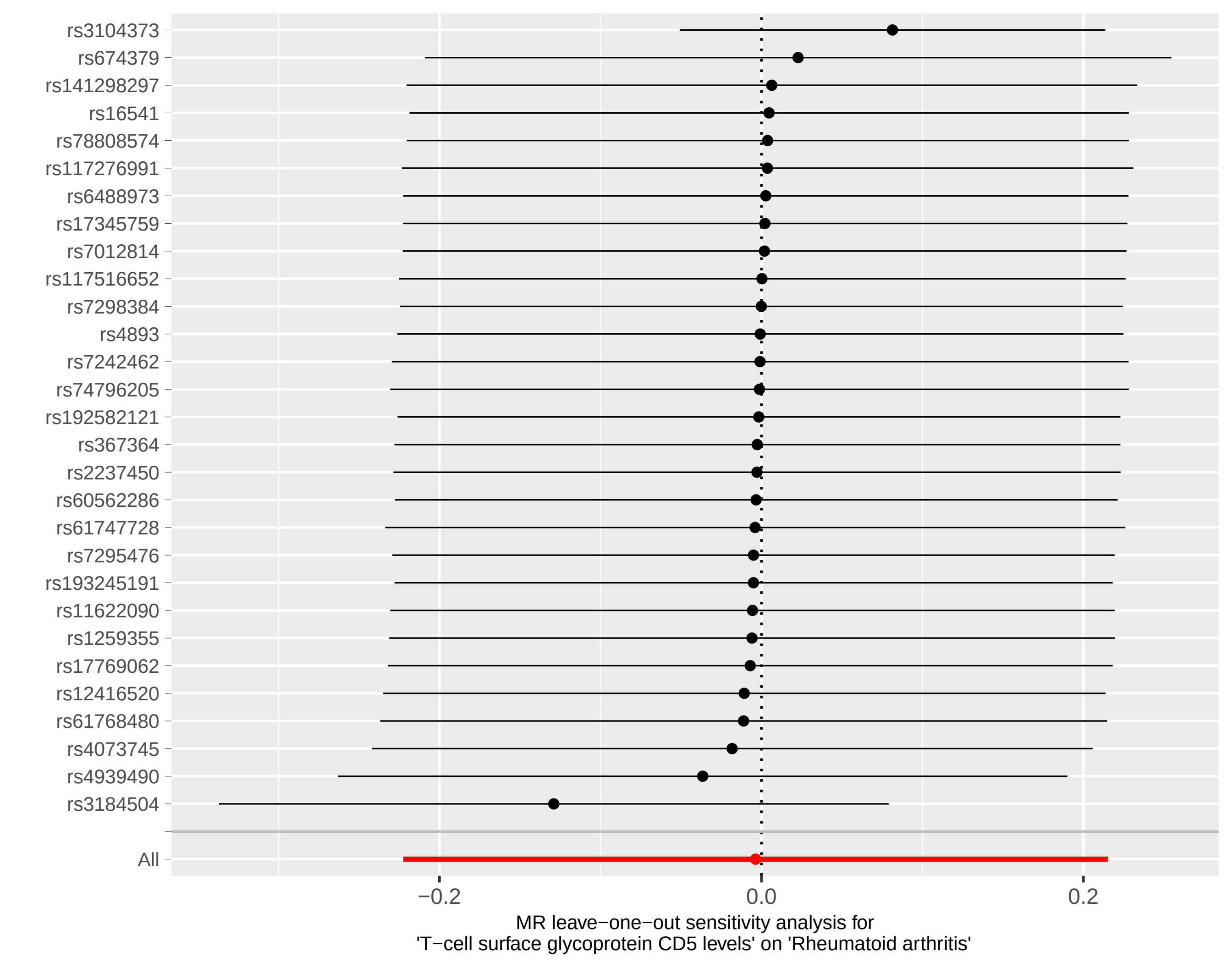
rs3104373
rs674379
rs141298297
rs16541
rs78808574
rs117276991
rs6488973
rs17345759
rs7012814
rs117516652
rs7298384
rs4893
rs7242462
rs74796205
rs192582121
rs367364
rs2237450
rs60562286
rs61747728
rs7295476
rs193245191
rs11622090
rs1259355
rs17769062
rs12416520
rs61768480
rs4073745
rs4939490
rs3184504
All
−0.2
0.0
0.2
MR leave−one−out sensitivity analysis for
'T−cell surface glycoprotein CD5 levels' on 'Rheumatoid arthritis'

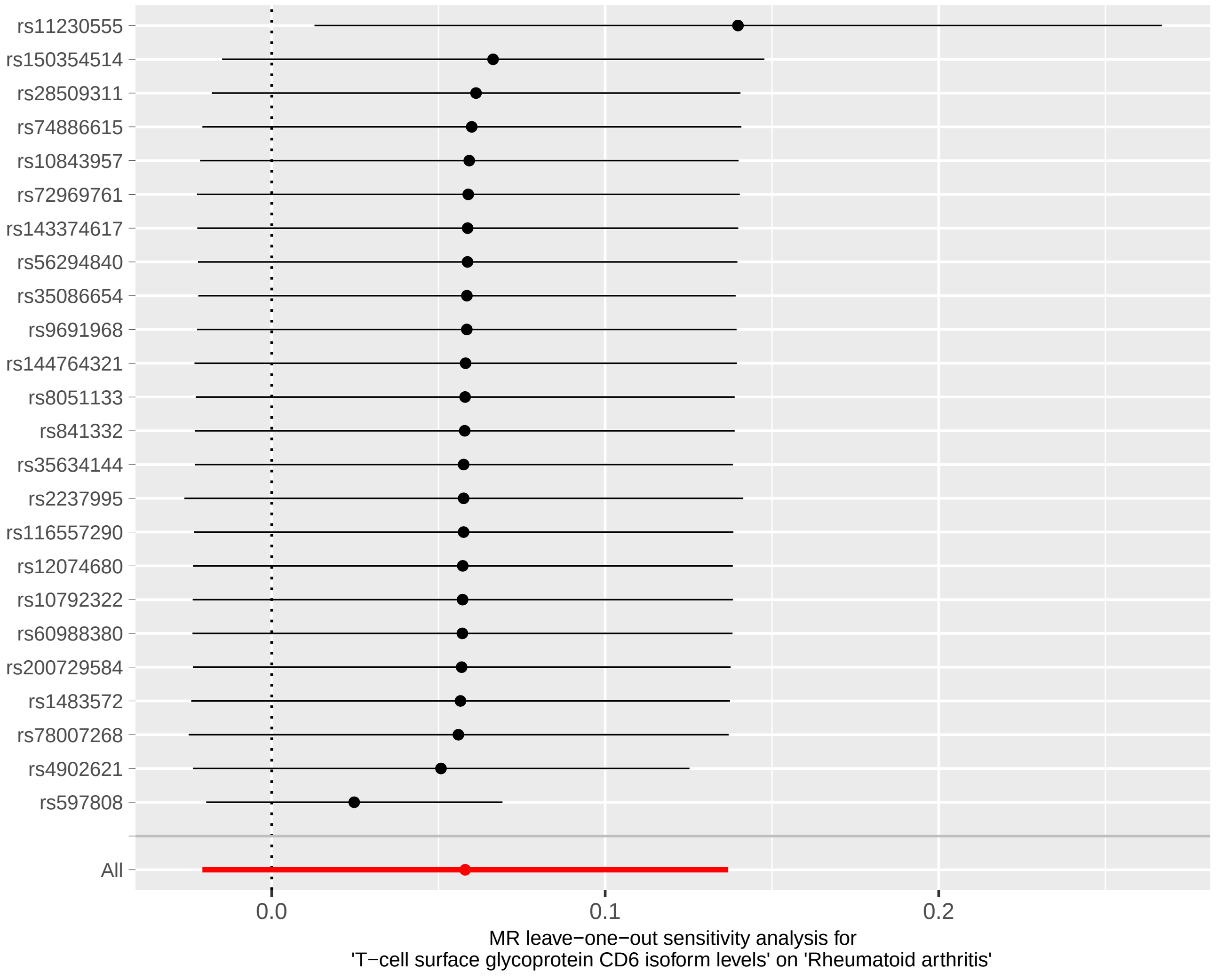
rs11230555
rs150354514
rs28509311
rs74886615
rs10843957
rs72969761
rs143374617
rs56294840
rs35086654
rs9691968
rs144764321
rs8051133
rs841332
rs35634144
rs2237995
rs116557290
rs12074680
rs10792322
rs60988380
rs200729584
rs1483572
rs78007268
rs4902621
rs597808
All
0.0
0.1
0.2
MR leave−one−out sensitivity analysis for
'T−cell surface glycoprotein CD6 isoform levels' on 'Rheumatoid arthritis'

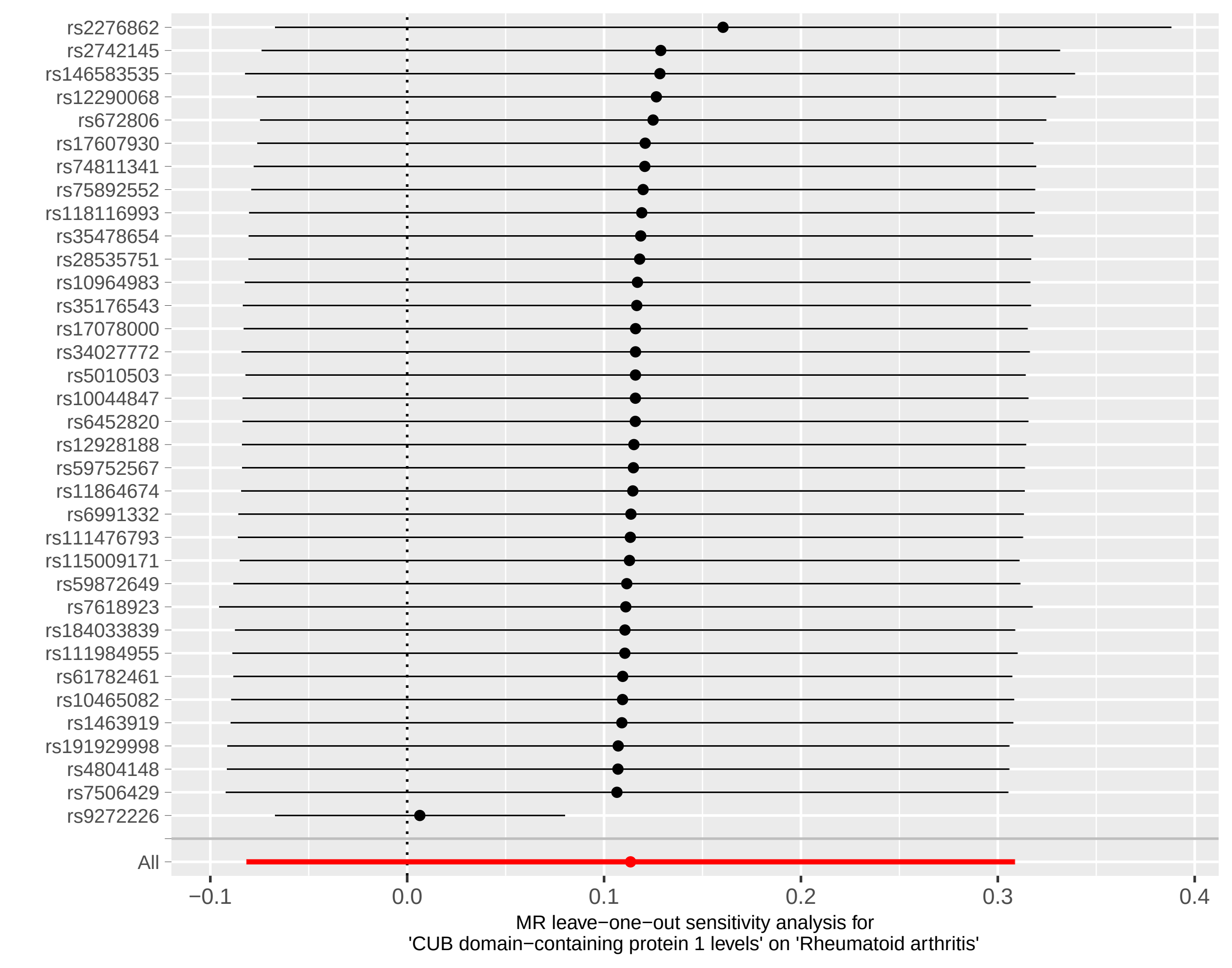
rs2276862
rs2742145
rs146583535
rs12290068
rs672806
rs17607930
rs74811341
rs75892552
rs118116993
rs35478654
rs28535751
rs10964983
rs35176543
rs17078000
rs34027772
rs5010503
rs10044847
rs6452820
rs12928188
rs59752567
rs11864674
rs6991332
rs111476793
rs115009171
rs59872649
rs7618923
rs184033839
rs111984955
rs61782461
rs10465082
rs1463919
rs191929998
rs4804148
rs7506429
rs9272226
All
−0.1
0.0
0.1
0.2
0.3
0.4
MR leave−one−out sensitivity analysis for
'CUB domain−containing protein 1 levels' on 'Rheumatoid arthritis'

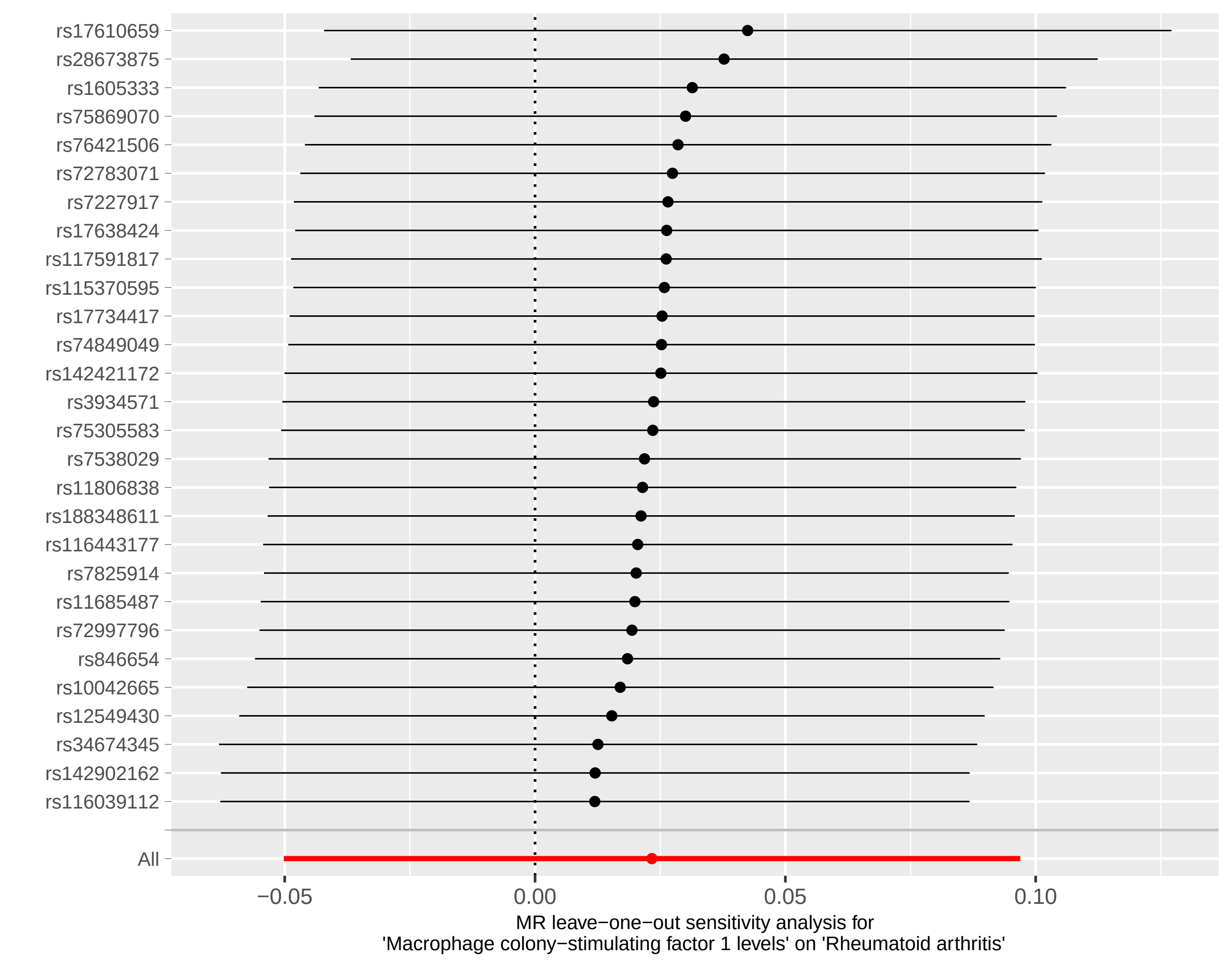

rs17610659
rs28673875
rs1605333
rs75869070
rs76421506
rs72783071
rs7227917
rs17638424
rs117591817
rs115370595
rs17734417
rs74849049
rs142421172
rs3934571
rs75305583
rs7538029
rs11806838
rs188348611
rs116443177
rs7825914
rs11685487
rs72997796
rs846654
rs10042665
rs12549430
rs34674345
rs142902162
rs116039112
All
−0.05
0.00
0.05
0.10
MR leave−one−out sensitivity analysis for
'Macrophage colony−stimulating factor 1 levels' on 'Rheumatoid arthritis'

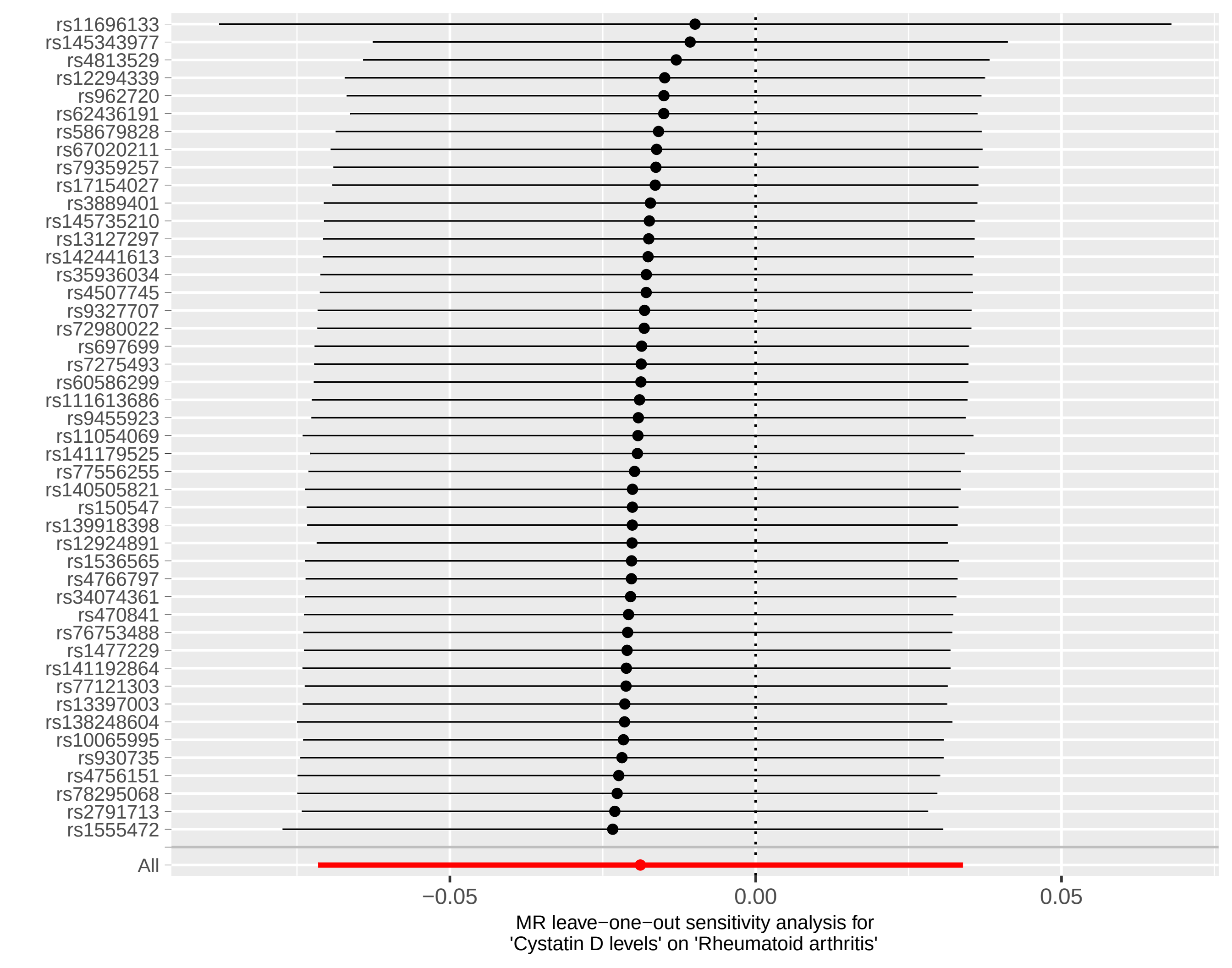
rs11696133
rs145343977
rs4813529
rs12294339
rs962720
rs62436191
rs58679828
rs67020211
rs79359257
rs17154027
rs3889401
rs145735210
rs13127297
rs142441613
rs35936034
rs4507745
rs9327707
rs72980022
rs697699
rs7275493
rs60586299
rs111613686
rs9455923
rs11054069
rs141179525
rs77556255
rs140505821
rs150547
rs139918398
rs12924891
rs1536565
rs4766797
rs34074361
rs470841
rs76753488
rs1477229
rs141192864
rs77121303
rs13397003
rs138248604
rs10065995
rs930735
rs4756151
rs78295068
rs2791713
rs1555472
All
−0.05
0.00
0.05
MR leave−one−out sensitivity analysis for
'Cystatin D levels' on 'Rheumatoid arthritis'

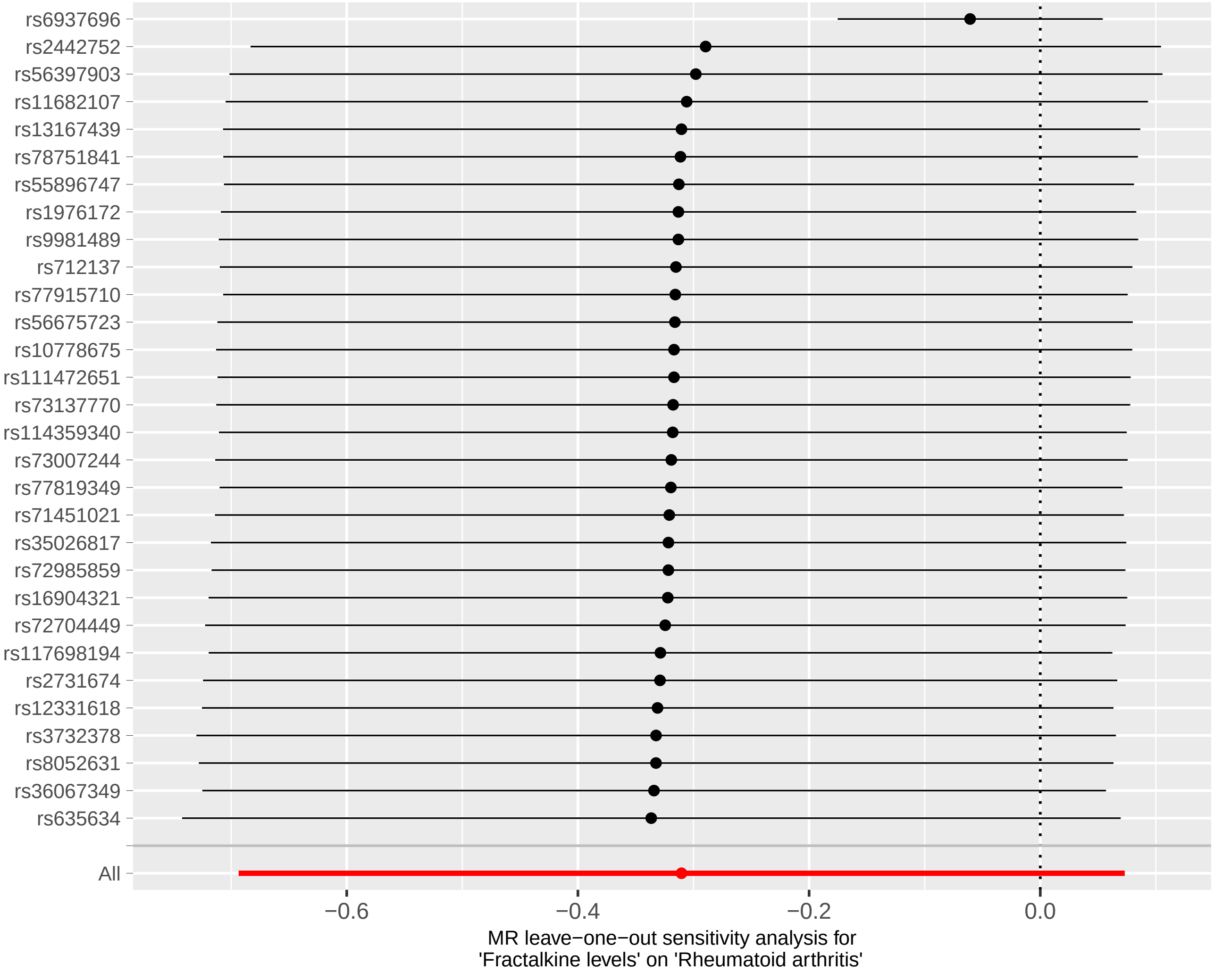
rs6937696
rs2442752
rs56397903
rs11682107
rs13167439
rs78751841
rs55896747
rs1976172
rs9981489
rs712137
rs77915710
rs56675723
rs10778675
rs111472651
rs73137770
rs114359340
rs73007244
rs77819349
rs71451021
rs35026817
rs72985859
rs16904321
rs72704449
rs117698194
rs2731674
rs12331618
rs3732378
rs8052631
rs36067349
rs635634
All
−0.6
−0.4
−0.2
0.0
MR leave−one−out sensitivity analysis for
'Fractalkine levels' on 'Rheumatoid arthritis'

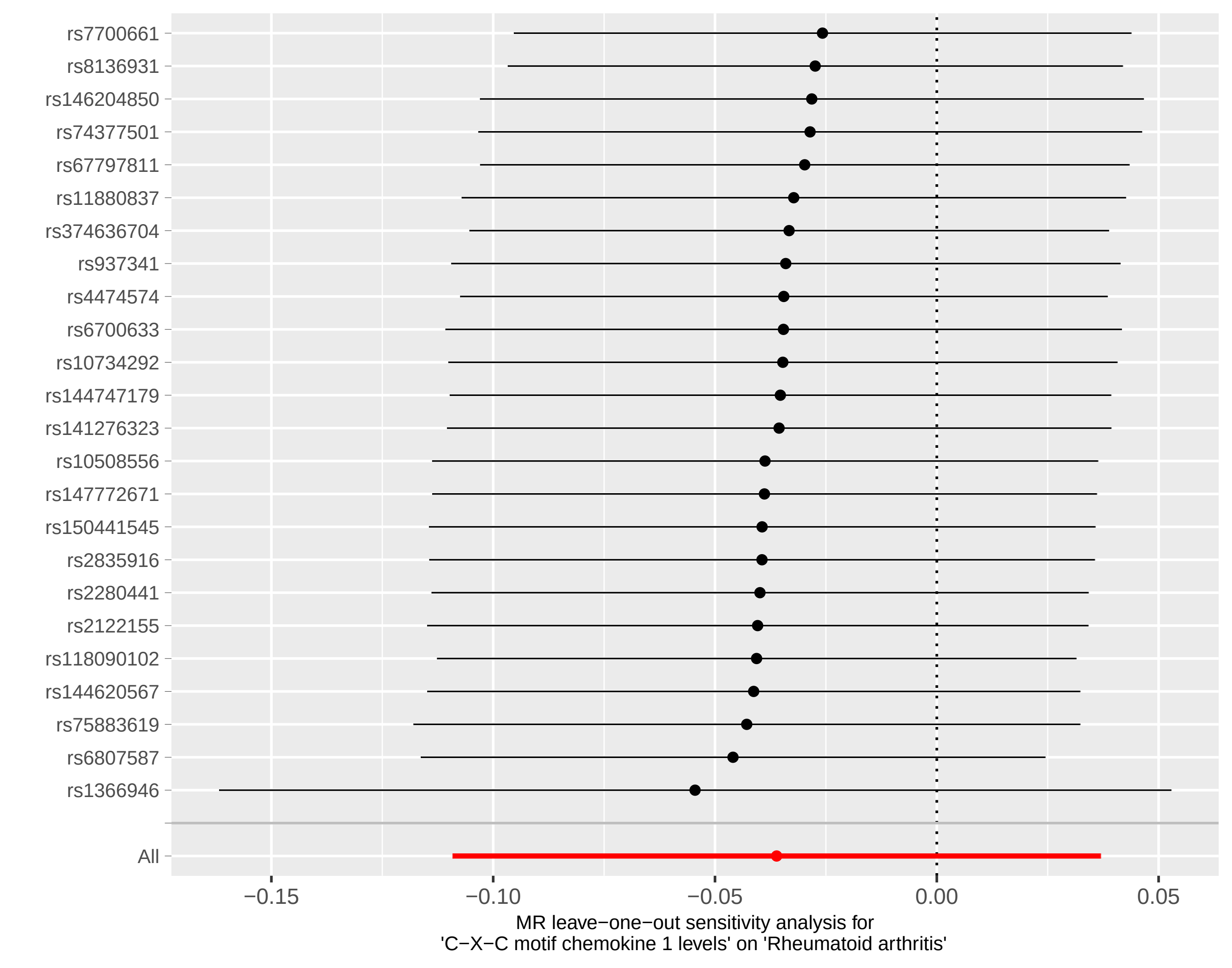
rs7700661
rs8136931
rs146204850
rs74377501
rs67797811
rs11880837
rs374636704
rs937341
rs4474574
rs6700633
rs10734292
rs144747179
rs141276323
rs10508556
rs147772671
rs150441545
rs2835916
rs2280441
rs2122155
rs118090102
rs144620567
rs75883619
rs6807587
rs1366946
All
−0.15
−0.10
−0.05
0.00
0.05
MR leave−one−out sensitivity analysis for
'C−X−C motif chemokine 1 levels' on 'Rheumatoid arthritis'

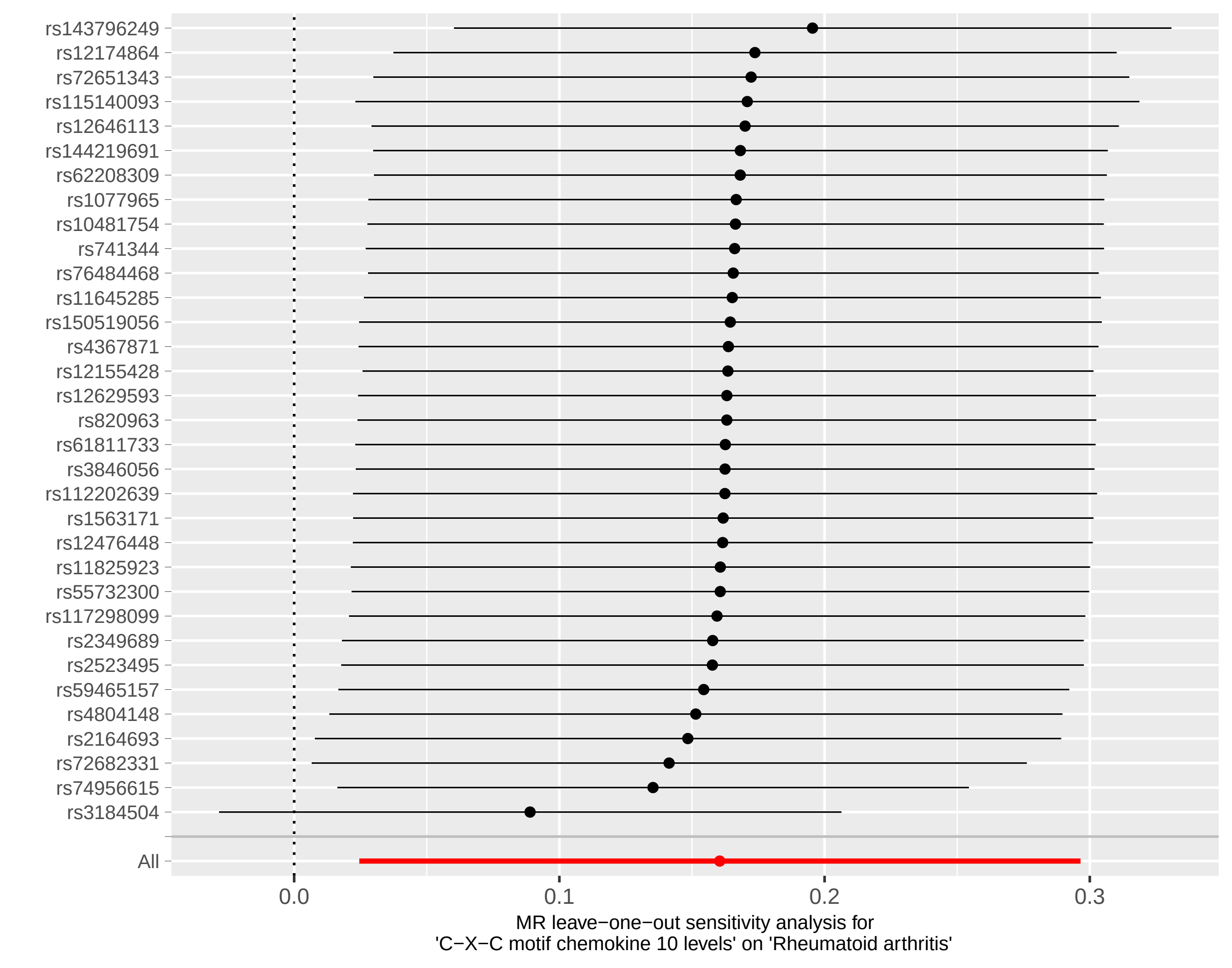
rs143796249
rs12174864
rs72651343
rs115140093
rs12646113
rs144219691
rs62208309
rs1077965
rs10481754
rs741344
rs76484468
rs11645285
rs150519056
rs4367871
rs12155428
rs12629593
rs820963
rs61811733
rs3846056
rs112202639
rs1563171
rs12476448
rs11825923
rs55732300
rs117298099
rs2349689
rs2523495
rs59465157
rs4804148
rs2164693
rs72682331
rs74956615
rs3184504
All
0.0
0.1
0.2
0.3
MR leave−one−out sensitivity analysis for
'C−X−C motif chemokine 10 levels' on 'Rheumatoid arthritis'

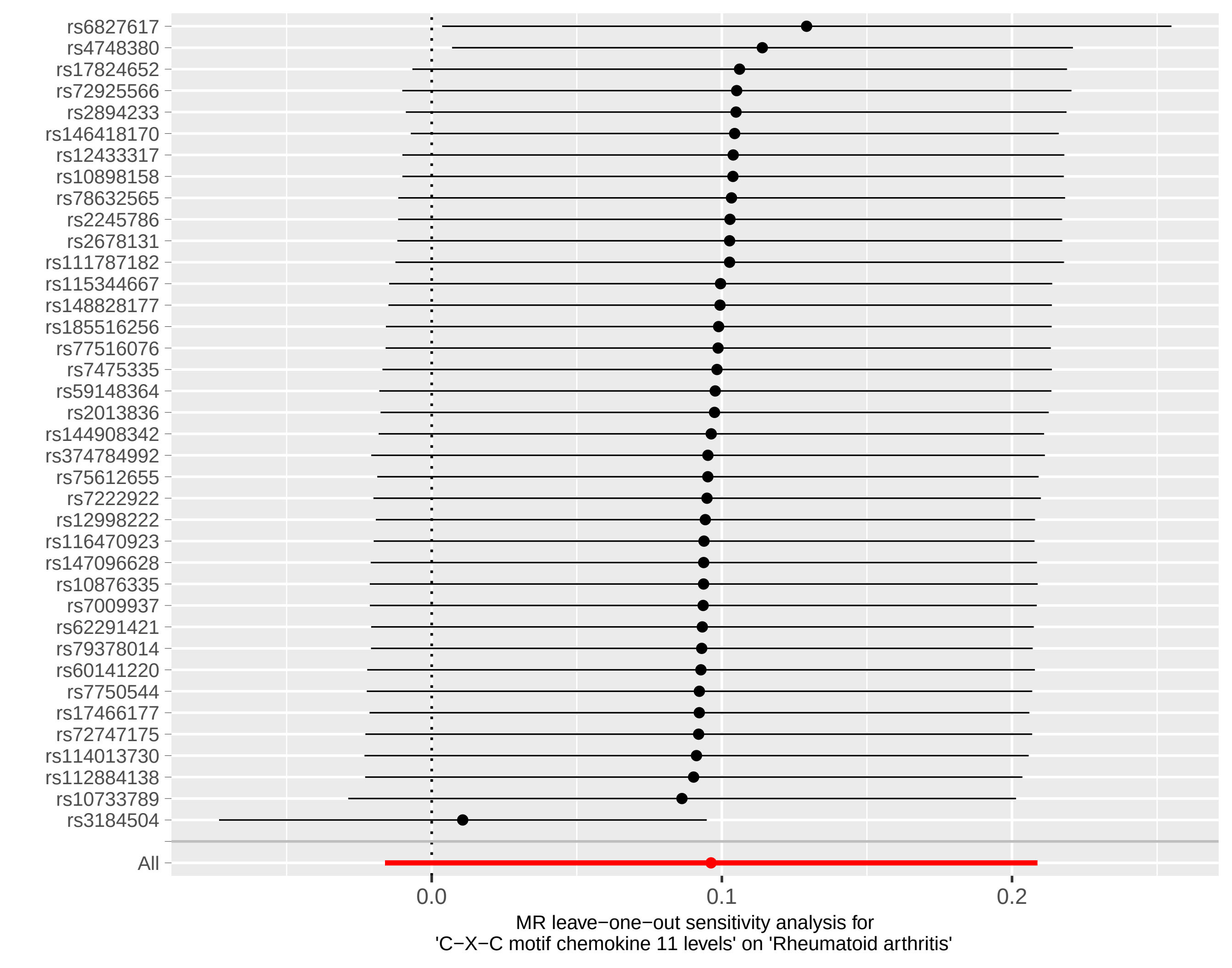

rs6827617
rs4748380
rs17824652
rs72925566
rs2894233
rs146418170
rs12433317
rs10898158
rs78632565
rs2245786
rs2678131
rs111787182
rs115344667
rs148828177
rs185516256
rs77516076
rs7475335
rs59148364
rs2013836
rs144908342
rs374784992
rs75612655
rs7222922
rs12998222
rs116470923
rs147096628
rs10876335
rs7009937
rs62291421
rs79378014
rs60141220
rs7750544
rs17466177
rs72747175
rs114013730
rs112884138
rs10733789
rs3184504
All
0.0
0.1
0.2
MR leave−one−out sensitivity analysis for
'C−X−C motif chemokine 11 levels' on 'Rheumatoid arthritis'

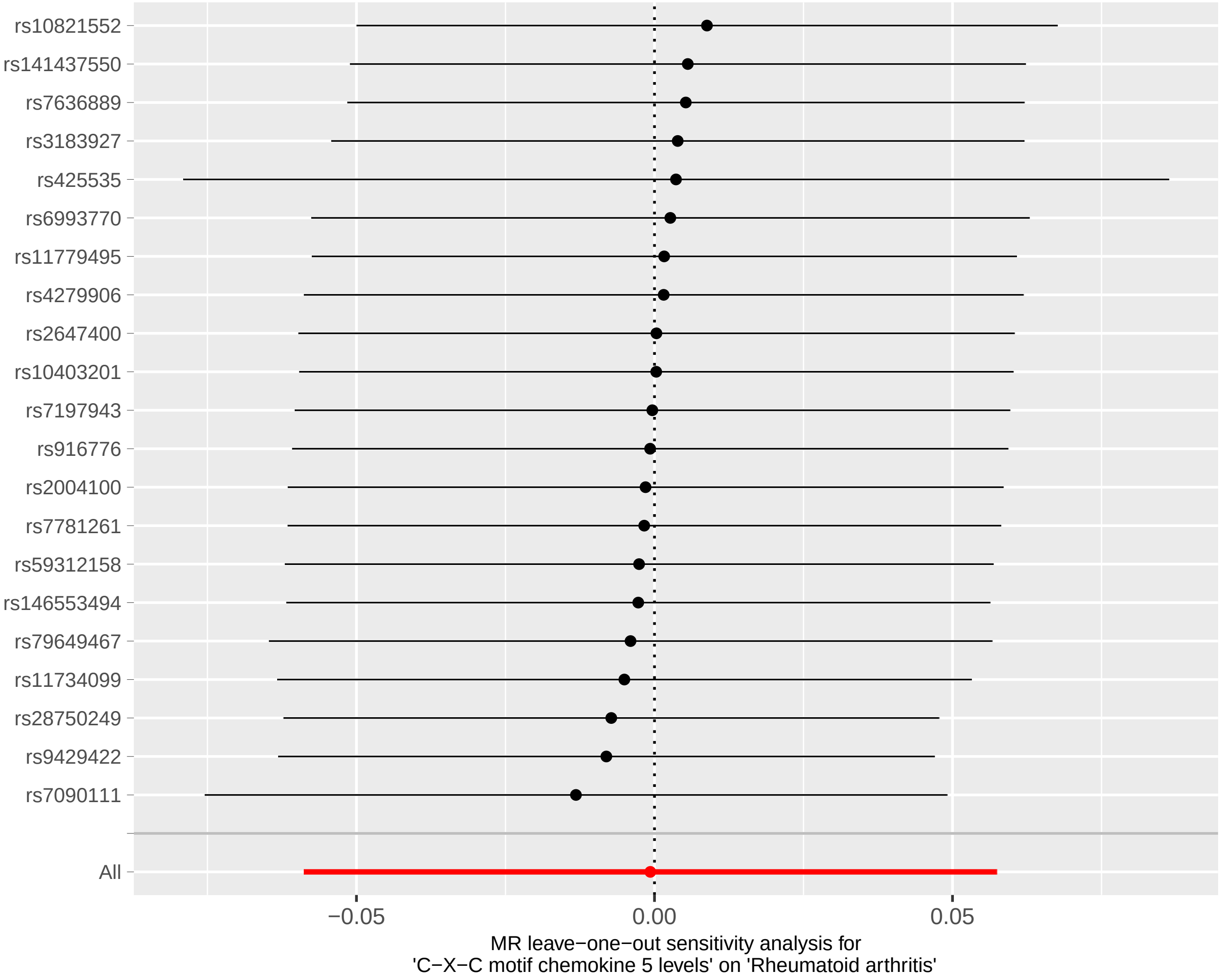

rs10821552
rs141437550
rs7636889
rs3183927
rs425535
rs6993770
rs11779495
rs4279906
rs2647400
rs10403201
rs7197943
rs916776
rs2004100
rs7781261
rs59312158
rs146553494
rs79649467
rs11734099
rs28750249
rs9429422
rs7090111
All
−0.05
0.00
0.05
MR leave−one−out sensitivity analysis for
'C−X−C motif chemokine 5 levels' on 'Rheumatoid arthritis'

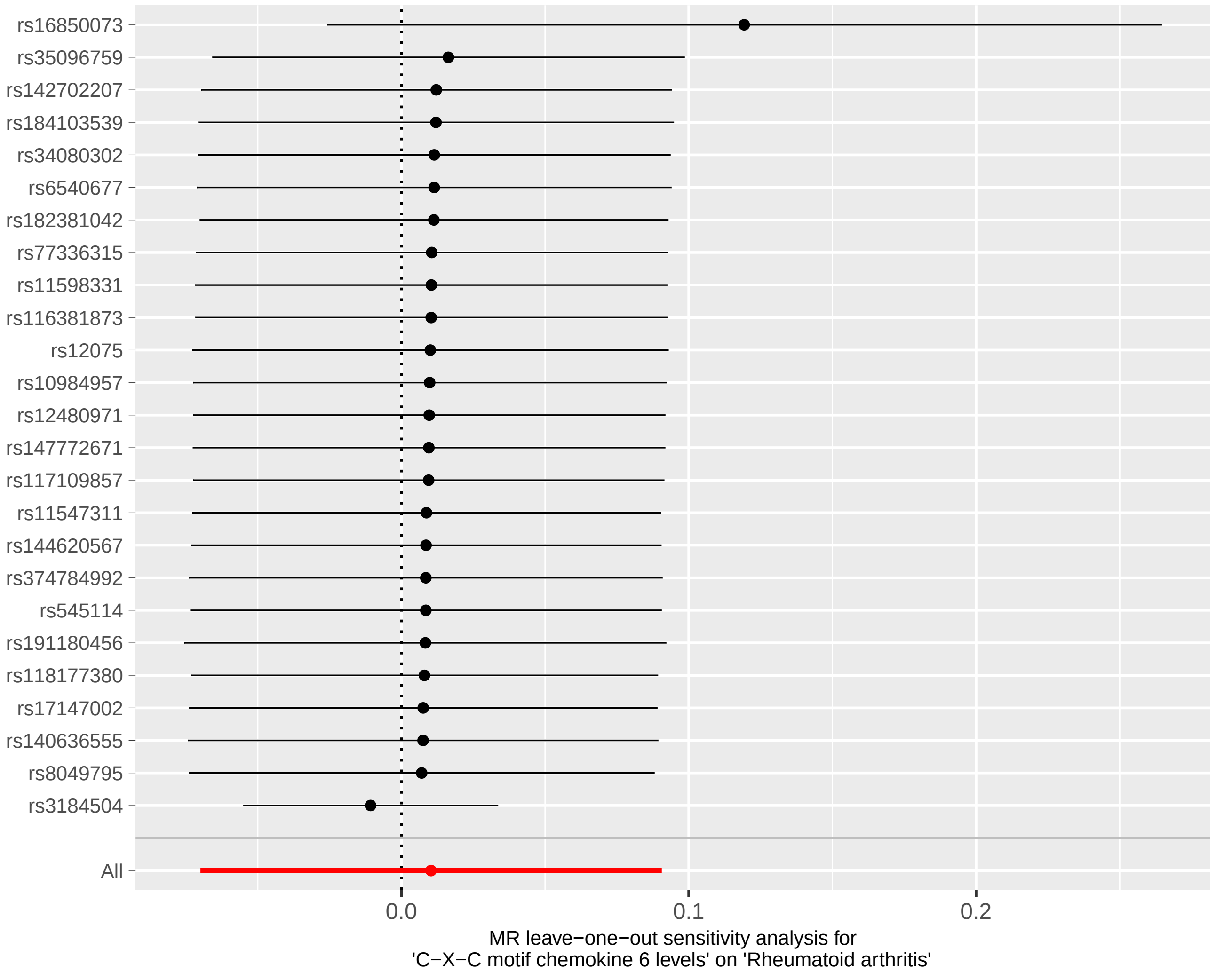
rs16850073
rs35096759
rs142702207
rs184103539
rs34080302
rs6540677
rs182381042
rs77336315
rs11598331
rs116381873
rs12075
rs10984957
rs12480971
rs147772671
rs117109857
rs11547311
rs144620567
rs374784992
rs545114
rs191180456
rs118177380
rs17147002
rs140636555
rs8049795
rs3184504
All
0.0
0.1
0.2
MR leave−one−out sensitivity analysis for
'C−X−C motif chemokine 6 levels' on 'Rheumatoid arthritis'

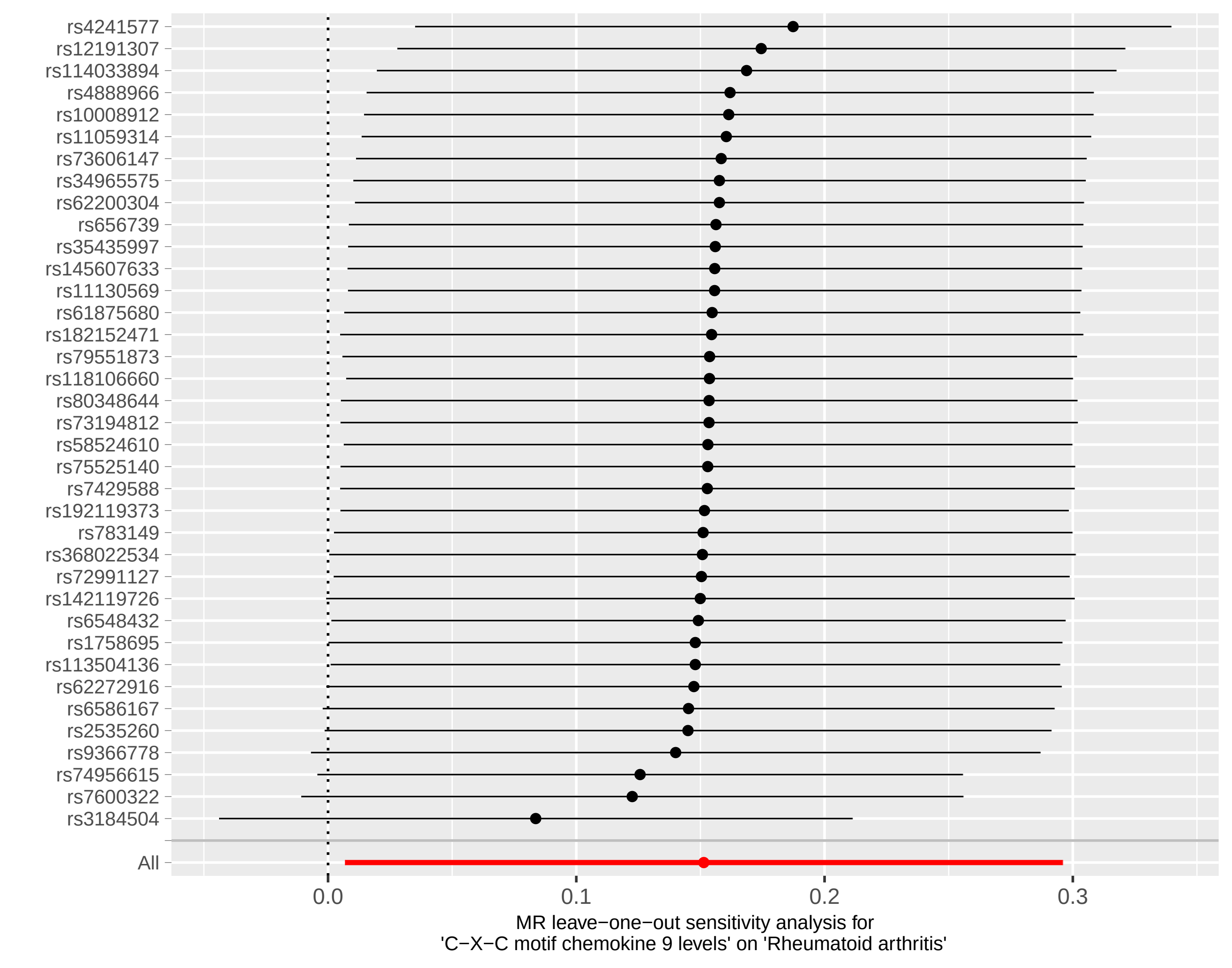

rs4241577
rs12191307
rs114033894
rs4888966
rs10008912
rs11059314
rs73606147
rs34965575
rs62200304
rs656739
rs35435997
rs145607633
rs11130569
rs61875680
rs182152471
rs79551873
rs118106660
rs80348644
rs73194812
rs58524610
rs75525140
rs7429588
rs192119373
rs783149
rs368022534
rs72991127
rs142119726
rs6548432
rs1758695
rs113504136
rs62272916
rs6586167
rs2535260
rs9366778
rs74956615
rs7600322
rs3184504
All
0.0
0.1
0.2
0.3
MR leave−one−out sensitivity analysis for
'C−X−C motif chemokine 9 levels' on 'Rheumatoid arthritis'

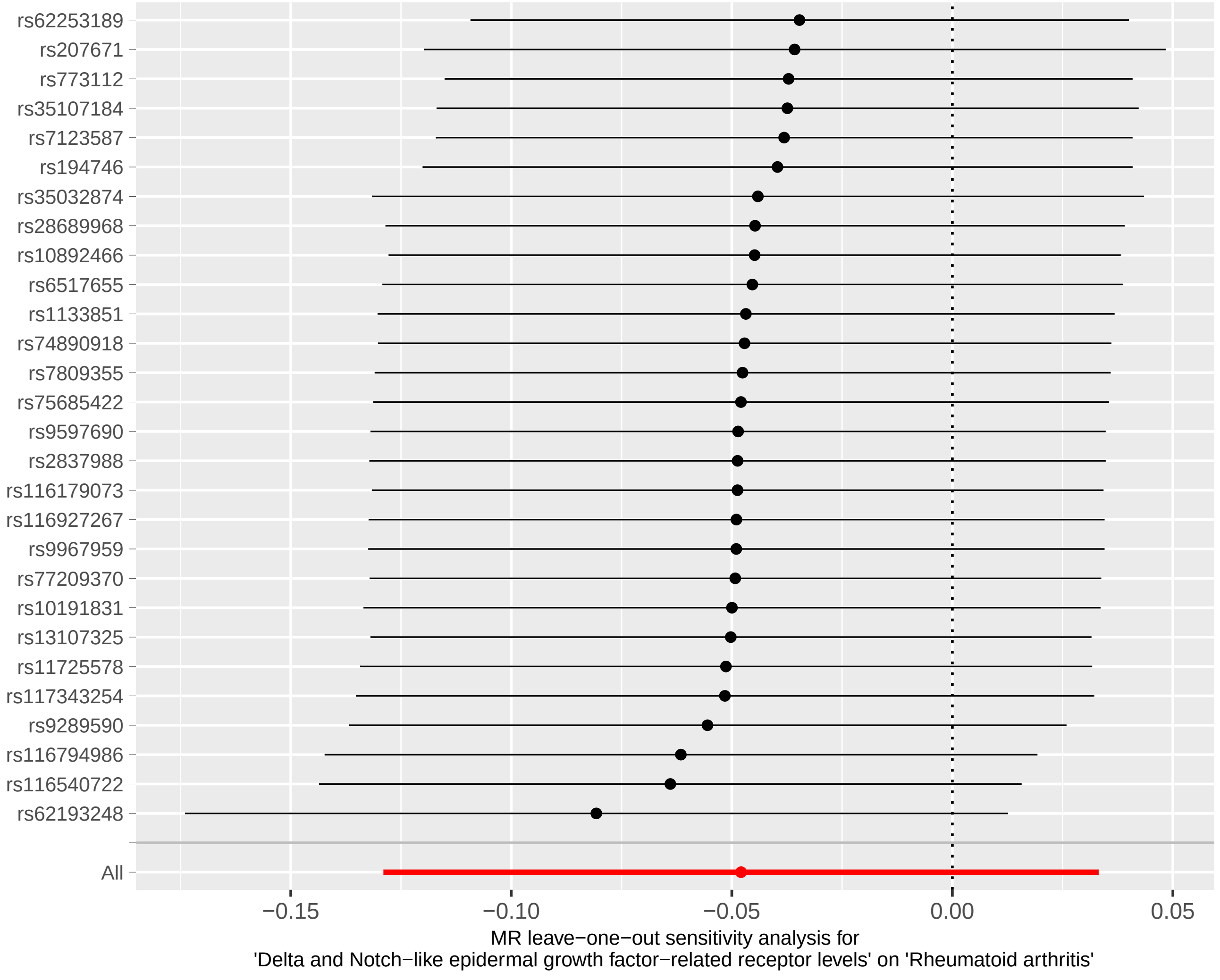
rs62253189
rs207671
rs773112
rs35107184
rs7123587
rs194746
rs35032874
rs28689968
rs10892466
rs6517655
rs1133851
rs74890918
rs7809355
rs75685422
rs9597690
rs2837988
rs116179073
rs116927267
rs9967959
rs77209370
rs10191831
rs13107325
rs11725578
rs117343254
rs9289590
rs116794986
rs116540722
rs62193248
All
−0.15
−0.10
−0.05
0.00
0.05
MR leave−one−out sensitivity analysis for
'Delta and Notch−like epidermal growth factor−related receptor levels' on 'Rheumatoid arthritis'

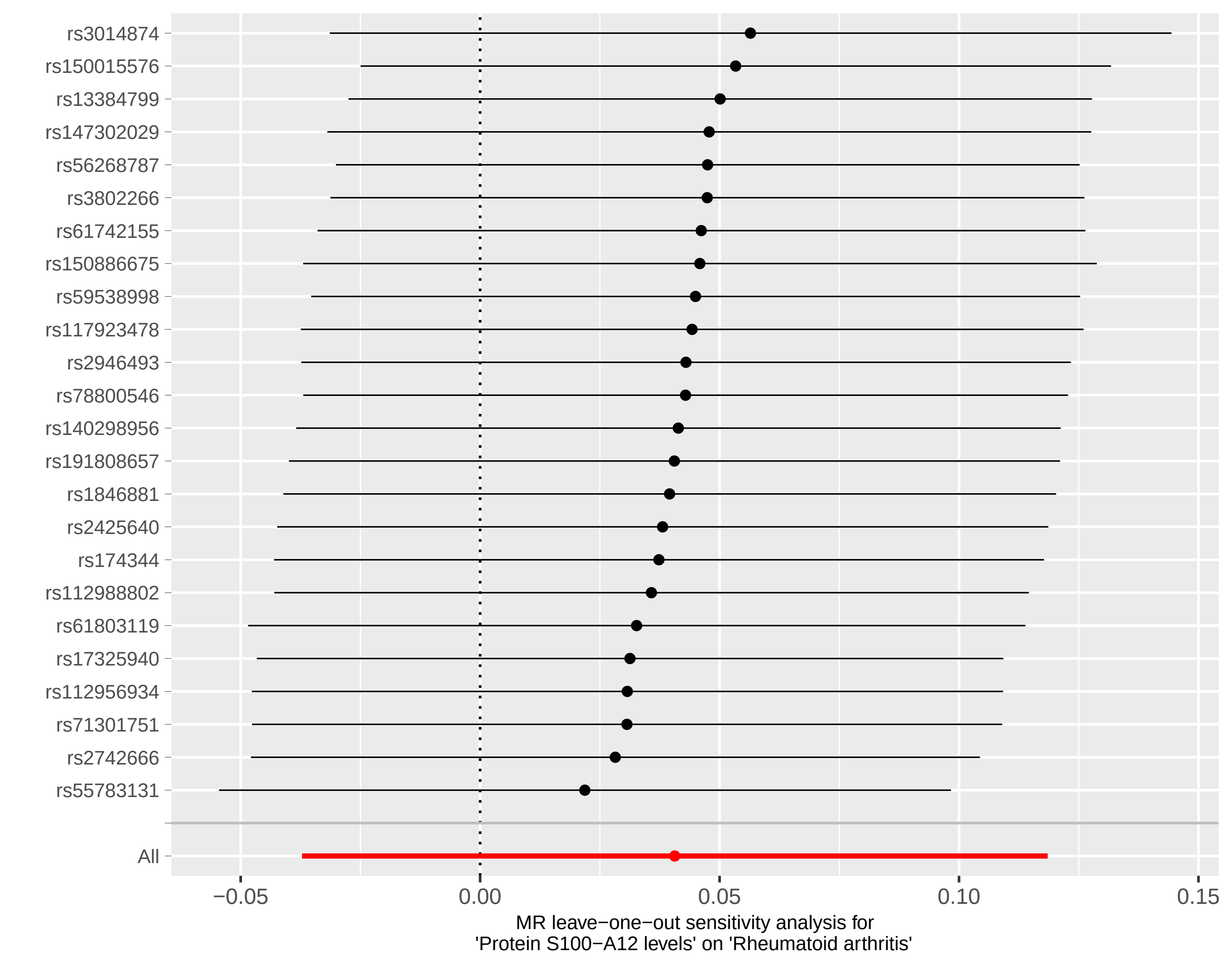

rs3014874
rs150015576
rs13384799
rs147302029
rs56268787
rs3802266
rs61742155
rs150886675
rs59538998
rs117923478
rs2946493
rs78800546
rs140298956
rs191808657
rs1846881
rs2425640
rs174344
rs112988802
rs61803119
rs17325940
rs112956934
rs71301751
rs2742666
rs55783131
All
−0.05
0.00
0.05
0.10
0.15
MR leave−one−out sensitivity analysis for
'Protein S100−A12 levels' on 'Rheumatoid arthritis'

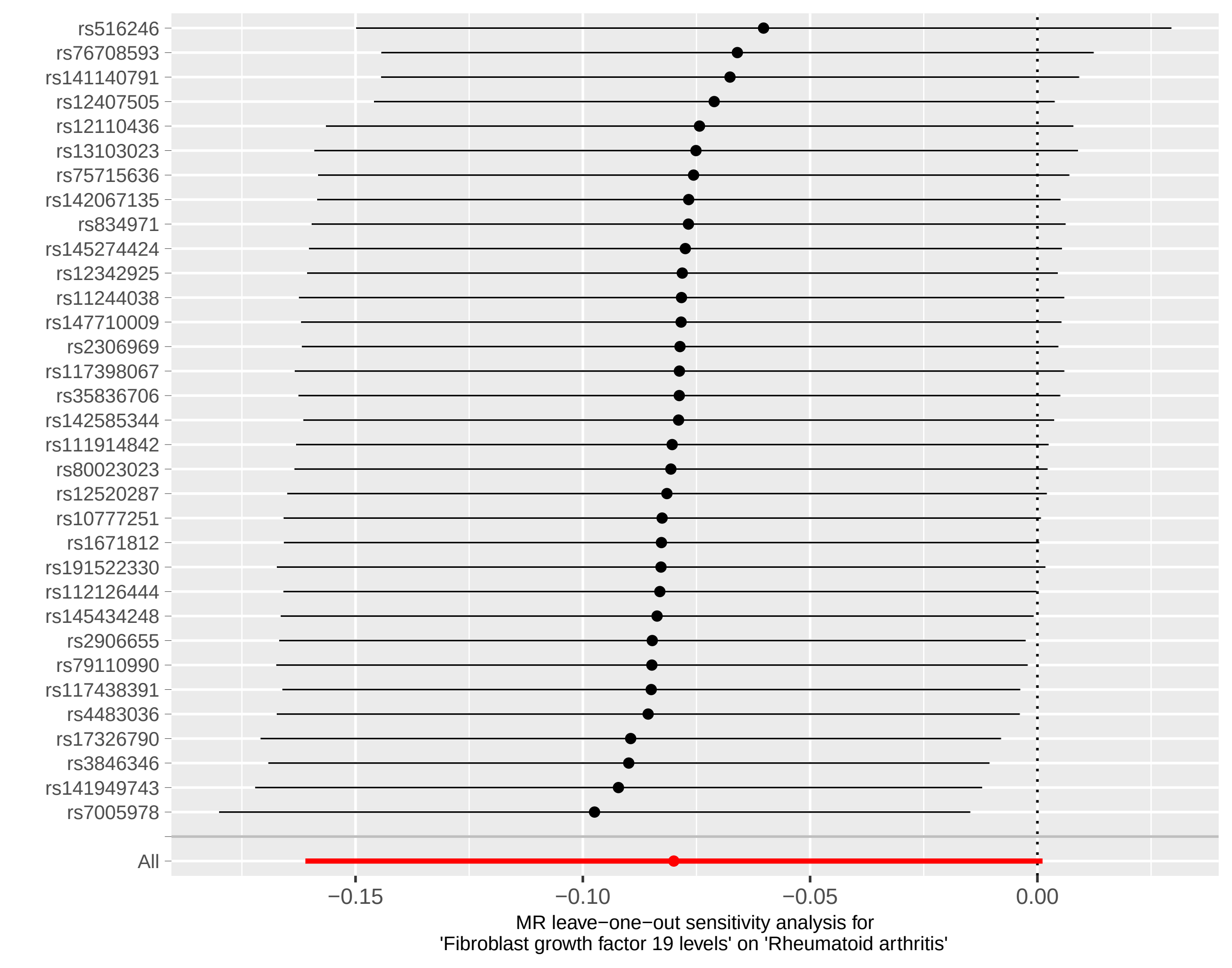

rs516246
rs76708593
rs141140791
rs12407505
rs12110436
rs13103023
rs75715636
rs142067135
rs834971
rs145274424
rs12342925
rs11244038
rs147710009
rs2306969
rs117398067
rs35836706
rs142585344
rs111914842
rs80023023
rs12520287
rs10777251
rs1671812
rs191522330
rs112126444
rs145434248
rs2906655
rs79110990
rs117438391
rs4483036
rs17326790
rs3846346
rs141949743
rs7005978
All
−0.15
−0.10
−0.05
0.00
MR leave−one−out sensitivity analysis for
'Fibroblast growth factor 19 levels' on 'Rheumatoid arthritis'

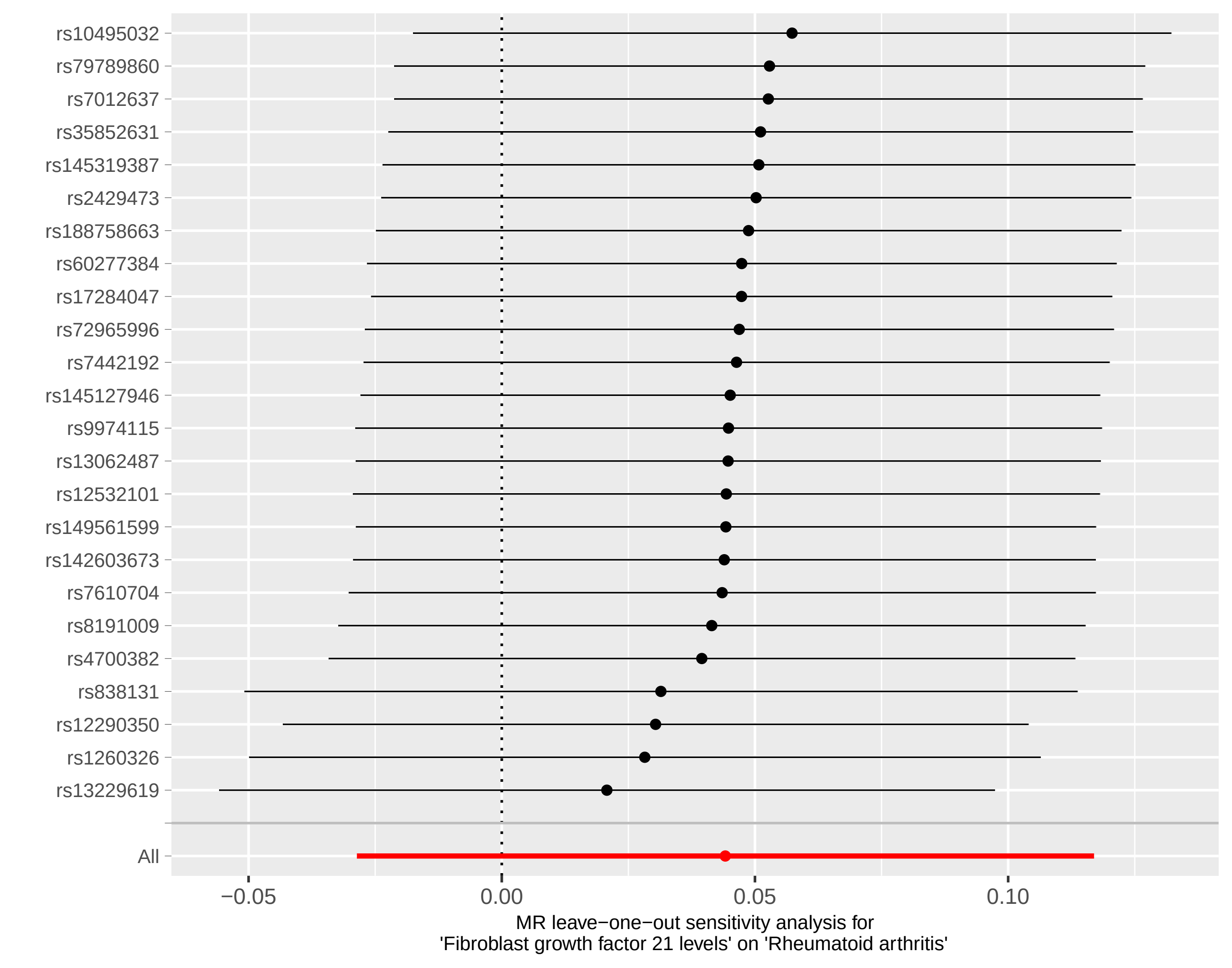

rs10495032
rs79789860
rs7012637
rs35852631
rs145319387
rs2429473
rs188758663
rs60277384
rs17284047
rs72965996
rs7442192
rs145127946
rs9974115
rs13062487
rs12532101
rs149561599
rs142603673
rs7610704
rs8191009
rs4700382
rs838131
rs12290350
rs1260326
rs13229619
All
−0.05
0.00
0.05
0.10
MR leave−one−out sensitivity analysis for
'Fibroblast growth factor 21 levels' on 'Rheumatoid arthritis'

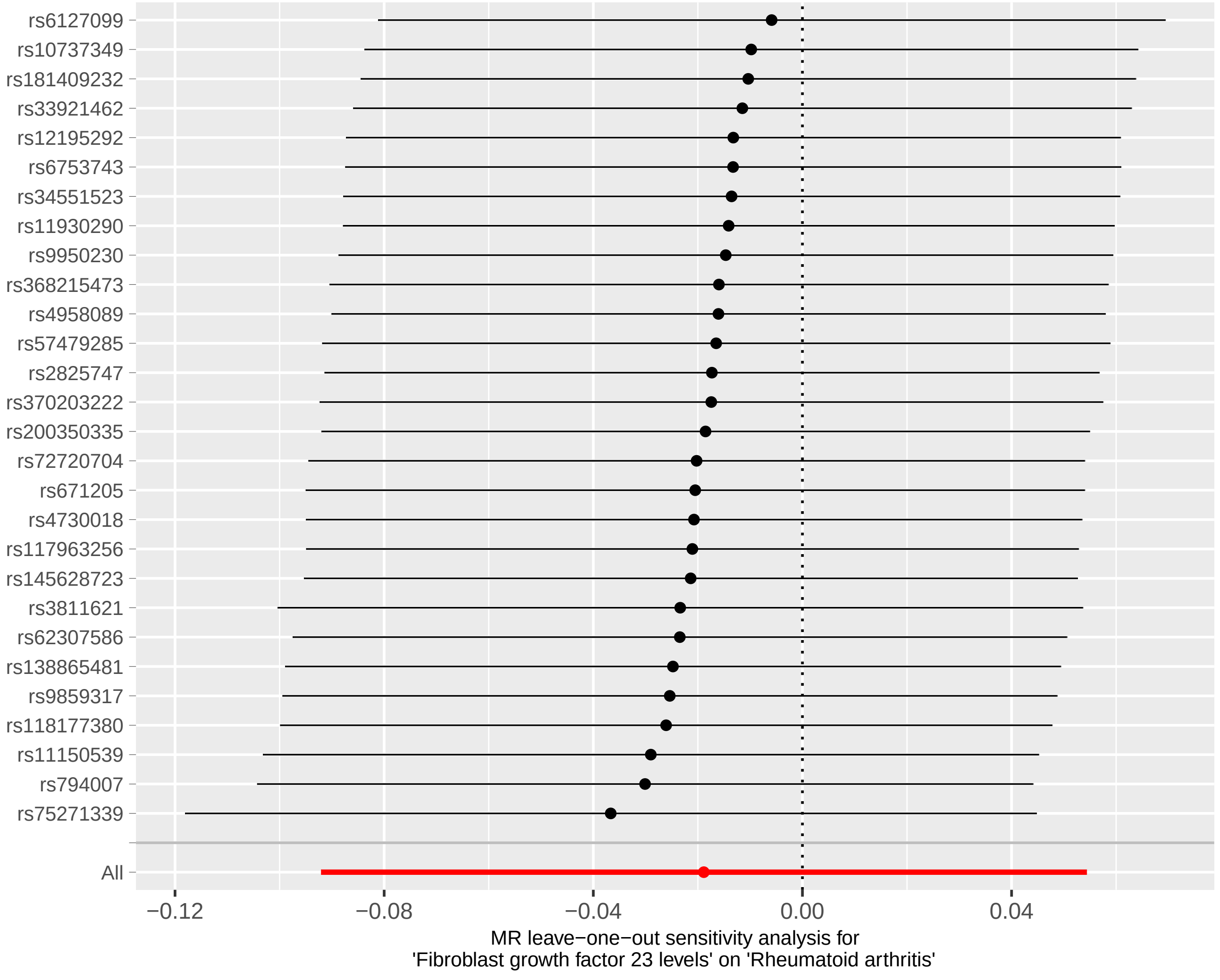
rs6127099
rs10737349
rs181409232
rs33921462
rs12195292
rs6753743
rs34551523
rs11930290
rs9950230
rs368215473
rs4958089
rs57479285
rs2825747
rs370203222
rs200350335
rs72720704
rs671205
rs4730018
rs117963256
rs145628723
rs3811621
rs62307586
rs138865481
rs9859317
rs118177380
rs11150539
rs794007
rs75271339
All
−0.12
−0.08
−0.04
0.00
0.04
MR leave−one−out sensitivity analysis for
'Fibroblast growth factor 23 levels' on 'Rheumatoid arthritis'

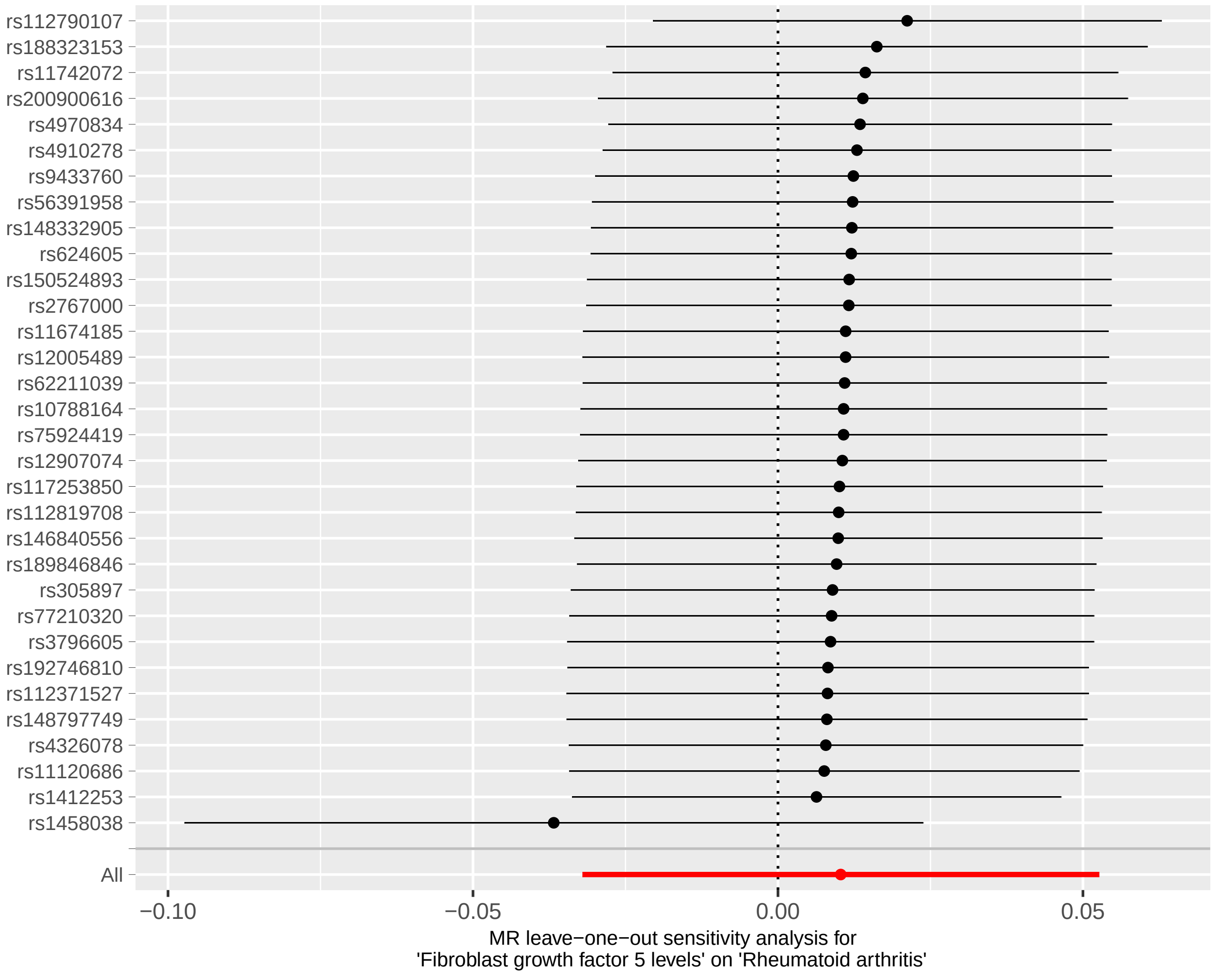
rs112790107
rs188323153
rs11742072
rs200900616
rs4970834
rs4910278
rs9433760
rs56391958
rs148332905
rs624605
rs150524893
rs2767000
rs11674185
rs12005489
rs62211039
rs10788164
rs75924419
rs12907074
rs117253850
rs112819708
rs146840556
rs189846846
rs305897
rs77210320
rs3796605
rs192746810
rs112371527
rs148797749
rs4326078
rs11120686
rs1412253
rs1458038
All
−0.10
−0.05
0.00
0.05
MR leave−one−out sensitivity analysis for
'Fibroblast growth factor 5 levels' on 'Rheumatoid arthritis'

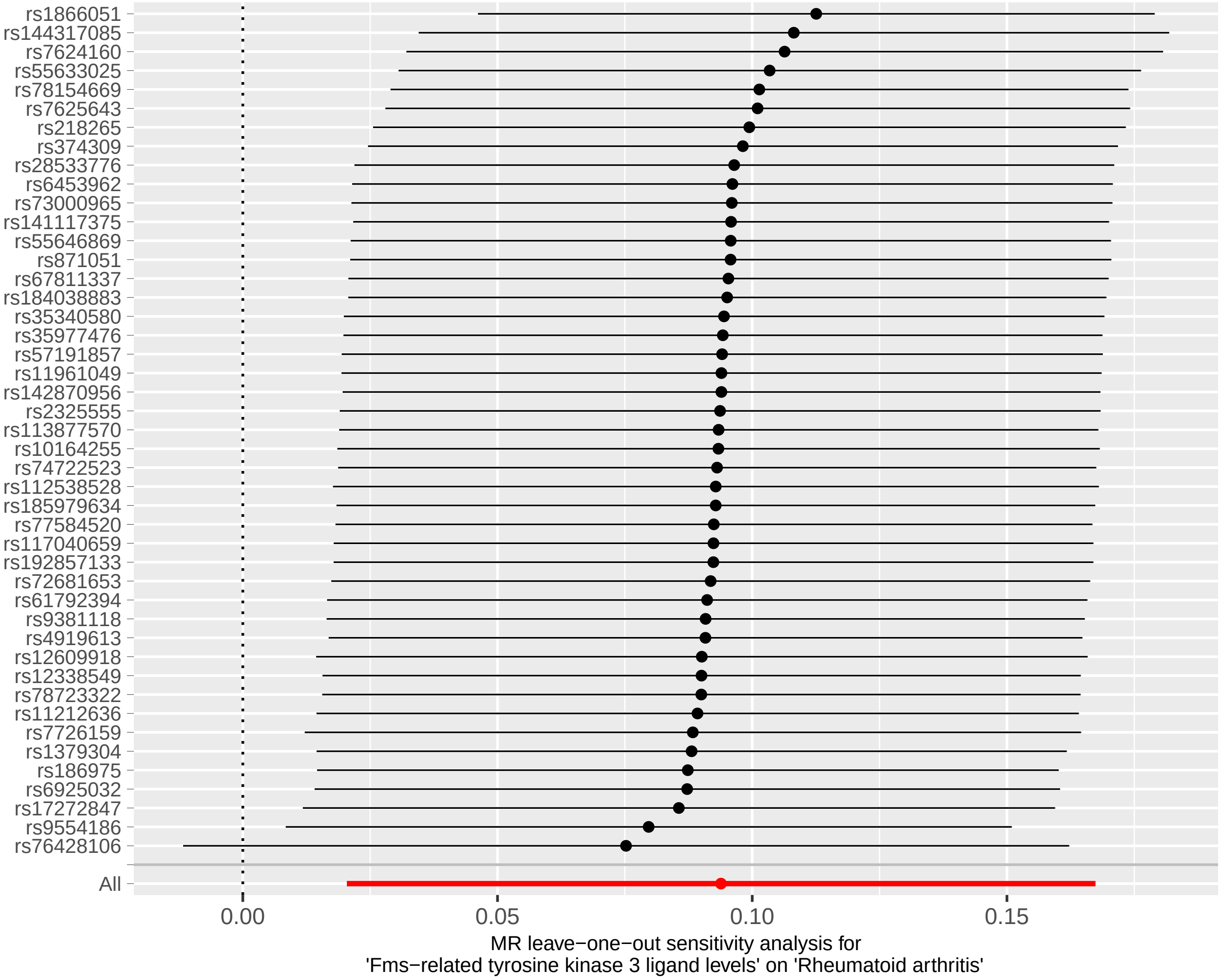
rs1866051
rs144317085
rs7624160
rs55633025
rs78154669
rs7625643
rs218265
rs374309
rs28533776
rs6453962
rs73000965
rs141117375
rs55646869
rs871051
rs67811337
rs184038883
rs35340580
rs35977476
rs57191857
rs11961049
rs142870956
rs2325555
rs113877570
rs10164255
rs74722523
rs112538528
rs185979634
rs77584520
rs117040659
rs192857133
rs72681653
rs61792394
rs9381118
rs4919613
rs12609918
rs12338549
rs78723322
rs11212636
rs7726159
rs1379304
rs186975
rs6925032
rs17272847
rs9554186
rs76428106
All
0.00
0.05
0.10
0.15
MR leave−one−out sensitivity analysis for
'Fms−related tyrosine kinase 3 ligand levels' on 'Rheumatoid arthritis'

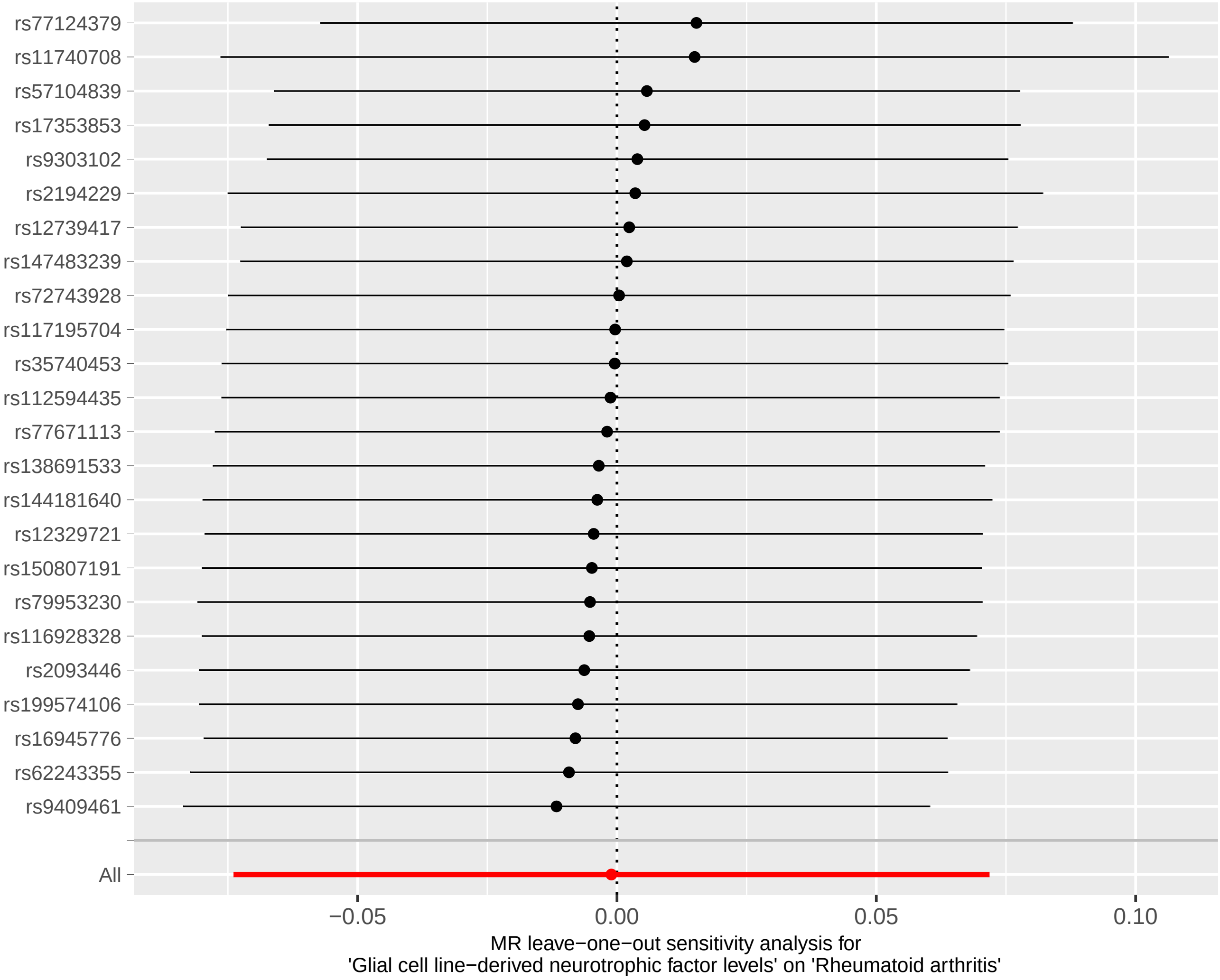
rs77124379
rs11740708
rs57104839
rs17353853
rs9303102
rs2194229
rs12739417
rs147483239
rs72743928
rs117195704
rs35740453
rs112594435
rs77671113
rs138691533
rs144181640
rs12329721
rs150807191
rs79953230
rs116928328
rs2093446
rs199574106
rs16945776
rs62243355
rs9409461
All
−0.05
0.00
0.05
0.10
MR leave−one−out sensitivity analysis for
'Glial cell line−derived neurotrophic factor levels' on 'Rheumatoid arthritis'

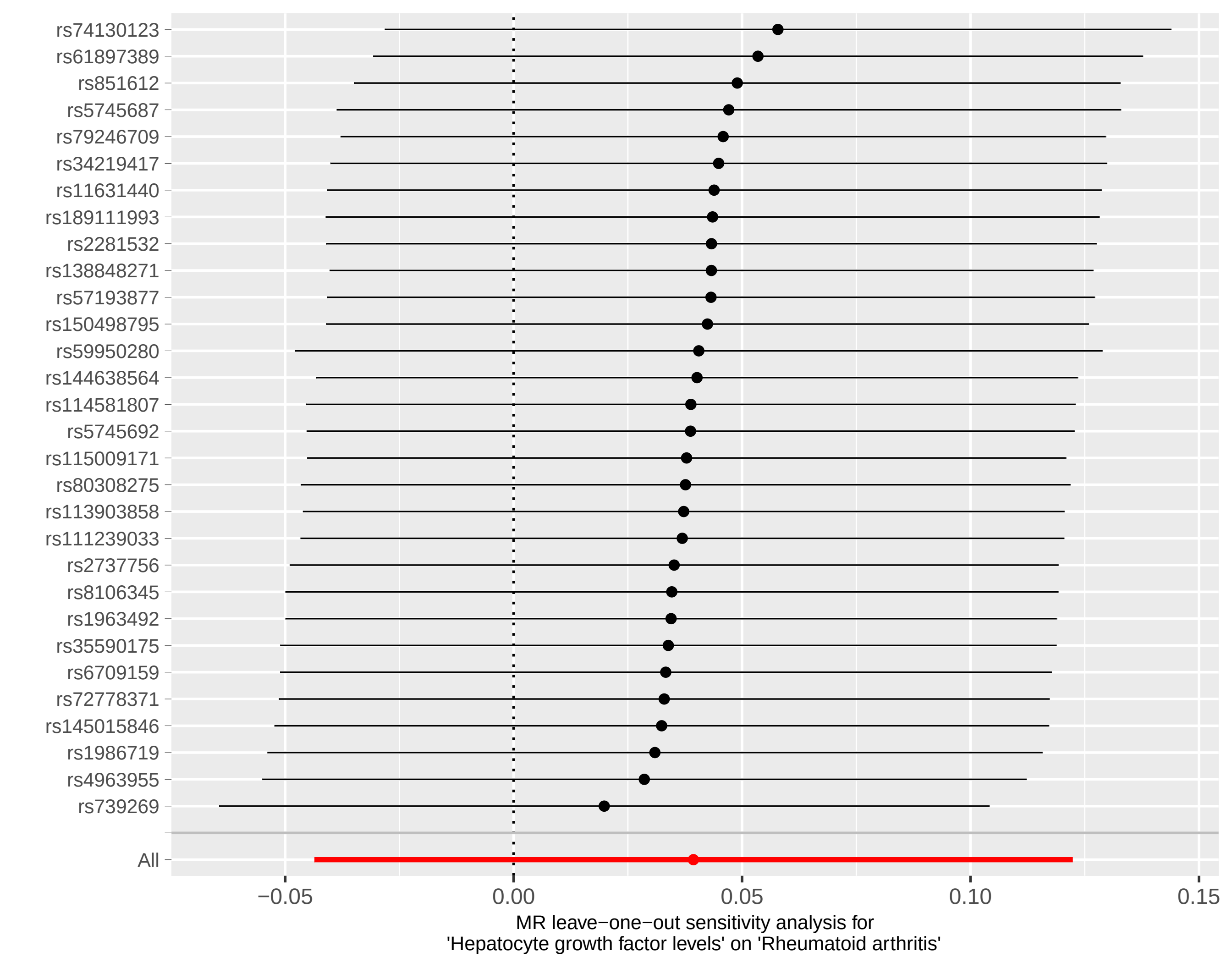
rs74130123
rs61897389
rs851612
rs5745687
rs79246709
rs34219417
rs11631440
rs189111993
rs2281532
rs138848271
rs57193877
rs150498795
rs59950280
rs144638564
rs114581807
rs5745692
rs115009171
rs80308275
rs113903858
rs111239033
rs2737756
rs8106345
rs1963492
rs35590175
rs6709159
rs72778371
rs145015846
rs1986719
rs4963955
rs739269
All
−0.05
0.00
0.05
0.10
0.15
MR leave−one−out sensitivity analysis for
'Hepatocyte growth factor levels' on 'Rheumatoid arthritis'

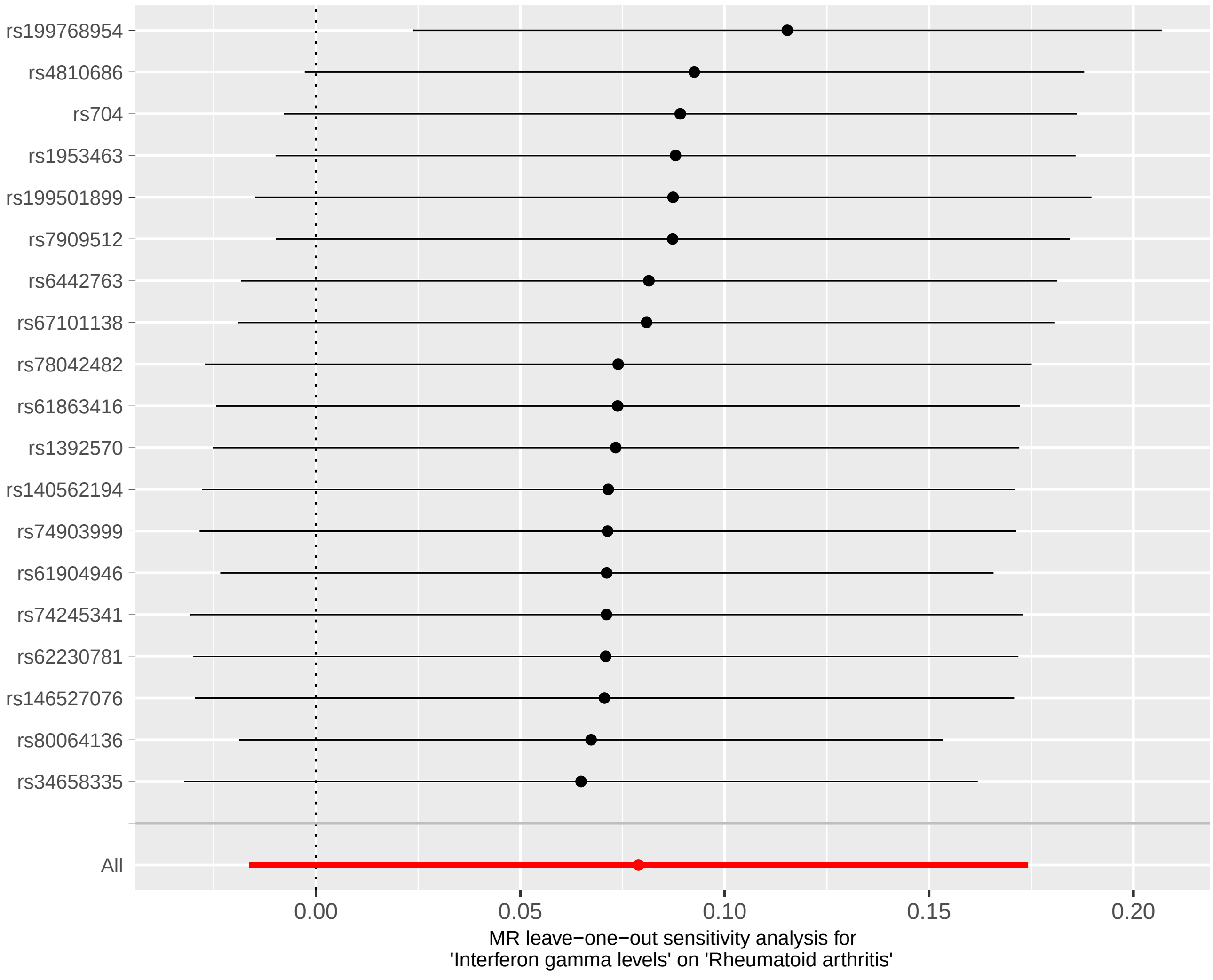
rs199768954
rs4810686
rs704
rs1953463
rs199501899
rs7909512
rs6442763
rs67101138
rs78042482
rs61863416
rs1392570
rs140562194
rs74903999
rs61904946
rs74245341
rs62230781
rs146527076
rs80064136
rs34658335
All
0.00
0.05
0.10
0.15
0.20
MR leave−one−out sensitivity analysis for
'Interferon gamma levels' on 'Rheumatoid arthritis'

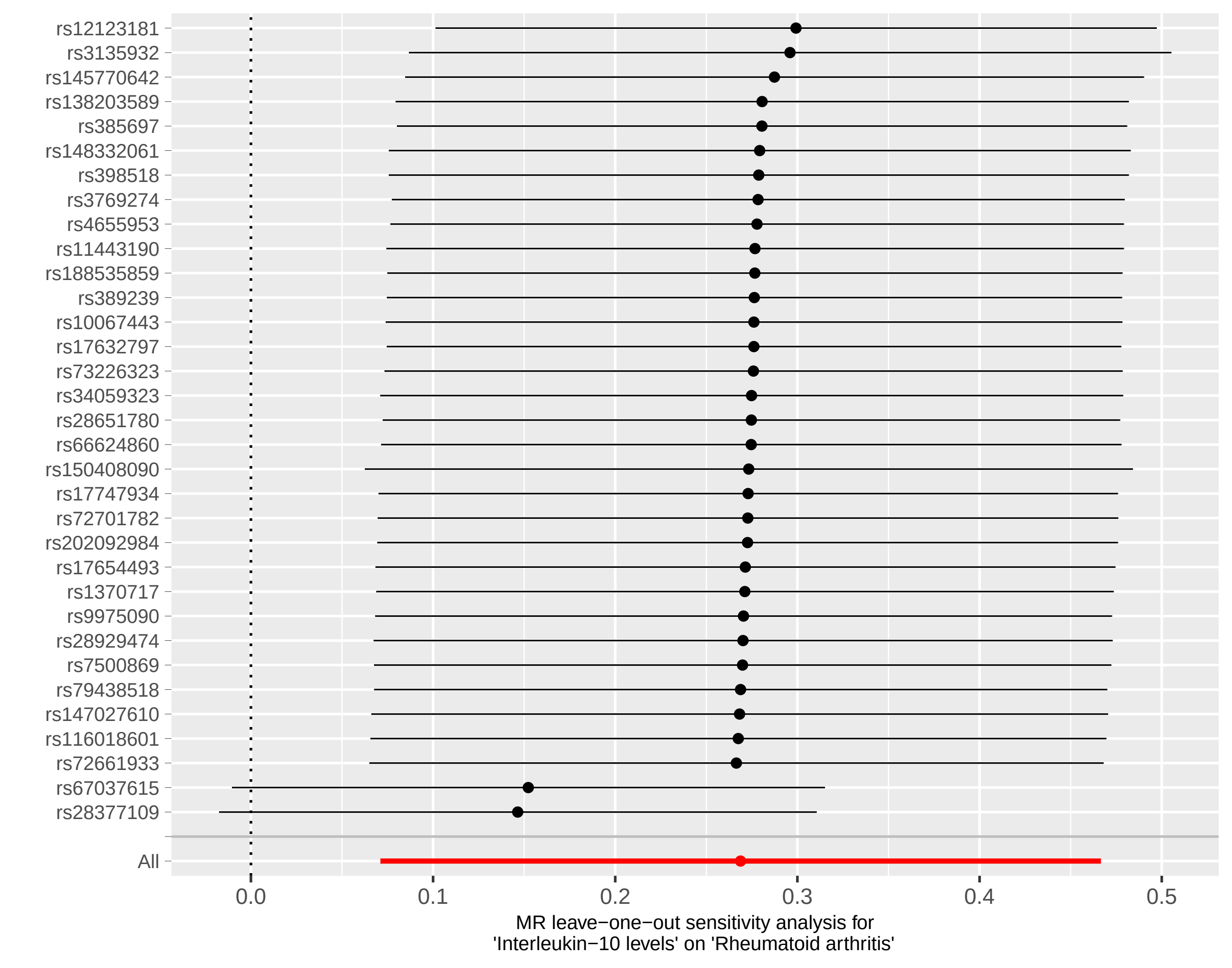

rs12123181
rs3135932
rs145770642
rs138203589
rs385697
rs148332061
rs398518
rs3769274
rs4655953
rs11443190
rs188535859
rs389239
rs10067443
rs17632797
rs73226323
rs34059323
rs28651780
rs66624860
rs150408090
rs17747934
rs72701782
rs202092984
rs17654493
rs1370717
rs9975090
rs28929474
rs7500869
rs79438518
rs147027610
rs116018601
rs72661933
rs67037615
rs28377109
All
0.0
0.1
0.2
0.3
0.4
0.5
MR leave−one−out sensitivity analysis for
'Interleukin−10 levels' on 'Rheumatoid arthritis'

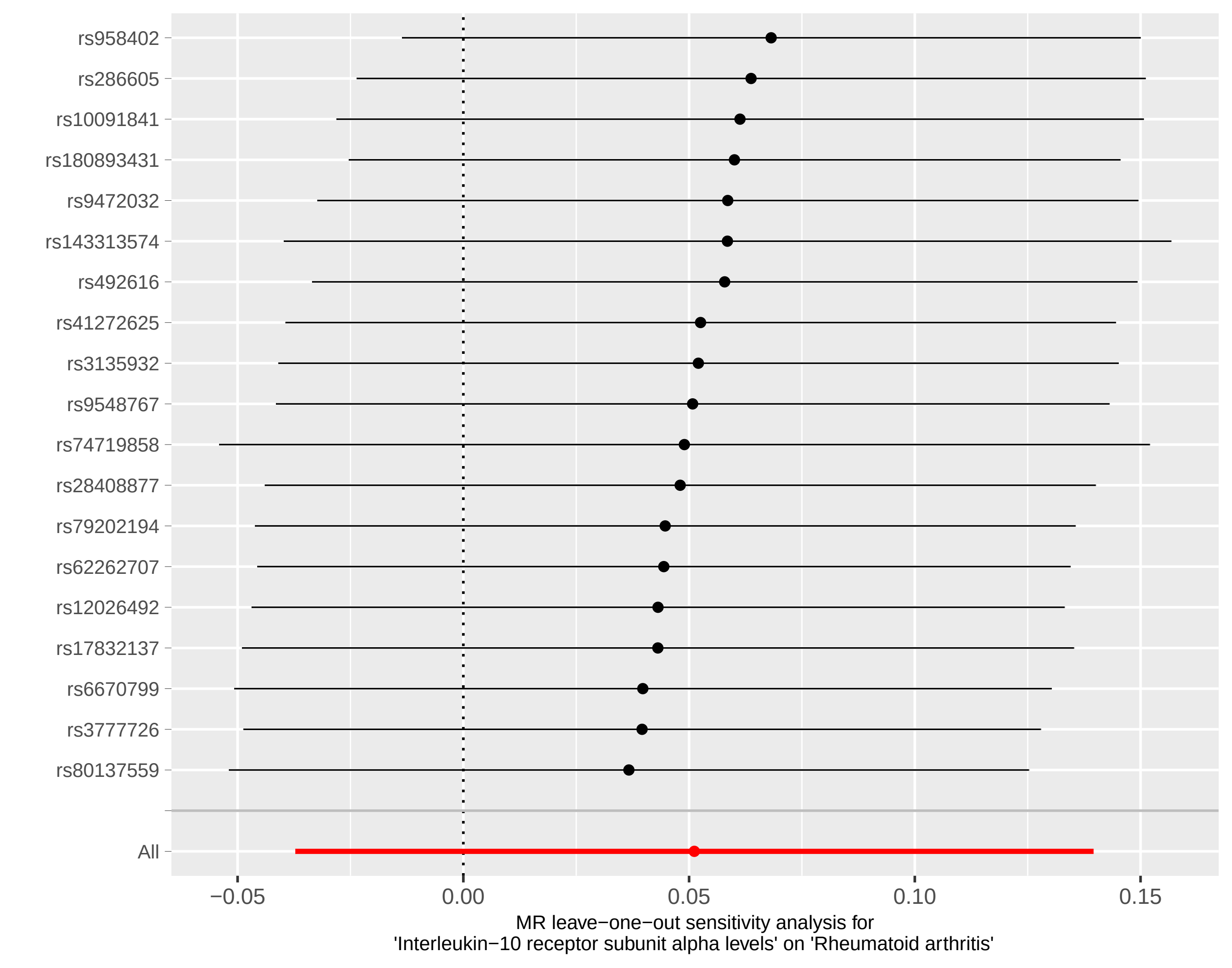
rs958402
rs286605
rs10091841
rs180893431
rs9472032
rs143313574
rs492616
rs41272625
rs3135932
rs9548767
rs74719858
rs28408877
rs79202194
rs62262707
rs12026492
rs17832137
rs6670799
rs3777726
rs80137559
All
−0.05
0.00
0.05
0.10
0.15
MR leave−one−out sensitivity analysis for
'Interleukin−10 receptor subunit alpha levels' on 'Rheumatoid arthritis'

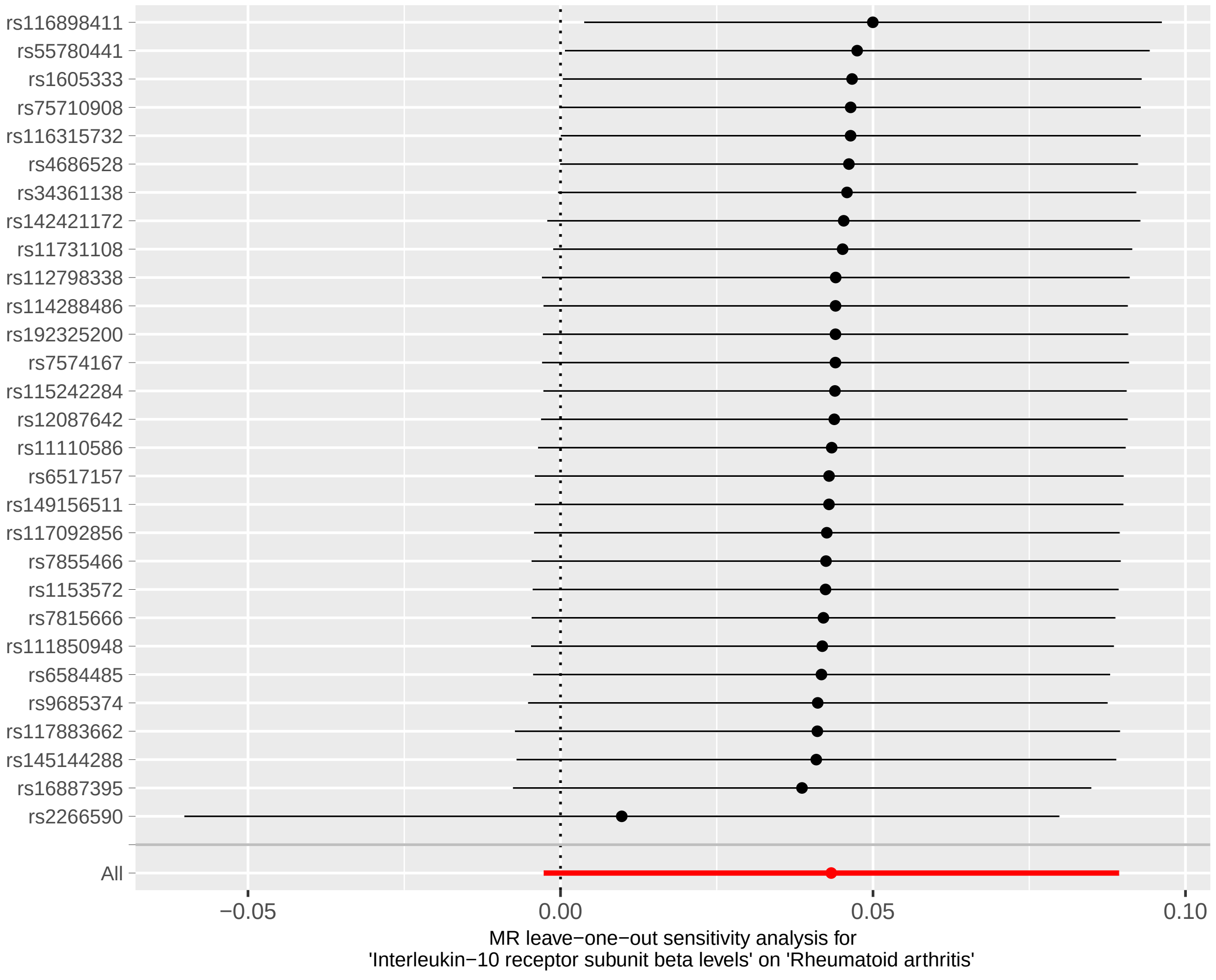
rs116898411
rs55780441
rs1605333
rs75710908
rs116315732
rs4686528
rs34361138
rs142421172
rs11731108
rs112798338
rs114288486
rs192325200
rs7574167
rs115242284
rs12087642
rs11110586
rs6517157
rs149156511
rs117092856
rs7855466
rs1153572
rs7815666
rs111850948
rs6584485
rs9685374
rs117883662
rs145144288
rs16887395
rs2266590
All
−0.05
0.00
0.05
0.10
MR leave−one−out sensitivity analysis for
'Interleukin−10 receptor subunit beta levels' on 'Rheumatoid arthritis'

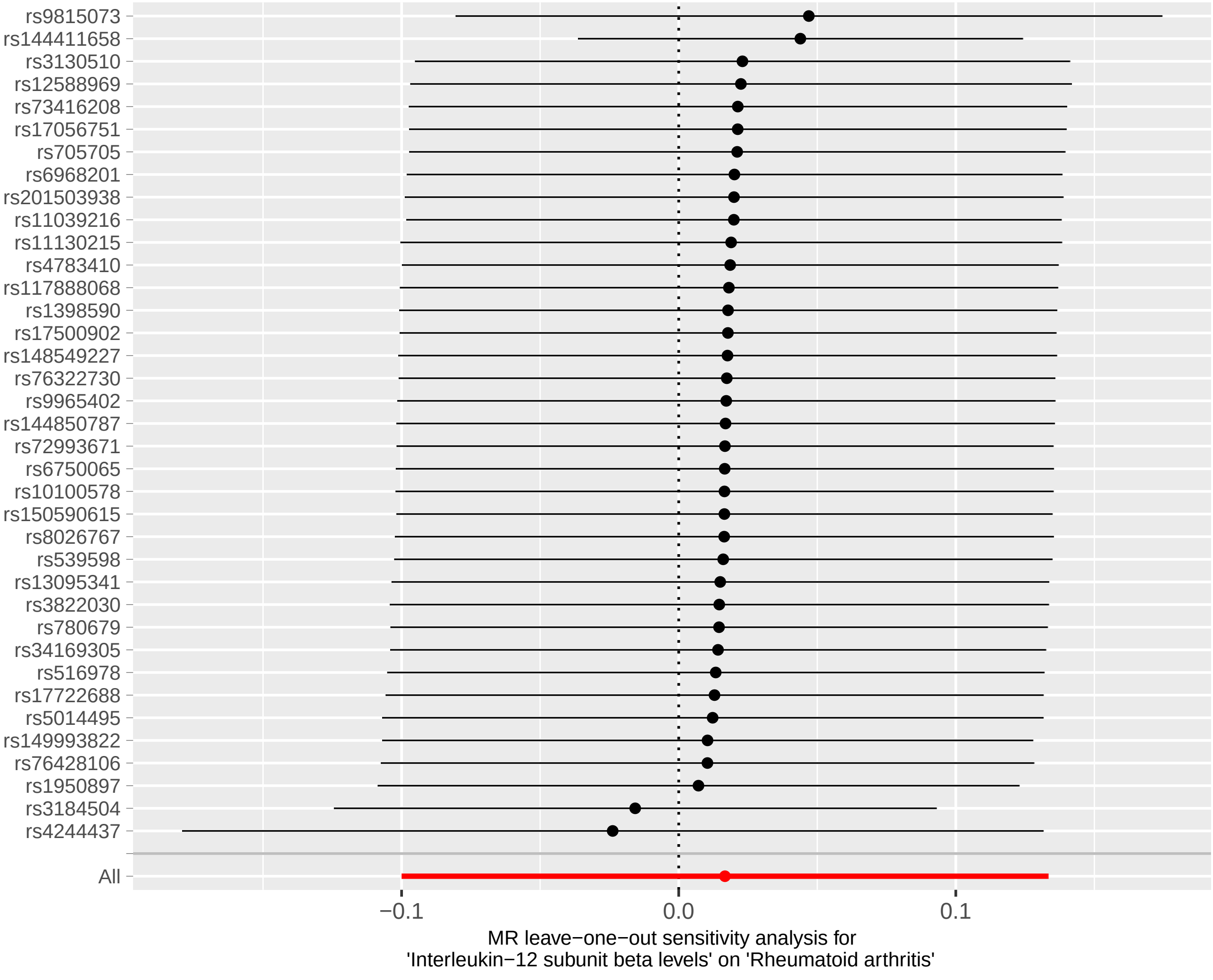
rs9815073
rs144411658
rs3130510
rs12588969
rs73416208
rs17056751
rs705705
rs6968201
rs201503938
rs11039216
rs11130215
rs4783410
rs117888068
rs1398590
rs17500902
rs148549227
rs76322730
rs9965402
rs144850787
rs72993671
rs6750065
rs10100578
rs150590615
rs8026767
rs539598
rs13095341
rs3822030
rs780679
rs34169305
rs516978
rs17722688
rs5014495
rs149993822
rs76428106
rs1950897
rs3184504
rs4244437
All
−0.1
0.0
0.1
MR leave−one−out sensitivity analysis for
'Interleukin−12 subunit beta levels' on 'Rheumatoid arthritis'

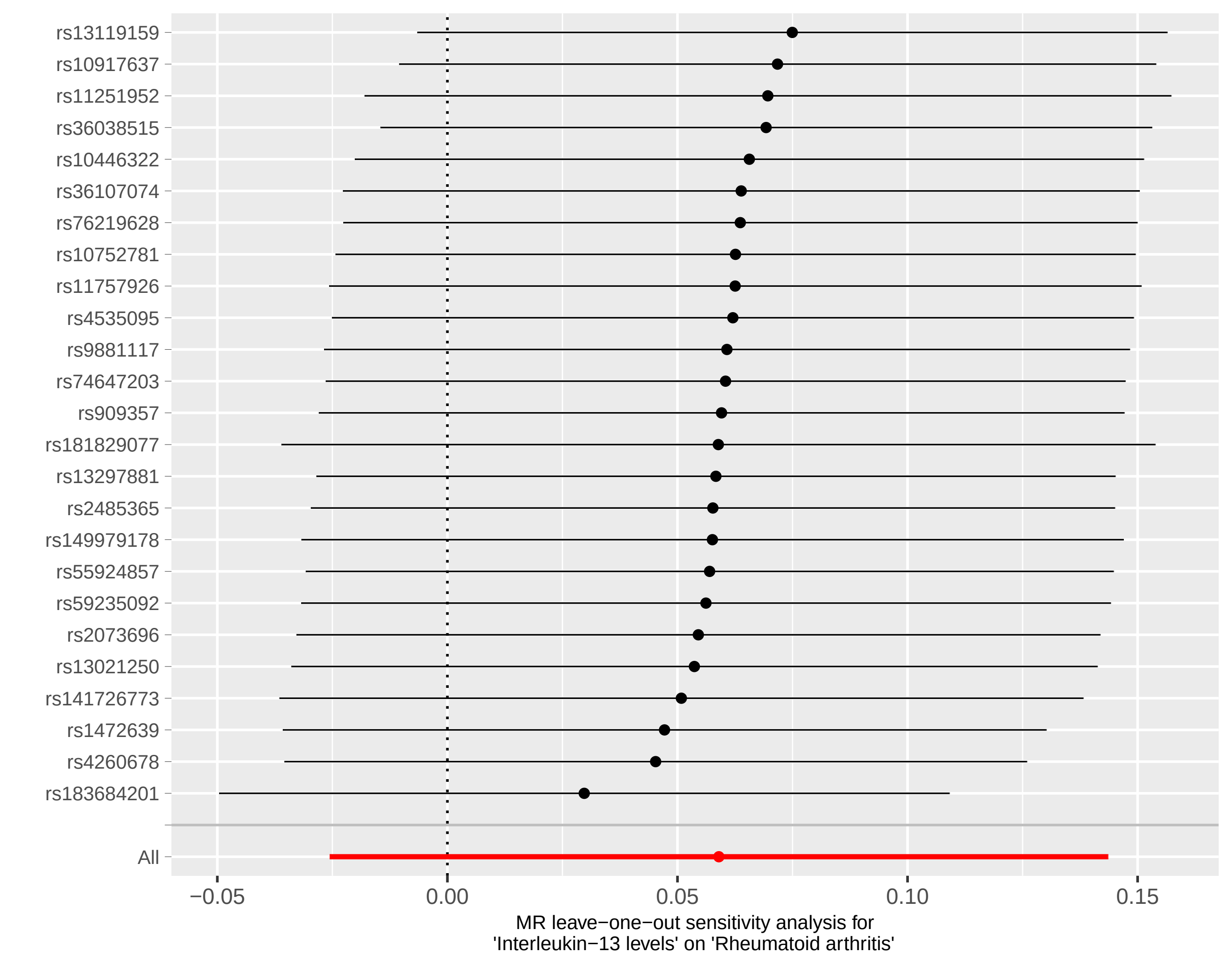
rs13119159
rs10917637
rs11251952
rs36038515
rs10446322
rs36107074
rs76219628
rs10752781
rs11757926
rs4535095
rs9881117
rs74647203
rs909357
rs181829077
rs13297881
rs2485365
rs149979178
rs55924857
rs59235092
rs2073696
rs13021250
rs141726773
rs1472639
rs4260678
rs183684201
All
−0.05
0.00
0.05
0.10
0.15
MR leave−one−out sensitivity analysis for
'Interleukin−13 levels' on 'Rheumatoid arthritis'

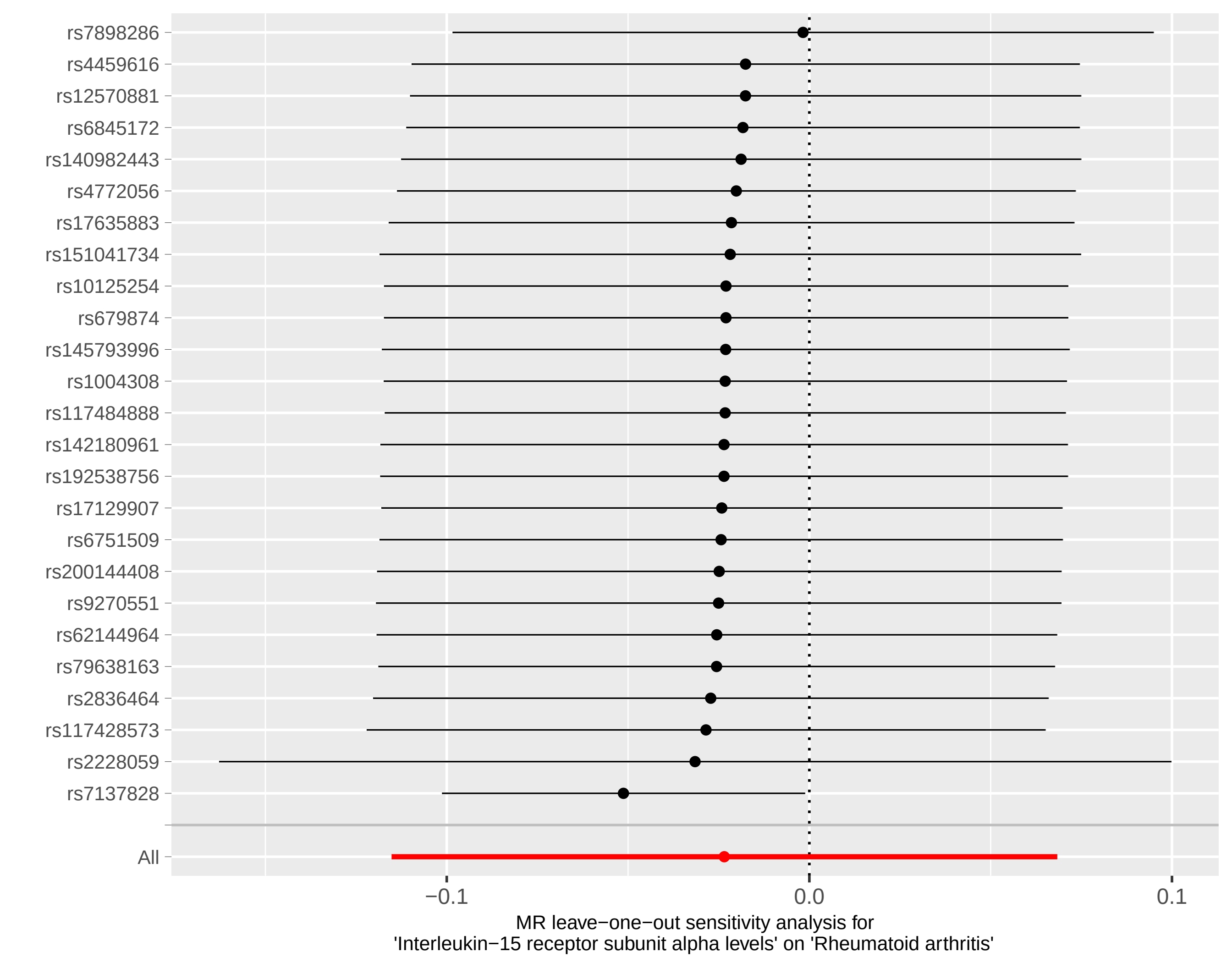
rs7898286
rs4459616
rs12570881
rs6845172
rs140982443
rs4772056
rs17635883
rs151041734
rs10125254
rs679874
rs145793996
rs1004308
rs117484888
rs142180961
rs192538756
rs17129907
rs6751509
rs200144408
rs9270551
rs62144964
rs79638163
rs2836464
rs117428573
rs2228059
rs7137828
All
−0.1
0.0
0.1
MR leave−one−out sensitivity analysis for
'Interleukin−15 receptor subunit alpha levels' on 'Rheumatoid arthritis'

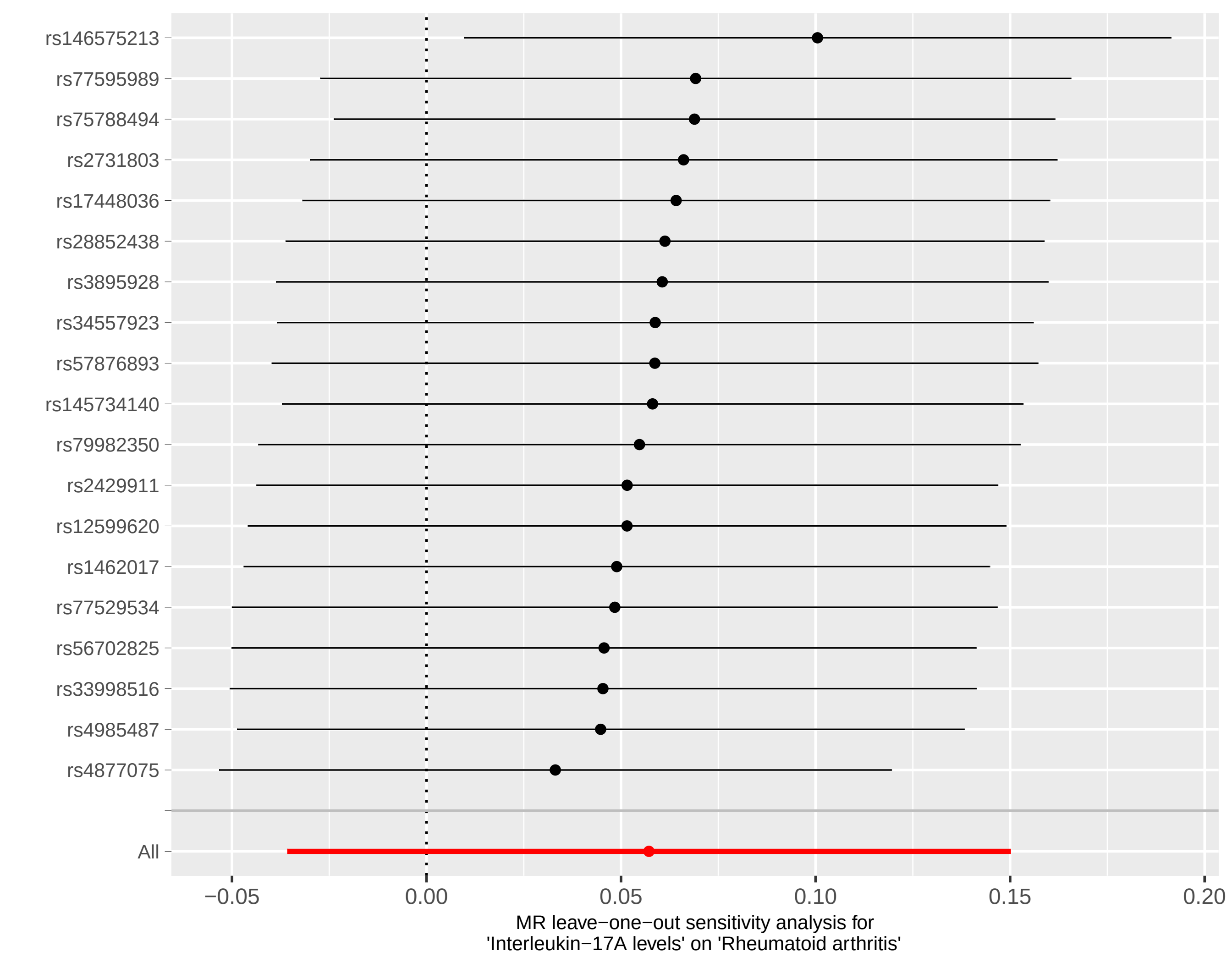

rs146575213
rs77595989
rs75788494
rs2731803
rs17448036
rs28852438
rs3895928
rs34557923
rs57876893
rs145734140
rs79982350
rs2429911
rs12599620
rs1462017
rs77529534
rs56702825
rs33998516
rs4985487
rs4877075
All
−0.05
0.00
0.05
0.10
0.15
0.20
MR leave−one−out sensitivity analysis for
'Interleukin−17A levels' on 'Rheumatoid arthritis'

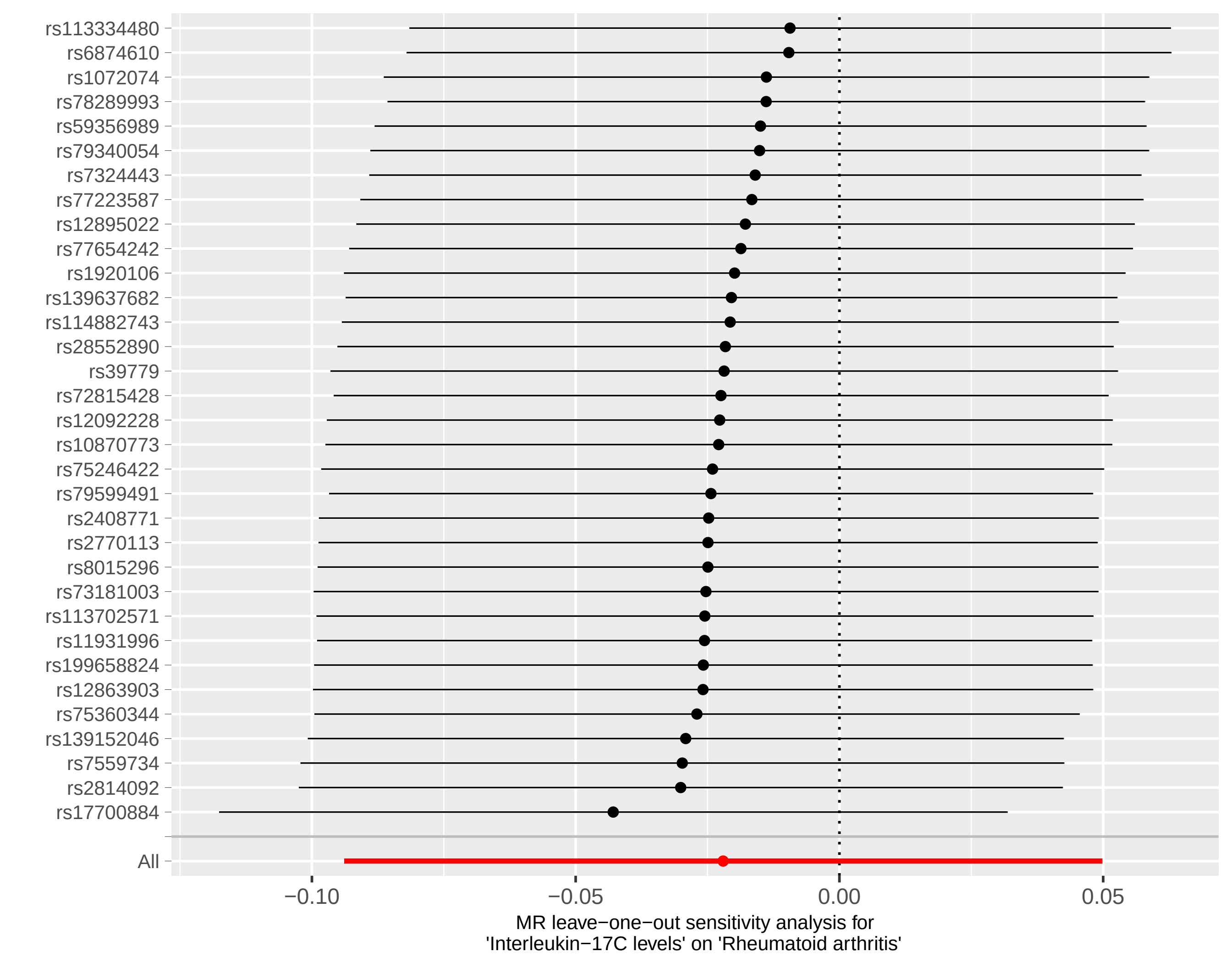

rs113334480
rs6874610
rs1072074
rs78289993
rs59356989
rs79340054
rs7324443
rs77223587
rs12895022
rs77654242
rs1920106
rs139637682
rs114882743
rs28552890
rs39779
rs72815428
rs12092228
rs10870773
rs75246422
rs79599491
rs2408771
rs2770113
rs8015296
rs73181003
rs113702571
rs11931996
rs199658824
rs12863903
rs75360344
rs139152046
rs7559734
rs2814092
rs17700884
All
−0.10
−0.05
0.00
0.05
MR leave−one−out sensitivity analysis for
'Interleukin−17C levels' on 'Rheumatoid arthritis'

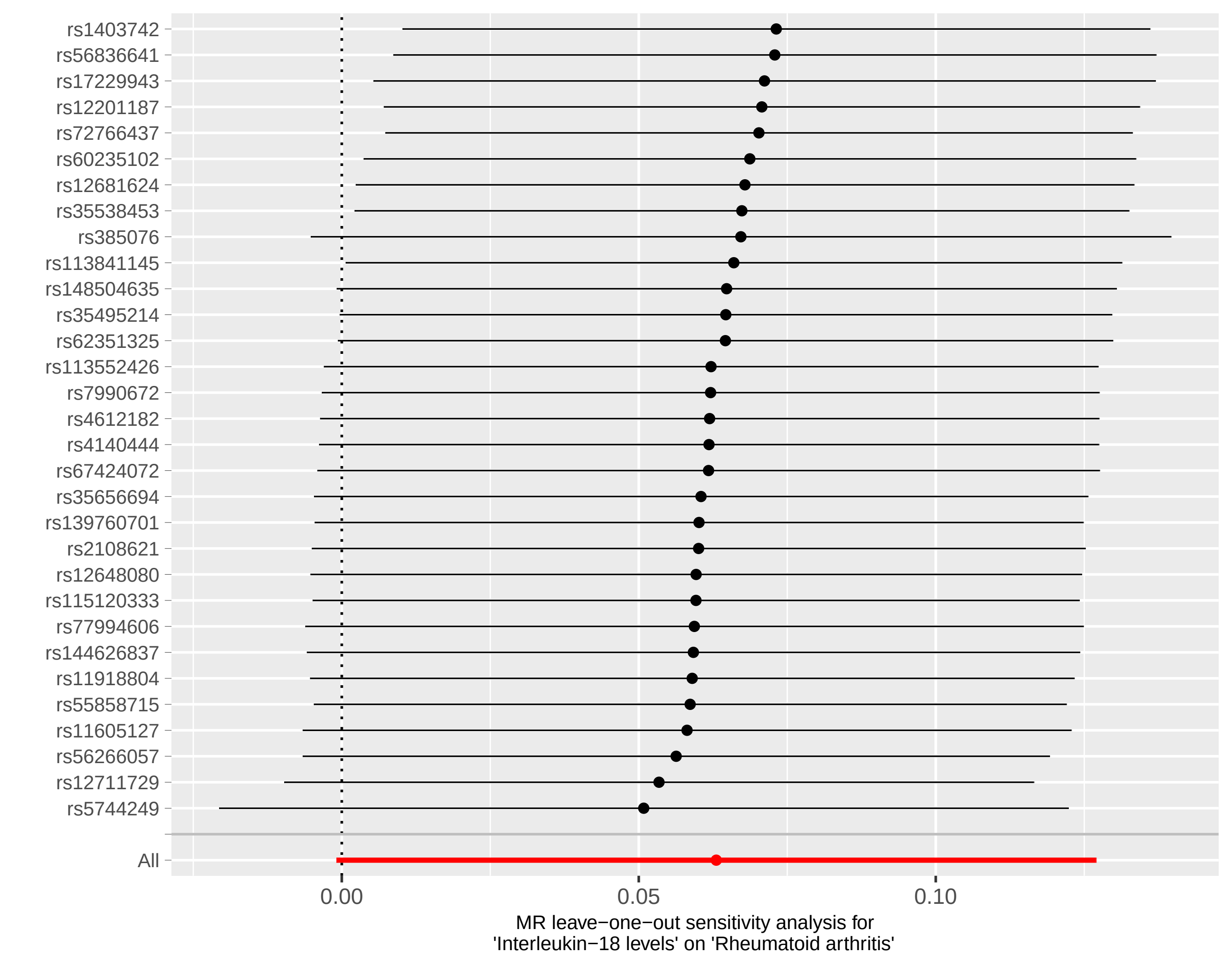
rs1403742
rs56836641
rs17229943
rs12201187
rs72766437
rs60235102
rs12681624
rs35538453
rs385076
rs113841145
rs148504635
rs35495214
rs62351325
rs113552426
rs7990672
rs4612182
rs4140444
rs67424072
rs35656694
rs139760701
rs2108621
rs12648080
rs115120333
rs77994606
rs144626837
rs11918804
rs55858715
rs11605127
rs56266057
rs12711729
rs5744249
All
0.00
0.05
0.10
MR leave−one−out sensitivity analysis for
'Interleukin−18 levels' on 'Rheumatoid arthritis'

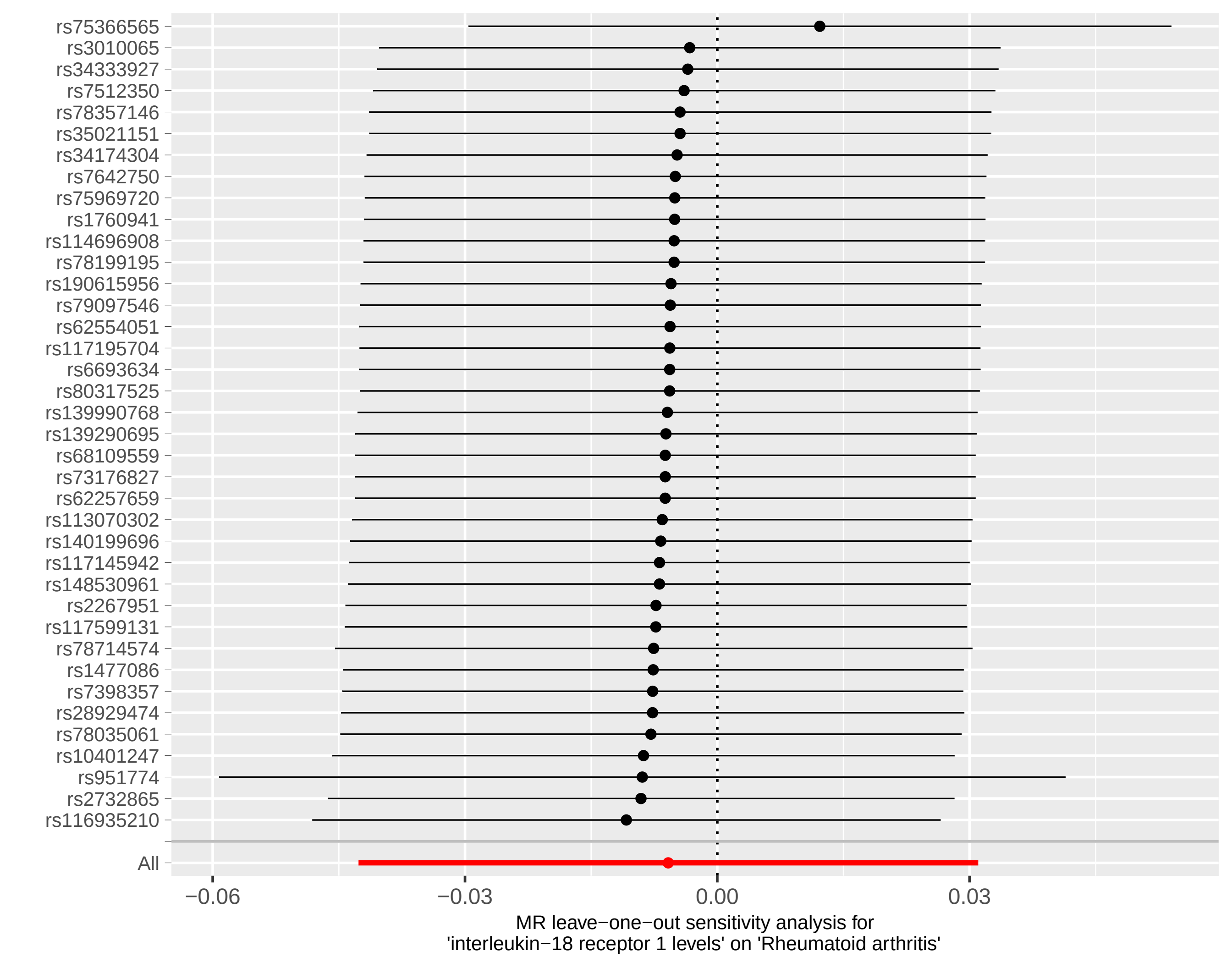
rs75366565
rs3010065
rs34333927
rs7512350
rs78357146
rs35021151
rs34174304
rs7642750
rs75969720
rs1760941
rs114696908
rs78199195
rs190615956
rs79097546
rs62554051
rs117195704
rs6693634
rs80317525
rs139990768
rs139290695
rs68109559
rs73176827
rs62257659
rs113070302
rs140199696
rs117145942
rs148530961
rs2267951
rs117599131
rs78714574
rs1477086
rs7398357
rs28929474
rs78035061
rs10401247
rs951774
rs2732865
rs116935210
All
−0.06
−0.03
0.00
0.03
MR leave−one−out sensitivity analysis for
'interleukin−18 receptor 1 levels' on 'Rheumatoid arthritis'

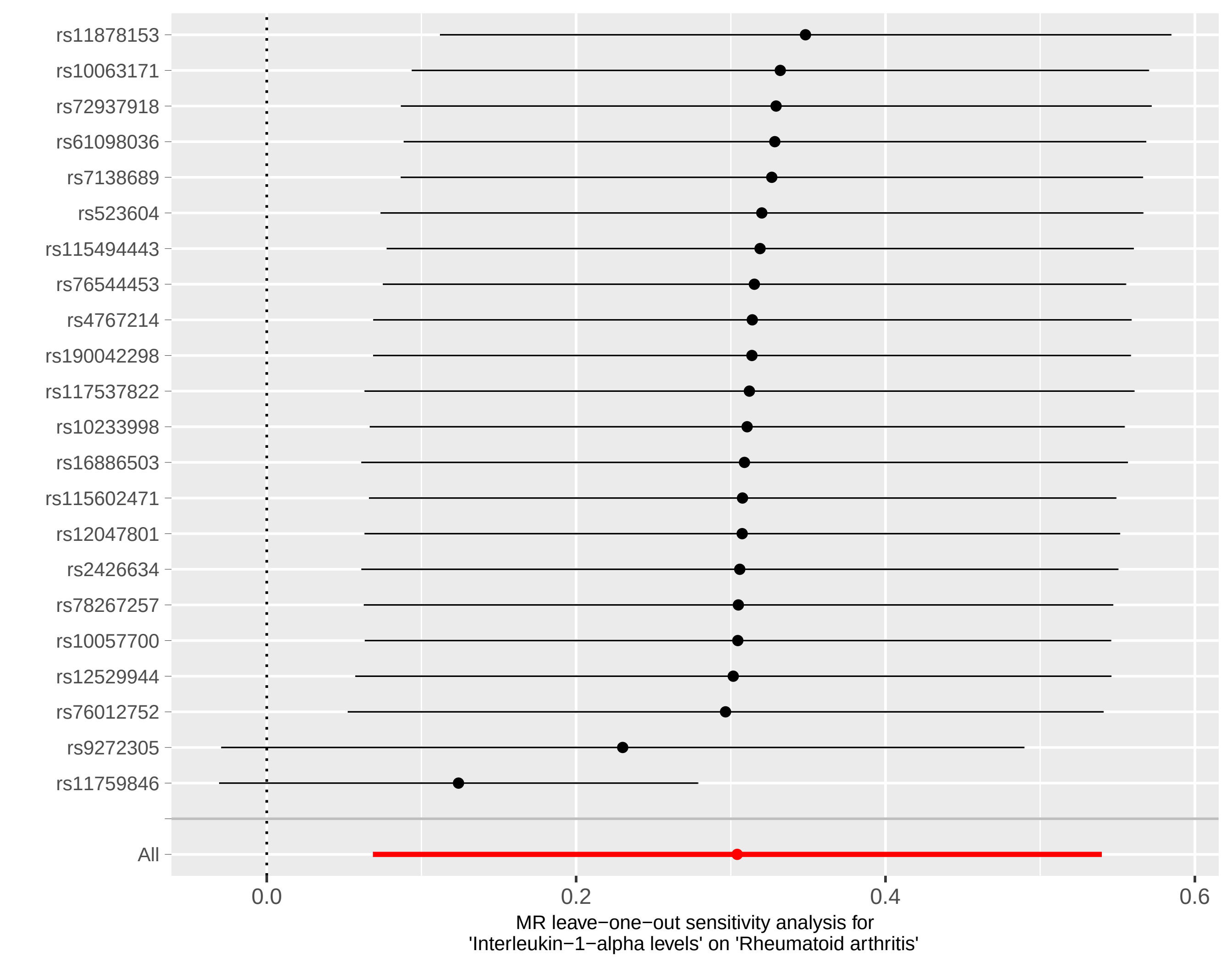

rs11878153
rs10063171
rs72937918
rs61098036
rs7138689
rs523604
rs115494443
rs76544453
rs4767214
rs190042298
rs117537822
rs10233998
rs16886503
rs115602471
rs12047801
rs2426634
rs78267257
rs10057700
rs12529944
rs76012752
rs9272305
rs11759846
All
0.0
0.2
0.4
0.6
MR leave−one−out sensitivity analysis for
'Interleukin−1−alpha levels' on 'Rheumatoid arthritis'

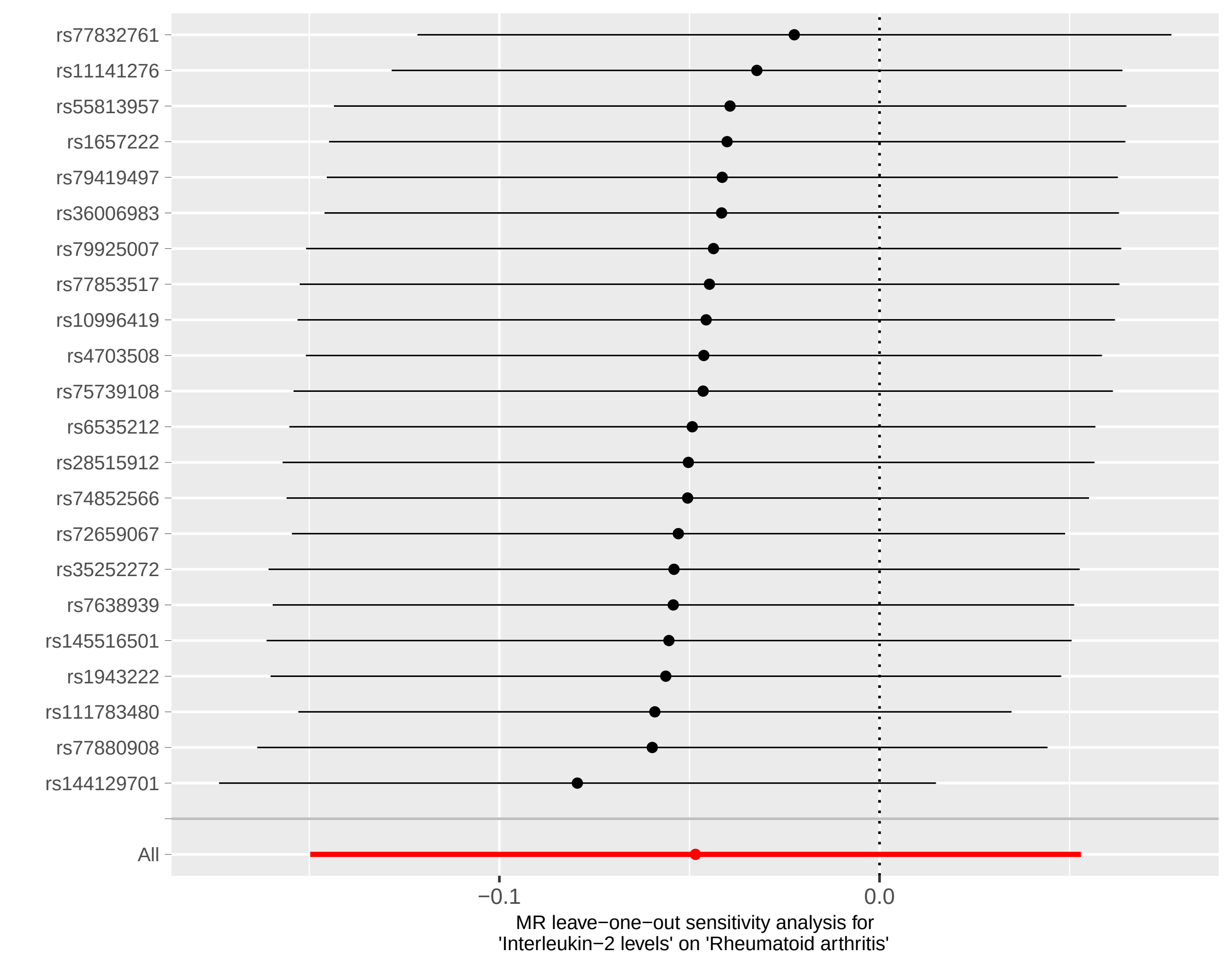
rs77832761
rs11141276
rs55813957
rs1657222
rs79419497
rs36006983
rs79925007
rs77853517
rs10996419
rs4703508
rs75739108
rs6535212
rs28515912
rs74852566
rs72659067
rs35252272
rs7638939
rs145516501
rs1943222
rs111783480
rs77880908
rs144129701
All
−0.1
0.0
MR leave−one−out sensitivity analysis for
'Interleukin−2 levels' on 'Rheumatoid arthritis'

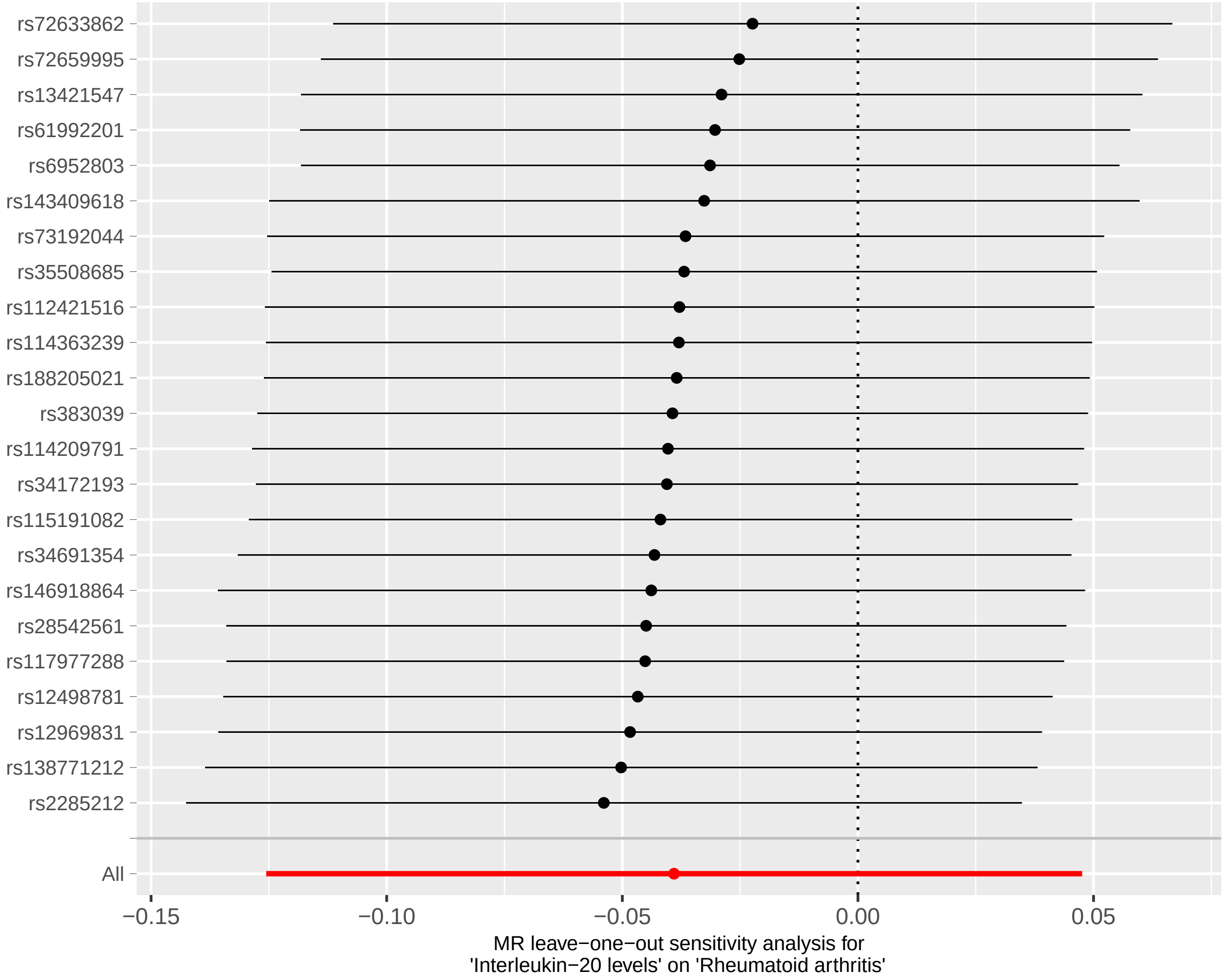
rs72633862
rs72659995
rs13421547
rs61992201
rs6952803
rs143409618
rs73192044
rs35508685
rs112421516
rs114363239
rs188205021
rs383039
rs114209791
rs34172193
rs115191082
rs34691354
rs146918864
rs28542561
rs117977288
rs12498781
rs12969831
rs138771212
rs2285212
All
−0.15
−0.10
−0.05
0.00
0.05
MR leave−one−out sensitivity analysis for
'Interleukin−20 levels' on 'Rheumatoid arthritis'

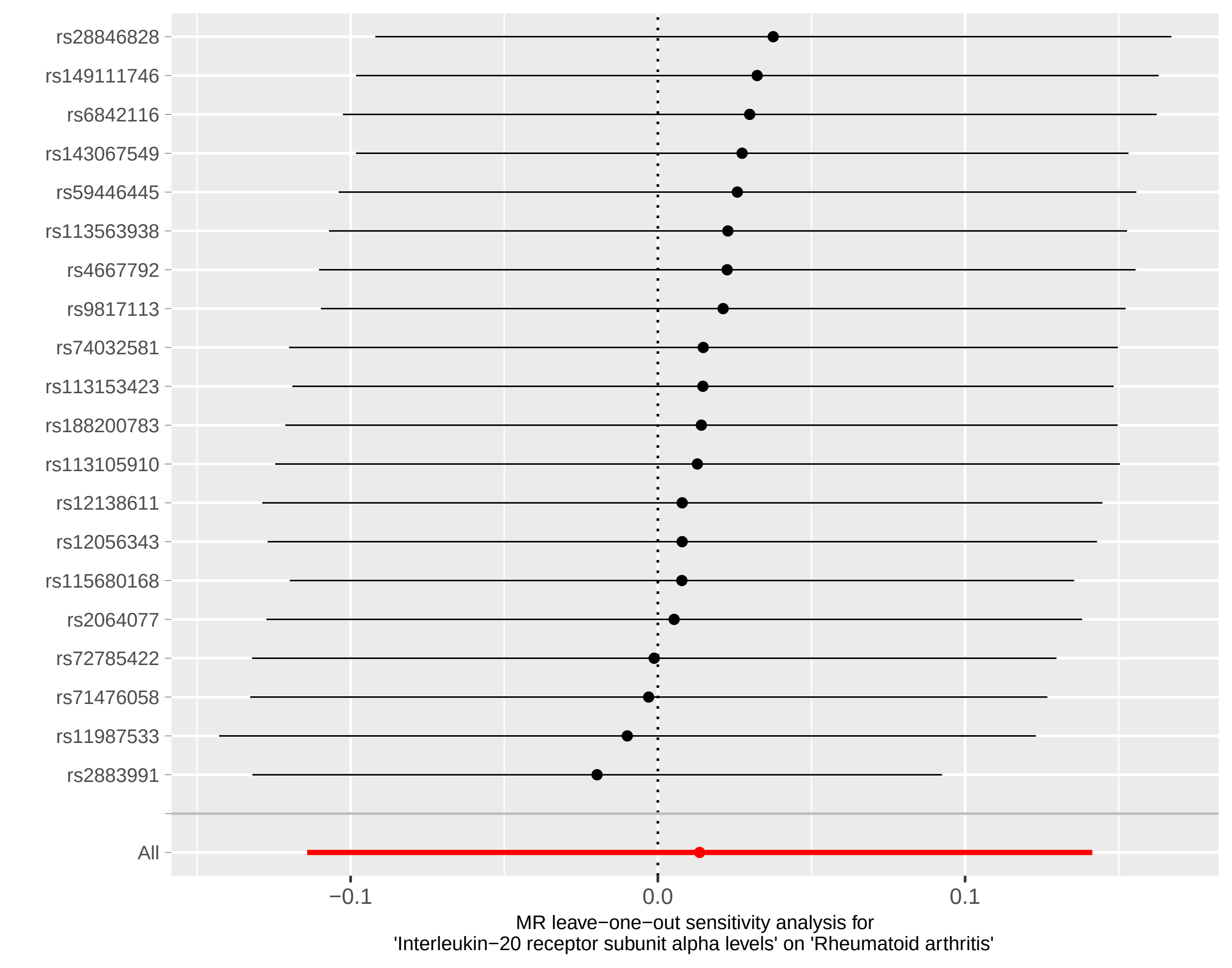
rs28846828
rs149111746
rs6842116
rs143067549
rs59446445
rs113563938
rs4667792
rs9817113
rs74032581
rs113153423
rs188200783
rs113105910
rs12138611
rs12056343
rs115680168
rs2064077
rs72785422
rs71476058
rs11987533
rs2883991
All
−0.1
0.0
0.1
MR leave−one−out sensitivity analysis for
'Interleukin−20 receptor subunit alpha levels' on 'Rheumatoid arthritis'

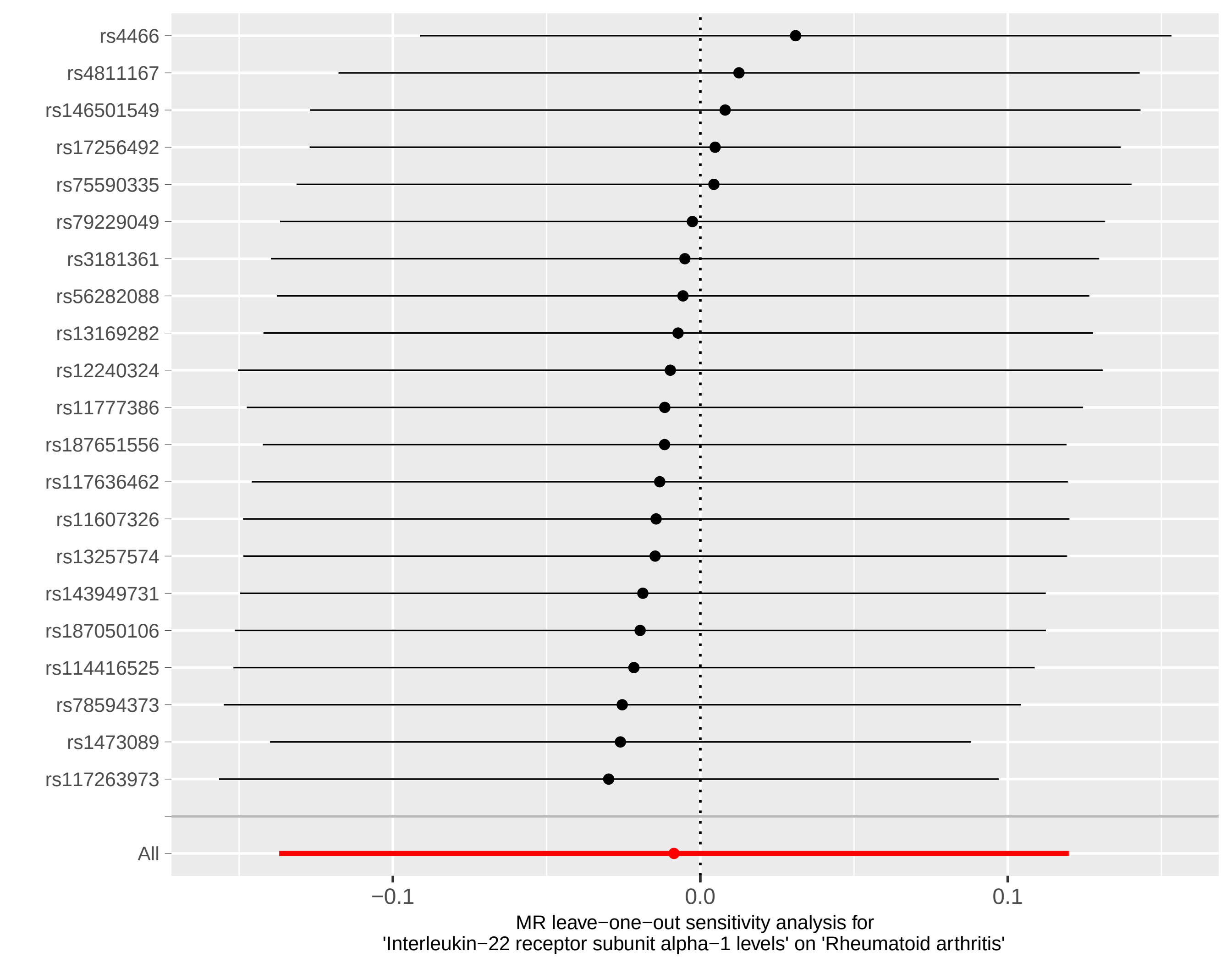

rs4466
rs4811167
rs146501549
rs17256492
rs75590335
rs79229049
rs3181361
rs56282088
rs13169282
rs12240324
rs11777386
rs187651556
rs117636462
rs11607326
rs13257574
rs143949731
rs187050106
rs114416525
rs78594373
rs1473089
rs117263973
All
−0.1
0.0
0.1
MR leave−one−out sensitivity analysis for
'Interleukin−22 receptor subunit alpha−1 levels' on 'Rheumatoid arthritis'

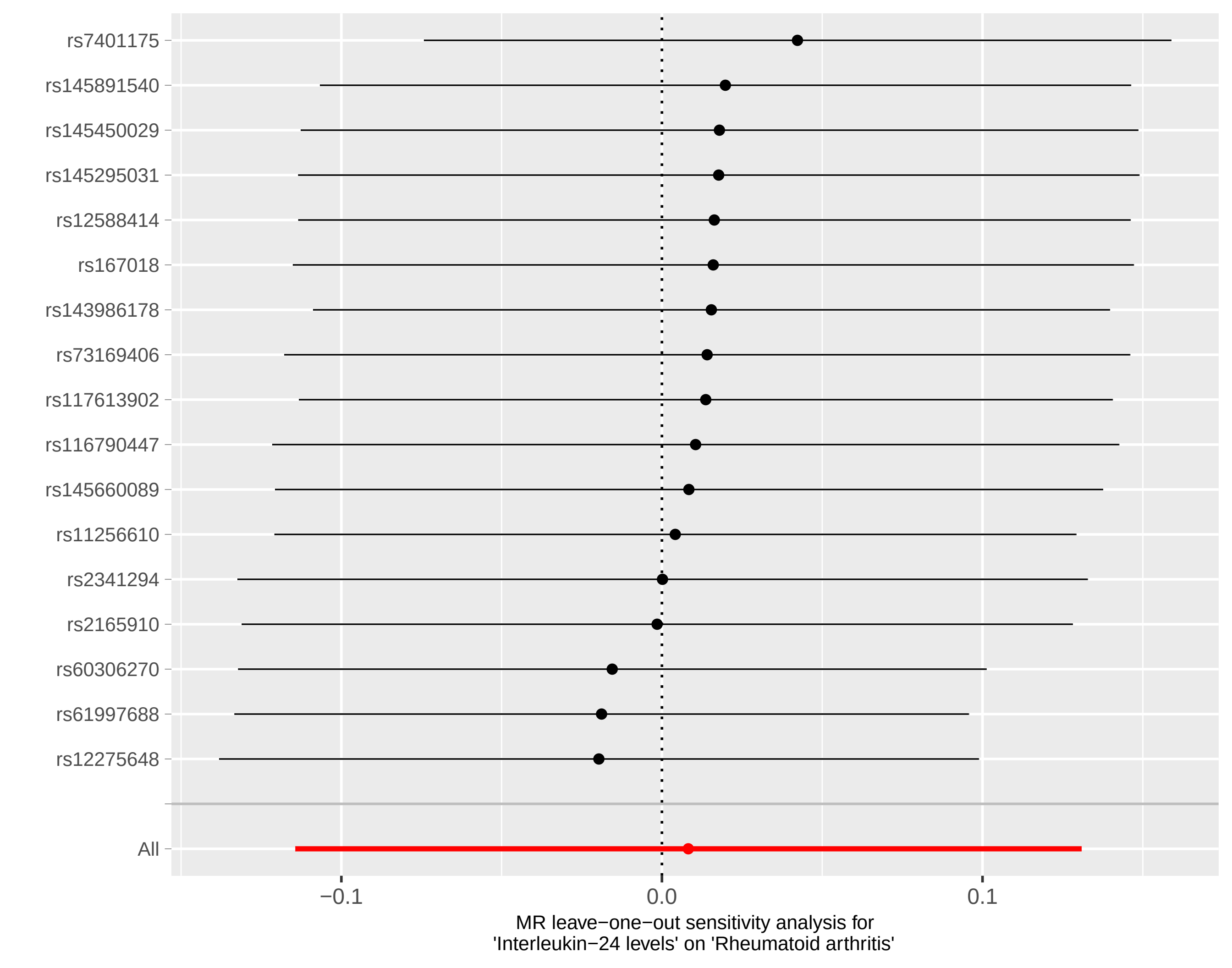
rs7401175
rs145891540
rs145450029
rs145295031
rs12588414
rs167018
rs143986178
rs73169406
rs117613902
rs116790447
rs145660089
rs11256610
rs2341294
rs2165910
rs60306270
rs61997688
rs12275648
All
−0.1
0.0
0.1
MR leave−one−out sensitivity analysis for
'Interleukin−24 levels' on 'Rheumatoid arthritis'

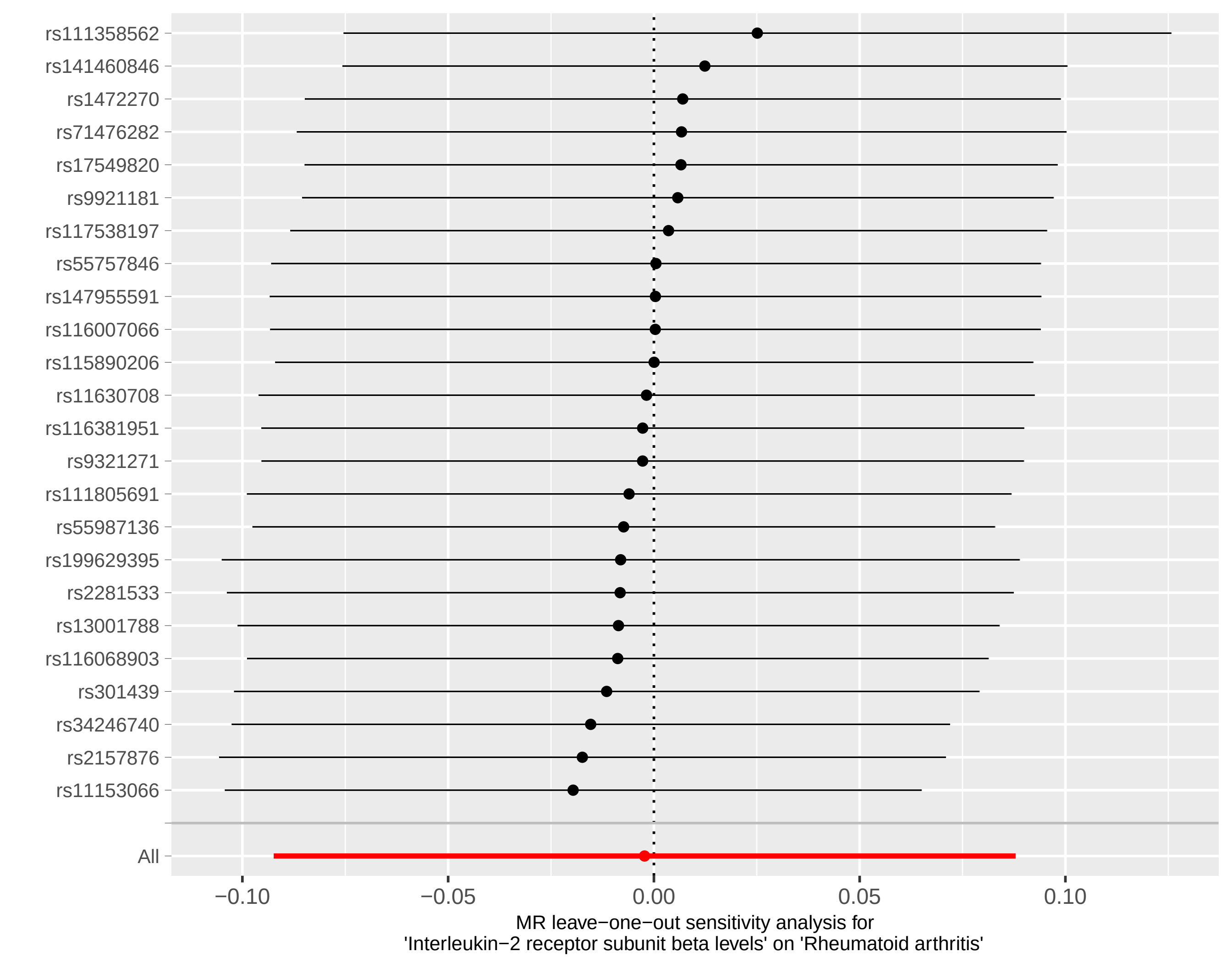

rs111358562
rs141460846
rs1472270
rs71476282
rs17549820
rs9921181
rs117538197
rs55757846
rs147955591
rs116007066
rs115890206
rs11630708
rs116381951
rs9321271
rs111805691
rs55987136
rs199629395
rs2281533
rs13001788
rs116068903
rs301439
rs34246740
rs2157876
rs11153066
All
−0.10
−0.05
0.00
0.05
0.10
MR leave−one−out sensitivity analysis for
'Interleukin−2 receptor subunit beta levels' on 'Rheumatoid arthritis'

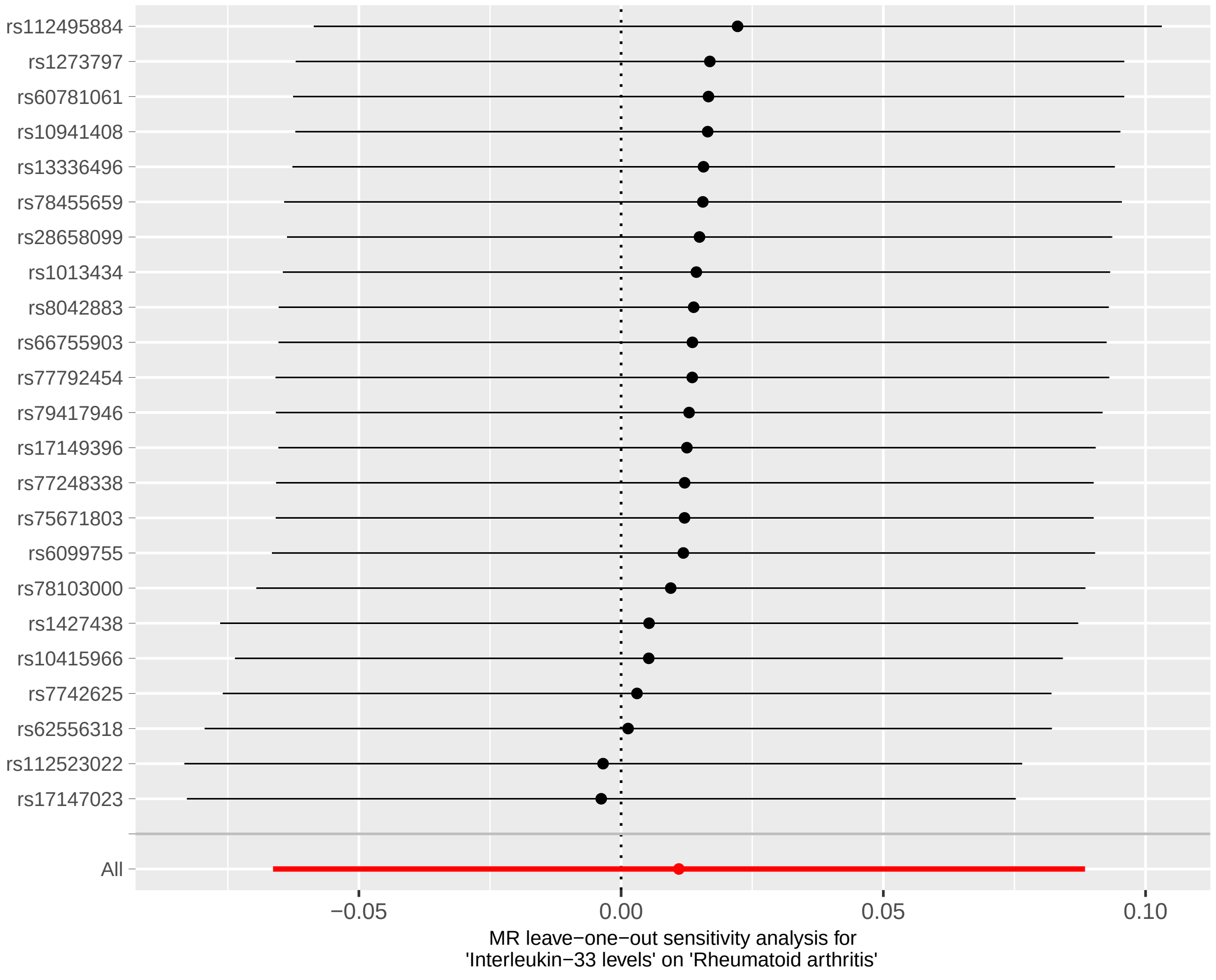
rs112495884
rs1273797
rs60781061
rs10941408
rs13336496
rs78455659
rs28658099
rs1013434
rs8042883
rs66755903
rs77792454
rs79417946
rs17149396
rs77248338
rs75671803
rs6099755
rs78103000
rs1427438
rs10415966
rs7742625
rs62556318
rs112523022
rs17147023
All
−0.05
0.00
0.05
0.10
MR leave−one−out sensitivity analysis for
'Interleukin−33 levels' on 'Rheumatoid arthritis'

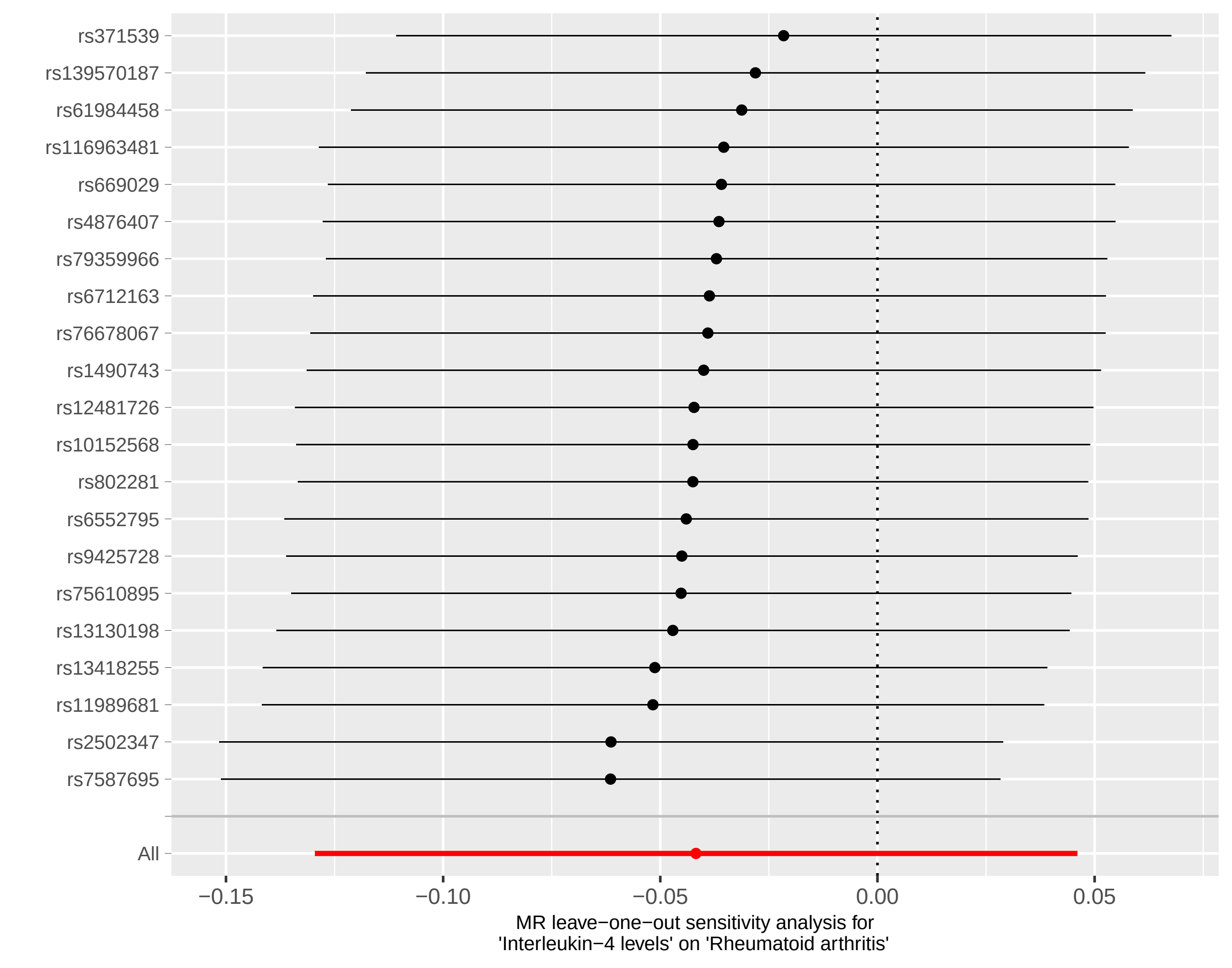
rs371539
rs139570187
rs61984458
rs116963481
rs669029
rs4876407
rs79359966
rs6712163
rs76678067
rs1490743
rs12481726
rs10152568
rs802281
rs6552795
rs9425728
rs75610895
rs13130198
rs13418255
rs11989681
rs2502347
rs7587695
All
−0.15
−0.10
−0.05
0.00
0.05
MR leave−one−out sensitivity analysis for
'Interleukin−4 levels' on 'Rheumatoid arthritis'

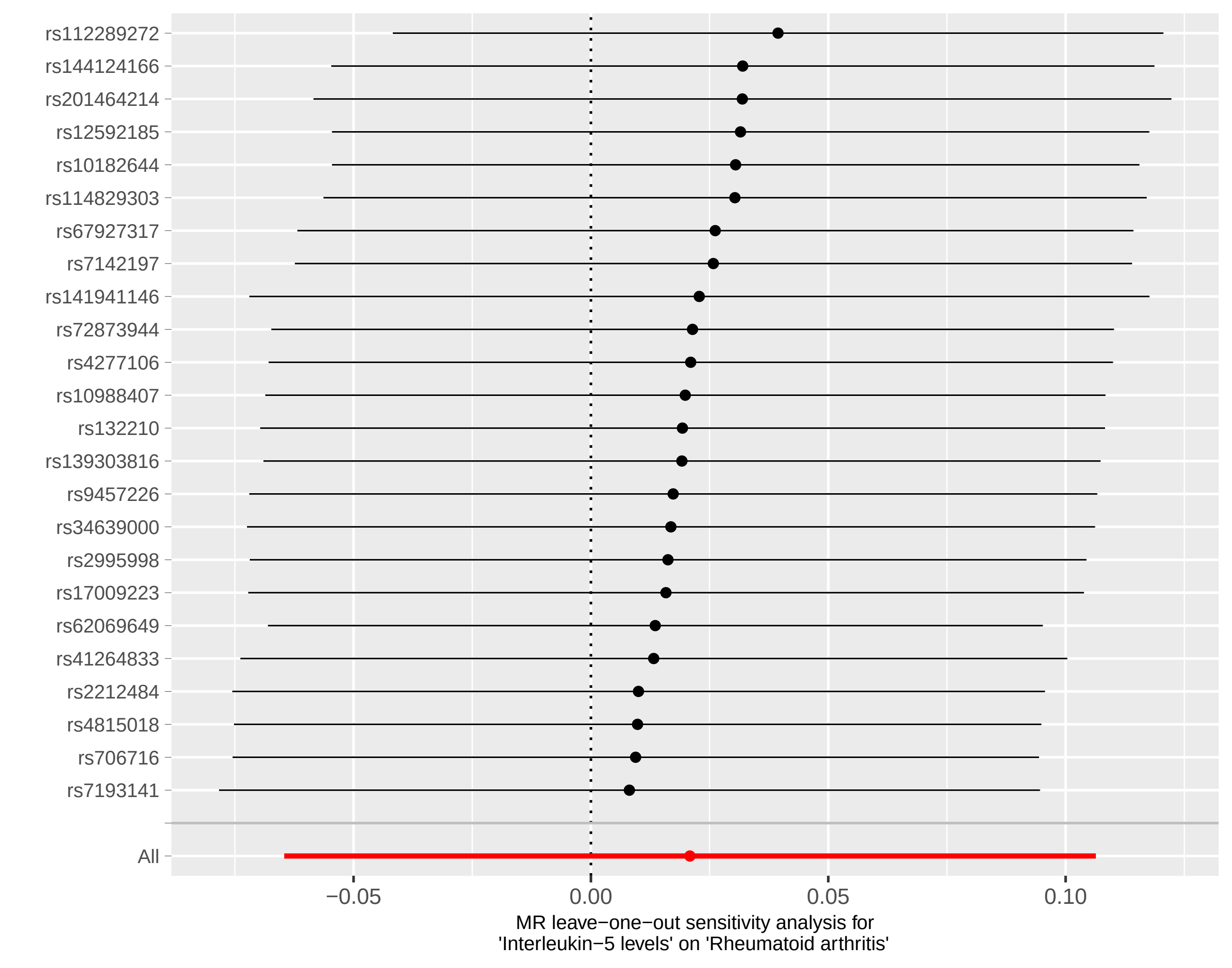
rs112289272
rs144124166
rs201464214
rs12592185
rs10182644
rs114829303
rs67927317
rs7142197
rs141941146
rs72873944
rs4277106
rs10988407
rs132210
rs139303816
rs9457226
rs34639000
rs2995998
rs17009223
rs62069649
rs41264833
rs2212484
rs4815018
rs706716
rs7193141
All
−0.05
0.00
0.05
0.10
MR leave−one−out sensitivity analysis for
'Interleukin−5 levels' on 'Rheumatoid arthritis'

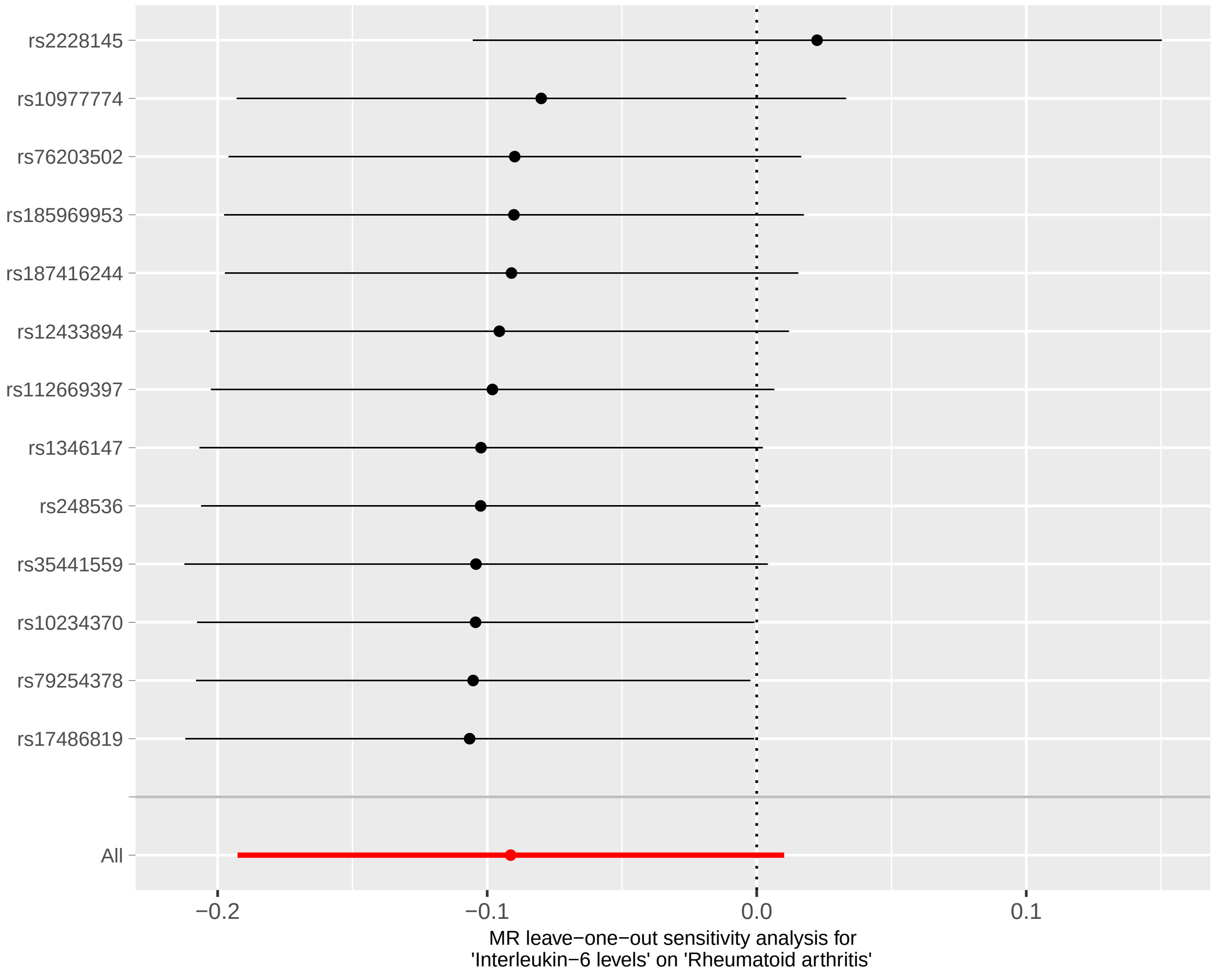

rs2228145
rs10977774
rs76203502
rs185969953
rs187416244
rs12433894
rs112669397
rs1346147
rs248536
rs35441559
rs10234370
rs79254378
rs17486819
All
−0.2
−0.1
0.0
0.1
MR leave−one−out sensitivity analysis for
'Interleukin−6 levels' on 'Rheumatoid arthritis'

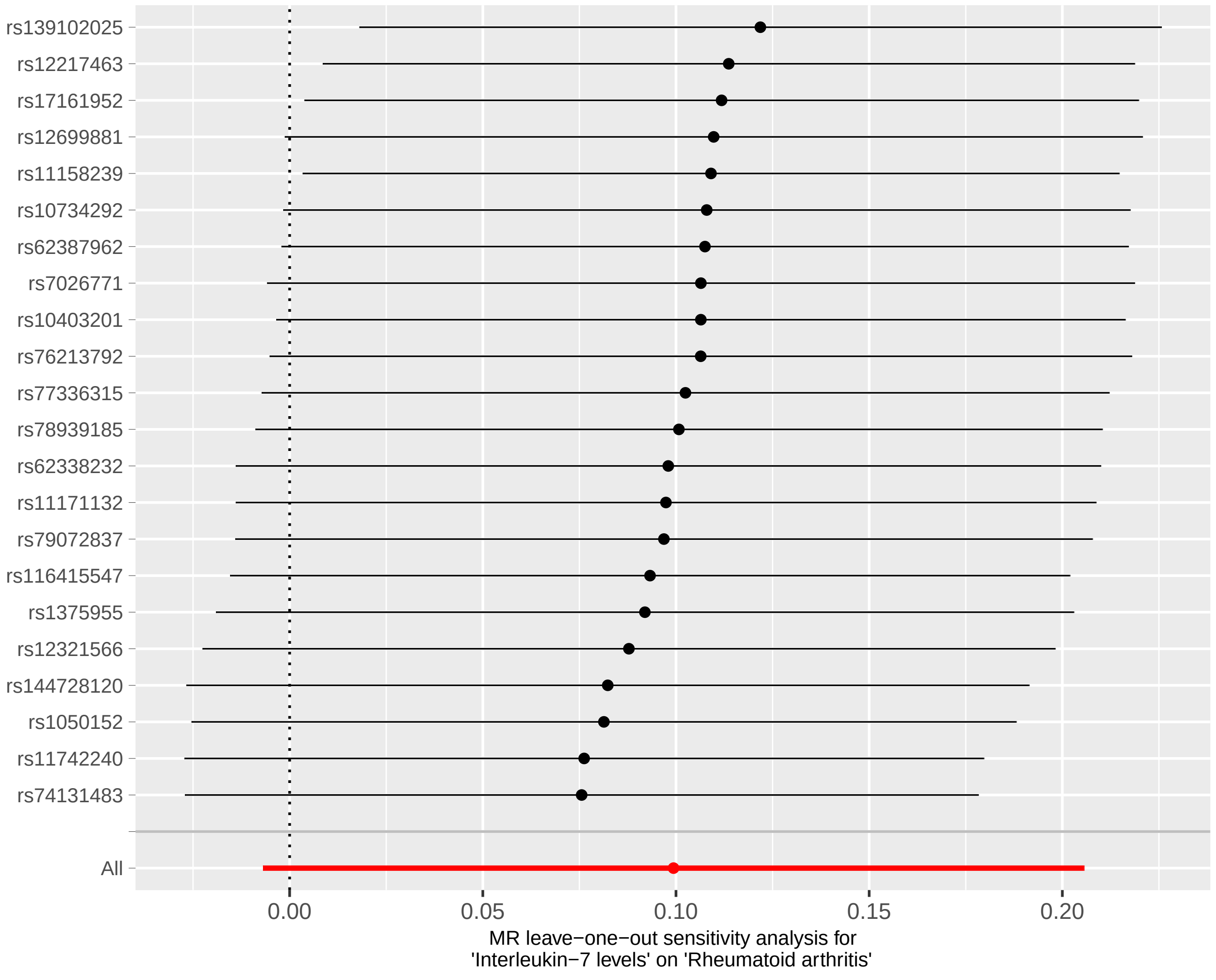

rs139102025
rs12217463
rs17161952
rs12699881
rs11158239
rs10734292
rs62387962
rs7026771
rs10403201
rs76213792
rs77336315
rs78939185
rs62338232
rs11171132
rs79072837
rs116415547
rs1375955
rs12321566
rs144728120
rs1050152
rs11742240
rs74131483
All
0.00
0.05
0.10
0.15
0.20
MR leave−one−out sensitivity analysis for
'Interleukin−7 levels' on 'Rheumatoid arthritis'

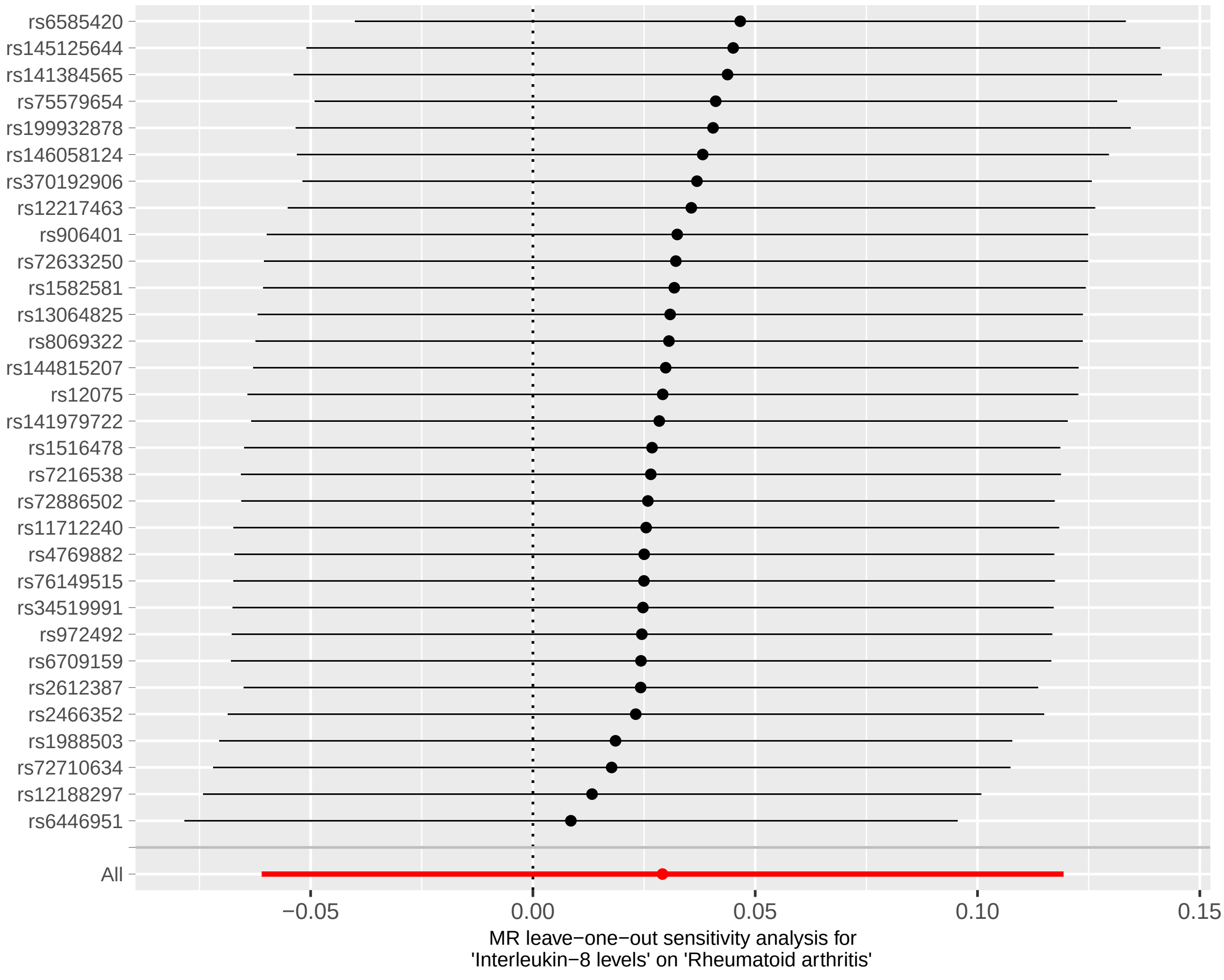
rs6585420
rs145125644
rs141384565
rs75579654
rs199932878
rs146058124
rs370192906
rs12217463
rs906401
rs72633250
rs1582581
rs13064825
rs8069322
rs144815207
rs12075
rs141979722
rs1516478
rs7216538
rs72886502
rs11712240
rs4769882
rs76149515
rs34519991
rs972492
rs6709159
rs2612387
rs2466352
rs1988503
rs72710634
rs12188297
rs6446951
All
−0.05
0.00
0.05
0.10
0.15
MR leave−one−out sensitivity analysis for
'Interleukin−8 levels' on 'Rheumatoid arthritis'

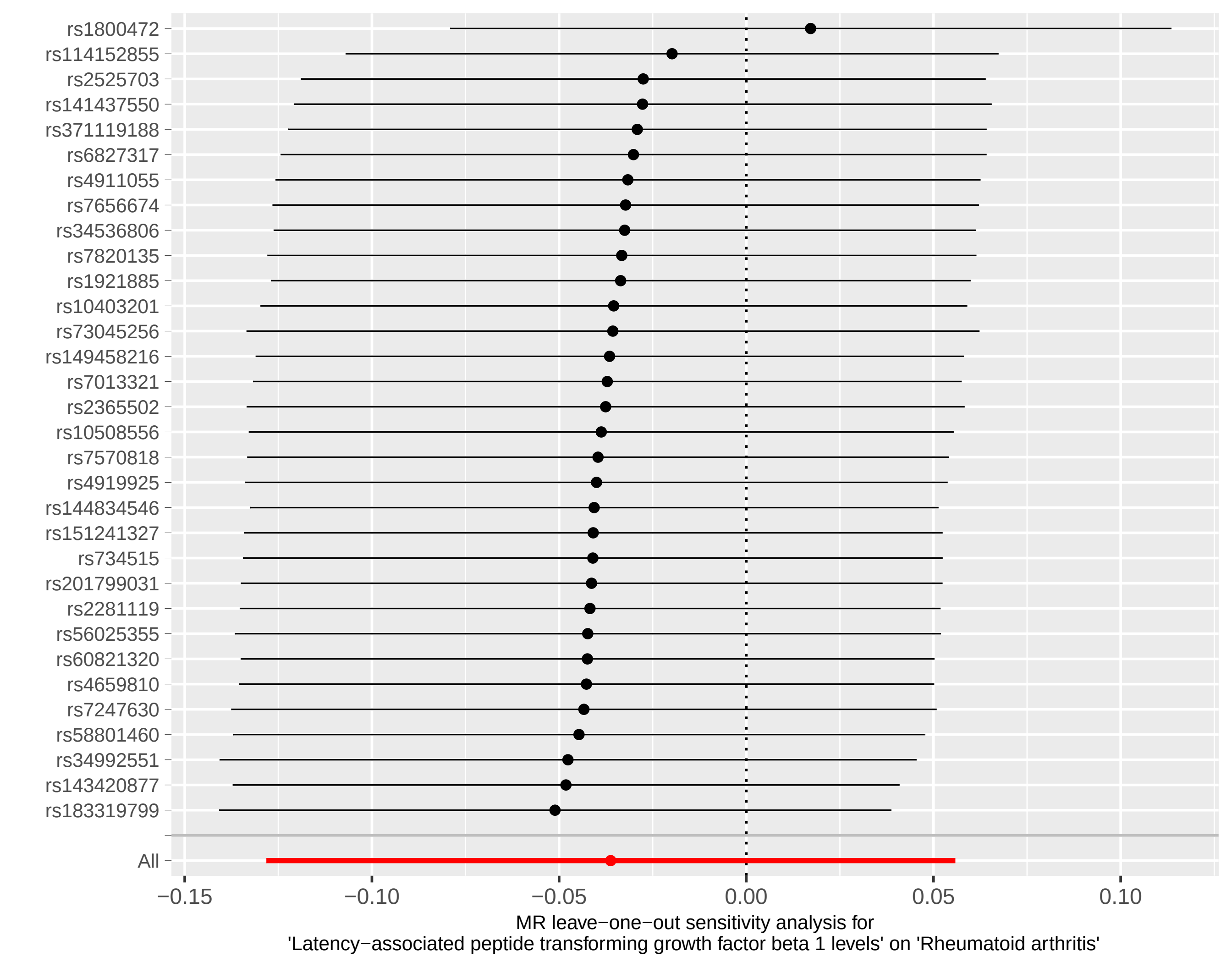

rs1800472
rs114152855
rs2525703
rs141437550
rs371119188
rs6827317
rs4911055
rs7656674
rs34536806
rs7820135
rs1921885
rs10403201
rs73045256
rs149458216
rs7013321
rs2365502
rs10508556
rs7570818
rs4919925
rs144834546
rs151241327
rs734515
rs201799031
rs2281119
rs56025355
rs60821320
rs4659810
rs7247630
rs58801460
rs34992551
rs143420877
rs183319799
All
−0.15
−0.10
−0.05
0.00
0.05
0.10
MR leave−one−out sensitivity analysis for
'Latency−associated peptide transforming growth factor beta 1 levels' on 'Rheumatoid arthritis'

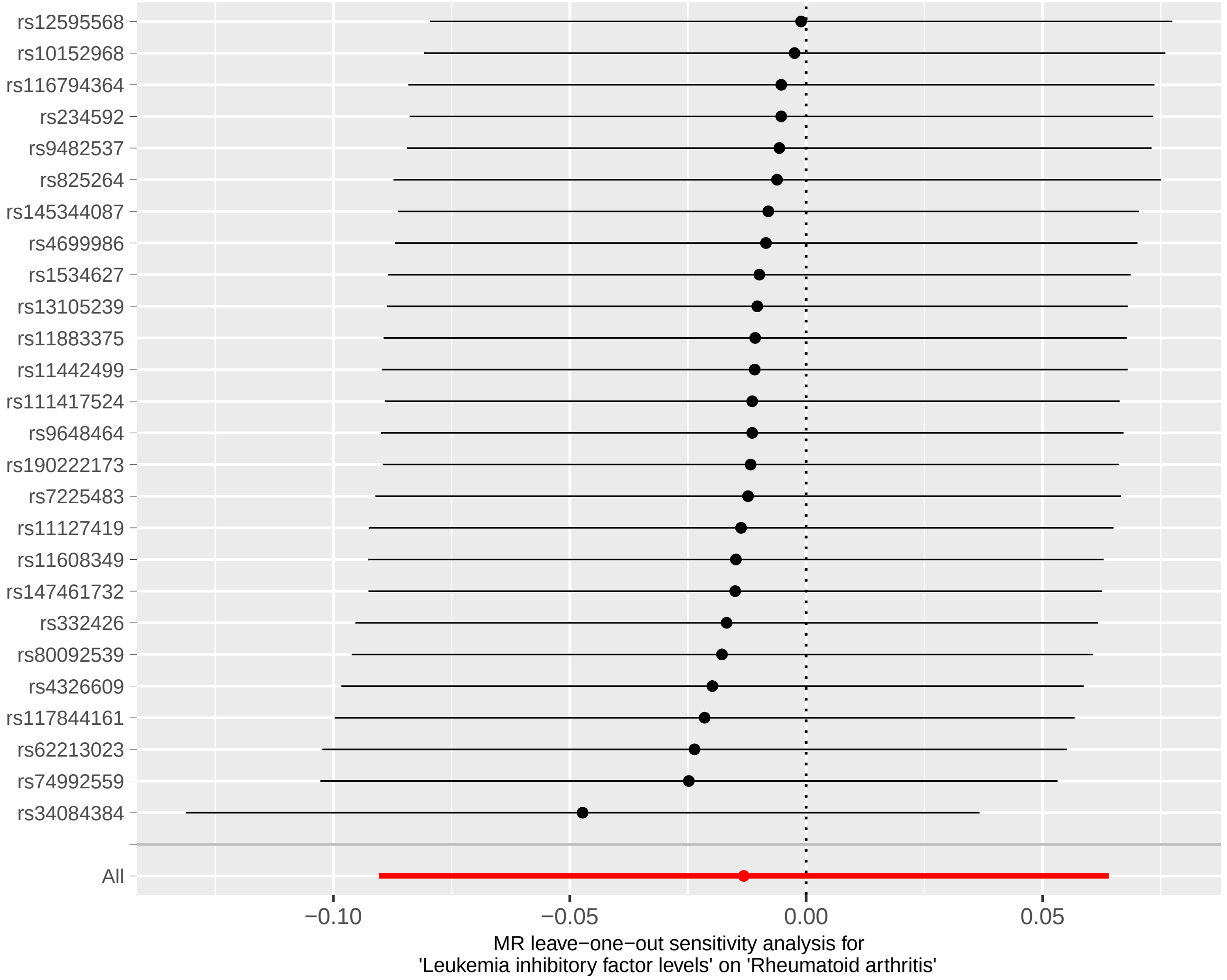
rs12595568
rs10152968
rs116794364
rs234592
rs9482537
rs825264
rs145344087
rs4699986
rs1534627
rs13105239
rs11883375
rs11442499
rs111417524
rs9648464
rs190222173
rs7225483
rs11127419
rs11608349
rs147461732
rs332426
rs80092539
rs4326609
rs117844161
rs62213023
rs74992559
rs34084384
All
−0.10
−0.05
0.00
0.05
MR leave−one−out sensitivity analysis for
'Leukemia inhibitory factor levels' on 'Rheumatoid arthritis'

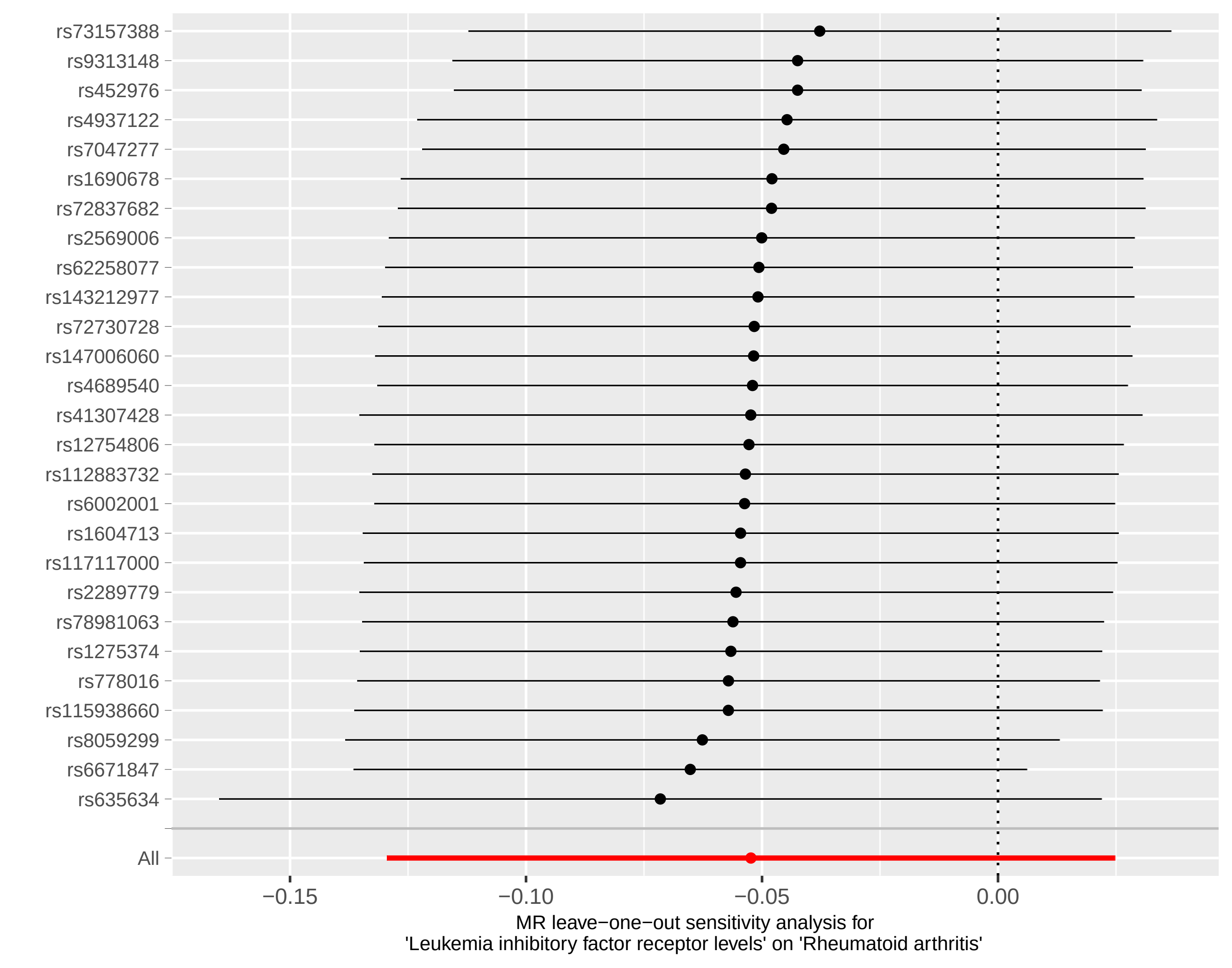
rs73157388
rs9313148
rs452976
rs4937122
rs7047277
rs1690678
rs72837682
rs2569006
rs62258077
rs143212977
rs72730728
rs147006060
rs4689540
rs41307428
rs12754806
rs112883732
rs6002001
rs1604713
rs117117000
rs2289779
rs78981063
rs1275374
rs778016
rs115938660
rs8059299
rs6671847
rs635634
All
−0.15
−0.10
−0.05
0.00
MR leave−one−out sensitivity analysis for
'Leukemia inhibitory factor receptor levels' on 'Rheumatoid arthritis'

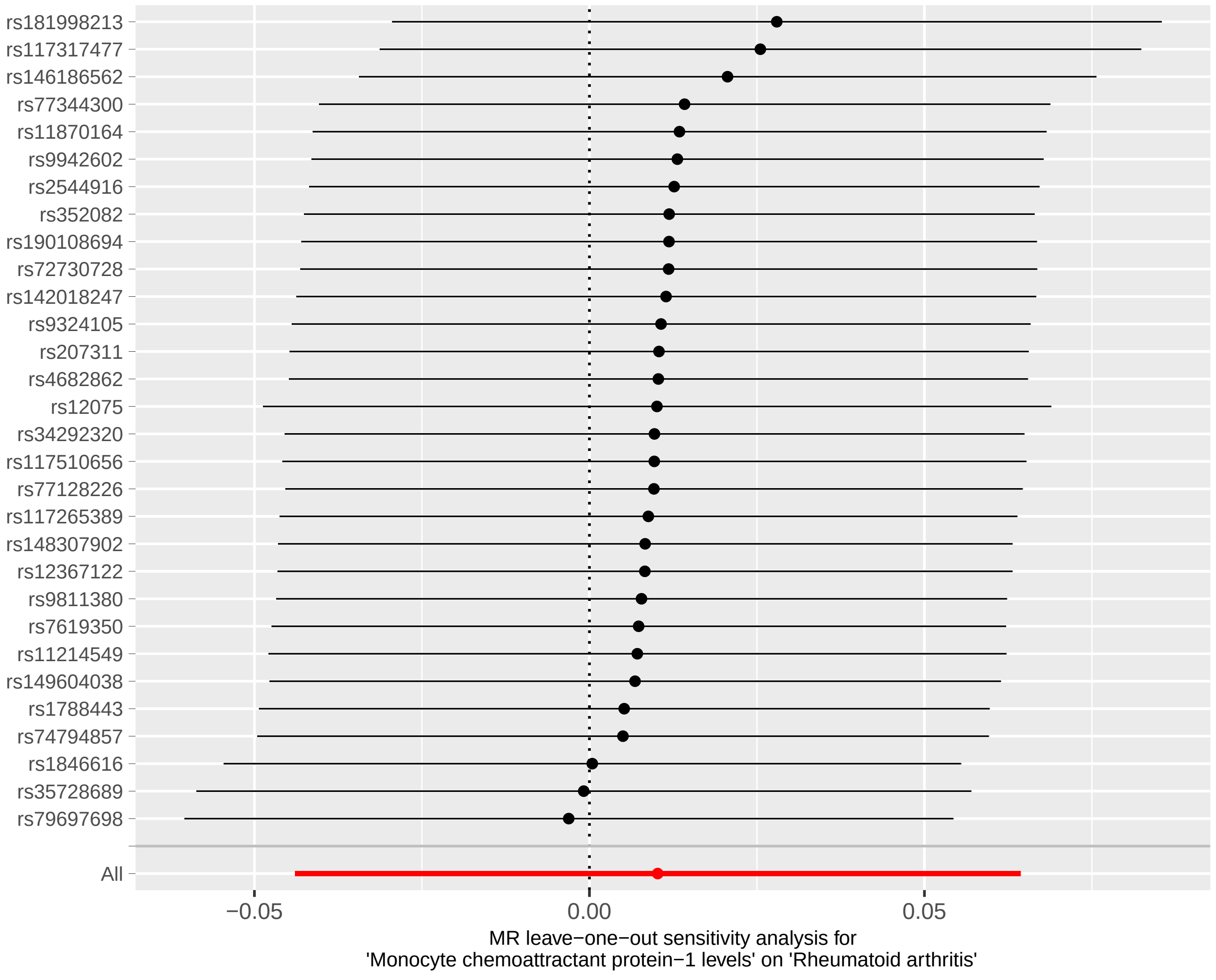
rs181998213
rs117317477
rs146186562
rs77344300
rs11870164
rs9942602
rs2544916
rs352082
rs190108694
rs72730728
rs142018247
rs9324105
rs207311
rs4682862
rs12075
rs34292320
rs117510656
rs77128226
rs117265389
rs148307902
rs12367122
rs9811380
rs7619350
rs11214549
rs149604038
rs1788443
rs74794857
rs1846616
rs35728689
rs79697698
All
−0.05
0.00
0.05
MR leave−one−out sensitivity analysis for
'Monocyte chemoattractant protein−1 levels' on 'Rheumatoid arthritis'

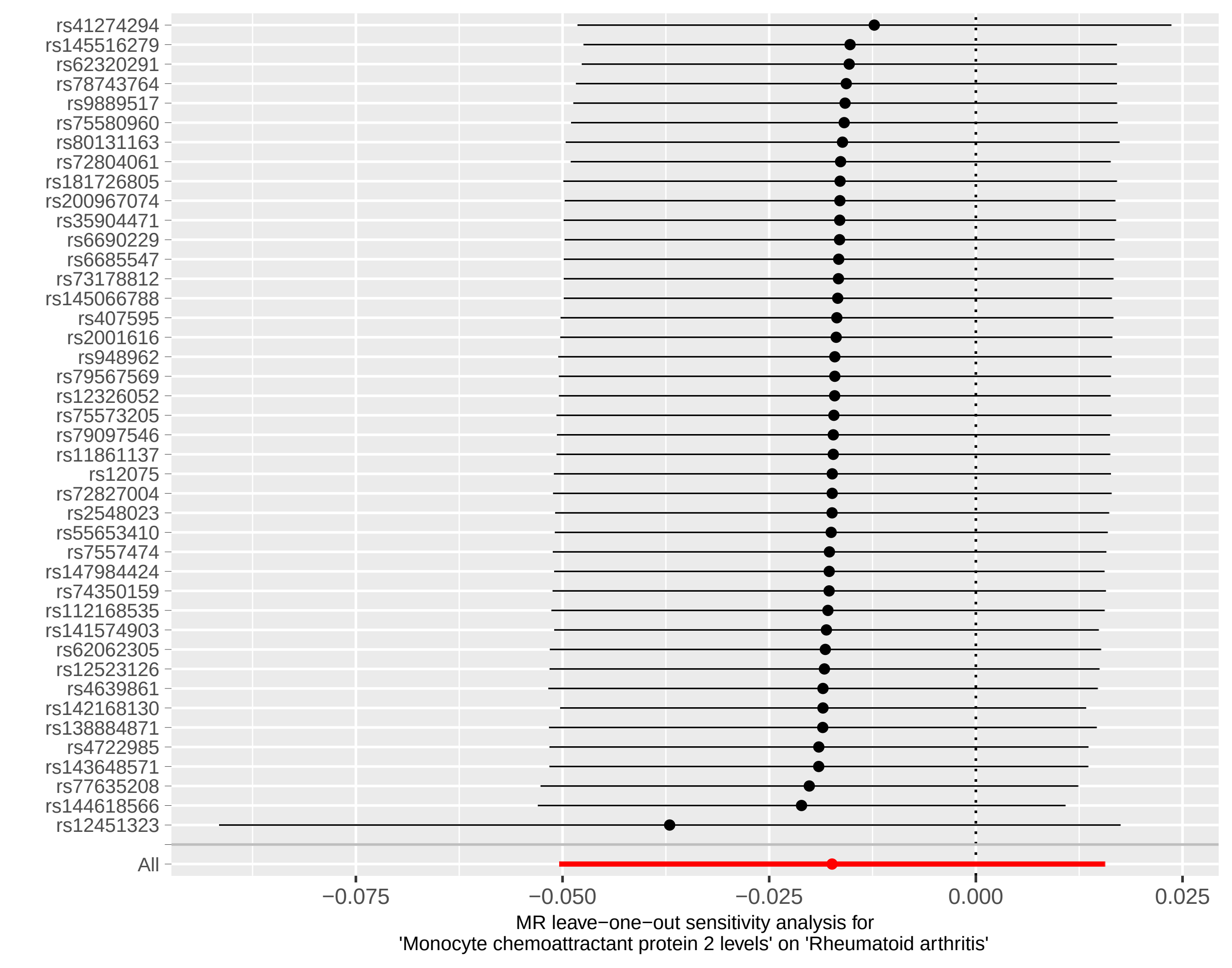
rs41274294
rs145516279
rs62320291
rs78743764
rs9889517
rs75580960
rs80131163
rs72804061
rs181726805
rs200967074
rs35904471
rs6690229
rs6685547
rs73178812
rs145066788
rs407595
rs2001616
rs948962
rs79567569
rs12326052
rs75573205
rs79097546
rs11861137
rs12075
rs72827004
rs2548023
rs55653410
rs7557474
rs147984424
rs74350159
rs112168535
rs141574903
rs62062305
rs12523126
rs4639861
rs142168130
rs138884871
rs4722985
rs143648571
rs77635208
rs144618566
rs12451323
All
−0.075
−0.050
−0.025
0.000
0.025
MR leave−one−out sensitivity analysis for
'Monocyte chemoattractant protein 2 levels' on 'Rheumatoid arthritis'

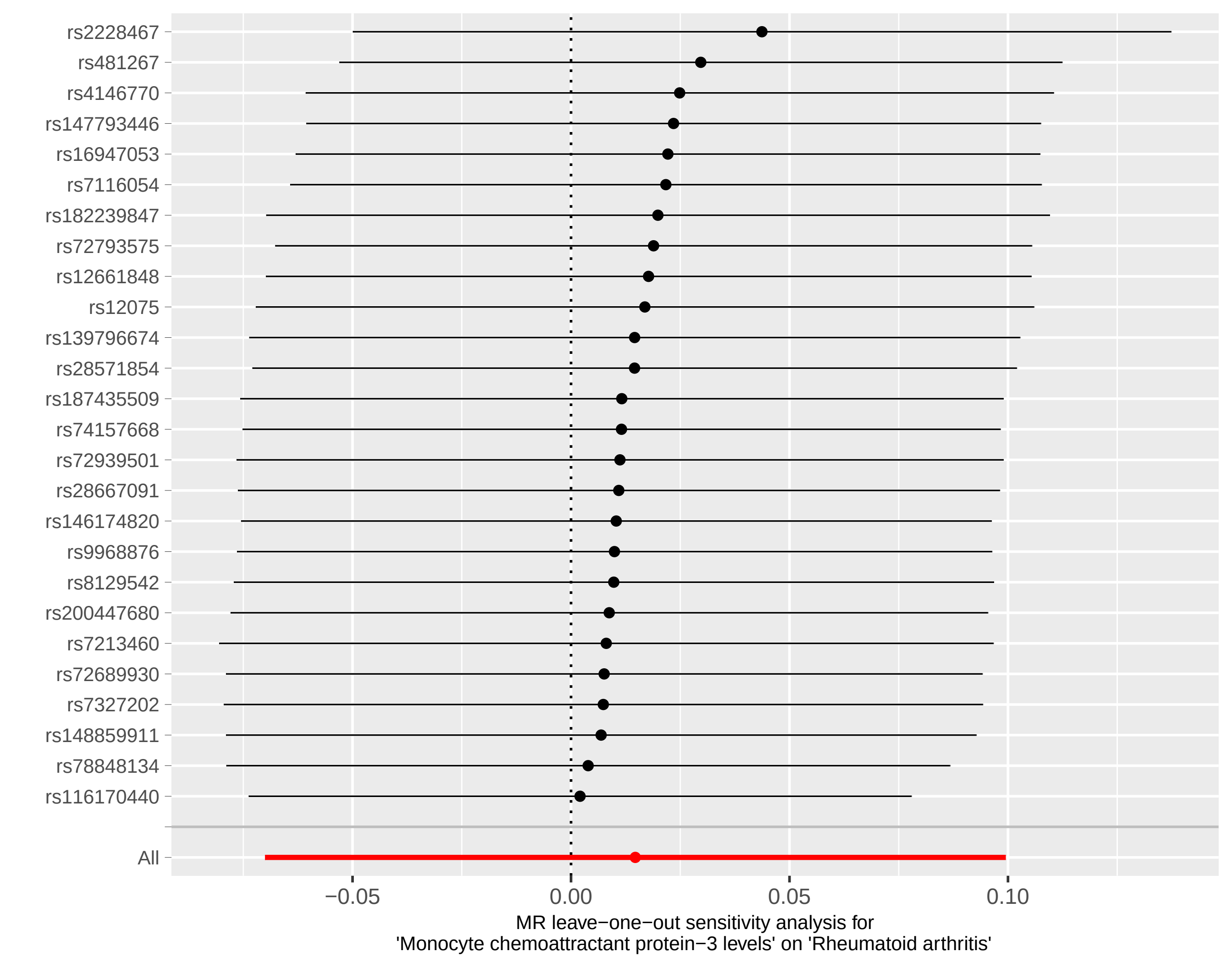
rs2228467
rs481267
rs4146770
rs147793446
rs16947053
rs7116054
rs182239847
rs72793575
rs12661848
rs12075
rs139796674
rs28571854
rs187435509
rs74157668
rs72939501
rs28667091
rs146174820
rs9968876
rs8129542
rs200447680
rs7213460
rs72689930
rs7327202
rs148859911
rs78848134
rs116170440
All
−0.05
0.00
0.05
0.10
MR leave−one−out sensitivity analysis for
'Monocyte chemoattractant protein−3 levels' on 'Rheumatoid arthritis'

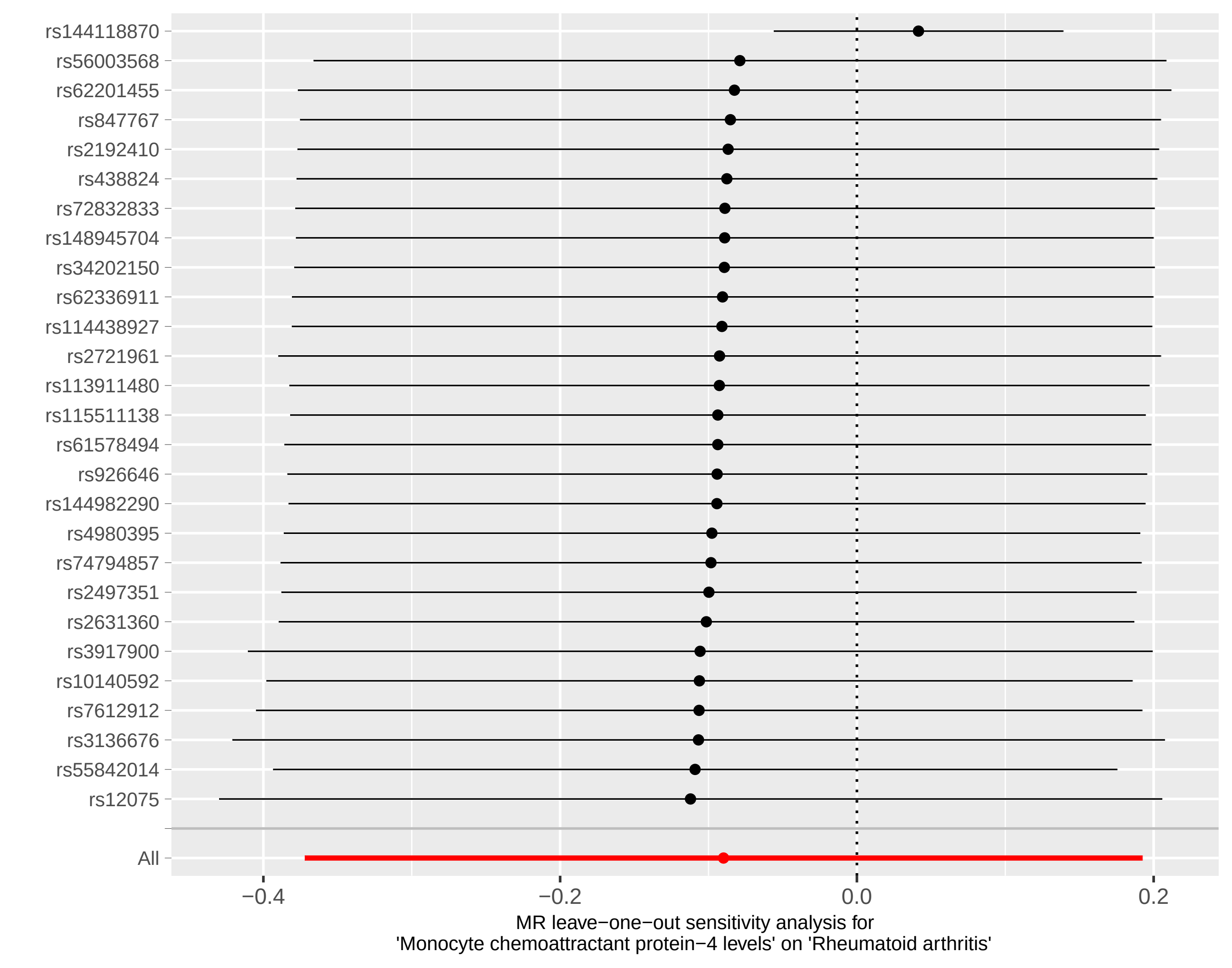
rs144118870
rs56003568
rs62201455
rs847767
rs2192410
rs438824
rs72832833
rs148945704
rs34202150
rs62336911
rs114438927
rs2721961
rs113911480
rs115511138
rs61578494
rs926646
rs144982290
rs4980395
rs74794857
rs2497351
rs2631360
rs3917900
rs10140592
rs7612912
rs3136676
rs55842014
rs12075
All
−0.4
−0.2
0.0
0.2
MR leave−one−out sensitivity analysis for
'Monocyte chemoattractant protein−4 levels' on 'Rheumatoid arthritis'

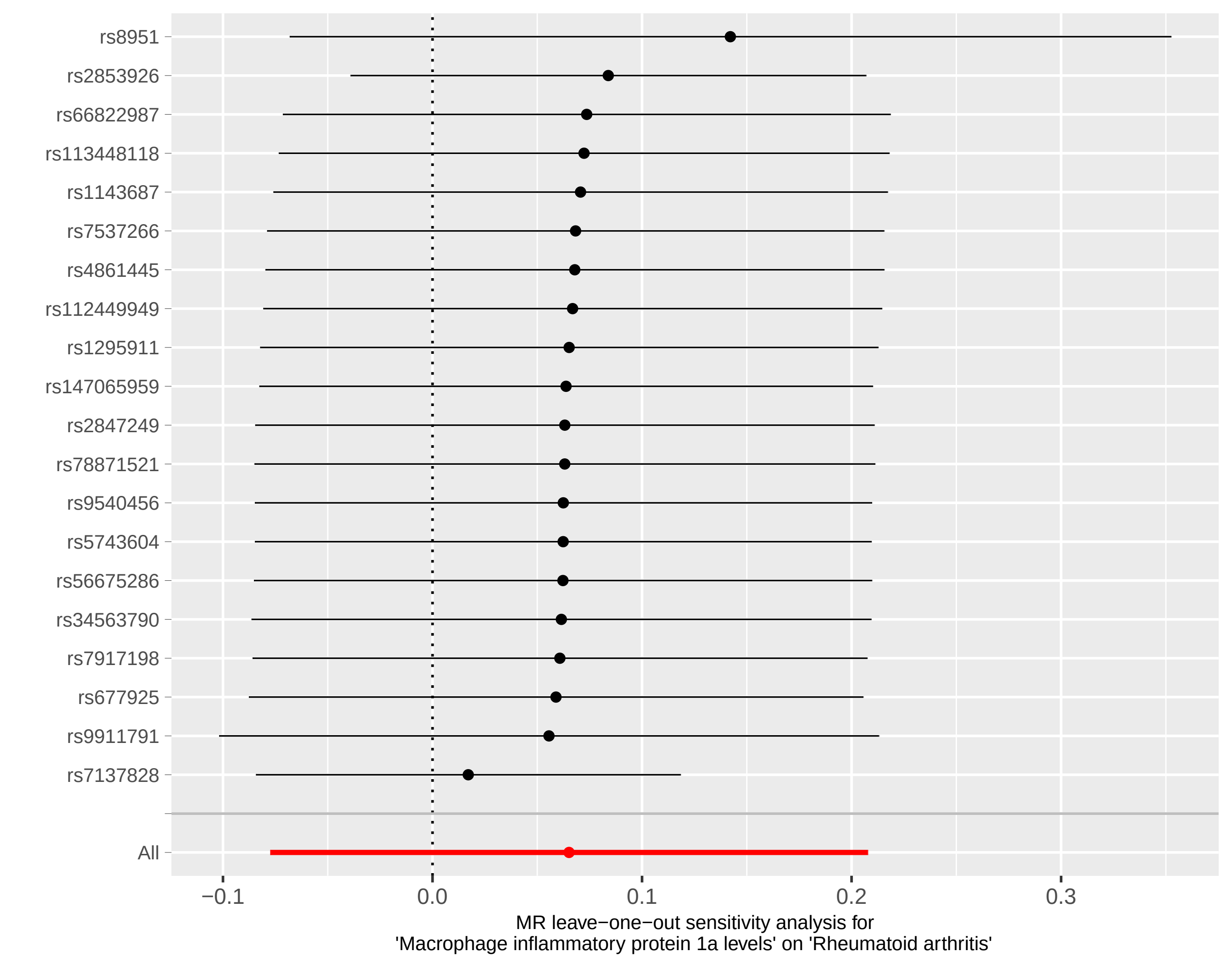
rs8951
rs2853926
rs66822987
rs113448118
rs1143687
rs7537266
rs4861445
rs112449949
rs1295911
rs147065959
rs2847249
rs78871521
rs9540456
rs5743604
rs56675286
rs34563790
rs7917198
rs677925
rs9911791
rs7137828
All
−0.1
0.0
0.1
0.2
0.3
MR leave−one−out sensitivity analysis for
'Macrophage inflammatory protein 1a levels' on 'Rheumatoid arthritis'

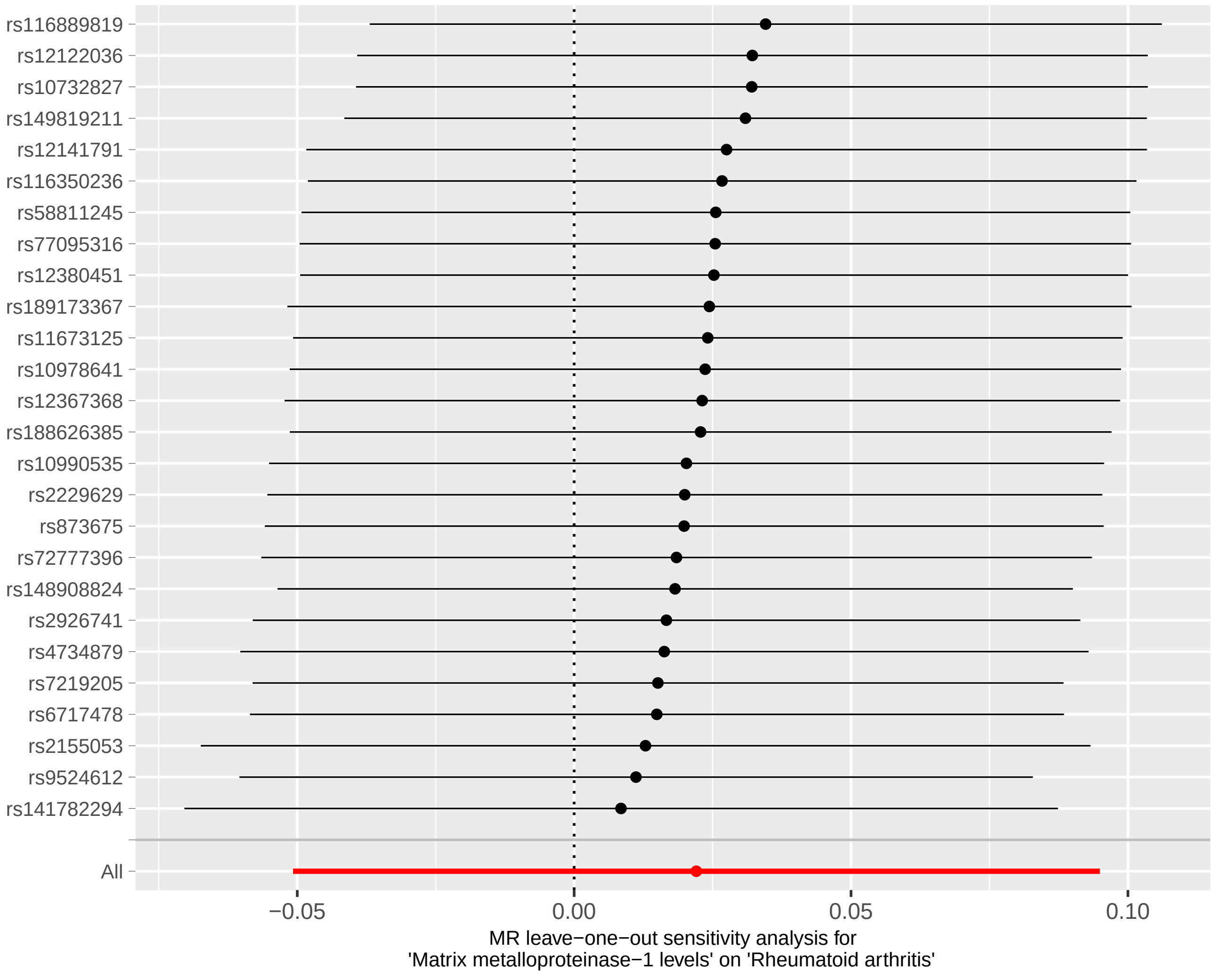
rs116889819
rs12122036
rs10732827
rs149819211
rs12141791
rs116350236
rs58811245
rs77095316
rs12380451
rs189173367
rs11673125
rs10978641
rs12367368
rs188626385
rs10990535
rs2229629
rs873675
rs72777396
rs148908824
rs2926741
rs4734879
rs7219205
rs6717478
rs2155053
rs9524612
rs141782294
All
−0.05
0.00
0.05
0.10
MR leave−one−out sensitivity analysis for
'Matrix metalloproteinase−1 levels' on 'Rheumatoid arthritis'

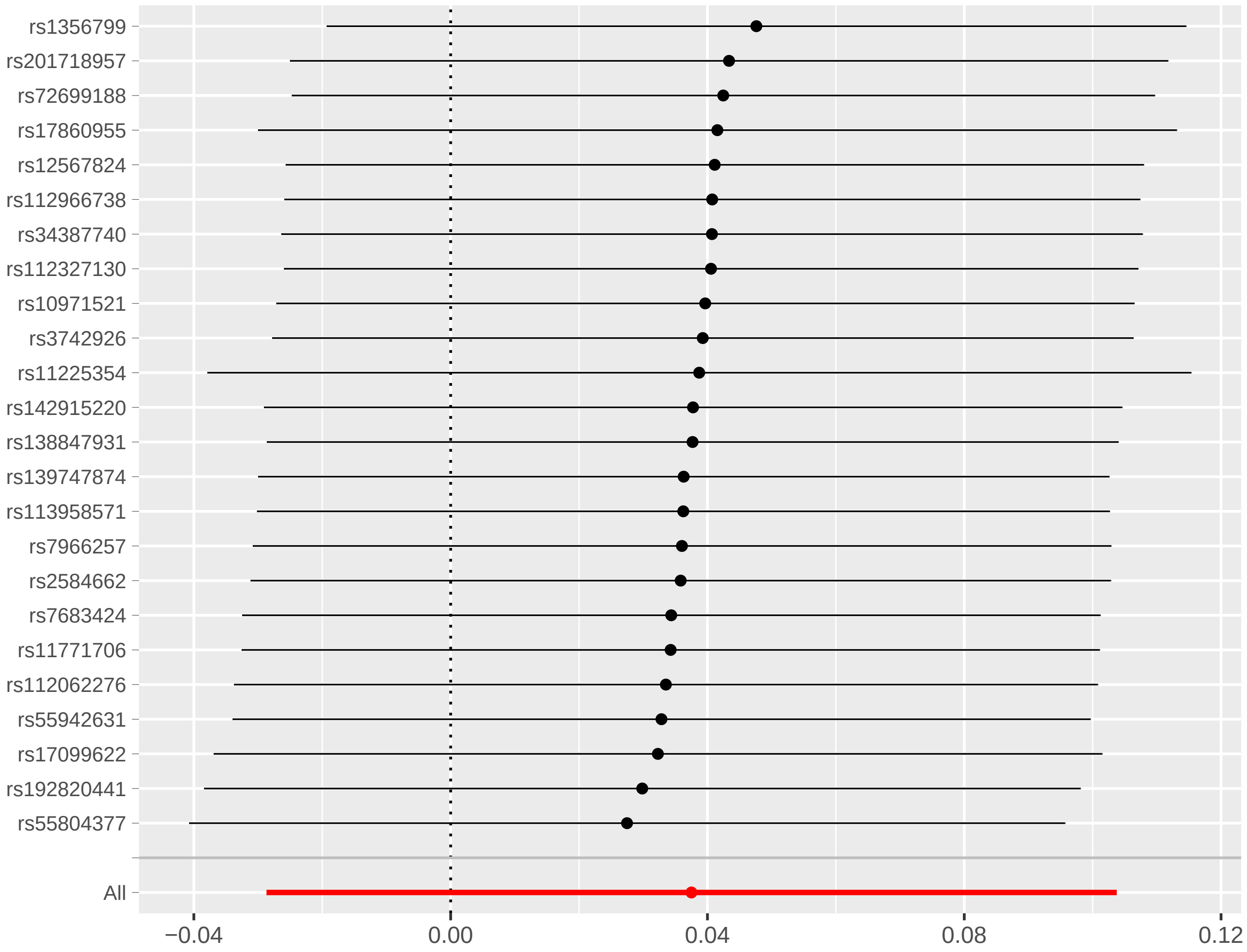

MR leave−one−out sensitivity analysis for
'Matrix metalloproteinase−10 levels' on 'Rheumatoid arthritis'

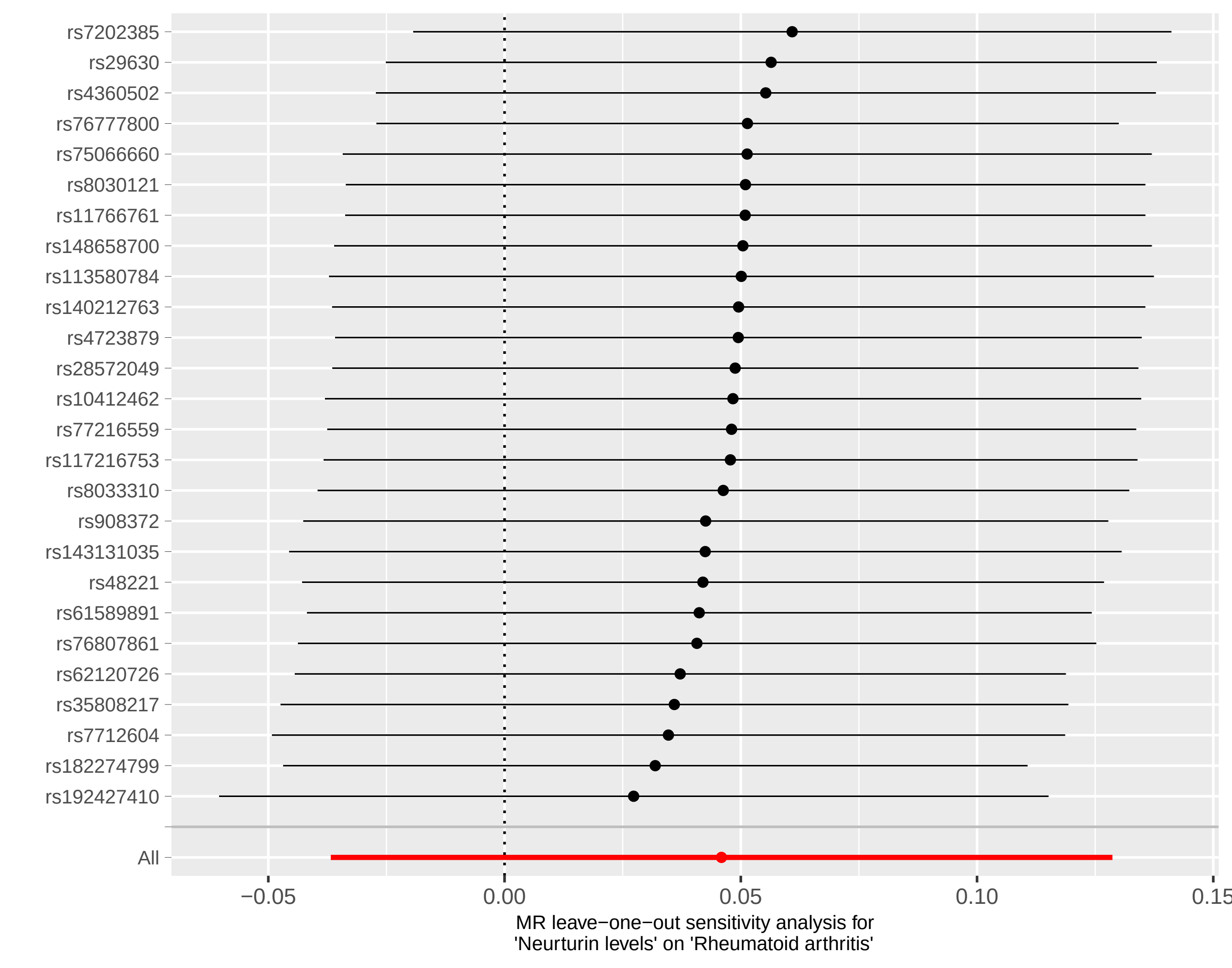
rs7202385
rs29630
rs4360502
rs76777800
rs75066660
rs8030121
rs11766761
rs148658700
rs113580784
rs140212763
rs4723879
rs28572049
rs10412462
rs77216559
rs117216753
rs8033310
rs908372
rs143131035
rs48221
rs61589891
rs76807861
rs62120726
rs35808217
rs7712604
rs182274799
rs192427410
All
−0.05
0.00
0.05
0.10
0.15
MR leave−one−out sensitivity analysis for
'Neurturin levels' on 'Rheumatoid arthritis'

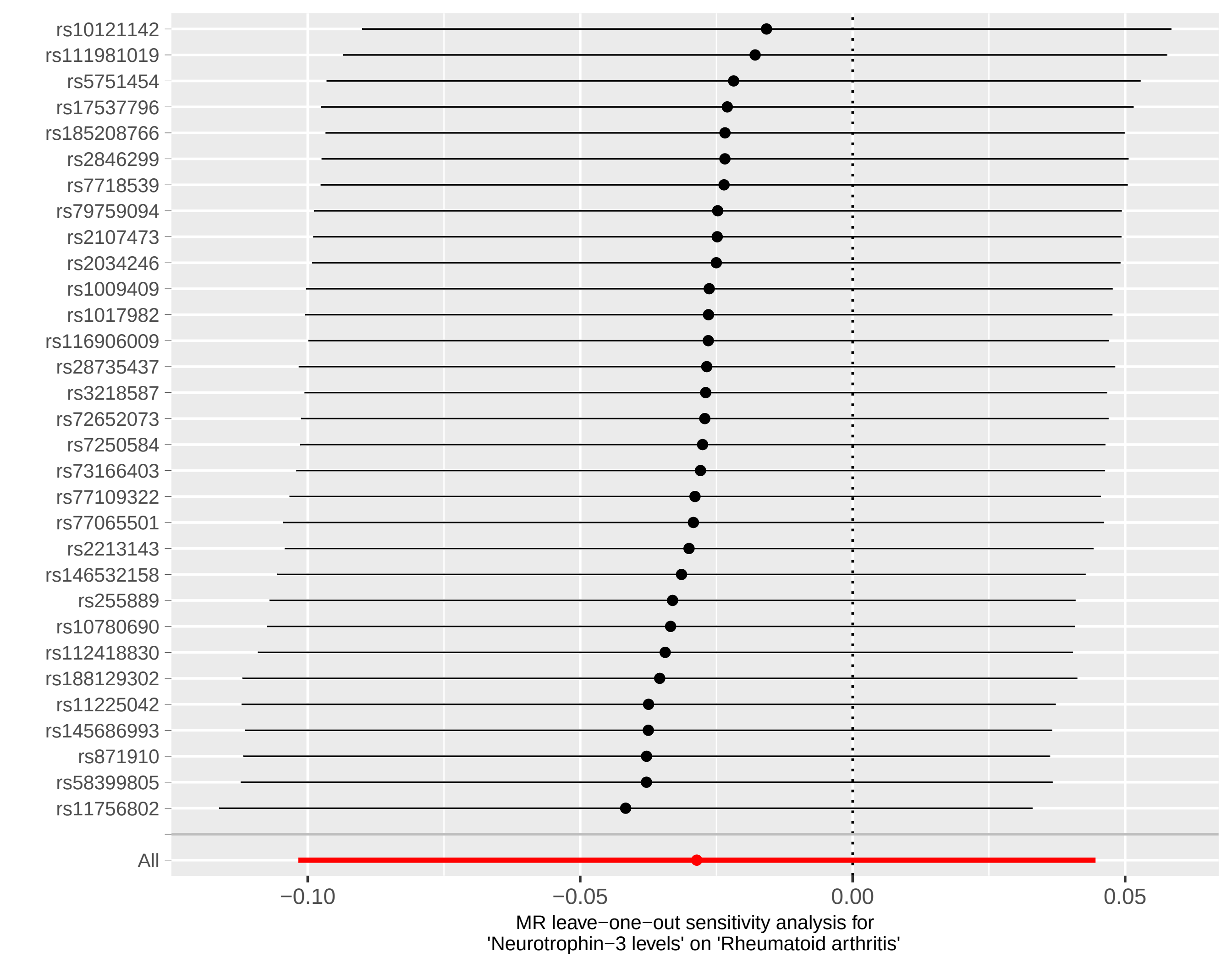

rs10121142
rs111981019
rs5751454
rs17537796
rs185208766
rs2846299
rs7718539
rs79759094
rs2107473
rs2034246
rs1009409
rs1017982
rs116906009
rs28735437
rs3218587
rs72652073
rs7250584
rs73166403
rs77109322
rs77065501
rs2213143
rs146532158
rs255889
rs10780690
rs112418830
rs188129302
rs11225042
rs145686993
rs871910
rs58399805
rs11756802
All
−0.10
−0.05
0.00
0.05
MR leave−one−out sensitivity analysis for
'Neurotrophin−3 levels' on 'Rheumatoid arthritis'

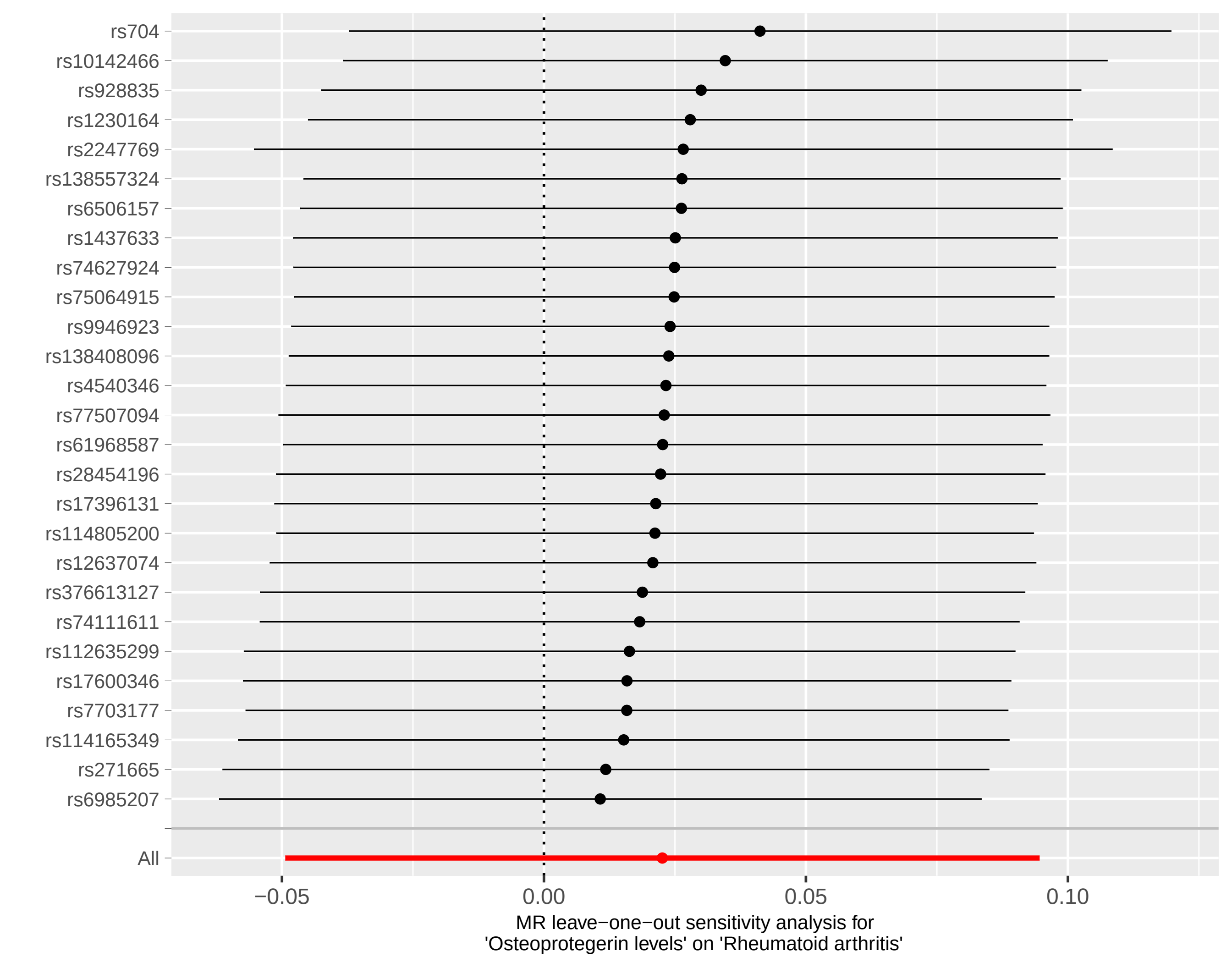

rs704
rs10142466
rs928835
rs1230164
rs2247769
rs138557324
rs6506157
rs1437633
rs74627924
rs75064915
rs9946923
rs138408096
rs4540346
rs77507094
rs61968587
rs28454196
rs17396131
rs114805200
rs12637074
rs376613127
rs74111611
rs112635299
rs17600346
rs7703177
rs114165349
rs271665
rs6985207
All
−0.05
0.00
0.05
0.10
MR leave−one−out sensitivity analysis for
'Osteoprotegerin levels' on 'Rheumatoid arthritis'

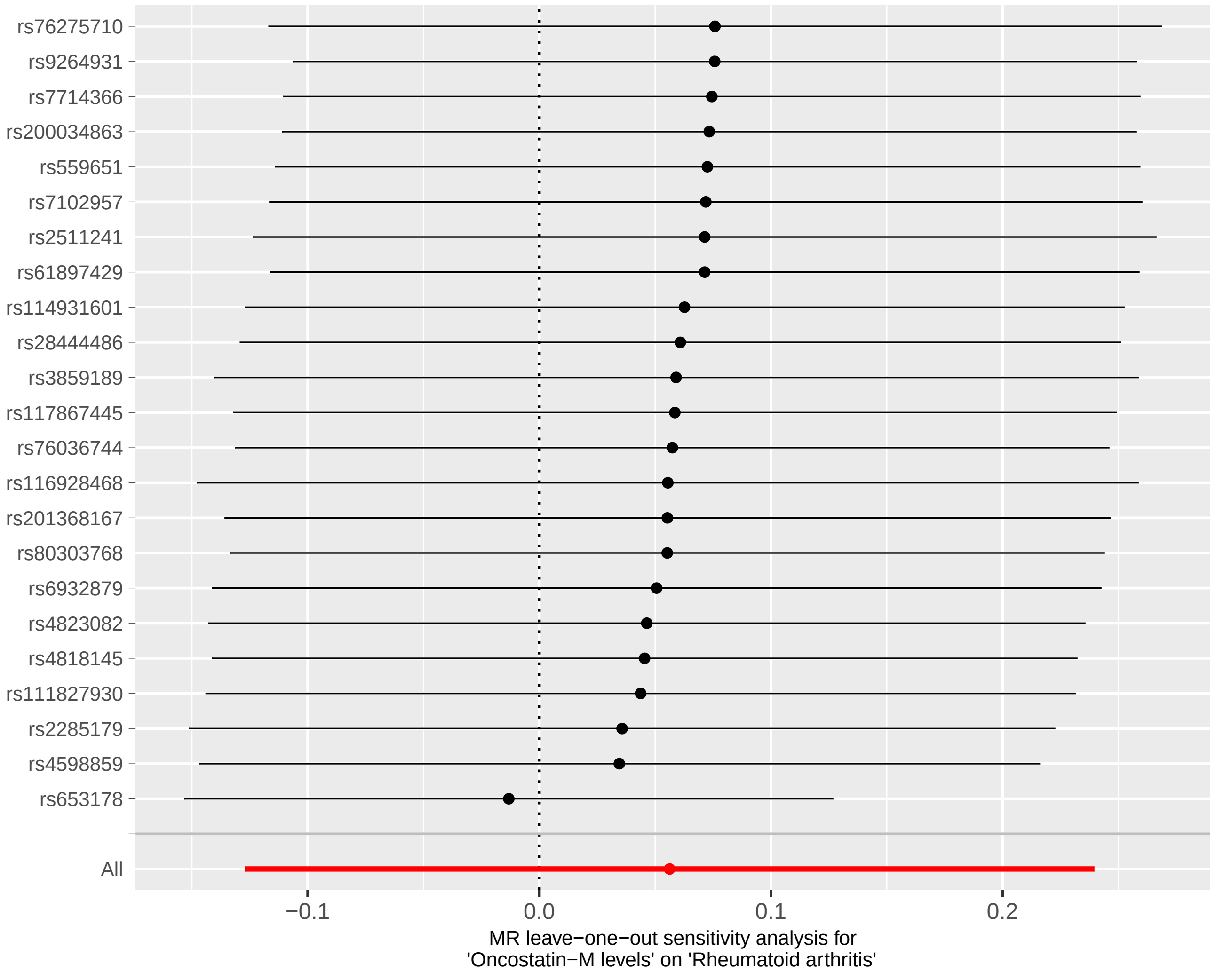

rs76275710
rs9264931
rs7714366
rs200034863
rs559651
rs7102957
rs2511241
rs61897429
rs114931601
rs28444486
rs3859189
rs117867445
rs76036744
rs116928468
rs201368167
rs80303768
rs6932879
rs4823082
rs4818145
rs111827930
rs2285179
rs4598859
rs653178
All
−0.1
0.0
0.1
0.2
MR leave−one−out sensitivity analysis for
'Oncostatin−M levels' on 'Rheumatoid arthritis'

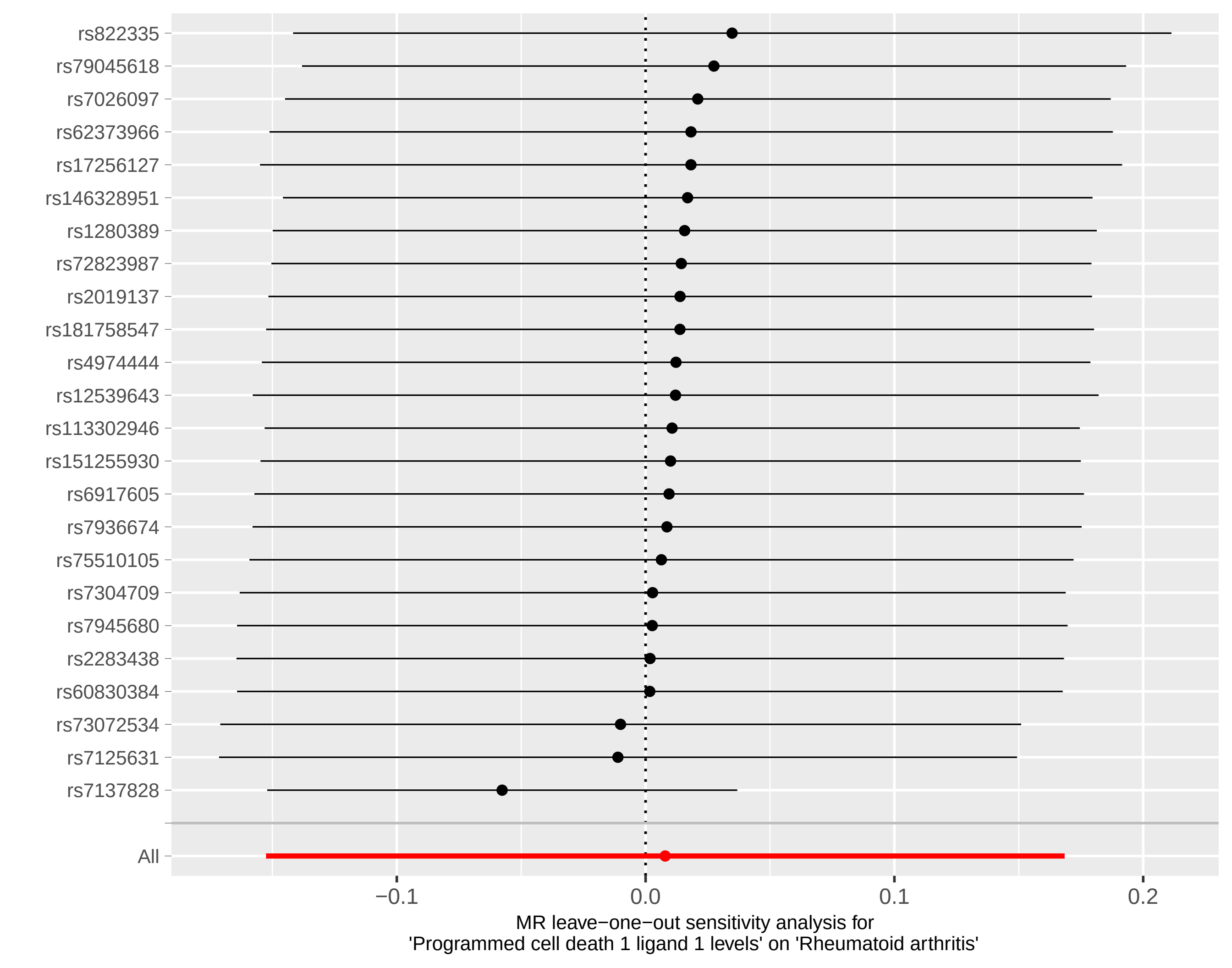
rs822335
rs79045618
rs7026097
rs62373966
rs17256127
rs146328951
rs1280389
rs72823987
rs2019137
rs181758547
rs4974444
rs12539643
rs113302946
rs151255930
rs6917605
rs7936674
rs75510105
rs7304709
rs7945680
rs2283438
rs60830384
rs73072534
rs7125631
rs7137828
All
−0.1
0.0
0.1
0.2
MR leave−one−out sensitivity analysis for
'Programmed cell death 1 ligand 1 levels' on 'Rheumatoid arthritis'

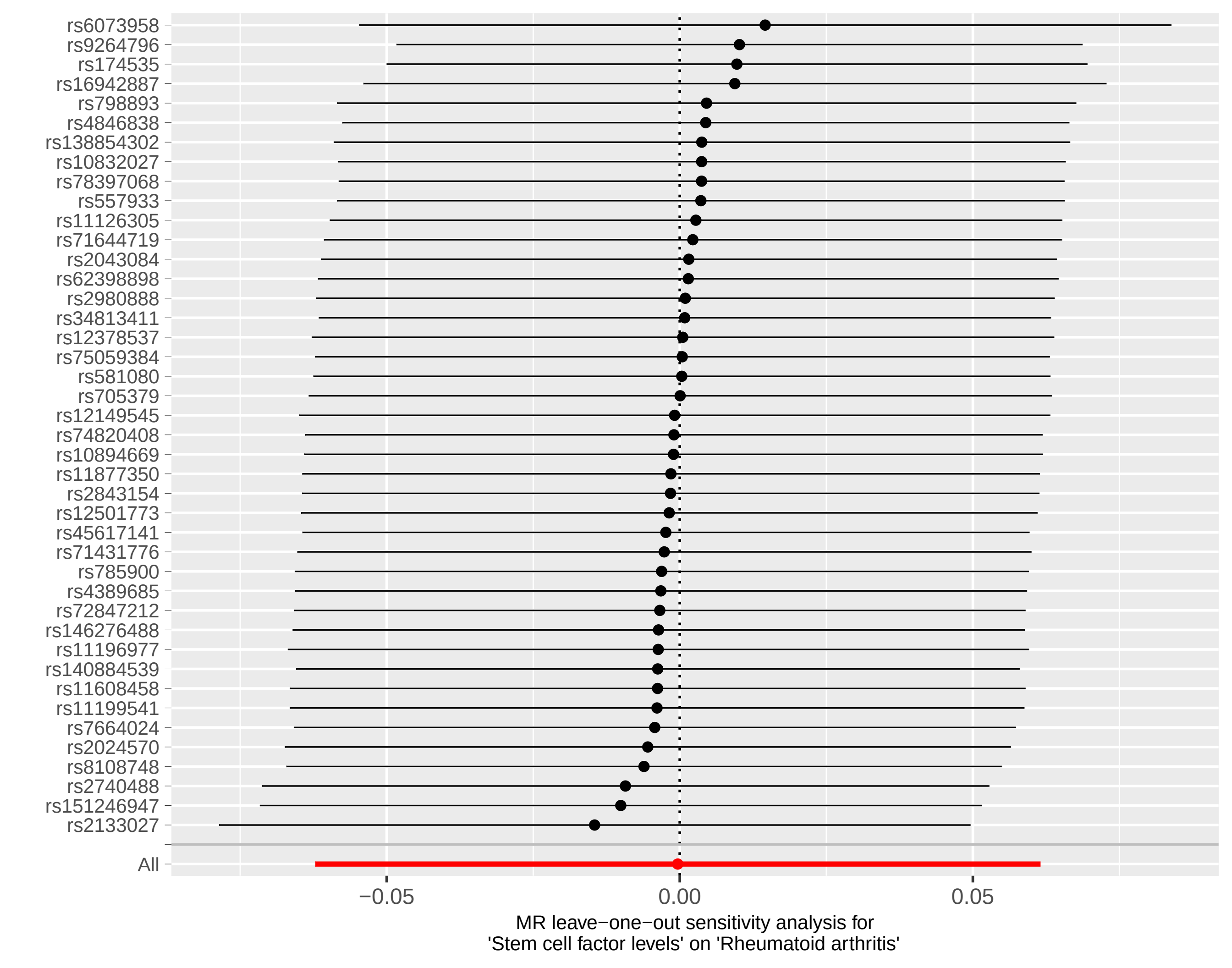
rs6073958
rs9264796
rs174535
rs16942887
rs798893
rs4846838
rs138854302
rs10832027
rs78397068
rs557933
rs11126305
rs71644719
rs2043084
rs62398898
rs2980888
rs34813411
rs12378537
rs75059384
rs581080
rs705379
rs12149545
rs74820408
rs10894669
rs11877350
rs2843154
rs12501773
rs45617141
rs71431776
rs785900
rs4389685
rs72847212
rs146276488
rs11196977
rs140884539
rs11608458
rs11199541
rs7664024
rs2024570
rs8108748
rs2740488
rs151246947
rs2133027
All
−0.05
0.00
0.05
MR leave−one−out sensitivity analysis for
'Stem cell factor levels' on 'Rheumatoid arthritis'

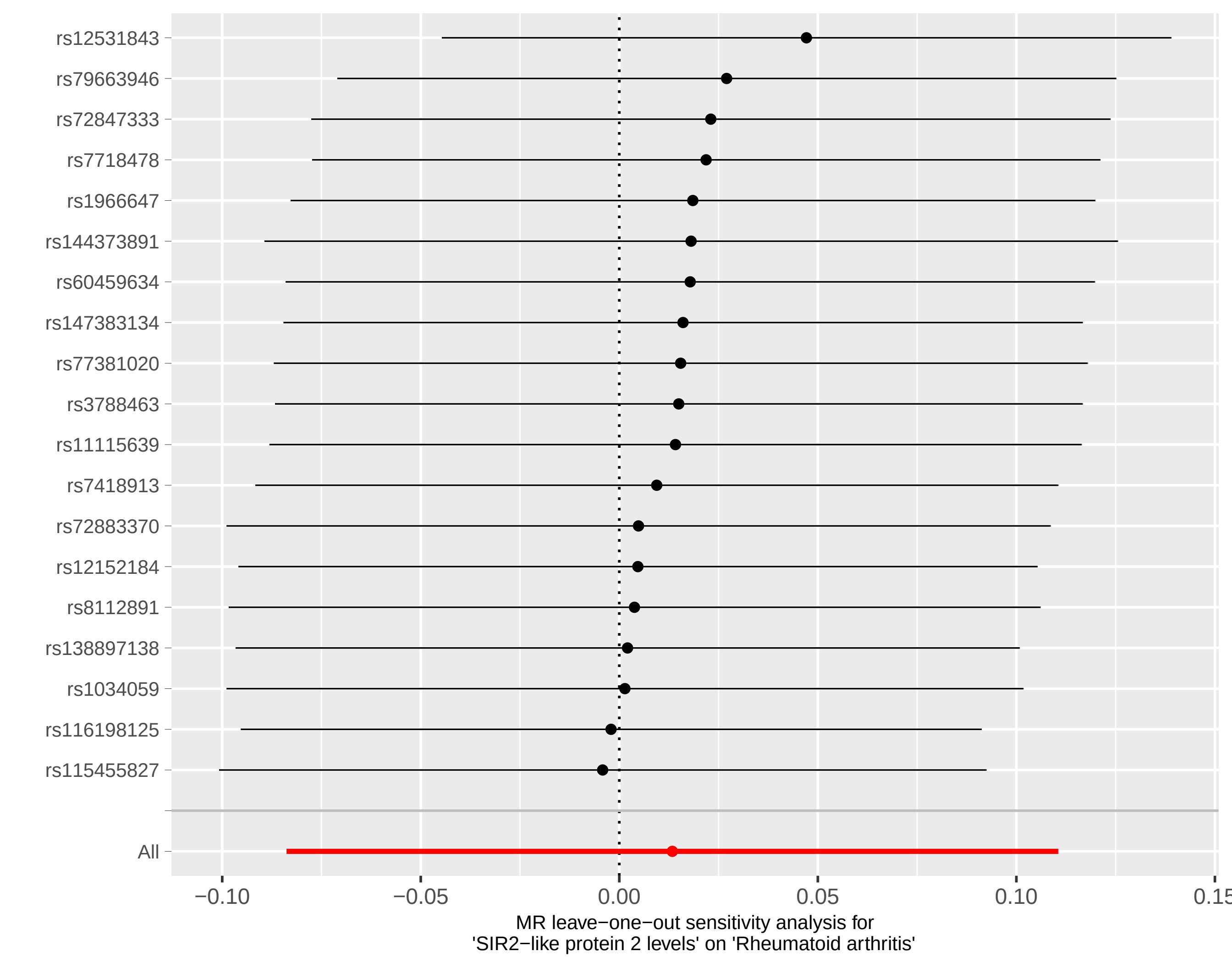

rs12531843
rs79663946
rs72847333
rs7718478
rs1966647
rs144373891
rs60459634
rs147383134
rs77381020
rs3788463
rs11115639
rs7418913
rs72883370
rs12152184
rs8112891
rs138897138
rs1034059
rs116198125
rs115455827
All
−0.10
−0.05
0.00
0.05
0.10
0.15
MR leave−one−out sensitivity analysis for
'SIR2−like protein 2 levels' on 'Rheumatoid arthritis'

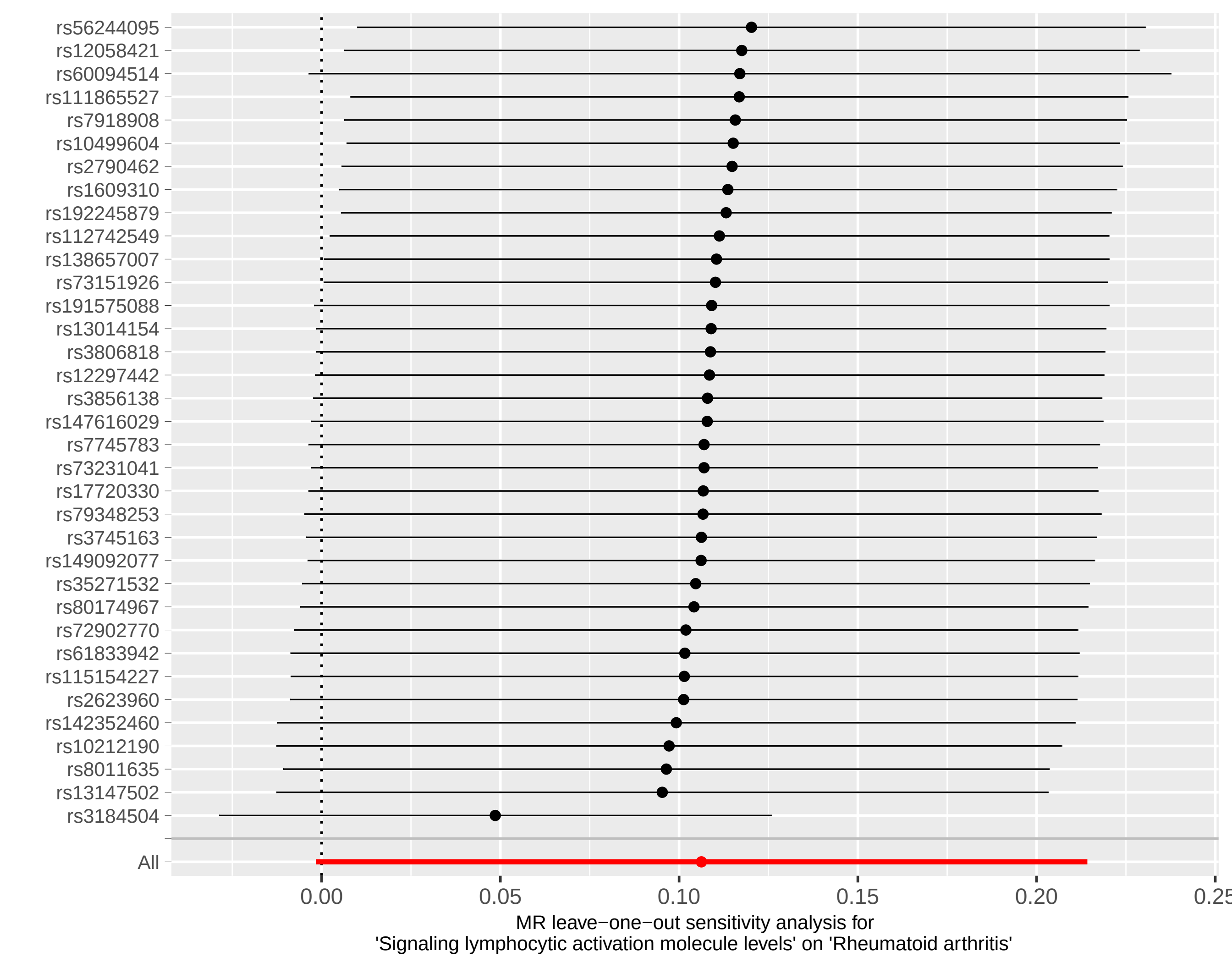
rs56244095
rs12058421
rs60094514
rs111865527
rs7918908
rs10499604
rs2790462
rs1609310
rs192245879
rs112742549
rs138657007
rs73151926
rs191575088
rs13014154
rs3806818
rs12297442
rs3856138
rs147616029
rs7745783
rs73231041
rs17720330
rs79348253
rs3745163
rs149092077
rs35271532
rs80174967
rs72902770
rs61833942
rs115154227
rs2623960
rs142352460
rs10212190
rs8011635
rs13147502
rs3184504
All
0.00
0.05
0.10
0.15
0.20
MR leave−one−out sensitivity analysis for
'Signaling lymphocytic activation molecule levels' on 'Rheumatoid arthritis'

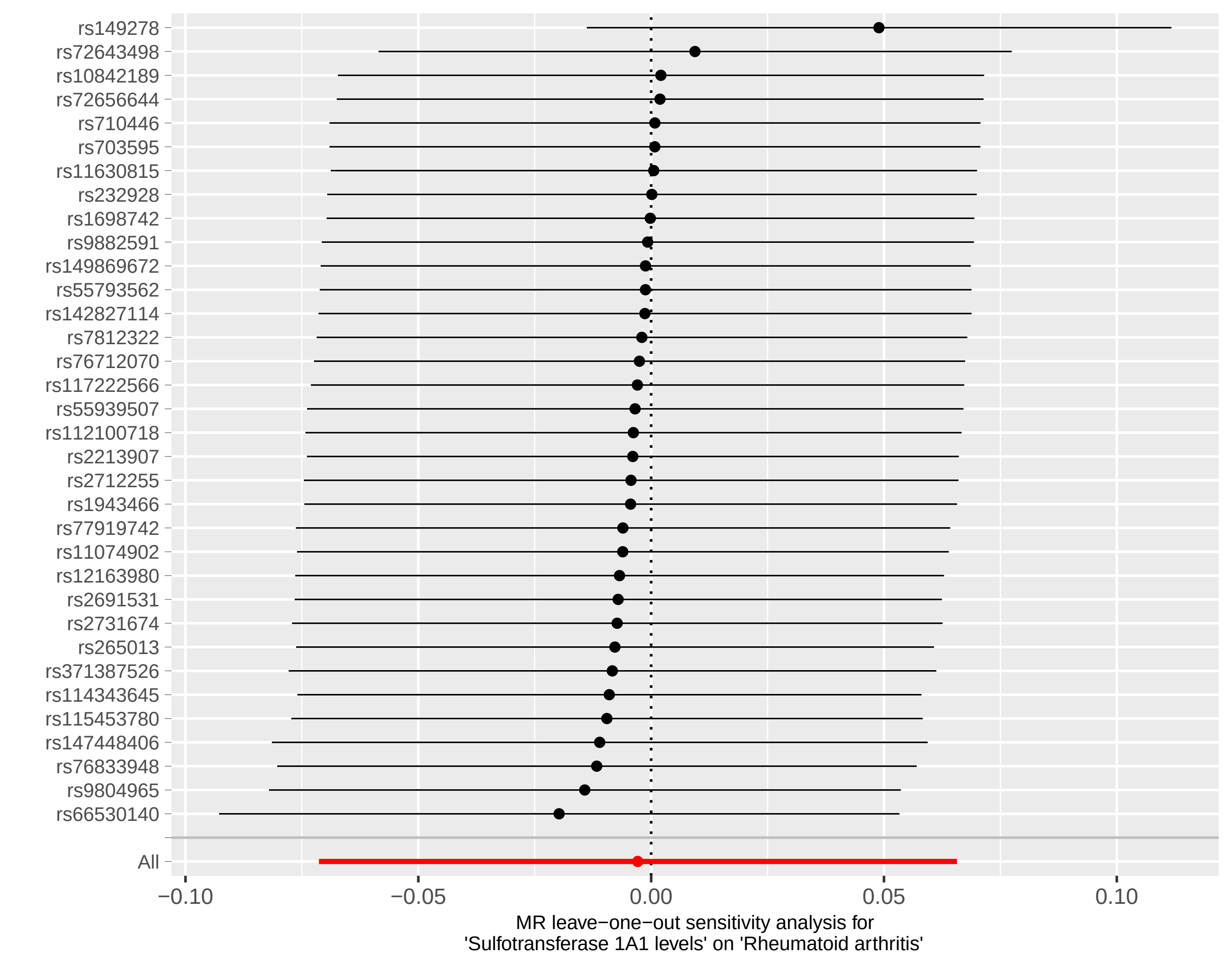
rs149278
rs72643498
rs10842189
rs72656644
rs710446
rs703595
rs11630815
rs232928
rs1698742
rs9882591
rs149869672
rs55793562
rs142827114
rs7812322
rs76712070
rs117222566
rs55939507
rs112100718
rs2213907
rs2712255
rs1943466
rs77919742
rs11074902
rs12163980
rs2691531
rs2731674
rs265013
rs371387526
rs114343645
rs115453780
rs147448406
rs76833948
rs9804965
rs66530140
All
−0.10
−0.05
0.00
0.05
0.10
MR leave−one−out sensitivity analysis for
'Sulfotransferase 1A1 levels' on 'Rheumatoid arthritis'

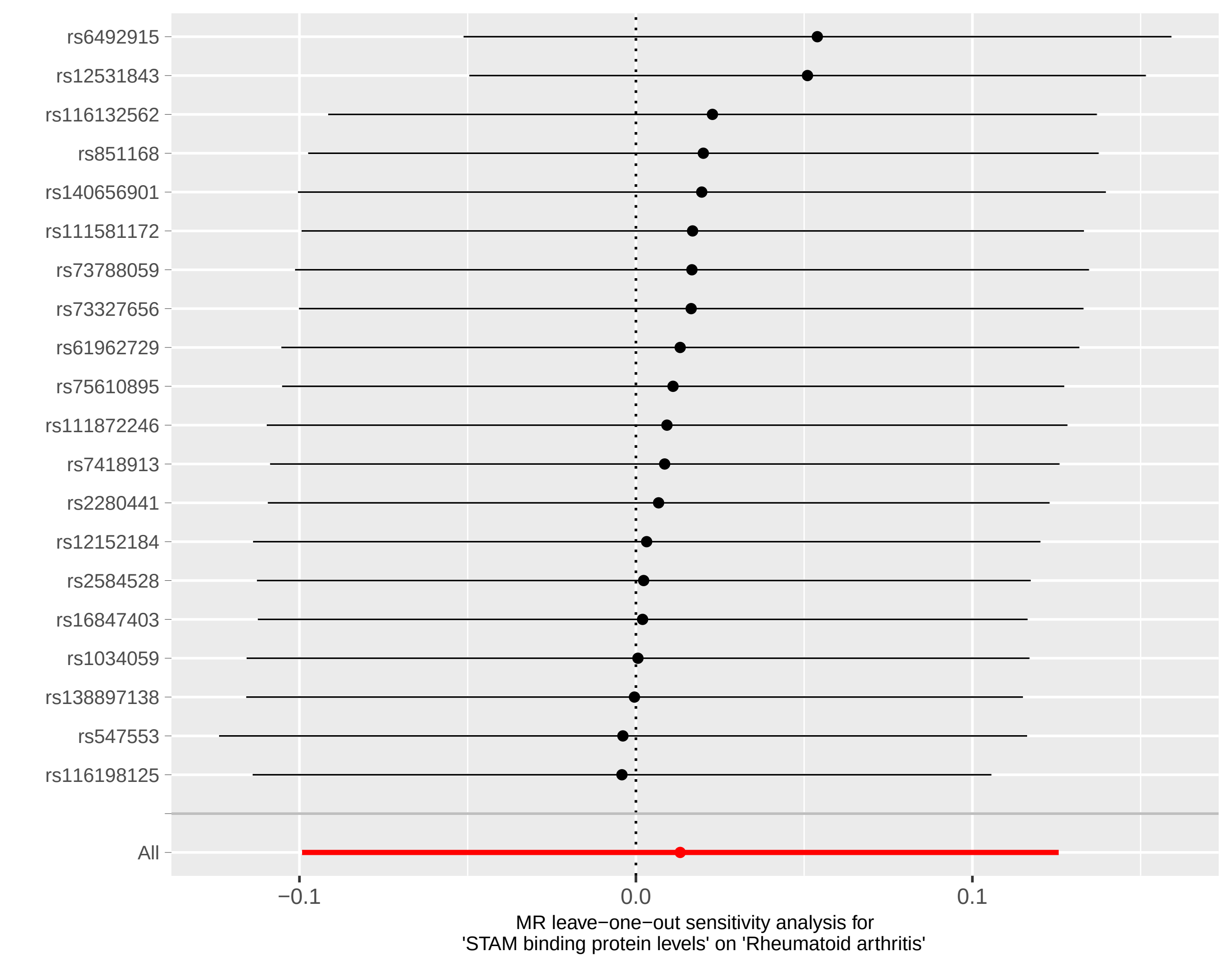

rs6492915
rs12531843
rs116132562
rs851168
rs140656901
rs111581172
rs73788059
rs73327656
rs61962729
rs75610895
rs111872246
rs7418913
rs2280441
rs12152184
rs2584528
rs16847403
rs1034059
rs138897138
rs547553
rs116198125
All
−0.1
0.0
0.1
MR leave−one−out sensitivity analysis for
'STAM binding protein levels' on 'Rheumatoid arthritis'

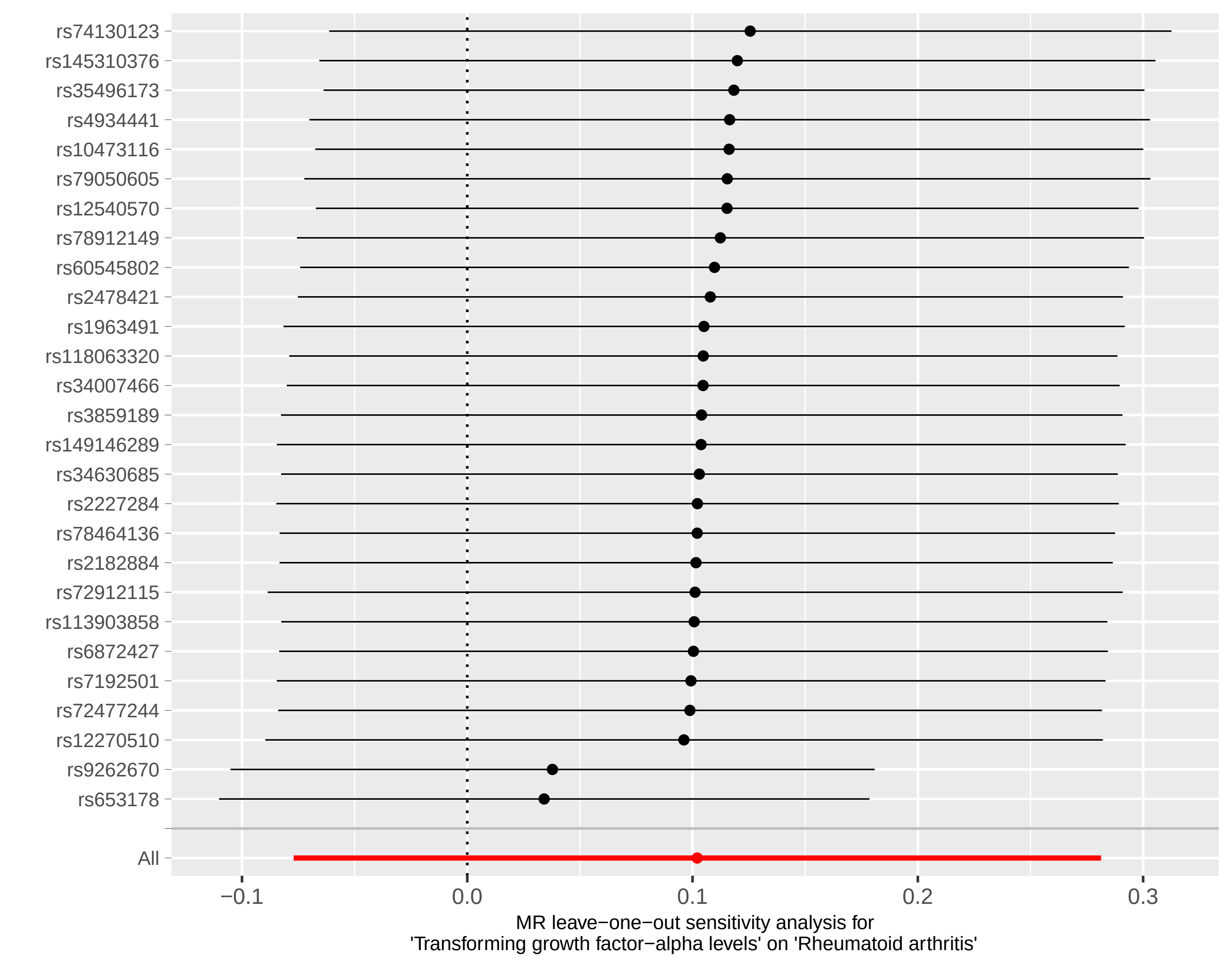
rs74130123
rs145310376
rs35496173
rs4934441
rs10473116
rs79050605
rs12540570
rs78912149
rs60545802
rs2478421
rs1963491
rs118063320
rs34007466
rs3859189
rs149146289
rs34630685
rs2227284
rs78464136
rs2182884
rs72912115
rs113903858
rs6872427
rs7192501
rs72477244
rs12270510
rs9262670
rs653178
All
−0.1
0.0
0.1
0.2
0.3
MR leave−one−out sensitivity analysis for
'Transforming growth factor−alpha levels' on 'Rheumatoid arthritis'

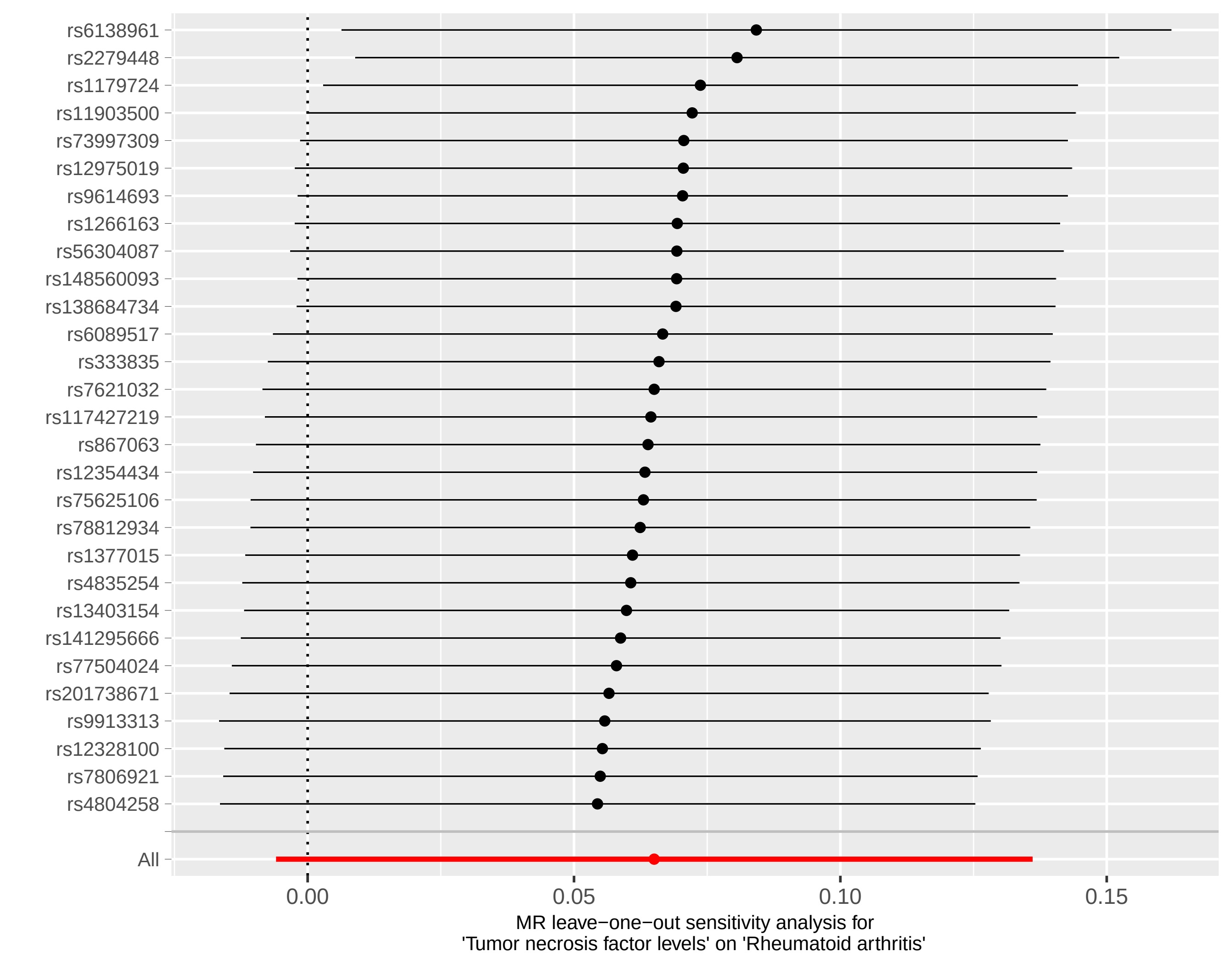
rs6138961
rs2279448
rs1179724
rs11903500
rs73997309
rs12975019
rs9614693
rs1266163
rs56304087
rs148560093
rs138684734
rs6089517
rs333835
rs7621032
rs117427219
rs867063
rs12354434
rs75625106
rs78812934
rs1377015
rs4835254
rs13403154
rs141295666
rs77504024
rs201738671
rs9913313
rs12328100
rs7806921
rs4804258
All
0.00
0.05
0.10
0.15
MR leave−one−out sensitivity analysis for
'Tumor necrosis factor levels' on 'Rheumatoid arthritis'

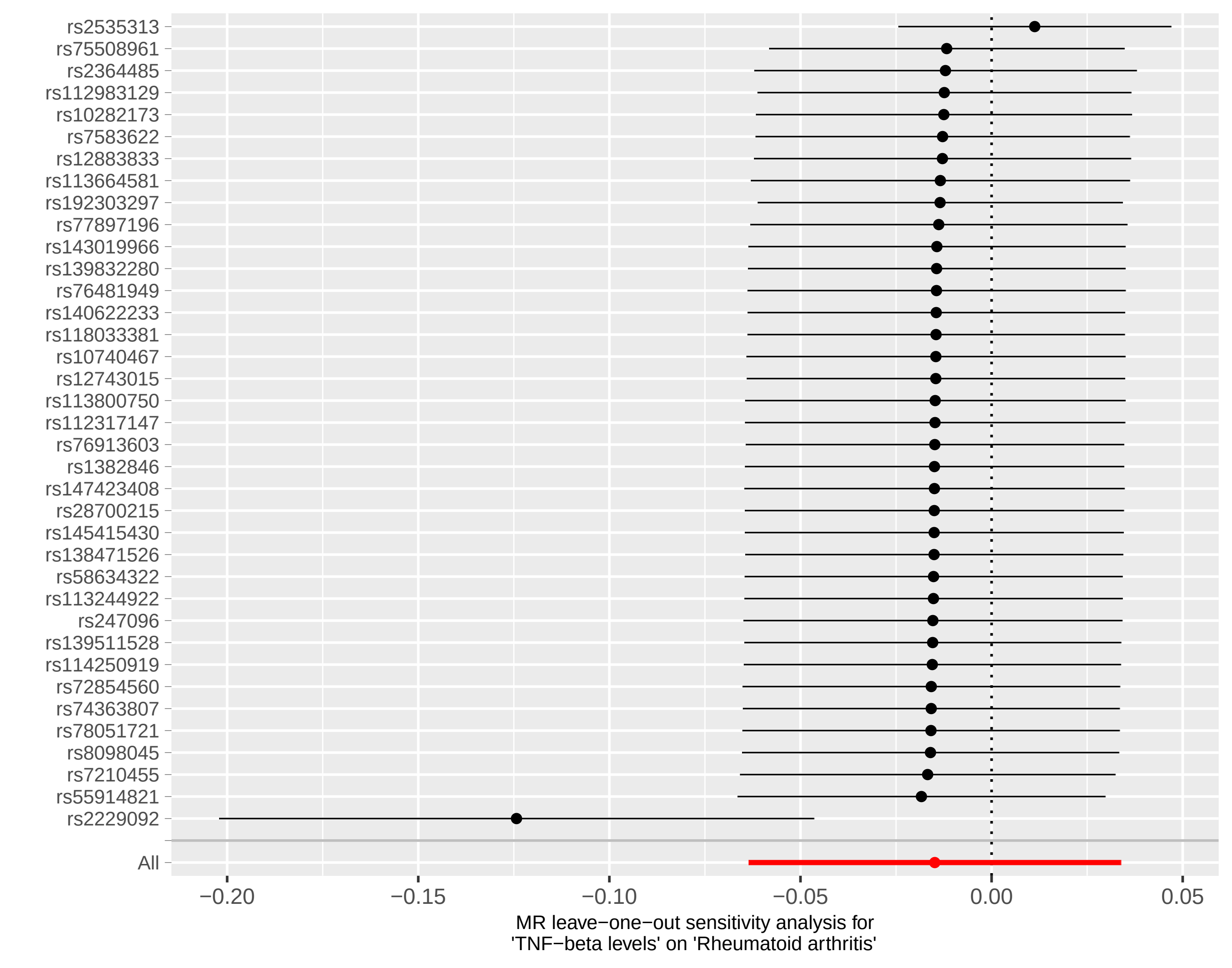
rs2535313
rs75508961
rs2364485
rs112983129
rs10282173
rs7583622
rs12883833
rs113664581
rs192303297
rs77897196
rs143019966
rs139832280
rs76481949
rs140622233
rs118033381
rs10740467
rs12743015
rs113800750
rs112317147
rs76913603
rs1382846
rs147423408
rs28700215
rs145415430
rs138471526
rs58634322
rs113244922
rs247096
rs139511528
rs114250919
rs72854560
rs74363807
rs78051721
rs8098045
rs7210455
rs55914821
rs2229092
All
−0.20
−0.15
−0.10
−0.05
0.00
0.05
MR leave−one−out sensitivity analysis for
'TNF−beta levels' on 'Rheumatoid arthritis'

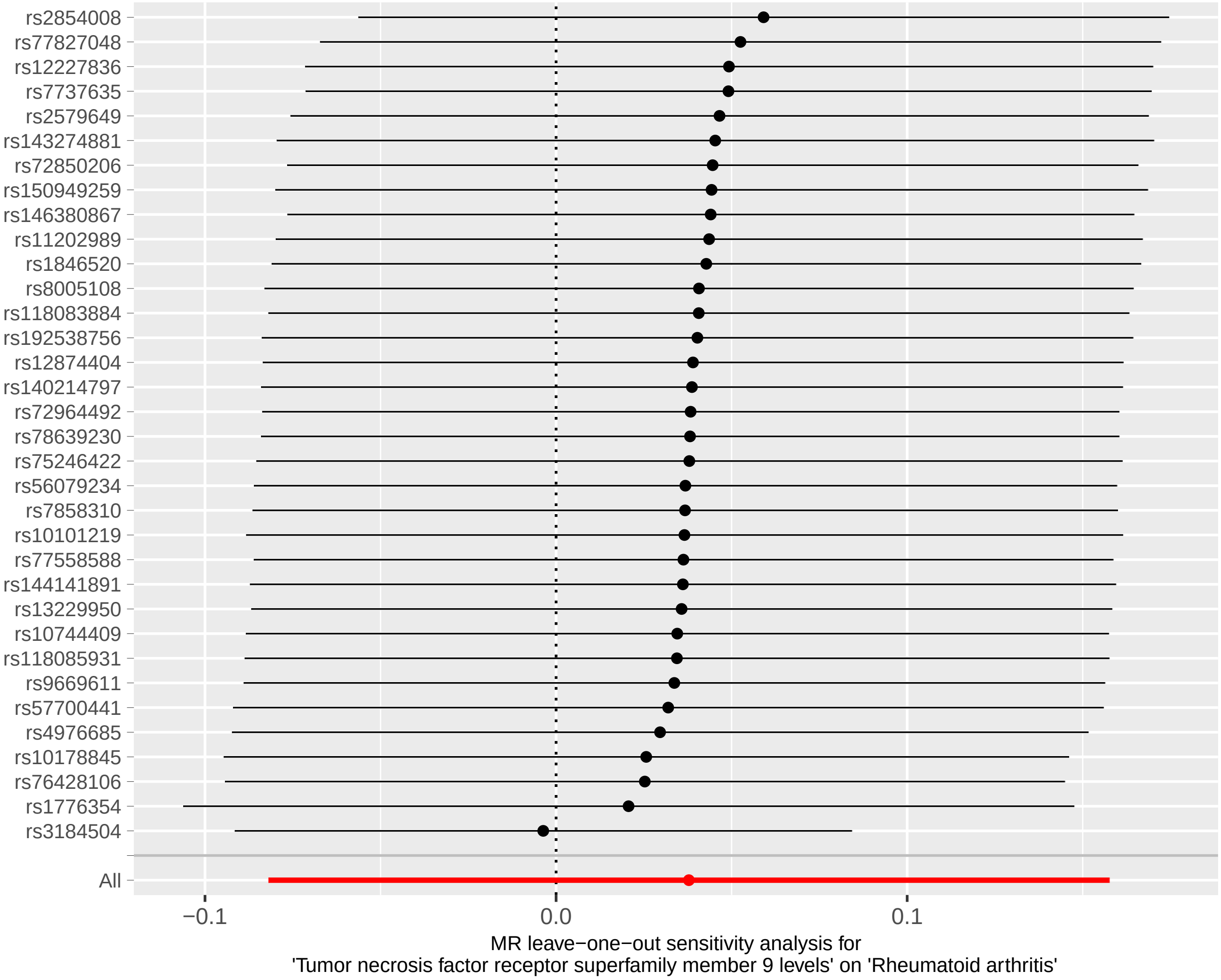

rs2854008
rs77827048
rs12227836
rs7737635
rs2579649
rs143274881
rs72850206
rs150949259
rs146380867
rs11202989
rs1846520
rs8005108
rs118083884
rs192538756
rs12874404
rs140214797
rs72964492
rs78639230
rs75246422
rs56079234
rs7858310
rs10101219
rs77558588
rs144141891
rs13229950
rs10744409
rs118085931
rs9669611
rs57700441
rs4976685
rs10178845
rs76428106
rs1776354
rs3184504
All
−0.1
0.0
0.1
MR leave−one−out sensitivity analysis for
'Tumor necrosis factor receptor superfamily member 9 levels' on 'Rheumatoid arthritis'

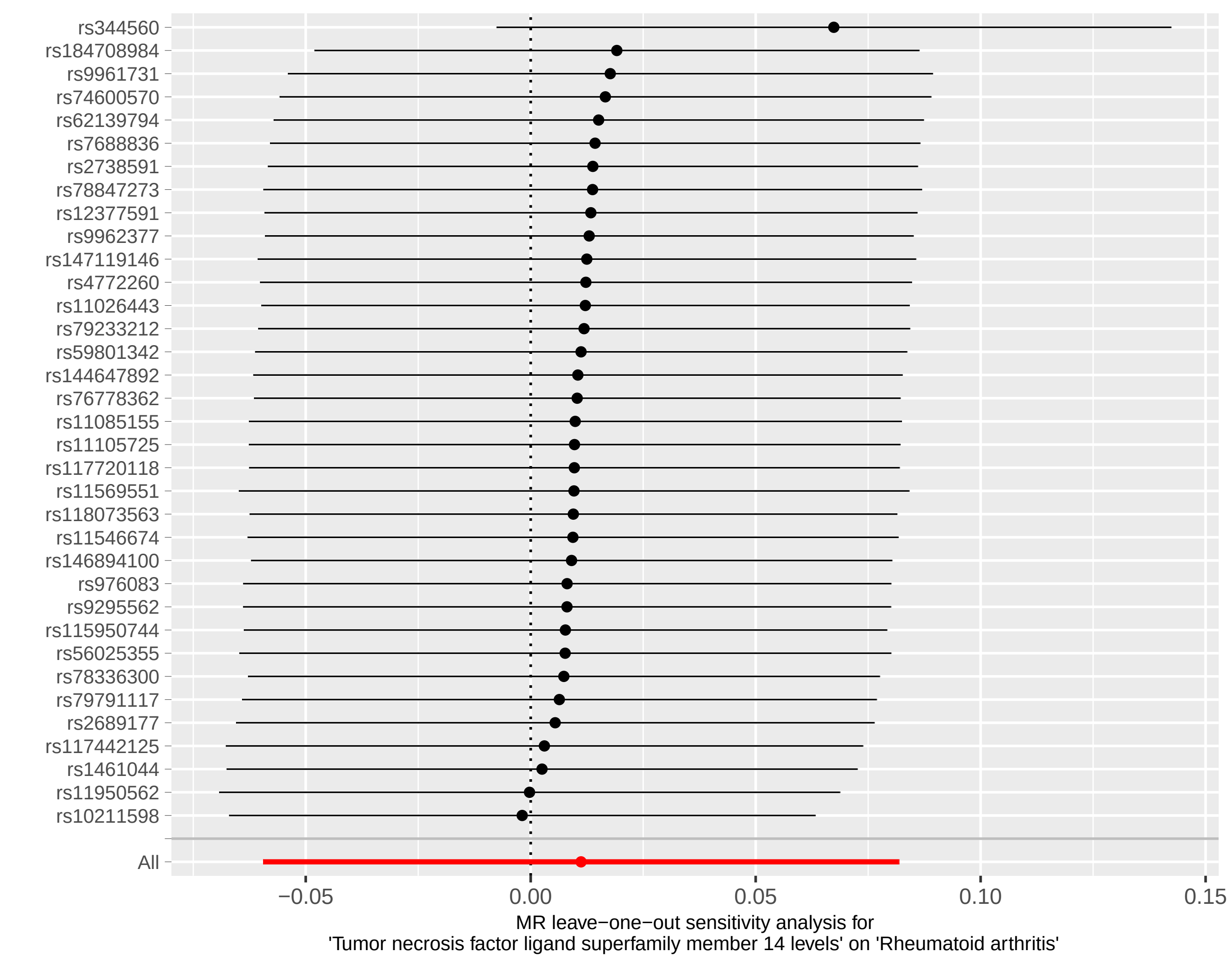
rs344560
rs184708984
rs9961731
rs74600570
rs62139794
rs7688836
rs2738591
rs78847273
rs12377591
rs9962377
rs147119146
rs4772260
rs11026443
rs79233212
rs59801342
rs144647892
rs76778362
rs11085155
rs11105725
rs117720118
rs11569551
rs118073563
rs11546674
rs146894100
rs976083
rs9295562
rs115950744
rs56025355
rs78336300
rs79791117
rs2689177
rs117442125
rs1461044
rs11950562
rs10211598
All
−0.05
0.00
0.05
0.10
0.15
MR leave−one−out sensitivity analysis for
'Tumor necrosis factor ligand superfamily member 14 levels' on 'Rheumatoid arthritis'

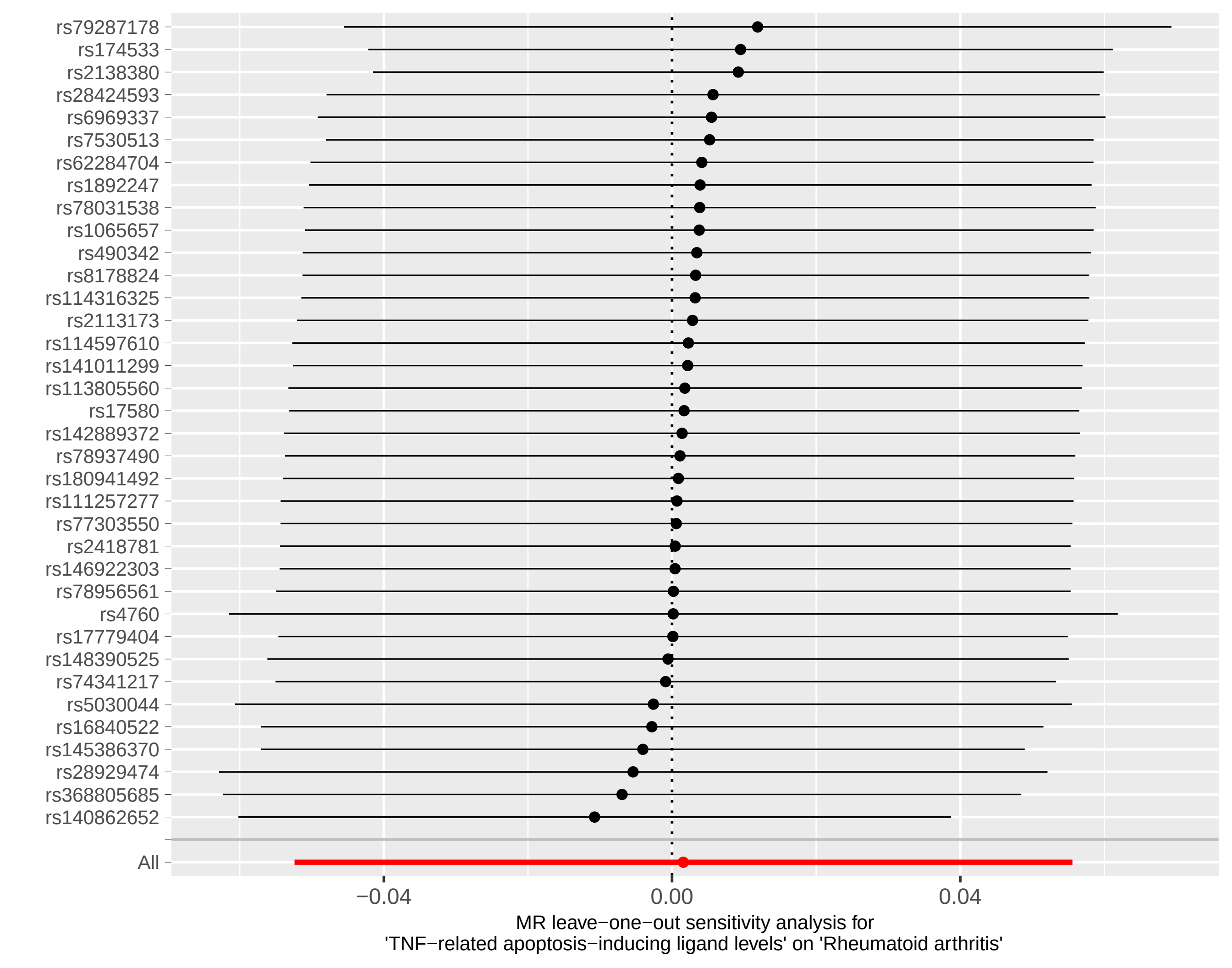
rs79287178
rs174533
rs2138380
rs28424593
rs6969337
rs7530513
rs62284704
rs1892247
rs78031538
rs1065657
rs490342
rs8178824
rs114316325
rs2113173
rs114597610
rs141011299
rs113805560
rs17580
rs142889372
rs78937490
rs180941492
rs111257277
rs77303550
rs2418781
rs146922303
rs78956561
rs4760
rs17779404
rs148390525
rs74341217
rs5030044
rs16840522
rs145386370
rs28929474
rs368805685
rs140862652
All
−0.04
0.00
0.04
MR leave−one−out sensitivity analysis for
'TNF−related apoptosis−inducing ligand levels' on 'Rheumatoid arthritis'

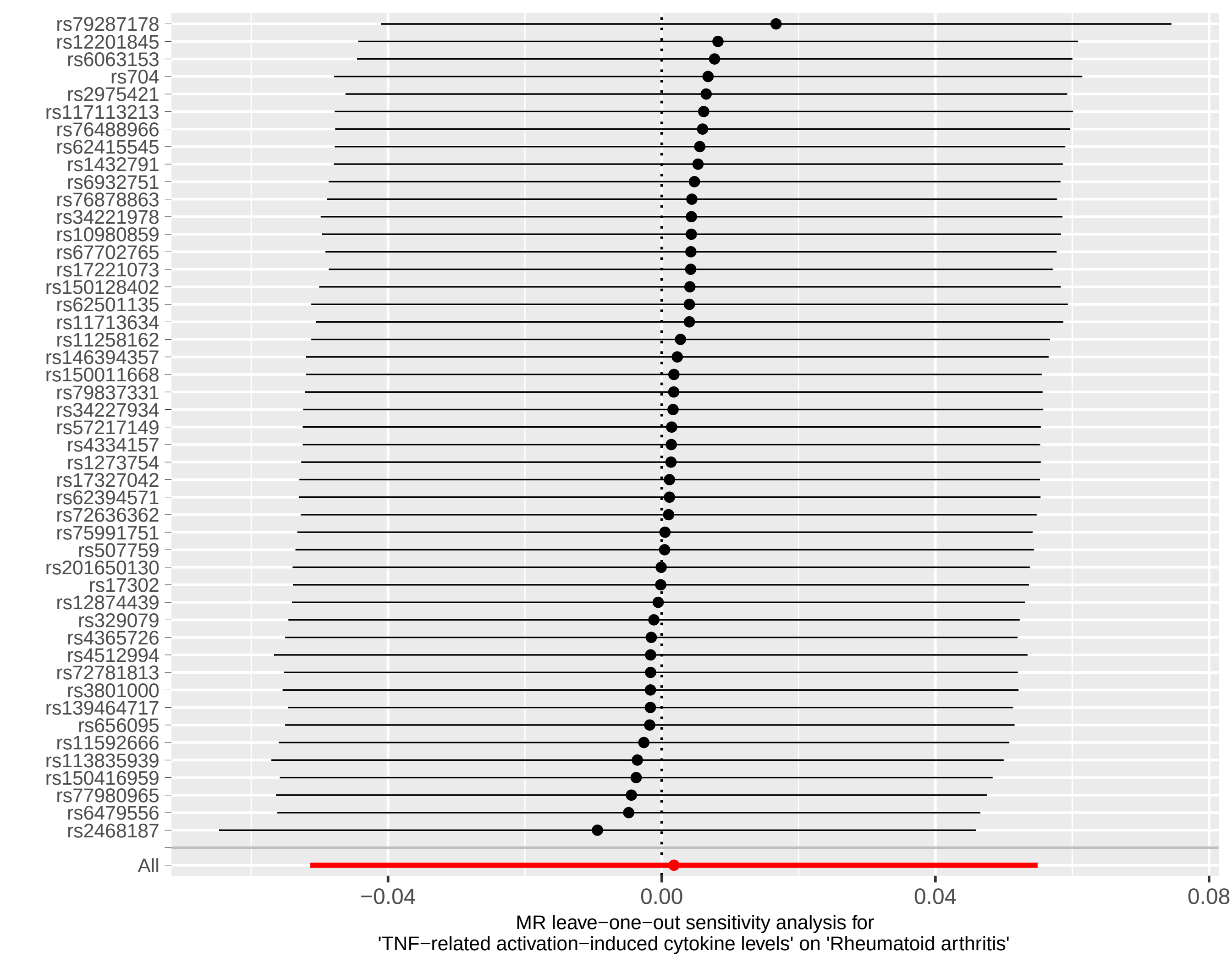

rs79287178
rs12201845
rs6063153
rs704
rs2975421
rs117113213
rs76488966
rs62415545
rs1432791
rs6932751
rs76878863
rs34221978
rs10980859
rs67702765
rs17221073
rs150128402
rs62501135
rs11713634
rs11258162
rs146394357
rs150011668
rs79837331
rs34227934
rs57217149
rs4334157
rs1273754
rs17327042
rs62394571
rs72636362
rs75991751
rs507759
rs201650130
rs17302
rs12874439
rs329079
rs4365726
rs4512994
rs72781813
rs3801000
rs139464717
rs656095
rs11592666
rs113835939
rs150416959
rs77980965
rs6479556
rs2468187
All
−0.04
0.00
0.04
0.08
MR leave−one−out sensitivity analysis for
'TNF−related activation−induced cytokine levels' on 'Rheumatoid arthritis'

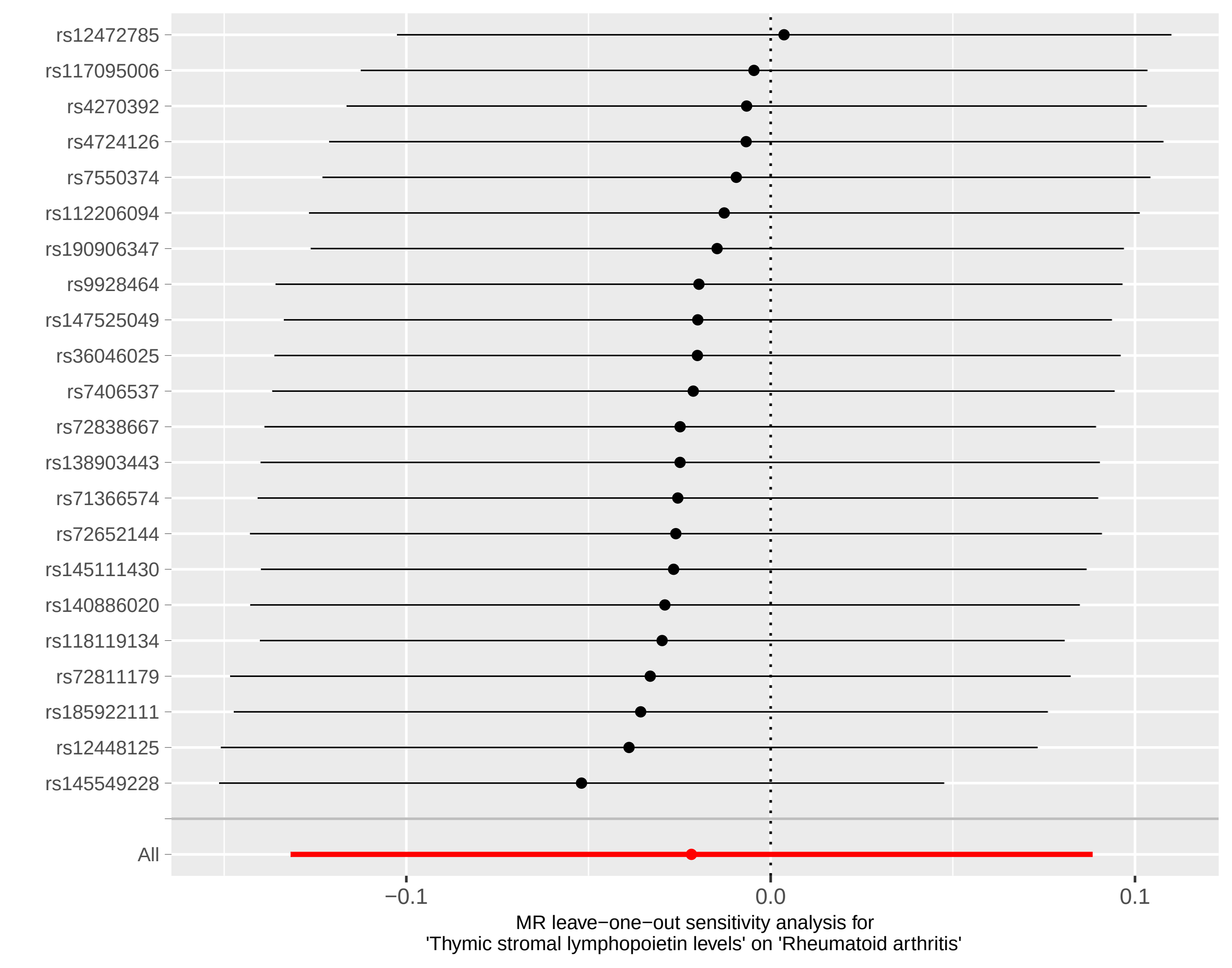
rs12472785
rs117095006
rs4270392
rs4724126
rs7550374
rs112206094
rs190906347
rs9928464
rs147525049
rs36046025
rs7406537
rs72838667
rs138903443
rs71366574
rs72652144
rs145111430
rs140886020
rs118119134
rs72811179
rs185922111
rs12448125
rs145549228
All
−0.1
0.0
0.1
MR leave−one−out sensitivity analysis for
'Thymic stromal lymphopoietin levels' on 'Rheumatoid arthritis'

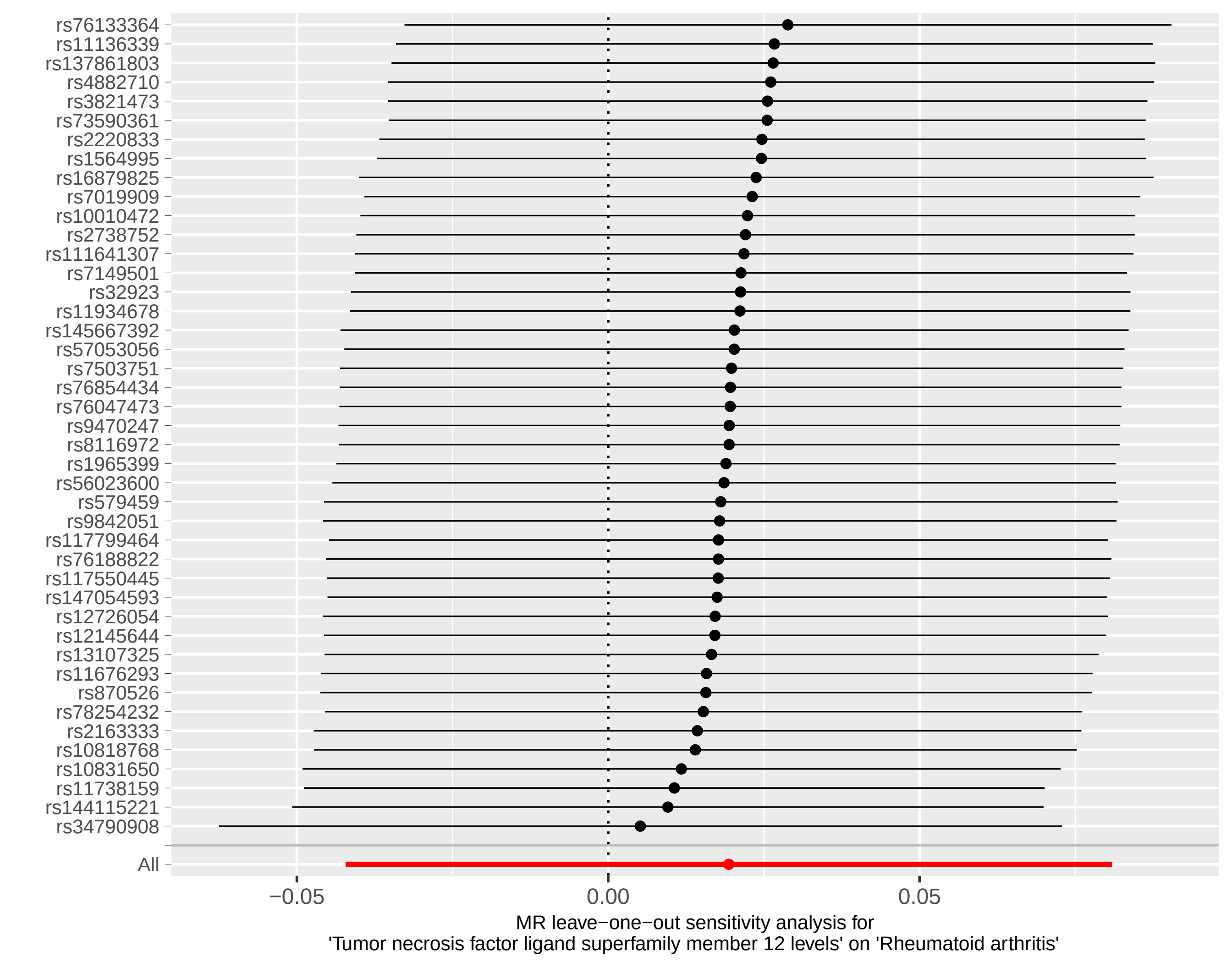
rs76133364
rs11136339
rs137861803
rs4882710
rs3821473
rs73590361
rs2220833
rs1564995
rs16879825
rs7019909
rs10010472
rs2738752
rs111641307
rs7149501
rs32923
rs11934678
rs145667392
rs57053056
rs7503751
rs76854434
rs76047473
rs9470247
rs8116972
rs1965399
rs56023600
rs579459
rs9842051
rs117799464
rs76188822
rs117550445
rs147054593
rs12726054
rs12145644
rs13107325
rs11676293
rs870526
rs78254232
rs2163333
rs10818768
rs10831650
rs11738159
rs144115221
rs34790908
All
−0.05
0.00
0.05
MR leave−one−out sensitivity analysis for
'Tumor necrosis factor ligand superfamily member 12 levels' on 'Rheumatoid arthritis'

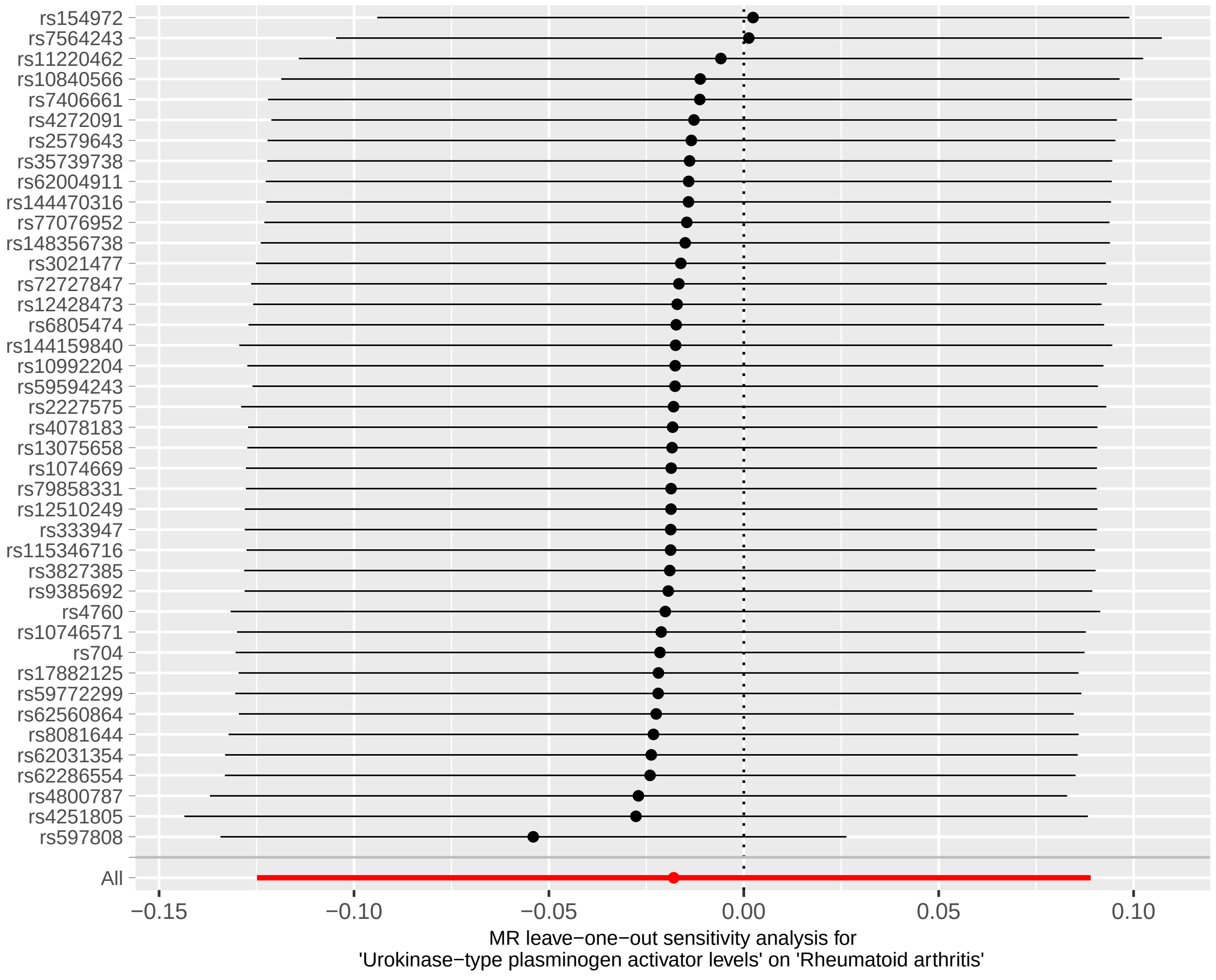

rs154972
rs7564243
rs11220462
rs10840566
rs7406661
rs4272091
rs2579643
rs35739738
rs62004911
rs144470316
rs77076952
rs148356738
rs3021477
rs72727847
rs12428473
rs6805474
rs144159840
rs10992204
rs59594243
rs2227575
rs4078183
rs13075658
rs1074669
rs79858331
rs12510249
rs333947
rs115346716
rs3827385
rs9385692
rs4760
rs10746571
rs704
rs17882125
rs59772299
rs62560864
rs8081644
rs62031354
rs62286554
rs4800787
rs4251805
rs597808
All
−0.15
−0.10
−0.05
0.00
0.05
0.10
MR leave−one−out sensitivity analysis for
'Urokinase−type plasminogen activator levels' on 'Rheumatoid arthritis'

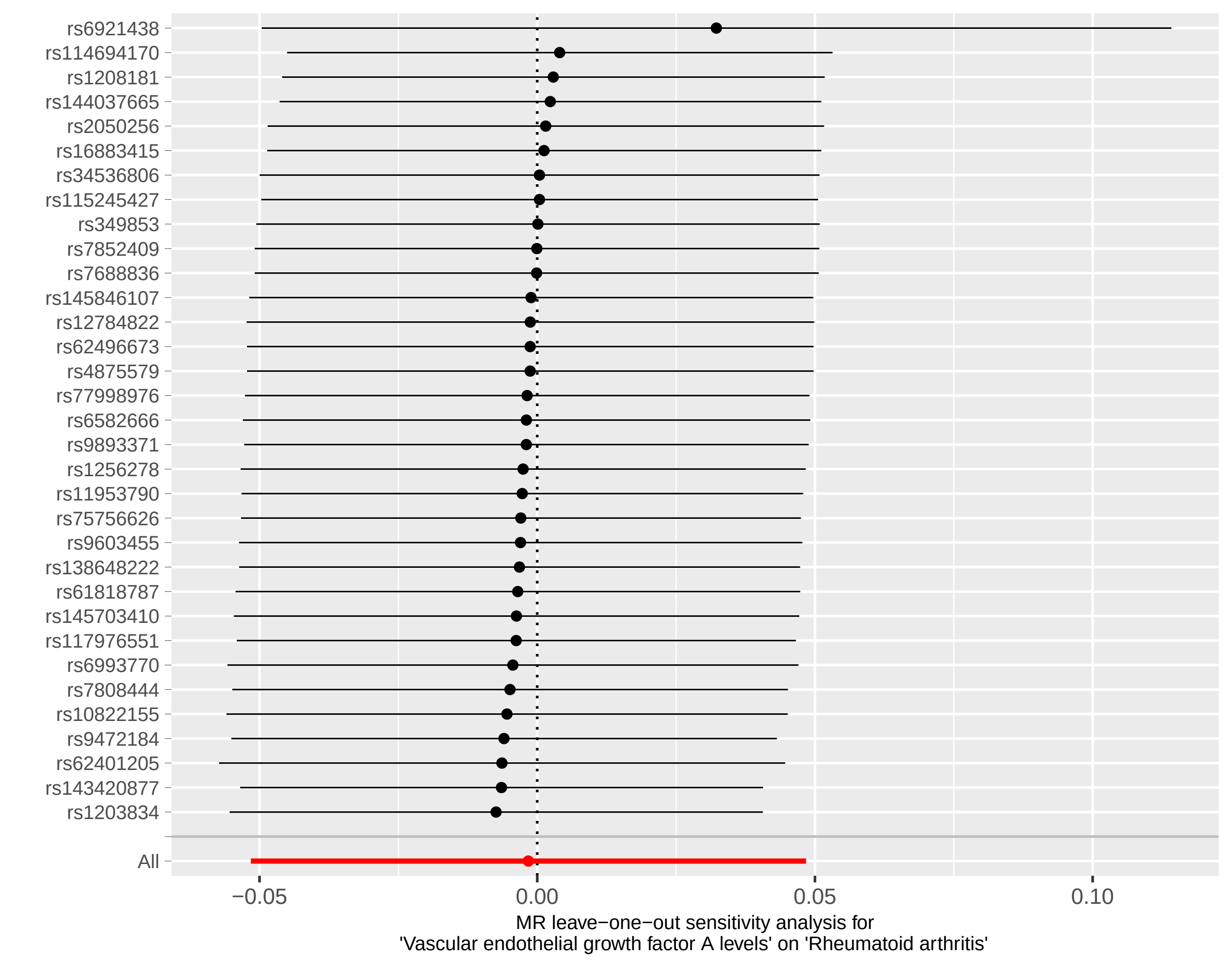

rs6921438
rs114694170
rs1208181
rs144037665
rs2050256
rs16883415
rs34536806
rs115245427
rs349853
rs7852409
rs7688836
rs145846107
rs12784822
rs62496673
rs4875579
rs77998976
rs6582666
rs9893371
rs1256278
rs11953790
rs75756626
rs9603455
rs138648222
rs61818787
rs145703410
rs117976551
rs6993770
rs7808444
rs10822155
rs9472184
rs62401205
rs143420877
rs1203834
All
−0.05
0.00
0.05
0.10
MR leave−one−out sensitivity analysis for
'Vascular endothelial growth factor A levels' on 'Rheumatoid arthritis'

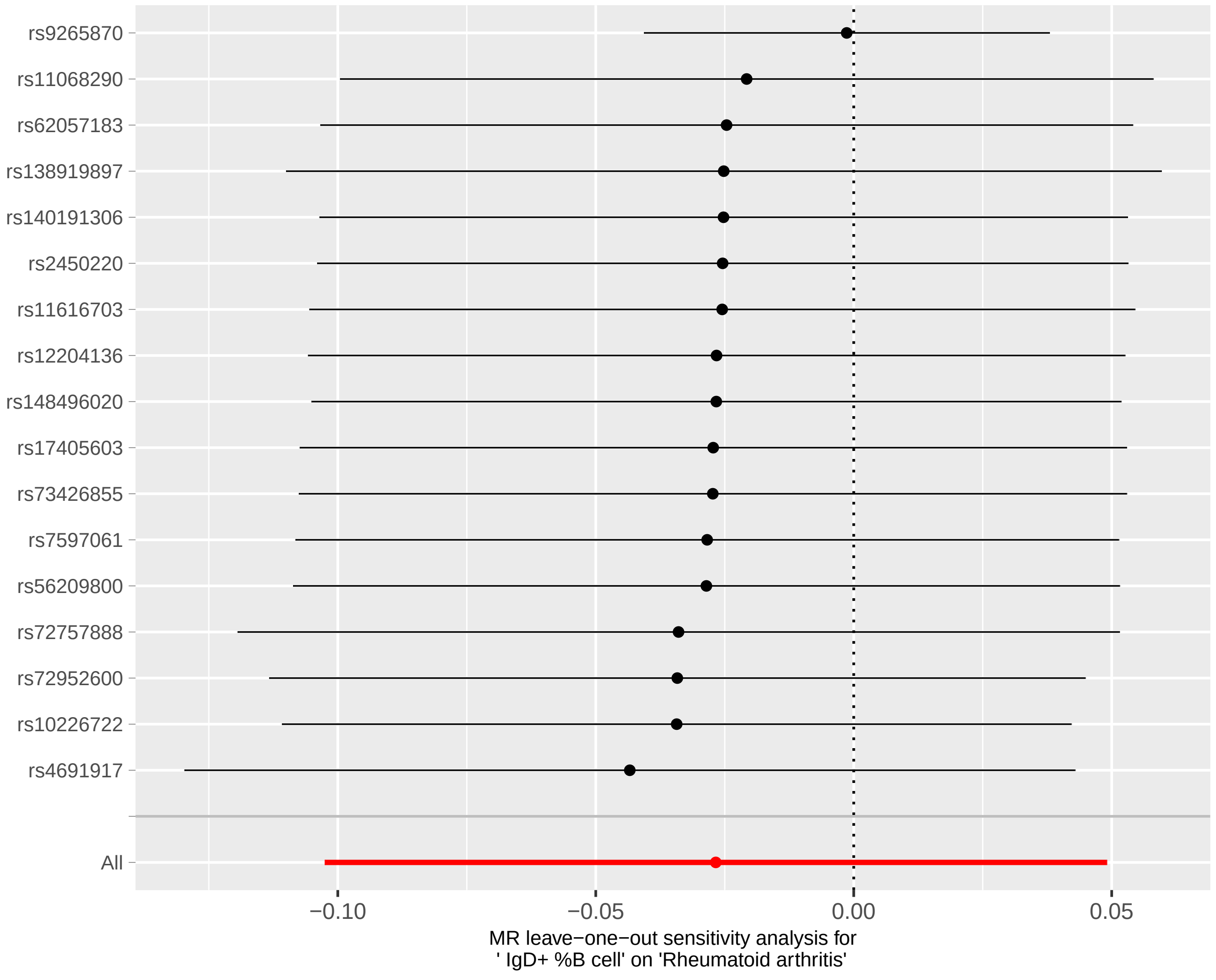
rs9265870
rs11068290
rs62057183
rs138919897
rs140191306
rs2450220
rs11616703
rs12204136
rs148496020
rs17405603
rs73426855
rs7597061
rs56209800
rs72757888
rs72952600
rs10226722
rs4691917
All
−0.10
−0.05
0.00
0.05
MR leave−one−out sensitivity analysis for
' IgD+ %B cell' on 'Rheumatoid arthritis'

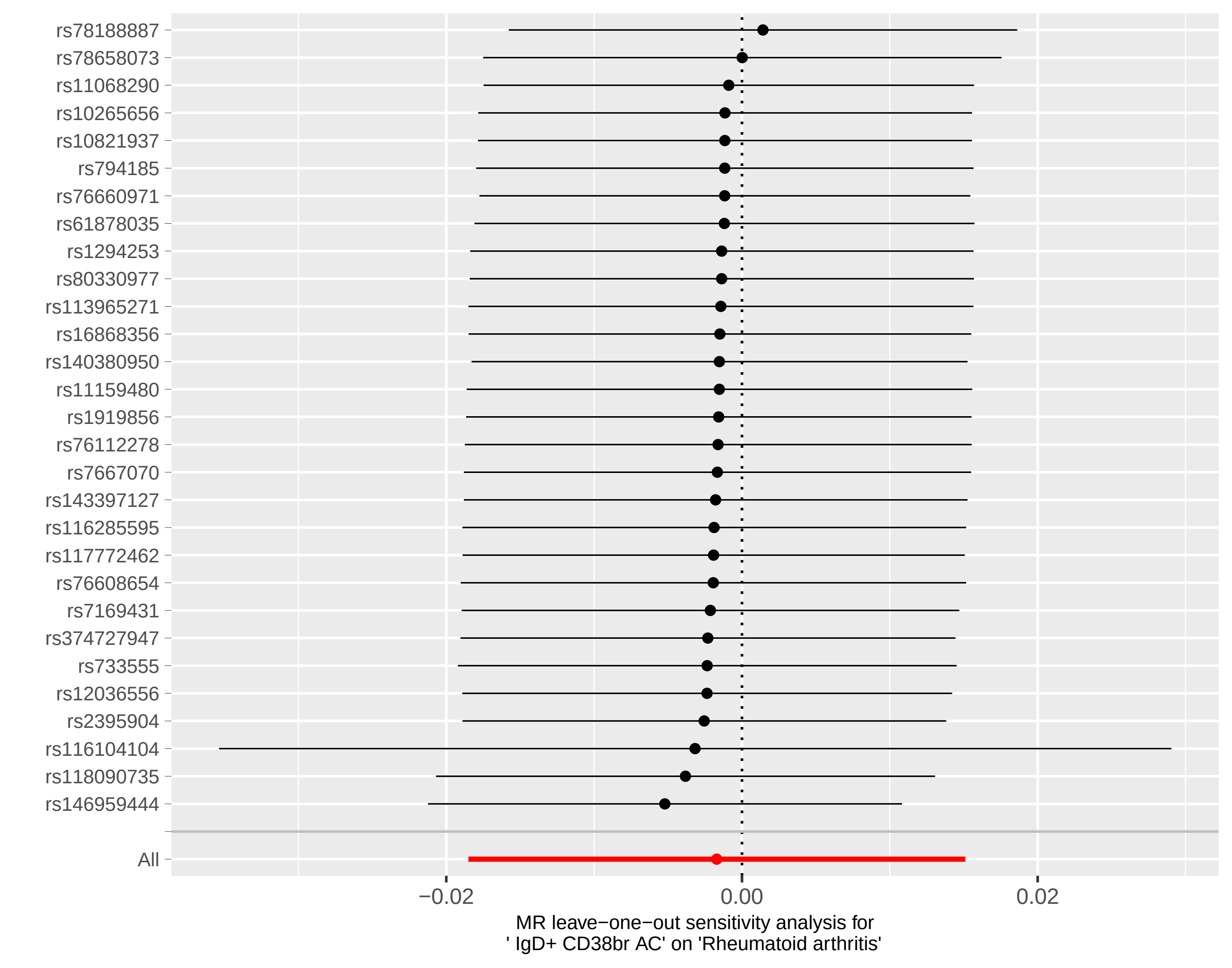
rs78188887
rs78658073
rs11068290
rs10265656
rs10821937
rs794185
rs76660971
rs61878035
rs1294253
rs80330977
rs113965271
rs16868356
rs140380950
rs11159480
rs1919856
rs76112278
rs7667070
rs143397127
rs116285595
rs117772462
rs76608654
rs7169431
rs374727947
rs733555
rs12036556
rs2395904
rs116104104
rs118090735
rs146959444
All
−0.02
0.00
0.02
MR leave−one−out sensitivity analysis for
' IgD+ CD38br AC' on 'Rheumatoid arthritis'

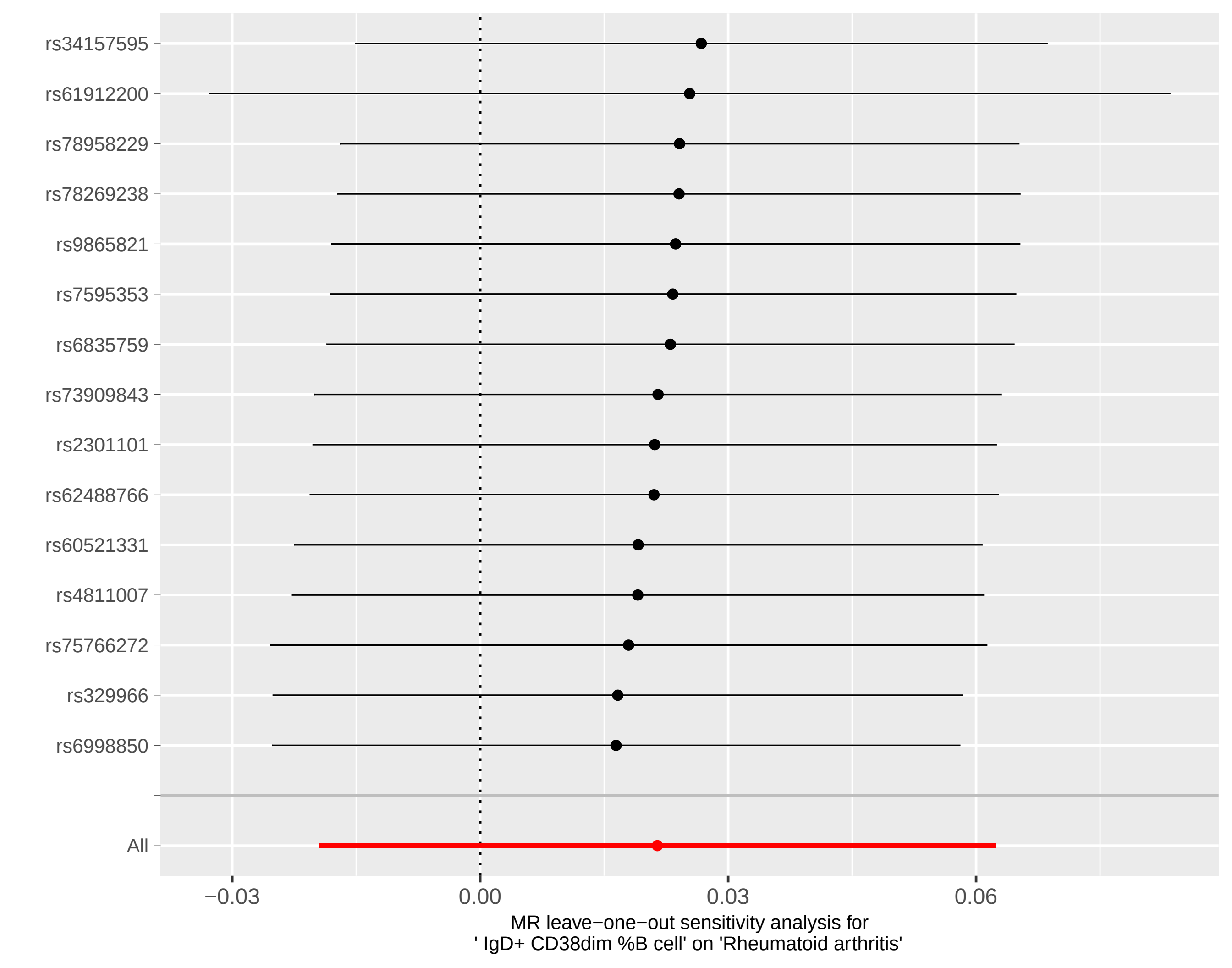

rs34157595
rs61912200
rs78958229
rs78269238
rs9865821
rs7595353
rs6835759
rs73909843
rs2301101
rs62488766
rs60521331
rs4811007
rs75766272
rs329966
rs6998850
All
−0.03
0.00
0.03
0.06
MR leave−one−out sensitivity analysis for
' IgD+ CD38dim %B cell' on 'Rheumatoid arthritis'

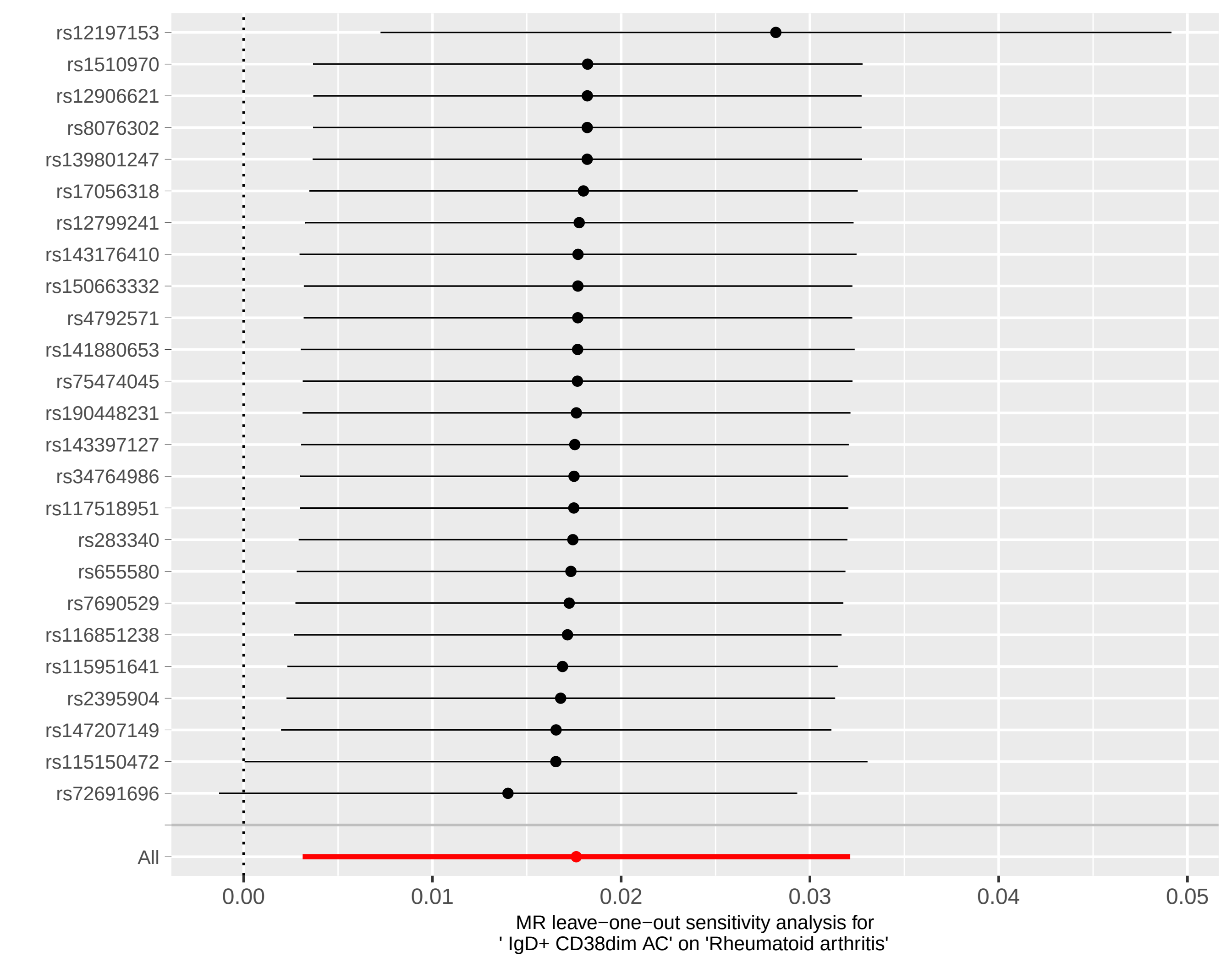
rs12197153
rs1510970
rs12906621
rs8076302
rs139801247
rs17056318
rs12799241
rs143176410
rs150663332
rs4792571
rs141880653
rs75474045
rs190448231
rs143397127
rs34764986
rs117518951
rs283340
rs655580
rs7690529
rs116851238
rs115951641
rs2395904
rs147207149
rs115150472
rs72691696
All
0.00
0.01
0.02
0.03
0.04
0.05
MR leave−one−out sensitivity analysis for
' IgD+ CD38dim AC' on 'Rheumatoid arthritis'

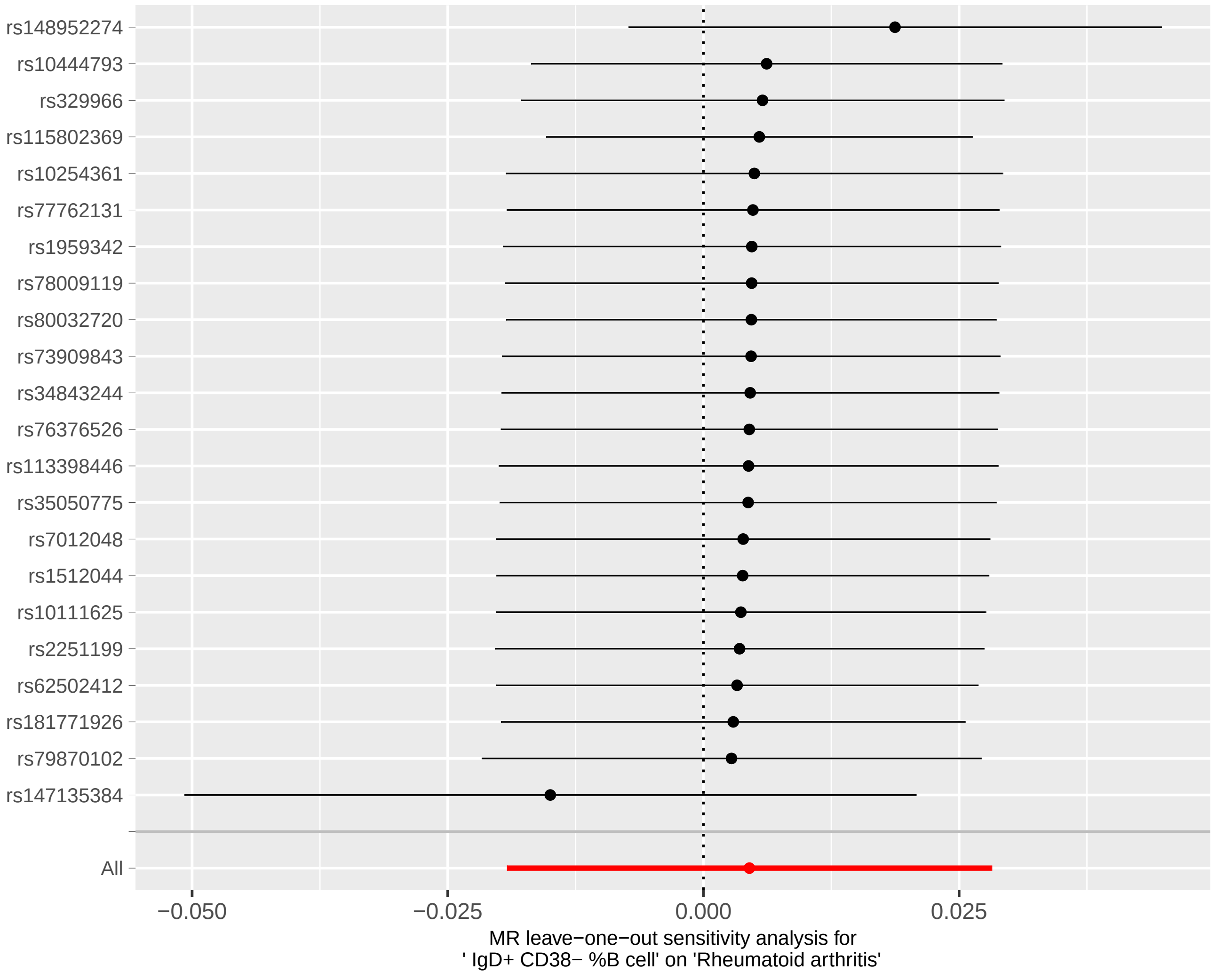
rs148952274
rs10444793
rs329966
rs115802369
rs10254361
rs77762131
rs1959342
rs78009119
rs80032720
rs73909843
rs34843244
rs76376526
rs113398446
rs35050775
rs7012048
rs1512044
rs10111625
rs2251199
rs62502412
rs181771926
rs79870102
rs147135384
All
−0.050
−0.025
0.000
0.025
MR leave−one−out sensitivity analysis for
' IgD+ CD38− %B cell' on 'Rheumatoid arthritis'

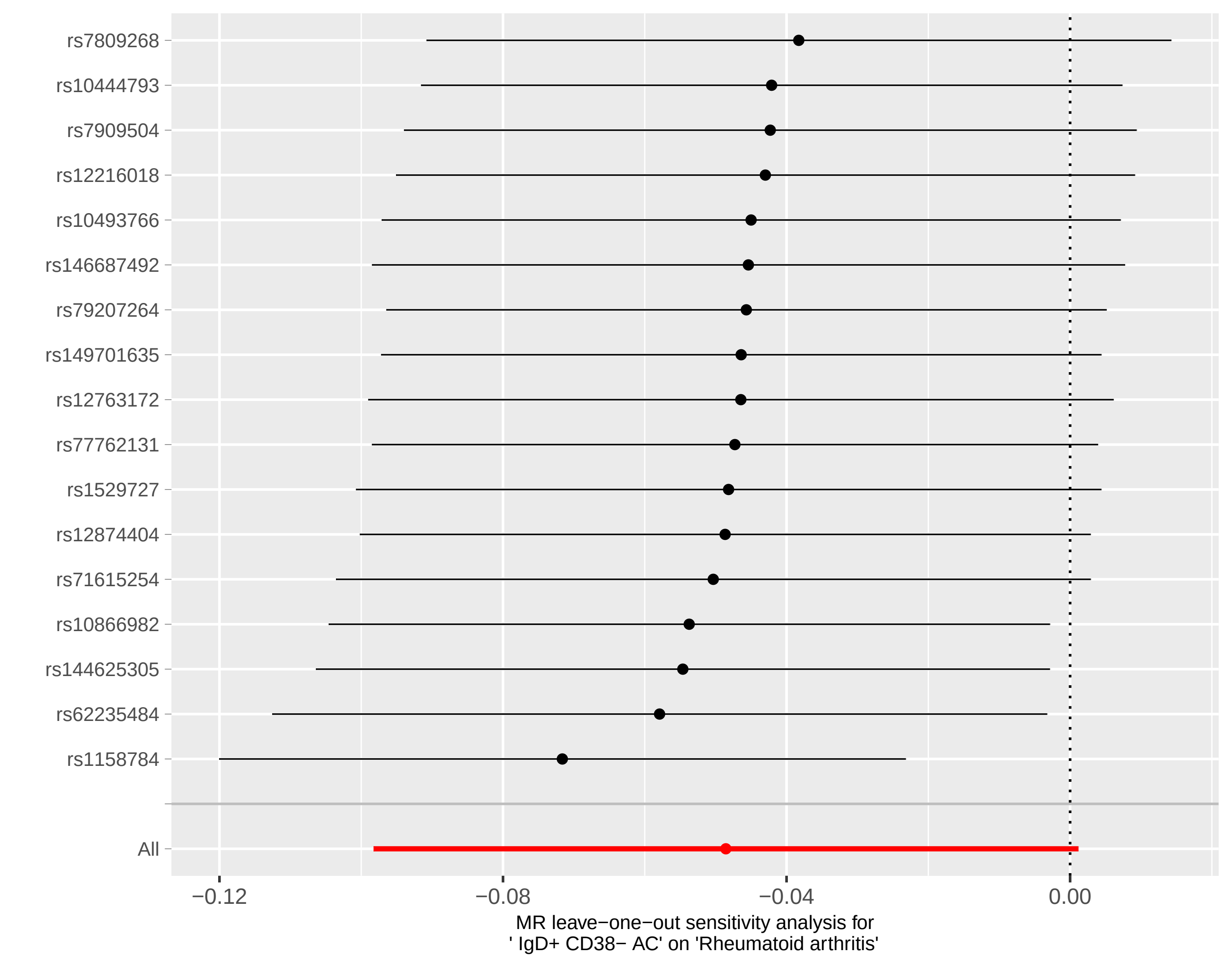
rs7809268
rs10444793
rs7909504
rs12216018
rs10493766
rs146687492
rs79207264
rs149701635
rs12763172
rs77762131
rs1529727
rs12874404
rs71615254
rs10866982
rs144625305
rs62235484
rs1158784
All
−0.12
−0.08
−0.04
0.00
MR leave−one−out sensitivity analysis for
' IgD+ CD38− AC' on 'Rheumatoid arthritis'

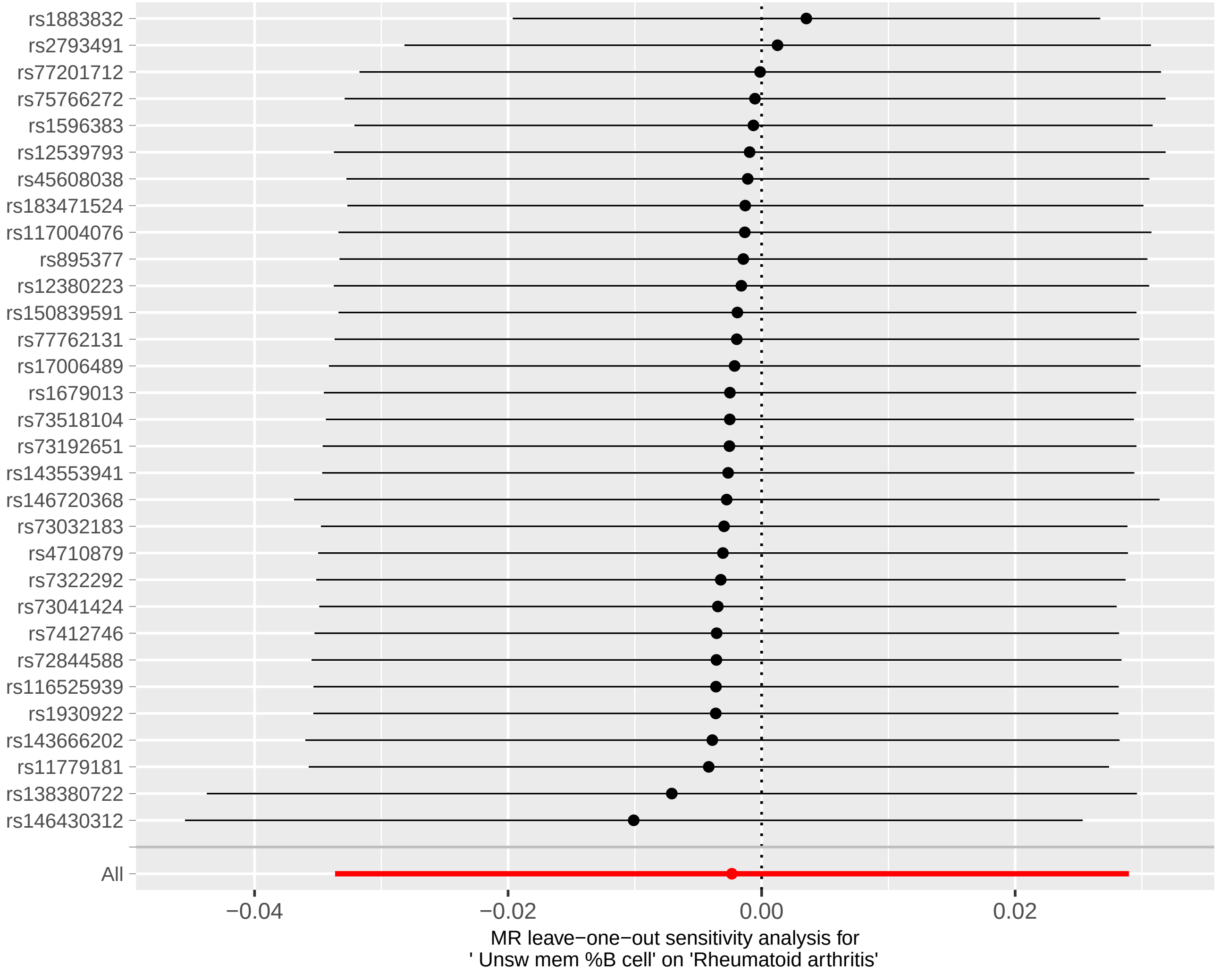
rs1883832
rs2793491
rs77201712
rs75766272
rs1596383
rs12539793
rs45608038
rs183471524
rs117004076
rs895377
rs12380223
rs150839591
rs77762131
rs17006489
rs1679013
rs73518104
rs73192651
rs143553941
rs146720368
rs73032183
rs4710879
rs7322292
rs73041424
rs7412746
rs72844588
rs116525939
rs1930922
rs143666202
rs11779181
rs138380722
rs146430312
All
−0.04
−0.02
0.00
0.02
MR leave−one−out sensitivity analysis for
' Unsw mem %B cell' on 'Rheumatoid arthritis'

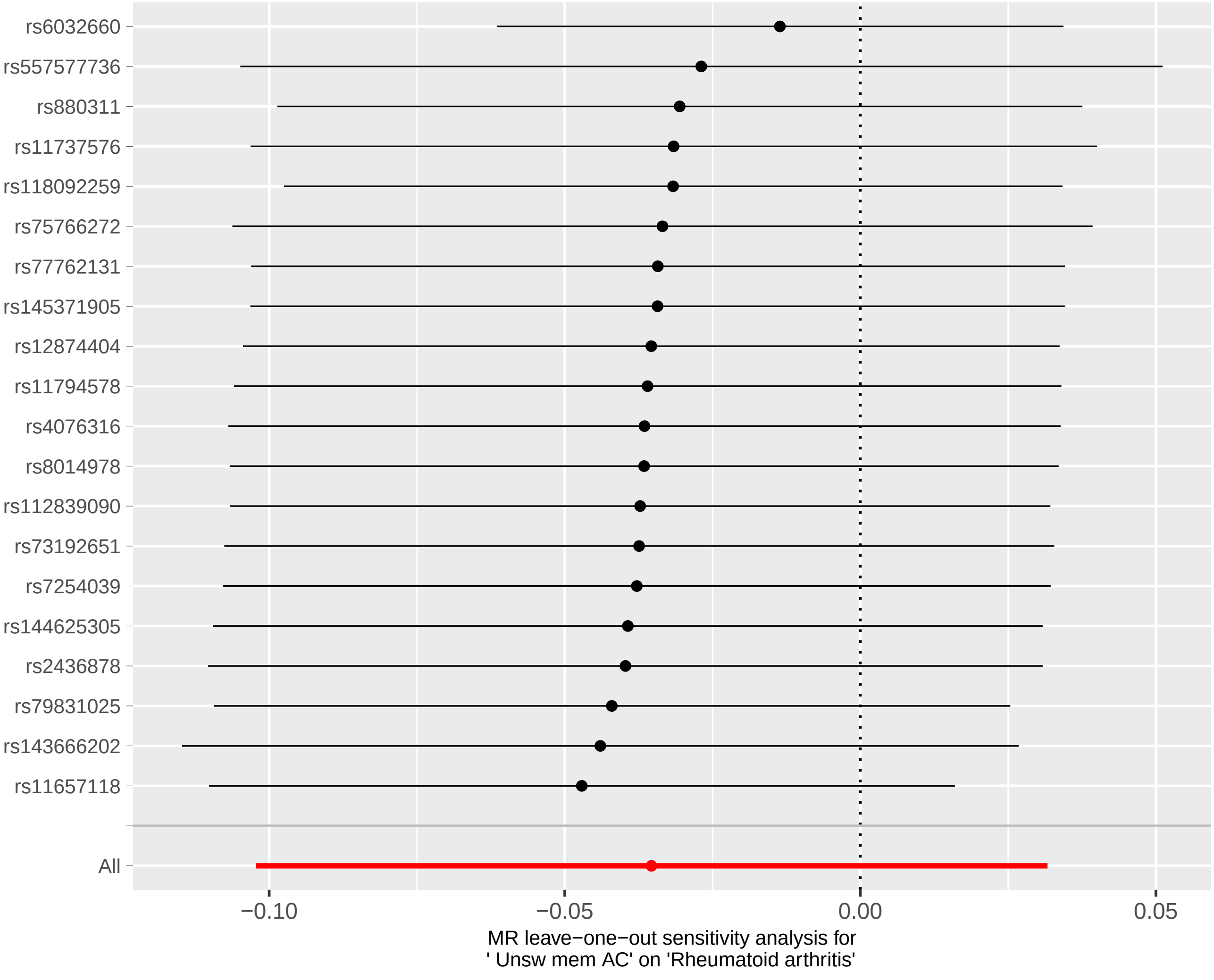
rs6032660
rs557577736
rs880311
rs11737576
rs118092259
rs75766272
rs77762131
rs145371905
rs12874404
rs11794578
rs4076316
rs8014978
rs112839090
rs73192651
rs7254039
rs144625305
rs2436878
rs79831025
rs143666202
rs11657118
All
−0.10
−0.05
0.00
0.05
MR leave−one−out sensitivity analysis for
' Unsw mem AC' on 'Rheumatoid arthritis'

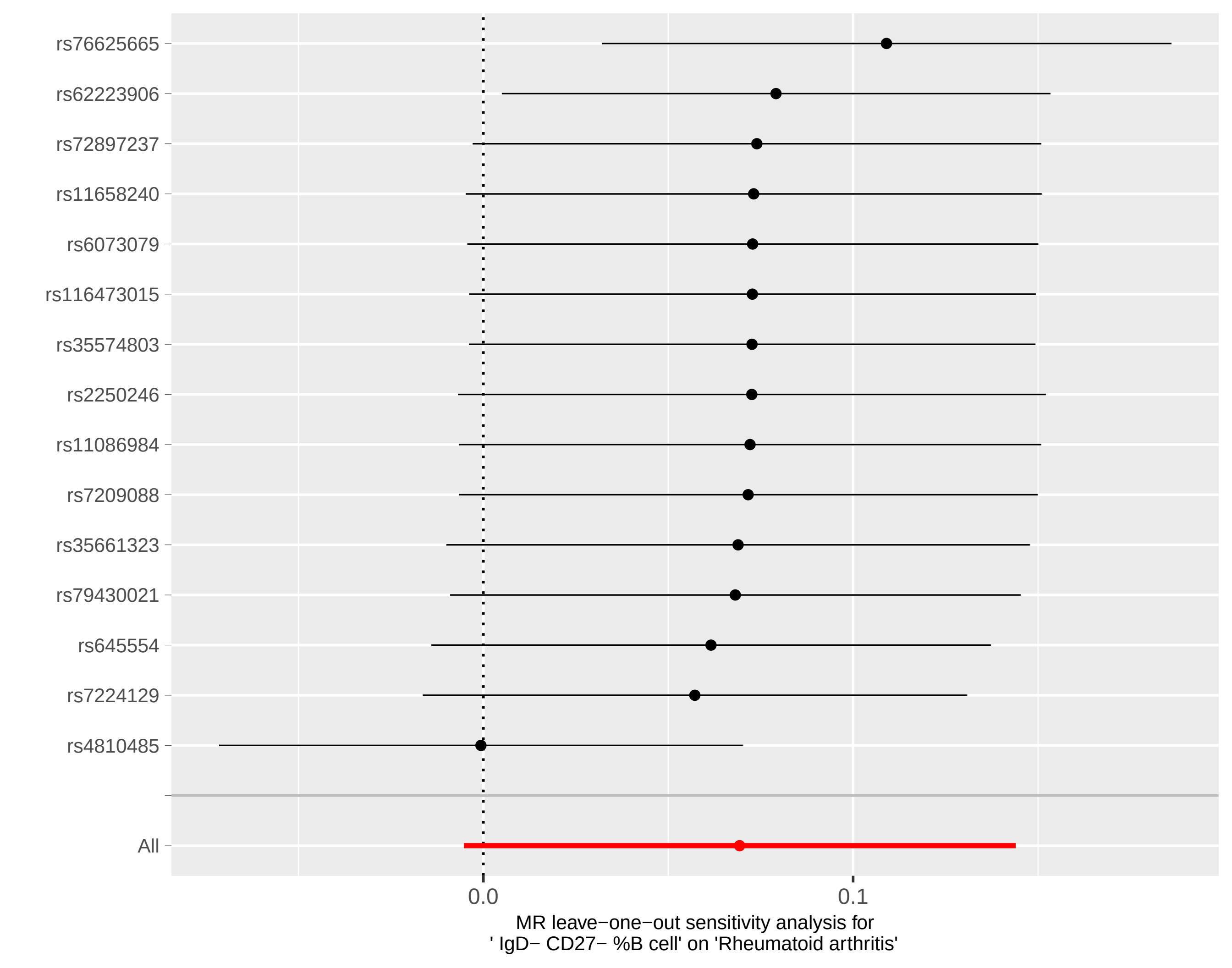

rs76625665
rs62223906
rs72897237
rs11658240
rs6073079
rs116473015
rs35574803
rs2250246
rs11086984
rs7209088
rs35661323
rs79430021
rs645554
rs7224129
rs4810485
All
0.0
0.1
MR leave−one−out sensitivity analysis for
' IgD− CD27− %B cell' on 'Rheumatoid arthritis'

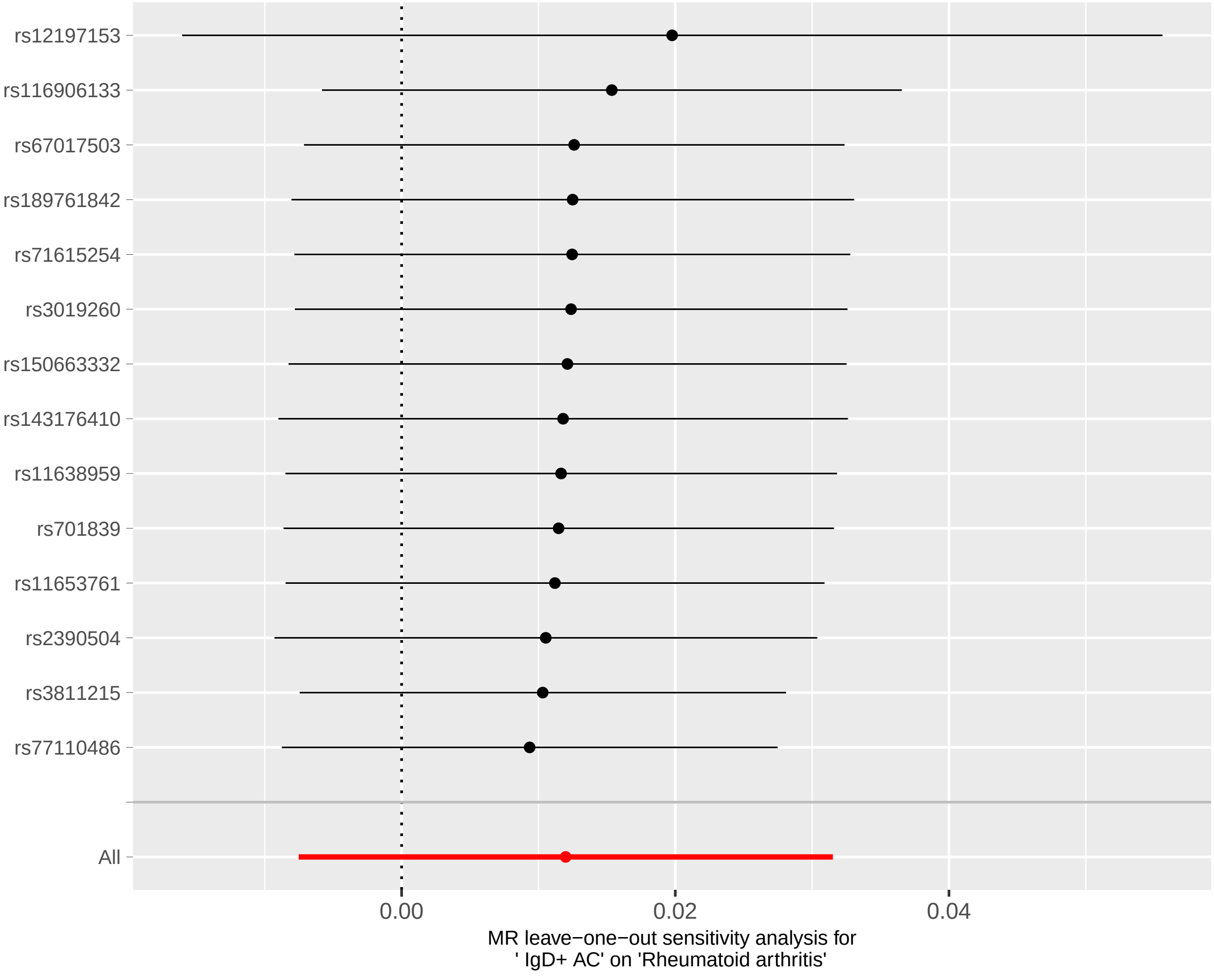
rs12197153
rs116906133
rs67017503
rs189761842
rs71615254
rs3019260
rs150663332
rs143176410
rs11638959
rs701839
rs11653761
rs2390504
rs3811215
rs77110486
All
0.00
0.02
0.04
MR leave−one−out sensitivity analysis for
' IgD+ AC' on 'Rheumatoid arthritis'

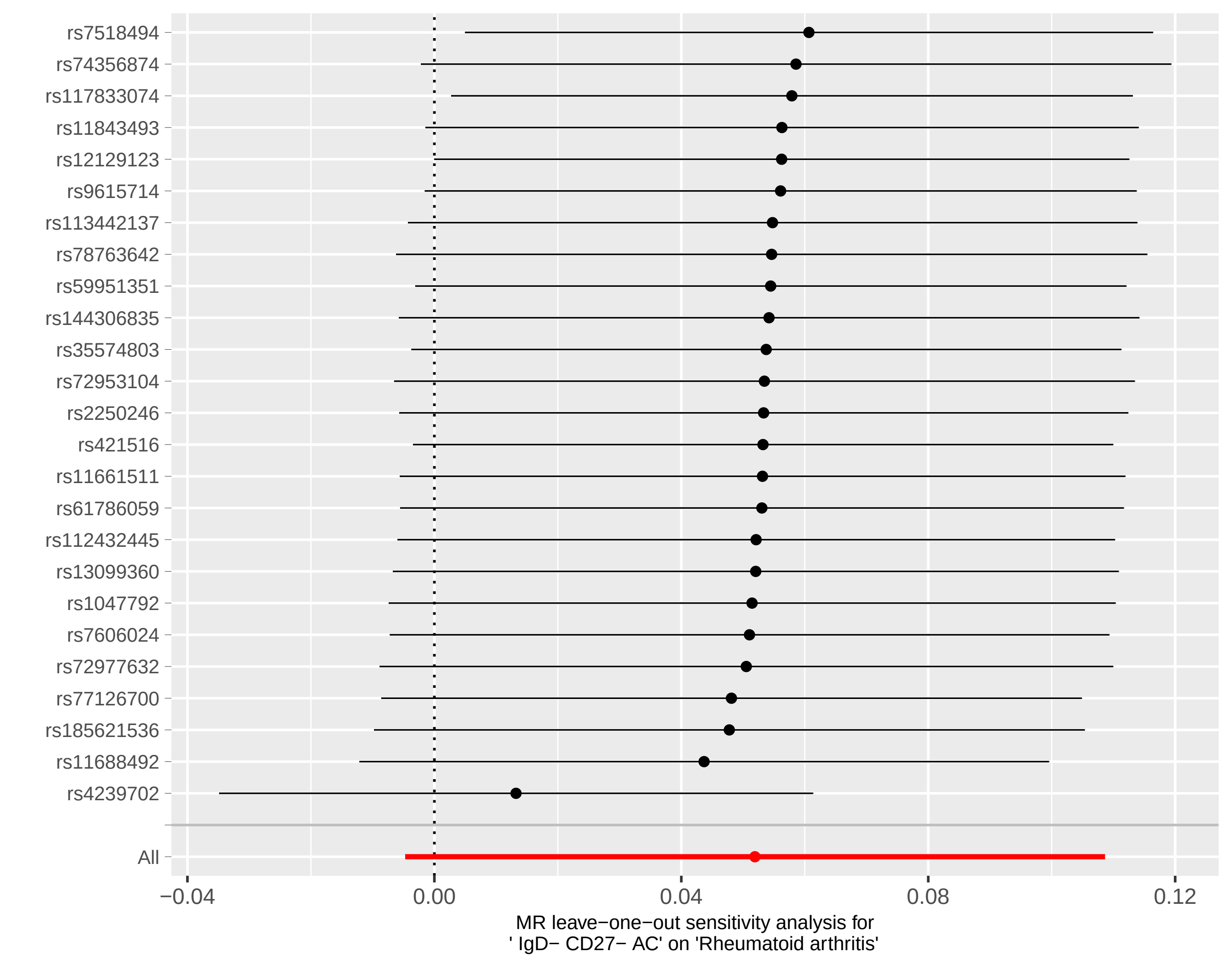

rs7518494
rs74356874
rs117833074
rs11843493
rs12129123
rs9615714
rs113442137
rs78763642
rs59951351
rs144306835
rs35574803
rs72953104
rs2250246
rs421516
rs11661511
rs61786059
rs112432445
rs13099360
rs1047792
rs7606024
rs72977632
rs77126700
rs185621536
rs11688492
rs4239702
All
−0.04
0.00
0.04
0.08
0.12
MR leave−one−out sensitivity analysis for
' IgD− CD27− AC' on 'Rheumatoid arthritis'

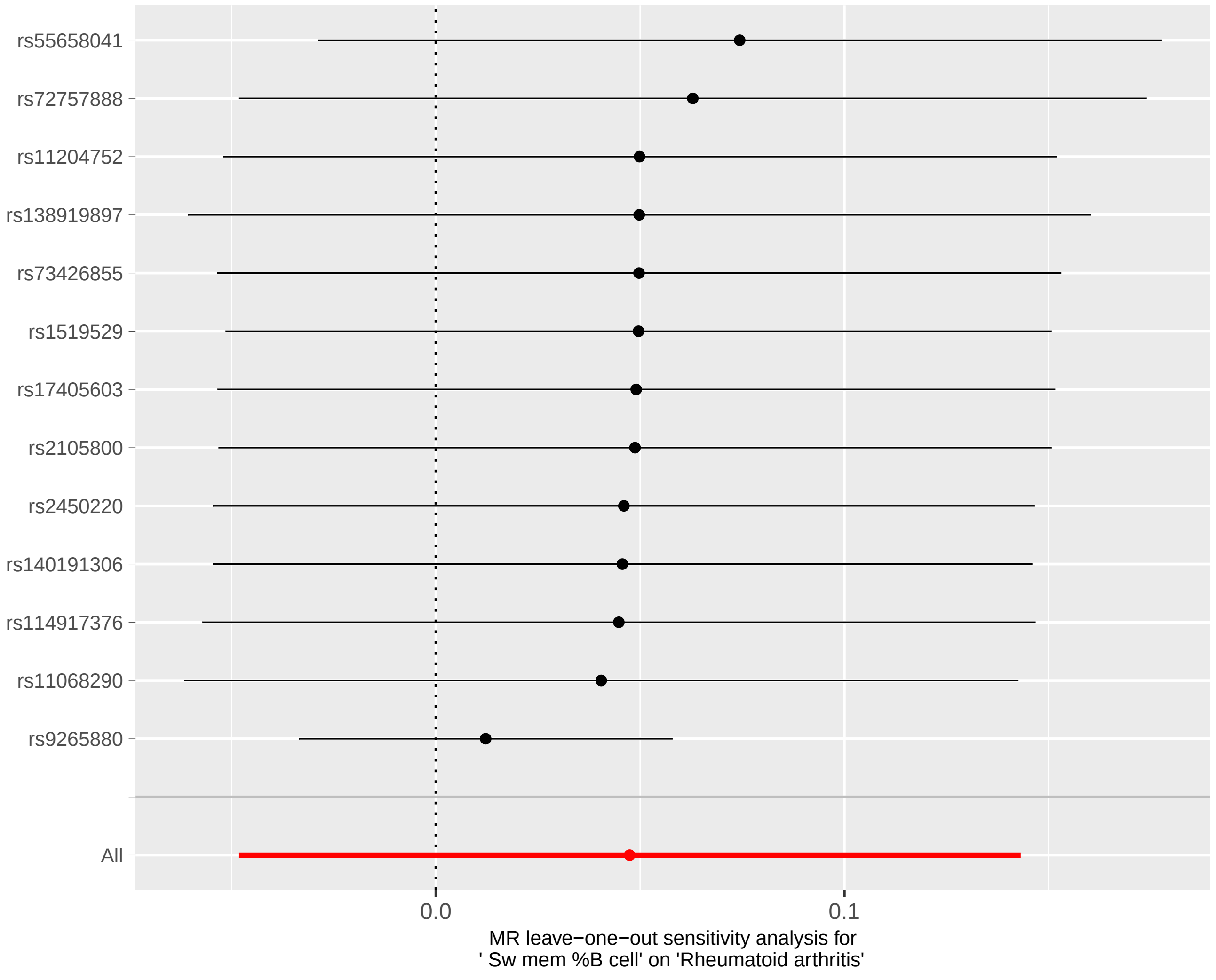

rs55658041
rs72757888
rs11204752
rs138919897
rs73426855
rs1519529
rs17405603
rs2105800
rs2450220
rs140191306
rs114917376
rs11068290
rs9265880
All
0.0
0.1
MR leave−one−out sensitivity analysis for
' Sw mem %B cell' on 'Rheumatoid arthritis'

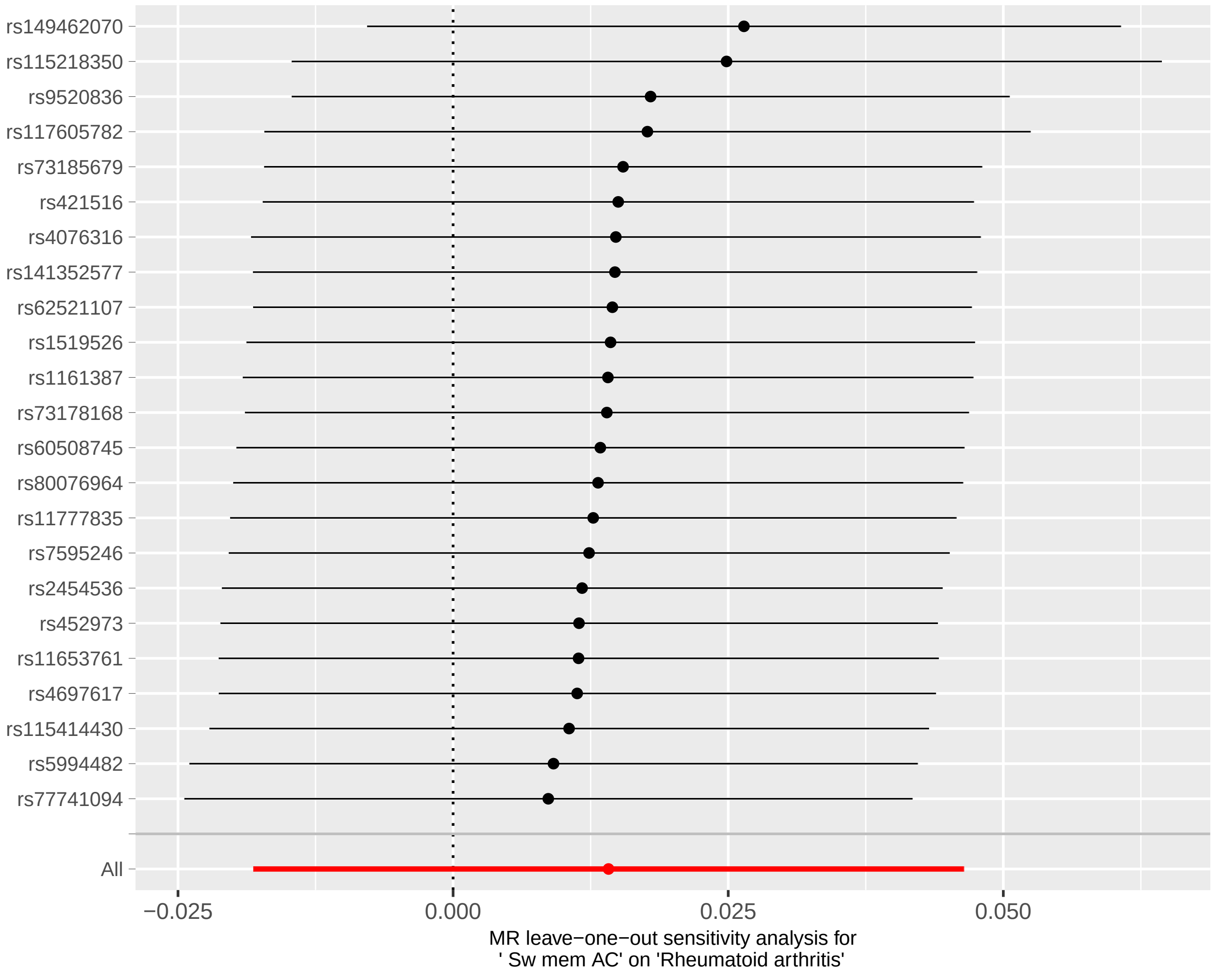
rs149462070
rs115218350
rs9520836
rs117605782
rs73185679
rs421516
rs4076316
rs141352577
rs62521107
rs1519526
rs1161387
rs73178168
rs60508745
rs80076964
rs11777835
rs7595246
rs2454536
rs452973
rs11653761
rs4697617
rs115414430
rs5994482
rs77741094
All
−0.025
0.000
0.025
0.050
MR leave−one−out sensitivity analysis for
' Sw mem AC' on 'Rheumatoid arthritis'

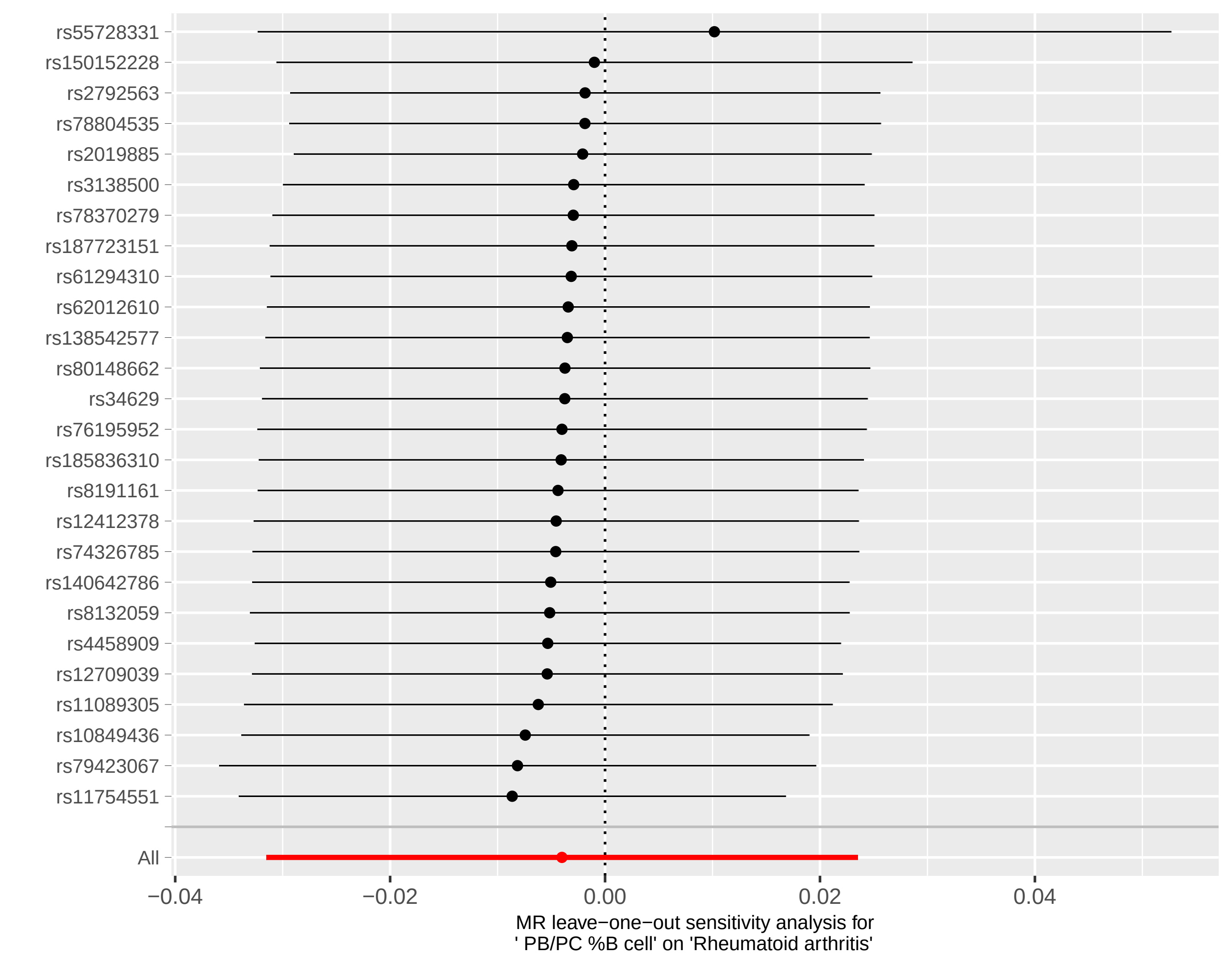

rs55728331
rs150152228
rs2792563
rs78804535
rs2019885
rs3138500
rs78370279
rs187723151
rs61294310
rs62012610
rs138542577
rs80148662
rs34629
rs76195952
rs185836310
rs8191161
rs12412378
rs74326785
rs140642786
rs8132059
rs4458909
rs12709039
rs11089305
rs10849436
rs79423067
rs11754551
All
−0.04
−0.02
0.00
0.02
0.04
MR leave−one−out sensitivity analysis for
' PB/PC %B cell' on 'Rheumatoid arthritis'

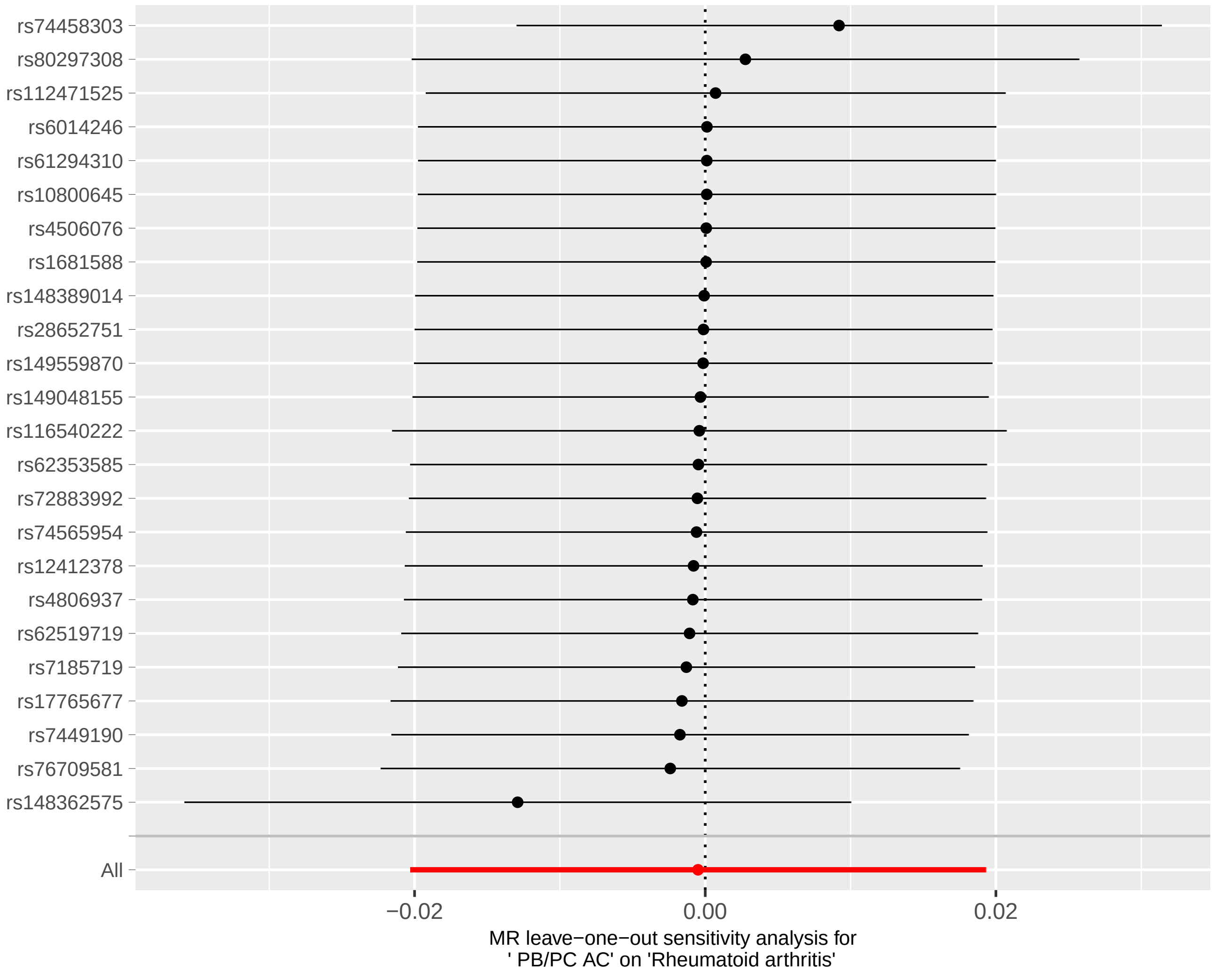
rs74458303
rs80297308
rs112471525
rs6014246
rs61294310
rs10800645
rs4506076
rs1681588
rs148389014
rs28652751
rs149559870
rs149048155
rs116540222
rs62353585
rs72883992
rs74565954
rs12412378
rs4806937
rs62519719
rs7185719
rs17765677
rs7449190
rs76709581
rs148362575
All
−0.02
0.00
0.02
MR leave−one−out sensitivity analysis for
' PB/PC AC' on 'Rheumatoid arthritis'

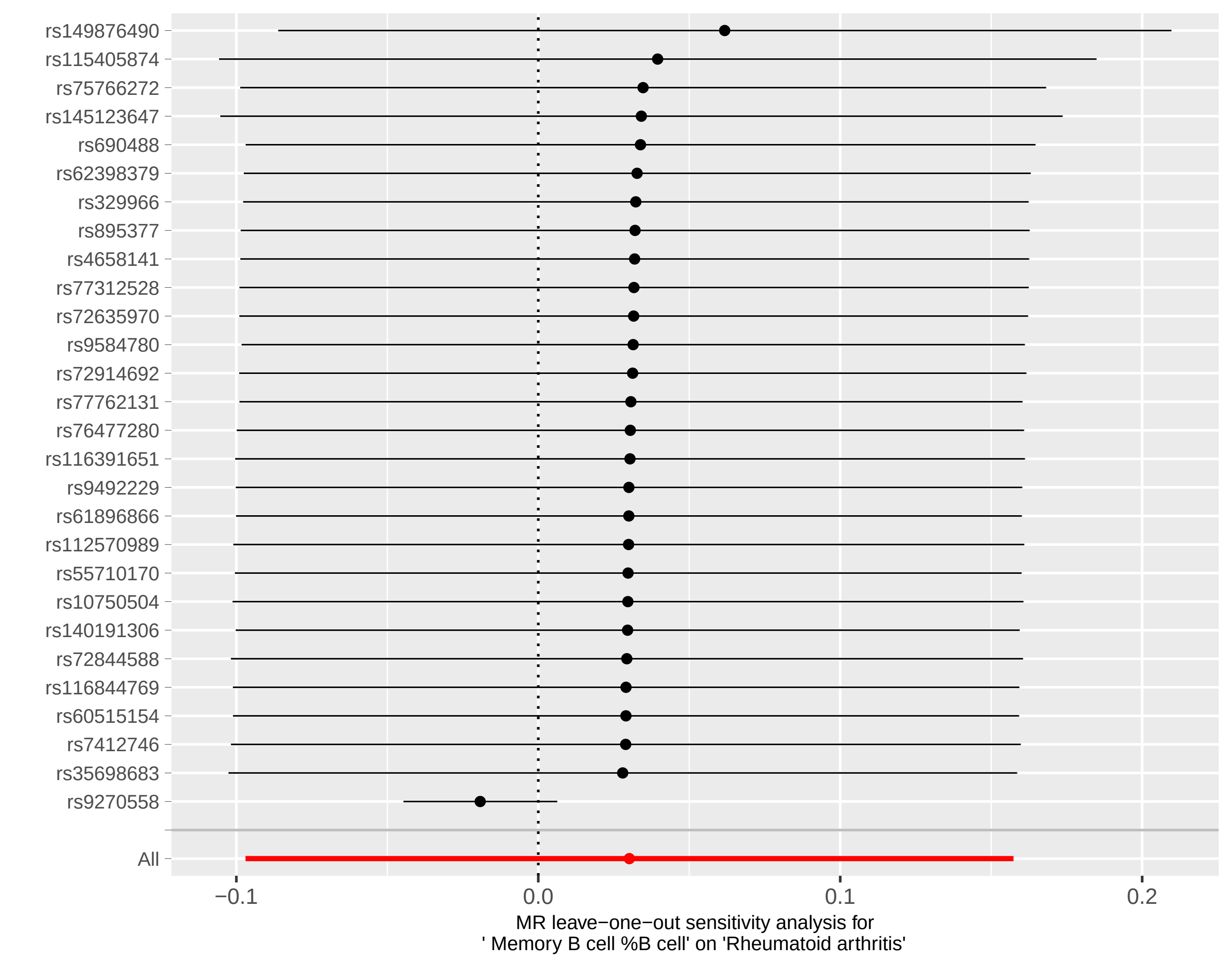

rs149876490
rs115405874
rs75766272
rs145123647
rs690488
rs62398379
rs329966
rs895377
rs4658141
rs77312528
rs72635970
rs9584780
rs72914692
rs77762131
rs76477280
rs116391651
rs9492229
rs61896866
rs112570989
rs55710170
rs10750504
rs140191306
rs72844588
rs116844769
rs60515154
rs7412746
rs35698683
rs9270558
All
−0.1
0.0
0.1
0.2
MR leave−one−out sensitivity analysis for
' Memory B cell %B cell' on 'Rheumatoid arthritis'

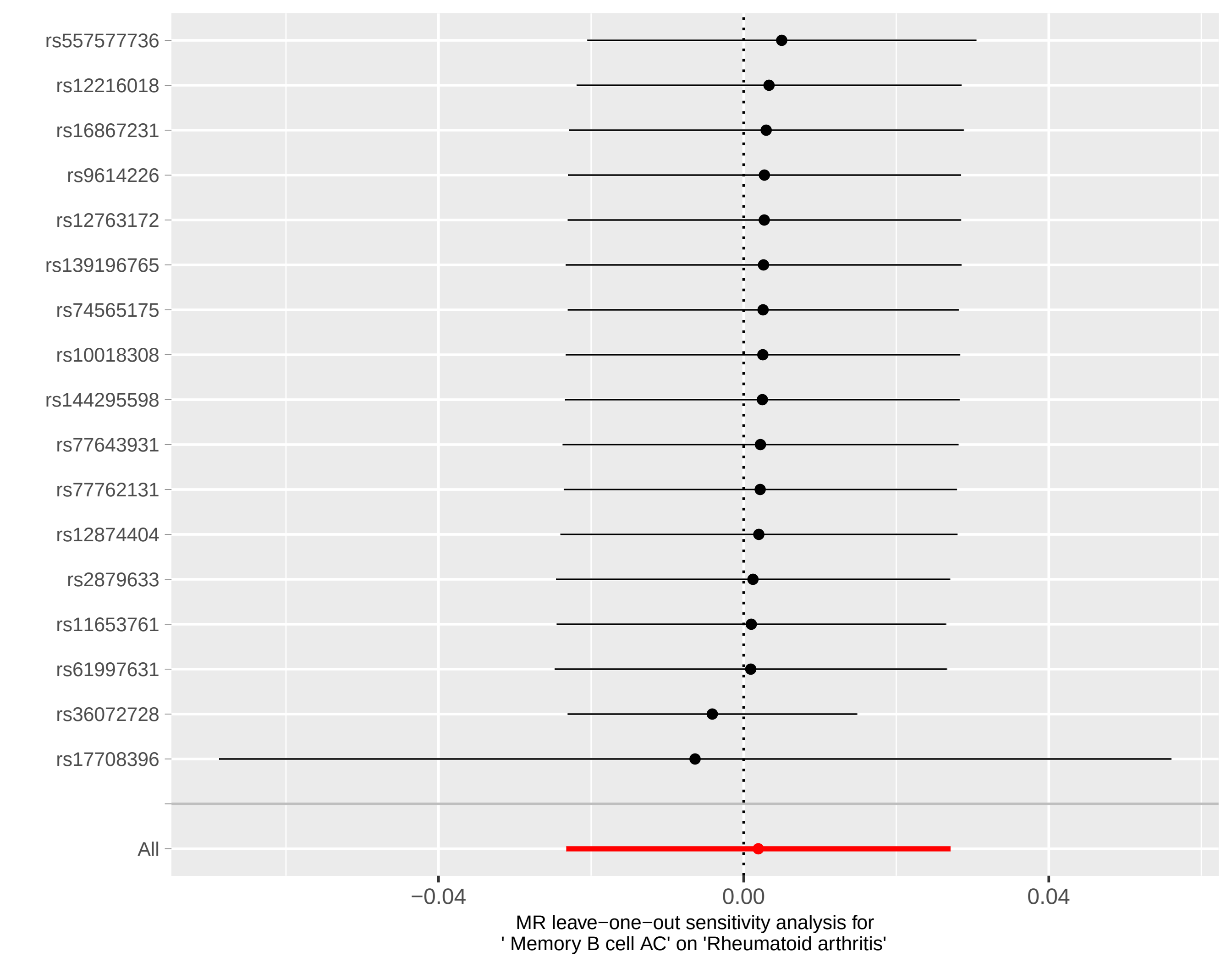
rs557577736
rs12216018
rs16867231
rs9614226
rs12763172
rs139196765
rs74565175
rs10018308
rs144295598
rs77643931
rs77762131
rs12874404
rs2879633
rs11653761
rs61997631
rs36072728
rs17708396
All
−0.04
0.00
0.04
MR leave−one−out sensitivity analysis for
' Memory B cell AC' on 'Rheumatoid arthritis'

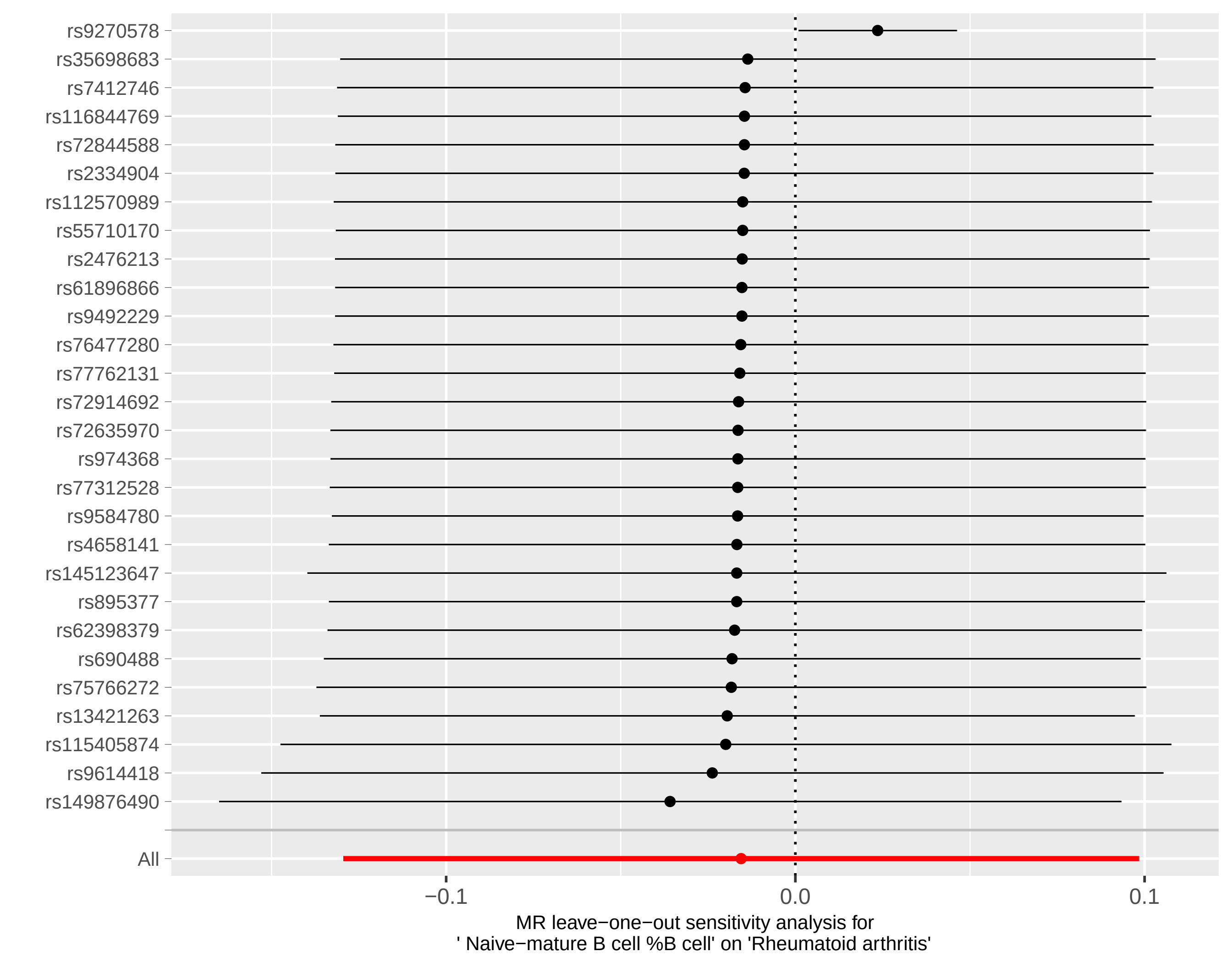
rs9270578
rs35698683
rs7412746
rs116844769
rs72844588
rs2334904
rs112570989
rs55710170
rs2476213
rs61896866
rs9492229
rs76477280
rs77762131
rs72914692
rs72635970
rs974368
rs77312528
rs9584780
rs4658141
rs145123647
rs895377
rs62398379
rs690488
rs75766272
rs13421263
rs115405874
rs9614418
rs149876490
All
−0.1
0.0
0.1
MR leave−one−out sensitivity analysis for
' Naive−mature B cell %B cell' on 'Rheumatoid arthritis'

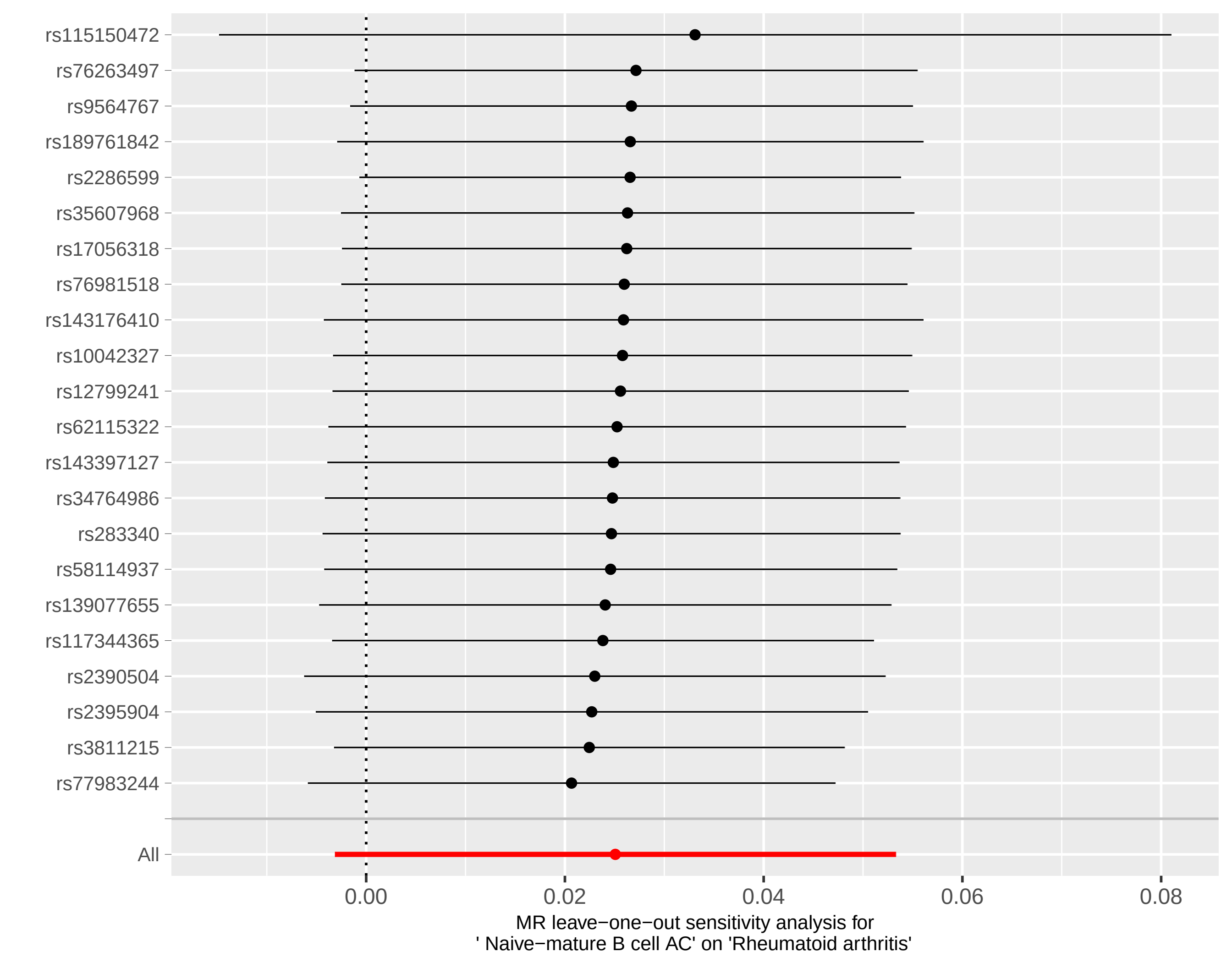

rs115150472
rs76263497
rs9564767
rs189761842
rs2286599
rs35607968
rs17056318
rs76981518
rs143176410
rs10042327
rs12799241
rs62115322
rs143397127
rs34764986
rs283340
rs58114937
rs139077655
rs117344365
rs2390504
rs2395904
rs3811215
rs77983244
All
0.00
0.02
0.04
0.06
0.08
MR leave−one−out sensitivity analysis for
' Naive−mature B cell AC' on 'Rheumatoid arthritis'

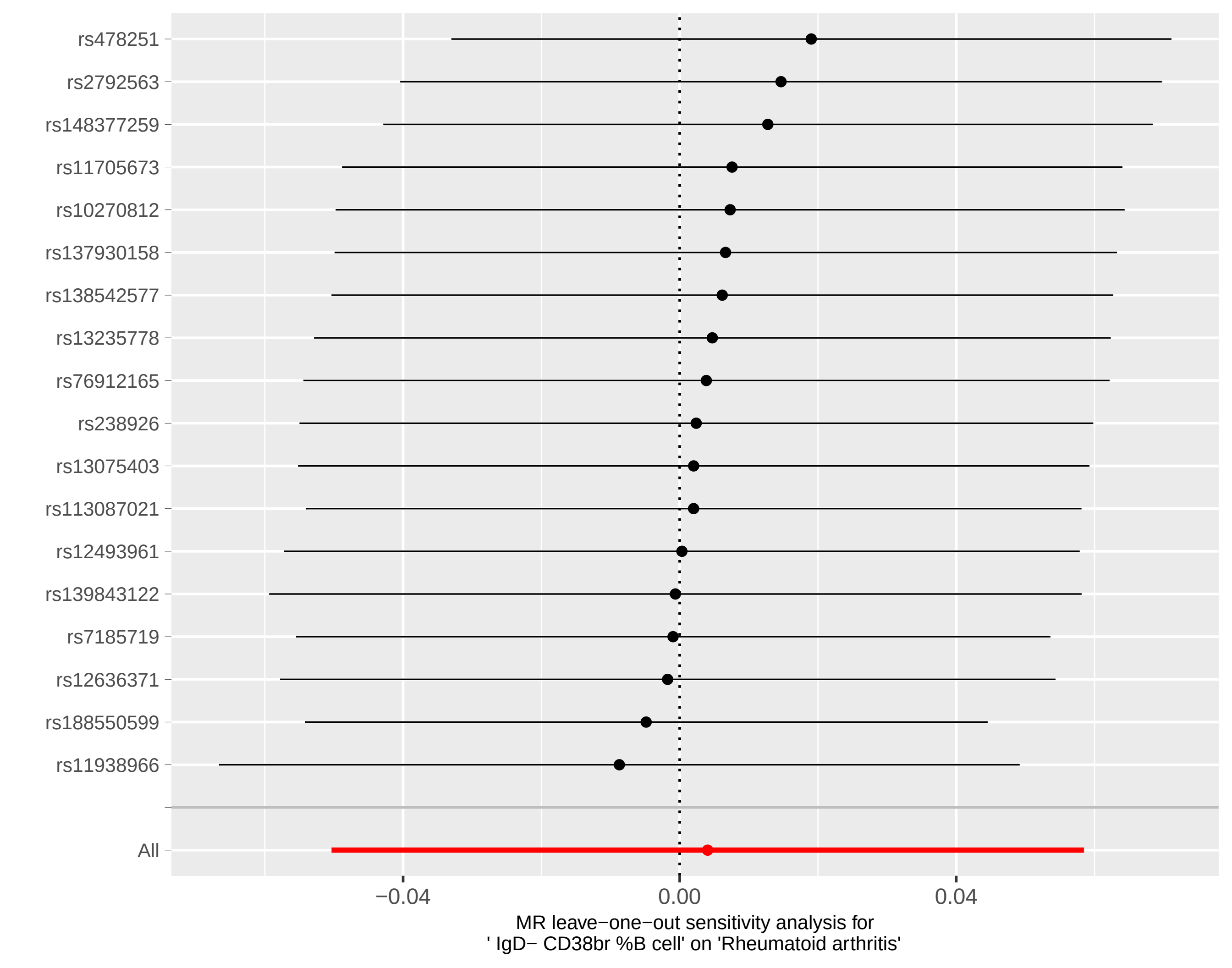
rs478251
rs2792563
rs148377259
rs11705673
rs10270812
rs137930158
rs138542577
rs13235778
rs76912165
rs238926
rs13075403
rs113087021
rs12493961
rs139843122
rs7185719
rs12636371
rs188550599
rs11938966
All
−0.04
0.00
0.04
MR leave−one−out sensitivity analysis for
' IgD− CD38br %B cell' on 'Rheumatoid arthritis'

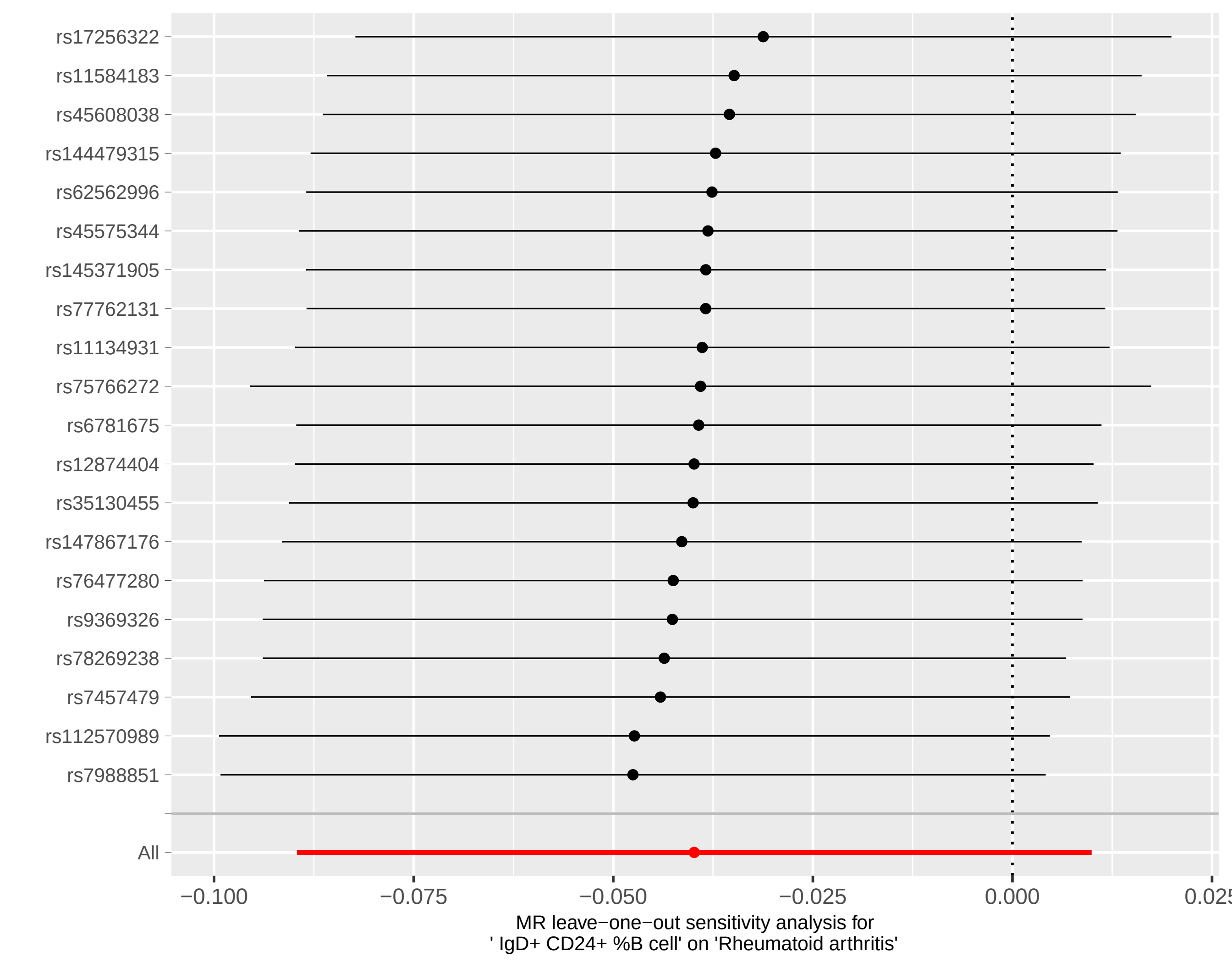

rs17256322
rs11584183
rs45608038
rs144479315
rs62562996
rs45575344
rs145371905
rs77762131
rs11134931
rs75766272
rs6781675
rs12874404
rs35130455
rs147867176
rs76477280
rs9369326
rs78269238
rs7457479
rs112570989
rs7988851
All
−0.100
−0.075
−0.050
−0.025
0.000
0.025
MR leave−one−out sensitivity analysis for
' IgD+ CD24+ %B cell' on 'Rheumatoid arthritis'

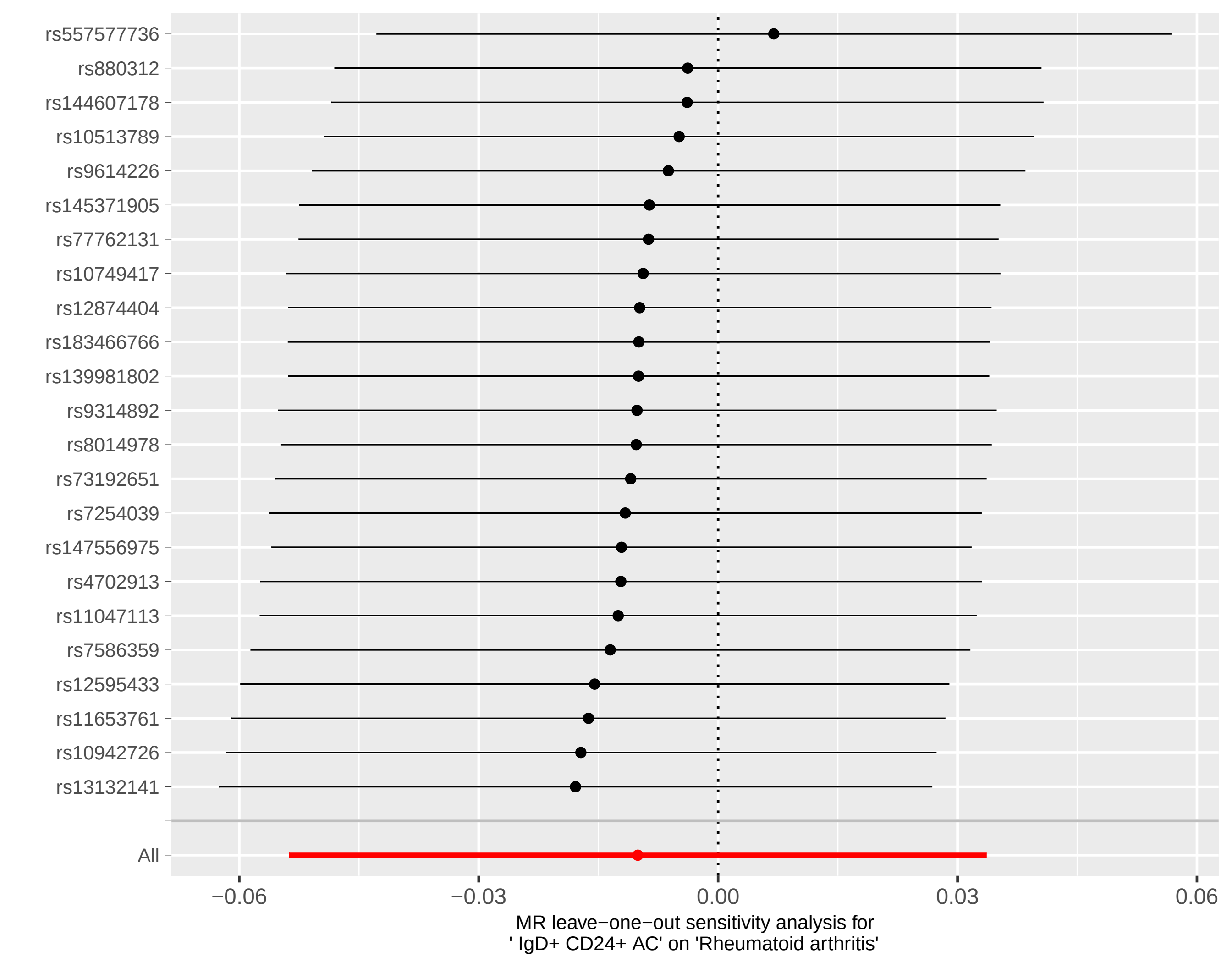
rs557577736
rs880312
rs144607178
rs10513789
rs9614226
rs145371905
rs77762131
rs10749417
rs12874404
rs183466766
rs139981802
rs9314892
rs8014978
rs73192651
rs7254039
rs147556975
rs4702913
rs11047113
rs7586359
rs12595433
rs11653761
rs10942726
rs13132141
All
−0.06
−0.03
0.00
0.03
0.06
MR leave−one−out sensitivity analysis for
' IgD+ CD24+ AC' on 'Rheumatoid arthritis'

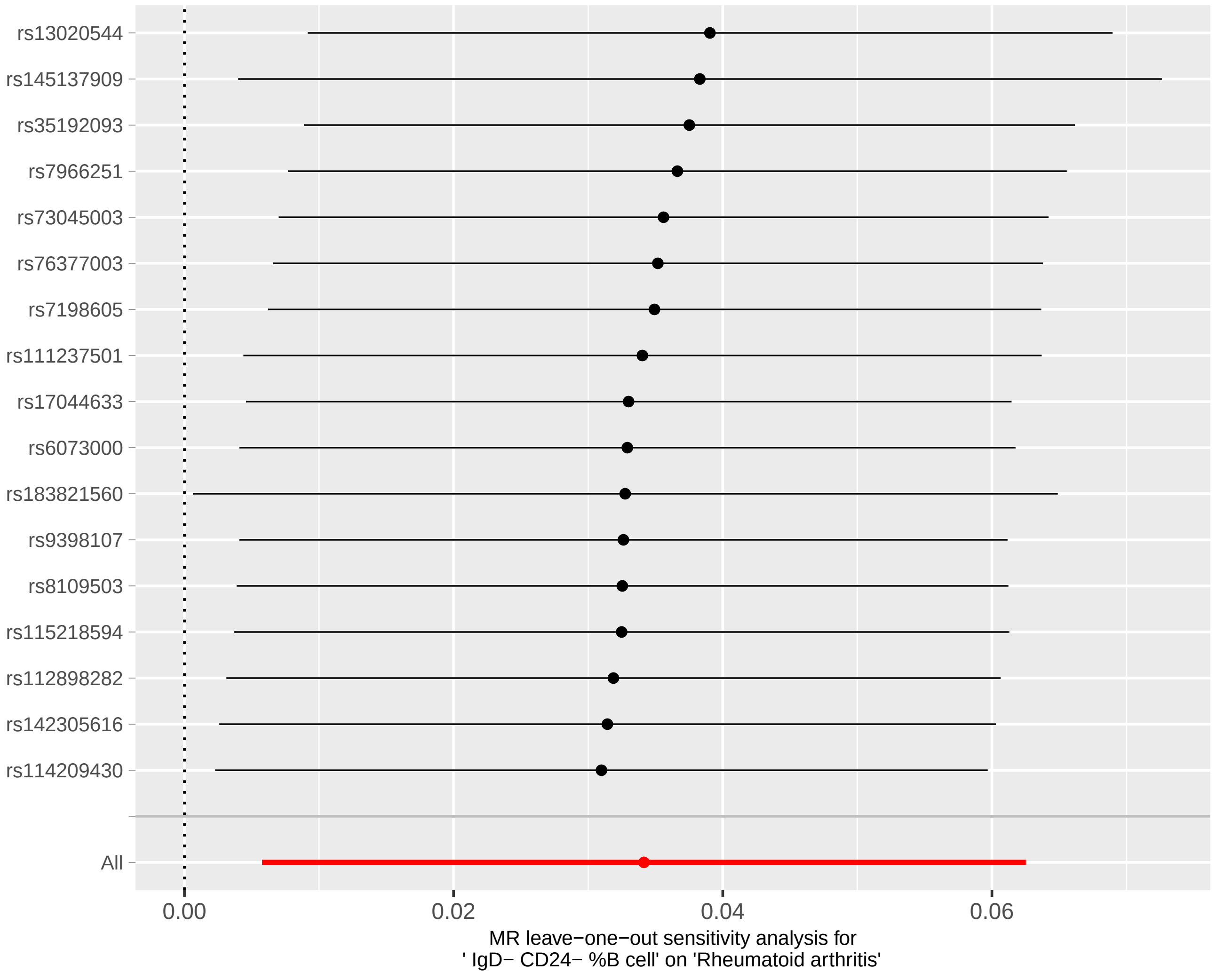
rs13020544
rs145137909
rs35192093
rs7966251
rs73045003
rs76377003
rs7198605
rs111237501
rs17044633
rs6073000
rs183821560
rs9398107
rs8109503
rs115218594
rs112898282
rs142305616
rs114209430
All
0.00
0.02
0.04
0.06
MR leave−one−out sensitivity analysis for
' IgD− CD24− %B cell' on 'Rheumatoid arthritis'

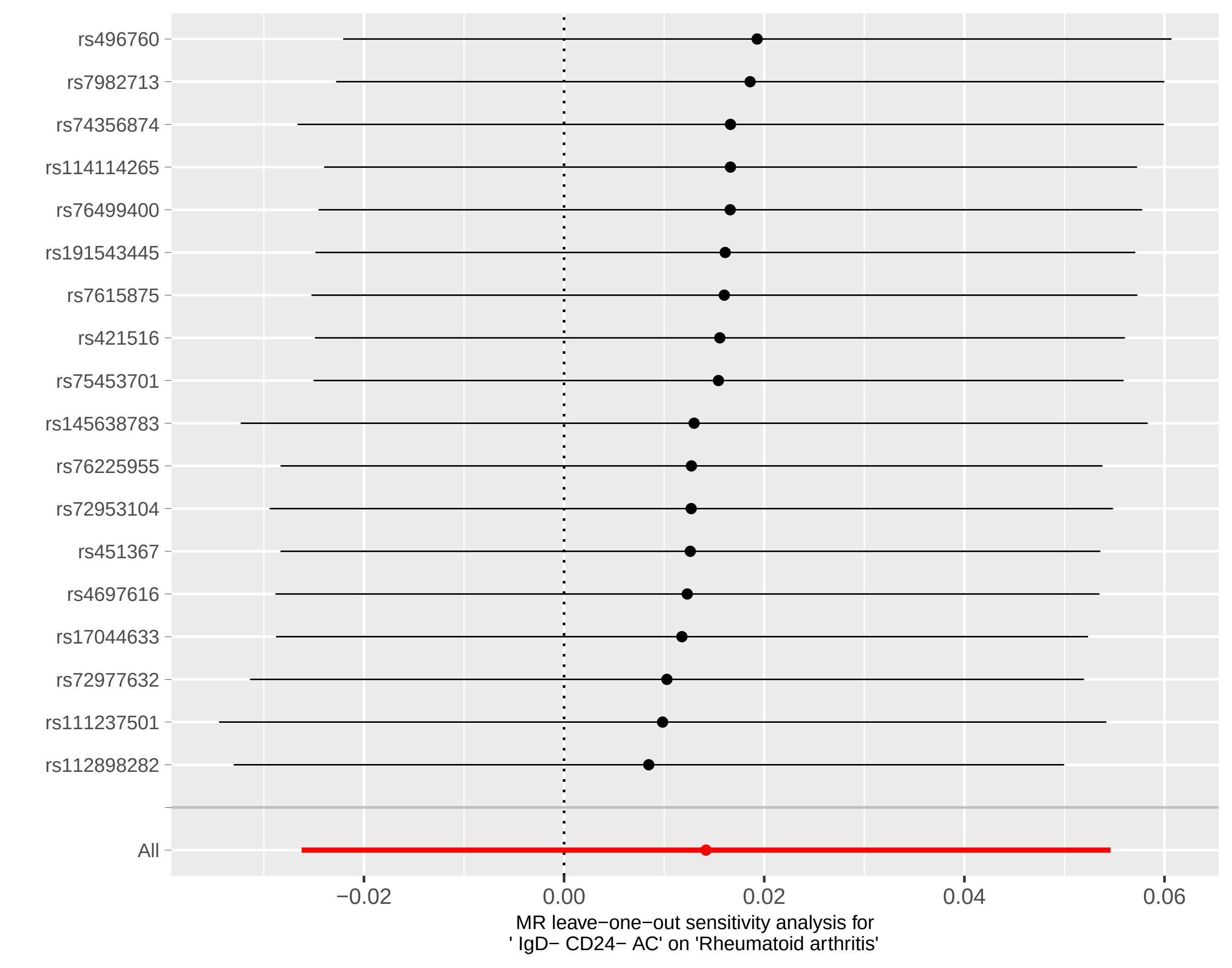
rs496760
rs7982713
rs74356874
rs114114265
rs76499400
rs191543445
rs7615875
rs421516
rs75453701
rs145638783
rs76225955
rs72953104
rs451367
rs4697616
rs17044633
rs72977632
rs111237501
rs112898282
All
−0.02
0.00
0.02
0.04
0.06
MR leave−one−out sensitivity analysis for
' IgD− CD24− AC' on 'Rheumatoid arthritis'

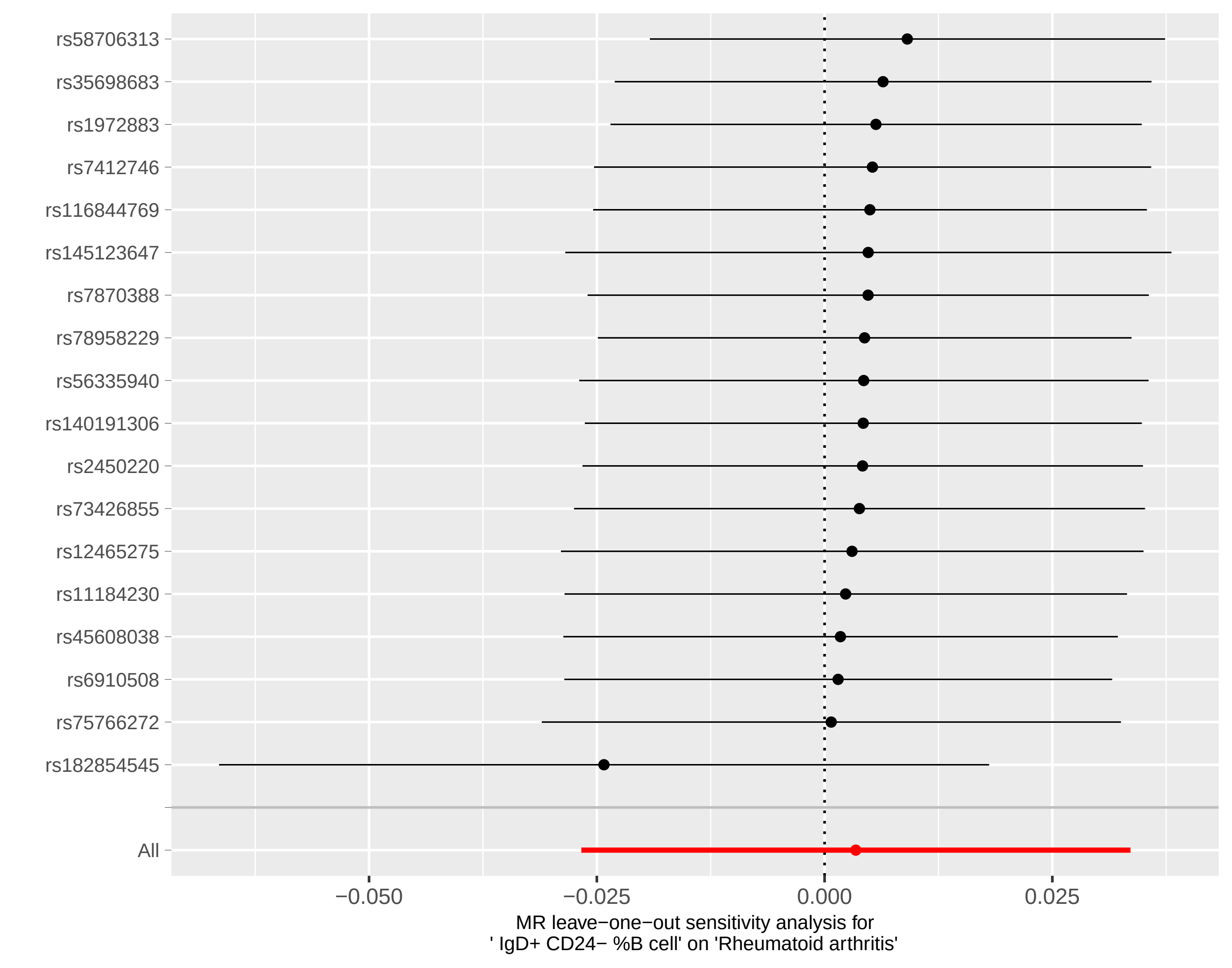
rs58706313
rs35698683
rs1972883
rs7412746
rs116844769
rs145123647
rs7870388
rs78958229
rs56335940
rs140191306
rs2450220
rs73426855
rs12465275
rs11184230
rs45608038
rs6910508
rs75766272
rs182854545
All
−0.050
−0.025
0.000
0.025
MR leave−one−out sensitivity analysis for
' IgD+ CD24− %B cell' on 'Rheumatoid arthritis'

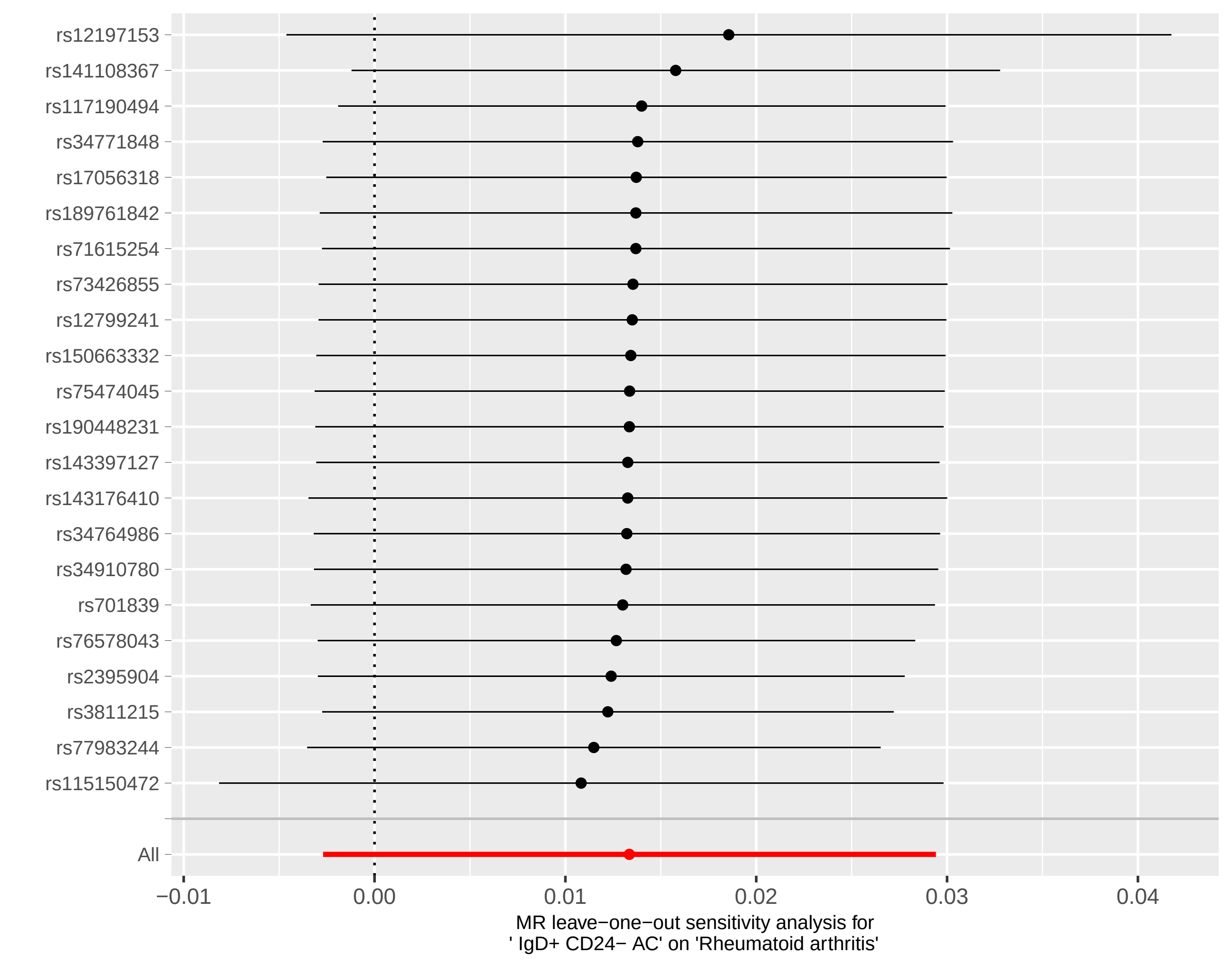
rs12197153
rs141108367
rs117190494
rs34771848
rs17056318
rs189761842
rs71615254
rs73426855
rs12799241
rs150663332
rs75474045
rs190448231
rs143397127
rs143176410
rs34764986
rs34910780
rs701839
rs76578043
rs2395904
rs3811215
rs77983244
rs115150472
All
−0.01
0.00
0.01
0.02
0.03
0.04
MR leave−one−out sensitivity analysis for
' IgD+ CD24− AC' on 'Rheumatoid arthritis'

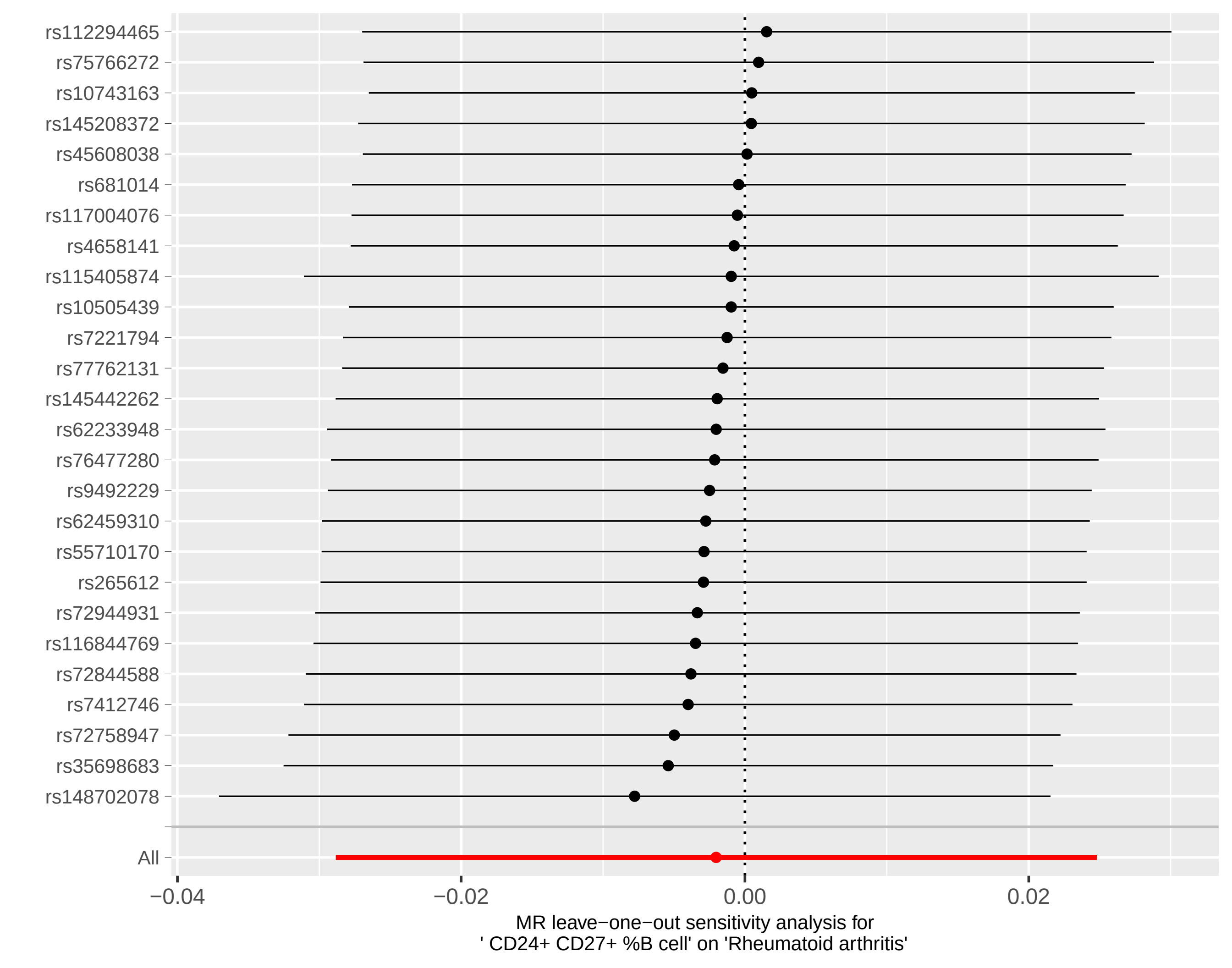
rs112294465
rs75766272
rs10743163
rs145208372
rs45608038
rs681014
rs117004076
rs4658141
rs115405874
rs10505439
rs7221794
rs77762131
rs145442262
rs62233948
rs76477280
rs9492229
rs62459310
rs55710170
rs265612
rs72944931
rs116844769
rs72844588
rs7412746
rs72758947
rs35698683
rs148702078
All
−0.04
−0.02
0.00
0.02
MR leave−one−out sensitivity analysis for
' CD24+ CD27+ %B cell' on 'Rheumatoid arthritis'

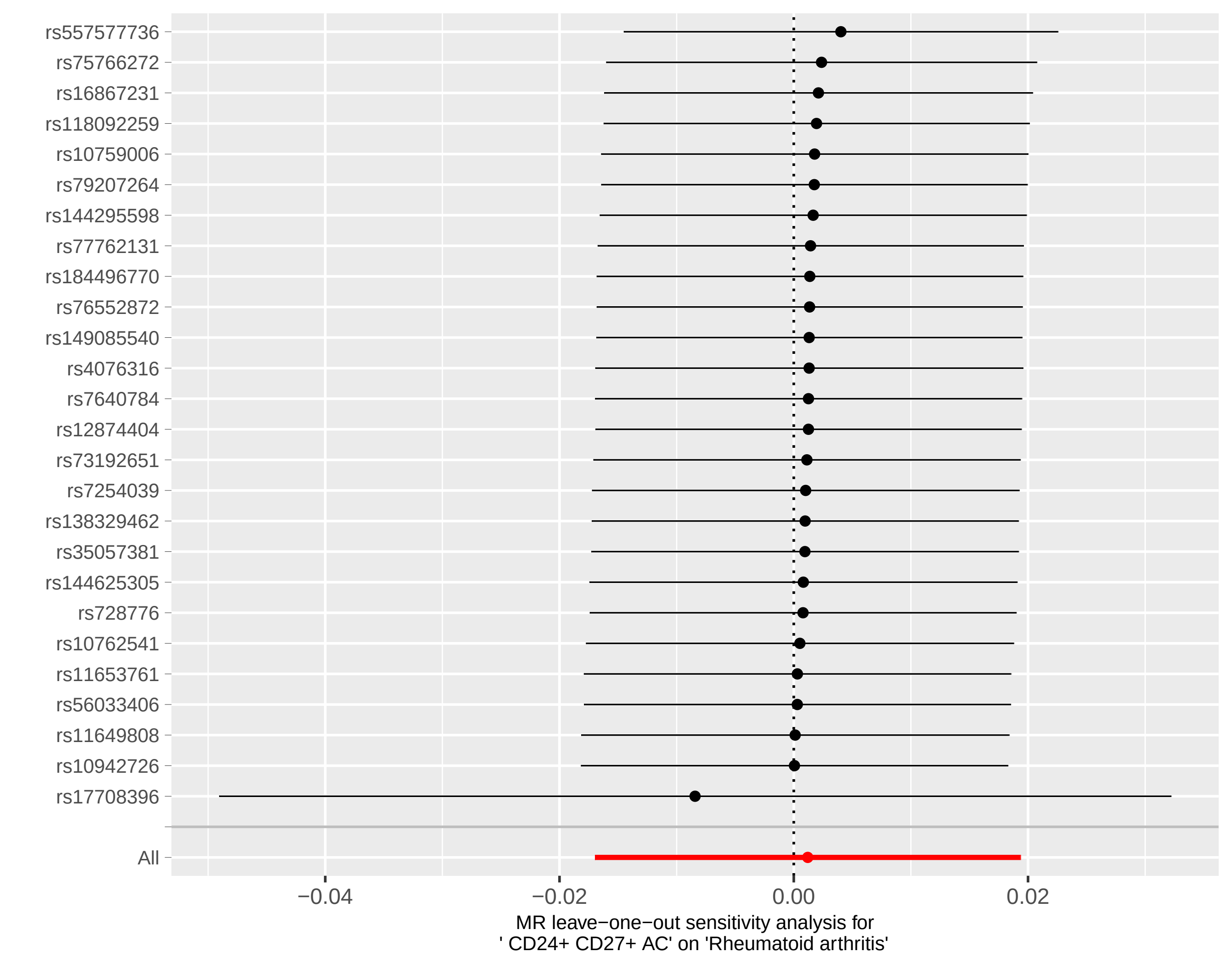

rs557577736
rs75766272
rs16867231
rs118092259
rs10759006
rs79207264
rs144295598
rs77762131
rs184496770
rs76552872
rs149085540
rs4076316
rs7640784
rs12874404
rs73192651
rs7254039
rs138329462
rs35057381
rs144625305
rs728776
rs10762541
rs11653761
rs56033406
rs11649808
rs10942726
rs17708396
All
−0.04
−0.02
0.00
0.02
MR leave−one−out sensitivity analysis for
' CD24+ CD27+ AC' on 'Rheumatoid arthritis'

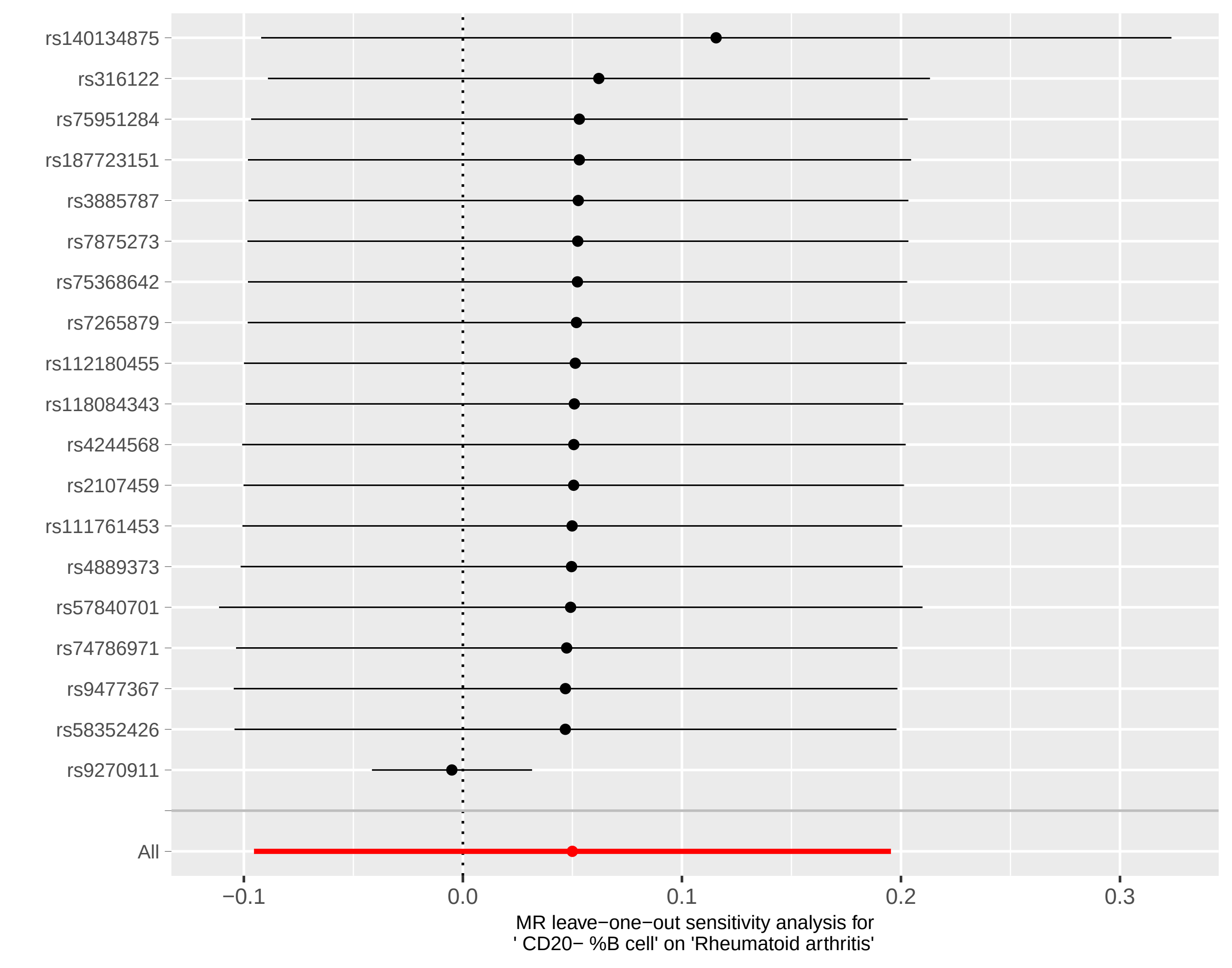

rs140134875
rs316122
rs75951284
rs187723151
rs3885787
rs7875273
rs75368642
rs7265879
rs112180455
rs118084343
rs4244568
rs2107459
rs111761453
rs4889373
rs57840701
rs74786971
rs9477367
rs58352426
rs9270911
All
−0.1
0.0
0.1
0.2
0.3
MR leave−one−out sensitivity analysis for
' CD20− %B cell' on 'Rheumatoid arthritis'

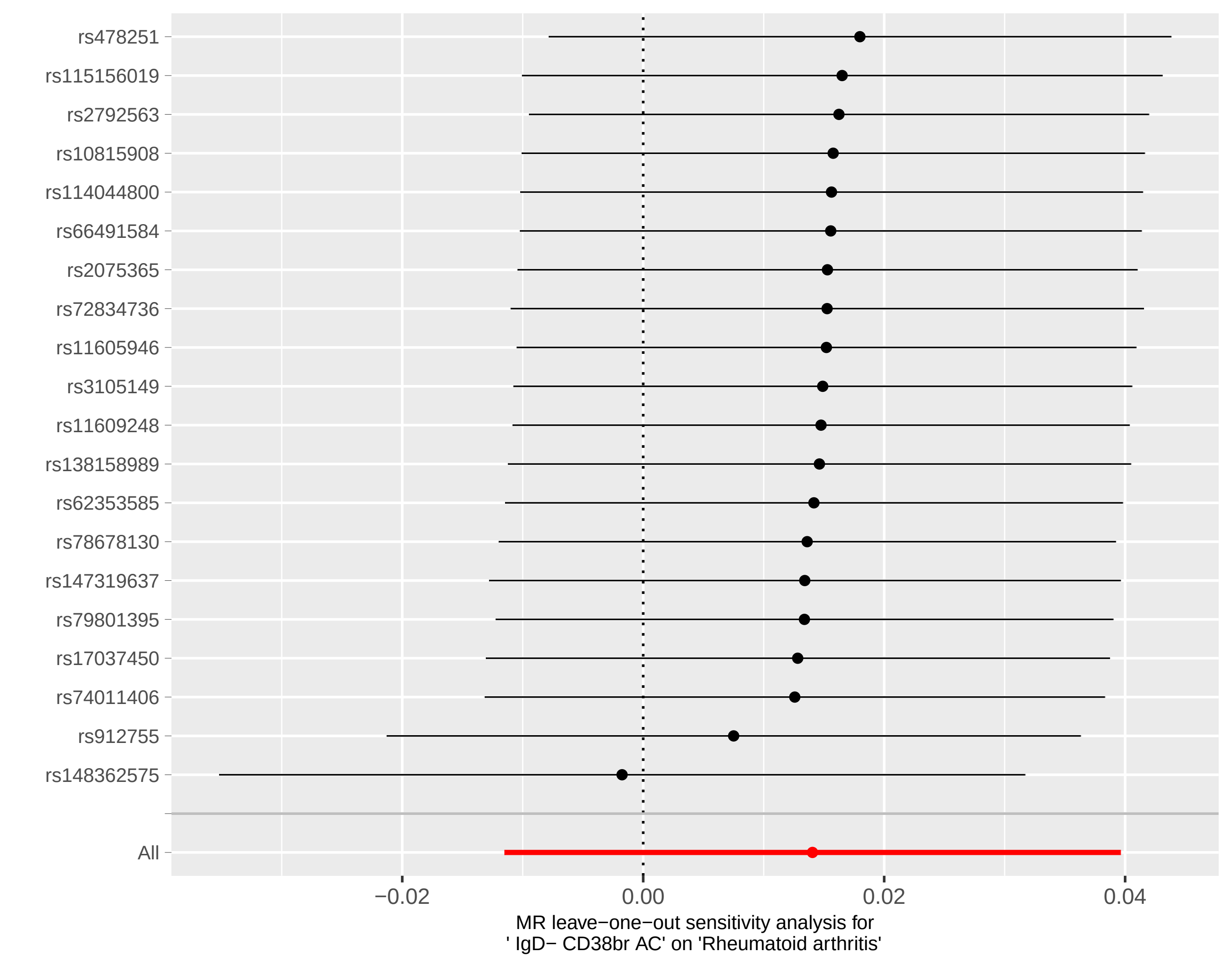
rs478251
rs115156019
rs2792563
rs10815908
rs114044800
rs66491584
rs2075365
rs72834736
rs11605946
rs3105149
rs11609248
rs138158989
rs62353585
rs78678130
rs147319637
rs79801395
rs17037450
rs74011406
rs912755
rs148362575
All
−0.02
0.00
0.02
0.04
MR leave−one−out sensitivity analysis for
' IgD− CD38br AC' on 'Rheumatoid arthritis'

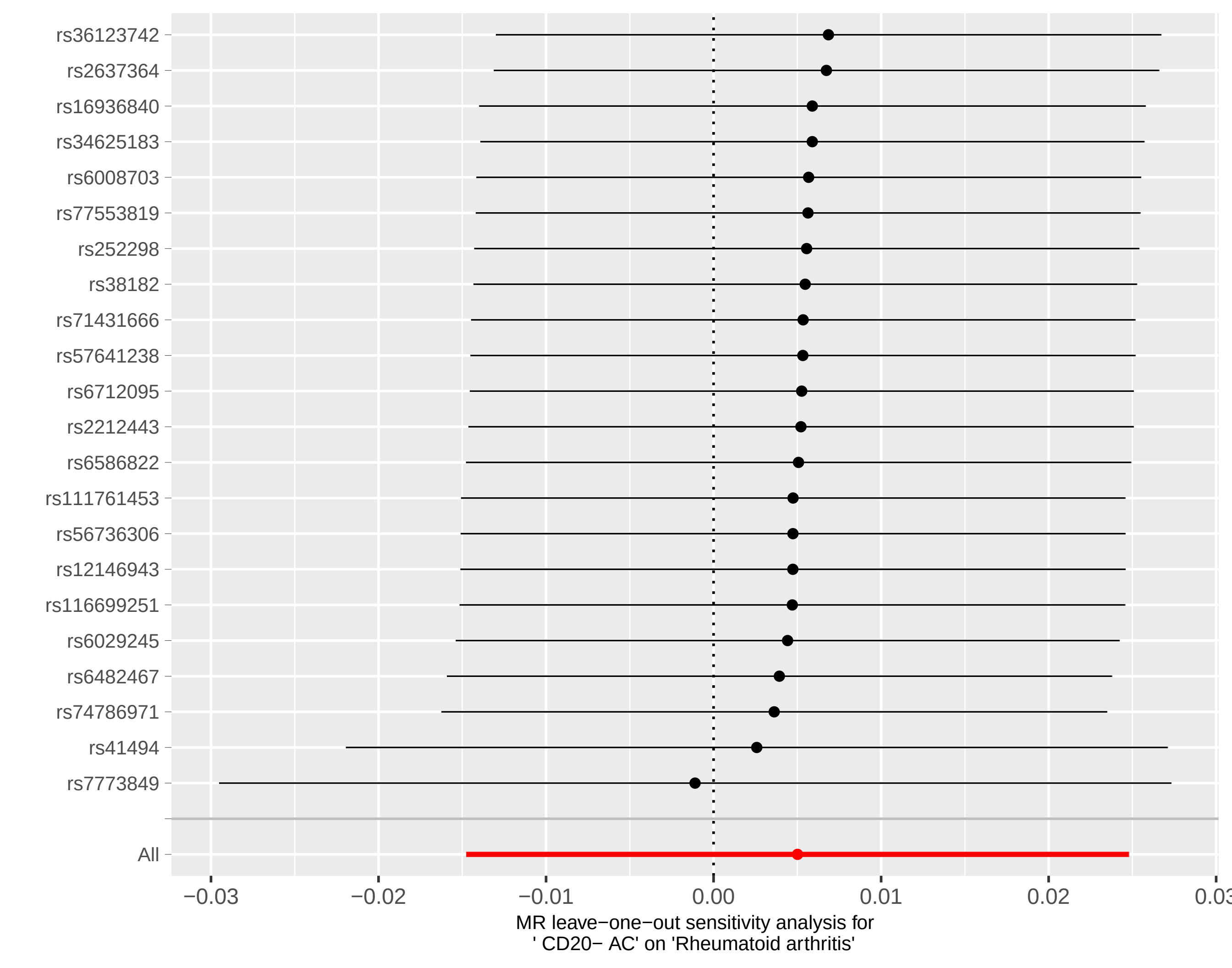
rs36123742
rs2637364
rs16936840
rs34625183
rs6008703
rs77553819
rs252298
rs38182
rs71431666
rs57641238
rs6712095
rs2212443
rs6586822
rs111761453
rs56736306
rs12146943
rs116699251
rs6029245
rs6482467
rs74786971
rs41494
rs7773849
All
−0.03
−0.02
−0.01
0.00
0.01
0.02
0.03
MR leave−one−out sensitivity analysis for
' CD20− AC' on 'Rheumatoid arthritis'

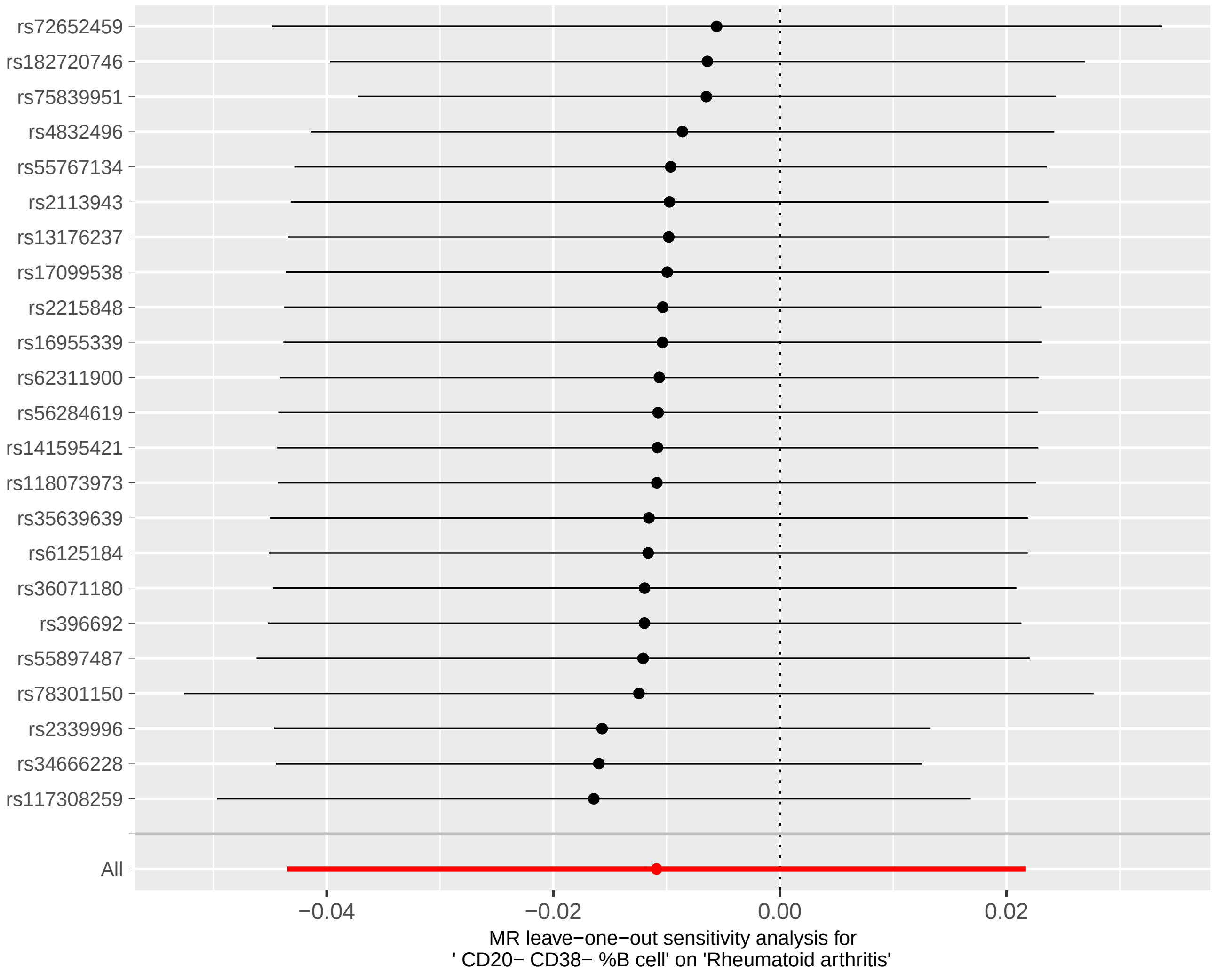
rs72652459
rs182720746
rs75839951
rs4832496
rs55767134
rs2113943
rs13176237
rs17099538
rs2215848
rs16955339
rs62311900
rs56284619
rs141595421
rs118073973
rs35639639
rs6125184
rs36071180
rs396692
rs55897487
rs78301150
rs2339996
rs34666228
rs117308259
All
−0.04
−0.02
0.00
0.02
MR leave−one−out sensitivity analysis for
' CD20− CD38− %B cell' on 'Rheumatoid arthritis'

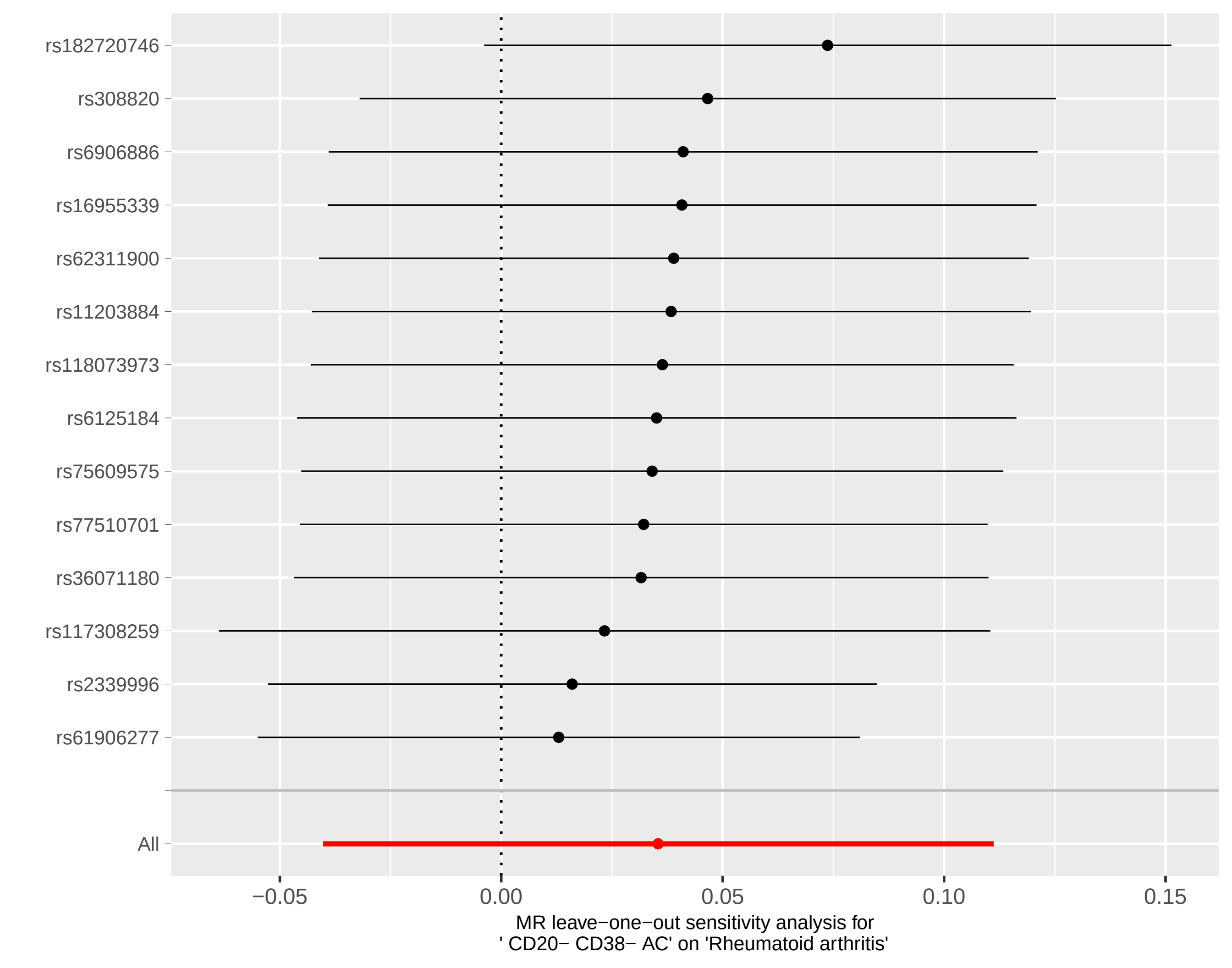

rs182720746
rs308820
rs6906886
rs16955339
rs62311900
rs11203884
rs118073973
rs6125184
rs75609575
rs77510701
rs36071180
rs117308259
rs2339996
rs61906277
All
−0.05
0.00
0.05
0.10
0.15
MR leave−one−out sensitivity analysis for
' CD20− CD38− AC' on 'Rheumatoid arthritis'

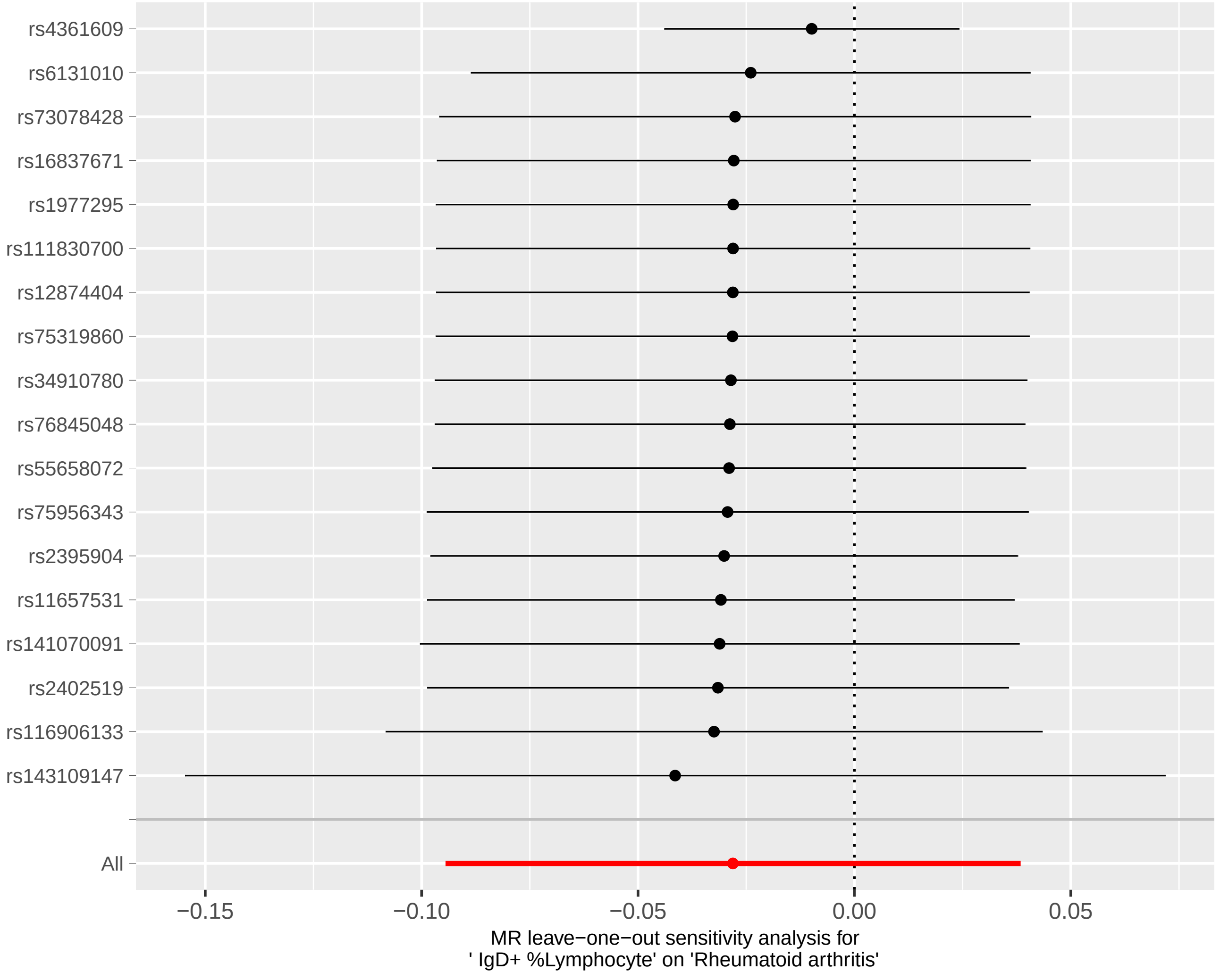
rs4361609
rs6131010
rs73078428
rs16837671
rs1977295
rs111830700
rs12874404
rs75319860
rs34910780
rs76845048
rs55658072
rs75956343
rs2395904
rs11657531
rs141070091
rs2402519
rs116906133
rs143109147
All
−0.15
−0.10
−0.05
0.00
0.05
MR leave−one−out sensitivity analysis for
' IgD+ %Lymphocyte' on 'Rheumatoid arthritis'

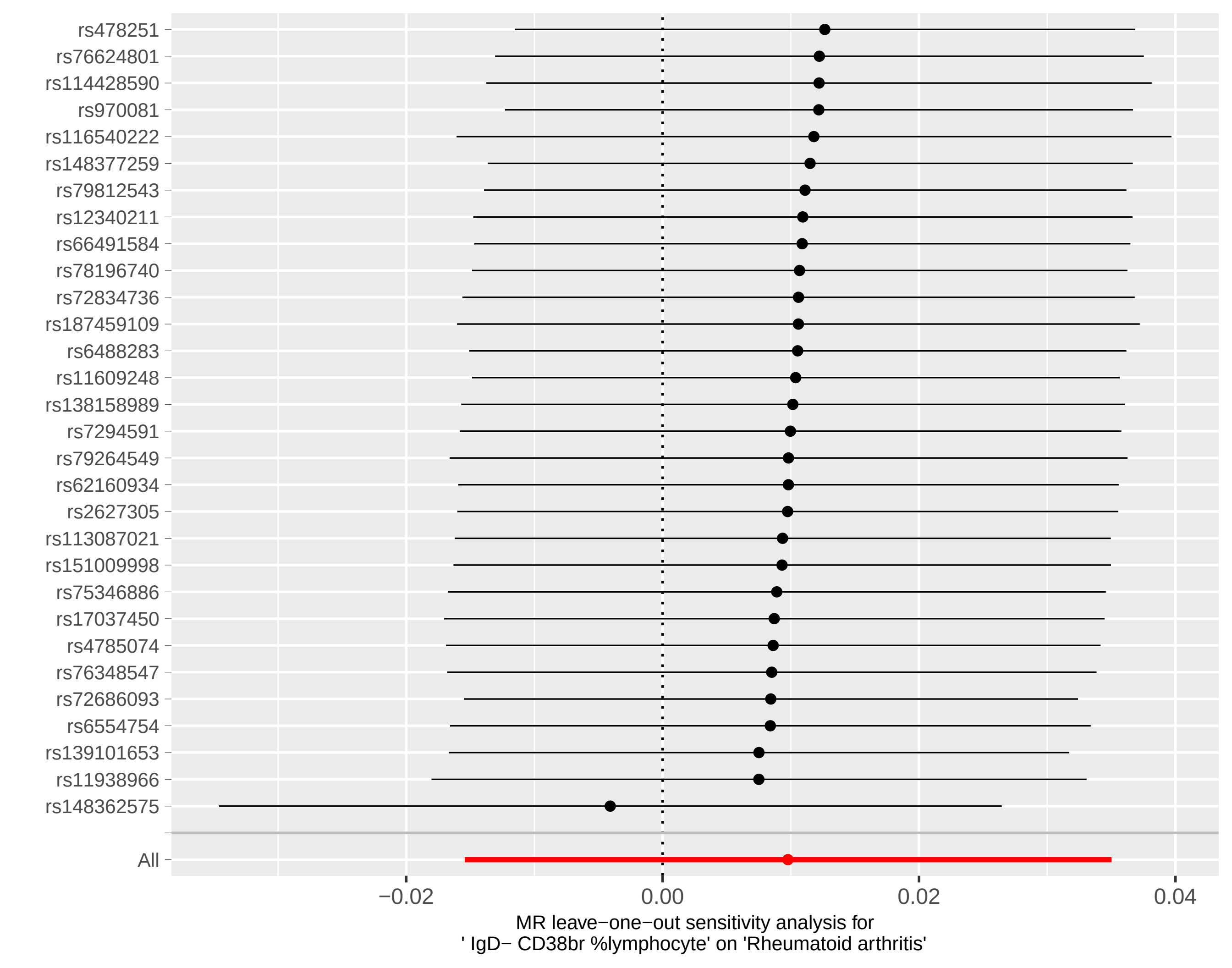
rs478251
rs76624801
rs114428590
rs970081
rs116540222
rs148377259
rs79812543
rs12340211
rs66491584
rs78196740
rs72834736
rs187459109
rs6488283
rs11609248
rs138158989
rs7294591
rs79264549
rs62160934
rs2627305
rs113087021
rs151009998
rs75346886
rs17037450
rs4785074
rs76348547
rs72686093
rs6554754
rs139101653
rs11938966
rs148362575
All
−0.02
0.00
0.02
0.04
MR leave−one−out sensitivity analysis for
' IgD− CD38br %lymphocyte' on 'Rheumatoid arthritis'

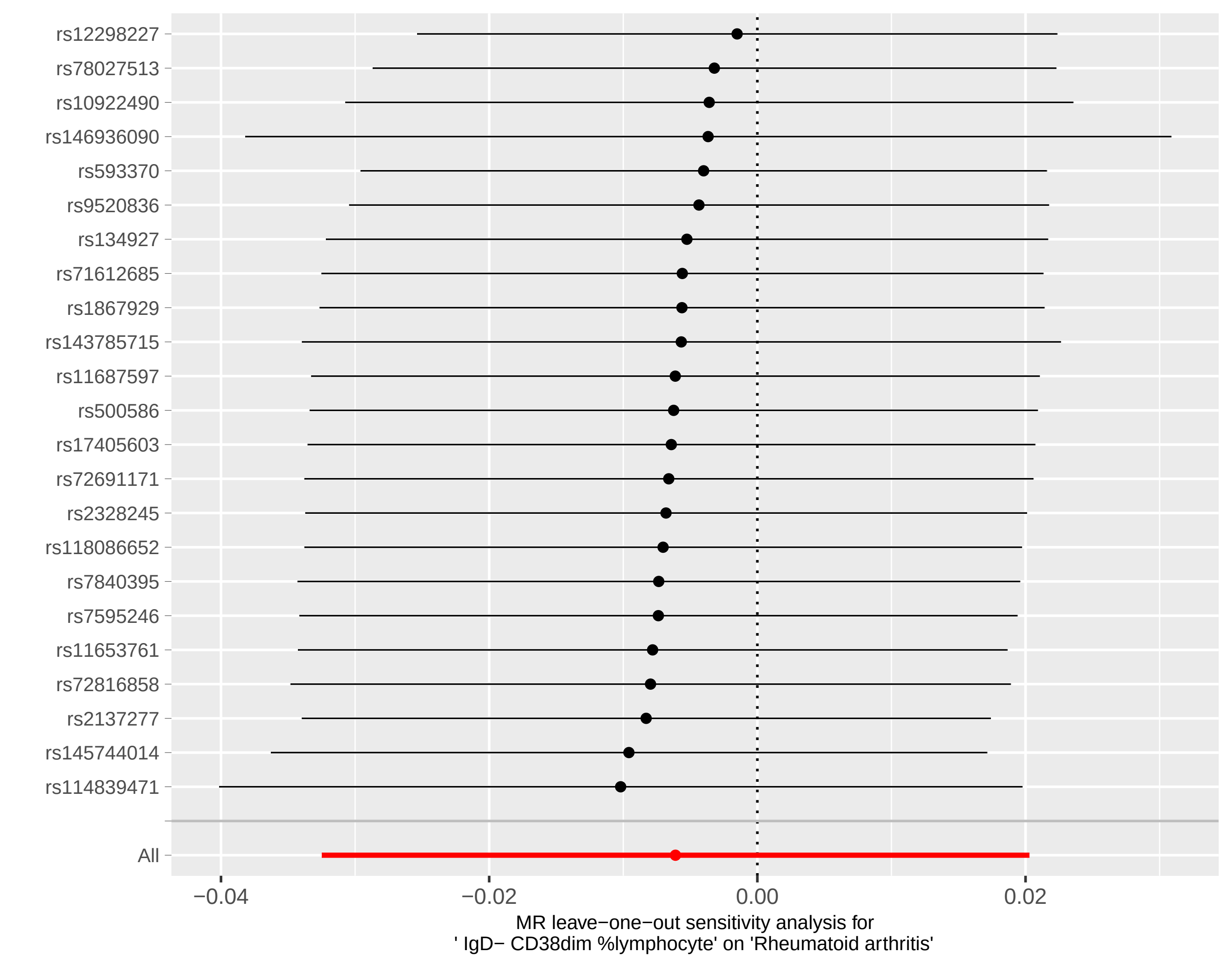
rs12298227
rs78027513
rs10922490
rs146936090
rs593370
rs9520836
rs134927
rs71612685
rs1867929
rs143785715
rs11687597
rs500586
rs17405603
rs72691171
rs2328245
rs118086652
rs7840395
rs7595246
rs11653761
rs72816858
rs2137277
rs145744014
rs114839471
All
−0.04
−0.02
0.00
0.02
MR leave−one−out sensitivity analysis for
' IgD− CD38dim %lymphocyte' on 'Rheumatoid arthritis'

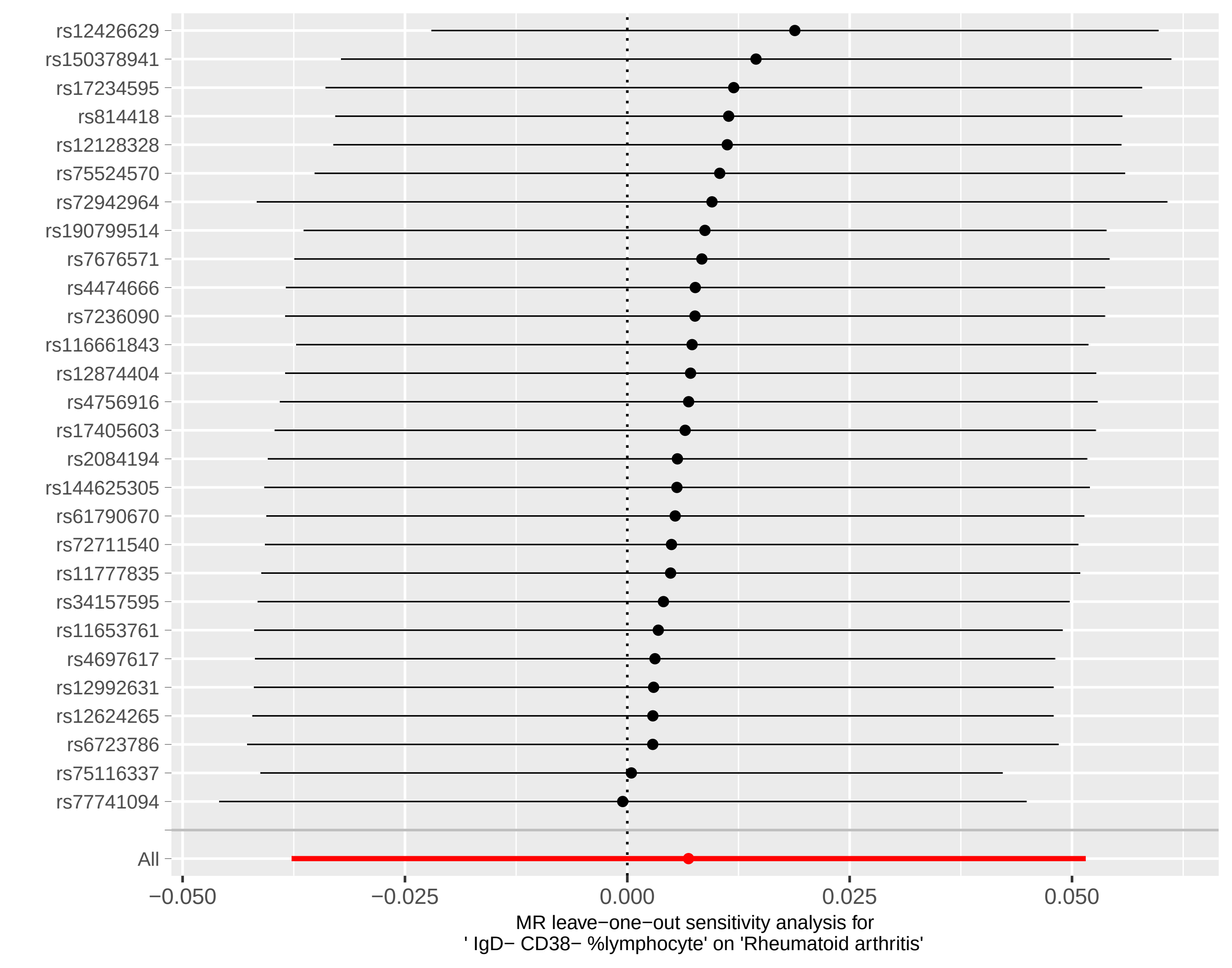

rs12426629
rs150378941
rs17234595
rs814418
rs12128328
rs75524570
rs72942964
rs190799514
rs7676571
rs4474666
rs7236090
rs116661843
rs12874404
rs4756916
rs17405603
rs2084194
rs144625305
rs61790670
rs72711540
rs11777835
rs34157595
rs11653761
rs4697617
rs12992631
rs12624265
rs6723786
rs75116337
rs77741094
All
−0.050
−0.025
0.000
0.025
0.050
MR leave−one−out sensitivity analysis for
' IgD− CD38− %lymphocyte' on 'Rheumatoid arthritis'

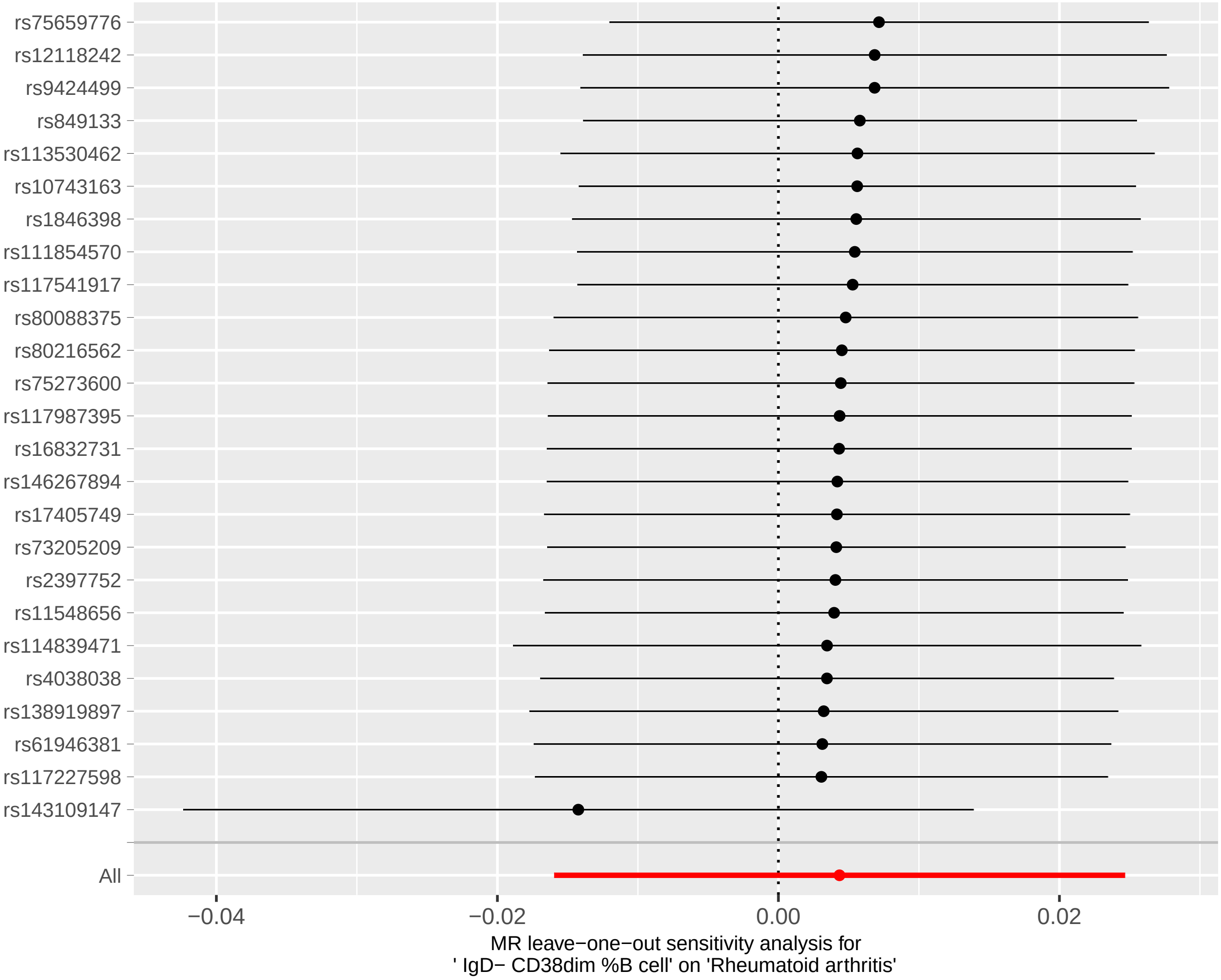
rs75659776
rs12118242
rs9424499
rs849133
rs113530462
rs10743163
rs1846398
rs111854570
rs117541917
rs80088375
rs80216562
rs75273600
rs117987395
rs16832731
rs146267894
rs17405749
rs73205209
rs2397752
rs11548656
rs114839471
rs4038038
rs138919897
rs61946381
rs117227598
rs143109147
All
−0.04
−0.02
0.00
0.02
MR leave−one−out sensitivity analysis for
' IgD− CD38dim %B cell' on 'Rheumatoid arthritis'

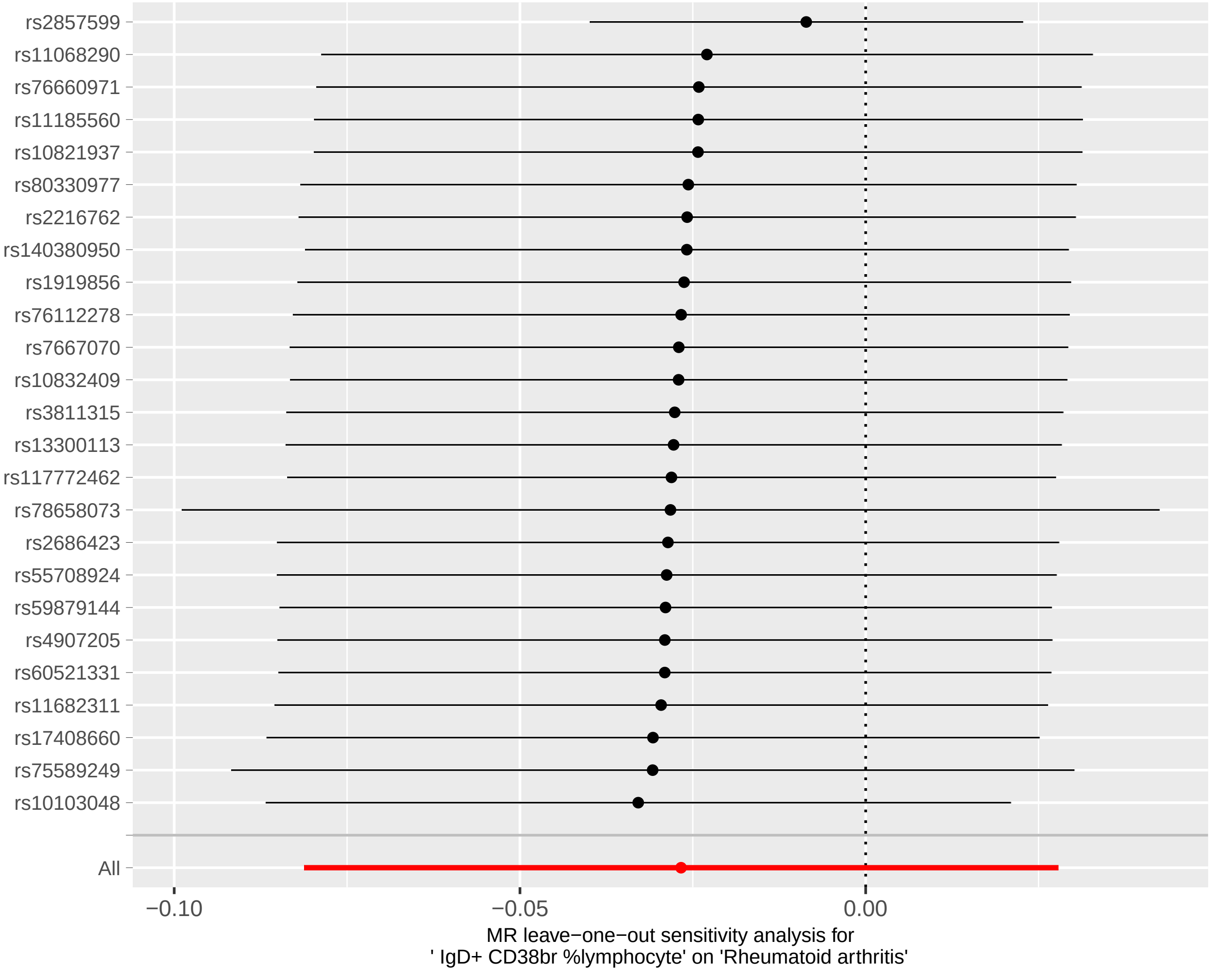
rs2857599
rs11068290
rs76660971
rs11185560
rs10821937
rs80330977
rs2216762
rs140380950
rs1919856
rs76112278
rs7667070
rs10832409
rs3811315
rs13300113
rs117772462
rs78658073
rs2686423
rs55708924
rs59879144
rs4907205
rs60521331
rs11682311
rs17408660
rs75589249
rs10103048
All
−0.10
−0.05
0.00
MR leave−one−out sensitivity analysis for
' IgD+ CD38br %lymphocyte' on 'Rheumatoid arthritis'

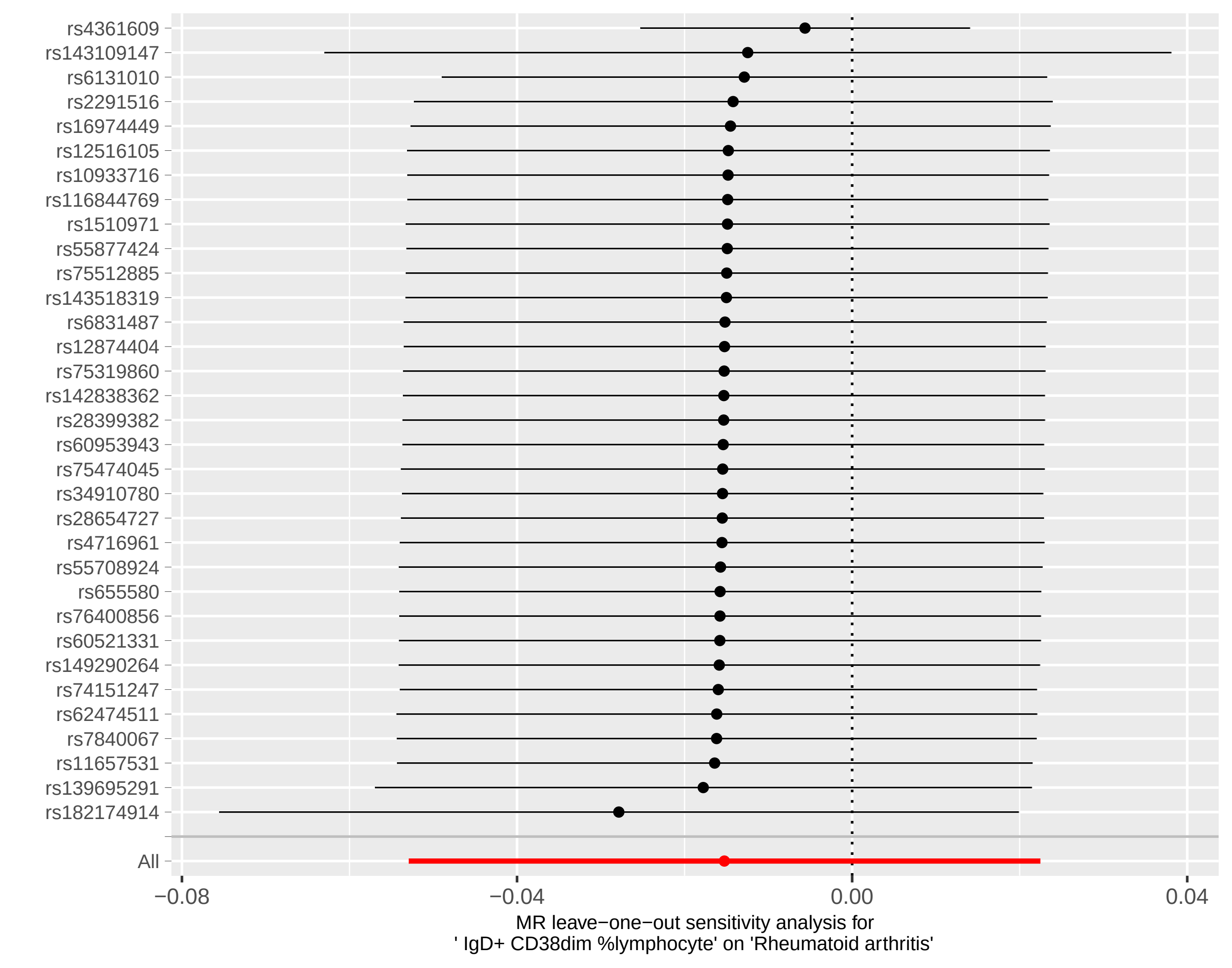
rs4361609
rs143109147
rs6131010
rs2291516
rs16974449
rs12516105
rs10933716
rs116844769
rs1510971
rs55877424
rs75512885
rs143518319
rs6831487
rs12874404
rs75319860
rs142838362
rs28399382
rs60953943
rs75474045
rs34910780
rs28654727
rs4716961
rs55708924
rs655580
rs76400856
rs60521331
rs149290264
rs74151247
rs62474511
rs7840067
rs11657531
rs139695291
rs182174914
All
−0.08
−0.04
0.00
0.04
MR leave−one−out sensitivity analysis for
' IgD+ CD38dim %lymphocyte' on 'Rheumatoid arthritis'

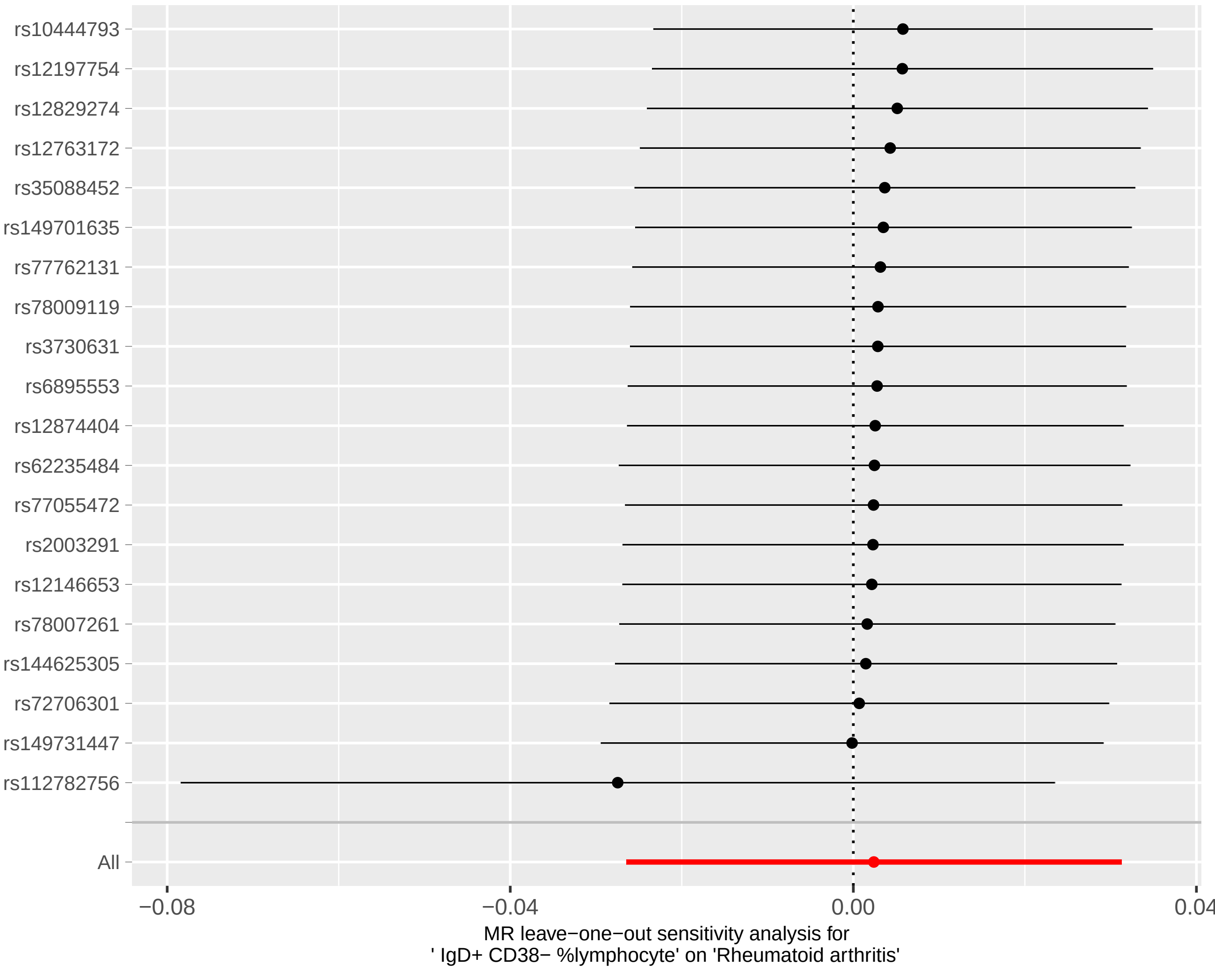

rs10444793
rs12197754
rs12829274
rs12763172
rs35088452
rs149701635
rs77762131
rs78009119
rs3730631
rs6895553
rs12874404
rs62235484
rs77055472
rs2003291
rs12146653
rs78007261
rs144625305
rs72706301
rs149731447
rs112782756
All
−0.08
−0.04
0.00
0.04
MR leave−one−out sensitivity analysis for
' IgD+ CD38− %lymphocyte' on 'Rheumatoid arthritis'

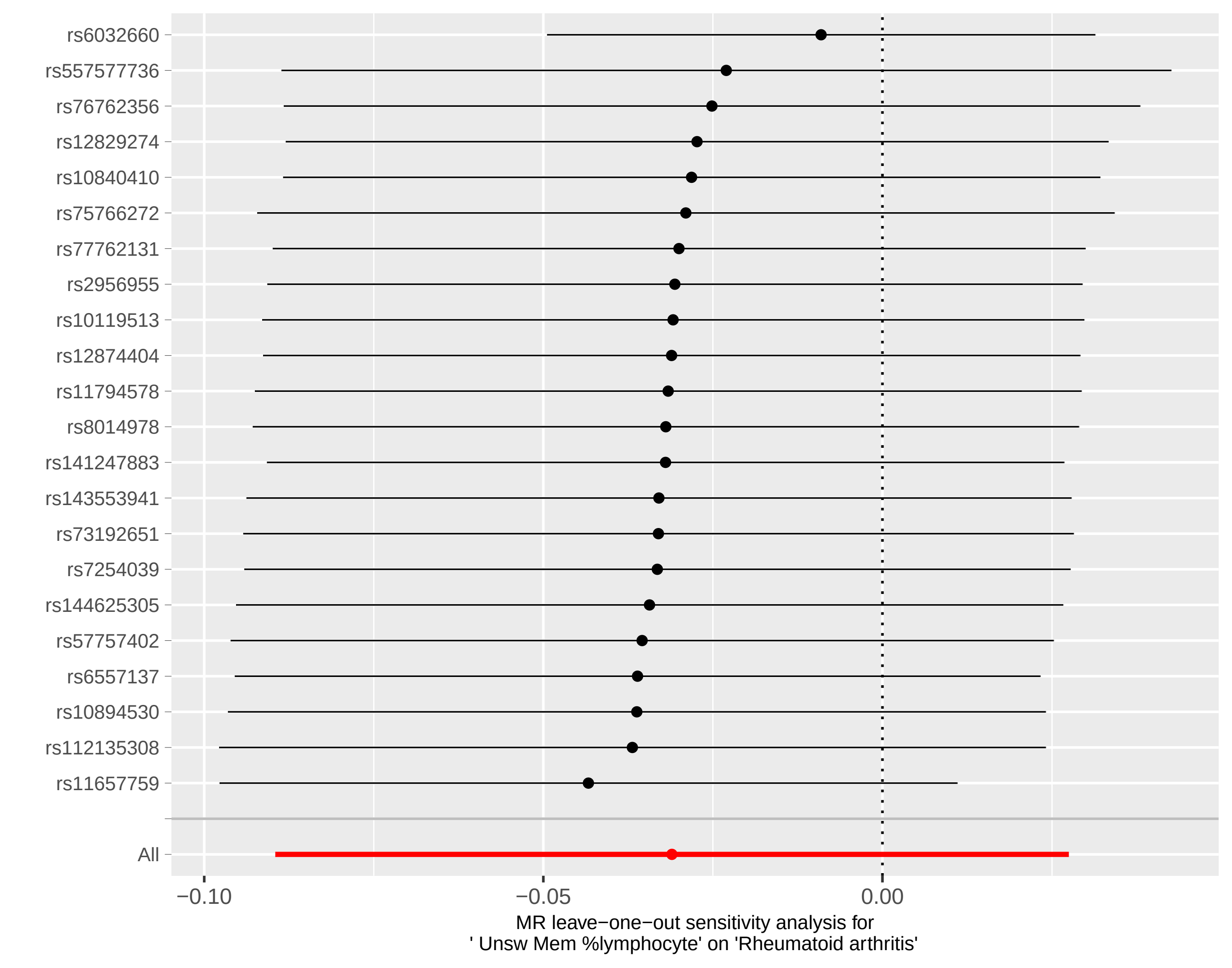
rs6032660
rs557577736
rs76762356
rs12829274
rs10840410
rs75766272
rs77762131
rs2956955
rs10119513
rs12874404
rs11794578
rs8014978
rs141247883
rs143553941
rs73192651
rs7254039
rs144625305
rs57757402
rs6557137
rs10894530
rs112135308
rs11657759
All
−0.10
−0.05
0.00
MR leave−one−out sensitivity analysis for
' Unsw Mem %lymphocyte' on 'Rheumatoid arthritis'

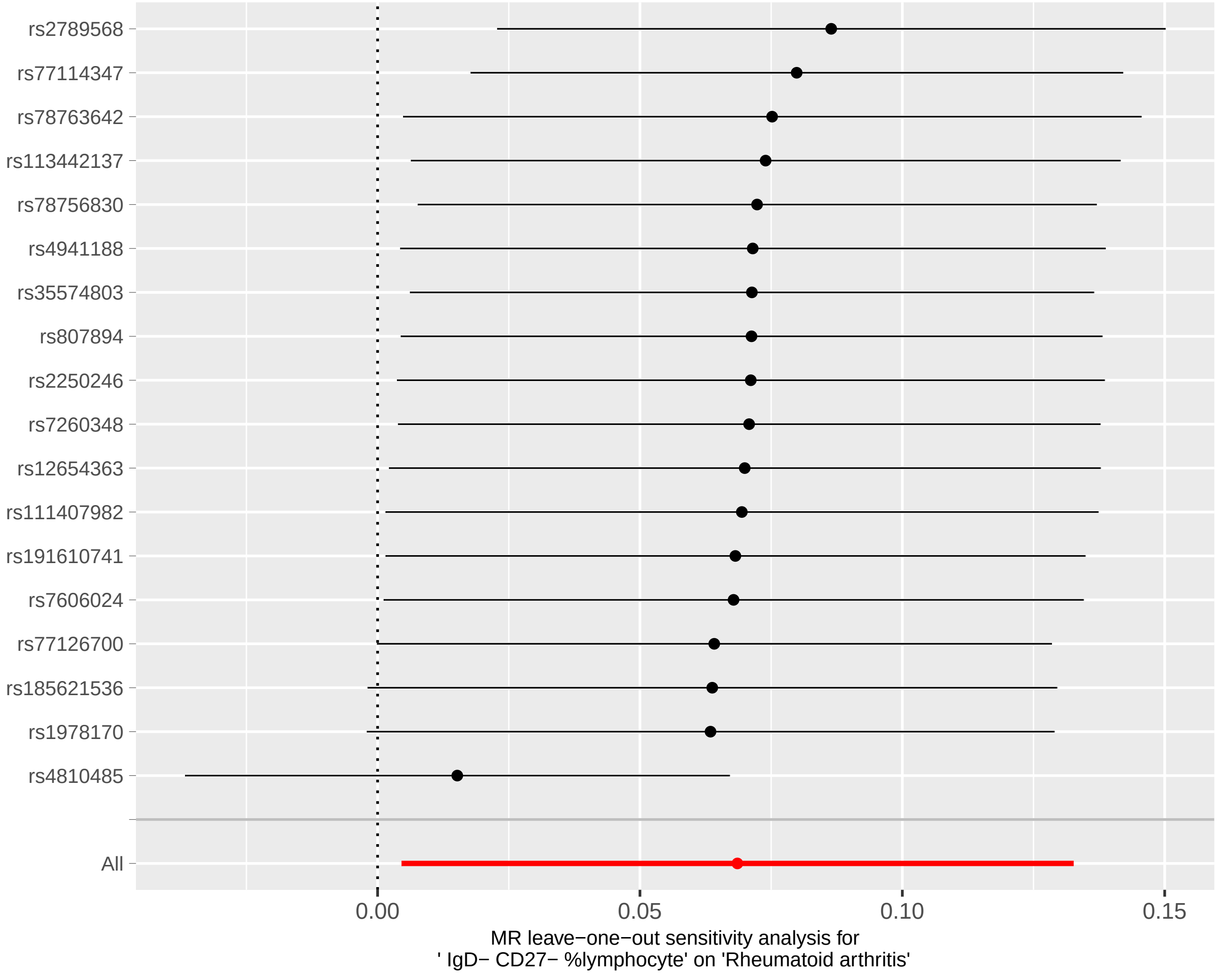
rs2789568
rs77114347
rs78763642
rs113442137
rs78756830
rs4941188
rs35574803
rs807894
rs2250246
rs7260348
rs12654363
rs111407982
rs191610741
rs7606024
rs77126700
rs185621536
rs1978170
rs4810485
All
0.00
0.05
0.10
0.15
MR leave−one−out sensitivity analysis for
' IgD− CD27− %lymphocyte' on 'Rheumatoid arthritis'

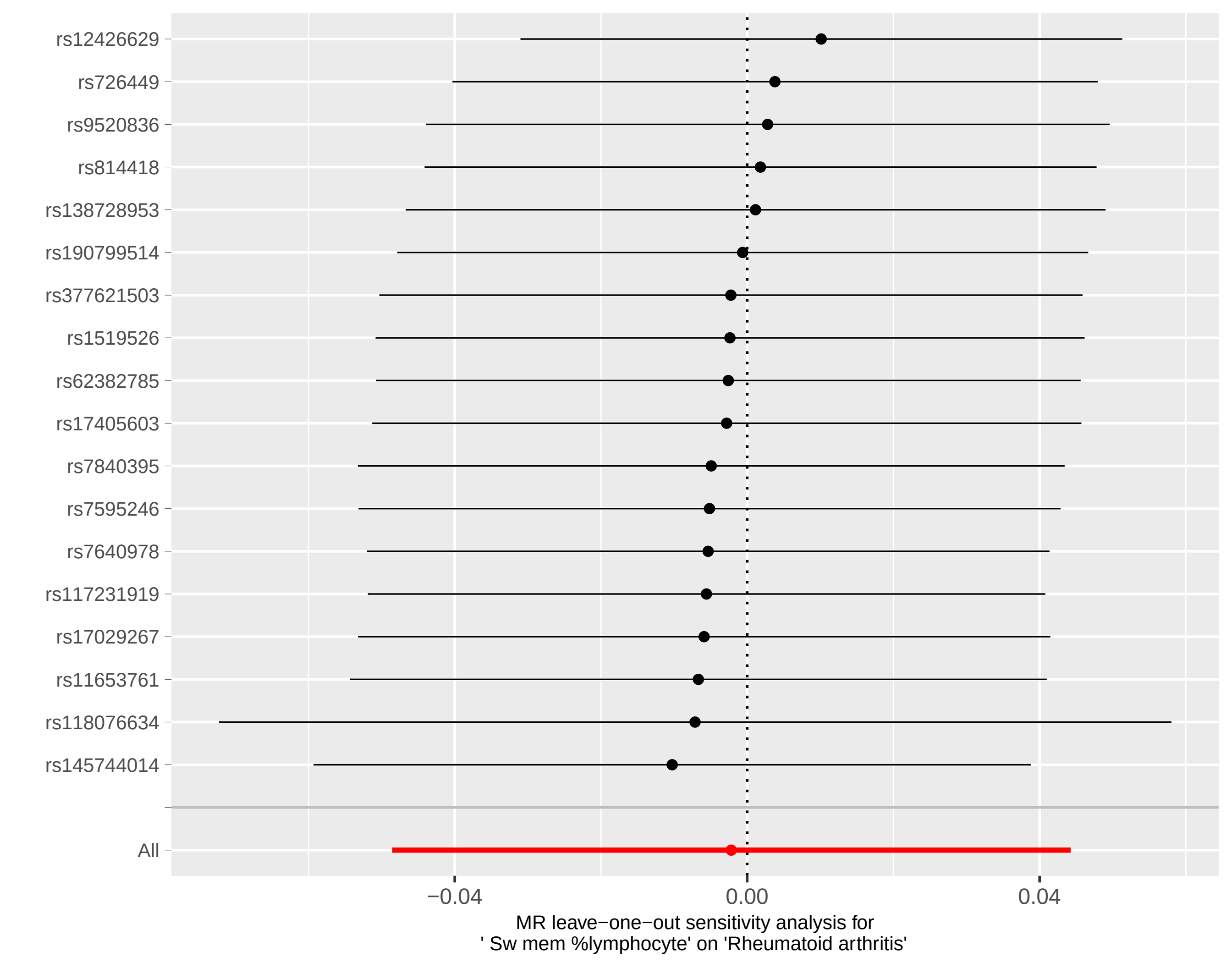
rs12426629
rs726449
rs9520836
rs814418
rs138728953
rs190799514
rs377621503
rs1519526
rs62382785
rs17405603
rs7840395
rs7595246
rs7640978
rs117231919
rs17029267
rs11653761
rs118076634
rs145744014
All
−0.04
0.00
0.04
MR leave−one−out sensitivity analysis for
' Sw mem %lymphocyte' on 'Rheumatoid arthritis'

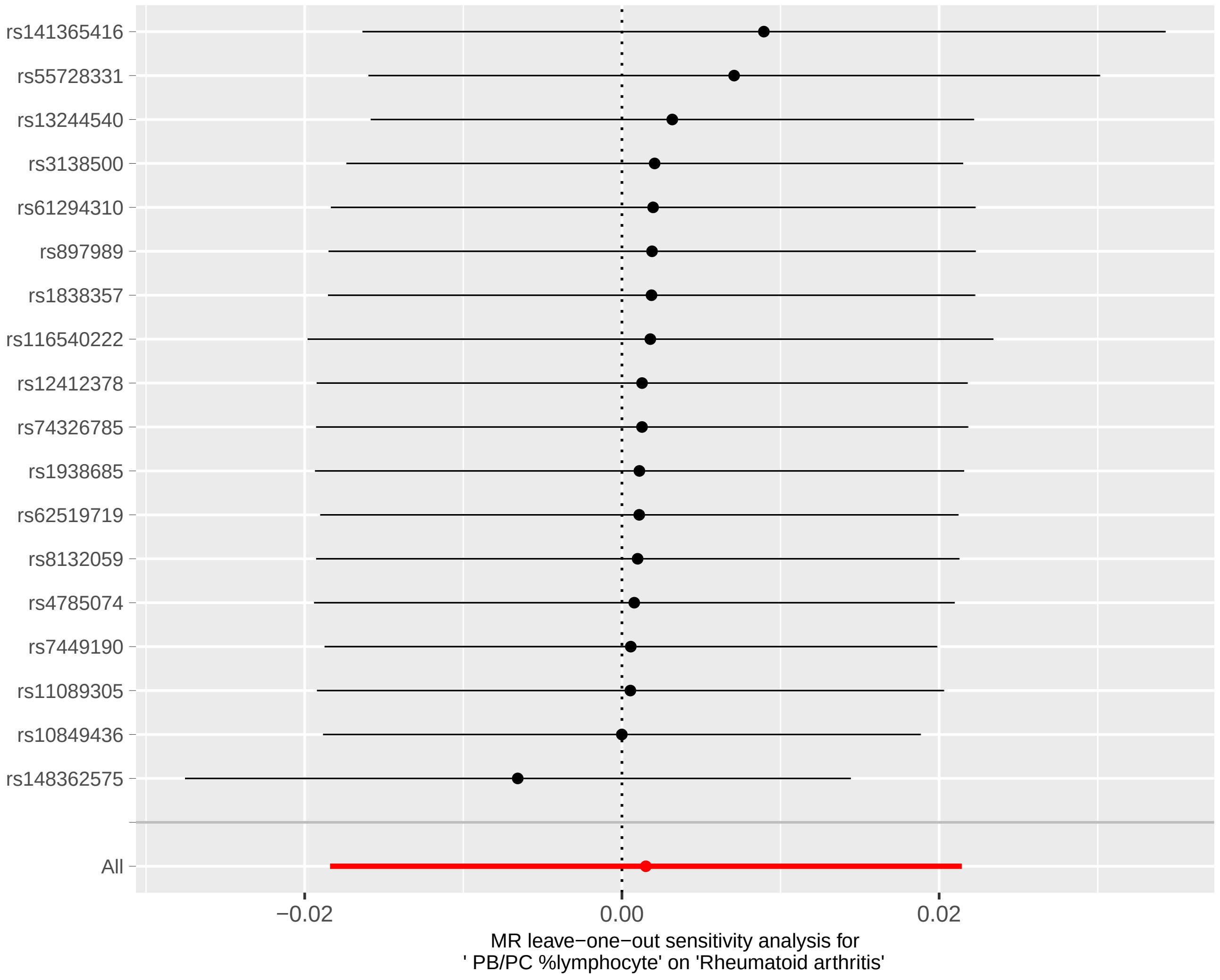
rs141365416
rs55728331
rs13244540
rs3138500
rs61294310
rs897989
rs1838357
rs116540222
rs12412378
rs74326785
rs1938685
rs62519719
rs8132059
rs4785074
rs7449190
rs11089305
rs10849436
rs148362575
All
−0.02
0.00
0.02
MR leave−one−out sensitivity analysis for
' PB/PC %lymphocyte' on 'Rheumatoid arthritis'

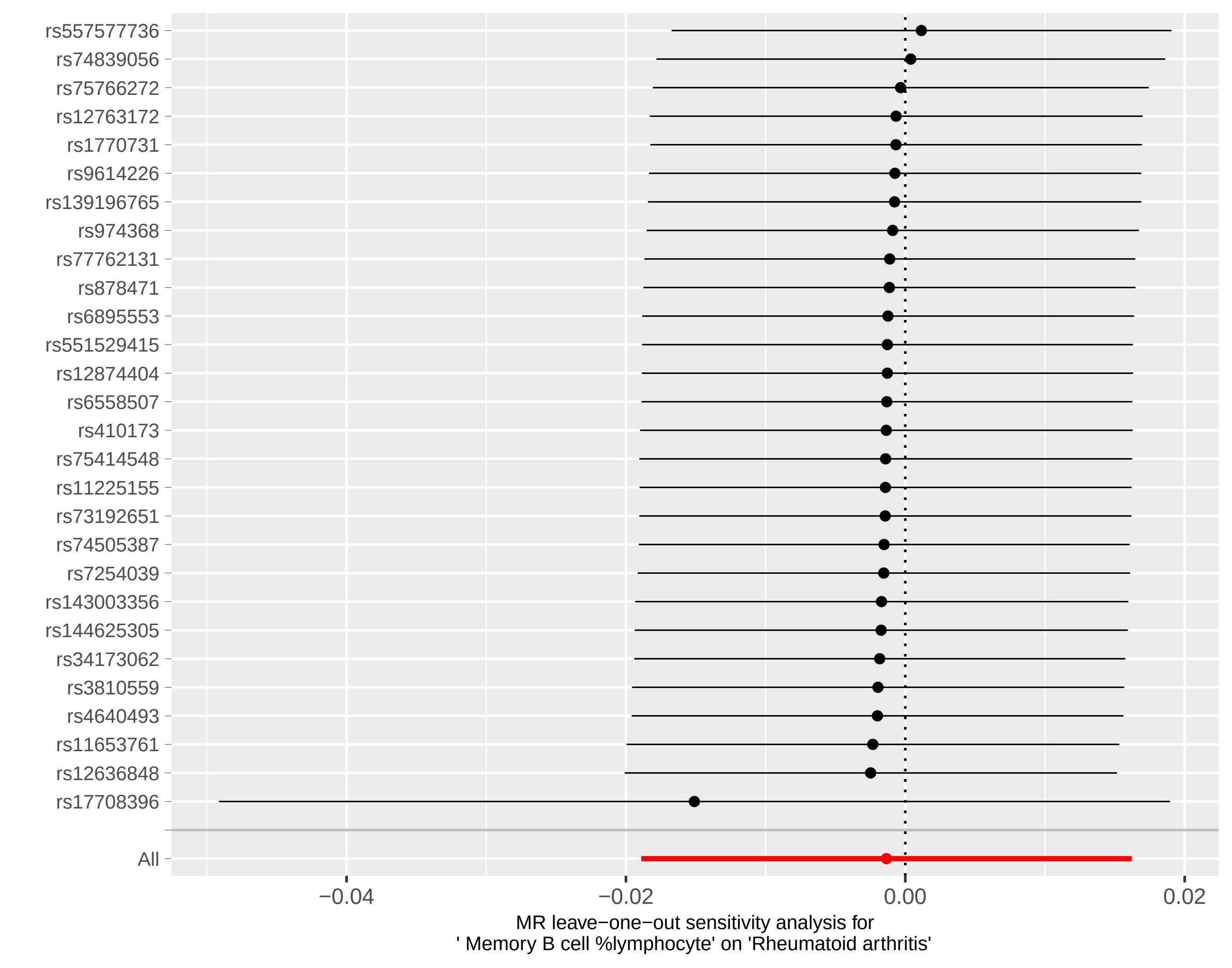
rs557577736
rs74839056
rs75766272
rs12763172
rs1770731
rs9614226
rs139196765
rs974368
rs77762131
rs878471
rs6895553
rs551529415
rs12874404
rs6558507
rs410173
rs75414548
rs11225155
rs73192651
rs74505387
rs7254039
rs143003356
rs144625305
rs34173062
rs3810559
rs4640493
rs11653761
rs12636848
rs17708396
All
−0.04
−0.02
0.00
0.02
MR leave−one−out sensitivity analysis for
' Memory B cell %lymphocyte' on 'Rheumatoid arthritis'

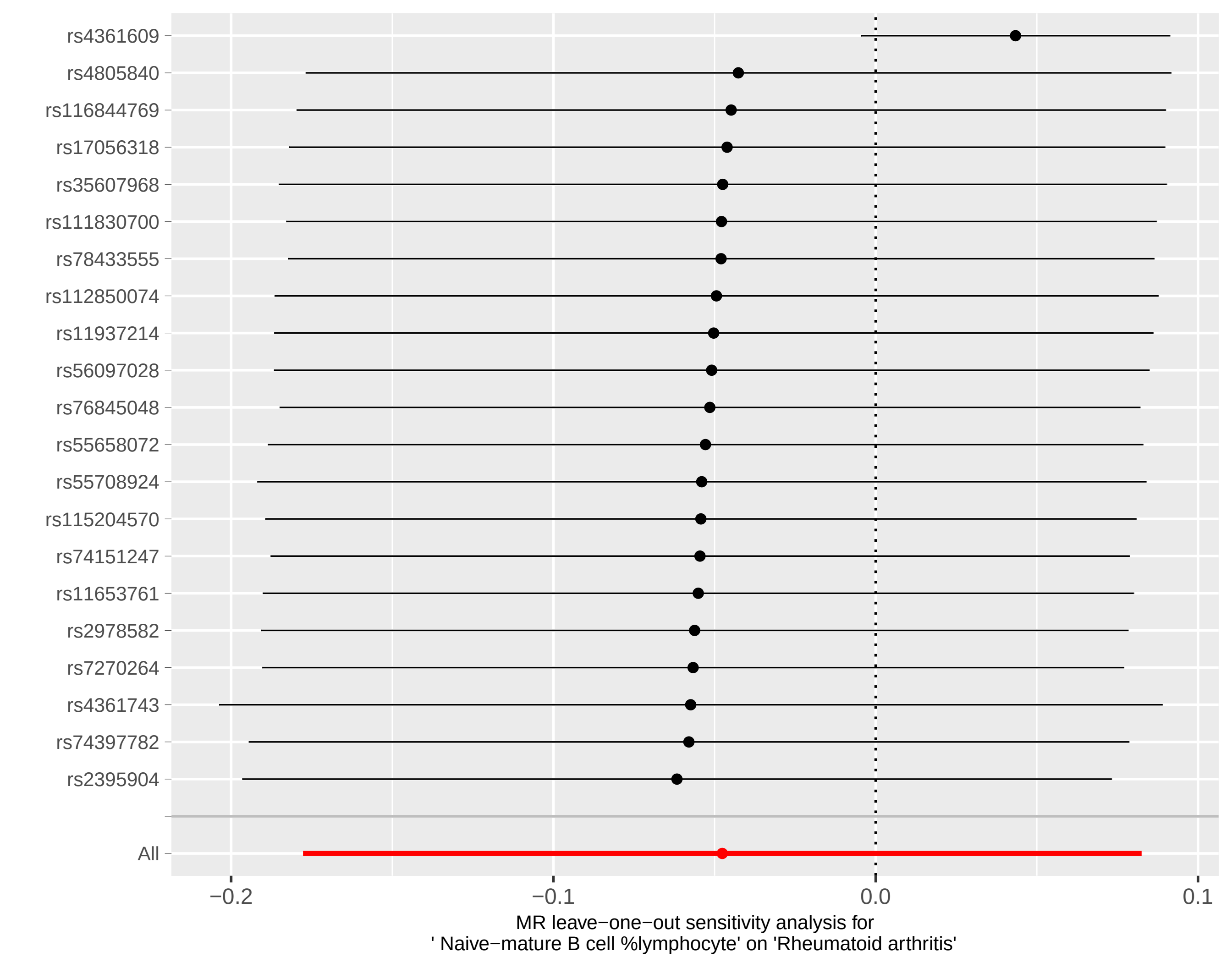
rs4361609
rs4805840
rs116844769
rs17056318
rs35607968
rs111830700
rs78433555
rs112850074
rs11937214
rs56097028
rs76845048
rs55658072
rs55708924
rs115204570
rs74151247
rs11653761
rs2978582
rs7270264
rs4361743
rs74397782
rs2395904
All
−0.2
−0.1
0.0
0.1
MR leave−one−out sensitivity analysis for
' Naive−mature B cell %lymphocyte' on 'Rheumatoid arthritis'

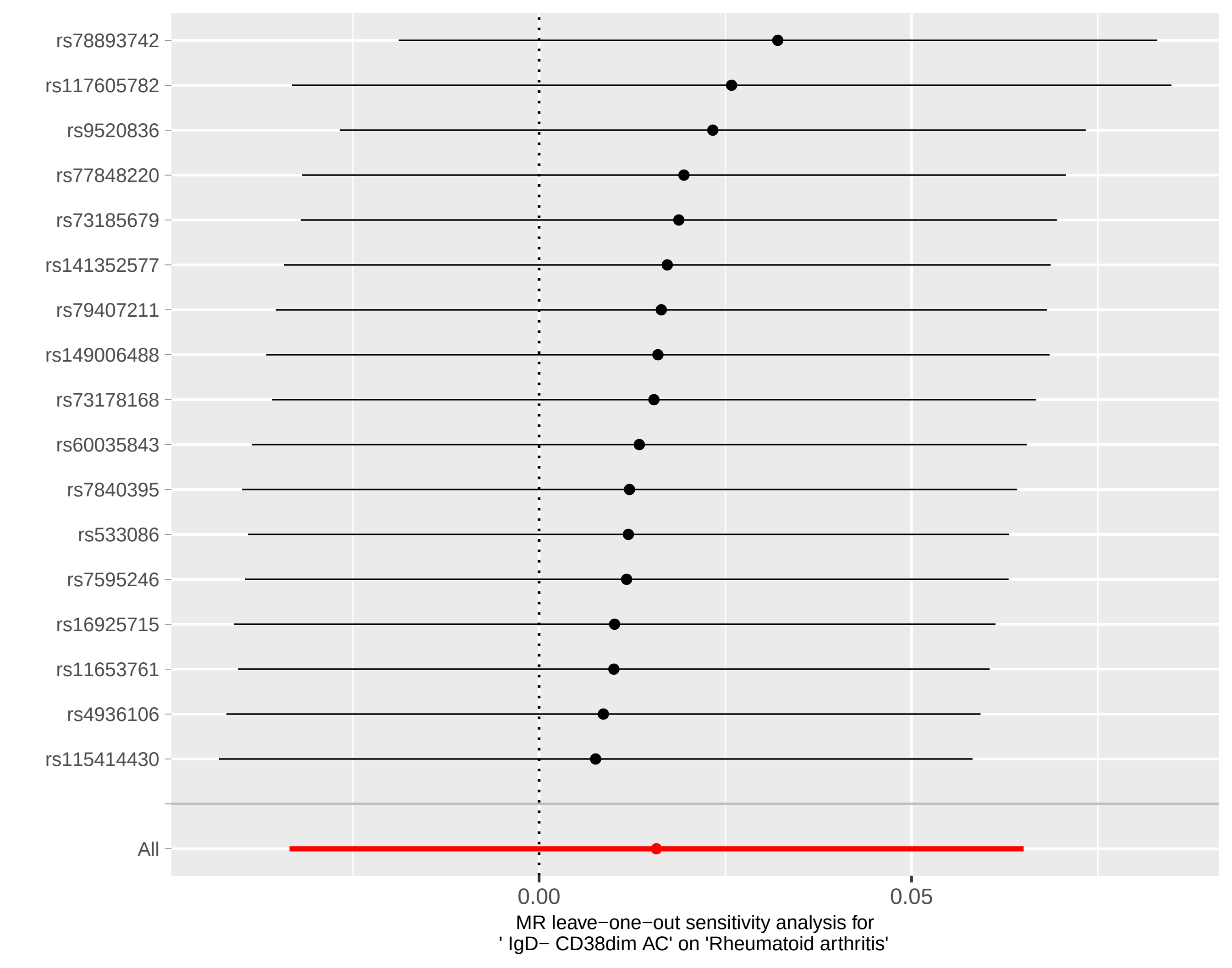
rs78893742
rs117605782
rs9520836
rs77848220
rs73185679
rs141352577
rs79407211
rs149006488
rs73178168
rs60035843
rs7840395
rs533086
rs7595246
rs16925715
rs11653761
rs4936106
rs115414430
All
0.00
0.05
MR leave−one−out sensitivity analysis for
' IgD− CD38dim AC' on 'Rheumatoid arthritis'

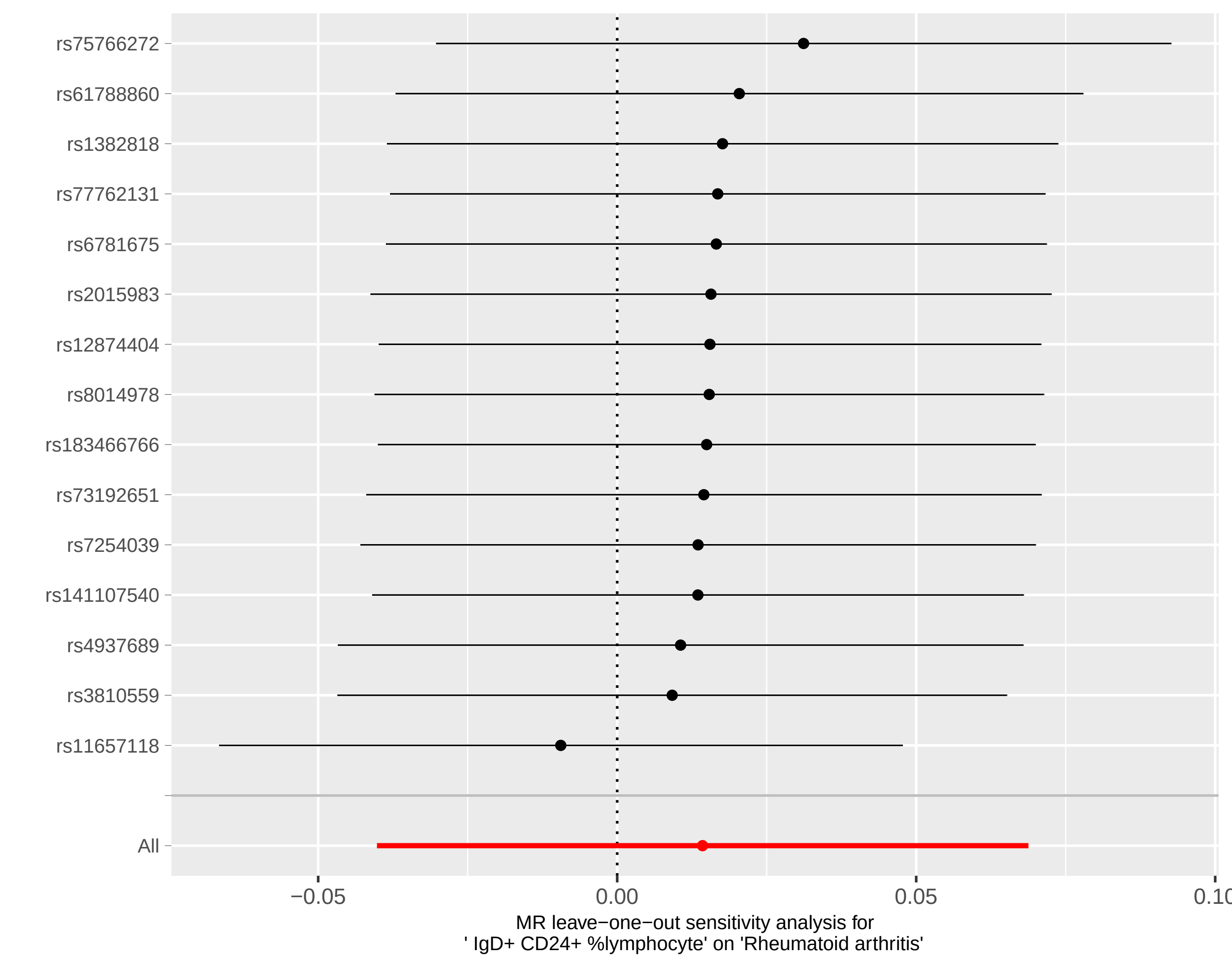
rs75766272
rs61788860
rs1382818
rs77762131
rs6781675
rs2015983
rs12874404
rs8014978
rs183466766
rs73192651
rs7254039
rs141107540
rs4937689
rs3810559
rs11657118
All
−0.05
0.00
0.05
0.10
MR leave−one−out sensitivity analysis for
' IgD+ CD24+ %lymphocyte' on 'Rheumatoid arthritis'

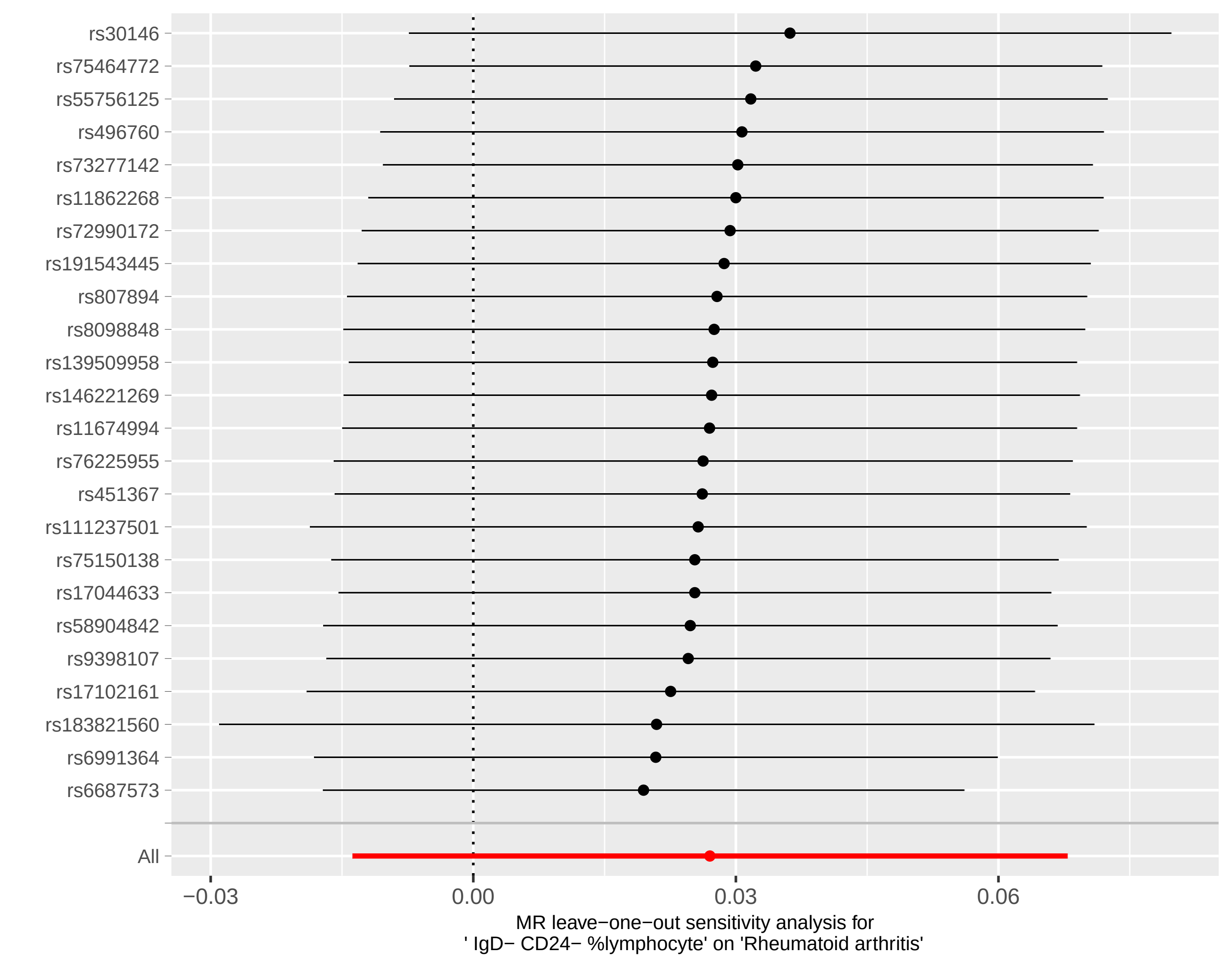
rs30146
rs75464772
rs55756125
rs496760
rs73277142
rs11862268
rs72990172
rs191543445
rs807894
rs8098848
rs139509958
rs146221269
rs11674994
rs76225955
rs451367
rs111237501
rs75150138
rs17044633
rs58904842
rs9398107
rs17102161
rs183821560
rs6991364
rs6687573
All
−0.03
0.00
0.03
0.06
MR leave−one−out sensitivity analysis for
' IgD− CD24− %lymphocyte' on 'Rheumatoid arthritis'

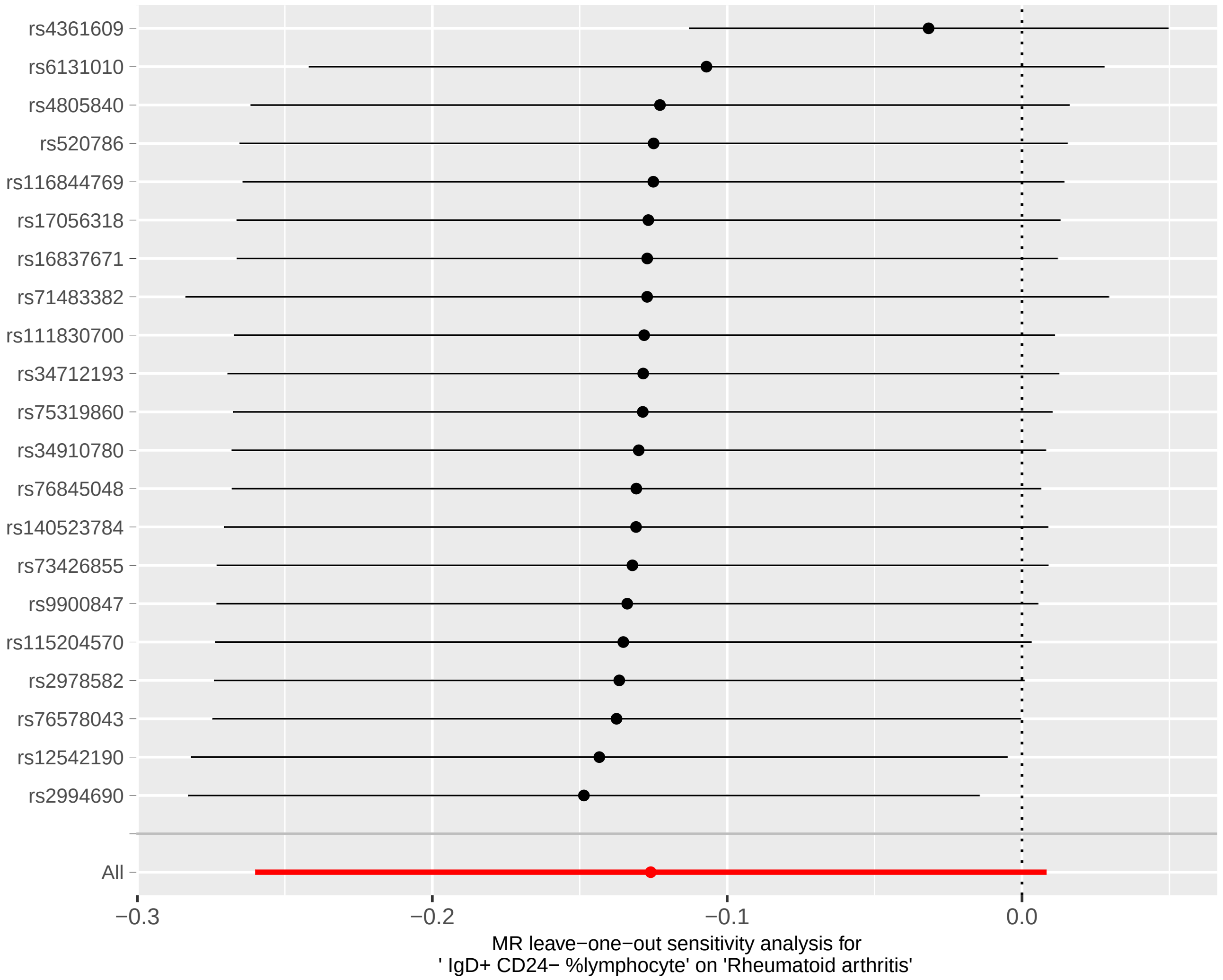
rs4361609
rs6131010
rs4805840
rs520786
rs116844769
rs17056318
rs16837671
rs71483382
rs111830700
rs34712193
rs75319860
rs34910780
rs76845048
rs140523784
rs73426855
rs9900847
rs115204570
rs2978582
rs76578043
rs12542190
rs2994690
All
−0.3
−0.2
−0.1
0.0
MR leave−one−out sensitivity analysis for
' IgD+ CD24− %lymphocyte' on 'Rheumatoid arthritis'

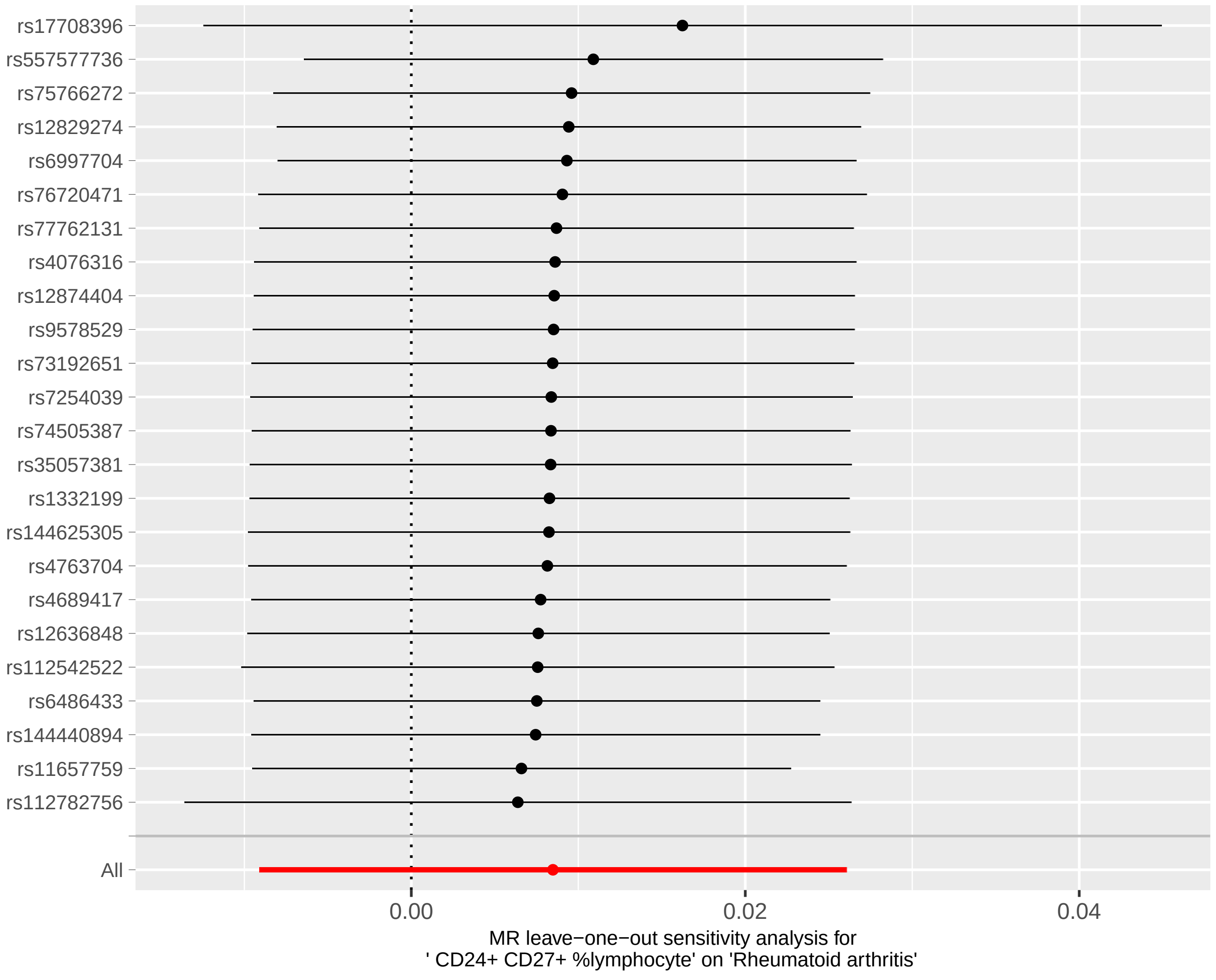
rs17708396
rs557577736
rs75766272
rs12829274
rs6997704
rs76720471
rs77762131
rs4076316
rs12874404
rs9578529
rs73192651
rs7254039
rs74505387
rs35057381
rs1332199
rs144625305
rs4763704
rs4689417
rs12636848
rs112542522
rs6486433
rs144440894
rs11657759
rs112782756
All
0.00
0.02
0.04
MR leave−one−out sensitivity analysis for
' CD24+ CD27+ %lymphocyte' on 'Rheumatoid arthritis'

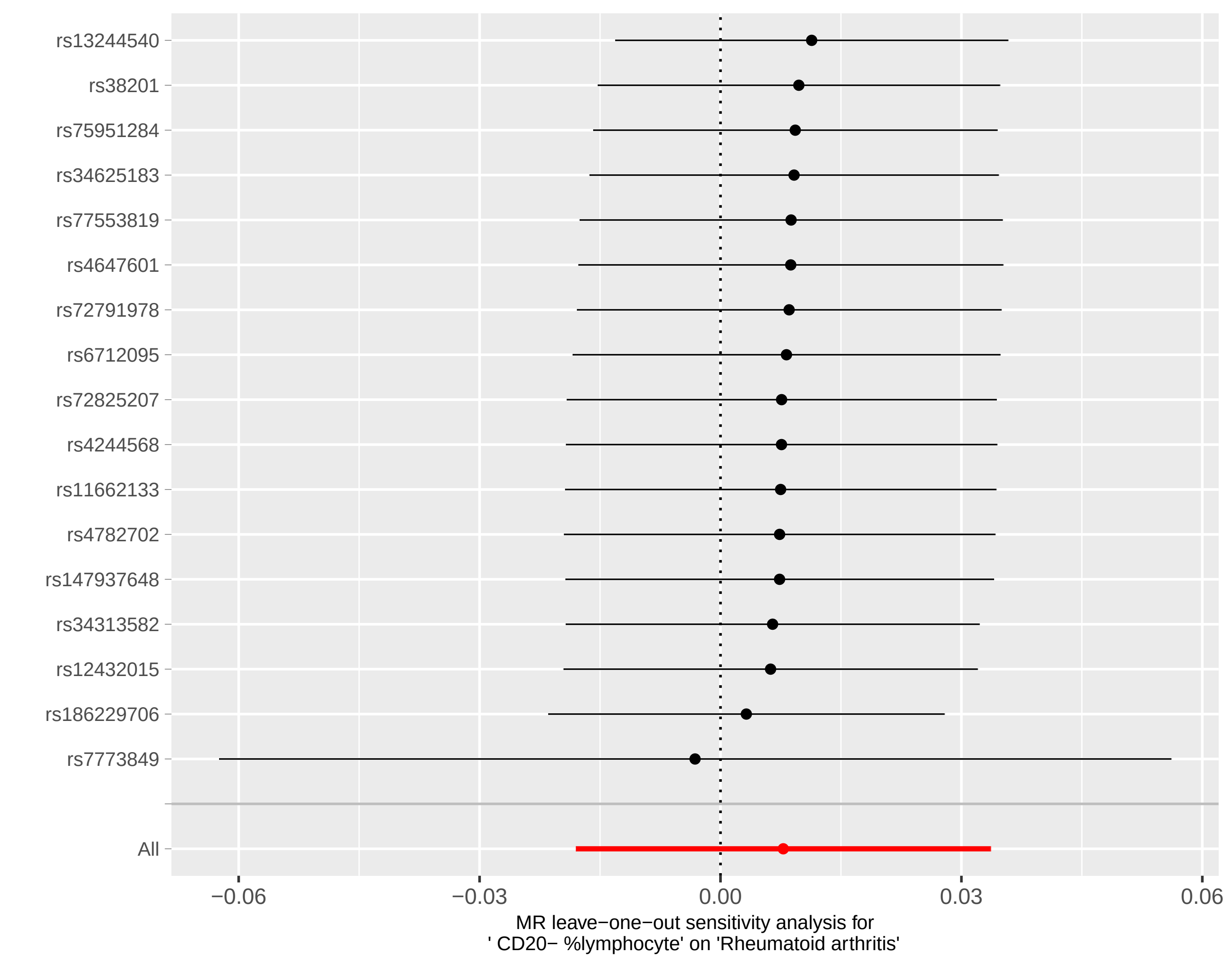
rs13244540
rs38201
rs75951284
rs34625183
rs77553819
rs4647601
rs72791978
rs6712095
rs72825207
rs4244568
rs11662133
rs4782702
rs147937648
rs34313582
rs12432015
rs186229706
rs7773849
All
−0.06
−0.03
0.00
0.03
0.06
MR leave−one−out sensitivity analysis for
' CD20− %lymphocyte' on 'Rheumatoid arthritis'

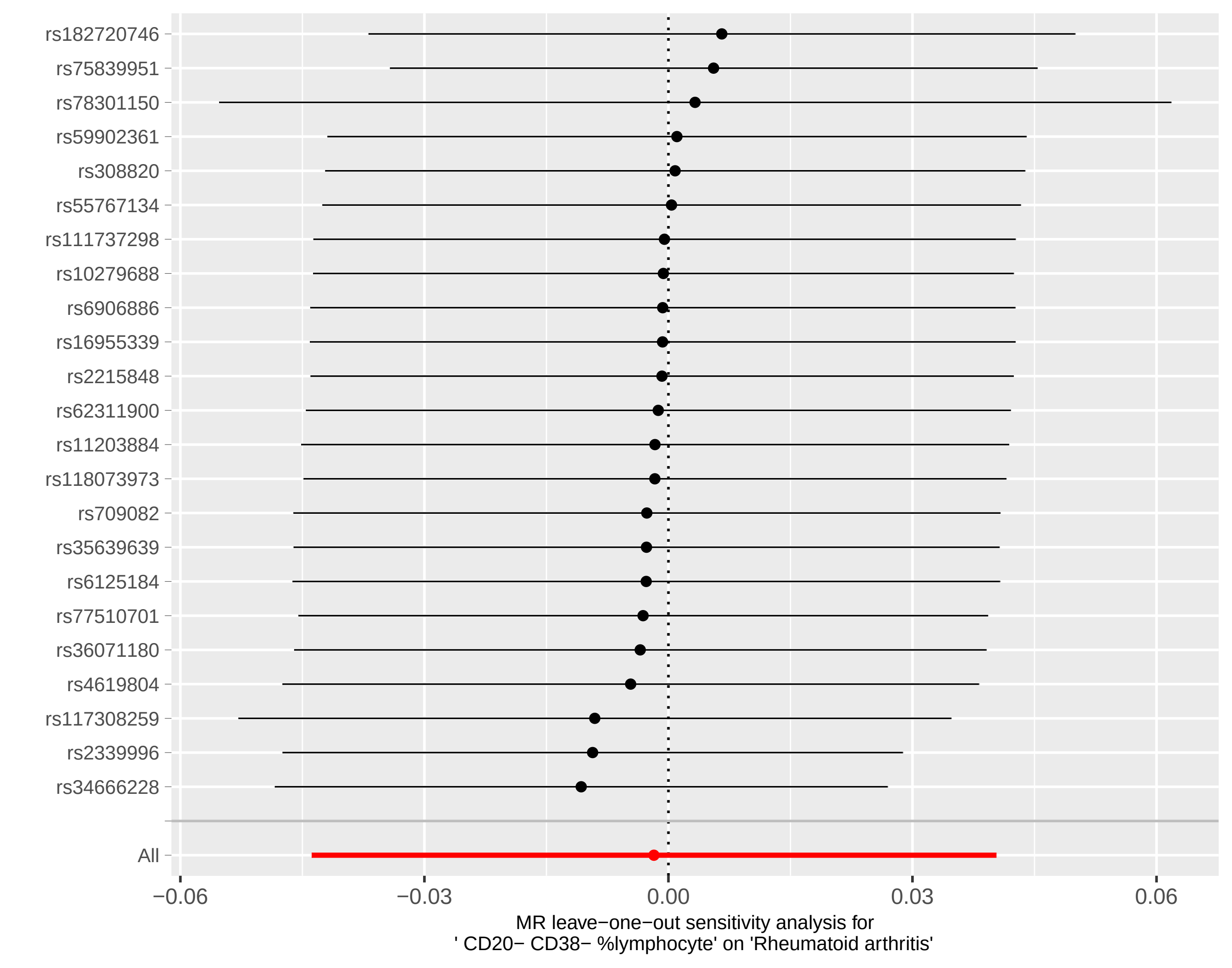
rs182720746
rs75839951
rs78301150
rs59902361
rs308820
rs55767134
rs111737298
rs10279688
rs6906886
rs16955339
rs2215848
rs62311900
rs11203884
rs118073973
rs709082
rs35639639
rs6125184
rs77510701
rs36071180
rs4619804
rs117308259
rs2339996
rs34666228
All
−0.06
−0.03
0.00
0.03
0.06
MR leave−one−out sensitivity analysis for
' CD20− CD38− %lymphocyte' on 'Rheumatoid arthritis'

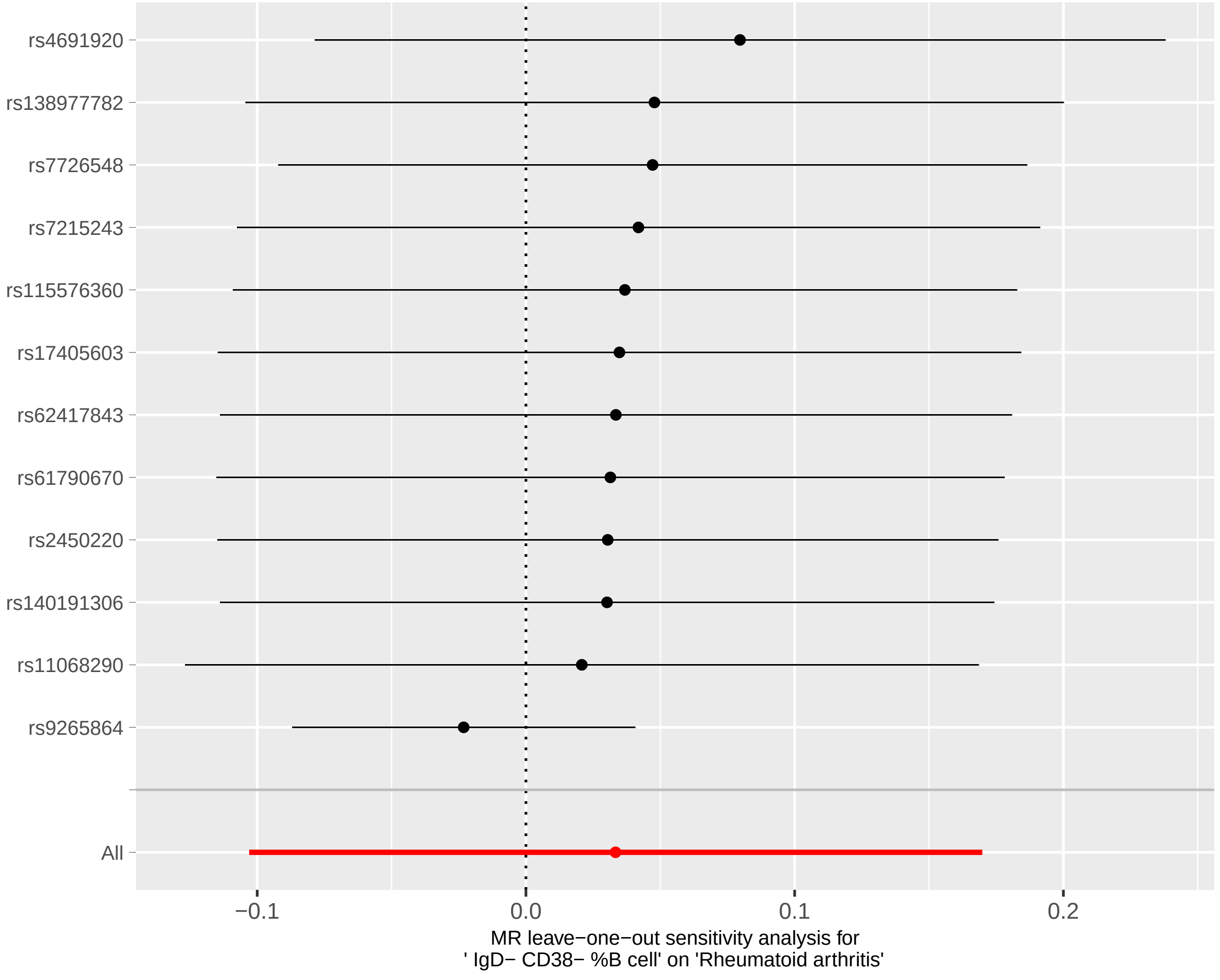
rs4691920
rs138977782
rs7726548
rs7215243
rs115576360
rs17405603
rs62417843
rs61790670
rs2450220
rs140191306
rs11068290
rs9265864
All
−0.1
0.0
0.1
0.2
MR leave−one−out sensitivity analysis for
' IgD− CD38− %B cell' on 'Rheumatoid arthritis'

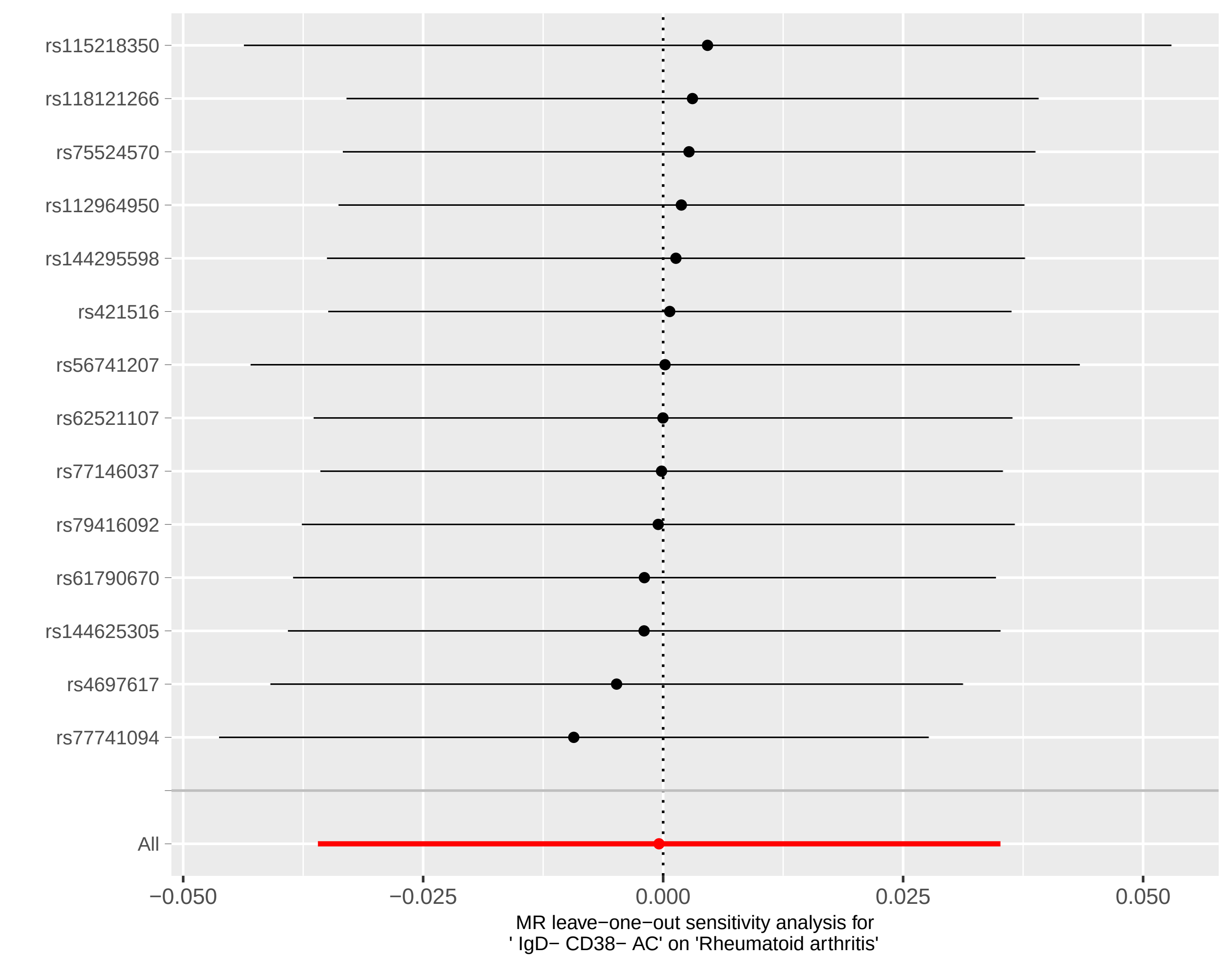
rs115218350
rs118121266
rs75524570
rs112964950
rs144295598
rs421516
rs56741207
rs62521107
rs77146037
rs79416092
rs61790670
rs144625305
rs4697617
rs77741094
All
−0.050
−0.025
0.000
0.025
0.050
MR leave−one−out sensitivity analysis for
' IgD− CD38− AC' on 'Rheumatoid arthritis'

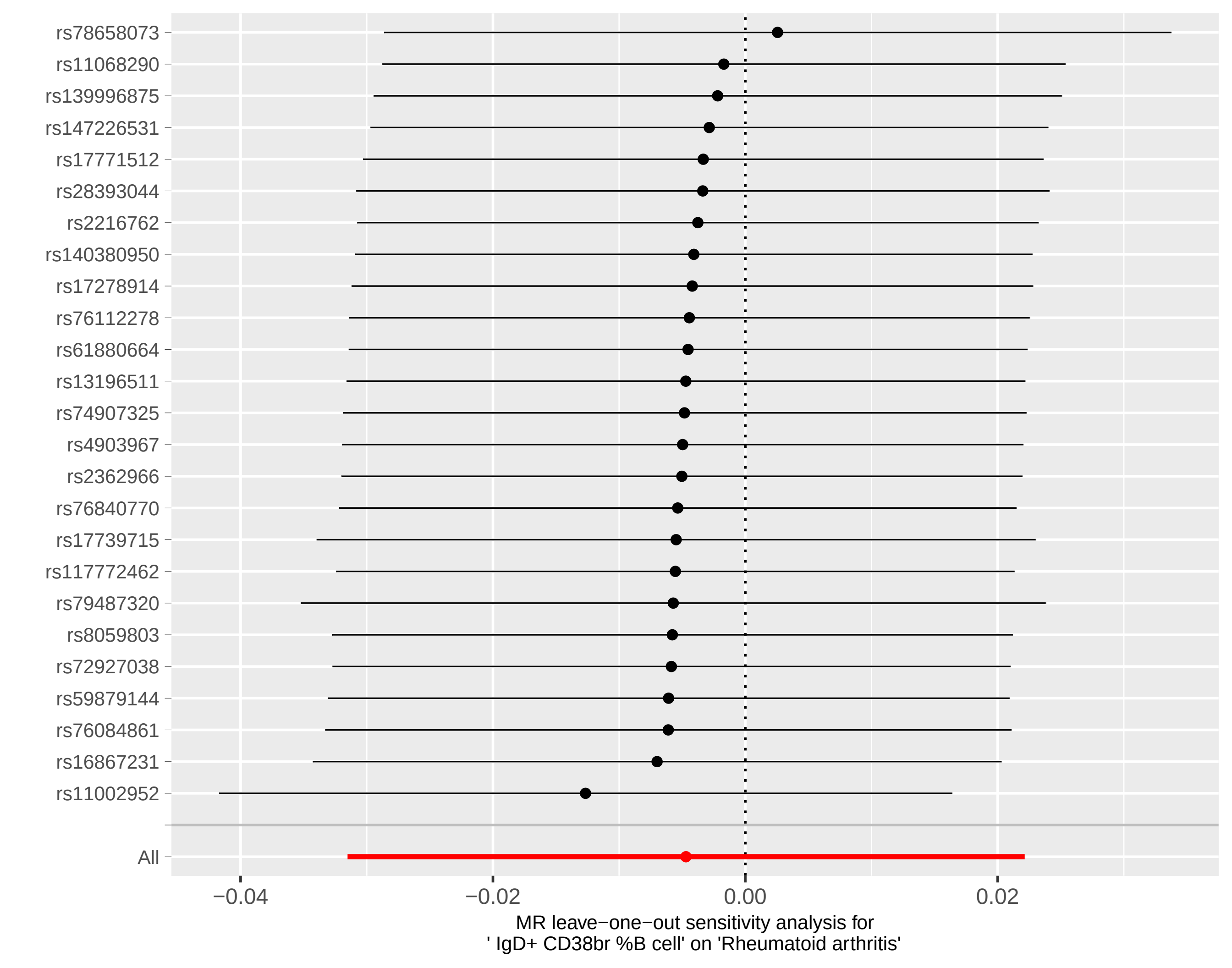
rs78658073
rs11068290
rs139996875
rs147226531
rs17771512
rs28393044
rs2216762
rs140380950
rs17278914
rs76112278
rs61880664
rs13196511
rs74907325
rs4903967
rs2362966
rs76840770
rs17739715
rs117772462
rs79487320
rs8059803
rs72927038
rs59879144
rs76084861
rs16867231
rs11002952
All
−0.04
−0.02
0.00
0.02
MR leave−one−out sensitivity analysis for
' IgD+ CD38br %B cell' on 'Rheumatoid arthritis'

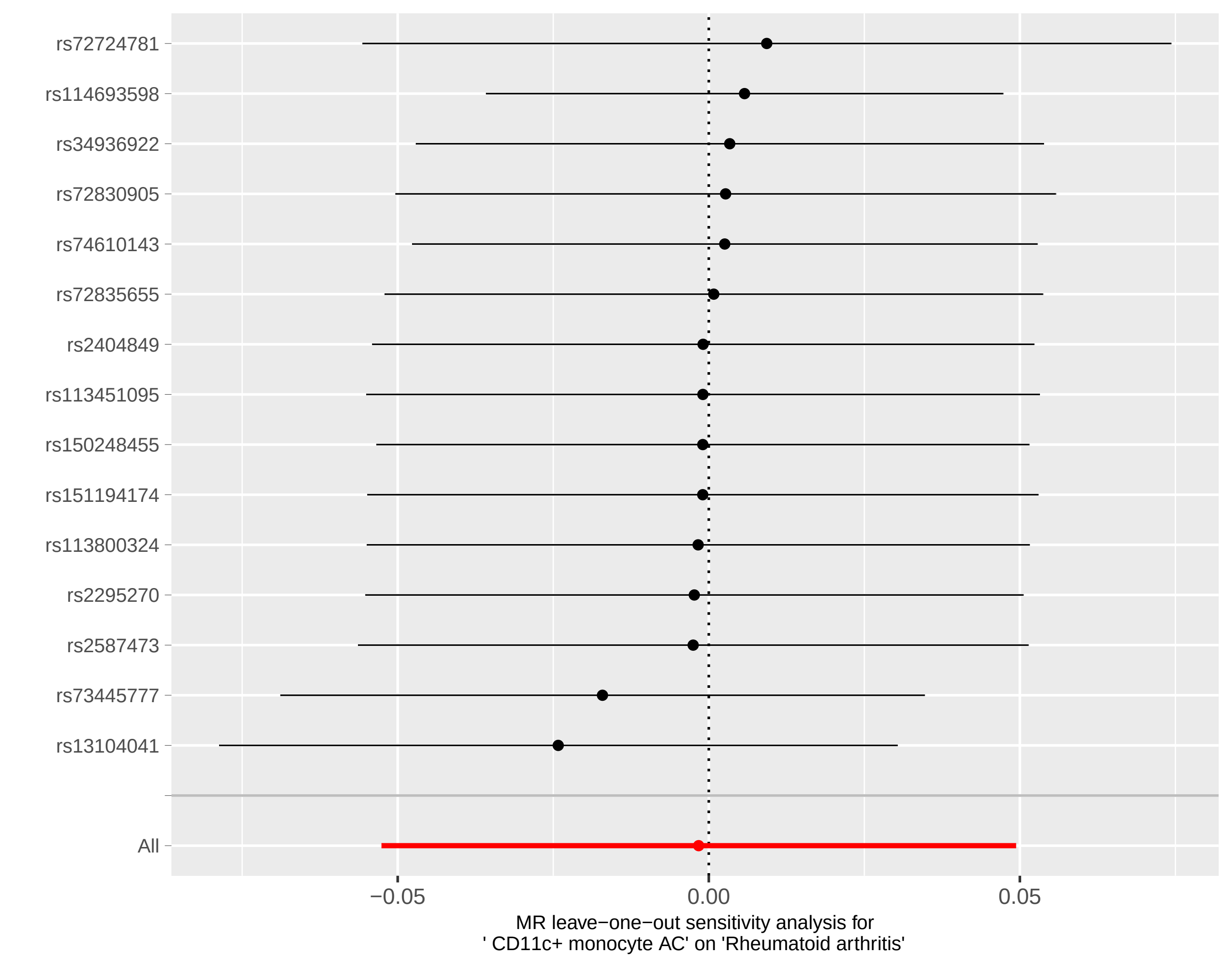
rs72724781
rs114693598
rs34936922
rs72830905
rs74610143
rs72835655
rs2404849
rs113451095
rs150248455
rs151194174
rs113800324
rs2295270
rs2587473
rs73445777
rs13104041
All
−0.05
0.00
0.05
MR leave−one−out sensitivity analysis for
' CD11c+ monocyte AC' on 'Rheumatoid arthritis'

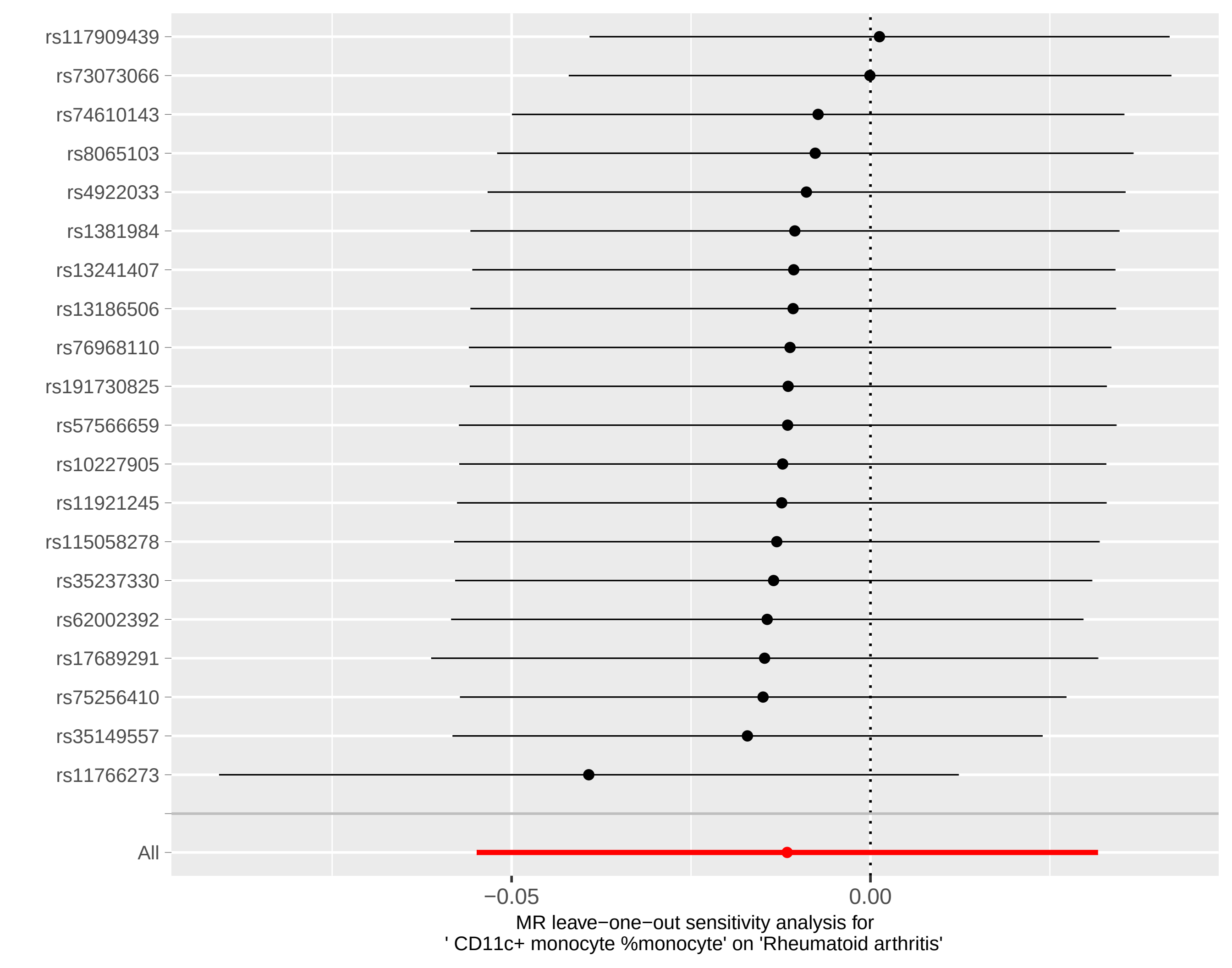
rs117909439
rs73073066
rs74610143
rs8065103
rs4922033
rs1381984
rs13241407
rs13186506
rs76968110
rs191730825
rs57566659
rs10227905
rs11921245
rs115058278
rs35237330
rs62002392
rs17689291
rs75256410
rs35149557
rs11766273
All
−0.05
0.00
MR leave−one−out sensitivity analysis for
' CD11c+ monocyte %monocyte' on 'Rheumatoid arthritis'

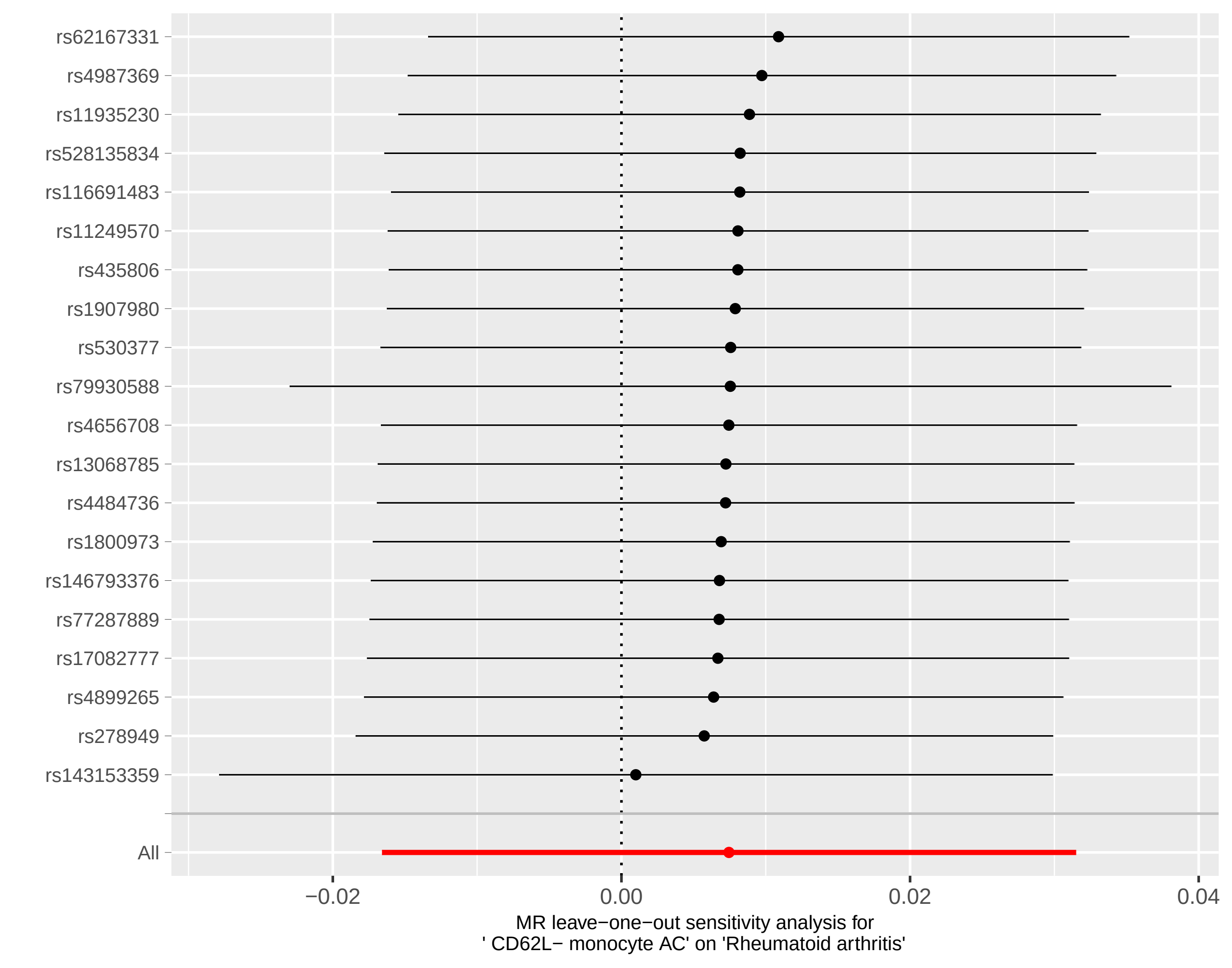

rs62167331
rs4987369
rs11935230
rs528135834
rs116691483
rs11249570
rs435806
rs1907980
rs530377
rs79930588
rs4656708
rs13068785
rs4484736
rs1800973
rs146793376
rs77287889
rs17082777
rs4899265
rs278949
rs143153359
All
−0.02
0.00
0.02
0.04
MR leave−one−out sensitivity analysis for
' CD62L− monocyte AC' on 'Rheumatoid arthritis'

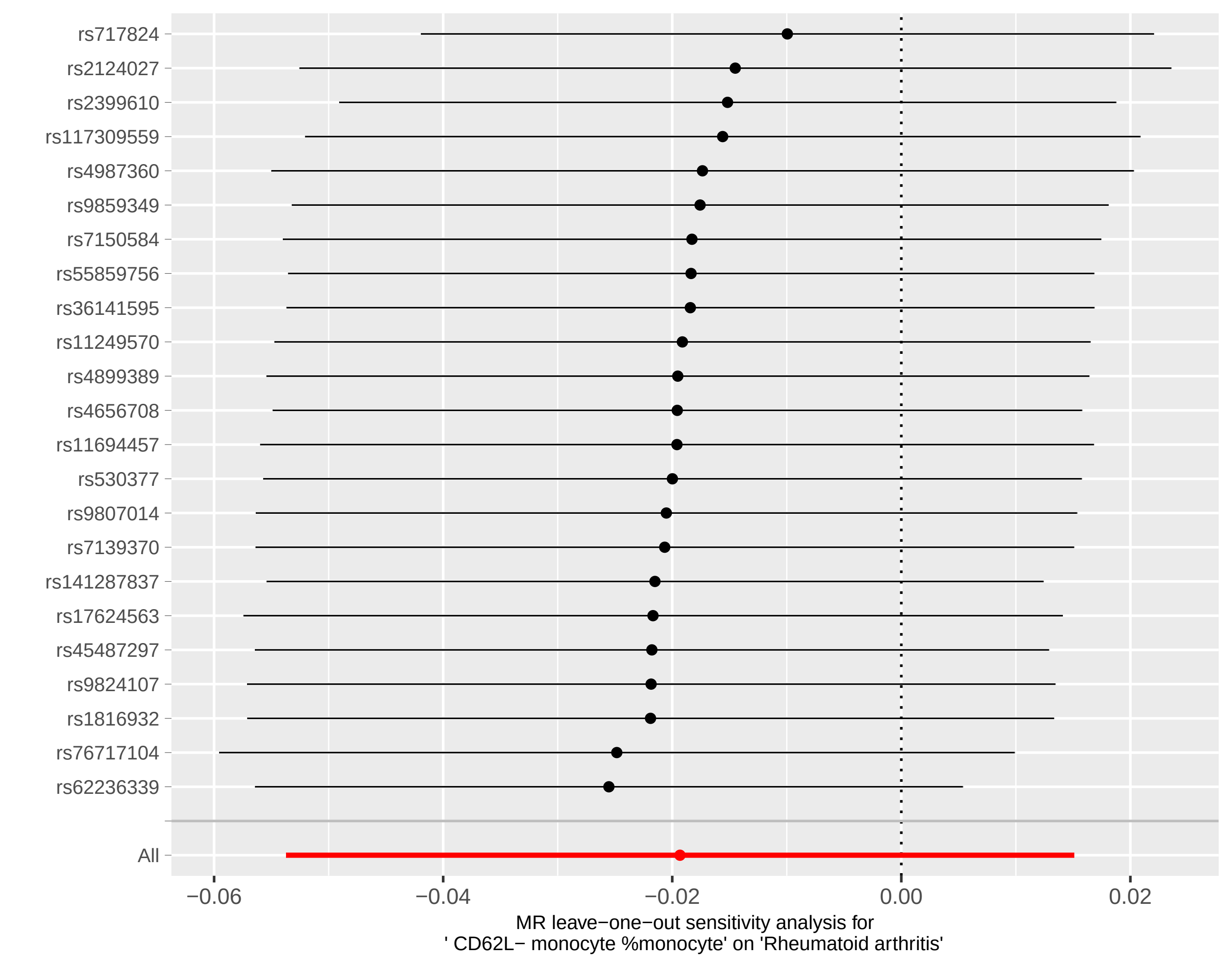
rs717824
rs2124027
rs2399610
rs117309559
rs4987360
rs9859349
rs7150584
rs55859756
rs36141595
rs11249570
rs4899389
rs4656708
rs11694457
rs530377
rs9807014
rs7139370
rs141287837
rs17624563
rs45487297
rs9824107
rs1816932
rs76717104
rs62236339
All
−0.06
−0.04
−0.02
0.00
0.02
MR leave−one−out sensitivity analysis for
' CD62L− monocyte %monocyte' on 'Rheumatoid arthritis'

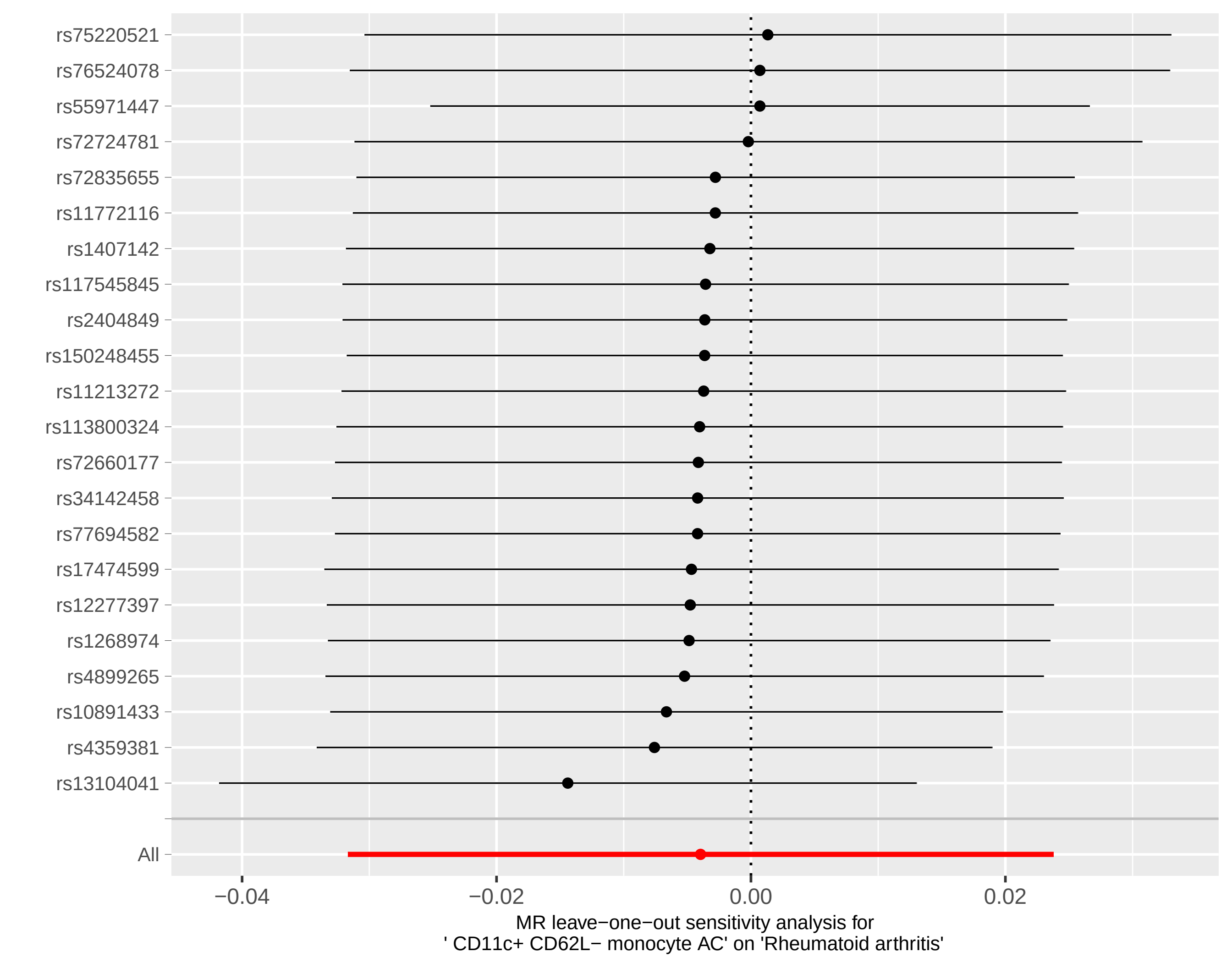
rs75220521
rs76524078
rs55971447
rs72724781
rs72835655
rs11772116
rs1407142
rs117545845
rs2404849
rs150248455
rs11213272
rs113800324
rs72660177
rs34142458
rs77694582
rs17474599
rs12277397
rs1268974
rs4899265
rs10891433
rs4359381
rs13104041
All
−0.04
−0.02
0.00
0.02
MR leave−one−out sensitivity analysis for
' CD11c+ CD62L− monocyte AC' on 'Rheumatoid arthritis'

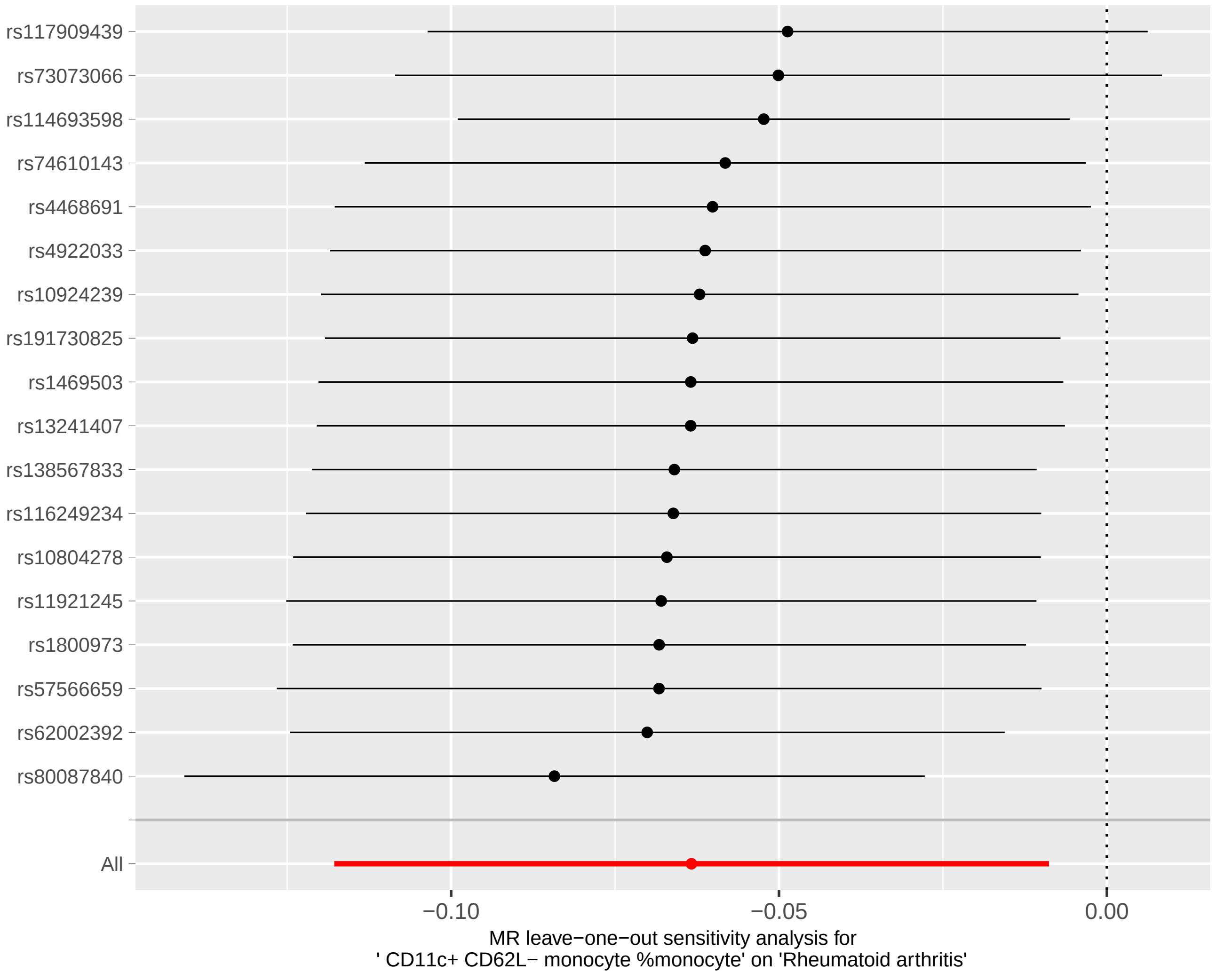
rs117909439
rs73073066
rs114693598
rs74610143
rs4468691
rs4922033
rs10924239
rs191730825
rs1469503
rs13241407
rs138567833
rs116249234
rs10804278
rs11921245
rs1800973
rs57566659
rs62002392
rs80087840
All
−0.10
−0.05
0.00
MR leave−one−out sensitivity analysis for
' CD11c+ CD62L− monocyte %monocyte' on 'Rheumatoid arthritis'

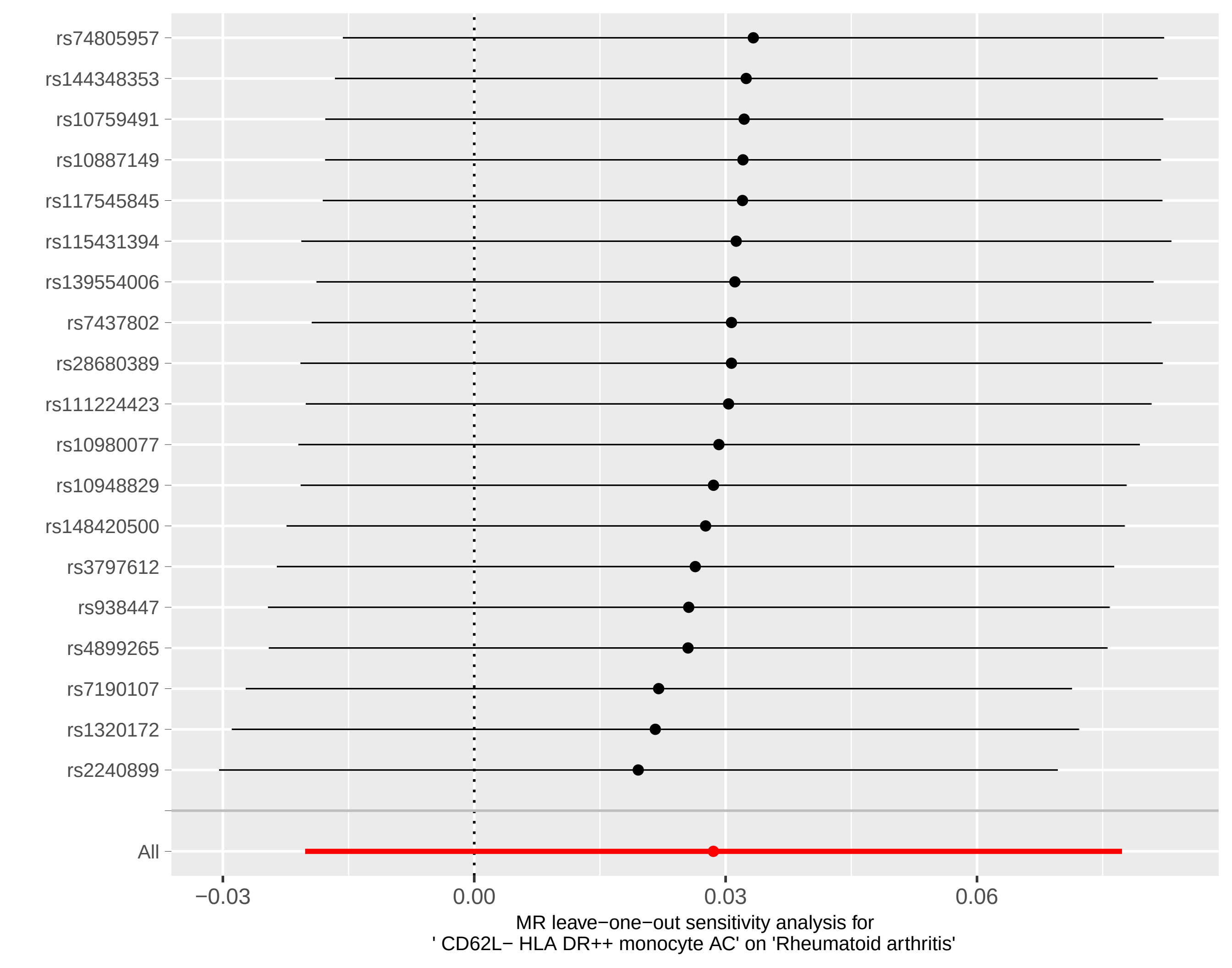
rs74805957
rs144348353
rs10759491
rs10887149
rs117545845
rs115431394
rs139554006
rs7437802
rs28680389
rs111224423
rs10980077
rs10948829
rs148420500
rs3797612
rs938447
rs4899265
rs7190107
rs1320172
rs2240899
All
−0.03
0.00
0.03
0.06
MR leave−one−out sensitivity analysis for
' CD62L− HLA DR++ monocyte AC' on 'Rheumatoid arthritis'

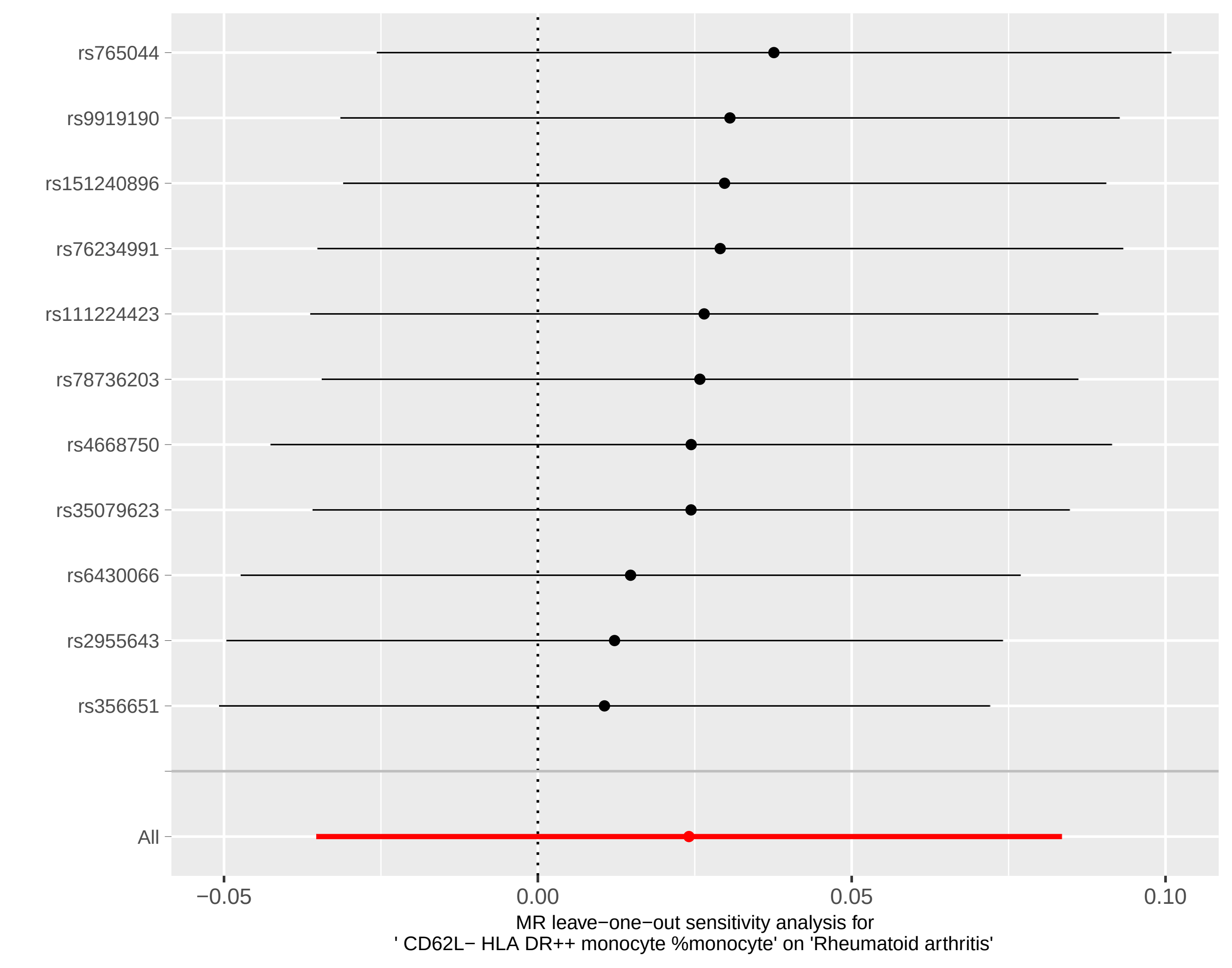
rs765044
rs9919190
rs151240896
rs76234991
rs111224423
rs78736203
rs4668750
rs35079623
rs6430066
rs2955643
rs356651
All
−0.05
0.00
0.05
0.10
MR leave−one−out sensitivity analysis for
' CD62L− HLA DR++ monocyte %monocyte' on 'Rheumatoid arthritis'

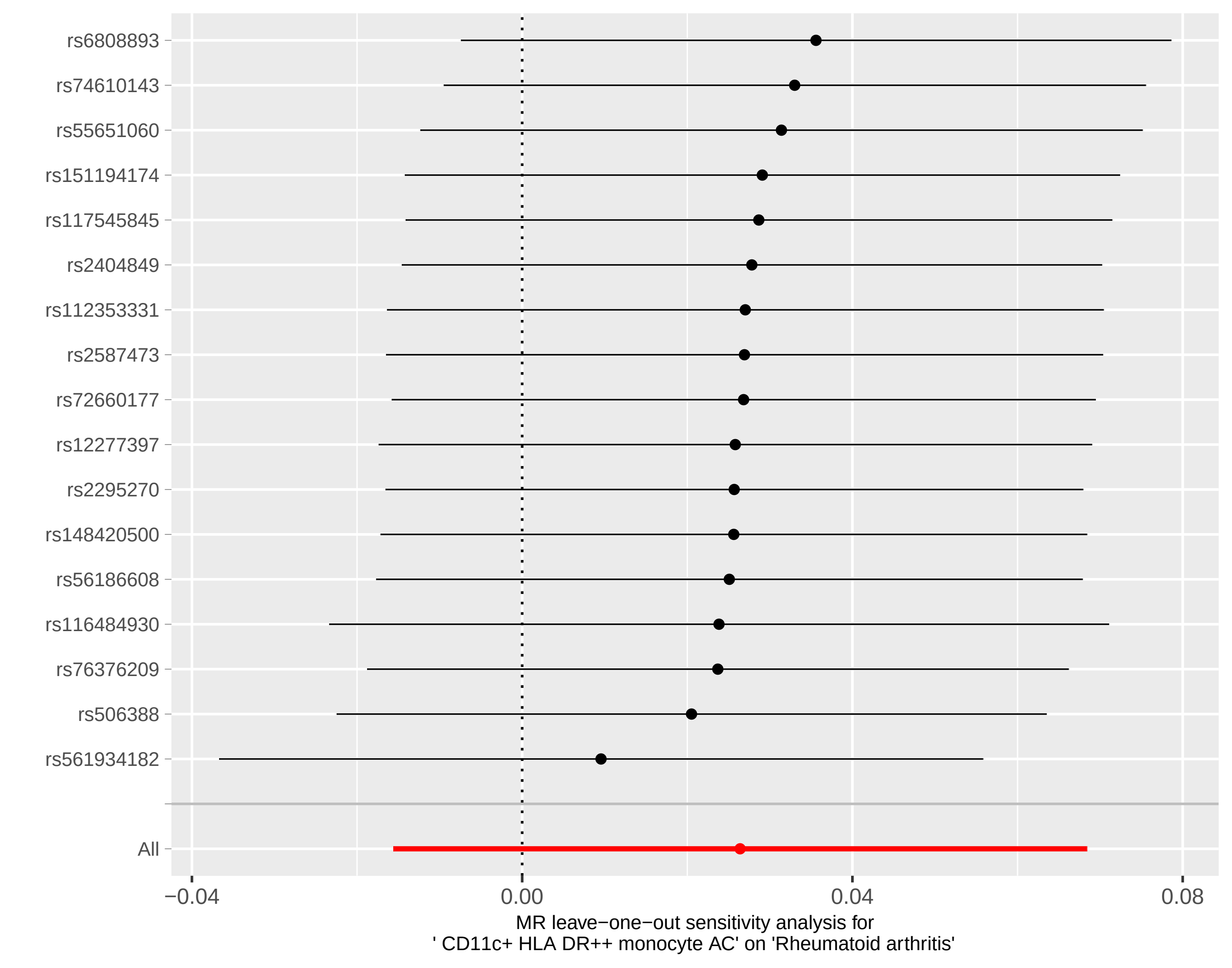
rs6808893
rs74610143
rs55651060
rs151194174
rs117545845
rs2404849
rs112353331
rs2587473
rs72660177
rs12277397
rs2295270
rs148420500
rs56186608
rs116484930
rs76376209
rs506388
rs561934182
All
−0.04
0.00
0.04
0.08
MR leave−one−out sensitivity analysis for
' CD11c+ HLA DR++ monocyte AC' on 'Rheumatoid arthritis'

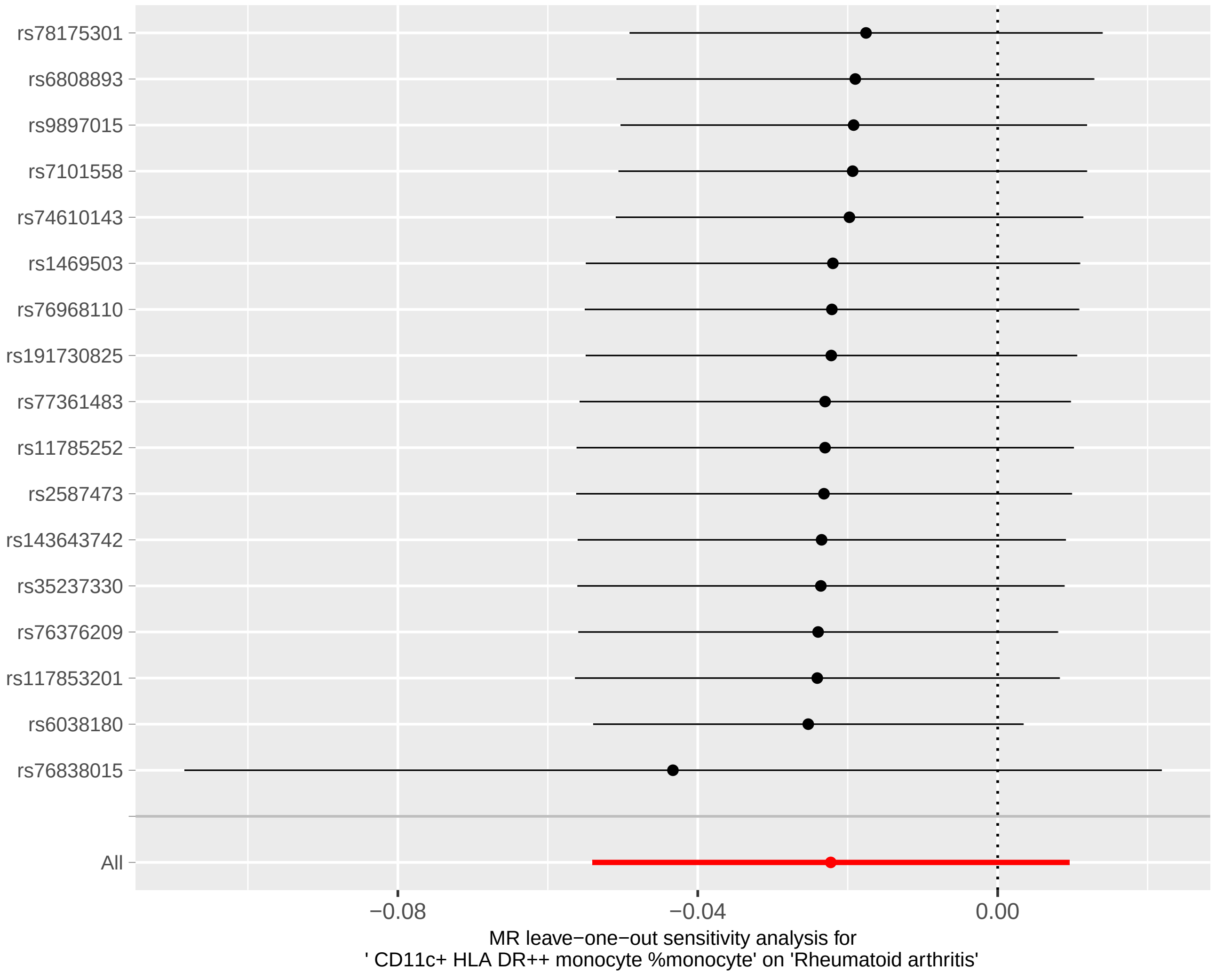
rs78175301
rs6808893
rs9897015
rs7101558
rs74610143
rs1469503
rs76968110
rs191730825
rs77361483
rs11785252
rs2587473
rs143643742
rs35237330
rs76376209
rs117853201
rs6038180
rs76838015
All
−0.08
−0.04
0.00
MR leave−one−out sensitivity analysis for
' CD11c+ HLA DR++ monocyte %monocyte' on 'Rheumatoid arthritis'

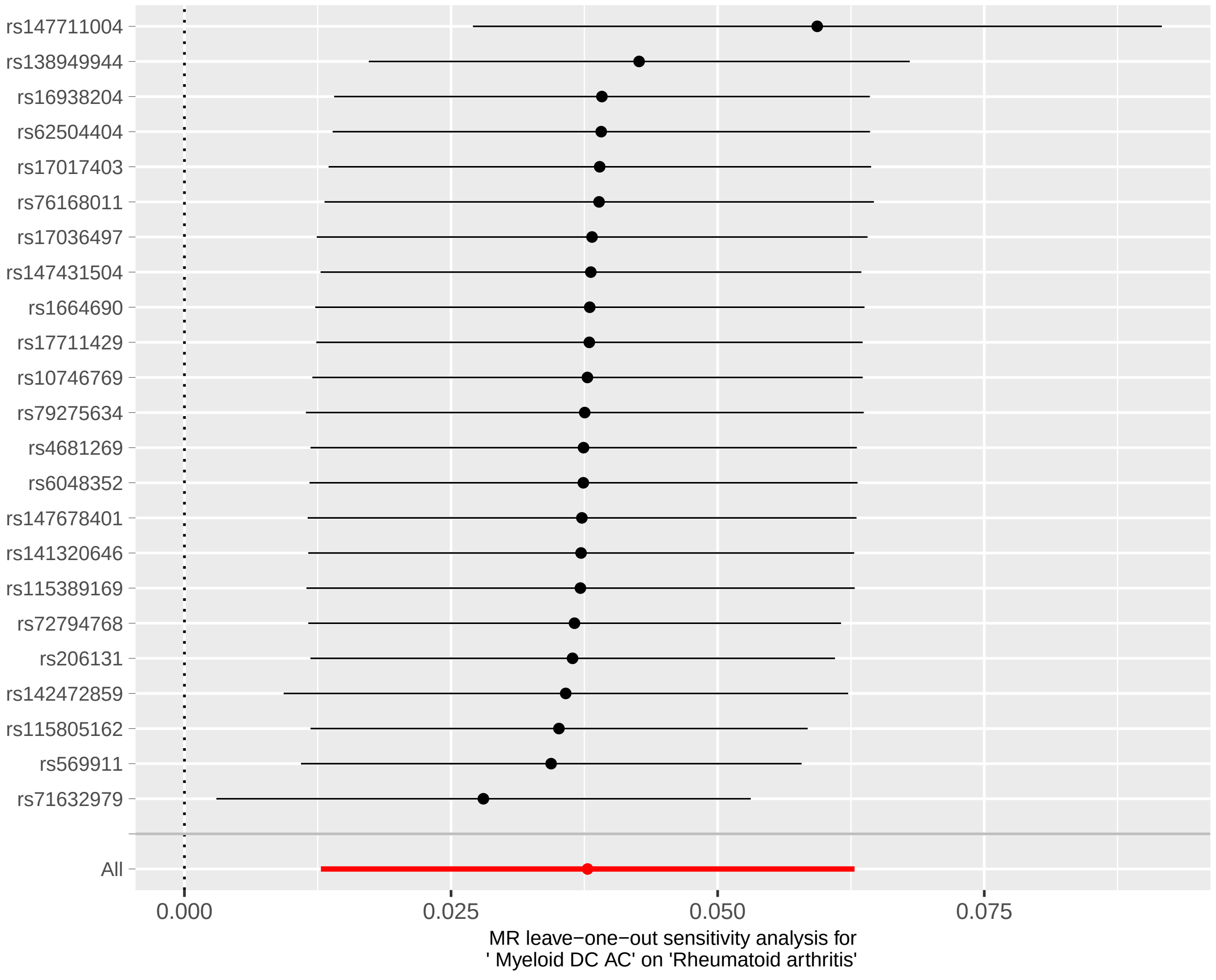

rs147711004
rs138949944
rs16938204
rs62504404
rs17017403
rs76168011
rs17036497
rs147431504
rs1664690
rs17711429
rs10746769
rs79275634
rs4681269
rs6048352
rs147678401
rs141320646
rs115389169
rs72794768
rs206131
rs142472859
rs115805162
rs569911
rs71632979
All
0.000
0.025
0.050
0.075
MR leave−one−out sensitivity analysis for
' Myeloid DC AC' on 'Rheumatoid arthritis'

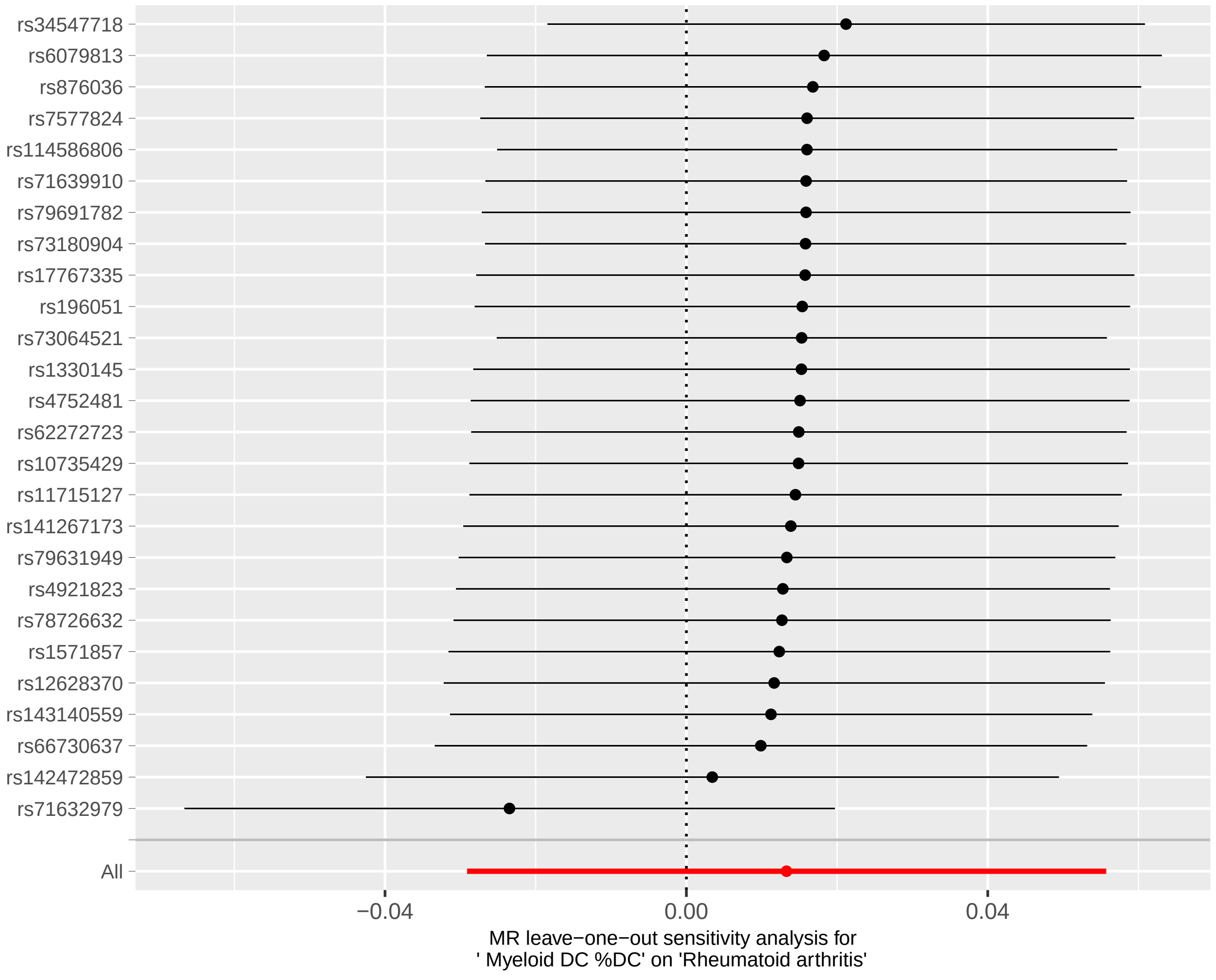
rs34547718
rs6079813
rs876036
rs7577824
rs114586806
rs71639910
rs79691782
rs73180904
rs17767335
rs196051
rs73064521
rs1330145
rs4752481
rs62272723
rs10735429
rs11715127
rs141267173
rs79631949
rs4921823
rs78726632
rs1571857
rs12628370
rs143140559
rs66730637
rs142472859
rs71632979
All
−0.04
0.00
0.04
MR leave−one−out sensitivity analysis for
' Myeloid DC %DC' on 'Rheumatoid arthritis'

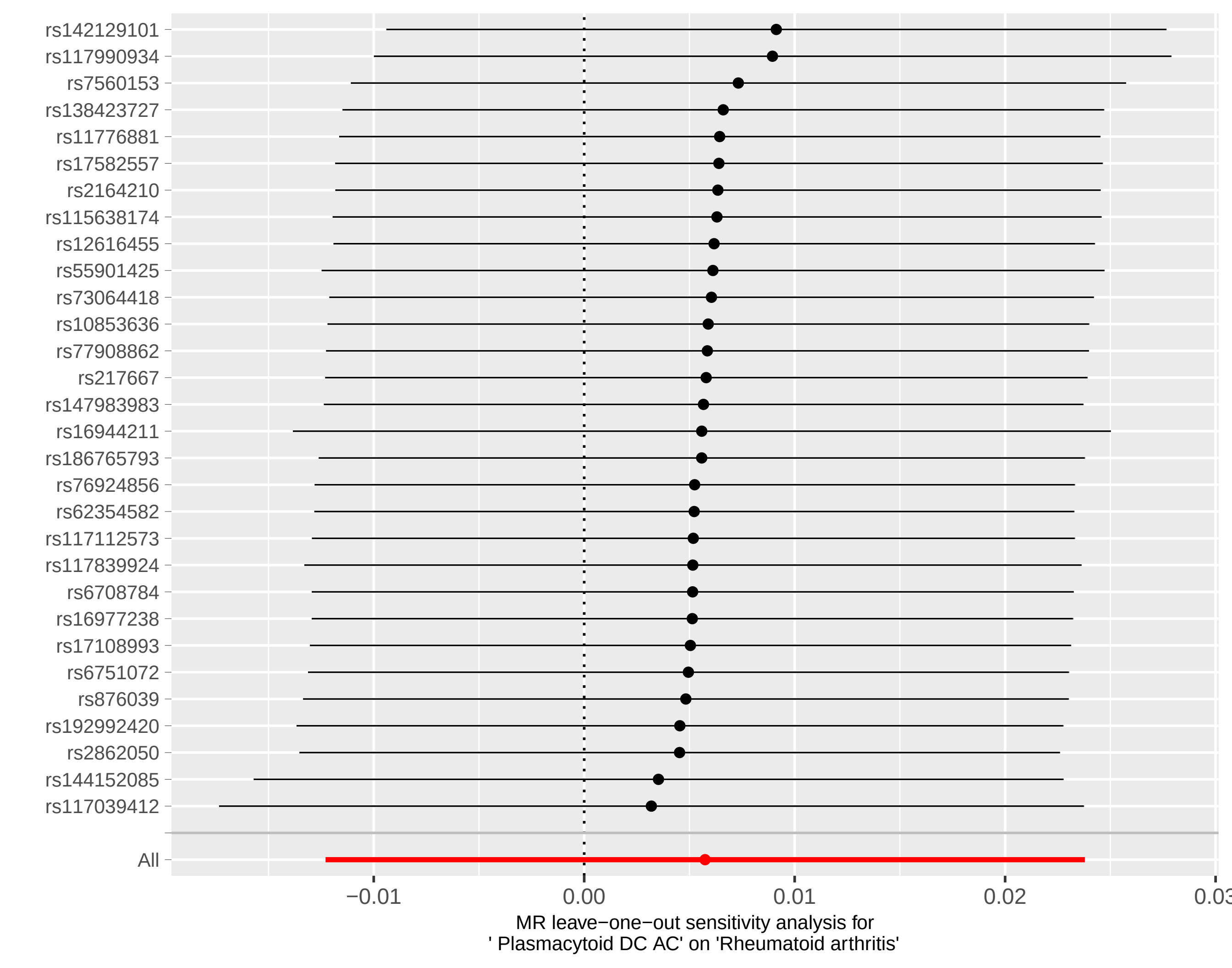
rs142129101
rs117990934
rs7560153
rs138423727
rs11776881
rs17582557
rs2164210
rs115638174
rs12616455
rs55901425
rs73064418
rs10853636
rs77908862
rs217667
rs147983983
rs16944211
rs186765793
rs76924856
rs62354582
rs117112573
rs117839924
rs6708784
rs16977238
rs17108993
rs6751072
rs876039
rs192992420
rs2862050
rs144152085
rs117039412
All
−0.01
0.00
0.01
0.02
MR leave−one−out sensitivity analysis for
' Plasmacytoid DC AC' on 'Rheumatoid arthritis'

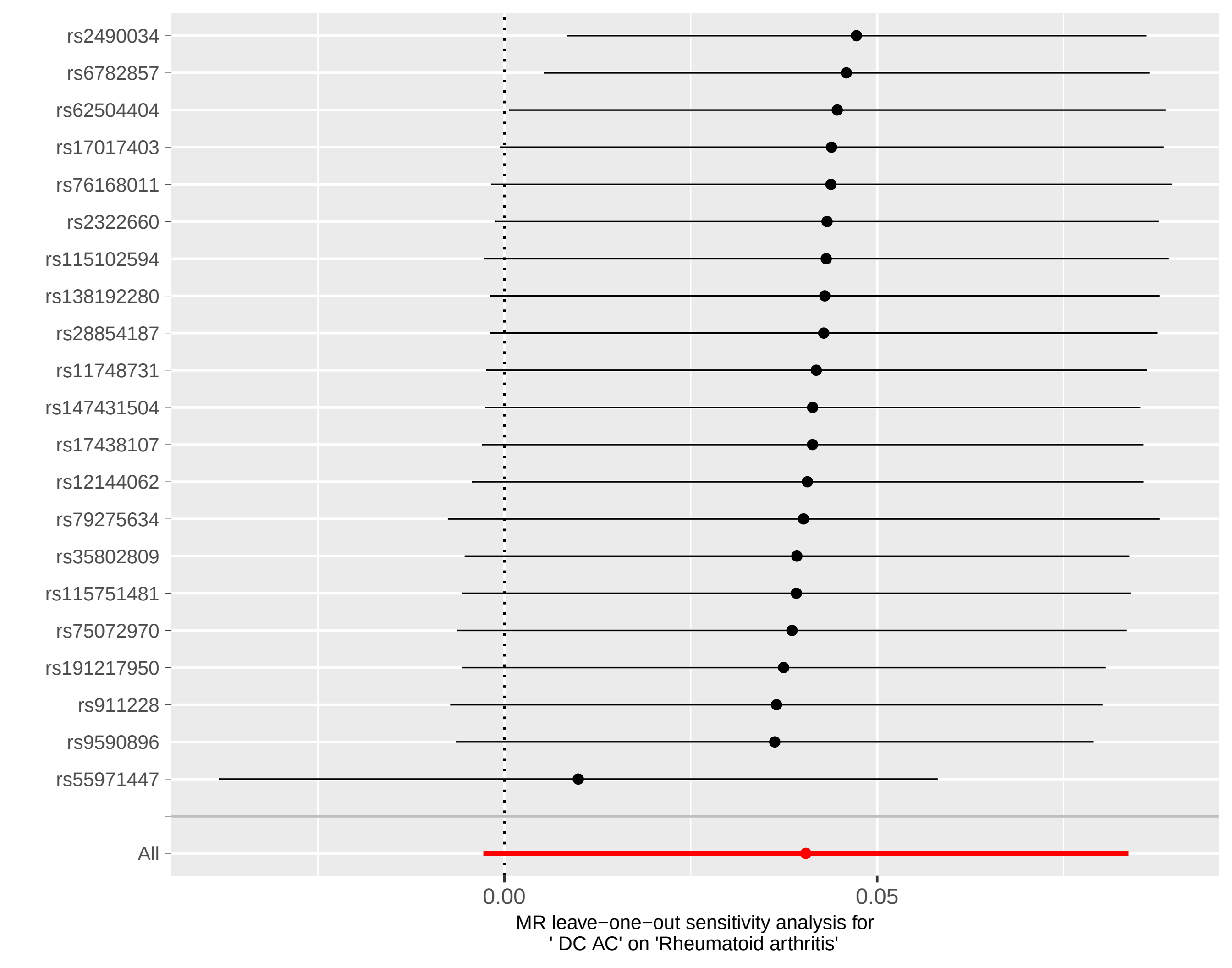
rs2490034
rs6782857
rs62504404
rs17017403
rs76168011
rs2322660
rs115102594
rs138192280
rs28854187
rs11748731
rs147431504
rs17438107
rs12144062
rs79275634
rs35802809
rs115751481
rs75072970
rs191217950
rs911228
rs9590896
rs55971447
All
0.00
0.05
MR leave−one−out sensitivity analysis for
' DC AC' on 'Rheumatoid arthritis'

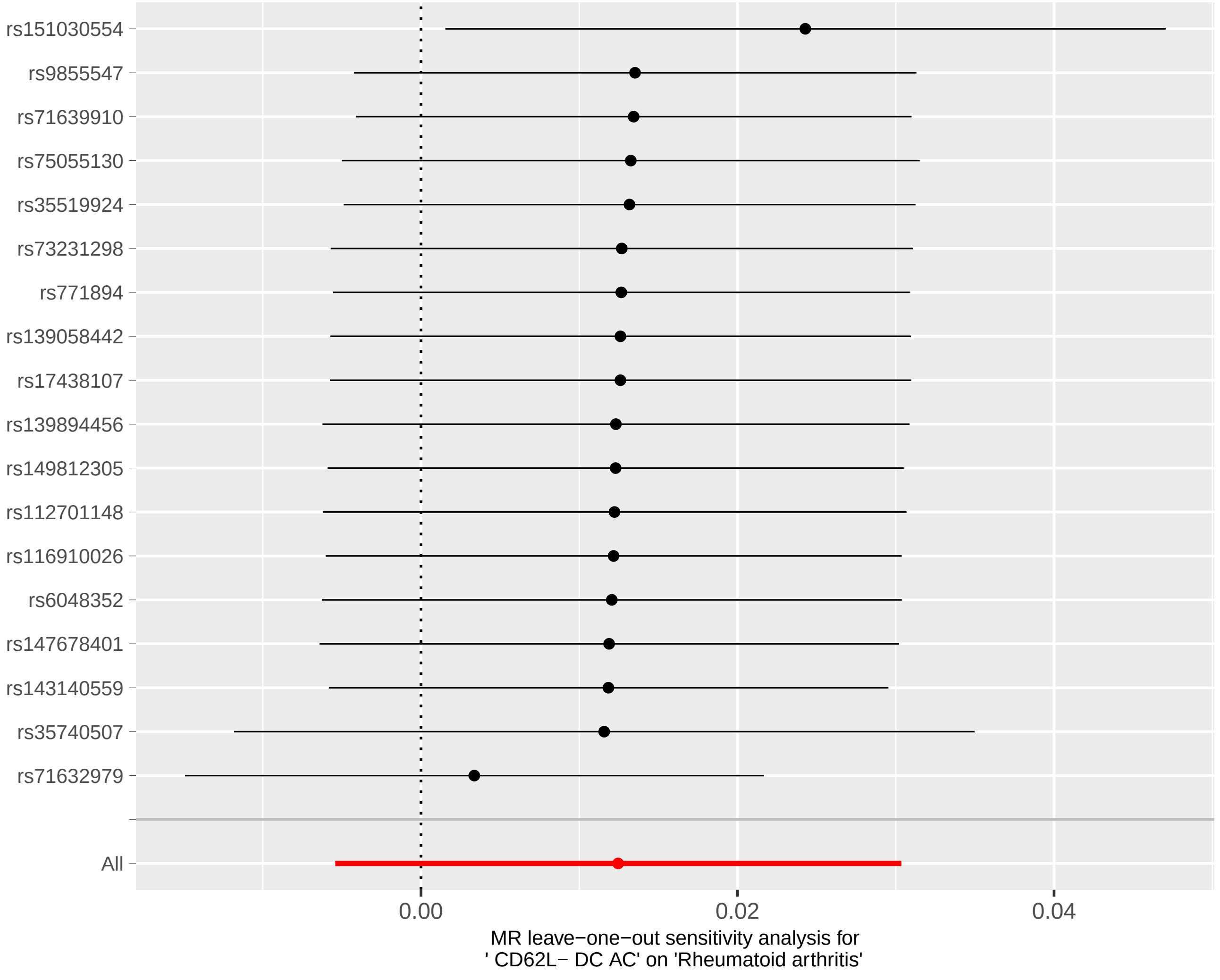

rs151030554
rs9855547
rs71639910
rs75055130
rs35519924
rs73231298
rs771894
rs139058442
rs17438107
rs139894456
rs149812305
rs112701148
rs116910026
rs6048352
rs147678401
rs143140559
rs35740507
rs71632979
All
0.00
0.02
0.04
MR leave−one−out sensitivity analysis for
' CD62L− DC AC' on 'Rheumatoid arthritis'

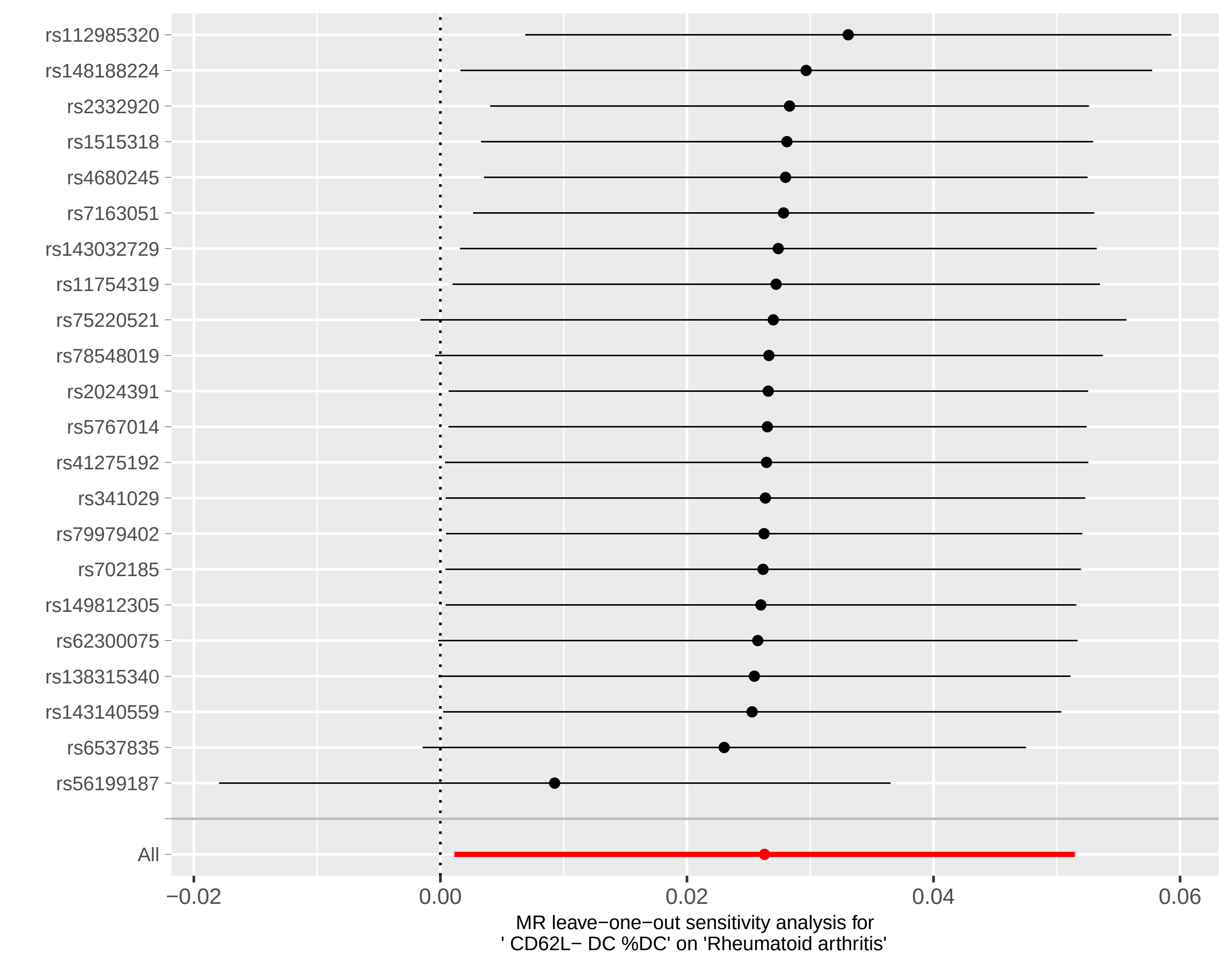
rs112985320
rs148188224
rs2332920
rs1515318
rs4680245
rs7163051
rs143032729
rs11754319
rs75220521
rs78548019
rs2024391
rs5767014
rs41275192
rs341029
rs79979402
rs702185
rs149812305
rs62300075
rs138315340
rs143140559
rs6537835
rs56199187
All
−0.02
0.00
0.02
0.04
0.06
MR leave−one−out sensitivity analysis for
' CD62L− DC %DC' on 'Rheumatoid arthritis'

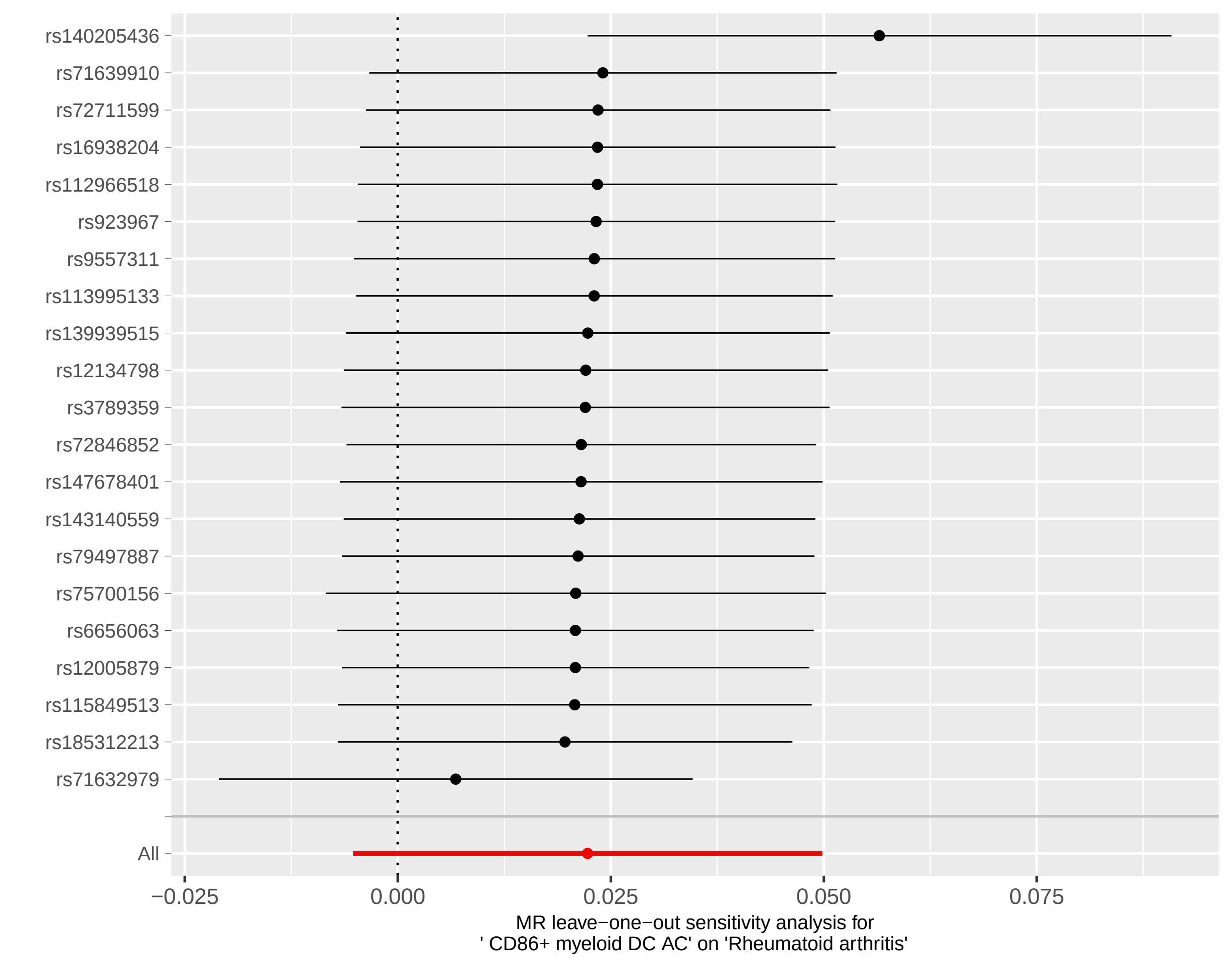
rs140205436
rs71639910
rs72711599
rs16938204
rs112966518
rs923967
rs9557311
rs113995133
rs139939515
rs12134798
rs3789359
rs72846852
rs147678401
rs143140559
rs79497887
rs75700156
rs6656063
rs12005879
rs115849513
rs185312213
rs71632979
All
−0.025
0.000
0.025
0.050
0.075
MR leave−one−out sensitivity analysis for
' CD86+ myeloid DC AC' on 'Rheumatoid arthritis'

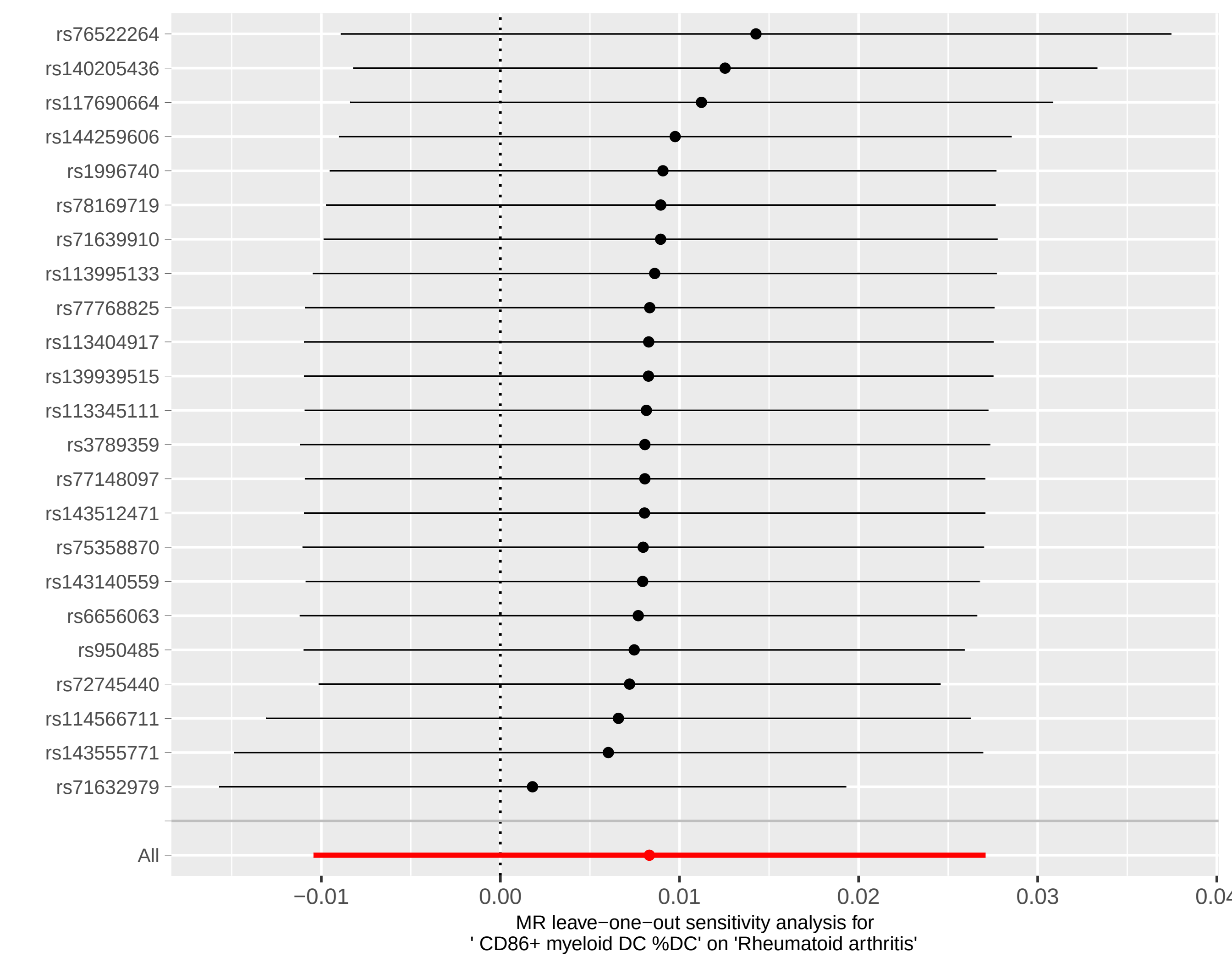
rs76522264
rs140205436
rs117690664
rs144259606
rs1996740
rs78169719
rs71639910
rs113995133
rs77768825
rs113404917
rs139939515
rs113345111
rs3789359
rs77148097
rs143512471
rs75358870
rs143140559
rs6656063
rs950485
rs72745440
rs114566711
rs143555771
rs71632979
All
−0.01
0.00
0.01
0.02
0.03
MR leave−one−out sensitivity analysis for
' CD86+ myeloid DC %DC' on 'Rheumatoid arthritis'

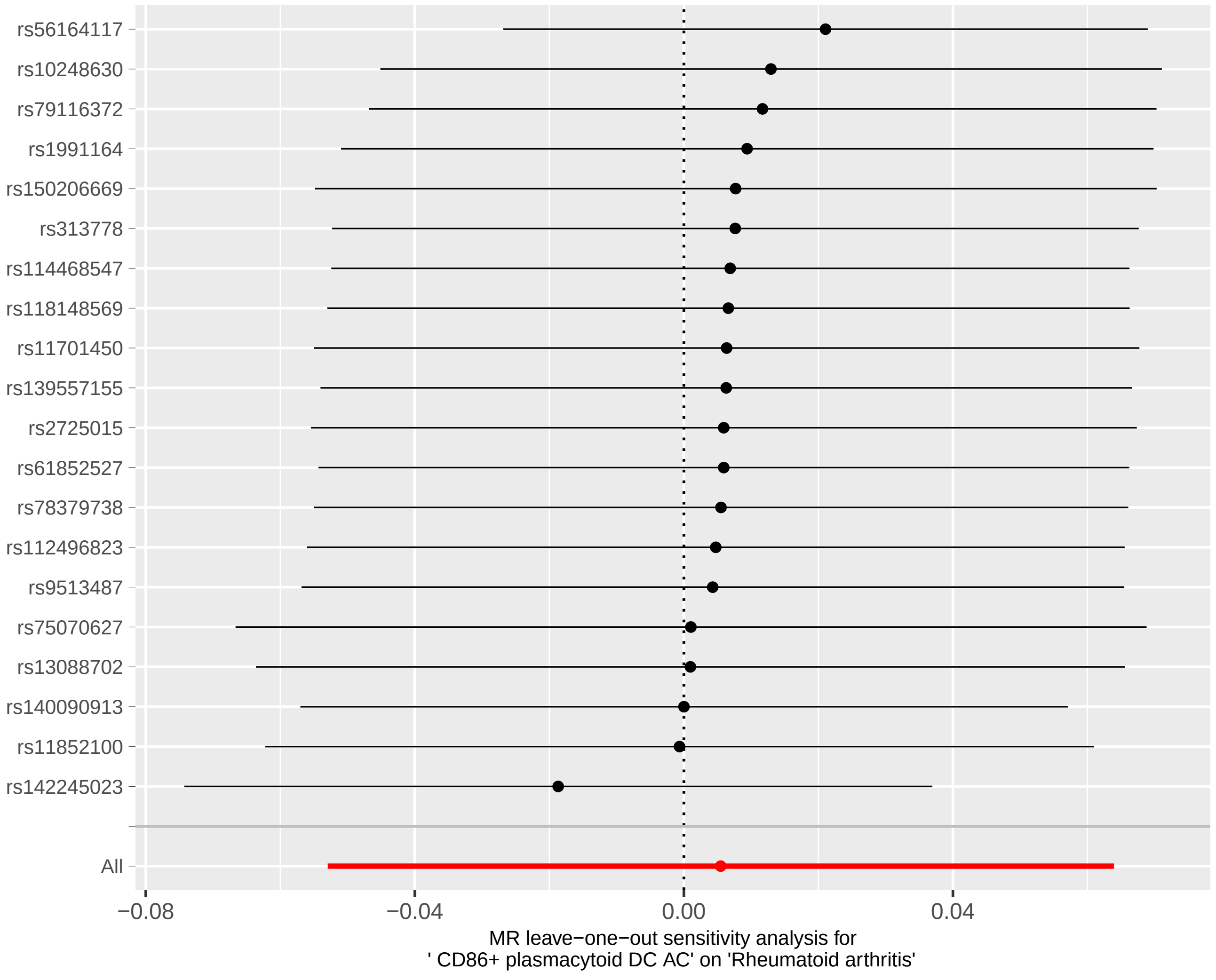
rs56164117
rs10248630
rs79116372
rs1991164
rs150206669
rs313778
rs114468547
rs118148569
rs11701450
rs139557155
rs2725015
rs61852527
rs78379738
rs112496823
rs9513487
rs75070627
rs13088702
rs140090913
rs11852100
rs142245023
All
−0.08
−0.04
0.00
0.04
MR leave−one−out sensitivity analysis for
' CD86+ plasmacytoid DC AC' on 'Rheumatoid arthritis'

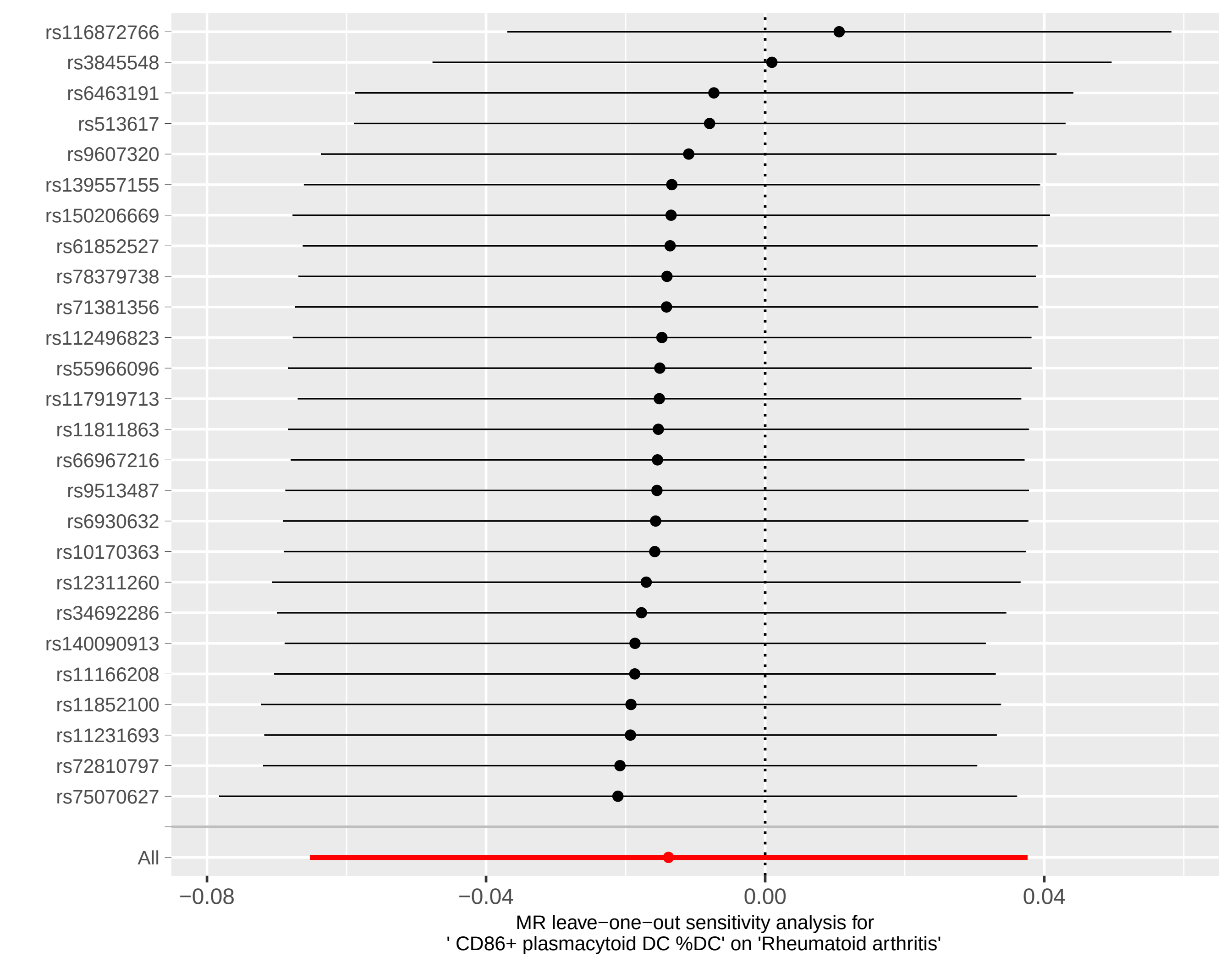
rs116872766
rs3845548
rs6463191
rs513617
rs9607320
rs139557155
rs150206669
rs61852527
rs78379738
rs71381356
rs112496823
rs55966096
rs117919713
rs11811863
rs66967216
rs9513487
rs6930632
rs10170363
rs12311260
rs34692286
rs140090913
rs11166208
rs11852100
rs11231693
rs72810797
rs75070627
All
−0.08
−0.04
0.00
0.04
MR leave−one−out sensitivity analysis for
' CD86+ plasmacytoid DC %DC' on 'Rheumatoid arthritis'

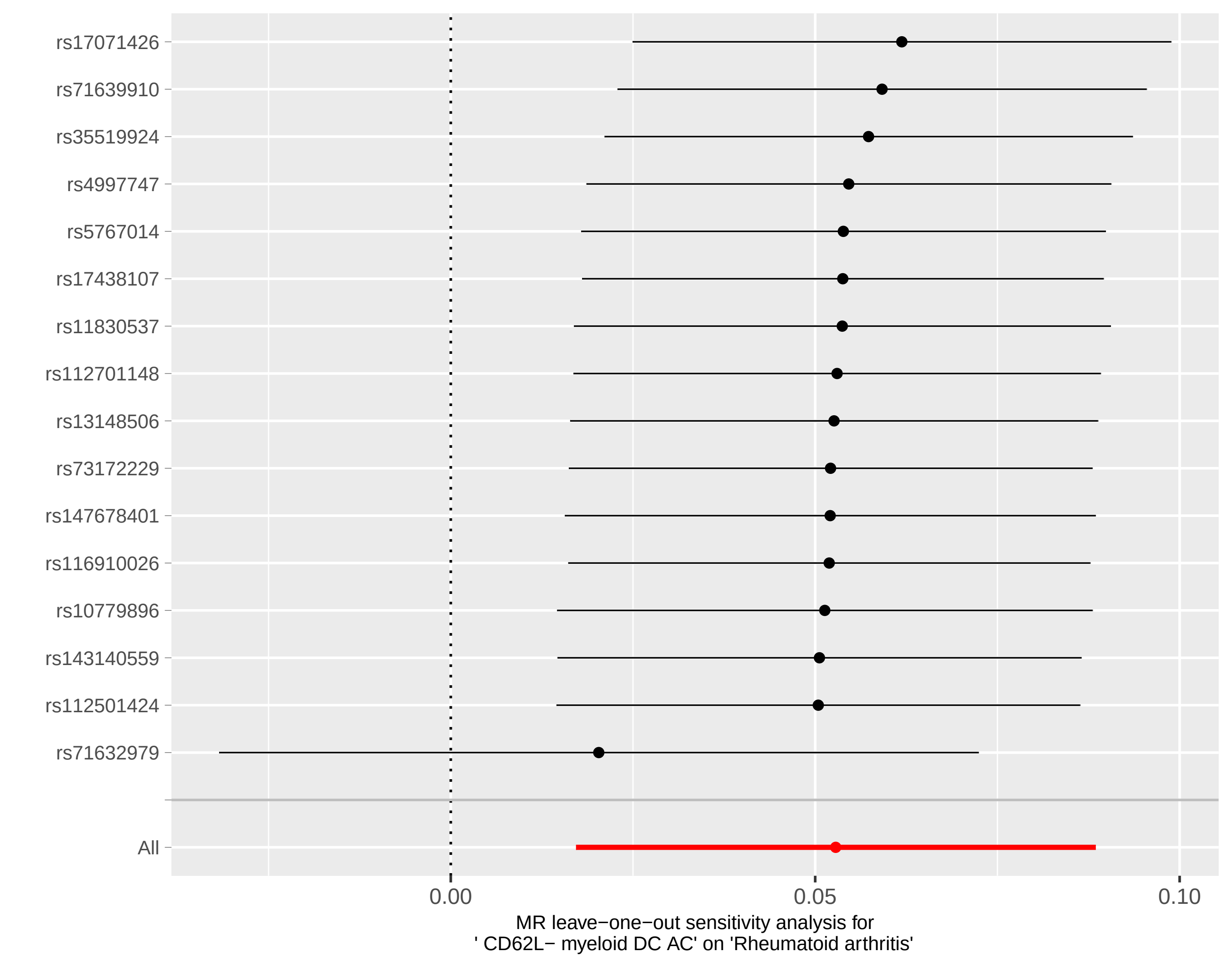

rs17071426
rs71639910
rs35519924
rs4997747
rs5767014
rs17438107
rs11830537
rs112701148
rs13148506
rs73172229
rs147678401
rs116910026
rs10779896
rs143140559
rs112501424
rs71632979
All
0.00
0.05
0.10
MR leave−one−out sensitivity analysis for
' CD62L− myeloid DC AC' on 'Rheumatoid arthritis'

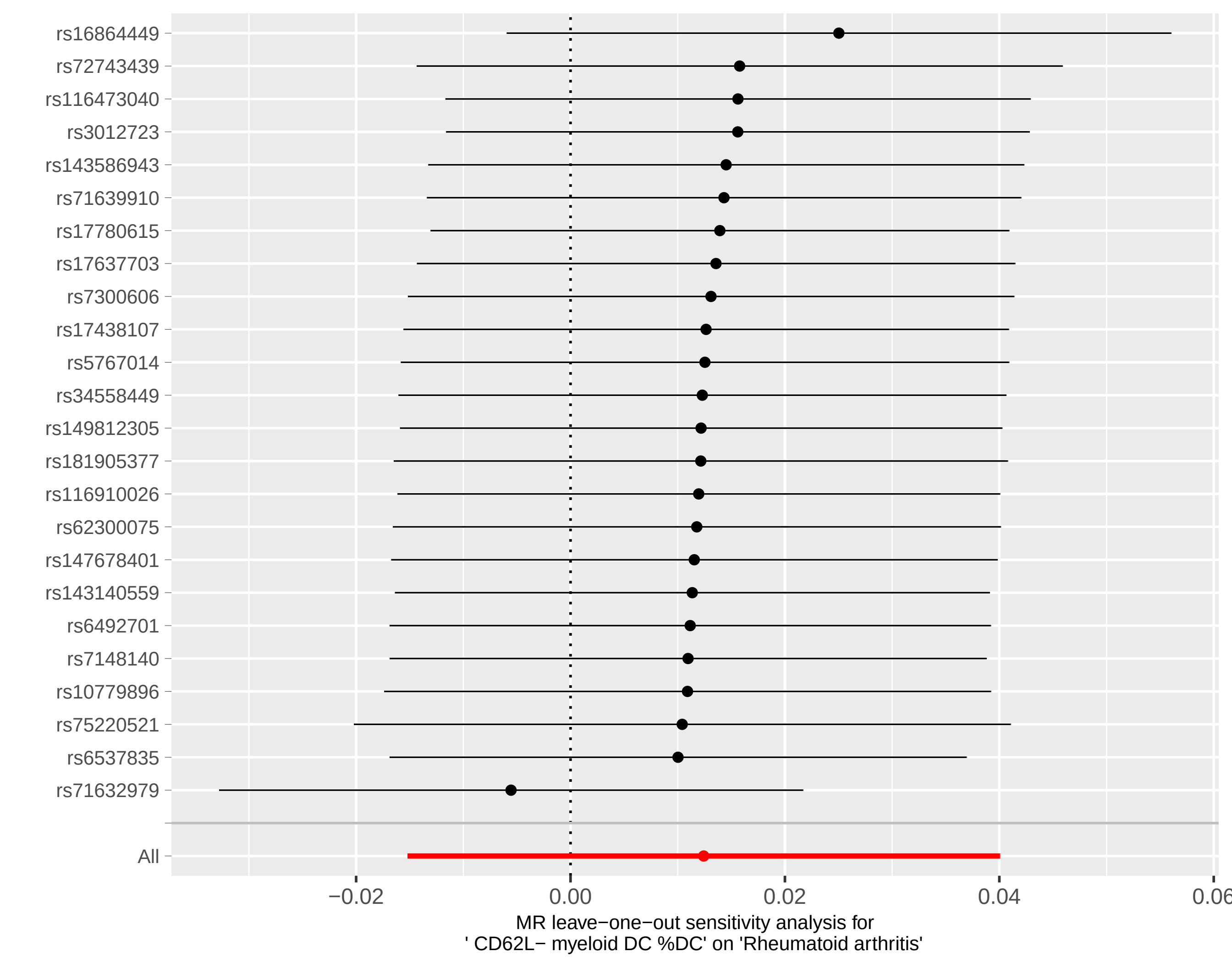
rs16864449
rs72743439
rs116473040
rs3012723
rs143586943
rs71639910
rs17780615
rs17637703
rs7300606
rs17438107
rs5767014
rs34558449
rs149812305
rs181905377
rs116910026
rs62300075
rs147678401
rs143140559
rs6492701
rs7148140
rs10779896
rs75220521
rs6537835
rs71632979
All
−0.02
0.00
0.02
0.04
0.06
MR leave−one−out sensitivity analysis for
' CD62L− myeloid DC %DC' on 'Rheumatoid arthritis'

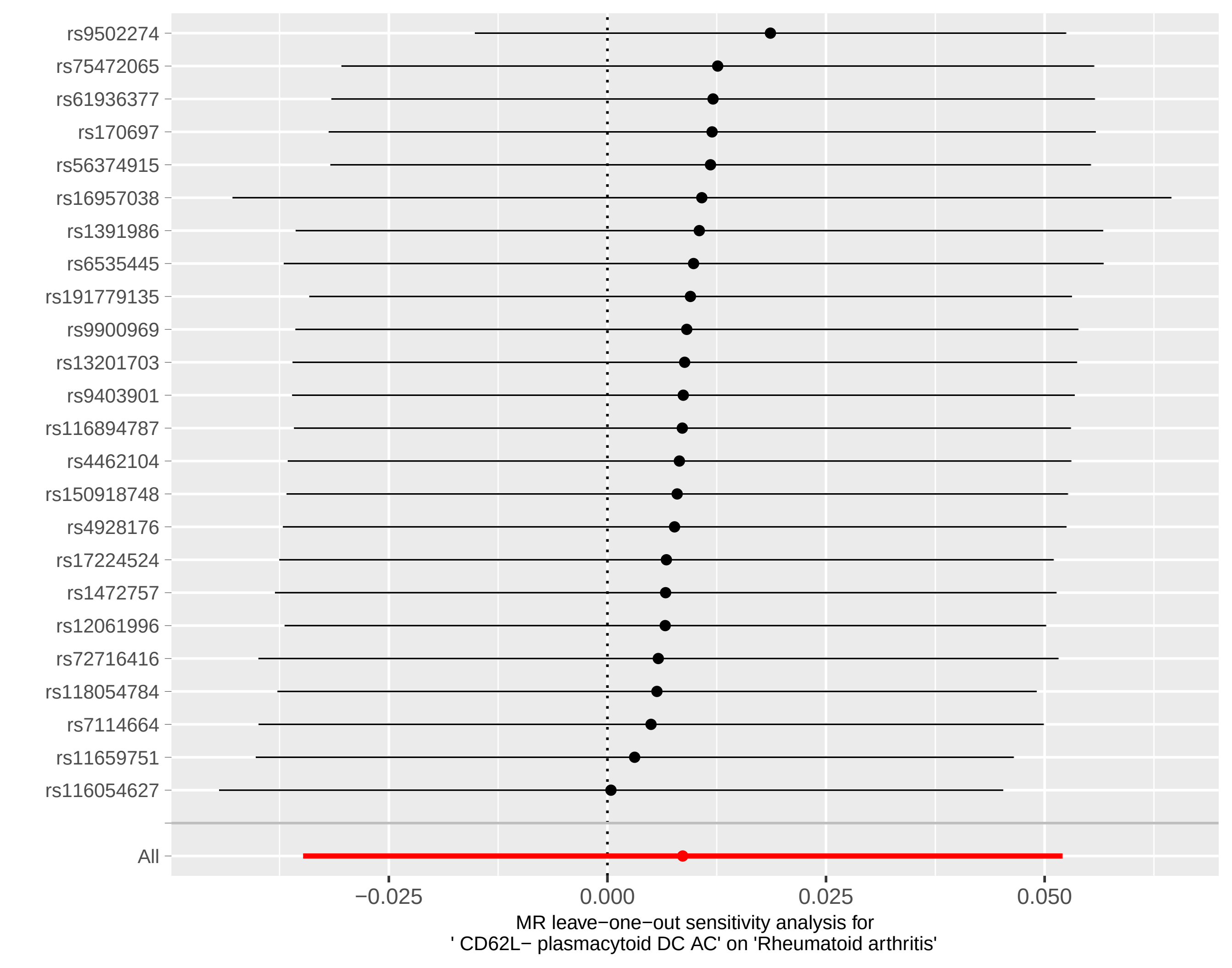

rs9502274
rs75472065
rs61936377
rs170697
rs56374915
rs16957038
rs1391986
rs6535445
rs191779135
rs9900969
rs13201703
rs9403901
rs116894787
rs4462104
rs150918748
rs4928176
rs17224524
rs1472757
rs12061996
rs72716416
rs118054784
rs7114664
rs11659751
rs116054627
All
−0.025
0.000
0.025
0.050
MR leave−one−out sensitivity analysis for
' CD62L− plasmacytoid DC AC' on 'Rheumatoid arthritis'

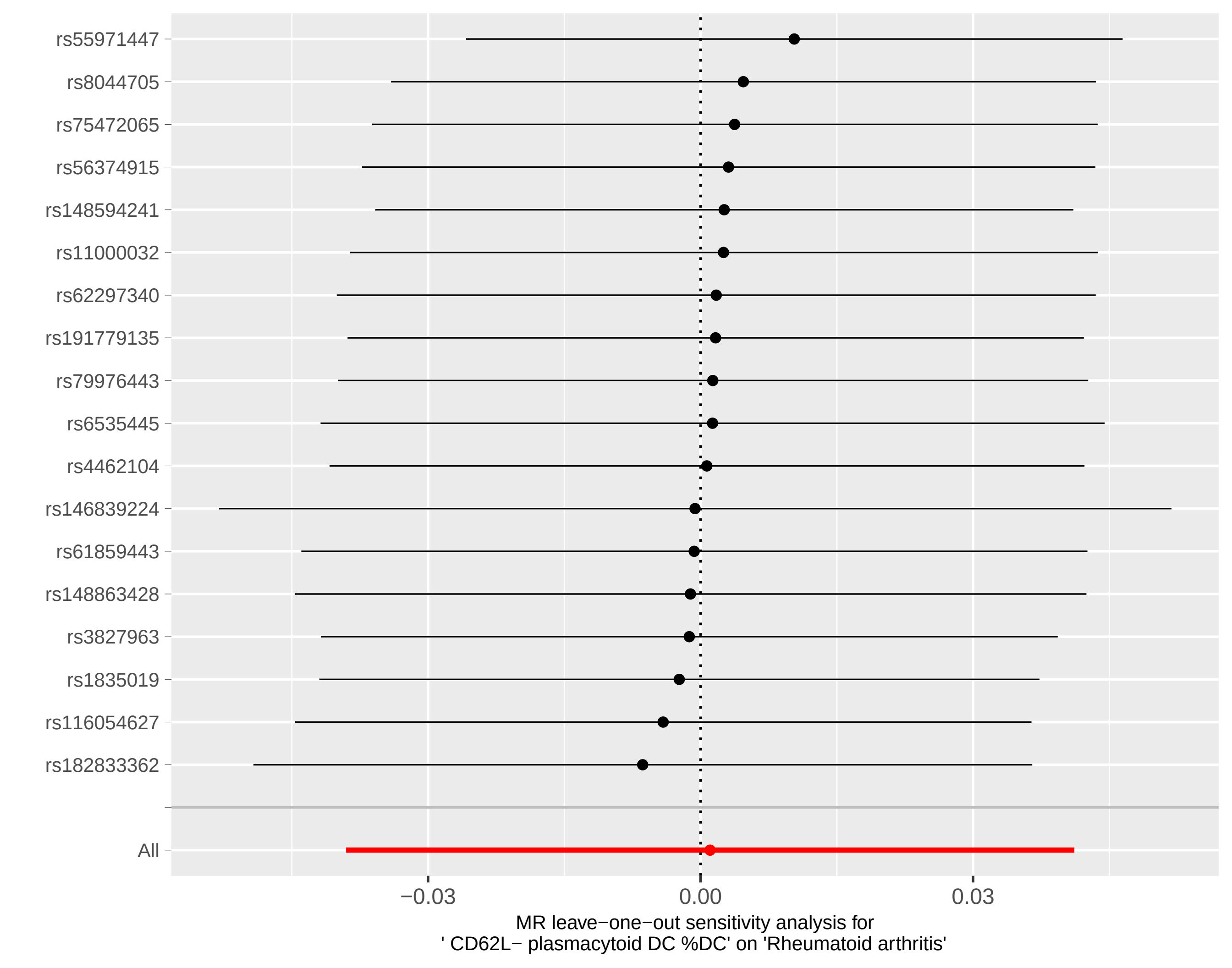

rs55971447
rs8044705
rs75472065
rs56374915
rs148594241
rs11000032
rs62297340
rs191779135
rs79976443
rs6535445
rs4462104
rs146839224
rs61859443
rs148863428
rs3827963
rs1835019
rs116054627
rs182833362
All
−0.03
0.00
0.03
MR leave−one−out sensitivity analysis for
' CD62L− plasmacytoid DC %DC' on 'Rheumatoid arthritis'

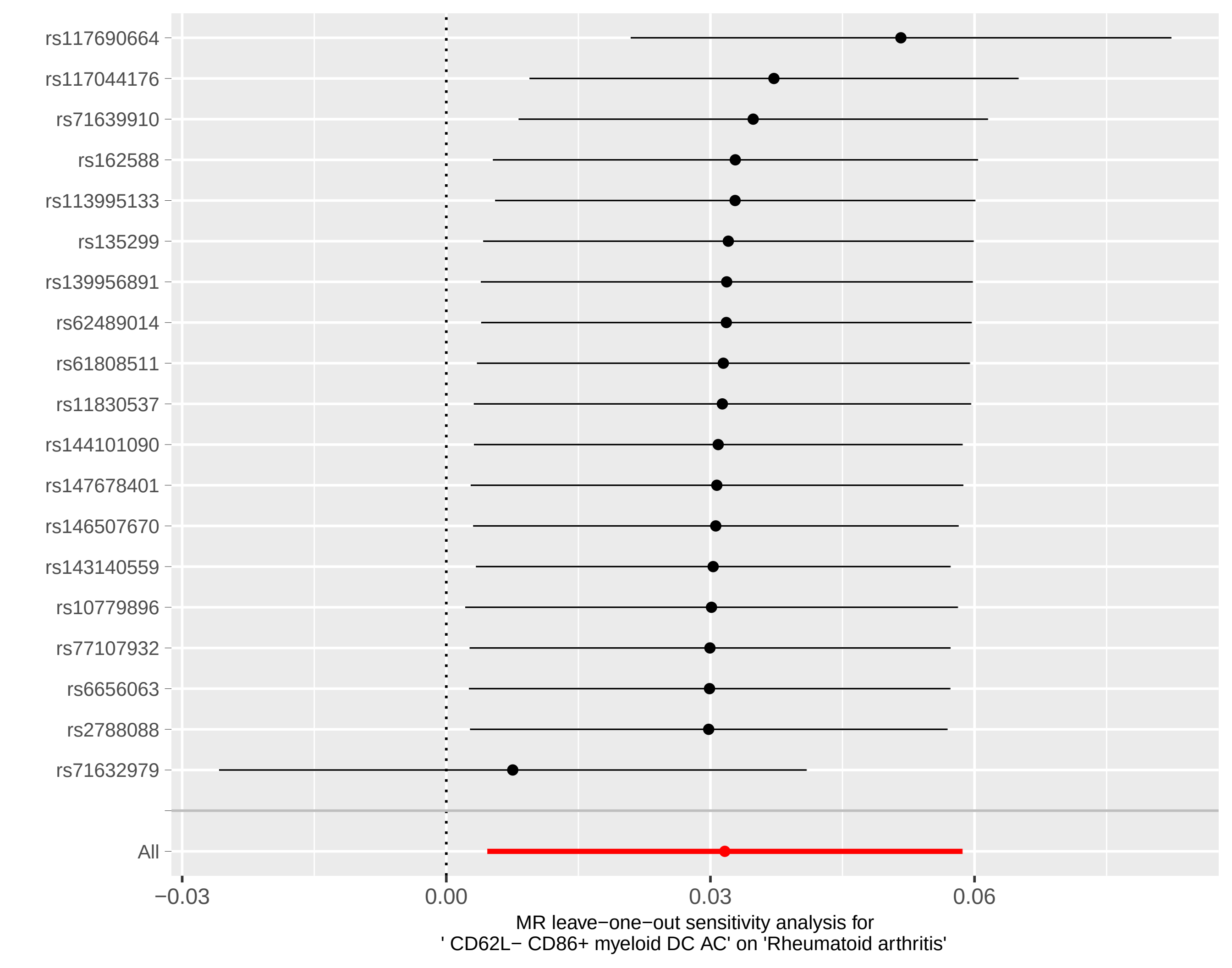
rs117690664
rs117044176
rs71639910
rs162588
rs113995133
rs135299
rs139956891
rs62489014
rs61808511
rs11830537
rs144101090
rs147678401
rs146507670
rs143140559
rs10779896
rs77107932
rs6656063
rs2788088
rs71632979
All
−0.03
0.00
0.03
0.06
MR leave−one−out sensitivity analysis for
' CD62L− CD86+ myeloid DC AC' on 'Rheumatoid arthritis'

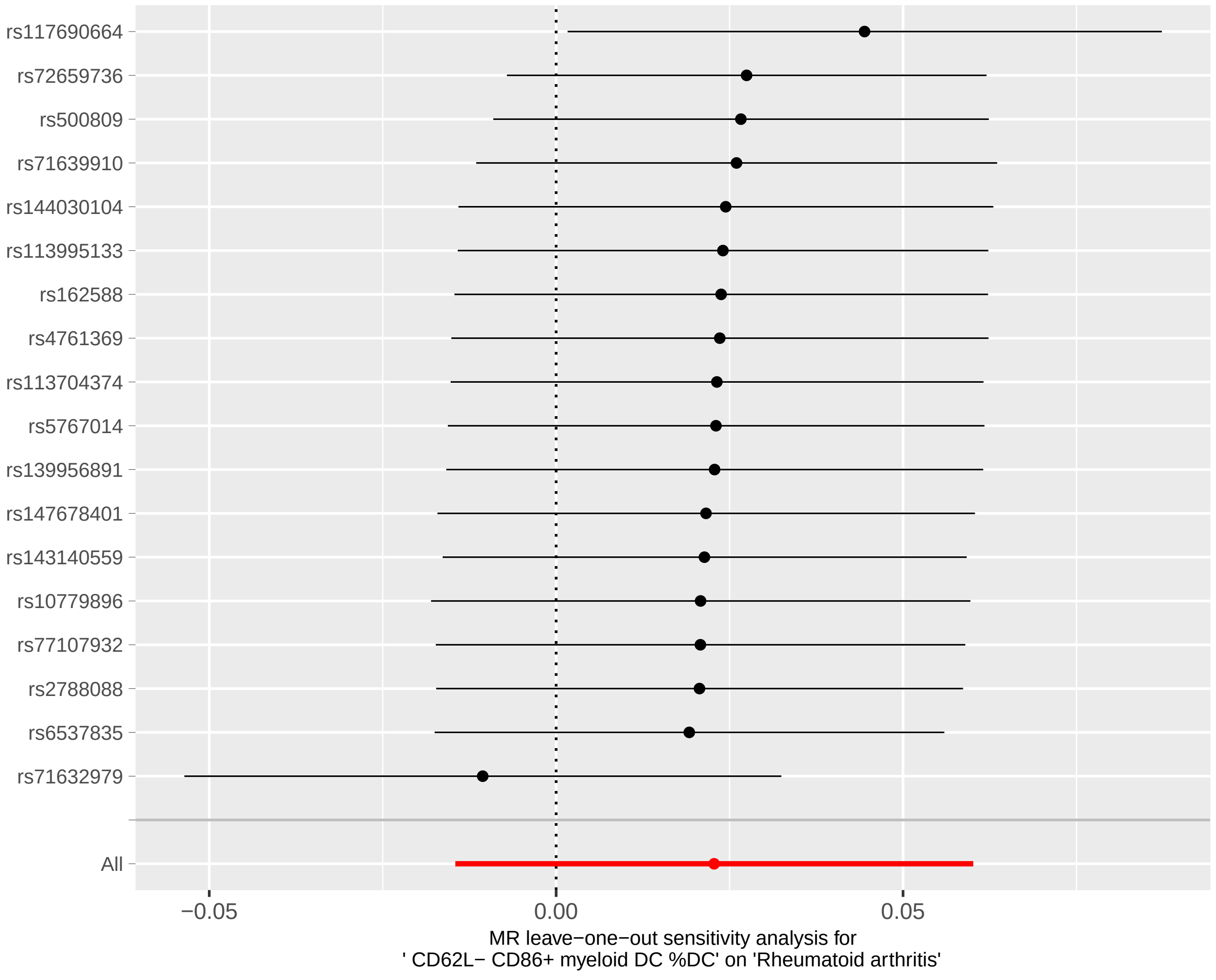
rs117690664
rs72659736
rs500809
rs71639910
rs144030104
rs113995133
rs162588
rs4761369
rs113704374
rs5767014
rs139956891
rs147678401
rs143140559
rs10779896
rs77107932
rs2788088
rs6537835
rs71632979
All
−0.05
0.00
0.05
MR leave−one−out sensitivity analysis for
' CD62L− CD86+ myeloid DC %DC' on 'Rheumatoid arthritis'

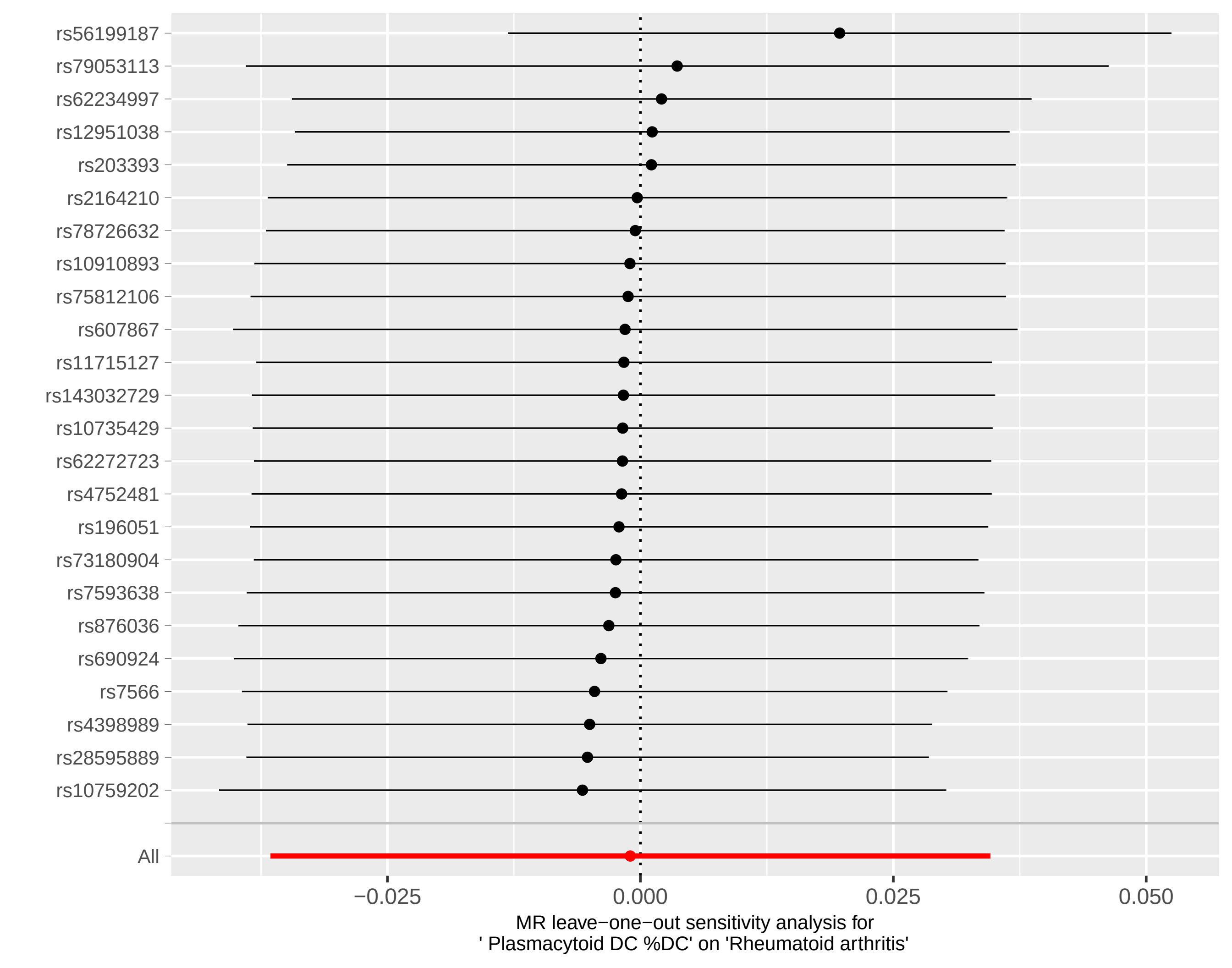
rs56199187
rs79053113
rs62234997
rs12951038
rs203393
rs2164210
rs78726632
rs10910893
rs75812106
rs607867
rs11715127
rs143032729
rs10735429
rs62272723
rs4752481
rs196051
rs73180904
rs7593638
rs876036
rs690924
rs7566
rs4398989
rs28595889
rs10759202
All
−0.025
0.000
0.025
0.050
MR leave−one−out sensitivity analysis for
' Plasmacytoid DC %DC' on 'Rheumatoid arthritis'

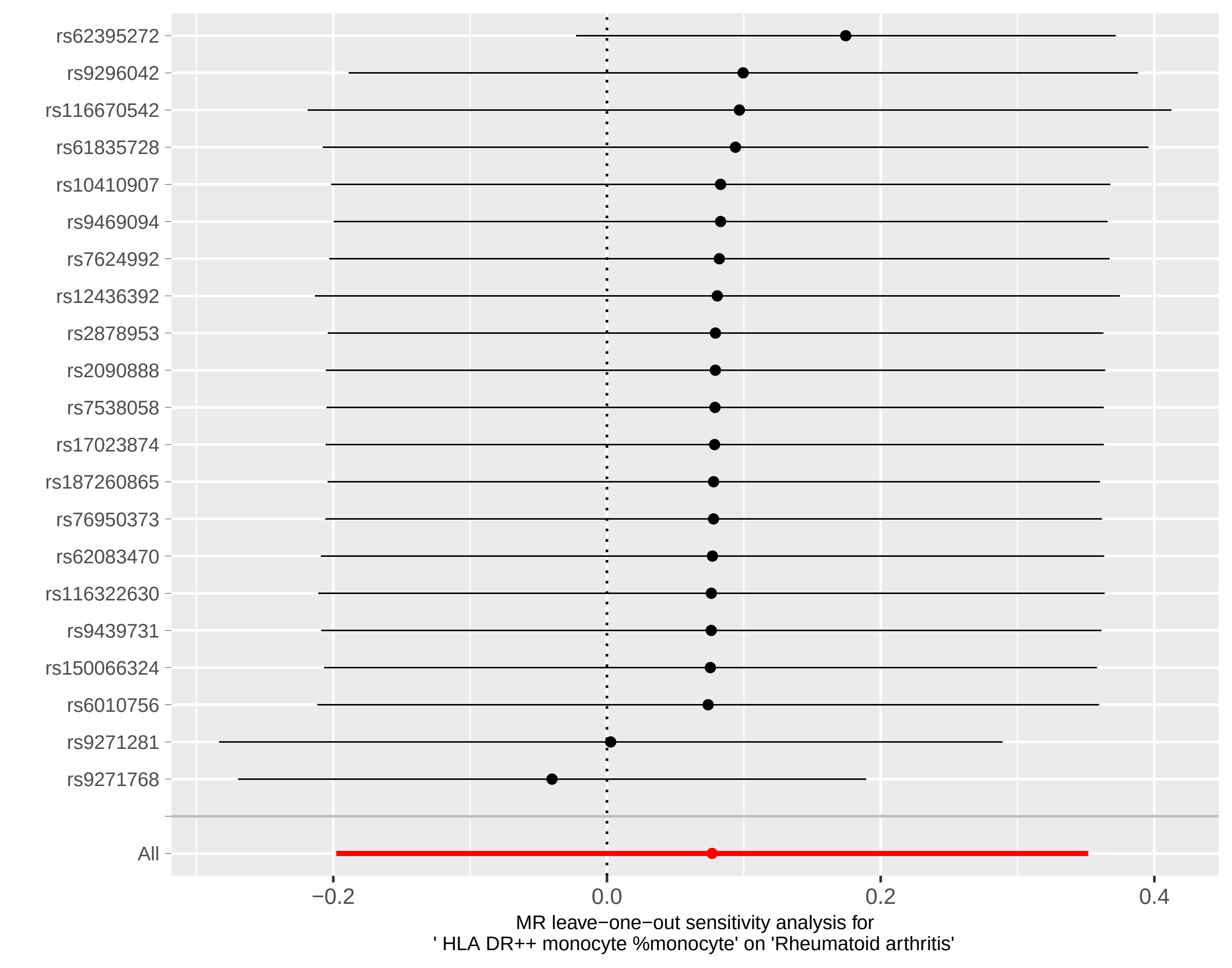

rs62395272
rs9296042
rs116670542
rs61835728
rs10410907
rs9469094
rs7624992
rs12436392
rs2878953
rs2090888
rs7538058
rs17023874
rs187260865
rs76950373
rs62083470
rs116322630
rs9439731
rs150066324
rs6010756
rs9271281
rs9271768
All
−0.2
0.0
0.2
0.4
MR leave−one−out sensitivity analysis for
' HLA DR++ monocyte %monocyte' on 'Rheumatoid arthritis'

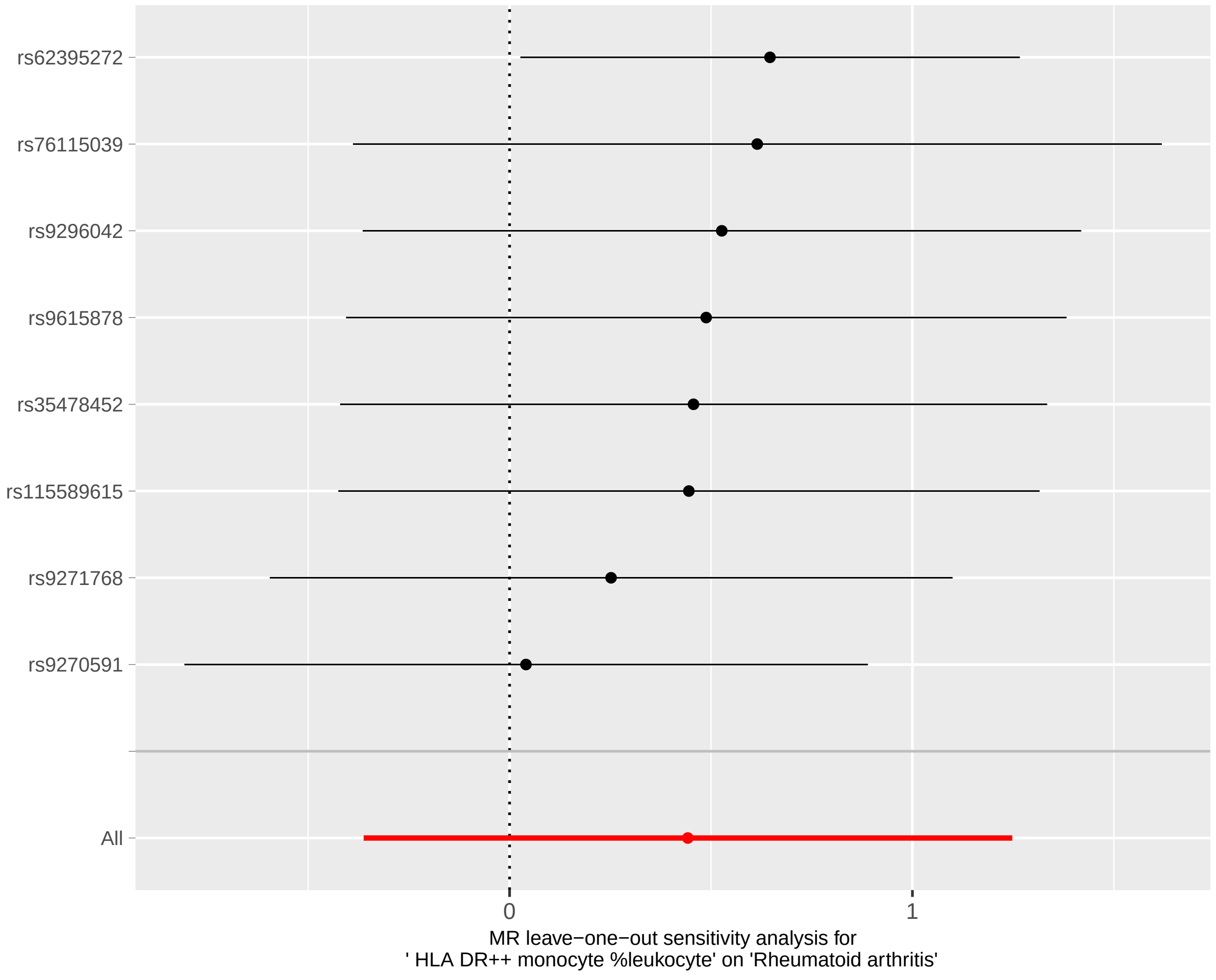

rs62395272
rs76115039
rs9296042
rs9615878
rs35478452
rs115589615
rs9271768
rs9270591
All
0
1
MR leave−one−out sensitivity analysis for
' HLA DR++ monocyte %leukocyte' on 'Rheumatoid arthritis'

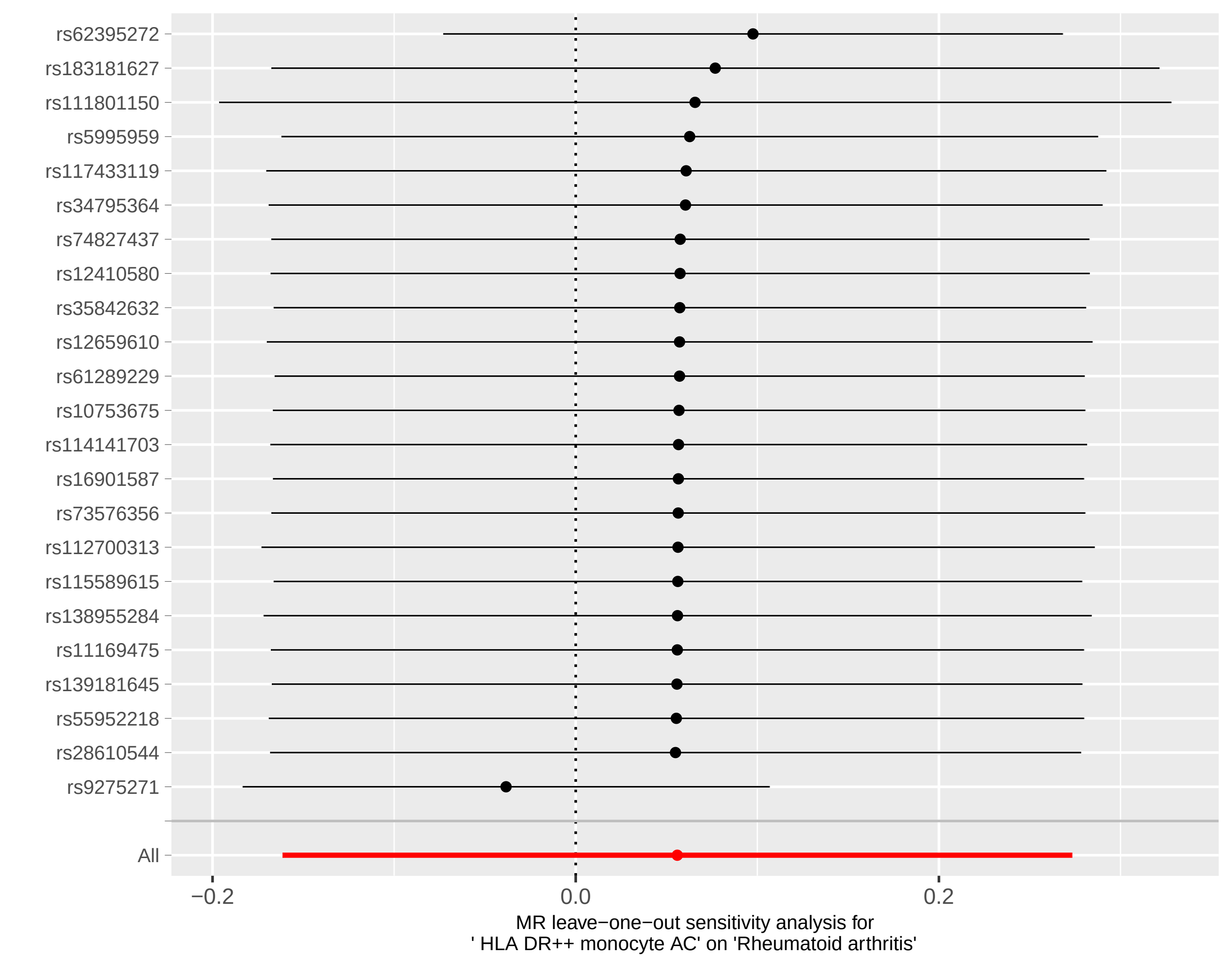
rs62395272
rs183181627
rs111801150
rs5995959
rs117433119
rs34795364
rs74827437
rs12410580
rs35842632
rs12659610
rs61289229
rs10753675
rs114141703
rs16901587
rs73576356
rs112700313
rs115589615
rs138955284
rs11169475
rs139181645
rs55952218
rs28610544
rs9275271
All
−0.2
0.0
0.2
MR leave−one−out sensitivity analysis for
' HLA DR++ monocyte AC' on 'Rheumatoid arthritis'

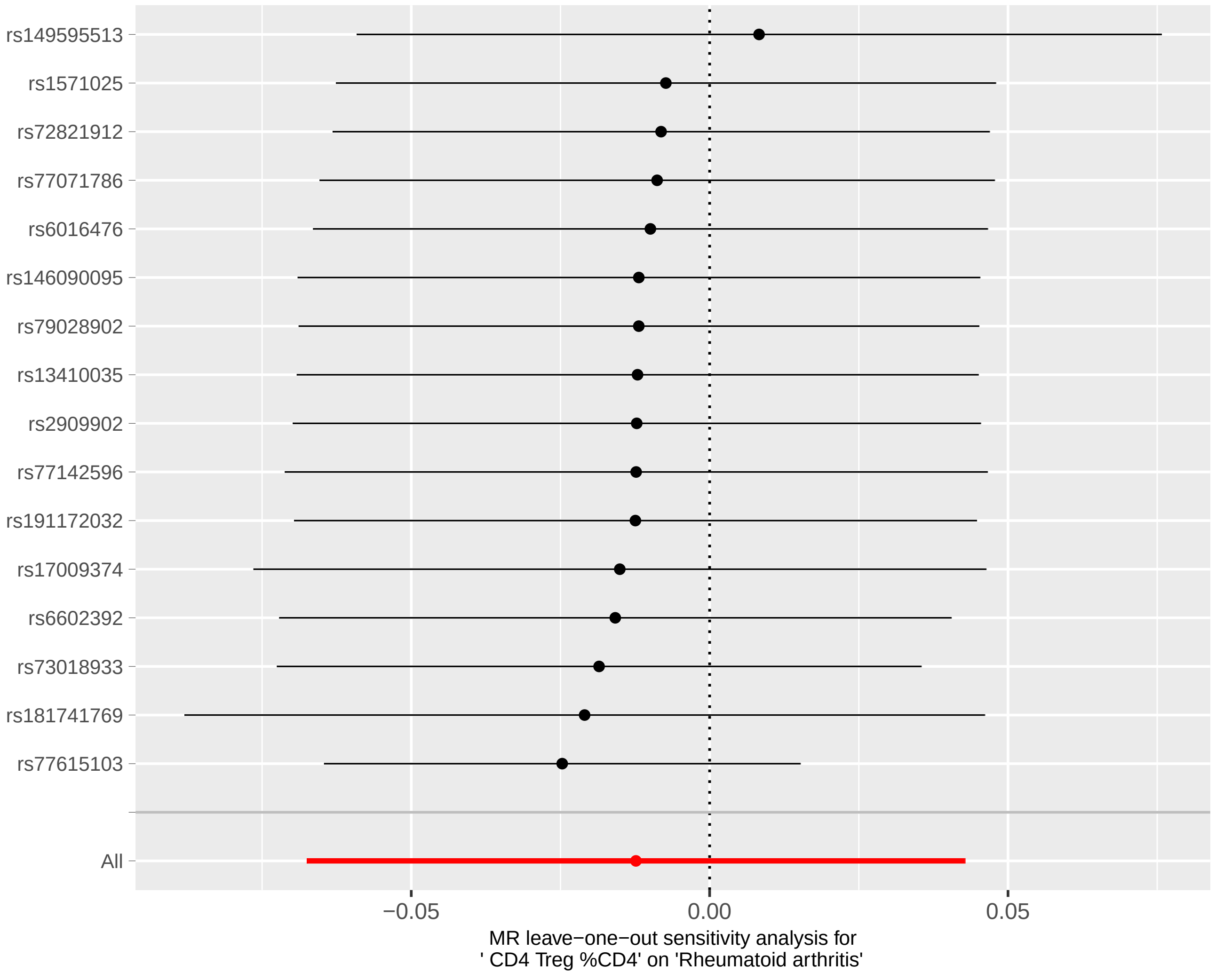

rs149595513
rs1571025
rs72821912
rs77071786
rs6016476
rs146090095
rs79028902
rs13410035
rs2909902
rs77142596
rs191172032
rs17009374
rs6602392
rs73018933
rs181741769
rs77615103
All
−0.05
0.00
0.05
MR leave−one−out sensitivity analysis for
' CD4 Treg %CD4' on 'Rheumatoid arthritis'

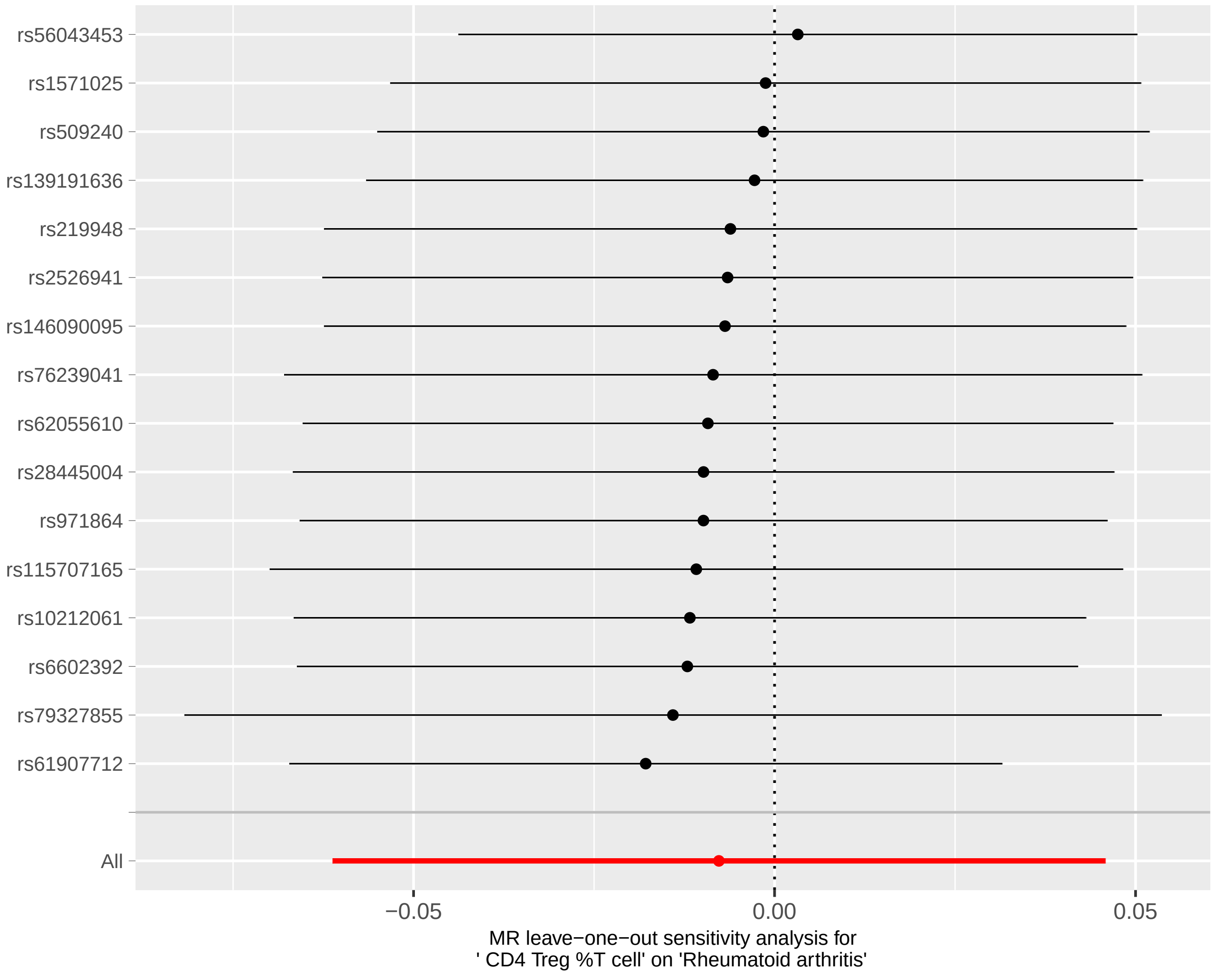
rs56043453
rs1571025
rs509240
rs139191636
rs219948
rs2526941
rs146090095
rs76239041
rs62055610
rs28445004
rs971864
rs115707165
rs10212061
rs6602392
rs79327855
rs61907712
All
−0.05
0.00
0.05
MR leave−one−out sensitivity analysis for
' CD4 Treg %T cell' on 'Rheumatoid arthritis'

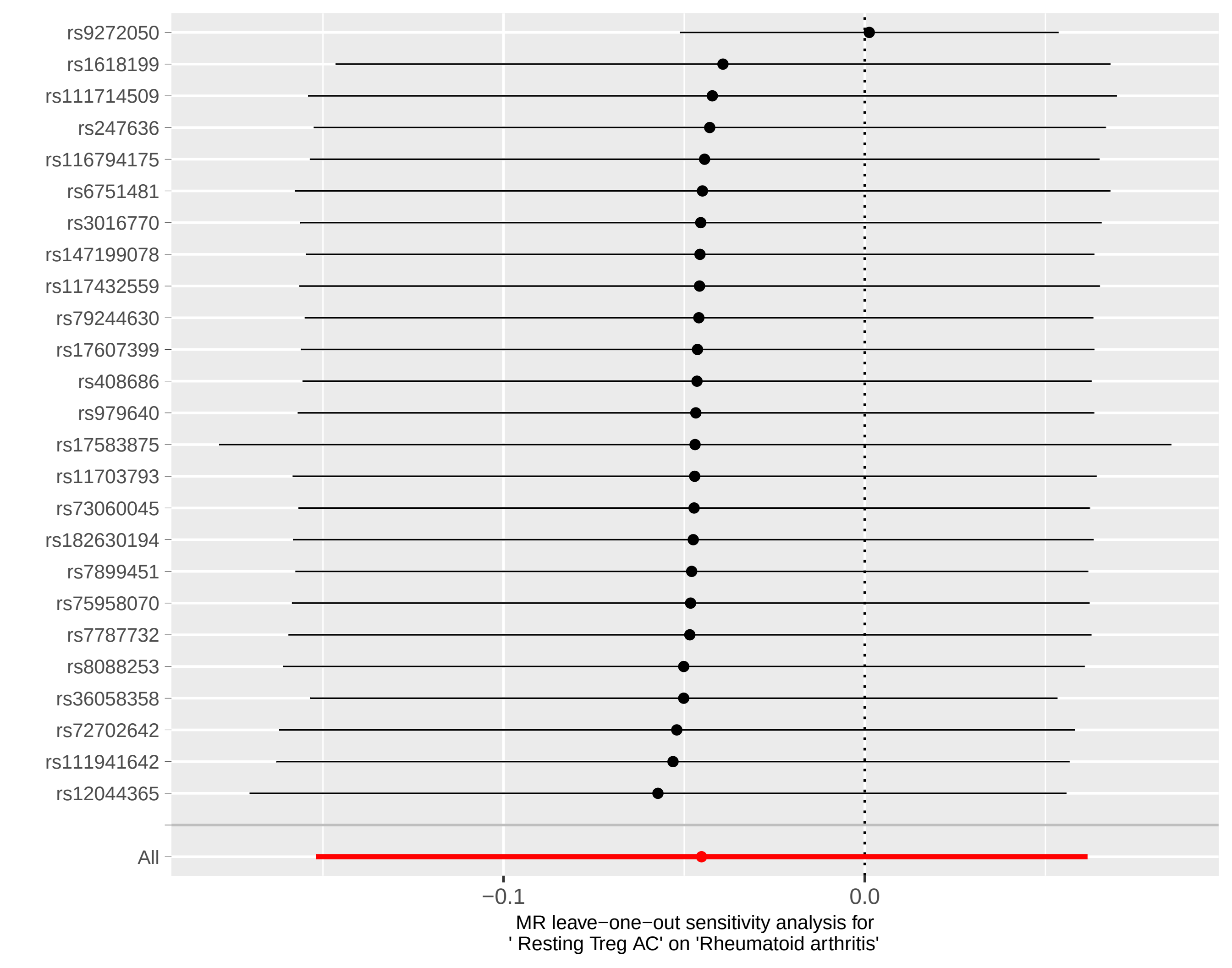
rs9272050
rs1618199
rs111714509
rs247636
rs116794175
rs6751481
rs3016770
rs147199078
rs117432559
rs79244630
rs17607399
rs408686
rs979640
rs17583875
rs11703793
rs73060045
rs182630194
rs7899451
rs75958070
rs7787732
rs8088253
rs36058358
rs72702642
rs111941642
rs12044365
All
−0.1
0.0
MR leave−one−out sensitivity analysis for
' Resting Treg AC' on 'Rheumatoid arthritis'

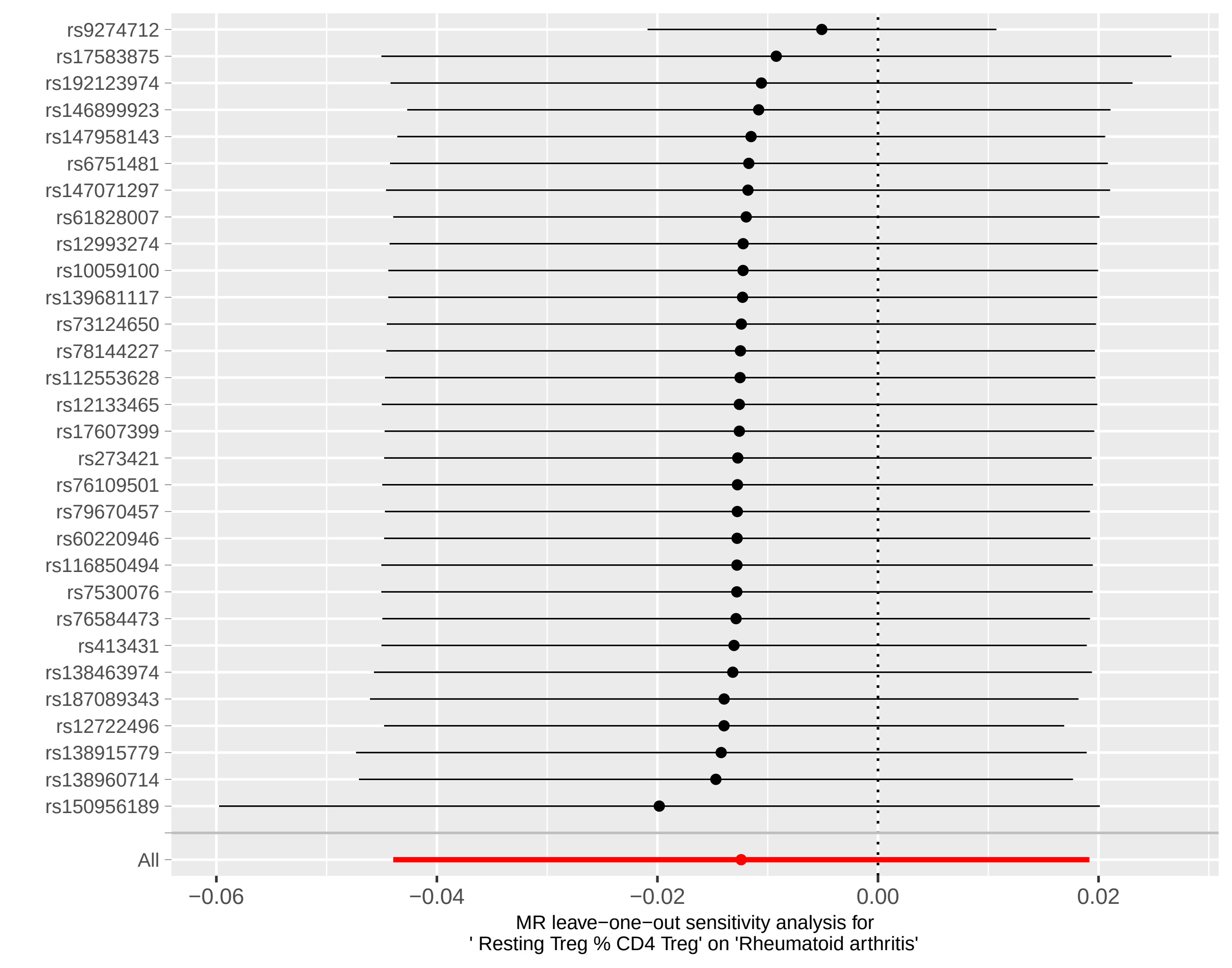
rs9274712
rs17583875
rs192123974
rs146899923
rs147958143
rs6751481
rs147071297
rs61828007
rs12993274
rs10059100
rs139681117
rs73124650
rs78144227
rs112553628
rs12133465
rs17607399
rs273421
rs76109501
rs79670457
rs60220946
rs116850494
rs7530076
rs76584473
rs413431
rs138463974
rs187089343
rs12722496
rs138915779
rs138960714
rs150956189
All
−0.06
−0.04
−0.02
0.00
0.02
MR leave−one−out sensitivity analysis for
' Resting Treg % CD4 Treg' on 'Rheumatoid arthritis'

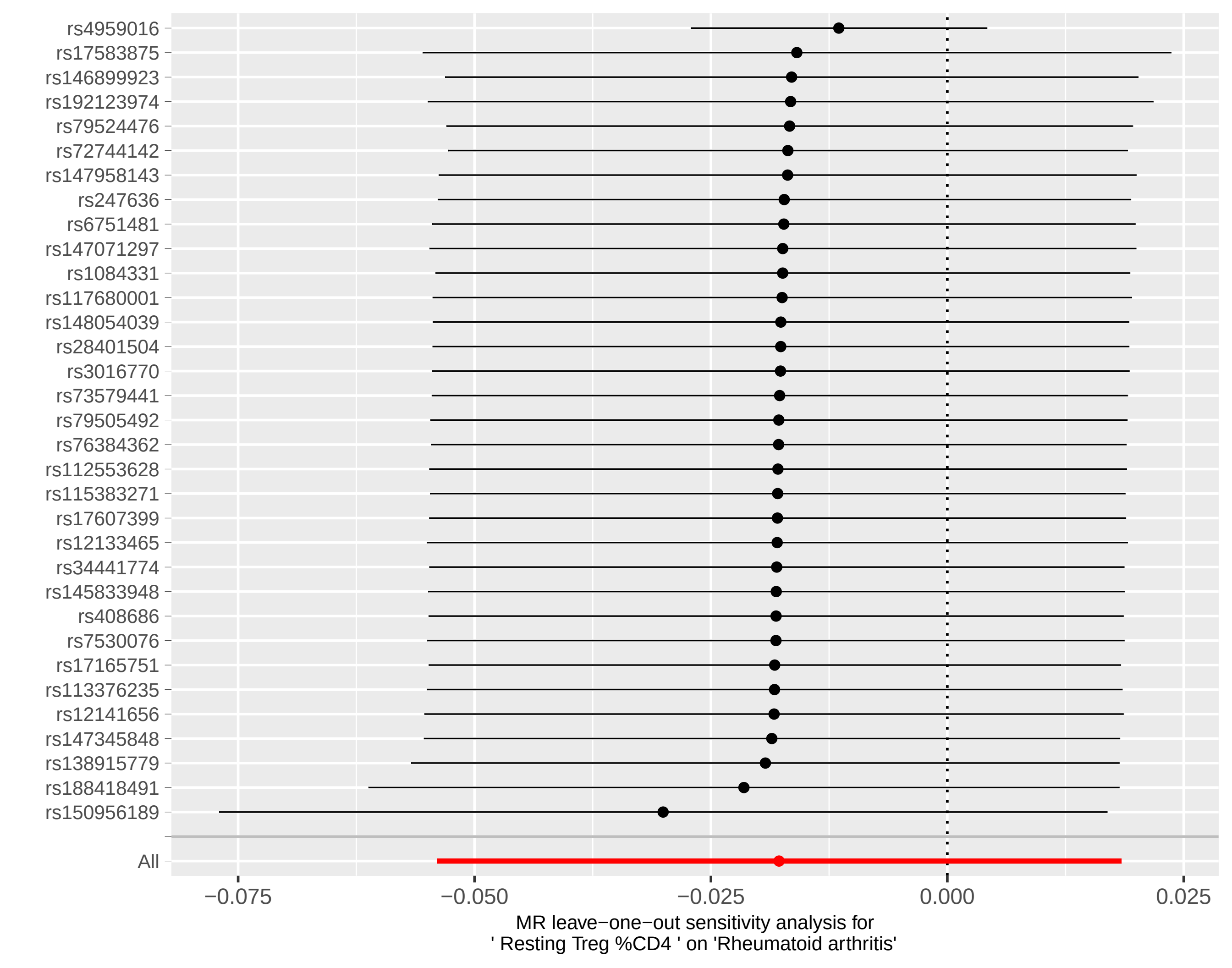
rs4959016
rs17583875
rs146899923
rs192123974
rs79524476
rs72744142
rs147958143
rs247636
rs6751481
rs147071297
rs1084331
rs117680001
rs148054039
rs28401504
rs3016770
rs73579441
rs79505492
rs76384362
rs112553628
rs115383271
rs17607399
rs12133465
rs34441774
rs145833948
rs408686
rs7530076
rs17165751
rs113376235
rs12141656
rs147345848
rs138915779
rs188418491
rs150956189
All
−0.075
−0.050
−0.025
0.000
0.025
MR leave−one−out sensitivity analysis for
' Resting Treg %CD4 ' on 'Rheumatoid arthritis'

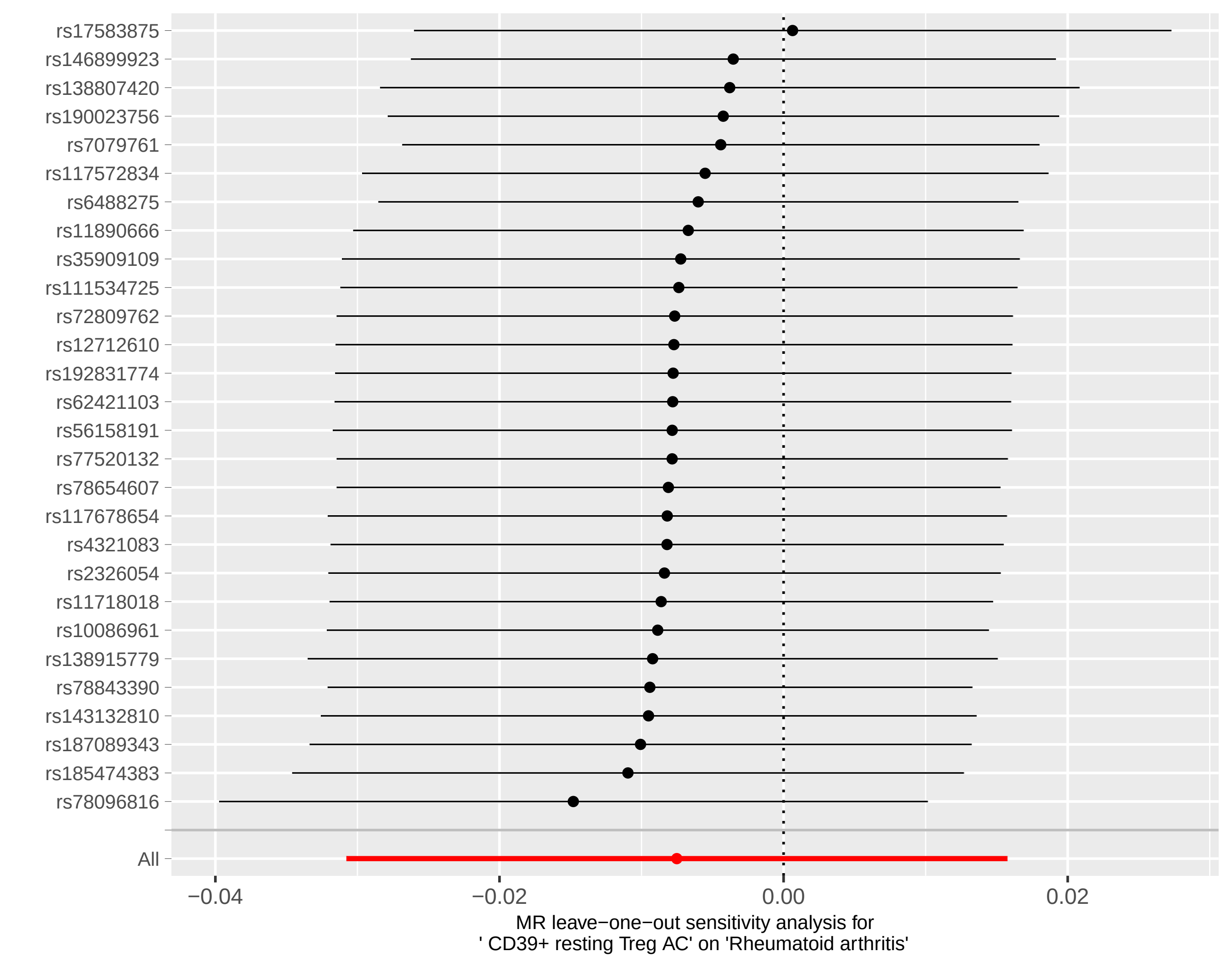

rs17583875
rs146899923
rs138807420
rs190023756
rs7079761
rs117572834
rs6488275
rs11890666
rs35909109
rs111534725
rs72809762
rs12712610
rs192831774
rs62421103
rs56158191
rs77520132
rs78654607
rs117678654
rs4321083
rs2326054
rs11718018
rs10086961
rs138915779
rs78843390
rs143132810
rs187089343
rs185474383
rs78096816
All
−0.04
−0.02
0.00
0.02
MR leave−one−out sensitivity analysis for
' CD39+ resting Treg AC' on 'Rheumatoid arthritis'

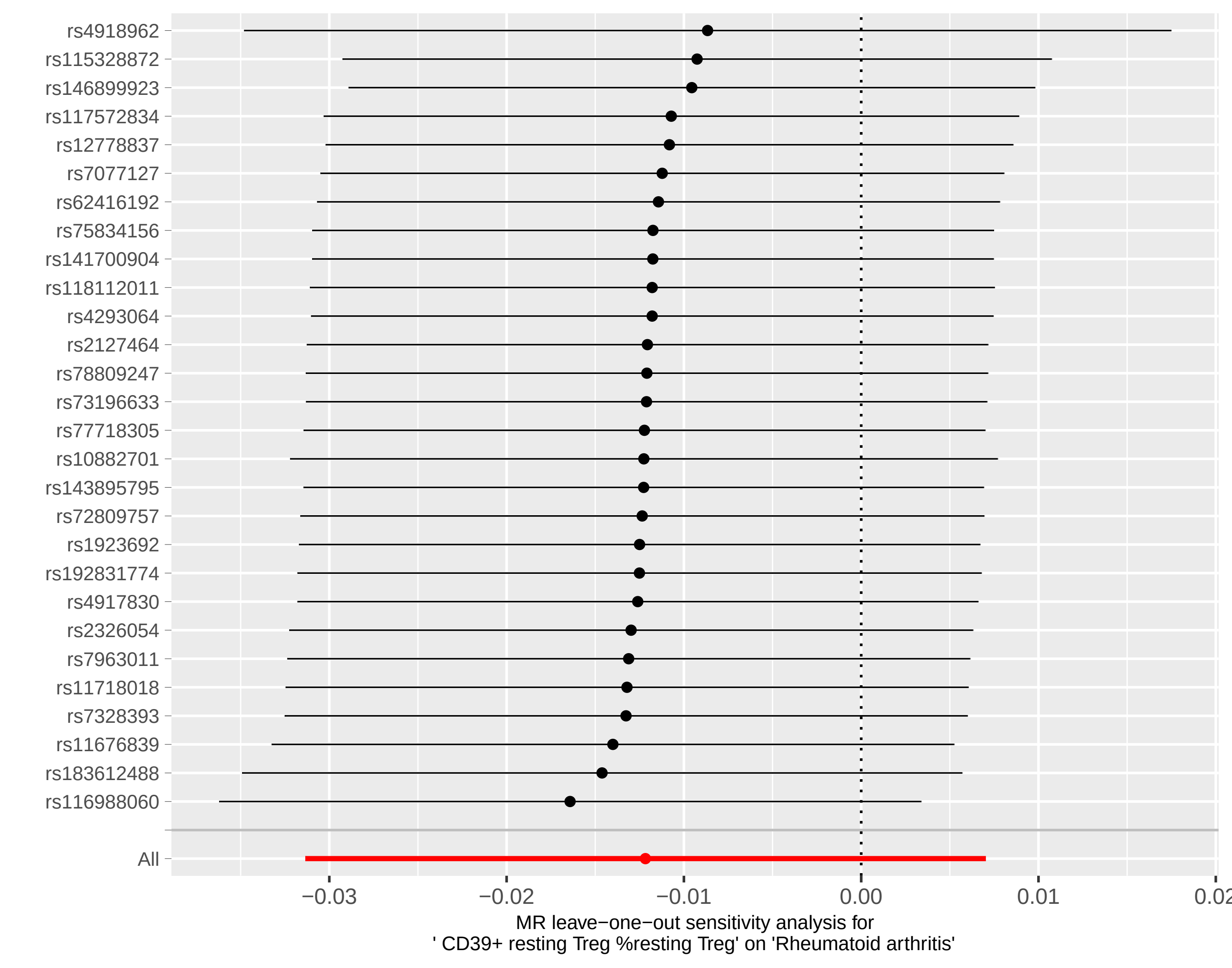

rs4918962
rs115328872
rs146899923
rs117572834
rs12778837
rs7077127
rs62416192
rs75834156
rs141700904
rs118112011
rs4293064
rs2127464
rs78809247
rs73196633
rs77718305
rs10882701
rs143895795
rs72809757
rs1923692
rs192831774
rs4917830
rs2326054
rs7963011
rs11718018
rs7328393
rs11676839
rs183612488
rs116988060
All
−0.03
−0.02
−0.01
0.00
0.01
MR leave−one−out sensitivity analysis for
' CD39+ resting Treg %resting Treg' on 'Rheumatoid arthritis'

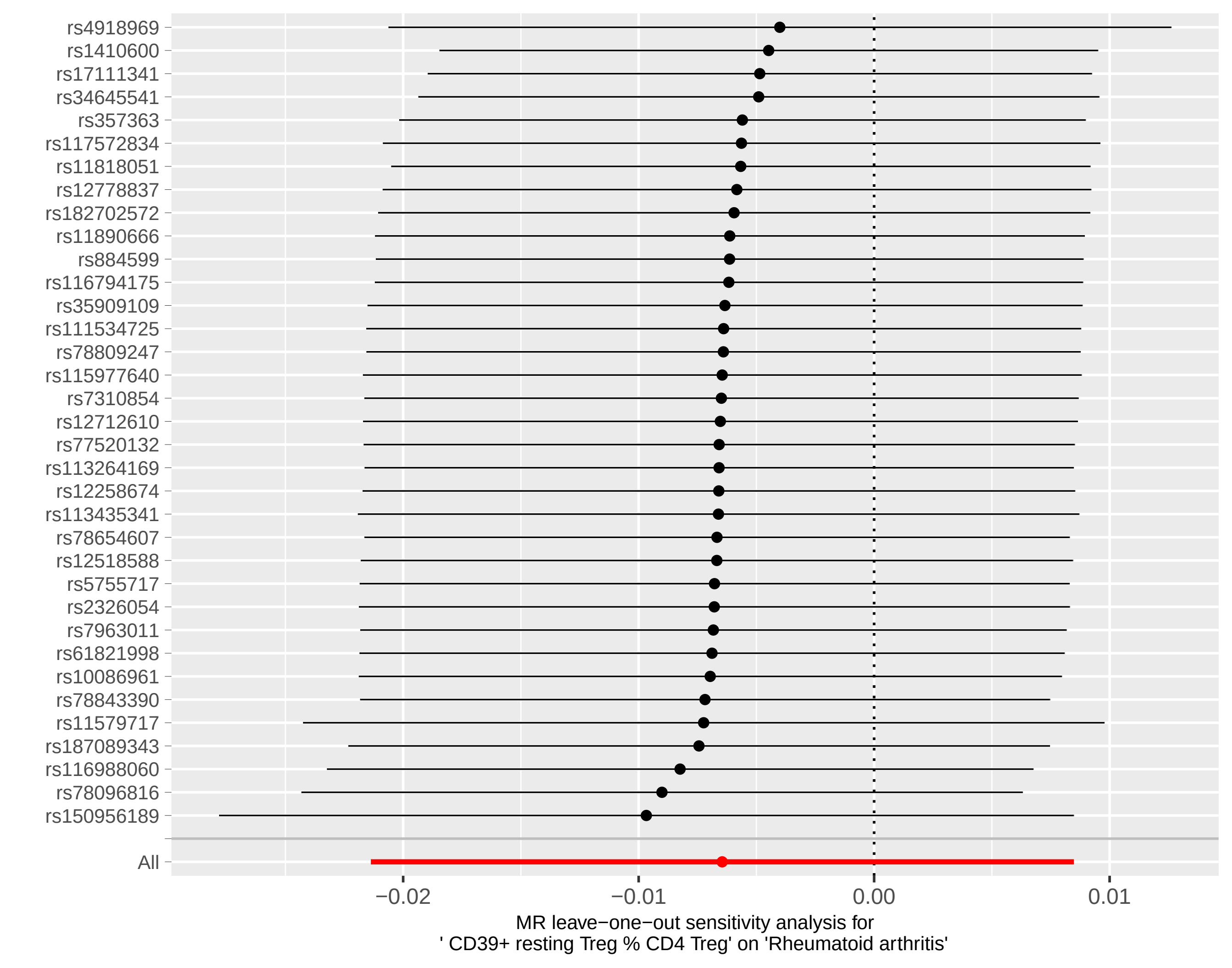
rs4918969
rs1410600
rs17111341
rs34645541
rs357363
rs117572834
rs11818051
rs12778837
rs182702572
rs11890666
rs884599
rs116794175
rs35909109
rs111534725
rs78809247
rs115977640
rs7310854
rs12712610
rs77520132
rs113264169
rs12258674
rs113435341
rs78654607
rs12518588
rs5755717
rs2326054
rs7963011
rs61821998
rs10086961
rs78843390
rs11579717
rs187089343
rs116988060
rs78096816
rs150956189
All
−0.02
−0.01
0.00
0.01
MR leave−one−out sensitivity analysis for
' CD39+ resting Treg % CD4 Treg' on 'Rheumatoid arthritis'

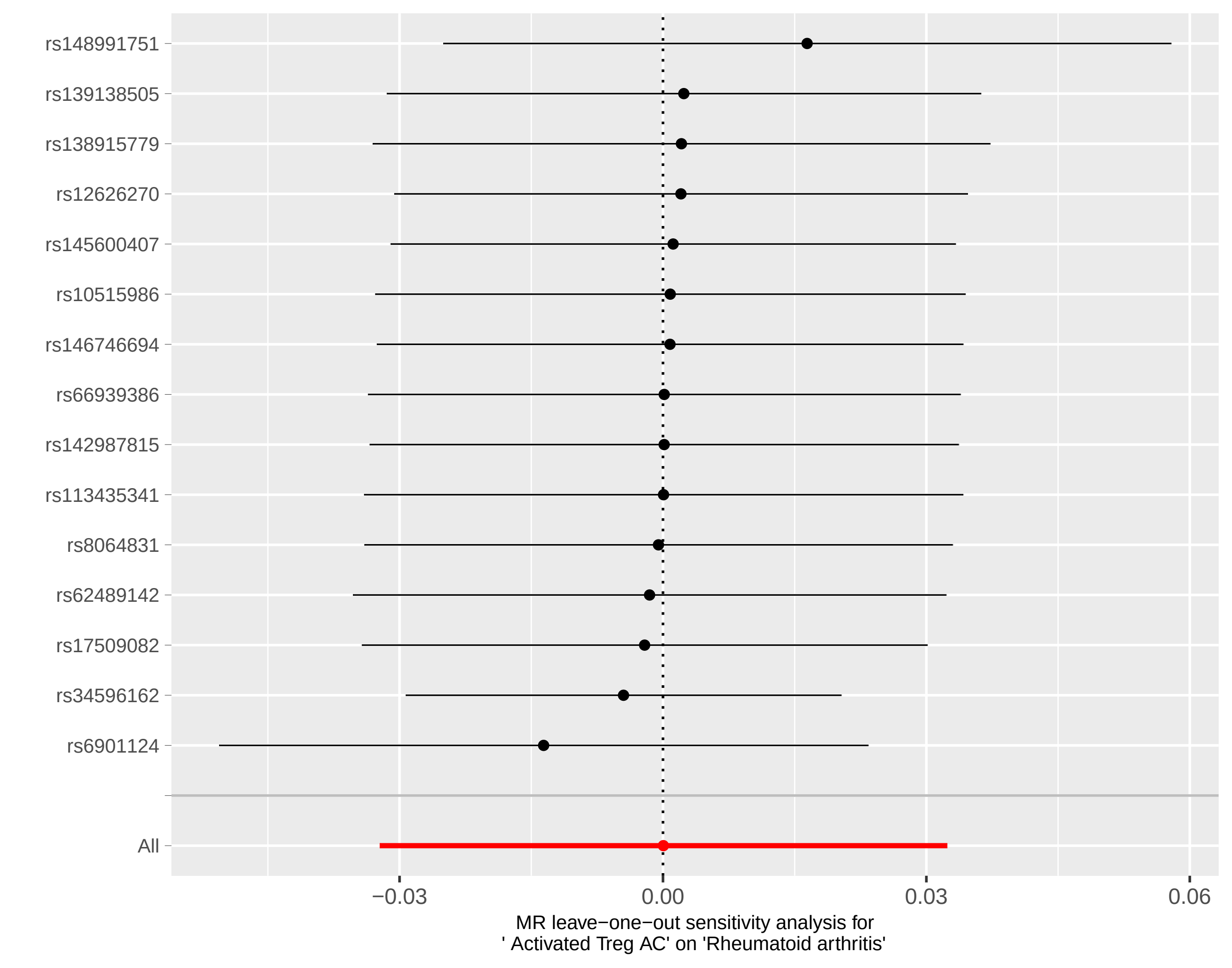

rs148991751
rs139138505
rs138915779
rs12626270
rs145600407
rs10515986
rs146746694
rs66939386
rs142987815
rs113435341
rs8064831
rs62489142
rs17509082
rs34596162
rs6901124
All
−0.03
0.00
0.03
0.06
MR leave−one−out sensitivity analysis for
' Activated Treg AC' on 'Rheumatoid arthritis'

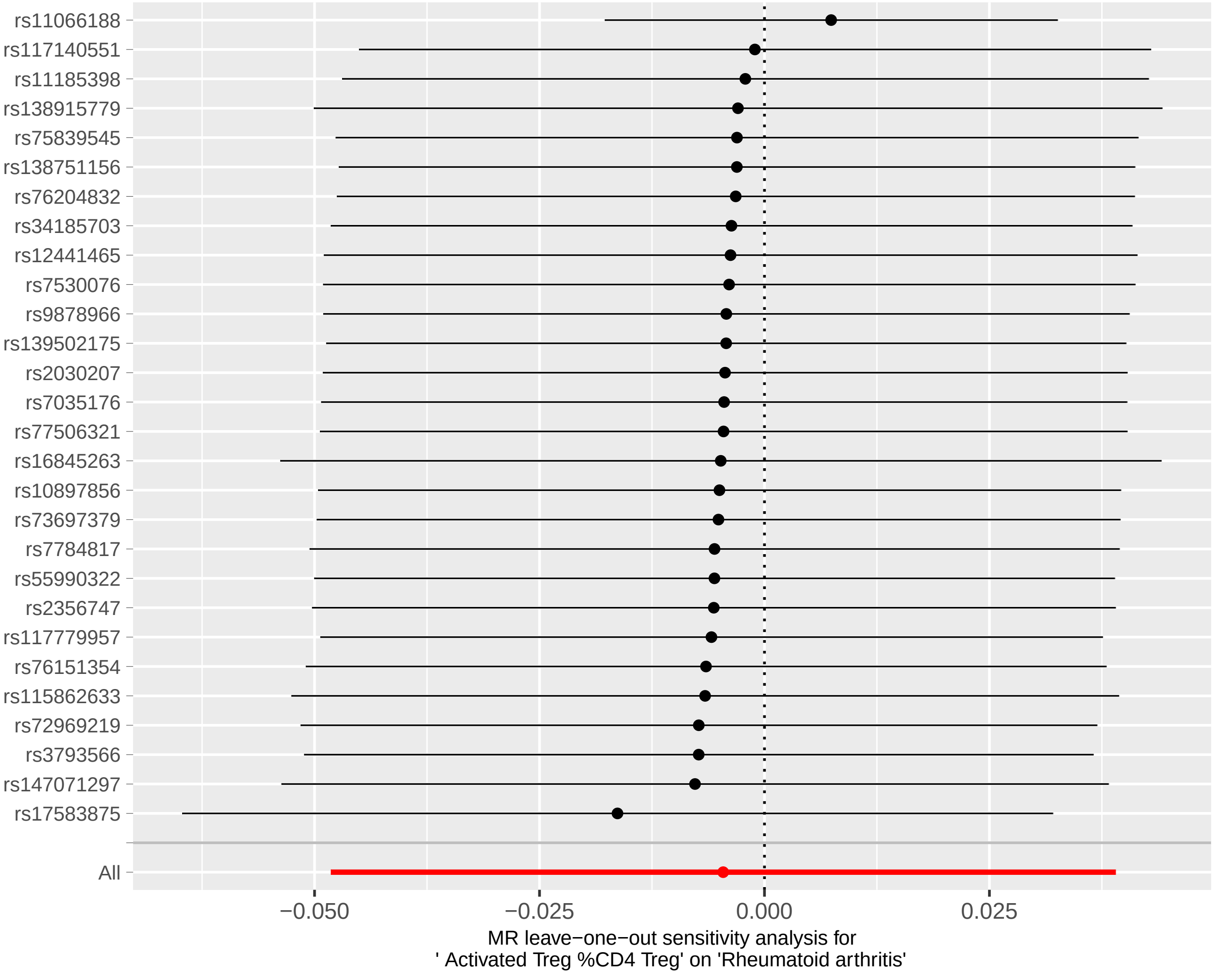
rs11066188
rs117140551
rs11185398
rs138915779
rs75839545
rs138751156
rs76204832
rs34185703
rs12441465
rs7530076
rs9878966
rs139502175
rs2030207
rs7035176
rs77506321
rs16845263
rs10897856
rs73697379
rs7784817
rs55990322
rs2356747
rs117779957
rs76151354
rs115862633
rs72969219
rs3793566
rs147071297
rs17583875
All
−0.050
−0.025
0.000
0.025
MR leave−one−out sensitivity analysis for
' Activated Treg %CD4 Treg' on 'Rheumatoid arthritis'

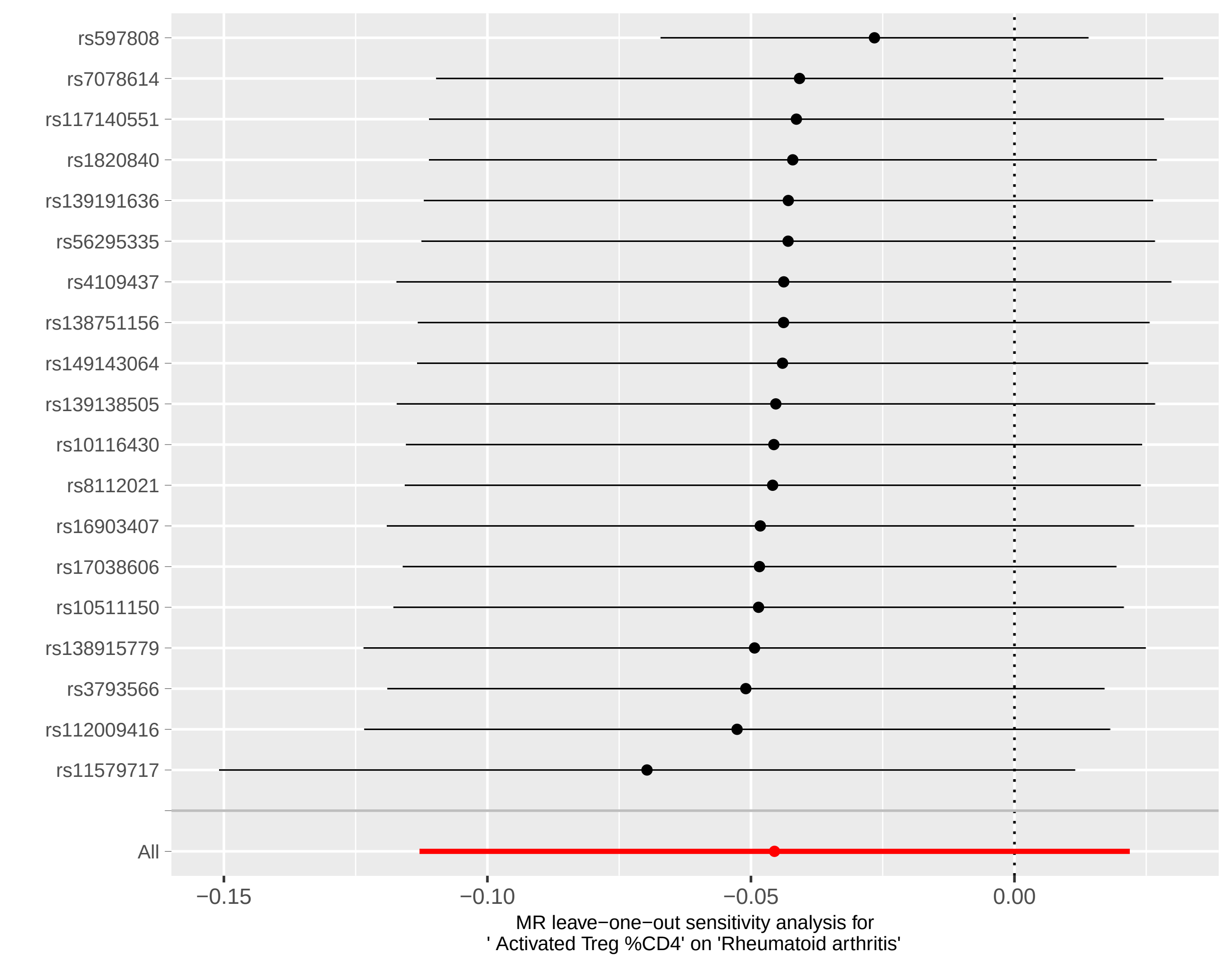

rs597808
rs7078614
rs117140551
rs1820840
rs139191636
rs56295335
rs4109437
rs138751156
rs149143064
rs139138505
rs10116430
rs8112021
rs16903407
rs17038606
rs10511150
rs138915779
rs3793566
rs112009416
rs11579717
All
−0.15
−0.10
−0.05
0.00
MR leave−one−out sensitivity analysis for
' Activated Treg %CD4' on 'Rheumatoid arthritis'

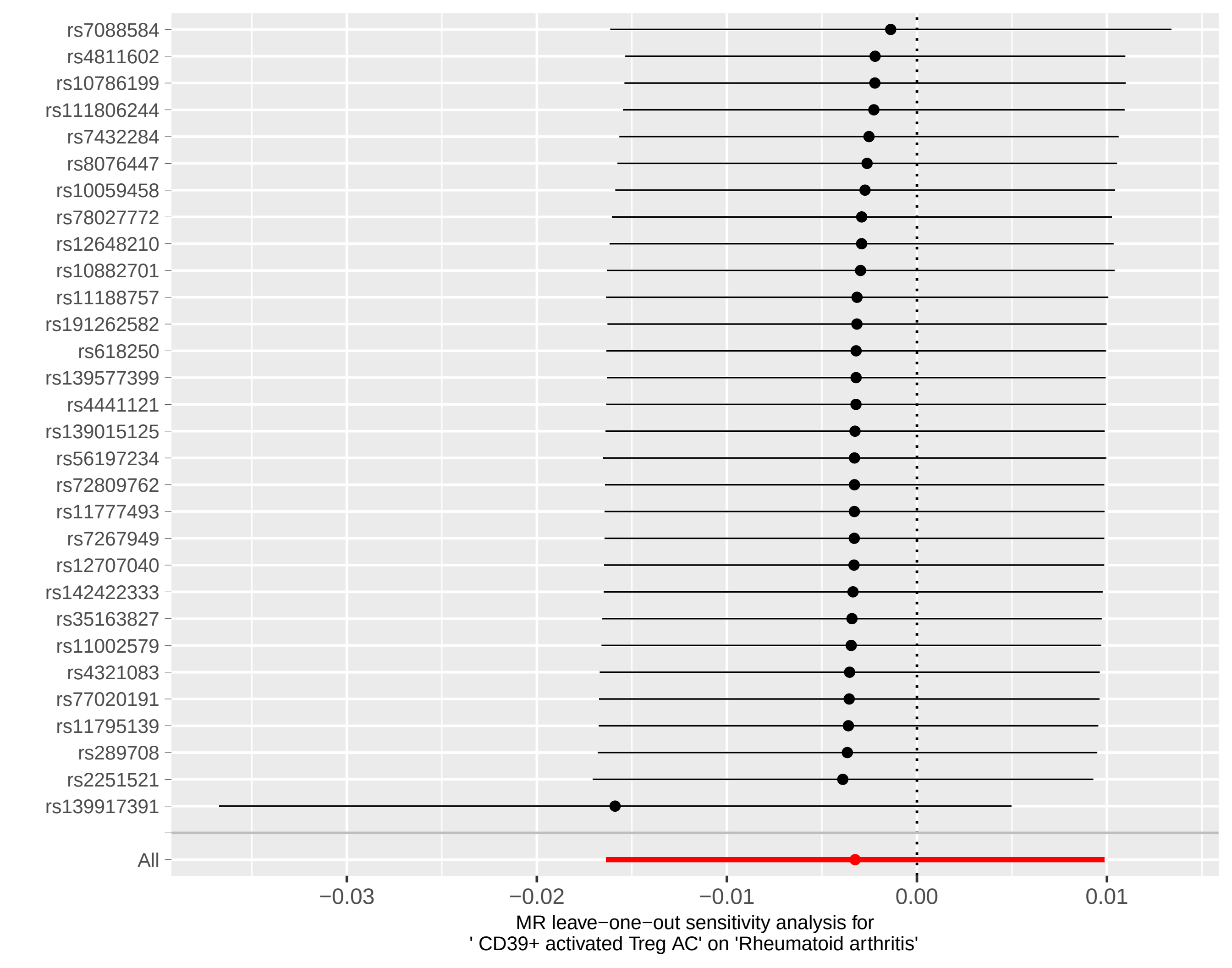

rs7088584
rs4811602
rs10786199
rs111806244
rs7432284
rs8076447
rs10059458
rs78027772
rs12648210
rs10882701
rs11188757
rs191262582
rs618250
rs139577399
rs4441121
rs139015125
rs56197234
rs72809762
rs11777493
rs7267949
rs12707040
rs142422333
rs35163827
rs11002579
rs4321083
rs77020191
rs11795139
rs289708
rs2251521
rs139917391
All
−0.03
−0.02
−0.01
0.00
0.01
MR leave−one−out sensitivity analysis for
' CD39+ activated Treg AC' on 'Rheumatoid arthritis'

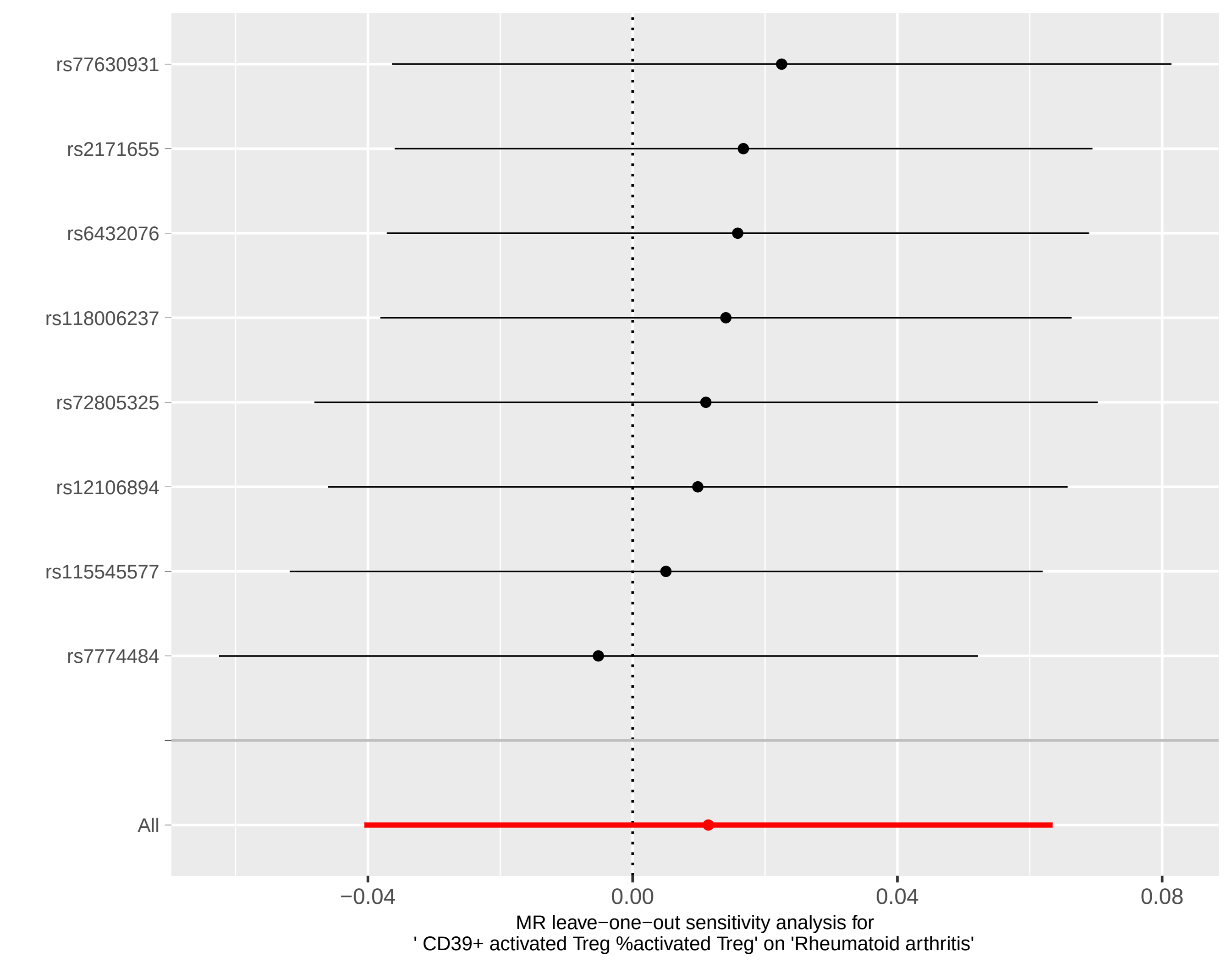
rs77630931
rs2171655
rs6432076
rs118006237
rs72805325
rs12106894
rs115545577
rs7774484
All
−0.04
0.00
0.04
0.08
MR leave−one−out sensitivity analysis for
' CD39+ activated Treg %activated Treg' on 'Rheumatoid arthritis'

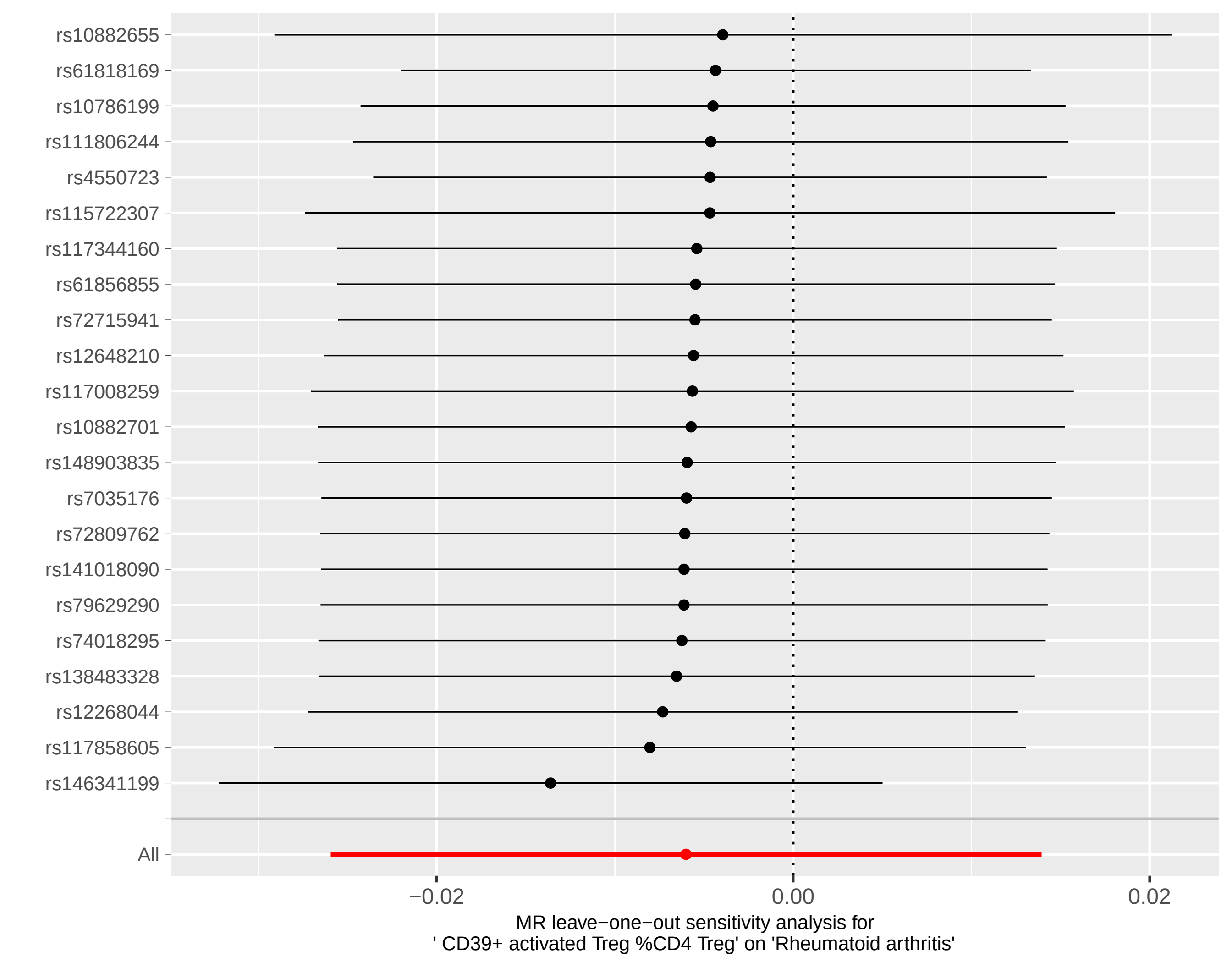
rs10882655
rs61818169
rs10786199
rs111806244
rs4550723
rs115722307
rs117344160
rs61856855
rs72715941
rs12648210
rs117008259
rs10882701
rs148903835
rs7035176
rs72809762
rs141018090
rs79629290
rs74018295
rs138483328
rs12268044
rs117858605
rs146341199
All
−0.02
0.00
0.02
MR leave−one−out sensitivity analysis for
' CD39+ activated Treg %CD4 Treg' on 'Rheumatoid arthritis'

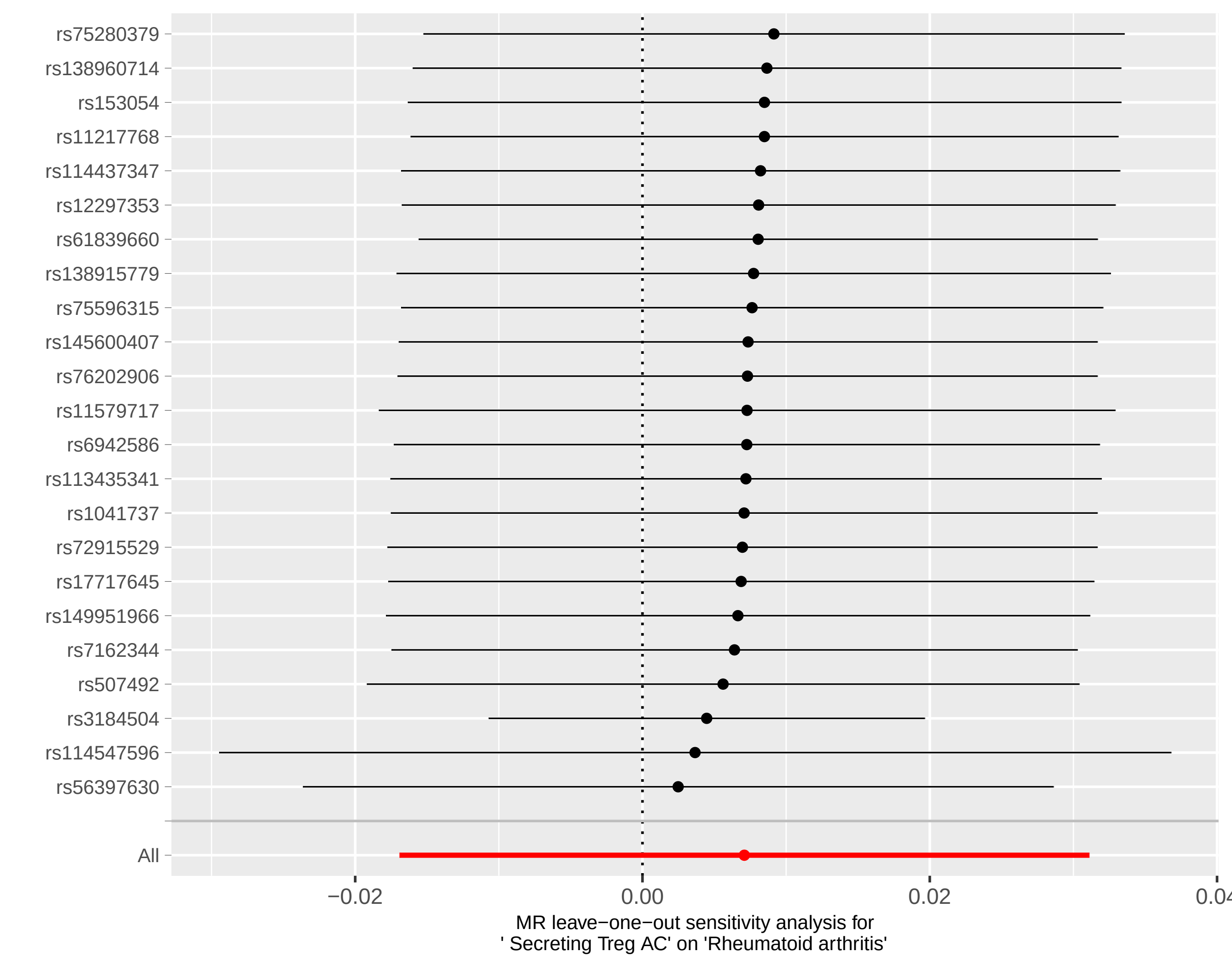

rs75280379
rs138960714
rs153054
rs11217768
rs114437347
rs12297353
rs61839660
rs138915779
rs75596315
rs145600407
rs76202906
rs11579717
rs6942586
rs113435341
rs1041737
rs72915529
rs17717645
rs149951966
rs7162344
rs507492
rs3184504
rs114547596
rs56397630
All
−0.02
0.00
0.02
MR leave−one−out sensitivity analysis for
' Secreting Treg AC' on 'Rheumatoid arthritis'

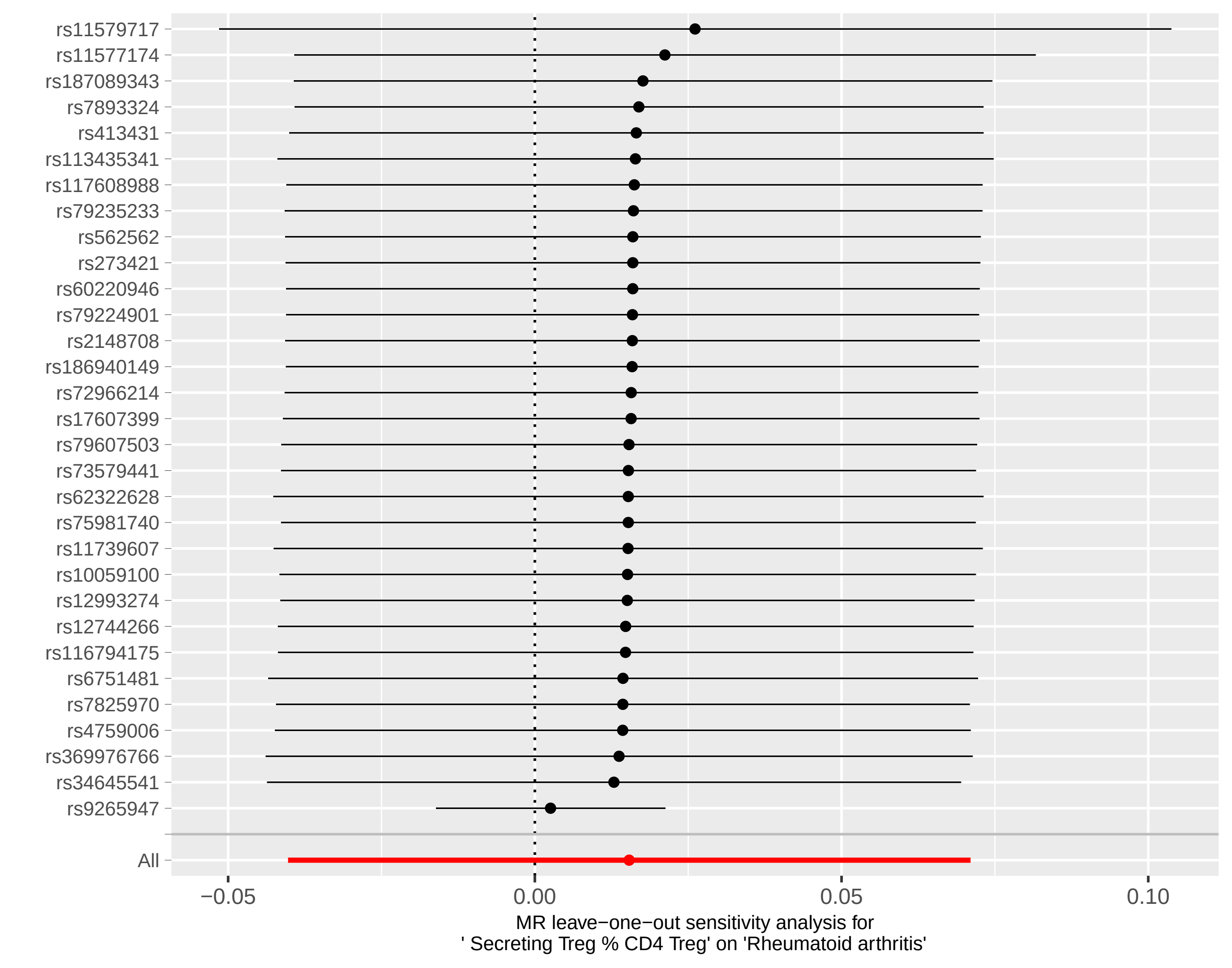
rs11579717
rs11577174
rs187089343
rs7893324
rs413431
rs113435341
rs117608988
rs79235233
rs562562
rs273421
rs60220946
rs79224901
rs2148708
rs186940149
rs72966214
rs17607399
rs79607503
rs73579441
rs62322628
rs75981740
rs11739607
rs10059100
rs12993274
rs12744266
rs116794175
rs6751481
rs7825970
rs4759006
rs369976766
rs34645541
rs9265947
All
−0.05
0.00
0.05
0.10
MR leave−one−out sensitivity analysis for
' Secreting Treg % CD4 Treg' on 'Rheumatoid arthritis'

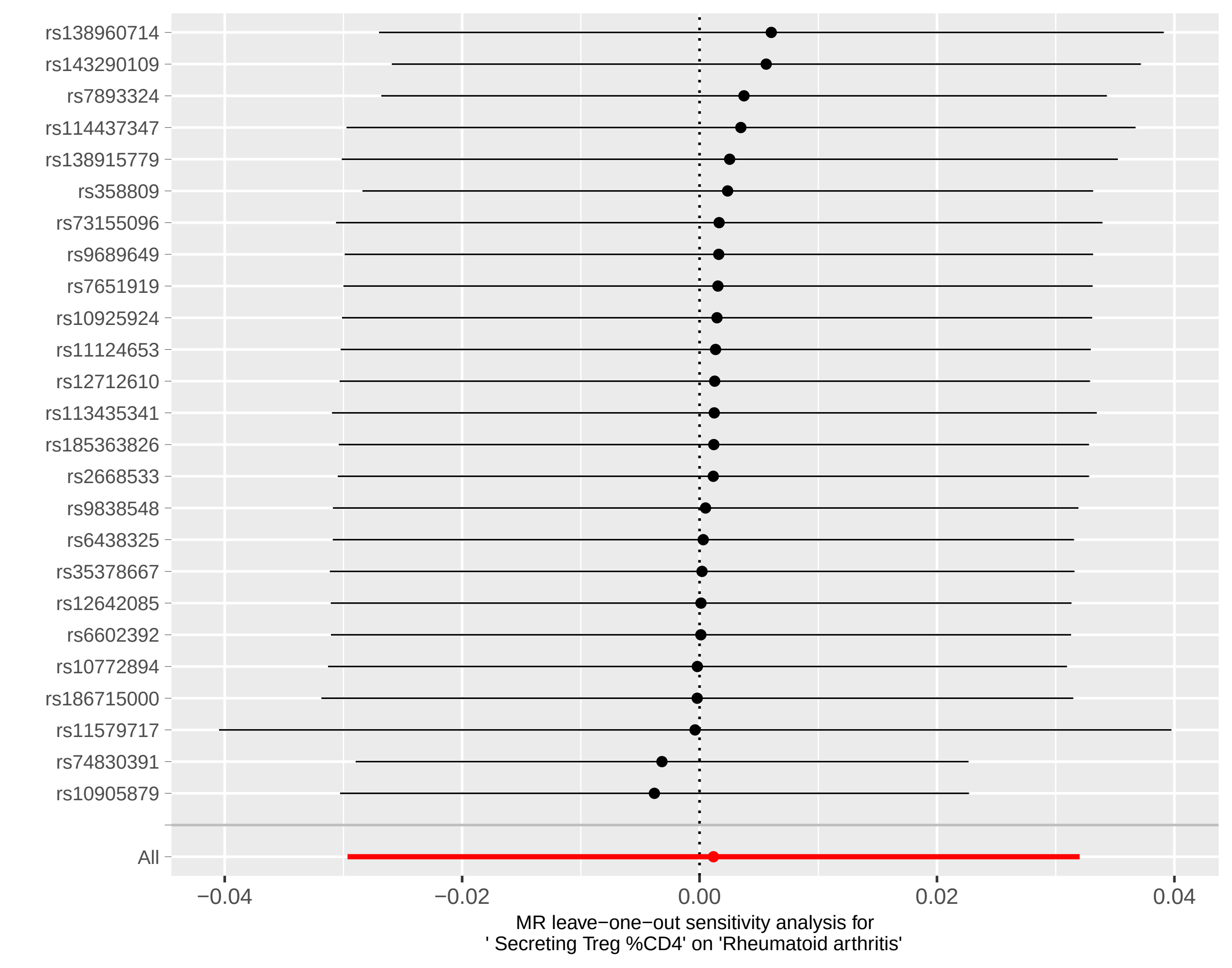

rs138960714
rs143290109
rs7893324
rs114437347
rs138915779
rs358809
rs73155096
rs9689649
rs7651919
rs10925924
rs11124653
rs12712610
rs113435341
rs185363826
rs2668533
rs9838548
rs6438325
rs35378667
rs12642085
rs6602392
rs10772894
rs186715000
rs11579717
rs74830391
rs10905879
All
−0.04
−0.02
0.00
0.02
0.04
MR leave−one−out sensitivity analysis for
' Secreting Treg %CD4' on 'Rheumatoid arthritis'

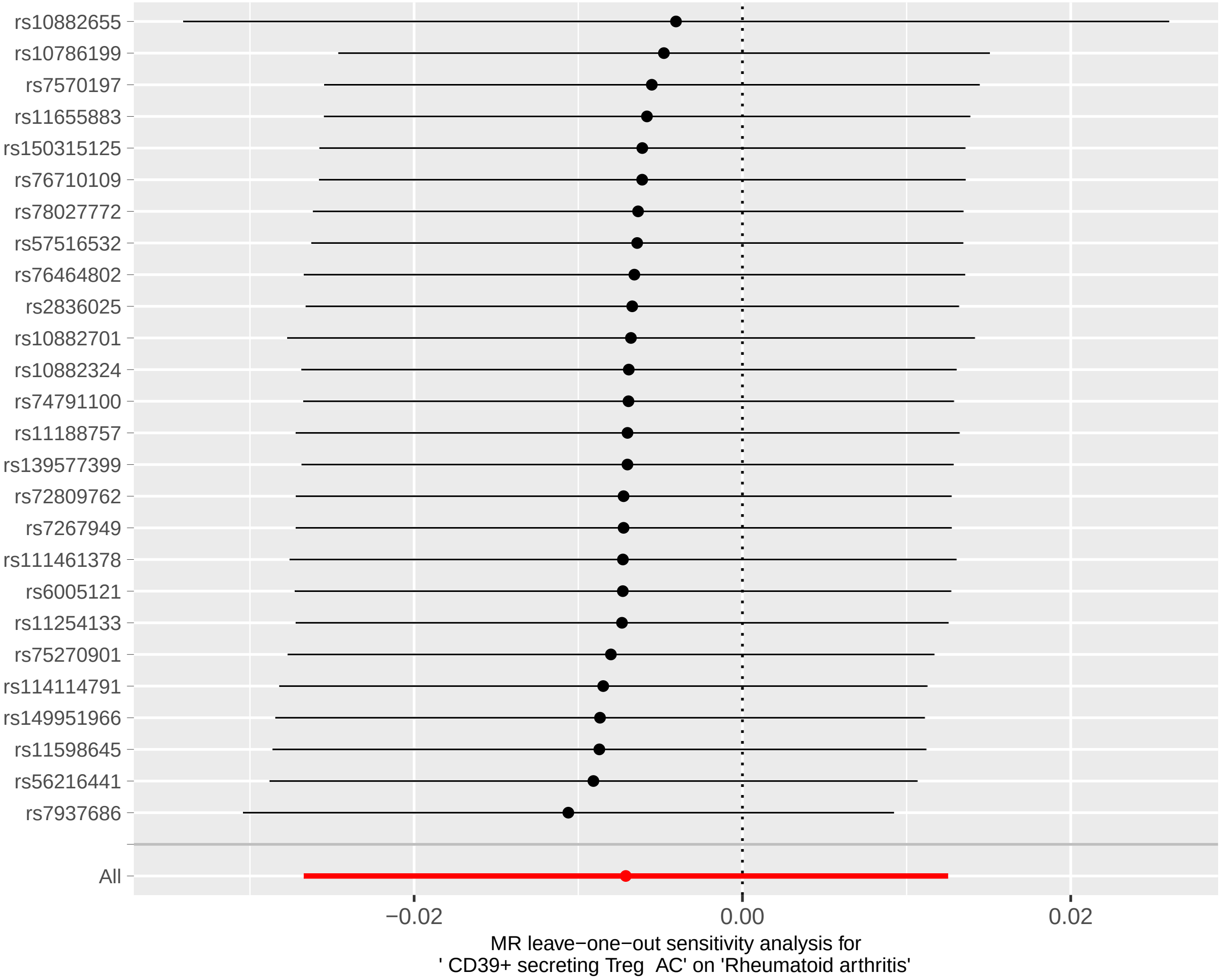
rs10882655
rs10786199
rs7570197
rs11655883
rs150315125
rs76710109
rs78027772
rs57516532
rs76464802
rs2836025
rs10882701
rs10882324
rs74791100
rs11188757
rs139577399
rs72809762
rs7267949
rs111461378
rs6005121
rs11254133
rs75270901
rs114114791
rs149951966
rs11598645
rs56216441
rs7937686
All
−0.02
0.00
0.02
MR leave−one−out sensitivity analysis for
' CD39+ secreting Treg AC' on 'Rheumatoid arthritis'

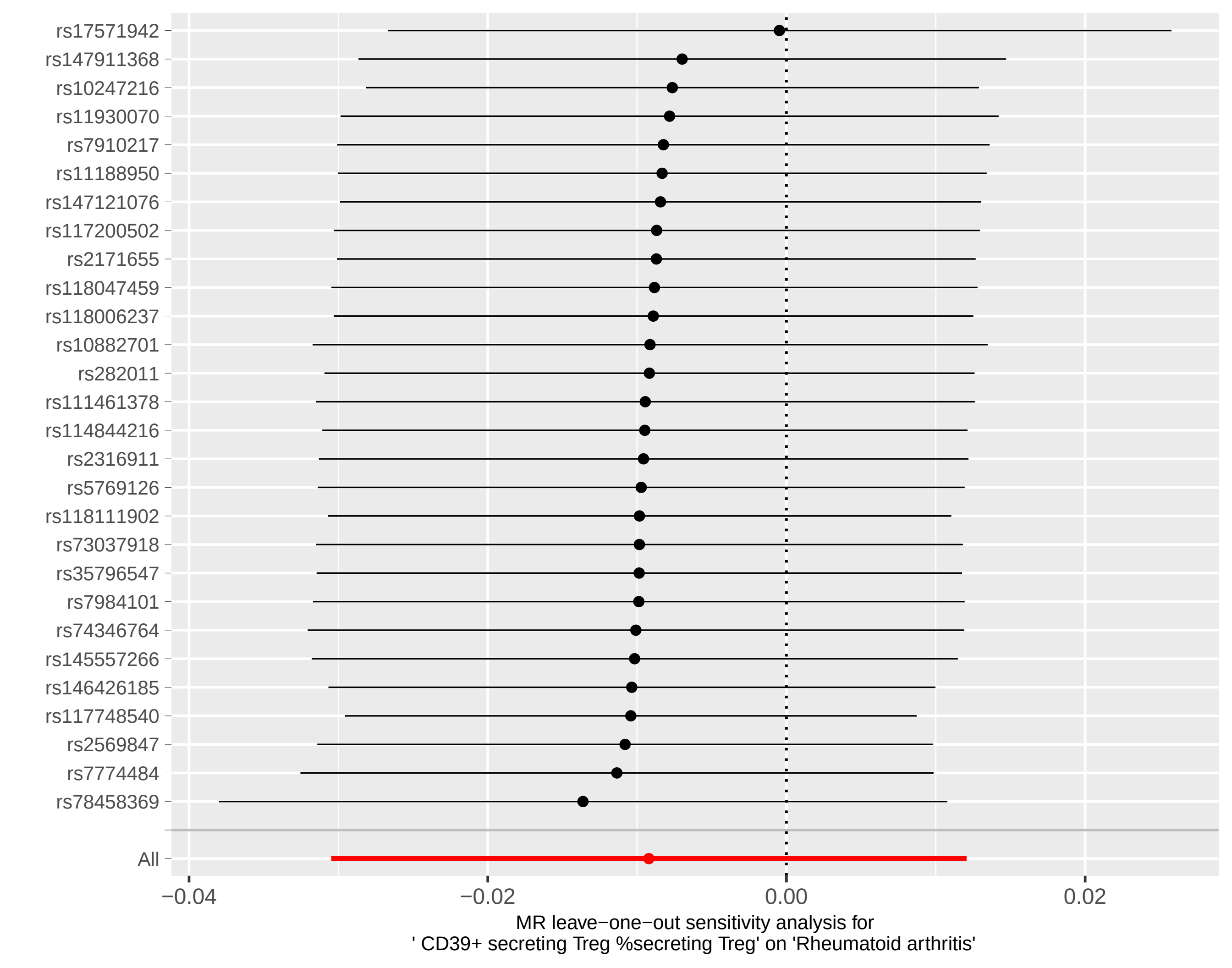
rs17571942
rs147911368
rs10247216
rs11930070
rs7910217
rs11188950
rs147121076
rs117200502
rs2171655
rs118047459
rs118006237
rs10882701
rs282011
rs111461378
rs114844216
rs2316911
rs5769126
rs118111902
rs73037918
rs35796547
rs7984101
rs74346764
rs145557266
rs146426185
rs117748540
rs2569847
rs7774484
rs78458369
All
−0.04
−0.02
0.00
0.02
MR leave−one−out sensitivity analysis for
' CD39+ secreting Treg %secreting Treg' on 'Rheumatoid arthritis'

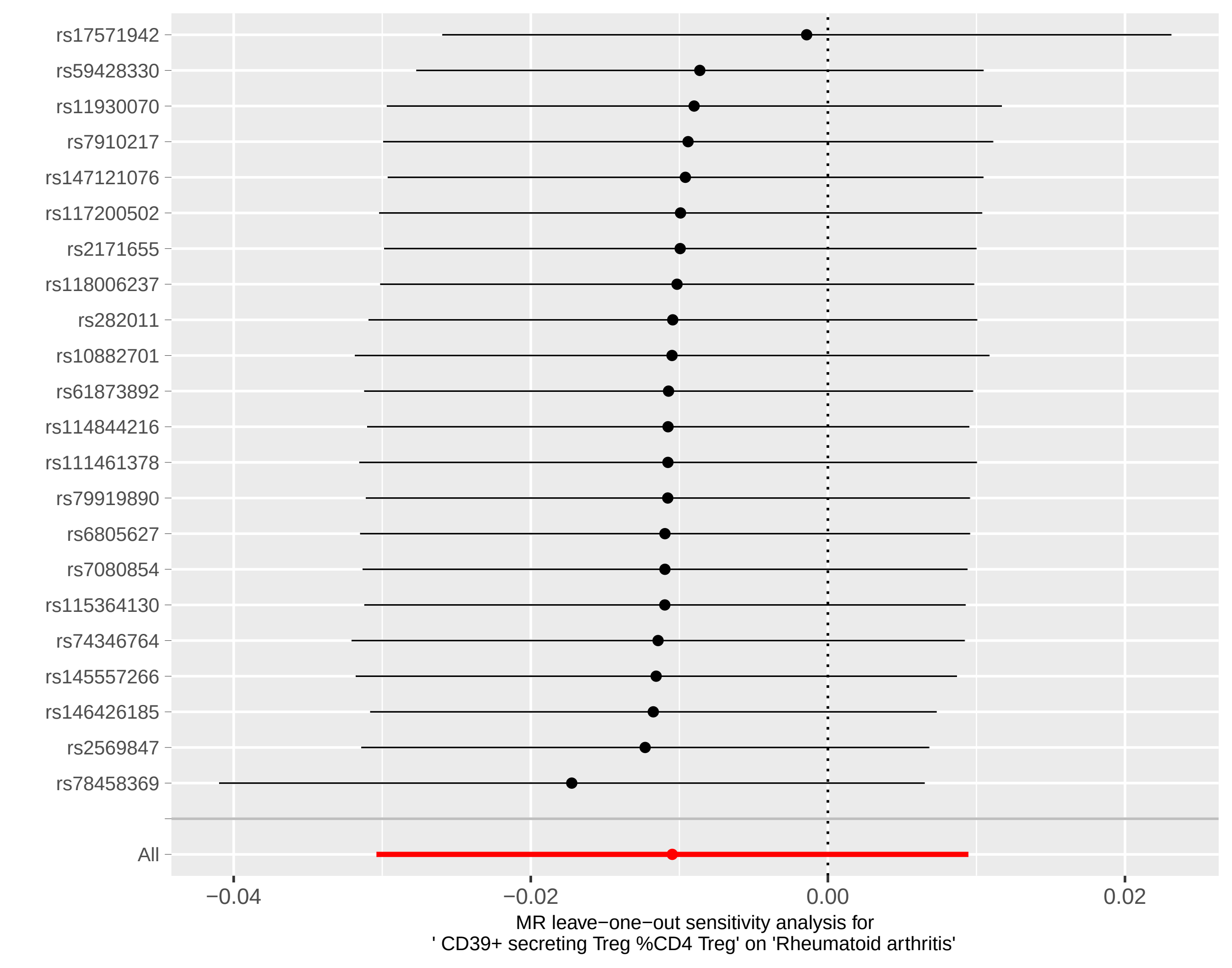

rs17571942
rs59428330
rs11930070
rs7910217
rs147121076
rs117200502
rs2171655
rs118006237
rs282011
rs10882701
rs61873892
rs114844216
rs111461378
rs79919890
rs6805627
rs7080854
rs115364130
rs74346764
rs145557266
rs146426185
rs2569847
rs78458369
All
−0.04
−0.02
0.00
0.02
MR leave−one−out sensitivity analysis for
' CD39+ secreting Treg %CD4 Treg' on 'Rheumatoid arthritis'

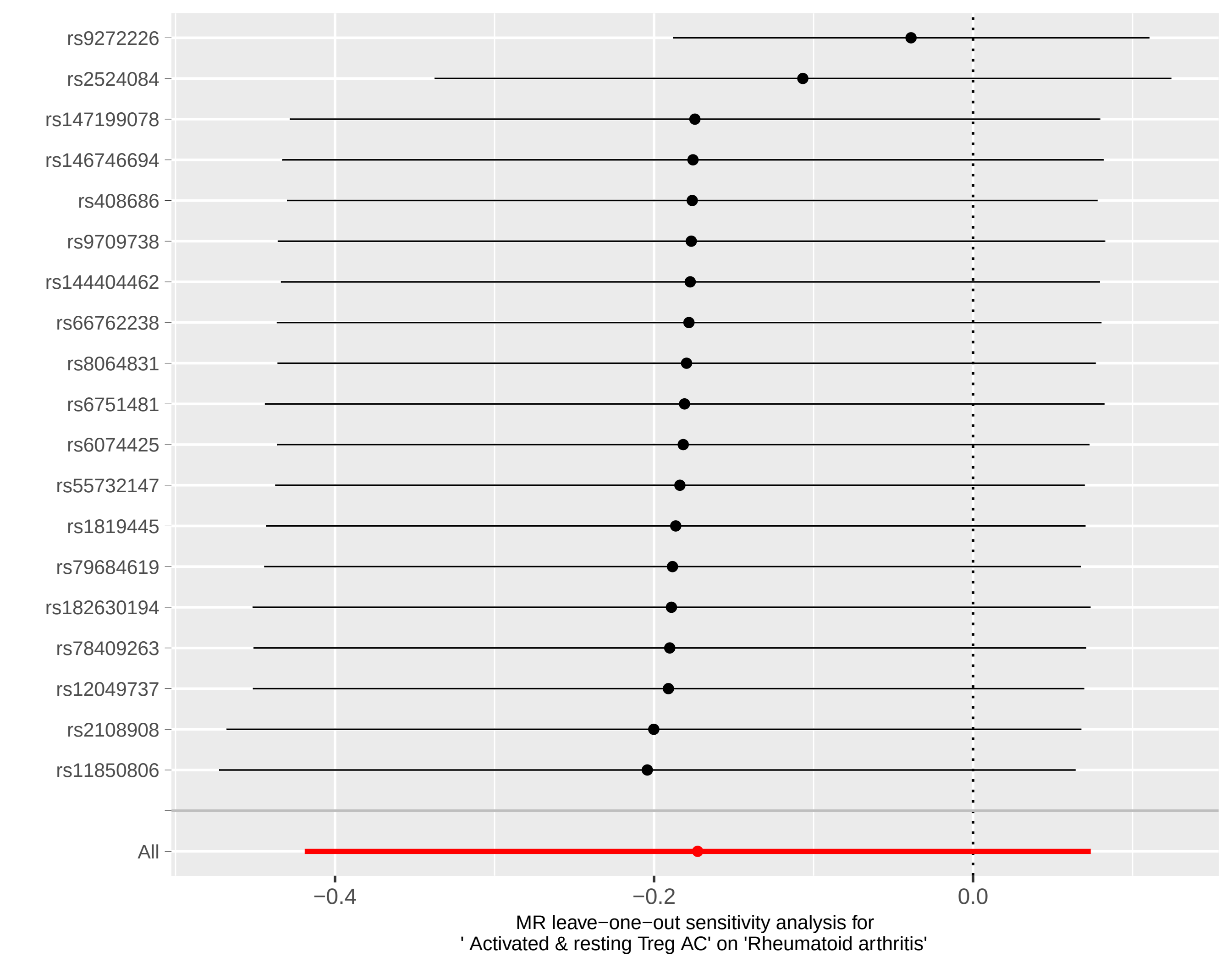
rs9272226
rs2524084
rs147199078
rs146746694
rs408686
rs9709738
rs144404462
rs66762238
rs8064831
rs6751481
rs6074425
rs55732147
rs1819445
rs79684619
rs182630194
rs78409263
rs12049737
rs2108908
rs11850806
All
−0.4
−0.2
0.0
MR leave−one−out sensitivity analysis for
' Activated & resting Treg AC' on 'Rheumatoid arthritis'

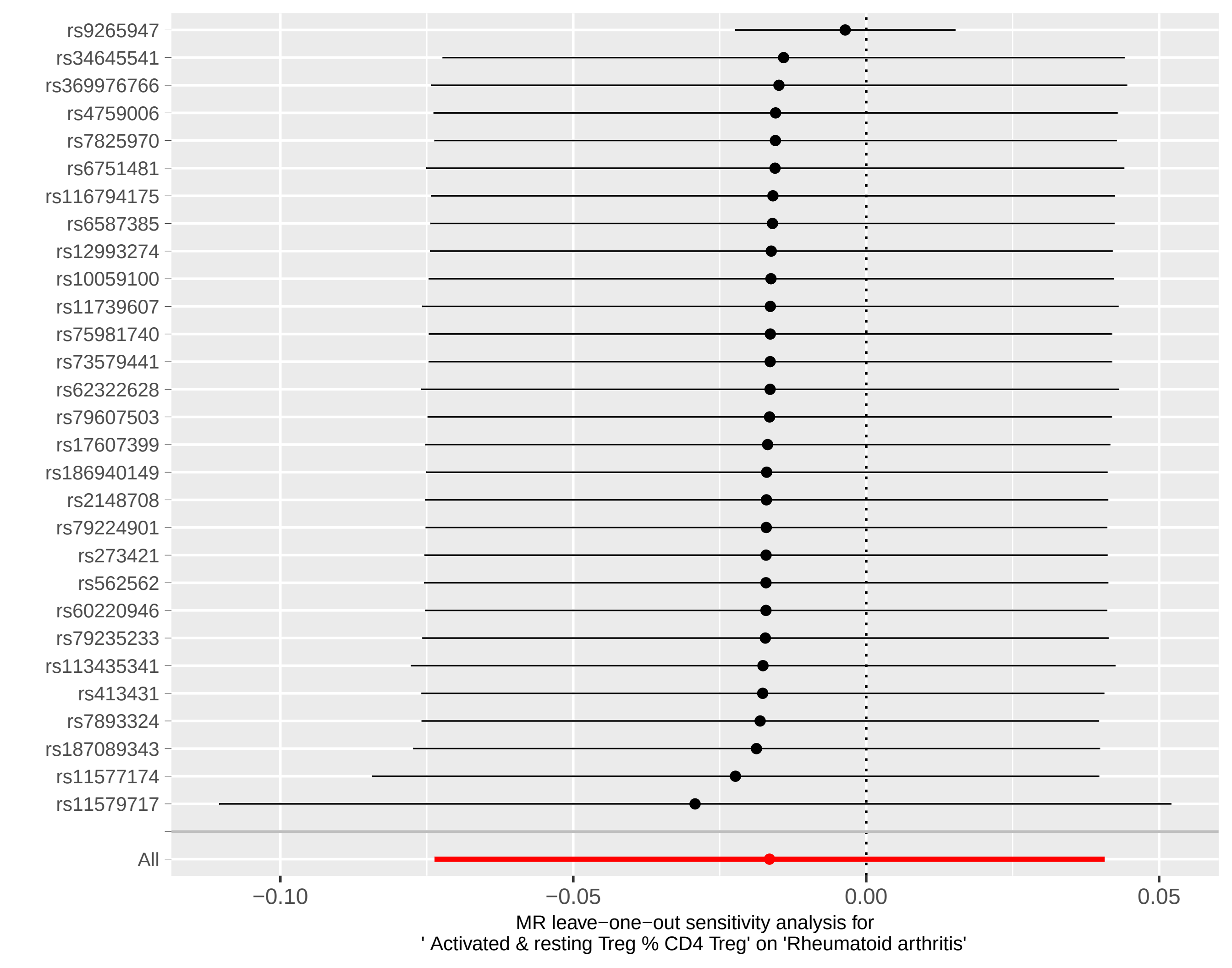
rs9265947
rs34645541
rs369976766
rs4759006
rs7825970
rs6751481
rs116794175
rs6587385
rs12993274
rs10059100
rs11739607
rs75981740
rs73579441
rs62322628
rs79607503
rs17607399
rs186940149
rs2148708
rs79224901
rs273421
rs562562
rs60220946
rs79235233
rs113435341
rs413431
rs7893324
rs187089343
rs11577174
rs11579717
All
−0.10
−0.05
0.00
0.05
MR leave−one−out sensitivity analysis for
' Activated & resting Treg % CD4 Treg' on 'Rheumatoid arthritis'

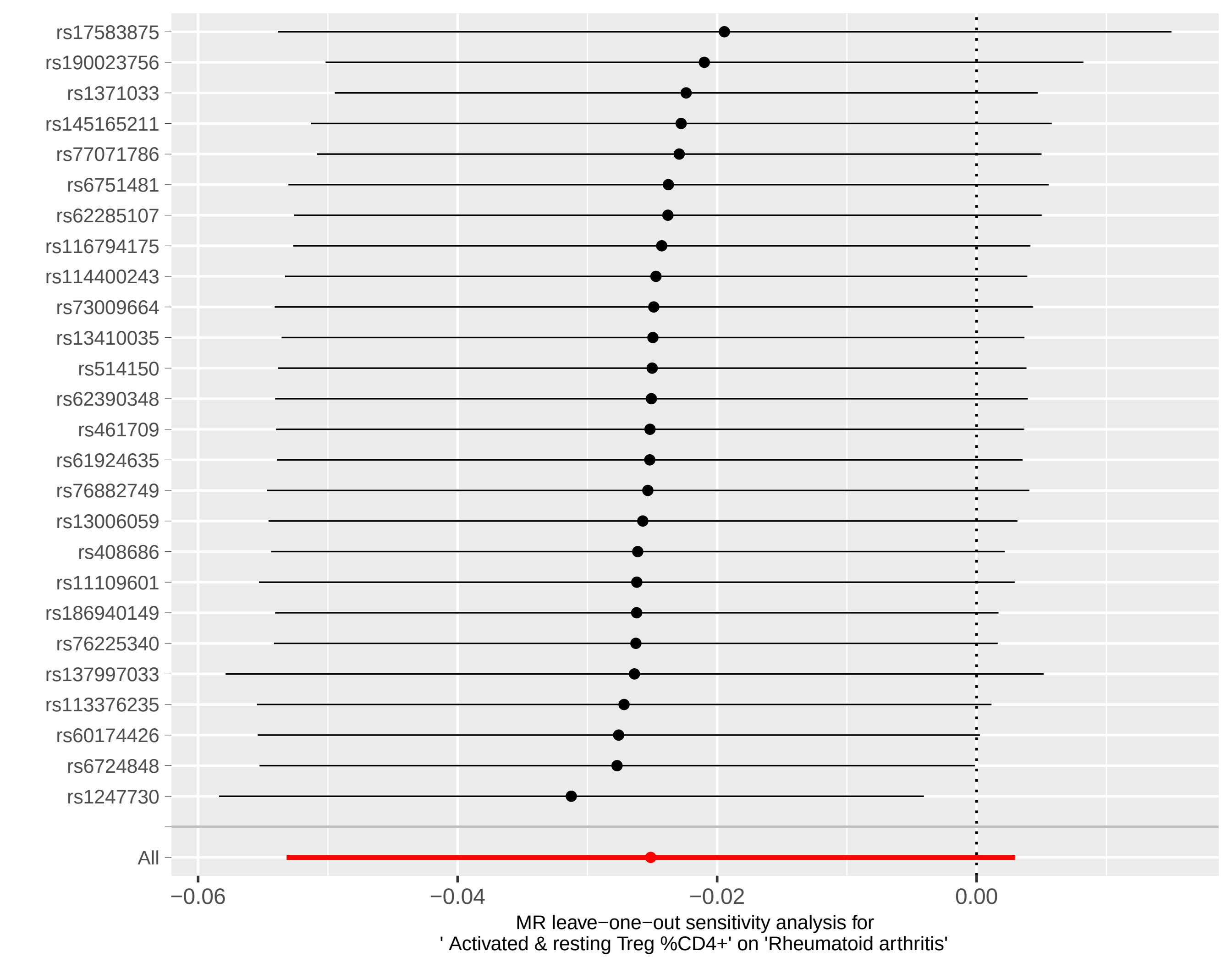
rs17583875
rs190023756
rs1371033
rs145165211
rs77071786
rs6751481
rs62285107
rs116794175
rs114400243
rs73009664
rs13410035
rs514150
rs62390348
rs461709
rs61924635
rs76882749
rs13006059
rs408686
rs11109601
rs186940149
rs76225340
rs137997033
rs113376235
rs60174426
rs6724848
rs1247730
All
−0.06
−0.04
−0.02
0.00
MR leave−one−out sensitivity analysis for
' Activated & resting Treg %CD4+' on 'Rheumatoid arthritis'

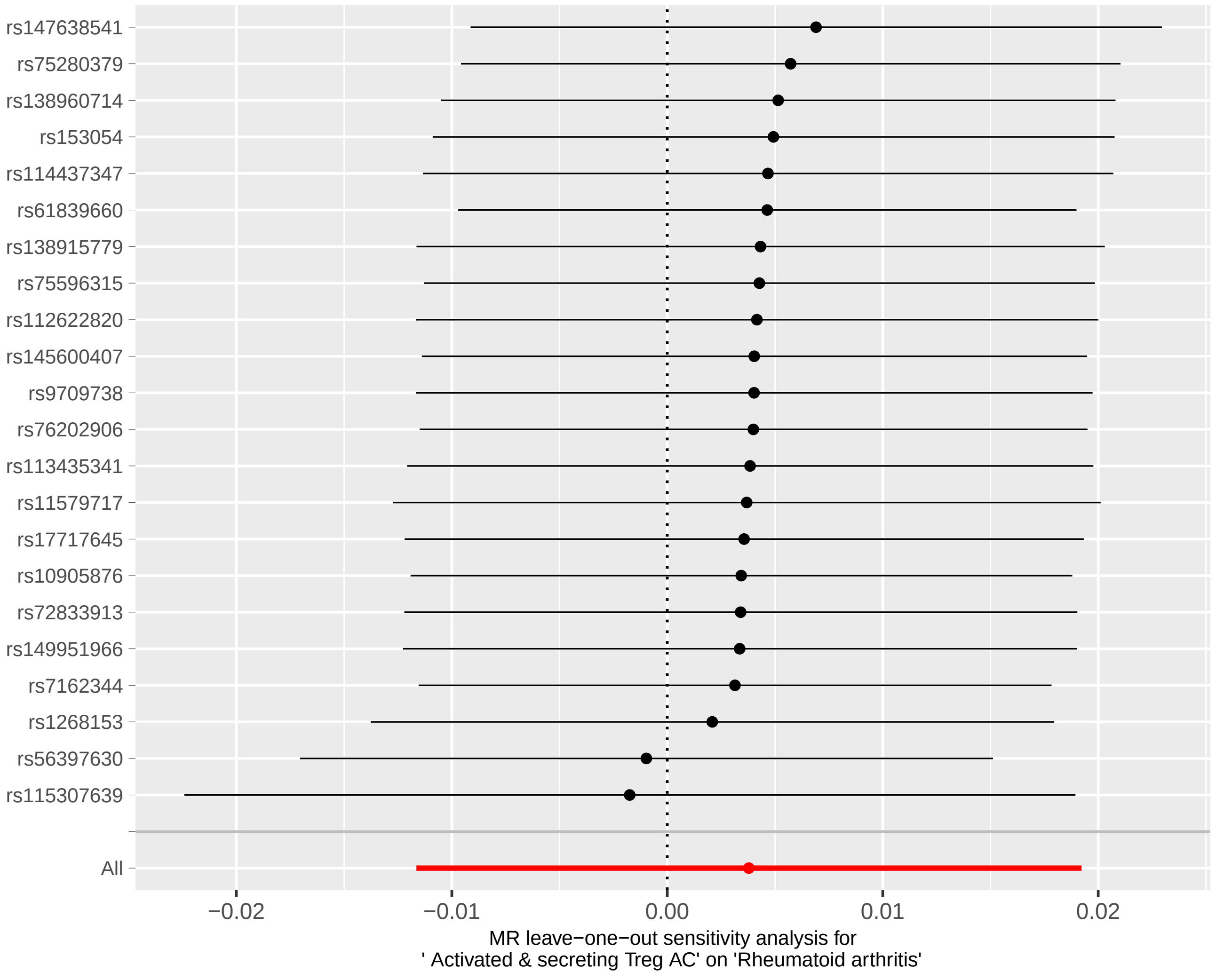
rs147638541
rs75280379
rs138960714
rs153054
rs114437347
rs61839660
rs138915779
rs75596315
rs112622820
rs145600407
rs9709738
rs76202906
rs113435341
rs11579717
rs17717645
rs10905876
rs72833913
rs149951966
rs7162344
rs1268153
rs56397630
rs115307639
All
−0.02
−0.01
0.00
0.01
0.02
MR leave−one−out sensitivity analysis for
' Activated & secreting Treg AC' on 'Rheumatoid arthritis'

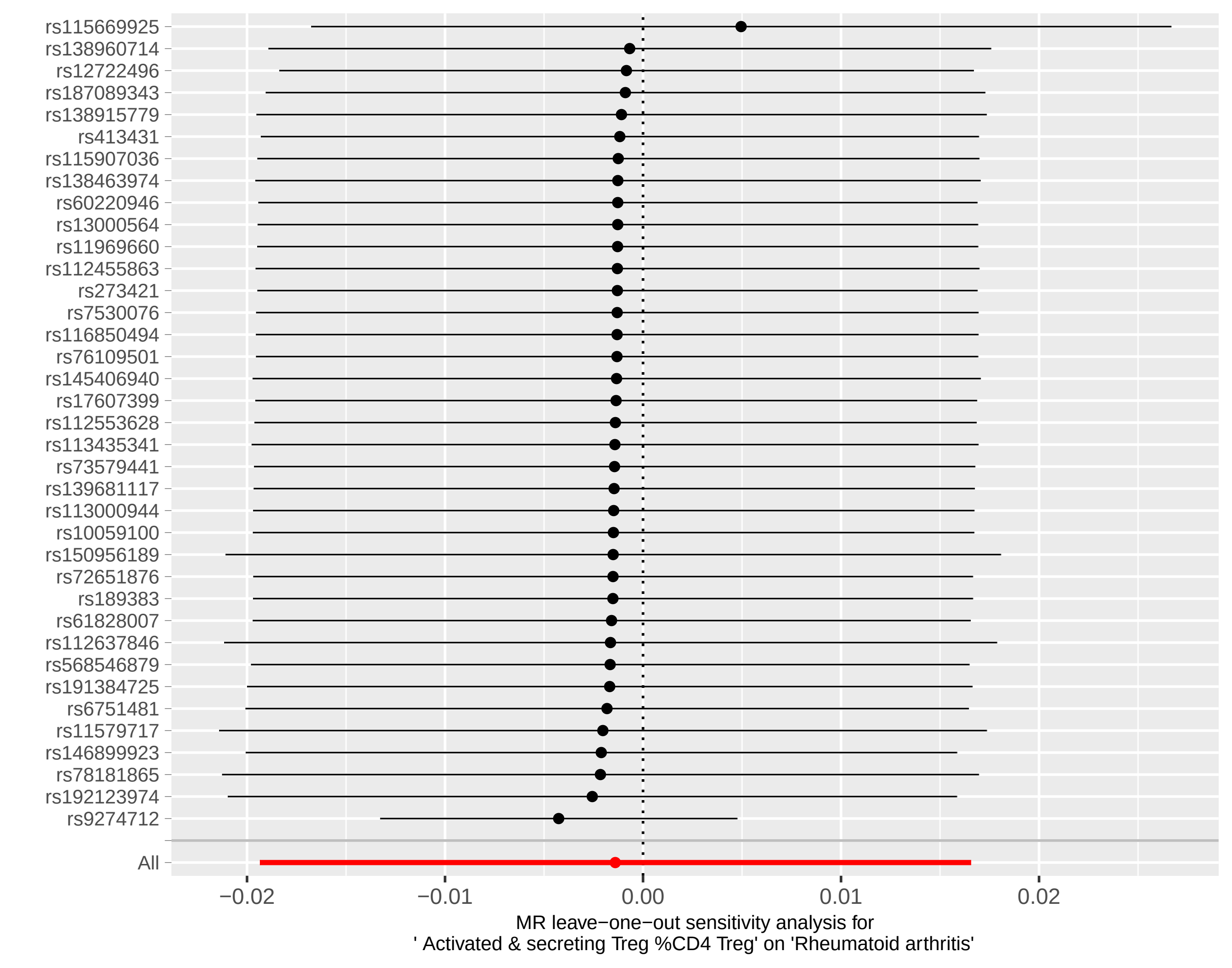

rs115669925
rs138960714
rs12722496
rs187089343
rs138915779
rs413431
rs115907036
rs138463974
rs60220946
rs13000564
rs11969660
rs112455863
rs273421
rs7530076
rs116850494
rs76109501
rs145406940
rs17607399
rs112553628
rs113435341
rs73579441
rs139681117
rs113000944
rs10059100
rs150956189
rs72651876
rs189383
rs61828007
rs112637846
rs568546879
rs191384725
rs6751481
rs11579717
rs146899923
rs78181865
rs192123974
rs9274712
All
−0.02
−0.01
0.00
0.01
0.02
MR leave−one−out sensitivity analysis for
' Activated & secreting Treg %CD4 Treg' on 'Rheumatoid arthritis'

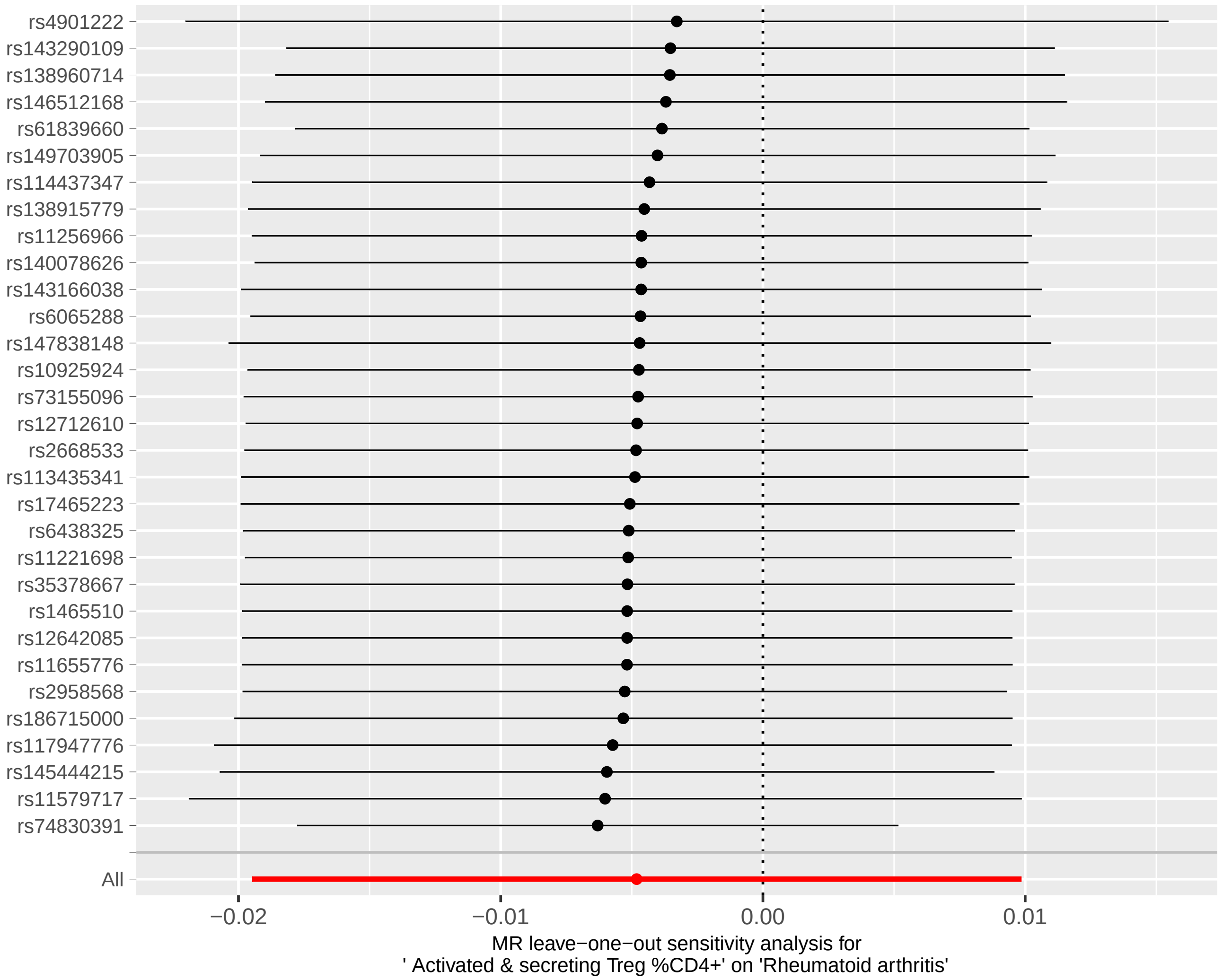
rs4901222
rs143290109
rs138960714
rs146512168
rs61839660
rs149703905
rs114437347
rs138915779
rs11256966
rs140078626
rs143166038
rs6065288
rs147838148
rs10925924
rs73155096
rs12712610
rs2668533
rs113435341
rs17465223
rs6438325
rs11221698
rs35378667
rs1465510
rs12642085
rs11655776
rs2958568
rs186715000
rs117947776
rs145444215
rs11579717
rs74830391
All
−0.02
−0.01
0.00
0.01
MR leave−one−out sensitivity analysis for
' Activated & secreting Treg %CD4+' on 'Rheumatoid arthritis'

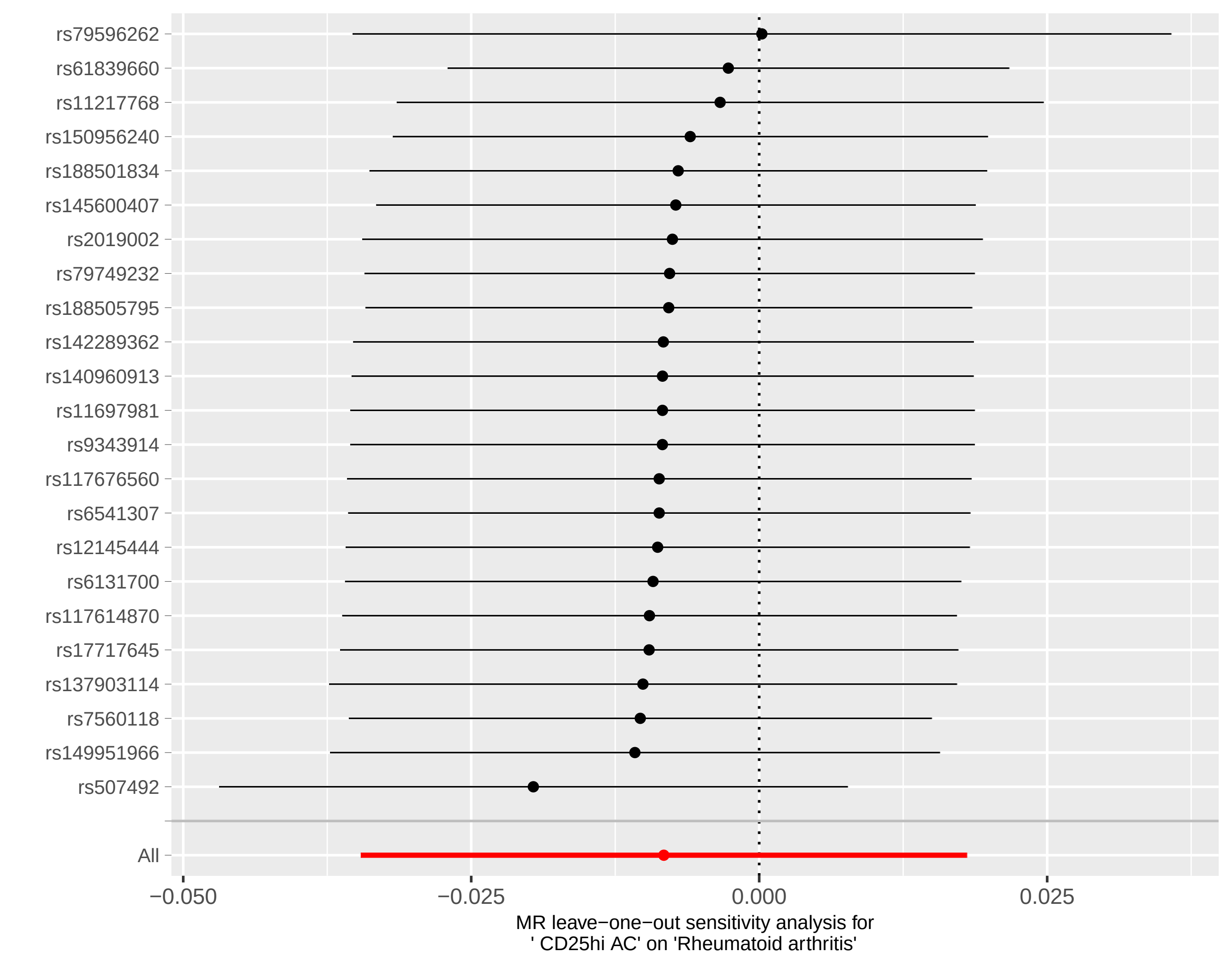
rs79596262
rs61839660
rs11217768
rs150956240
rs188501834
rs145600407
rs2019002
rs79749232
rs188505795
rs142289362
rs140960913
rs11697981
rs9343914
rs117676560
rs6541307
rs12145444
rs6131700
rs117614870
rs17717645
rs137903114
rs7560118
rs149951966
rs507492
All
−0.050
−0.025
0.000
0.025
MR leave−one−out sensitivity analysis for
' CD25hi AC' on 'Rheumatoid arthritis'

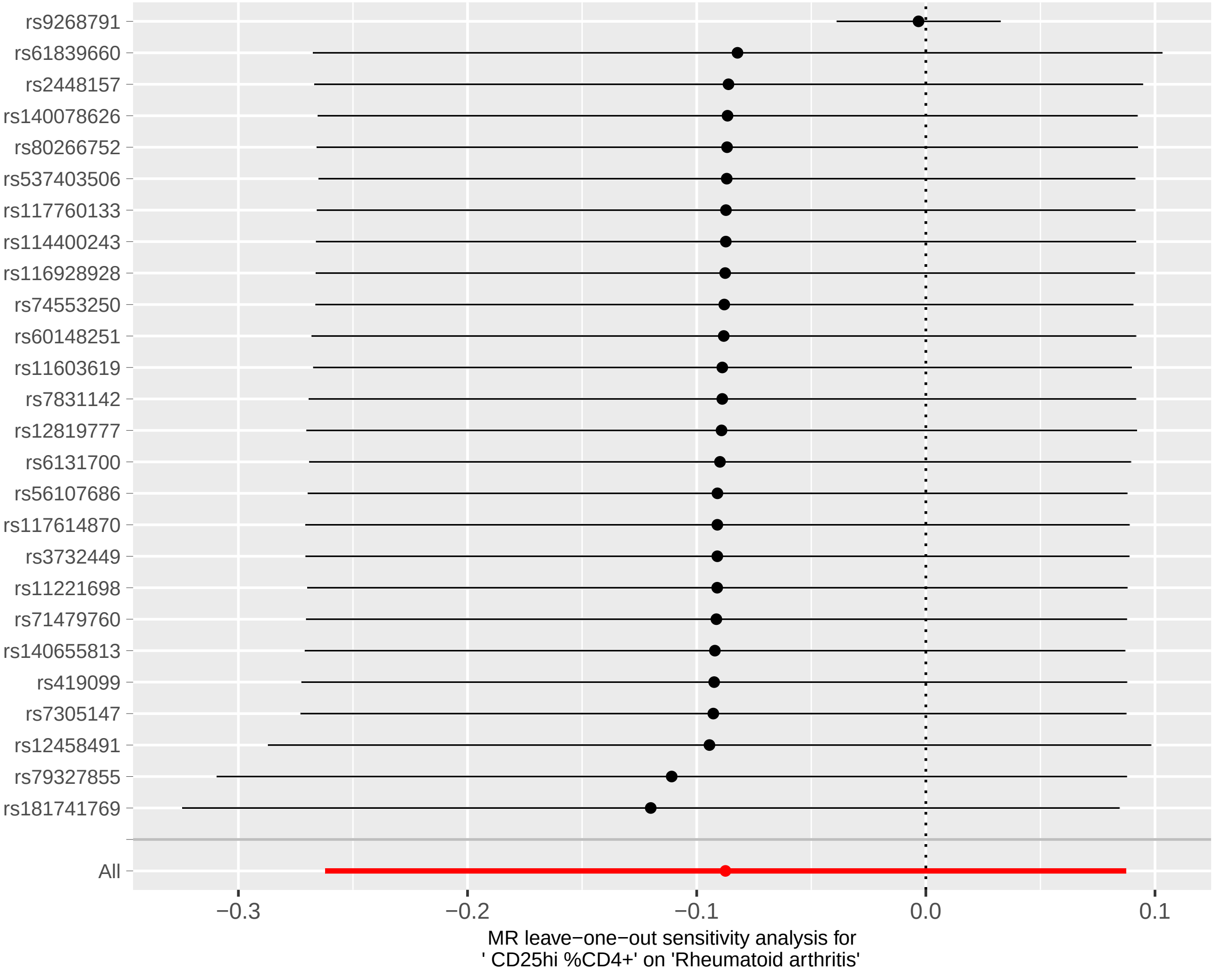
rs9268791
rs61839660
rs2448157
rs140078626
rs80266752
rs537403506
rs117760133
rs114400243
rs116928928
rs74553250
rs60148251
rs11603619
rs7831142
rs12819777
rs6131700
rs56107686
rs117614870
rs3732449
rs11221698
rs71479760
rs140655813
rs419099
rs7305147
rs12458491
rs79327855
rs181741769
All
−0.3
−0.2
−0.1
0.0
0.1
MR leave−one−out sensitivity analysis for
' CD25hi %CD4+' on 'Rheumatoid arthritis'

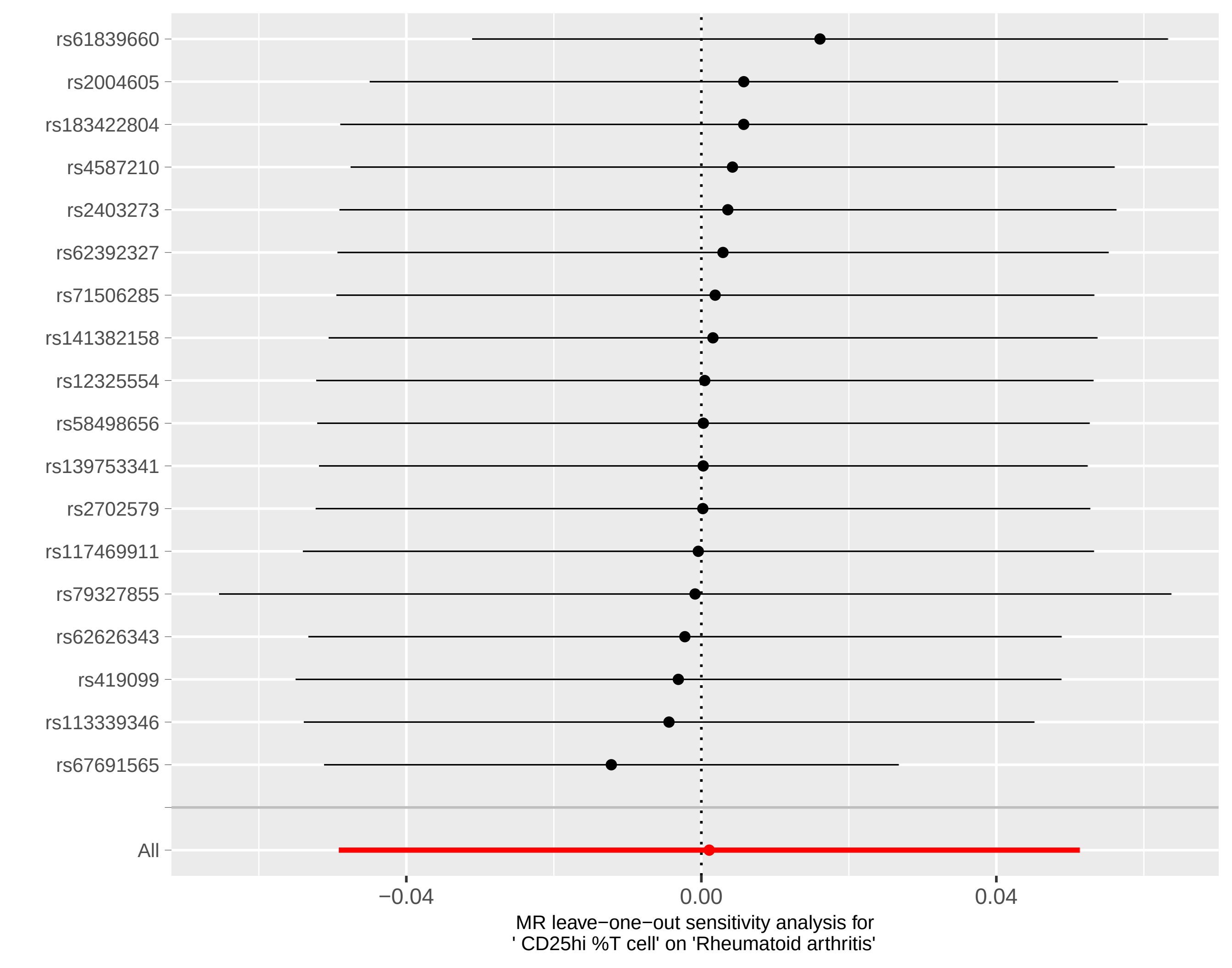

rs61839660
rs2004605
rs183422804
rs4587210
rs2403273
rs62392327
rs71506285
rs141382158
rs12325554
rs58498656
rs139753341
rs2702579
rs117469911
rs79327855
rs62626343
rs419099
rs113339346
rs67691565
All
−0.04
0.00
0.04
MR leave−one−out sensitivity analysis for
' CD25hi %T cell' on 'Rheumatoid arthritis'

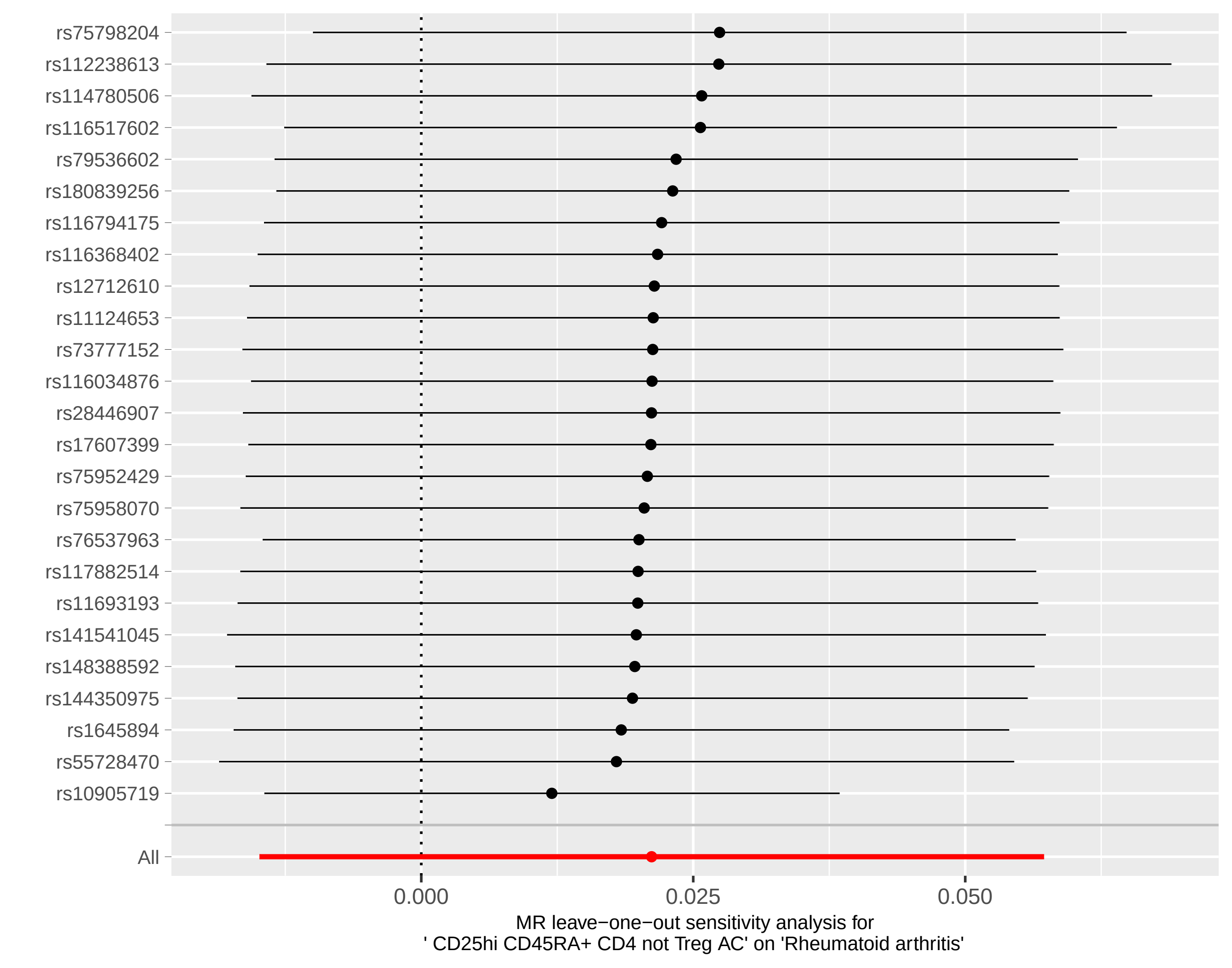

rs75798204
rs112238613
rs114780506
rs116517602
rs79536602
rs180839256
rs116794175
rs116368402
rs12712610
rs11124653
rs73777152
rs116034876
rs28446907
rs17607399
rs75952429
rs75958070
rs76537963
rs117882514
rs11693193
rs141541045
rs148388592
rs144350975
rs1645894
rs55728470
rs10905719
All
0.000
0.025
0.050
MR leave−one−out sensitivity analysis for
' CD25hi CD45RA+ CD4 not Treg AC' on 'Rheumatoid arthritis'

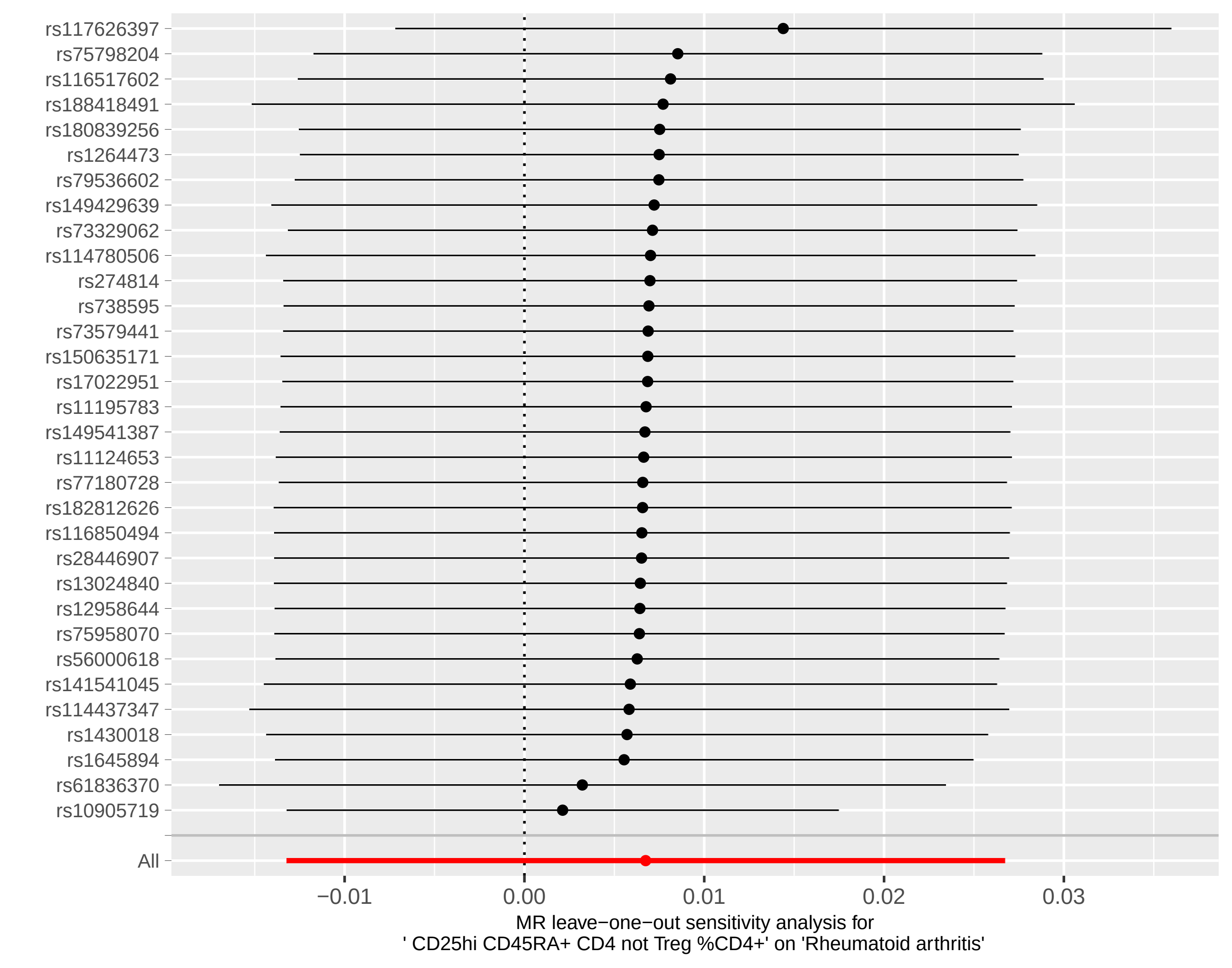

rs117626397
rs75798204
rs116517602
rs188418491
rs180839256
rs1264473
rs79536602
rs149429639
rs73329062
rs114780506
rs274814
rs738595
rs73579441
rs150635171
rs17022951
rs11195783
rs149541387
rs11124653
rs77180728
rs182812626
rs116850494
rs28446907
rs13024840
rs12958644
rs75958070
rs56000618
rs141541045
rs114437347
rs1430018
rs1645894
rs61836370
rs10905719
All
−0.01
0.00
0.01
0.02
0.03
MR leave−one−out sensitivity analysis for
' CD25hi CD45RA+ CD4 not Treg %CD4+' on 'Rheumatoid arthritis'

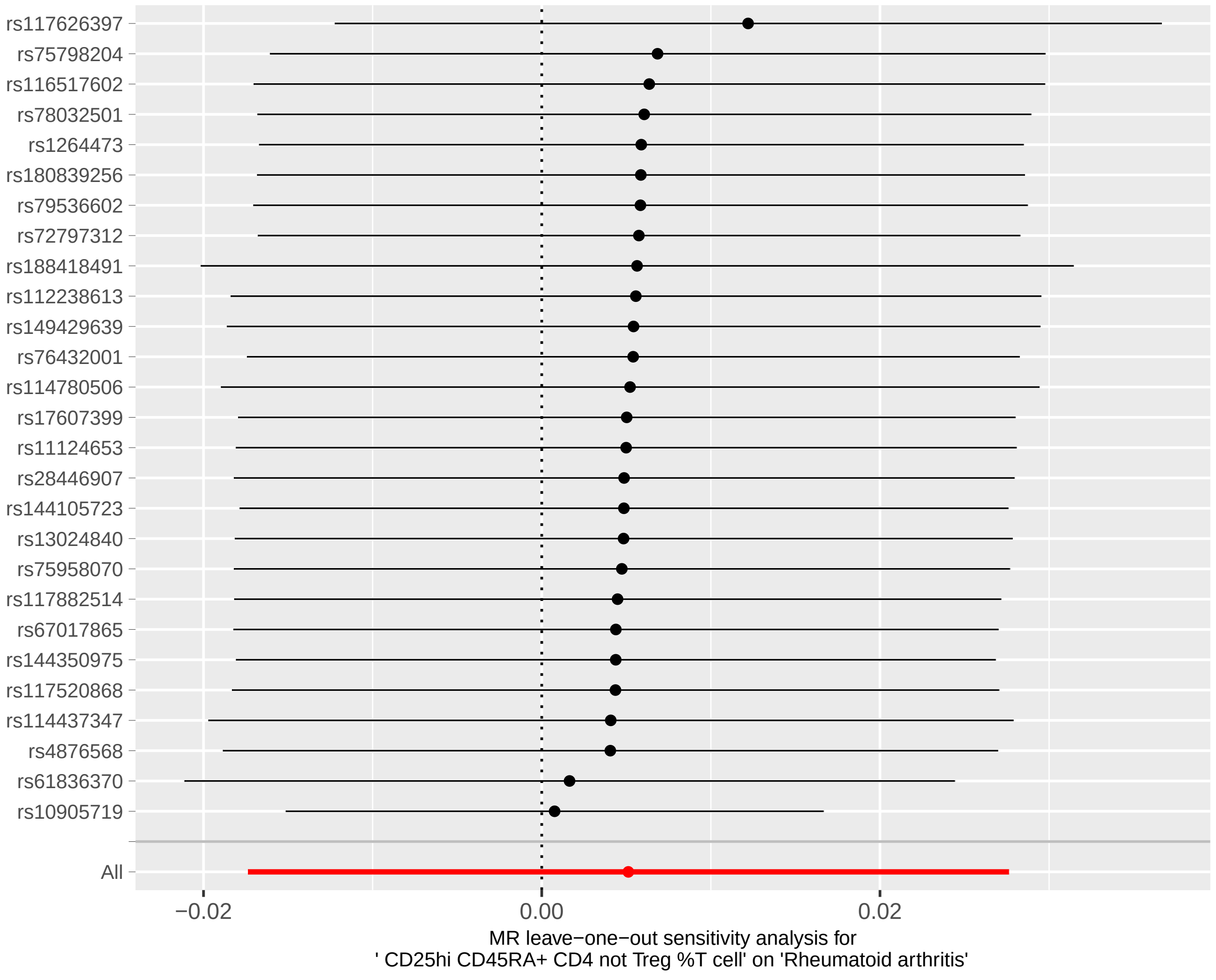
rs117626397
rs75798204
rs116517602
rs78032501
rs1264473
rs180839256
rs79536602
rs72797312
rs188418491
rs112238613
rs149429639
rs76432001
rs114780506
rs17607399
rs11124653
rs28446907
rs144105723
rs13024840
rs75958070
rs117882514
rs67017865
rs144350975
rs117520868
rs114437347
rs4876568
rs61836370
rs10905719
All
−0.02
0.00
0.02
MR leave−one−out sensitivity analysis for
' CD25hi CD45RA+ CD4 not Treg %T cell' on 'Rheumatoid arthritis'

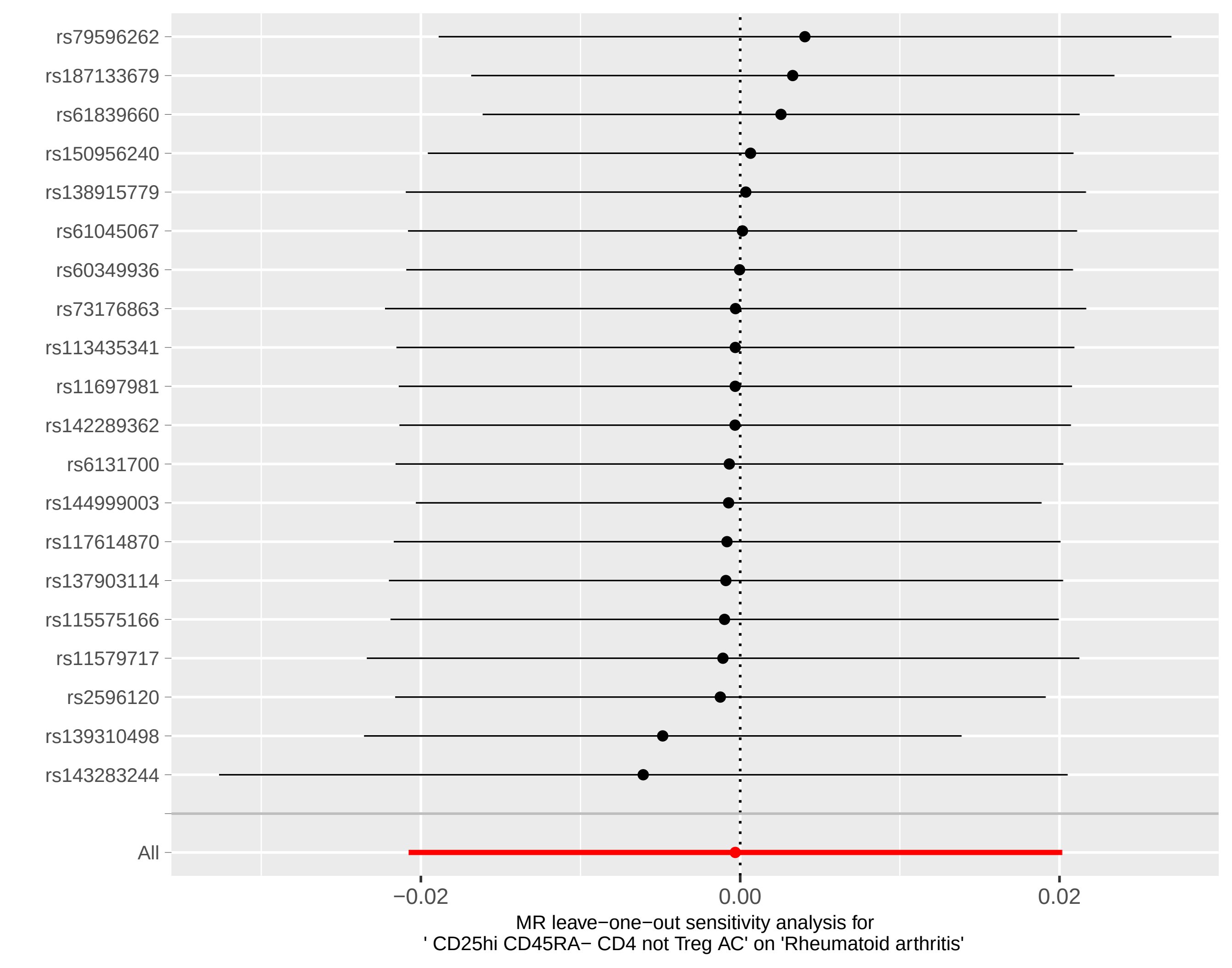
rs79596262
rs187133679
rs61839660
rs150956240
rs138915779
rs61045067
rs60349936
rs73176863
rs113435341
rs11697981
rs142289362
rs6131700
rs144999003
rs117614870
rs137903114
rs115575166
rs11579717
rs2596120
rs139310498
rs143283244
All
−0.02
0.00
0.02
MR leave−one−out sensitivity analysis for
' CD25hi CD45RA− CD4 not Treg AC' on 'Rheumatoid arthritis'

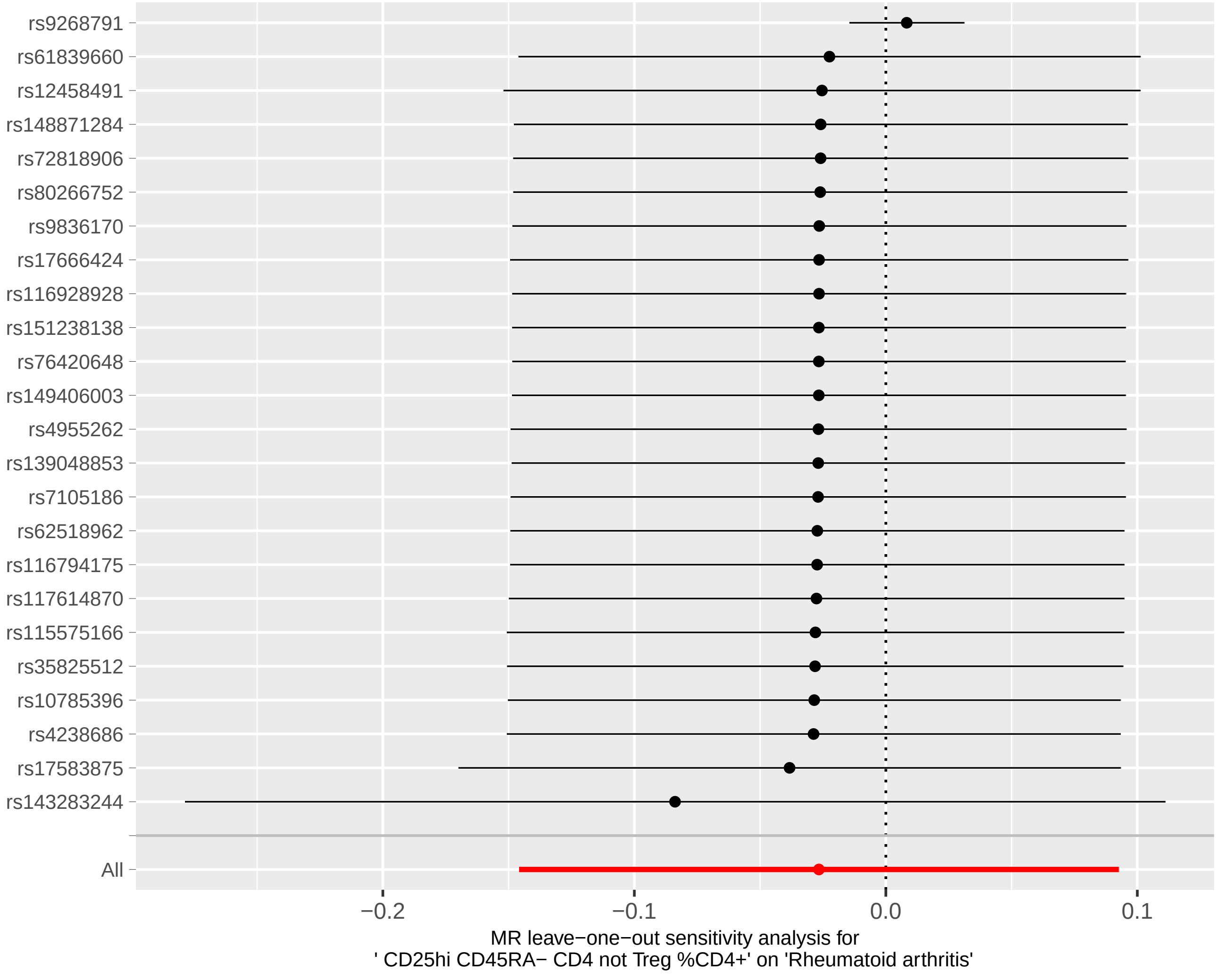
rs9268791
rs61839660
rs12458491
rs148871284
rs72818906
rs80266752
rs9836170
rs17666424
rs116928928
rs151238138
rs76420648
rs149406003
rs4955262
rs139048853
rs7105186
rs62518962
rs116794175
rs117614870
rs115575166
rs35825512
rs10785396
rs4238686
rs17583875
rs143283244
All
−0.2
−0.1
0.0
0.1
MR leave−one−out sensitivity analysis for
' CD25hi CD45RA− CD4 not Treg %CD4+' on 'Rheumatoid arthritis'

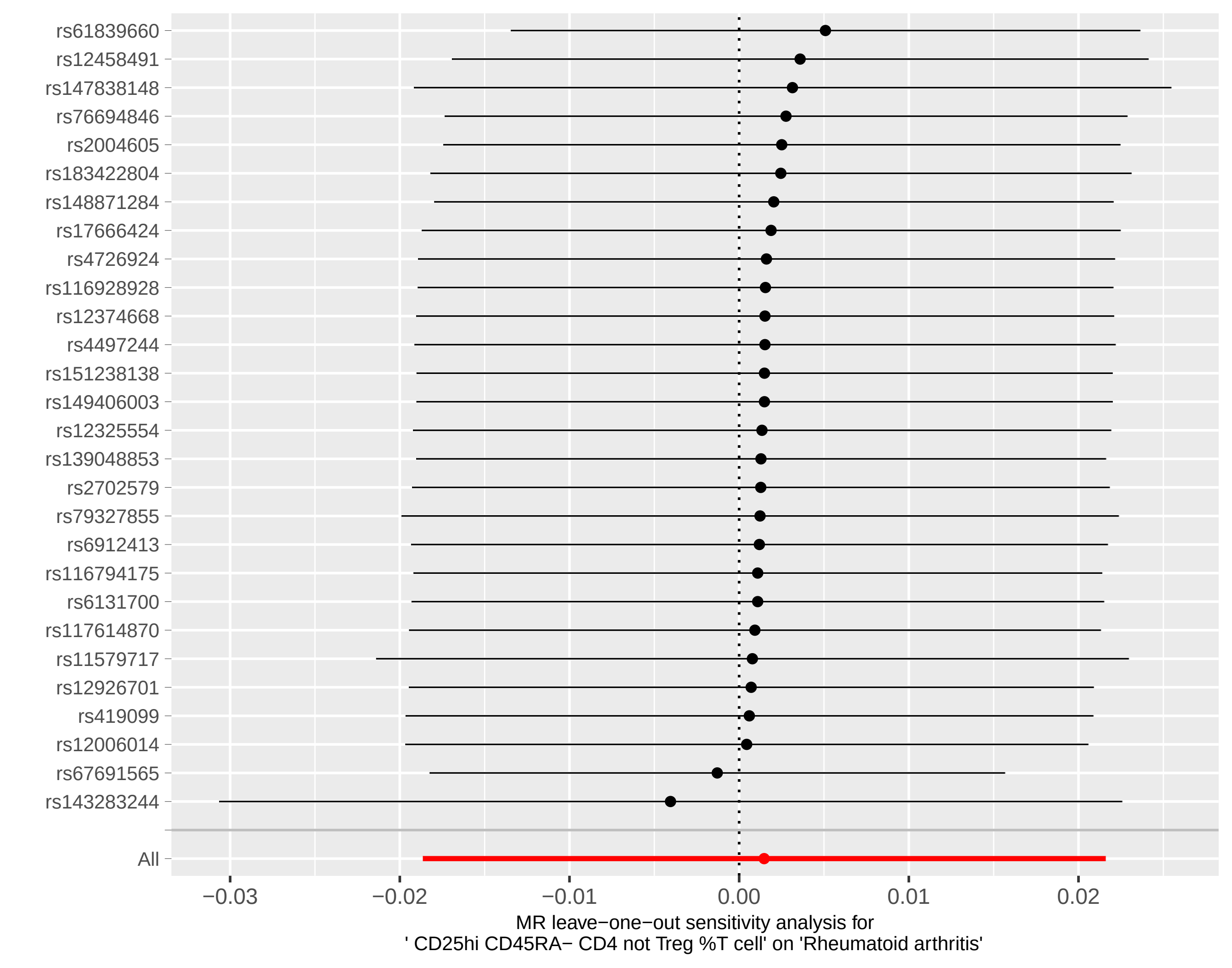
rs61839660
rs12458491
rs147838148
rs76694846
rs2004605
rs183422804
rs148871284
rs17666424
rs4726924
rs116928928
rs12374668
rs4497244
rs151238138
rs149406003
rs12325554
rs139048853
rs2702579
rs79327855
rs6912413
rs116794175
rs6131700
rs117614870
rs11579717
rs12926701
rs419099
rs12006014
rs67691565
rs143283244
All
−0.03
−0.02
−0.01
0.00
0.01
0.02
MR leave−one−out sensitivity analysis for
' CD25hi CD45RA− CD4 not Treg %T cell' on 'Rheumatoid arthritis'

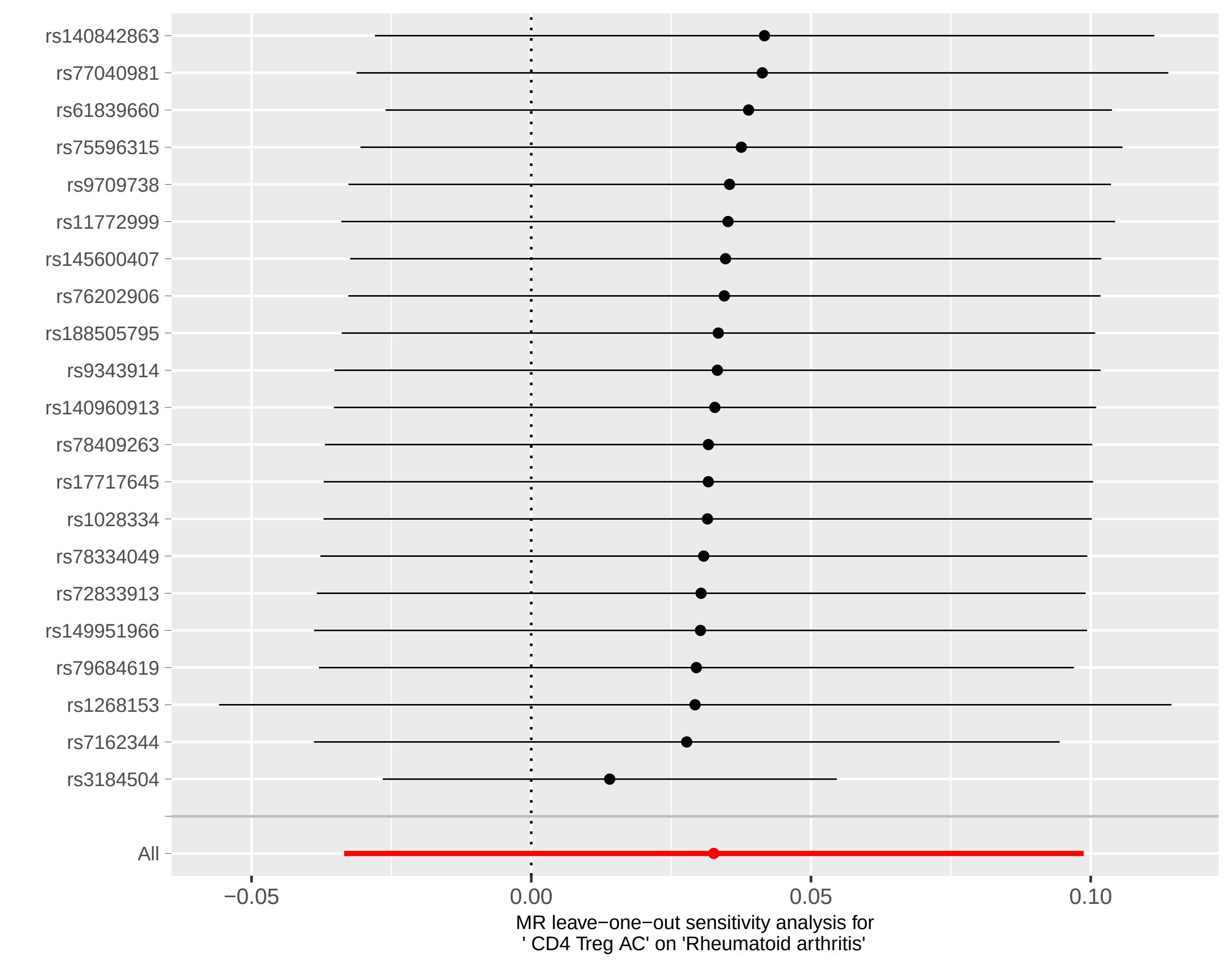
rs140842863
rs77040981
rs61839660
rs75596315
rs9709738
rs11772999
rs145600407
rs76202906
rs188505795
rs9343914
rs140960913
rs78409263
rs17717645
rs1028334
rs78334049
rs72833913
rs149951966
rs79684619
rs1268153
rs7162344
rs3184504
All
−0.05
0.00
0.05
0.10
MR leave−one−out sensitivity analysis for
' CD4 Treg AC' on 'Rheumatoid arthritis'

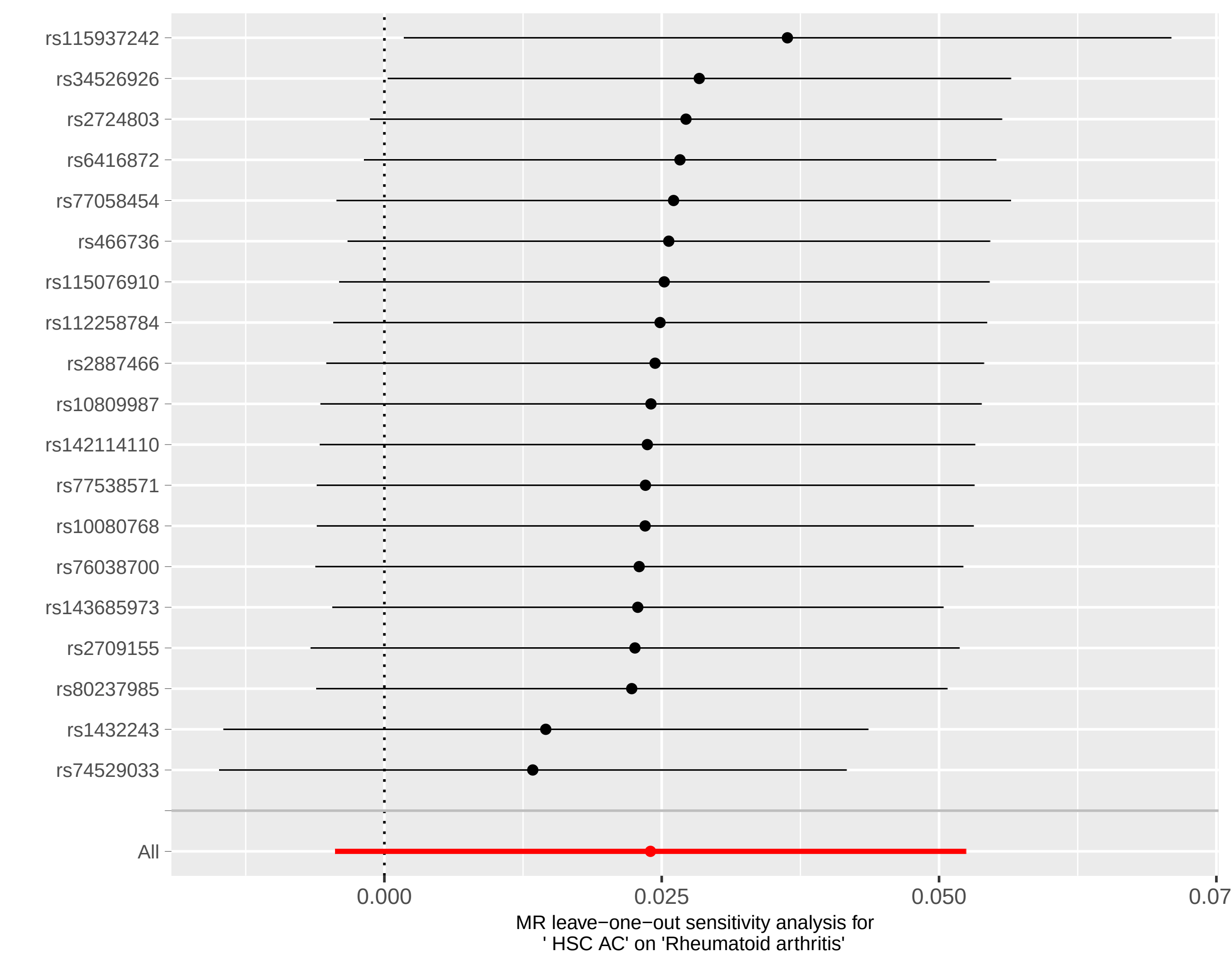
rs115937242
rs34526926
rs2724803
rs6416872
rs77058454
rs466736
rs115076910
rs112258784
rs2887466
rs10809987
rs142114110
rs77538571
rs10080768
rs76038700
rs143685973
rs2709155
rs80237985
rs1432243
rs74529033
All
0.000
0.025
0.050
0.07
MR leave−one−out sensitivity analysis for
' HSC AC' on 'Rheumatoid arthritis'

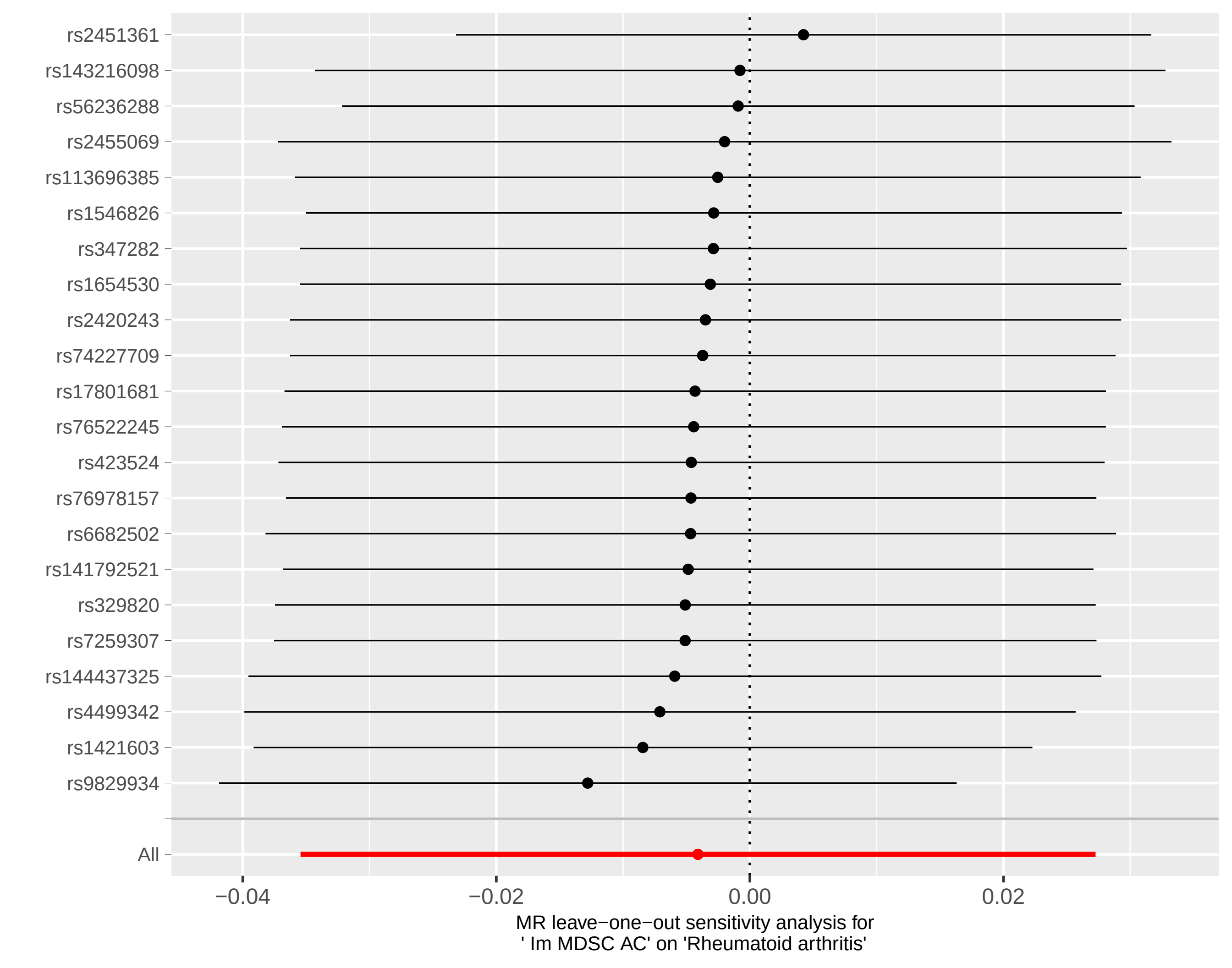

rs2451361
rs143216098
rs56236288
rs2455069
rs113696385
rs1546826
rs347282
rs1654530
rs2420243
rs74227709
rs17801681
rs76522245
rs423524
rs76978157
rs6682502
rs141792521
rs329820
rs7259307
rs144437325
rs4499342
rs1421603
rs9829934
All
−0.04
−0.02
0.00
0.02
MR leave−one−out sensitivity analysis for
' Im MDSC AC' on 'Rheumatoid arthritis'

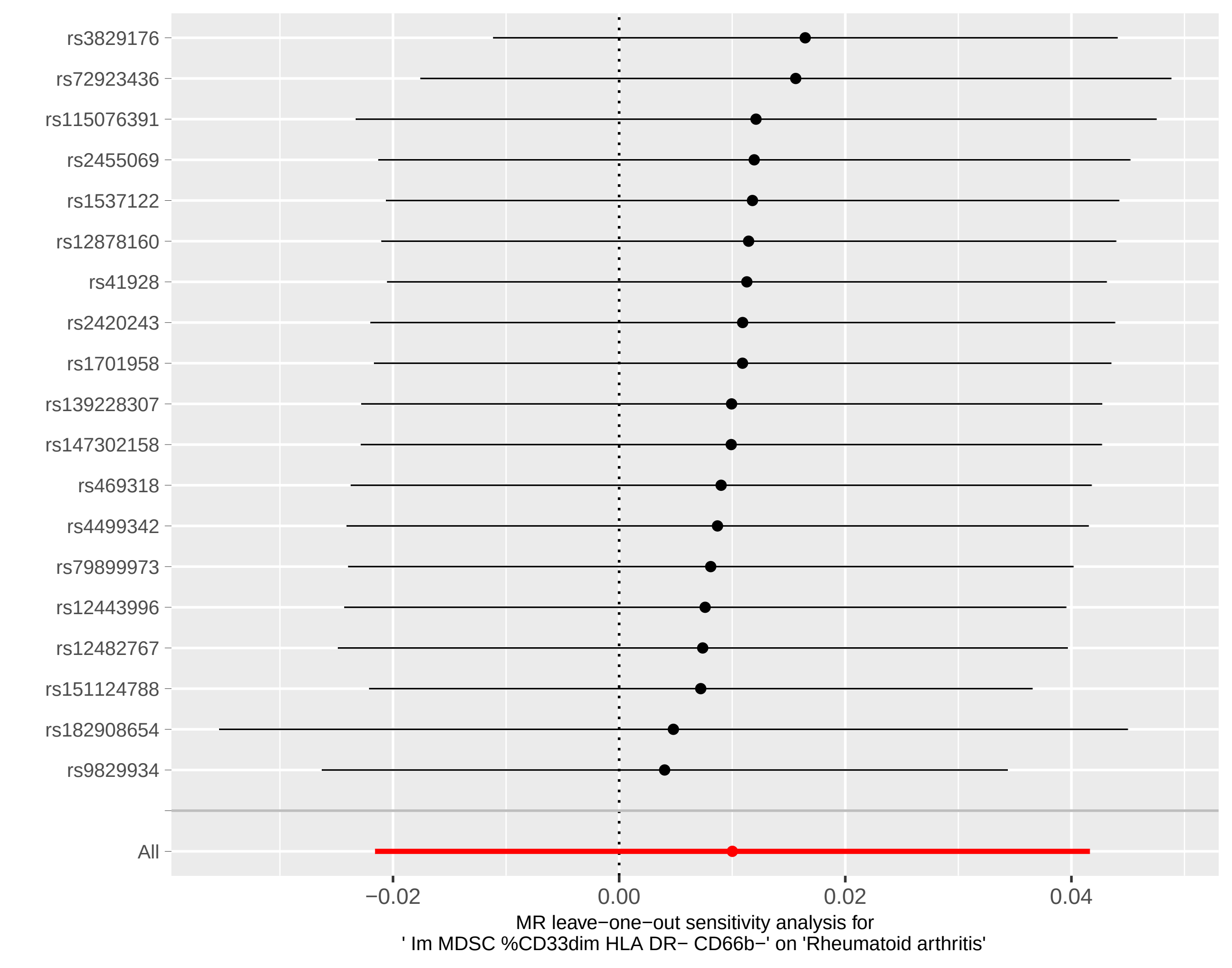
rs3829176
rs72923436
rs115076391
rs2455069
rs1537122
rs12878160
rs41928
rs2420243
rs1701958
rs139228307
rs147302158
rs469318
rs4499342
rs79899973
rs12443996
rs12482767
rs151124788
rs182908654
rs9829934
All
−0.02
0.00
0.02
0.04
MR leave−one−out sensitivity analysis for
' Im MDSC %CD33dim HLA DR− CD66b−' on 'Rheumatoid arthritis'

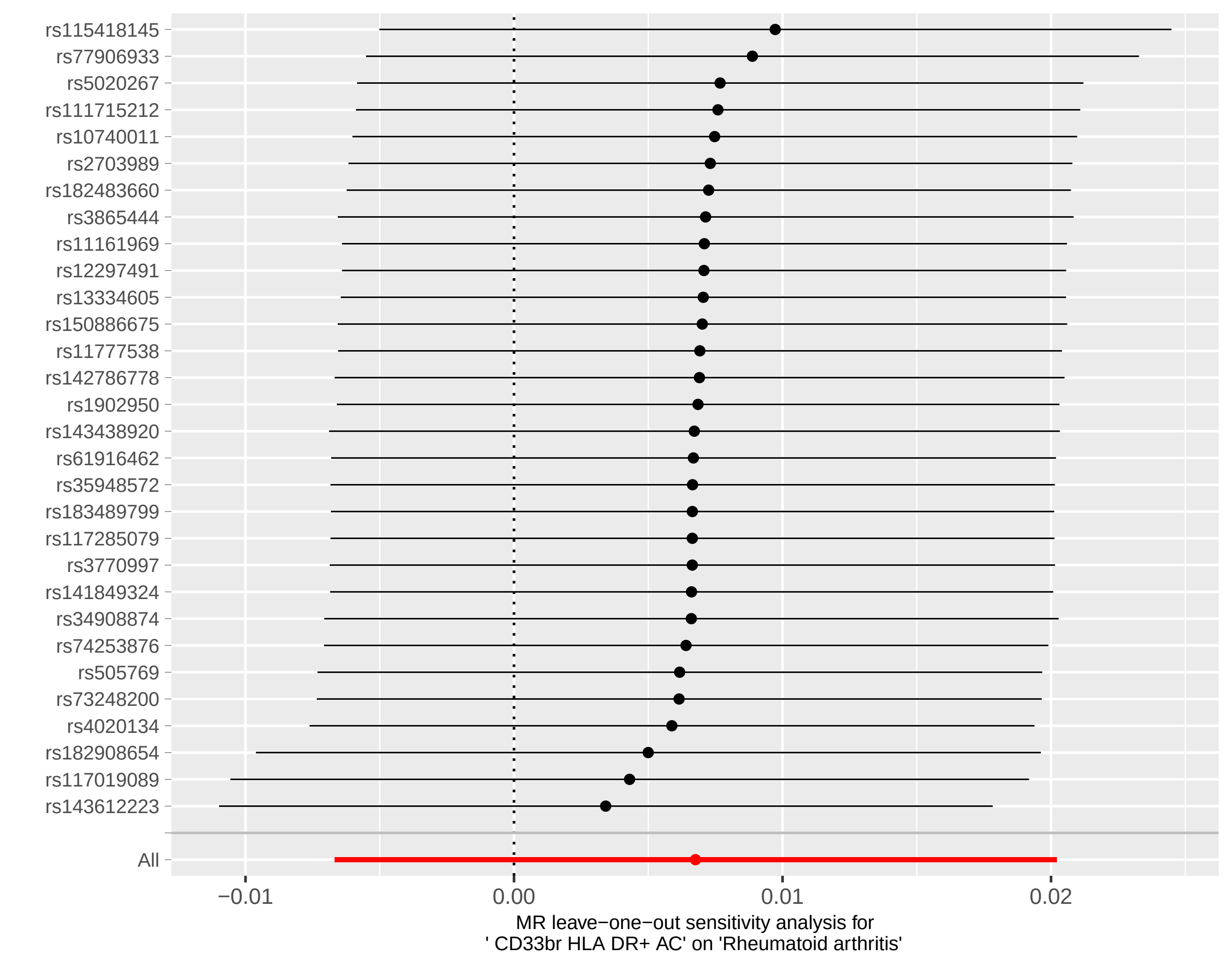

rs115418145
rs77906933
rs5020267
rs111715212
rs10740011
rs2703989
rs182483660
rs3865444
rs11161969
rs12297491
rs13334605
rs150886675
rs11777538
rs142786778
rs1902950
rs143438920
rs61916462
rs35948572
rs183489799
rs117285079
rs3770997
rs141849324
rs34908874
rs74253876
rs505769
rs73248200
rs4020134
rs182908654
rs117019089
rs143612223
All
−0.01
0.00
0.01
0.02
MR leave−one−out sensitivity analysis for
' CD33br HLA DR+ AC' on 'Rheumatoid arthritis'

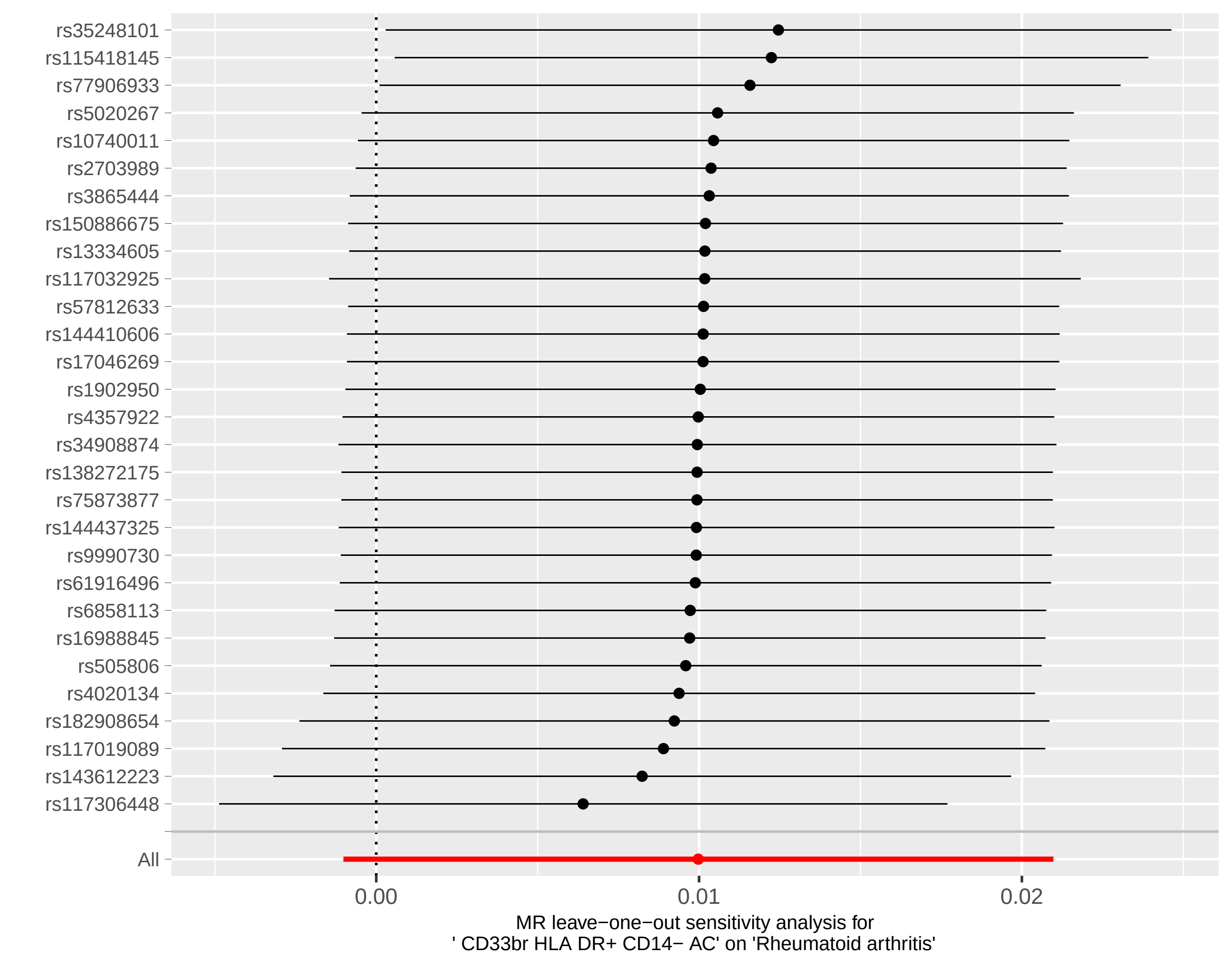
rs35248101
rs115418145
rs77906933
rs5020267
rs10740011
rs2703989
rs3865444
rs150886675
rs13334605
rs117032925
rs57812633
rs144410606
rs17046269
rs1902950
rs4357922
rs34908874
rs138272175
rs75873877
rs144437325
rs9990730
rs61916496
rs6858113
rs16988845
rs505806
rs4020134
rs182908654
rs117019089
rs143612223
rs117306448
All
0.00
0.01
0.02
MR leave−one−out sensitivity analysis for
' CD33br HLA DR+ CD14− AC' on 'Rheumatoid arthritis'

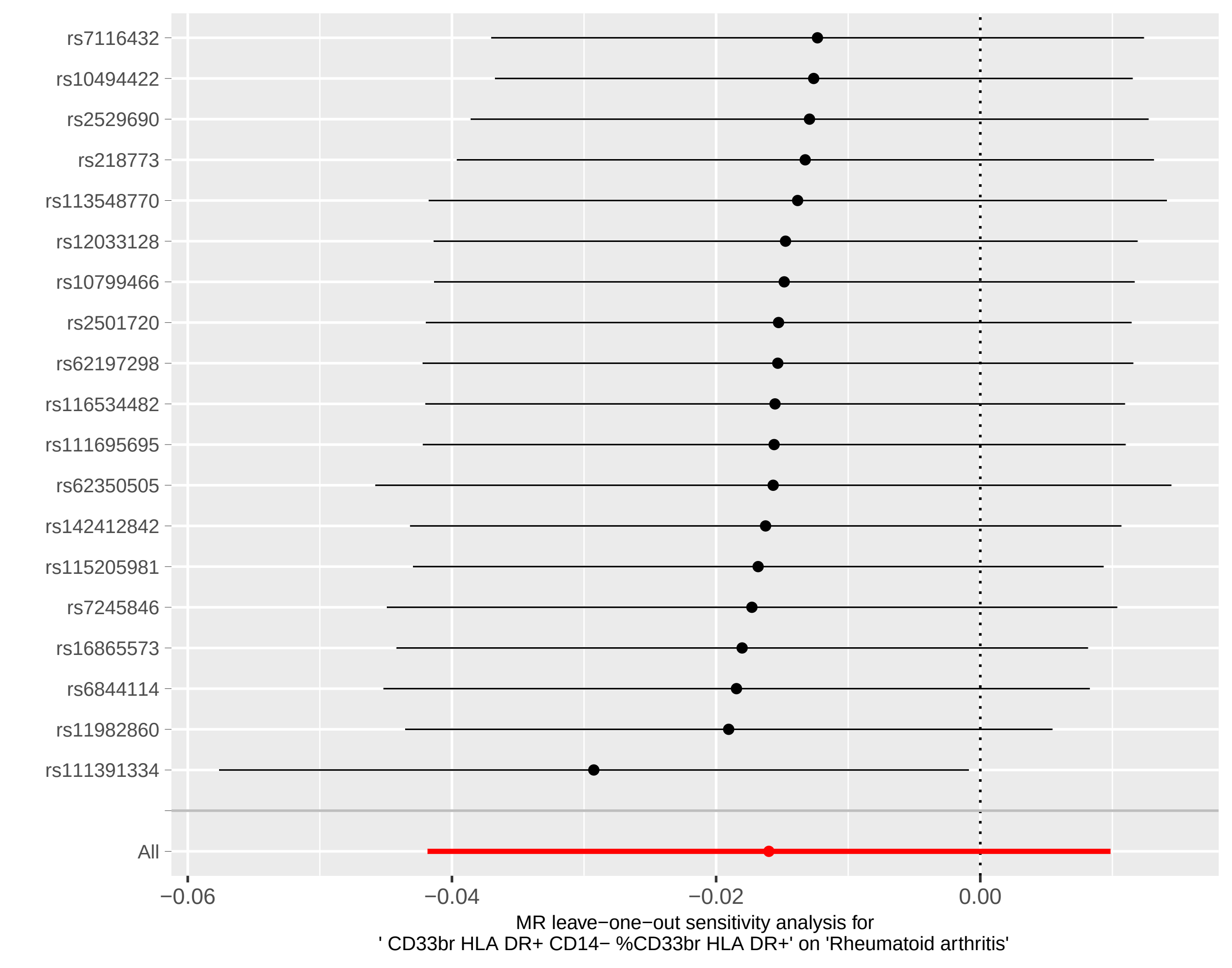
rs7116432
rs10494422
rs2529690
rs218773
rs113548770
rs12033128
rs10799466
rs2501720
rs62197298
rs116534482
rs111695695
rs62350505
rs142412842
rs115205981
rs7245846
rs16865573
rs6844114
rs11982860
rs111391334
All
−0.06
−0.04
−0.02
0.00
MR leave−one−out sensitivity analysis for
' CD33br HLA DR+ CD14− %CD33br HLA DR+' on 'Rheumatoid arthritis'

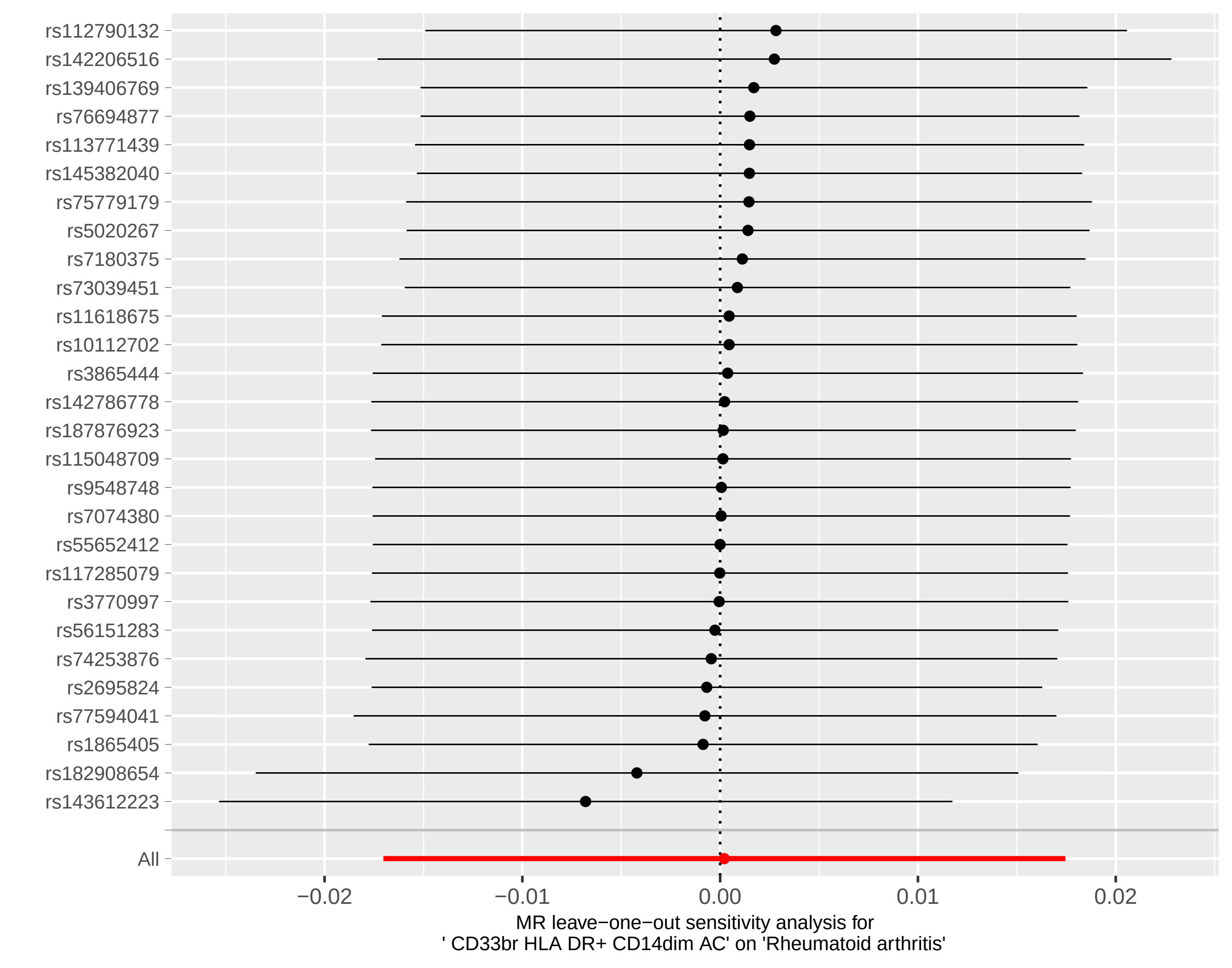
rs112790132
rs142206516
rs139406769
rs76694877
rs113771439
rs145382040
rs75779179
rs5020267
rs7180375
rs73039451
rs11618675
rs10112702
rs3865444
rs142786778
rs187876923
rs115048709
rs9548748
rs7074380
rs55652412
rs117285079
rs3770997
rs56151283
rs74253876
rs2695824
rs77594041
rs1865405
rs182908654
rs143612223
All
−0.02
−0.01
0.00
0.01
0.02
MR leave−one−out sensitivity analysis for
' CD33br HLA DR+ CD14dim AC' on 'Rheumatoid arthritis'

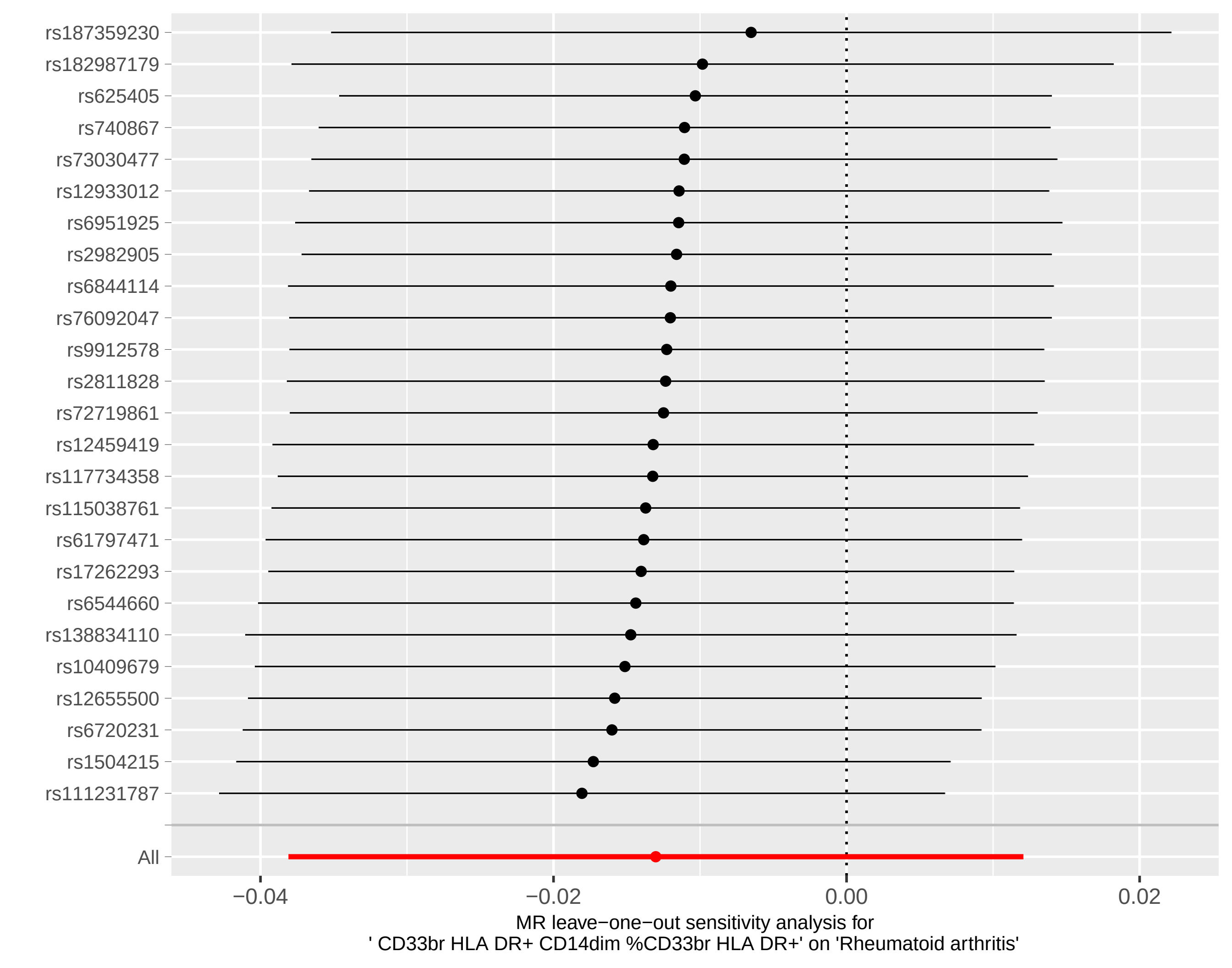
rs187359230
rs182987179
rs625405
rs740867
rs73030477
rs12933012
rs6951925
rs2982905
rs6844114
rs76092047
rs9912578
rs2811828
rs72719861
rs12459419
rs117734358
rs115038761
rs61797471
rs17262293
rs6544660
rs138834110
rs10409679
rs12655500
rs6720231
rs1504215
rs111231787
All
−0.04
−0.02
0.00
0.02
MR leave−one−out sensitivity analysis for
' CD33br HLA DR+ CD14dim %CD33br HLA DR+' on 'Rheumatoid arthritis'

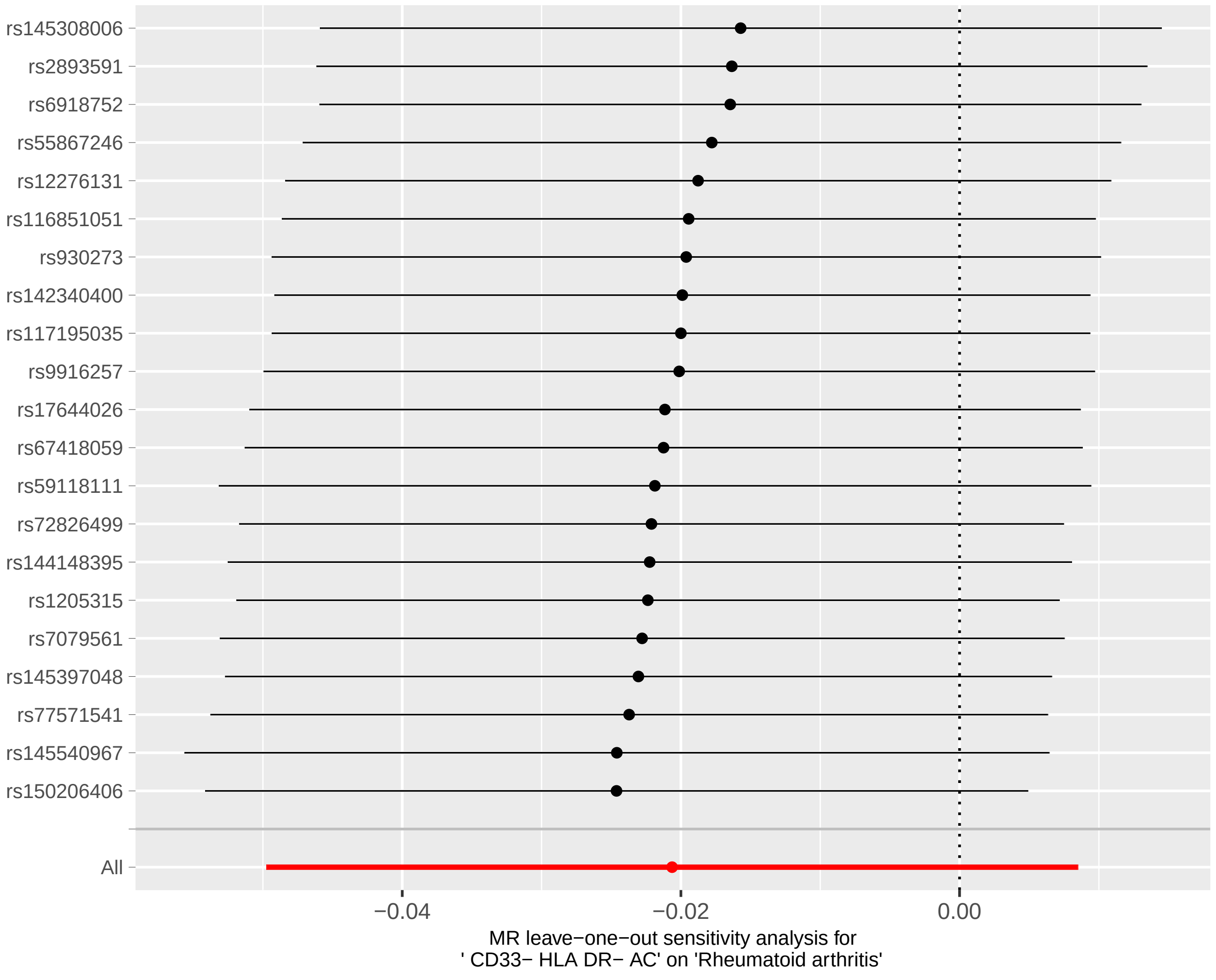
rs145308006
rs2893591
rs6918752
rs55867246
rs12276131
rs116851051
rs930273
rs142340400
rs117195035
rs9916257
rs17644026
rs67418059
rs59118111
rs72826499
rs144148395
rs1205315
rs7079561
rs145397048
rs77571541
rs145540967
rs150206406
All
−0.04
−0.02
0.00
MR leave−one−out sensitivity analysis for
' CD33− HLA DR− AC' on 'Rheumatoid arthritis'

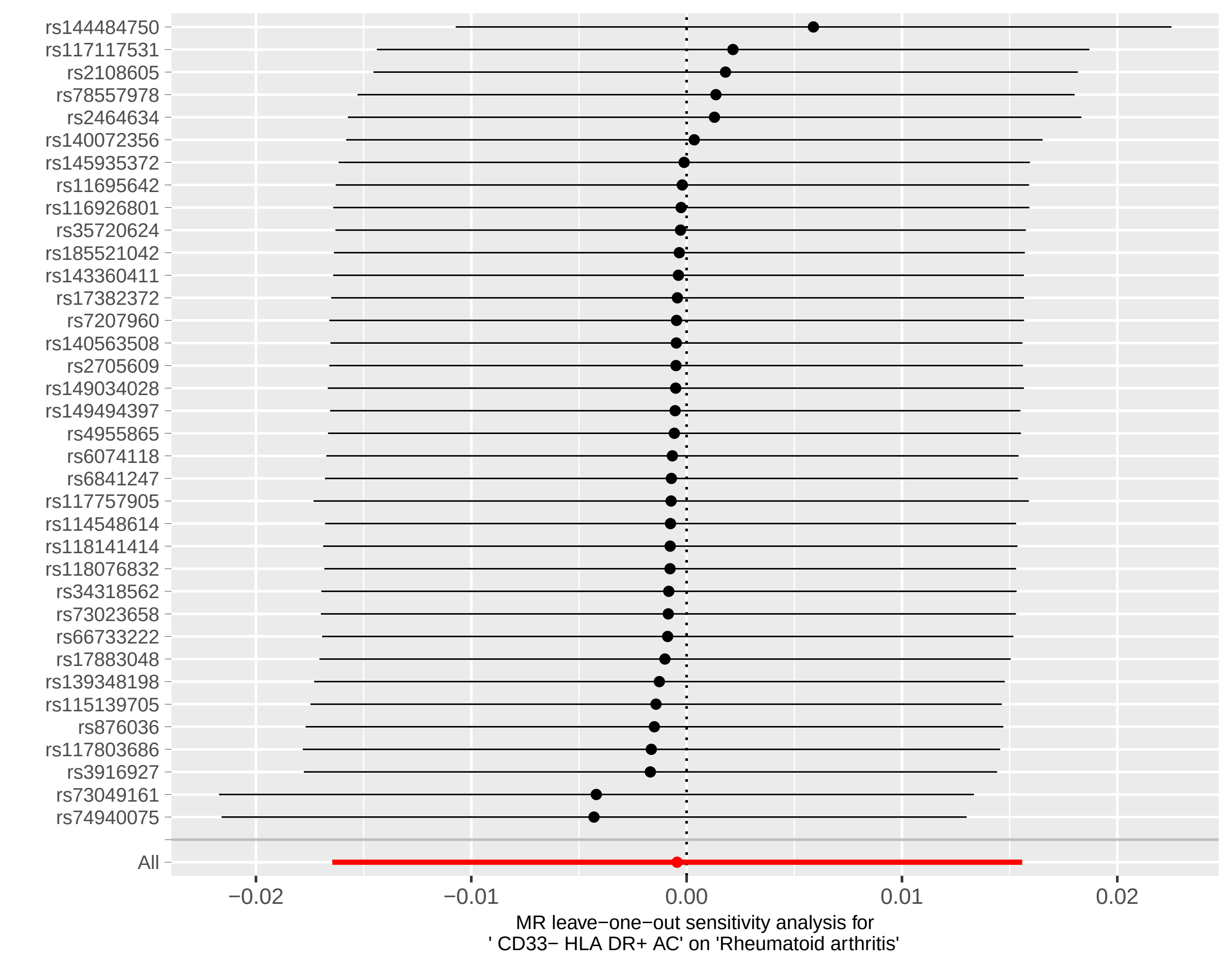

rs144484750
rs117117531
rs2108605
rs78557978
rs2464634
rs140072356
rs145935372
rs11695642
rs116926801
rs35720624
rs185521042
rs143360411
rs17382372
rs7207960
rs140563508
rs2705609
rs149034028
rs149494397
rs4955865
rs6074118
rs6841247
rs117757905
rs114548614
rs118141414
rs118076832
rs34318562
rs73023658
rs66733222
rs17883048
rs139348198
rs115139705
rs876036
rs117803686
rs3916927
rs73049161
rs74940075
All
−0.02
−0.01
0.00
0.01
0.02
MR leave−one−out sensitivity analysis for
' CD33− HLA DR+ AC' on 'Rheumatoid arthritis'

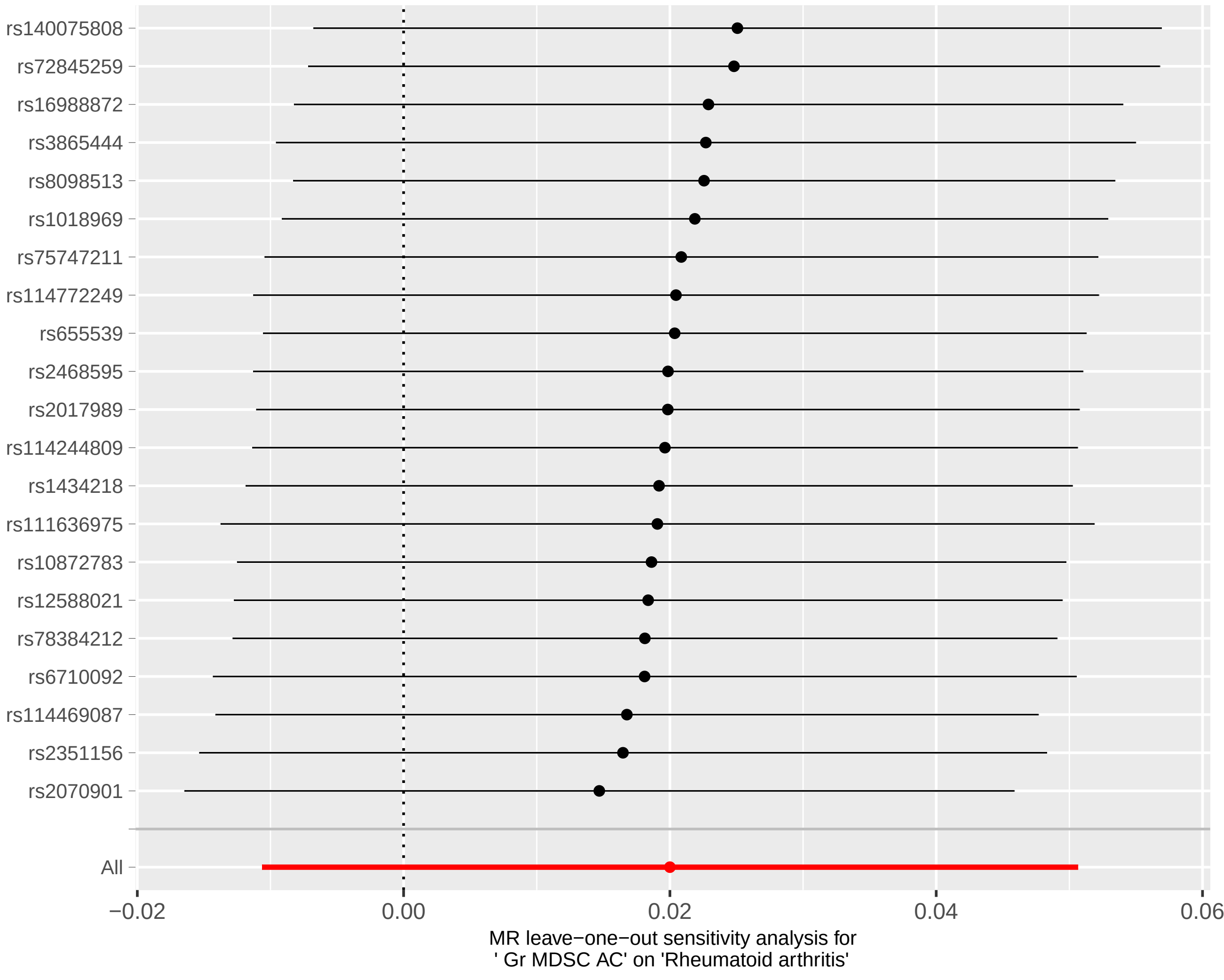
rs140075808
rs72845259
rs16988872
rs3865444
rs8098513
rs1018969
rs75747211
rs114772249
rs655539
rs2468595
rs2017989
rs114244809
rs1434218
rs111636975
rs10872783
rs12588021
rs78384212
rs6710092
rs114469087
rs2351156
rs2070901
All
−0.02
0.00
0.02
0.04
0.06
MR leave−one−out sensitivity analysis for
' Gr MDSC AC' on 'Rheumatoid arthritis'

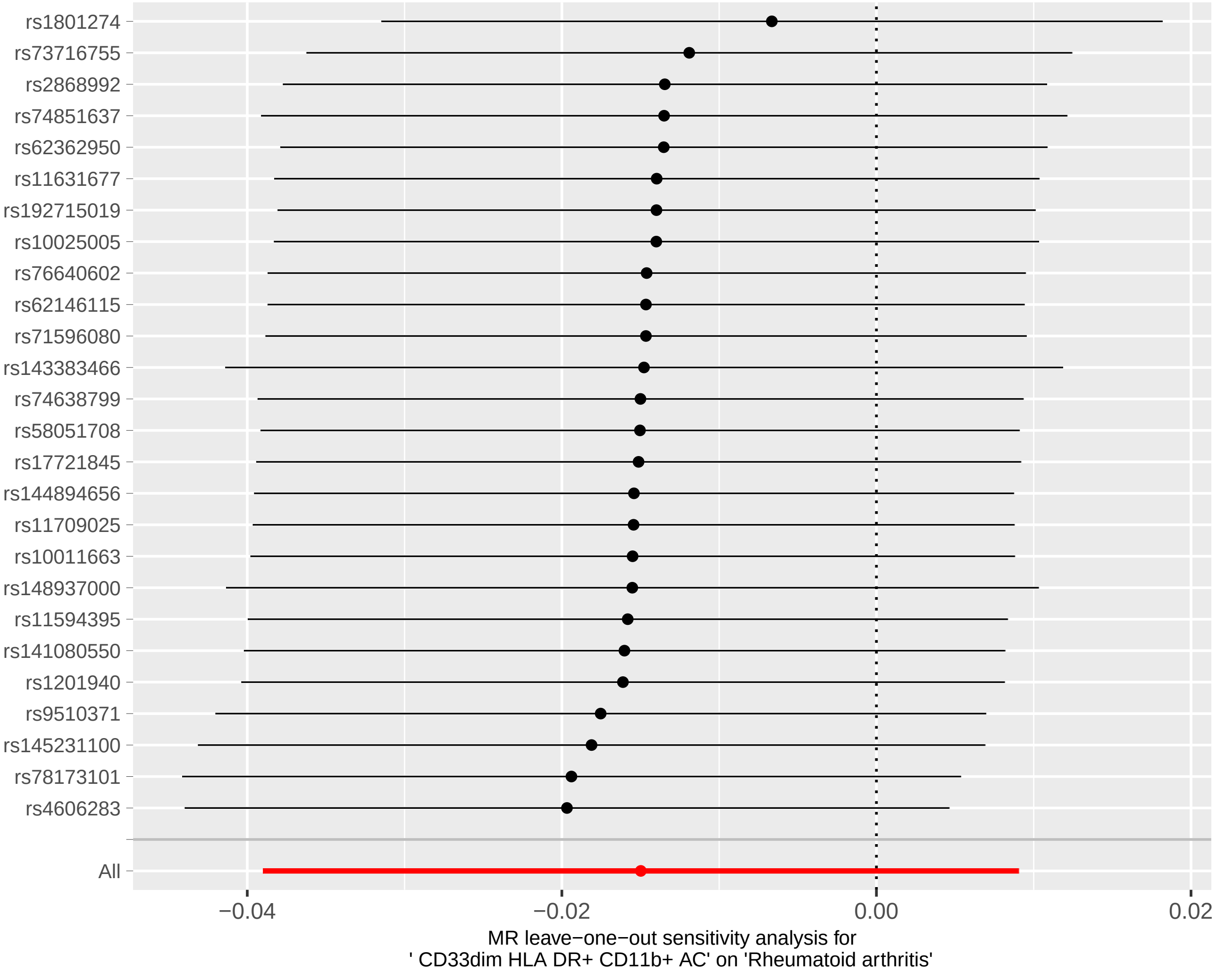

rs1801274
rs73716755
rs2868992
rs74851637
rs62362950
rs11631677
rs192715019
rs10025005
rs76640602
rs62146115
rs71596080
rs143383466
rs74638799
rs58051708
rs17721845
rs144894656
rs11709025
rs10011663
rs148937000
rs11594395
rs141080550
rs1201940
rs9510371
rs145231100
rs78173101
rs4606283
All
−0.04
−0.02
0.00
0.02
MR leave−one−out sensitivity analysis for
' CD33dim HLA DR+ CD11b+ AC' on 'Rheumatoid arthritis'

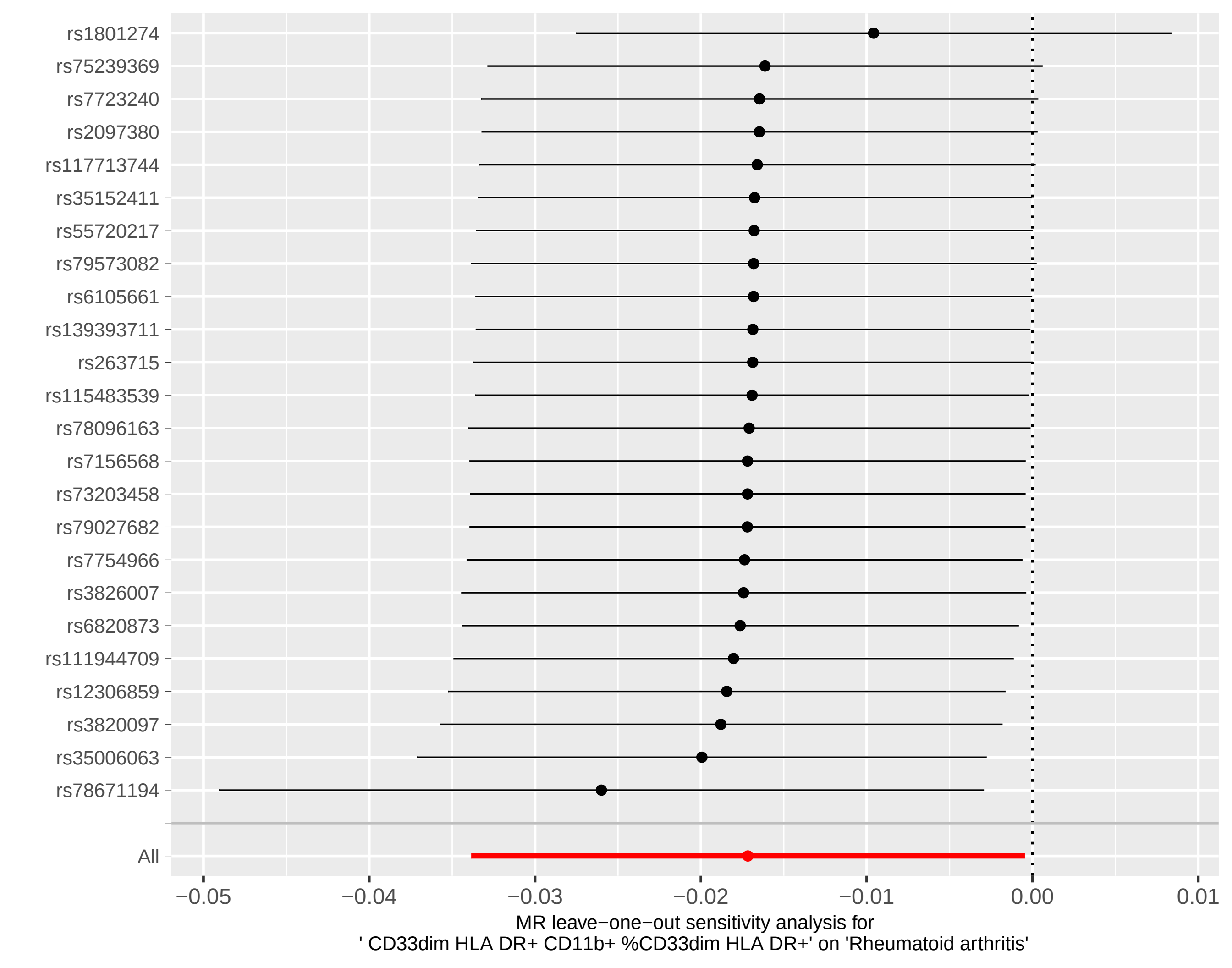
rs1801274
rs75239369
rs7723240
rs2097380
rs117713744
rs35152411
rs55720217
rs79573082
rs6105661
rs139393711
rs263715
rs115483539
rs78096163
rs7156568
rs73203458
rs79027682
rs7754966
rs3826007
rs6820873
rs111944709
rs12306859
rs3820097
rs35006063
rs78671194
All
−0.05
−0.04
−0.03
−0.02
−0.01
0.00
0.01
MR leave−one−out sensitivity analysis for
' CD33dim HLA DR+ CD11b+ %CD33dim HLA DR+' on 'Rheumatoid arthritis'

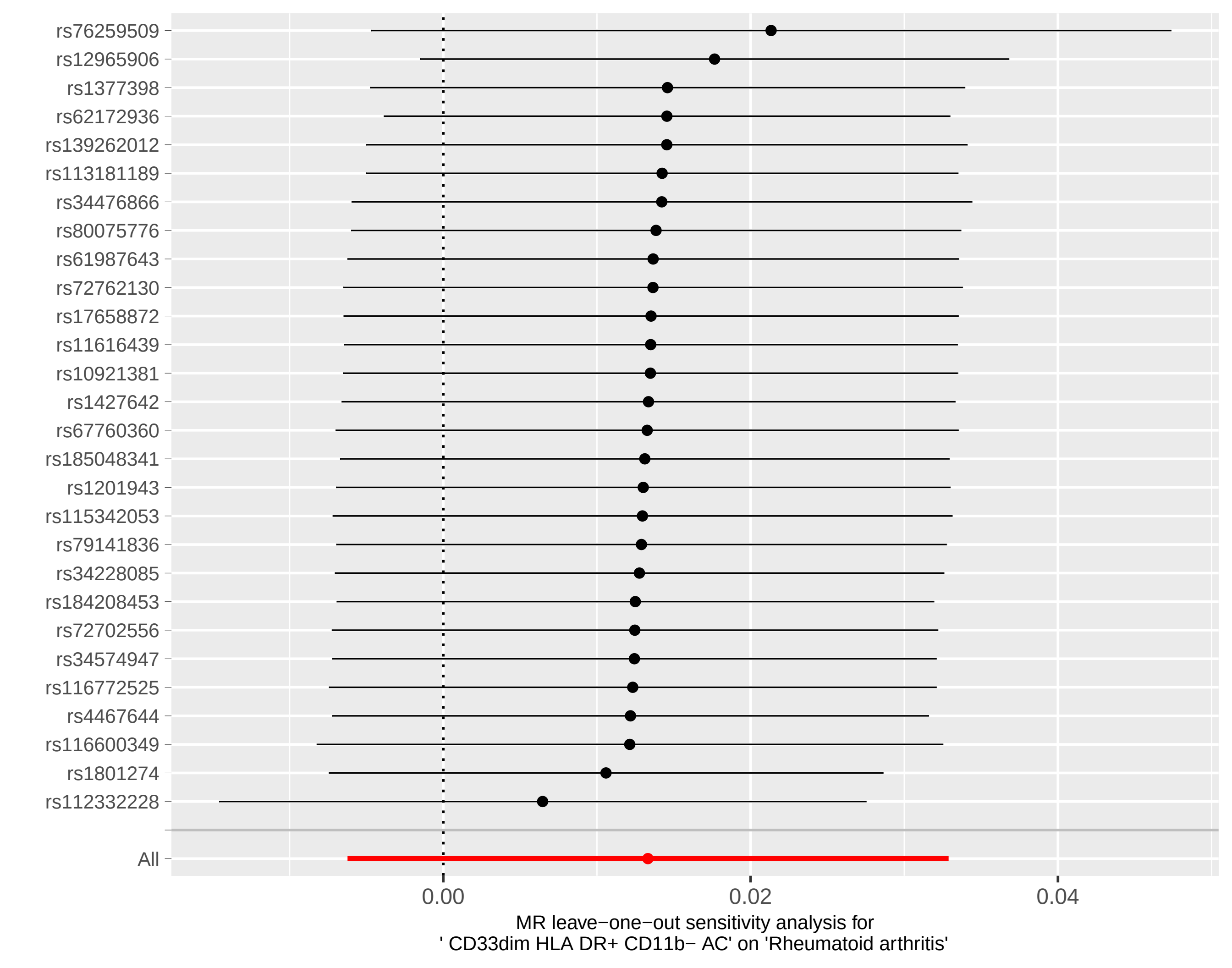
rs76259509
rs12965906
rs1377398
rs62172936
rs139262012
rs113181189
rs34476866
rs80075776
rs61987643
rs72762130
rs17658872
rs11616439
rs10921381
rs1427642
rs67760360
rs185048341
rs1201943
rs115342053
rs79141836
rs34228085
rs184208453
rs72702556
rs34574947
rs116772525
rs4467644
rs116600349
rs1801274
rs112332228
All
0.00
0.02
0.04
MR leave−one−out sensitivity analysis for
' CD33dim HLA DR+ CD11b− AC' on 'Rheumatoid arthritis'

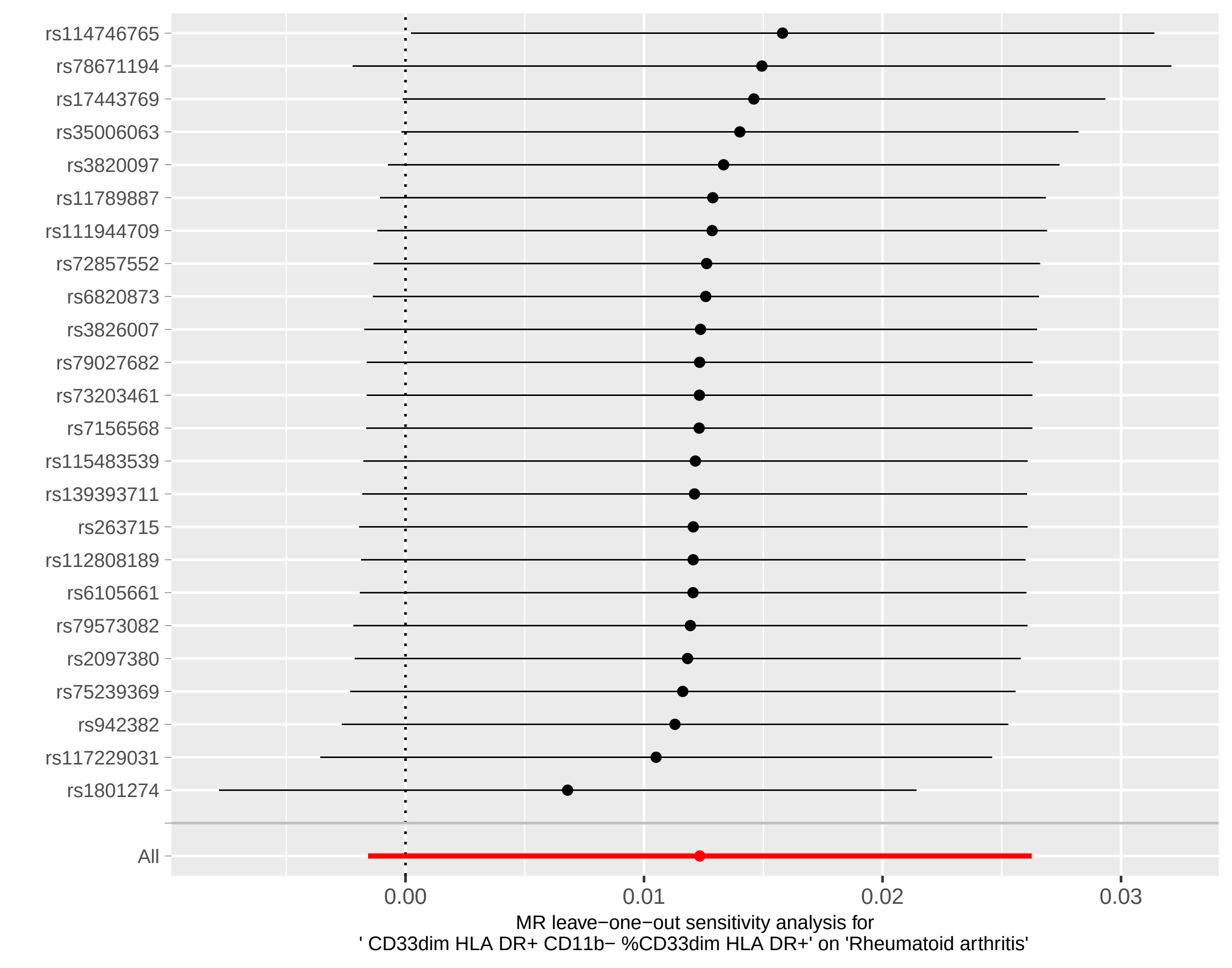
rs114746765
rs78671194
rs17443769
rs35006063
rs3820097
rs11789887
rs111944709
rs72857552
rs6820873
rs3826007
rs79027682
rs73203461
rs7156568
rs115483539
rs139393711
rs263715
rs112808189
rs6105661
rs79573082
rs2097380
rs75239369
rs942382
rs117229031
rs1801274
All
0.00
0.01
0.02
0.03
MR leave−one−out sensitivity analysis for
' CD33dim HLA DR+ CD11b− %CD33dim HLA DR+' on 'Rheumatoid arthritis'

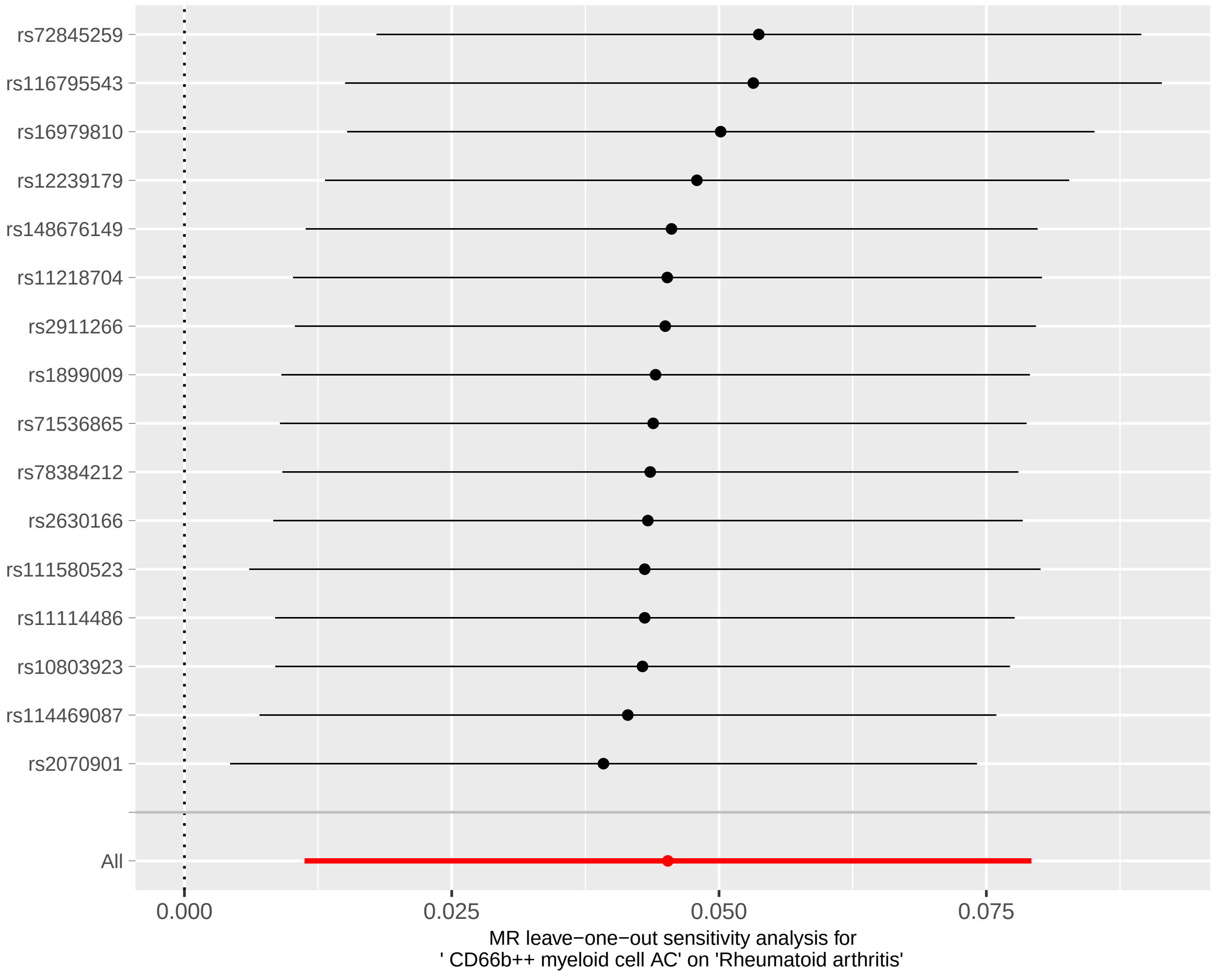

rs72845259
rs116795543
rs16979810
rs12239179
rs148676149
rs11218704
rs2911266
rs1899009
rs71536865
rs78384212
rs2630166
rs111580523
rs11114486
rs10803923
rs114469087
rs2070901
All
0.000
0.025
0.050
0.075
MR leave−one−out sensitivity analysis for
' CD66b++ myeloid cell AC' on 'Rheumatoid arthritis'

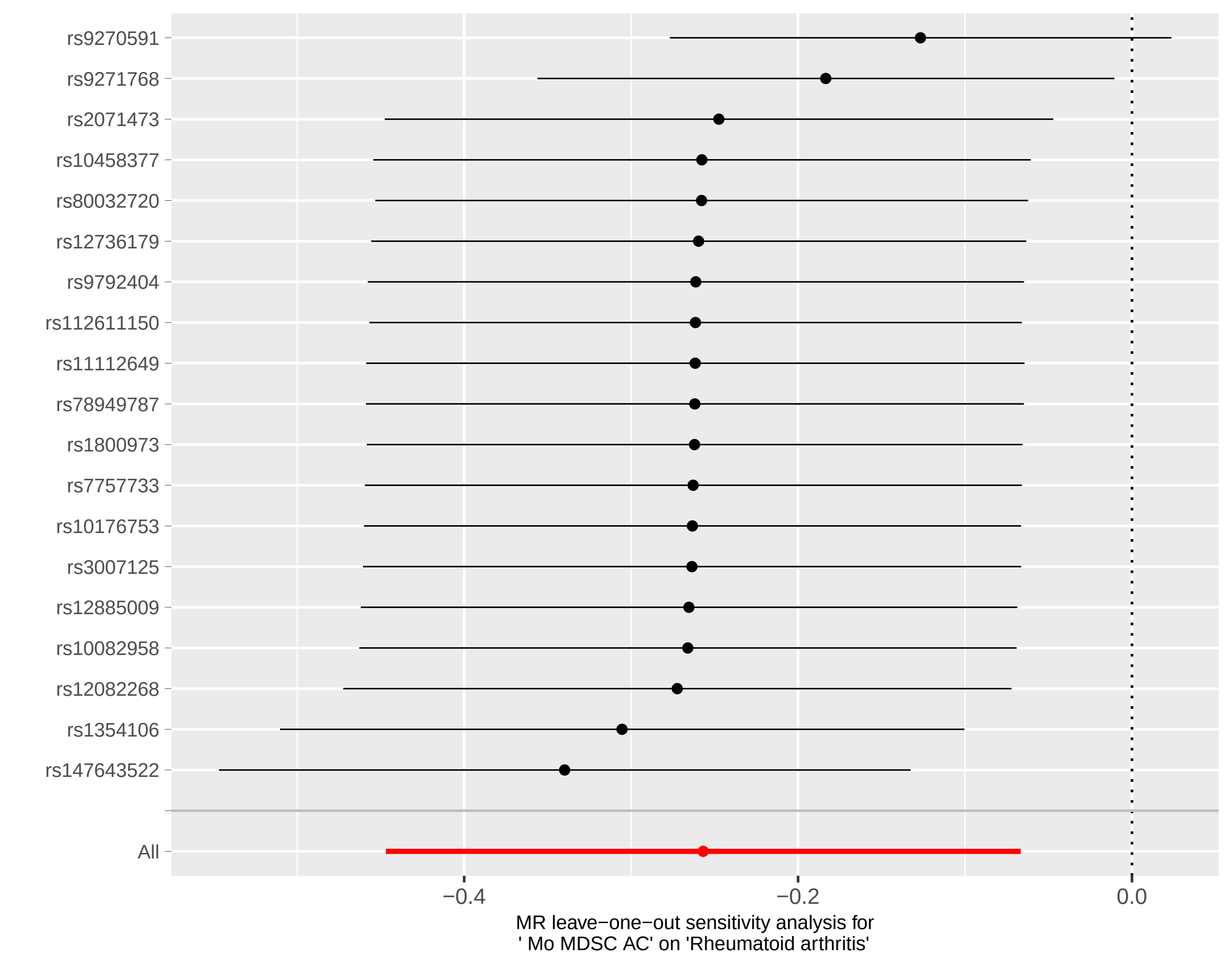
rs9270591
rs9271768
rs2071473
rs10458377
rs80032720
rs12736179
rs9792404
rs112611150
rs11112649
rs78949787
rs1800973
rs7757733
rs10176753
rs3007125
rs12885009
rs10082958
rs12082268
rs1354106
rs147643522
All
−0.4
−0.2
0.0
MR leave−one−out sensitivity analysis for
' Mo MDSC AC' on 'Rheumatoid arthritis'

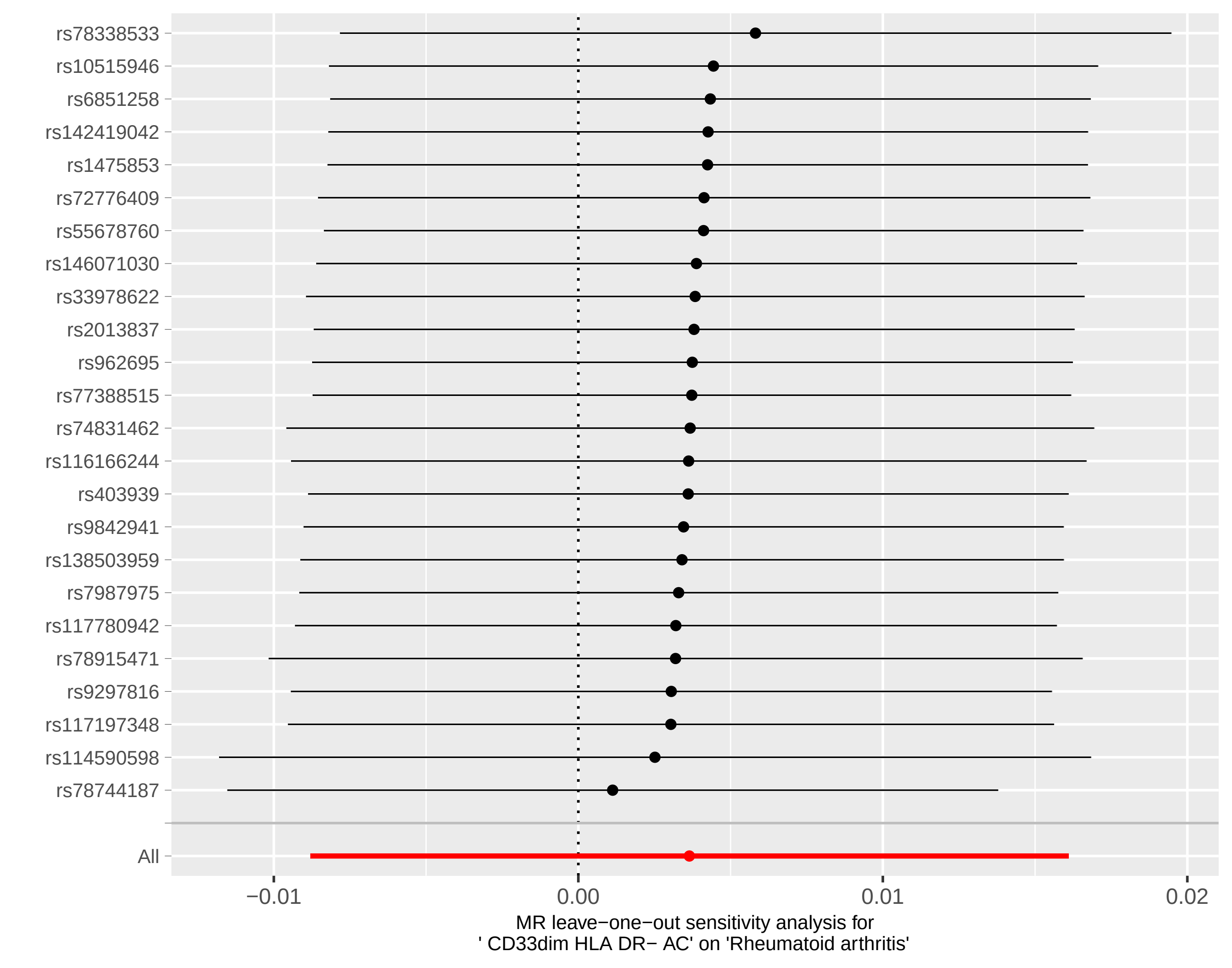
rs78338533
rs10515946
rs6851258
rs142419042
rs1475853
rs72776409
rs55678760
rs146071030
rs33978622
rs2013837
rs962695
rs77388515
rs74831462
rs116166244
rs403939
rs9842941
rs138503959
rs7987975
rs117780942
rs78915471
rs9297816
rs117197348
rs114590598
rs78744187
All
−0.01
0.00
0.01
0.02
MR leave−one−out sensitivity analysis for
' CD33dim HLA DR− AC' on 'Rheumatoid arthritis'

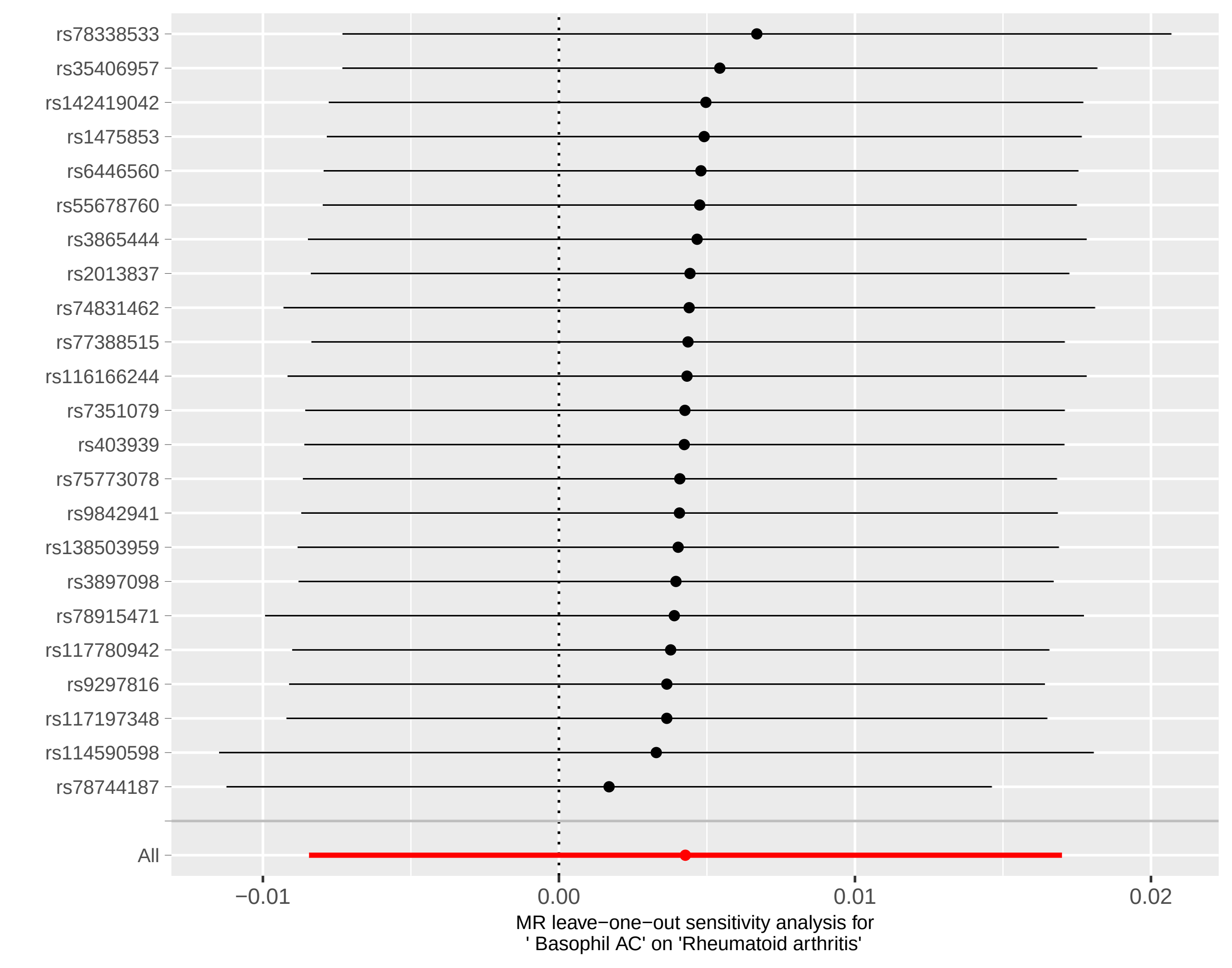
rs78338533
rs35406957
rs142419042
rs1475853
rs6446560
rs55678760
rs3865444
rs2013837
rs74831462
rs77388515
rs116166244
rs7351079
rs403939
rs75773078
rs9842941
rs138503959
rs3897098
rs78915471
rs117780942
rs9297816
rs117197348
rs114590598
rs78744187
All
−0.01
0.00
0.01
0.02
MR leave−one−out sensitivity analysis for
' Basophil AC' on 'Rheumatoid arthritis'

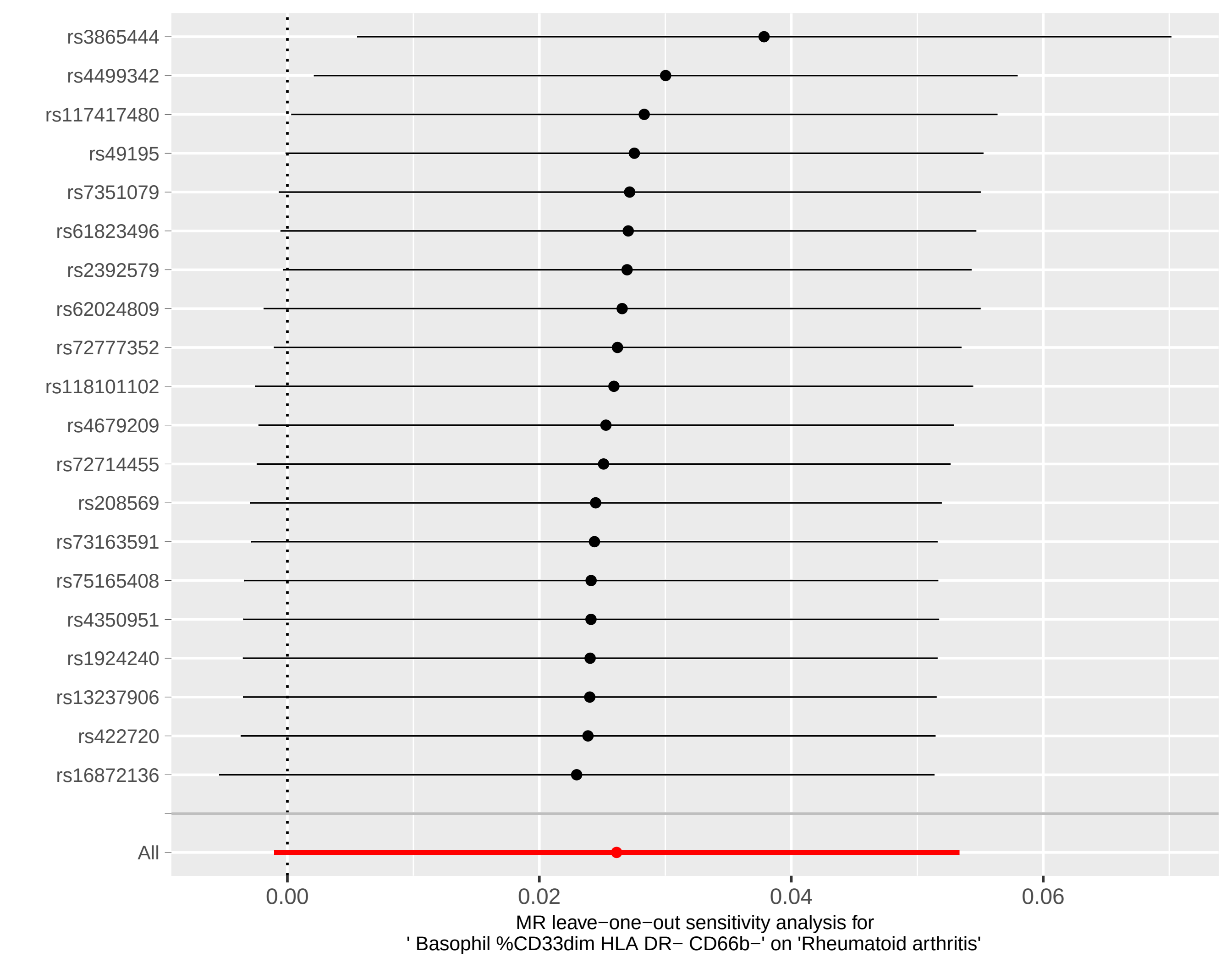

rs3865444
rs4499342
rs117417480
rs49195
rs7351079
rs61823496
rs2392579
rs62024809
rs72777352
rs118101102
rs4679209
rs72714455
rs208569
rs73163591
rs75165408
rs4350951
rs1924240
rs13237906
rs422720
rs16872136
All
0.00
0.02
0.04
0.06
MR leave−one−out sensitivity analysis for
' Basophil %CD33dim HLA DR− CD66b−' on 'Rheumatoid arthritis'

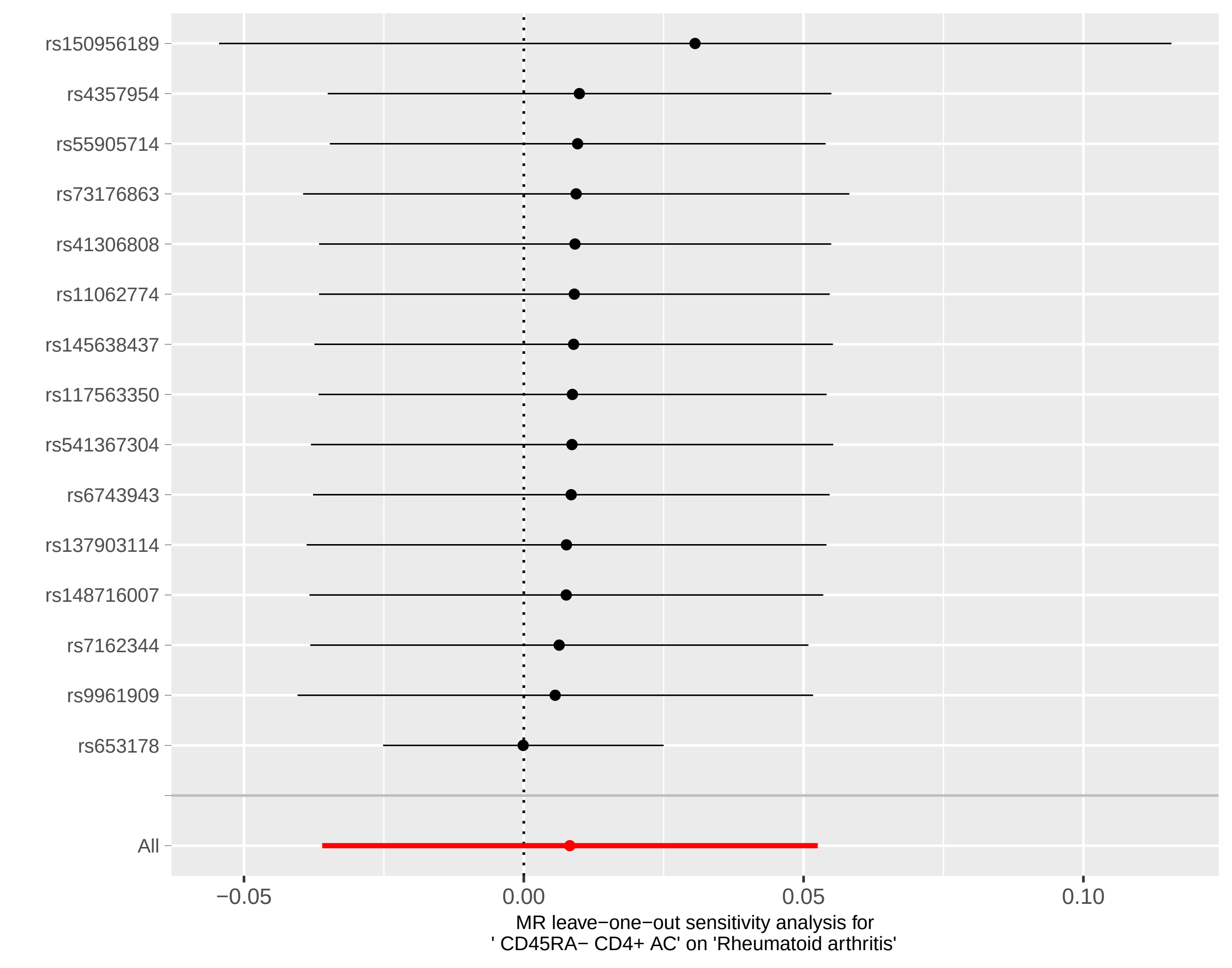
rs150956189
rs4357954
rs55905714
rs73176863
rs41306808
rs11062774
rs145638437
rs117563350
rs541367304
rs6743943
rs137903114
rs148716007
rs7162344
rs9961909
rs653178
All
−0.05
0.00
0.05
0.10
MR leave−one−out sensitivity analysis for
' CD45RA− CD4+ AC' on 'Rheumatoid arthritis'

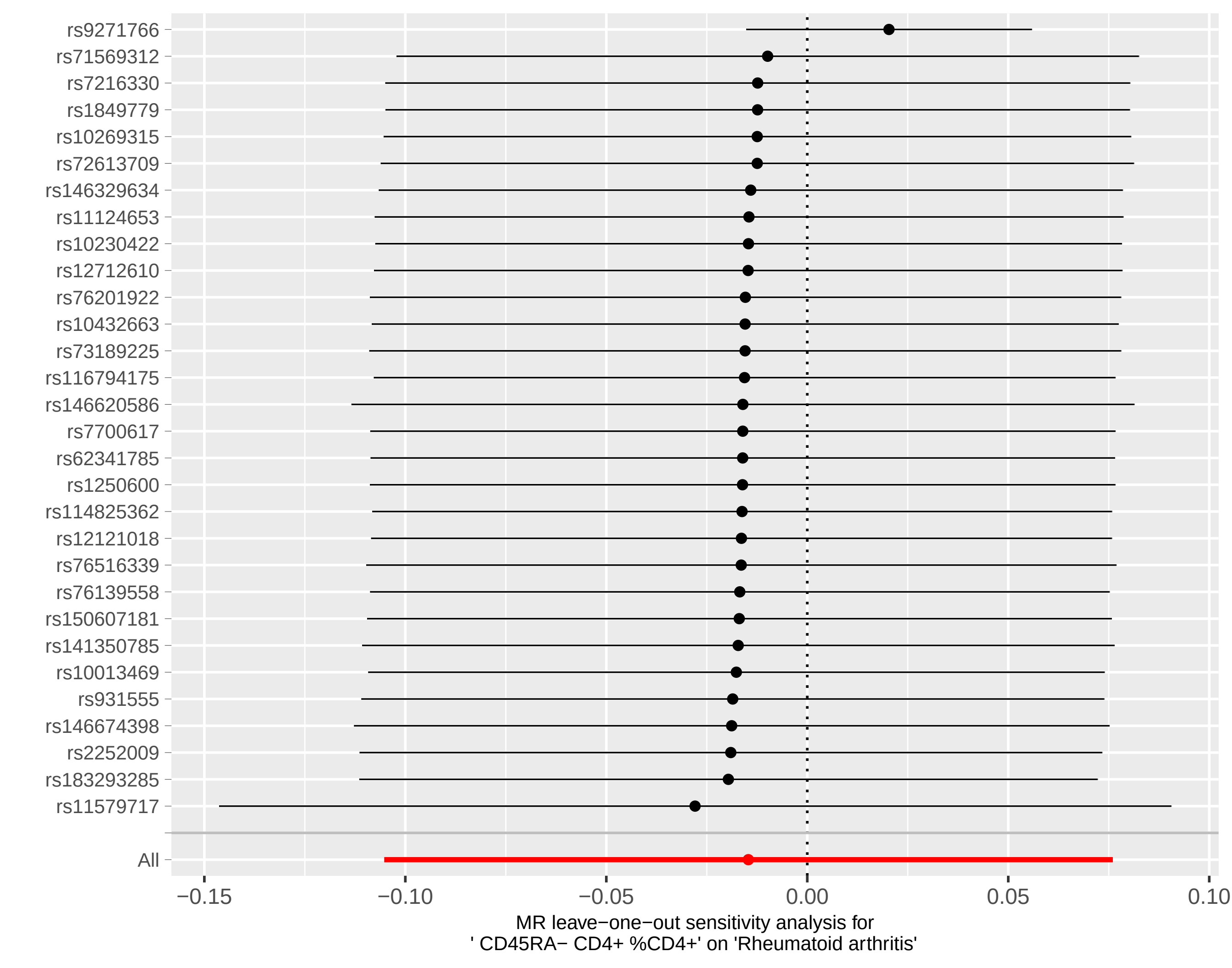

rs9271766
rs71569312
rs7216330
rs1849779
rs10269315
rs72613709
rs146329634
rs11124653
rs10230422
rs12712610
rs76201922
rs10432663
rs73189225
rs116794175
rs146620586
rs7700617
rs62341785
rs1250600
rs114825362
rs12121018
rs76516339
rs76139558
rs150607181
rs141350785
rs10013469
rs931555
rs146674398
rs2252009
rs183293285
rs11579717
All
−0.15
−0.10
−0.05
0.00
0.05
0.10
MR leave−one−out sensitivity analysis for
' CD45RA− CD4+ %CD4+' on 'Rheumatoid arthritis'

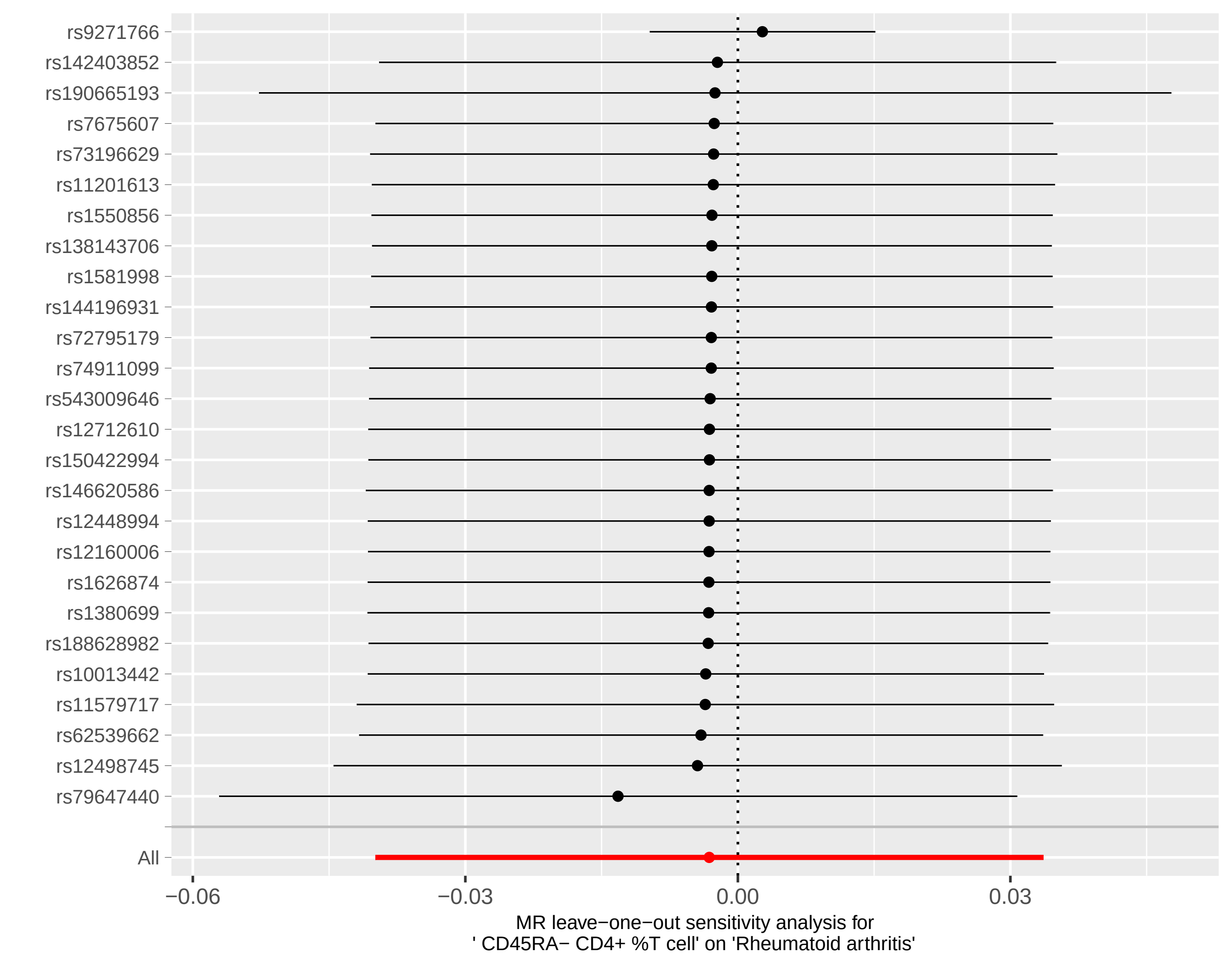

rs9271766
rs142403852
rs190665193
rs7675607
rs73196629
rs11201613
rs1550856
rs138143706
rs1581998
rs144196931
rs72795179
rs74911099
rs543009646
rs12712610
rs150422994
rs146620586
rs12448994
rs12160006
rs1626874
rs1380699
rs188628982
rs10013442
rs11579717
rs62539662
rs12498745
rs79647440
All
−0.06
−0.03
0.00
0.03
MR leave−one−out sensitivity analysis for
' CD45RA− CD4+ %T cell' on 'Rheumatoid arthritis'

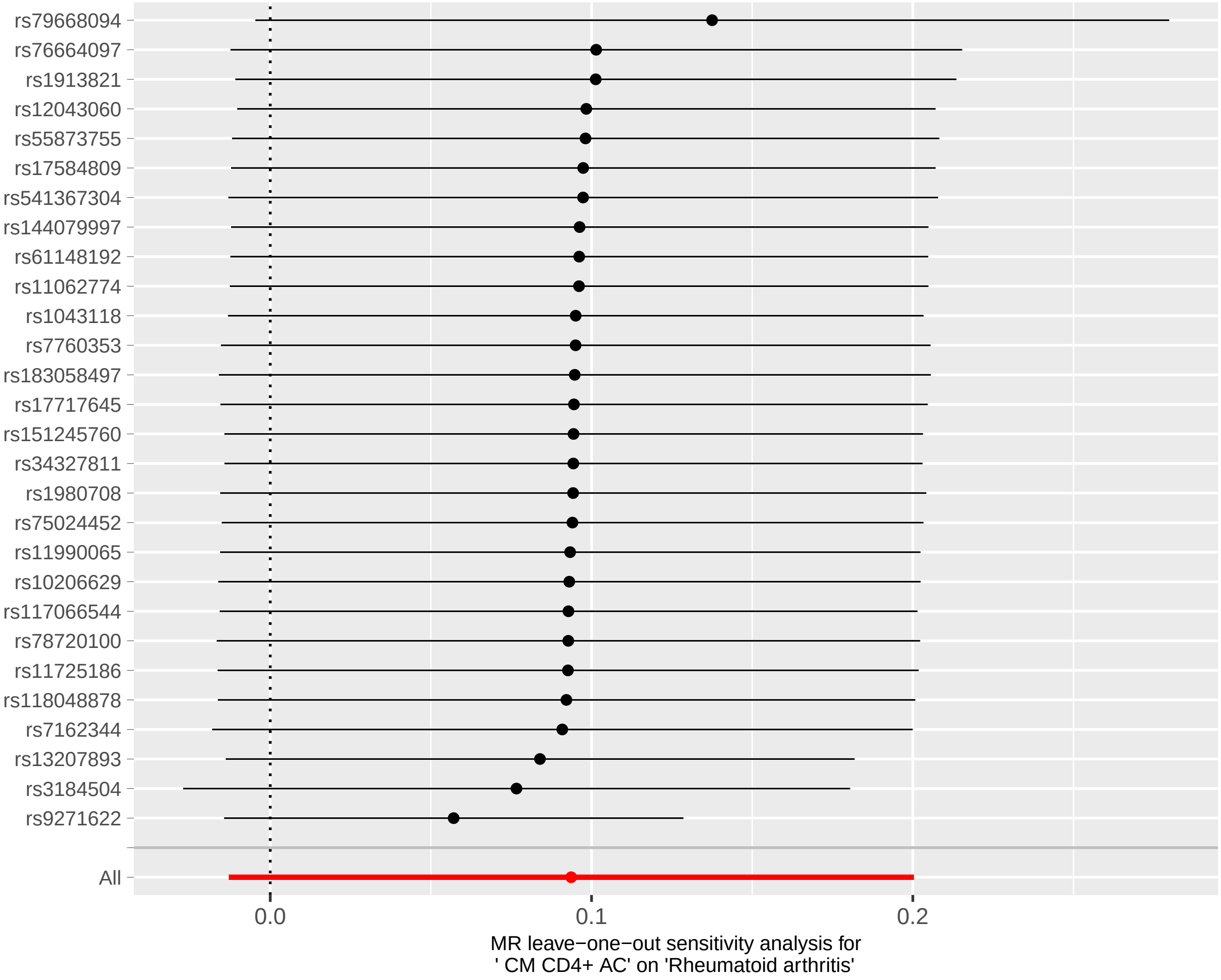

rs79668094
rs76664097
rs1913821
rs12043060
rs55873755
rs17584809
rs541367304
rs144079997
rs61148192
rs11062774
rs1043118
rs7760353
rs183058497
rs17717645
rs151245760
rs34327811
rs1980708
rs75024452
rs11990065
rs10206629
rs117066544
rs78720100
rs11725186
rs118048878
rs7162344
rs13207893
rs3184504
rs9271622
All
0.0
0.1
0.2
MR leave−one−out sensitivity analysis for
' CM CD4+ AC' on 'Rheumatoid arthritis'

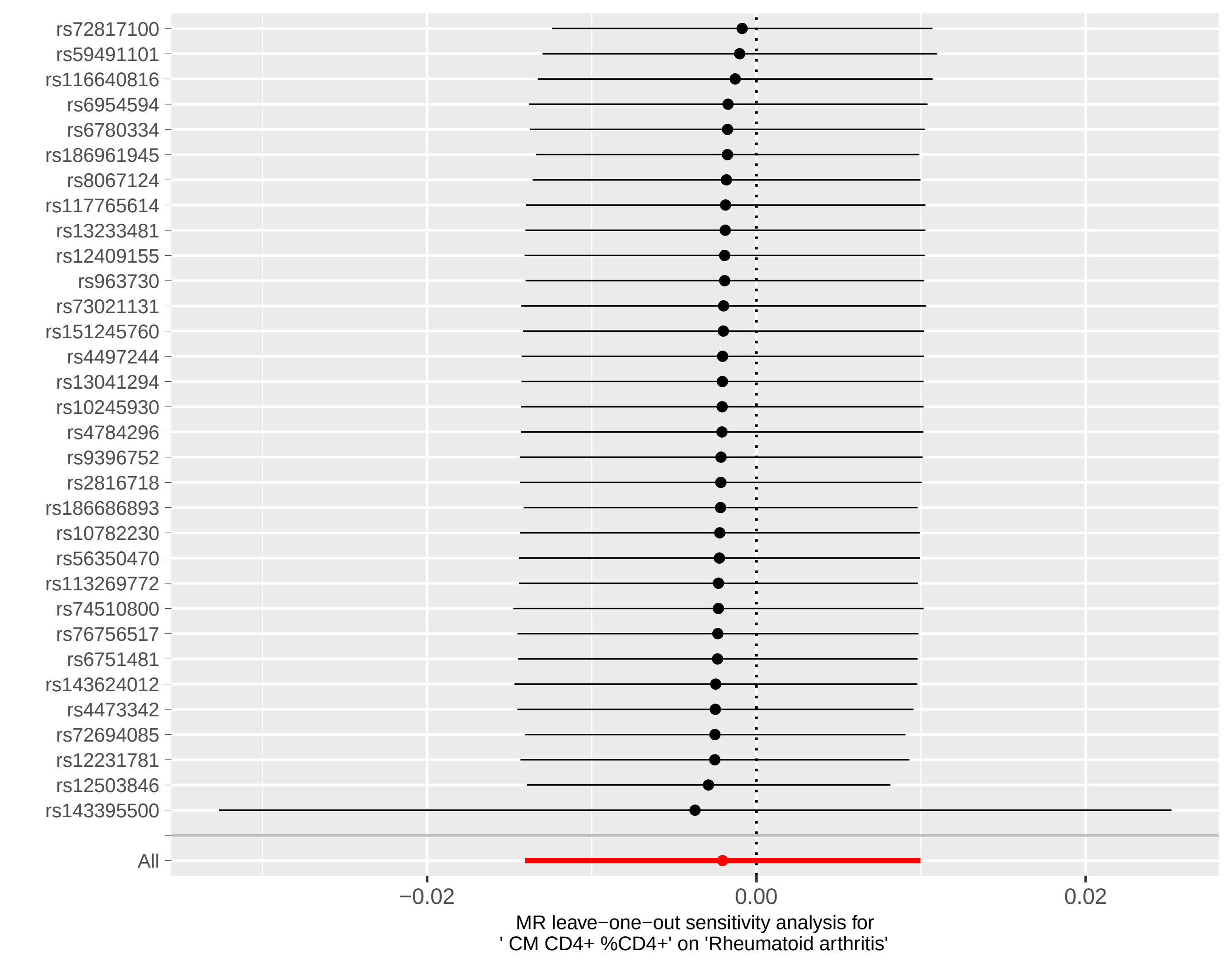

rs72817100
rs59491101
rs116640816
rs6954594
rs6780334
rs186961945
rs8067124
rs117765614
rs13233481
rs12409155
rs963730
rs73021131
rs151245760
rs4497244
rs13041294
rs10245930
rs4784296
rs9396752
rs2816718
rs186686893
rs10782230
rs56350470
rs113269772
rs74510800
rs76756517
rs6751481
rs143624012
rs4473342
rs72694085
rs12231781
rs12503846
rs143395500
All
−0.02
0.00
0.02
MR leave−one−out sensitivity analysis for
' CM CD4+ %CD4+' on 'Rheumatoid arthritis'

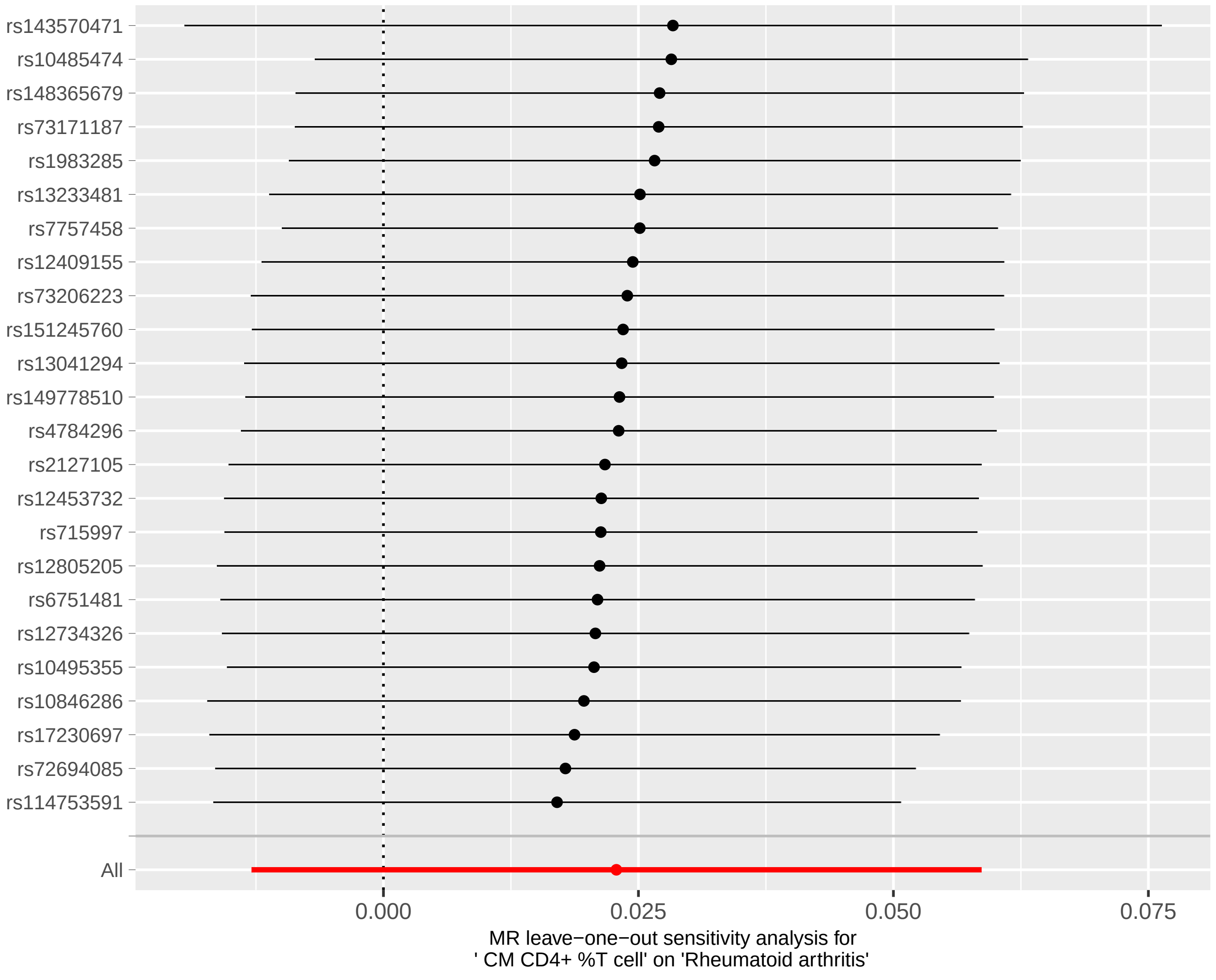
rs143570471
rs10485474
rs148365679
rs73171187
rs1983285
rs13233481
rs7757458
rs12409155
rs73206223
rs151245760
rs13041294
rs149778510
rs4784296
rs2127105
rs12453732
rs715997
rs12805205
rs6751481
rs12734326
rs10495355
rs10846286
rs17230697
rs72694085
rs114753591
All
0.000
0.025
0.050
0.075
MR leave−one−out sensitivity analysis for
' CM CD4+ %T cell' on 'Rheumatoid arthritis'

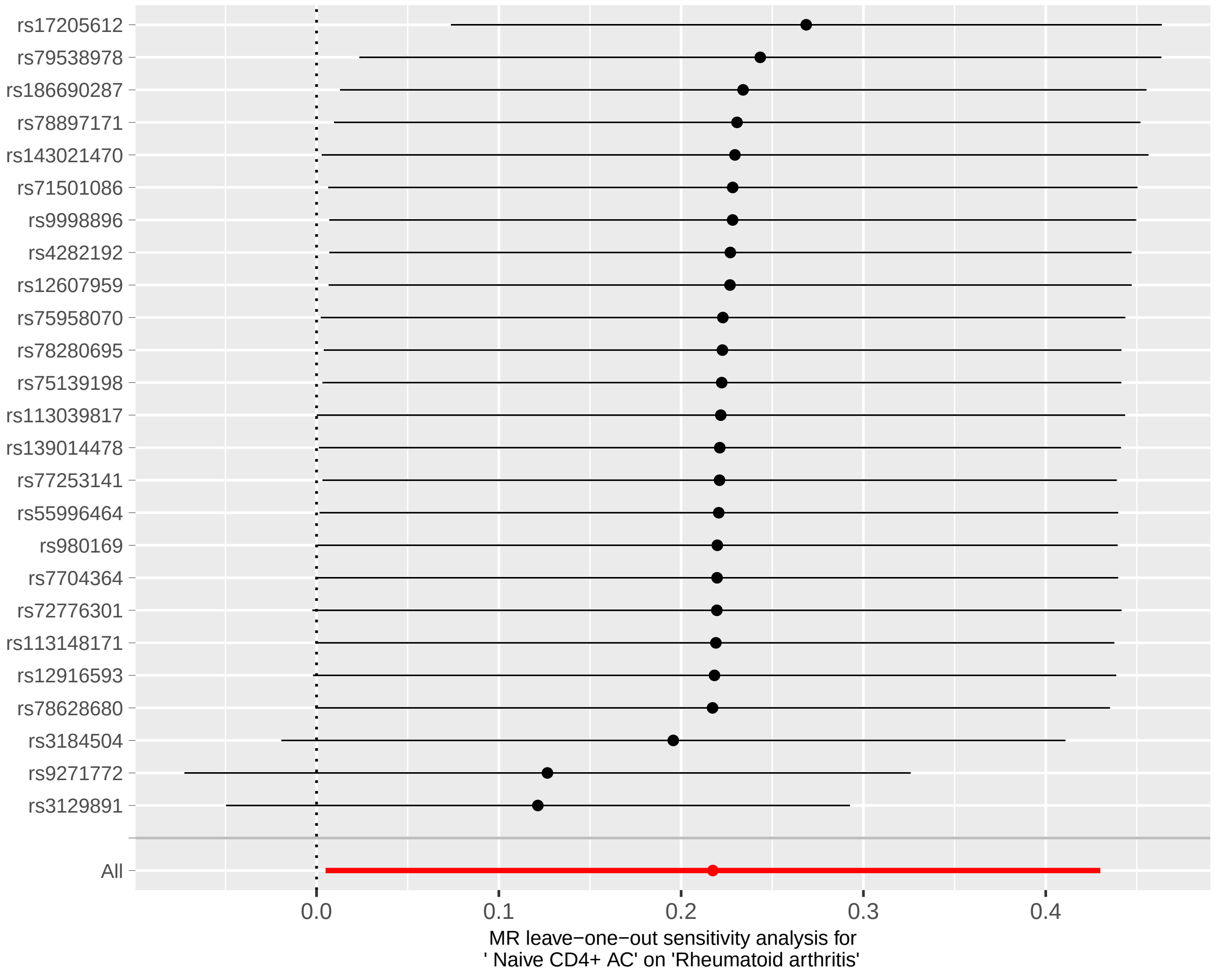
rs17205612
rs79538978
rs186690287
rs78897171
rs143021470
rs71501086
rs9998896
rs4282192
rs12607959
rs75958070
rs78280695
rs75139198
rs113039817
rs139014478
rs77253141
rs55996464
rs980169
rs7704364
rs72776301
rs113148171
rs12916593
rs78628680
rs3184504
rs9271772
rs3129891
All
0.0
0.1
0.2
0.3
0.4
MR leave−one−out sensitivity analysis for
' Naive CD4+ AC' on 'Rheumatoid arthritis'

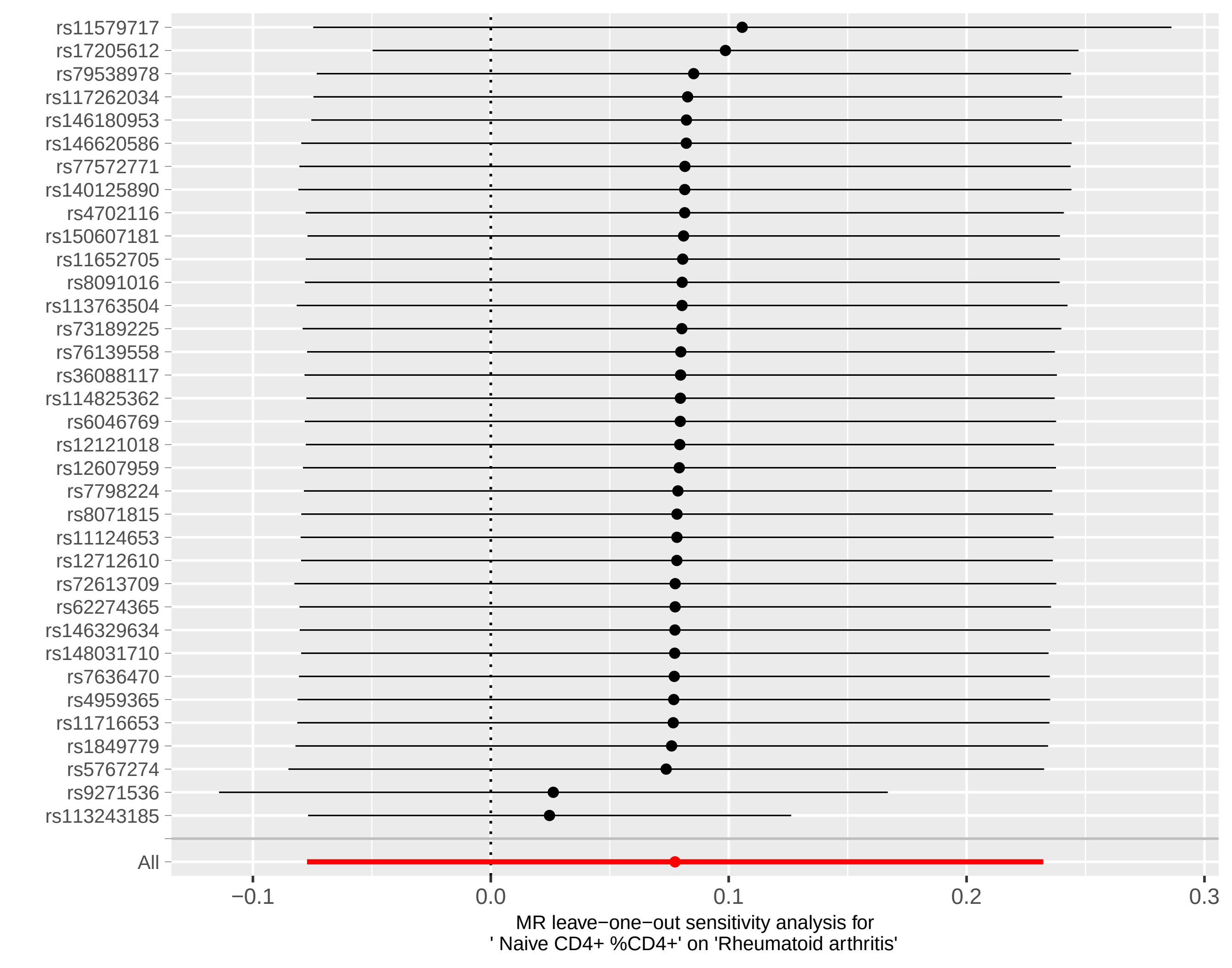
rs11579717
rs17205612
rs79538978
rs117262034
rs146180953
rs146620586
rs77572771
rs140125890
rs4702116
rs150607181
rs11652705
rs8091016
rs113763504
rs73189225
rs76139558
rs36088117
rs114825362
rs6046769
rs12121018
rs12607959
rs7798224
rs8071815
rs11124653
rs12712610
rs72613709
rs62274365
rs146329634
rs148031710
rs7636470
rs4959365
rs11716653
rs1849779
rs5767274
rs9271536
rs113243185
All
−0.1
0.0
0.1
0.2
0.3
MR leave−one−out sensitivity analysis for
' Naive CD4+ %CD4+' on 'Rheumatoid arthritis'

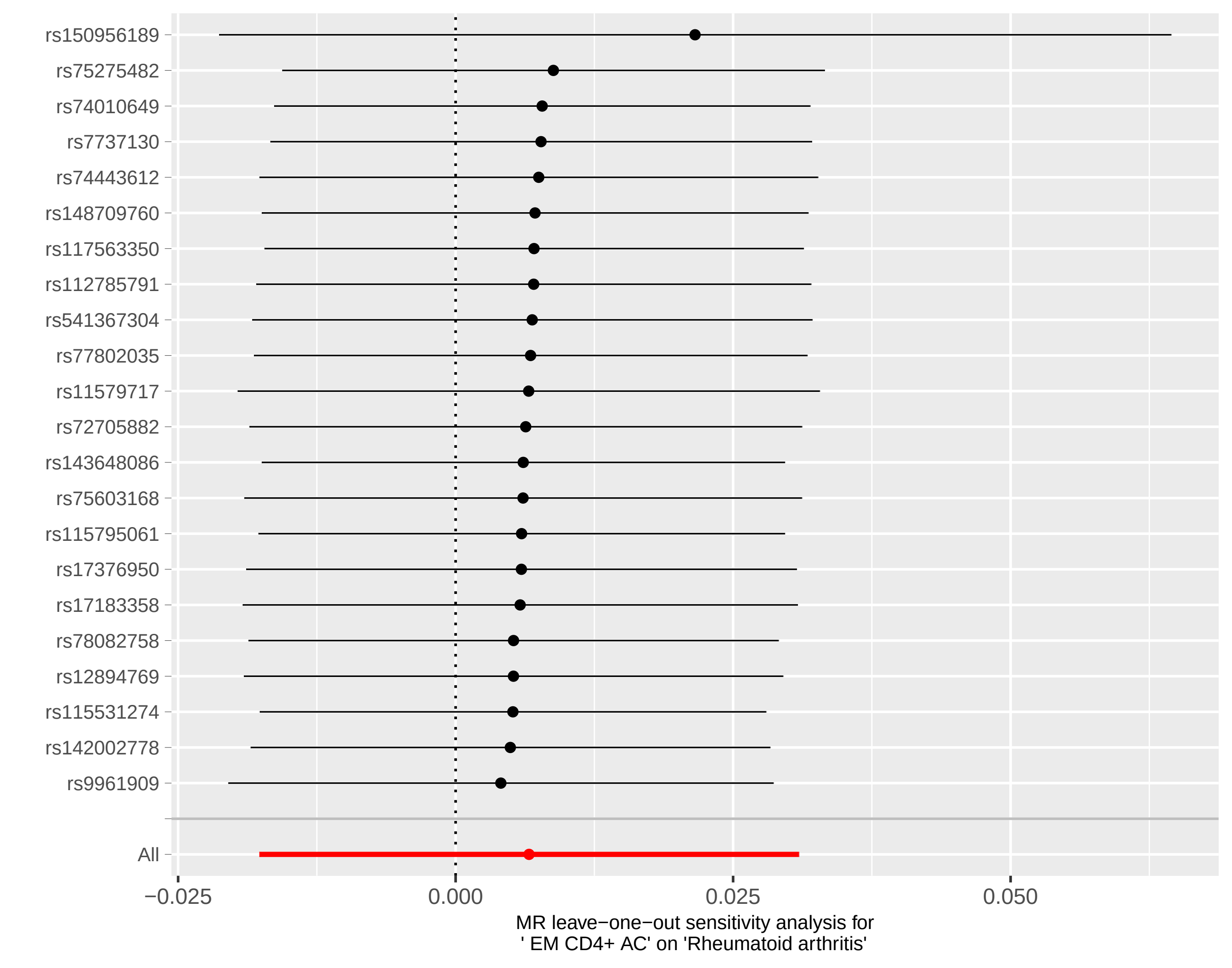
rs150956189
rs75275482
rs74010649
rs7737130
rs74443612
rs148709760
rs117563350
rs112785791
rs541367304
rs77802035
rs11579717
rs72705882
rs143648086
rs75603168
rs115795061
rs17376950
rs17183358
rs78082758
rs12894769
rs115531274
rs142002778
rs9961909
All
−0.025
0.000
0.025
0.050
MR leave−one−out sensitivity analysis for
' EM CD4+ AC' on 'Rheumatoid arthritis'

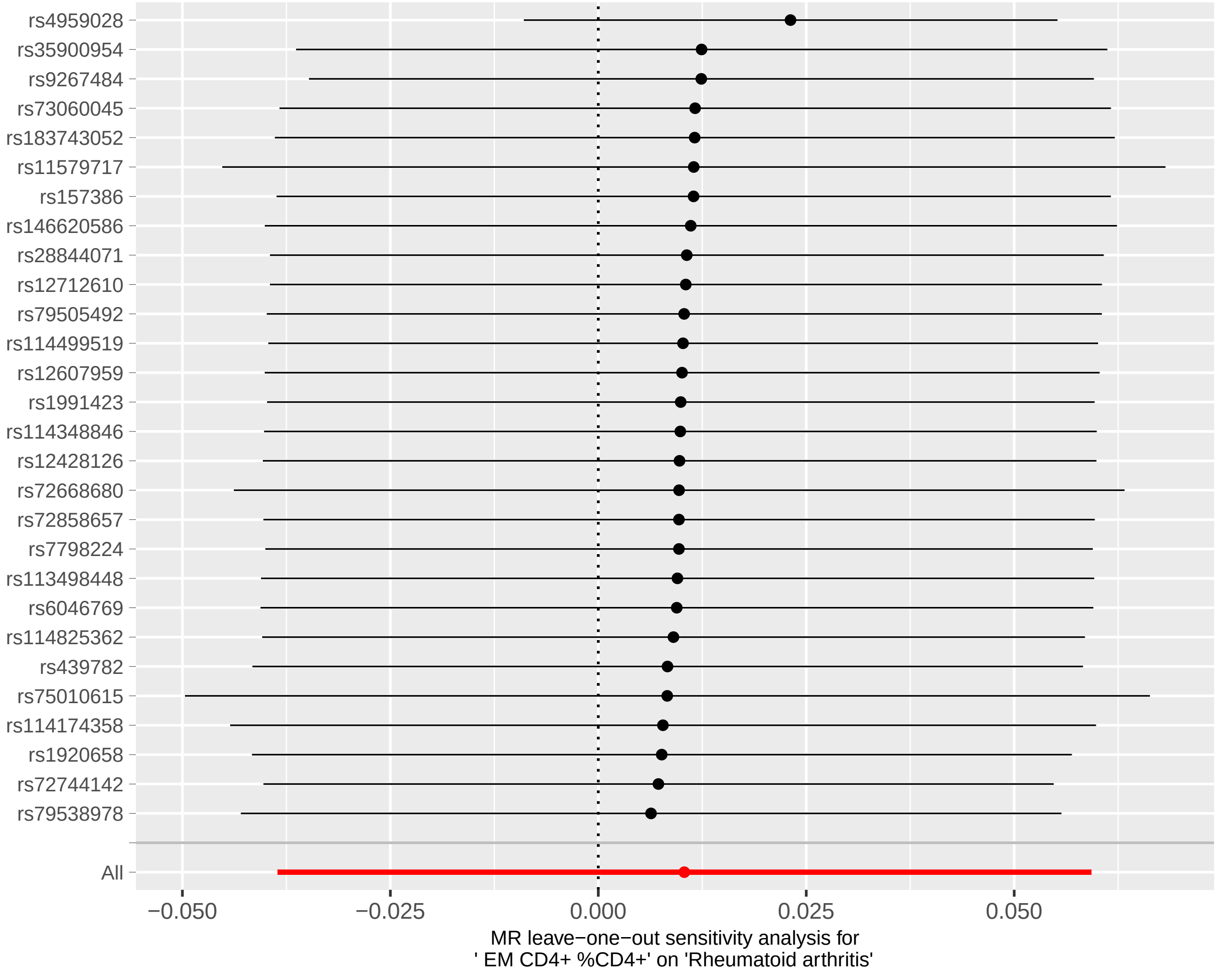

rs4959028
rs35900954
rs9267484
rs73060045
rs183743052
rs11579717
rs157386
rs146620586
rs28844071
rs12712610
rs79505492
rs114499519
rs12607959
rs1991423
rs114348846
rs12428126
rs72668680
rs72858657
rs7798224
rs113498448
rs6046769
rs114825362
rs439782
rs75010615
rs114174358
rs1920658
rs72744142
rs79538978
All
−0.050
−0.025
0.000
0.025
0.050
MR leave−one−out sensitivity analysis for
' EM CD4+ %CD4+' on 'Rheumatoid arthritis'

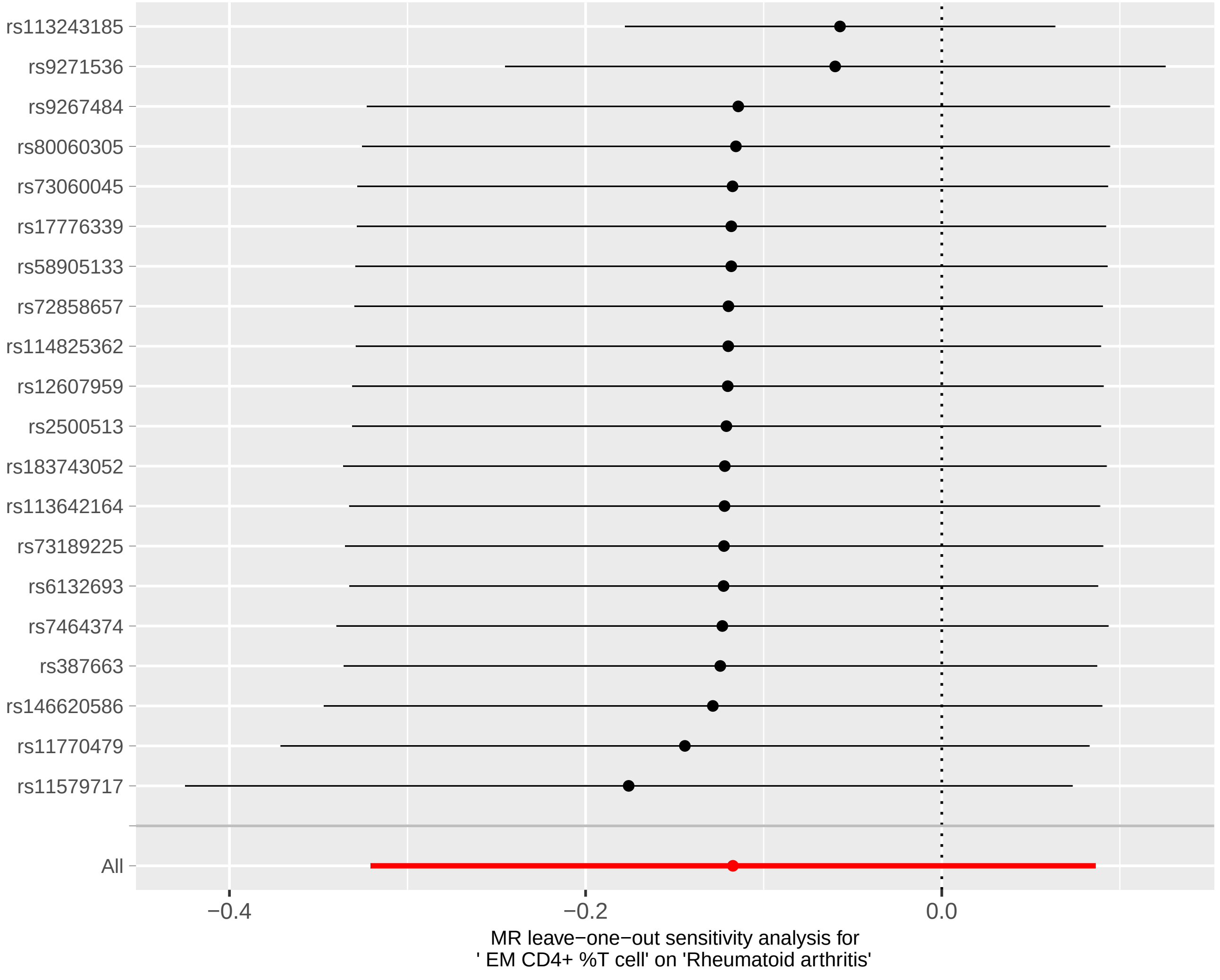
rs113243185
rs9271536
rs9267484
rs80060305
rs73060045
rs17776339
rs58905133
rs72858657
rs114825362
rs12607959
rs2500513
rs183743052
rs113642164
rs73189225
rs6132693
rs7464374
rs387663
rs146620586
rs11770479
rs11579717
All
−0.4
−0.2
0.0
MR leave−one−out sensitivity analysis for
' EM CD4+ %T cell' on 'Rheumatoid arthritis'

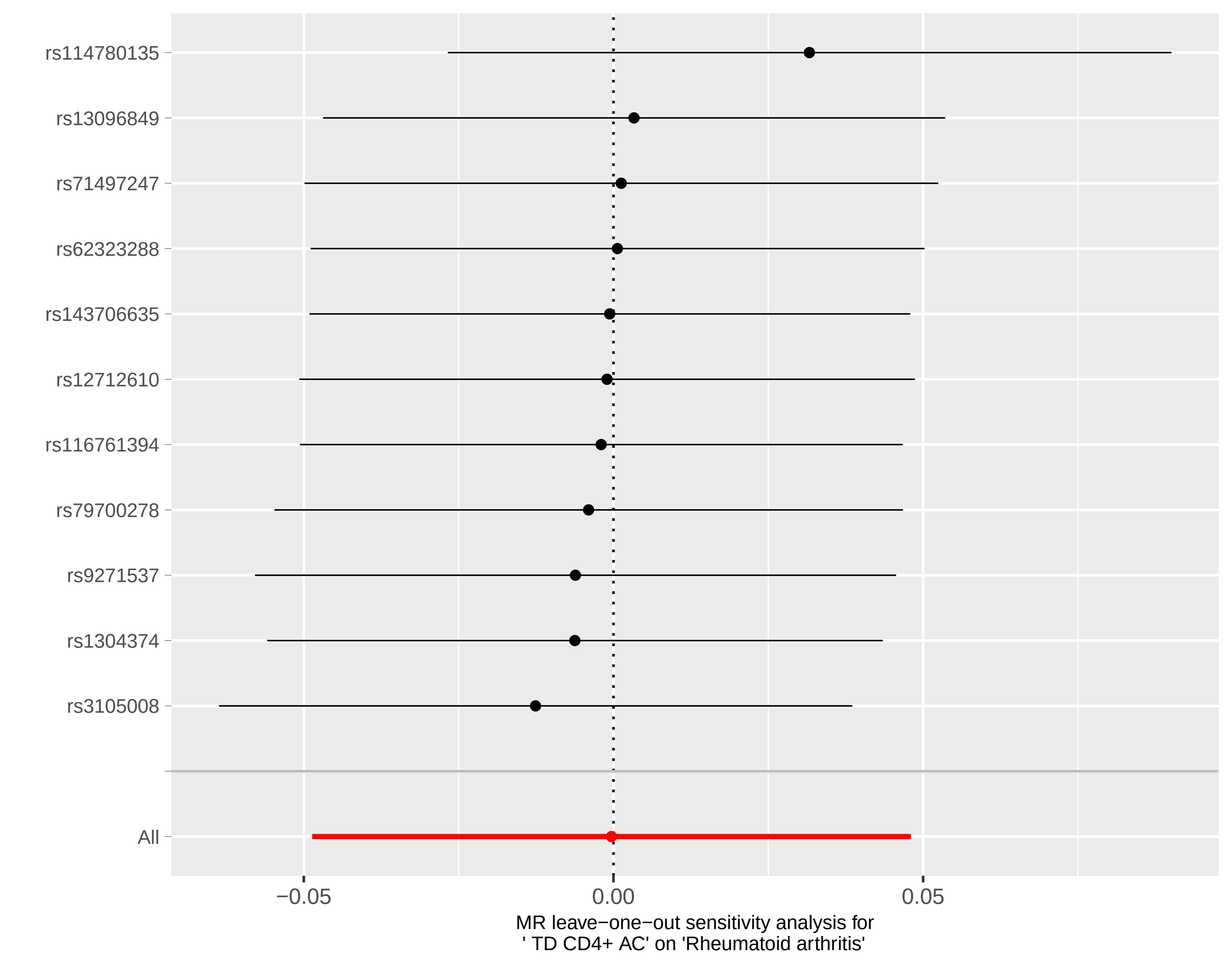
rs114780135
rs13096849
rs71497247
rs62323288
rs143706635
rs12712610
rs116761394
rs79700278
rs9271537
rs1304374
rs3105008
All
−0.05
0.00
0.05
MR leave−one−out sensitivity analysis for
' TD CD4+ AC' on 'Rheumatoid arthritis'

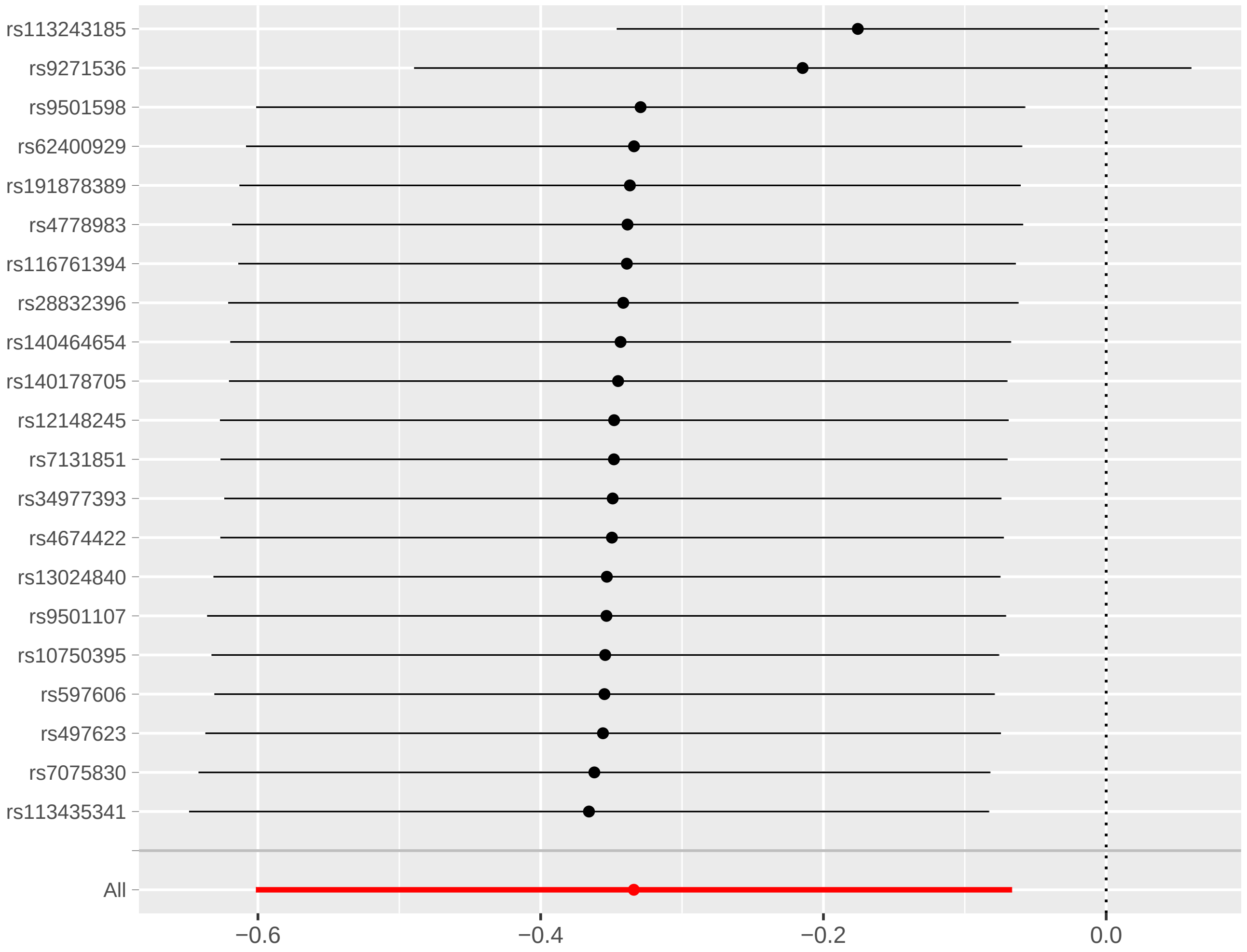

MR leave−one−out sensitivity analysis for
' TD CD4+ %CD4+' on 'Rheumatoid arthritis'

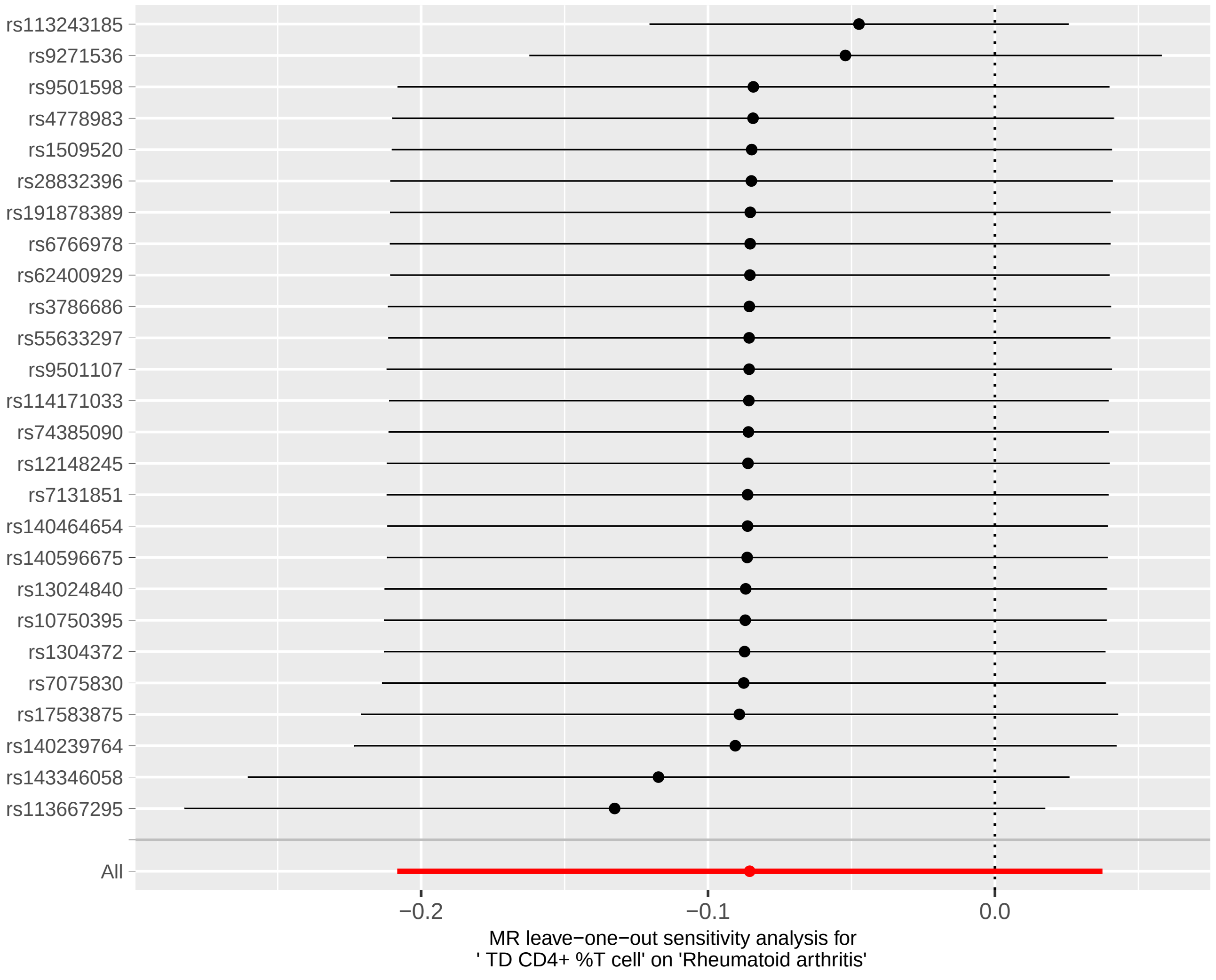

rs113243185
rs9271536
rs9501598
rs4778983
rs1509520
rs28832396
rs191878389
rs6766978
rs62400929
rs3786686
rs55633297
rs9501107
rs114171033
rs74385090
rs12148245
rs7131851
rs140464654
rs140596675
rs13024840
rs10750395
rs1304372
rs7075830
rs17583875
rs140239764
rs143346058
rs113667295
All
−0.2
−0.1
0.0
MR leave−one−out sensitivity analysis for
' TD CD4+ %T cell' on 'Rheumatoid arthritis'

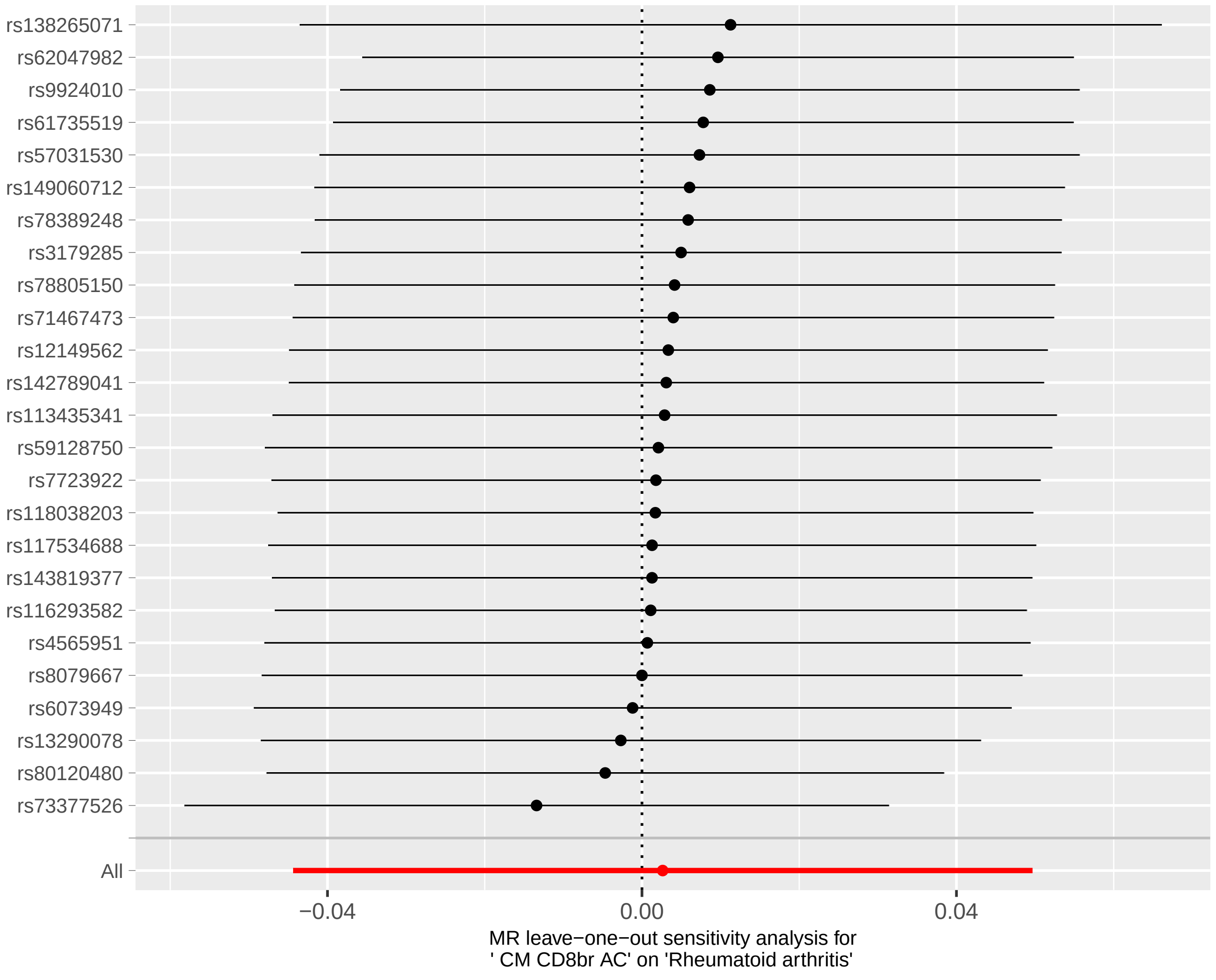
rs138265071
rs62047982
rs9924010
rs61735519
rs57031530
rs149060712
rs78389248
rs3179285
rs78805150
rs71467473
rs12149562
rs142789041
rs113435341
rs59128750
rs7723922
rs118038203
rs117534688
rs143819377
rs116293582
rs4565951
rs8079667
rs6073949
rs13290078
rs80120480
rs73377526
All
−0.04
0.00
0.04
MR leave−one−out sensitivity analysis for
' CM CD8br AC' on 'Rheumatoid arthritis'

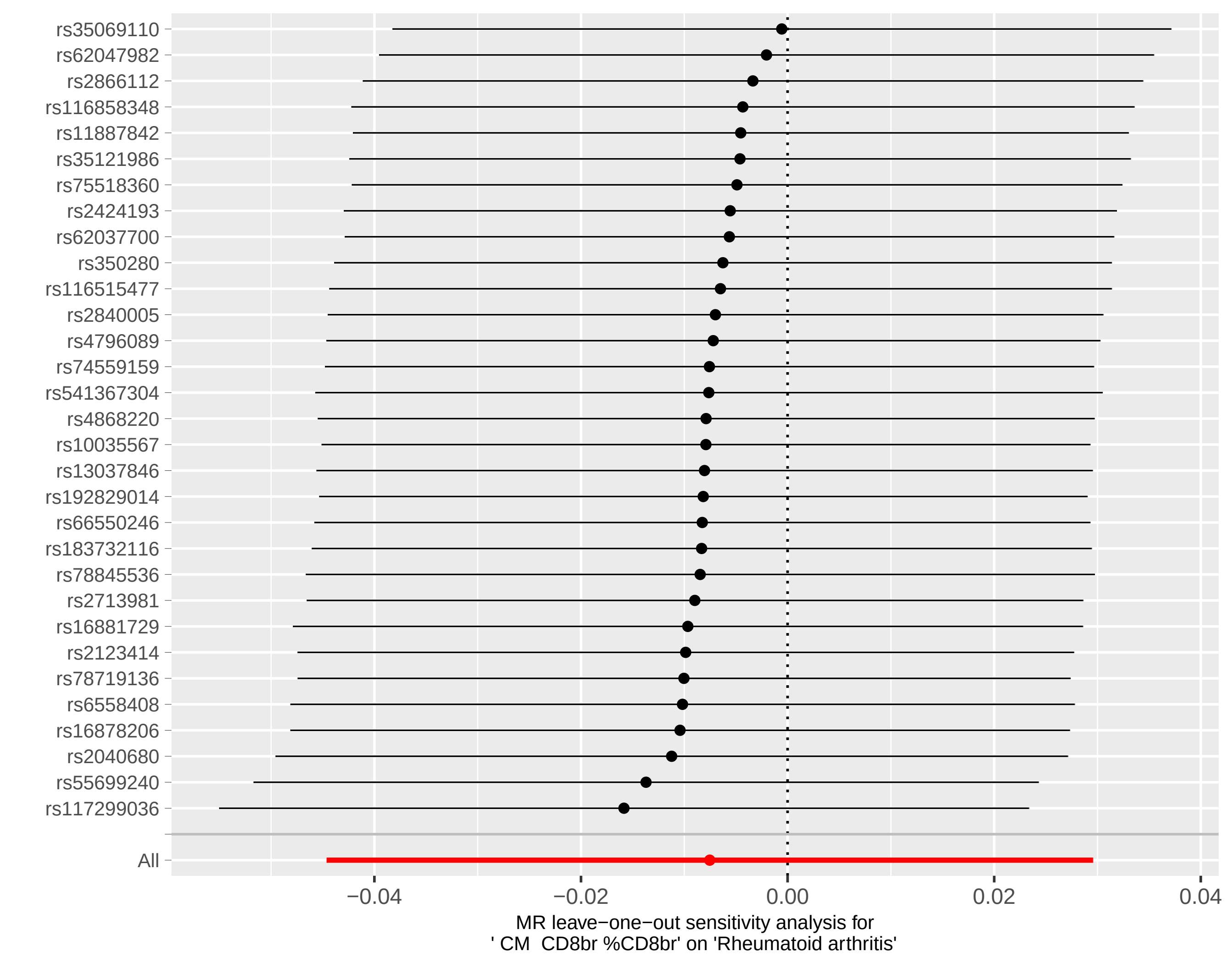
rs35069110
rs62047982
rs2866112
rs116858348
rs11887842
rs35121986
rs75518360
rs2424193
rs62037700
rs350280
rs116515477
rs2840005
rs4796089
rs74559159
rs541367304
rs4868220
rs10035567
rs13037846
rs192829014
rs66550246
rs183732116
rs78845536
rs2713981
rs16881729
rs2123414
rs78719136
rs6558408
rs16878206
rs2040680
rs55699240
rs117299036
All
−0.04
−0.02
0.00
0.02
0.04
MR leave−one−out sensitivity analysis for
' CM CD8br %CD8br' on 'Rheumatoid arthritis'

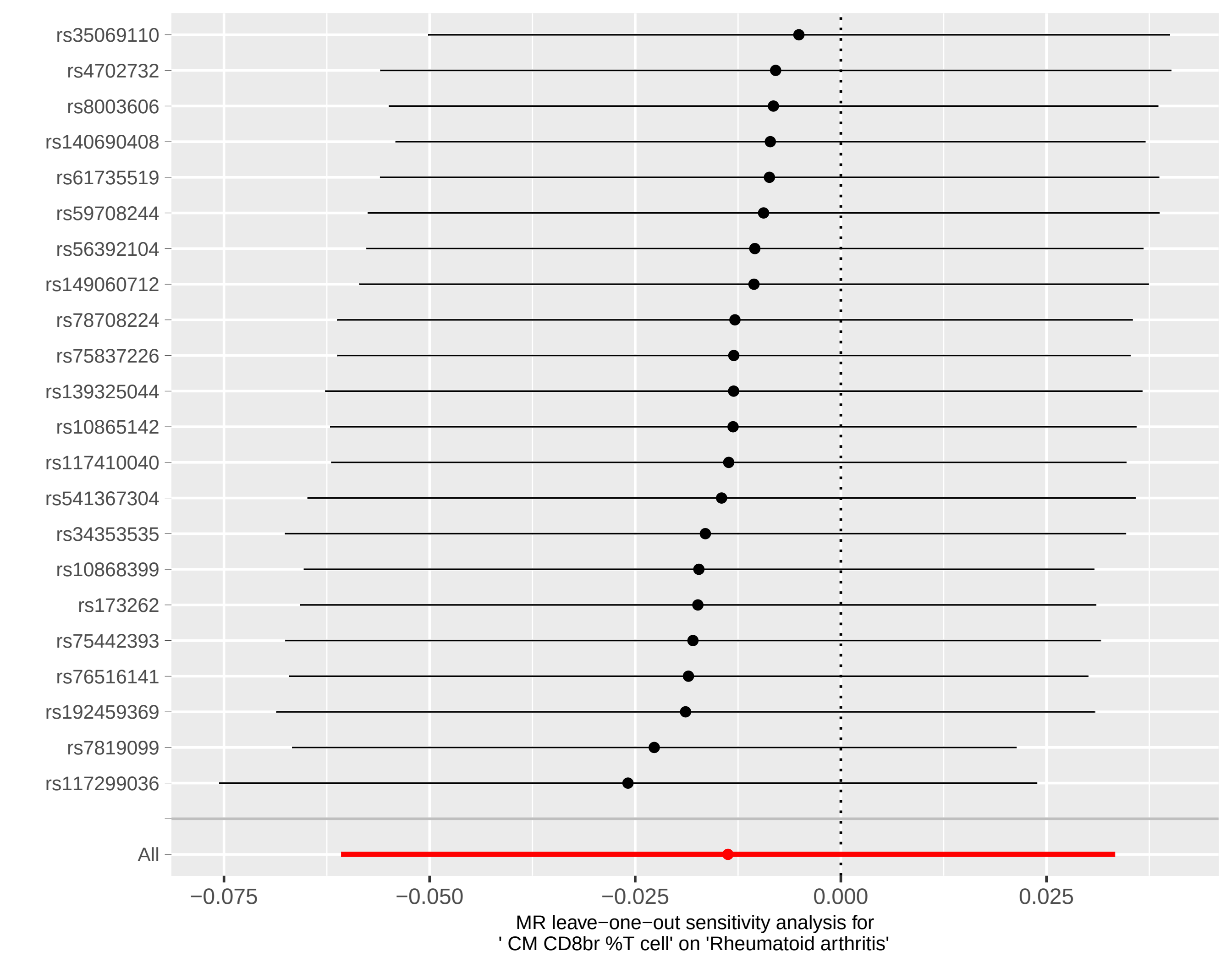
rs35069110
rs4702732
rs8003606
rs140690408
rs61735519
rs59708244
rs56392104
rs149060712
rs78708224
rs75837226
rs139325044
rs10865142
rs117410040
rs541367304
rs34353535
rs10868399
rs173262
rs75442393
rs76516141
rs192459369
rs7819099
rs117299036
All
−0.075
−0.050
−0.025
0.000
0.025
MR leave−one−out sensitivity analysis for
' CM CD8br %T cell' on 'Rheumatoid arthritis'

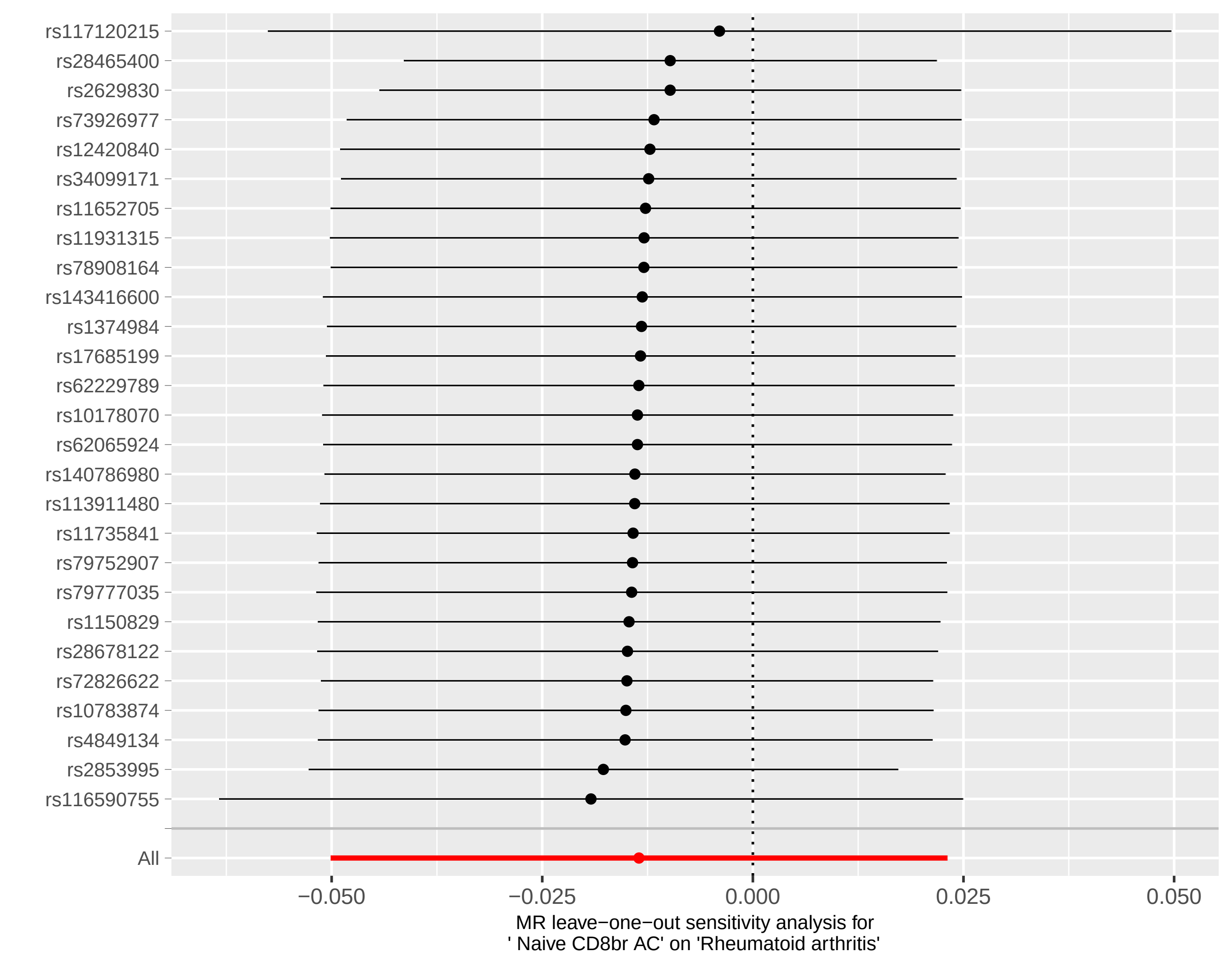
rs117120215
rs28465400
rs2629830
rs73926977
rs12420840
rs34099171
rs11652705
rs11931315
rs78908164
rs143416600
rs1374984
rs17685199
rs62229789
rs10178070
rs62065924
rs140786980
rs113911480
rs11735841
rs79752907
rs79777035
rs1150829
rs28678122
rs72826622
rs10783874
rs4849134
rs2853995
rs116590755
All
−0.050
−0.025
0.000
0.025
0.050
MR leave−one−out sensitivity analysis for
' Naive CD8br AC' on 'Rheumatoid arthritis'

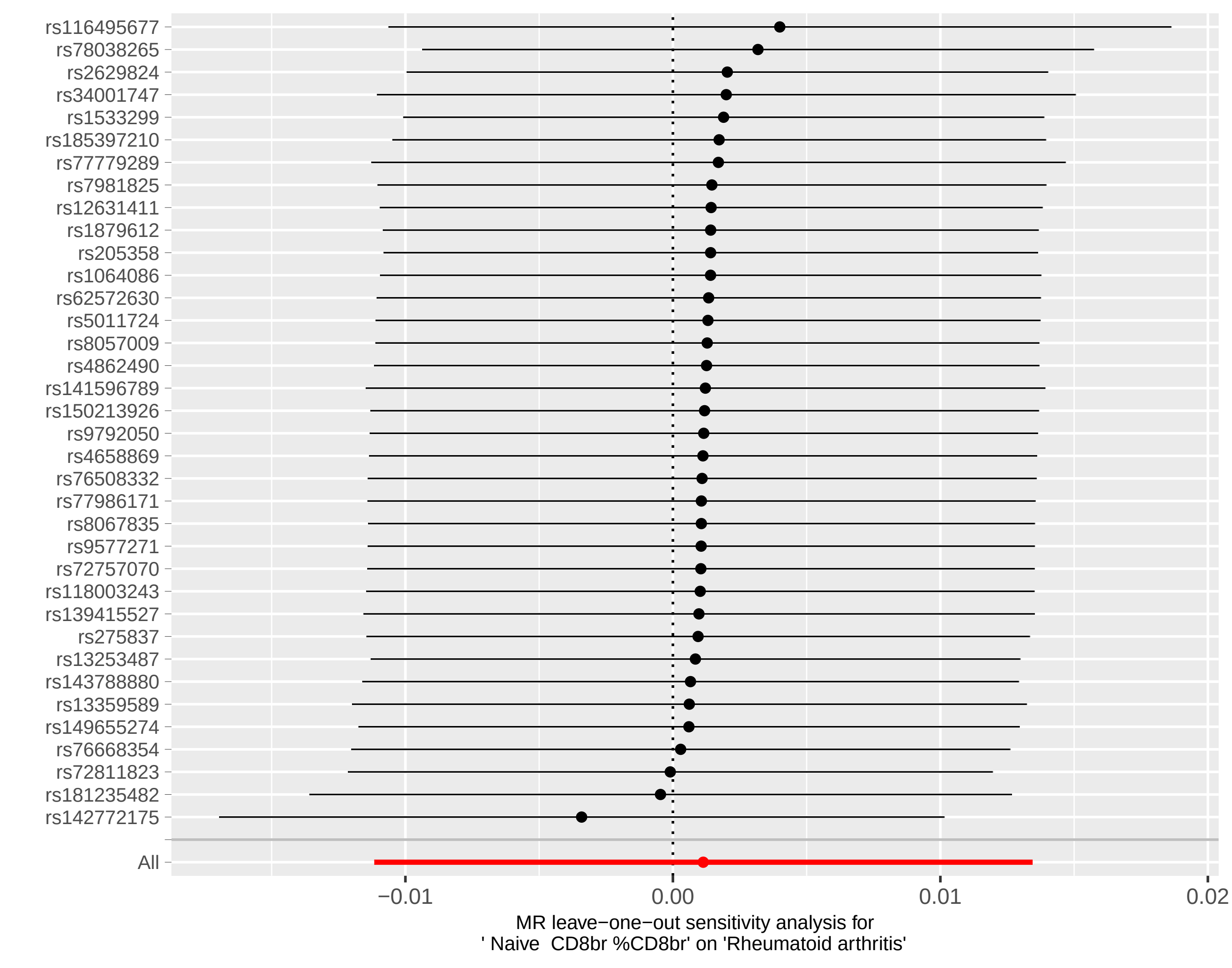
rs116495677
rs78038265
rs2629824
rs34001747
rs1533299
rs185397210
rs77779289
rs7981825
rs12631411
rs1879612
rs205358
rs1064086
rs62572630
rs5011724
rs8057009
rs4862490
rs141596789
rs150213926
rs9792050
rs4658869
rs76508332
rs77986171
rs8067835
rs9577271
rs72757070
rs118003243
rs139415527
rs275837
rs13253487
rs143788880
rs13359589
rs149655274
rs76668354
rs72811823
rs181235482
rs142772175
All
−0.01
0.00
0.01
0.02
MR leave−one−out sensitivity analysis for
' Naive CD8br %CD8br' on 'Rheumatoid arthritis'

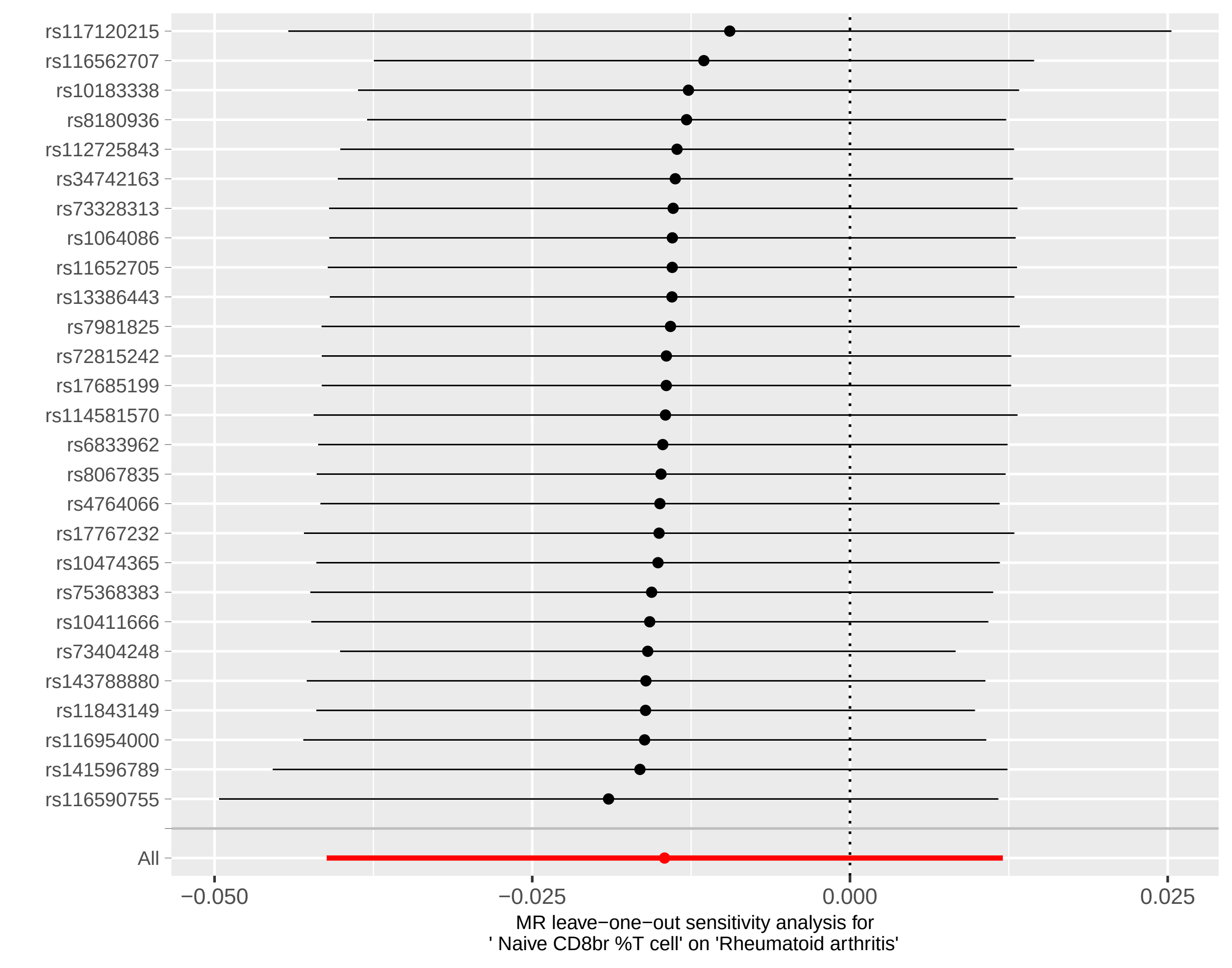

rs117120215
rs116562707
rs10183338
rs8180936
rs112725843
rs34742163
rs73328313
rs1064086
rs11652705
rs13386443
rs7981825
rs72815242
rs17685199
rs114581570
rs6833962
rs8067835
rs4764066
rs17767232
rs10474365
rs75368383
rs10411666
rs73404248
rs143788880
rs11843149
rs116954000
rs141596789
rs116590755
All
−0.050
−0.025
0.000
0.025
MR leave−one−out sensitivity analysis for
' Naive CD8br %T cell' on 'Rheumatoid arthritis'

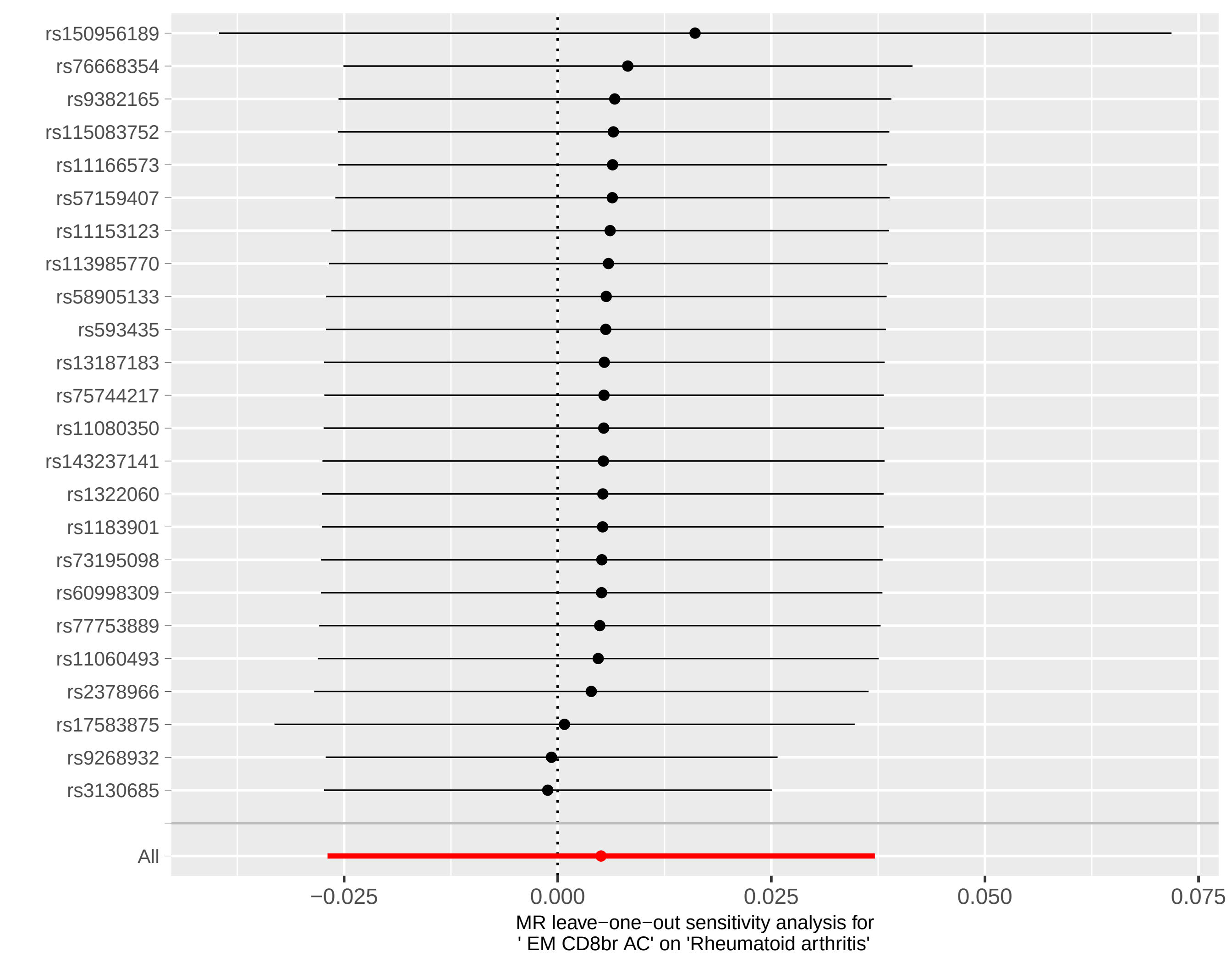

rs150956189
rs76668354
rs9382165
rs115083752
rs11166573
rs57159407
rs11153123
rs113985770
rs58905133
rs593435
rs13187183
rs75744217
rs11080350
rs143237141
rs1322060
rs1183901
rs73195098
rs60998309
rs77753889
rs11060493
rs2378966
rs17583875
rs9268932
rs3130685
All
−0.025
0.000
0.025
0.050
0.075
MR leave−one−out sensitivity analysis for
' EM CD8br AC' on 'Rheumatoid arthritis'

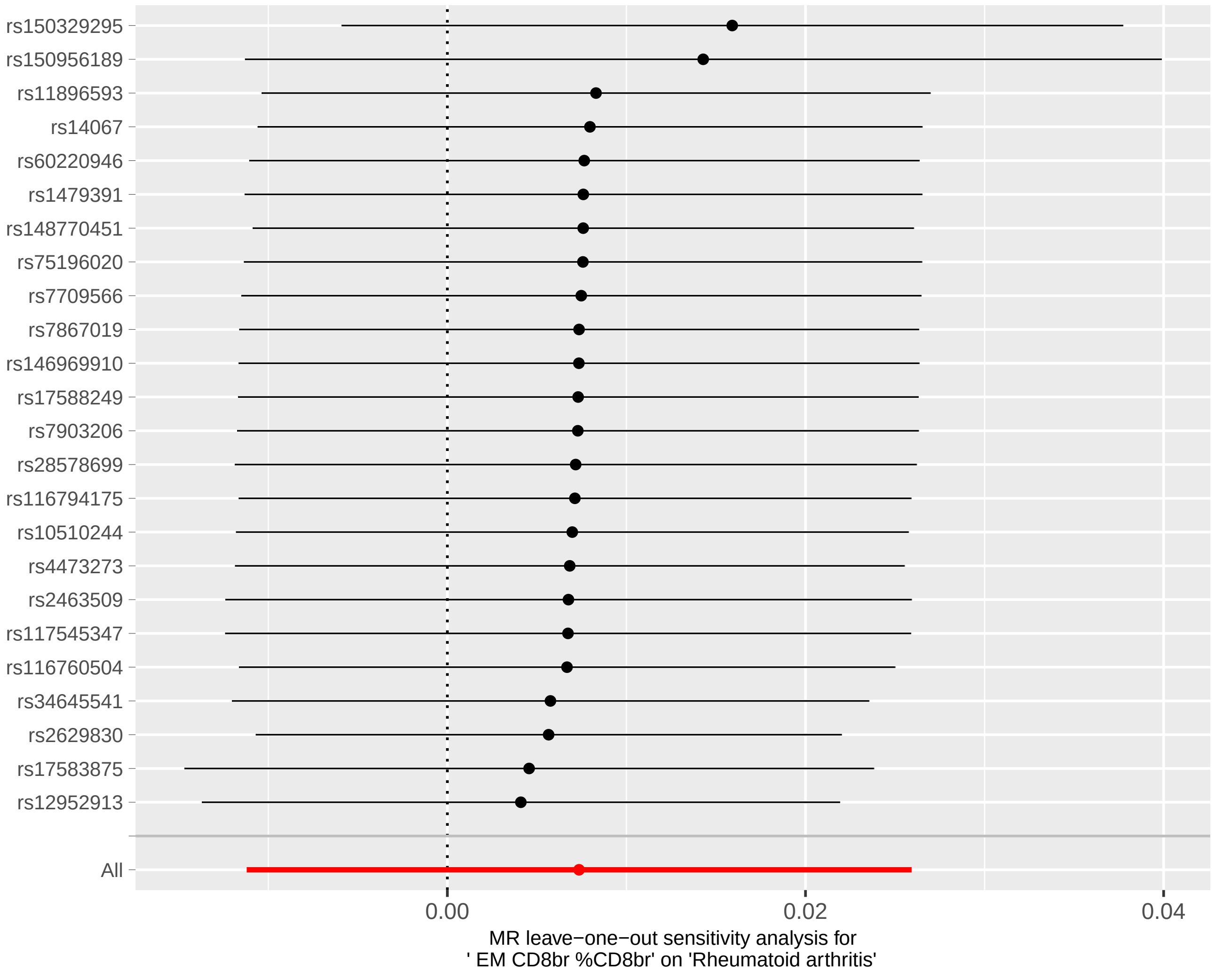
rs150329295
rs150956189
rs11896593
rs14067
rs60220946
rs1479391
rs148770451
rs75196020
rs7709566
rs7867019
rs146969910
rs17588249
rs7903206
rs28578699
rs116794175
rs10510244
rs4473273
rs2463509
rs117545347
rs116760504
rs34645541
rs2629830
rs17583875
rs12952913
All
0.00
0.02
0.04
MR leave−one−out sensitivity analysis for
' EM CD8br %CD8br' on 'Rheumatoid arthritis'

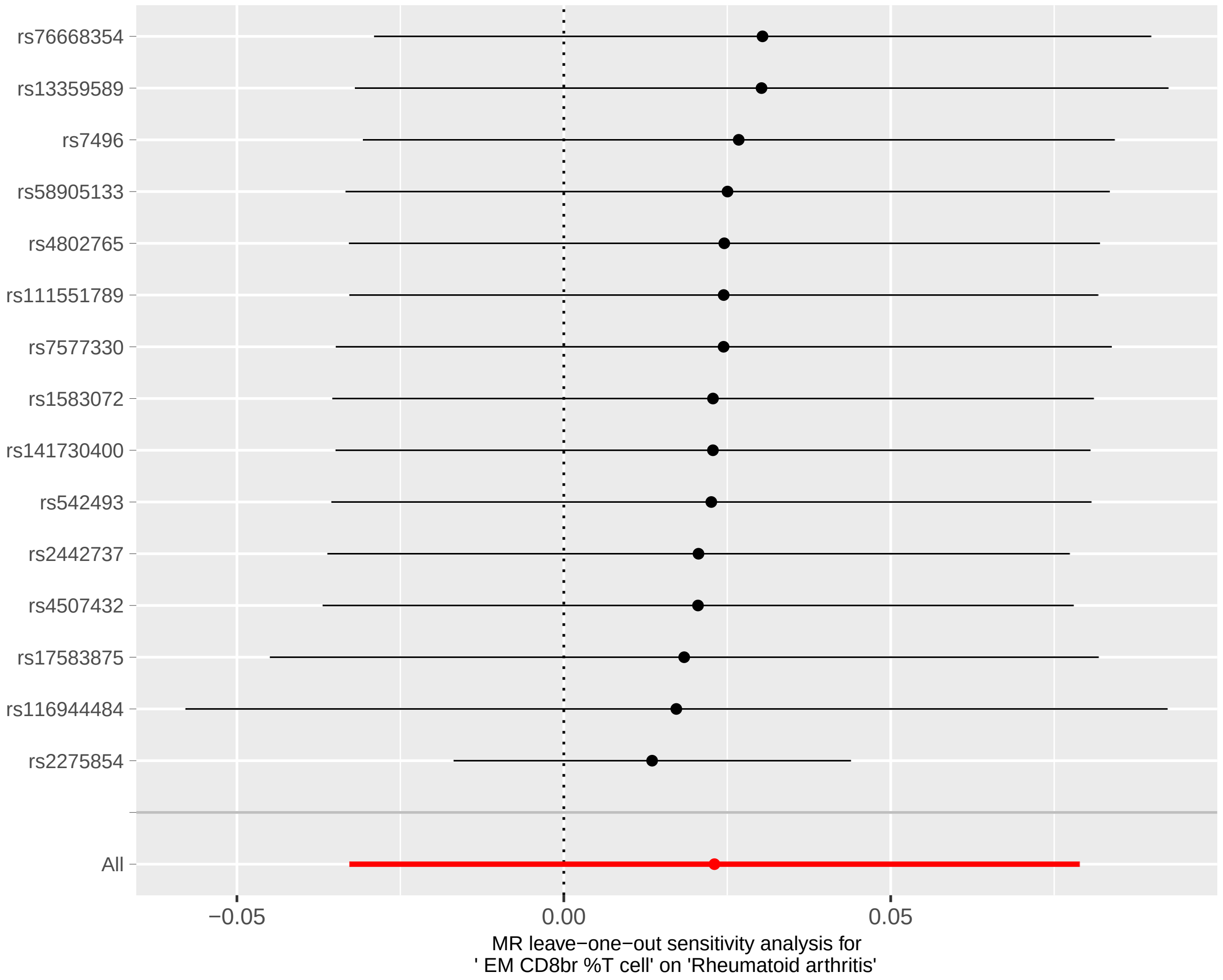

rs76668354
rs13359589
rs7496
rs58905133
rs4802765
rs111551789
rs7577330
rs1583072
rs141730400
rs542493
rs2442737
rs4507432
rs17583875
rs116944484
rs2275854
All
−0.05
0.00
0.05
MR leave−one−out sensitivity analysis for
' EM CD8br %T cell' on 'Rheumatoid arthritis'

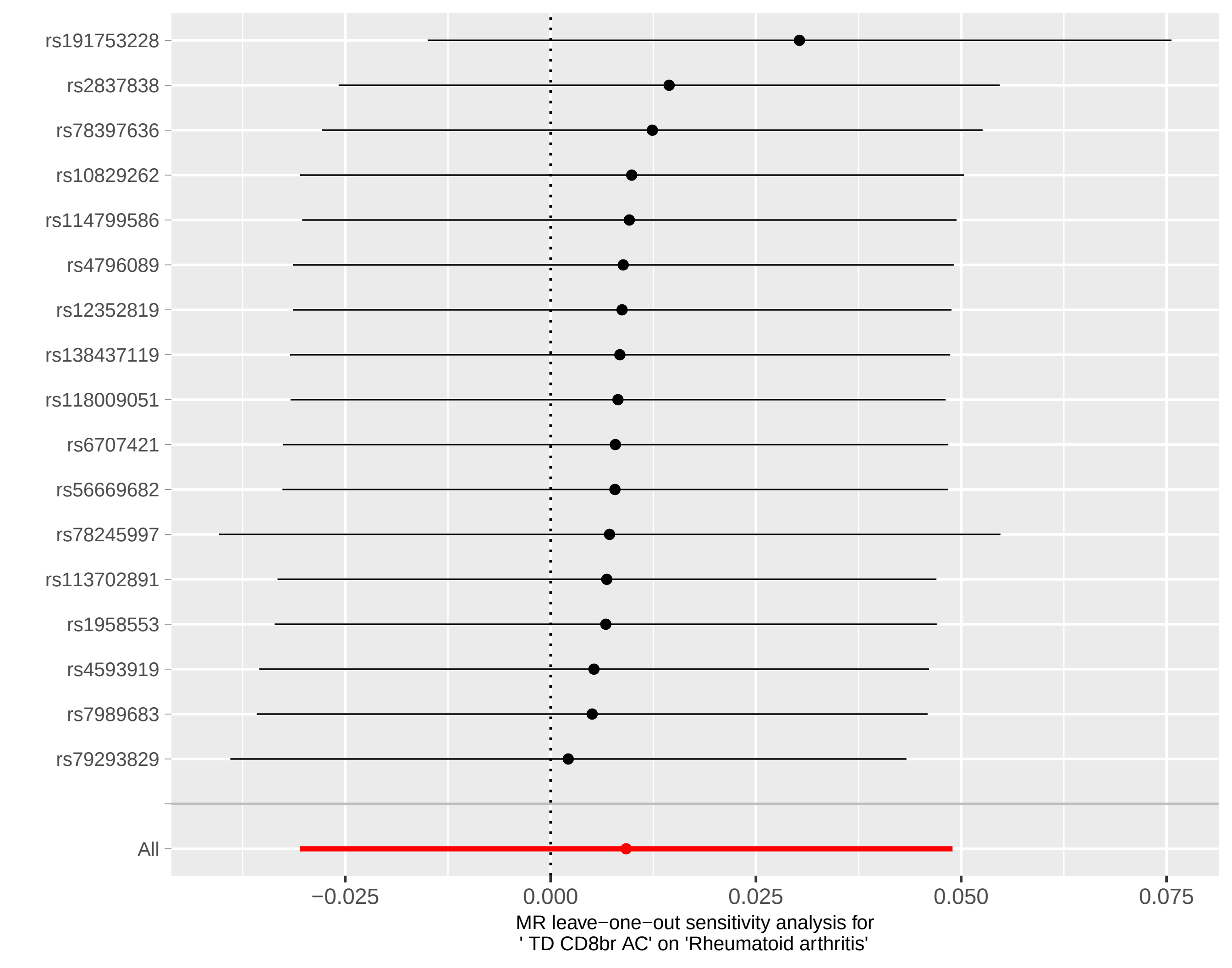
rs191753228
rs2837838
rs78397636
rs10829262
rs114799586
rs4796089
rs12352819
rs138437119
rs118009051
rs6707421
rs56669682
rs78245997
rs113702891
rs1958553
rs4593919
rs7989683
rs79293829
All
−0.025
0.000
0.025
0.050
0.075
MR leave−one−out sensitivity analysis for
' TD CD8br AC' on 'Rheumatoid arthritis'

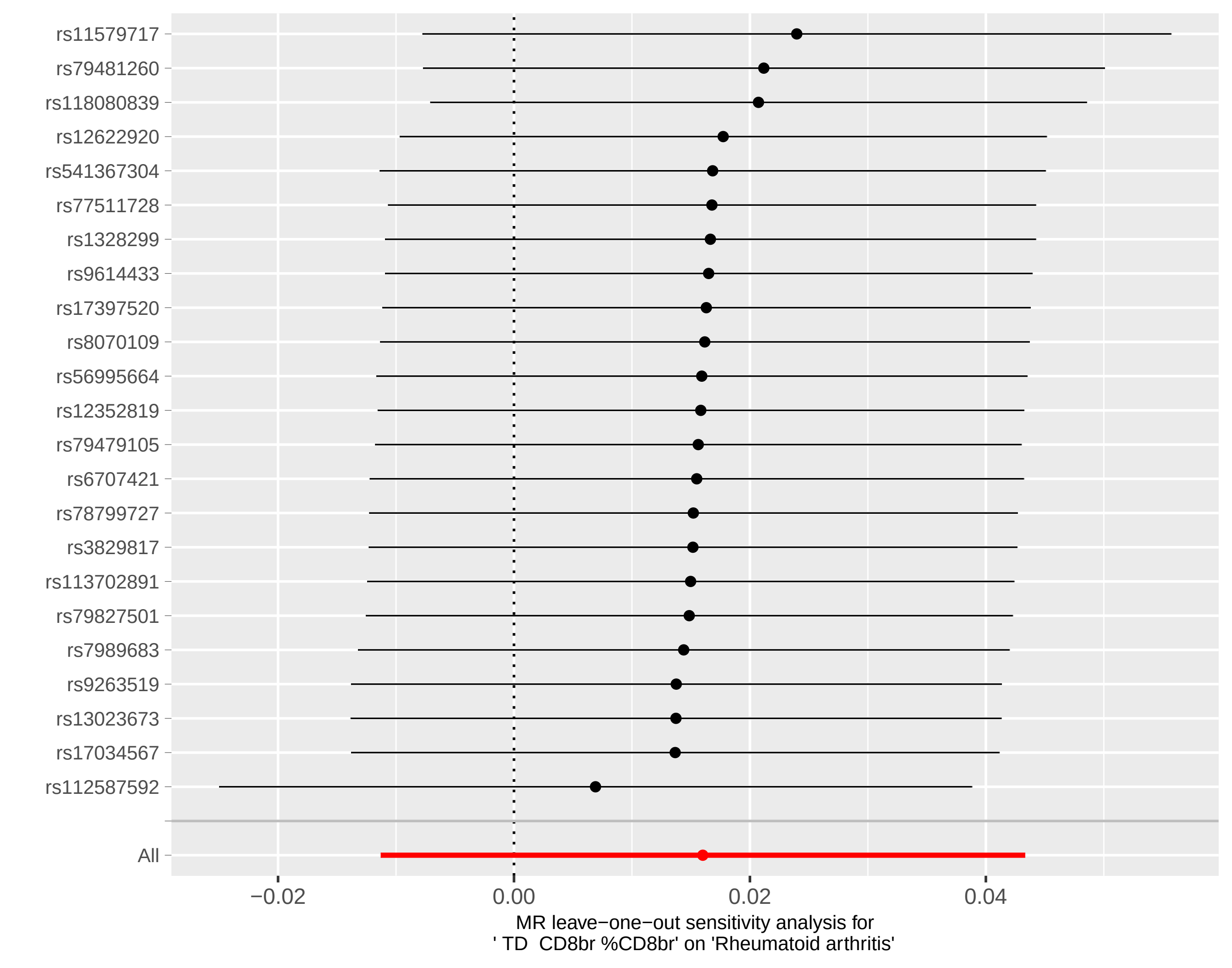

rs11579717
rs79481260
rs118080839
rs12622920
rs541367304
rs77511728
rs1328299
rs9614433
rs17397520
rs8070109
rs56995664
rs12352819
rs79479105
rs6707421
rs78799727
rs3829817
rs113702891
rs79827501
rs7989683
rs9263519
rs13023673
rs17034567
rs112587592
All
−0.02
0.00
0.02
0.04
MR leave−one−out sensitivity analysis for
' TD CD8br %CD8br' on 'Rheumatoid arthritis'

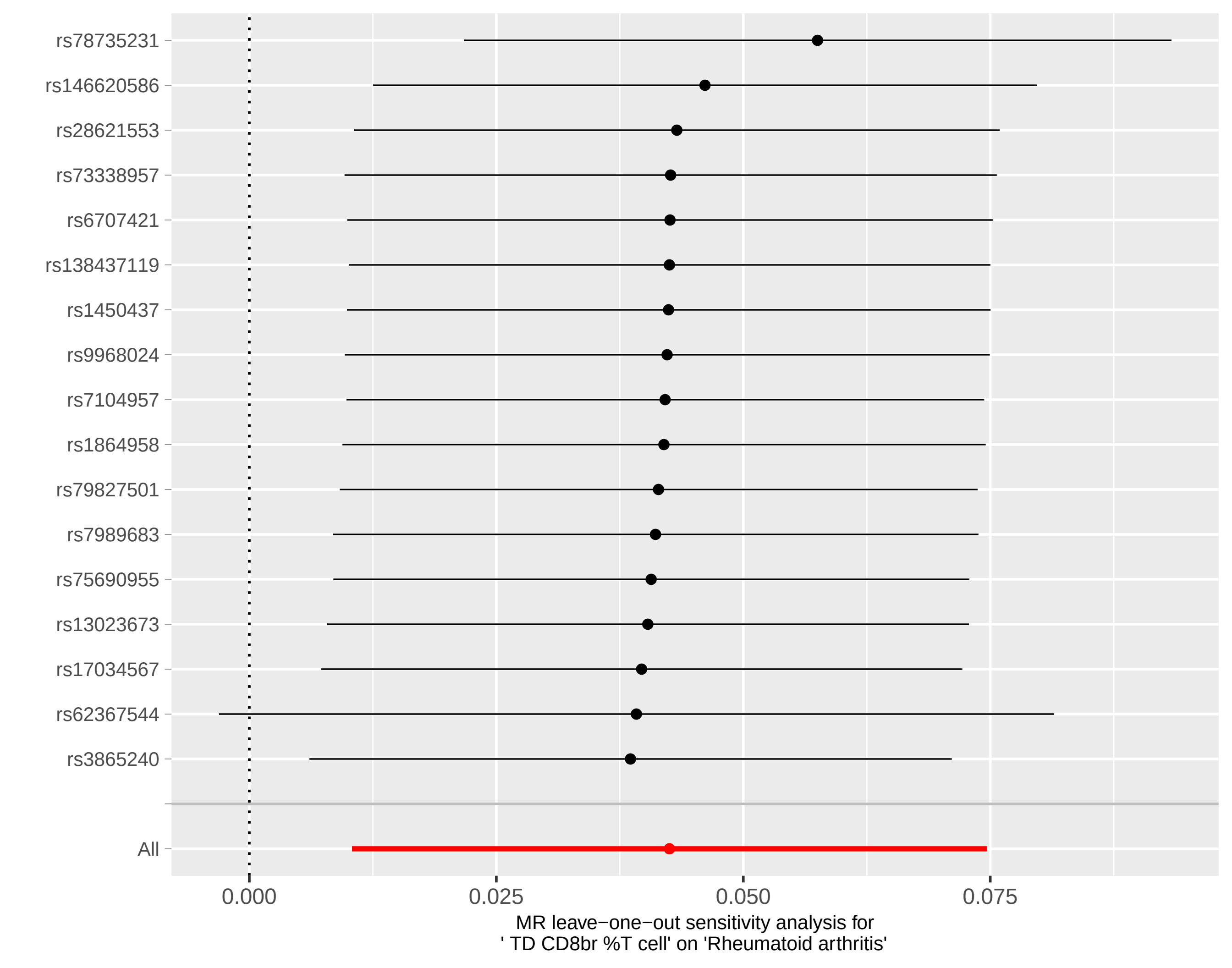

rs78735231
rs146620586
rs28621553
rs73338957
rs6707421
rs138437119
rs1450437
rs9968024
rs7104957
rs1864958
rs79827501
rs7989683
rs75690955
rs13023673
rs17034567
rs62367544
rs3865240
All
0.000
0.025
0.050
0.075
MR leave−one−out sensitivity analysis for
' TD CD8br %T cell' on 'Rheumatoid arthritis'

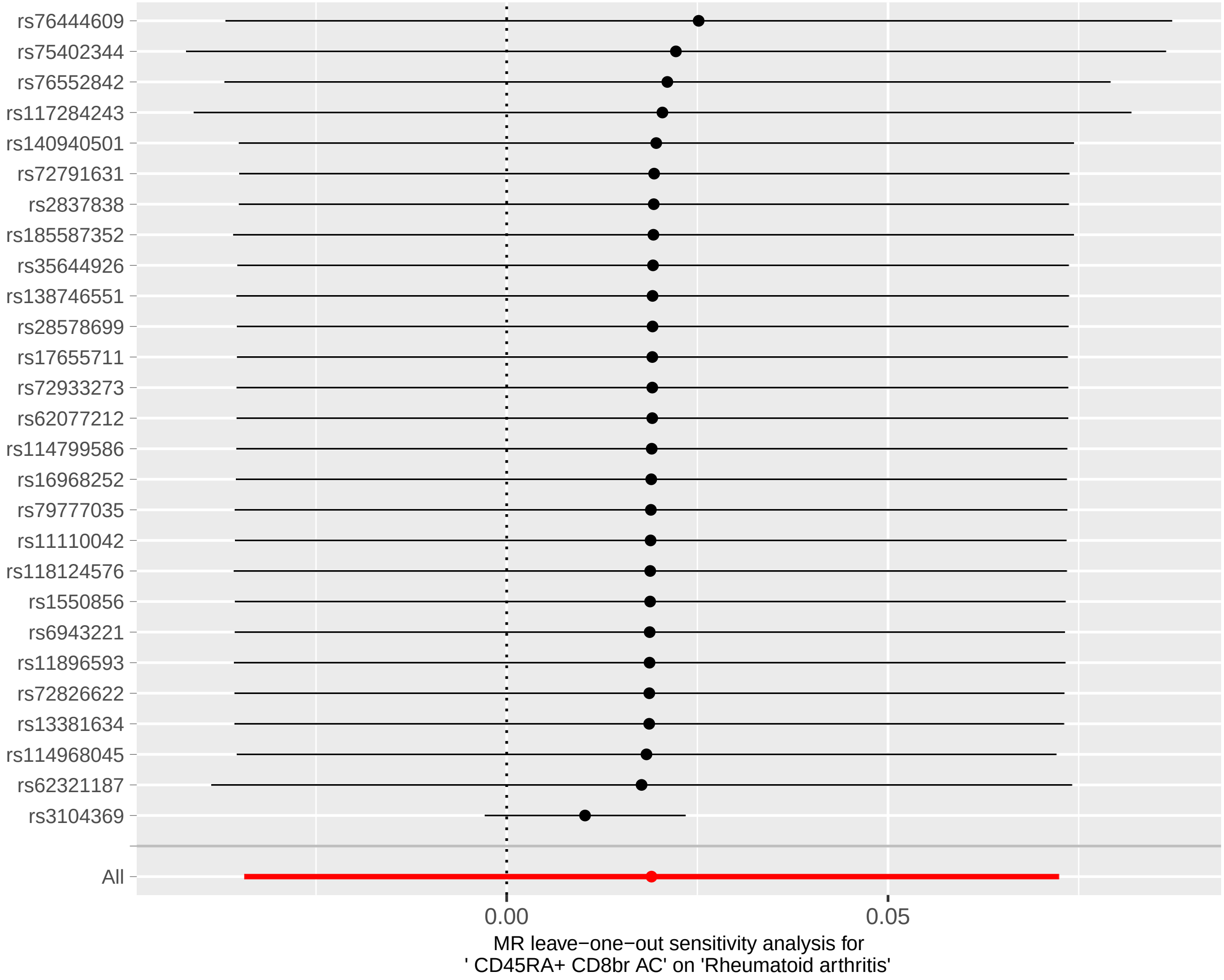

rs76444609
rs75402344
rs76552842
rs117284243
rs140940501
rs72791631
rs2837838
rs185587352
rs35644926
rs138746551
rs28578699
rs17655711
rs72933273
rs62077212
rs114799586
rs16968252
rs79777035
rs11110042
rs118124576
rs1550856
rs6943221
rs11896593
rs72826622
rs13381634
rs114968045
rs62321187
rs3104369
All
0.00
0.05
MR leave−one−out sensitivity analysis for
' CD45RA+ CD8br AC' on 'Rheumatoid arthritis'

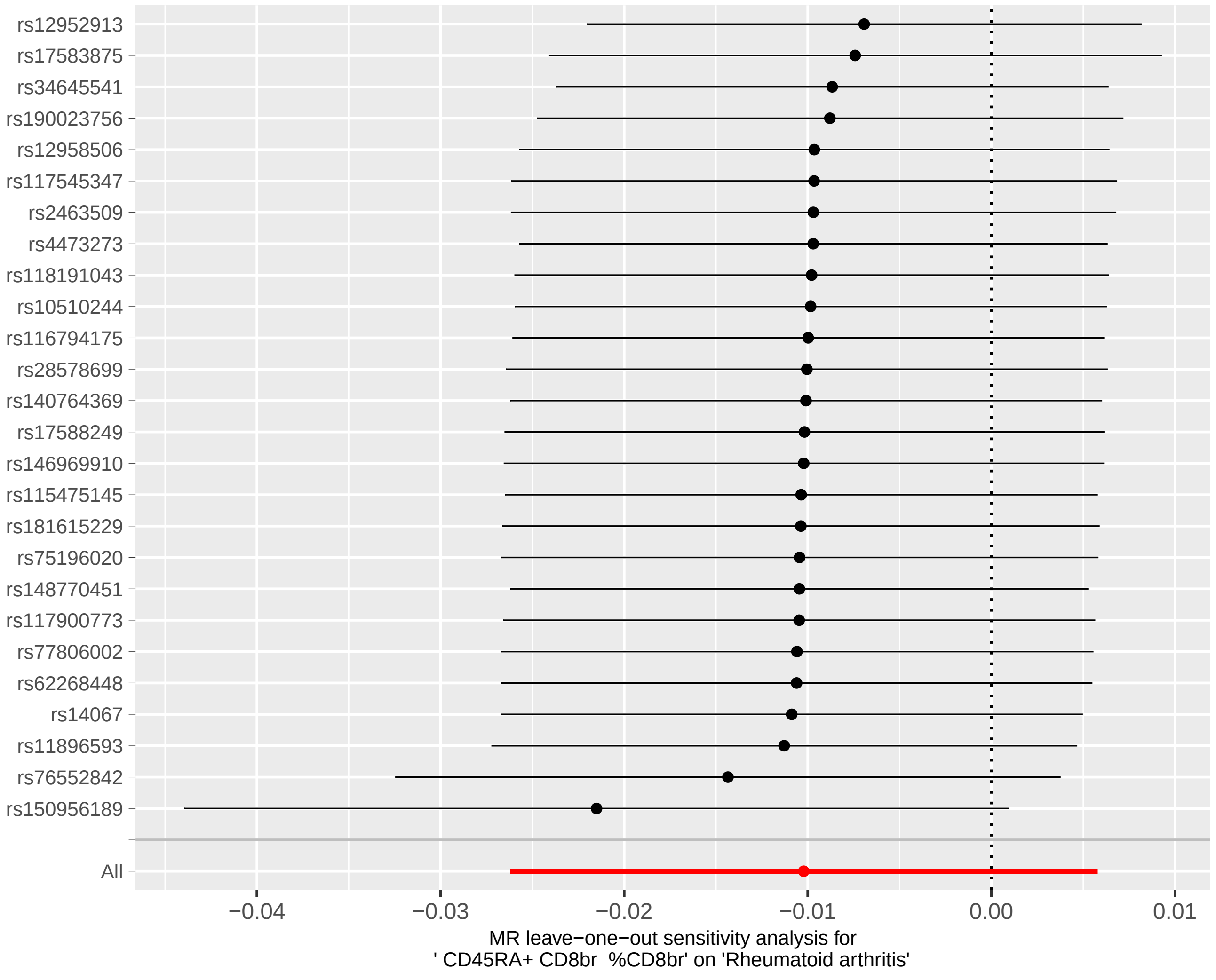
rs12952913
rs17583875
rs34645541
rs190023756
rs12958506
rs117545347
rs2463509
rs4473273
rs118191043
rs10510244
rs116794175
rs28578699
rs140764369
rs17588249
rs146969910
rs115475145
rs181615229
rs75196020
rs148770451
rs117900773
rs77806002
rs62268448
rs14067
rs11896593
rs76552842
rs150956189
All
−0.04
−0.03
−0.02
−0.01
0.00
0.01
MR leave−one−out sensitivity analysis for
' CD45RA+ CD8br %CD8br' on 'Rheumatoid arthritis'

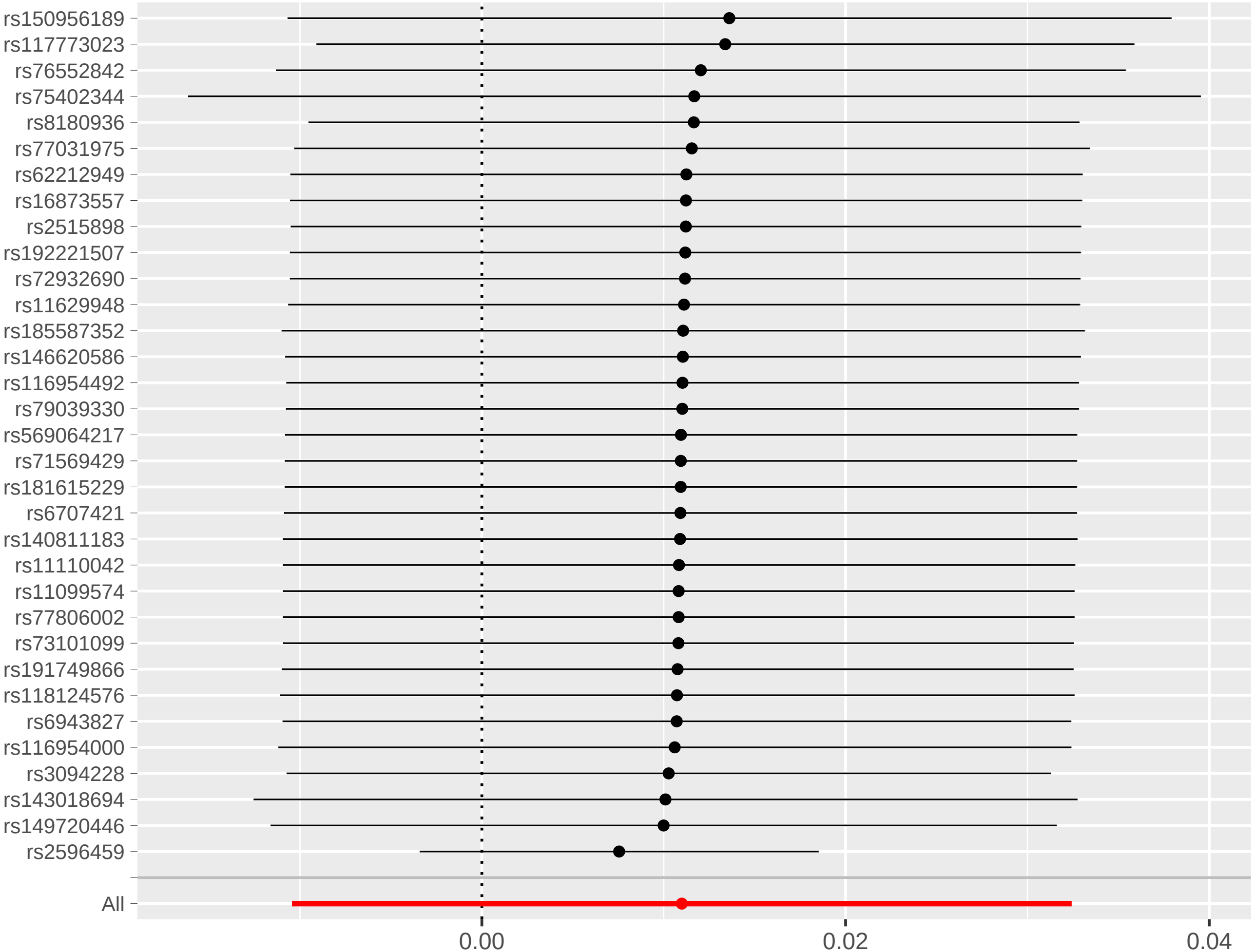

MR leave−one−out sensitivity analysis for
' CD45RA+ CD8br %T cell' on 'Rheumatoid arthritis'

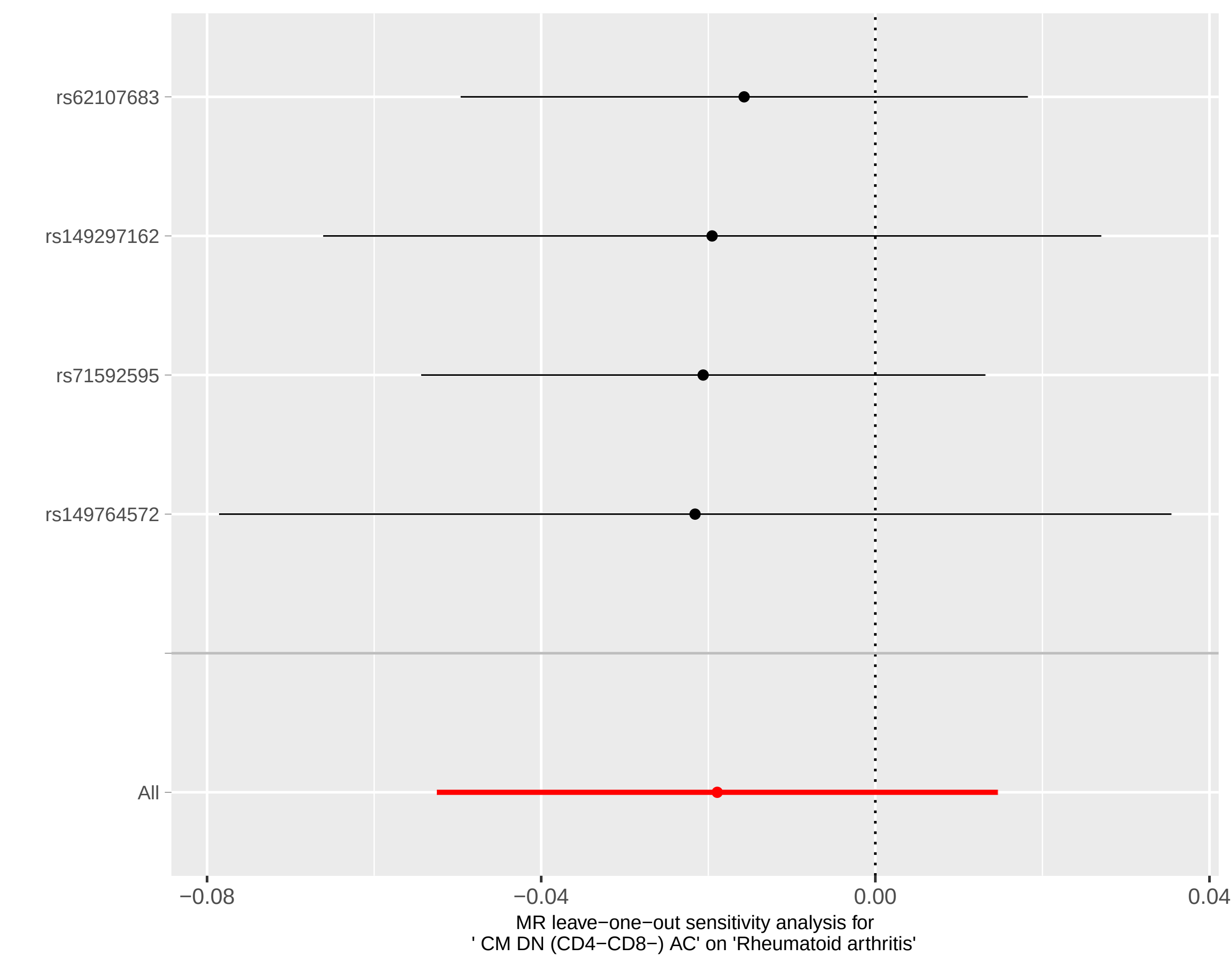
rs62107683
rs149297162
rs71592595
rs149764572
All
−0.08
−0.04
0.00
0.04
MR leave−one−out sensitivity analysis for
' CM DN (CD4−CD8−) AC' on 'Rheumatoid arthritis'

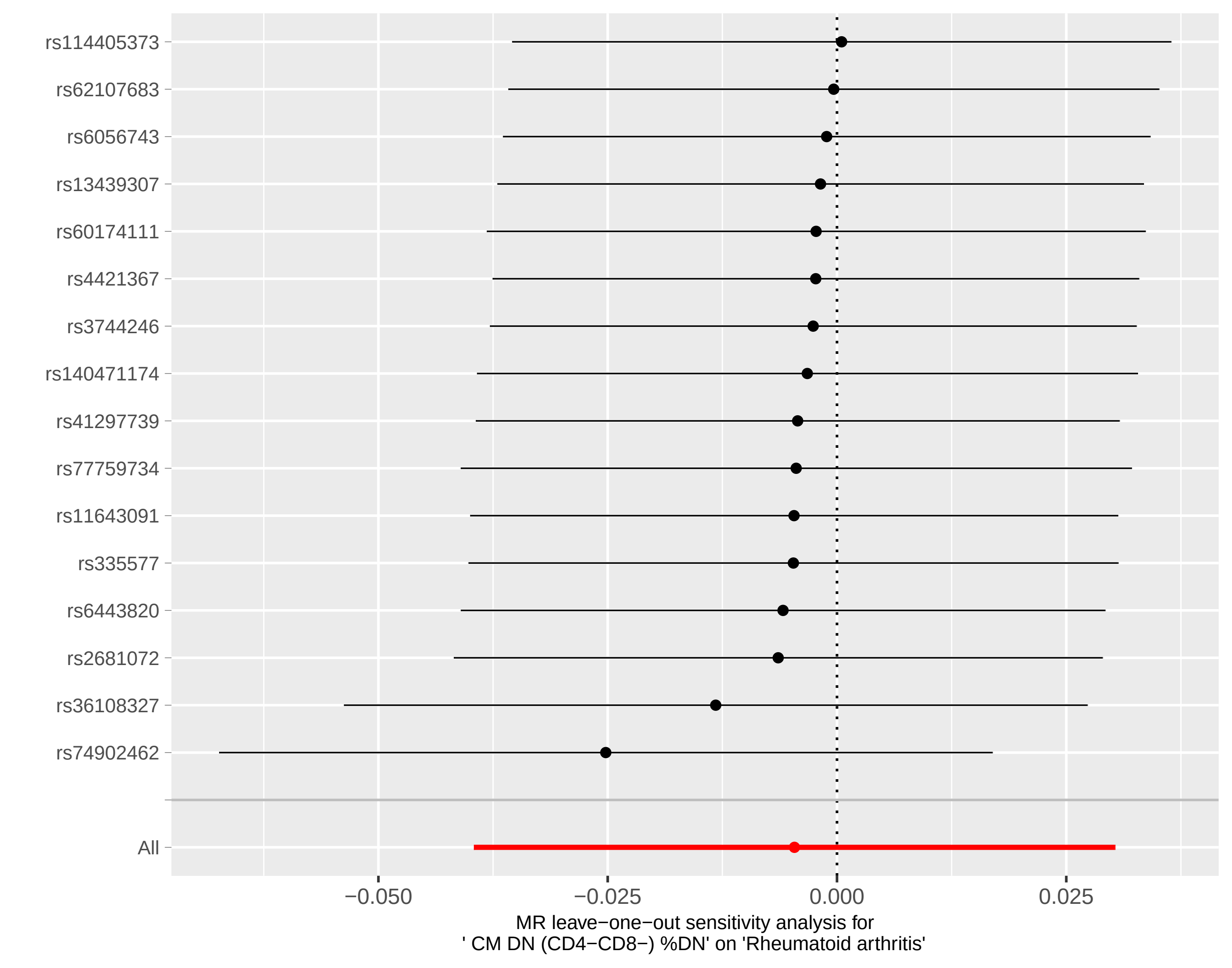

rs114405373
rs62107683
rs6056743
rs13439307
rs60174111
rs4421367
rs3744246
rs140471174
rs41297739
rs77759734
rs11643091
rs335577
rs6443820
rs2681072
rs36108327
rs74902462
All
−0.050
−0.025
0.000
0.025
MR leave−one−out sensitivity analysis for
' CM DN (CD4−CD8−) %DN' on 'Rheumatoid arthritis'

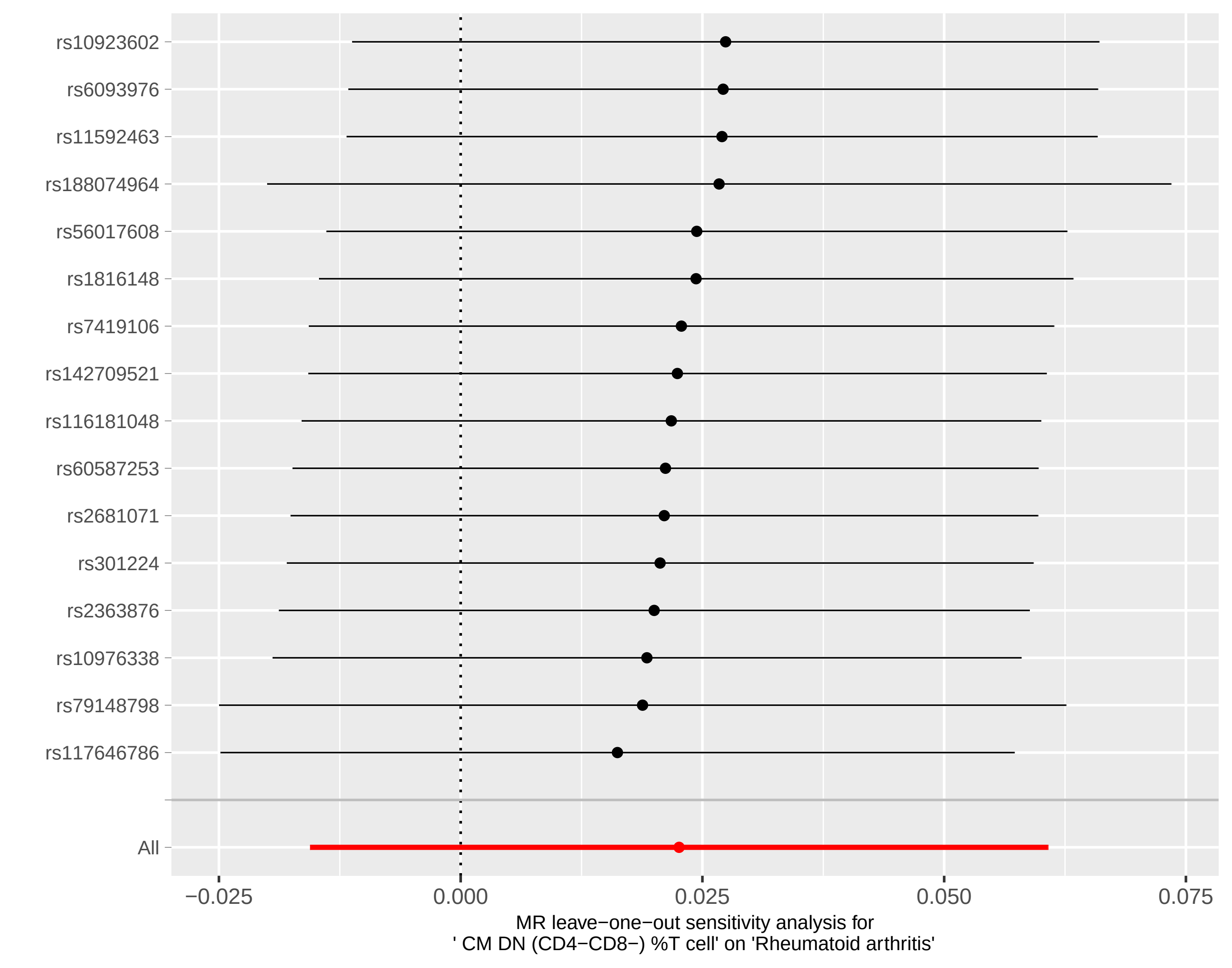

rs10923602
rs6093976
rs11592463
rs188074964
rs56017608
rs1816148
rs7419106
rs142709521
rs116181048
rs60587253
rs2681071
rs301224
rs2363876
rs10976338
rs79148798
rs117646786
All
−0.025
0.000
0.025
0.050
0.075
MR leave−one−out sensitivity analysis for
' CM DN (CD4−CD8−) %T cell' on 'Rheumatoid arthritis'

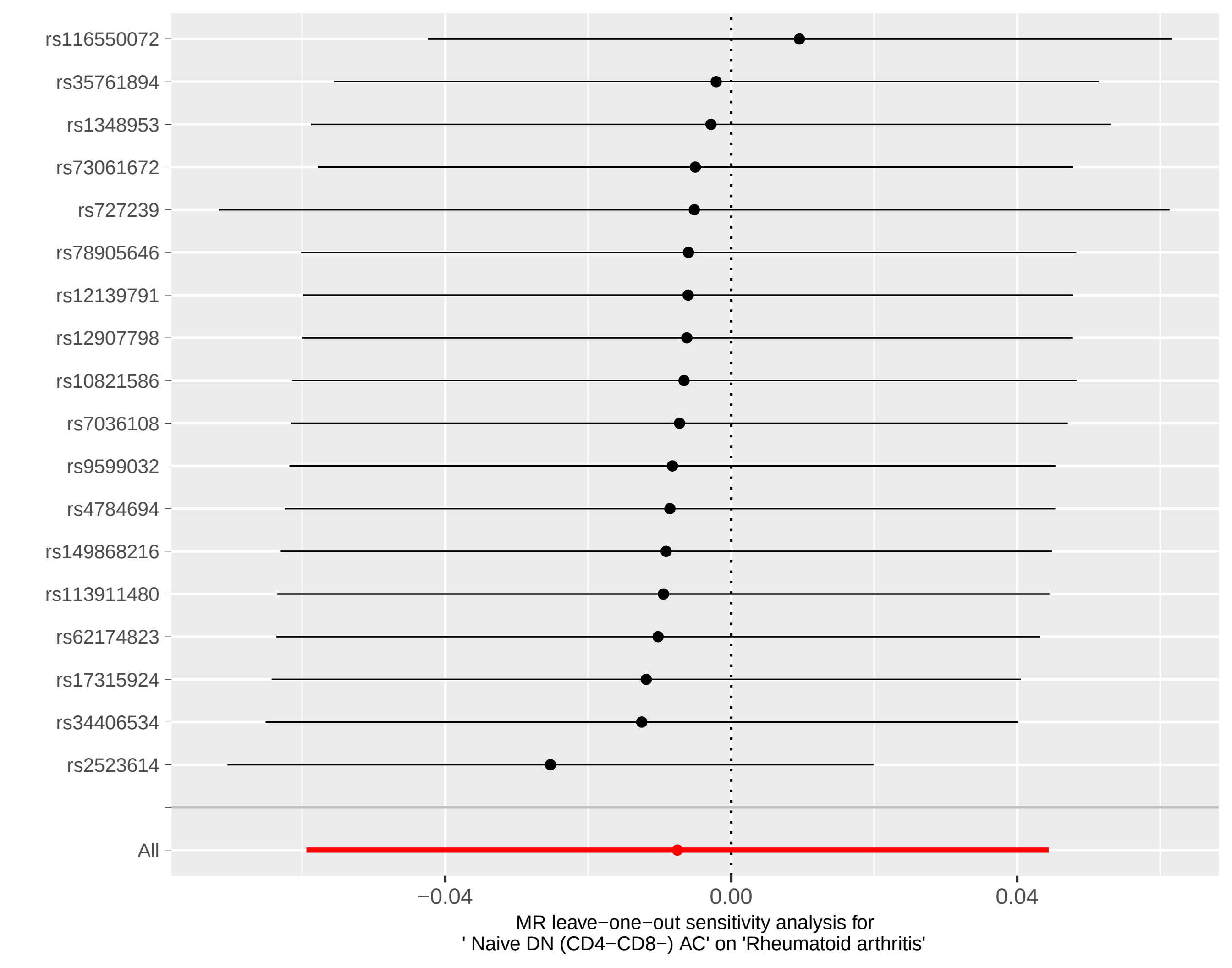
rs116550072
rs35761894
rs1348953
rs73061672
rs727239
rs78905646
rs12139791
rs12907798
rs10821586
rs7036108
rs9599032
rs4784694
rs149868216
rs113911480
rs62174823
rs17315924
rs34406534
rs2523614
All
−0.04
0.00
0.04
MR leave−one−out sensitivity analysis for
' Naive DN (CD4−CD8−) AC' on 'Rheumatoid arthritis'

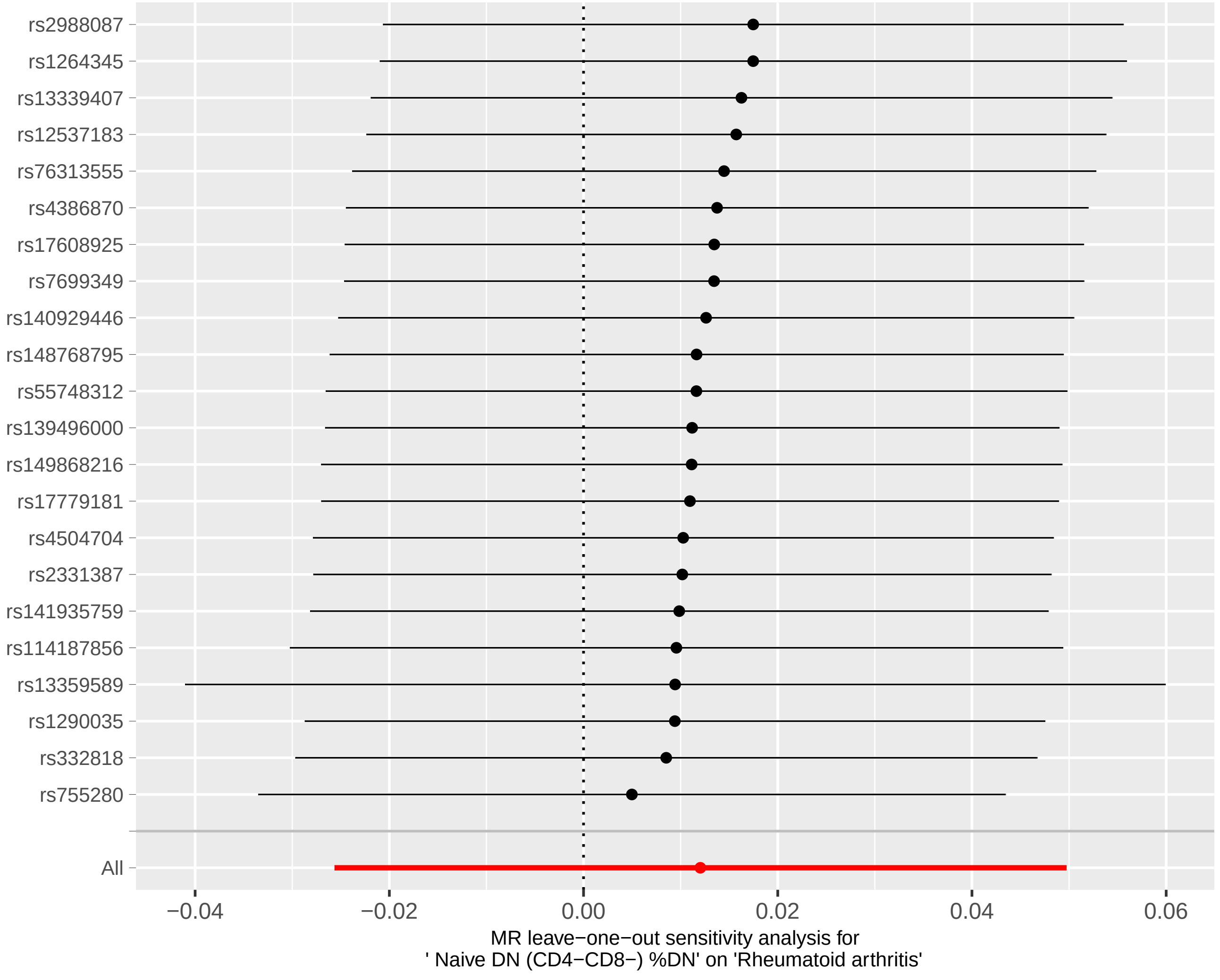
rs2988087
rs1264345
rs13339407
rs12537183
rs76313555
rs4386870
rs17608925
rs7699349
rs140929446
rs148768795
rs55748312
rs139496000
rs149868216
rs17779181
rs4504704
rs2331387
rs141935759
rs114187856
rs13359589
rs1290035
rs332818
rs755280
All
−0.04
−0.02
0.00
0.02
0.04
0.06
MR leave−one−out sensitivity analysis for
' Naive DN (CD4−CD8−) %DN' on 'Rheumatoid arthritis'

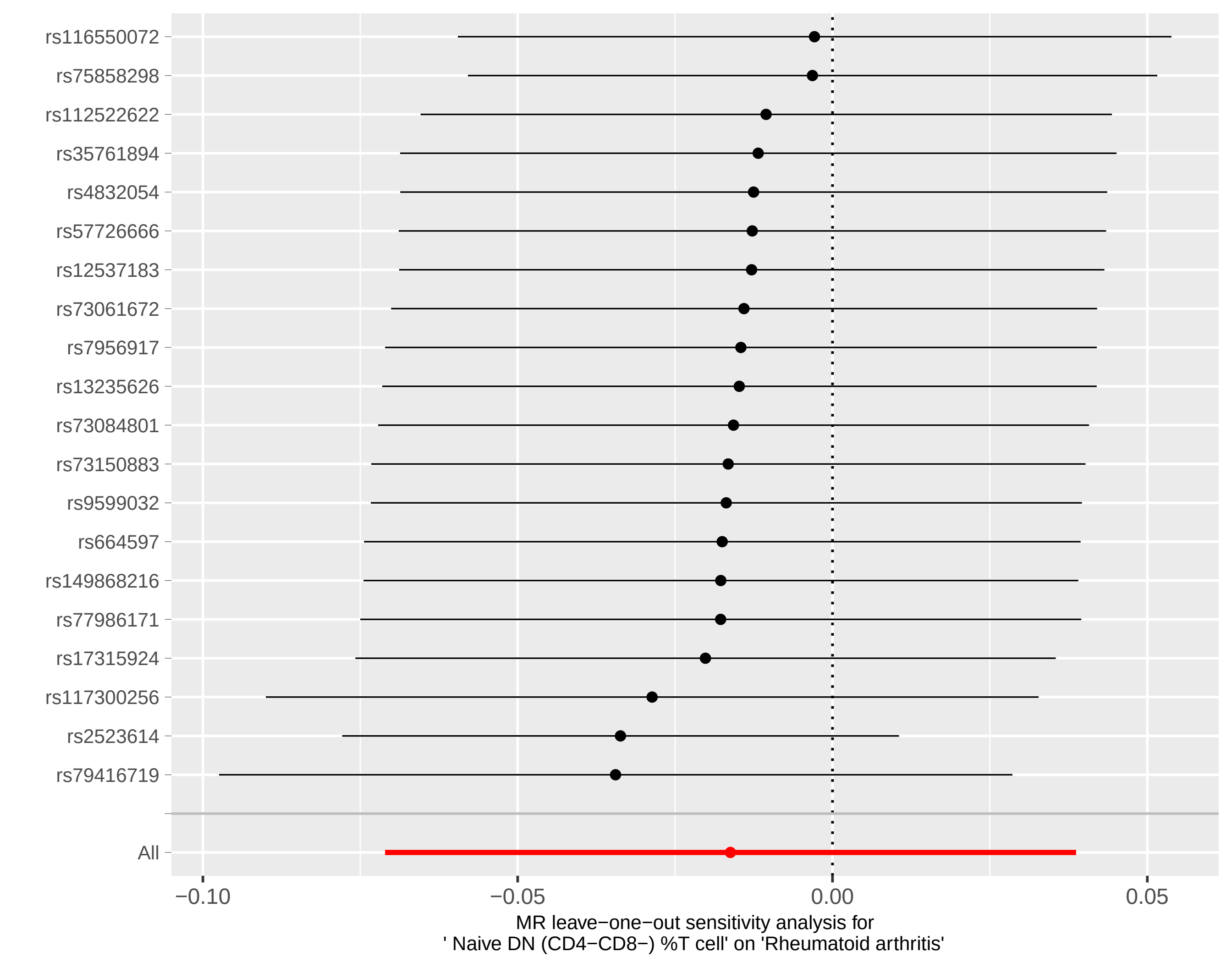
rs116550072
rs75858298
rs112522622
rs35761894
rs4832054
rs57726666
rs12537183
rs73061672
rs7956917
rs13235626
rs73084801
rs73150883
rs9599032
rs664597
rs149868216
rs77986171
rs17315924
rs117300256
rs2523614
rs79416719
All
−0.10
−0.05
0.00
0.05
MR leave−one−out sensitivity analysis for
' Naive DN (CD4−CD8−) %T cell' on 'Rheumatoid arthritis'

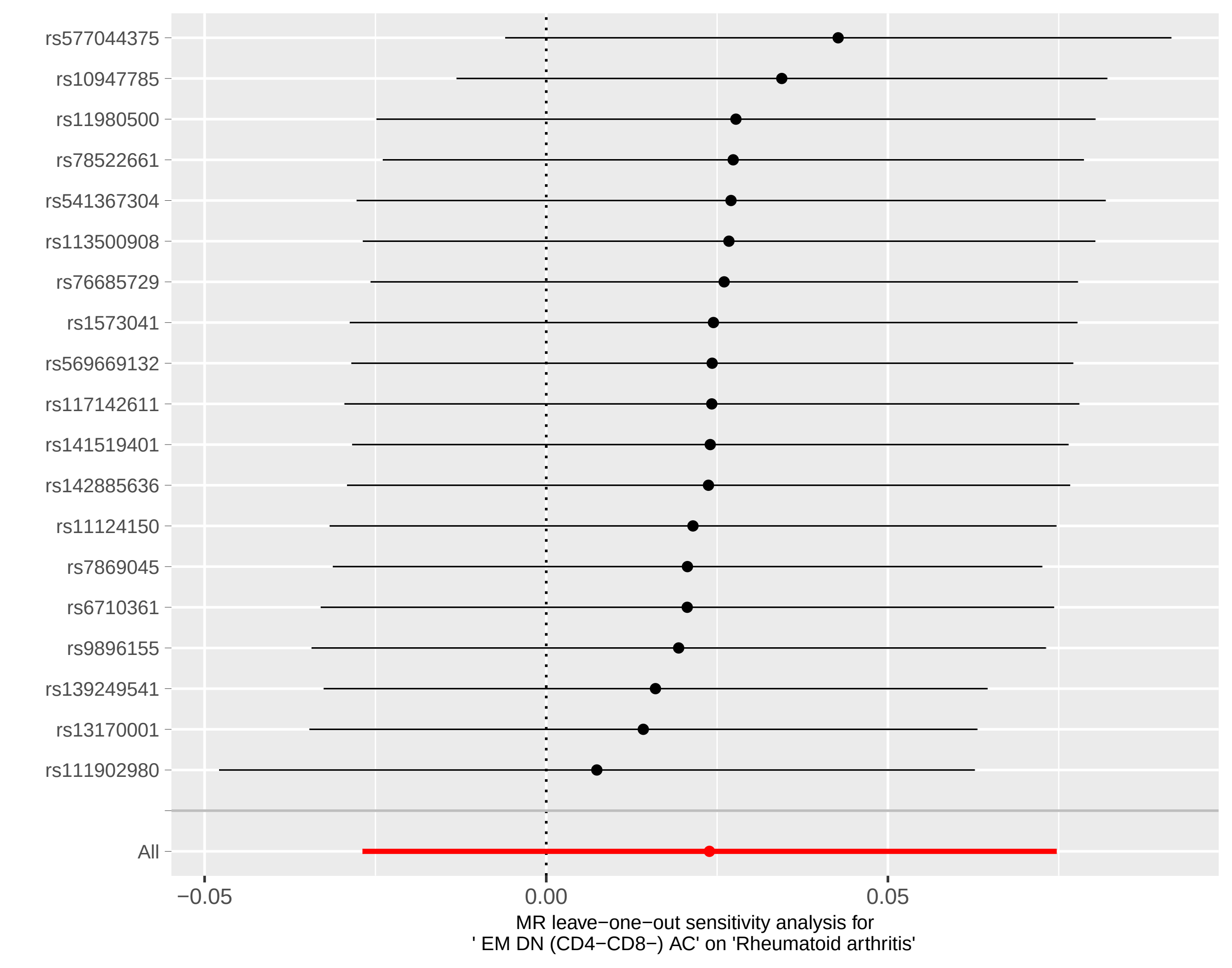
rs577044375
rs10947785
rs11980500
rs78522661
rs541367304
rs113500908
rs76685729
rs1573041
rs569669132
rs117142611
rs141519401
rs142885636
rs11124150
rs7869045
rs6710361
rs9896155
rs139249541
rs13170001
rs111902980
All
−0.05
0.00
0.05
MR leave−one−out sensitivity analysis for
' EM DN (CD4−CD8−) AC' on 'Rheumatoid arthritis'

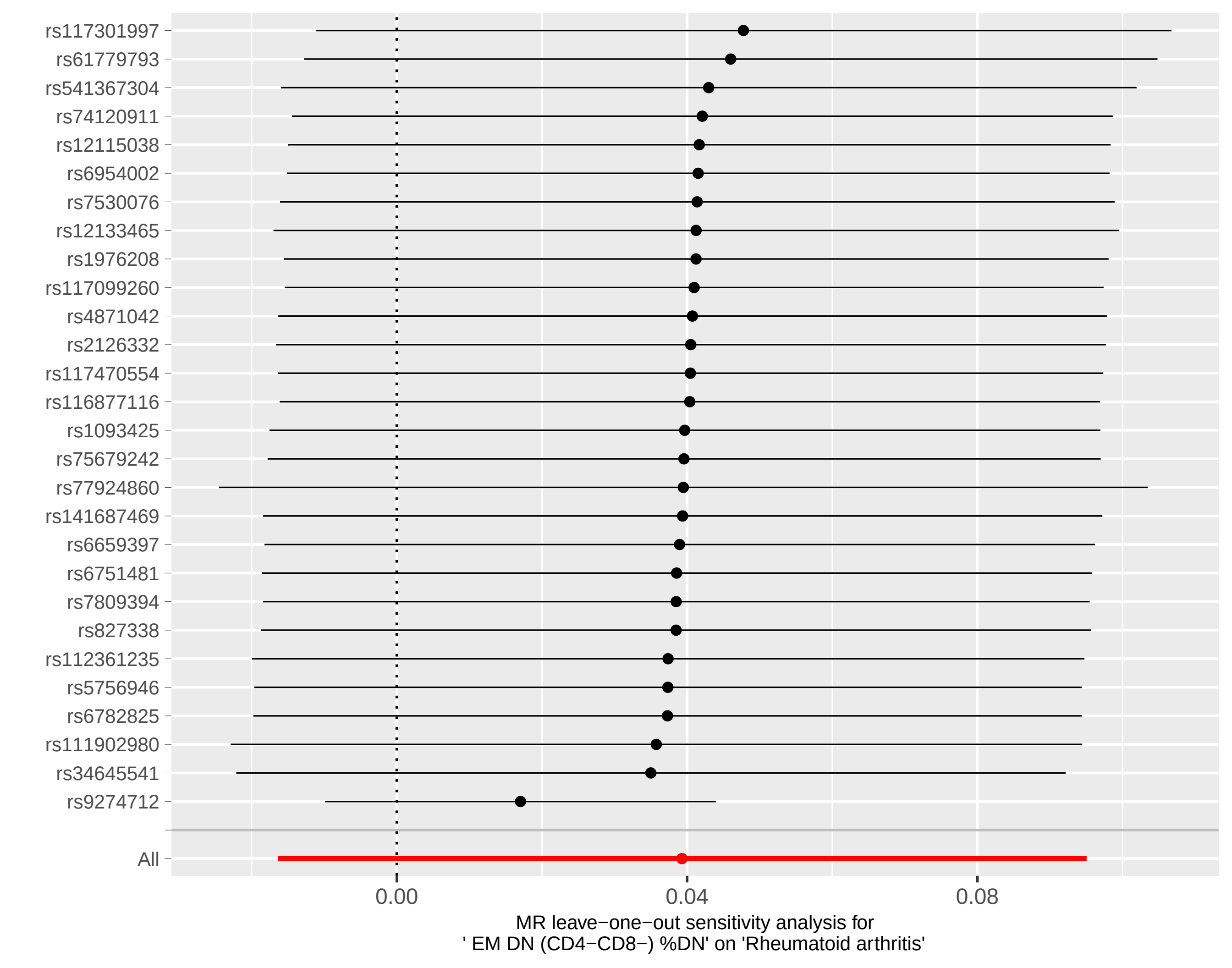

rs117301997
rs61779793
rs541367304
rs74120911
rs12115038
rs6954002
rs7530076
rs12133465
rs1976208
rs117099260
rs4871042
rs2126332
rs117470554
rs116877116
rs1093425
rs75679242
rs77924860
rs141687469
rs6659397
rs6751481
rs7809394
rs827338
rs112361235
rs5756946
rs6782825
rs111902980
rs34645541
rs9274712
All
0.00
0.04
0.08
MR leave−one−out sensitivity analysis for
' EM DN (CD4−CD8−) %DN' on 'Rheumatoid arthritis'

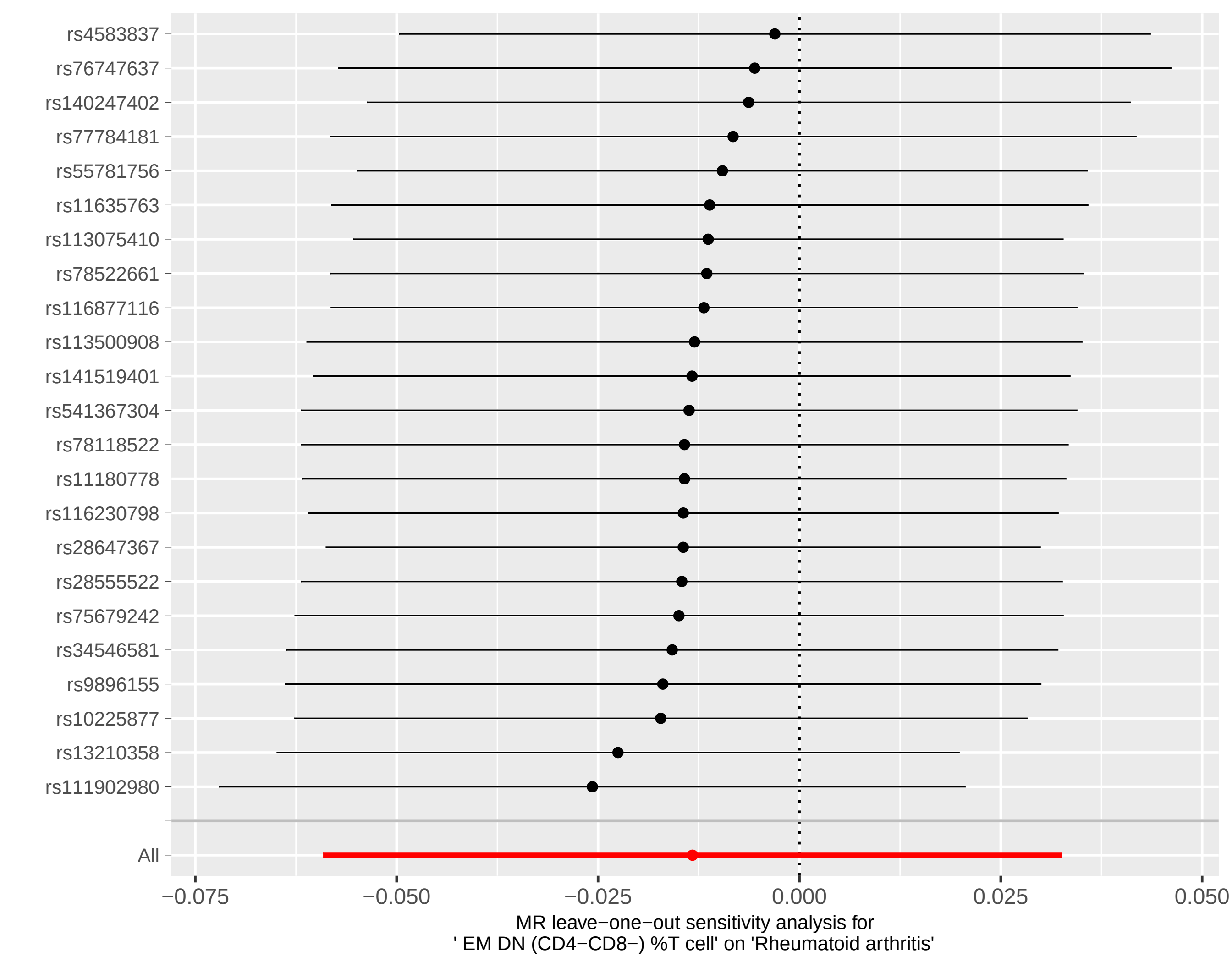
rs4583837
rs76747637
rs140247402
rs77784181
rs55781756
rs11635763
rs113075410
rs78522661
rs116877116
rs113500908
rs141519401
rs541367304
rs78118522
rs11180778
rs116230798
rs28647367
rs28555522
rs75679242
rs34546581
rs9896155
rs10225877
rs13210358
rs111902980
All
−0.075
−0.050
−0.025
0.000
0.025
0.050
MR leave−one−out sensitivity analysis for
' EM DN (CD4−CD8−) %T cell' on 'Rheumatoid arthritis'

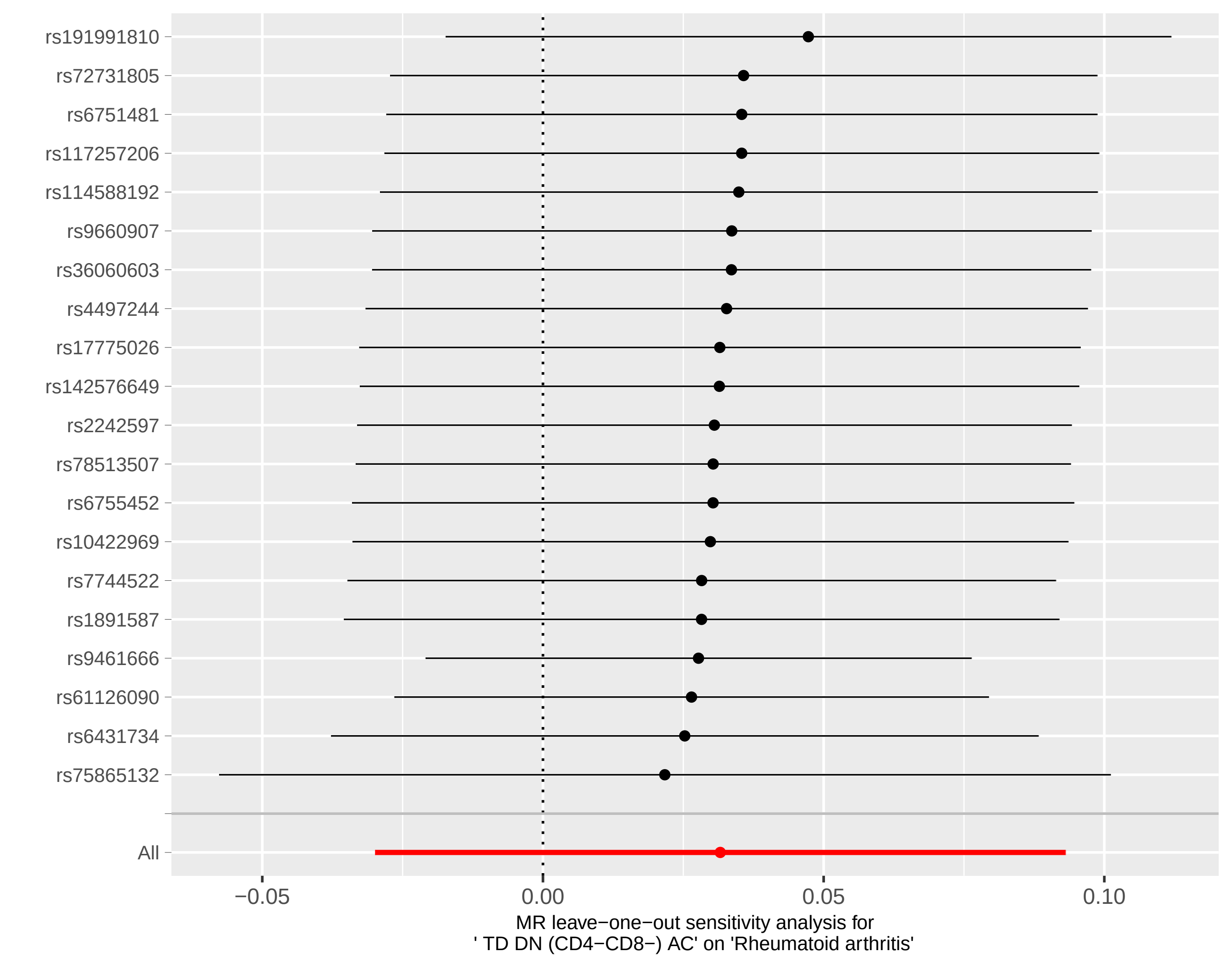
rs191991810
rs72731805
rs6751481
rs117257206
rs114588192
rs9660907
rs36060603
rs4497244
rs17775026
rs142576649
rs2242597
rs78513507
rs6755452
rs10422969
rs7744522
rs1891587
rs9461666
rs61126090
rs6431734
rs75865132
All
−0.05
0.00
0.05
0.10
MR leave−one−out sensitivity analysis for
' TD DN (CD4−CD8−) AC' on 'Rheumatoid arthritis'

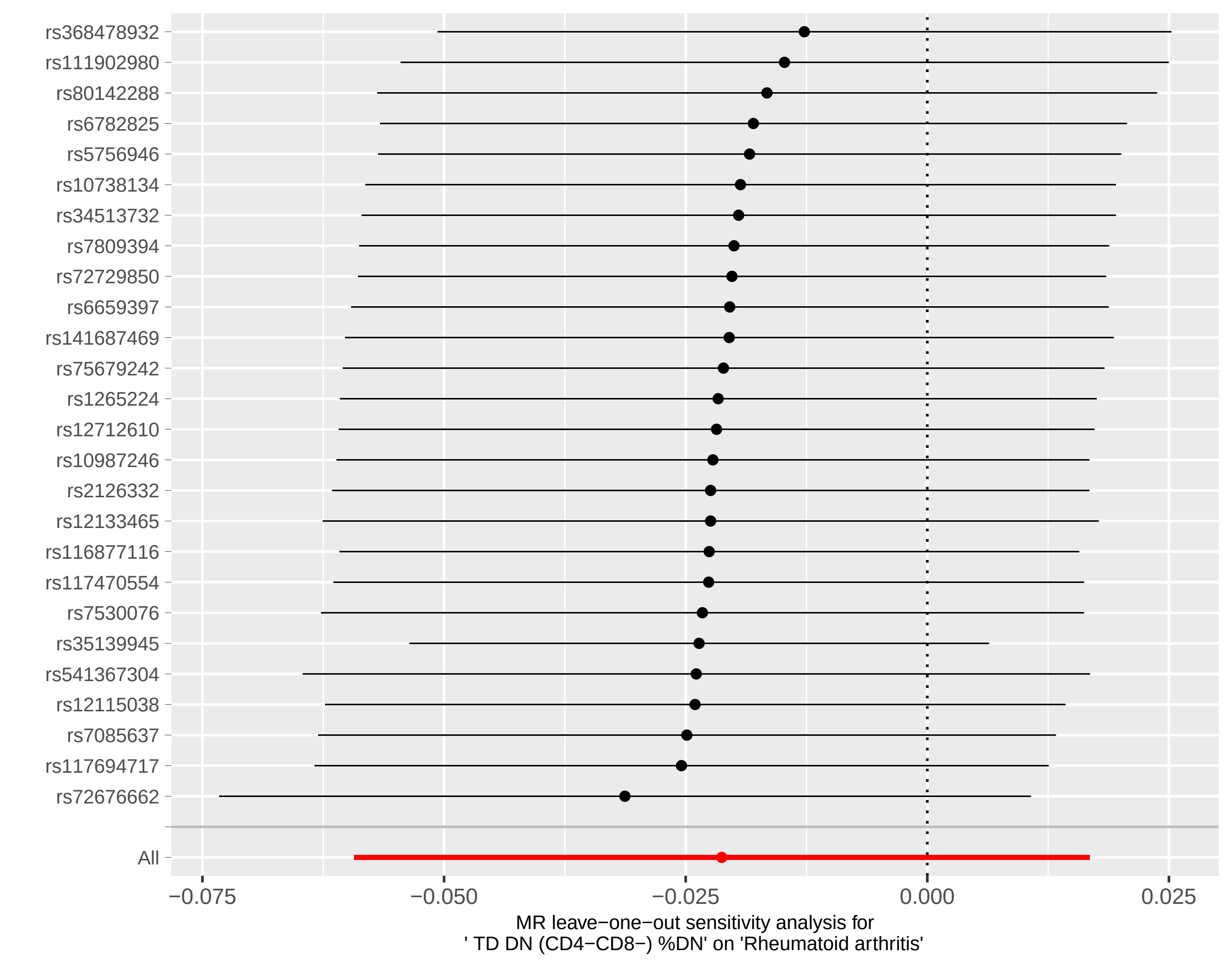
rs368478932
rs111902980
rs80142288
rs6782825
rs5756946
rs10738134
rs34513732
rs7809394
rs72729850
rs6659397
rs141687469
rs75679242
rs1265224
rs12712610
rs10987246
rs2126332
rs12133465
rs116877116
rs117470554
rs7530076
rs35139945
rs541367304
rs12115038
rs7085637
rs117694717
rs72676662
All
−0.075
−0.050
−0.025
0.000
0.025
MR leave−one−out sensitivity analysis for
' TD DN (CD4−CD8−) %DN' on 'Rheumatoid arthritis'

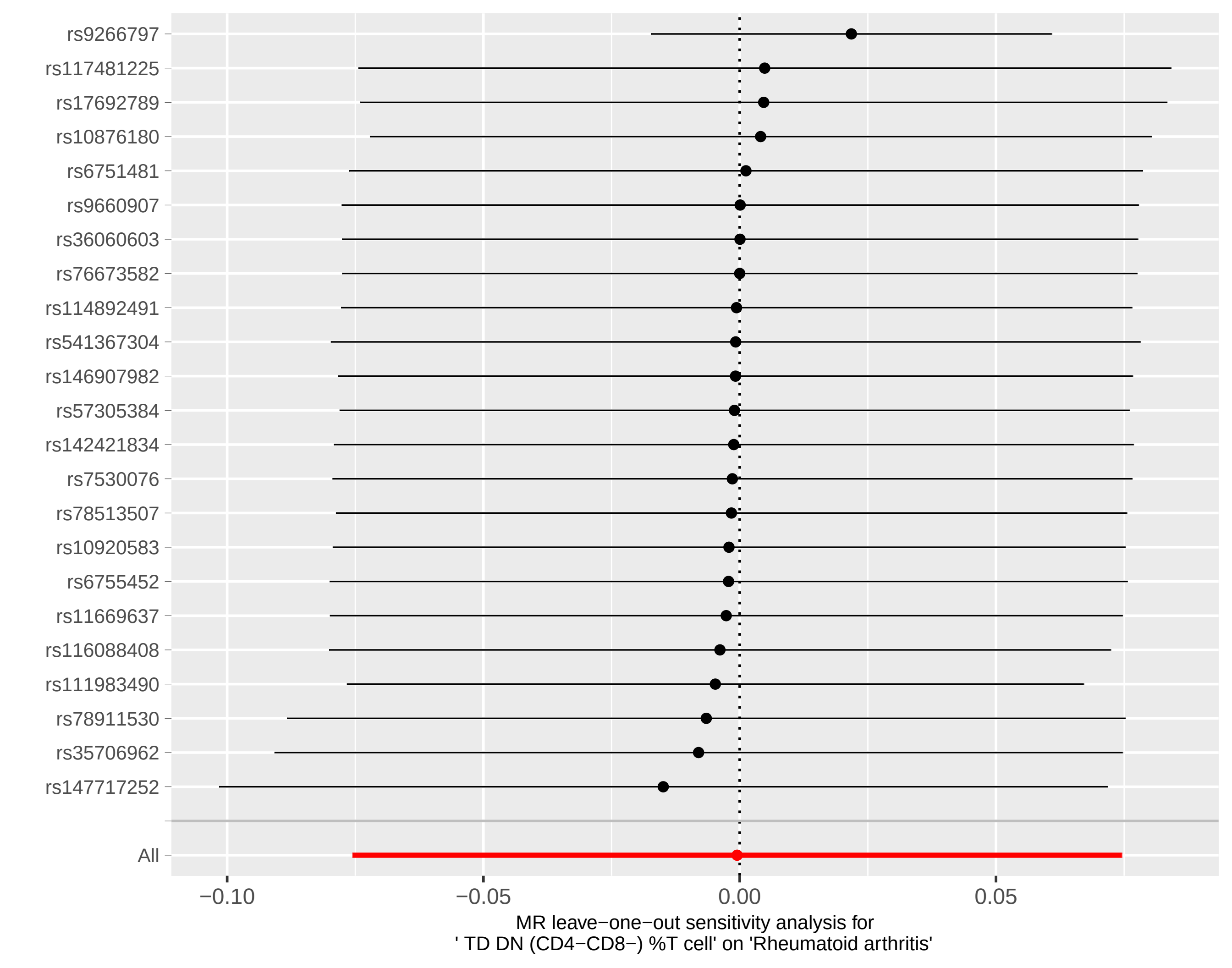
rs9266797
rs117481225
rs17692789
rs10876180
rs6751481
rs9660907
rs36060603
rs76673582
rs114892491
rs541367304
rs146907982
rs57305384
rs142421834
rs7530076
rs78513507
rs10920583
rs6755452
rs11669637
rs116088408
rs111983490
rs78911530
rs35706962
rs147717252
All
−0.10
−0.05
0.00
0.05
MR leave−one−out sensitivity analysis for
' TD DN (CD4−CD8−) %T cell' on 'Rheumatoid arthritis'

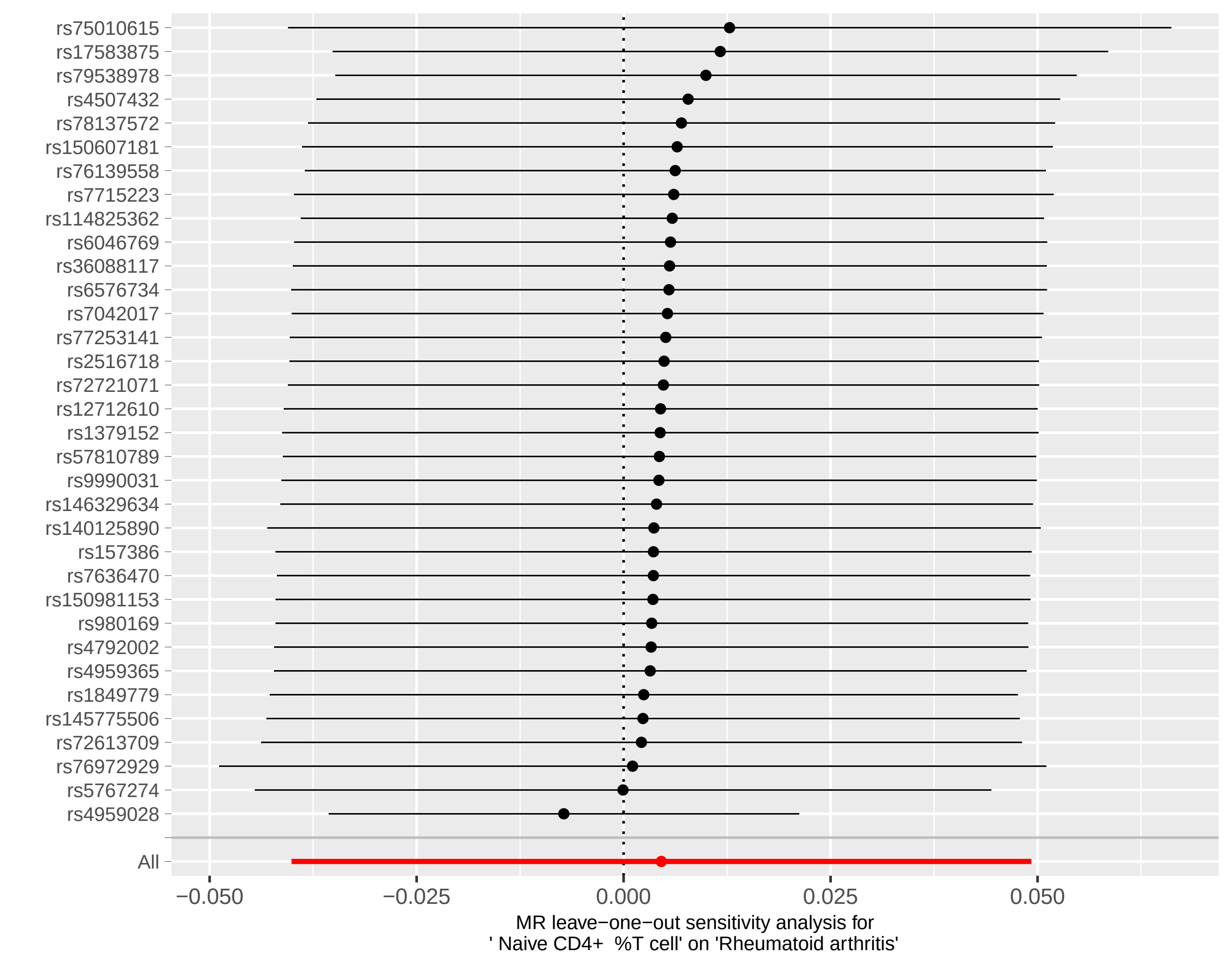
rs75010615
rs17583875
rs79538978
rs4507432
rs78137572
rs150607181
rs76139558
rs7715223
rs114825362
rs6046769
rs36088117
rs6576734
rs7042017
rs77253141
rs2516718
rs72721071
rs12712610
rs1379152
rs57810789
rs9990031
rs146329634
rs140125890
rs157386
rs7636470
rs150981153
rs980169
rs4792002
rs4959365
rs1849779
rs145775506
rs72613709
rs76972929
rs5767274
rs4959028
All
−0.050
−0.025
0.000
0.025
0.050
MR leave−one−out sensitivity analysis for
' Naive CD4+ %T cell' on 'Rheumatoid arthritis'

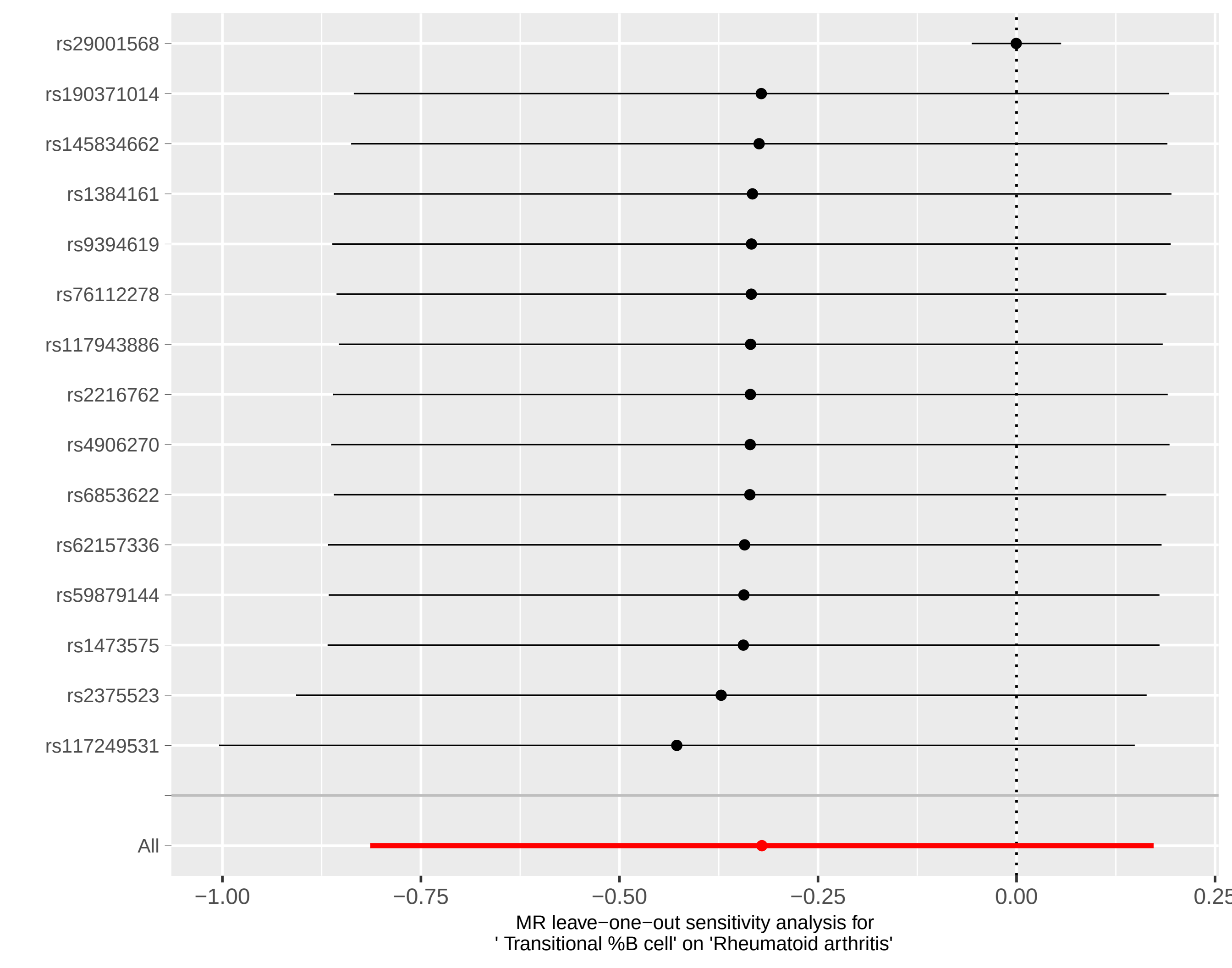

rs29001568
rs190371014
rs145834662
rs1384161
rs9394619
rs76112278
rs117943886
rs2216762
rs4906270
rs6853622
rs62157336
rs59879144
rs1473575
rs2375523
rs117249531
All
−1.00
−0.75
−0.50
−0.25
0.00
0.25
MR leave−one−out sensitivity analysis for
' Transitional %B cell' on 'Rheumatoid arthritis'

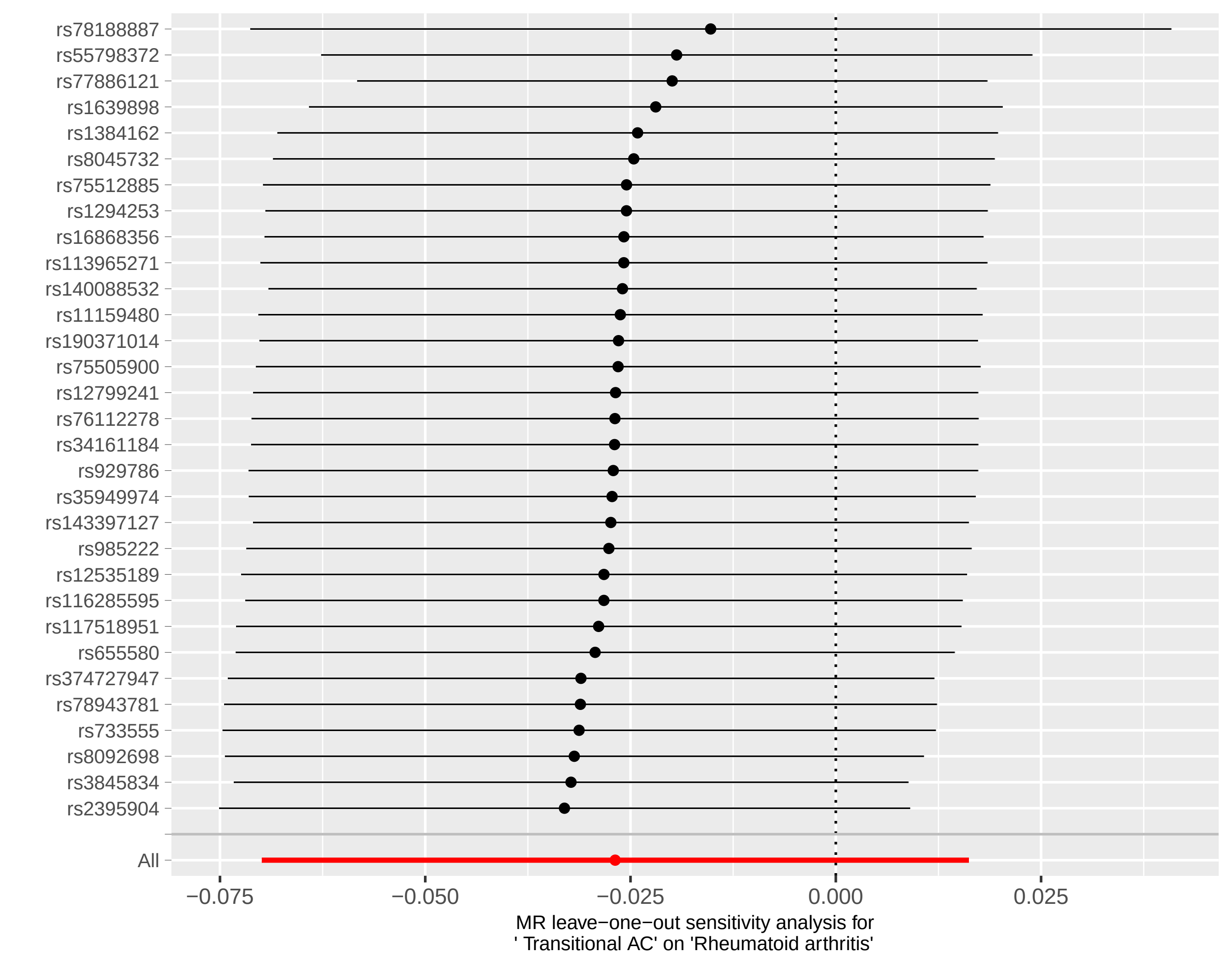

rs78188887
rs55798372
rs77886121
rs1639898
rs1384162
rs8045732
rs75512885
rs1294253
rs16868356
rs113965271
rs140088532
rs11159480
rs190371014
rs75505900
rs12799241
rs76112278
rs34161184
rs929786
rs35949974
rs143397127
rs985222
rs12535189
rs116285595
rs117518951
rs655580
rs374727947
rs78943781
rs733555
rs8092698
rs3845834
rs2395904
All
−0.075
−0.050
−0.025
0.000
0.025
MR leave−one−out sensitivity analysis for
' Transitional AC' on 'Rheumatoid arthritis'

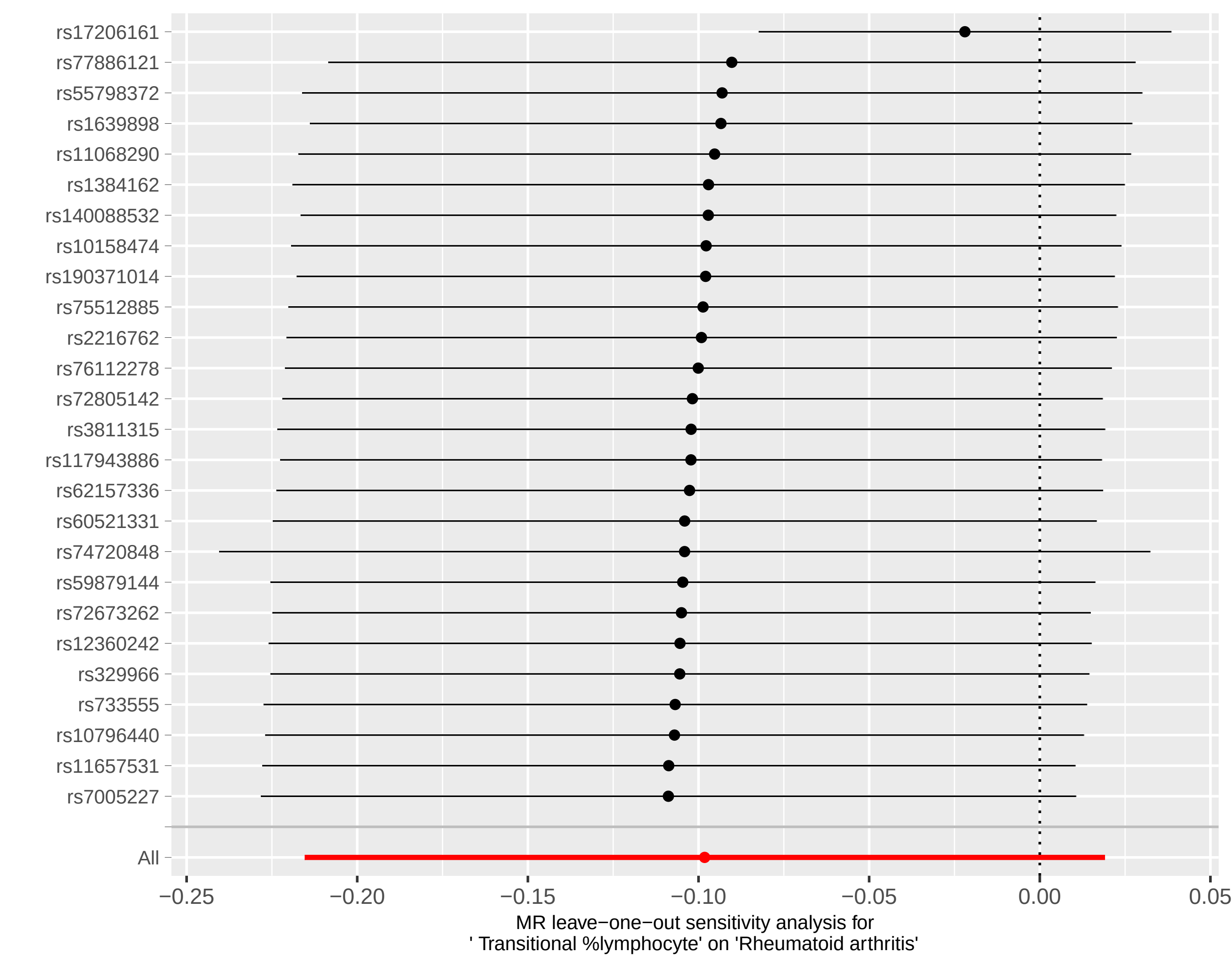
rs17206161
rs77886121
rs55798372
rs1639898
rs11068290
rs1384162
rs140088532
rs10158474
rs190371014
rs75512885
rs2216762
rs76112278
rs72805142
rs3811315
rs117943886
rs62157336
rs60521331
rs74720848
rs59879144
rs72673262
rs12360242
rs329966
rs733555
rs10796440
rs11657531
rs7005227
All
−0.25
−0.20
−0.15
−0.10
−0.05
0.00
0.05
MR leave−one−out sensitivity analysis for
' Transitional %lymphocyte' on 'Rheumatoid arthritis'

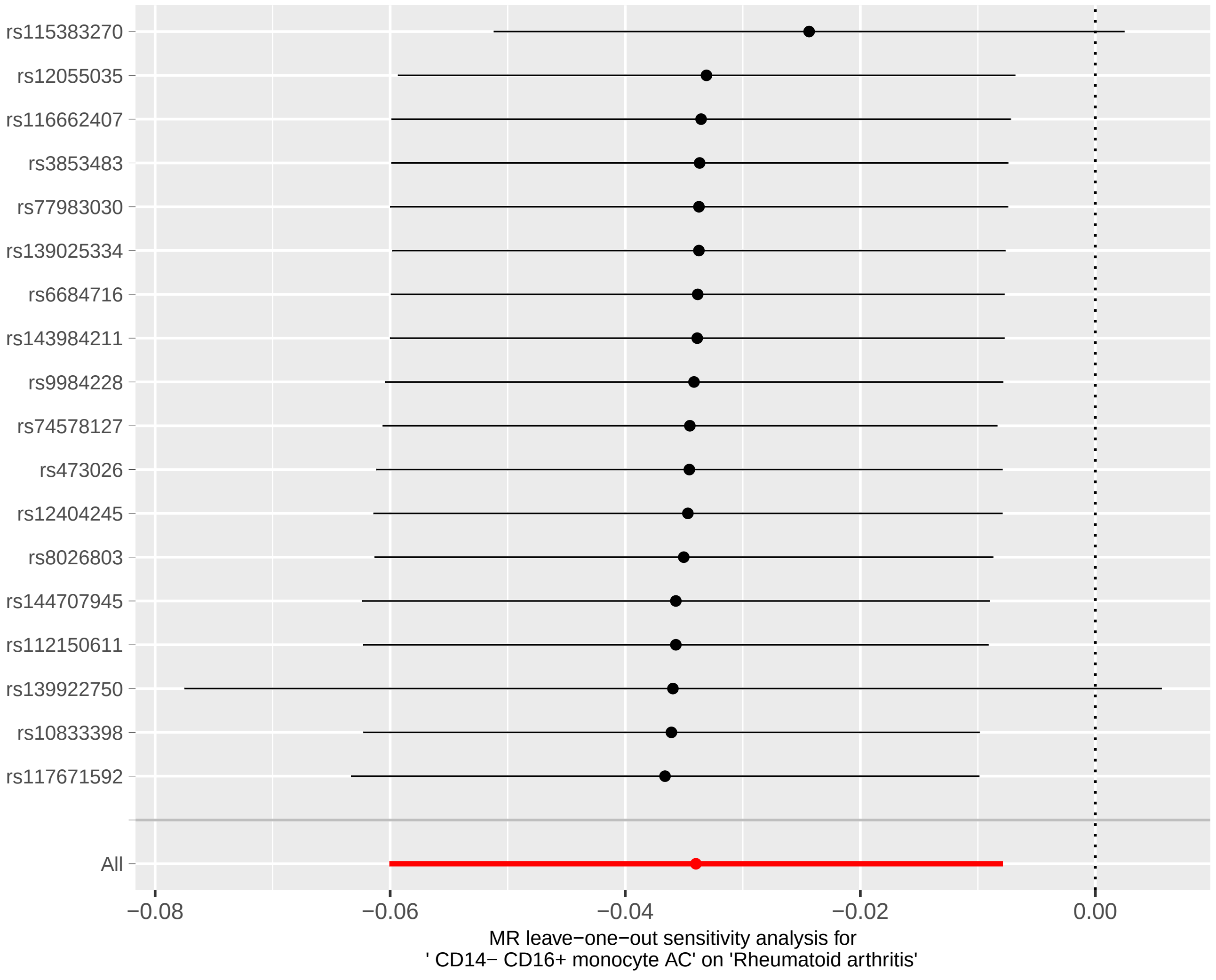
rs115383270
rs12055035
rs116662407
rs3853483
rs77983030
rs139025334
rs6684716
rs143984211
rs9984228
rs74578127
rs473026
rs12404245
rs8026803
rs144707945
rs112150611
rs139922750
rs10833398
rs117671592
All
−0.08
−0.06
−0.04
−0.02
0.00
MR leave−one−out sensitivity analysis for
' CD14− CD16+ monocyte AC' on 'Rheumatoid arthritis'

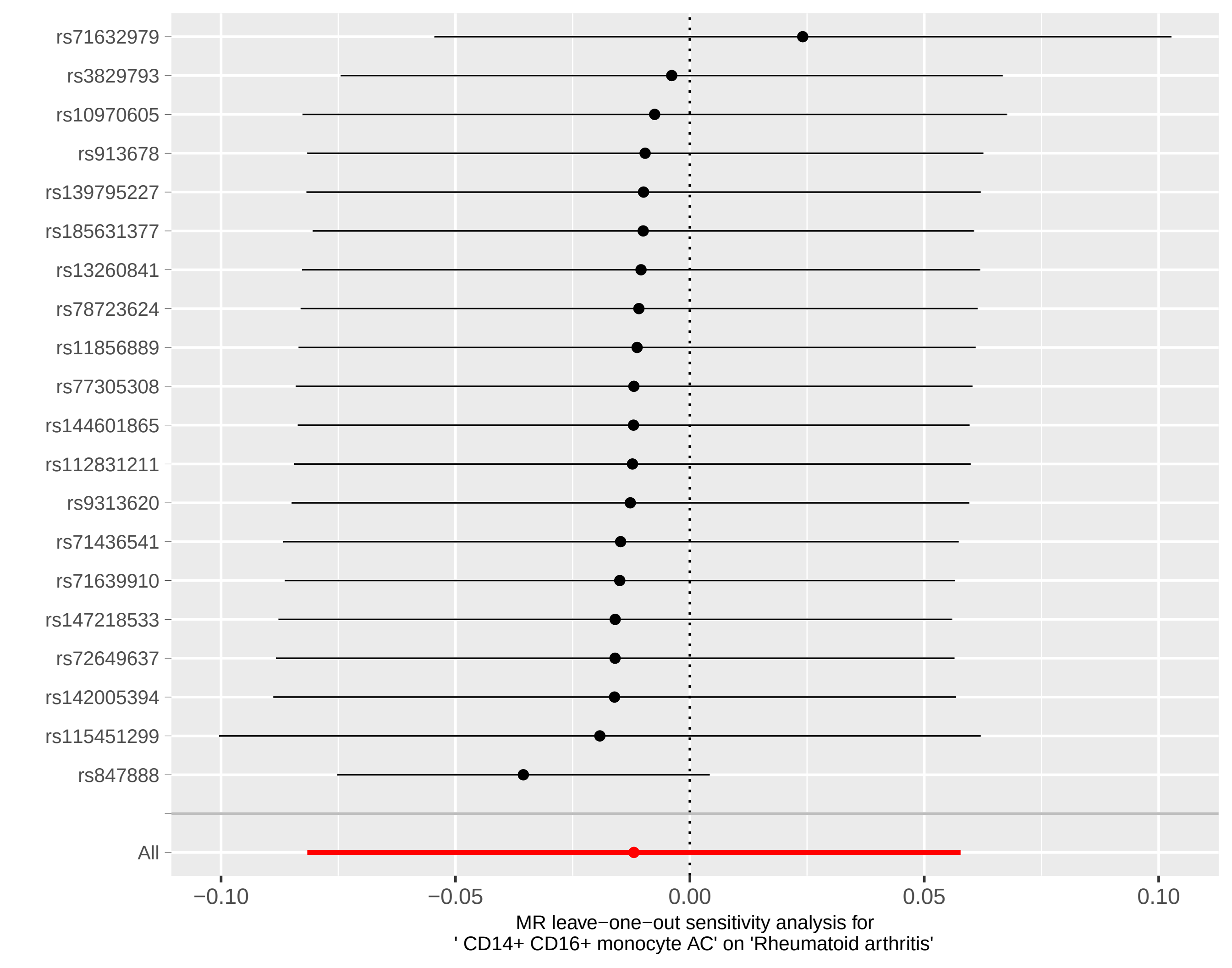

rs71632979
rs3829793
rs10970605
rs913678
rs139795227
rs185631377
rs13260841
rs78723624
rs11856889
rs77305308
rs144601865
rs112831211
rs9313620
rs71436541
rs71639910
rs147218533
rs72649637
rs142005394
rs115451299
rs847888
All
−0.10
−0.05
0.00
0.05
0.10
MR leave−one−out sensitivity analysis for
' CD14+ CD16+ monocyte AC' on 'Rheumatoid arthritis'

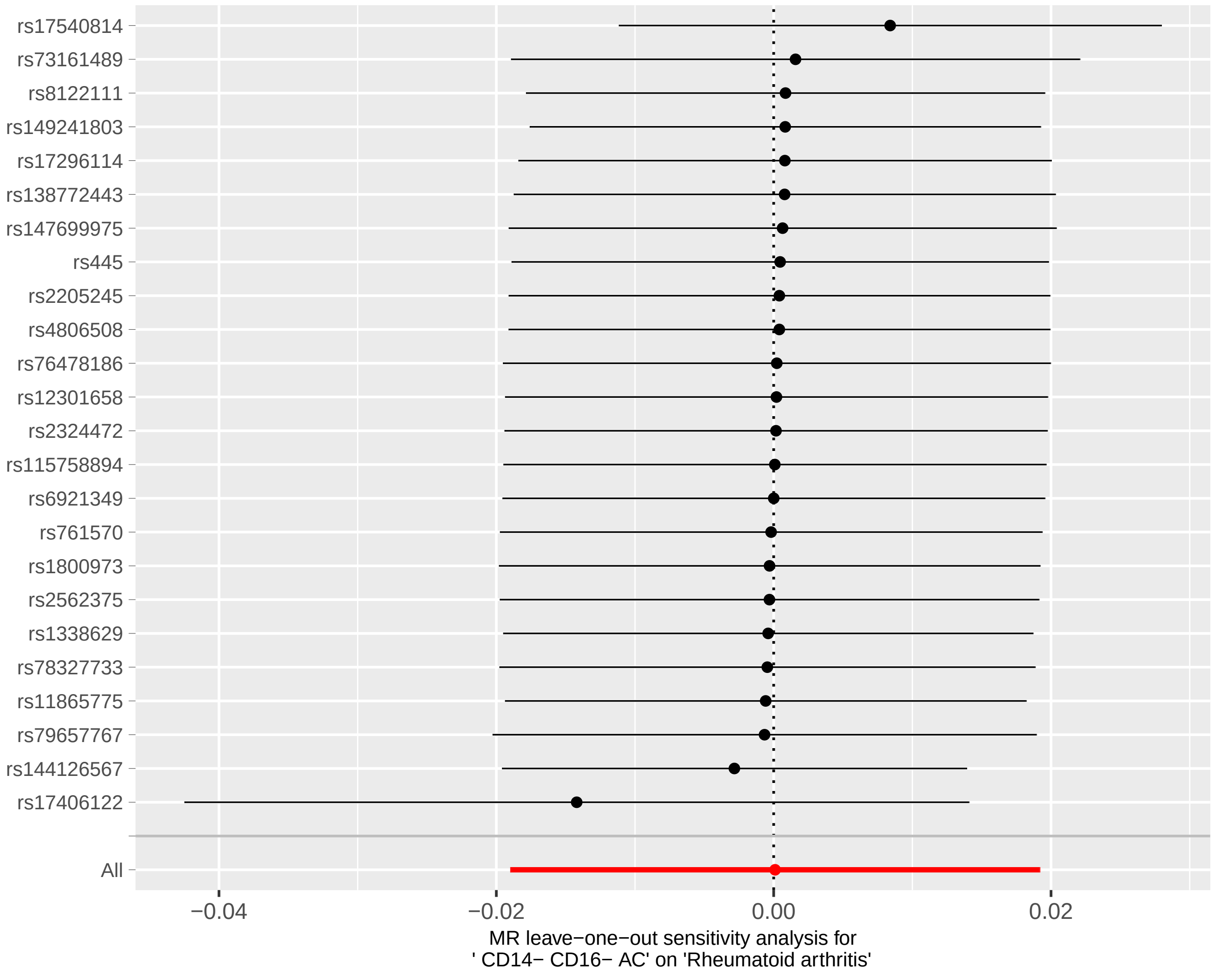
rs17540814
rs73161489
rs8122111
rs149241803
rs17296114
rs138772443
rs147699975
rs445
rs2205245
rs4806508
rs76478186
rs12301658
rs2324472
rs115758894
rs6921349
rs761570
rs1800973
rs2562375
rs1338629
rs78327733
rs11865775
rs79657767
rs144126567
rs17406122
All
−0.04
−0.02
0.00
0.02
MR leave−one−out sensitivity analysis for
' CD14− CD16− AC' on 'Rheumatoid arthritis'

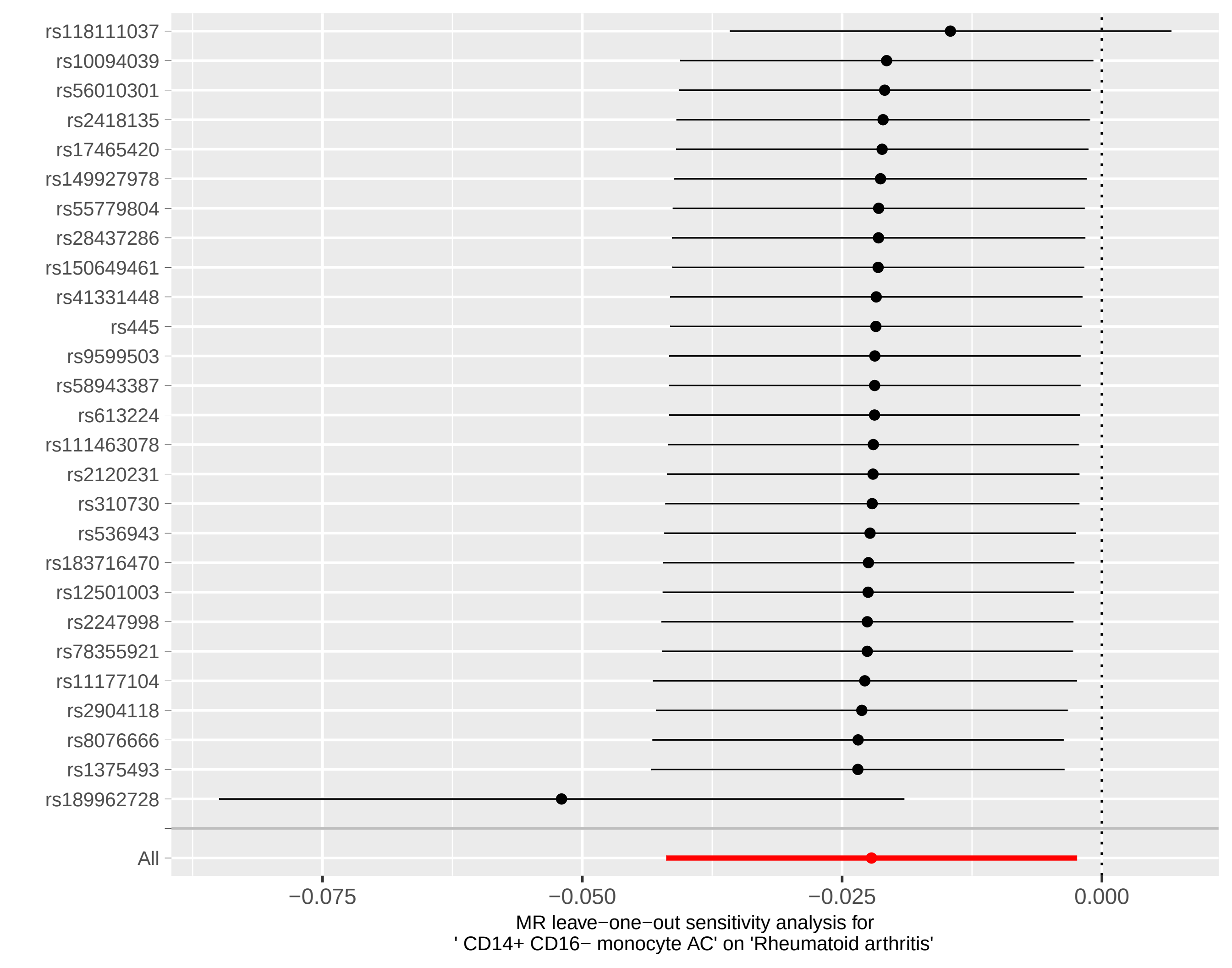

rs118111037
rs10094039
rs56010301
rs2418135
rs17465420
rs149927978
rs55779804
rs28437286
rs150649461
rs41331448
rs445
rs9599503
rs58943387
rs613224
rs111463078
rs2120231
rs310730
rs536943
rs183716470
rs12501003
rs2247998
rs78355921
rs11177104
rs2904118
rs8076666
rs1375493
rs189962728
All
−0.075
−0.050
−0.025
0.000
MR leave−one−out sensitivity analysis for
' CD14+ CD16− monocyte AC' on 'Rheumatoid arthritis'

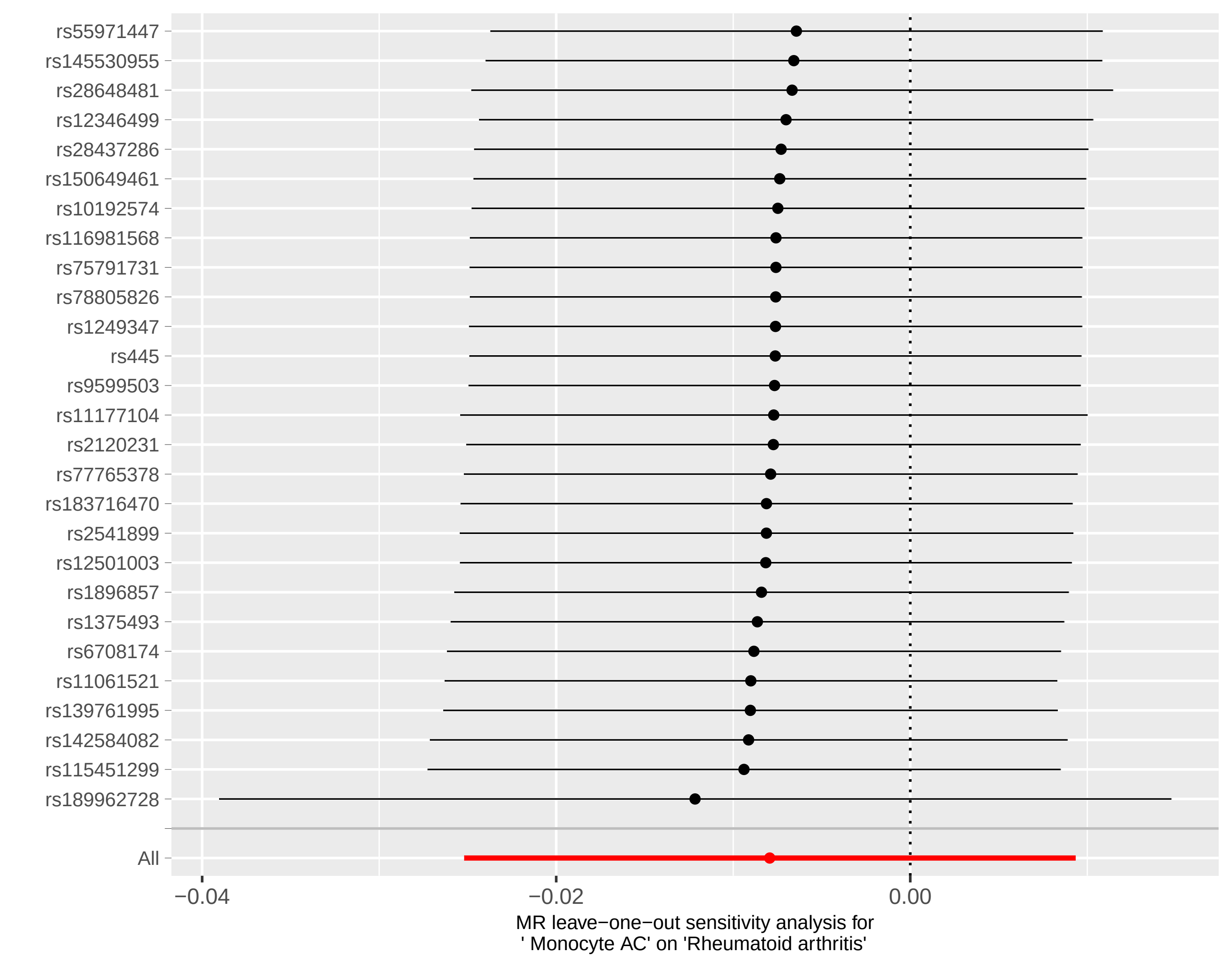
rs55971447
rs145530955
rs28648481
rs12346499
rs28437286
rs150649461
rs10192574
rs116981568
rs75791731
rs78805826
rs1249347
rs445
rs9599503
rs11177104
rs2120231
rs77765378
rs183716470
rs2541899
rs12501003
rs1896857
rs1375493
rs6708174
rs11061521
rs139761995
rs142584082
rs115451299
rs189962728
All
−0.04
−0.02
0.00
MR leave−one−out sensitivity analysis for
' Monocyte AC' on 'Rheumatoid arthritis'

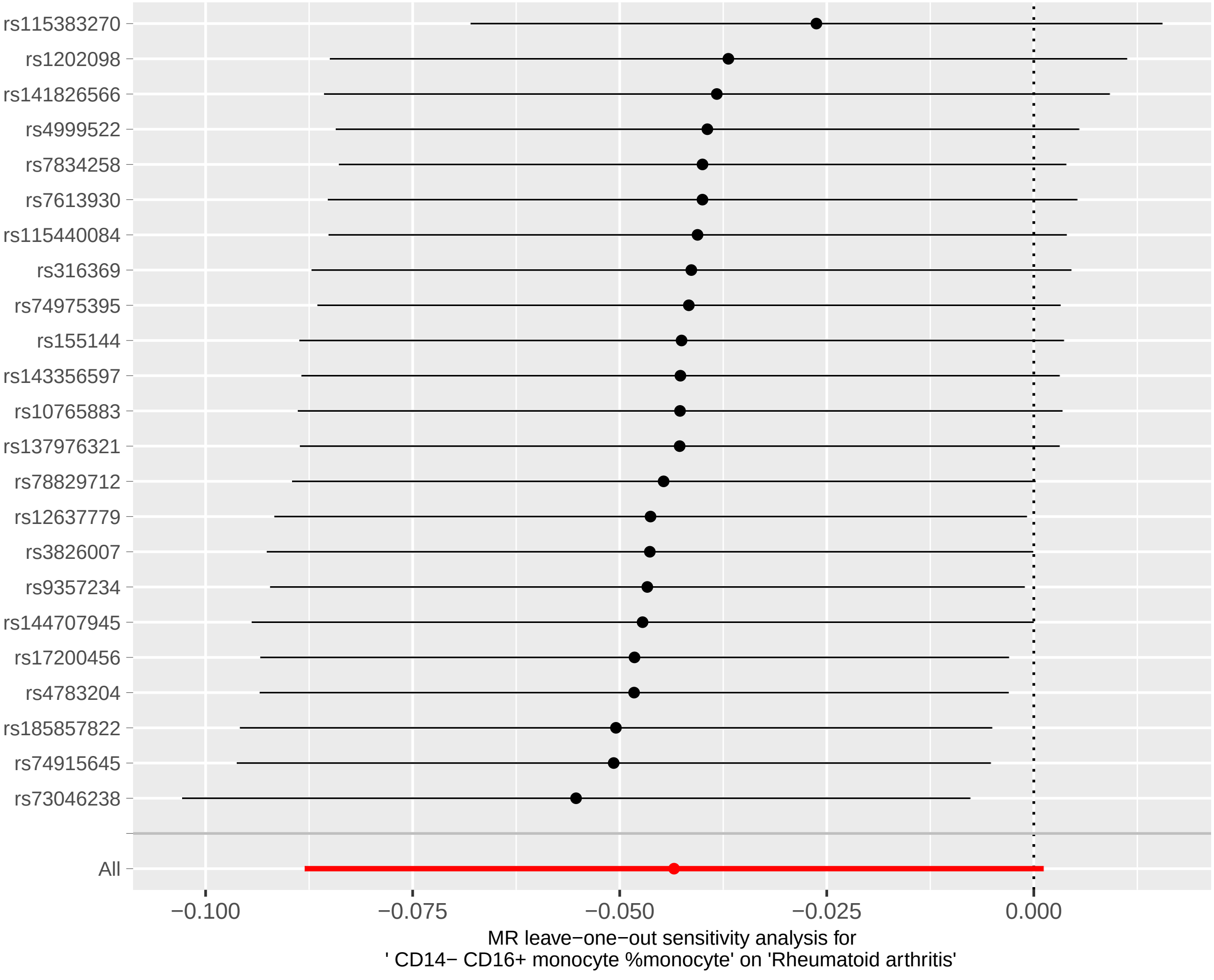
rs115383270
rs1202098
rs141826566
rs4999522
rs7834258
rs7613930
rs115440084
rs316369
rs74975395
rs155144
rs143356597
rs10765883
rs137976321
rs78829712
rs12637779
rs3826007
rs9357234
rs144707945
rs17200456
rs4783204
rs185857822
rs74915645
rs73046238
All
−0.100
−0.075
−0.050
−0.025
0.000
MR leave−one−out sensitivity analysis for
' CD14− CD16+ monocyte %monocyte' on 'Rheumatoid arthritis'

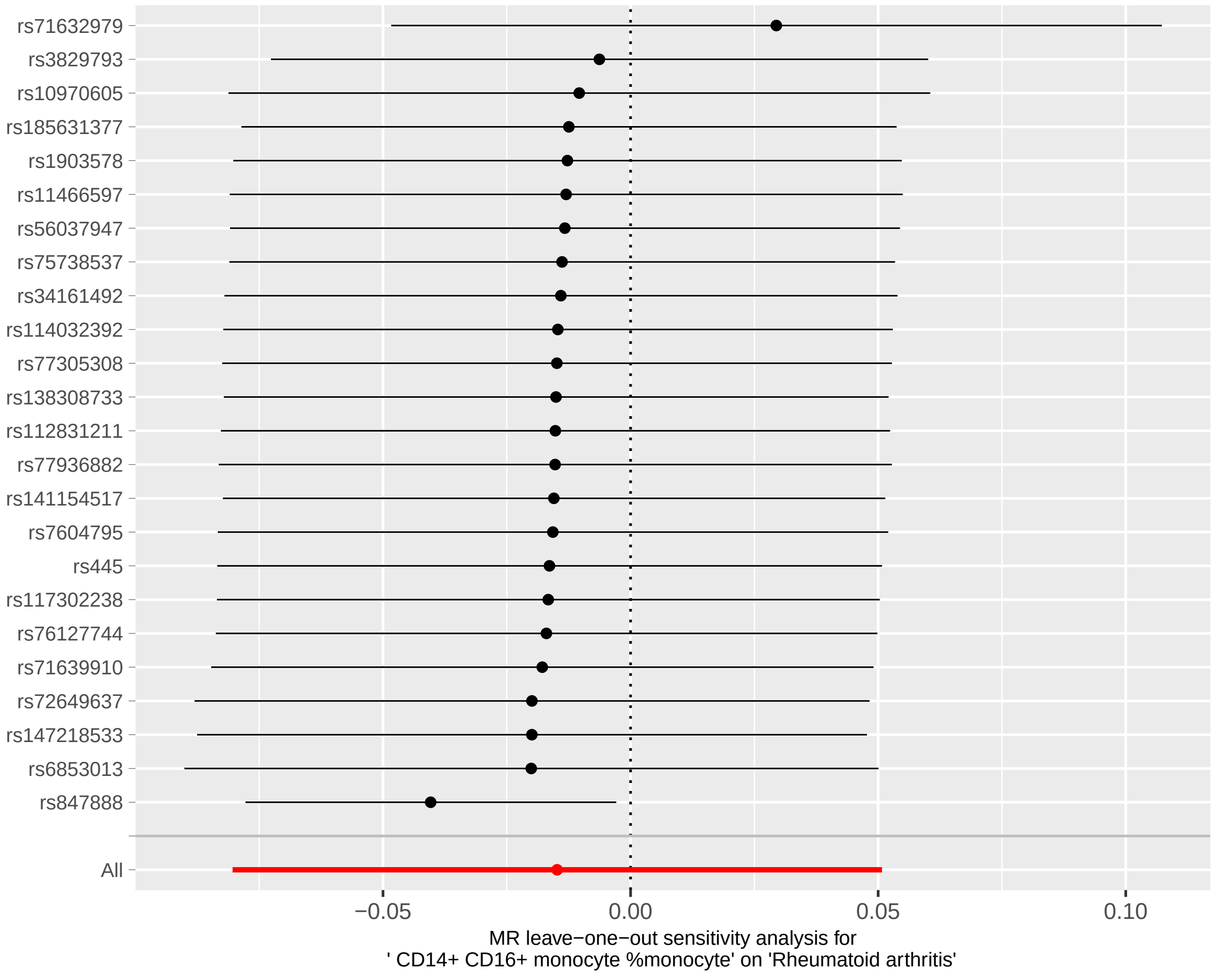
rs71632979
rs3829793
rs10970605
rs185631377
rs1903578
rs11466597
rs56037947
rs75738537
rs34161492
rs114032392
rs77305308
rs138308733
rs112831211
rs77936882
rs141154517
rs7604795
rs445
rs117302238
rs76127744
rs71639910
rs72649637
rs147218533
rs6853013
rs847888
All
−0.05
0.00
0.05
0.10
MR leave−one−out sensitivity analysis for
' CD14+ CD16+ monocyte %monocyte' on 'Rheumatoid arthritis'

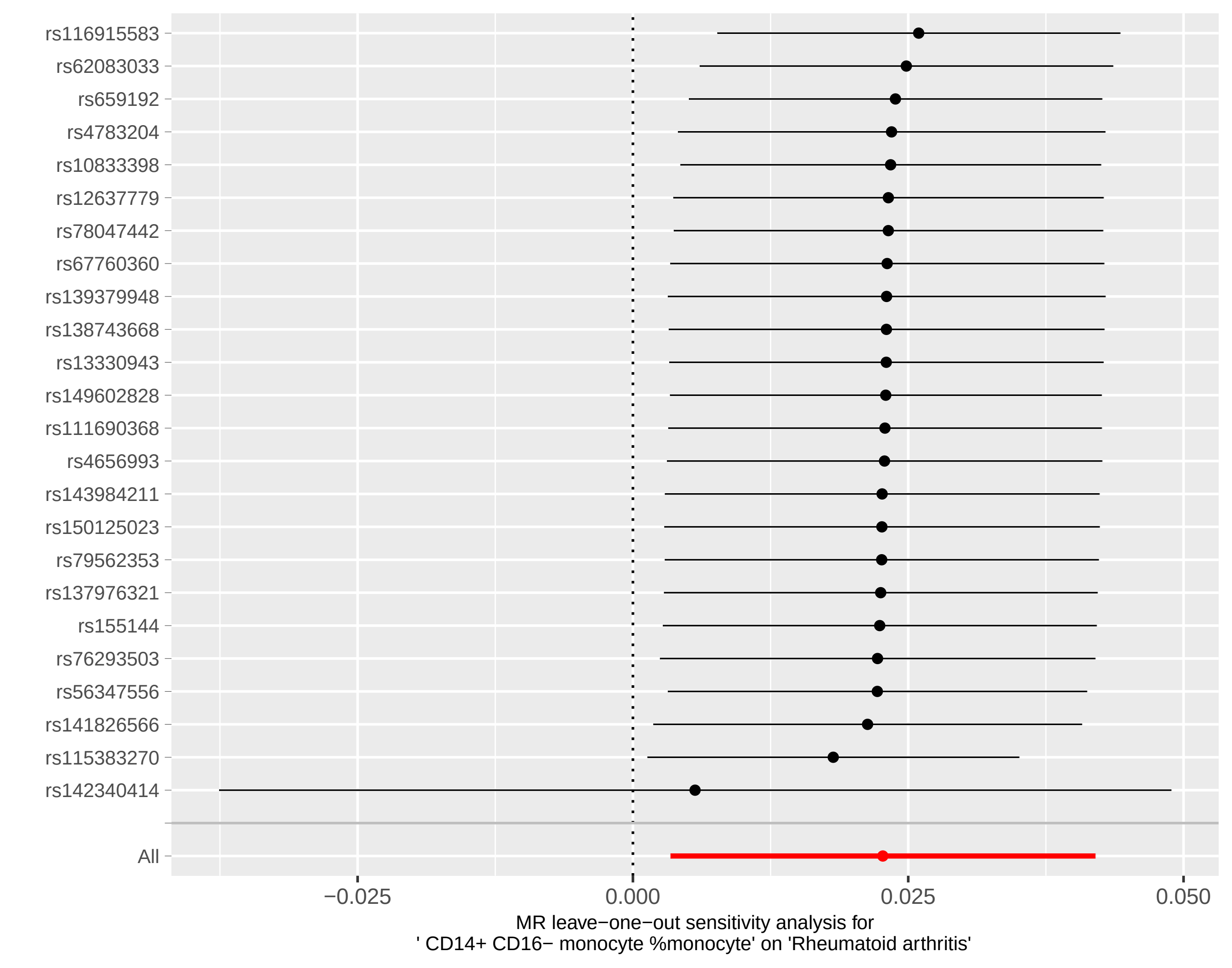
rs116915583
rs62083033
rs659192
rs4783204
rs10833398
rs12637779
rs78047442
rs67760360
rs139379948
rs138743668
rs13330943
rs149602828
rs111690368
rs4656993
rs143984211
rs150125023
rs79562353
rs137976321
rs155144
rs76293503
rs56347556
rs141826566
rs115383270
rs142340414
All
−0.025
0.000
0.025
0.050
MR leave−one−out sensitivity analysis for
' CD14+ CD16− monocyte %monocyte' on 'Rheumatoid arthritis'

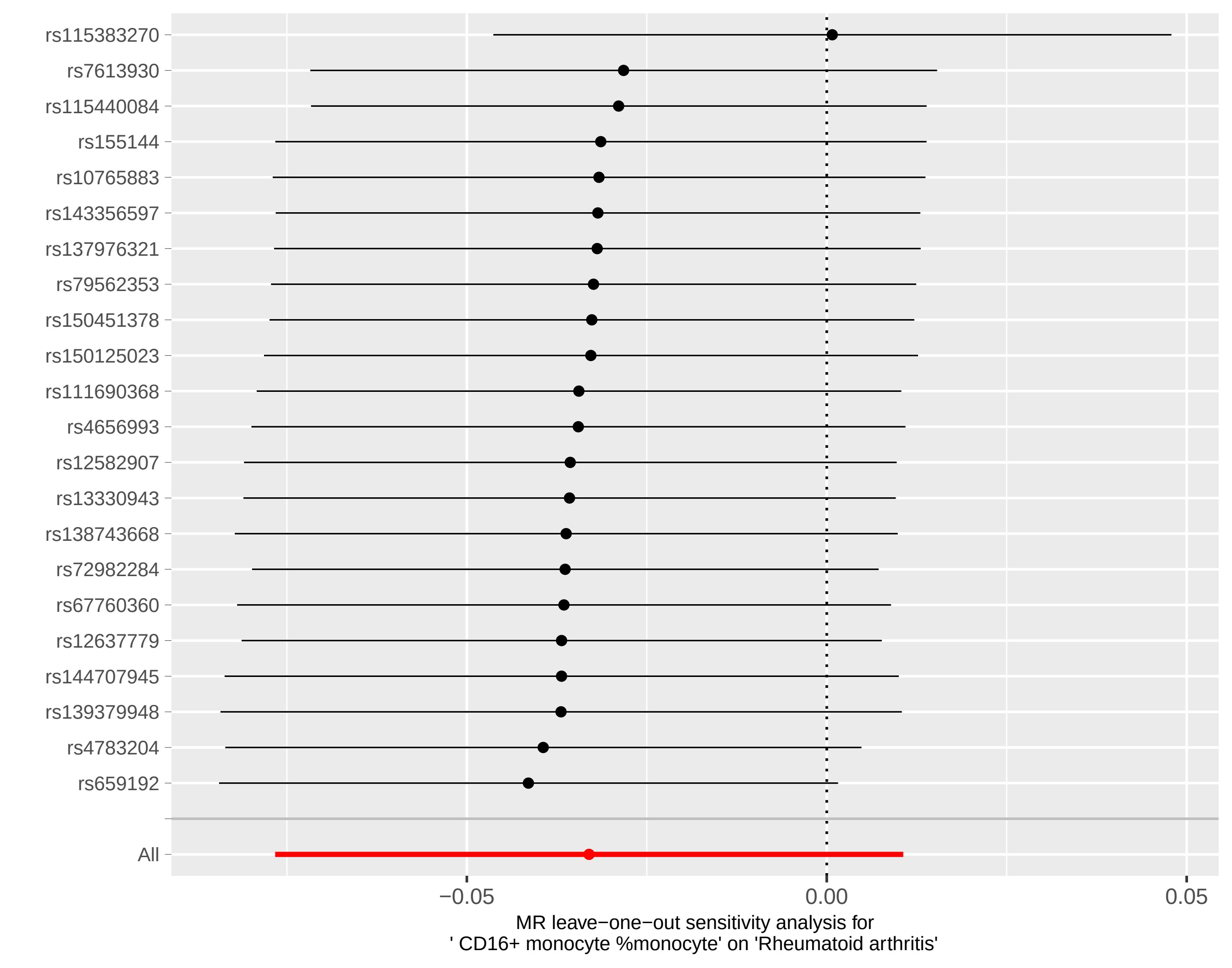
rs115383270
rs7613930
rs115440084
rs155144
rs10765883
rs143356597
rs137976321
rs79562353
rs150451378
rs150125023
rs111690368
rs4656993
rs12582907
rs13330943
rs138743668
rs72982284
rs67760360
rs12637779
rs144707945
rs139379948
rs4783204
rs659192
All
−0.05
0.00
0.05
MR leave−one−out sensitivity analysis for
' CD16+ monocyte %monocyte' on 'Rheumatoid arthritis'

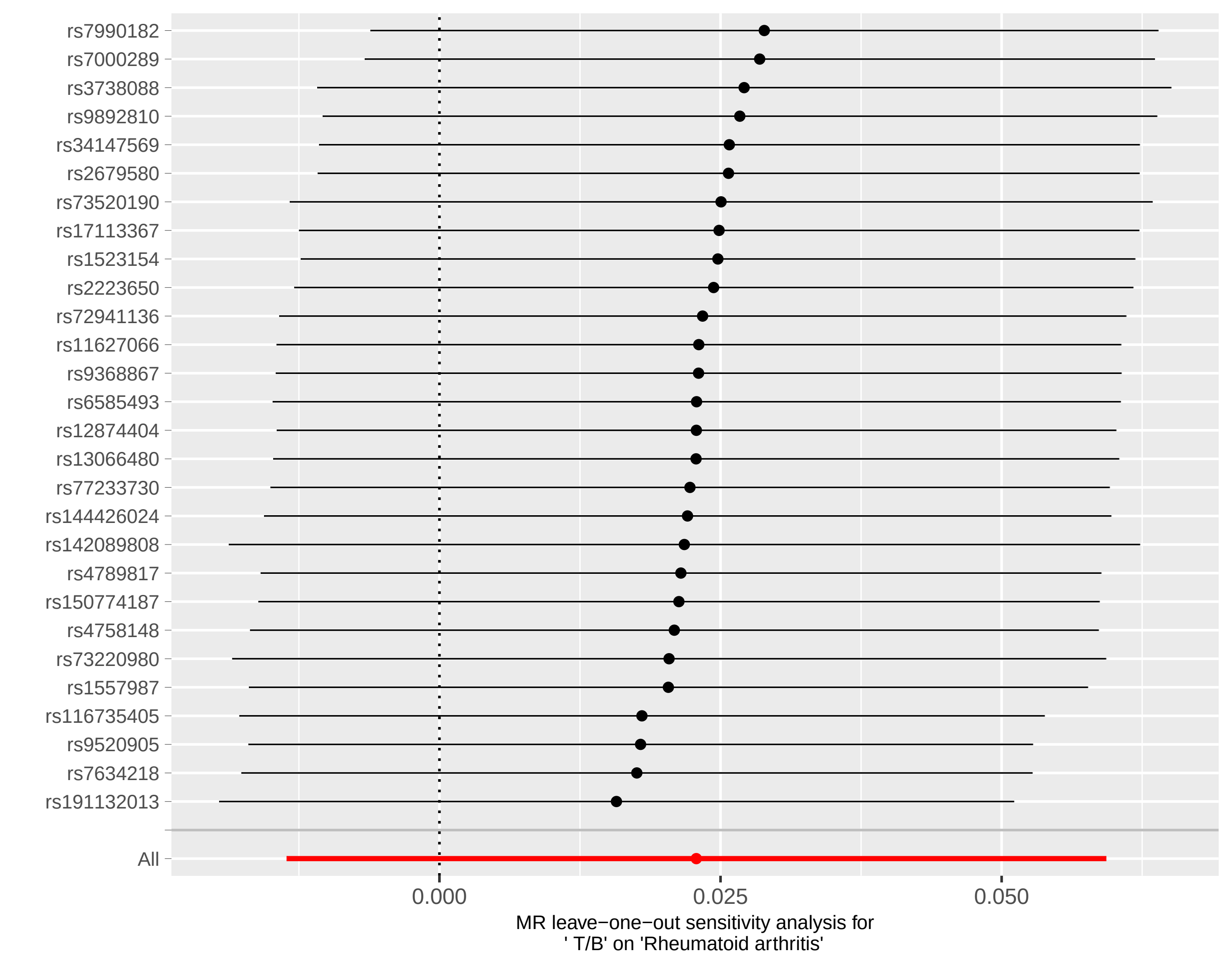
rs7990182
rs7000289
rs3738088
rs9892810
rs34147569
rs2679580
rs73520190
rs17113367
rs1523154
rs2223650
rs72941136
rs11627066
rs9368867
rs6585493
rs12874404
rs13066480
rs77233730
rs144426024
rs142089808
rs4789817
rs150774187
rs4758148
rs73220980
rs1557987
rs116735405
rs9520905
rs7634218
rs191132013
All
0.000
0.025
0.050
MR leave−one−out sensitivity analysis for
' T/B' on 'Rheumatoid arthritis'

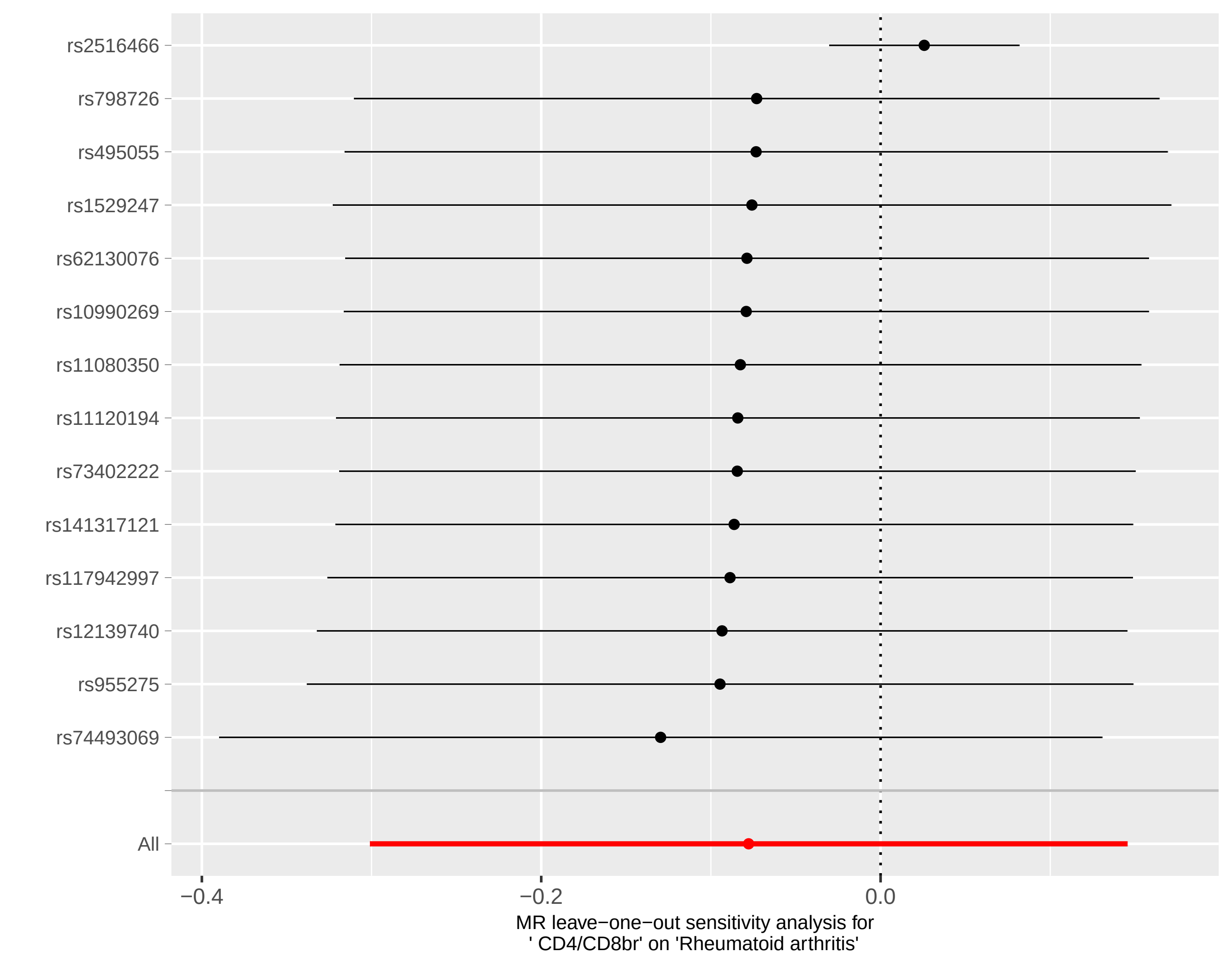

rs2516466
rs798726
rs495055
rs1529247
rs62130076
rs10990269
rs11080350
rs11120194
rs73402222
rs141317121
rs117942997
rs12139740
rs955275
rs74493069
All
−0.4
−0.2
0.0
MR leave−one−out sensitivity analysis for
' CD4/CD8br' on 'Rheumatoid arthritis'

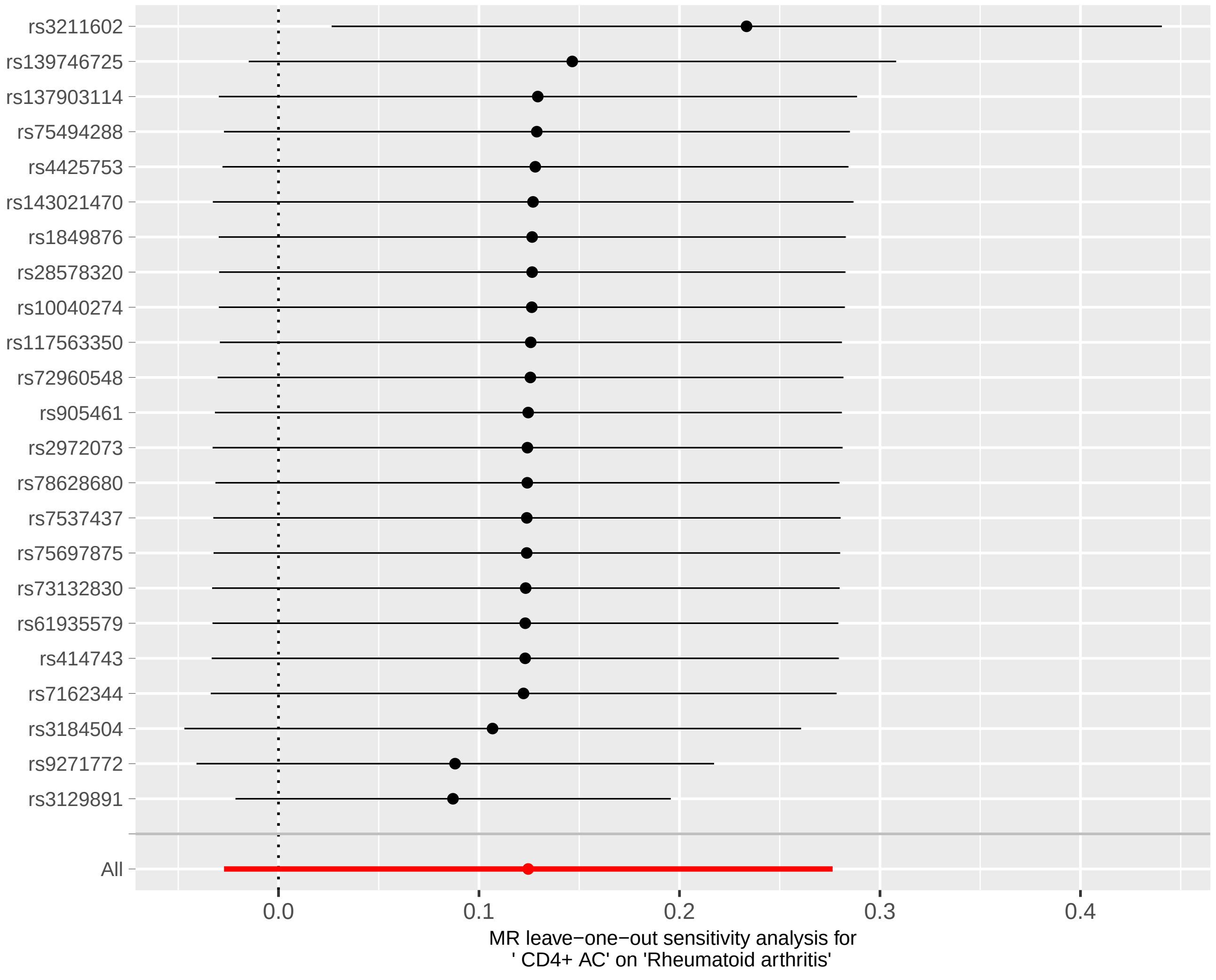
rs3211602
rs139746725
rs137903114
rs75494288
rs4425753
rs143021470
rs1849876
rs28578320
rs10040274
rs117563350
rs72960548
rs905461
rs2972073
rs78628680
rs7537437
rs75697875
rs73132830
rs61935579
rs414743
rs7162344
rs3184504
rs9271772
rs3129891
All
0.0
0.1
0.2
0.3
0.4
MR leave−one−out sensitivity analysis for
' CD4+ AC' on 'Rheumatoid arthritis'

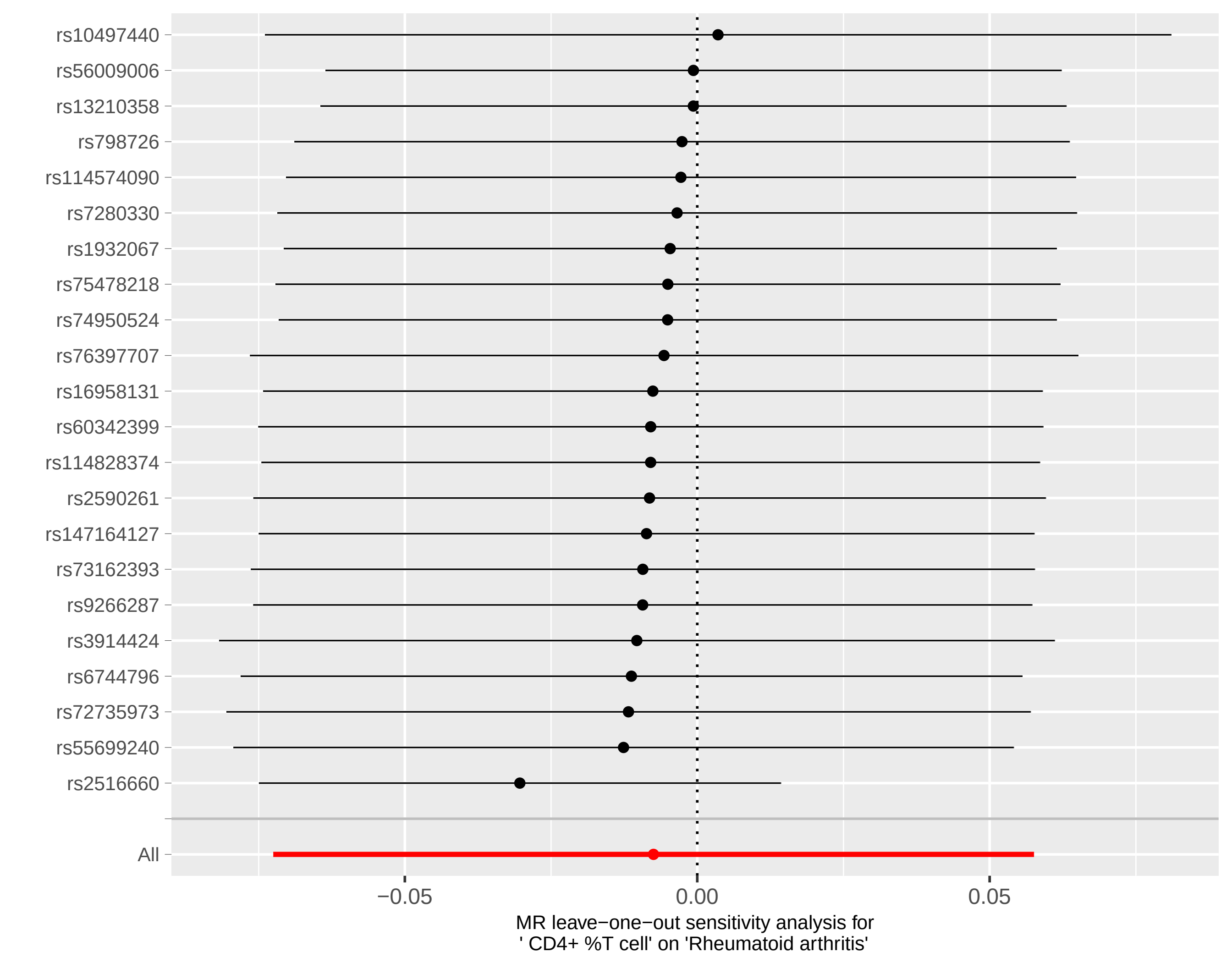

rs10497440
rs56009006
rs13210358
rs798726
rs114574090
rs7280330
rs1932067
rs75478218
rs74950524
rs76397707
rs16958131
rs60342399
rs114828374
rs2590261
rs147164127
rs73162393
rs9266287
rs3914424
rs6744796
rs72735973
rs55699240
rs2516660
All
−0.05
0.00
0.05
MR leave−one−out sensitivity analysis for
' CD4+ %T cell' on 'Rheumatoid arthritis'

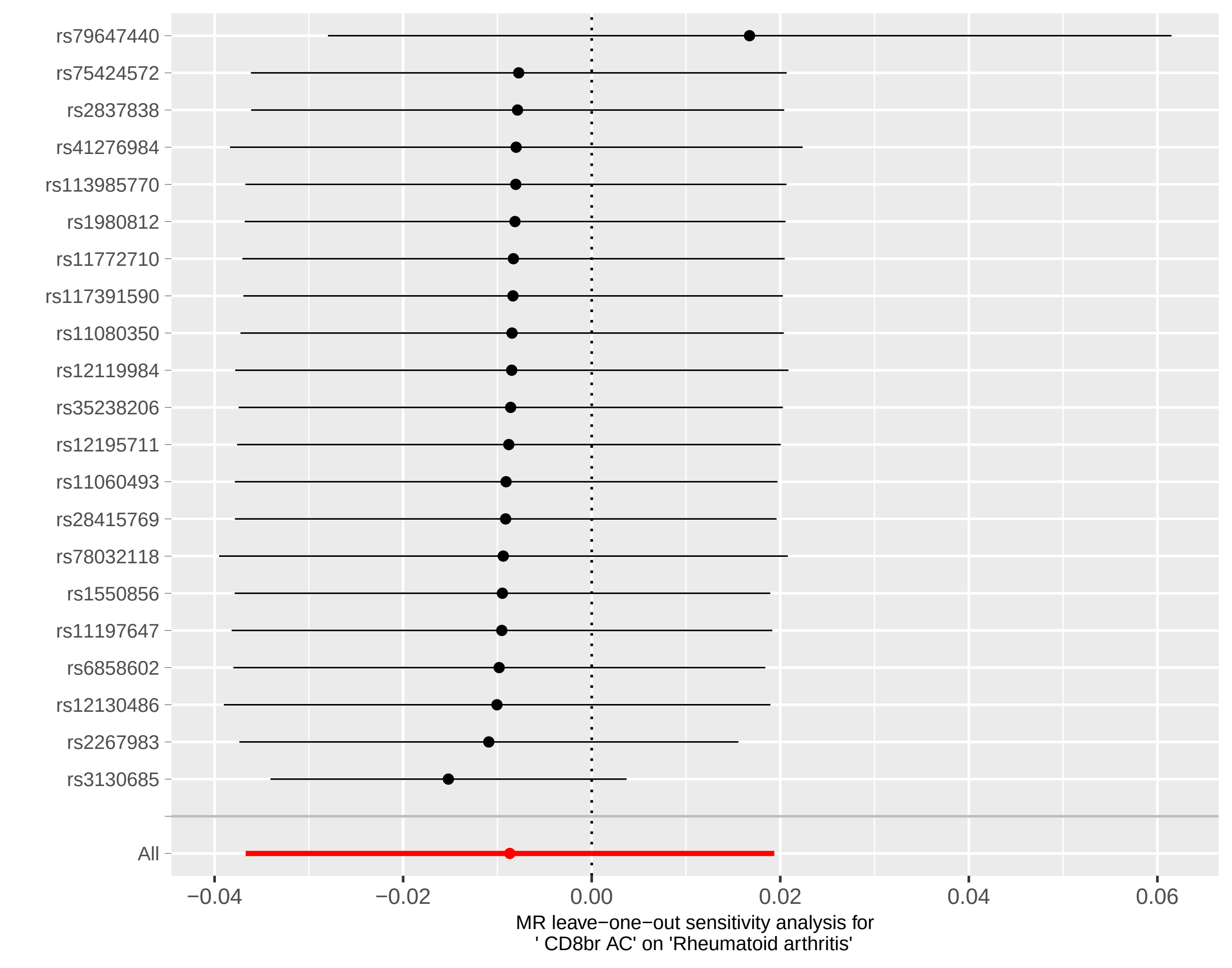
rs79647440
rs75424572
rs2837838
rs41276984
rs113985770
rs1980812
rs11772710
rs117391590
rs11080350
rs12119984
rs35238206
rs12195711
rs11060493
rs28415769
rs78032118
rs1550856
rs11197647
rs6858602
rs12130486
rs2267983
rs3130685
All
−0.04
−0.02
0.00
0.02
0.04
0.06
MR leave−one−out sensitivity analysis for
' CD8br AC' on 'Rheumatoid arthritis'

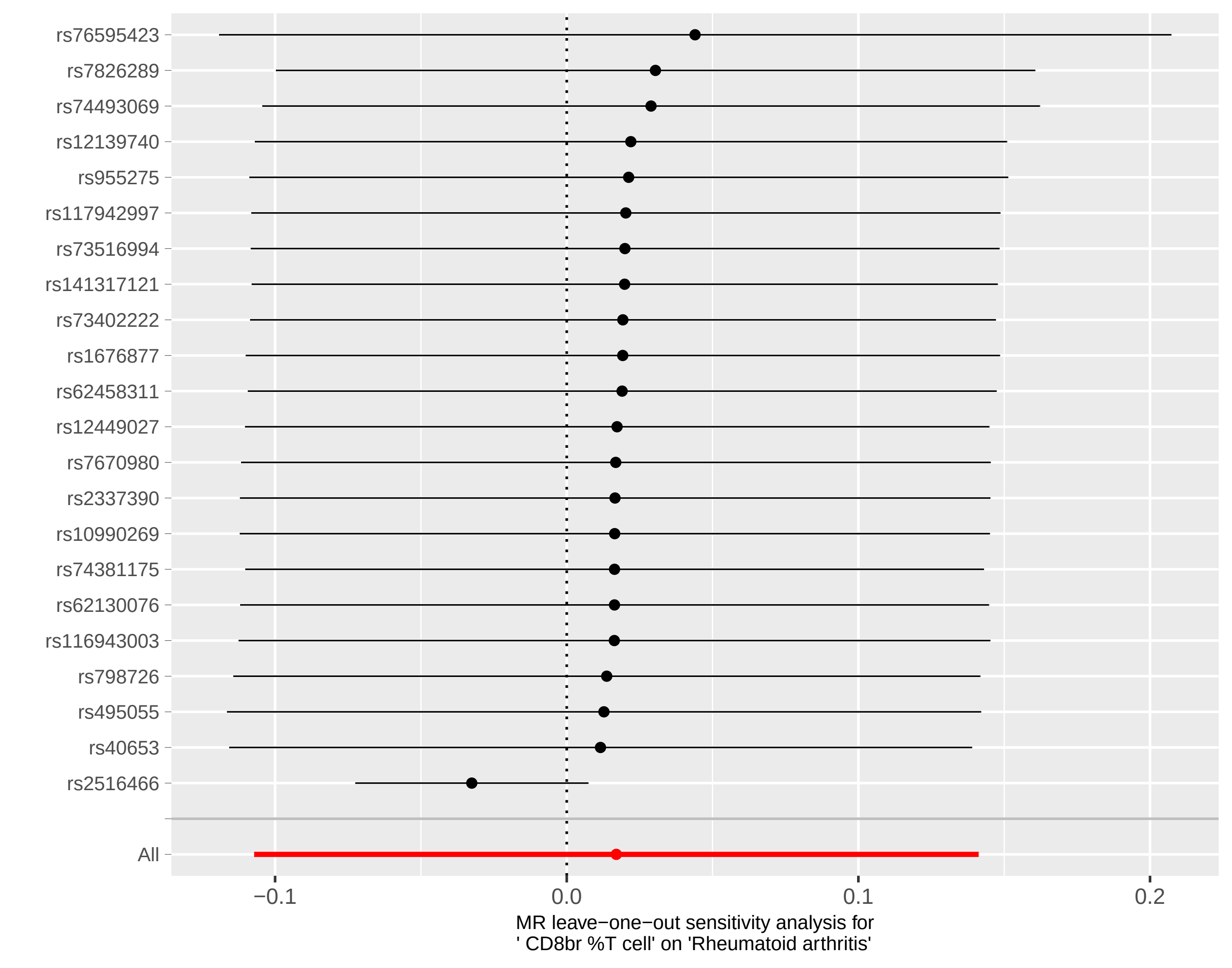

rs76595423
rs7826289
rs74493069
rs12139740
rs955275
rs117942997
rs73516994
rs141317121
rs73402222
rs1676877
rs62458311
rs12449027
rs7670980
rs2337390
rs10990269
rs74381175
rs62130076
rs116943003
rs798726
rs495055
rs40653
rs2516466
All
−0.1
0.0
0.1
0.2
MR leave−one−out sensitivity analysis for
' CD8br %T cell' on 'Rheumatoid arthritis'

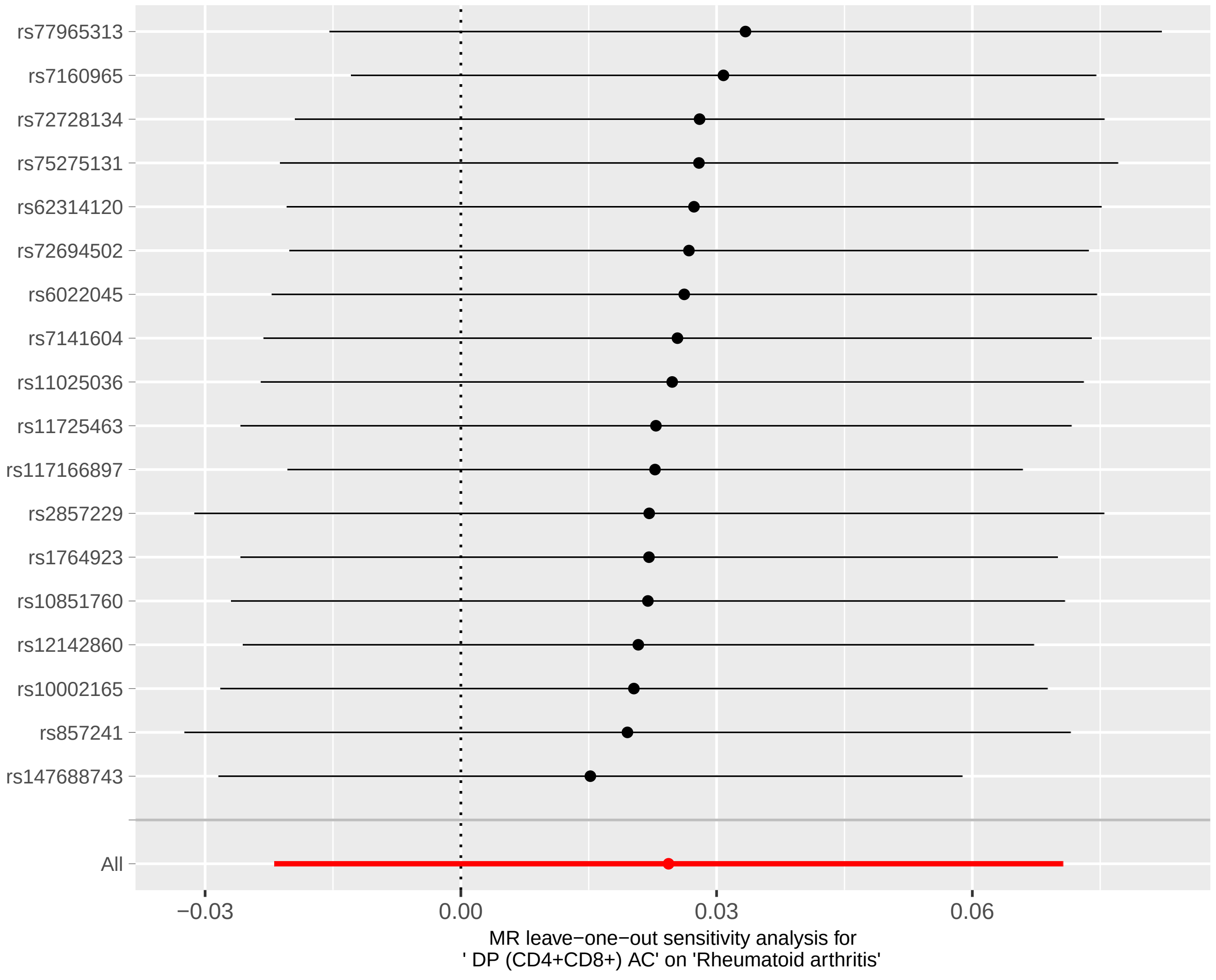

rs77965313
rs7160965
rs72728134
rs75275131
rs62314120
rs72694502
rs6022045
rs7141604
rs11025036
rs11725463
rs117166897
rs2857229
rs1764923
rs10851760
rs12142860
rs10002165
rs857241
rs147688743
All
−0.03
0.00
0.03
0.06
MR leave−one−out sensitivity analysis for
' DP (CD4+CD8+) AC' on 'Rheumatoid arthritis'

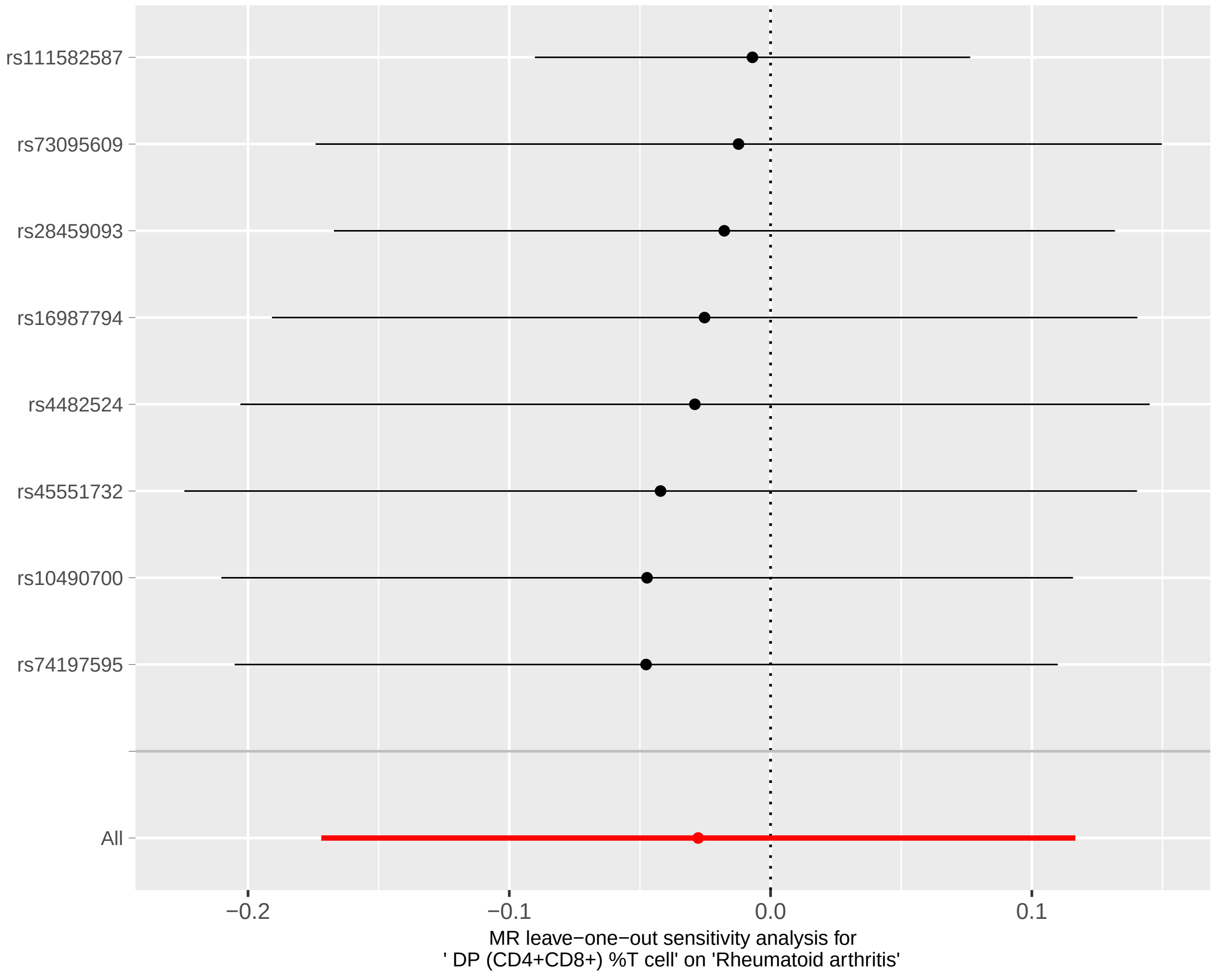
rs111582587
rs73095609
rs28459093
rs16987794
rs4482524
rs45551732
rs10490700
rs74197595
All
−0.2
−0.1
0.0
0.1
MR leave−one−out sensitivity analysis for
' DP (CD4+CD8+) %T cell' on 'Rheumatoid arthritis'

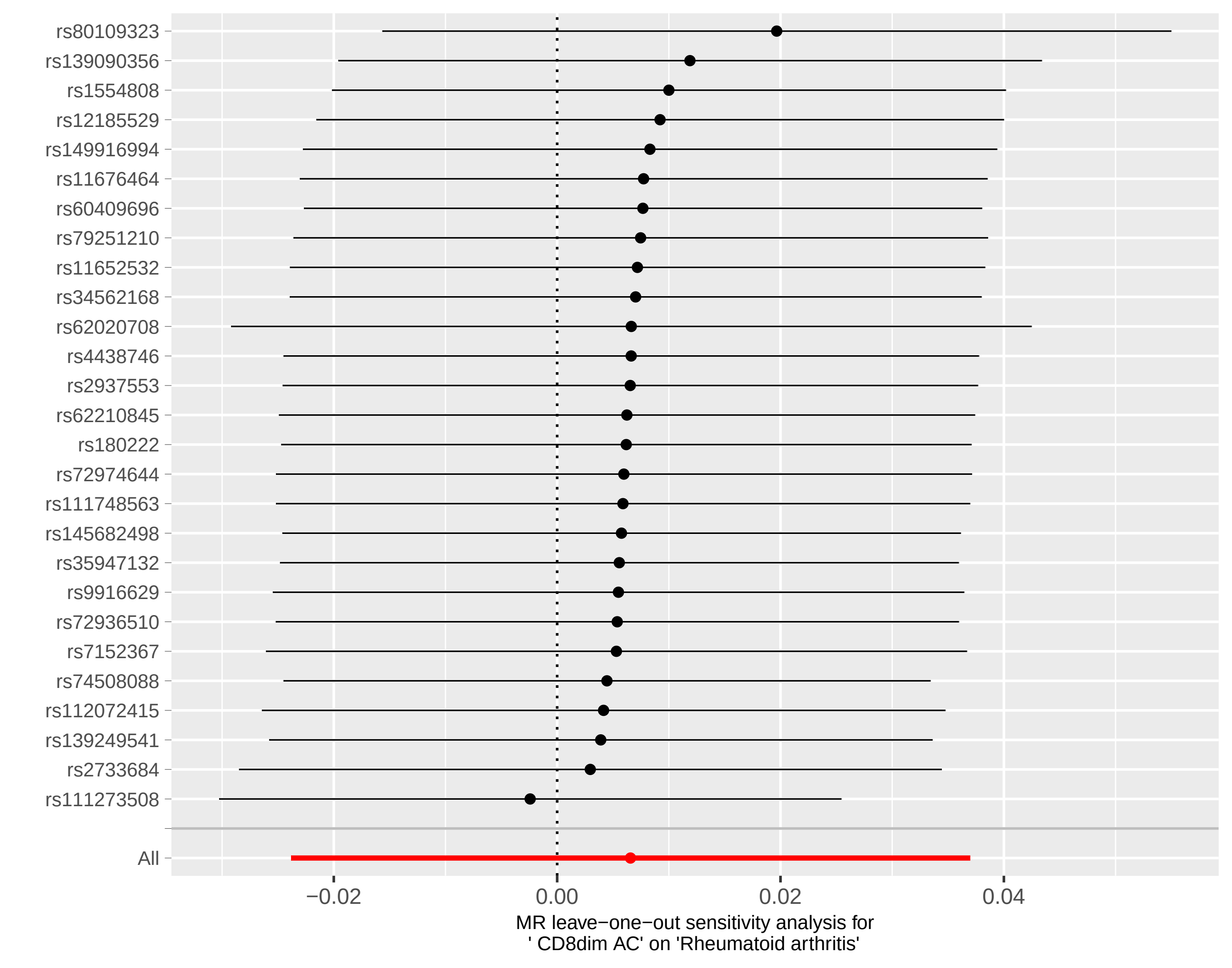

rs80109323
rs139090356
rs1554808
rs12185529
rs149916994
rs11676464
rs60409696
rs79251210
rs11652532
rs34562168
rs62020708
rs4438746
rs2937553
rs62210845
rs180222
rs72974644
rs111748563
rs145682498
rs35947132
rs9916629
rs72936510
rs7152367
rs74508088
rs112072415
rs139249541
rs2733684
rs111273508
All
−0.02
0.00
0.02
0.04
MR leave−one−out sensitivity analysis for
' CD8dim AC' on 'Rheumatoid arthritis'

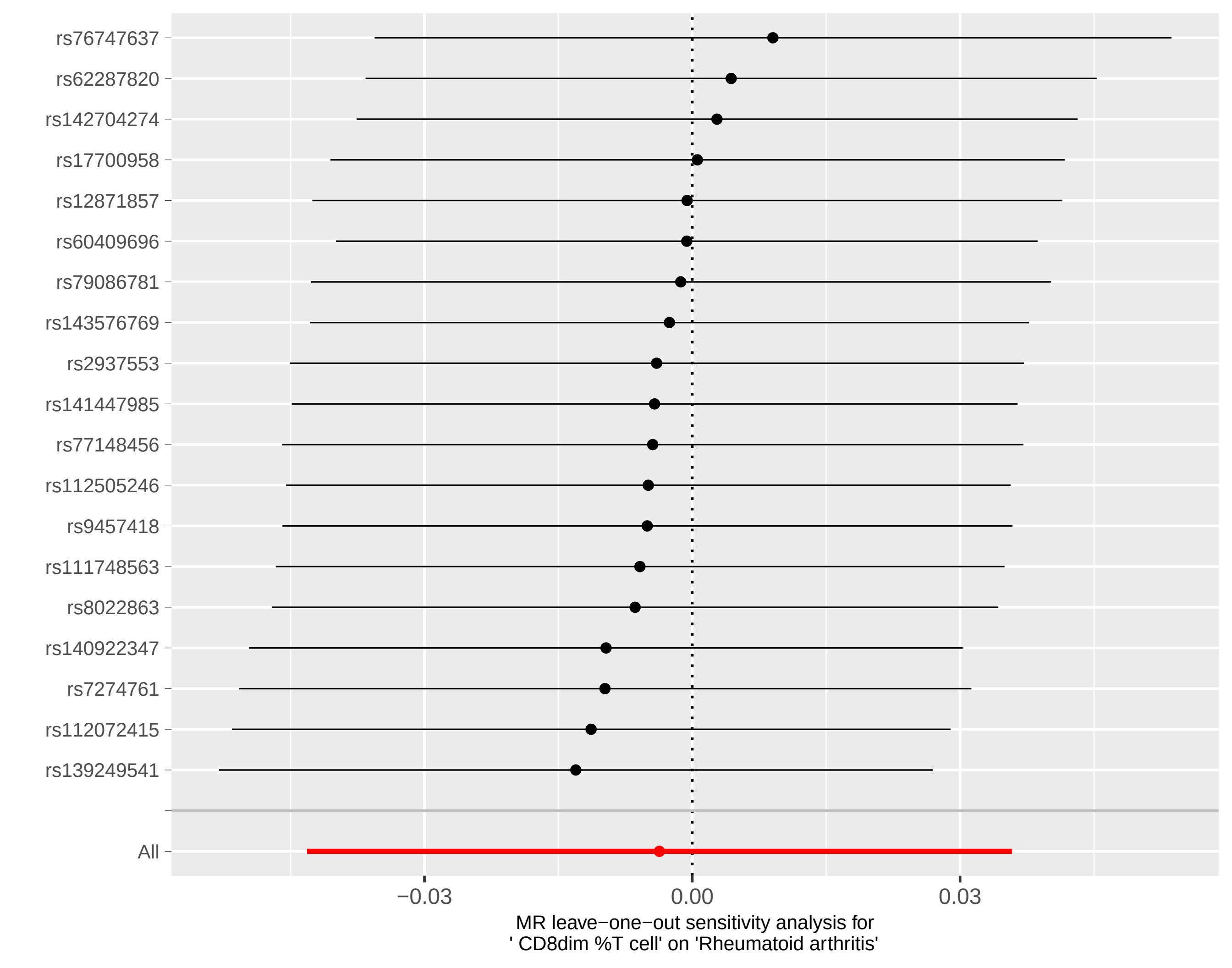
rs76747637
rs62287820
rs142704274
rs17700958
rs12871857
rs60409696
rs79086781
rs143576769
rs2937553
rs141447985
rs77148456
rs112505246
rs9457418
rs111748563
rs8022863
rs140922347
rs7274761
rs112072415
rs139249541
All
−0.03
0.00
0.03
MR leave−one−out sensitivity analysis for
' CD8dim %T cell' on 'Rheumatoid arthritis'

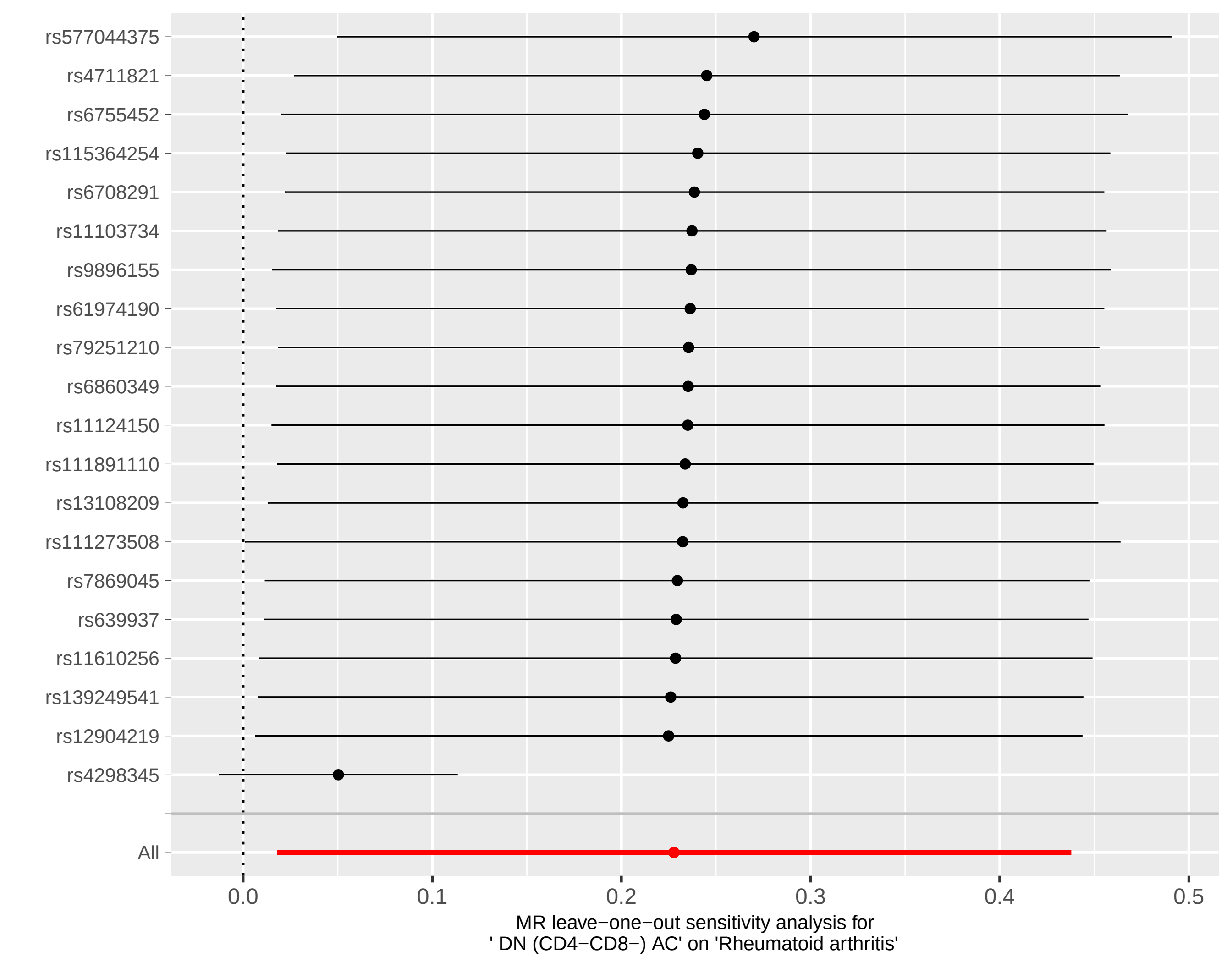

rs577044375
rs4711821
rs6755452
rs115364254
rs6708291
rs11103734
rs9896155
rs61974190
rs79251210
rs6860349
rs11124150
rs111891110
rs13108209
rs111273508
rs7869045
rs639937
rs11610256
rs139249541
rs12904219
rs4298345
All
0.0
0.1
0.2
0.3
0.4
0.5
MR leave−one−out sensitivity analysis for
' DN (CD4−CD8−) AC' on 'Rheumatoid arthritis'

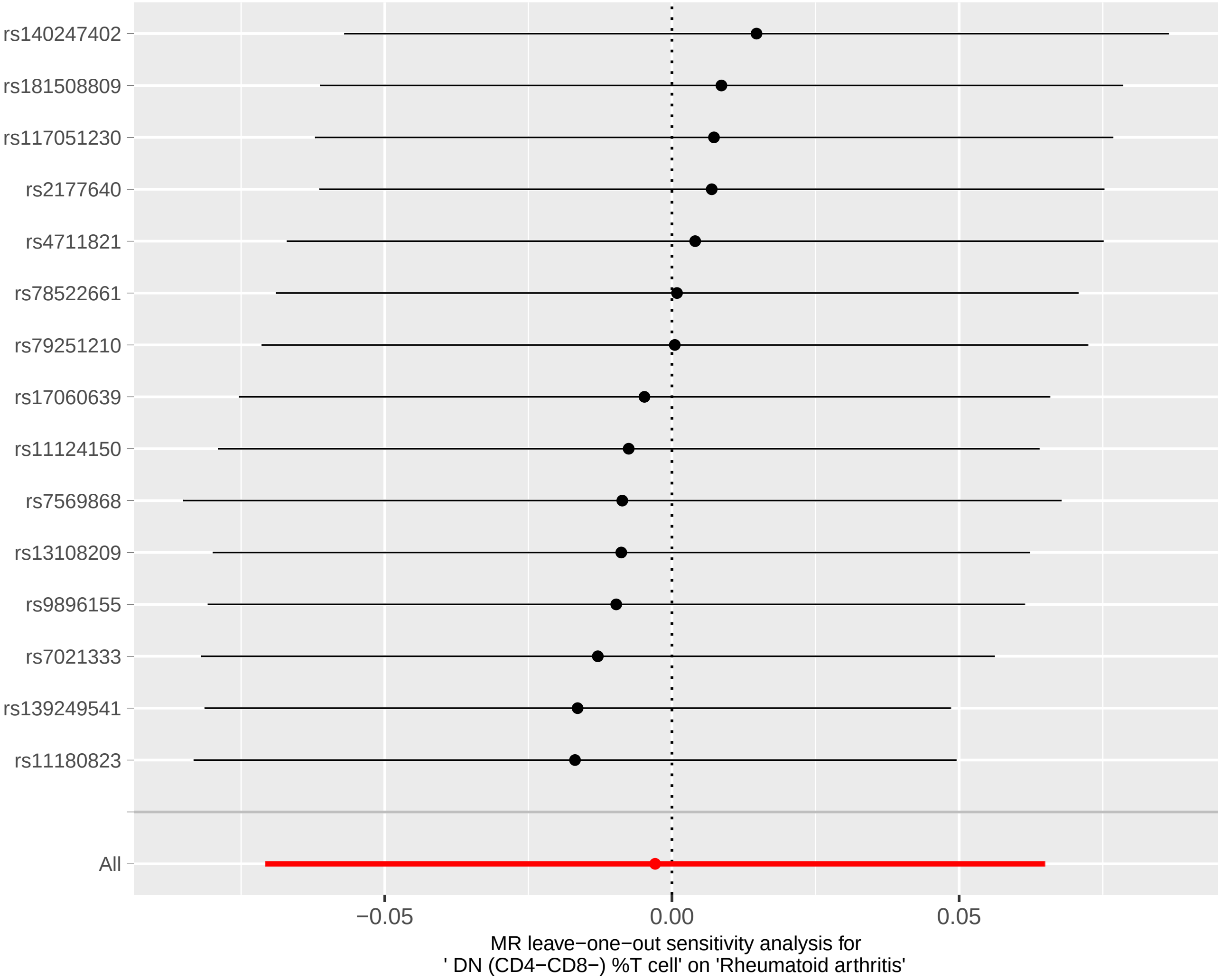
rs140247402
rs181508809
rs117051230
rs2177640
rs4711821
rs78522661
rs79251210
rs17060639
rs11124150
rs7569868
rs13108209
rs9896155
rs7021333
rs139249541
rs11180823
All
−0.05
0.00
0.05
MR leave−one−out sensitivity analysis for
' DN (CD4−CD8−) %T cell' on 'Rheumatoid arthritis'

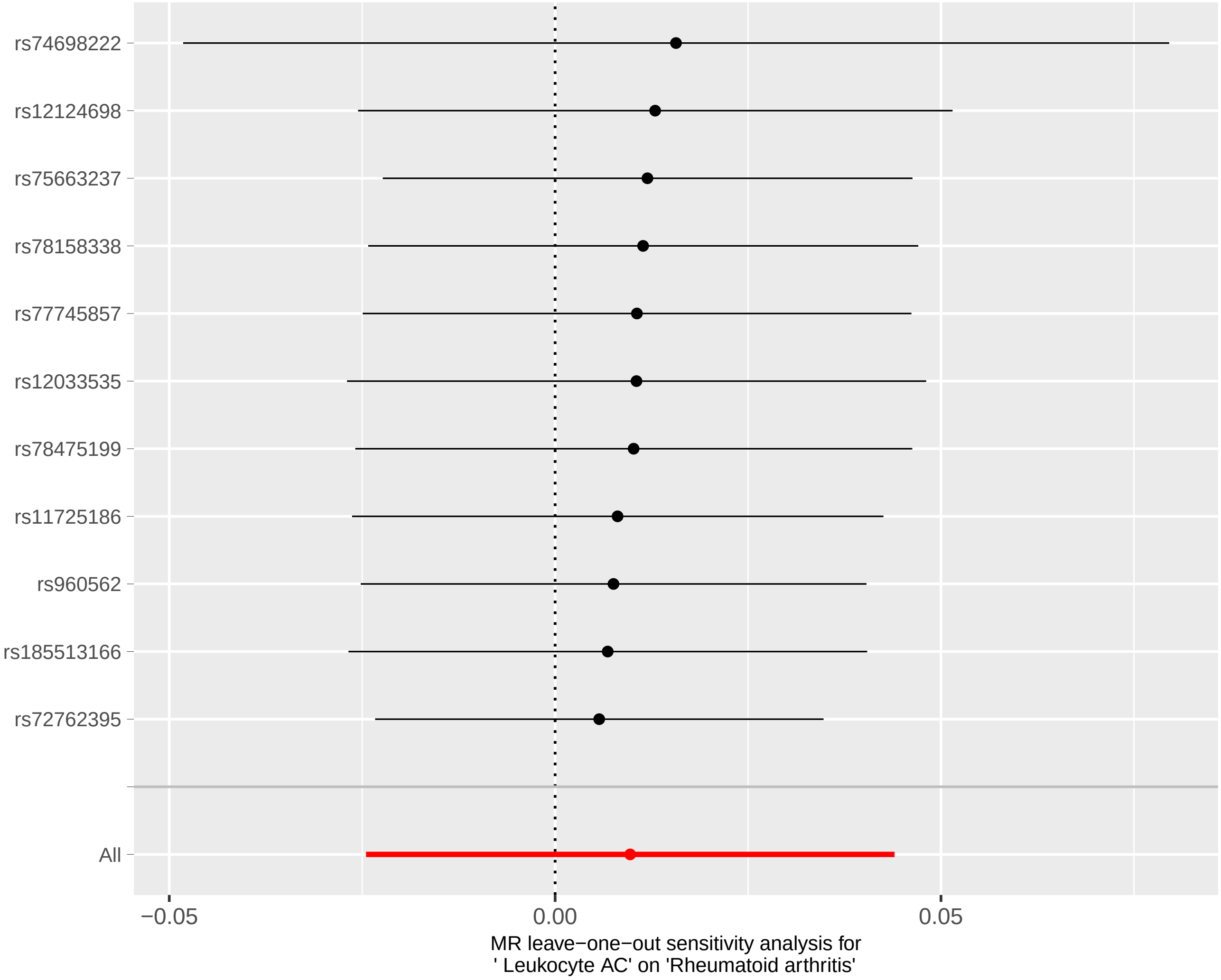
rs74698222
rs12124698
rs75663237
rs78158338
rs77745857
rs12033535
rs78475199
rs11725186
rs960562
rs185513166
rs72762395
All
−0.05
0.00
0.05
MR leave−one−out sensitivity analysis for
' Leukocyte AC' on 'Rheumatoid arthritis'

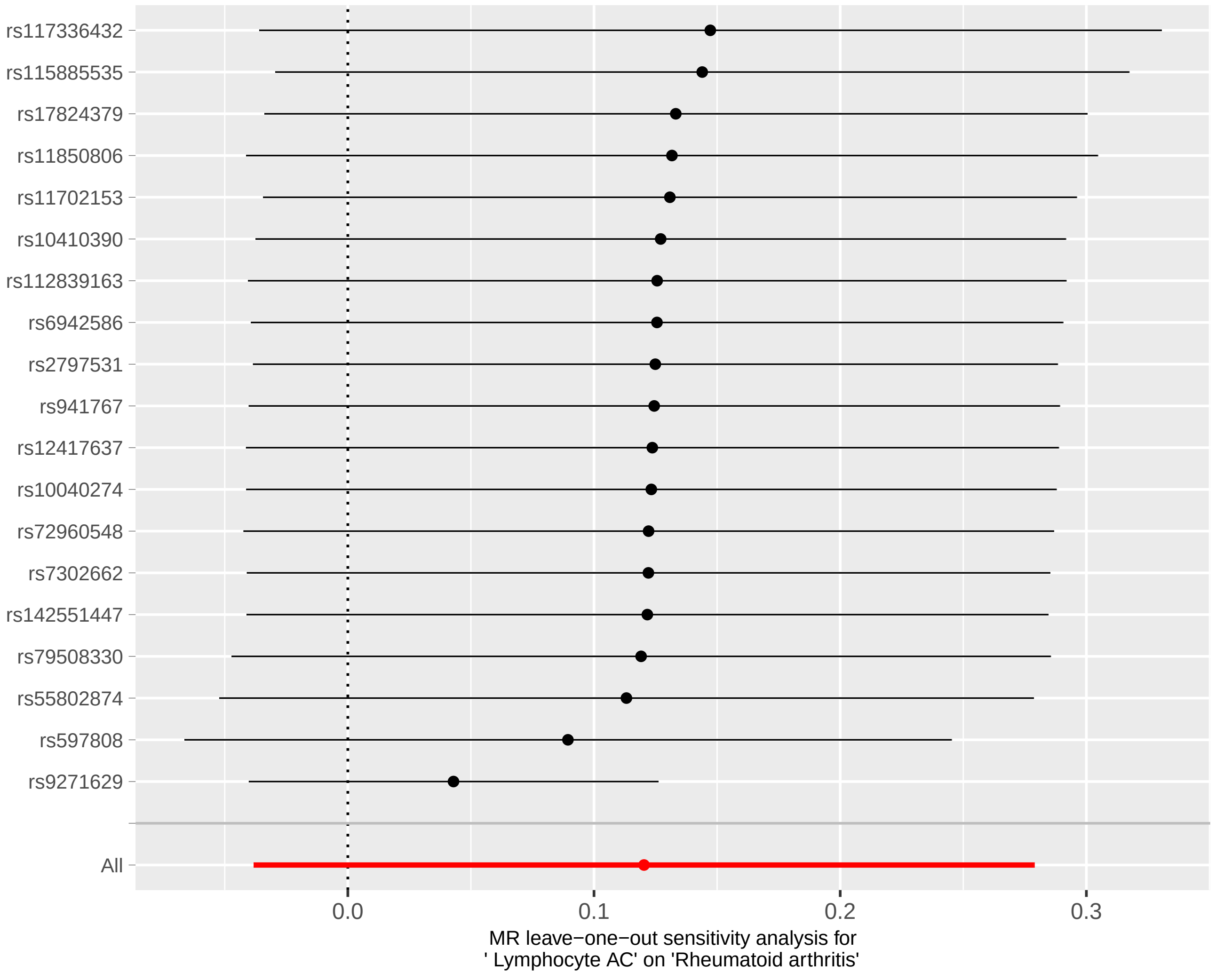
rs117336432
rs115885535
rs17824379
rs11850806
rs11702153
rs10410390
rs112839163
rs6942586
rs2797531
rs941767
rs12417637
rs10040274
rs72960548
rs7302662
rs142551447
rs79508330
rs55802874
rs597808
rs9271629
All
0.0
0.1
0.2
0.3
MR leave−one−out sensitivity analysis for
' Lymphocyte AC' on 'Rheumatoid arthritis'

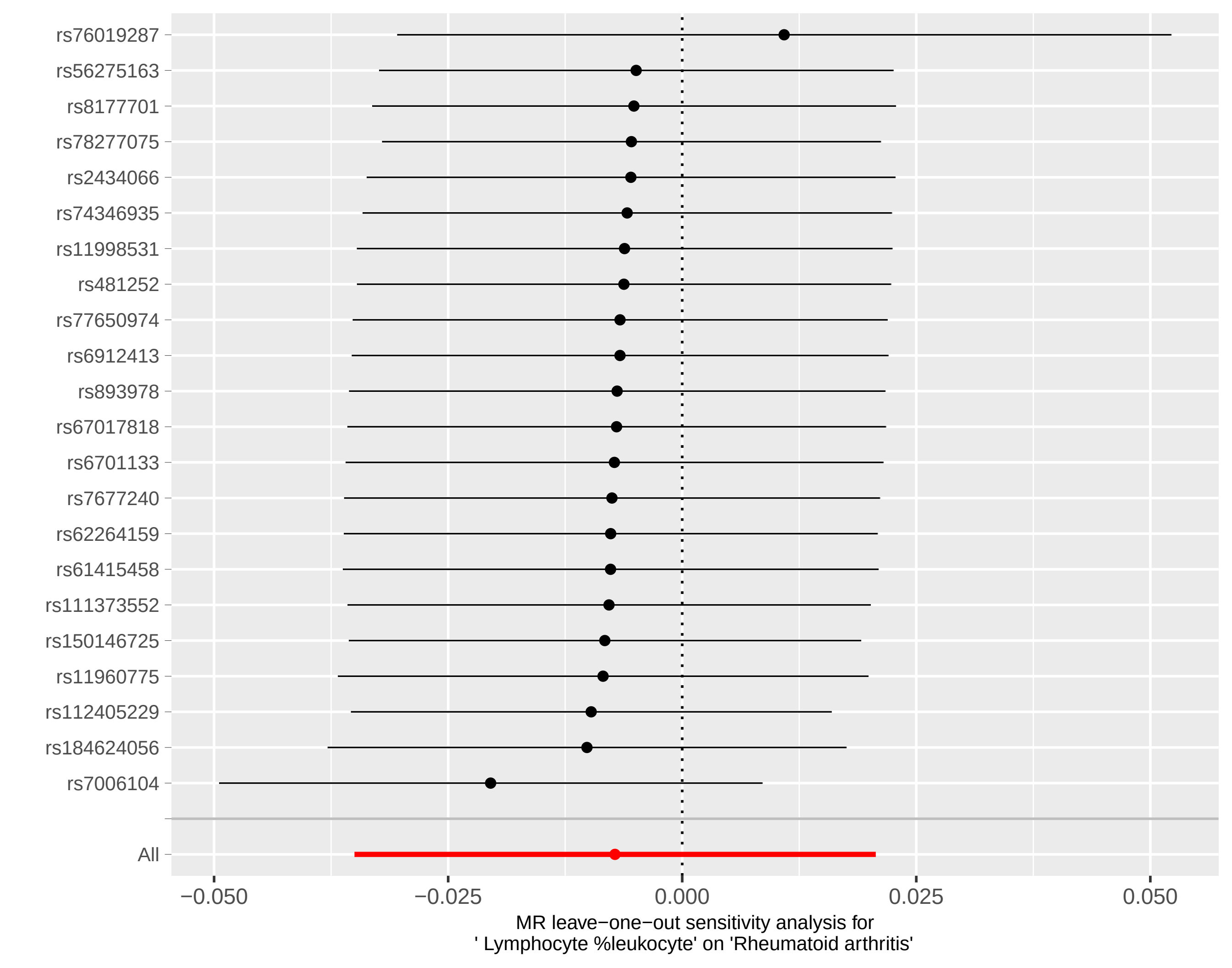
rs76019287
rs56275163
rs8177701
rs78277075
rs2434066
rs74346935
rs11998531
rs481252
rs77650974
rs6912413
rs893978
rs67017818
rs6701133
rs7677240
rs62264159
rs61415458
rs111373552
rs150146725
rs11960775
rs112405229
rs184624056
rs7006104
All
−0.050
−0.025
0.000
0.025
0.050
MR leave−one−out sensitivity analysis for
' Lymphocyte %leukocyte' on 'Rheumatoid arthritis'

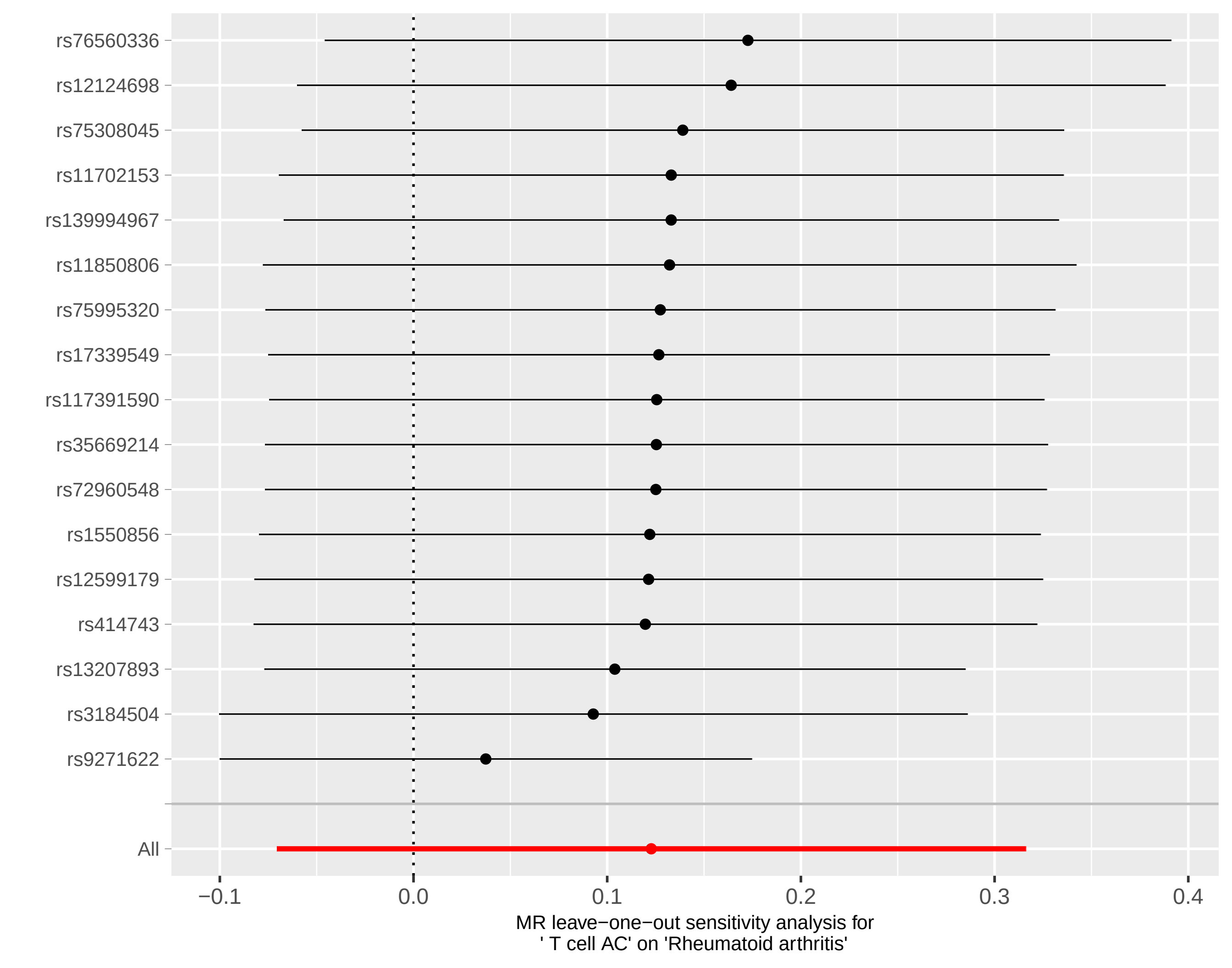
rs76560336
rs12124698
rs75308045
rs11702153
rs139994967
rs11850806
rs75995320
rs17339549
rs117391590
rs35669214
rs72960548
rs1550856
rs12599179
rs414743
rs13207893
rs3184504
rs9271622
All
−0.1
0.0
0.1
0.2
0.3
0.4
MR leave−one−out sensitivity analysis for
' T cell AC' on 'Rheumatoid arthritis'

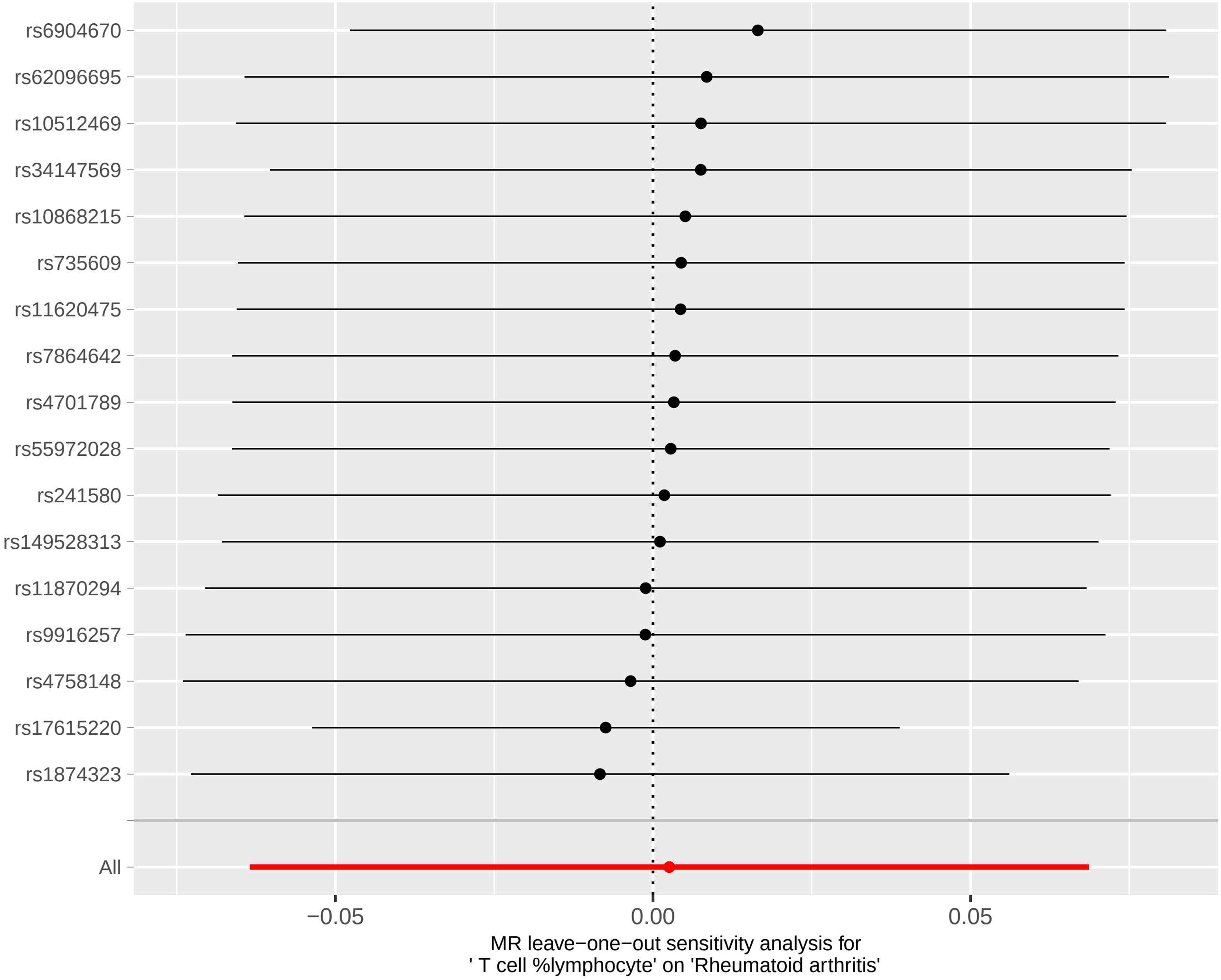

rs6904670
rs62096695
rs10512469
rs34147569
rs10868215
rs735609
rs11620475
rs7864642
rs4701789
rs55972028
rs241580
rs149528313
rs11870294
rs9916257
rs4758148
rs17615220
rs1874323
All
−0.05
0.00
0.05
MR leave−one−out sensitivity analysis for
' T cell %lymphocyte' on 'Rheumatoid arthritis'

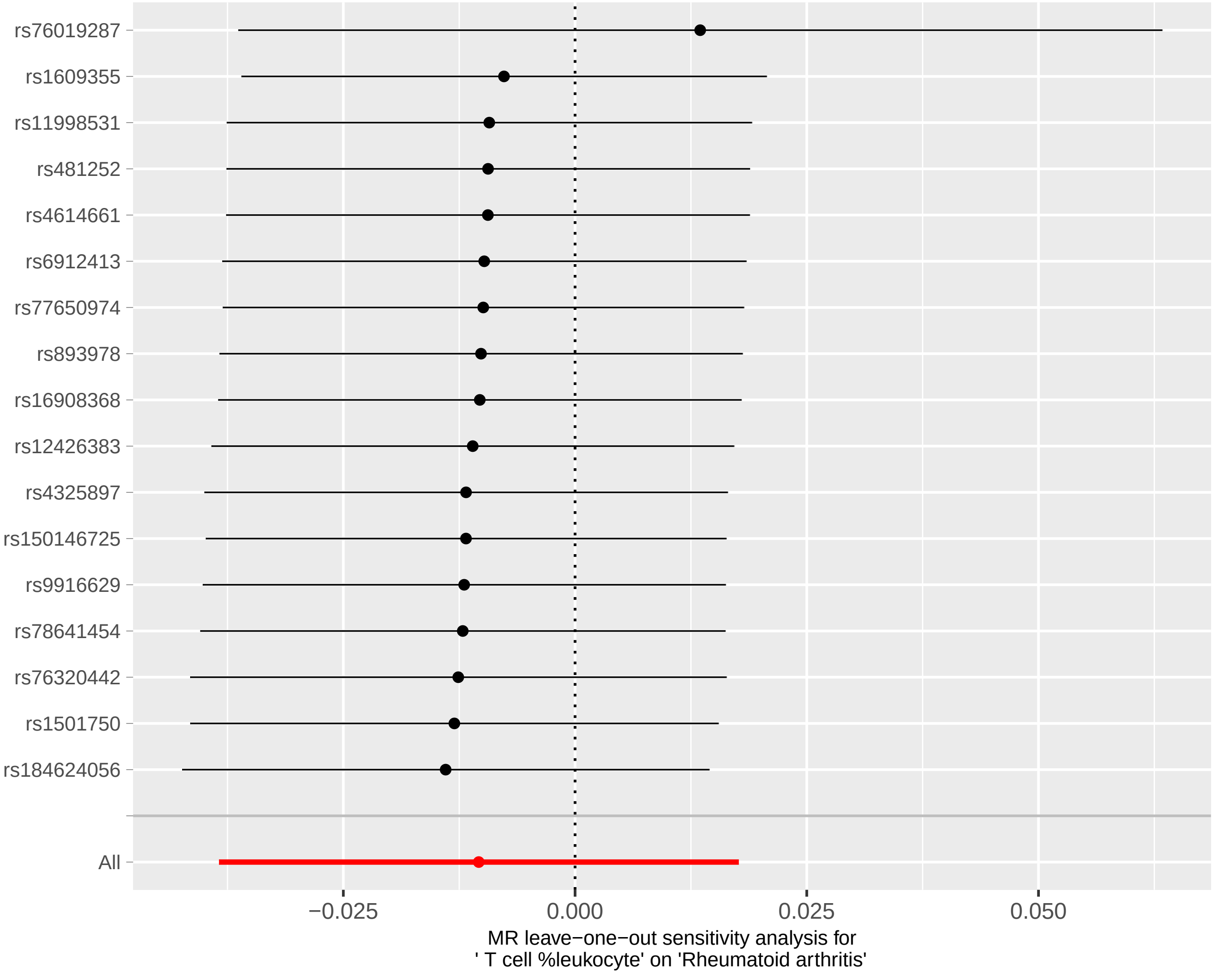
rs76019287
rs1609355
rs11998531
rs481252
rs4614661
rs6912413
rs77650974
rs893978
rs16908368
rs12426383
rs4325897
rs150146725
rs9916629
rs78641454
rs76320442
rs1501750
rs184624056
All
−0.025
0.000
0.025
0.050
MR leave−one−out sensitivity analysis for
' T cell %leukocyte' on 'Rheumatoid arthritis'

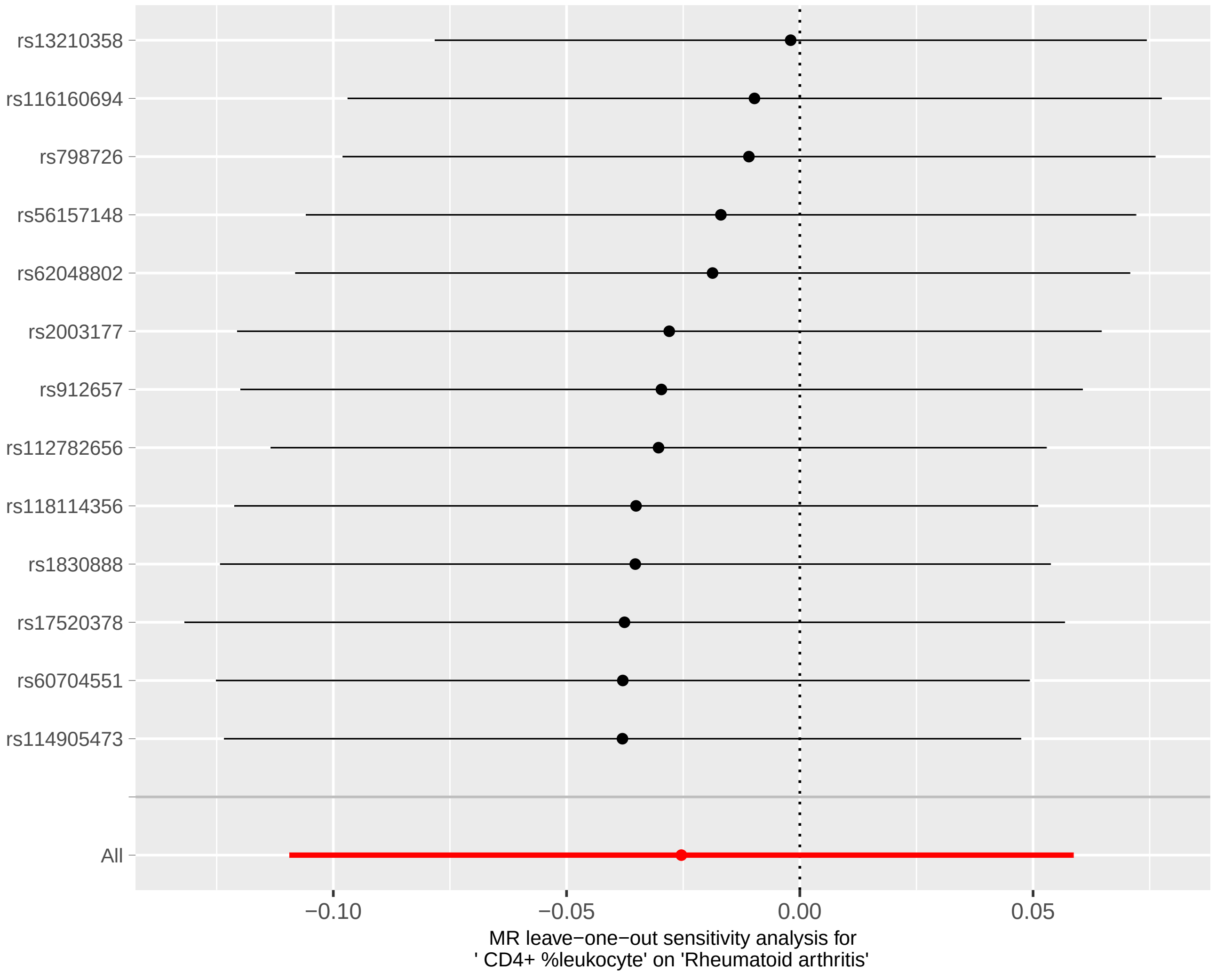

rs13210358
rs116160694
rs798726
rs56157148
rs62048802
rs2003177
rs912657
rs112782656
rs118114356
rs1830888
rs17520378
rs60704551
rs114905473
All
−0.10
−0.05
0.00
0.05
MR leave−one−out sensitivity analysis for
' CD4+ %leukocyte' on 'Rheumatoid arthritis'

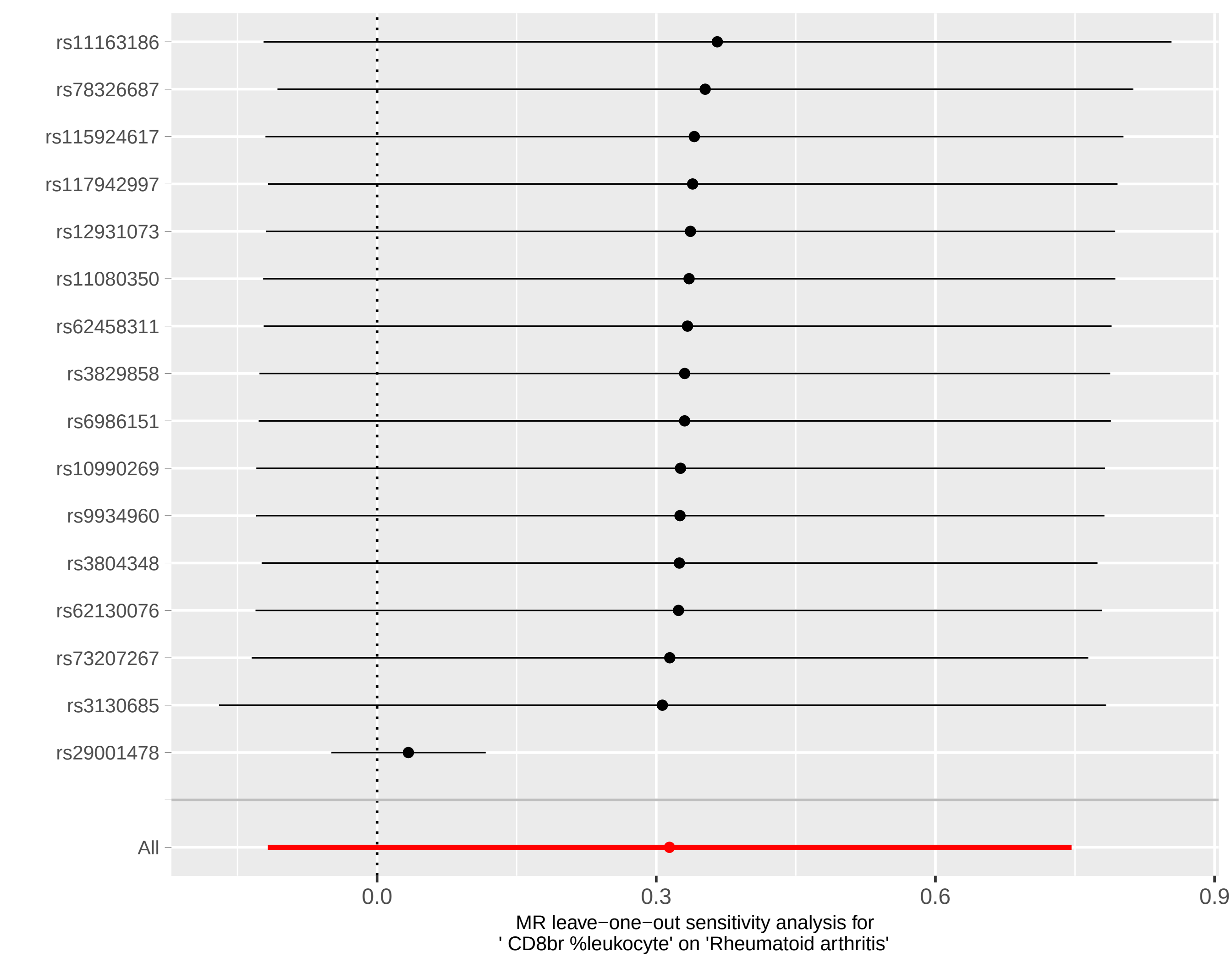
rs11163186
rs78326687
rs115924617
rs117942997
rs12931073
rs11080350
rs62458311
rs3829858
rs6986151
rs10990269
rs9934960
rs3804348
rs62130076
rs73207267
rs3130685
rs29001478
All
0.0
0.3
0.6
0.9
MR leave−one−out sensitivity analysis for
' CD8br %leukocyte' on 'Rheumatoid arthritis'

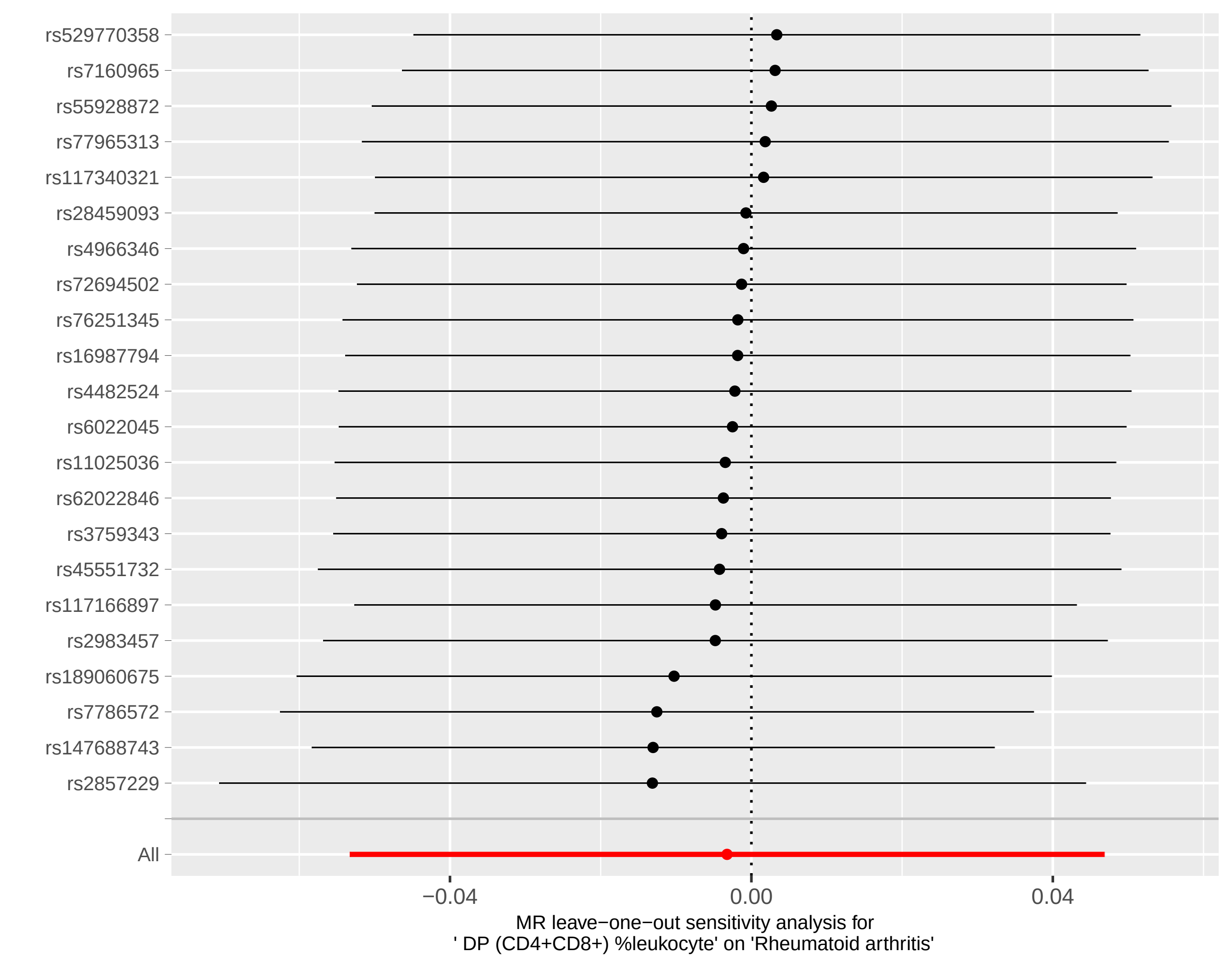
rs529770358
rs7160965
rs55928872
rs77965313
rs117340321
rs28459093
rs4966346
rs72694502
rs76251345
rs16987794
rs4482524
rs6022045
rs11025036
rs62022846
rs3759343
rs45551732
rs117166897
rs2983457
rs189060675
rs7786572
rs147688743
rs2857229
All
−0.04
0.00
0.04
MR leave−one−out sensitivity analysis for
' DP (CD4+CD8+) %leukocyte' on 'Rheumatoid arthritis'

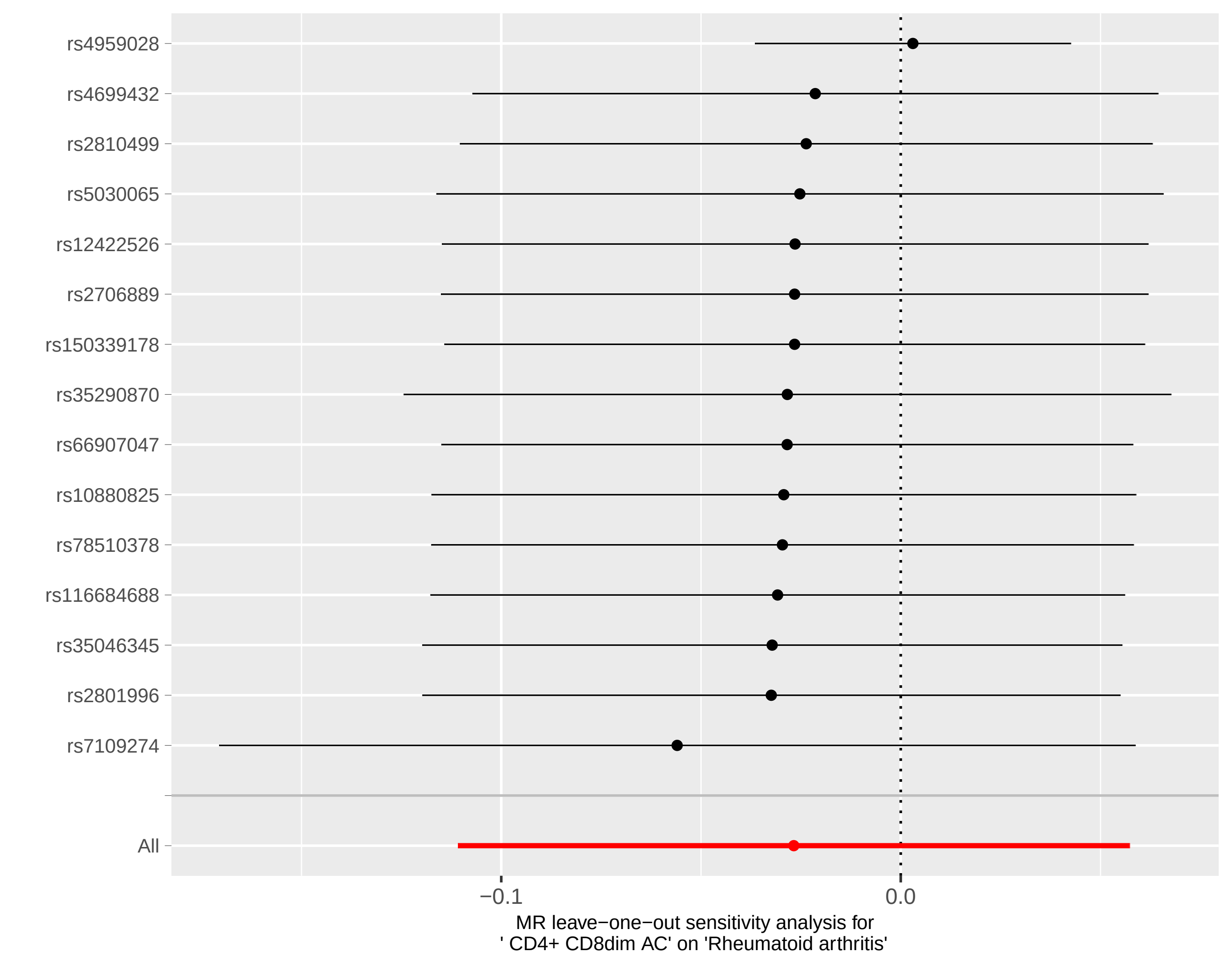
rs4959028
rs4699432
rs2810499
rs5030065
rs12422526
rs2706889
rs150339178
rs35290870
rs66907047
rs10880825
rs78510378
rs116684688
rs35046345
rs2801996
rs7109274
All
−0.1
0.0
MR leave−one−out sensitivity analysis for
' CD4+ CD8dim AC' on 'Rheumatoid arthritis'

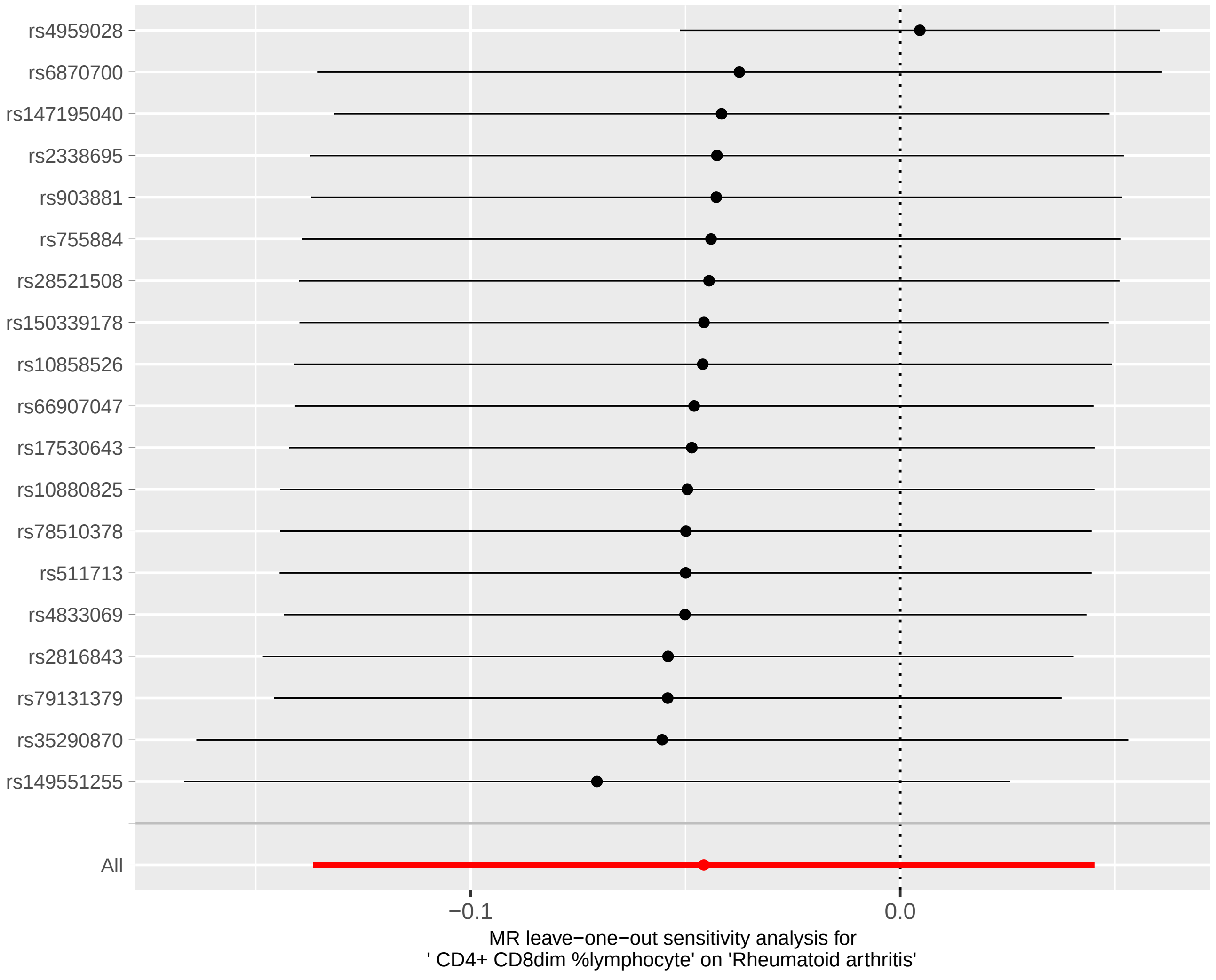
rs4959028
rs6870700
rs147195040
rs2338695
rs903881
rs755884
rs28521508
rs150339178
rs10858526
rs66907047
rs17530643
rs10880825
rs78510378
rs511713
rs4833069
rs2816843
rs79131379
rs35290870
rs149551255
All
−0.1
0.0
MR leave−one−out sensitivity analysis for
' CD4+ CD8dim %lymphocyte' on 'Rheumatoid arthritis'

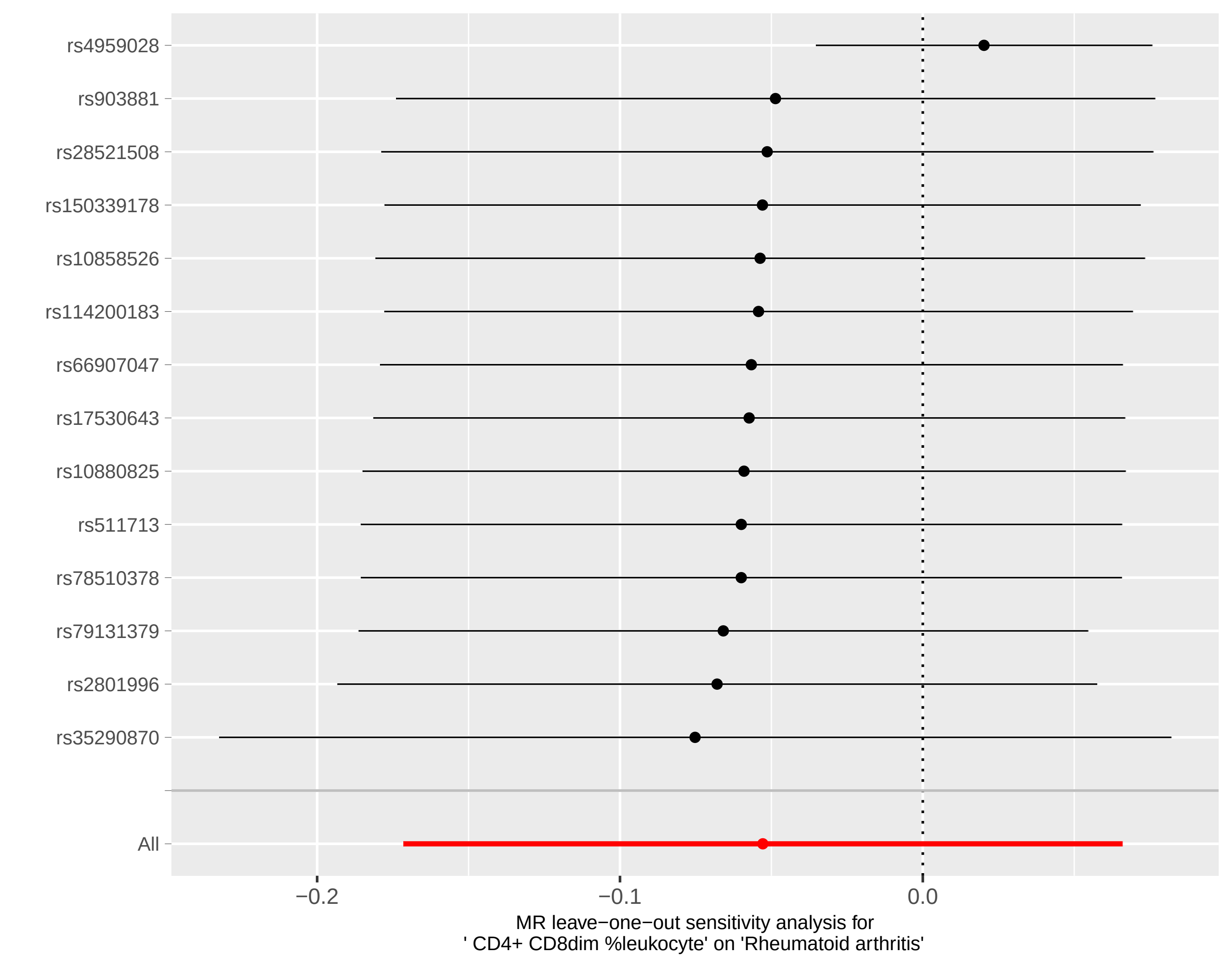
rs4959028
rs903881
rs28521508
rs150339178
rs10858526
rs114200183
rs66907047
rs17530643
rs10880825
rs511713
rs78510378
rs79131379
rs2801996
rs35290870
All
−0.2
−0.1
0.0
MR leave−one−out sensitivity analysis for
' CD4+ CD8dim %leukocyte' on 'Rheumatoid arthritis'

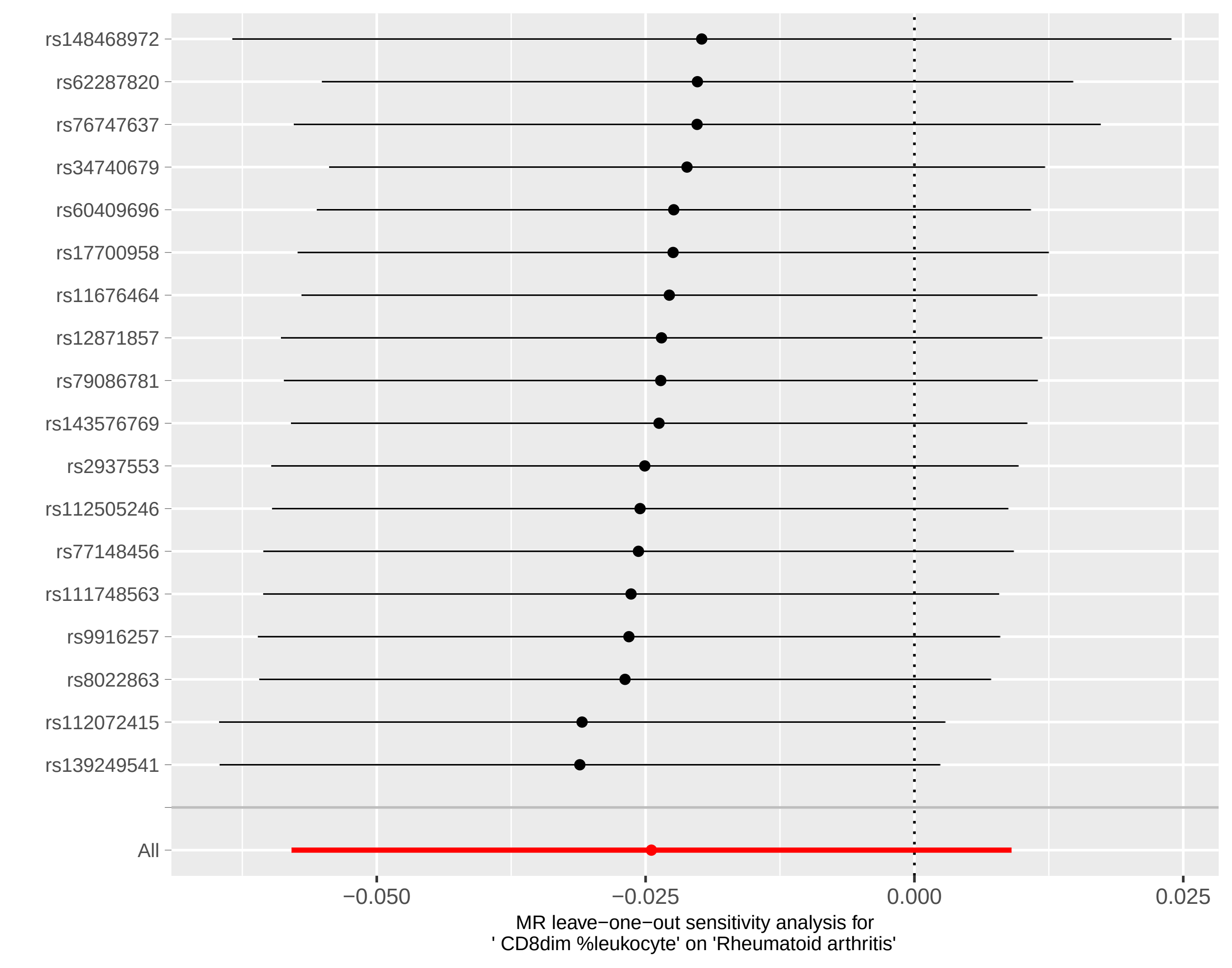

rs148468972
rs62287820
rs76747637
rs34740679
rs60409696
rs17700958
rs11676464
rs12871857
rs79086781
rs143576769
rs2937553
rs112505246
rs77148456
rs111748563
rs9916257
rs8022863
rs112072415
rs139249541
All
−0.050
−0.025
0.000
0.025
MR leave−one−out sensitivity analysis for
' CD8dim %leukocyte' on 'Rheumatoid arthritis'

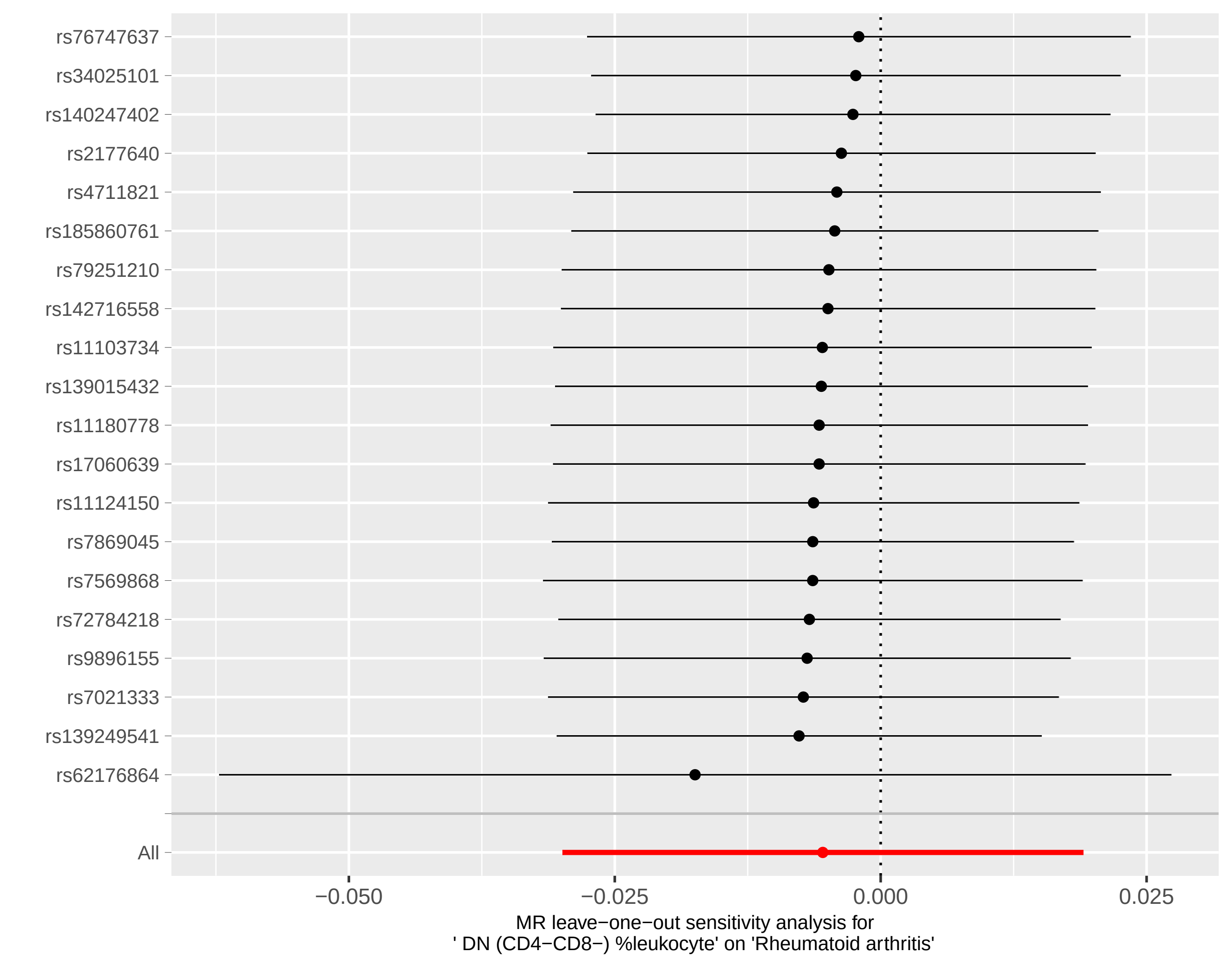
rs76747637
rs34025101
rs140247402
rs2177640
rs4711821
rs185860761
rs79251210
rs142716558
rs11103734
rs139015432
rs11180778
rs17060639
rs11124150
rs7869045
rs7569868
rs72784218
rs9896155
rs7021333
rs139249541
rs62176864
All
−0.050
−0.025
0.000
0.025
MR leave−one−out sensitivity analysis for
' DN (CD4−CD8−) %leukocyte' on 'Rheumatoid arthritis'

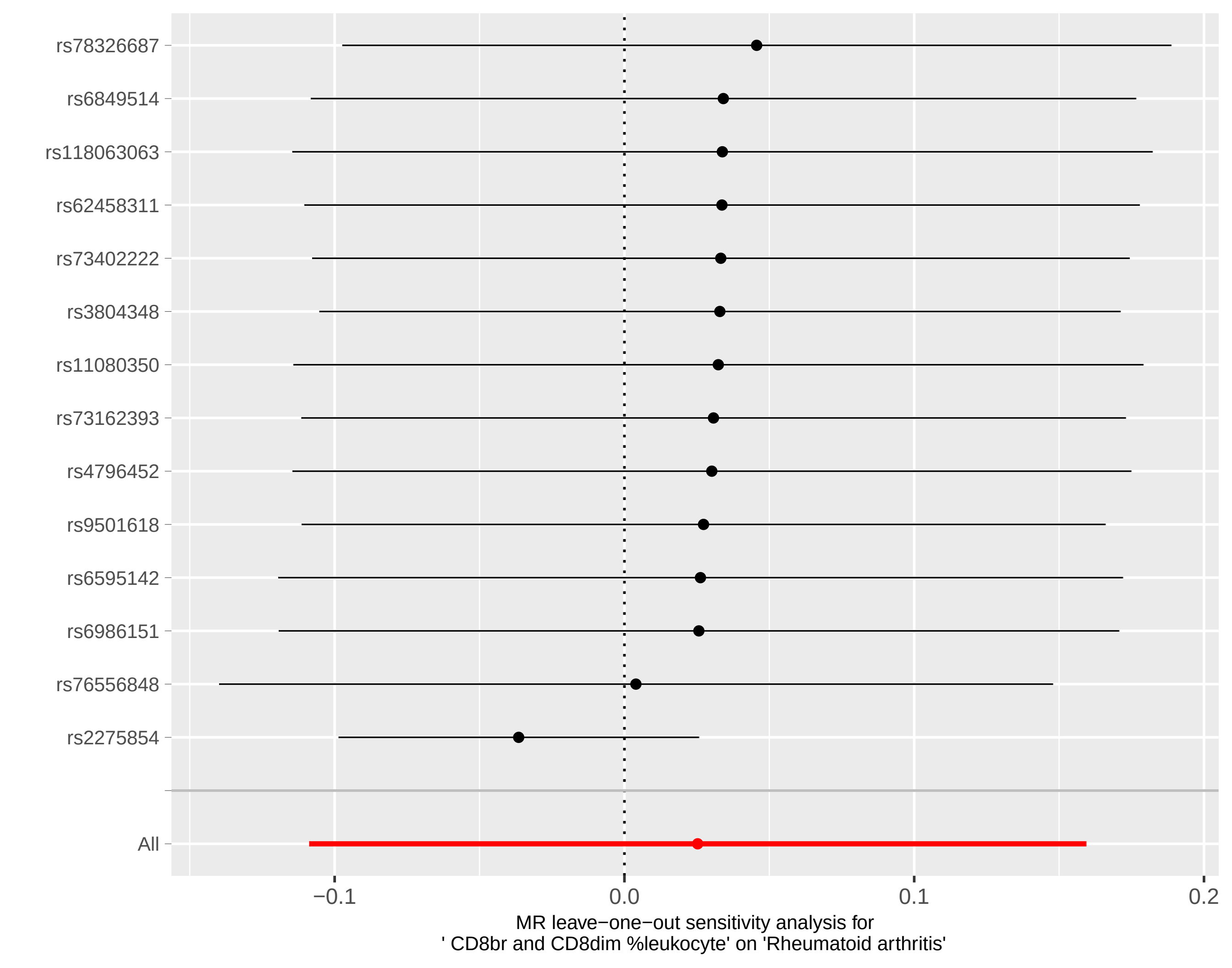
rs78326687
rs6849514
rs118063063
rs62458311
rs73402222
rs3804348
rs11080350
rs73162393
rs4796452
rs9501618
rs6595142
rs6986151
rs76556848
rs2275854
All
−0.1
0.0
0.1
0.2
MR leave−one−out sensitivity analysis for
' CD8br and CD8dim %leukocyte' on 'Rheumatoid arthritis'

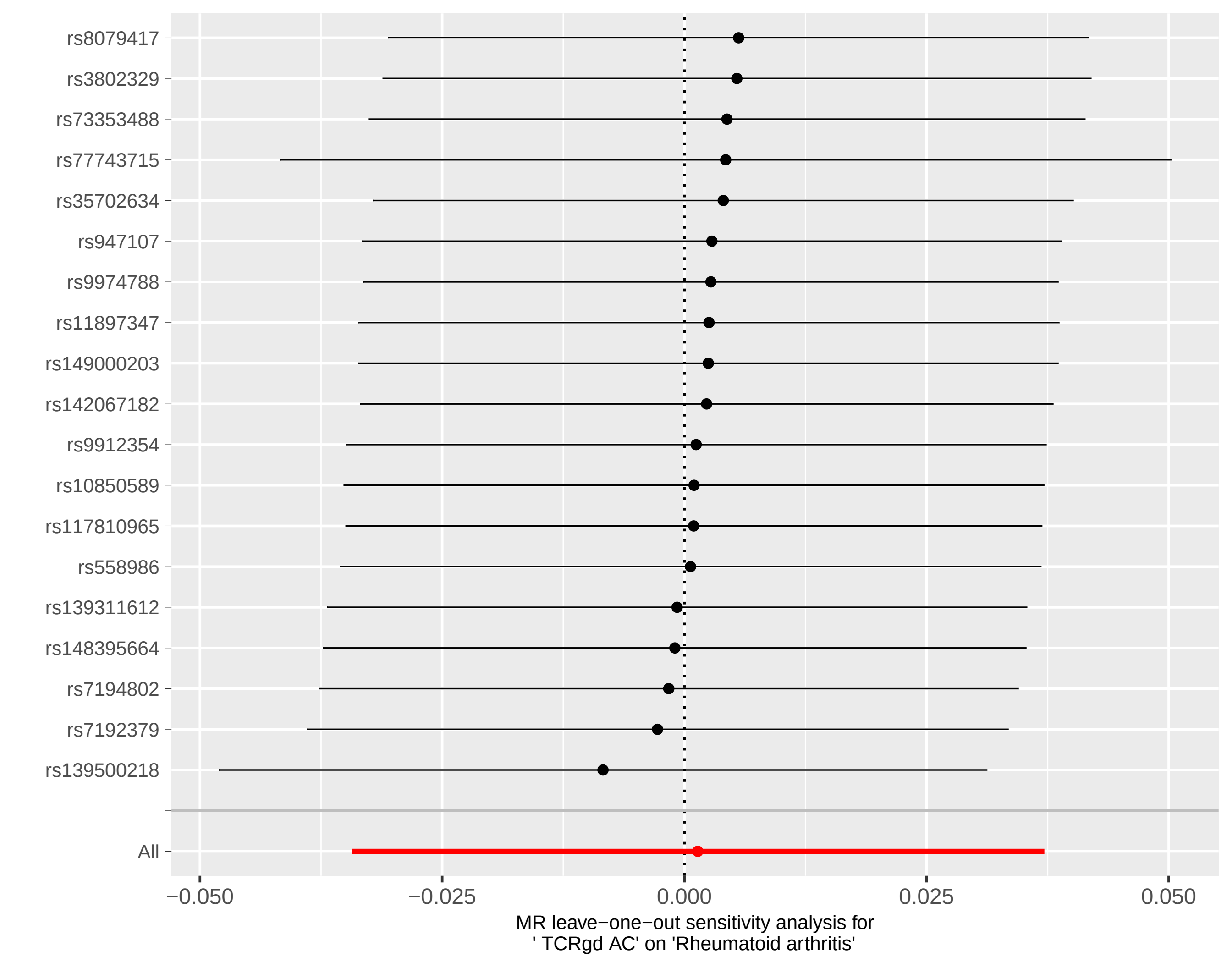
rs8079417
rs3802329
rs73353488
rs77743715
rs35702634
rs947107
rs9974788
rs11897347
rs149000203
rs142067182
rs9912354
rs10850589
rs117810965
rs558986
rs139311612
rs148395664
rs7194802
rs7192379
rs139500218
All
−0.050
−0.025
0.000
0.025
0.050
MR leave−one−out sensitivity analysis for
' TCRgd AC' on 'Rheumatoid arthritis'

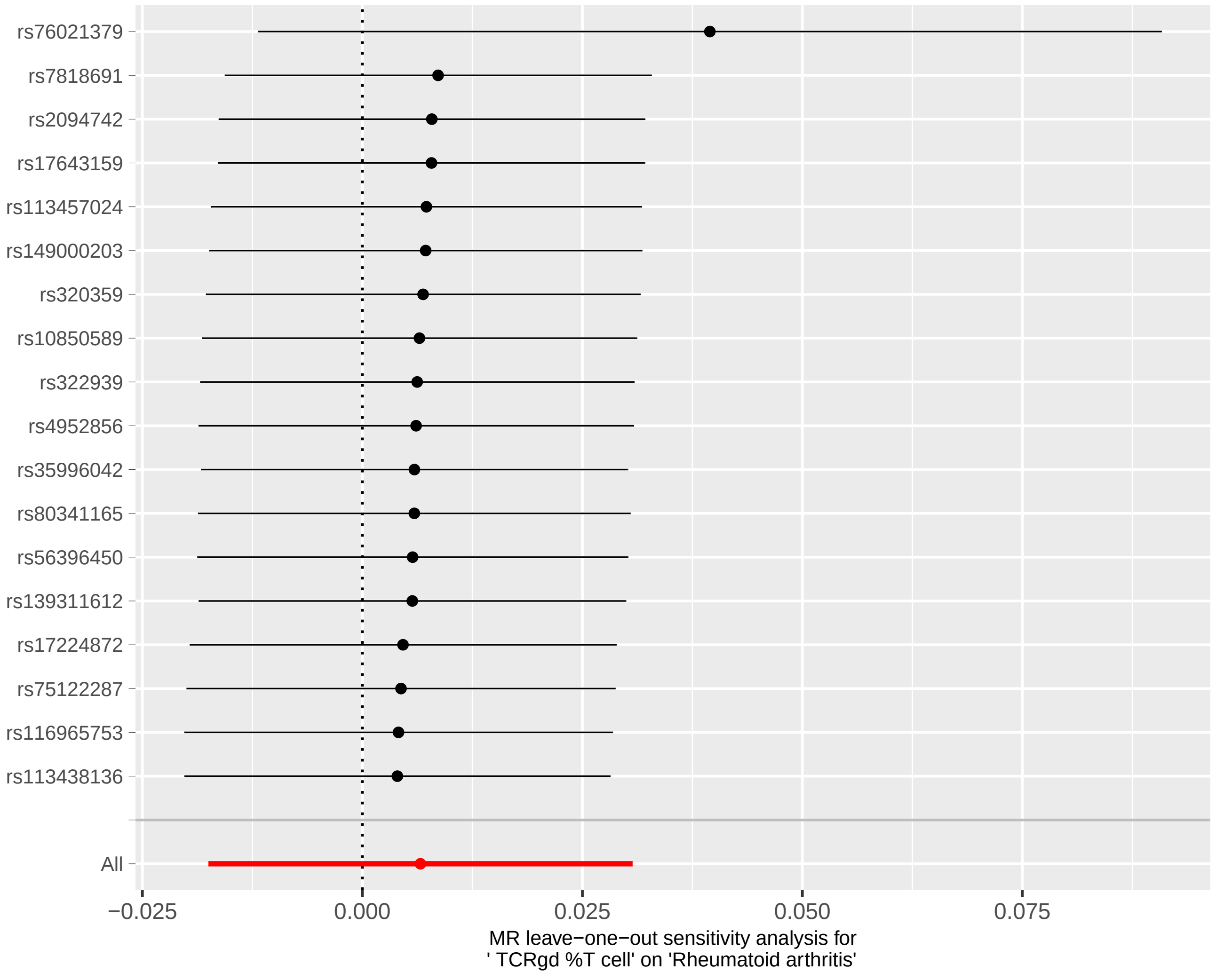
rs76021379
rs7818691
rs2094742
rs17643159
rs113457024
rs149000203
rs320359
rs10850589
rs322939
rs4952856
rs35996042
rs80341165
rs56396450
rs139311612
rs17224872
rs75122287
rs116965753
rs113438136
All
−0.025
0.000
0.025
0.050
0.075
MR leave−one−out sensitivity analysis for
' TCRgd %T cell' on 'Rheumatoid arthritis'

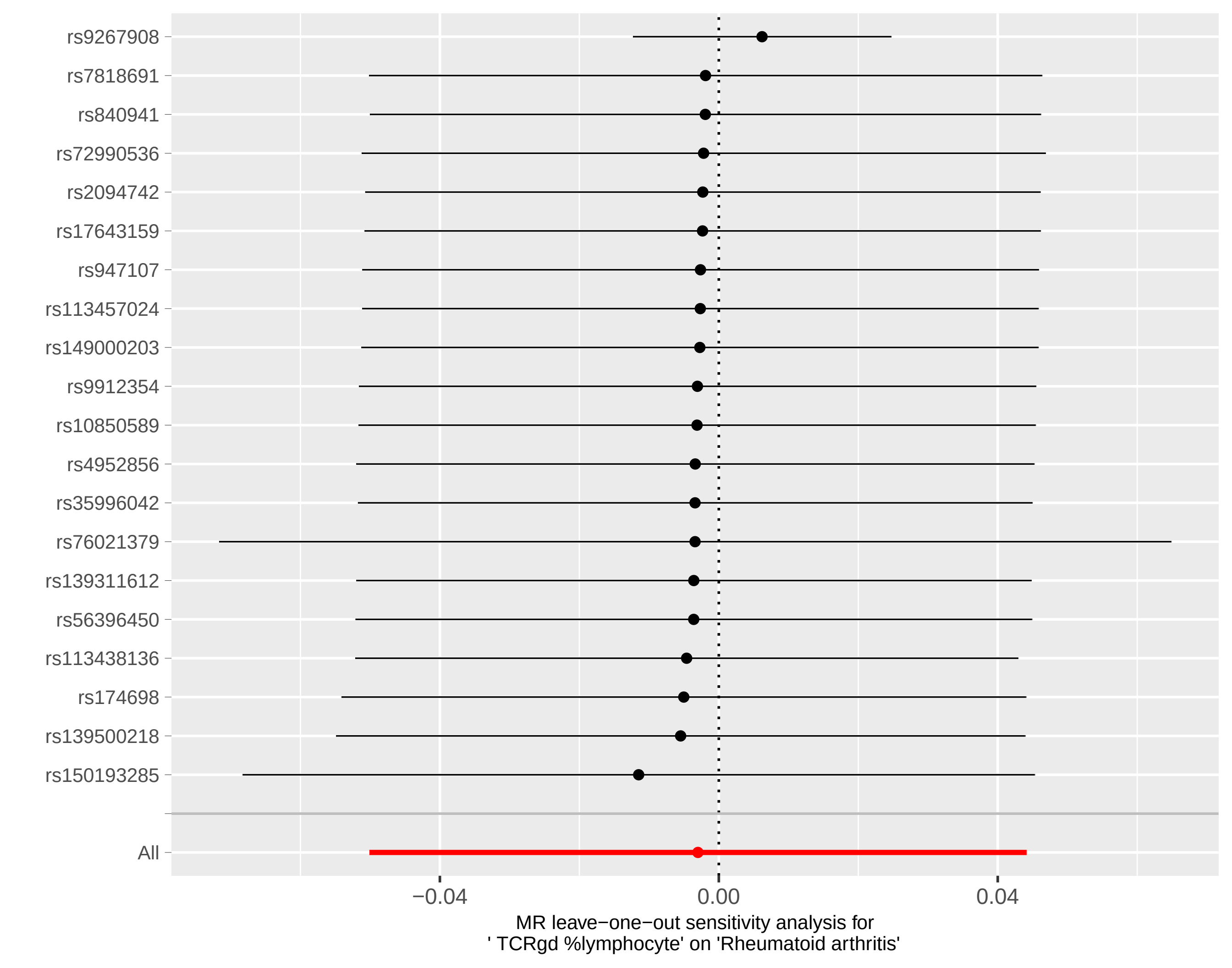
rs9267908
rs7818691
rs840941
rs72990536
rs2094742
rs17643159
rs947107
rs113457024
rs149000203
rs9912354
rs10850589
rs4952856
rs35996042
rs76021379
rs139311612
rs56396450
rs113438136
rs174698
rs139500218
rs150193285
All
−0.04
0.00
0.04
MR leave−one−out sensitivity analysis for
' TCRgd %lymphocyte' on 'Rheumatoid arthritis'

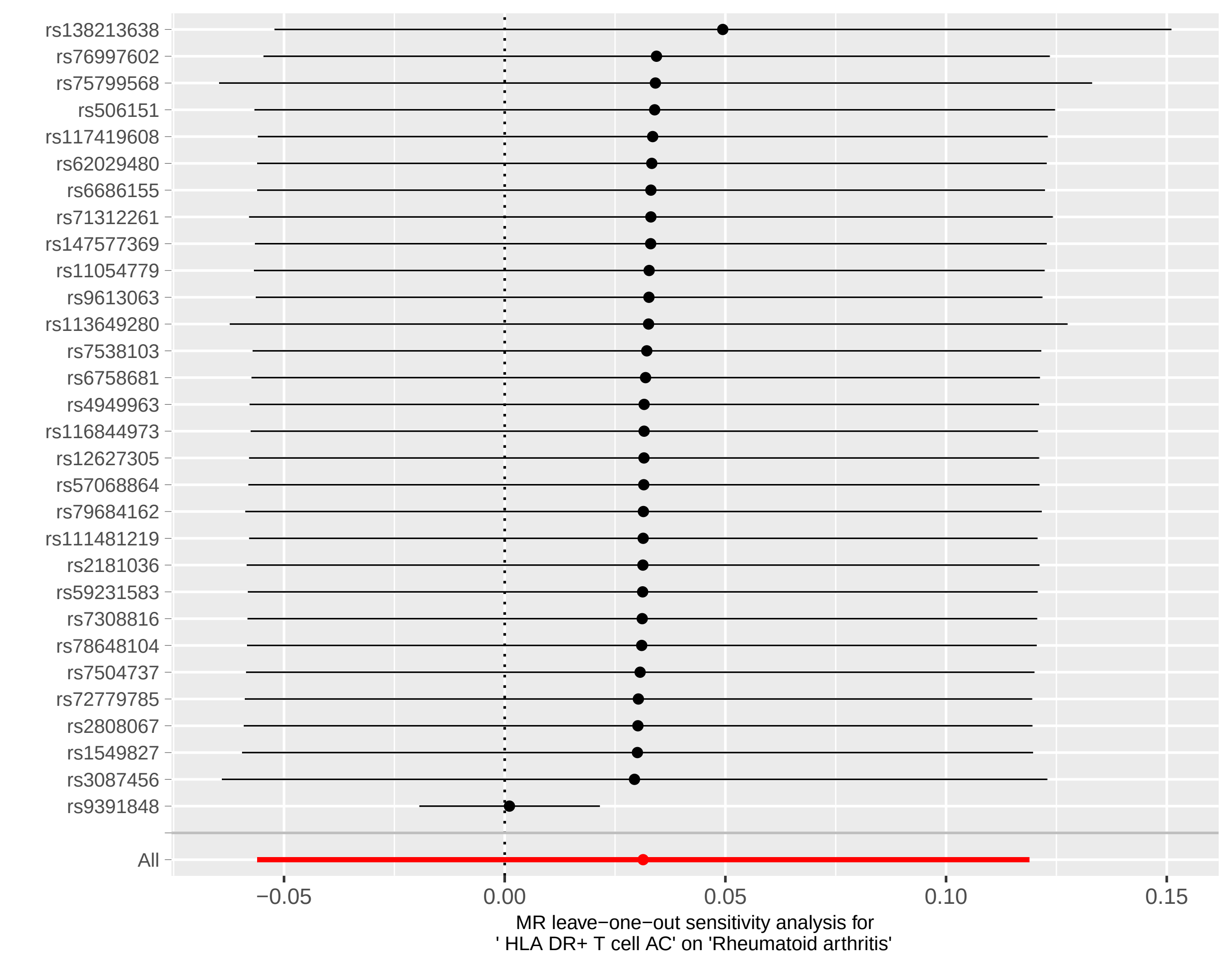

rs138213638
rs76997602
rs75799568
rs506151
rs117419608
rs62029480
rs6686155
rs71312261
rs147577369
rs11054779
rs9613063
rs113649280
rs7538103
rs6758681
rs4949963
rs116844973
rs12627305
rs57068864
rs79684162
rs111481219
rs2181036
rs59231583
rs7308816
rs78648104
rs7504737
rs72779785
rs2808067
rs1549827
rs3087456
rs9391848
All
−0.05
0.00
0.05
0.10
0.15
MR leave−one−out sensitivity analysis for
' HLA DR+ T cell AC' on 'Rheumatoid arthritis'

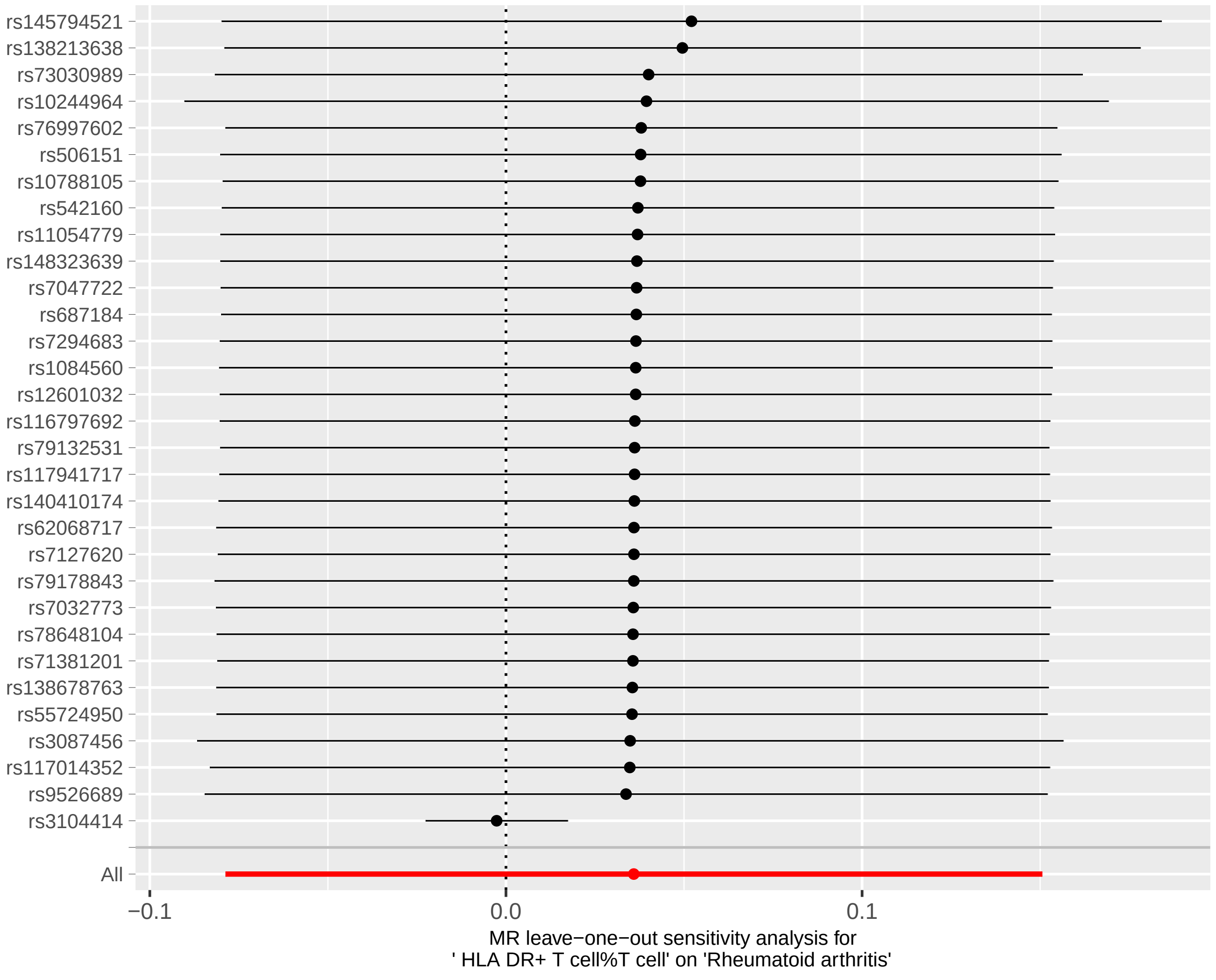
rs145794521
rs138213638
rs73030989
rs10244964
rs76997602
rs506151
rs10788105
rs542160
rs11054779
rs148323639
rs7047722
rs687184
rs7294683
rs1084560
rs12601032
rs116797692
rs79132531
rs117941717
rs140410174
rs62068717
rs7127620
rs79178843
rs7032773
rs78648104
rs71381201
rs138678763
rs55724950
rs3087456
rs117014352
rs9526689
rs3104414
All
−0.1
0.0
0.1
MR leave−one−out sensitivity analysis for
' HLA DR+ T cell%T cell' on 'Rheumatoid arthritis'

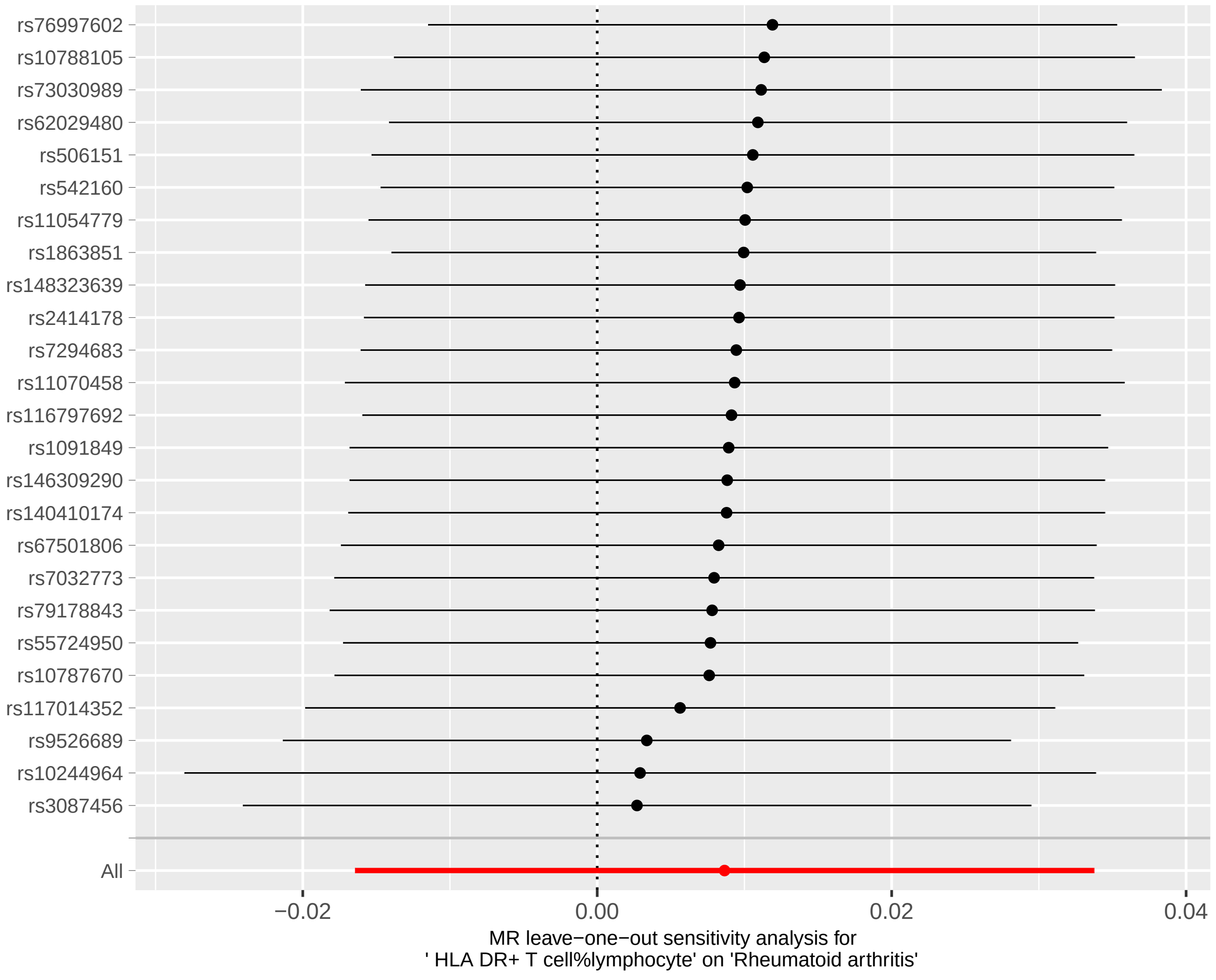
rs76997602
rs10788105
rs73030989
rs62029480
rs506151
rs542160
rs11054779
rs1863851
rs148323639
rs2414178
rs7294683
rs11070458
rs116797692
rs1091849
rs146309290
rs140410174
rs67501806
rs7032773
rs79178843
rs55724950
rs10787670
rs117014352
rs9526689
rs10244964
rs3087456
All
−0.02
0.00
0.02
0.04
MR leave−one−out sensitivity analysis for
' HLA DR+ T cell%lymphocyte' on 'Rheumatoid arthritis'

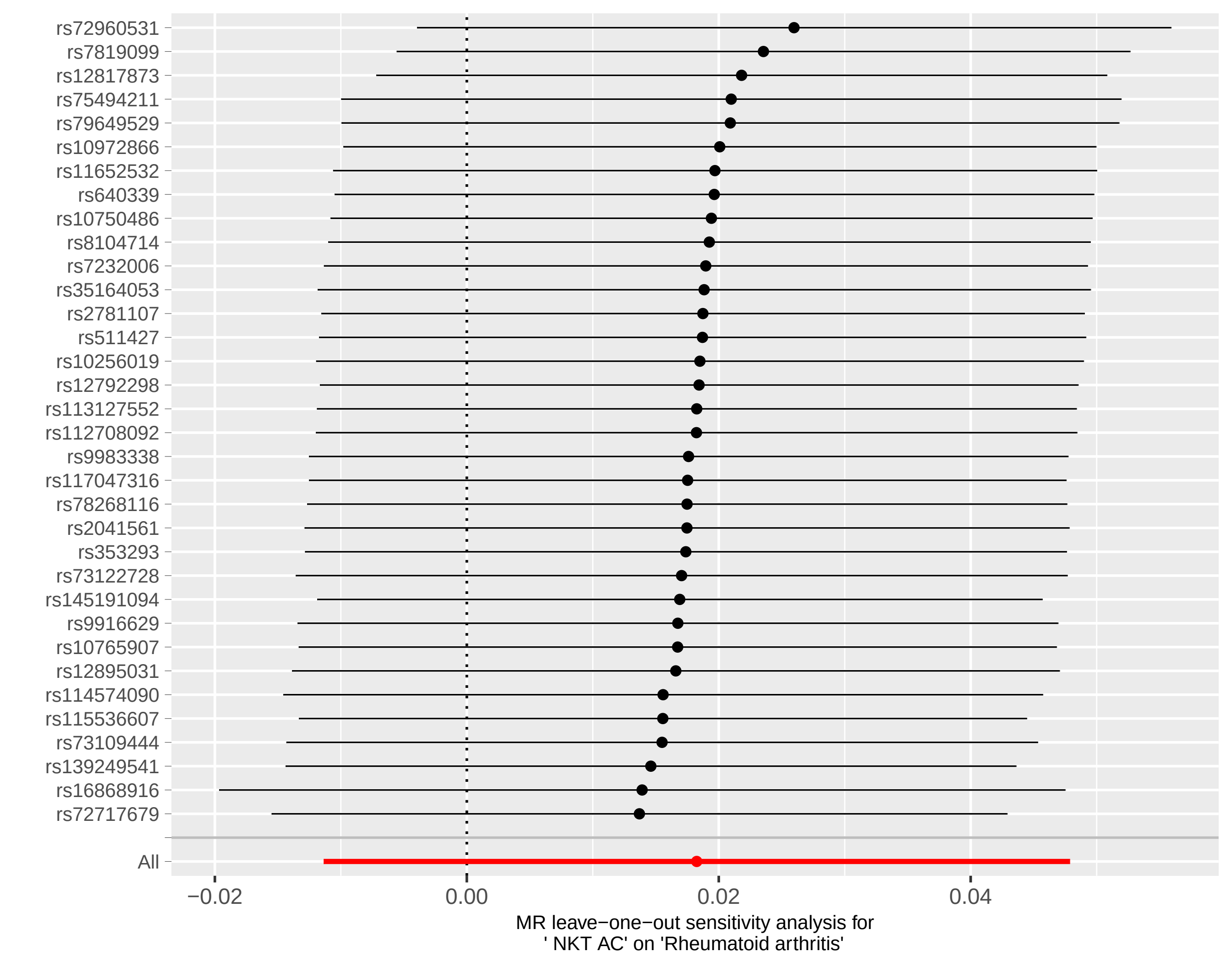

rs72960531
rs7819099
rs12817873
rs75494211
rs79649529
rs10972866
rs11652532
rs640339
rs10750486
rs8104714
rs7232006
rs35164053
rs2781107
rs511427
rs10256019
rs12792298
rs113127552
rs112708092
rs9983338
rs117047316
rs78268116
rs2041561
rs353293
rs73122728
rs145191094
rs9916629
rs10765907
rs12895031
rs114574090
rs115536607
rs73109444
rs139249541
rs16868916
rs72717679
All
−0.02
0.00
0.02
0.04
MR leave−one−out sensitivity analysis for
' NKT AC' on 'Rheumatoid arthritis'

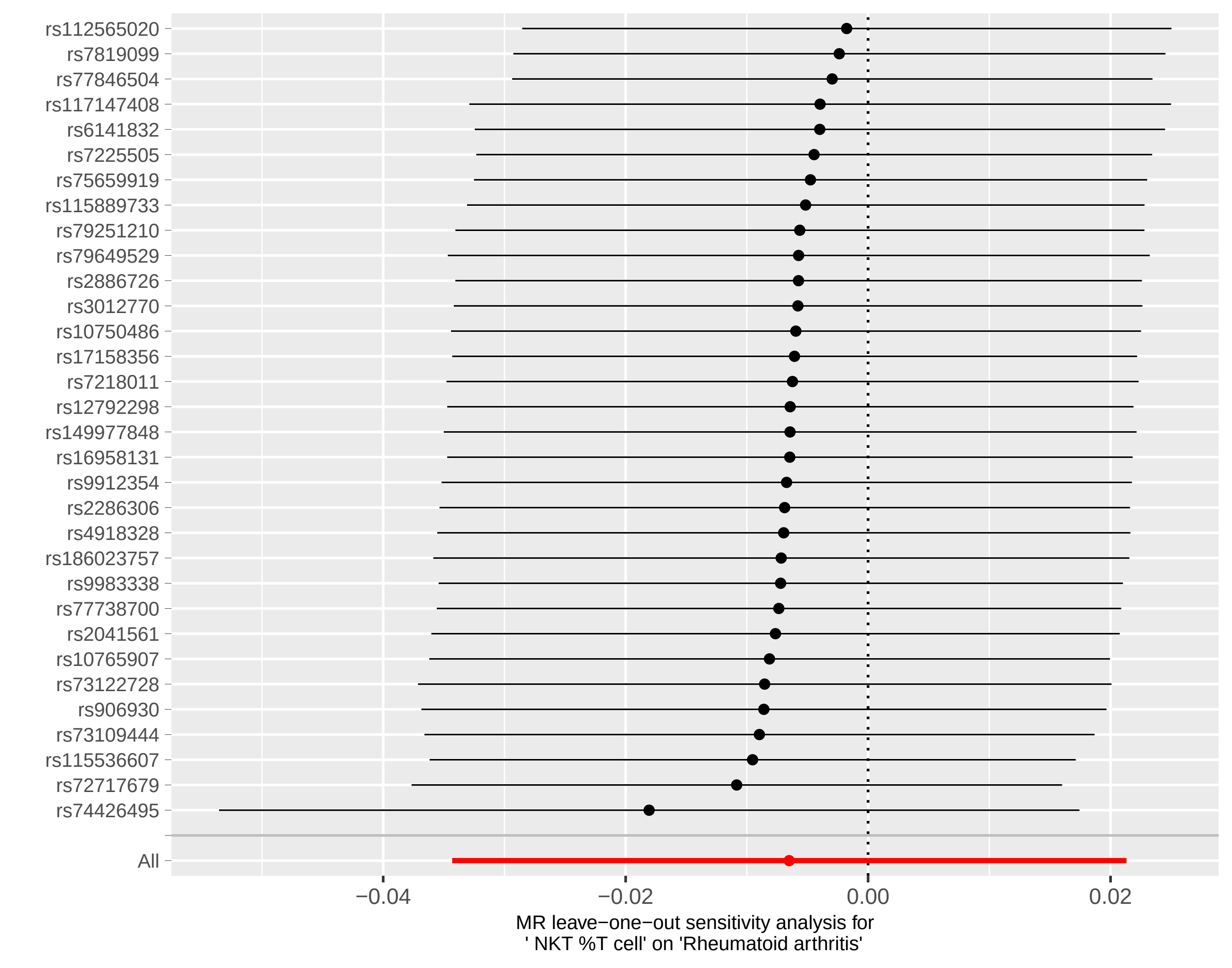
rs112565020
rs7819099
rs77846504
rs117147408
rs6141832
rs7225505
rs75659919
rs115889733
rs79251210
rs79649529
rs2886726
rs3012770
rs10750486
rs17158356
rs7218011
rs12792298
rs149977848
rs16958131
rs9912354
rs2286306
rs4918328
rs186023757
rs9983338
rs77738700
rs2041561
rs10765907
rs73122728
rs906930
rs73109444
rs115536607
rs72717679
rs74426495
All
−0.04
−0.02
0.00
0.02
MR leave−one−out sensitivity analysis for
' NKT %T cell' on 'Rheumatoid arthritis'

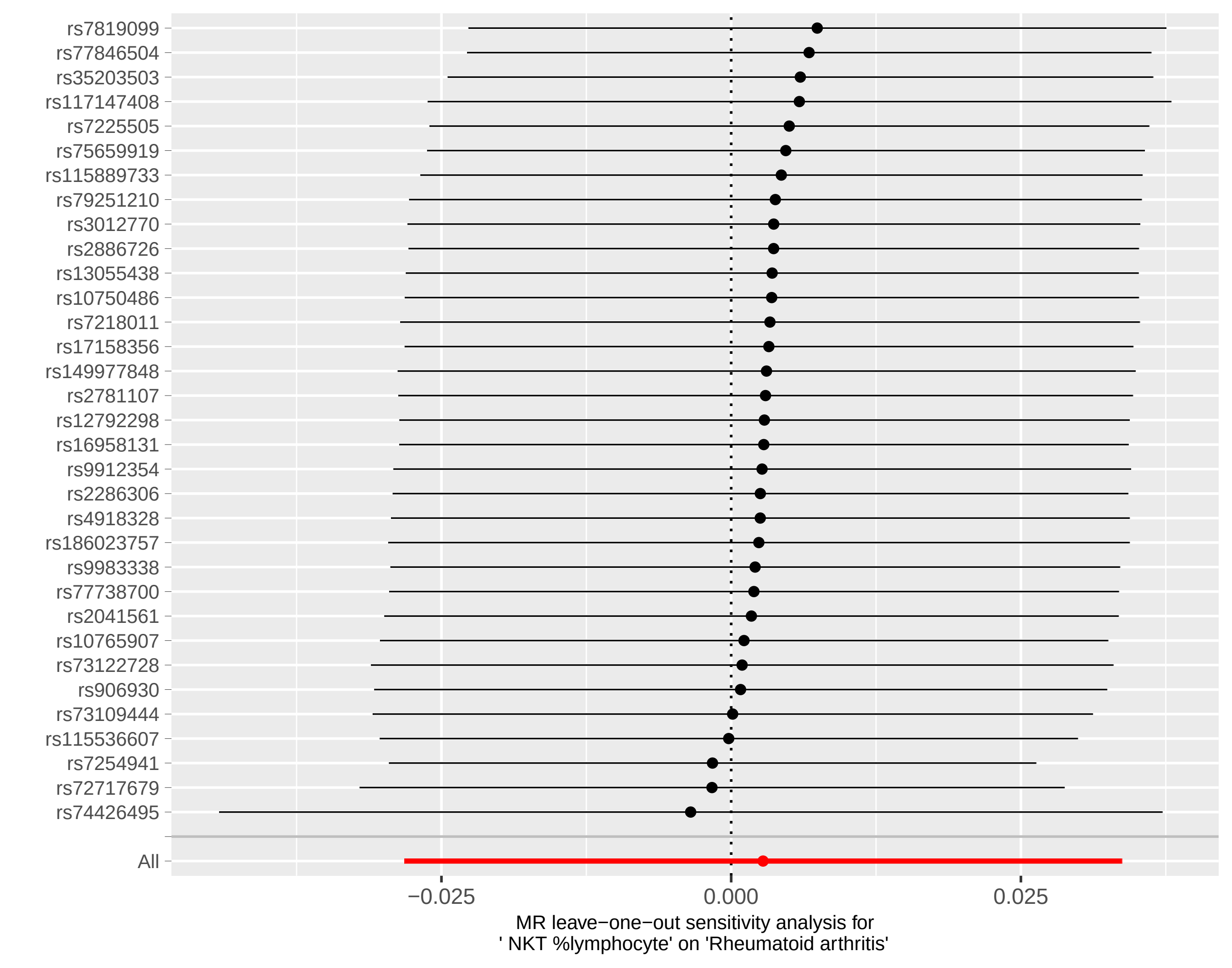

rs7819099
rs77846504
rs35203503
rs117147408
rs7225505
rs75659919
rs115889733
rs79251210
rs3012770
rs2886726
rs13055438
rs10750486
rs7218011
rs17158356
rs149977848
rs2781107
rs12792298
rs16958131
rs9912354
rs2286306
rs4918328
rs186023757
rs9983338
rs77738700
rs2041561
rs10765907
rs73122728
rs906930
rs73109444
rs115536607
rs7254941
rs72717679
rs74426495
All
−0.025
0.000
0.025
MR leave−one−out sensitivity analysis for
' NKT %lymphocyte' on 'Rheumatoid arthritis'

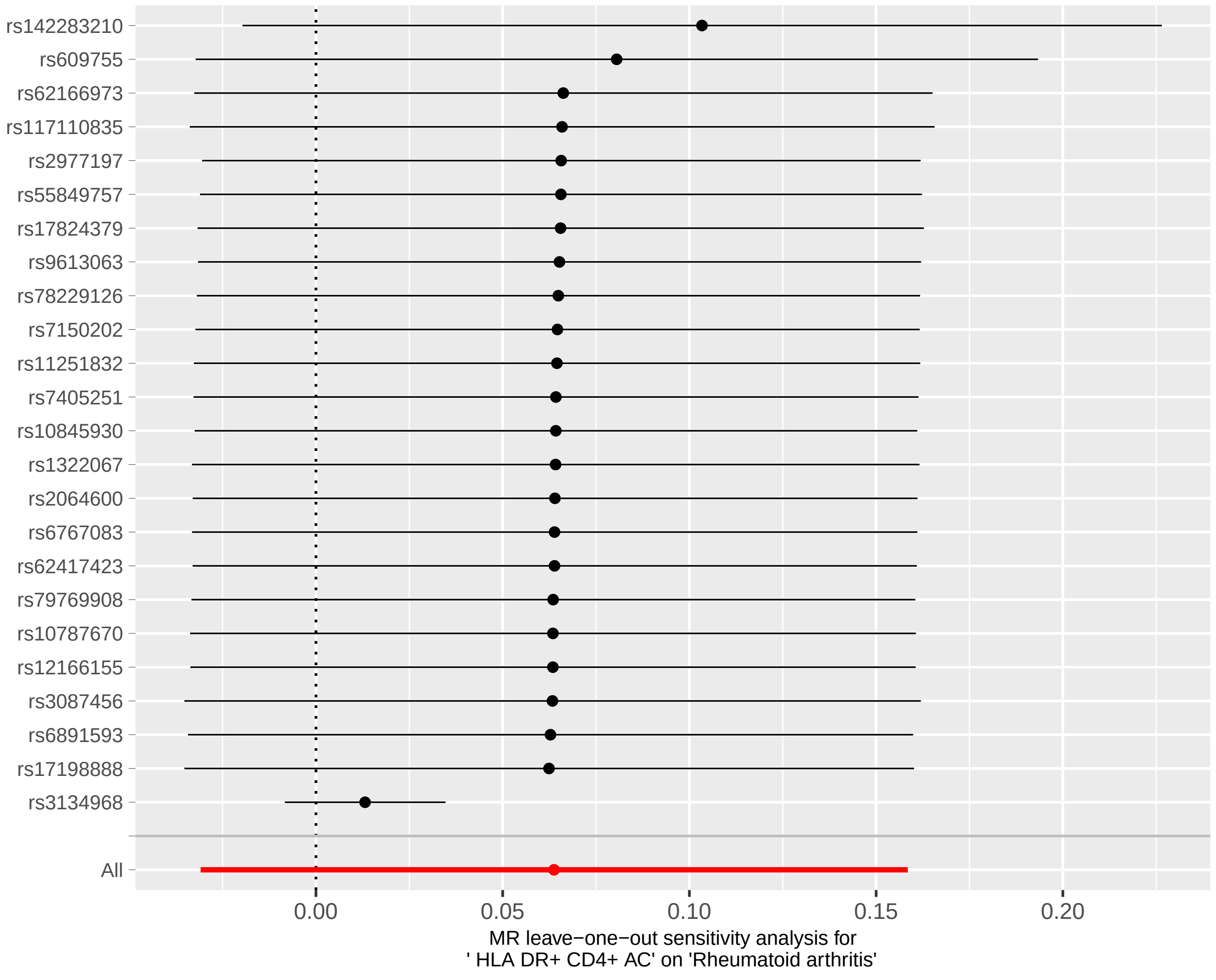
rs142283210
rs609755
rs62166973
rs117110835
rs2977197
rs55849757
rs17824379
rs9613063
rs78229126
rs7150202
rs11251832
rs7405251
rs10845930
rs1322067
rs2064600
rs6767083
rs62417423
rs79769908
rs10787670
rs12166155
rs3087456
rs6891593
rs17198888
rs3134968
All
0.00
0.05
0.10
0.15
0.20
MR leave−one−out sensitivity analysis for
' HLA DR+ CD4+ AC' on 'Rheumatoid arthritis'

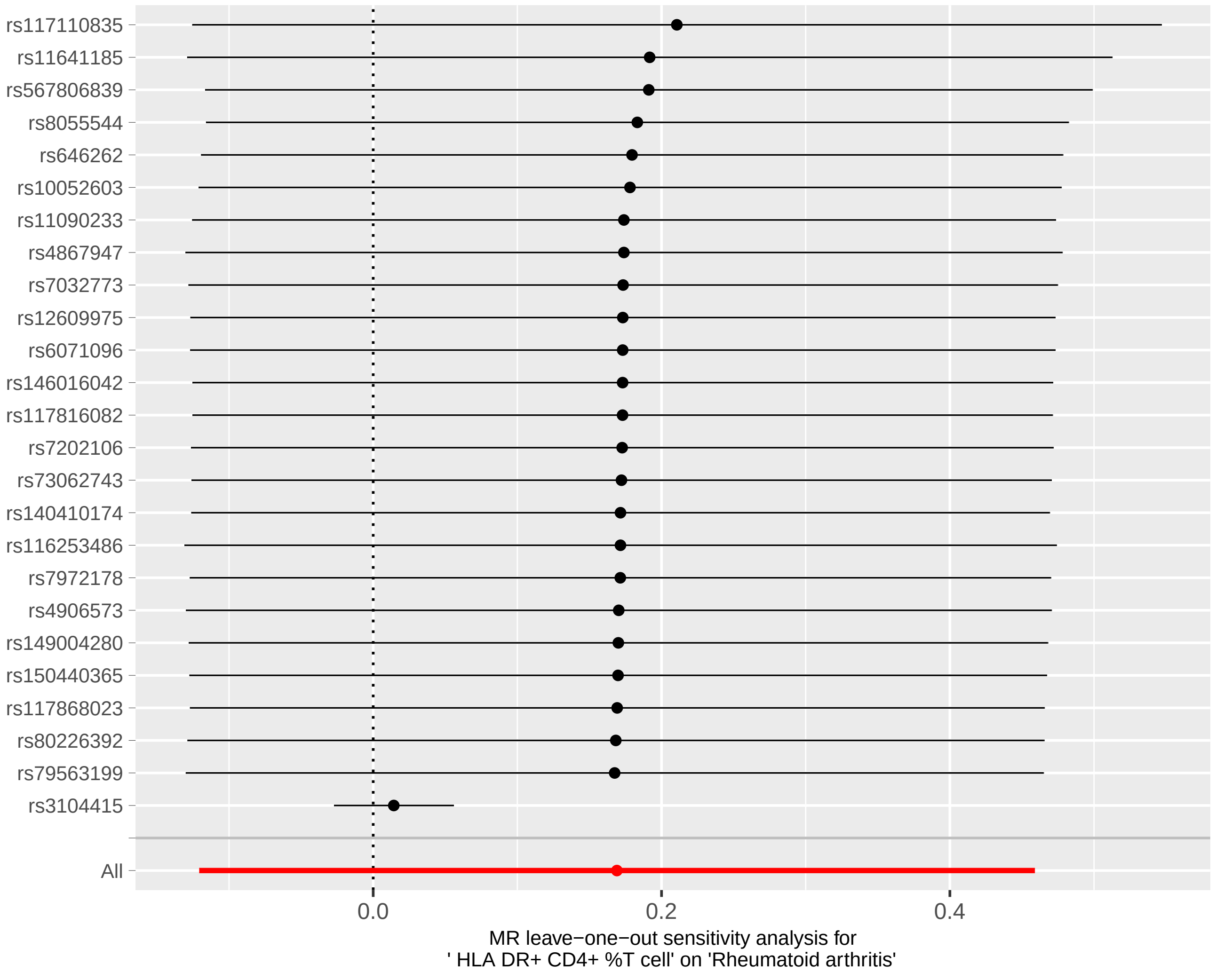
rs117110835
rs11641185
rs567806839
rs8055544
rs646262
rs10052603
rs11090233
rs4867947
rs7032773
rs12609975
rs6071096
rs146016042
rs117816082
rs7202106
rs73062743
rs140410174
rs116253486
rs7972178
rs4906573
rs149004280
rs150440365
rs117868023
rs80226392
rs79563199
rs3104415
All
0.0
0.2
0.4
MR leave−one−out sensitivity analysis for
' HLA DR+ CD4+ %T cell' on 'Rheumatoid arthritis'

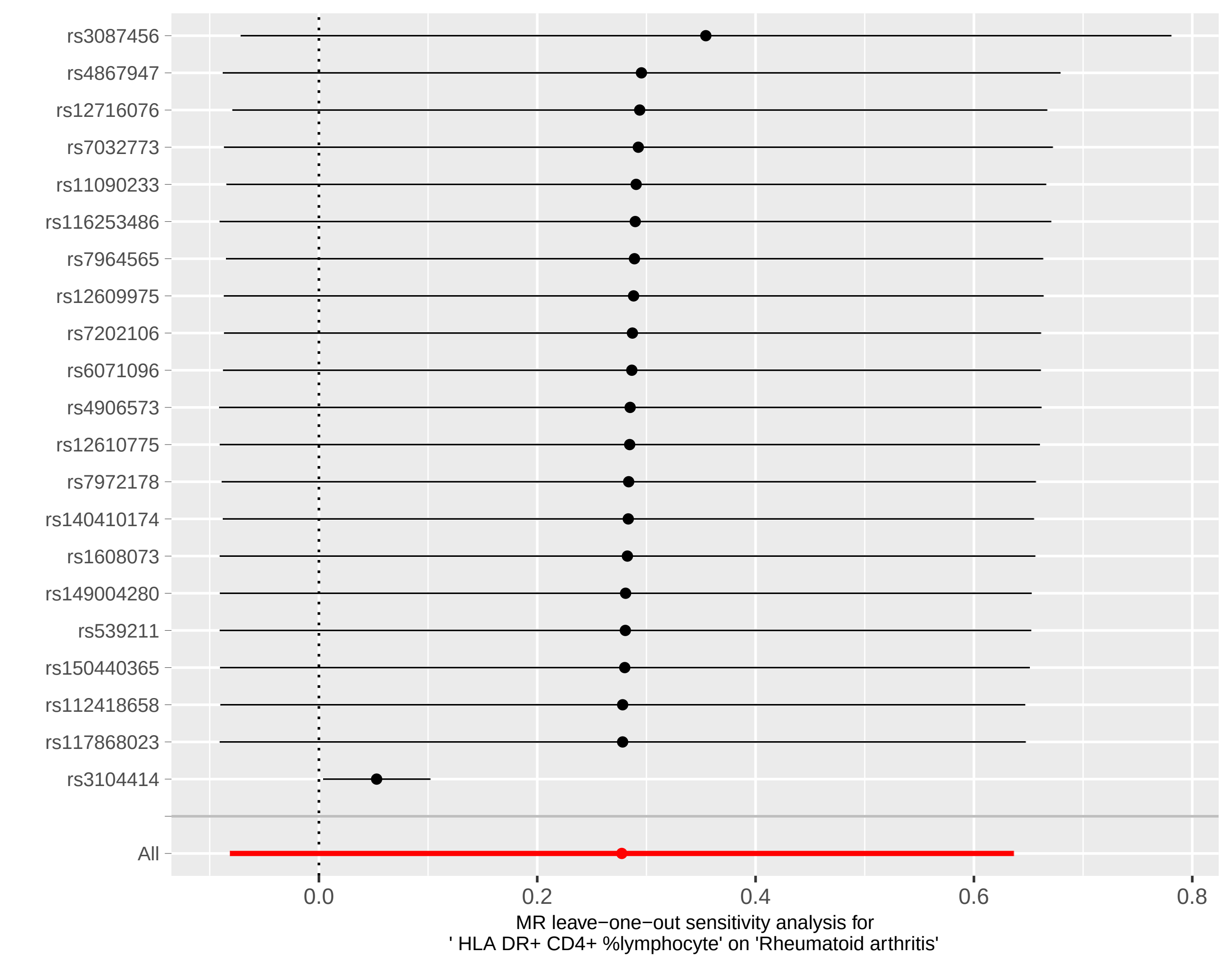
rs3087456
rs4867947
rs12716076
rs7032773
rs11090233
rs116253486
rs7964565
rs12609975
rs7202106
rs6071096
rs4906573
rs12610775
rs7972178
rs140410174
rs1608073
rs149004280
rs539211
rs150440365
rs112418658
rs117868023
rs3104414
All
0.0
0.2
0.4
0.6
0.8
MR leave−one−out sensitivity analysis for
' HLA DR+ CD4+ %lymphocyte' on 'Rheumatoid arthritis'

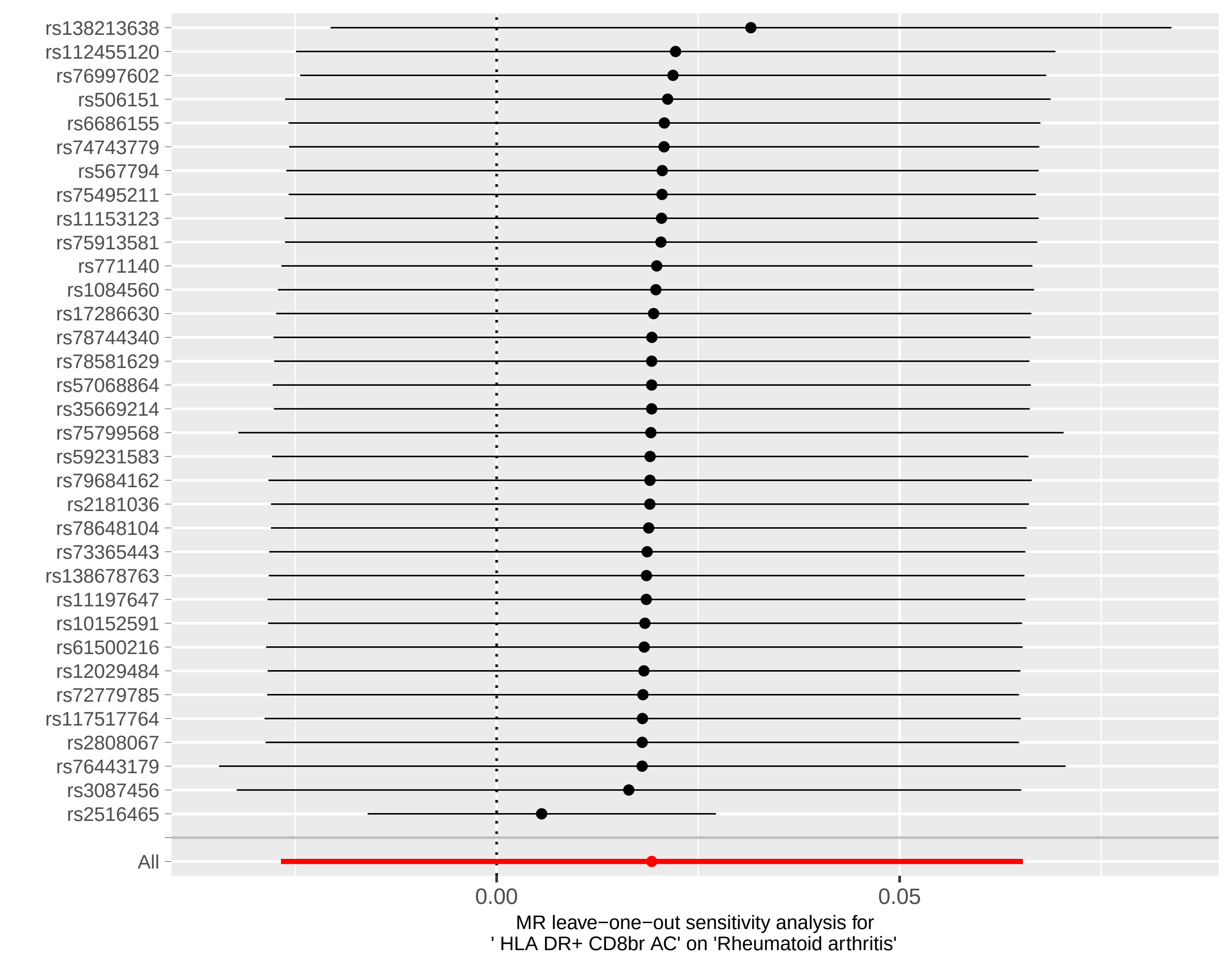

rs138213638
rs112455120
rs76997602
rs506151
rs6686155
rs74743779
rs567794
rs75495211
rs11153123
rs75913581
rs771140
rs1084560
rs17286630
rs78744340
rs78581629
rs57068864
rs35669214
rs75799568
rs59231583
rs79684162
rs2181036
rs78648104
rs73365443
rs138678763
rs11197647
rs10152591
rs61500216
rs12029484
rs72779785
rs117517764
rs2808067
rs76443179
rs3087456
rs2516465
All
0.00
0.05
MR leave−one−out sensitivity analysis for
' HLA DR+ CD8br AC' on 'Rheumatoid arthritis'

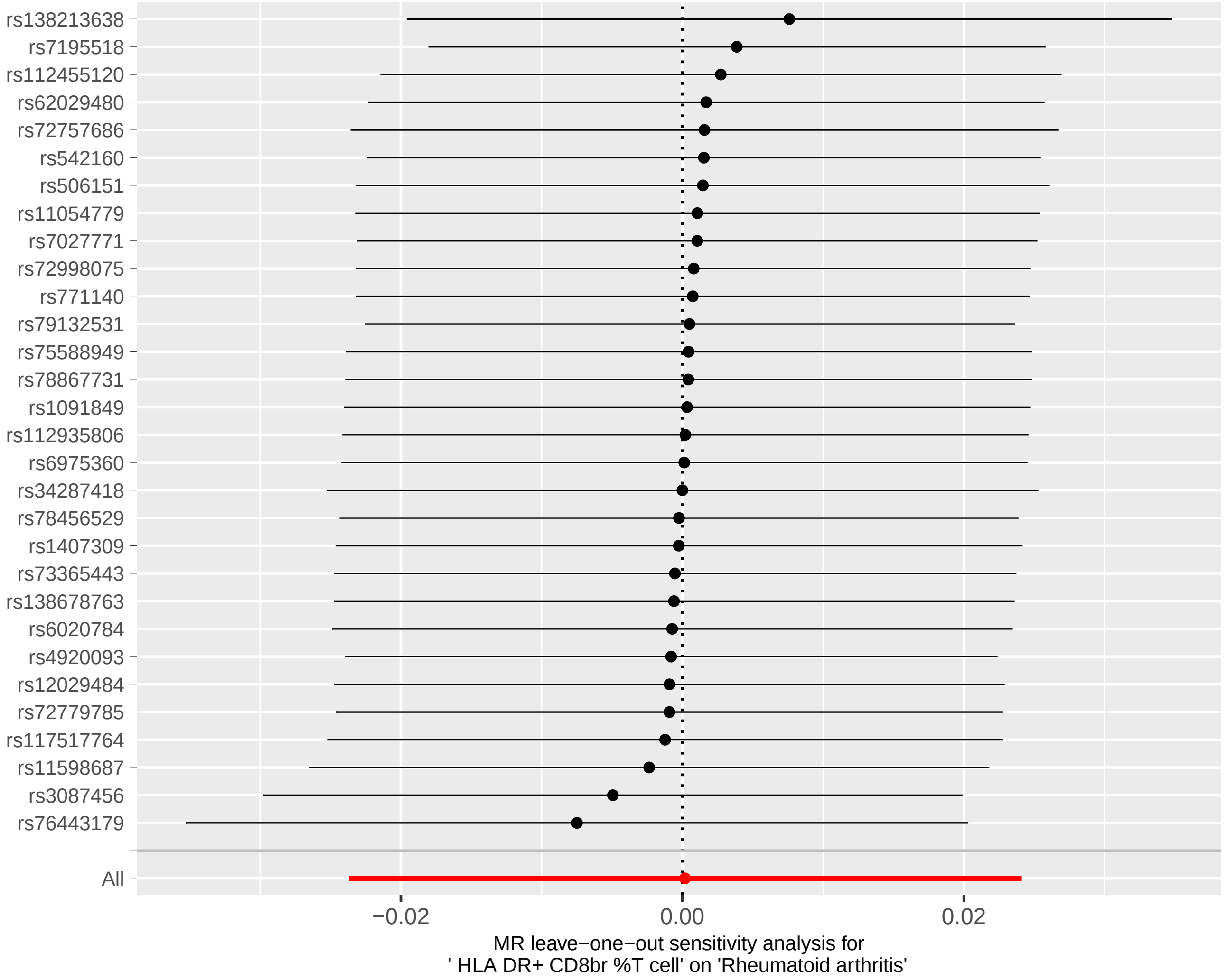

rs138213638
rs7195518
rs112455120
rs62029480
rs72757686
rs542160
rs506151
rs11054779
rs7027771
rs72998075
rs771140
rs79132531
rs75588949
rs78867731
rs1091849
rs112935806
rs6975360
rs34287418
rs78456529
rs1407309
rs73365443
rs138678763
rs6020784
rs4920093
rs12029484
rs72779785
rs117517764
rs11598687
rs3087456
rs76443179
All
−0.02
0.00
0.02
MR leave−one−out sensitivity analysis for
' HLA DR+ CD8br %T cell' on 'Rheumatoid arthritis'

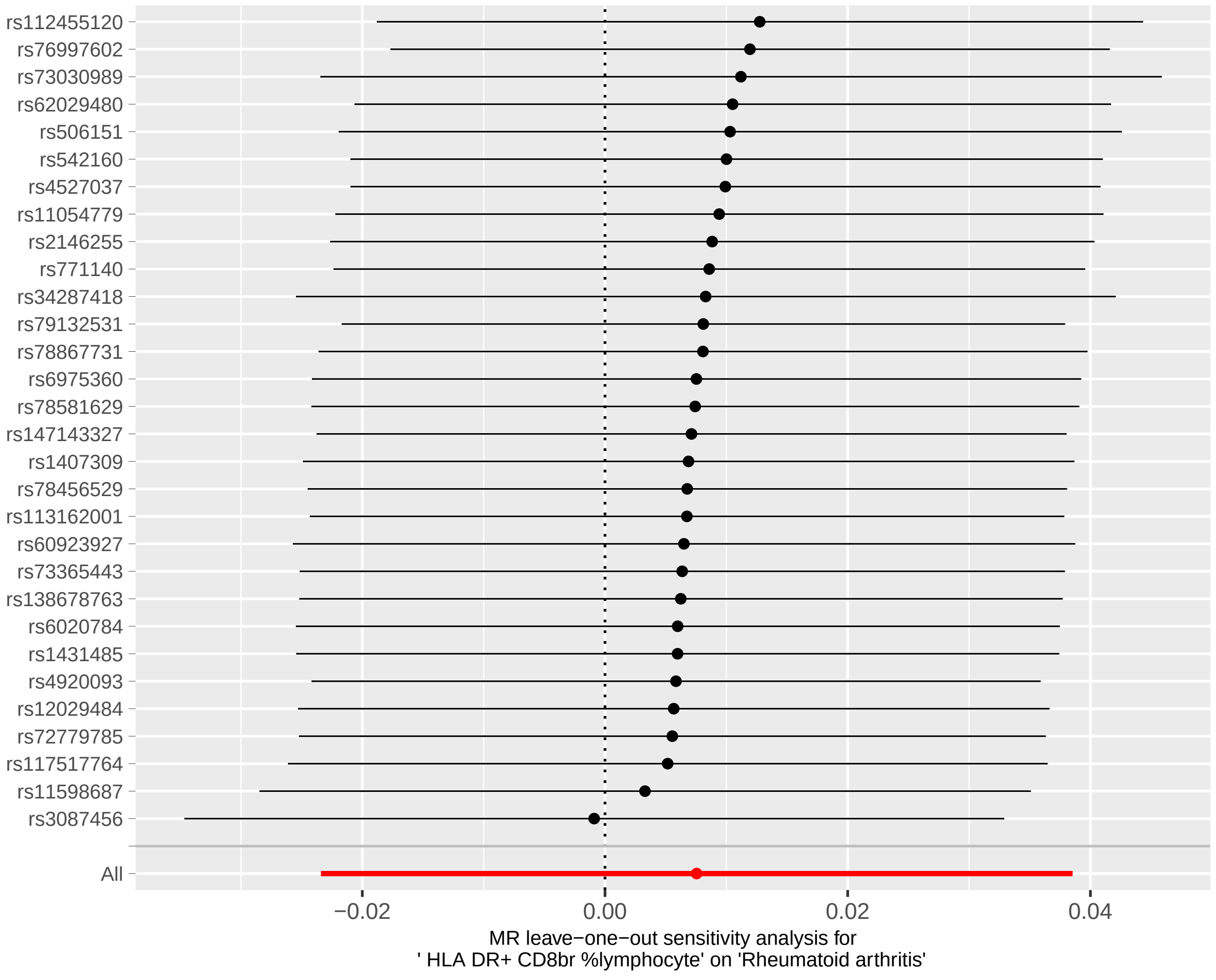

rs112455120
rs76997602
rs73030989
rs62029480
rs506151
rs542160
rs4527037
rs11054779
rs2146255
rs771140
rs34287418
rs79132531
rs78867731
rs6975360
rs78581629
rs147143327
rs1407309
rs78456529
rs113162001
rs60923927
rs73365443
rs138678763
rs6020784
rs1431485
rs4920093
rs12029484
rs72779785
rs117517764
rs11598687
rs3087456
All
−0.02
0.00
0.02
0.04
MR leave−one−out sensitivity analysis for
' HLA DR+ CD8br %lymphocyte' on 'Rheumatoid arthritis'

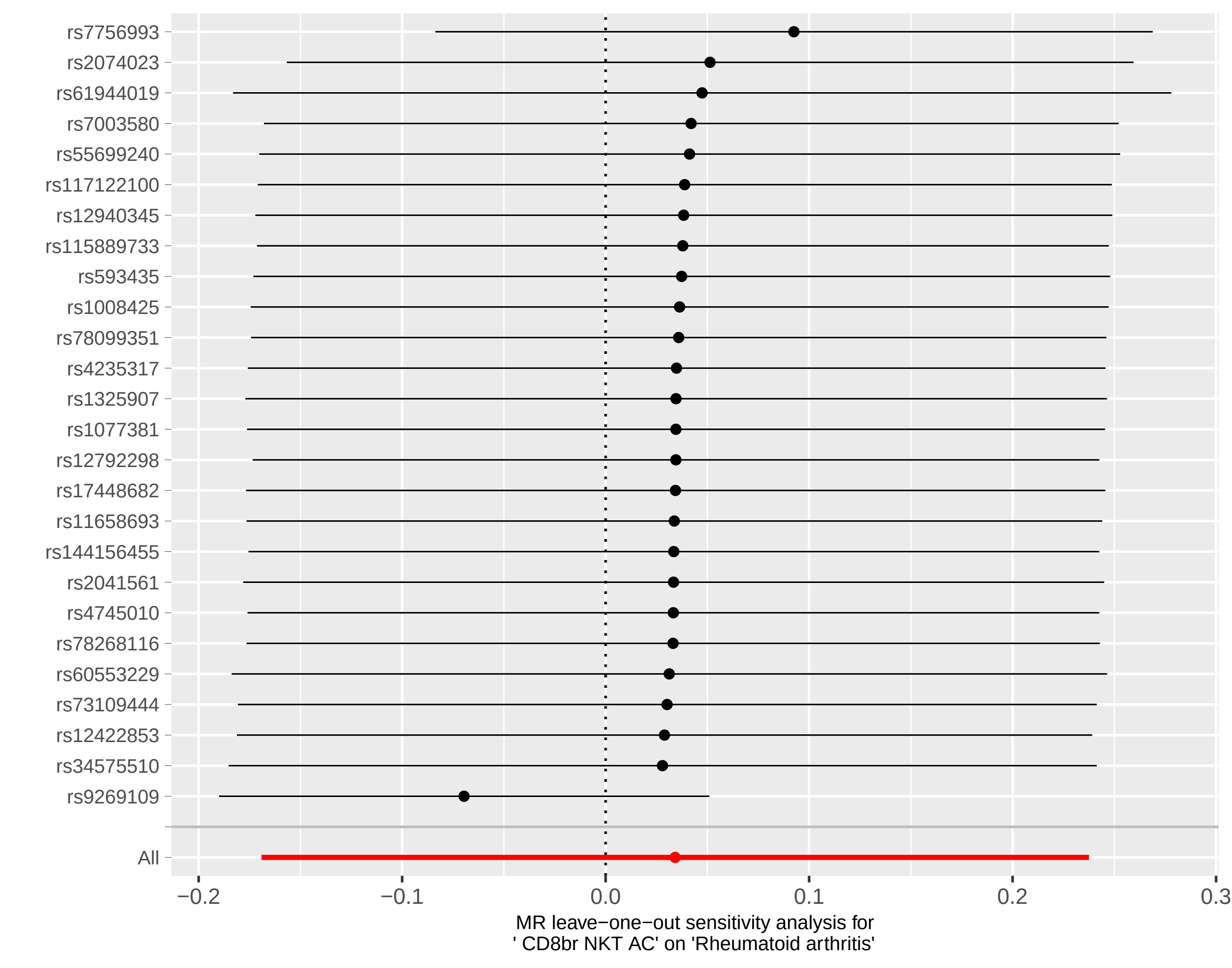
rs7756993
rs2074023
rs61944019
rs7003580
rs55699240
rs117122100
rs12940345
rs115889733
rs593435
rs1008425
rs78099351
rs4235317
rs1325907
rs1077381
rs12792298
rs17448682
rs11658693
rs144156455
rs2041561
rs4745010
rs78268116
rs60553229
rs73109444
rs12422853
rs34575510
rs9269109
All
−0.2
−0.1
0.0
0.1
0.2
0.3
MR leave−one−out sensitivity analysis for
' CD8br NKT AC' on 'Rheumatoid arthritis'

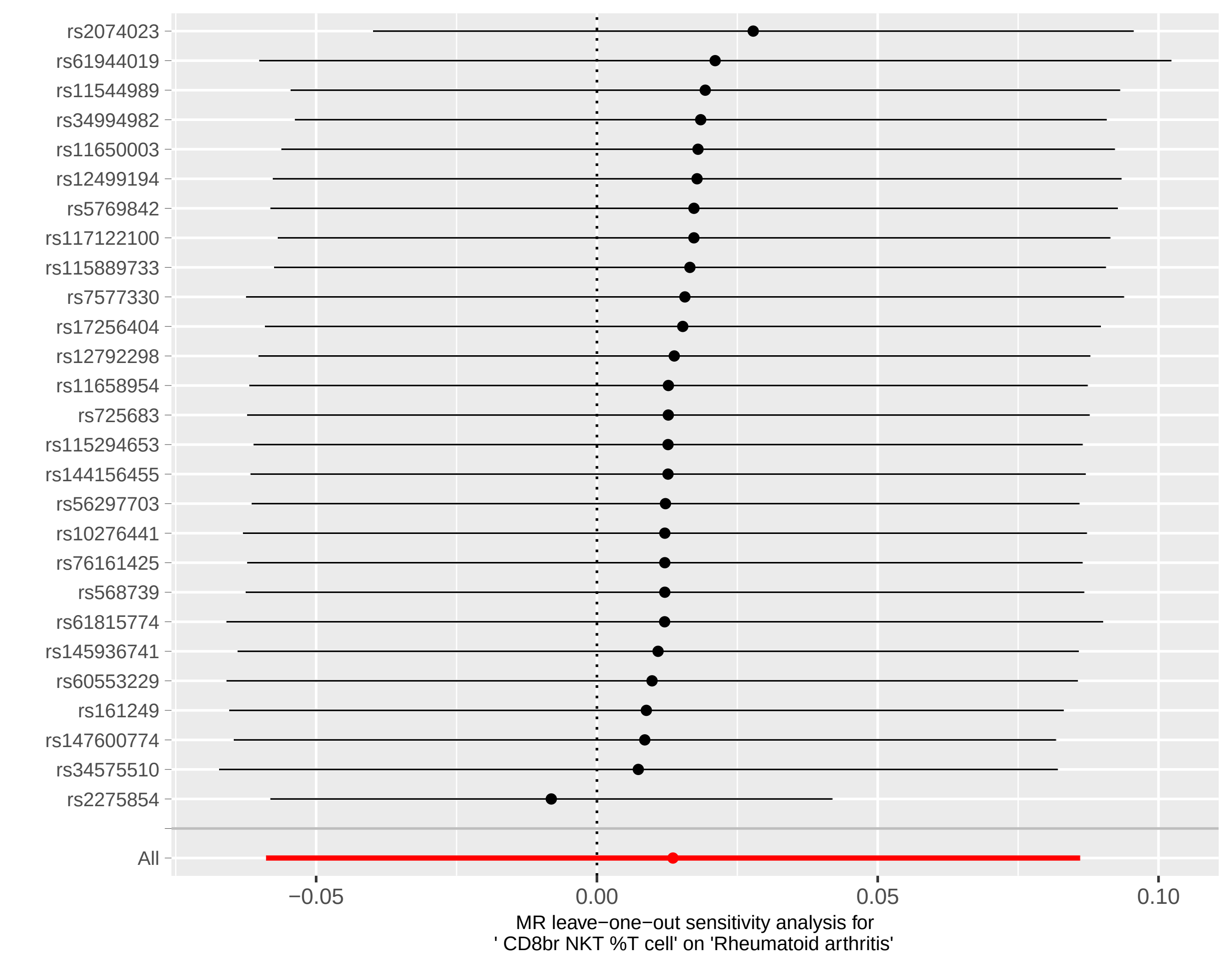

rs2074023
rs61944019
rs11544989
rs34994982
rs11650003
rs12499194
rs5769842
rs117122100
rs115889733
rs7577330
rs17256404
rs12792298
rs11658954
rs725683
rs115294653
rs144156455
rs56297703
rs10276441
rs76161425
rs568739
rs61815774
rs145936741
rs60553229
rs161249
rs147600774
rs34575510
rs2275854
All
−0.05
0.00
0.05
0.10
MR leave−one−out sensitivity analysis for
' CD8br NKT %T cell' on 'Rheumatoid arthritis'

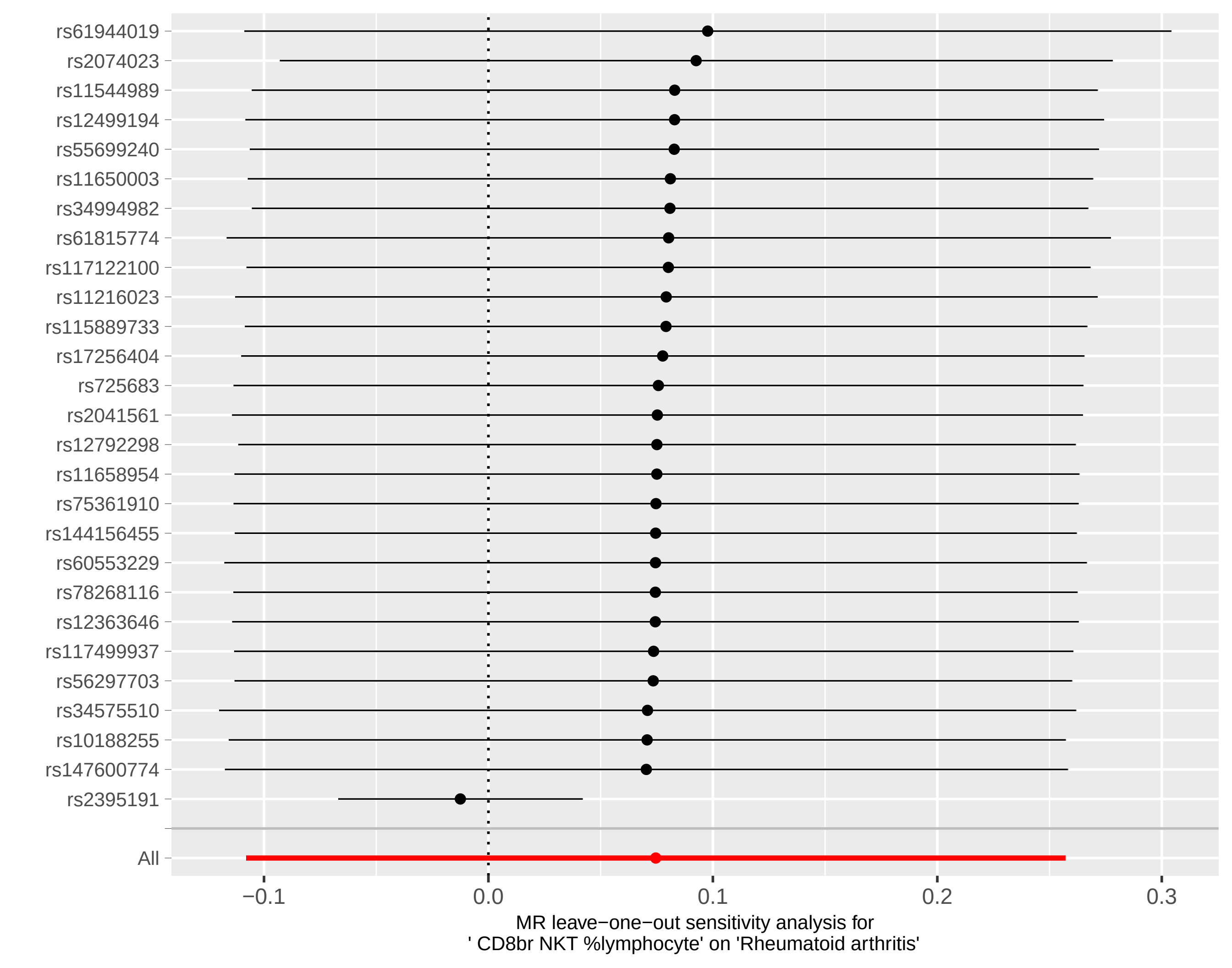
rs61944019
rs2074023
rs11544989
rs12499194
rs55699240
rs11650003
rs34994982
rs61815774
rs117122100
rs11216023
rs115889733
rs17256404
rs725683
rs2041561
rs12792298
rs11658954
rs75361910
rs144156455
rs60553229
rs78268116
rs12363646
rs117499937
rs56297703
rs34575510
rs10188255
rs147600774
rs2395191
All
−0.1
0.0
0.1
0.2
0.3
MR leave−one−out sensitivity analysis for
' CD8br NKT %lymphocyte' on 'Rheumatoid arthritis'

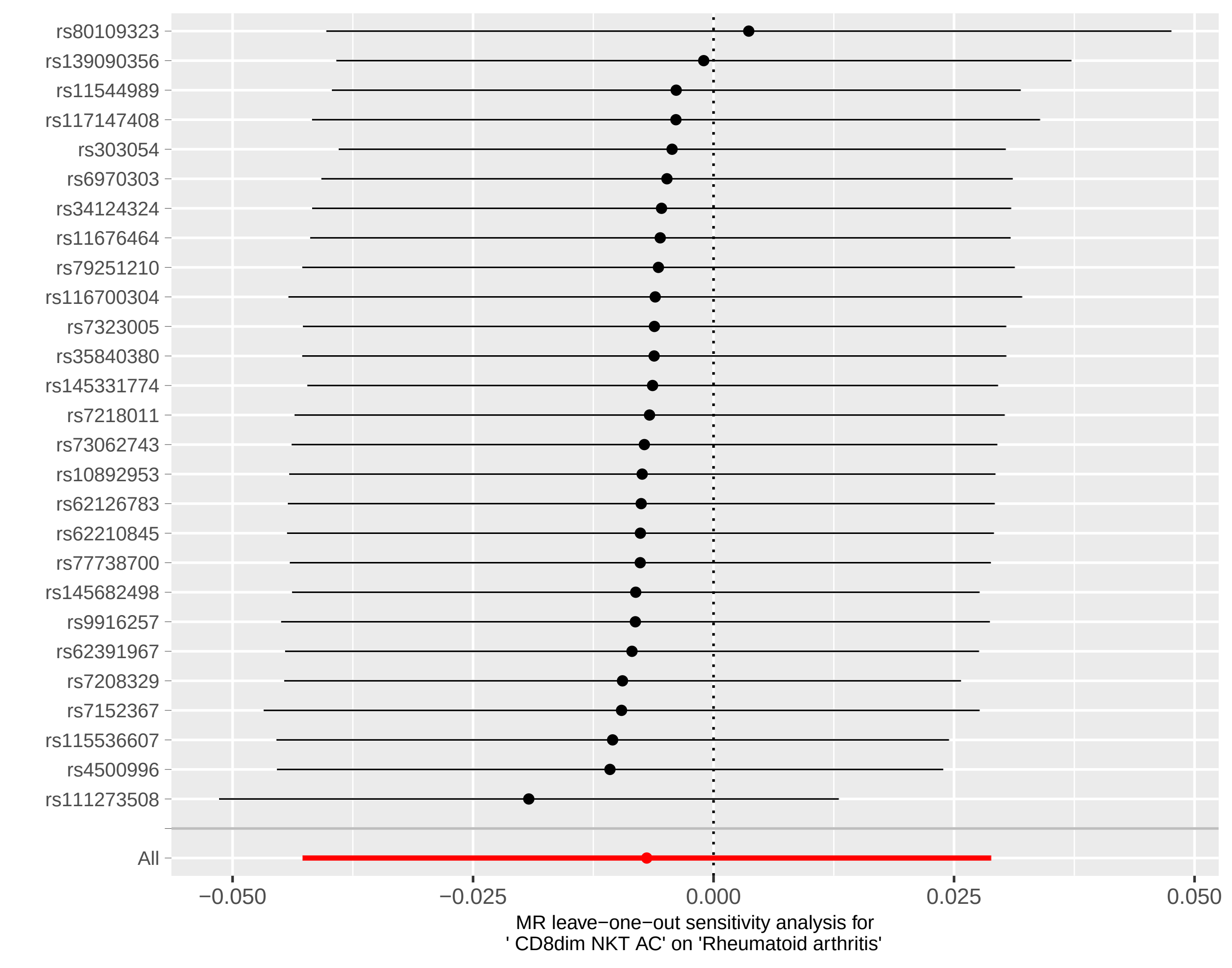
rs80109323
rs139090356
rs11544989
rs117147408
rs303054
rs6970303
rs34124324
rs11676464
rs79251210
rs116700304
rs7323005
rs35840380
rs145331774
rs7218011
rs73062743
rs10892953
rs62126783
rs62210845
rs77738700
rs145682498
rs9916257
rs62391967
rs7208329
rs7152367
rs115536607
rs4500996
rs111273508
All
−0.050
−0.025
0.000
0.025
0.050
MR leave−one−out sensitivity analysis for
' CD8dim NKT AC' on 'Rheumatoid arthritis'

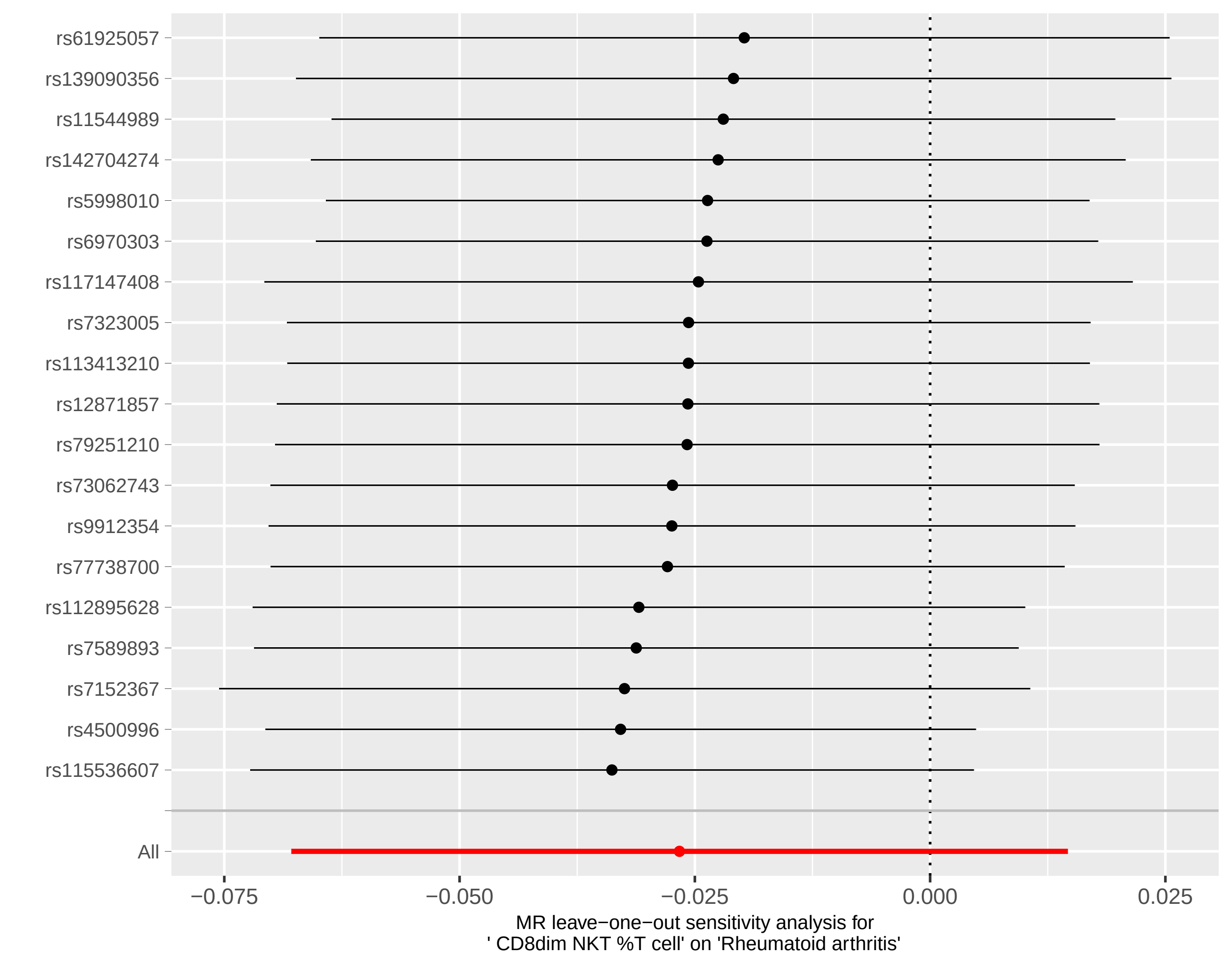
rs61925057
rs139090356
rs11544989
rs142704274
rs5998010
rs6970303
rs117147408
rs7323005
rs113413210
rs12871857
rs79251210
rs73062743
rs9912354
rs77738700
rs112895628
rs7589893
rs7152367
rs4500996
rs115536607
All
−0.075
−0.050
−0.025
0.000
0.025
MR leave−one−out sensitivity analysis for
' CD8dim NKT %T cell' on 'Rheumatoid arthritis'

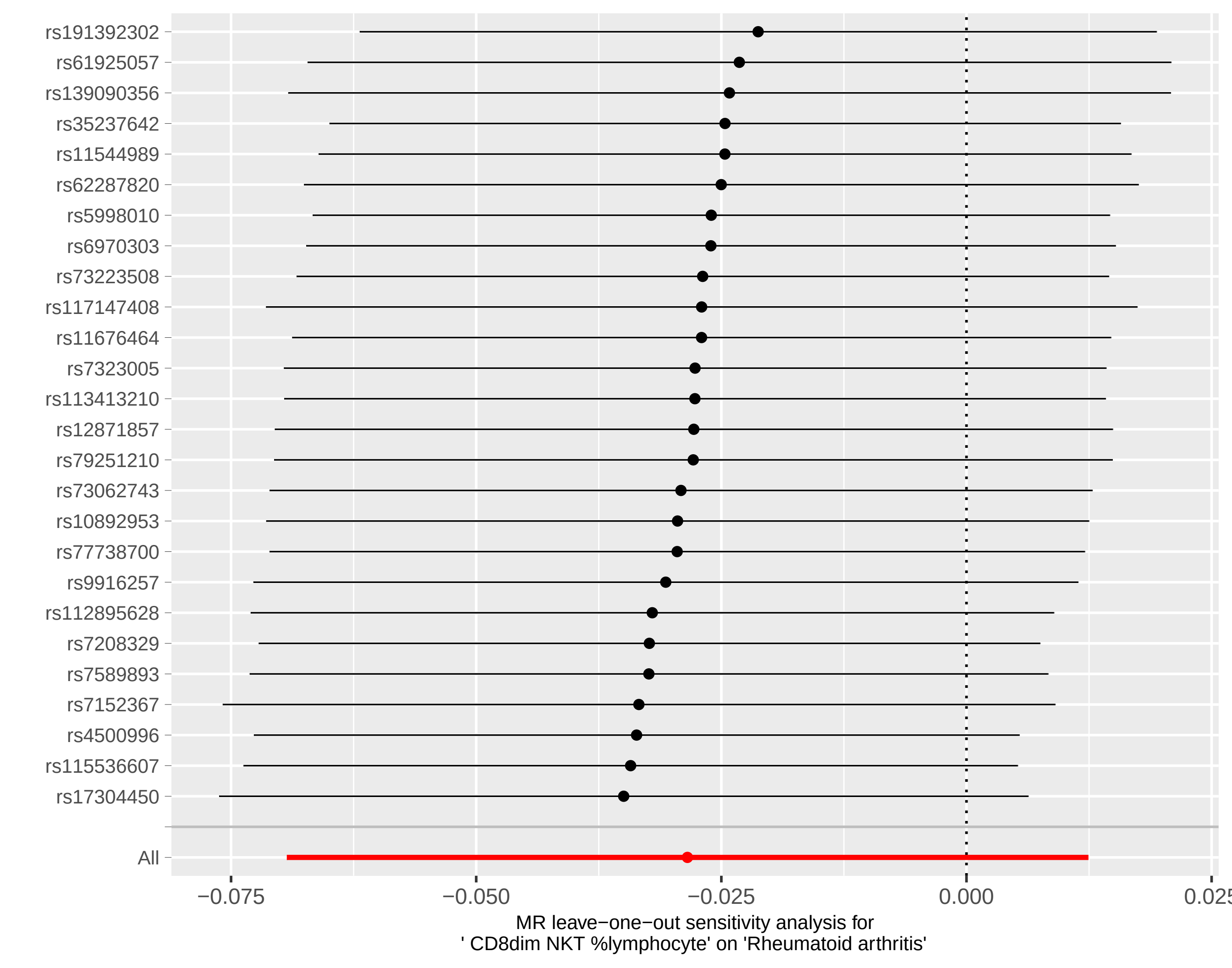
rs191392302
rs61925057
rs139090356
rs35237642
rs11544989
rs62287820
rs5998010
rs6970303
rs73223508
rs117147408
rs11676464
rs7323005
rs113413210
rs12871857
rs79251210
rs73062743
rs10892953
rs77738700
rs9916257
rs112895628
rs7208329
rs7589893
rs7152367
rs4500996
rs115536607
rs17304450
All
−0.075
−0.050
−0.025
0.000
MR leave−one−out sensitivity analysis for
' CD8dim NKT %lymphocyte' on 'Rheumatoid arthritis'

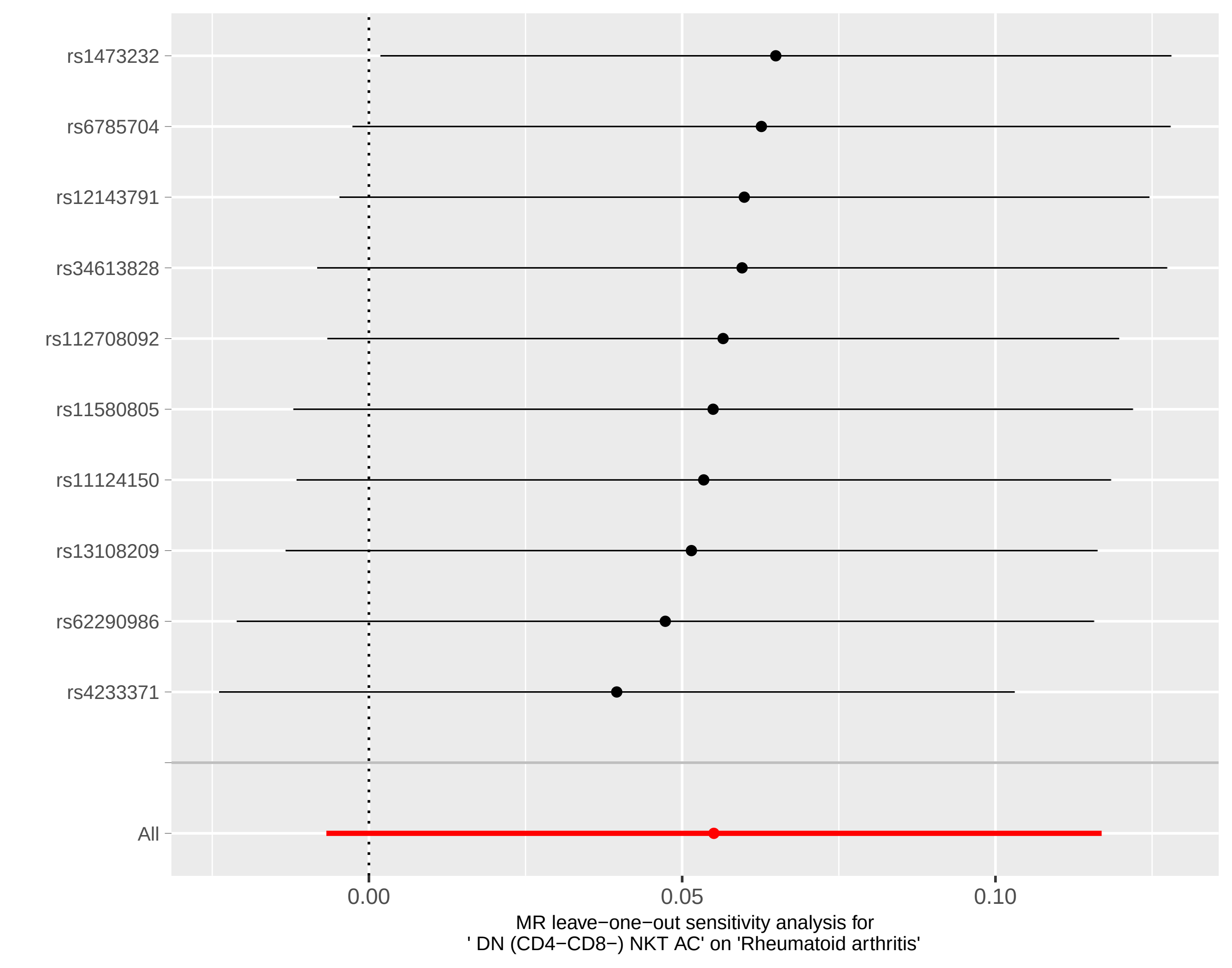
rs1473232
rs6785704
rs12143791
rs34613828
rs112708092
rs11580805
rs11124150
rs13108209
rs62290986
rs4233371
All
0.00
0.05
0.10
MR leave−one−out sensitivity analysis for
' DN (CD4−CD8−) NKT AC' on 'Rheumatoid arthritis'

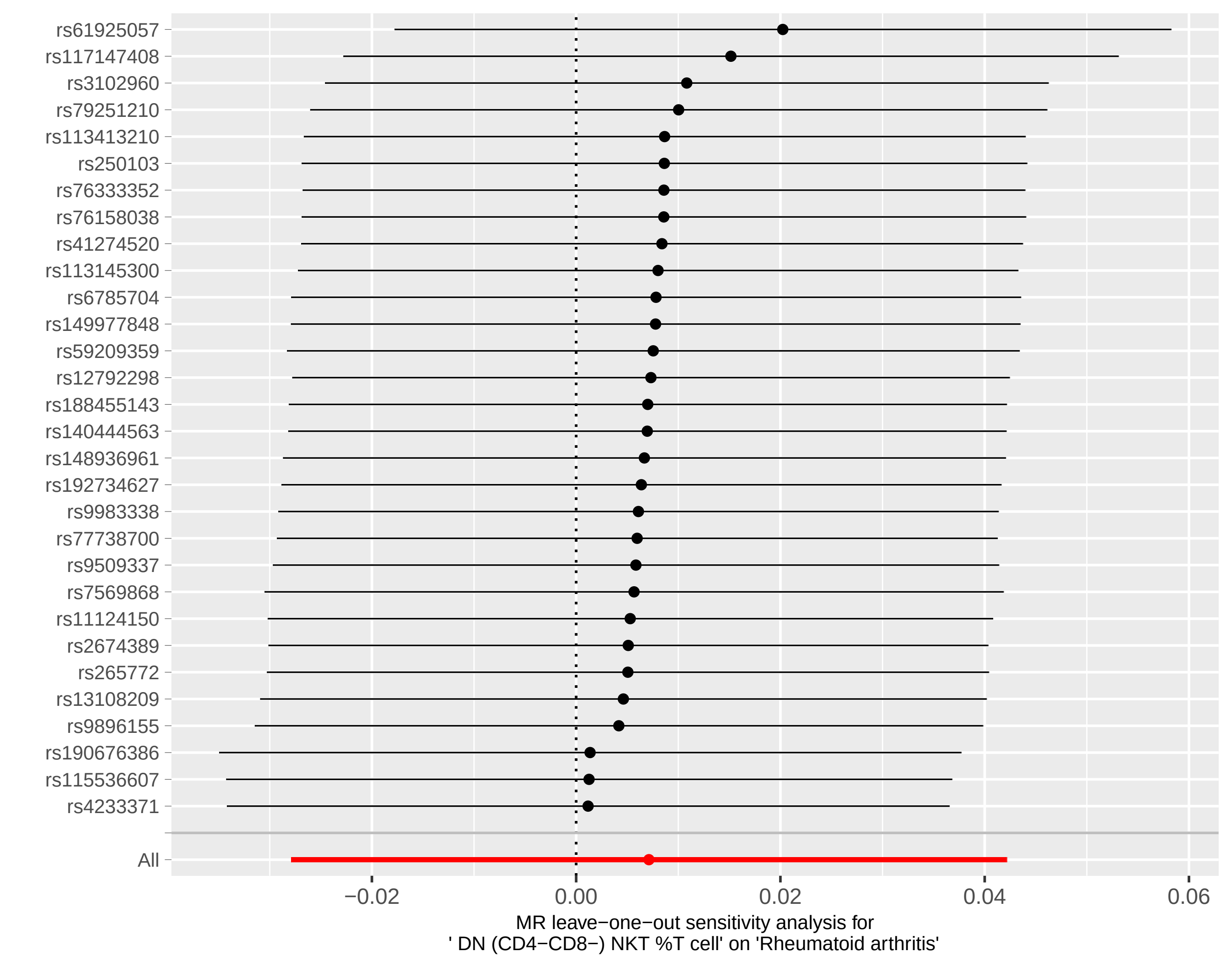
rs61925057
rs117147408
rs3102960
rs79251210
rs113413210
rs250103
rs76333352
rs76158038
rs41274520
rs113145300
rs6785704
rs149977848
rs59209359
rs12792298
rs188455143
rs140444563
rs148936961
rs192734627
rs9983338
rs77738700
rs9509337
rs7569868
rs11124150
rs2674389
rs265772
rs13108209
rs9896155
rs190676386
rs115536607
rs4233371
All
−0.02
0.00
0.02
0.04
0.06
MR leave−one−out sensitivity analysis for
' DN (CD4−CD8−) NKT %T cell' on 'Rheumatoid arthritis'

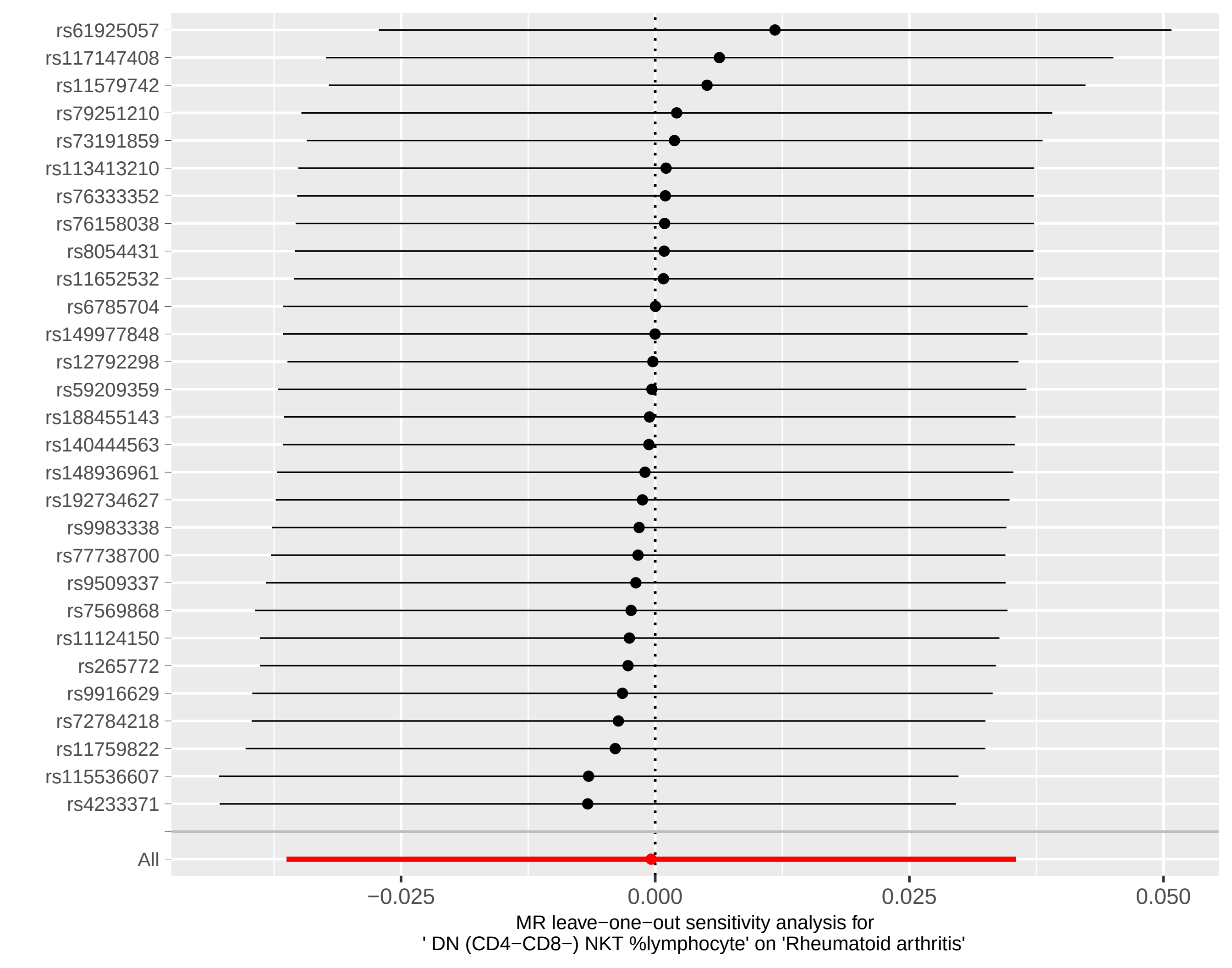
rs61925057
rs117147408
rs11579742
rs79251210
rs73191859
rs113413210
rs76333352
rs76158038
rs8054431
rs11652532
rs6785704
rs149977848
rs12792298
rs59209359
rs188455143
rs140444563
rs148936961
rs192734627
rs9983338
rs77738700
rs9509337
rs7569868
rs11124150
rs265772
rs9916629
rs72784218
rs11759822
rs115536607
rs4233371
All
−0.025
0.000
0.025
0.050
MR leave−one−out sensitivity analysis for
' DN (CD4−CD8−) NKT %lymphocyte' on 'Rheumatoid arthritis'

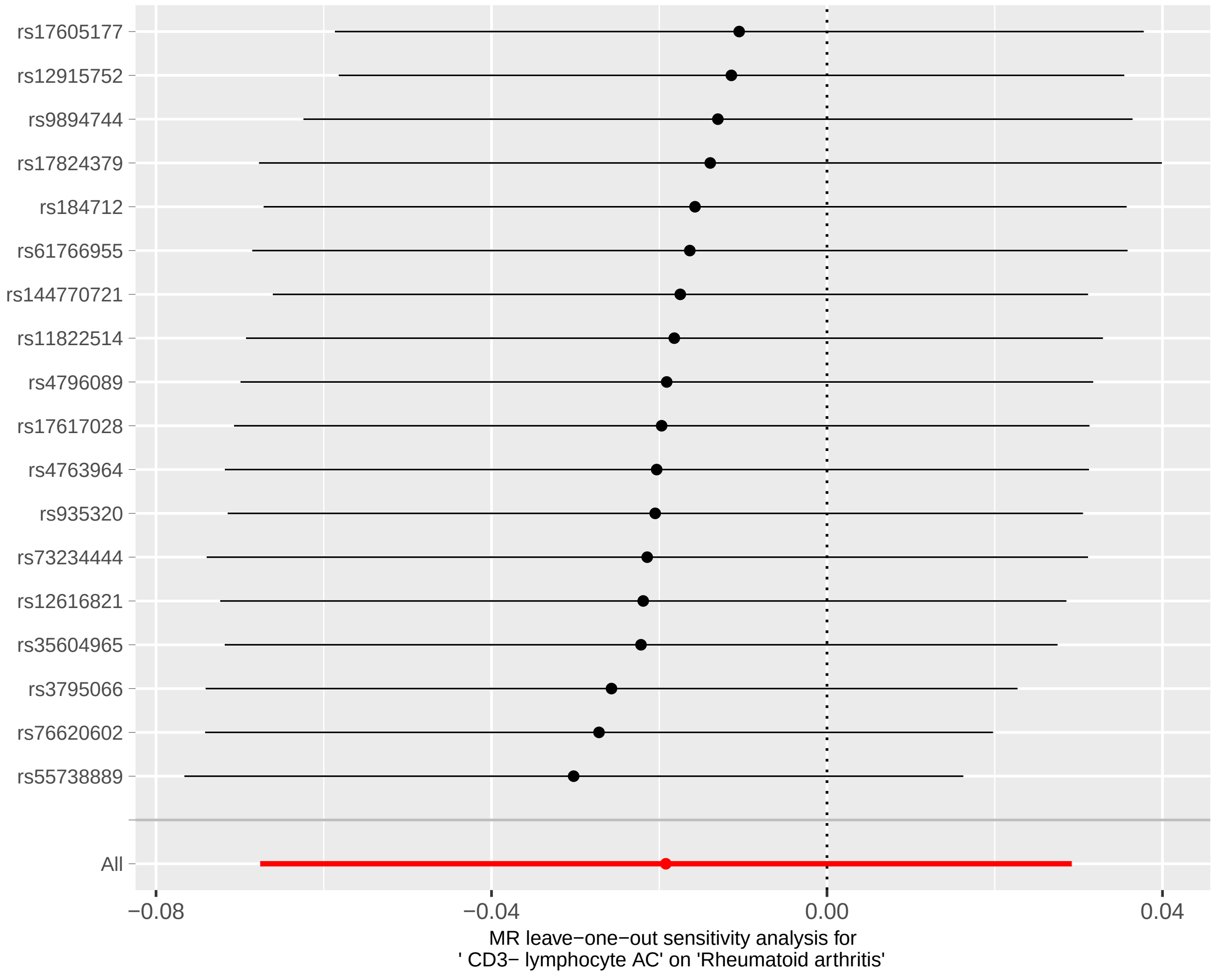
rs17605177
rs12915752
rs9894744
rs17824379
rs184712
rs61766955
rs144770721
rs11822514
rs4796089
rs17617028
rs4763964
rs935320
rs73234444
rs12616821
rs35604965
rs3795066
rs76620602
rs55738889
All
−0.08
−0.04
0.00
0.04
MR leave−one−out sensitivity analysis for
' CD3− lymphocyte AC' on 'Rheumatoid arthritis'

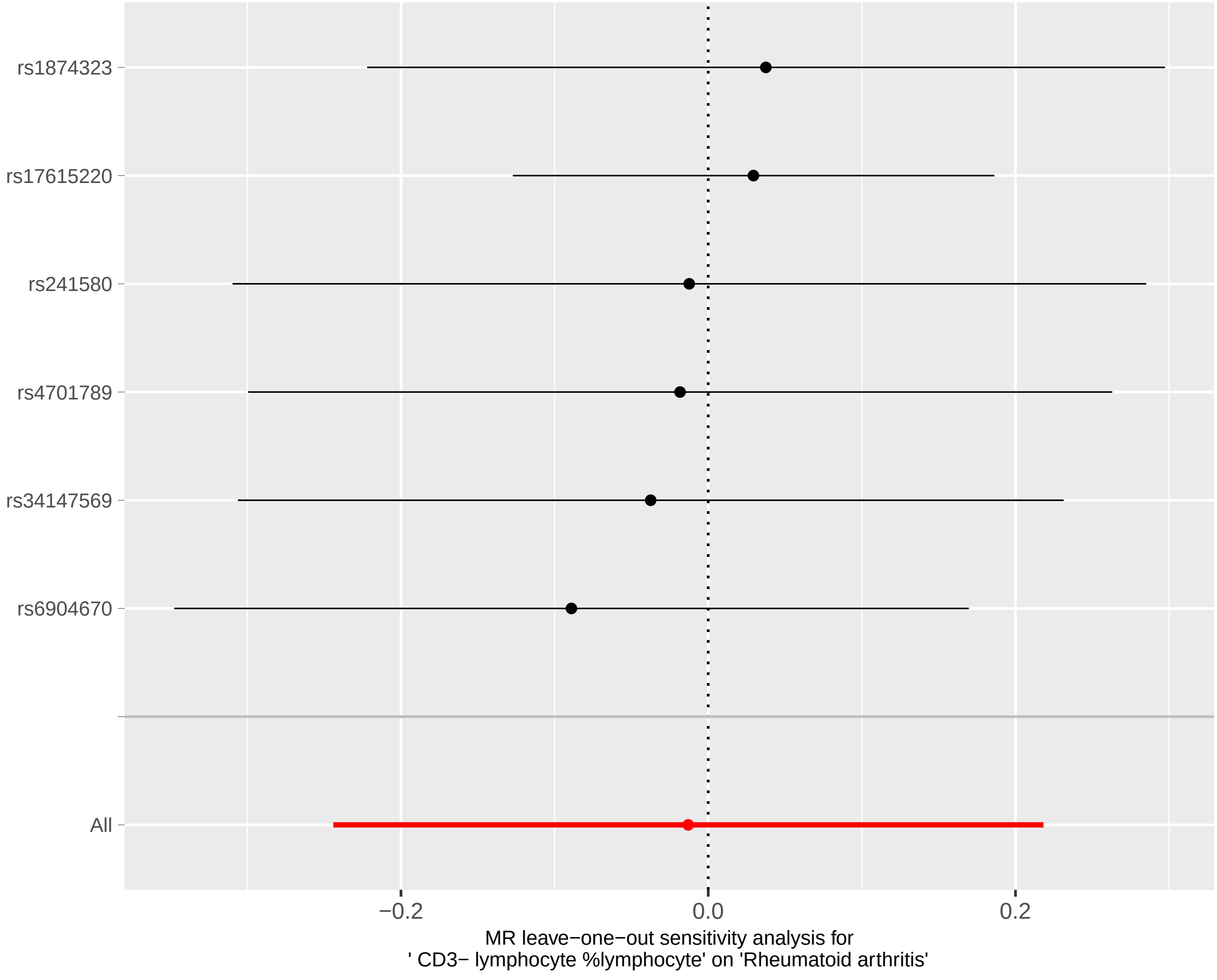

rs1874323
rs17615220
rs241580
rs4701789
rs34147569
rs6904670
All
−0.2
0.0
0.2
MR leave−one−out sensitivity analysis for
' CD3− lymphocyte %lymphocyte' on 'Rheumatoid arthritis'

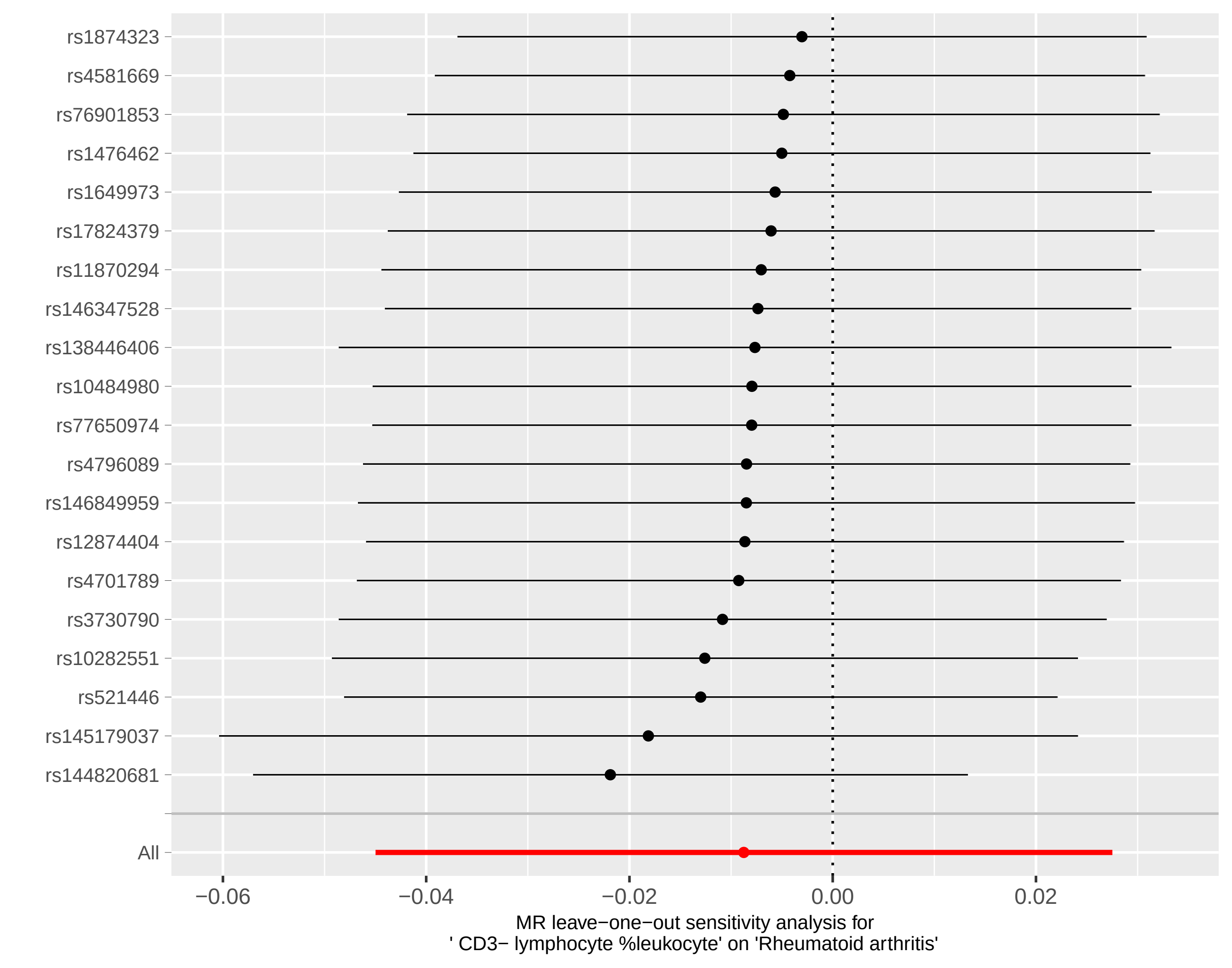
rs1874323
rs4581669
rs76901853
rs1476462
rs1649973
rs17824379
rs11870294
rs146347528
rs138446406
rs10484980
rs77650974
rs4796089
rs146849959
rs12874404
rs4701789
rs3730790
rs10282551
rs521446
rs145179037
rs144820681
All
−0.06
−0.04
−0.02
0.00
0.02
MR leave−one−out sensitivity analysis for
' CD3− lymphocyte %leukocyte' on 'Rheumatoid arthritis'

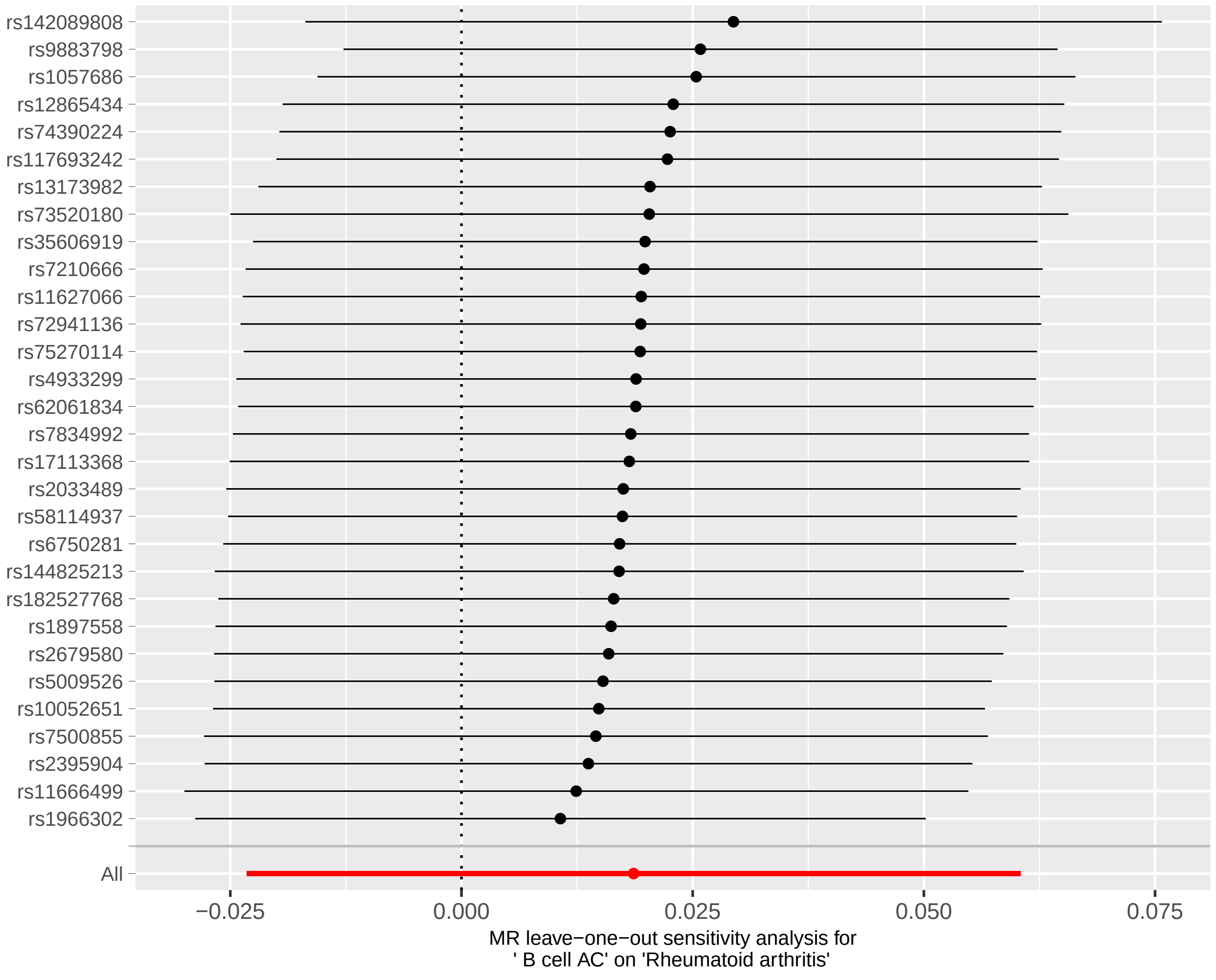

rs142089808
rs9883798
rs1057686
rs12865434
rs74390224
rs117693242
rs13173982
rs73520180
rs35606919
rs7210666
rs11627066
rs72941136
rs75270114
rs4933299
rs62061834
rs7834992
rs17113368
rs2033489
rs58114937
rs6750281
rs144825213
rs182527768
rs1897558
rs2679580
rs5009526
rs10052651
rs7500855
rs2395904
rs11666499
rs1966302
All
−0.025
0.000
0.025
0.050
0.075
MR leave−one−out sensitivity analysis for
' B cell AC' on 'Rheumatoid arthritis'

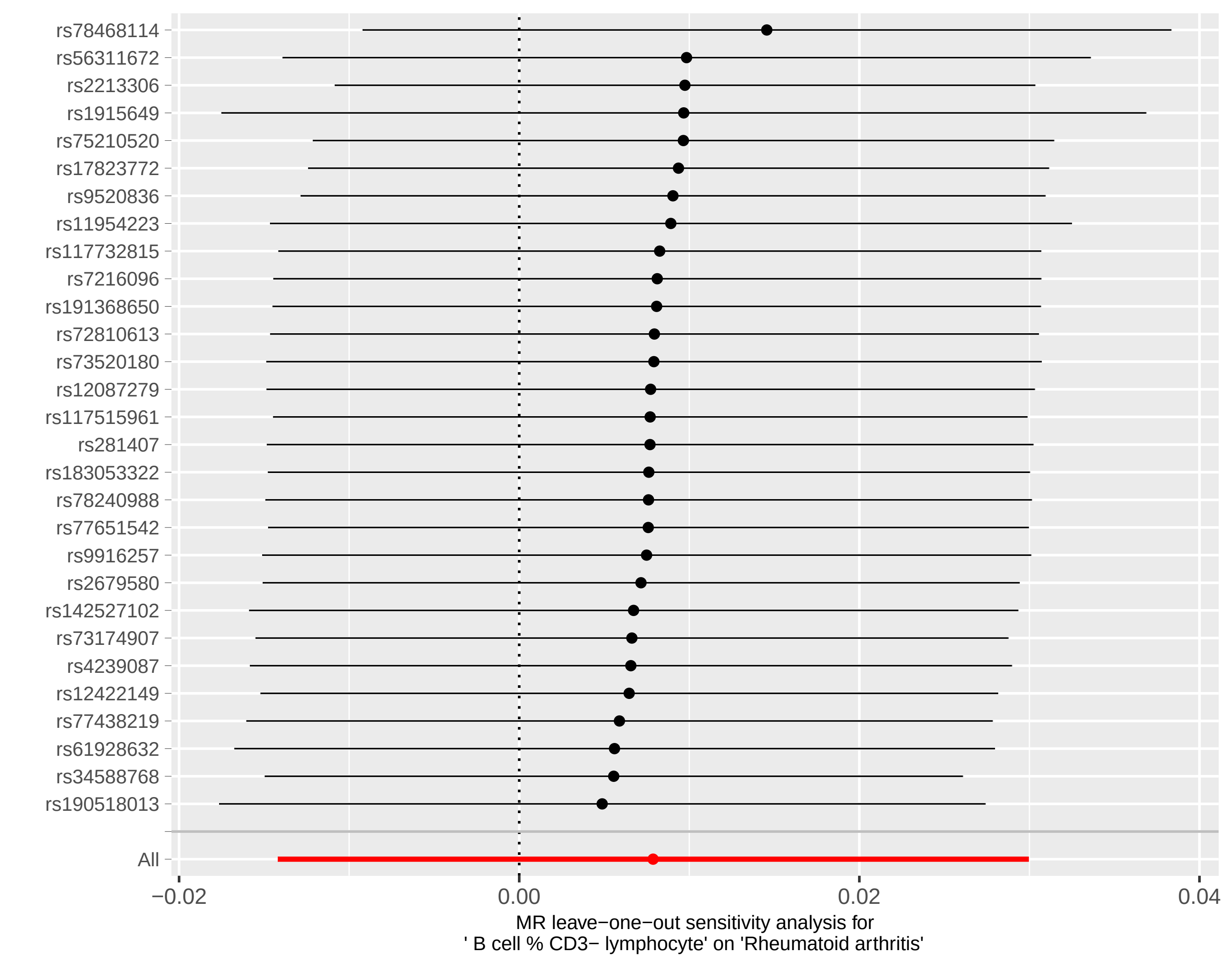
rs78468114
rs56311672
rs2213306
rs1915649
rs75210520
rs17823772
rs9520836
rs11954223
rs117732815
rs7216096
rs191368650
rs72810613
rs73520180
rs12087279
rs117515961
rs281407
rs183053322
rs78240988
rs77651542
rs9916257
rs2679580
rs142527102
rs73174907
rs4239087
rs12422149
rs77438219
rs61928632
rs34588768
rs190518013
All
−0.02
0.00
0.02
0.04
MR leave−one−out sensitivity analysis for
' B cell % CD3− lymphocyte' on 'Rheumatoid arthritis'

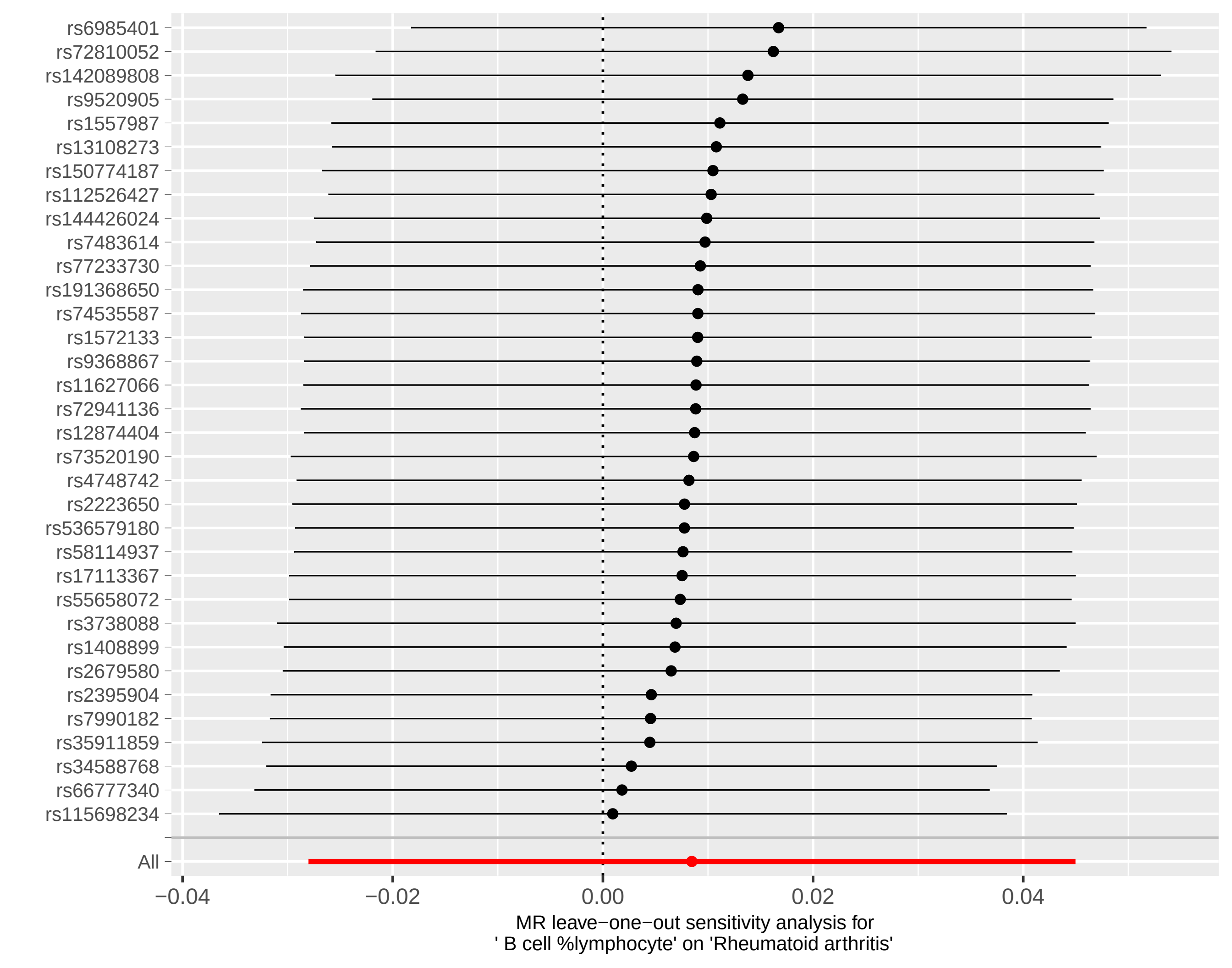
rs6985401
rs72810052
rs142089808
rs9520905
rs1557987
rs13108273
rs150774187
rs112526427
rs144426024
rs7483614
rs77233730
rs191368650
rs74535587
rs1572133
rs9368867
rs11627066
rs72941136
rs12874404
rs73520190
rs4748742
rs2223650
rs536579180
rs58114937
rs17113367
rs55658072
rs3738088
rs1408899
rs2679580
rs2395904
rs7990182
rs35911859
rs34588768
rs66777340
rs115698234
All
−0.04
−0.02
0.00
0.02
0.04
MR leave−one−out sensitivity analysis for
' B cell %lymphocyte' on 'Rheumatoid arthritis'

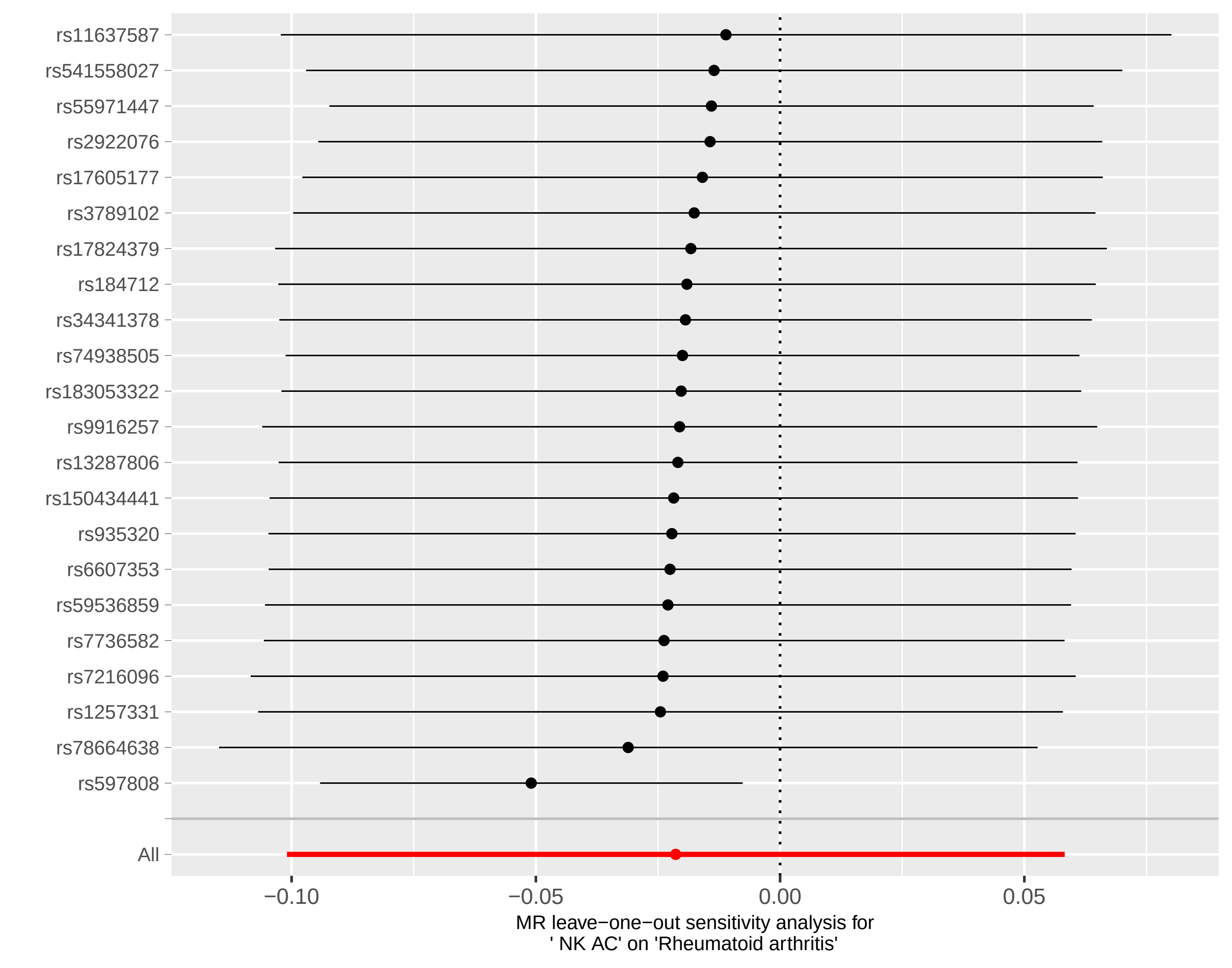
rs11637587
rs541558027
rs55971447
rs2922076
rs17605177
rs3789102
rs17824379
rs184712
rs34341378
rs74938505
rs183053322
rs9916257
rs13287806
rs150434441
rs935320
rs6607353
rs59536859
rs7736582
rs7216096
rs1257331
rs78664638
rs597808
All
−0.10
−0.05
0.00
0.05
MR leave−one−out sensitivity analysis for
' NK AC' on 'Rheumatoid arthritis'

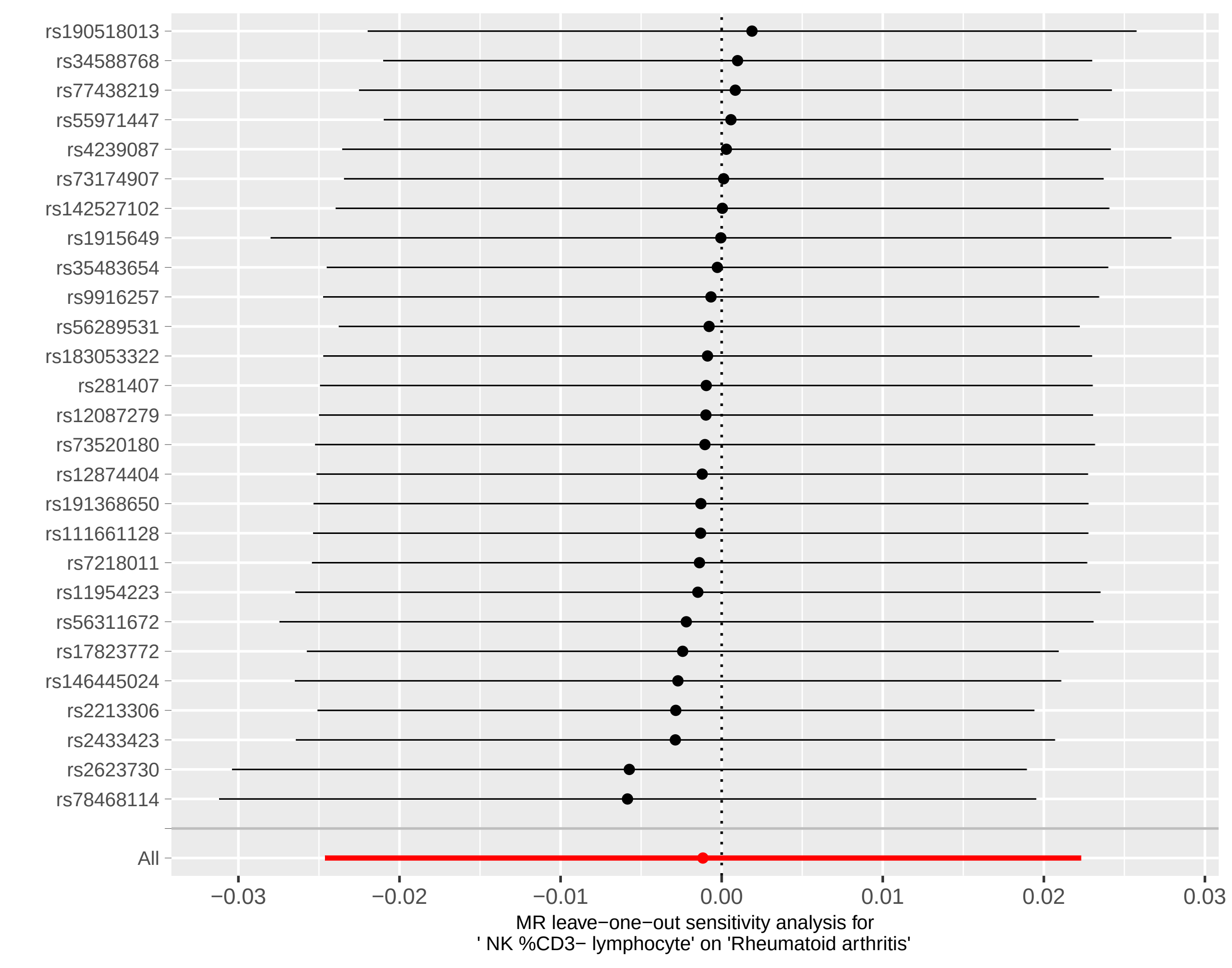
rs190518013
rs34588768
rs77438219
rs55971447
rs4239087
rs73174907
rs142527102
rs1915649
rs35483654
rs9916257
rs56289531
rs183053322
rs281407
rs12087279
rs73520180
rs12874404
rs191368650
rs111661128
rs7218011
rs11954223
rs56311672
rs17823772
rs146445024
rs2213306
rs2433423
rs2623730
rs78468114
All
−0.03
−0.02
−0.01
0.00
0.01
0.02
0.03
MR leave−one−out sensitivity analysis for
' NK %CD3− lymphocyte' on 'Rheumatoid arthritis'

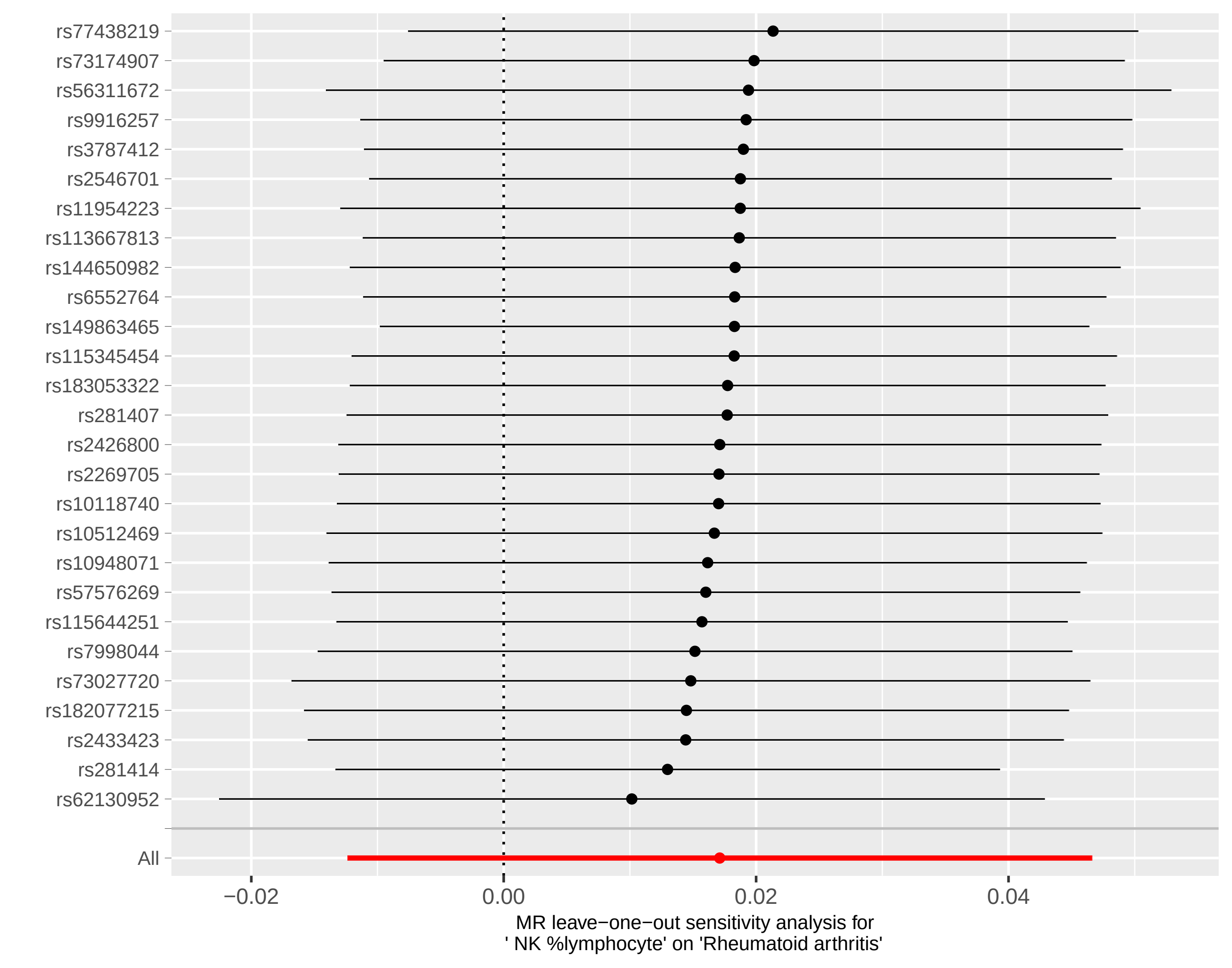

rs77438219
rs73174907
rs56311672
rs9916257
rs3787412
rs2546701
rs11954223
rs113667813
rs144650982
rs6552764
rs149863465
rs115345454
rs183053322
rs281407
rs2426800
rs2269705
rs10118740
rs10512469
rs10948071
rs57576269
rs115644251
rs7998044
rs73027720
rs182077215
rs2433423
rs281414
rs62130952
All
−0.02
0.00
0.02
0.04
MR leave−one−out sensitivity analysis for
' NK %lymphocyte' on 'Rheumatoid arthritis'

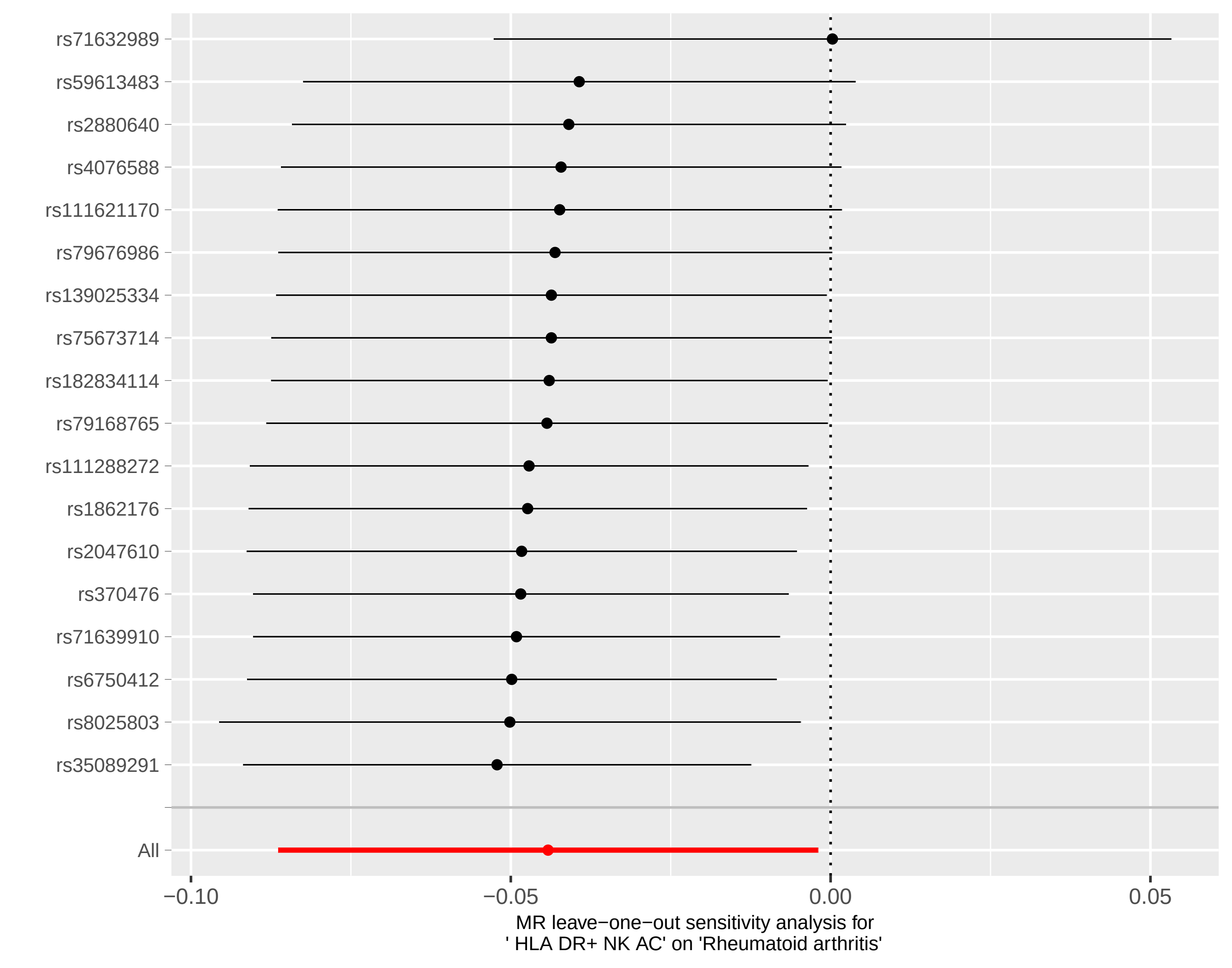

rs71632989
rs59613483
rs2880640
rs4076588
rs111621170
rs79676986
rs139025334
rs75673714
rs182834114
rs79168765
rs111288272
rs1862176
rs2047610
rs370476
rs71639910
rs6750412
rs8025803
rs35089291
All
−0.10
−0.05
0.00
0.05
MR leave−one−out sensitivity analysis for
' HLA DR+ NK AC' on 'Rheumatoid arthritis'

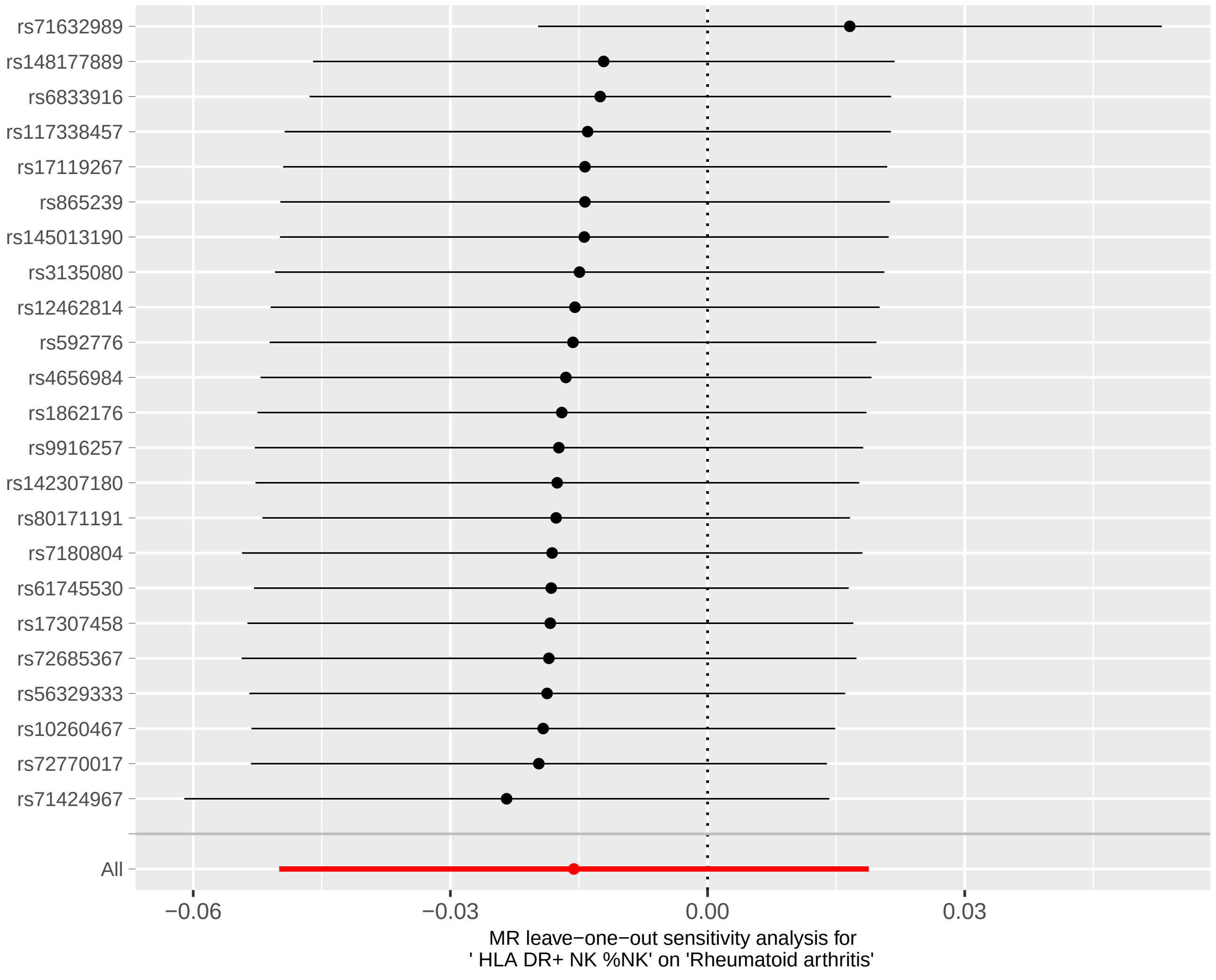
rs71632989
rs148177889
rs6833916
rs117338457
rs17119267
rs865239
rs145013190
rs3135080
rs12462814
rs592776
rs4656984
rs1862176
rs9916257
rs142307180
rs80171191
rs7180804
rs61745530
rs17307458
rs72685367
rs56329333
rs10260467
rs72770017
rs71424967
All
−0.06
−0.03
0.00
0.03
MR leave−one−out sensitivity analysis for
' HLA DR+ NK %NK' on 'Rheumatoid arthritis'

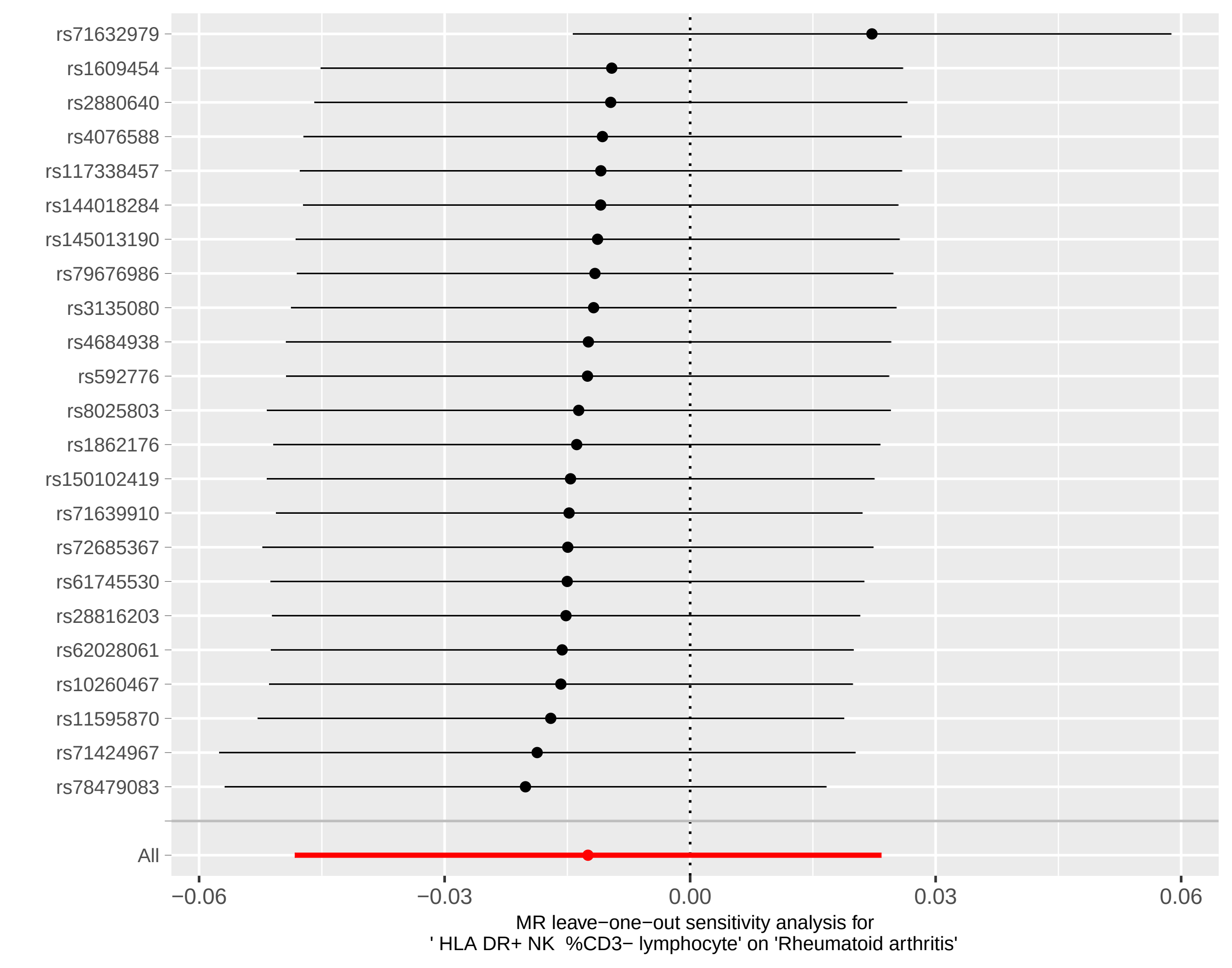

rs71632979
rs1609454
rs2880640
rs4076588
rs117338457
rs144018284
rs145013190
rs79676986
rs3135080
rs4684938
rs592776
rs8025803
rs1862176
rs150102419
rs71639910
rs72685367
rs61745530
rs28816203
rs62028061
rs10260467
rs11595870
rs71424967
rs78479083
All
−0.06
−0.03
0.00
0.03
0.06
MR leave−one−out sensitivity analysis for
' HLA DR+ NK %CD3− lymphocyte' on 'Rheumatoid arthritis'

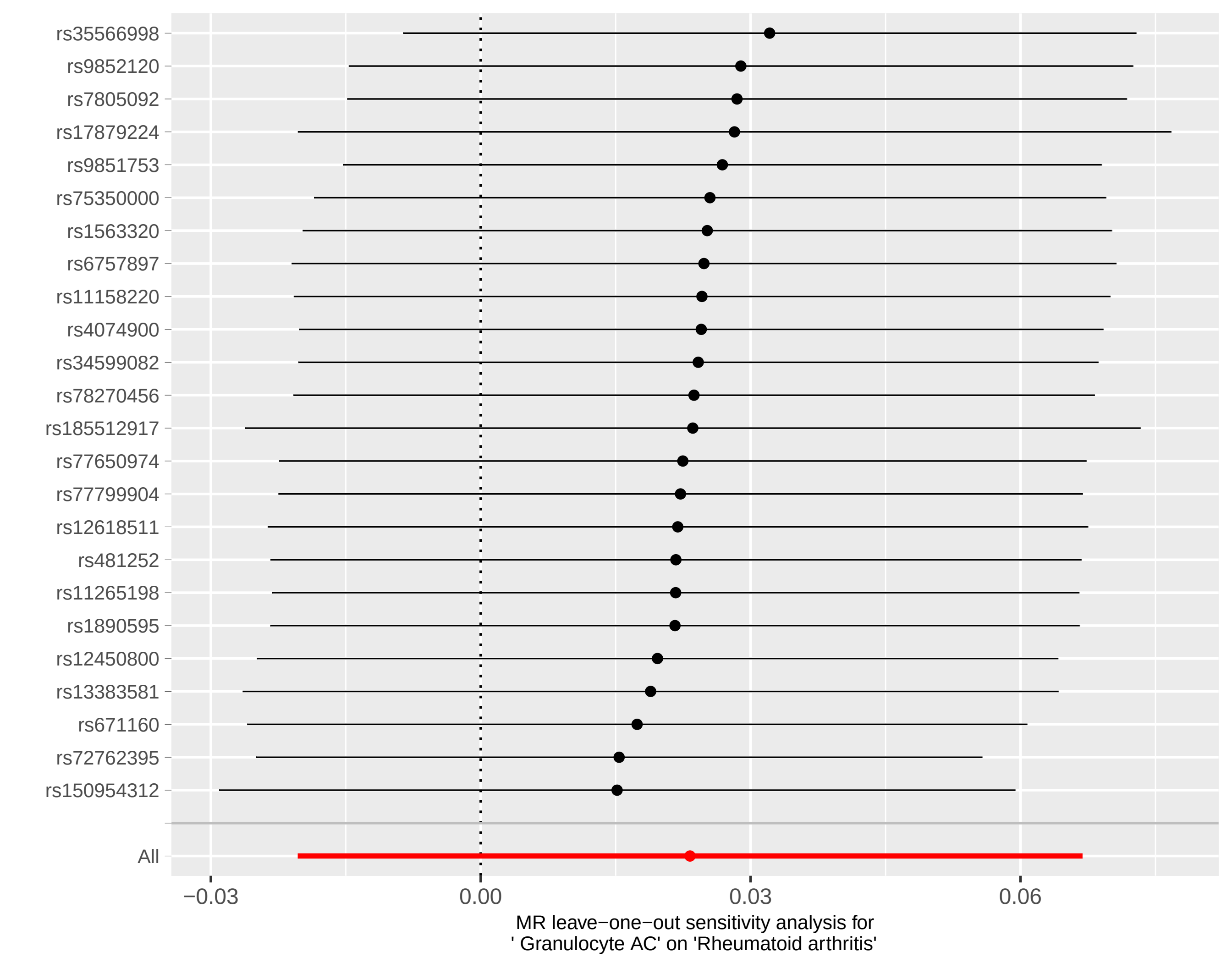

rs35566998
rs9852120
rs7805092
rs17879224
rs9851753
rs75350000
rs1563320
rs6757897
rs11158220
rs4074900
rs34599082
rs78270456
rs185512917
rs77650974
rs77799904
rs12618511
rs481252
rs11265198
rs1890595
rs12450800
rs13383581
rs671160
rs72762395
rs150954312
All
−0.03
0.00
0.03
0.06
MR leave−one−out sensitivity analysis for
' Granulocyte AC' on 'Rheumatoid arthritis'

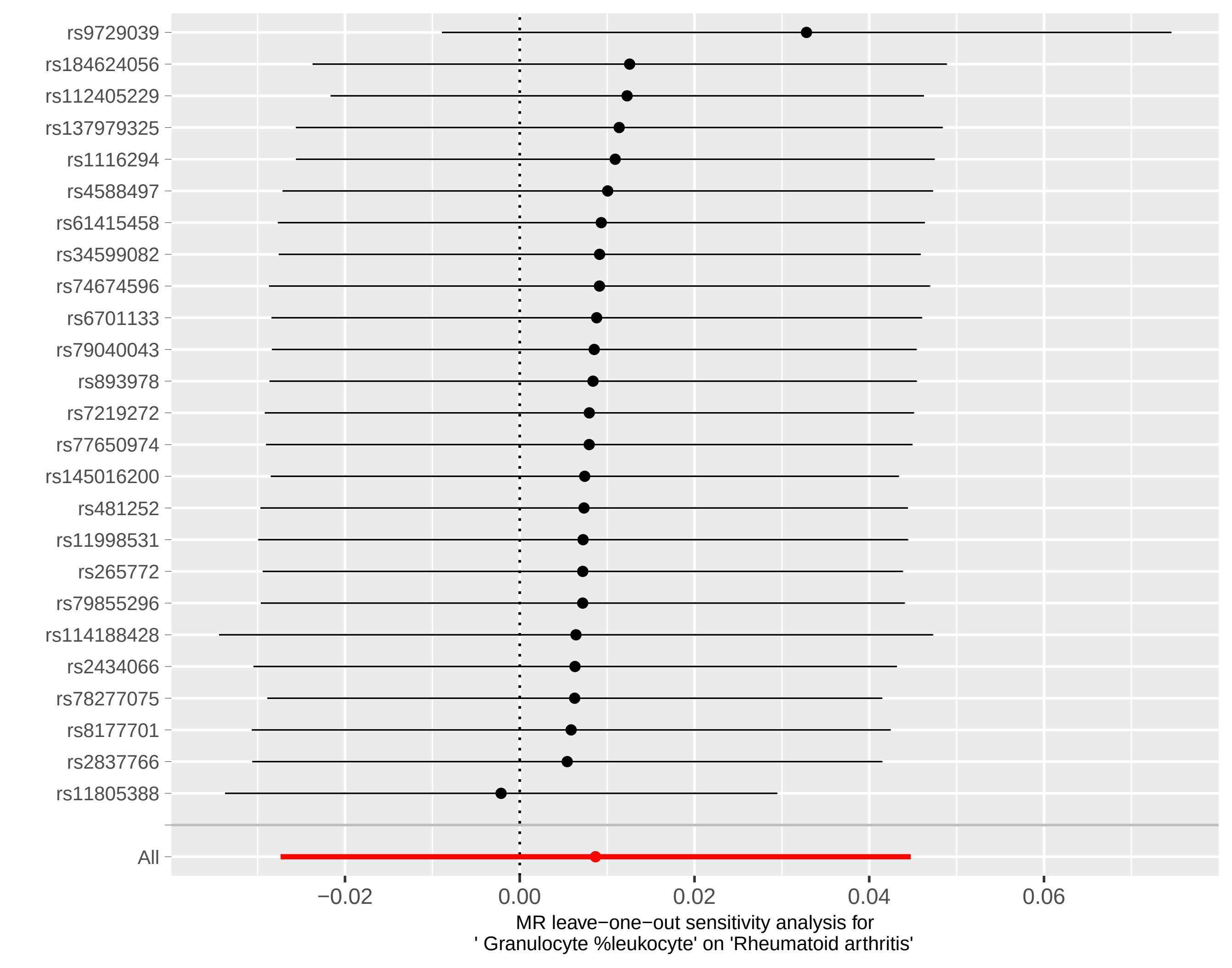
rs9729039
rs184624056
rs112405229
rs137979325
rs1116294
rs4588497
rs61415458
rs34599082
rs74674596
rs6701133
rs79040043
rs893978
rs7219272
rs77650974
rs145016200
rs481252
rs11998531
rs265772
rs79855296
rs114188428
rs2434066
rs78277075
rs8177701
rs2837766
rs11805388
All
−0.02
0.00
0.02
0.04
0.06
MR leave−one−out sensitivity analysis for
' Granulocyte %leukocyte' on 'Rheumatoid arthritis'

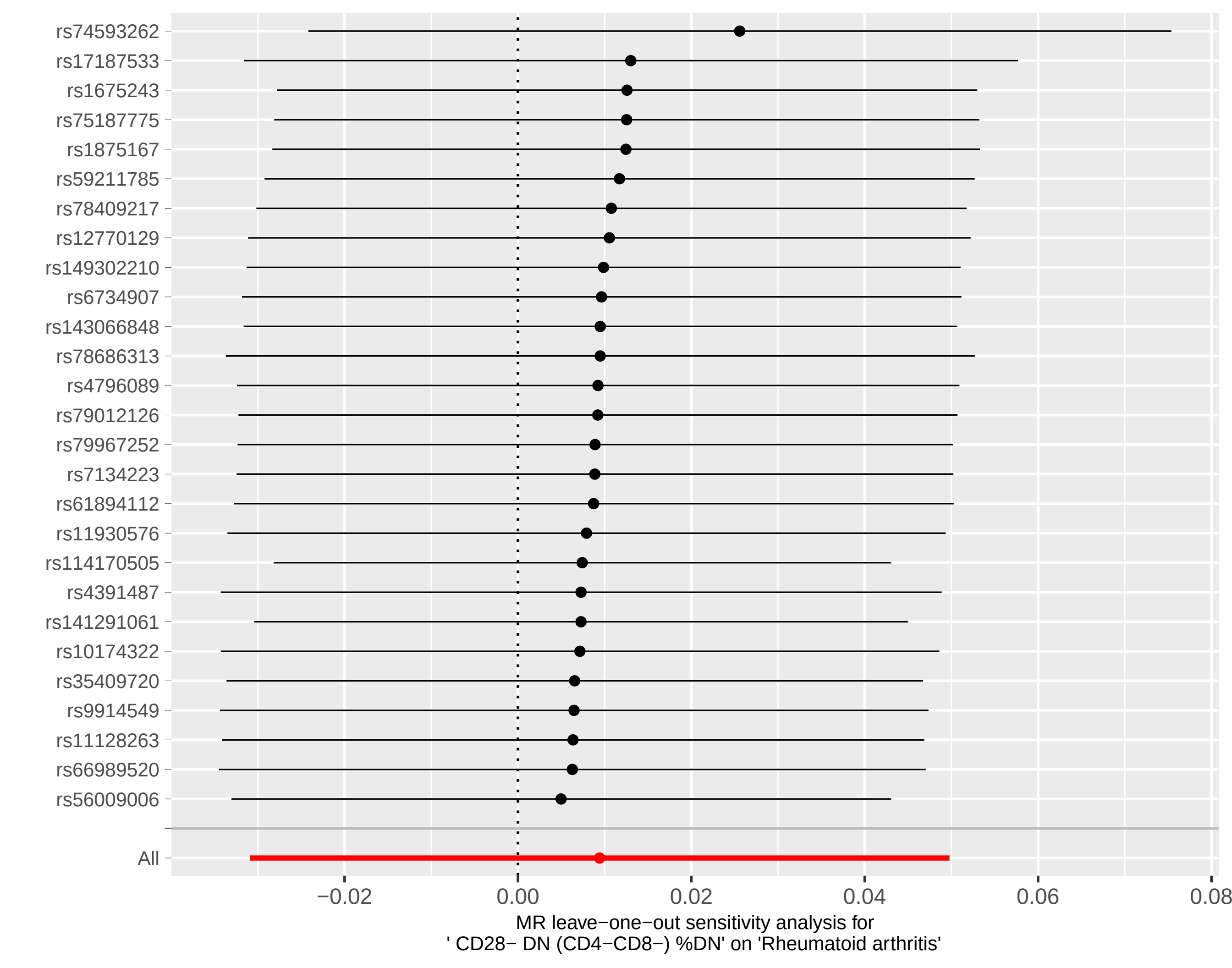
rs74593262
rs17187533
rs1675243
rs75187775
rs1875167
rs59211785
rs78409217
rs12770129
rs149302210
rs6734907
rs143066848
rs78686313
rs4796089
rs79012126
rs79967252
rs7134223
rs61894112
rs11930576
rs114170505
rs4391487
rs141291061
rs10174322
rs35409720
rs9914549
rs11128263
rs66989520
rs56009006
All
−0.02
0.00
0.02
0.04
0.06
0.08
MR leave−one−out sensitivity analysis for
' CD28− DN (CD4−CD8−) %DN' on 'Rheumatoid arthritis'

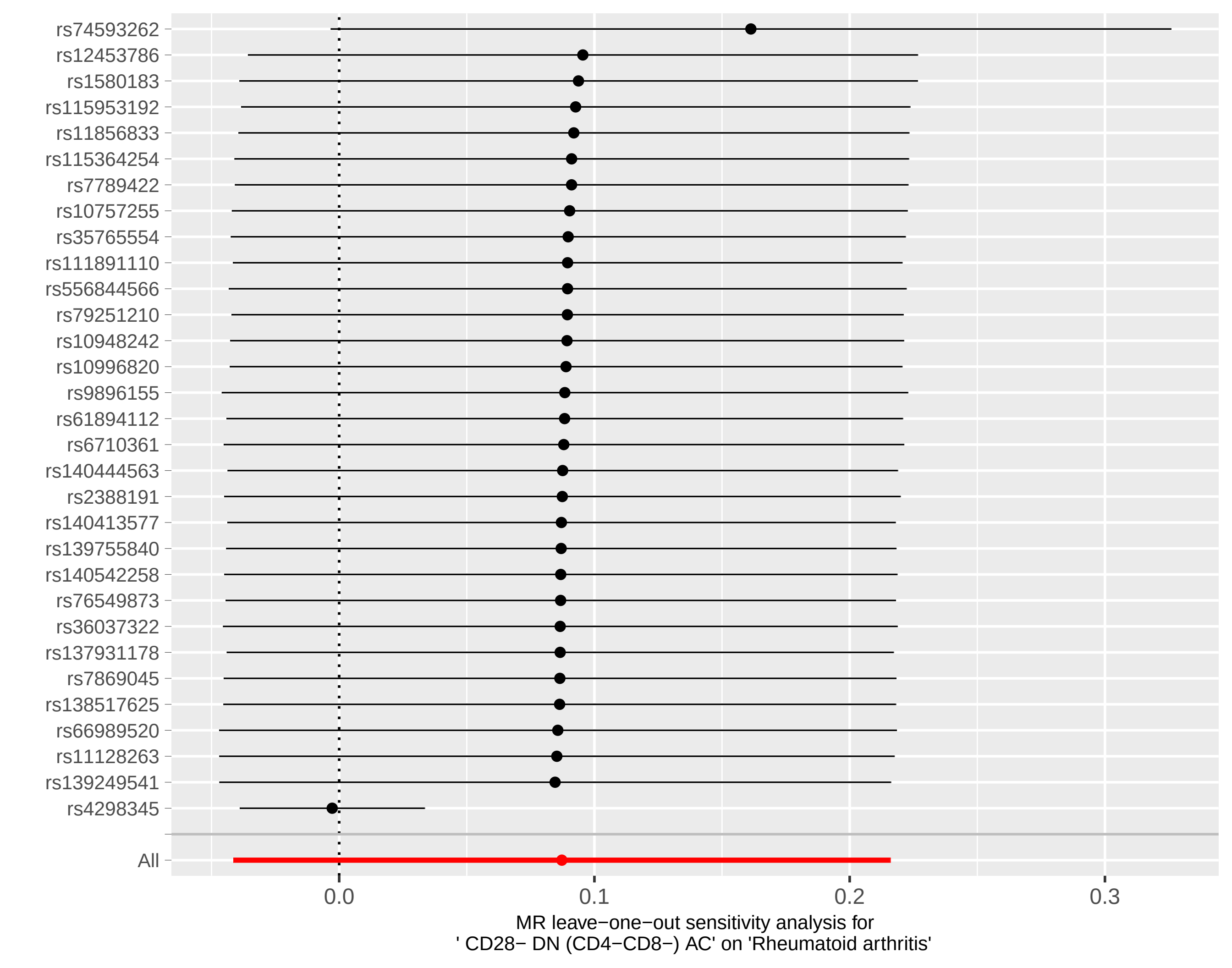
rs74593262
rs12453786
rs1580183
rs115953192
rs11856833
rs115364254
rs7789422
rs10757255
rs35765554
rs111891110
rs556844566
rs79251210
rs10948242
rs10996820
rs9896155
rs61894112
rs6710361
rs140444563
rs2388191
rs140413577
rs139755840
rs140542258
rs76549873
rs36037322
rs137931178
rs7869045
rs138517625
rs66989520
rs11128263
rs139249541
rs4298345
All
0.0
0.1
0.2
0.3
MR leave−one−out sensitivity analysis for
' CD28− DN (CD4−CD8−) AC' on 'Rheumatoid arthritis'

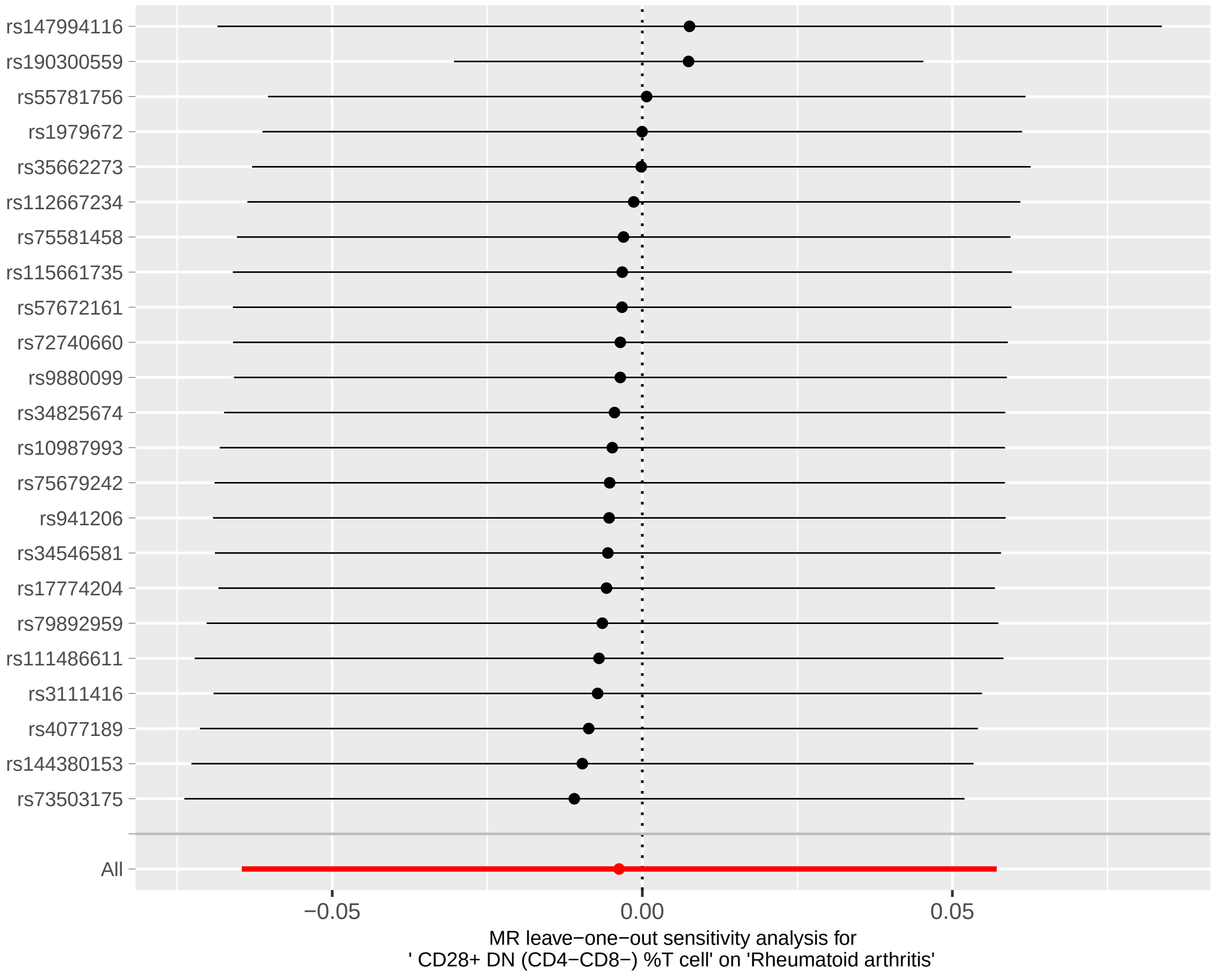
rs147994116
rs190300559
rs55781756
rs1979672
rs35662273
rs112667234
rs75581458
rs115661735
rs57672161
rs72740660
rs9880099
rs34825674
rs10987993
rs75679242
rs941206
rs34546581
rs17774204
rs79892959
rs111486611
rs3111416
rs4077189
rs144380153
rs73503175
All
−0.05
0.00
0.05
MR leave−one−out sensitivity analysis for
' CD28+ DN (CD4−CD8−) %T cell' on 'Rheumatoid arthritis'

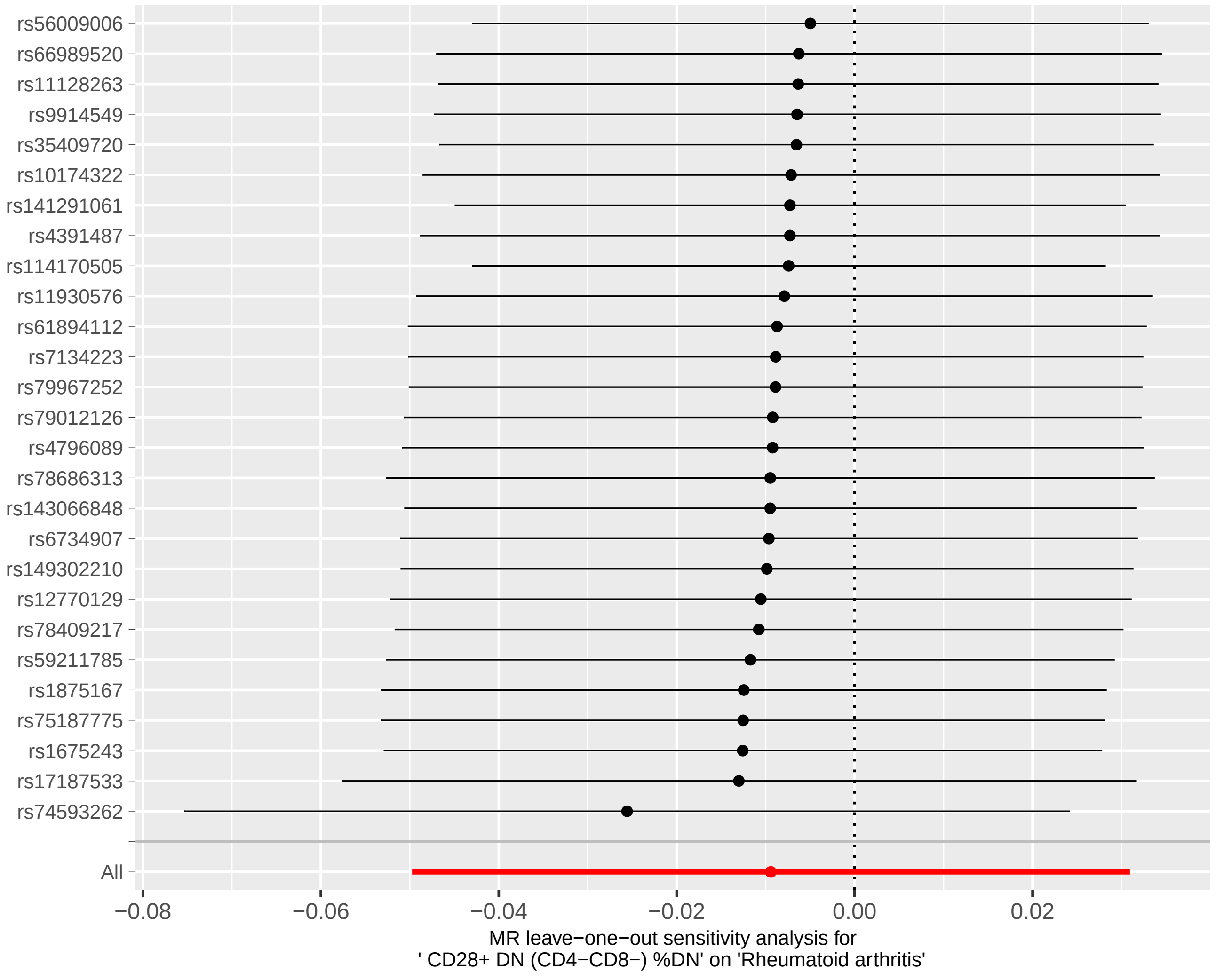

rs56009006
rs66989520
rs11128263
rs9914549
rs35409720
rs10174322
rs141291061
rs4391487
rs114170505
rs11930576
rs61894112
rs7134223
rs79967252
rs79012126
rs4796089
rs78686313
rs143066848
rs6734907
rs149302210
rs12770129
rs78409217
rs59211785
rs1875167
rs75187775
rs1675243
rs17187533
rs74593262
All
−0.08
−0.06
−0.04
−0.02
0.00
0.02
MR leave−one−out sensitivity analysis for
' CD28+ DN (CD4−CD8−) %DN' on 'Rheumatoid arthritis'

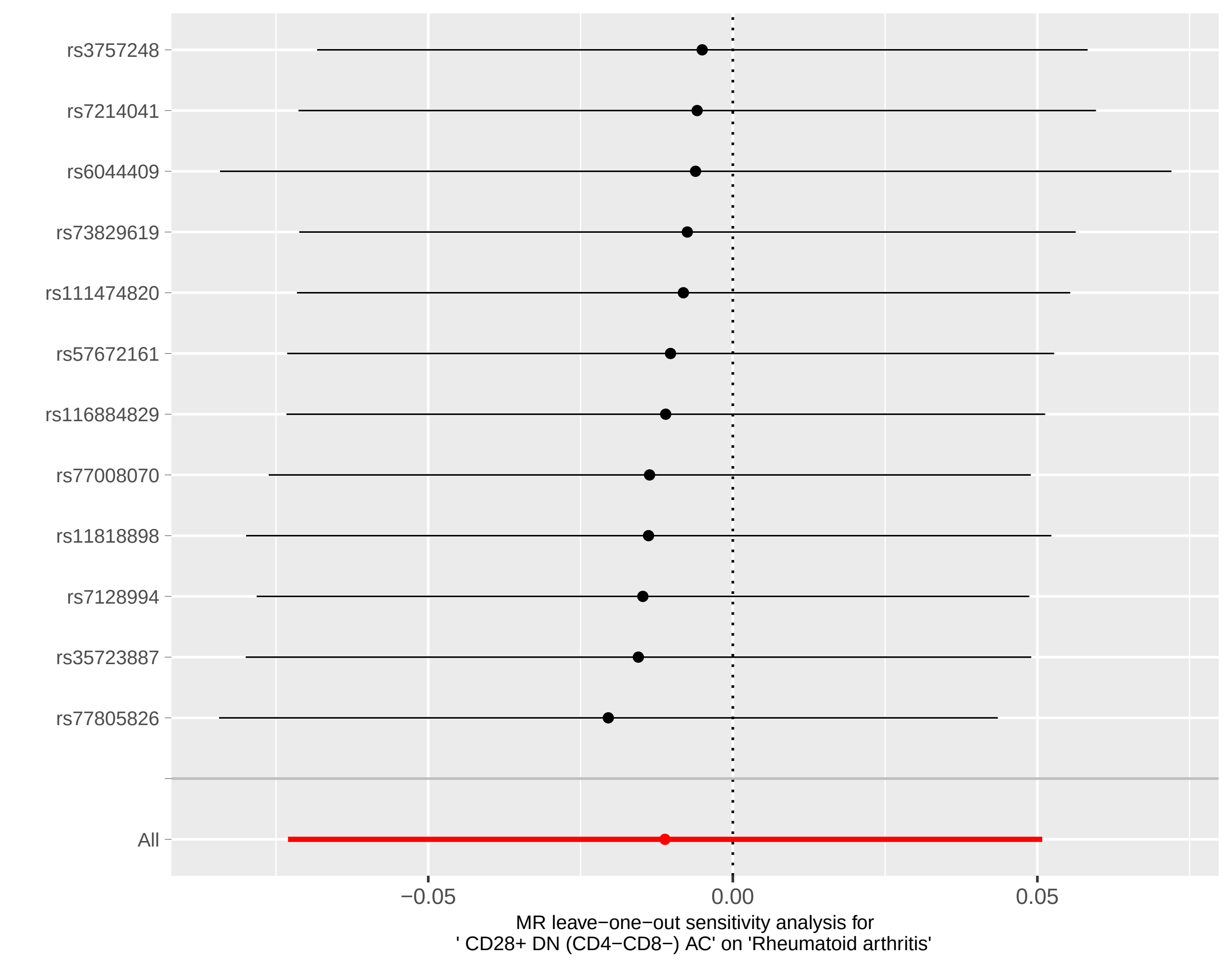
rs3757248
rs7214041
rs6044409
rs73829619
rs111474820
rs57672161
rs116884829
rs77008070
rs11818898
rs7128994
rs35723887
rs77805826
All
−0.05
0.00
0.05
MR leave−one−out sensitivity analysis for
' CD28+ DN (CD4−CD8−) AC' on 'Rheumatoid arthritis'

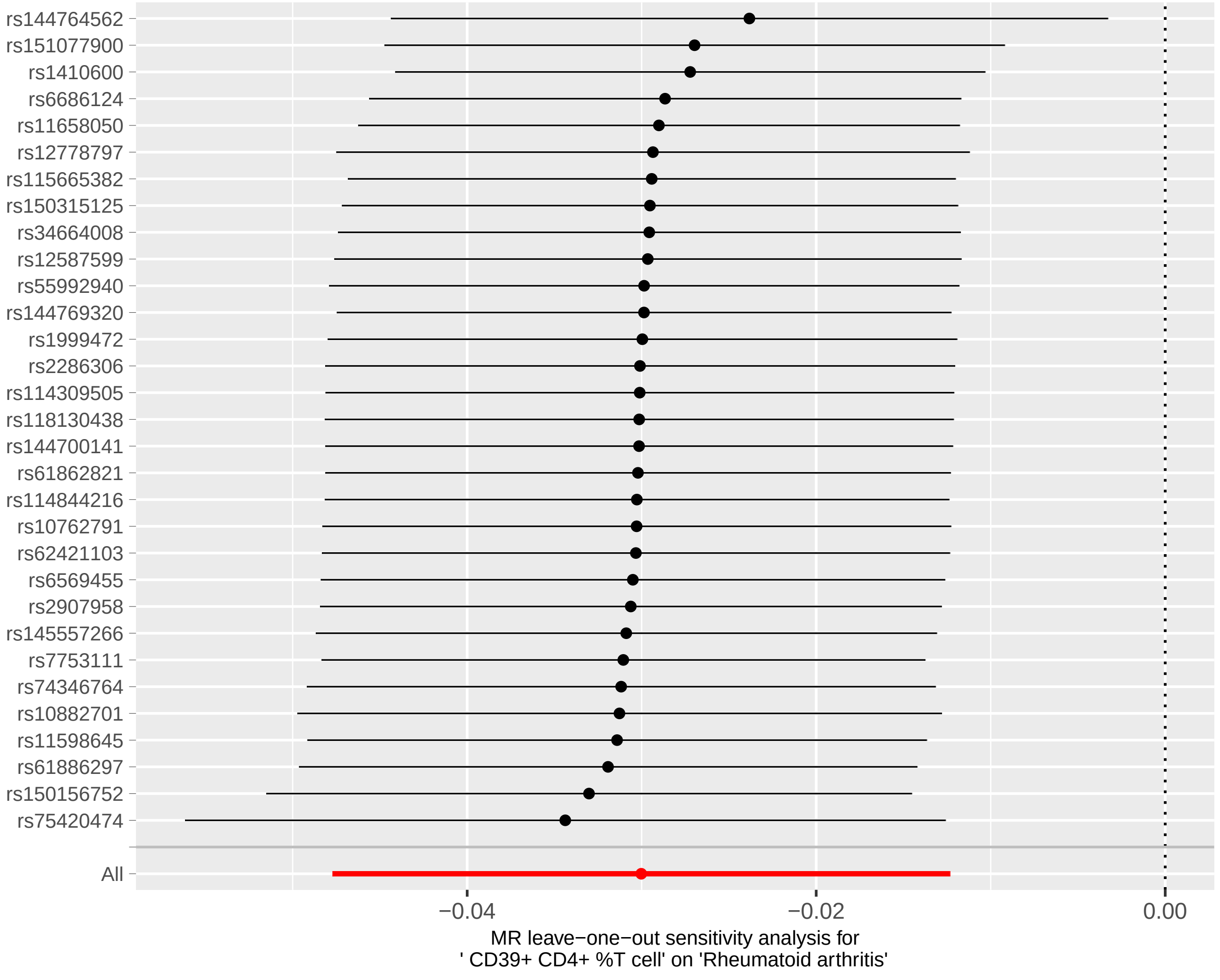
rs144764562
rs151077900
rs1410600
rs6686124
rs11658050
rs12778797
rs115665382
rs150315125
rs34664008
rs12587599
rs55992940
rs144769320
rs1999472
rs2286306
rs114309505
rs118130438
rs144700141
rs61862821
rs114844216
rs10762791
rs62421103
rs6569455
rs2907958
rs145557266
rs7753111
rs74346764
rs10882701
rs11598645
rs61886297
rs150156752
rs75420474
All
−0.04
−0.02
0.00
MR leave−one−out sensitivity analysis for
' CD39+ CD4+ %T cell' on 'Rheumatoid arthritis'

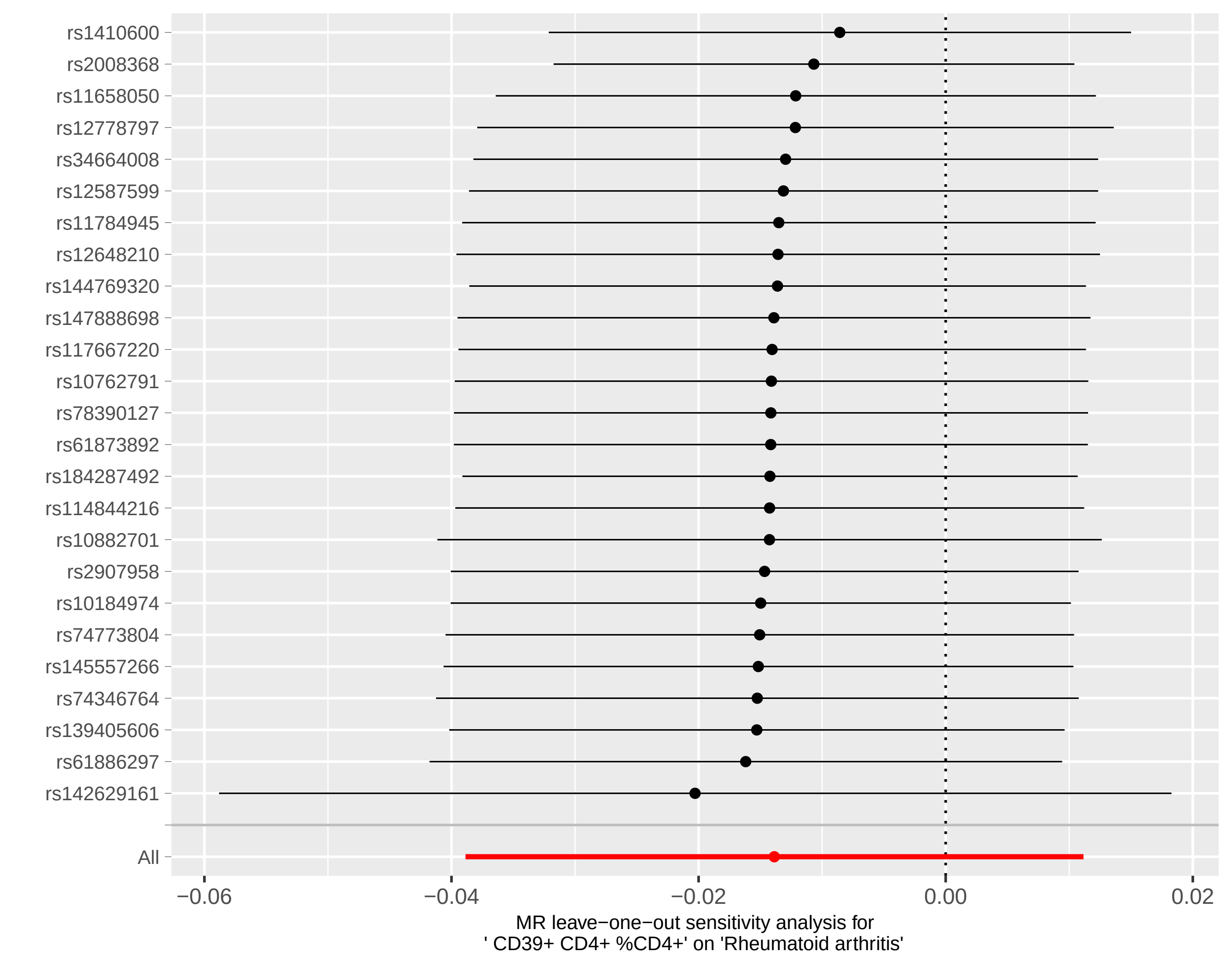
rs1410600
rs2008368
rs11658050
rs12778797
rs34664008
rs12587599
rs11784945
rs12648210
rs144769320
rs147888698
rs117667220
rs10762791
rs78390127
rs61873892
rs184287492
rs114844216
rs10882701
rs2907958
rs10184974
rs74773804
rs145557266
rs74346764
rs139405606
rs61886297
rs142629161
All
−0.06
−0.04
−0.02
0.00
0.02
MR leave−one−out sensitivity analysis for
' CD39+ CD4+ %CD4+' on 'Rheumatoid arthritis'

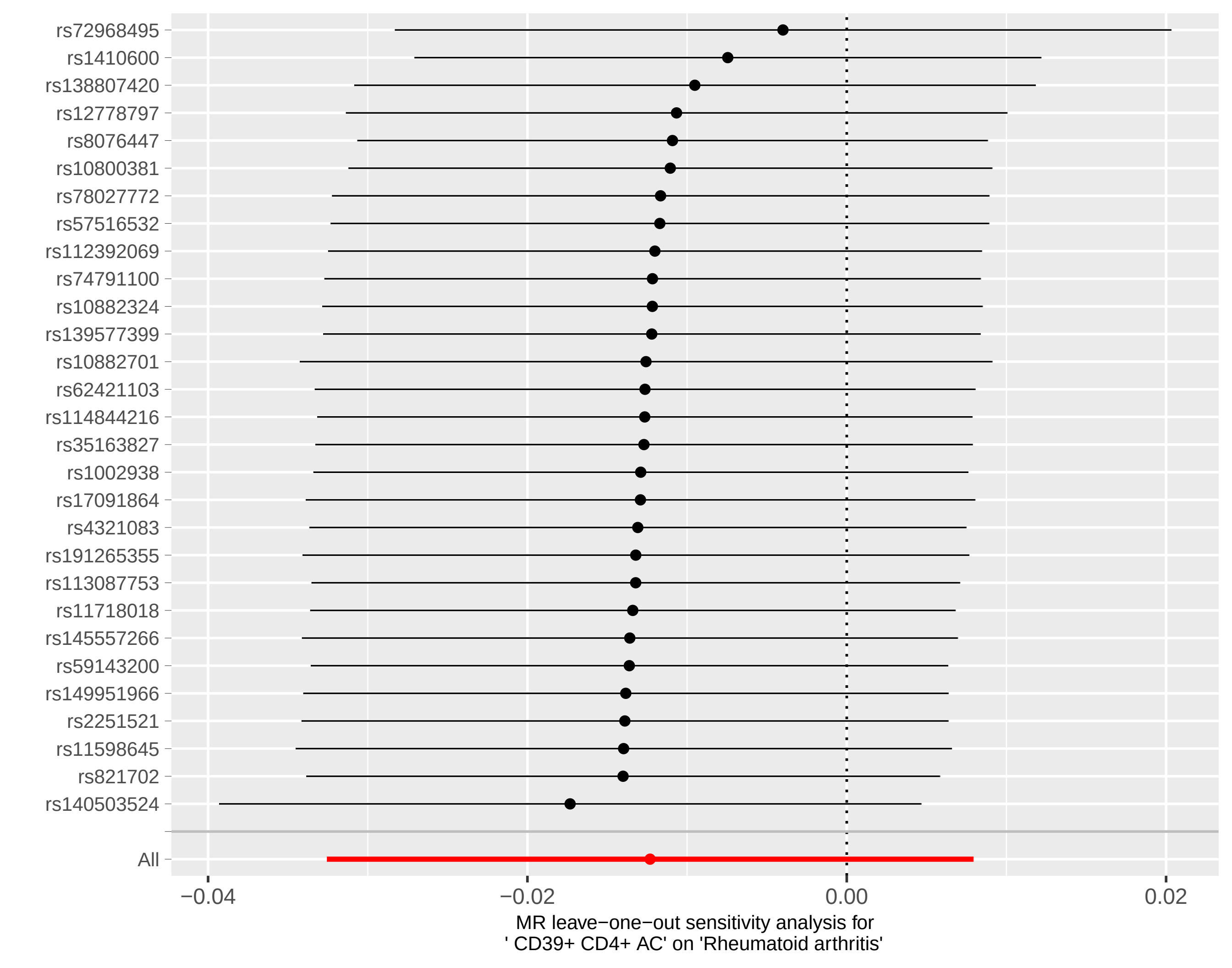
rs72968495
rs1410600
rs138807420
rs12778797
rs8076447
rs10800381
rs78027772
rs57516532
rs112392069
rs74791100
rs10882324
rs139577399
rs10882701
rs62421103
rs114844216
rs35163827
rs1002938
rs17091864
rs4321083
rs191265355
rs113087753
rs11718018
rs145557266
rs59143200
rs149951966
rs2251521
rs11598645
rs821702
rs140503524
All
−0.04
−0.02
0.00
0.02
MR leave−one−out sensitivity analysis for
' CD39+ CD4+ AC' on 'Rheumatoid arthritis'

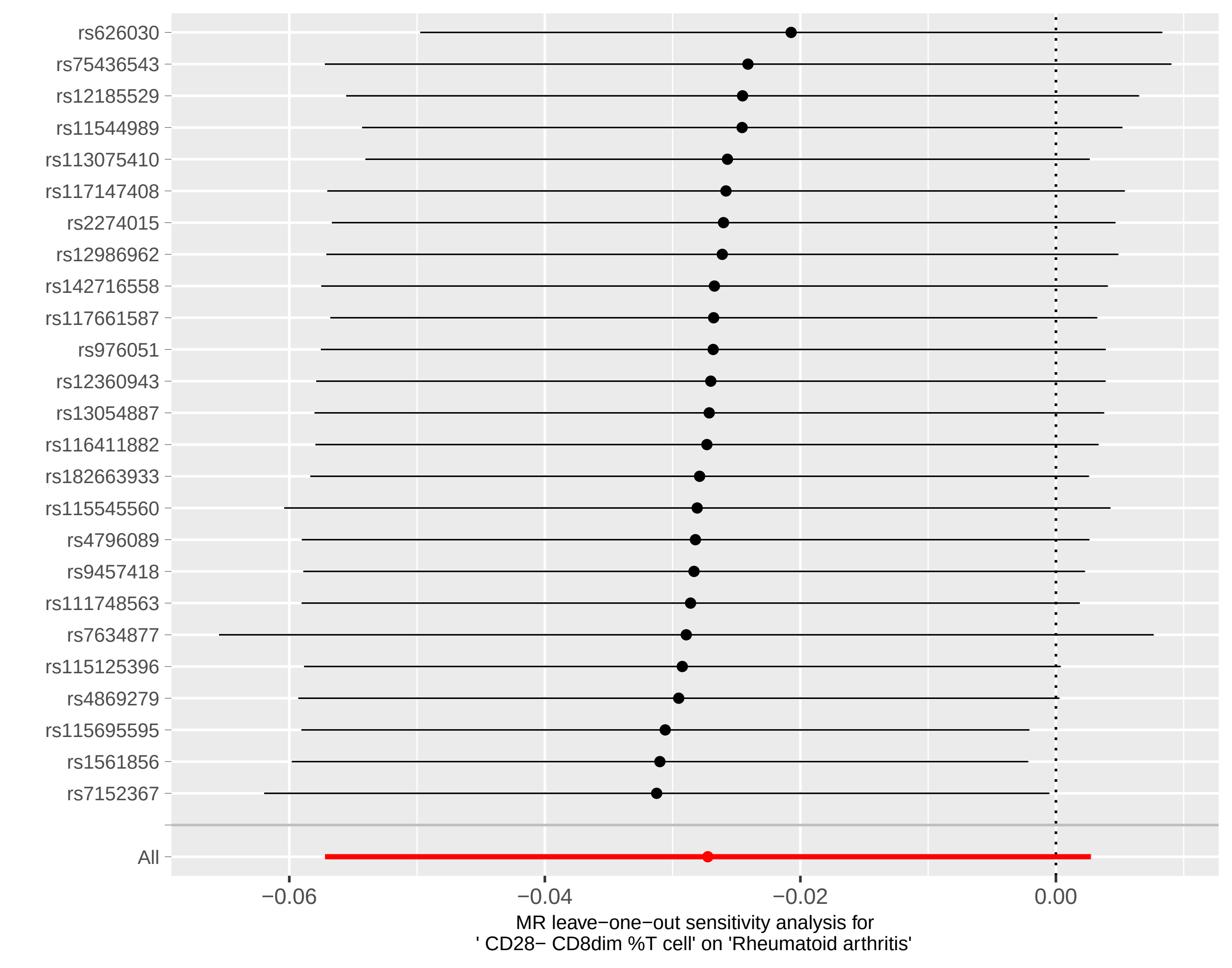
rs626030
rs75436543
rs12185529
rs11544989
rs113075410
rs117147408
rs2274015
rs12986962
rs142716558
rs117661587
rs976051
rs12360943
rs13054887
rs116411882
rs182663933
rs115545560
rs4796089
rs9457418
rs111748563
rs7634877
rs115125396
rs4869279
rs115695595
rs1561856
rs7152367
All
−0.06
−0.04
−0.02
0.00
MR leave−one−out sensitivity analysis for
' CD28− CD8dim %T cell' on 'Rheumatoid arthritis'

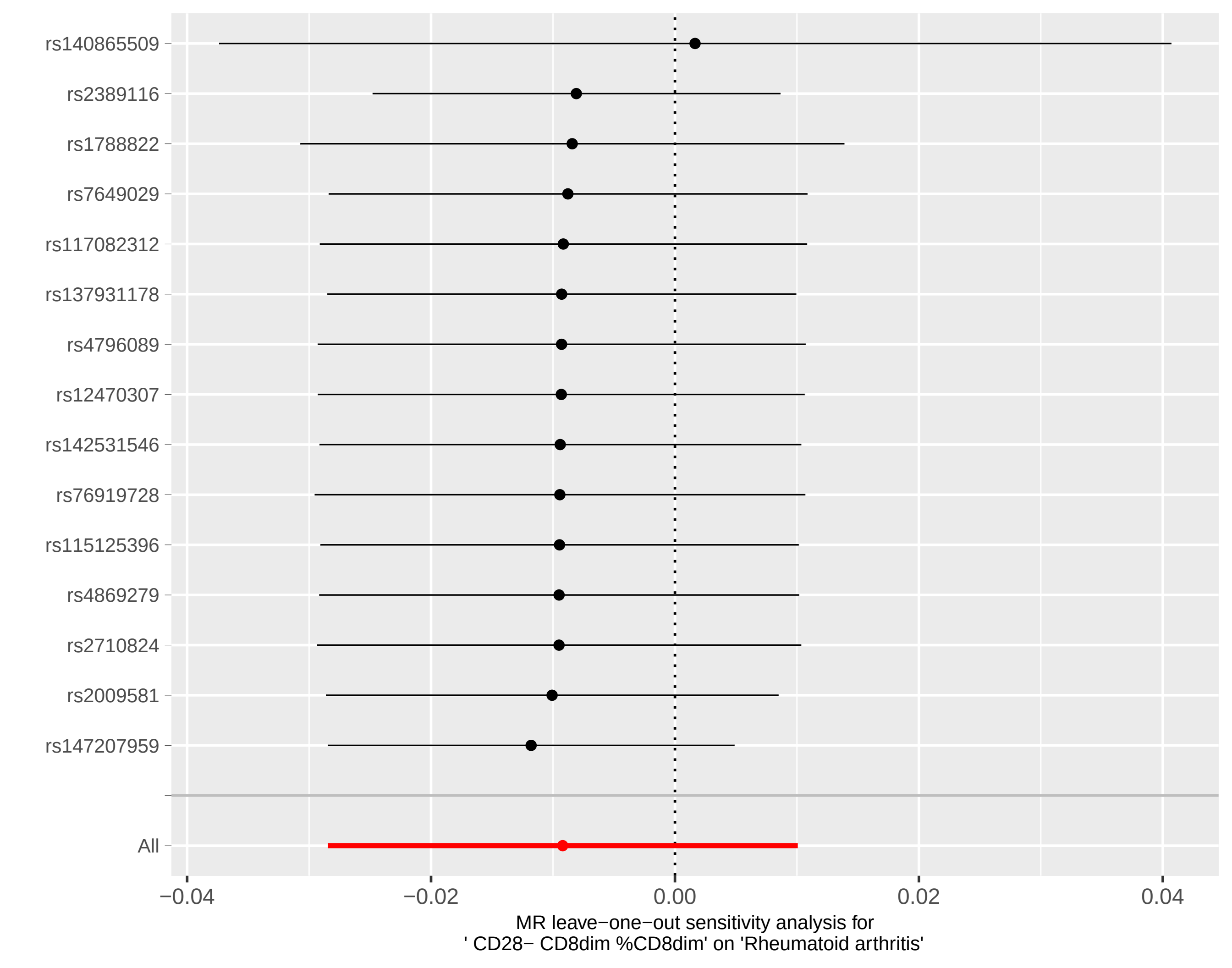

rs140865509
rs2389116
rs1788822
rs7649029
rs117082312
rs137931178
rs4796089
rs12470307
rs142531546
rs76919728
rs115125396
rs4869279
rs2710824
rs2009581
rs147207959
All
−0.04
−0.02
0.00
0.02
0.04
MR leave−one−out sensitivity analysis for
' CD28− CD8dim %CD8dim' on 'Rheumatoid arthritis'

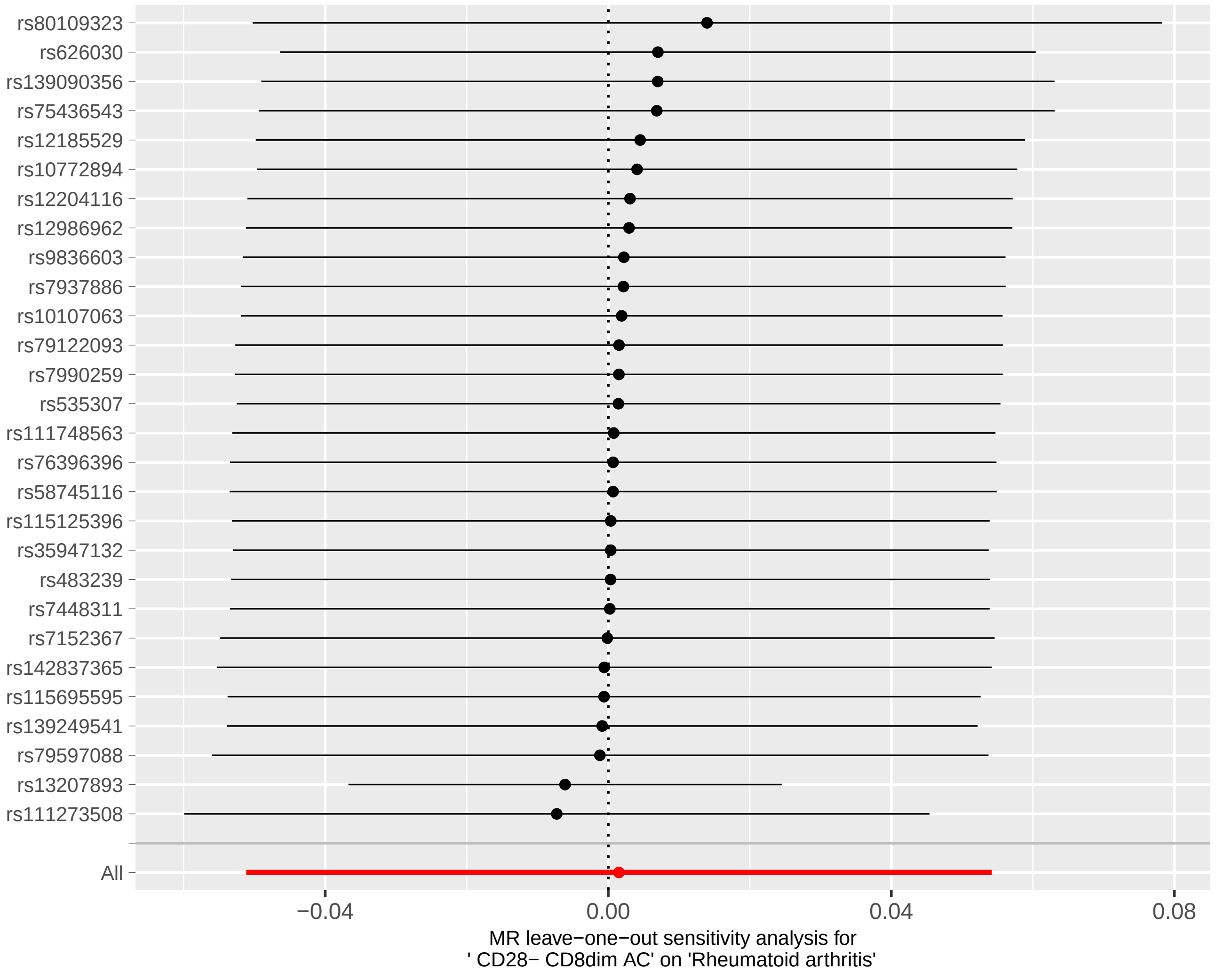
rs80109323
rs626030
rs139090356
rs75436543
rs12185529
rs10772894
rs12204116
rs12986962
rs9836603
rs7937886
rs10107063
rs79122093
rs7990259
rs535307
rs111748563
rs76396396
rs58745116
rs115125396
rs35947132
rs483239
rs7448311
rs7152367
rs142837365
rs115695595
rs139249541
rs79597088
rs13207893
rs111273508
All
−0.04
0.00
0.04
0.08
MR leave−one−out sensitivity analysis for
' CD28− CD8dim AC' on 'Rheumatoid arthritis'

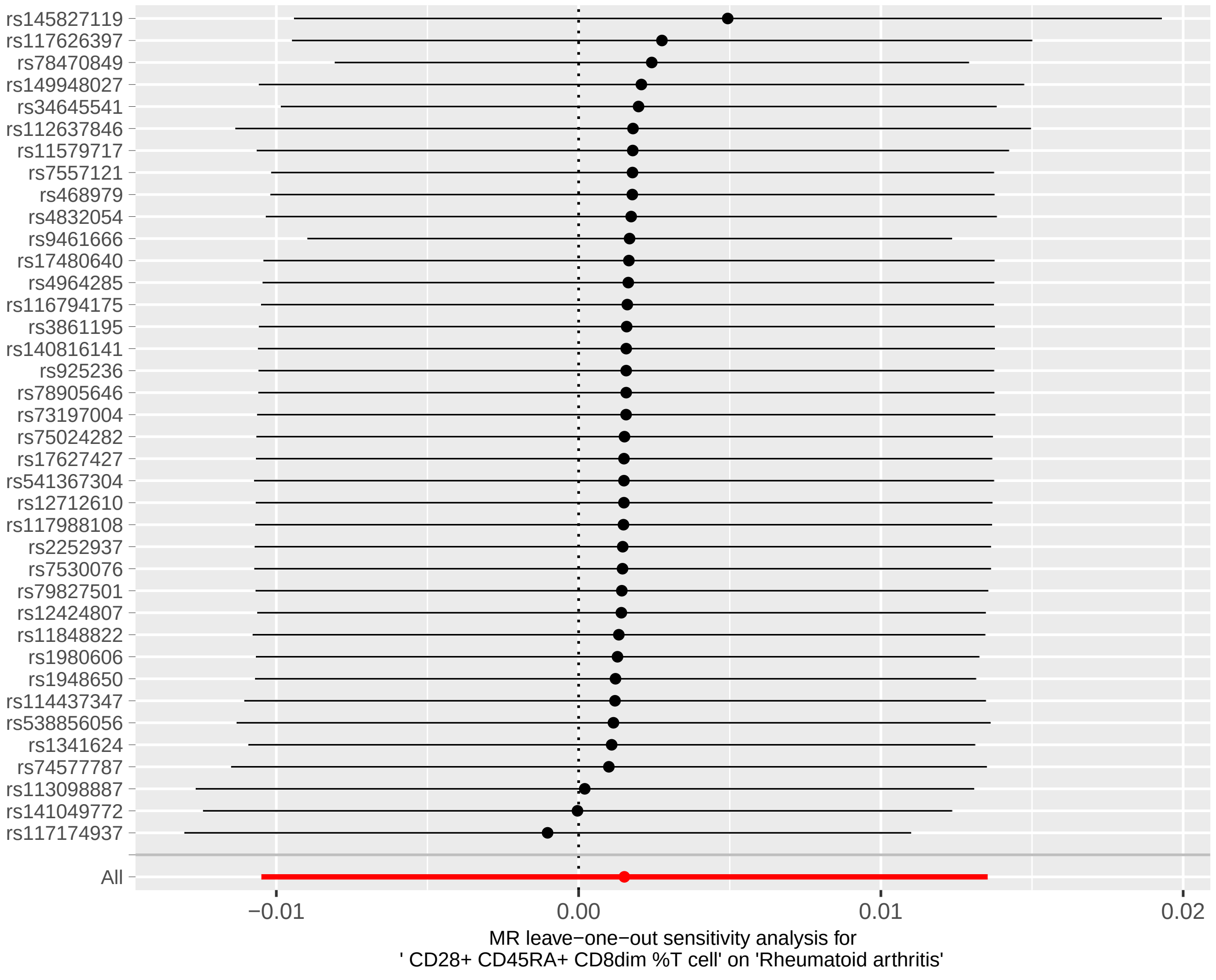
rs145827119
rs117626397
rs78470849
rs149948027
rs34645541
rs112637846
rs11579717
rs7557121
rs468979
rs4832054
rs9461666
rs17480640
rs4964285
rs116794175
rs3861195
rs140816141
rs925236
rs78905646
rs73197004
rs75024282
rs17627427
rs541367304
rs12712610
rs117988108
rs2252937
rs7530076
rs79827501
rs12424807
rs11848822
rs1980606
rs1948650
rs114437347
rs538856056
rs1341624
rs74577787
rs113098887
rs141049772
rs117174937
All
−0.01
0.00
0.01
0.02
MR leave−one−out sensitivity analysis for
' CD28+ CD45RA+ CD8dim %T cell' on 'Rheumatoid arthritis'

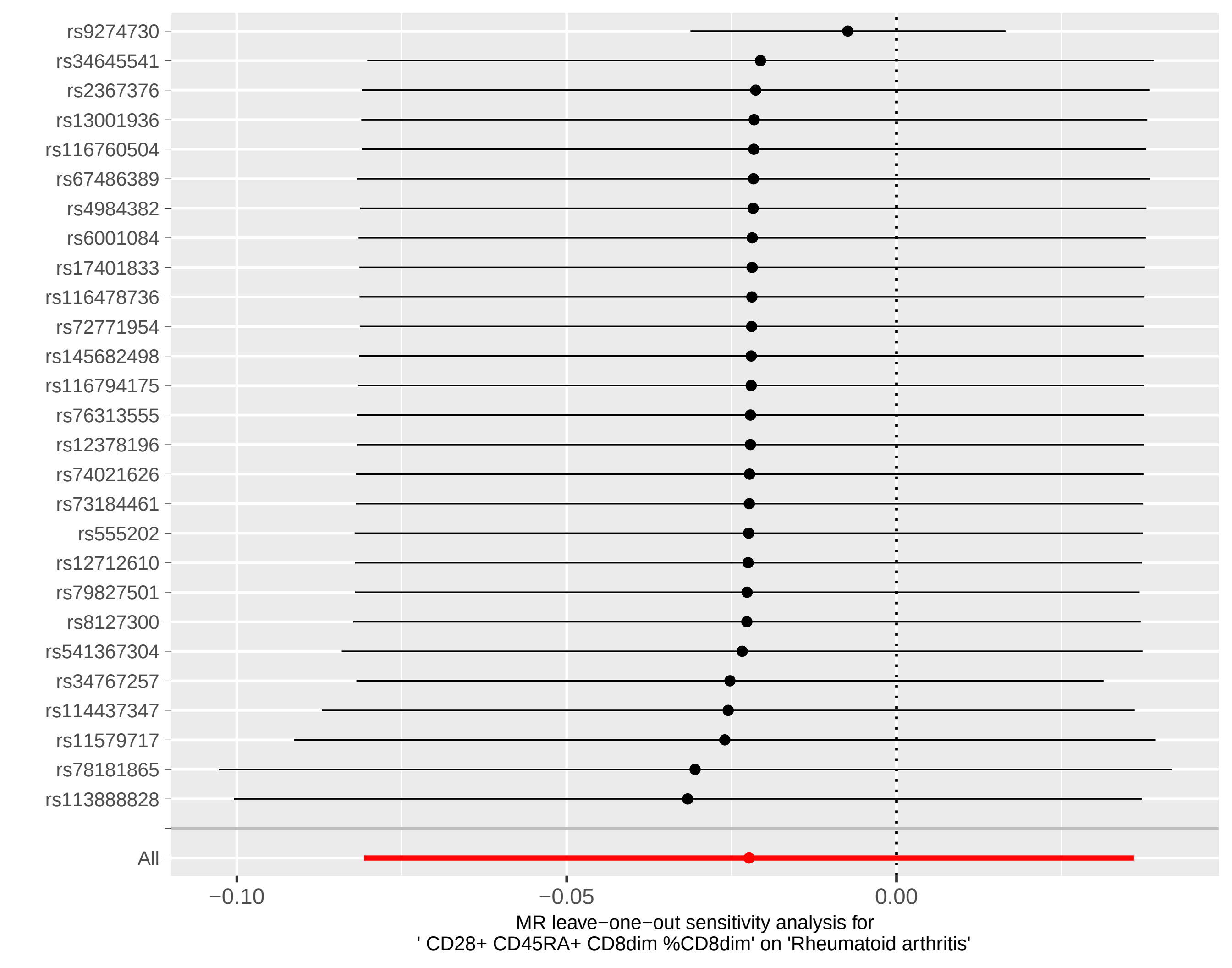
rs9274730
rs34645541
rs2367376
rs13001936
rs116760504
rs67486389
rs4984382
rs6001084
rs17401833
rs116478736
rs72771954
rs145682498
rs116794175
rs76313555
rs12378196
rs74021626
rs73184461
rs555202
rs12712610
rs79827501
rs8127300
rs541367304
rs34767257
rs114437347
rs11579717
rs78181865
rs113888828
All
−0.10
−0.05
0.00
MR leave−one−out sensitivity analysis for
' CD28+ CD45RA+ CD8dim %CD8dim' on 'Rheumatoid arthritis'

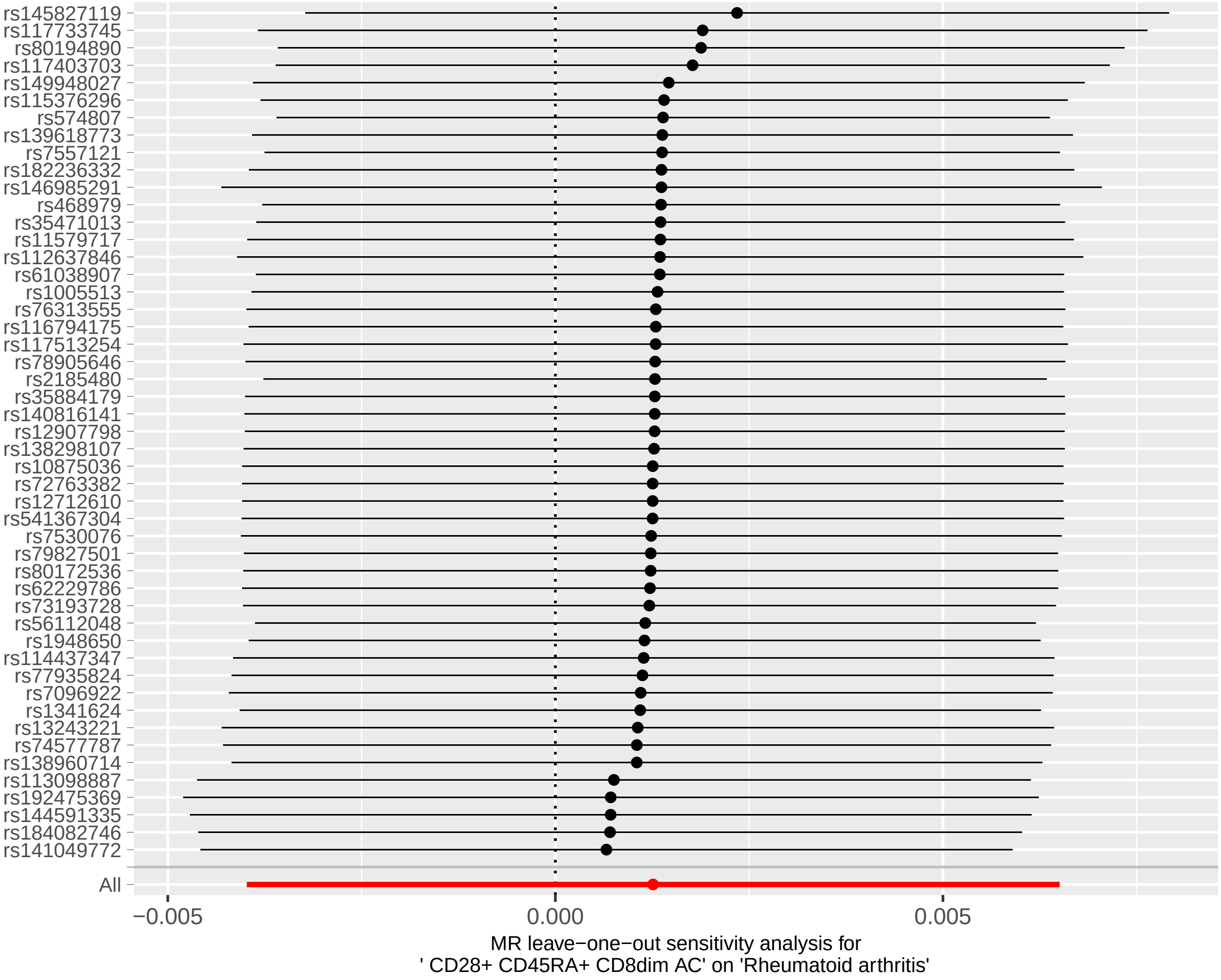
rs145827119
rs117733745
rs80194890
rs117403703
rs149948027
rs115376296
rs574807
rs139618773
rs7557121
rs182236332
rs146985291
rs468979
rs35471013
rs11579717
rs112637846
rs61038907
rs1005513
rs76313555
rs116794175
rs117513254
rs78905646
rs2185480
rs35884179
rs140816141
rs12907798
rs138298107
rs10875036
rs72763382
rs12712610
rs541367304
rs7530076
rs79827501
rs80172536
rs62229786
rs73193728
rs56112048
rs1948650
rs114437347
rs77935824
rs7096922
rs1341624
rs13243221
rs74577787
rs138960714
rs113098887
rs192475369
rs144591335
rs184082746
rs141049772
All
−0.005
0.000
0.005
MR leave−one−out sensitivity analysis for
' CD28+ CD45RA+ CD8dim AC' on 'Rheumatoid arthritis'

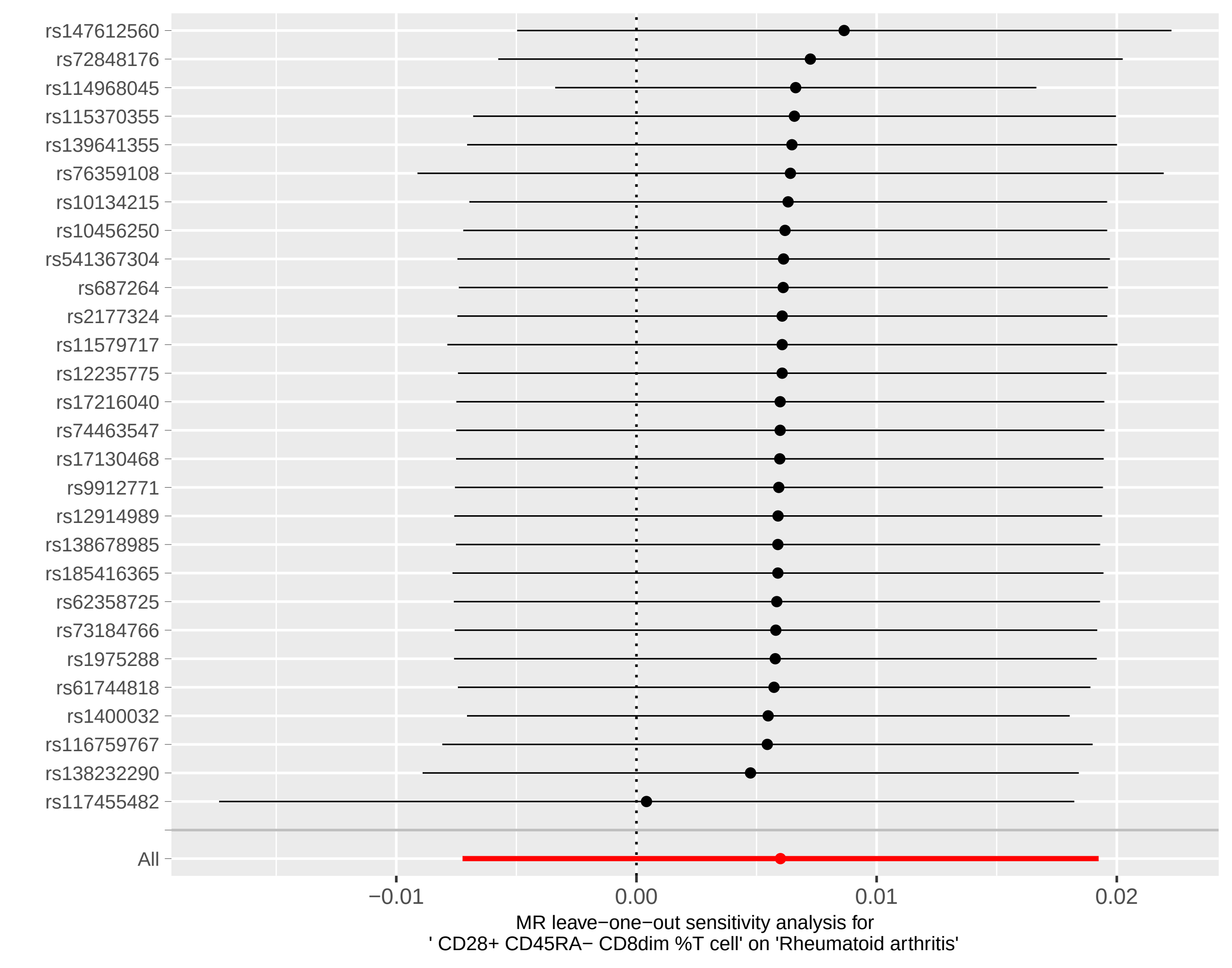
rs147612560
rs72848176
rs114968045
rs115370355
rs139641355
rs76359108
rs10134215
rs10456250
rs541367304
rs687264
rs2177324
rs11579717
rs12235775
rs17216040
rs74463547
rs17130468
rs9912771
rs12914989
rs138678985
rs185416365
rs62358725
rs73184766
rs1975288
rs61744818
rs1400032
rs116759767
rs138232290
rs117455482
All
−0.01
0.00
0.01
0.02
MR leave−one−out sensitivity analysis for
' CD28+ CD45RA− CD8dim %T cell' on 'Rheumatoid arthritis'

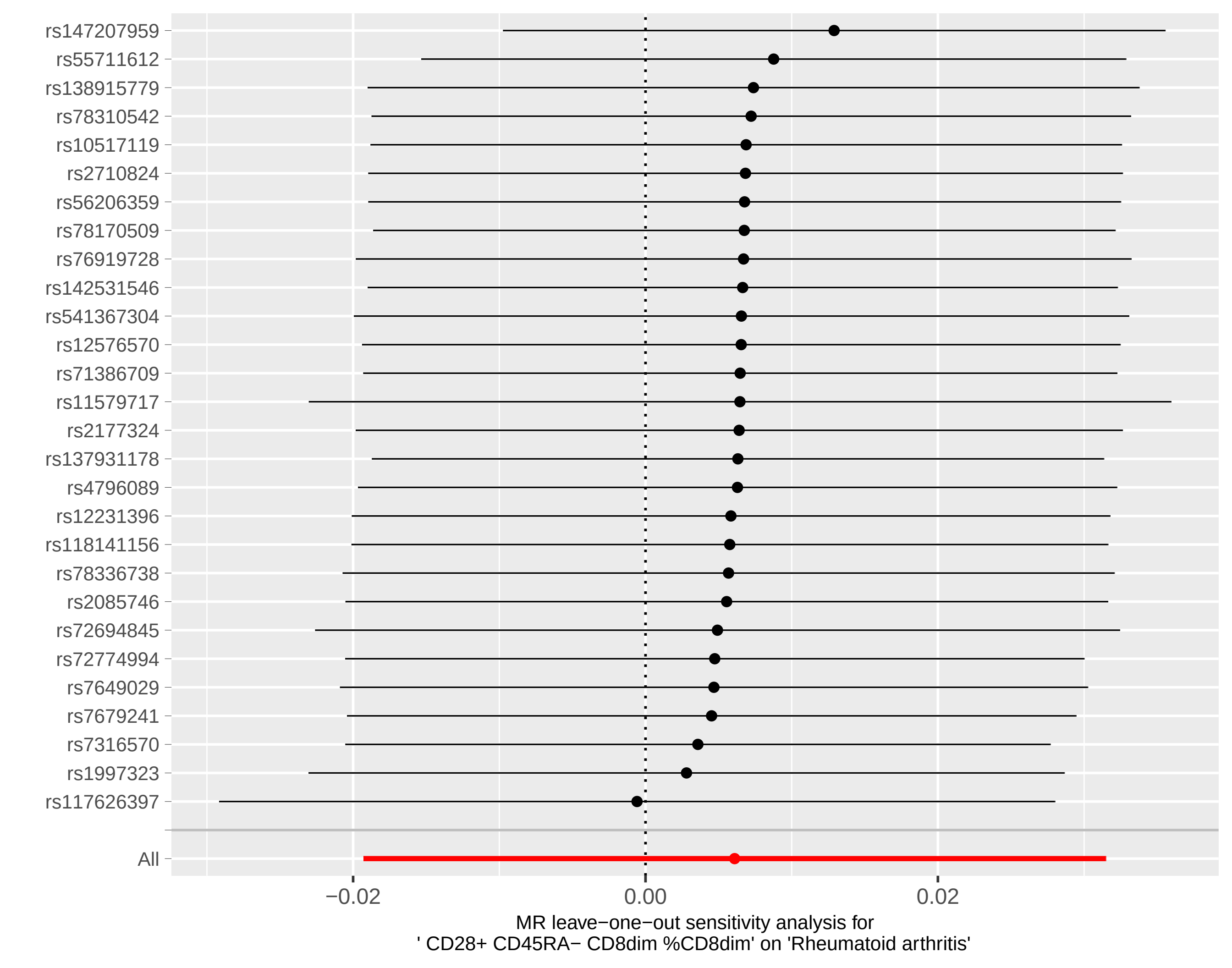
rs147207959
rs55711612
rs138915779
rs78310542
rs10517119
rs2710824
rs56206359
rs78170509
rs76919728
rs142531546
rs541367304
rs12576570
rs71386709
rs11579717
rs2177324
rs137931178
rs4796089
rs12231396
rs118141156
rs78336738
rs2085746
rs72694845
rs72774994
rs7649029
rs7679241
rs7316570
rs1997323
rs117626397
All
−0.02
0.00
0.02
MR leave−one−out sensitivity analysis for
' CD28+ CD45RA− CD8dim %CD8dim' on 'Rheumatoid arthritis'

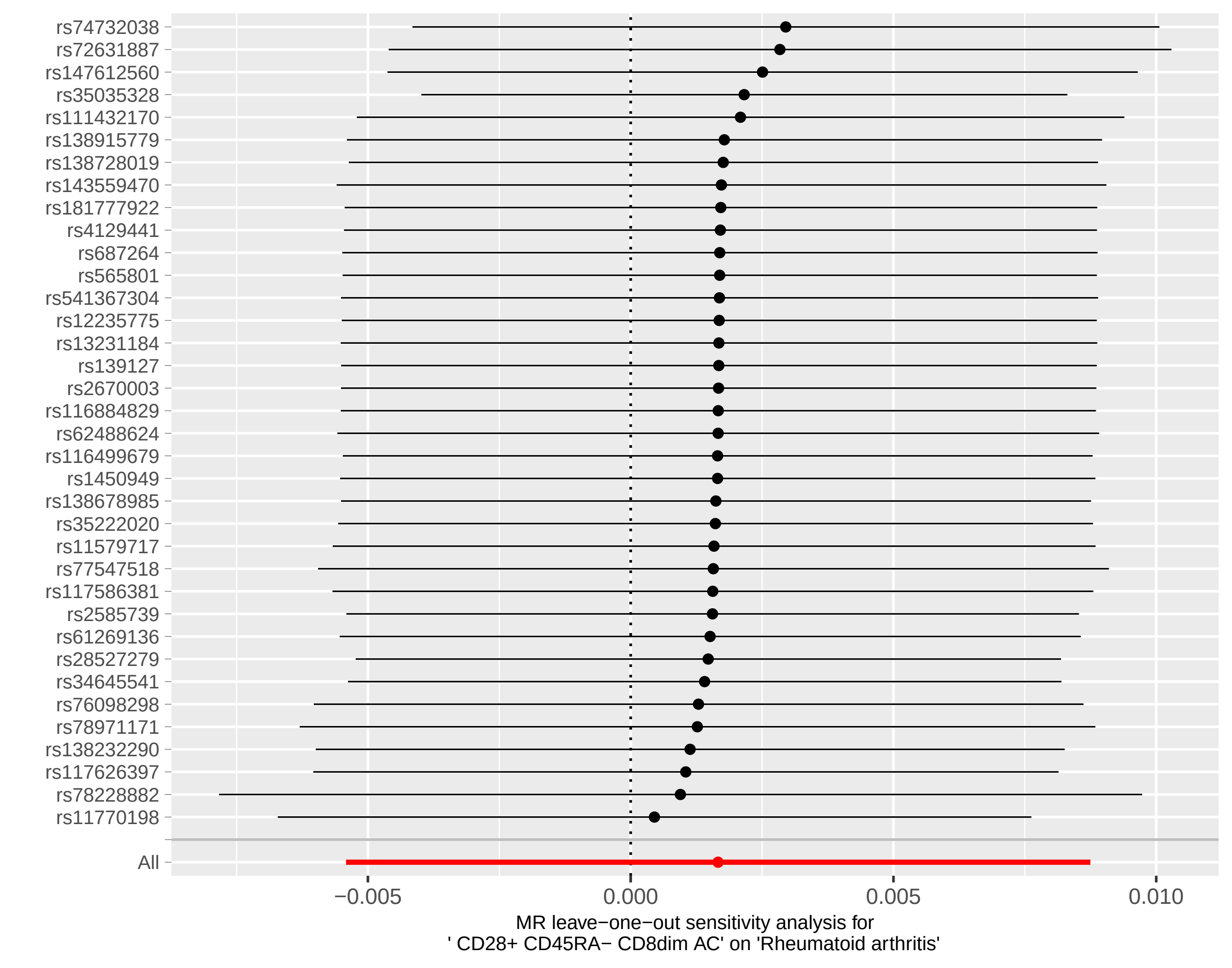

rs74732038
rs72631887
rs147612560
rs35035328
rs111432170
rs138915779
rs138728019
rs143559470
rs181777922
rs4129441
rs687264
rs565801
rs541367304
rs12235775
rs13231184
rs139127
rs2670003
rs116884829
rs62488624
rs116499679
rs1450949
rs138678985
rs35222020
rs11579717
rs77547518
rs117586381
rs2585739
rs61269136
rs28527279
rs34645541
rs76098298
rs78971171
rs138232290
rs117626397
rs78228882
rs11770198
All
−0.005
0.000
0.005
0.010
MR leave−one−out sensitivity analysis for
' CD28+ CD45RA− CD8dim AC' on 'Rheumatoid arthritis'

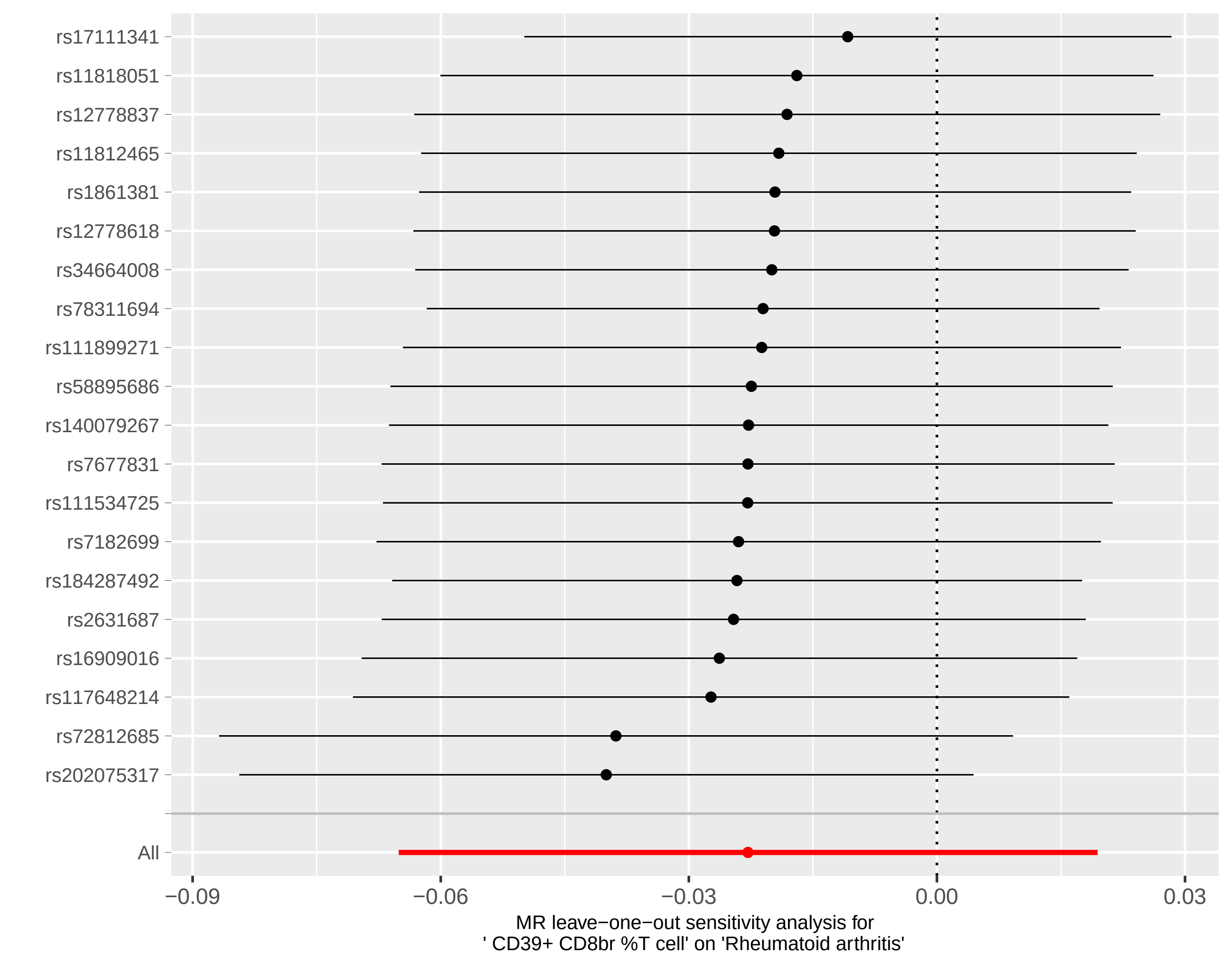
rs17111341
rs11818051
rs12778837
rs11812465
rs1861381
rs12778618
rs34664008
rs78311694
rs111899271
rs58895686
rs140079267
rs7677831
rs111534725
rs7182699
rs184287492
rs2631687
rs16909016
rs117648214
rs72812685
rs202075317
All
−0.09
−0.06
−0.03
0.00
0.03
MR leave−one−out sensitivity analysis for
' CD39+ CD8br %T cell' on 'Rheumatoid arthritis'

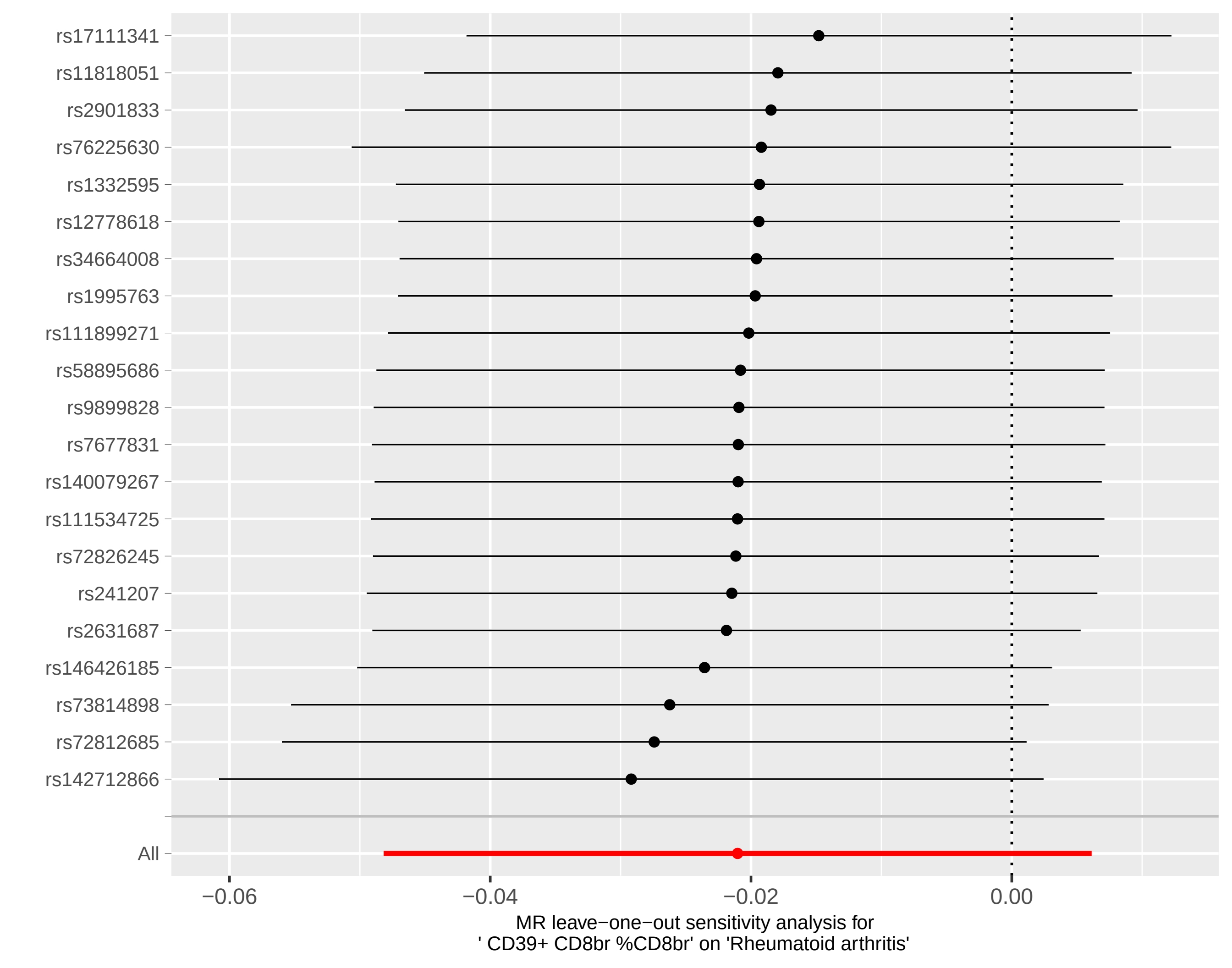
rs17111341
rs11818051
rs2901833
rs76225630
rs1332595
rs12778618
rs34664008
rs1995763
rs111899271
rs58895686
rs9899828
rs7677831
rs140079267
rs111534725
rs72826245
rs241207
rs2631687
rs146426185
rs73814898
rs72812685
rs142712866
All
−0.06
−0.04
−0.02
0.00
MR leave−one−out sensitivity analysis for
' CD39+ CD8br %CD8br' on 'Rheumatoid arthritis'

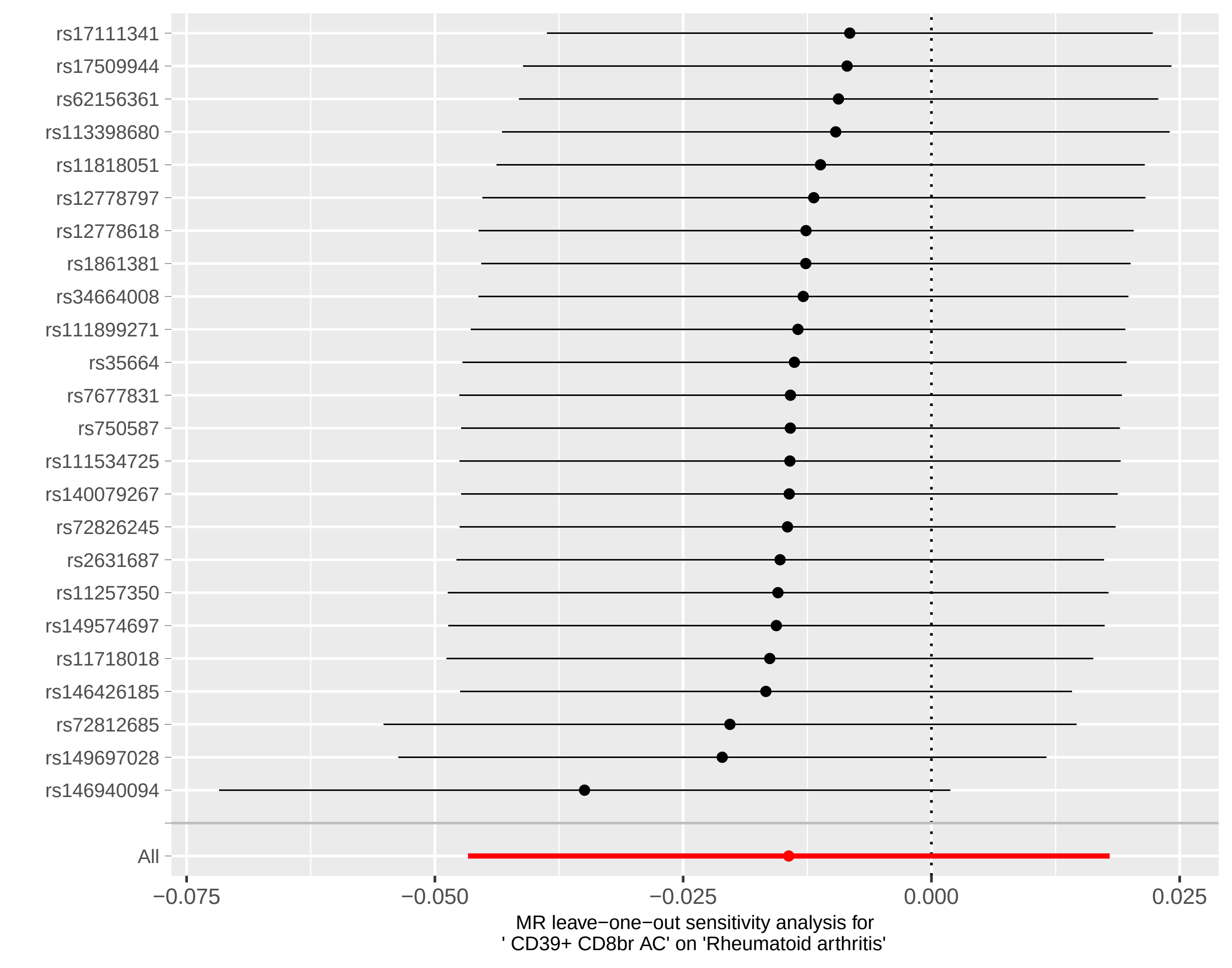
rs17111341
rs17509944
rs62156361
rs113398680
rs11818051
rs12778797
rs12778618
rs1861381
rs34664008
rs111899271
rs35664
rs7677831
rs750587
rs111534725
rs140079267
rs72826245
rs2631687
rs11257350
rs149574697
rs11718018
rs146426185
rs72812685
rs149697028
rs146940094
All
−0.075
−0.050
−0.025
0.000
0.025
MR leave−one−out sensitivity analysis for
' CD39+ CD8br AC' on 'Rheumatoid arthritis'

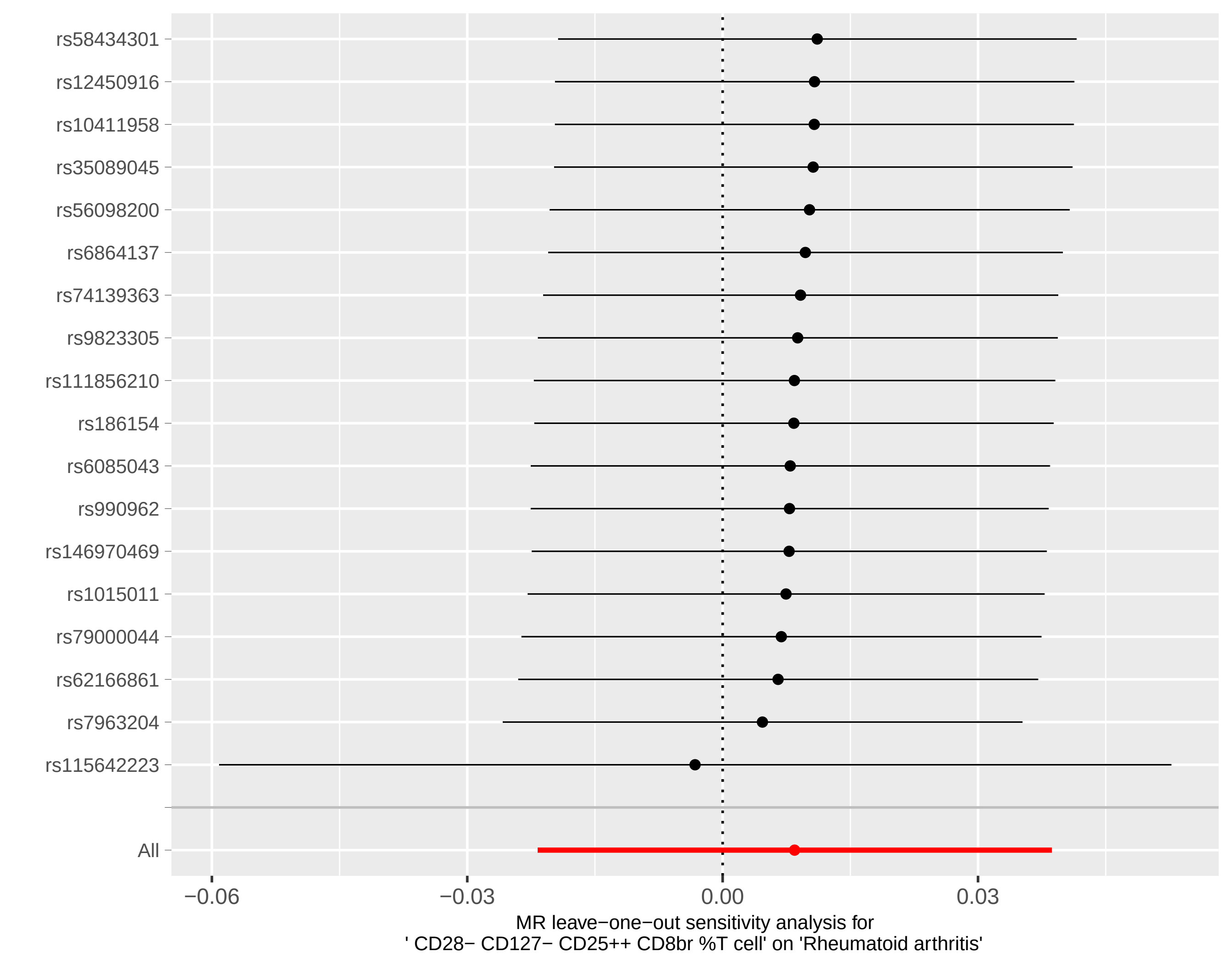

rs58434301
rs12450916
rs10411958
rs35089045
rs56098200
rs6864137
rs74139363
rs9823305
rs111856210
rs186154
rs6085043
rs990962
rs146970469
rs1015011
rs79000044
rs62166861
rs7963204
rs115642223
All
−0.06
−0.03
0.00
0.03
MR leave−one−out sensitivity analysis for
' CD28− CD127− CD25++ CD8br %T cell' on 'Rheumatoid arthritis'

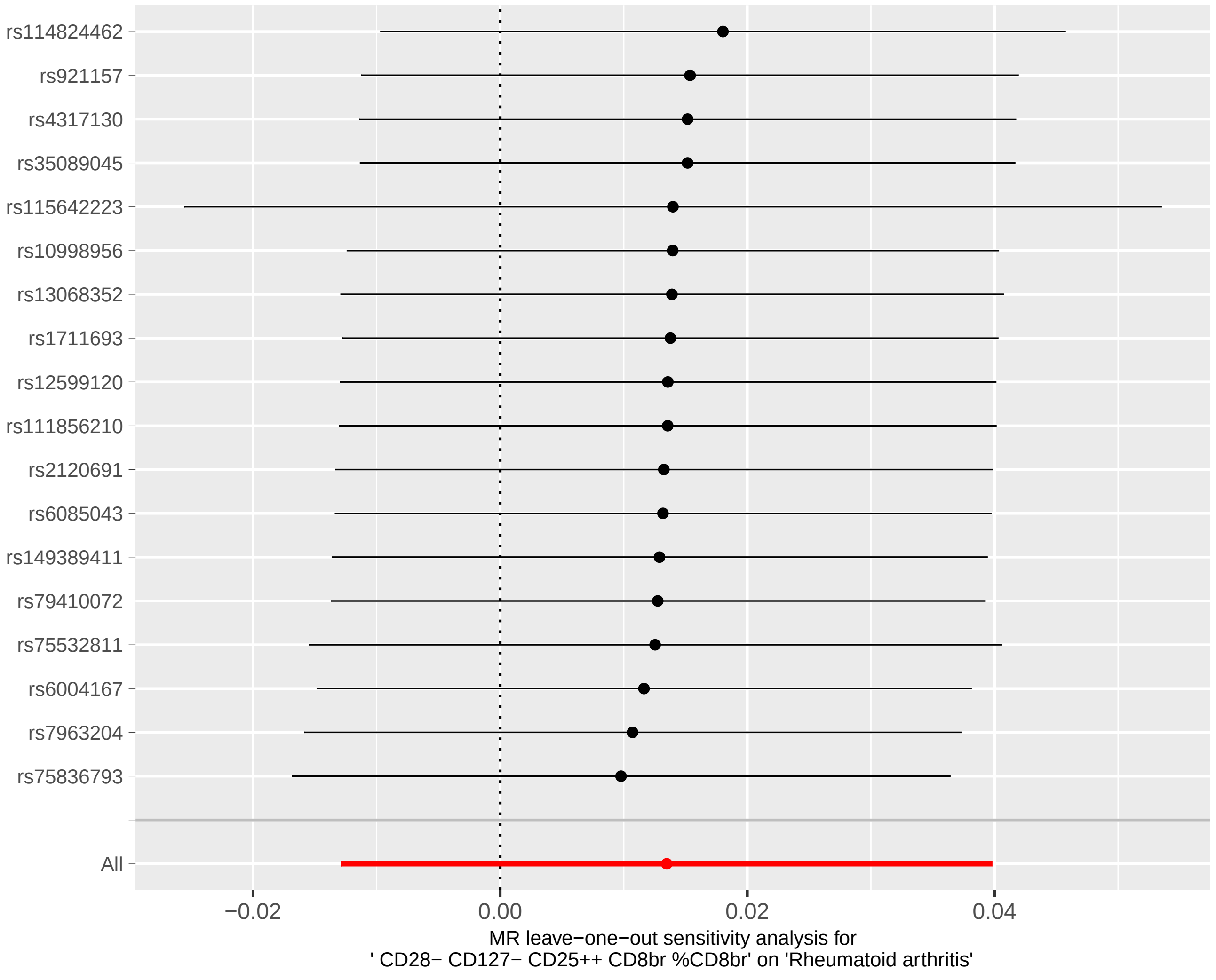
rs114824462
rs921157
rs4317130
rs35089045
rs115642223
rs10998956
rs13068352
rs1711693
rs12599120
rs111856210
rs2120691
rs6085043
rs149389411
rs79410072
rs75532811
rs6004167
rs7963204
rs75836793
All
−0.02
0.00
0.02
0.04
MR leave−one−out sensitivity analysis for
' CD28− CD127− CD25++ CD8br %CD8br' on 'Rheumatoid arthritis'

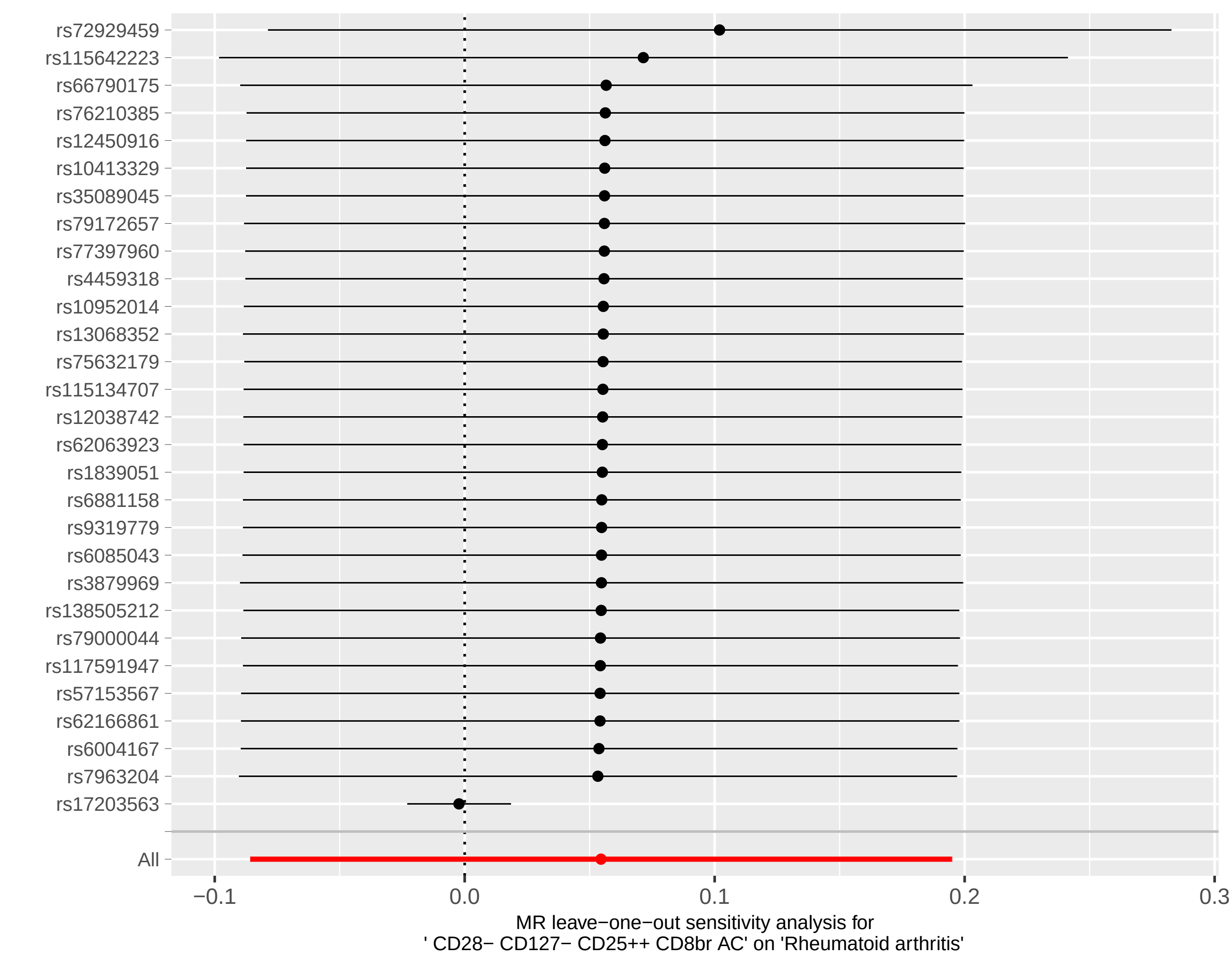
rs72929459
rs115642223
rs66790175
rs76210385
rs12450916
rs10413329
rs35089045
rs79172657
rs77397960
rs4459318
rs10952014
rs13068352
rs75632179
rs115134707
rs12038742
rs62063923
rs1839051
rs6881158
rs9319779
rs6085043
rs3879969
rs138505212
rs79000044
rs117591947
rs57153567
rs62166861
rs6004167
rs7963204
rs17203563
All
−0.1
0.0
0.1
0.2
0.3
MR leave−one−out sensitivity analysis for
' CD28− CD127− CD25++ CD8br AC' on 'Rheumatoid arthritis'

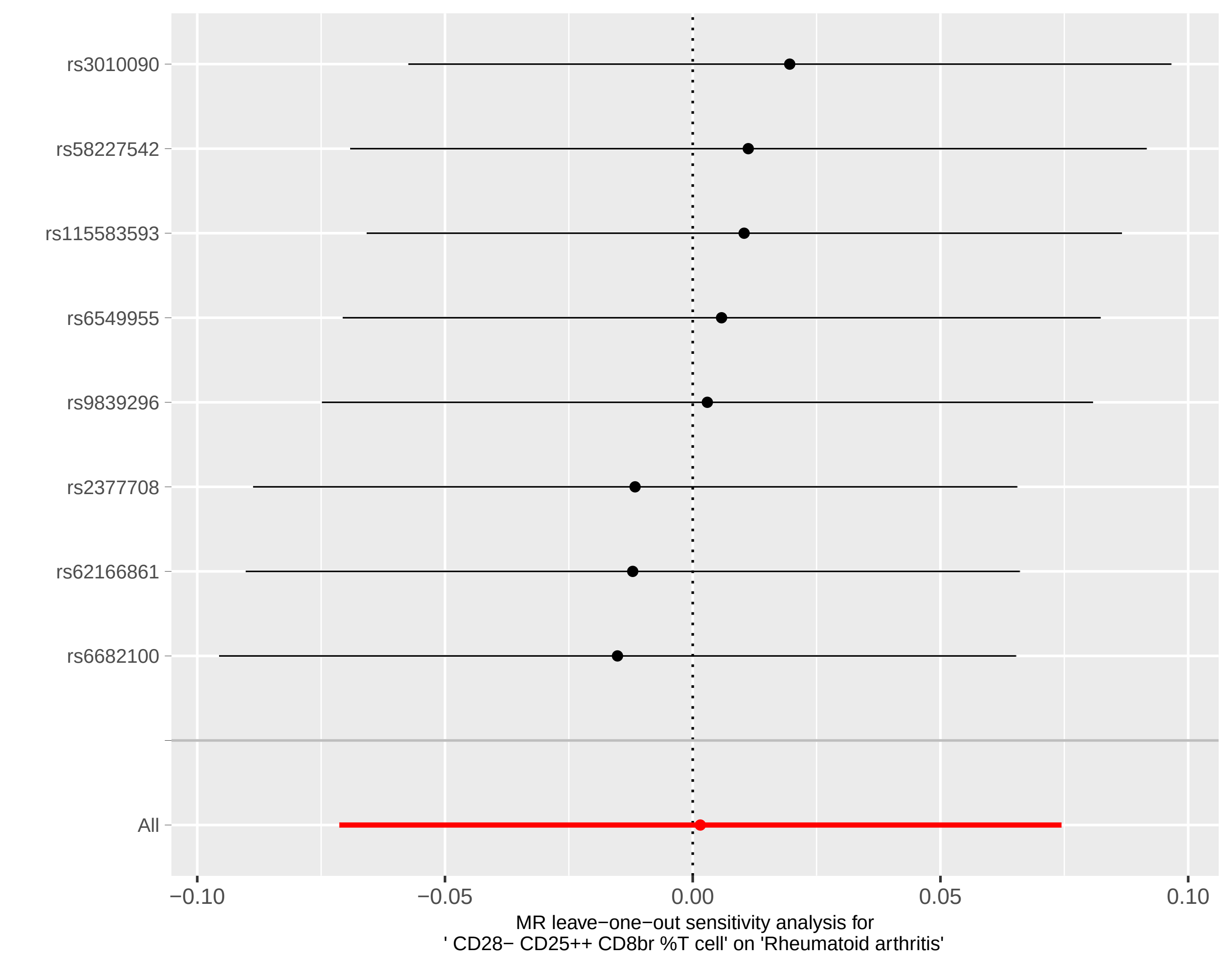
rs3010090
rs58227542
rs115583593
rs6549955
rs9839296
rs2377708
rs62166861
rs6682100
All
−0.10
−0.05
0.00
0.05
0.10
MR leave−one−out sensitivity analysis for
' CD28− CD25++ CD8br %T cell' on 'Rheumatoid arthritis'

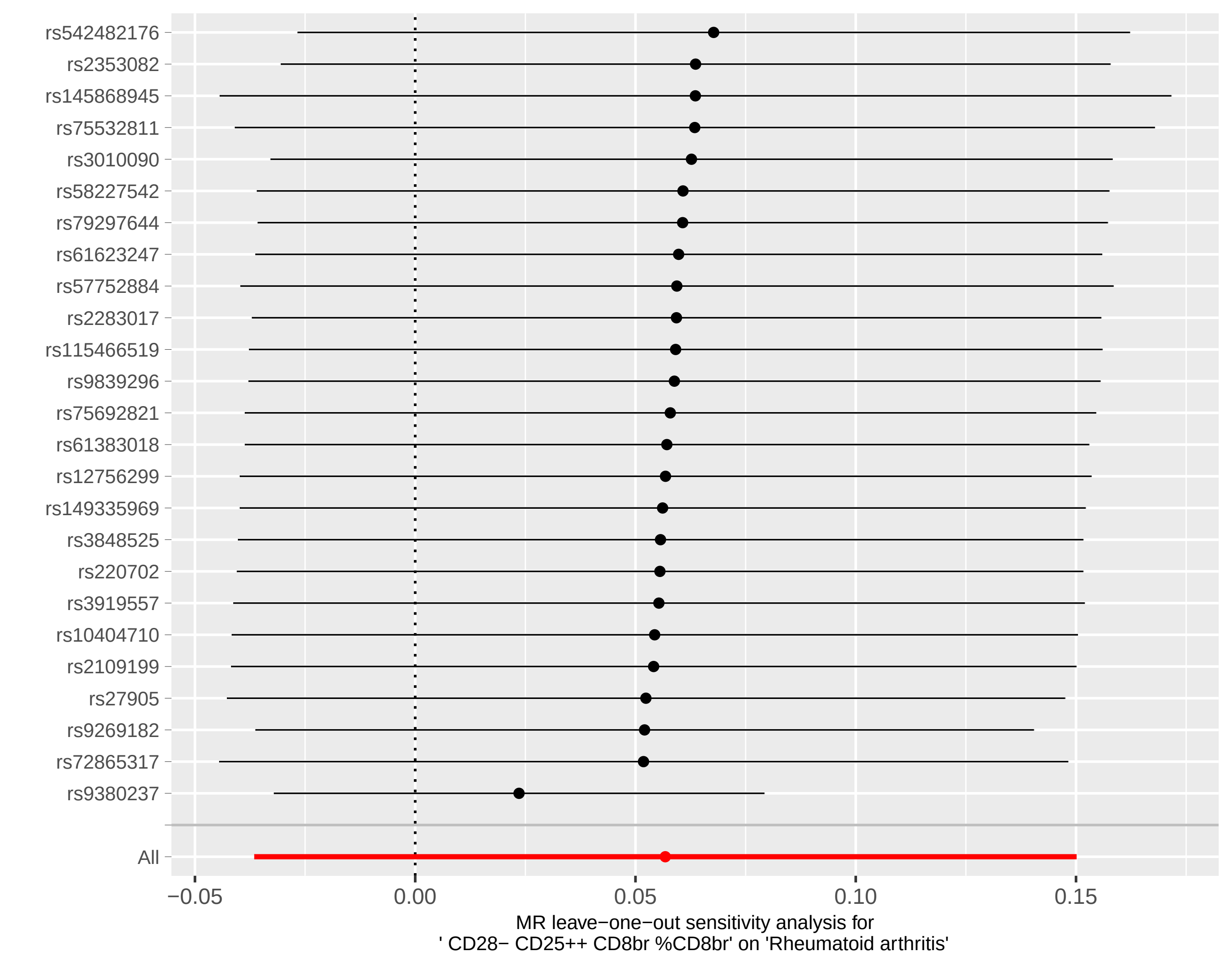
rs542482176
rs2353082
rs145868945
rs75532811
rs3010090
rs58227542
rs79297644
rs61623247
rs57752884
rs2283017
rs115466519
rs9839296
rs75692821
rs61383018
rs12756299
rs149335969
rs3848525
rs220702
rs3919557
rs10404710
rs2109199
rs27905
rs9269182
rs72865317
rs9380237
All
−0.05
0.00
0.05
0.10
0.15
MR leave−one−out sensitivity analysis for
' CD28− CD25++ CD8br %CD8br' on 'Rheumatoid arthritis'

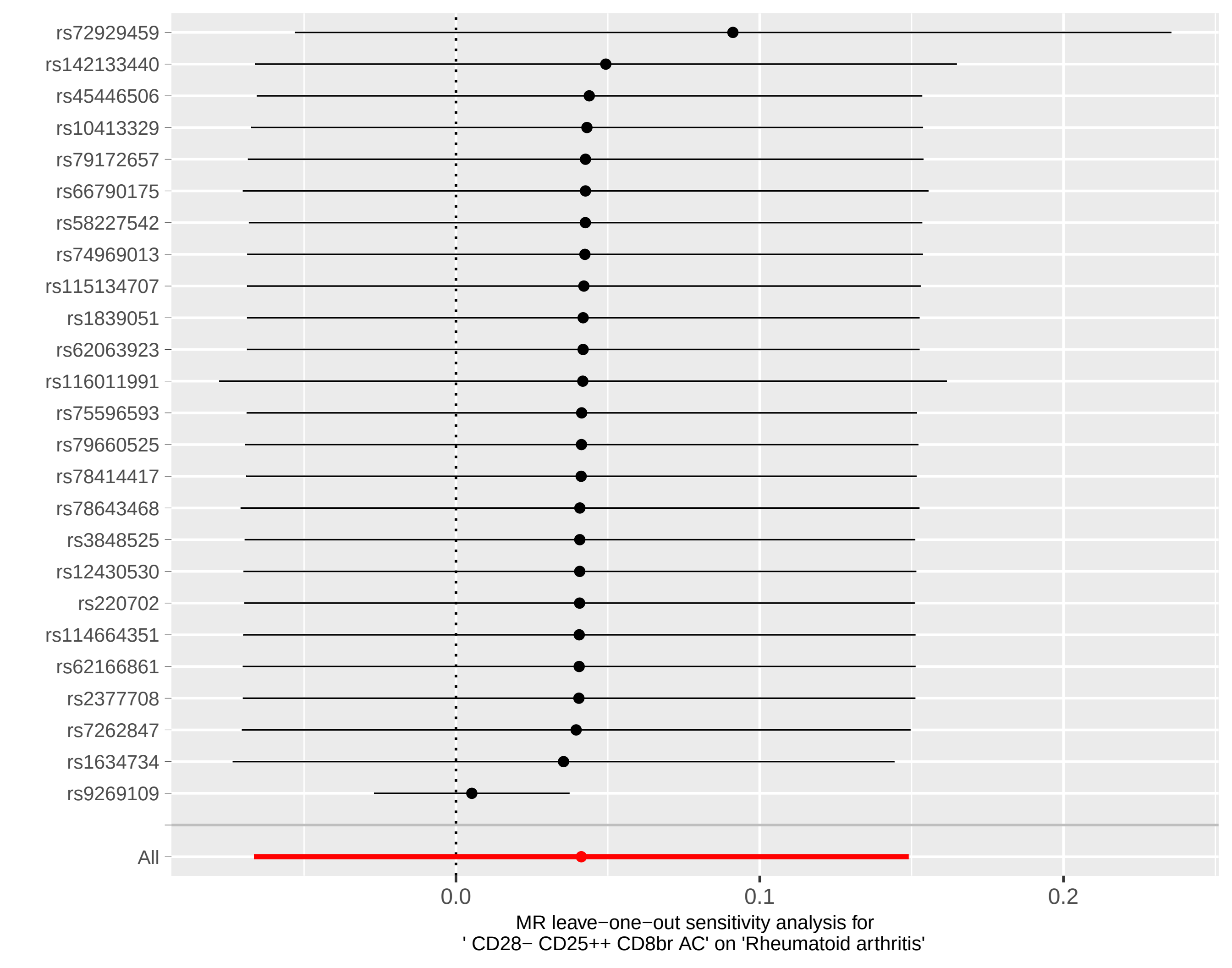

rs72929459
rs142133440
rs45446506
rs10413329
rs79172657
rs66790175
rs58227542
rs74969013
rs115134707
rs1839051
rs62063923
rs116011991
rs75596593
rs79660525
rs78414417
rs78643468
rs3848525
rs12430530
rs220702
rs114664351
rs62166861
rs2377708
rs7262847
rs1634734
rs9269109
All
0.0
0.1
0.2
MR leave−one−out sensitivity analysis for
' CD28− CD25++ CD8br AC' on 'Rheumatoid arthritis'

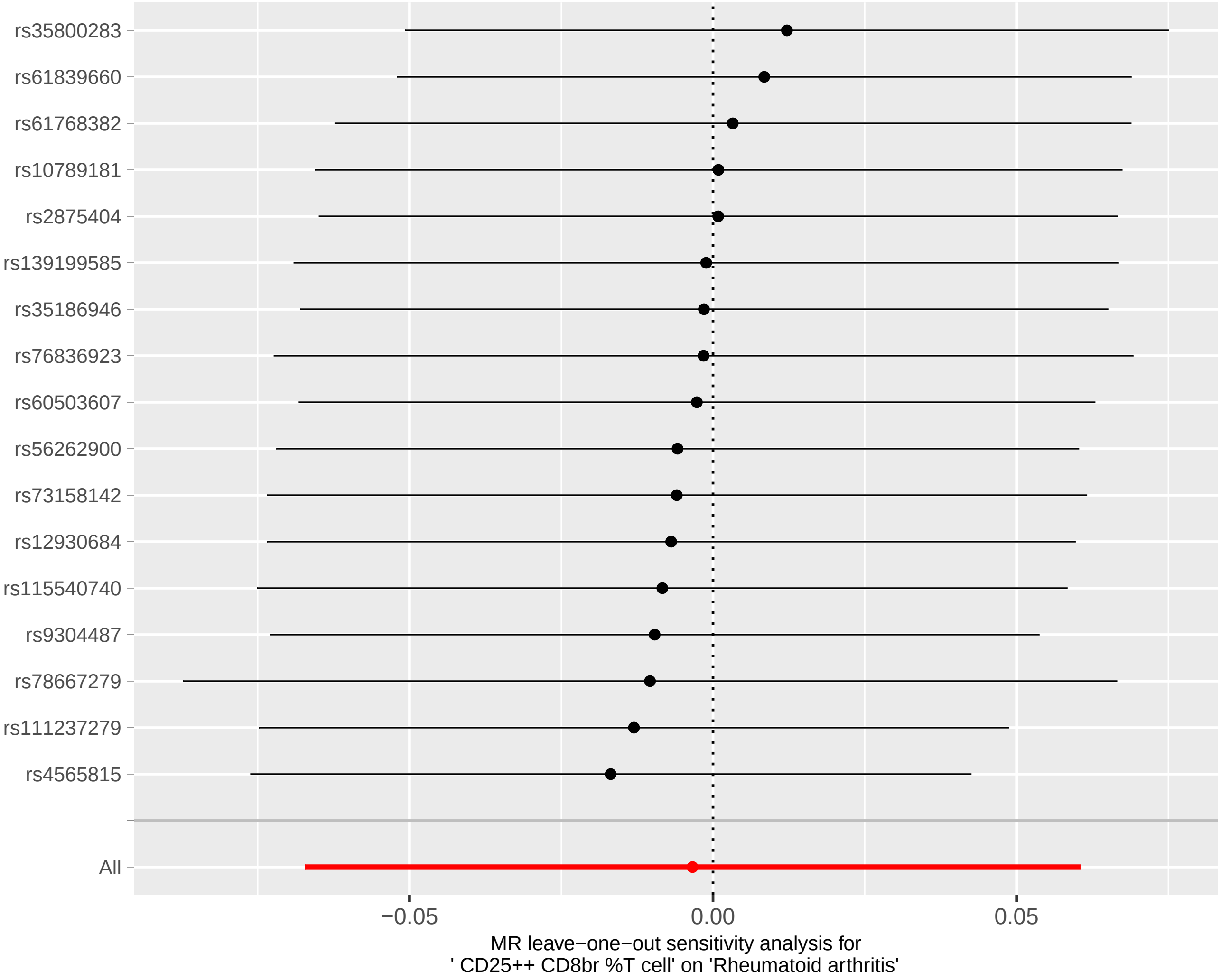
rs35800283
rs61839660
rs61768382
rs10789181
rs2875404
rs139199585
rs35186946
rs76836923
rs60503607
rs56262900
rs73158142
rs12930684
rs115540740
rs9304487
rs78667279
rs111237279
rs4565815
All
−0.05
0.00
0.05
MR leave−one−out sensitivity analysis for
' CD25++ CD8br %T cell' on 'Rheumatoid arthritis'

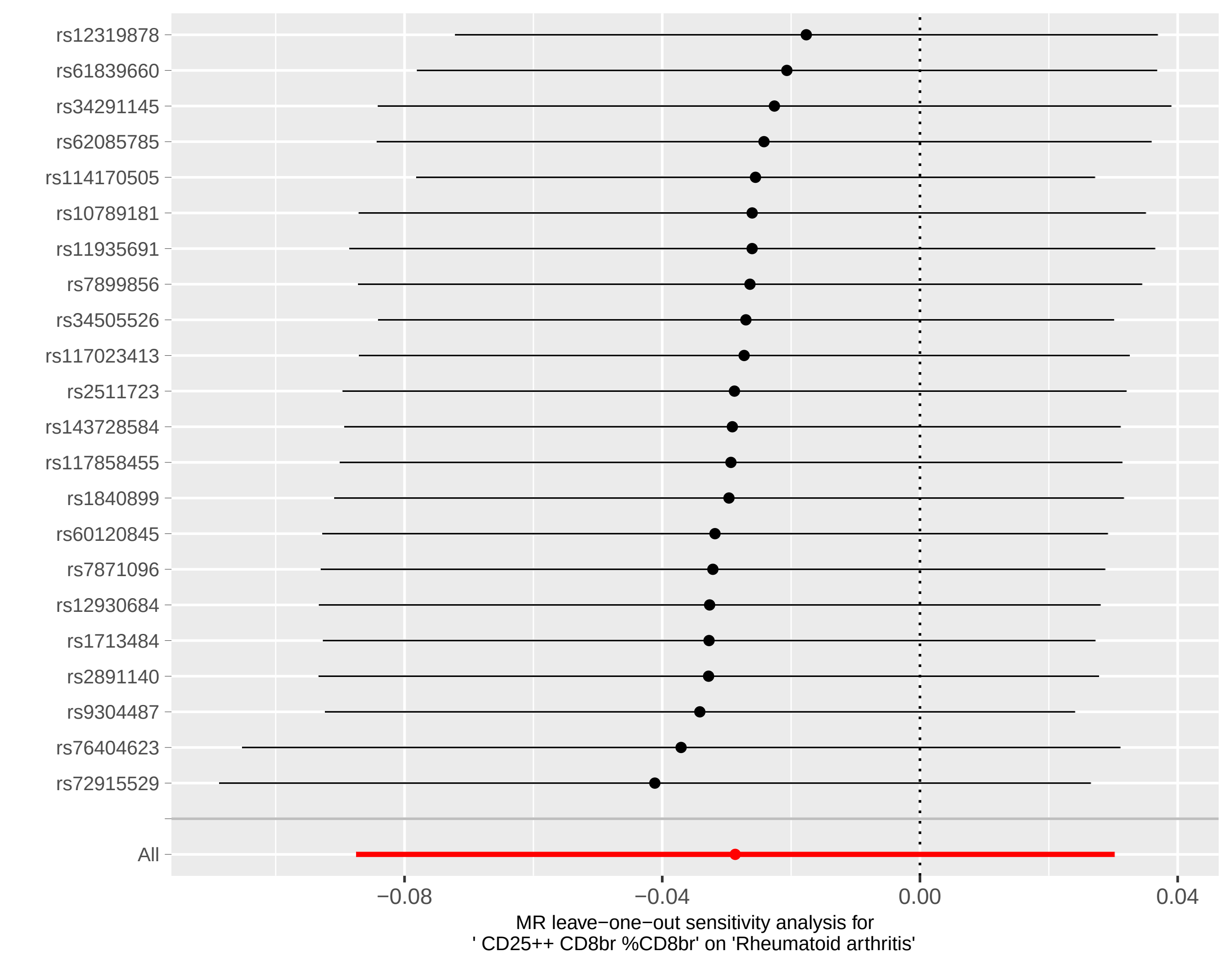
rs12319878
rs61839660
rs34291145
rs62085785
rs114170505
rs10789181
rs11935691
rs7899856
rs34505526
rs117023413
rs2511723
rs143728584
rs117858455
rs1840899
rs60120845
rs7871096
rs12930684
rs1713484
rs2891140
rs9304487
rs76404623
rs72915529
All
−0.08
−0.04
0.00
0.04
MR leave−one−out sensitivity analysis for
' CD25++ CD8br %CD8br' on 'Rheumatoid arthritis'

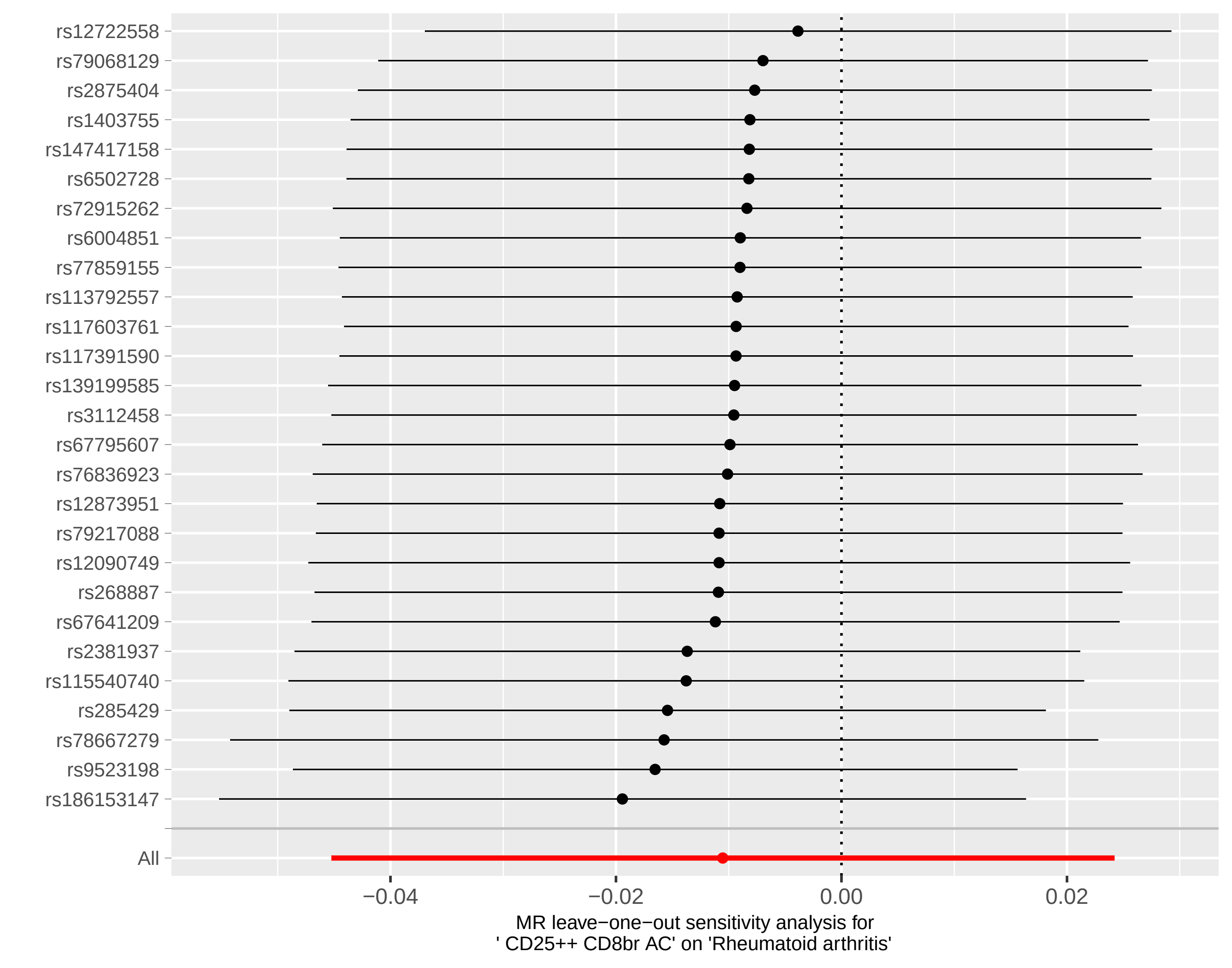
rs12722558
rs79068129
rs2875404
rs1403755
rs147417158
rs6502728
rs72915262
rs6004851
rs77859155
rs113792557
rs117603761
rs117391590
rs139199585
rs3112458
rs67795607
rs76836923
rs12873951
rs79217088
rs12090749
rs268887
rs67641209
rs2381937
rs115540740
rs285429
rs78667279
rs9523198
rs186153147
All
−0.04
−0.02
0.00
0.02
MR leave−one−out sensitivity analysis for
' CD25++ CD8br AC' on 'Rheumatoid arthritis'

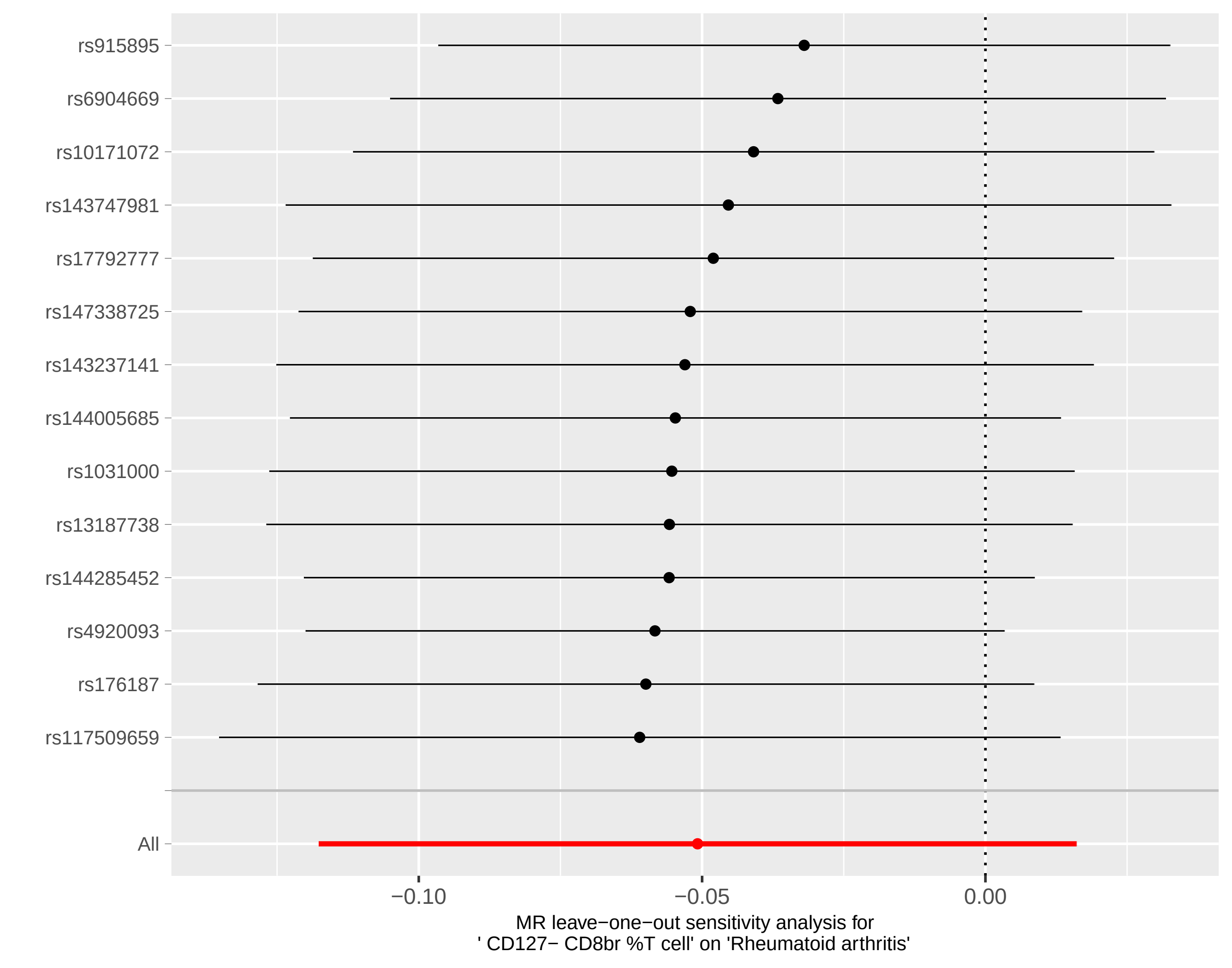
rs915895
rs6904669
rs10171072
rs143747981
rs17792777
rs147338725
rs143237141
rs144005685
rs1031000
rs13187738
rs144285452
rs4920093
rs176187
rs117509659
All
−0.10
−0.05
0.00
MR leave−one−out sensitivity analysis for
' CD127− CD8br %T cell' on 'Rheumatoid arthritis'

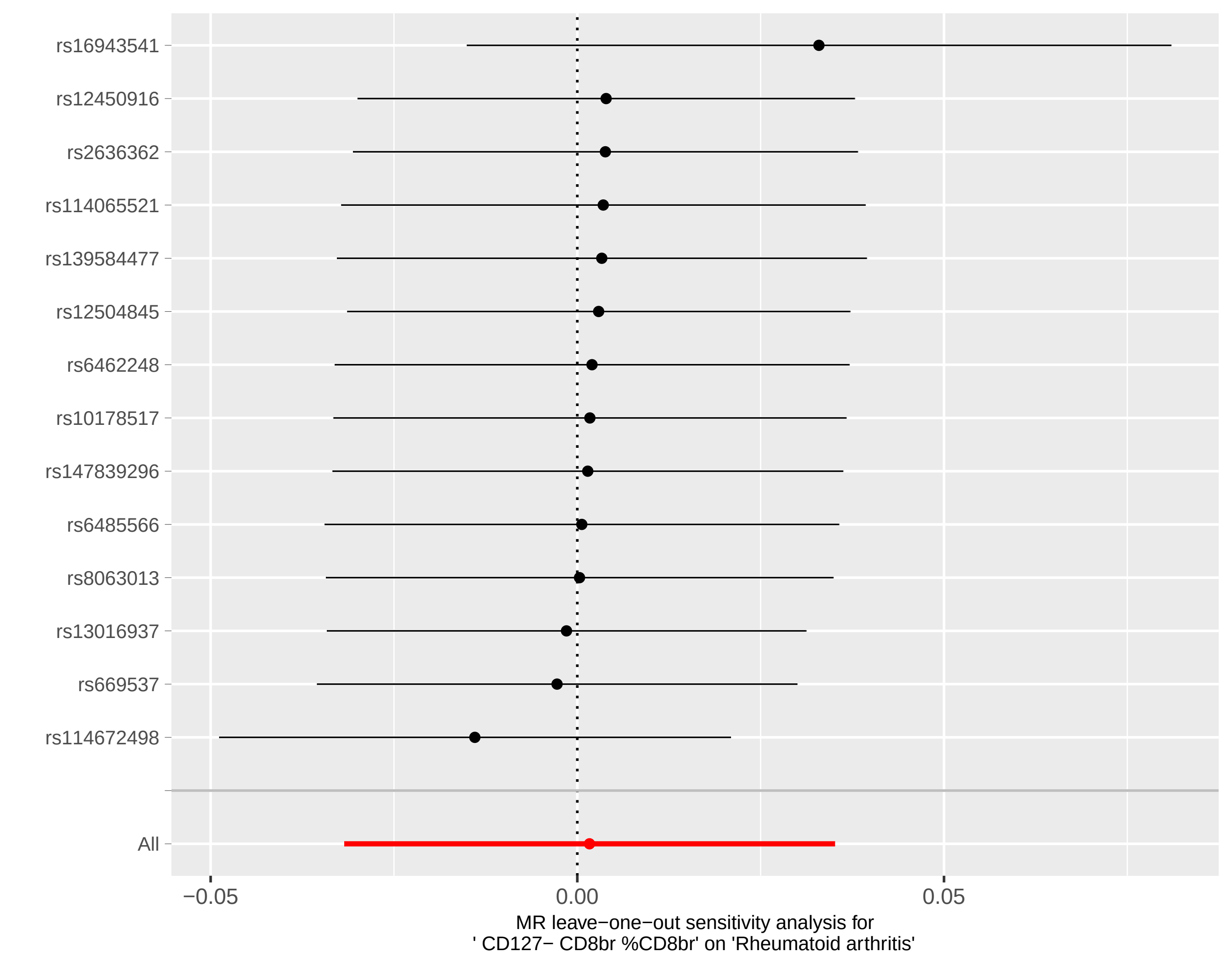
rs16943541
rs12450916
rs2636362
rs114065521
rs139584477
rs12504845
rs6462248
rs10178517
rs147839296
rs6485566
rs8063013
rs13016937
rs669537
rs114672498
All
−0.05
0.00
0.05
MR leave−one−out sensitivity analysis for
' CD127− CD8br %CD8br' on 'Rheumatoid arthritis'

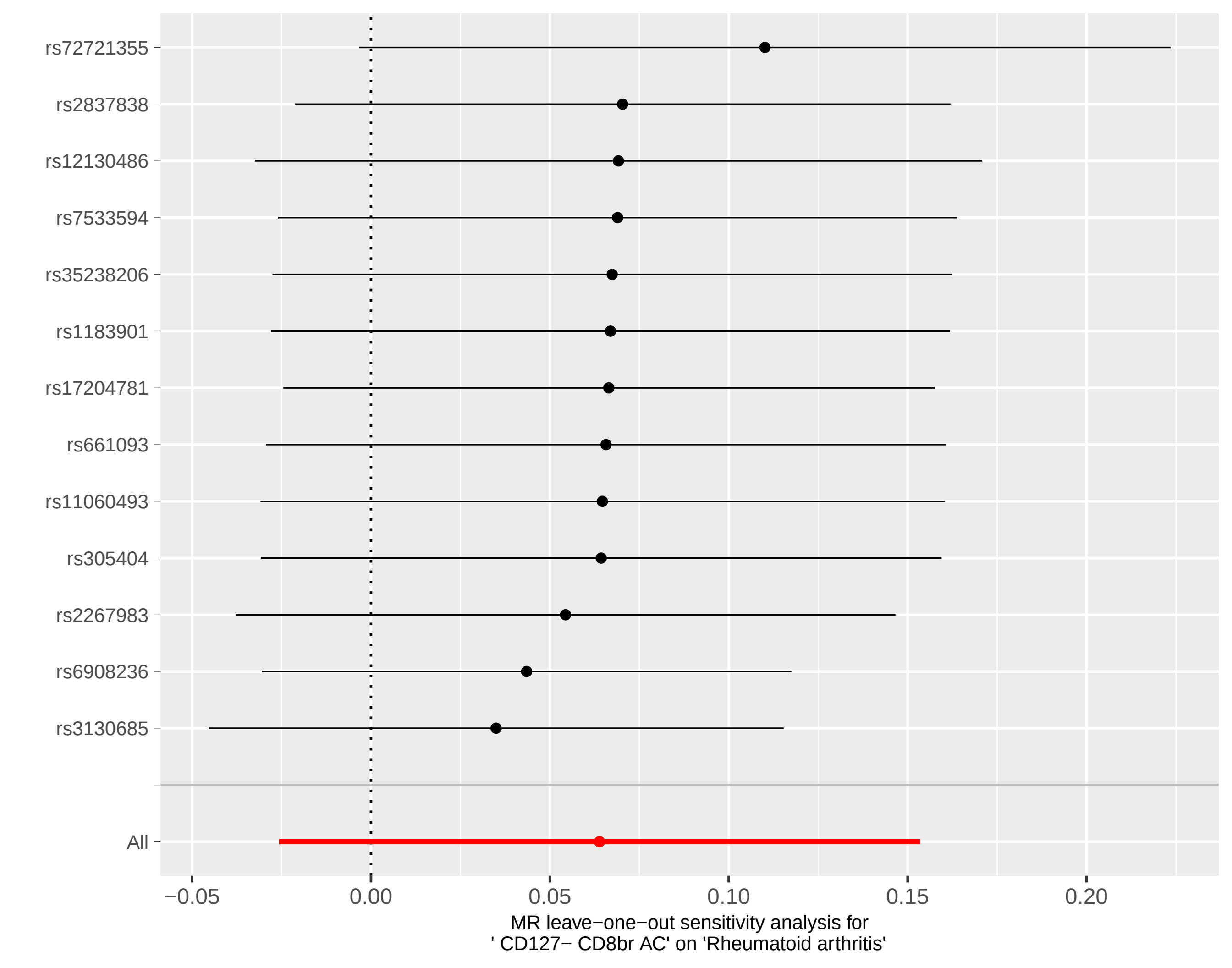
rs72721355
rs2837838
rs12130486
rs7533594
rs35238206
rs1183901
rs17204781
rs661093
rs11060493
rs305404
rs2267983
rs6908236
rs3130685
All
−0.05
0.00
0.05
0.10
0.15
0.20
MR leave−one−out sensitivity analysis for
' CD127− CD8br AC' on 'Rheumatoid arthritis'

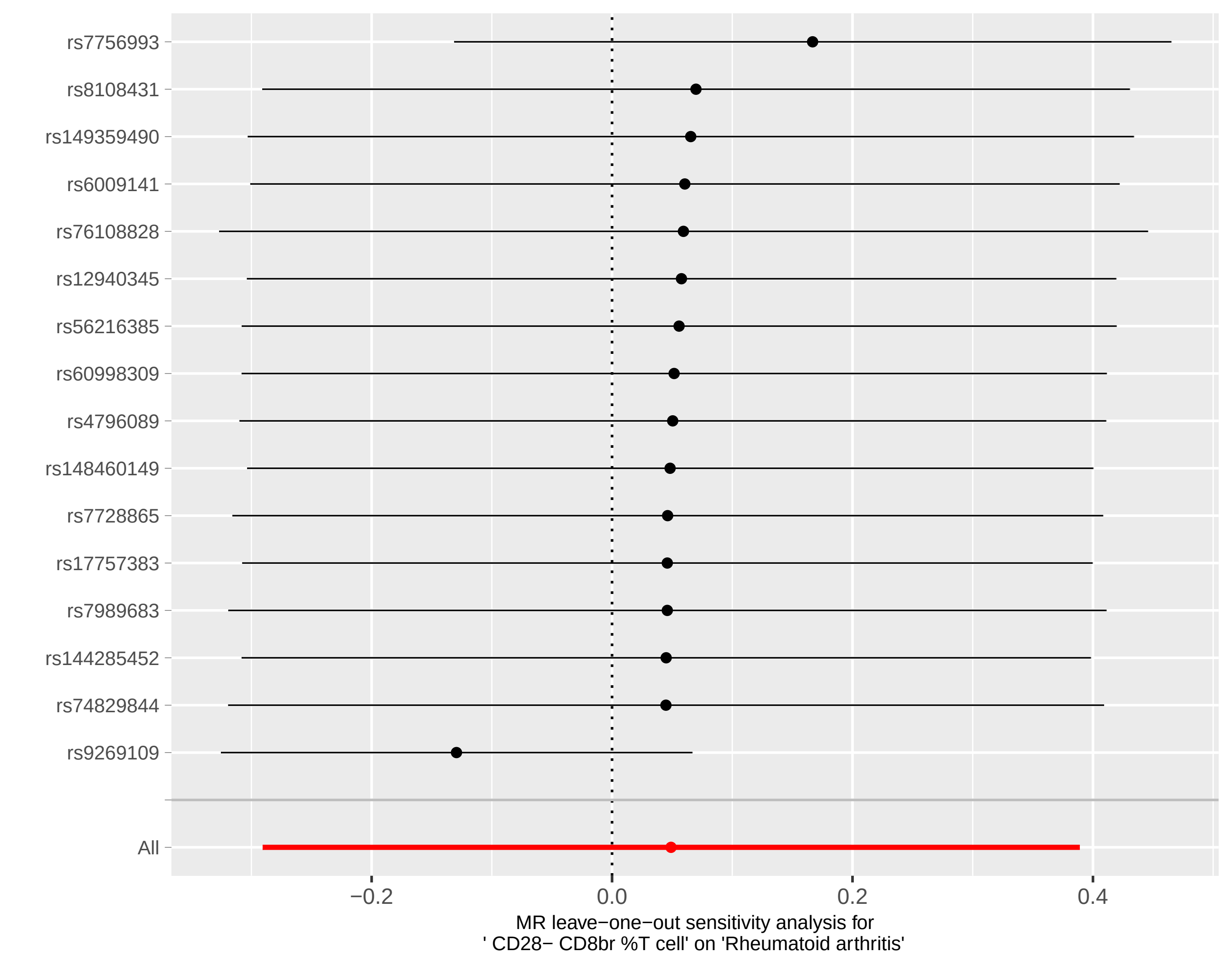
rs7756993
rs8108431
rs149359490
rs6009141
rs76108828
rs12940345
rs56216385
rs60998309
rs4796089
rs148460149
rs7728865
rs17757383
rs7989683
rs144285452
rs74829844
rs9269109
All
−0.2
0.0
0.2
0.4
MR leave−one−out sensitivity analysis for
' CD28− CD8br %T cell' on 'Rheumatoid arthritis'

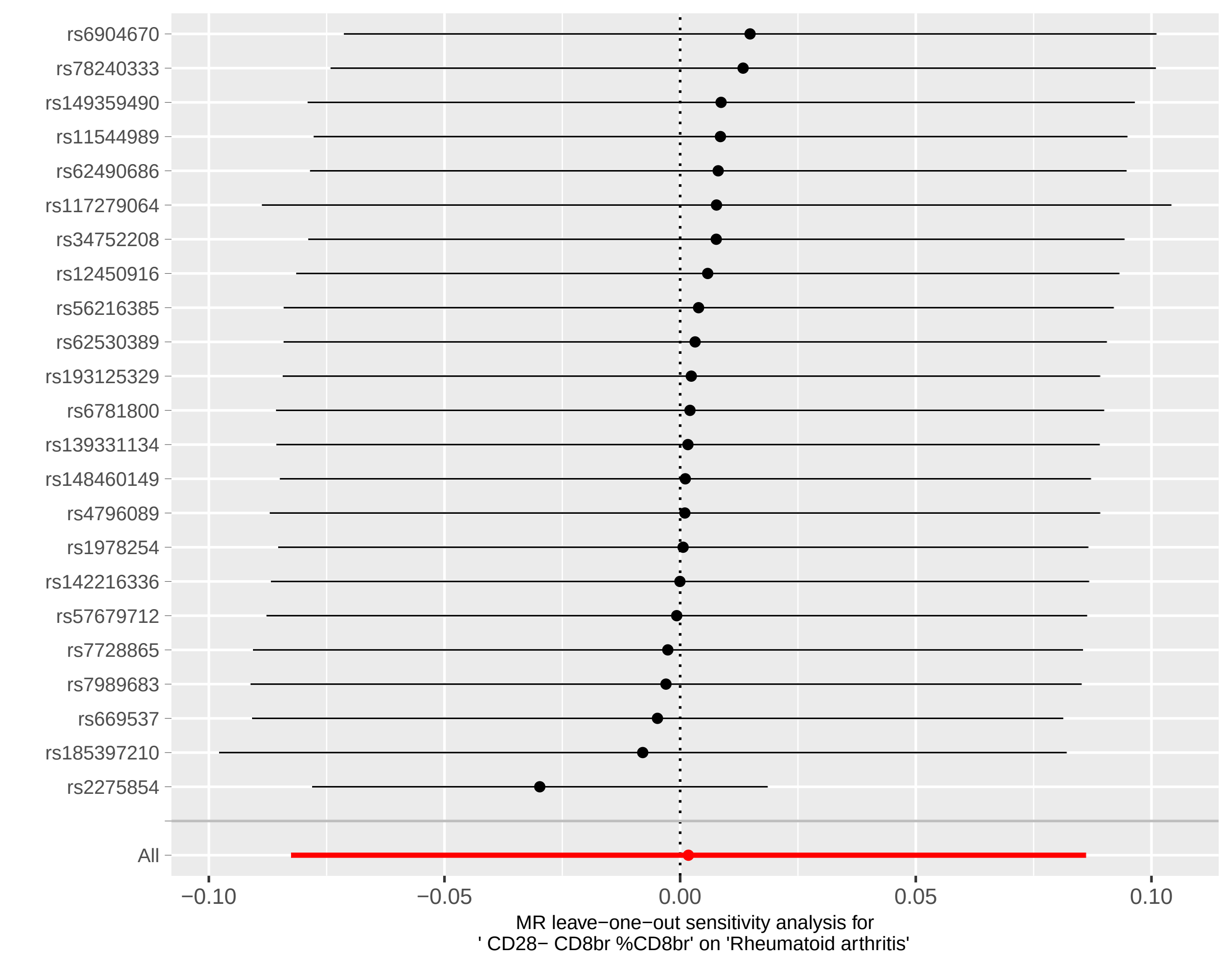
rs6904670
rs78240333
rs149359490
rs11544989
rs62490686
rs117279064
rs34752208
rs12450916
rs56216385
rs62530389
rs193125329
rs6781800
rs139331134
rs148460149
rs4796089
rs1978254
rs142216336
rs57679712
rs7728865
rs7989683
rs669537
rs185397210
rs2275854
All
−0.10
−0.05
0.00
0.05
0.10
MR leave−one−out sensitivity analysis for
' CD28− CD8br %CD8br' on 'Rheumatoid arthritis'

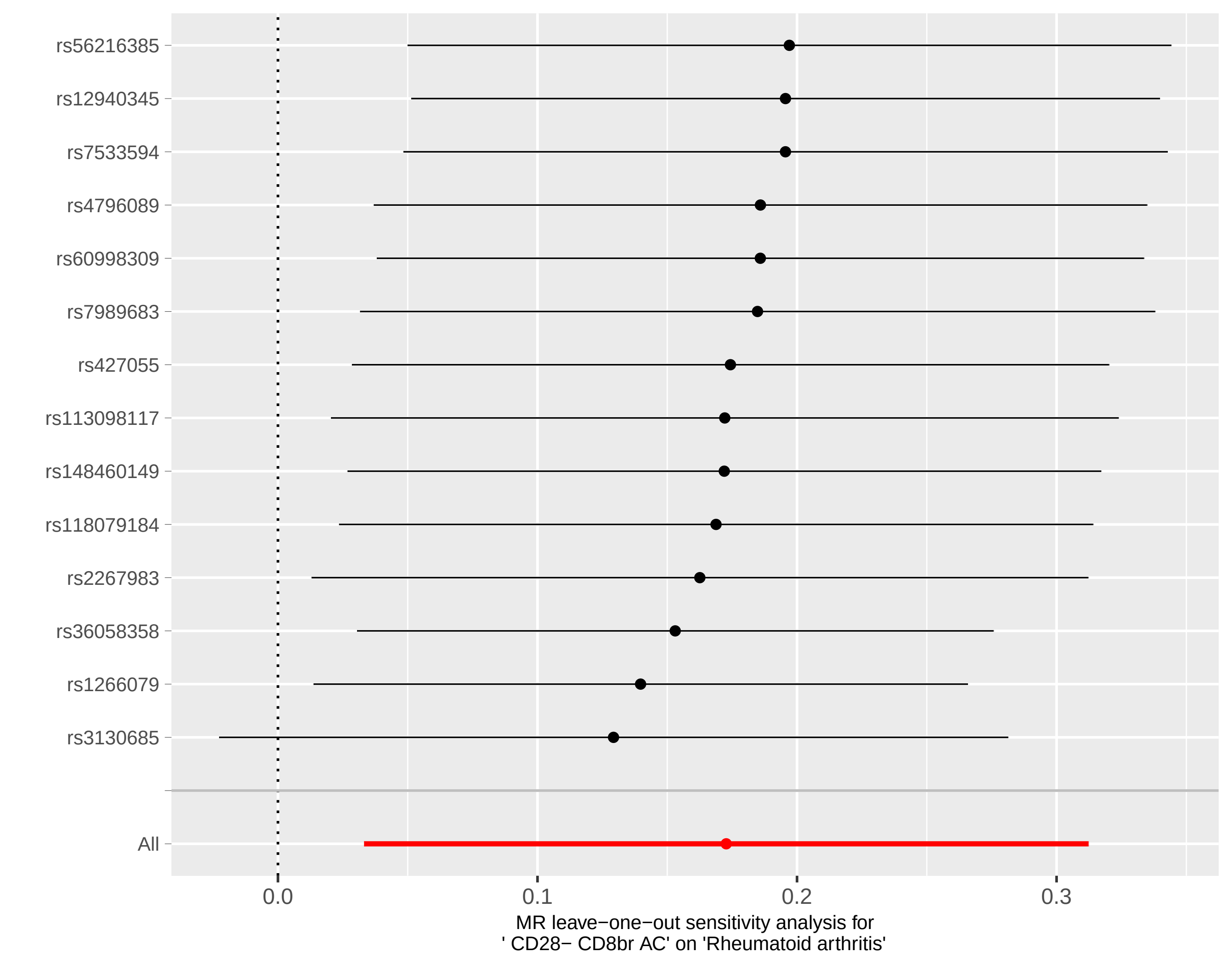
rs56216385
rs12940345
rs7533594
rs4796089
rs60998309
rs7989683
rs427055
rs113098117
rs148460149
rs118079184
rs2267983
rs36058358
rs1266079
rs3130685
All
0.0
0.1
0.2
0.3
MR leave−one−out sensitivity analysis for
' CD28− CD8br AC' on 'Rheumatoid arthritis'

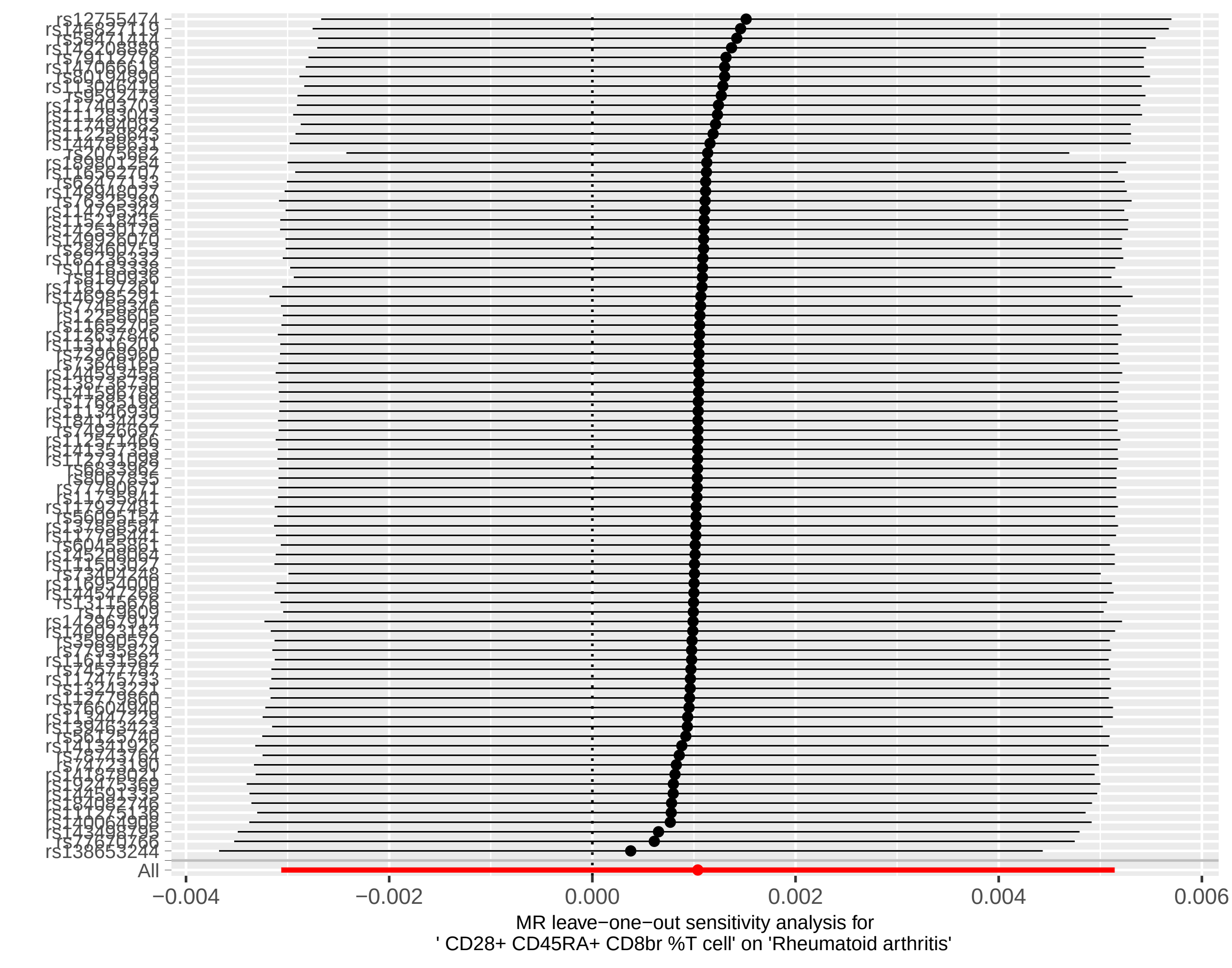
rs12755474
rs138653244
All
−0.004
−0.002
0.000
0.002
0.004
0.006
MR leave−one−out sensitivity analysis for
' CD28+ CD45RA+ CD8br %T cell' on 'Rheumatoid arthritis'

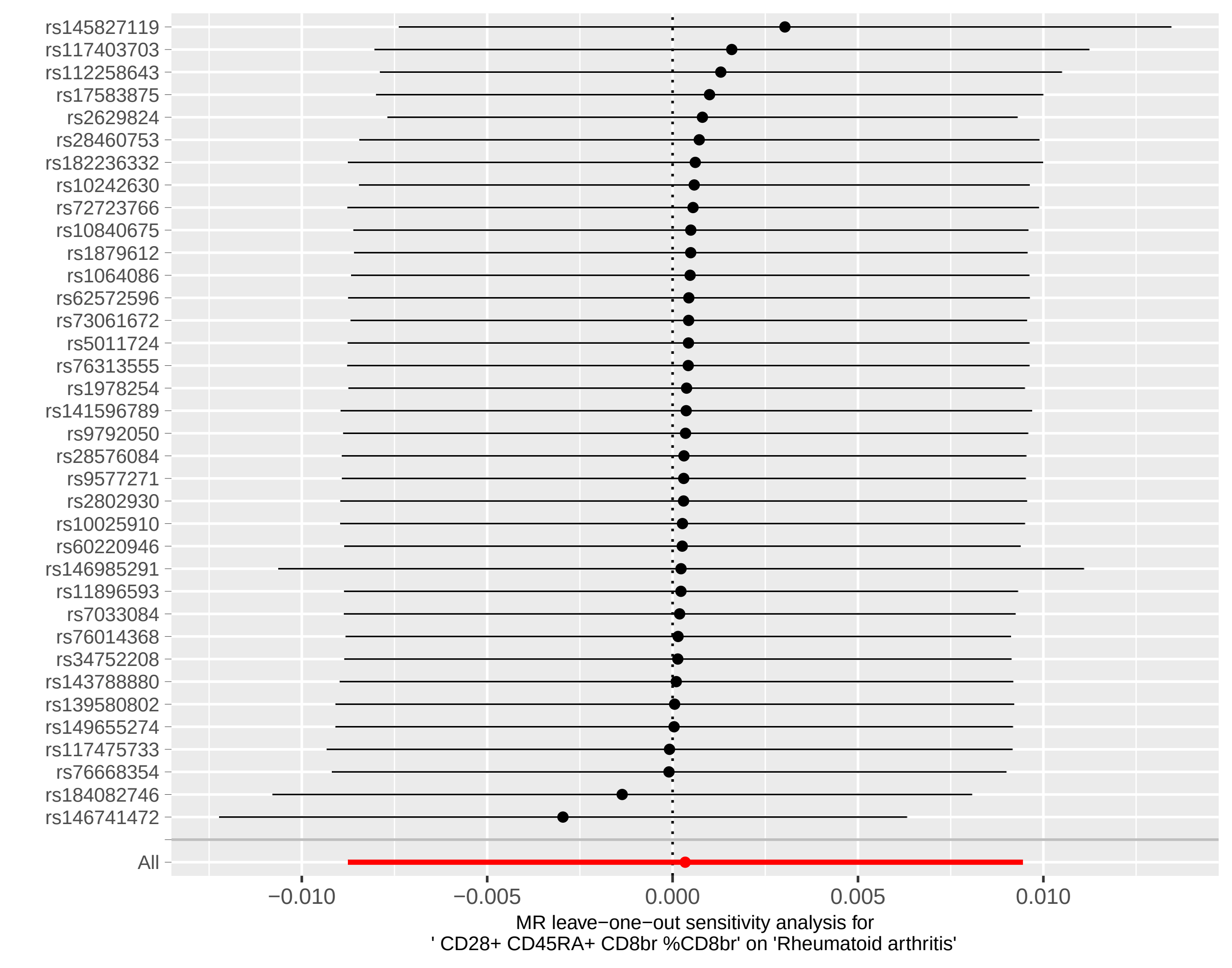

rs145827119
rs117403703
rs112258643
rs17583875
rs2629824
rs28460753
rs182236332
rs10242630
rs72723766
rs10840675
rs1879612
rs1064086
rs62572596
rs73061672
rs5011724
rs76313555
rs1978254
rs141596789
rs9792050
rs28576084
rs9577271
rs2802930
rs10025910
rs60220946
rs146985291
rs11896593
rs7033084
rs76014368
rs34752208
rs143788880
rs139580802
rs149655274
rs117475733
rs76668354
rs184082746
rs146741472
All
−0.010
−0.005
0.000
0.005
0.010
MR leave−one−out sensitivity analysis for
' CD28+ CD45RA+ CD8br %CD8br' on 'Rheumatoid arthritis'

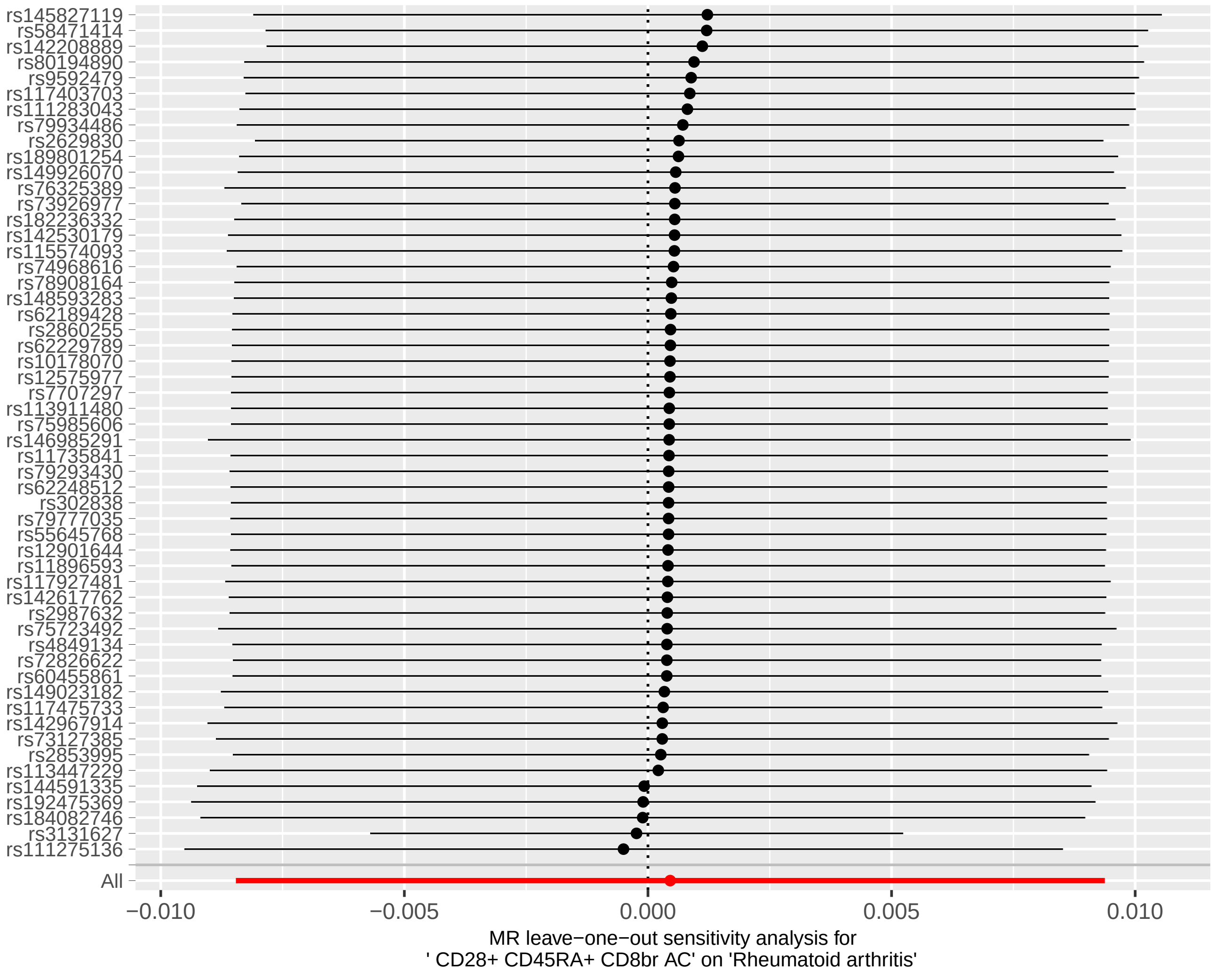

rs145827119
rs58471414
rs142208889
rs80194890
rs9592479
rs117403703
rs111283043
rs79934486
rs2629830
rs189801254
rs149926070
rs76325389
rs73926977
rs182236332
rs142530179
rs115574093
rs74968616
rs78908164
rs148593283
rs62189428
rs2860255
rs62229789
rs10178070
rs12575977
rs7707297
rs113911480
rs75985606
rs146985291
rs11735841
rs79293430
rs62248512
rs302838
rs79777035
rs55645768
rs12901644
rs11896593
rs117927481
rs142617762
rs2987632
rs75723492
rs4849134
rs72826622
rs60455861
rs149023182
rs117475733
rs142967914
rs73127385
rs2853995
rs113447229
rs144591335
rs192475369
rs184082746
rs3131627
rs111275136
All
−0.010
−0.005
0.000
0.005
0.010
MR leave−one−out sensitivity analysis for
' CD28+ CD45RA+ CD8br AC' on 'Rheumatoid arthritis'

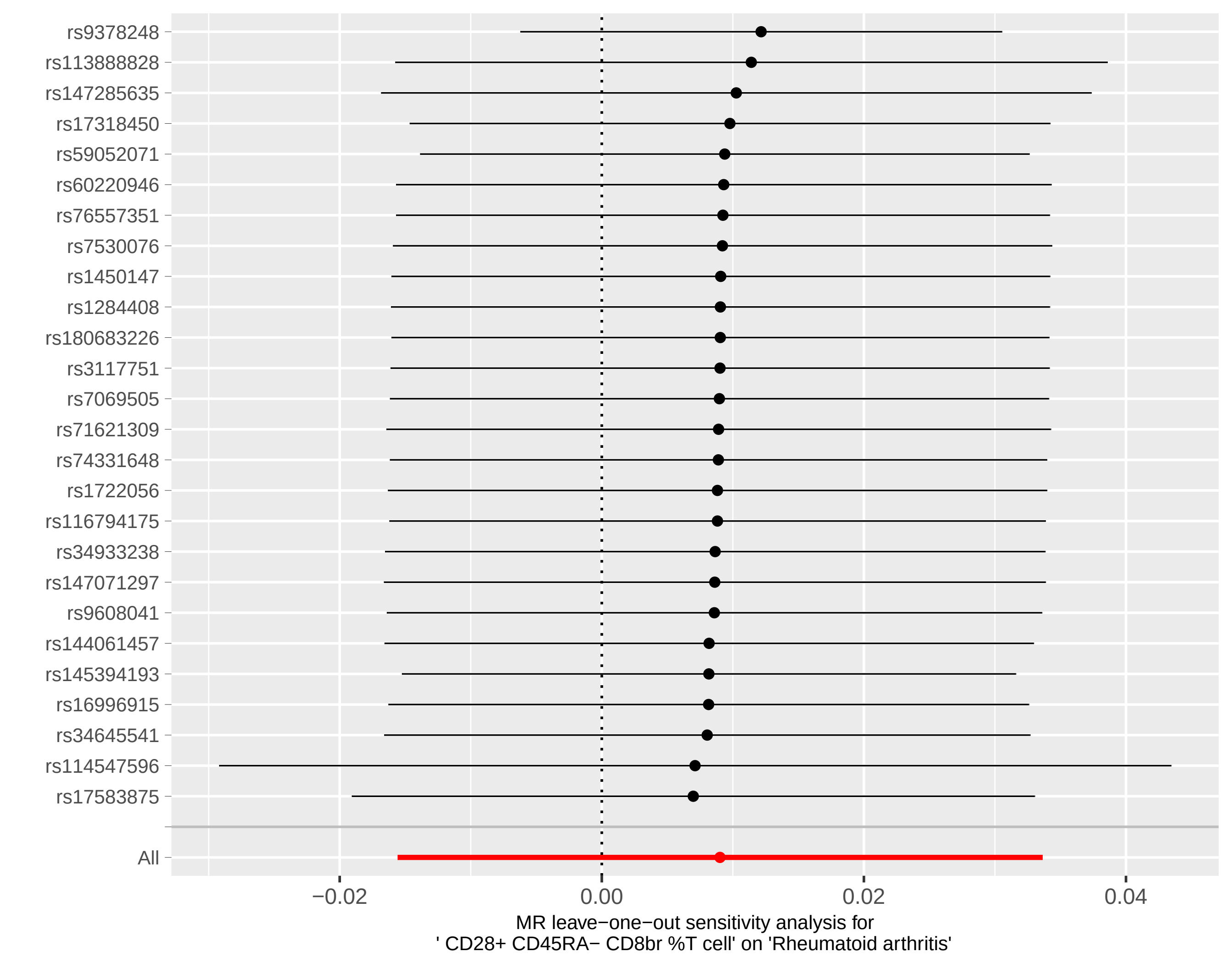
rs9378248
rs113888828
rs147285635
rs17318450
rs59052071
rs60220946
rs76557351
rs7530076
rs1450147
rs1284408
rs180683226
rs3117751
rs7069505
rs71621309
rs74331648
rs1722056
rs116794175
rs34933238
rs147071297
rs9608041
rs144061457
rs145394193
rs16996915
rs34645541
rs114547596
rs17583875
All
−0.02
0.00
0.02
0.04
MR leave−one−out sensitivity analysis for
' CD28+ CD45RA− CD8br %T cell' on 'Rheumatoid arthritis'

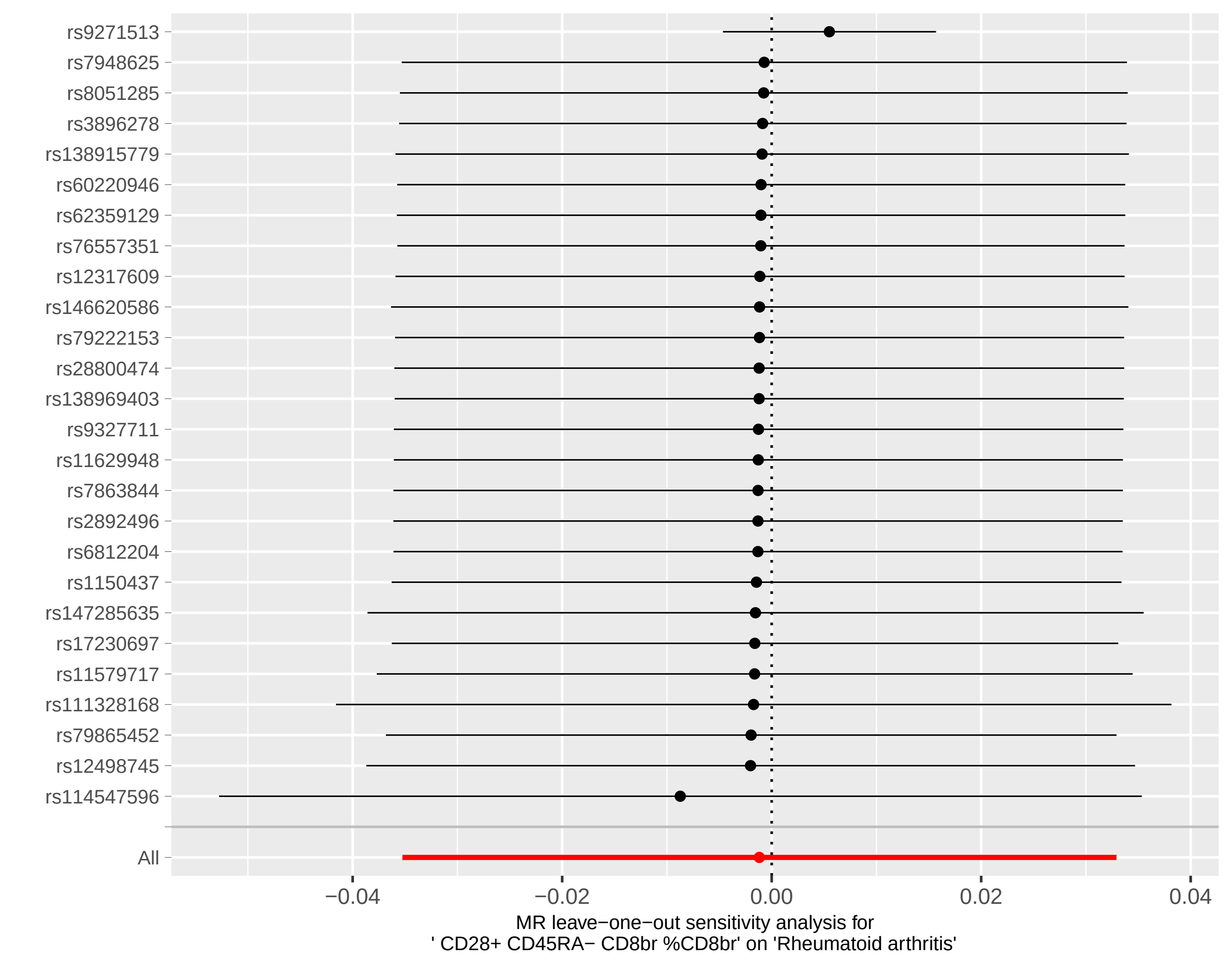
rs9271513
rs7948625
rs8051285
rs3896278
rs138915779
rs60220946
rs62359129
rs76557351
rs12317609
rs146620586
rs79222153
rs28800474
rs138969403
rs9327711
rs11629948
rs7863844
rs2892496
rs6812204
rs1150437
rs147285635
rs17230697
rs11579717
rs111328168
rs79865452
rs12498745
rs114547596
All
−0.04
−0.02
0.00
0.02
0.04
MR leave−one−out sensitivity analysis for
' CD28+ CD45RA− CD8br %CD8br' on 'Rheumatoid arthritis'

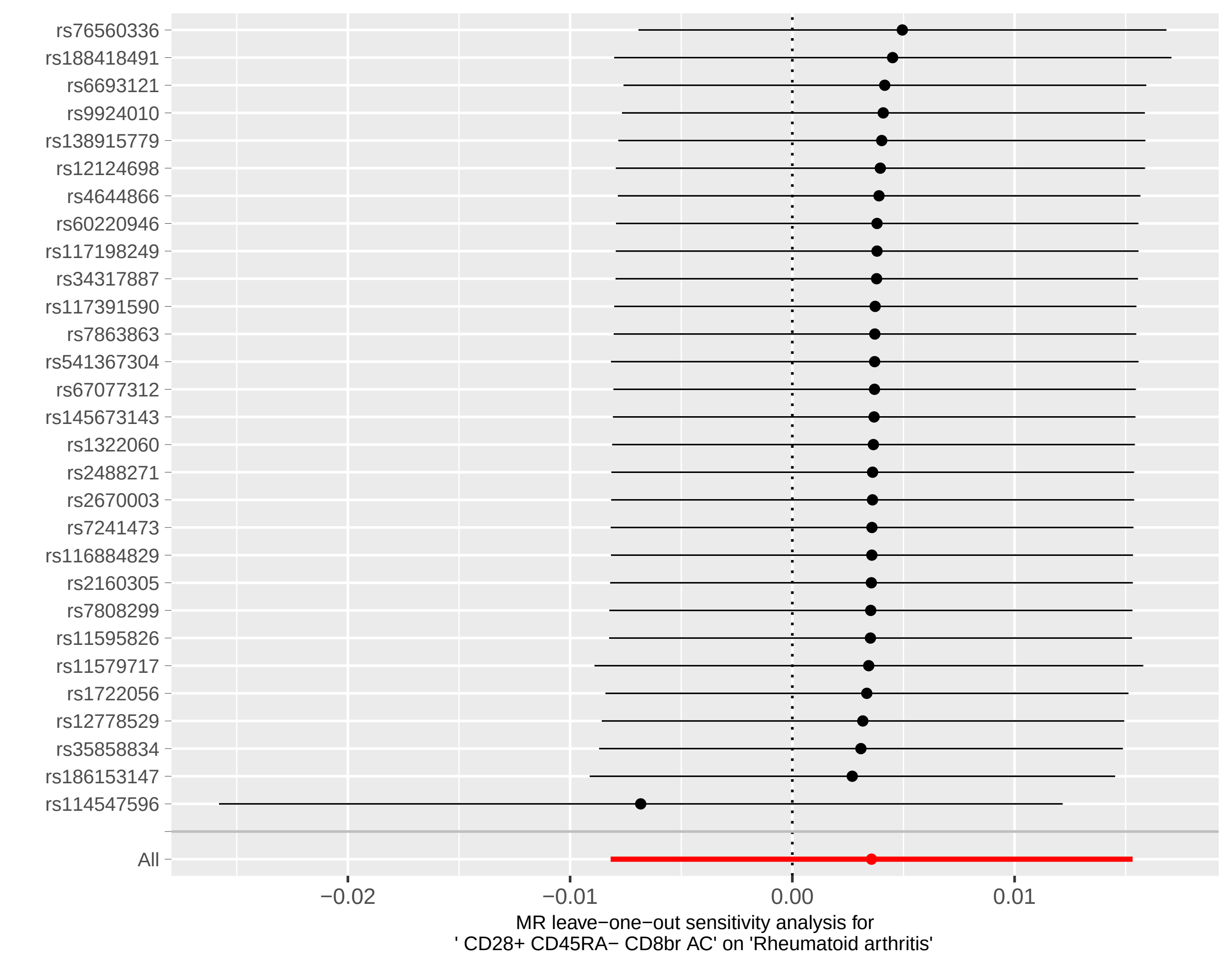
rs76560336
rs188418491
rs6693121
rs9924010
rs138915779
rs12124698
rs4644866
rs60220946
rs117198249
rs34317887
rs117391590
rs7863863
rs541367304
rs67077312
rs145673143
rs1322060
rs2488271
rs2670003
rs7241473
rs116884829
rs2160305
rs7808299
rs11595826
rs11579717
rs1722056
rs12778529
rs35858834
rs186153147
rs114547596
All
−0.02
−0.01
0.00
0.01
MR leave−one−out sensitivity analysis for
' CD28+ CD45RA− CD8br AC' on 'Rheumatoid arthritis'

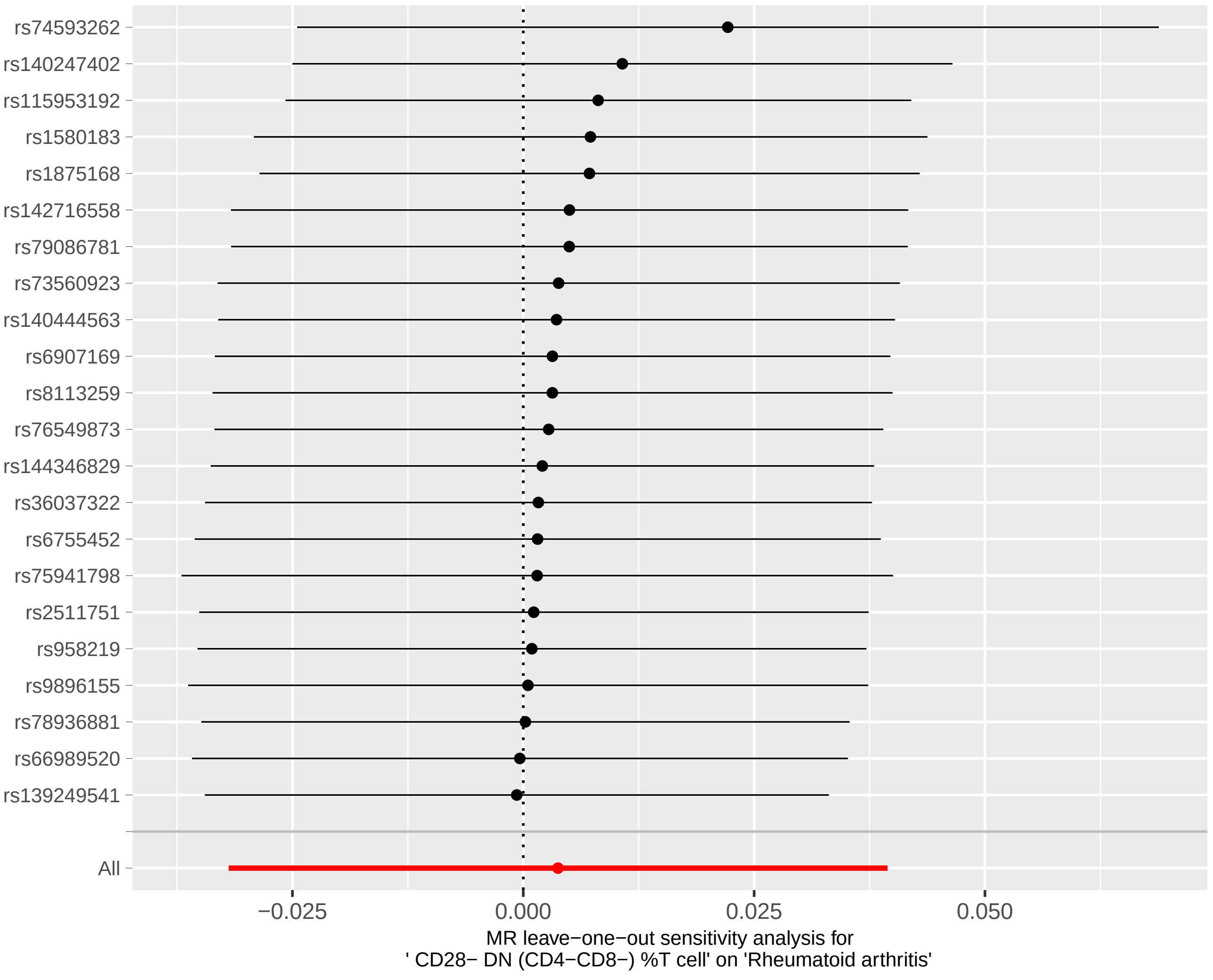
rs74593262
rs140247402
rs115953192
rs1580183
rs1875168
rs142716558
rs79086781
rs73560923
rs140444563
rs6907169
rs8113259
rs76549873
rs144346829
rs36037322
rs6755452
rs75941798
rs2511751
rs958219
rs9896155
rs78936881
rs66989520
rs139249541
All
−0.025
0.000
0.025
0.050
MR leave−one−out sensitivity analysis for
' CD28− DN (CD4−CD8−) %T cell' on 'Rheumatoid arthritis'

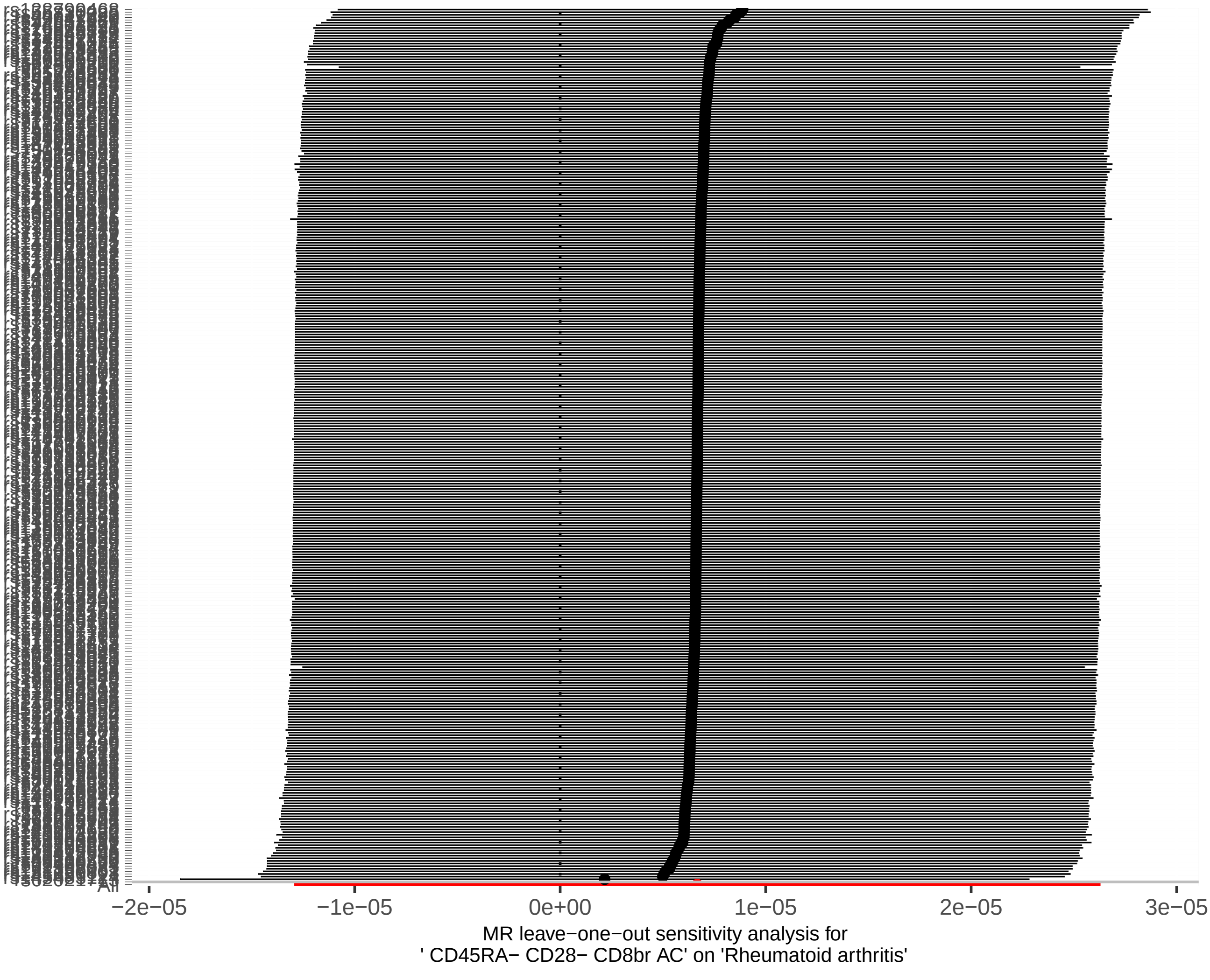

All
−2e−05
−1e−05
0e+00
1e−05
2e−05
3e−05
MR leave−one−out sensitivity analysis for
' CD45RA− CD28− CD8br AC' on 'Rheumatoid arthritis'

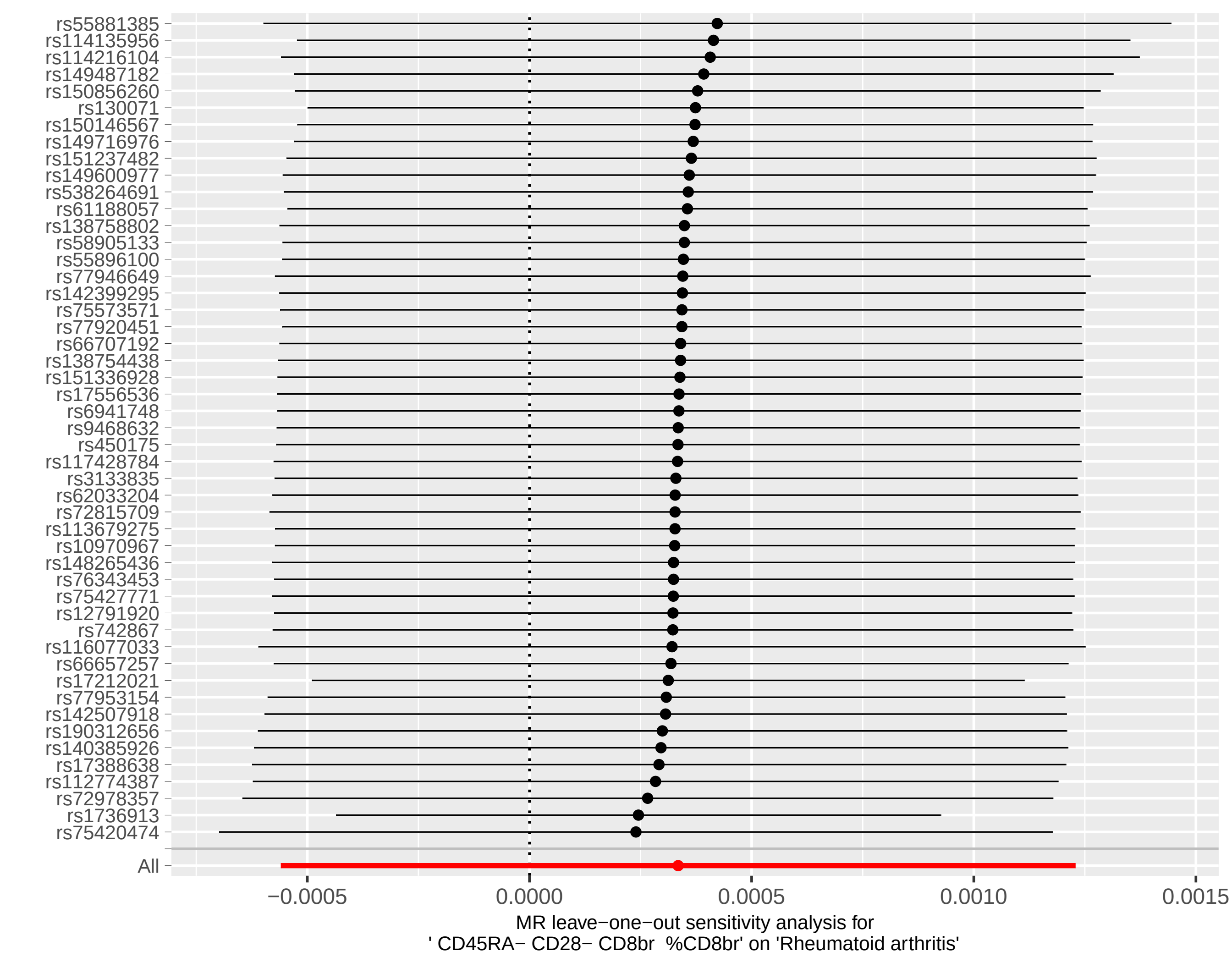
rs55881385
rs114135956
rs114216104
rs149487182
rs150856260
rs130071
rs150146567
rs149716976
rs151237482
rs149600977
rs538264691
rs61188057
rs138758802
rs58905133
rs55896100
rs77946649
rs142399295
rs75573571
rs77920451
rs66707192
rs138754438
rs151336928
rs17556536
rs6941748
rs9468632
rs450175
rs117428784
rs3133835
rs62033204
rs72815709
rs113679275
rs10970967
rs148265436
rs76343453
rs75427771
rs12791920
rs742867
rs116077033
rs66657257
rs17212021
rs77953154
rs142507918
rs190312656
rs140385926
rs17388638
rs112774387
rs72978357
rs1736913
rs75420474
All
−0.0005
0.0000
0.0005
0.0010
0.0015
MR leave−one−out sensitivity analysis for
' CD45RA− CD28− CD8br %CD8br' on 'Rheumatoid arthritis'

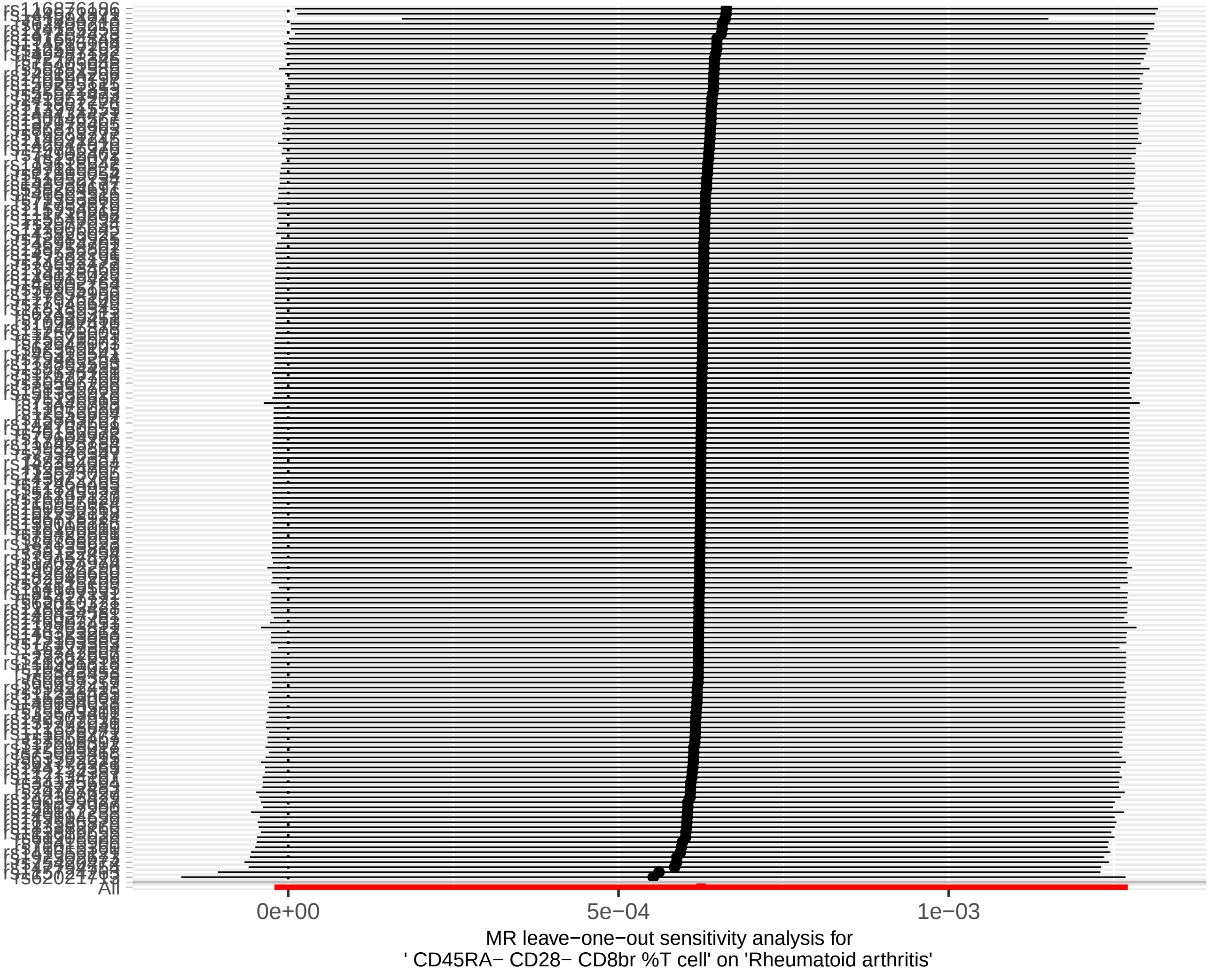

All
0e+00
5e−04
1e−03
MR leave−one−out sensitivity analysis for
' CD45RA− CD28− CD8br %T cell' on 'Rheumatoid arthritis'

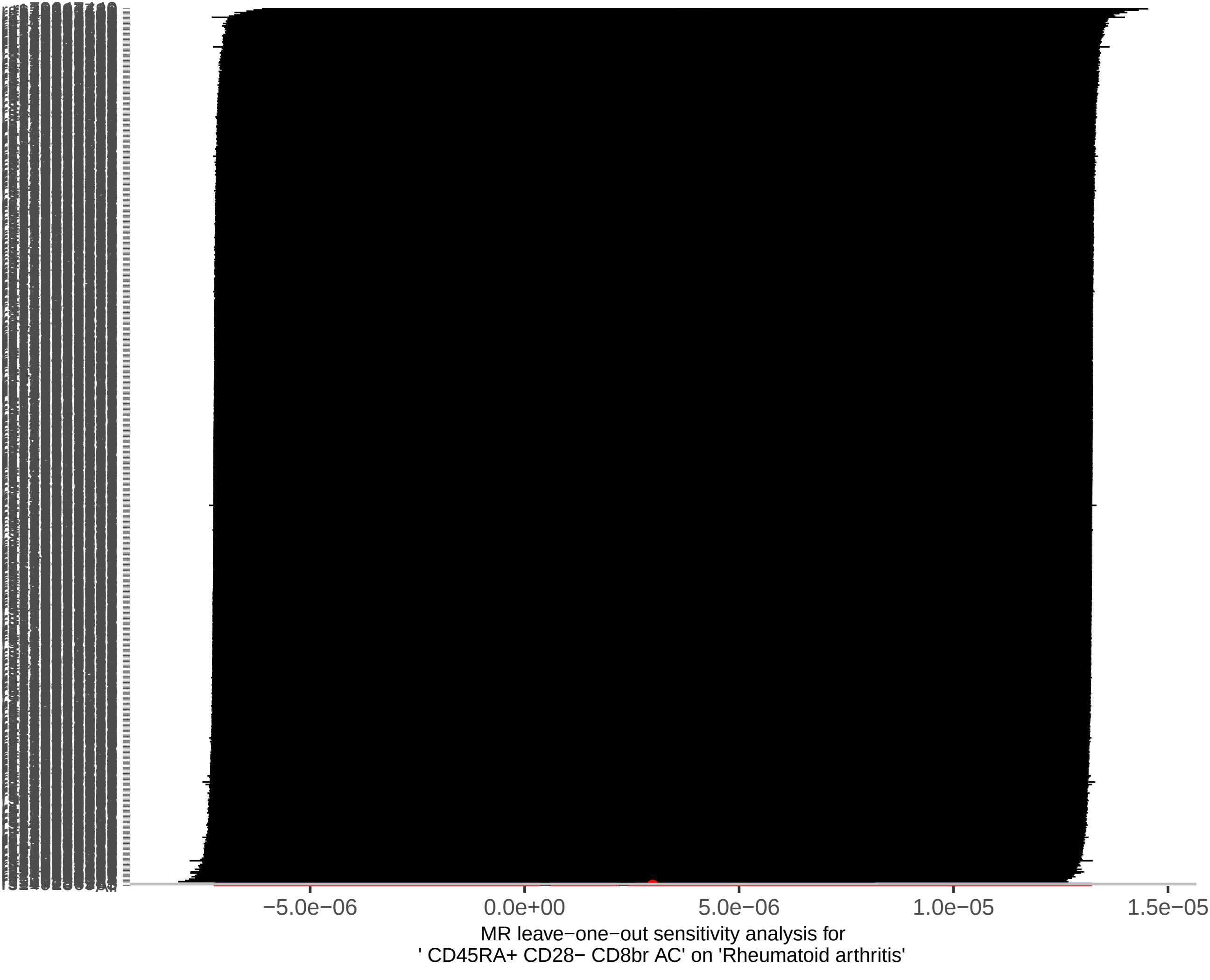

−5.0e−06
0.0e+00
5.0e−06
1.0e−05
1.5e−05
MR leave−one−out sensitivity analysis for
' CD45RA+ CD28− CD8br AC' on 'Rheumatoid arthritis'

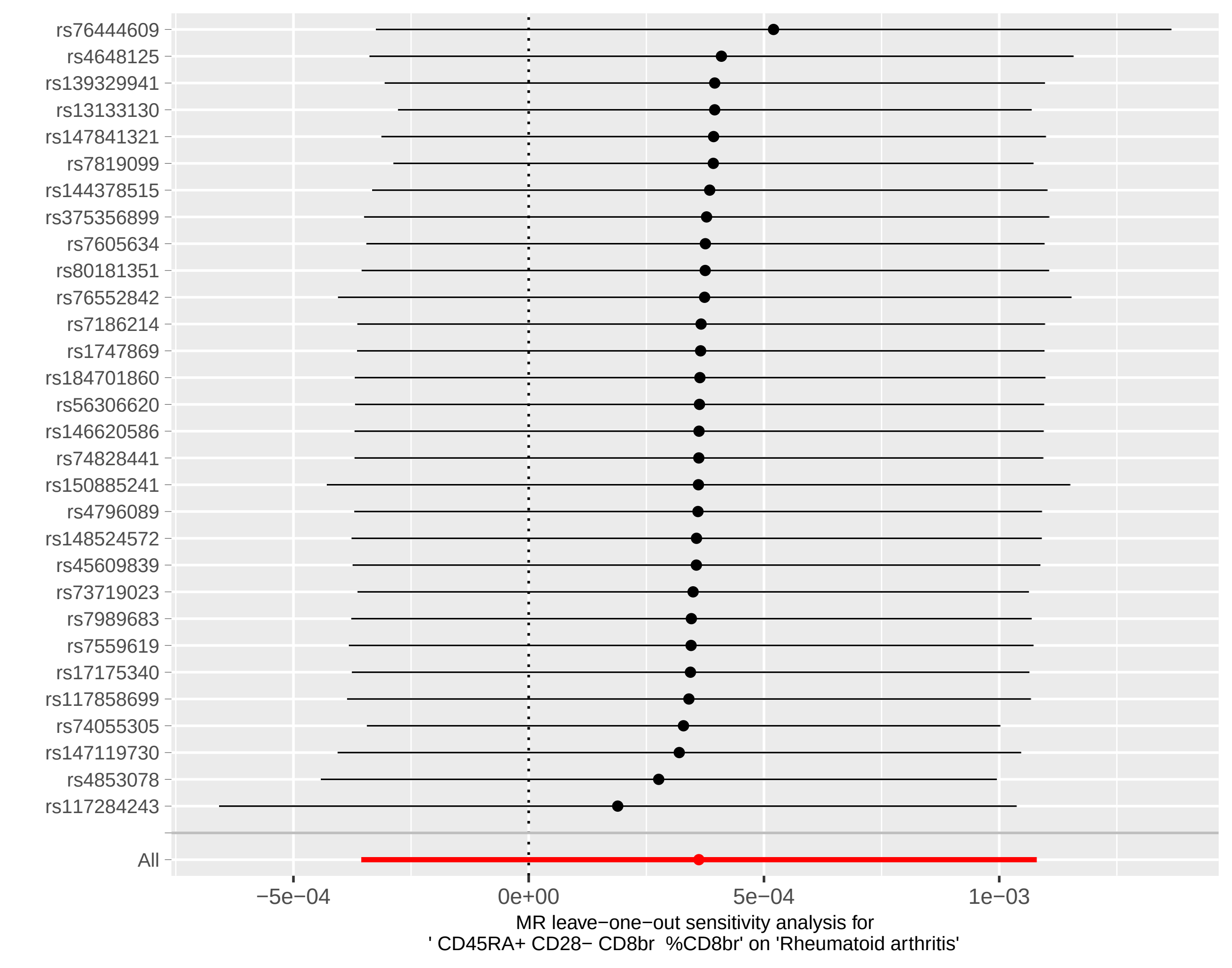
rs76444609
rs4648125
rs139329941
rs13133130
rs147841321
rs7819099
rs144378515
rs375356899
rs7605634
rs80181351
rs76552842
rs7186214
rs1747869
rs184701860
rs56306620
rs146620586
rs74828441
rs150885241
rs4796089
rs148524572
rs45609839
rs73719023
rs7989683
rs7559619
rs17175340
rs117858699
rs74055305
rs147119730
rs4853078
rs117284243
All
−5e−04
0e+00
5e−04
1e−03
MR leave−one−out sensitivity analysis for
' CD45RA+ CD28− CD8br %CD8br' on 'Rheumatoid arthritis'

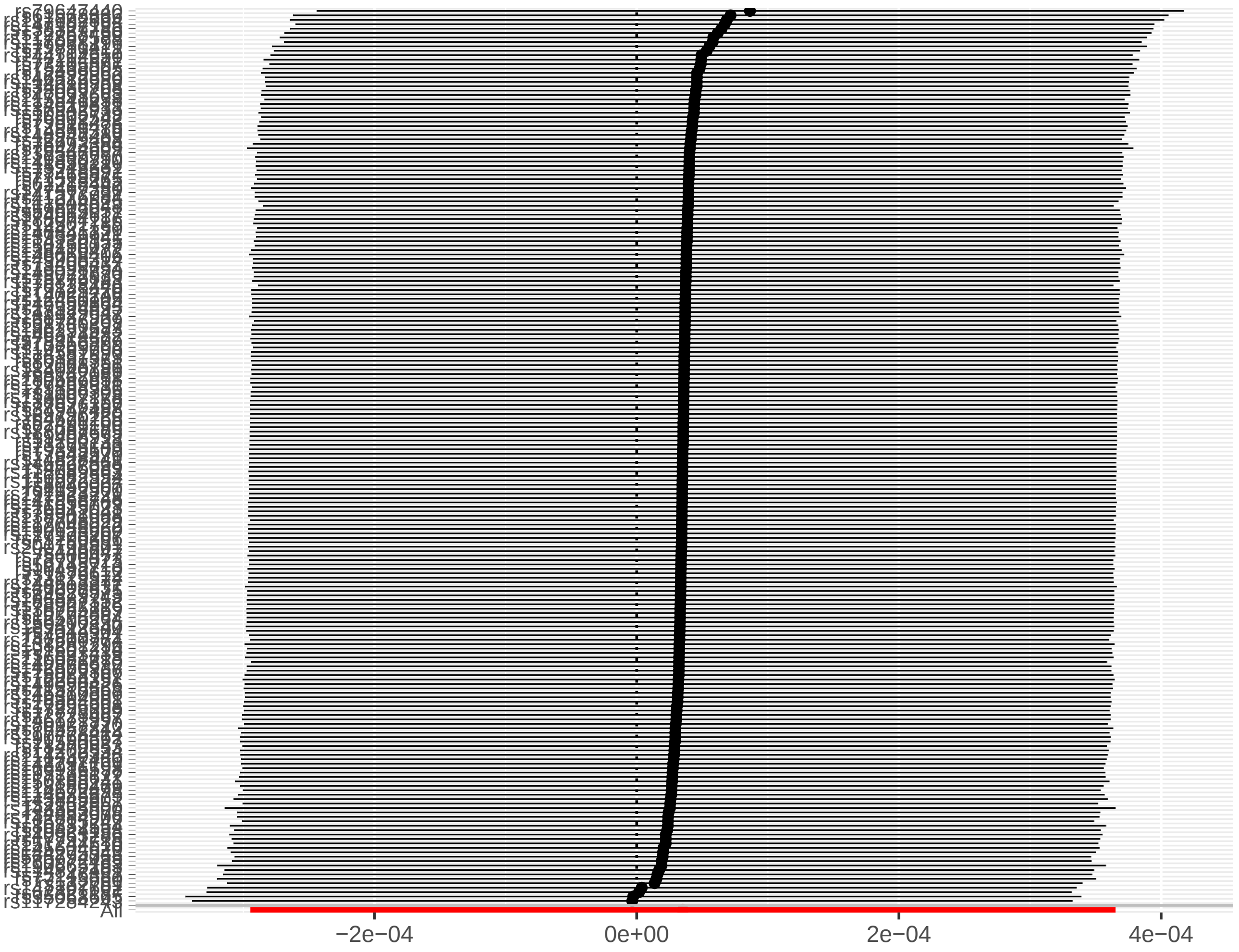

MR leave−one−out sensitivity analysis for
' CD45RA+ CD28− CD8br %T cell' on 'Rheumatoid arthritis'

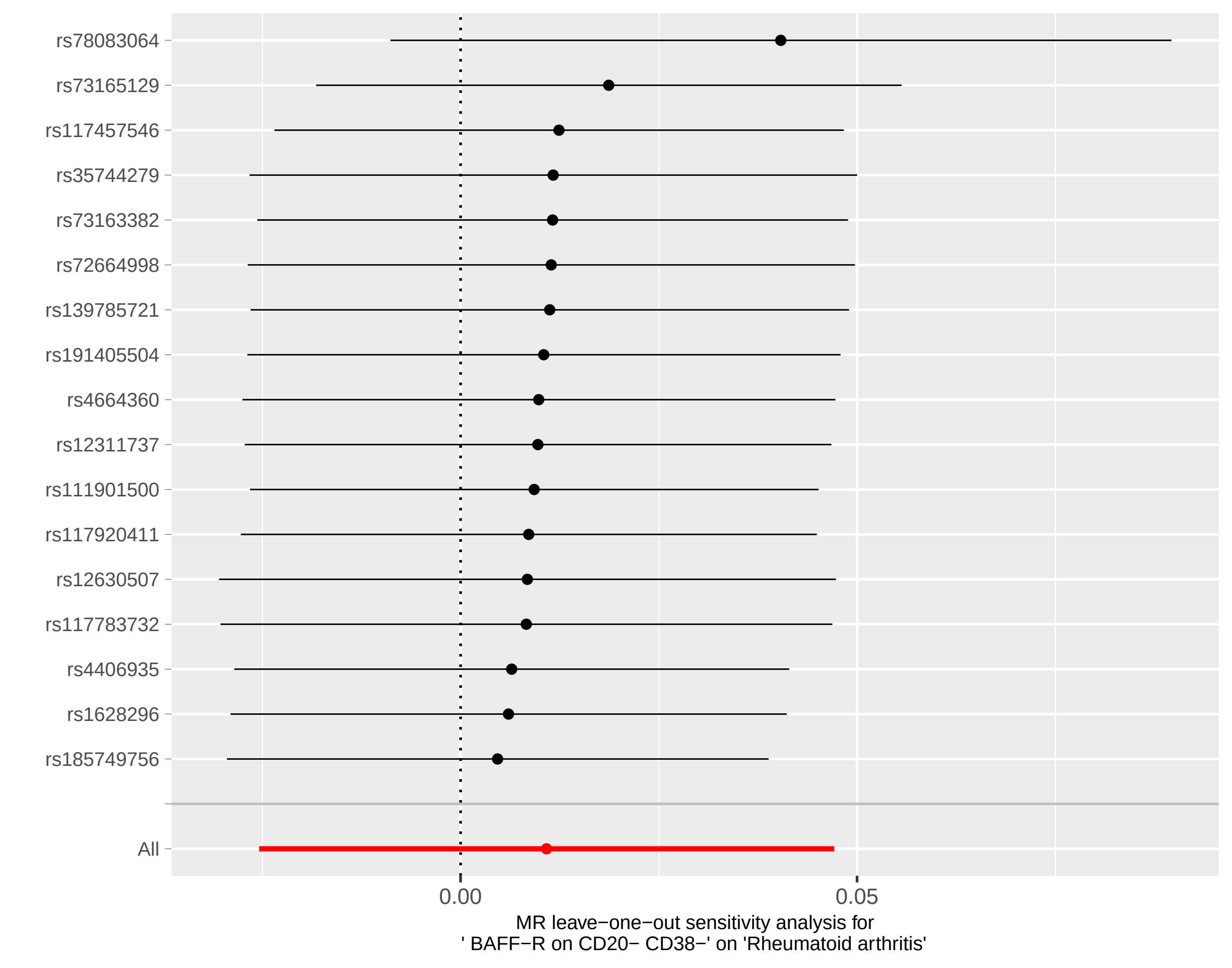

rs78083064
rs73165129
rs117457546
rs35744279
rs73163382
rs72664998
rs139785721
rs191405504
rs4664360
rs12311737
rs111901500
rs117920411
rs12630507
rs117783732
rs4406935
rs1628296
rs185749756
All
0.00
0.05
MR leave−one−out sensitivity analysis for
' BAFF−R on CD20− CD38−' on 'Rheumatoid arthritis'

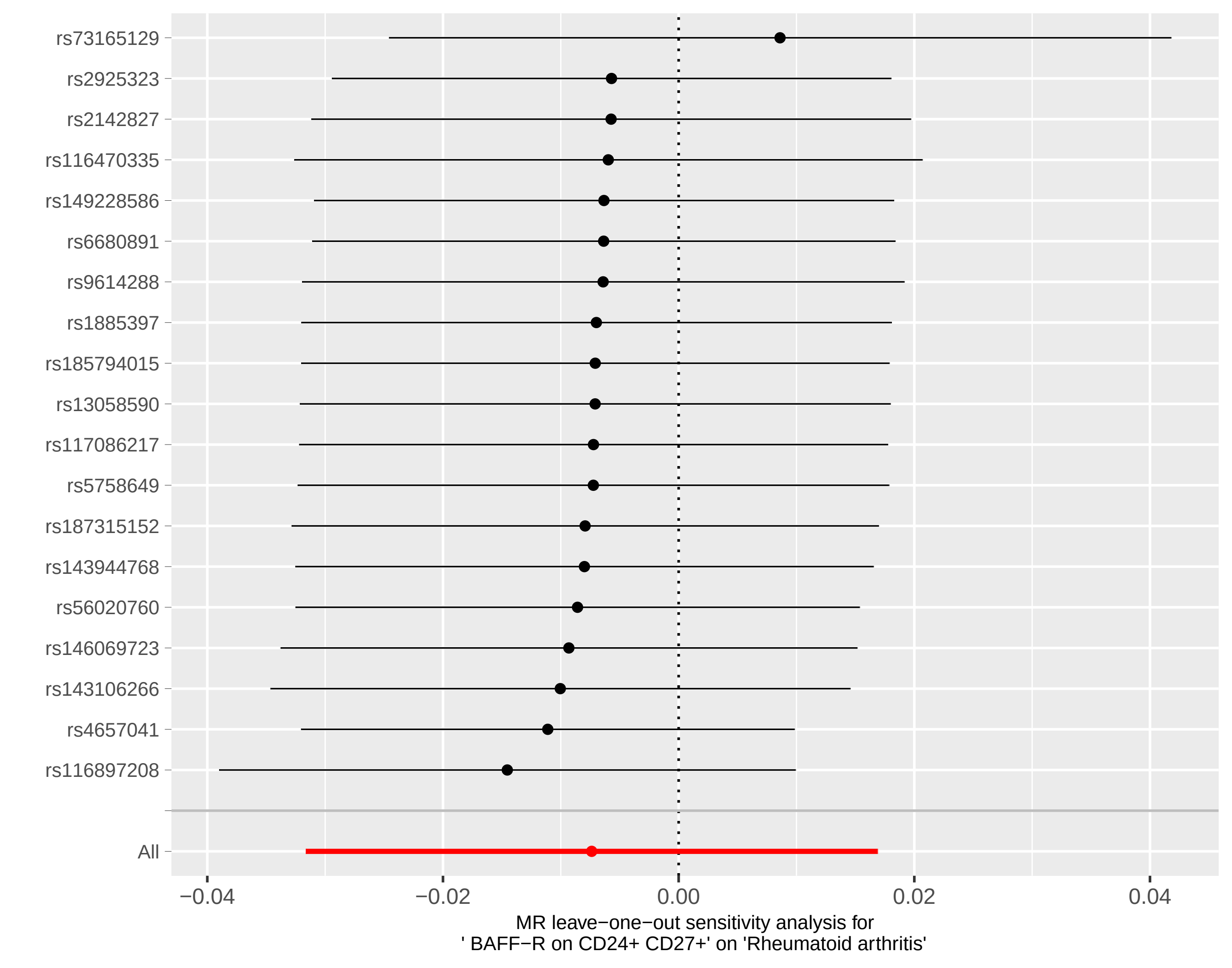
rs73165129
rs2925323
rs2142827
rs116470335
rs149228586
rs6680891
rs9614288
rs1885397
rs185794015
rs13058590
rs117086217
rs5758649
rs187315152
rs143944768
rs56020760
rs146069723
rs143106266
rs4657041
rs116897208
All
−0.04
−0.02
0.00
0.02
0.04
MR leave−one−out sensitivity analysis for
' BAFF−R on CD24+ CD27+' on 'Rheumatoid arthritis'

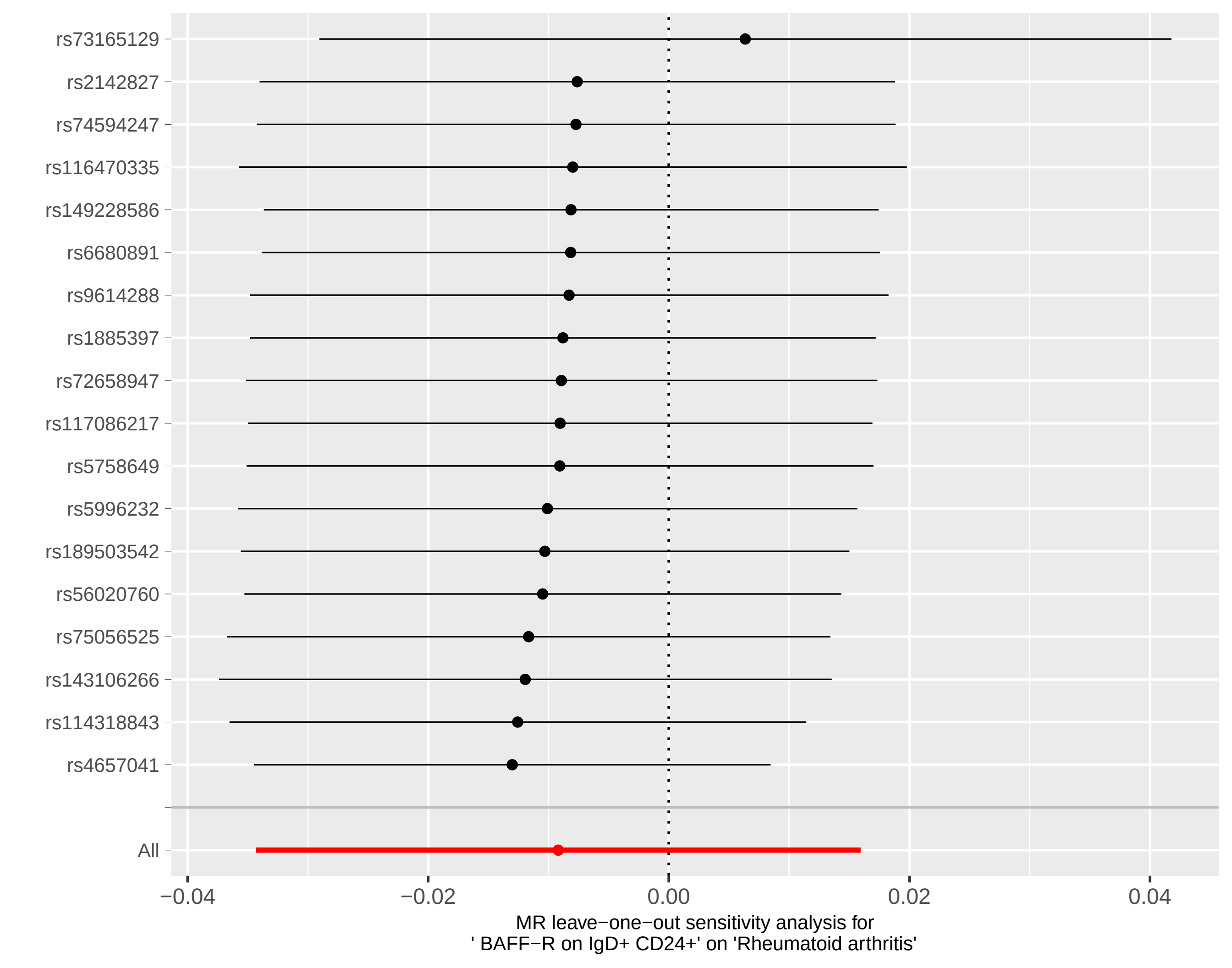
rs73165129
rs2142827
rs74594247
rs116470335
rs149228586
rs6680891
rs9614288
rs1885397
rs72658947
rs117086217
rs5758649
rs5996232
rs189503542
rs56020760
rs75056525
rs143106266
rs114318843
rs4657041
All
−0.04
−0.02
0.00
0.02
0.04
MR leave−one−out sensitivity analysis for
' BAFF−R on IgD+ CD24+' on 'Rheumatoid arthritis'

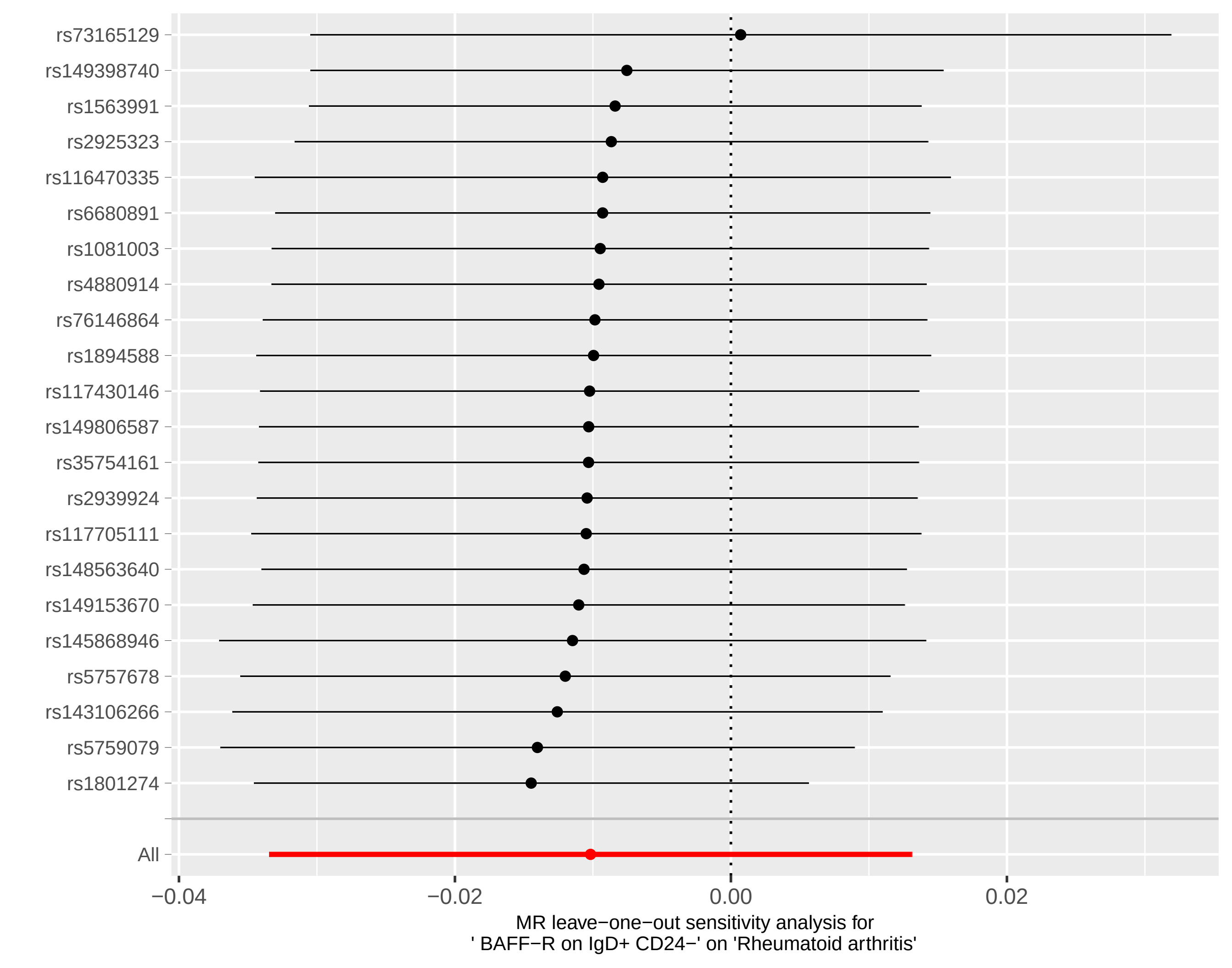
rs73165129
rs149398740
rs1563991
rs2925323
rs116470335
rs6680891
rs1081003
rs4880914
rs76146864
rs1894588
rs117430146
rs149806587
rs35754161
rs2939924
rs117705111
rs148563640
rs149153670
rs145868946
rs5757678
rs143106266
rs5759079
rs1801274
All
−0.04
−0.02
0.00
0.02
MR leave−one−out sensitivity analysis for
' BAFF−R on IgD+ CD24−' on 'Rheumatoid arthritis'

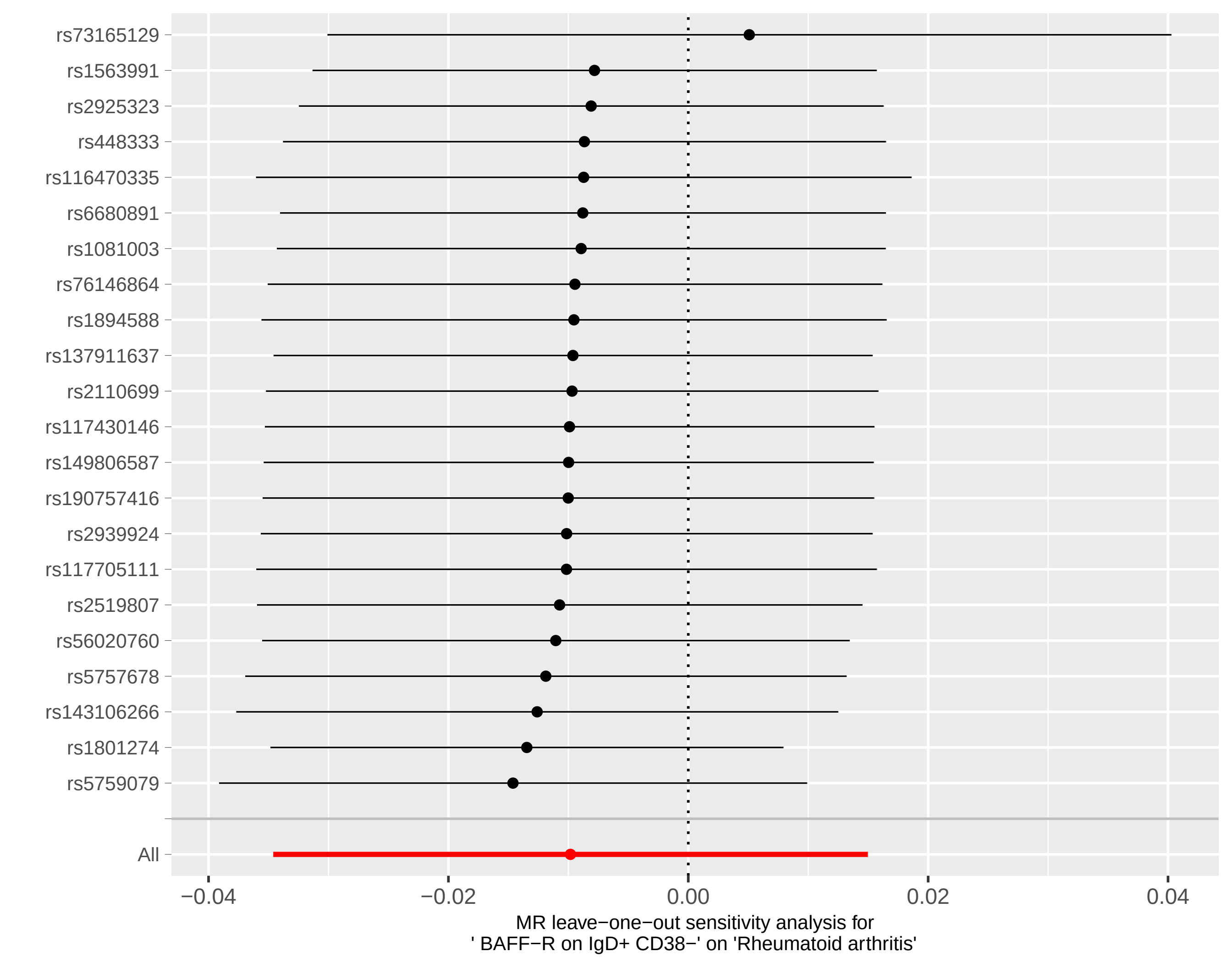
rs73165129
rs1563991
rs2925323
rs448333
rs116470335
rs6680891
rs1081003
rs76146864
rs1894588
rs137911637
rs2110699
rs117430146
rs149806587
rs190757416
rs2939924
rs117705111
rs2519807
rs56020760
rs5757678
rs143106266
rs1801274
rs5759079
All
−0.04
−0.02
0.00
0.02
0.04
MR leave−one−out sensitivity analysis for
' BAFF−R on IgD+ CD38−' on 'Rheumatoid arthritis'

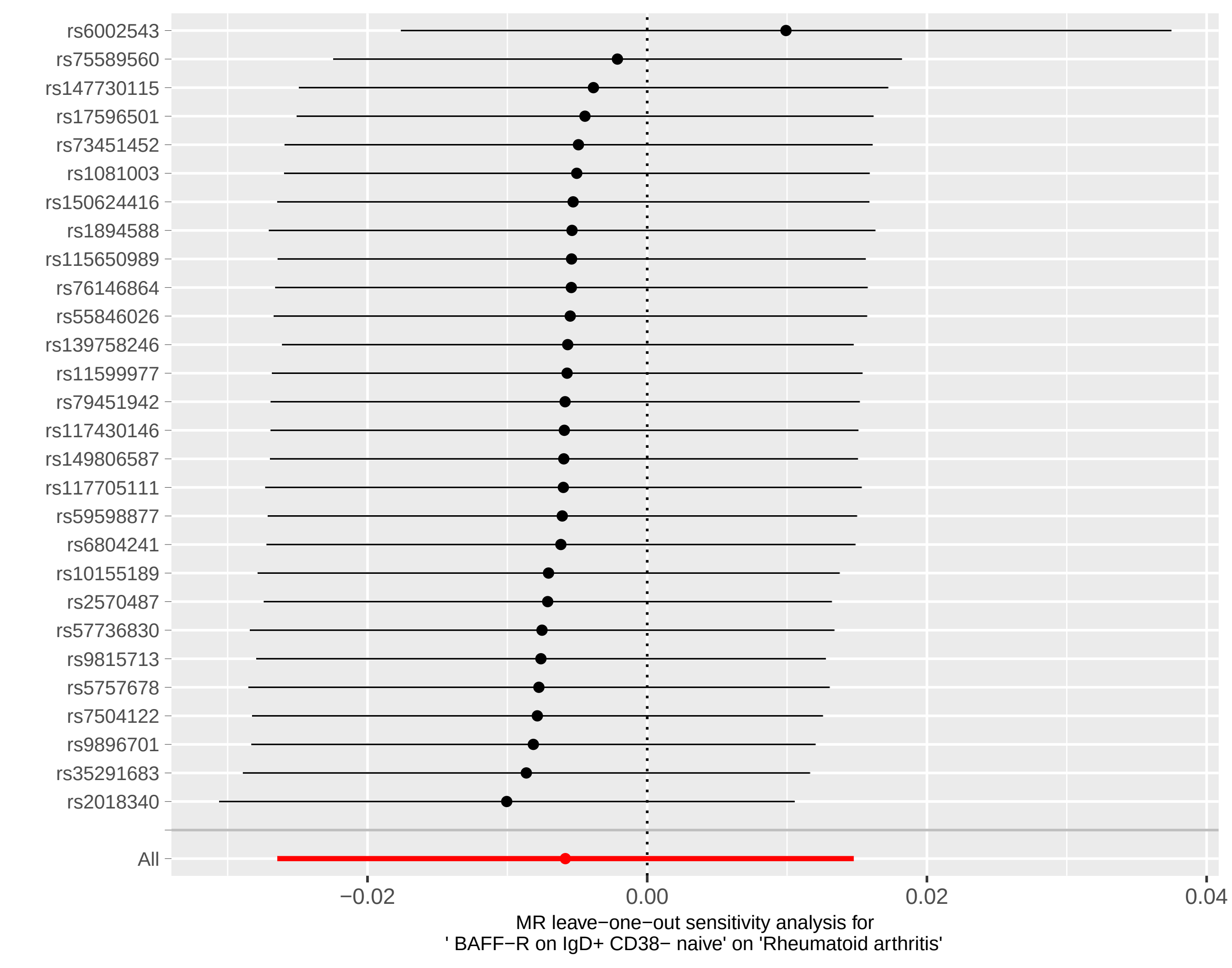
rs6002543
rs75589560
rs147730115
rs17596501
rs73451452
rs1081003
rs150624416
rs1894588
rs115650989
rs76146864
rs55846026
rs139758246
rs11599977
rs79451942
rs117430146
rs149806587
rs117705111
rs59598877
rs6804241
rs10155189
rs2570487
rs57736830
rs9815713
rs5757678
rs7504122
rs9896701
rs35291683
rs2018340
All
−0.02
0.00
0.02
0.04
MR leave−one−out sensitivity analysis for
' BAFF−R on IgD+ CD38− naive' on 'Rheumatoid arthritis'

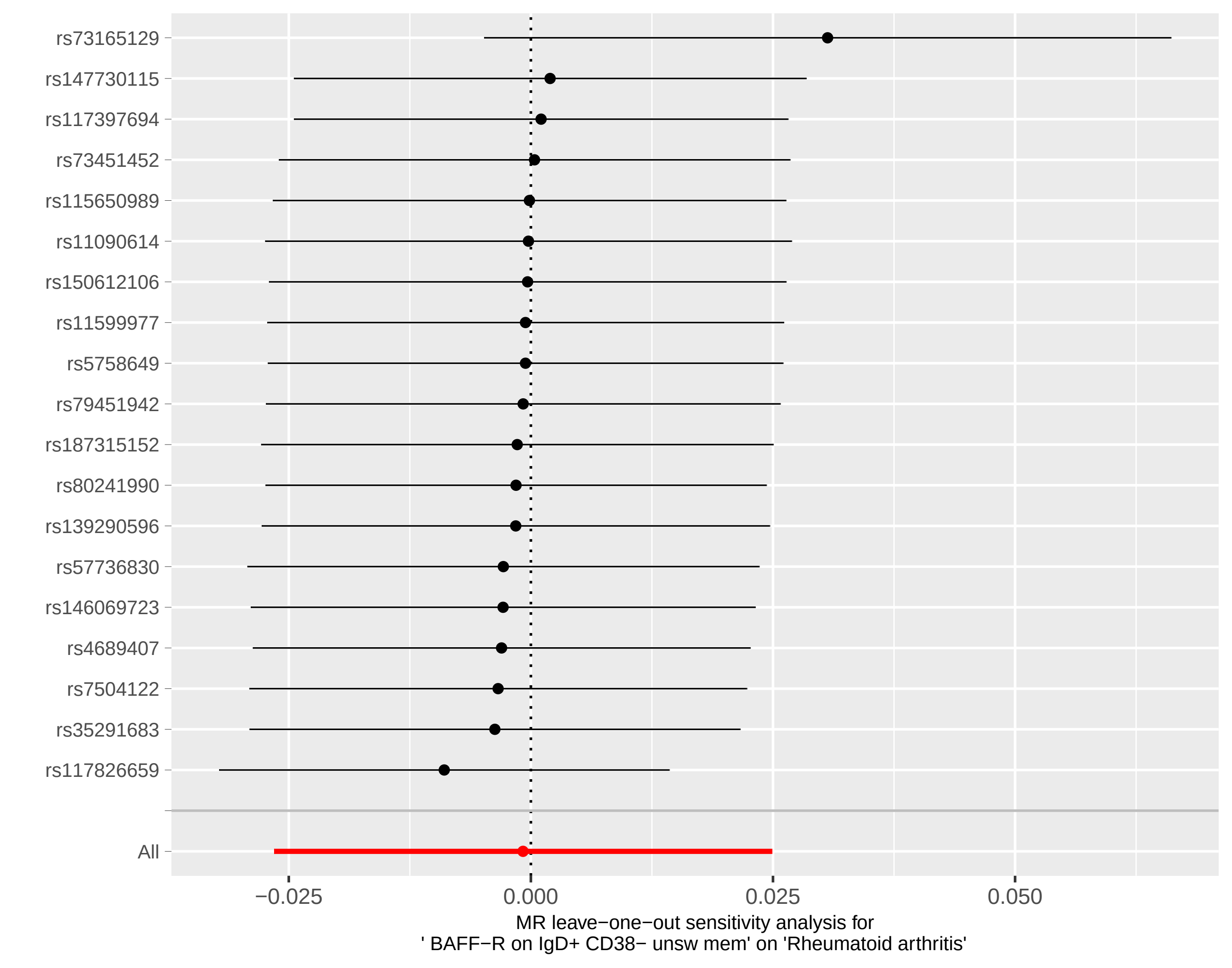
rs73165129
rs147730115
rs117397694
rs73451452
rs115650989
rs11090614
rs150612106
rs11599977
rs5758649
rs79451942
rs187315152
rs80241990
rs139290596
rs57736830
rs146069723
rs4689407
rs7504122
rs35291683
rs117826659
All
−0.025
0.000
0.025
0.050
MR leave−one−out sensitivity analysis for
' BAFF−R on IgD+ CD38− unsw mem' on 'Rheumatoid arthritis'

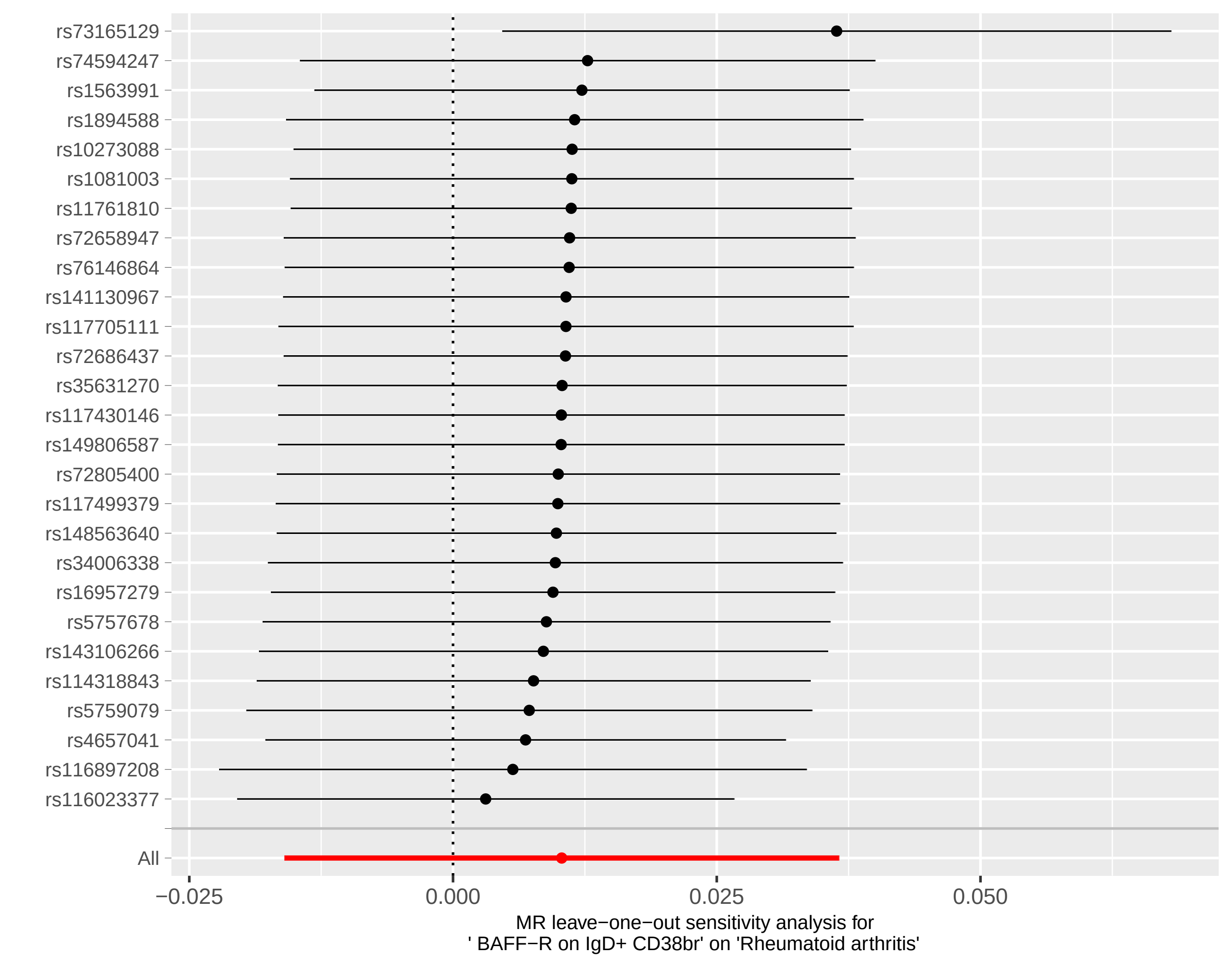

rs73165129
rs74594247
rs1563991
rs1894588
rs10273088
rs1081003
rs11761810
rs72658947
rs76146864
rs141130967
rs117705111
rs72686437
rs35631270
rs117430146
rs149806587
rs72805400
rs117499379
rs148563640
rs34006338
rs16957279
rs5757678
rs143106266
rs114318843
rs5759079
rs4657041
rs116897208
rs116023377
All
−0.025
0.000
0.025
0.050
MR leave−one−out sensitivity analysis for
' BAFF−R on IgD+ CD38br' on 'Rheumatoid arthritis'

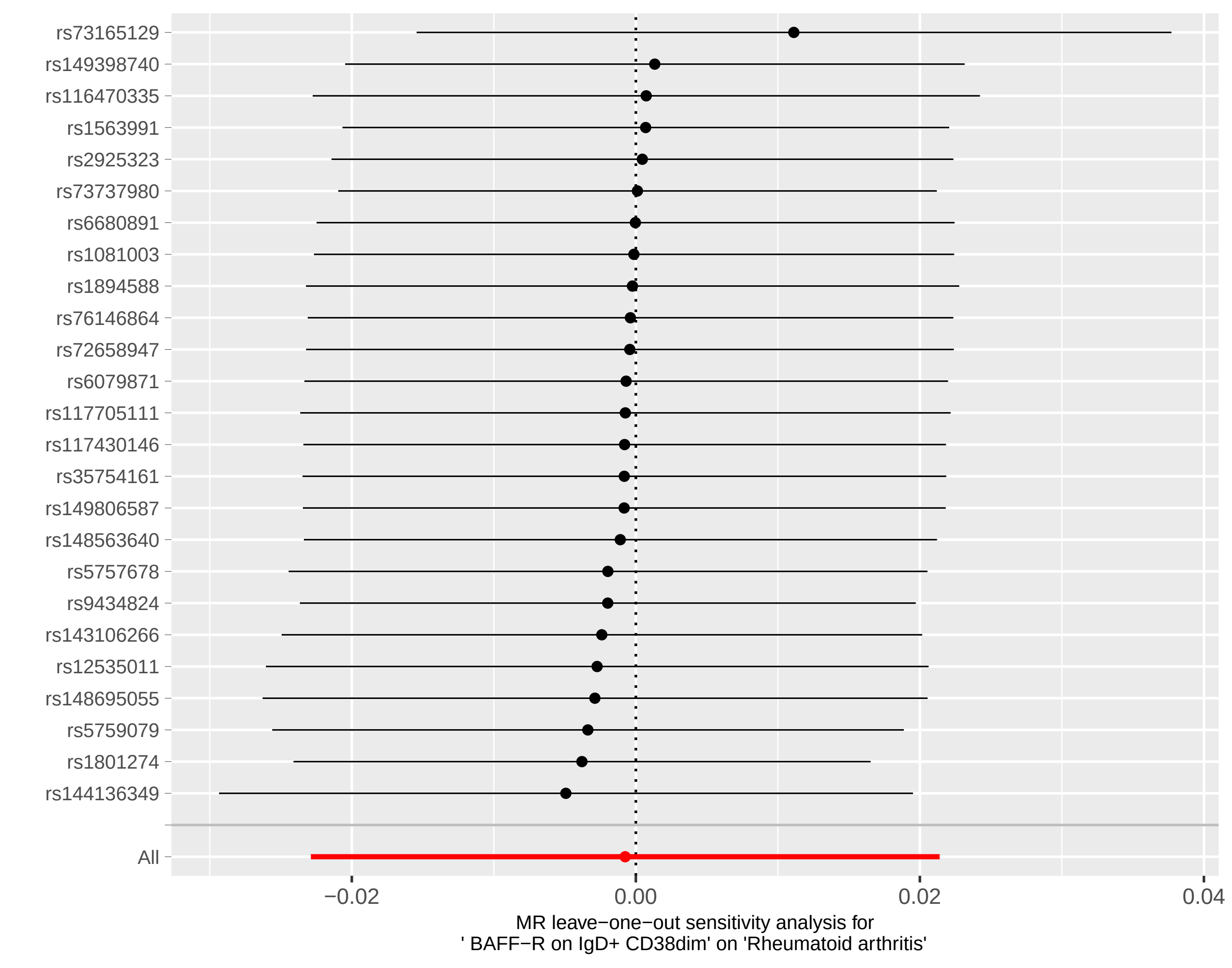

rs73165129
rs149398740
rs116470335
rs1563991
rs2925323
rs73737980
rs6680891
rs1081003
rs1894588
rs76146864
rs72658947
rs6079871
rs117705111
rs117430146
rs35754161
rs149806587
rs148563640
rs5757678
rs9434824
rs143106266
rs12535011
rs148695055
rs5759079
rs1801274
rs144136349
All
−0.02
0.00
0.02
0.04
MR leave−one−out sensitivity analysis for
' BAFF−R on IgD+ CD38dim' on 'Rheumatoid arthritis'

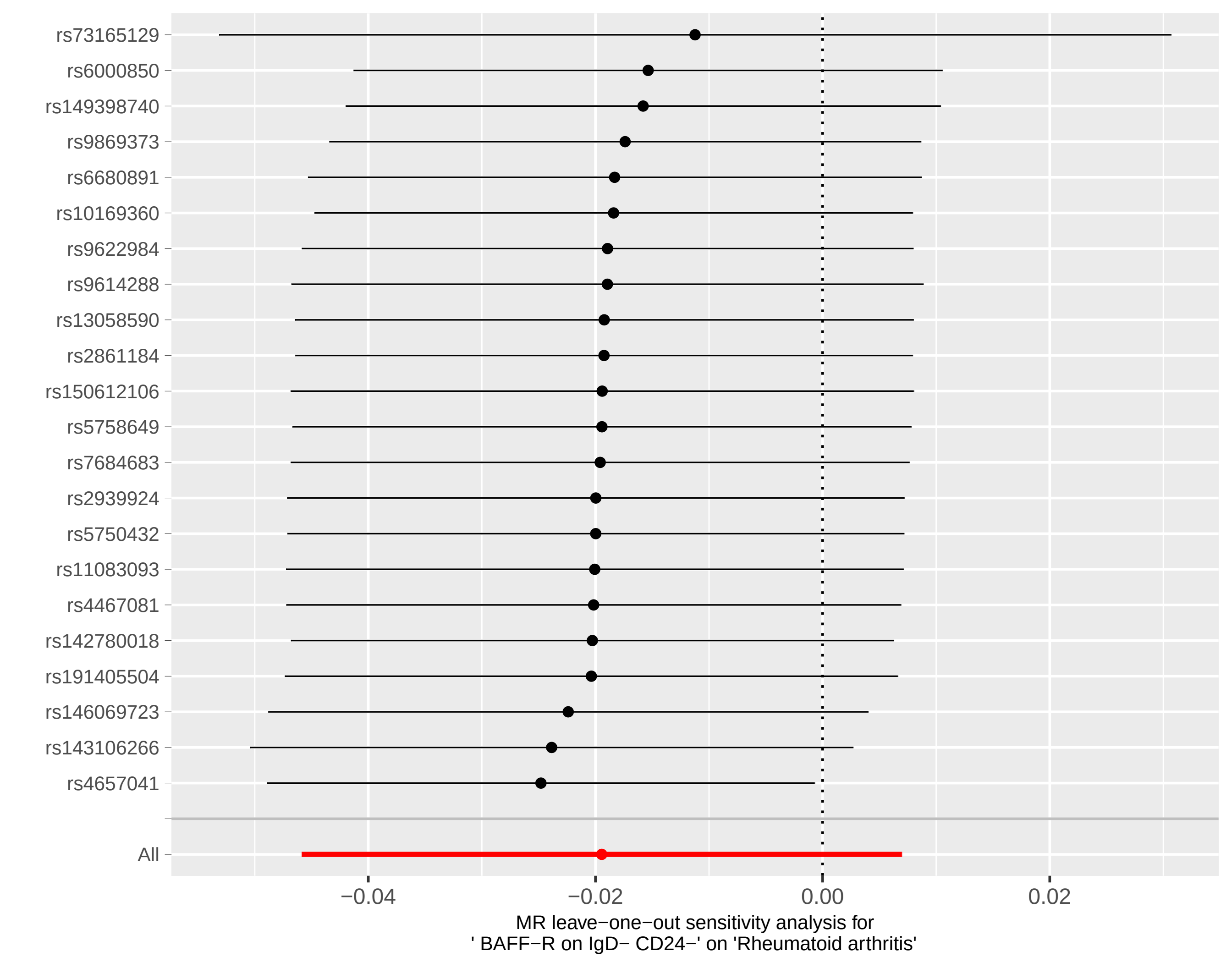
rs73165129
rs6000850
rs149398740
rs9869373
rs6680891
rs10169360
rs9622984
rs9614288
rs13058590
rs2861184
rs150612106
rs5758649
rs7684683
rs2939924
rs5750432
rs11083093
rs4467081
rs142780018
rs191405504
rs146069723
rs143106266
rs4657041
All
−0.04
−0.02
0.00
0.02
MR leave−one−out sensitivity analysis for
' BAFF−R on IgD− CD24−' on 'Rheumatoid arthritis'

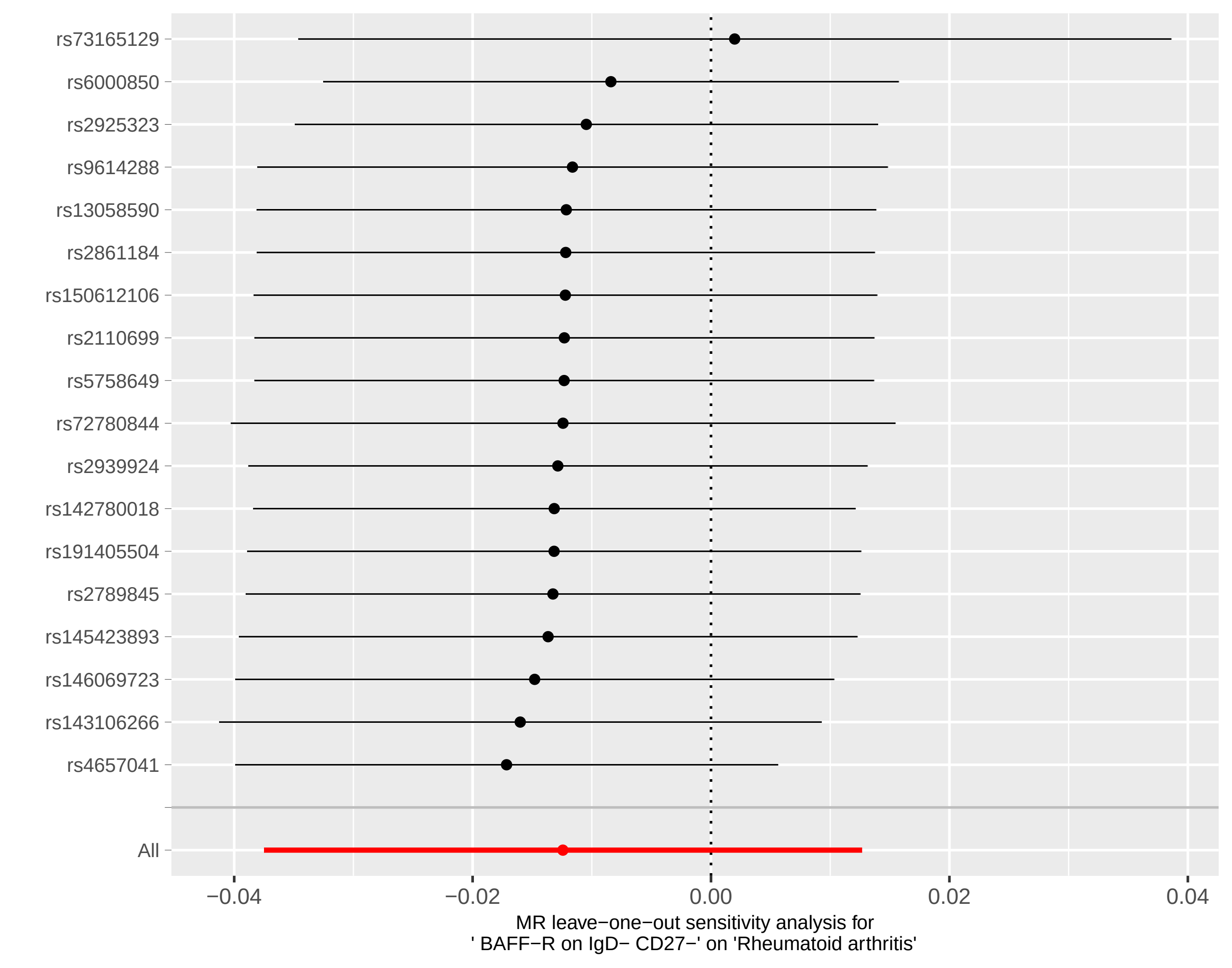
rs73165129
rs6000850
rs2925323
rs9614288
rs13058590
rs2861184
rs150612106
rs2110699
rs5758649
rs72780844
rs2939924
rs142780018
rs191405504
rs2789845
rs145423893
rs146069723
rs143106266
rs4657041
All
−0.04
−0.02
0.00
0.02
0.04
MR leave−one−out sensitivity analysis for
' BAFF−R on IgD− CD27−' on 'Rheumatoid arthritis'

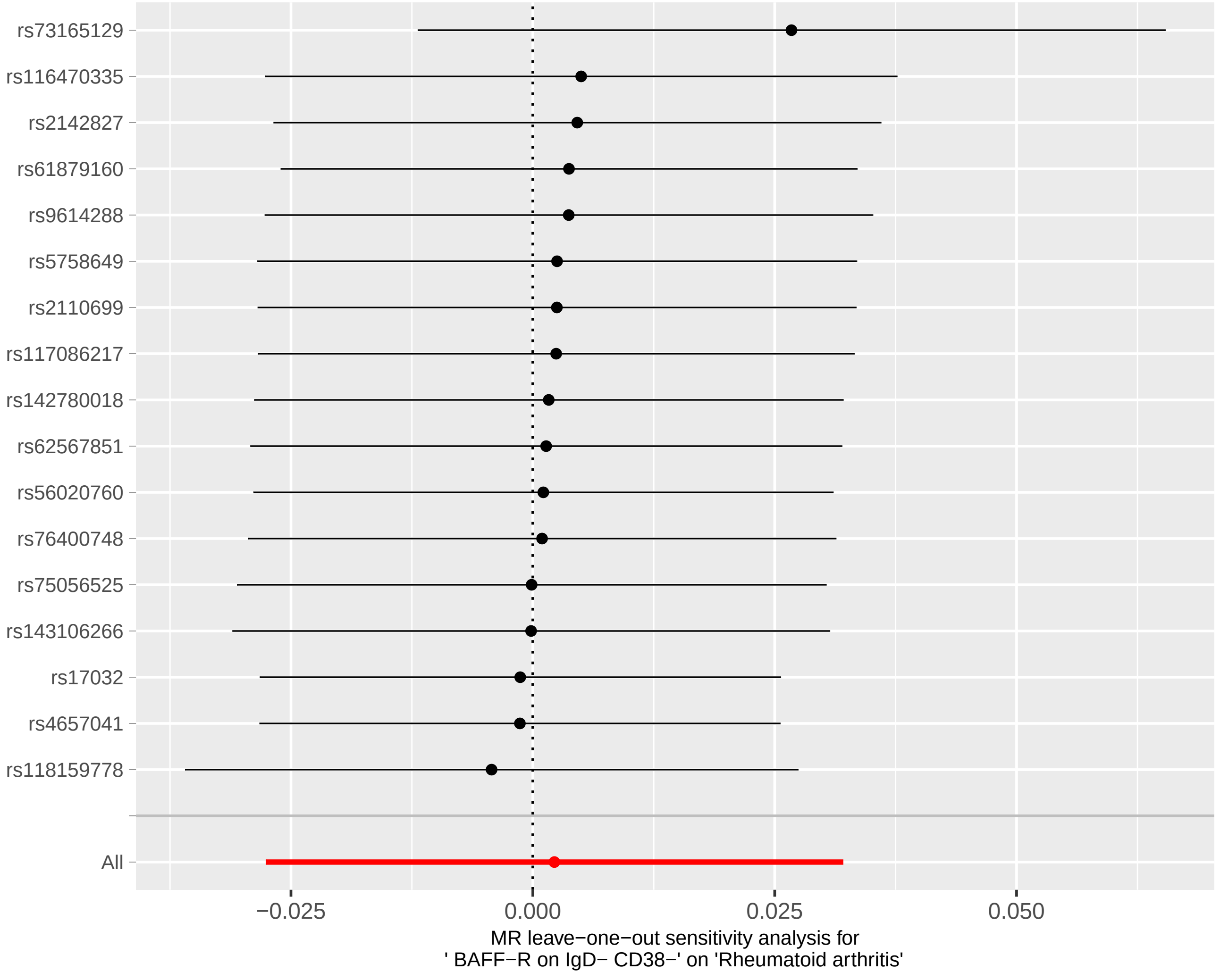
rs73165129
rs116470335
rs2142827
rs61879160
rs9614288
rs5758649
rs2110699
rs117086217
rs142780018
rs62567851
rs56020760
rs76400748
rs75056525
rs143106266
rs17032
rs4657041
rs118159778
All
−0.025
0.000
0.025
0.050
MR leave−one−out sensitivity analysis for
' BAFF−R on IgD− CD38−' on 'Rheumatoid arthritis'

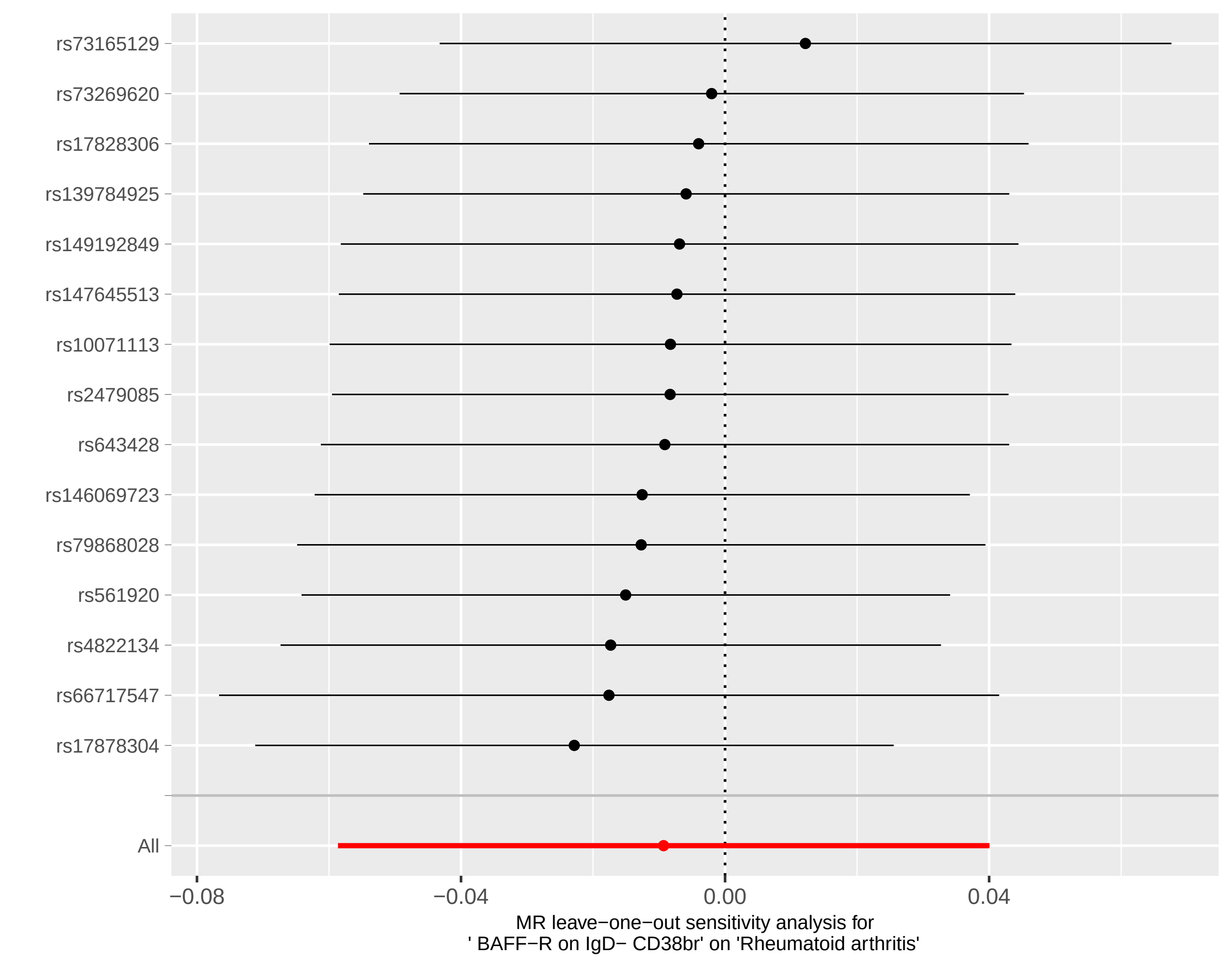
rs73165129
rs73269620
rs17828306
rs139784925
rs149192849
rs147645513
rs10071113
rs2479085
rs643428
rs146069723
rs79868028
rs561920
rs4822134
rs66717547
rs17878304
All
−0.08
−0.04
0.00
0.04
MR leave−one−out sensitivity analysis for
' BAFF−R on IgD− CD38br' on 'Rheumatoid arthritis'

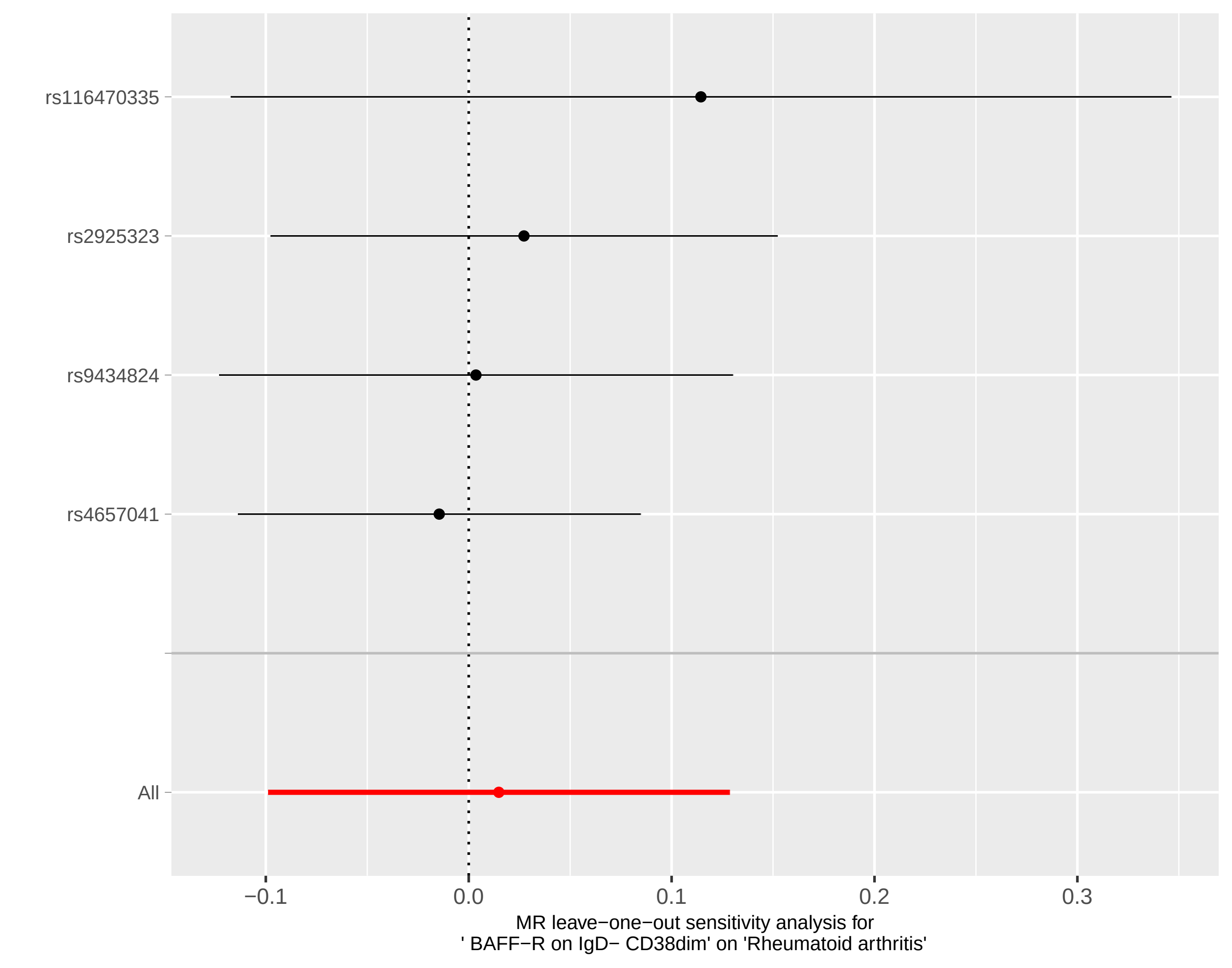
rs116470335
rs2925323
rs9434824
rs4657041
All
−0.1
0.0
0.1
0.2
0.3
MR leave−one−out sensitivity analysis for
' BAFF−R on IgD− CD38dim' on 'Rheumatoid arthritis'

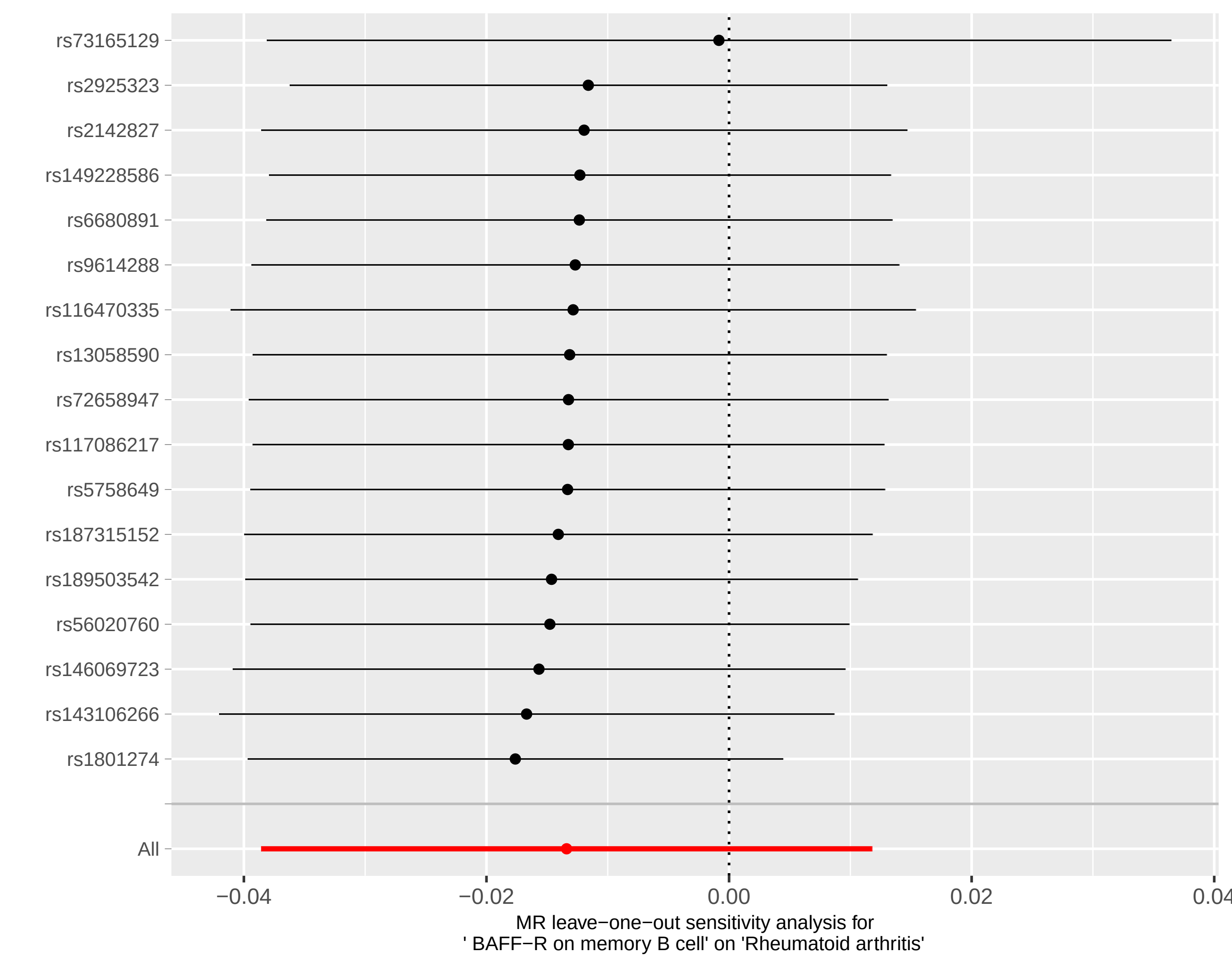
rs73165129
rs2925323
rs2142827
rs149228586
rs6680891
rs9614288
rs116470335
rs13058590
rs72658947
rs117086217
rs5758649
rs187315152
rs189503542
rs56020760
rs146069723
rs143106266
rs1801274
All
−0.04
−0.02
0.00
0.02
MR leave−one−out sensitivity analysis for
' BAFF−R on memory B cell' on 'Rheumatoid arthritis'

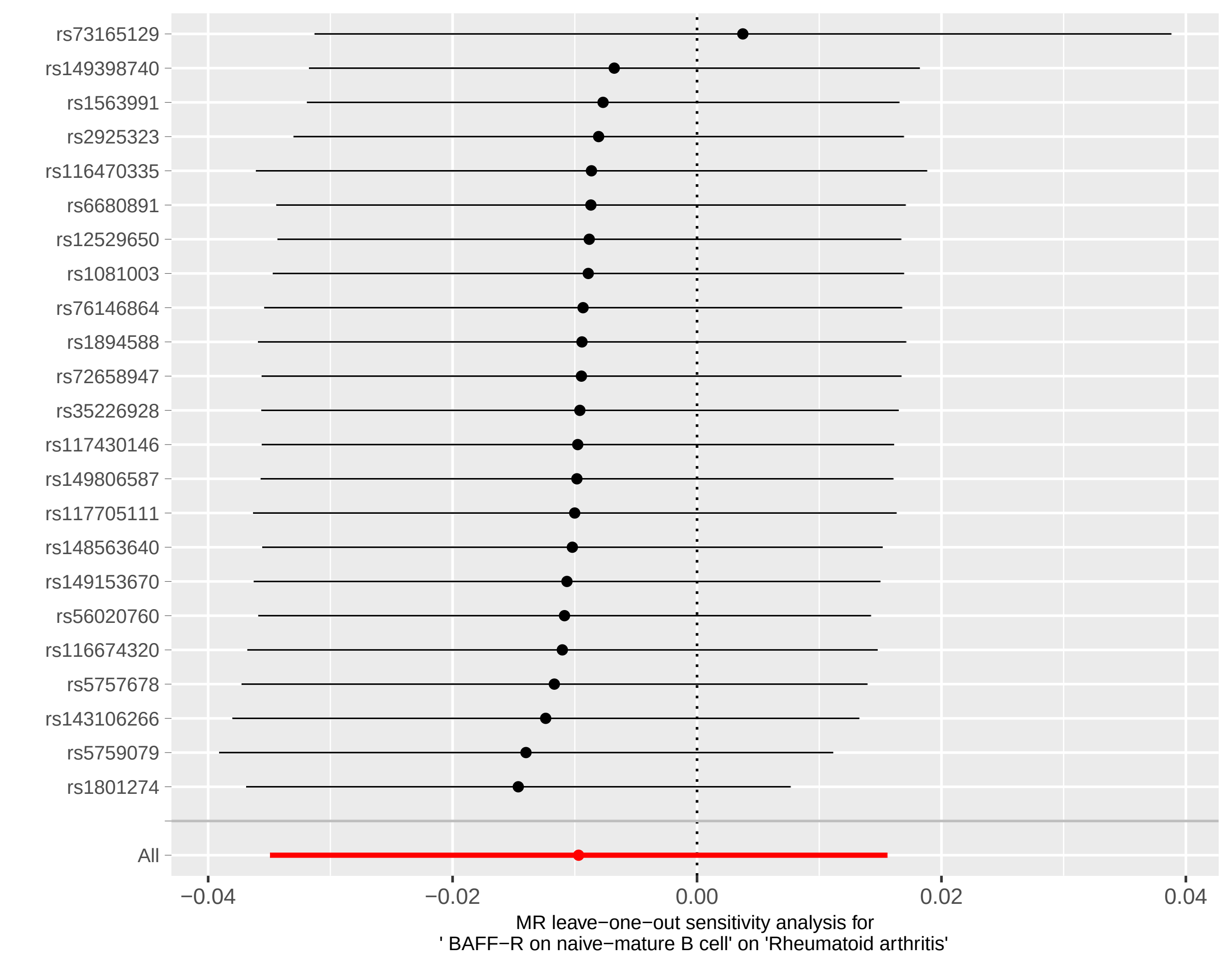

rs73165129
rs149398740
rs1563991
rs2925323
rs116470335
rs6680891
rs12529650
rs1081003
rs76146864
rs1894588
rs72658947
rs35226928
rs117430146
rs149806587
rs117705111
rs148563640
rs149153670
rs56020760
rs116674320
rs5757678
rs143106266
rs5759079
rs1801274
All
−0.04
−0.02
0.00
0.02
0.04
MR leave−one−out sensitivity analysis for
' BAFF−R on naive−mature B cell' on 'Rheumatoid arthritis'

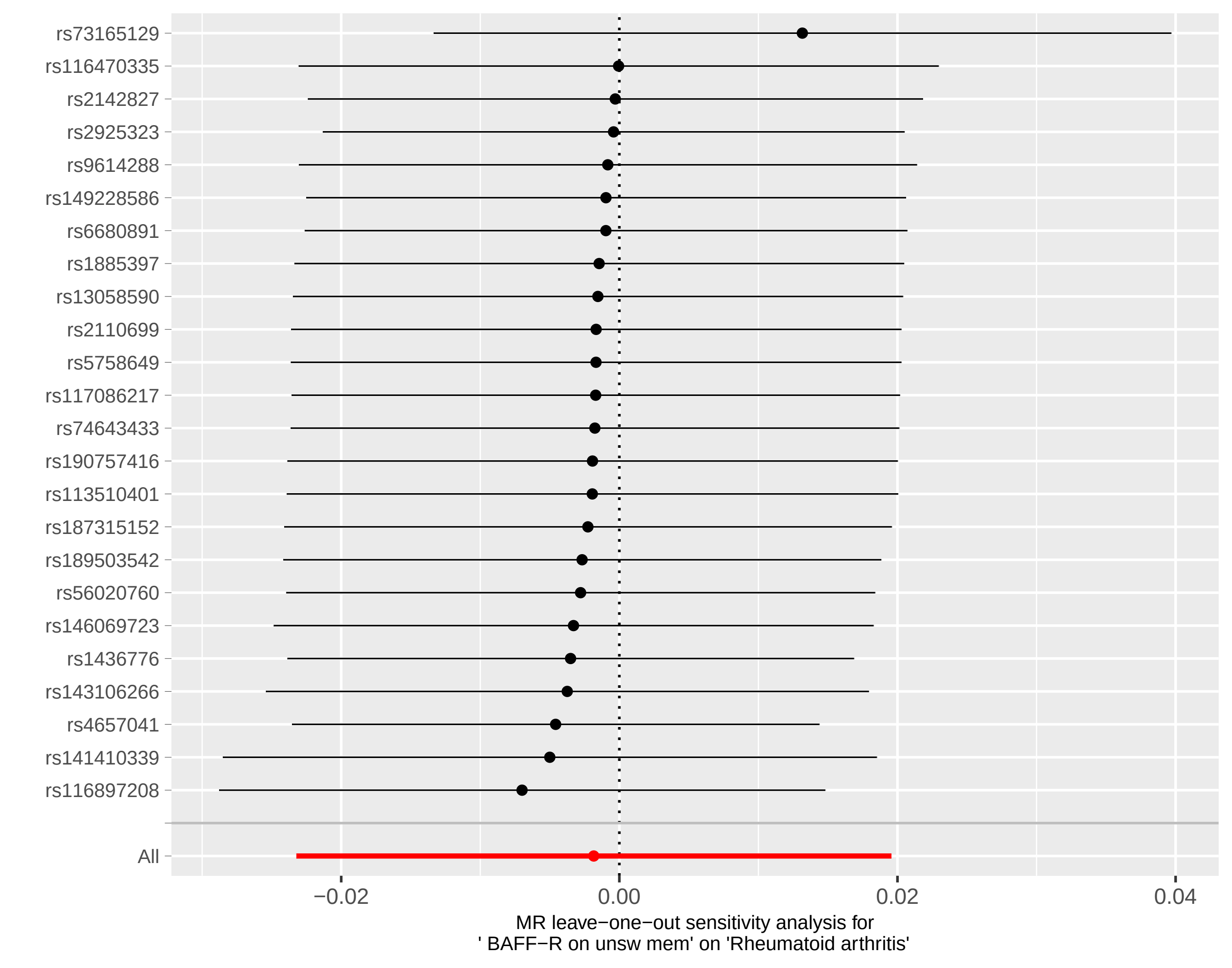
rs73165129
rs116470335
rs2142827
rs2925323
rs9614288
rs149228586
rs6680891
rs1885397
rs13058590
rs2110699
rs5758649
rs117086217
rs74643433
rs190757416
rs113510401
rs187315152
rs189503542
rs56020760
rs146069723
rs1436776
rs143106266
rs4657041
rs141410339
rs116897208
All
−0.02
0.00
0.02
0.04
MR leave−one−out sensitivity analysis for
' BAFF−R on unsw mem' on 'Rheumatoid arthritis'

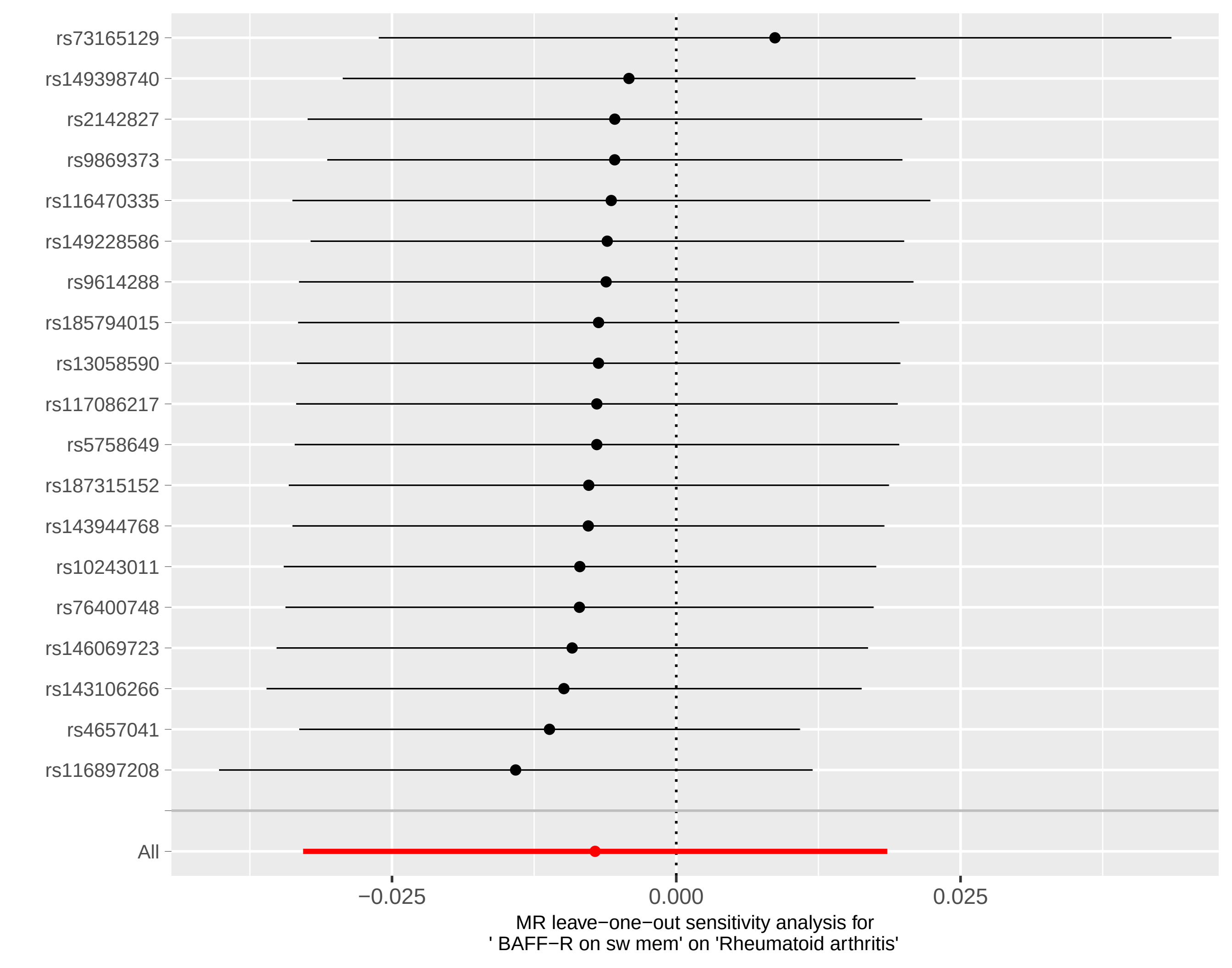
rs73165129
rs149398740
rs2142827
rs9869373
rs116470335
rs149228586
rs9614288
rs185794015
rs13058590
rs117086217
rs5758649
rs187315152
rs143944768
rs10243011
rs76400748
rs146069723
rs143106266
rs4657041
rs116897208
All
−0.025
0.000
0.025
MR leave−one−out sensitivity analysis for
' BAFF−R on sw mem' on 'Rheumatoid arthritis'

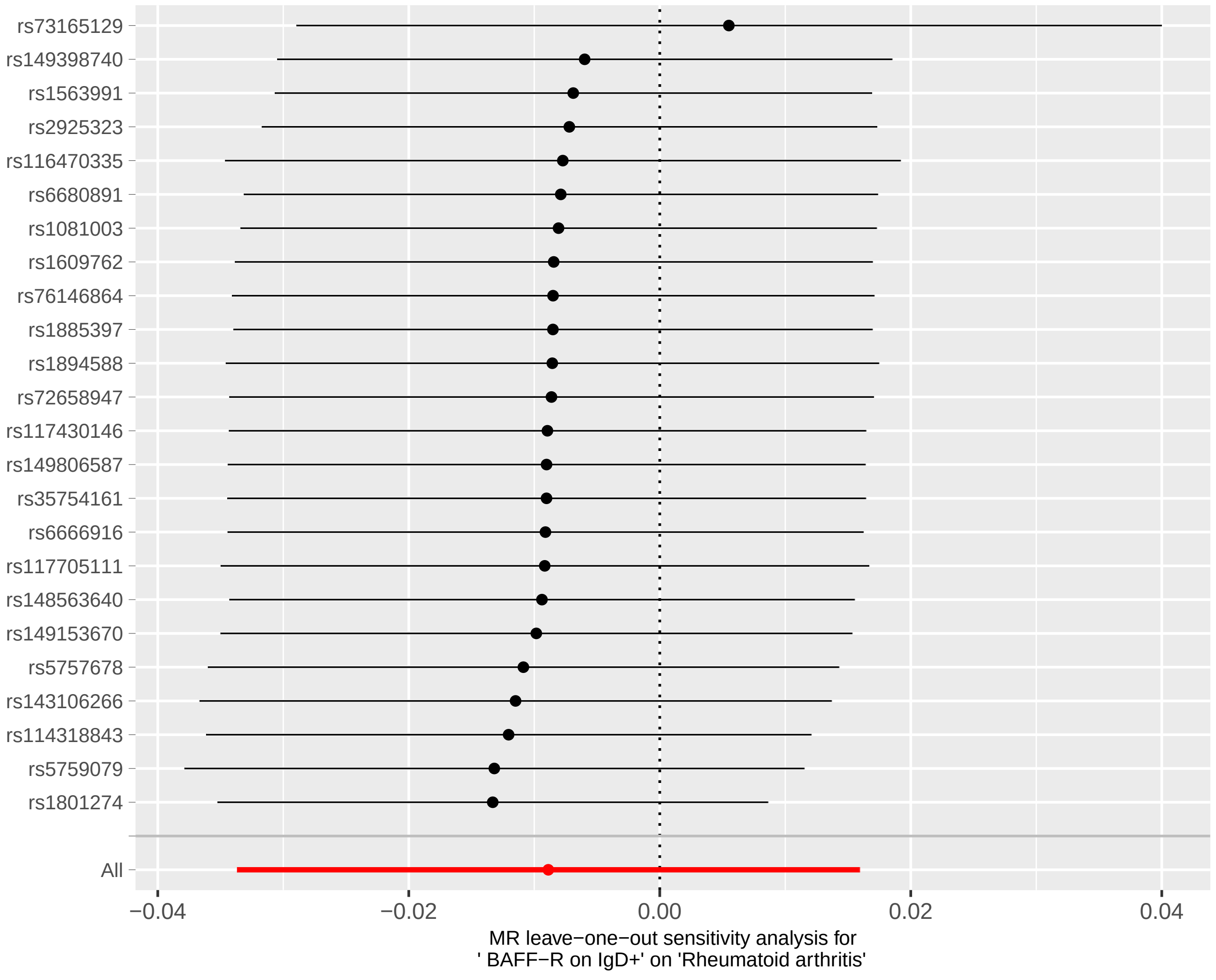
rs73165129
rs149398740
rs1563991
rs2925323
rs116470335
rs6680891
rs1081003
rs1609762
rs76146864
rs1885397
rs1894588
rs72658947
rs117430146
rs149806587
rs35754161
rs6666916
rs117705111
rs148563640
rs149153670
rs5757678
rs143106266
rs114318843
rs5759079
rs1801274
All
−0.04
−0.02
0.00
0.02
0.04
MR leave−one−out sensitivity analysis for
' BAFF−R on IgD+' on 'Rheumatoid arthritis'

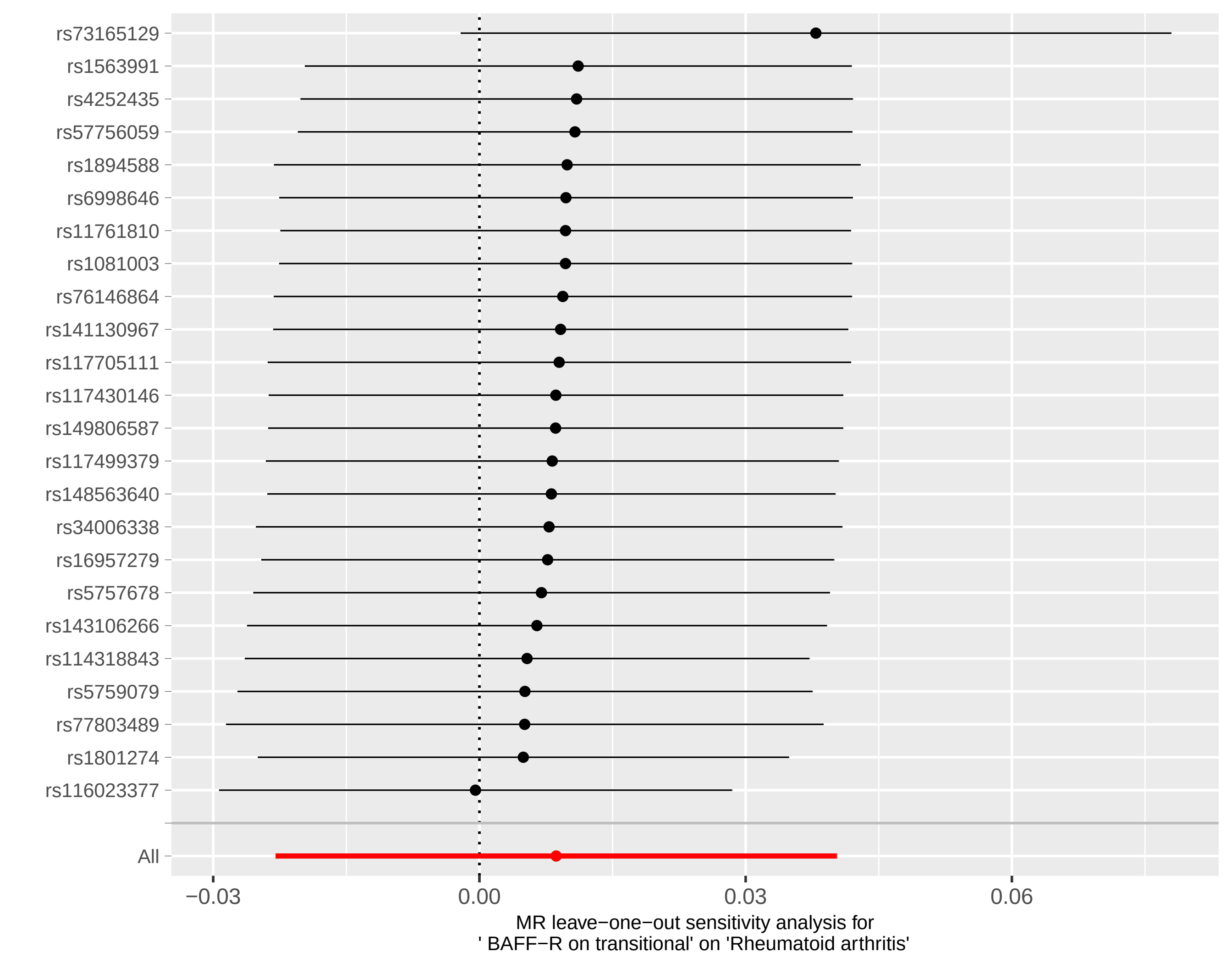
rs73165129
rs1563991
rs4252435
rs57756059
rs1894588
rs6998646
rs11761810
rs1081003
rs76146864
rs141130967
rs117705111
rs117430146
rs149806587
rs117499379
rs148563640
rs34006338
rs16957279
rs5757678
rs143106266
rs114318843
rs5759079
rs77803489
rs1801274
rs116023377
All
−0.03
0.00
0.03
0.06
MR leave−one−out sensitivity analysis for
' BAFF−R on transitional' on 'Rheumatoid arthritis'

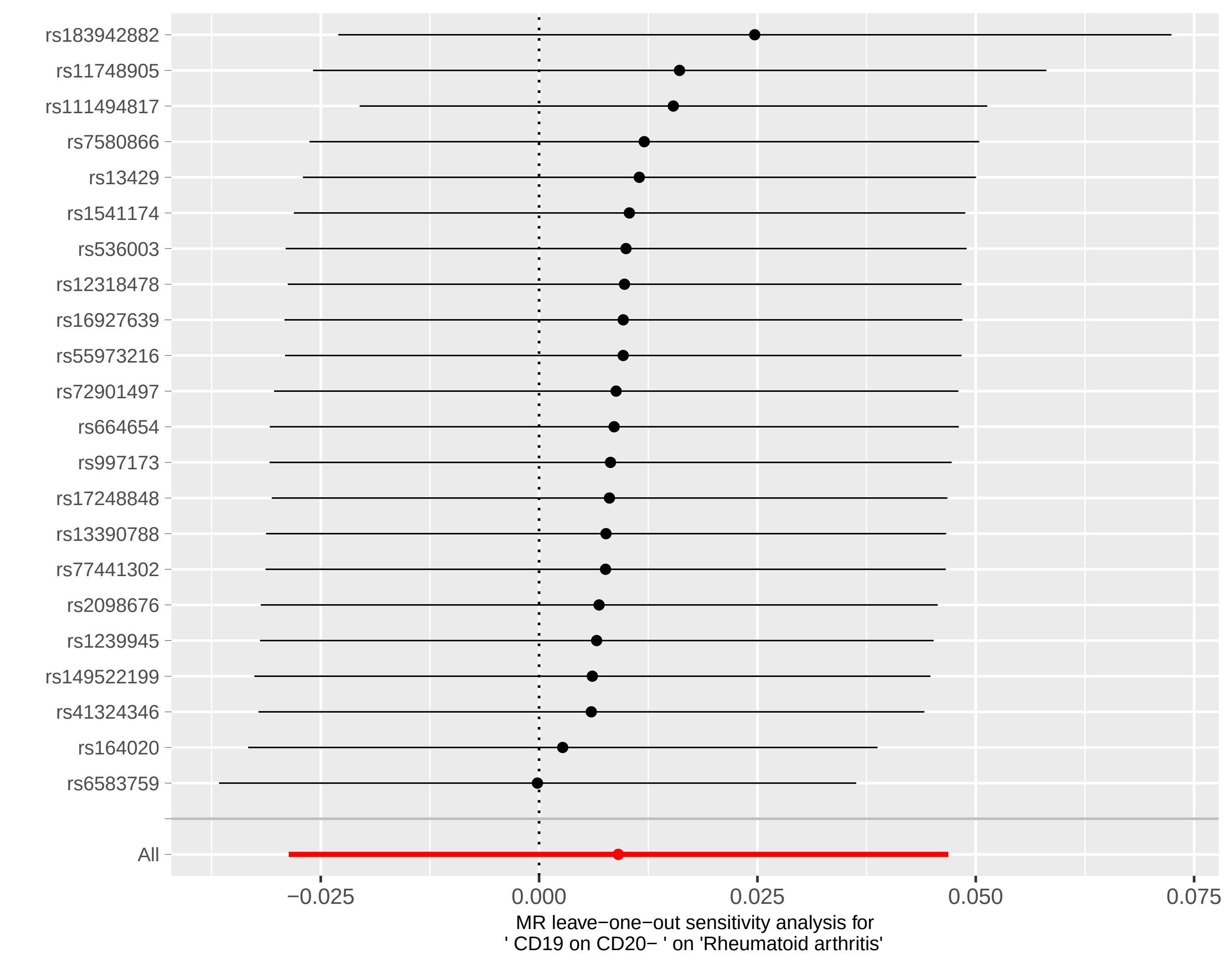

rs183942882
rs11748905
rs111494817
rs7580866
rs13429
rs1541174
rs536003
rs12318478
rs16927639
rs55973216
rs72901497
rs664654
rs997173
rs17248848
rs13390788
rs77441302
rs2098676
rs1239945
rs149522199
rs41324346
rs164020
rs6583759
All
−0.025
0.000
0.025
0.050
0.075
MR leave−one−out sensitivity analysis for
' CD19 on CD20− ' on 'Rheumatoid arthritis'

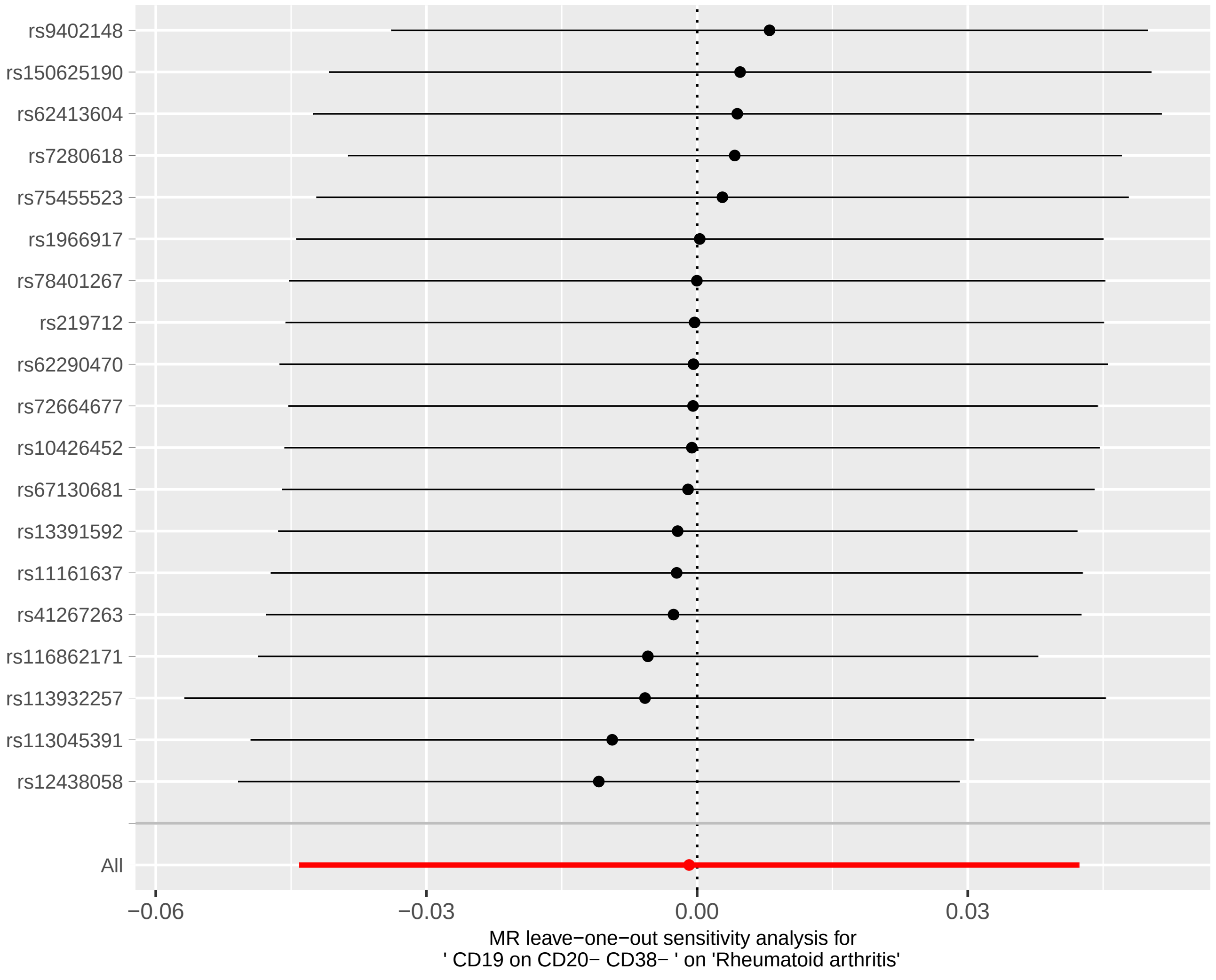

rs9402148
rs150625190
rs62413604
rs7280618
rs75455523
rs1966917
rs78401267
rs219712
rs62290470
rs72664677
rs10426452
rs67130681
rs13391592
rs11161637
rs41267263
rs116862171
rs113932257
rs113045391
rs12438058
All
−0.06
−0.03
0.00
0.03
MR leave−one−out sensitivity analysis for
' CD19 on CD20− CD38− ' on 'Rheumatoid arthritis'

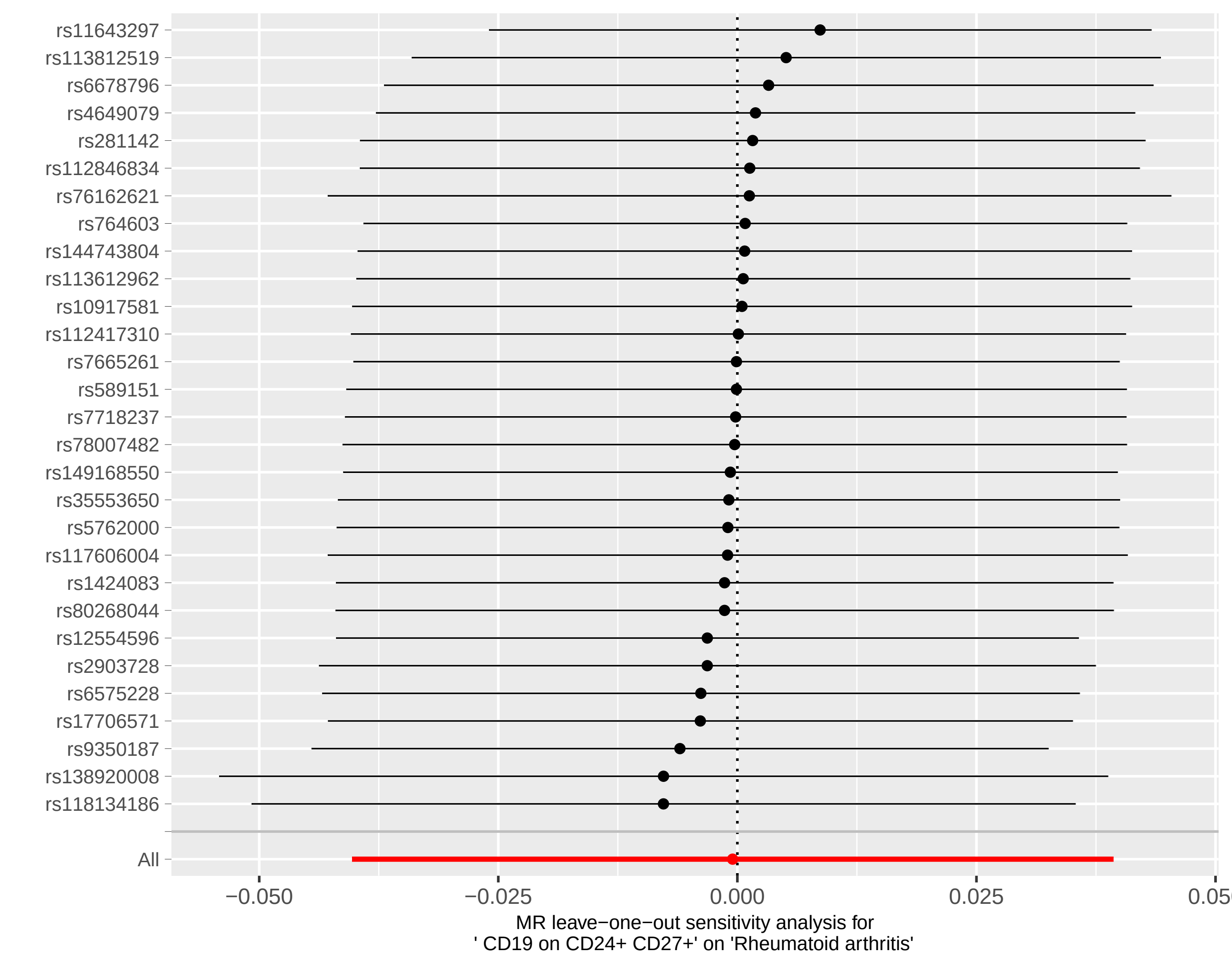
rs11643297
rs113812519
rs6678796
rs4649079
rs281142
rs112846834
rs76162621
rs764603
rs144743804
rs113612962
rs10917581
rs112417310
rs7665261
rs589151
rs7718237
rs78007482
rs149168550
rs35553650
rs5762000
rs117606004
rs1424083
rs80268044
rs12554596
rs2903728
rs6575228
rs17706571
rs9350187
rs138920008
rs118134186
All
−0.050
−0.025
0.000
0.025
MR leave−one−out sensitivity analysis for
' CD19 on CD24+ CD27+' on 'Rheumatoid arthritis'

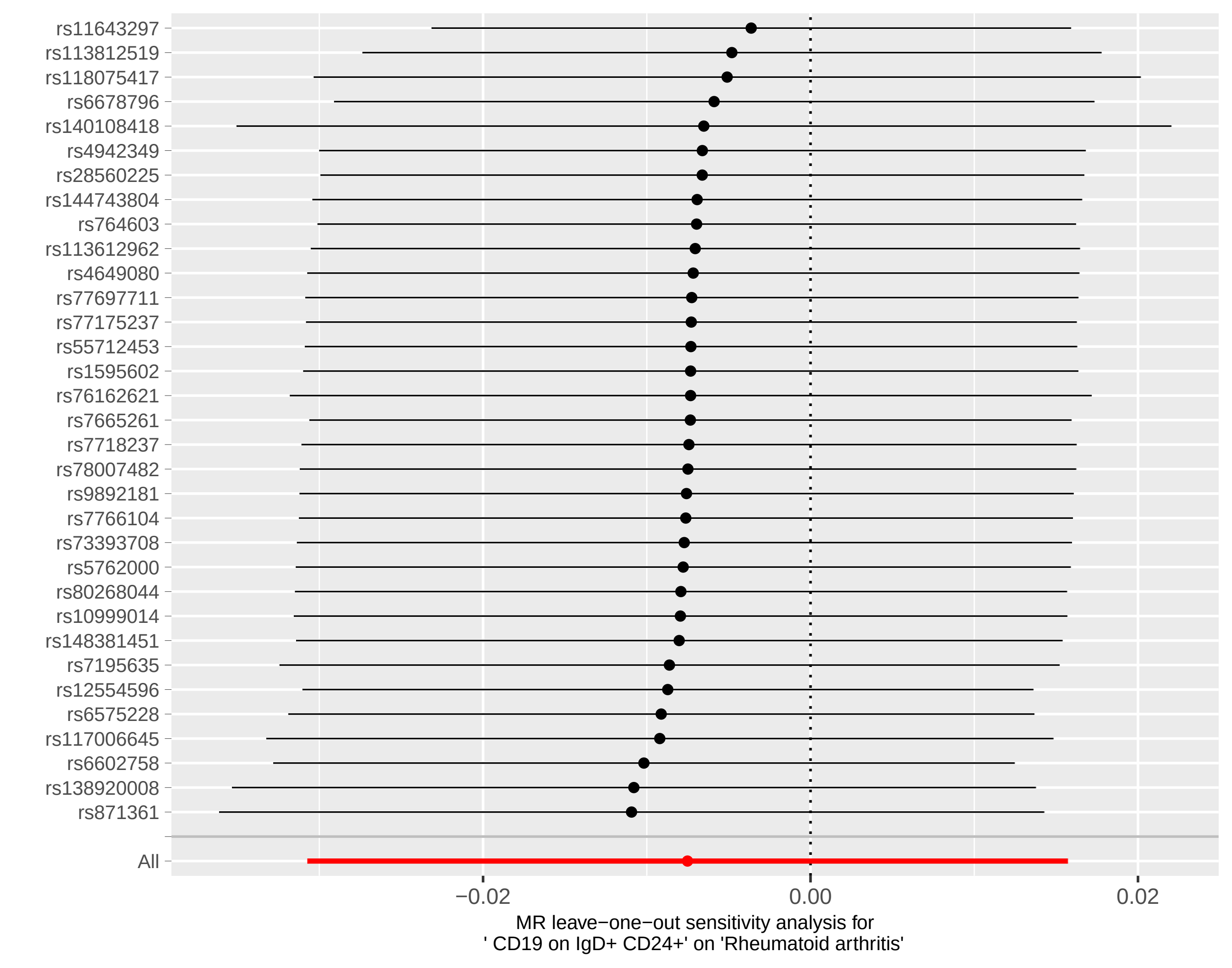
rs11643297
rs113812519
rs118075417
rs6678796
rs140108418
rs4942349
rs28560225
rs144743804
rs764603
rs113612962
rs4649080
rs77697711
rs77175237
rs55712453
rs1595602
rs76162621
rs7665261
rs7718237
rs78007482
rs9892181
rs7766104
rs73393708
rs5762000
rs80268044
rs10999014
rs148381451
rs7195635
rs12554596
rs6575228
rs117006645
rs6602758
rs138920008
rs871361
All
−0.02
0.00
0.02
MR leave−one−out sensitivity analysis for
' CD19 on IgD+ CD24+' on 'Rheumatoid arthritis'

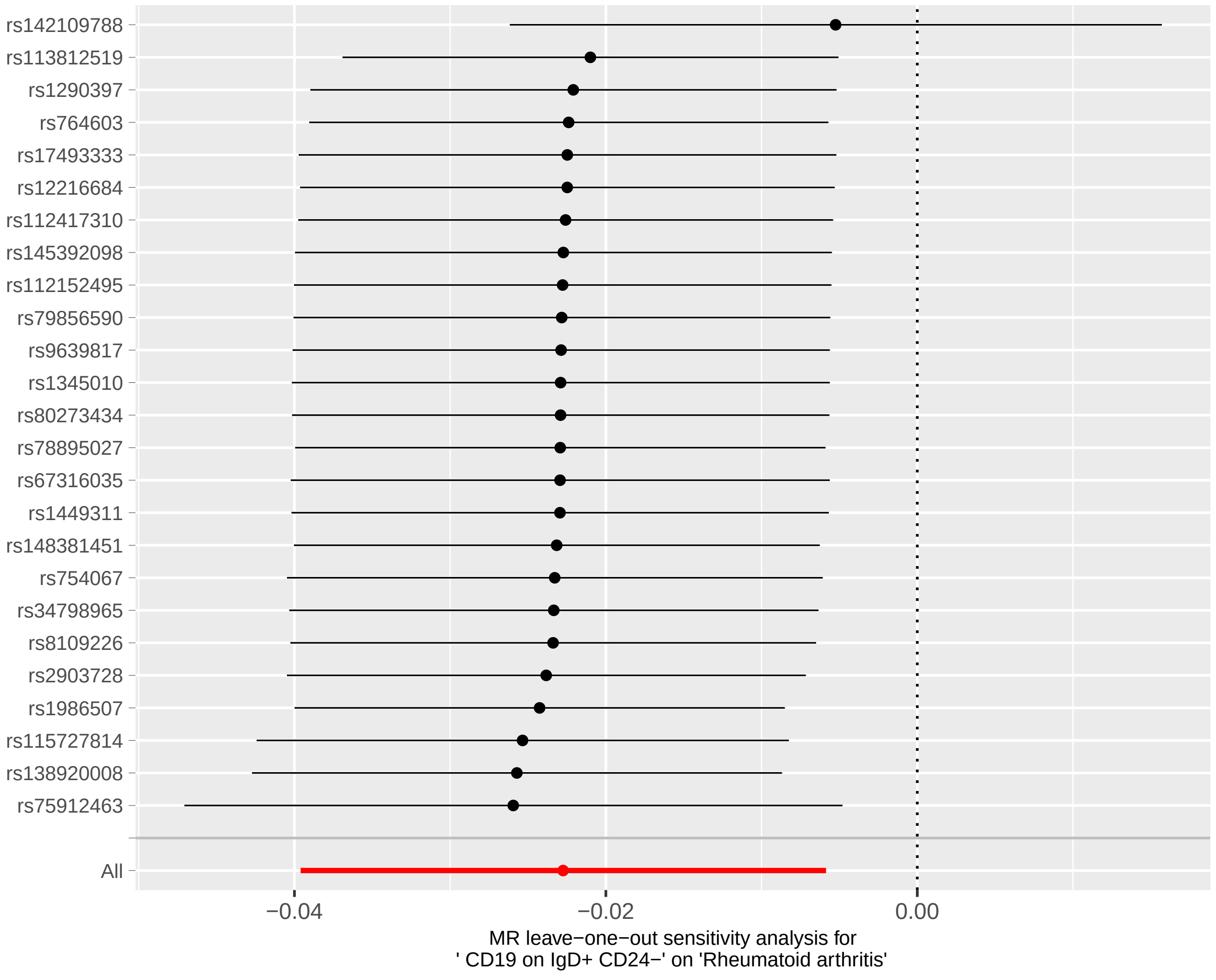
rs142109788
rs113812519
rs1290397
rs764603
rs17493333
rs12216684
rs112417310
rs145392098
rs112152495
rs79856590
rs9639817
rs1345010
rs80273434
rs78895027
rs67316035
rs1449311
rs148381451
rs754067
rs34798965
rs8109226
rs2903728
rs1986507
rs115727814
rs138920008
rs75912463
All
−0.04
−0.02
0.00
MR leave−one−out sensitivity analysis for
' CD19 on IgD+ CD24−' on 'Rheumatoid arthritis'

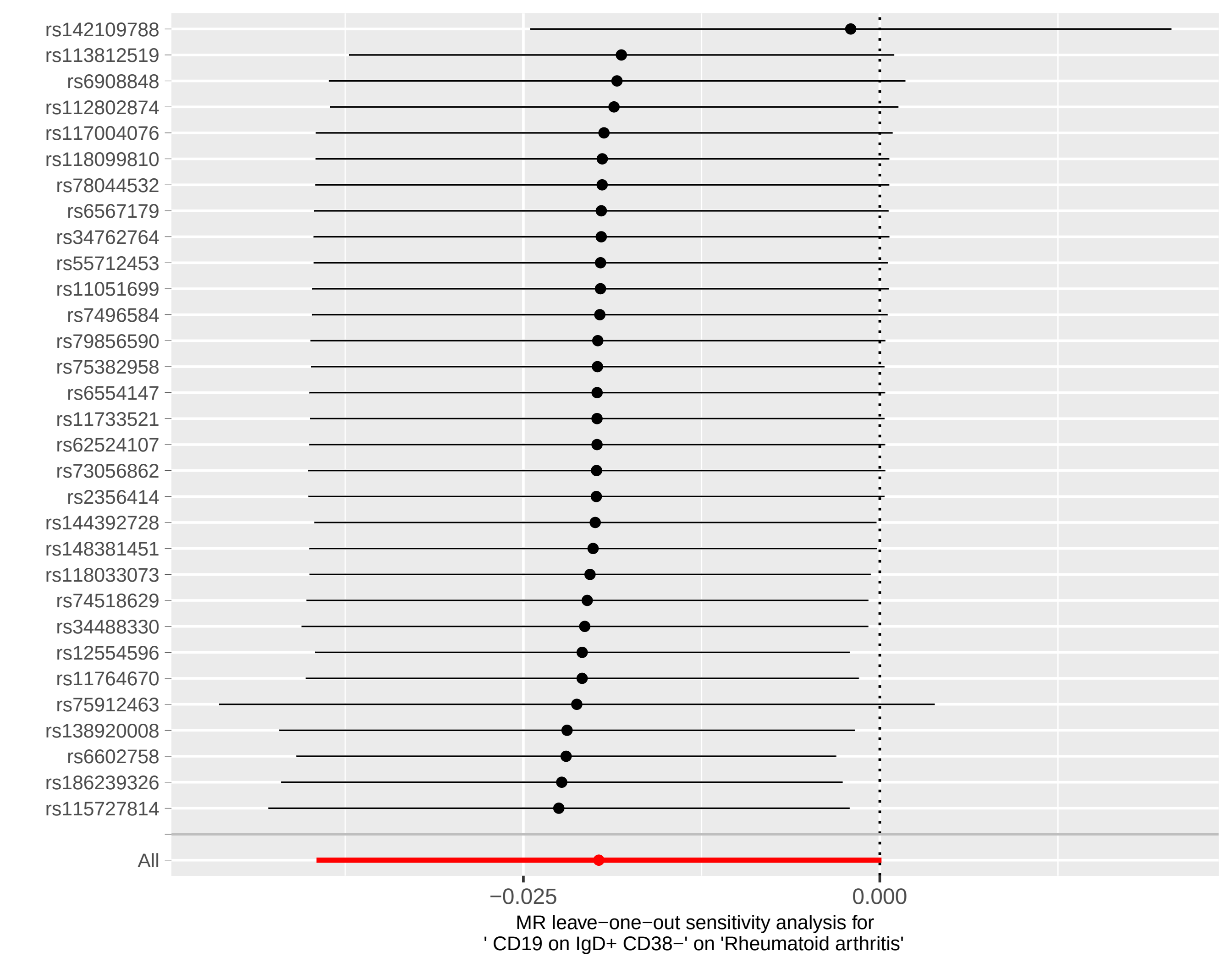

rs142109788
rs113812519
rs6908848
rs112802874
rs117004076
rs118099810
rs78044532
rs6567179
rs34762764
rs55712453
rs11051699
rs7496584
rs79856590
rs75382958
rs6554147
rs11733521
rs62524107
rs73056862
rs2356414
rs144392728
rs148381451
rs118033073
rs74518629
rs34488330
rs12554596
rs11764670
rs75912463
rs138920008
rs6602758
rs186239326
rs115727814
All
−0.025
0.000
MR leave−one−out sensitivity analysis for
' CD19 on IgD+ CD38−' on 'Rheumatoid arthritis'

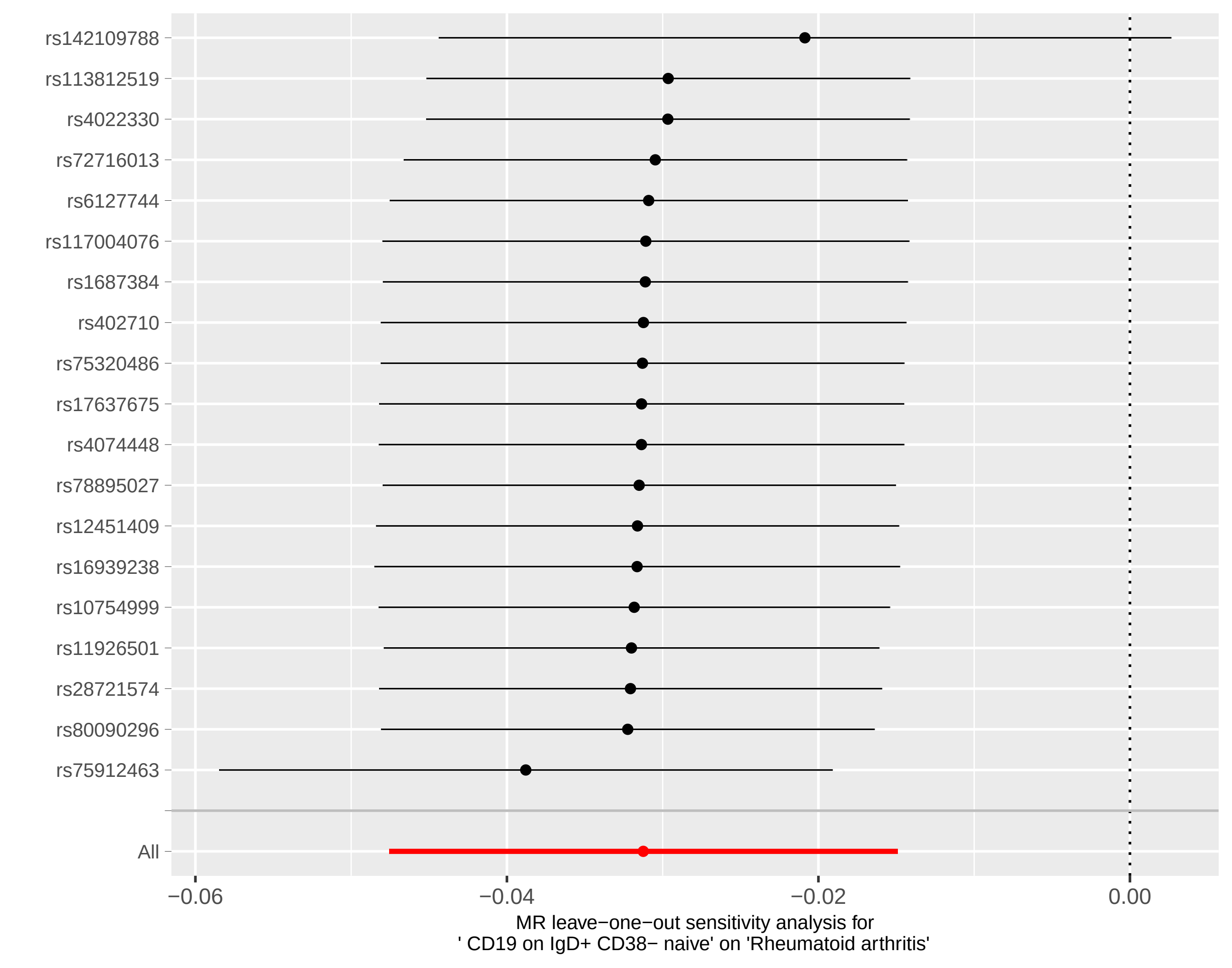
rs142109788
rs113812519
rs4022330
rs72716013
rs6127744
rs117004076
rs1687384
rs402710
rs75320486
rs17637675
rs4074448
rs78895027
rs12451409
rs16939238
rs10754999
rs11926501
rs28721574
rs80090296
rs75912463
All
−0.06
−0.04
−0.02
0.00
MR leave−one−out sensitivity analysis for
' CD19 on IgD+ CD38− naive' on 'Rheumatoid arthritis'

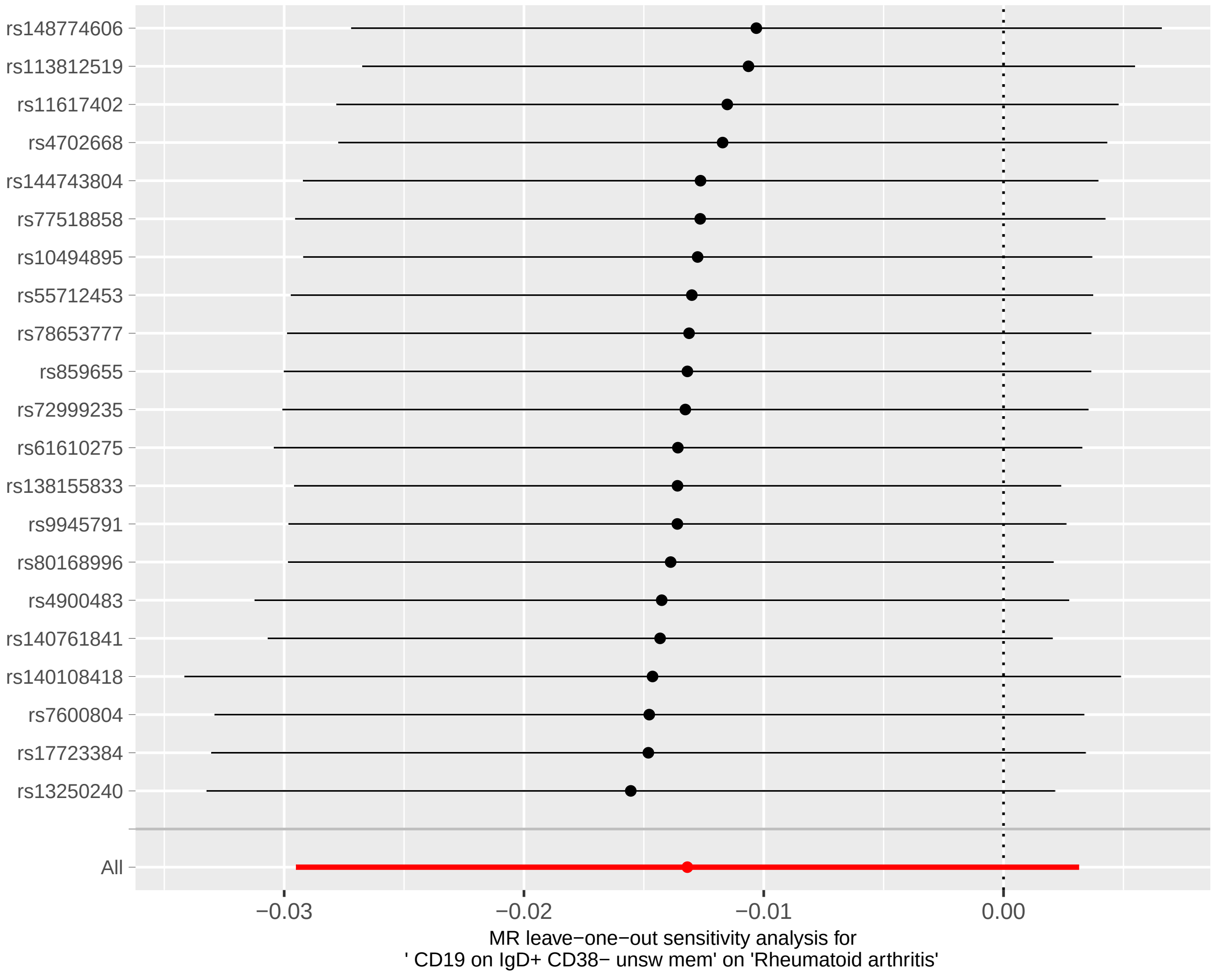
rs148774606
rs113812519
rs11617402
rs4702668
rs144743804
rs77518858
rs10494895
rs55712453
rs78653777
rs859655
rs72999235
rs61610275
rs138155833
rs9945791
rs80168996
rs4900483
rs140761841
rs140108418
rs7600804
rs17723384
rs13250240
All
−0.03
−0.02
−0.01
0.00
MR leave−one−out sensitivity analysis for
' CD19 on IgD+ CD38− unsw mem' on 'Rheumatoid arthritis'

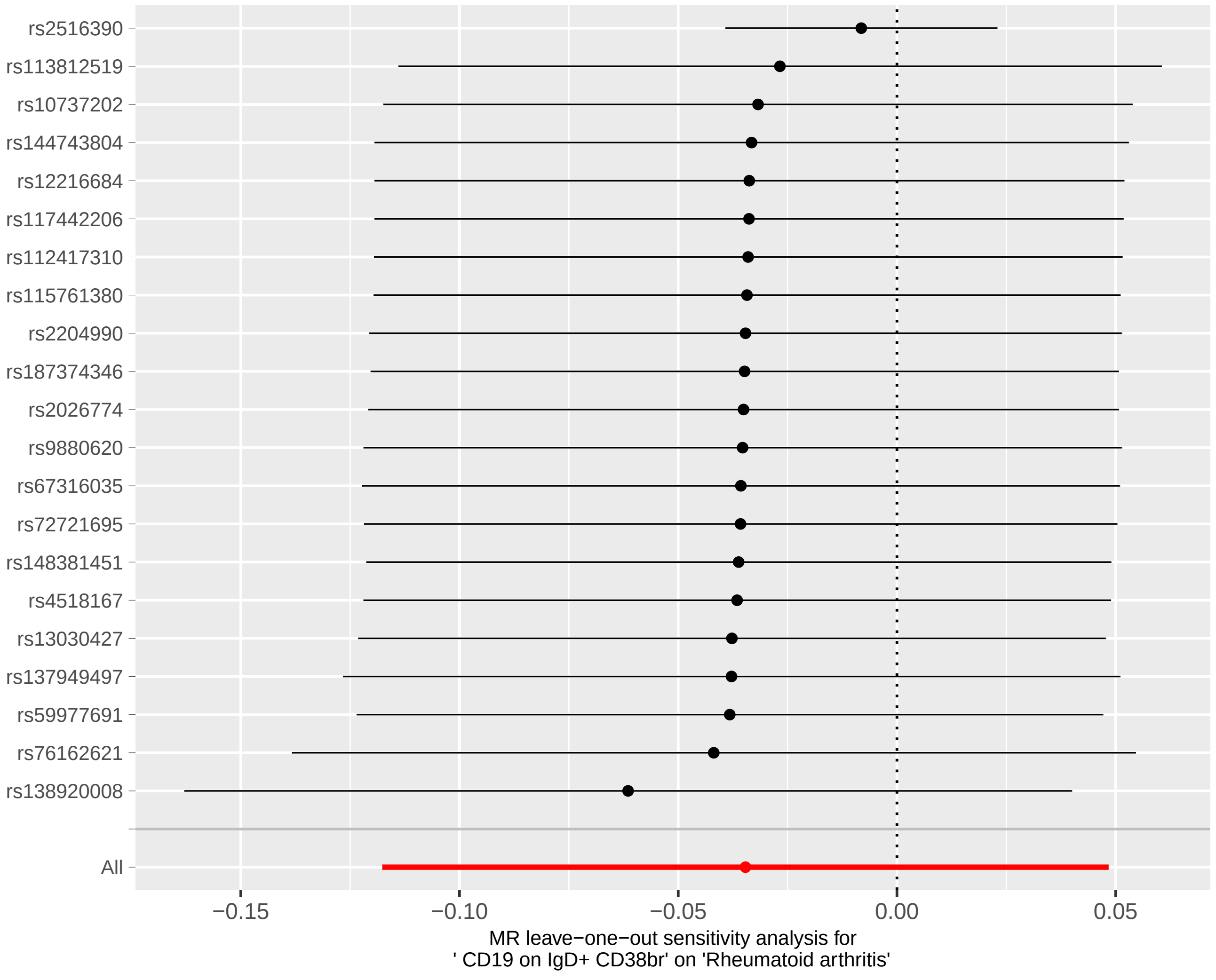

rs2516390
rs113812519
rs10737202
rs144743804
rs12216684
rs117442206
rs112417310
rs115761380
rs2204990
rs187374346
rs2026774
rs9880620
rs67316035
rs72721695
rs148381451
rs4518167
rs13030427
rs137949497
rs59977691
rs76162621
rs138920008
All
−0.15
−0.10
−0.05
0.00
0.05
MR leave−one−out sensitivity analysis for
' CD19 on IgD+ CD38br' on 'Rheumatoid arthritis'

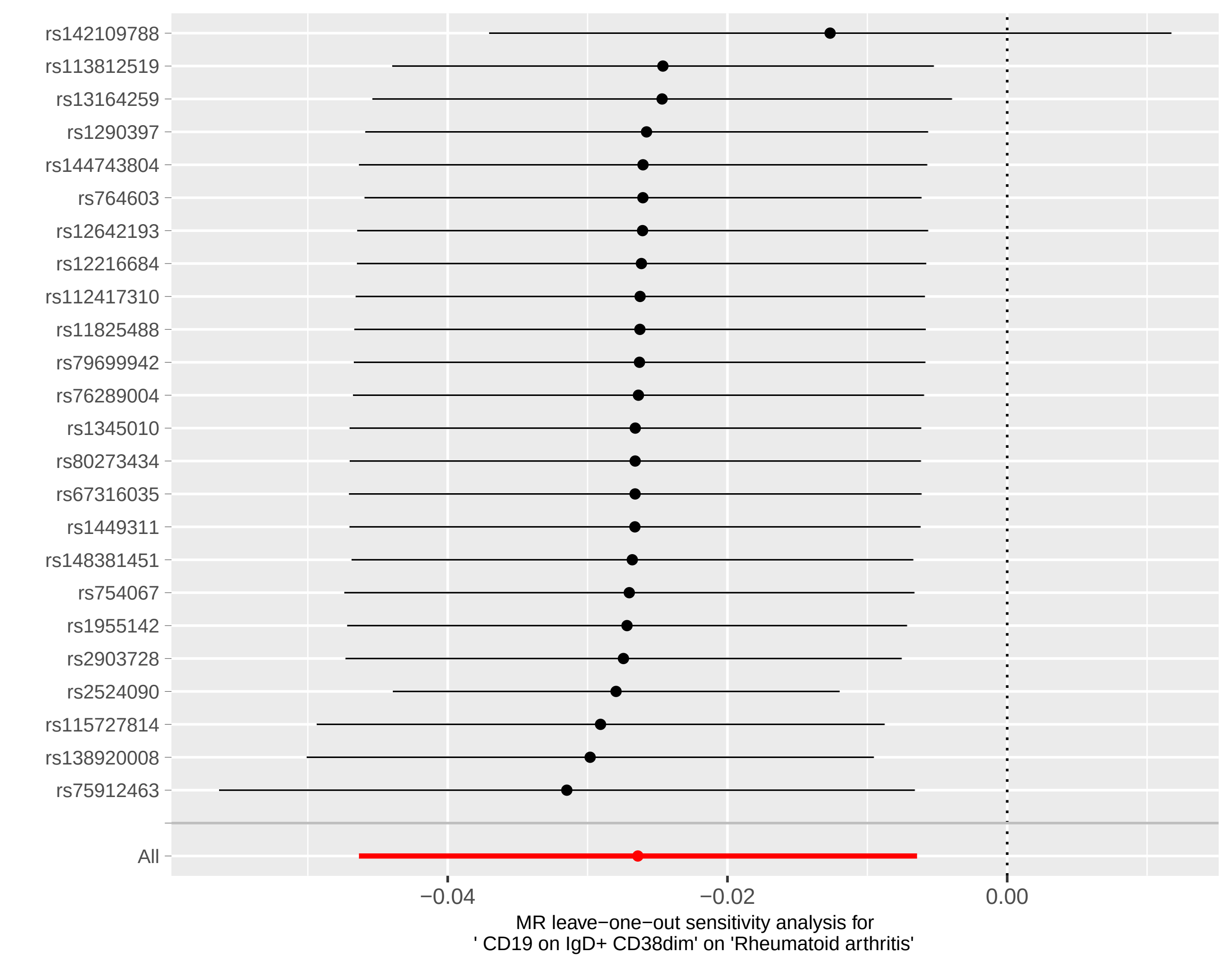

rs142109788
rs113812519
rs13164259
rs1290397
rs144743804
rs764603
rs12642193
rs12216684
rs112417310
rs11825488
rs79699942
rs76289004
rs1345010
rs80273434
rs67316035
rs1449311
rs148381451
rs754067
rs1955142
rs2903728
rs2524090
rs115727814
rs138920008
rs75912463
All
−0.04
−0.02
0.00
MR leave−one−out sensitivity analysis for
' CD19 on IgD+ CD38dim' on 'Rheumatoid arthritis'

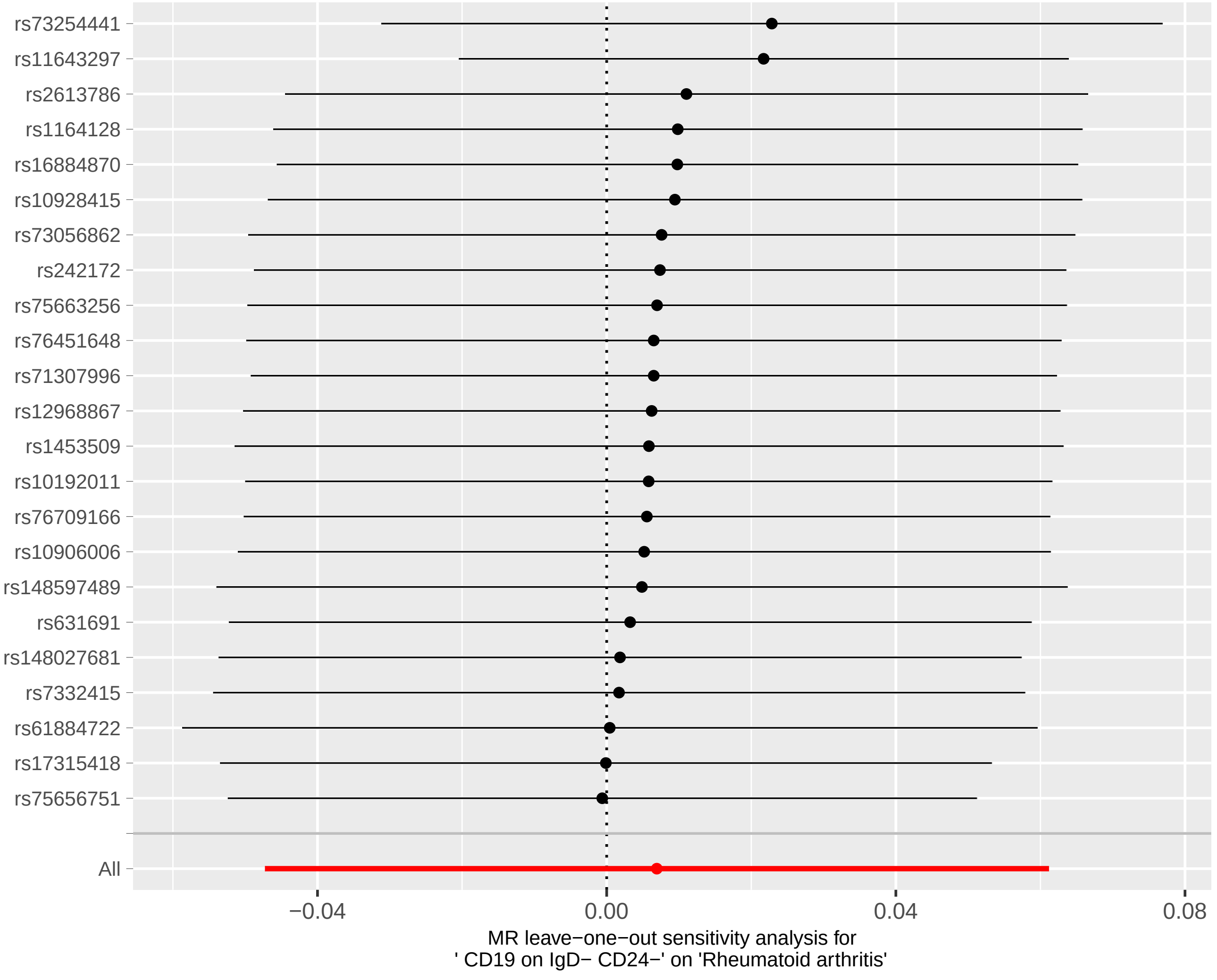
rs73254441
rs11643297
rs2613786
rs1164128
rs16884870
rs10928415
rs73056862
rs242172
rs75663256
rs76451648
rs71307996
rs12968867
rs1453509
rs10192011
rs76709166
rs10906006
rs148597489
rs631691
rs148027681
rs7332415
rs61884722
rs17315418
rs75656751
All
−0.04
0.00
0.04
0.08
MR leave−one−out sensitivity analysis for
' CD19 on IgD− CD24−' on 'Rheumatoid arthritis'

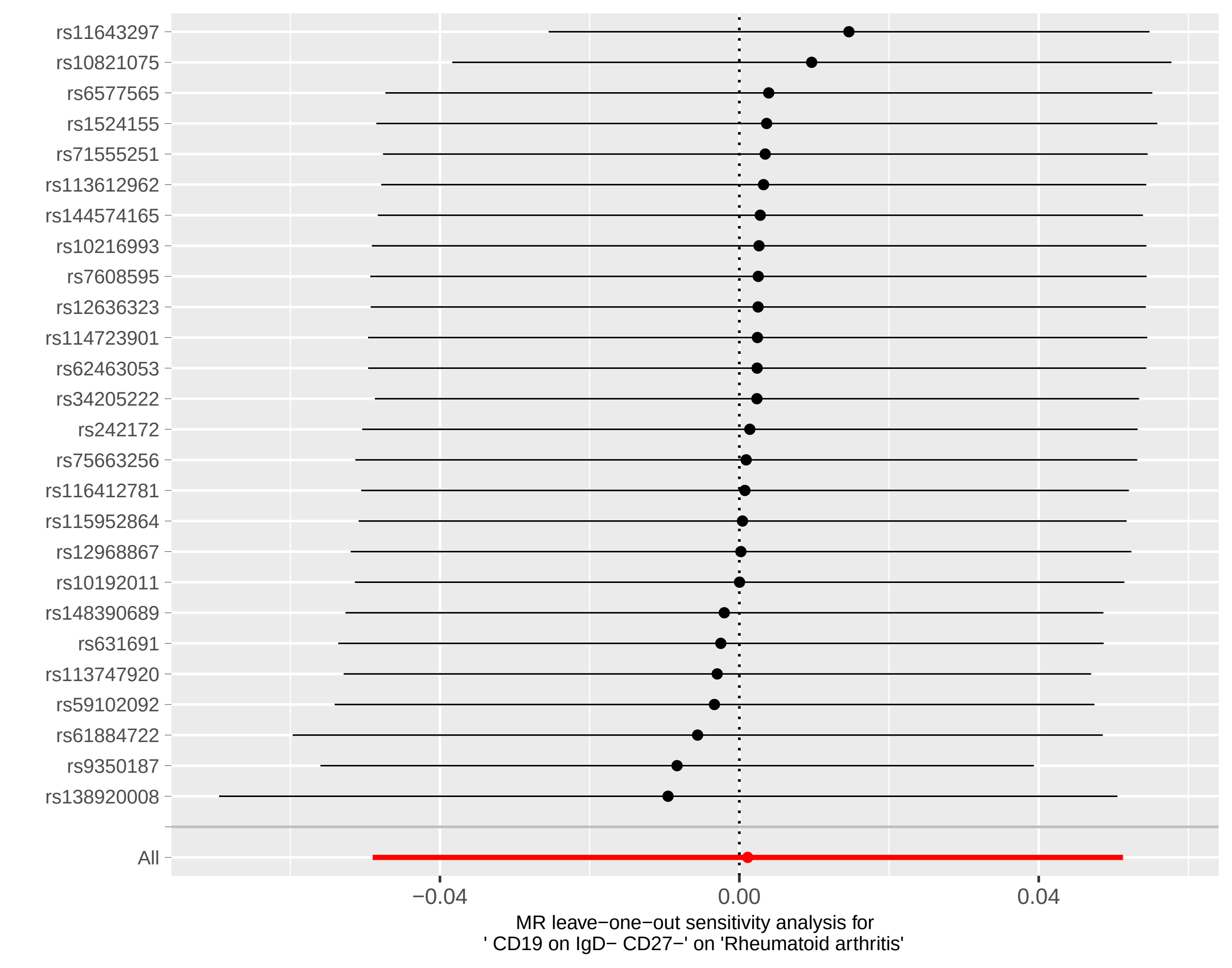
rs11643297
rs10821075
rs6577565
rs1524155
rs71555251
rs113612962
rs144574165
rs10216993
rs7608595
rs12636323
rs114723901
rs62463053
rs34205222
rs242172
rs75663256
rs116412781
rs115952864
rs12968867
rs10192011
rs148390689
rs631691
rs113747920
rs59102092
rs61884722
rs9350187
rs138920008
All
−0.04
0.00
0.04
MR leave−one−out sensitivity analysis for
' CD19 on IgD− CD27−' on 'Rheumatoid arthritis'

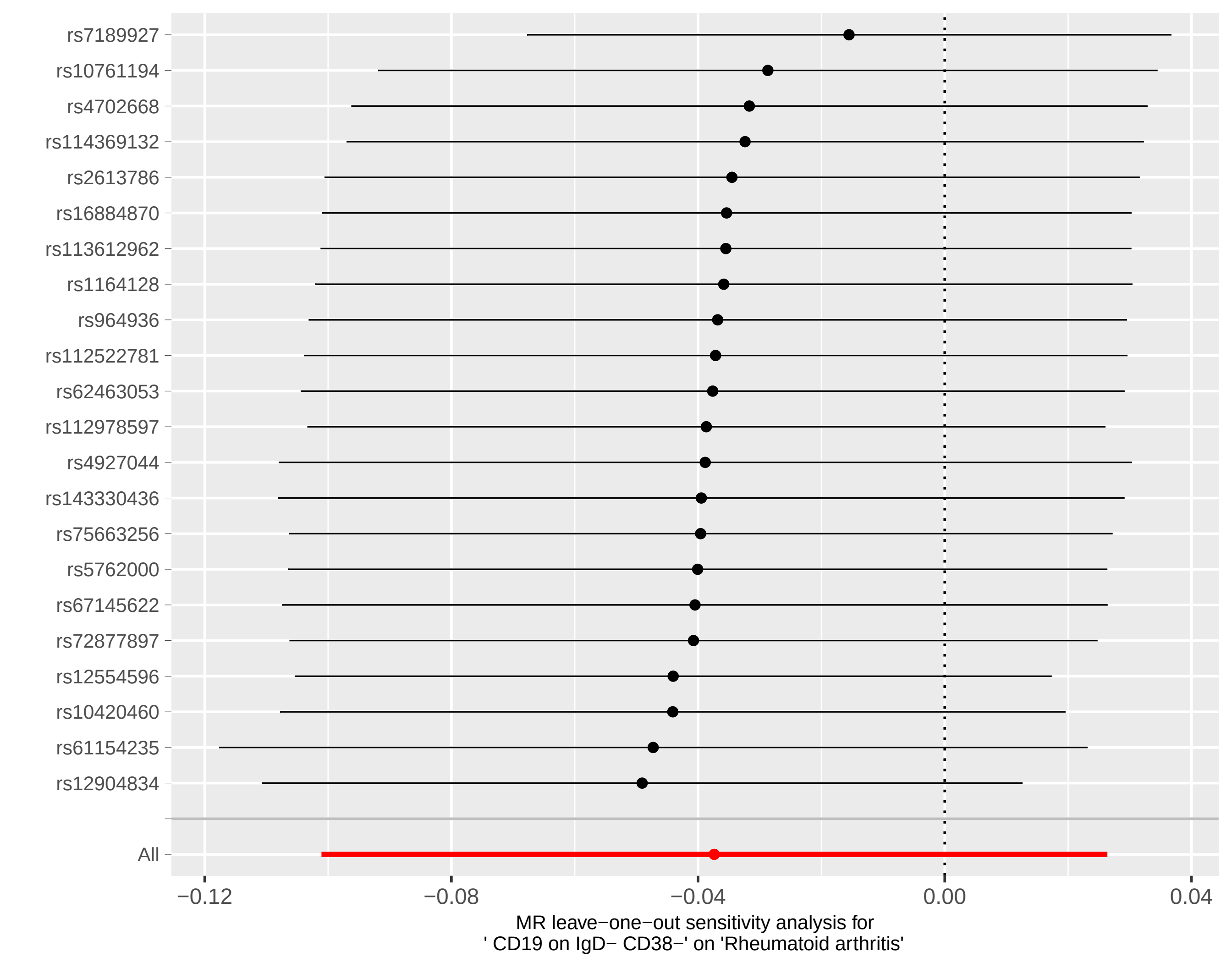
rs7189927
rs10761194
rs4702668
rs114369132
rs2613786
rs16884870
rs113612962
rs1164128
rs964936
rs112522781
rs62463053
rs112978597
rs4927044
rs143330436
rs75663256
rs5762000
rs67145622
rs72877897
rs12554596
rs10420460
rs61154235
rs12904834
All
−0.12
−0.08
−0.04
0.00
0.04
MR leave−one−out sensitivity analysis for
' CD19 on IgD− CD38−' on 'Rheumatoid arthritis'

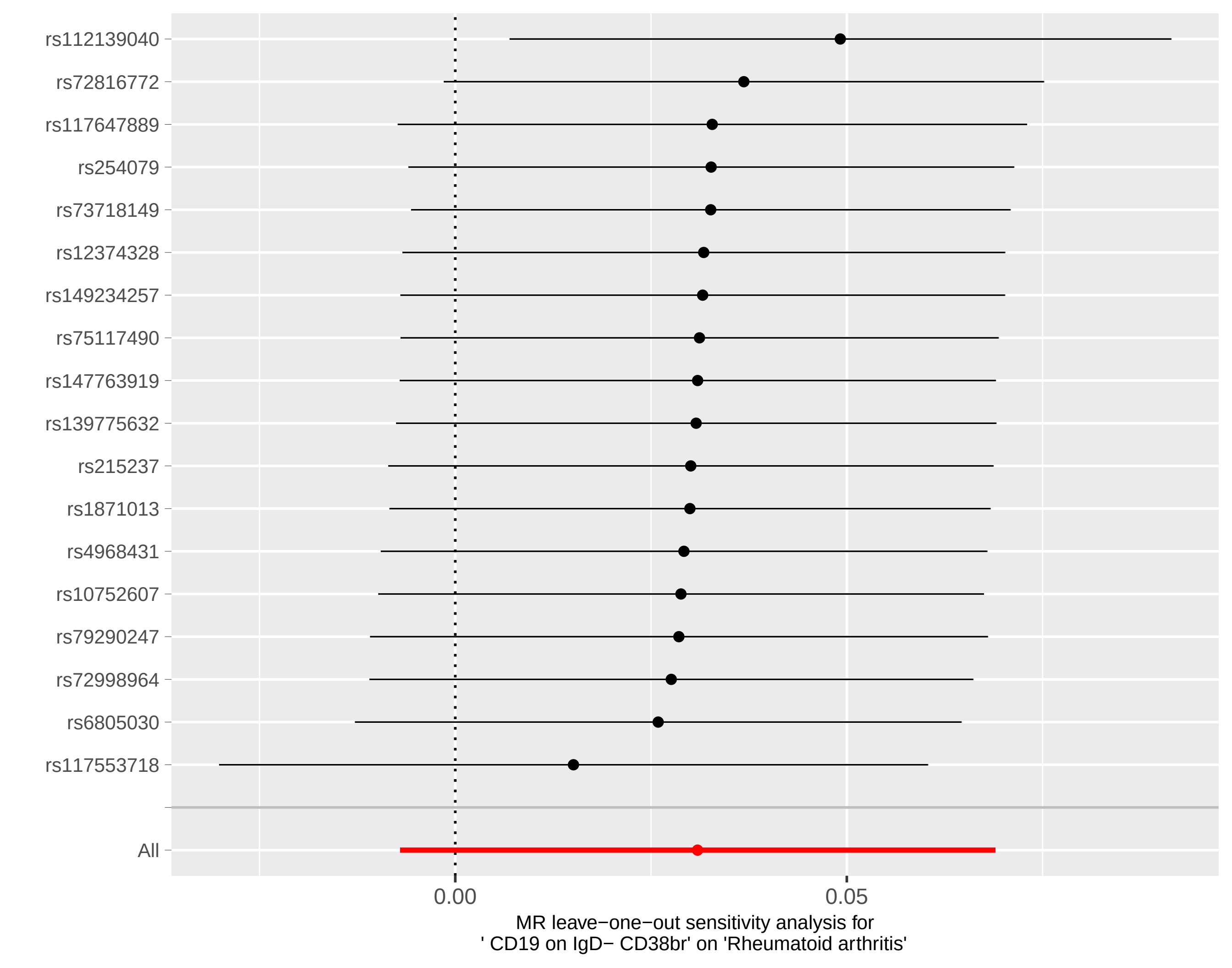
rs112139040
rs72816772
rs117647889
rs254079
rs73718149
rs12374328
rs149234257
rs75117490
rs147763919
rs139775632
rs215237
rs1871013
rs4968431
rs10752607
rs79290247
rs72998964
rs6805030
rs117553718
All
0.00
0.05
MR leave−one−out sensitivity analysis for
' CD19 on IgD− CD38br' on 'Rheumatoid arthritis'

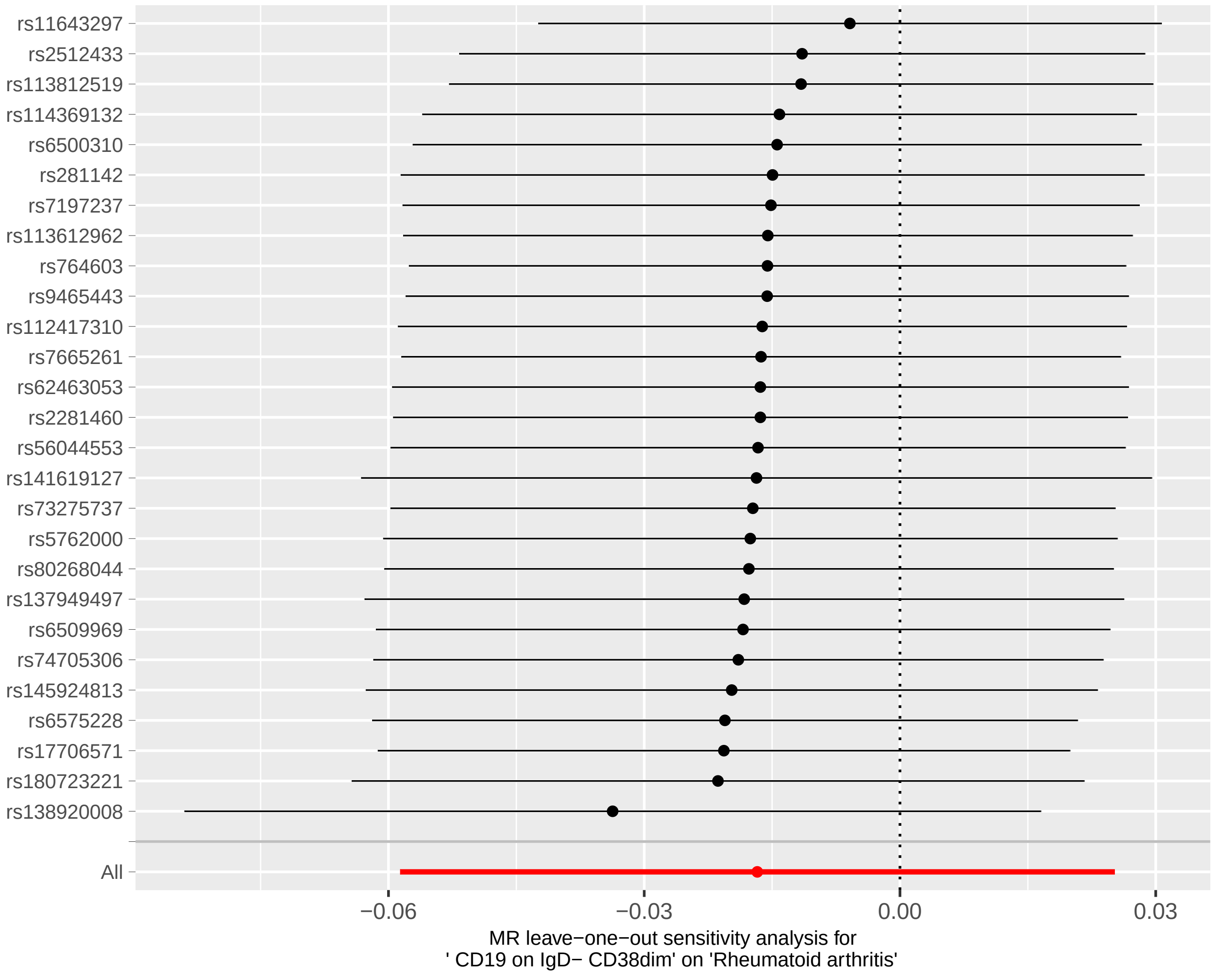
rs11643297
rs2512433
rs113812519
rs114369132
rs6500310
rs281142
rs7197237
rs113612962
rs764603
rs9465443
rs112417310
rs7665261
rs62463053
rs2281460
rs56044553
rs141619127
rs73275737
rs5762000
rs80268044
rs137949497
rs6509969
rs74705306
rs145924813
rs6575228
rs17706571
rs180723221
rs138920008
All
−0.06
−0.03
0.00
0.03
MR leave−one−out sensitivity analysis for
' CD19 on IgD− CD38dim' on 'Rheumatoid arthritis'

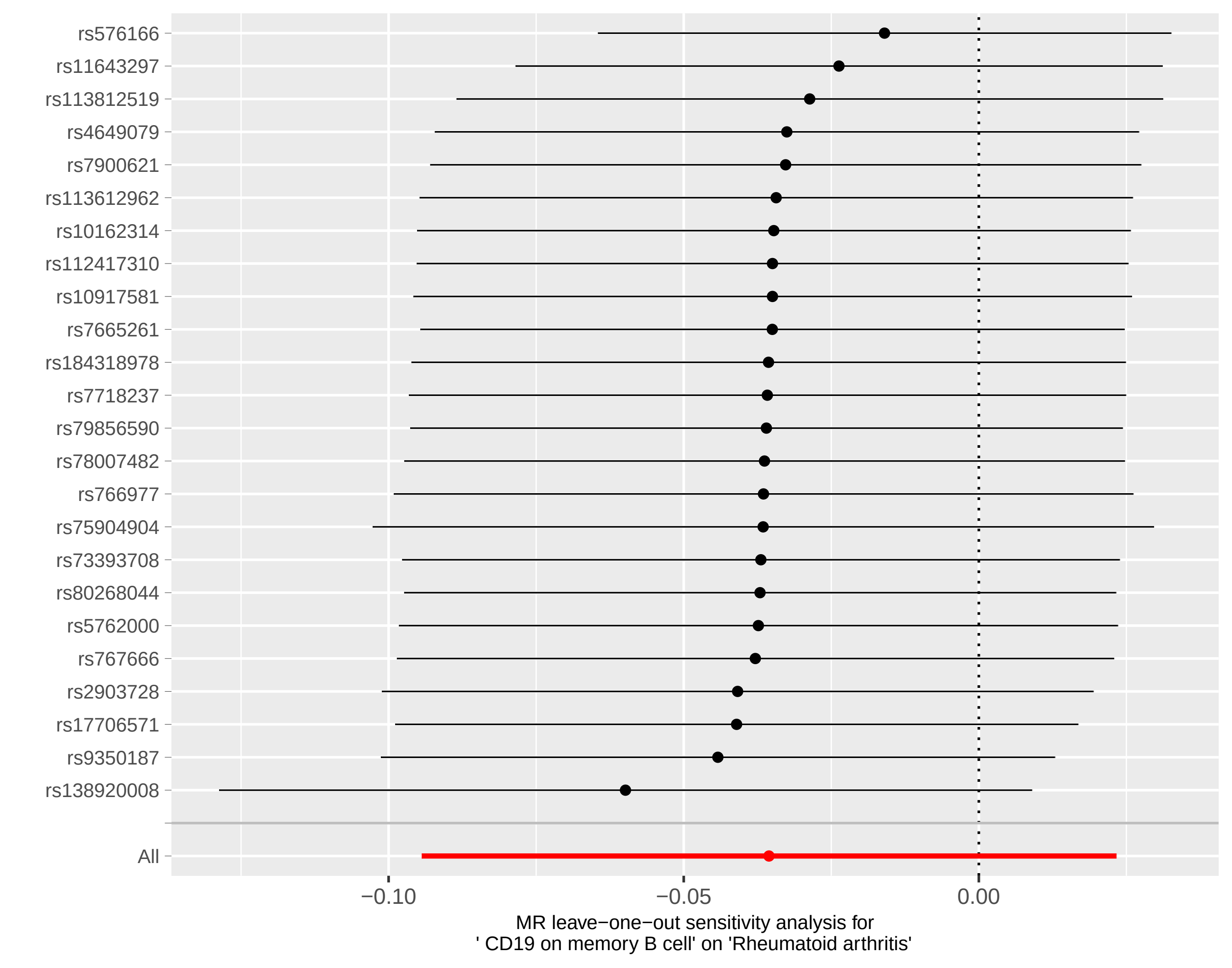
rs576166
rs11643297
rs113812519
rs4649079
rs7900621
rs113612962
rs10162314
rs112417310
rs10917581
rs7665261
rs184318978
rs7718237
rs79856590
rs78007482
rs766977
rs75904904
rs73393708
rs80268044
rs5762000
rs767666
rs2903728
rs17706571
rs9350187
rs138920008
All
−0.10
−0.05
0.00
MR leave−one−out sensitivity analysis for
' CD19 on memory B cell' on 'Rheumatoid arthritis'

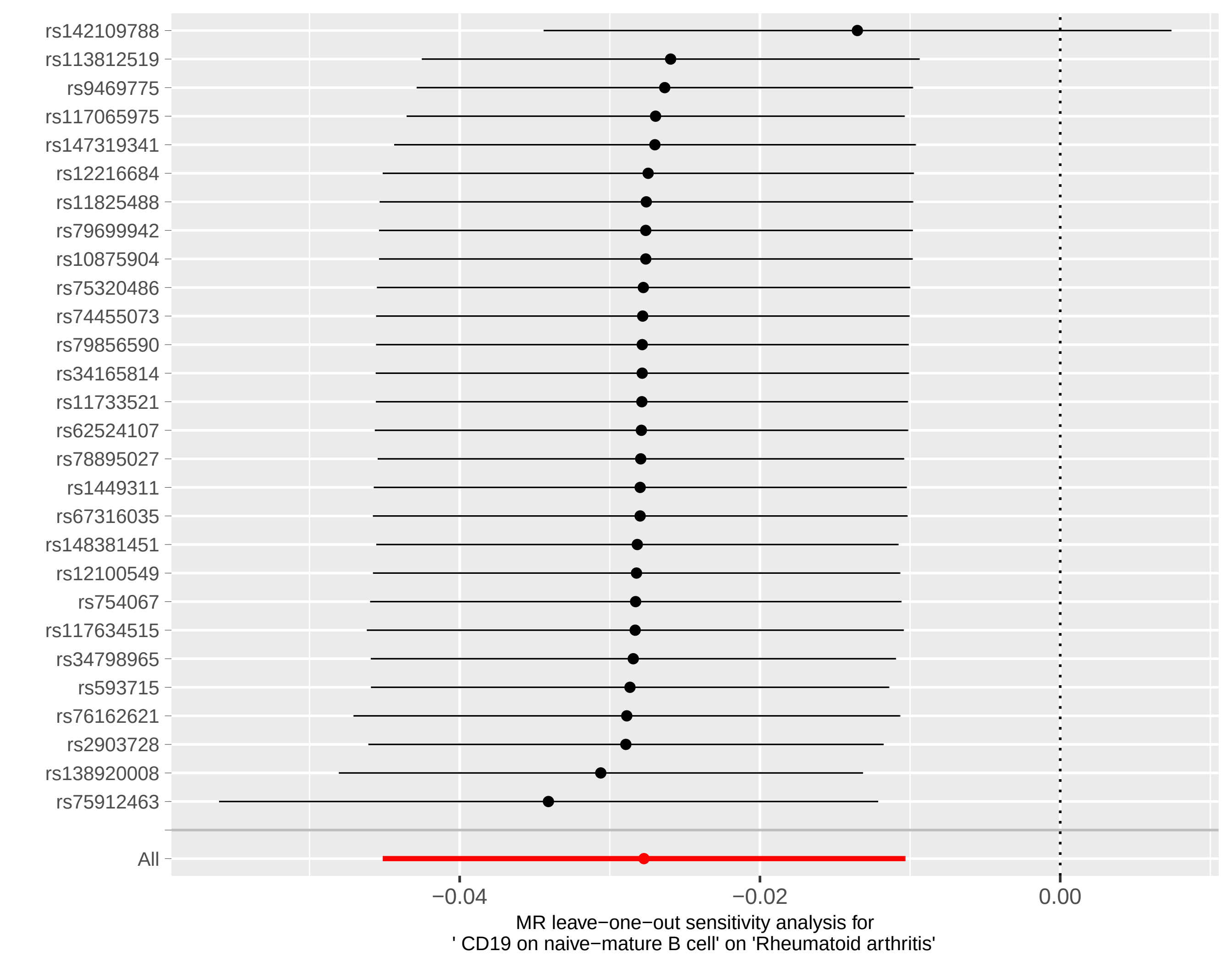
rs142109788
rs113812519
rs9469775
rs117065975
rs147319341
rs12216684
rs11825488
rs79699942
rs10875904
rs75320486
rs74455073
rs79856590
rs34165814
rs11733521
rs62524107
rs78895027
rs1449311
rs67316035
rs148381451
rs12100549
rs754067
rs117634515
rs34798965
rs593715
rs76162621
rs2903728
rs138920008
rs75912463
All
−0.04
−0.02
0.00
MR leave−one−out sensitivity analysis for
' CD19 on naive−mature B cell' on 'Rheumatoid arthritis'

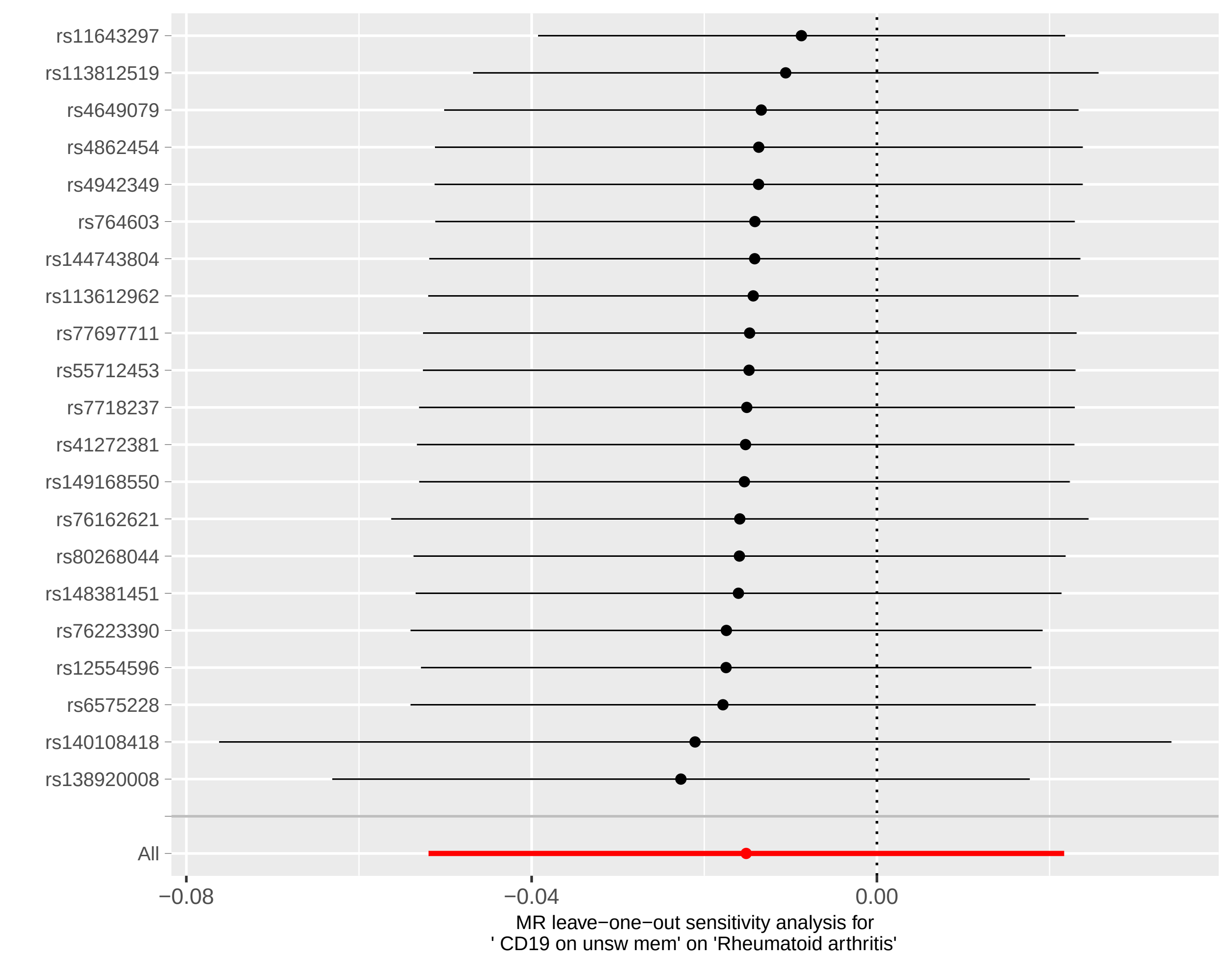

rs11643297
rs113812519
rs4649079
rs4862454
rs4942349
rs764603
rs144743804
rs113612962
rs77697711
rs55712453
rs7718237
rs41272381
rs149168550
rs76162621
rs80268044
rs148381451
rs76223390
rs12554596
rs6575228
rs140108418
rs138920008
All
−0.08
−0.04
0.00
MR leave−one−out sensitivity analysis for
' CD19 on unsw mem' on 'Rheumatoid arthritis'

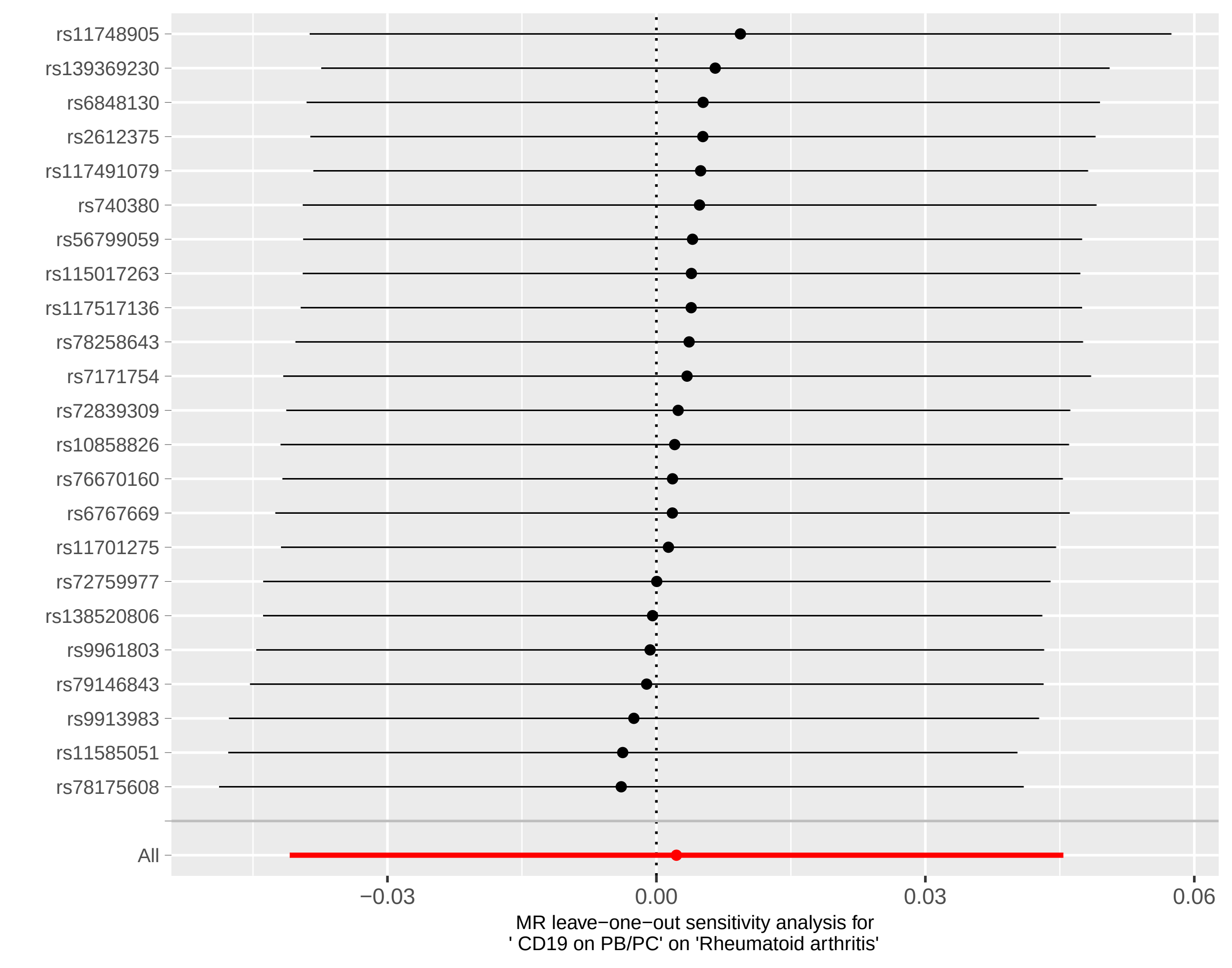

rs11748905
rs139369230
rs6848130
rs2612375
rs117491079
rs740380
rs56799059
rs115017263
rs117517136
rs78258643
rs7171754
rs72839309
rs10858826
rs76670160
rs6767669
rs11701275
rs72759977
rs138520806
rs9961803
rs79146843
rs9913983
rs11585051
rs78175608
All
−0.03
0.00
0.03
0.06
MR leave−one−out sensitivity analysis for
' CD19 on PB/PC' on 'Rheumatoid arthritis'

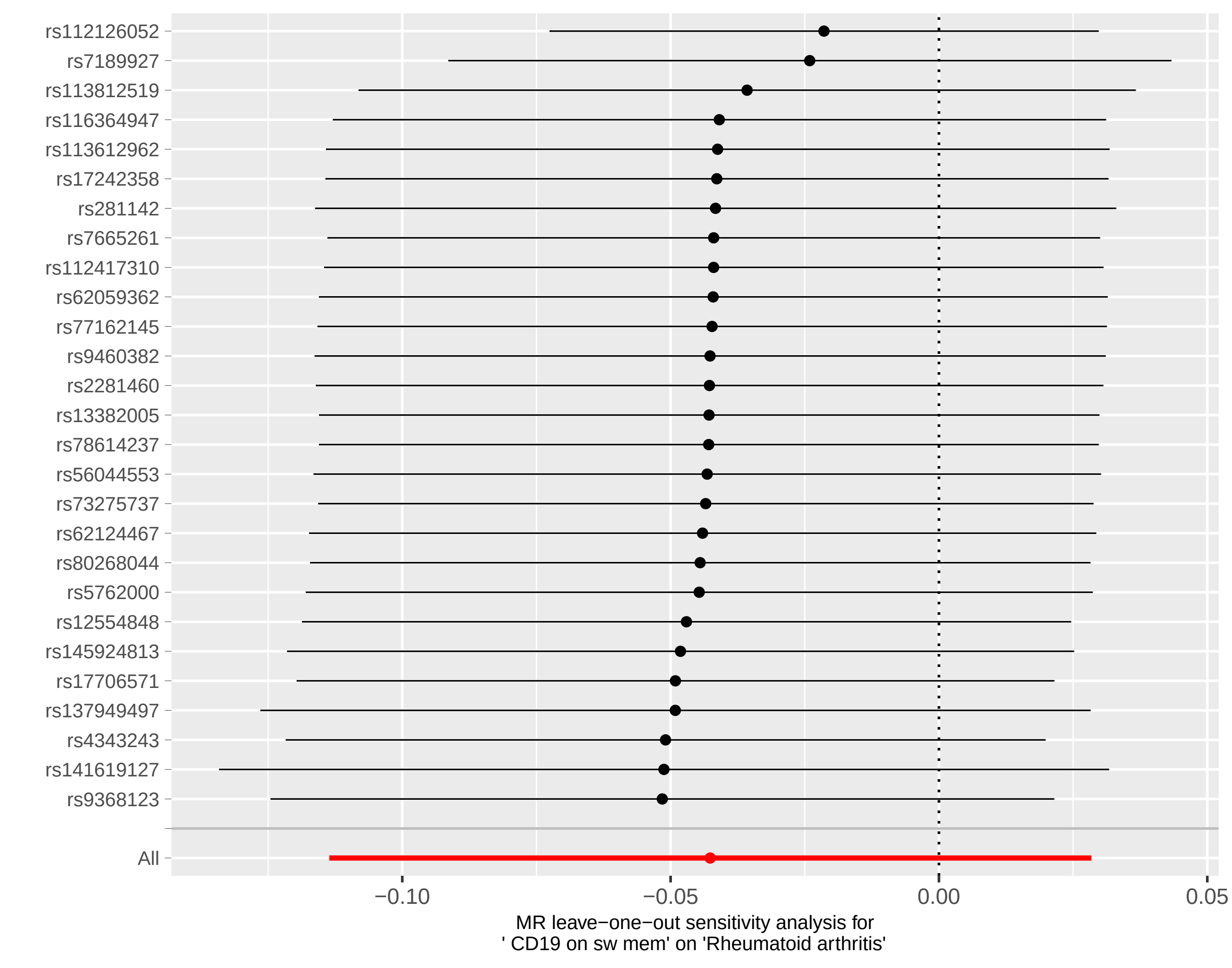
rs112126052
rs7189927
rs113812519
rs116364947
rs113612962
rs17242358
rs281142
rs7665261
rs112417310
rs62059362
rs77162145
rs9460382
rs2281460
rs13382005
rs78614237
rs56044553
rs73275737
rs62124467
rs80268044
rs5762000
rs12554848
rs145924813
rs17706571
rs137949497
rs4343243
rs141619127
rs9368123
All
−0.10
−0.05
0.00
0.05
MR leave−one−out sensitivity analysis for
' CD19 on sw mem' on 'Rheumatoid arthritis'

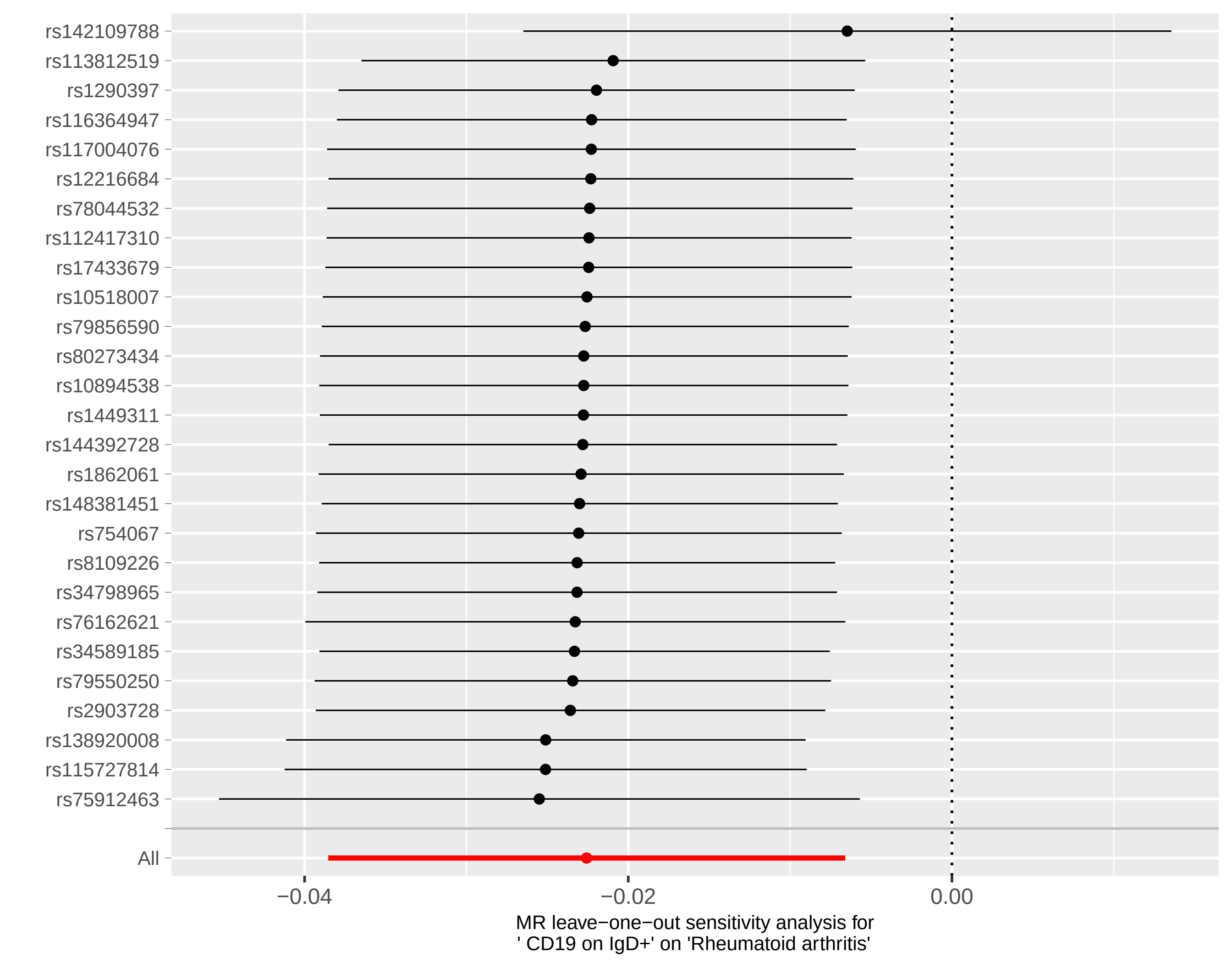
rs142109788
rs113812519
rs1290397
rs116364947
rs117004076
rs12216684
rs78044532
rs112417310
rs17433679
rs10518007
rs79856590
rs80273434
rs10894538
rs1449311
rs144392728
rs1862061
rs148381451
rs754067
rs8109226
rs34798965
rs76162621
rs34589185
rs79550250
rs2903728
rs138920008
rs115727814
rs75912463
All
−0.04
−0.02
0.00
MR leave−one−out sensitivity analysis for
' CD19 on IgD+' on 'Rheumatoid arthritis'

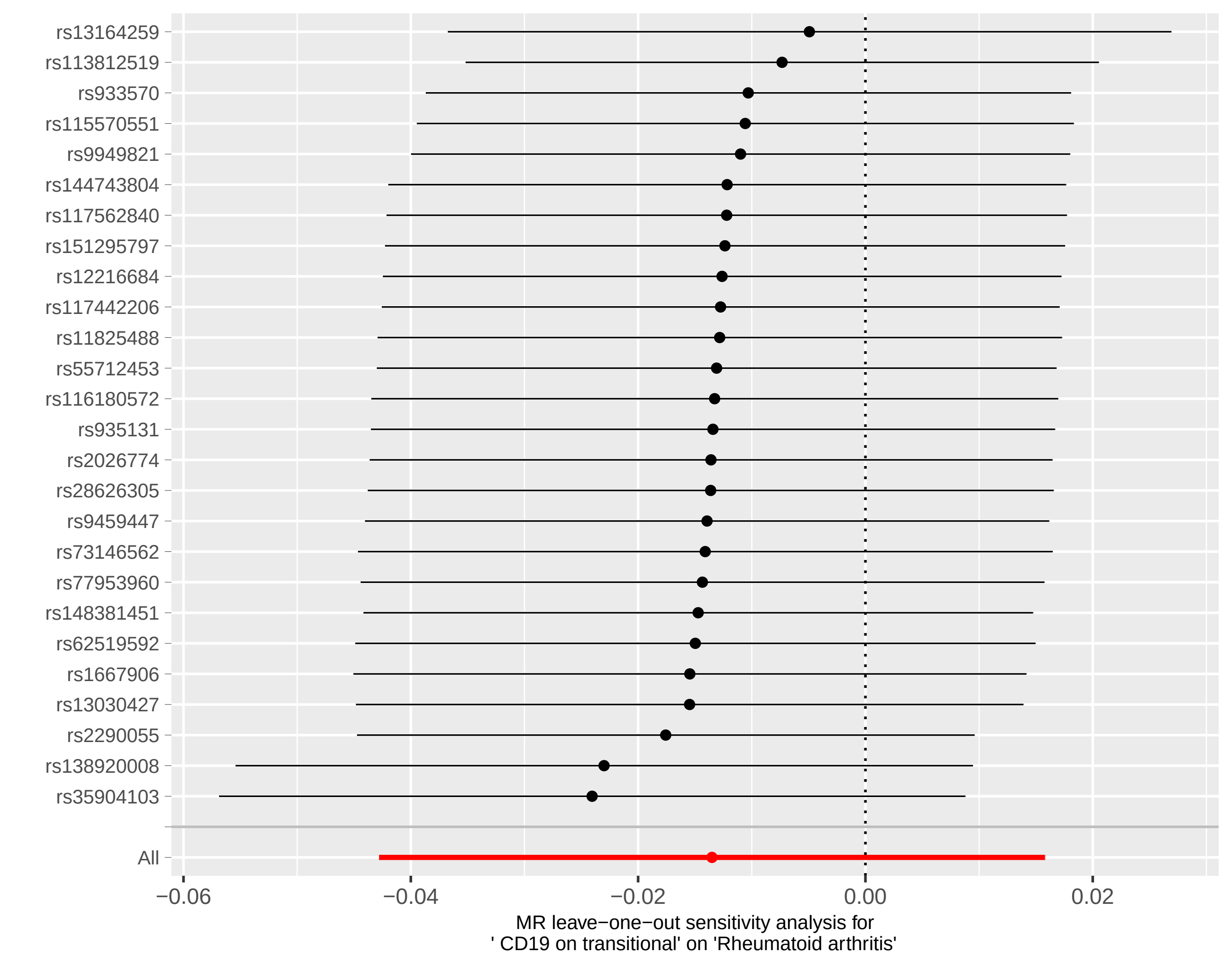
rs13164259
rs113812519
rs933570
rs115570551
rs9949821
rs144743804
rs117562840
rs151295797
rs12216684
rs117442206
rs11825488
rs55712453
rs116180572
rs935131
rs2026774
rs28626305
rs9459447
rs73146562
rs77953960
rs148381451
rs62519592
rs1667906
rs13030427
rs2290055
rs138920008
rs35904103
All
−0.06
−0.04
−0.02
0.00
0.02
MR leave−one−out sensitivity analysis for
' CD19 on transitional' on 'Rheumatoid arthritis'

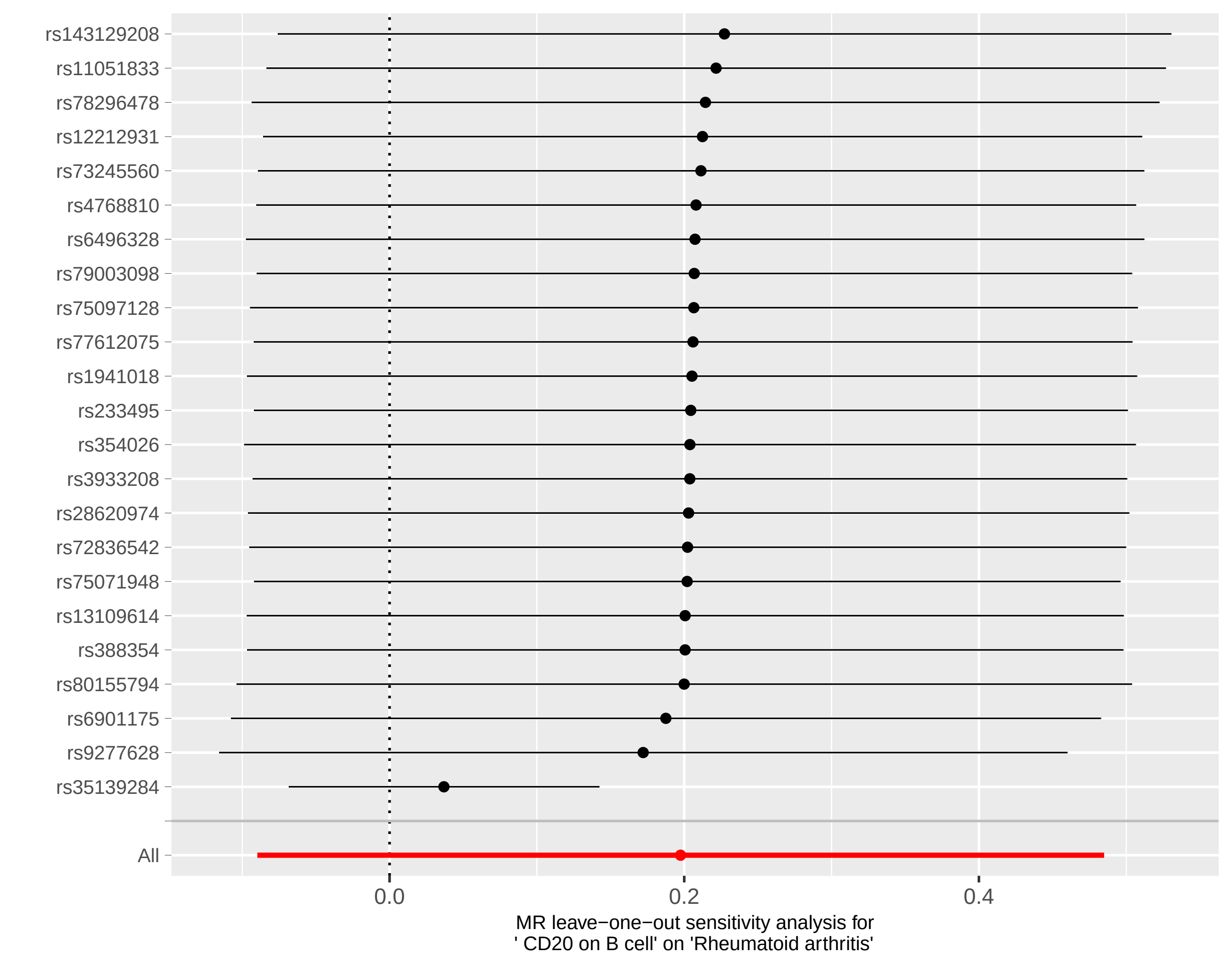
rs143129208
rs11051833
rs78296478
rs12212931
rs73245560
rs4768810
rs6496328
rs79003098
rs75097128
rs77612075
rs1941018
rs233495
rs354026
rs3933208
rs28620974
rs72836542
rs75071948
rs13109614
rs388354
rs80155794
rs6901175
rs9277628
rs35139284
All
0.0
0.2
0.4
MR leave−one−out sensitivity analysis for
' CD20 on B cell' on 'Rheumatoid arthritis'

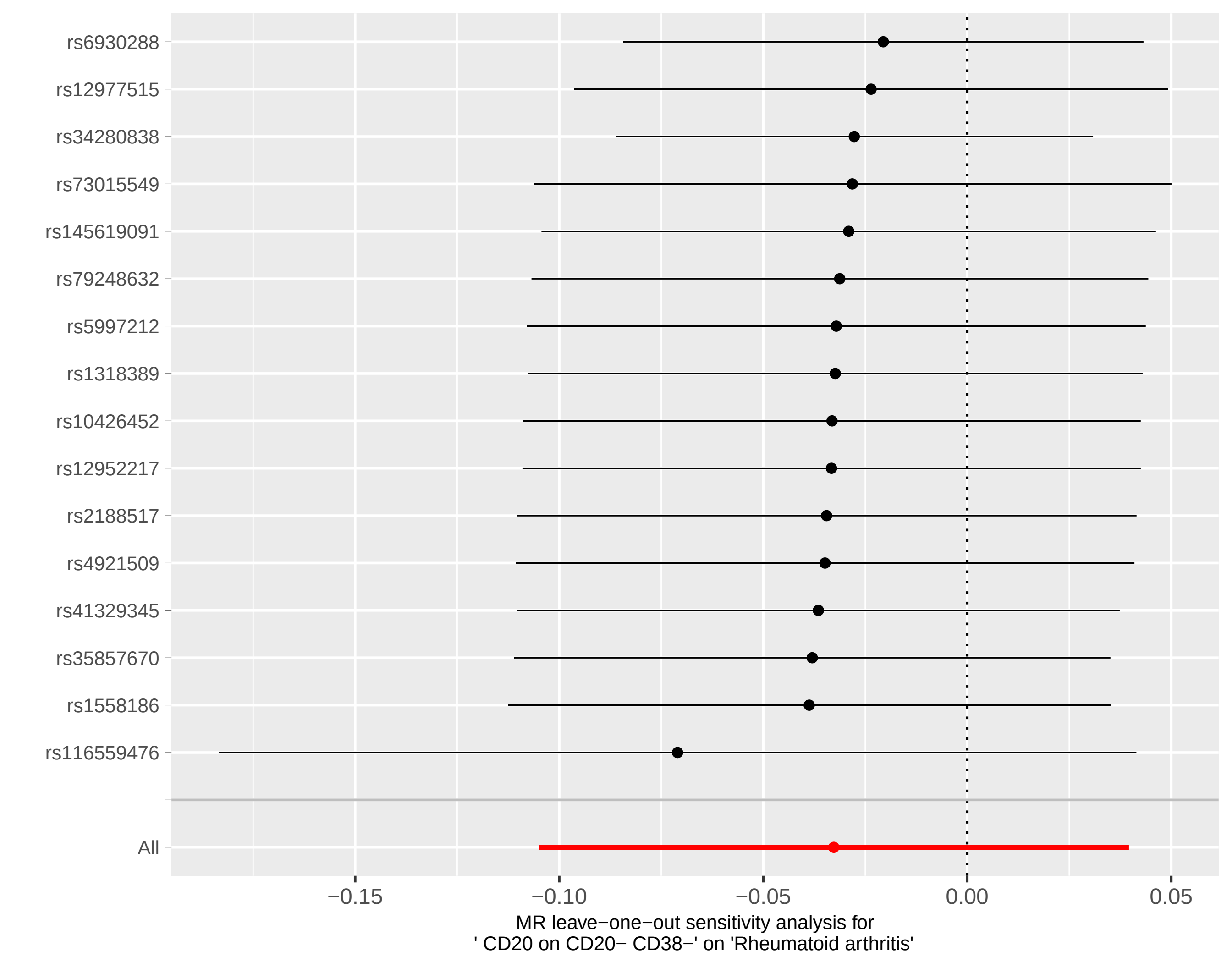
rs6930288
rs12977515
rs34280838
rs73015549
rs145619091
rs79248632
rs5997212
rs1318389
rs10426452
rs12952217
rs2188517
rs4921509
rs41329345
rs35857670
rs1558186
rs116559476
All
−0.15
−0.10
−0.05
0.00
0.05
MR leave−one−out sensitivity analysis for
' CD20 on CD20− CD38−' on 'Rheumatoid arthritis'

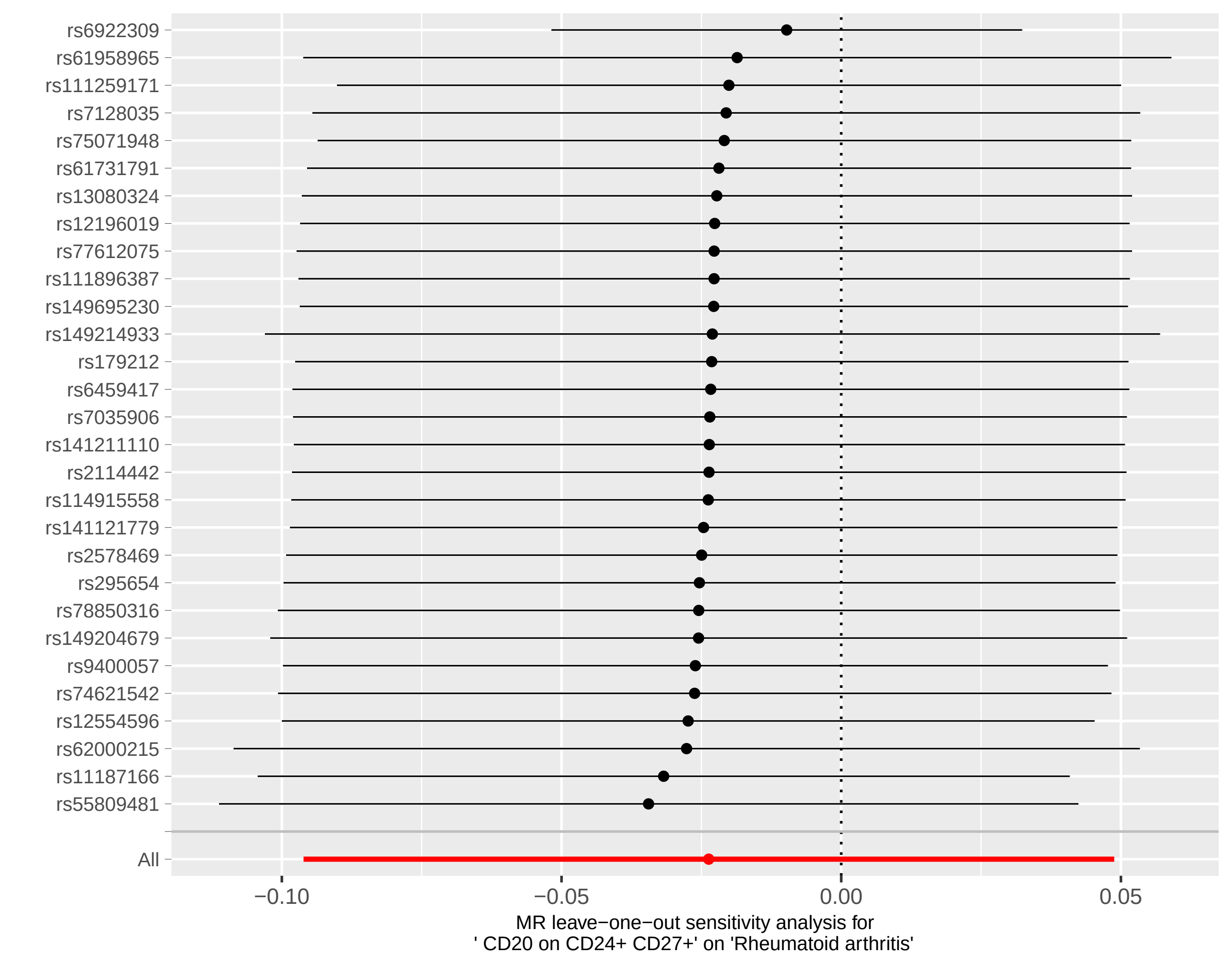
rs6922309
rs61958965
rs111259171
rs7128035
rs75071948
rs61731791
rs13080324
rs12196019
rs77612075
rs111896387
rs149695230
rs149214933
rs179212
rs6459417
rs7035906
rs141211110
rs2114442
rs114915558
rs141121779
rs2578469
rs295654
rs78850316
rs149204679
rs9400057
rs74621542
rs12554596
rs62000215
rs11187166
rs55809481
All
−0.10
−0.05
0.00
0.05
MR leave−one−out sensitivity analysis for
' CD20 on CD24+ CD27+' on 'Rheumatoid arthritis'

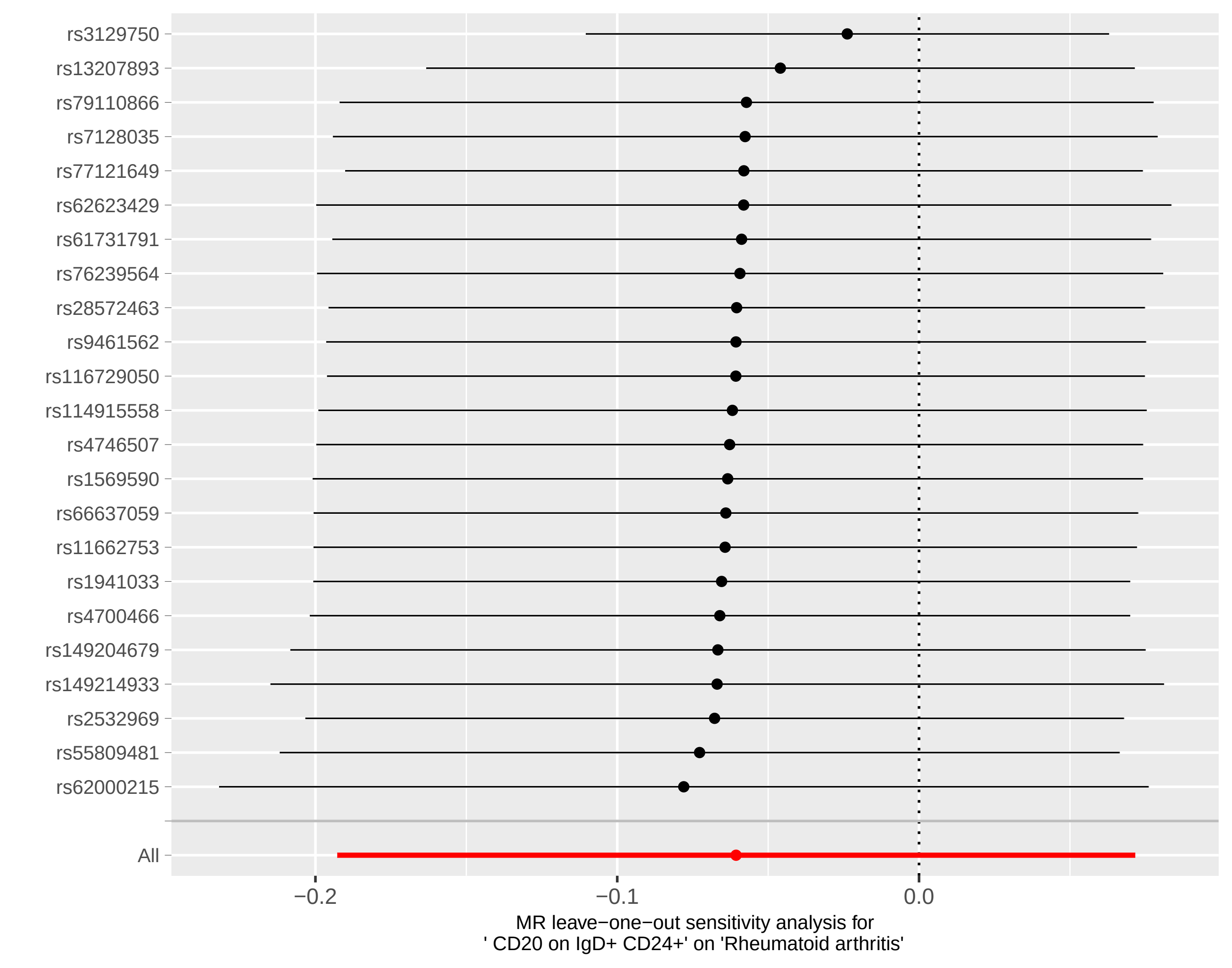

rs3129750
rs13207893
rs79110866
rs7128035
rs77121649
rs62623429
rs61731791
rs76239564
rs28572463
rs9461562
rs116729050
rs114915558
rs4746507
rs1569590
rs66637059
rs11662753
rs1941033
rs4700466
rs149204679
rs149214933
rs2532969
rs55809481
rs62000215
All
−0.2
−0.1
0.0
MR leave−one−out sensitivity analysis for
' CD20 on IgD+ CD24+' on 'Rheumatoid arthritis'

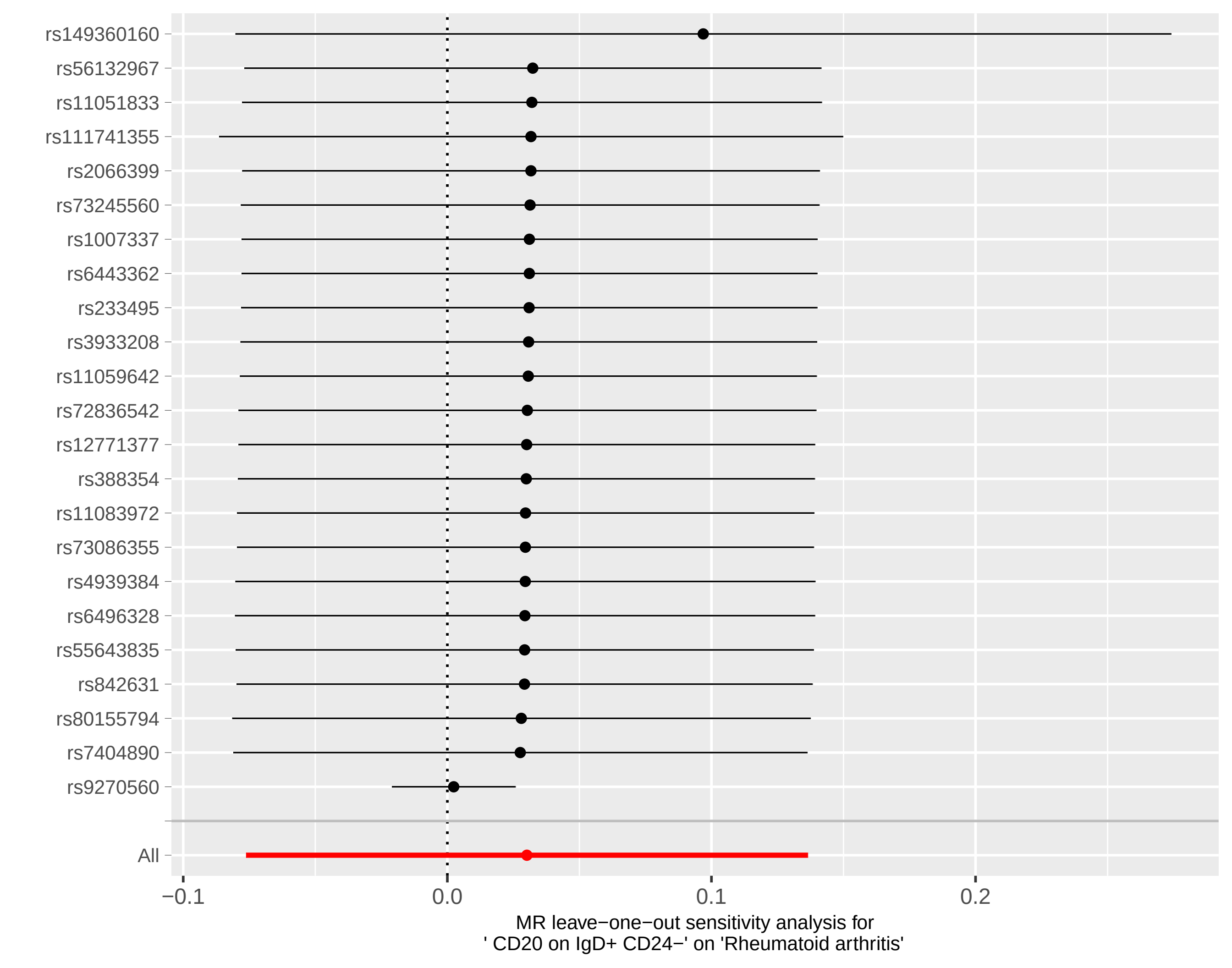
rs149360160
rs56132967
rs11051833
rs111741355
rs2066399
rs73245560
rs1007337
rs6443362
rs233495
rs3933208
rs11059642
rs72836542
rs12771377
rs388354
rs11083972
rs73086355
rs4939384
rs6496328
rs55643835
rs842631
rs80155794
rs7404890
rs9270560
All
−0.1
0.0
0.1
0.2
MR leave−one−out sensitivity analysis for
' CD20 on IgD+ CD24−' on 'Rheumatoid arthritis'

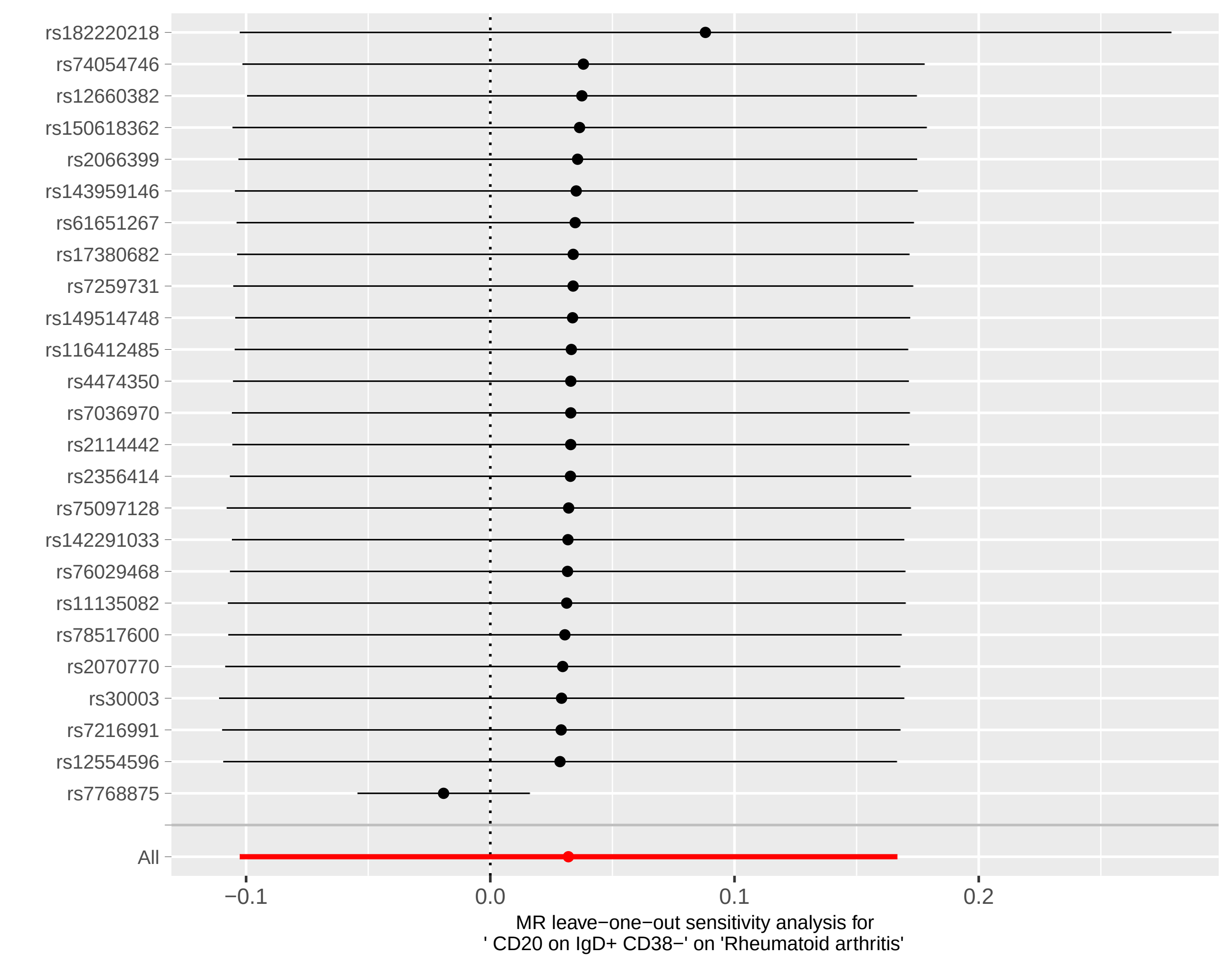
rs182220218
rs74054746
rs12660382
rs150618362
rs2066399
rs143959146
rs61651267
rs17380682
rs7259731
rs149514748
rs116412485
rs4474350
rs7036970
rs2114442
rs2356414
rs75097128
rs142291033
rs76029468
rs11135082
rs78517600
rs2070770
rs30003
rs7216991
rs12554596
rs7768875
All
−0.1
0.0
0.1
0.2
MR leave−one−out sensitivity analysis for
' CD20 on IgD+ CD38−' on 'Rheumatoid arthritis'

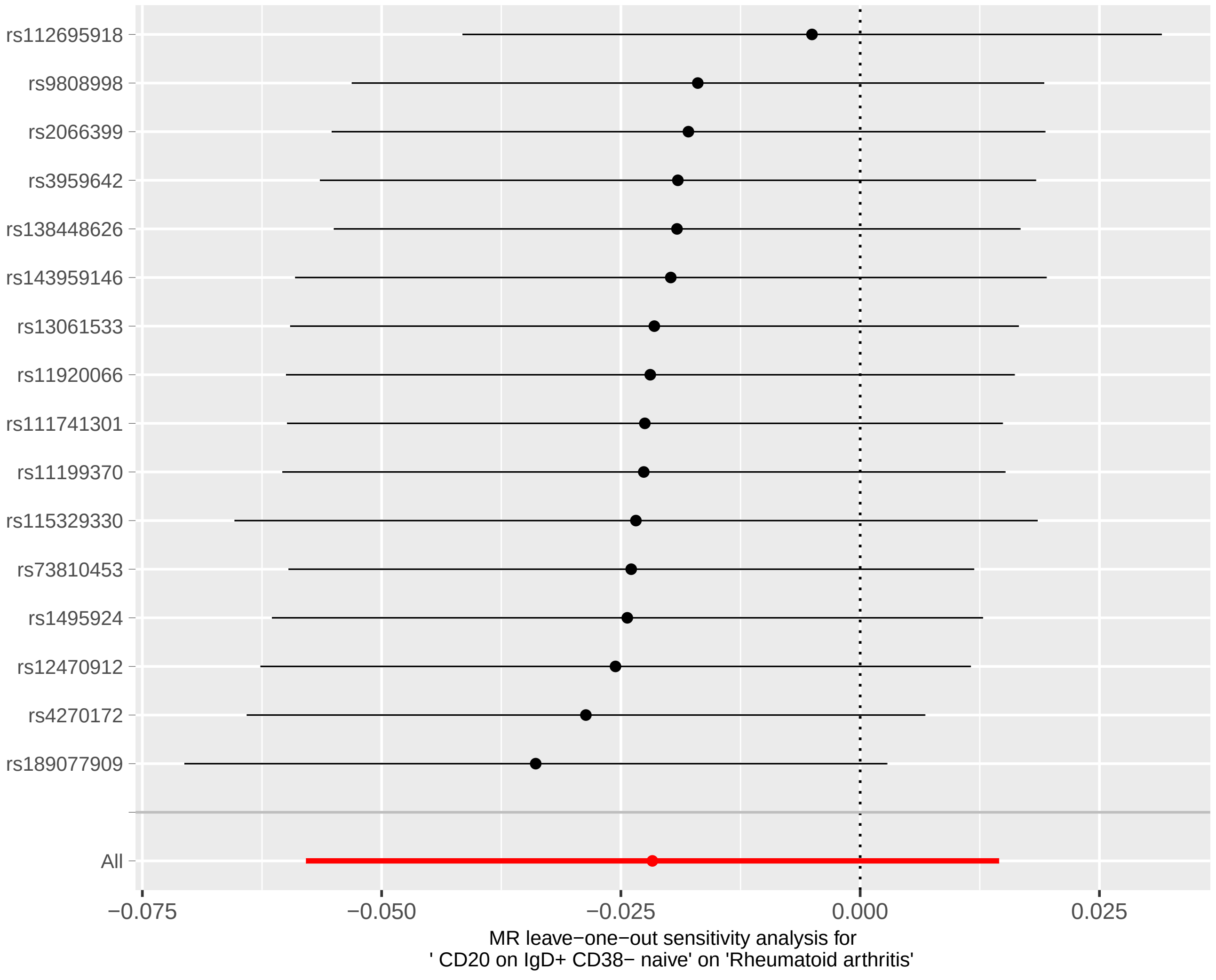
rs112695918
rs9808998
rs2066399
rs3959642
rs138448626
rs143959146
rs13061533
rs11920066
rs111741301
rs11199370
rs115329330
rs73810453
rs1495924
rs12470912
rs4270172
rs189077909
All
−0.075
−0.050
−0.025
0.000
0.025
MR leave−one−out sensitivity analysis for
' CD20 on IgD+ CD38− naive' on 'Rheumatoid arthritis'

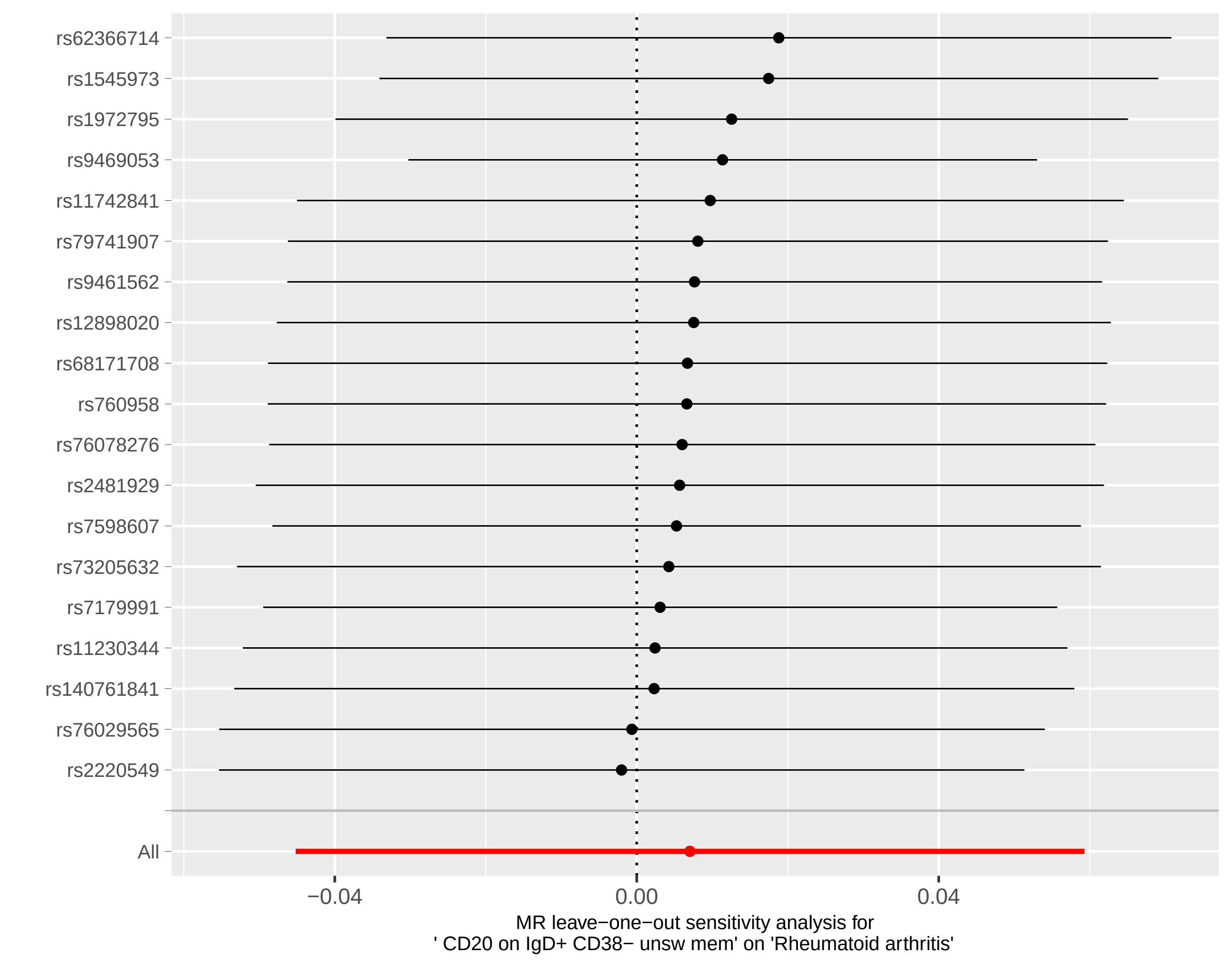
rs62366714
rs1545973
rs1972795
rs9469053
rs11742841
rs79741907
rs9461562
rs12898020
rs68171708
rs760958
rs76078276
rs2481929
rs7598607
rs73205632
rs7179991
rs11230344
rs140761841
rs76029565
rs2220549
All
−0.04
0.00
0.04
MR leave−one−out sensitivity analysis for
' CD20 on IgD+ CD38− unsw mem' on 'Rheumatoid arthritis'

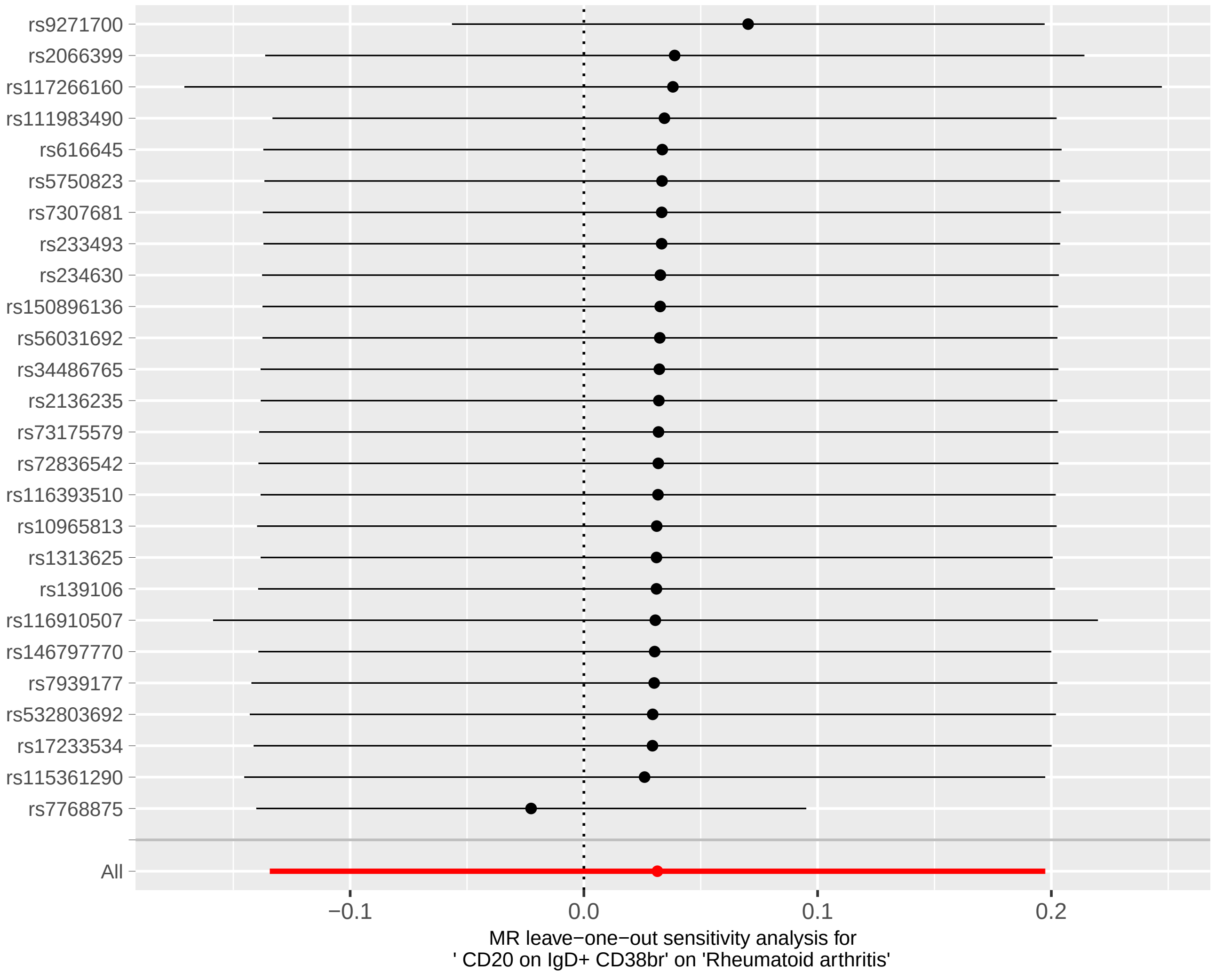
rs9271700
rs2066399
rs117266160
rs111983490
rs616645
rs5750823
rs7307681
rs233493
rs234630
rs150896136
rs56031692
rs34486765
rs2136235
rs73175579
rs72836542
rs116393510
rs10965813
rs1313625
rs139106
rs116910507
rs146797770
rs7939177
rs532803692
rs17233534
rs115361290
rs7768875
All
−0.1
0.0
0.1
0.2
MR leave−one−out sensitivity analysis for
' CD20 on IgD+ CD38br' on 'Rheumatoid arthritis'

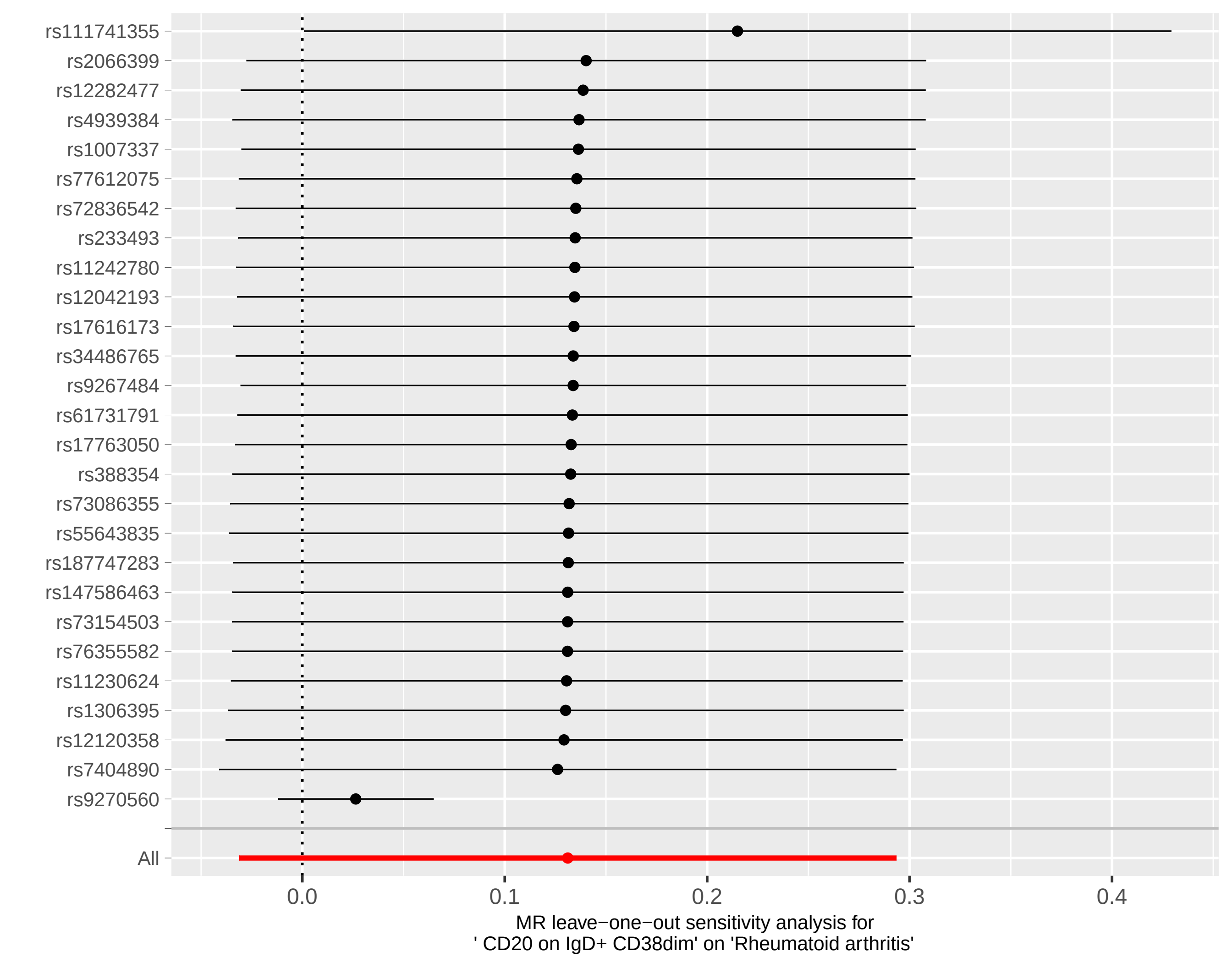
rs111741355
rs2066399
rs12282477
rs4939384
rs1007337
rs77612075
rs72836542
rs233493
rs11242780
rs12042193
rs17616173
rs34486765
rs9267484
rs61731791
rs17763050
rs388354
rs73086355
rs55643835
rs187747283
rs147586463
rs73154503
rs76355582
rs11230624
rs1306395
rs12120358
rs7404890
rs9270560
All
0.0
0.1
0.2
0.3
0.4
MR leave−one−out sensitivity analysis for
' CD20 on IgD+ CD38dim' on 'Rheumatoid arthritis'

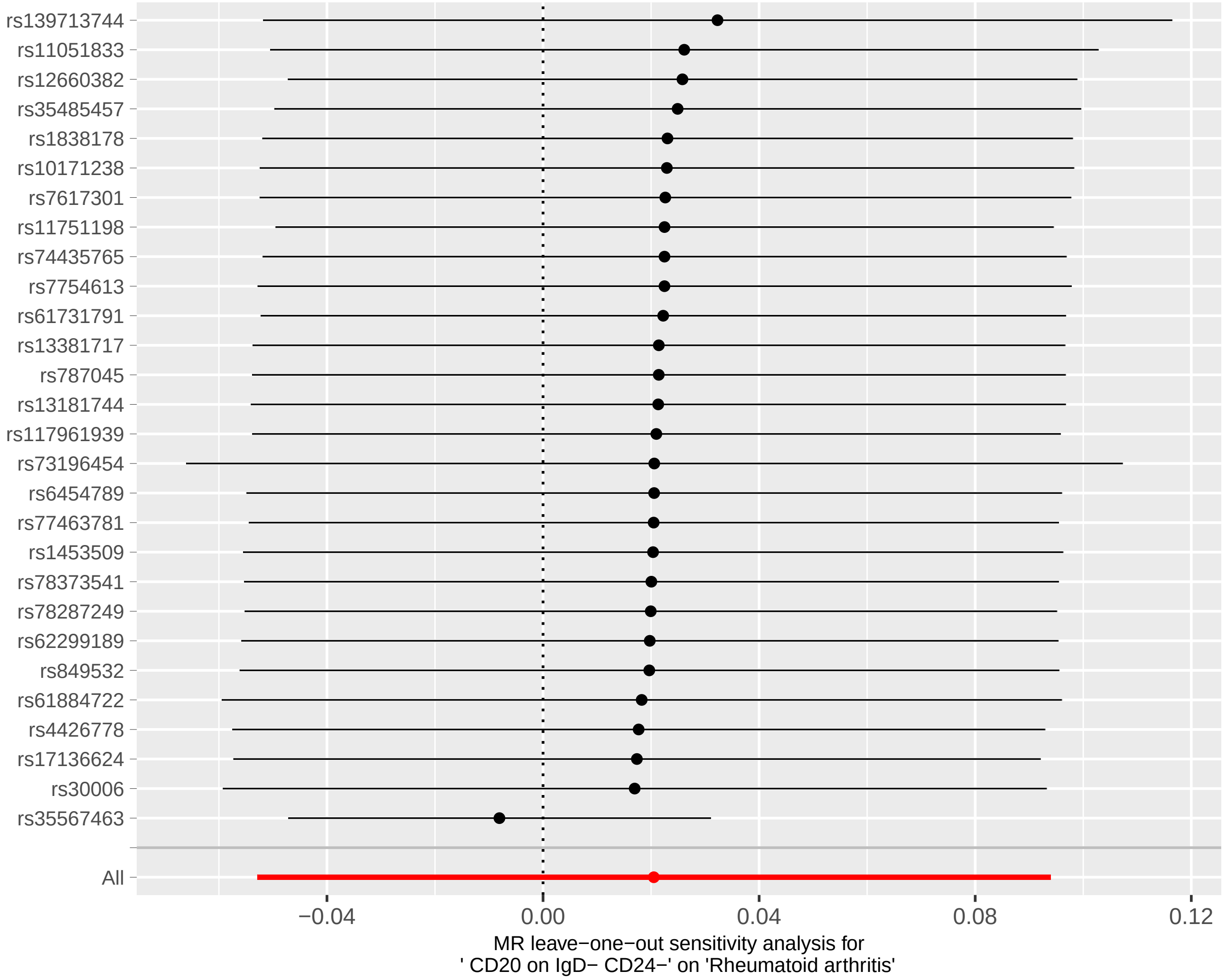
rs139713744
rs11051833
rs12660382
rs35485457
rs1838178
rs10171238
rs7617301
rs11751198
rs74435765
rs7754613
rs61731791
rs13381717
rs787045
rs13181744
rs117961939
rs73196454
rs6454789
rs77463781
rs1453509
rs78373541
rs78287249
rs62299189
rs849532
rs61884722
rs4426778
rs17136624
rs30006
rs35567463
All
−0.04
0.00
0.04
0.08
0.12
MR leave−one−out sensitivity analysis for
' CD20 on IgD− CD24−' on 'Rheumatoid arthritis'

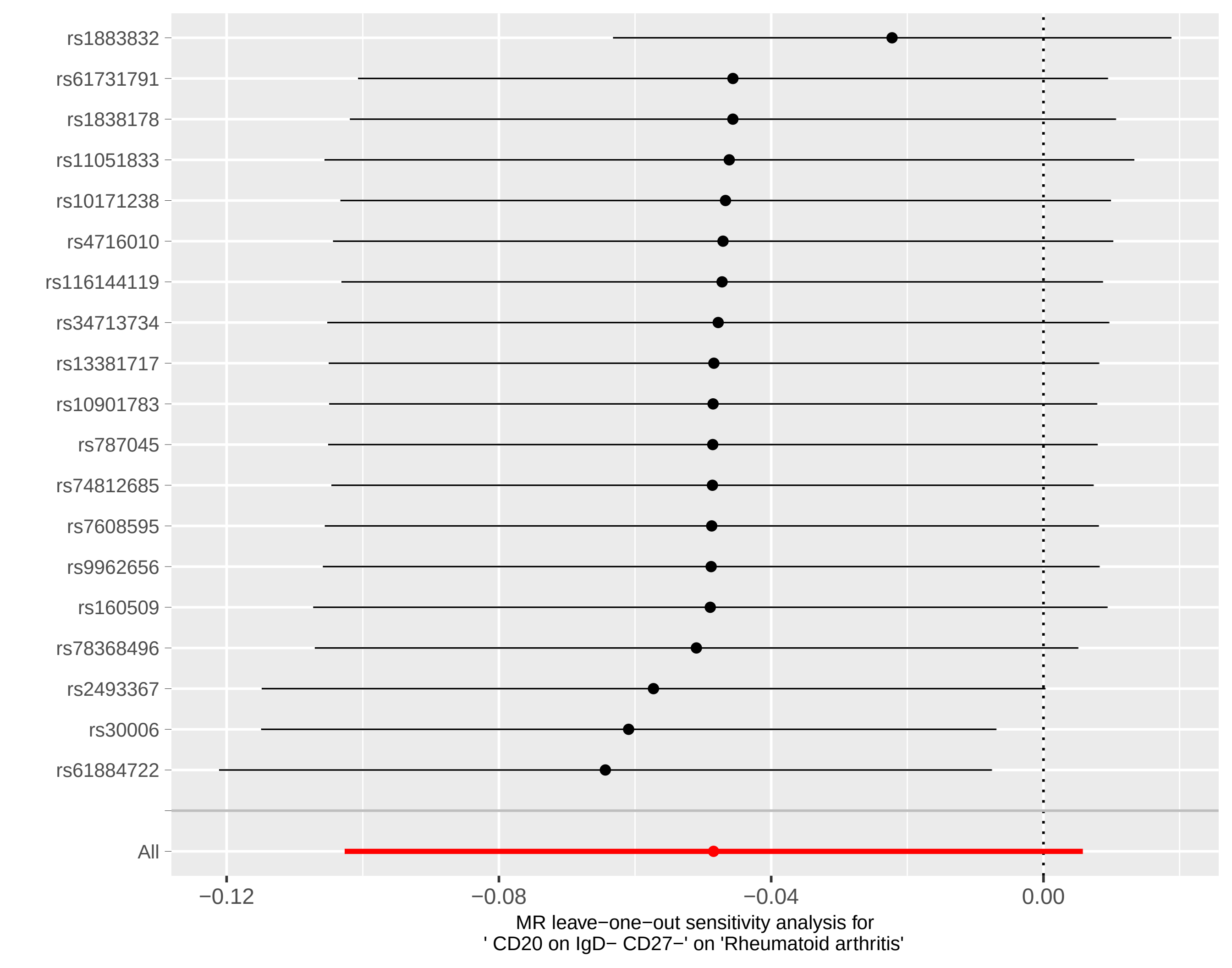

rs1883832
rs61731791
rs1838178
rs11051833
rs10171238
rs4716010
rs116144119
rs34713734
rs13381717
rs10901783
rs787045
rs74812685
rs7608595
rs9962656
rs160509
rs78368496
rs2493367
rs30006
rs61884722
All
−0.12
−0.08
−0.04
0.00
MR leave−one−out sensitivity analysis for
' CD20 on IgD− CD27−' on 'Rheumatoid arthritis'

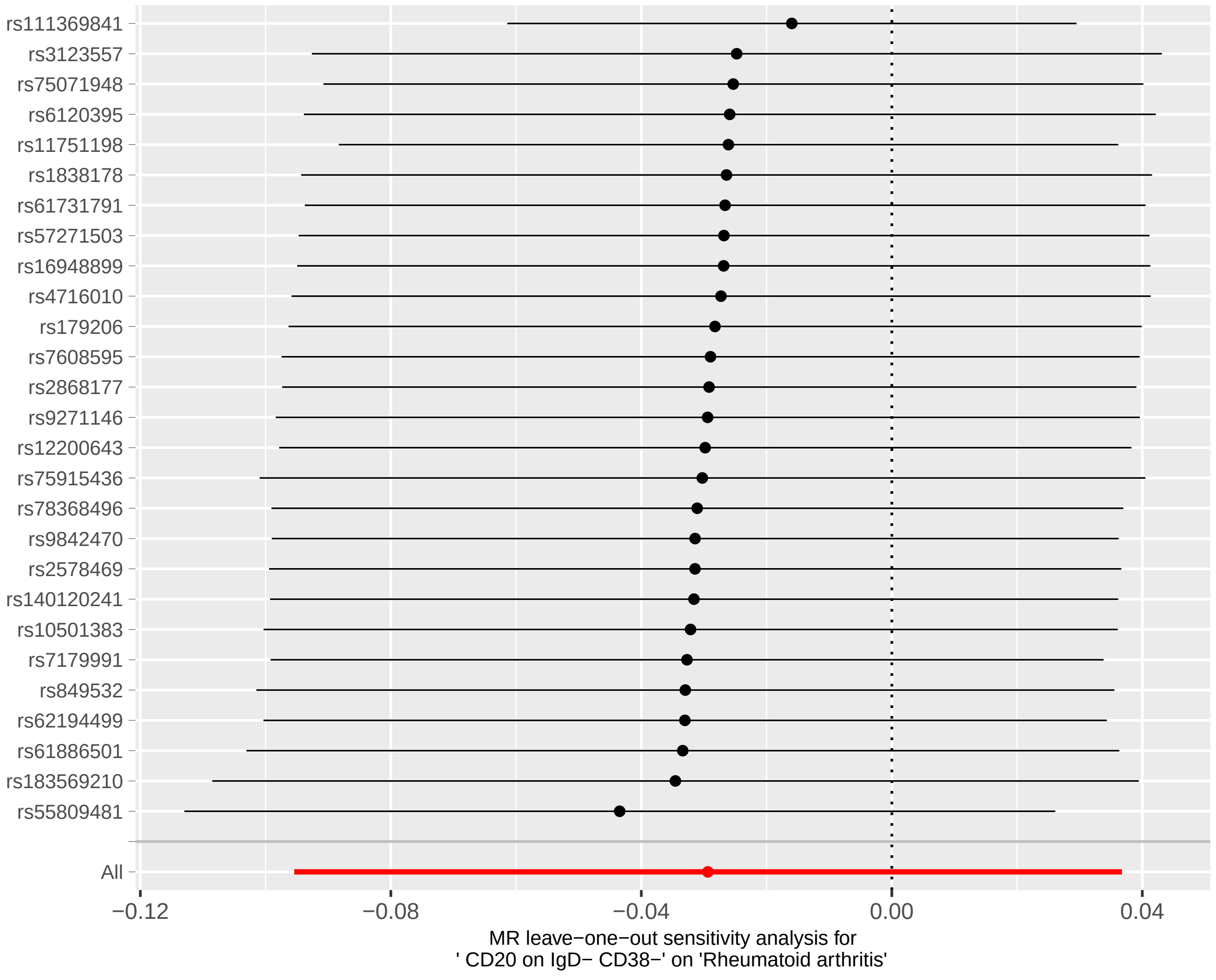

rs111369841
rs3123557
rs75071948
rs6120395
rs11751198
rs1838178
rs61731791
rs57271503
rs16948899
rs4716010
rs179206
rs7608595
rs2868177
rs9271146
rs12200643
rs75915436
rs78368496
rs9842470
rs2578469
rs140120241
rs10501383
rs7179991
rs849532
rs62194499
rs61886501
rs183569210
rs55809481
All
−0.12
−0.08
−0.04
0.00
0.04
MR leave−one−out sensitivity analysis for
' CD20 on IgD− CD38−' on 'Rheumatoid arthritis'

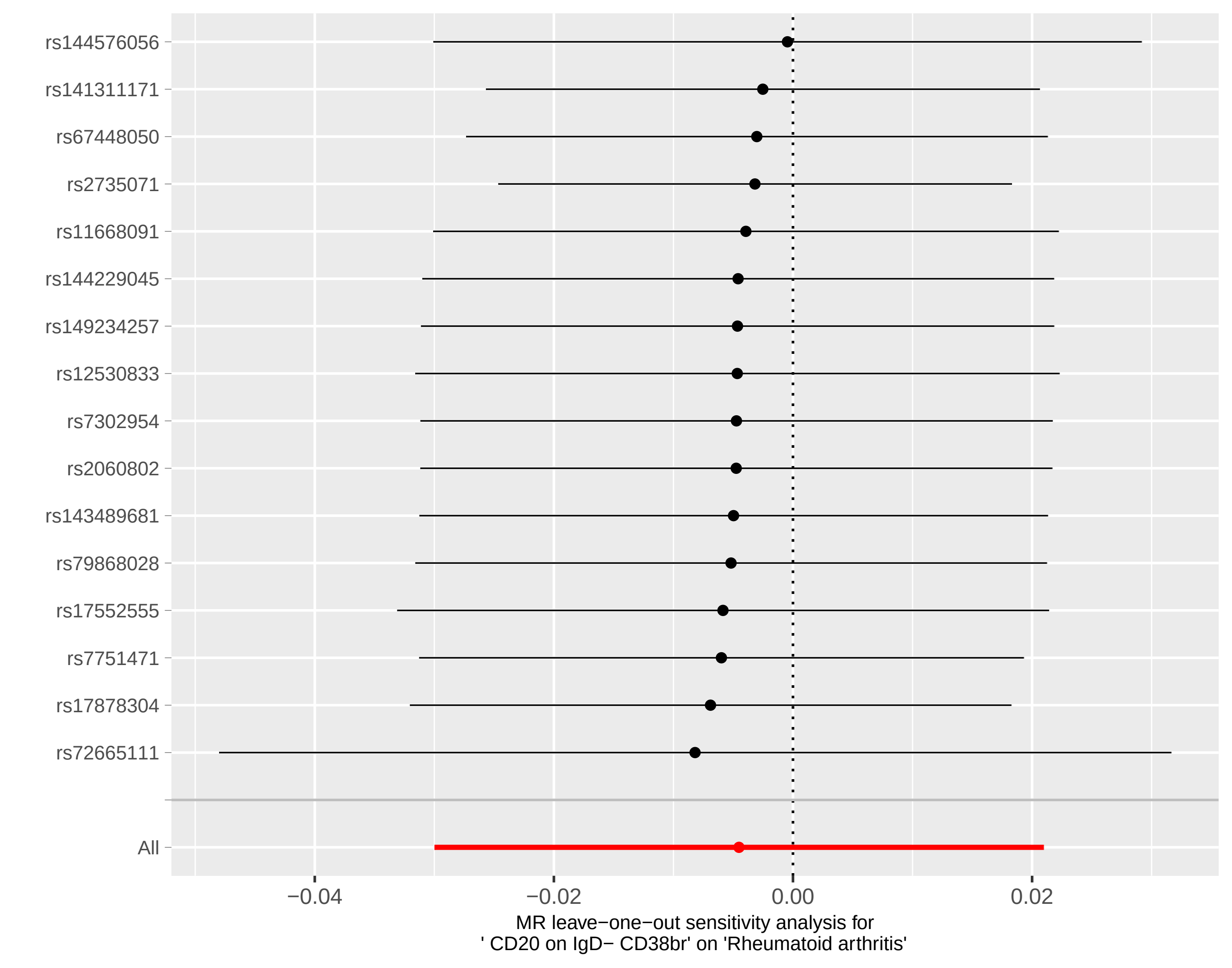

rs144576056
rs141311171
rs67448050
rs2735071
rs11668091
rs144229045
rs149234257
rs12530833
rs7302954
rs2060802
rs143489681
rs79868028
rs17552555
rs7751471
rs17878304
rs72665111
All
−0.04
−0.02
0.00
0.02
MR leave−one−out sensitivity analysis for
' CD20 on IgD− CD38br' on 'Rheumatoid arthritis'

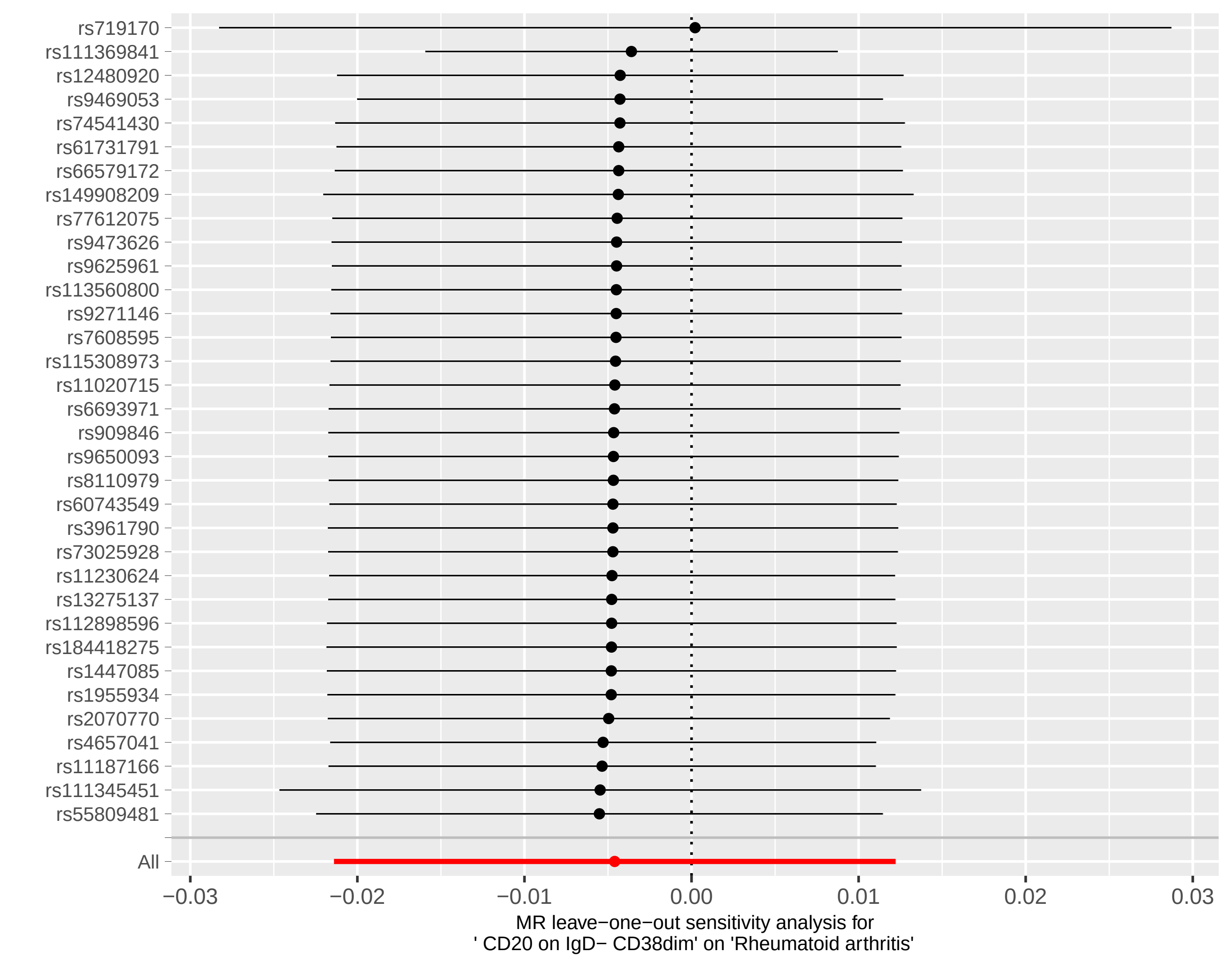
rs719170
rs111369841
rs12480920
rs9469053
rs74541430
rs61731791
rs66579172
rs149908209
rs77612075
rs9473626
rs9625961
rs113560800
rs9271146
rs7608595
rs115308973
rs11020715
rs6693971
rs909846
rs9650093
rs8110979
rs60743549
rs3961790
rs73025928
rs11230624
rs13275137
rs112898596
rs184418275
rs1447085
rs1955934
rs2070770
rs4657041
rs11187166
rs111345451
rs55809481
All
−0.03
−0.02
−0.01
0.00
0.01
0.02
0.03
MR leave−one−out sensitivity analysis for
' CD20 on IgD− CD38dim' on 'Rheumatoid arthritis'

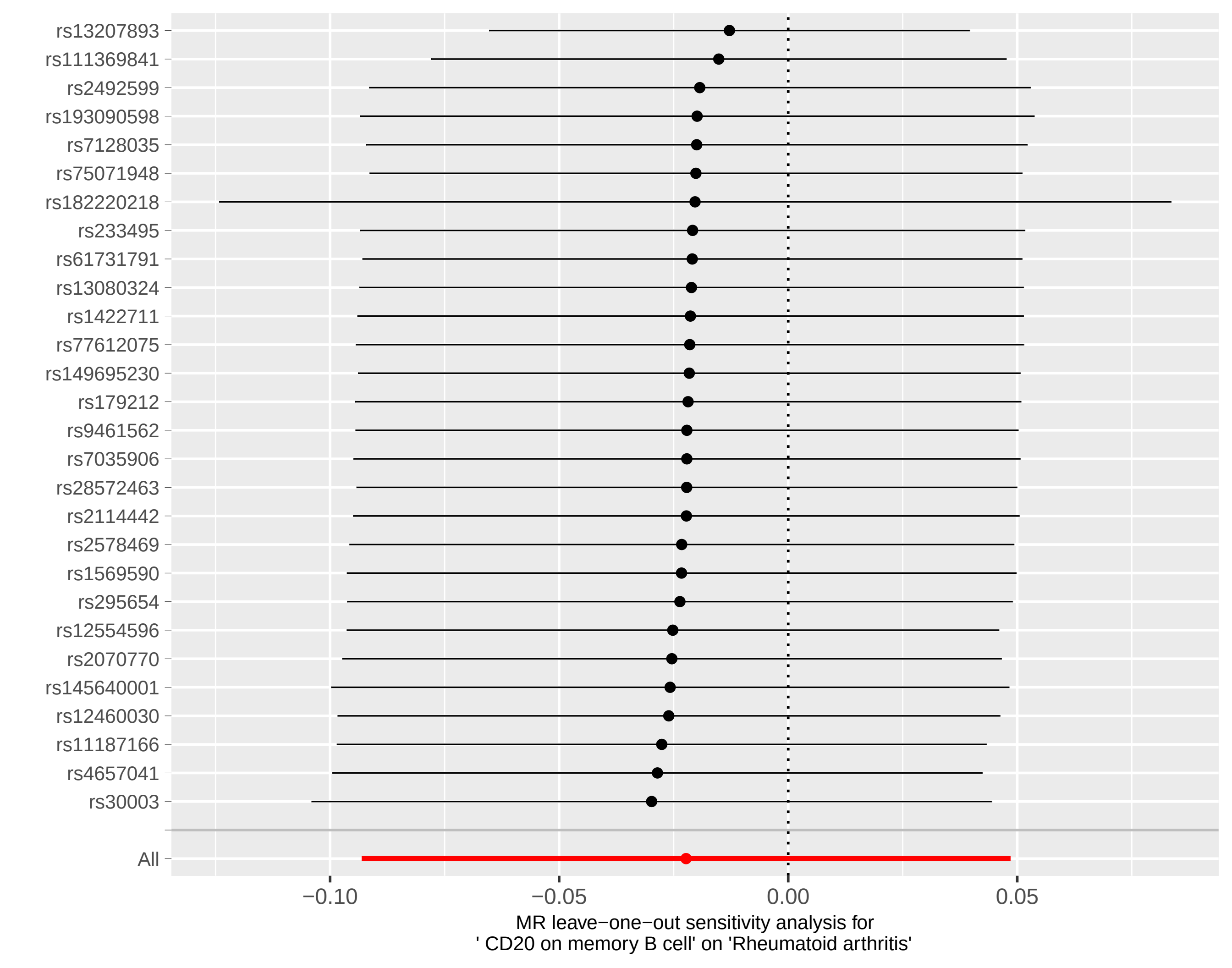
rs13207893
rs111369841
rs2492599
rs193090598
rs7128035
rs75071948
rs182220218
rs233495
rs61731791
rs13080324
rs1422711
rs77612075
rs149695230
rs179212
rs9461562
rs7035906
rs28572463
rs2114442
rs2578469
rs1569590
rs295654
rs12554596
rs2070770
rs145640001
rs12460030
rs11187166
rs4657041
rs30003
All
−0.10
−0.05
0.00
0.05
MR leave−one−out sensitivity analysis for
' CD20 on memory B cell' on 'Rheumatoid arthritis'

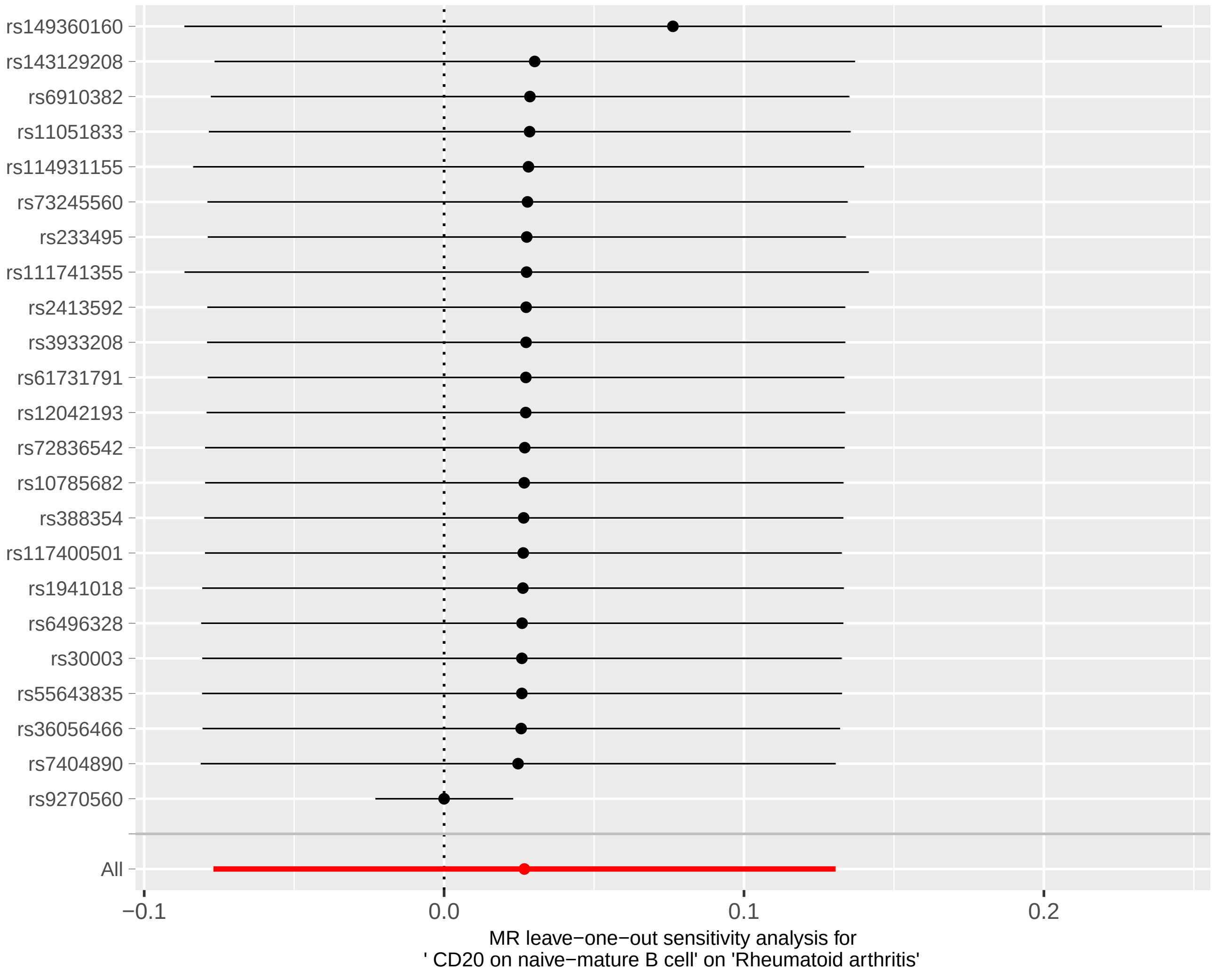

rs149360160
rs143129208
rs6910382
rs11051833
rs114931155
rs73245560
rs233495
rs111741355
rs2413592
rs3933208
rs61731791
rs12042193
rs72836542
rs10785682
rs388354
rs117400501
rs1941018
rs6496328
rs30003
rs55643835
rs36056466
rs7404890
rs9270560
All
−0.1
0.0
0.1
0.2
MR leave−one−out sensitivity analysis for
' CD20 on naive−mature B cell' on 'Rheumatoid arthritis'

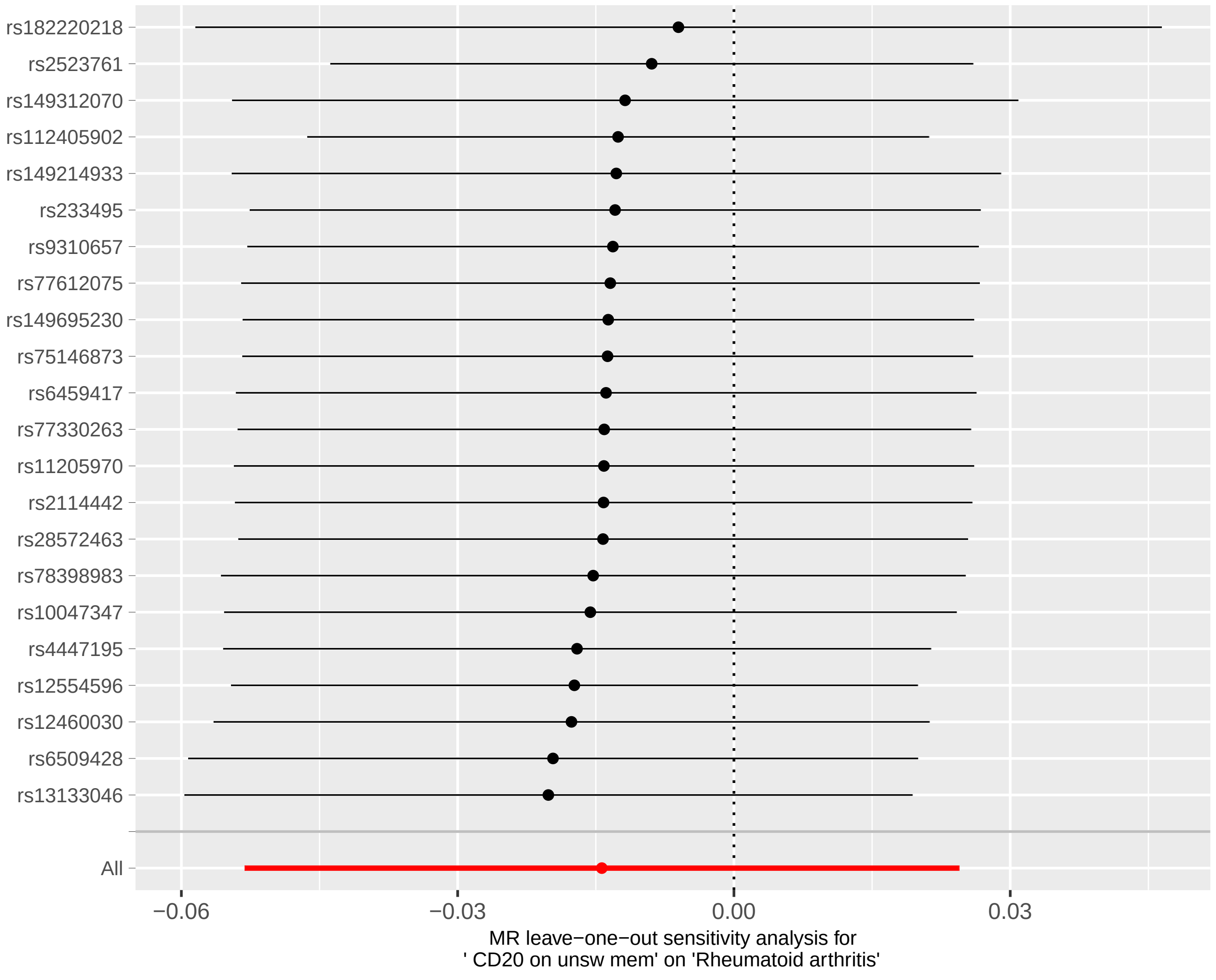

rs182220218
rs2523761
rs149312070
rs112405902
rs149214933
rs233495
rs9310657
rs77612075
rs149695230
rs75146873
rs6459417
rs77330263
rs11205970
rs2114442
rs28572463
rs78398983
rs10047347
rs4447195
rs12554596
rs12460030
rs6509428
rs13133046
All
−0.06
−0.03
0.00
0.03
MR leave−one−out sensitivity analysis for
' CD20 on unsw mem' on 'Rheumatoid arthritis'

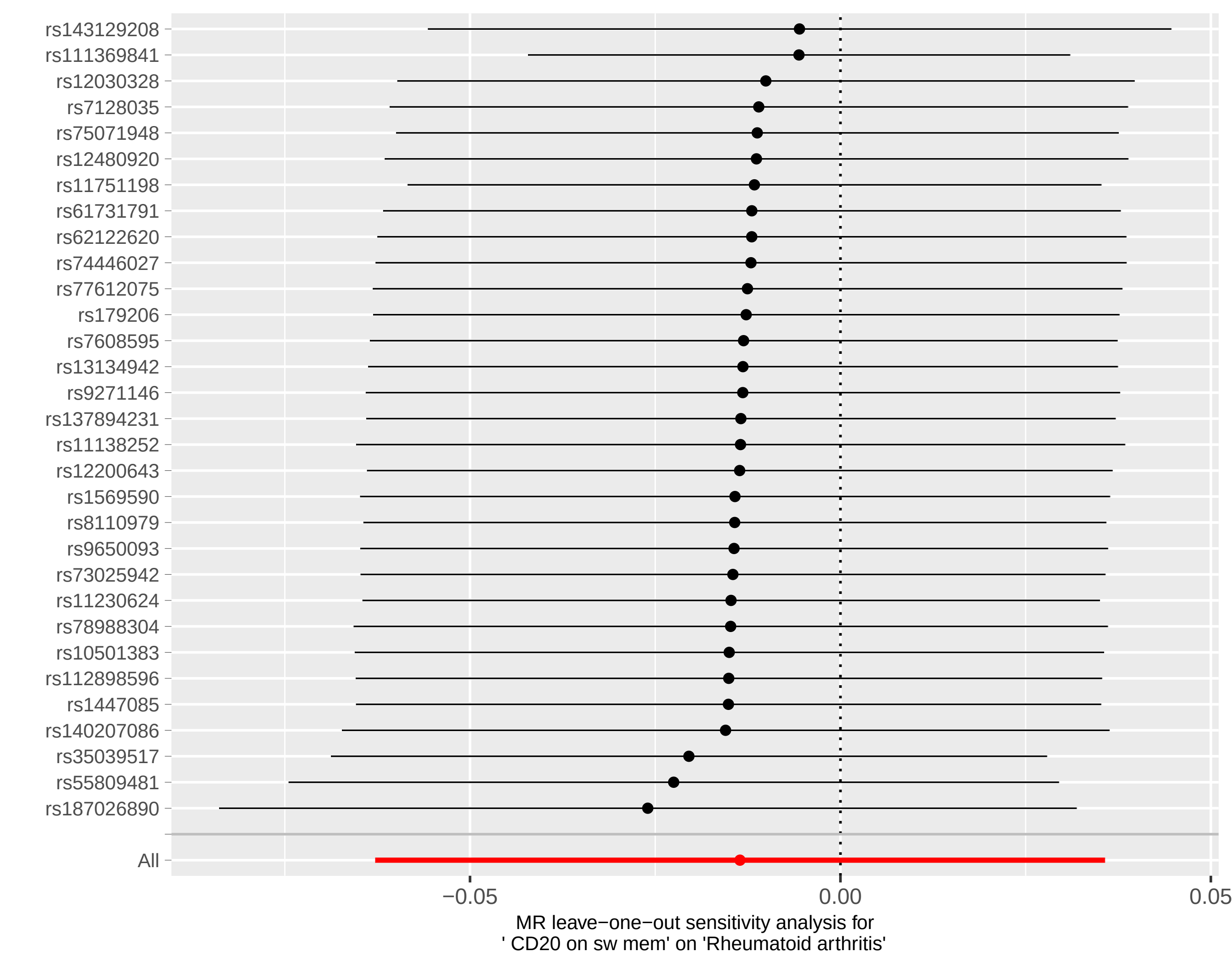
rs143129208
rs111369841
rs12030328
rs7128035
rs75071948
rs12480920
rs11751198
rs61731791
rs62122620
rs74446027
rs77612075
rs179206
rs7608595
rs13134942
rs9271146
rs137894231
rs11138252
rs12200643
rs1569590
rs8110979
rs9650093
rs73025942
rs11230624
rs78988304
rs10501383
rs112898596
rs1447085
rs140207086
rs35039517
rs55809481
rs187026890
All
−0.05
0.00
0.05
MR leave−one−out sensitivity analysis for
' CD20 on sw mem' on 'Rheumatoid arthritis'

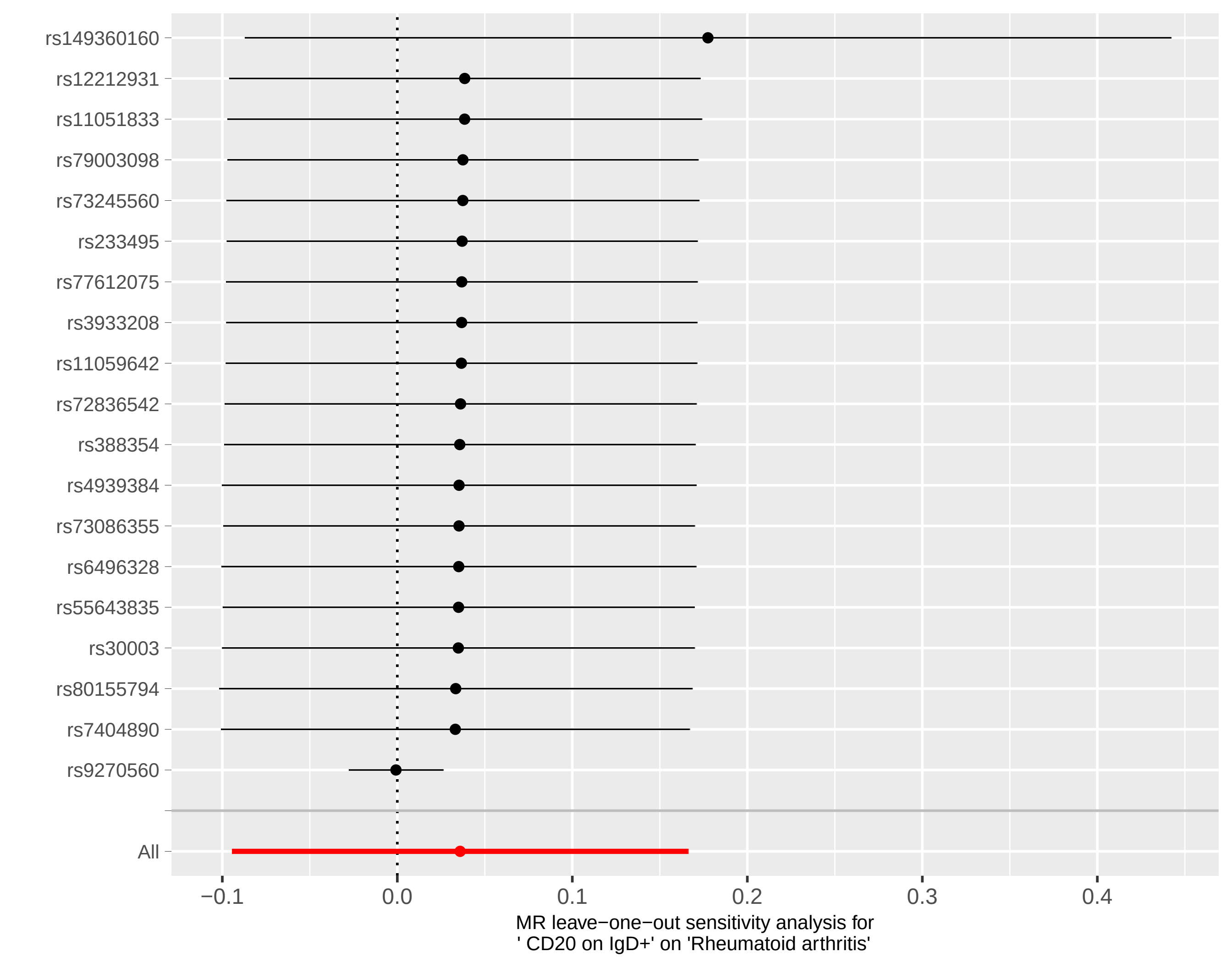

rs149360160
rs12212931
rs11051833
rs79003098
rs73245560
rs233495
rs77612075
rs3933208
rs11059642
rs72836542
rs388354
rs4939384
rs73086355
rs6496328
rs55643835
rs30003
rs80155794
rs7404890
rs9270560
All
−0.1
0.0
0.1
0.2
0.3
0.4
MR leave−one−out sensitivity analysis for
' CD20 on IgD+' on 'Rheumatoid arthritis'

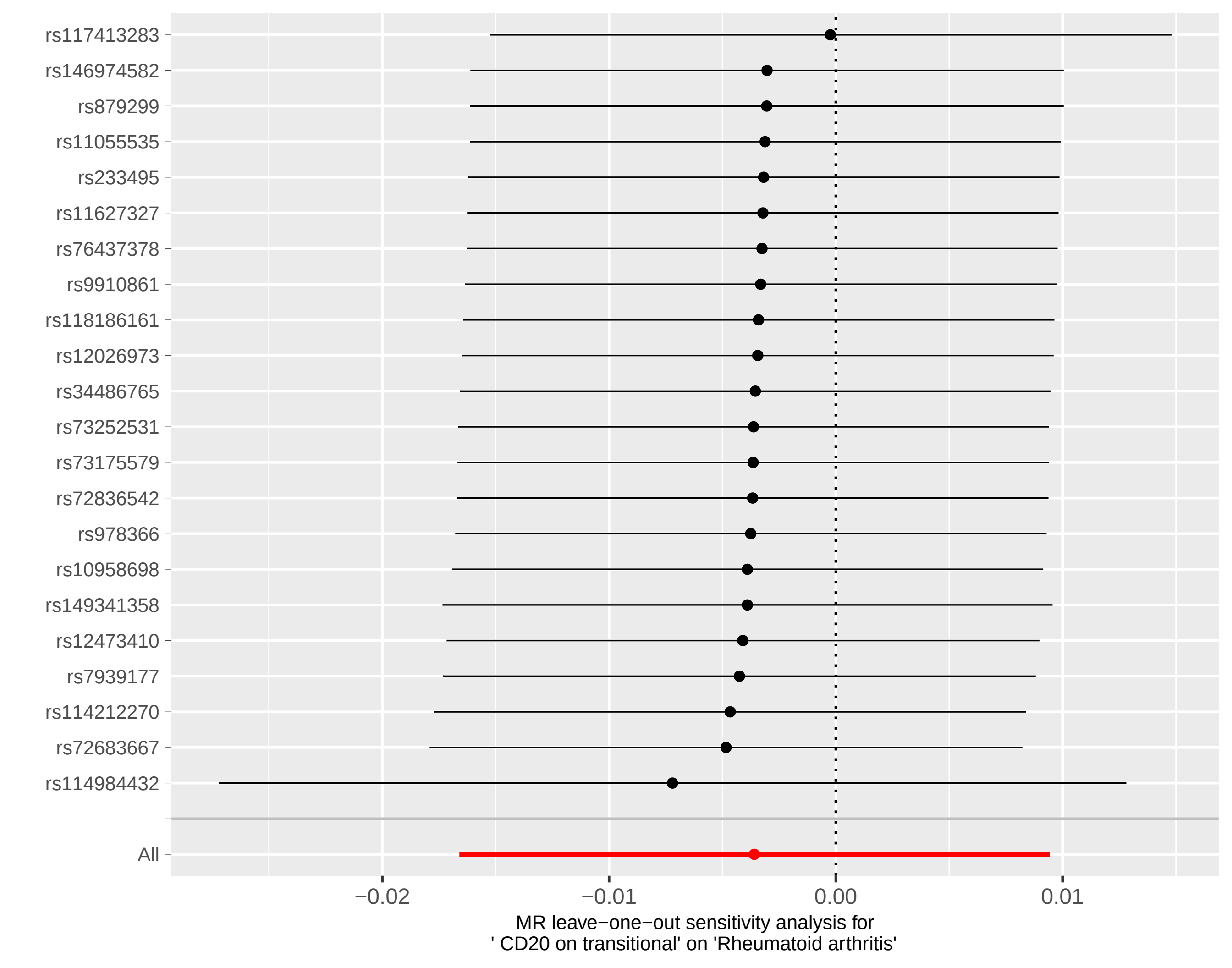

rs117413283
rs146974582
rs879299
rs11055535
rs233495
rs11627327
rs76437378
rs9910861
rs118186161
rs12026973
rs34486765
rs73252531
rs73175579
rs72836542
rs978366
rs10958698
rs149341358
rs12473410
rs7939177
rs114212270
rs72683667
rs114984432
All
−0.02
−0.01
0.00
0.01
MR leave−one−out sensitivity analysis for
' CD20 on transitional' on 'Rheumatoid arthritis'

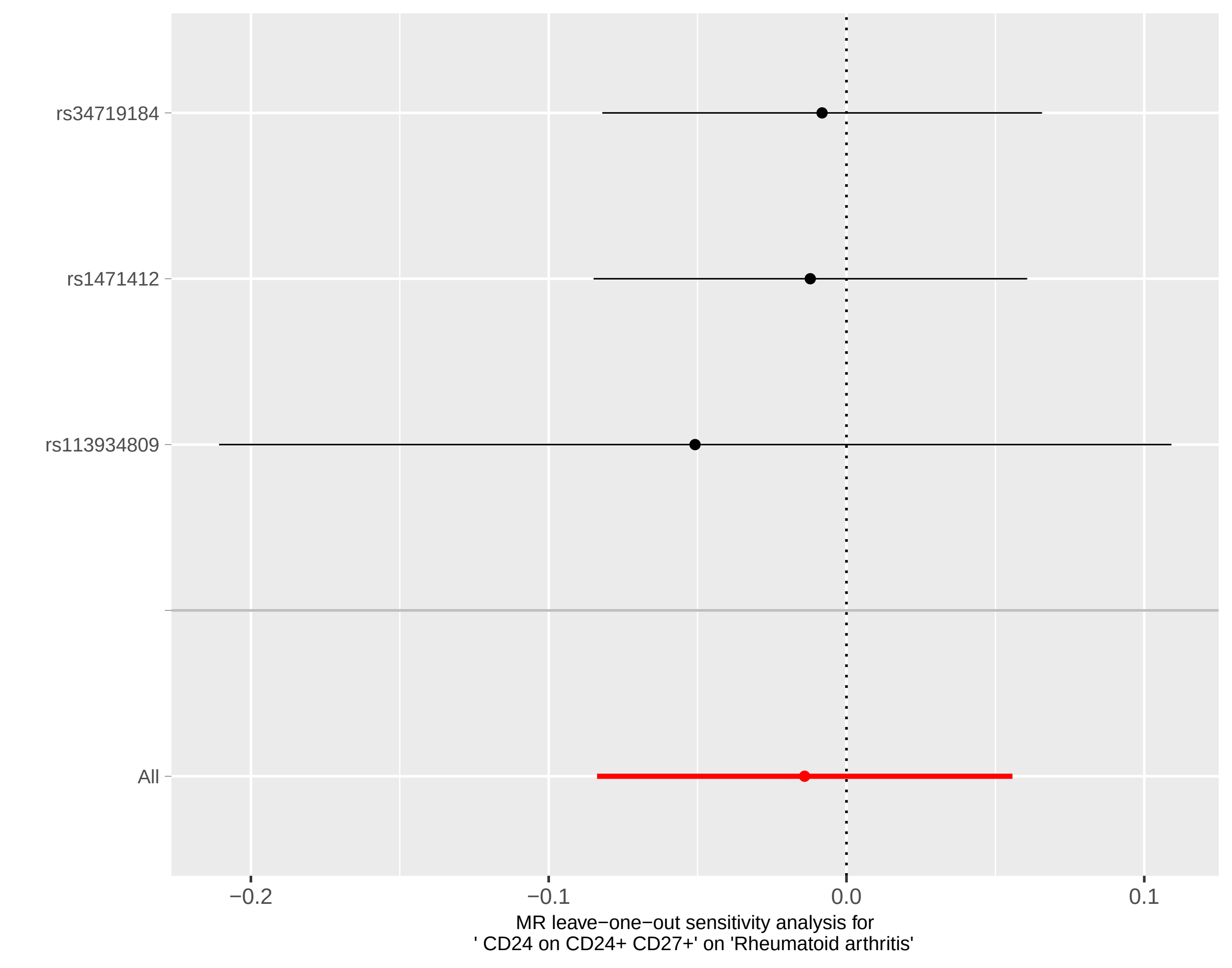
rs34719184
rs1471412
rs113934809
All
−0.2
−0.1
0.0
0.1
MR leave−one−out sensitivity analysis for
' CD24 on CD24+ CD27+' on 'Rheumatoid arthritis'

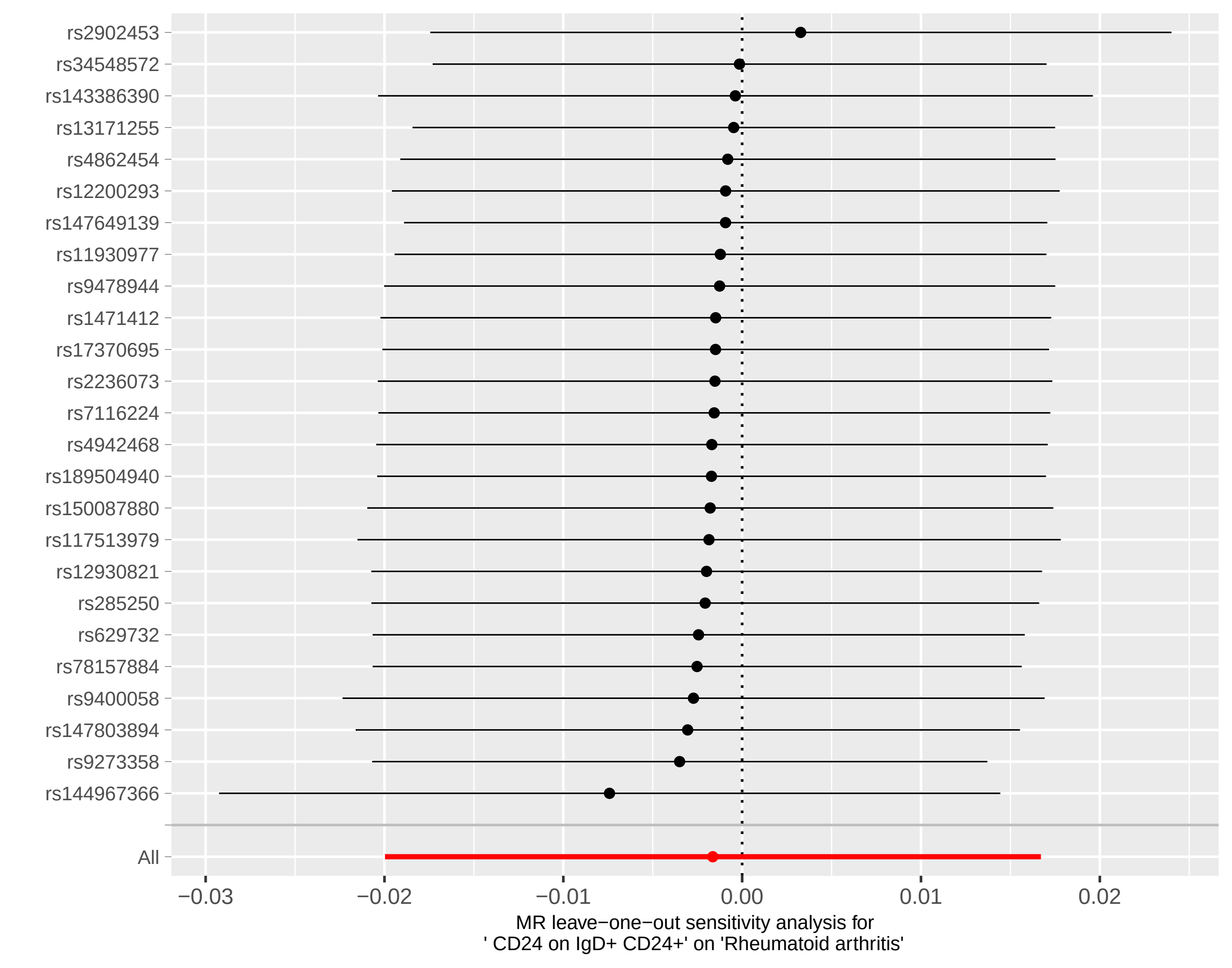
rs2902453
rs34548572
rs143386390
rs13171255
rs4862454
rs12200293
rs147649139
rs11930977
rs9478944
rs1471412
rs17370695
rs2236073
rs7116224
rs4942468
rs189504940
rs150087880
rs117513979
rs12930821
rs285250
rs629732
rs78157884
rs9400058
rs147803894
rs9273358
rs144967366
All
−0.03
−0.02
−0.01
0.00
0.01
0.02
MR leave−one−out sensitivity analysis for
' CD24 on IgD+ CD24+' on 'Rheumatoid arthritis'

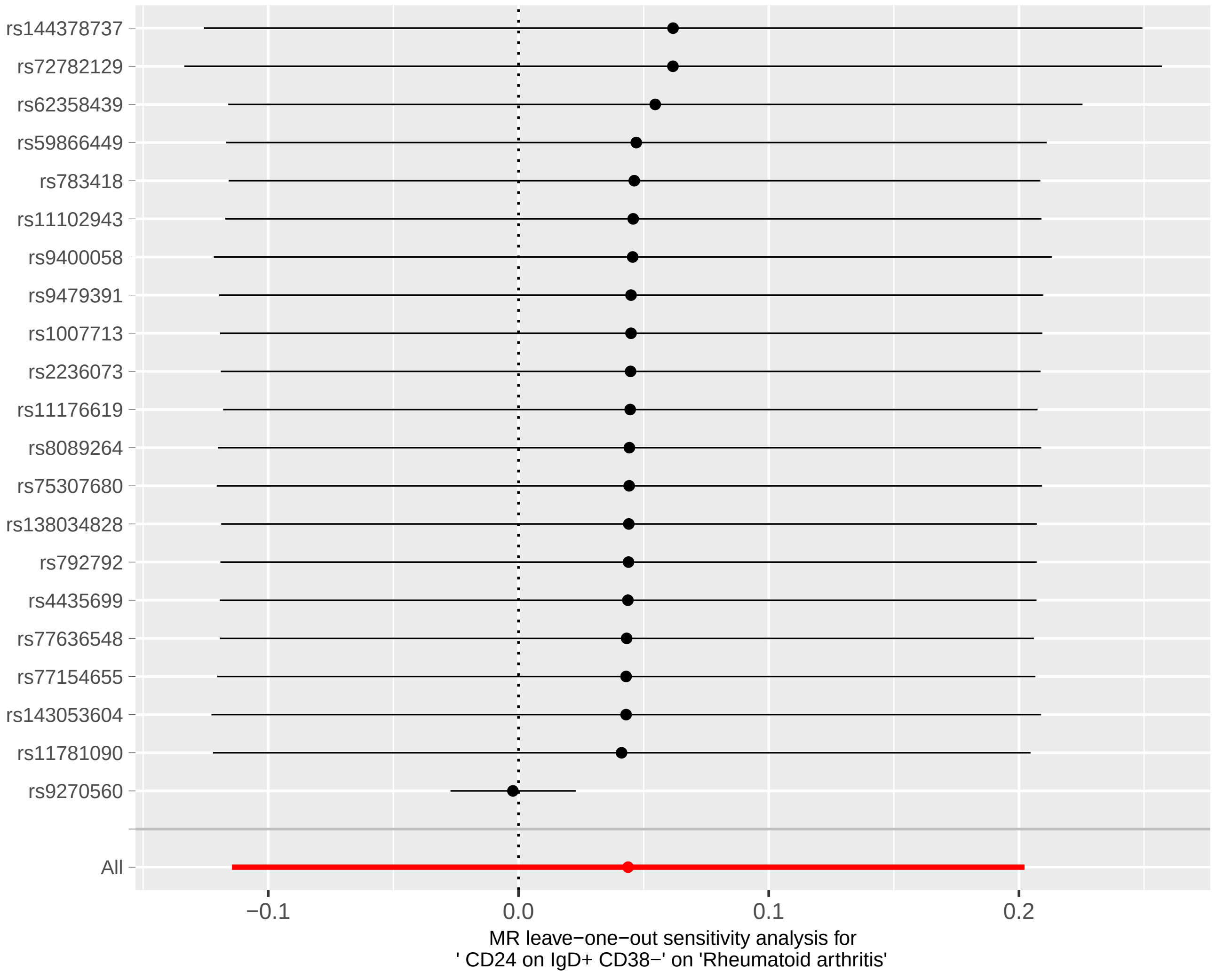
rs144378737
rs72782129
rs62358439
rs59866449
rs783418
rs11102943
rs9400058
rs9479391
rs1007713
rs2236073
rs11176619
rs8089264
rs75307680
rs138034828
rs792792
rs4435699
rs77636548
rs77154655
rs143053604
rs11781090
rs9270560
All
−0.1
0.0
0.1
0.2
MR leave−one−out sensitivity analysis for
' CD24 on IgD+ CD38−' on 'Rheumatoid arthritis'

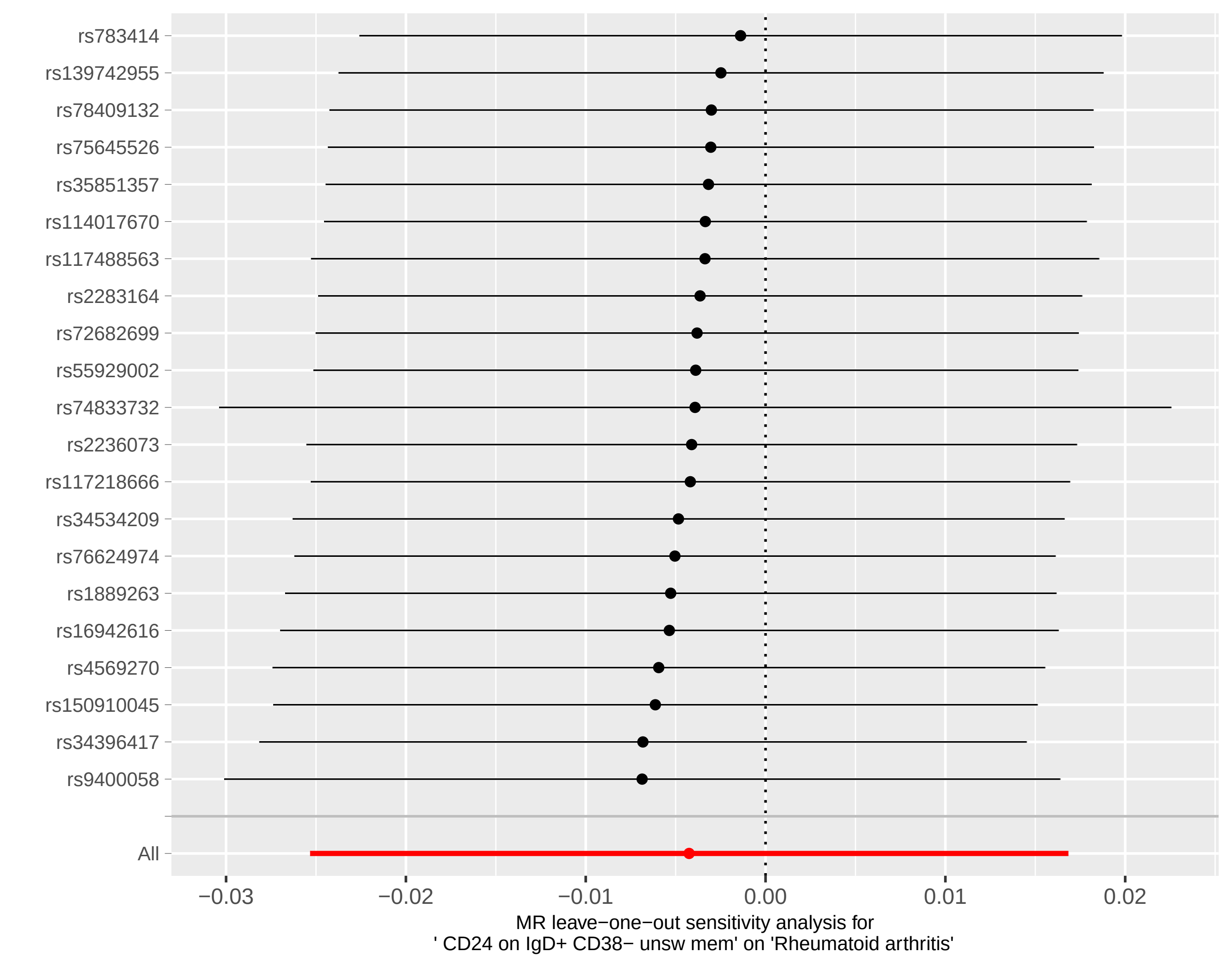
rs783414
rs139742955
rs78409132
rs75645526
rs35851357
rs114017670
rs117488563
rs2283164
rs72682699
rs55929002
rs74833732
rs2236073
rs117218666
rs34534209
rs76624974
rs1889263
rs16942616
rs4569270
rs150910045
rs34396417
rs9400058
All
−0.03
−0.02
−0.01
0.00
0.01
0.02
MR leave−one−out sensitivity analysis for
' CD24 on IgD+ CD38− unsw mem' on 'Rheumatoid arthritis'

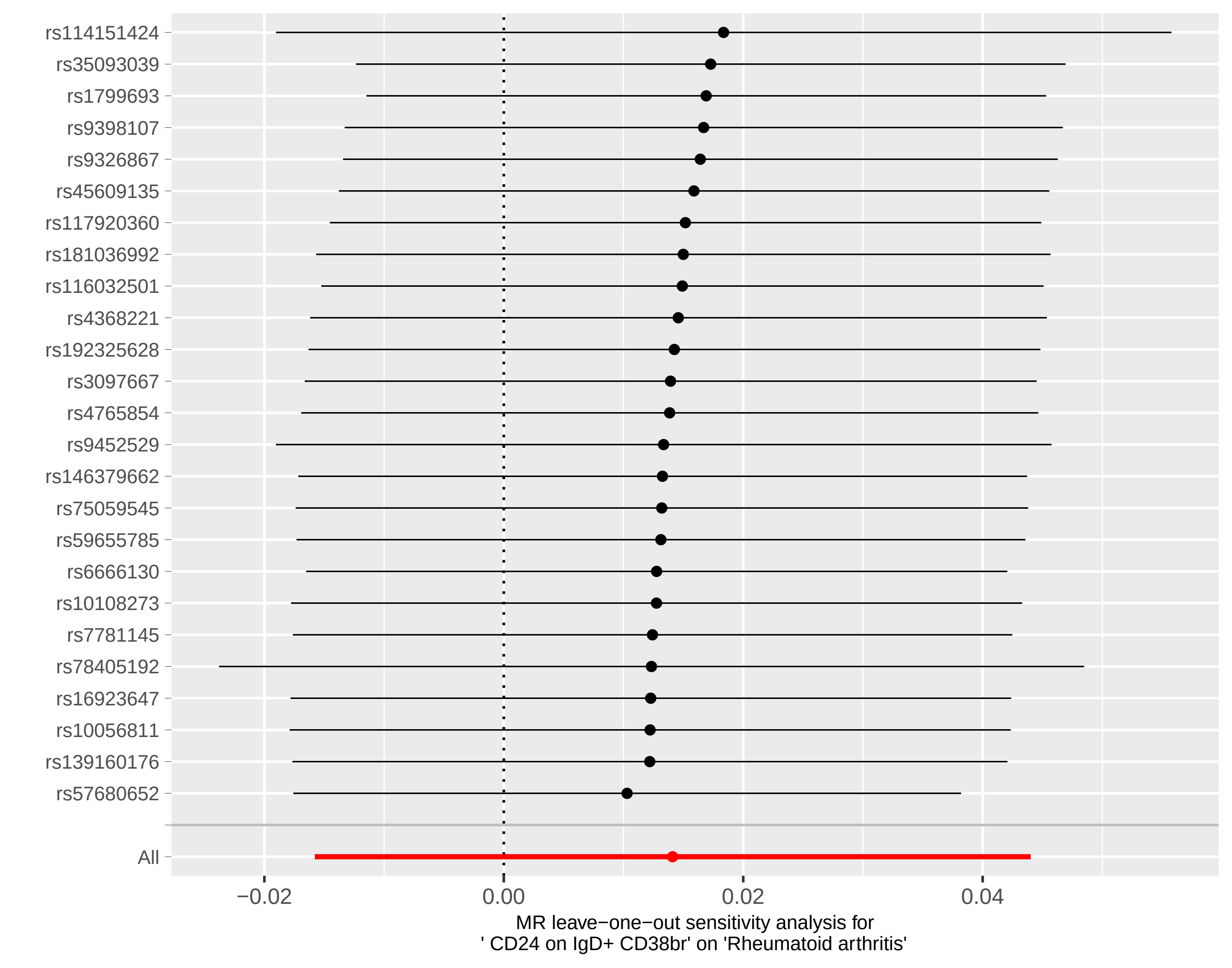
rs114151424
rs35093039
rs1799693
rs9398107
rs9326867
rs45609135
rs117920360
rs181036992
rs116032501
rs4368221
rs192325628
rs3097667
rs4765854
rs9452529
rs146379662
rs75059545
rs59655785
rs6666130
rs10108273
rs7781145
rs78405192
rs16923647
rs10056811
rs139160176
rs57680652
All
−0.02
0.00
0.02
0.04
MR leave−one−out sensitivity analysis for
' CD24 on IgD+ CD38br' on 'Rheumatoid arthritis'

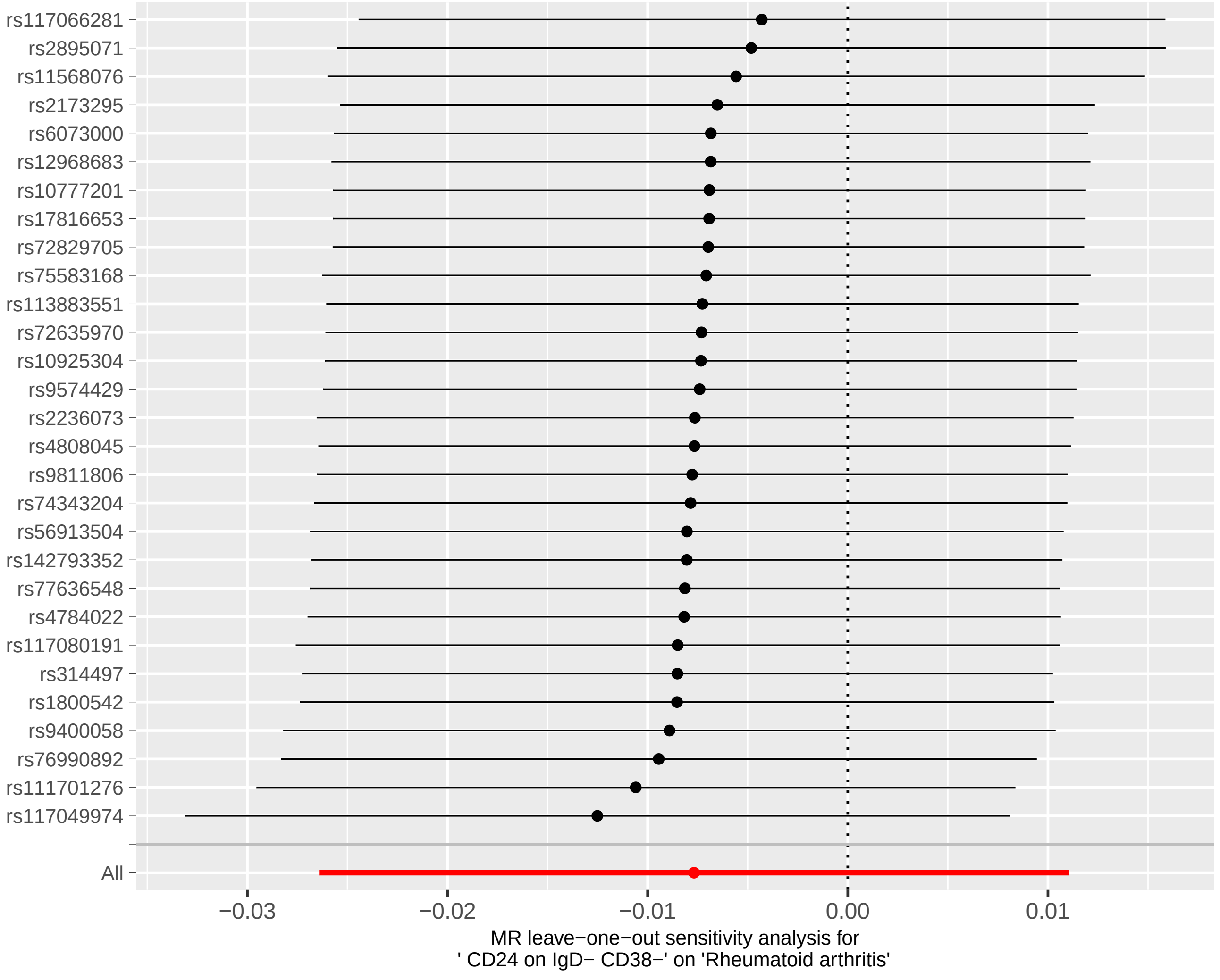

rs117066281
rs2895071
rs11568076
rs2173295
rs6073000
rs12968683
rs10777201
rs17816653
rs72829705
rs75583168
rs113883551
rs72635970
rs10925304
rs9574429
rs2236073
rs4808045
rs9811806
rs74343204
rs56913504
rs142793352
rs77636548
rs4784022
rs117080191
rs314497
rs1800542
rs9400058
rs76990892
rs111701276
rs117049974
All
−0.03
−0.02
−0.01
0.00
0.01
MR leave−one−out sensitivity analysis for
' CD24 on IgD− CD38−' on 'Rheumatoid arthritis'

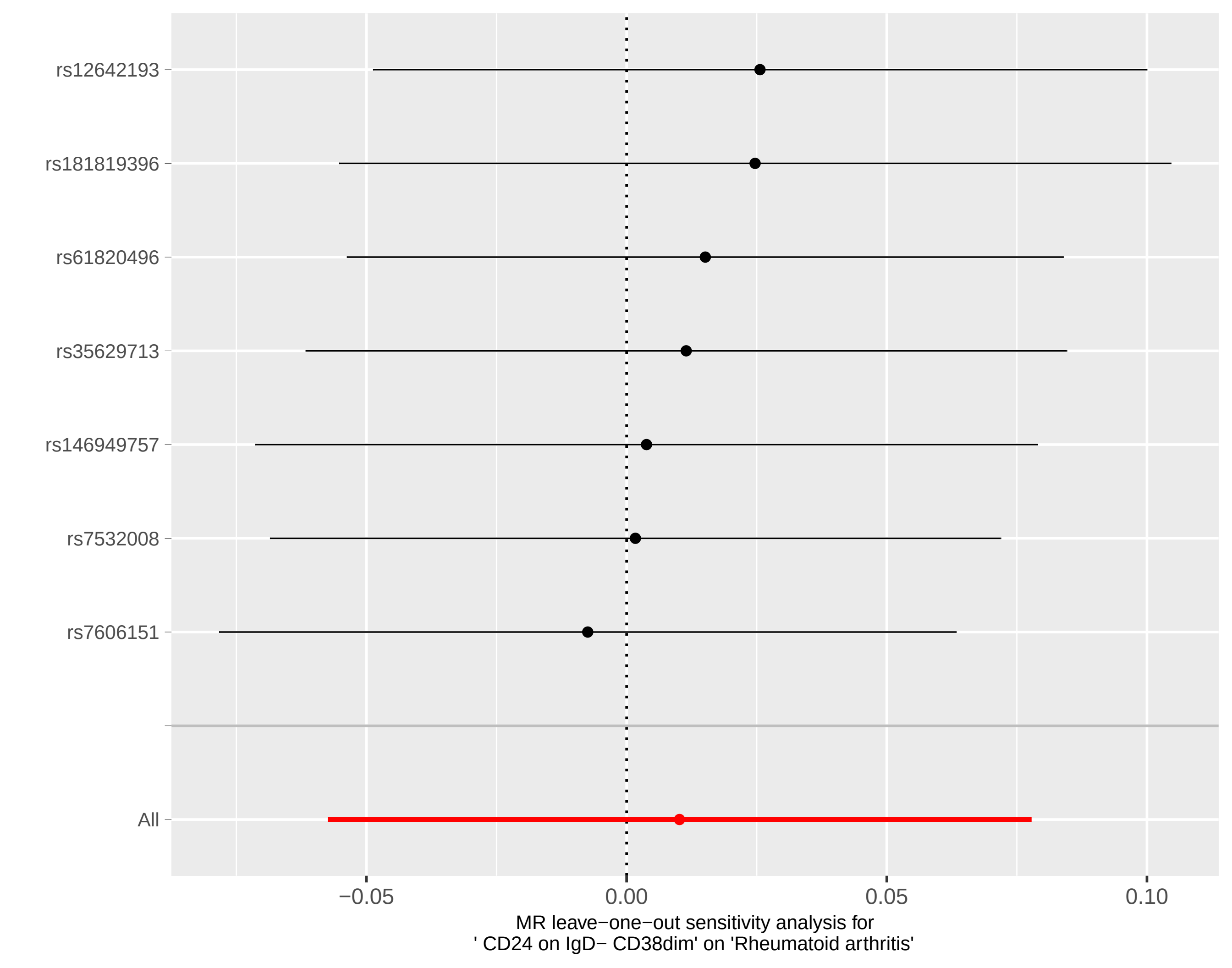
rs12642193
rs181819396
rs61820496
rs35629713
rs146949757
rs7532008
rs7606151
All
−0.05
0.00
0.05
0.10
MR leave−one−out sensitivity analysis for
' CD24 on IgD− CD38dim' on 'Rheumatoid arthritis'

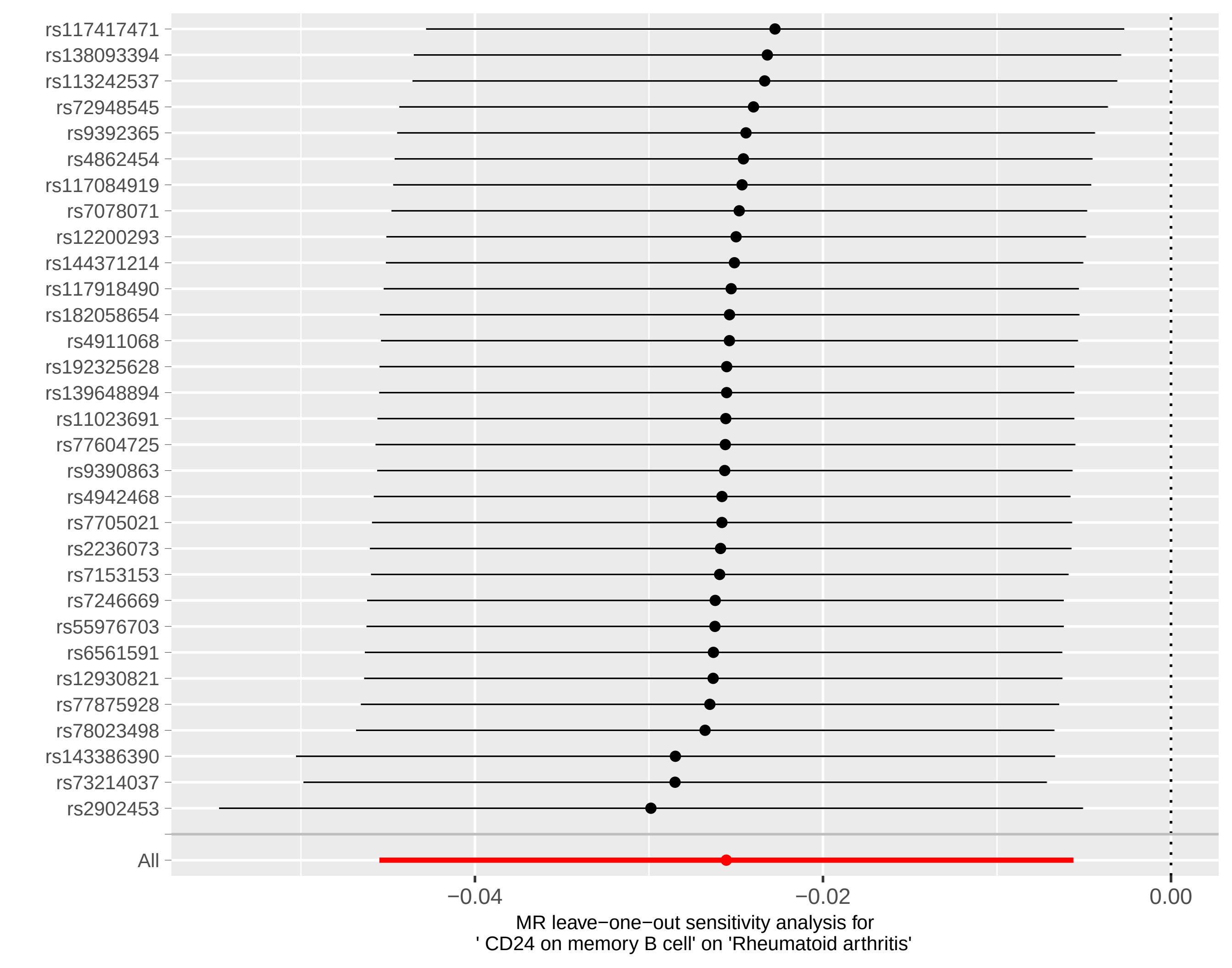
rs117417471
rs138093394
rs113242537
rs72948545
rs9392365
rs4862454
rs117084919
rs7078071
rs12200293
rs144371214
rs117918490
rs182058654
rs4911068
rs192325628
rs139648894
rs11023691
rs77604725
rs9390863
rs4942468
rs7705021
rs2236073
rs7153153
rs7246669
rs55976703
rs6561591
rs12930821
rs77875928
rs78023498
rs143386390
rs73214037
rs2902453
All
−0.04
−0.02
0.00
MR leave−one−out sensitivity analysis for
' CD24 on memory B cell' on 'Rheumatoid arthritis'

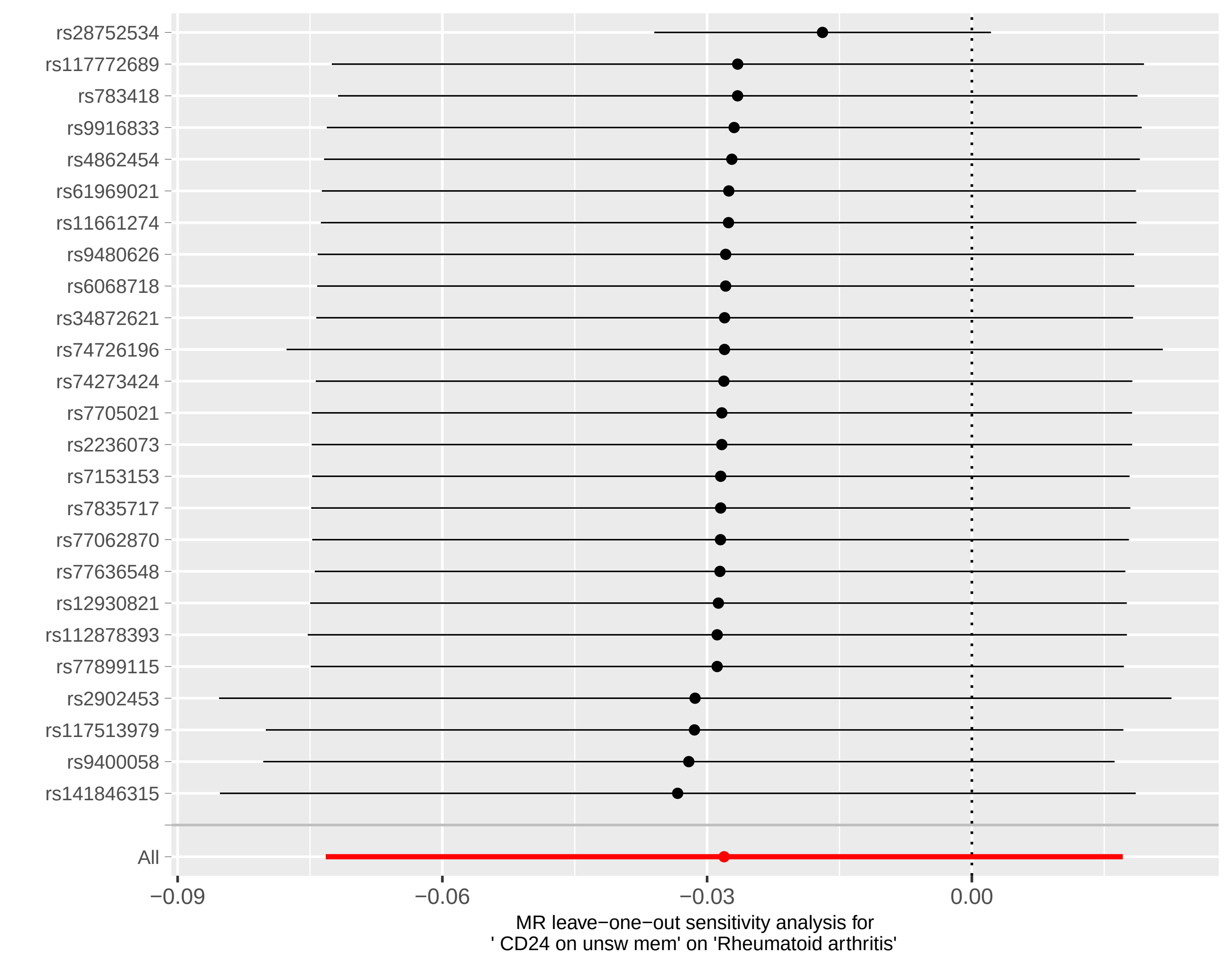

rs28752534
rs117772689
rs783418
rs9916833
rs4862454
rs61969021
rs11661274
rs9480626
rs6068718
rs34872621
rs74726196
rs74273424
rs7705021
rs2236073
rs7153153
rs7835717
rs77062870
rs77636548
rs12930821
rs112878393
rs77899115
rs2902453
rs117513979
rs9400058
rs141846315
All
−0.09
−0.06
−0.03
0.00
MR leave−one−out sensitivity analysis for
' CD24 on unsw mem' on 'Rheumatoid arthritis'

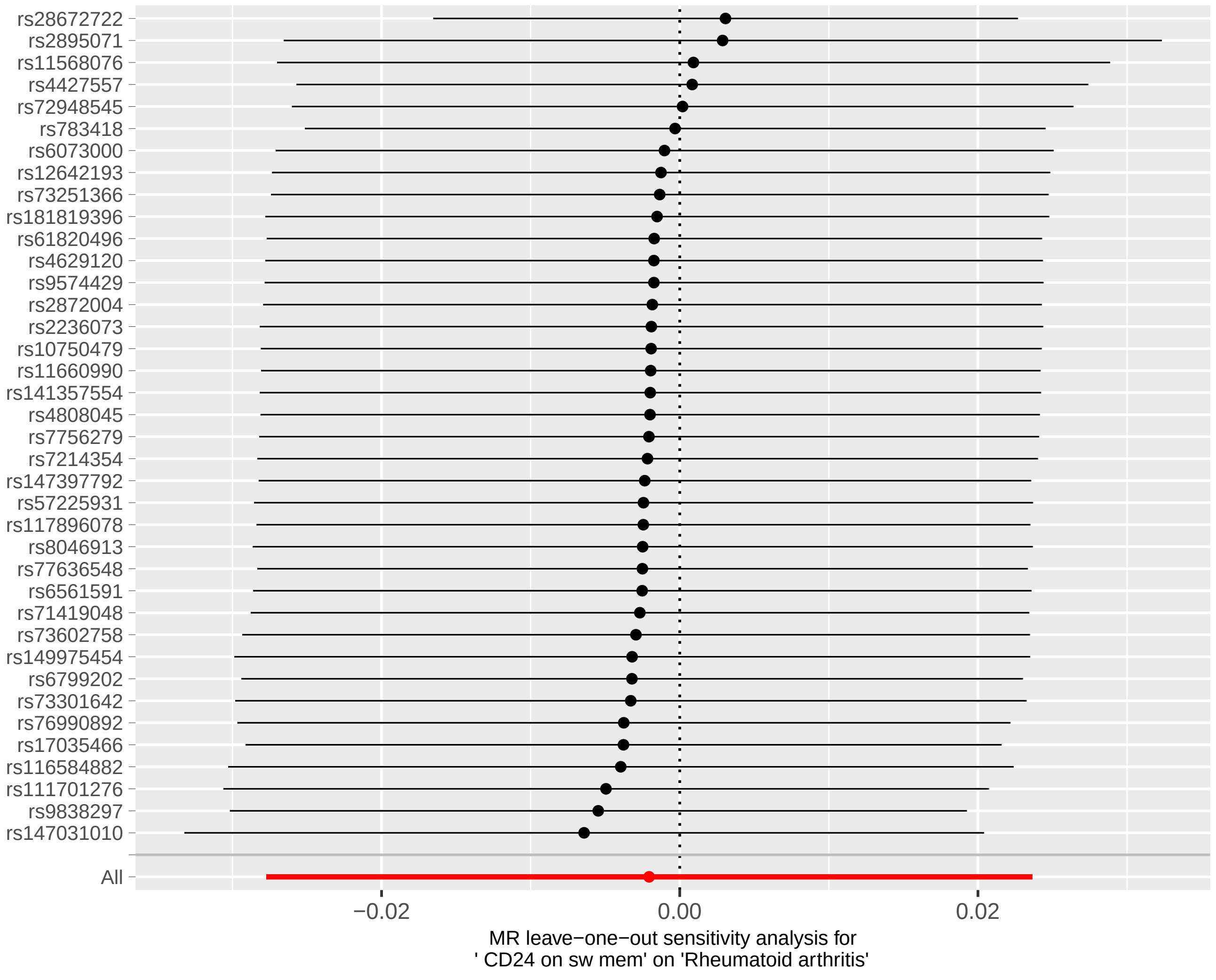

rs28672722
rs2895071
rs11568076
rs4427557
rs72948545
rs783418
rs6073000
rs12642193
rs73251366
rs181819396
rs61820496
rs4629120
rs9574429
rs2872004
rs2236073
rs10750479
rs11660990
rs141357554
rs4808045
rs7756279
rs7214354
rs147397792
rs57225931
rs117896078
rs8046913
rs77636548
rs6561591
rs71419048
rs73602758
rs149975454
rs6799202
rs73301642
rs76990892
rs17035466
rs116584882
rs111701276
rs9838297
rs147031010
All
−0.02
0.00
0.02
MR leave−one−out sensitivity analysis for
' CD24 on sw mem' on 'Rheumatoid arthritis'

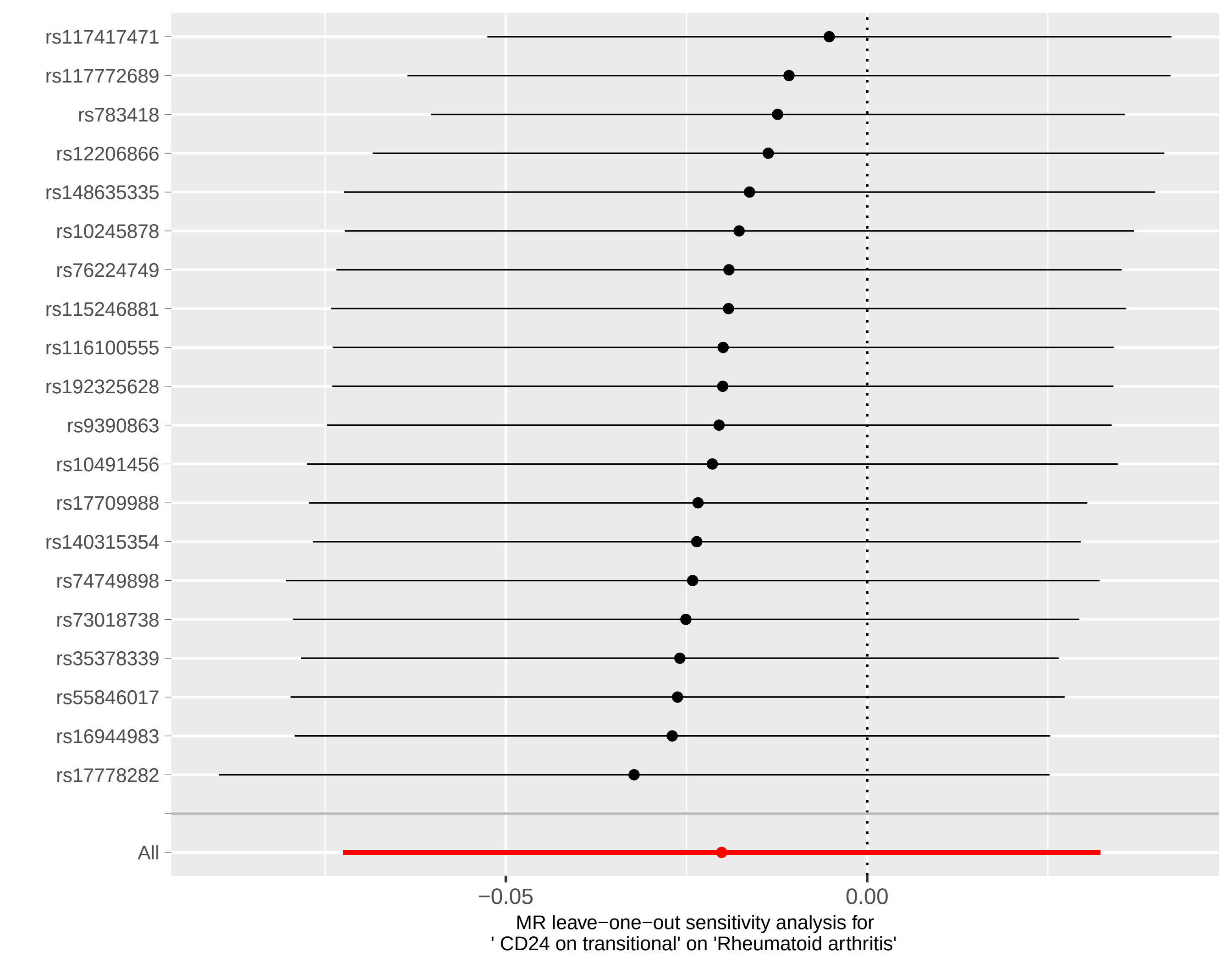
rs117417471
rs117772689
rs783418
rs12206866
rs148635335
rs10245878
rs76224749
rs115246881
rs116100555
rs192325628
rs9390863
rs10491456
rs17709988
rs140315354
rs74749898
rs73018738
rs35378339
rs55846017
rs16944983
rs17778282
All
−0.05
0.00
MR leave−one−out sensitivity analysis for
' CD24 on transitional' on 'Rheumatoid arthritis'

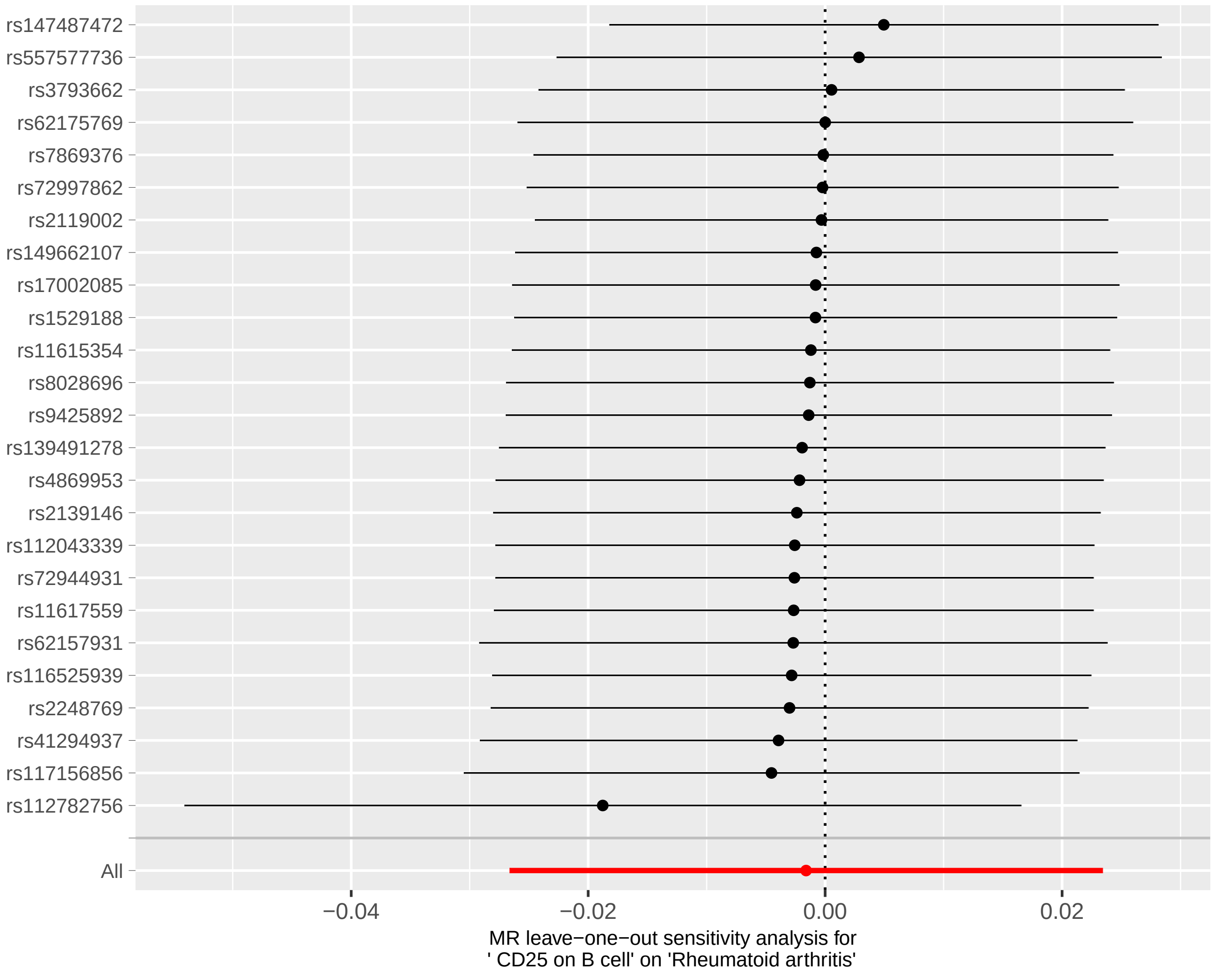
rs147487472
rs557577736
rs3793662
rs62175769
rs7869376
rs72997862
rs2119002
rs149662107
rs17002085
rs1529188
rs11615354
rs8028696
rs9425892
rs139491278
rs4869953
rs2139146
rs112043339
rs72944931
rs11617559
rs62157931
rs116525939
rs2248769
rs41294937
rs117156856
rs112782756
All
−0.04
−0.02
0.00
0.02
MR leave−one−out sensitivity analysis for
' CD25 on B cell' on 'Rheumatoid arthritis'

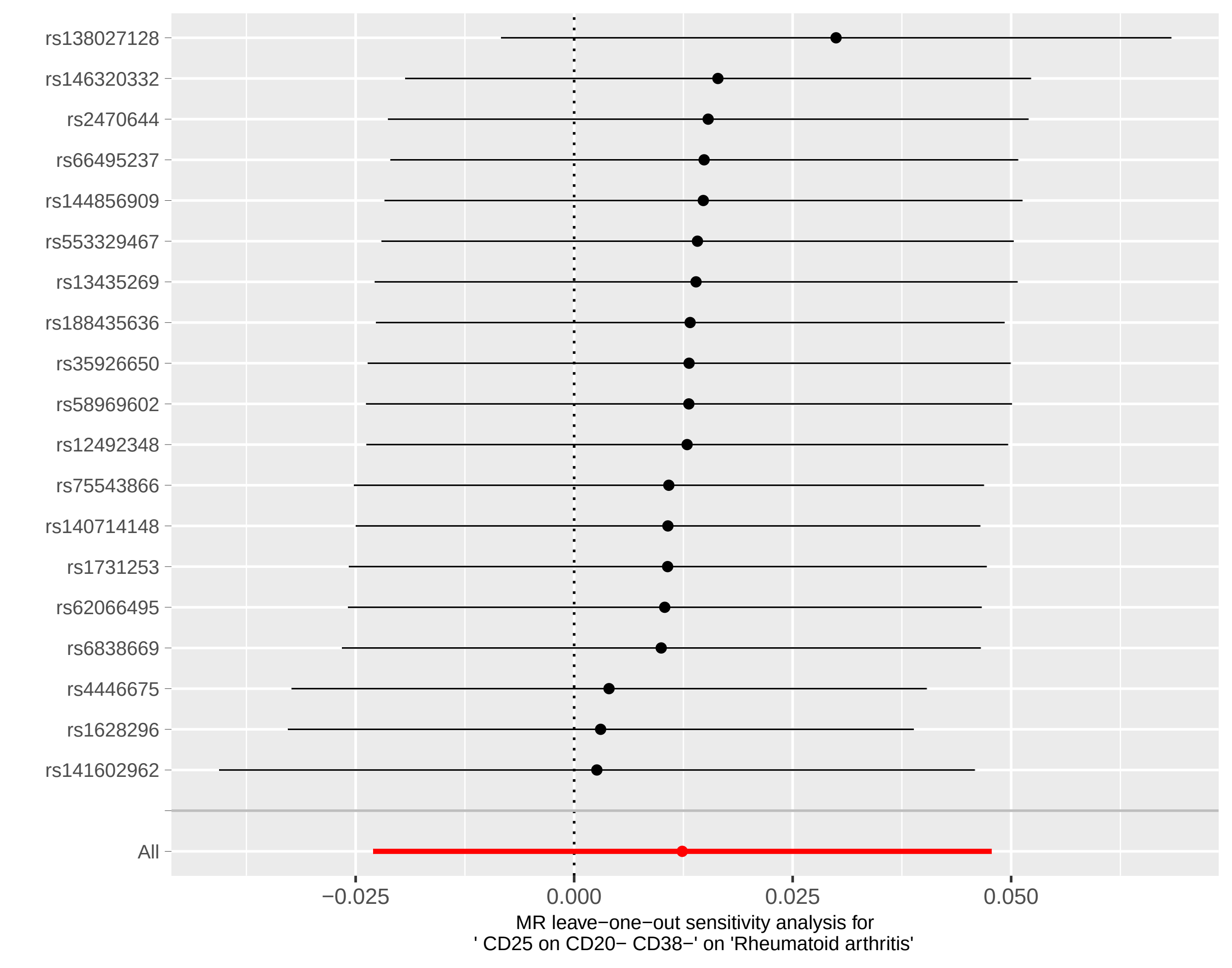
rs138027128
rs146320332
rs2470644
rs66495237
rs144856909
rs553329467
rs13435269
rs188435636
rs35926650
rs58969602
rs12492348
rs75543866
rs140714148
rs1731253
rs62066495
rs6838669
rs4446675
rs1628296
rs141602962
All
−0.025
0.000
0.025
0.050
MR leave−one−out sensitivity analysis for
' CD25 on CD20− CD38−' on 'Rheumatoid arthritis'

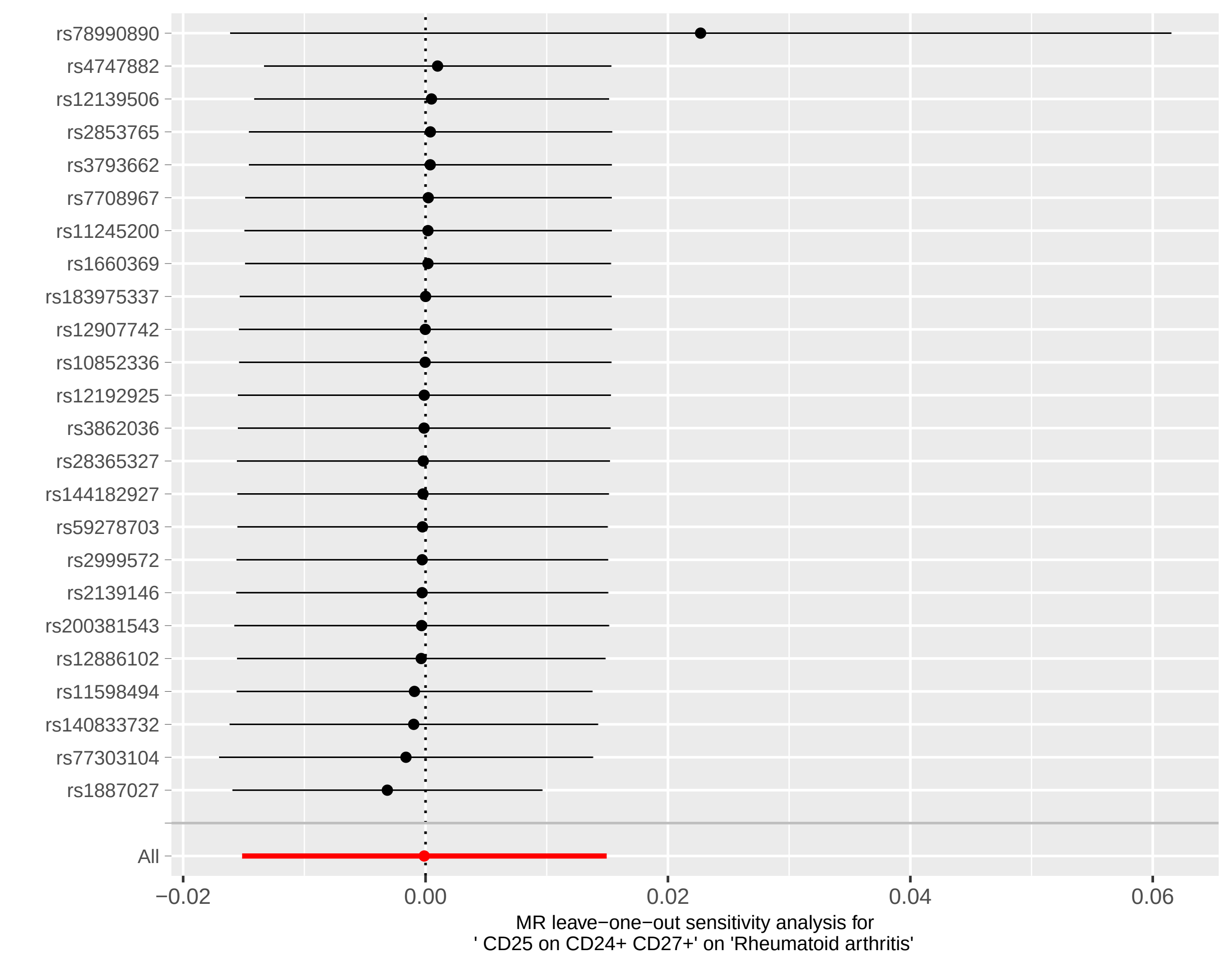
rs78990890
rs4747882
rs12139506
rs2853765
rs3793662
rs7708967
rs11245200
rs1660369
rs183975337
rs12907742
rs10852336
rs12192925
rs3862036
rs28365327
rs144182927
rs59278703
rs2999572
rs2139146
rs200381543
rs12886102
rs11598494
rs140833732
rs77303104
rs1887027
All
−0.02
0.00
0.02
0.04
0.06
MR leave−one−out sensitivity analysis for
' CD25 on CD24+ CD27+' on 'Rheumatoid arthritis'

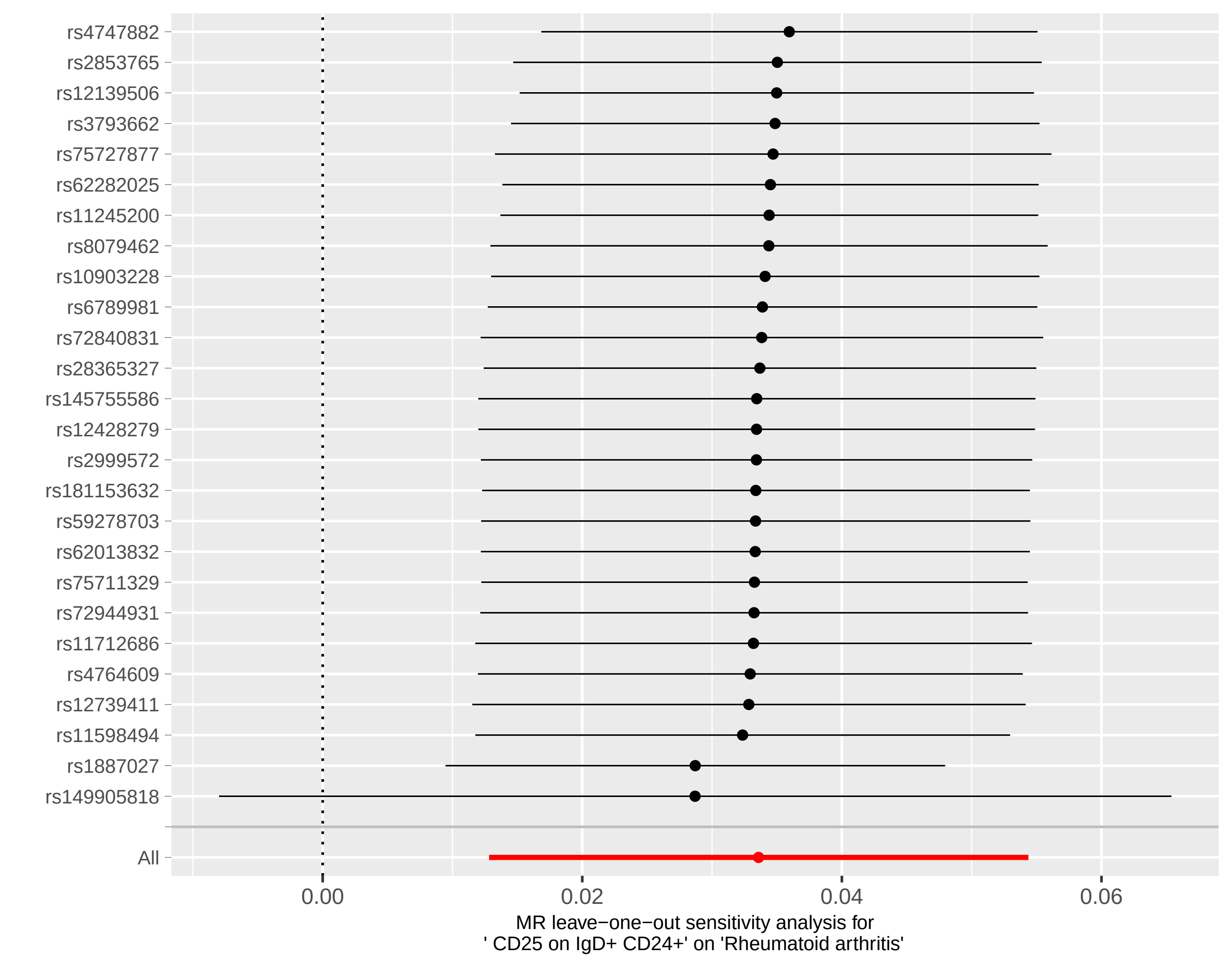

rs4747882
rs2853765
rs12139506
rs3793662
rs75727877
rs62282025
rs11245200
rs8079462
rs10903228
rs6789981
rs72840831
rs28365327
rs145755586
rs12428279
rs2999572
rs181153632
rs59278703
rs62013832
rs75711329
rs72944931
rs11712686
rs4764609
rs12739411
rs11598494
rs1887027
rs149905818
All
0.00
0.02
0.04
0.06
MR leave−one−out sensitivity analysis for
' CD25 on IgD+ CD24+' on 'Rheumatoid arthritis'

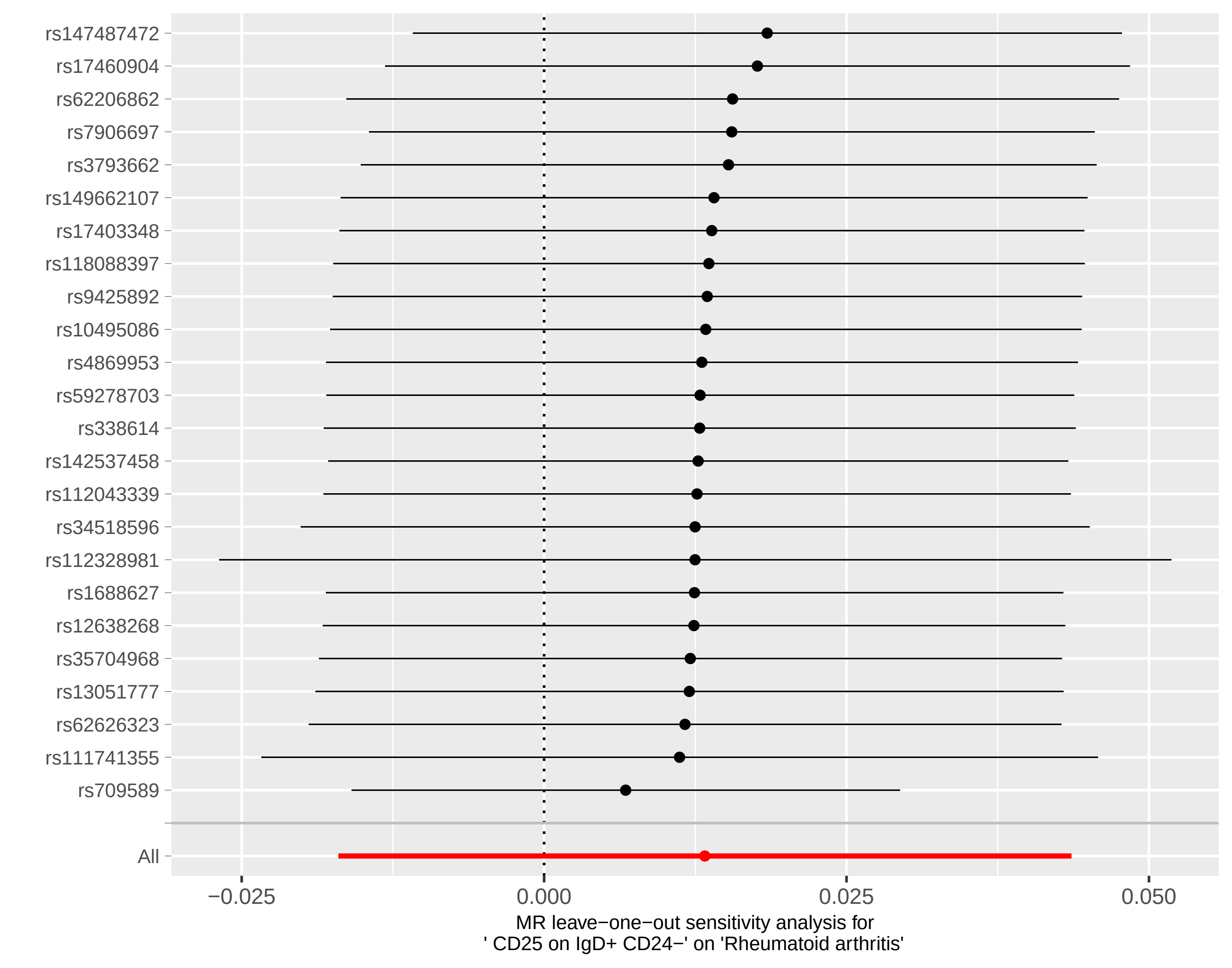
rs147487472
rs17460904
rs62206862
rs7906697
rs3793662
rs149662107
rs17403348
rs118088397
rs9425892
rs10495086
rs4869953
rs59278703
rs338614
rs142537458
rs112043339
rs34518596
rs112328981
rs1688627
rs12638268
rs35704968
rs13051777
rs62626323
rs111741355
rs709589
All
−0.025
0.000
0.025
0.050
MR leave−one−out sensitivity analysis for
' CD25 on IgD+ CD24−' on 'Rheumatoid arthritis'

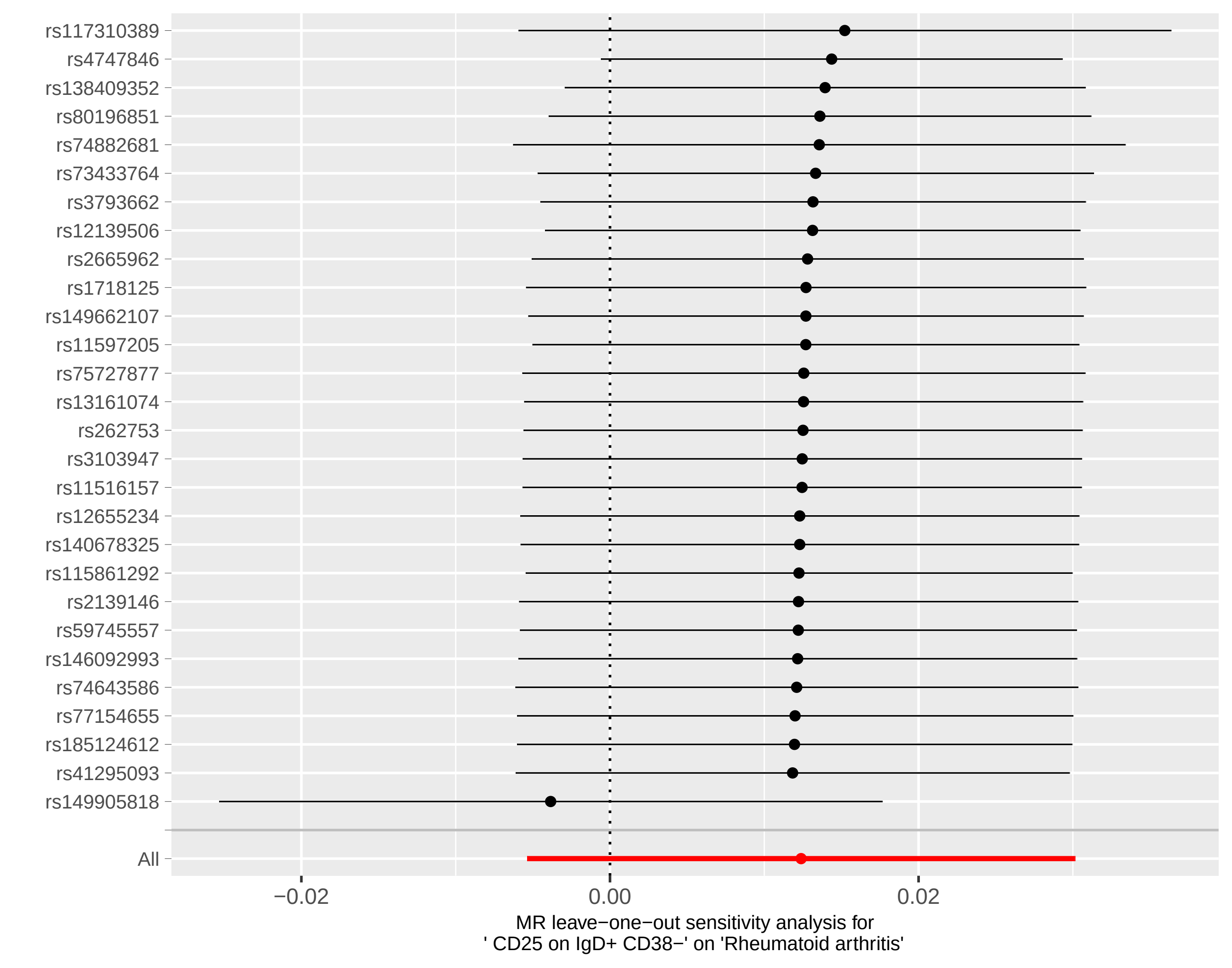
rs117310389
rs4747846
rs138409352
rs80196851
rs74882681
rs73433764
rs3793662
rs12139506
rs2665962
rs1718125
rs149662107
rs11597205
rs75727877
rs13161074
rs262753
rs3103947
rs11516157
rs12655234
rs140678325
rs115861292
rs2139146
rs59745557
rs146092993
rs74643586
rs77154655
rs185124612
rs41295093
rs149905818
All
−0.02
0.00
0.02
MR leave−one−out sensitivity analysis for
' CD25 on IgD+ CD38−' on 'Rheumatoid arthritis'

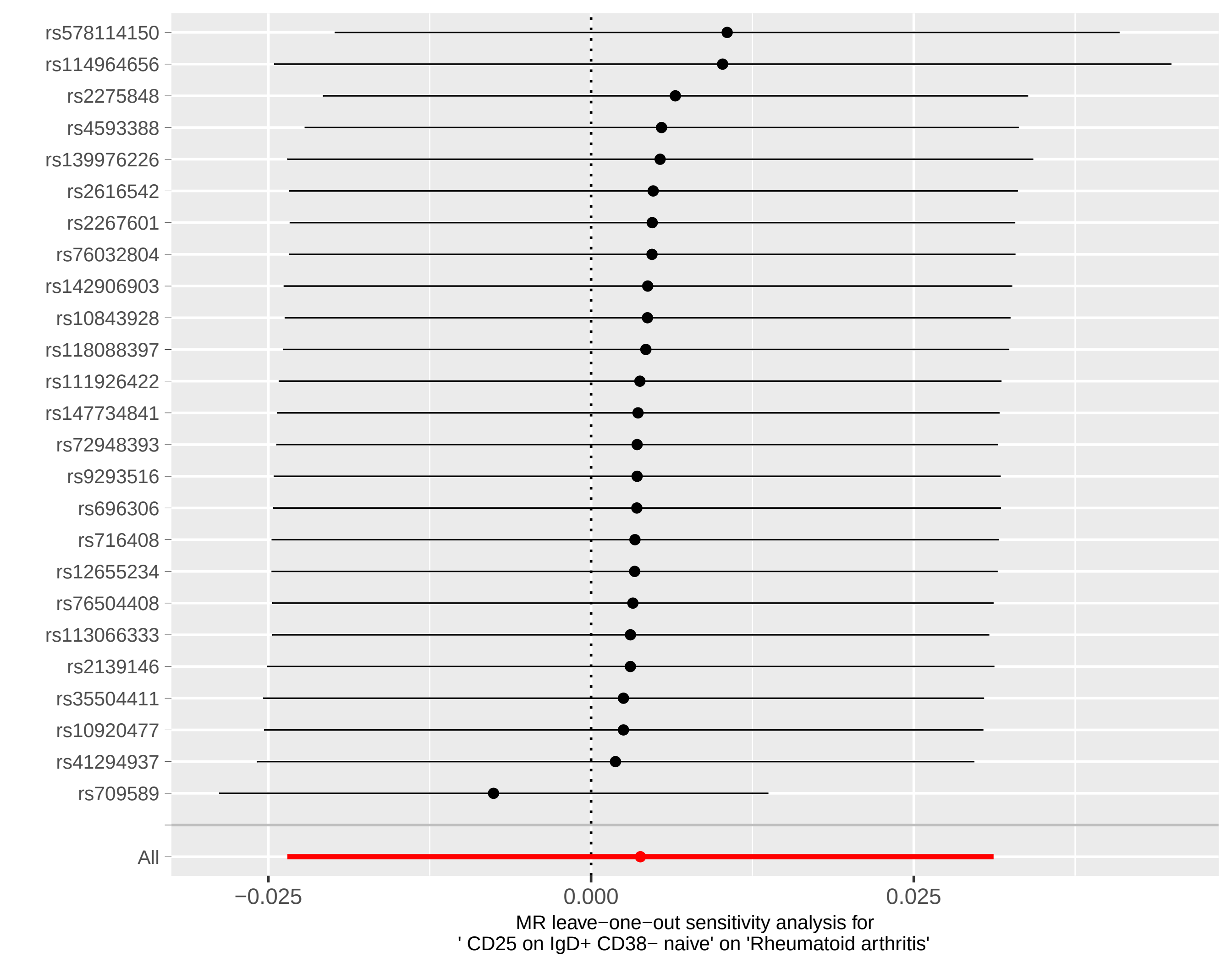
rs578114150
rs114964656
rs2275848
rs4593388
rs139976226
rs2616542
rs2267601
rs76032804
rs142906903
rs10843928
rs118088397
rs111926422
rs147734841
rs72948393
rs9293516
rs696306
rs716408
rs12655234
rs76504408
rs113066333
rs2139146
rs35504411
rs10920477
rs41294937
rs709589
All
−0.025
0.000
0.025
MR leave−one−out sensitivity analysis for
' CD25 on IgD+ CD38− naive' on 'Rheumatoid arthritis'

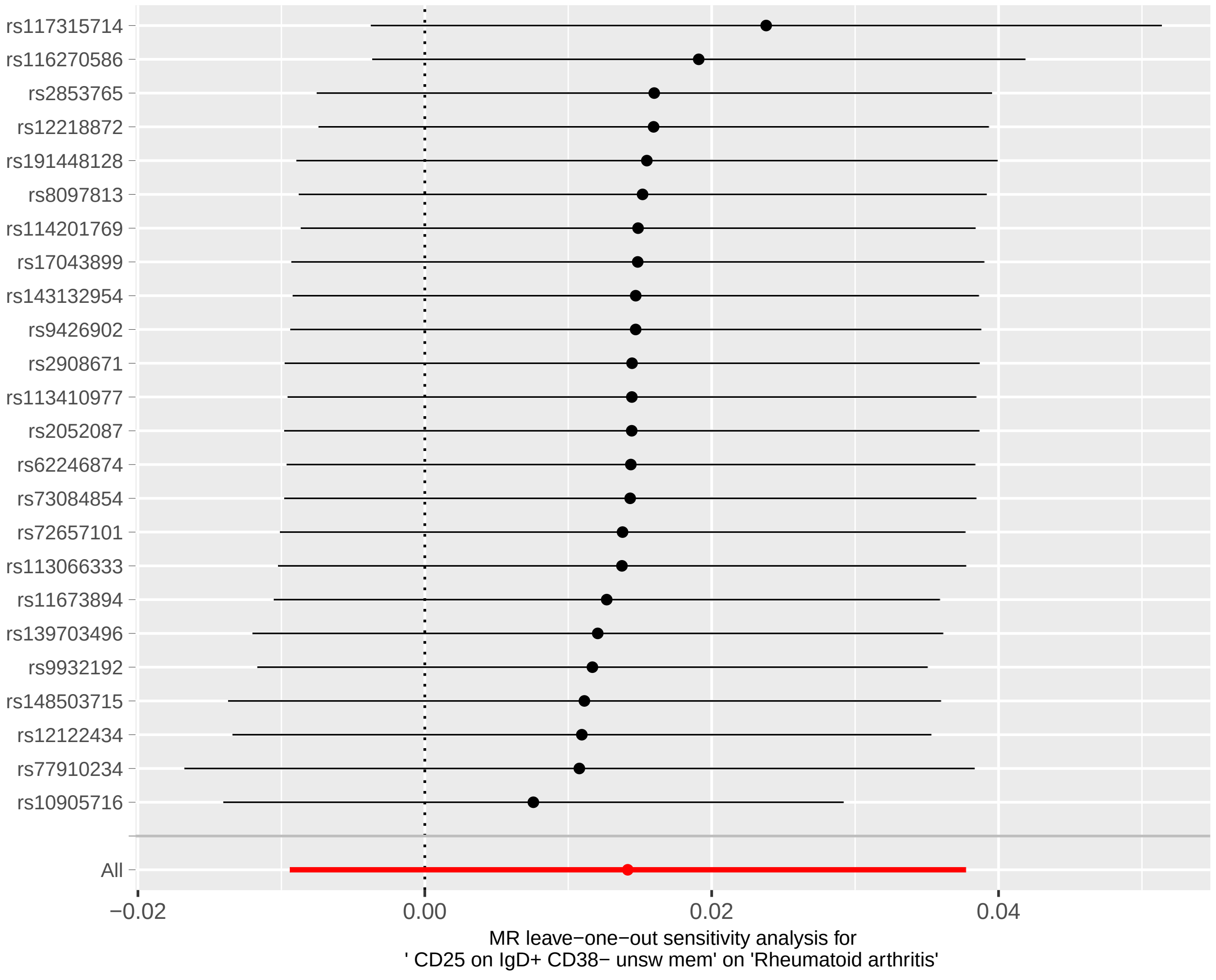
rs117315714
rs116270586
rs2853765
rs12218872
rs191448128
rs8097813
rs114201769
rs17043899
rs143132954
rs9426902
rs2908671
rs113410977
rs2052087
rs62246874
rs73084854
rs72657101
rs113066333
rs11673894
rs139703496
rs9932192
rs148503715
rs12122434
rs77910234
rs10905716
All
−0.02
0.00
0.02
0.04
MR leave−one−out sensitivity analysis for
' CD25 on IgD+ CD38− unsw mem' on 'Rheumatoid arthritis'

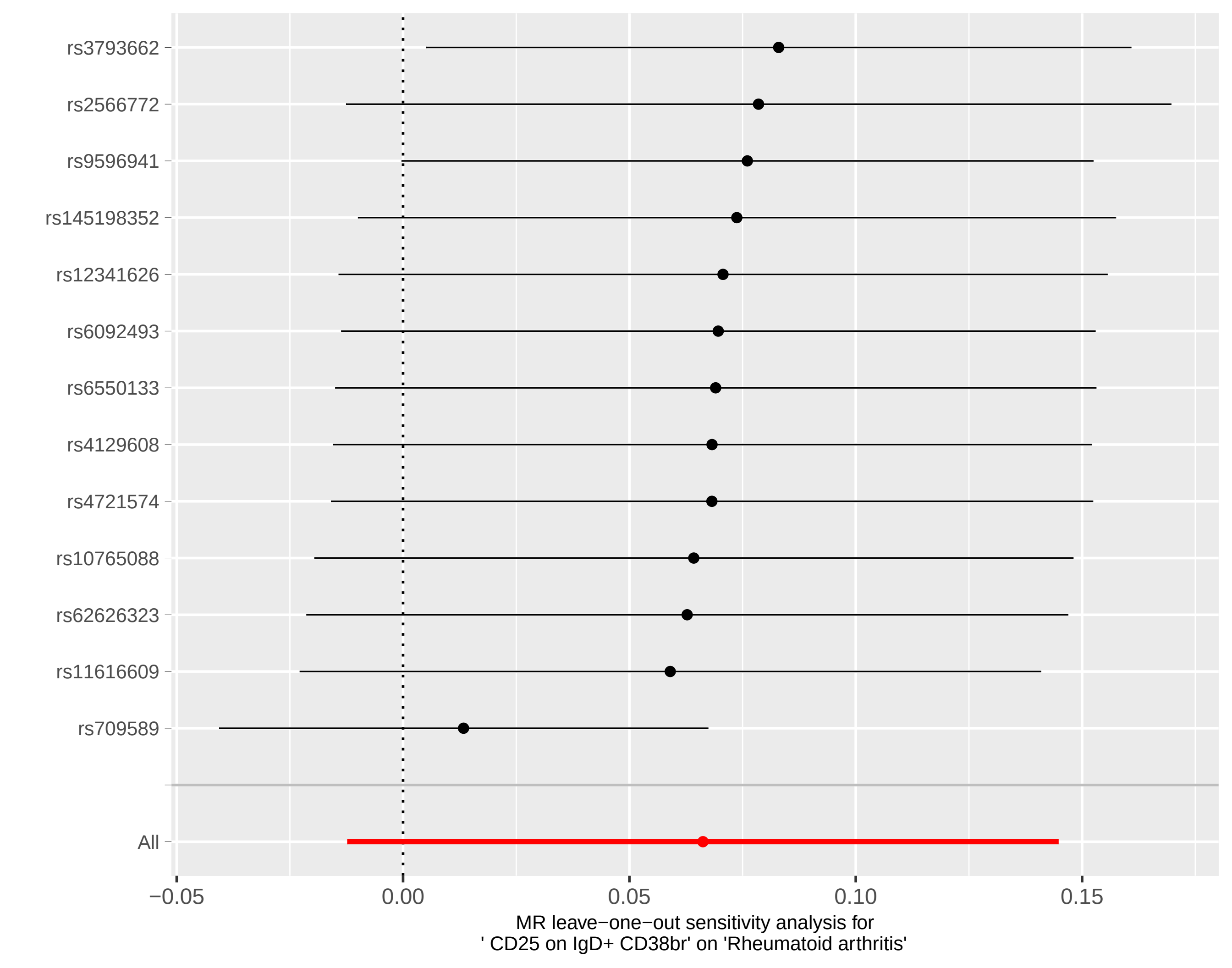
rs3793662
rs2566772
rs9596941
rs145198352
rs12341626
rs6092493
rs6550133
rs4129608
rs4721574
rs10765088
rs62626323
rs11616609
rs709589
All
−0.05
0.00
0.05
0.10
0.15
MR leave−one−out sensitivity analysis for
' CD25 on IgD+ CD38br' on 'Rheumatoid arthritis'

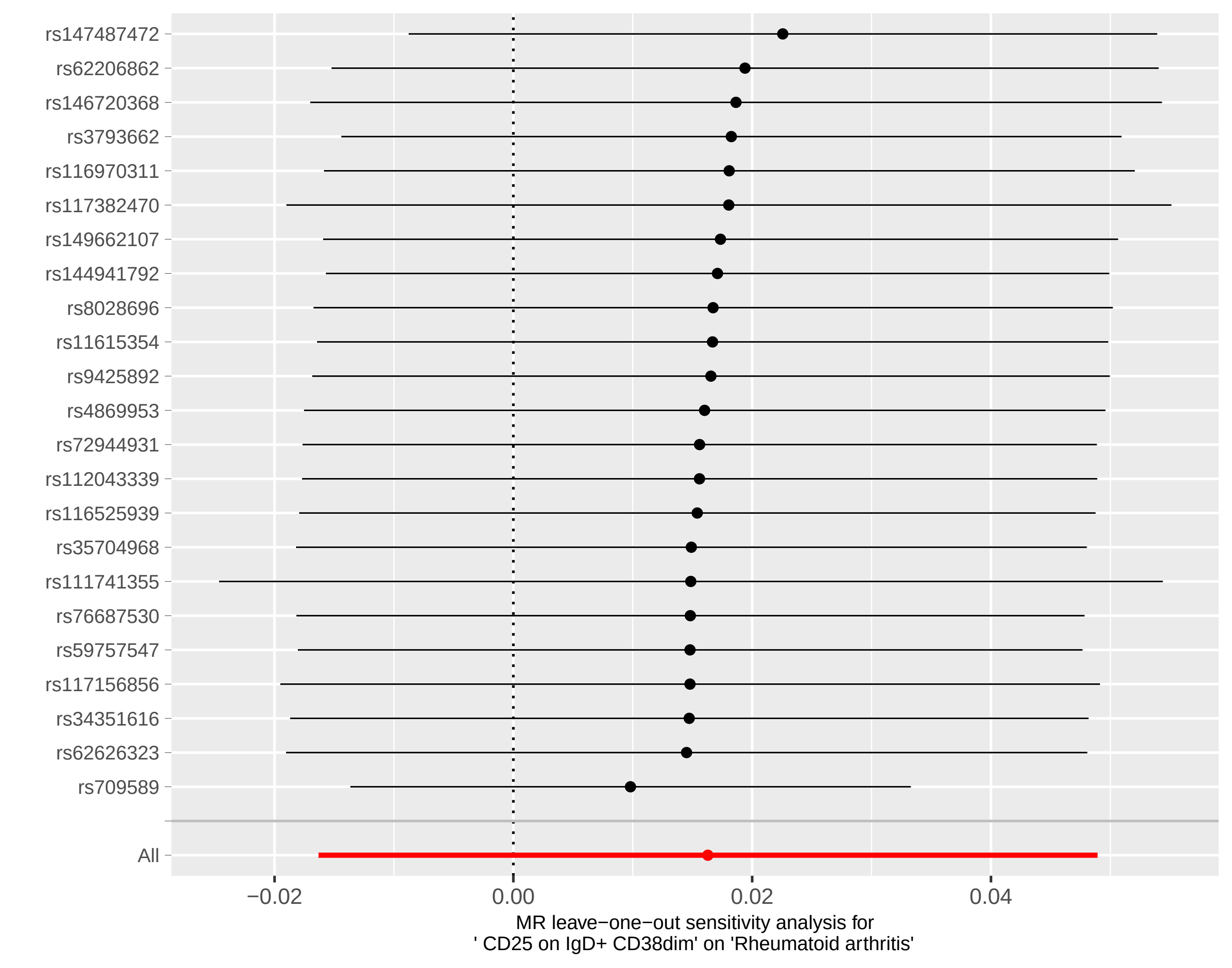
rs147487472
rs62206862
rs146720368
rs3793662
rs116970311
rs117382470
rs149662107
rs144941792
rs8028696
rs11615354
rs9425892
rs4869953
rs72944931
rs112043339
rs116525939
rs35704968
rs111741355
rs76687530
rs59757547
rs117156856
rs34351616
rs62626323
rs709589
All
−0.02
0.00
0.02
0.04
MR leave−one−out sensitivity analysis for
' CD25 on IgD+ CD38dim' on 'Rheumatoid arthritis'

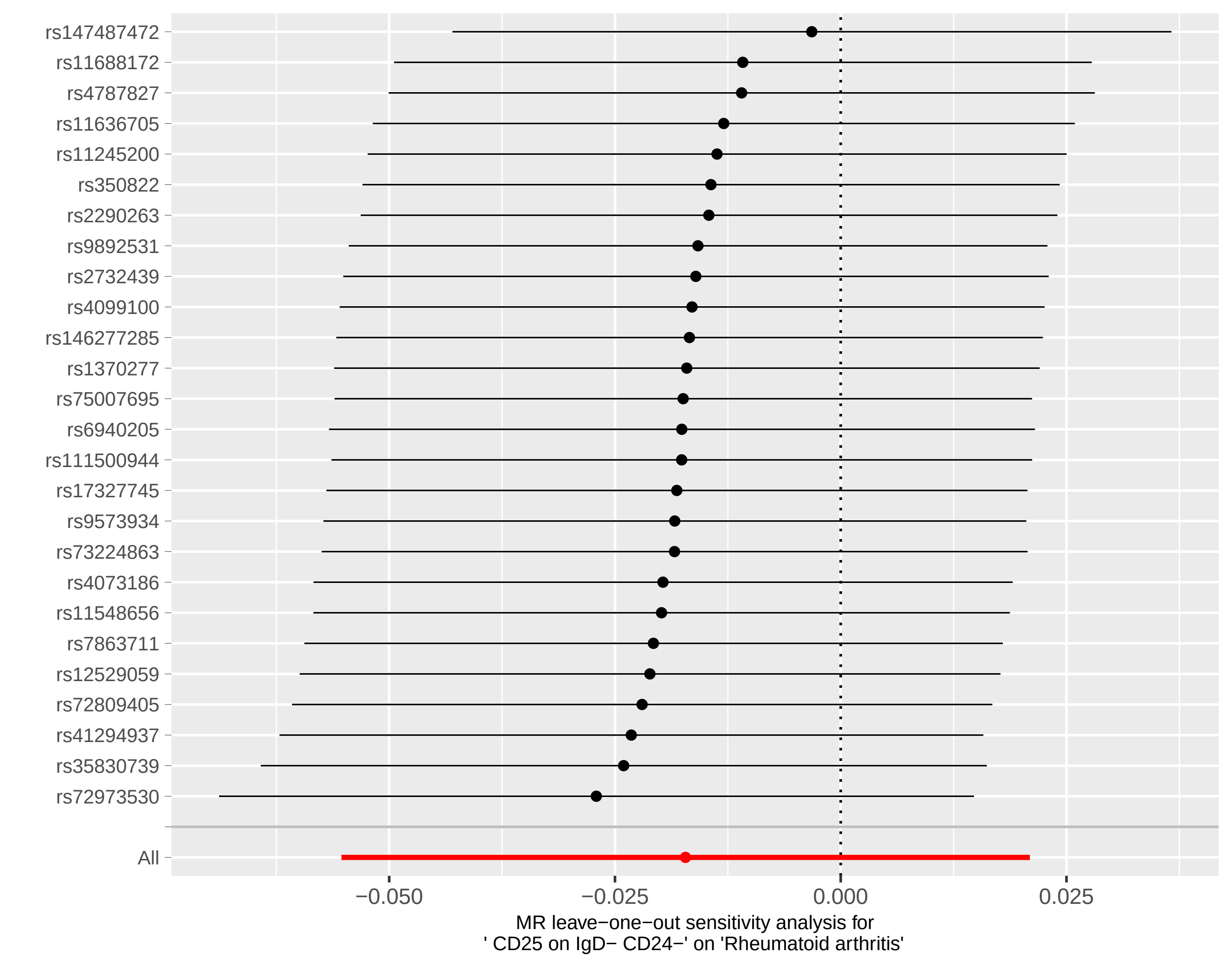

rs147487472
rs11688172
rs4787827
rs11636705
rs11245200
rs350822
rs2290263
rs9892531
rs2732439
rs4099100
rs146277285
rs1370277
rs75007695
rs6940205
rs111500944
rs17327745
rs9573934
rs73224863
rs4073186
rs11548656
rs7863711
rs12529059
rs72809405
rs41294937
rs35830739
rs72973530
All
−0.050
−0.025
0.000
0.025
MR leave−one−out sensitivity analysis for
' CD25 on IgD− CD24−' on 'Rheumatoid arthritis'

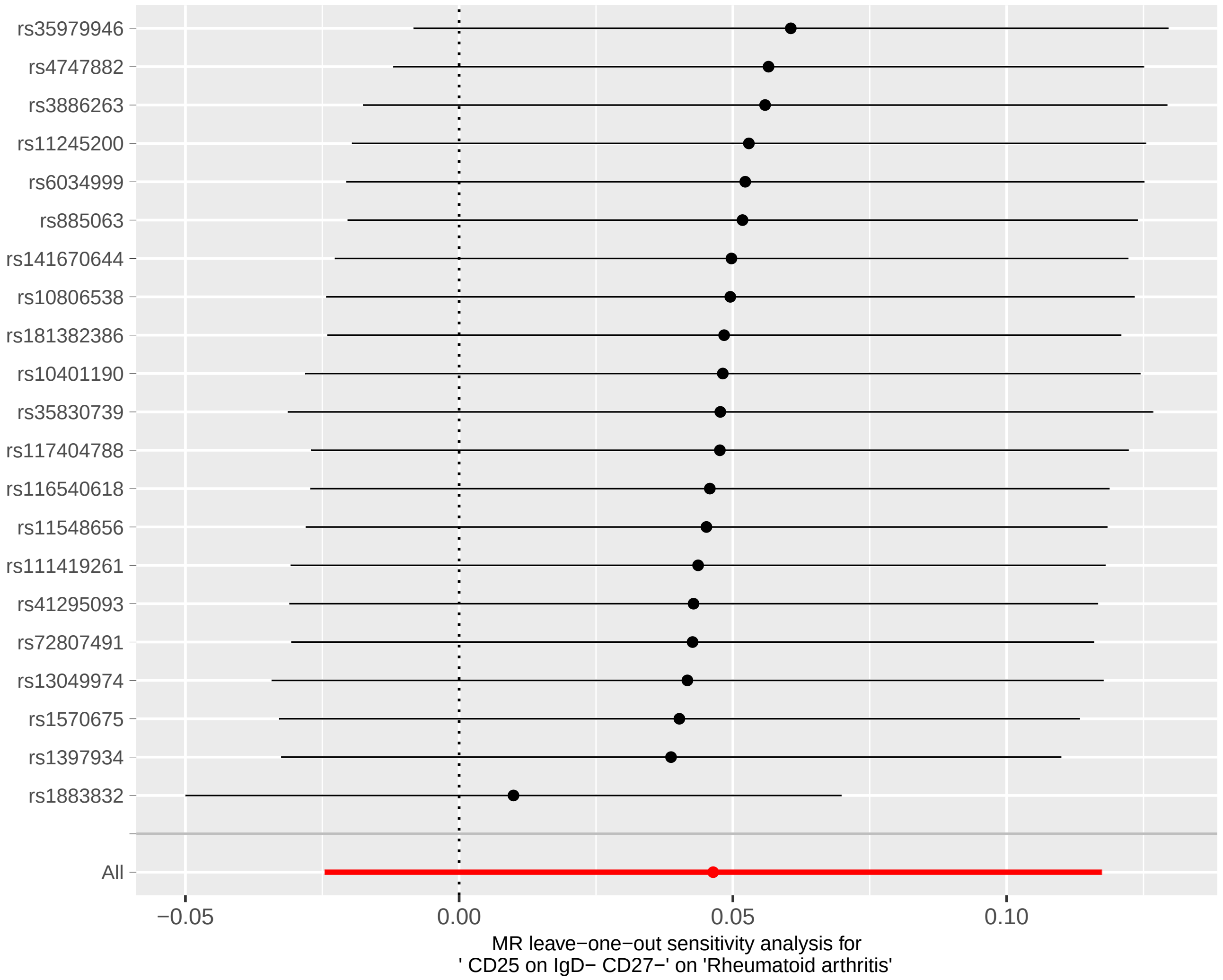
rs35979946
rs4747882
rs3886263
rs11245200
rs6034999
rs885063
rs141670644
rs10806538
rs181382386
rs10401190
rs35830739
rs117404788
rs116540618
rs11548656
rs111419261
rs41295093
rs72807491
rs13049974
rs1570675
rs1397934
rs1883832
All
−0.05
0.00
0.05
0.10
MR leave−one−out sensitivity analysis for
' CD25 on IgD− CD27−' on 'Rheumatoid arthritis'

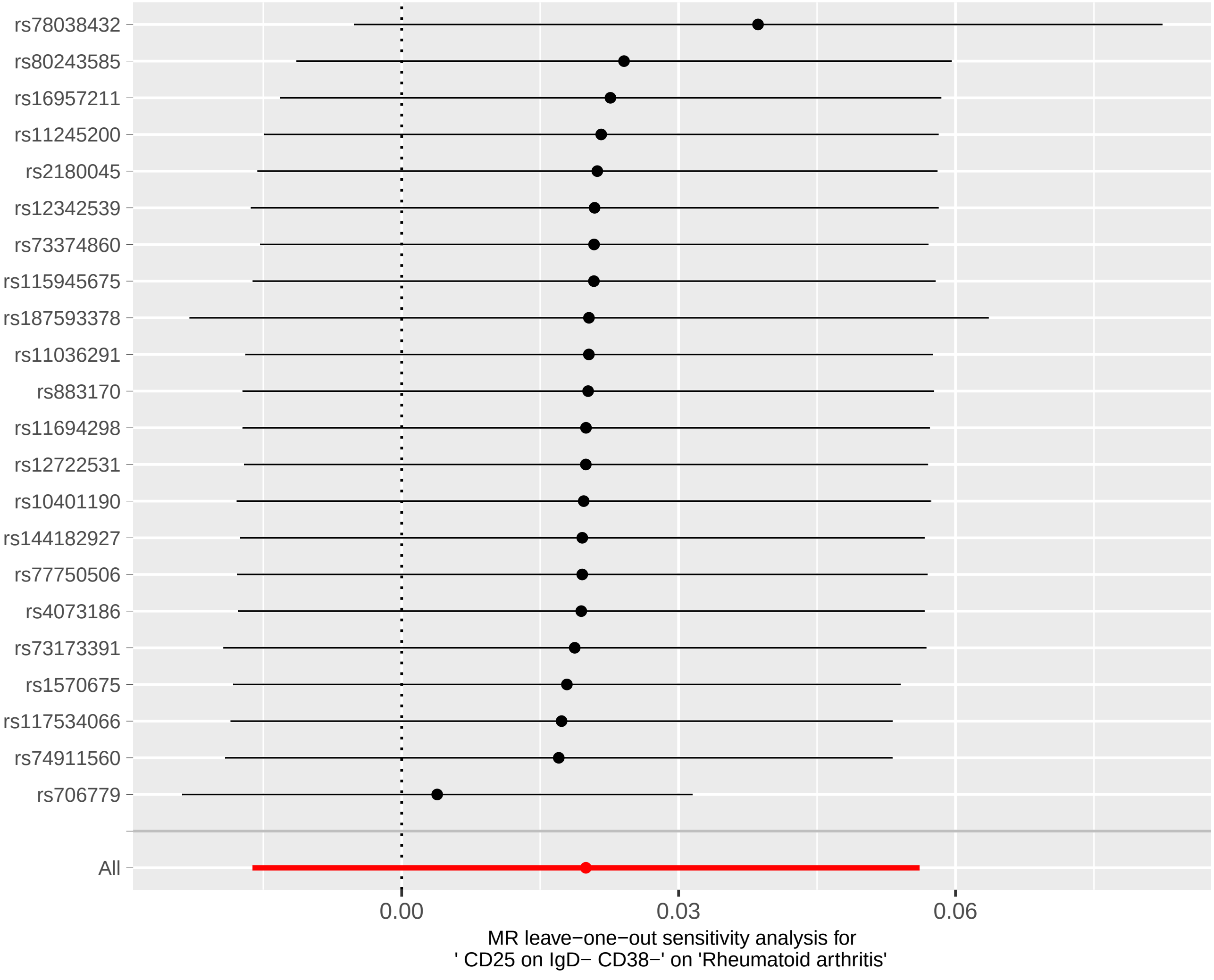
rs78038432
rs80243585
rs16957211
rs11245200
rs2180045
rs12342539
rs73374860
rs115945675
rs187593378
rs11036291
rs883170
rs11694298
rs12722531
rs10401190
rs144182927
rs77750506
rs4073186
rs73173391
rs1570675
rs117534066
rs74911560
rs706779
All
0.00
0.03
0.06
MR leave−one−out sensitivity analysis for
' CD25 on IgD− CD38−' on 'Rheumatoid arthritis'

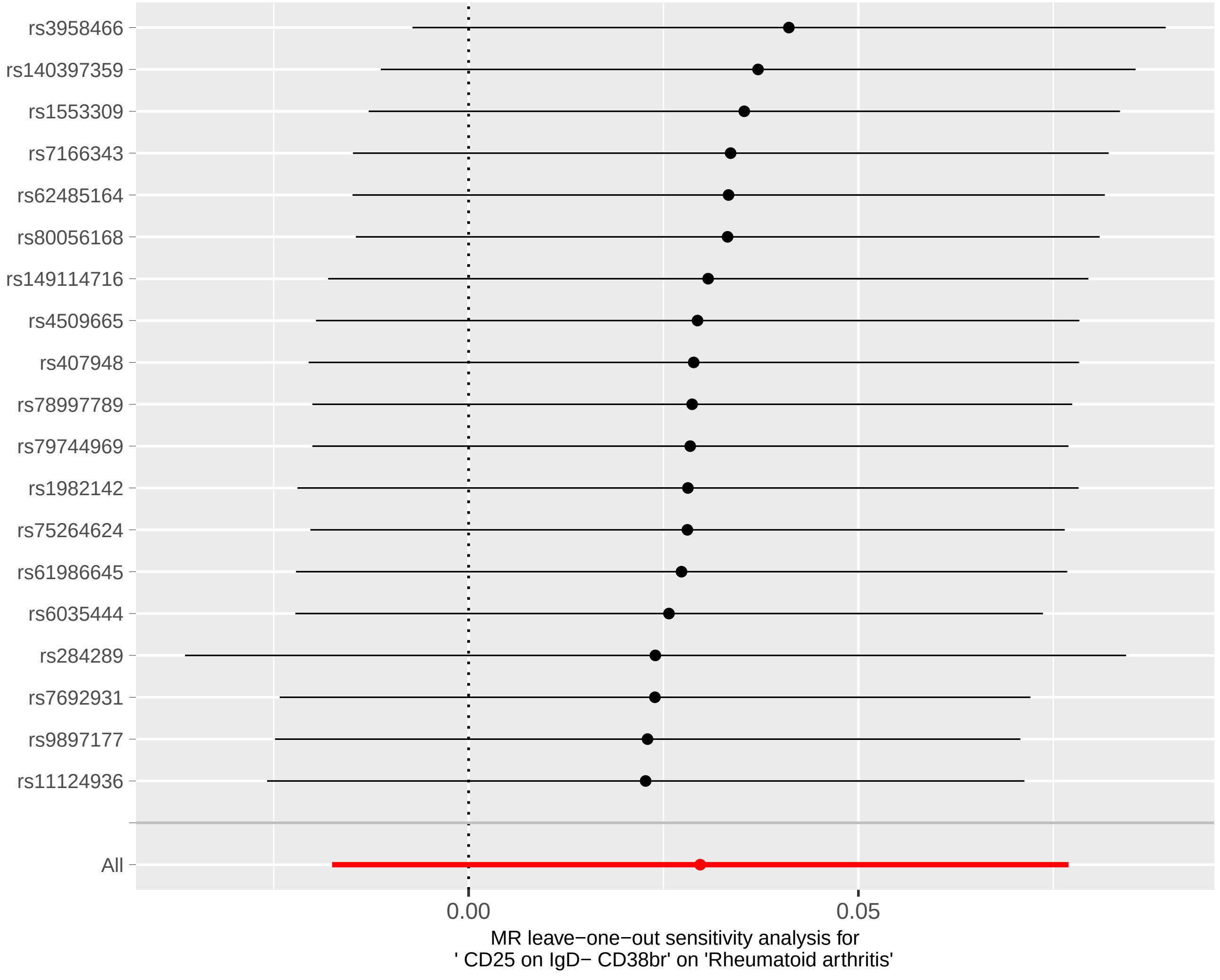
rs3958466
rs140397359
rs1553309
rs7166343
rs62485164
rs80056168
rs149114716
rs4509665
rs407948
rs78997789
rs79744969
rs1982142
rs75264624
rs61986645
rs6035444
rs284289
rs7692931
rs9897177
rs11124936
All
0.00
0.05
MR leave−one−out sensitivity analysis for
' CD25 on IgD− CD38br' on 'Rheumatoid arthritis'

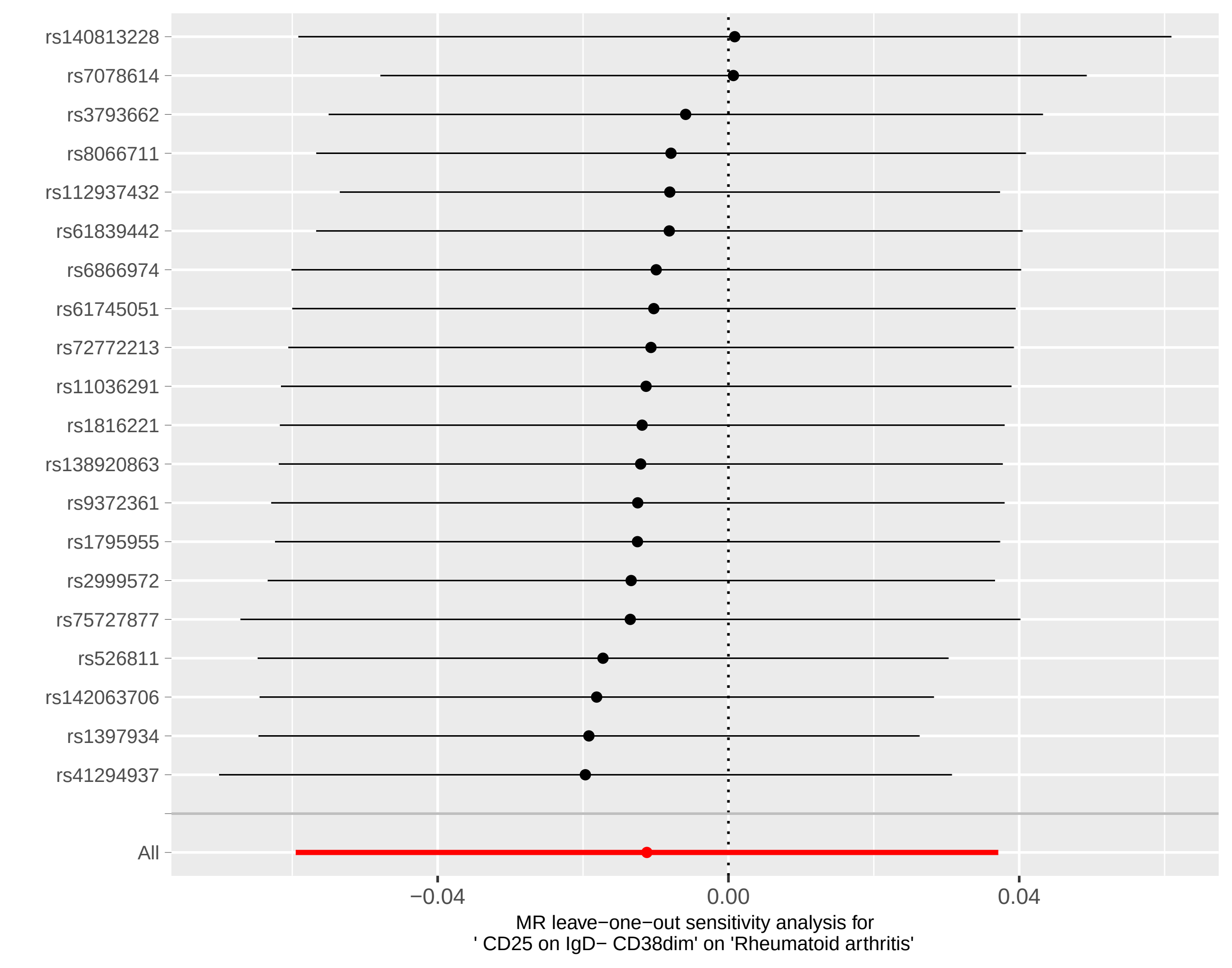
rs140813228
rs7078614
rs3793662
rs8066711
rs112937432
rs61839442
rs6866974
rs61745051
rs72772213
rs11036291
rs1816221
rs138920863
rs9372361
rs1795955
rs2999572
rs75727877
rs526811
rs142063706
rs1397934
rs41294937
All
−0.04
0.00
0.04
MR leave−one−out sensitivity analysis for
' CD25 on IgD− CD38dim' on 'Rheumatoid arthritis'

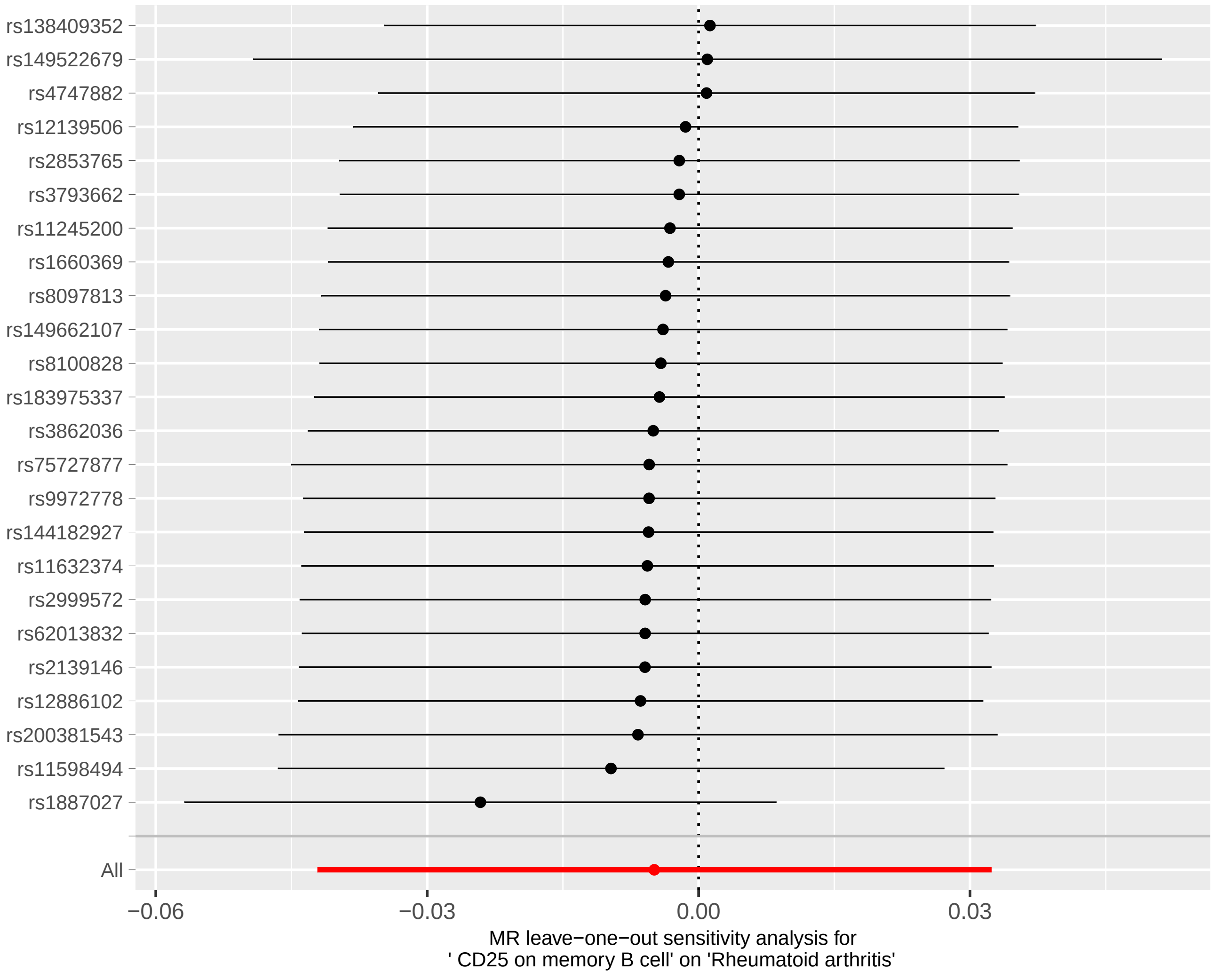
rs138409352
rs149522679
rs4747882
rs12139506
rs2853765
rs3793662
rs11245200
rs1660369
rs8097813
rs149662107
rs8100828
rs183975337
rs3862036
rs75727877
rs9972778
rs144182927
rs11632374
rs2999572
rs62013832
rs2139146
rs12886102
rs200381543
rs11598494
rs1887027
All
−0.06
−0.03
0.00
0.03
MR leave−one−out sensitivity analysis for
' CD25 on memory B cell' on 'Rheumatoid arthritis'

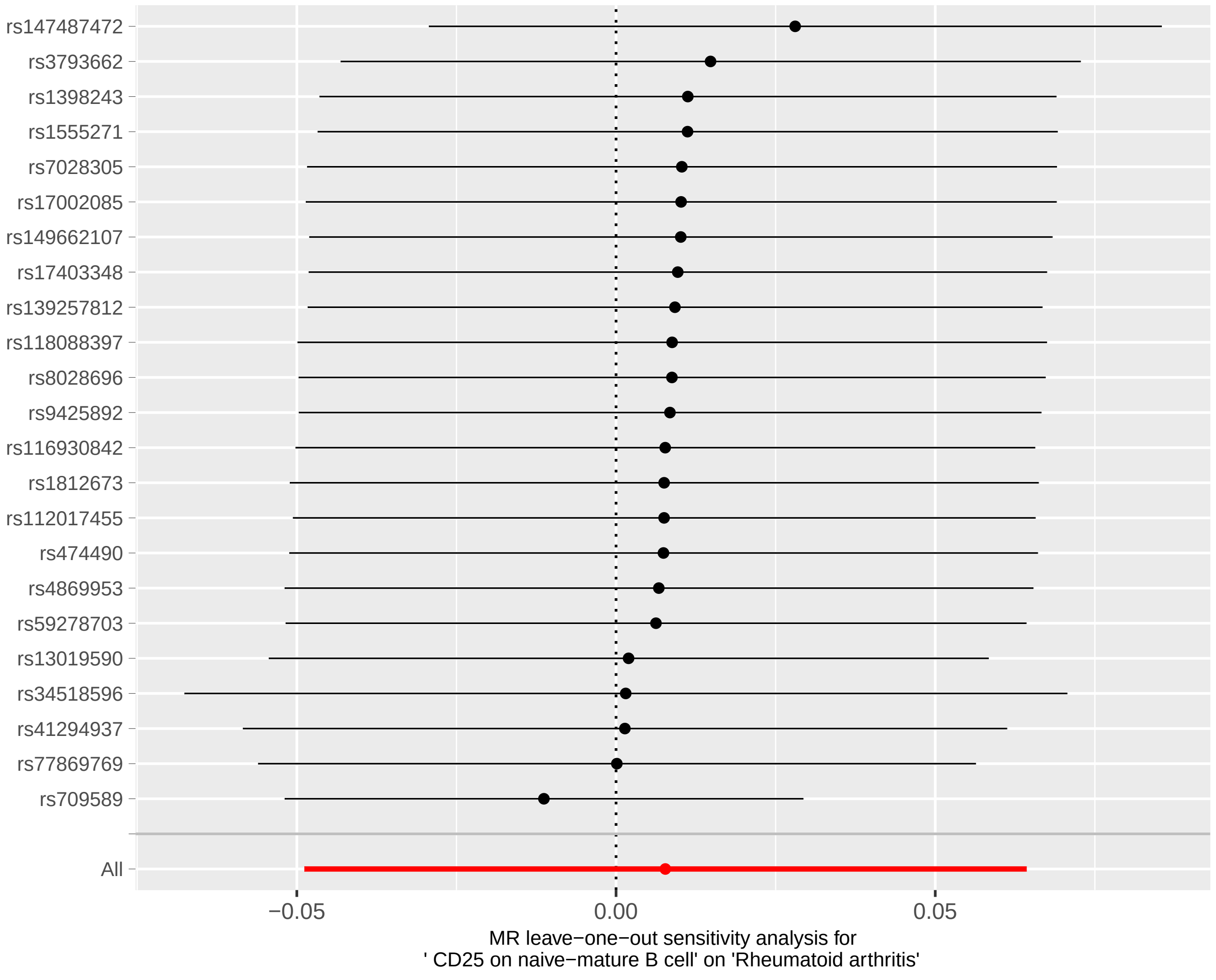
rs147487472
rs3793662
rs1398243
rs1555271
rs7028305
rs17002085
rs149662107
rs17403348
rs139257812
rs118088397
rs8028696
rs9425892
rs116930842
rs1812673
rs112017455
rs474490
rs4869953
rs59278703
rs13019590
rs34518596
rs41294937
rs77869769
rs709589
All
−0.05
0.00
0.05
MR leave−one−out sensitivity analysis for
' CD25 on naive−mature B cell' on 'Rheumatoid arthritis'

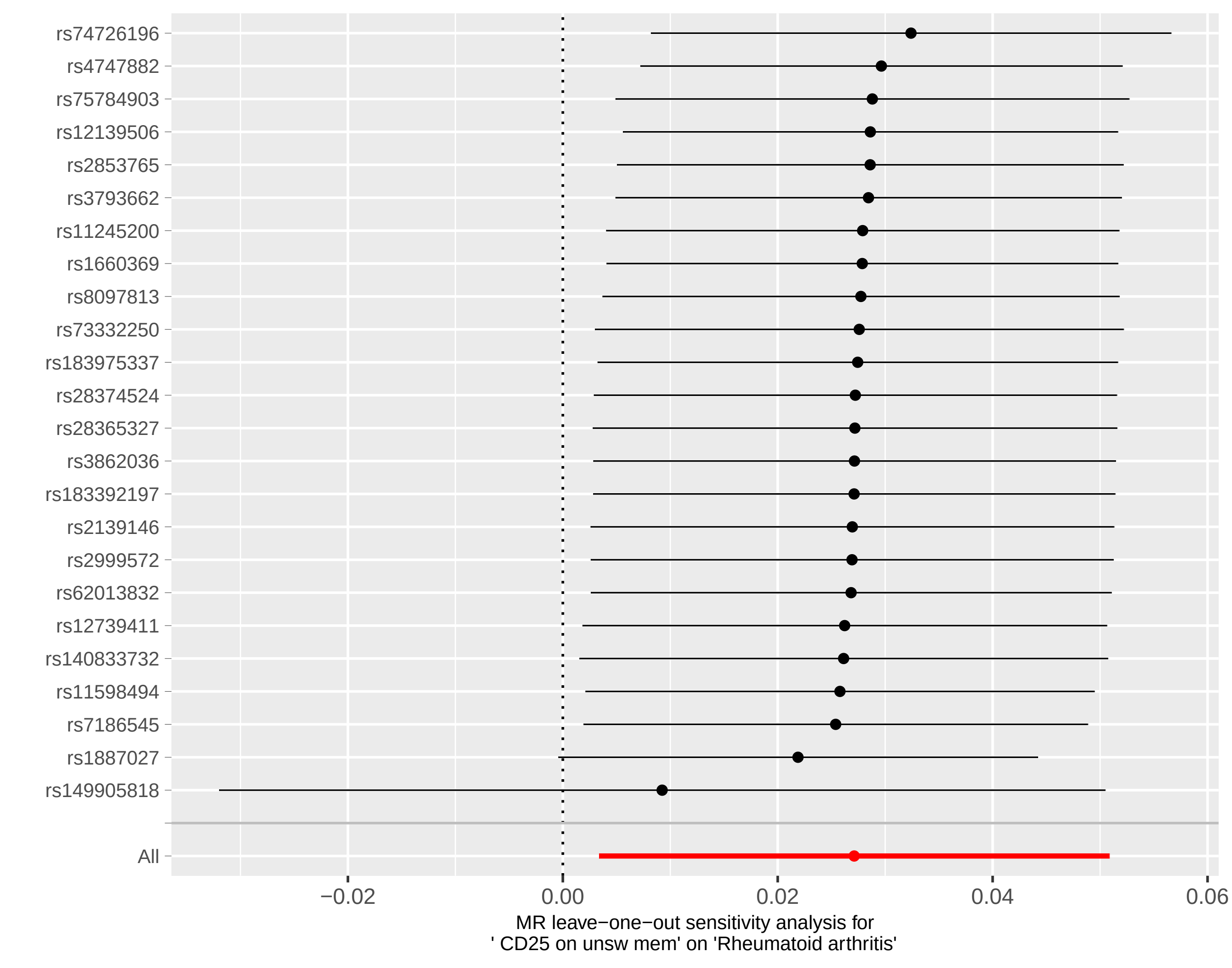
rs74726196
rs4747882
rs75784903
rs12139506
rs2853765
rs3793662
rs11245200
rs1660369
rs8097813
rs73332250
rs183975337
rs28374524
rs28365327
rs3862036
rs183392197
rs2139146
rs2999572
rs62013832
rs12739411
rs140833732
rs11598494
rs7186545
rs1887027
rs149905818
All
−0.02
0.00
0.02
0.04
0.06
MR leave−one−out sensitivity analysis for
' CD25 on unsw mem' on 'Rheumatoid arthritis'

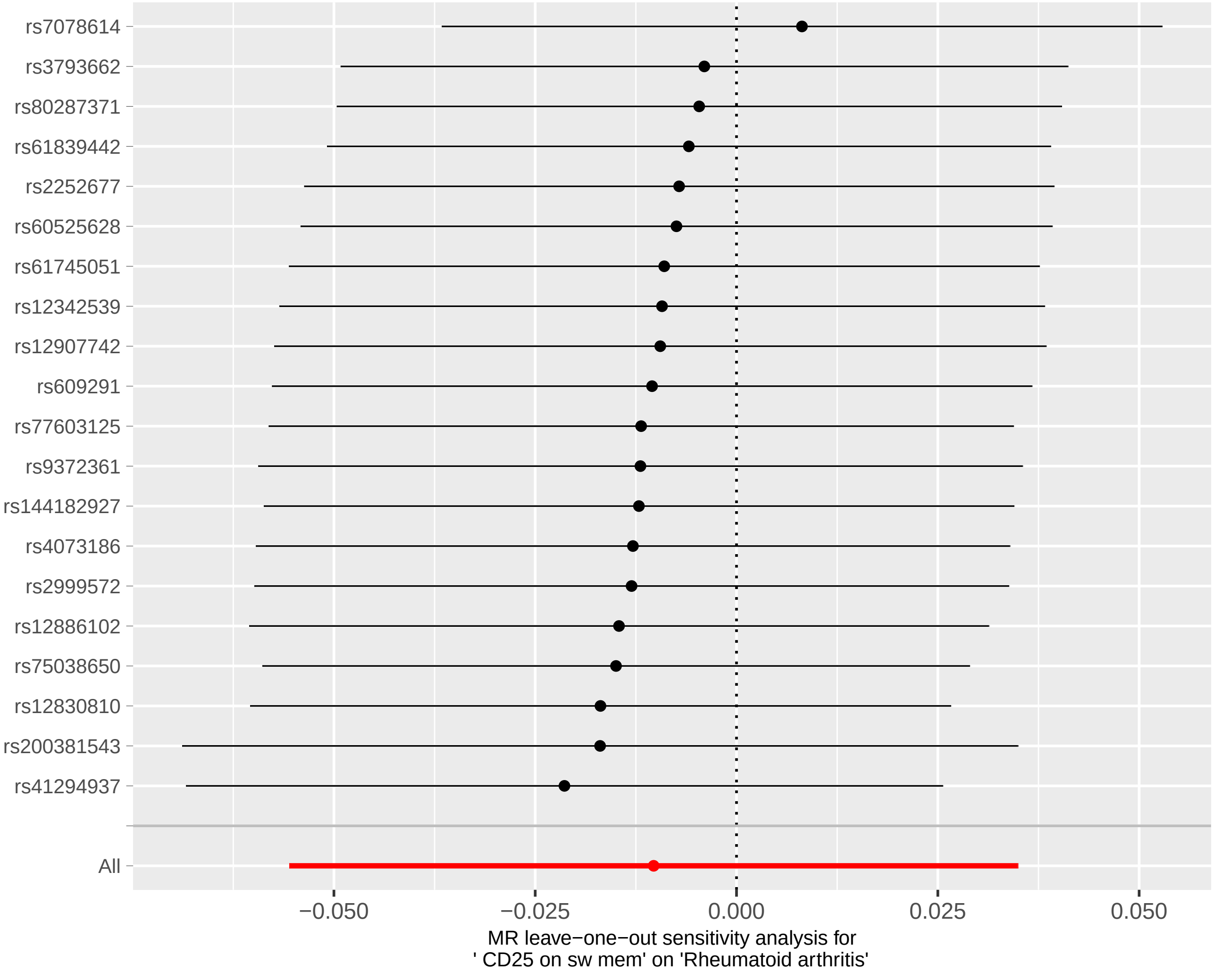
rs7078614
rs3793662
rs80287371
rs61839442
rs2252677
rs60525628
rs61745051
rs12342539
rs12907742
rs609291
rs77603125
rs9372361
rs144182927
rs4073186
rs2999572
rs12886102
rs75038650
rs12830810
rs200381543
rs41294937
All
−0.050
−0.025
0.000
0.025
0.050
MR leave−one−out sensitivity analysis for
' CD25 on sw mem' on 'Rheumatoid arthritis'

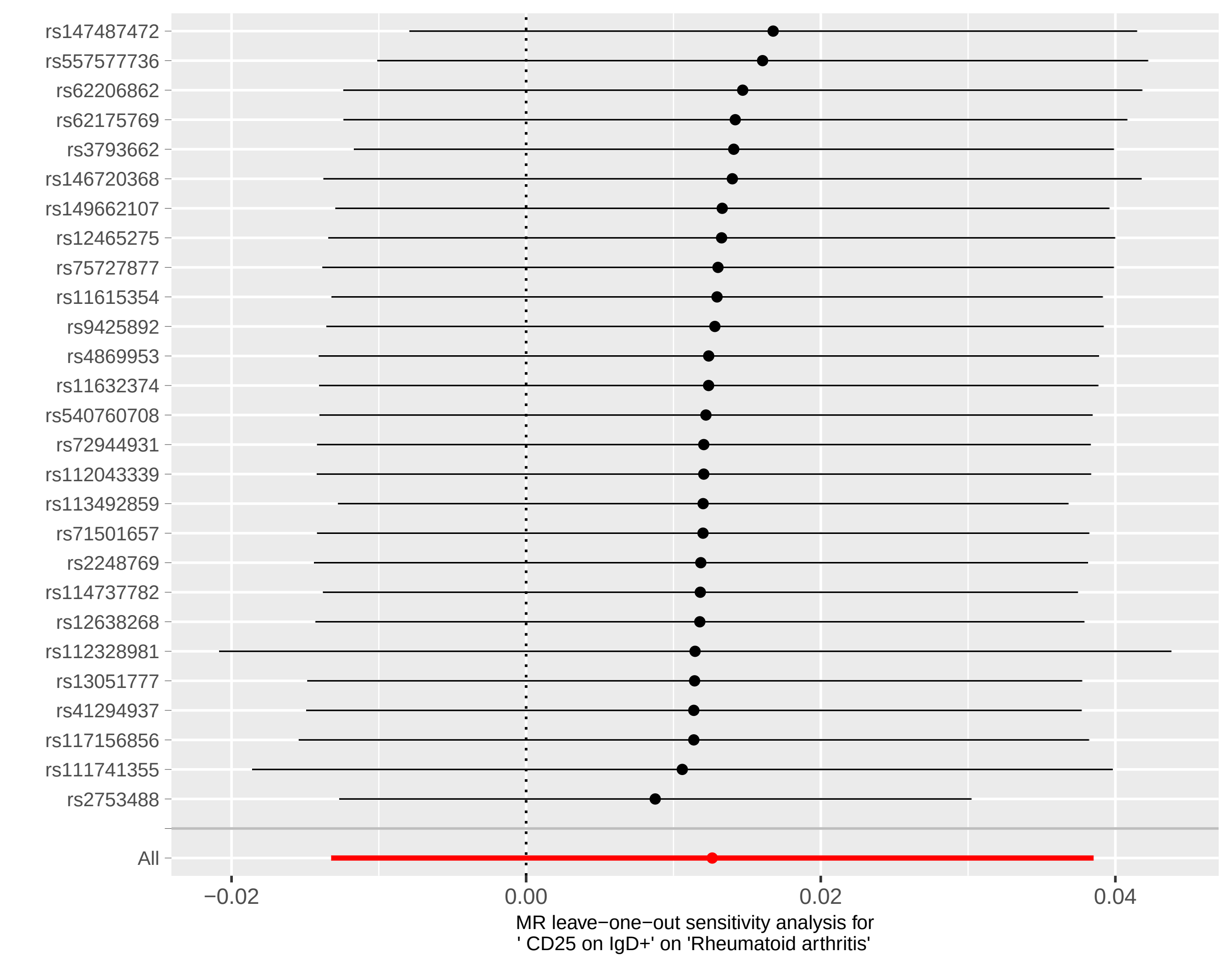
rs147487472
rs557577736
rs62206862
rs62175769
rs3793662
rs146720368
rs149662107
rs12465275
rs75727877
rs11615354
rs9425892
rs4869953
rs11632374
rs540760708
rs72944931
rs112043339
rs113492859
rs71501657
rs2248769
rs114737782
rs12638268
rs112328981
rs13051777
rs41294937
rs117156856
rs111741355
rs2753488
All
−0.02
0.00
0.02
0.04
MR leave−one−out sensitivity analysis for
' CD25 on IgD+' on 'Rheumatoid arthritis'

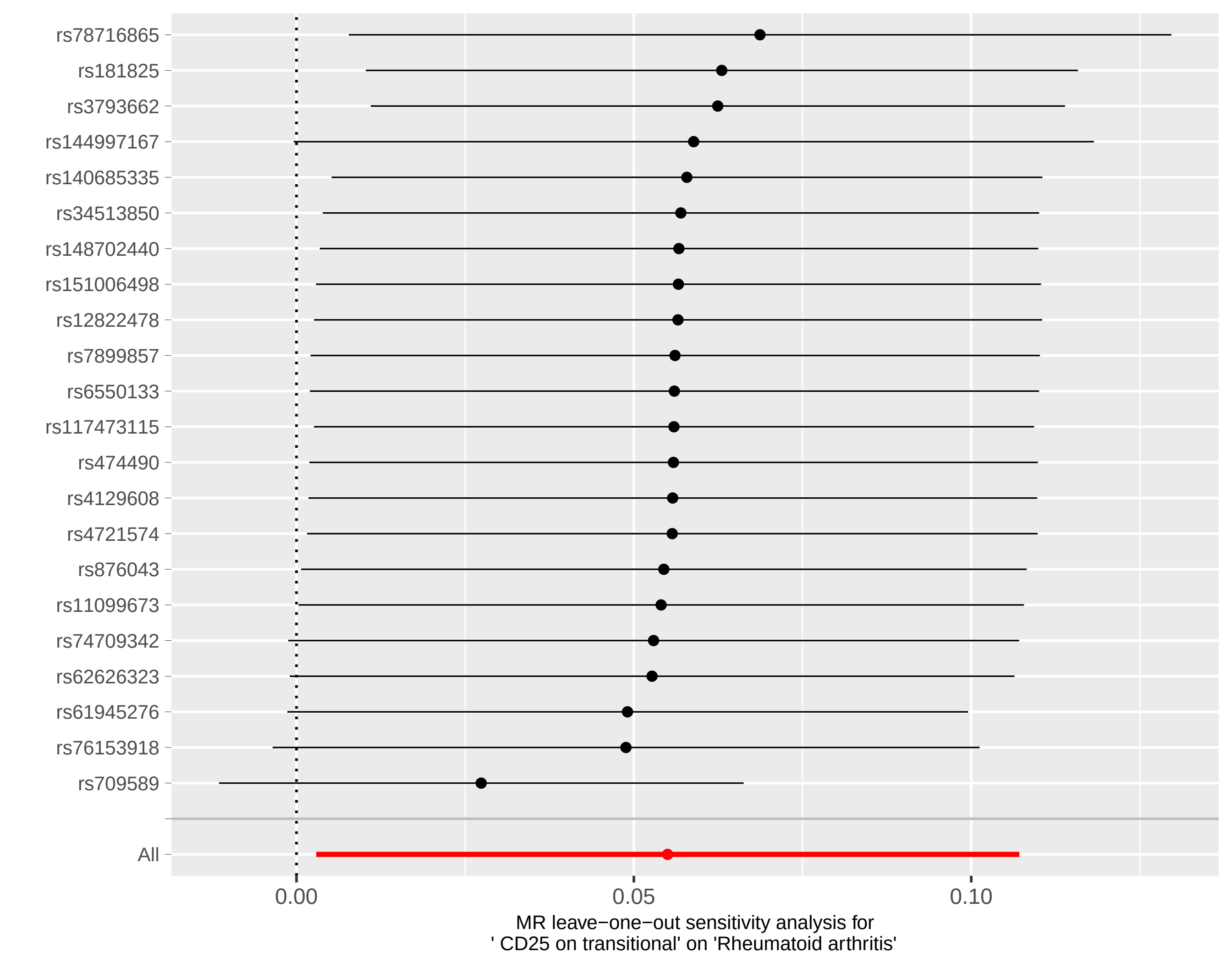

rs78716865
rs181825
rs3793662
rs144997167
rs140685335
rs34513850
rs148702440
rs151006498
rs12822478
rs7899857
rs6550133
rs117473115
rs474490
rs4129608
rs4721574
rs876043
rs11099673
rs74709342
rs62626323
rs61945276
rs76153918
rs709589
All
0.00
0.05
0.10
MR leave−one−out sensitivity analysis for
' CD25 on transitional' on 'Rheumatoid arthritis'

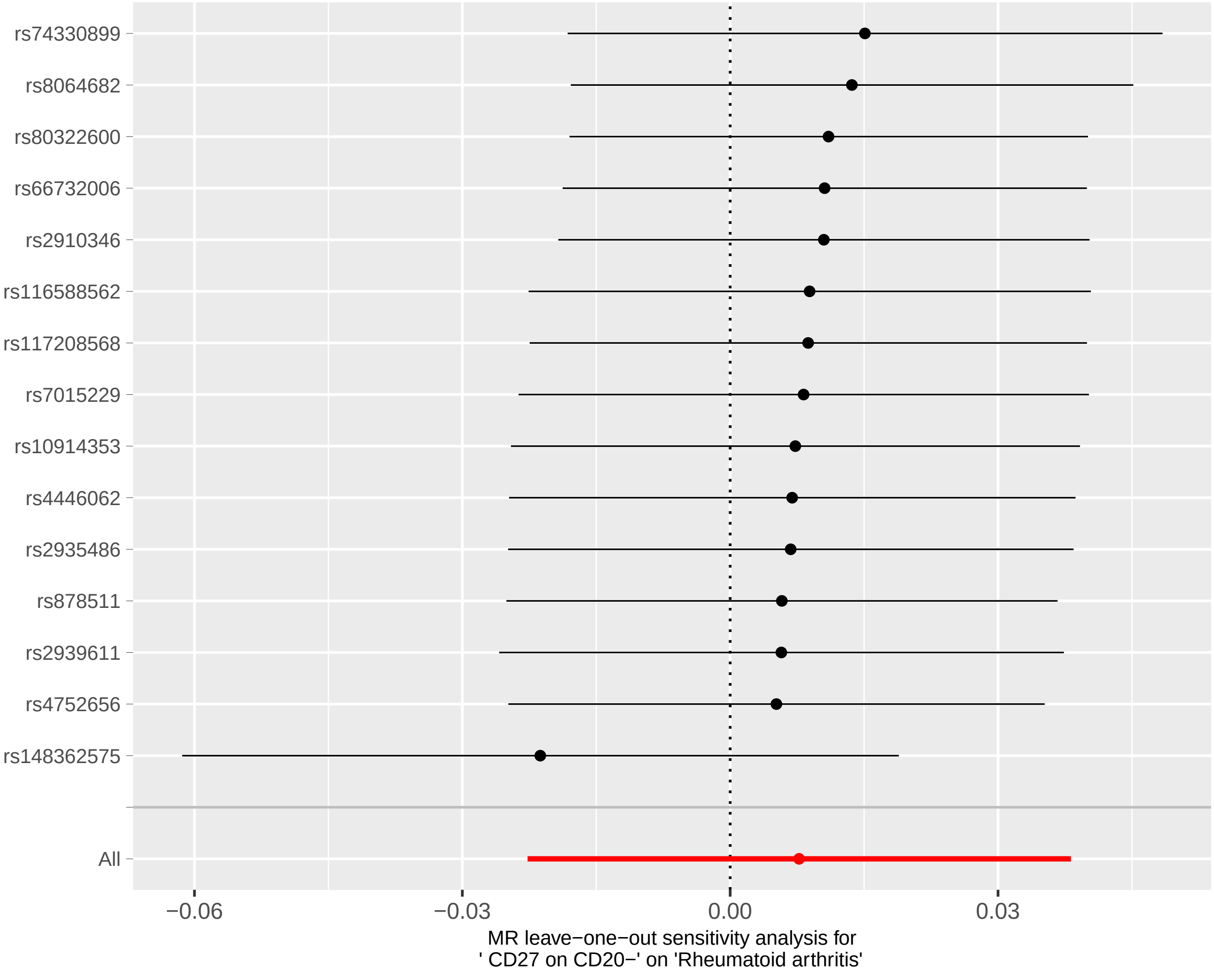
rs74330899
rs8064682
rs80322600
rs66732006
rs2910346
rs116588562
rs117208568
rs7015229
rs10914353
rs4446062
rs2935486
rs878511
rs2939611
rs4752656
rs148362575
All
−0.06
−0.03
0.00
0.03
MR leave−one−out sensitivity analysis for
' CD27 on CD20−' on 'Rheumatoid arthritis'

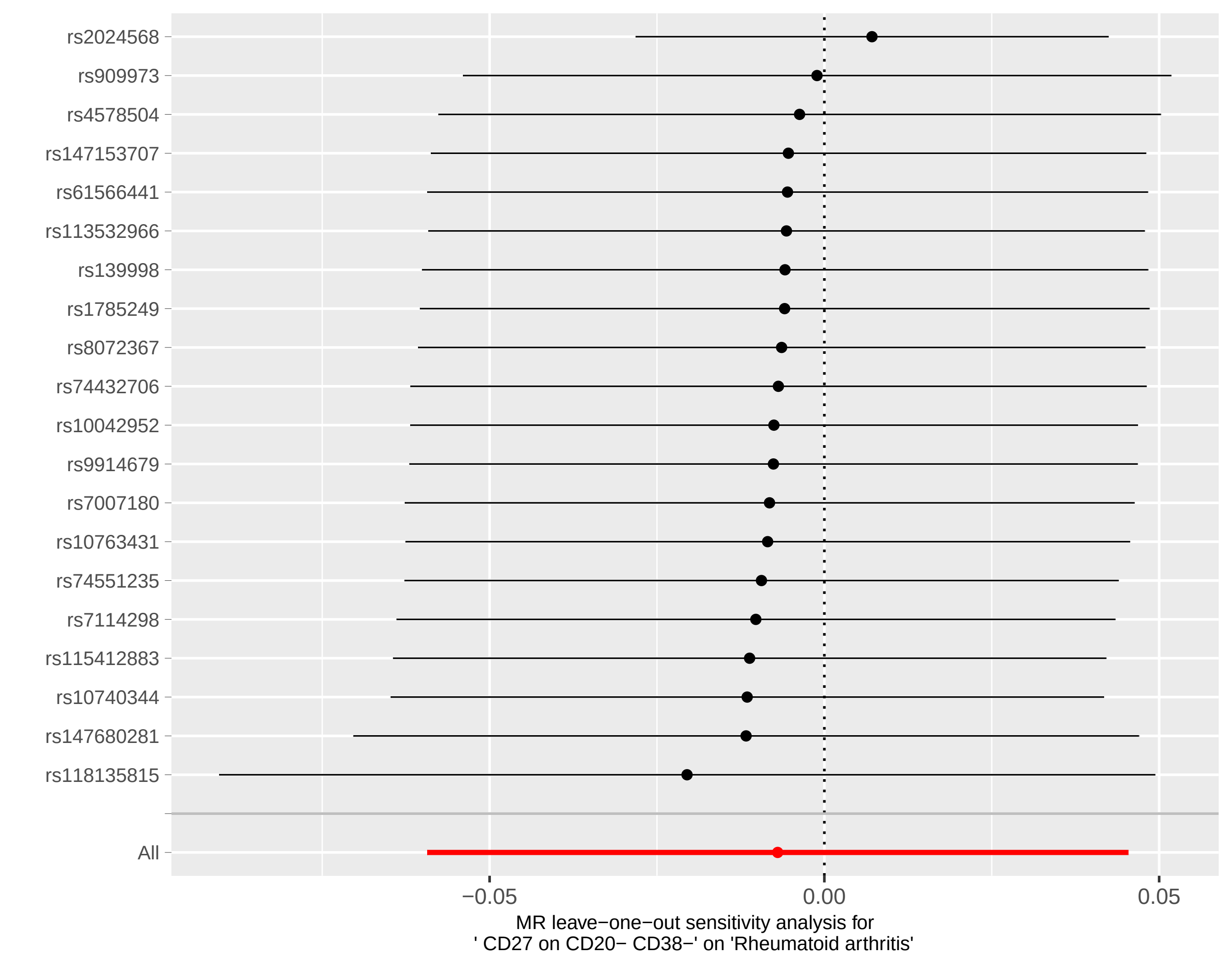
rs2024568
rs909973
rs4578504
rs147153707
rs61566441
rs113532966
rs139998
rs1785249
rs8072367
rs74432706
rs10042952
rs9914679
rs7007180
rs10763431
rs74551235
rs7114298
rs115412883
rs10740344
rs147680281
rs118135815
All
−0.05
0.00
0.05
MR leave−one−out sensitivity analysis for
' CD27 on CD20− CD38−' on 'Rheumatoid arthritis'

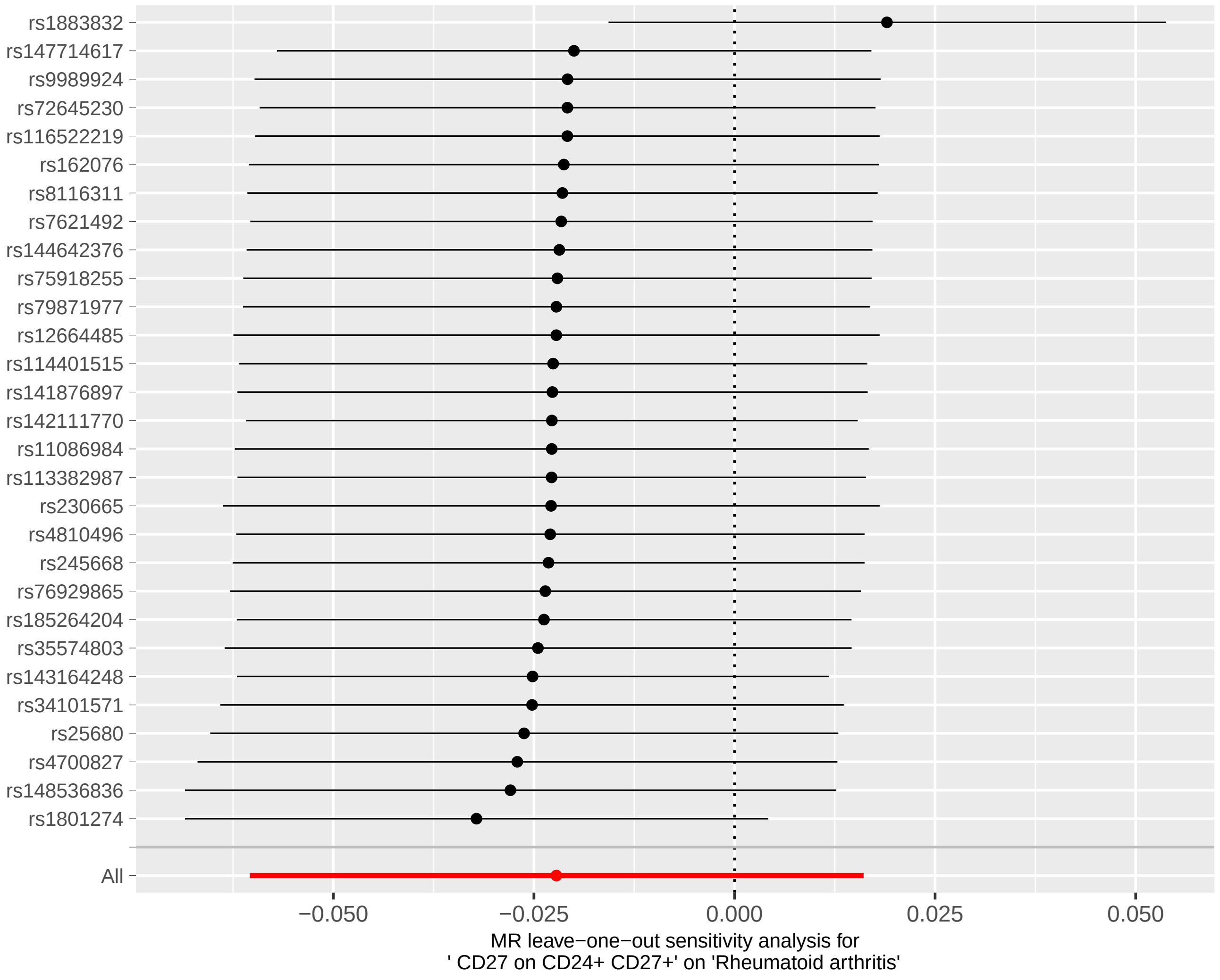

rs1883832
rs147714617
rs9989924
rs72645230
rs116522219
rs162076
rs8116311
rs7621492
rs144642376
rs75918255
rs79871977
rs12664485
rs114401515
rs141876897
rs142111770
rs11086984
rs113382987
rs230665
rs4810496
rs245668
rs76929865
rs185264204
rs35574803
rs143164248
rs34101571
rs25680
rs4700827
rs148536836
rs1801274
All
−0.050
−0.025
0.000
0.025
0.050
MR leave−one−out sensitivity analysis for
' CD27 on CD24+ CD27+' on 'Rheumatoid arthritis'

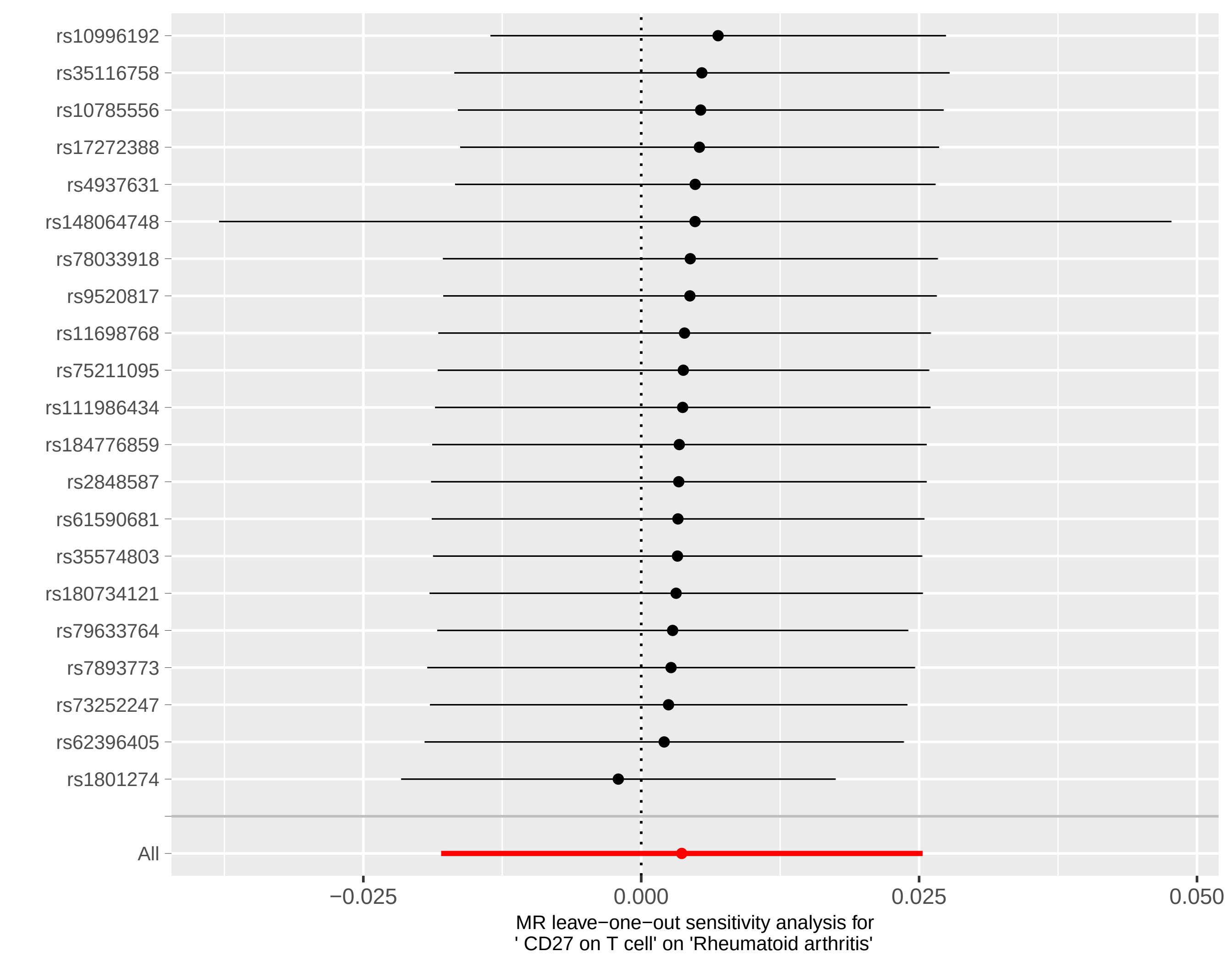
rs10996192
rs35116758
rs10785556
rs17272388
rs4937631
rs148064748
rs78033918
rs9520817
rs11698768
rs75211095
rs111986434
rs184776859
rs2848587
rs61590681
rs35574803
rs180734121
rs79633764
rs7893773
rs73252247
rs62396405
rs1801274
All
−0.025
0.000
0.025
0.050
MR leave−one−out sensitivity analysis for
' CD27 on T cell' on 'Rheumatoid arthritis'

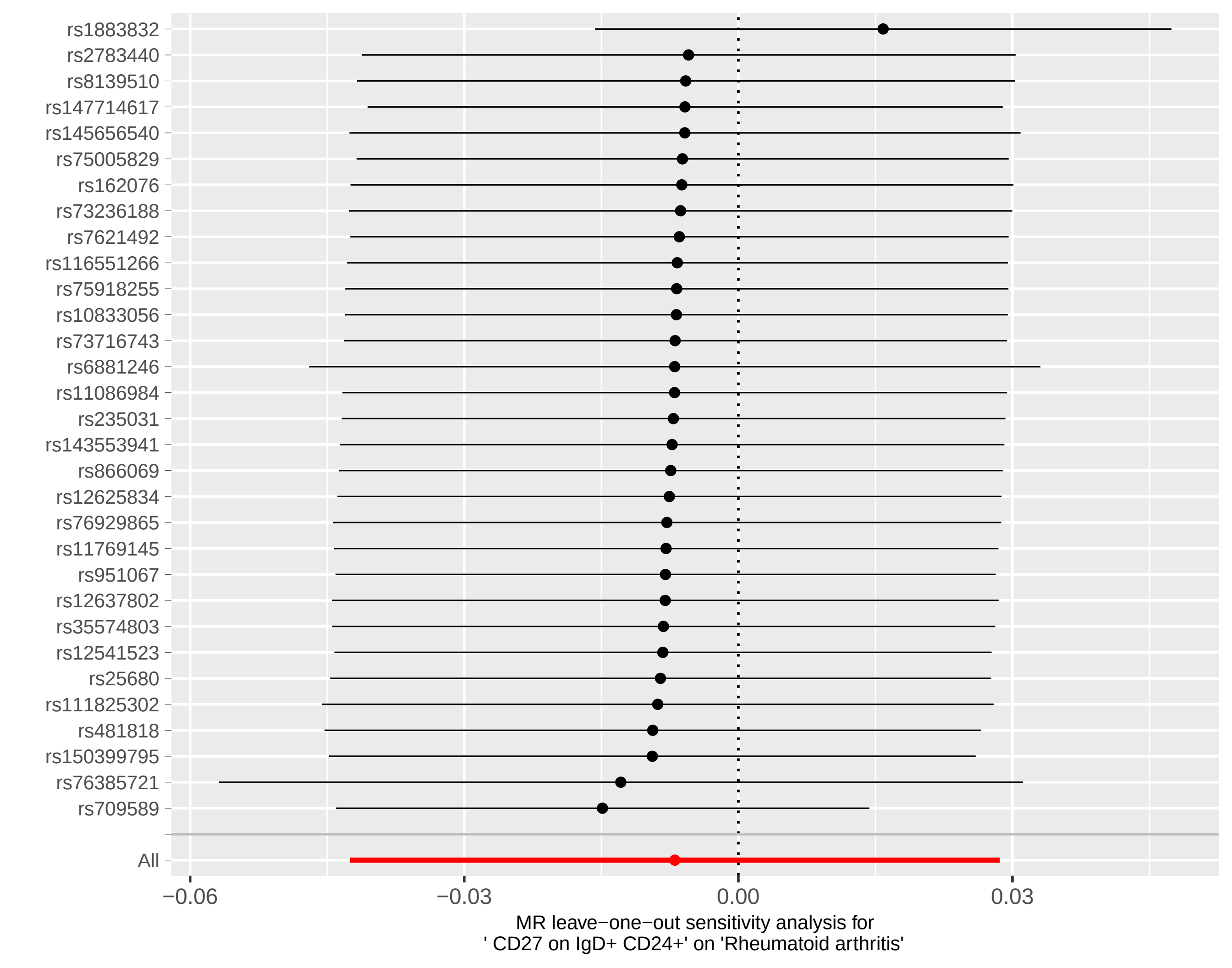
rs1883832
rs2783440
rs8139510
rs147714617
rs145656540
rs75005829
rs162076
rs73236188
rs7621492
rs116551266
rs75918255
rs10833056
rs73716743
rs6881246
rs11086984
rs235031
rs143553941
rs866069
rs12625834
rs76929865
rs11769145
rs951067
rs12637802
rs35574803
rs12541523
rs25680
rs111825302
rs481818
rs150399795
rs76385721
rs709589
All
−0.06
−0.03
0.00
0.03
MR leave−one−out sensitivity analysis for
' CD27 on IgD+ CD24+' on 'Rheumatoid arthritis'

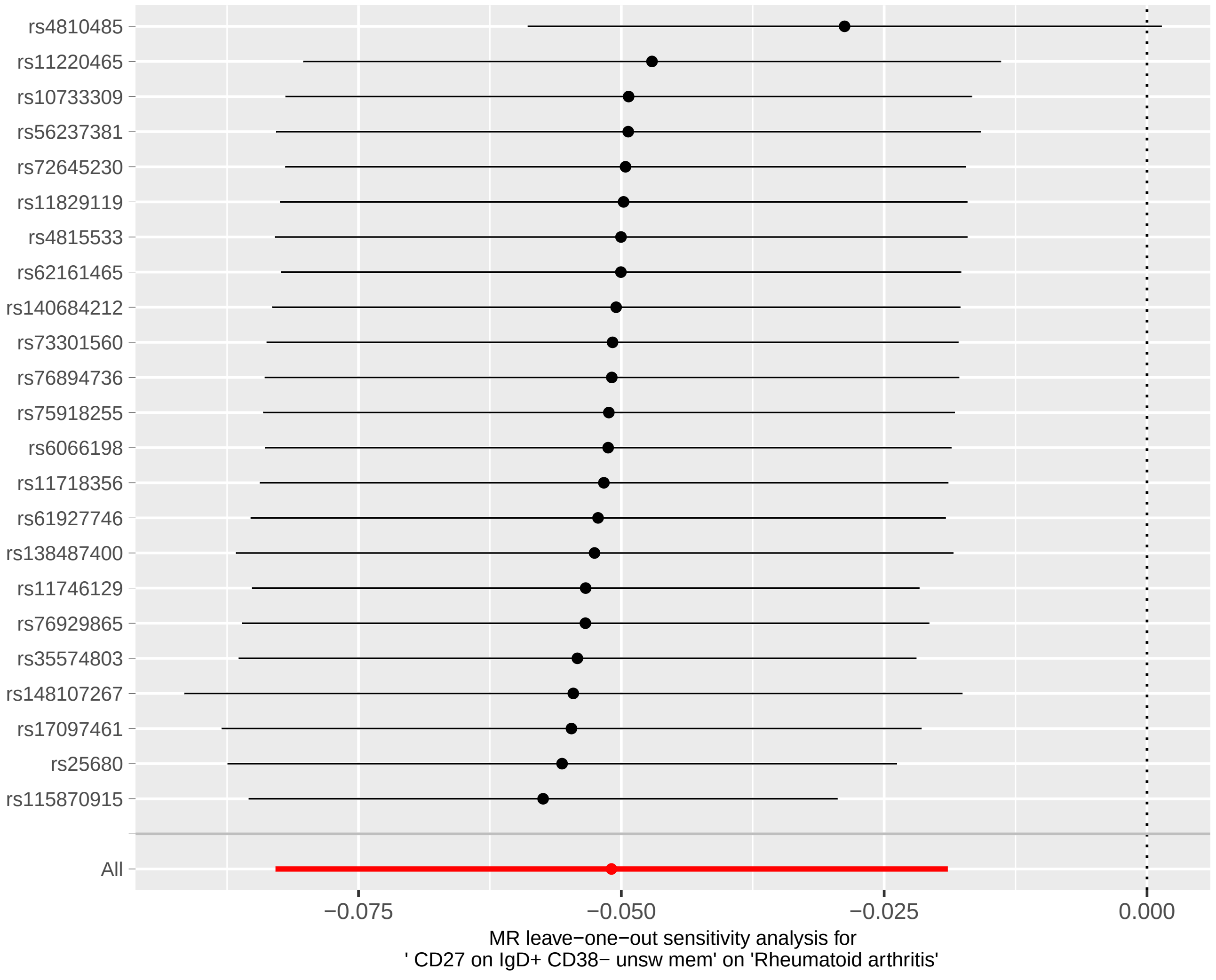
rs4810485
rs11220465
rs10733309
rs56237381
rs72645230
rs11829119
rs4815533
rs62161465
rs140684212
rs73301560
rs76894736
rs75918255
rs6066198
rs11718356
rs61927746
rs138487400
rs11746129
rs76929865
rs35574803
rs148107267
rs17097461
rs25680
rs115870915
All
−0.075
−0.050
−0.025
0.000
MR leave−one−out sensitivity analysis for
' CD27 on IgD+ CD38− unsw mem' on 'Rheumatoid arthritis'

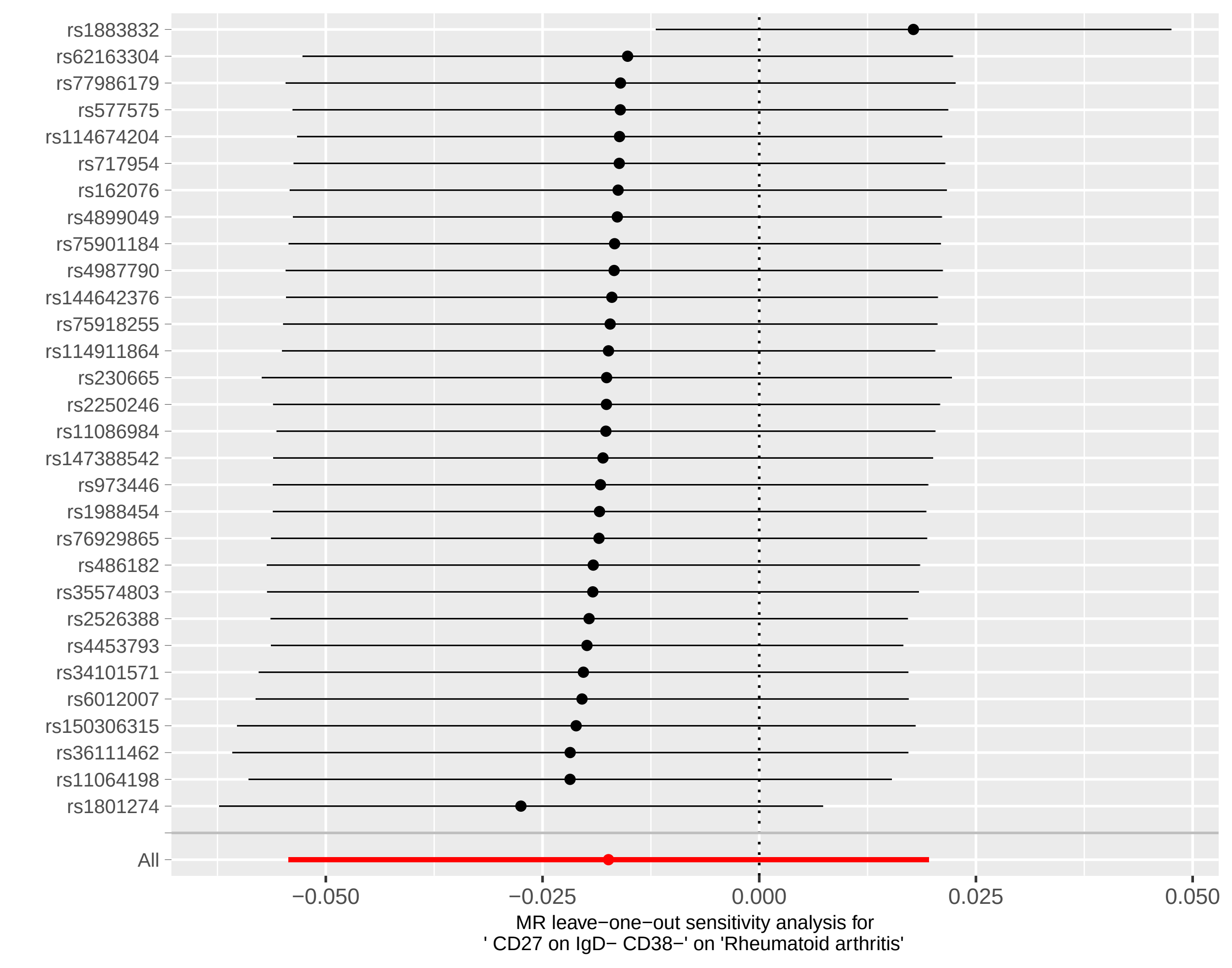
rs1883832
rs62163304
rs77986179
rs577575
rs114674204
rs717954
rs162076
rs4899049
rs75901184
rs4987790
rs144642376
rs75918255
rs114911864
rs230665
rs2250246
rs11086984
rs147388542
rs973446
rs1988454
rs76929865
rs486182
rs35574803
rs2526388
rs4453793
rs34101571
rs6012007
rs150306315
rs36111462
rs11064198
rs1801274
All
−0.050
−0.025
0.000
0.025
0.050
MR leave−one−out sensitivity analysis for
' CD27 on IgD− CD38−' on 'Rheumatoid arthritis'

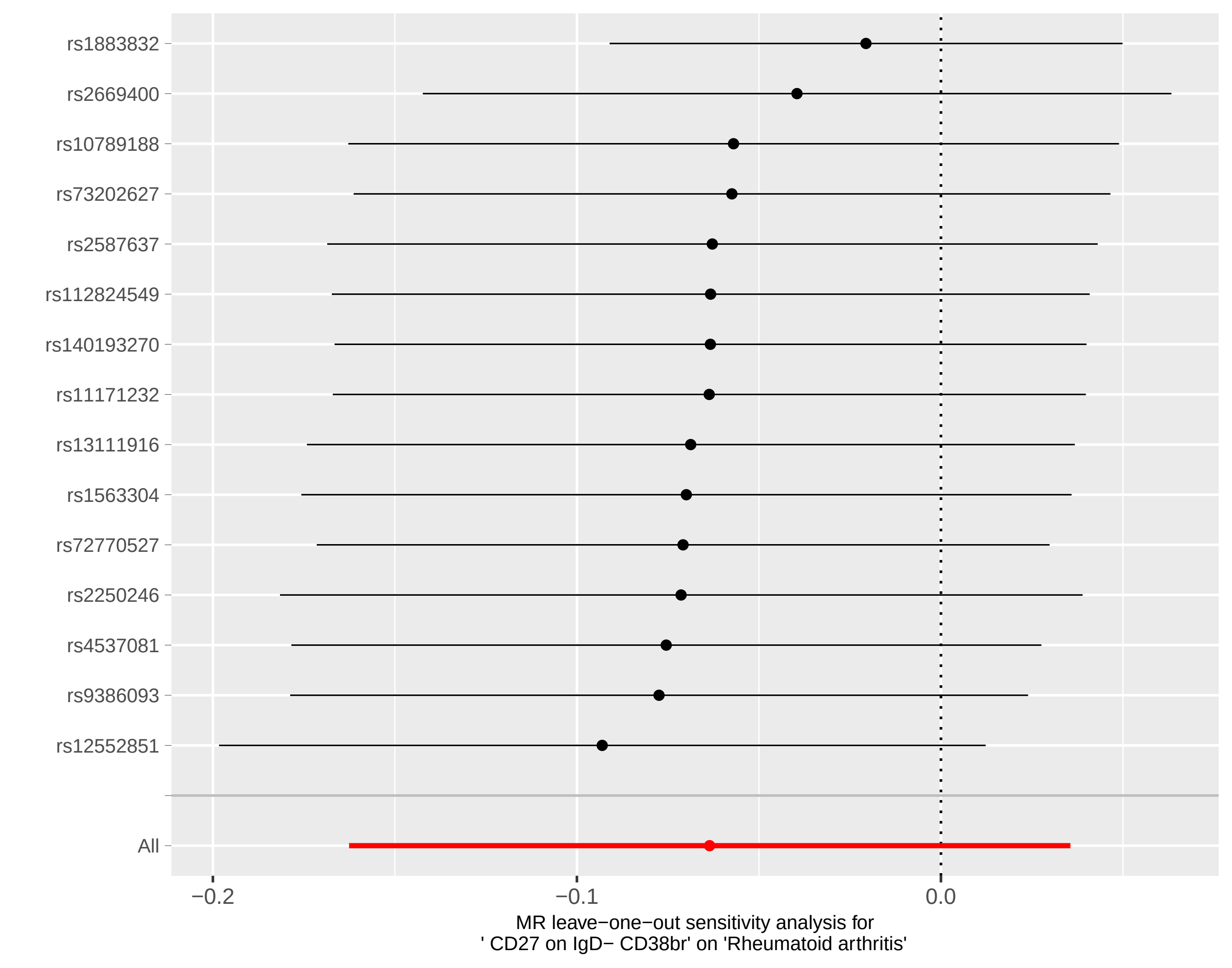
rs1883832
rs2669400
rs10789188
rs73202627
rs2587637
rs112824549
rs140193270
rs11171232
rs13111916
rs1563304
rs72770527
rs2250246
rs4537081
rs9386093
rs12552851
All
−0.2
−0.1
0.0
MR leave−one−out sensitivity analysis for
' CD27 on IgD− CD38br' on 'Rheumatoid arthritis'

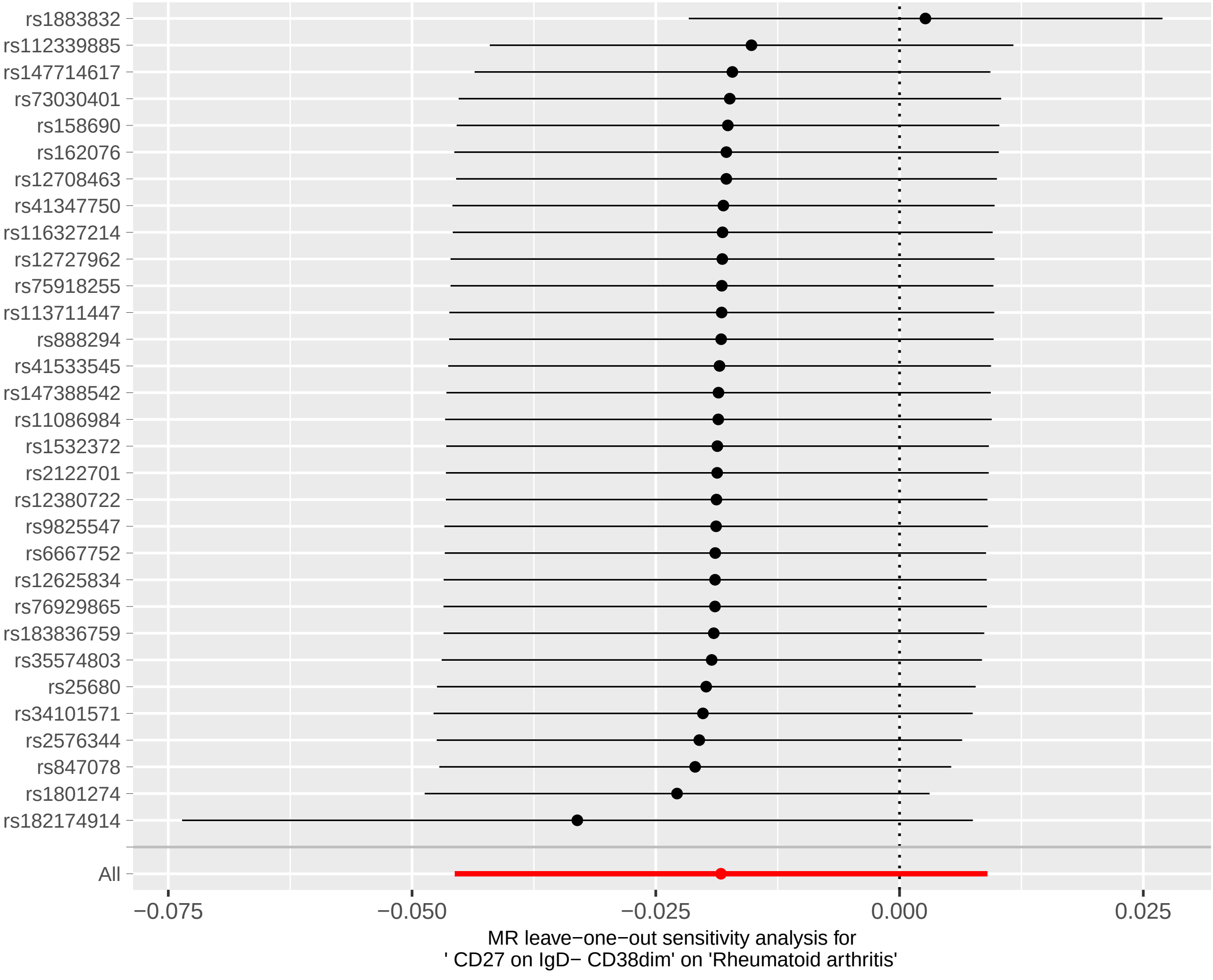

rs1883832
rs112339885
rs147714617
rs73030401
rs158690
rs162076
rs12708463
rs41347750
rs116327214
rs12727962
rs75918255
rs113711447
rs888294
rs41533545
rs147388542
rs11086984
rs1532372
rs2122701
rs12380722
rs9825547
rs6667752
rs12625834
rs76929865
rs183836759
rs35574803
rs25680
rs34101571
rs2576344
rs847078
rs1801274
rs182174914
All
−0.075
−0.050
−0.025
0.000
0.025
MR leave−one−out sensitivity analysis for
' CD27 on IgD− CD38dim' on 'Rheumatoid arthritis'

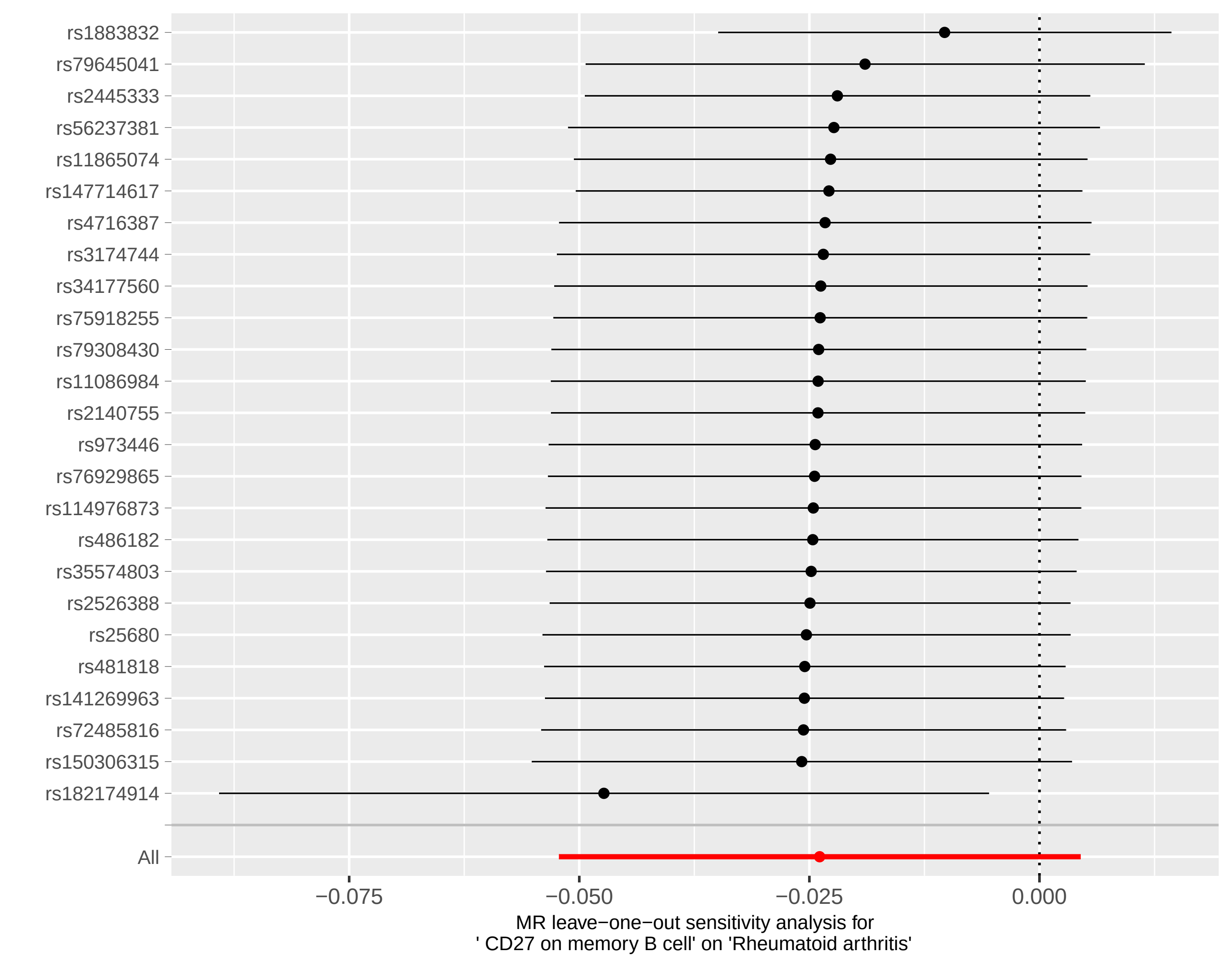
rs1883832
rs79645041
rs2445333
rs56237381
rs11865074
rs147714617
rs4716387
rs3174744
rs34177560
rs75918255
rs79308430
rs11086984
rs2140755
rs973446
rs76929865
rs114976873
rs486182
rs35574803
rs2526388
rs25680
rs481818
rs141269963
rs72485816
rs150306315
rs182174914
All
−0.075
−0.050
−0.025
0.000
MR leave−one−out sensitivity analysis for
' CD27 on memory B cell' on 'Rheumatoid arthritis'

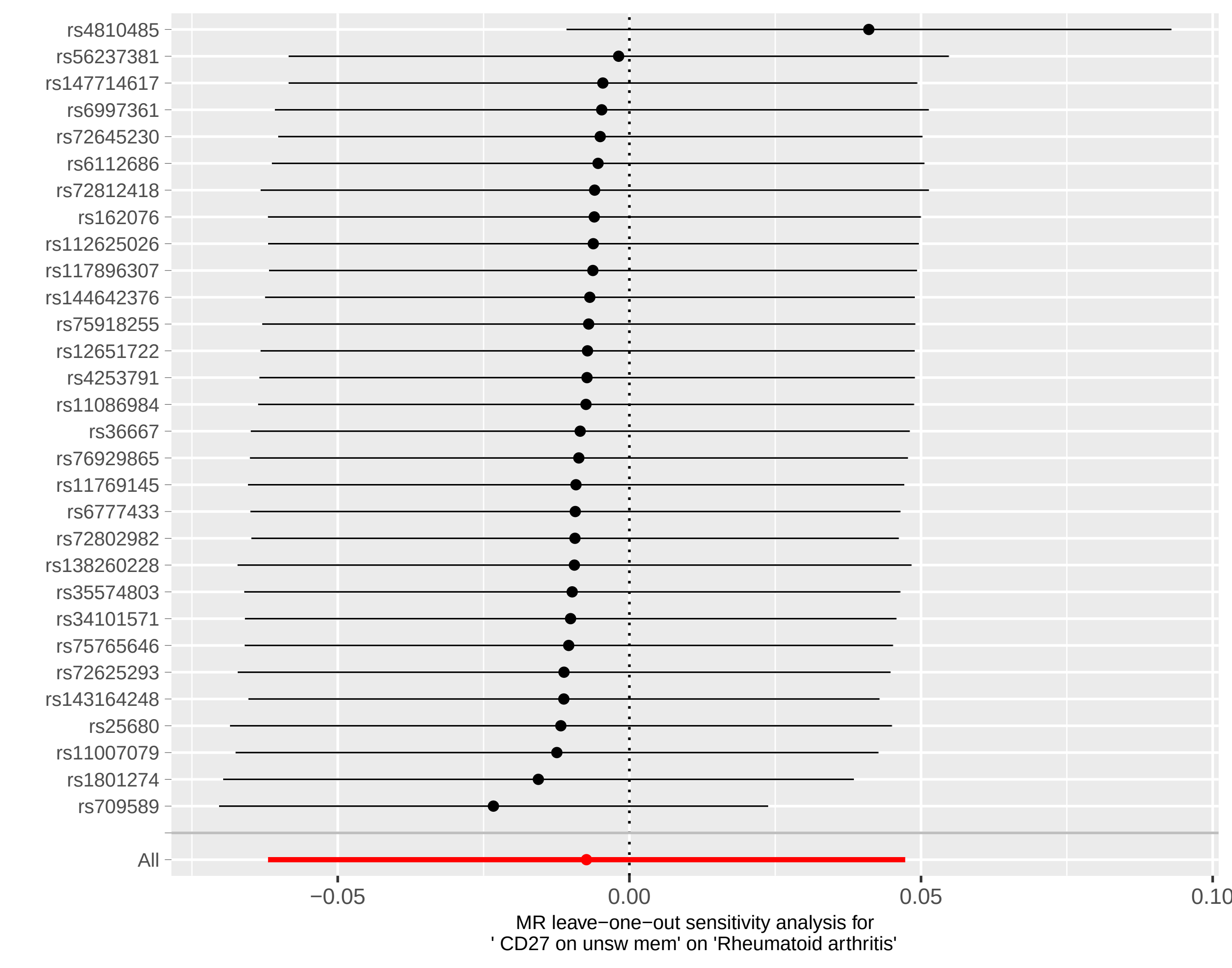
rs4810485
rs56237381
rs147714617
rs6997361
rs72645230
rs6112686
rs72812418
rs162076
rs112625026
rs117896307
rs144642376
rs75918255
rs12651722
rs4253791
rs11086984
rs36667
rs76929865
rs11769145
rs6777433
rs72802982
rs138260228
rs35574803
rs34101571
rs75765646
rs72625293
rs143164248
rs25680
rs11007079
rs1801274
rs709589
All
−0.05
0.00
0.05
0.10
MR leave−one−out sensitivity analysis for
' CD27 on unsw mem' on 'Rheumatoid arthritis'

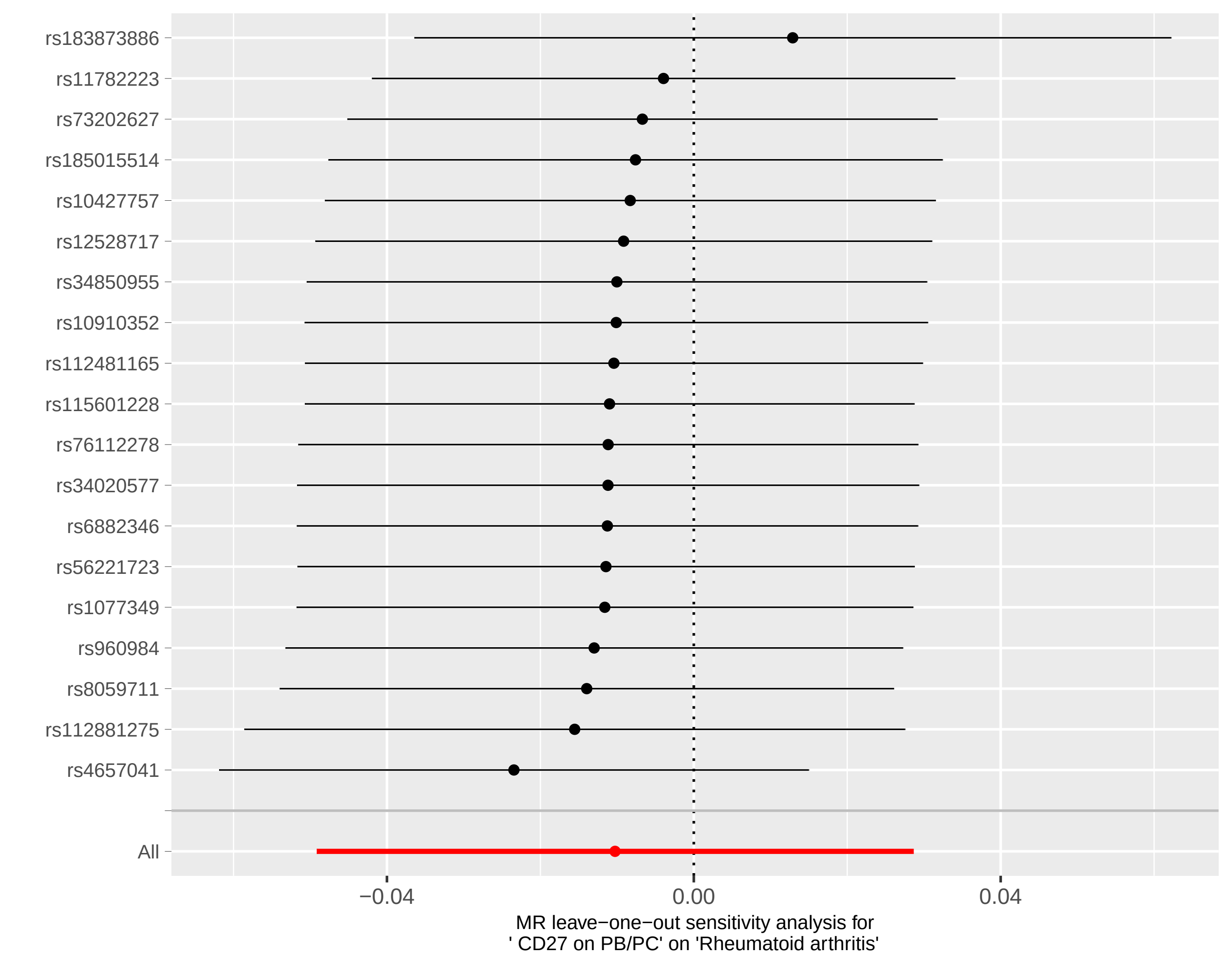
rs183873886
rs11782223
rs73202627
rs185015514
rs10427757
rs12528717
rs34850955
rs10910352
rs112481165
rs115601228
rs76112278
rs34020577
rs6882346
rs56221723
rs1077349
rs960984
rs8059711
rs112881275
rs4657041
All
−0.04
0.00
0.04
MR leave−one−out sensitivity analysis for
' CD27 on PB/PC' on 'Rheumatoid arthritis'

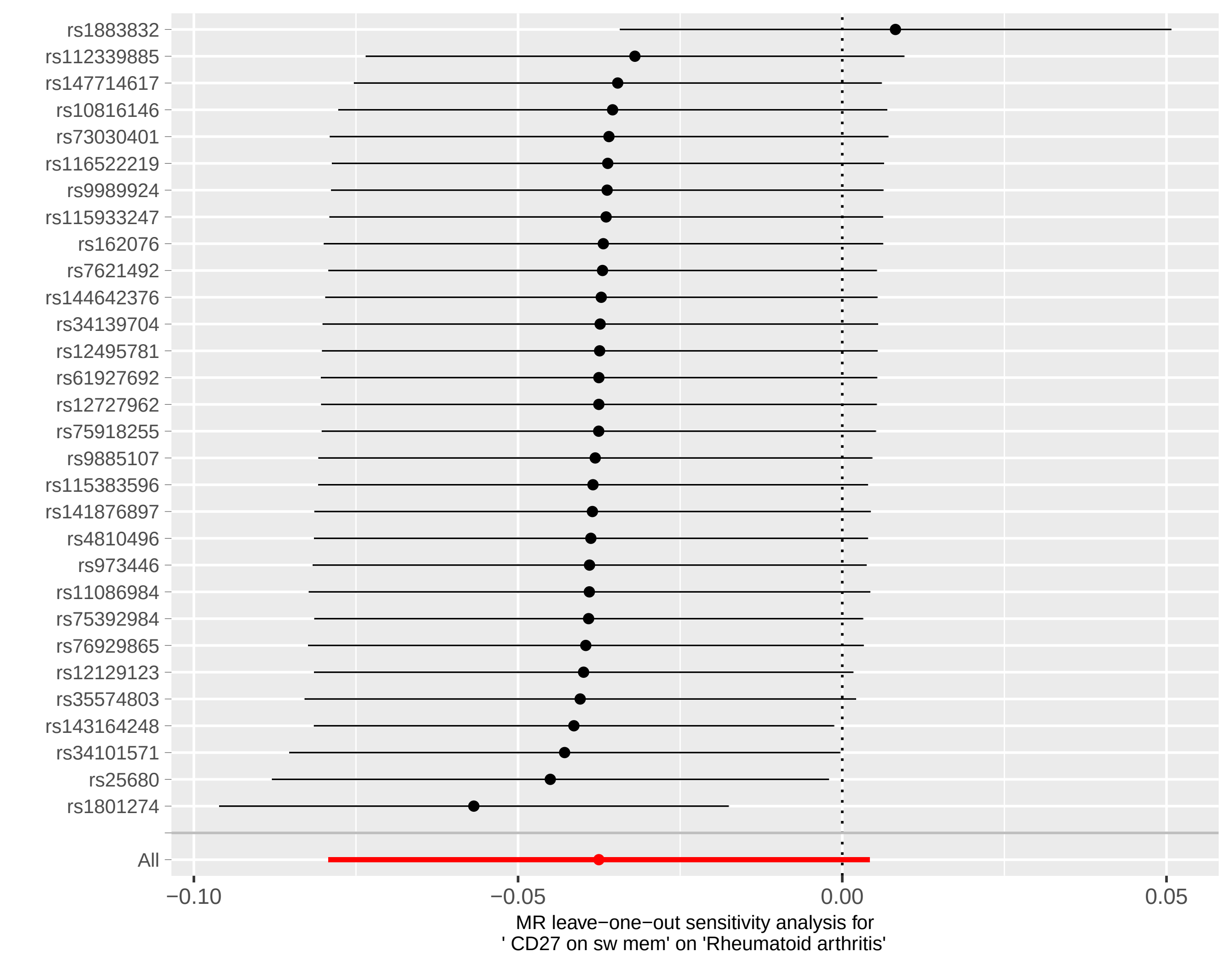
rs1883832
rs112339885
rs147714617
rs10816146
rs73030401
rs116522219
rs9989924
rs115933247
rs162076
rs7621492
rs144642376
rs34139704
rs12495781
rs61927692
rs12727962
rs75918255
rs9885107
rs115383596
rs141876897
rs4810496
rs973446
rs11086984
rs75392984
rs76929865
rs12129123
rs35574803
rs143164248
rs34101571
rs25680
rs1801274
All
−0.10
−0.05
0.00
0.05
MR leave−one−out sensitivity analysis for
' CD27 on sw mem' on 'Rheumatoid arthritis'

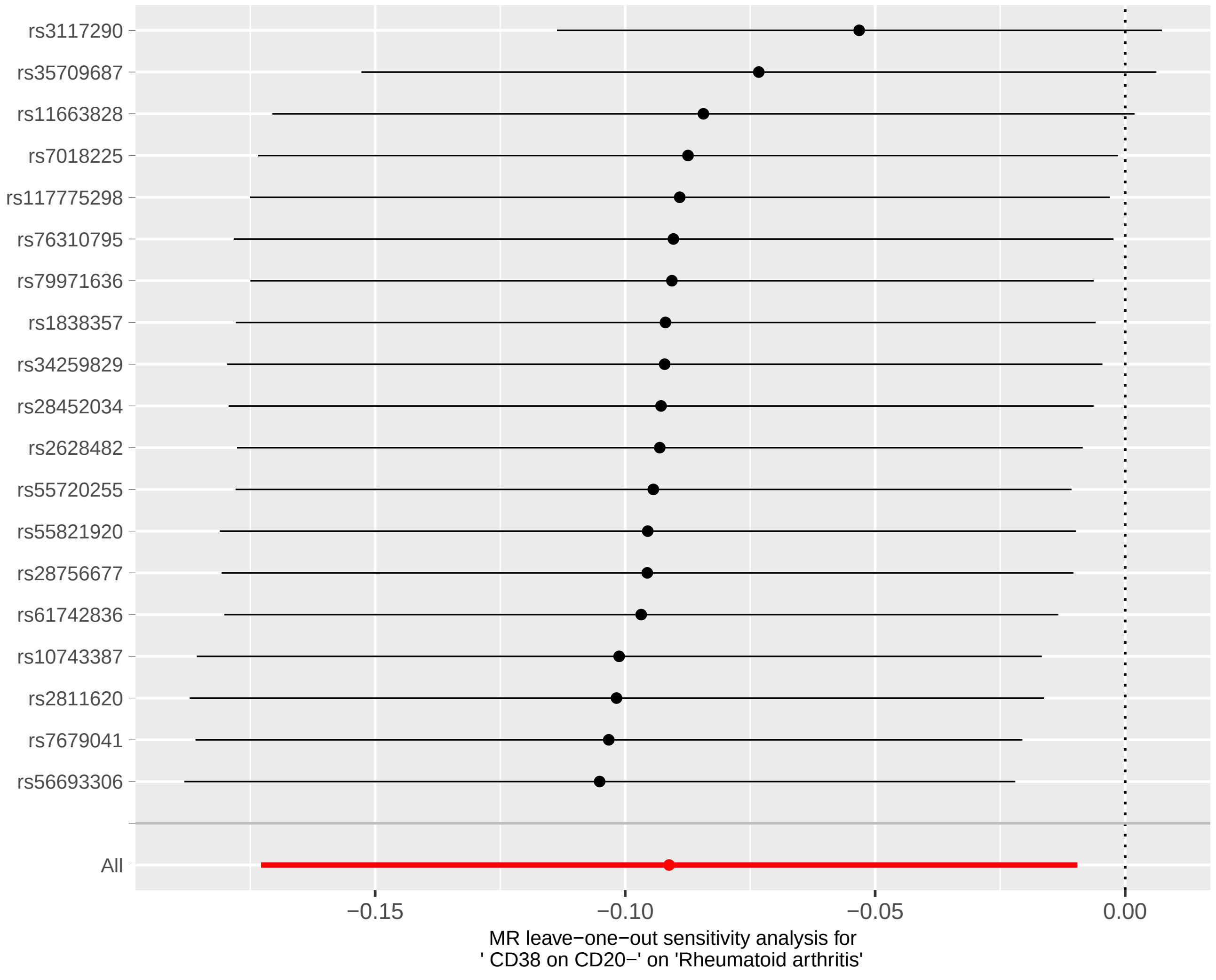
rs3117290
rs35709687
rs11663828
rs7018225
rs117775298
rs76310795
rs79971636
rs1838357
rs34259829
rs28452034
rs2628482
rs55720255
rs55821920
rs28756677
rs61742836
rs10743387
rs2811620
rs7679041
rs56693306
All
−0.15
−0.10
−0.05
0.00
MR leave−one−out sensitivity analysis for
' CD38 on CD20−' on 'Rheumatoid arthritis'

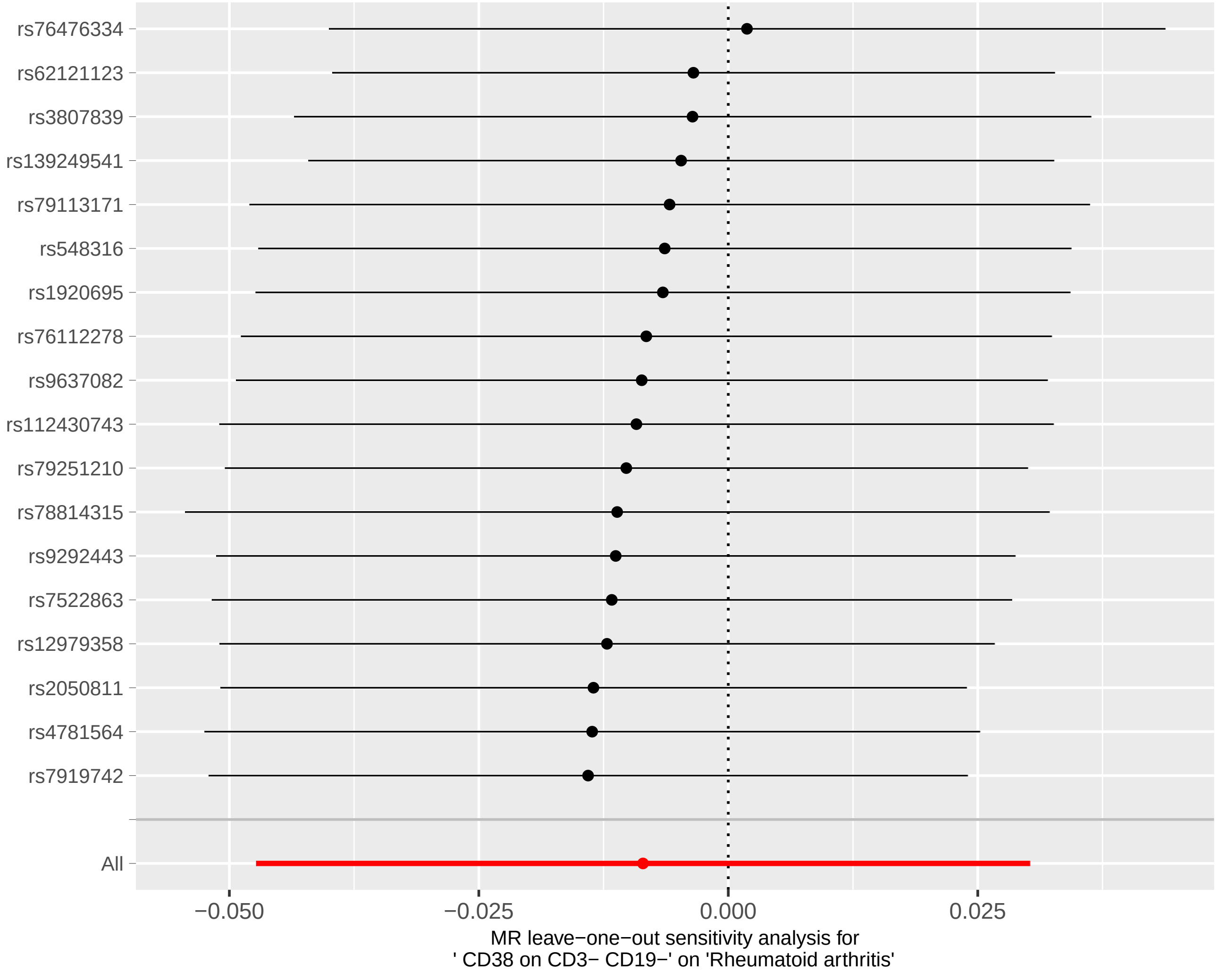
rs76476334
rs62121123
rs3807839
rs139249541
rs79113171
rs548316
rs1920695
rs76112278
rs9637082
rs112430743
rs79251210
rs78814315
rs9292443
rs7522863
rs12979358
rs2050811
rs4781564
rs7919742
All
−0.050
−0.025
0.000
0.025
MR leave−one−out sensitivity analysis for
' CD38 on CD3− CD19−' on 'Rheumatoid arthritis'

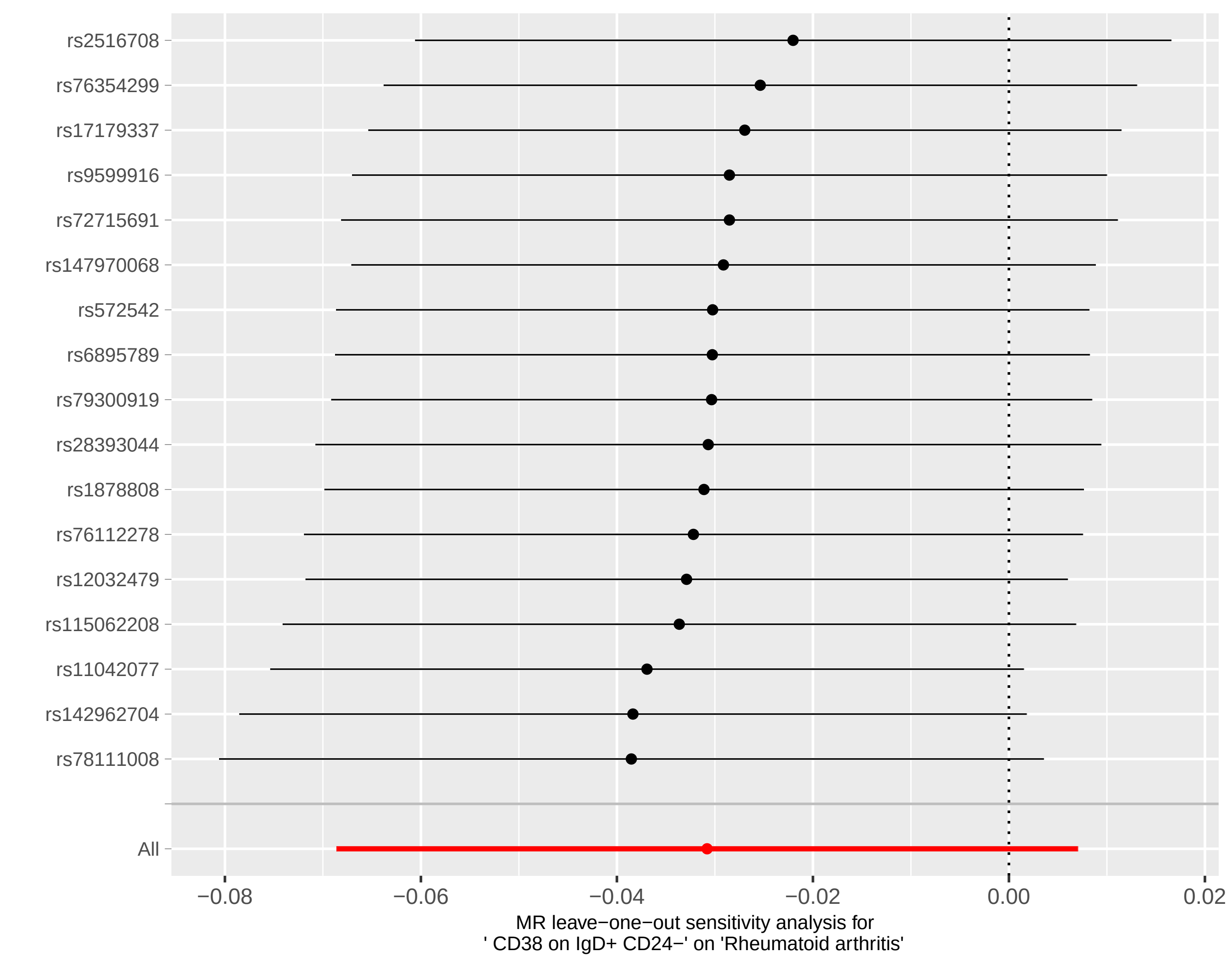
rs2516708
rs76354299
rs17179337
rs9599916
rs72715691
rs147970068
rs572542
rs6895789
rs79300919
rs28393044
rs1878808
rs76112278
rs12032479
rs115062208
rs11042077
rs142962704
rs78111008
All
−0.08
−0.06
−0.04
−0.02
0.00
0.02
MR leave−one−out sensitivity analysis for
' CD38 on IgD+ CD24−' on 'Rheumatoid arthritis'

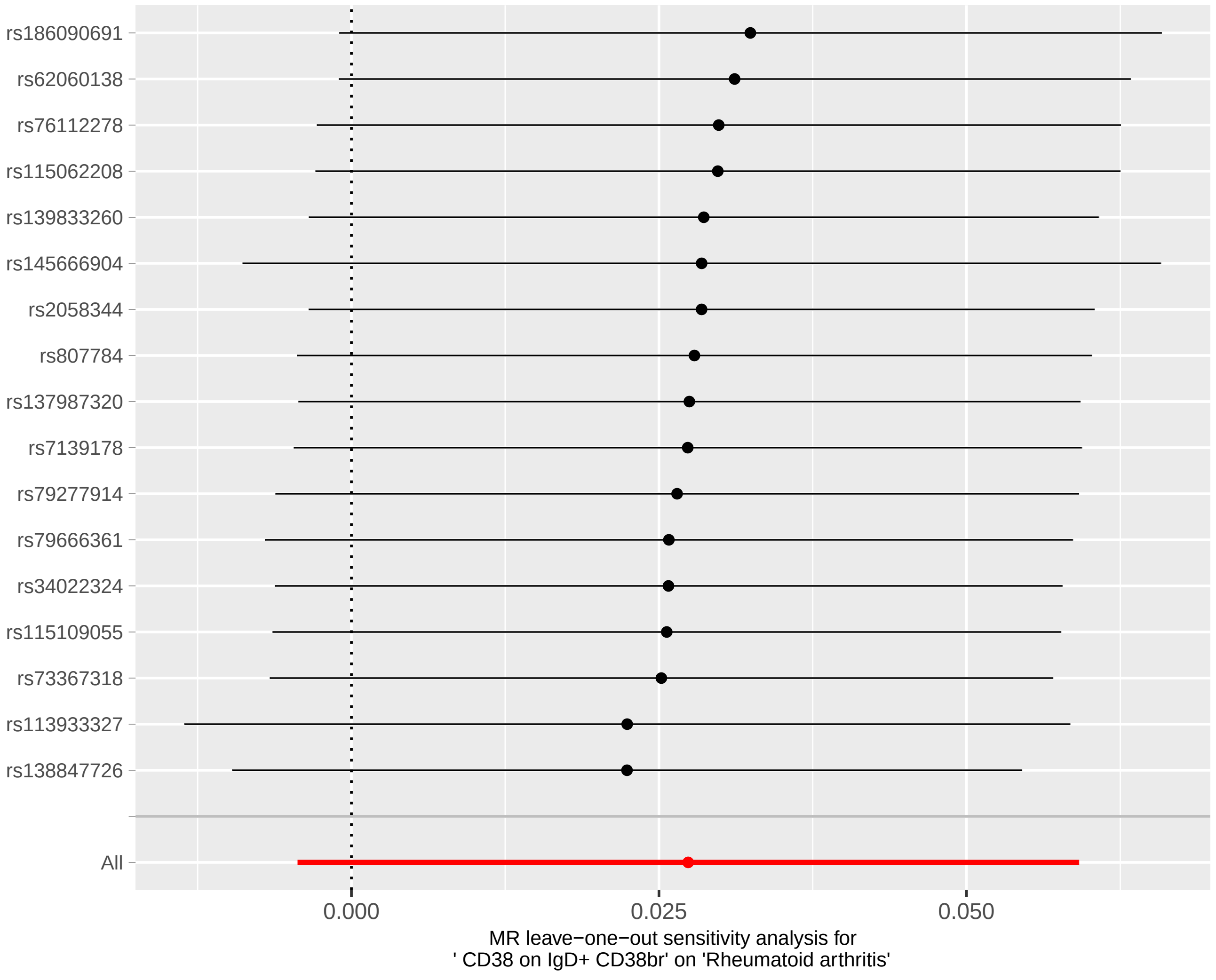
rs186090691
rs62060138
rs76112278
rs115062208
rs139833260
rs145666904
rs2058344
rs807784
rs137987320
rs7139178
rs79277914
rs79666361
rs34022324
rs115109055
rs73367318
rs113933327
rs138847726
All
0.000
0.025
0.050
MR leave−one−out sensitivity analysis for
' CD38 on IgD+ CD38br' on 'Rheumatoid arthritis'

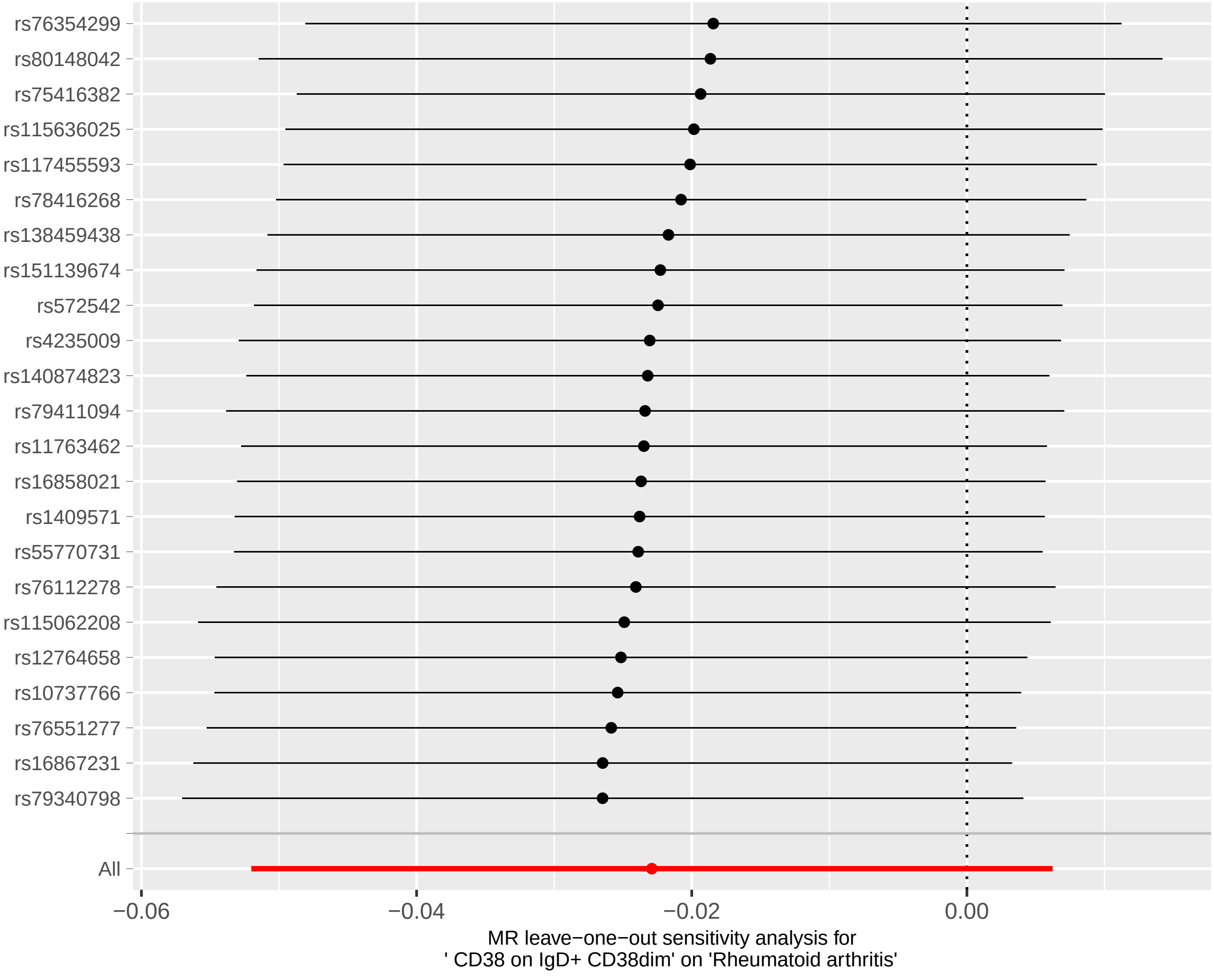
rs76354299
rs80148042
rs75416382
rs115636025
rs117455593
rs78416268
rs138459438
rs151139674
rs572542
rs4235009
rs140874823
rs79411094
rs11763462
rs16858021
rs1409571
rs55770731
rs76112278
rs115062208
rs12764658
rs10737766
rs76551277
rs16867231
rs79340798
All
−0.06
−0.04
−0.02
0.00
MR leave−one−out sensitivity analysis for
' CD38 on IgD+ CD38dim' on 'Rheumatoid arthritis'

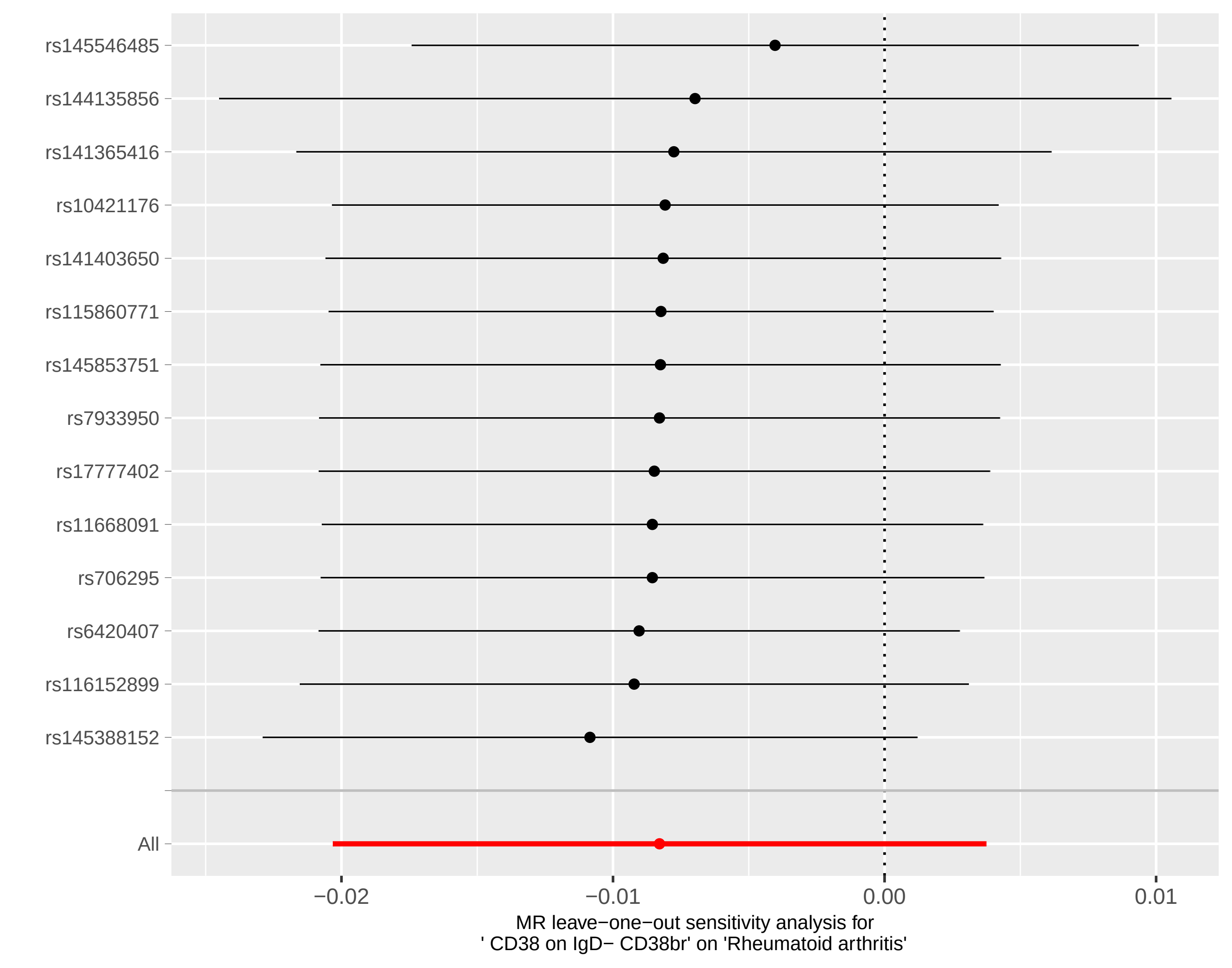

rs145546485
rs144135856
rs141365416
rs10421176
rs141403650
rs115860771
rs145853751
rs7933950
rs17777402
rs11668091
rs706295
rs6420407
rs116152899
rs145388152
All
−0.02
−0.01
0.00
0.01
MR leave−one−out sensitivity analysis for
' CD38 on IgD− CD38br' on 'Rheumatoid arthritis'

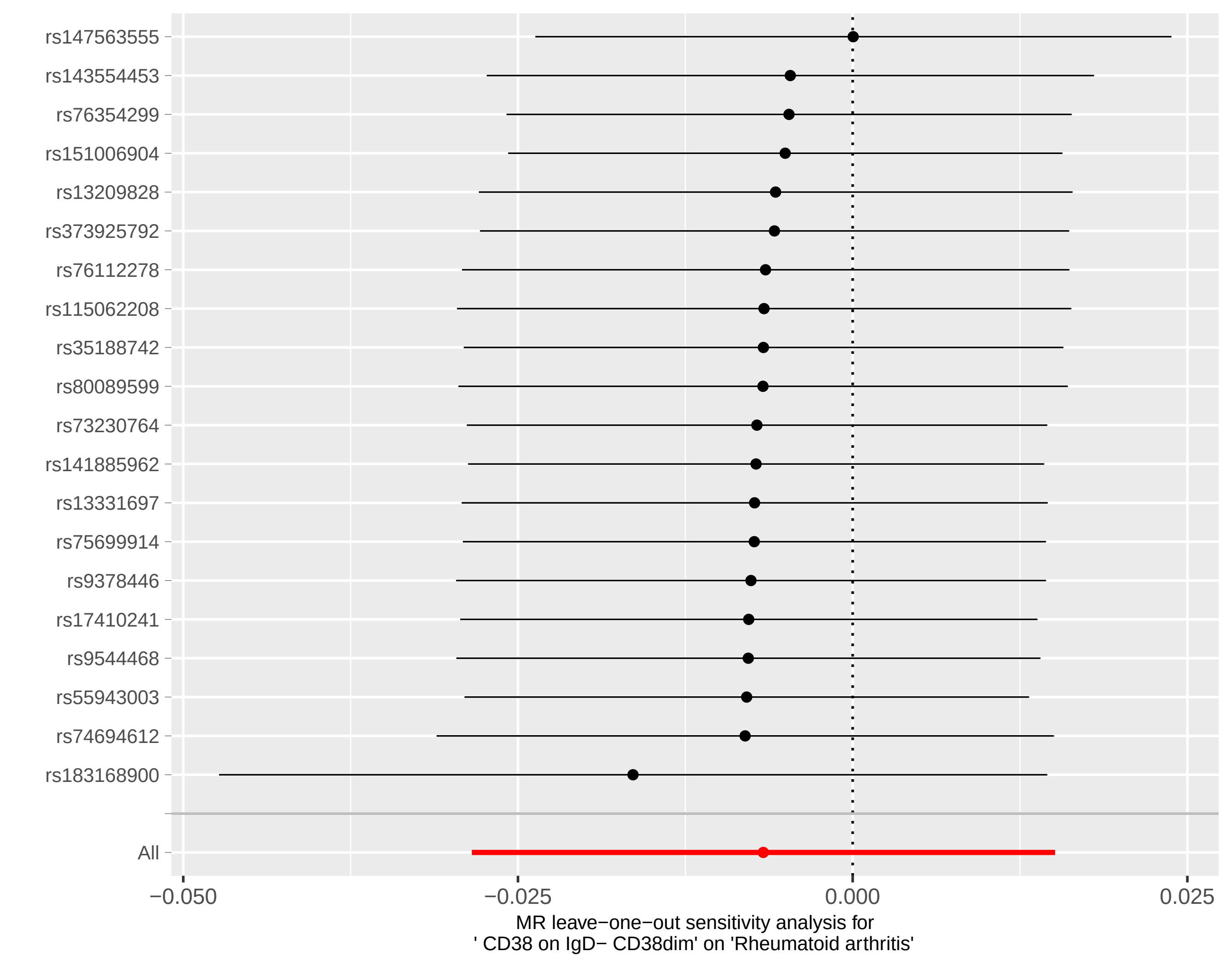
rs147563555
rs143554453
rs76354299
rs151006904
rs13209828
rs373925792
rs76112278
rs115062208
rs35188742
rs80089599
rs73230764
rs141885962
rs13331697
rs75699914
rs9378446
rs17410241
rs9544468
rs55943003
rs74694612
rs183168900
All
−0.050
−0.025
0.000
0.025
MR leave−one−out sensitivity analysis for
' CD38 on IgD− CD38dim' on 'Rheumatoid arthritis'

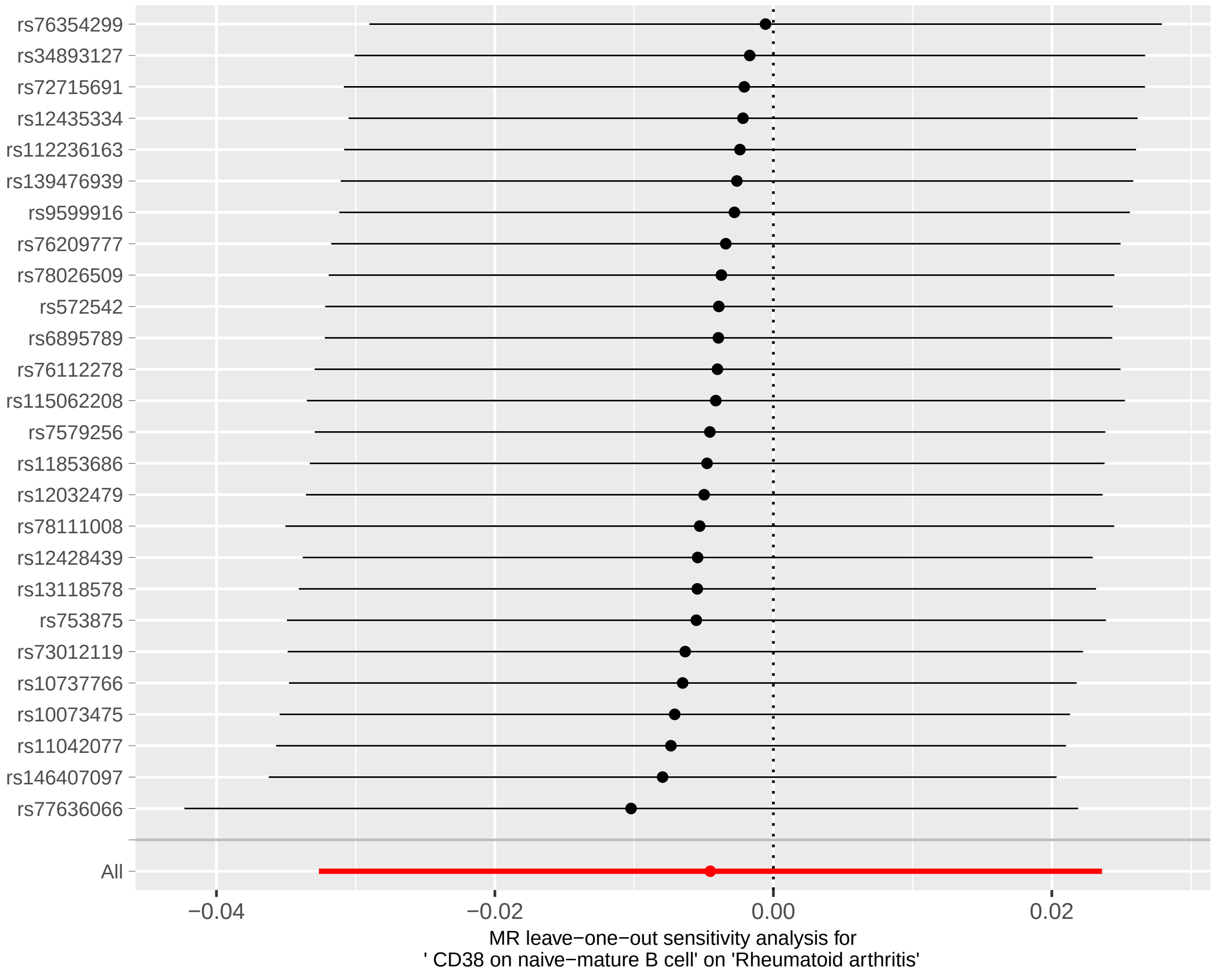
rs76354299
rs34893127
rs72715691
rs12435334
rs112236163
rs139476939
rs9599916
rs76209777
rs78026509
rs572542
rs6895789
rs76112278
rs115062208
rs7579256
rs11853686
rs12032479
rs78111008
rs12428439
rs13118578
rs753875
rs73012119
rs10737766
rs10073475
rs11042077
rs146407097
rs77636066
All
−0.04
−0.02
0.00
0.02
MR leave−one−out sensitivity analysis for
' CD38 on naive−mature B cell' on 'Rheumatoid arthritis'

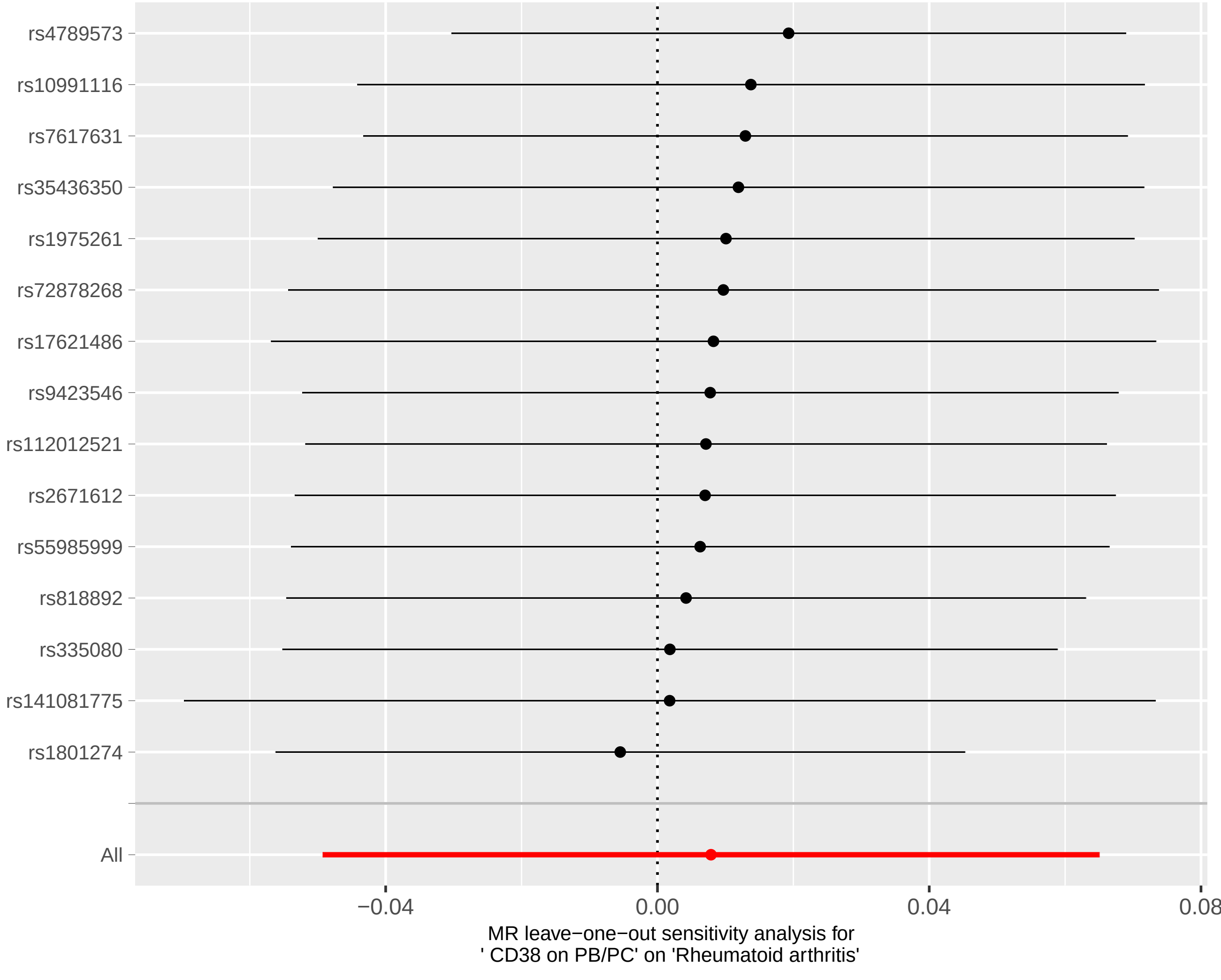
rs4789573
rs10991116
rs7617631
rs35436350
rs1975261
rs72878268
rs17621486
rs9423546
rs112012521
rs2671612
rs55985999
rs818892
rs335080
rs141081775
rs1801274
All
−0.04
0.00
0.04
0.08
MR leave−one−out sensitivity analysis for
' CD38 on PB/PC' on 'Rheumatoid arthritis'

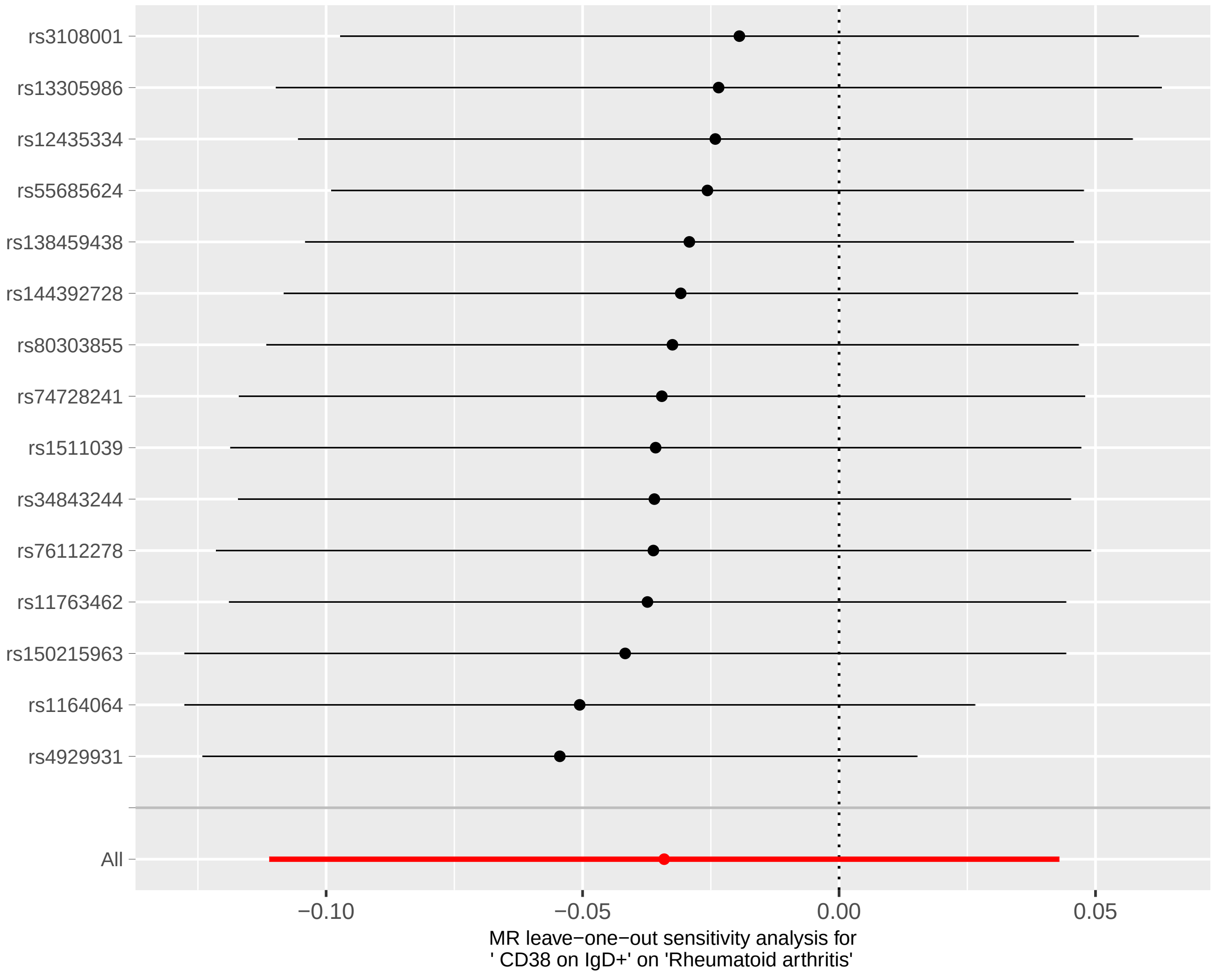
rs3108001
rs13305986
rs12435334
rs55685624
rs138459438
rs144392728
rs80303855
rs74728241
rs1511039
rs34843244
rs76112278
rs11763462
rs150215963
rs1164064
rs4929931
All
−0.10
−0.05
0.00
0.05
MR leave−one−out sensitivity analysis for
' CD38 on IgD+' on 'Rheumatoid arthritis'

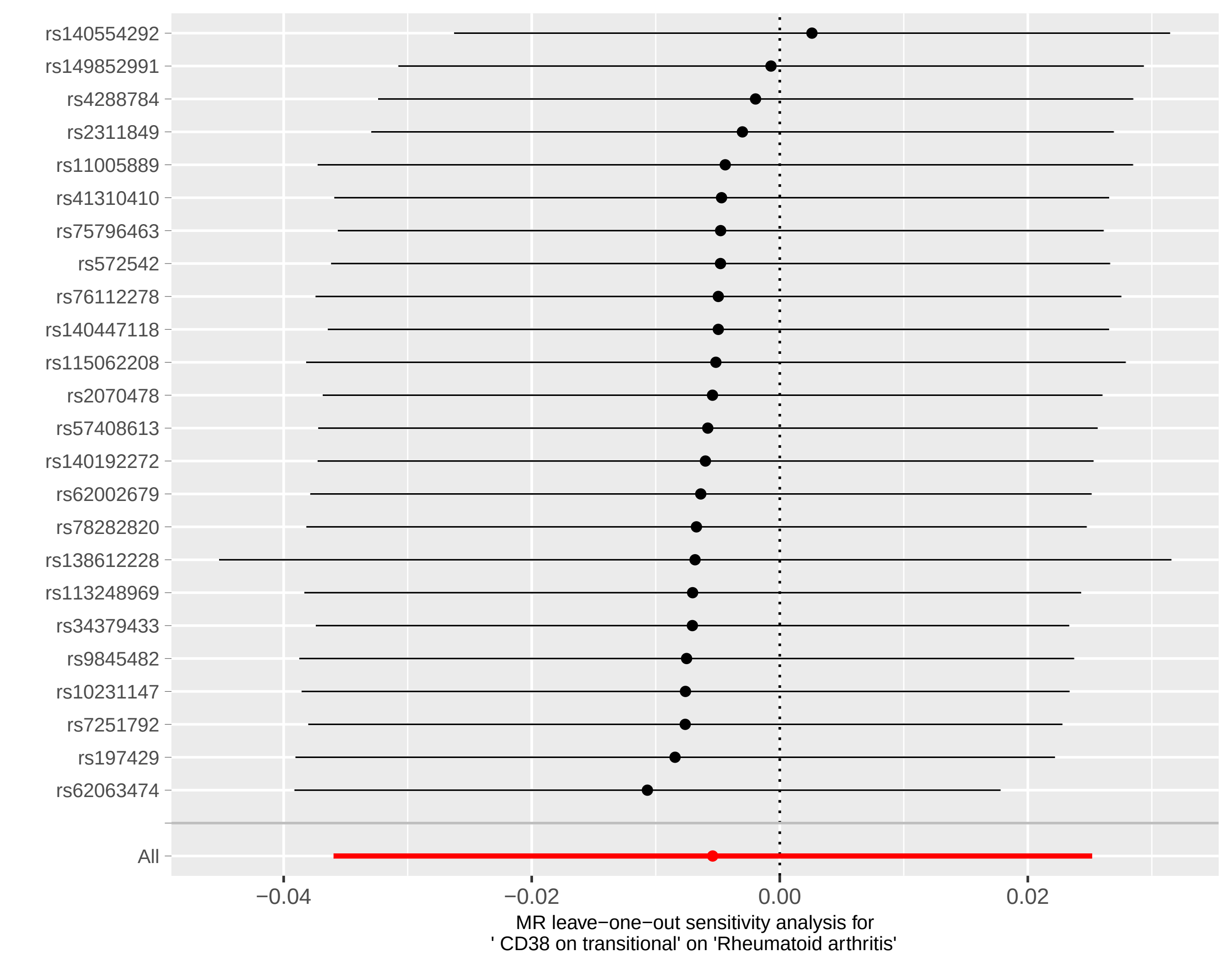
rs140554292
rs149852991
rs4288784
rs2311849
rs11005889
rs41310410
rs75796463
rs572542
rs76112278
rs140447118
rs115062208
rs2070478
rs57408613
rs140192272
rs62002679
rs78282820
rs138612228
rs113248969
rs34379433
rs9845482
rs10231147
rs7251792
rs197429
rs62063474
All
−0.04
−0.02
0.00
0.02
MR leave−one−out sensitivity analysis for
' CD38 on transitional' on 'Rheumatoid arthritis'

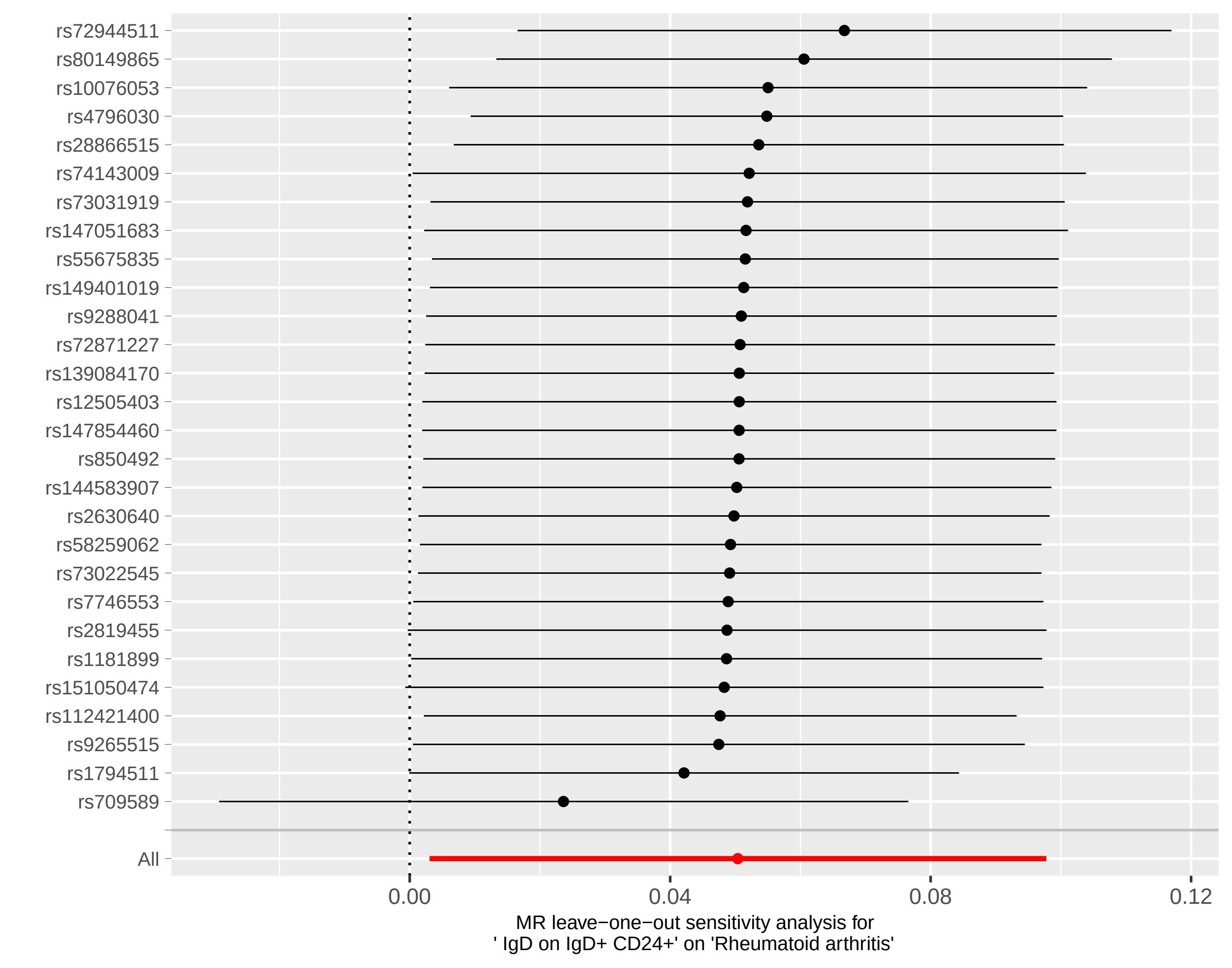
rs72944511
rs80149865
rs10076053
rs4796030
rs28866515
rs74143009
rs73031919
rs147051683
rs55675835
rs149401019
rs9288041
rs72871227
rs139084170
rs12505403
rs147854460
rs850492
rs144583907
rs2630640
rs58259062
rs73022545
rs7746553
rs2819455
rs1181899
rs151050474
rs112421400
rs9265515
rs1794511
rs709589
All
0.00
0.04
0.08
0.12
MR leave−one−out sensitivity analysis for
' IgD on IgD+ CD24+' on 'Rheumatoid arthritis'

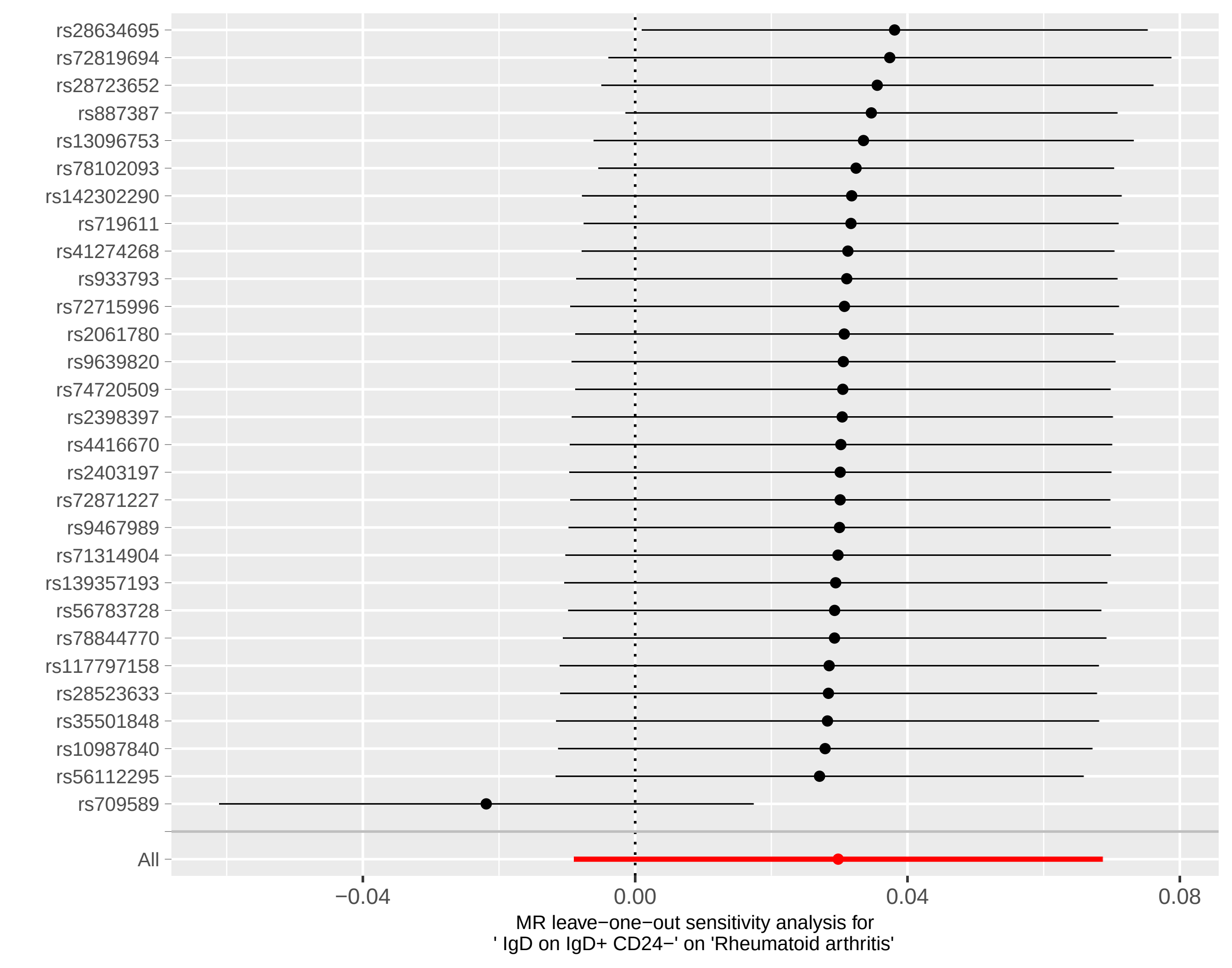
rs28634695
rs72819694
rs28723652
rs887387
rs13096753
rs78102093
rs142302290
rs719611
rs41274268
rs933793
rs72715996
rs2061780
rs9639820
rs74720509
rs2398397
rs4416670
rs2403197
rs72871227
rs9467989
rs71314904
rs139357193
rs56783728
rs78844770
rs117797158
rs28523633
rs35501848
rs10987840
rs56112295
rs709589
All
−0.04
0.00
0.04
0.08
MR leave−one−out sensitivity analysis for
' IgD on IgD+ CD24−' on 'Rheumatoid arthritis'

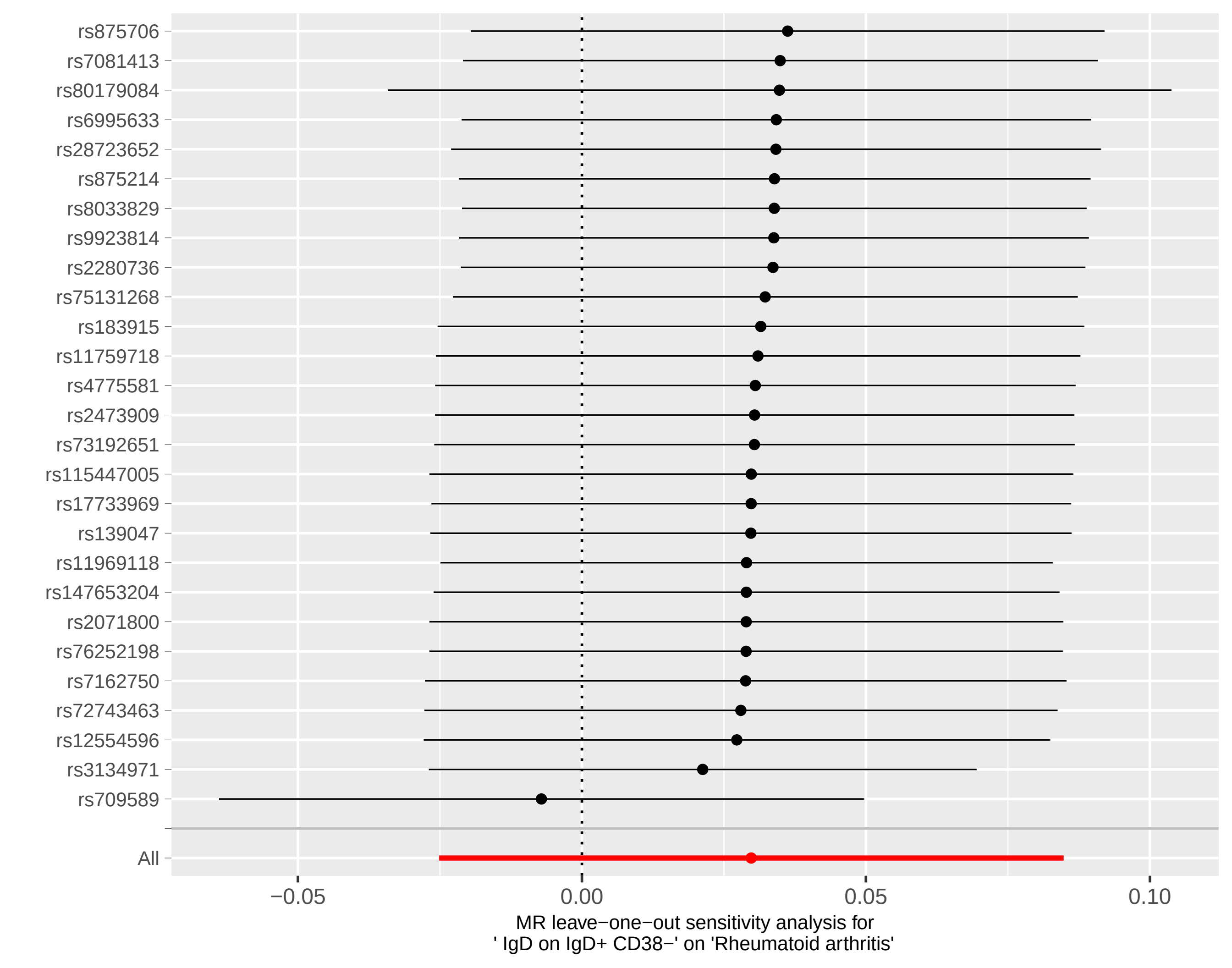
rs875706
rs7081413
rs80179084
rs6995633
rs28723652
rs875214
rs8033829
rs9923814
rs2280736
rs75131268
rs183915
rs11759718
rs4775581
rs2473909
rs73192651
rs115447005
rs17733969
rs139047
rs11969118
rs147653204
rs2071800
rs76252198
rs7162750
rs72743463
rs12554596
rs3134971
rs709589
All
−0.05
0.00
0.05
0.10
MR leave−one−out sensitivity analysis for
' IgD on IgD+ CD38−' on 'Rheumatoid arthritis'

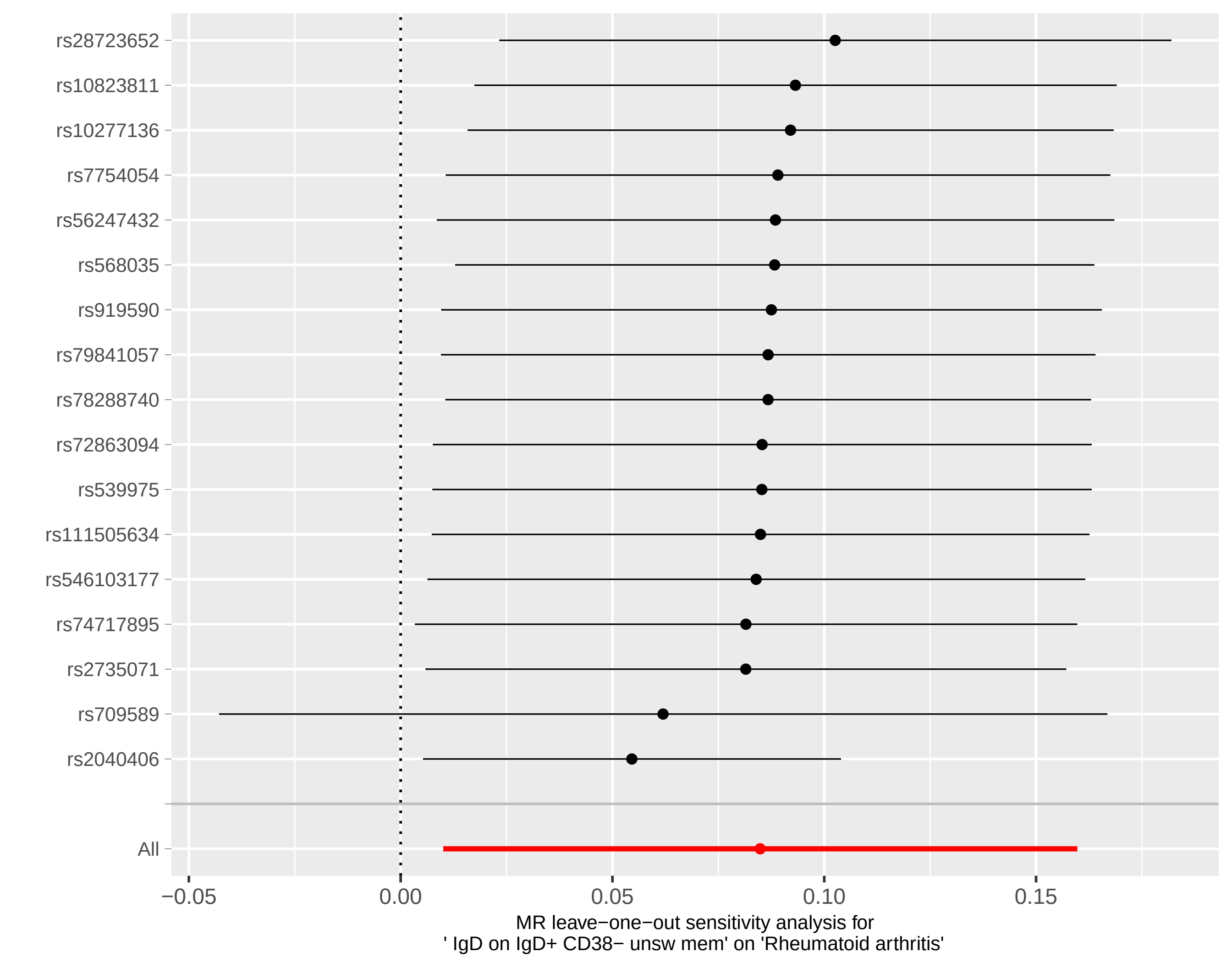

rs28723652
rs10823811
rs10277136
rs7754054
rs56247432
rs568035
rs919590
rs79841057
rs78288740
rs72863094
rs539975
rs111505634
rs546103177
rs74717895
rs2735071
rs709589
rs2040406
All
−0.05
0.00
0.05
0.10
0.15
MR leave−one−out sensitivity analysis for
' IgD on IgD+ CD38− unsw mem' on 'Rheumatoid arthritis'

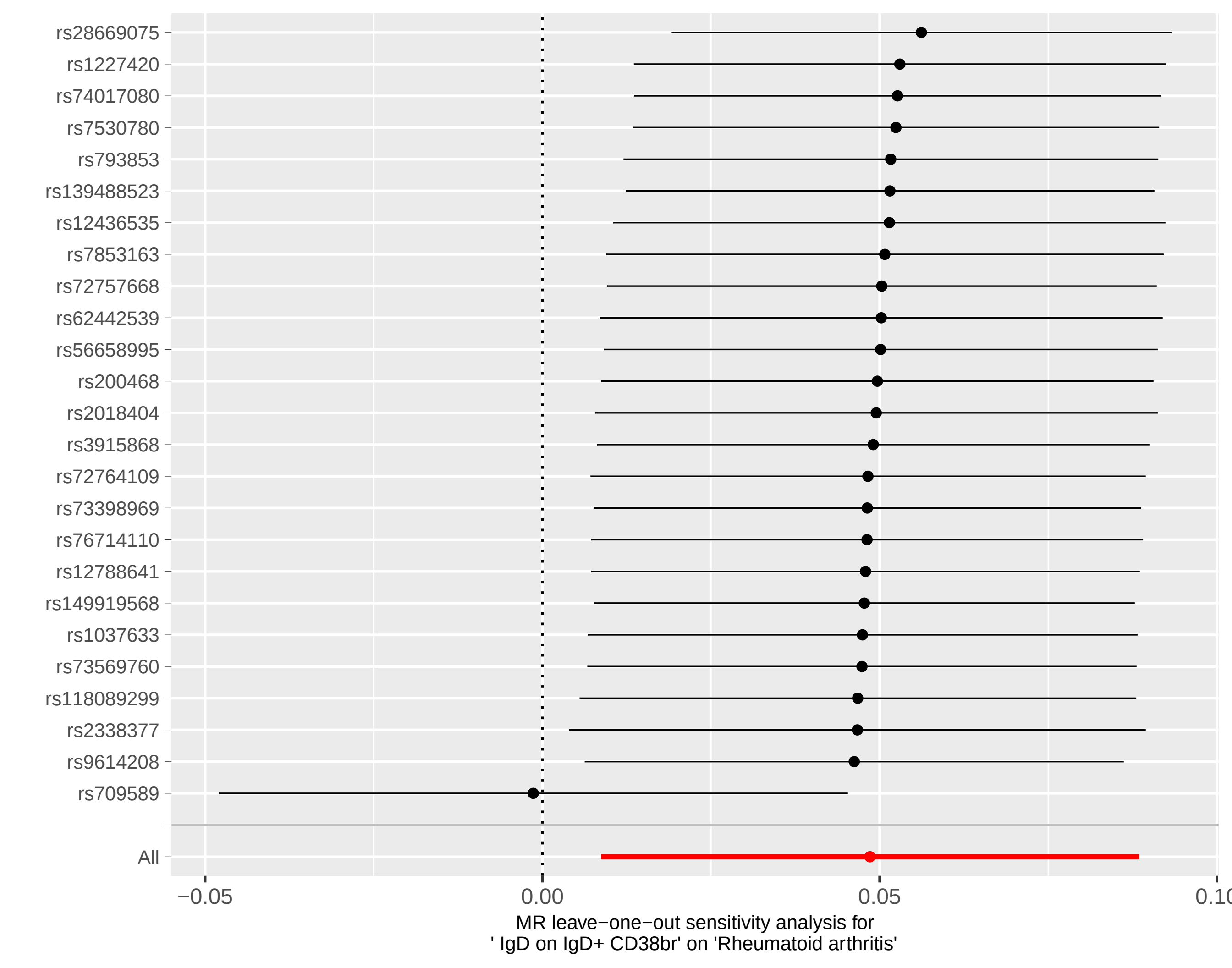
rs28669075
rs1227420
rs74017080
rs7530780
rs793853
rs139488523
rs12436535
rs7853163
rs72757668
rs62442539
rs56658995
rs200468
rs2018404
rs3915868
rs72764109
rs73398969
rs76714110
rs12788641
rs149919568
rs1037633
rs73569760
rs118089299
rs2338377
rs9614208
rs709589
All
−0.05
0.00
0.05
MR leave−one−out sensitivity analysis for
' IgD on IgD+ CD38br' on 'Rheumatoid arthritis'

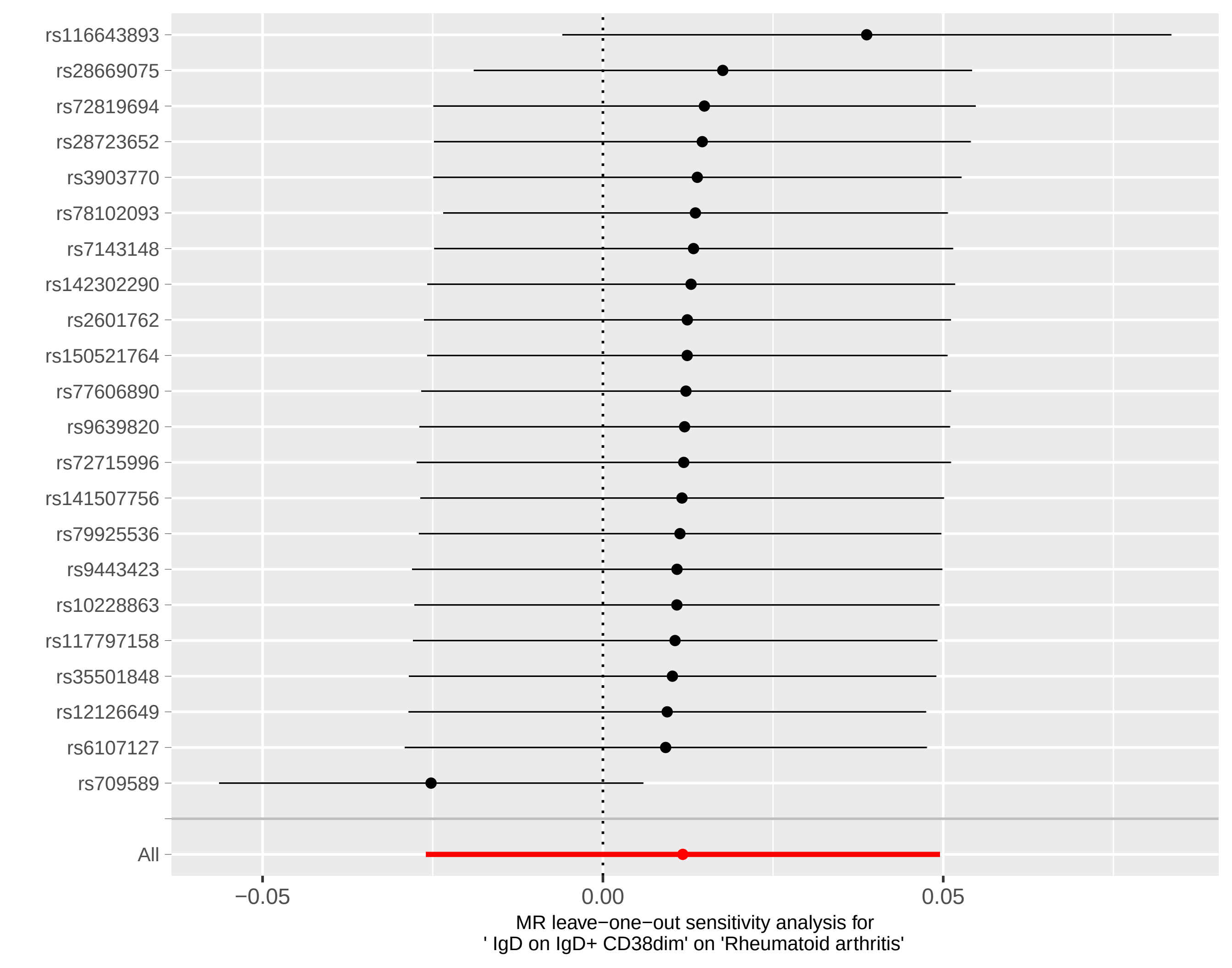
rs116643893
rs28669075
rs72819694
rs28723652
rs3903770
rs78102093
rs7143148
rs142302290
rs2601762
rs150521764
rs77606890
rs9639820
rs72715996
rs141507756
rs79925536
rs9443423
rs10228863
rs117797158
rs35501848
rs12126649
rs6107127
rs709589
All
−0.05
0.00
0.05
MR leave−one−out sensitivity analysis for
' IgD on IgD+ CD38dim' on 'Rheumatoid arthritis'

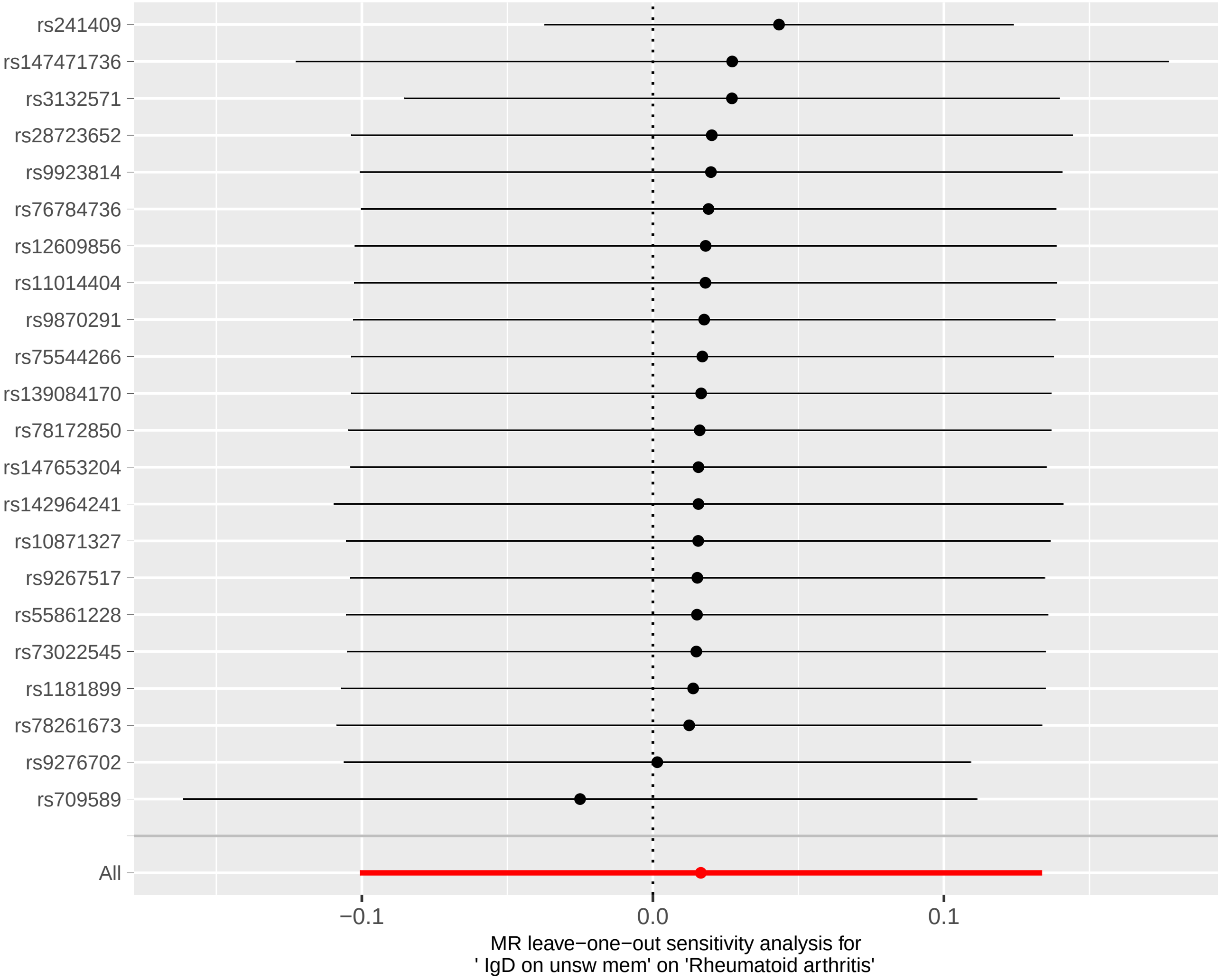

rs241409
rs147471736
rs3132571
rs28723652
rs9923814
rs76784736
rs12609856
rs11014404
rs9870291
rs75544266
rs139084170
rs78172850
rs147653204
rs142964241
rs10871327
rs9267517
rs55861228
rs73022545
rs1181899
rs78261673
rs9276702
rs709589
All
−0.1
0.0
0.1
MR leave−one−out sensitivity analysis for
' IgD on unsw mem' on 'Rheumatoid arthritis'

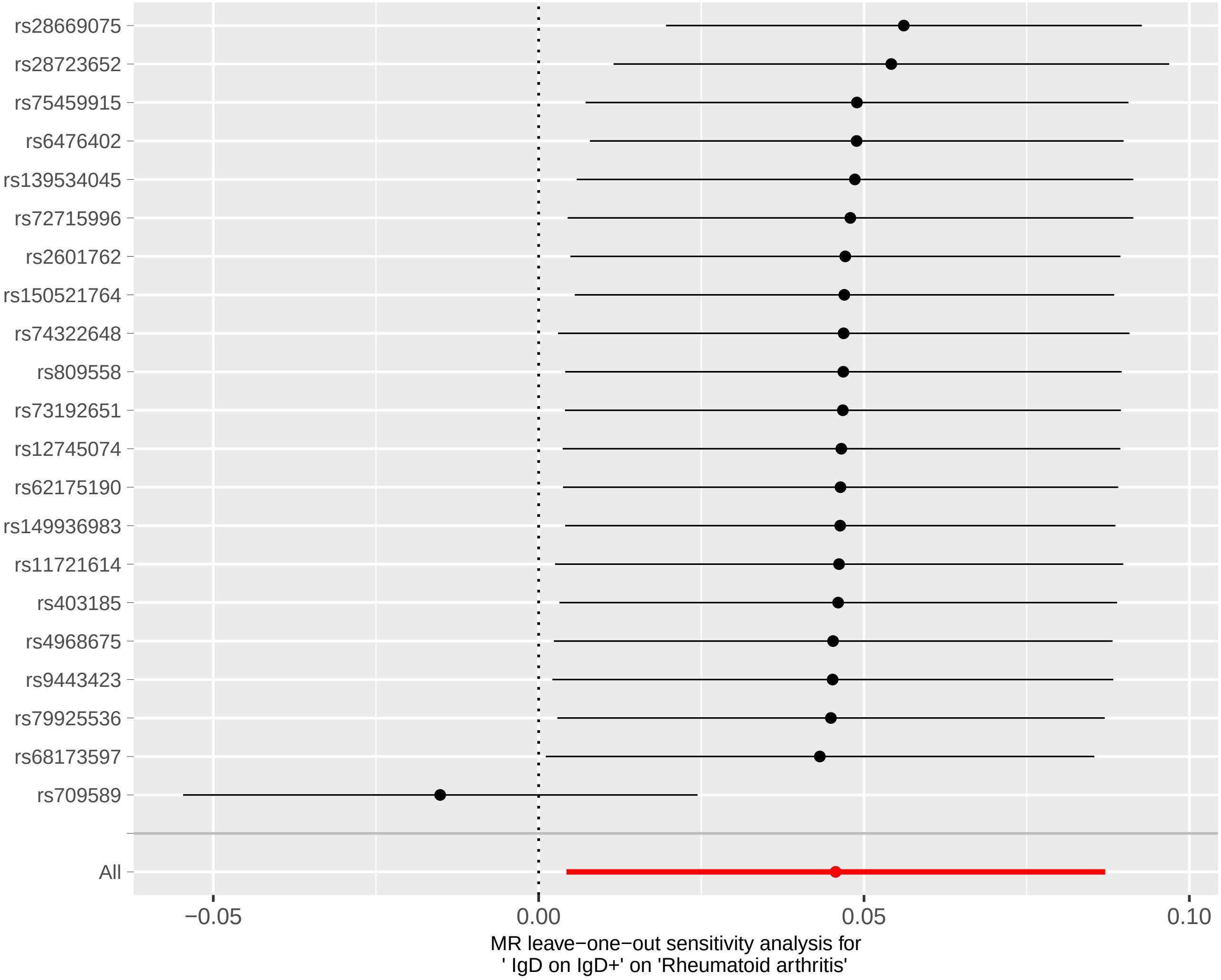

rs28669075
rs28723652
rs75459915
rs6476402
rs139534045
rs72715996
rs2601762
rs150521764
rs74322648
rs809558
rs73192651
rs12745074
rs62175190
rs149936983
rs11721614
rs403185
rs4968675
rs9443423
rs79925536
rs68173597
rs709589
All
−0.05
0.00
0.05
0.10
MR leave−one−out sensitivity analysis for
' IgD on IgD+' on 'Rheumatoid arthritis'

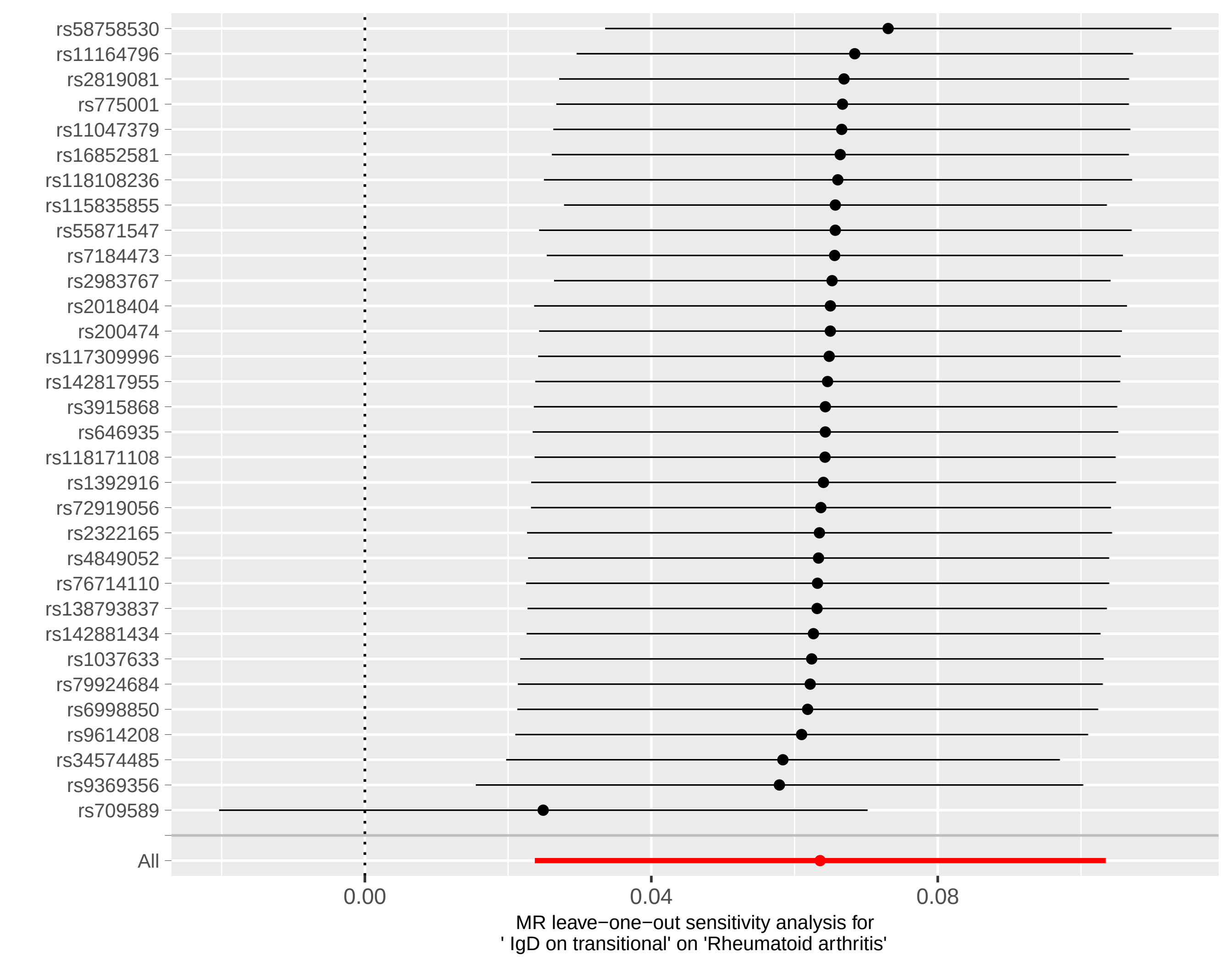

rs58758530
rs11164796
rs2819081
rs775001
rs11047379
rs16852581
rs118108236
rs115835855
rs55871547
rs7184473
rs2983767
rs2018404
rs200474
rs117309996
rs142817955
rs3915868
rs646935
rs118171108
rs1392916
rs72919056
rs2322165
rs4849052
rs76714110
rs138793837
rs142881434
rs1037633
rs79924684
rs6998850
rs9614208
rs34574485
rs9369356
rs709589
All
0.00
0.04
0.08
MR leave−one−out sensitivity analysis for
' IgD on transitional' on 'Rheumatoid arthritis'

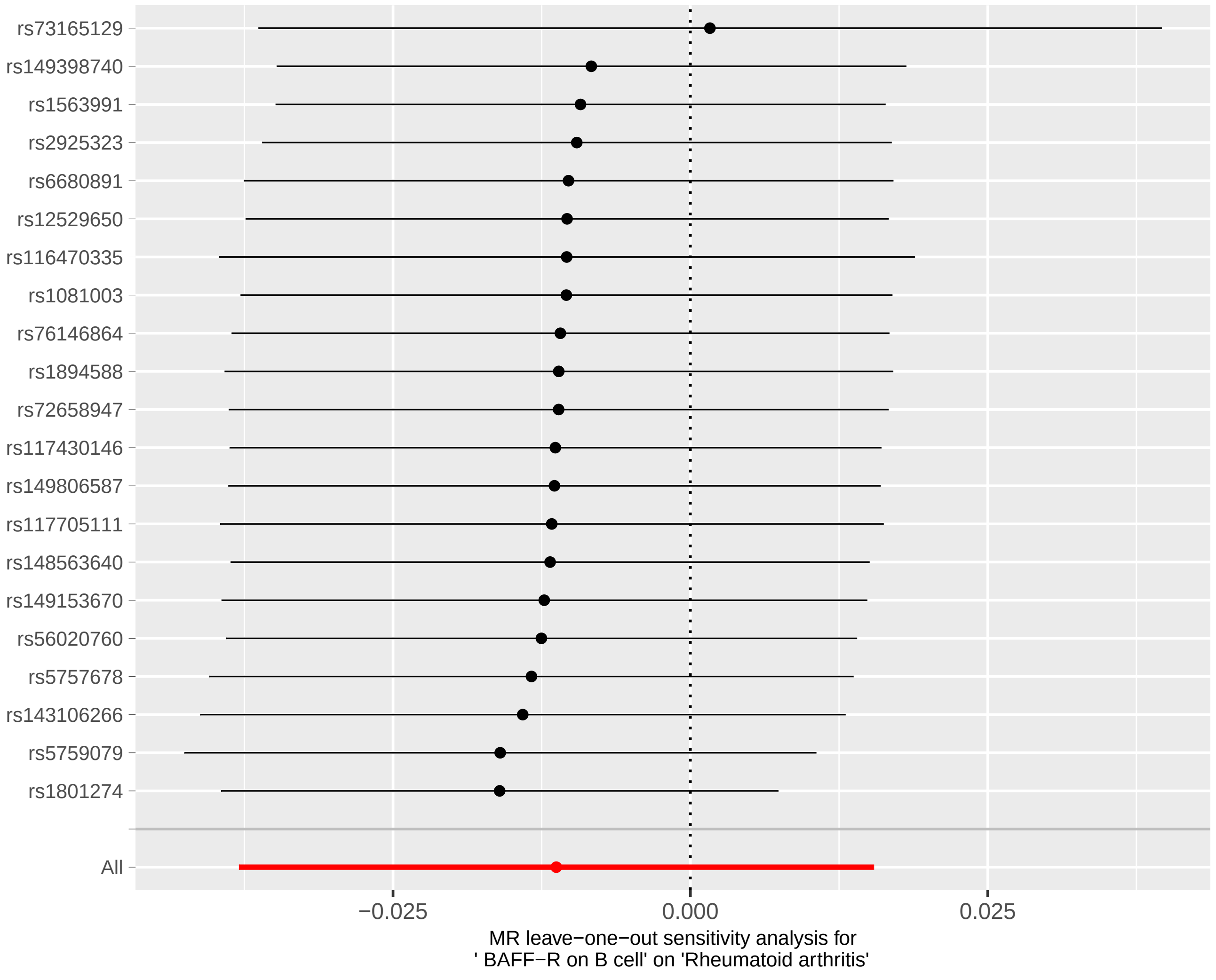

rs73165129
rs149398740
rs1563991
rs2925323
rs6680891
rs12529650
rs116470335
rs1081003
rs76146864
rs1894588
rs72658947
rs117430146
rs149806587
rs117705111
rs148563640
rs149153670
rs56020760
rs5757678
rs143106266
rs5759079
rs1801274
All
−0.025
0.000
0.025
MR leave−one−out sensitivity analysis for
' BAFF−R on B cell' on 'Rheumatoid arthritis'

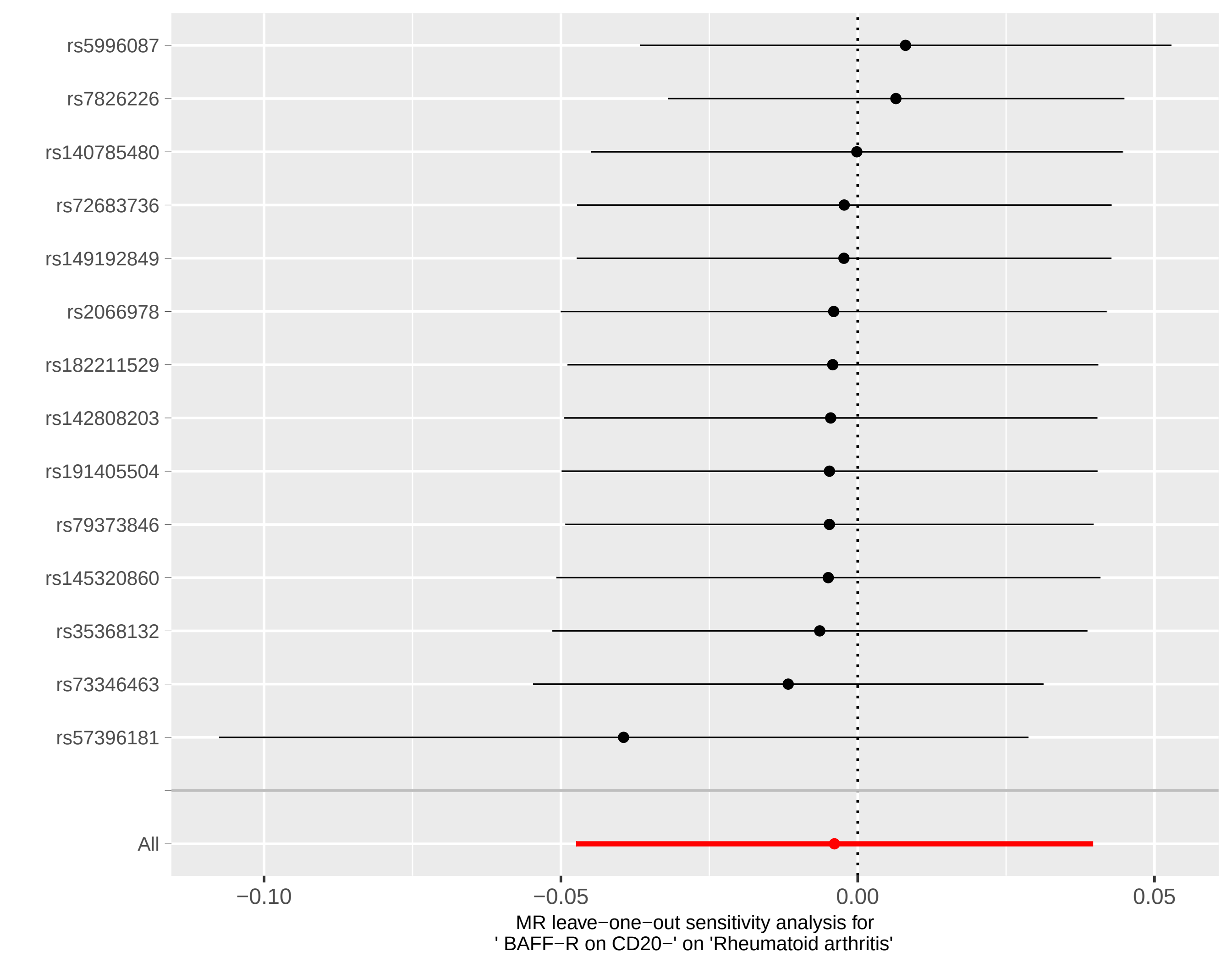
rs5996087
rs7826226
rs140785480
rs72683736
rs149192849
rs2066978
rs182211529
rs142808203
rs191405504
rs79373846
rs145320860
rs35368132
rs73346463
rs57396181
All
−0.10
−0.05
0.00
0.05
MR leave−one−out sensitivity analysis for
' BAFF−R on CD20−' on 'Rheumatoid arthritis'

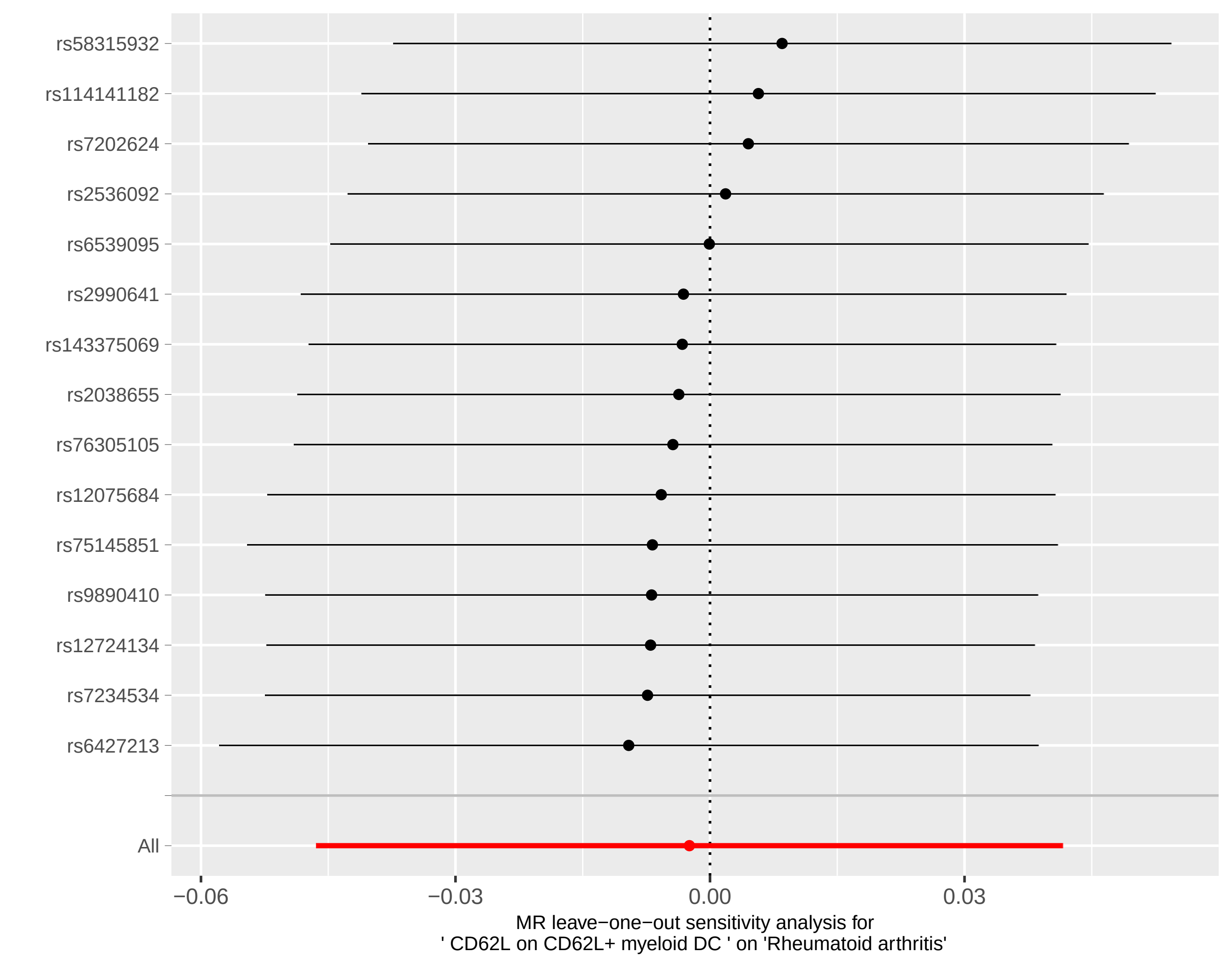
rs58315932
rs114141182
rs7202624
rs2536092
rs6539095
rs2990641
rs143375069
rs2038655
rs76305105
rs12075684
rs75145851
rs9890410
rs12724134
rs7234534
rs6427213
All
−0.06
−0.03
0.00
0.03
MR leave−one−out sensitivity analysis for
' CD62L on CD62L+ myeloid DC ' on 'Rheumatoid arthritis'

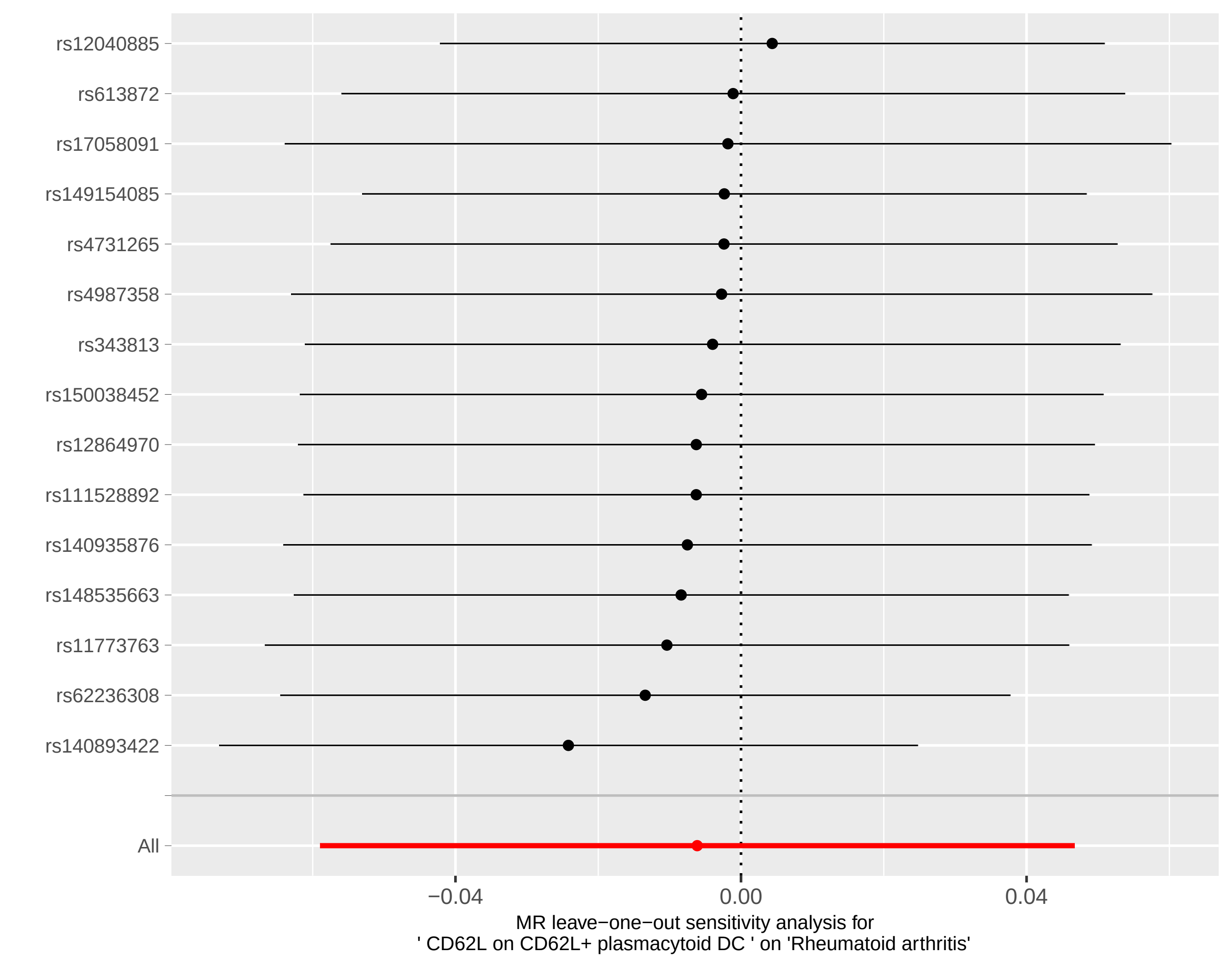
rs12040885
rs613872
rs17058091
rs149154085
rs4731265
rs4987358
rs343813
rs150038452
rs12864970
rs111528892
rs140935876
rs148535663
rs11773763
rs62236308
rs140893422
All
−0.04
0.00
0.04
MR leave−one−out sensitivity analysis for
' CD62L on CD62L+ plasmacytoid DC ' on 'Rheumatoid arthritis'

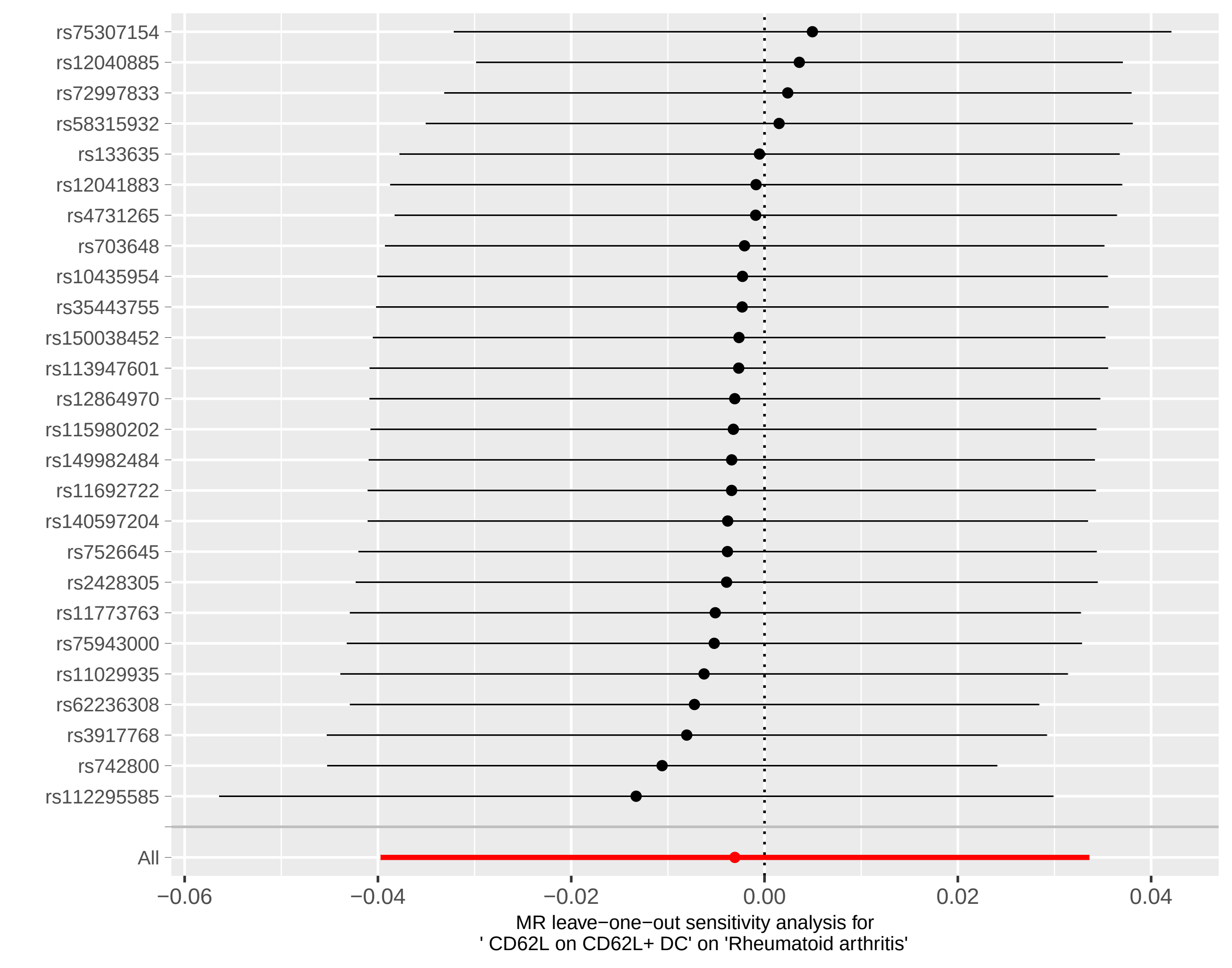
rs75307154
rs12040885
rs72997833
rs58315932
rs133635
rs12041883
rs4731265
rs703648
rs10435954
rs35443755
rs150038452
rs113947601
rs12864970
rs115980202
rs149982484
rs11692722
rs140597204
rs7526645
rs2428305
rs11773763
rs75943000
rs11029935
rs62236308
rs3917768
rs742800
rs112295585
All
−0.06
−0.04
−0.02
0.00
0.02
0.04
MR leave−one−out sensitivity analysis for
' CD62L on CD62L+ DC' on 'Rheumatoid arthritis'

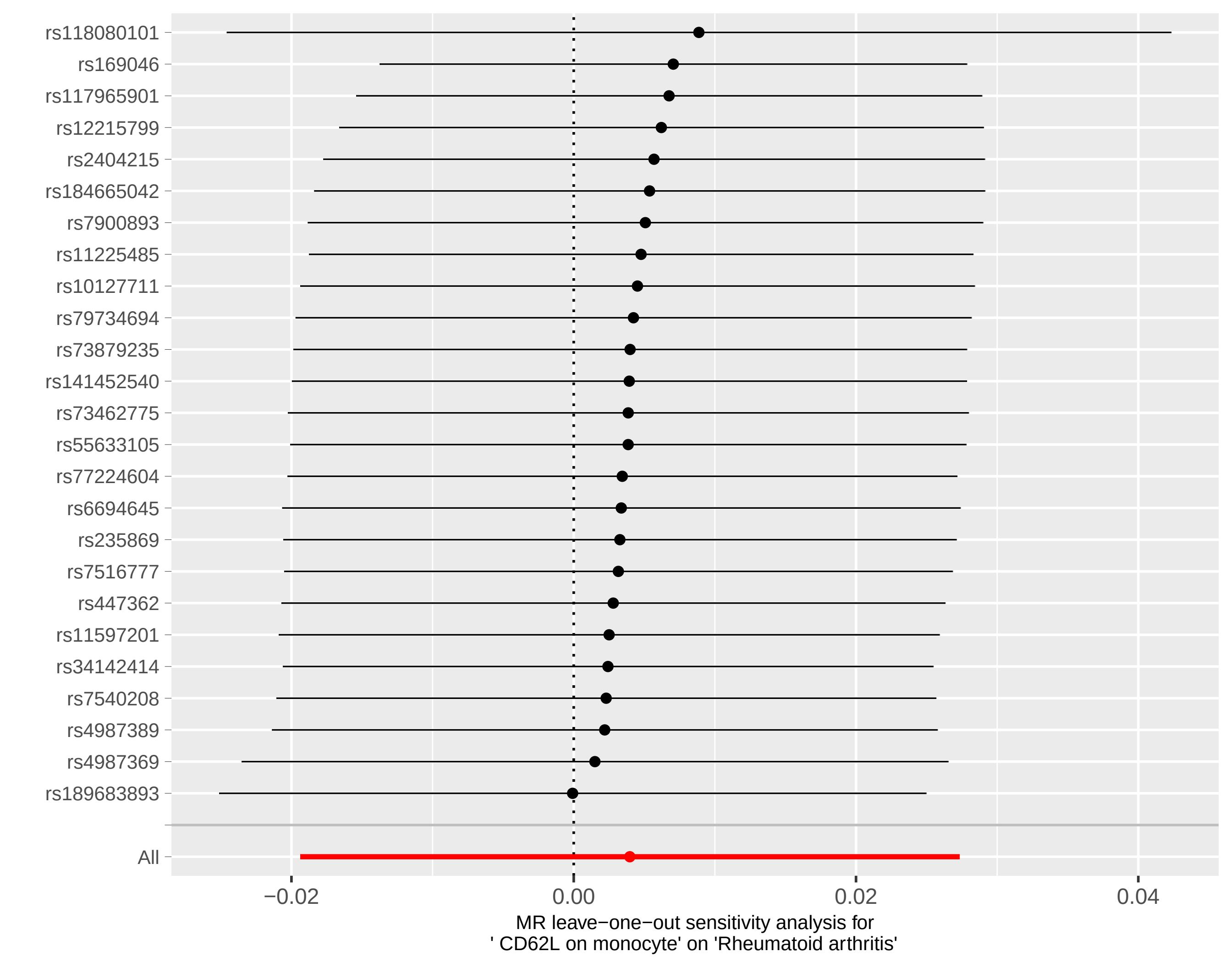
rs118080101
rs169046
rs117965901
rs12215799
rs2404215
rs184665042
rs7900893
rs11225485
rs10127711
rs79734694
rs73879235
rs141452540
rs73462775
rs55633105
rs77224604
rs6694645
rs235869
rs7516777
rs447362
rs11597201
rs34142414
rs7540208
rs4987389
rs4987369
rs189683893
All
−0.02
0.00
0.02
0.04
MR leave−one−out sensitivity analysis for
' CD62L on monocyte' on 'Rheumatoid arthritis'

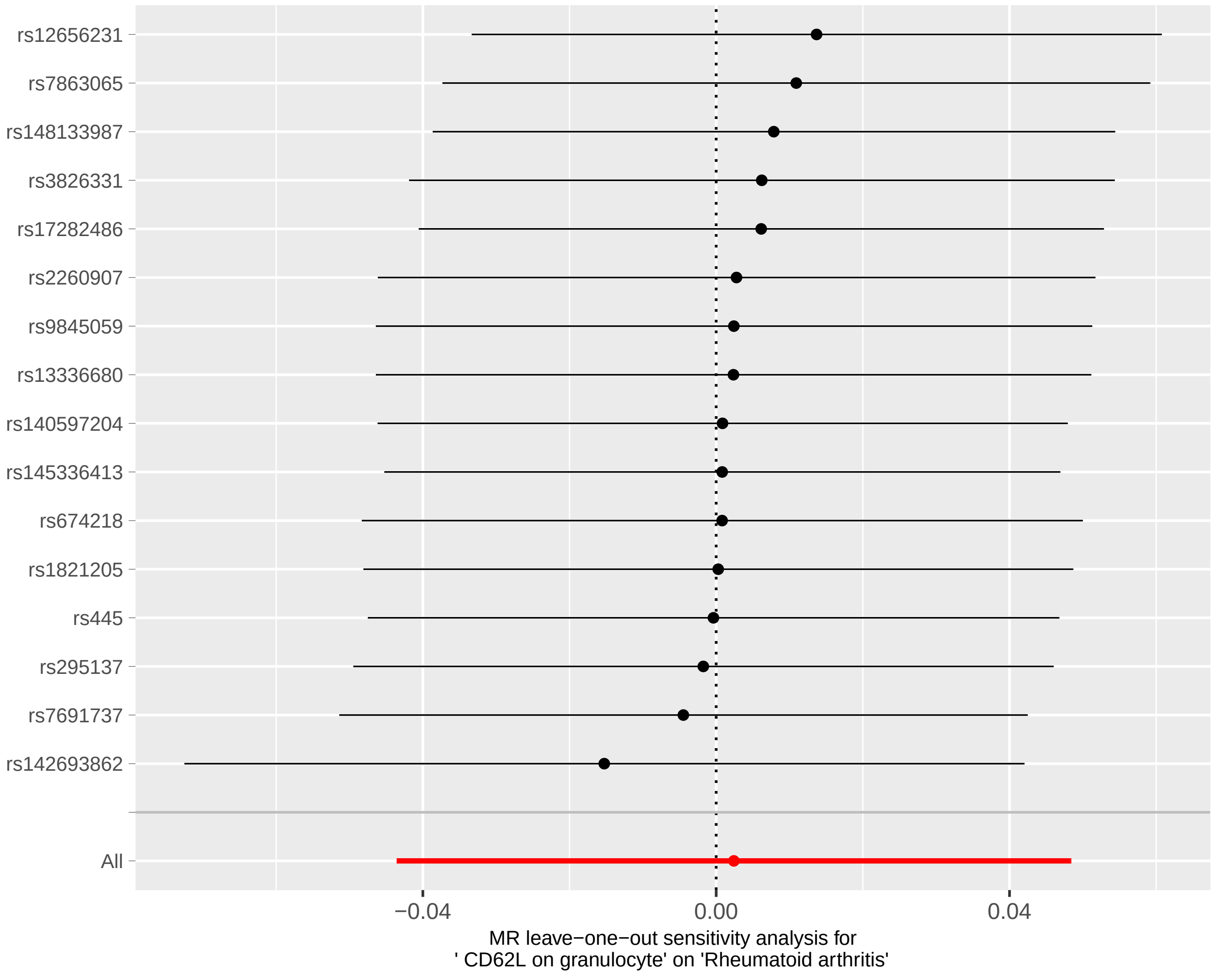
rs12656231
rs7863065
rs148133987
rs3826331
rs17282486
rs2260907
rs9845059
rs13336680
rs140597204
rs145336413
rs674218
rs1821205
rs445
rs295137
rs7691737
rs142693862
All
−0.04
0.00
0.04
MR leave−one−out sensitivity analysis for
' CD62L on granulocyte' on 'Rheumatoid arthritis'

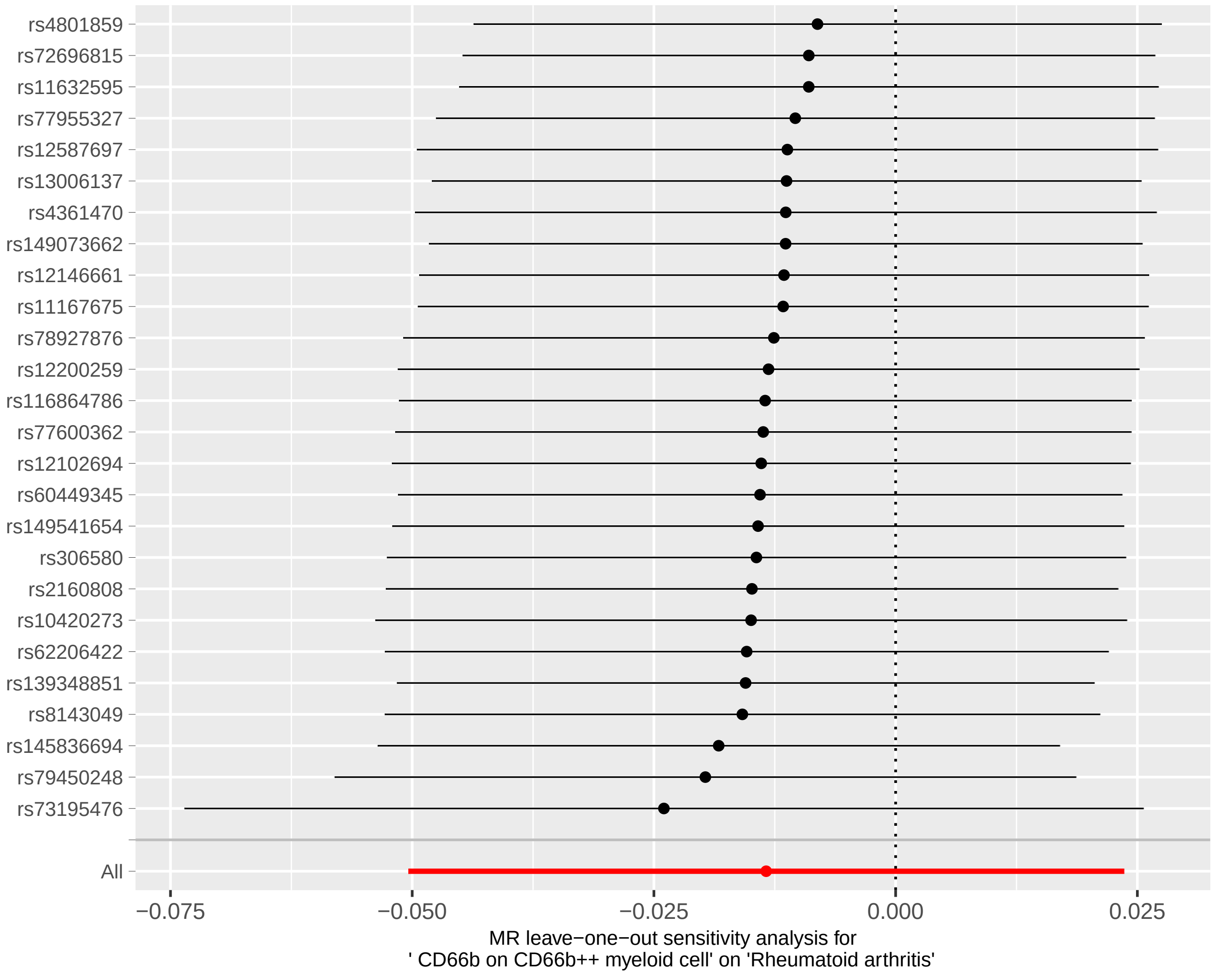
rs4801859
rs72696815
rs11632595
rs77955327
rs12587697
rs13006137
rs4361470
rs149073662
rs12146661
rs11167675
rs78927876
rs12200259
rs116864786
rs77600362
rs12102694
rs60449345
rs149541654
rs306580
rs2160808
rs10420273
rs62206422
rs139348851
rs8143049
rs145836694
rs79450248
rs73195476
All
−0.075
−0.050
−0.025
0.000
0.025
MR leave−one−out sensitivity analysis for
' CD66b on CD66b++ myeloid cell' on 'Rheumatoid arthritis'

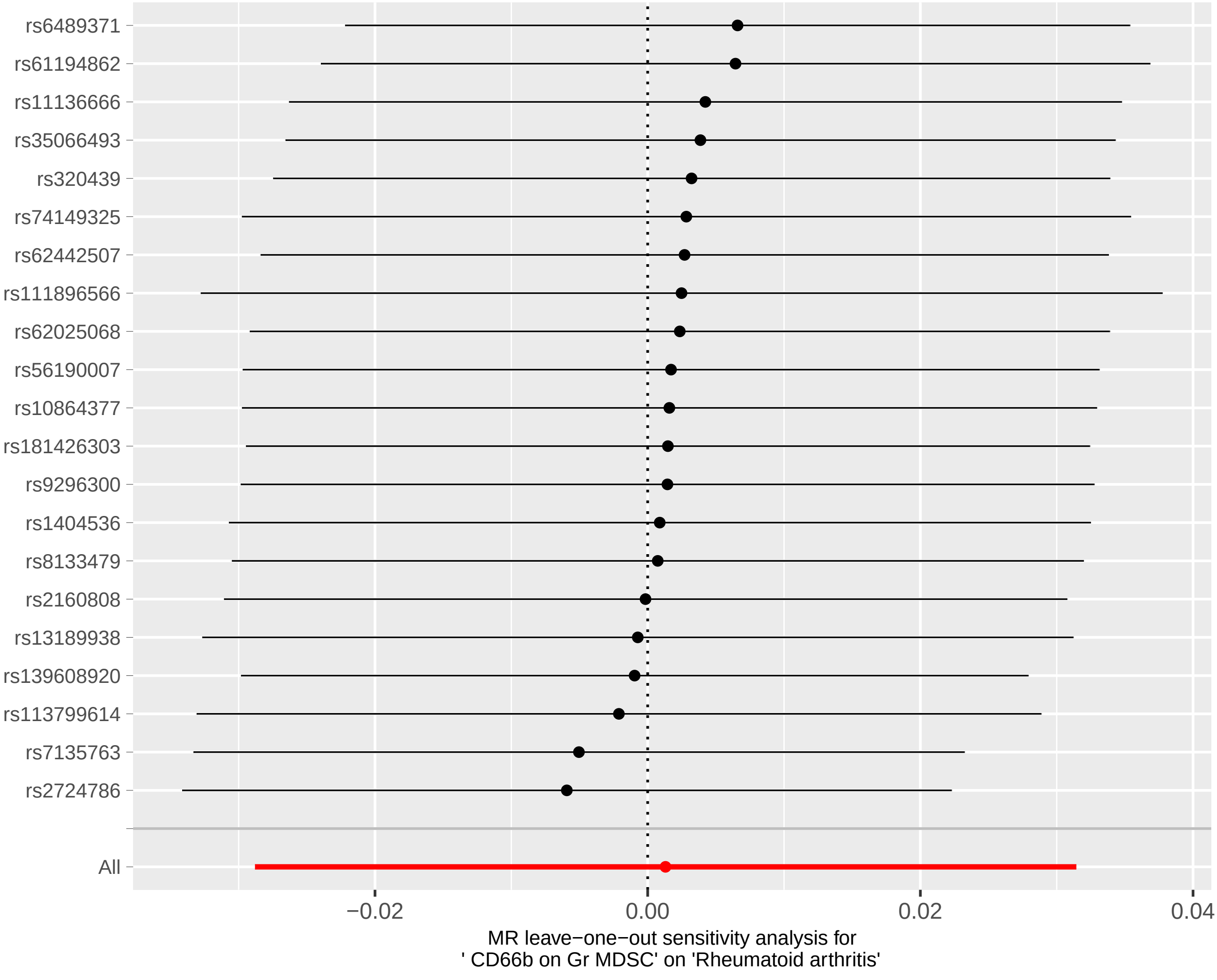
rs6489371
rs61194862
rs11136666
rs35066493
rs320439
rs74149325
rs62442507
rs111896566
rs62025068
rs56190007
rs10864377
rs181426303
rs9296300
rs1404536
rs8133479
rs2160808
rs13189938
rs139608920
rs113799614
rs7135763
rs2724786
All
−0.02
0.00
0.02
0.04
MR leave−one−out sensitivity analysis for
' CD66b on Gr MDSC' on 'Rheumatoid arthritis'

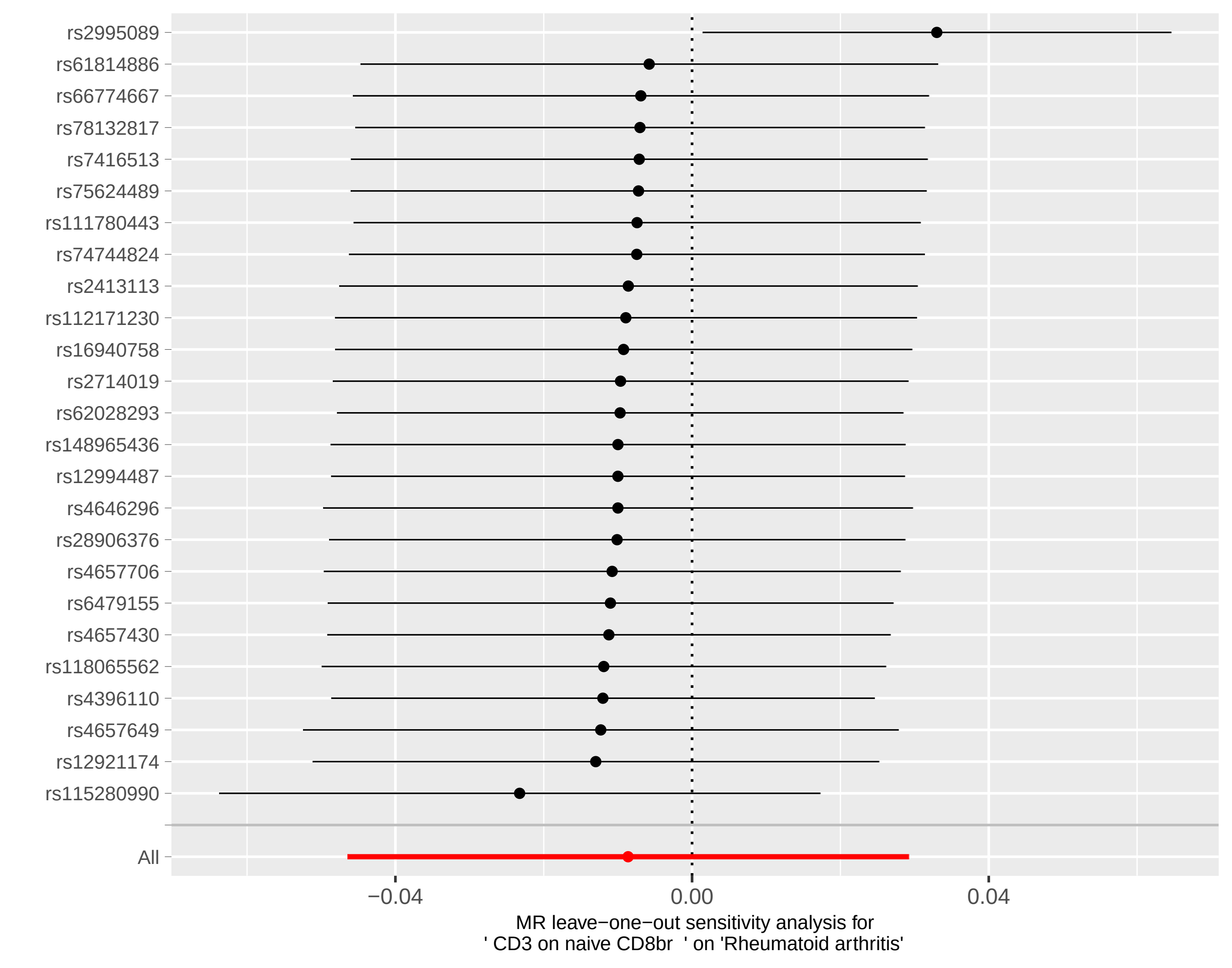

rs2995089
rs61814886
rs66774667
rs78132817
rs7416513
rs75624489
rs111780443
rs74744824
rs2413113
rs112171230
rs16940758
rs2714019
rs62028293
rs148965436
rs12994487
rs4646296
rs28906376
rs4657706
rs6479155
rs4657430
rs118065562
rs4396110
rs4657649
rs12921174
rs115280990
All
−0.04
0.00
0.04
MR leave−one−out sensitivity analysis for
' CD3 on naive CD8br ' on 'Rheumatoid arthritis'

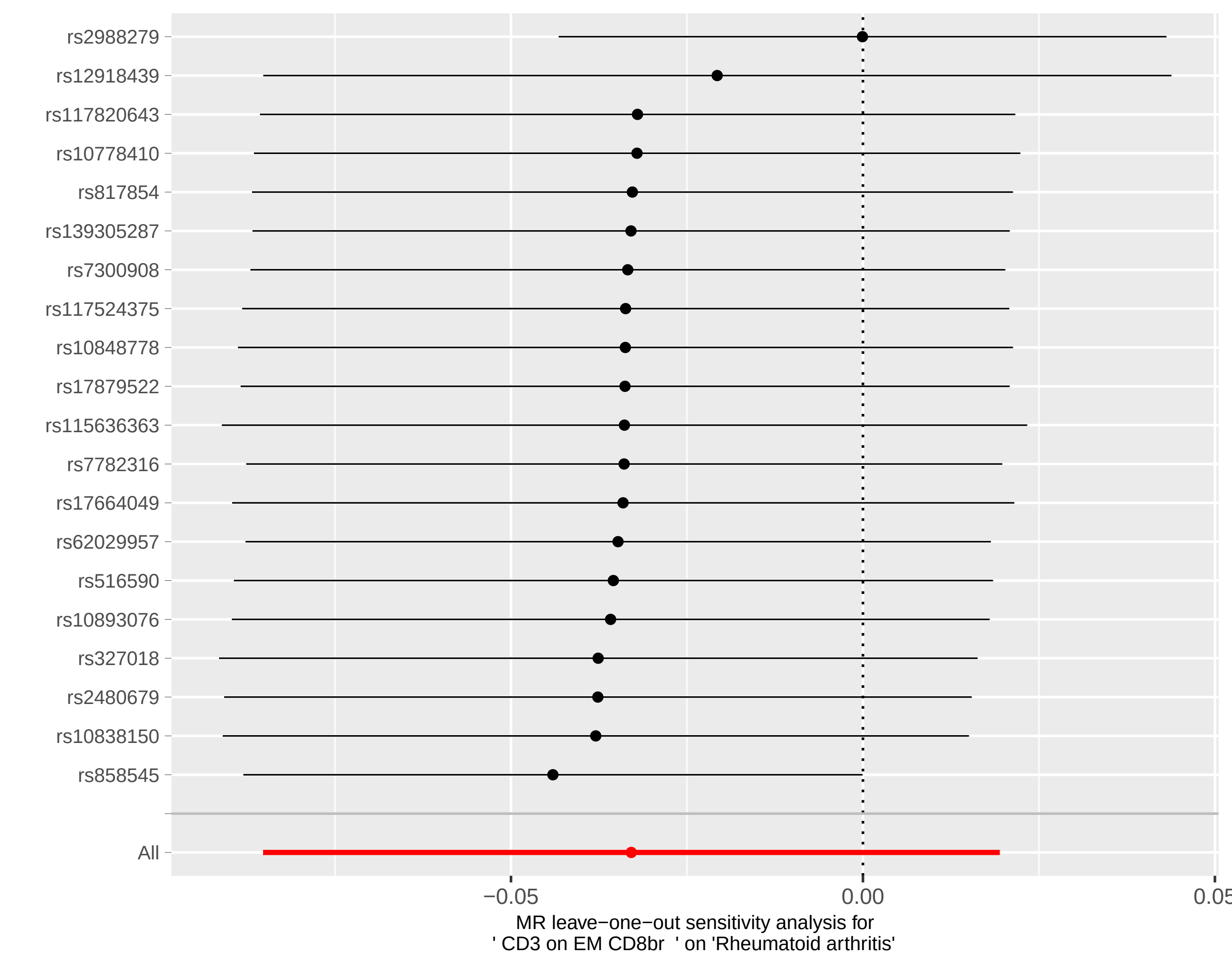

rs2988279
rs12918439
rs117820643
rs10778410
rs817854
rs139305287
rs7300908
rs117524375
rs10848778
rs17879522
rs115636363
rs7782316
rs17664049
rs62029957
rs516590
rs10893076
rs327018
rs2480679
rs10838150
rs858545
All
−0.05
0.00
0.05
MR leave−one−out sensitivity analysis for
' CD3 on EM CD8br ' on 'Rheumatoid arthritis'

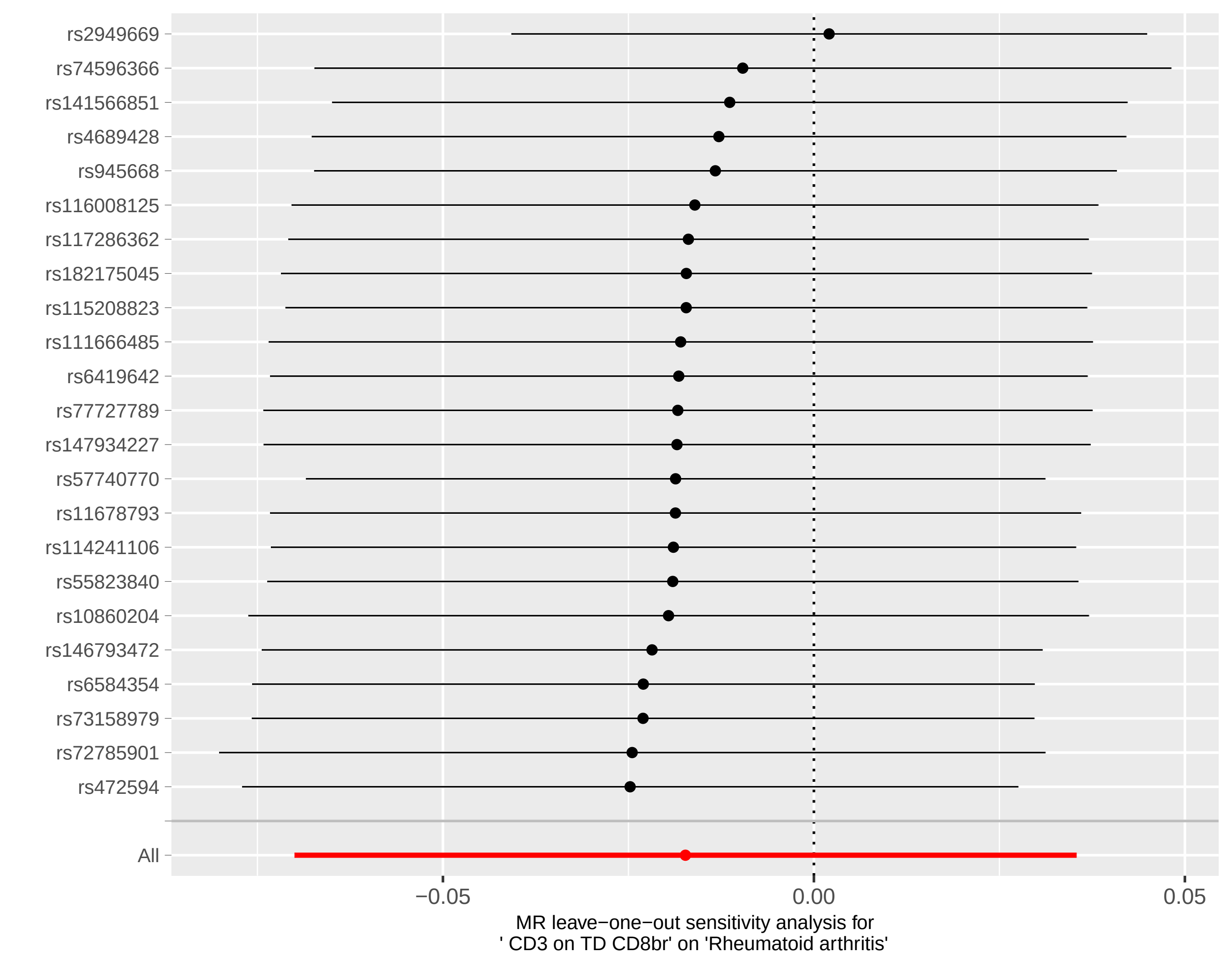
rs2949669
rs74596366
rs141566851
rs4689428
rs945668
rs116008125
rs117286362
rs182175045
rs115208823
rs111666485
rs6419642
rs77727789
rs147934227
rs57740770
rs11678793
rs114241106
rs55823840
rs10860204
rs146793472
rs6584354
rs73158979
rs72785901
rs472594
All
−0.05
0.00
0.05
MR leave−one−out sensitivity analysis for
' CD3 on TD CD8br' on 'Rheumatoid arthritis'

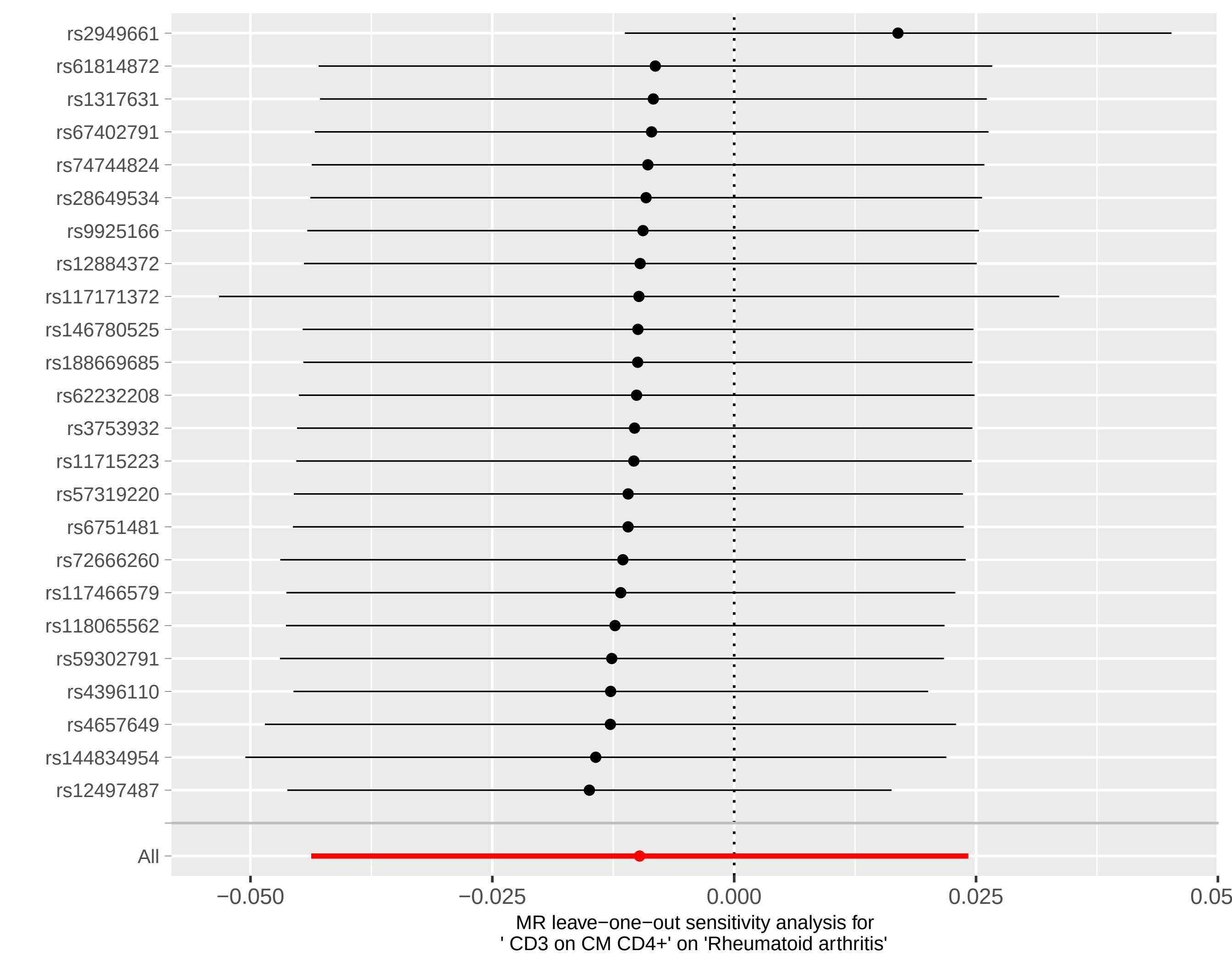

rs2949661
rs61814872
rs1317631
rs67402791
rs74744824
rs28649534
rs9925166
rs12884372
rs117171372
rs146780525
rs188669685
rs62232208
rs3753932
rs11715223
rs57319220
rs6751481
rs72666260
rs117466579
rs118065562
rs59302791
rs4396110
rs4657649
rs144834954
rs12497487
All
−0.050
−0.025
0.000
0.025
0.05
MR leave−one−out sensitivity analysis for
' CD3 on CM CD4+' on 'Rheumatoid arthritis'

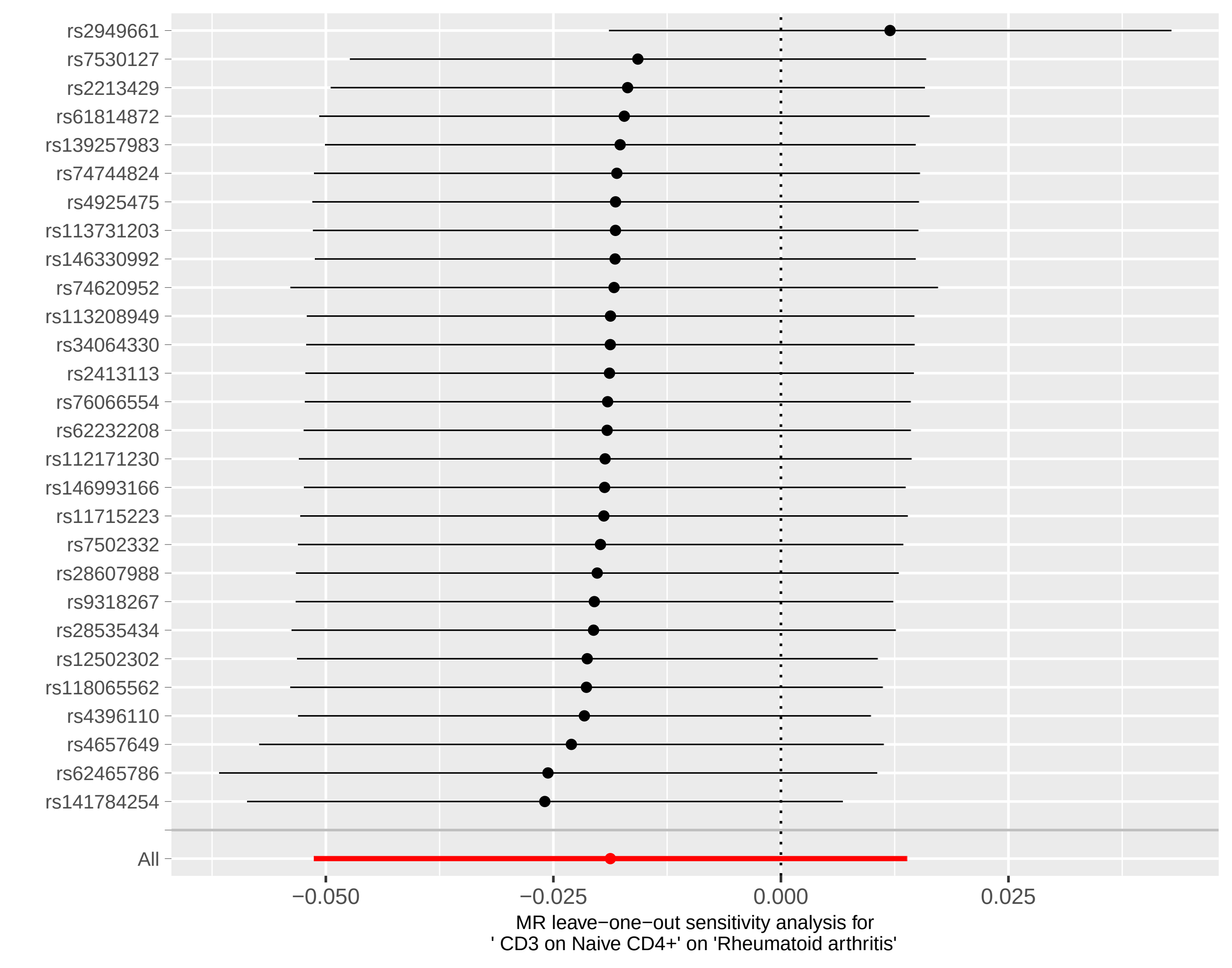
rs2949661
rs7530127
rs2213429
rs61814872
rs139257983
rs74744824
rs4925475
rs113731203
rs146330992
rs74620952
rs113208949
rs34064330
rs2413113
rs76066554
rs62232208
rs112171230
rs146993166
rs11715223
rs7502332
rs28607988
rs9318267
rs28535434
rs12502302
rs118065562
rs4396110
rs4657649
rs62465786
rs141784254
All
−0.050
−0.025
0.000
0.025
MR leave−one−out sensitivity analysis for
' CD3 on Naive CD4+' on 'Rheumatoid arthritis'

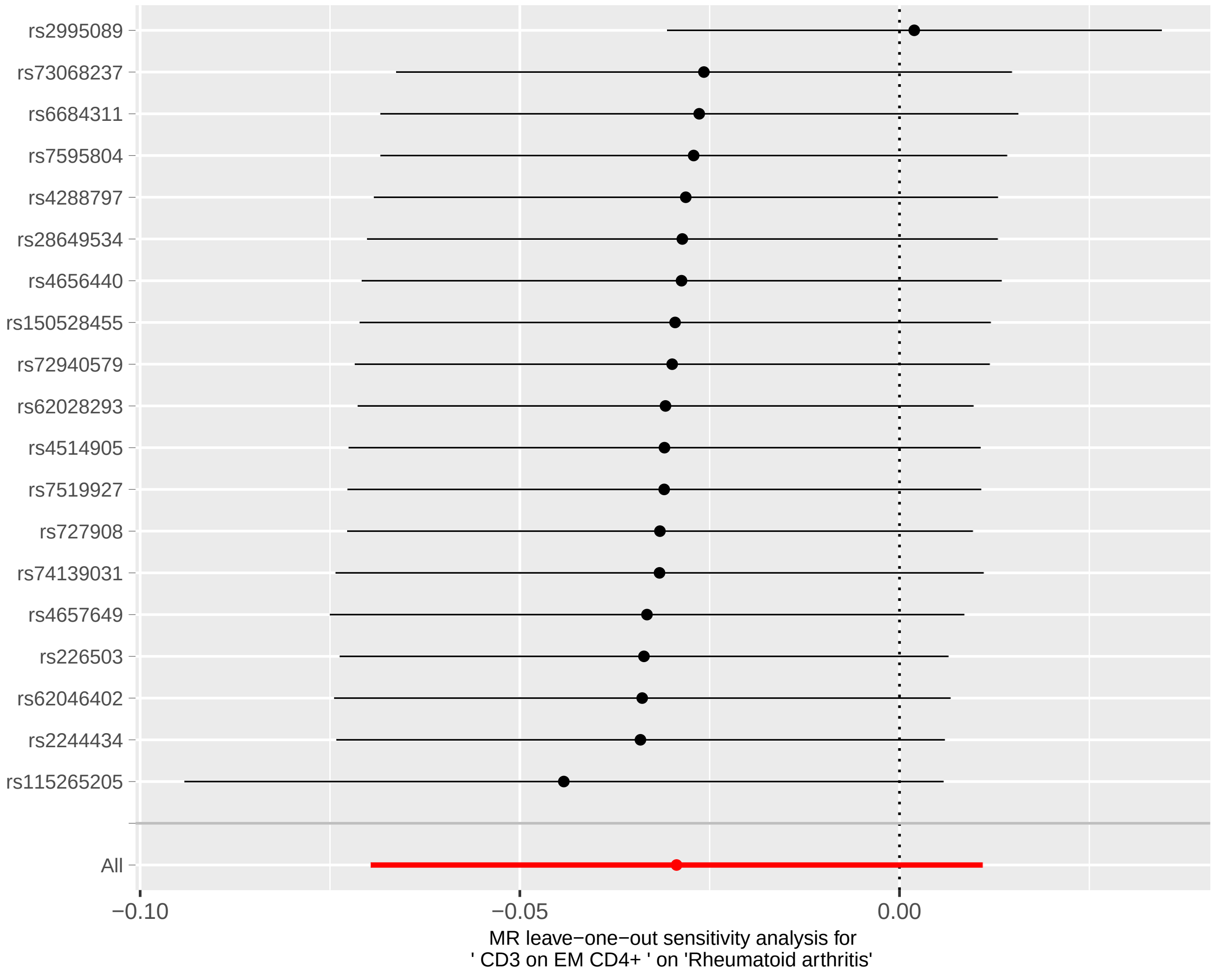
rs2995089
rs73068237
rs6684311
rs7595804
rs4288797
rs28649534
rs4656440
rs150528455
rs72940579
rs62028293
rs4514905
rs7519927
rs727908
rs74139031
rs4657649
rs226503
rs62046402
rs2244434
rs115265205
All
−0.10
−0.05
0.00
MR leave−one−out sensitivity analysis for
' CD3 on EM CD4+ ' on 'Rheumatoid arthritis'

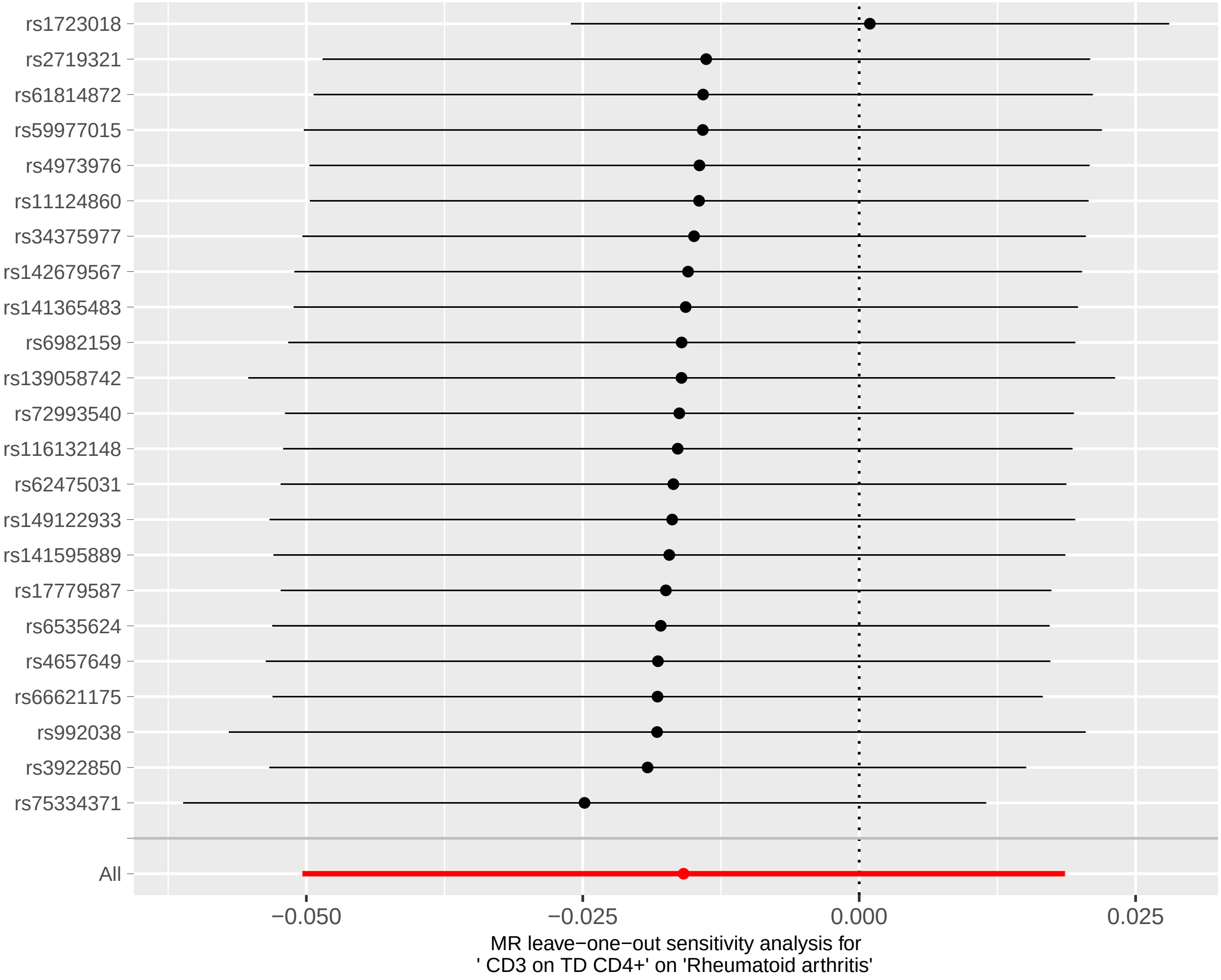
rs1723018
rs2719321
rs61814872
rs59977015
rs4973976
rs11124860
rs34375977
rs142679567
rs141365483
rs6982159
rs139058742
rs72993540
rs116132148
rs62475031
rs149122933
rs141595889
rs17779587
rs6535624
rs4657649
rs66621175
rs992038
rs3922850
rs75334371
All
−0.050
−0.025
0.000
0.025
MR leave−one−out sensitivity analysis for
' CD3 on TD CD4+' on 'Rheumatoid arthritis'

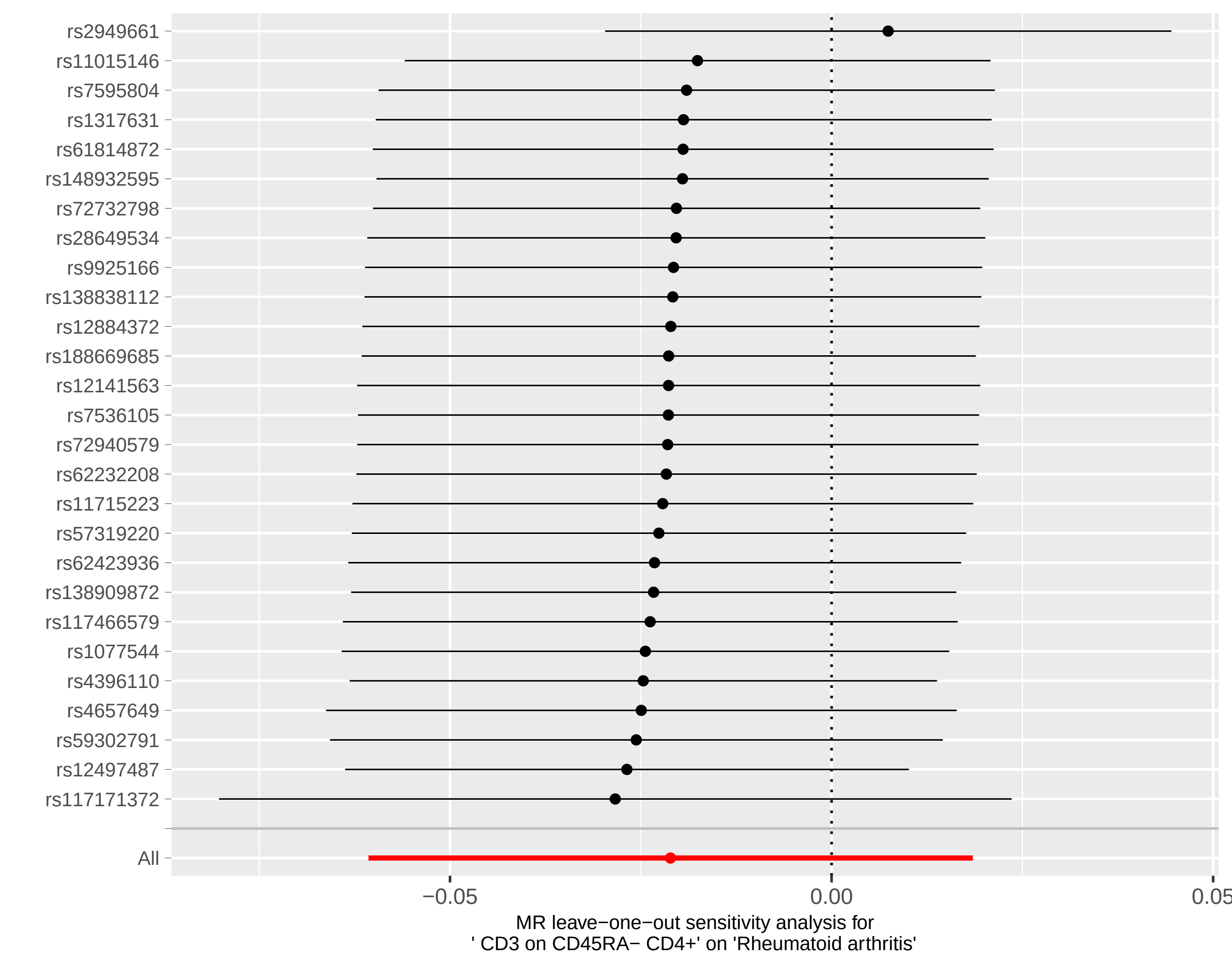
rs2949661
rs11015146
rs7595804
rs1317631
rs61814872
rs148932595
rs72732798
rs28649534
rs9925166
rs138838112
rs12884372
rs188669685
rs12141563
rs7536105
rs72940579
rs62232208
rs11715223
rs57319220
rs62423936
rs138909872
rs117466579
rs1077544
rs4396110
rs4657649
rs59302791
rs12497487
rs117171372
All
−0.05
0.00
0.05
MR leave−one−out sensitivity analysis for
' CD3 on CD45RA− CD4+' on 'Rheumatoid arthritis'

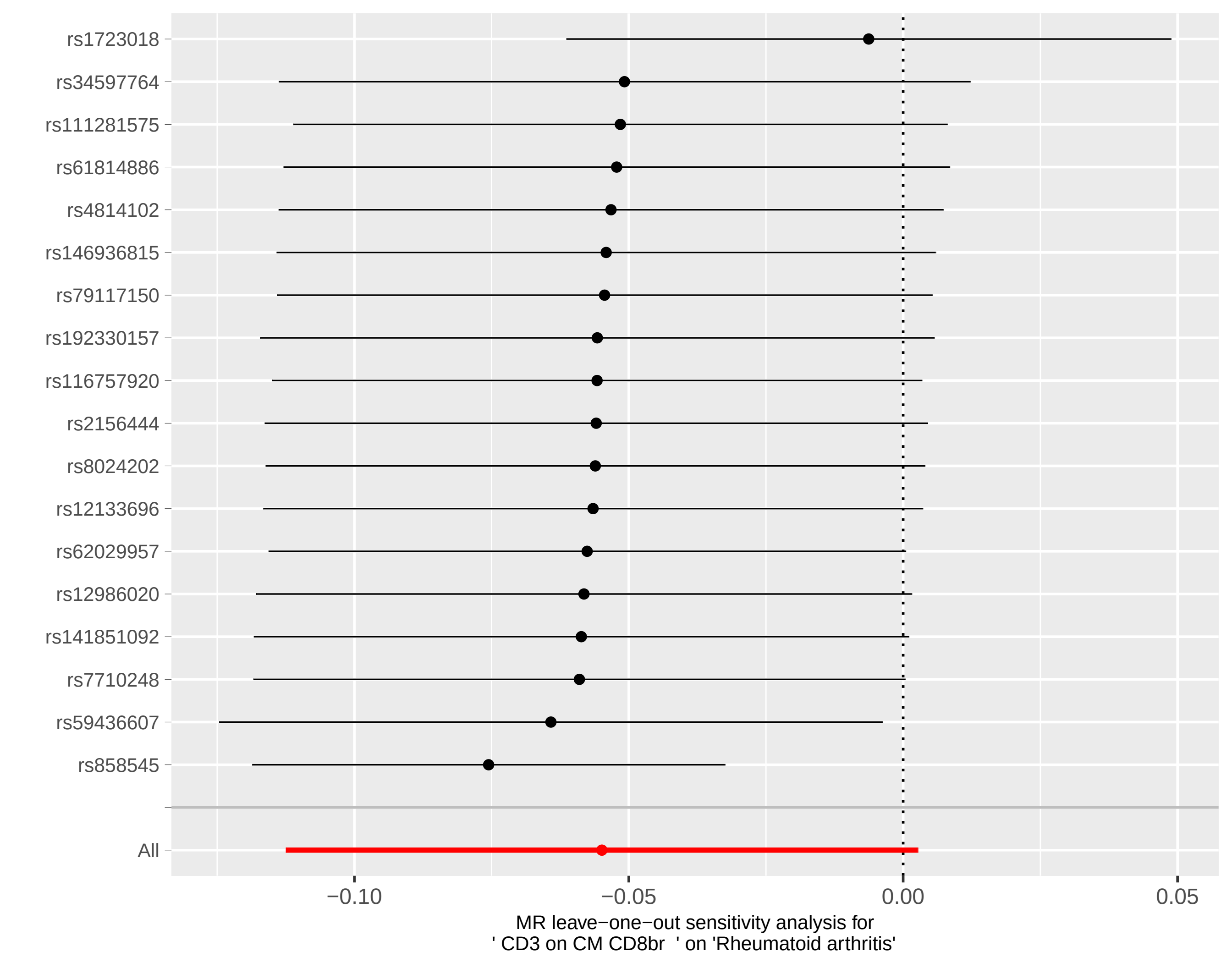
rs1723018
rs34597764
rs111281575
rs61814886
rs4814102
rs146936815
rs79117150
rs192330157
rs116757920
rs2156444
rs8024202
rs12133696
rs62029957
rs12986020
rs141851092
rs7710248
rs59436607
rs858545
All
−0.10
−0.05
0.00
0.05
MR leave−one−out sensitivity analysis for
' CD3 on CM CD8br ' on 'Rheumatoid arthritis'

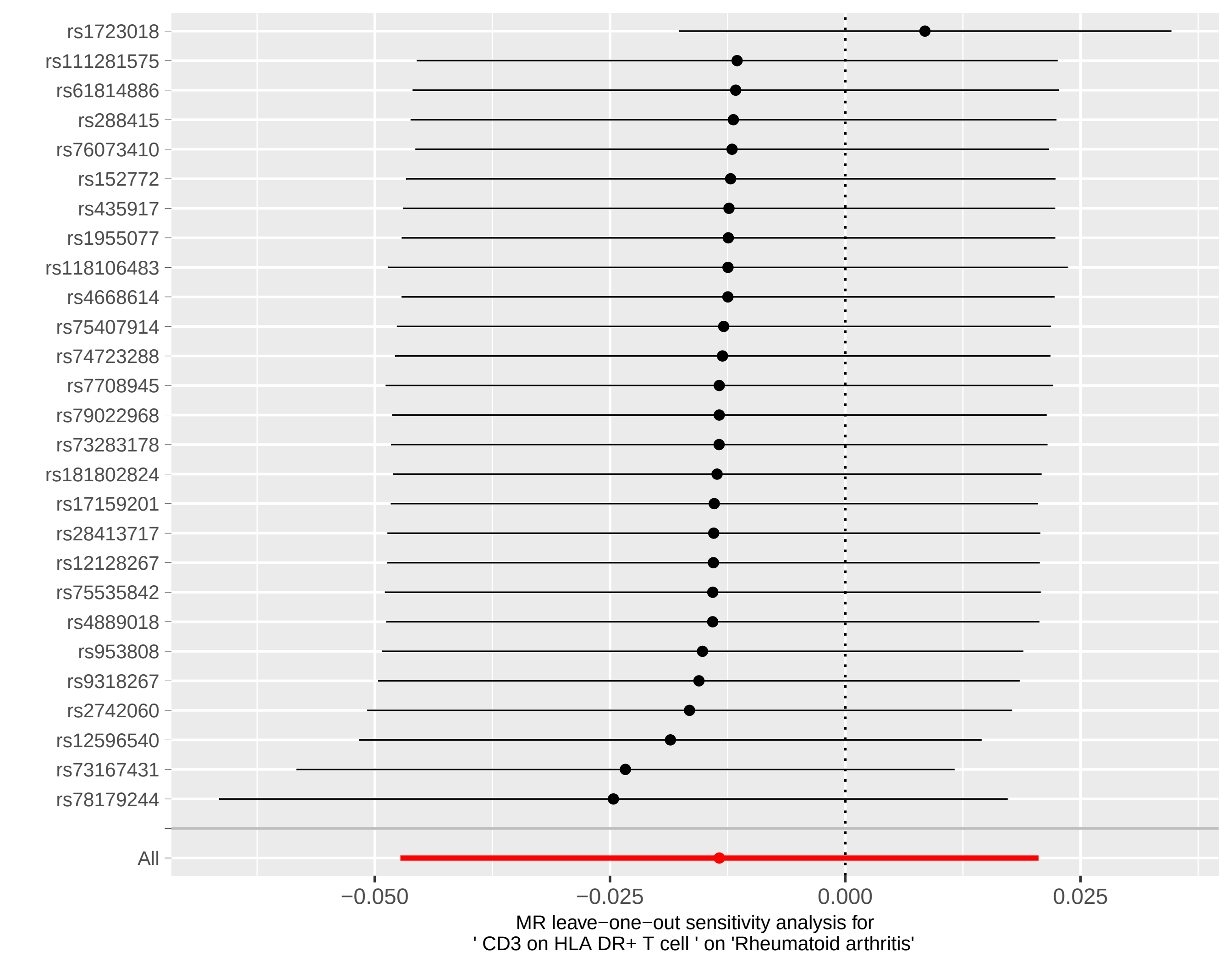
rs1723018
rs111281575
rs61814886
rs288415
rs76073410
rs152772
rs435917
rs1955077
rs118106483
rs4668614
rs75407914
rs74723288
rs7708945
rs79022968
rs73283178
rs181802824
rs17159201
rs28413717
rs12128267
rs75535842
rs4889018
rs953808
rs9318267
rs2742060
rs12596540
rs73167431
rs78179244
All
−0.050
−0.025
0.000
0.025
MR leave−one−out sensitivity analysis for
' CD3 on HLA DR+ T cell ' on 'Rheumatoid arthritis'

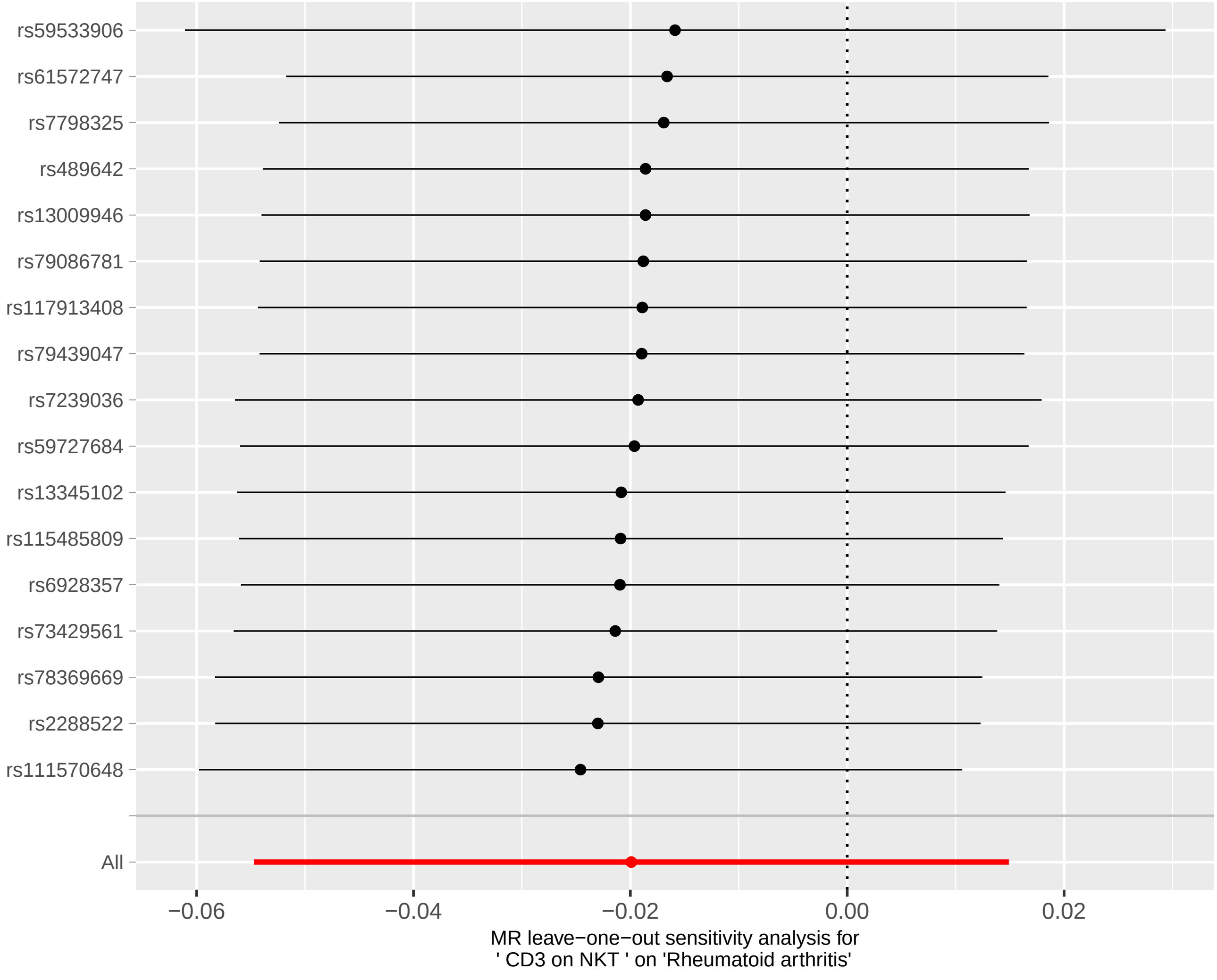

rs59533906
rs61572747
rs7798325
rs489642
rs13009946
rs79086781
rs117913408
rs79439047
rs7239036
rs59727684
rs13345102
rs115485809
rs6928357
rs73429561
rs78369669
rs2288522
rs111570648
All
−0.06
−0.04
−0.02
0.00
0.02
MR leave−one−out sensitivity analysis for
' CD3 on NKT ' on 'Rheumatoid arthritis'

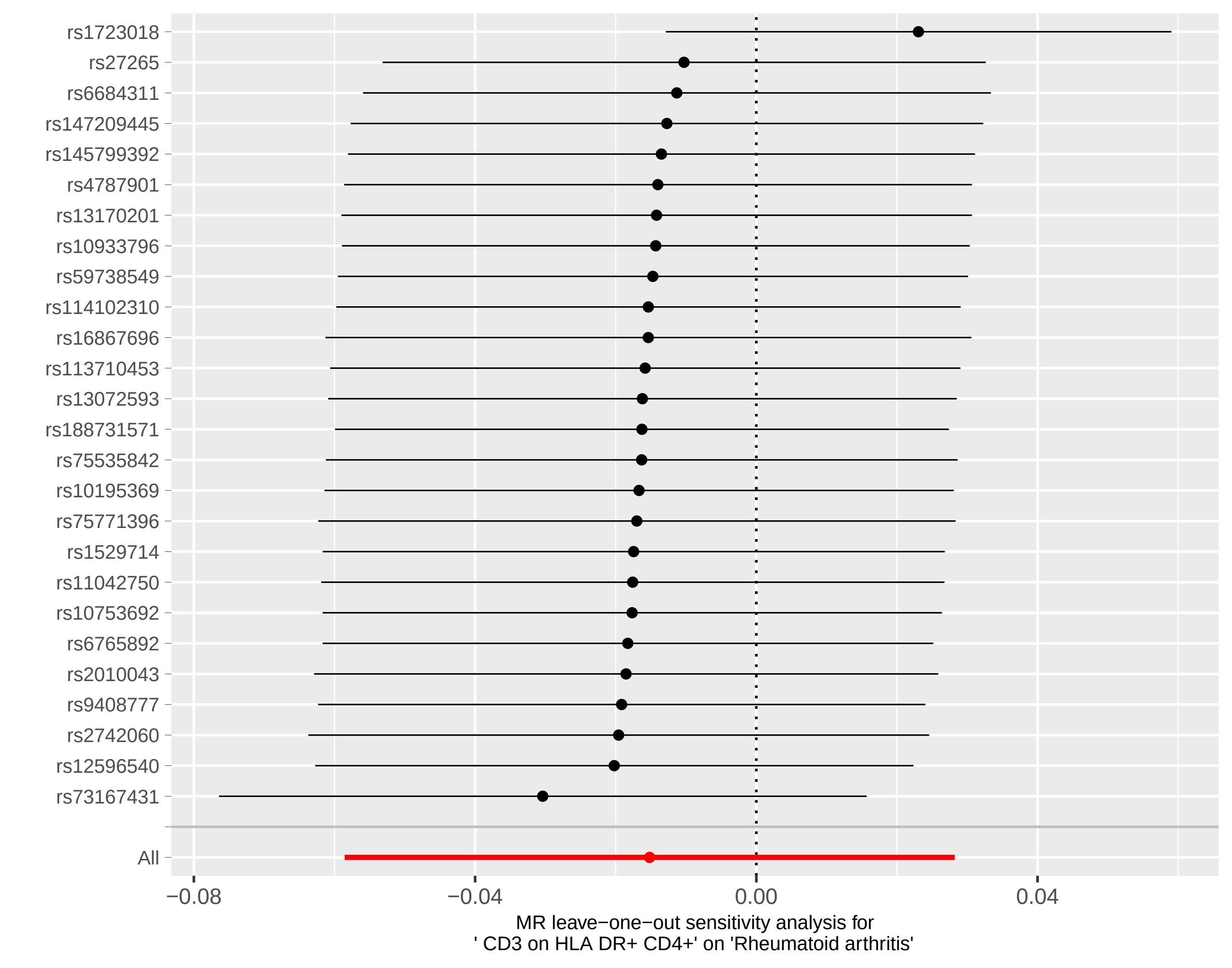

rs1723018
rs27265
rs6684311
rs147209445
rs145799392
rs4787901
rs13170201
rs10933796
rs59738549
rs114102310
rs16867696
rs113710453
rs13072593
rs188731571
rs75535842
rs10195369
rs75771396
rs1529714
rs11042750
rs10753692
rs6765892
rs2010043
rs9408777
rs2742060
rs12596540
rs73167431
All
−0.08
−0.04
0.00
0.04
MR leave−one−out sensitivity analysis for
' CD3 on HLA DR+ CD4+' on 'Rheumatoid arthritis'

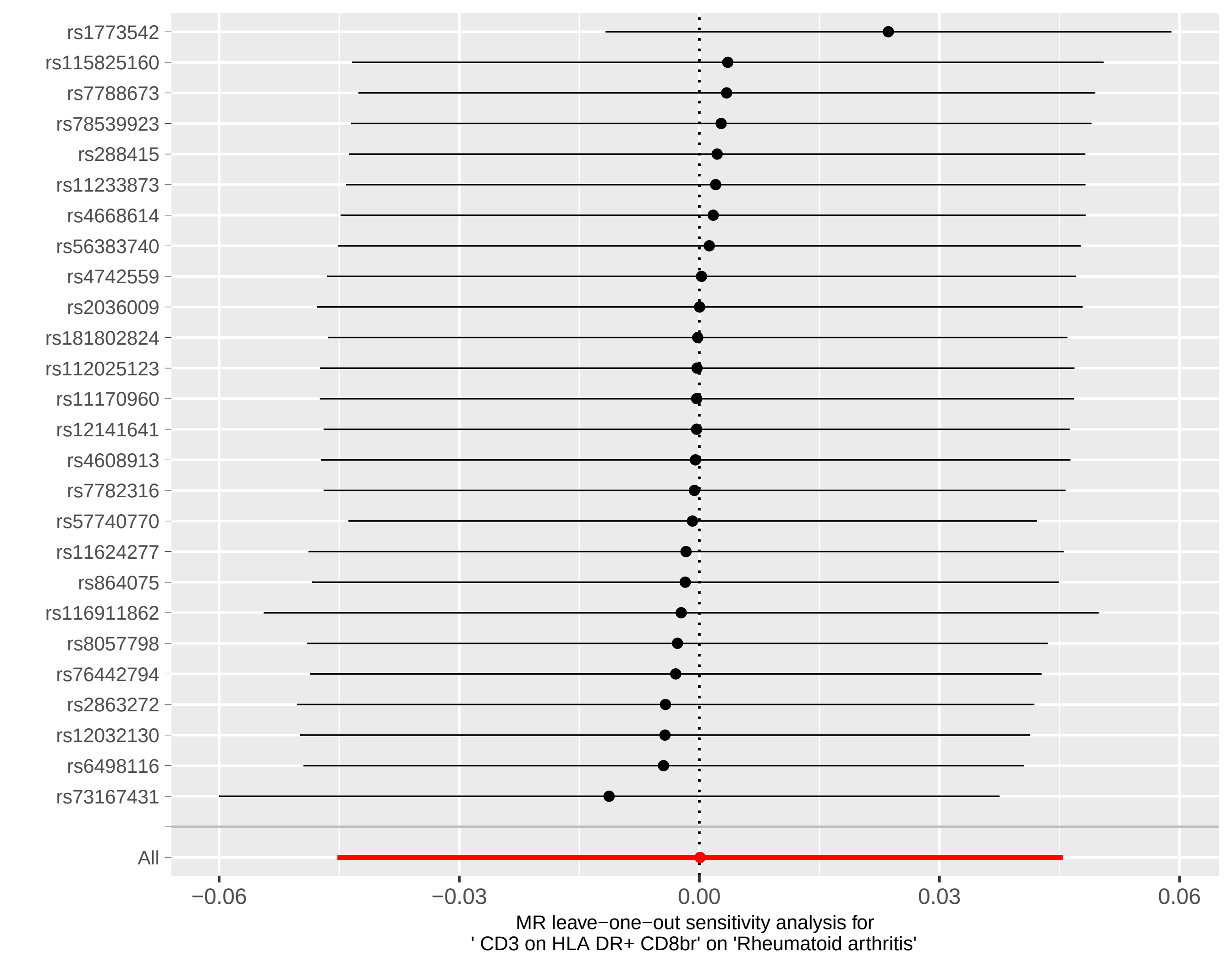
rs1773542
rs115825160
rs7788673
rs78539923
rs288415
rs11233873
rs4668614
rs56383740
rs4742559
rs2036009
rs181802824
rs112025123
rs11170960
rs12141641
rs4608913
rs7782316
rs57740770
rs11624277
rs864075
rs116911862
rs8057798
rs76442794
rs2863272
rs12032130
rs6498116
rs73167431
All
−0.06
−0.03
0.00
0.03
0.06
MR leave−one−out sensitivity analysis for
' CD3 on HLA DR+ CD8br' on 'Rheumatoid arthritis'

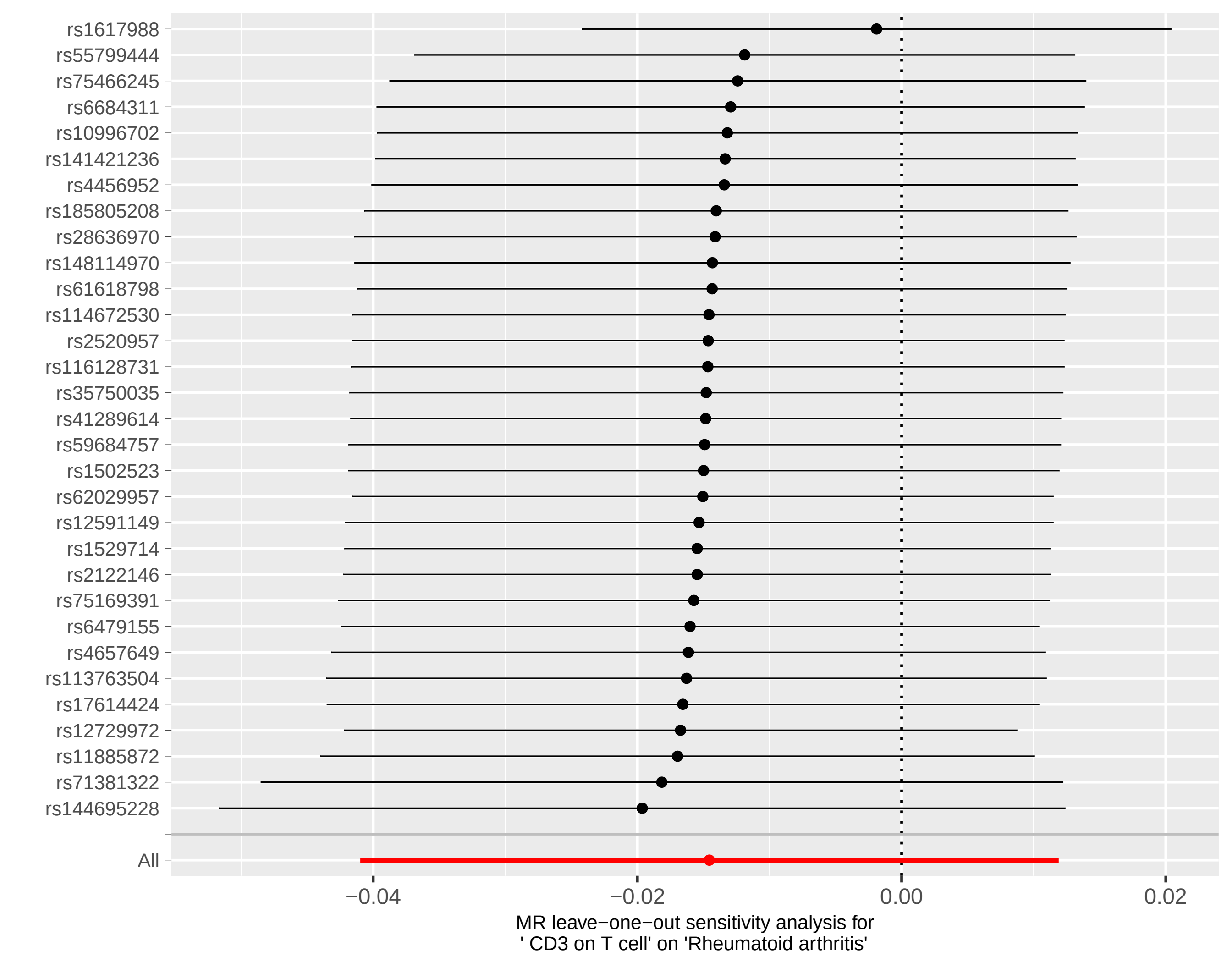
rs1617988
rs55799444
rs75466245
rs6684311
rs10996702
rs141421236
rs4456952
rs185805208
rs28636970
rs148114970
rs61618798
rs114672530
rs2520957
rs116128731
rs35750035
rs41289614
rs59684757
rs1502523
rs62029957
rs12591149
rs1529714
rs2122146
rs75169391
rs6479155
rs4657649
rs113763504
rs17614424
rs12729972
rs11885872
rs71381322
rs144695228
All
−0.04
−0.02
0.00
0.02
MR leave−one−out sensitivity analysis for
' CD3 on T cell' on 'Rheumatoid arthritis'

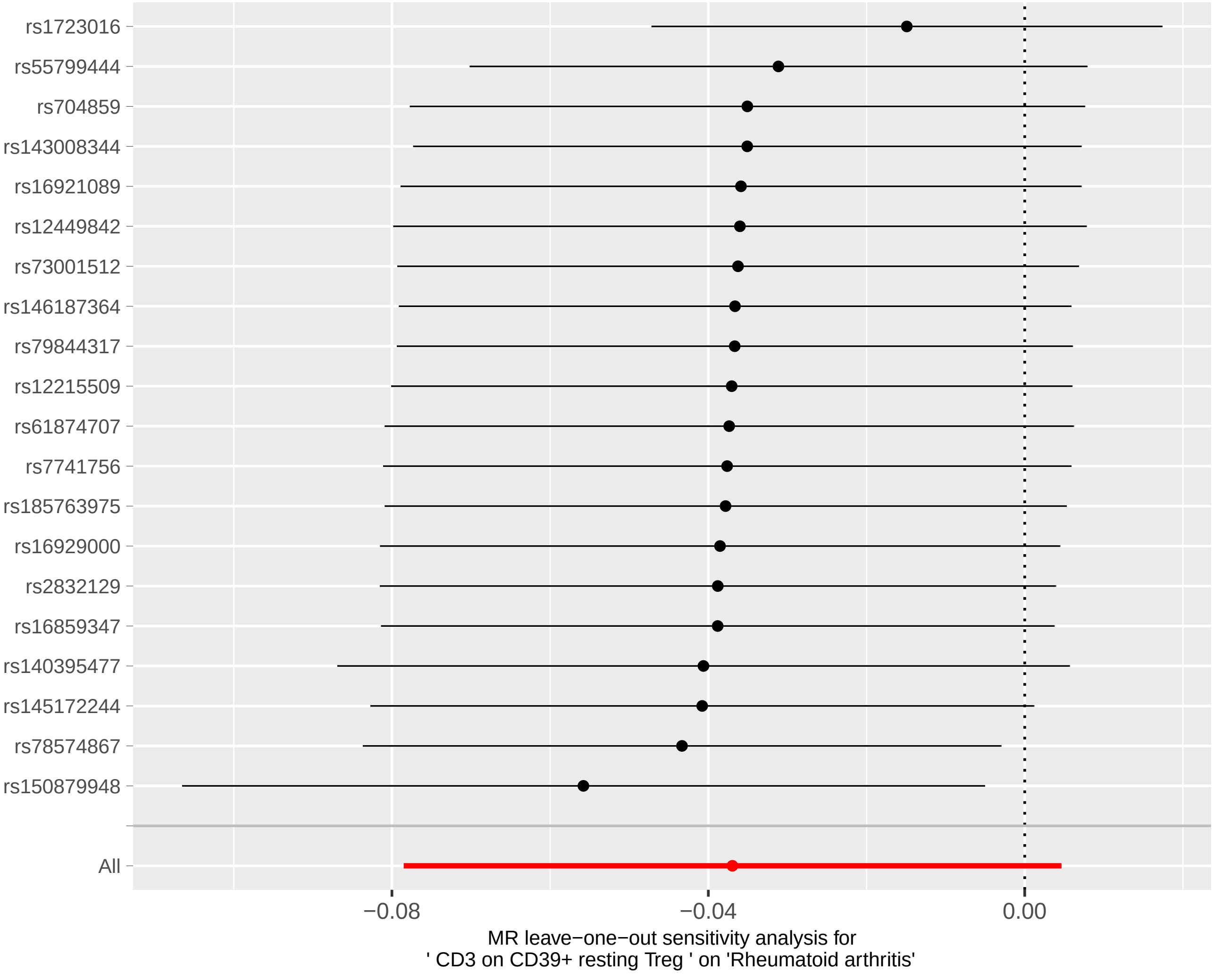

rs1723016
rs55799444
rs704859
rs143008344
rs16921089
rs12449842
rs73001512
rs146187364
rs79844317
rs12215509
rs61874707
rs7741756
rs185763975
rs16929000
rs2832129
rs16859347
rs140395477
rs145172244
rs78574867
rs150879948
All
−0.08
−0.04
0.00
MR leave−one−out sensitivity analysis for
' CD3 on CD39+ resting Treg ' on 'Rheumatoid arthritis'

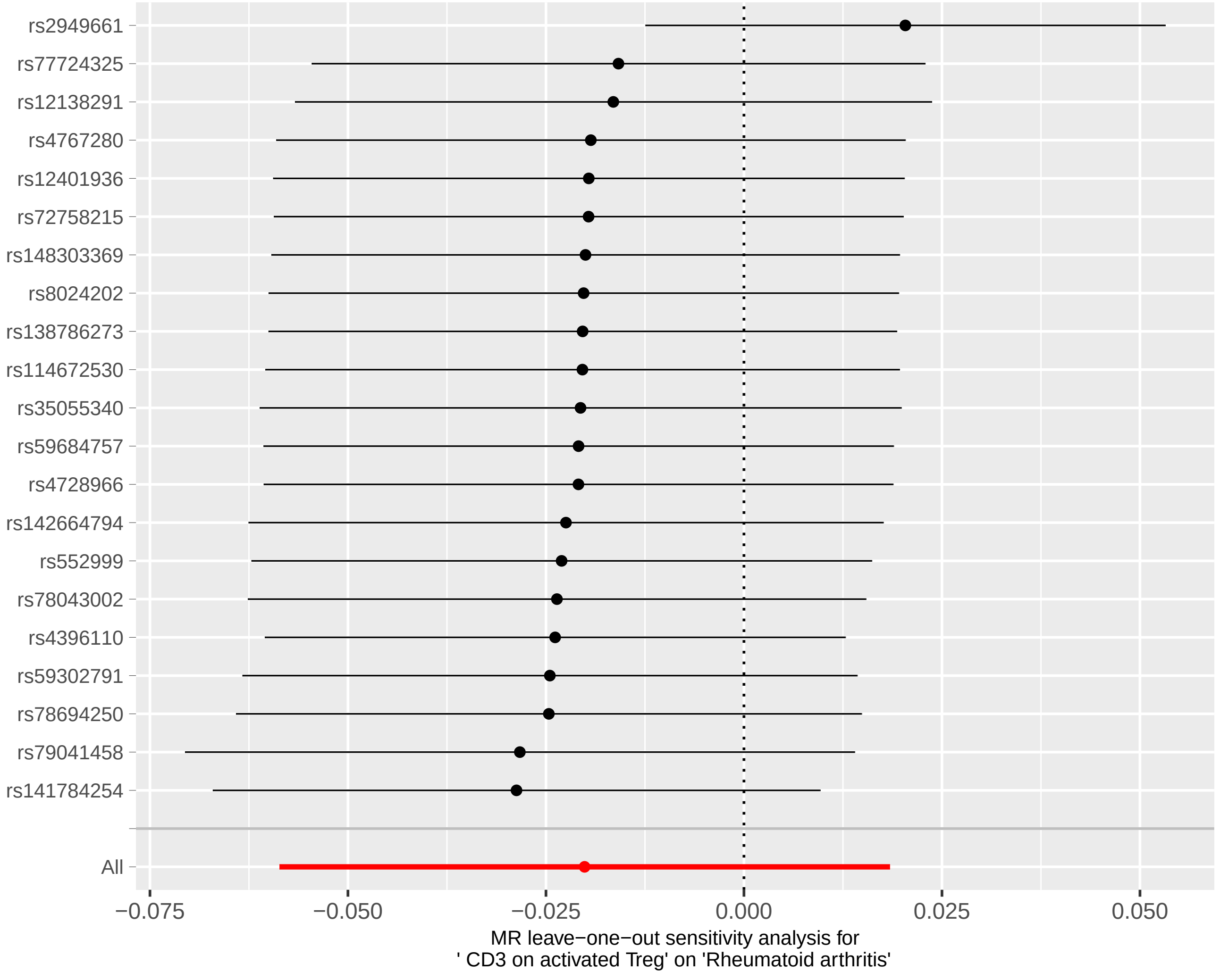
rs2949661
rs77724325
rs12138291
rs4767280
rs12401936
rs72758215
rs148303369
rs8024202
rs138786273
rs114672530
rs35055340
rs59684757
rs4728966
rs142664794
rs552999
rs78043002
rs4396110
rs59302791
rs78694250
rs79041458
rs141784254
All
−0.075
−0.050
−0.025
0.000
0.025
0.050
MR leave−one−out sensitivity analysis for
' CD3 on activated Treg' on 'Rheumatoid arthritis'

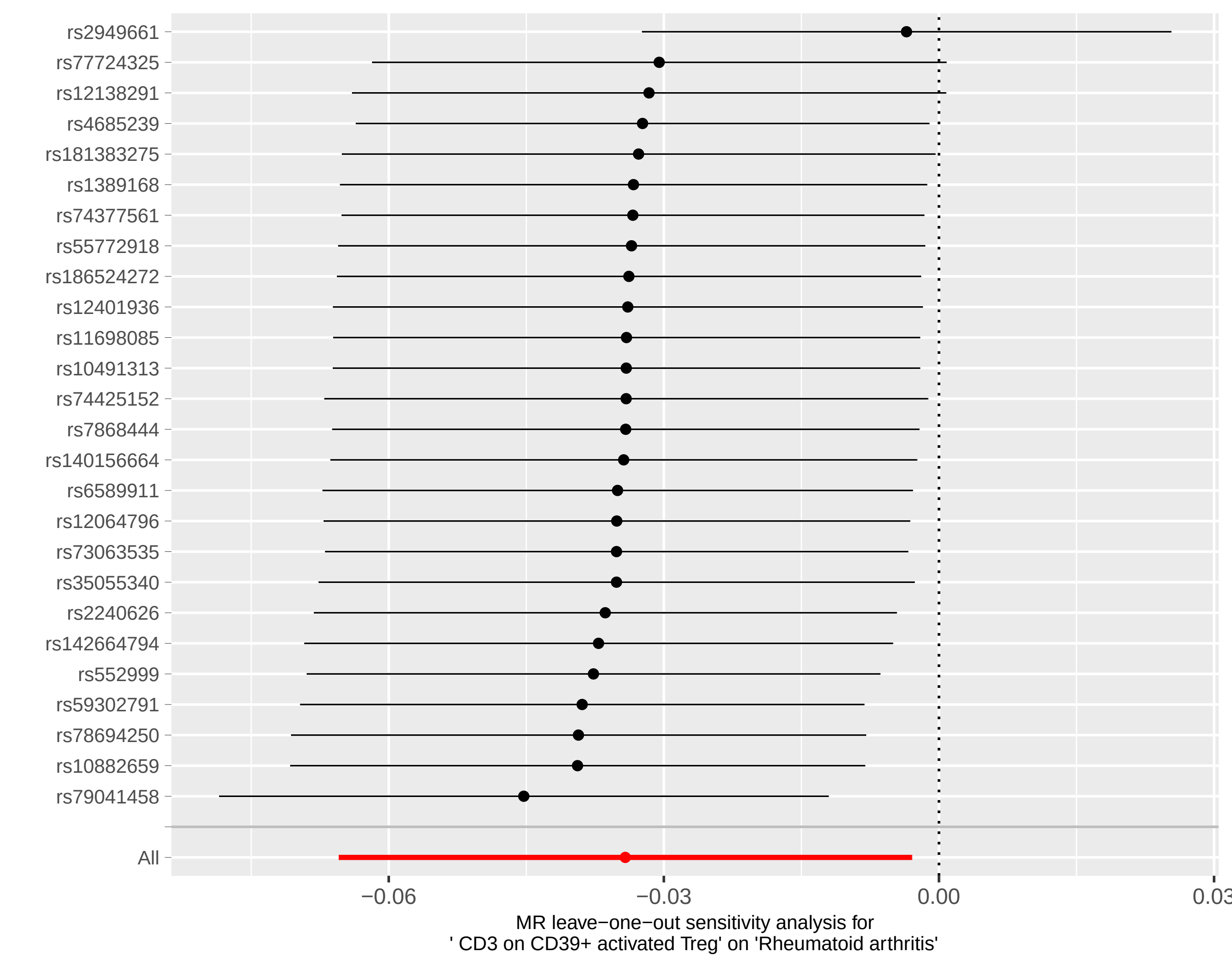

rs2949661
rs77724325
rs12138291
rs4685239
rs181383275
rs1389168
rs74377561
rs55772918
rs186524272
rs12401936
rs11698085
rs10491313
rs74425152
rs7868444
rs140156664
rs6589911
rs12064796
rs73063535
rs35055340
rs2240626
rs142664794
rs552999
rs59302791
rs78694250
rs10882659
rs79041458
All
−0.06
−0.03
0.00
MR leave−one−out sensitivity analysis for
' CD3 on CD39+ activated Treg' on 'Rheumatoid arthritis'

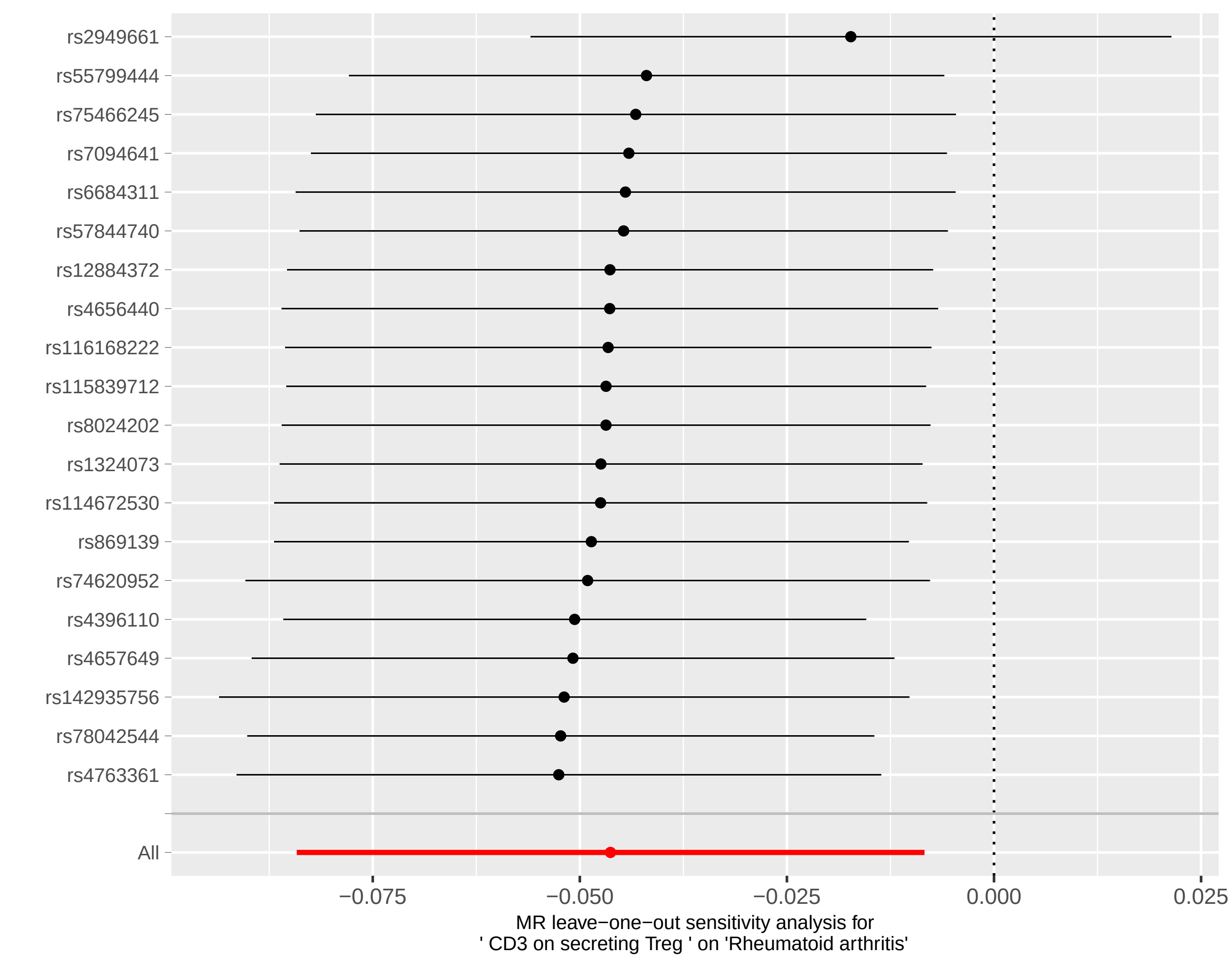
rs2949661
rs55799444
rs75466245
rs7094641
rs6684311
rs57844740
rs12884372
rs4656440
rs116168222
rs115839712
rs8024202
rs1324073
rs114672530
rs869139
rs74620952
rs4396110
rs4657649
rs142935756
rs78042544
rs4763361
All
−0.075
−0.050
−0.025
0.000
0.025
MR leave−one−out sensitivity analysis for
' CD3 on secreting Treg ' on 'Rheumatoid arthritis'

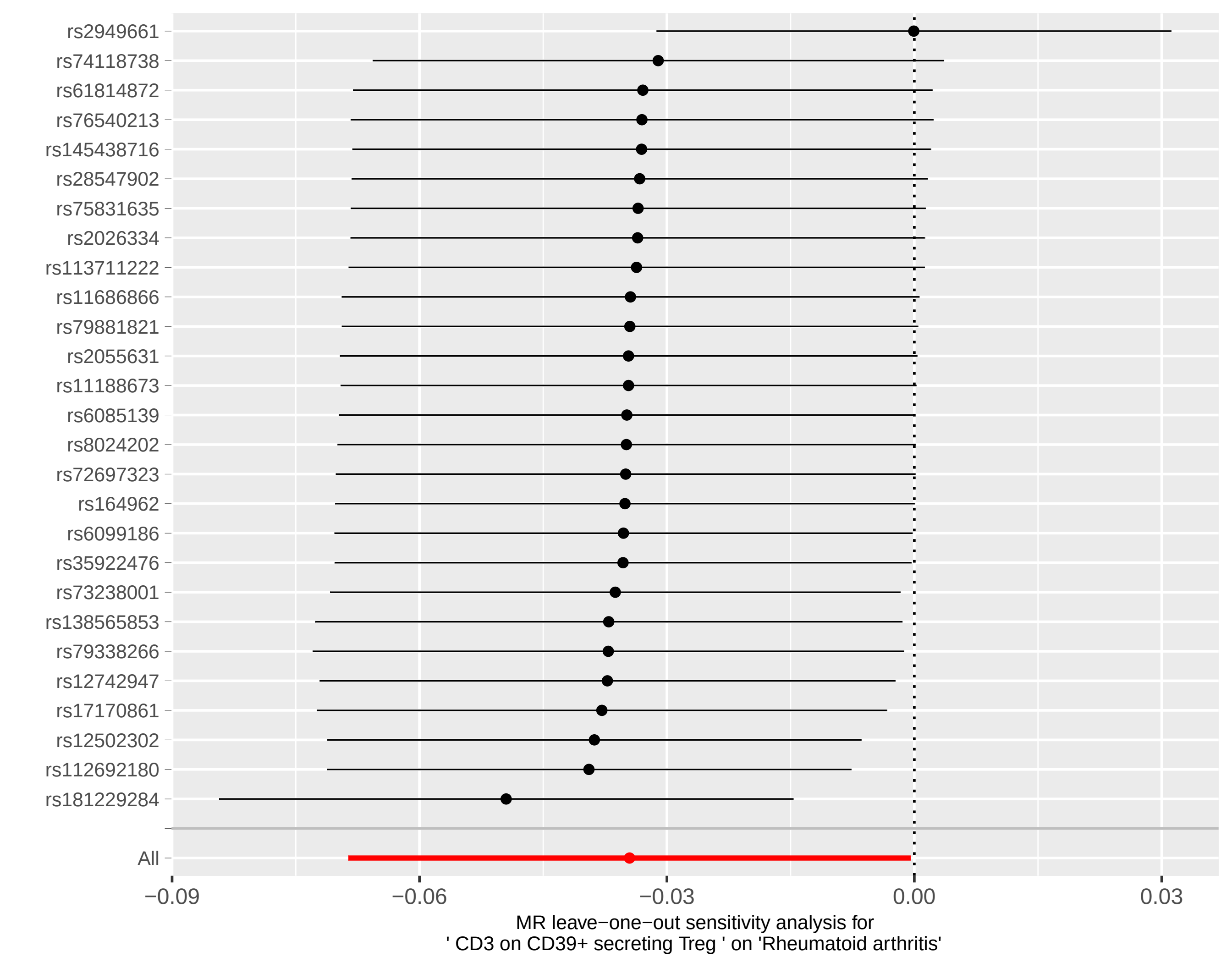

rs2949661
rs74118738
rs61814872
rs76540213
rs145438716
rs28547902
rs75831635
rs2026334
rs113711222
rs11686866
rs79881821
rs2055631
rs11188673
rs6085139
rs8024202
rs72697323
rs164962
rs6099186
rs35922476
rs73238001
rs138565853
rs79338266
rs12742947
rs17170861
rs12502302
rs112692180
rs181229284
All
−0.09
−0.06
−0.03
0.00
0.03
MR leave−one−out sensitivity analysis for
' CD3 on CD39+ secreting Treg ' on 'Rheumatoid arthritis'

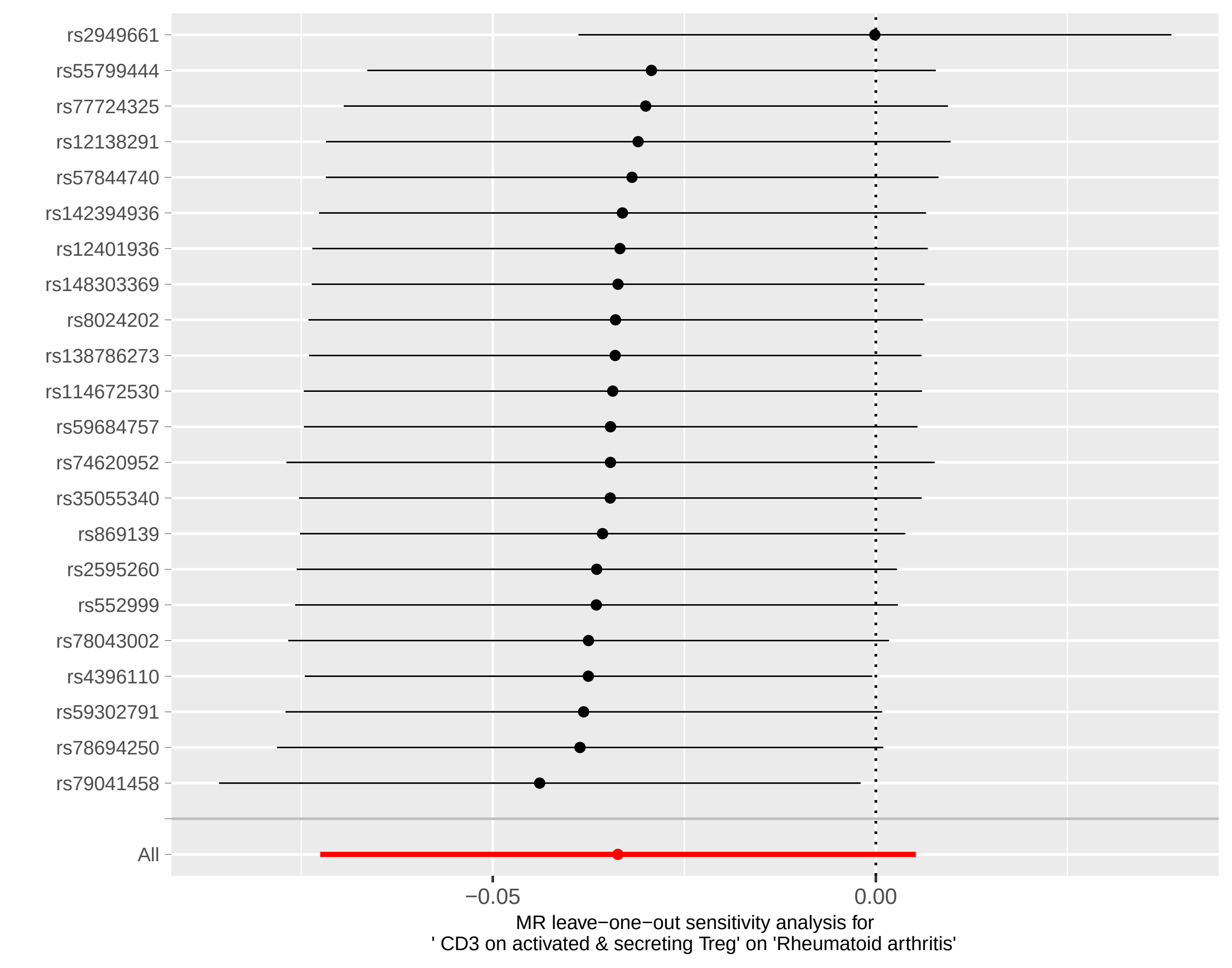
rs2949661
rs55799444
rs77724325
rs12138291
rs57844740
rs142394936
rs12401936
rs148303369
rs8024202
rs138786273
rs114672530
rs59684757
rs74620952
rs35055340
rs869139
rs2595260
rs552999
rs78043002
rs4396110
rs59302791
rs78694250
rs79041458
All
−0.05
0.00
MR leave−one−out sensitivity analysis for
' CD3 on activated & secreting Treg' on 'Rheumatoid arthritis'

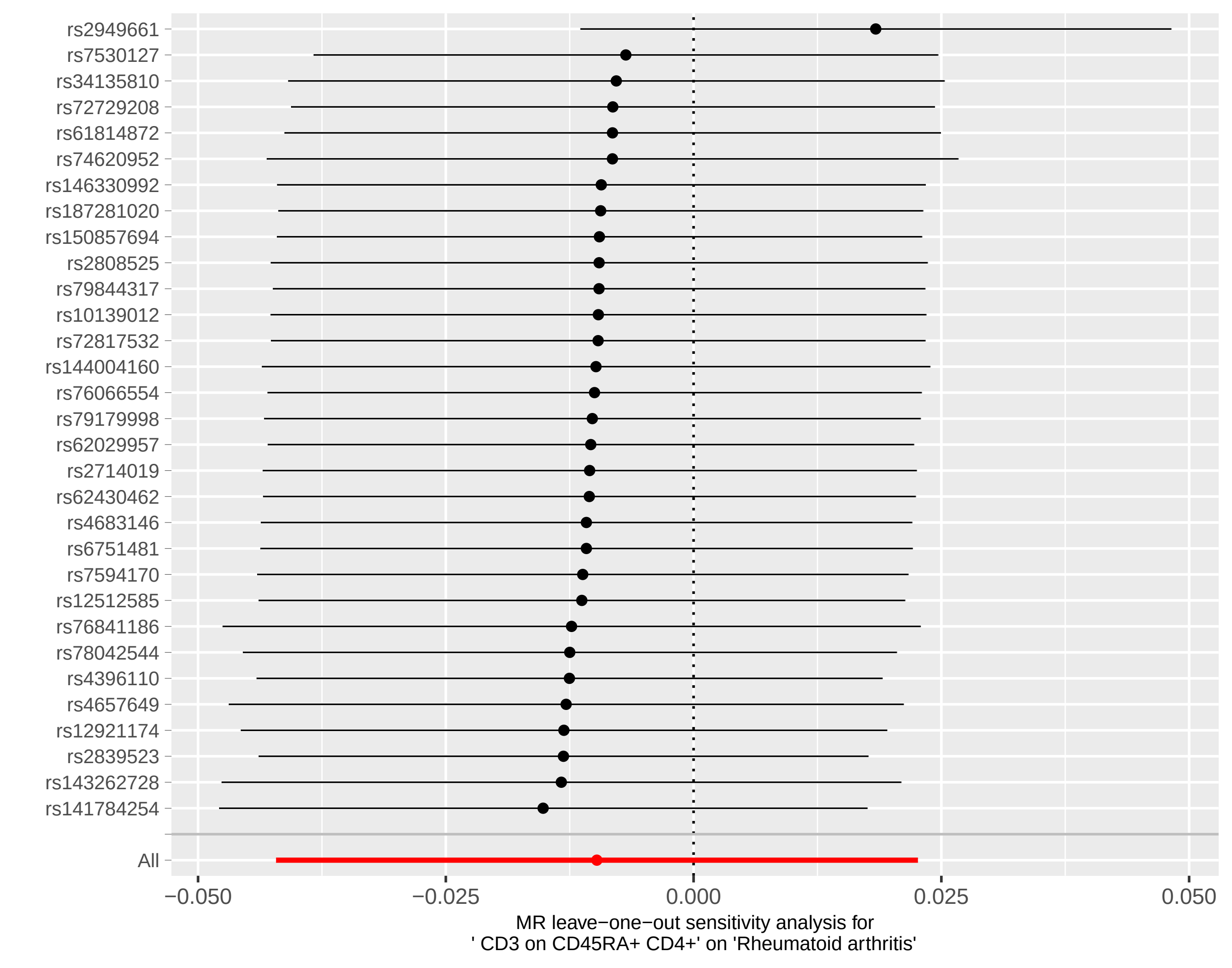
rs2949661
rs7530127
rs34135810
rs72729208
rs61814872
rs74620952
rs146330992
rs187281020
rs150857694
rs2808525
rs79844317
rs10139012
rs72817532
rs144004160
rs76066554
rs79179998
rs62029957
rs2714019
rs62430462
rs4683146
rs6751481
rs7594170
rs12512585
rs76841186
rs78042544
rs4396110
rs4657649
rs12921174
rs2839523
rs143262728
rs141784254
All
−0.050
−0.025
0.000
0.025
0.050
MR leave−one−out sensitivity analysis for
' CD3 on CD45RA+ CD4+' on 'Rheumatoid arthritis'

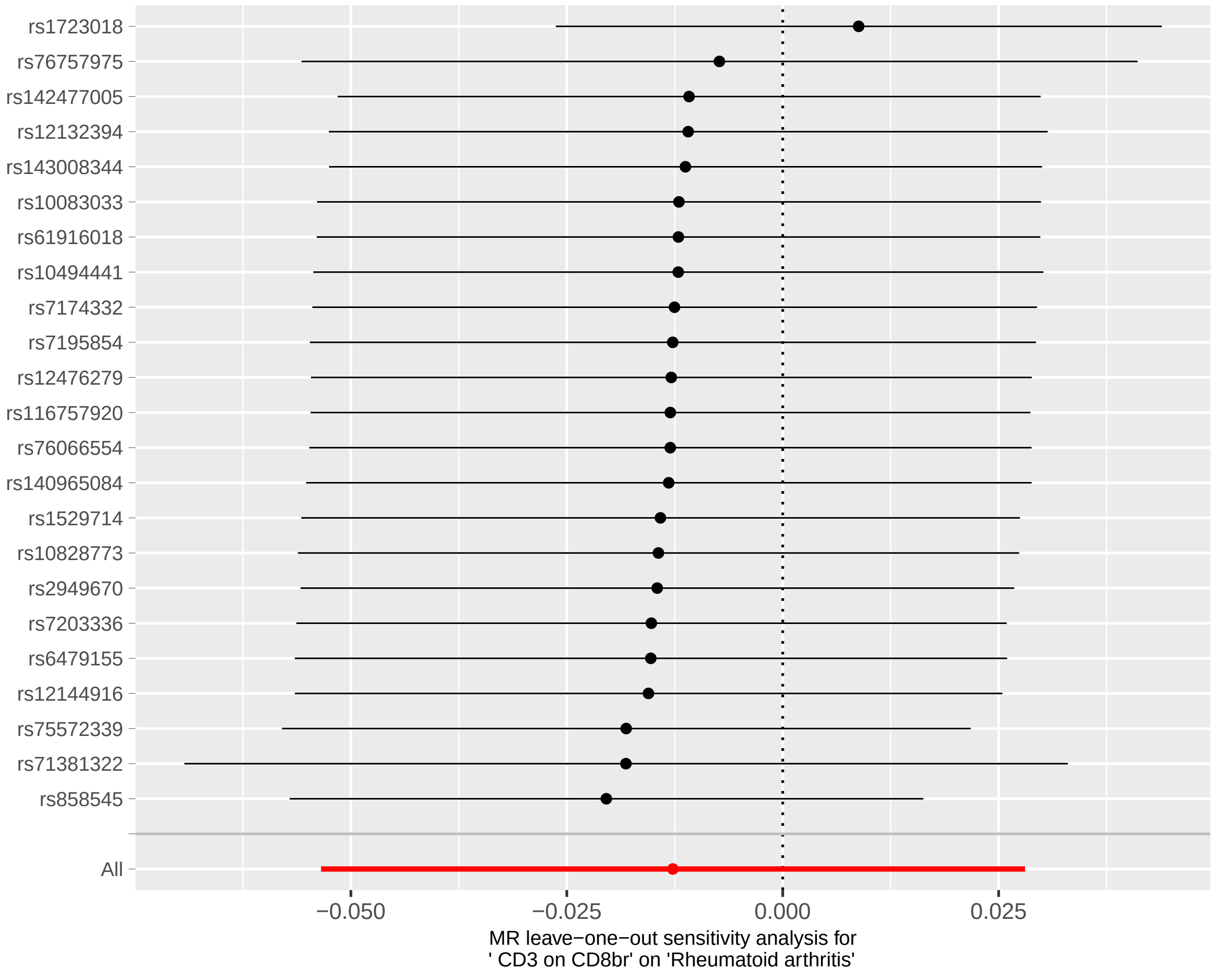

rs1723018
rs76757975
rs142477005
rs12132394
rs143008344
rs10083033
rs61916018
rs10494441
rs7174332
rs7195854
rs12476279
rs116757920
rs76066554
rs140965084
rs1529714
rs10828773
rs2949670
rs7203336
rs6479155
rs12144916
rs75572339
rs71381322
rs858545
All
−0.050
−0.025
0.000
0.025
MR leave−one−out sensitivity analysis for
' CD3 on CD8br' on 'Rheumatoid arthritis'

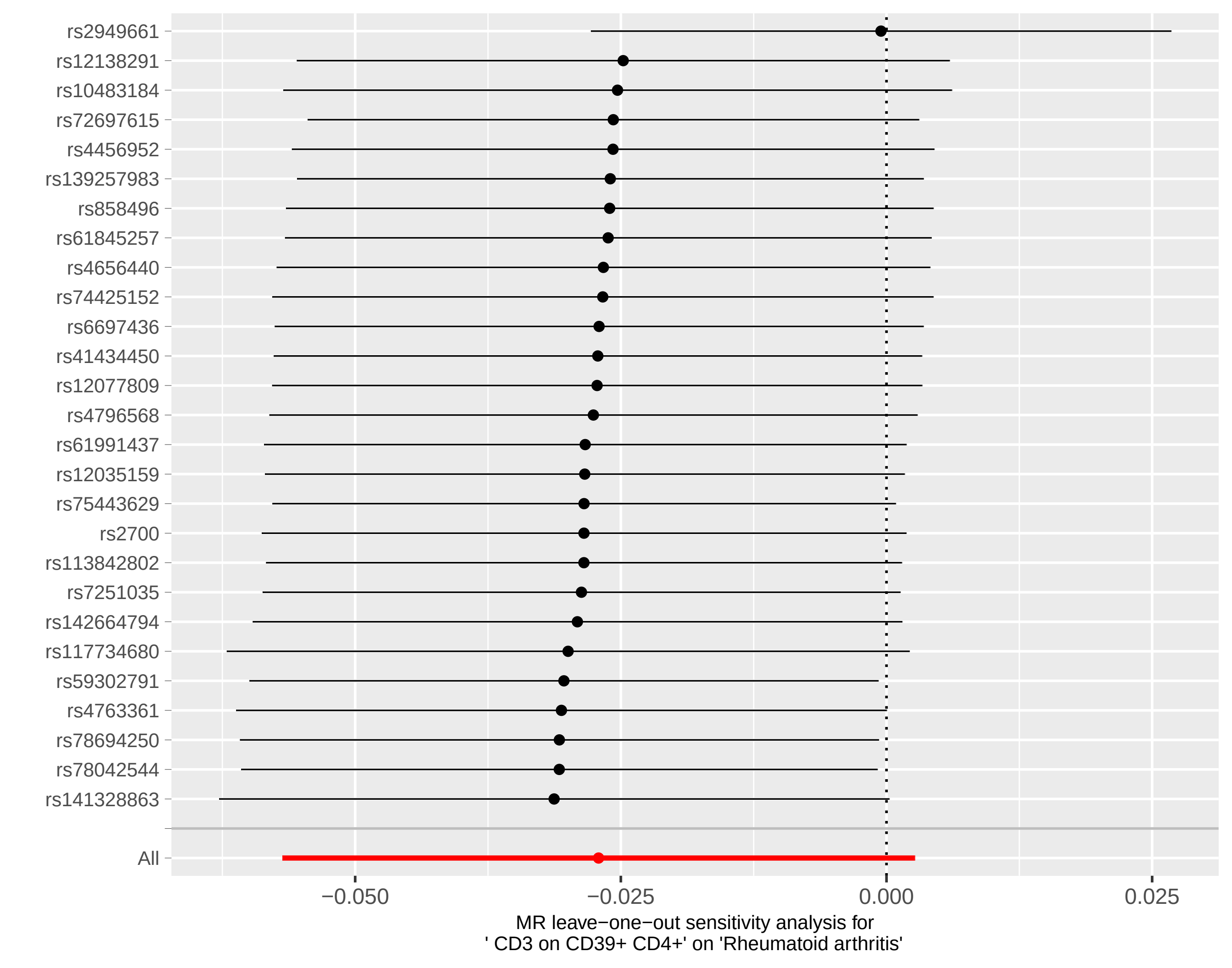

rs2949661
rs12138291
rs10483184
rs72697615
rs4456952
rs139257983
rs858496
rs61845257
rs4656440
rs74425152
rs6697436
rs41434450
rs12077809
rs4796568
rs61991437
rs12035159
rs75443629
rs2700
rs113842802
rs7251035
rs142664794
rs117734680
rs59302791
rs4763361
rs78694250
rs78042544
rs141328863
All
−0.050
−0.025
0.000
0.025
MR leave−one−out sensitivity analysis for
' CD3 on CD39+ CD4+' on 'Rheumatoid arthritis'

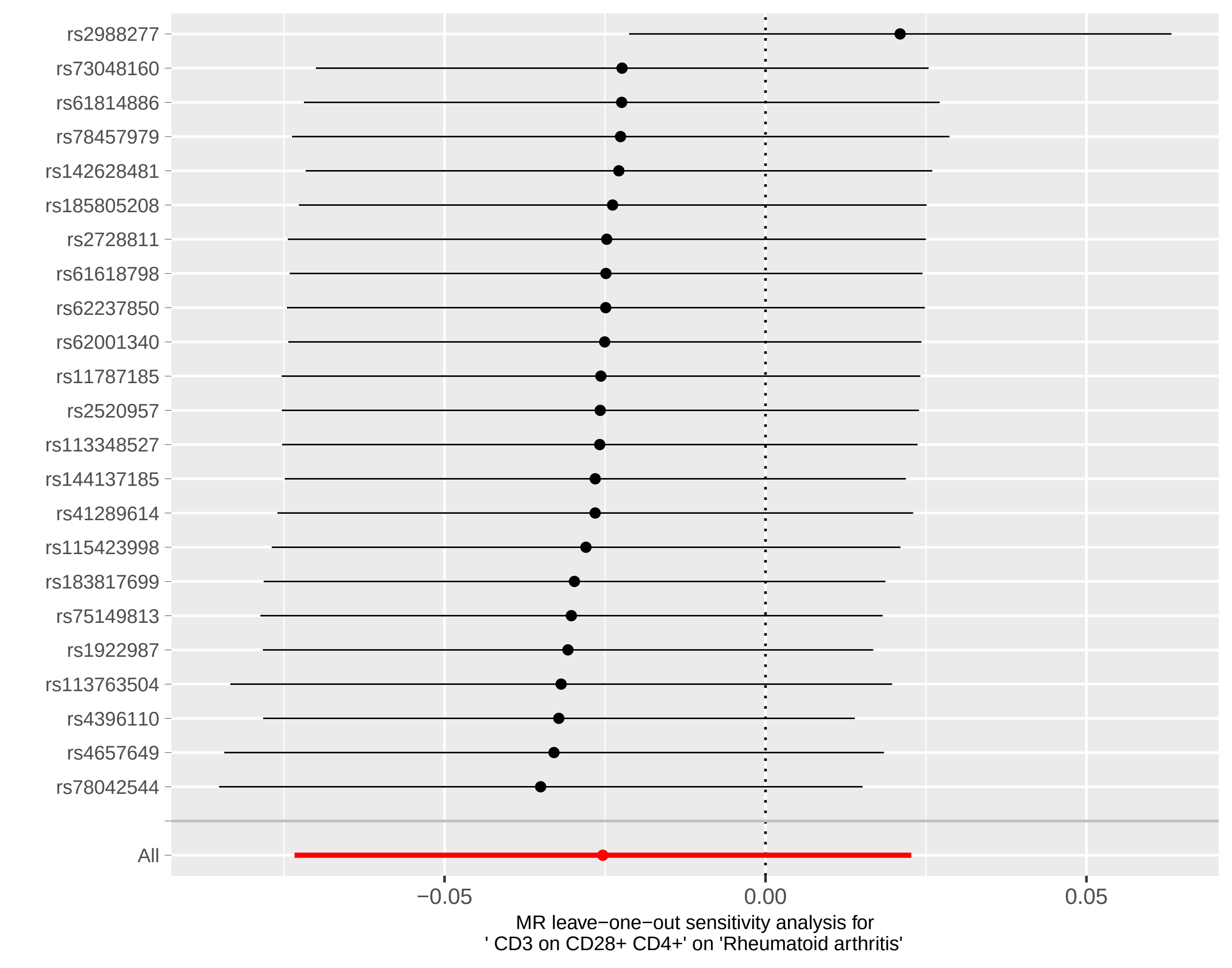
rs2988277
rs73048160
rs61814886
rs78457979
rs142628481
rs185805208
rs2728811
rs61618798
rs62237850
rs62001340
rs11787185
rs2520957
rs113348527
rs144137185
rs41289614
rs115423998
rs183817699
rs75149813
rs1922987
rs113763504
rs4396110
rs4657649
rs78042544
All
−0.05
0.00
0.05
MR leave−one−out sensitivity analysis for
' CD3 on CD28+ CD4+' on 'Rheumatoid arthritis'

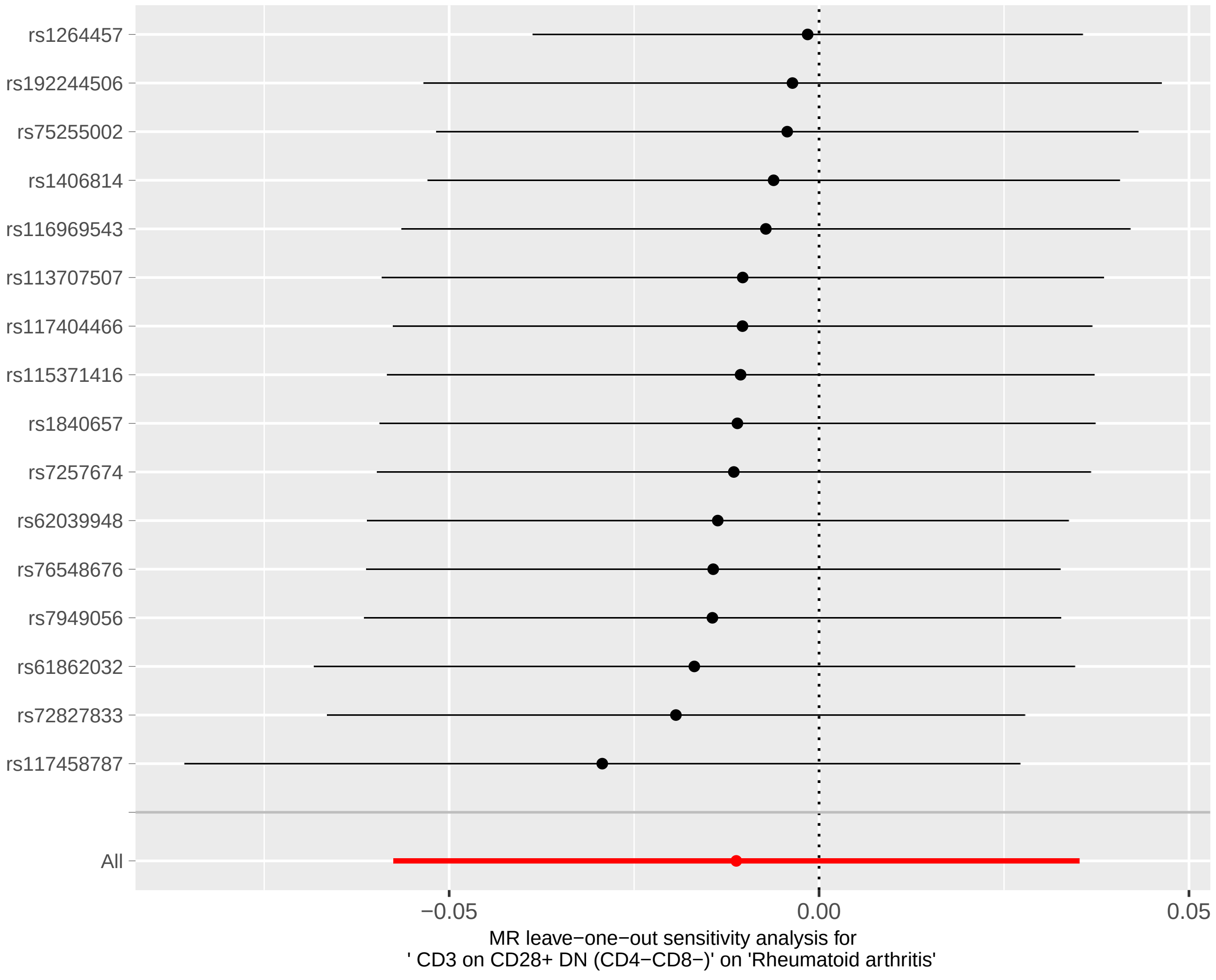
rs1264457
rs192244506
rs75255002
rs1406814
rs116969543
rs113707507
rs117404466
rs115371416
rs1840657
rs7257674
rs62039948
rs76548676
rs7949056
rs61862032
rs72827833
rs117458787
All
−0.05
0.00
0.05
MR leave−one−out sensitivity analysis for
' CD3 on CD28+ DN (CD4−CD8−)' on 'Rheumatoid arthritis'

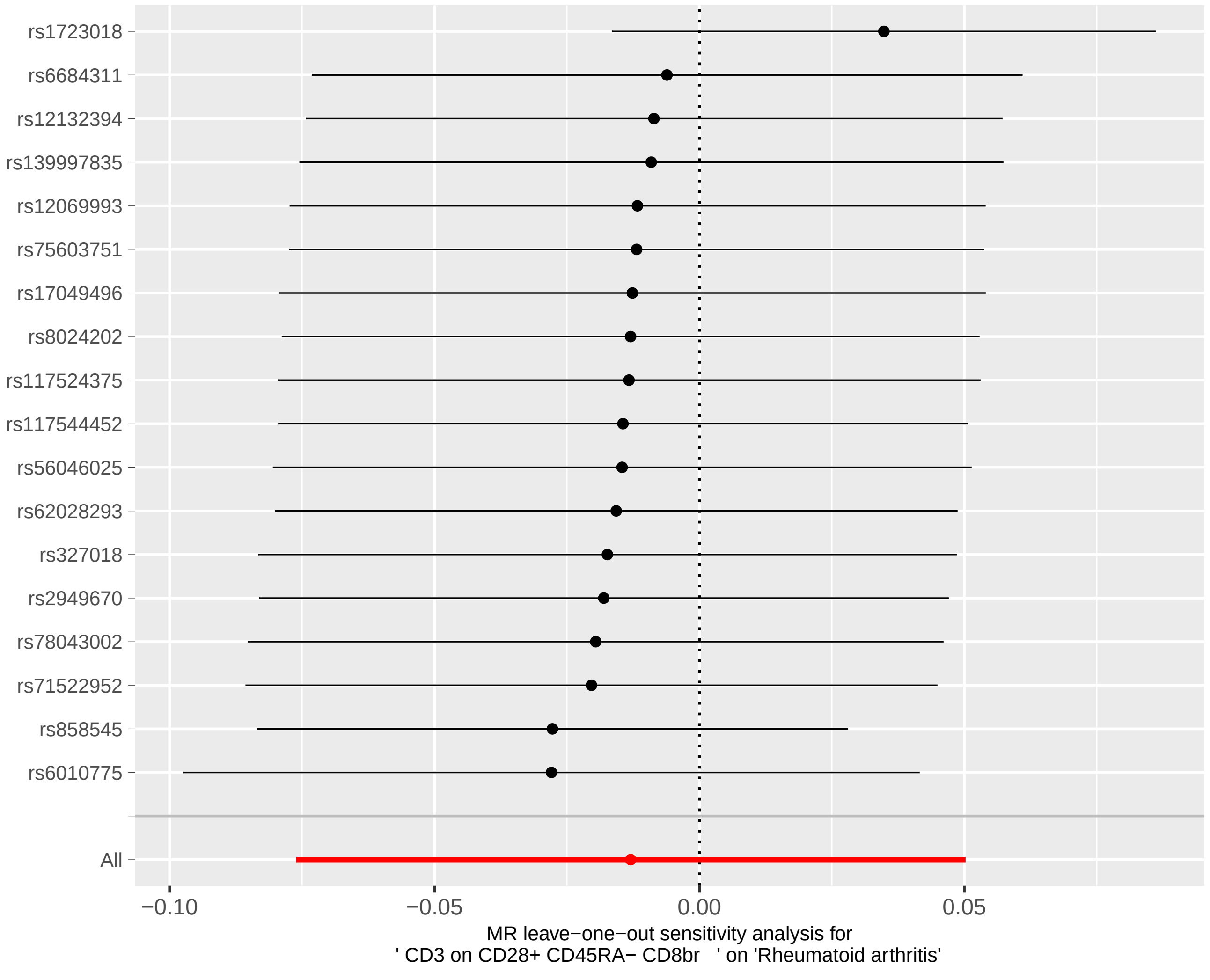
rs1723018
rs6684311
rs12132394
rs139997835
rs12069993
rs75603751
rs17049496
rs8024202
rs117524375
rs117544452
rs56046025
rs62028293
rs327018
rs2949670
rs78043002
rs71522952
rs858545
rs6010775
All
−0.10
−0.05
0.00
0.05
MR leave−one−out sensitivity analysis for
' CD3 on CD28+ CD45RA− CD8br ' on 'Rheumatoid arthritis'

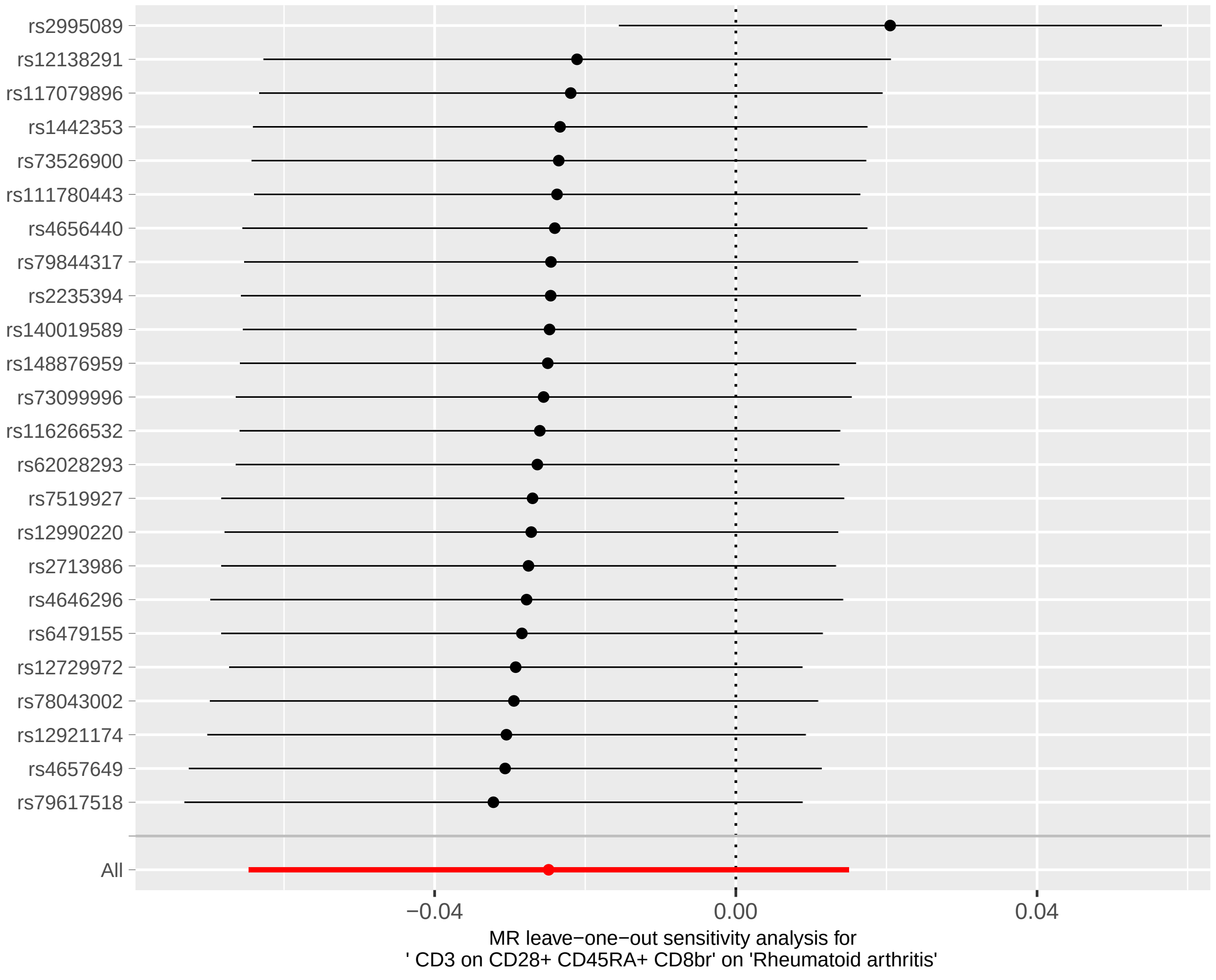
rs2995089
rs12138291
rs117079896
rs1442353
rs73526900
rs111780443
rs4656440
rs79844317
rs2235394
rs140019589
rs148876959
rs73099996
rs116266532
rs62028293
rs7519927
rs12990220
rs2713986
rs4646296
rs6479155
rs12729972
rs78043002
rs12921174
rs4657649
rs79617518
All
−0.04
0.00
0.04
MR leave−one−out sensitivity analysis for
' CD3 on CD28+ CD45RA+ CD8br' on 'Rheumatoid arthritis'

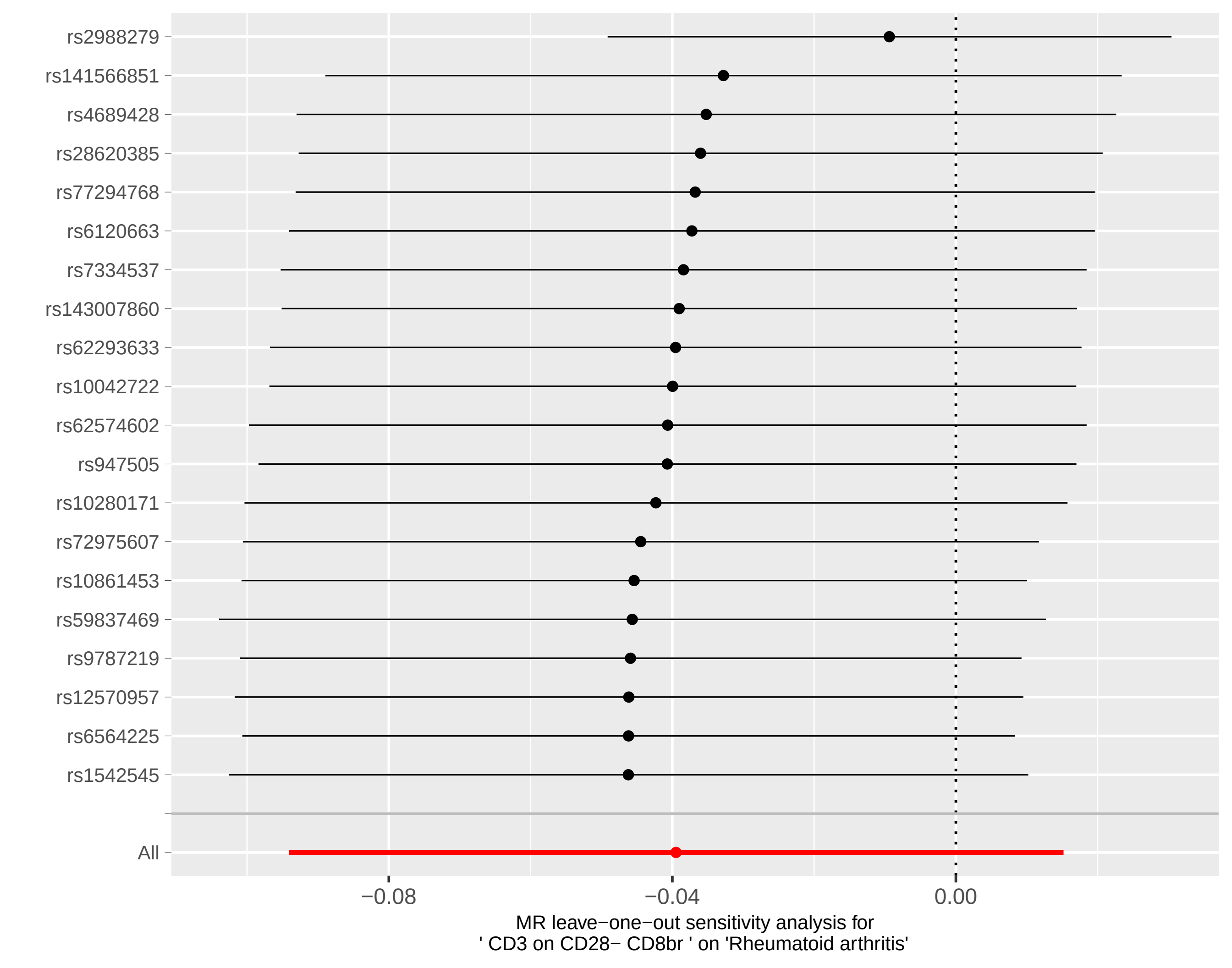
rs2988279
rs141566851
rs4689428
rs28620385
rs77294768
rs6120663
rs7334537
rs143007860
rs62293633
rs10042722
rs62574602
rs947505
rs10280171
rs72975607
rs10861453
rs59837469
rs9787219
rs12570957
rs6564225
rs1542545
All
−0.08
−0.04
0.00
MR leave−one−out sensitivity analysis for
' CD3 on CD28− CD8br ' on 'Rheumatoid arthritis'

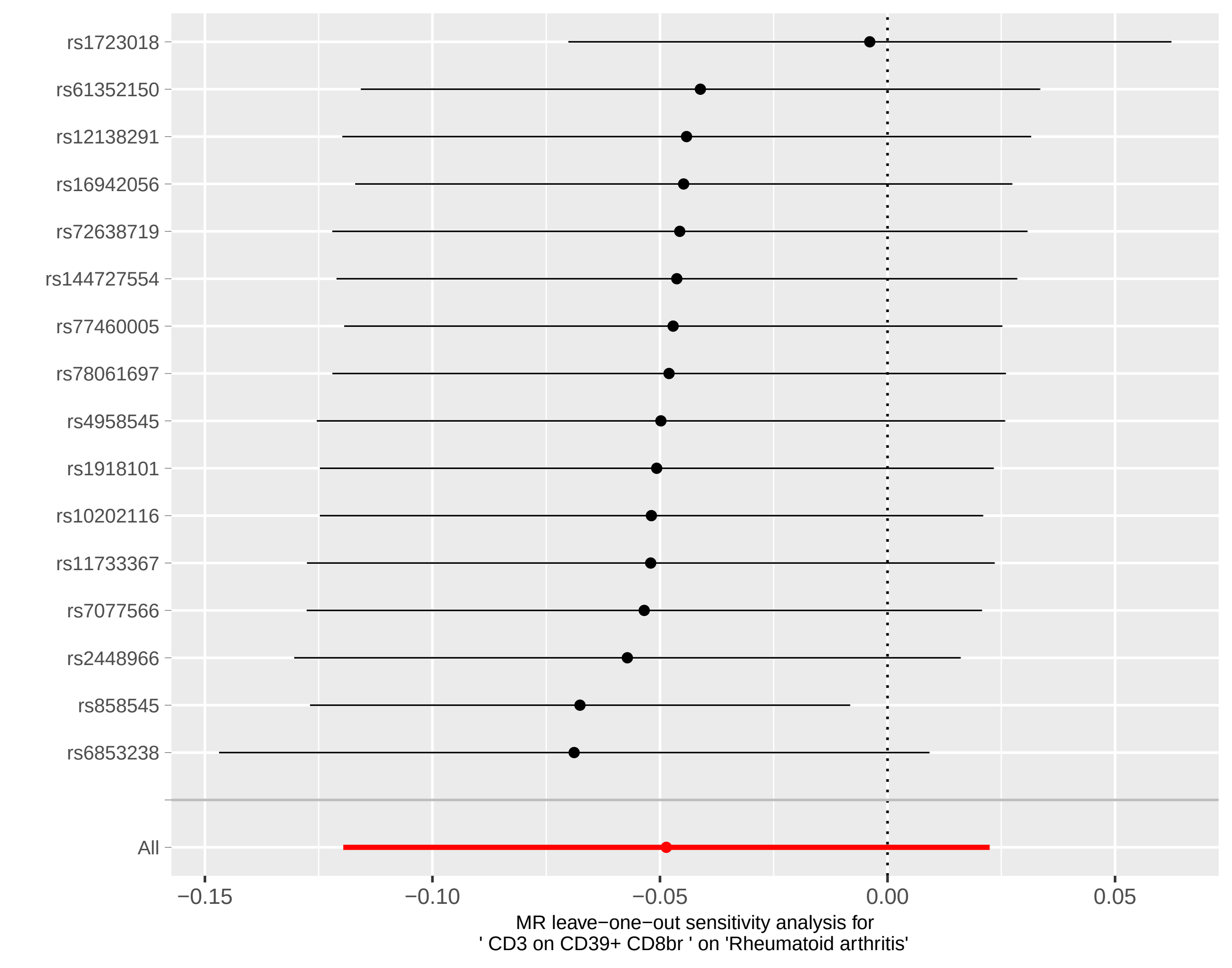
rs1723018
rs61352150
rs12138291
rs16942056
rs72638719
rs144727554
rs77460005
rs78061697
rs4958545
rs1918101
rs10202116
rs11733367
rs7077566
rs2448966
rs858545
rs6853238
All
−0.15
−0.10
−0.05
0.00
0.05
MR leave−one−out sensitivity analysis for
' CD3 on CD39+ CD8br ' on 'Rheumatoid arthritis'

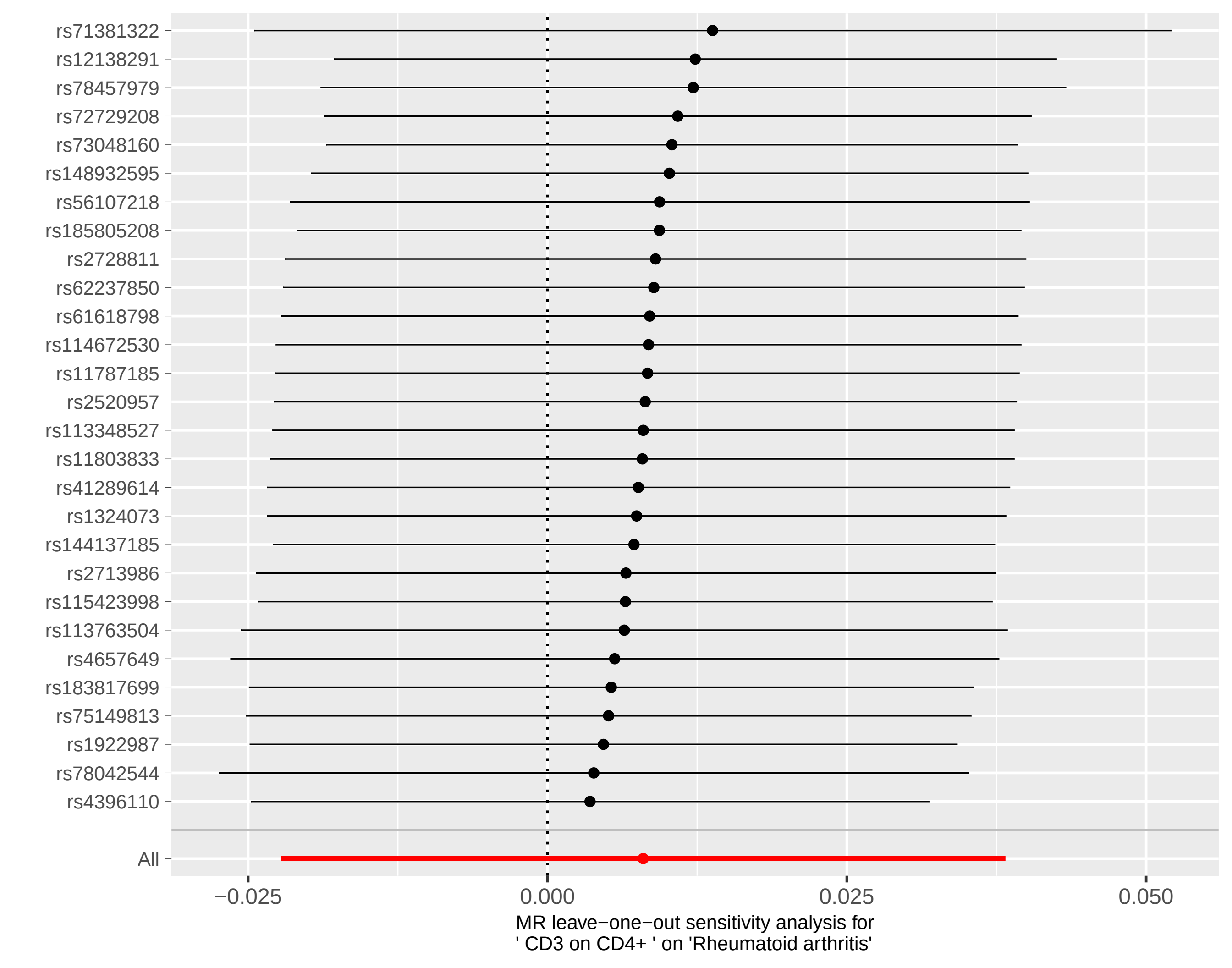

rs71381322
rs12138291
rs78457979
rs72729208
rs73048160
rs148932595
rs56107218
rs185805208
rs2728811
rs62237850
rs61618798
rs114672530
rs11787185
rs2520957
rs113348527
rs11803833
rs41289614
rs1324073
rs144137185
rs2713986
rs115423998
rs113763504
rs4657649
rs183817699
rs75149813
rs1922987
rs78042544
rs4396110
All
−0.025
0.000
0.025
0.050
MR leave−one−out sensitivity analysis for
' CD3 on CD4+ ' on 'Rheumatoid arthritis'

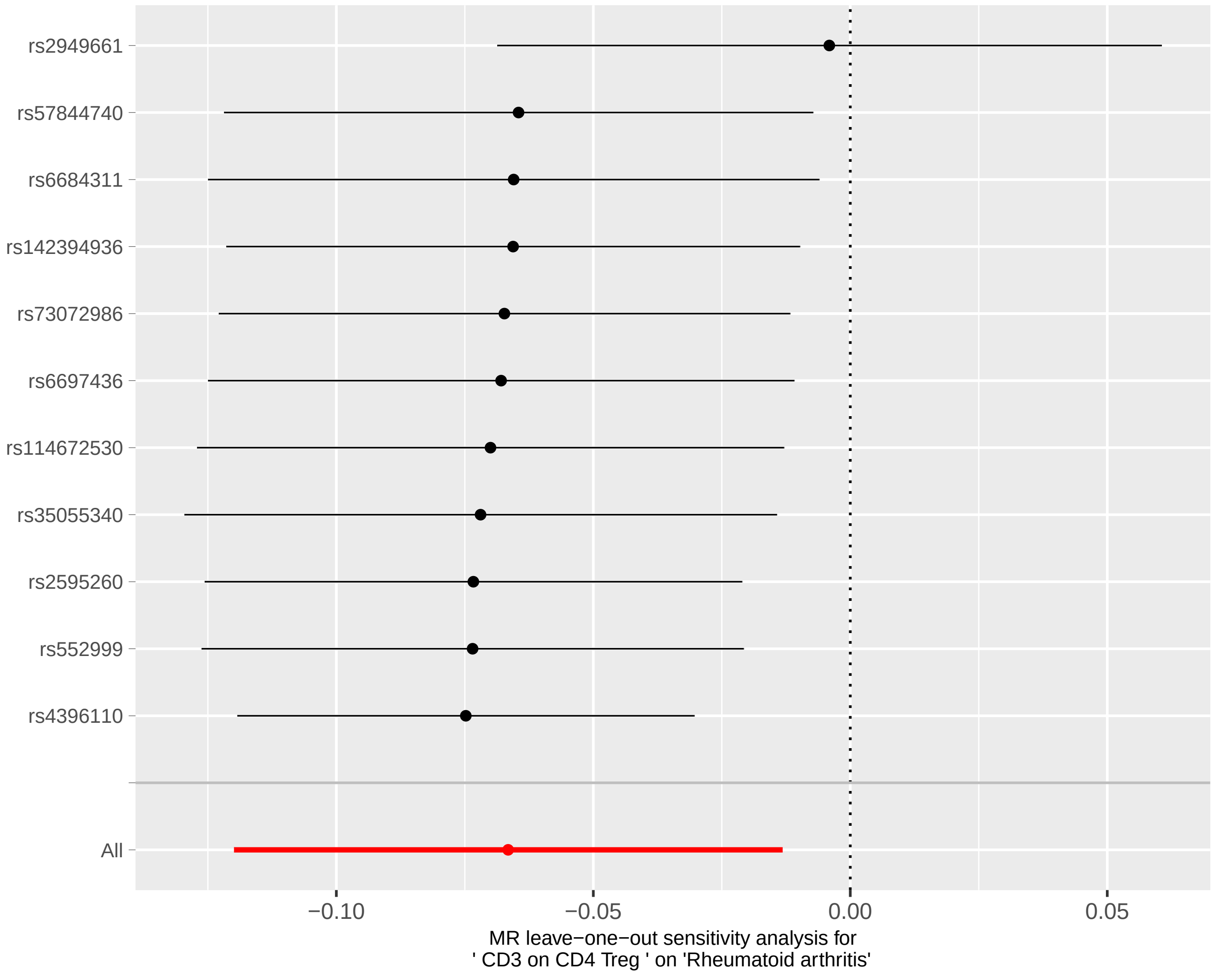
rs2949661
rs57844740
rs6684311
rs142394936
rs73072986
rs6697436
rs114672530
rs35055340
rs2595260
rs552999
rs4396110
All
−0.10
−0.05
0.00
0.05
MR leave−one−out sensitivity analysis for
' CD3 on CD4 Treg ' on 'Rheumatoid arthritis'

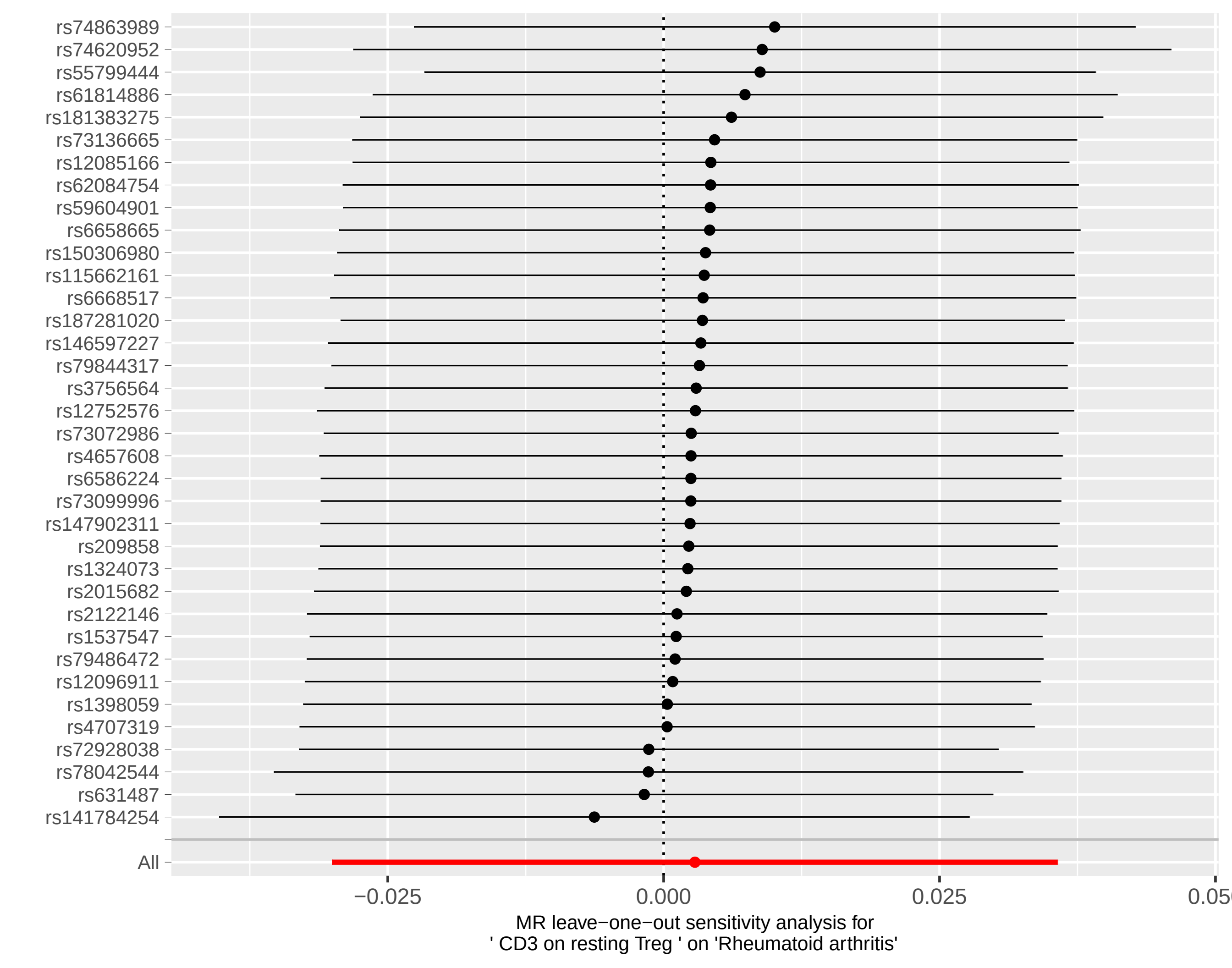

rs74863989
rs74620952
rs55799444
rs61814886
rs181383275
rs73136665
rs12085166
rs62084754
rs59604901
rs6658665
rs150306980
rs115662161
rs6668517
rs187281020
rs146597227
rs79844317
rs3756564
rs12752576
rs73072986
rs4657608
rs6586224
rs73099996
rs147902311
rs209858
rs1324073
rs2015682
rs2122146
rs1537547
rs79486472
rs12096911
rs1398059
rs4707319
rs72928038
rs78042544
rs631487
rs141784254
All
−0.025
0.000
0.025
MR leave−one−out sensitivity analysis for
' CD3 on resting Treg ' on 'Rheumatoid arthritis'

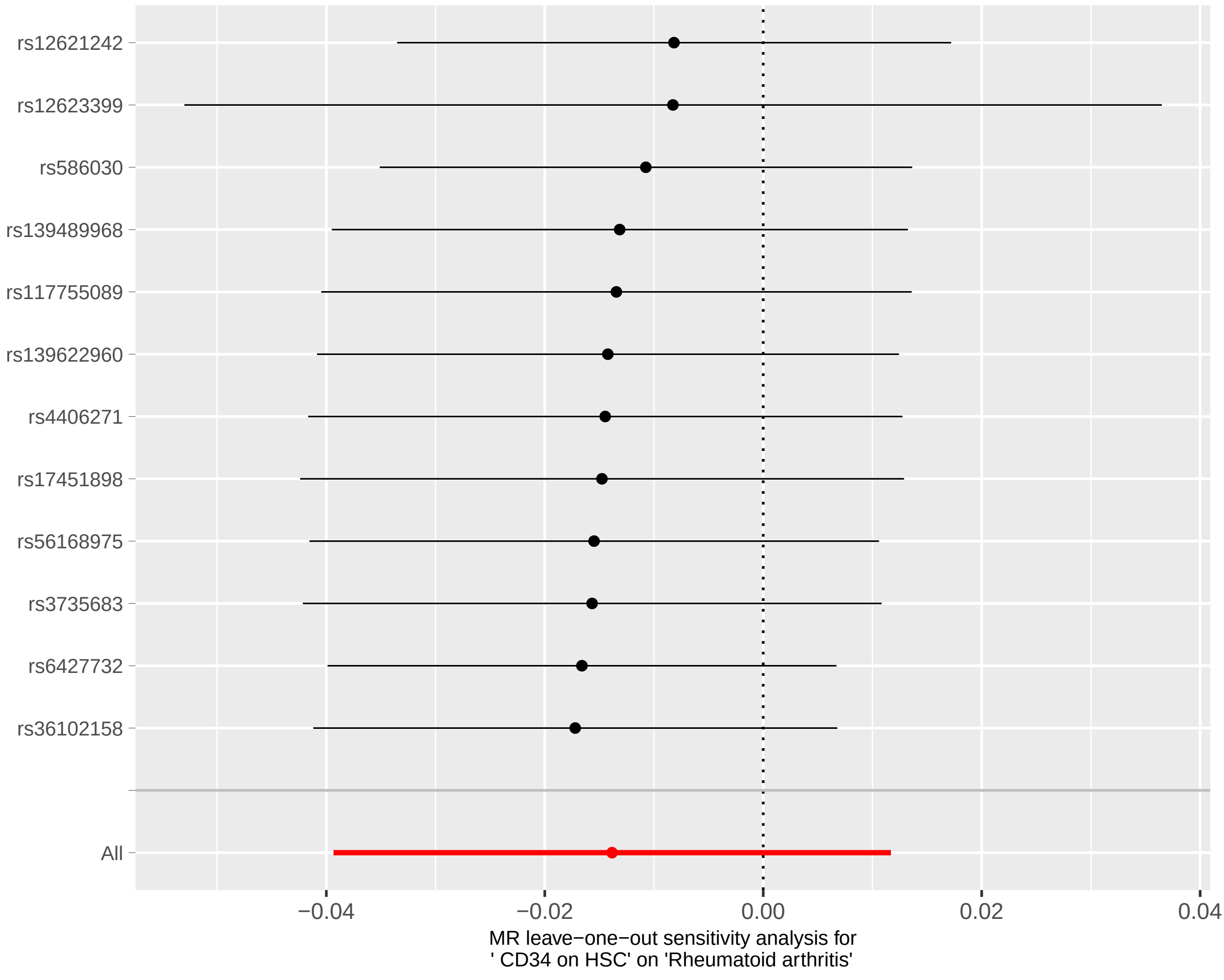
rs12621242
rs12623399
rs586030
rs139489968
rs117755089
rs139622960
rs4406271
rs17451898
rs56168975
rs3735683
rs6427732
rs36102158
All
−0.04
−0.02
0.00
0.02
0.04
MR leave−one−out sensitivity analysis for
' CD34 on HSC' on 'Rheumatoid arthritis'

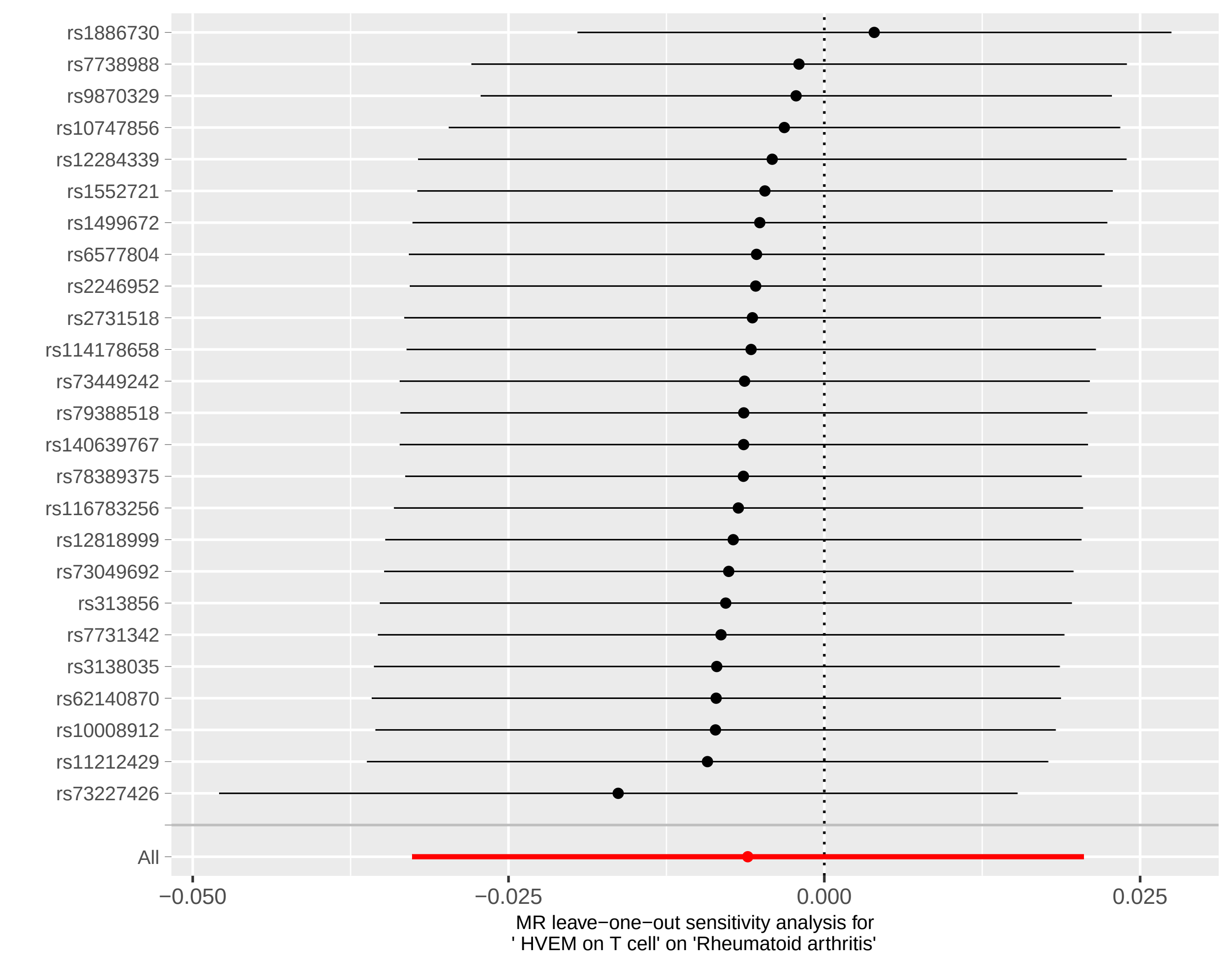

rs1886730
rs7738988
rs9870329
rs10747856
rs12284339
rs1552721
rs1499672
rs6577804
rs2246952
rs2731518
rs114178658
rs73449242
rs79388518
rs140639767
rs78389375
rs116783256
rs12818999
rs73049692
rs313856
rs7731342
rs3138035
rs62140870
rs10008912
rs11212429
rs73227426
All
−0.050
−0.025
0.000
0.025
MR leave−one−out sensitivity analysis for
' HVEM on T cell' on 'Rheumatoid arthritis'

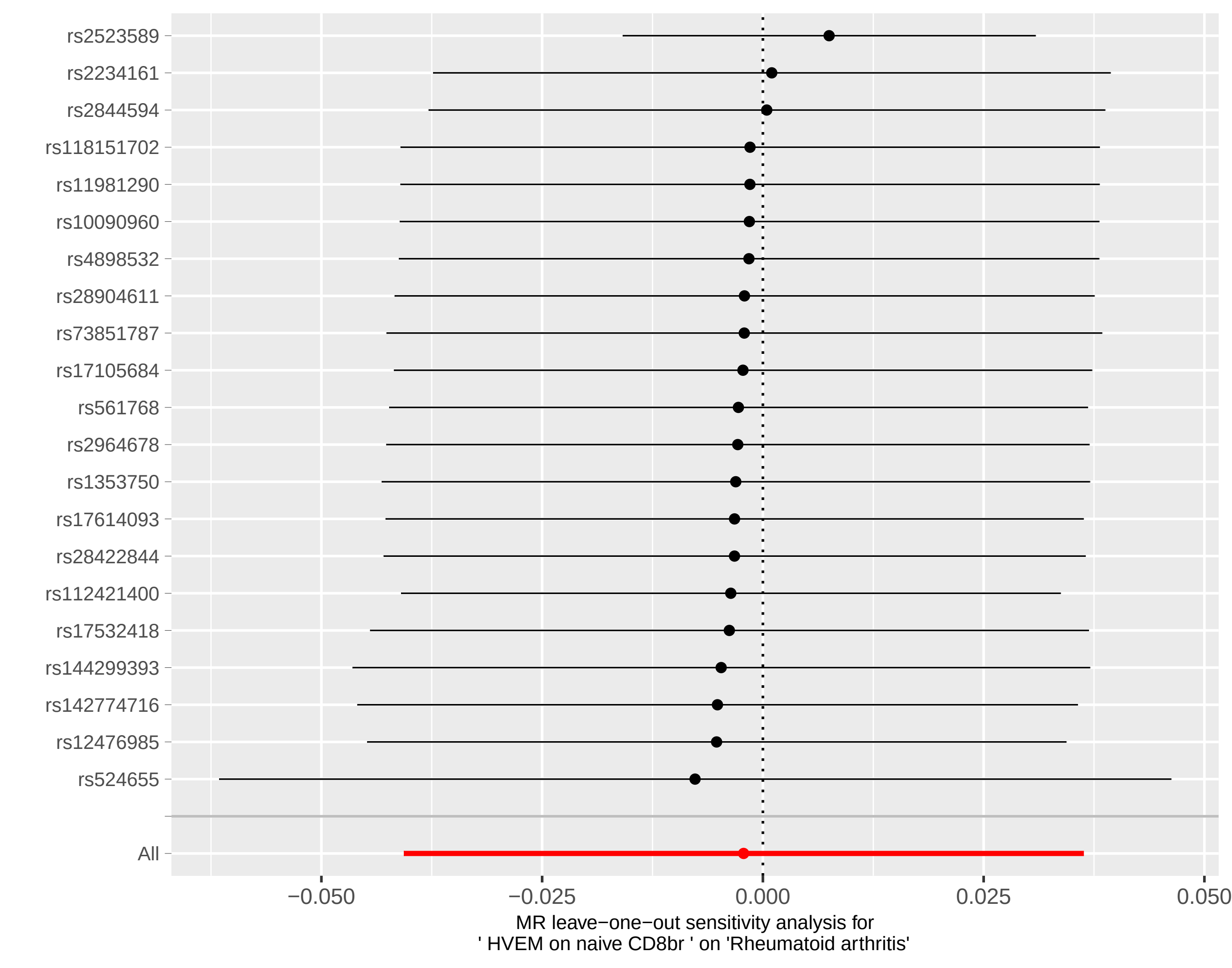

rs2523589
rs2234161
rs2844594
rs118151702
rs11981290
rs10090960
rs4898532
rs28904611
rs73851787
rs17105684
rs561768
rs2964678
rs1353750
rs17614093
rs28422844
rs112421400
rs17532418
rs144299393
rs142774716
rs12476985
rs524655
All
−0.050
−0.025
0.000
0.025
0.050
MR leave−one−out sensitivity analysis for
' HVEM on naive CD8br ' on 'Rheumatoid arthritis'

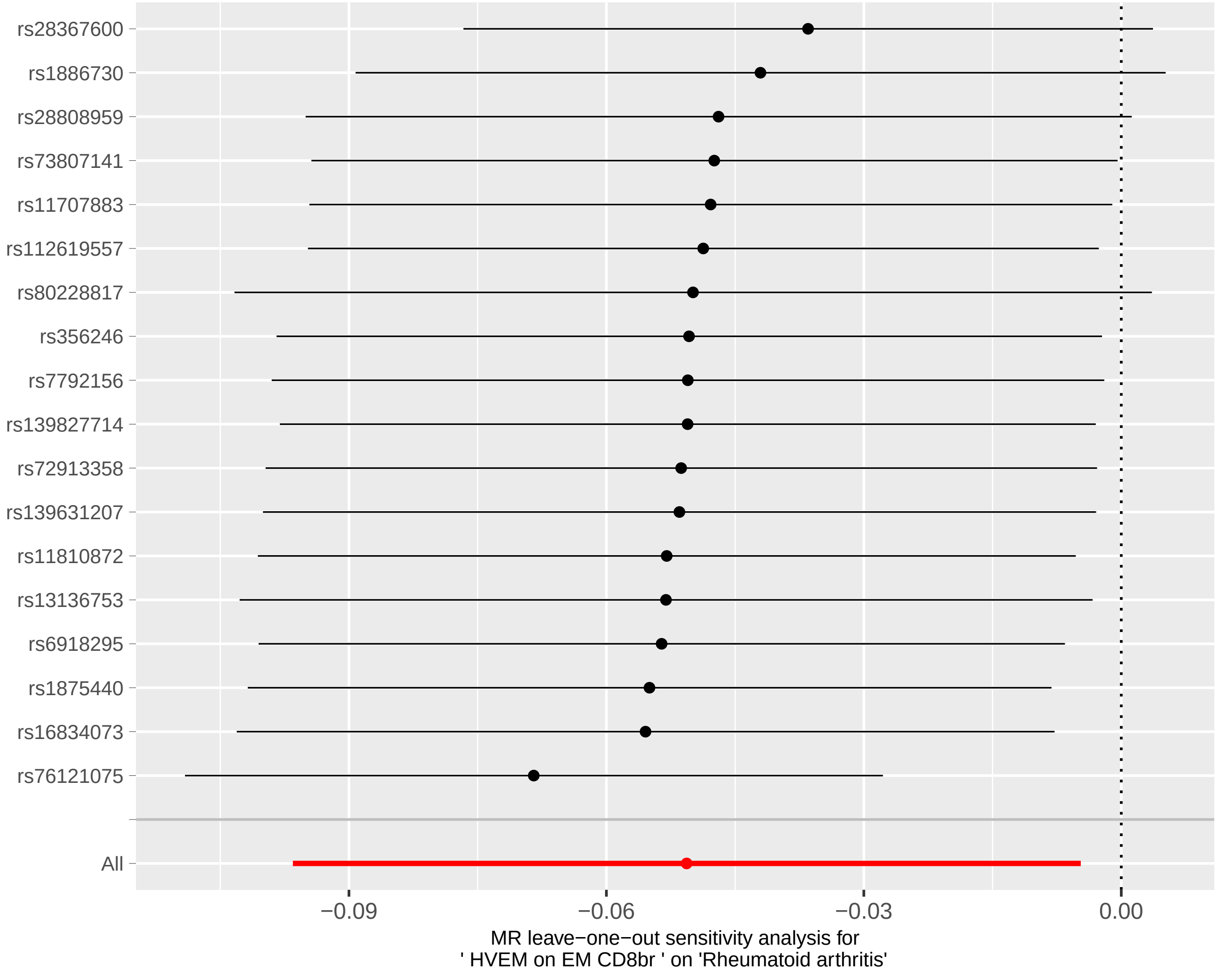
rs28367600
rs1886730
rs28808959
rs73807141
rs11707883
rs112619557
rs80228817
rs356246
rs7792156
rs139827714
rs72913358
rs139631207
rs11810872
rs13136753
rs6918295
rs1875440
rs16834073
rs76121075
All
−0.09
−0.06
−0.03
0.00
MR leave−one−out sensitivity analysis for
' HVEM on EM CD8br ' on 'Rheumatoid arthritis'

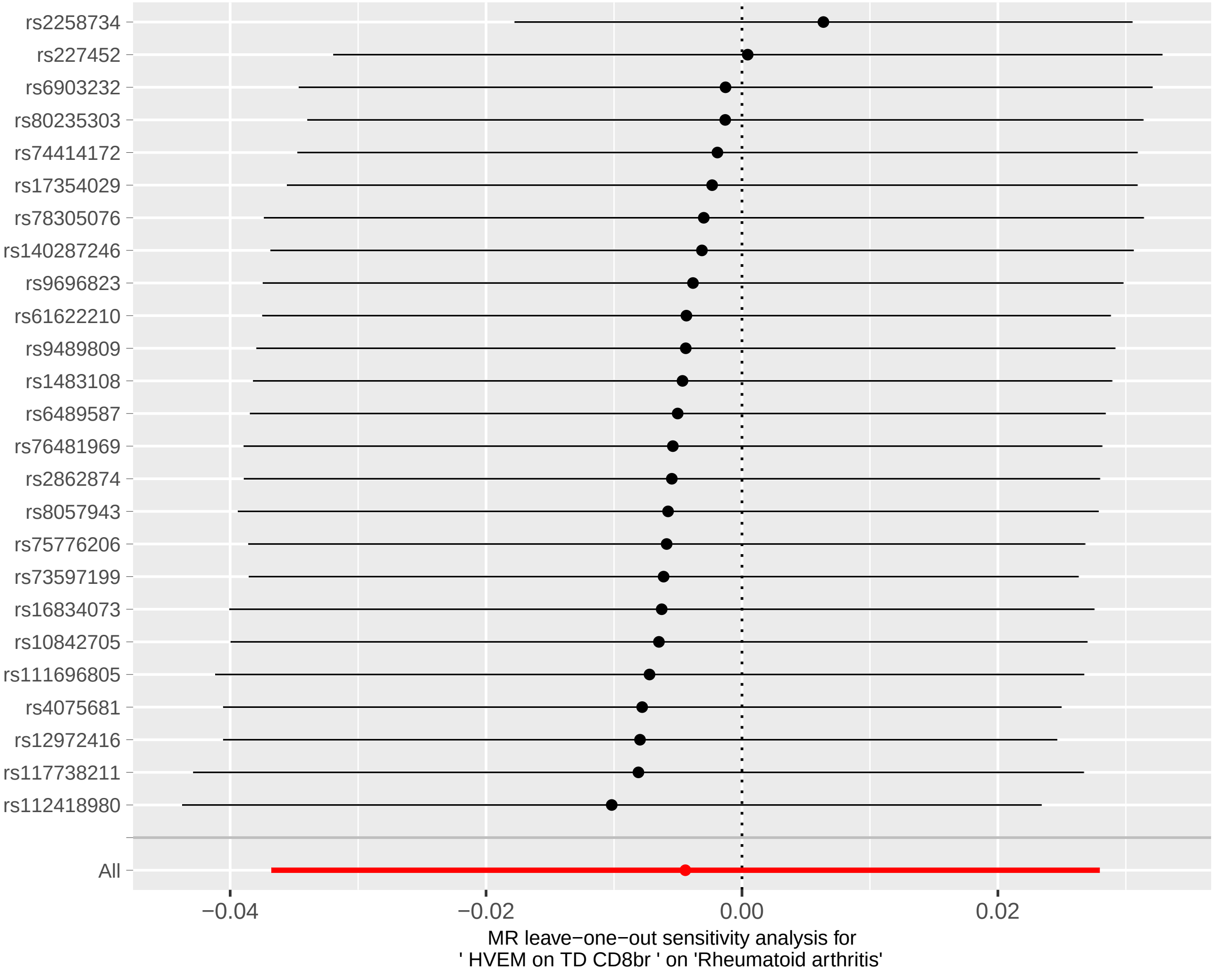

rs2258734
rs227452
rs6903232
rs80235303
rs74414172
rs17354029
rs78305076
rs140287246
rs9696823
rs61622210
rs9489809
rs1483108
rs6489587
rs76481969
rs2862874
rs8057943
rs75776206
rs73597199
rs16834073
rs10842705
rs111696805
rs4075681
rs12972416
rs117738211
rs112418980
All
−0.04
−0.02
0.00
0.02
MR leave−one−out sensitivity analysis for
' HVEM on TD CD8br ' on 'Rheumatoid arthritis'

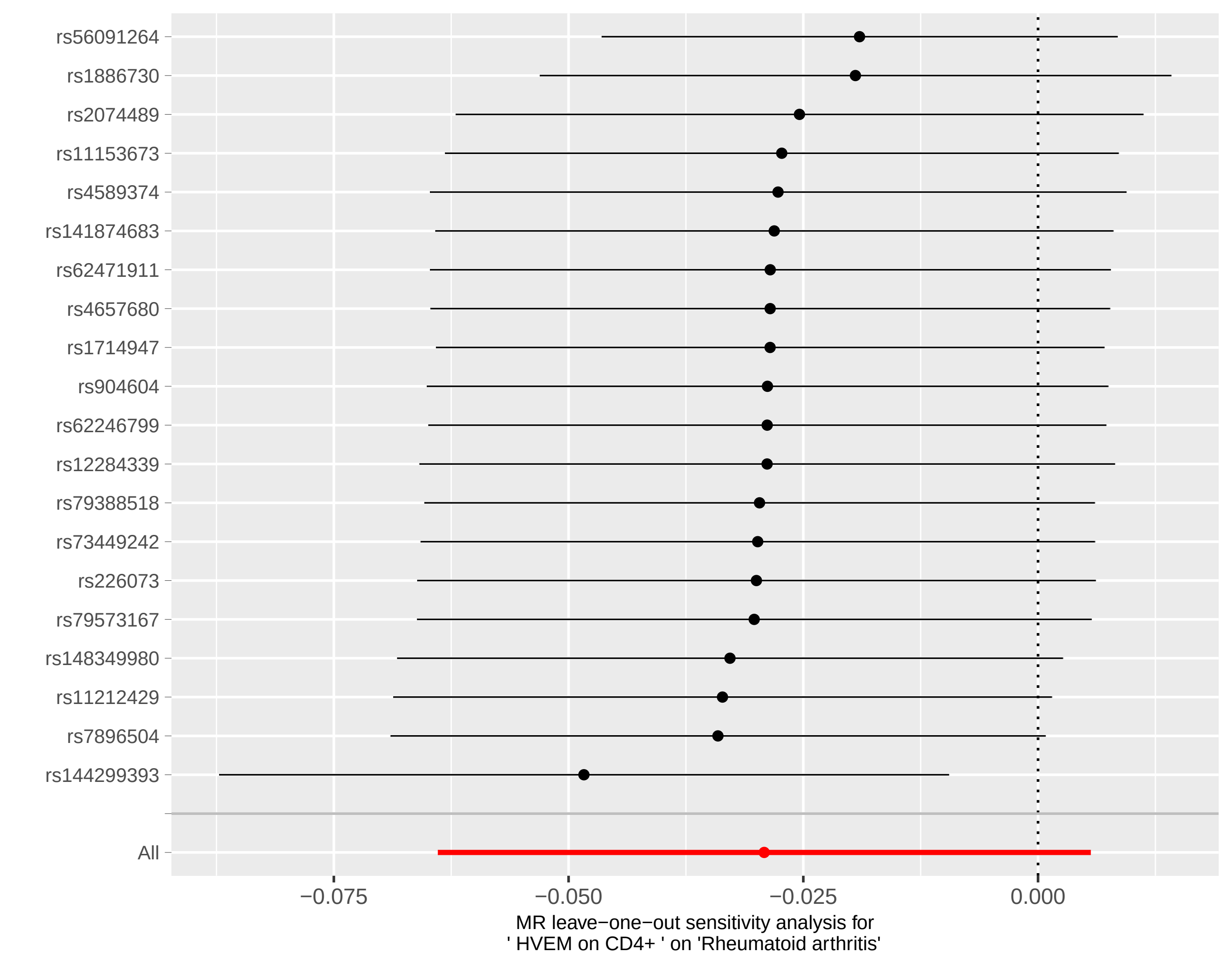

rs56091264
rs1886730
rs2074489
rs11153673
rs4589374
rs141874683
rs62471911
rs4657680
rs1714947
rs904604
rs62246799
rs12284339
rs79388518
rs73449242
rs226073
rs79573167
rs148349980
rs11212429
rs7896504
rs144299393
All
−0.075
−0.050
−0.025
0.000
MR leave−one−out sensitivity analysis for
' HVEM on CD4+ ' on 'Rheumatoid arthritis'

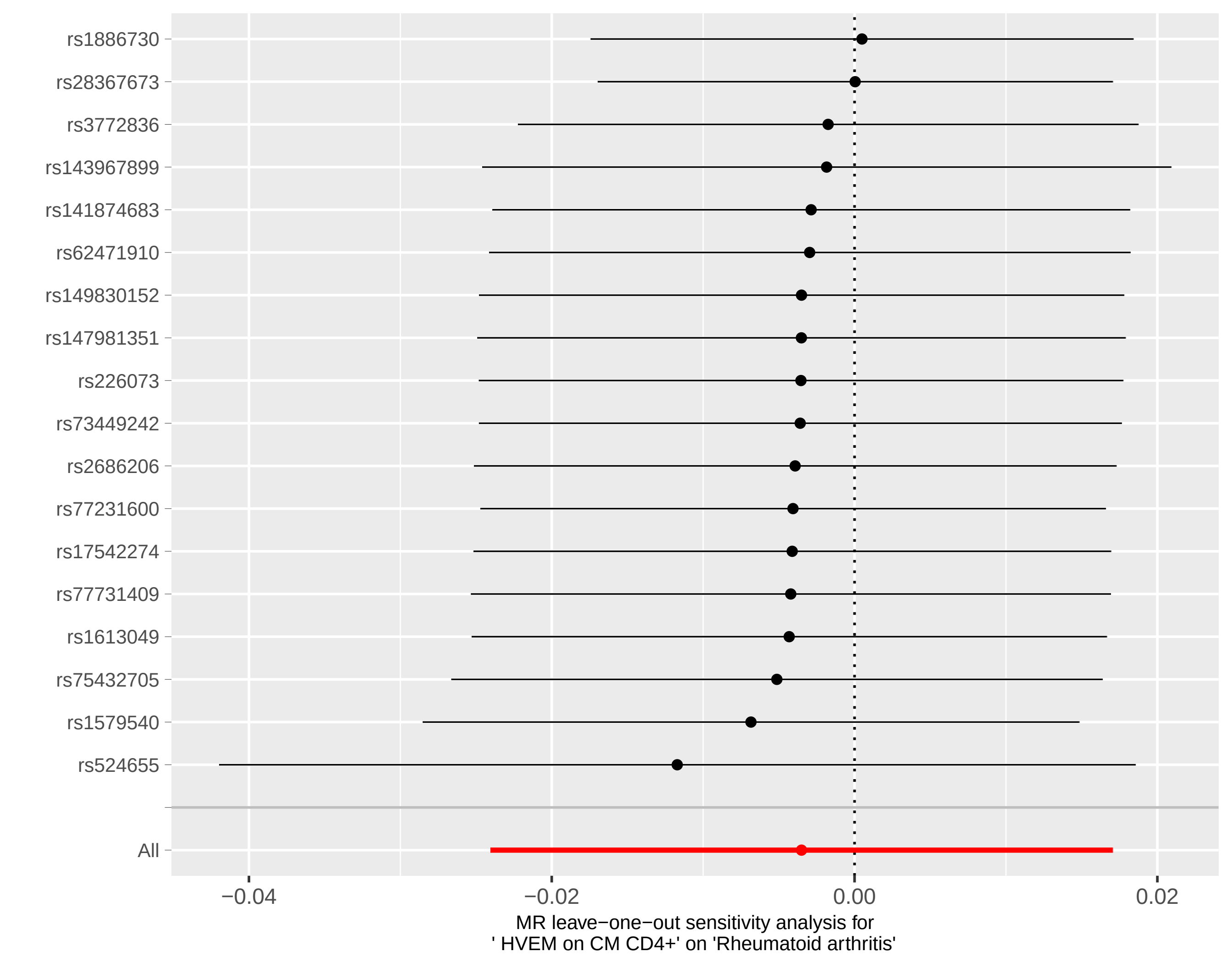
rs1886730
rs28367673
rs3772836
rs143967899
rs141874683
rs62471910
rs149830152
rs147981351
rs226073
rs73449242
rs2686206
rs77231600
rs17542274
rs77731409
rs1613049
rs75432705
rs1579540
rs524655
All
−0.04
−0.02
0.00
0.02
MR leave−one−out sensitivity analysis for
' HVEM on CM CD4+' on 'Rheumatoid arthritis'

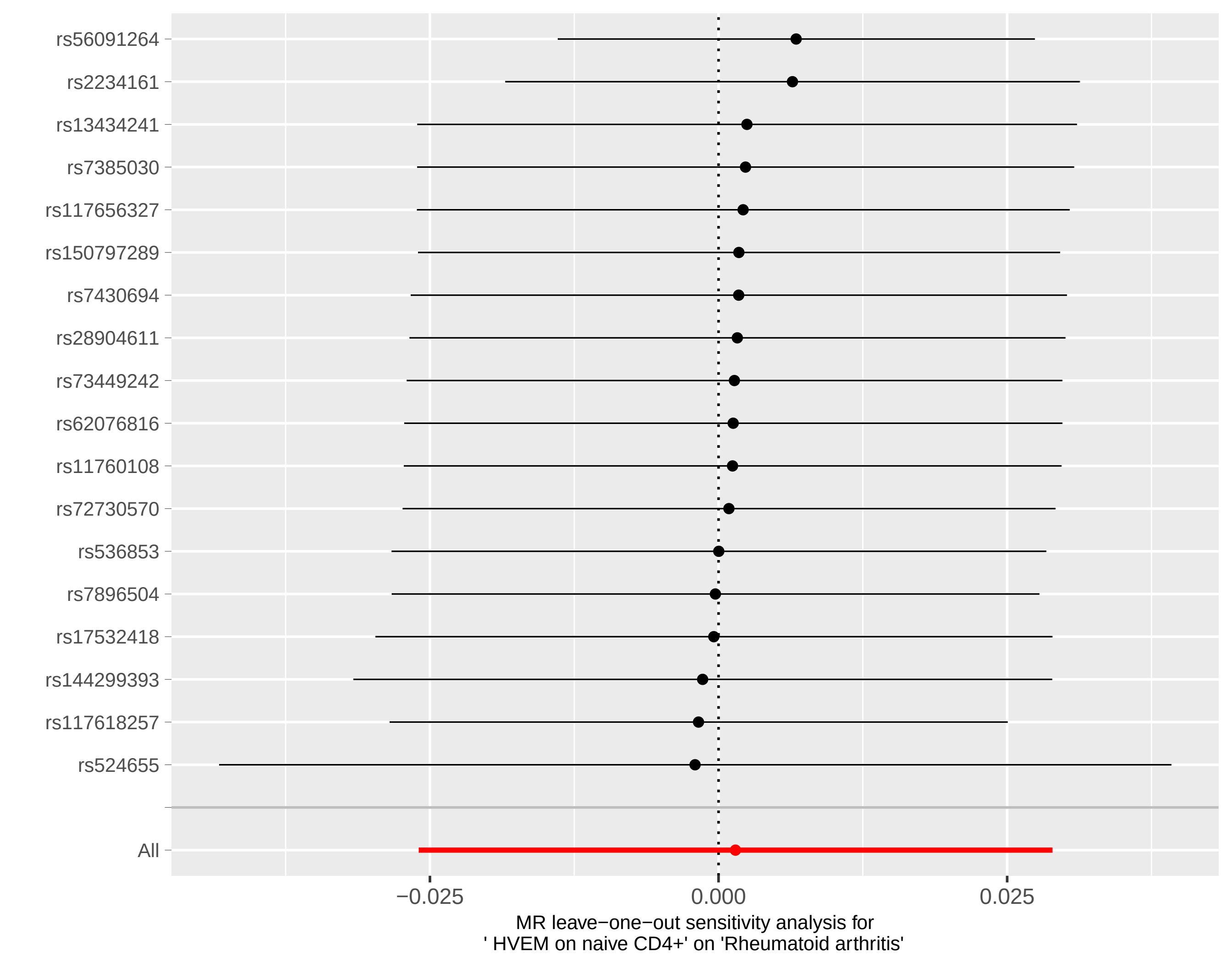
rs56091264
rs2234161
rs13434241
rs7385030
rs117656327
rs150797289
rs7430694
rs28904611
rs73449242
rs62076816
rs11760108
rs72730570
rs536853
rs7896504
rs17532418
rs144299393
rs117618257
rs524655
All
−0.025
0.000
0.025
MR leave−one−out sensitivity analysis for
' HVEM on naive CD4+' on 'Rheumatoid arthritis'

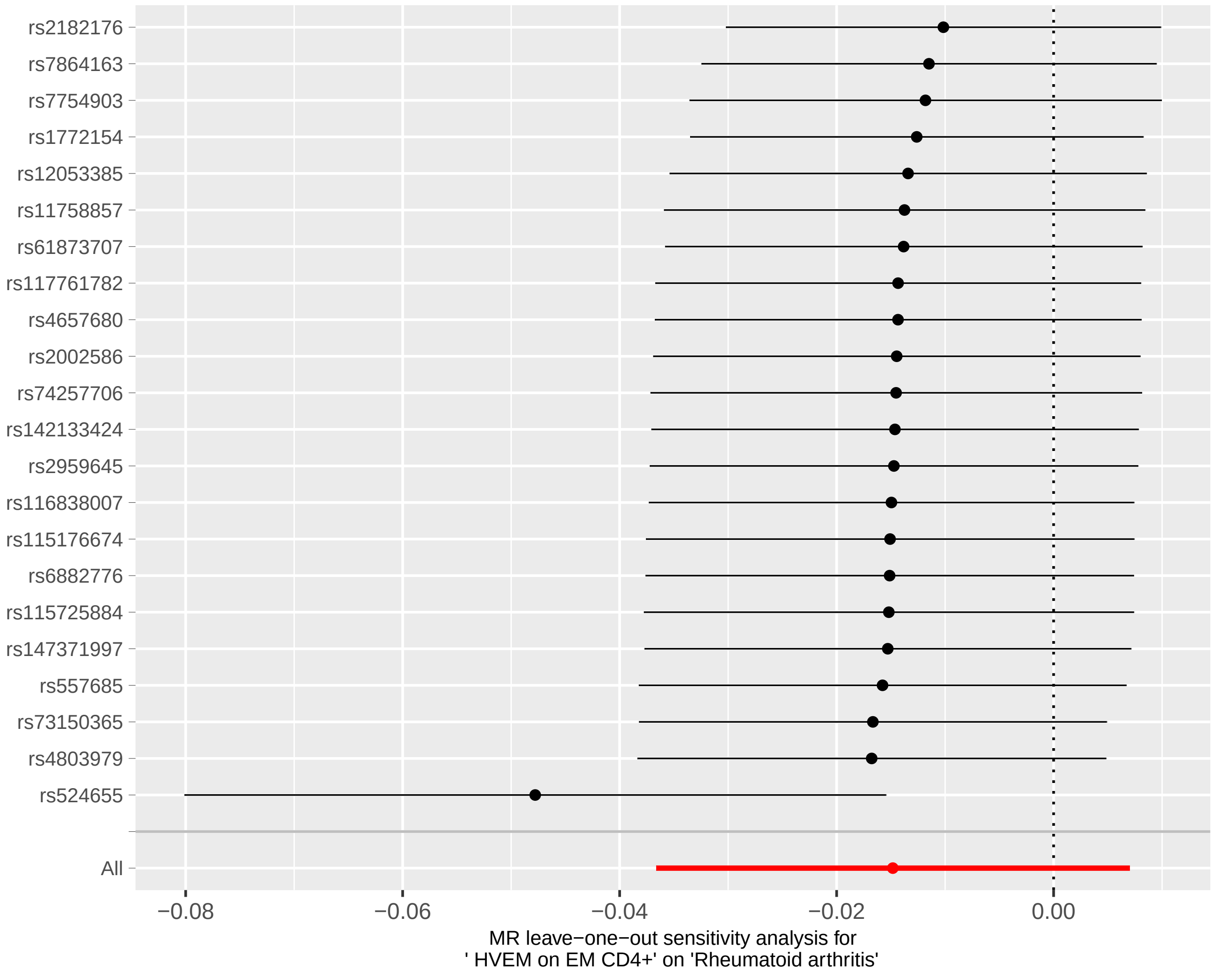

rs2182176
rs7864163
rs7754903
rs1772154
rs12053385
rs11758857
rs61873707
rs117761782
rs4657680
rs2002586
rs74257706
rs142133424
rs2959645
rs116838007
rs115176674
rs6882776
rs115725884
rs147371997
rs557685
rs73150365
rs4803979
rs524655
All
−0.08
−0.06
−0.04
−0.02
0.00
MR leave−one−out sensitivity analysis for
' HVEM on EM CD4+' on 'Rheumatoid arthritis'

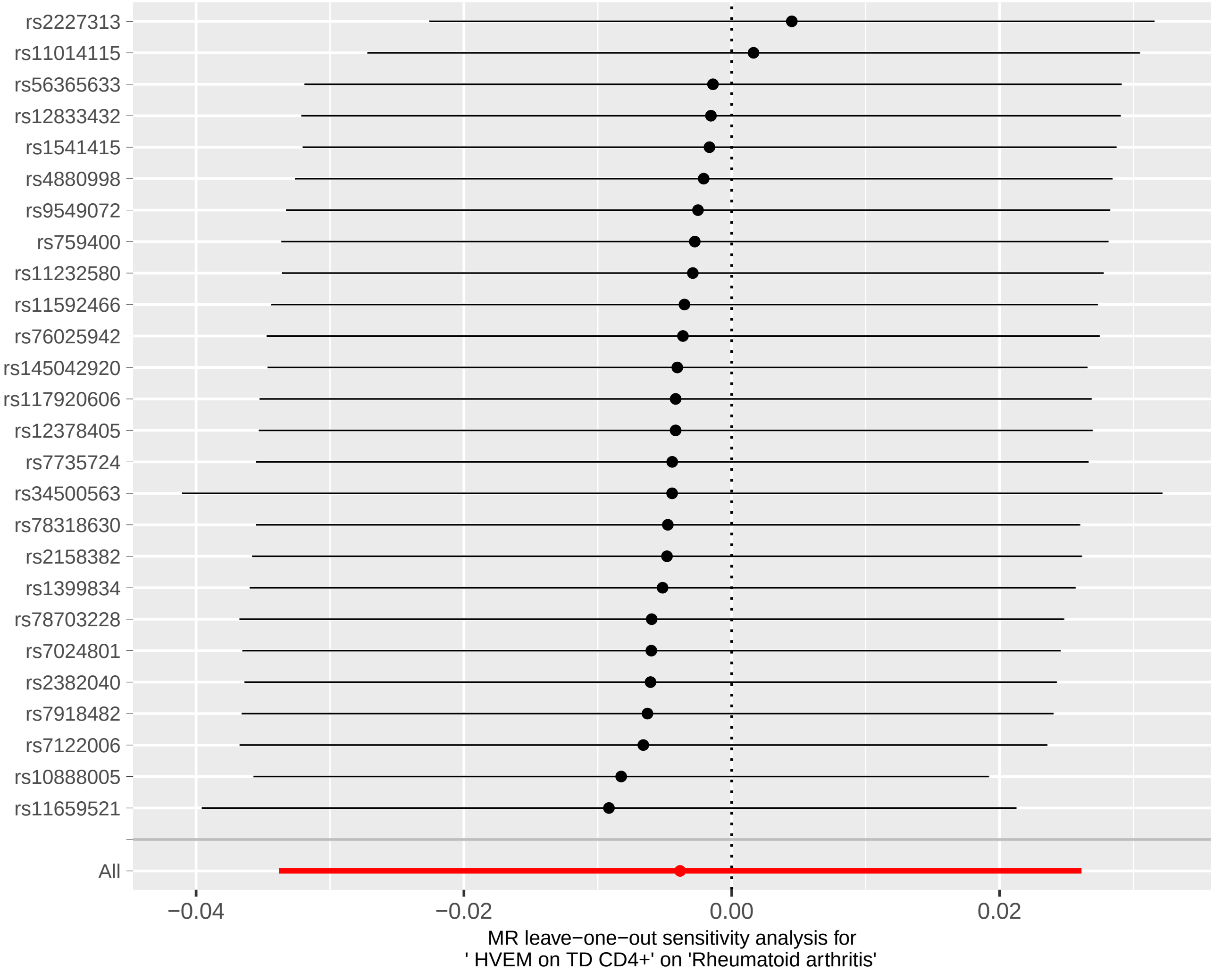
rs2227313
rs11014115
rs56365633
rs12833432
rs1541415
rs4880998
rs9549072
rs759400
rs11232580
rs11592466
rs76025942
rs145042920
rs117920606
rs12378405
rs7735724
rs34500563
rs78318630
rs2158382
rs1399834
rs78703228
rs7024801
rs2382040
rs7918482
rs7122006
rs10888005
rs11659521
All
−0.04
−0.02
0.00
0.02
MR leave−one−out sensitivity analysis for
' HVEM on TD CD4+' on 'Rheumatoid arthritis'

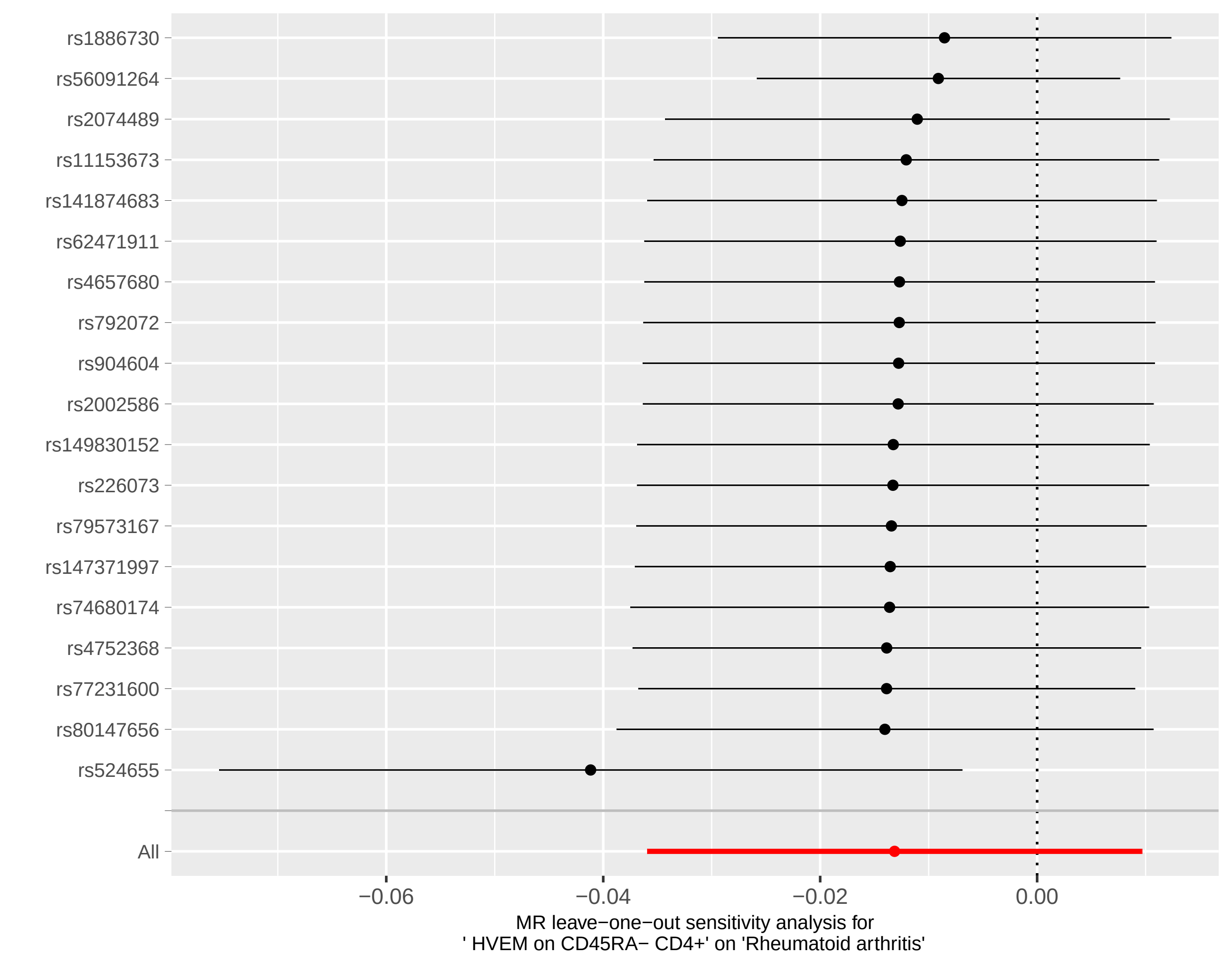

rs1886730
rs56091264
rs2074489
rs11153673
rs141874683
rs62471911
rs4657680
rs792072
rs904604
rs2002586
rs149830152
rs226073
rs79573167
rs147371997
rs74680174
rs4752368
rs77231600
rs80147656
rs524655
All
−0.06
−0.04
−0.02
0.00
MR leave−one−out sensitivity analysis for
' HVEM on CD45RA− CD4+' on 'Rheumatoid arthritis'

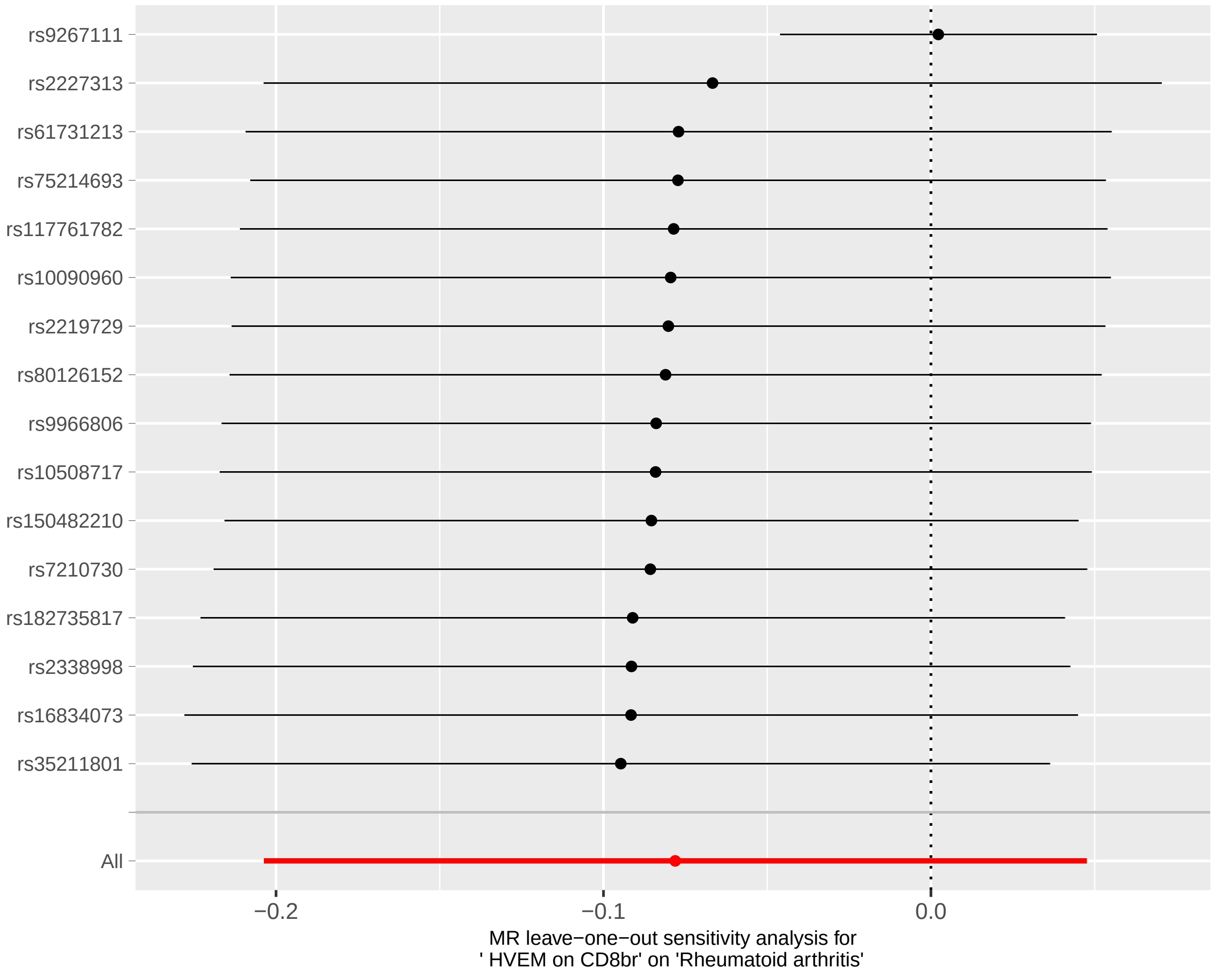
rs9267111
rs2227313
rs61731213
rs75214693
rs117761782
rs10090960
rs2219729
rs80126152
rs9966806
rs10508717
rs150482210
rs7210730
rs182735817
rs2338998
rs16834073
rs35211801
All
−0.2
−0.1
0.0
MR leave−one−out sensitivity analysis for
' HVEM on CD8br' on 'Rheumatoid arthritis'

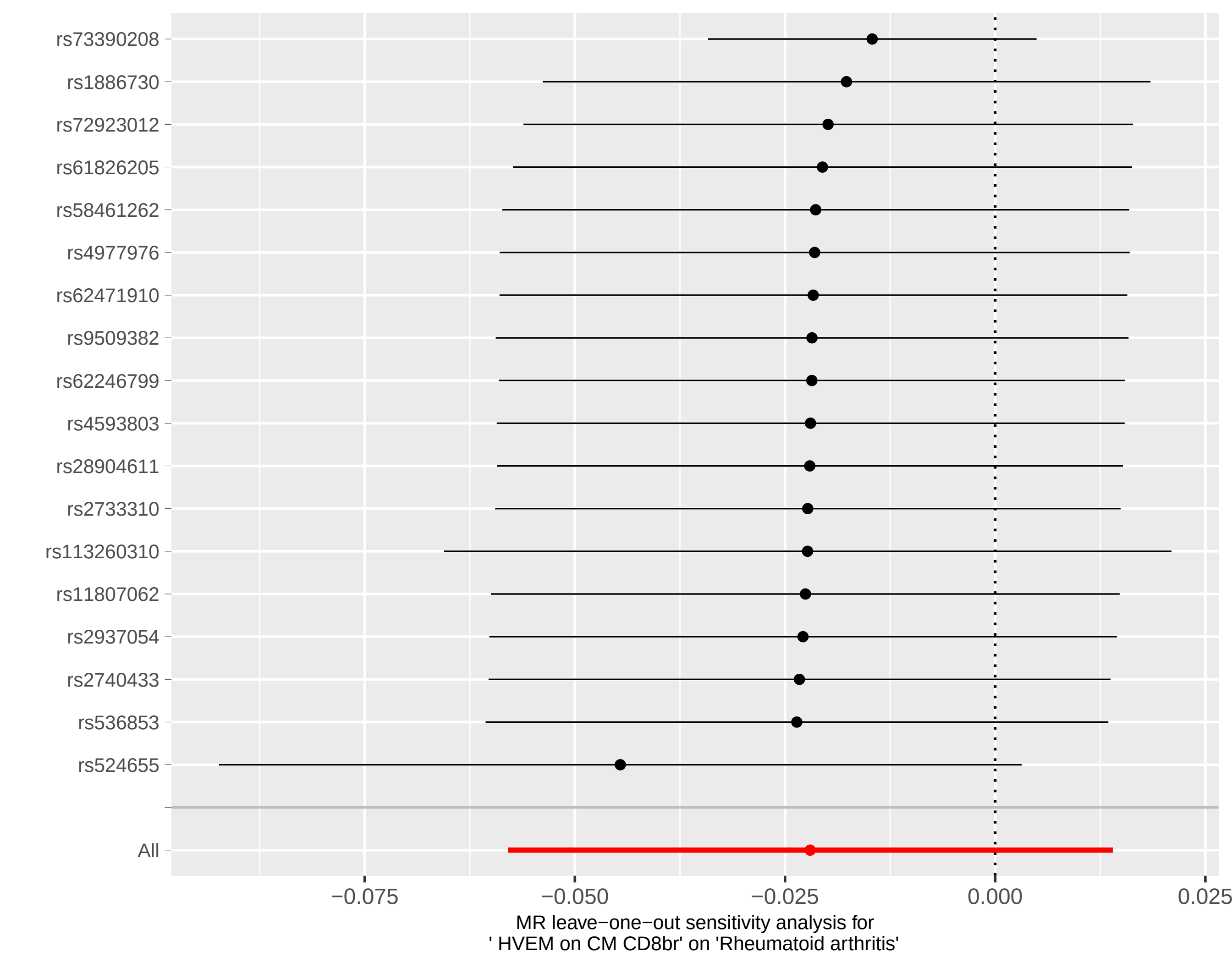

rs73390208
rs1886730
rs72923012
rs61826205
rs58461262
rs4977976
rs62471910
rs9509382
rs62246799
rs4593803
rs28904611
rs2733310
rs113260310
rs11807062
rs2937054
rs2740433
rs536853
rs524655
All
−0.075
−0.050
−0.025
0.000
0.025
MR leave−one−out sensitivity analysis for
' HVEM on CM CD8br' on 'Rheumatoid arthritis'

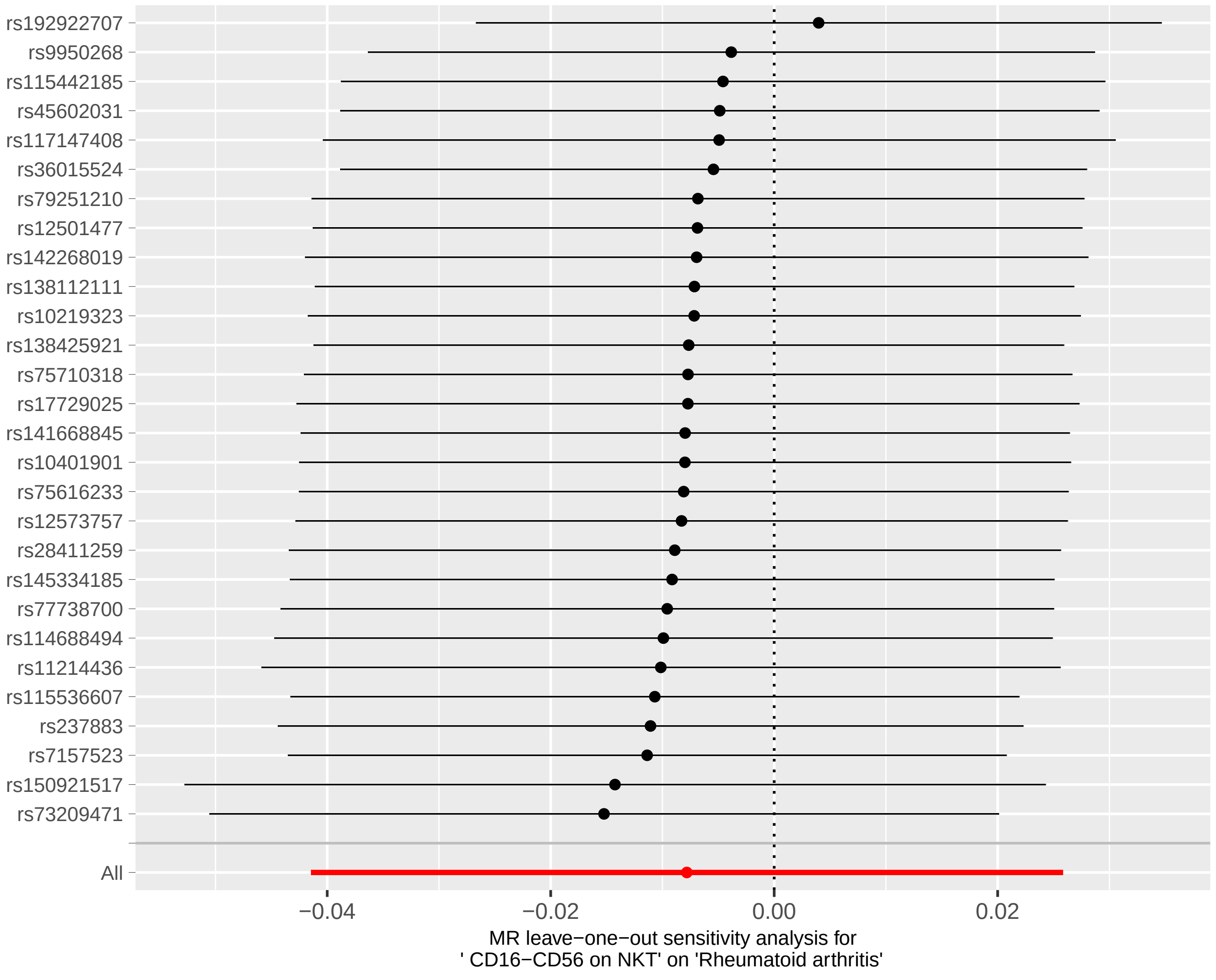

rs192922707
rs9950268
rs115442185
rs45602031
rs117147408
rs36015524
rs79251210
rs12501477
rs142268019
rs138112111
rs10219323
rs138425921
rs75710318
rs17729025
rs141668845
rs10401901
rs75616233
rs12573757
rs28411259
rs145334185
rs77738700
rs114688494
rs11214436
rs115536607
rs237883
rs7157523
rs150921517
rs73209471
All
−0.04
−0.02
0.00
0.02
MR leave−one−out sensitivity analysis for
' CD16−CD56 on NKT' on 'Rheumatoid arthritis'

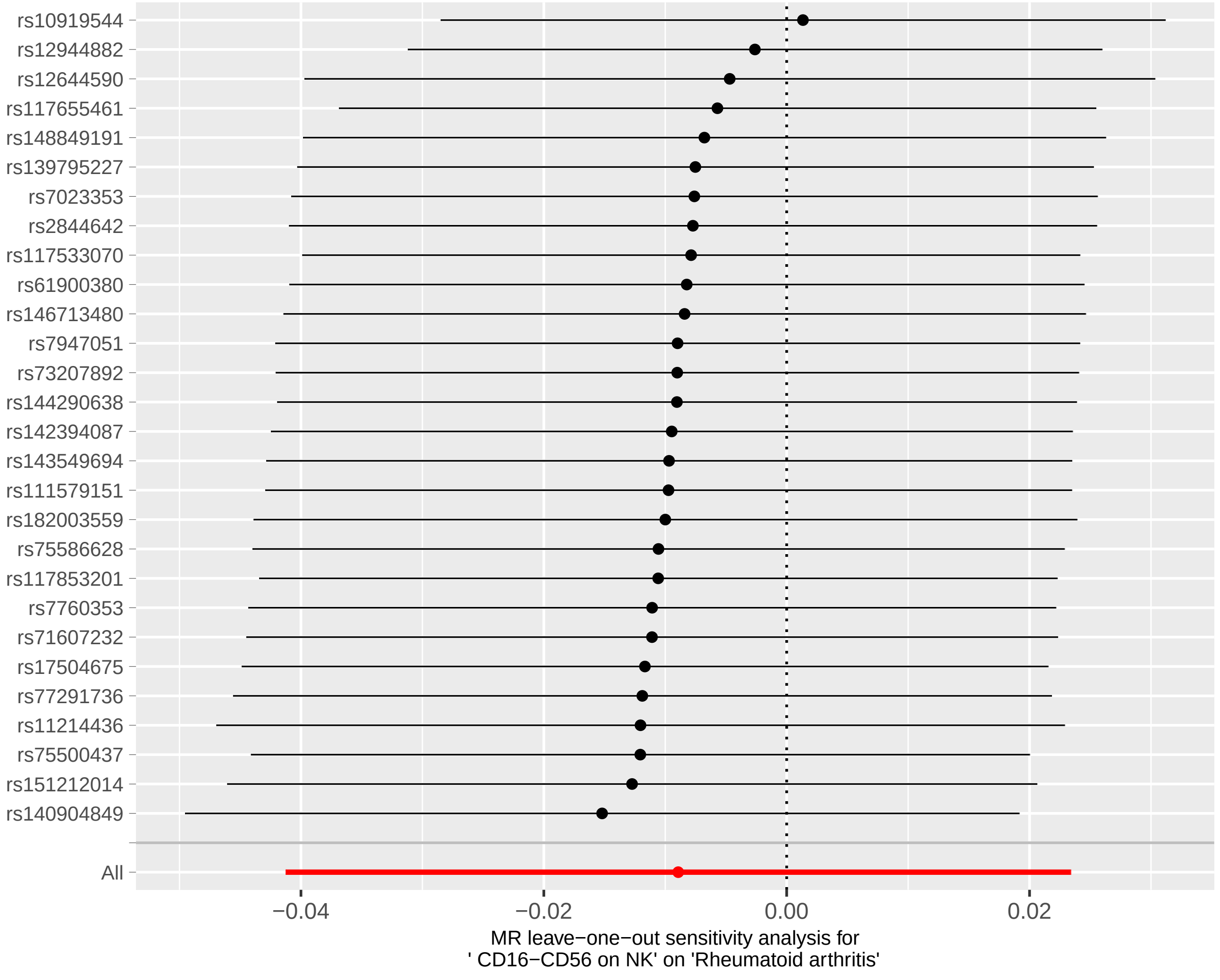
rs10919544
rs12944882
rs12644590
rs117655461
rs148849191
rs139795227
rs7023353
rs2844642
rs117533070
rs61900380
rs146713480
rs7947051
rs73207892
rs144290638
rs142394087
rs143549694
rs111579151
rs182003559
rs75586628
rs117853201
rs7760353
rs71607232
rs17504675
rs77291736
rs11214436
rs75500437
rs151212014
rs140904849
All
−0.04
−0.02
0.00
0.02
MR leave−one−out sensitivity analysis for
' CD16−CD56 on NK' on 'Rheumatoid arthritis'

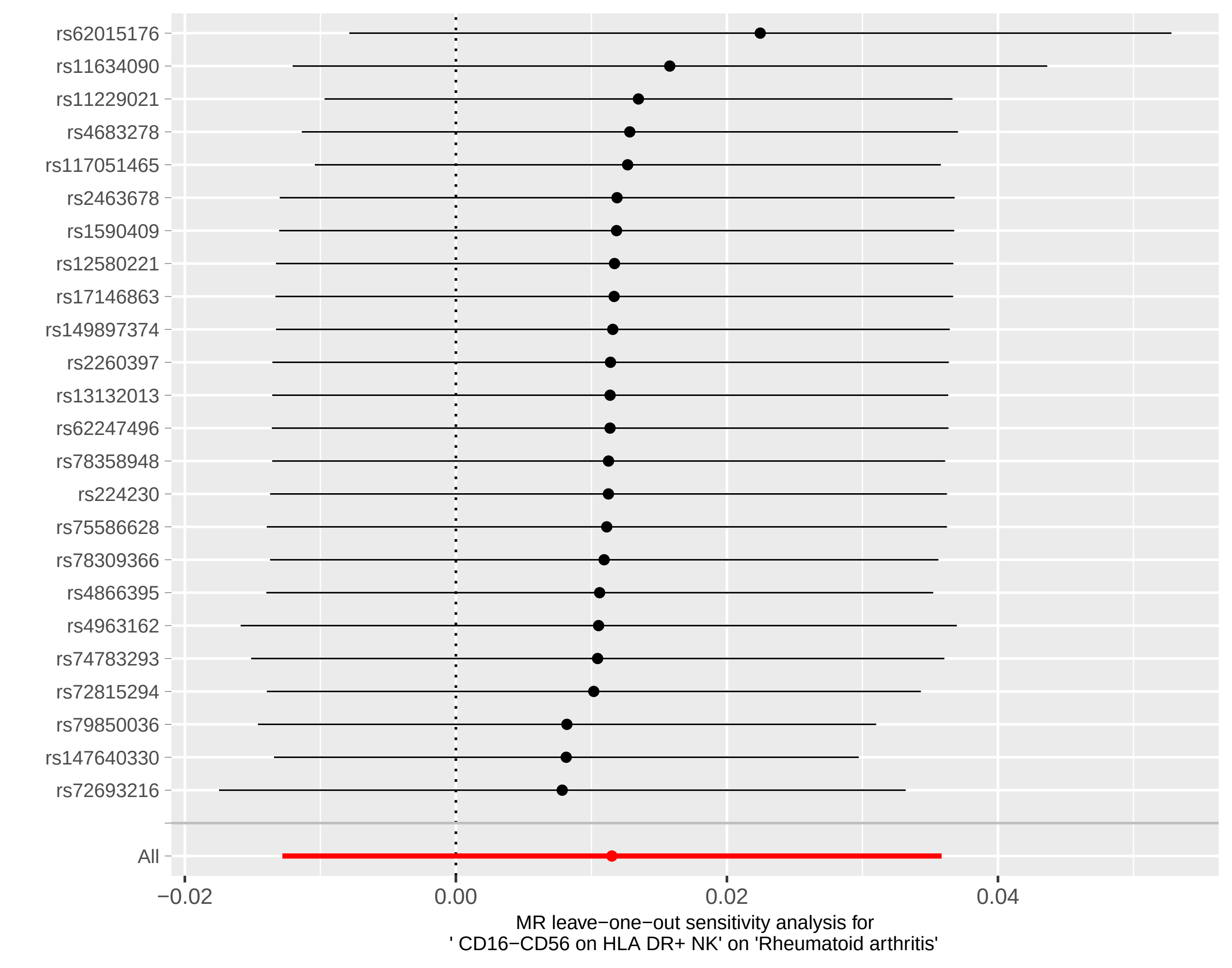
rs62015176
rs11634090
rs11229021
rs4683278
rs117051465
rs2463678
rs1590409
rs12580221
rs17146863
rs149897374
rs2260397
rs13132013
rs62247496
rs78358948
rs224230
rs75586628
rs78309366
rs4866395
rs4963162
rs74783293
rs72815294
rs79850036
rs147640330
rs72693216
All
−0.02
0.00
0.02
0.04
MR leave−one−out sensitivity analysis for
' CD16−CD56 on HLA DR+ NK' on 'Rheumatoid arthritis'

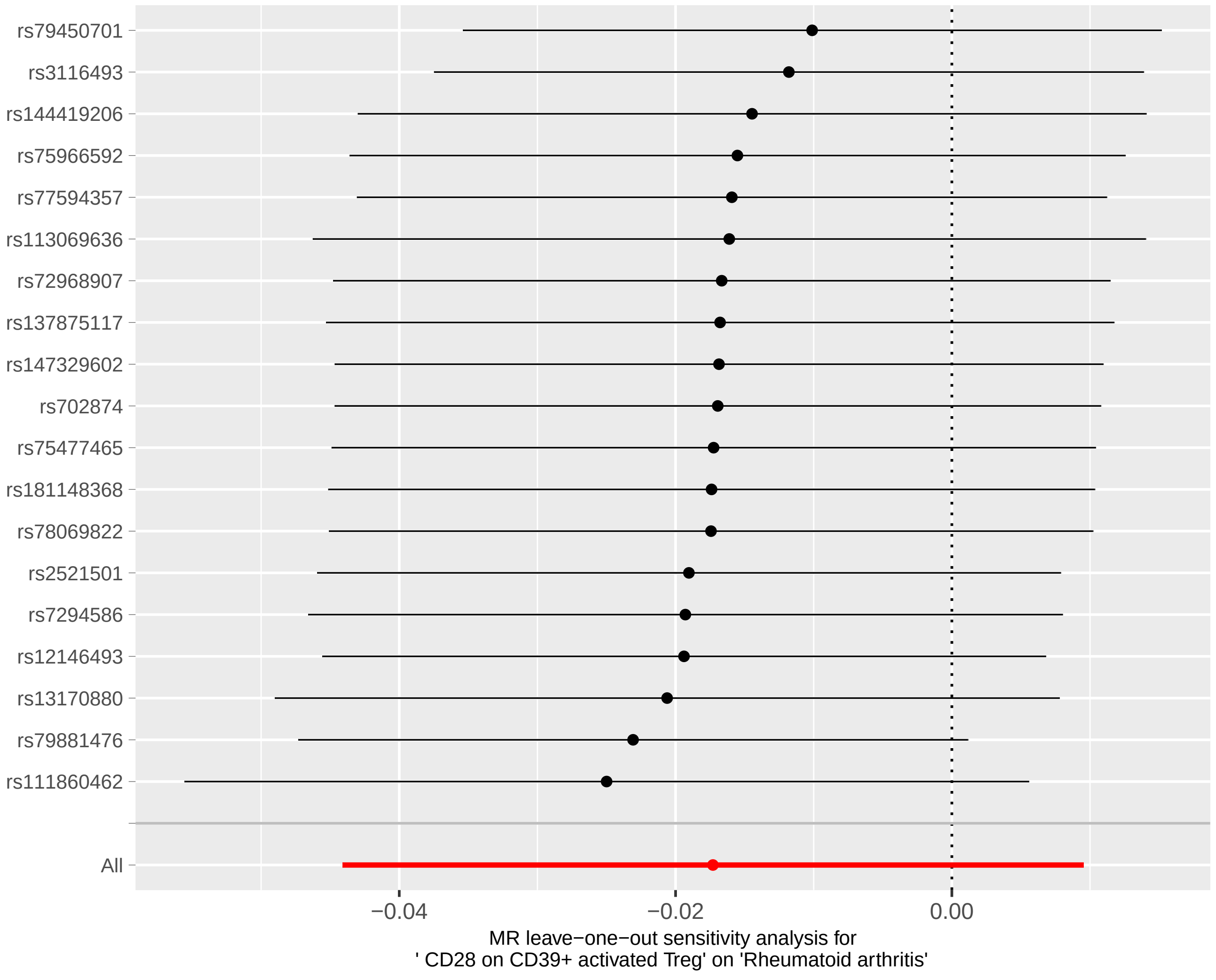
rs79450701
rs3116493
rs144419206
rs75966592
rs77594357
rs113069636
rs72968907
rs137875117
rs147329602
rs702874
rs75477465
rs181148368
rs78069822
rs2521501
rs7294586
rs12146493
rs13170880
rs79881476
rs111860462
All
−0.04
−0.02
0.00
MR leave−one−out sensitivity analysis for
' CD28 on CD39+ activated Treg' on 'Rheumatoid arthritis'

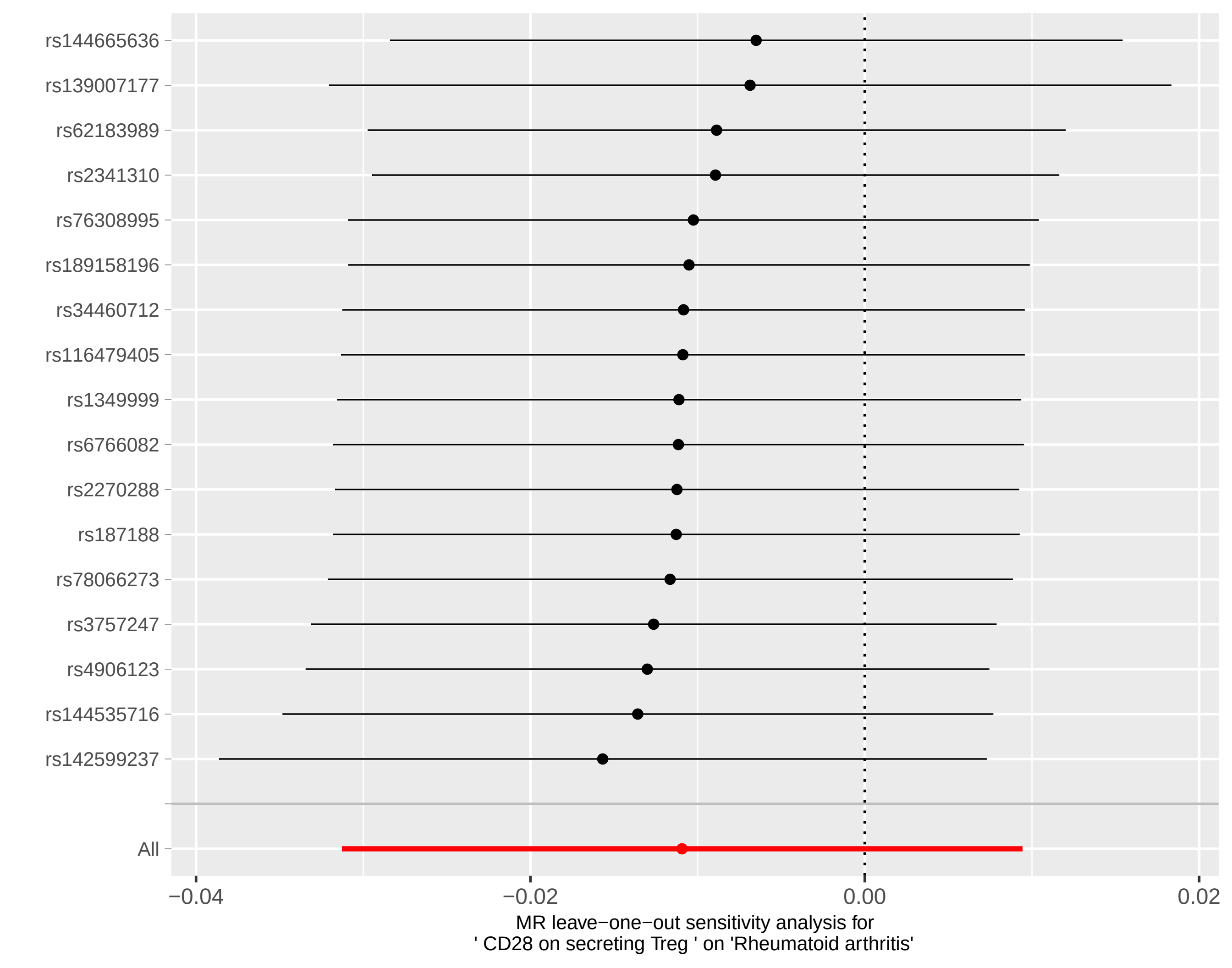
rs144665636
rs139007177
rs62183989
rs2341310
rs76308995
rs189158196
rs34460712
rs116479405
rs1349999
rs6766082
rs2270288
rs187188
rs78066273
rs3757247
rs4906123
rs144535716
rs142599237
All
−0.04
−0.02
0.00
0.02
MR leave−one−out sensitivity analysis for
' CD28 on secreting Treg ' on 'Rheumatoid arthritis'

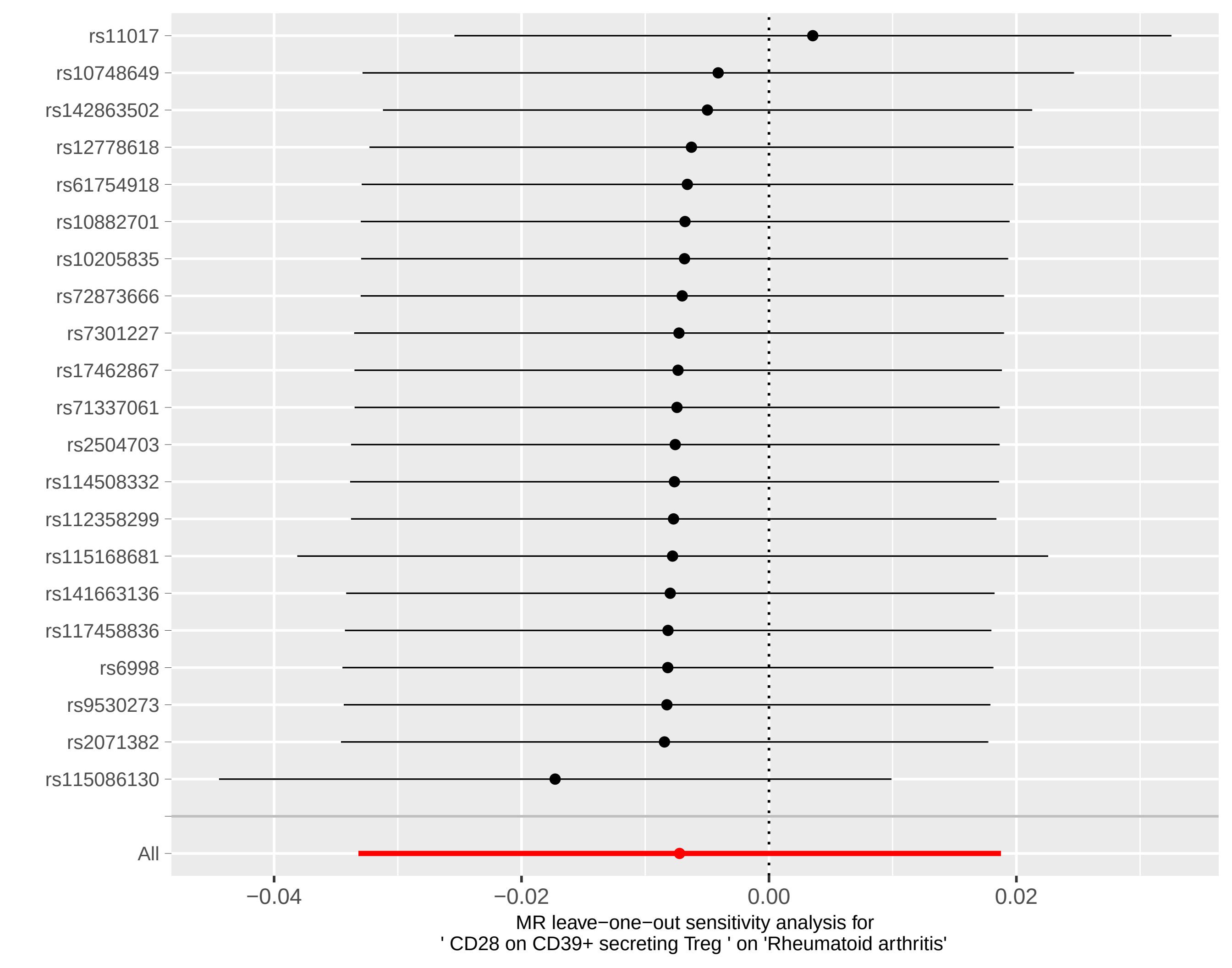
rs11017
rs10748649
rs142863502
rs12778618
rs61754918
rs10882701
rs10205835
rs72873666
rs7301227
rs17462867
rs71337061
rs2504703
rs114508332
rs112358299
rs115168681
rs141663136
rs117458836
rs6998
rs9530273
rs2071382
rs115086130
All
−0.04
−0.02
0.00
0.02
MR leave−one−out sensitivity analysis for
' CD28 on CD39+ secreting Treg ' on 'Rheumatoid arthritis'

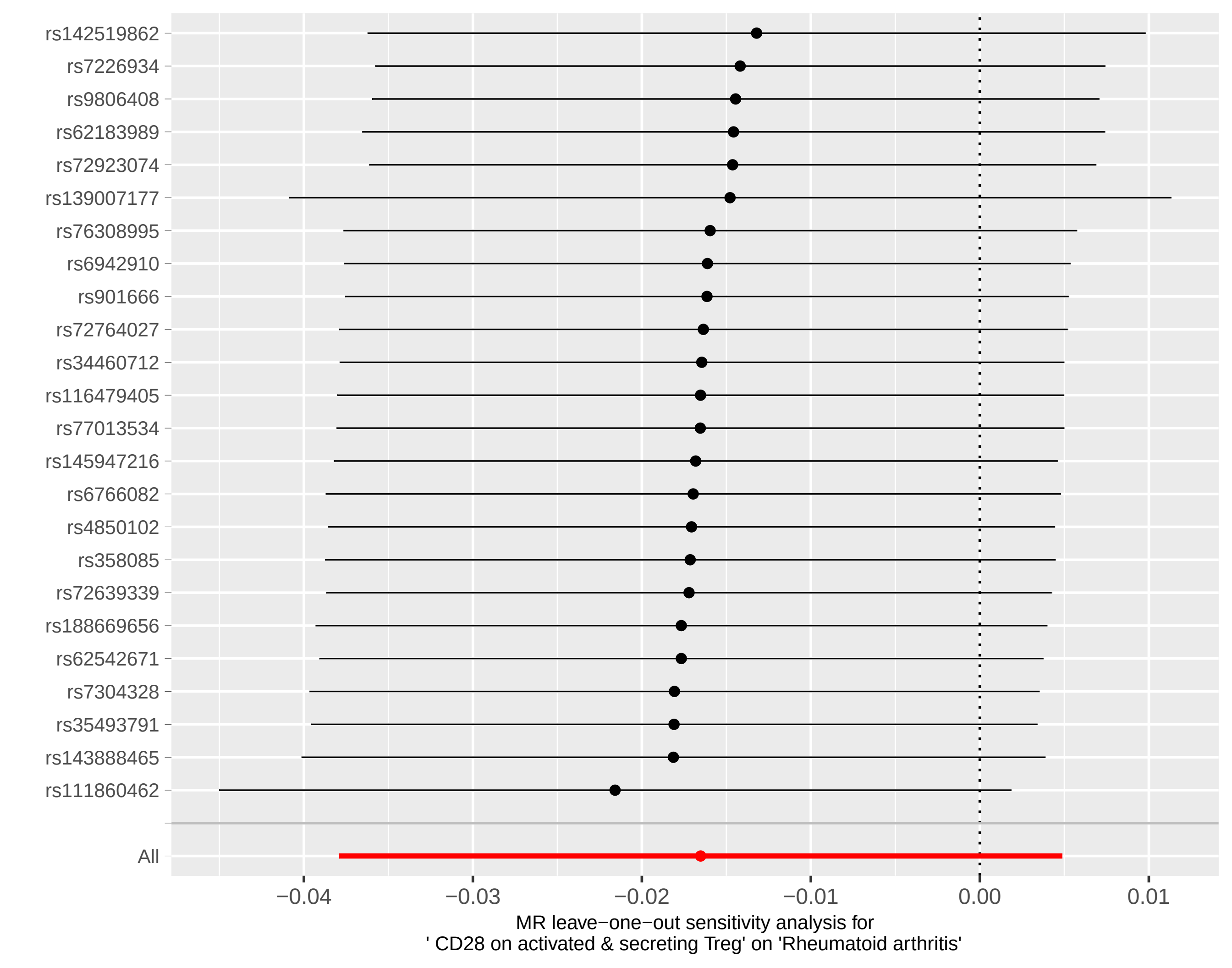

rs142519862
rs7226934
rs9806408
rs62183989
rs72923074
rs139007177
rs76308995
rs6942910
rs901666
rs72764027
rs34460712
rs116479405
rs77013534
rs145947216
rs6766082
rs4850102
rs358085
rs72639339
rs188669656
rs62542671
rs7304328
rs35493791
rs143888465
rs111860462
All
−0.04
−0.03
−0.02
−0.01
0.00
0.01
MR leave−one−out sensitivity analysis for
' CD28 on activated & secreting Treg' on 'Rheumatoid arthritis'

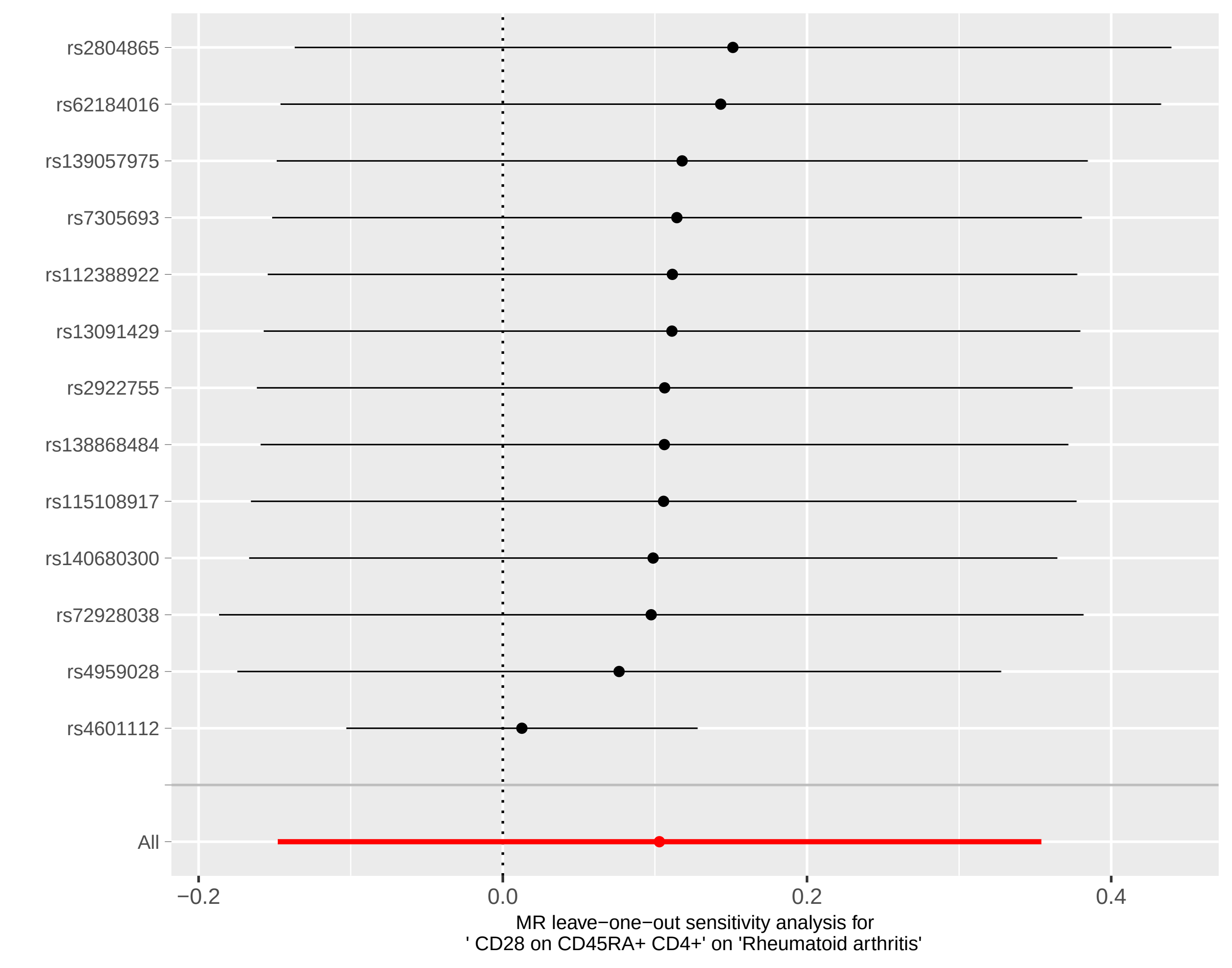
rs2804865
rs62184016
rs139057975
rs7305693
rs112388922
rs13091429
rs2922755
rs138868484
rs115108917
rs140680300
rs72928038
rs4959028
rs4601112
All
−0.2
0.0
0.2
0.4
MR leave−one−out sensitivity analysis for
' CD28 on CD45RA+ CD4+' on 'Rheumatoid arthritis'

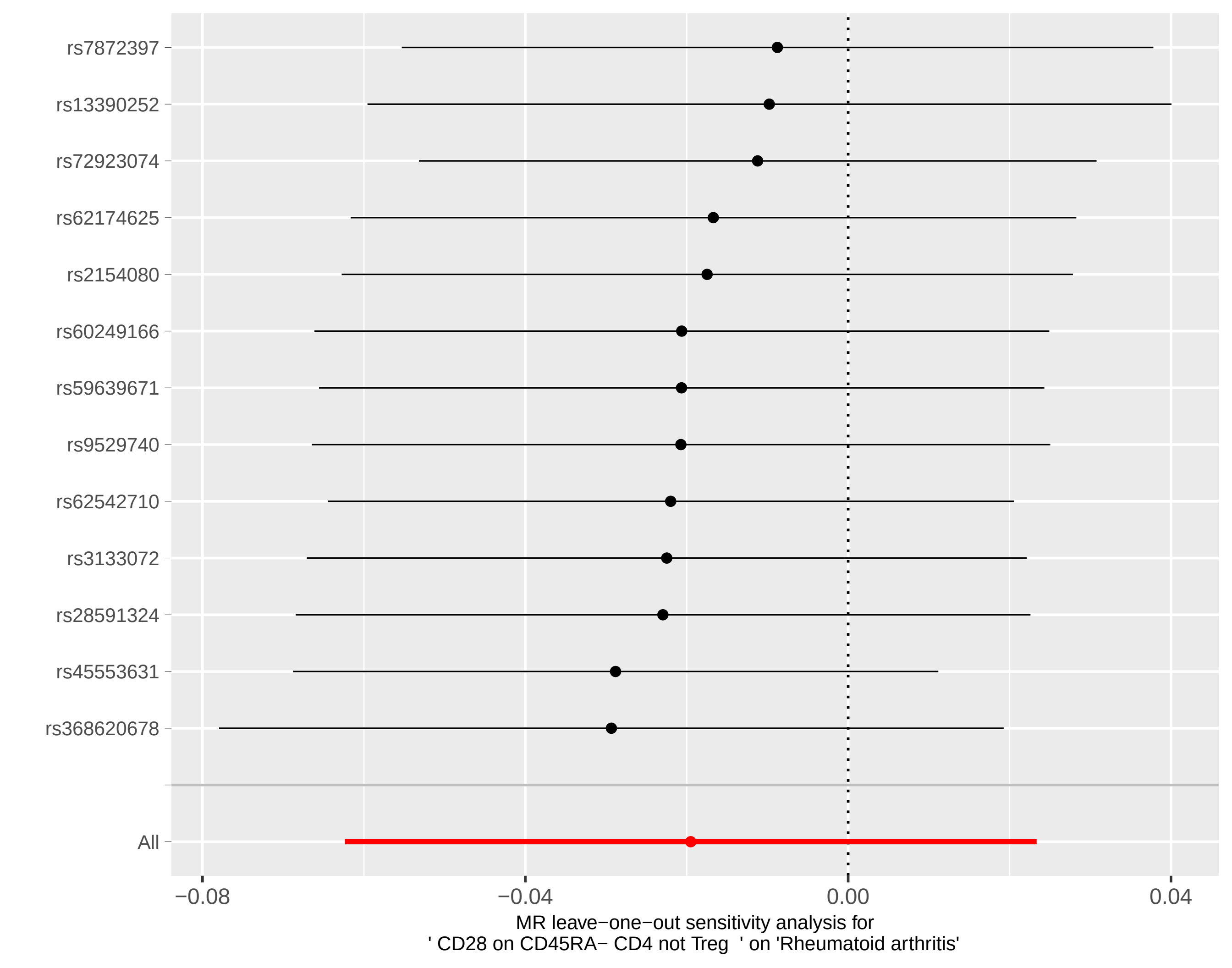
rs7872397
rs13390252
rs72923074
rs62174625
rs2154080
rs60249166
rs59639671
rs9529740
rs62542710
rs3133072
rs28591324
rs45553631
rs368620678
All
−0.08
−0.04
0.00
0.04
MR leave−one−out sensitivity analysis for
' CD28 on CD45RA− CD4 not Treg ' on 'Rheumatoid arthritis'

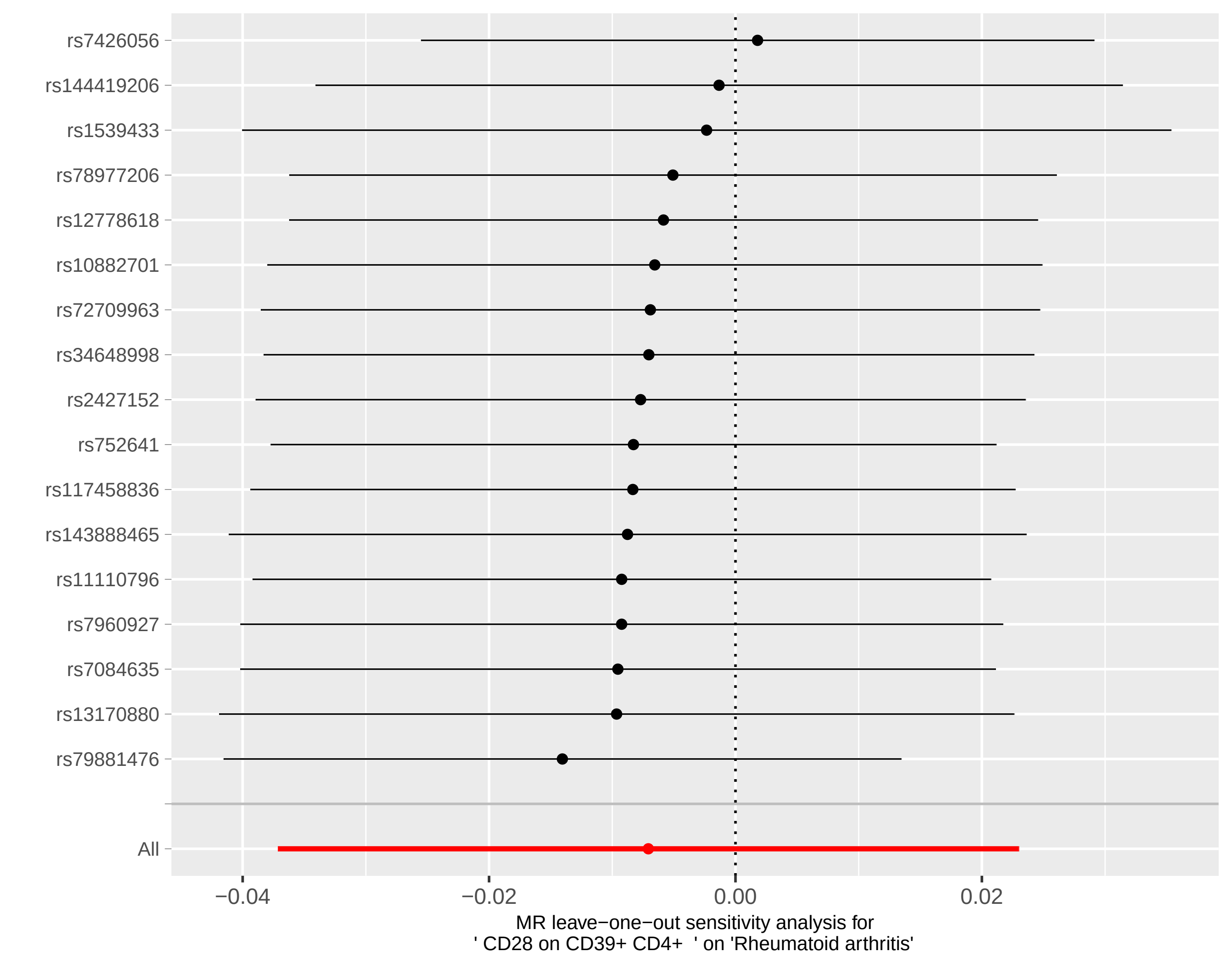
rs7426056
rs144419206
rs1539433
rs78977206
rs12778618
rs10882701
rs72709963
rs34648998
rs2427152
rs752641
rs117458836
rs143888465
rs11110796
rs7960927
rs7084635
rs13170880
rs79881476
All
−0.04
−0.02
0.00
0.02
MR leave−one−out sensitivity analysis for
' CD28 on CD39+ CD4+ ' on 'Rheumatoid arthritis'

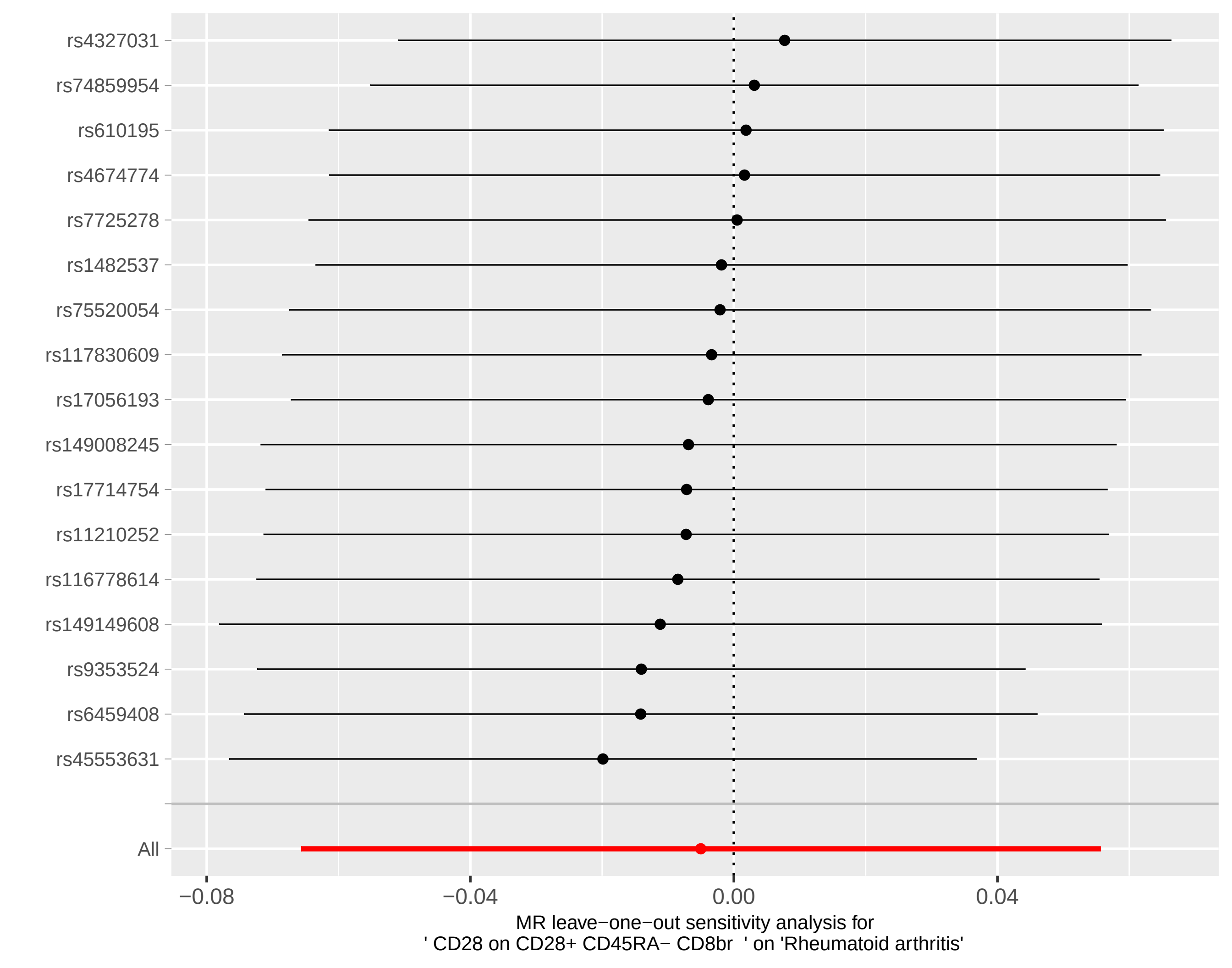
rs4327031
rs74859954
rs610195
rs4674774
rs7725278
rs1482537
rs75520054
rs117830609
rs17056193
rs149008245
rs17714754
rs11210252
rs116778614
rs149149608
rs9353524
rs6459408
rs45553631
All
−0.08
−0.04
0.00
0.04
MR leave−one−out sensitivity analysis for
' CD28 on CD28+ CD45RA− CD8br ' on 'Rheumatoid arthritis'

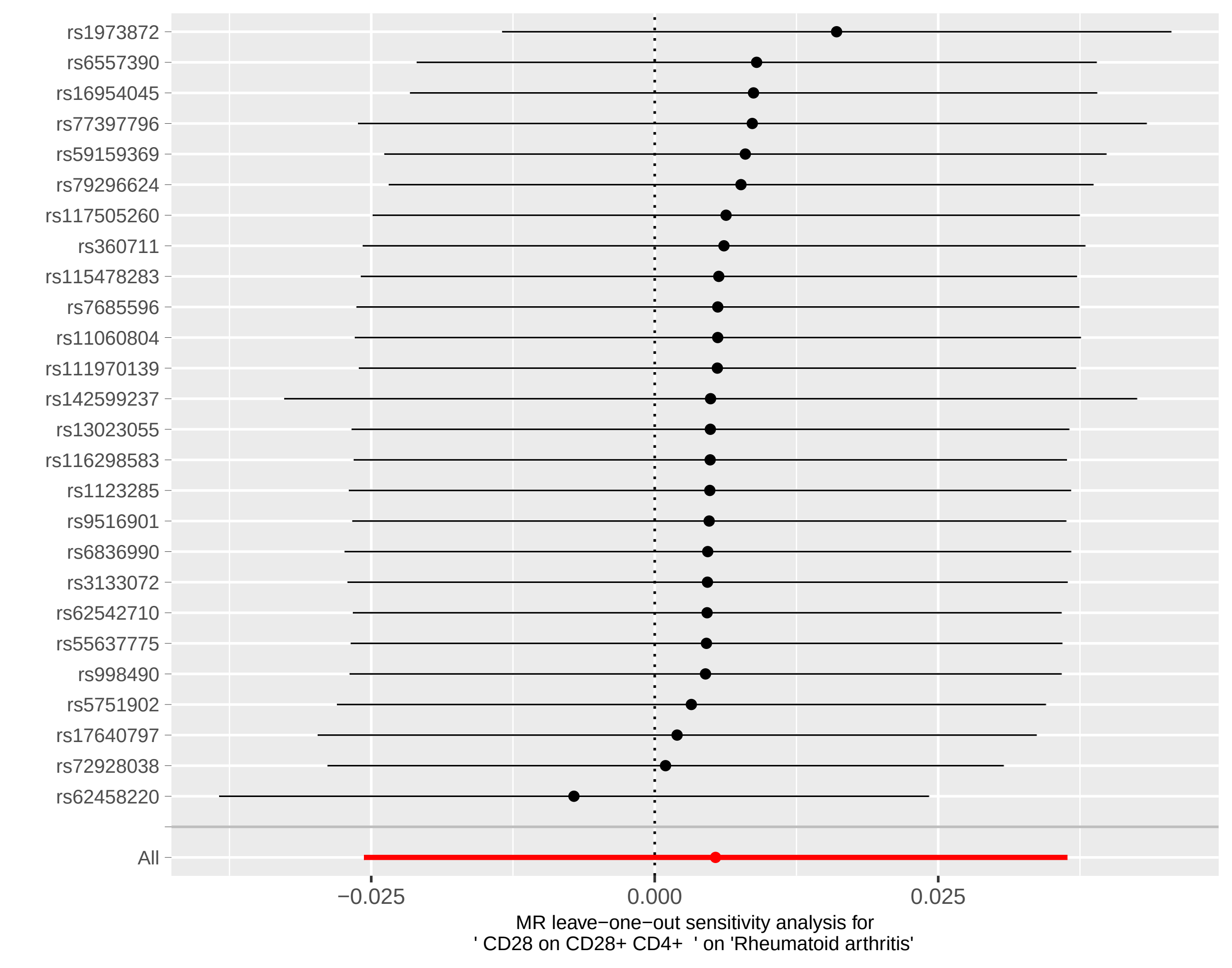
rs1973872
rs6557390
rs16954045
rs77397796
rs59159369
rs79296624
rs117505260
rs360711
rs115478283
rs7685596
rs11060804
rs111970139
rs142599237
rs13023055
rs116298583
rs1123285
rs9516901
rs6836990
rs3133072
rs62542710
rs55637775
rs998490
rs5751902
rs17640797
rs72928038
rs62458220
All
−0.025
0.000
0.025
MR leave−one−out sensitivity analysis for
' CD28 on CD28+ CD4+ ' on 'Rheumatoid arthritis'

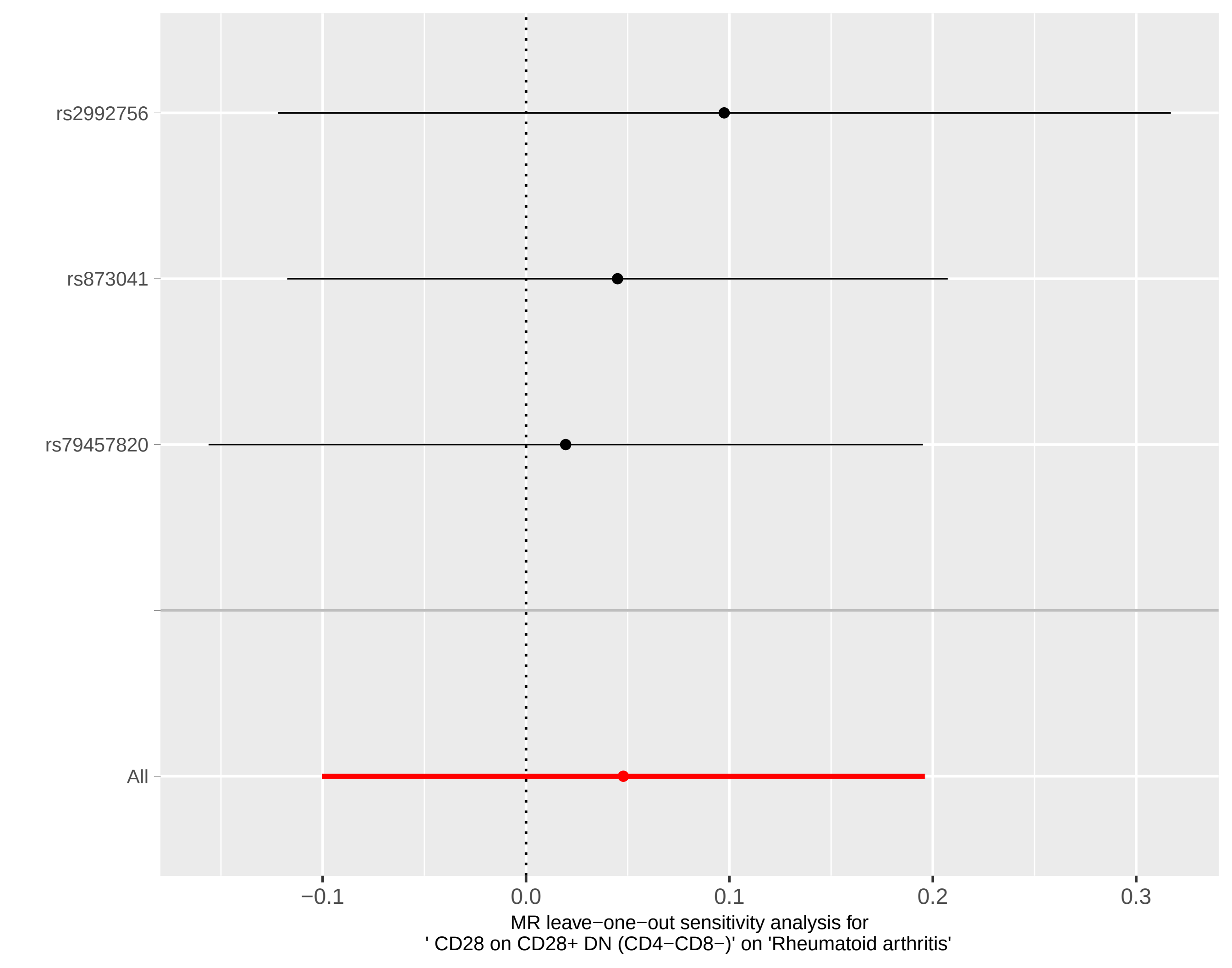

rs2992756
rs873041
rs79457820
All
−0.1
0.0
0.1
0.2
0.3
MR leave−one−out sensitivity analysis for
' CD28 on CD28+ DN (CD4−CD8−)' on 'Rheumatoid arthritis'

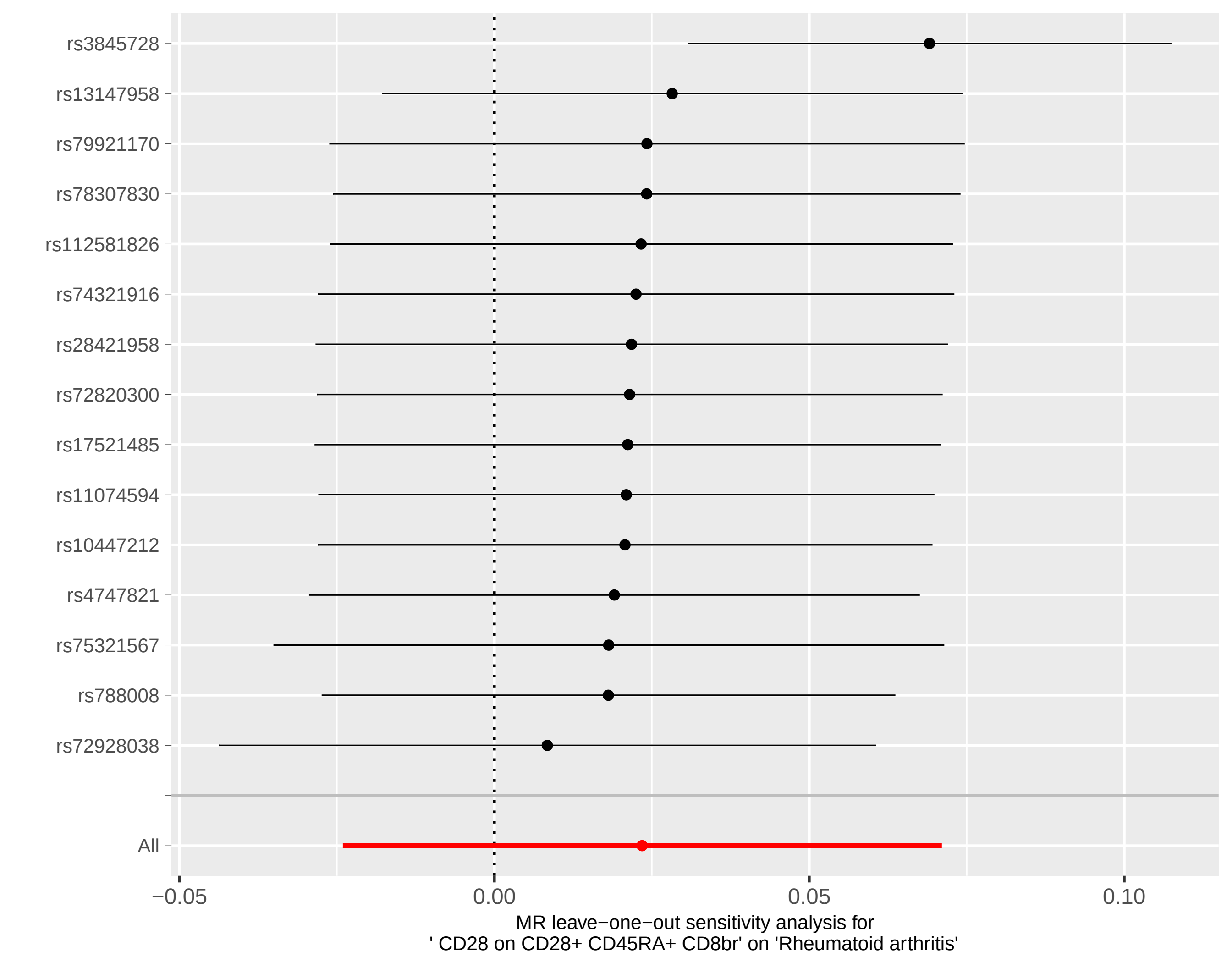

rs3845728
rs13147958
rs79921170
rs78307830
rs112581826
rs74321916
rs28421958
rs72820300
rs17521485
rs11074594
rs10447212
rs4747821
rs75321567
rs788008
rs72928038
All
−0.05
0.00
0.05
0.10
MR leave−one−out sensitivity analysis for
' CD28 on CD28+ CD45RA+ CD8br' on 'Rheumatoid arthritis'

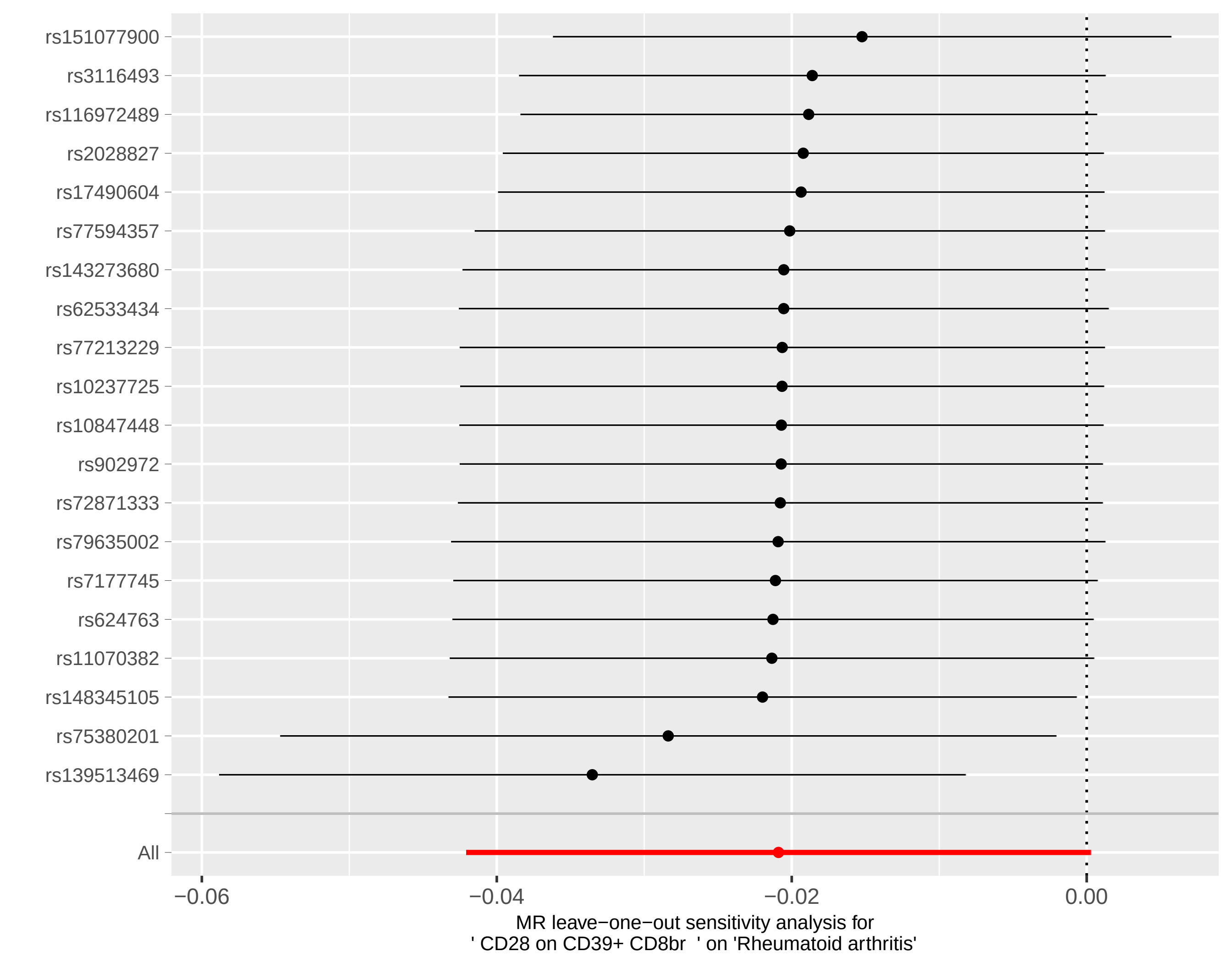

rs151077900
rs3116493
rs116972489
rs2028827
rs17490604
rs77594357
rs143273680
rs62533434
rs77213229
rs10237725
rs10847448
rs902972
rs72871333
rs79635002
rs7177745
rs624763
rs11070382
rs148345105
rs75380201
rs139513469
All
−0.06
−0.04
−0.02
0.00
MR leave−one−out sensitivity analysis for
' CD28 on CD39+ CD8br ' on 'Rheumatoid arthritis'

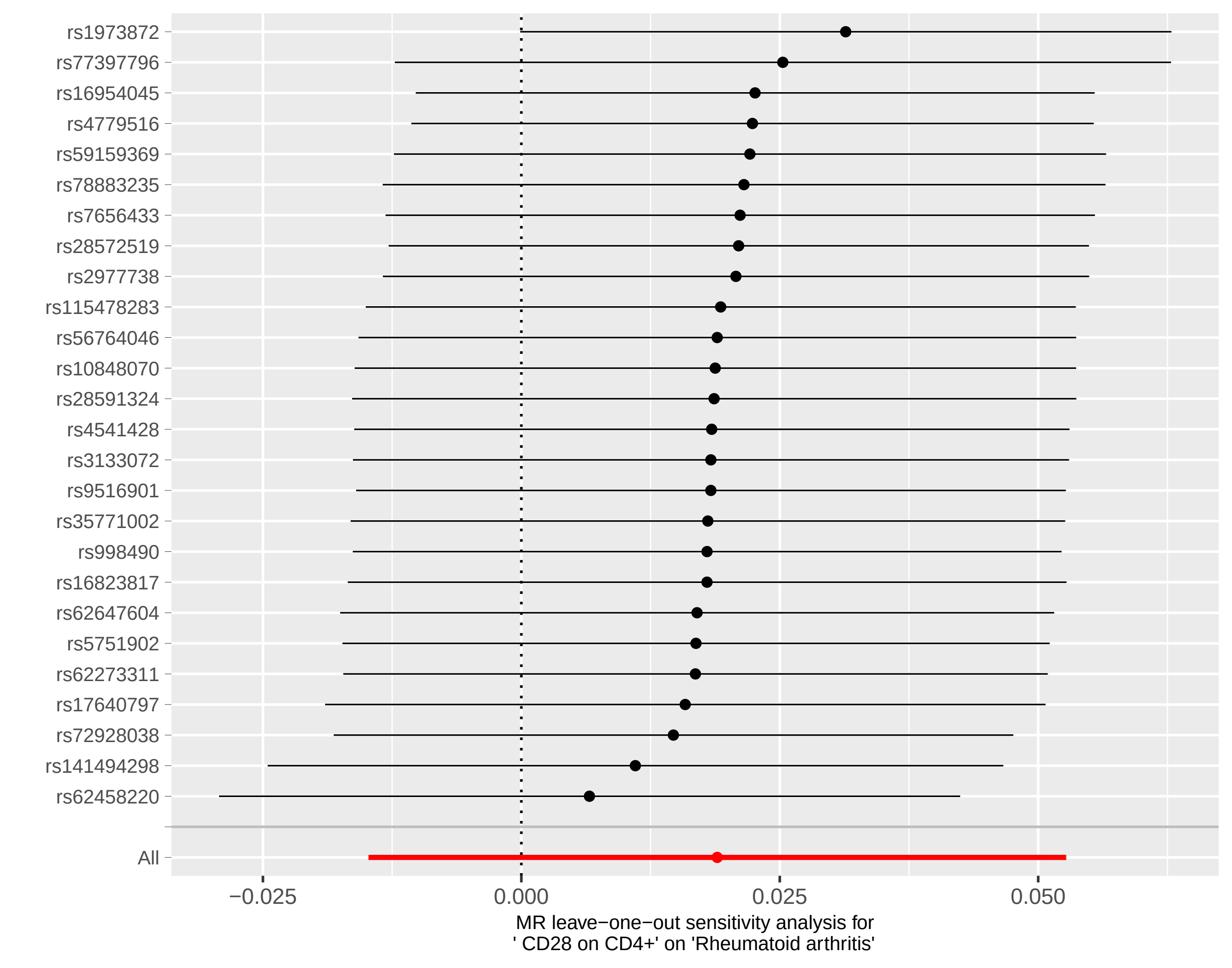
rs1973872
rs77397796
rs16954045
rs4779516
rs59159369
rs78883235
rs7656433
rs28572519
rs2977738
rs115478283
rs56764046
rs10848070
rs28591324
rs4541428
rs3133072
rs9516901
rs35771002
rs998490
rs16823817
rs62647604
rs5751902
rs62273311
rs17640797
rs72928038
rs141494298
rs62458220
All
−0.025
0.000
0.025
0.050
MR leave−one−out sensitivity analysis for
' CD28 on CD4+' on 'Rheumatoid arthritis'

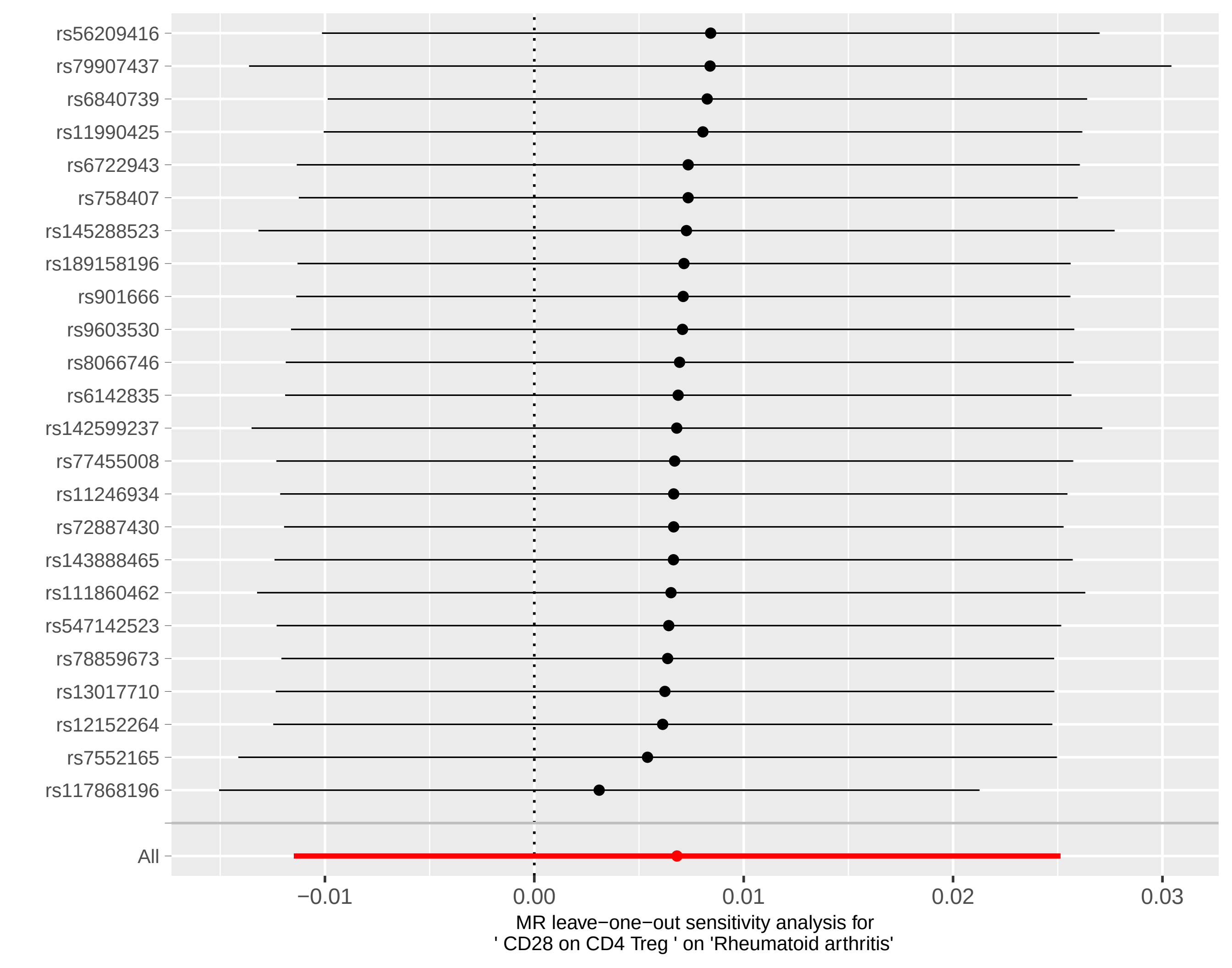
rs56209416
rs79907437
rs6840739
rs11990425
rs6722943
rs758407
rs145288523
rs189158196
rs901666
rs9603530
rs8066746
rs6142835
rs142599237
rs77455008
rs11246934
rs72887430
rs143888465
rs111860462
rs547142523
rs78859673
rs13017710
rs12152264
rs7552165
rs117868196
All
−0.01
0.00
0.01
0.02
0.03
MR leave−one−out sensitivity analysis for
' CD28 on CD4 Treg ' on 'Rheumatoid arthritis'

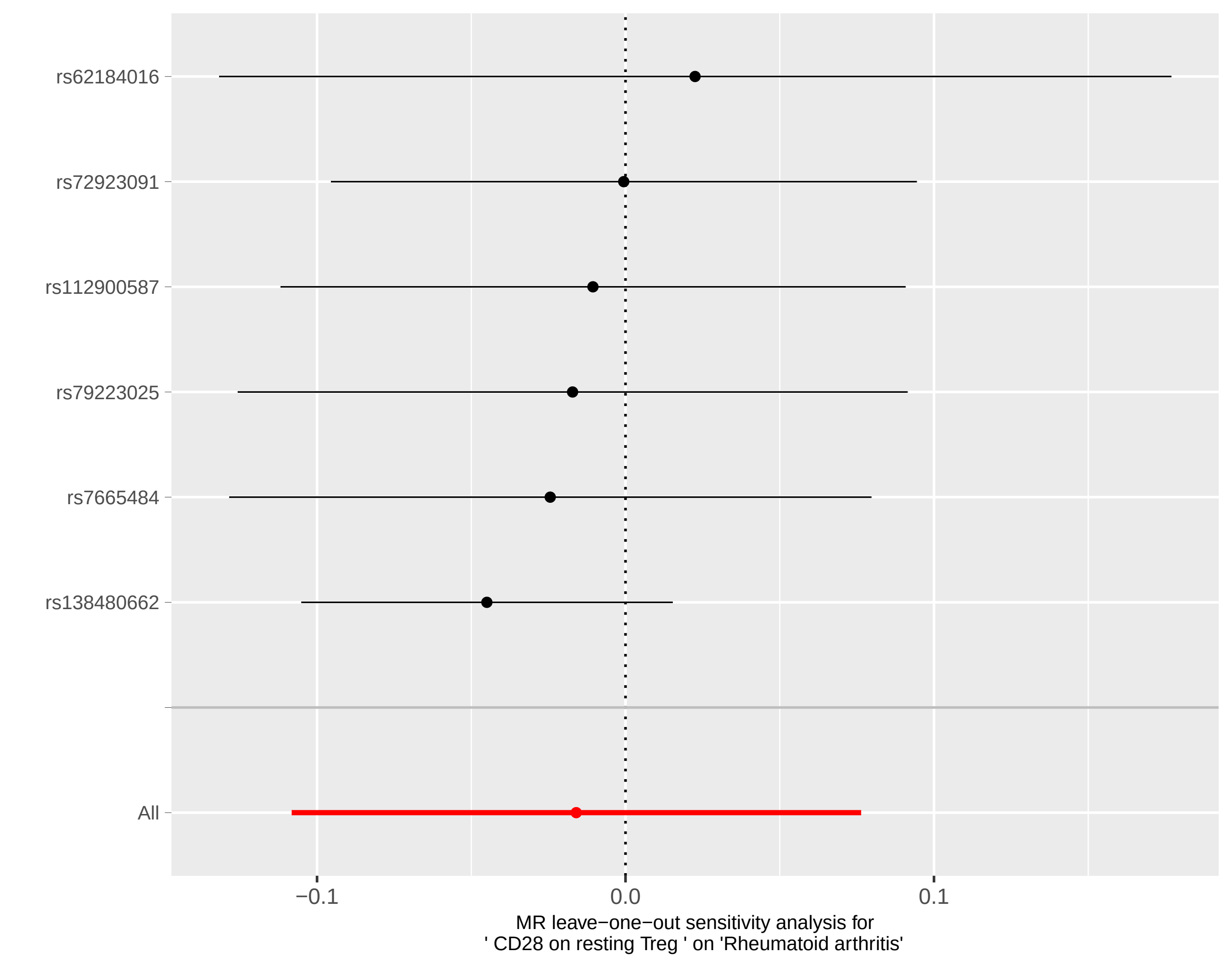
rs62184016
rs72923091
rs112900587
rs79223025
rs7665484
rs138480662
All
−0.1
0.0
0.1
MR leave−one−out sensitivity analysis for
' CD28 on resting Treg ' on 'Rheumatoid arthritis'

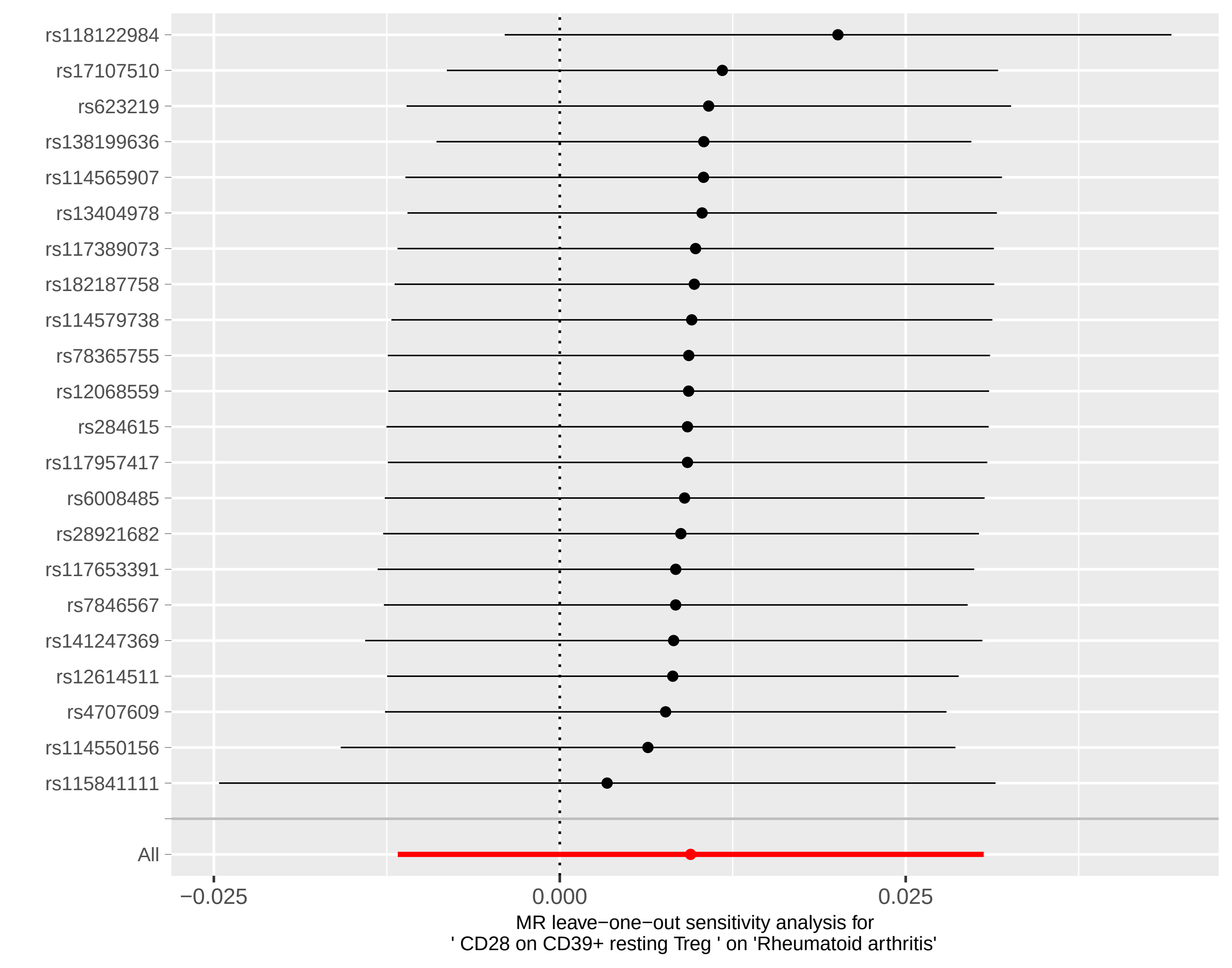

rs118122984
rs17107510
rs623219
rs138199636
rs114565907
rs13404978
rs117389073
rs182187758
rs114579738
rs78365755
rs12068559
rs284615
rs117957417
rs6008485
rs28921682
rs117653391
rs7846567
rs141247369
rs12614511
rs4707609
rs114550156
rs115841111
All
−0.025
0.000
0.025
MR leave−one−out sensitivity analysis for
' CD28 on CD39+ resting Treg ' on 'Rheumatoid arthritis'

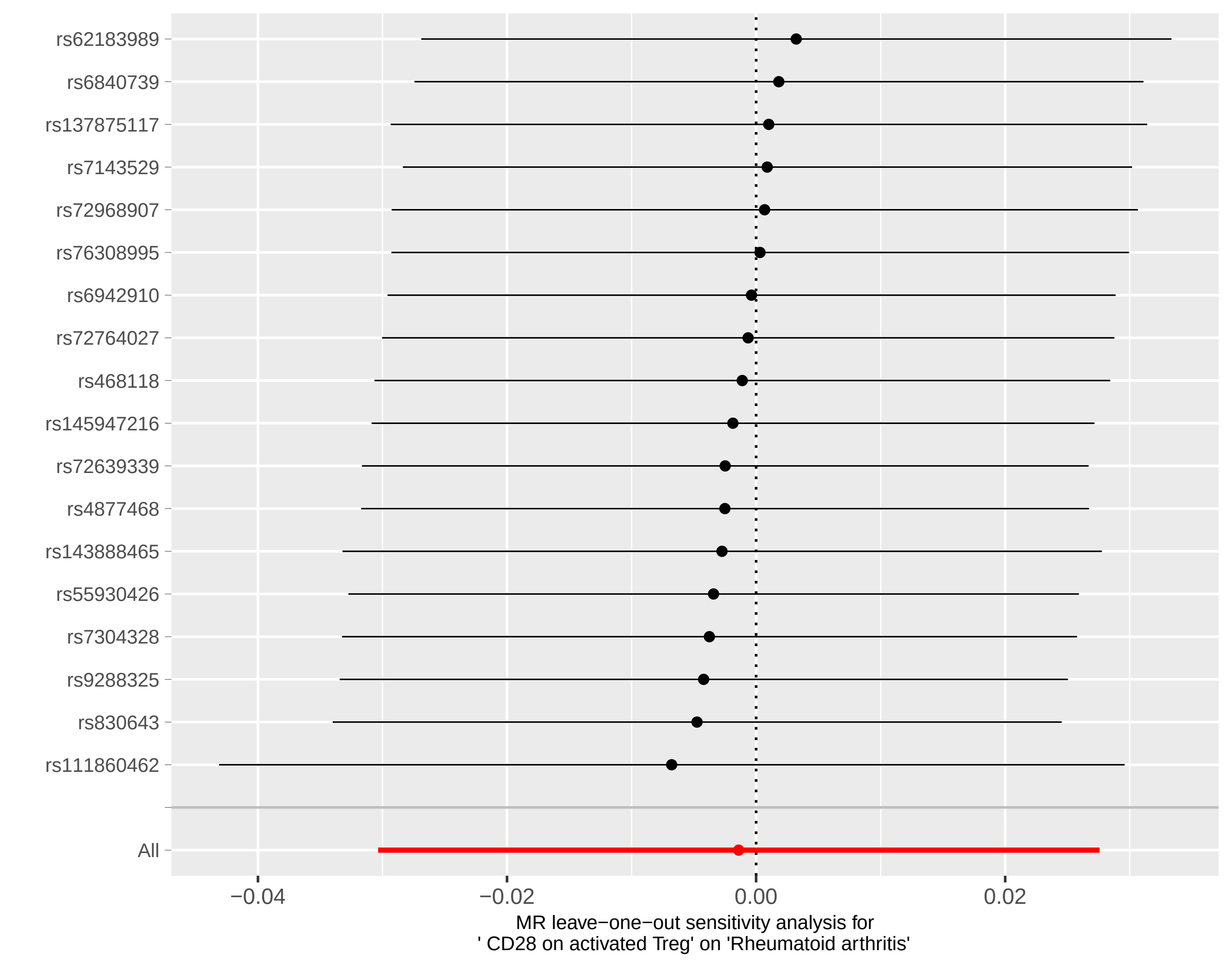

rs62183989
rs6840739
rs137875117
rs7143529
rs72968907
rs76308995
rs6942910
rs72764027
rs468118
rs145947216
rs72639339
rs4877468
rs143888465
rs55930426
rs7304328
rs9288325
rs830643
rs111860462
All
−0.04
−0.02
0.00
0.02
MR leave−one−out sensitivity analysis for
' CD28 on activated Treg' on 'Rheumatoid arthritis'

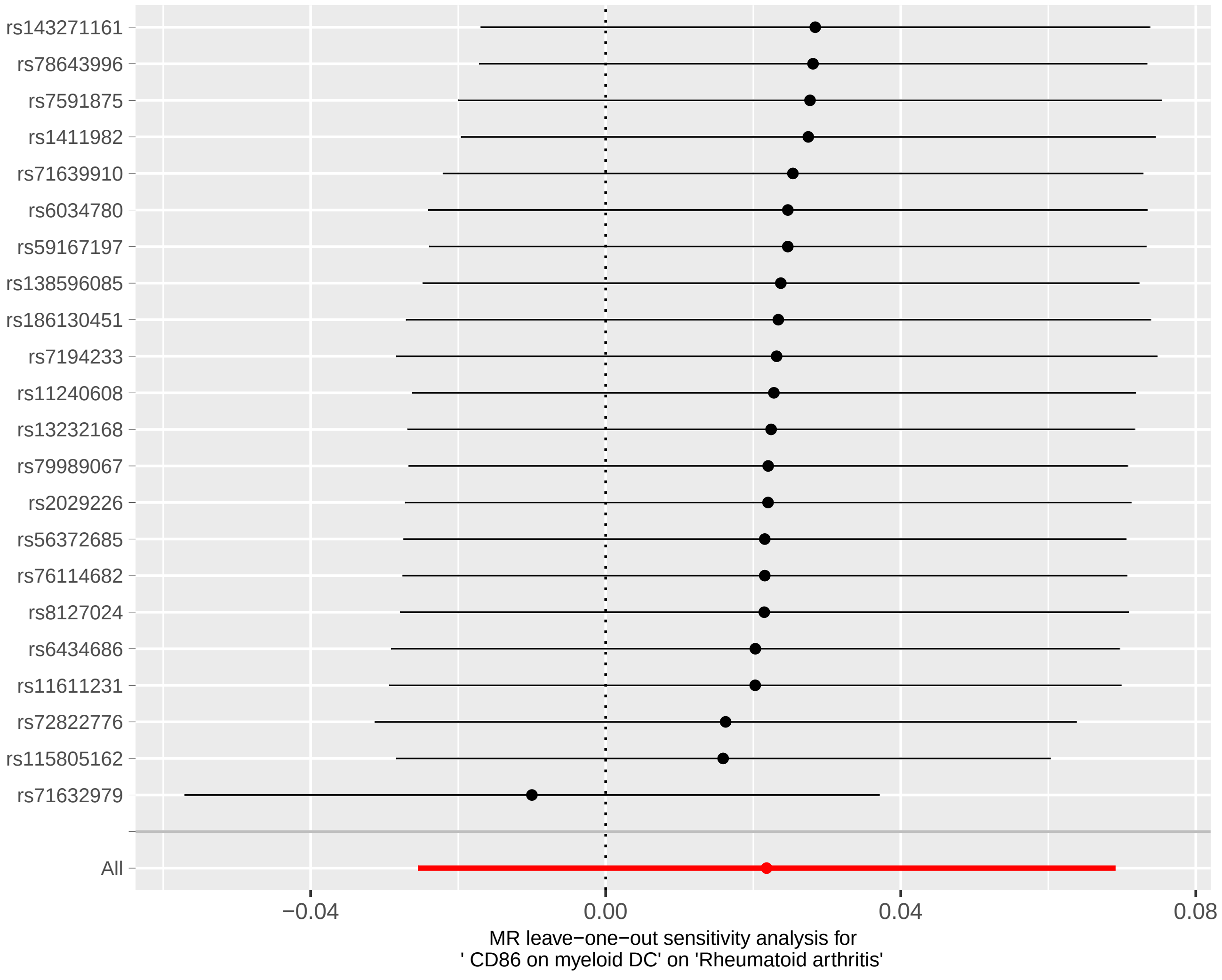
rs143271161
rs78643996
rs7591875
rs1411982
rs71639910
rs6034780
rs59167197
rs138596085
rs186130451
rs7194233
rs11240608
rs13232168
rs79989067
rs2029226
rs56372685
rs76114682
rs8127024
rs6434686
rs11611231
rs72822776
rs115805162
rs71632979
All
−0.04
0.00
0.04
0.08
MR leave−one−out sensitivity analysis for
' CD86 on myeloid DC' on 'Rheumatoid arthritis'

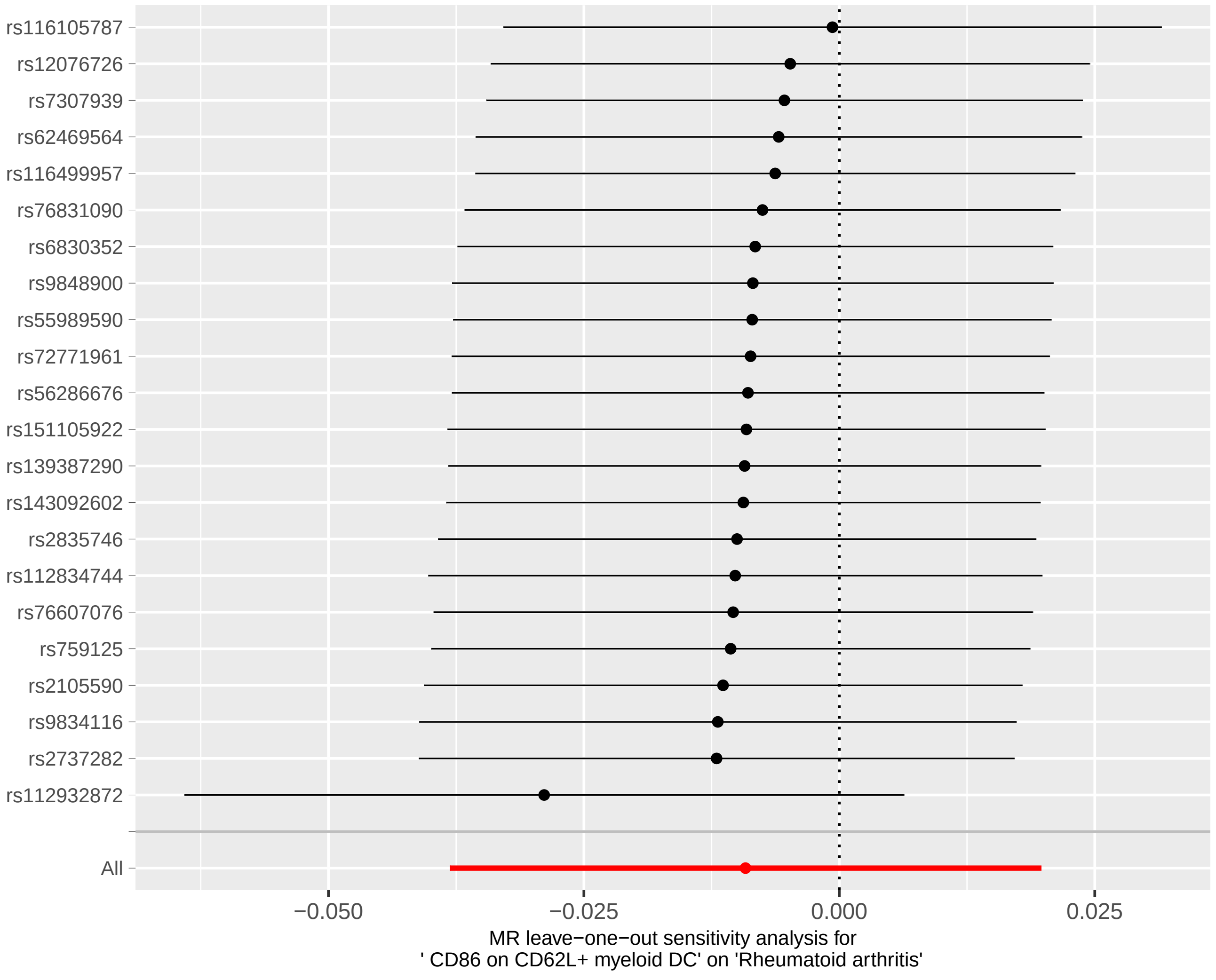
rs116105787
rs12076726
rs7307939
rs62469564
rs116499957
rs76831090
rs6830352
rs9848900
rs55989590
rs72771961
rs56286676
rs151105922
rs139387290
rs143092602
rs2835746
rs112834744
rs76607076
rs759125
rs2105590
rs9834116
rs2737282
rs112932872
All
−0.050
−0.025
0.000
0.025
MR leave−one−out sensitivity analysis for
' CD86 on CD62L+ myeloid DC' on 'Rheumatoid arthritis'

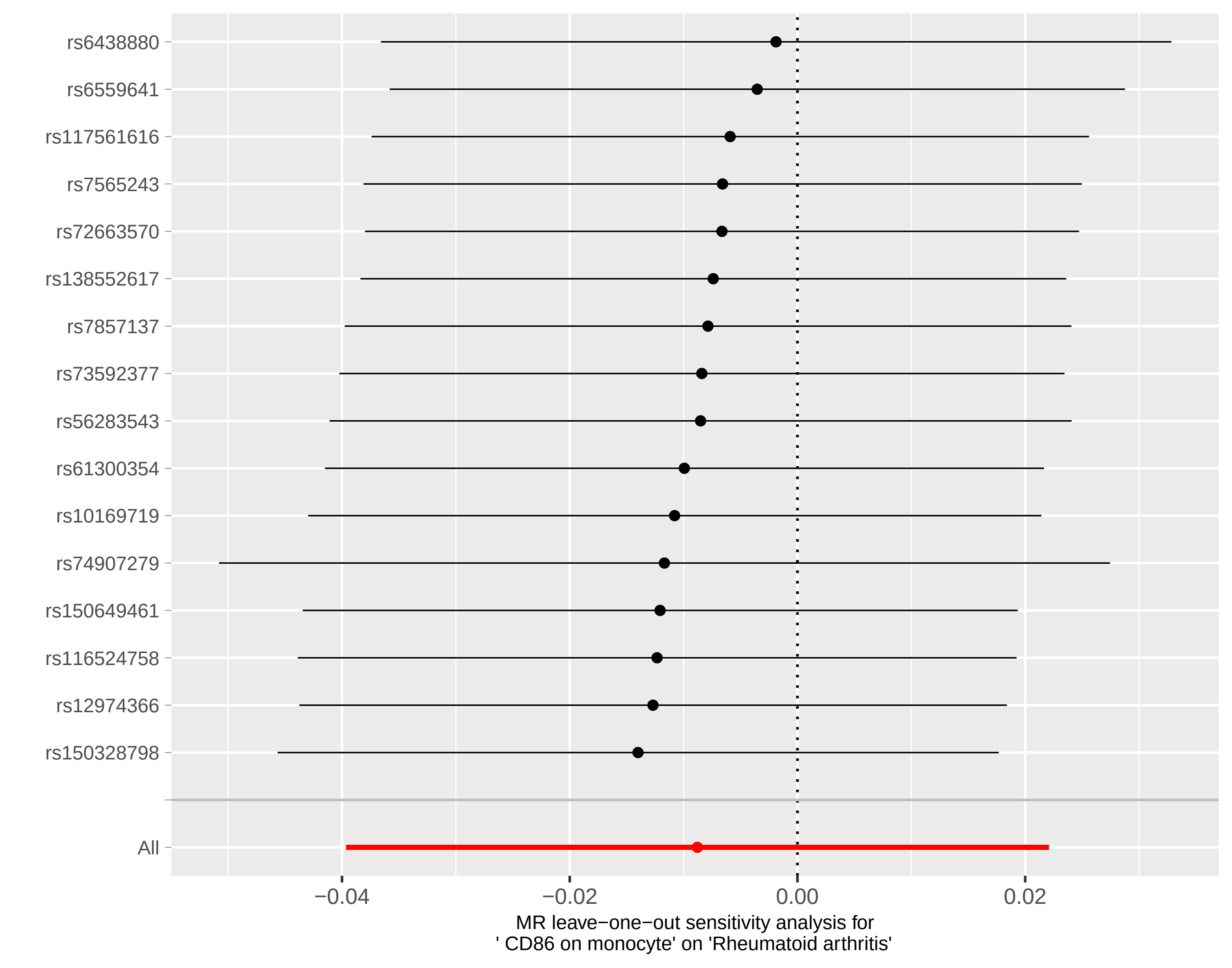

rs6438880
rs6559641
rs117561616
rs7565243
rs72663570
rs138552617
rs7857137
rs73592377
rs56283543
rs61300354
rs10169719
rs74907279
rs150649461
rs116524758
rs12974366
rs150328798
All
−0.04
−0.02
0.00
0.02
MR leave−one−out sensitivity analysis for
' CD86 on monocyte' on 'Rheumatoid arthritis'

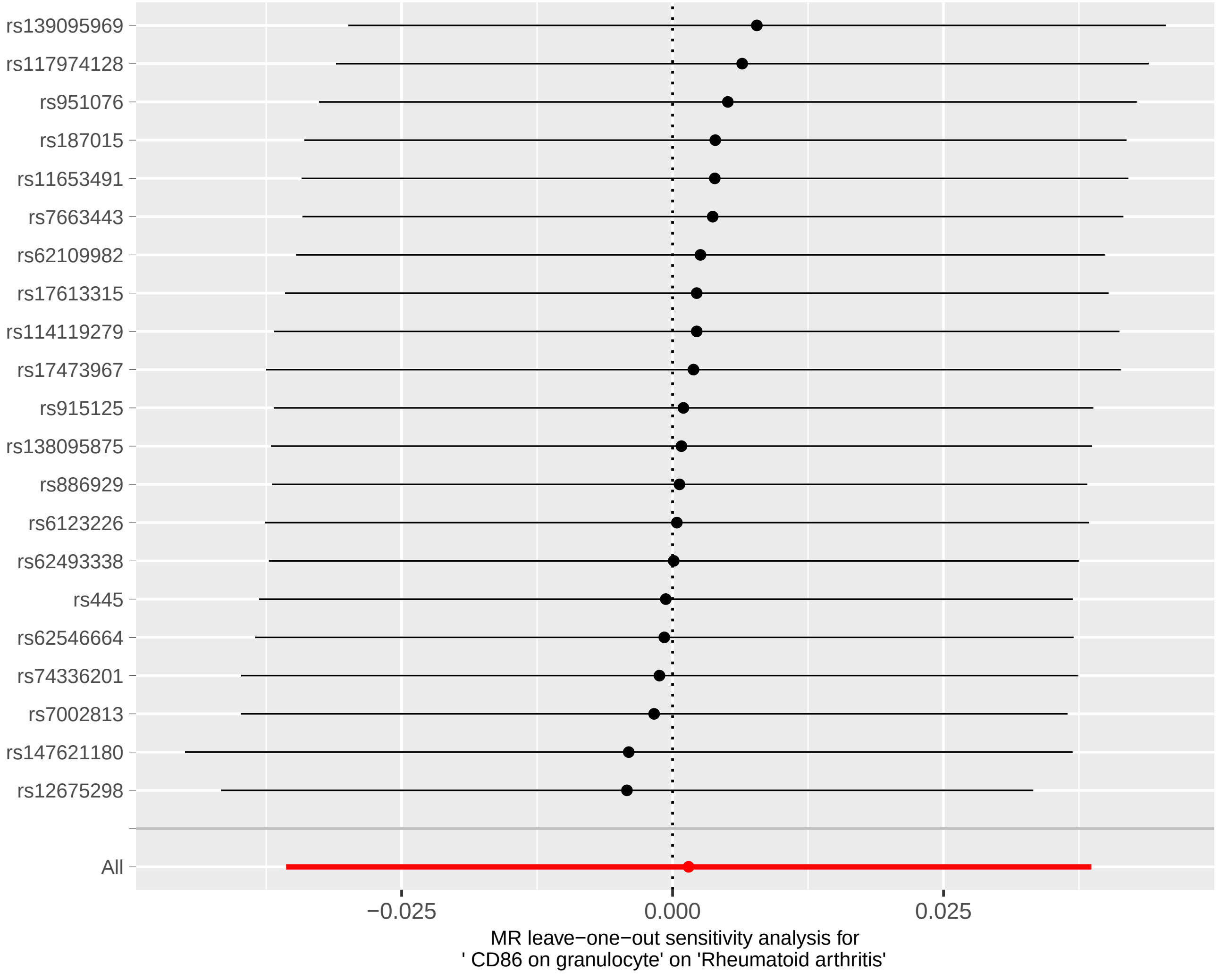

rs139095969
rs117974128
rs951076
rs187015
rs11653491
rs7663443
rs62109982
rs17613315
rs114119279
rs17473967
rs915125
rs138095875
rs886929
rs6123226
rs62493338
rs445
rs62546664
rs74336201
rs7002813
rs147621180
rs12675298
All
−0.025
0.000
0.025
MR leave−one−out sensitivity analysis for
' CD86 on granulocyte' on 'Rheumatoid arthritis'

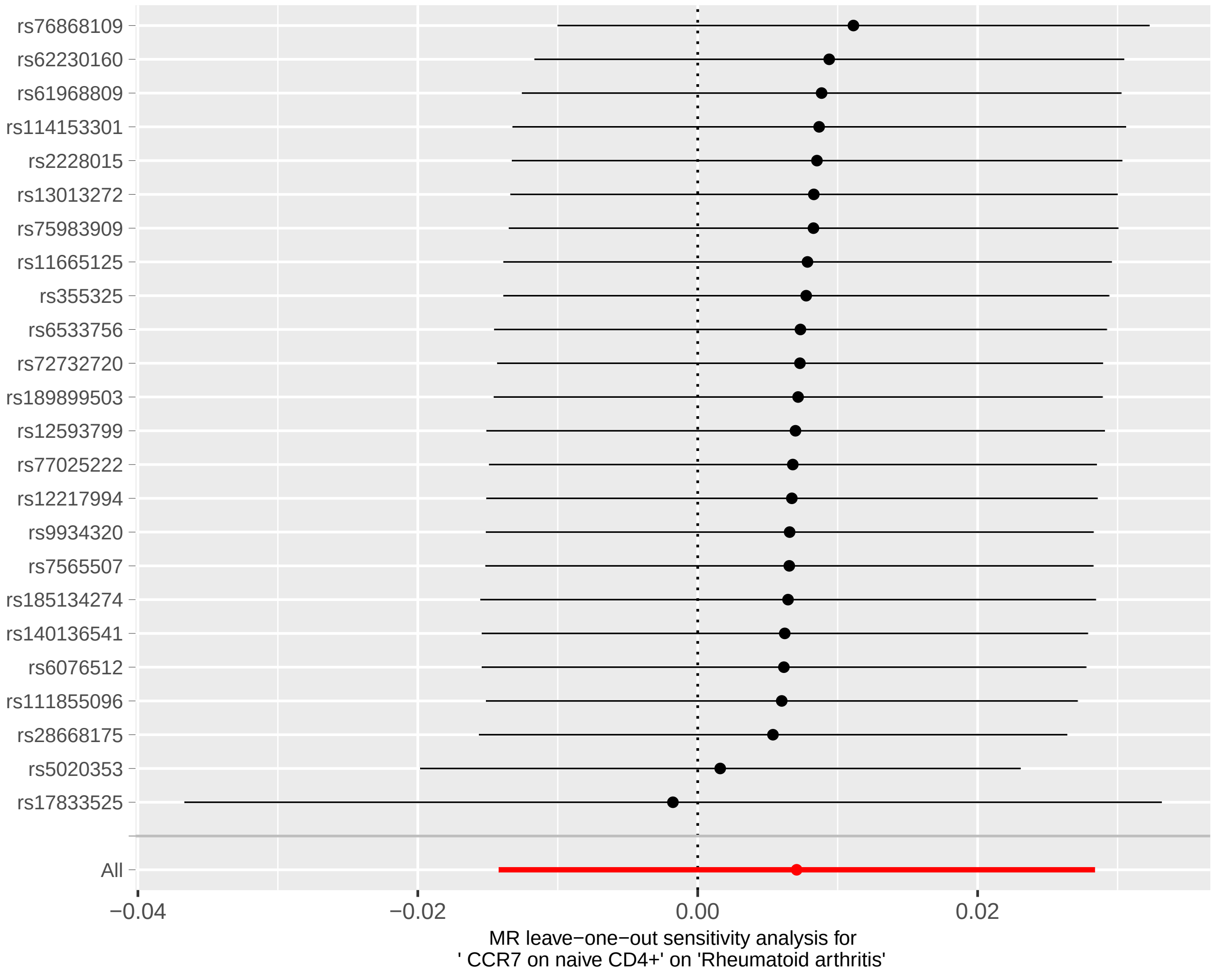

rs76868109
rs62230160
rs61968809
rs114153301
rs2228015
rs13013272
rs75983909
rs11665125
rs355325
rs6533756
rs72732720
rs189899503
rs12593799
rs77025222
rs12217994
rs9934320
rs7565507
rs185134274
rs140136541
rs6076512
rs111855096
rs28668175
rs5020353
rs17833525
All
−0.04
−0.02
0.00
0.02
MR leave−one−out sensitivity analysis for
' CCR7 on naive CD4+' on 'Rheumatoid arthritis'

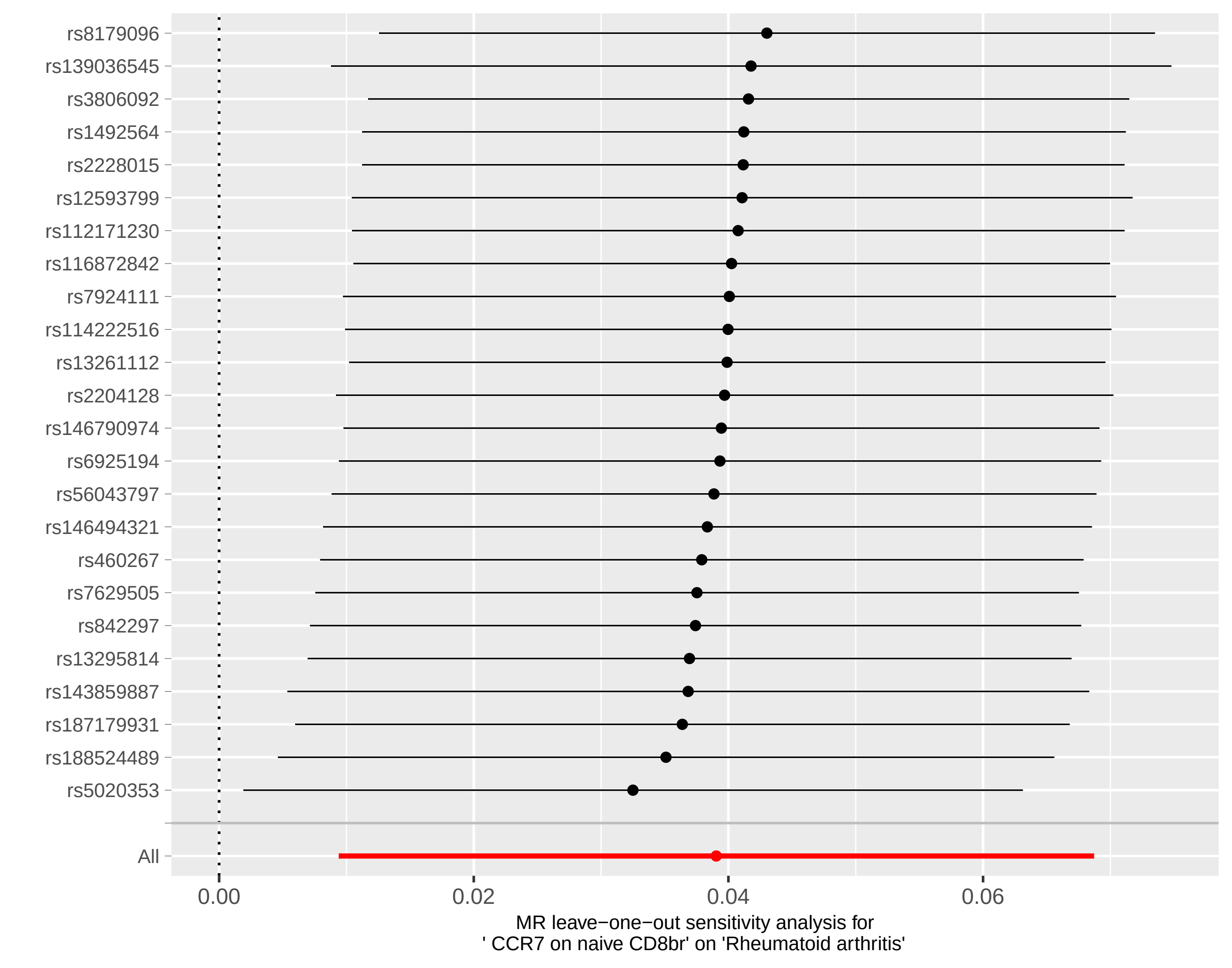
rs8179096
rs139036545
rs3806092
rs1492564
rs2228015
rs12593799
rs112171230
rs116872842
rs7924111
rs114222516
rs13261112
rs2204128
rs146790974
rs6925194
rs56043797
rs146494321
rs460267
rs7629505
rs842297
rs13295814
rs143859887
rs187179931
rs188524489
rs5020353
All
0.00
0.02
0.04
0.06
MR leave−one−out sensitivity analysis for
' CCR7 on naive CD8br' on 'Rheumatoid arthritis'

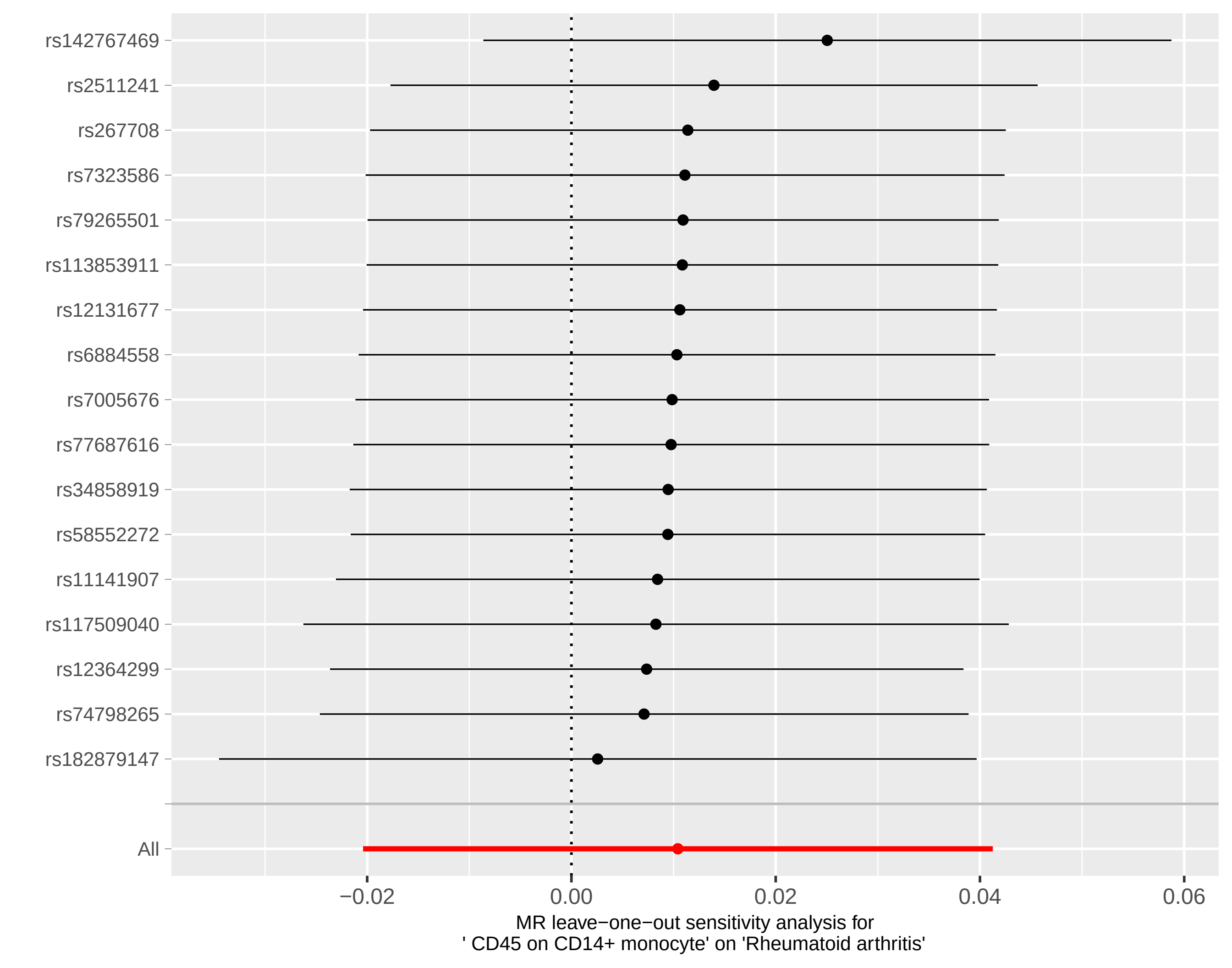

rs142767469
rs2511241
rs267708
rs7323586
rs79265501
rs113853911
rs12131677
rs6884558
rs7005676
rs77687616
rs34858919
rs58552272
rs11141907
rs117509040
rs12364299
rs74798265
rs182879147
All
−0.02
0.00
0.02
0.04
0.06
MR leave−one−out sensitivity analysis for
' CD45 on CD14+ monocyte' on 'Rheumatoid arthritis'

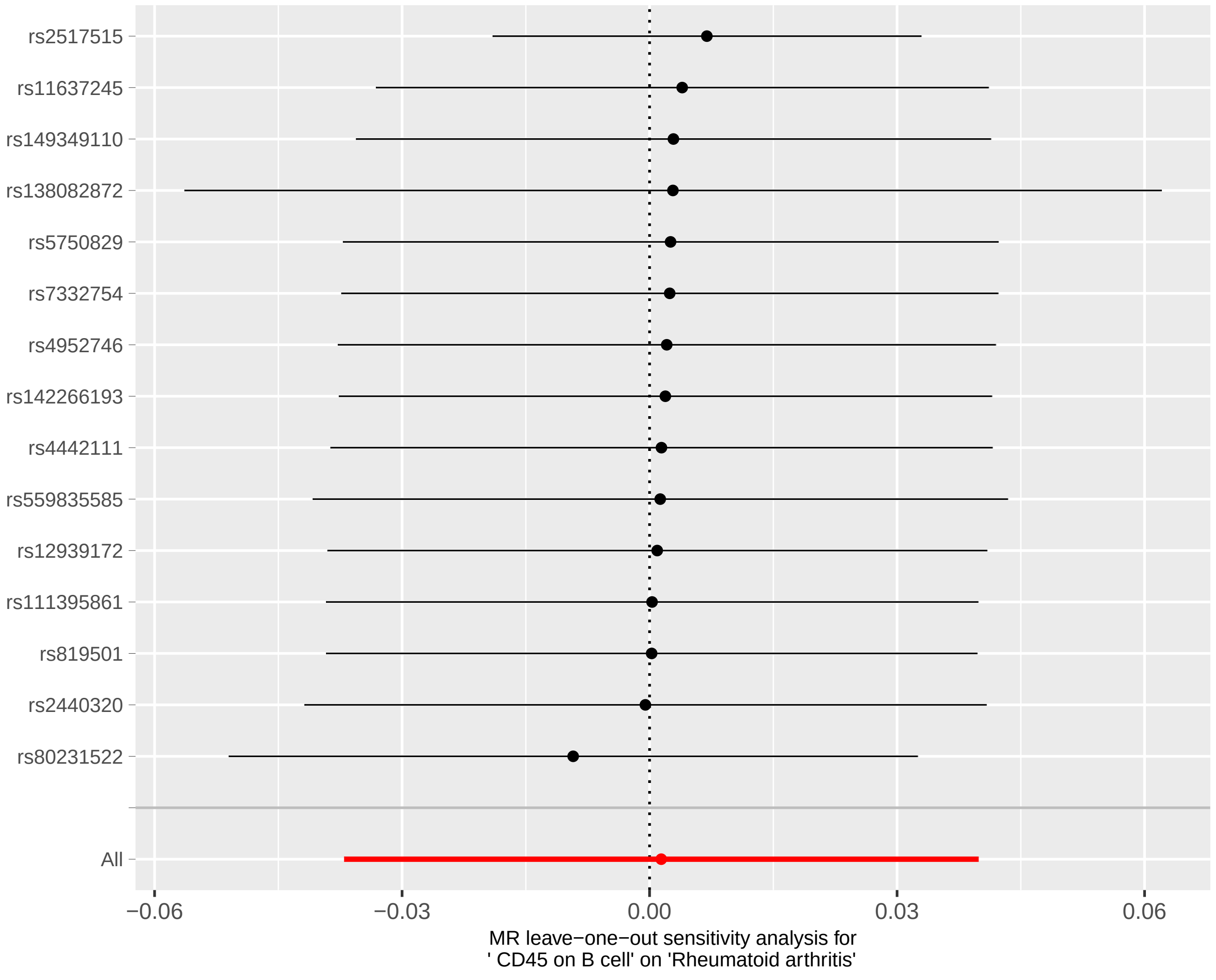
rs2517515
rs11637245
rs149349110
rs138082872
rs5750829
rs7332754
rs4952746
rs142266193
rs4442111
rs559835585
rs12939172
rs111395861
rs819501
rs2440320
rs80231522
All
−0.06
−0.03
0.00
0.03
0.06
MR leave−one−out sensitivity analysis for
' CD45 on B cell' on 'Rheumatoid arthritis'

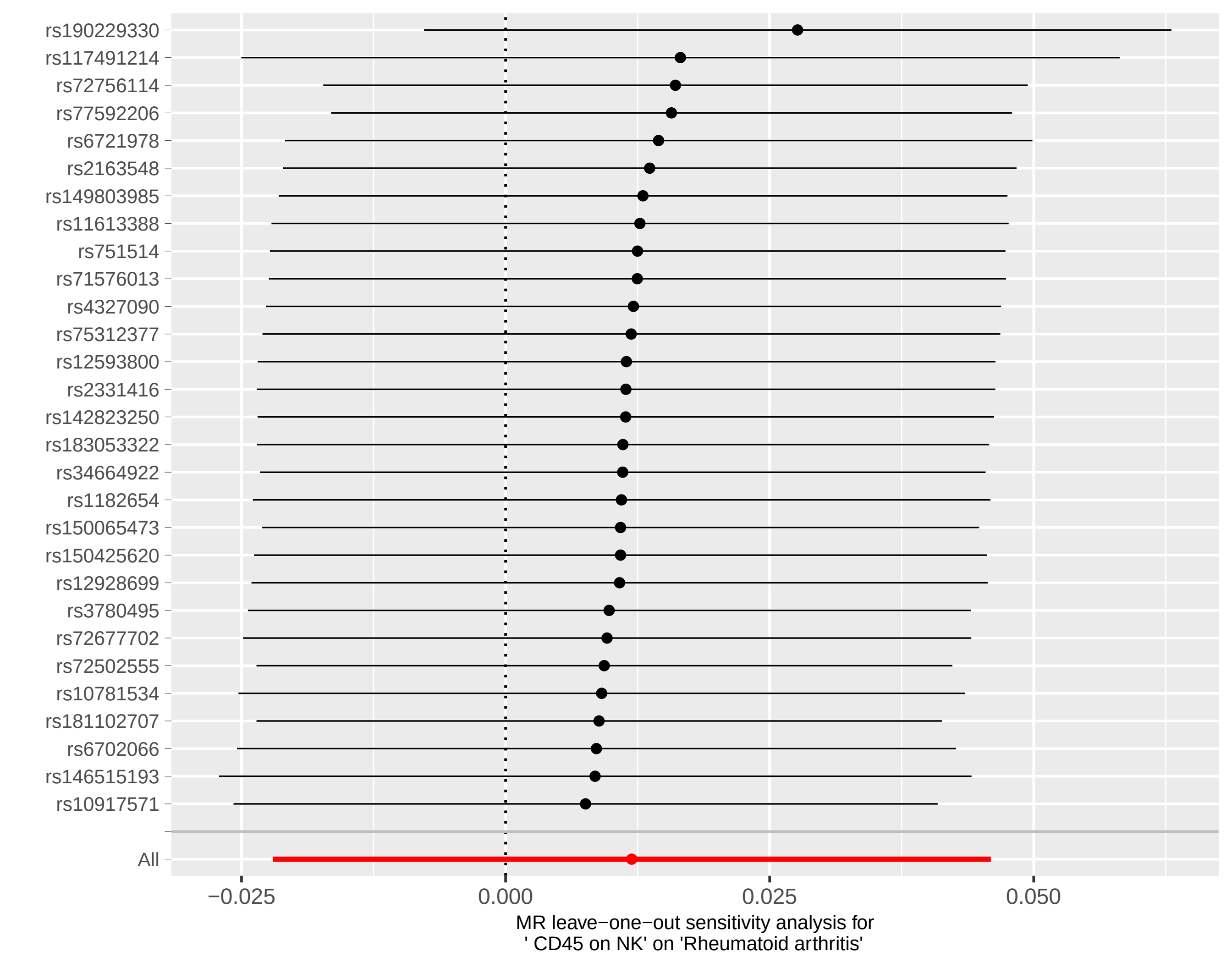
rs190229330
rs117491214
rs72756114
rs77592206
rs6721978
rs2163548
rs149803985
rs11613388
rs751514
rs71576013
rs4327090
rs75312377
rs12593800
rs2331416
rs142823250
rs183053322
rs34664922
rs1182654
rs150065473
rs150425620
rs12928699
rs3780495
rs72677702
rs72502555
rs10781534
rs181102707
rs6702066
rs146515193
rs10917571
All
−0.025
0.000
0.025
0.050
MR leave−one−out sensitivity analysis for
' CD45 on NK' on 'Rheumatoid arthritis'

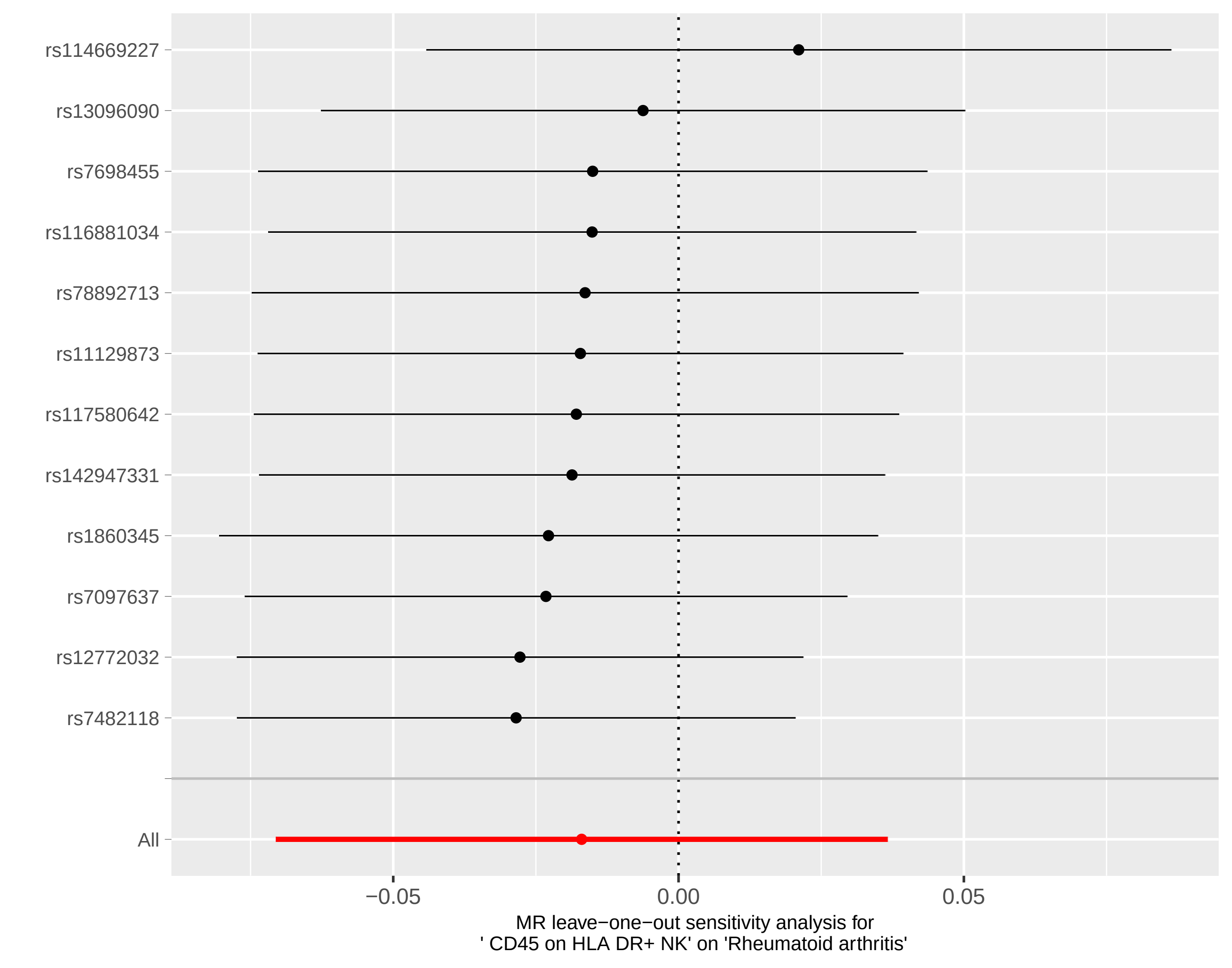

rs114669227
rs13096090
rs7698455
rs116881034
rs78892713
rs11129873
rs117580642
rs142947331
rs1860345
rs7097637
rs12772032
rs7482118
All
−0.05
0.00
0.05
MR leave−one−out sensitivity analysis for
' CD45 on HLA DR+ NK' on 'Rheumatoid arthritis'

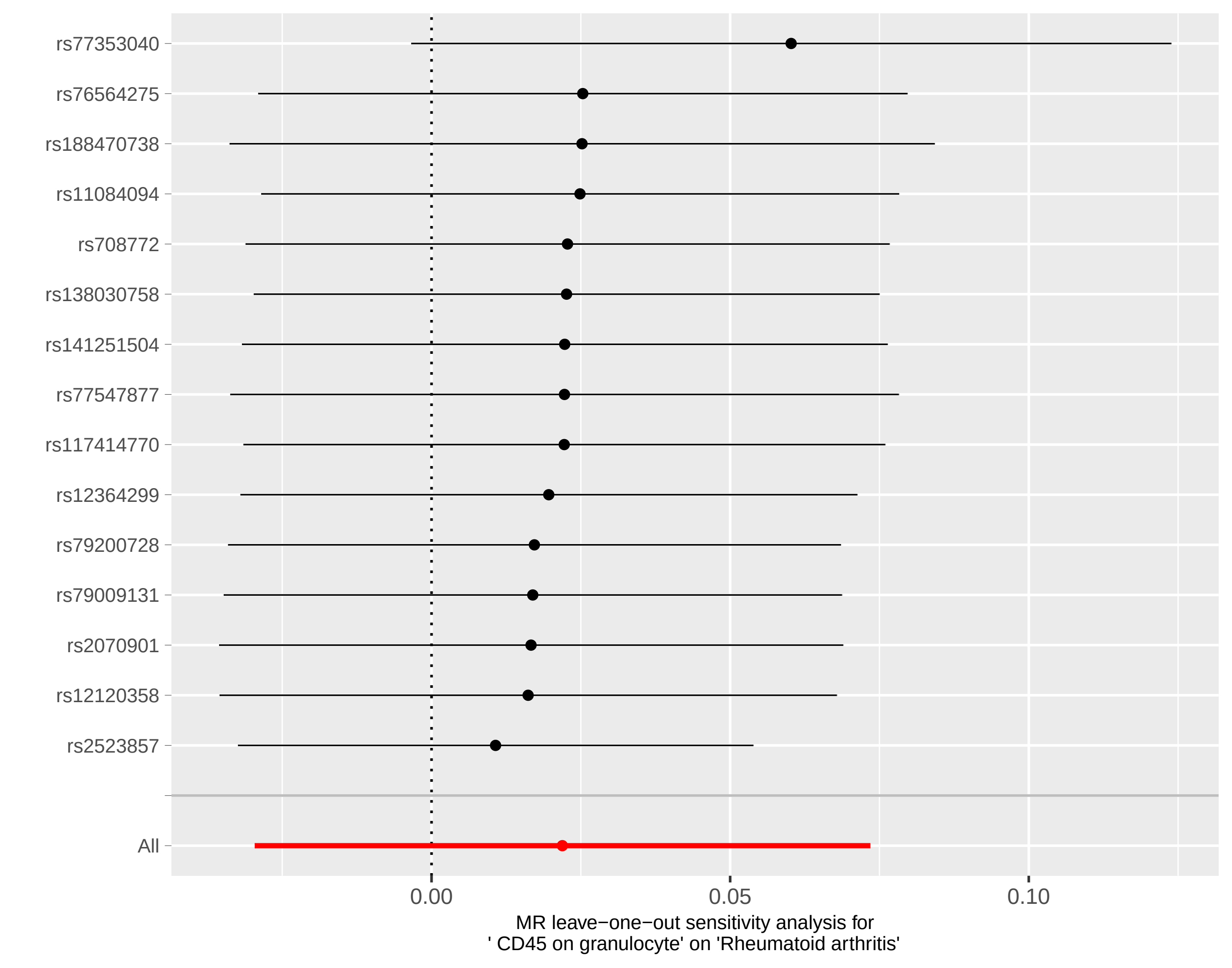
rs77353040
rs76564275
rs188470738
rs11084094
rs708772
rs138030758
rs141251504
rs77547877
rs117414770
rs12364299
rs79200728
rs79009131
rs2070901
rs12120358
rs2523857
All
0.00
0.05
0.10
MR leave−one−out sensitivity analysis for
' CD45 on granulocyte' on 'Rheumatoid arthritis'

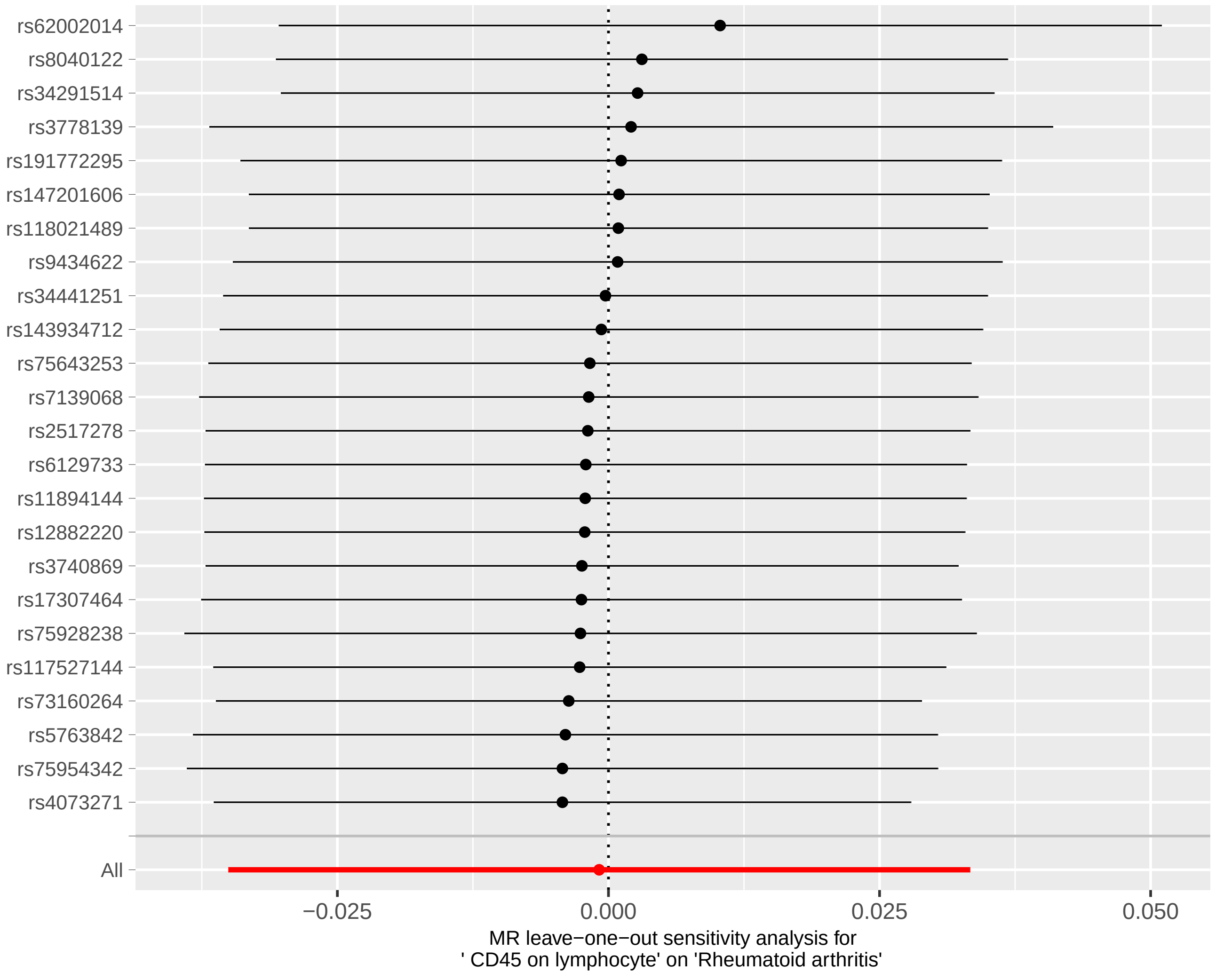
rs62002014
rs8040122
rs34291514
rs3778139
rs191772295
rs147201606
rs118021489
rs9434622
rs34441251
rs143934712
rs75643253
rs7139068
rs2517278
rs6129733
rs11894144
rs12882220
rs3740869
rs17307464
rs75928238
rs117527144
rs73160264
rs5763842
rs75954342
rs4073271
All
−0.025
0.000
0.025
0.050
MR leave−one−out sensitivity analysis for
' CD45 on lymphocyte' on 'Rheumatoid arthritis'

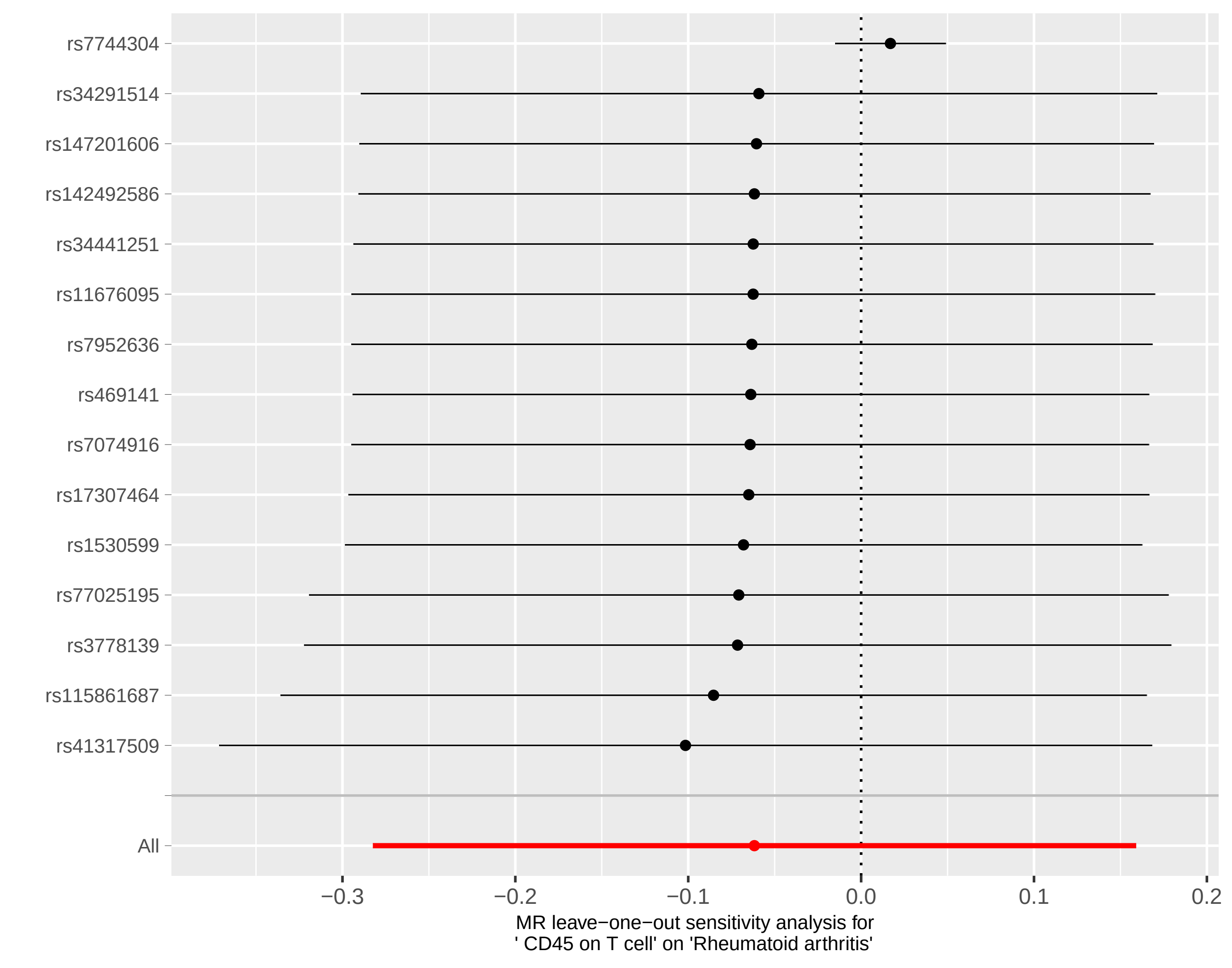

rs7744304
rs34291514
rs147201606
rs142492586
rs34441251
rs11676095
rs7952636
rs469141
rs7074916
rs17307464
rs1530599
rs77025195
rs3778139
rs115861687
rs41317509
All
−0.3
−0.2
−0.1
0.0
0.1
0.2
MR leave−one−out sensitivity analysis for
' CD45 on T cell' on 'Rheumatoid arthritis'

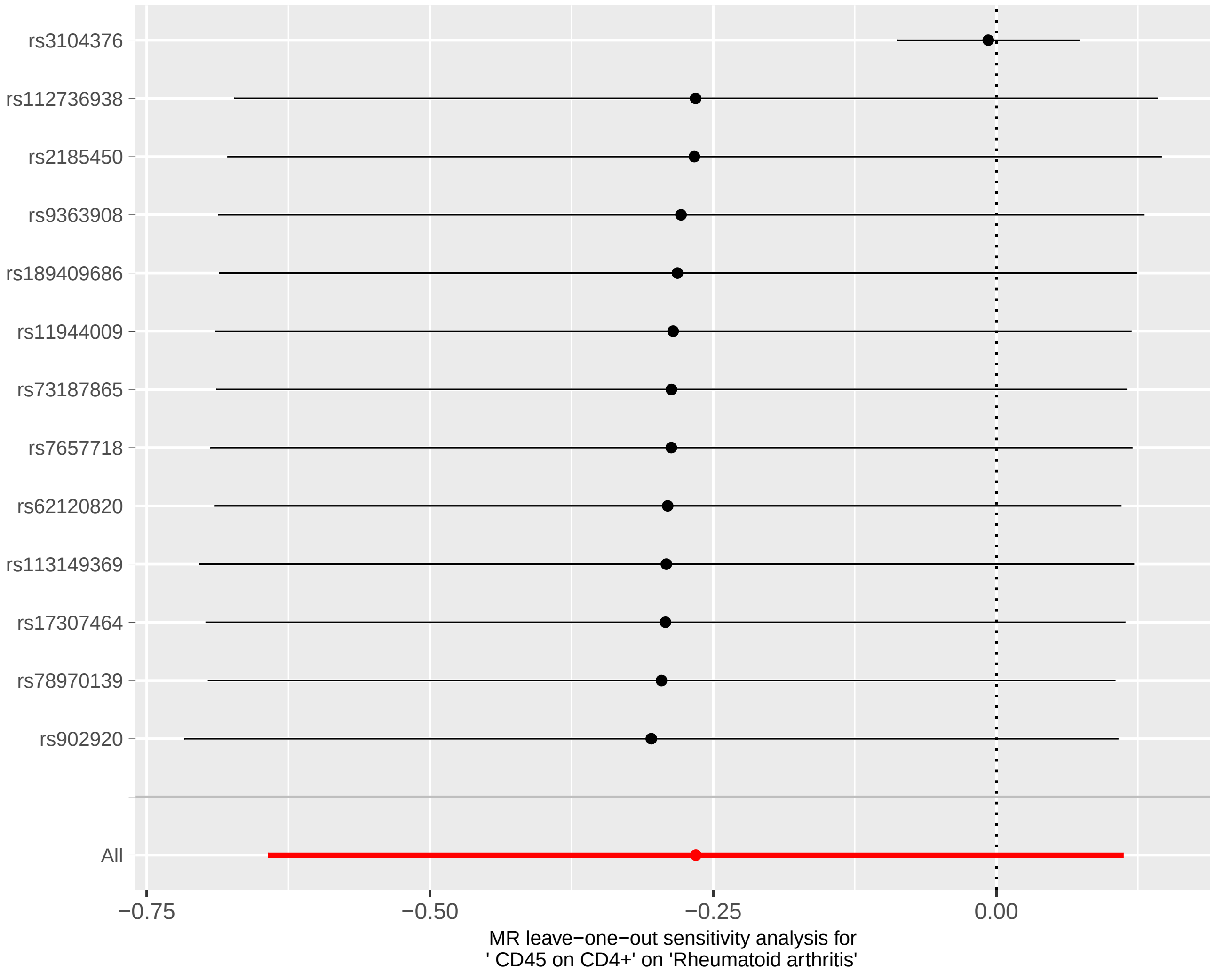
rs3104376
rs112736938
rs2185450
rs9363908
rs189409686
rs11944009
rs73187865
rs7657718
rs62120820
rs113149369
rs17307464
rs78970139
rs902920
All
−0.75
−0.50
−0.25
0.00
MR leave−one−out sensitivity analysis for
' CD45 on CD4+' on 'Rheumatoid arthritis'

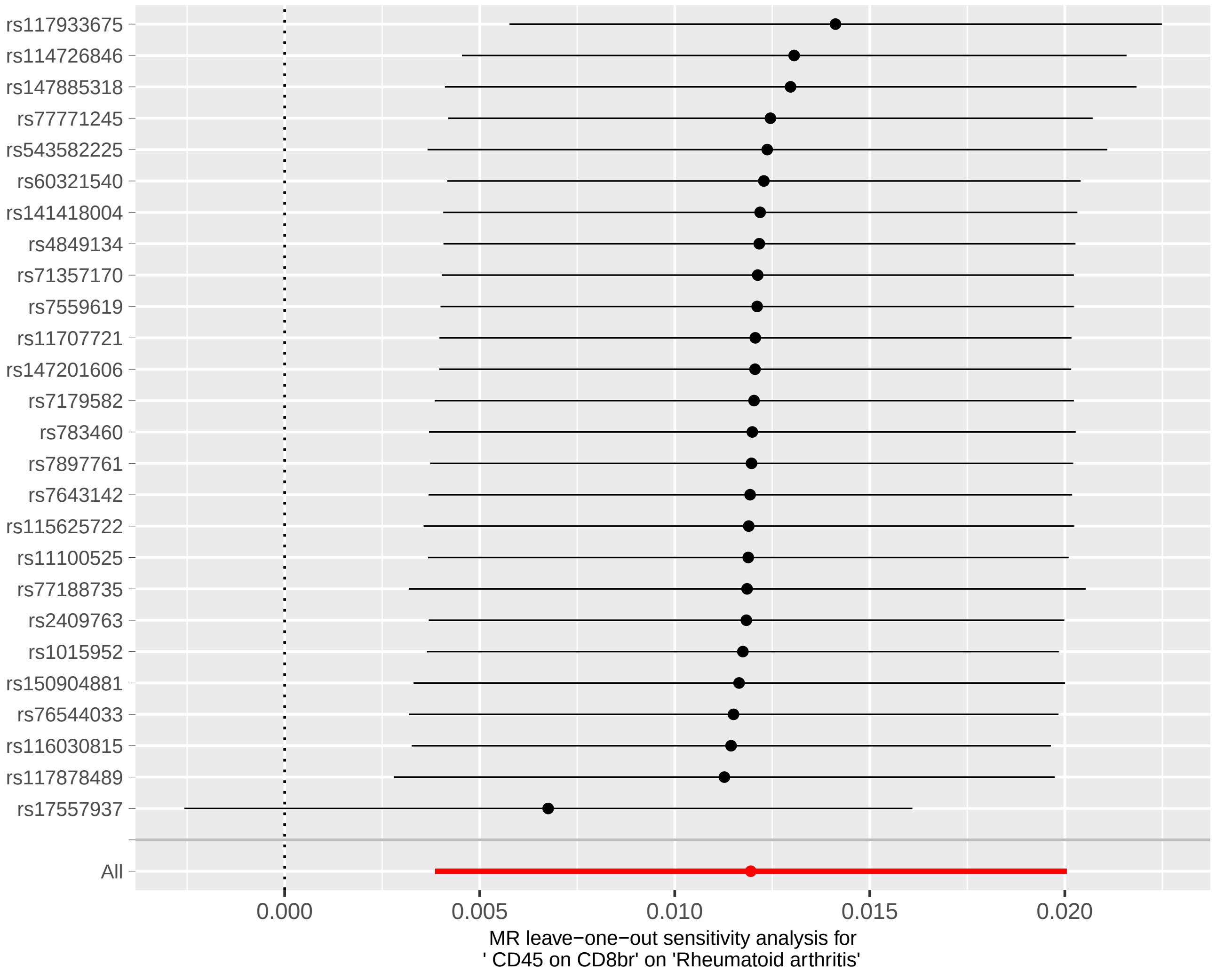
rs117933675
rs114726846
rs147885318
rs77771245
rs543582225
rs60321540
rs141418004
rs4849134
rs71357170
rs7559619
rs11707721
rs147201606
rs7179582
rs783460
rs7897761
rs7643142
rs115625722
rs11100525
rs77188735
rs2409763
rs1015952
rs150904881
rs76544033
rs116030815
rs117878489
rs17557937
All
0.000
0.005
0.010
0.015
0.020
MR leave−one−out sensitivity analysis for
' CD45 on CD8br' on 'Rheumatoid arthritis'

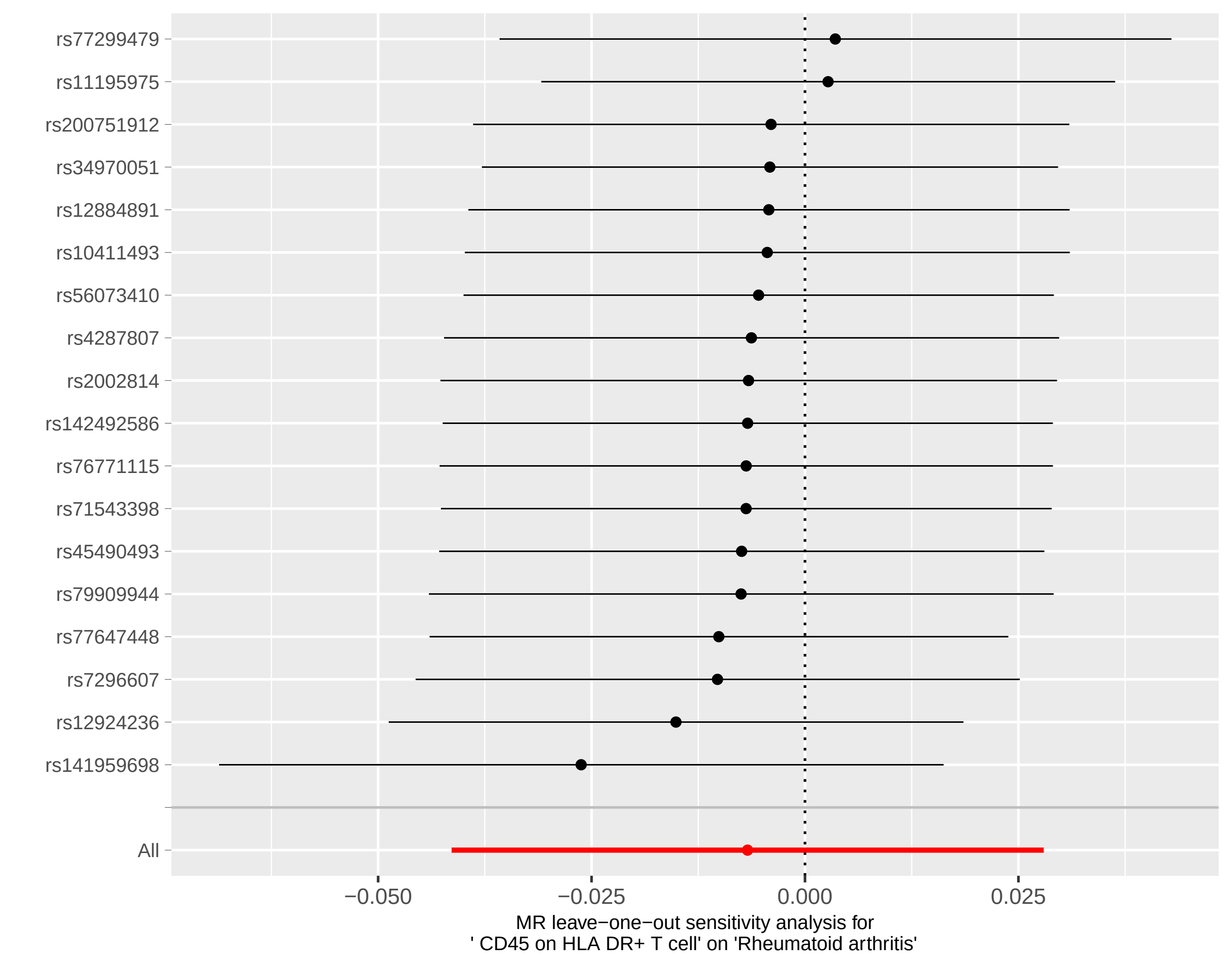
rs77299479
rs11195975
rs200751912
rs34970051
rs12884891
rs10411493
rs56073410
rs4287807
rs2002814
rs142492586
rs76771115
rs71543398
rs45490493
rs79909944
rs77647448
rs7296607
rs12924236
rs141959698
All
−0.050
−0.025
0.000
0.025
MR leave−one−out sensitivity analysis for
' CD45 on HLA DR+ T cell' on 'Rheumatoid arthritis'

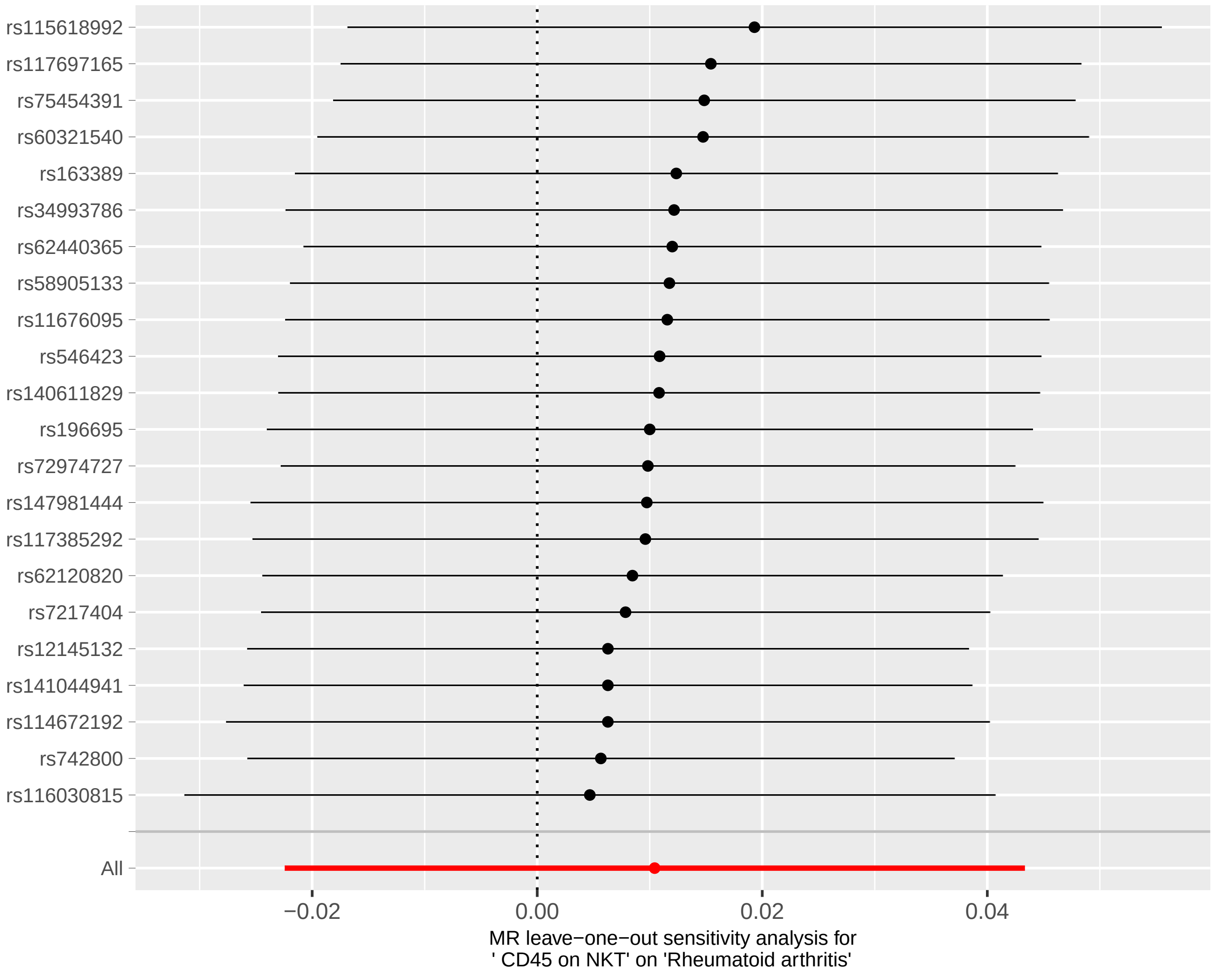

rs115618992
rs117697165
rs75454391
rs60321540
rs163389
rs34993786
rs62440365
rs58905133
rs11676095
rs546423
rs140611829
rs196695
rs72974727
rs147981444
rs117385292
rs62120820
rs7217404
rs12145132
rs141044941
rs114672192
rs742800
rs116030815
All
−0.02
0.00
0.02
0.04
MR leave−one−out sensitivity analysis for
' CD45 on NKT' on 'Rheumatoid arthritis'

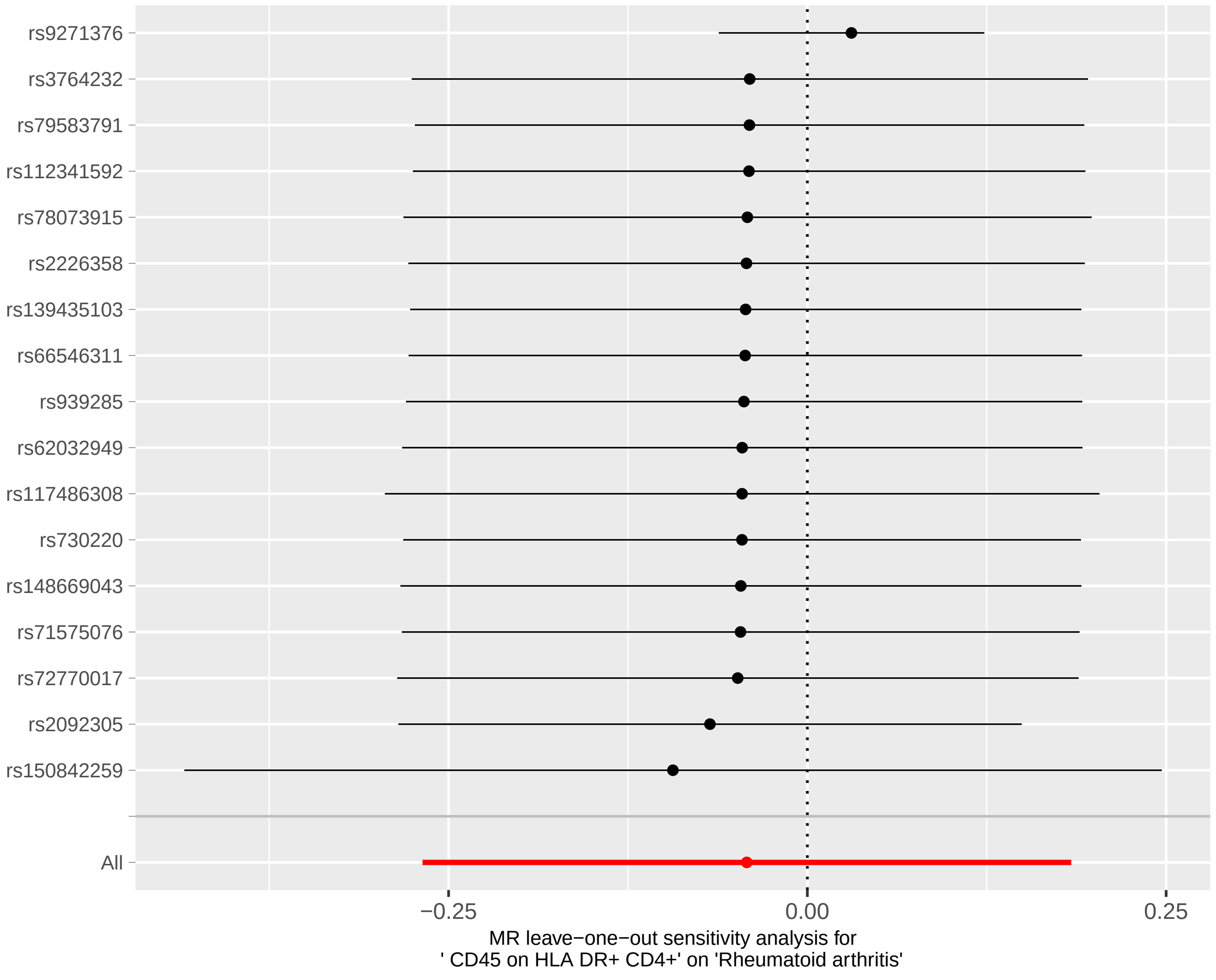
rs9271376
rs3764232
rs79583791
rs112341592
rs78073915
rs2226358
rs139435103
rs66546311
rs939285
rs62032949
rs117486308
rs730220
rs148669043
rs71575076
rs72770017
rs2092305
rs150842259
All
−0.25
0.00
0.25
MR leave−one−out sensitivity analysis for
' CD45 on HLA DR+ CD4+' on 'Rheumatoid arthritis'

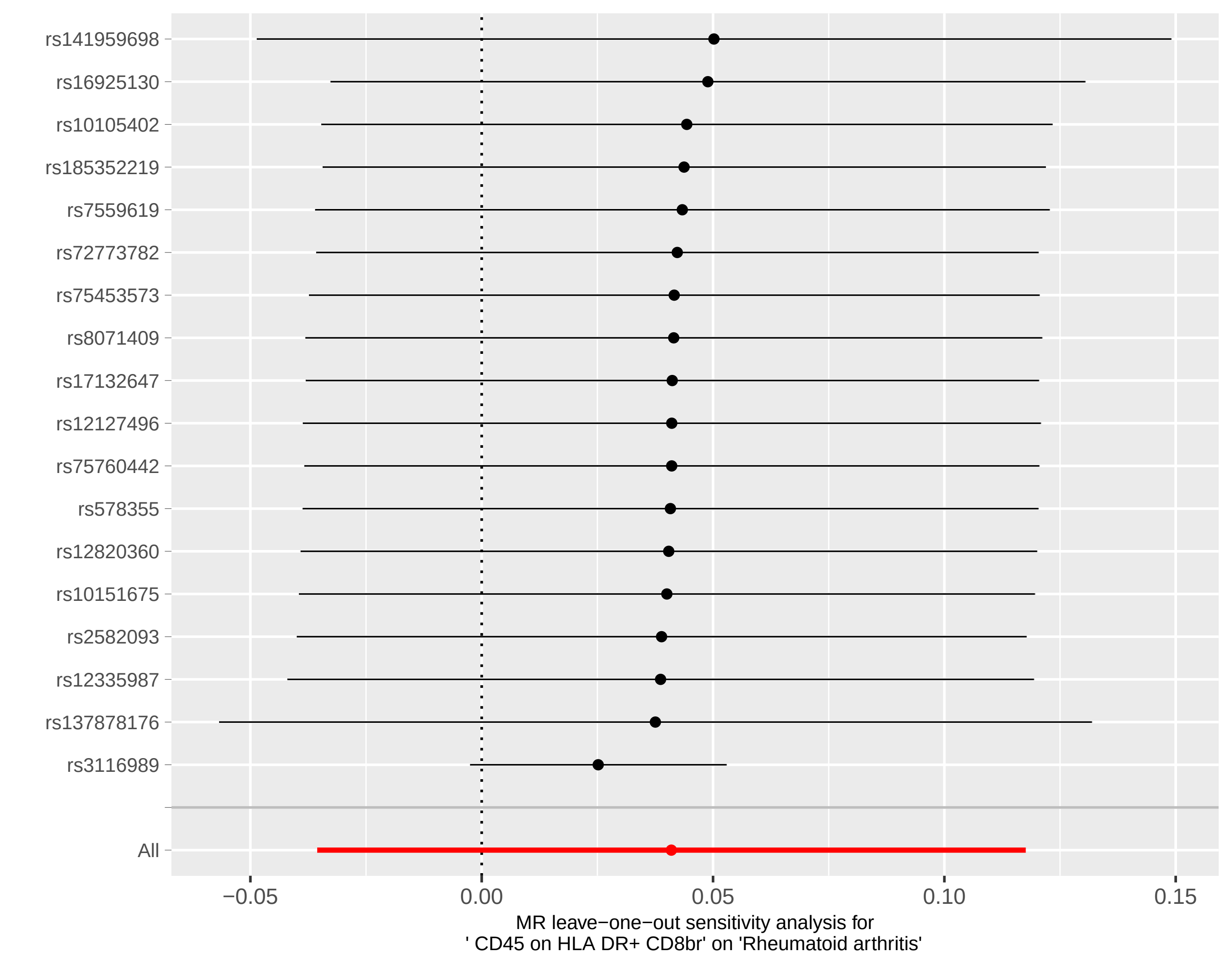

rs141959698
rs16925130
rs10105402
rs185352219
rs7559619
rs72773782
rs75453573
rs8071409
rs17132647
rs12127496
rs75760442
rs578355
rs12820360
rs10151675
rs2582093
rs12335987
rs137878176
rs3116989
All
−0.05
0.00
0.05
0.10
0.15
MR leave−one−out sensitivity analysis for
' CD45 on HLA DR+ CD8br' on 'Rheumatoid arthritis'

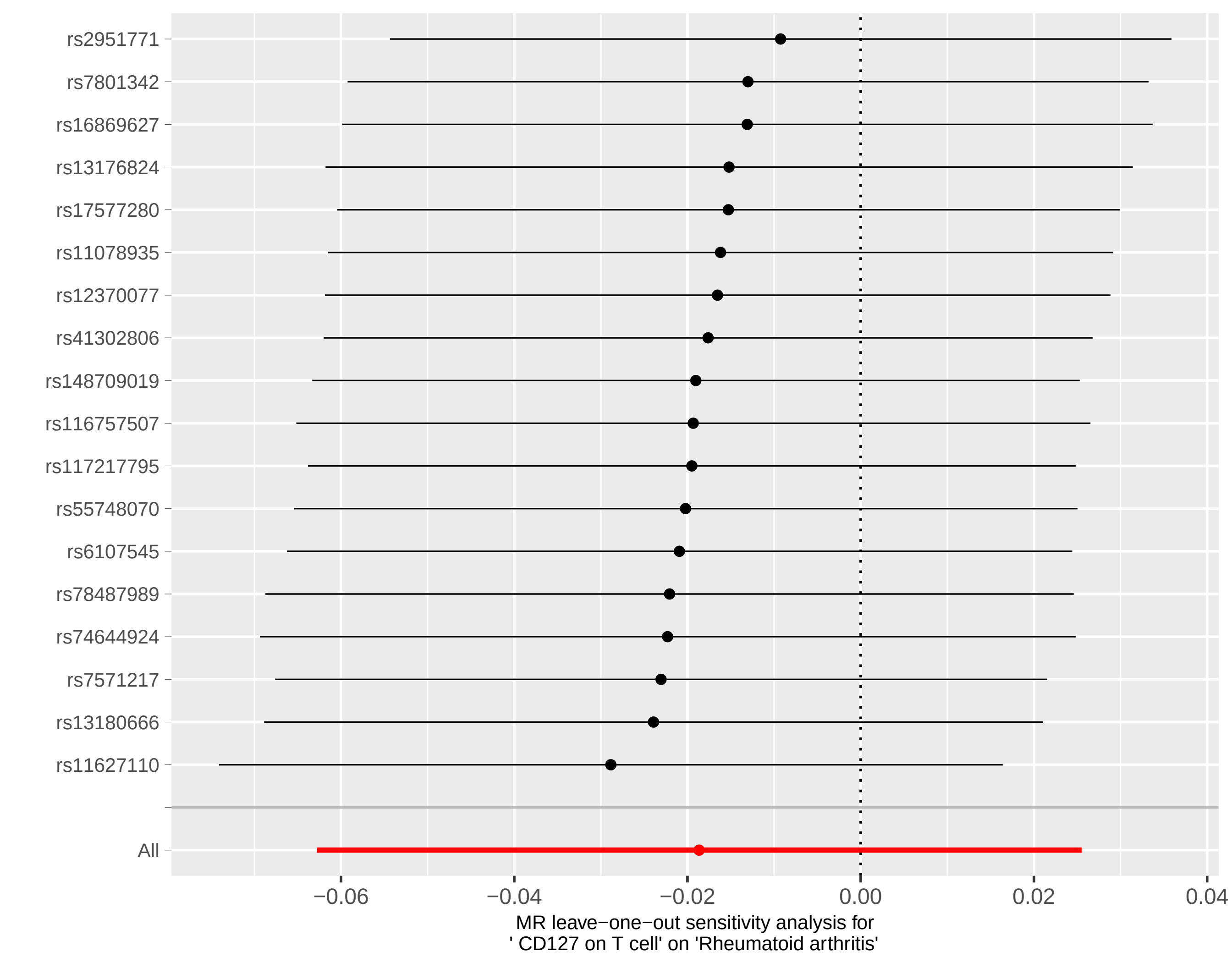
rs2951771
rs7801342
rs16869627
rs13176824
rs17577280
rs11078935
rs12370077
rs41302806
rs148709019
rs116757507
rs117217795
rs55748070
rs6107545
rs78487989
rs74644924
rs7571217
rs13180666
rs11627110
All
−0.06
−0.04
−0.02
0.00
0.02
0.04
MR leave−one−out sensitivity analysis for
' CD127 on T cell' on 'Rheumatoid arthritis'

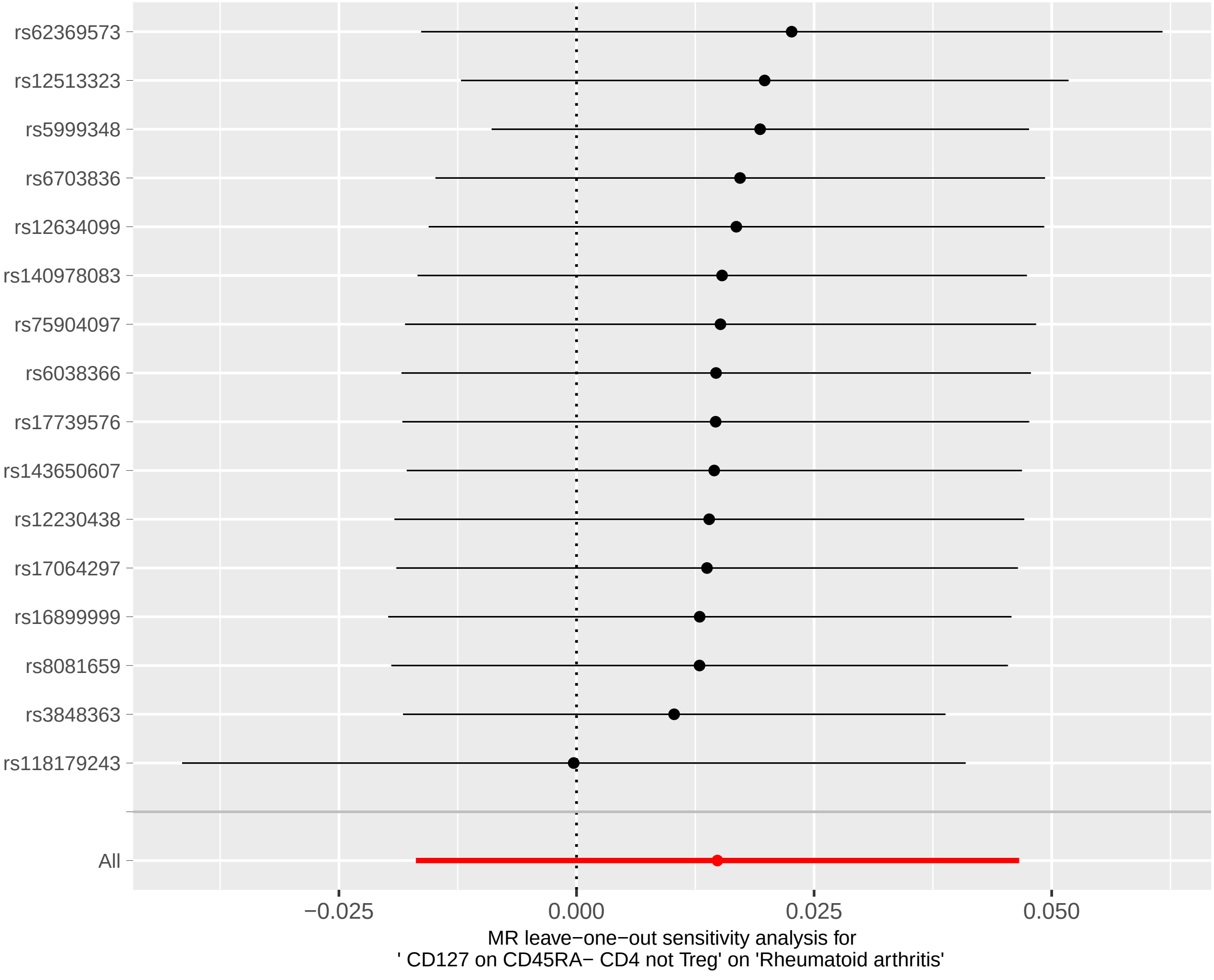
rs62369573
rs12513323
rs5999348
rs6703836
rs12634099
rs140978083
rs75904097
rs6038366
rs17739576
rs143650607
rs12230438
rs17064297
rs16899999
rs8081659
rs3848363
rs118179243
All
−0.025
0.000
0.025
0.050
MR leave−one−out sensitivity analysis for
' CD127 on CD45RA− CD4 not Treg' on 'Rheumatoid arthritis'

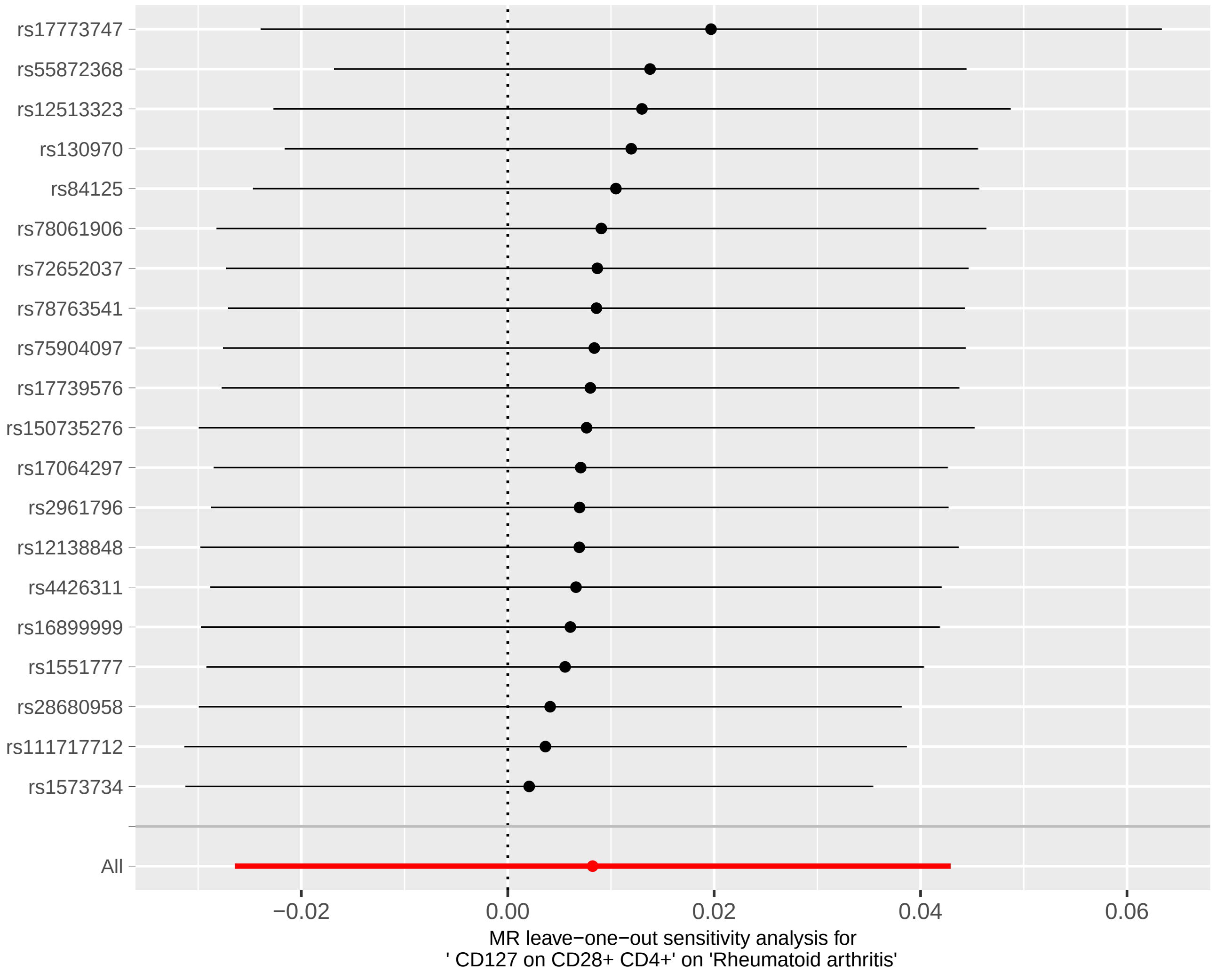
rs17773747
rs55872368
rs12513323
rs130970
rs84125
rs78061906
rs72652037
rs78763541
rs75904097
rs17739576
rs150735276
rs17064297
rs2961796
rs12138848
rs4426311
rs16899999
rs1551777
rs28680958
rs111717712
rs1573734
All
−0.02
0.00
0.02
0.04
0.06
MR leave−one−out sensitivity analysis for
' CD127 on CD28+ CD4+' on 'Rheumatoid arthritis'

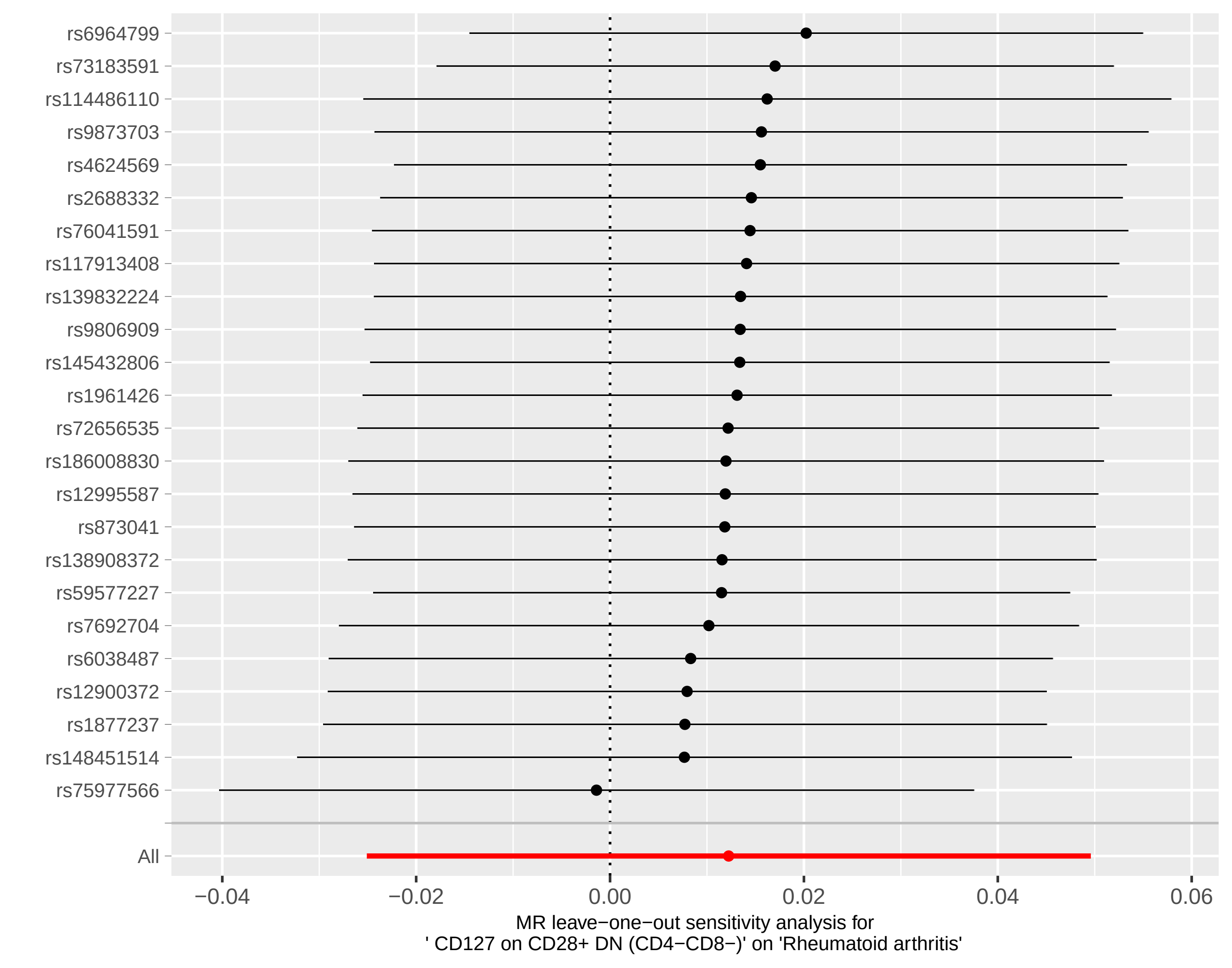
rs6964799
rs73183591
rs114486110
rs9873703
rs4624569
rs2688332
rs76041591
rs117913408
rs139832224
rs9806909
rs145432806
rs1961426
rs72656535
rs186008830
rs12995587
rs873041
rs138908372
rs59577227
rs7692704
rs6038487
rs12900372
rs1877237
rs148451514
rs75977566
All
−0.04
−0.02
0.00
0.02
0.04
0.06
MR leave−one−out sensitivity analysis for
' CD127 on CD28+ DN (CD4−CD8−)' on 'Rheumatoid arthritis'

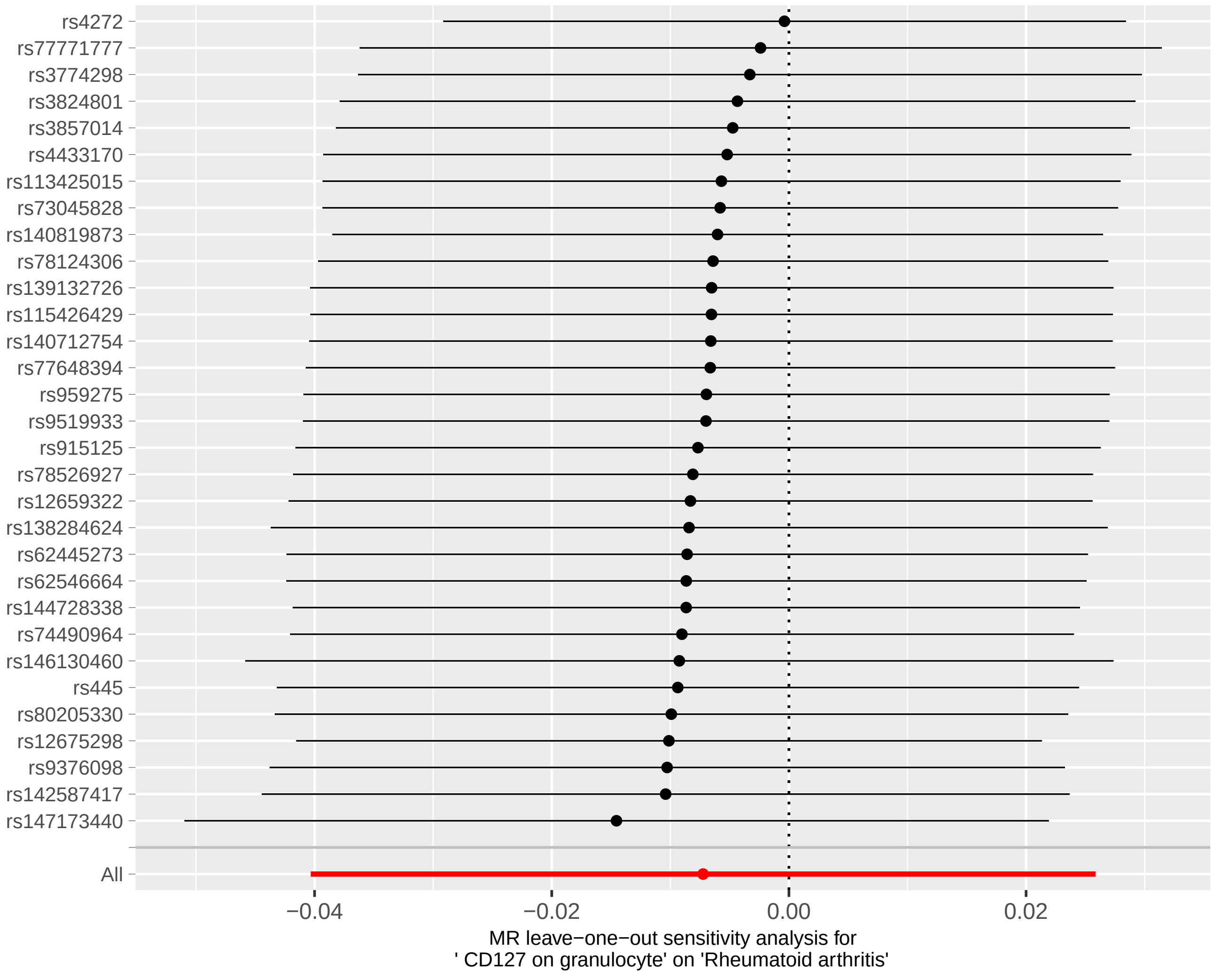
rs4272
rs77771777
rs3774298
rs3824801
rs3857014
rs4433170
rs113425015
rs73045828
rs140819873
rs78124306
rs139132726
rs115426429
rs140712754
rs77648394
rs959275
rs9519933
rs915125
rs78526927
rs12659322
rs138284624
rs62445273
rs62546664
rs144728338
rs74490964
rs146130460
rs445
rs80205330
rs12675298
rs9376098
rs142587417
rs147173440
All
−0.04
−0.02
0.00
0.02
MR leave−one−out sensitivity analysis for
' CD127 on granulocyte' on 'Rheumatoid arthritis'

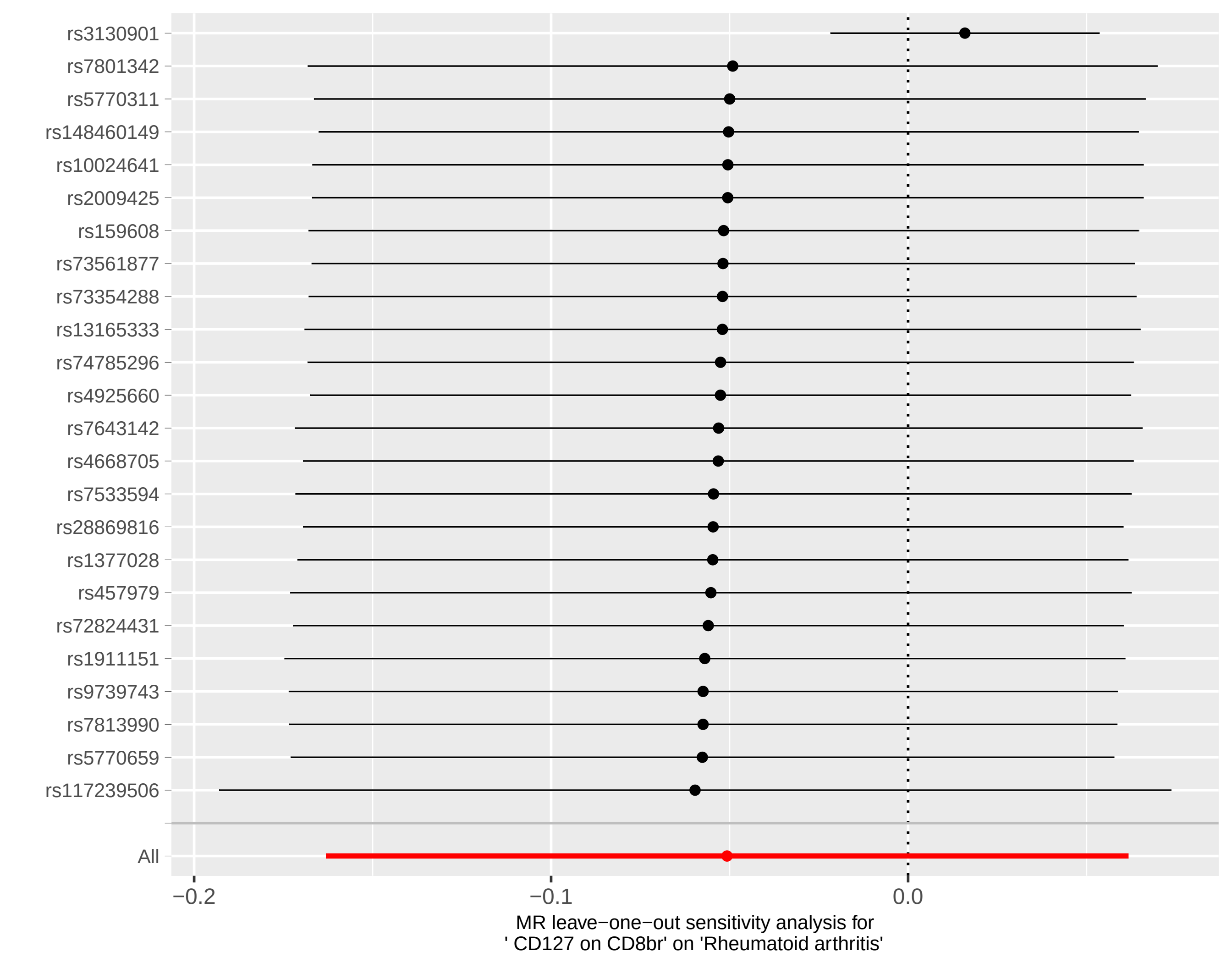
rs3130901
rs7801342
rs5770311
rs148460149
rs10024641
rs2009425
rs159608
rs73561877
rs73354288
rs13165333
rs74785296
rs4925660
rs7643142
rs4668705
rs7533594
rs28869816
rs1377028
rs457979
rs72824431
rs1911151
rs9739743
rs7813990
rs5770659
rs117239506
All
−0.2
−0.1
0.0
MR leave−one−out sensitivity analysis for
' CD127 on CD8br' on 'Rheumatoid arthritis'

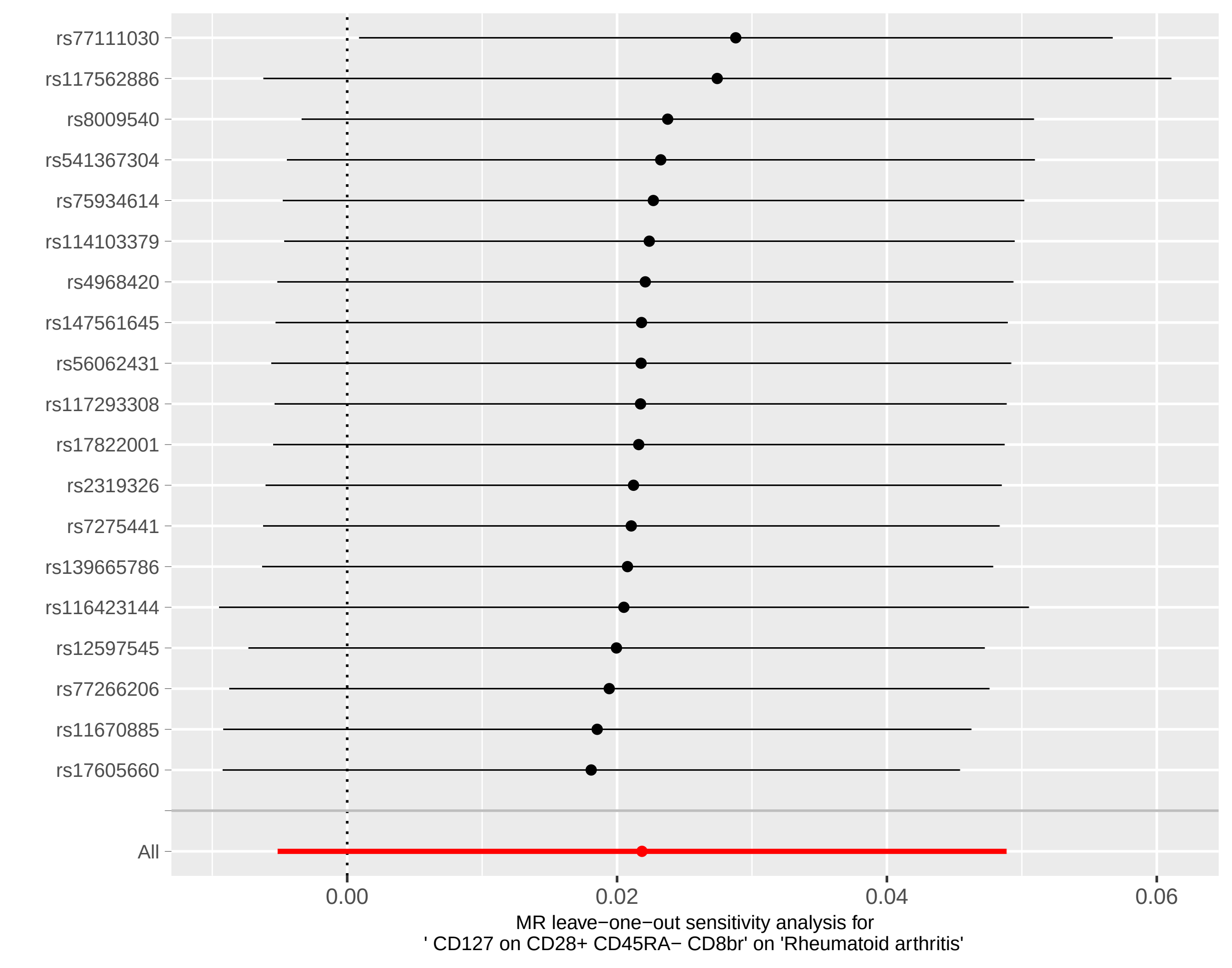
rs77111030
rs117562886
rs8009540
rs541367304
rs75934614
rs114103379
rs4968420
rs147561645
rs56062431
rs117293308
rs17822001
rs2319326
rs7275441
rs139665786
rs116423144
rs12597545
rs77266206
rs11670885
rs17605660
All
0.00
0.02
0.04
0.06
MR leave−one−out sensitivity analysis for
' CD127 on CD28+ CD45RA− CD8br' on 'Rheumatoid arthritis'

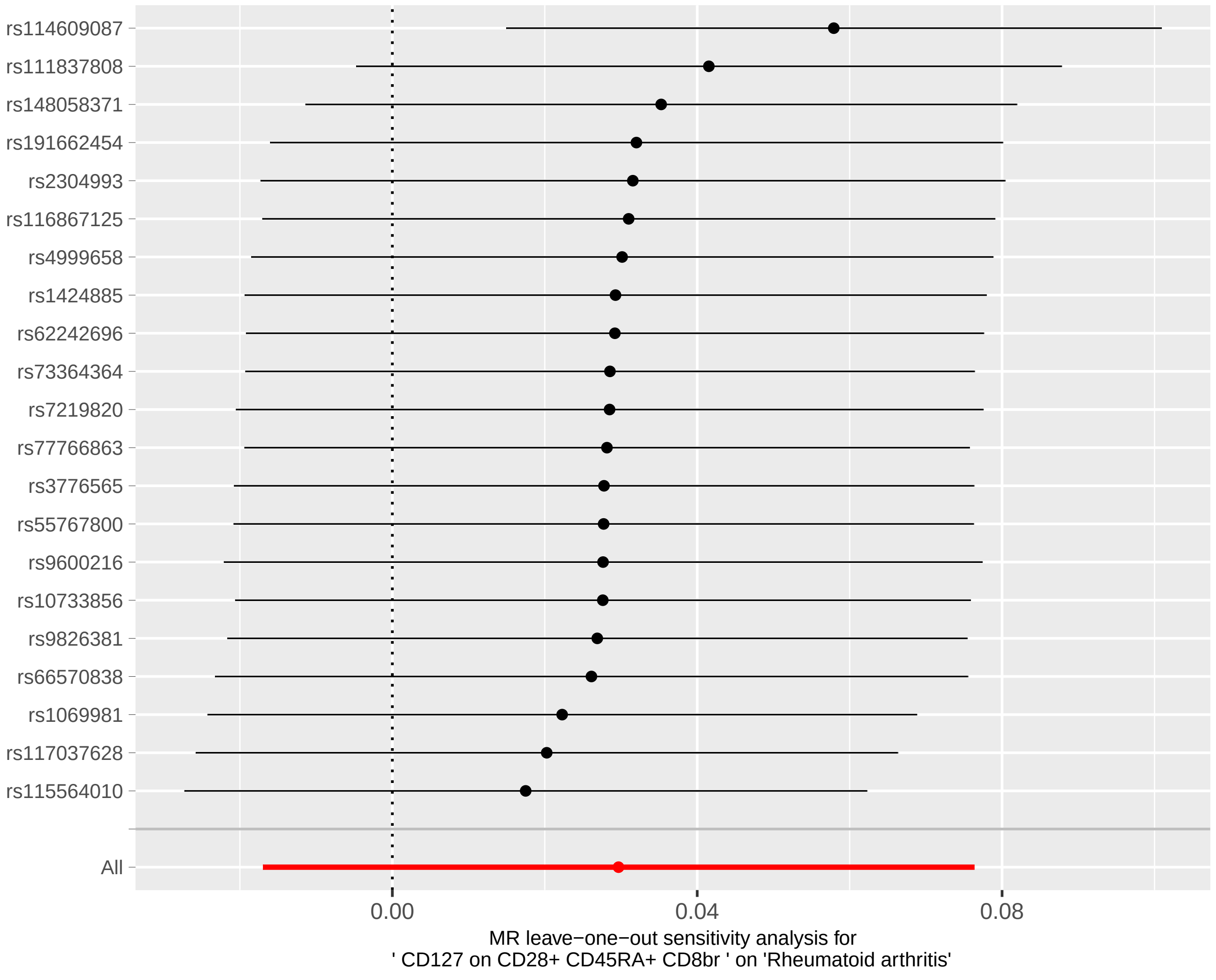
rs114609087
rs111837808
rs148058371
rs191662454
rs2304993
rs116867125
rs4999658
rs1424885
rs62242696
rs73364364
rs7219820
rs77766863
rs3776565
rs55767800
rs9600216
rs10733856
rs9826381
rs66570838
rs1069981
rs117037628
rs115564010
All
0.00
0.04
0.08
MR leave−one−out sensitivity analysis for
' CD127 on CD28+ CD45RA+ CD8br ' on 'Rheumatoid arthritis'

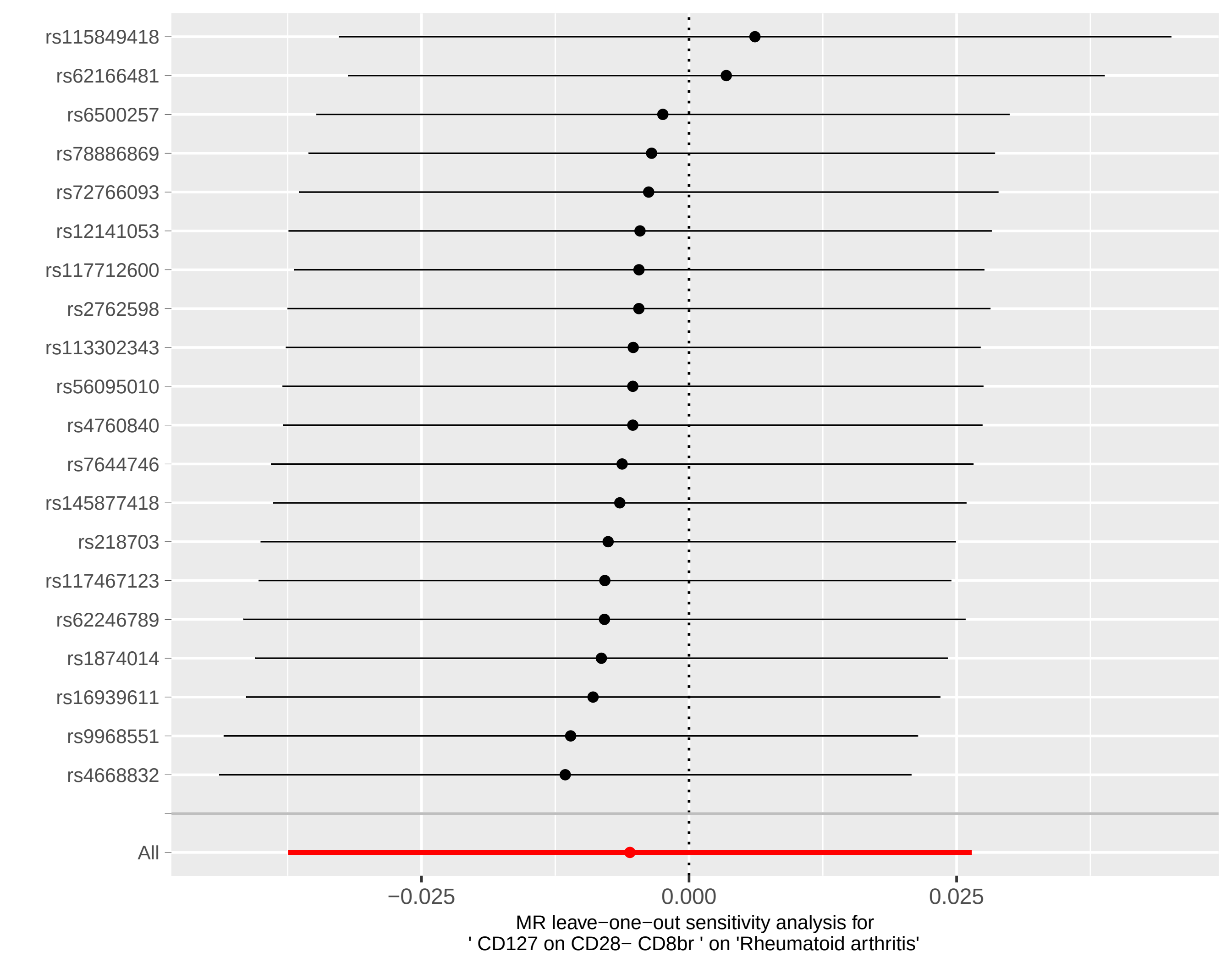
rs115849418
rs62166481
rs6500257
rs78886869
rs72766093
rs12141053
rs117712600
rs2762598
rs113302343
rs56095010
rs4760840
rs7644746
rs145877418
rs218703
rs117467123
rs62246789
rs1874014
rs16939611
rs9968551
rs4668832
All
−0.025
0.000
0.025
MR leave−one−out sensitivity analysis for
' CD127 on CD28− CD8br ' on 'Rheumatoid arthritis'

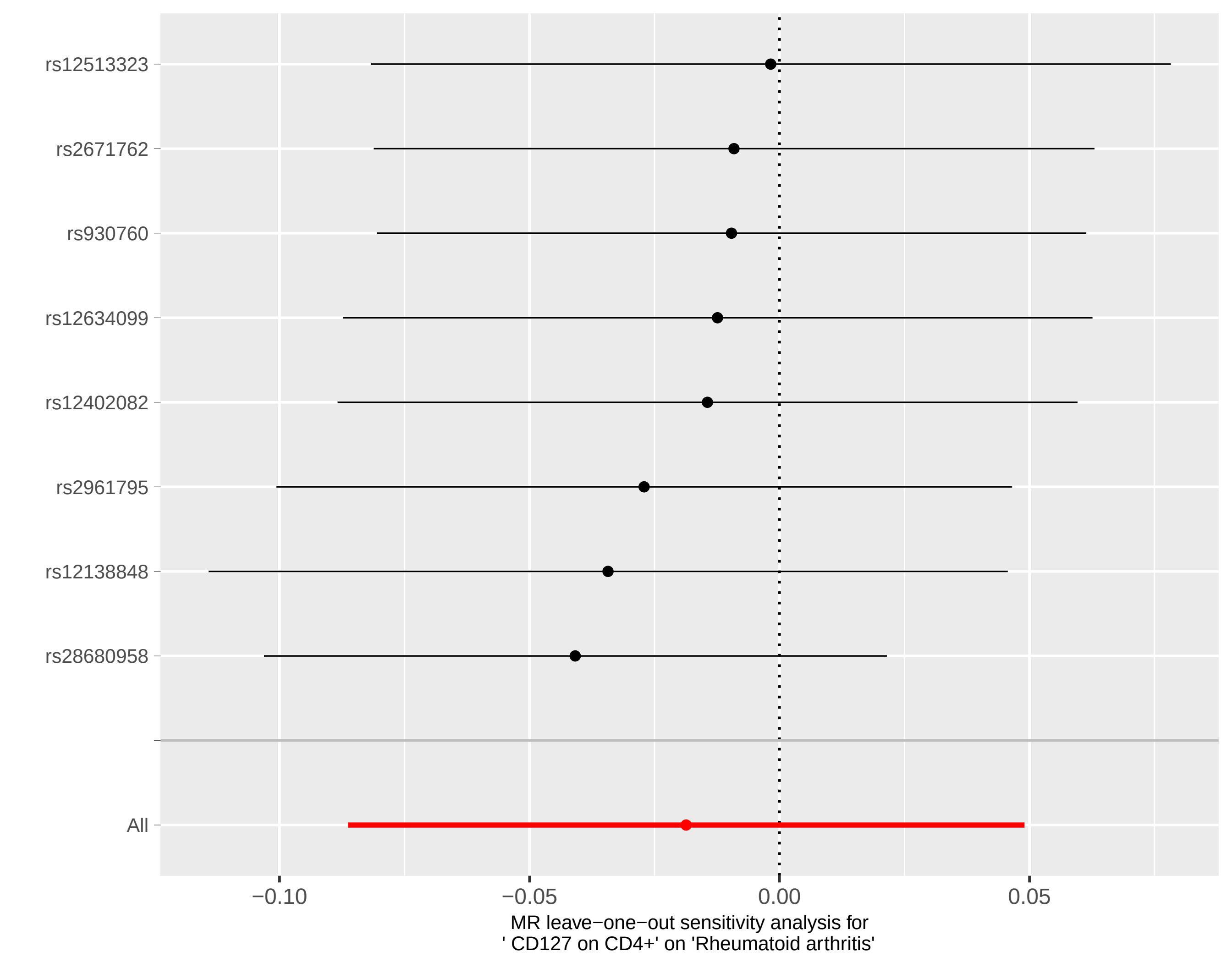
rs12513323
rs2671762
rs930760
rs12634099
rs12402082
rs2961795
rs12138848
rs28680958
All
−0.10
−0.05
0.00
0.05
MR leave−one−out sensitivity analysis for
' CD127 on CD4+' on 'Rheumatoid arthritis'

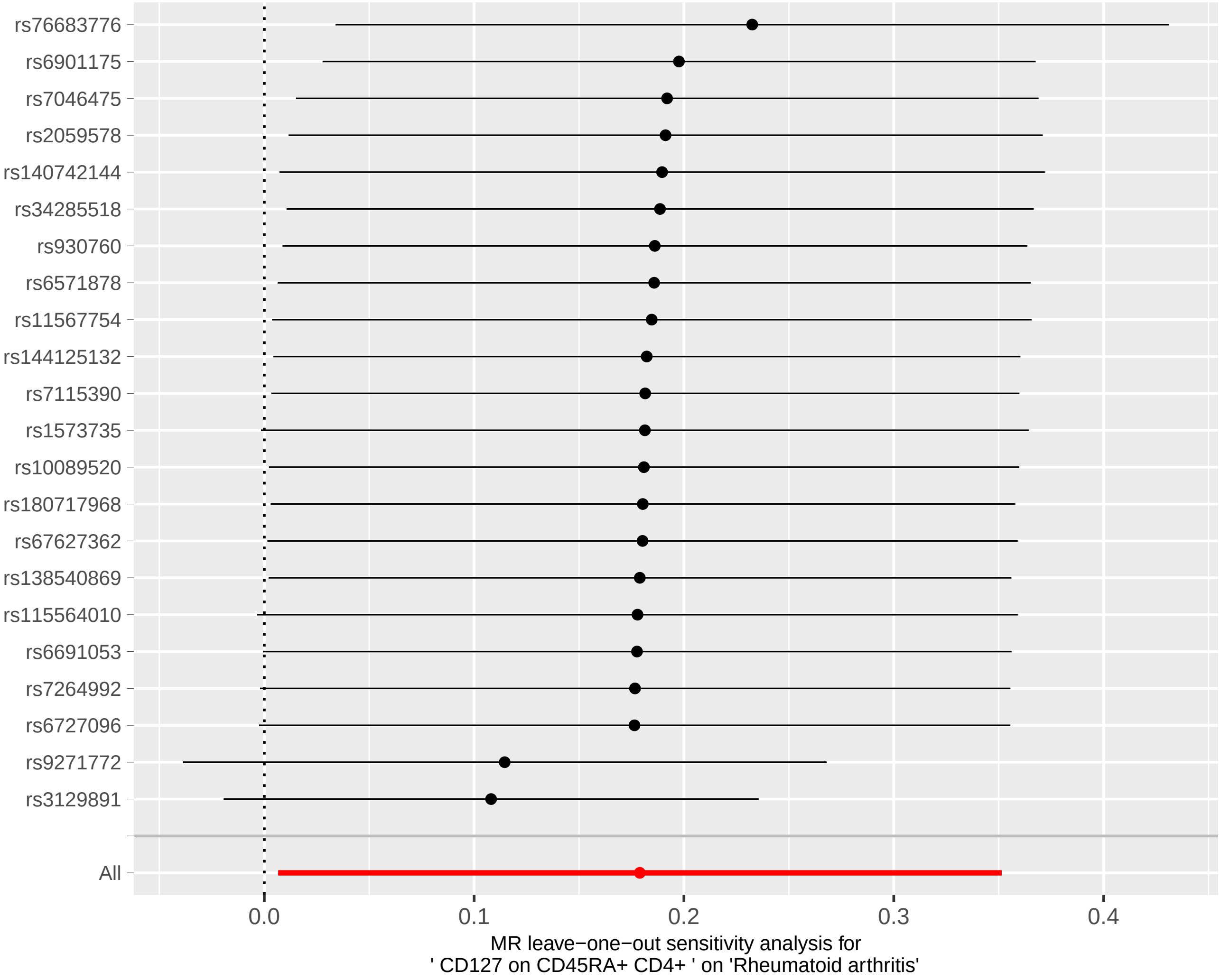
rs76683776
rs6901175
rs7046475
rs2059578
rs140742144
rs34285518
rs930760
rs6571878
rs11567754
rs144125132
rs7115390
rs1573735
rs10089520
rs180717968
rs67627362
rs138540869
rs115564010
rs6691053
rs7264992
rs6727096
rs9271772
rs3129891
All
0.0
0.1
0.2
0.3
0.4
MR leave−one−out sensitivity analysis for
' CD127 on CD45RA+ CD4+ ' on 'Rheumatoid arthritis'

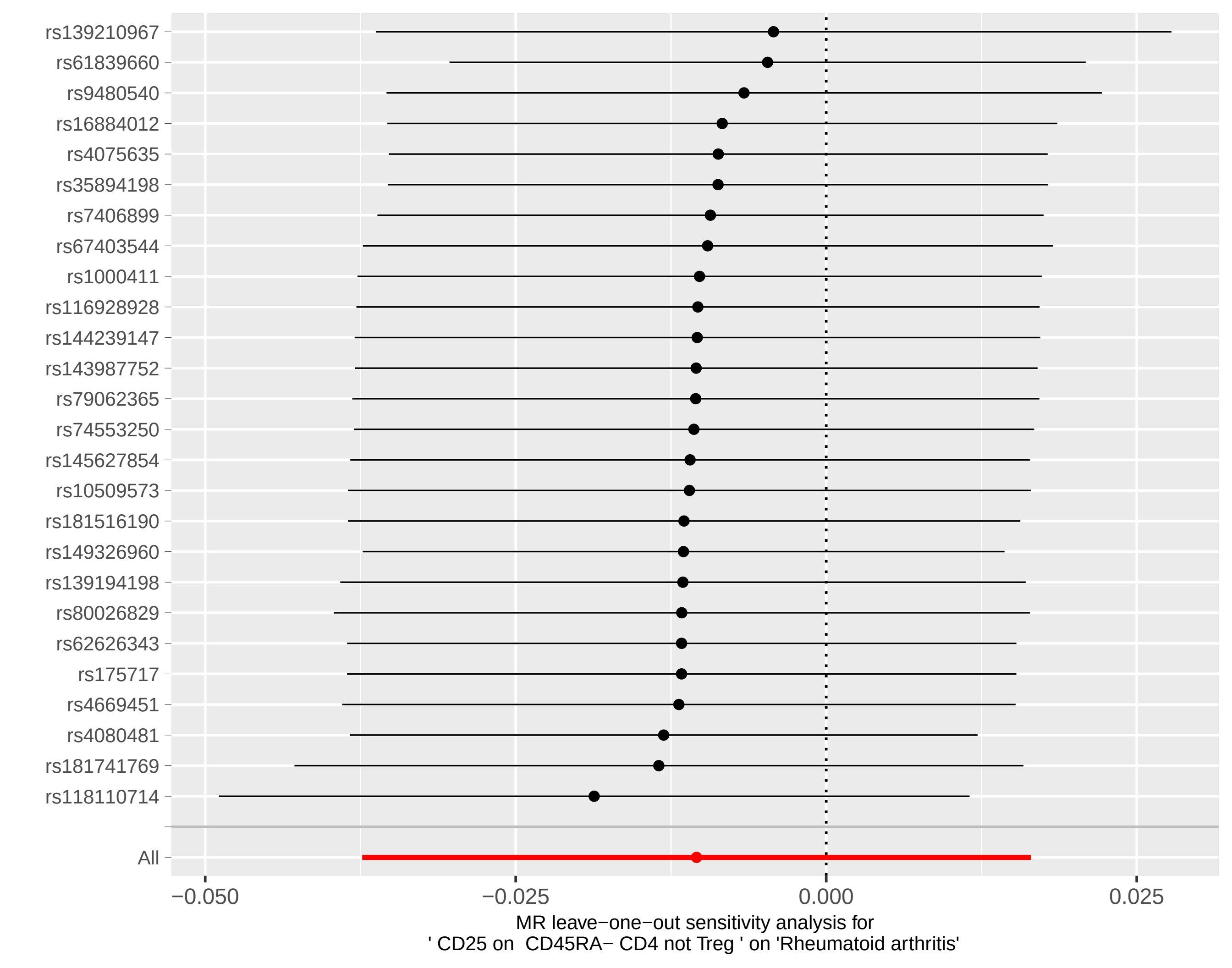
rs139210967
rs61839660
rs9480540
rs16884012
rs4075635
rs35894198
rs7406899
rs67403544
rs1000411
rs116928928
rs144239147
rs143987752
rs79062365
rs74553250
rs145627854
rs10509573
rs181516190
rs149326960
rs139194198
rs80026829
rs62626343
rs175717
rs4669451
rs4080481
rs181741769
rs118110714
All
−0.050
−0.025
0.000
0.025
MR leave−one−out sensitivity analysis for
' CD25 on CD45RA− CD4 not Treg ' on 'Rheumatoid arthritis'

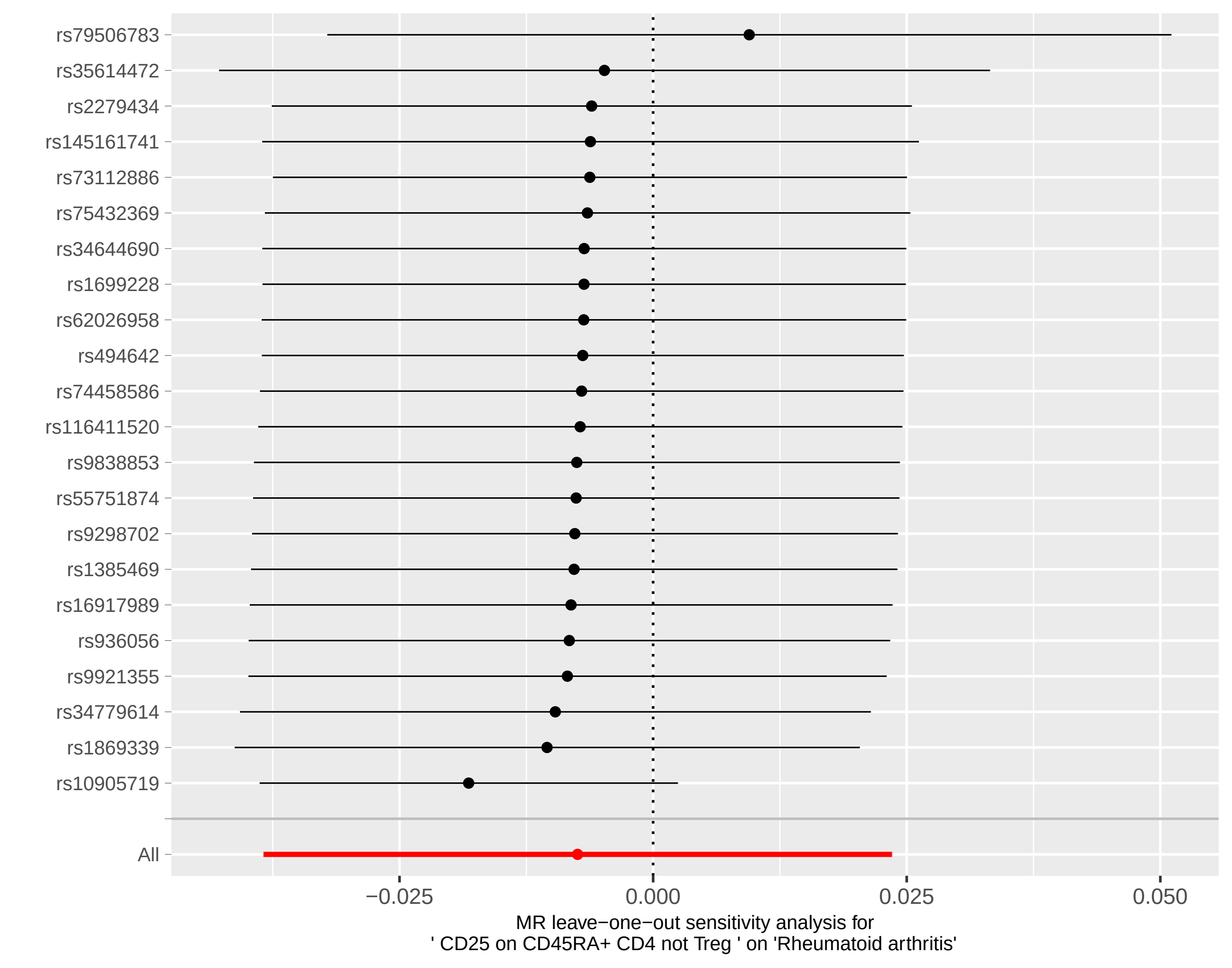
rs79506783
rs35614472
rs2279434
rs145161741
rs73112886
rs75432369
rs34644690
rs1699228
rs62026958
rs494642
rs74458586
rs116411520
rs9838853
rs55751874
rs9298702
rs1385469
rs16917989
rs936056
rs9921355
rs34779614
rs1869339
rs10905719
All
−0.025
0.000
0.025
0.050
MR leave−one−out sensitivity analysis for
' CD25 on CD45RA+ CD4 not Treg ' on 'Rheumatoid arthritis'

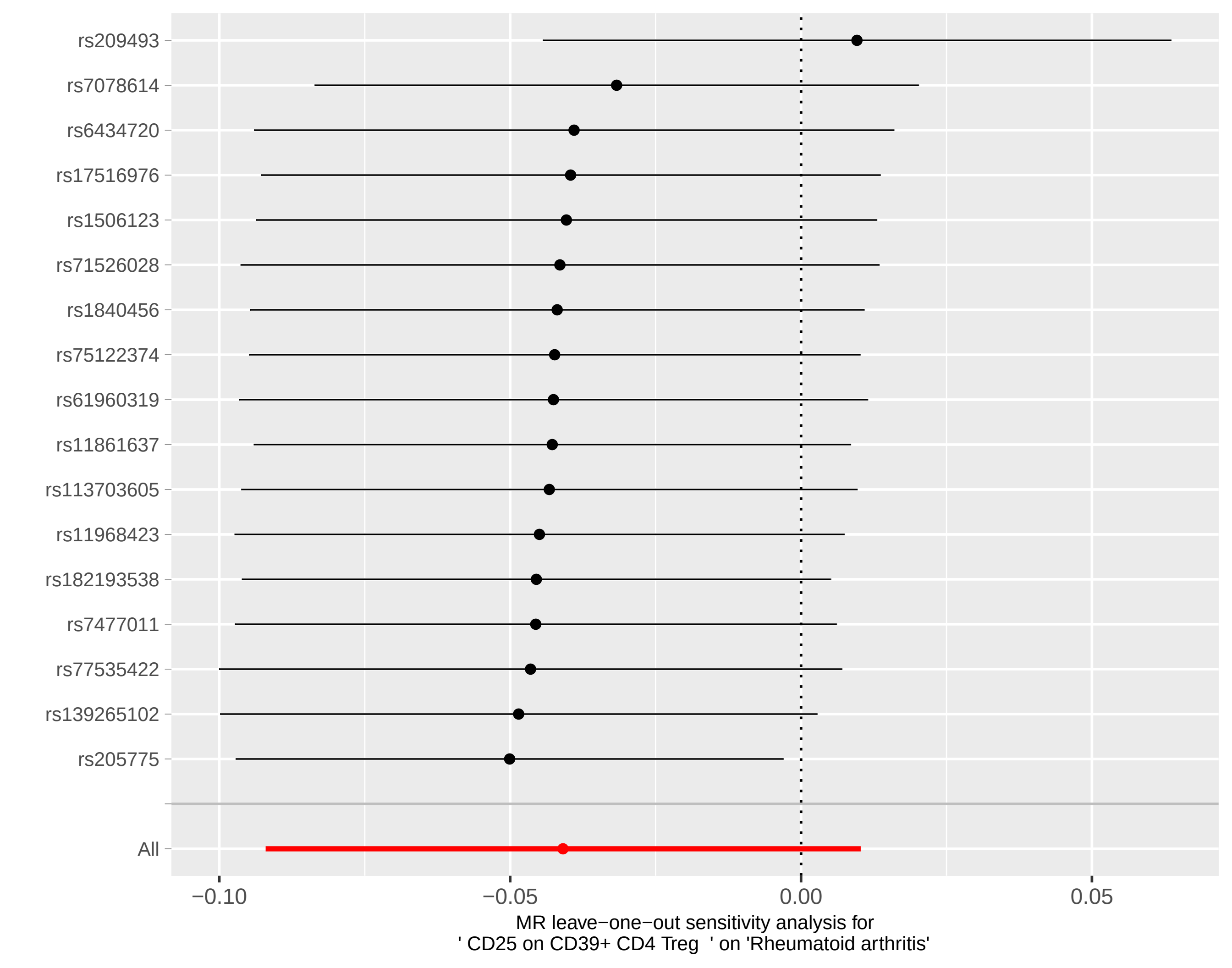
rs209493
rs7078614
rs6434720
rs17516976
rs1506123
rs71526028
rs1840456
rs75122374
rs61960319
rs11861637
rs113703605
rs11968423
rs182193538
rs7477011
rs77535422
rs139265102
rs205775
All
−0.10
−0.05
0.00
0.05
MR leave−one−out sensitivity analysis for
' CD25 on CD39+ CD4 Treg ' on 'Rheumatoid arthritis'

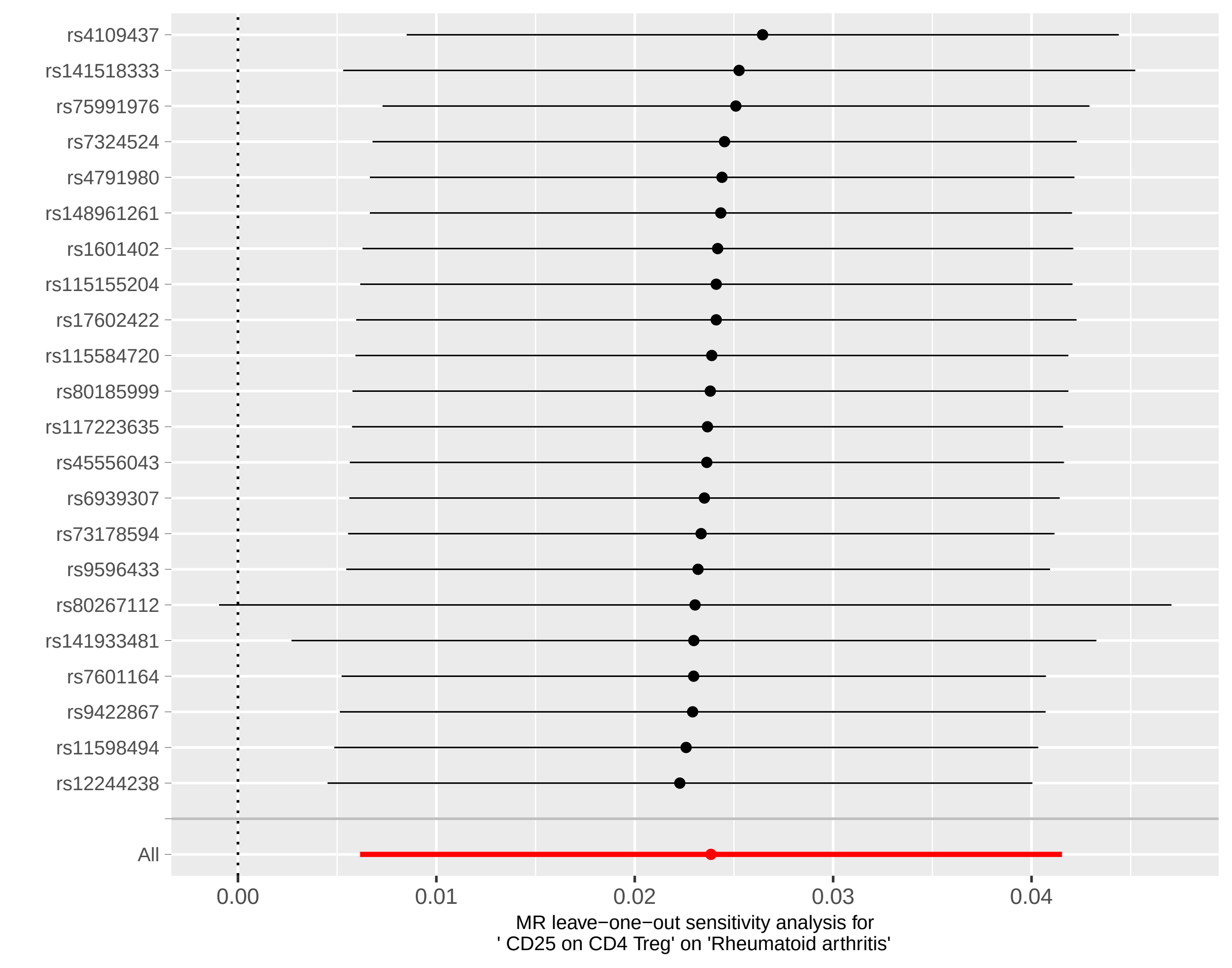
rs4109437
rs141518333
rs75991976
rs7324524
rs4791980
rs148961261
rs1601402
rs115155204
rs17602422
rs115584720
rs80185999
rs117223635
rs45556043
rs6939307
rs73178594
rs9596433
rs80267112
rs141933481
rs7601164
rs9422867
rs11598494
rs12244238
All
0.00
0.01
0.02
0.03
0.04
MR leave−one−out sensitivity analysis for
' CD25 on CD4 Treg' on 'Rheumatoid arthritis'

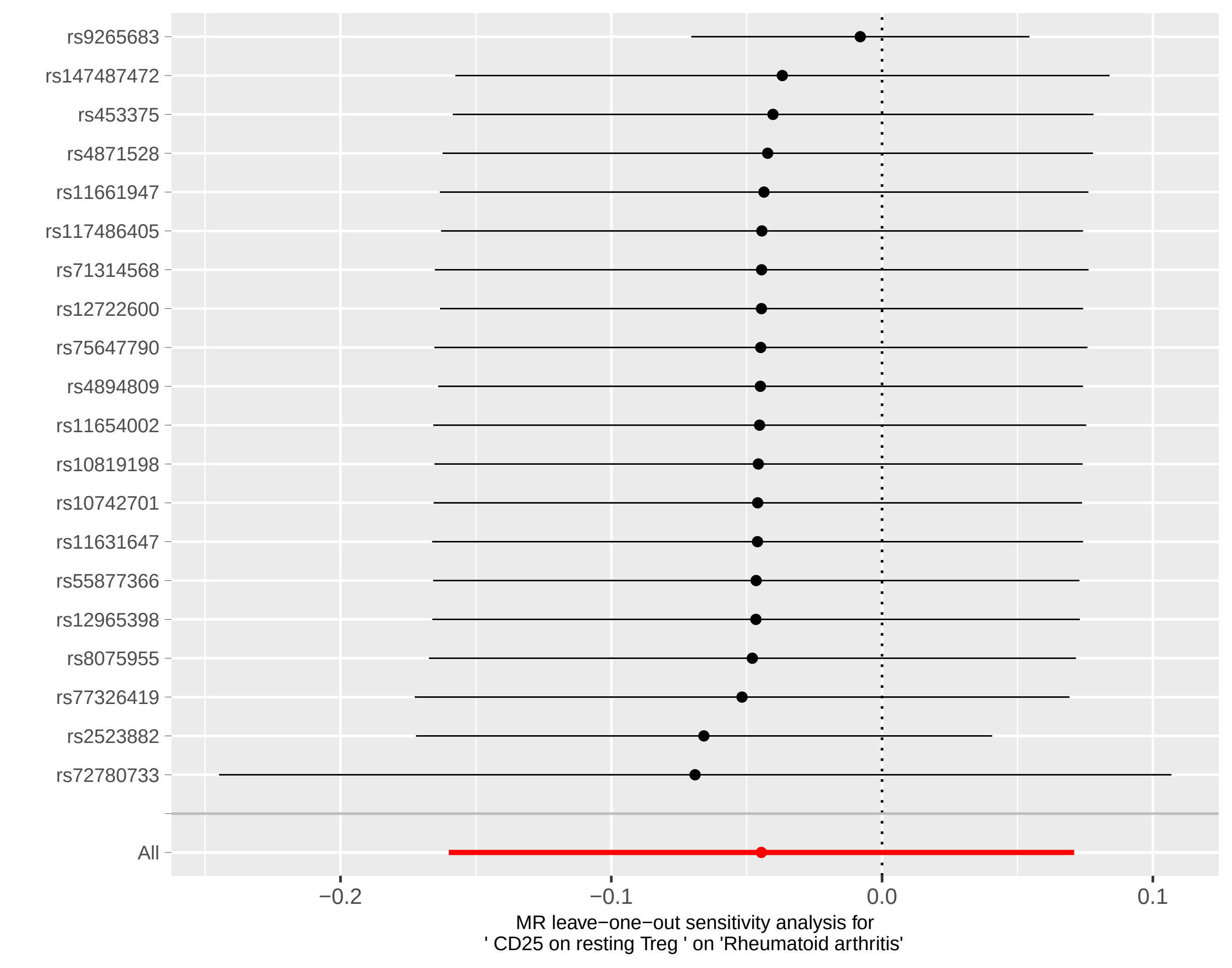

rs9265683
rs147487472
rs453375
rs4871528
rs11661947
rs117486405
rs71314568
rs12722600
rs75647790
rs4894809
rs11654002
rs10819198
rs10742701
rs11631647
rs55877366
rs12965398
rs8075955
rs77326419
rs2523882
rs72780733
All
−0.2
−0.1
0.0
0.1
MR leave−one−out sensitivity analysis for
' CD25 on resting Treg ' on 'Rheumatoid arthritis'

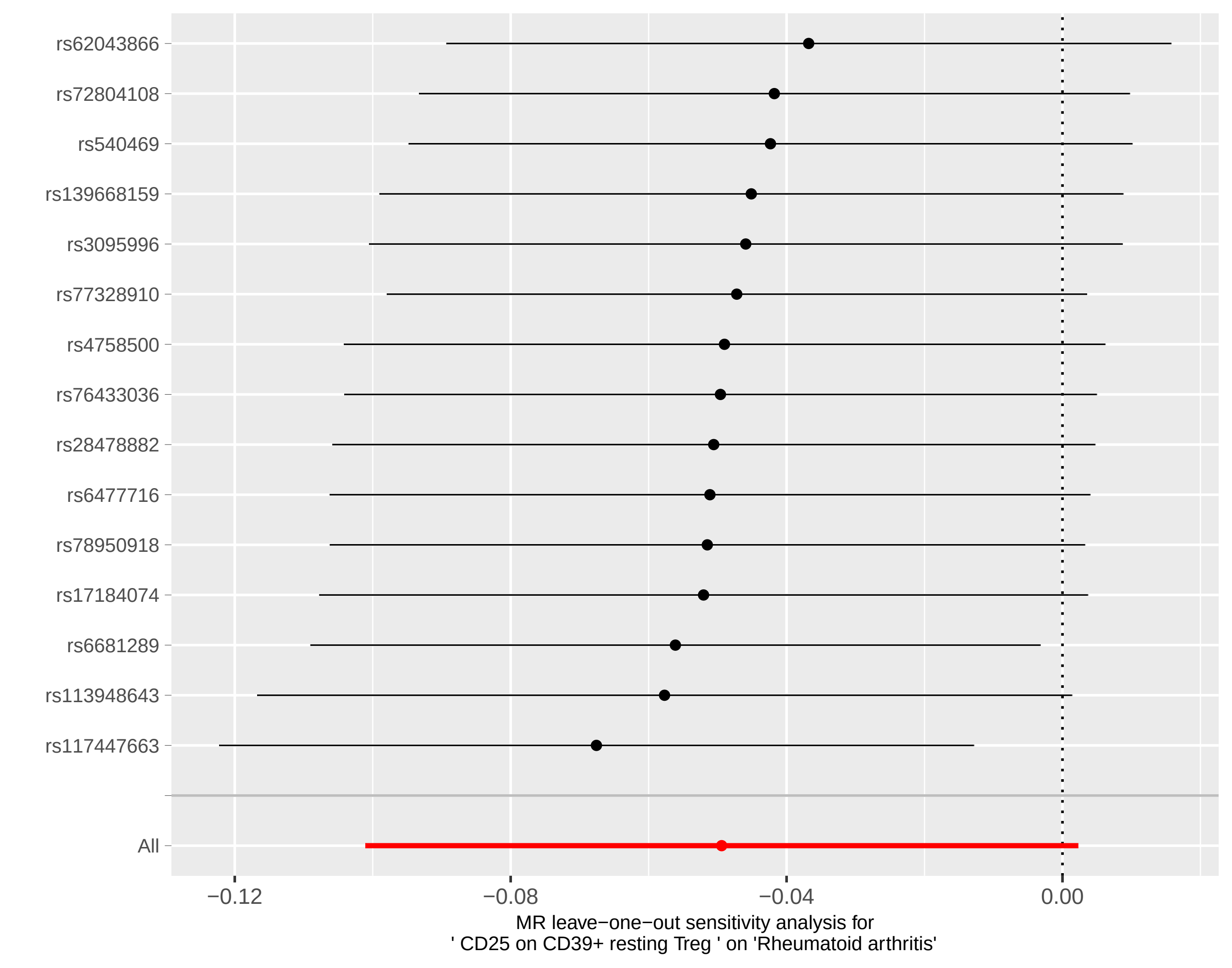
rs62043866
rs72804108
rs540469
rs139668159
rs3095996
rs77328910
rs4758500
rs76433036
rs28478882
rs6477716
rs78950918
rs17184074
rs6681289
rs113948643
rs117447663
All
−0.12
−0.08
−0.04
0.00
MR leave−one−out sensitivity analysis for
' CD25 on CD39+ resting Treg ' on 'Rheumatoid arthritis'

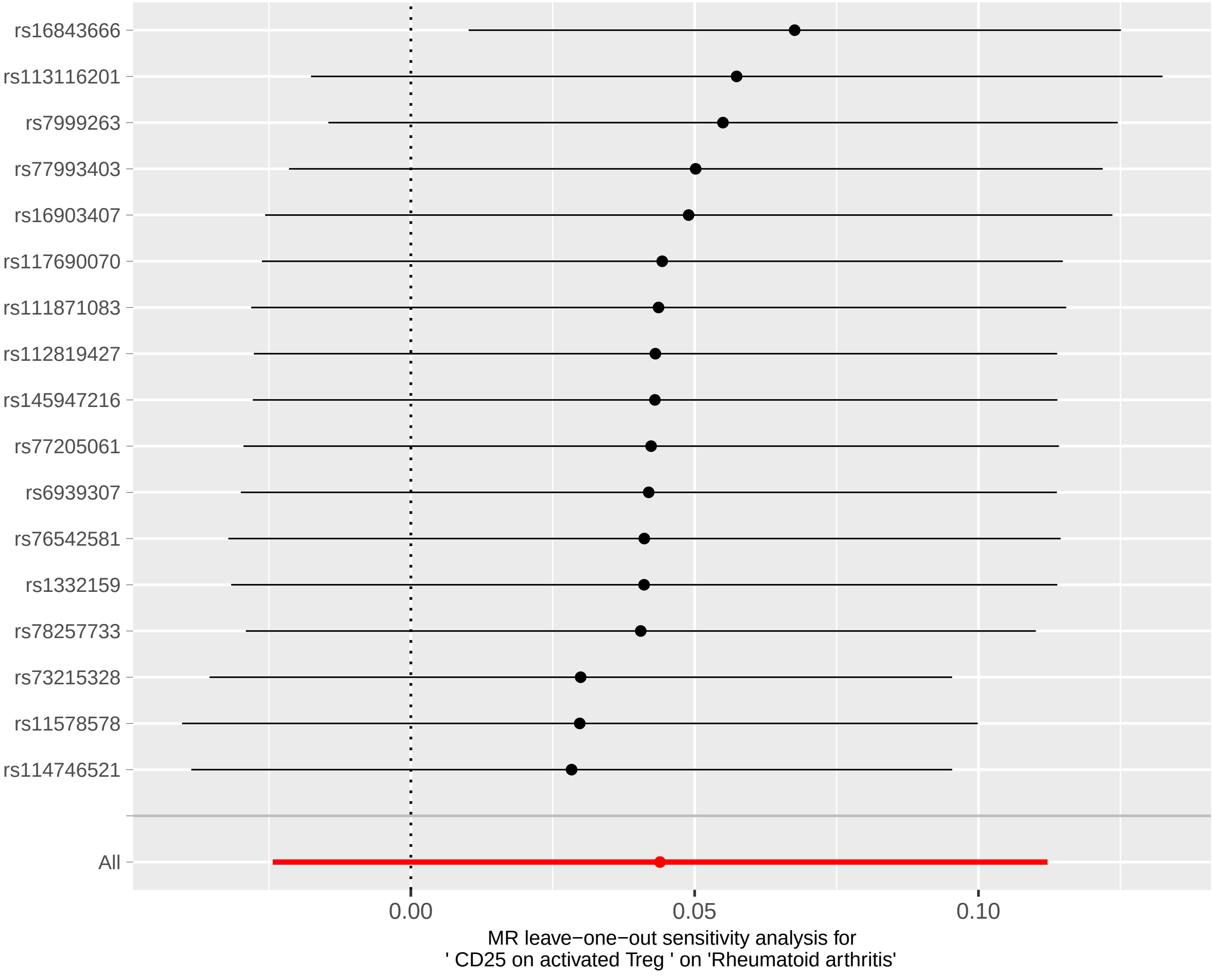
rs16843666
rs113116201
rs7999263
rs77993403
rs16903407
rs117690070
rs111871083
rs112819427
rs145947216
rs77205061
rs6939307
rs76542581
rs1332159
rs78257733
rs73215328
rs11578578
rs114746521
All
0.00
0.05
0.10
MR leave−one−out sensitivity analysis for
' CD25 on activated Treg ' on 'Rheumatoid arthritis'

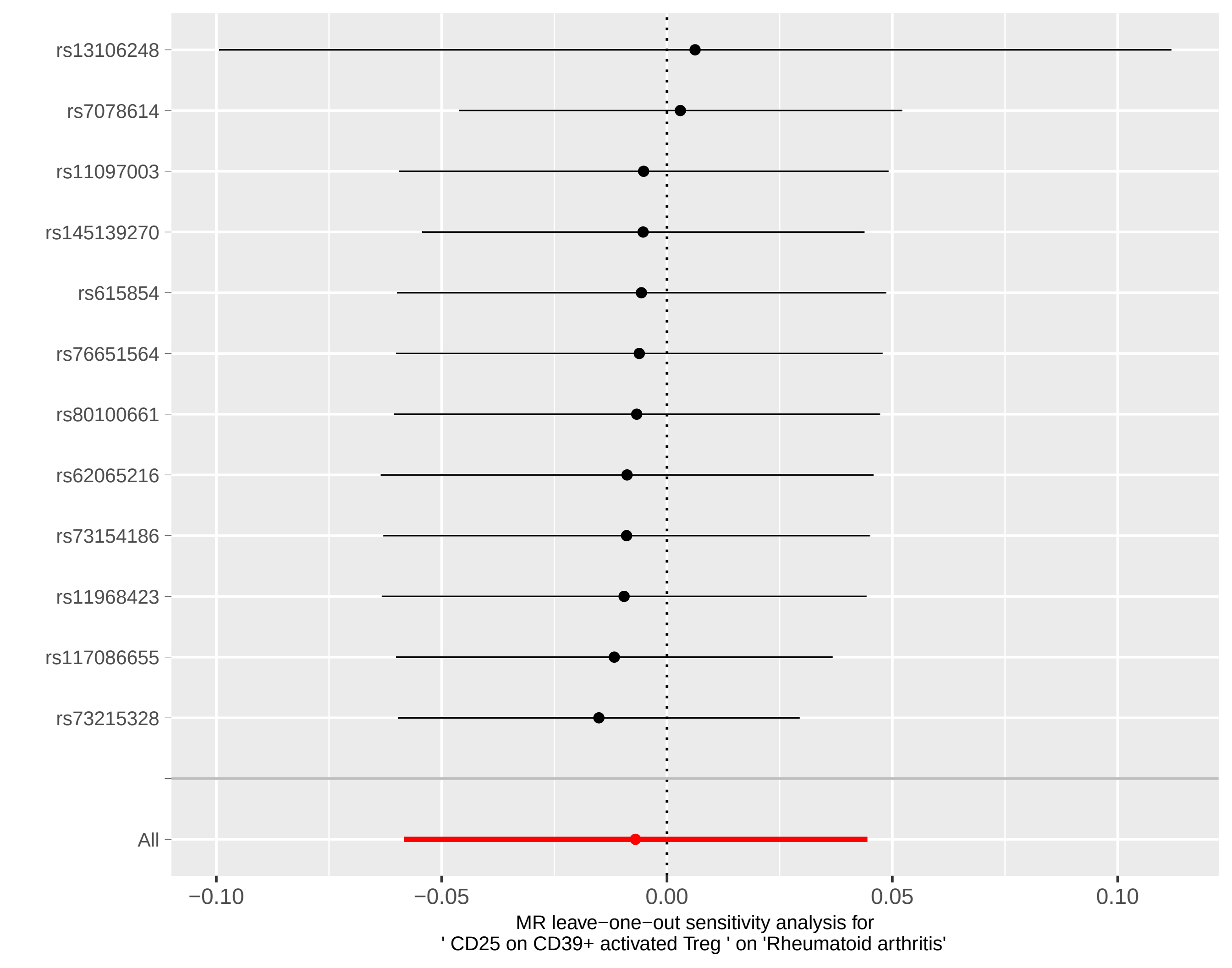
rs13106248
rs7078614
rs11097003
rs145139270
rs615854
rs76651564
rs80100661
rs62065216
rs73154186
rs11968423
rs117086655
rs73215328
All
−0.10
−0.05
0.00
0.05
0.10
MR leave−one−out sensitivity analysis for
' CD25 on CD39+ activated Treg ' on 'Rheumatoid arthritis'

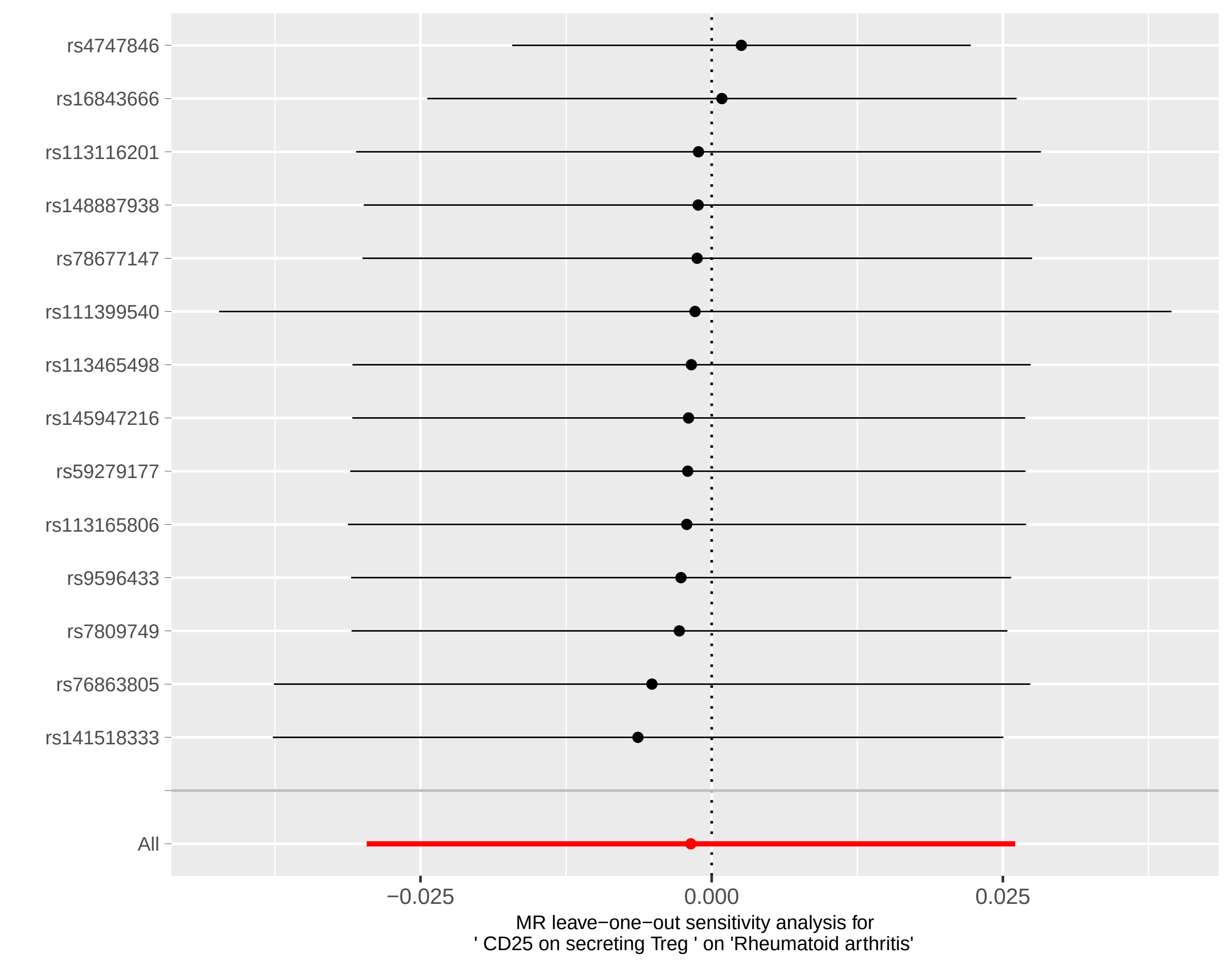

rs4747846
rs16843666
rs113116201
rs148887938
rs78677147
rs111399540
rs113465498
rs145947216
rs59279177
rs113165806
rs9596433
rs7809749
rs76863805
rs141518333
All
−0.025
0.000
0.025
MR leave−one−out sensitivity analysis for
' CD25 on secreting Treg ' on 'Rheumatoid arthritis'

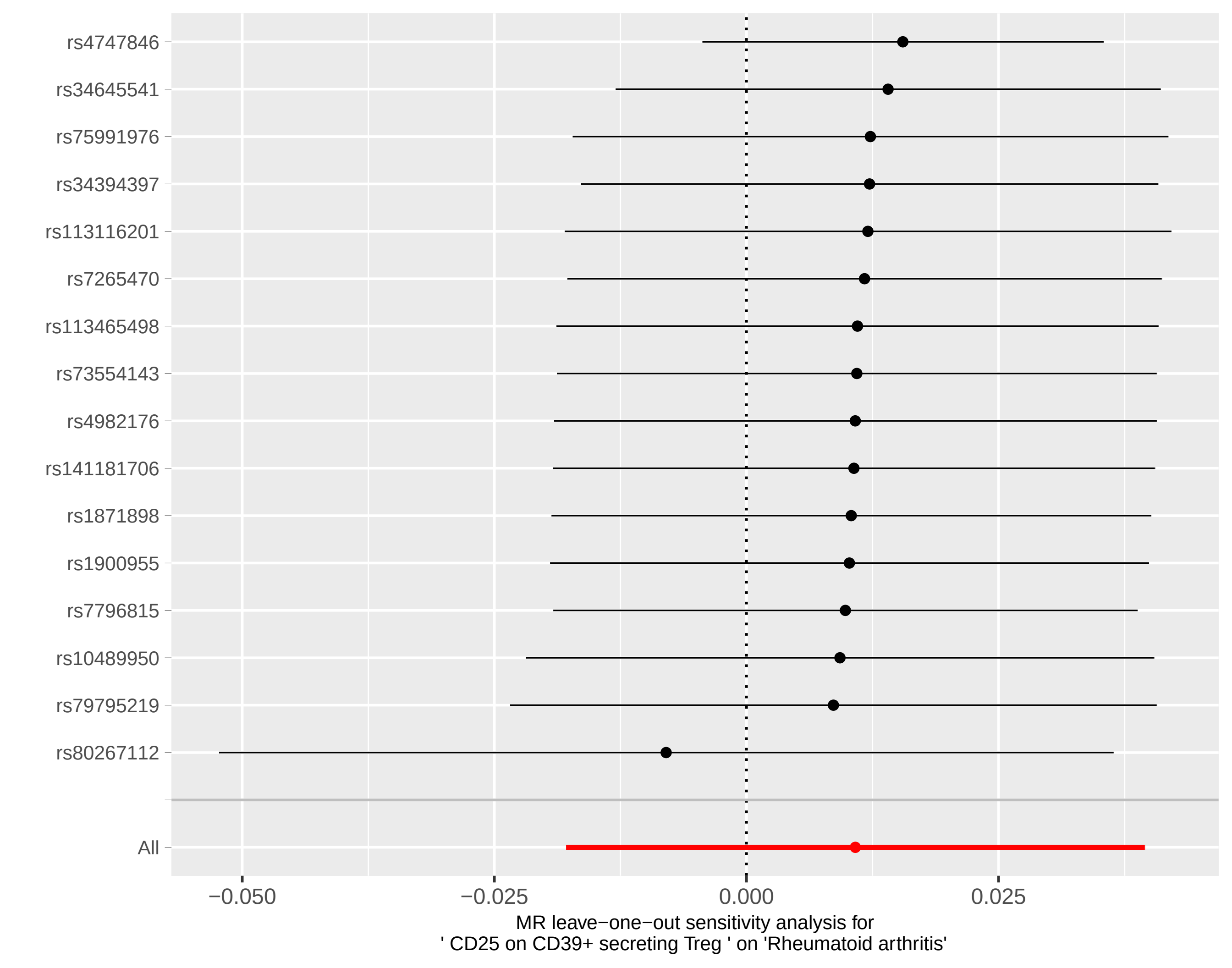
rs4747846
rs34645541
rs75991976
rs34394397
rs113116201
rs7265470
rs113465498
rs73554143
rs4982176
rs141181706
rs1871898
rs1900955
rs7796815
rs10489950
rs79795219
rs80267112
All
−0.050
−0.025
0.000
0.025
MR leave−one−out sensitivity analysis for
' CD25 on CD39+ secreting Treg ' on 'Rheumatoid arthritis'

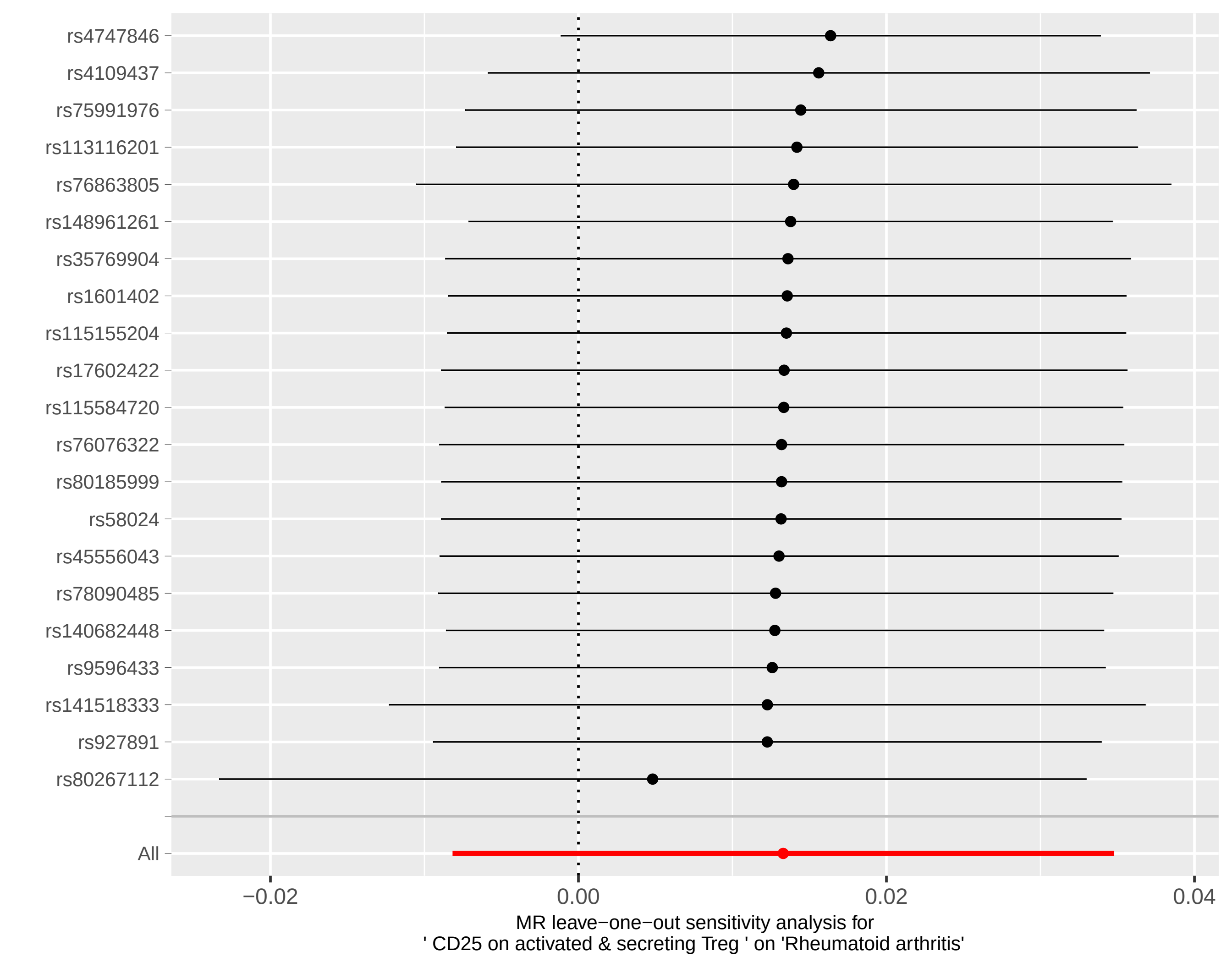

rs4747846
rs4109437
rs75991976
rs113116201
rs76863805
rs148961261
rs35769904
rs1601402
rs115155204
rs17602422
rs115584720
rs76076322
rs80185999
rs58024
rs45556043
rs78090485
rs140682448
rs9596433
rs141518333
rs927891
rs80267112
All
−0.02
0.00
0.02
0.04
MR leave−one−out sensitivity analysis for
' CD25 on activated & secreting Treg ' on 'Rheumatoid arthritis'

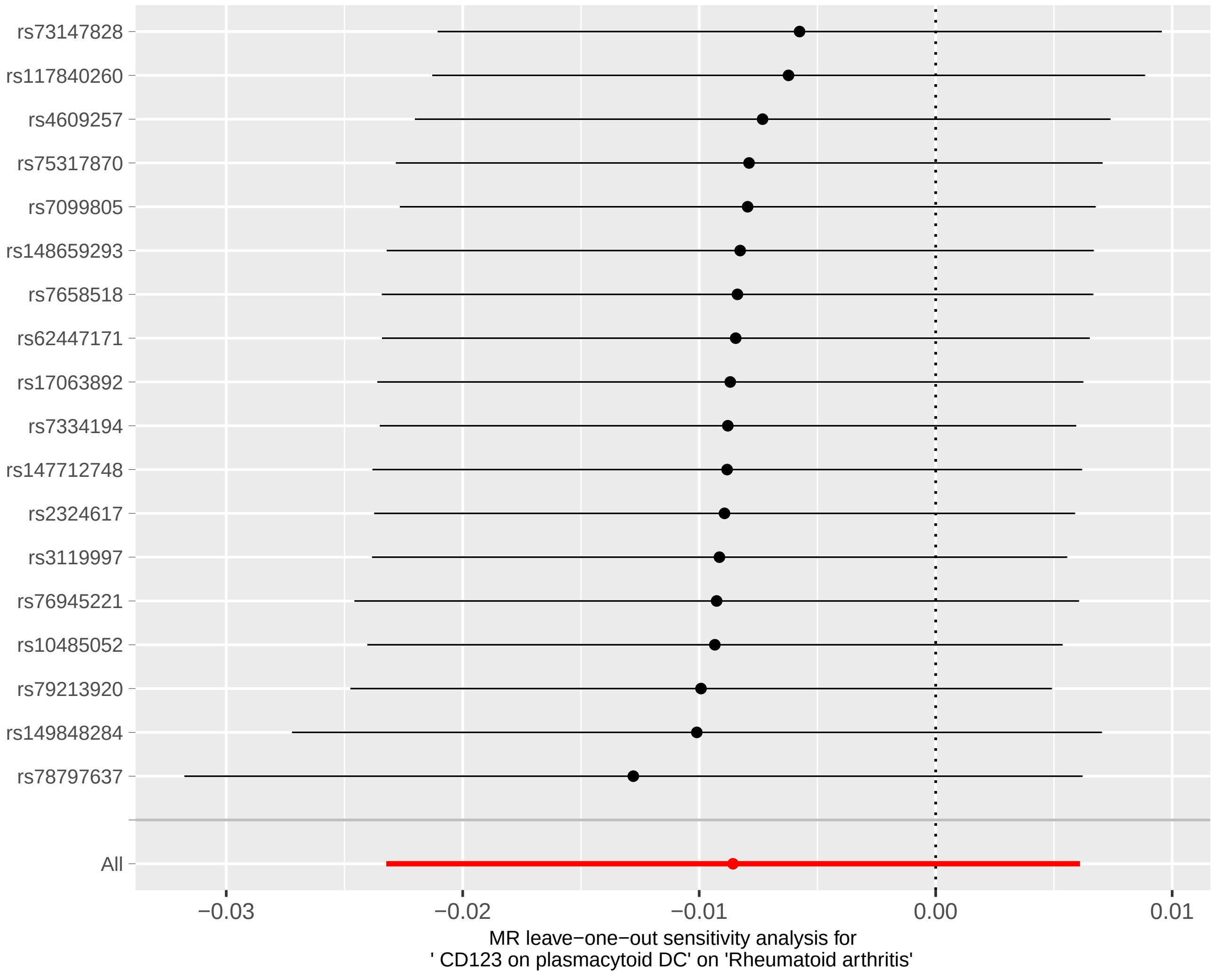
rs73147828
rs117840260
rs4609257
rs75317870
rs7099805
rs148659293
rs7658518
rs62447171
rs17063892
rs7334194
rs147712748
rs2324617
rs3119997
rs76945221
rs10485052
rs79213920
rs149848284
rs78797637
All
−0.03
−0.02
−0.01
0.00
0.01
MR leave−one−out sensitivity analysis for
' CD123 on plasmacytoid DC' on 'Rheumatoid arthritis'

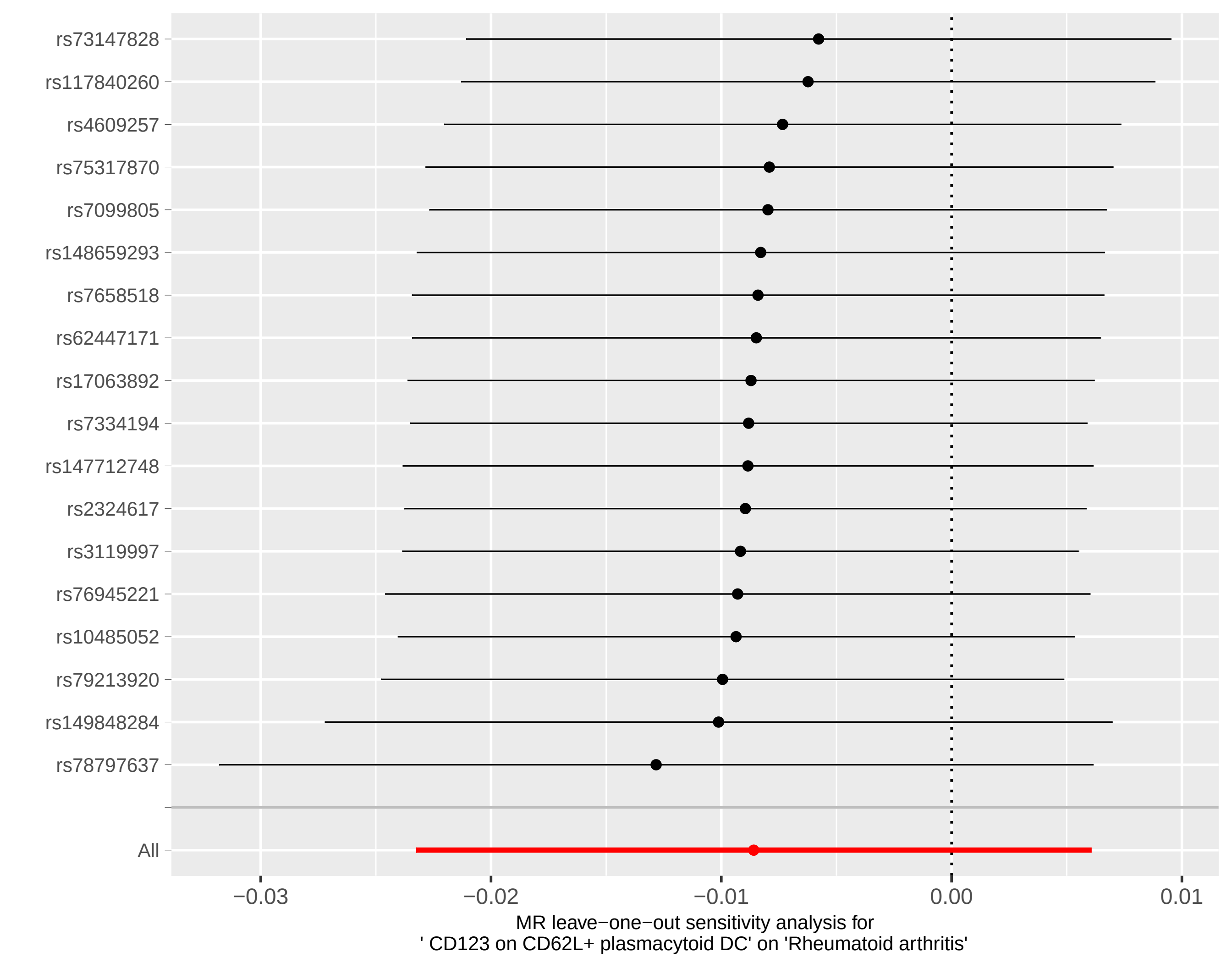
rs73147828
rs117840260
rs4609257
rs75317870
rs7099805
rs148659293
rs7658518
rs62447171
rs17063892
rs7334194
rs147712748
rs2324617
rs3119997
rs76945221
rs10485052
rs79213920
rs149848284
rs78797637
All
−0.03
−0.02
−0.01
0.00
0.01
MR leave−one−out sensitivity analysis for
' CD123 on CD62L+ plasmacytoid DC' on 'Rheumatoid arthritis'

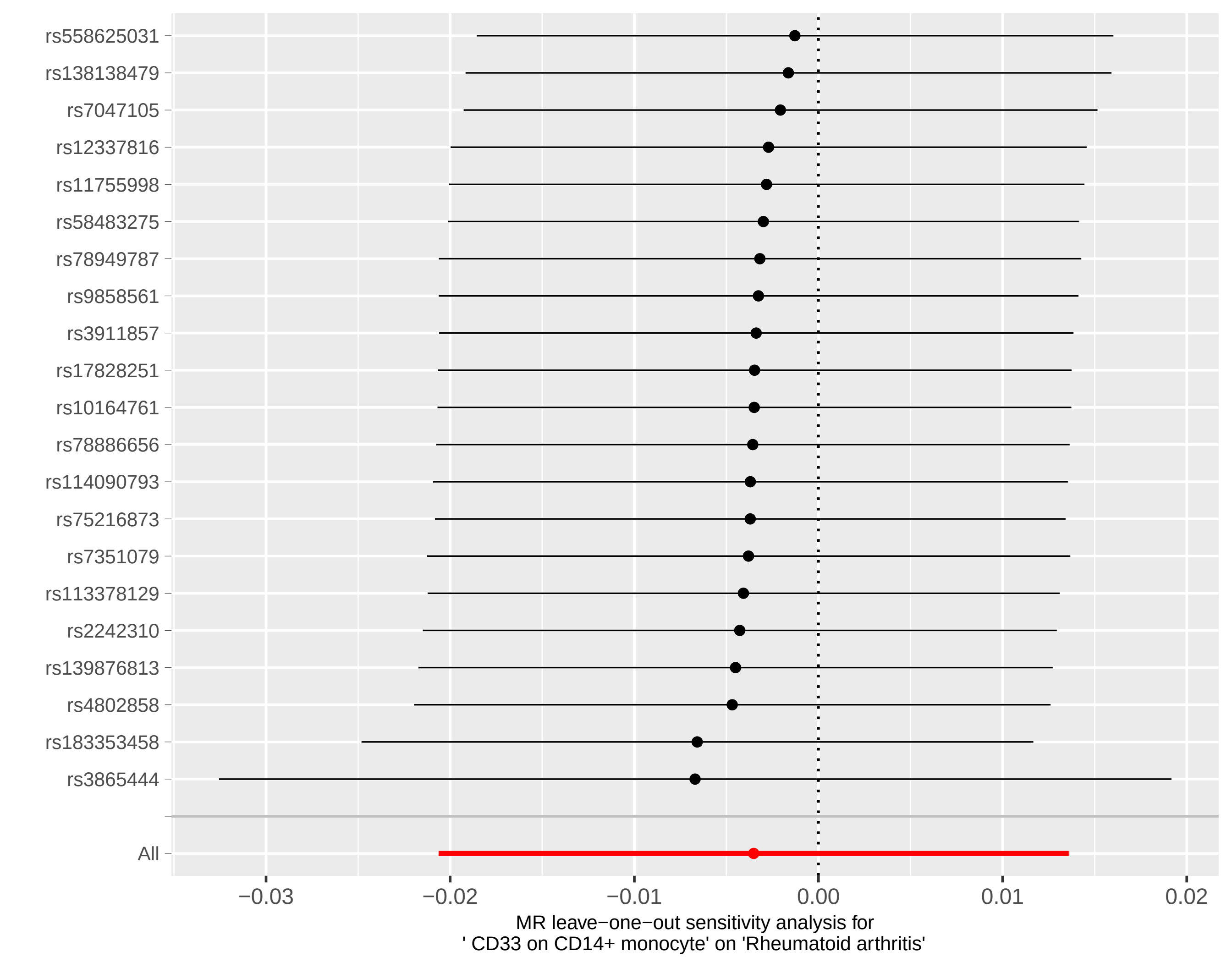
rs558625031
rs138138479
rs7047105
rs12337816
rs11755998
rs58483275
rs78949787
rs9858561
rs3911857
rs17828251
rs10164761
rs78886656
rs114090793
rs75216873
rs7351079
rs113378129
rs2242310
rs139876813
rs4802858
rs183353458
rs3865444
All
−0.03
−0.02
−0.01
0.00
0.01
0.02
MR leave−one−out sensitivity analysis for
' CD33 on CD14+ monocyte' on 'Rheumatoid arthritis'

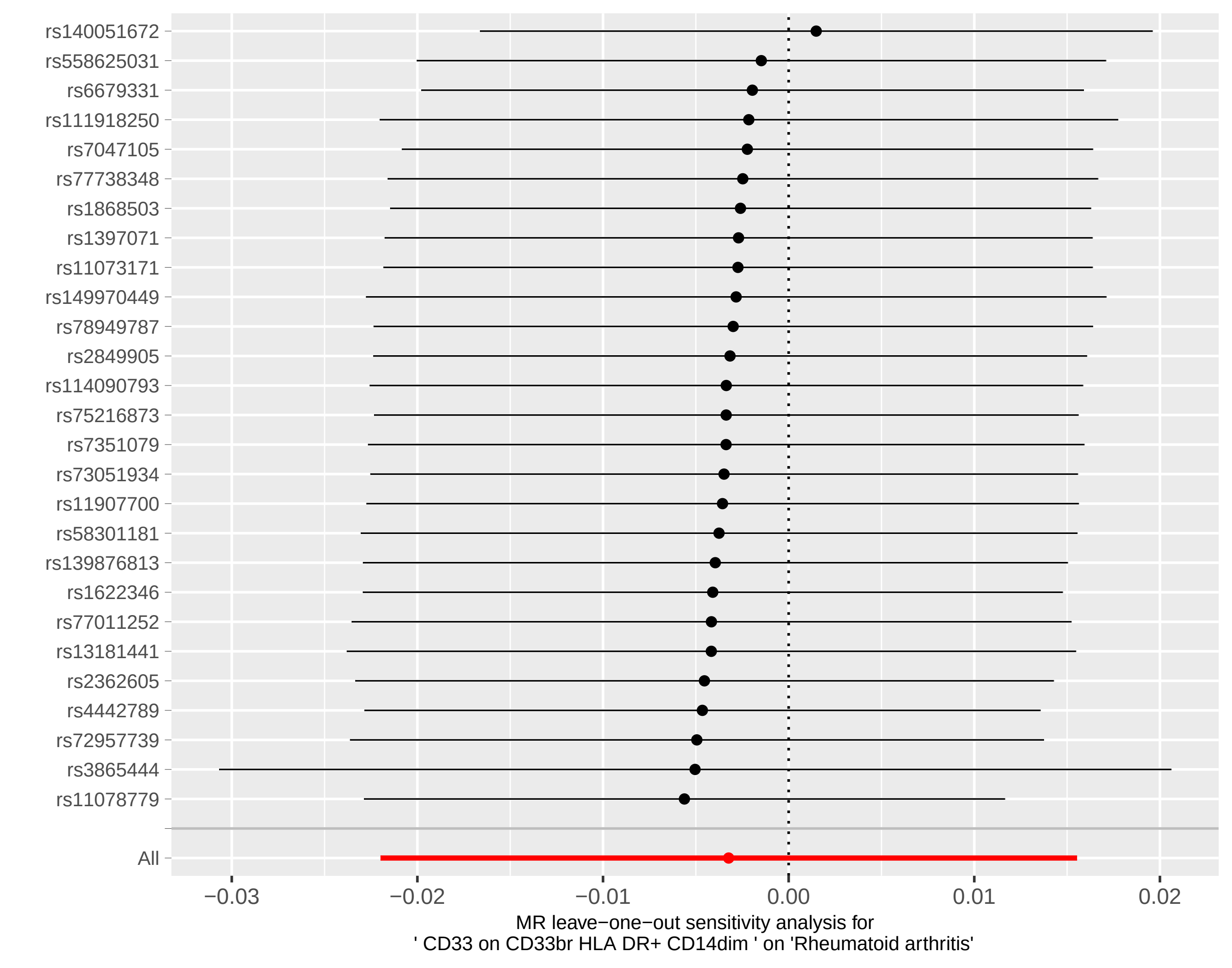
rs140051672
rs558625031
rs6679331
rs111918250
rs7047105
rs77738348
rs1868503
rs1397071
rs11073171
rs149970449
rs78949787
rs2849905
rs114090793
rs75216873
rs7351079
rs73051934
rs11907700
rs58301181
rs139876813
rs1622346
rs77011252
rs13181441
rs2362605
rs4442789
rs72957739
rs3865444
rs11078779
All
−0.03
−0.02
−0.01
0.00
0.01
0.02
MR leave−one−out sensitivity analysis for
' CD33 on CD33br HLA DR+ CD14dim ' on 'Rheumatoid arthritis'

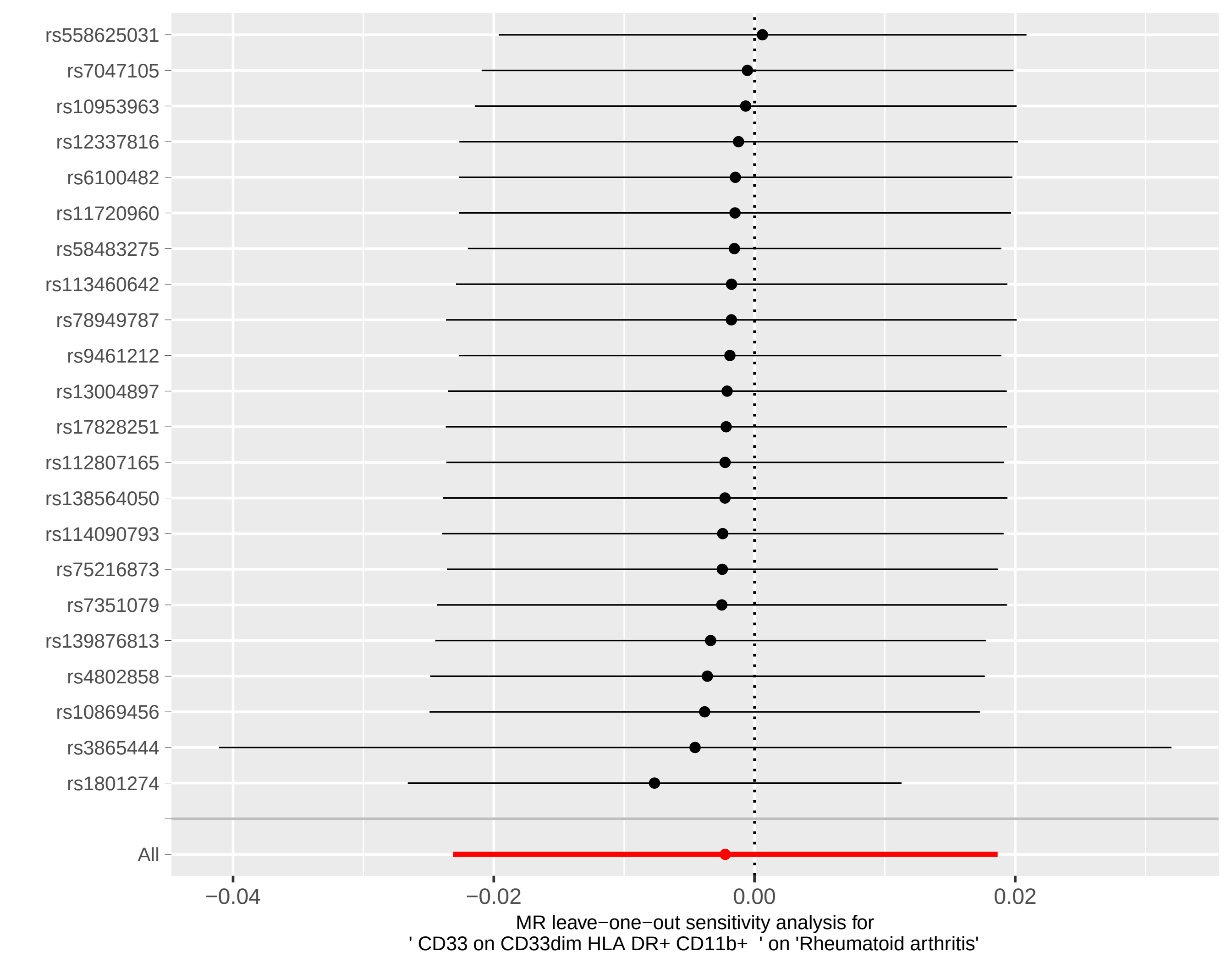
rs558625031
rs7047105
rs10953963
rs12337816
rs6100482
rs11720960
rs58483275
rs113460642
rs78949787
rs9461212
rs13004897
rs17828251
rs112807165
rs138564050
rs114090793
rs75216873
rs7351079
rs139876813
rs4802858
rs10869456
rs3865444
rs1801274
All
−0.04
−0.02
0.00
0.02
MR leave−one−out sensitivity analysis for
' CD33 on CD33dim HLA DR+ CD11b+ ' on 'Rheumatoid arthritis'

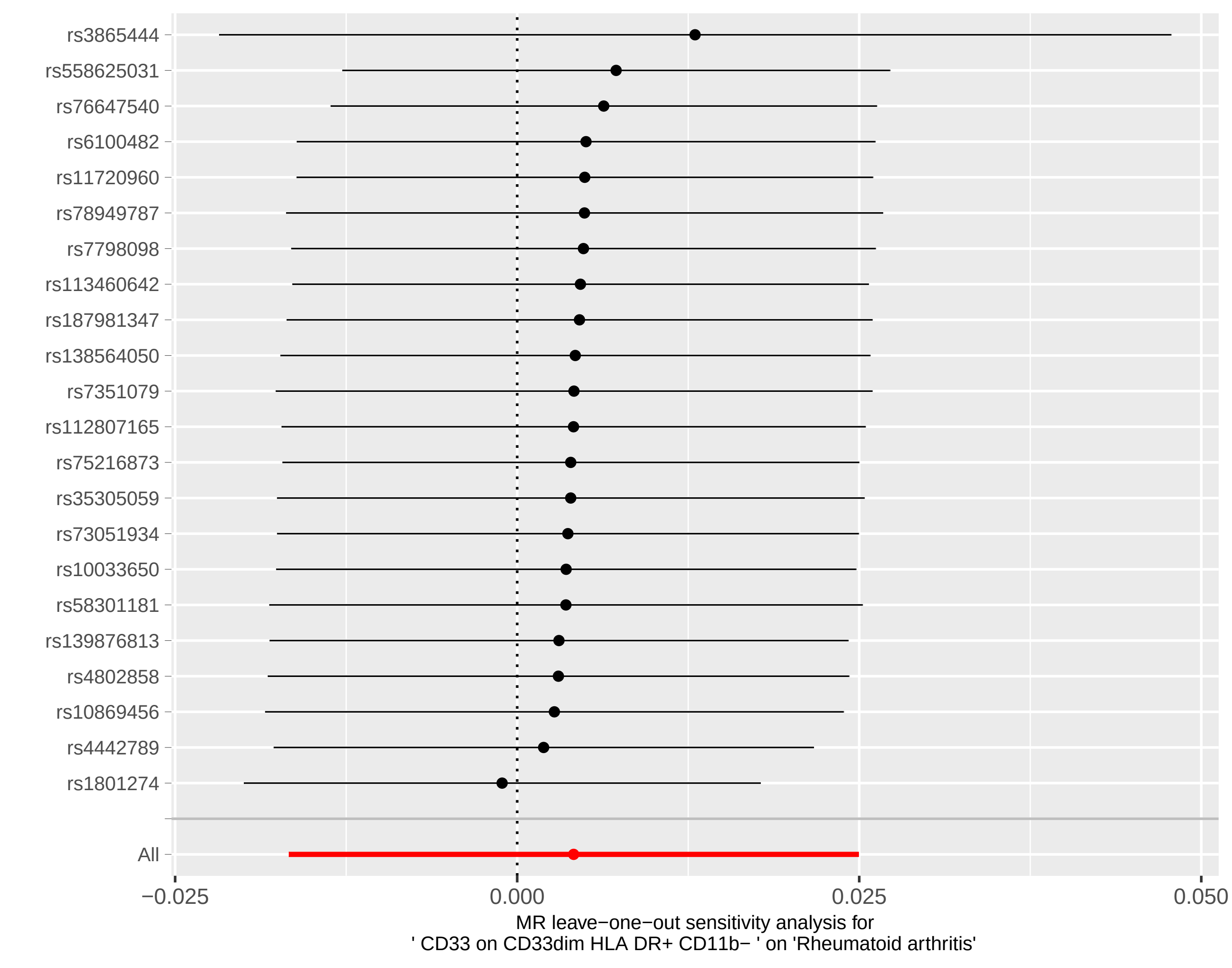
rs3865444
rs558625031
rs76647540
rs6100482
rs11720960
rs78949787
rs7798098
rs113460642
rs187981347
rs138564050
rs7351079
rs112807165
rs75216873
rs35305059
rs73051934
rs10033650
rs58301181
rs139876813
rs4802858
rs10869456
rs4442789
rs1801274
All
−0.025
0.000
0.025
0.050
MR leave−one−out sensitivity analysis for
' CD33 on CD33dim HLA DR+ CD11b− ' on 'Rheumatoid arthritis'

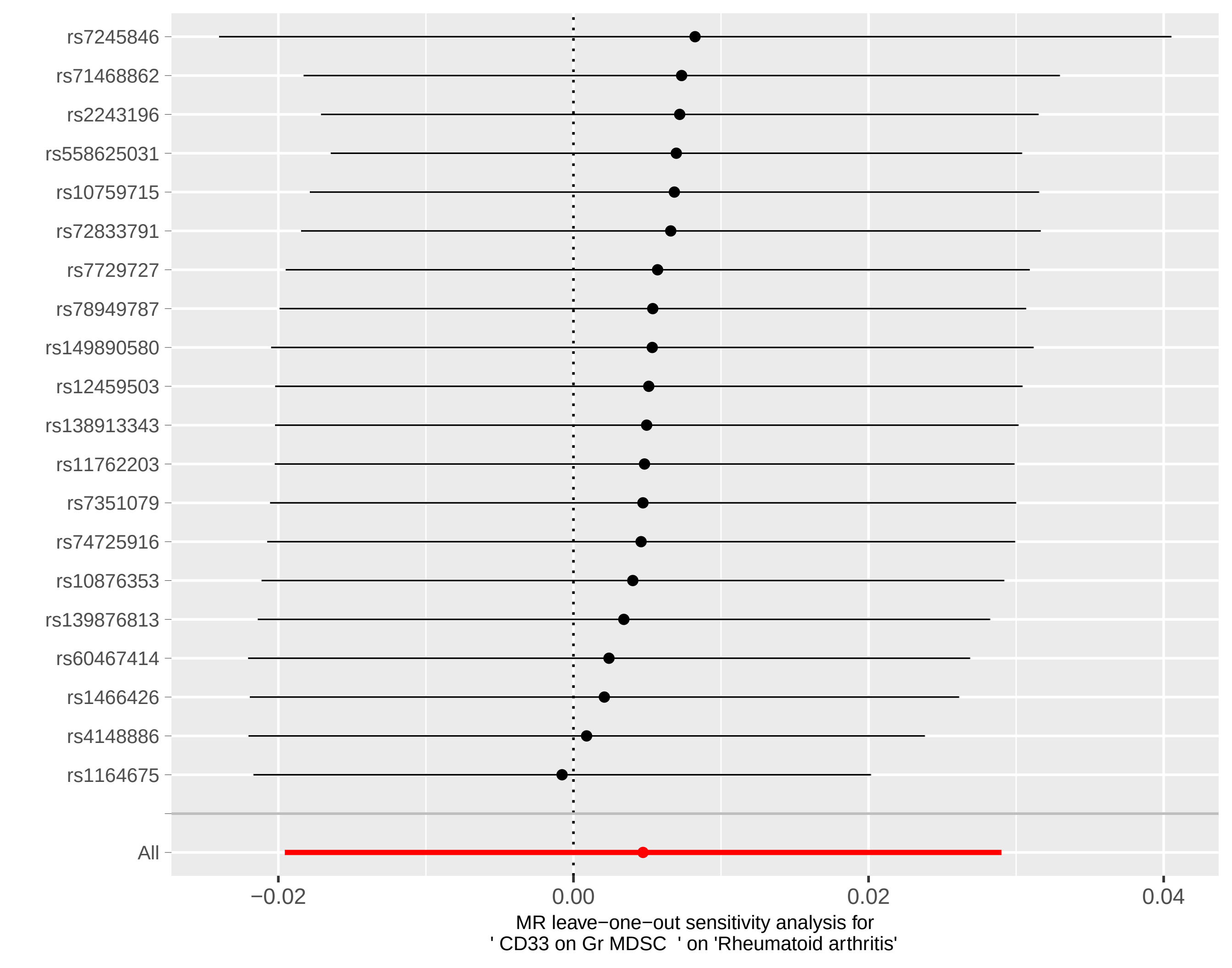
rs7245846
rs71468862
rs2243196
rs558625031
rs10759715
rs72833791
rs7729727
rs78949787
rs149890580
rs12459503
rs138913343
rs11762203
rs7351079
rs74725916
rs10876353
rs139876813
rs60467414
rs1466426
rs4148886
rs1164675
All
−0.02
0.00
0.02
0.04
MR leave−one−out sensitivity analysis for
' CD33 on Gr MDSC ' on 'Rheumatoid arthritis'

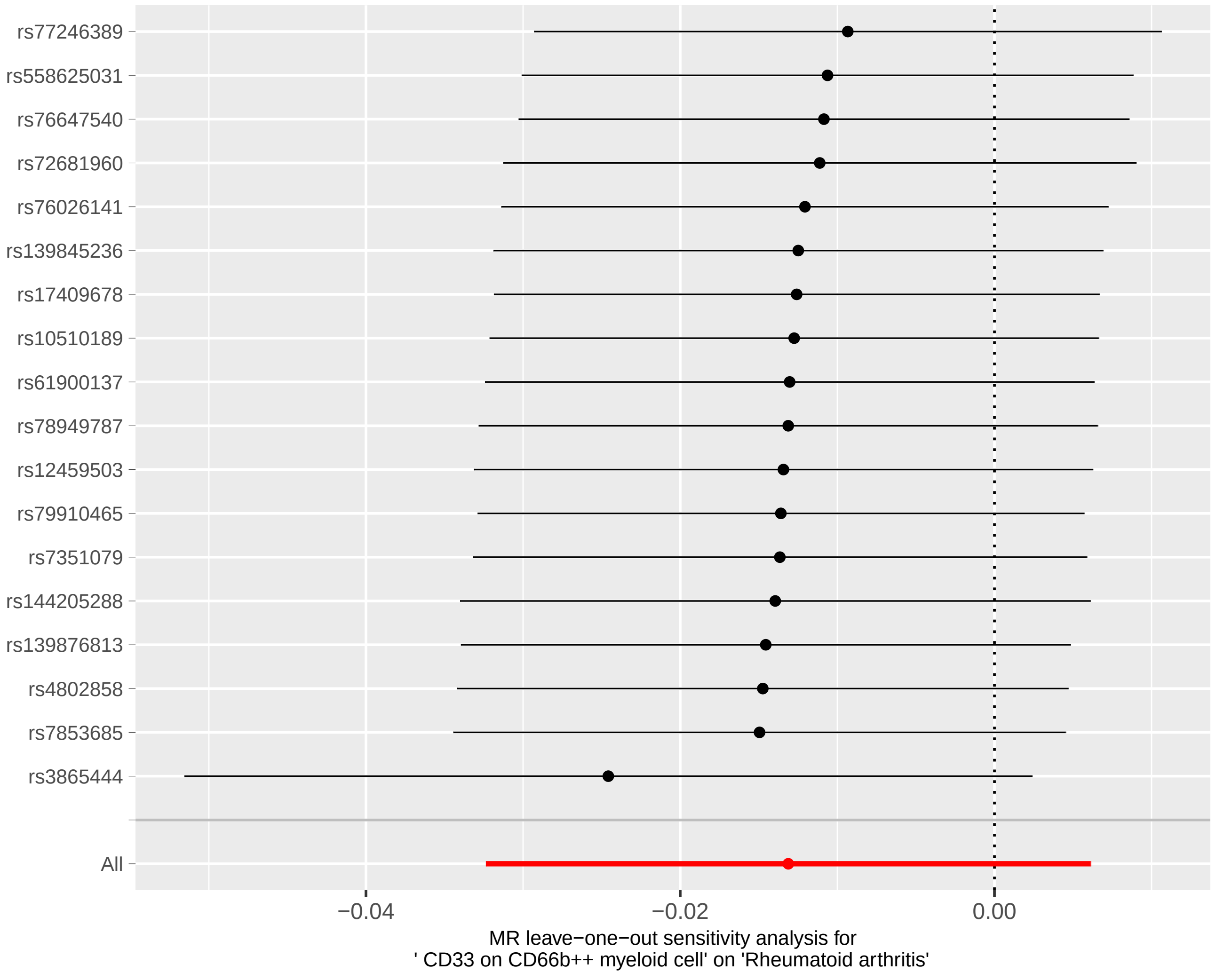
rs77246389
rs558625031
rs76647540
rs72681960
rs76026141
rs139845236
rs17409678
rs10510189
rs61900137
rs78949787
rs12459503
rs79910465
rs7351079
rs144205288
rs139876813
rs4802858
rs7853685
rs3865444
All
−0.04
−0.02
0.00
MR leave−one−out sensitivity analysis for
' CD33 on CD66b++ myeloid cell' on 'Rheumatoid arthritis'

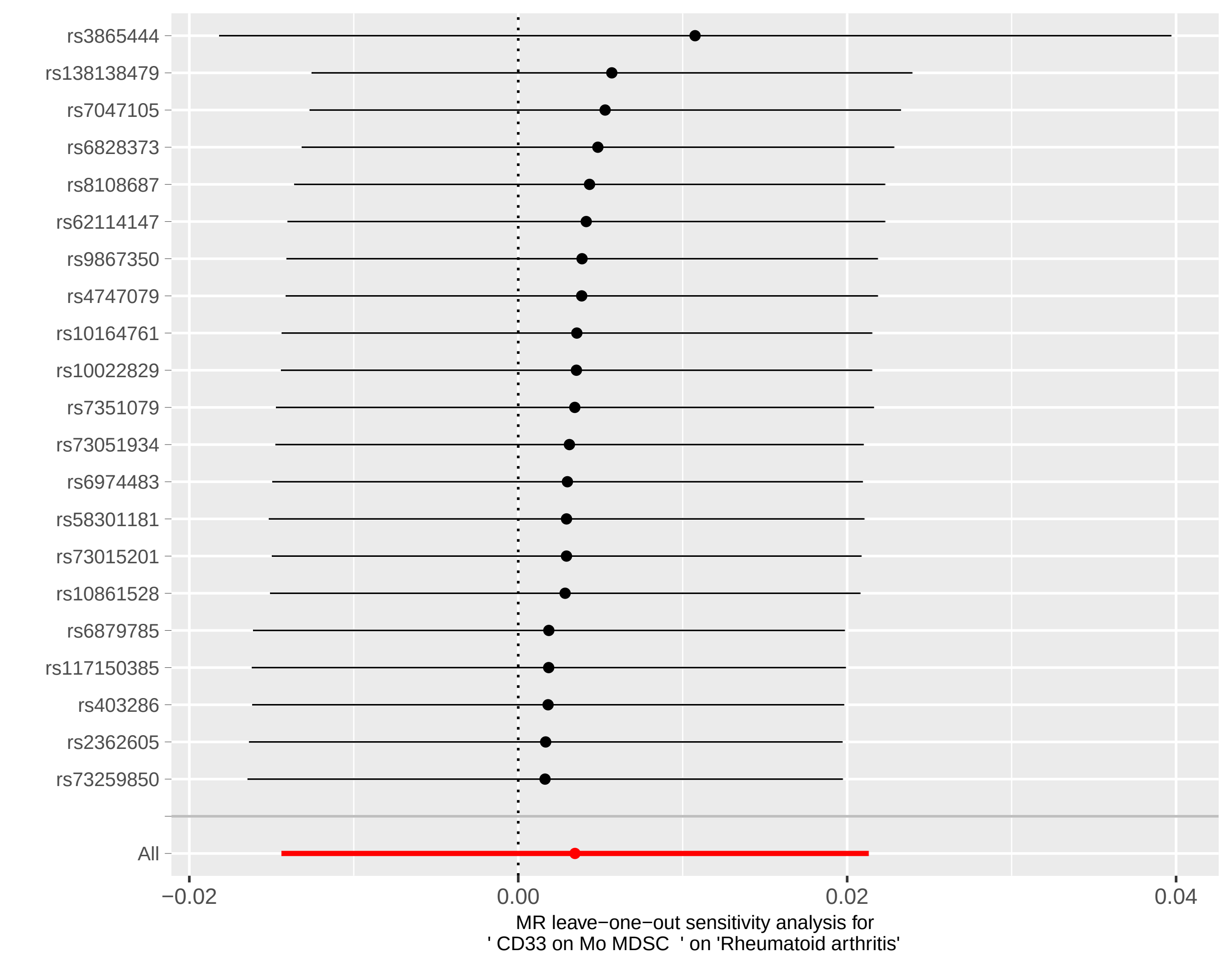
rs3865444
rs138138479
rs7047105
rs6828373
rs8108687
rs62114147
rs9867350
rs4747079
rs10164761
rs10022829
rs7351079
rs73051934
rs6974483
rs58301181
rs73015201
rs10861528
rs6879785
rs117150385
rs403286
rs2362605
rs73259850
All
−0.02
0.00
0.02
0.04
MR leave−one−out sensitivity analysis for
' CD33 on Mo MDSC ' on 'Rheumatoid arthritis'

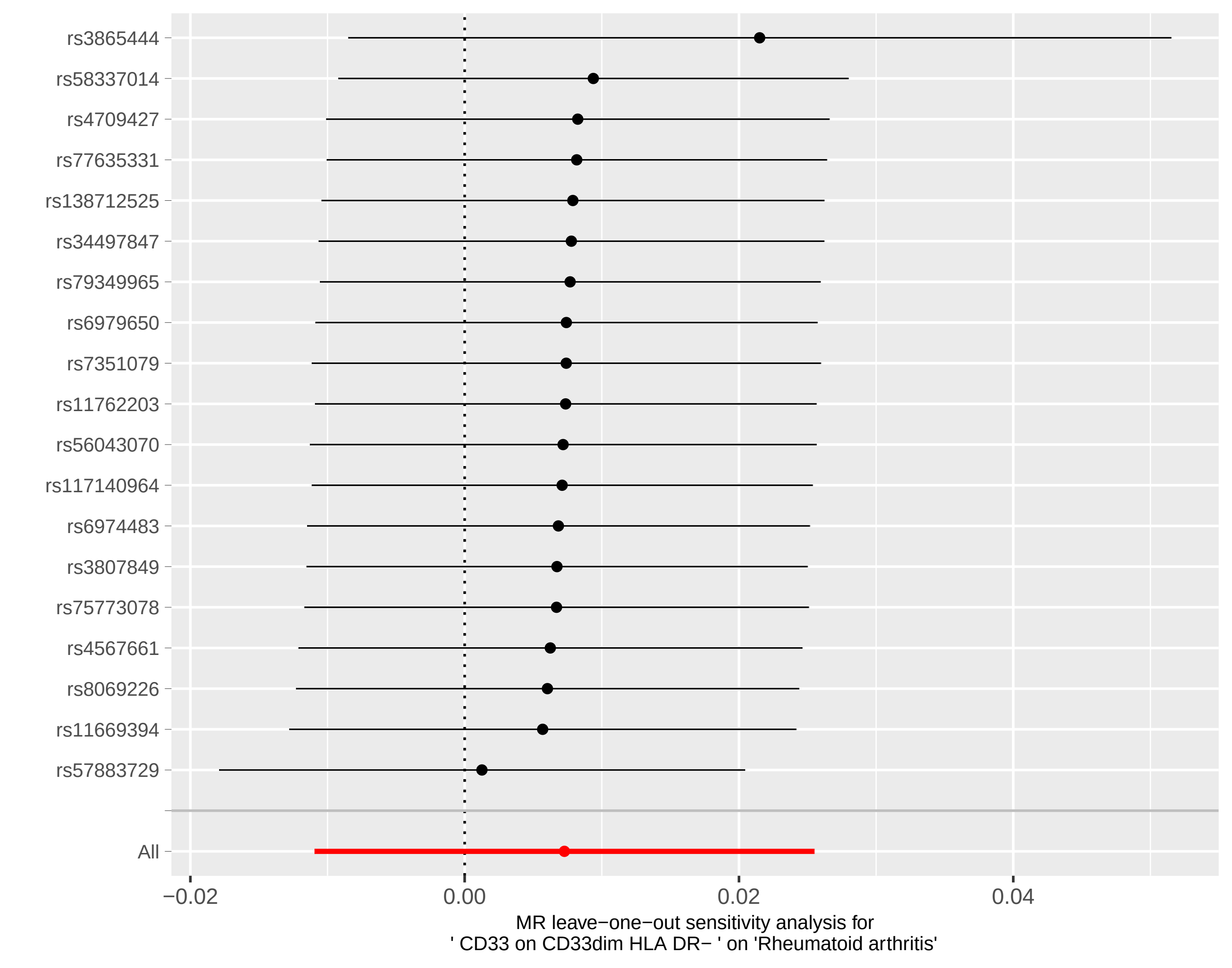

rs3865444
rs58337014
rs4709427
rs77635331
rs138712525
rs34497847
rs79349965
rs6979650
rs7351079
rs11762203
rs56043070
rs117140964
rs6974483
rs3807849
rs75773078
rs4567661
rs8069226
rs11669394
rs57883729
All
−0.02
0.00
0.02
0.04
MR leave−one−out sensitivity analysis for
' CD33 on CD33dim HLA DR− ' on 'Rheumatoid arthritis'

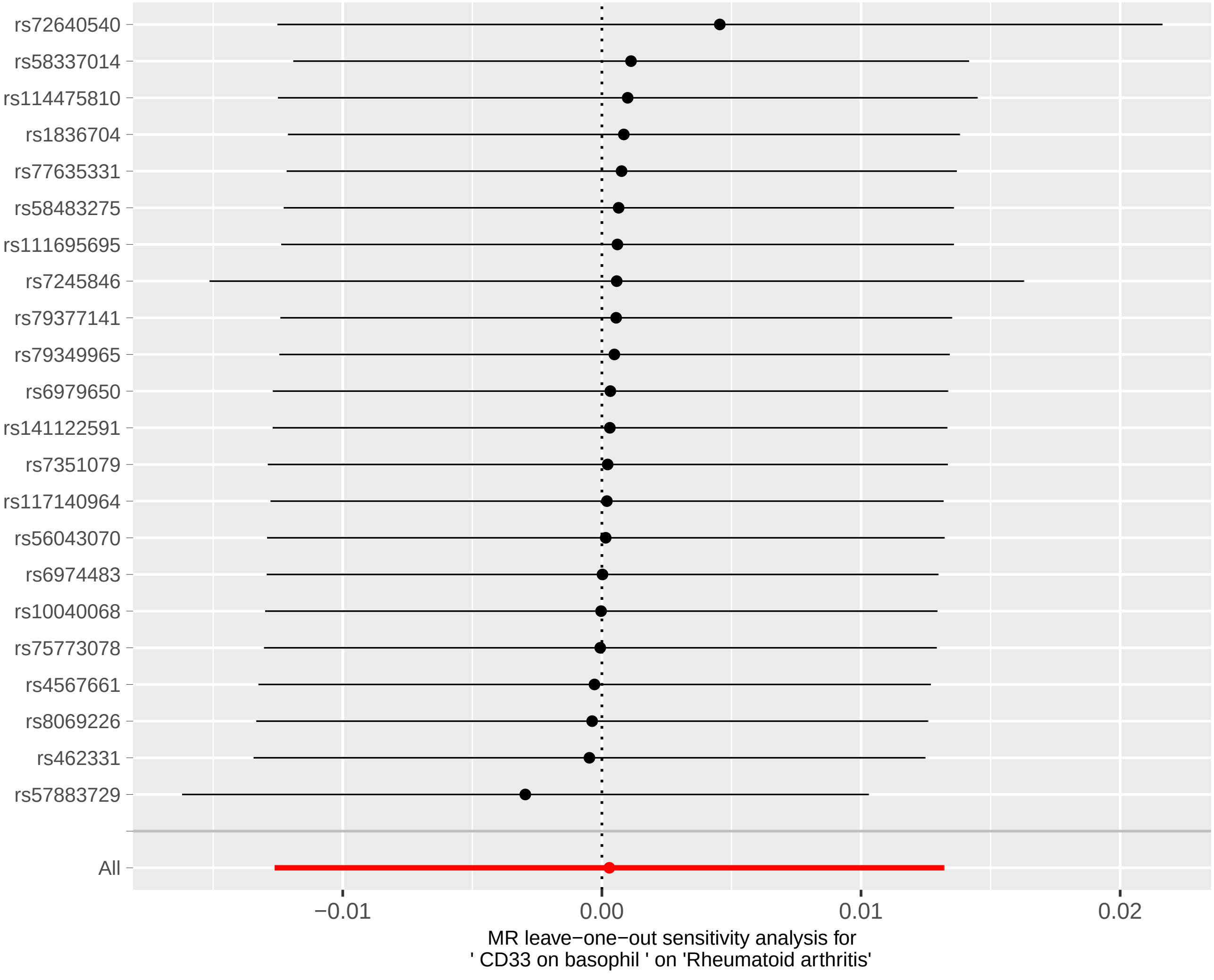
rs72640540
rs58337014
rs114475810
rs1836704
rs77635331
rs58483275
rs111695695
rs7245846
rs79377141
rs79349965
rs6979650
rs141122591
rs7351079
rs117140964
rs56043070
rs6974483
rs10040068
rs75773078
rs4567661
rs8069226
rs462331
rs57883729
All
−0.01
0.00
0.01
0.02
MR leave−one−out sensitivity analysis for
' CD33 on basophil ' on 'Rheumatoid arthritis'

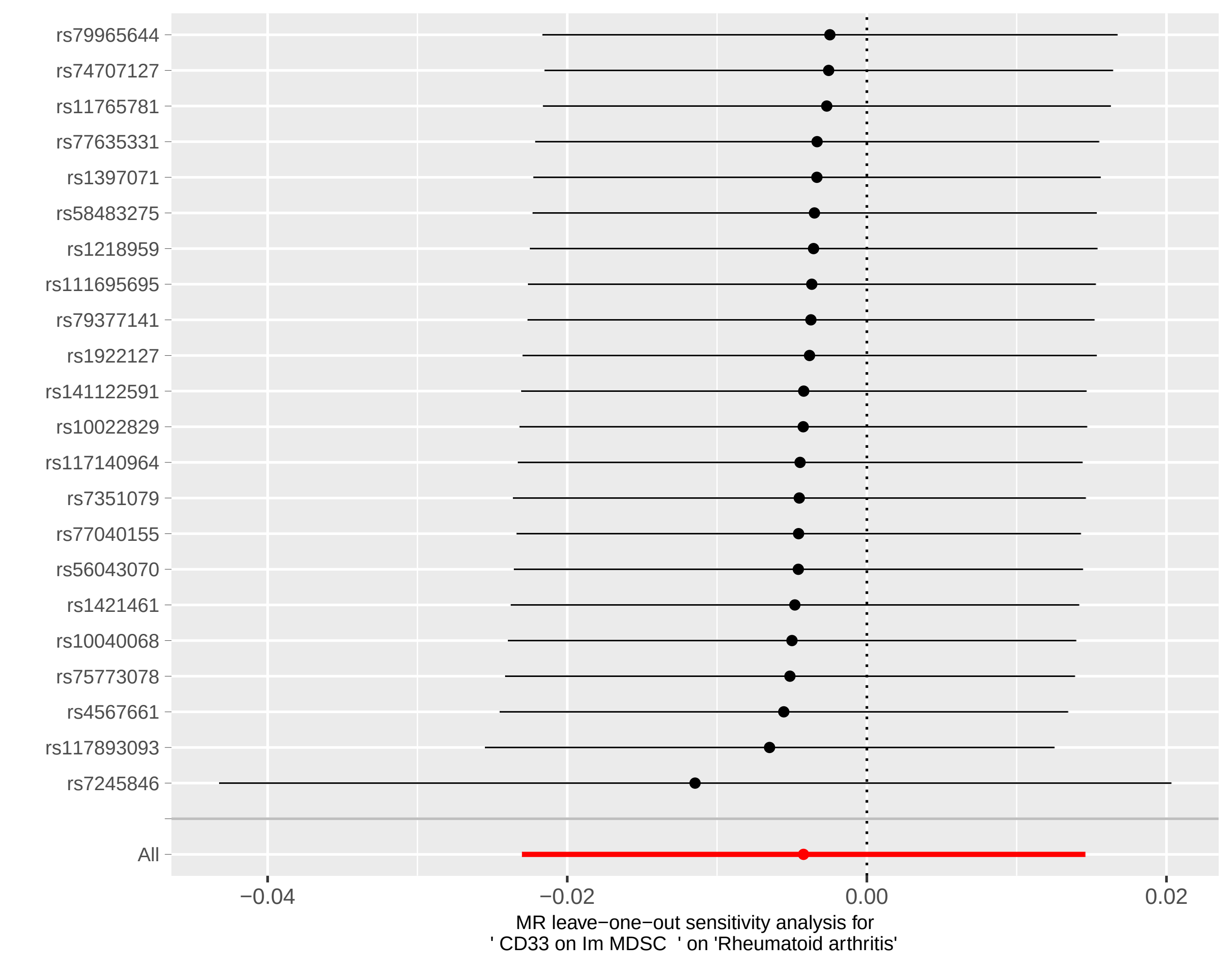
rs79965644
rs74707127
rs11765781
rs77635331
rs1397071
rs58483275
rs1218959
rs111695695
rs79377141
rs1922127
rs141122591
rs10022829
rs117140964
rs7351079
rs77040155
rs56043070
rs1421461
rs10040068
rs75773078
rs4567661
rs117893093
rs7245846
All
−0.04
−0.02
0.00
0.02
MR leave−one−out sensitivity analysis for
' CD33 on Im MDSC ' on 'Rheumatoid arthritis'

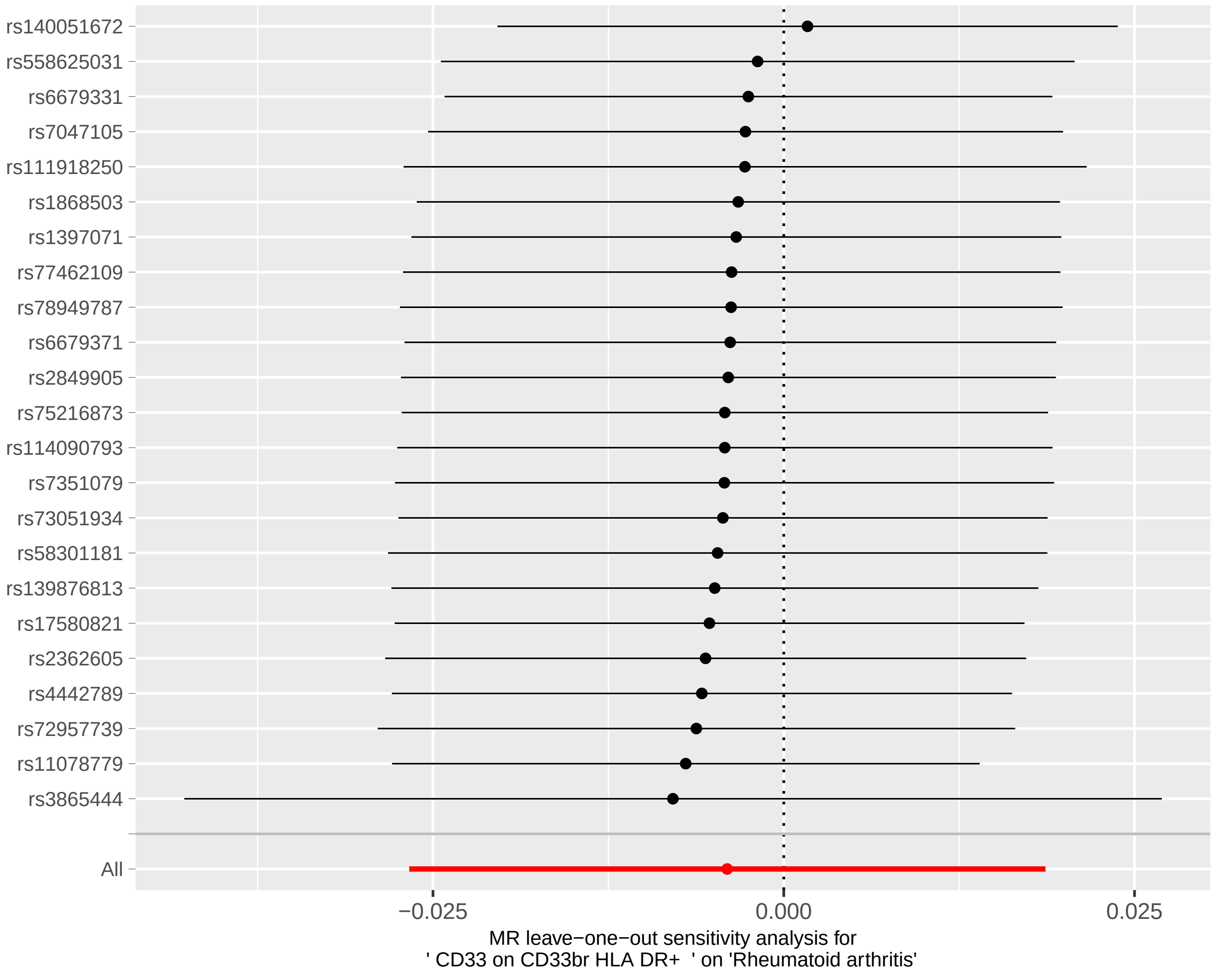
rs140051672
rs558625031
rs6679331
rs7047105
rs111918250
rs1868503
rs1397071
rs77462109
rs78949787
rs6679371
rs2849905
rs75216873
rs114090793
rs7351079
rs73051934
rs58301181
rs139876813
rs17580821
rs2362605
rs4442789
rs72957739
rs11078779
rs3865444
All
−0.025
0.000
0.025
MR leave−one−out sensitivity analysis for
' CD33 on CD33br HLA DR+ ' on 'Rheumatoid arthritis'

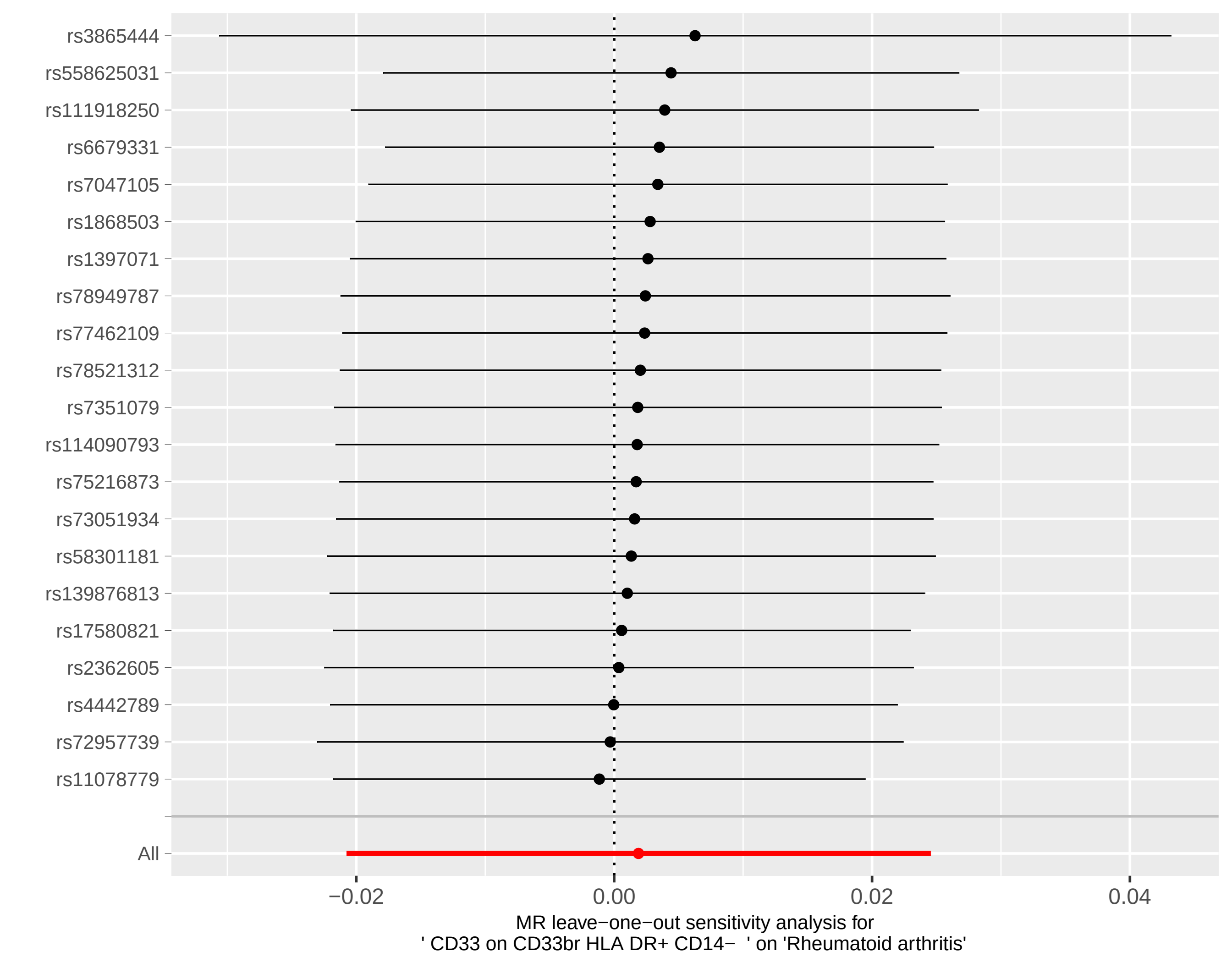
rs3865444
rs558625031
rs111918250
rs6679331
rs7047105
rs1868503
rs1397071
rs78949787
rs77462109
rs78521312
rs7351079
rs114090793
rs75216873
rs73051934
rs58301181
rs139876813
rs17580821
rs2362605
rs4442789
rs72957739
rs11078779
All
−0.02
0.00
0.02
0.04
MR leave−one−out sensitivity analysis for
' CD33 on CD33br HLA DR+ CD14− ' on 'Rheumatoid arthritis'

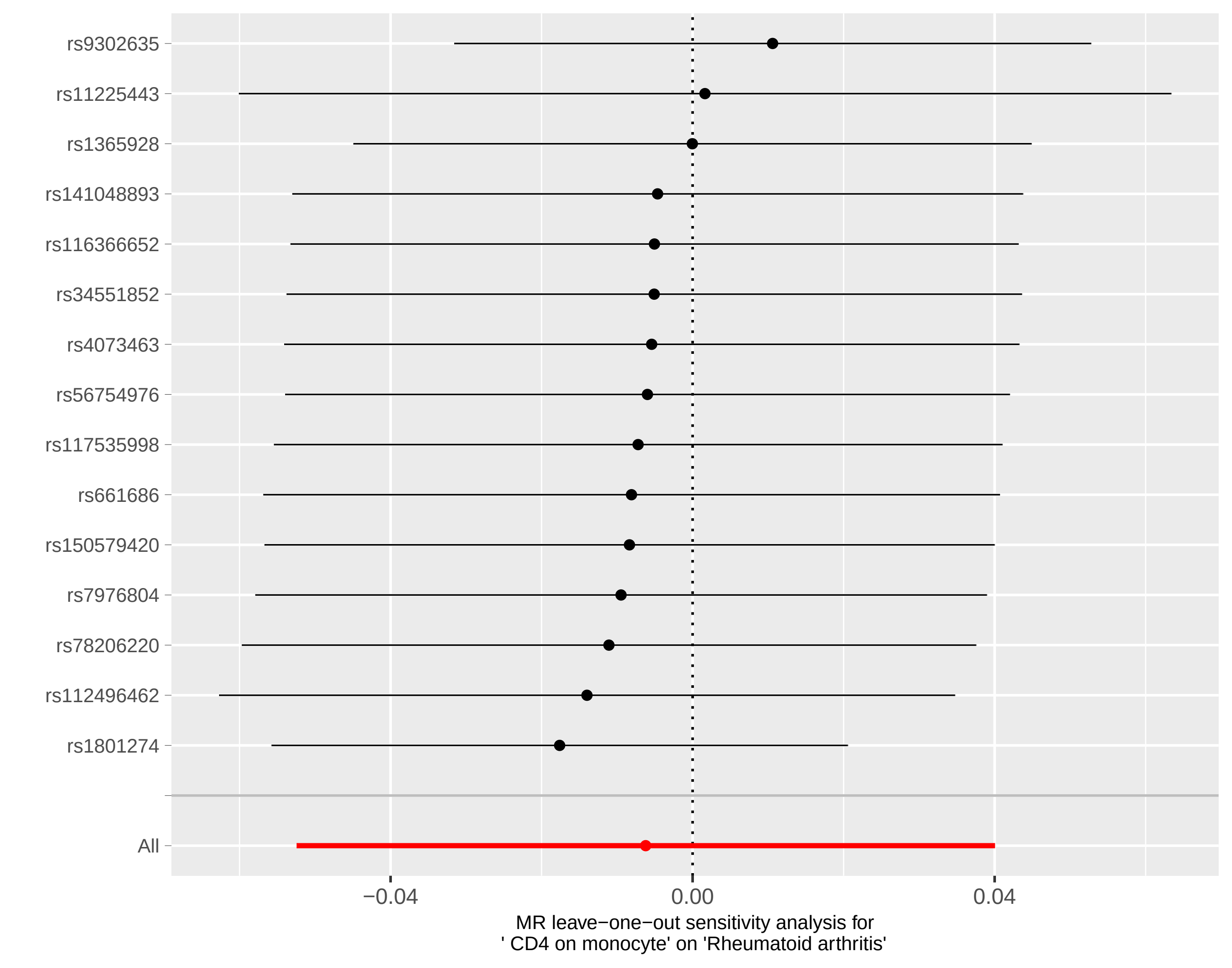
rs9302635
rs11225443
rs1365928
rs141048893
rs116366652
rs34551852
rs4073463
rs56754976
rs117535998
rs661686
rs150579420
rs7976804
rs78206220
rs112496462
rs1801274
All
−0.04
0.00
0.04
MR leave−one−out sensitivity analysis for
' CD4 on monocyte' on 'Rheumatoid arthritis'

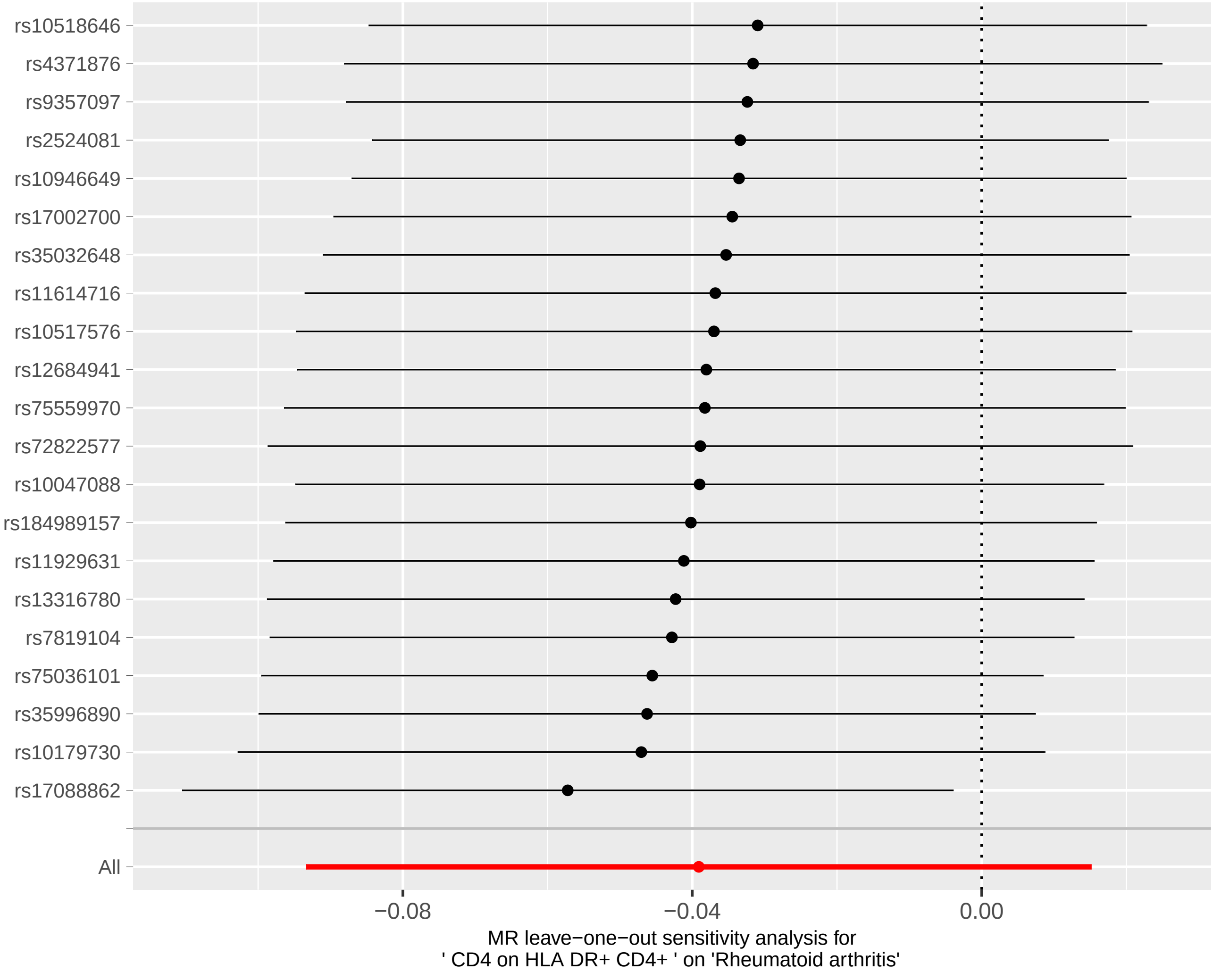

rs10518646
rs4371876
rs9357097
rs2524081
rs10946649
rs17002700
rs35032648
rs11614716
rs10517576
rs12684941
rs75559970
rs72822577
rs10047088
rs184989157
rs11929631
rs13316780
rs7819104
rs75036101
rs35996890
rs10179730
rs17088862
All
−0.08
−0.04
0.00
MR leave−one−out sensitivity analysis for
' CD4 on HLA DR+ CD4+ ' on 'Rheumatoid arthritis'

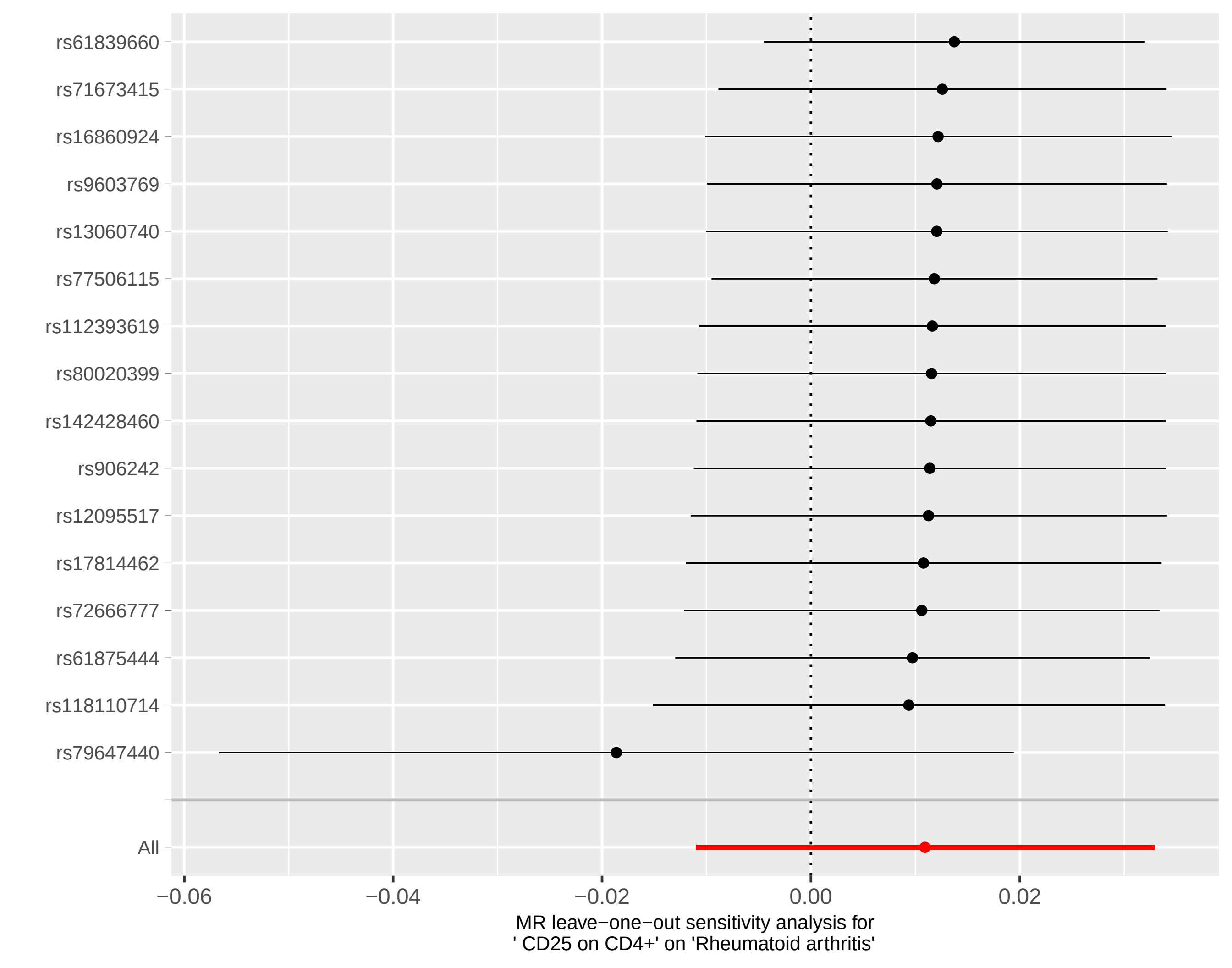

rs61839660
rs71673415
rs16860924
rs9603769
rs13060740
rs77506115
rs112393619
rs80020399
rs142428460
rs906242
rs12095517
rs17814462
rs72666777
rs61875444
rs118110714
rs79647440
All
−0.06
−0.04
−0.02
0.00
0.02
MR leave−one−out sensitivity analysis for
' CD25 on CD4+' on 'Rheumatoid arthritis'

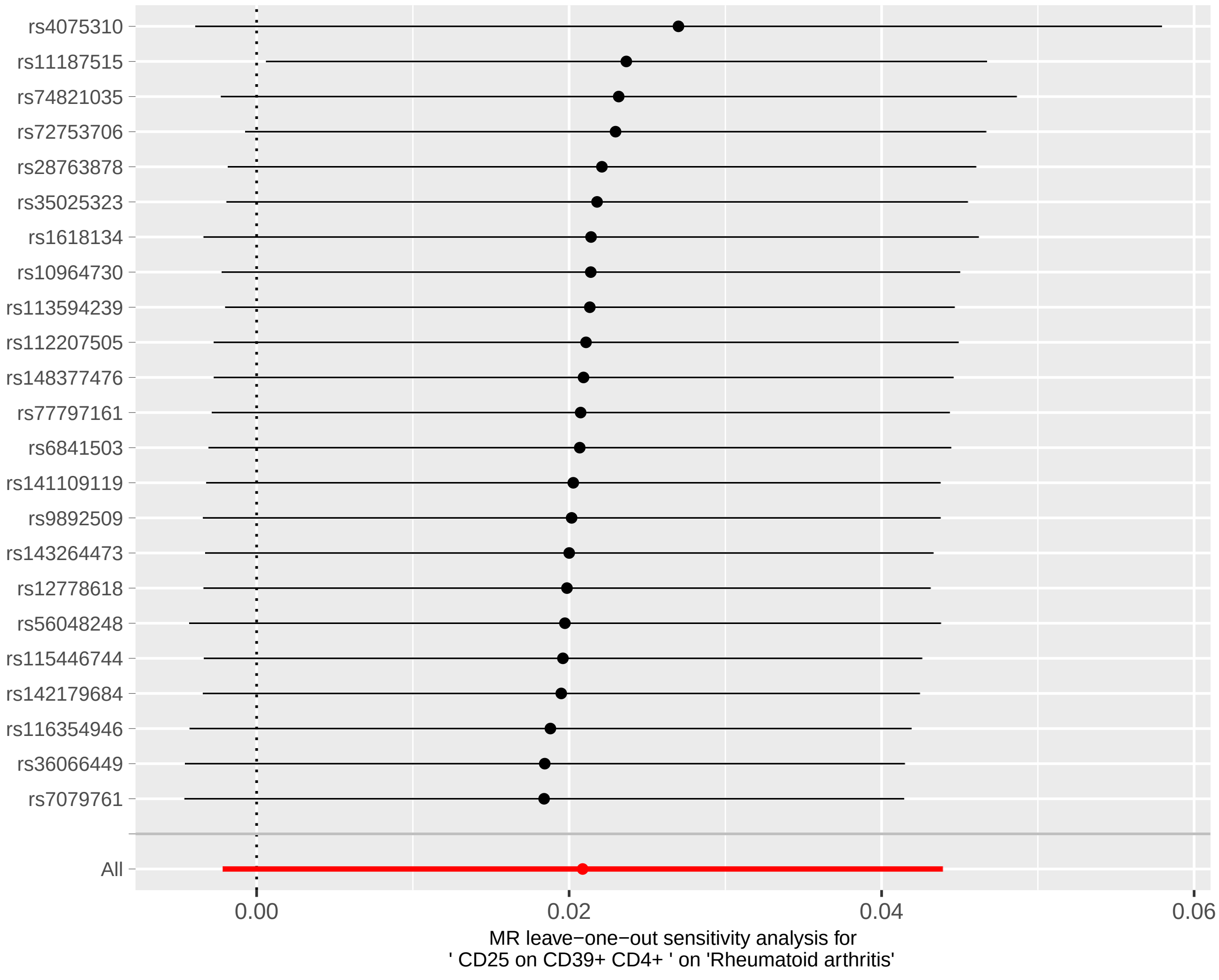

rs4075310
rs11187515
rs74821035
rs72753706
rs28763878
rs35025323
rs1618134
rs10964730
rs113594239
rs112207505
rs148377476
rs77797161
rs6841503
rs141109119
rs9892509
rs143264473
rs12778618
rs56048248
rs115446744
rs142179684
rs116354946
rs36066449
rs7079761
All
0.00
0.02
0.04
0.06
MR leave−one−out sensitivity analysis for
' CD25 on CD39+ CD4+ ' on 'Rheumatoid arthritis'

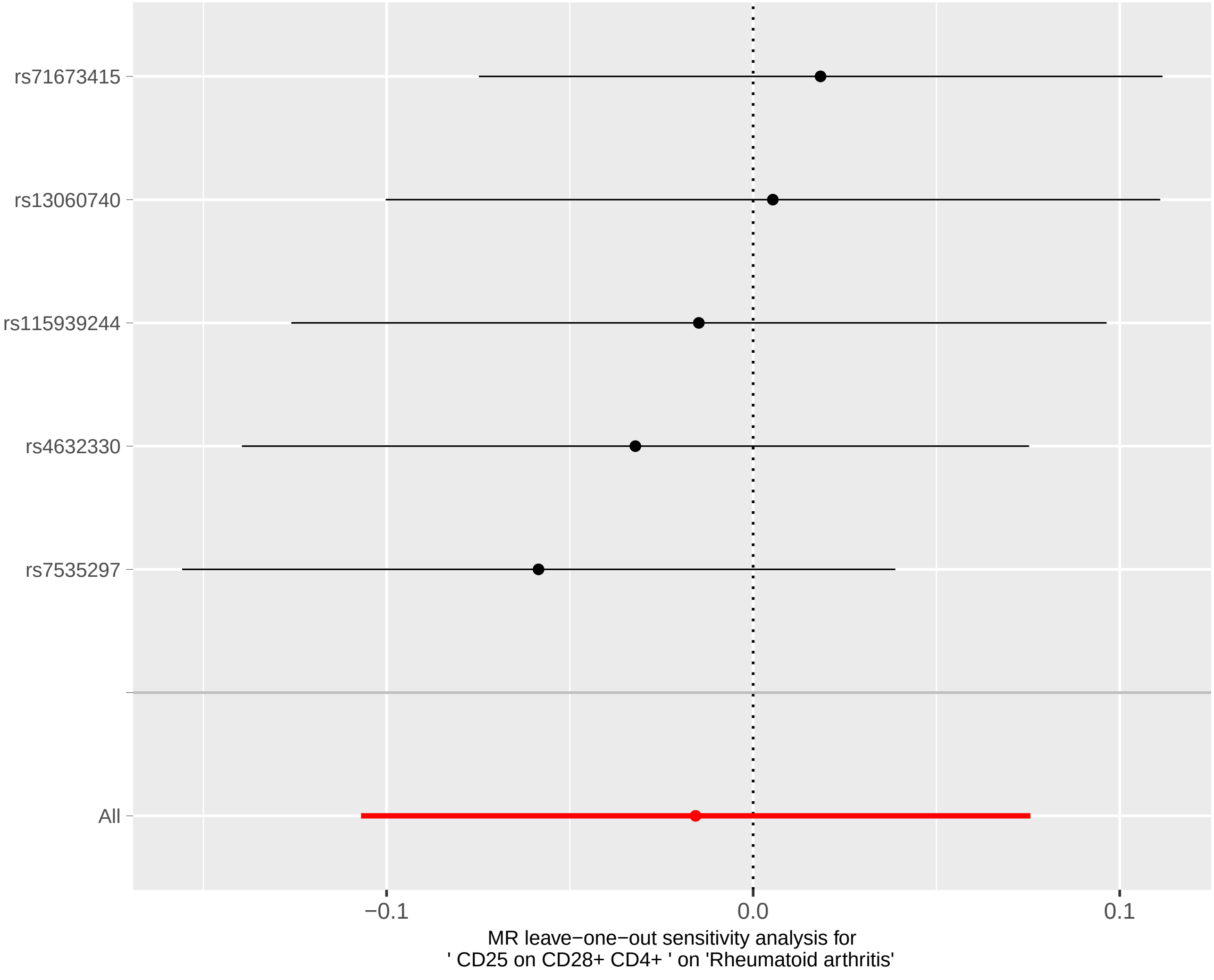

rs71673415
rs13060740
rs115939244
rs4632330
rs7535297
All
−0.1
0.0
0.1
MR leave−one−out sensitivity analysis for
' CD25 on CD28+ CD4+ ' on 'Rheumatoid arthritis'

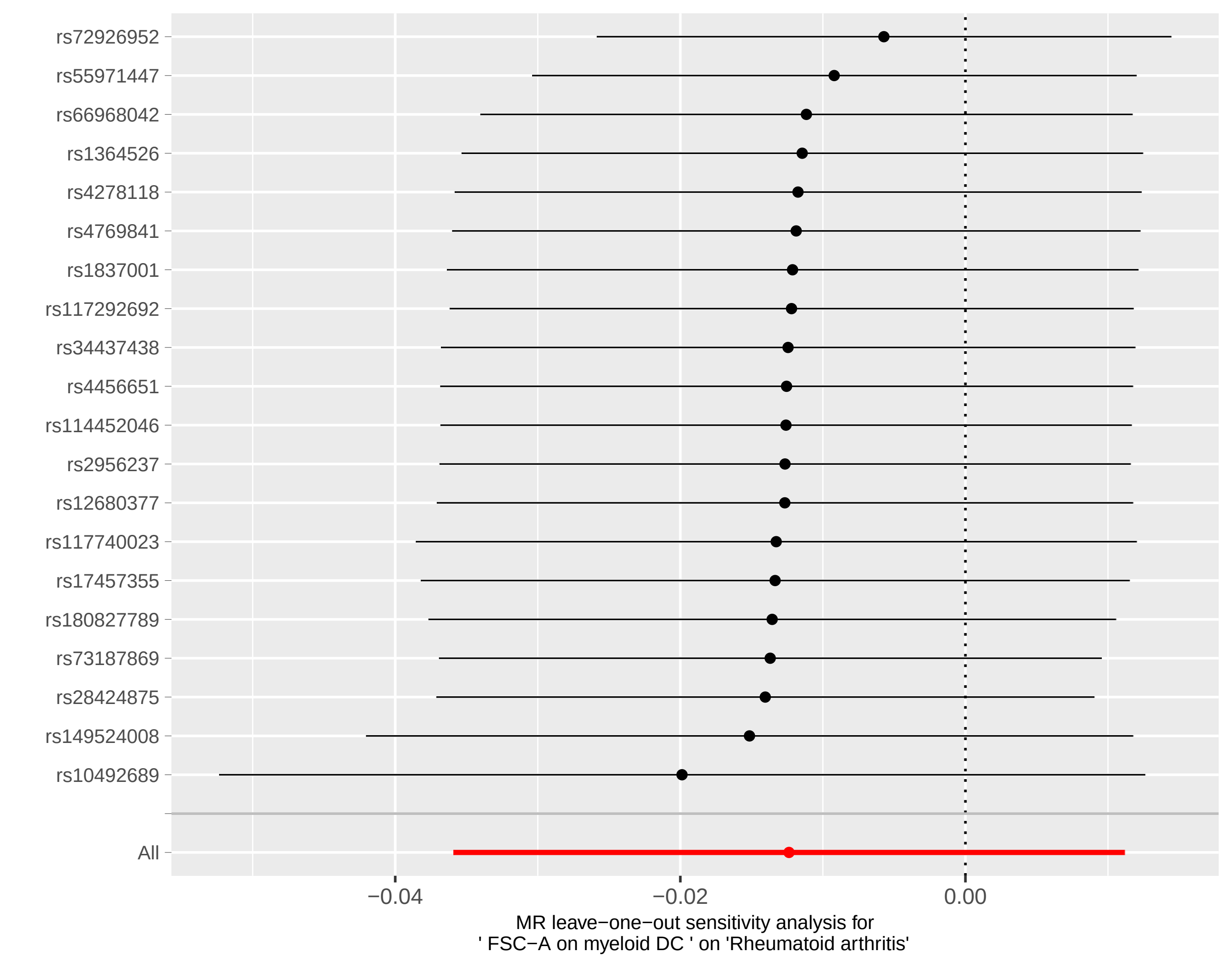
rs72926952
rs55971447
rs66968042
rs1364526
rs4278118
rs4769841
rs1837001
rs117292692
rs34437438
rs4456651
rs114452046
rs2956237
rs12680377
rs117740023
rs17457355
rs180827789
rs73187869
rs28424875
rs149524008
rs10492689
All
−0.04
−0.02
0.00
MR leave−one−out sensitivity analysis for
' FSC−A on myeloid DC ' on 'Rheumatoid arthritis'

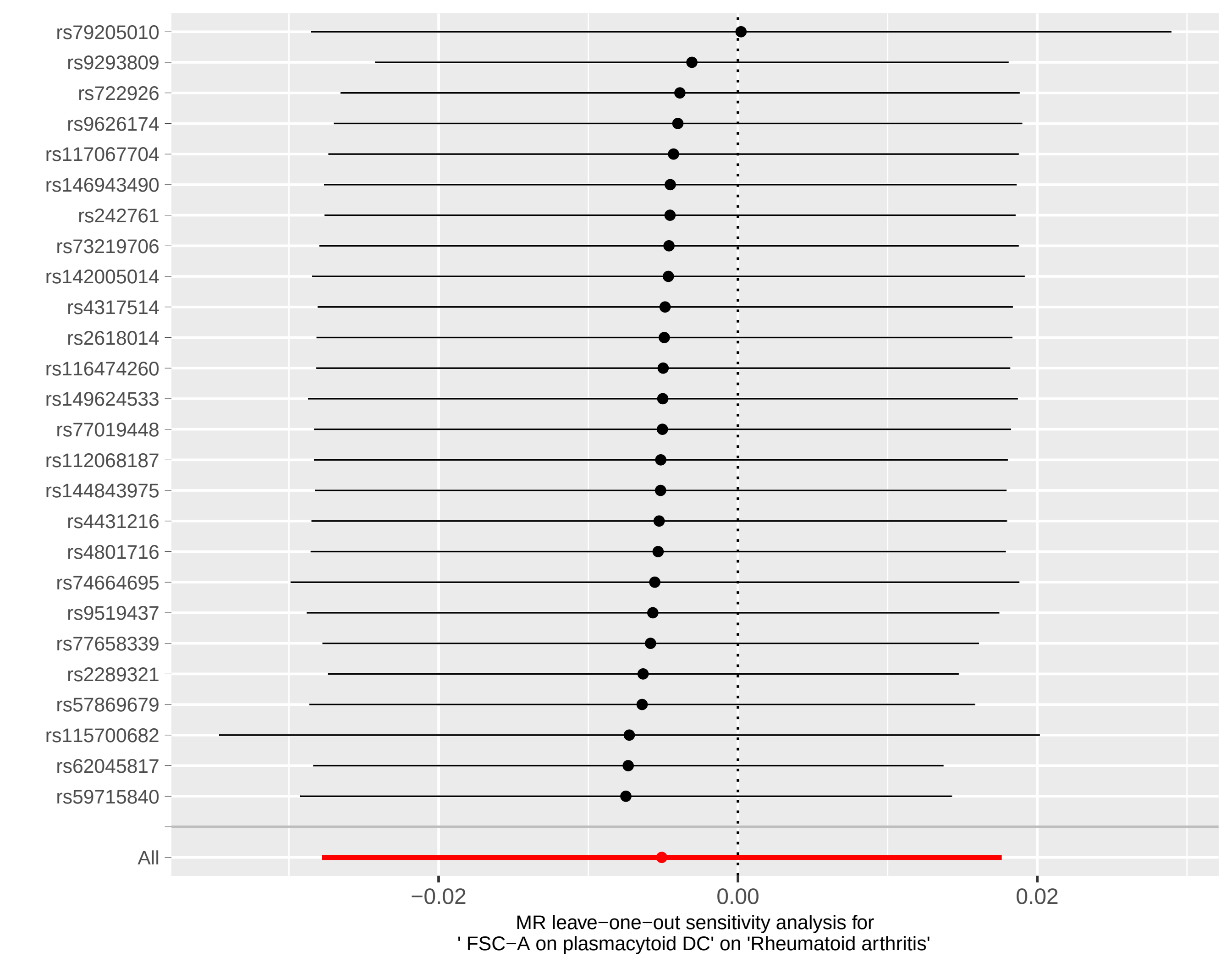

rs79205010
rs9293809
rs722926
rs9626174
rs117067704
rs146943490
rs242761
rs73219706
rs142005014
rs4317514
rs2618014
rs116474260
rs149624533
rs77019448
rs112068187
rs144843975
rs4431216
rs4801716
rs74664695
rs9519437
rs77658339
rs2289321
rs57869679
rs115700682
rs62045817
rs59715840
All
−0.02
0.00
0.02
MR leave−one−out sensitivity analysis for
' FSC−A on plasmacytoid DC' on 'Rheumatoid arthritis'

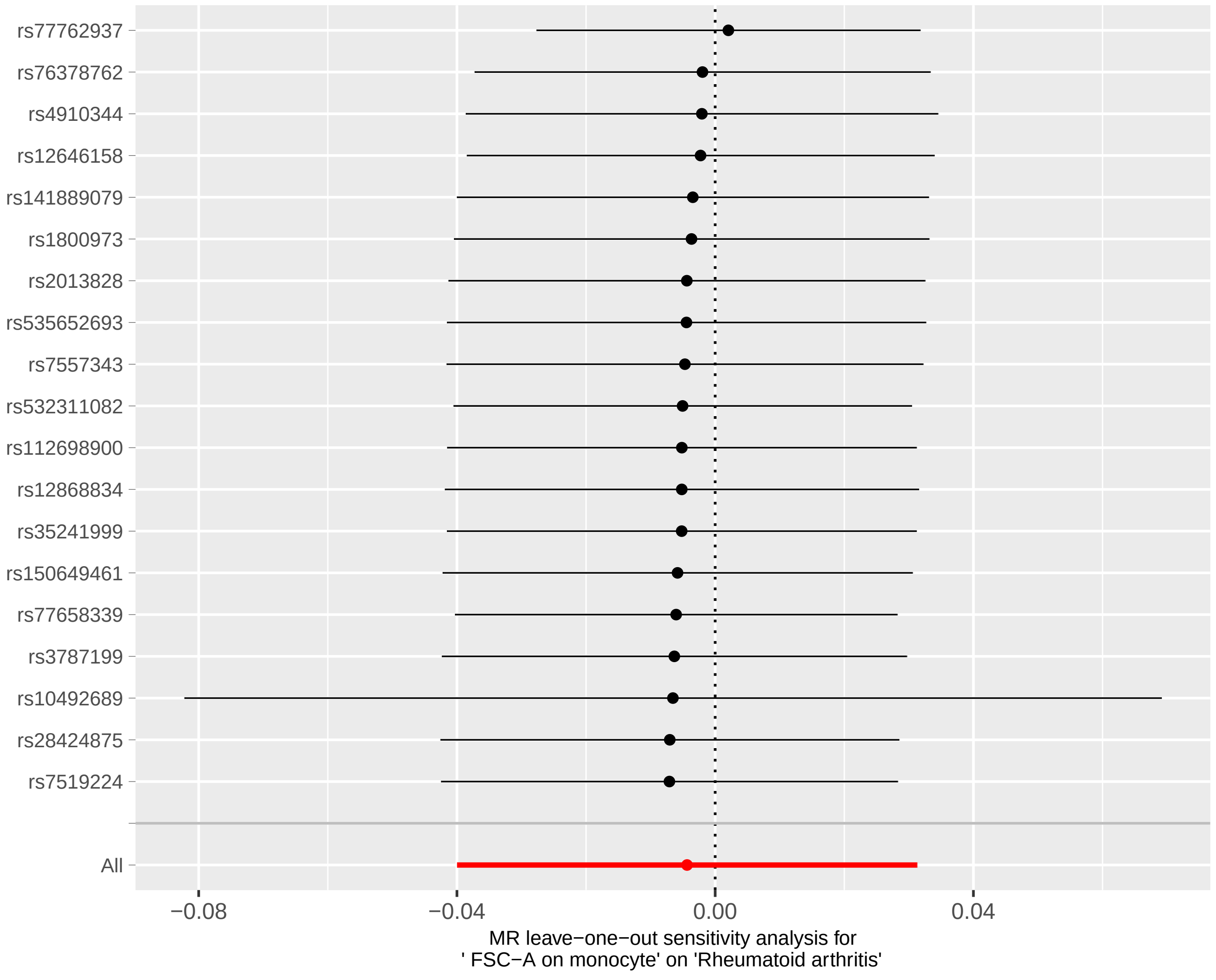
rs77762937
rs76378762
rs4910344
rs12646158
rs141889079
rs1800973
rs2013828
rs535652693
rs7557343
rs532311082
rs112698900
rs12868834
rs35241999
rs150649461
rs77658339
rs3787199
rs10492689
rs28424875
rs7519224
All
−0.08
−0.04
0.00
0.04
MR leave−one−out sensitivity analysis for
' FSC−A on monocyte' on 'Rheumatoid arthritis'

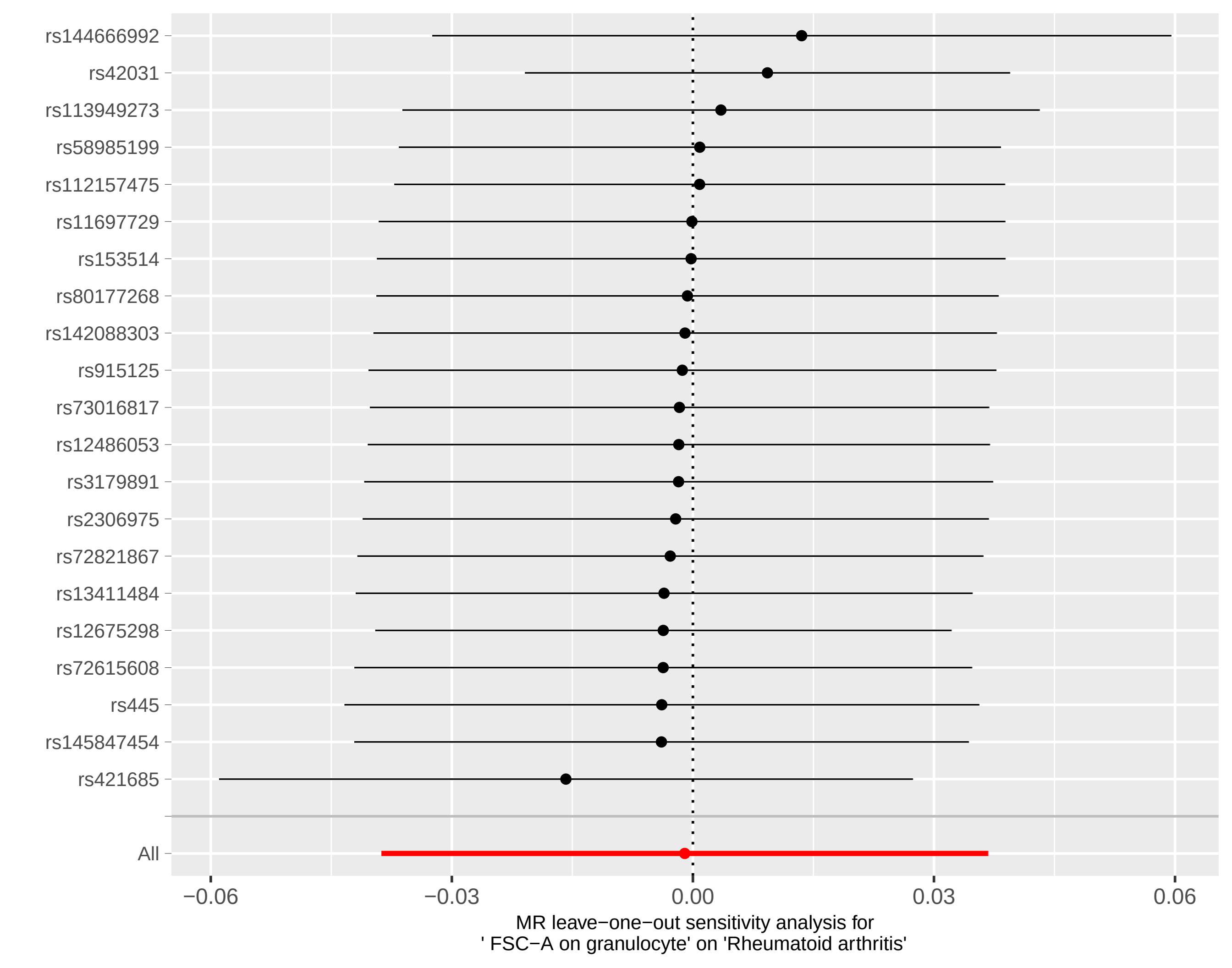

rs144666992
rs42031
rs113949273
rs58985199
rs112157475
rs11697729
rs153514
rs80177268
rs142088303
rs915125
rs73016817
rs12486053
rs3179891
rs2306975
rs72821867
rs13411484
rs12675298
rs72615608
rs445
rs145847454
rs421685
All
−0.06
−0.03
0.00
0.03
0.06
MR leave−one−out sensitivity analysis for
' FSC−A on granulocyte' on 'Rheumatoid arthritis'

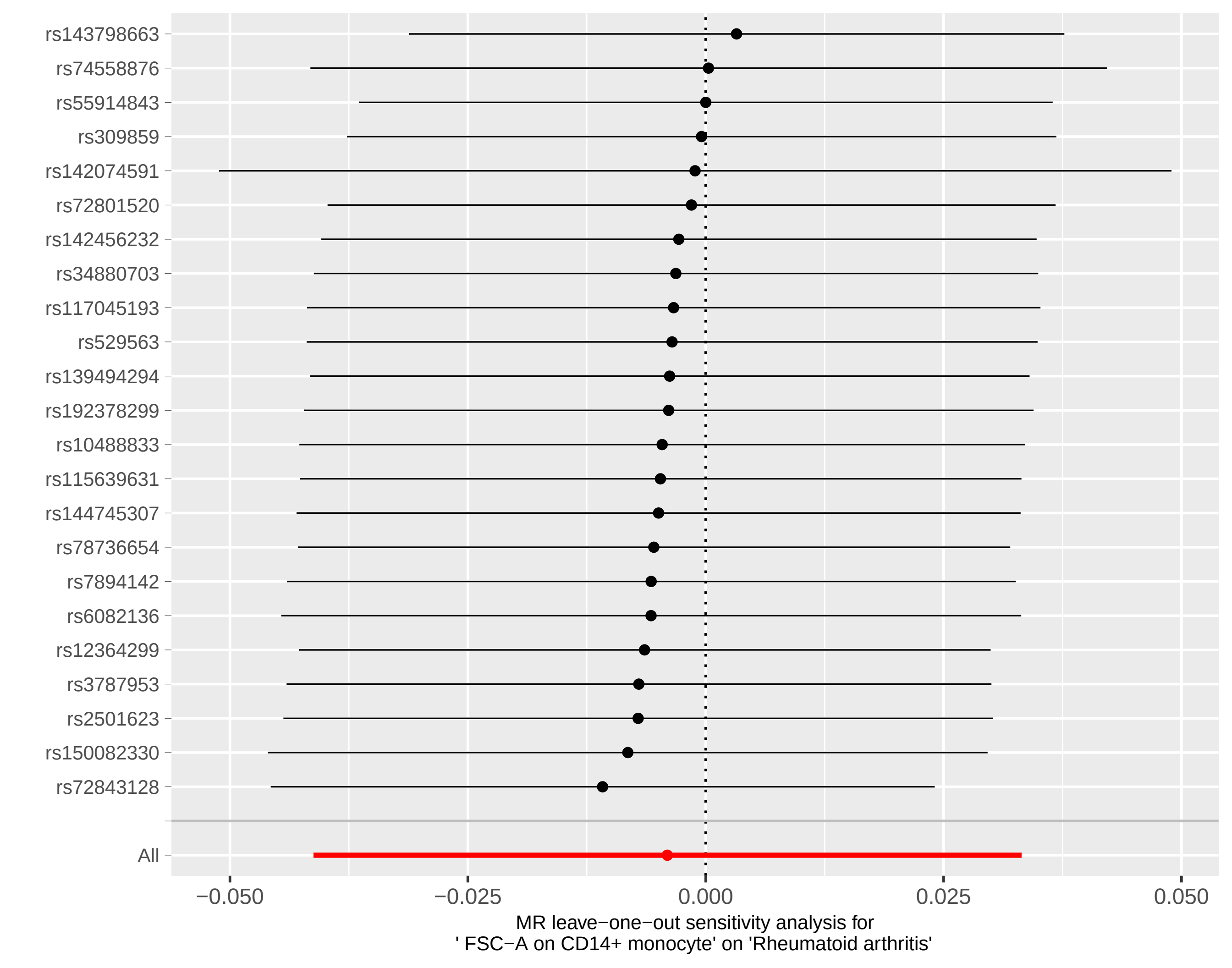
rs143798663
rs74558876
rs55914843
rs309859
rs142074591
rs72801520
rs142456232
rs34880703
rs117045193
rs529563
rs139494294
rs192378299
rs10488833
rs115639631
rs144745307
rs78736654
rs7894142
rs6082136
rs12364299
rs3787953
rs2501623
rs150082330
rs72843128
All
−0.050
−0.025
0.000
0.025
0.050
MR leave−one−out sensitivity analysis for
' FSC−A on CD14+ monocyte' on 'Rheumatoid arthritis'

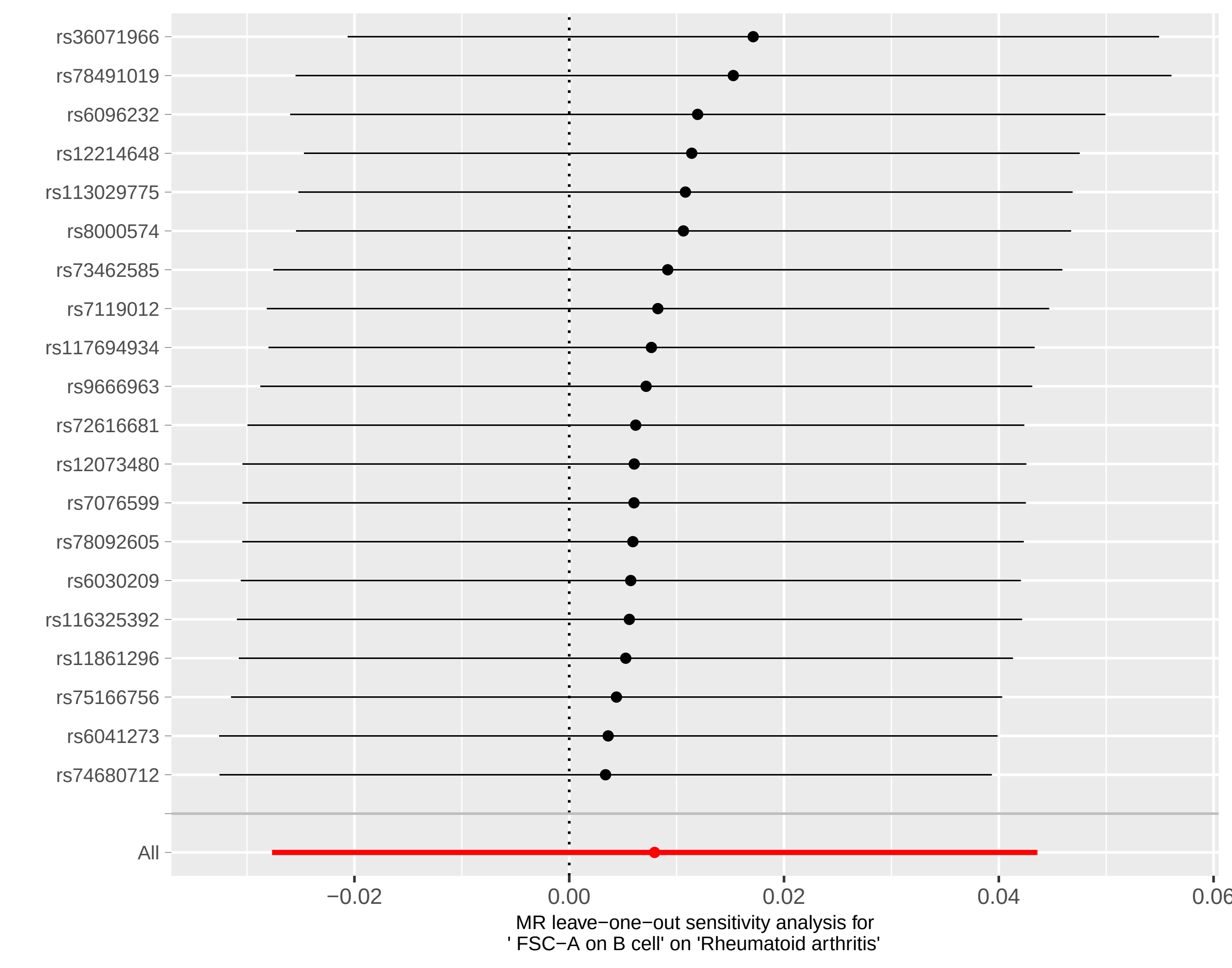
rs36071966
rs78491019
rs6096232
rs12214648
rs113029775
rs8000574
rs73462585
rs7119012
rs117694934
rs9666963
rs72616681
rs12073480
rs7076599
rs78092605
rs6030209
rs116325392
rs11861296
rs75166756
rs6041273
rs74680712
All
−0.02
0.00
0.02
0.04
0.06
MR leave−one−out sensitivity analysis for
' FSC−A on B cell' on 'Rheumatoid arthritis'

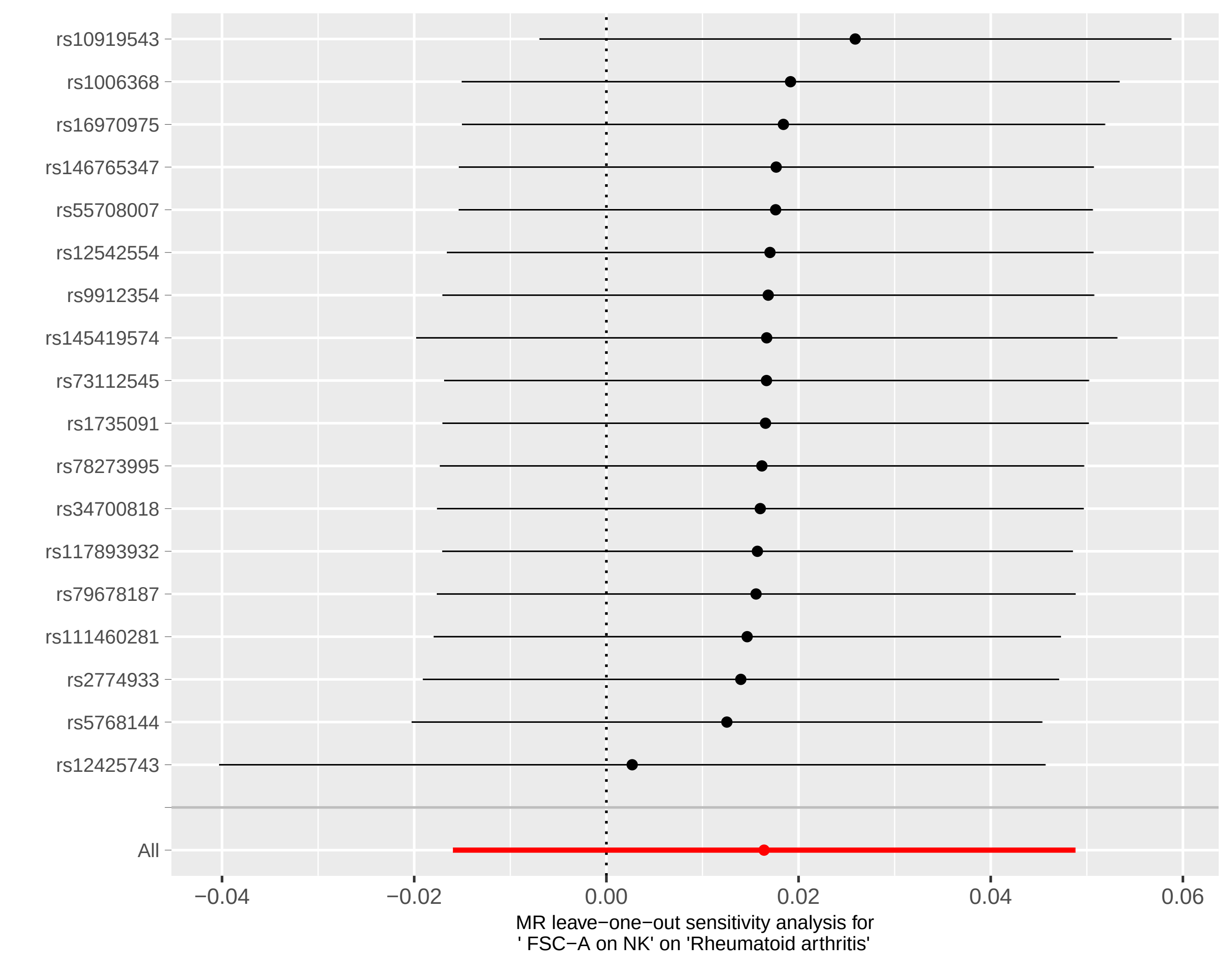
rs10919543
rs1006368
rs16970975
rs146765347
rs55708007
rs12542554
rs9912354
rs145419574
rs73112545
rs1735091
rs78273995
rs34700818
rs117893932
rs79678187
rs111460281
rs2774933
rs5768144
rs12425743
All
−0.04
−0.02
0.00
0.02
0.04
0.06
MR leave−one−out sensitivity analysis for
' FSC−A on NK' on 'Rheumatoid arthritis'

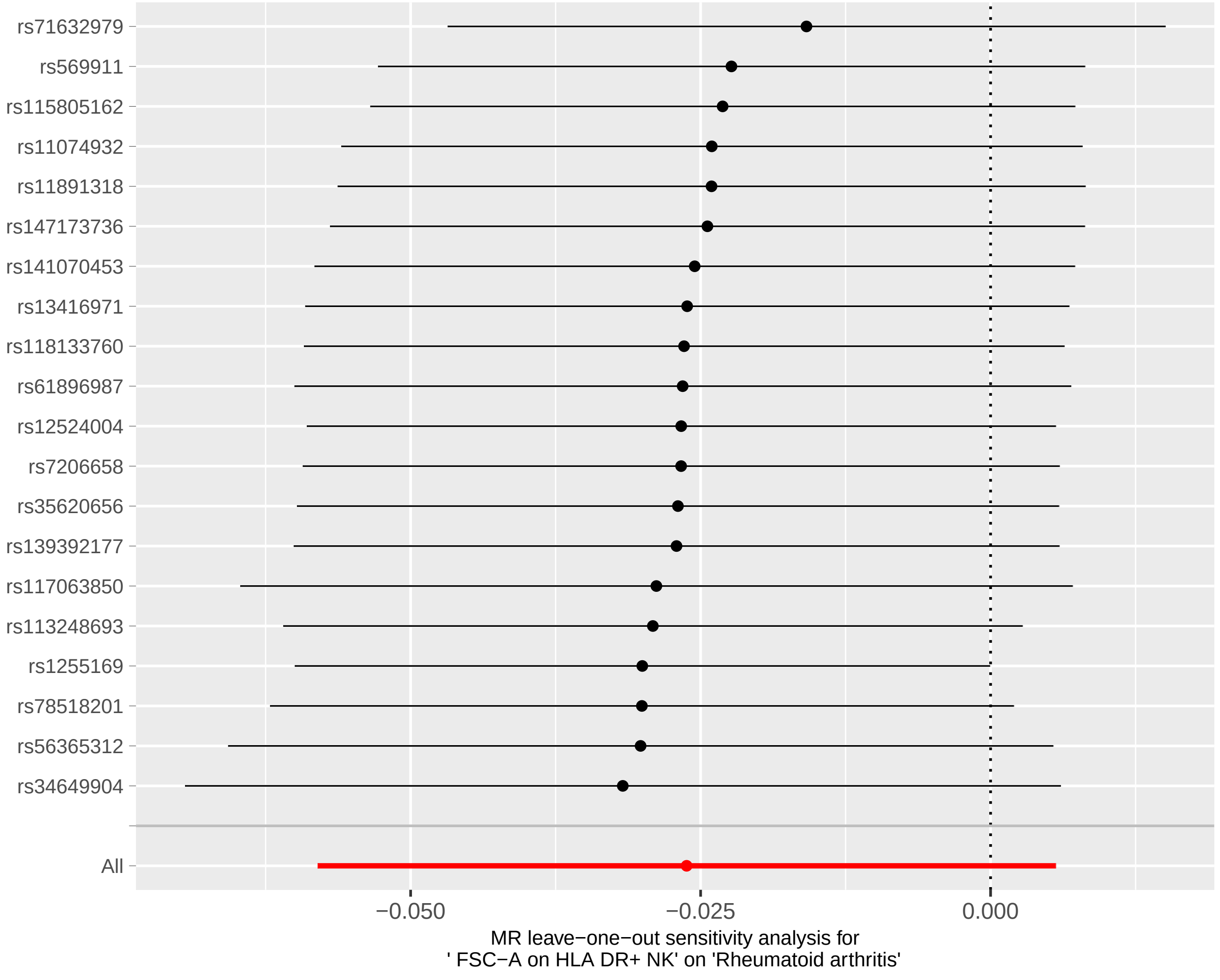
rs71632979
rs569911
rs115805162
rs11074932
rs11891318
rs147173736
rs141070453
rs13416971
rs118133760
rs61896987
rs12524004
rs7206658
rs35620656
rs139392177
rs117063850
rs113248693
rs1255169
rs78518201
rs56365312
rs34649904
All
−0.050
−0.025
0.000
MR leave−one−out sensitivity analysis for
' FSC−A on HLA DR+ NK' on 'Rheumatoid arthritis'

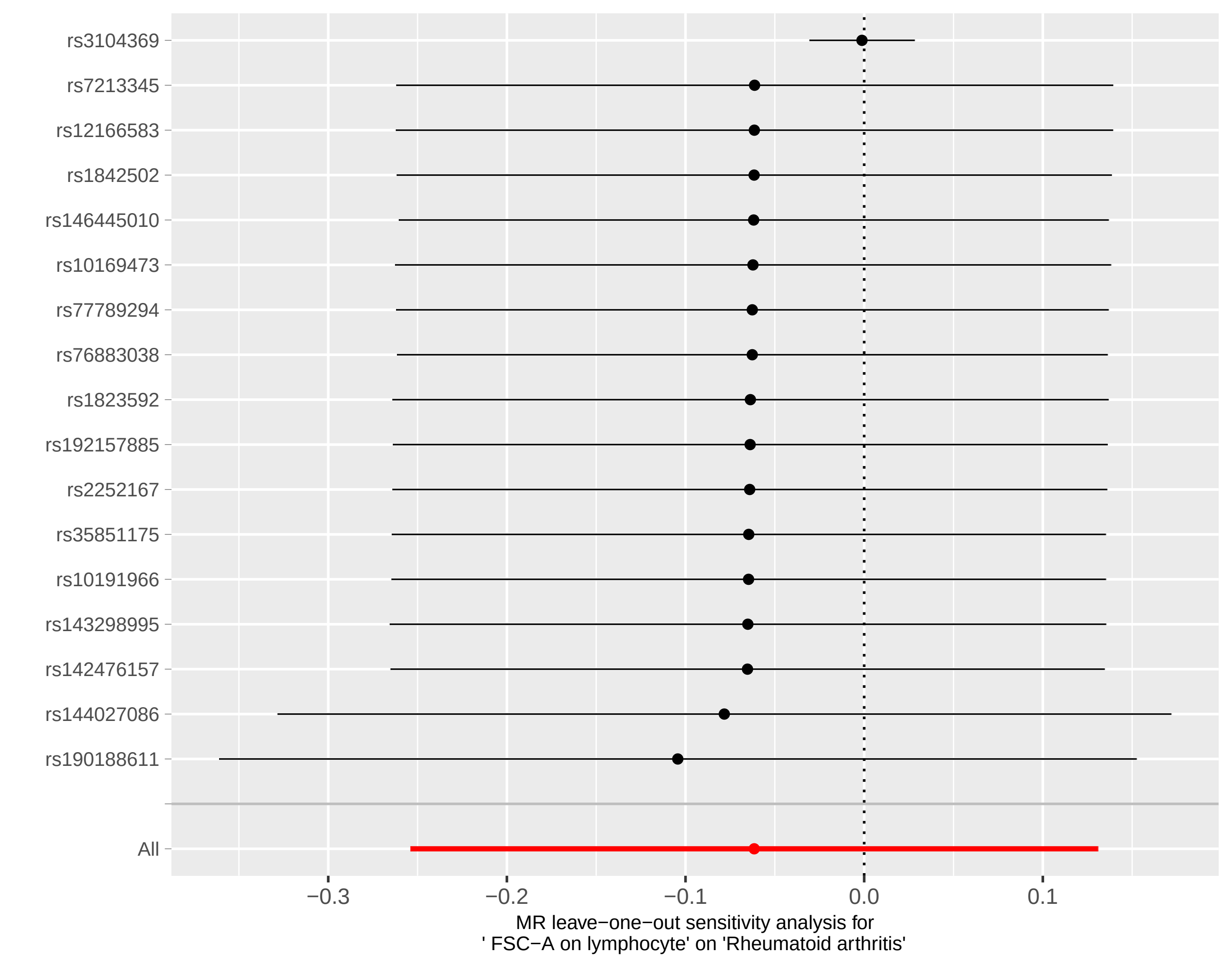
rs3104369
rs7213345
rs12166583
rs1842502
rs146445010
rs10169473
rs77789294
rs76883038
rs1823592
rs192157885
rs2252167
rs35851175
rs10191966
rs143298995
rs142476157
rs144027086
rs190188611
All
−0.3
−0.2
−0.1
0.0
0.1
MR leave−one−out sensitivity analysis for
' FSC−A on lymphocyte' on 'Rheumatoid arthritis'

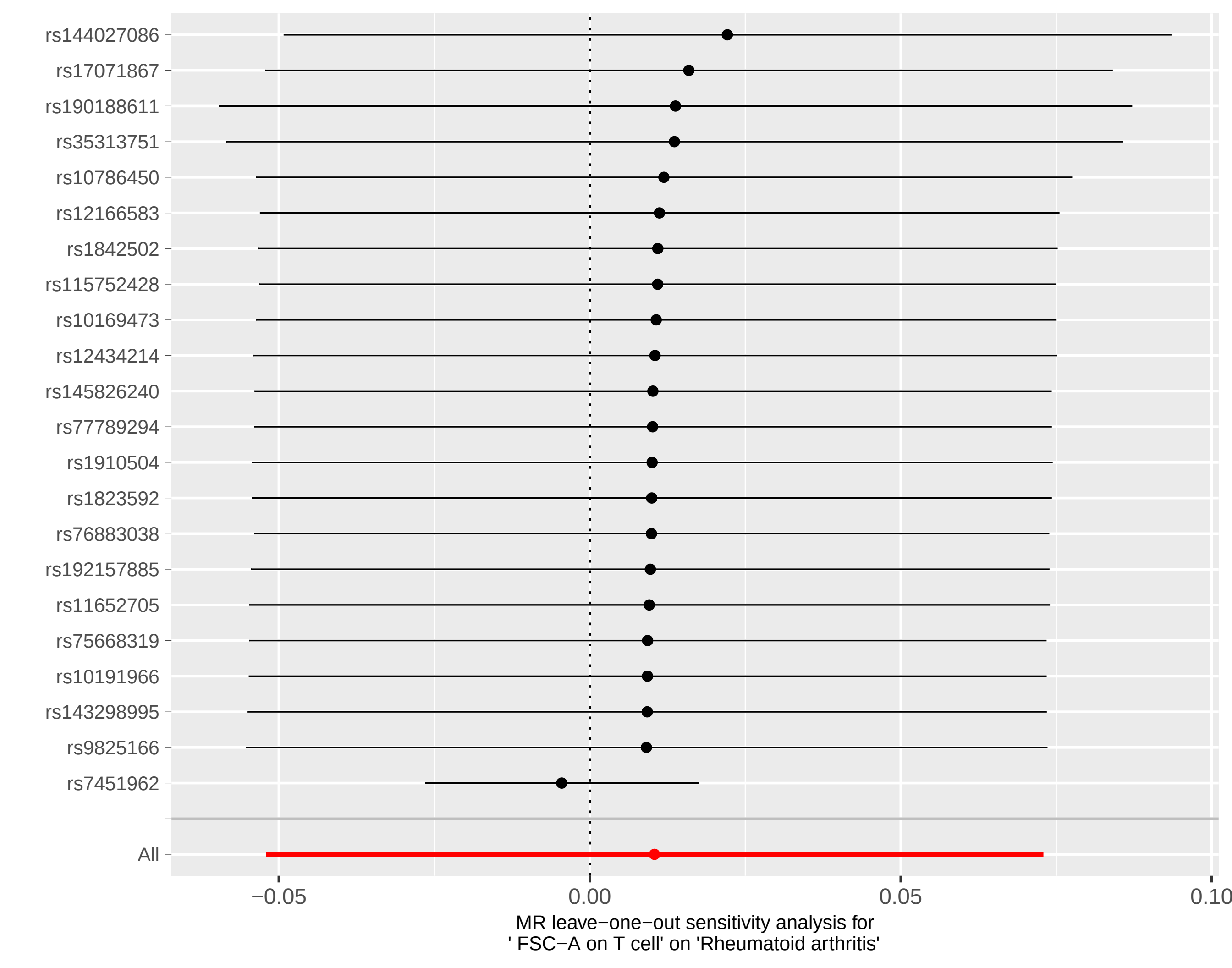
rs144027086
rs17071867
rs190188611
rs35313751
rs10786450
rs12166583
rs1842502
rs115752428
rs10169473
rs12434214
rs145826240
rs77789294
rs1910504
rs1823592
rs76883038
rs192157885
rs11652705
rs75668319
rs10191966
rs143298995
rs9825166
rs7451962
All
−0.05
0.00
0.05
0.10
MR leave−one−out sensitivity analysis for
' FSC−A on T cell' on 'Rheumatoid arthritis'

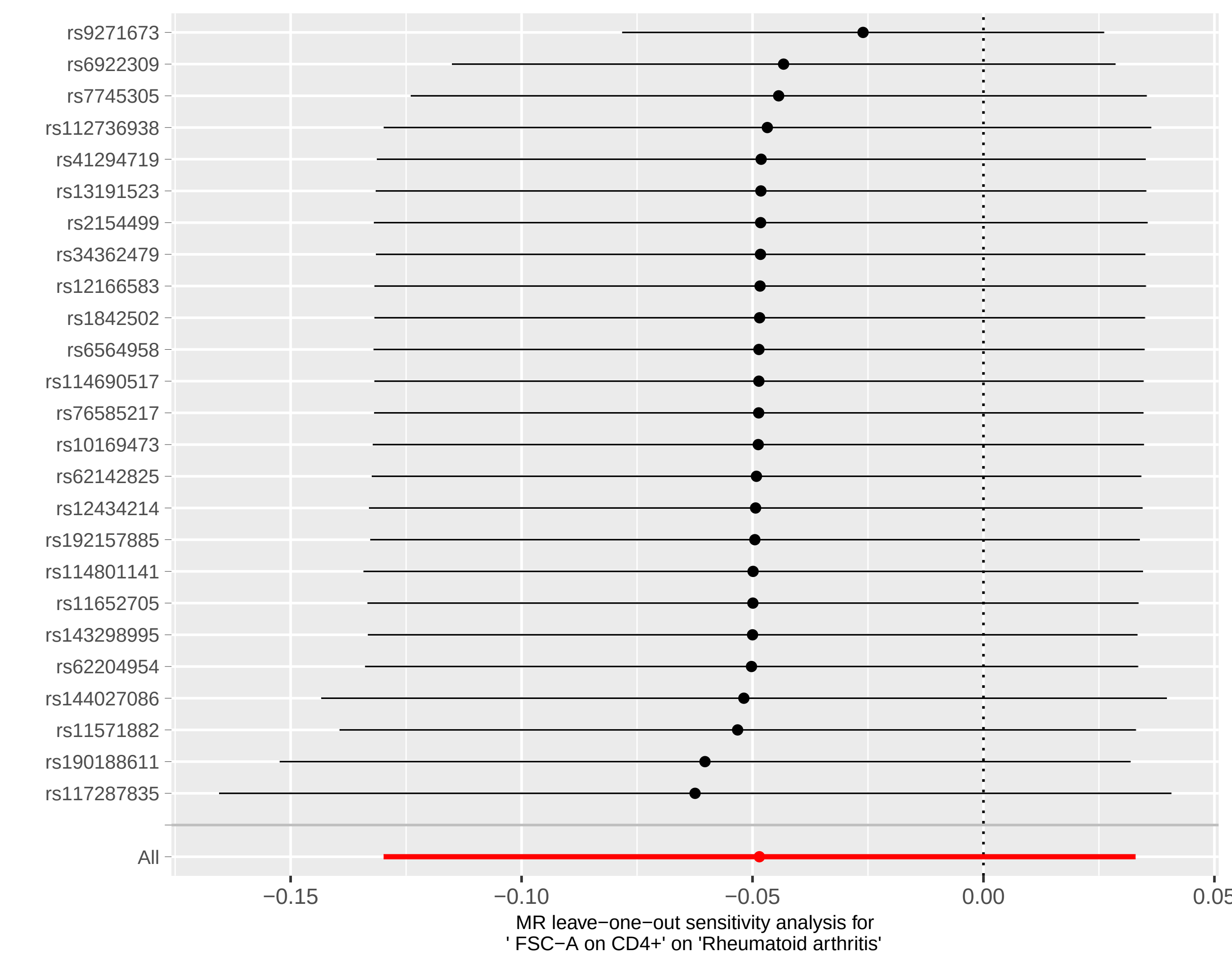
rs9271673
rs6922309
rs7745305
rs112736938
rs41294719
rs13191523
rs2154499
rs34362479
rs12166583
rs1842502
rs6564958
rs114690517
rs76585217
rs10169473
rs62142825
rs12434214
rs192157885
rs114801141
rs11652705
rs143298995
rs62204954
rs144027086
rs11571882
rs190188611
rs117287835
All
−0.15
−0.10
−0.05
0.00
0.05
MR leave−one−out sensitivity analysis for
' FSC−A on CD4+' on 'Rheumatoid arthritis'

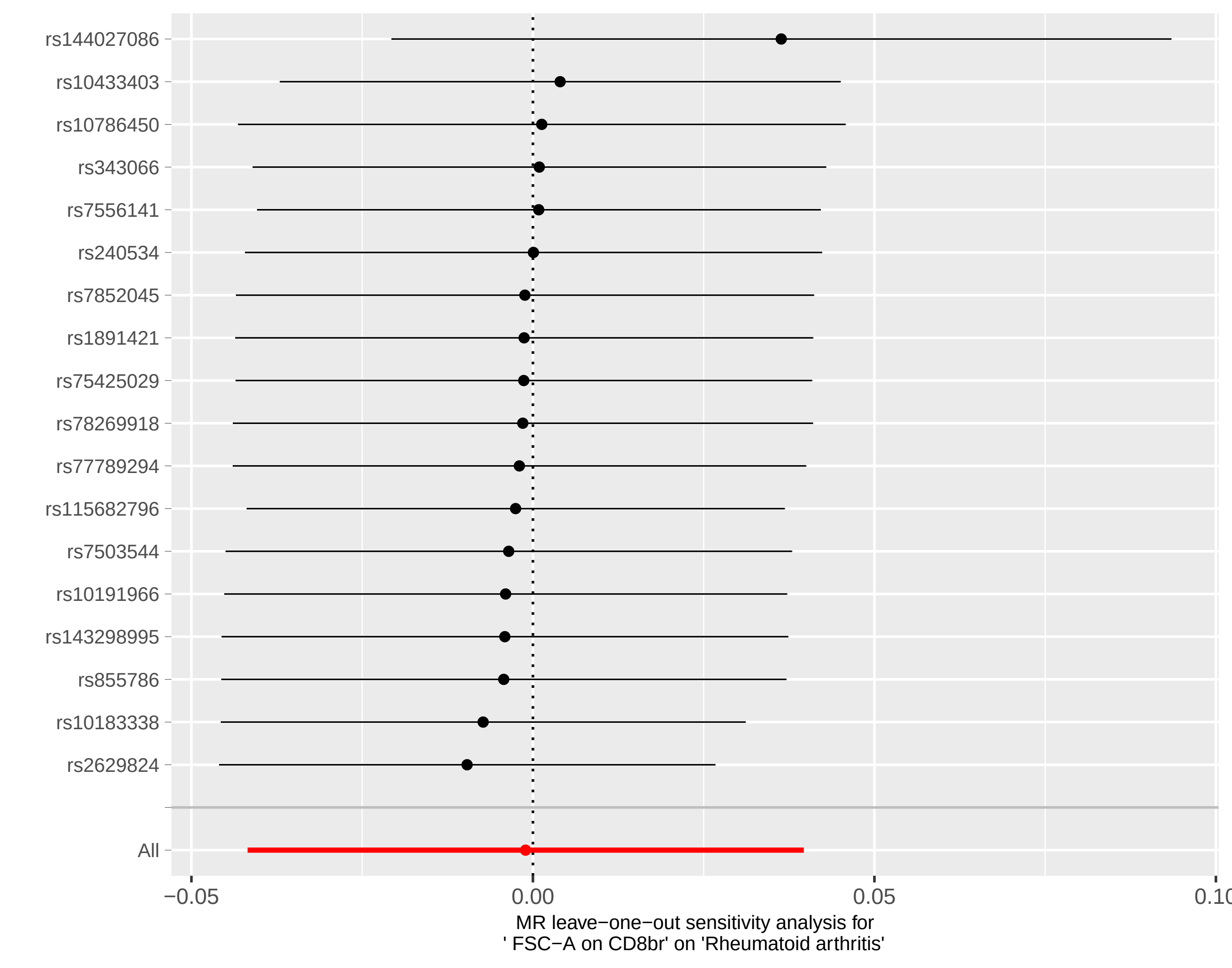
rs144027086
rs10433403
rs10786450
rs343066
rs7556141
rs240534
rs7852045
rs1891421
rs75425029
rs78269918
rs77789294
rs115682796
rs7503544
rs10191966
rs143298995
rs855786
rs10183338
rs2629824
All
−0.05
0.00
0.05
0.10
MR leave−one−out sensitivity analysis for
' FSC−A on CD8br' on 'Rheumatoid arthritis'

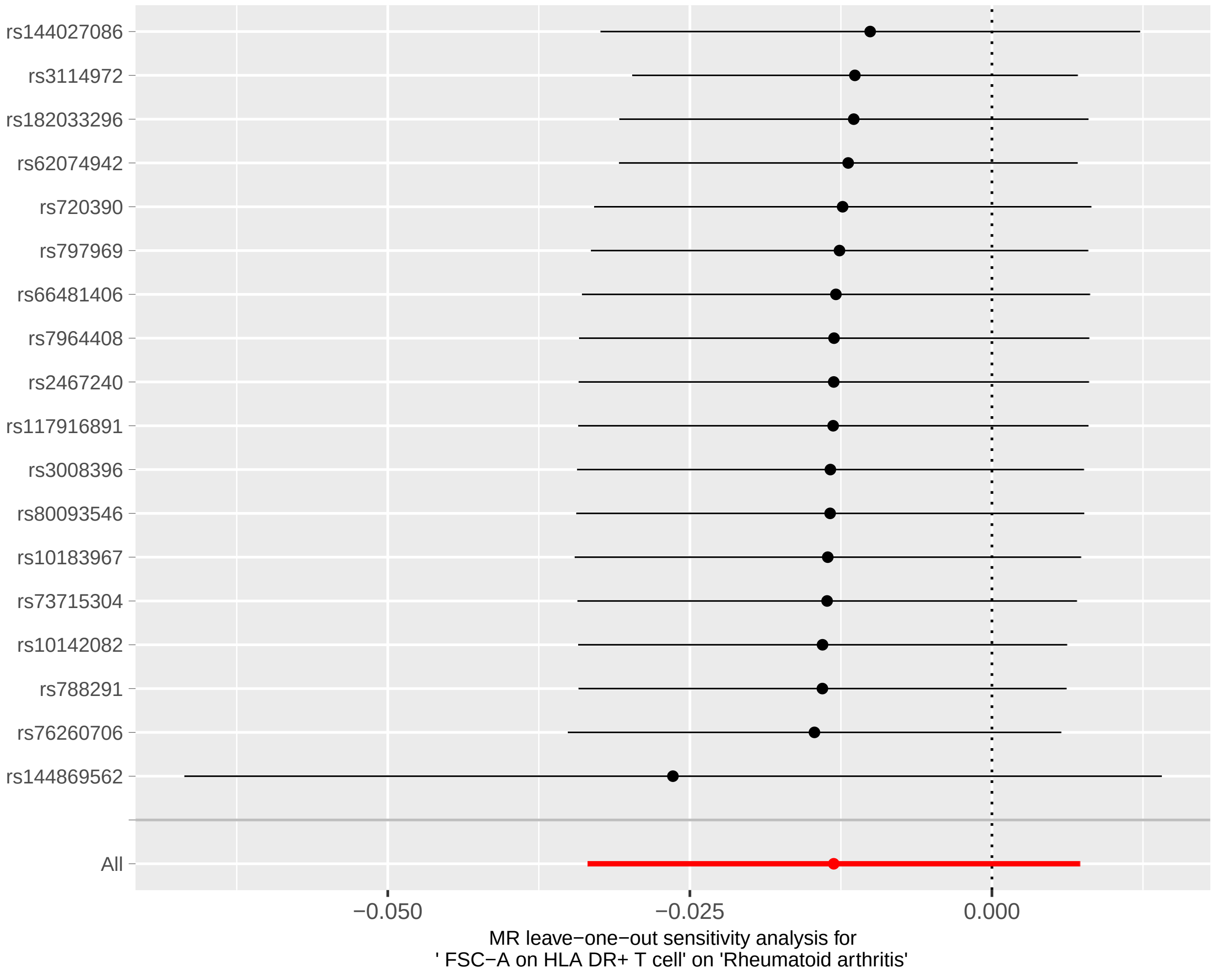
rs144027086
rs3114972
rs182033296
rs62074942
rs720390
rs797969
rs66481406
rs7964408
rs2467240
rs117916891
rs3008396
rs80093546
rs10183967
rs73715304
rs10142082
rs788291
rs76260706
rs144869562
All
−0.050
−0.025
0.000
MR leave−one−out sensitivity analysis for
' FSC−A on HLA DR+ T cell' on 'Rheumatoid arthritis'

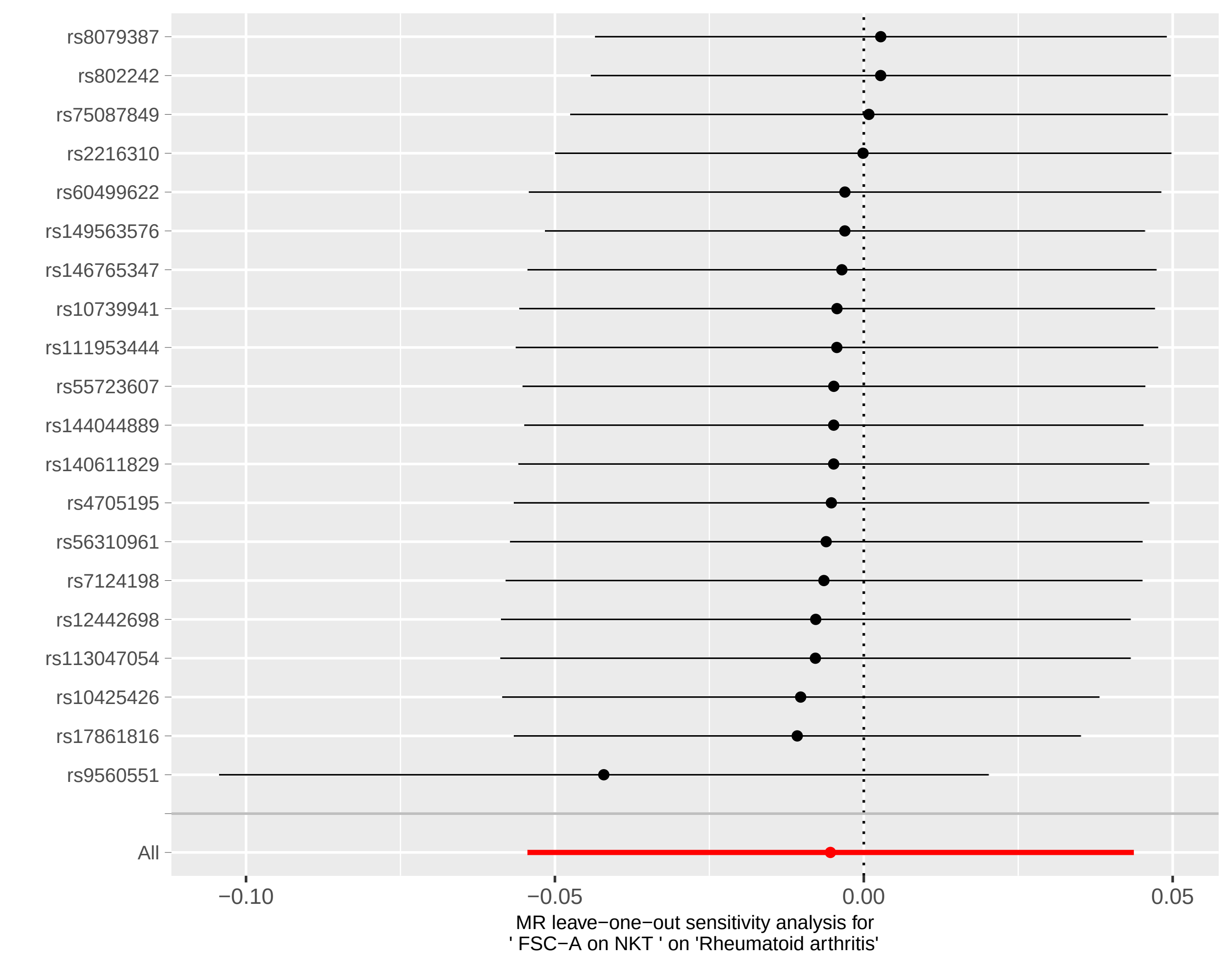
rs8079387
rs802242
rs75087849
rs2216310
rs60499622
rs149563576
rs146765347
rs10739941
rs111953444
rs55723607
rs144044889
rs140611829
rs4705195
rs56310961
rs7124198
rs12442698
rs113047054
rs10425426
rs17861816
rs9560551
All
−0.10
−0.05
0.00
0.05
MR leave−one−out sensitivity analysis for
' FSC−A on NKT ' on 'Rheumatoid arthritis'

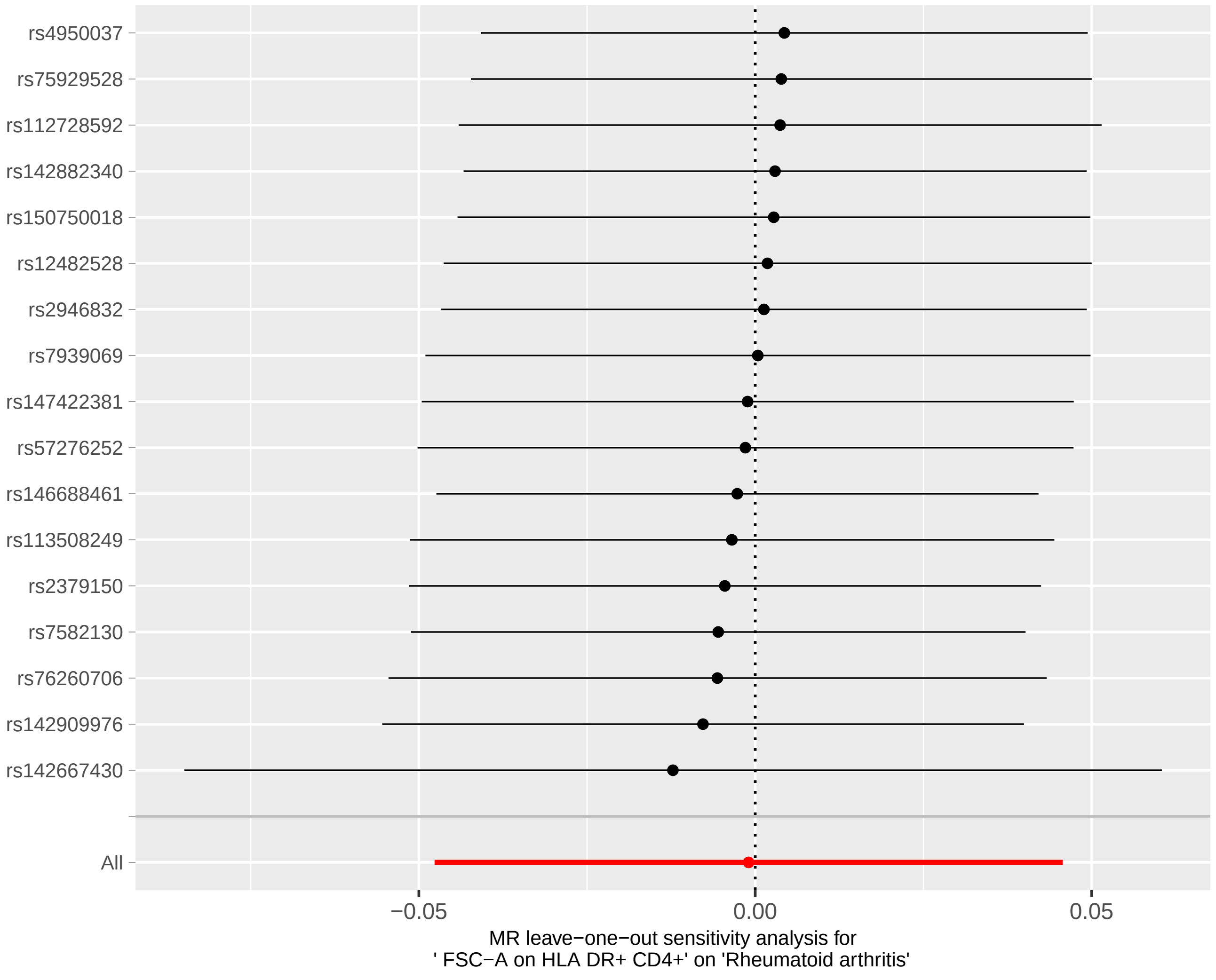

rs4950037
rs75929528
rs112728592
rs142882340
rs150750018
rs12482528
rs2946832
rs7939069
rs147422381
rs57276252
rs146688461
rs113508249
rs2379150
rs7582130
rs76260706
rs142909976
rs142667430
All
−0.05
0.00
0.05
MR leave−one−out sensitivity analysis for
' FSC−A on HLA DR+ CD4+' on 'Rheumatoid arthritis'

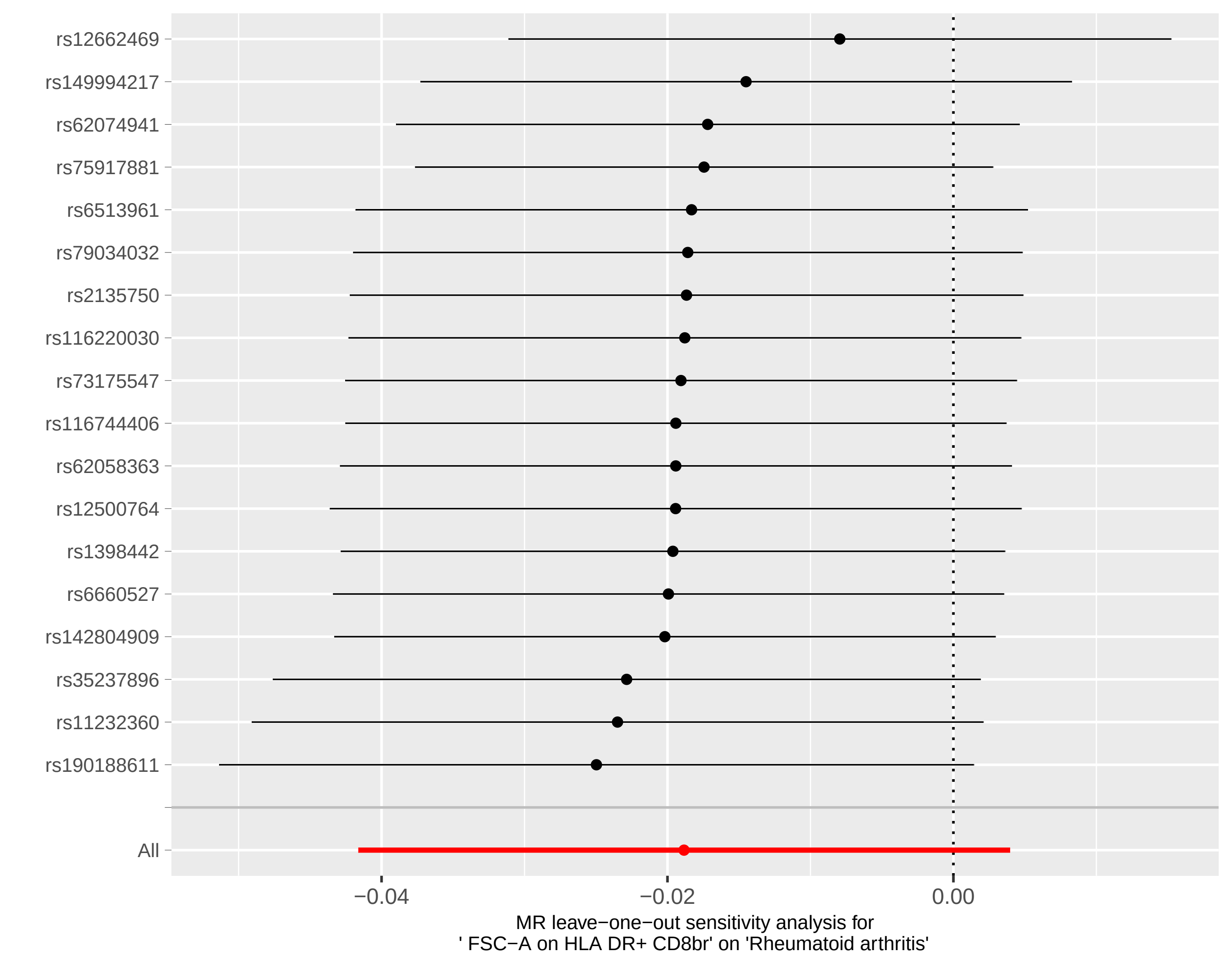

rs12662469
rs149994217
rs62074941
rs75917881
rs6513961
rs79034032
rs2135750
rs116220030
rs73175547
rs116744406
rs62058363
rs12500764
rs1398442
rs6660527
rs142804909
rs35237896
rs11232360
rs190188611
All
−0.04
−0.02
0.00
MR leave−one−out sensitivity analysis for
' FSC−A on HLA DR+ CD8br' on 'Rheumatoid arthritis'

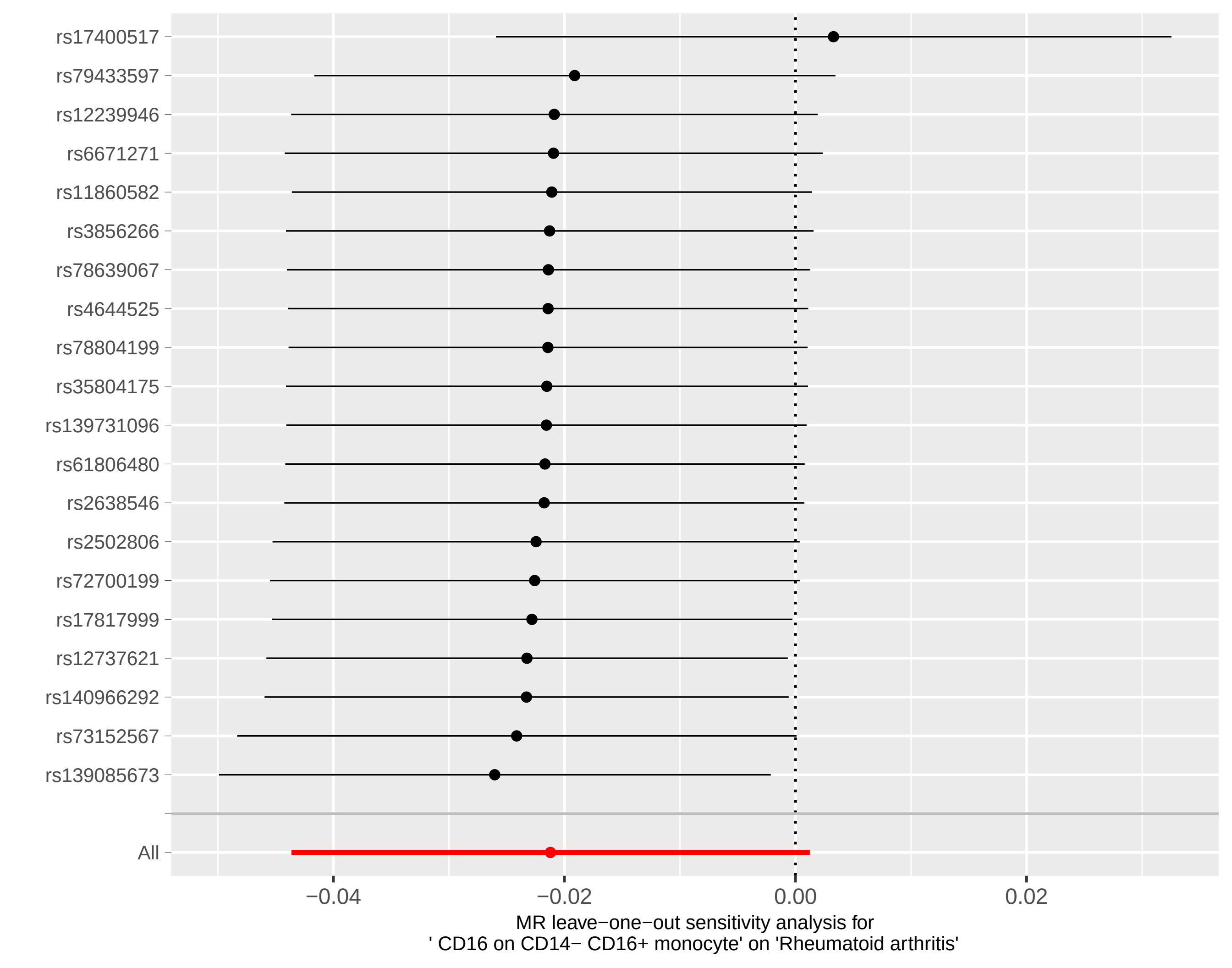
rs17400517
rs79433597
rs12239946
rs6671271
rs11860582
rs3856266
rs78639067
rs4644525
rs78804199
rs35804175
rs139731096
rs61806480
rs2638546
rs2502806
rs72700199
rs17817999
rs12737621
rs140966292
rs73152567
rs139085673
All
−0.04
−0.02
0.00
0.02
MR leave−one−out sensitivity analysis for
' CD16 on CD14− CD16+ monocyte' on 'Rheumatoid arthritis'

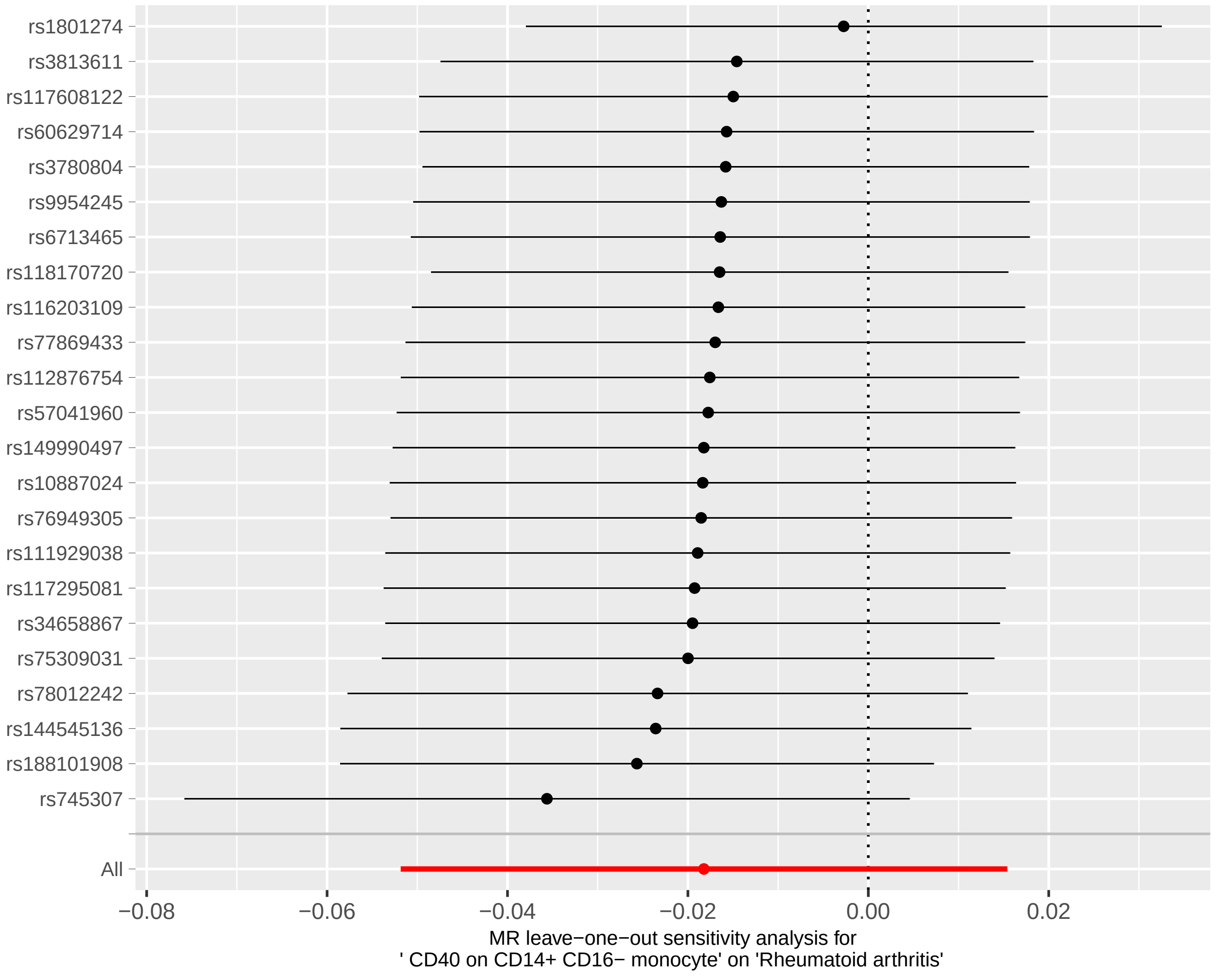
rs1801274
rs3813611
rs117608122
rs60629714
rs3780804
rs9954245
rs6713465
rs118170720
rs116203109
rs77869433
rs112876754
rs57041960
rs149990497
rs10887024
rs76949305
rs111929038
rs117295081
rs34658867
rs75309031
rs78012242
rs144545136
rs188101908
rs745307
All
−0.08
−0.06
−0.04
−0.02
0.00
0.02
MR leave−one−out sensitivity analysis for
' CD40 on CD14+ CD16− monocyte' on 'Rheumatoid arthritis'

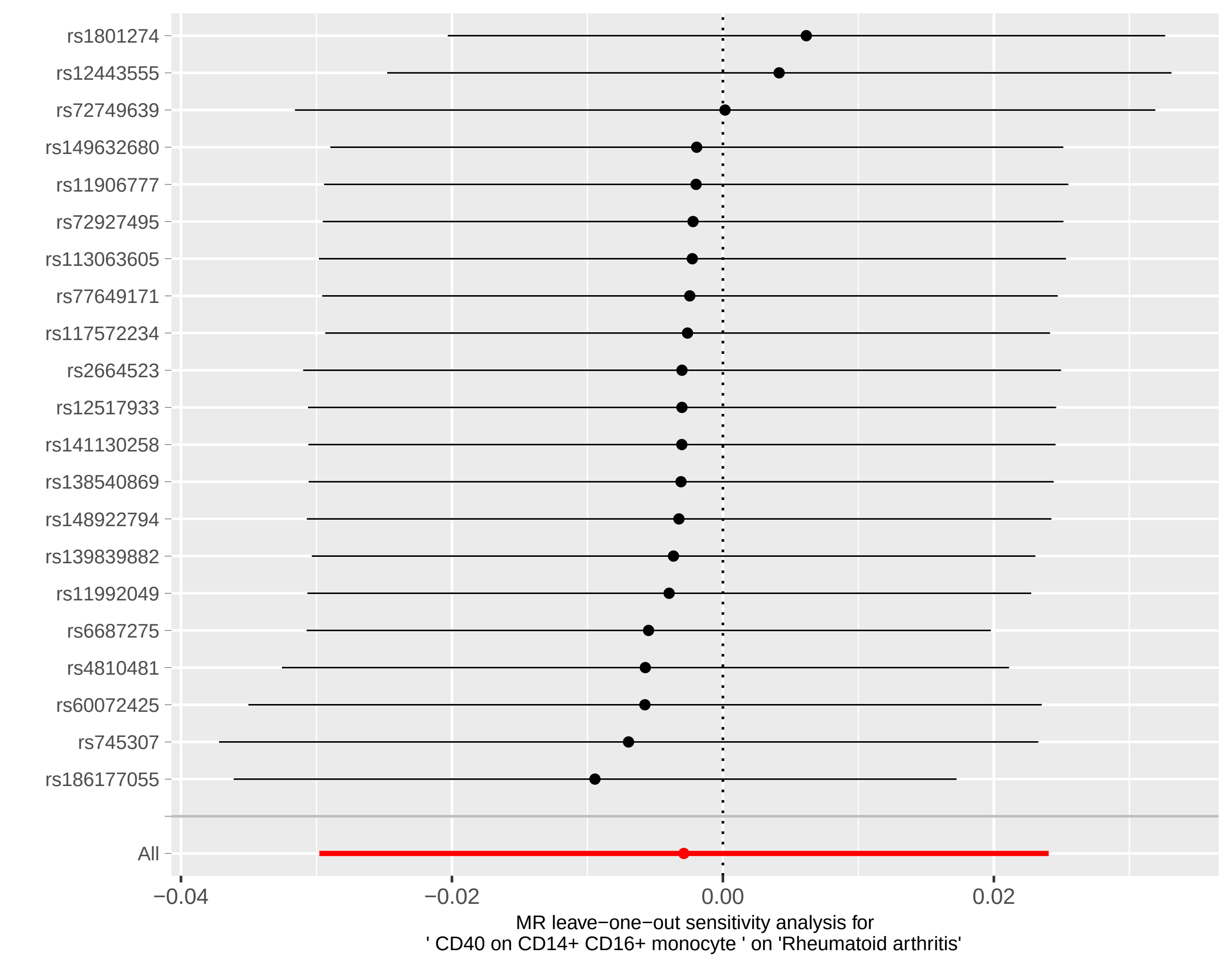

rs1801274
rs12443555
rs72749639
rs149632680
rs11906777
rs72927495
rs113063605
rs77649171
rs117572234
rs2664523
rs12517933
rs141130258
rs138540869
rs148922794
rs139839882
rs11992049
rs6687275
rs4810481
rs60072425
rs745307
rs186177055
All
−0.04
−0.02
0.00
0.02
MR leave−one−out sensitivity analysis for
' CD40 on CD14+ CD16+ monocyte ' on 'Rheumatoid arthritis'

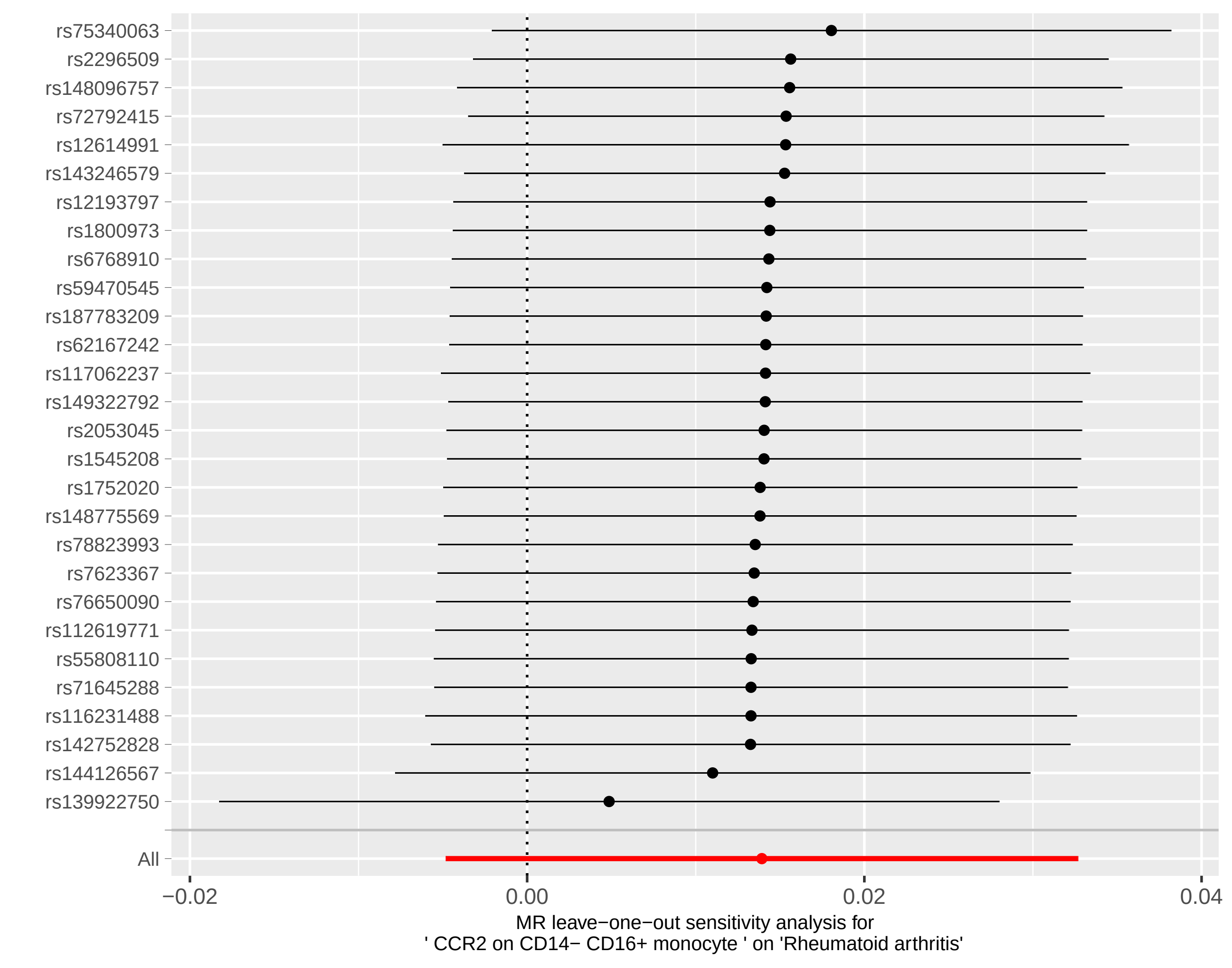
rs75340063
rs2296509
rs148096757
rs72792415
rs12614991
rs143246579
rs12193797
rs1800973
rs6768910
rs59470545
rs187783209
rs62167242
rs117062237
rs149322792
rs2053045
rs1545208
rs1752020
rs148775569
rs78823993
rs7623367
rs76650090
rs112619771
rs55808110
rs71645288
rs116231488
rs142752828
rs144126567
rs139922750
All
−0.02
0.00
0.02
0.04
MR leave−one−out sensitivity analysis for
' CCR2 on CD14− CD16+ monocyte ' on 'Rheumatoid arthritis'

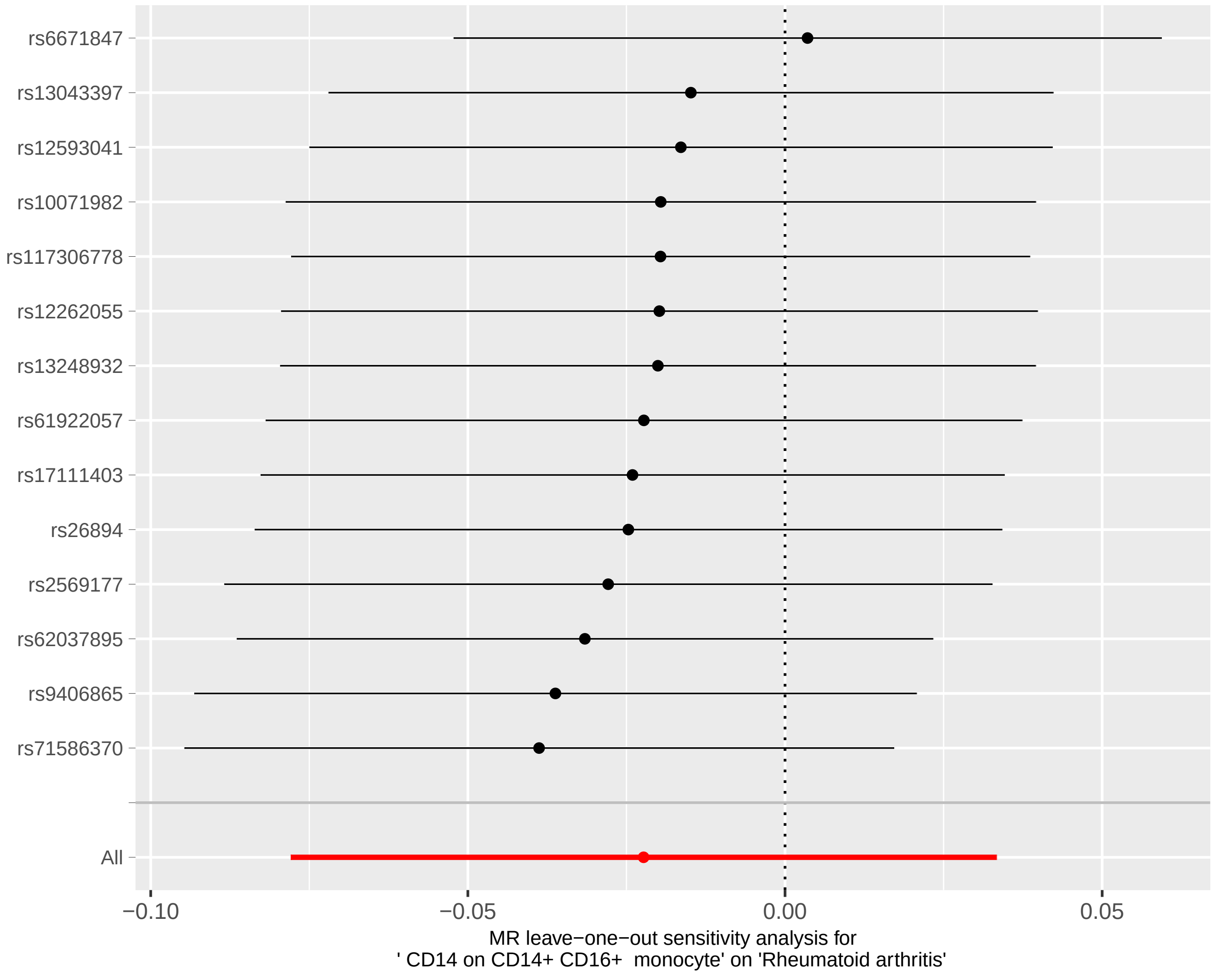

rs6671847
rs13043397
rs12593041
rs10071982
rs117306778
rs12262055
rs13248932
rs61922057
rs17111403
rs26894
rs2569177
rs62037895
rs9406865
rs71586370
All
−0.10
−0.05
0.00
0.05
MR leave−one−out sensitivity analysis for
' CD14 on CD14+ CD16+ monocyte' on 'Rheumatoid arthritis'

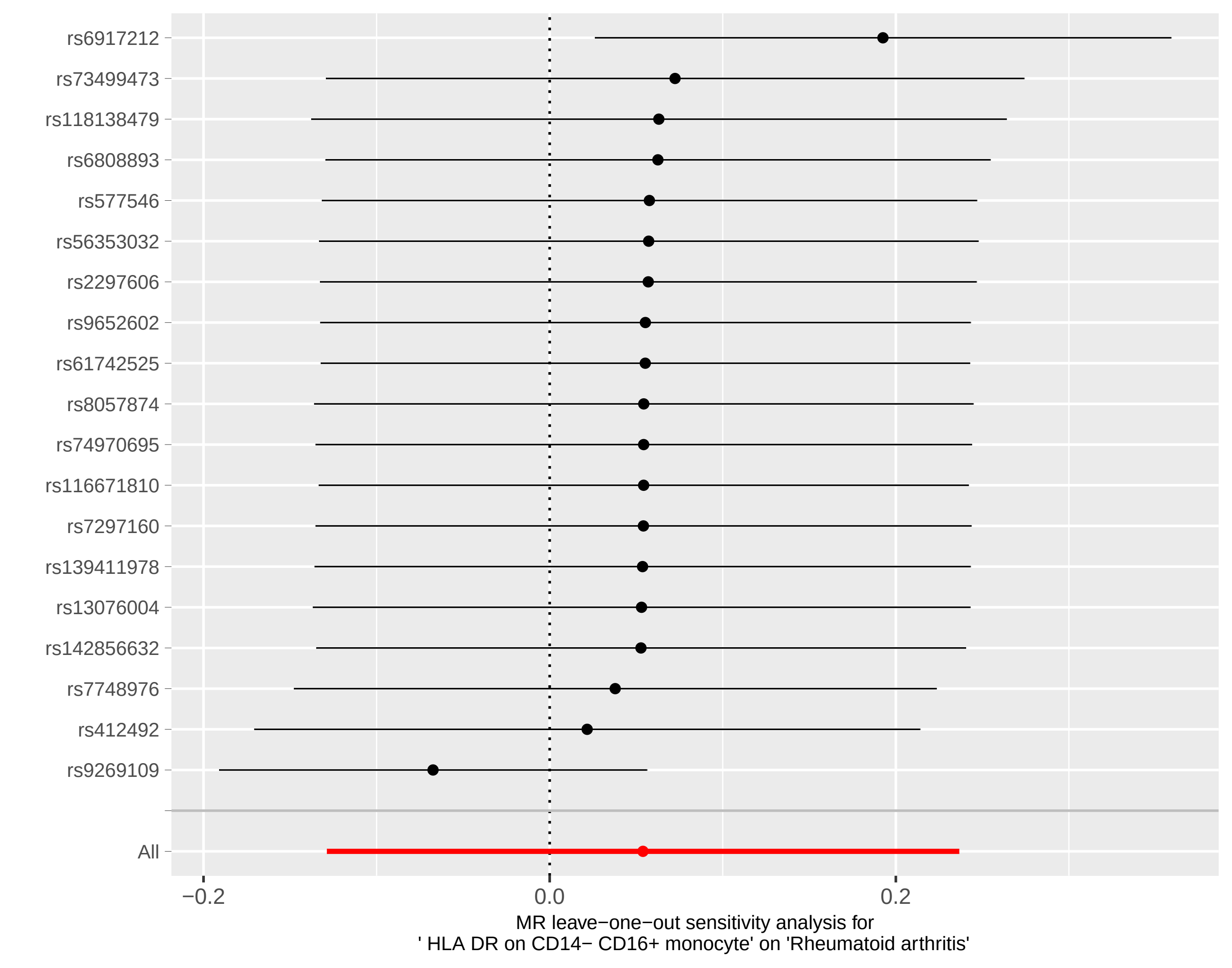
rs6917212
rs73499473
rs118138479
rs6808893
rs577546
rs56353032
rs2297606
rs9652602
rs61742525
rs8057874
rs74970695
rs116671810
rs7297160
rs139411978
rs13076004
rs142856632
rs7748976
rs412492
rs9269109
All
−0.2
0.0
0.2
MR leave−one−out sensitivity analysis for
' HLA DR on CD14− CD16+ monocyte' on 'Rheumatoid arthritis'

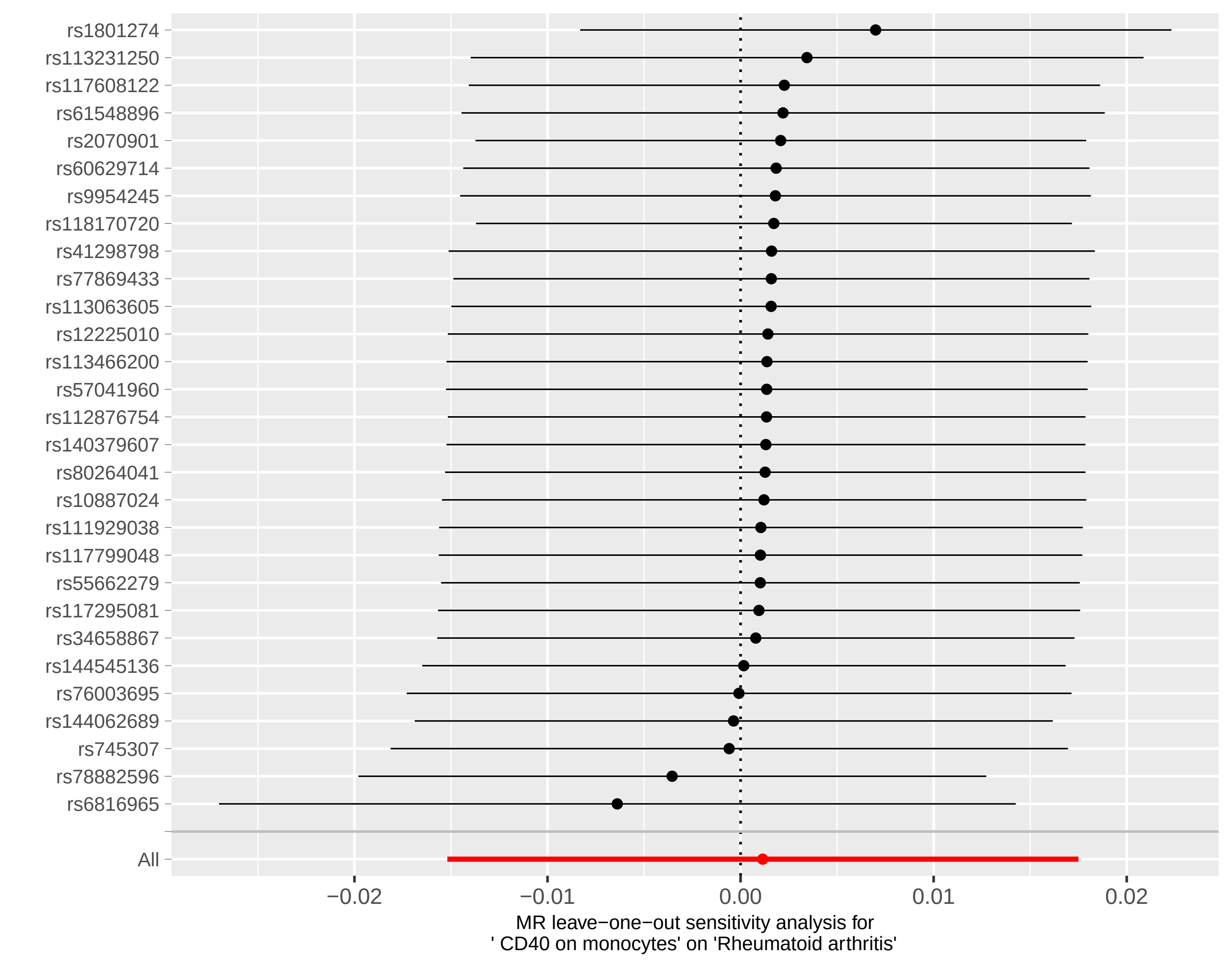
rs1801274
rs113231250
rs117608122
rs61548896
rs2070901
rs60629714
rs9954245
rs118170720
rs41298798
rs77869433
rs113063605
rs12225010
rs113466200
rs57041960
rs112876754
rs140379607
rs80264041
rs10887024
rs111929038
rs117799048
rs55662279
rs117295081
rs34658867
rs144545136
rs76003695
rs144062689
rs745307
rs78882596
rs6816965
All
−0.02
−0.01
0.00
0.01
0.02
MR leave−one−out sensitivity analysis for
' CD40 on monocytes' on 'Rheumatoid arthritis'

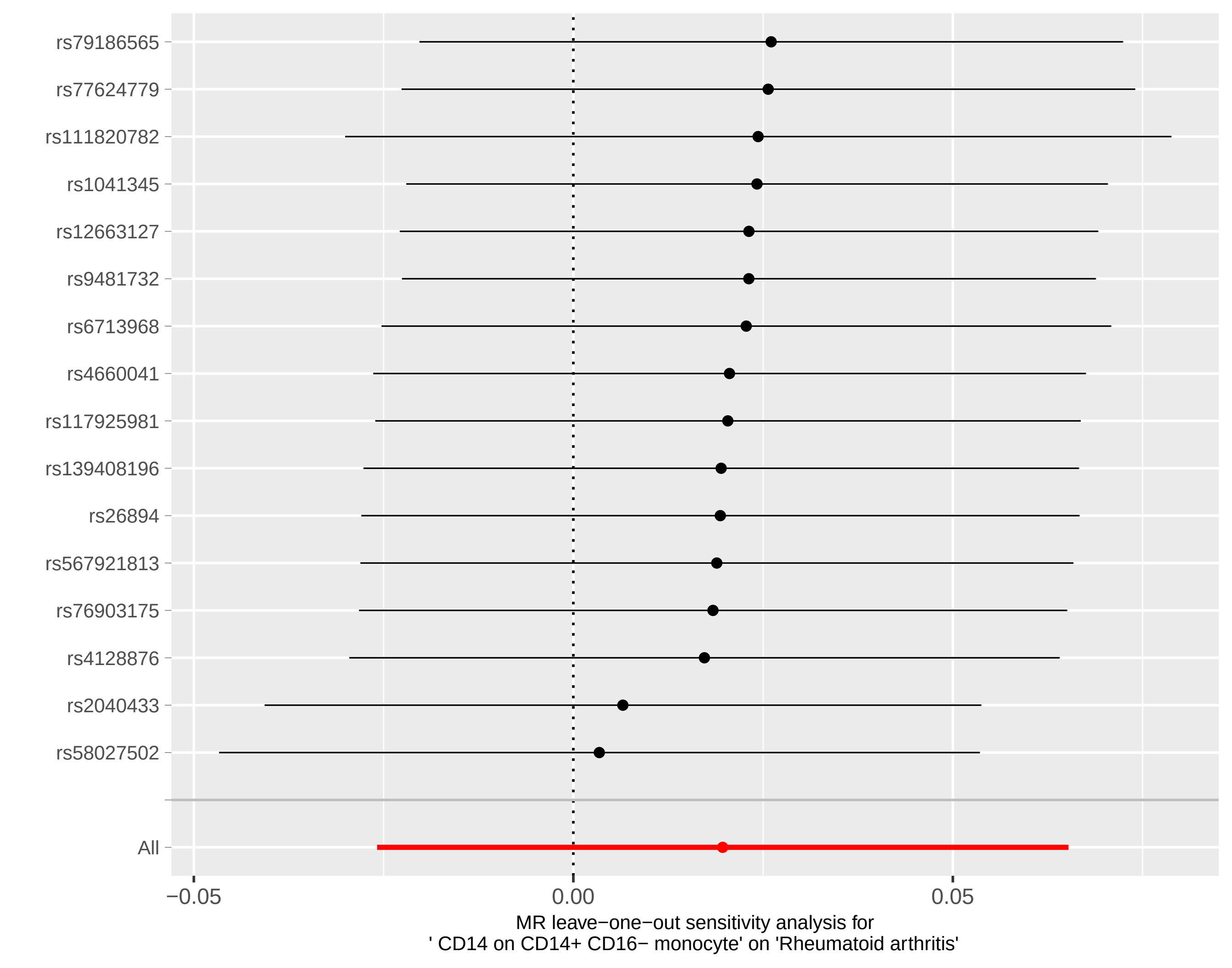

rs79186565
rs77624779
rs111820782
rs1041345
rs12663127
rs9481732
rs6713968
rs4660041
rs117925981
rs139408196
rs26894
rs567921813
rs76903175
rs4128876
rs2040433
rs58027502
All
−0.05
0.00
0.05
MR leave−one−out sensitivity analysis for
' CD14 on CD14+ CD16− monocyte' on 'Rheumatoid arthritis'

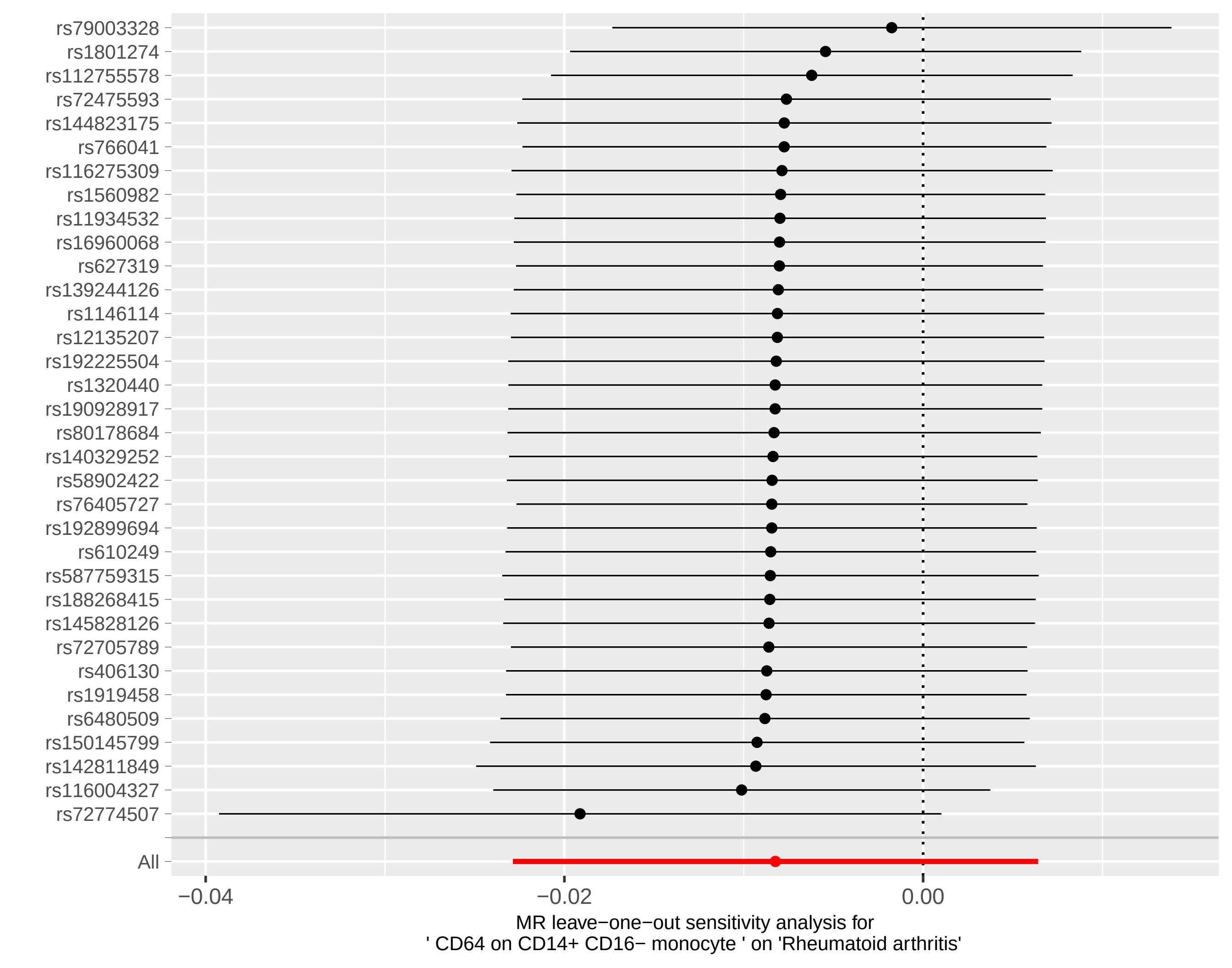
rs79003328
rs1801274
rs112755578
rs72475593
rs144823175
rs766041
rs116275309
rs1560982
rs11934532
rs16960068
rs627319
rs139244126
rs1146114
rs12135207
rs192225504
rs1320440
rs190928917
rs80178684
rs140329252
rs58902422
rs76405727
rs192899694
rs610249
rs587759315
rs188268415
rs145828126
rs72705789
rs406130
rs1919458
rs6480509
rs150145799
rs142811849
rs116004327
rs72774507
All
−0.04
−0.02
0.00
MR leave−one−out sensitivity analysis for
' CD64 on CD14+ CD16− monocyte ' on 'Rheumatoid arthritis'

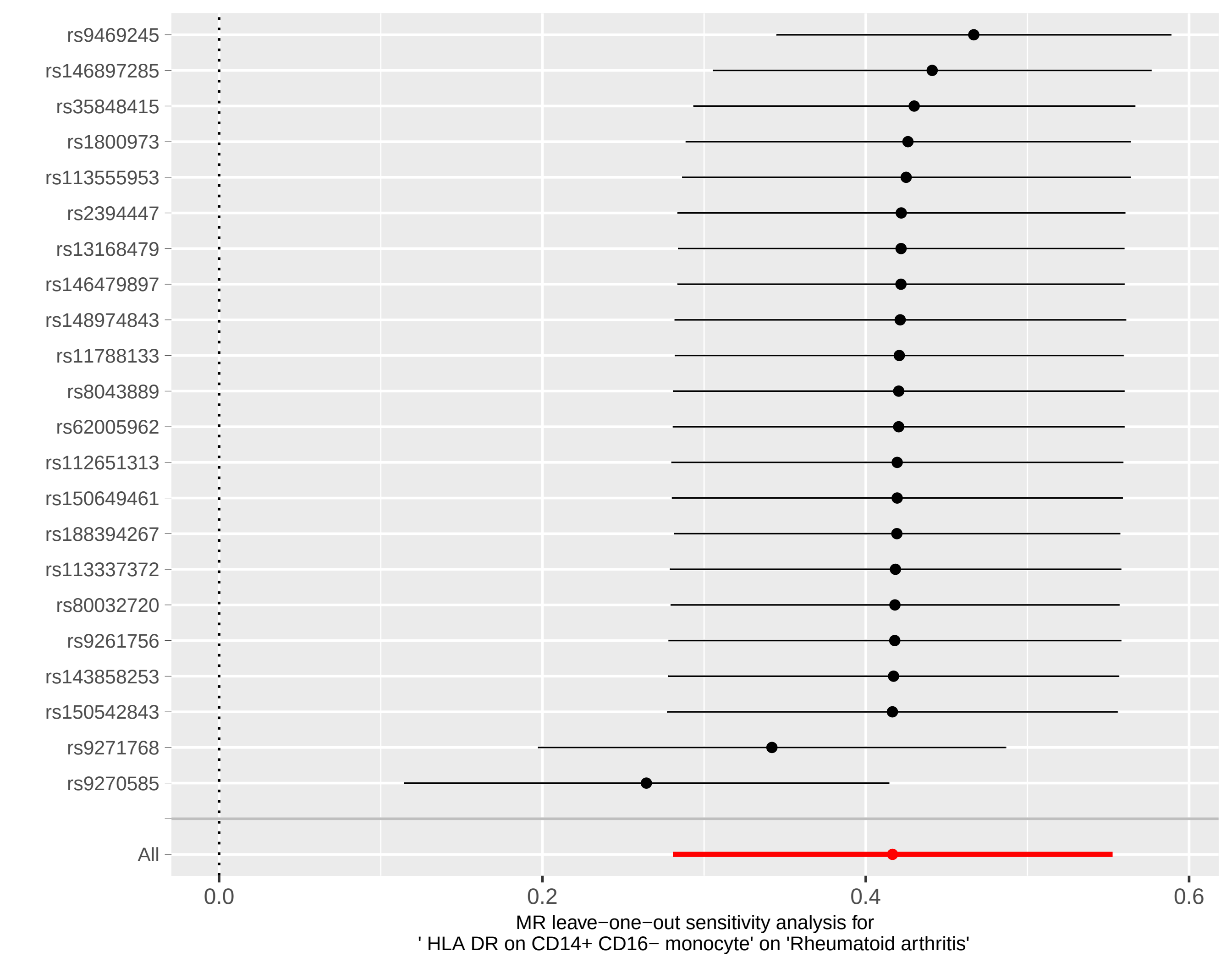
rs9469245
rs146897285
rs35848415
rs1800973
rs113555953
rs2394447
rs13168479
rs146479897
rs148974843
rs11788133
rs8043889
rs62005962
rs112651313
rs150649461
rs188394267
rs113337372
rs80032720
rs9261756
rs143858253
rs150542843
rs9271768
rs9270585
All
0.0
0.2
0.4
0.6
MR leave−one−out sensitivity analysis for
' HLA DR on CD14+ CD16− monocyte' on 'Rheumatoid arthritis'

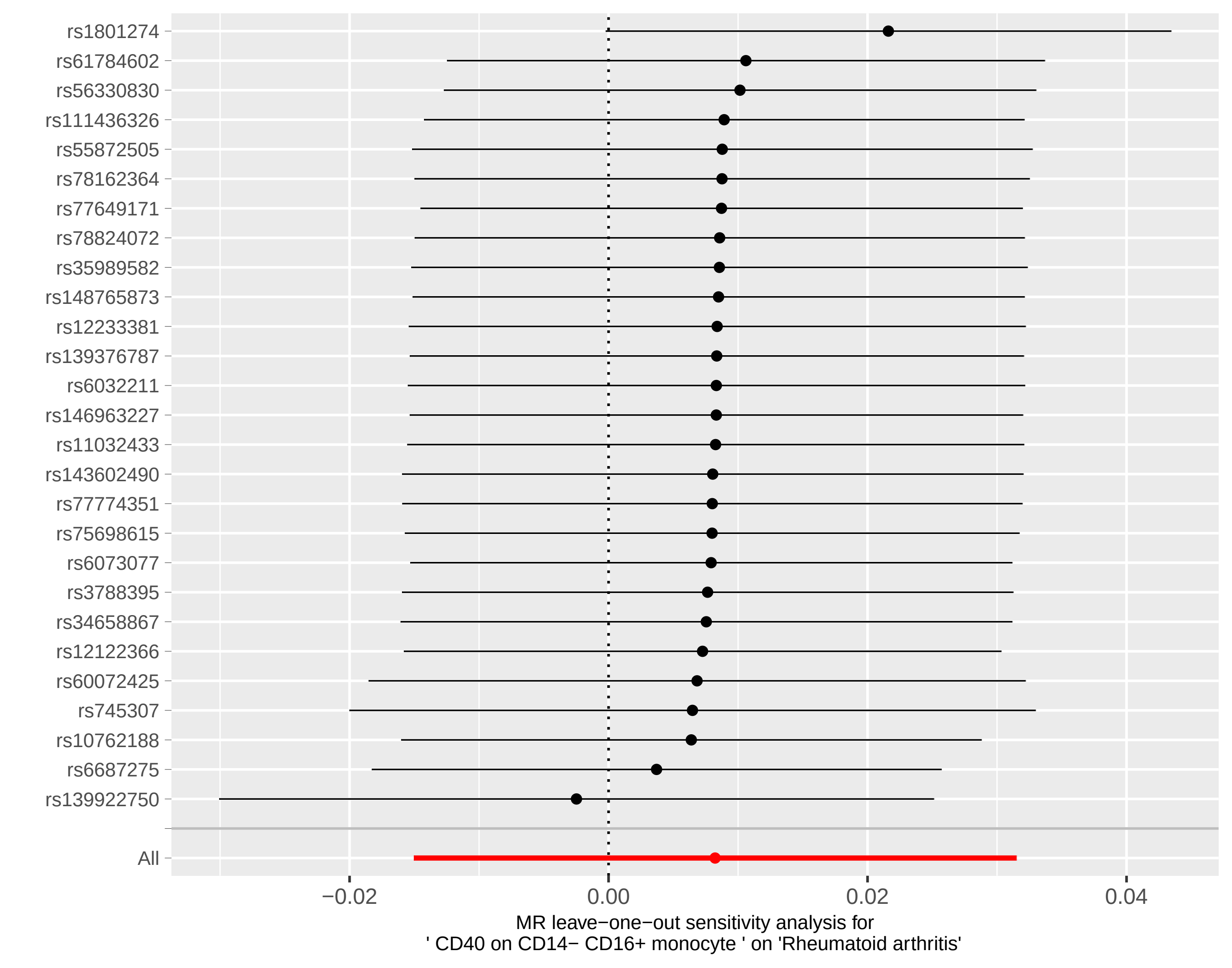
rs1801274
rs61784602
rs56330830
rs111436326
rs55872505
rs78162364
rs77649171
rs78824072
rs35989582
rs148765873
rs12233381
rs139376787
rs6032211
rs146963227
rs11032433
rs143602490
rs77774351
rs75698615
rs6073077
rs3788395
rs34658867
rs12122366
rs60072425
rs745307
rs10762188
rs6687275
rs139922750
All
−0.02
0.00
0.02
0.04
MR leave−one−out sensitivity analysis for
' CD40 on CD14− CD16+ monocyte ' on 'Rheumatoid arthritis'

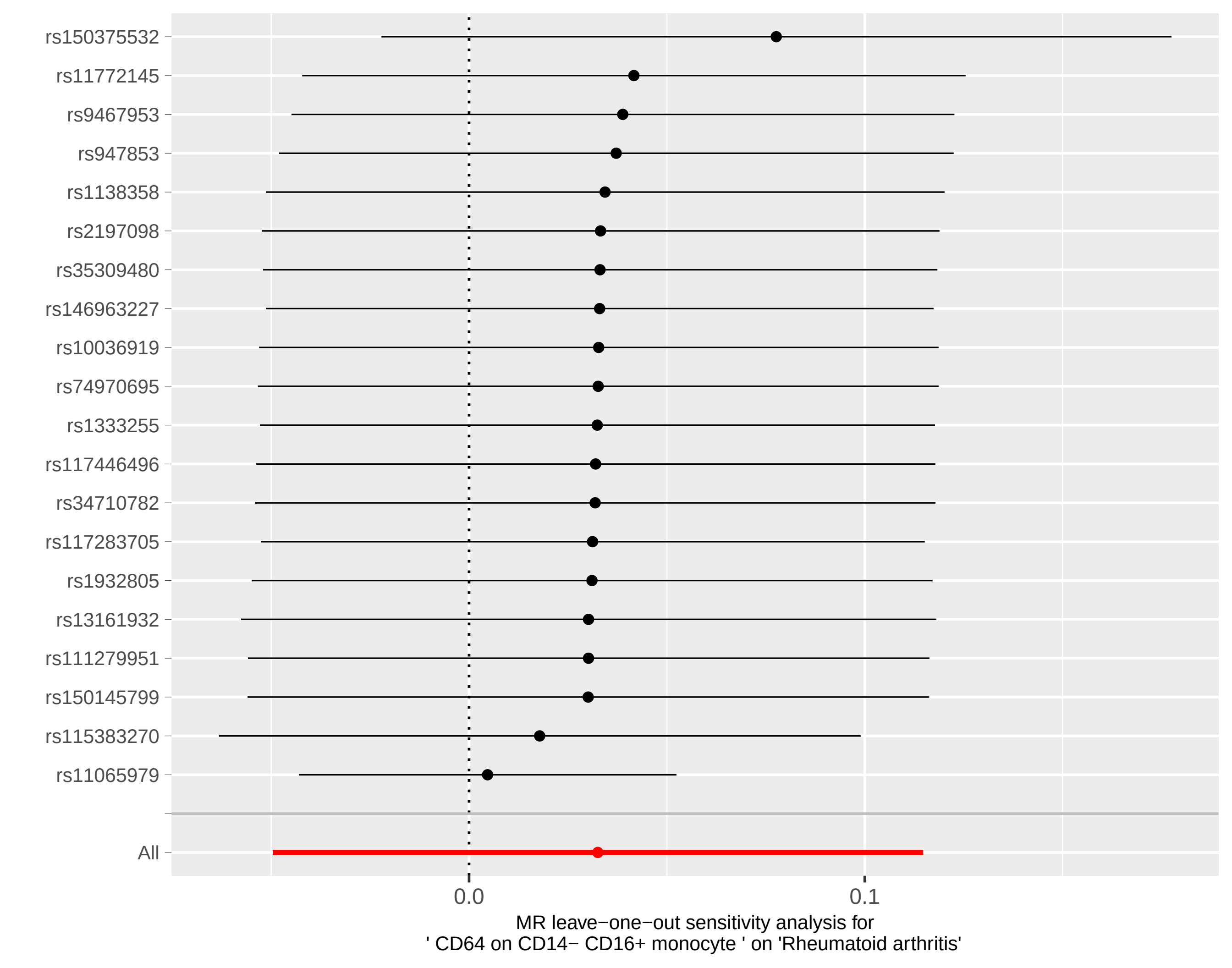
rs150375532
rs11772145
rs9467953
rs947853
rs1138358
rs2197098
rs35309480
rs146963227
rs10036919
rs74970695
rs1333255
rs117446496
rs34710782
rs117283705
rs1932805
rs13161932
rs111279951
rs150145799
rs115383270
rs11065979
All
0.0
0.1
MR leave−one−out sensitivity analysis for
' CD64 on CD14− CD16+ monocyte ' on 'Rheumatoid arthritis'

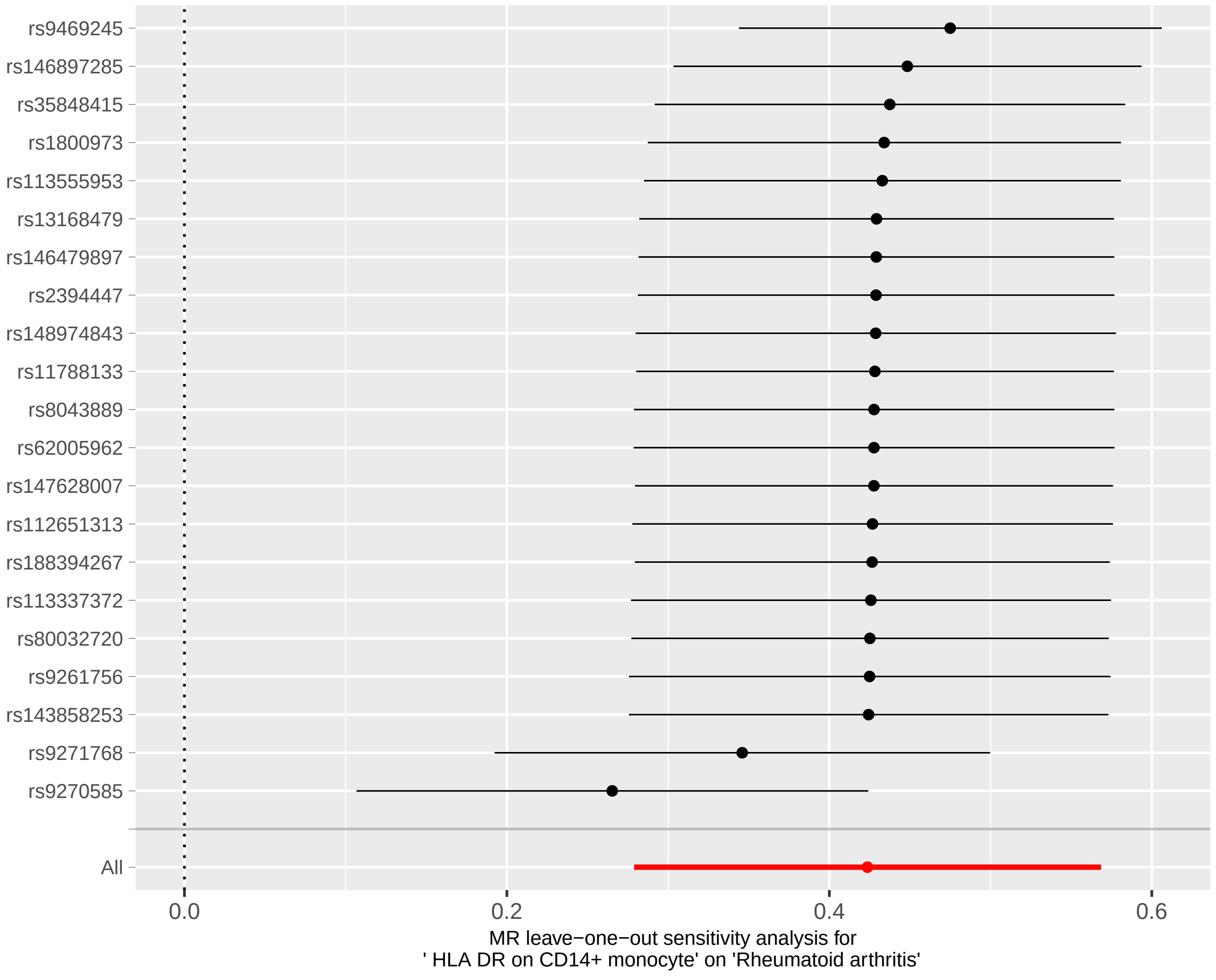
rs9469245
rs146897285
rs35848415
rs1800973
rs113555953
rs13168479
rs146479897
rs2394447
rs148974843
rs11788133
rs8043889
rs62005962
rs147628007
rs112651313
rs188394267
rs113337372
rs80032720
rs9261756
rs143858253
rs9271768
rs9270585
All
0.0
0.2
0.4
0.6
MR leave−one−out sensitivity analysis for
' HLA DR on CD14+ monocyte' on 'Rheumatoid arthritis'

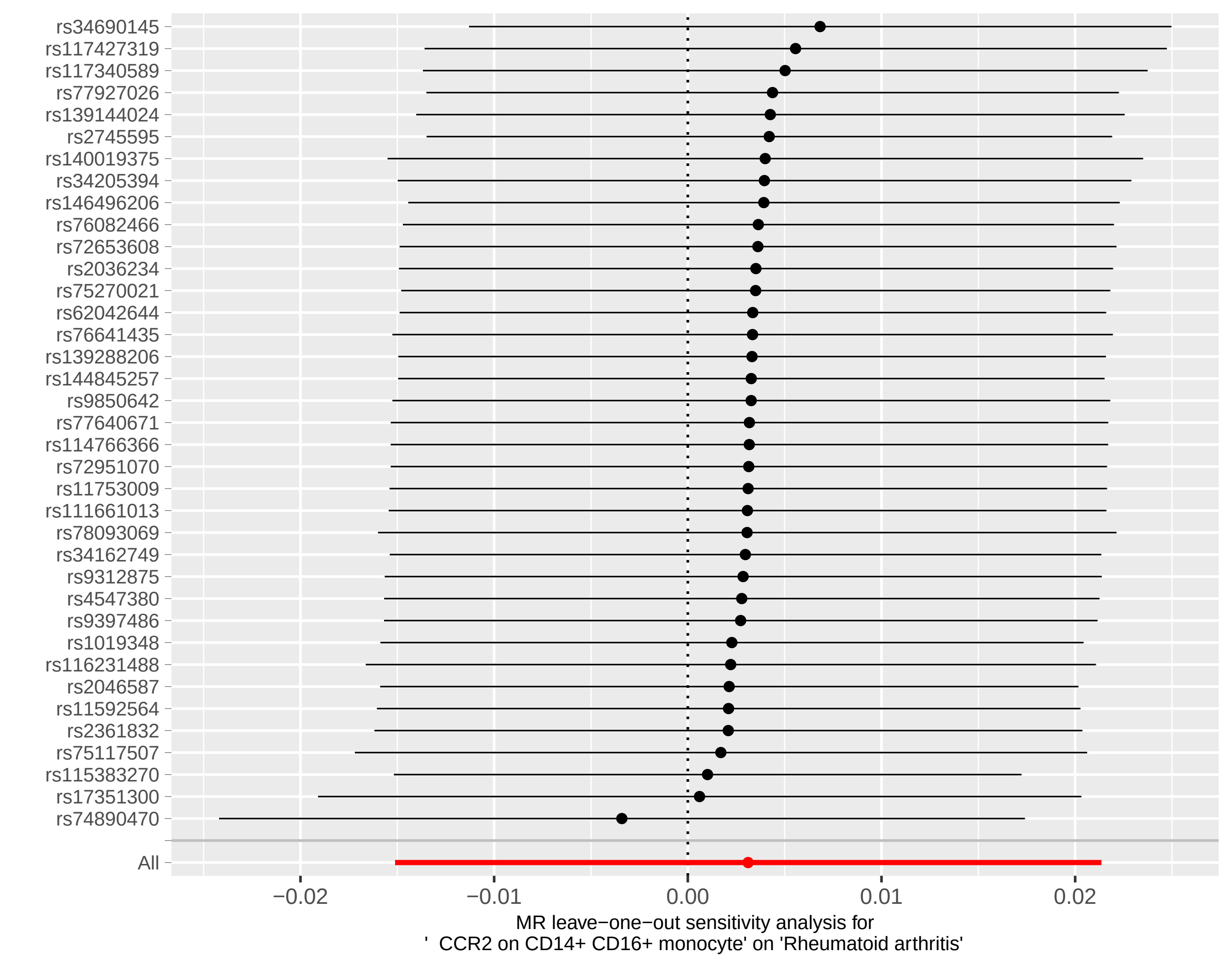
rs34690145
rs117427319
rs117340589
rs77927026
rs139144024
rs2745595
rs140019375
rs34205394
rs146496206
rs76082466
rs72653608
rs2036234
rs75270021
rs62042644
rs76641435
rs139288206
rs144845257
rs9850642
rs77640671
rs114766366
rs72951070
rs11753009
rs111661013
rs78093069
rs34162749
rs9312875
rs4547380
rs9397486
rs1019348
rs116231488
rs2046587
rs11592564
rs2361832
rs75117507
rs115383270
rs17351300
rs74890470
All
−0.02
−0.01
0.00
0.01
0.02
MR leave−one−out sensitivity analysis for
' CCR2 on CD14+ CD16+ monocyte' on 'Rheumatoid arthritis'

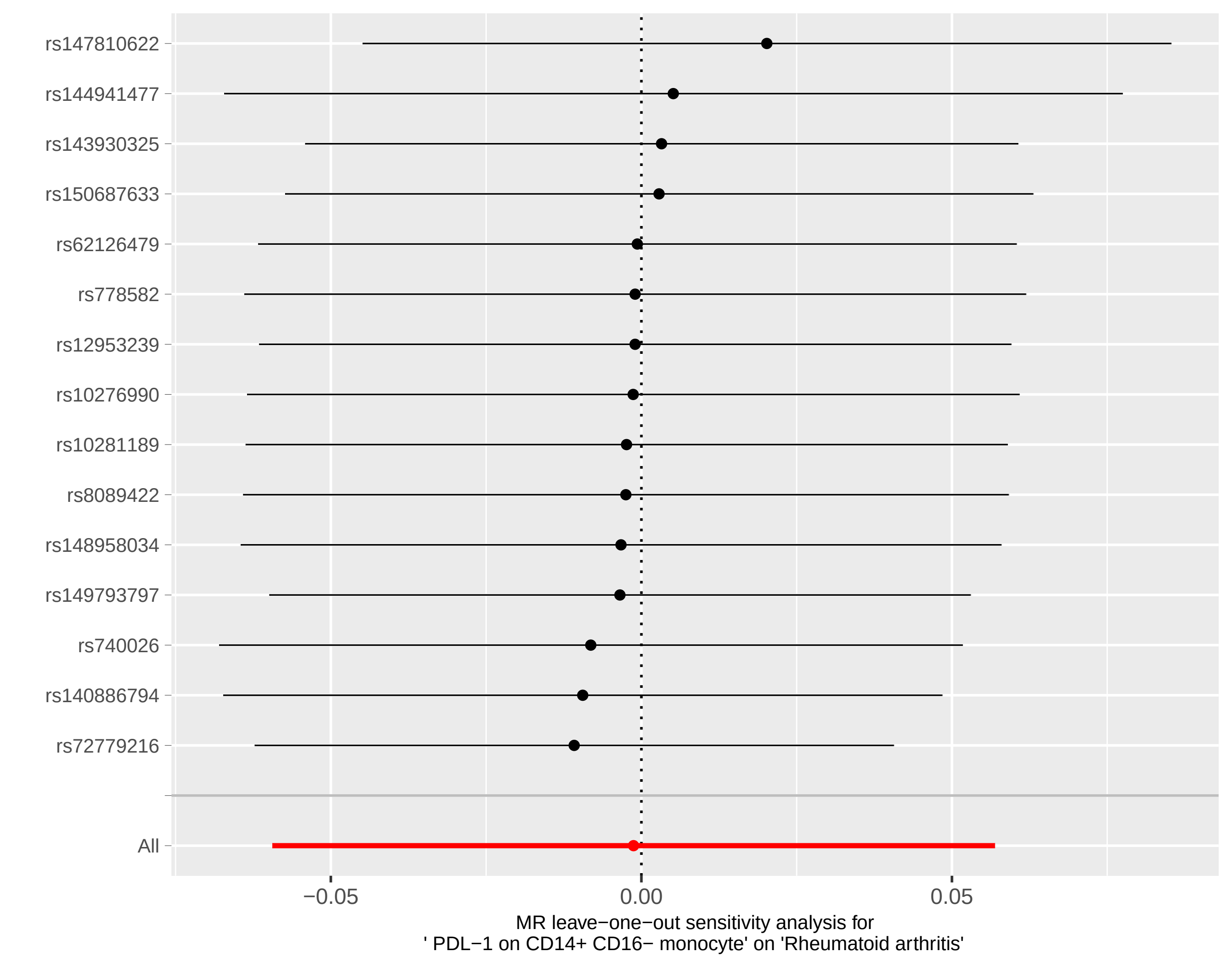
rs147810622
rs144941477
rs143930325
rs150687633
rs62126479
rs778582
rs12953239
rs10276990
rs10281189
rs8089422
rs148958034
rs149793797
rs740026
rs140886794
rs72779216
All
−0.05
0.00
0.05
MR leave−one−out sensitivity analysis for
' PDL−1 on CD14+ CD16− monocyte' on 'Rheumatoid arthritis'

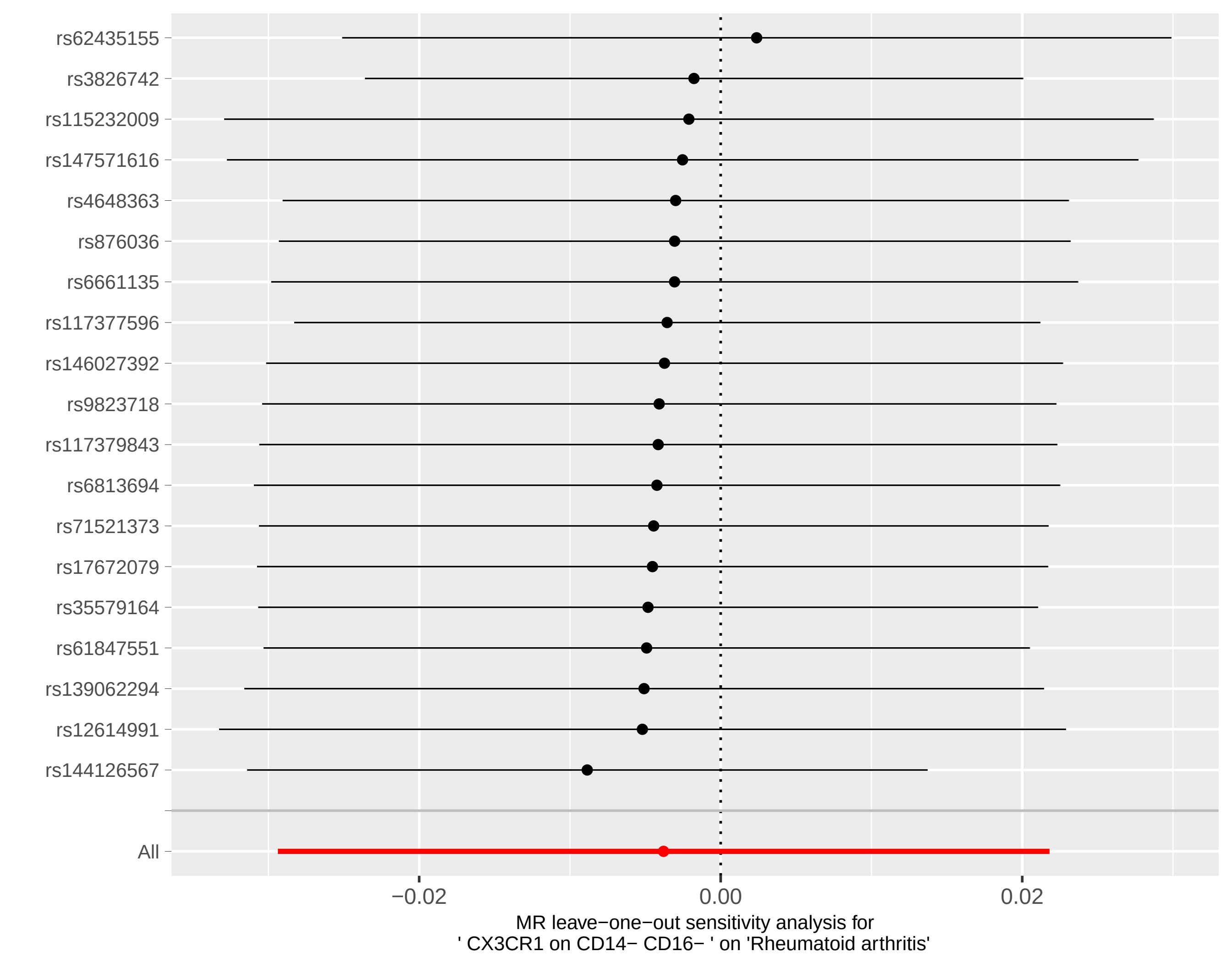

rs62435155
rs3826742
rs115232009
rs147571616
rs4648363
rs876036
rs6661135
rs117377596
rs146027392
rs9823718
rs117379843
rs6813694
rs71521373
rs17672079
rs35579164
rs61847551
rs139062294
rs12614991
rs144126567
All
−0.02
0.00
0.02
MR leave−one−out sensitivity analysis for
' CX3CR1 on CD14− CD16− ' on 'Rheumatoid arthritis'

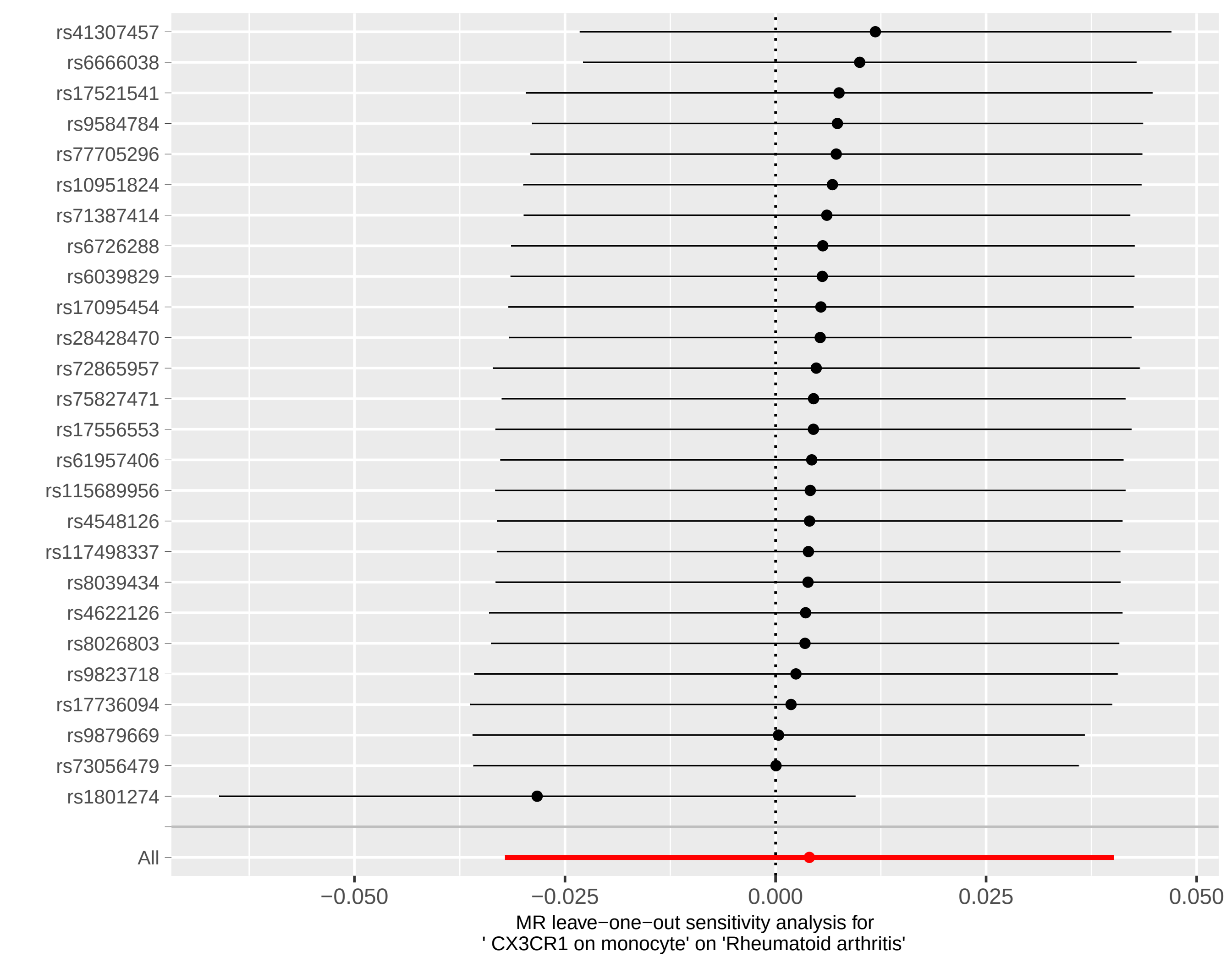
rs41307457
rs6666038
rs17521541
rs9584784
rs77705296
rs10951824
rs71387414
rs6726288
rs6039829
rs17095454
rs28428470
rs72865957
rs75827471
rs17556553
rs61957406
rs115689956
rs4548126
rs117498337
rs8039434
rs4622126
rs8026803
rs9823718
rs17736094
rs9879669
rs73056479
rs1801274
All
−0.050
−0.025
0.000
0.025
0.050
MR leave−one−out sensitivity analysis for
' CX3CR1 on monocyte' on 'Rheumatoid arthritis'

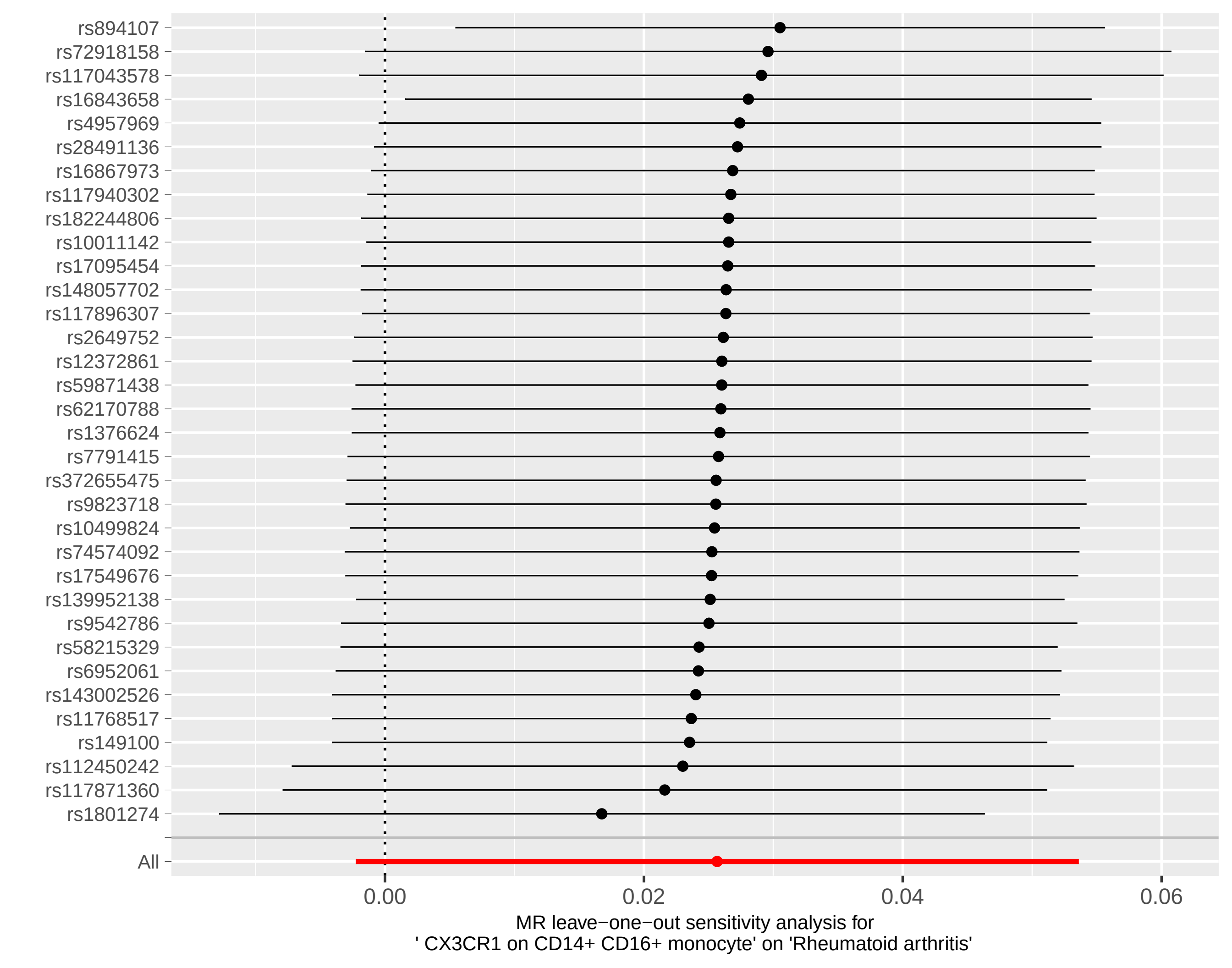
rs894107
rs72918158
rs117043578
rs16843658
rs4957969
rs28491136
rs16867973
rs117940302
rs182244806
rs10011142
rs17095454
rs148057702
rs117896307
rs2649752
rs12372861
rs59871438
rs62170788
rs1376624
rs7791415
rs372655475
rs9823718
rs10499824
rs74574092
rs17549676
rs139952138
rs9542786
rs58215329
rs6952061
rs143002526
rs11768517
rs149100
rs112450242
rs117871360
rs1801274
All
0.00
0.02
0.04
0.06
MR leave−one−out sensitivity analysis for
' CX3CR1 on CD14+ CD16+ monocyte' on 'Rheumatoid arthritis'

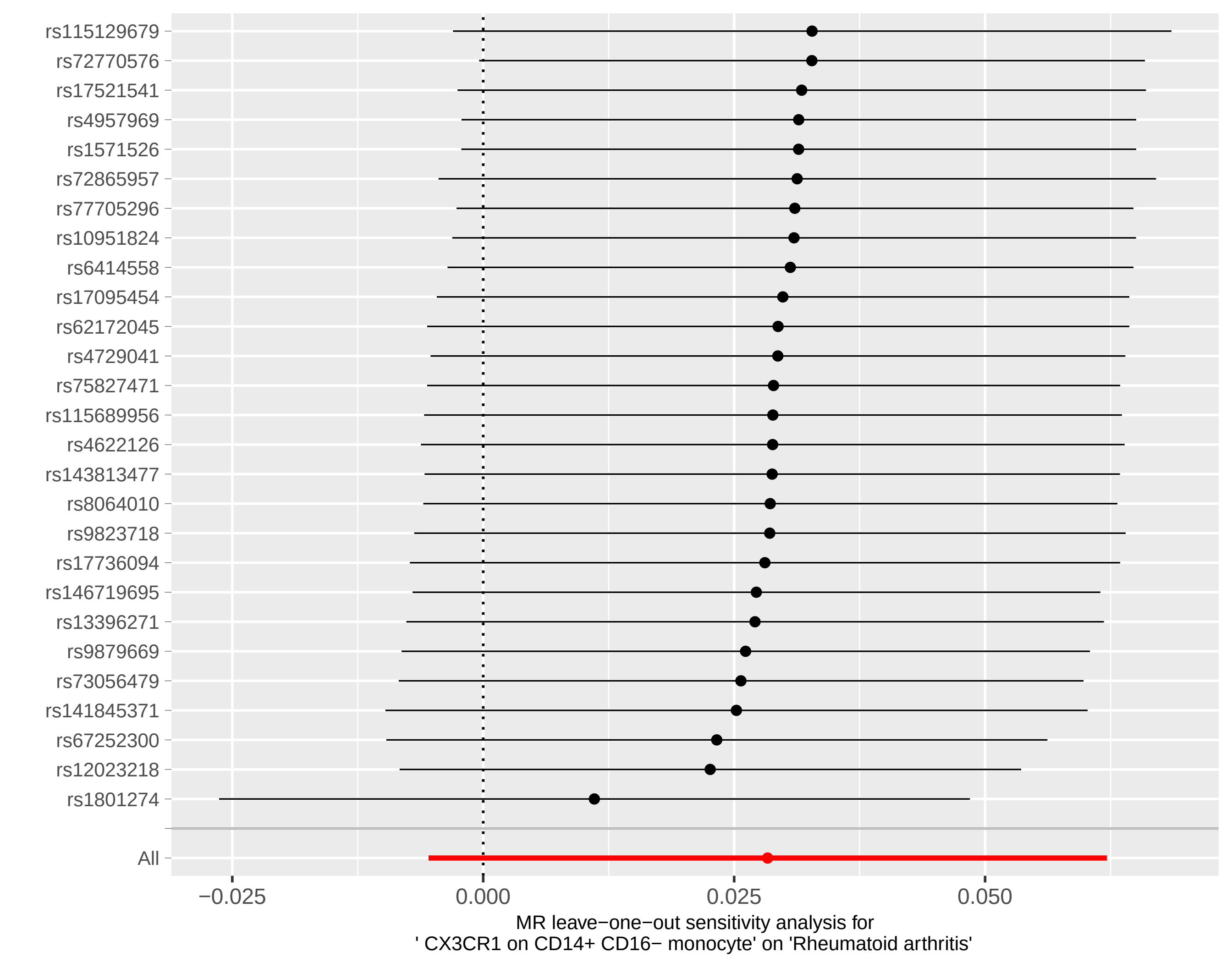
rs115129679
rs72770576
rs17521541
rs4957969
rs1571526
rs72865957
rs77705296
rs10951824
rs6414558
rs17095454
rs62172045
rs4729041
rs75827471
rs115689956
rs4622126
rs143813477
rs8064010
rs9823718
rs17736094
rs146719695
rs13396271
rs9879669
rs73056479
rs141845371
rs67252300
rs12023218
rs1801274
All
−0.025
0.000
0.025
0.050
MR leave−one−out sensitivity analysis for
' CX3CR1 on CD14+ CD16− monocyte' on 'Rheumatoid arthritis'

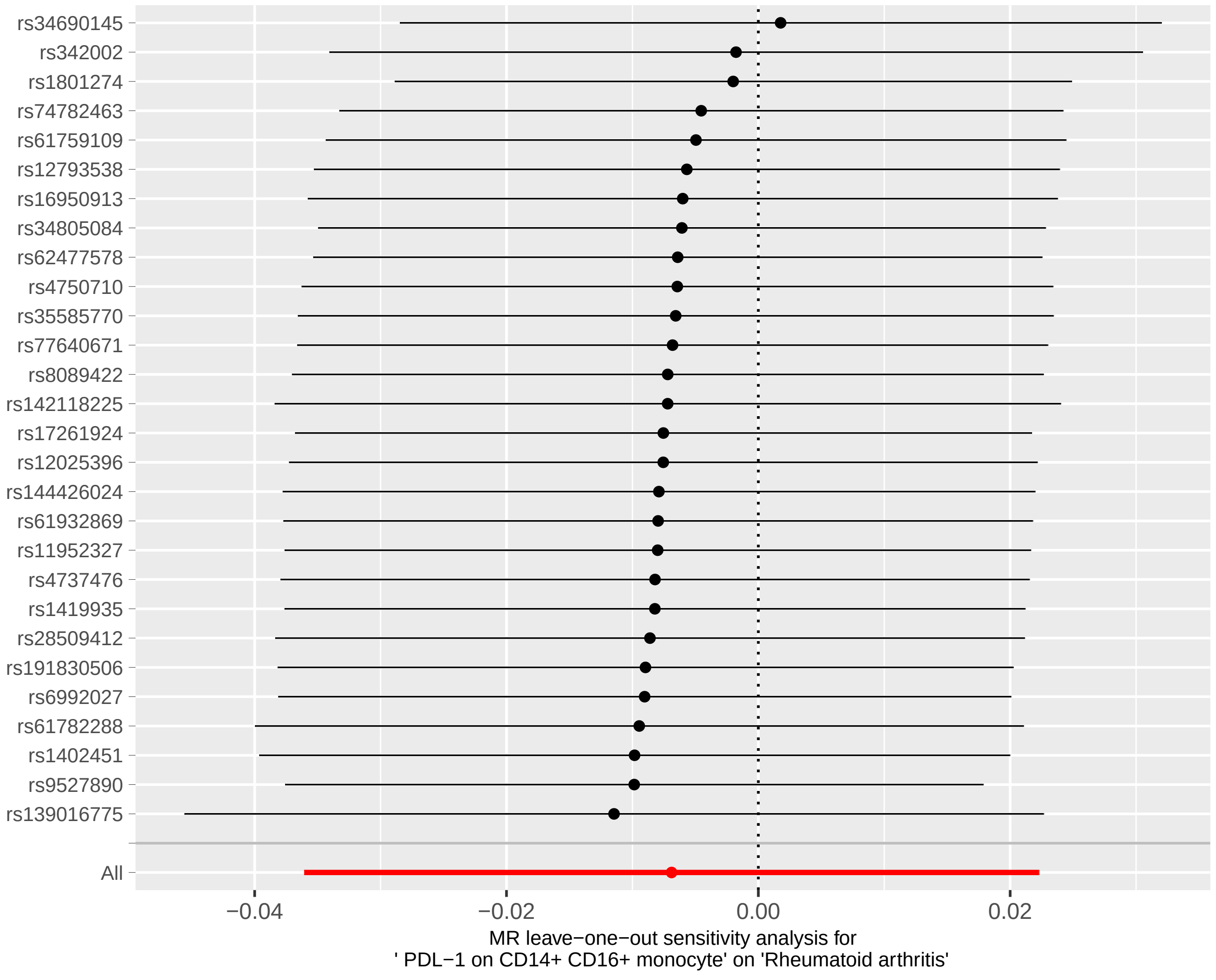

rs34690145
rs342002
rs1801274
rs74782463
rs61759109
rs12793538
rs16950913
rs34805084
rs62477578
rs4750710
rs35585770
rs77640671
rs8089422
rs142118225
rs17261924
rs12025396
rs144426024
rs61932869
rs11952327
rs4737476
rs1419935
rs28509412
rs191830506
rs6992027
rs61782288
rs1402451
rs9527890
rs139016775
All
−0.04
−0.02
0.00
0.02
MR leave−one−out sensitivity analysis for
' PDL−1 on CD14+ CD16+ monocyte' on 'Rheumatoid arthritis'

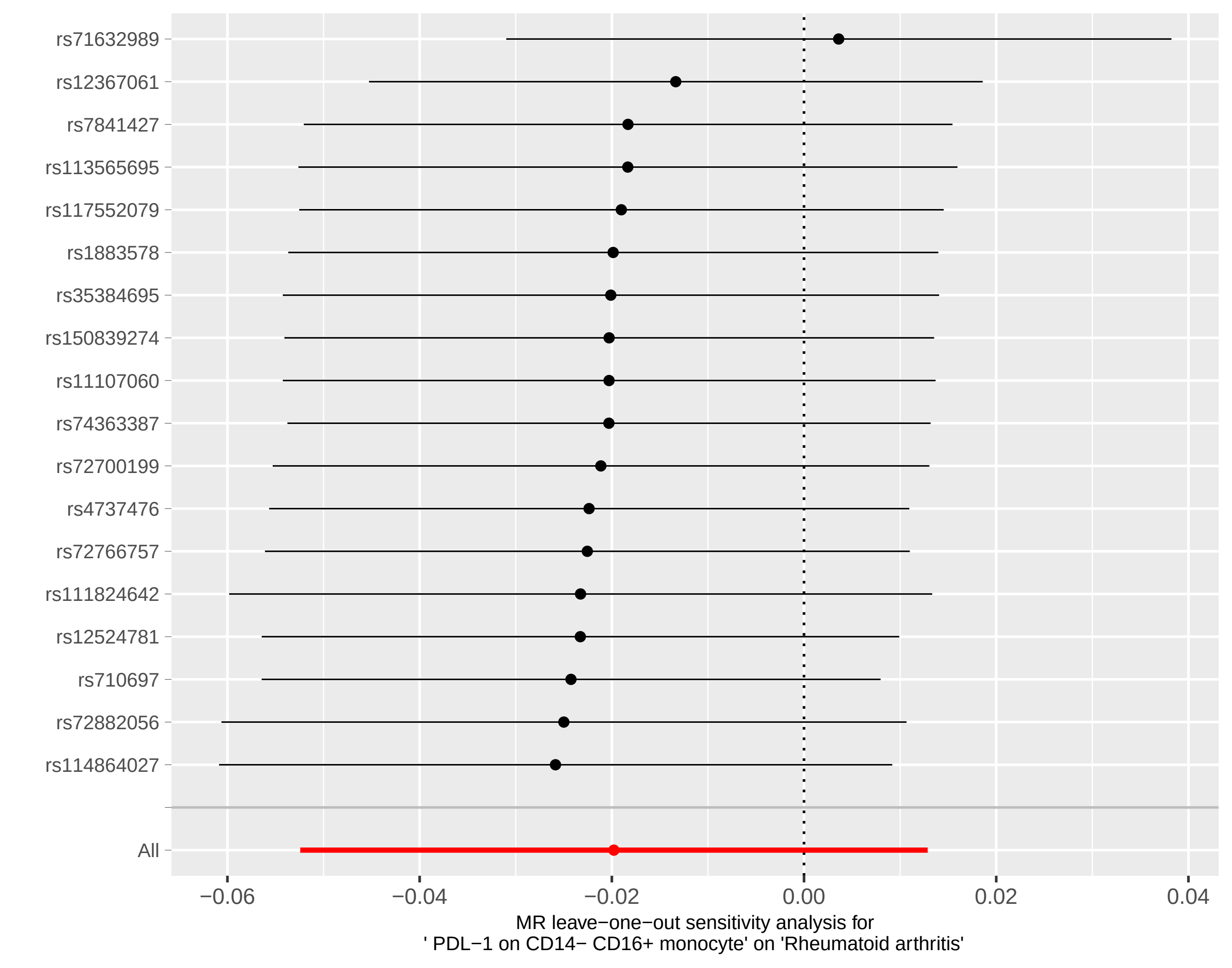

rs71632989
rs12367061
rs7841427
rs113565695
rs117552079
rs1883578
rs35384695
rs150839274
rs11107060
rs74363387
rs72700199
rs4737476
rs72766757
rs111824642
rs12524781
rs710697
rs72882056
rs114864027
All
−0.06
−0.04
−0.02
0.00
0.02
0.04
MR leave−one−out sensitivity analysis for
' PDL−1 on CD14− CD16+ monocyte' on 'Rheumatoid arthritis'

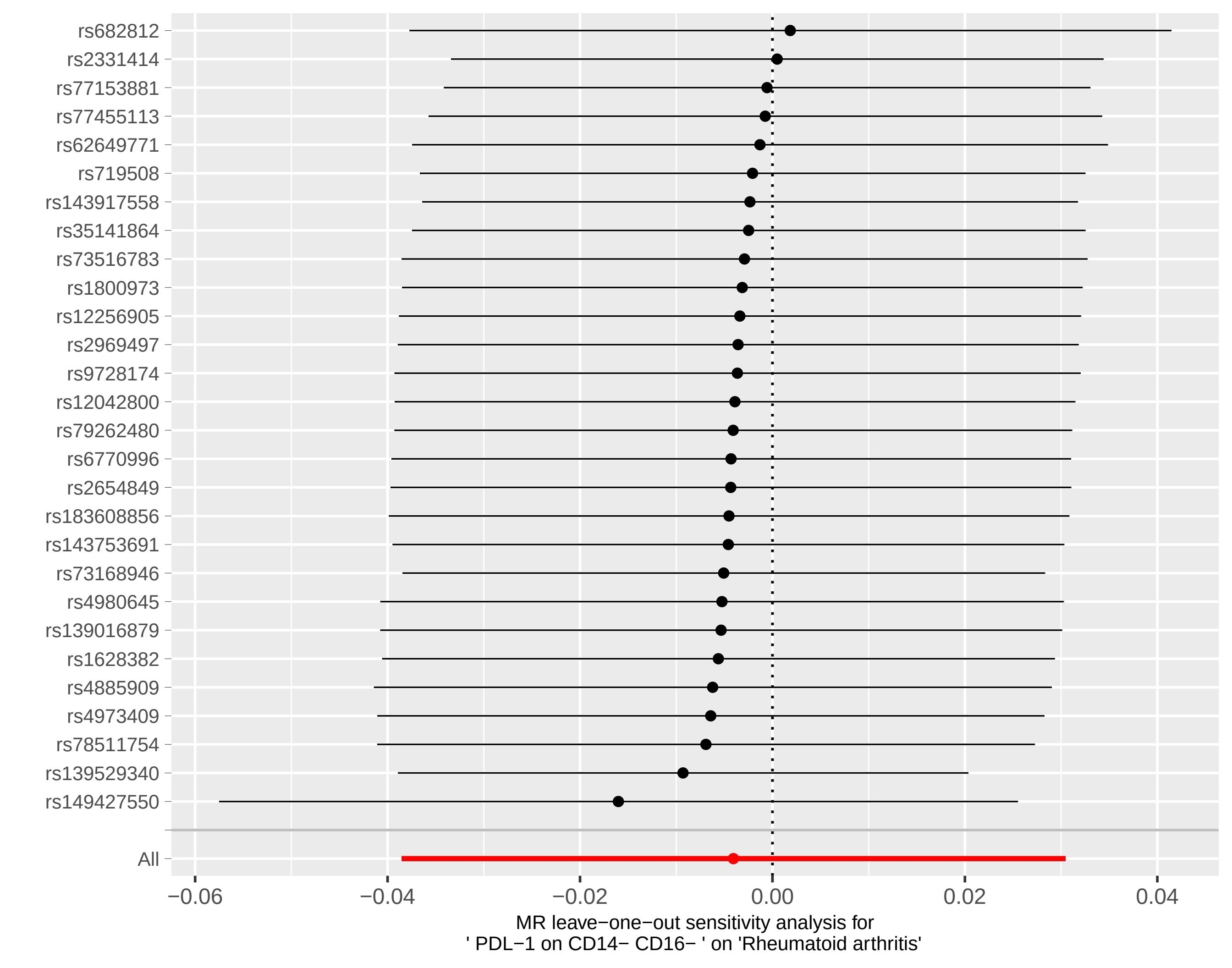
rs682812
rs2331414
rs77153881
rs77455113
rs62649771
rs719508
rs143917558
rs35141864
rs73516783
rs1800973
rs12256905
rs2969497
rs9728174
rs12042800
rs79262480
rs6770996
rs2654849
rs183608856
rs143753691
rs73168946
rs4980645
rs139016879
rs1628382
rs4885909
rs4973409
rs78511754
rs139529340
rs149427550
All
−0.06
−0.04
−0.02
0.00
0.02
0.04
MR leave−one−out sensitivity analysis for
' PDL−1 on CD14− CD16− ' on 'Rheumatoid arthritis'

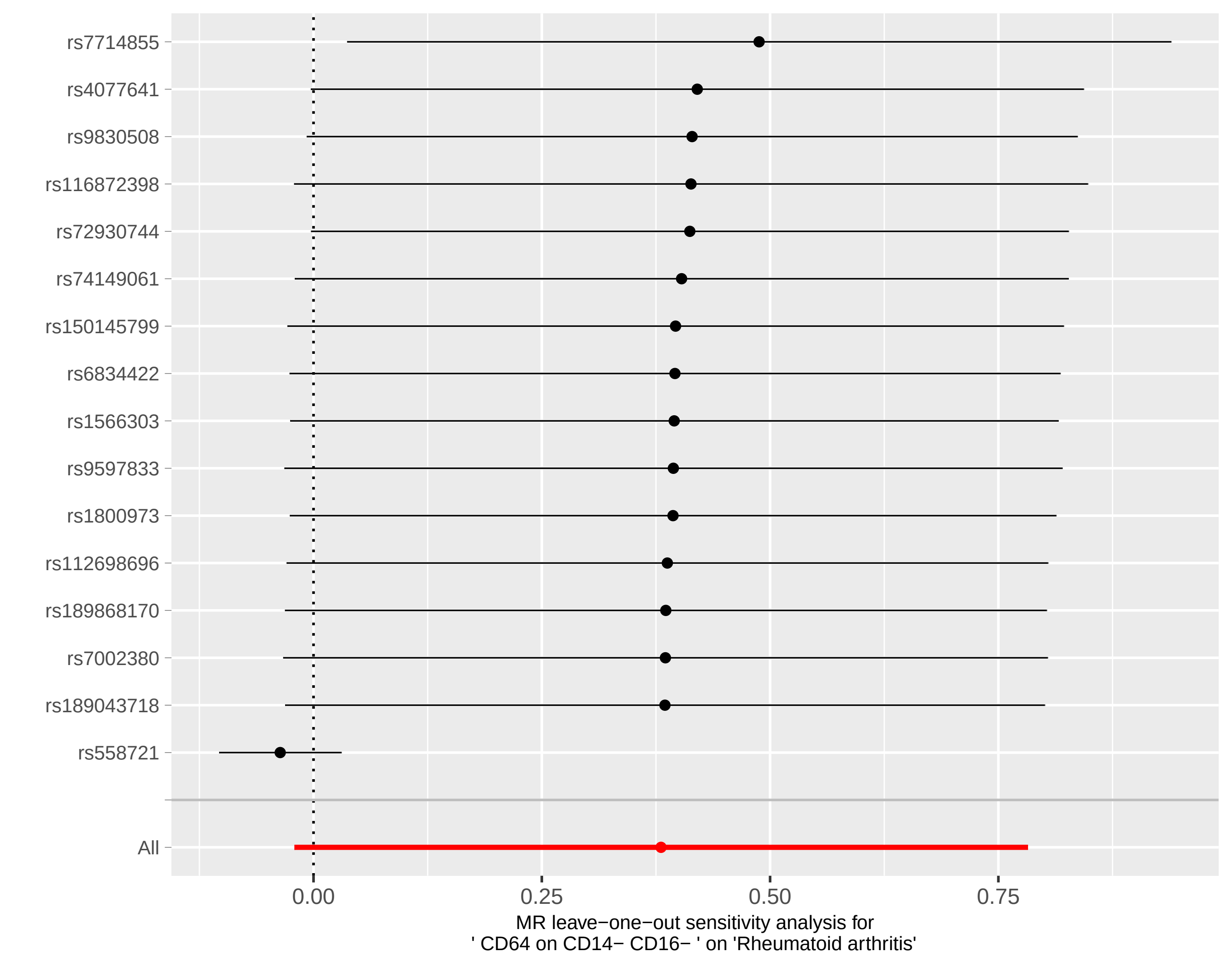
rs7714855
rs4077641
rs9830508
rs116872398
rs72930744
rs74149061
rs150145799
rs6834422
rs1566303
rs9597833
rs1800973
rs112698696
rs189868170
rs7002380
rs189043718
rs558721
All
0.00
0.25
0.50
0.75
MR leave−one−out sensitivity analysis for
' CD64 on CD14− CD16− ' on 'Rheumatoid arthritis'

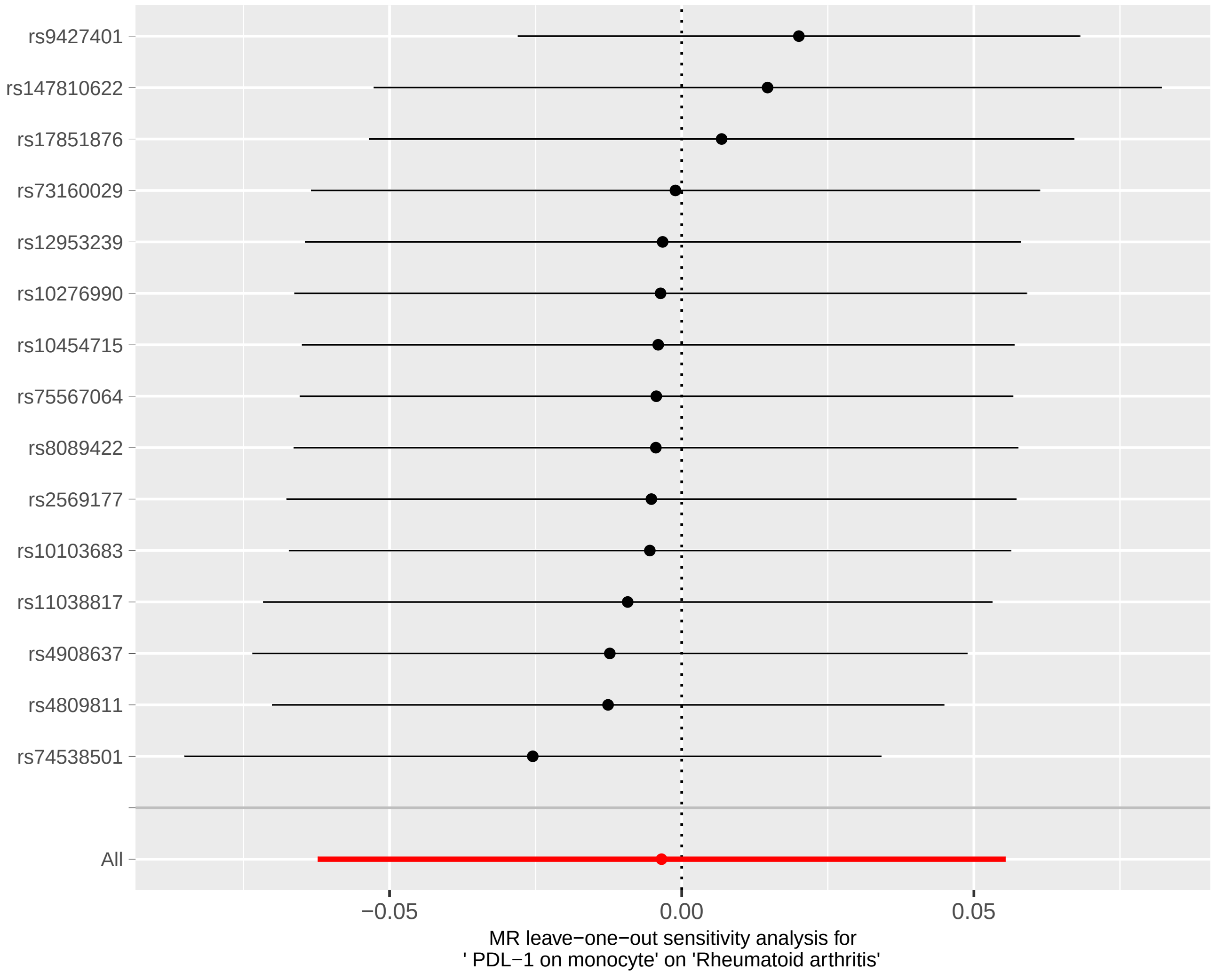
rs9427401
rs147810622
rs17851876
rs73160029
rs12953239
rs10276990
rs10454715
rs75567064
rs8089422
rs2569177
rs10103683
rs11038817
rs4908637
rs4809811
rs74538501
All
−0.05
0.00
0.05
MR leave−one−out sensitivity analysis for
' PDL−1 on monocyte' on 'Rheumatoid arthritis'

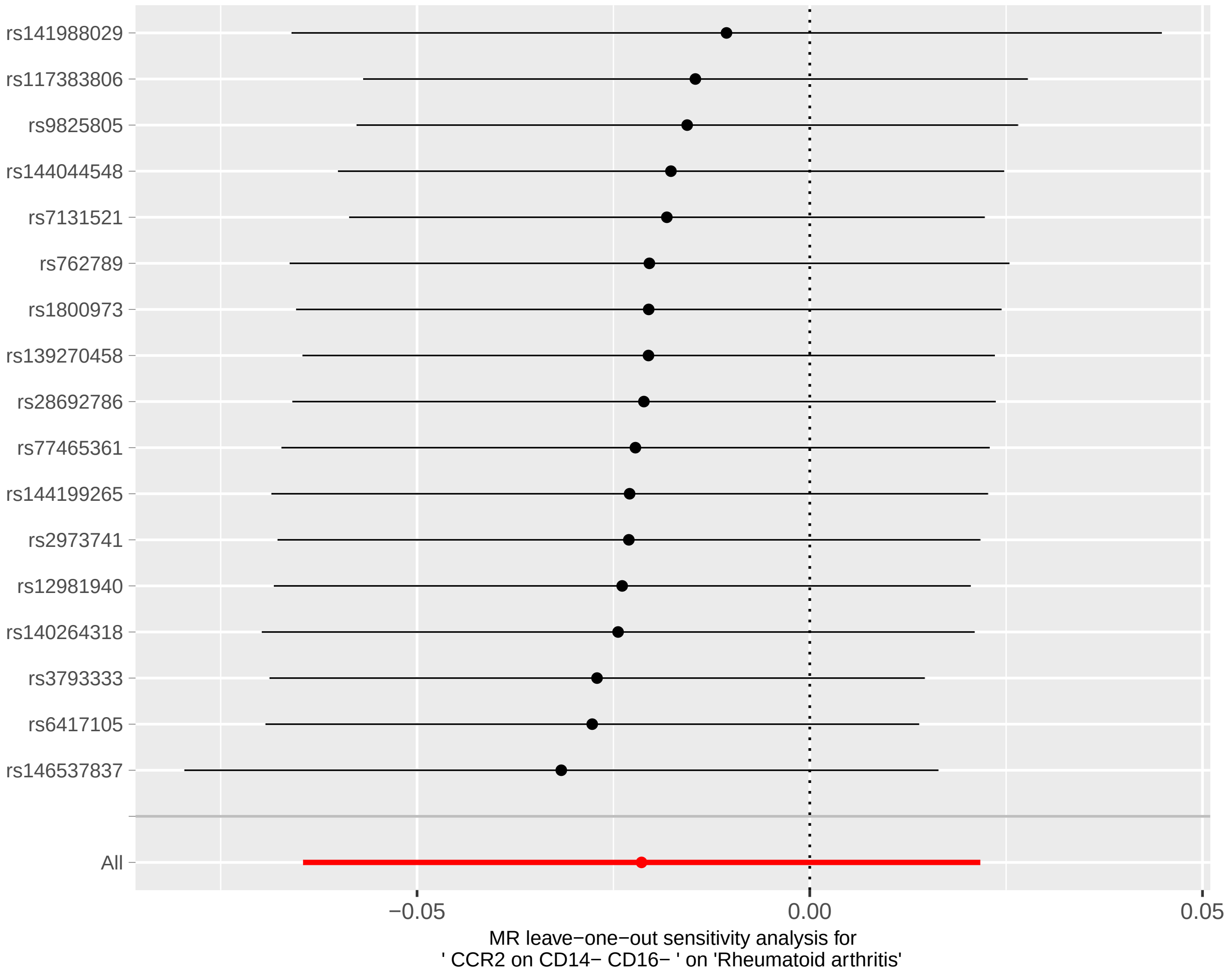
rs141988029
rs117383806
rs9825805
rs144044548
rs7131521
rs762789
rs1800973
rs139270458
rs28692786
rs77465361
rs144199265
rs2973741
rs12981940
rs140264318
rs3793333
rs6417105
rs146537837
All
−0.05
0.00
0.05
MR leave−one−out sensitivity analysis for
' CCR2 on CD14− CD16− ' on 'Rheumatoid arthritis'

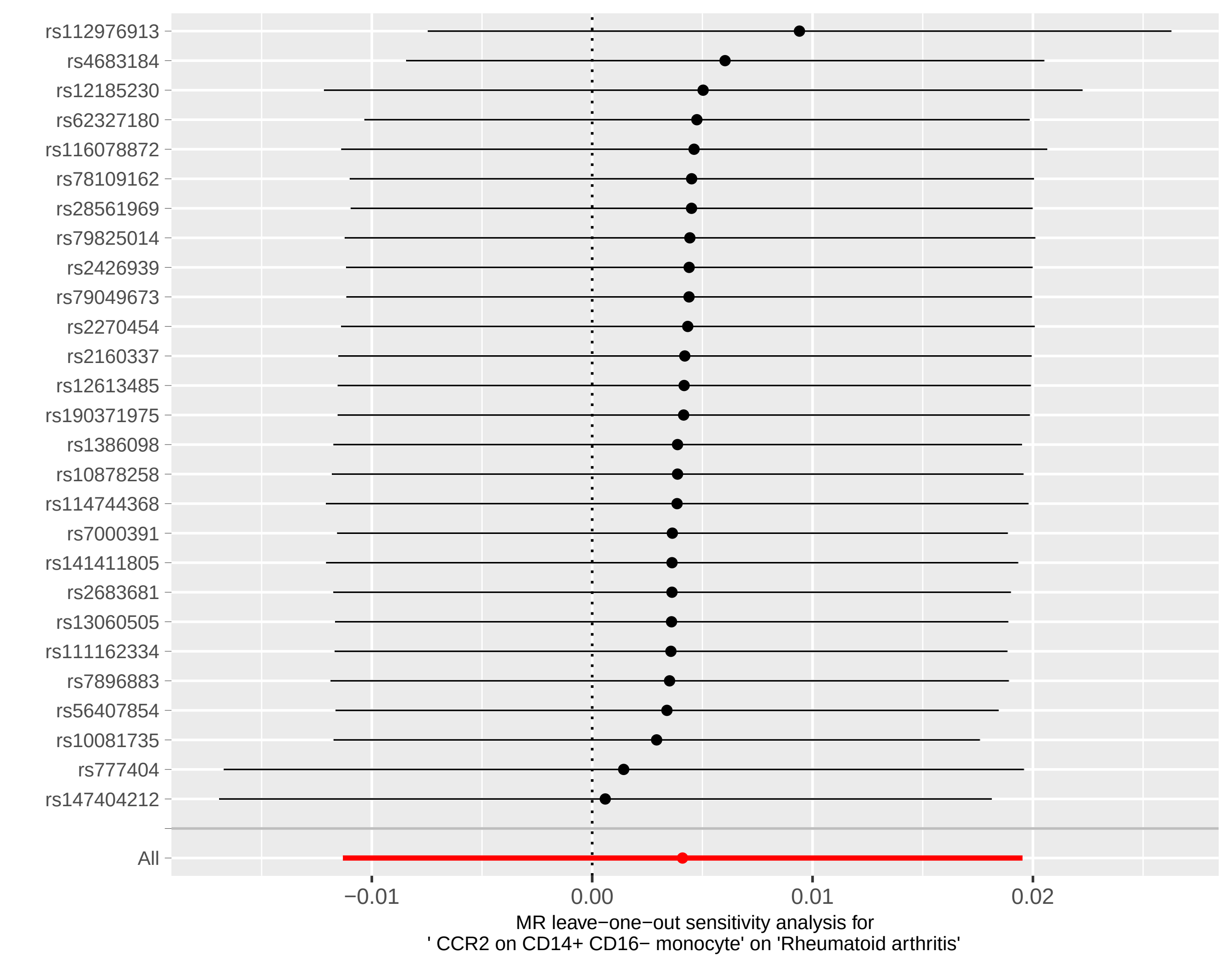
rs112976913
rs4683184
rs12185230
rs62327180
rs116078872
rs78109162
rs28561969
rs79825014
rs2426939
rs79049673
rs2270454
rs2160337
rs12613485
rs190371975
rs1386098
rs10878258
rs114744368
rs7000391
rs141411805
rs2683681
rs13060505
rs111162334
rs7896883
rs56407854
rs10081735
rs777404
rs147404212
All
−0.01
0.00
0.01
0.02
MR leave−one−out sensitivity analysis for
' CCR2 on CD14+ CD16− monocyte' on 'Rheumatoid arthritis'

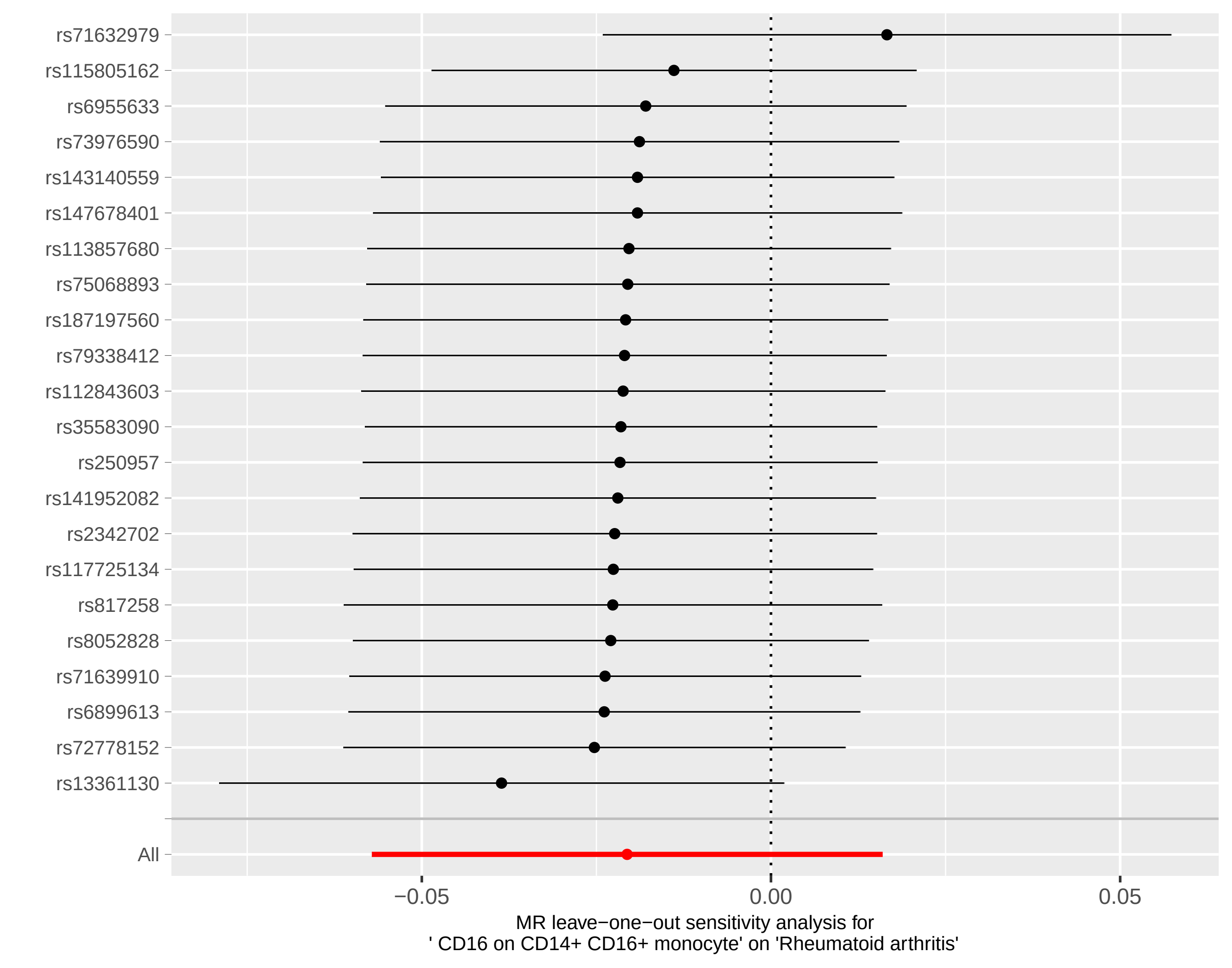
rs71632979
rs115805162
rs6955633
rs73976590
rs143140559
rs147678401
rs113857680
rs75068893
rs187197560
rs79338412
rs112843603
rs35583090
rs250957
rs141952082
rs2342702
rs117725134
rs817258
rs8052828
rs71639910
rs6899613
rs72778152
rs13361130
All
−0.05
0.00
0.05
MR leave−one−out sensitivity analysis for
' CD16 on CD14+ CD16+ monocyte' on 'Rheumatoid arthritis'

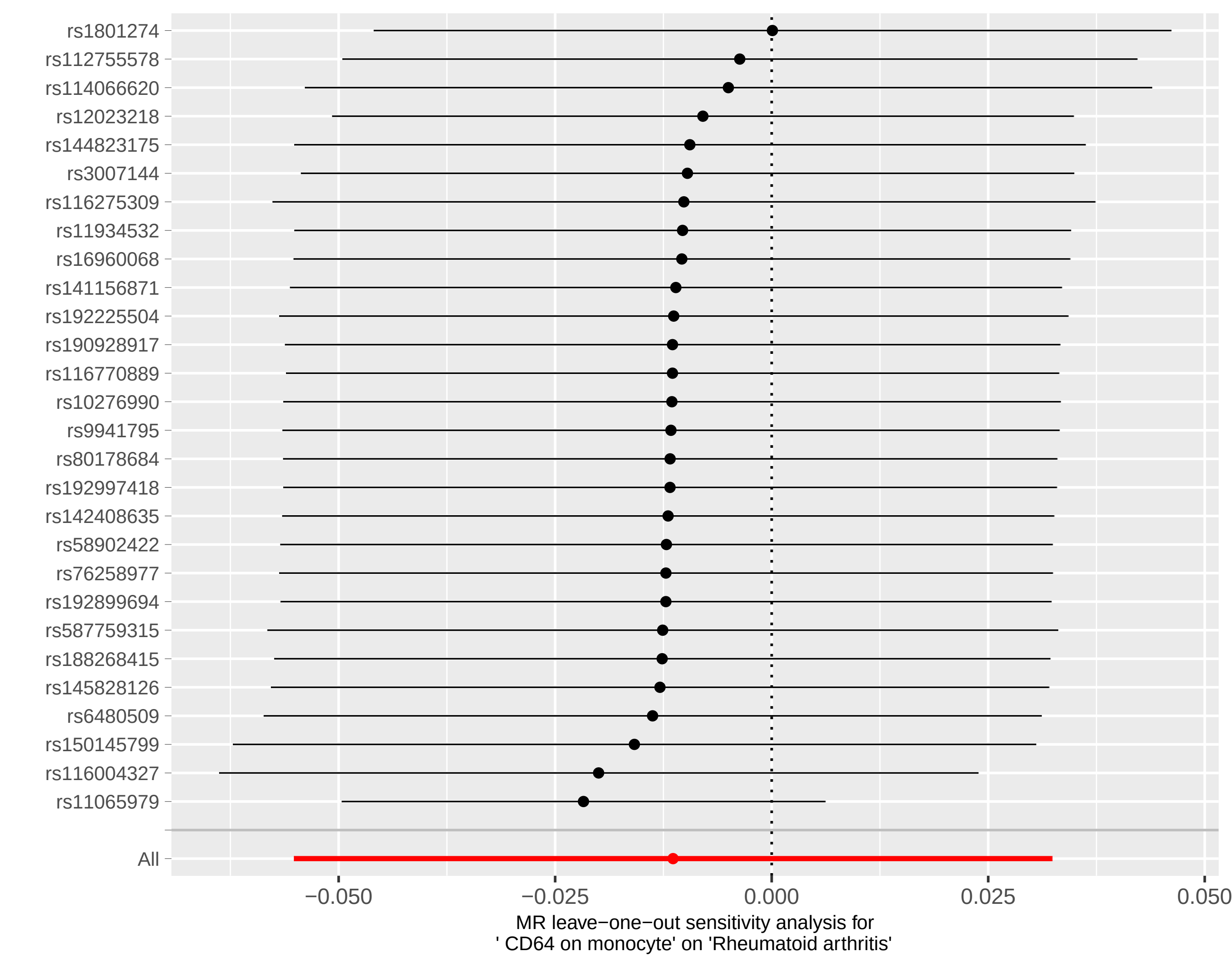

rs1801274
rs112755578
rs114066620
rs12023218
rs144823175
rs3007144
rs116275309
rs11934532
rs16960068
rs141156871
rs192225504
rs190928917
rs116770889
rs10276990
rs9941795
rs80178684
rs192997418
rs142408635
rs58902422
rs76258977
rs192899694
rs587759315
rs188268415
rs145828126
rs6480509
rs150145799
rs116004327
rs11065979
All
−0.050
−0.025
0.000
0.025
0.050
MR leave−one−out sensitivity analysis for
' CD64 on monocyte' on 'Rheumatoid arthritis'

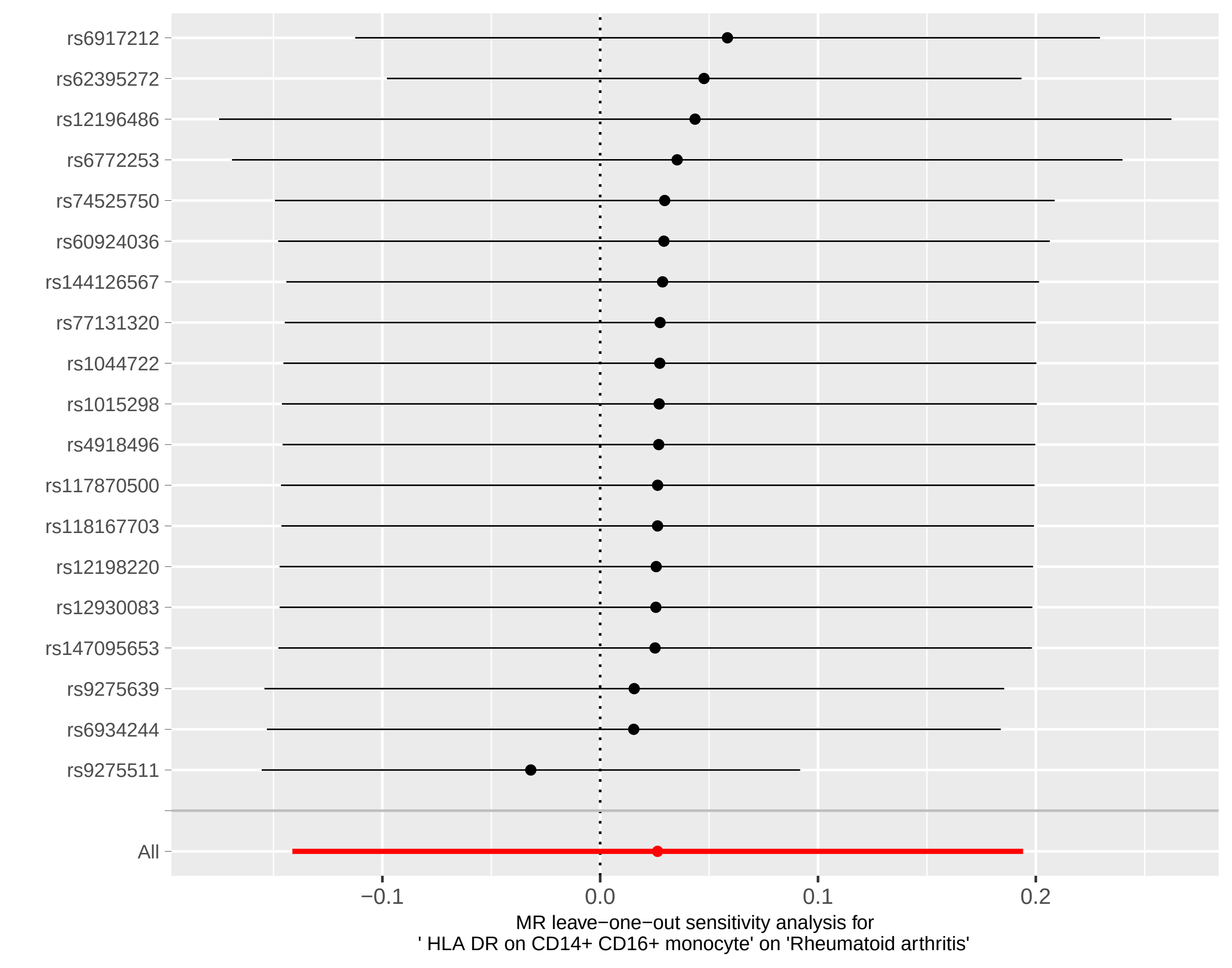
rs6917212
rs62395272
rs12196486
rs6772253
rs74525750
rs60924036
rs144126567
rs77131320
rs1044722
rs1015298
rs4918496
rs117870500
rs118167703
rs12198220
rs12930083
rs147095653
rs9275639
rs6934244
rs9275511
All
−0.1
0.0
0.1
0.2
MR leave−one−out sensitivity analysis for
' HLA DR on CD14+ CD16+ monocyte' on 'Rheumatoid arthritis'

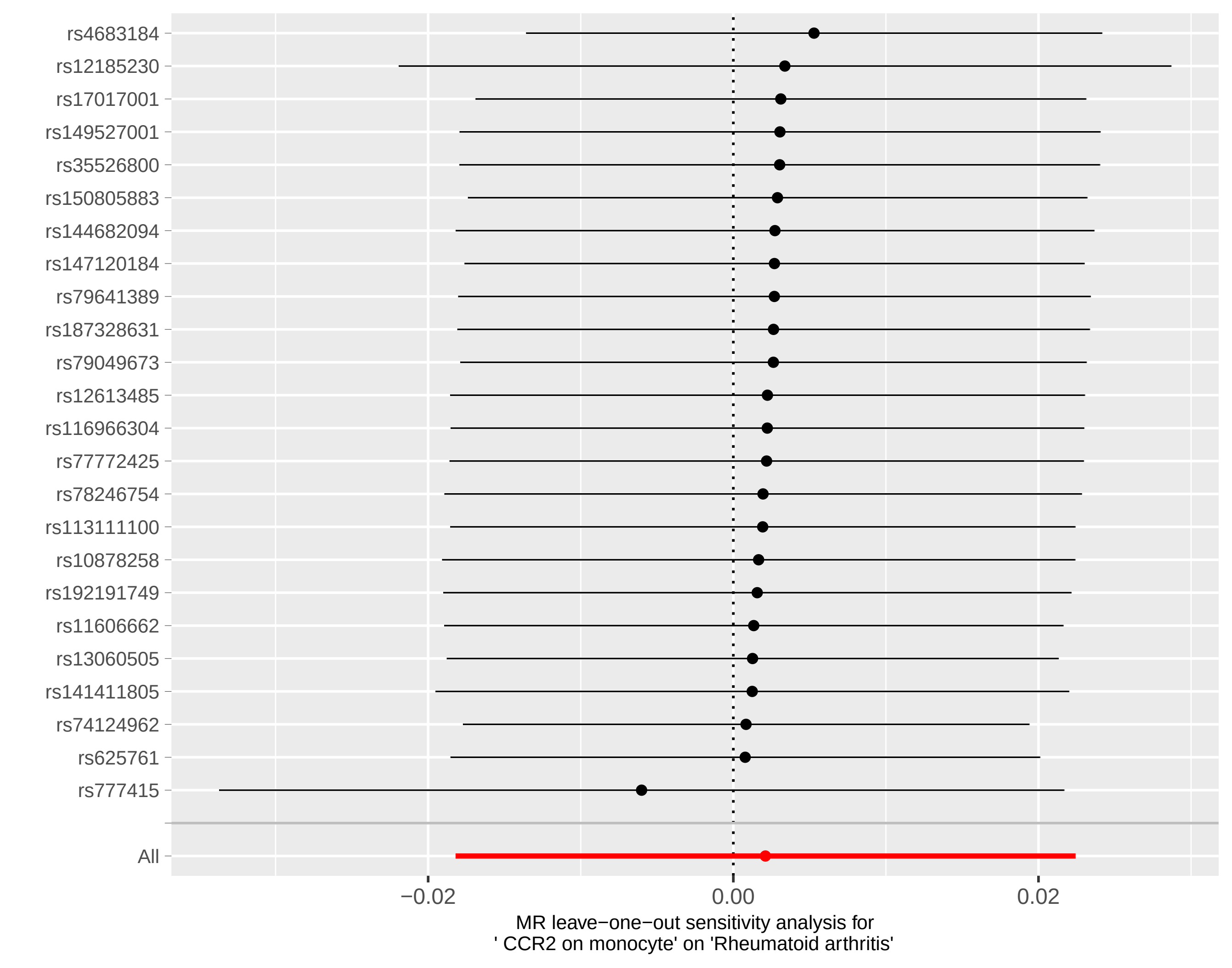
rs4683184
rs12185230
rs17017001
rs149527001
rs35526800
rs150805883
rs144682094
rs147120184
rs79641389
rs187328631
rs79049673
rs12613485
rs116966304
rs77772425
rs78246754
rs113111100
rs10878258
rs192191749
rs11606662
rs13060505
rs141411805
rs74124962
rs625761
rs777415
All
−0.02
0.00
0.02
MR leave−one−out sensitivity analysis for
' CCR2 on monocyte' on 'Rheumatoid arthritis'

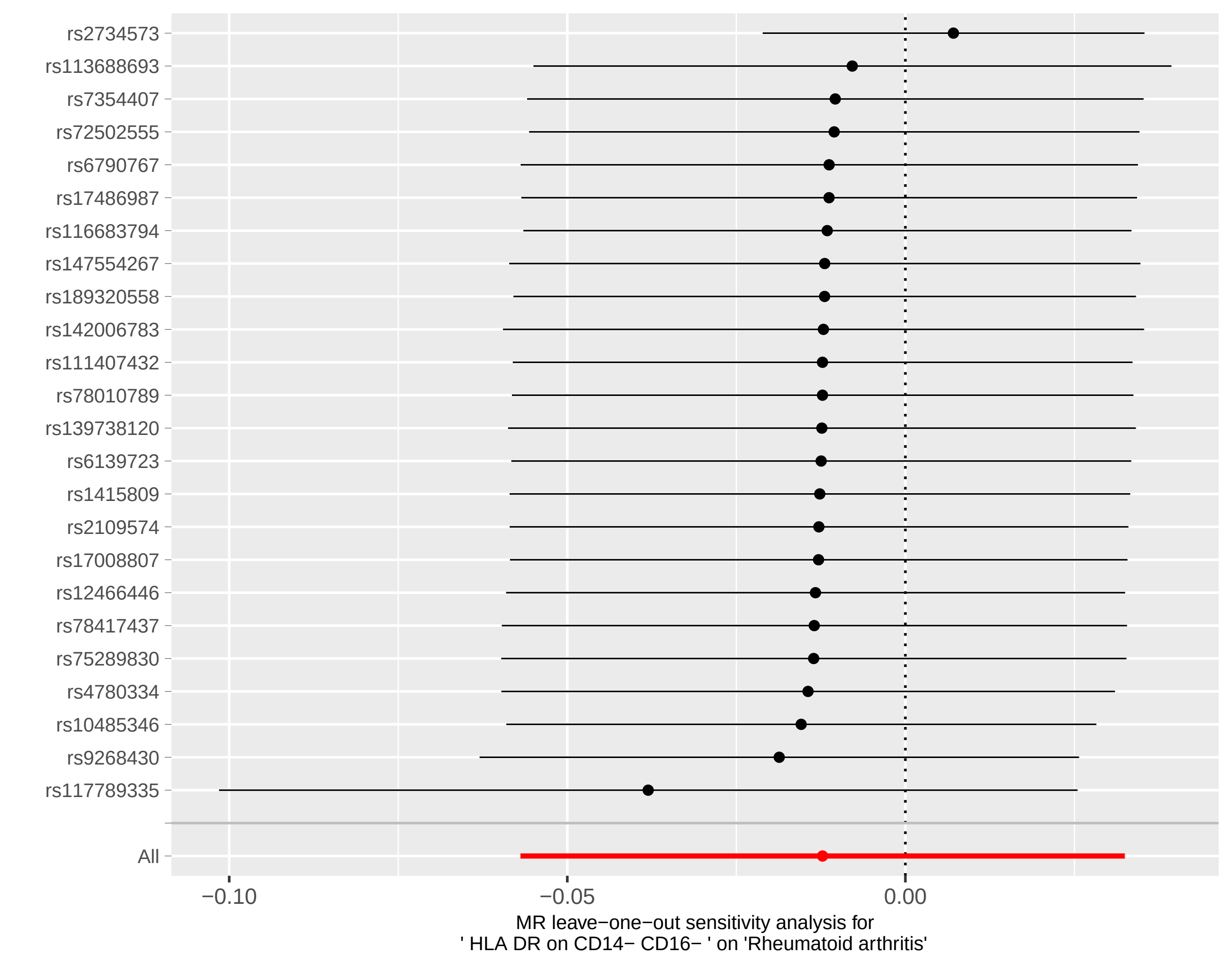

rs2734573
rs113688693
rs7354407
rs72502555
rs6790767
rs17486987
rs116683794
rs147554267
rs189320558
rs142006783
rs111407432
rs78010789
rs139738120
rs6139723
rs1415809
rs2109574
rs17008807
rs12466446
rs78417437
rs75289830
rs4780334
rs10485346
rs9268430
rs117789335
All
−0.10
−0.05
0.00
MR leave−one−out sensitivity analysis for
' HLA DR on CD14− CD16− ' on 'Rheumatoid arthritis'

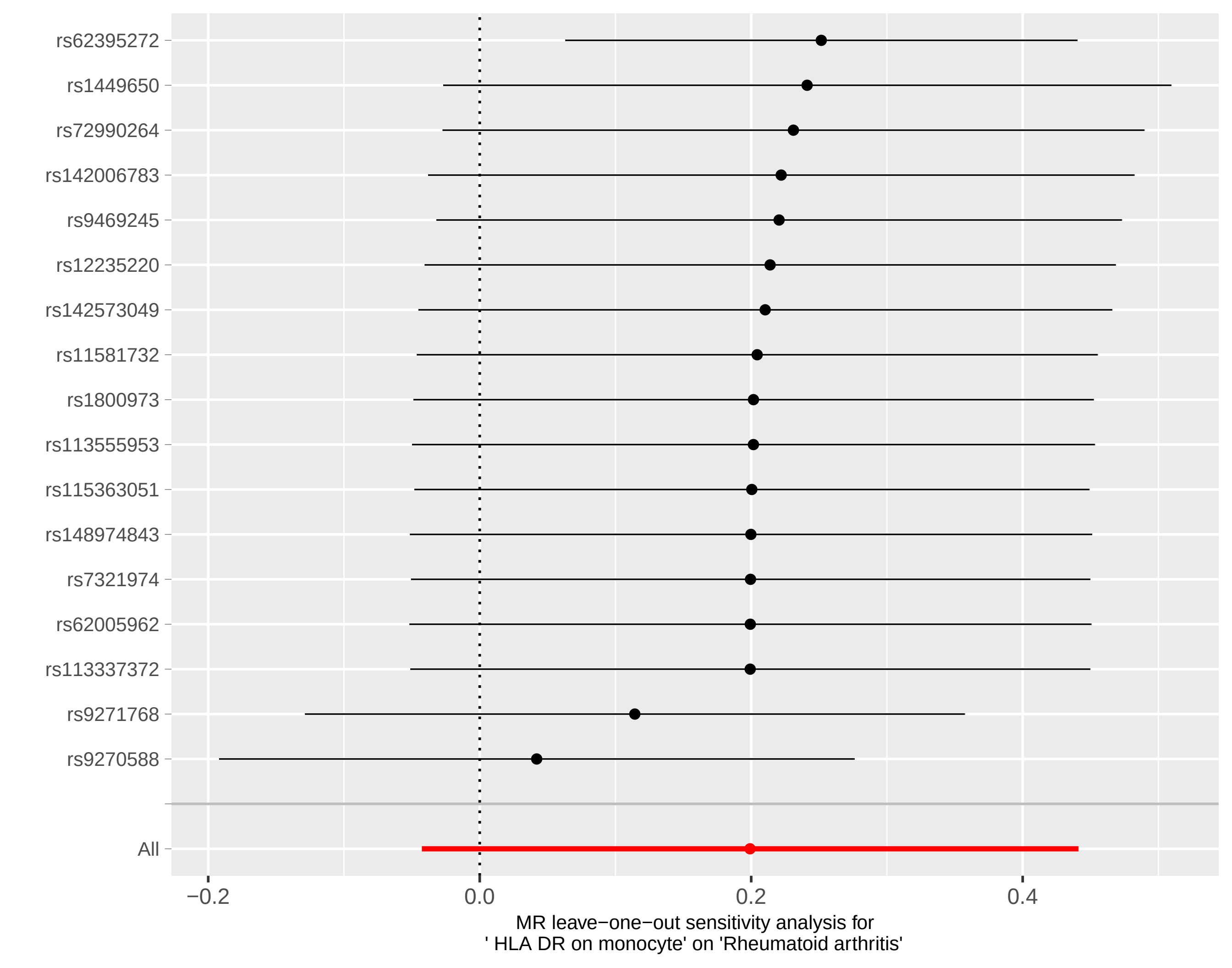
rs62395272
rs1449650
rs72990264
rs142006783
rs9469245
rs12235220
rs142573049
rs11581732
rs1800973
rs113555953
rs115363051
rs148974843
rs7321974
rs62005962
rs113337372
rs9271768
rs9270588
All
−0.2
0.0
0.2
0.4
MR leave−one−out sensitivity analysis for
' HLA DR on monocyte' on 'Rheumatoid arthritis'

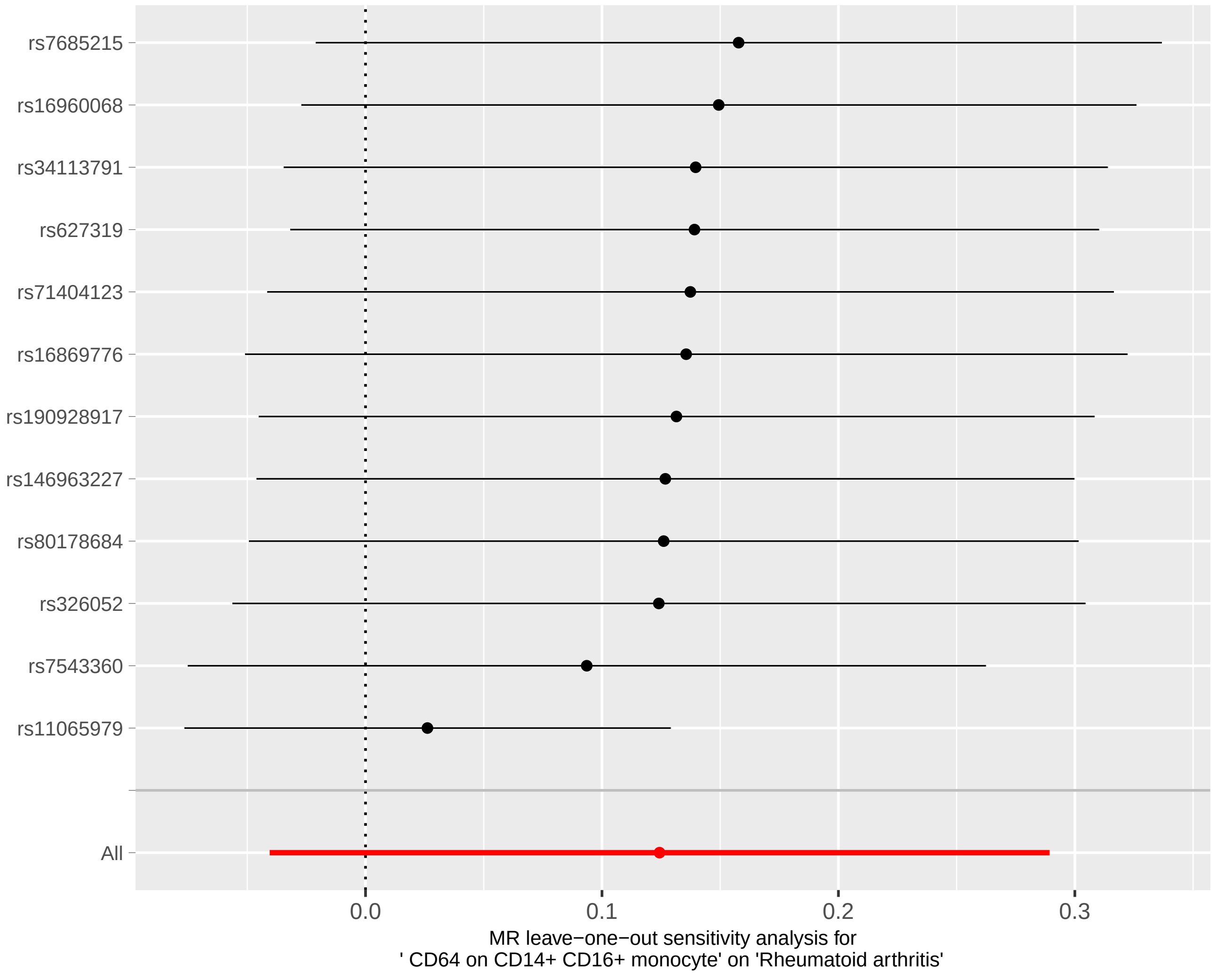

rs7685215
rs16960068
rs34113791
rs627319
rs71404123
rs16869776
rs190928917
rs146963227
rs80178684
rs326052
rs7543360
rs11065979
All
0.0
0.1
0.2
0.3
MR leave−one−out sensitivity analysis for
' CD64 on CD14+ CD16+ monocyte' on 'Rheumatoid arthritis'

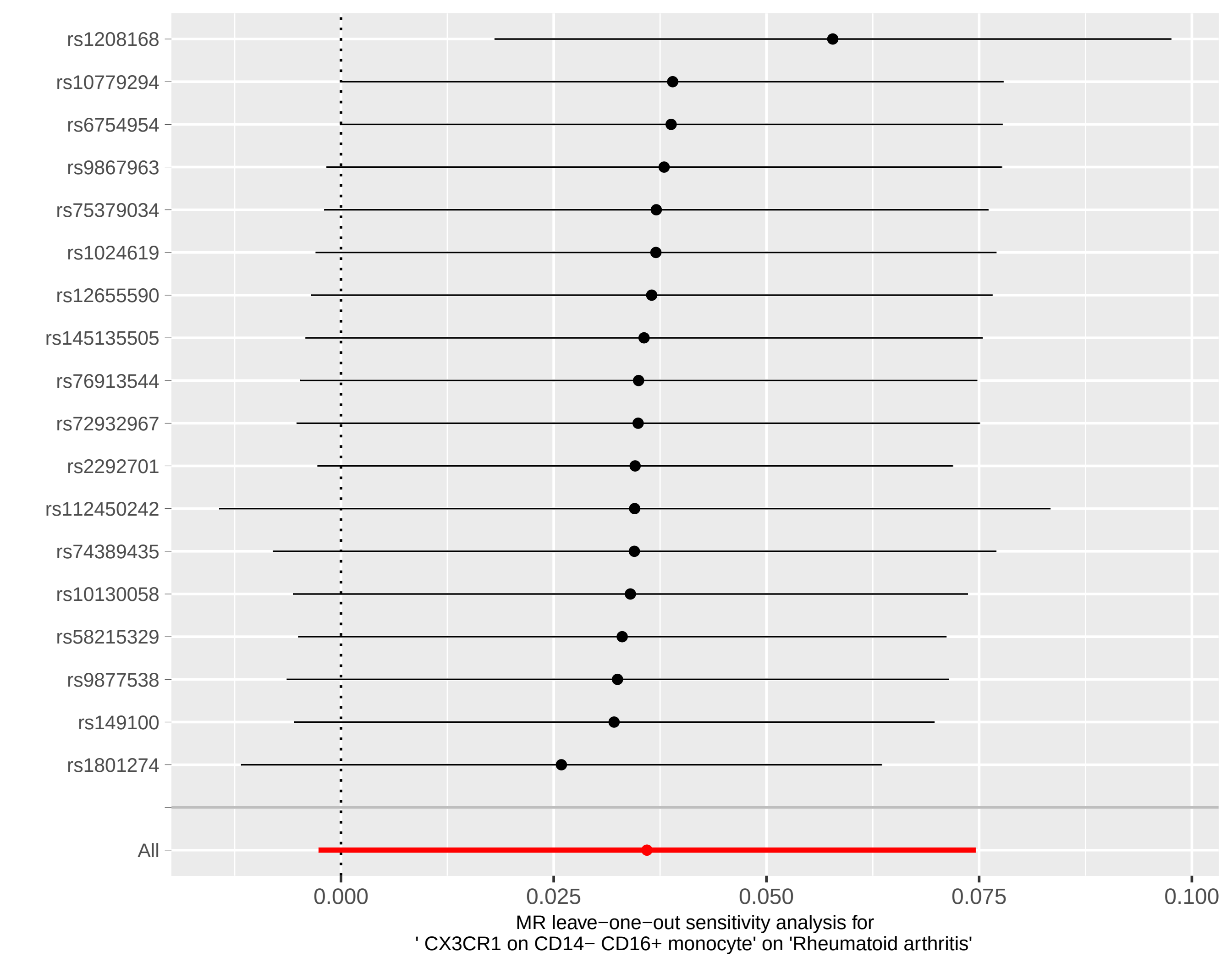
rs1208168
rs10779294
rs6754954
rs9867963
rs75379034
rs1024619
rs12655590
rs145135505
rs76913544
rs72932967
rs2292701
rs112450242
rs74389435
rs10130058
rs58215329
rs9877538
rs149100
rs1801274
All
0.000
0.025
0.050
0.075
0.100
MR leave−one−out sensitivity analysis for
' CX3CR1 on CD14− CD16+ monocyte' on 'Rheumatoid arthritis'

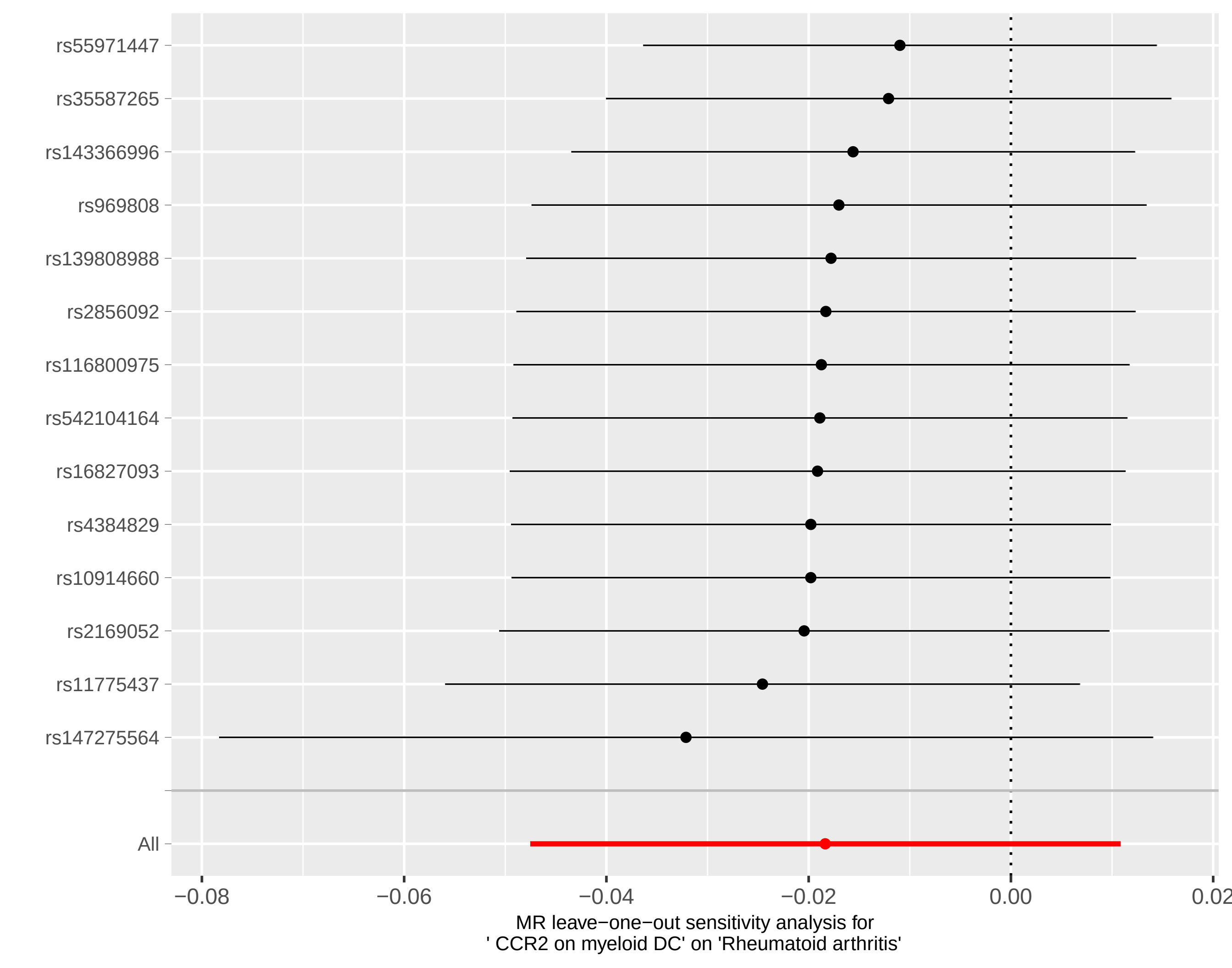

rs55971447
rs35587265
rs143366996
rs969808
rs139808988
rs2856092
rs116800975
rs542104164
rs16827093
rs4384829
rs10914660
rs2169052
rs11775437
rs147275564
All
−0.08
−0.06
−0.04
−0.02
0.00
0.02
MR leave−one−out sensitivity analysis for
' CCR2 on myeloid DC' on 'Rheumatoid arthritis'

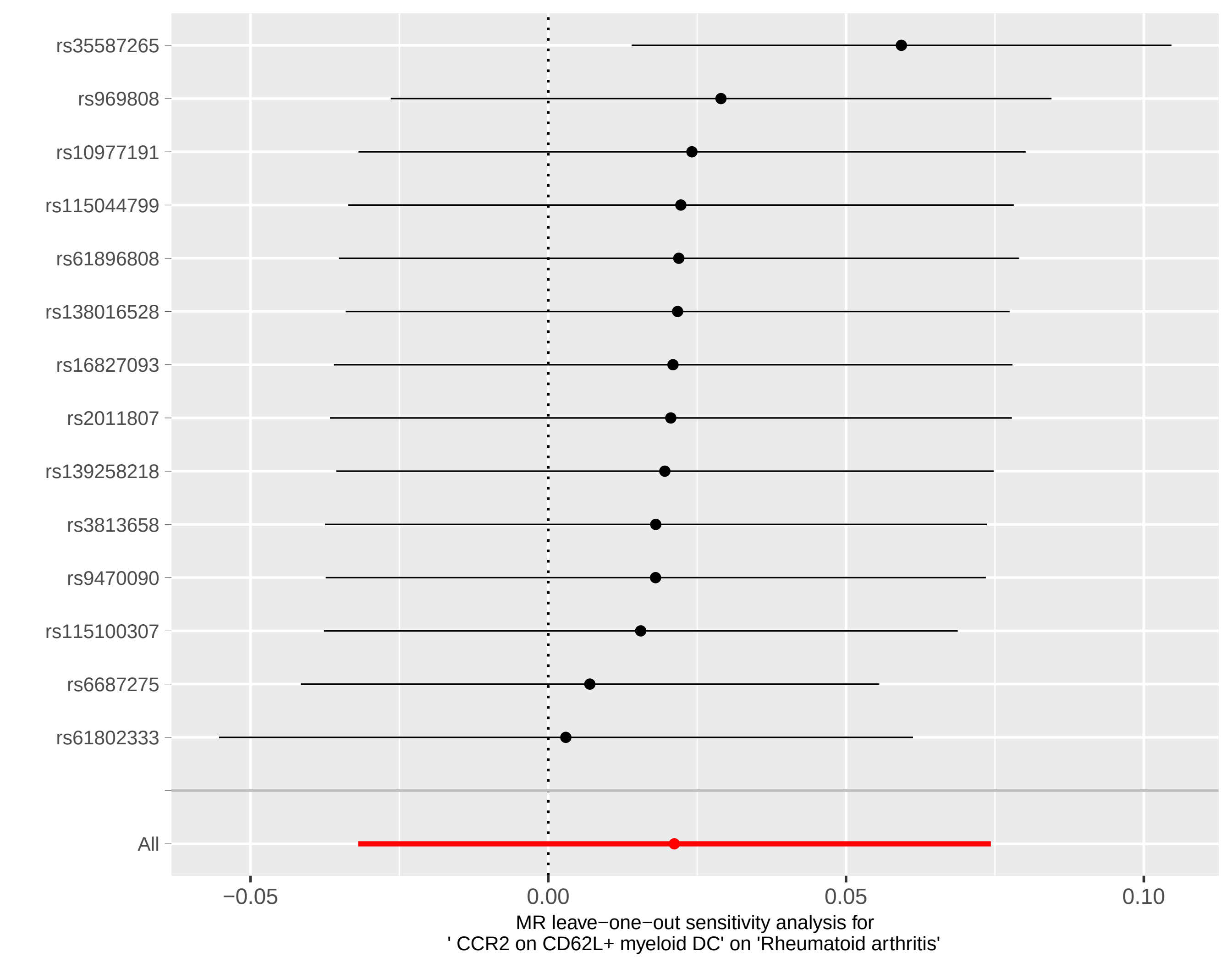
rs35587265
rs969808
rs10977191
rs115044799
rs61896808
rs138016528
rs16827093
rs2011807
rs139258218
rs3813658
rs9470090
rs115100307
rs6687275
rs61802333
All
−0.05
0.00
0.05
0.10
MR leave−one−out sensitivity analysis for
' CCR2 on CD62L+ myeloid DC' on 'Rheumatoid arthritis'

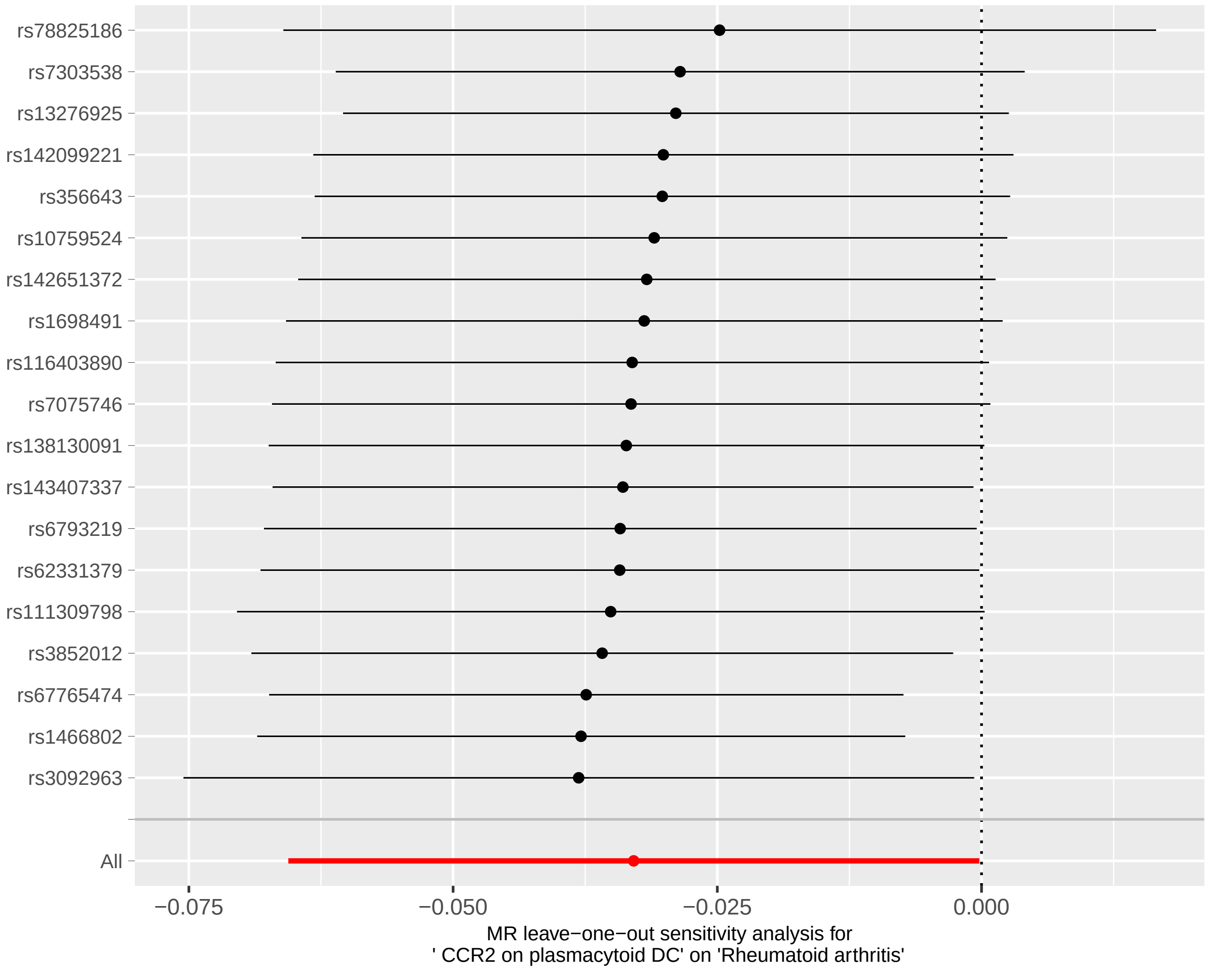
rs78825186
rs7303538
rs13276925
rs142099221
rs356643
rs10759524
rs142651372
rs1698491
rs116403890
rs7075746
rs138130091
rs143407337
rs6793219
rs62331379
rs111309798
rs3852012
rs67765474
rs1466802
rs3092963
All
−0.075
−0.050
−0.025
0.000
MR leave−one−out sensitivity analysis for
' CCR2 on plasmacytoid DC' on 'Rheumatoid arthritis'

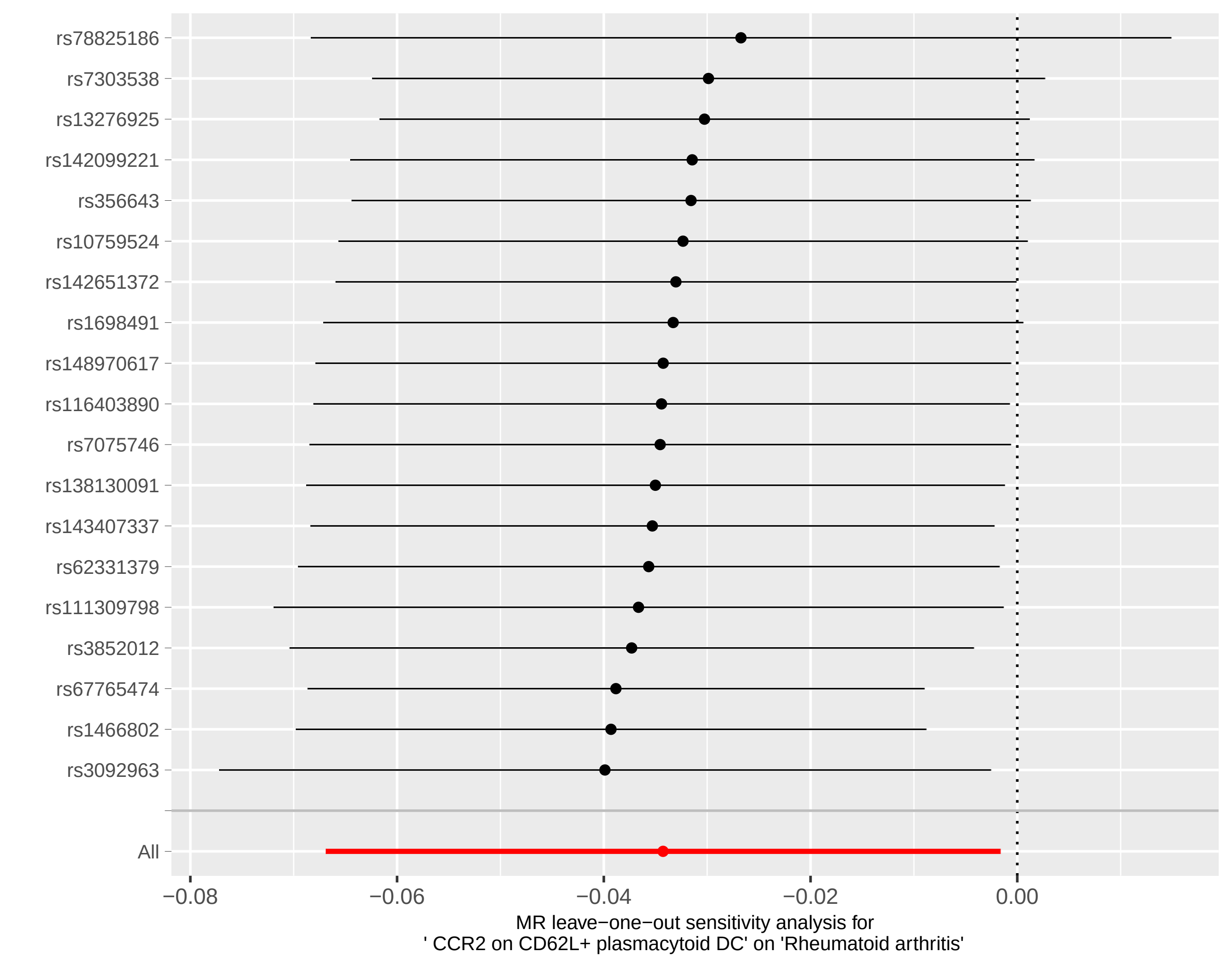

rs78825186
rs7303538
rs13276925
rs142099221
rs356643
rs10759524
rs142651372
rs1698491
rs148970617
rs116403890
rs7075746
rs138130091
rs143407337
rs62331379
rs111309798
rs3852012
rs67765474
rs1466802
rs3092963
All
−0.08
−0.06
−0.04
−0.02
0.00
MR leave−one−out sensitivity analysis for
' CCR2 on CD62L+ plasmacytoid DC' on 'Rheumatoid arthritis'

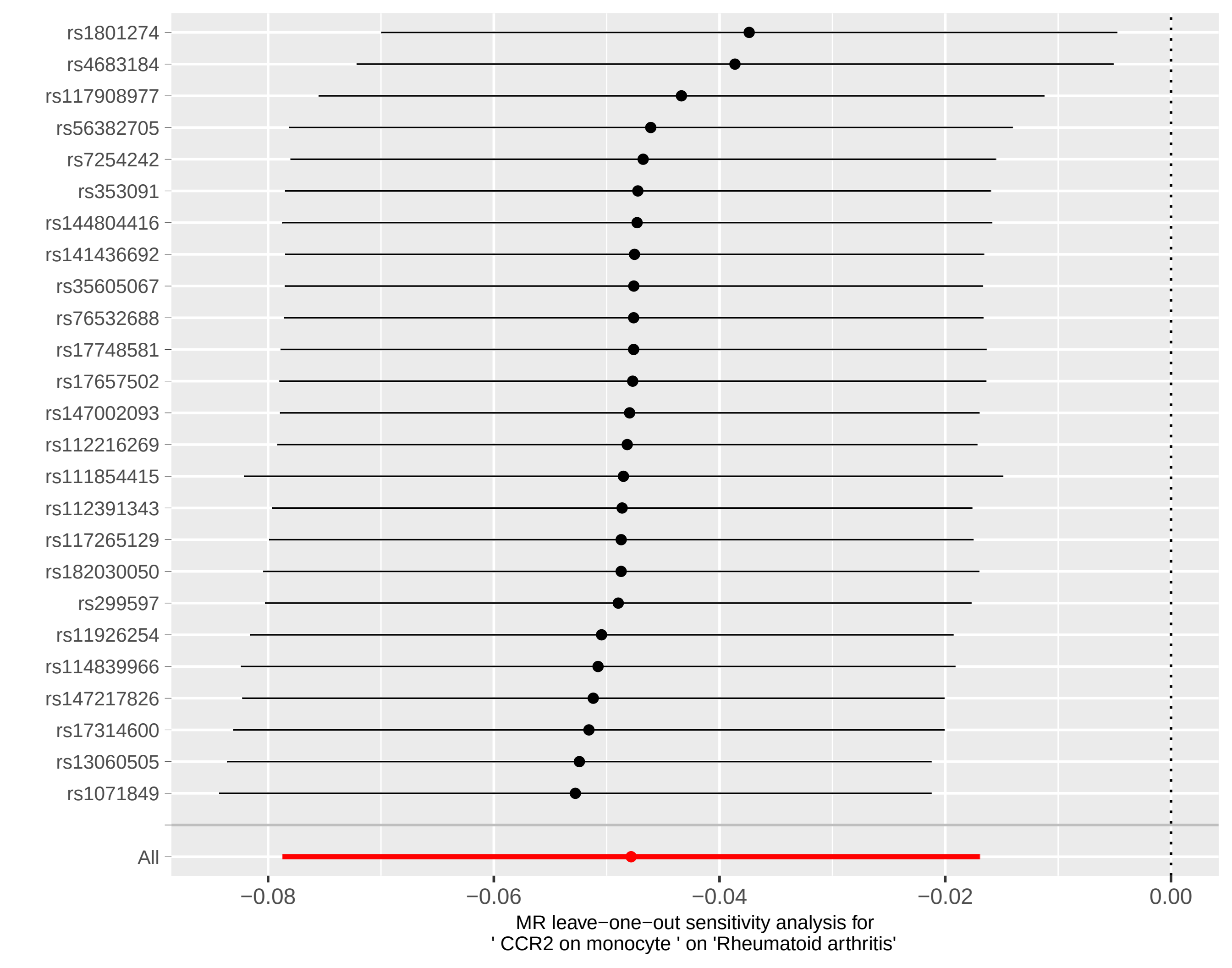
rs1801274
rs4683184
rs117908977
rs56382705
rs7254242
rs353091
rs144804416
rs141436692
rs35605067
rs76532688
rs17748581
rs17657502
rs147002093
rs112216269
rs111854415
rs112391343
rs117265129
rs182030050
rs299597
rs11926254
rs114839966
rs147217826
rs17314600
rs13060505
rs1071849
All
−0.08
−0.06
−0.04
−0.02
0.00
MR leave−one−out sensitivity analysis for
' CCR2 on monocyte ' on 'Rheumatoid arthritis'

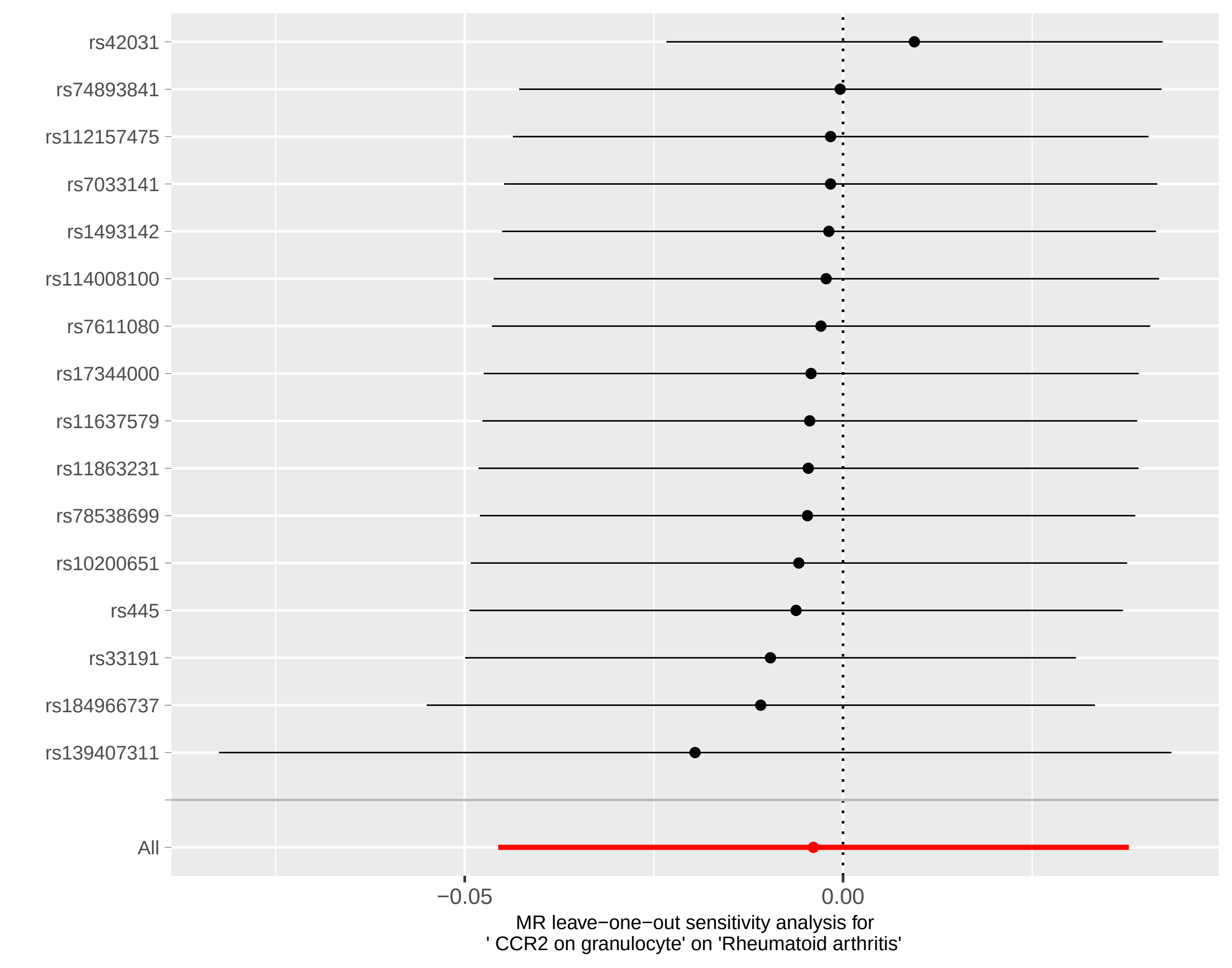
rs42031
rs74893841
rs112157475
rs7033141
rs1493142
rs114008100
rs7611080
rs17344000
rs11637579
rs11863231
rs78538699
rs10200651
rs445
rs33191
rs184966737
rs139407311
All
−0.05
0.00
MR leave−one−out sensitivity analysis for
' CCR2 on granulocyte' on 'Rheumatoid arthritis'

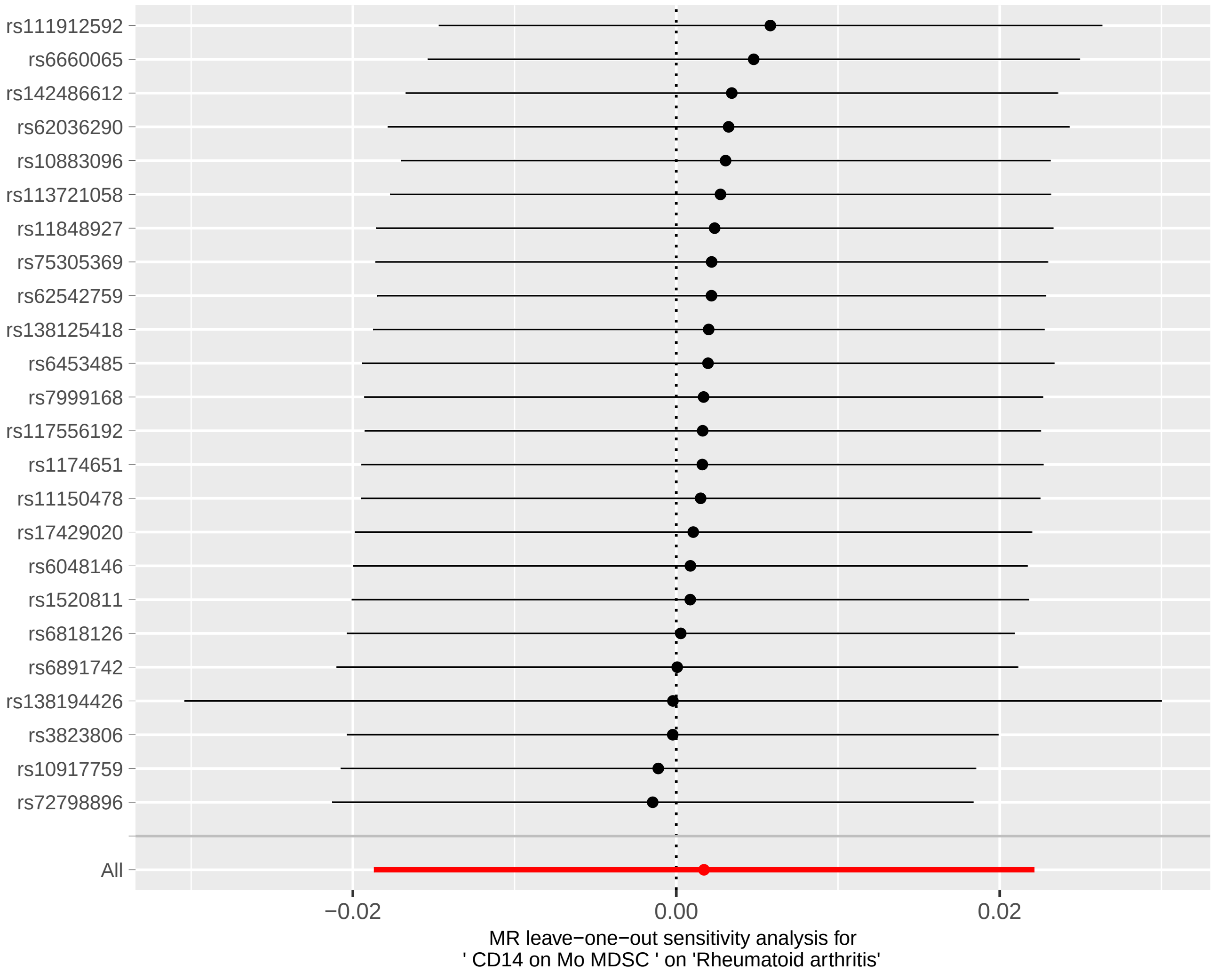
rs111912592
rs6660065
rs142486612
rs62036290
rs10883096
rs113721058
rs11848927
rs75305369
rs62542759
rs138125418
rs6453485
rs7999168
rs117556192
rs1174651
rs11150478
rs17429020
rs6048146
rs1520811
rs6818126
rs6891742
rs138194426
rs3823806
rs10917759
rs72798896
All
−0.02
0.00
0.02
MR leave−one−out sensitivity analysis for
' CD14 on Mo MDSC ' on 'Rheumatoid arthritis'

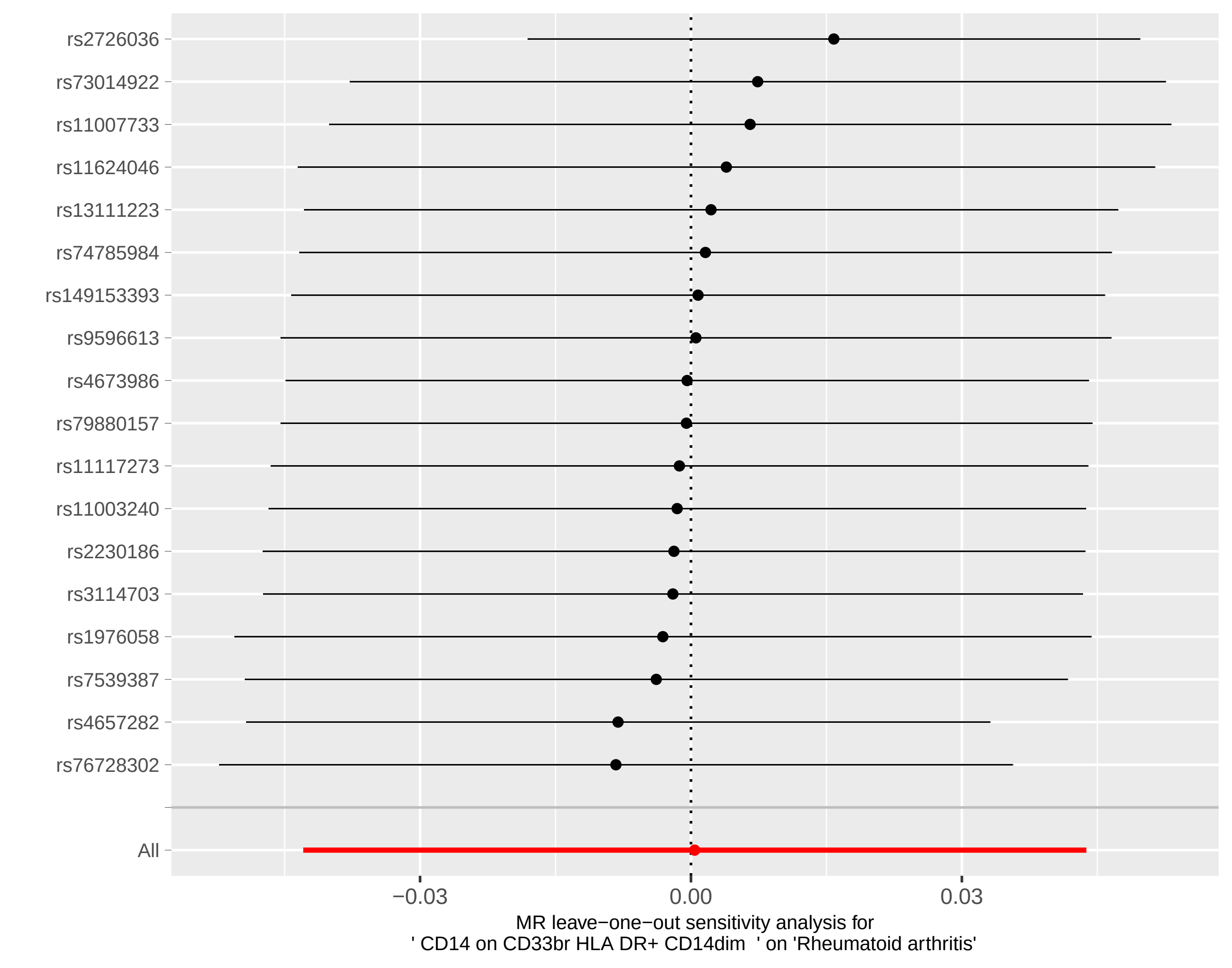

rs2726036
rs73014922
rs11007733
rs11624046
rs13111223
rs74785984
rs149153393
rs9596613
rs4673986
rs79880157
rs11117273
rs11003240
rs2230186
rs3114703
rs1976058
rs7539387
rs4657282
rs76728302
All
−0.03
0.00
0.03
MR leave−one−out sensitivity analysis for
' CD14 on CD33br HLA DR+ CD14dim ' on 'Rheumatoid arthritis'

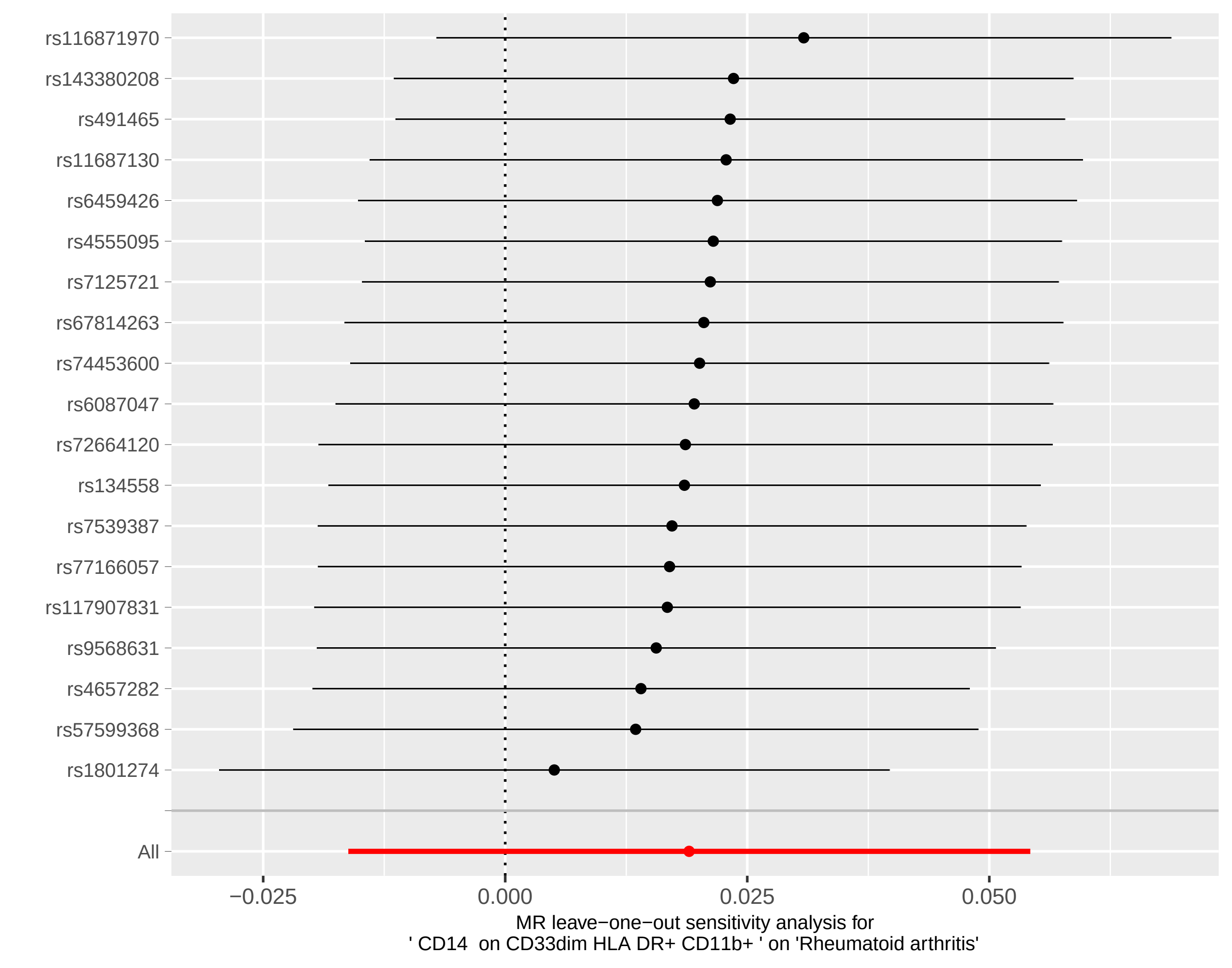

rs116871970
rs143380208
rs491465
rs11687130
rs6459426
rs4555095
rs7125721
rs67814263
rs74453600
rs6087047
rs72664120
rs134558
rs7539387
rs77166057
rs117907831
rs9568631
rs4657282
rs57599368
rs1801274
All
−0.025
0.000
0.025
0.050
MR leave−one−out sensitivity analysis for
' CD14 on CD33dim HLA DR+ CD11b+ ' on 'Rheumatoid arthritis'

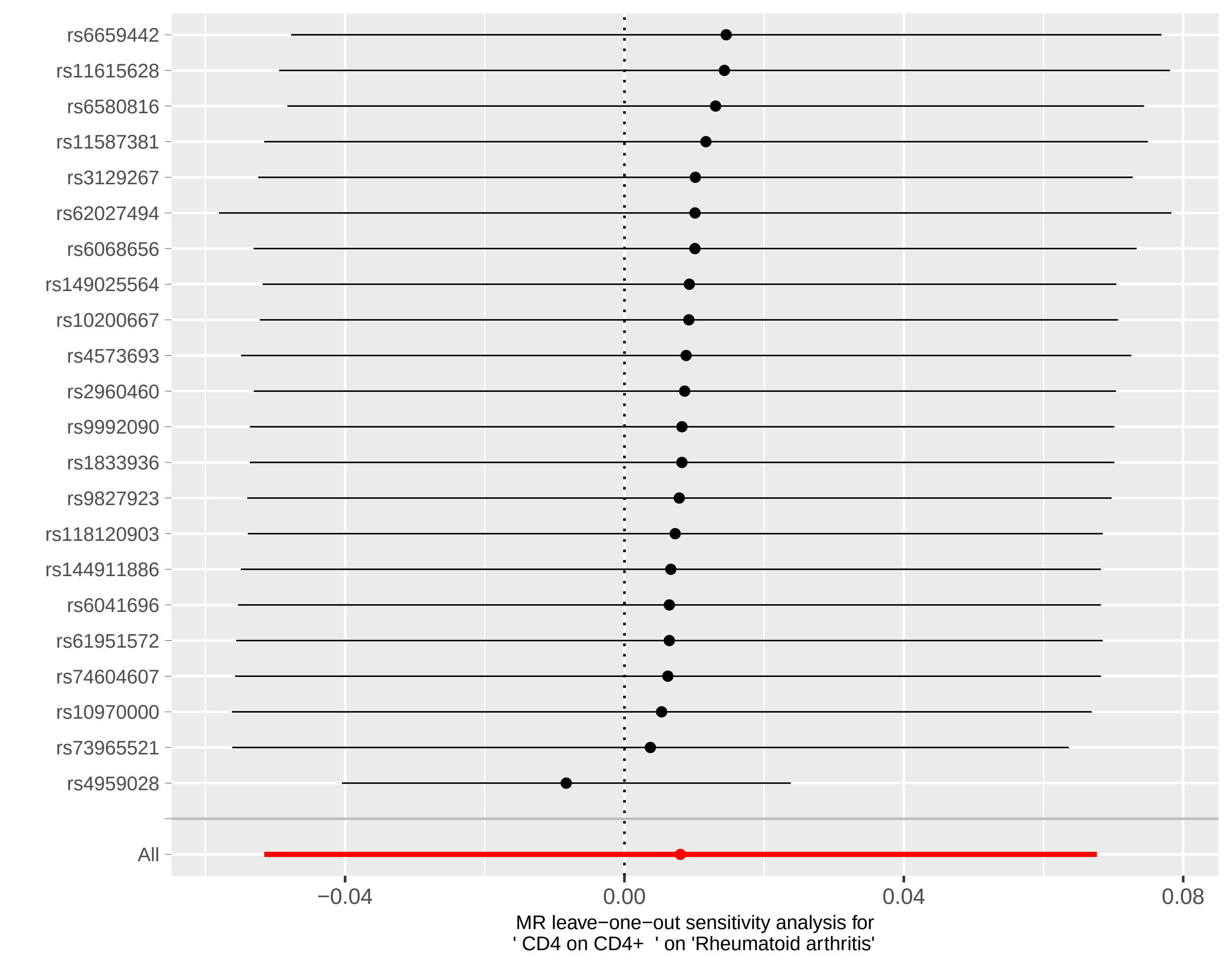

rs6659442
rs11615628
rs6580816
rs11587381
rs3129267
rs62027494
rs6068656
rs149025564
rs10200667
rs4573693
rs2960460
rs9992090
rs1833936
rs9827923
rs118120903
rs144911886
rs6041696
rs61951572
rs74604607
rs10970000
rs73965521
rs4959028
All
−0.04
0.00
0.04
0.08
MR leave−one−out sensitivity analysis for
' CD4 on CD4+ ' on 'Rheumatoid arthritis'

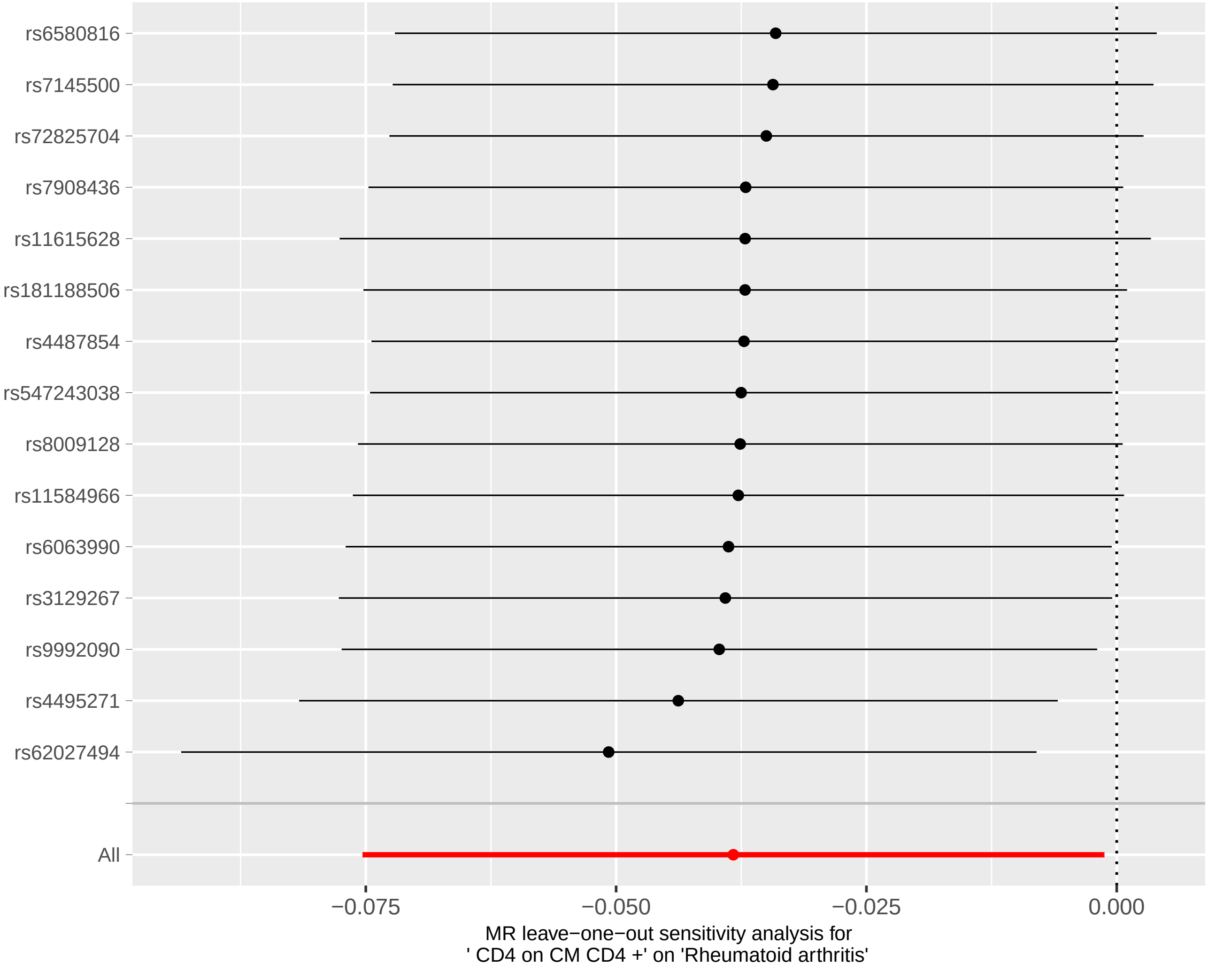
rs6580816
rs7145500
rs72825704
rs7908436
rs11615628
rs181188506
rs4487854
rs547243038
rs8009128
rs11584966
rs6063990
rs3129267
rs9992090
rs4495271
rs62027494
All
−0.075
−0.050
−0.025
0.000
MR leave−one−out sensitivity analysis for
' CD4 on CM CD4 +' on 'Rheumatoid arthritis'

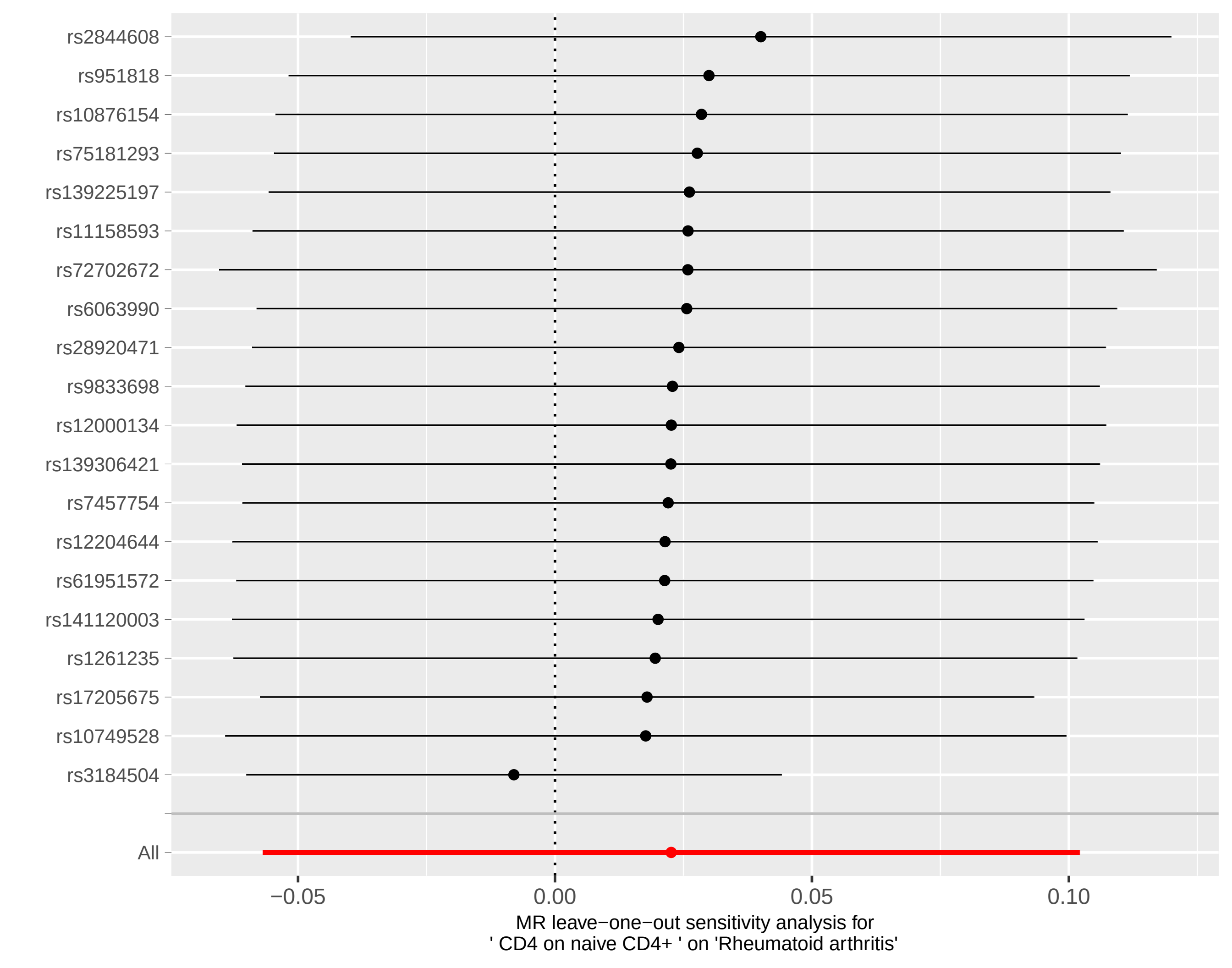

rs2844608
rs951818
rs10876154
rs75181293
rs139225197
rs11158593
rs72702672
rs6063990
rs28920471
rs9833698
rs12000134
rs139306421
rs7457754
rs12204644
rs61951572
rs141120003
rs1261235
rs17205675
rs10749528
rs3184504
All
−0.05
0.00
0.05
0.10
MR leave−one−out sensitivity analysis for
' CD4 on naive CD4+ ' on 'Rheumatoid arthritis'

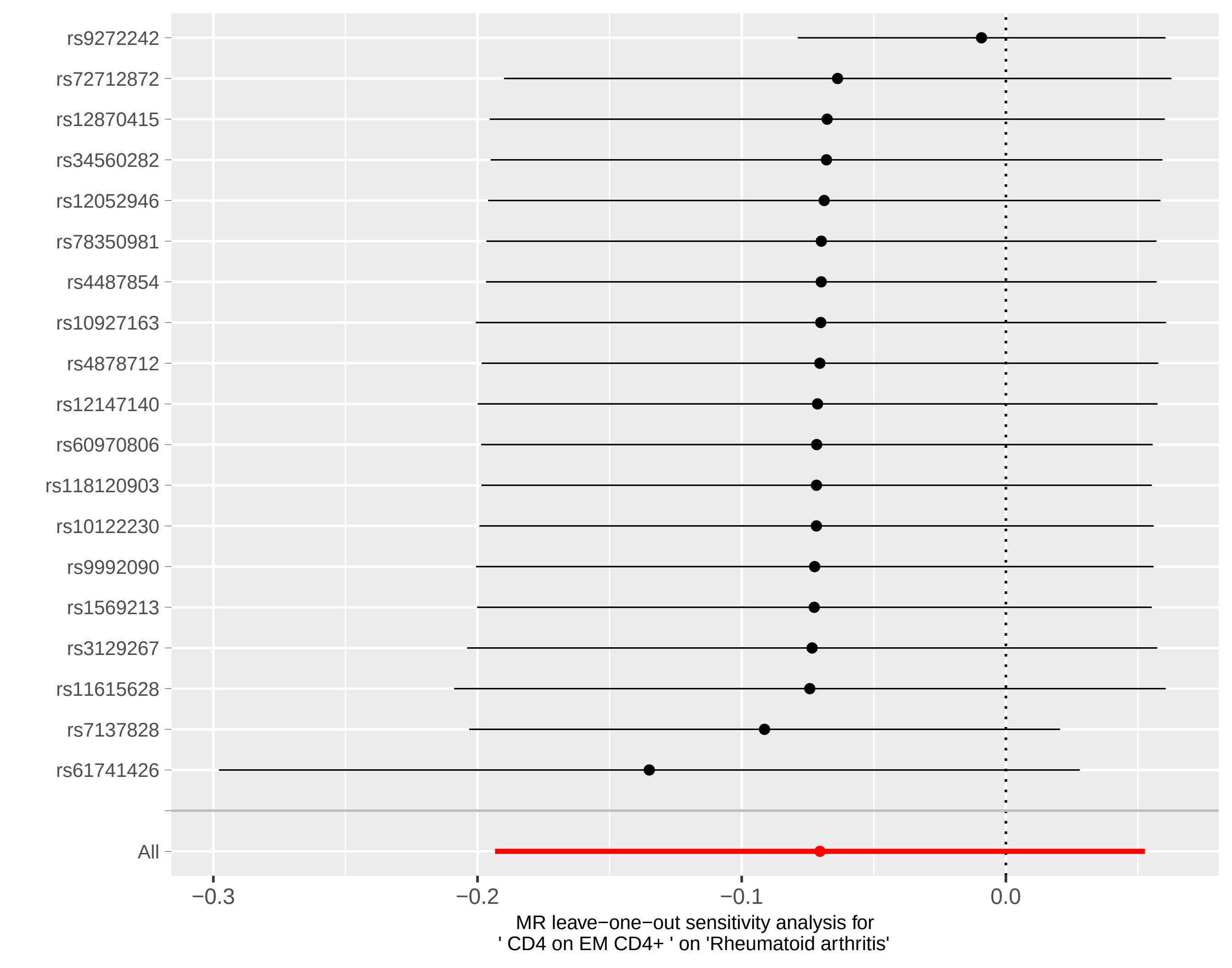
rs9272242
rs72712872
rs12870415
rs34560282
rs12052946
rs78350981
rs4487854
rs10927163
rs4878712
rs12147140
rs60970806
rs118120903
rs10122230
rs9992090
rs1569213
rs3129267
rs11615628
rs7137828
rs61741426
All
−0.3
−0.2
−0.1
0.0
MR leave−one−out sensitivity analysis for
' CD4 on EM CD4+ ' on 'Rheumatoid arthritis'

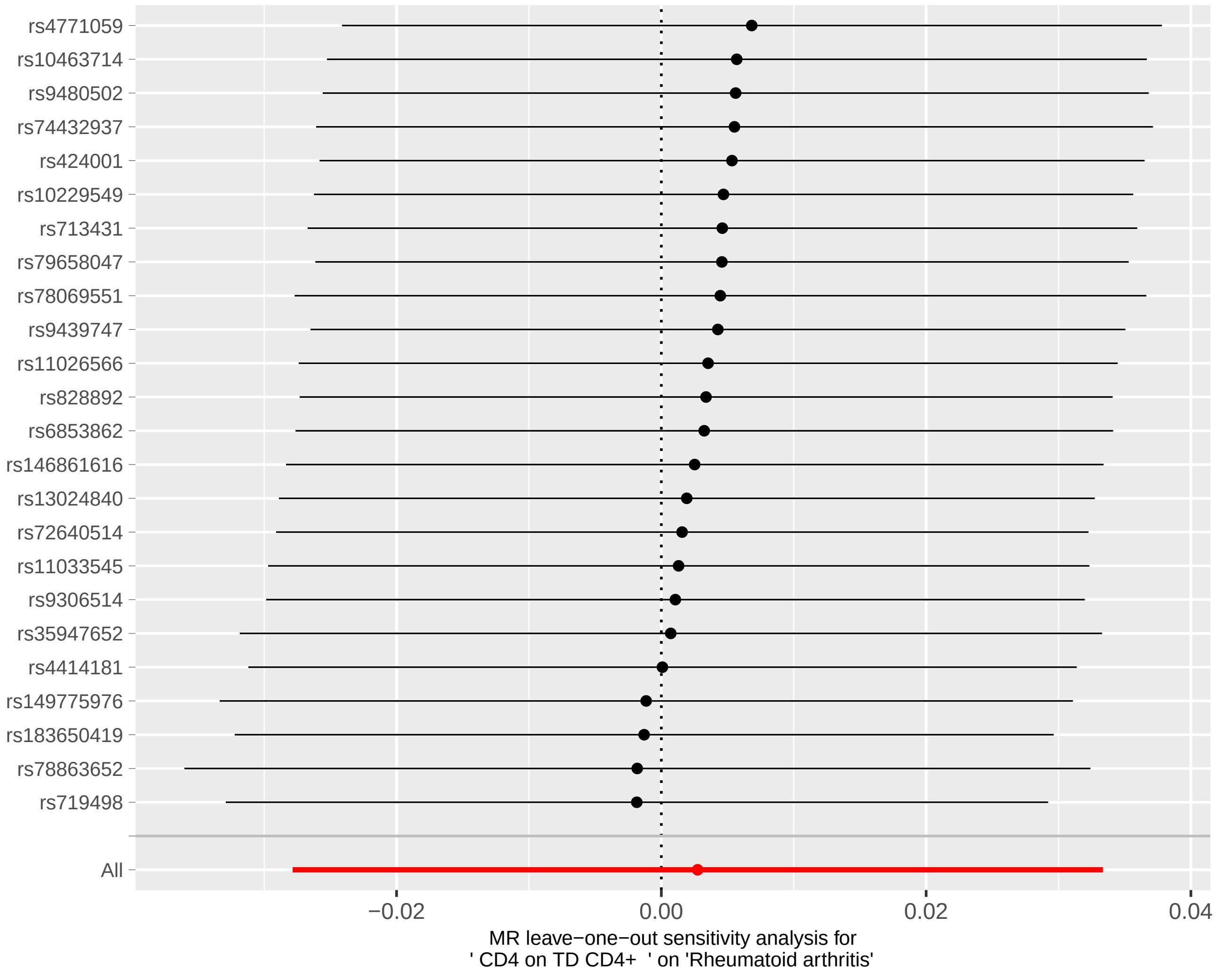
rs4771059
rs10463714
rs9480502
rs74432937
rs424001
rs10229549
rs713431
rs79658047
rs78069551
rs9439747
rs11026566
rs828892
rs6853862
rs146861616
rs13024840
rs72640514
rs11033545
rs9306514
rs35947652
rs4414181
rs149775976
rs183650419
rs78863652
rs719498
All
−0.02
0.00
0.02
0.04
MR leave−one−out sensitivity analysis for
' CD4 on TD CD4+ ' on 'Rheumatoid arthritis'

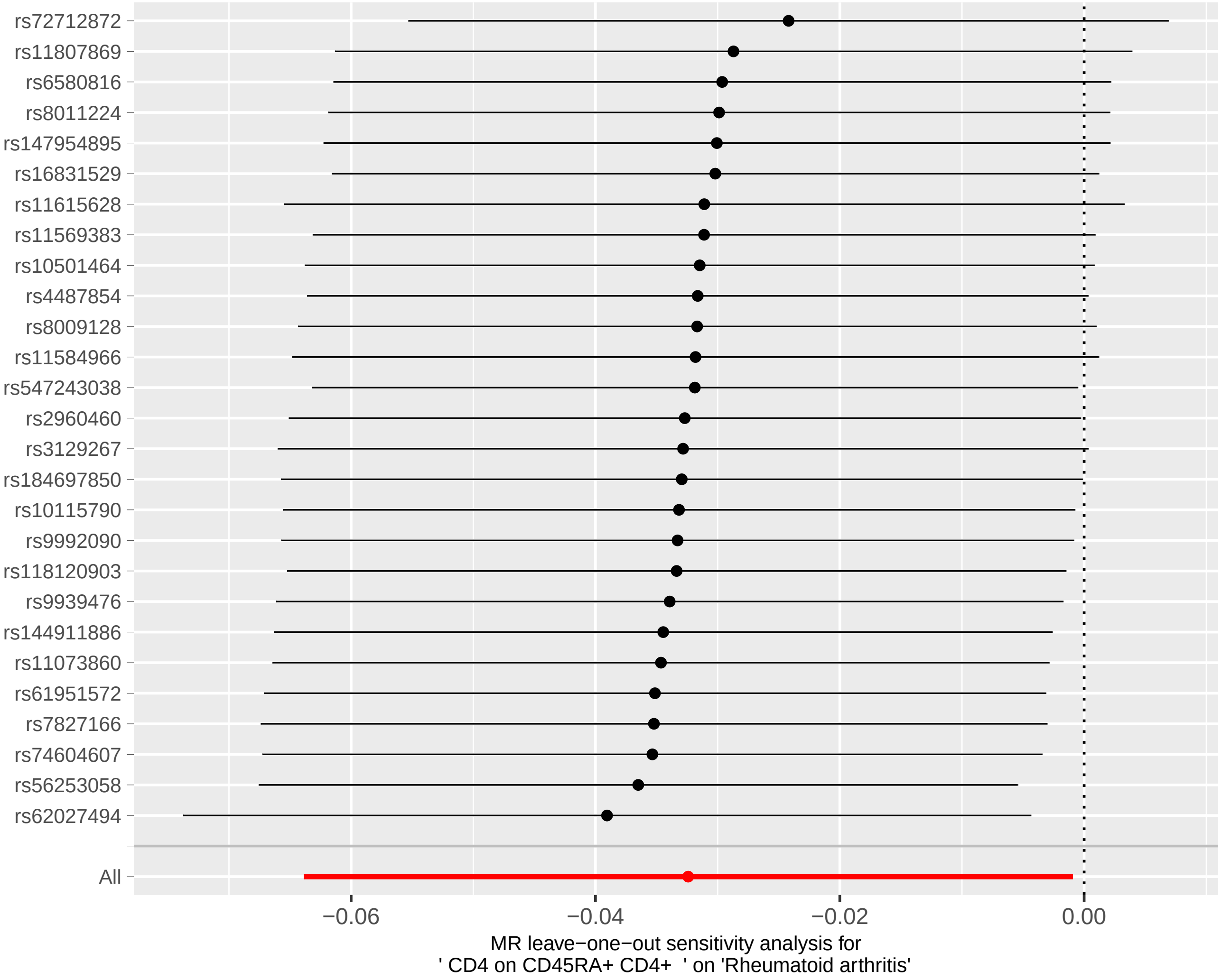
rs72712872
rs11807869
rs6580816
rs8011224
rs147954895
rs16831529
rs11615628
rs11569383
rs10501464
rs4487854
rs8009128
rs11584966
rs547243038
rs2960460
rs3129267
rs184697850
rs10115790
rs9992090
rs118120903
rs9939476
rs144911886
rs11073860
rs61951572
rs7827166
rs74604607
rs56253058
rs62027494
All
−0.06
−0.04
−0.02
0.00
MR leave−one−out sensitivity analysis for
' CD4 on CD45RA+ CD4+ ' on 'Rheumatoid arthritis'

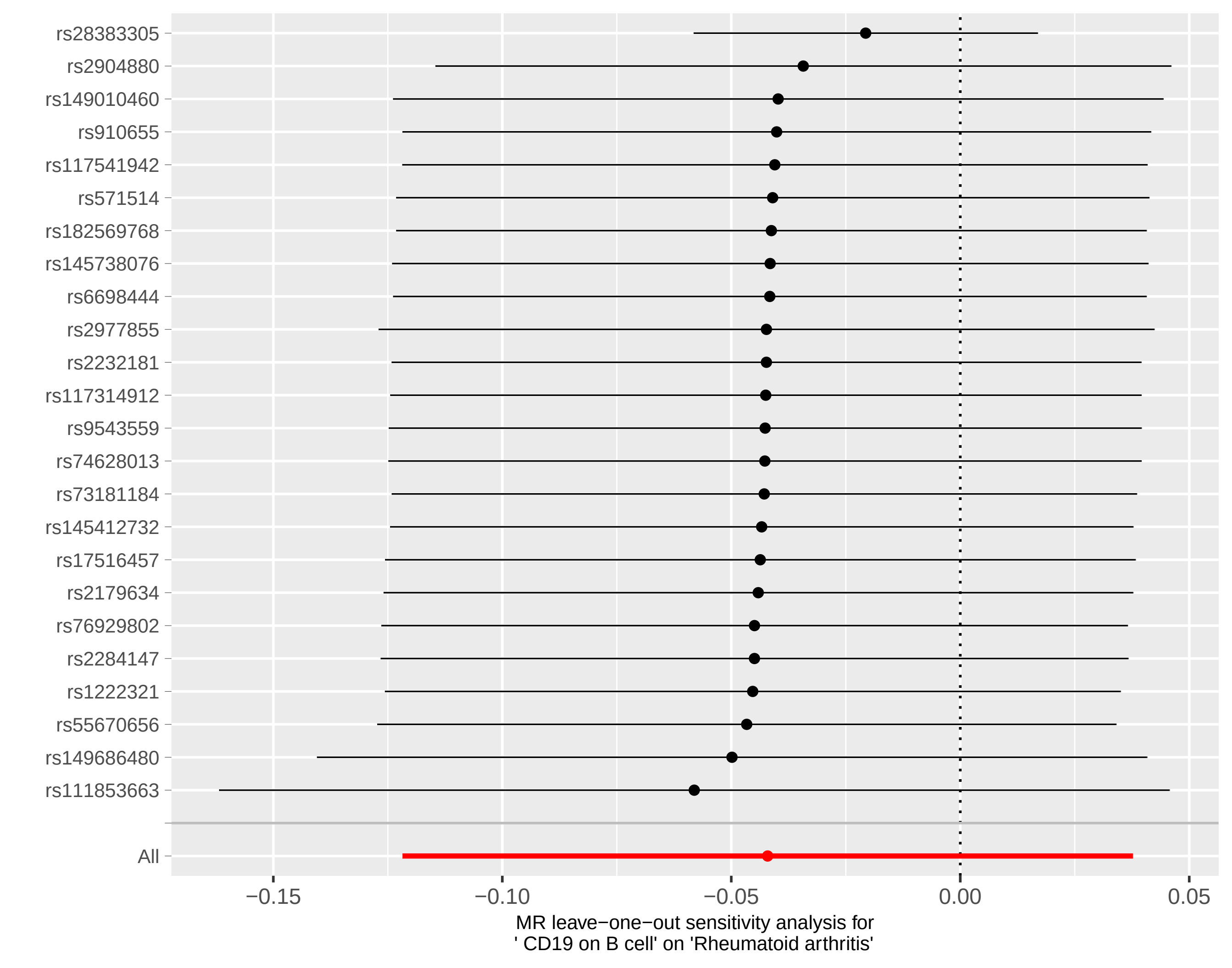
rs28383305
rs2904880
rs149010460
rs910655
rs117541942
rs571514
rs182569768
rs145738076
rs6698444
rs2977855
rs2232181
rs117314912
rs9543559
rs74628013
rs73181184
rs145412732
rs17516457
rs2179634
rs76929802
rs2284147
rs1222321
rs55670656
rs149686480
rs111853663
All
−0.15
−0.10
−0.05
0.00
0.05
MR leave−one−out sensitivity analysis for
' CD19 on B cell' on 'Rheumatoid arthritis'

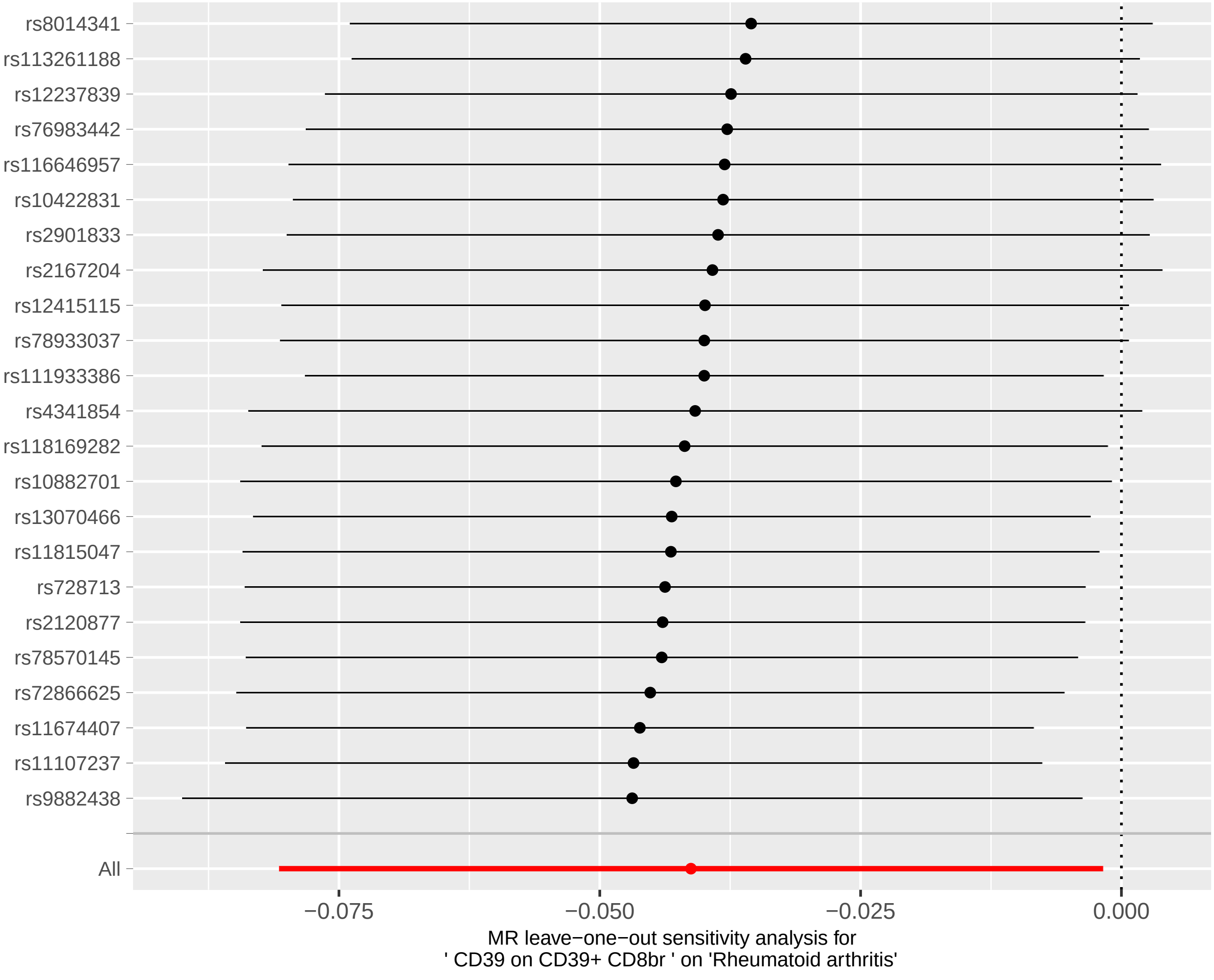
rs8014341
rs113261188
rs12237839
rs76983442
rs116646957
rs10422831
rs2901833
rs2167204
rs12415115
rs78933037
rs111933386
rs4341854
rs118169282
rs10882701
rs13070466
rs11815047
rs728713
rs2120877
rs78570145
rs72866625
rs11674407
rs11107237
rs9882438
All
−0.075
−0.050
−0.025
0.000
MR leave−one−out sensitivity analysis for
' CD39 on CD39+ CD8br ' on 'Rheumatoid arthritis'

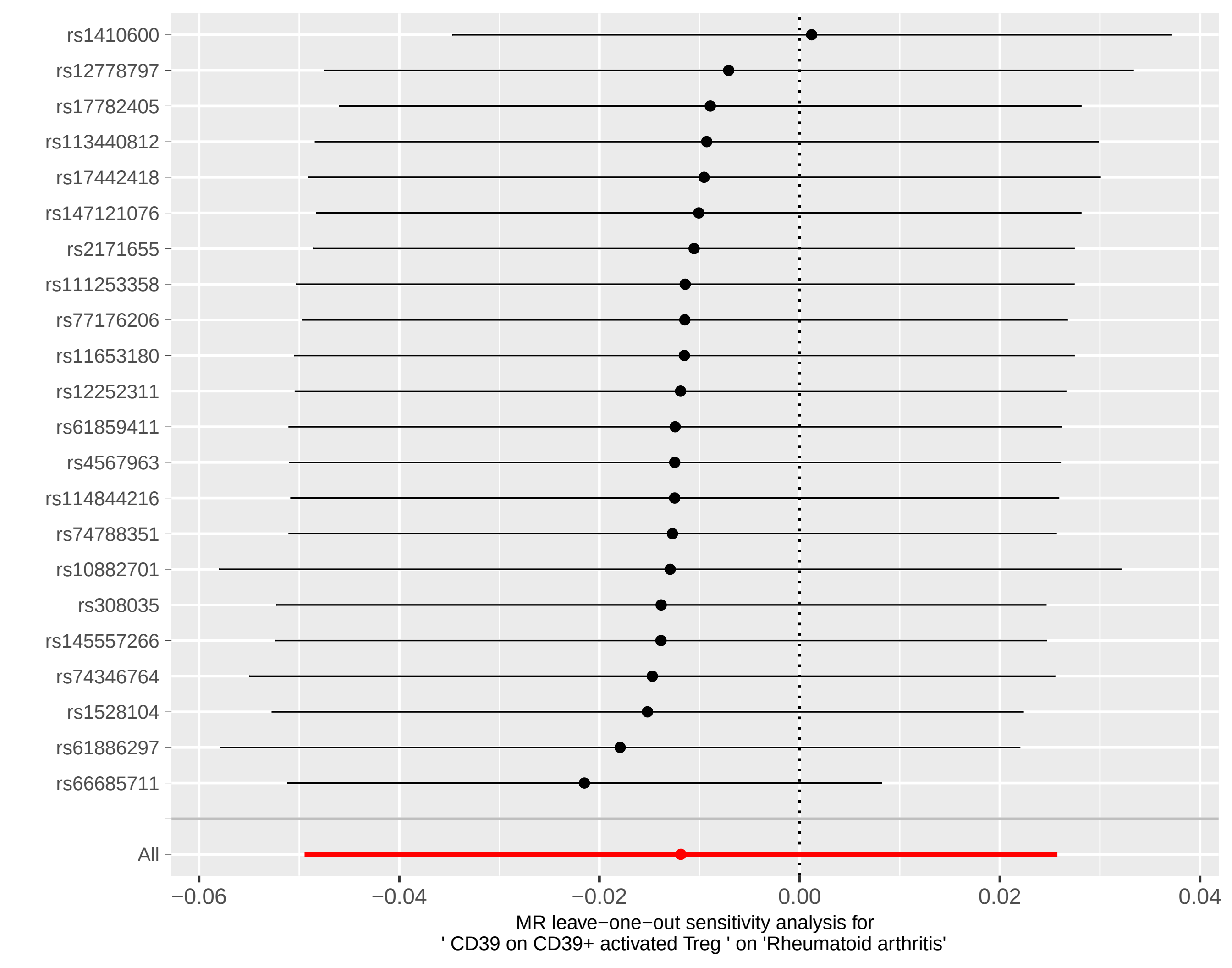

rs1410600
rs12778797
rs17782405
rs113440812
rs17442418
rs147121076
rs2171655
rs111253358
rs77176206
rs11653180
rs12252311
rs61859411
rs4567963
rs114844216
rs74788351
rs10882701
rs308035
rs145557266
rs74346764
rs1528104
rs61886297
rs66685711
All
−0.06
−0.04
−0.02
0.00
0.02
0.04
MR leave−one−out sensitivity analysis for
' CD39 on CD39+ activated Treg ' on 'Rheumatoid arthritis'

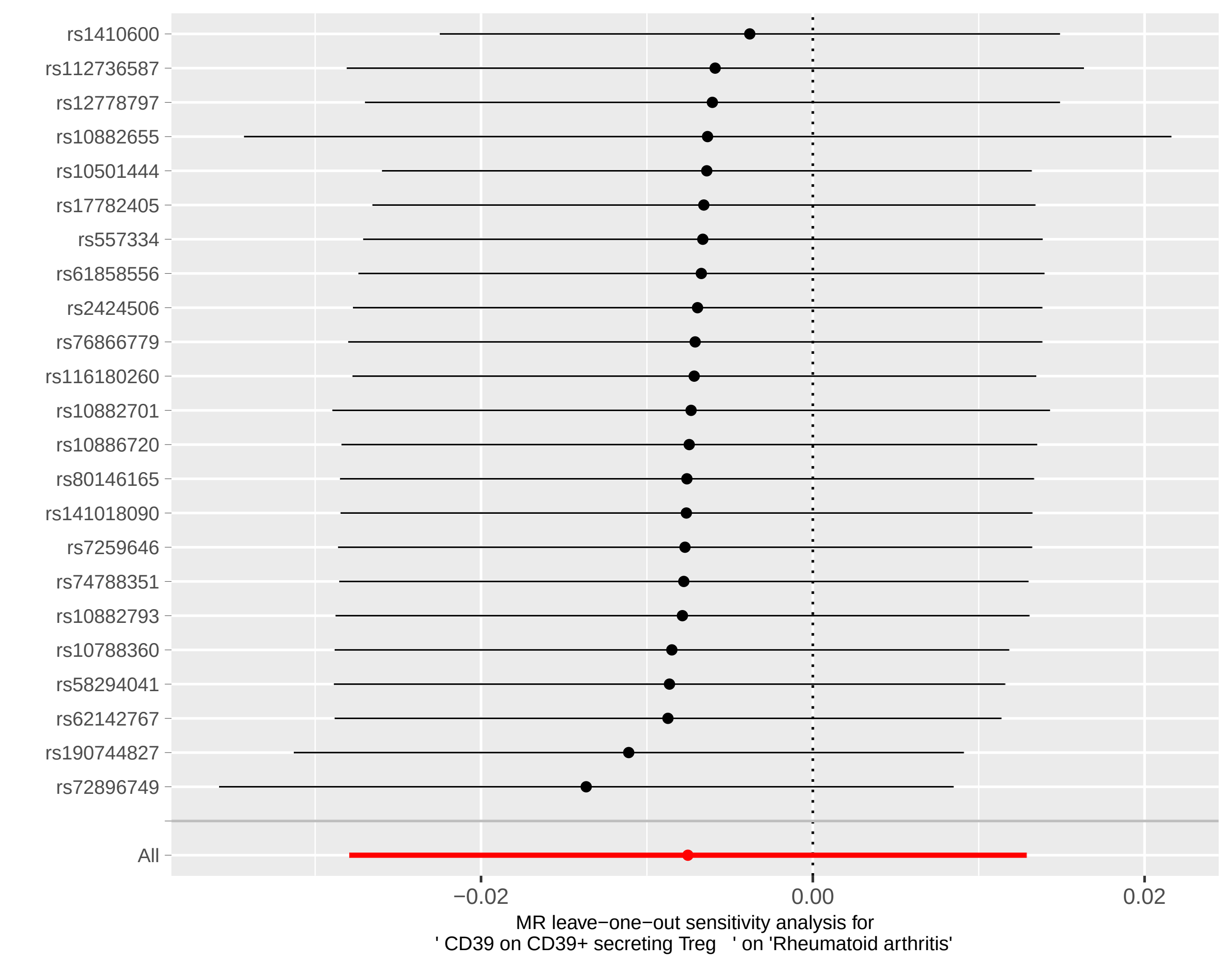
rs1410600
rs112736587
rs12778797
rs10882655
rs10501444
rs17782405
rs557334
rs61858556
rs2424506
rs76866779
rs116180260
rs10882701
rs10886720
rs80146165
rs141018090
rs7259646
rs74788351
rs10882793
rs10788360
rs58294041
rs62142767
rs190744827
rs72896749
All
−0.02
0.00
0.02
MR leave−one−out sensitivity analysis for
' CD39 on CD39+ secreting Treg ' on 'Rheumatoid arthritis'

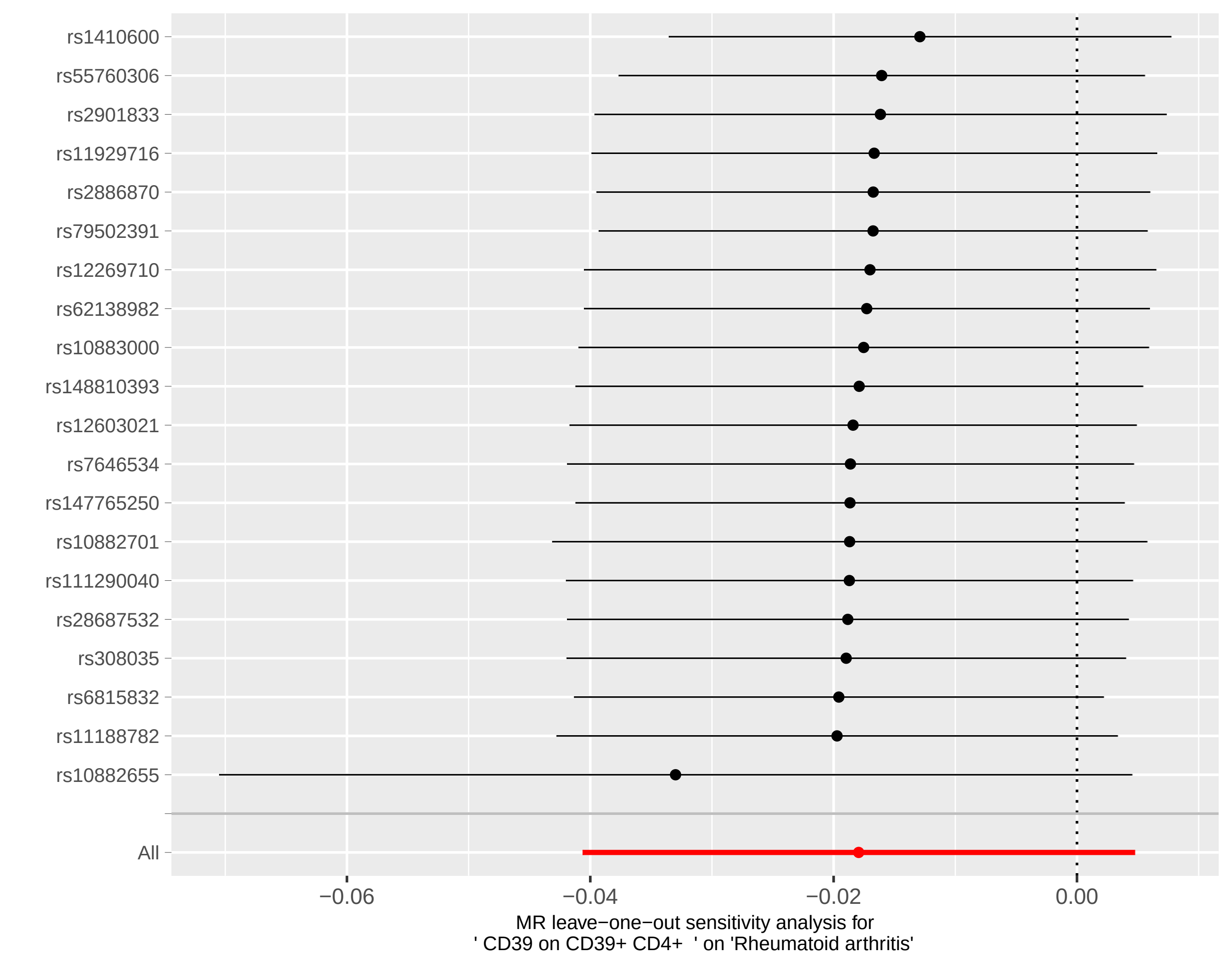

rs1410600
rs55760306
rs2901833
rs11929716
rs2886870
rs79502391
rs12269710
rs62138982
rs10883000
rs148810393
rs12603021
rs7646534
rs147765250
rs10882701
rs111290040
rs28687532
rs308035
rs6815832
rs11188782
rs10882655
All
−0.06
−0.04
−0.02
0.00
MR leave−one−out sensitivity analysis for
' CD39 on CD39+ CD4+ ' on 'Rheumatoid arthritis'

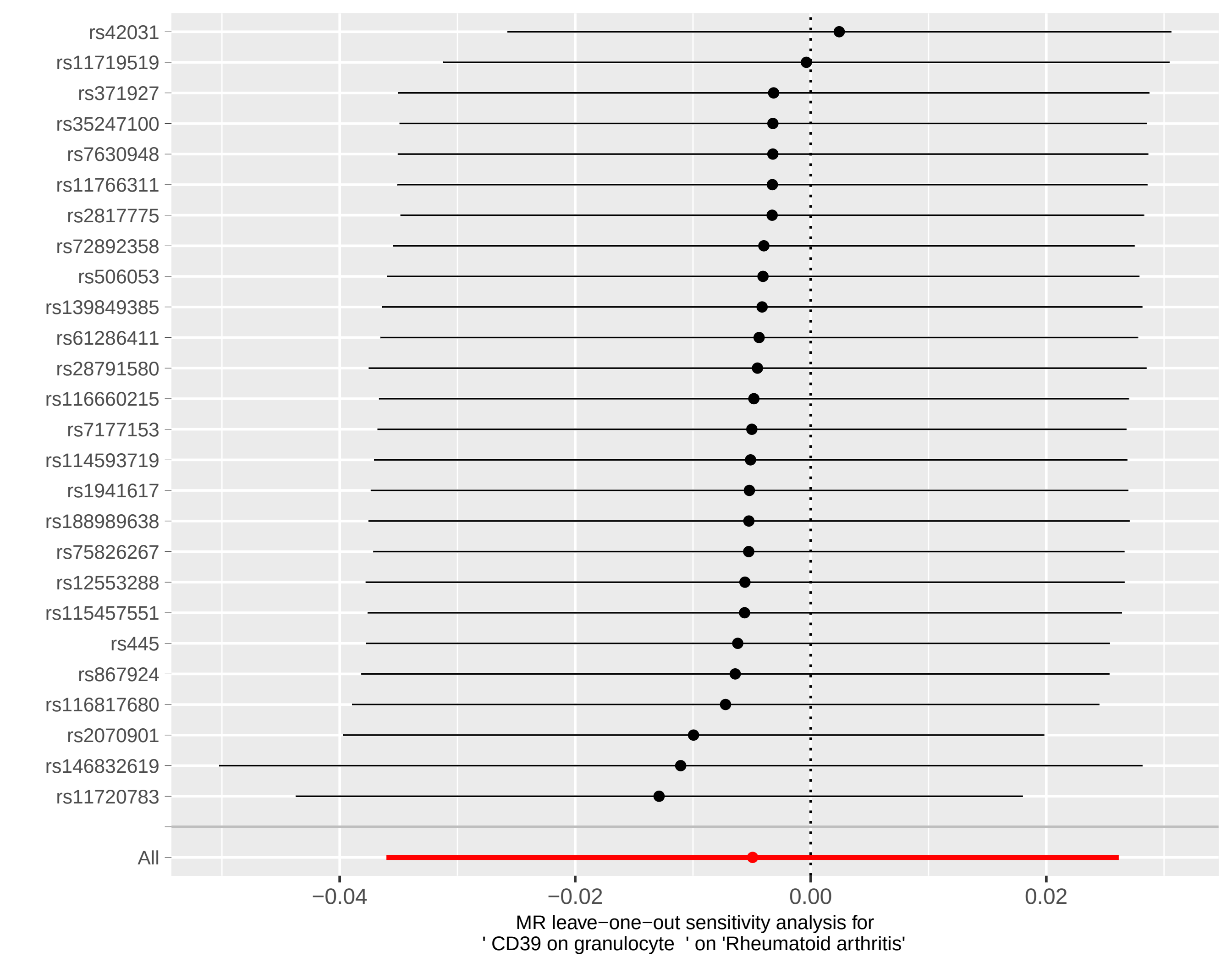
rs42031
rs11719519
rs371927
rs35247100
rs7630948
rs11766311
rs2817775
rs72892358
rs506053
rs139849385
rs61286411
rs28791580
rs116660215
rs7177153
rs114593719
rs1941617
rs188989638
rs75826267
rs12553288
rs115457551
rs445
rs867924
rs116817680
rs2070901
rs146832619
rs11720783
All
−0.04
−0.02
0.00
0.02
MR leave−one−out sensitivity analysis for
' CD39 on granulocyte ' on 'Rheumatoid arthritis'

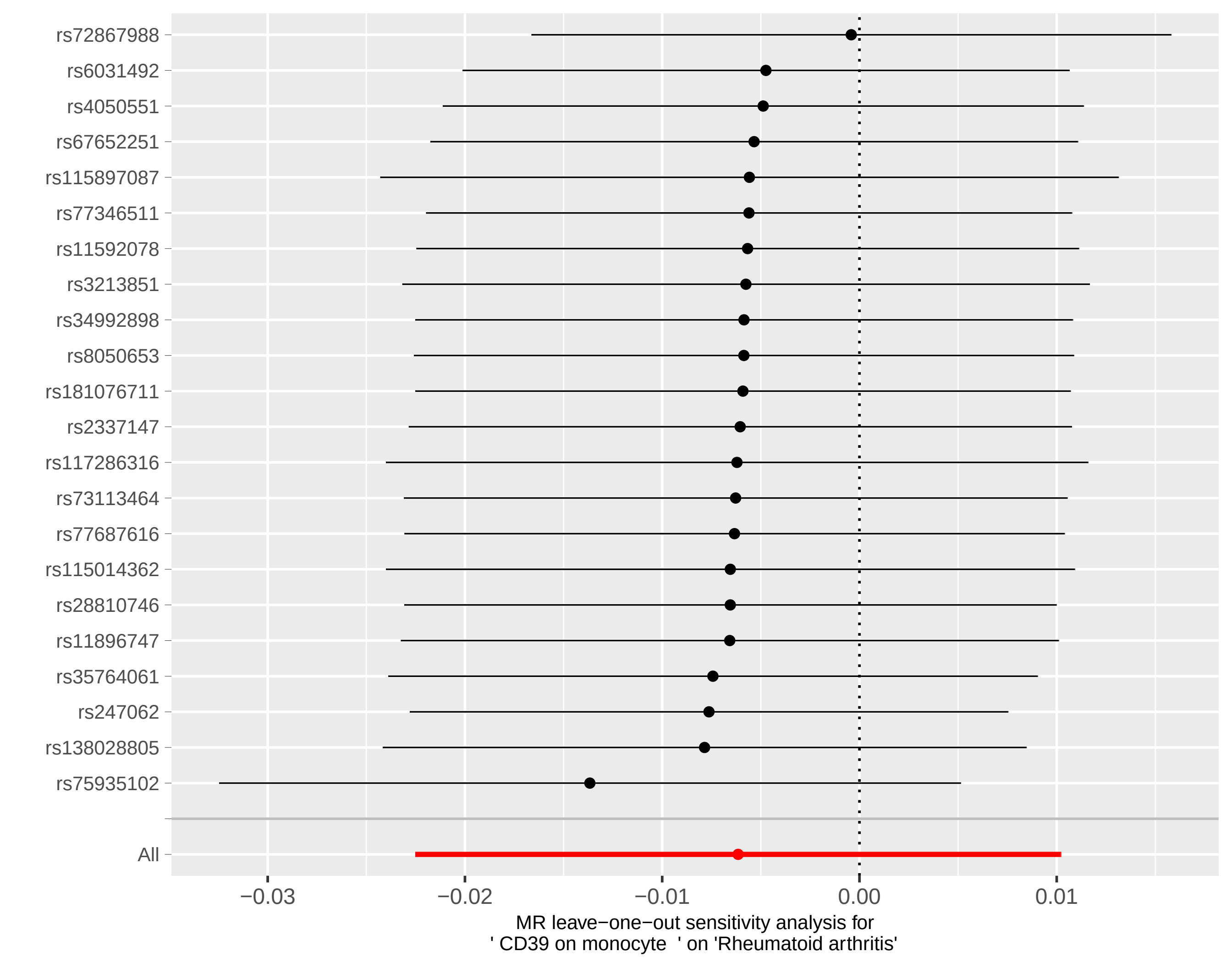
rs72867988
rs6031492
rs4050551
rs67652251
rs115897087
rs77346511
rs11592078
rs3213851
rs34992898
rs8050653
rs181076711
rs2337147
rs117286316
rs73113464
rs77687616
rs115014362
rs28810746
rs11896747
rs35764061
rs247062
rs138028805
rs75935102
All
−0.03
−0.02
−0.01
0.00
0.01
MR leave−one−out sensitivity analysis for
' CD39 on monocyte ' on 'Rheumatoid arthritis'

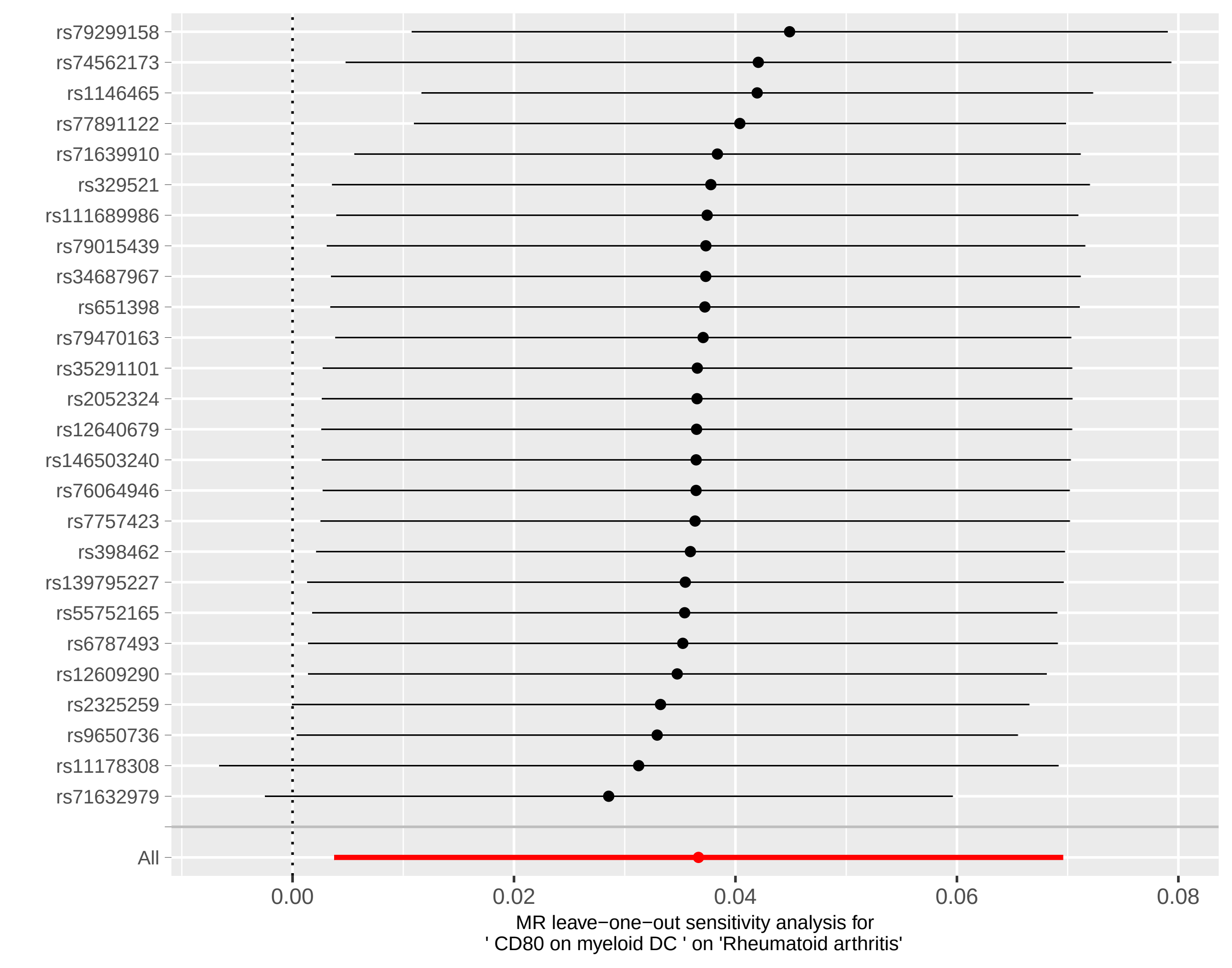

rs79299158
rs74562173
rs1146465
rs77891122
rs71639910
rs329521
rs111689986
rs79015439
rs34687967
rs651398
rs79470163
rs35291101
rs2052324
rs12640679
rs146503240
rs76064946
rs7757423
rs398462
rs139795227
rs55752165
rs6787493
rs12609290
rs2325259
rs9650736
rs11178308
rs71632979
All
0.00
0.02
0.04
0.06
0.08
MR leave−one−out sensitivity analysis for
' CD80 on myeloid DC ' on 'Rheumatoid arthritis'

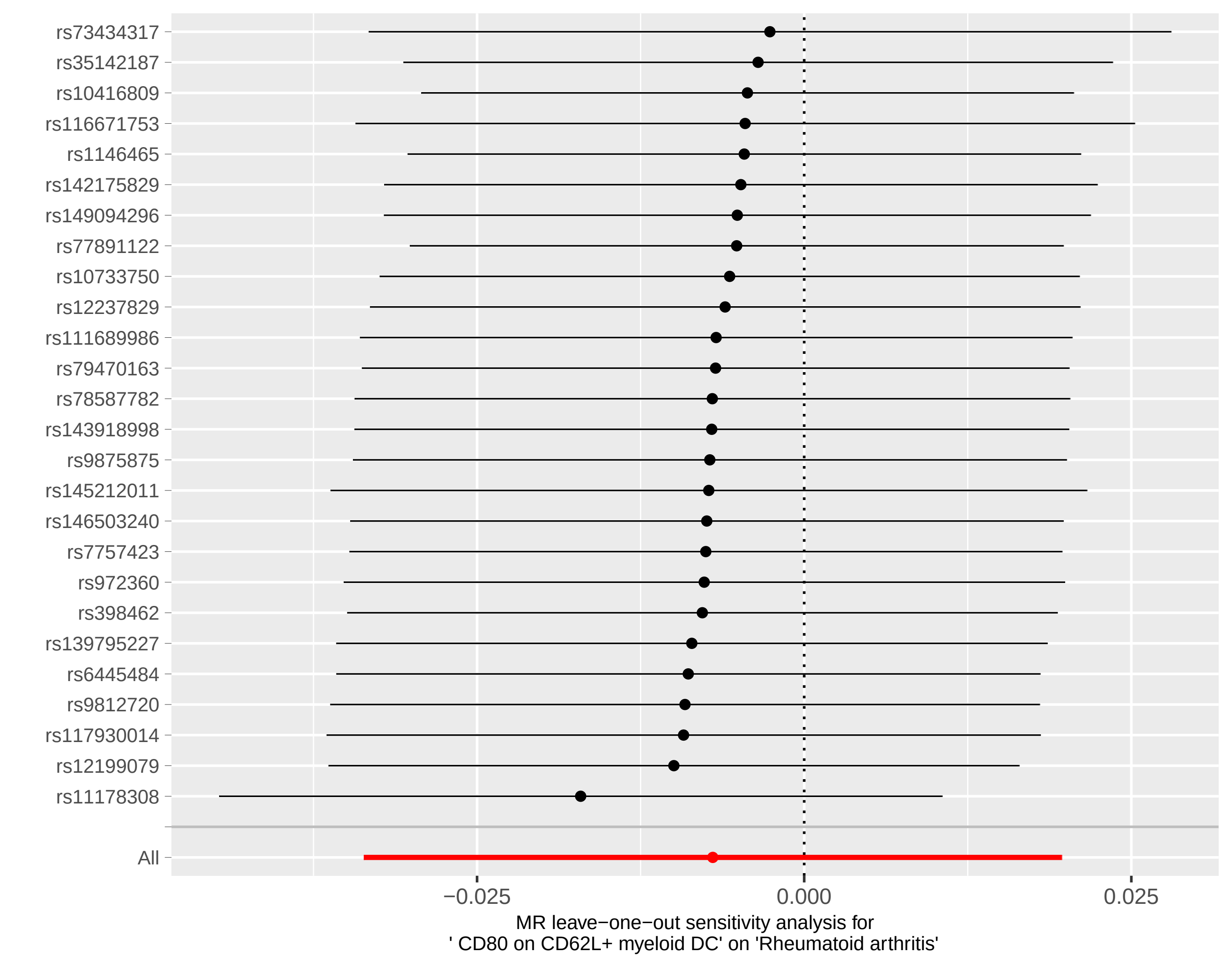

rs73434317
rs35142187
rs10416809
rs116671753
rs1146465
rs142175829
rs149094296
rs77891122
rs10733750
rs12237829
rs111689986
rs79470163
rs78587782
rs143918998
rs9875875
rs145212011
rs146503240
rs7757423
rs972360
rs398462
rs139795227
rs6445484
rs9812720
rs117930014
rs12199079
rs11178308
All
−0.025
0.000
0.025
MR leave−one−out sensitivity analysis for
' CD80 on CD62L+ myeloid DC' on 'Rheumatoid arthritis'

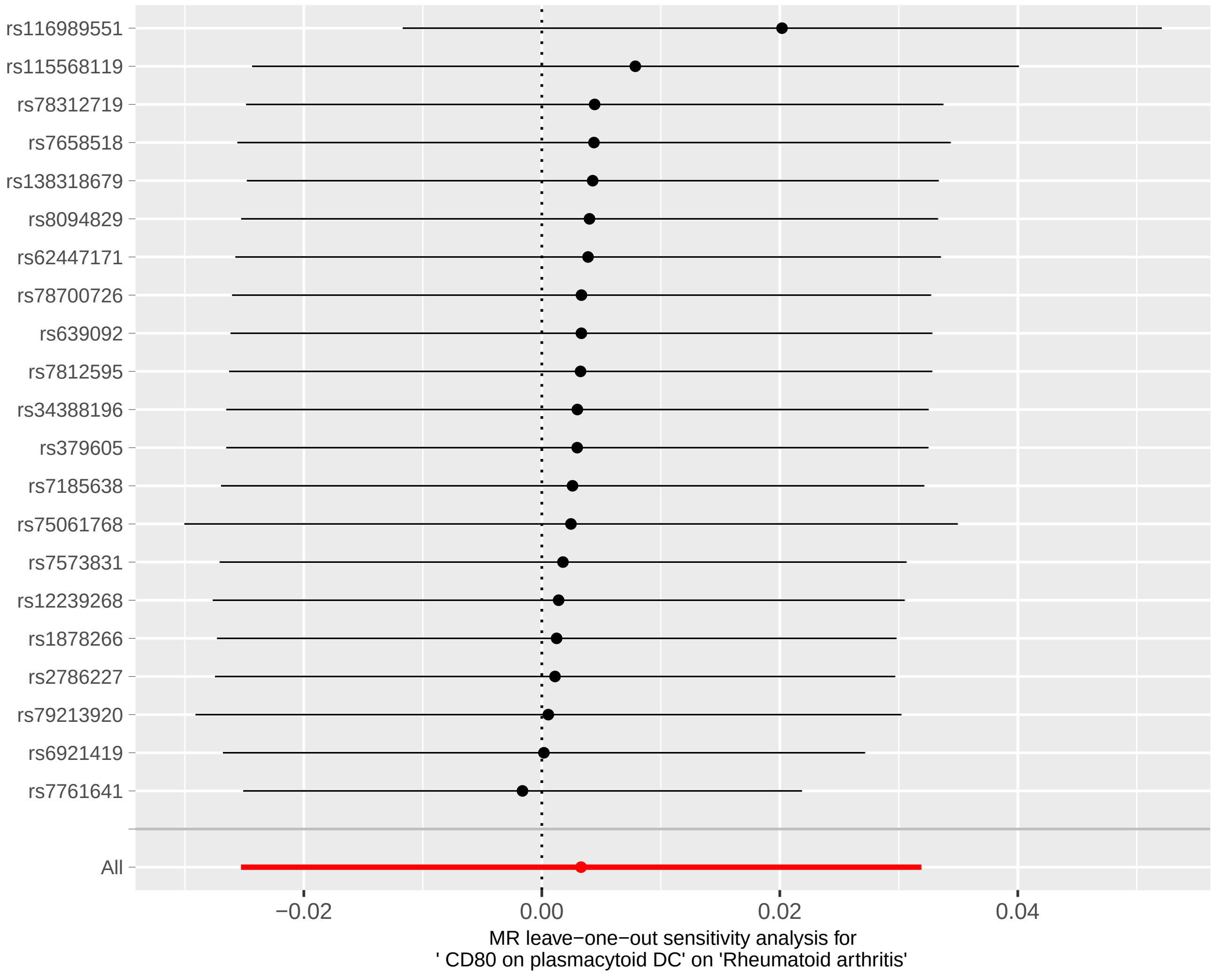
rs116989551
rs115568119
rs78312719
rs7658518
rs138318679
rs8094829
rs62447171
rs78700726
rs639092
rs7812595
rs34388196
rs379605
rs7185638
rs75061768
rs7573831
rs12239268
rs1878266
rs2786227
rs79213920
rs6921419
rs7761641
All
−0.02
0.00
0.02
0.04
MR leave−one−out sensitivity analysis for
' CD80 on plasmacytoid DC' on 'Rheumatoid arthritis'

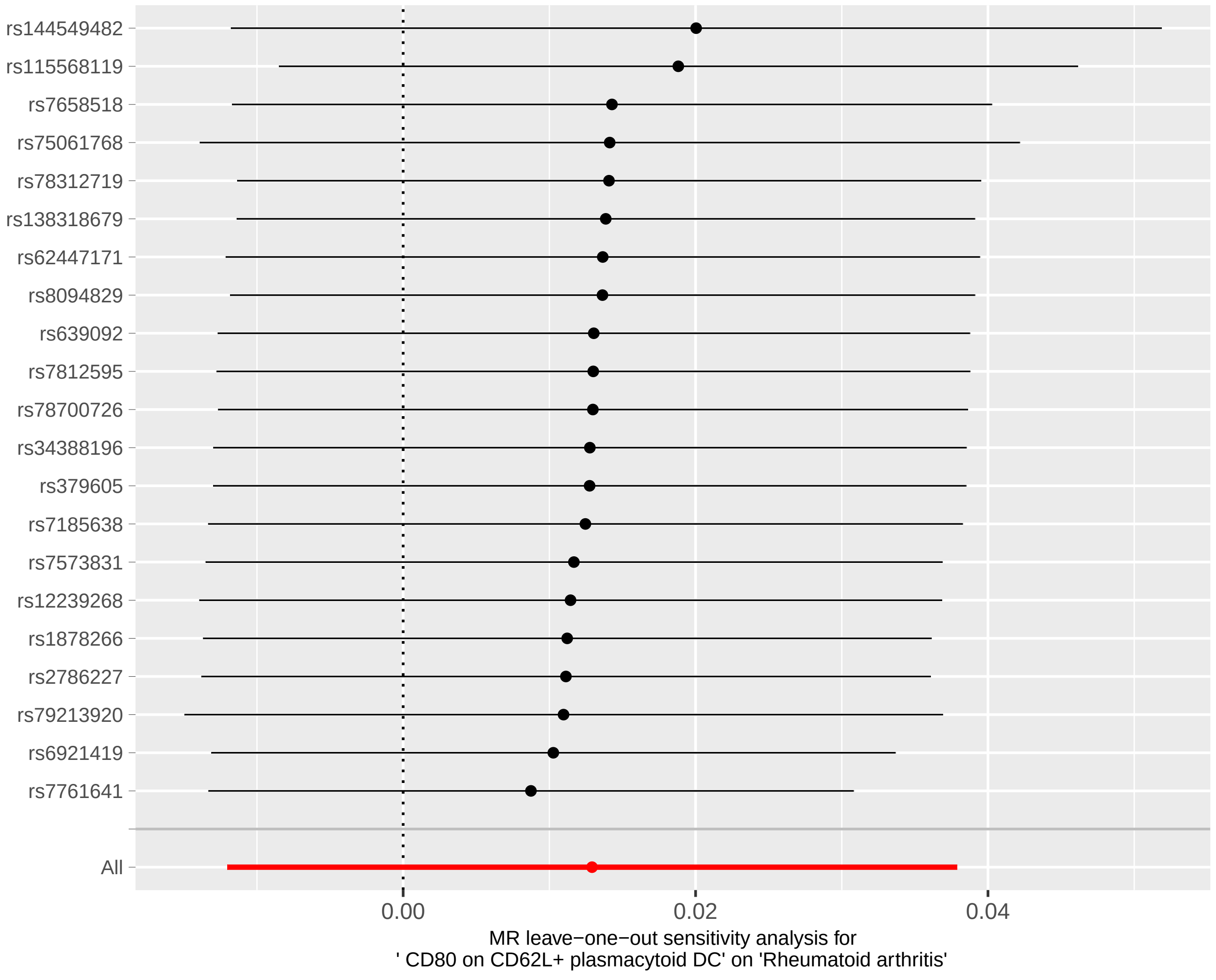
rs144549482
rs115568119
rs7658518
rs75061768
rs78312719
rs138318679
rs62447171
rs8094829
rs639092
rs7812595
rs78700726
rs34388196
rs379605
rs7185638
rs7573831
rs12239268
rs1878266
rs2786227
rs79213920
rs6921419
rs7761641
All
0.00
0.02
0.04
MR leave−one−out sensitivity analysis for
' CD80 on CD62L+ plasmacytoid DC' on 'Rheumatoid arthritis'

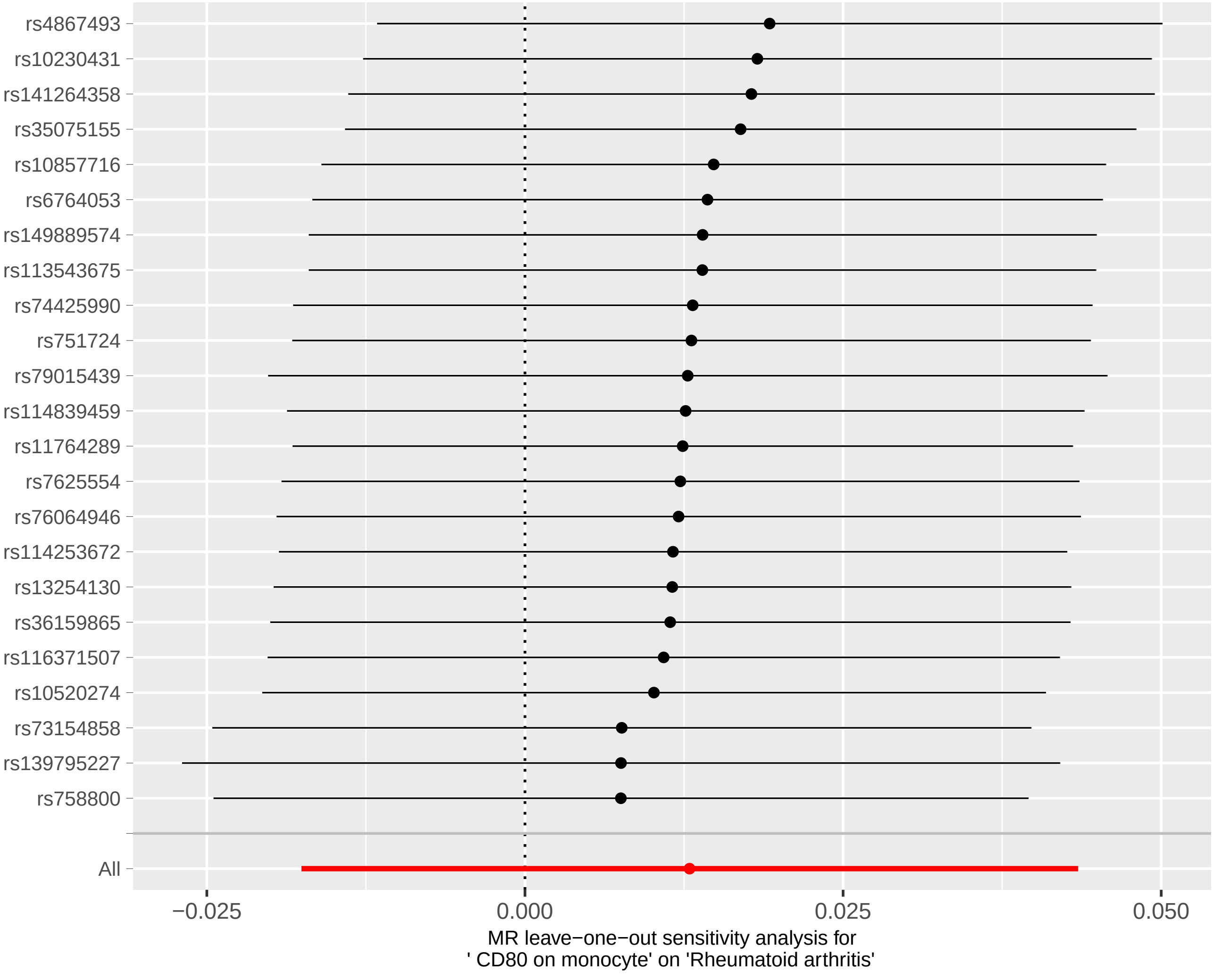

rs4867493
rs10230431
rs141264358
rs35075155
rs10857716
rs6764053
rs149889574
rs113543675
rs74425990
rs751724
rs79015439
rs114839459
rs11764289
rs7625554
rs76064946
rs114253672
rs13254130
rs36159865
rs116371507
rs10520274
rs73154858
rs139795227
rs758800
All
−0.025
0.000
0.025
0.050
MR leave−one−out sensitivity analysis for
' CD80 on monocyte' on 'Rheumatoid arthritis'

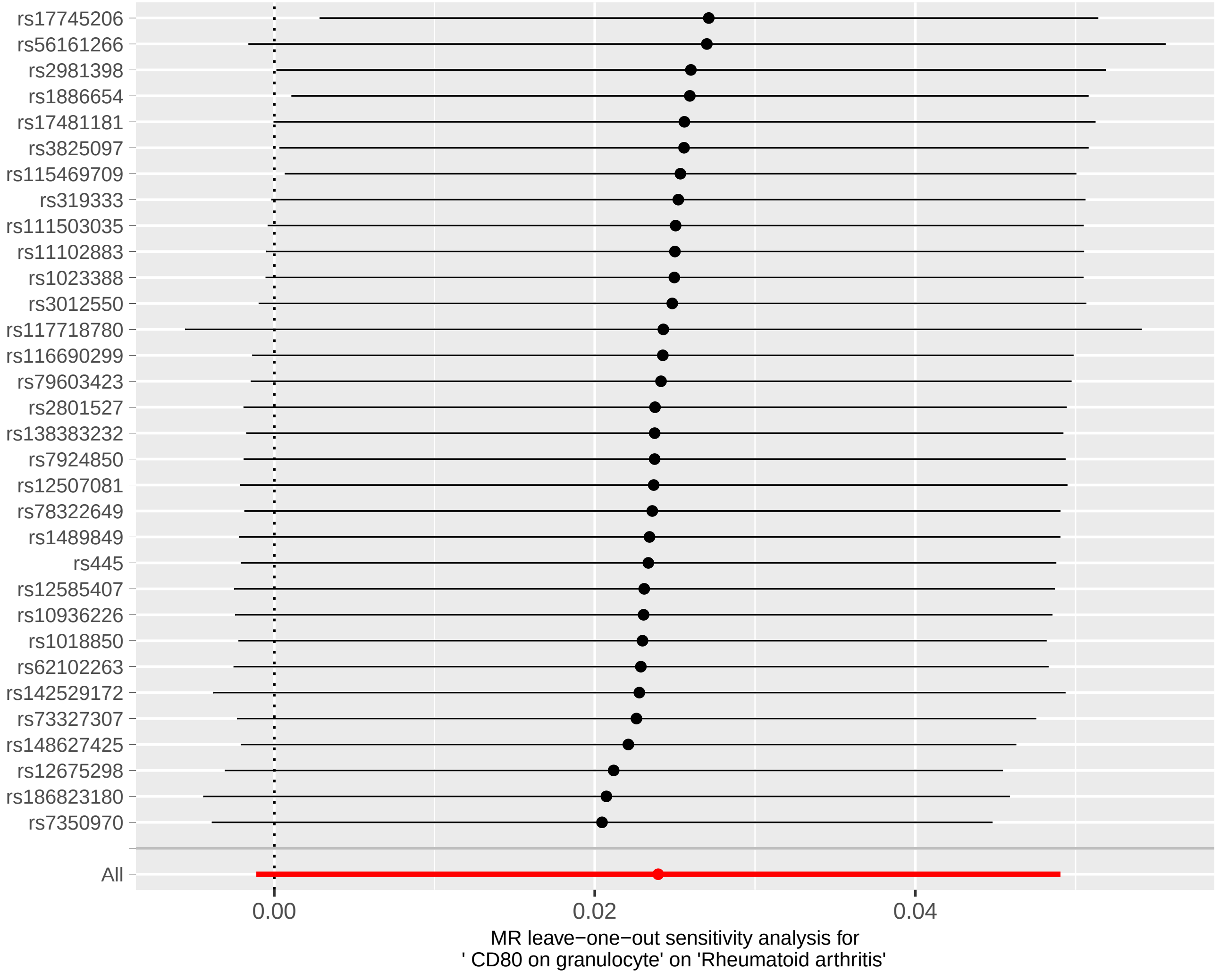
rs17745206
rs56161266
rs2981398
rs1886654
rs17481181
rs3825097
rs115469709
rs319333
rs111503035
rs11102883
rs1023388
rs3012550
rs117718780
rs116690299
rs79603423
rs2801527
rs138383232
rs7924850
rs12507081
rs78322649
rs1489849
rs445
rs12585407
rs10936226
rs1018850
rs62102263
rs142529172
rs73327307
rs148627425
rs12675298
rs186823180
rs7350970
All
0.00
0.02
0.04
MR leave−one−out sensitivity analysis for
' CD80 on granulocyte' on 'Rheumatoid arthritis'

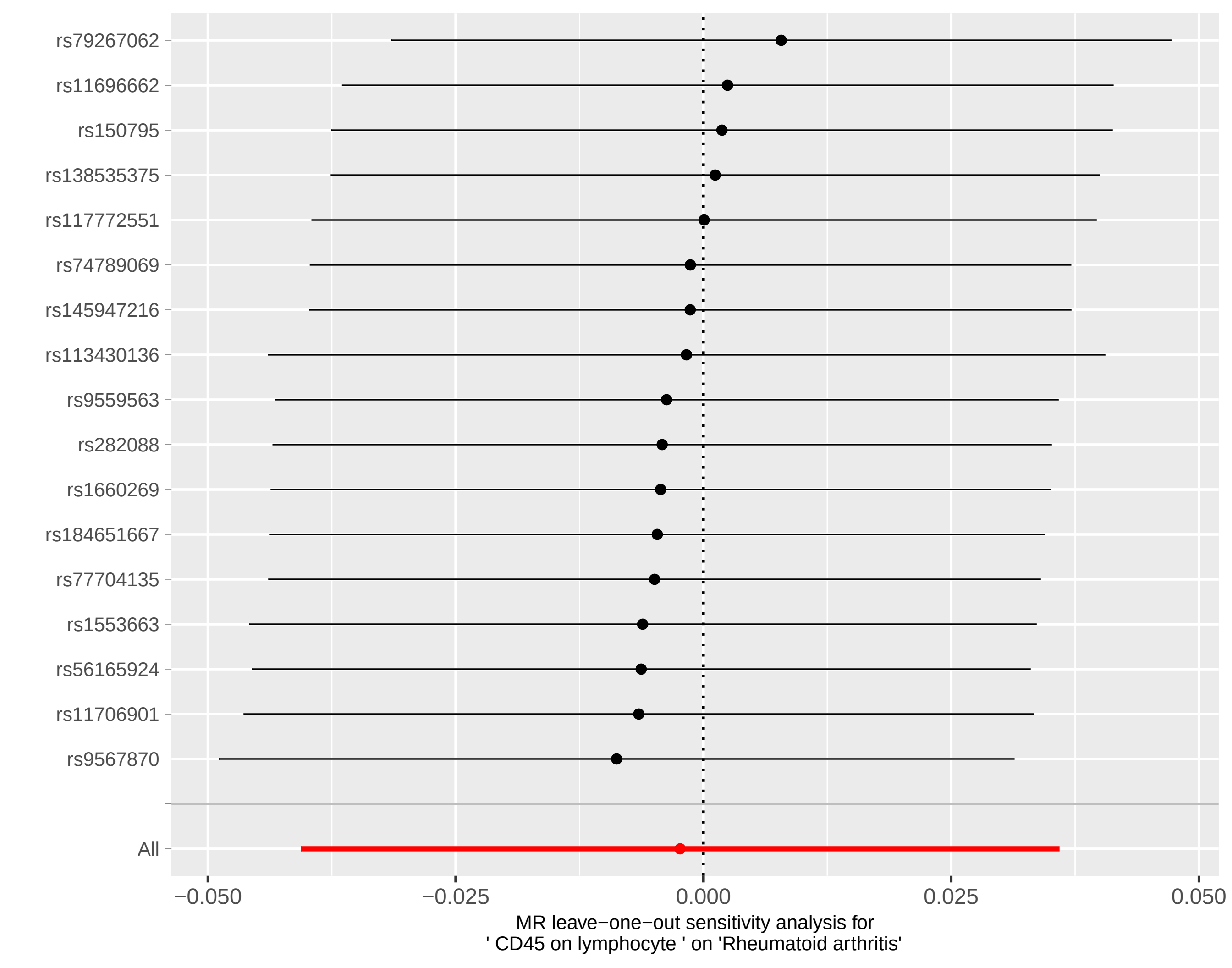

rs79267062
rs11696662
rs150795
rs138535375
rs117772551
rs74789069
rs145947216
rs113430136
rs9559563
rs282088
rs1660269
rs184651667
rs77704135
rs1553663
rs56165924
rs11706901
rs9567870
All
−0.050
−0.025
0.000
0.025
0.050
MR leave−one−out sensitivity analysis for
' CD45 on lymphocyte ' on 'Rheumatoid arthritis'

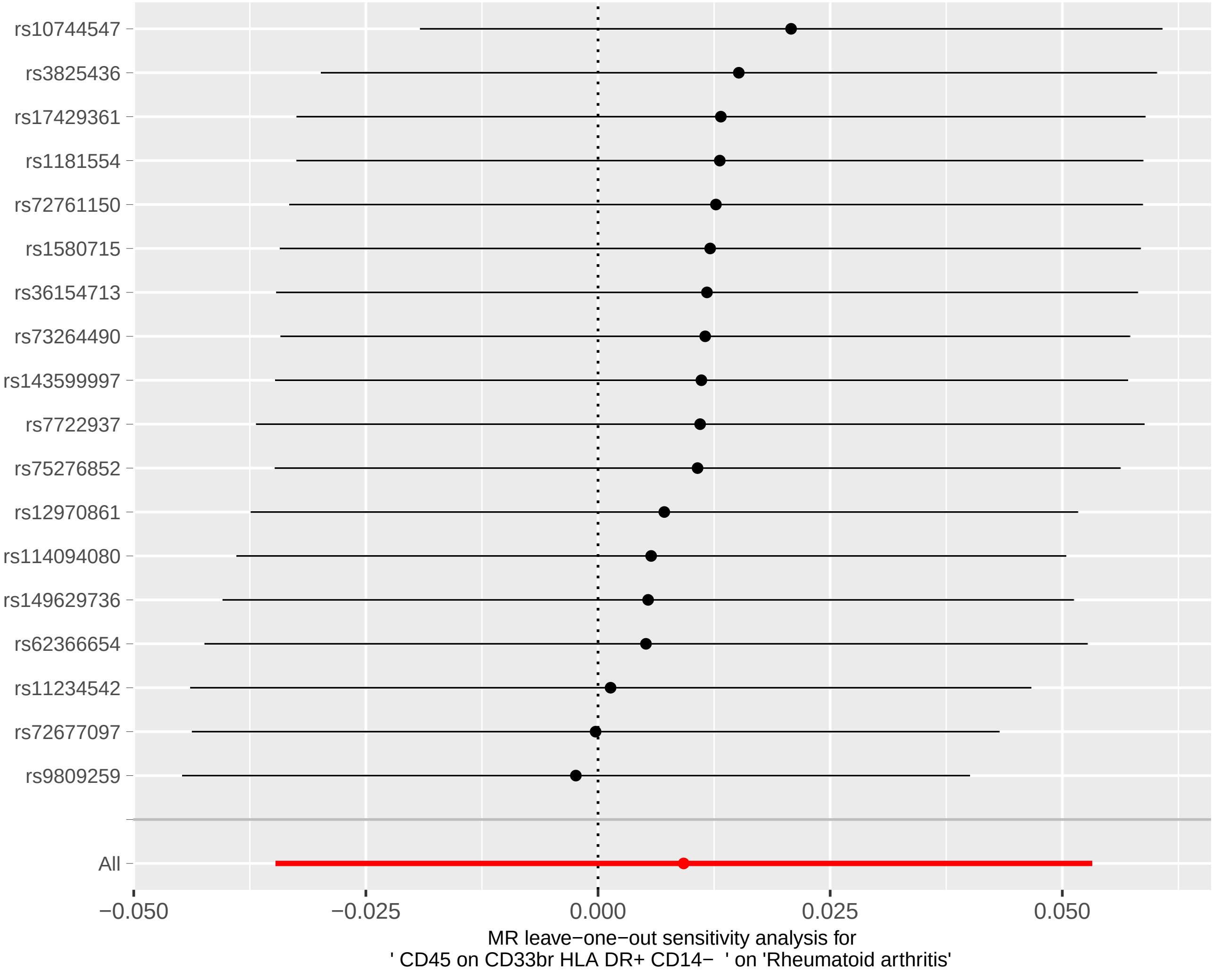

rs10744547
rs3825436
rs17429361
rs1181554
rs72761150
rs1580715
rs36154713
rs73264490
rs143599997
rs7722937
rs75276852
rs12970861
rs114094080
rs149629736
rs62366654
rs11234542
rs72677097
rs9809259
All
−0.050
−0.025
0.000
0.025
0.050
MR leave−one−out sensitivity analysis for
' CD45 on CD33br HLA DR+ CD14− ' on 'Rheumatoid arthritis'

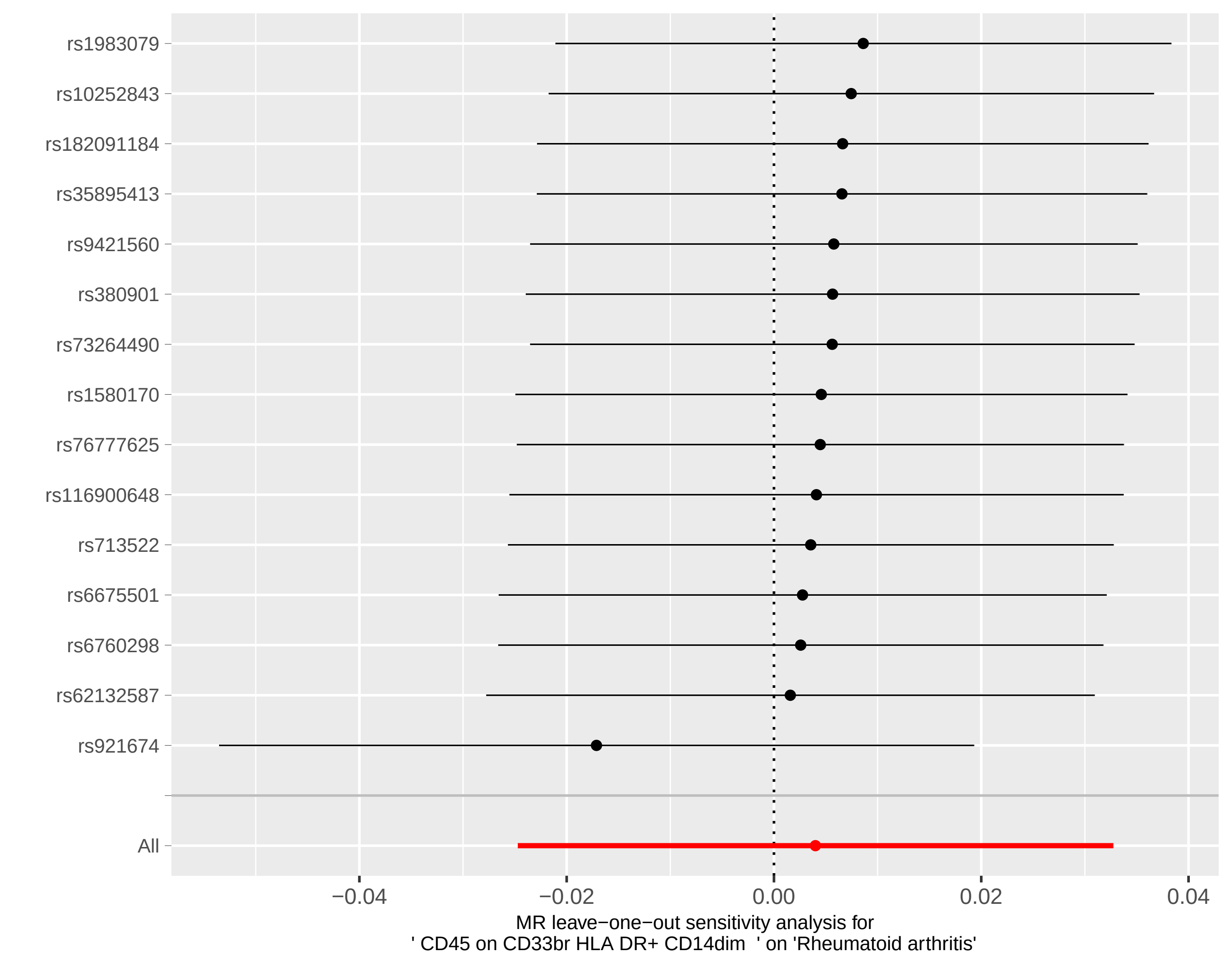

rs1983079
rs10252843
rs182091184
rs35895413
rs9421560
rs380901
rs73264490
rs1580170
rs76777625
rs116900648
rs713522
rs6675501
rs6760298
rs62132587
rs921674
All
−0.04
−0.02
0.00
0.02
0.04
MR leave−one−out sensitivity analysis for
' CD45 on CD33br HLA DR+ CD14dim ' on 'Rheumatoid arthritis'

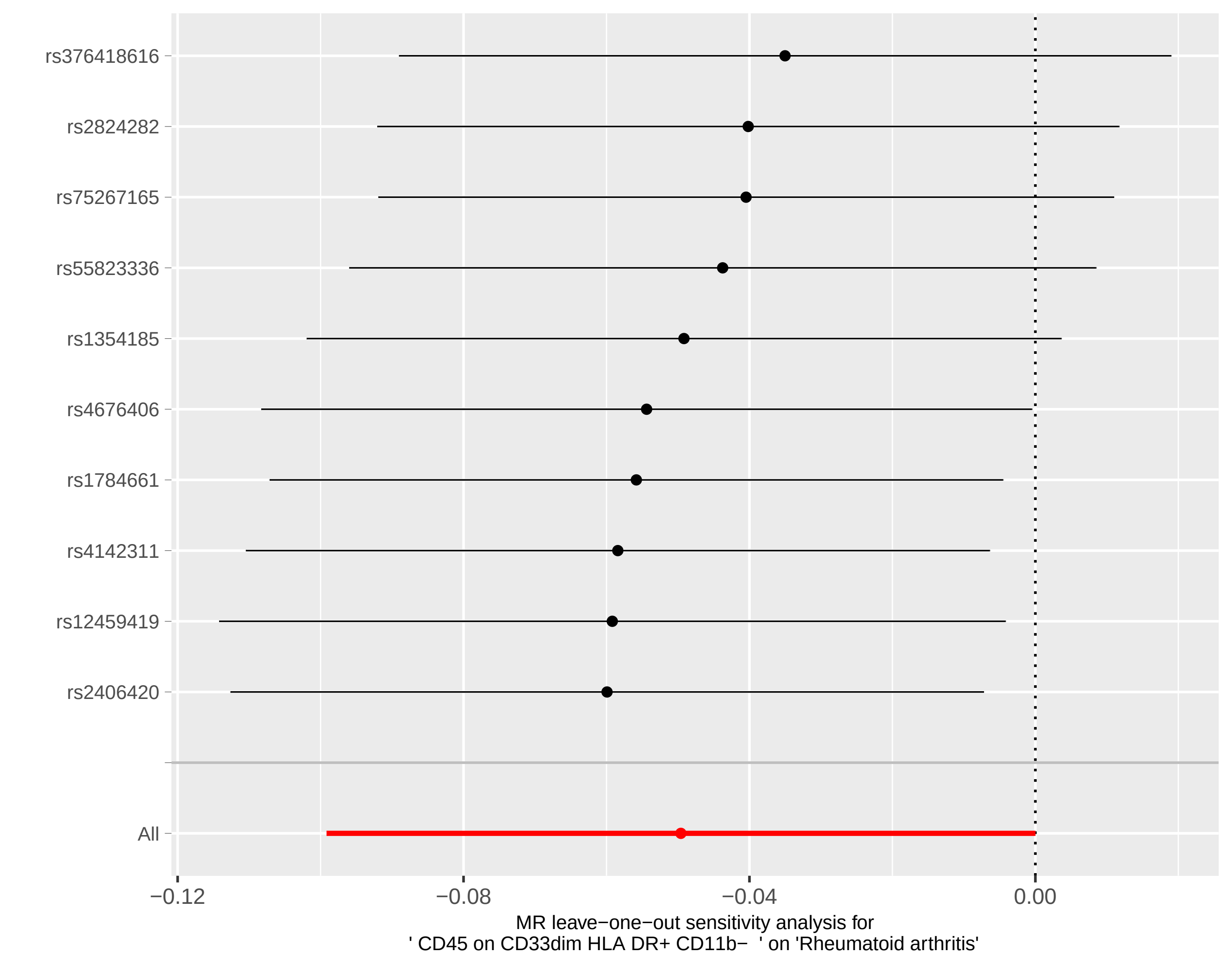

rs376418616
rs2824282
rs75267165
rs55823336
rs1354185
rs4676406
rs1784661
rs4142311
rs12459419
rs2406420
All
−0.12
−0.08
−0.04
0.00
MR leave−one−out sensitivity analysis for
' CD45 on CD33dim HLA DR+ CD11b− ' on 'Rheumatoid arthritis'

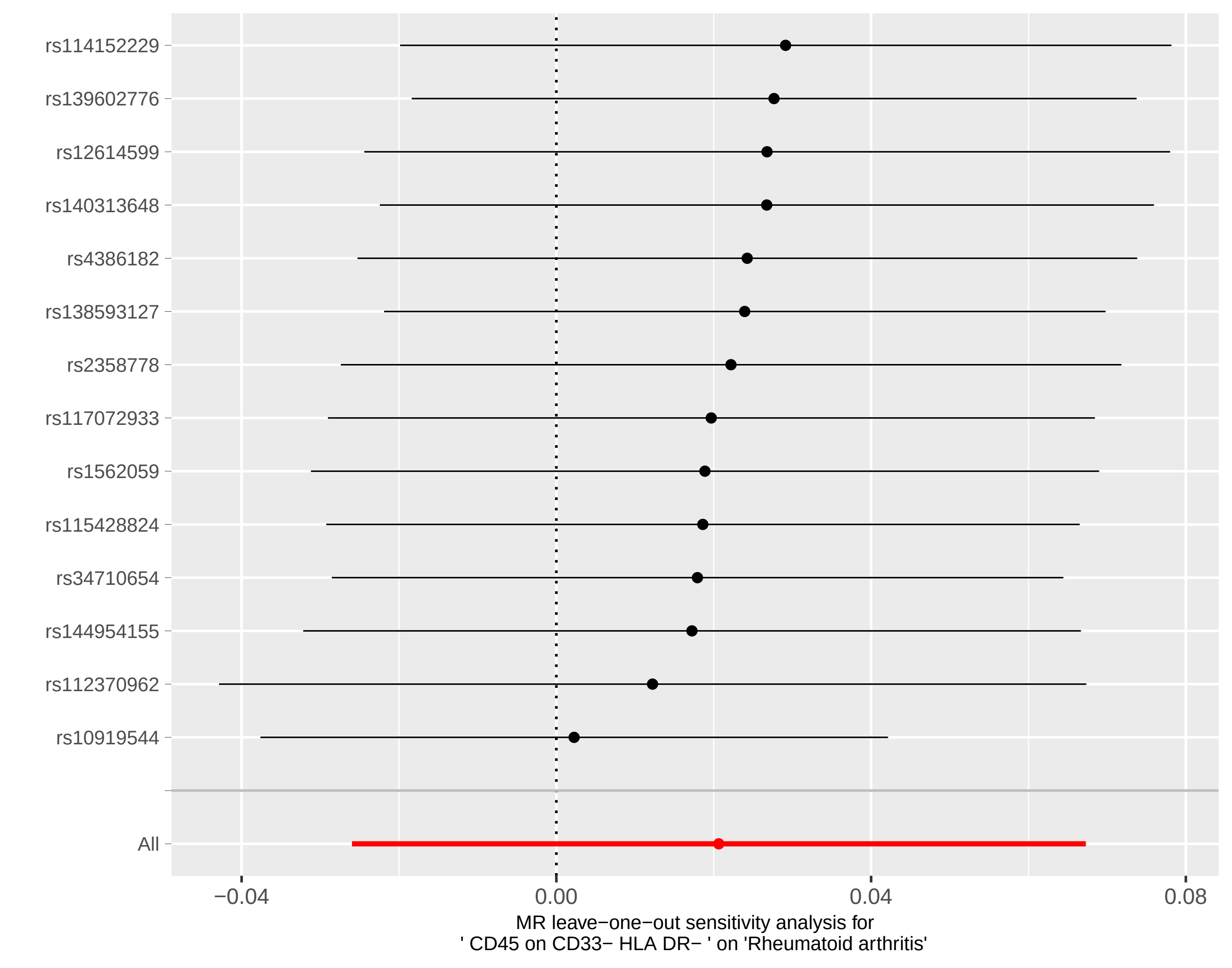
rs114152229
rs139602776
rs12614599
rs140313648
rs4386182
rs138593127
rs2358778
rs117072933
rs1562059
rs115428824
rs34710654
rs144954155
rs112370962
rs10919544
All
−0.04
0.00
0.04
0.08
MR leave−one−out sensitivity analysis for
' CD45 on CD33− HLA DR− ' on 'Rheumatoid arthritis'

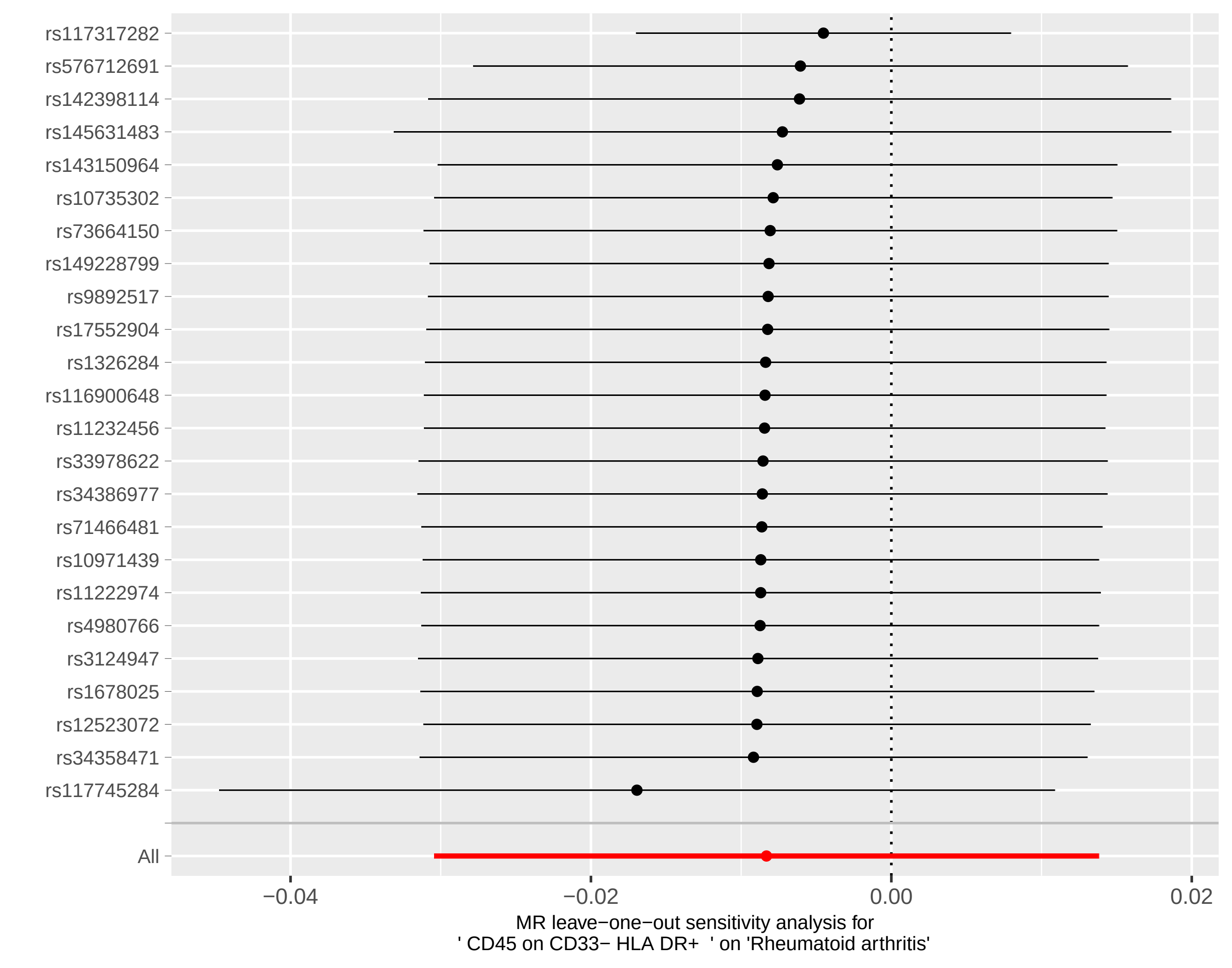

rs117317282
rs576712691
rs142398114
rs145631483
rs143150964
rs10735302
rs73664150
rs149228799
rs9892517
rs17552904
rs1326284
rs116900648
rs11232456
rs33978622
rs34386977
rs71466481
rs10971439
rs11222974
rs4980766
rs3124947
rs1678025
rs12523072
rs34358471
rs117745284
All
−0.04
−0.02
0.00
0.02
MR leave−one−out sensitivity analysis for
' CD45 on CD33− HLA DR+ ' on 'Rheumatoid arthritis'

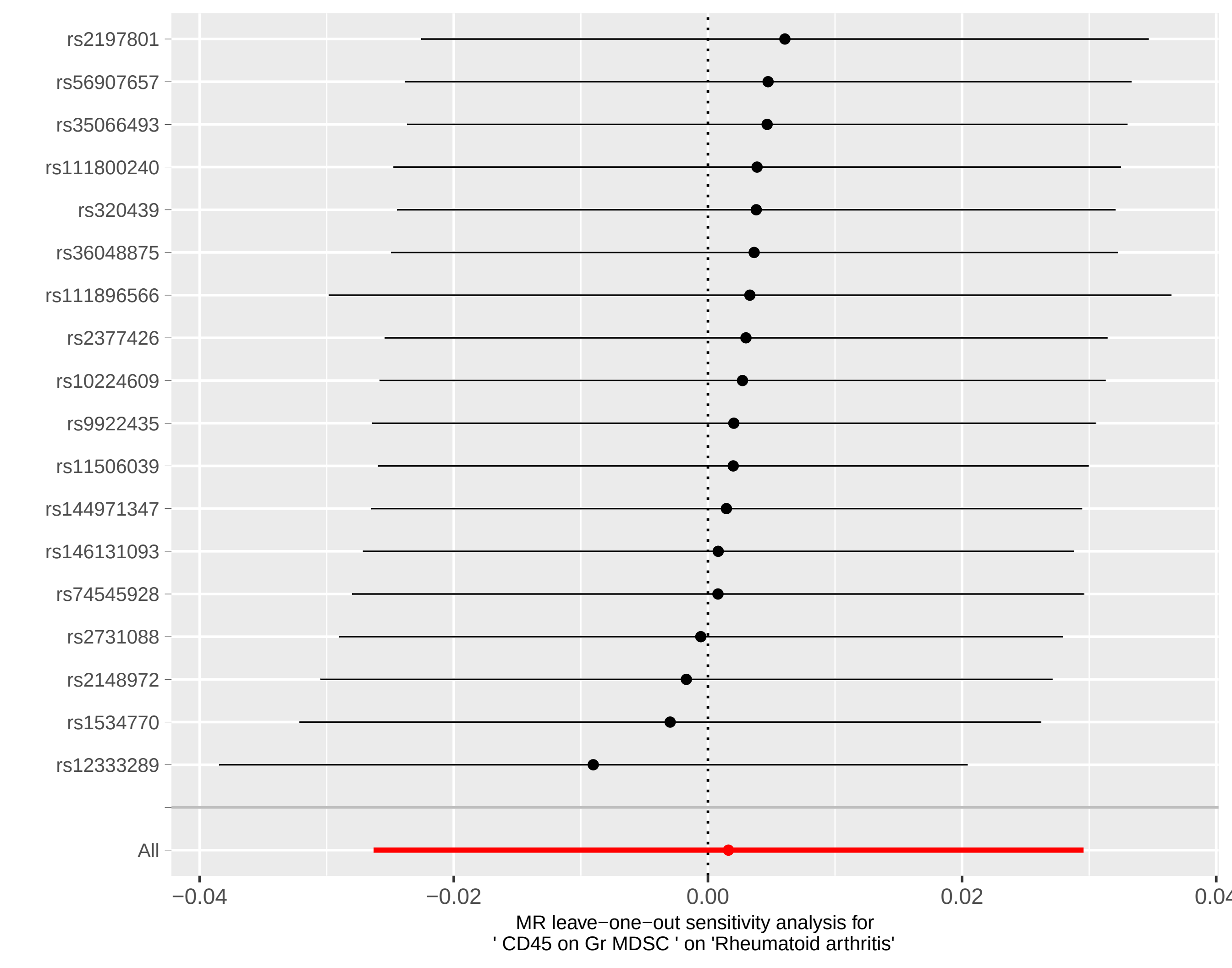

rs2197801
rs56907657
rs35066493
rs111800240
rs320439
rs36048875
rs111896566
rs2377426
rs10224609
rs9922435
rs11506039
rs144971347
rs146131093
rs74545928
rs2731088
rs2148972
rs1534770
rs12333289
All
−0.04
−0.02
0.00
0.02
0.04
MR leave−one−out sensitivity analysis for
' CD45 on Gr MDSC ' on 'Rheumatoid arthritis'

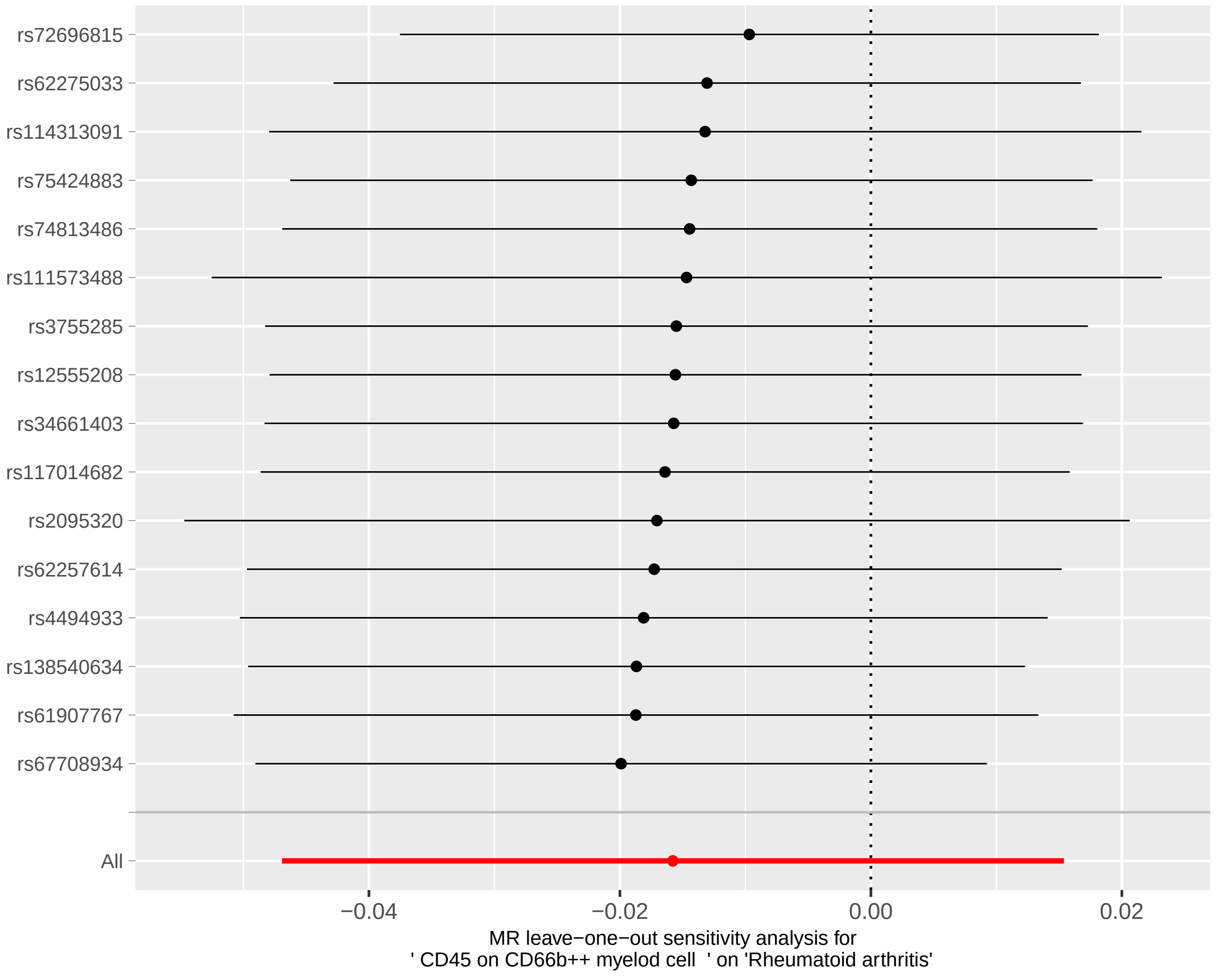
rs72696815
rs62275033
rs114313091
rs75424883
rs74813486
rs111573488
rs3755285
rs12555208
rs34661403
rs117014682
rs2095320
rs62257614
rs4494933
rs138540634
rs61907767
rs67708934
All
−0.04
−0.02
0.00
0.02
MR leave−one−out sensitivity analysis for
' CD45 on CD66b++ myelod cell ' on 'Rheumatoid arthritis'

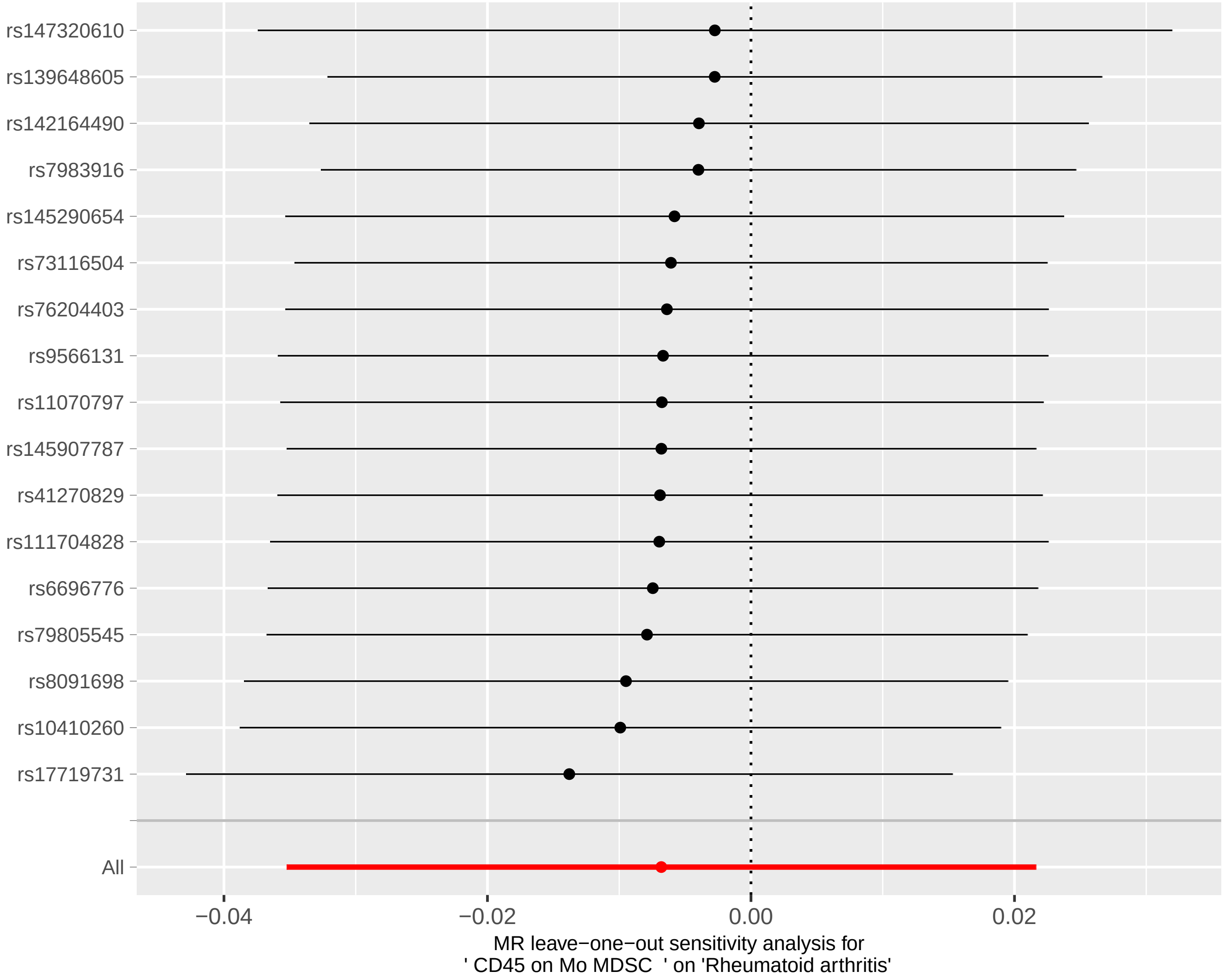
rs147320610
rs139648605
rs142164490
rs7983916
rs145290654
rs73116504
rs76204403
rs9566131
rs11070797
rs145907787
rs41270829
rs111704828
rs6696776
rs79805545
rs8091698
rs10410260
rs17719731
All
−0.04
−0.02
0.00
0.02
MR leave−one−out sensitivity analysis for
' CD45 on Mo MDSC ' on 'Rheumatoid arthritis'

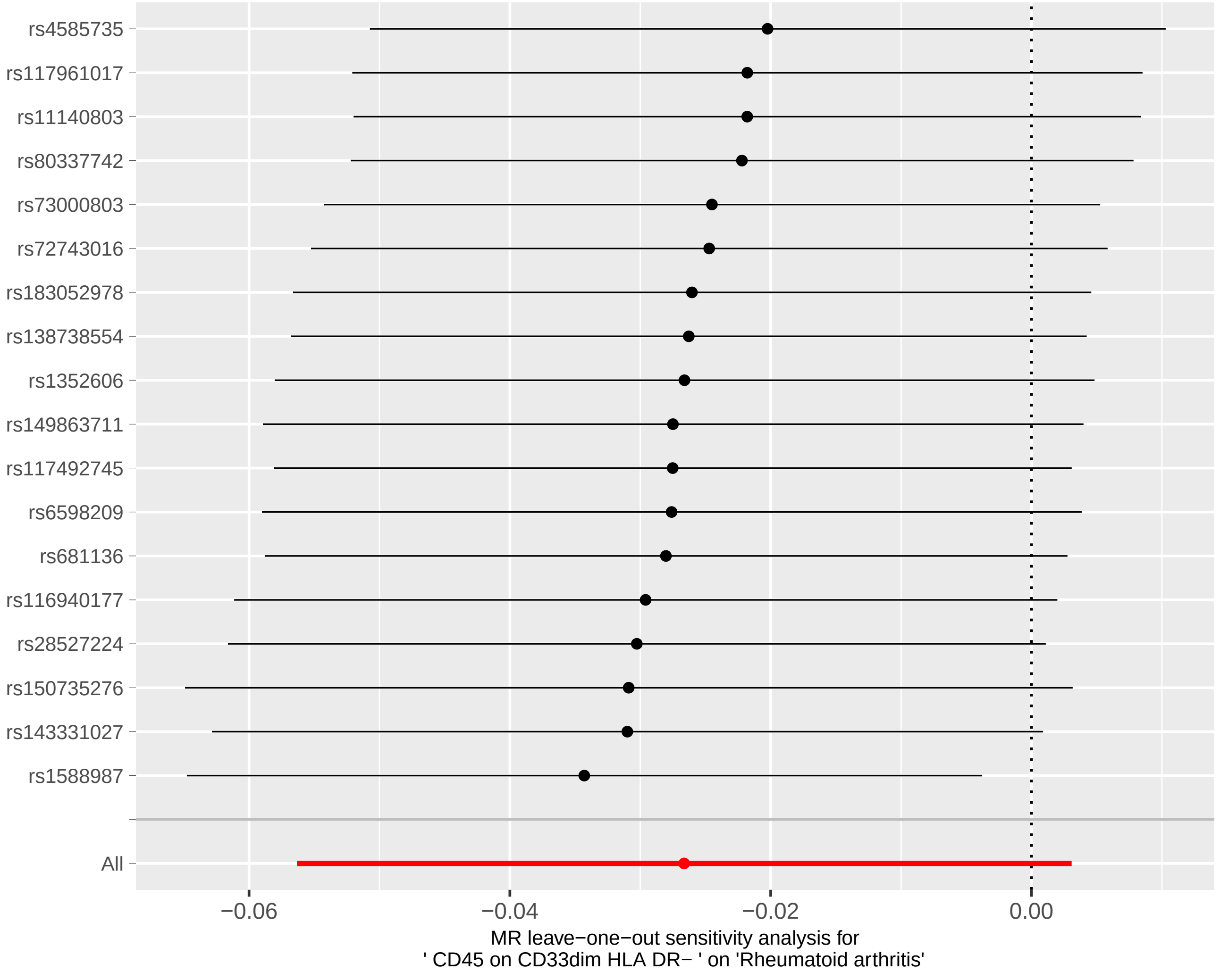
rs4585735
rs117961017
rs11140803
rs80337742
rs73000803
rs72743016
rs183052978
rs138738554
rs1352606
rs149863711
rs117492745
rs6598209
rs681136
rs116940177
rs28527224
rs150735276
rs143331027
rs1588987
All
−0.06
−0.04
−0.02
0.00
MR leave−one−out sensitivity analysis for
' CD45 on CD33dim HLA DR− ' on 'Rheumatoid arthritis'

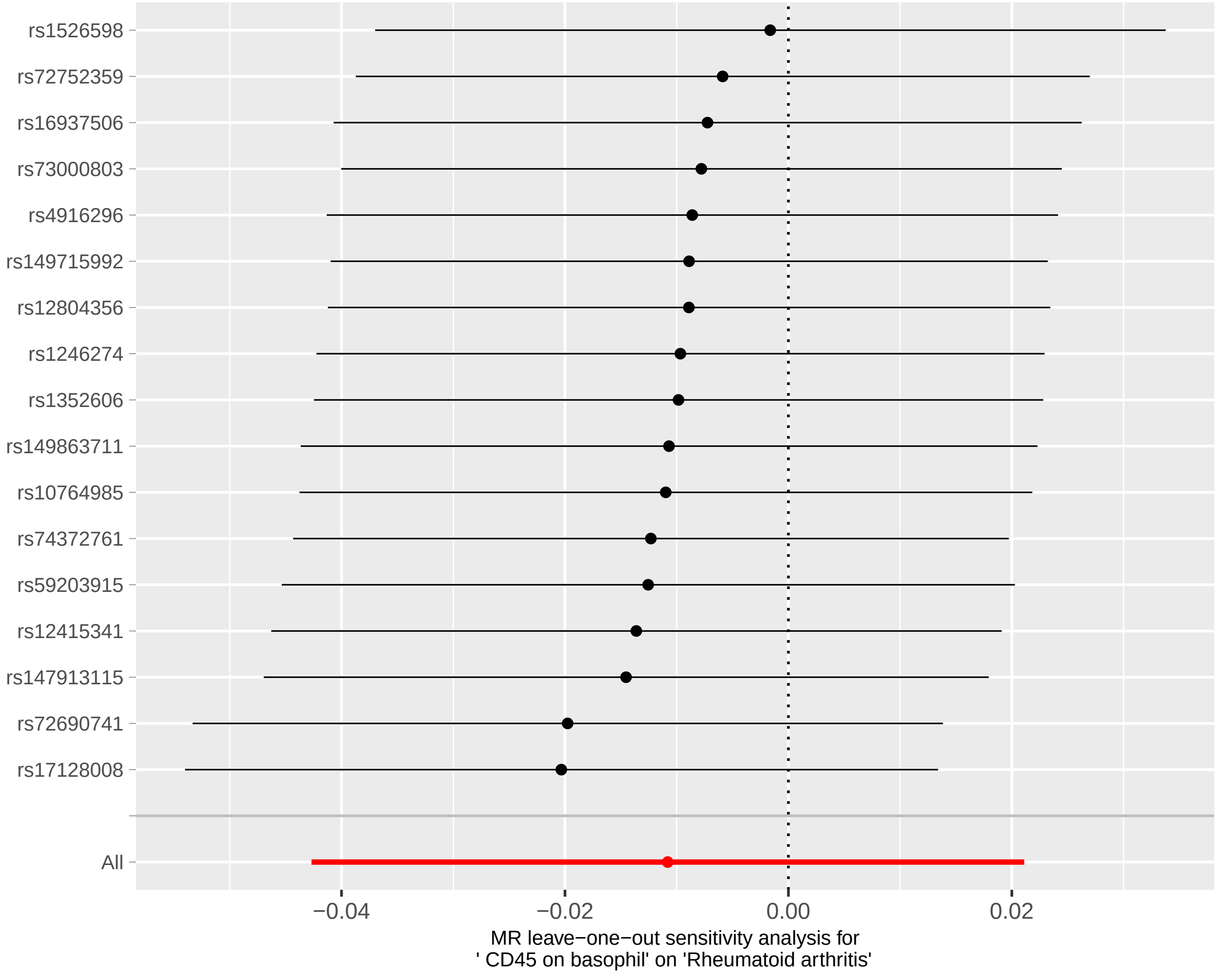
rs1526598
rs72752359
rs16937506
rs73000803
rs4916296
rs149715992
rs12804356
rs1246274
rs1352606
rs149863711
rs10764985
rs74372761
rs59203915
rs12415341
rs147913115
rs72690741
rs17128008
All
−0.04
−0.02
0.00
0.02
MR leave−one−out sensitivity analysis for
' CD45 on basophil' on 'Rheumatoid arthritis'

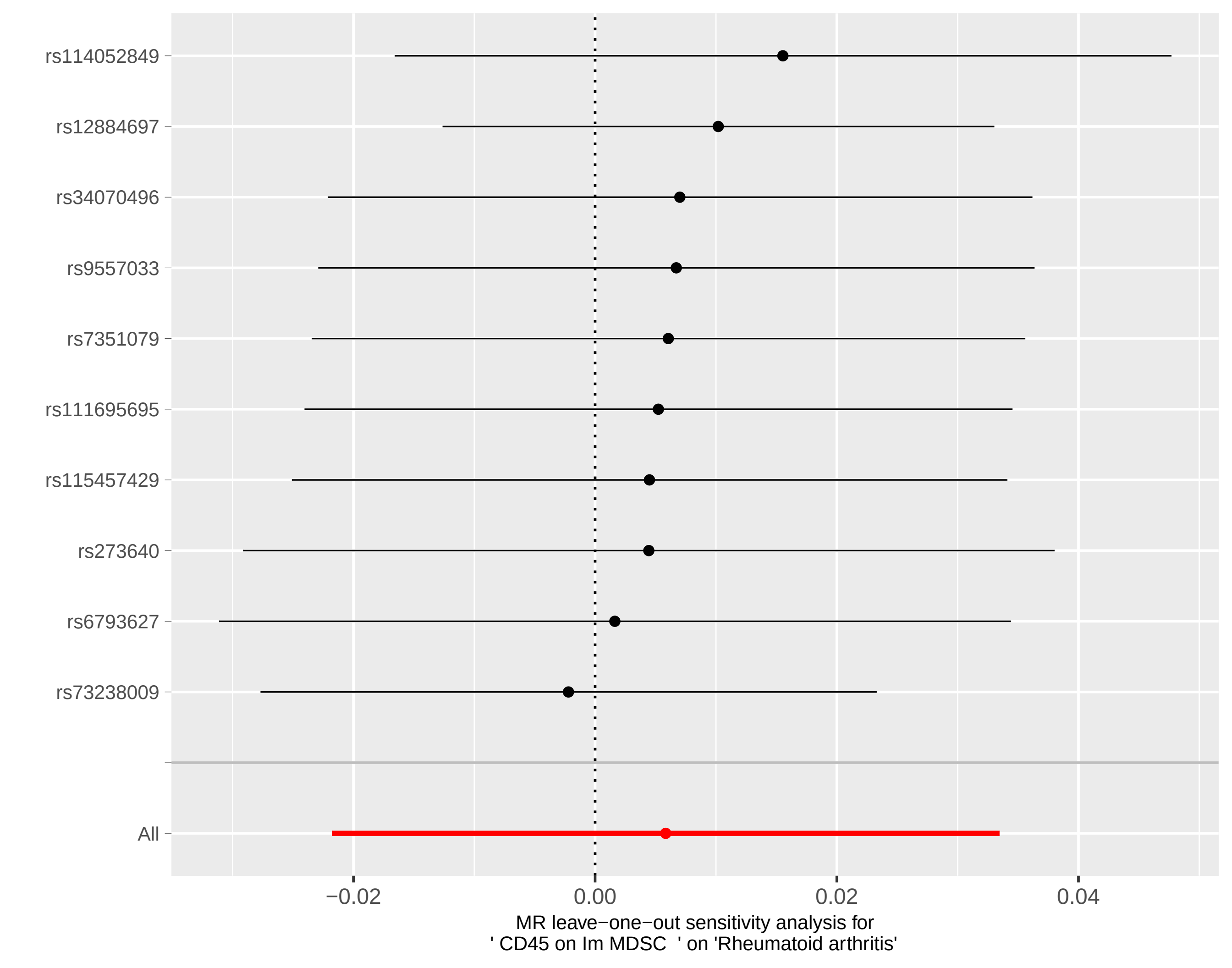
rs114052849
rs12884697
rs34070496
rs9557033
rs7351079
rs111695695
rs115457429
rs273640
rs6793627
rs73238009
All
−0.02
0.00
0.02
0.04
MR leave−one−out sensitivity analysis for
' CD45 on Im MDSC ' on 'Rheumatoid arthritis'

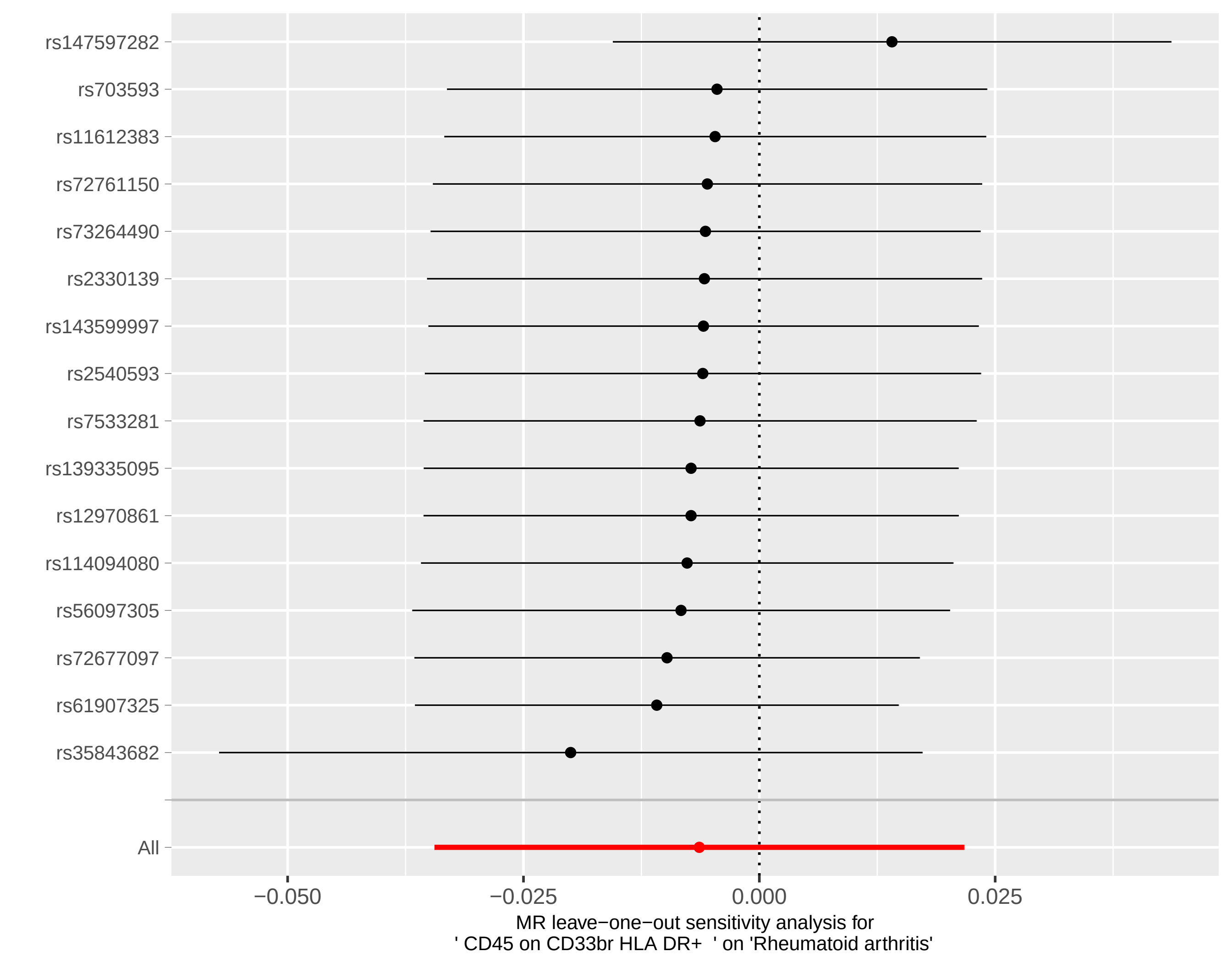
rs147597282
rs703593
rs11612383
rs72761150
rs73264490
rs2330139
rs143599997
rs2540593
rs7533281
rs139335095
rs12970861
rs114094080
rs56097305
rs72677097
rs61907325
rs35843682
All
−0.050
−0.025
0.000
0.025
MR leave−one−out sensitivity analysis for
' CD45 on CD33br HLA DR+ ' on 'Rheumatoid arthritis'

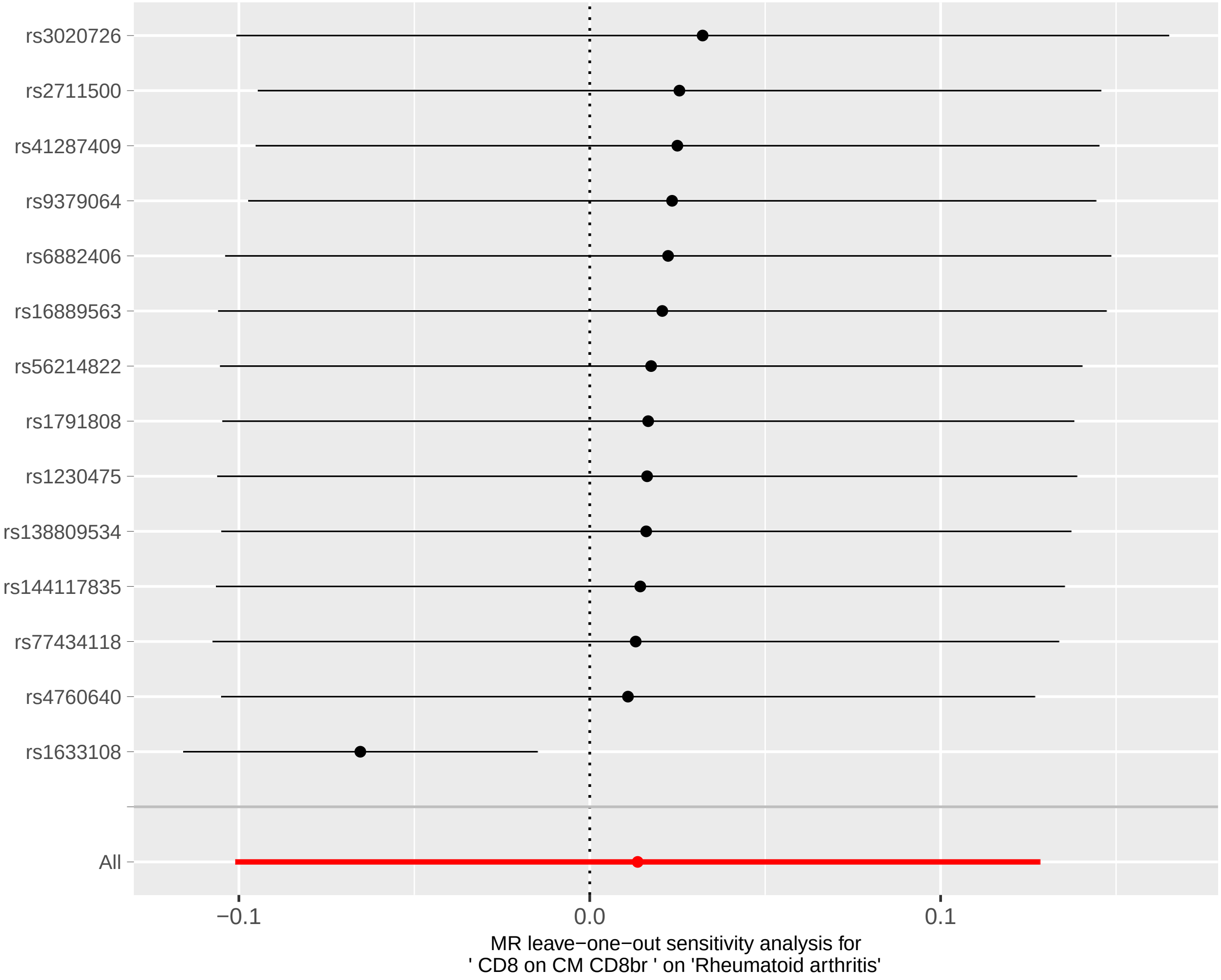

rs3020726
rs2711500
rs41287409
rs9379064
rs6882406
rs16889563
rs56214822
rs1791808
rs1230475
rs138809534
rs144117835
rs77434118
rs4760640
rs1633108
All
−0.1
0.0
0.1
MR leave−one−out sensitivity analysis for
' CD8 on CM CD8br ' on 'Rheumatoid arthritis'

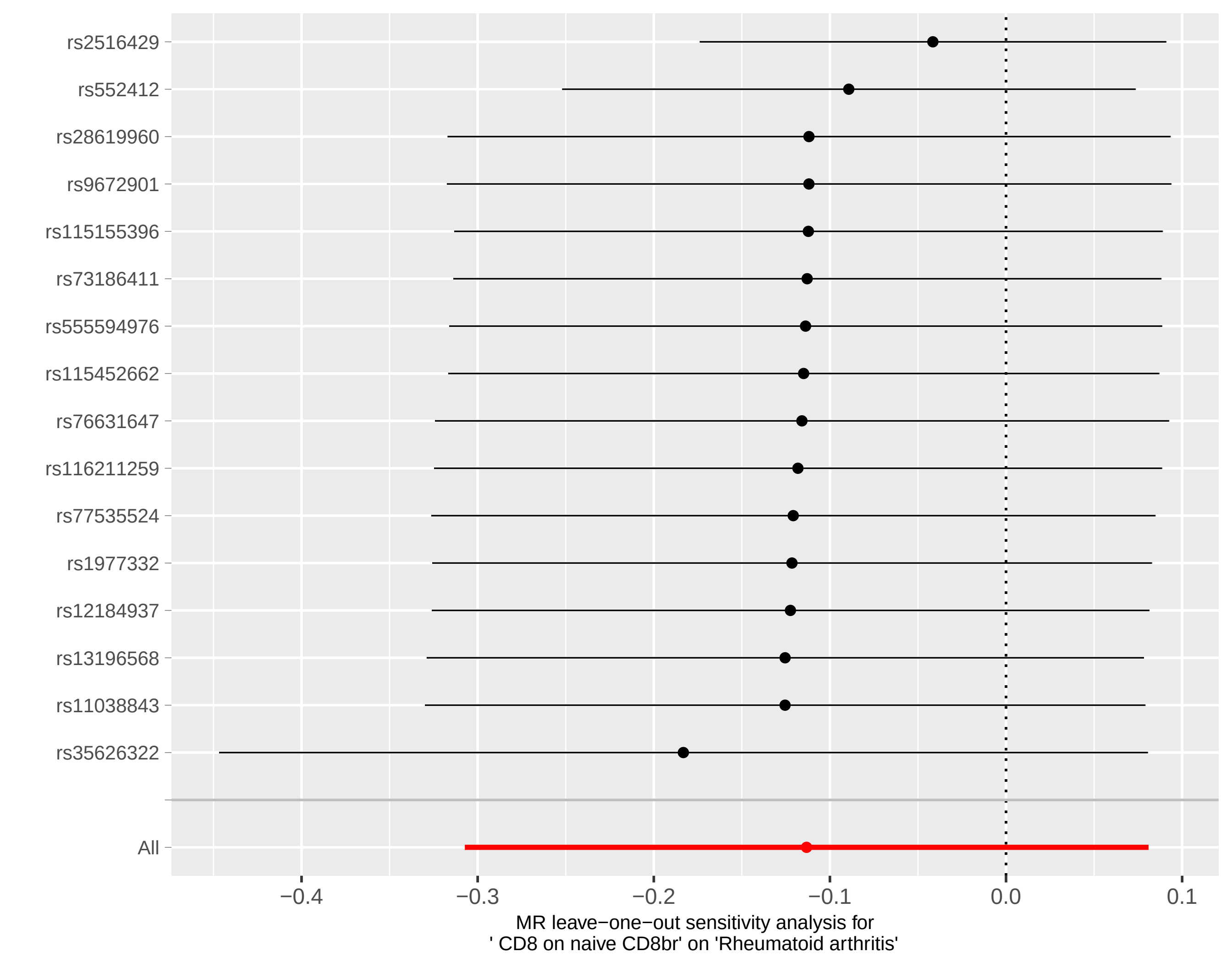

rs2516429
rs552412
rs28619960
rs9672901
rs115155396
rs73186411
rs555594976
rs115452662
rs76631647
rs116211259
rs77535524
rs1977332
rs12184937
rs13196568
rs11038843
rs35626322
All
−0.4
−0.3
−0.2
−0.1
0.0
0.1
MR leave−one−out sensitivity analysis for
' CD8 on naive CD8br' on 'Rheumatoid arthritis'

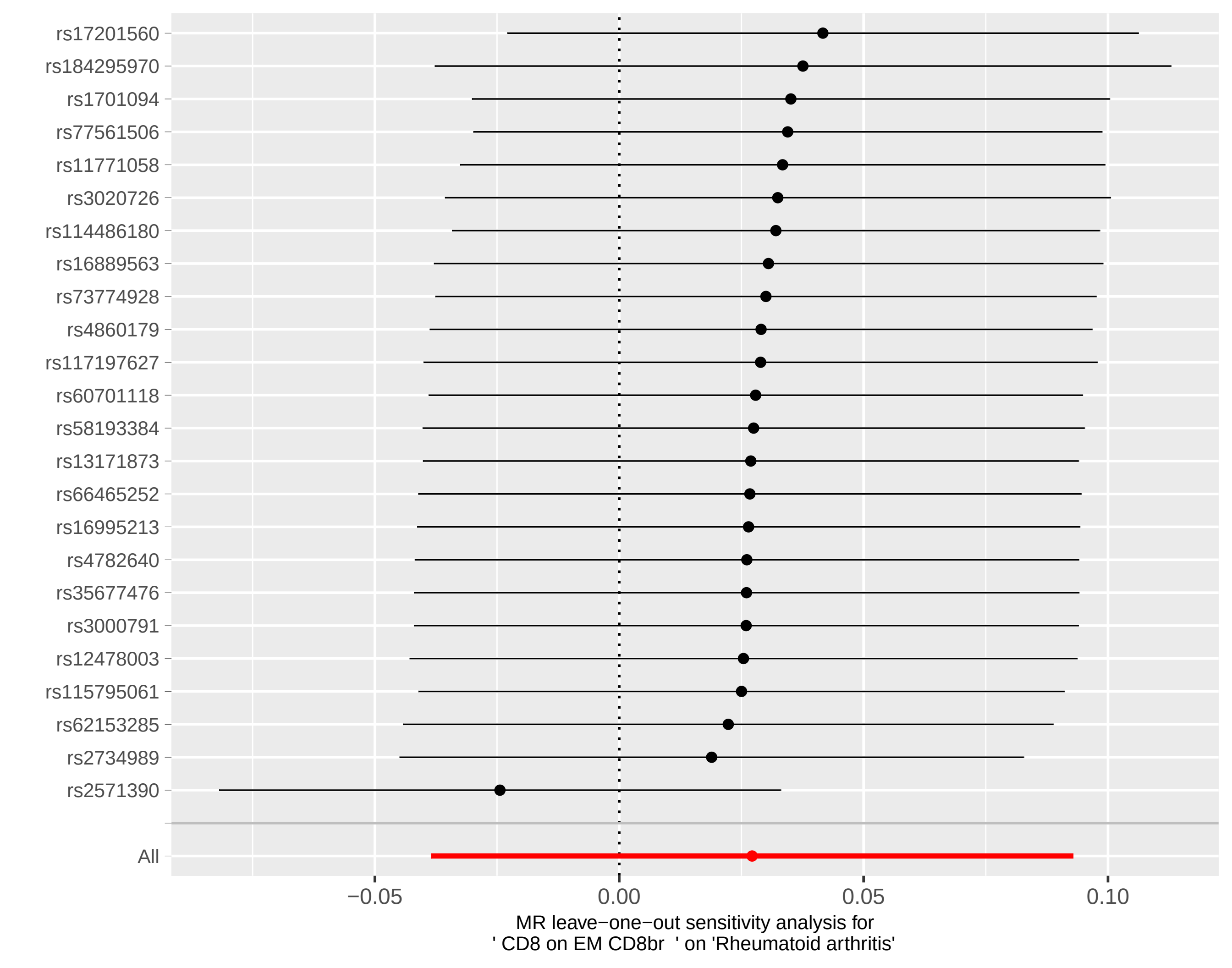
rs17201560
rs184295970
rs1701094
rs77561506
rs11771058
rs3020726
rs114486180
rs16889563
rs73774928
rs4860179
rs117197627
rs60701118
rs58193384
rs13171873
rs66465252
rs16995213
rs4782640
rs35677476
rs3000791
rs12478003
rs115795061
rs62153285
rs2734989
rs2571390
All
−0.05
0.00
0.05
0.10
MR leave−one−out sensitivity analysis for
' CD8 on EM CD8br ' on 'Rheumatoid arthritis'

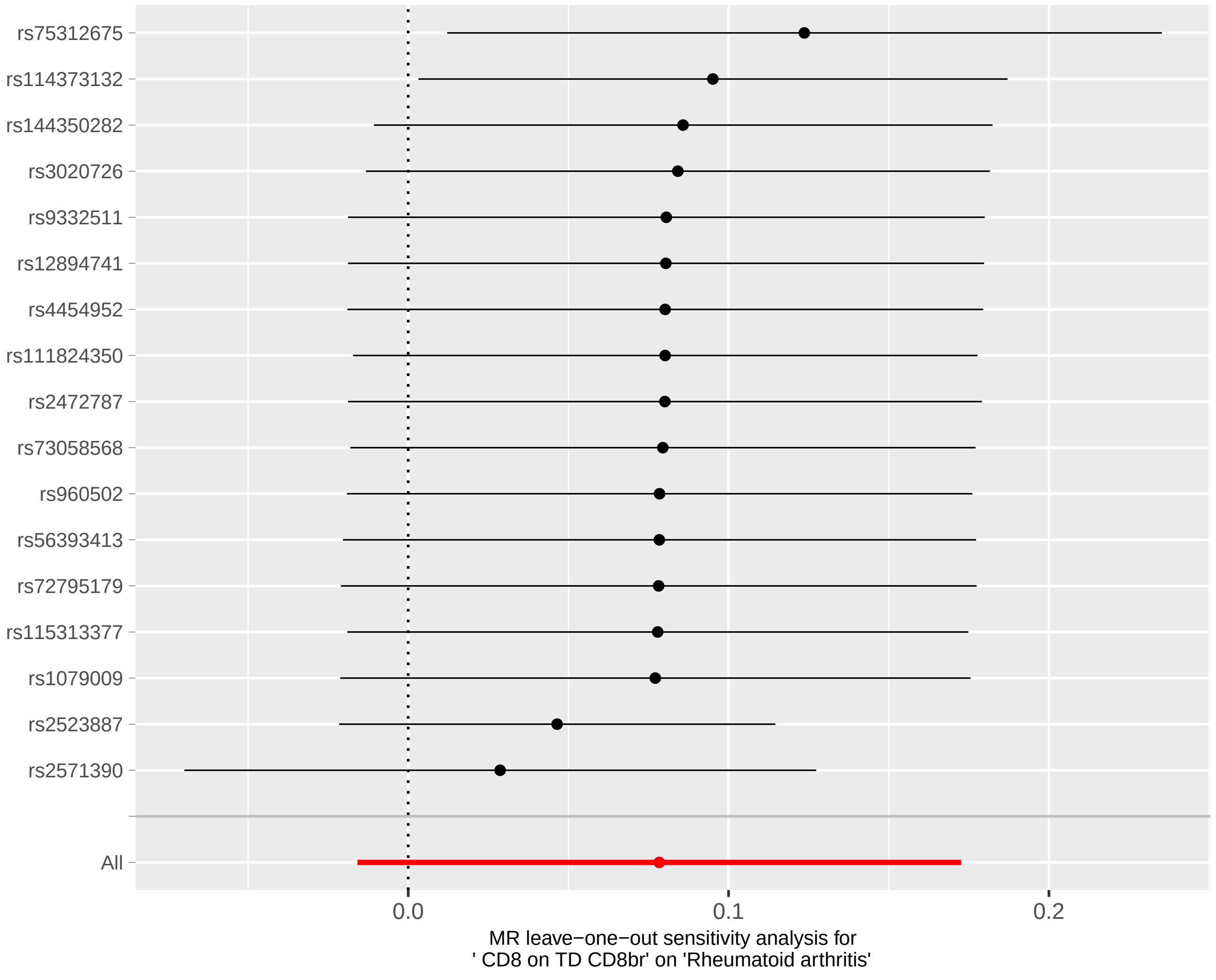
rs75312675
rs114373132
rs144350282
rs3020726
rs9332511
rs12894741
rs4454952
rs111824350
rs2472787
rs73058568
rs960502
rs56393413
rs72795179
rs115313377
rs1079009
rs2523887
rs2571390
All
0.0
0.1
0.2
MR leave−one−out sensitivity analysis for
' CD8 on TD CD8br' on 'Rheumatoid arthritis'

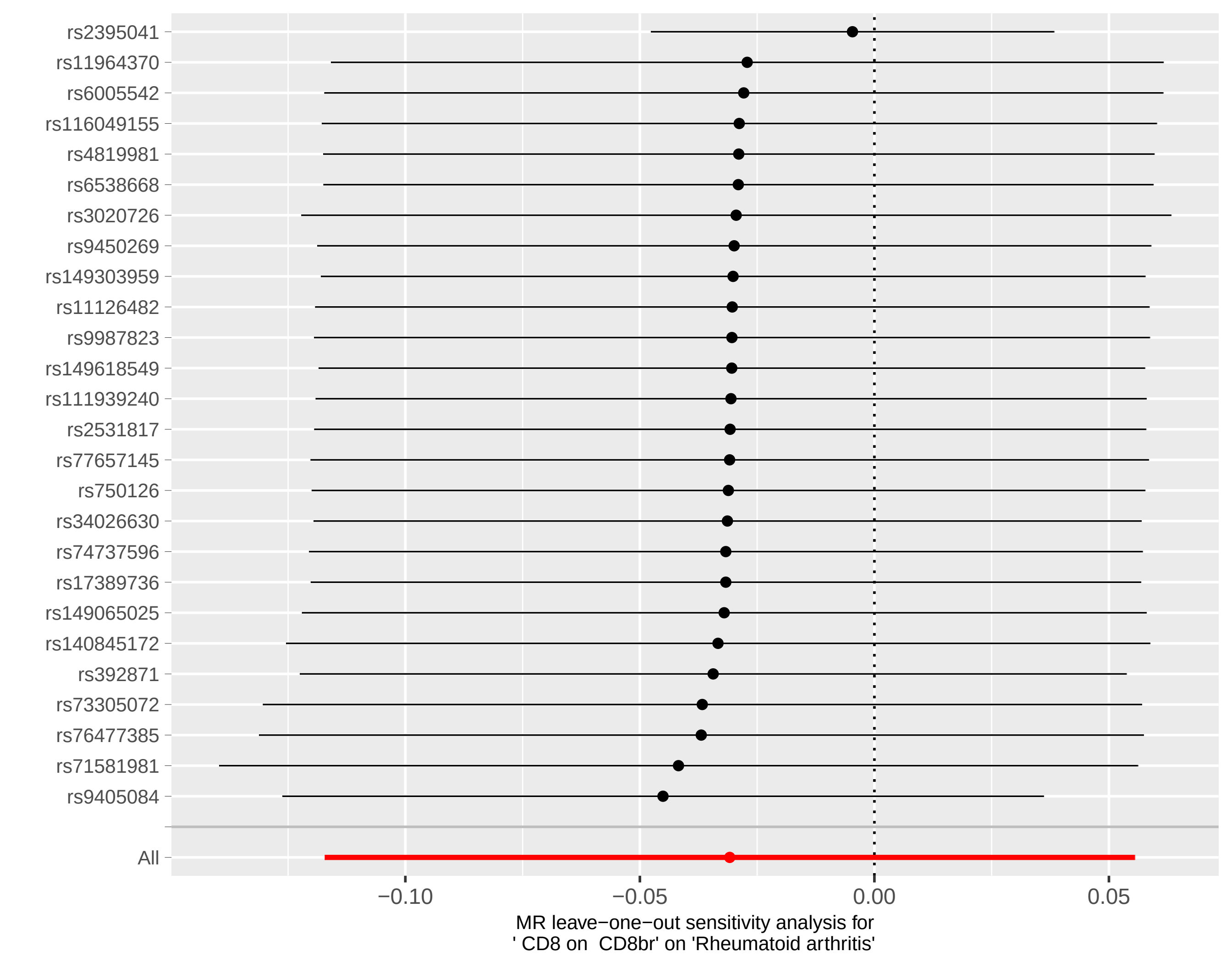

rs2395041
rs11964370
rs6005542
rs116049155
rs4819981
rs6538668
rs3020726
rs9450269
rs149303959
rs11126482
rs9987823
rs149618549
rs111939240
rs2531817
rs77657145
rs750126
rs34026630
rs74737596
rs17389736
rs149065025
rs140845172
rs392871
rs73305072
rs76477385
rs71581981
rs9405084
All
−0.10
−0.05
0.00
0.05
MR leave−one−out sensitivity analysis for
' CD8 on CD8br' on 'Rheumatoid arthritis'

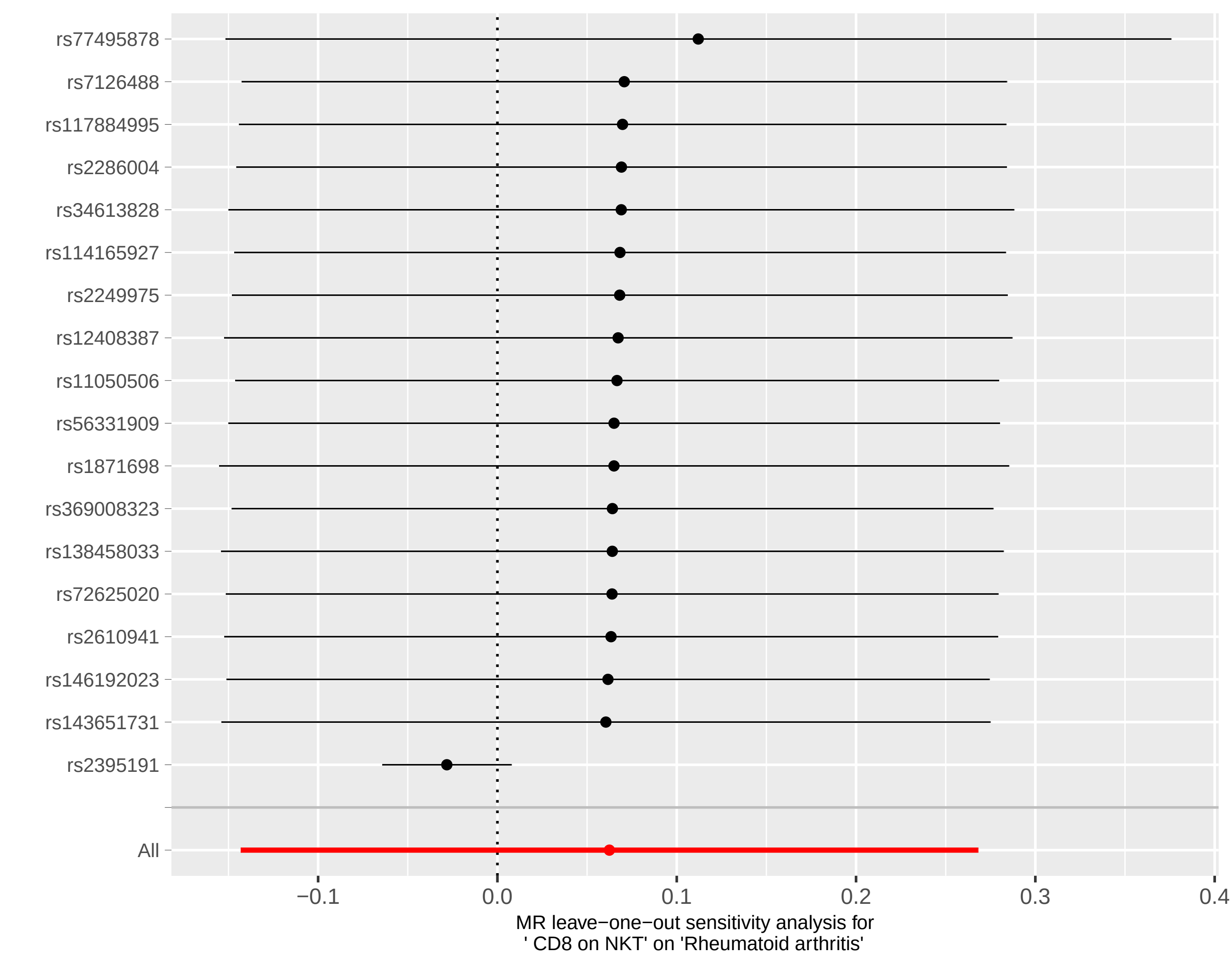
rs77495878
rs7126488
rs117884995
rs2286004
rs34613828
rs114165927
rs2249975
rs12408387
rs11050506
rs56331909
rs1871698
rs369008323
rs138458033
rs72625020
rs2610941
rs146192023
rs143651731
rs2395191
All
−0.1
0.0
0.1
0.2
0.3
0.4
MR leave−one−out sensitivity analysis for
' CD8 on NKT' on 'Rheumatoid arthritis'

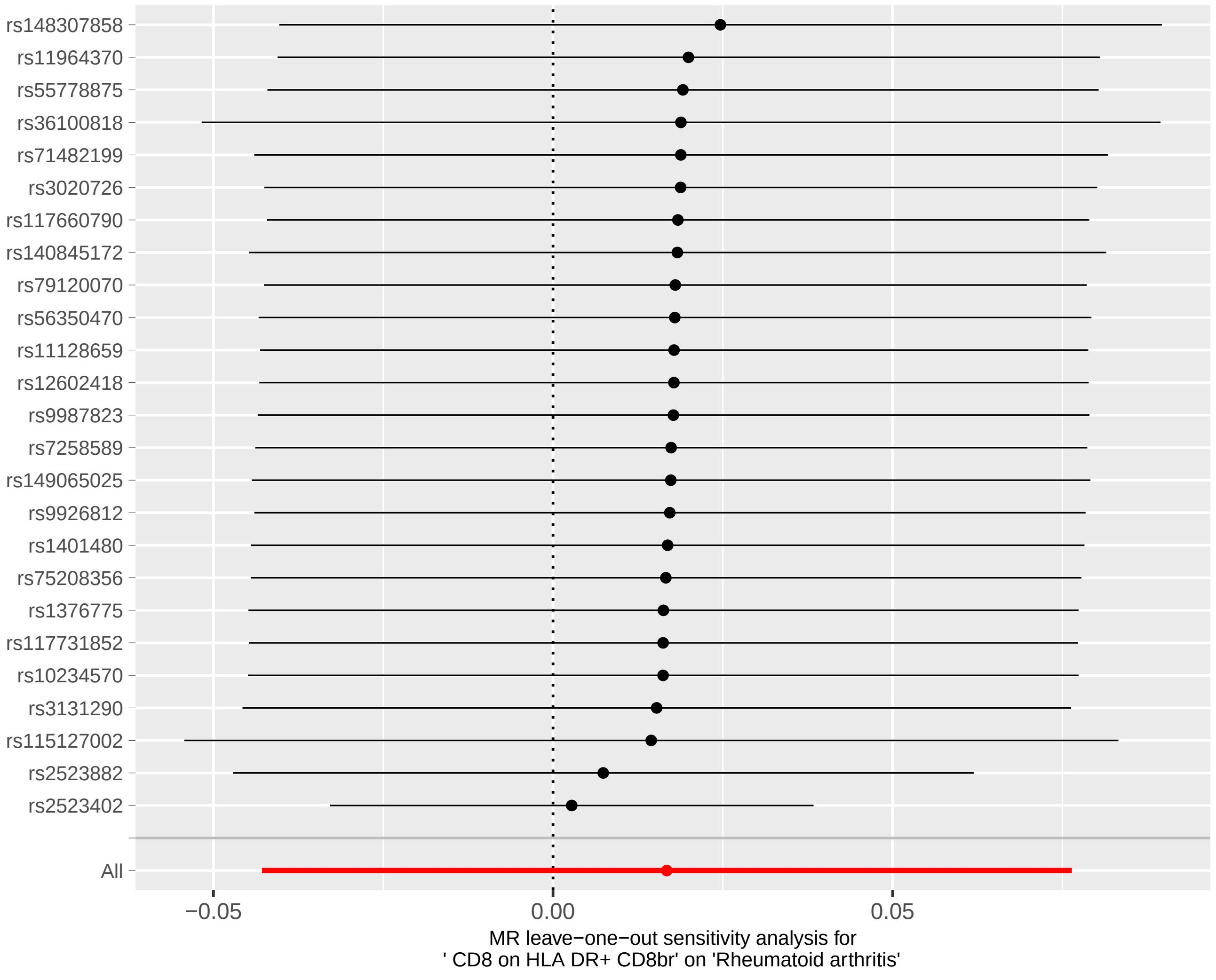
rs148307858
rs11964370
rs55778875
rs36100818
rs71482199
rs3020726
rs117660790
rs140845172
rs79120070
rs56350470
rs11128659
rs12602418
rs9987823
rs7258589
rs149065025
rs9926812
rs1401480
rs75208356
rs1376775
rs117731852
rs10234570
rs3131290
rs115127002
rs2523882
rs2523402
All
−0.05
0.00
0.05
MR leave−one−out sensitivity analysis for
' CD8 on HLA DR+ CD8br' on 'Rheumatoid arthritis'

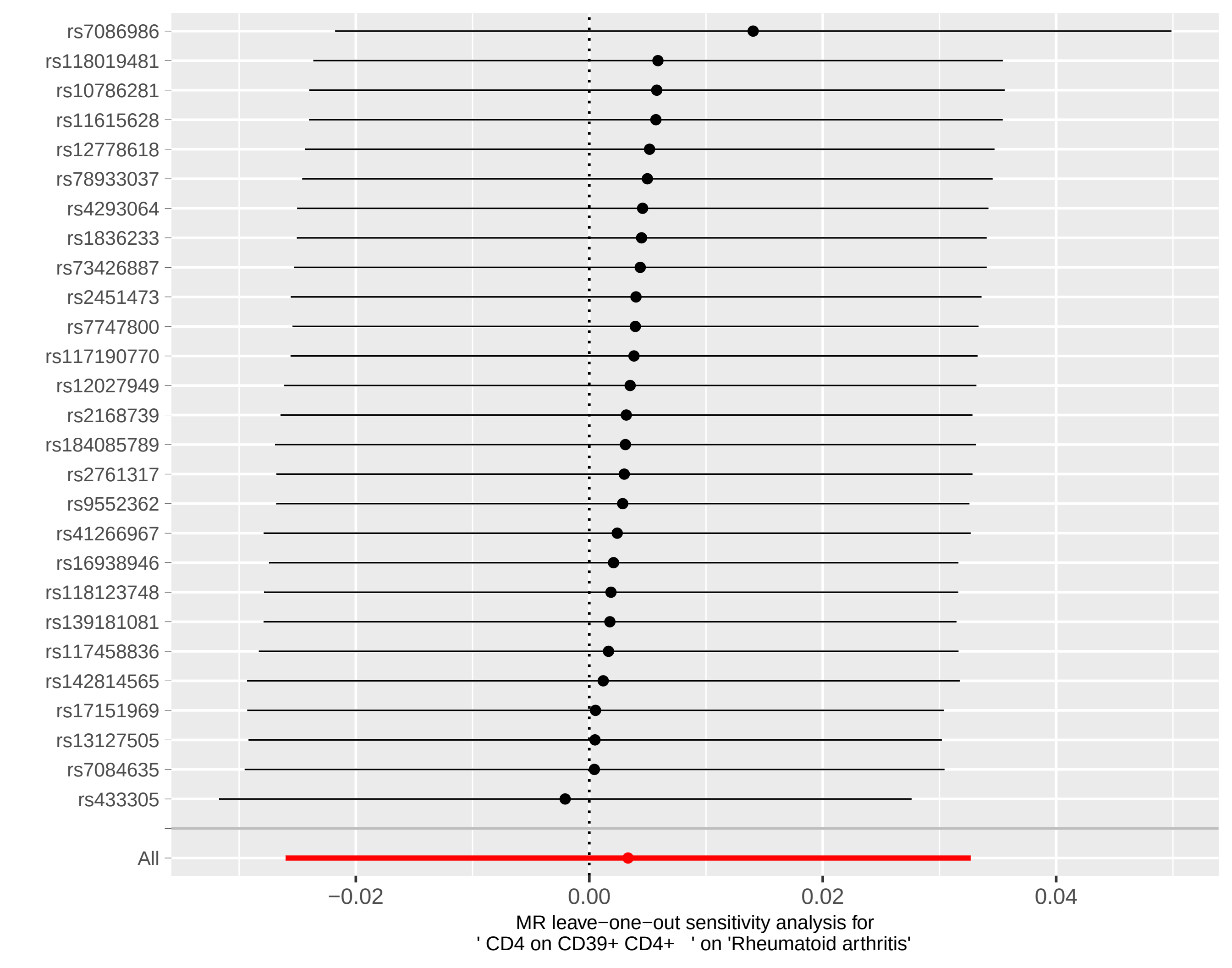

rs7086986
rs118019481
rs10786281
rs11615628
rs12778618
rs78933037
rs4293064
rs1836233
rs73426887
rs2451473
rs7747800
rs117190770
rs12027949
rs2168739
rs184085789
rs2761317
rs9552362
rs41266967
rs16938946
rs118123748
rs139181081
rs117458836
rs142814565
rs17151969
rs13127505
rs7084635
rs433305
All
−0.02
0.00
0.02
0.04
MR leave−one−out sensitivity analysis for
' CD4 on CD39+ CD4+ ' on 'Rheumatoid arthritis'

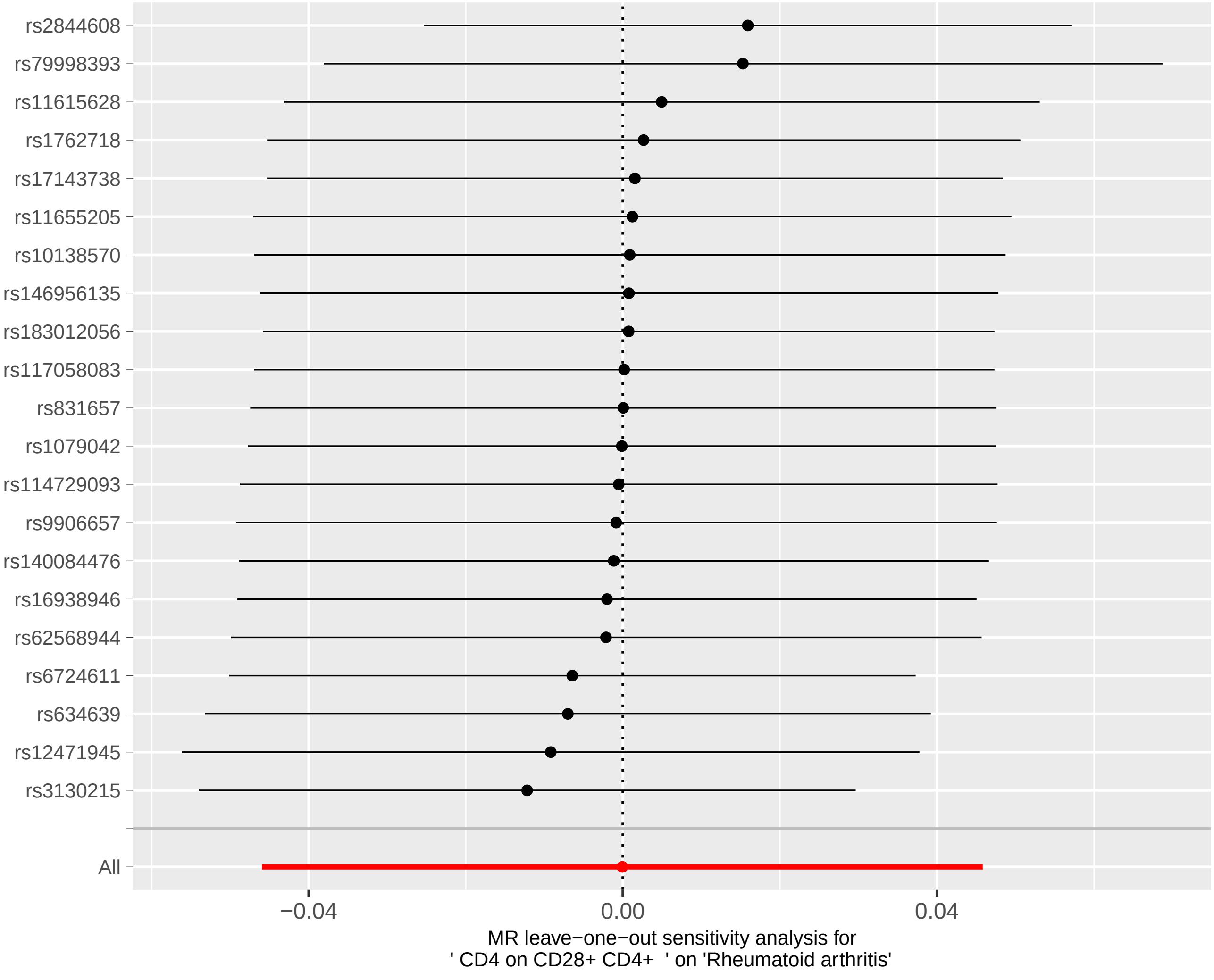
rs2844608
rs79998393
rs11615628
rs1762718
rs17143738
rs11655205
rs10138570
rs146956135
rs183012056
rs117058083
rs831657
rs1079042
rs114729093
rs9906657
rs140084476
rs16938946
rs62568944
rs6724611
rs634639
rs12471945
rs3130215
All
−0.04
0.00
0.04
MR leave−one−out sensitivity analysis for
' CD4 on CD28+ CD4+ ' on 'Rheumatoid arthritis'

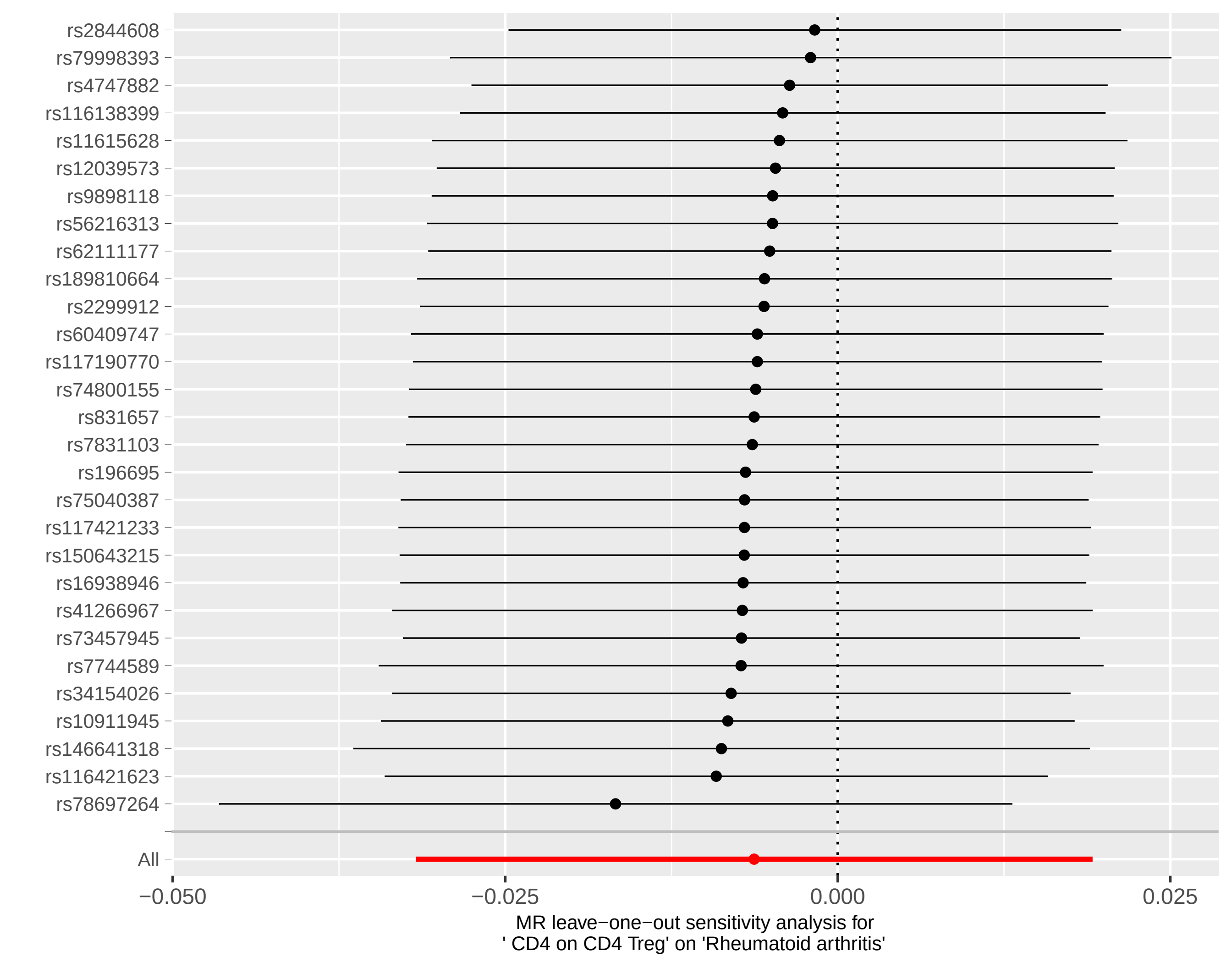

rs2844608
rs79998393
rs4747882
rs116138399
rs11615628
rs12039573
rs9898118
rs56216313
rs62111177
rs189810664
rs2299912
rs60409747
rs117190770
rs74800155
rs831657
rs7831103
rs196695
rs75040387
rs117421233
rs150643215
rs16938946
rs41266967
rs73457945
rs7744589
rs34154026
rs10911945
rs146641318
rs116421623
rs78697264
All
−0.050
−0.025
0.000
0.025
MR leave−one−out sensitivity analysis for
' CD4 on CD4 Treg' on 'Rheumatoid arthritis'

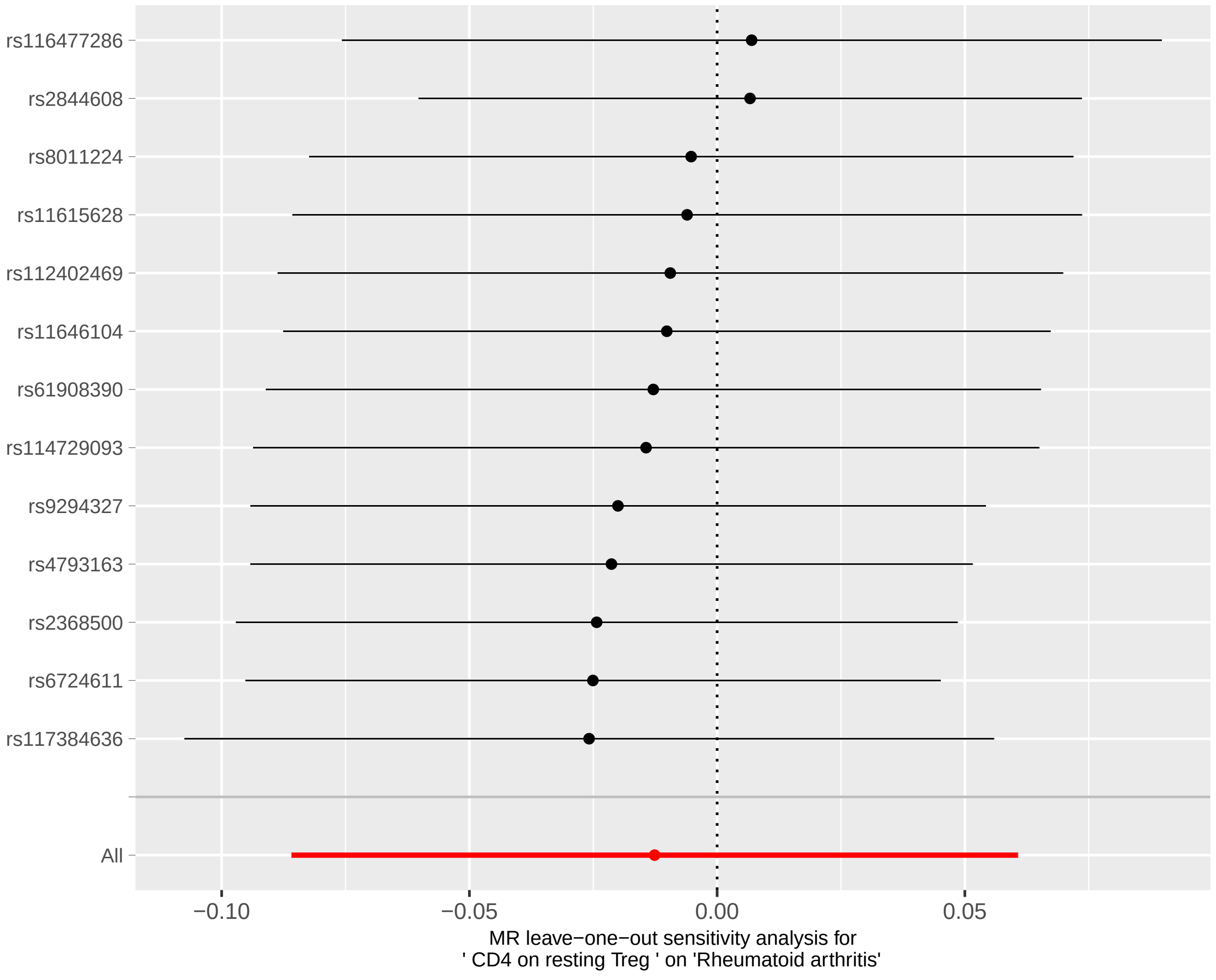
rs116477286
rs2844608
rs8011224
rs11615628
rs112402469
rs11646104
rs61908390
rs114729093
rs9294327
rs4793163
rs2368500
rs6724611
rs117384636
All
−0.10
−0.05
0.00
0.05
MR leave−one−out sensitivity analysis for
' CD4 on resting Treg ' on 'Rheumatoid arthritis'

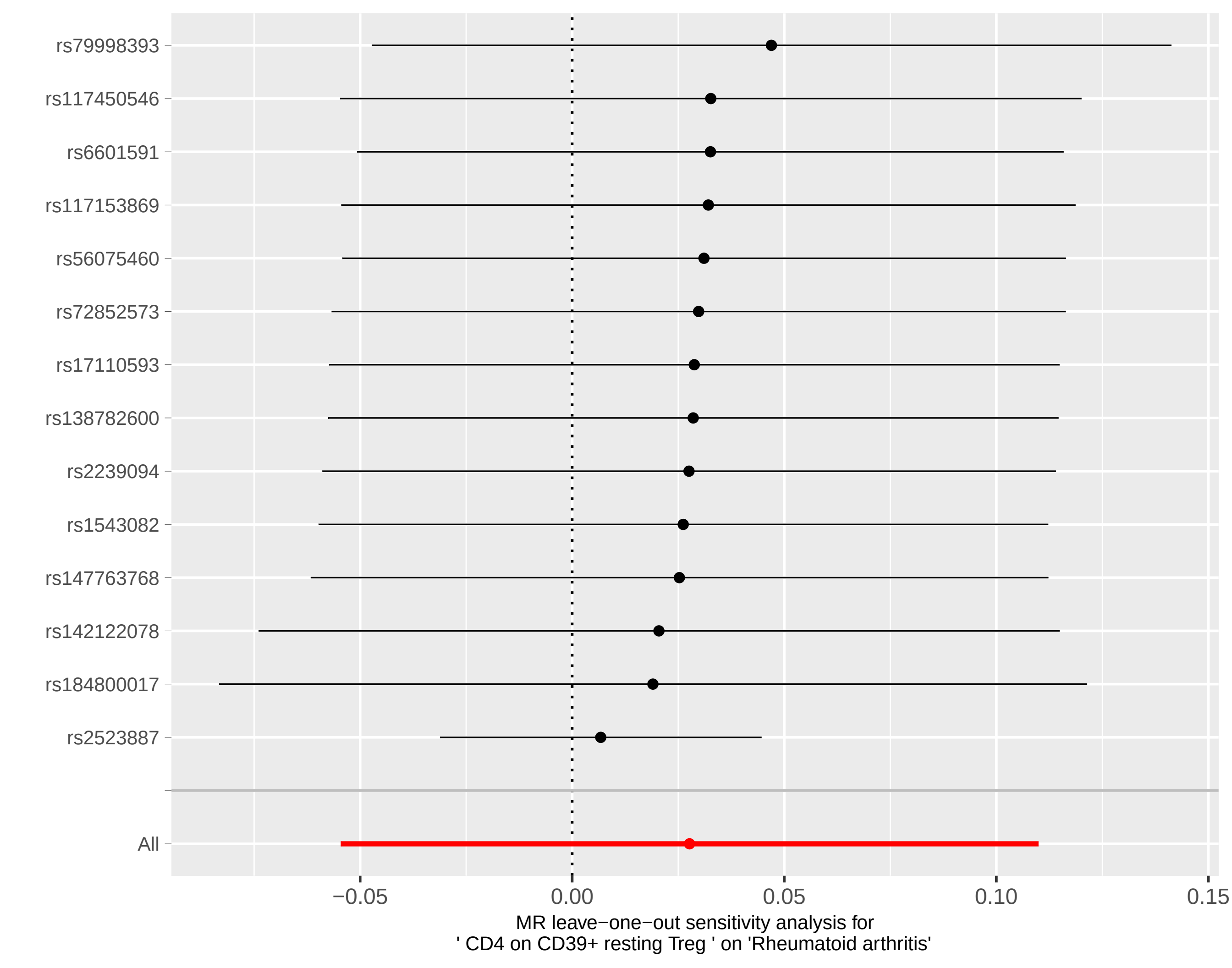

rs79998393
rs117450546
rs6601591
rs117153869
rs56075460
rs72852573
rs17110593
rs138782600
rs2239094
rs1543082
rs147763768
rs142122078
rs184800017
rs2523887
All
−0.05
0.00
0.05
0.10
0.15
MR leave−one−out sensitivity analysis for
' CD4 on CD39+ resting Treg ' on 'Rheumatoid arthritis'

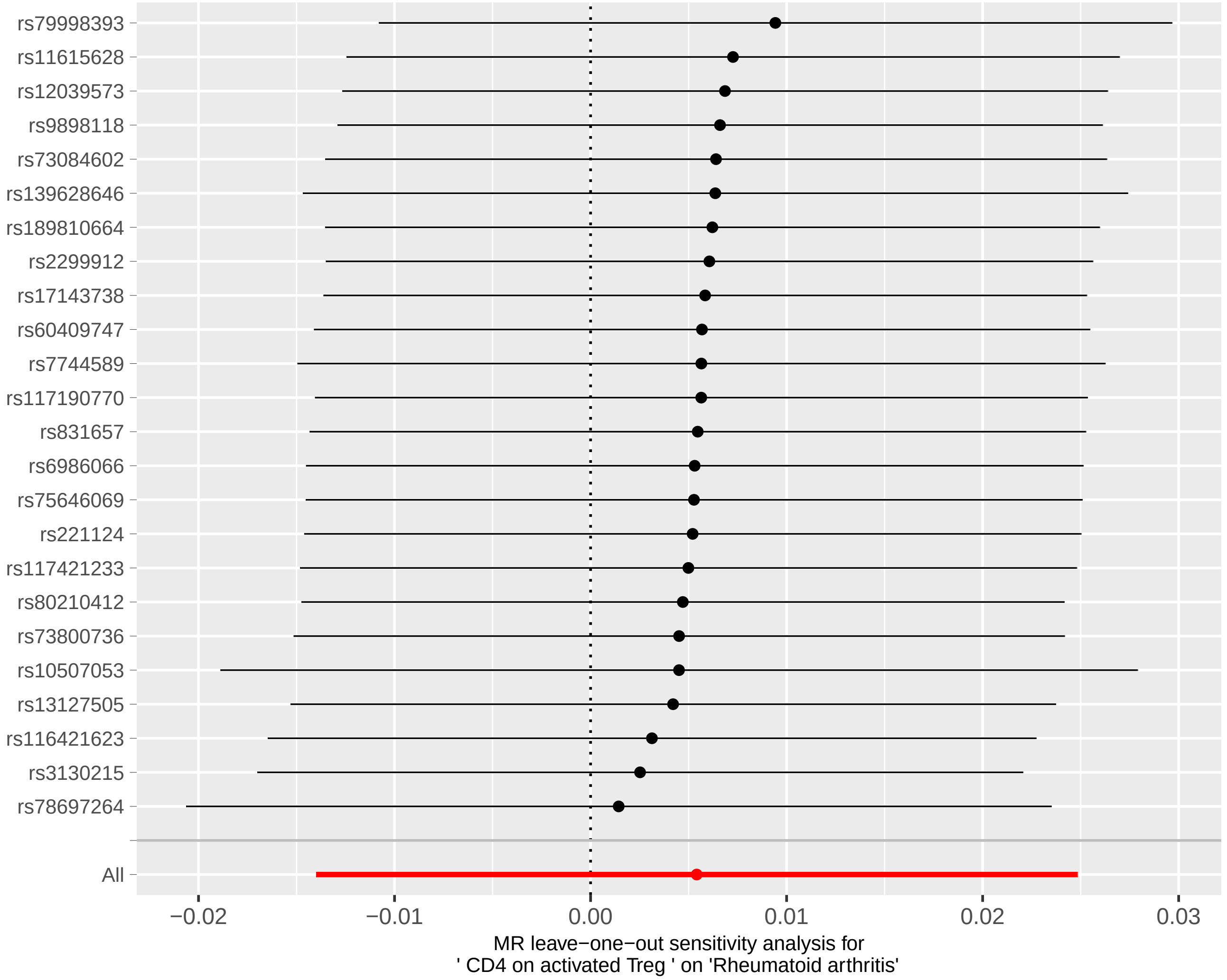
rs79998393
rs11615628
rs12039573
rs9898118
rs73084602
rs139628646
rs189810664
rs2299912
rs17143738
rs60409747
rs7744589
rs117190770
rs831657
rs6986066
rs75646069
rs221124
rs117421233
rs80210412
rs73800736
rs10507053
rs13127505
rs116421623
rs3130215
rs78697264
All
−0.02
−0.01
0.00
0.01
0.02
0.03
MR leave−one−out sensitivity analysis for
' CD4 on activated Treg ' on 'Rheumatoid arthritis'

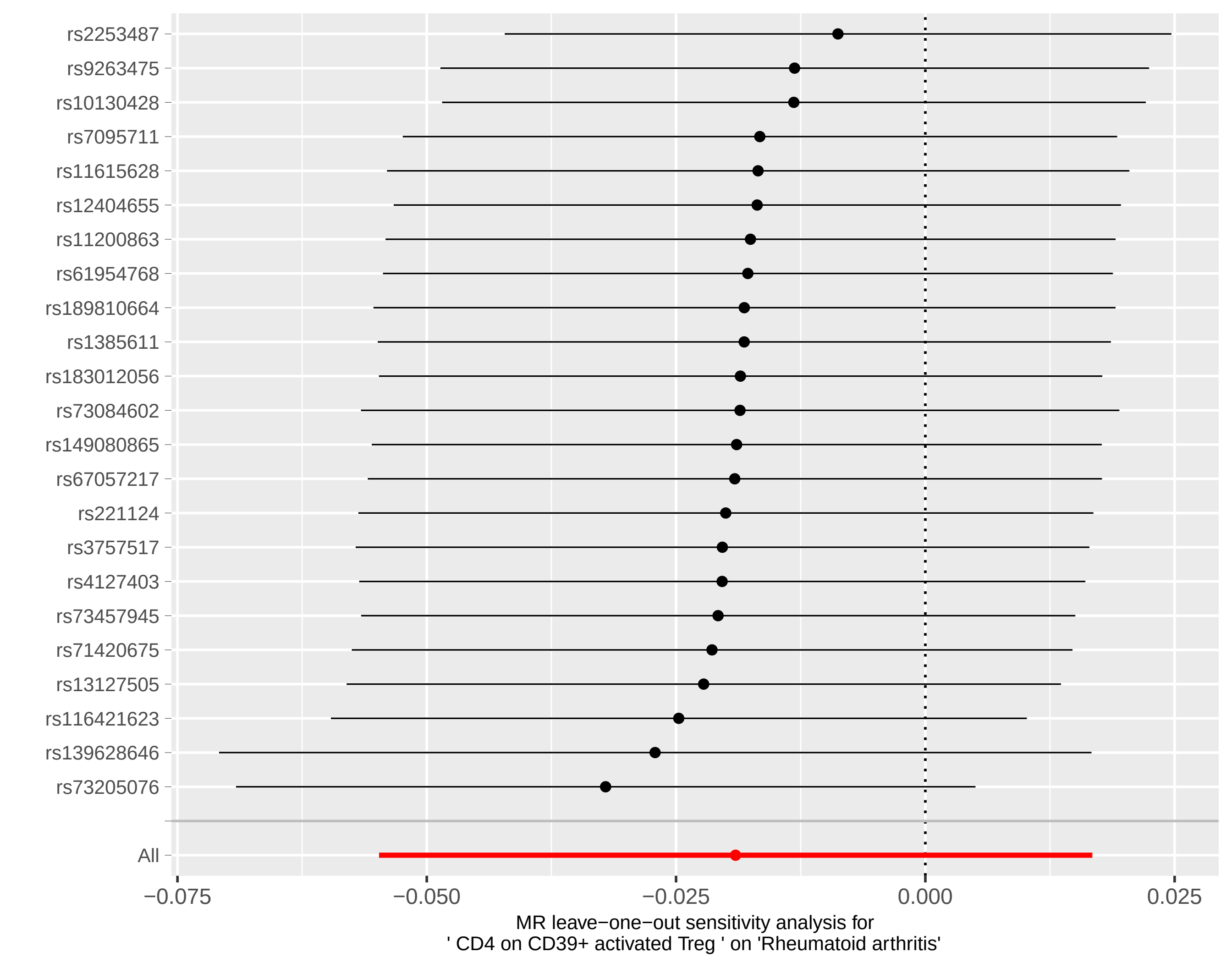

rs2253487
rs9263475
rs10130428
rs7095711
rs11615628
rs12404655
rs11200863
rs61954768
rs189810664
rs1385611
rs183012056
rs73084602
rs149080865
rs67057217
rs221124
rs3757517
rs4127403
rs73457945
rs71420675
rs13127505
rs116421623
rs139628646
rs73205076
All
−0.075
−0.050
−0.025
0.000
0.025
MR leave−one−out sensitivity analysis for
' CD4 on CD39+ activated Treg ' on 'Rheumatoid arthritis'

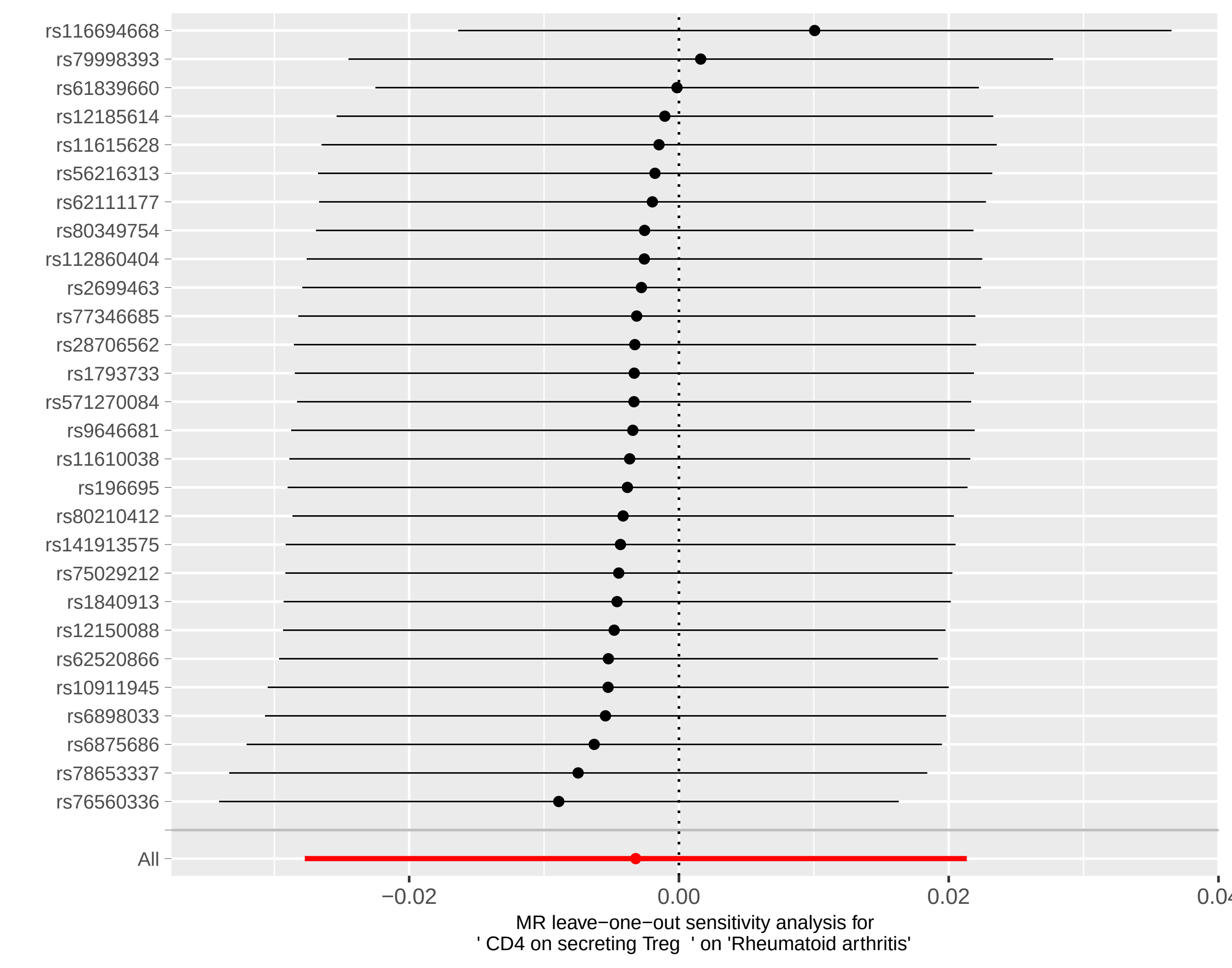
rs116694668
rs79998393
rs61839660
rs12185614
rs11615628
rs56216313
rs62111177
rs80349754
rs112860404
rs2699463
rs77346685
rs28706562
rs1793733
rs571270084
rs9646681
rs11610038
rs196695
rs80210412
rs141913575
rs75029212
rs1840913
rs12150088
rs62520866
rs10911945
rs6898033
rs6875686
rs78653337
rs76560336
All
−0.02
0.00
0.02
MR leave−one−out sensitivity analysis for
' CD4 on secreting Treg ' on 'Rheumatoid arthritis'

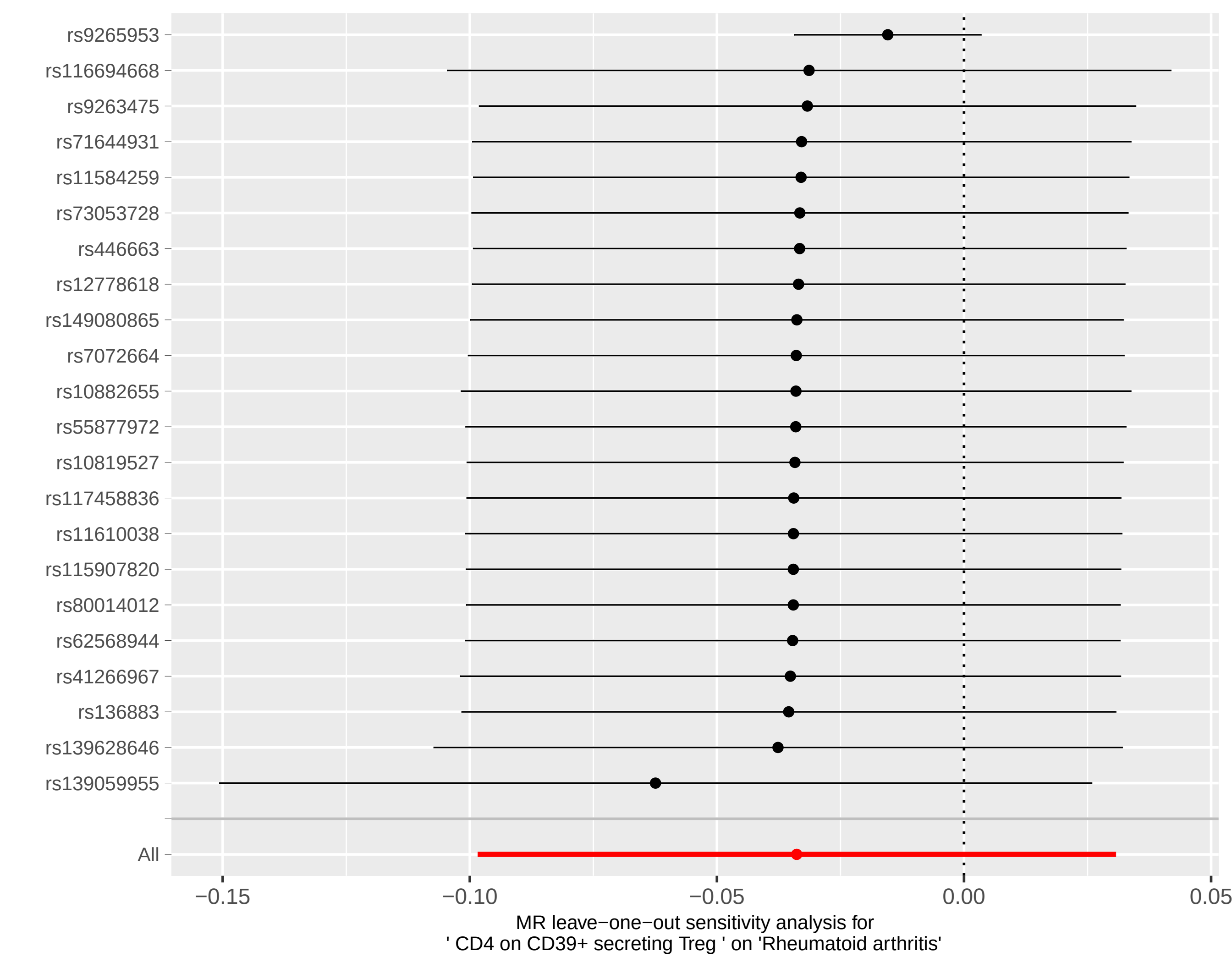
rs9265953
rs116694668
rs9263475
rs71644931
rs11584259
rs73053728
rs446663
rs12778618
rs149080865
rs7072664
rs10882655
rs55877972
rs10819527
rs117458836
rs11610038
rs115907820
rs80014012
rs62568944
rs41266967
rs136883
rs139628646
rs139059955
All
−0.15
−0.10
−0.05
0.00
0.05
MR leave−one−out sensitivity analysis for
' CD4 on CD39+ secreting Treg ' on 'Rheumatoid arthritis'

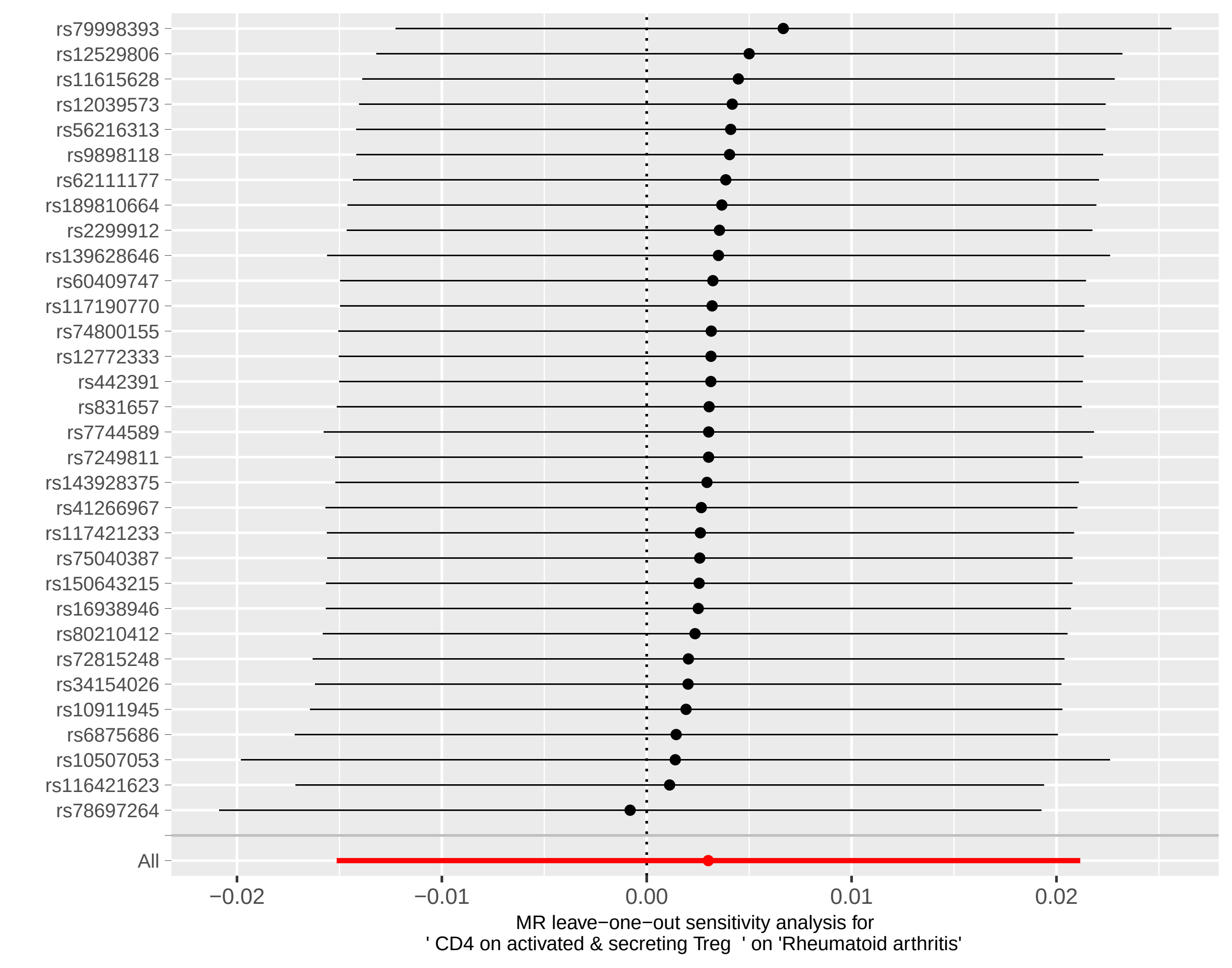
rs79998393
rs12529806
rs11615628
rs12039573
rs56216313
rs9898118
rs62111177
rs189810664
rs2299912
rs139628646
rs60409747
rs117190770
rs74800155
rs12772333
rs442391
rs831657
rs7744589
rs7249811
rs143928375
rs41266967
rs117421233
rs75040387
rs150643215
rs16938946
rs80210412
rs72815248
rs34154026
rs10911945
rs6875686
rs10507053
rs116421623
rs78697264
All
−0.02
−0.01
0.00
0.01
0.02
MR leave−one−out sensitivity analysis for
' CD4 on activated & secreting Treg ' on 'Rheumatoid arthritis'

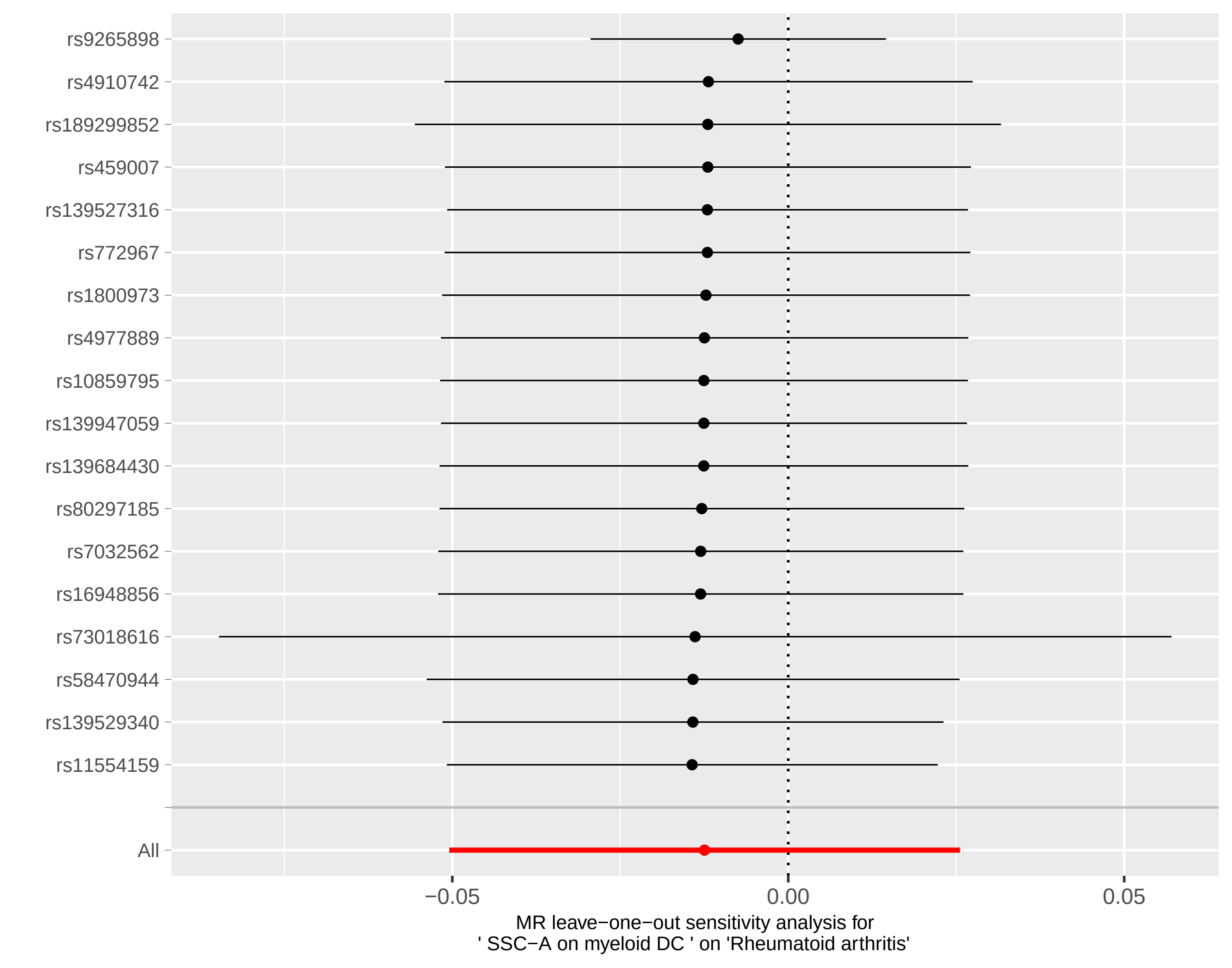
rs9265898
rs4910742
rs189299852
rs459007
rs139527316
rs772967
rs1800973
rs4977889
rs10859795
rs139947059
rs139684430
rs80297185
rs7032562
rs16948856
rs73018616
rs58470944
rs139529340
rs11554159
All
−0.05
0.00
0.05
MR leave−one−out sensitivity analysis for
' SSC−A on myeloid DC ' on 'Rheumatoid arthritis'

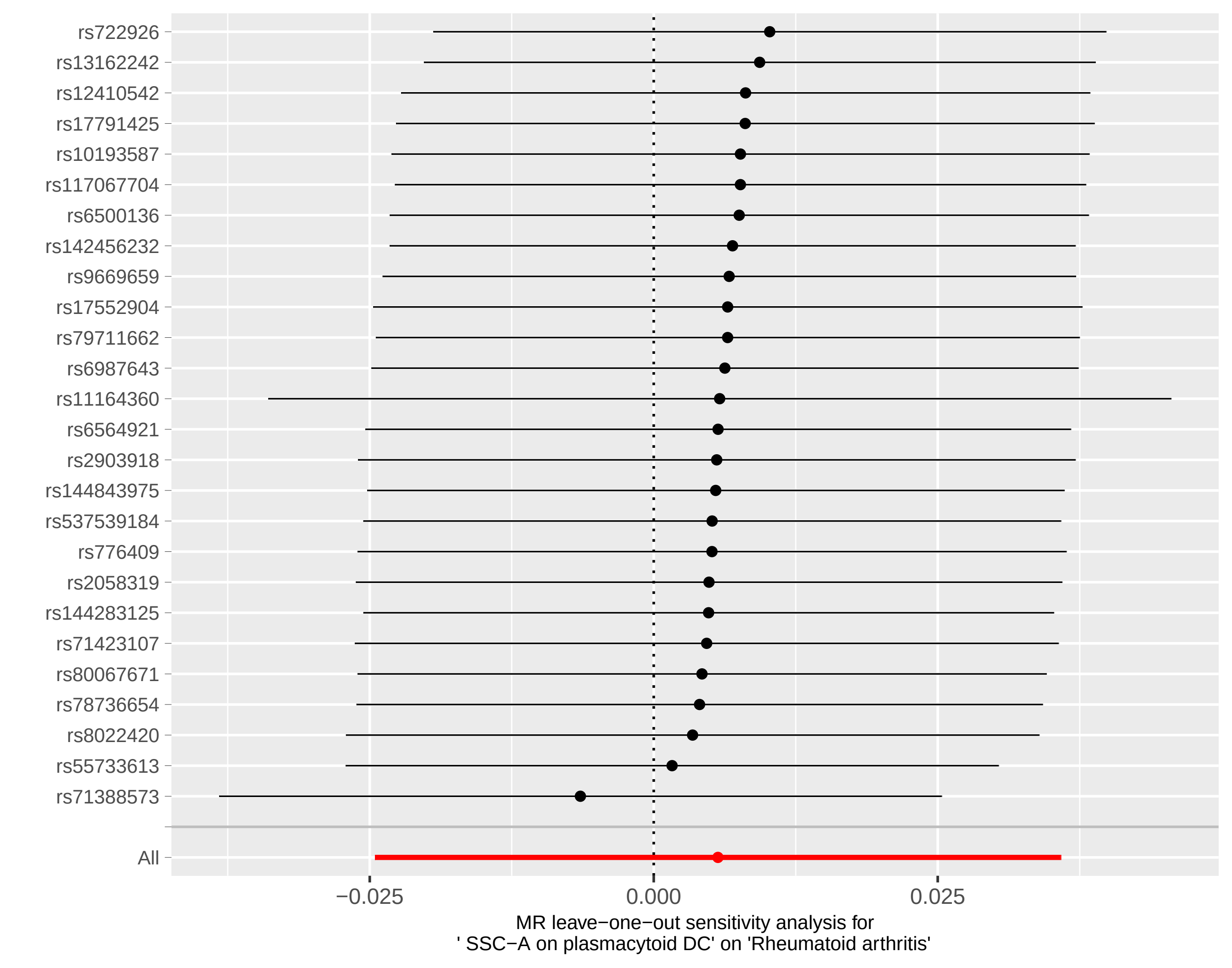
rs722926
rs13162242
rs12410542
rs17791425
rs10193587
rs117067704
rs6500136
rs142456232
rs9669659
rs17552904
rs79711662
rs6987643
rs11164360
rs6564921
rs2903918
rs144843975
rs537539184
rs776409
rs2058319
rs144283125
rs71423107
rs80067671
rs78736654
rs8022420
rs55733613
rs71388573
All
−0.025
0.000
0.025
MR leave−one−out sensitivity analysis for
' SSC−A on plasmacytoid DC' on 'Rheumatoid arthritis'

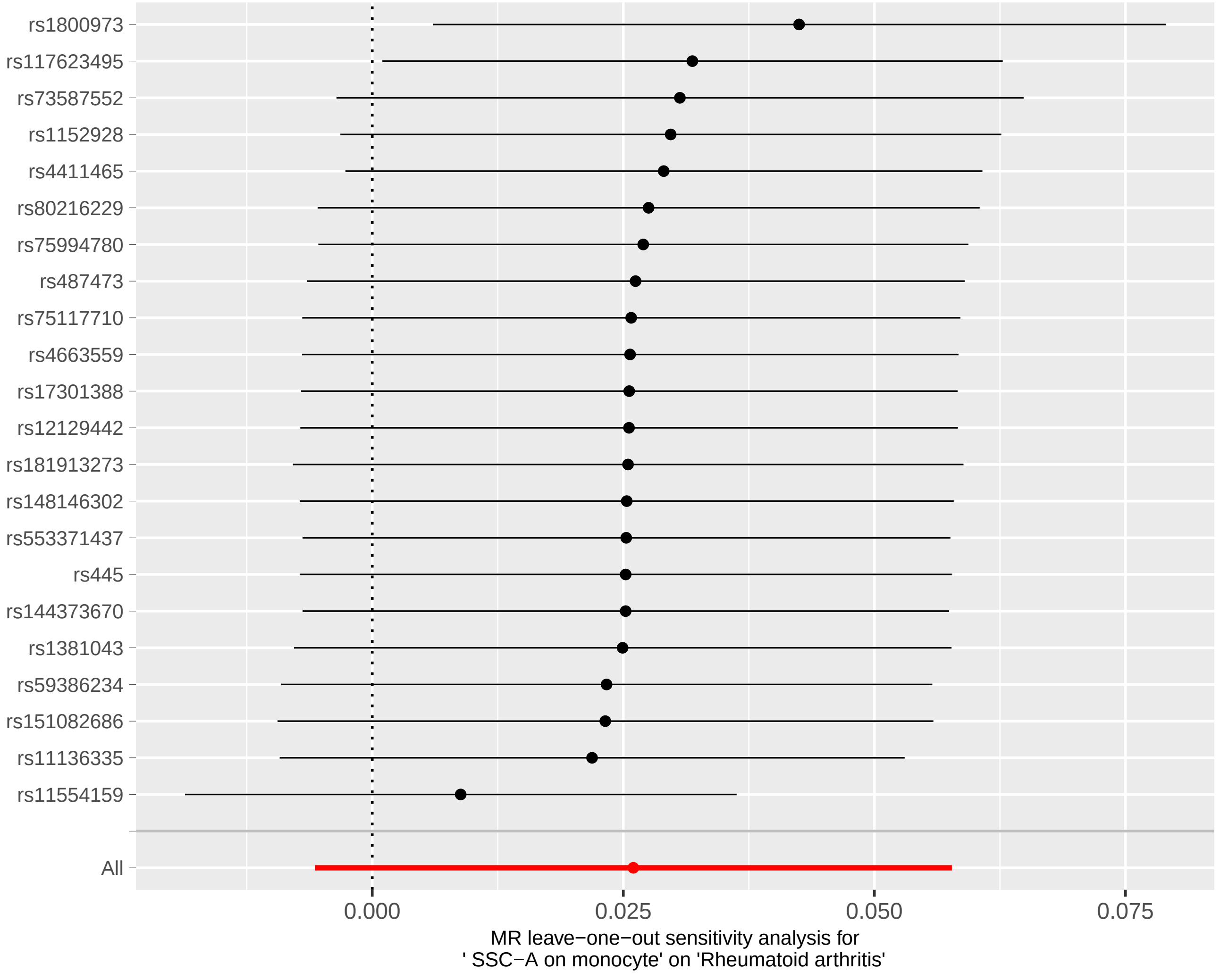
rs1800973
rs117623495
rs73587552
rs1152928
rs4411465
rs80216229
rs75994780
rs487473
rs75117710
rs4663559
rs17301388
rs12129442
rs181913273
rs148146302
rs553371437
rs445
rs144373670
rs1381043
rs59386234
rs151082686
rs11136335
rs11554159
All
0.000
0.025
0.050
0.075
MR leave−one−out sensitivity analysis for
' SSC−A on monocyte' on 'Rheumatoid arthritis'

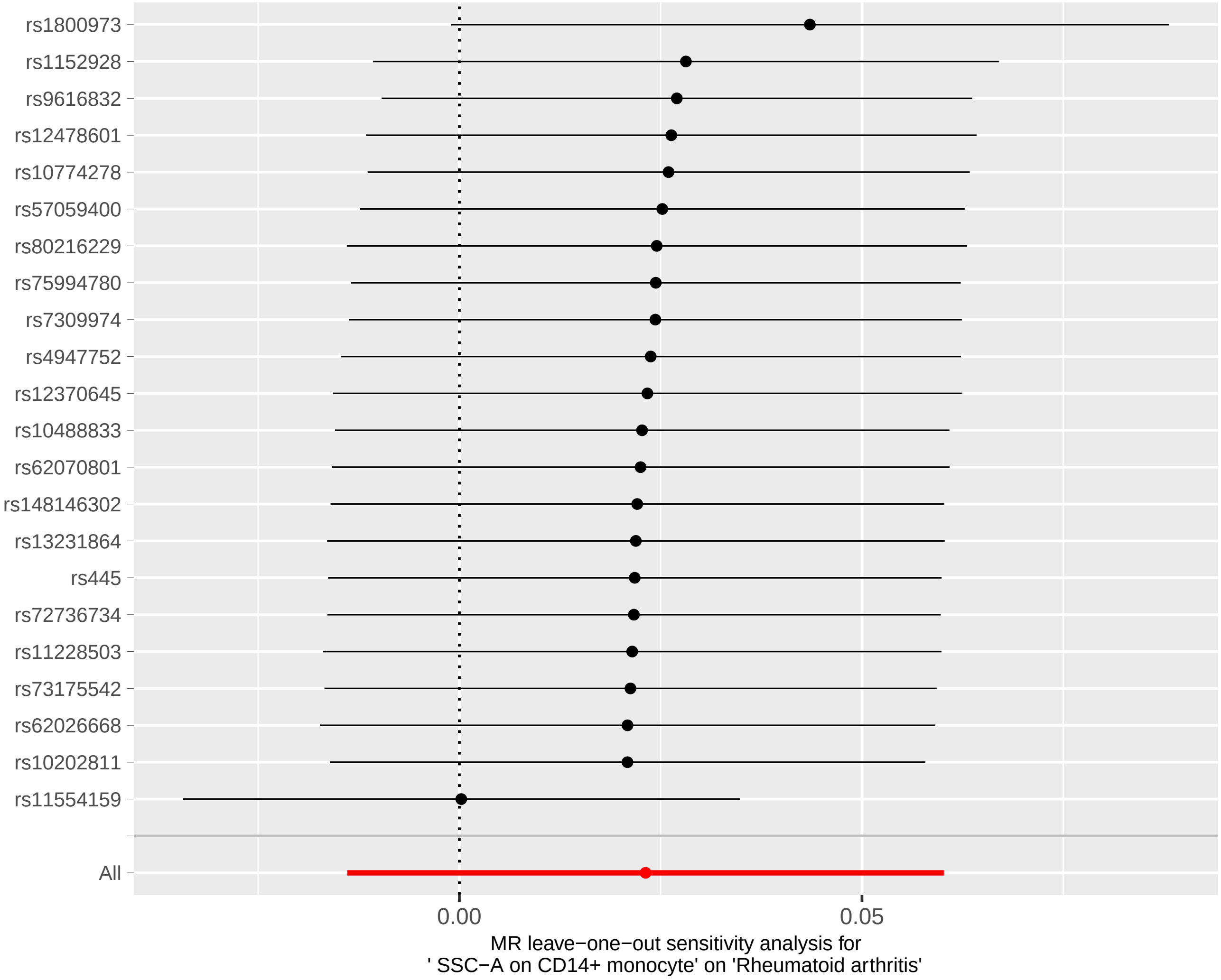
rs1800973
rs1152928
rs9616832
rs12478601
rs10774278
rs57059400
rs80216229
rs75994780
rs7309974
rs4947752
rs12370645
rs10488833
rs62070801
rs148146302
rs13231864
rs445
rs72736734
rs11228503
rs73175542
rs62026668
rs10202811
rs11554159
All
0.00
0.05
MR leave−one−out sensitivity analysis for
' SSC−A on CD14+ monocyte' on 'Rheumatoid arthritis'

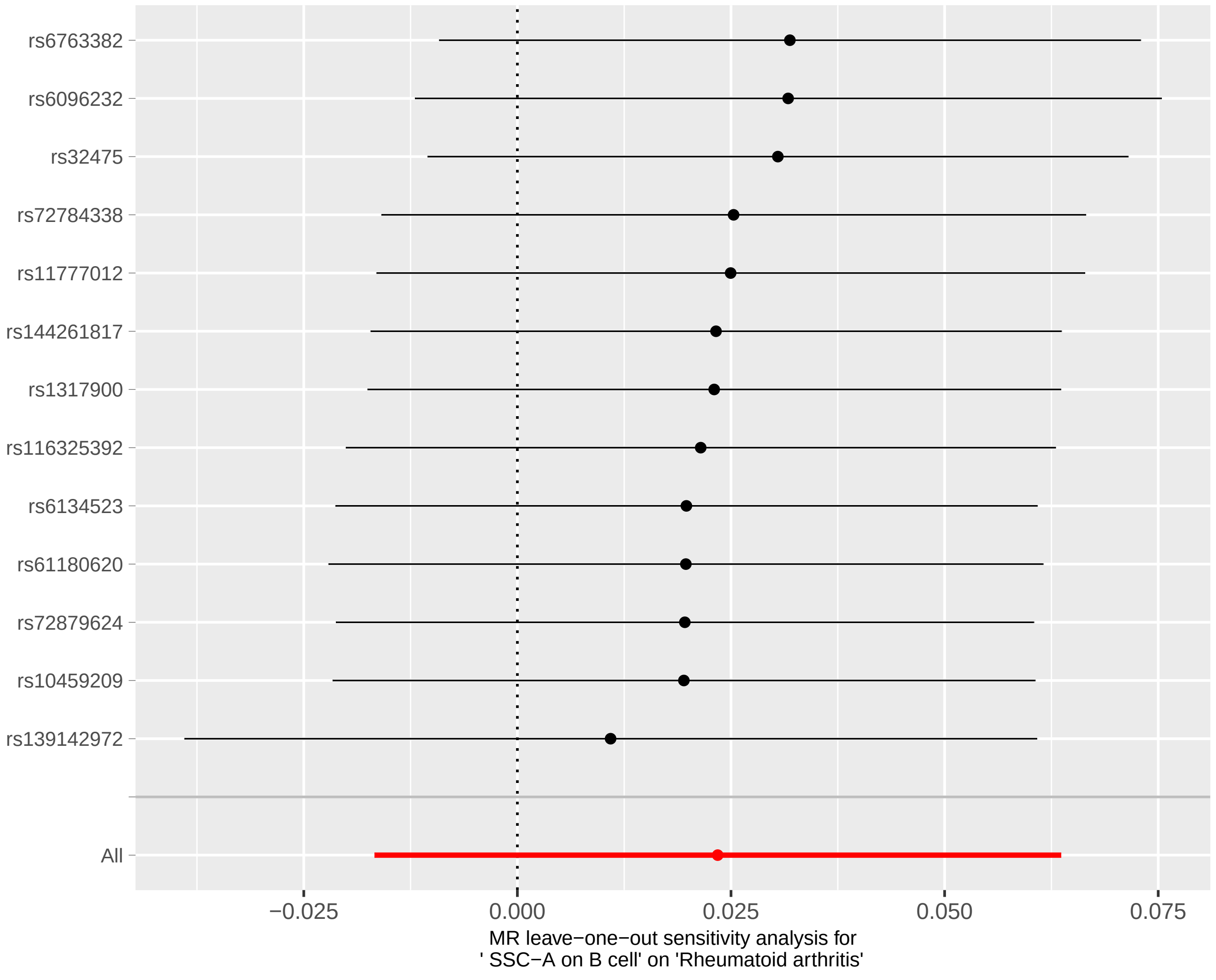
rs6763382
rs6096232
rs32475
rs72784338
rs11777012
rs144261817
rs1317900
rs116325392
rs6134523
rs61180620
rs72879624
rs10459209
rs139142972
All
−0.025
0.000
0.025
0.050
0.075
MR leave−one−out sensitivity analysis for
' SSC−A on B cell' on 'Rheumatoid arthritis'

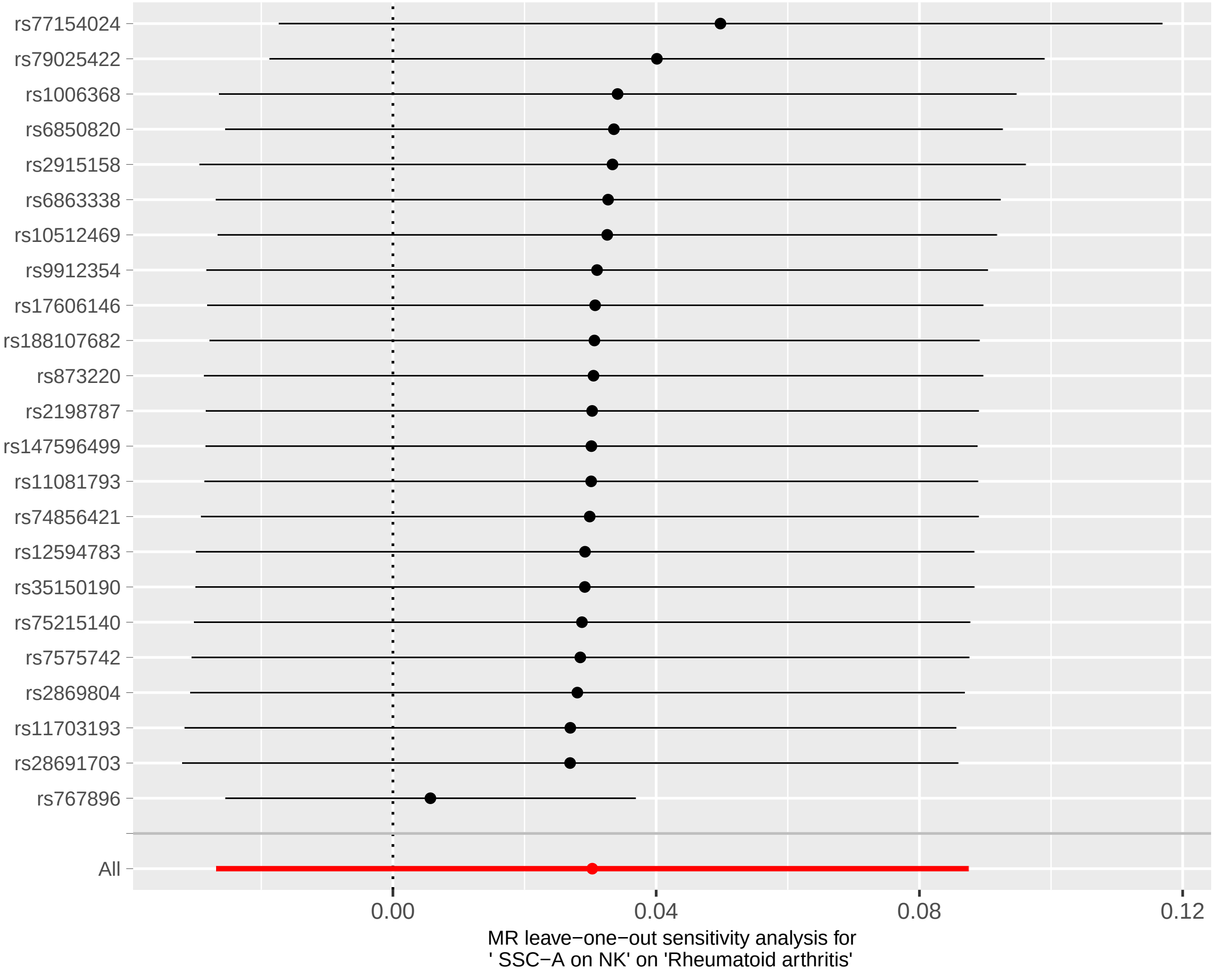

rs77154024
rs79025422
rs1006368
rs6850820
rs2915158
rs6863338
rs10512469
rs9912354
rs17606146
rs188107682
rs873220
rs2198787
rs147596499
rs11081793
rs74856421
rs12594783
rs35150190
rs75215140
rs7575742
rs2869804
rs11703193
rs28691703
rs767896
All
0.00
0.04
0.08
0.12
MR leave−one−out sensitivity analysis for
' SSC−A on NK' on 'Rheumatoid arthritis'

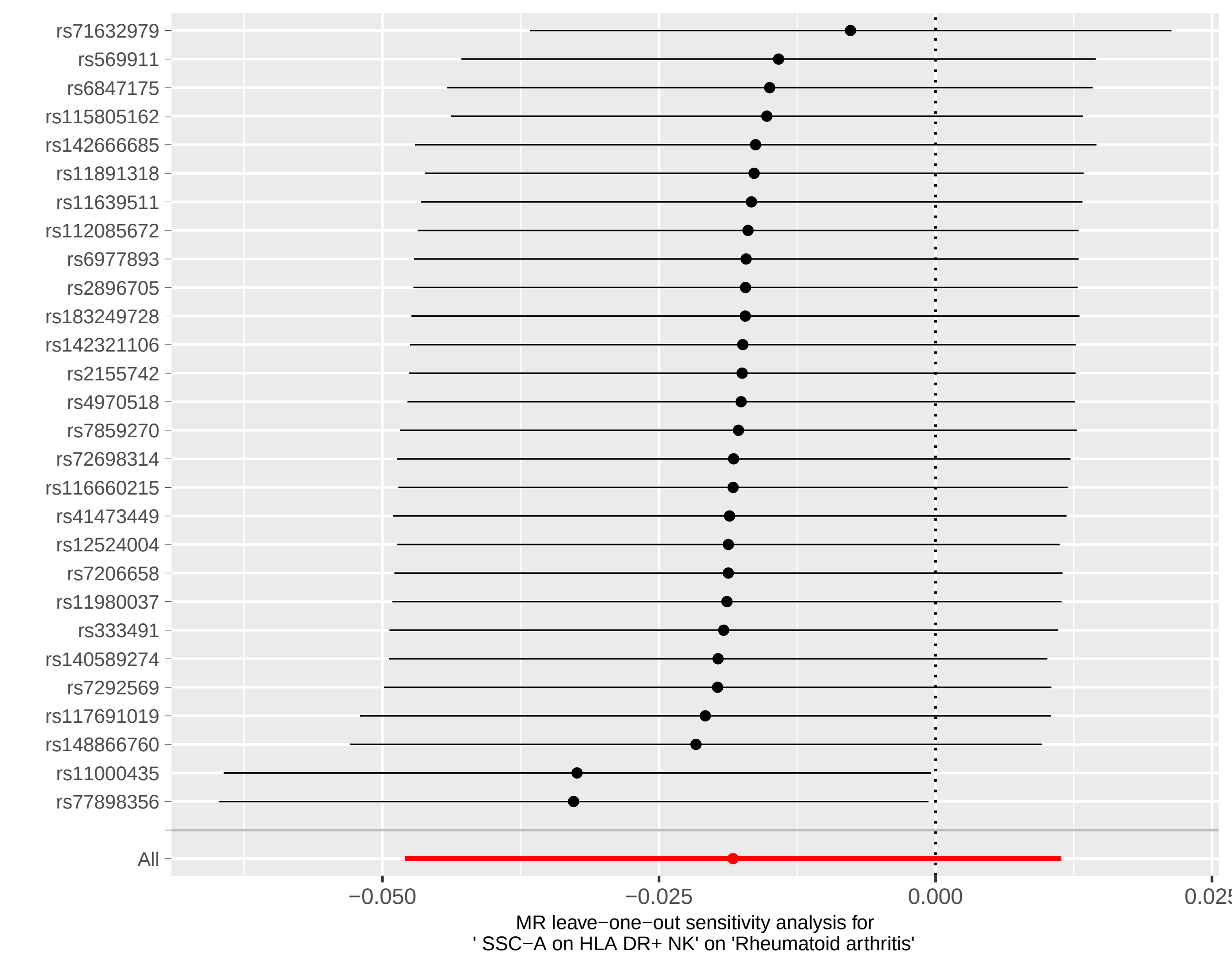

rs71632979
rs569911
rs6847175
rs115805162
rs142666685
rs11891318
rs11639511
rs112085672
rs6977893
rs2896705
rs183249728
rs142321106
rs2155742
rs4970518
rs7859270
rs72698314
rs116660215
rs41473449
rs12524004
rs7206658
rs11980037
rs333491
rs140589274
rs7292569
rs117691019
rs148866760
rs11000435
rs77898356
All
−0.050
−0.025
0.000
0.025
MR leave−one−out sensitivity analysis for
' SSC−A on HLA DR+ NK' on 'Rheumatoid arthritis'

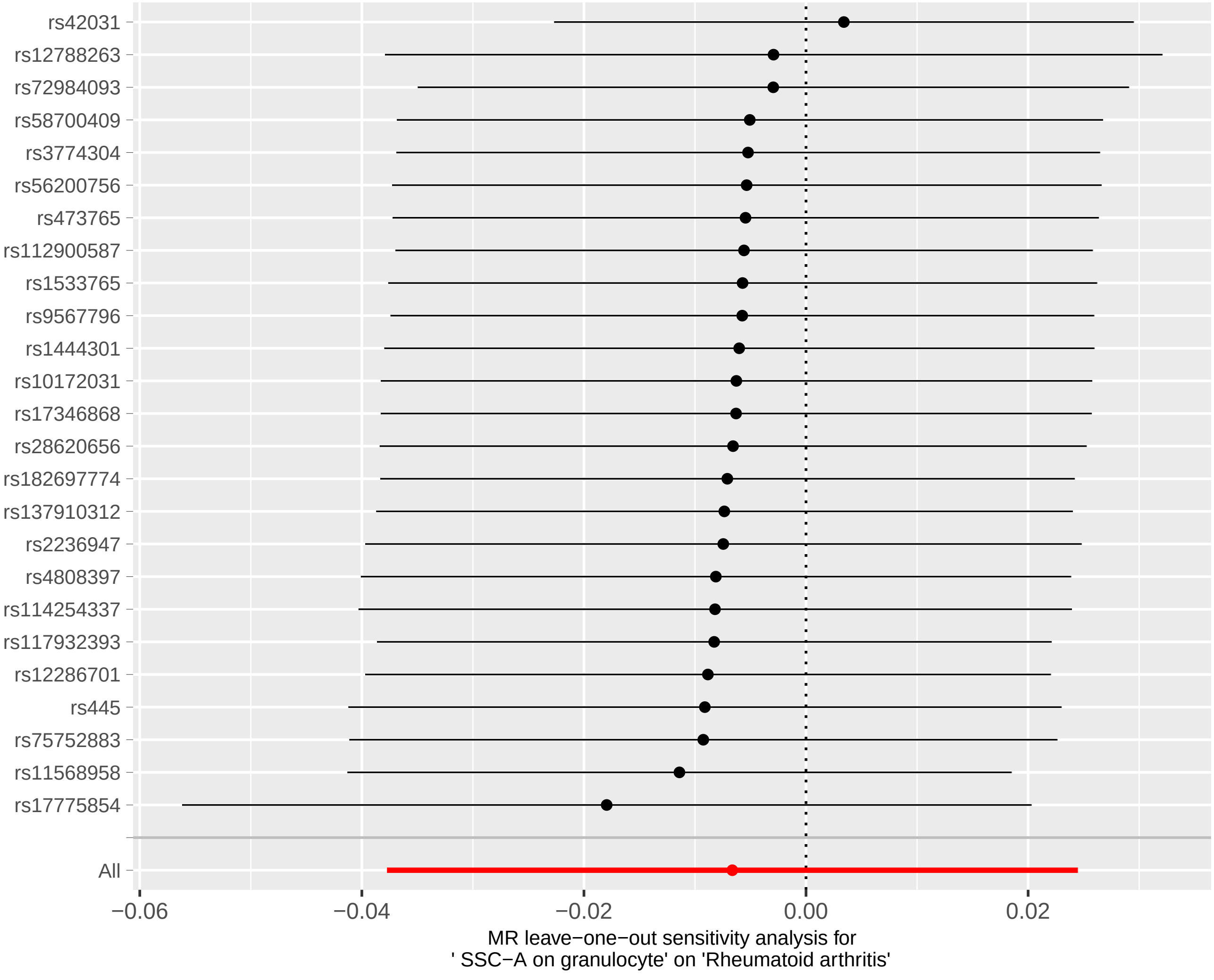
rs42031
rs12788263
rs72984093
rs58700409
rs3774304
rs56200756
rs473765
rs112900587
rs1533765
rs9567796
rs1444301
rs10172031
rs17346868
rs28620656
rs182697774
rs137910312
rs2236947
rs4808397
rs114254337
rs117932393
rs12286701
rs445
rs75752883
rs11568958
rs17775854
All
−0.06
−0.04
−0.02
0.00
0.02
MR leave−one−out sensitivity analysis for
' SSC−A on granulocyte' on 'Rheumatoid arthritis'

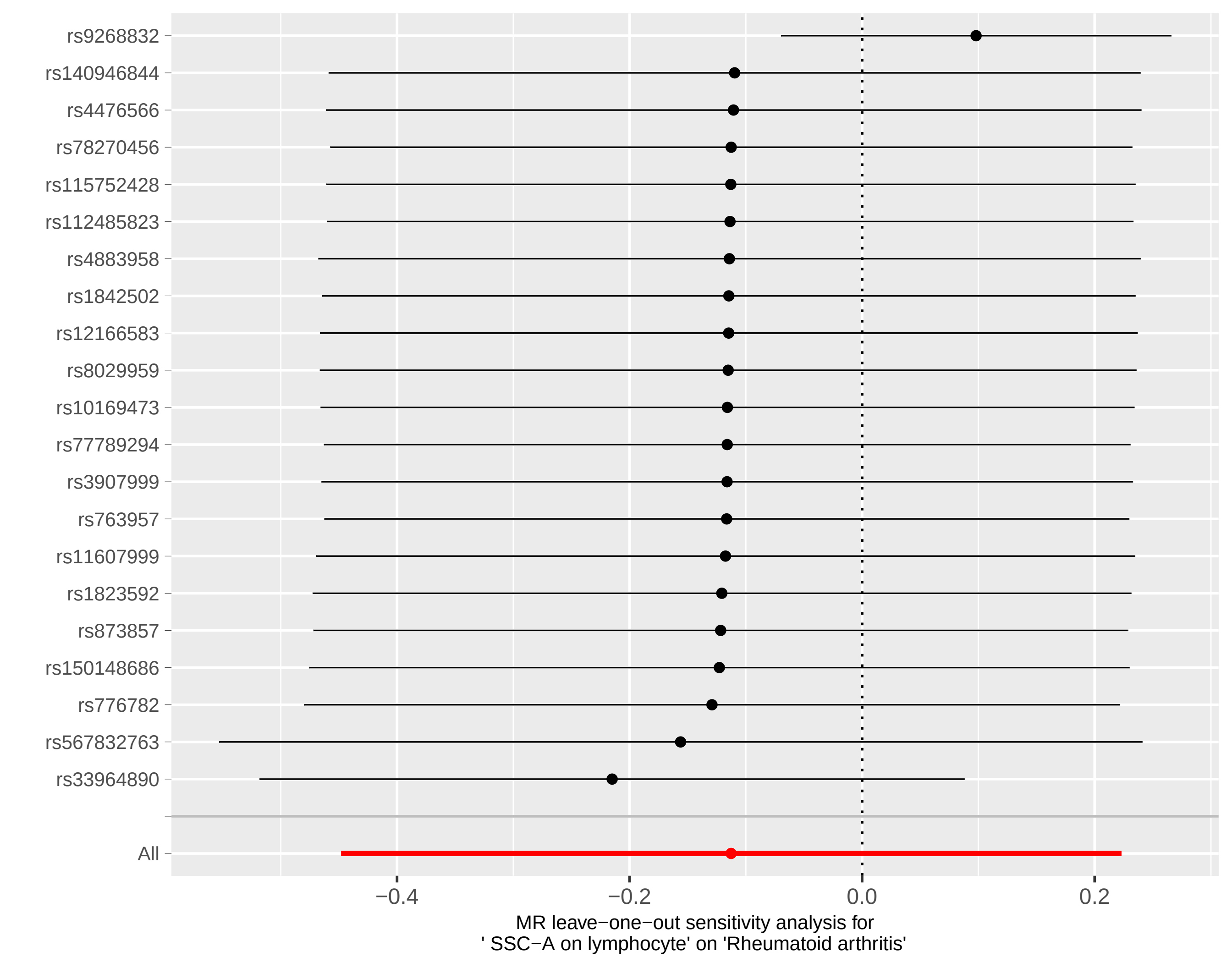
rs9268832
rs140946844
rs4476566
rs78270456
rs115752428
rs112485823
rs4883958
rs1842502
rs12166583
rs8029959
rs10169473
rs77789294
rs3907999
rs763957
rs11607999
rs1823592
rs873857
rs150148686
rs776782
rs567832763
rs33964890
All
−0.4
−0.2
0.0
0.2
MR leave−one−out sensitivity analysis for
' SSC−A on lymphocyte' on 'Rheumatoid arthritis'

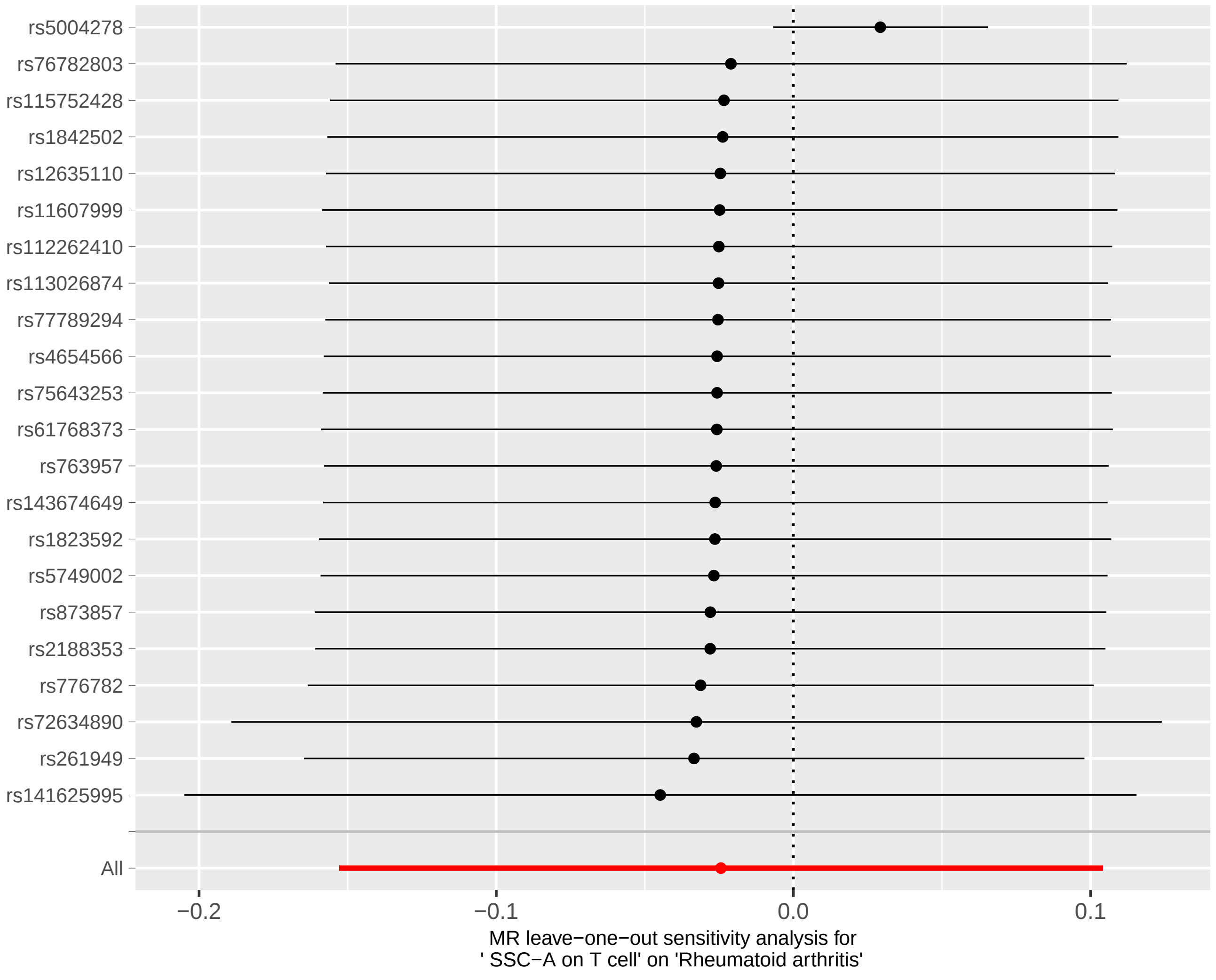

rs5004278
rs76782803
rs115752428
rs1842502
rs12635110
rs11607999
rs112262410
rs113026874
rs77789294
rs4654566
rs75643253
rs61768373
rs763957
rs143674649
rs1823592
rs5749002
rs873857
rs2188353
rs776782
rs72634890
rs261949
rs141625995
All
−0.2
−0.1
0.0
0.1
MR leave−one−out sensitivity analysis for
' SSC−A on T cell' on 'Rheumatoid arthritis'

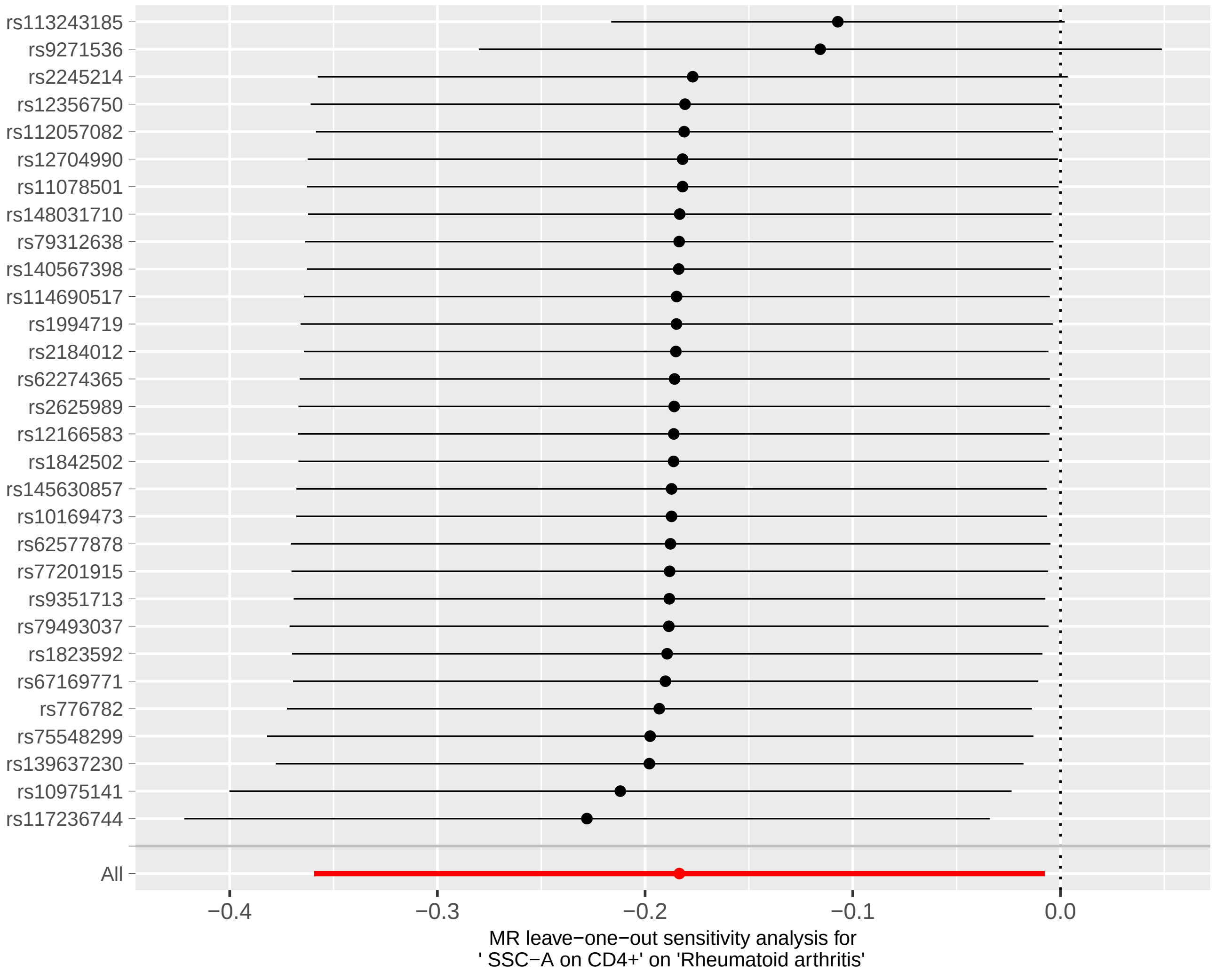
rs113243185
rs9271536
rs2245214
rs12356750
rs112057082
rs12704990
rs11078501
rs148031710
rs79312638
rs140567398
rs114690517
rs1994719
rs2184012
rs62274365
rs2625989
rs12166583
rs1842502
rs145630857
rs10169473
rs62577878
rs77201915
rs9351713
rs79493037
rs1823592
rs67169771
rs776782
rs75548299
rs139637230
rs10975141
rs117236744
All
−0.4
−0.3
−0.2
−0.1
0.0
MR leave−one−out sensitivity analysis for
' SSC−A on CD4+' on 'Rheumatoid arthritis'

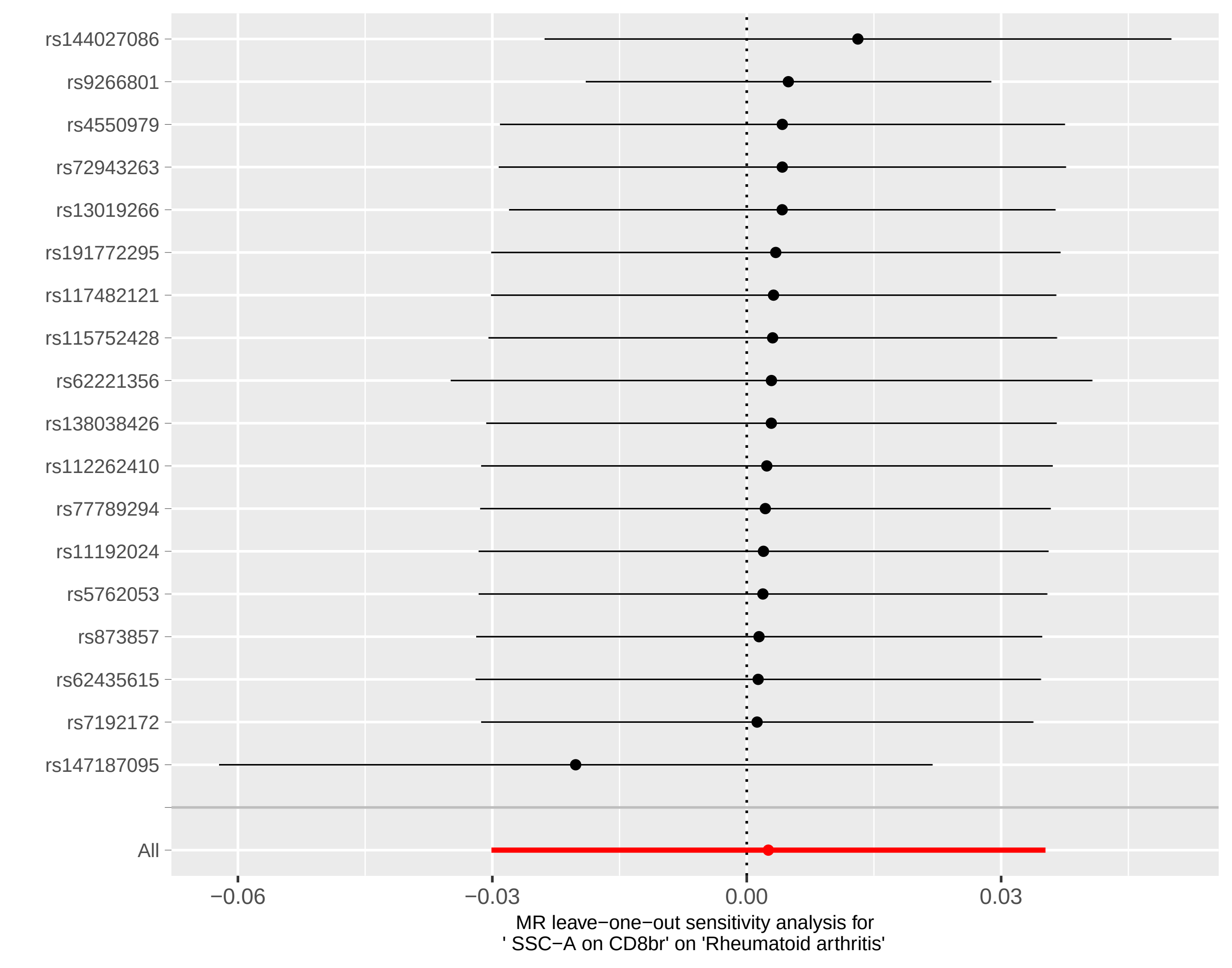

rs144027086
rs9266801
rs4550979
rs72943263
rs13019266
rs191772295
rs117482121
rs115752428
rs62221356
rs138038426
rs112262410
rs77789294
rs11192024
rs5762053
rs873857
rs62435615
rs7192172
rs147187095
All
−0.06
−0.03
0.00
0.03
MR leave−one−out sensitivity analysis for
' SSC−A on CD8br' on 'Rheumatoid arthritis'

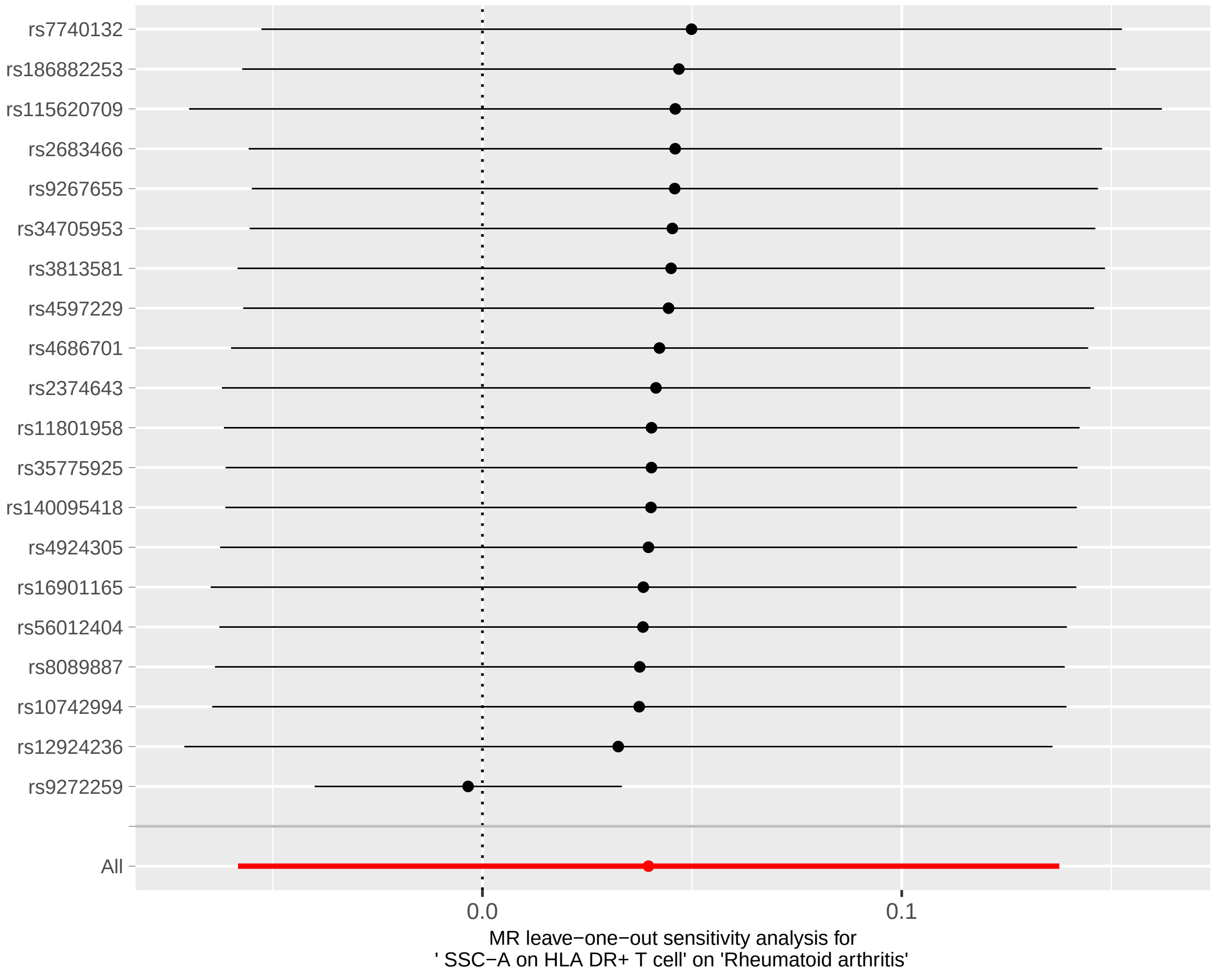

rs7740132
rs186882253
rs115620709
rs2683466
rs9267655
rs34705953
rs3813581
rs4597229
rs4686701
rs2374643
rs11801958
rs35775925
rs140095418
rs4924305
rs16901165
rs56012404
rs8089887
rs10742994
rs12924236
rs9272259
All
0.0
0.1
MR leave−one−out sensitivity analysis for
' SSC−A on HLA DR+ T cell' on 'Rheumatoid arthritis'

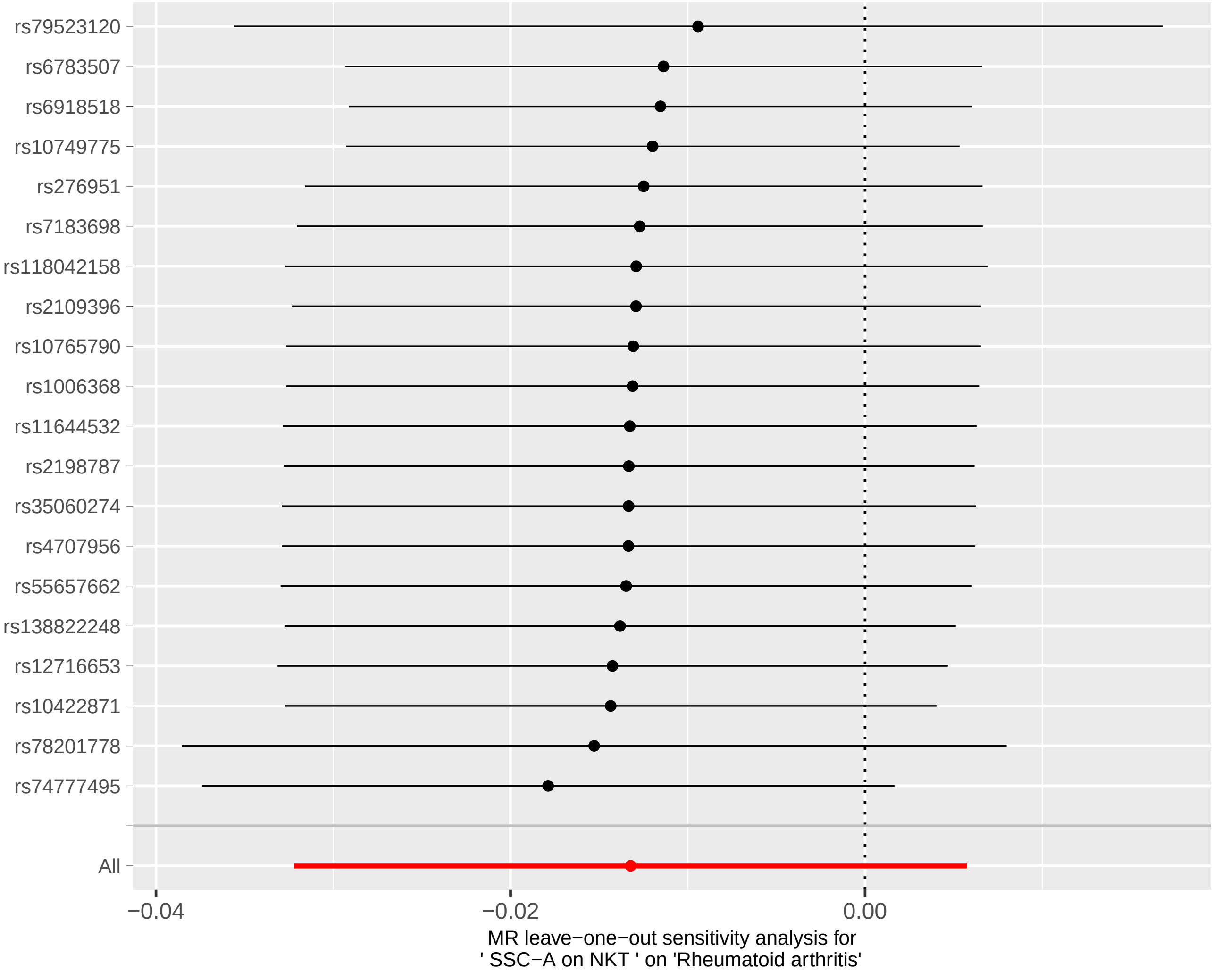

rs79523120
rs6783507
rs6918518
rs10749775
rs276951
rs7183698
rs118042158
rs2109396
rs10765790
rs1006368
rs11644532
rs2198787
rs35060274
rs4707956
rs55657662
rs138822248
rs12716653
rs10422871
rs78201778
rs74777495
All
−0.04
−0.02
0.00
MR leave−one−out sensitivity analysis for
' SSC−A on NKT ' on 'Rheumatoid arthritis'

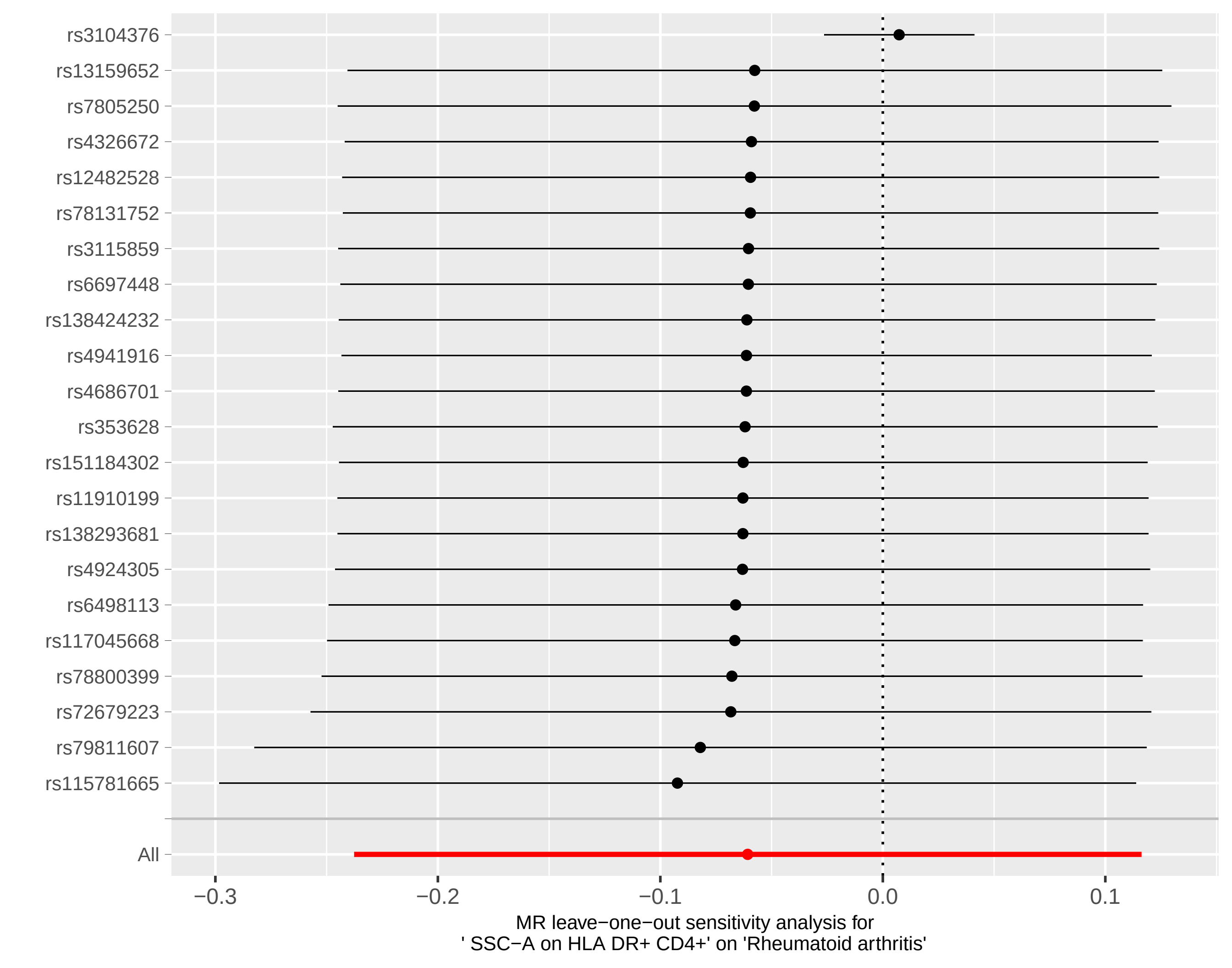
rs3104376
rs13159652
rs7805250
rs4326672
rs12482528
rs78131752
rs3115859
rs6697448
rs138424232
rs4941916
rs4686701
rs353628
rs151184302
rs11910199
rs138293681
rs4924305
rs6498113
rs117045668
rs78800399
rs72679223
rs79811607
rs115781665
All
−0.3
−0.2
−0.1
0.0
0.1
MR leave−one−out sensitivity analysis for
' SSC−A on HLA DR+ CD4+' on 'Rheumatoid arthritis'

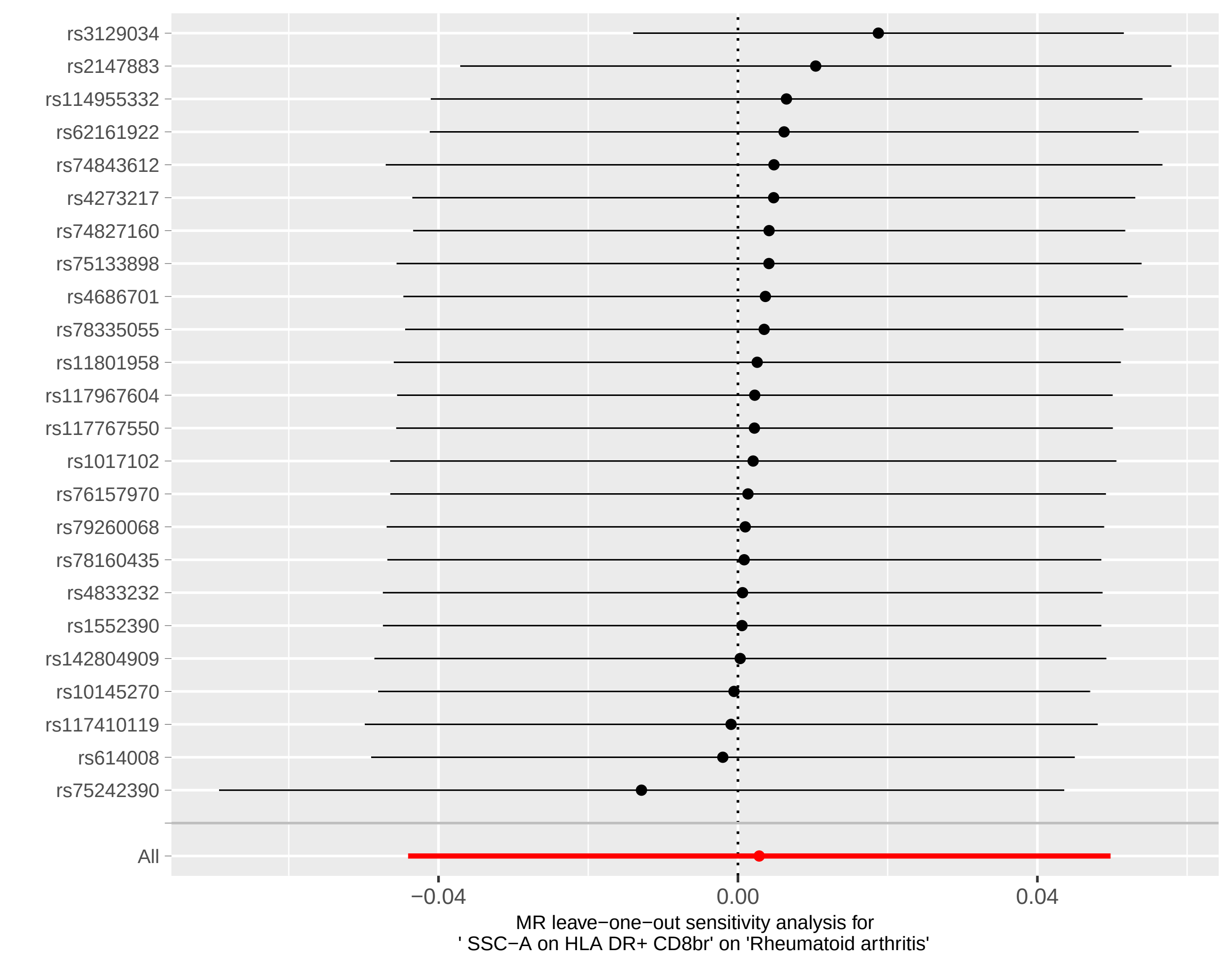

rs3129034
rs2147883
rs114955332
rs62161922
rs74843612
rs4273217
rs74827160
rs75133898
rs4686701
rs78335055
rs11801958
rs117967604
rs117767550
rs1017102
rs76157970
rs79260068
rs78160435
rs4833232
rs1552390
rs142804909
rs10145270
rs117410119
rs614008
rs75242390
All
−0.04
0.00
0.04
MR leave−one−out sensitivity analysis for
' SSC−A on HLA DR+ CD8br' on 'Rheumatoid arthritis'

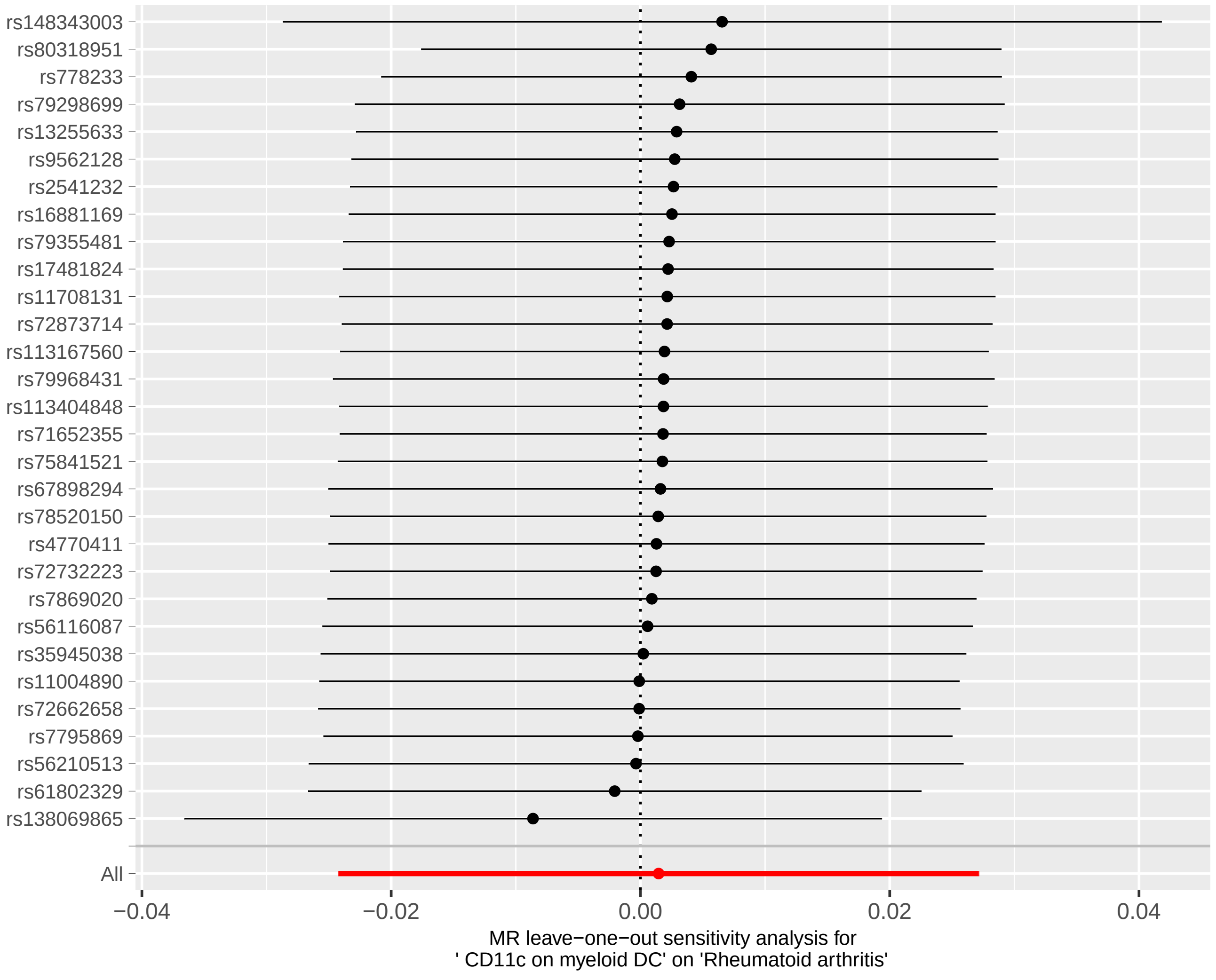
rs148343003
rs80318951
rs778233
rs79298699
rs13255633
rs9562128
rs2541232
rs16881169
rs79355481
rs17481824
rs11708131
rs72873714
rs113167560
rs79968431
rs113404848
rs71652355
rs75841521
rs67898294
rs78520150
rs4770411
rs72732223
rs7869020
rs56116087
rs35945038
rs11004890
rs72662658
rs7795869
rs56210513
rs61802329
rs138069865
All
−0.04
−0.02
0.00
0.02
0.04
MR leave−one−out sensitivity analysis for
' CD11c on myeloid DC' on 'Rheumatoid arthritis'

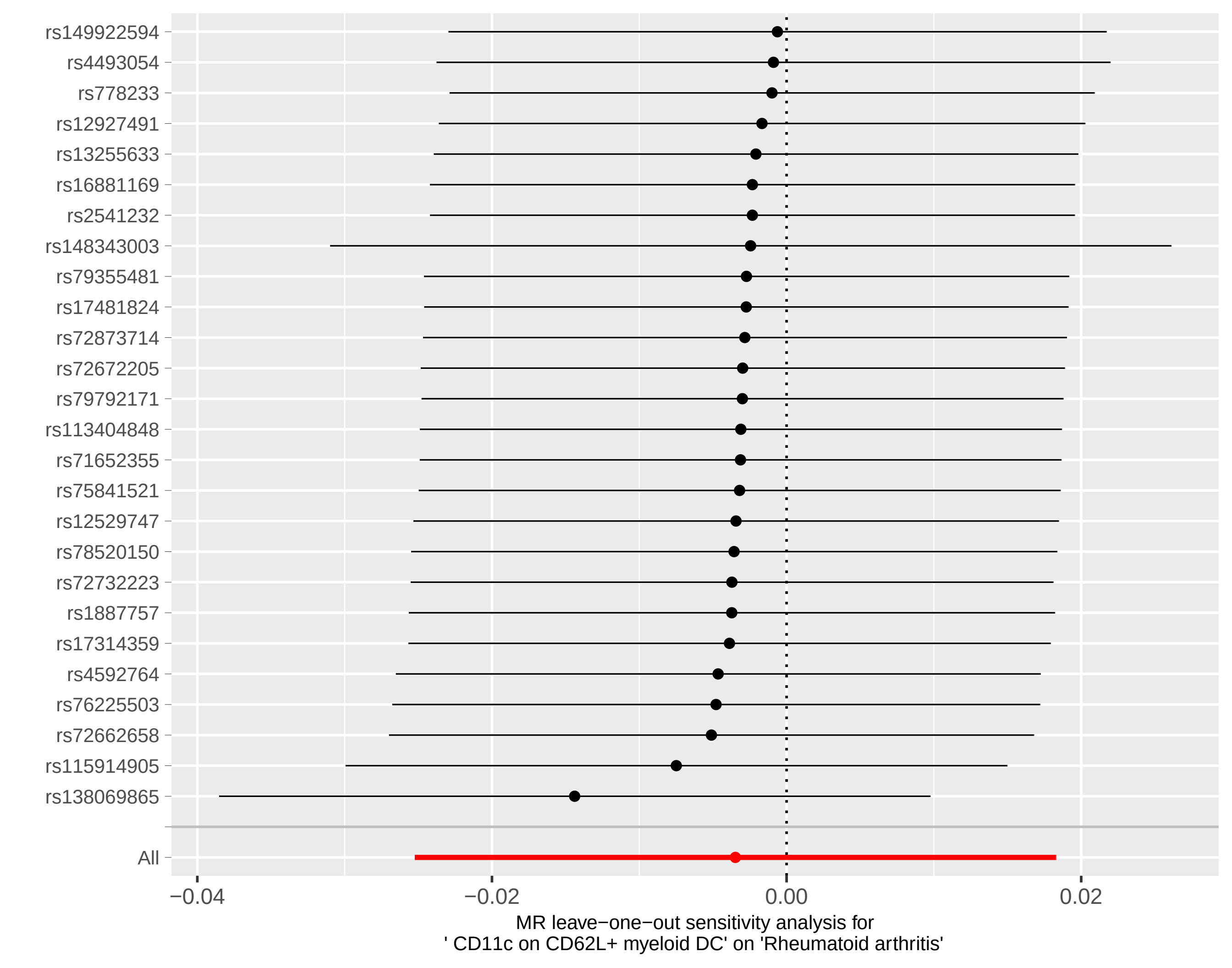
rs149922594
rs4493054
rs778233
rs12927491
rs13255633
rs16881169
rs2541232
rs148343003
rs79355481
rs17481824
rs72873714
rs72672205
rs79792171
rs113404848
rs71652355
rs75841521
rs12529747
rs78520150
rs72732223
rs1887757
rs17314359
rs4592764
rs76225503
rs72662658
rs115914905
rs138069865
All
−0.04
−0.02
0.00
0.02
MR leave−one−out sensitivity analysis for
' CD11c on CD62L+ myeloid DC' on 'Rheumatoid arthritis'

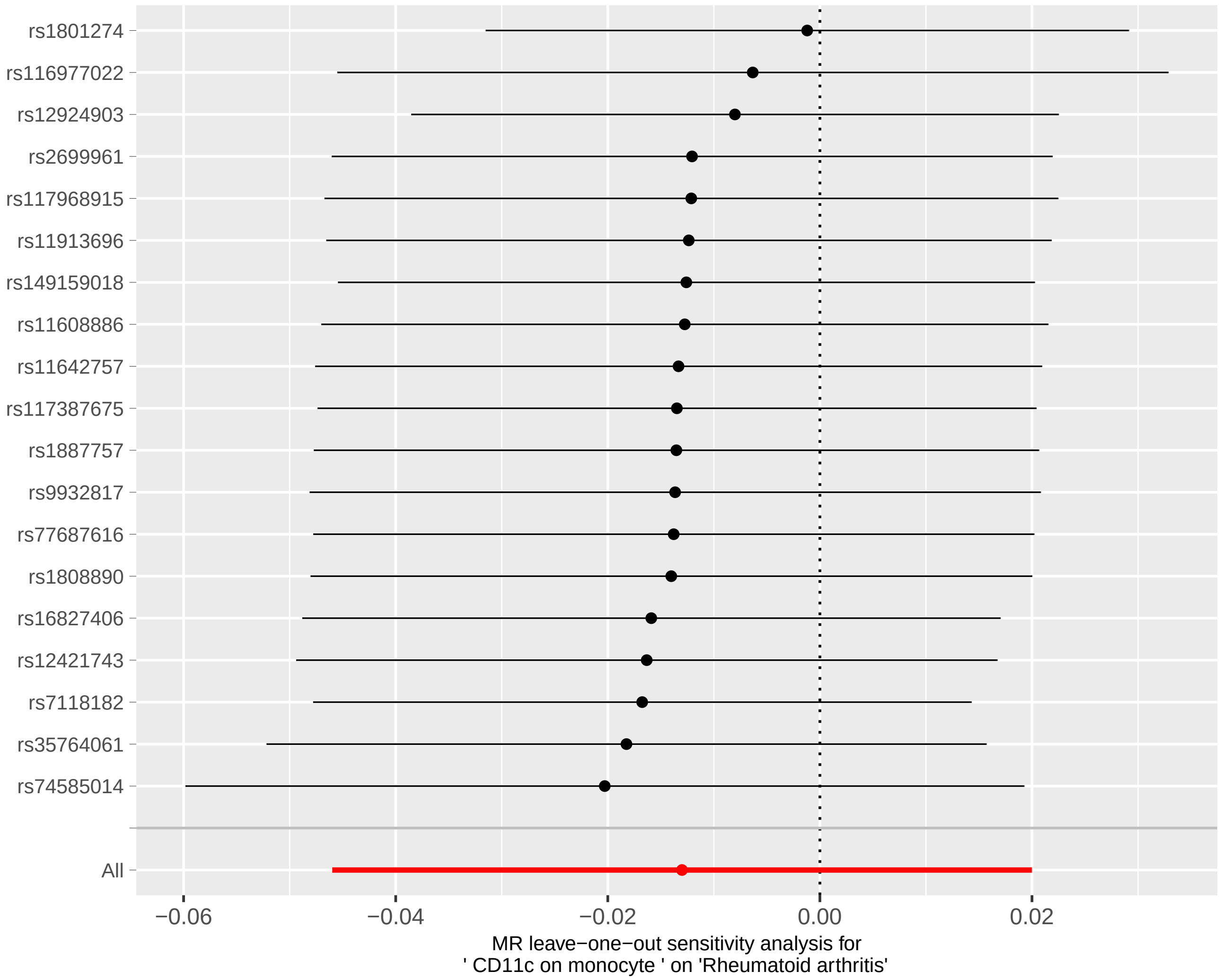
rs1801274
rs116977022
rs12924903
rs2699961
rs117968915
rs11913696
rs149159018
rs11608886
rs11642757
rs117387675
rs1887757
rs9932817
rs77687616
rs1808890
rs16827406
rs12421743
rs7118182
rs35764061
rs74585014
All
−0.06
−0.04
−0.02
0.00
0.02
MR leave−one−out sensitivity analysis for
' CD11c on monocyte ' on 'Rheumatoid arthritis'

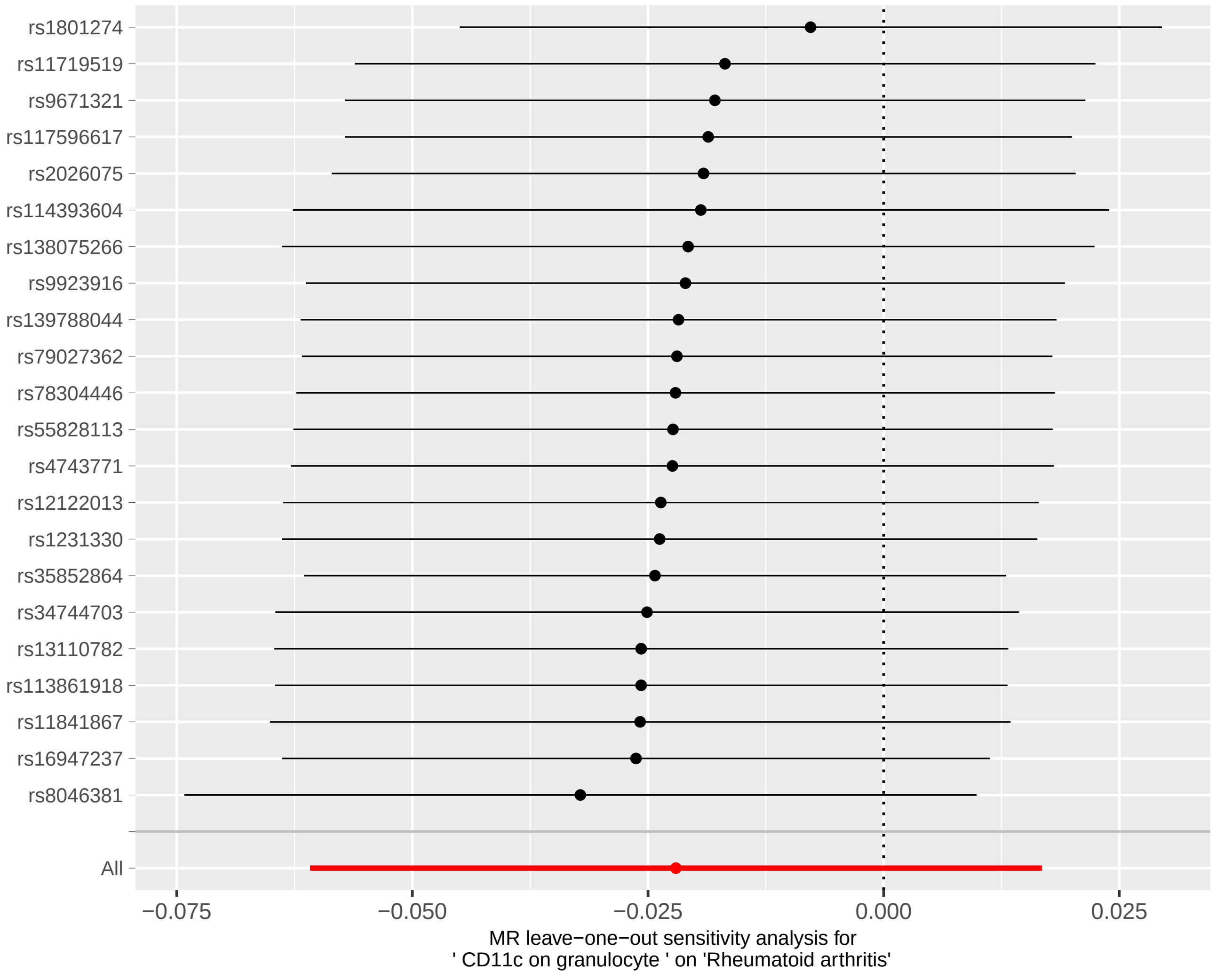
rs1801274
rs11719519
rs9671321
rs117596617
rs2026075
rs114393604
rs138075266
rs9923916
rs139788044
rs79027362
rs78304446
rs55828113
rs4743771
rs12122013
rs1231330
rs35852864
rs34744703
rs13110782
rs113861918
rs11841867
rs16947237
rs8046381
All
−0.075
−0.050
−0.025
0.000
0.025
MR leave−one−out sensitivity analysis for
' CD11c on granulocyte ' on 'Rheumatoid arthritis'

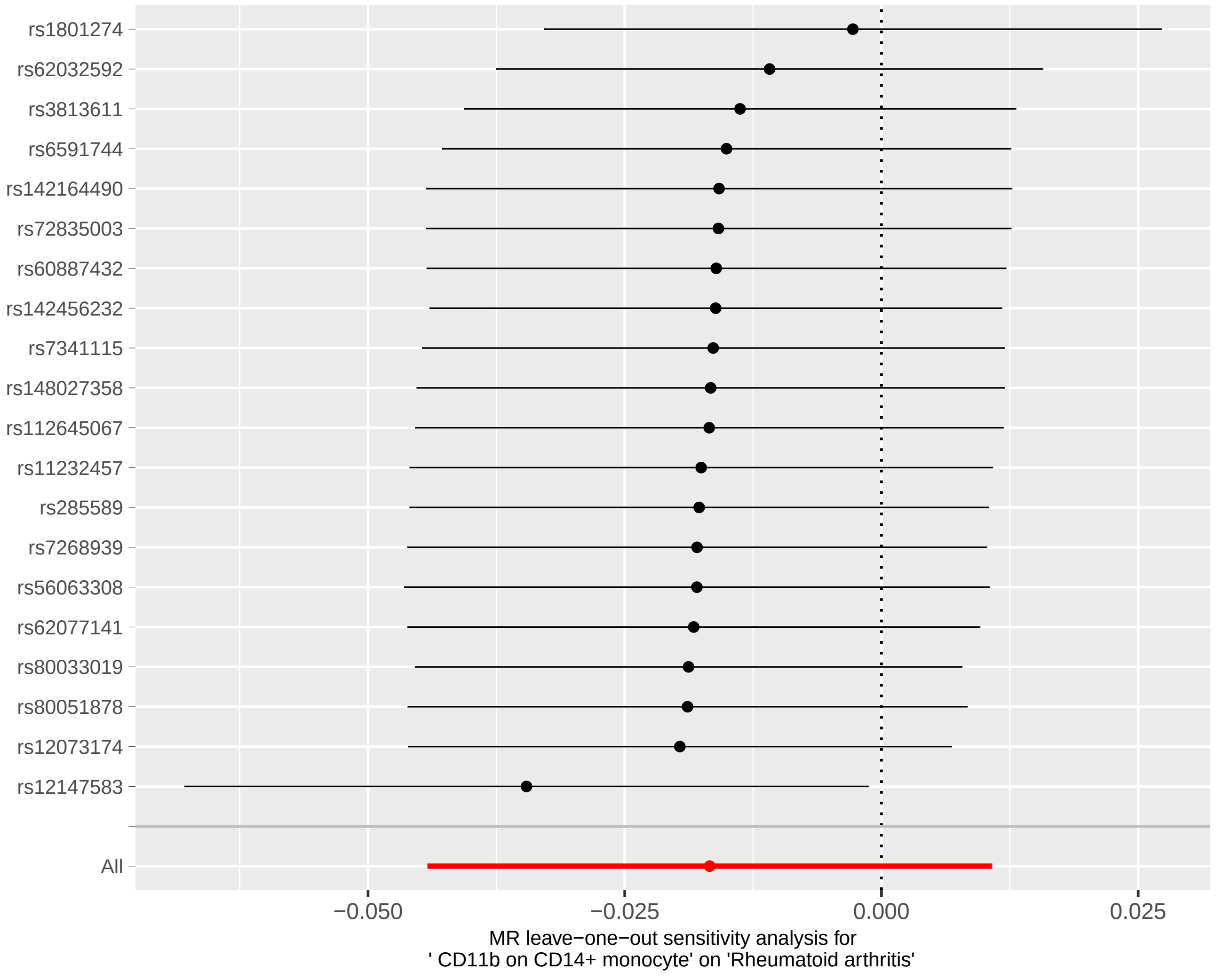
rs1801274
rs62032592
rs3813611
rs6591744
rs142164490
rs72835003
rs60887432
rs142456232
rs7341115
rs148027358
rs112645067
rs11232457
rs285589
rs7268939
rs56063308
rs62077141
rs80033019
rs80051878
rs12073174
rs12147583
All
−0.050
−0.025
0.000
0.025
MR leave−one−out sensitivity analysis for
' CD11b on CD14+ monocyte' on 'Rheumatoid arthritis'

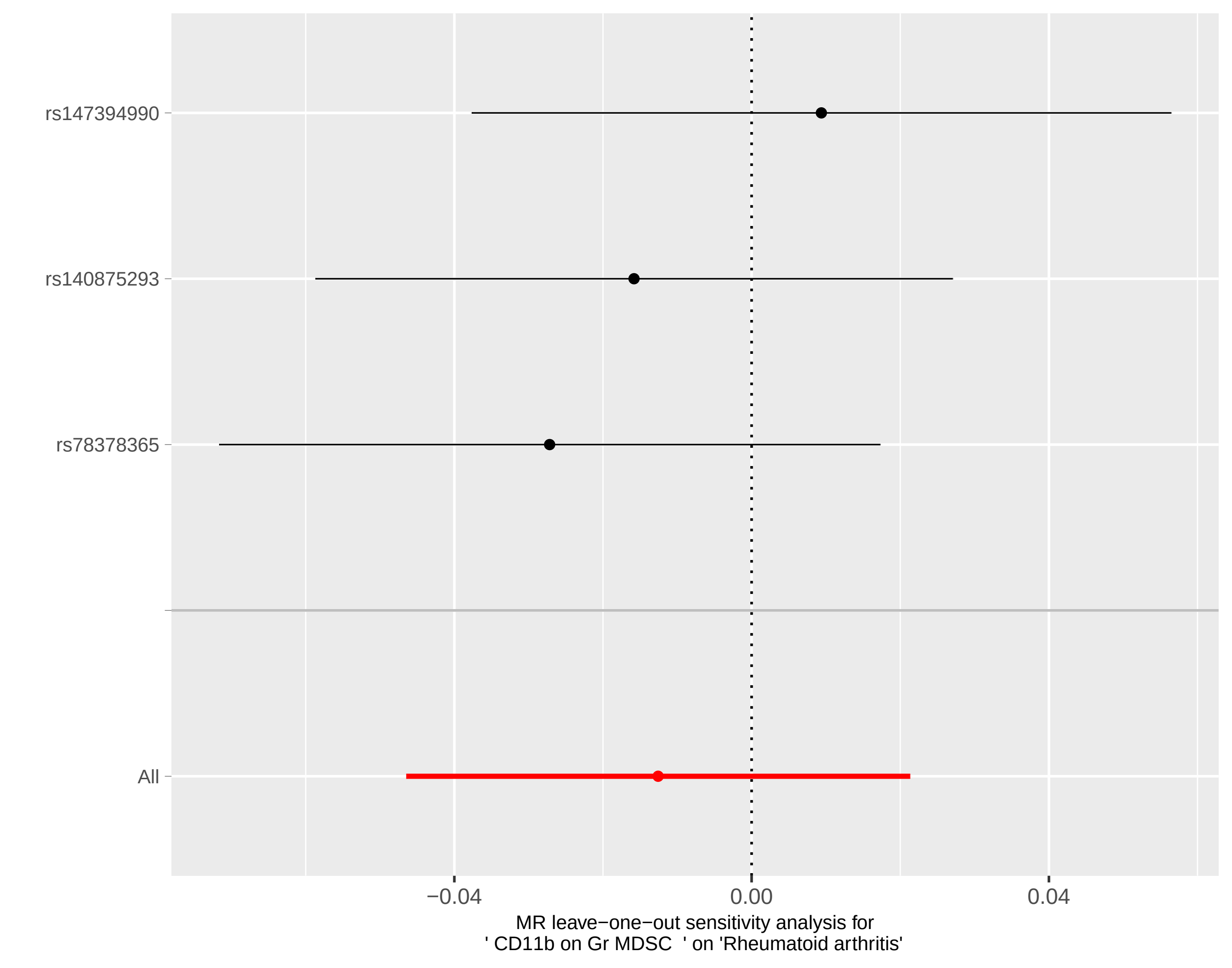

rs147394990
rs140875293
rs78378365
All
−0.04
0.00
0.04
MR leave−one−out sensitivity analysis for
' CD11b on Gr MDSC ' on 'Rheumatoid arthritis'

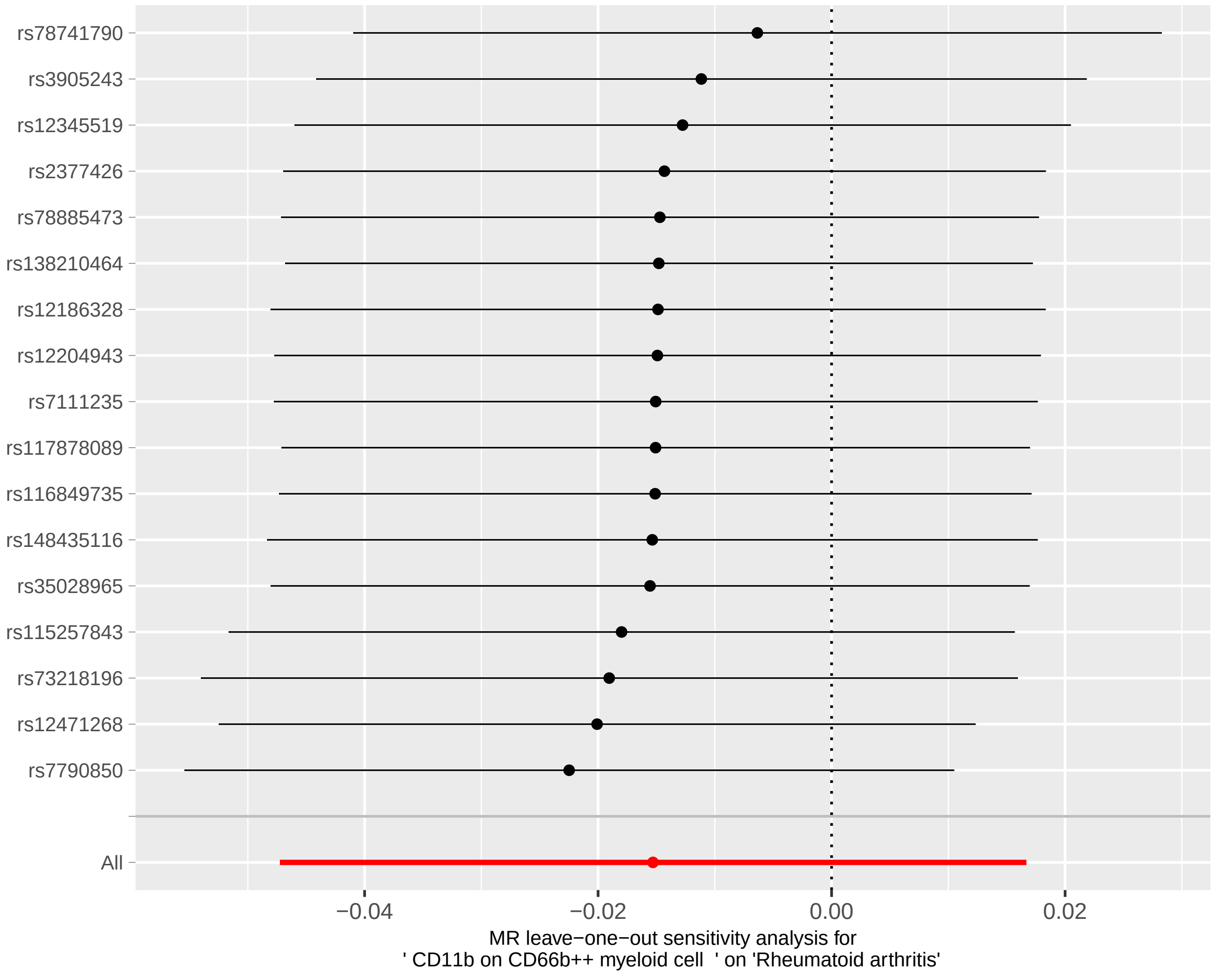
rs78741790
rs3905243
rs12345519
rs2377426
rs78885473
rs138210464
rs12186328
rs12204943
rs7111235
rs117878089
rs116849735
rs148435116
rs35028965
rs115257843
rs73218196
rs12471268
rs7790850
All
−0.04
−0.02
0.00
0.02
MR leave−one−out sensitivity analysis for
' CD11b on CD66b++ myeloid cell ' on 'Rheumatoid arthritis'

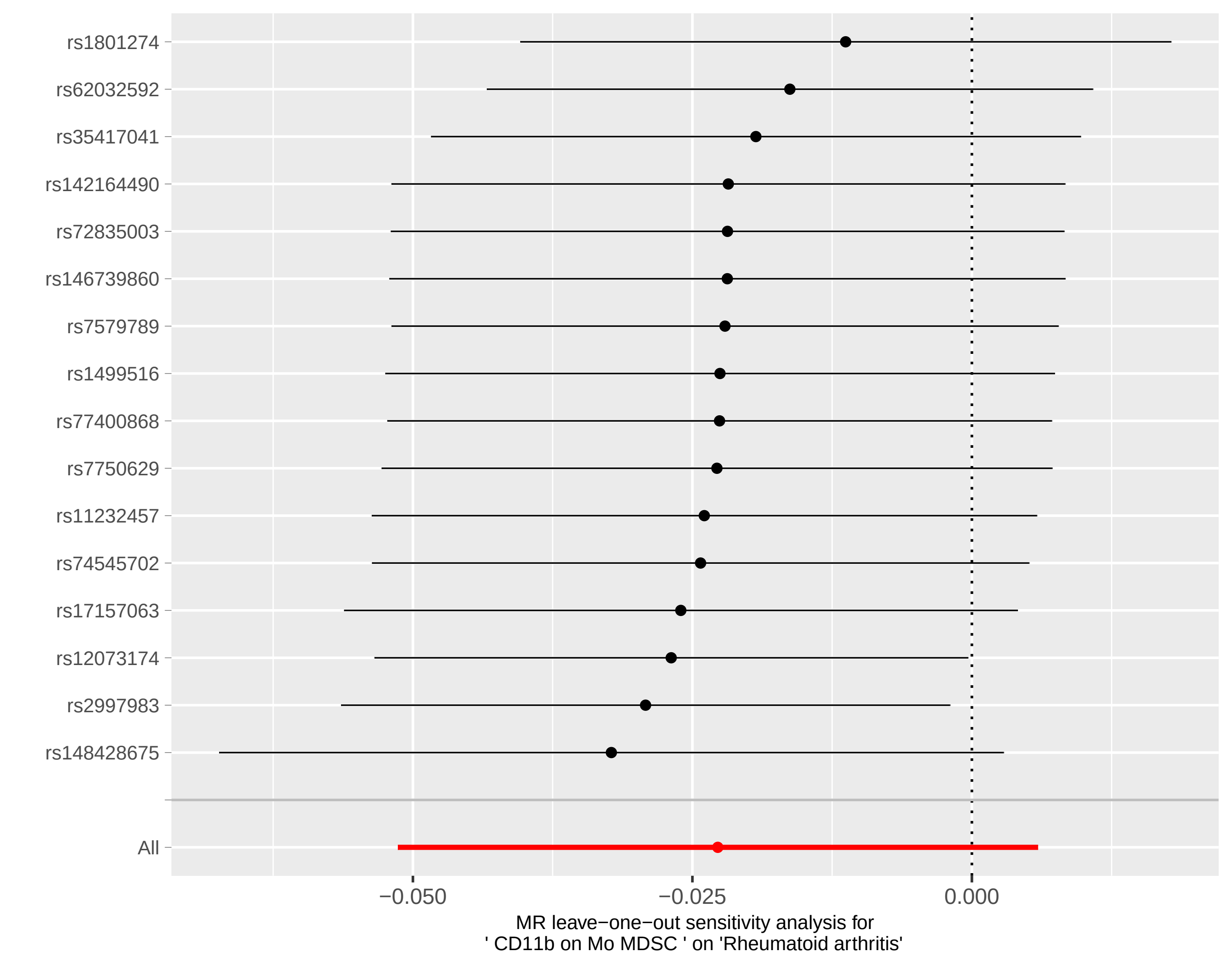
rs1801274
rs62032592
rs35417041
rs142164490
rs72835003
rs146739860
rs7579789
rs1499516
rs77400868
rs7750629
rs11232457
rs74545702
rs17157063
rs12073174
rs2997983
rs148428675
All
−0.050
−0.025
0.000
MR leave−one−out sensitivity analysis for
' CD11b on Mo MDSC ' on 'Rheumatoid arthritis'

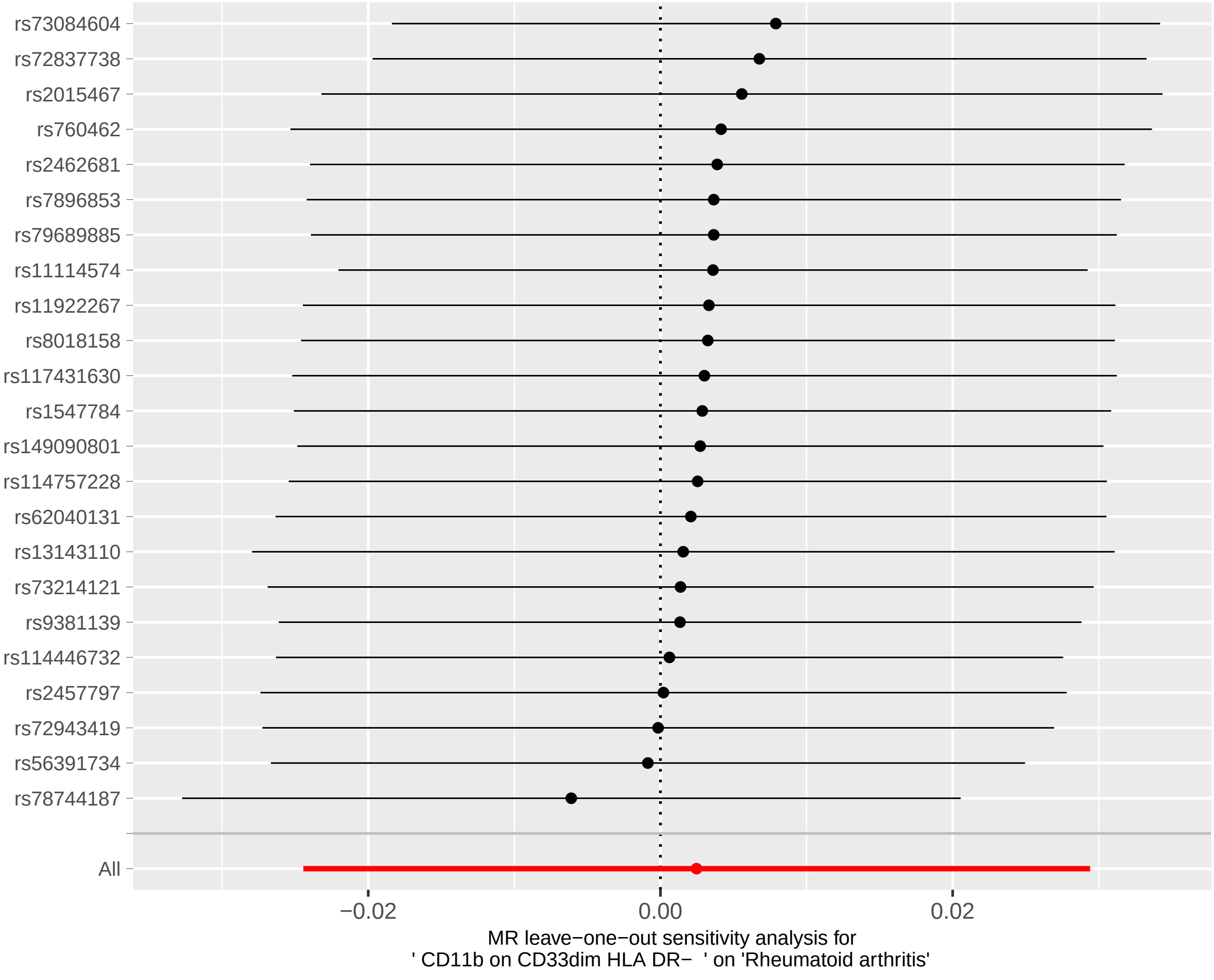
rs73084604
rs72837738
rs2015467
rs760462
rs2462681
rs7896853
rs79689885
rs11114574
rs11922267
rs8018158
rs117431630
rs1547784
rs149090801
rs114757228
rs62040131
rs13143110
rs73214121
rs9381139
rs114446732
rs2457797
rs72943419
rs56391734
rs78744187
All
−0.02
0.00
0.02
MR leave−one−out sensitivity analysis for
' CD11b on CD33dim HLA DR− ' on 'Rheumatoid arthritis'

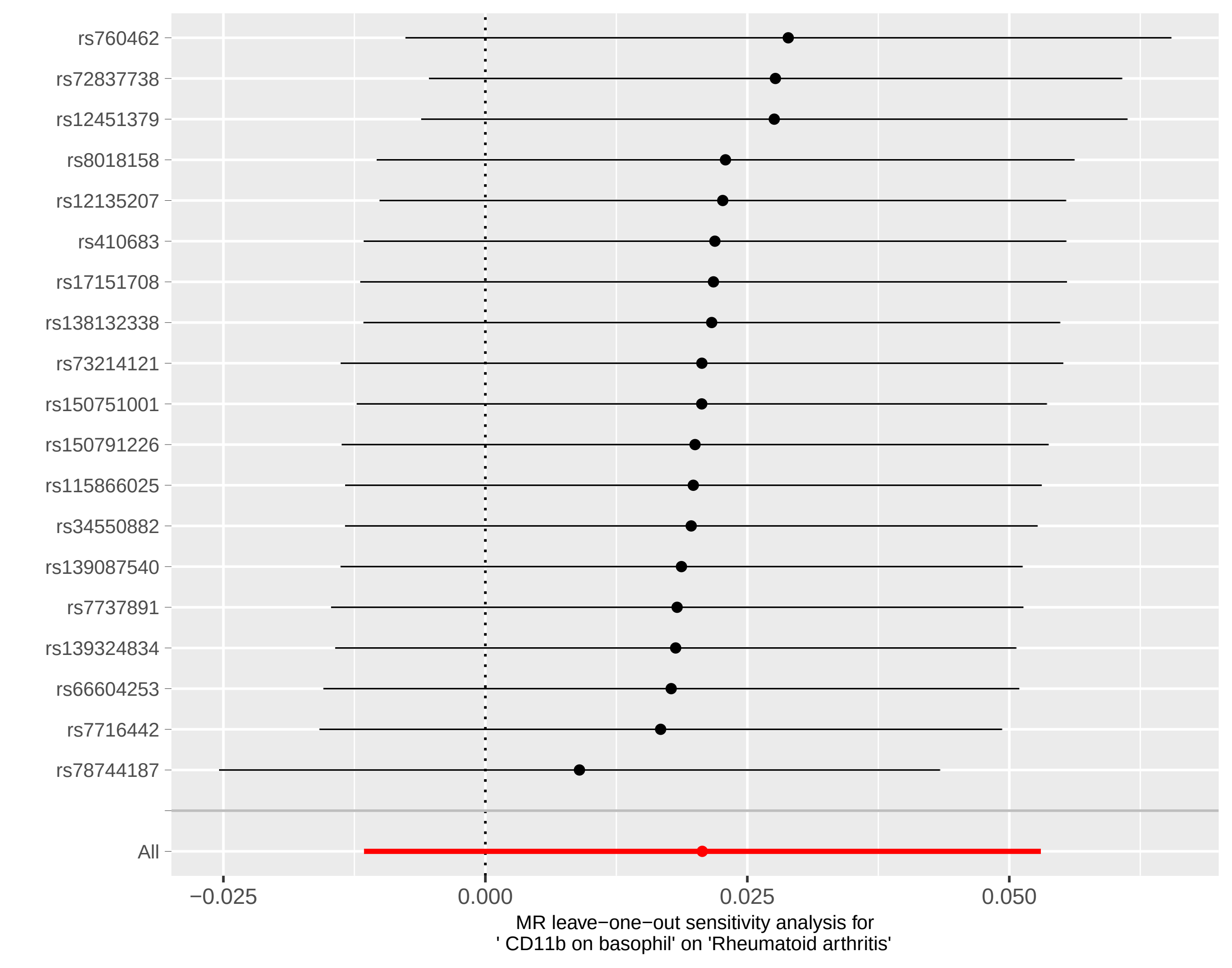
rs760462
rs72837738
rs12451379
rs8018158
rs12135207
rs410683
rs17151708
rs138132338
rs73214121
rs150751001
rs150791226
rs115866025
rs34550882
rs139087540
rs7737891
rs139324834
rs66604253
rs7716442
rs78744187
All
−0.025
0.000
0.025
0.050
MR leave−one−out sensitivity analysis for
' CD11b on basophil' on 'Rheumatoid arthritis'

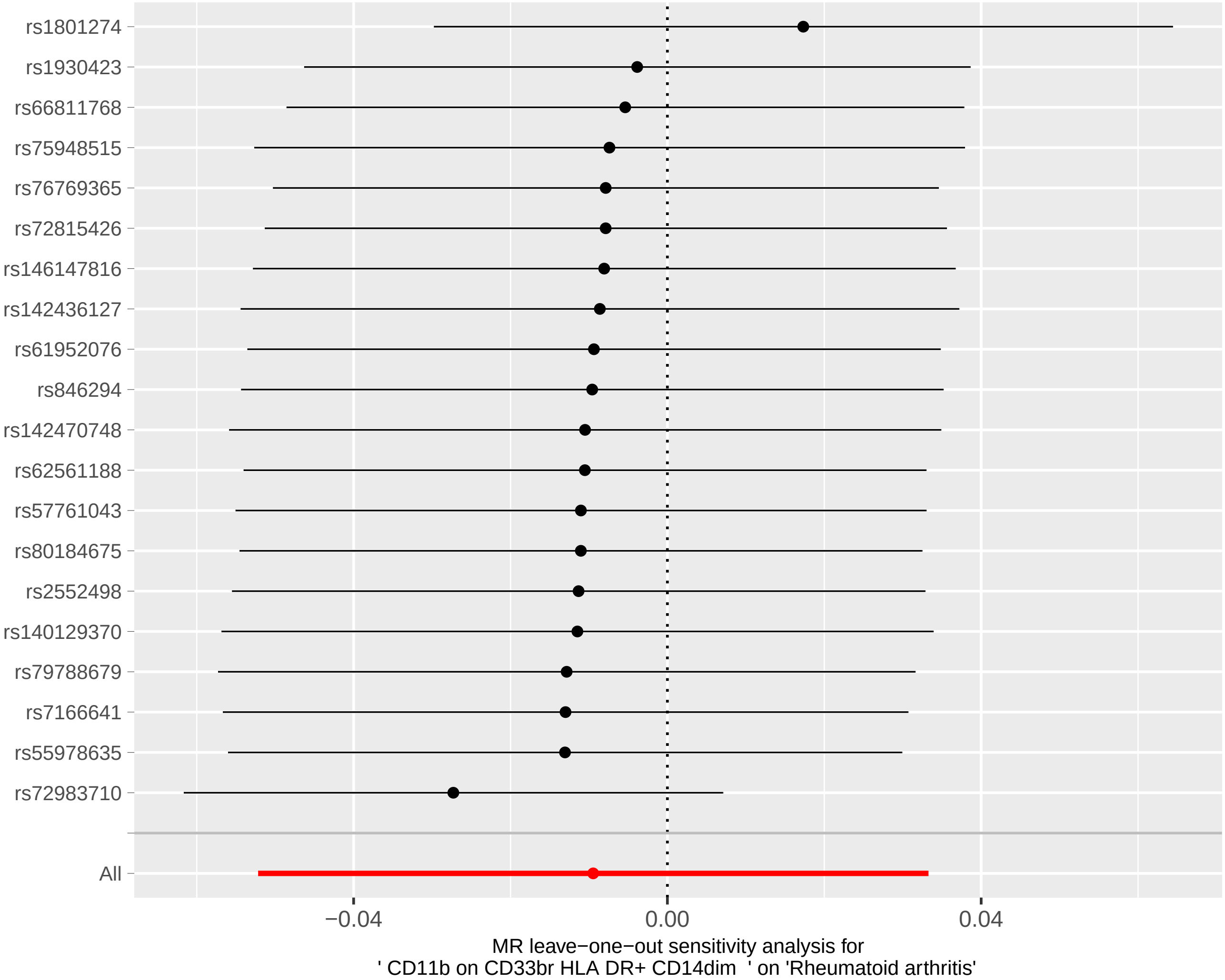
rs1801274
rs1930423
rs66811768
rs75948515
rs76769365
rs72815426
rs146147816
rs142436127
rs61952076
rs846294
rs142470748
rs62561188
rs57761043
rs80184675
rs2552498
rs140129370
rs79788679
rs7166641
rs55978635
rs72983710
All
−0.04
0.00
0.04
MR leave−one−out sensitivity analysis for
' CD11b on CD33br HLA DR+ CD14dim ' on 'Rheumatoid arthritis'

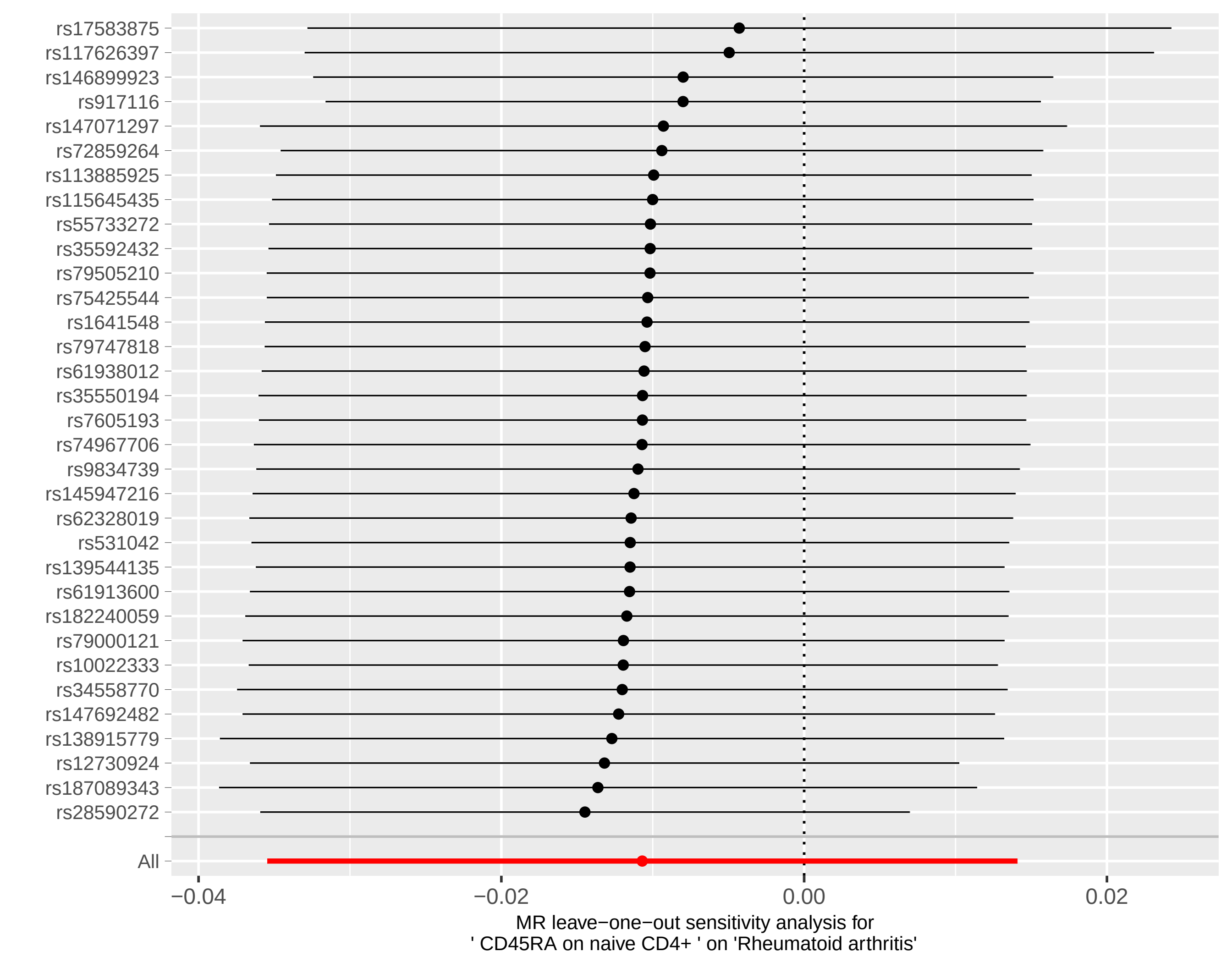
rs17583875
rs117626397
rs146899923
rs917116
rs147071297
rs72859264
rs113885925
rs115645435
rs55733272
rs35592432
rs79505210
rs75425544
rs1641548
rs79747818
rs61938012
rs35550194
rs7605193
rs74967706
rs9834739
rs145947216
rs62328019
rs531042
rs139544135
rs61913600
rs182240059
rs79000121
rs10022333
rs34558770
rs147692482
rs138915779
rs12730924
rs187089343
rs28590272
All
−0.04
−0.02
0.00
0.02
MR leave−one−out sensitivity analysis for
' CD45RA on naive CD4+ ' on 'Rheumatoid arthritis'

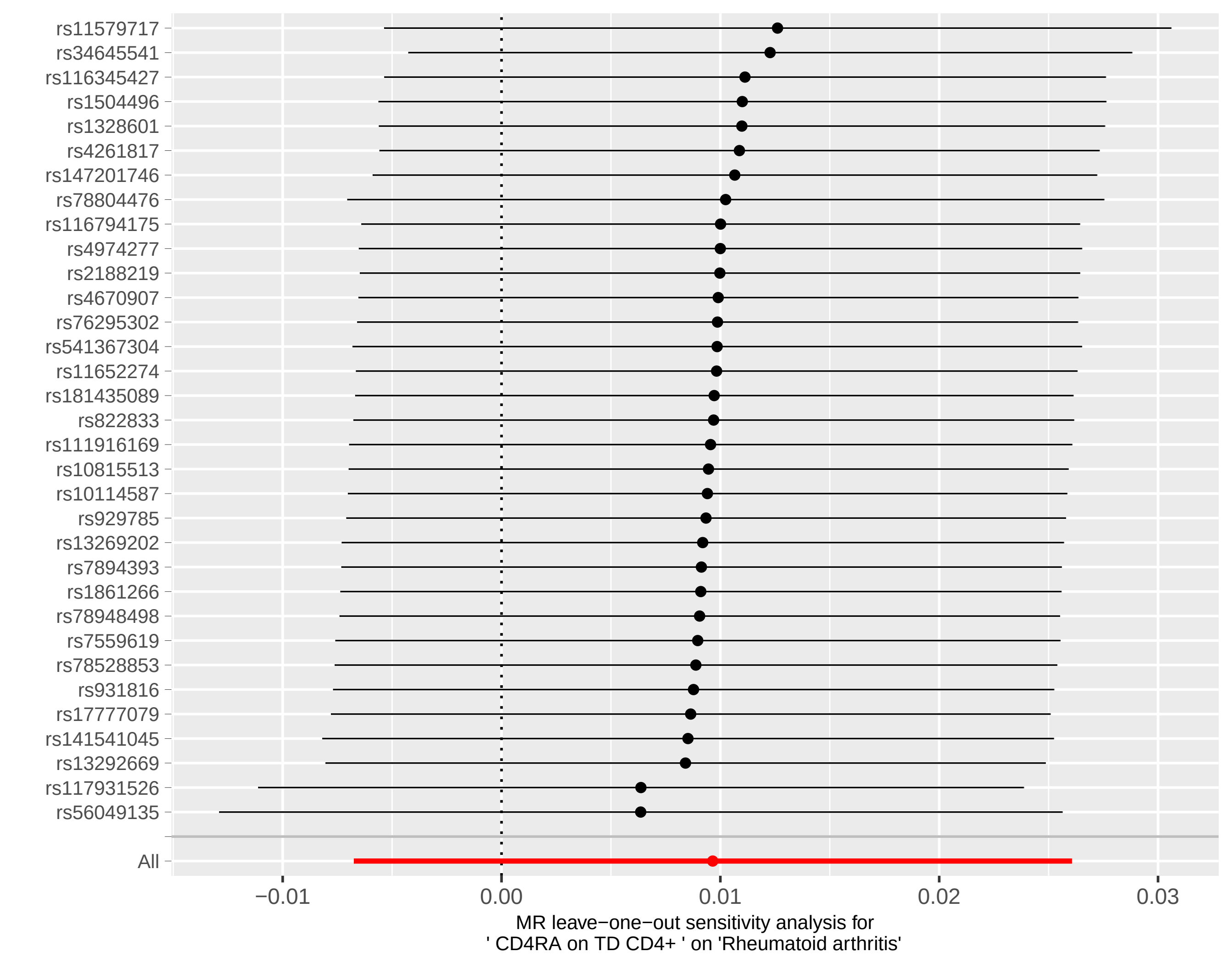

rs11579717
rs34645541
rs116345427
rs1504496
rs1328601
rs4261817
rs147201746
rs78804476
rs116794175
rs4974277
rs2188219
rs4670907
rs76295302
rs541367304
rs11652274
rs181435089
rs822833
rs111916169
rs10815513
rs10114587
rs929785
rs13269202
rs7894393
rs1861266
rs78948498
rs7559619
rs78528853
rs931816
rs17777079
rs141541045
rs13292669
rs117931526
rs56049135
All
−0.01
0.00
0.01
0.02
0.03
MR leave−one−out sensitivity analysis for
' CD4RA on TD CD4+ ' on 'Rheumatoid arthritis'

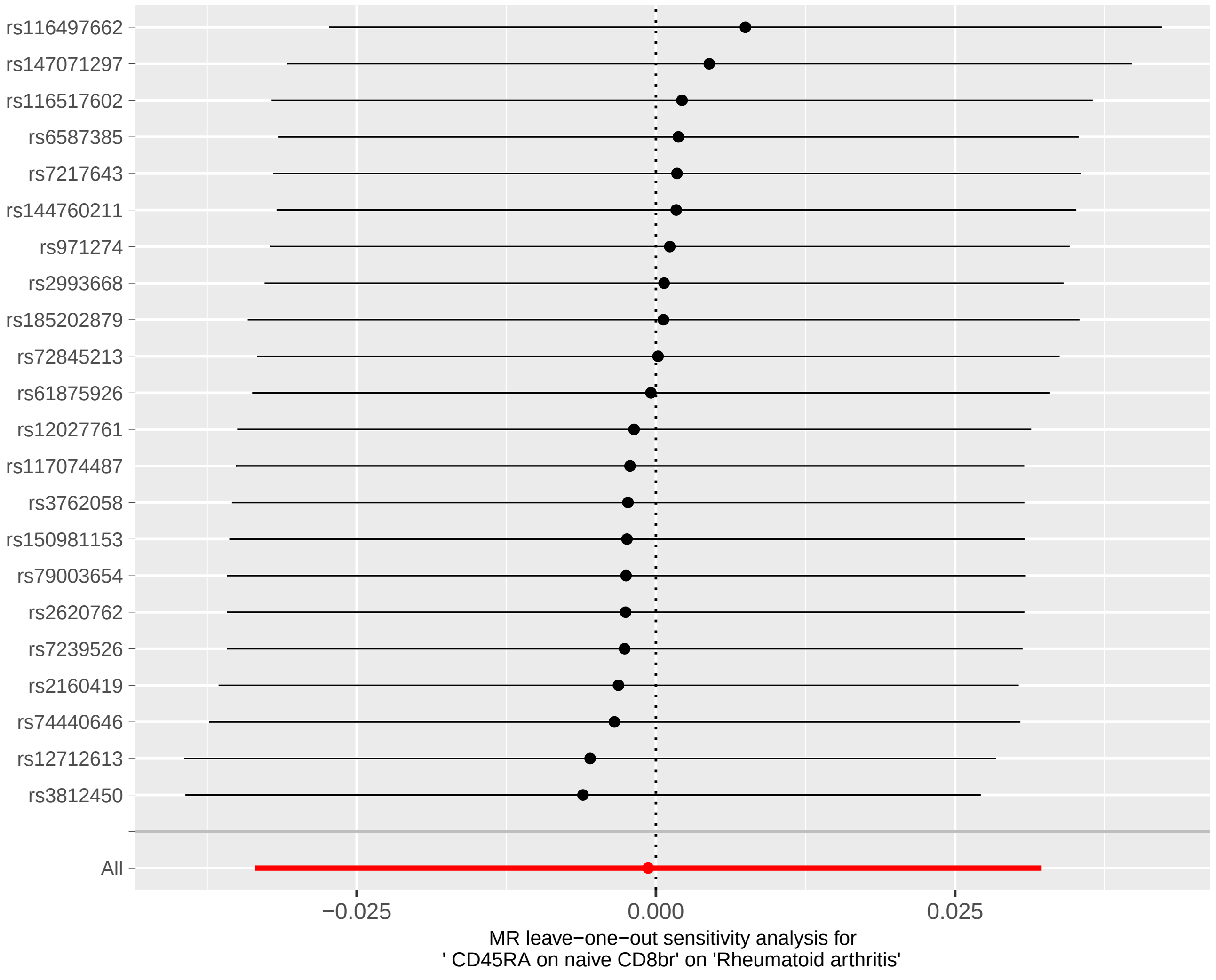

rs116497662
rs147071297
rs116517602
rs6587385
rs7217643
rs144760211
rs971274
rs2993668
rs185202879
rs72845213
rs61875926
rs12027761
rs117074487
rs3762058
rs150981153
rs79003654
rs2620762
rs7239526
rs2160419
rs74440646
rs12712613
rs3812450
All
−0.025
0.000
0.025
MR leave−one−out sensitivity analysis for
' CD45RA on naive CD8br' on 'Rheumatoid arthritis'

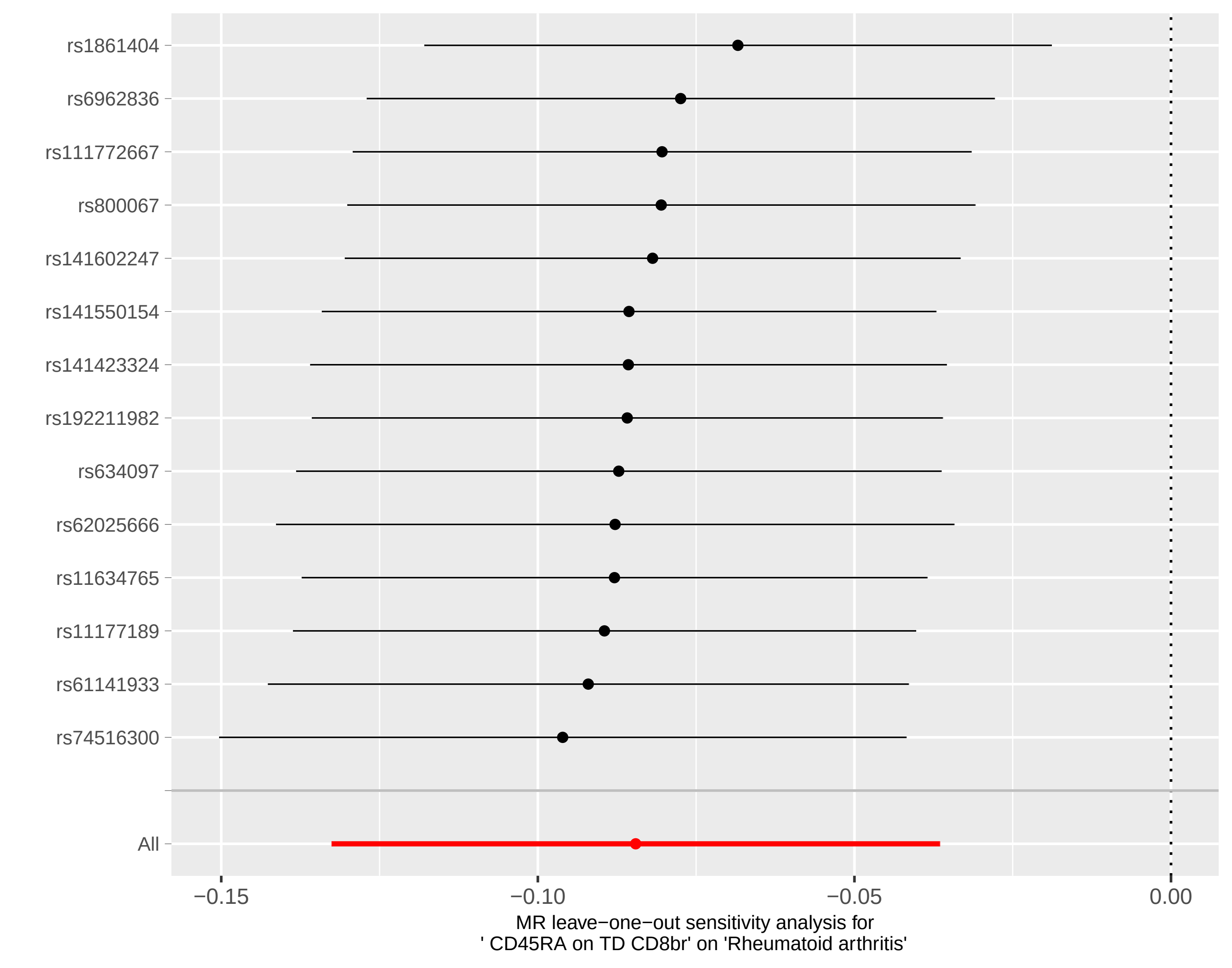
rs1861404
rs6962836
rs111772667
rs800067
rs141602247
rs141550154
rs141423324
rs192211982
rs634097
rs62025666
rs11634765
rs11177189
rs61141933
rs74516300
All
−0.15
−0.10
−0.05
0.00
MR leave−one−out sensitivity analysis for
' CD45RA on TD CD8br' on 'Rheumatoid arthritis'

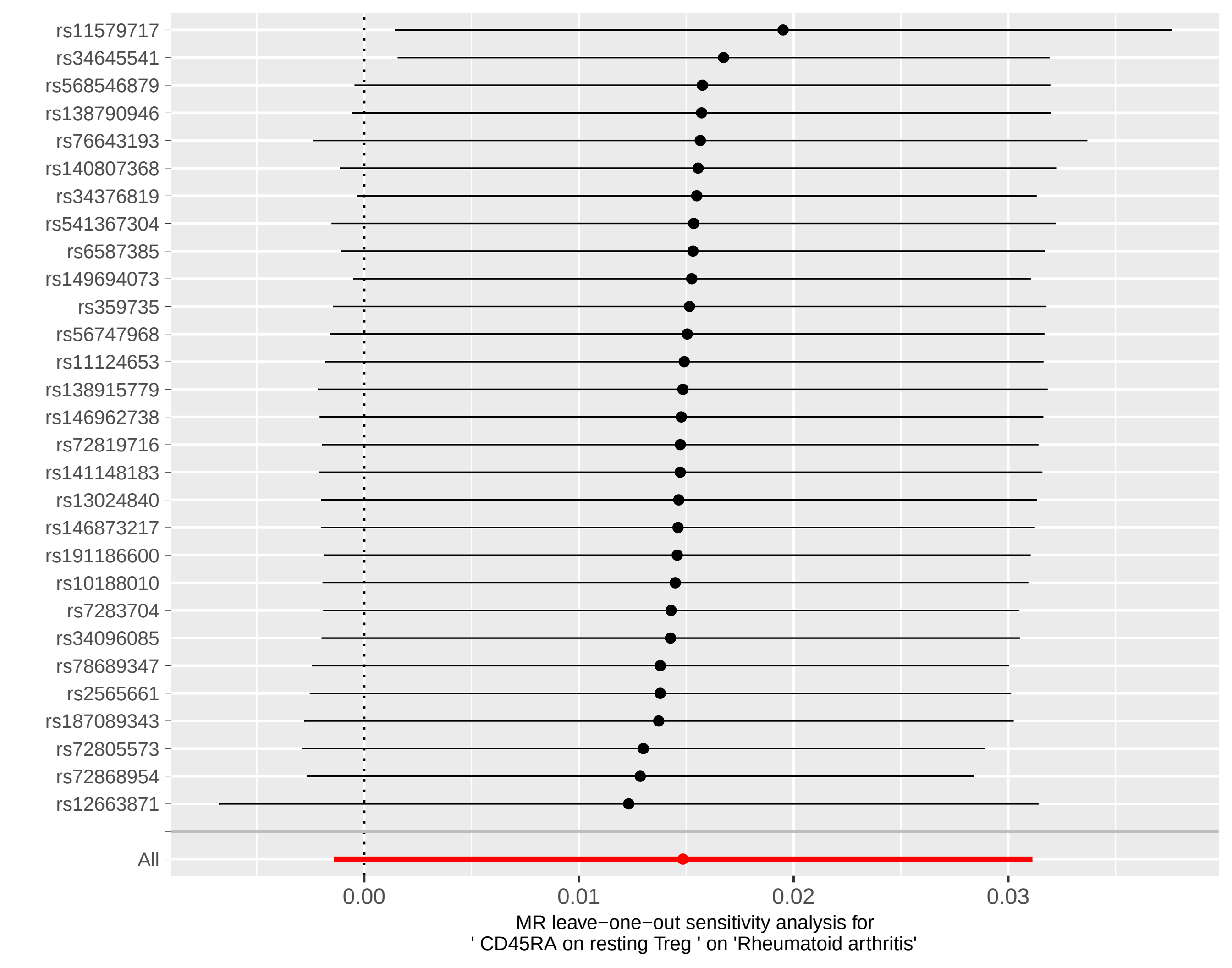

rs11579717
rs34645541
rs568546879
rs138790946
rs76643193
rs140807368
rs34376819
rs541367304
rs6587385
rs149694073
rs359735
rs56747968
rs11124653
rs138915779
rs146962738
rs72819716
rs141148183
rs13024840
rs146873217
rs191186600
rs10188010
rs7283704
rs34096085
rs78689347
rs2565661
rs187089343
rs72805573
rs72868954
rs12663871
All
0.00
0.01
0.02
0.03
MR leave−one−out sensitivity analysis for
' CD45RA on resting Treg ' on 'Rheumatoid arthritis'

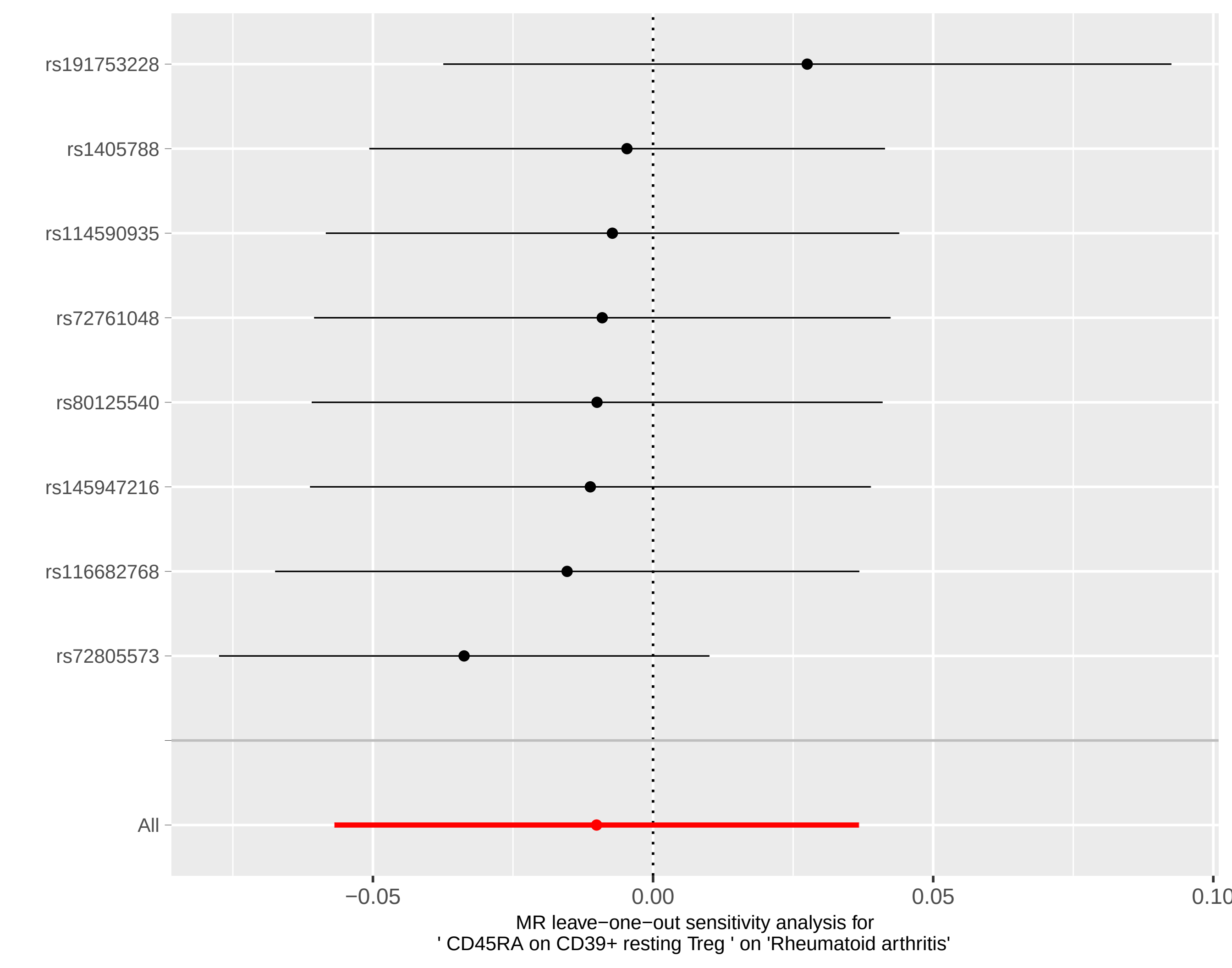

rs191753228
rs1405788
rs114590935
rs72761048
rs80125540
rs145947216
rs116682768
rs72805573
All
−0.05
0.00
0.05
0.10
MR leave−one−out sensitivity analysis for
' CD45RA on CD39+ resting Treg ' on 'Rheumatoid arthritis'

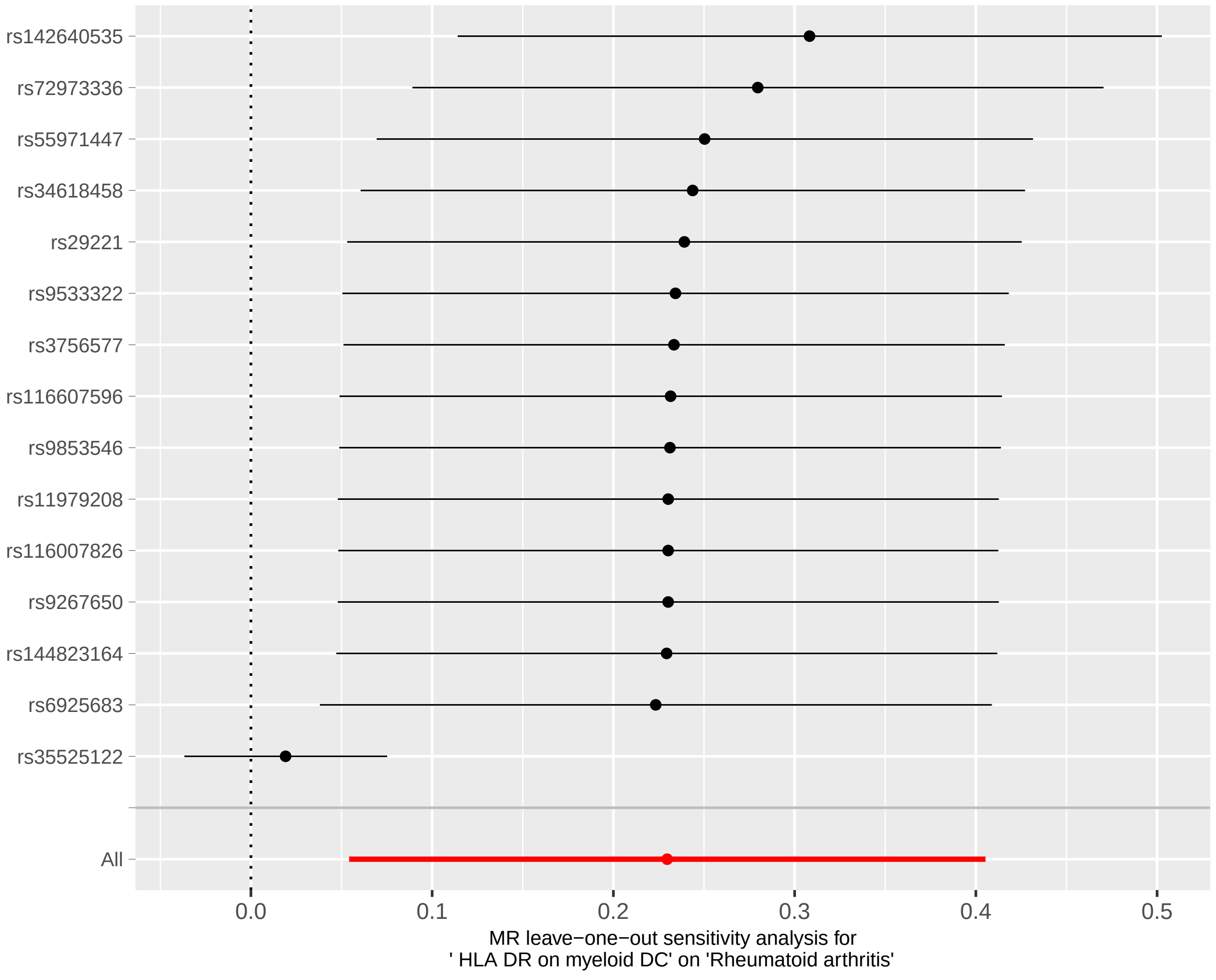

rs142640535
rs72973336
rs55971447
rs34618458
rs29221
rs9533322
rs3756577
rs116607596
rs9853546
rs11979208
rs116007826
rs9267650
rs144823164
rs6925683
rs35525122
All
0.0
0.1
0.2
0.3
0.4
0.5
MR leave−one−out sensitivity analysis for
' HLA DR on myeloid DC' on 'Rheumatoid arthritis'

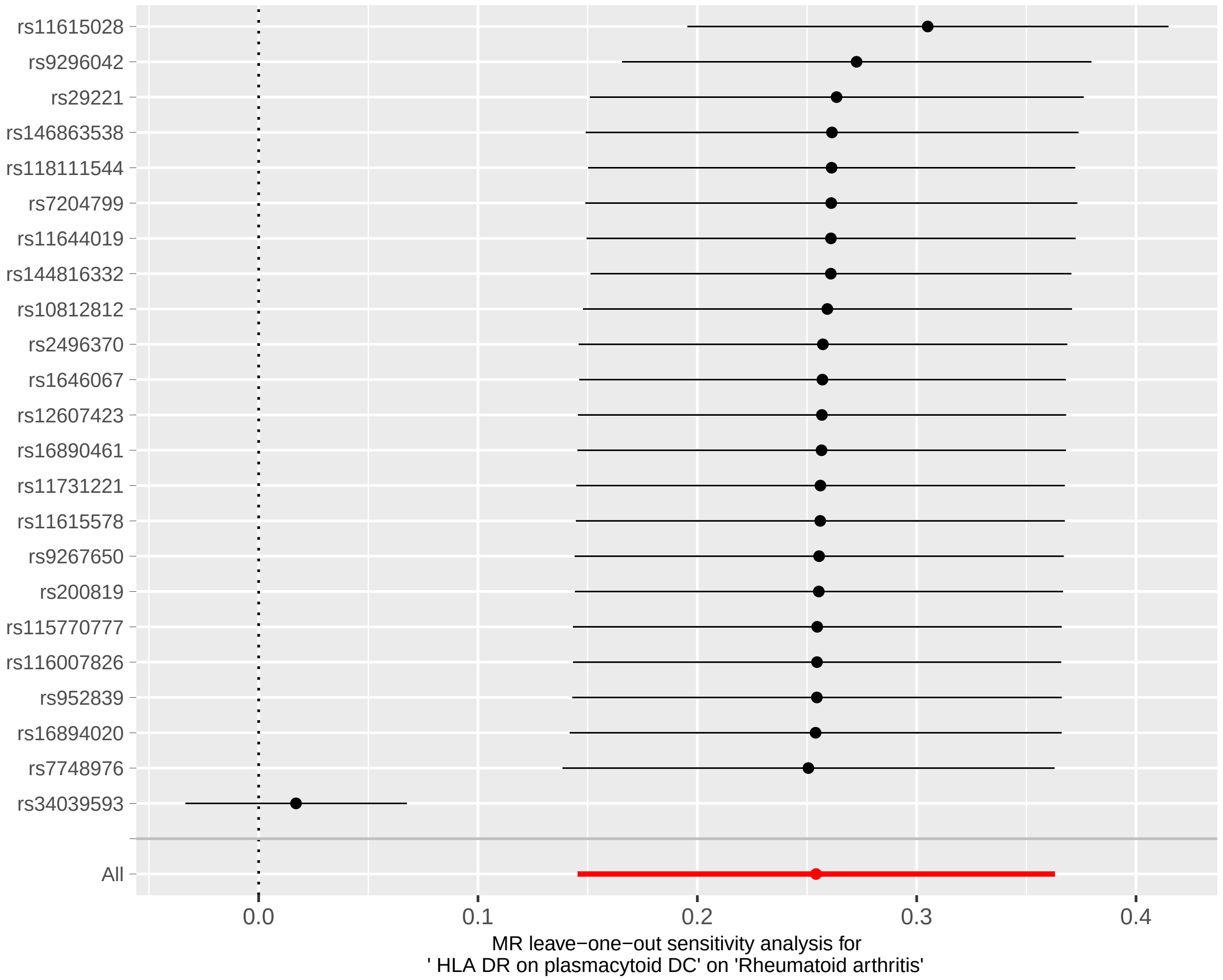
rs11615028
rs9296042
rs29221
rs146863538
rs118111544
rs7204799
rs11644019
rs144816332
rs10812812
rs2496370
rs1646067
rs12607423
rs16890461
rs11731221
rs11615578
rs9267650
rs200819
rs115770777
rs116007826
rs952839
rs16894020
rs7748976
rs34039593
All
0.0
0.1
0.2
0.3
0.4
MR leave−one−out sensitivity analysis for
' HLA DR on plasmacytoid DC' on 'Rheumatoid arthritis'

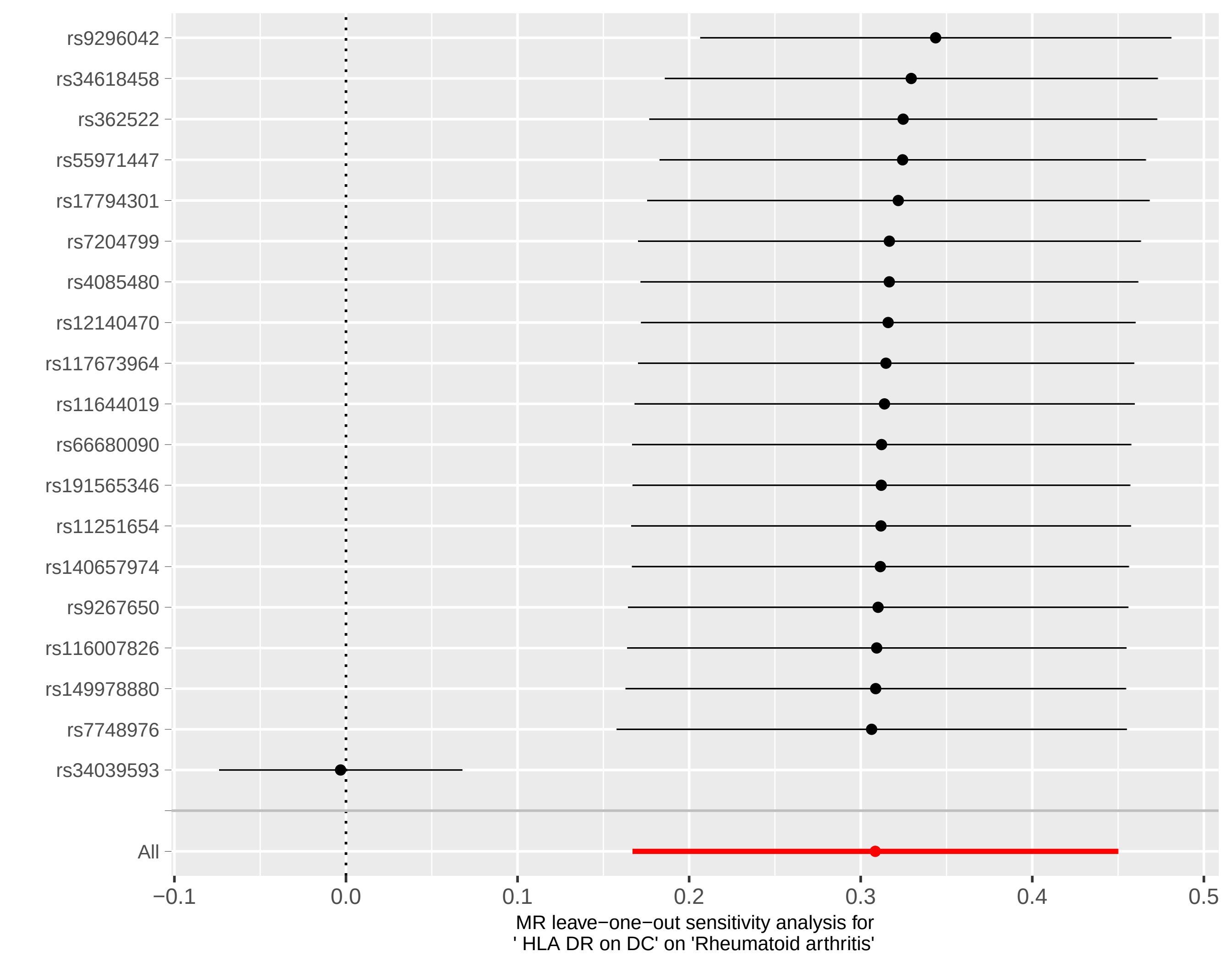

rs9296042
rs34618458
rs362522
rs55971447
rs17794301
rs7204799
rs4085480
rs12140470
rs117673964
rs11644019
rs66680090
rs191565346
rs11251654
rs140657974
rs9267650
rs116007826
rs149978880
rs7748976
rs34039593
All
−0.1
0.0
0.1
0.2
0.3
0.4
0.5
MR leave−one−out sensitivity analysis for
' HLA DR on DC' on 'Rheumatoid arthritis'

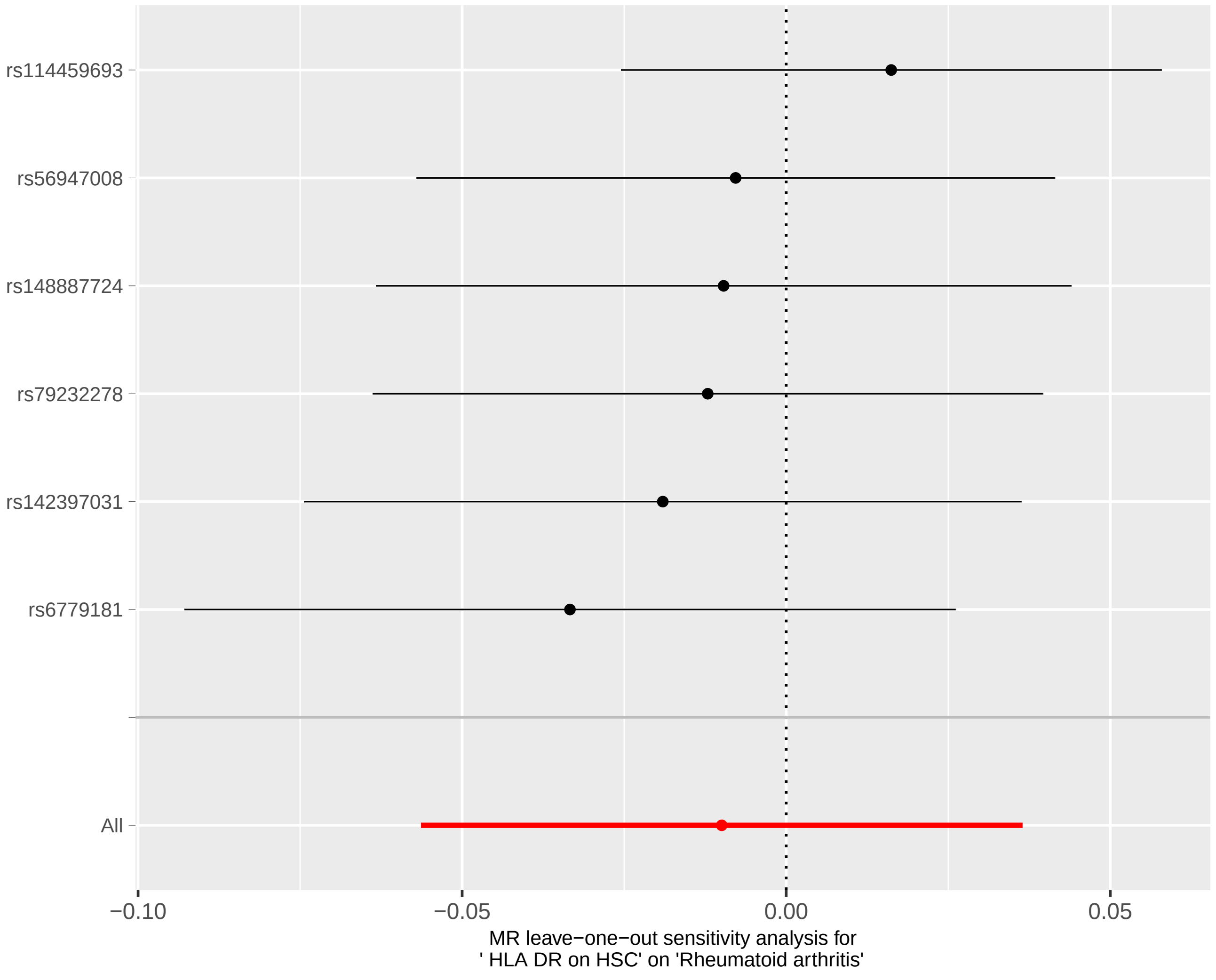
rs114459693
rs56947008
rs148887724
rs79232278
rs142397031
rs6779181
All
−0.10
−0.05
0.00
0.05
MR leave−one−out sensitivity analysis for
' HLA DR on HSC' on 'Rheumatoid arthritis'

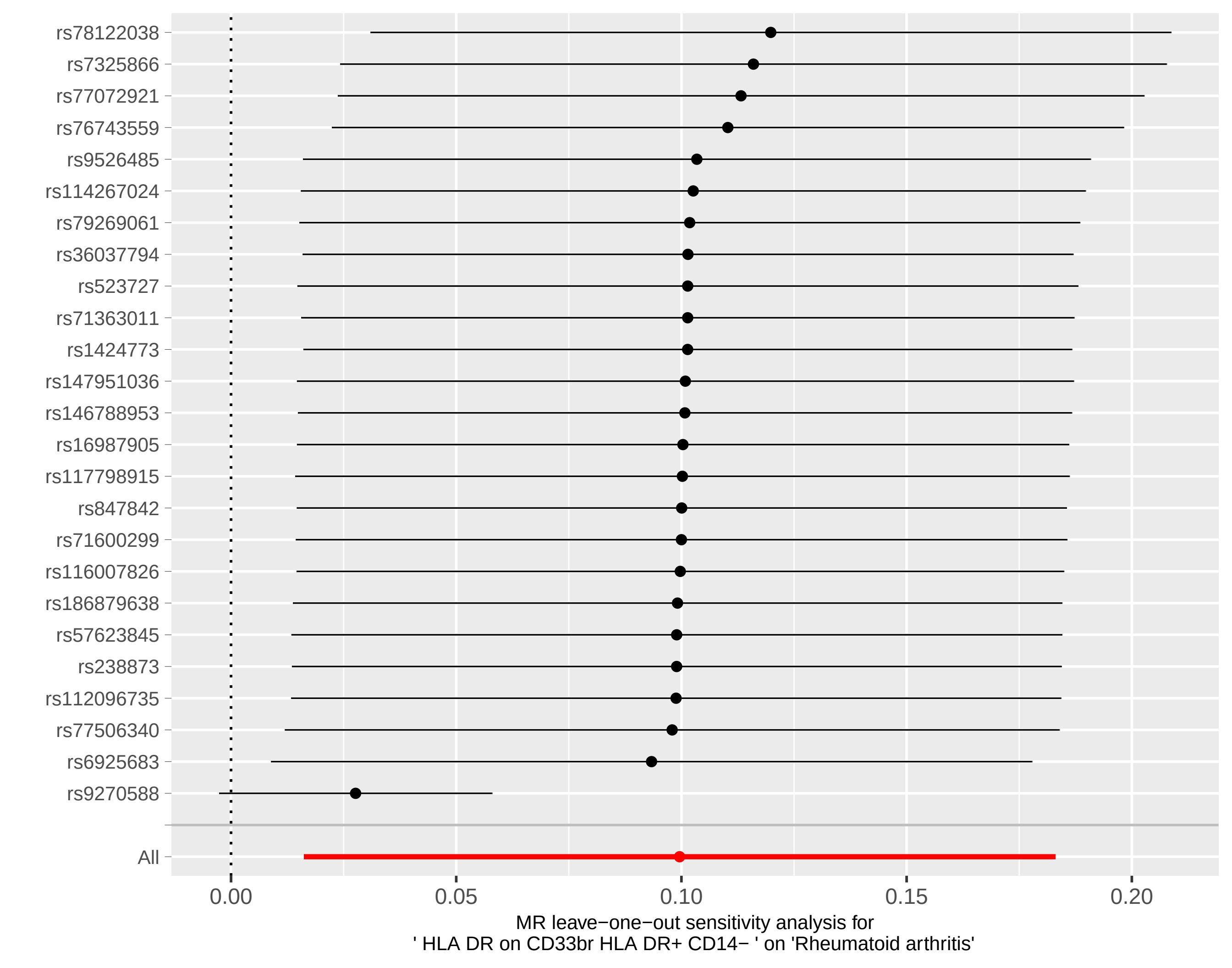
rs78122038
rs7325866
rs77072921
rs76743559
rs9526485
rs114267024
rs79269061
rs36037794
rs523727
rs71363011
rs1424773
rs147951036
rs146788953
rs16987905
rs117798915
rs847842
rs71600299
rs116007826
rs186879638
rs57623845
rs238873
rs112096735
rs77506340
rs6925683
rs9270588
All
0.00
0.05
0.10
0.15
0.20
MR leave−one−out sensitivity analysis for
' HLA DR on CD33br HLA DR+ CD14− ' on 'Rheumatoid arthritis'

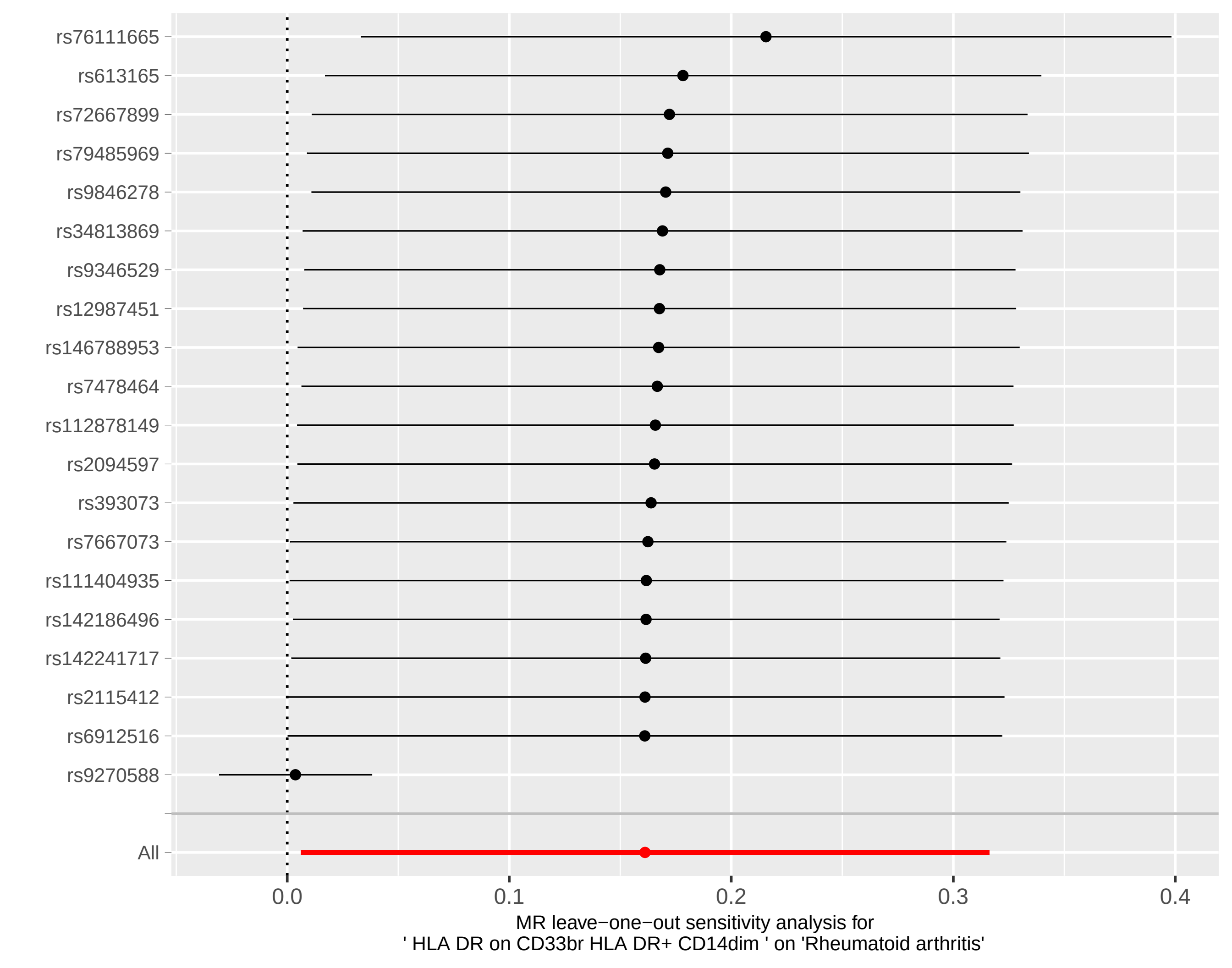

rs76111665
rs613165
rs72667899
rs79485969
rs9846278
rs34813869
rs9346529
rs12987451
rs146788953
rs7478464
rs112878149
rs2094597
rs393073
rs7667073
rs111404935
rs142186496
rs142241717
rs2115412
rs6912516
rs9270588
All
0.0
0.1
0.2
0.3
0.4
MR leave−one−out sensitivity analysis for
' HLA DR on CD33br HLA DR+ CD14dim ' on 'Rheumatoid arthritis'

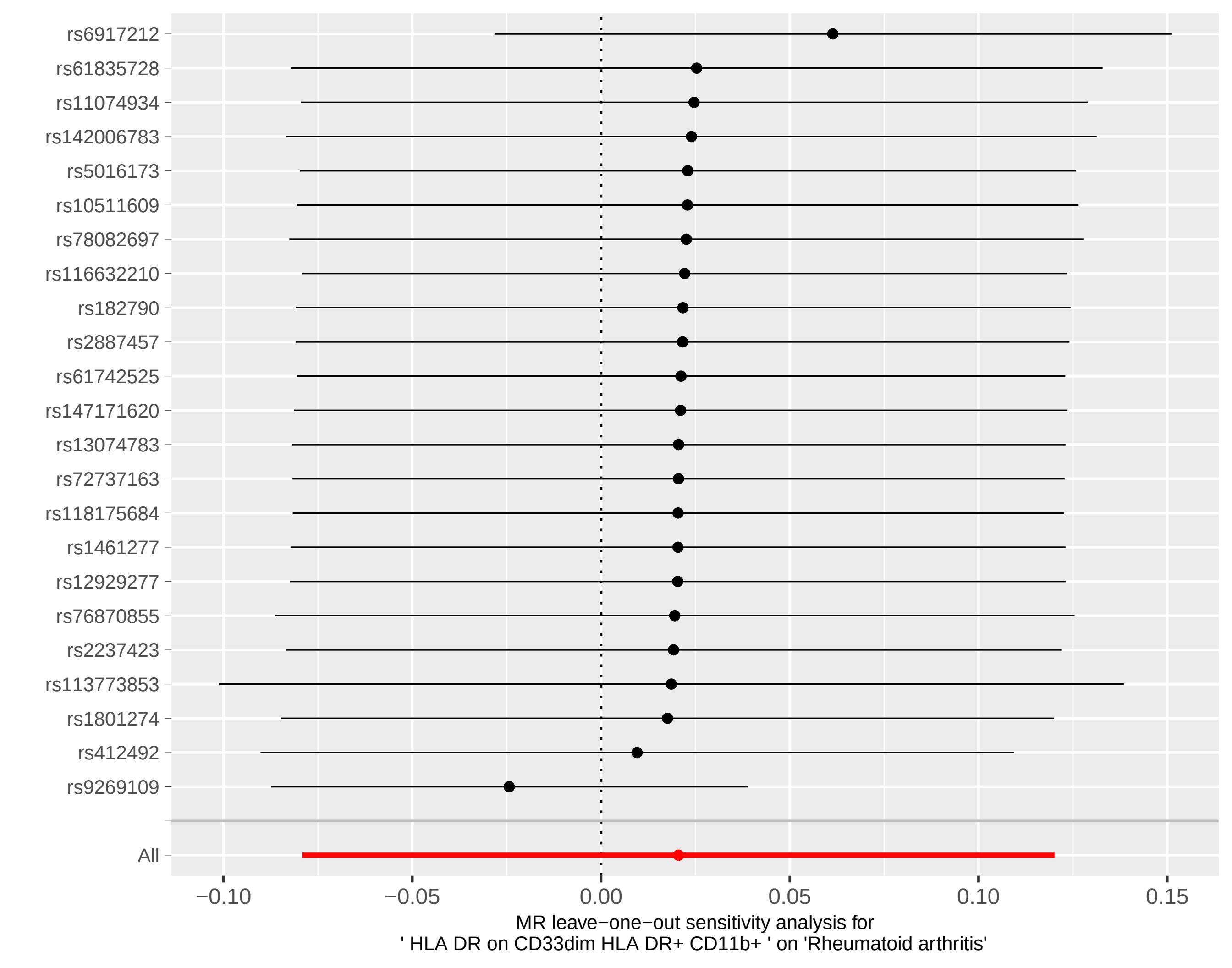
rs6917212
rs61835728
rs11074934
rs142006783
rs5016173
rs10511609
rs78082697
rs116632210
rs182790
rs2887457
rs61742525
rs147171620
rs13074783
rs72737163
rs118175684
rs1461277
rs12929277
rs76870855
rs2237423
rs113773853
rs1801274
rs412492
rs9269109
All
−0.10
−0.05
0.00
0.05
0.10
0.15
MR leave−one−out sensitivity analysis for
' HLA DR on CD33dim HLA DR+ CD11b+ ' on 'Rheumatoid arthritis'

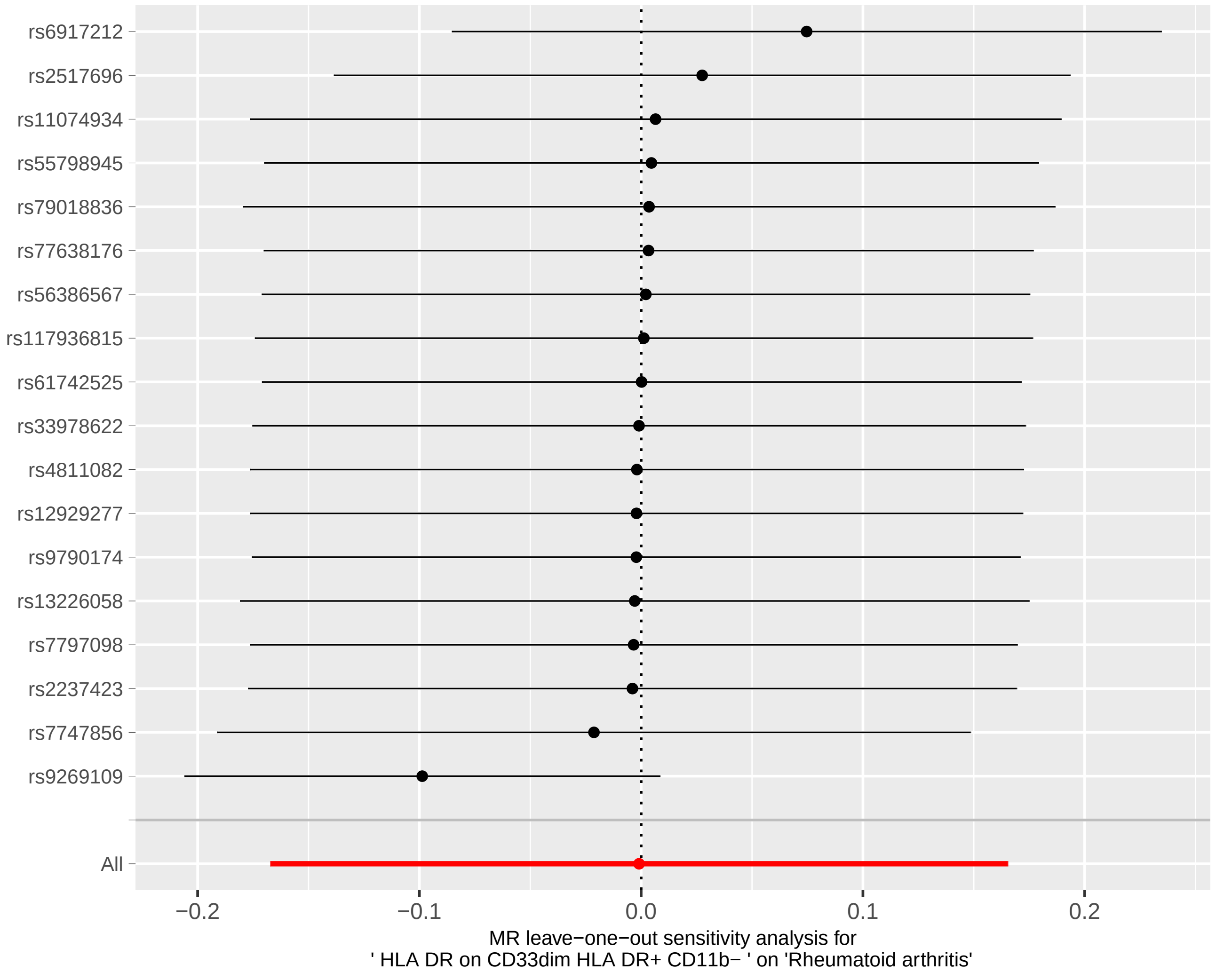
rs6917212
rs2517696
rs11074934
rs55798945
rs79018836
rs77638176
rs56386567
rs117936815
rs61742525
rs33978622
rs4811082
rs12929277
rs9790174
rs13226058
rs7797098
rs2237423
rs7747856
rs9269109
All
−0.2
−0.1
0.0
0.1
0.2
MR leave−one−out sensitivity analysis for
' HLA DR on CD33dim HLA DR+ CD11b− ' on 'Rheumatoid arthritis'

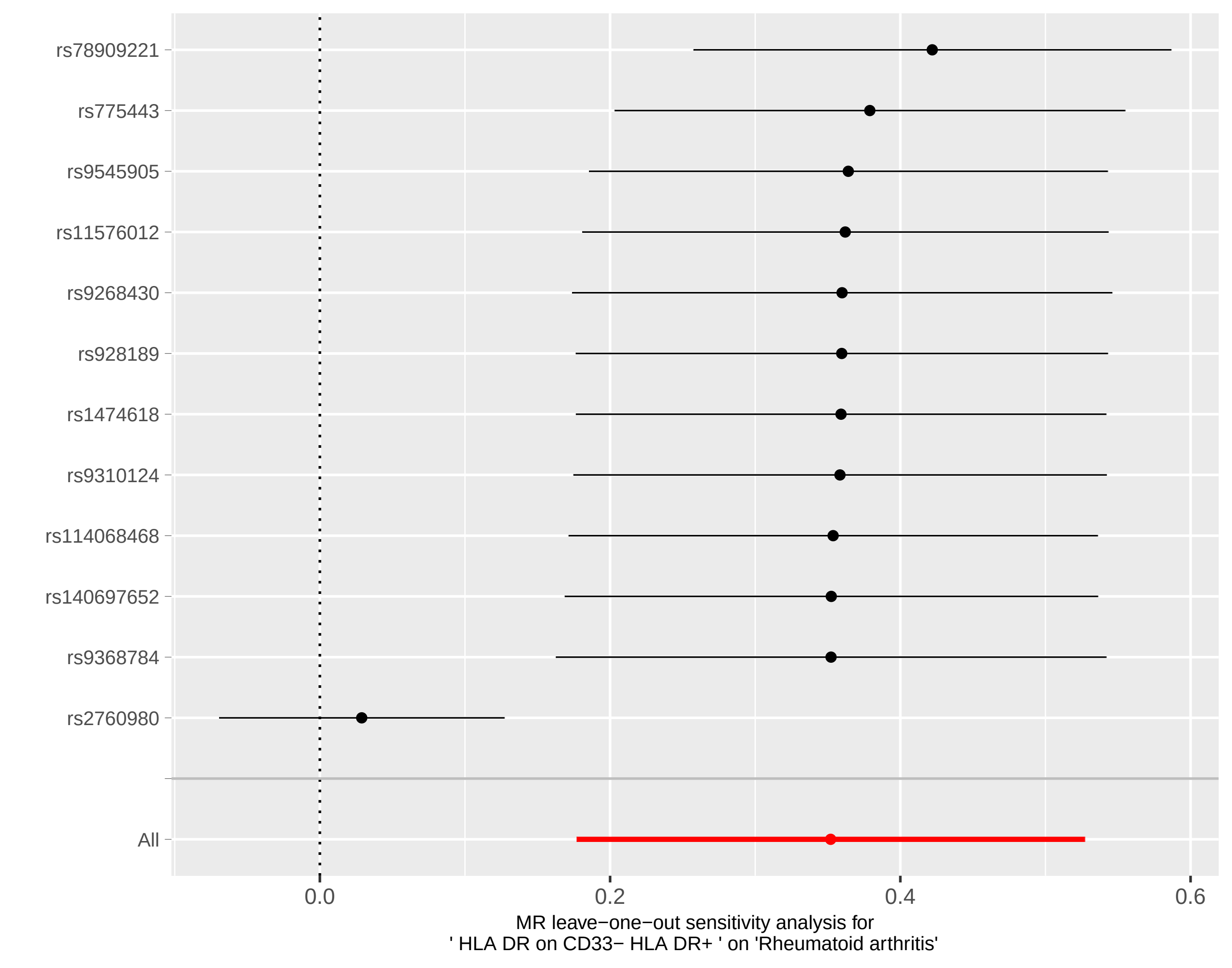

rs78909221
rs775443
rs9545905
rs11576012
rs9268430
rs928189
rs1474618
rs9310124
rs114068468
rs140697652
rs9368784
rs2760980
All
0.0
0.2
0.4
0.6
MR leave−one−out sensitivity analysis for
' HLA DR on CD33− HLA DR+ ' on 'Rheumatoid arthritis'

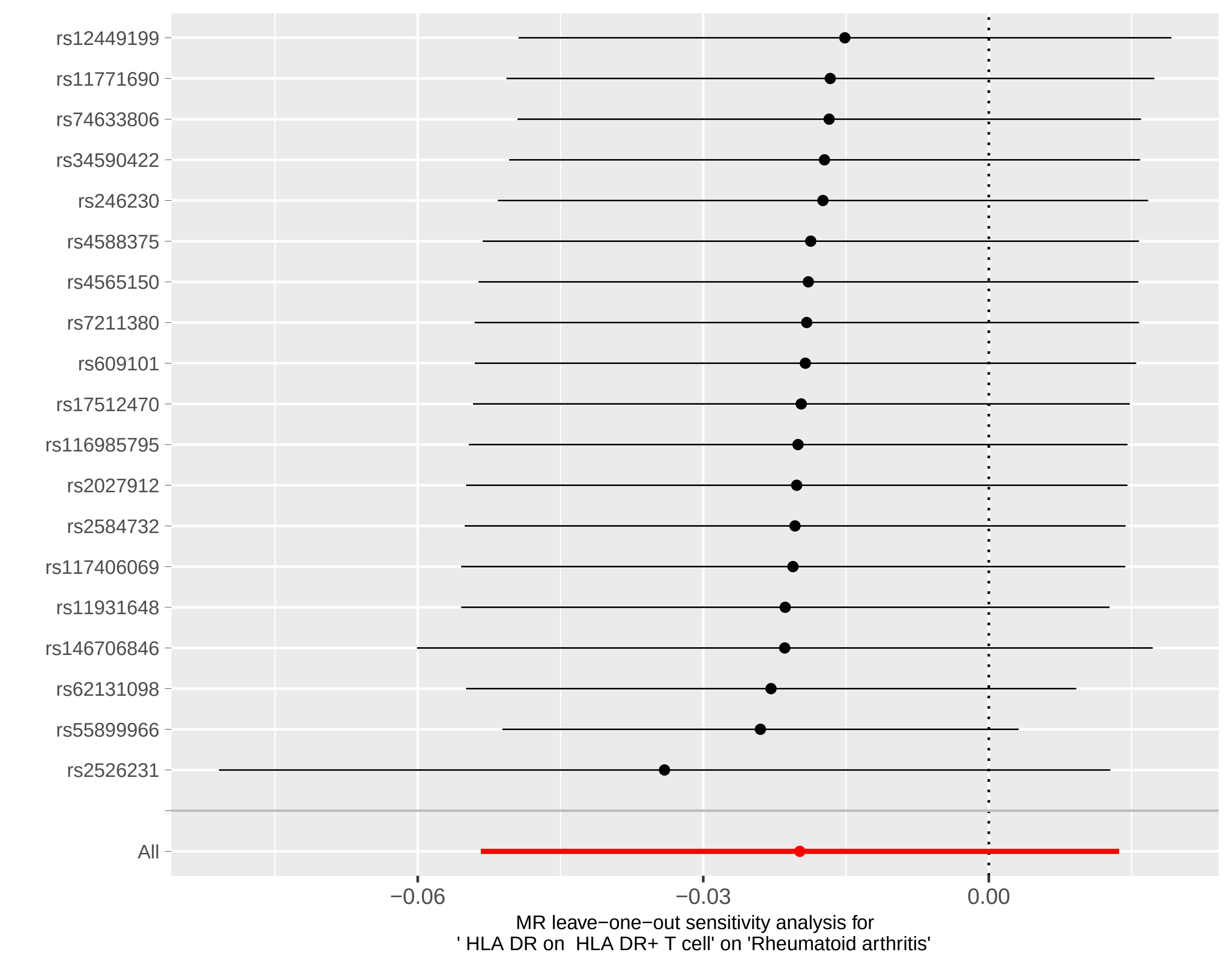
rs12449199
rs11771690
rs74633806
rs34590422
rs246230
rs4588375
rs4565150
rs7211380
rs609101
rs17512470
rs116985795
rs2027912
rs2584732
rs117406069
rs11931648
rs146706846
rs62131098
rs55899966
rs2526231
All
−0.06
−0.03
0.00
MR leave−one−out sensitivity analysis for
' HLA DR on HLA DR+ T cell' on 'Rheumatoid arthritis'

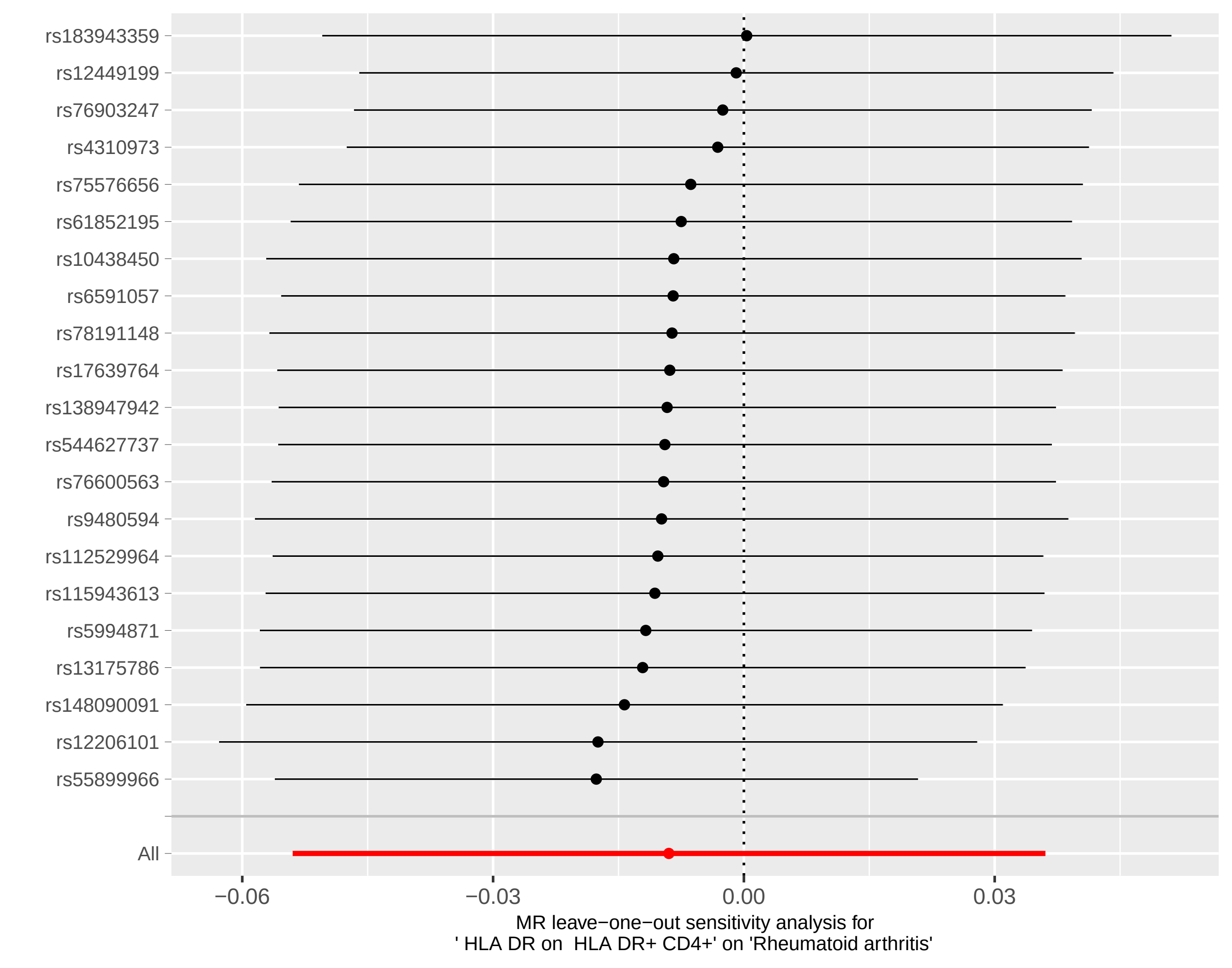
rs183943359
rs12449199
rs76903247
rs4310973
rs75576656
rs61852195
rs10438450
rs6591057
rs78191148
rs17639764
rs138947942
rs544627737
rs76600563
rs9480594
rs112529964
rs115943613
rs5994871
rs13175786
rs148090091
rs12206101
rs55899966
All
−0.06
−0.03
0.00
0.03
MR leave−one−out sensitivity analysis for
' HLA DR on HLA DR+ CD4+' on 'Rheumatoid arthritis'

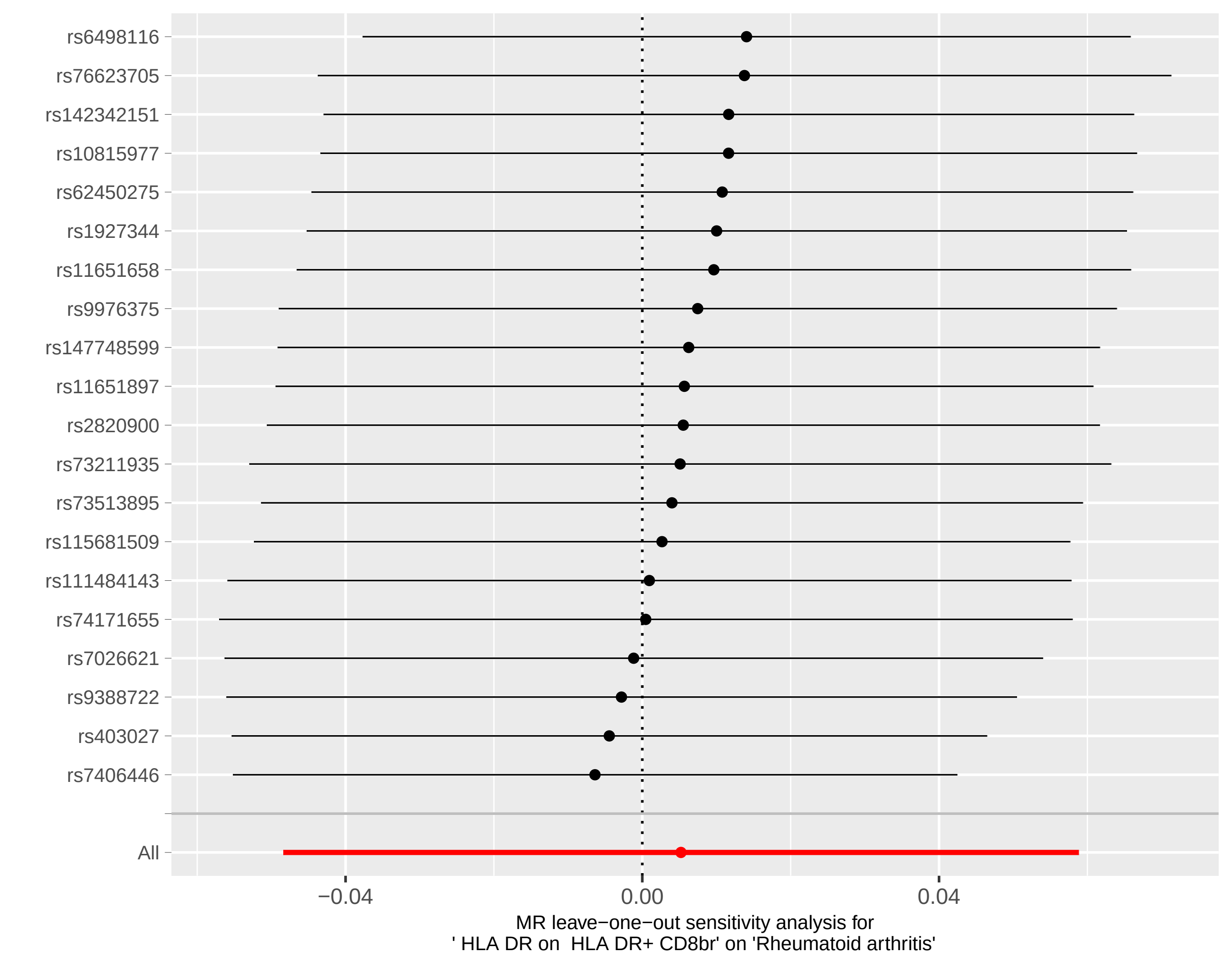
rs6498116
rs76623705
rs142342151
rs10815977
rs62450275
rs1927344
rs11651658
rs9976375
rs147748599
rs11651897
rs2820900
rs73211935
rs73513895
rs115681509
rs111484143
rs74171655
rs7026621
rs9388722
rs403027
rs7406446
All
−0.04
0.00
0.04
MR leave−one−out sensitivity analysis for
' HLA DR on HLA DR+ CD8br' on 'Rheumatoid arthritis'

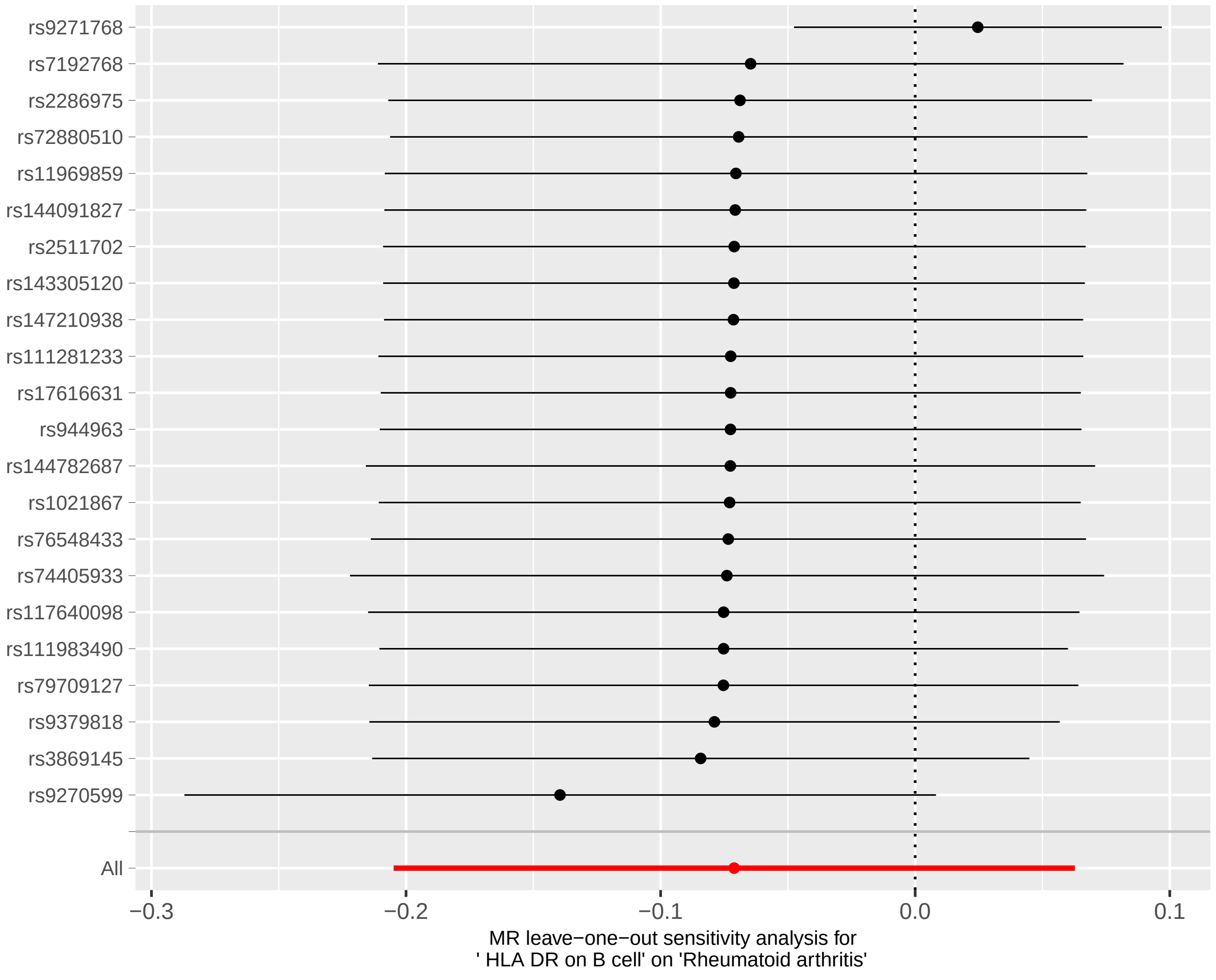
rs9271768
rs7192768
rs2286975
rs72880510
rs11969859
rs144091827
rs2511702
rs143305120
rs147210938
rs111281233
rs17616631
rs944963
rs144782687
rs1021867
rs76548433
rs74405933
rs117640098
rs111983490
rs79709127
rs9379818
rs3869145
rs9270599
All
−0.3
−0.2
−0.1
0.0
0.1
MR leave−one−out sensitivity analysis for
' HLA DR on B cell' on 'Rheumatoid arthritis'

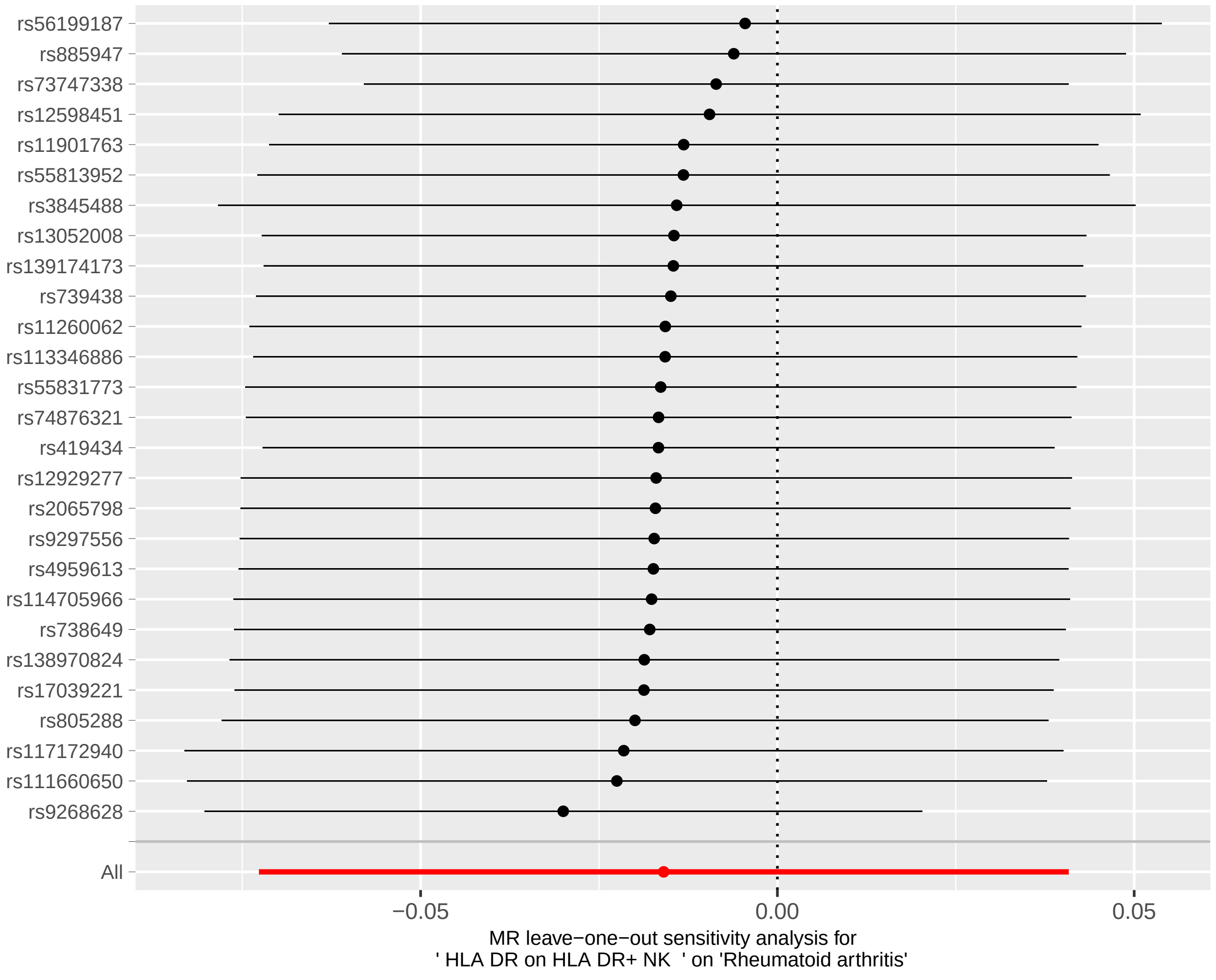

rs56199187
rs885947
rs73747338
rs12598451
rs11901763
rs55813952
rs3845488
rs13052008
rs139174173
rs739438
rs11260062
rs113346886
rs55831773
rs74876321
rs419434
rs12929277
rs2065798
rs9297556
rs4959613
rs114705966
rs738649
rs138970824
rs17039221
rs805288
rs117172940
rs111660650
rs9268628
All
−0.05
0.00
0.05
MR leave−one−out sensitivity analysis for
' HLA DR on HLA DR+ NK ' on 'Rheumatoid arthritis'

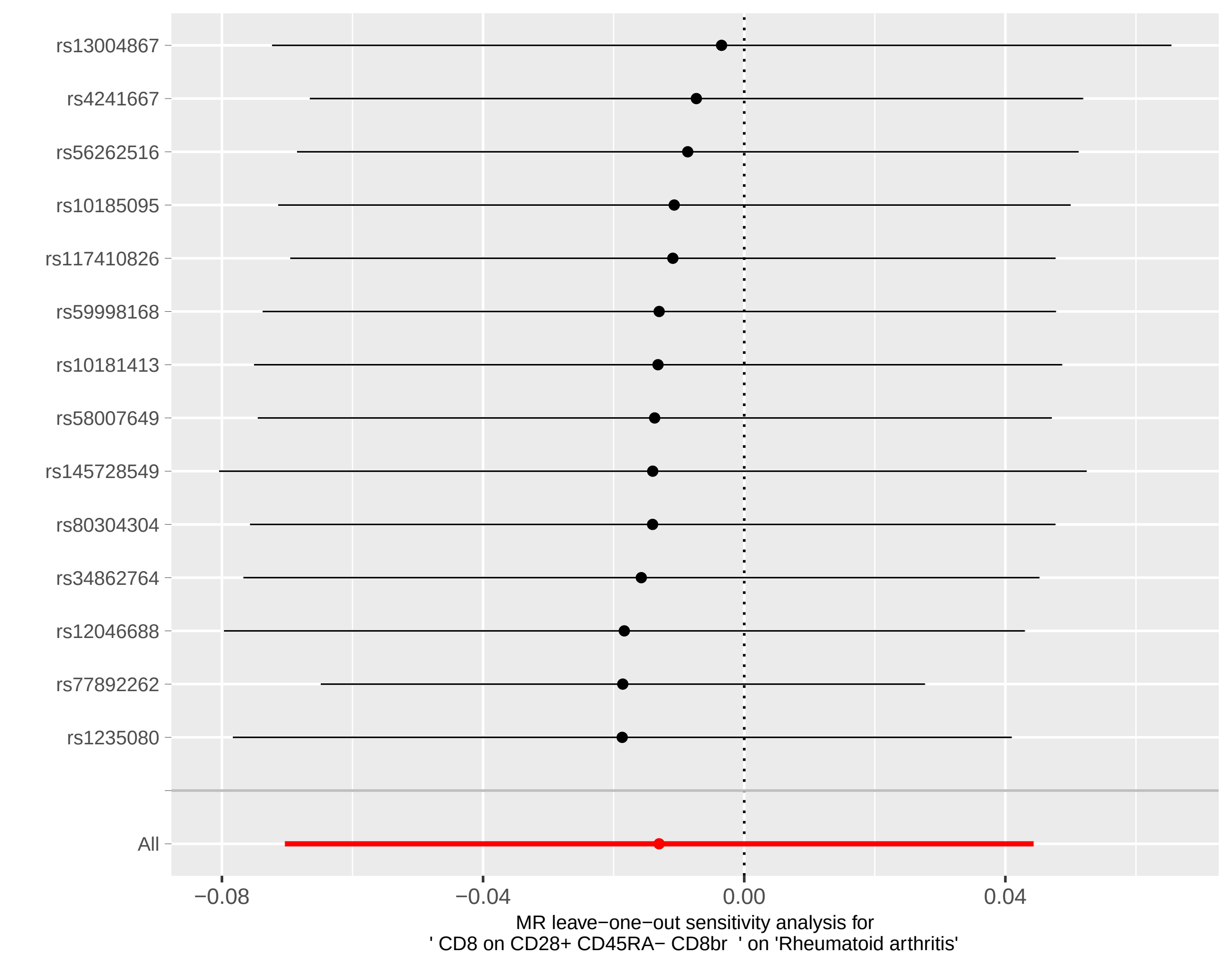

rs13004867
rs4241667
rs56262516
rs10185095
rs117410826
rs59998168
rs10181413
rs58007649
rs145728549
rs80304304
rs34862764
rs12046688
rs77892262
rs1235080
All
−0.08
−0.04
0.00
0.04
MR leave−one−out sensitivity analysis for
' CD8 on CD28+ CD45RA− CD8br ' on 'Rheumatoid arthritis'

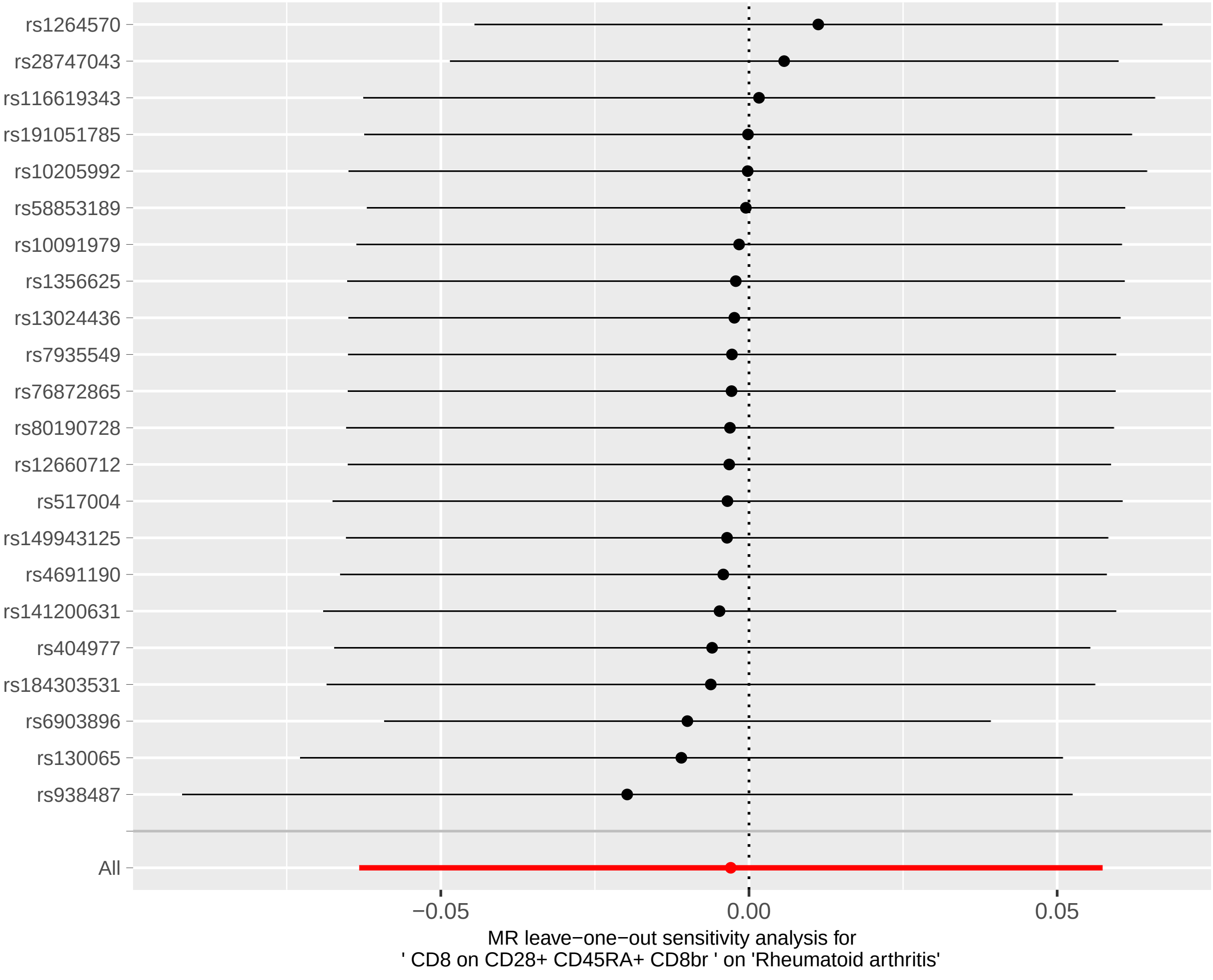
rs1264570
rs28747043
rs116619343
rs191051785
rs10205992
rs58853189
rs10091979
rs1356625
rs13024436
rs7935549
rs76872865
rs80190728
rs12660712
rs517004
rs149943125
rs4691190
rs141200631
rs404977
rs184303531
rs6903896
rs130065
rs938487
All
−0.05
0.00
0.05
MR leave−one−out sensitivity analysis for
' CD8 on CD28+ CD45RA+ CD8br ' on 'Rheumatoid arthritis'

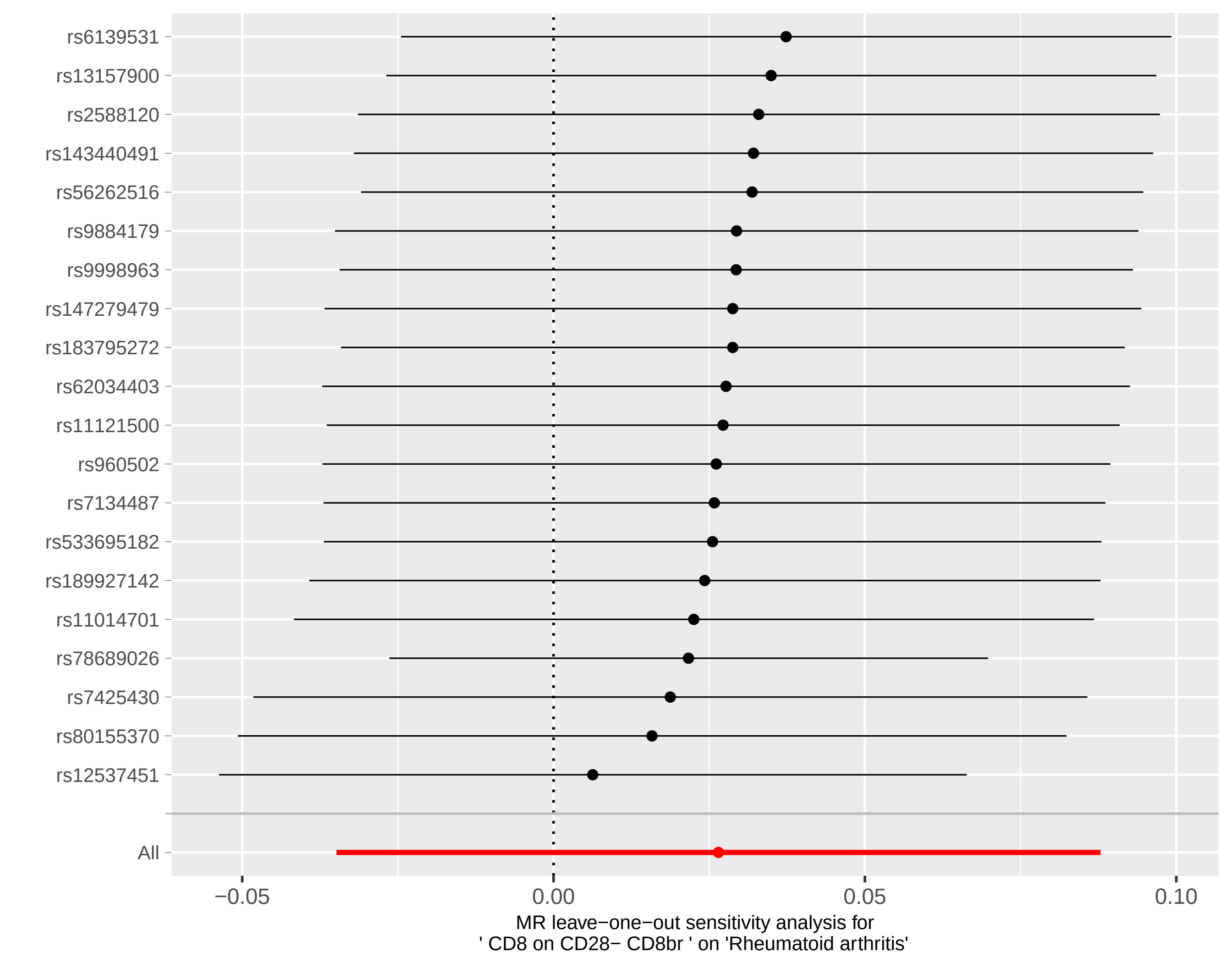
rs6139531
rs13157900
rs2588120
rs143440491
rs56262516
rs9884179
rs9998963
rs147279479
rs183795272
rs62034403
rs11121500
rs960502
rs7134487
rs533695182
rs189927142
rs11014701
rs78689026
rs7425430
rs80155370
rs12537451
All
−0.05
0.00
0.05
0.10
MR leave−one−out sensitivity analysis for
' CD8 on CD28− CD8br ' on 'Rheumatoid arthritis'

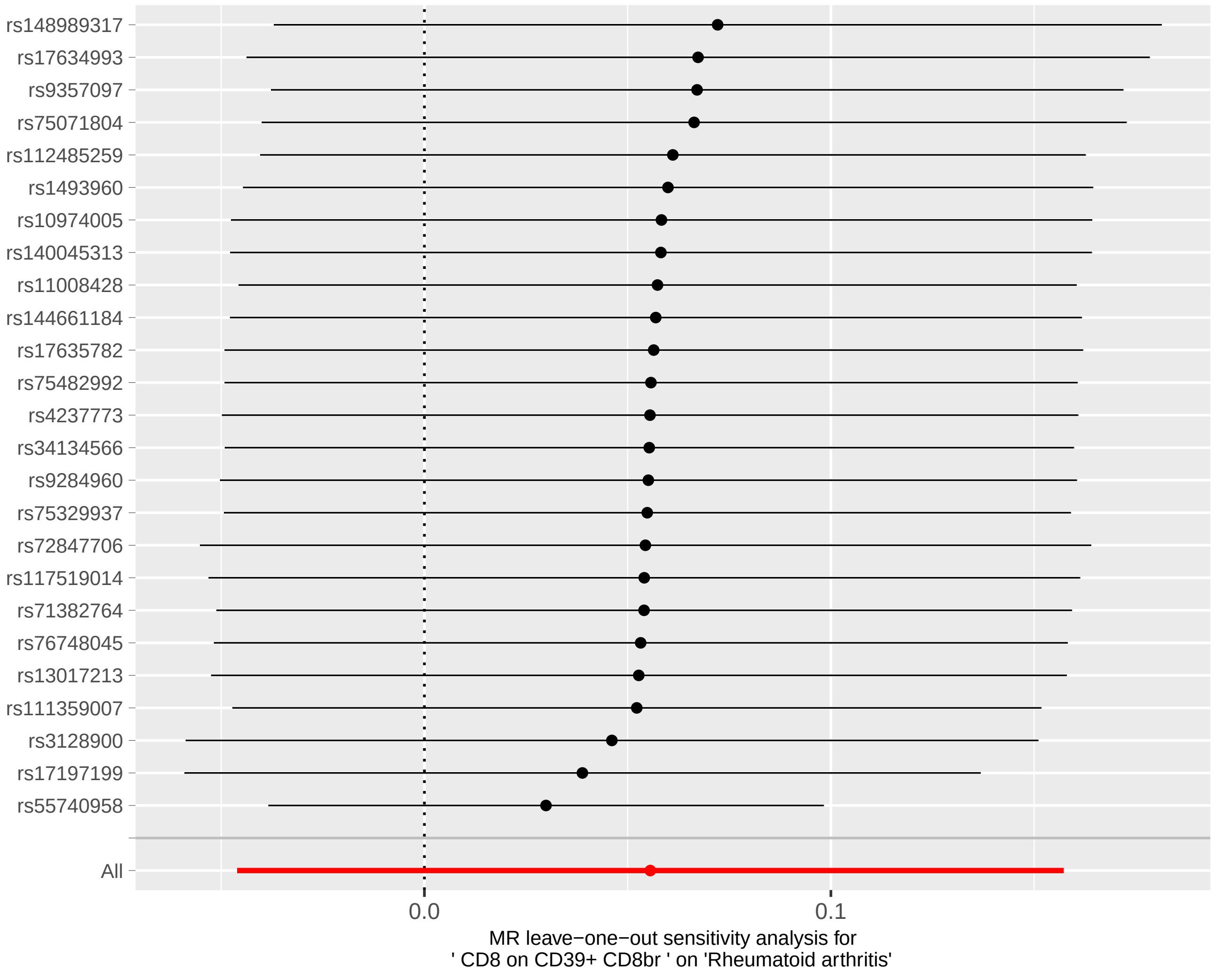

rs148989317
rs17634993
rs9357097
rs75071804
rs112485259
rs1493960
rs10974005
rs140045313
rs11008428
rs144661184
rs17635782
rs75482992
rs4237773
rs34134566
rs9284960
rs75329937
rs72847706
rs117519014
rs71382764
rs76748045
rs13017213
rs111359007
rs3128900
rs17197199
rs55740958
All
0.0
0.1
MR leave−one−out sensitivity analysis for
' CD8 on CD39+ CD8br ' on 'Rheumatoid arthritis'

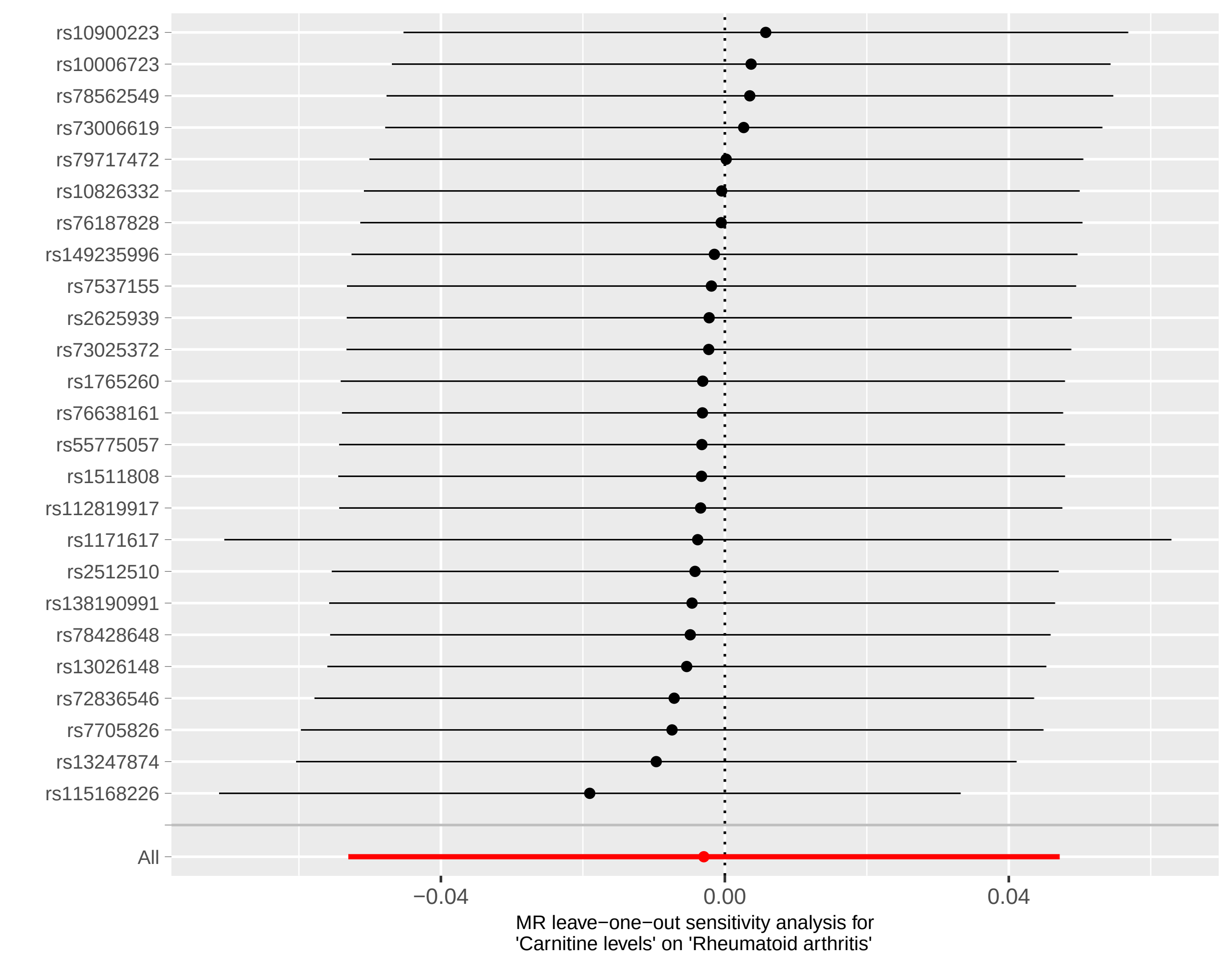
rs10900223
rs10006723
rs78562549
rs73006619
rs79717472
rs10826332
rs76187828
rs149235996
rs7537155
rs2625939
rs73025372
rs1765260
rs76638161
rs55775057
rs1511808
rs112819917
rs1171617
rs2512510
rs138190991
rs78428648
rs13026148
rs72836546
rs7705826
rs13247874
rs115168226
All
−0.04
0.00
0.04
MR leave−one−out sensitivity analysis for
'Carnitine levels' on 'Rheumatoid arthritis'

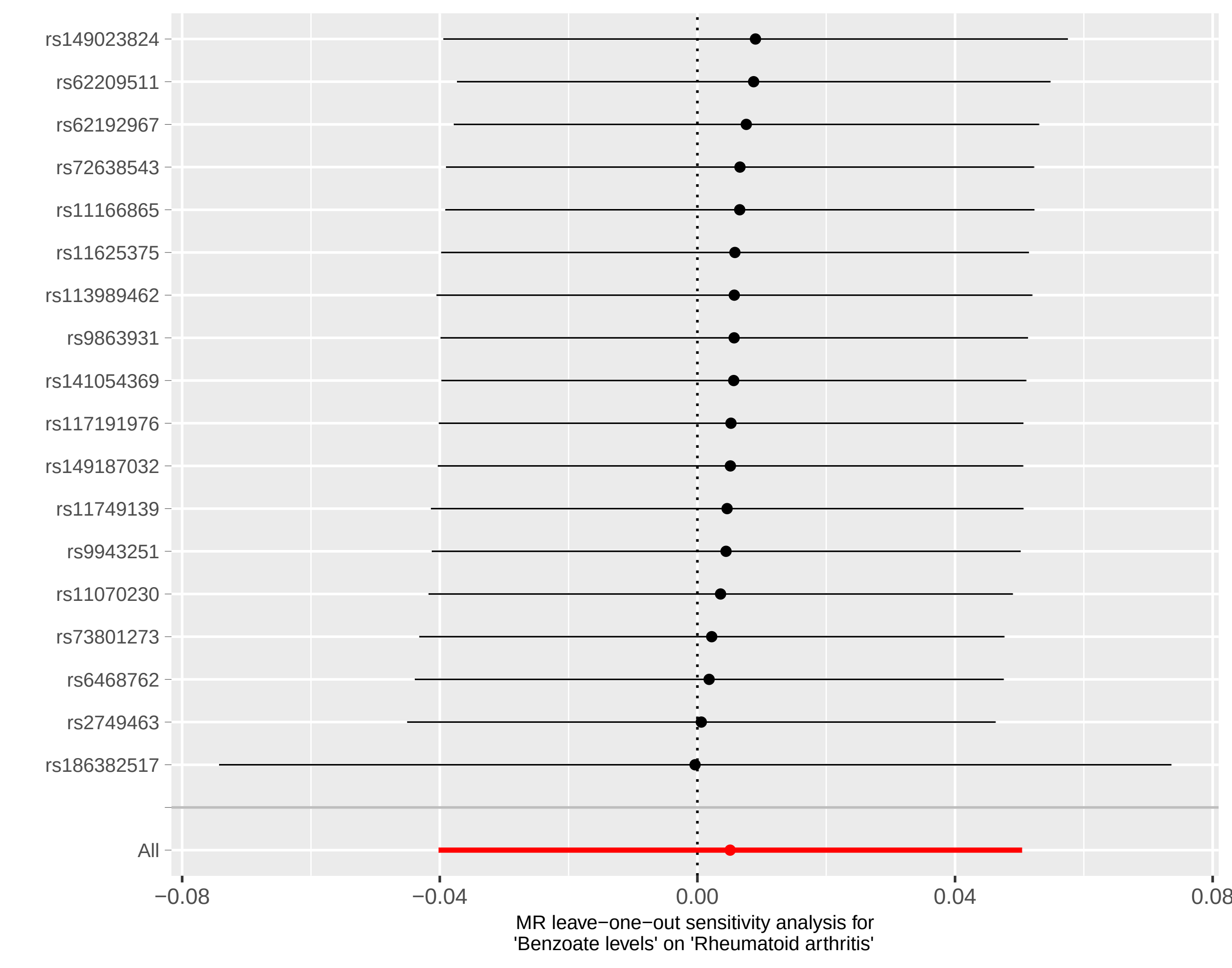
rs149023824
rs62209511
rs62192967
rs72638543
rs11166865
rs11625375
rs113989462
rs9863931
rs141054369
rs117191976
rs149187032
rs11749139
rs9943251
rs11070230
rs73801273
rs6468762
rs2749463
rs186382517
All
−0.08
−0.04
0.00
0.04
0.08
MR leave−one−out sensitivity analysis for
'Benzoate levels' on 'Rheumatoid arthritis'

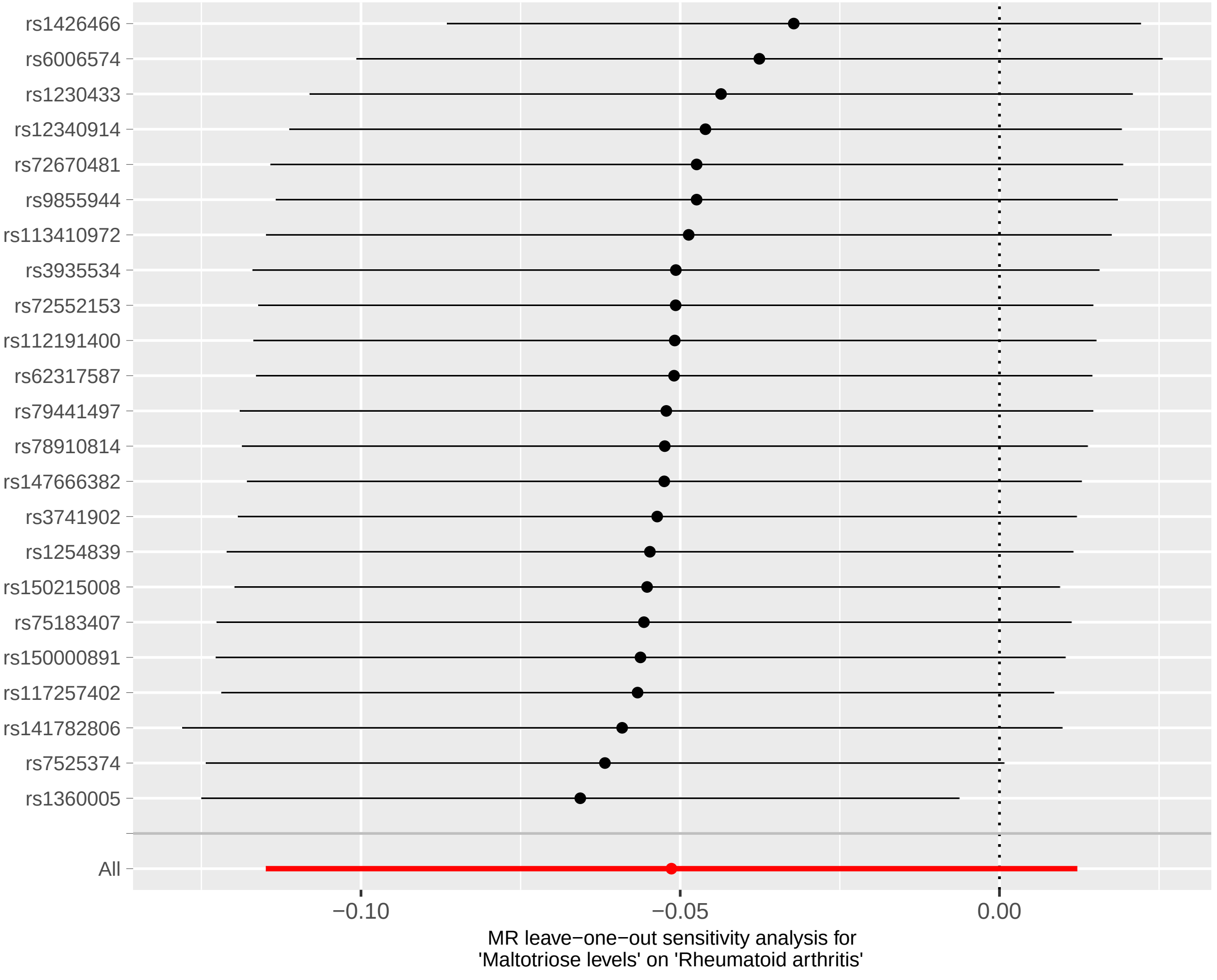

rs1426466
rs6006574
rs1230433
rs12340914
rs72670481
rs9855944
rs113410972
rs3935534
rs72552153
rs112191400
rs62317587
rs79441497
rs78910814
rs147666382
rs3741902
rs1254839
rs150215008
rs75183407
rs150000891
rs117257402
rs141782806
rs7525374
rs1360005
All
−0.10
−0.05
0.00
MR leave−one−out sensitivity analysis for
'Maltotriose levels' on 'Rheumatoid arthritis'

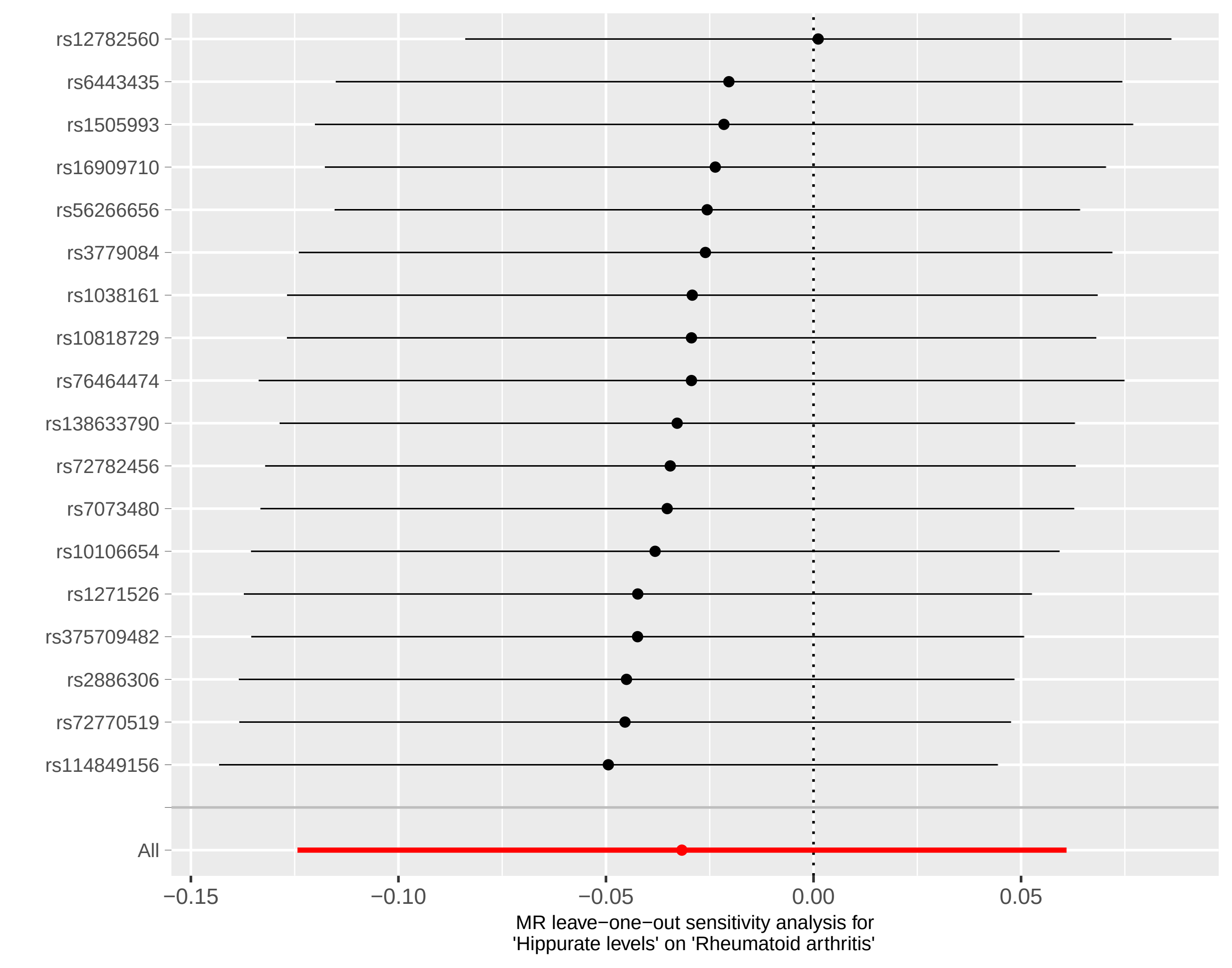
rs12782560
rs6443435
rs1505993
rs16909710
rs56266656
rs3779084
rs1038161
rs10818729
rs76464474
rs138633790
rs72782456
rs7073480
rs10106654
rs1271526
rs375709482
rs2886306
rs72770519
rs114849156
All
−0.15
−0.10
−0.05
0.00
0.05
MR leave−one−out sensitivity analysis for
'Hippurate levels' on 'Rheumatoid arthritis'

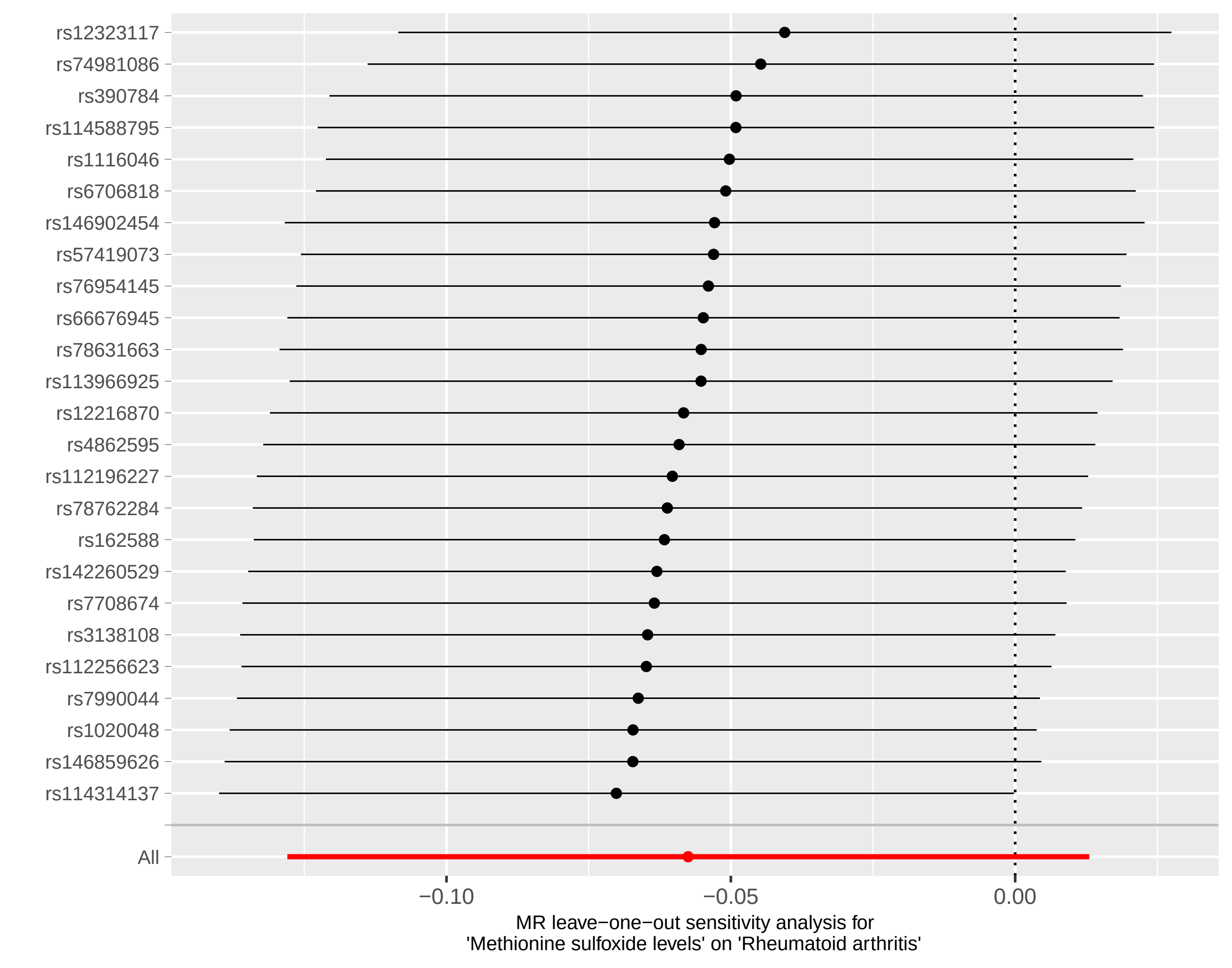
rs12323117
rs74981086
rs390784
rs114588795
rs1116046
rs6706818
rs146902454
rs57419073
rs76954145
rs66676945
rs78631663
rs113966925
rs12216870
rs4862595
rs112196227
rs78762284
rs162588
rs142260529
rs7708674
rs3138108
rs112256623
rs7990044
rs1020048
rs146859626
rs114314137
All
−0.10
−0.05
0.00
MR leave−one−out sensitivity analysis for
'Methionine sulfoxide levels' on 'Rheumatoid arthritis'

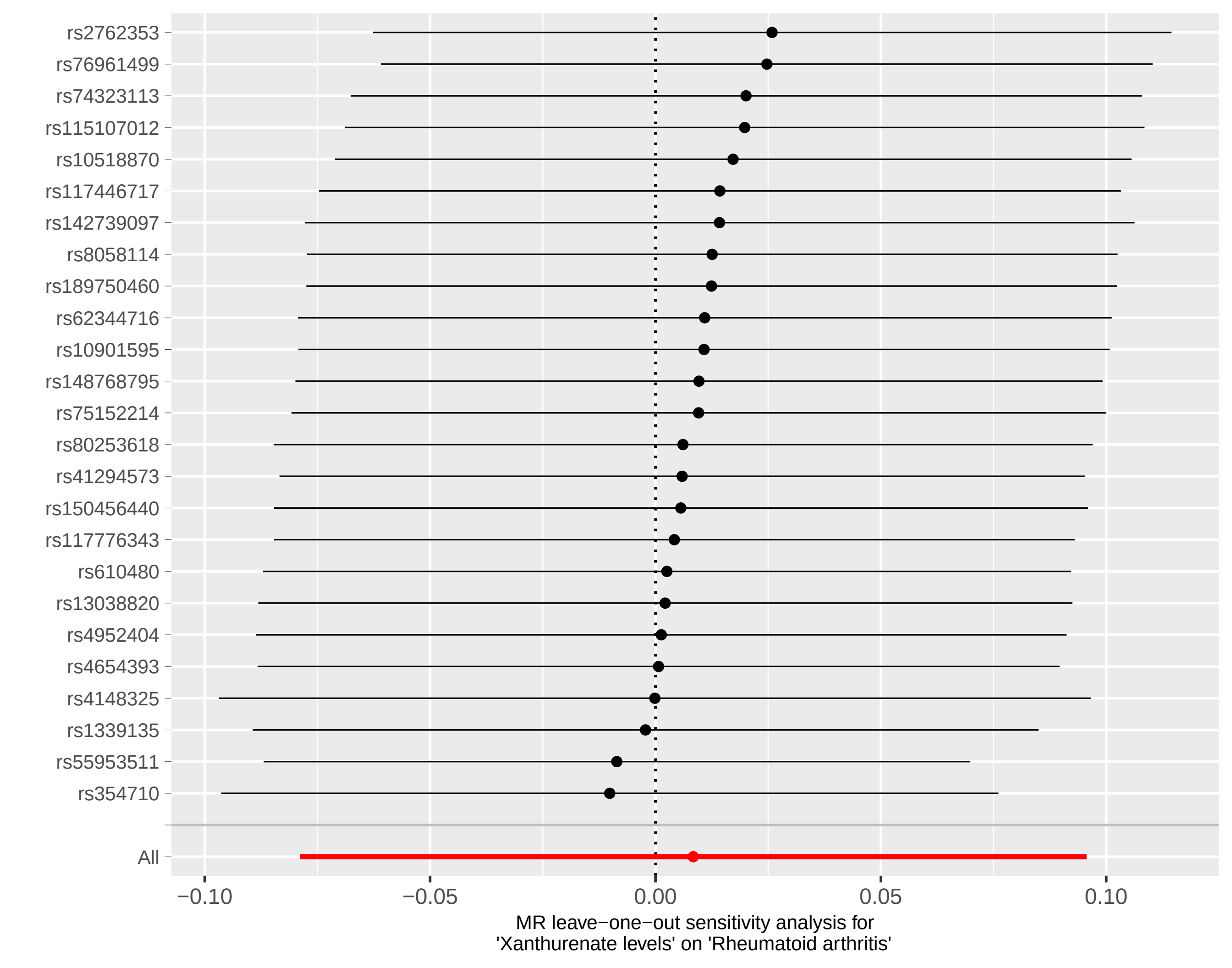
rs2762353
rs76961499
rs74323113
rs115107012
rs10518870
rs117446717
rs142739097
rs8058114
rs189750460
rs62344716
rs10901595
rs148768795
rs75152214
rs80253618
rs41294573
rs150456440
rs117776343
rs610480
rs13038820
rs4952404
rs4654393
rs4148325
rs1339135
rs55953511
rs354710
All
−0.10
−0.05
0.00
0.05
0.10
MR leave−one−out sensitivity analysis for
'Xanthurenate levels' on 'Rheumatoid arthritis'

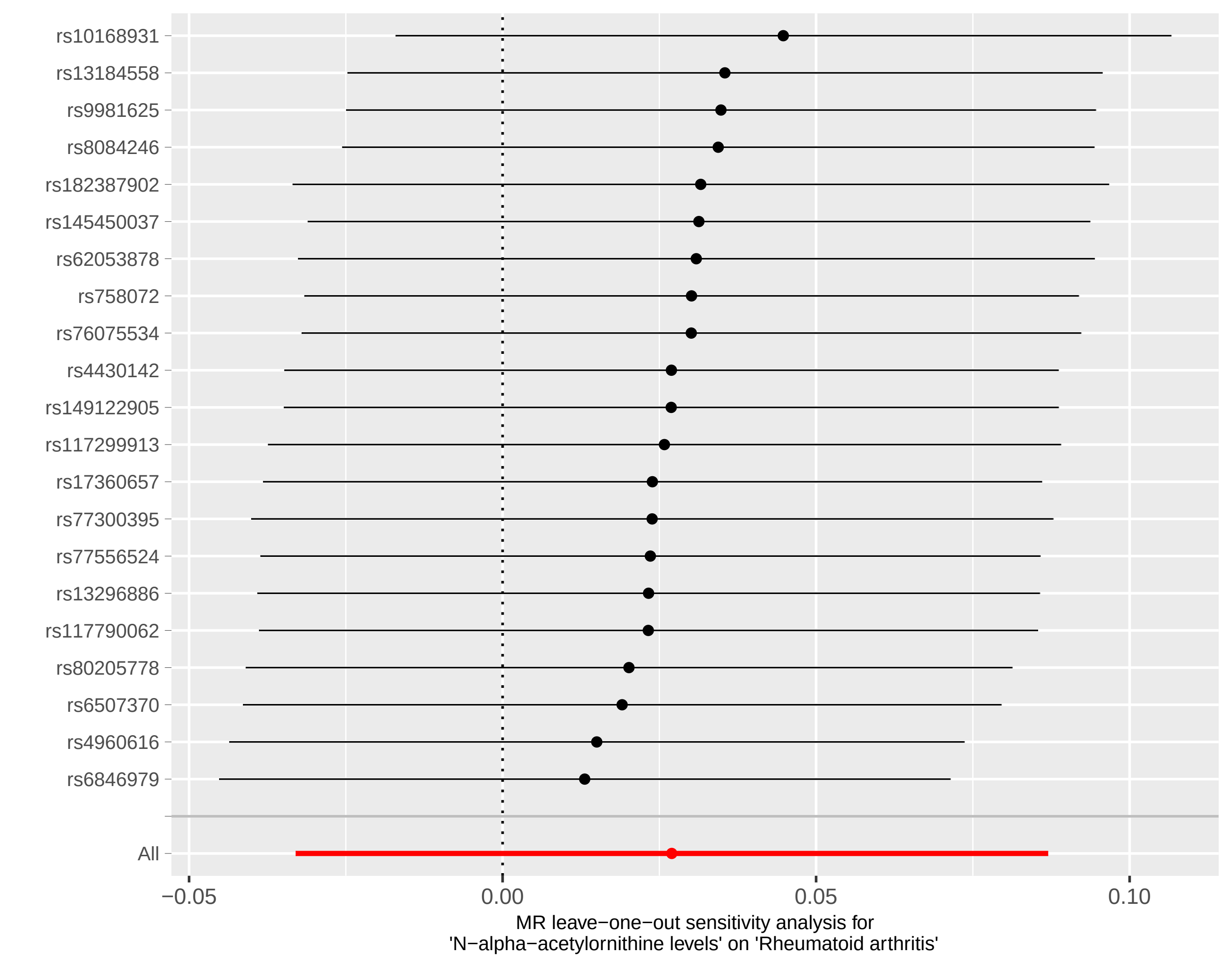

rs10168931
rs13184558
rs9981625
rs8084246
rs182387902
rs145450037
rs62053878
rs758072
rs76075534
rs4430142
rs149122905
rs117299913
rs17360657
rs77300395
rs77556524
rs13296886
rs117790062
rs80205778
rs6507370
rs4960616
rs6846979
All
−0.05
0.00
0.05
0.10
MR leave−one−out sensitivity analysis for
'N−alpha−acetylornithine levels' on 'Rheumatoid arthritis'

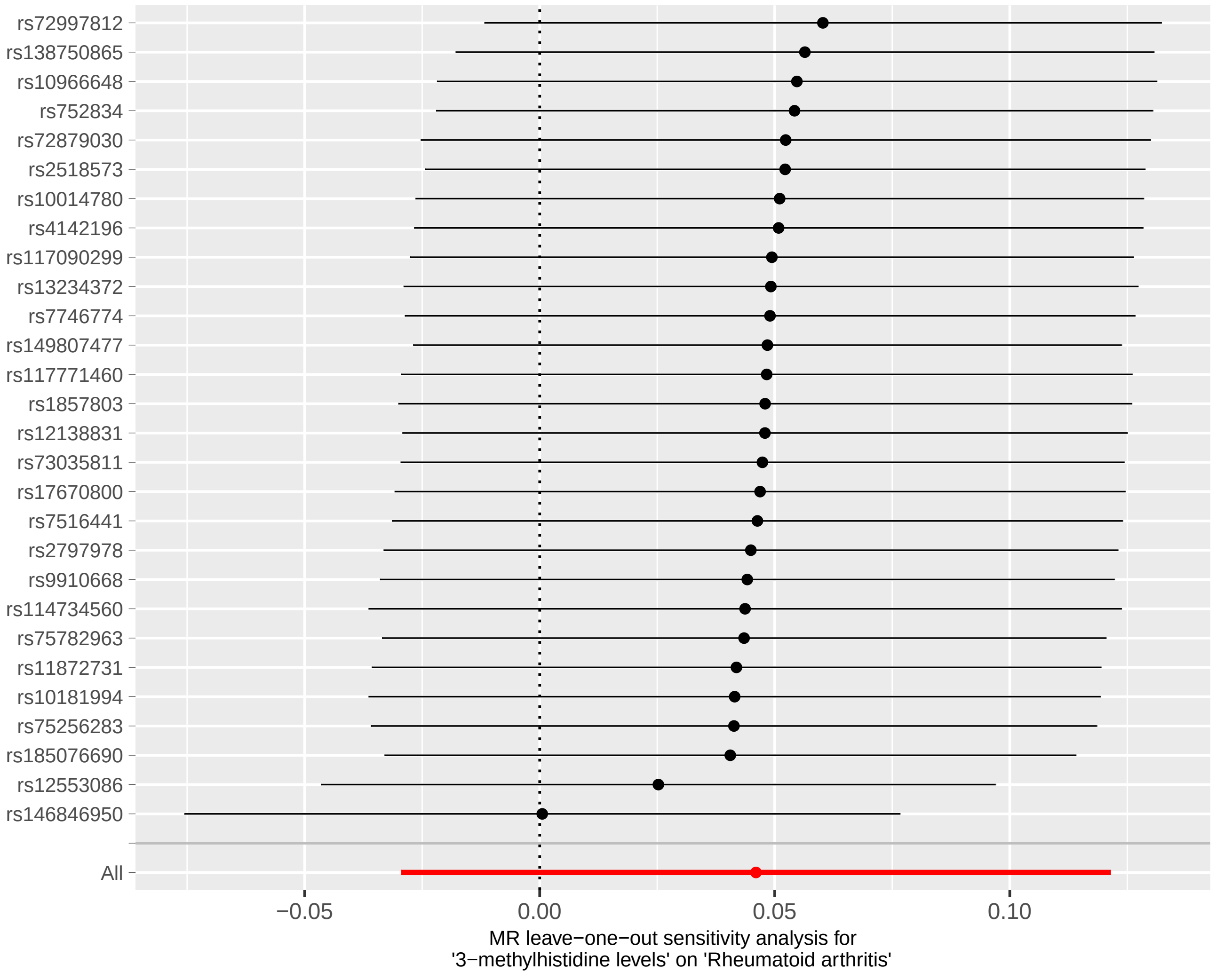
rs72997812
rs138750865
rs10966648
rs752834
rs72879030
rs2518573
rs10014780
rs4142196
rs117090299
rs13234372
rs7746774
rs149807477
rs117771460
rs1857803
rs12138831
rs73035811
rs17670800
rs7516441
rs2797978
rs9910668
rs114734560
rs75782963
rs11872731
rs10181994
rs75256283
rs185076690
rs12553086
rs146846950
All
−0.05
0.00
0.05
0.10
MR leave−one−out sensitivity analysis for
'3−methylhistidine levels' on 'Rheumatoid arthritis'

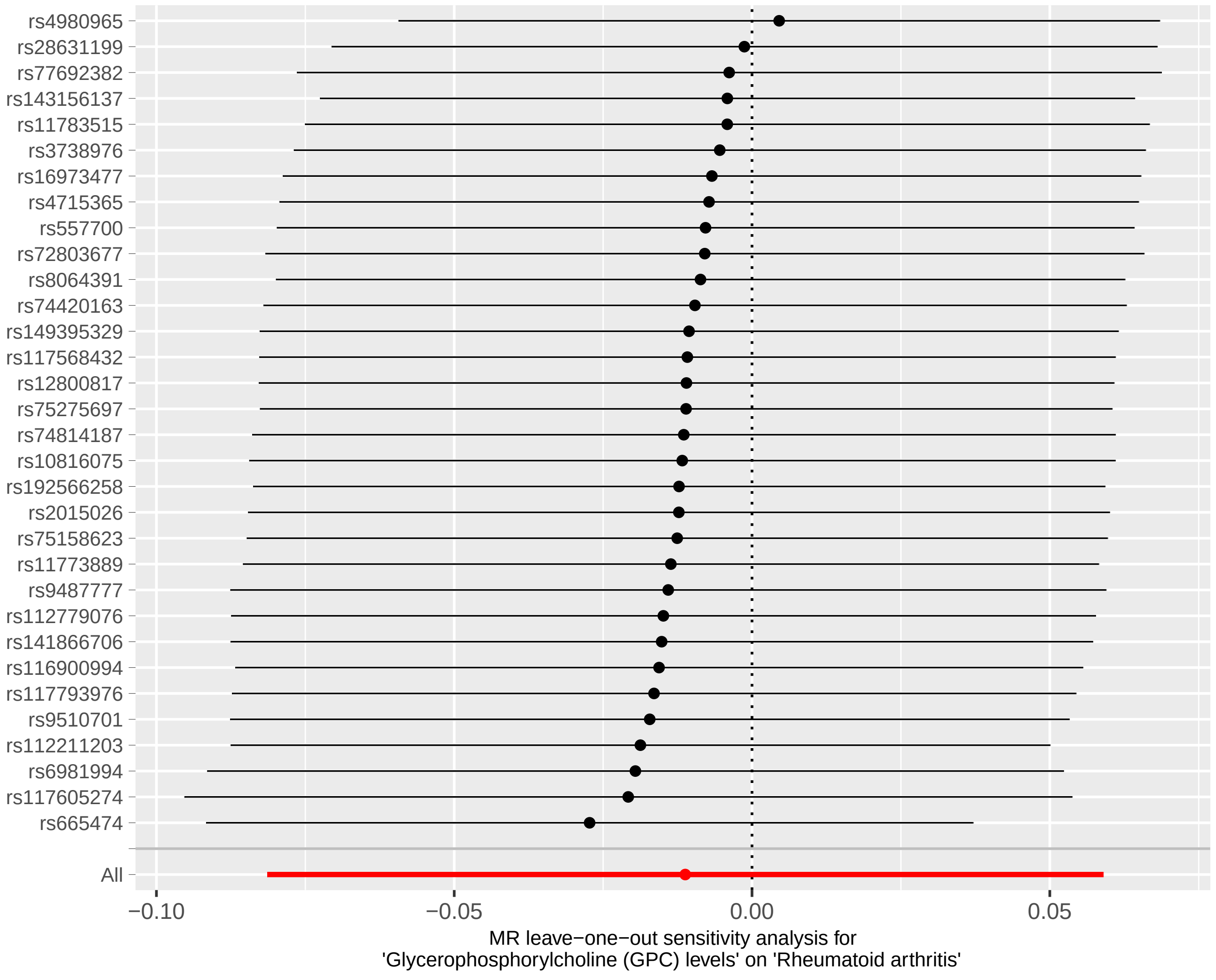
rs4980965
rs28631199
rs77692382
rs143156137
rs11783515
rs3738976
rs16973477
rs4715365
rs557700
rs72803677
rs8064391
rs74420163
rs149395329
rs117568432
rs12800817
rs75275697
rs74814187
rs10816075
rs192566258
rs2015026
rs75158623
rs11773889
rs9487777
rs112779076
rs141866706
rs116900994
rs117793976
rs9510701
rs112211203
rs6981994
rs117605274
rs665474
All
−0.10
−0.05
0.00
0.05
MR leave−one−out sensitivity analysis for
'Glycerophosphorylcholine (GPC) levels' on 'Rheumatoid arthritis'

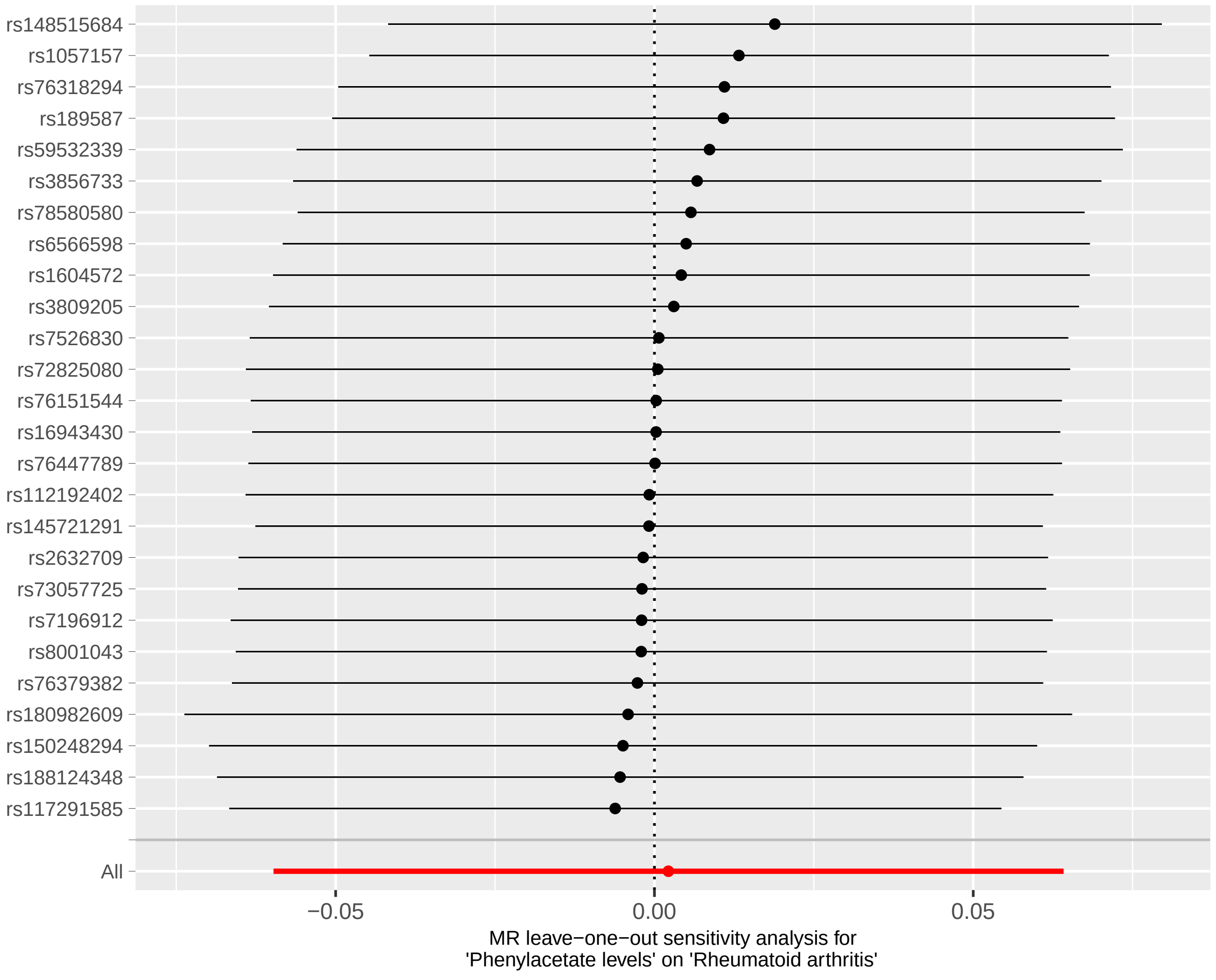
rs148515684
rs1057157
rs76318294
rs189587
rs59532339
rs3856733
rs78580580
rs6566598
rs1604572
rs3809205
rs7526830
rs72825080
rs76151544
rs16943430
rs76447789
rs112192402
rs145721291
rs2632709
rs73057725
rs7196912
rs8001043
rs76379382
rs180982609
rs150248294
rs188124348
rs117291585
All
−0.05
0.00
0.05
MR leave−one−out sensitivity analysis for
'Phenylacetate levels' on 'Rheumatoid arthritis'

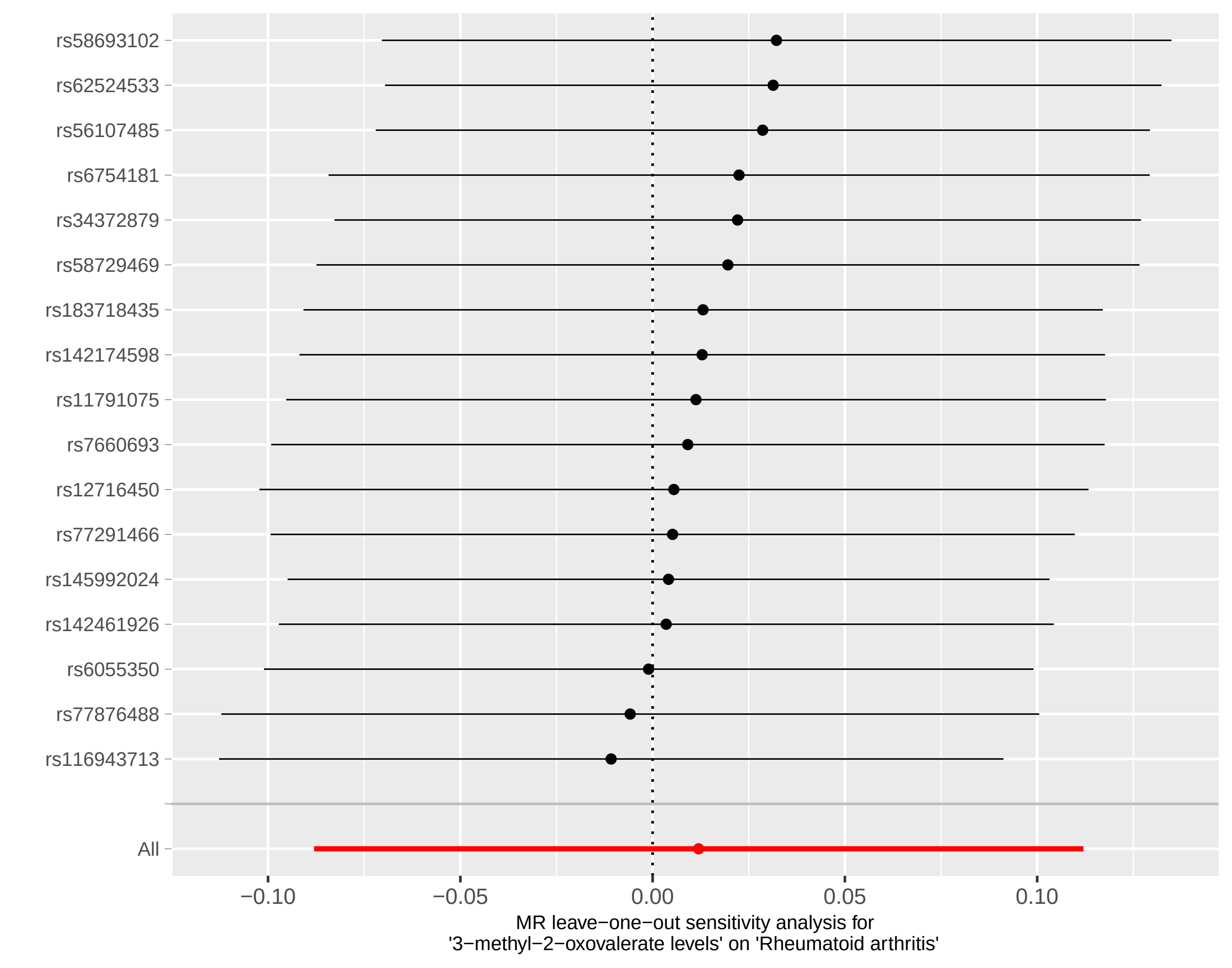

rs58693102
rs62524533
rs56107485
rs6754181
rs34372879
rs58729469
rs183718435
rs142174598
rs11791075
rs7660693
rs12716450
rs77291466
rs145992024
rs142461926
rs6055350
rs77876488
rs116943713
All
−0.10
−0.05
0.00
0.05
0.10
MR leave−one−out sensitivity analysis for
'3−methyl−2−oxovalerate levels' on 'Rheumatoid arthritis'

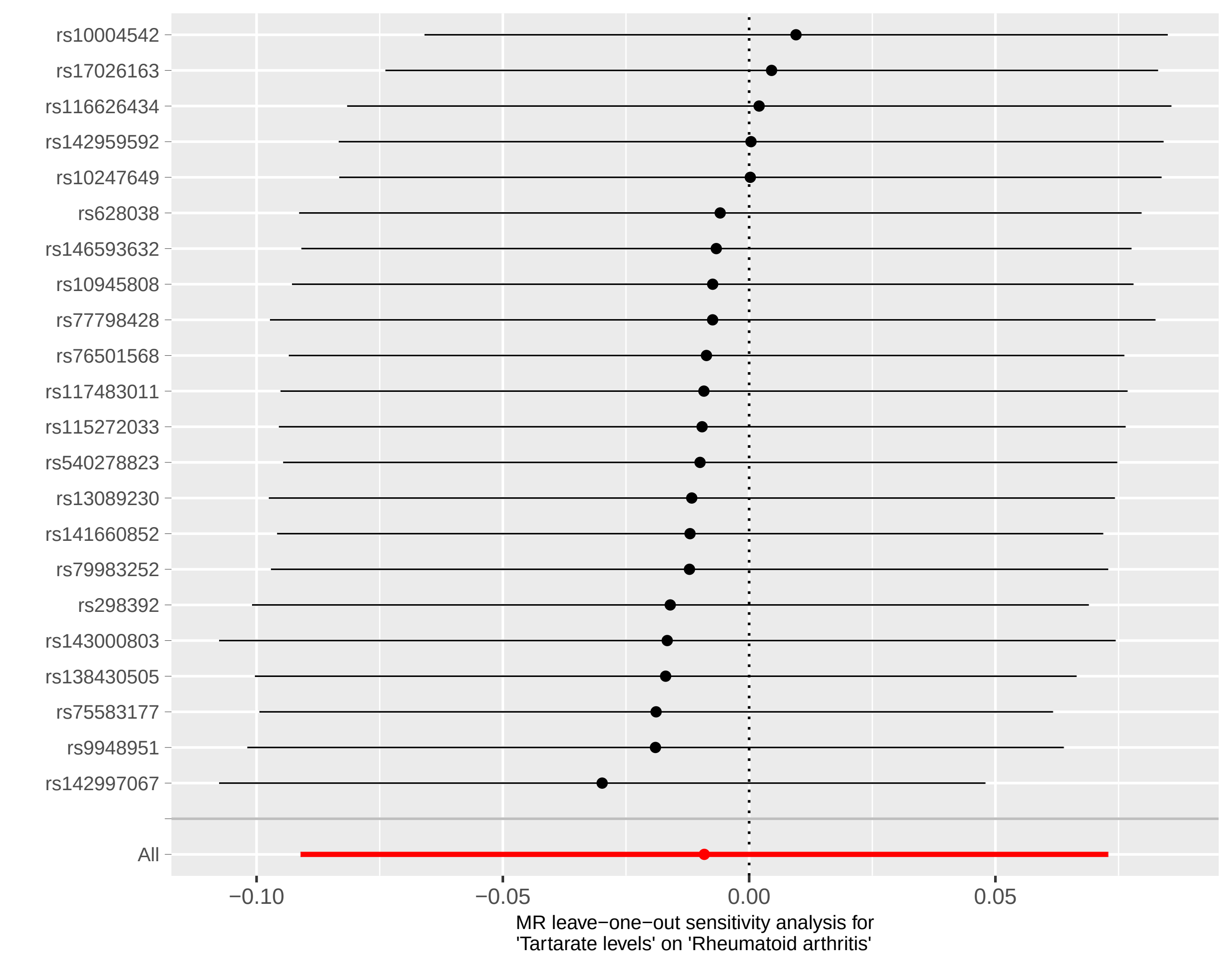
rs10004542
rs17026163
rs116626434
rs142959592
rs10247649
rs628038
rs146593632
rs10945808
rs77798428
rs76501568
rs117483011
rs115272033
rs540278823
rs13089230
rs141660852
rs79983252
rs298392
rs143000803
rs138430505
rs75583177
rs9948951
rs142997067
All
−0.10
−0.05
0.00
0.05
MR leave−one−out sensitivity analysis for
'Tartarate levels' on 'Rheumatoid arthritis'

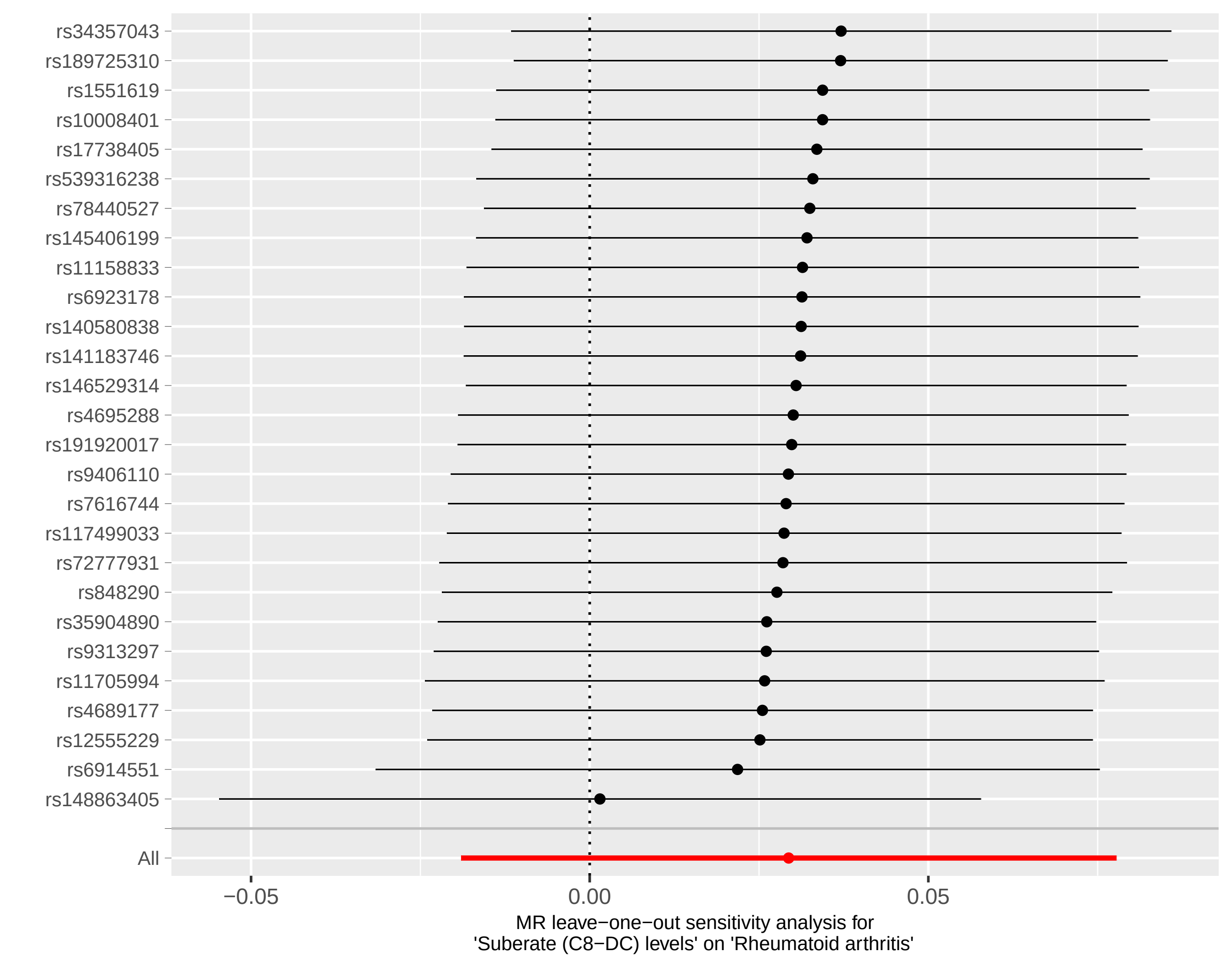

rs34357043
rs189725310
rs1551619
rs10008401
rs17738405
rs539316238
rs78440527
rs145406199
rs11158833
rs6923178
rs140580838
rs141183746
rs146529314
rs4695288
rs191920017
rs9406110
rs7616744
rs117499033
rs72777931
rs848290
rs35904890
rs9313297
rs11705994
rs4689177
rs12555229
rs6914551
rs148863405
All
−0.05
0.00
0.05
MR leave−one−out sensitivity analysis for
'Suberate (C8−DC) levels' on 'Rheumatoid arthritis'

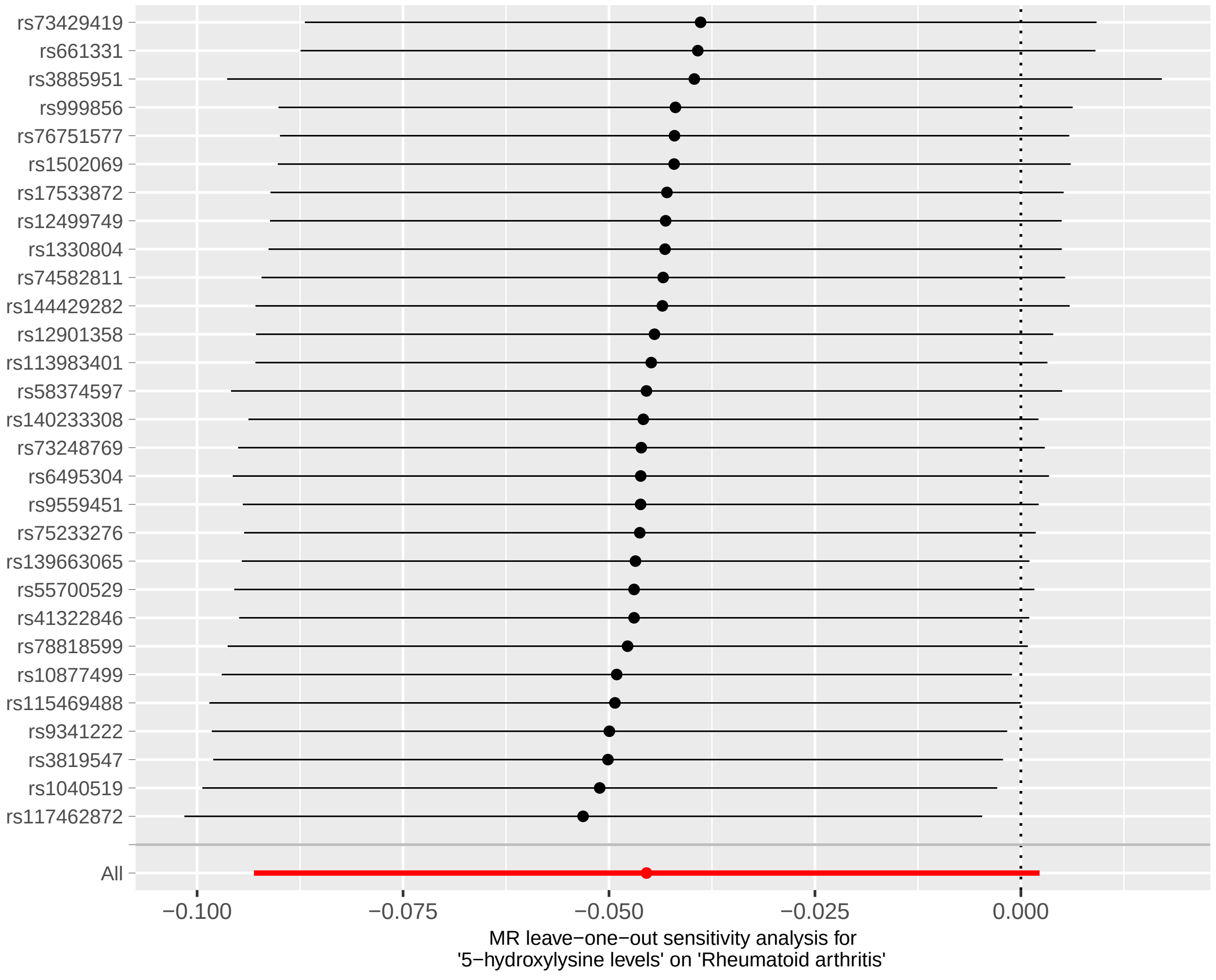
rs73429419
rs661331
rs3885951
rs999856
rs76751577
rs1502069
rs17533872
rs12499749
rs1330804
rs74582811
rs144429282
rs12901358
rs113983401
rs58374597
rs140233308
rs73248769
rs6495304
rs9559451
rs75233276
rs139663065
rs55700529
rs41322846
rs78818599
rs10877499
rs115469488
rs9341222
rs3819547
rs1040519
rs117462872
All
−0.100
−0.075
−0.050
−0.025
0.000
MR leave−one−out sensitivity analysis for
'5−hydroxylysine levels' on 'Rheumatoid arthritis'

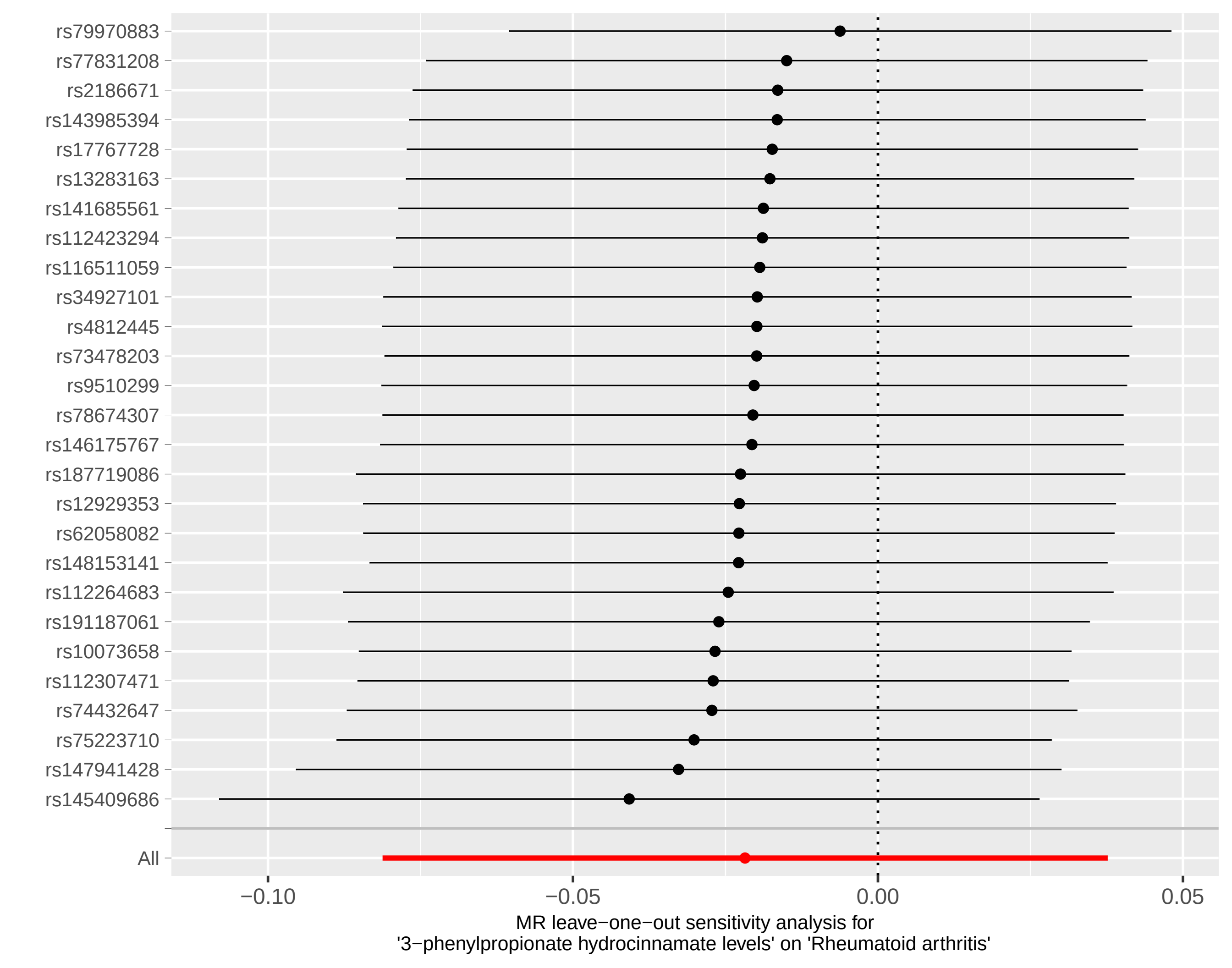
rs79970883
rs77831208
rs2186671
rs143985394
rs17767728
rs13283163
rs141685561
rs112423294
rs116511059
rs34927101
rs4812445
rs73478203
rs9510299
rs78674307
rs146175767
rs187719086
rs12929353
rs62058082
rs148153141
rs112264683
rs191187061
rs10073658
rs112307471
rs74432647
rs75223710
rs147941428
rs145409686
All
−0.10
−0.05
0.00
0.05
MR leave−one−out sensitivity analysis for
'3−phenylpropionate hydrocinnamate levels' on 'Rheumatoid arthritis'

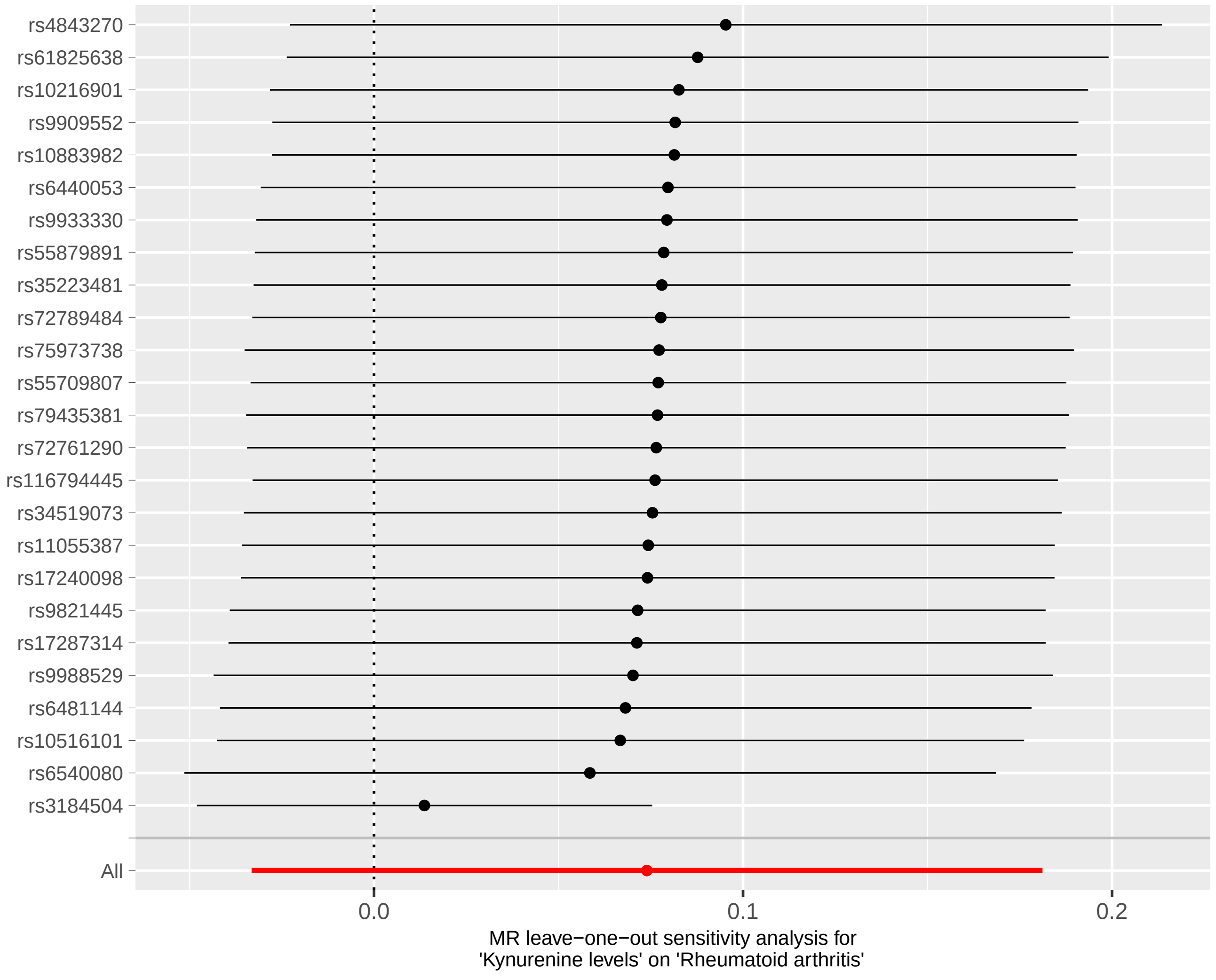

rs4843270
rs61825638
rs10216901
rs9909552
rs10883982
rs6440053
rs9933330
rs55879891
rs35223481
rs72789484
rs75973738
rs55709807
rs79435381
rs72761290
rs116794445
rs34519073
rs11055387
rs17240098
rs9821445
rs17287314
rs9988529
rs6481144
rs10516101
rs6540080
rs3184504
All
0.0
0.1
0.2
MR leave−one−out sensitivity analysis for
'Kynurenine levels' on 'Rheumatoid arthritis'

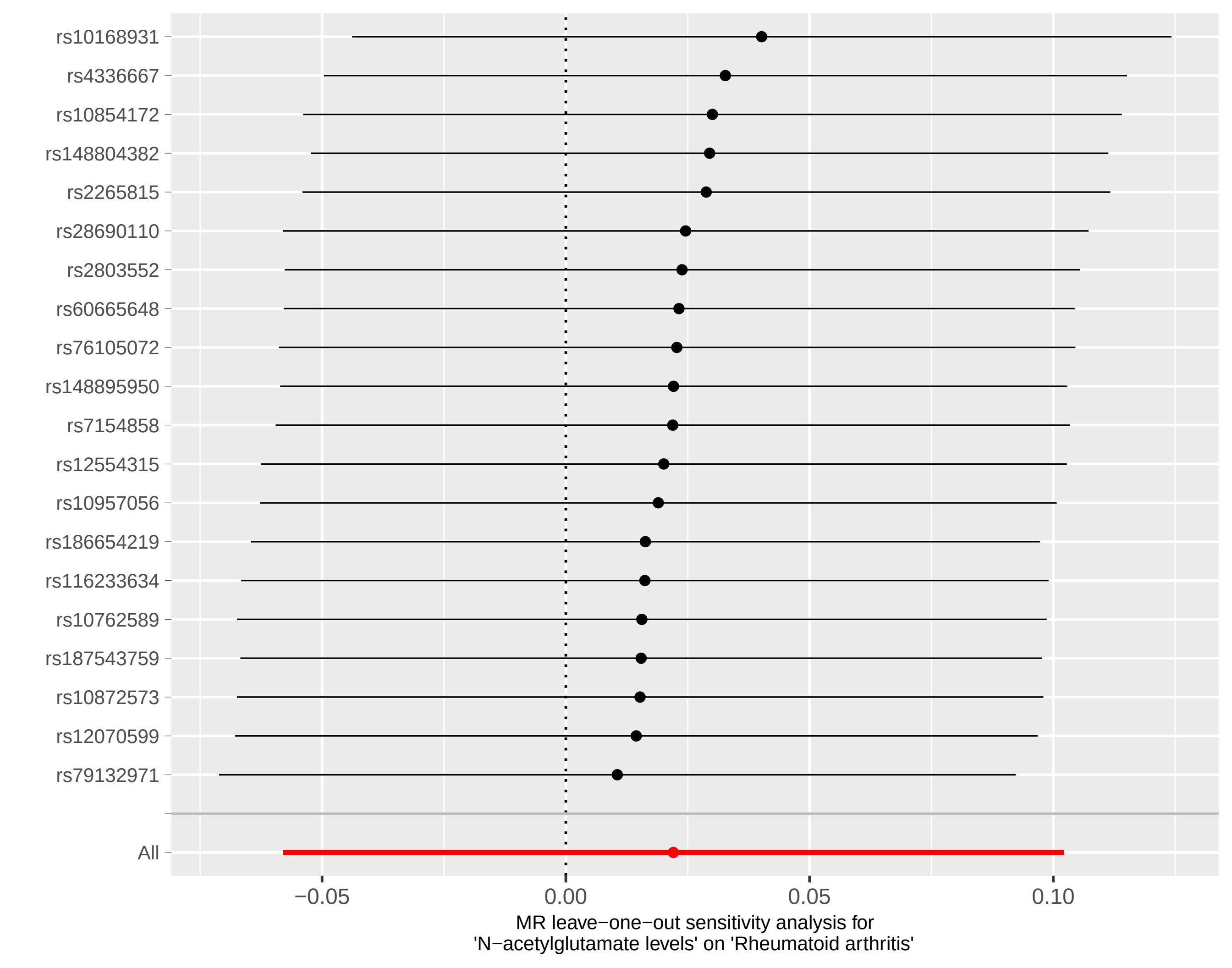
rs10168931
rs4336667
rs10854172
rs148804382
rs2265815
rs28690110
rs2803552
rs60665648
rs76105072
rs148895950
rs7154858
rs12554315
rs10957056
rs186654219
rs116233634
rs10762589
rs187543759
rs10872573
rs12070599
rs79132971
All
−0.05
0.00
0.05
0.10
MR leave−one−out sensitivity analysis for
'N−acetylglutamate levels' on 'Rheumatoid arthritis'

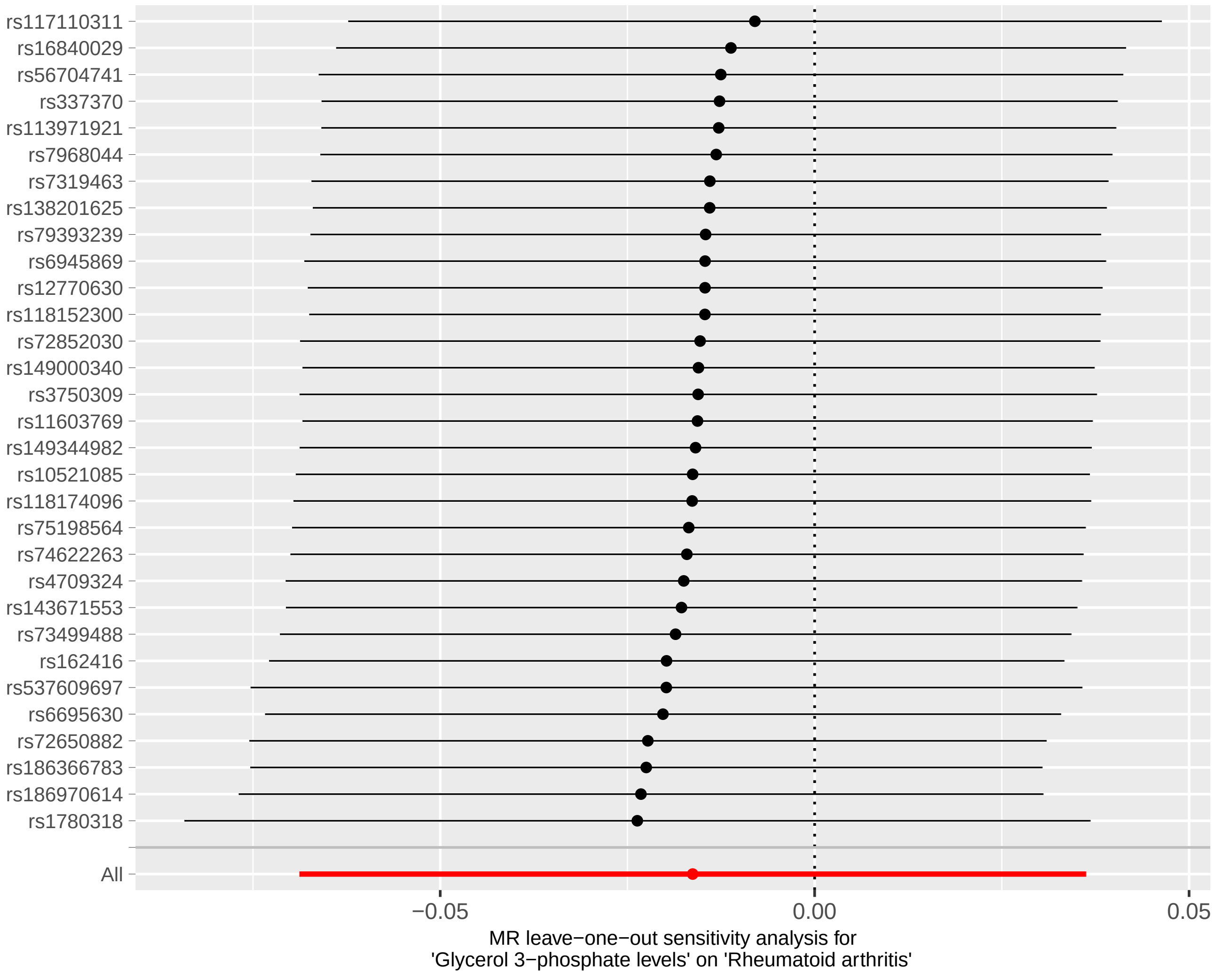
rs117110311
rs16840029
rs56704741
rs337370
rs113971921
rs7968044
rs7319463
rs138201625
rs79393239
rs6945869
rs12770630
rs118152300
rs72852030
rs149000340
rs3750309
rs11603769
rs149344982
rs10521085
rs118174096
rs75198564
rs74622263
rs4709324
rs143671553
rs73499488
rs162416
rs537609697
rs6695630
rs72650882
rs186366783
rs186970614
rs1780318
All
−0.05
0.00
0.05
MR leave−one−out sensitivity analysis for
'Glycerol 3−phosphate levels' on 'Rheumatoid arthritis'

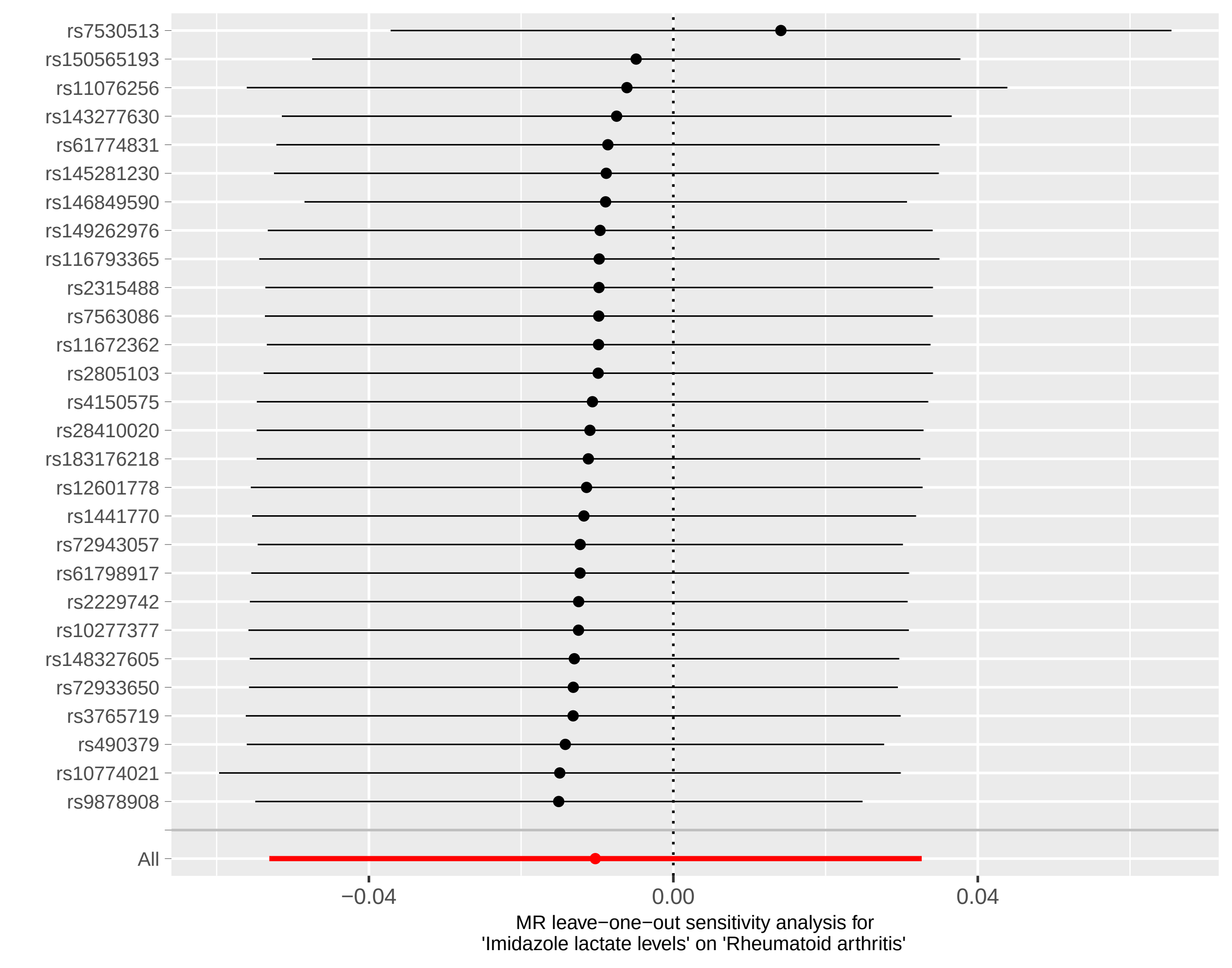
rs7530513
rs150565193
rs11076256
rs143277630
rs61774831
rs145281230
rs146849590
rs149262976
rs116793365
rs2315488
rs7563086
rs11672362
rs2805103
rs4150575
rs28410020
rs183176218
rs12601778
rs1441770
rs72943057
rs61798917
rs2229742
rs10277377
rs148327605
rs72933650
rs3765719
rs490379
rs10774021
rs9878908
All
−0.04
0.00
0.04
MR leave−one−out sensitivity analysis for
'Imidazole lactate levels' on 'Rheumatoid arthritis'

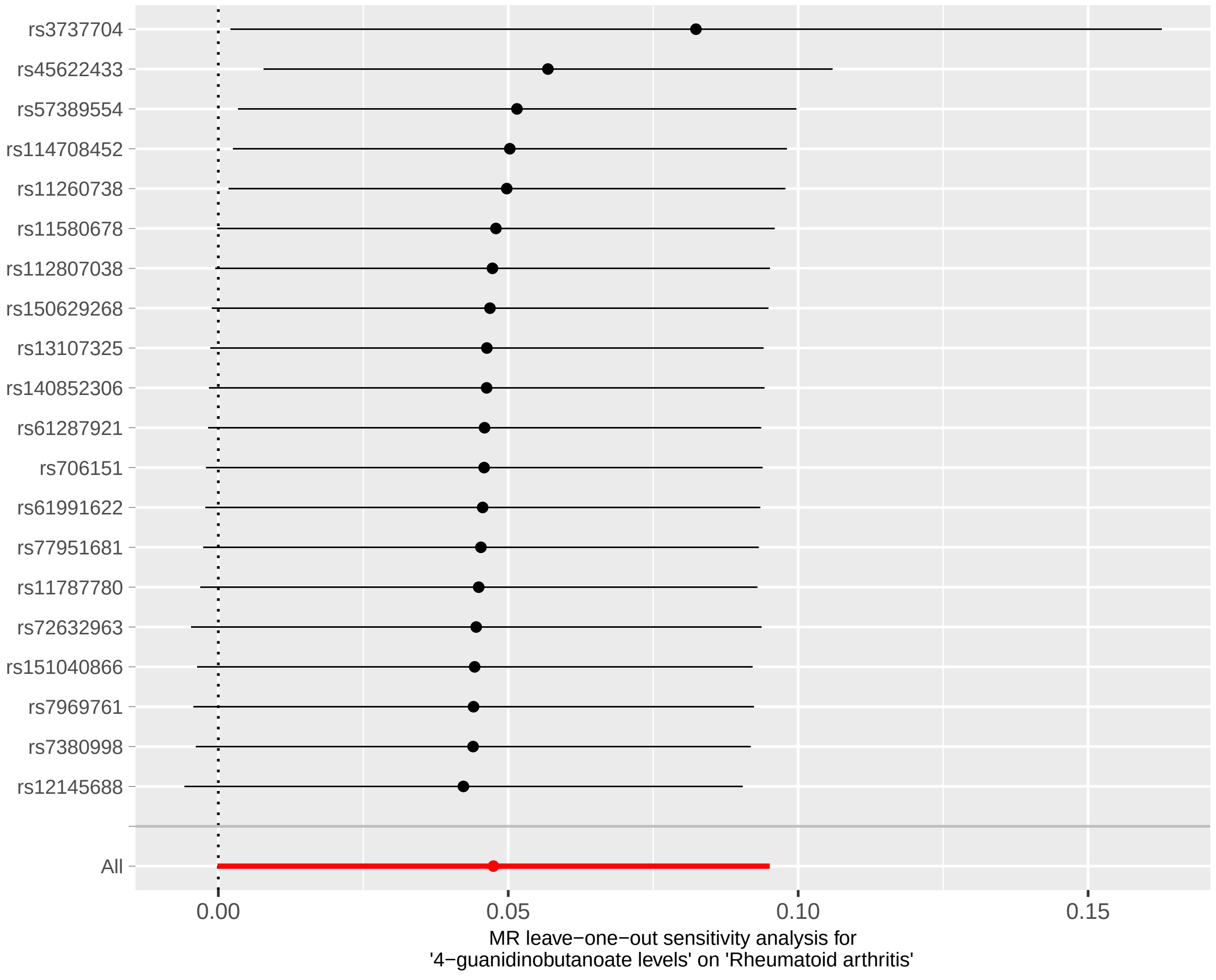
rs3737704
rs45622433
rs57389554
rs114708452
rs11260738
rs11580678
rs112807038
rs150629268
rs13107325
rs140852306
rs61287921
rs706151
rs61991622
rs77951681
rs11787780
rs72632963
rs151040866
rs7969761
rs7380998
rs12145688
All
0.00
0.05
0.10
0.15
MR leave−one−out sensitivity analysis for
'4−guanidinobutanoate levels' on 'Rheumatoid arthritis'

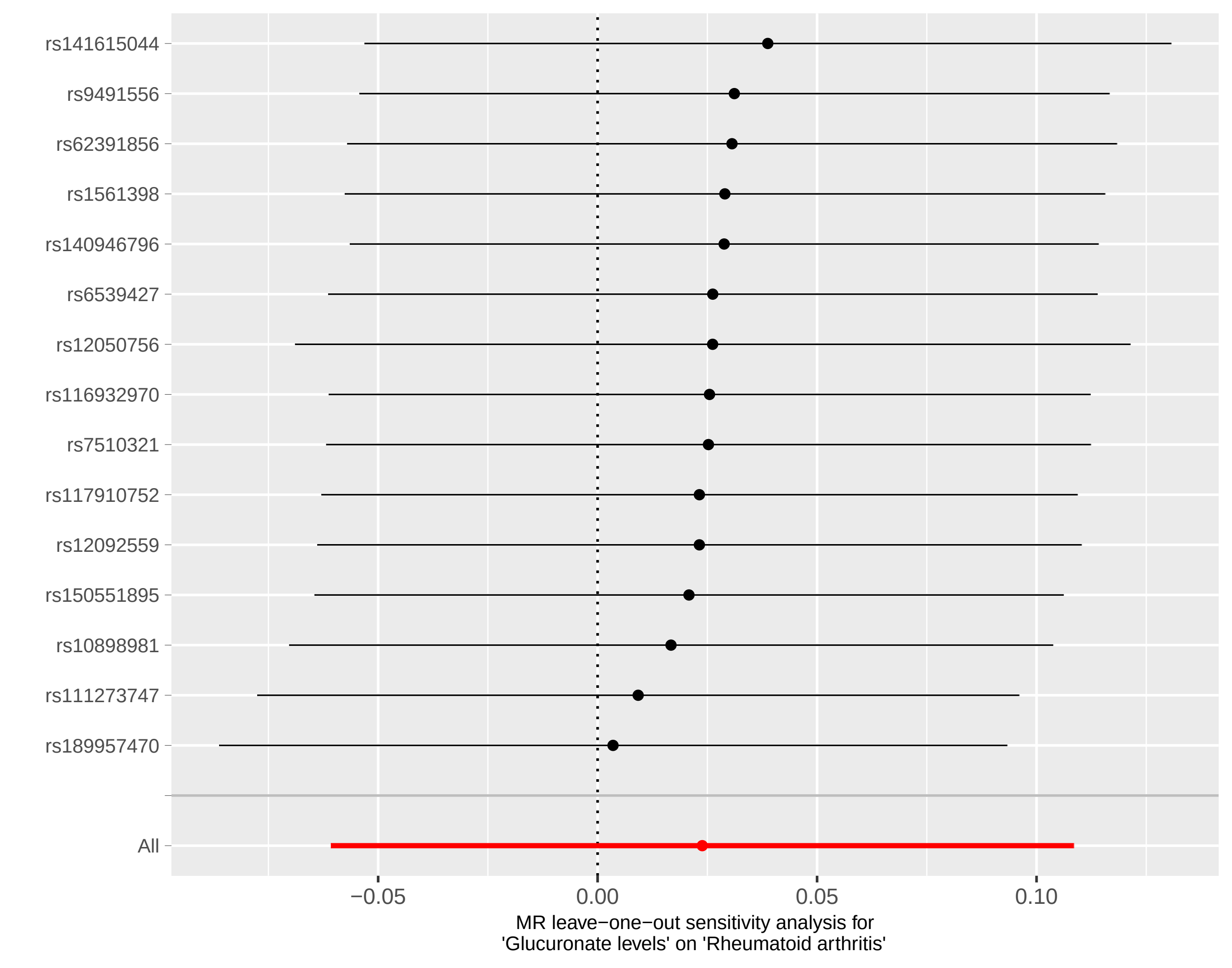
rs141615044
rs9491556
rs62391856
rs1561398
rs140946796
rs6539427
rs12050756
rs116932970
rs7510321
rs117910752
rs12092559
rs150551895
rs10898981
rs111273747
rs189957470
All
−0.05
0.00
0.05
0.10
MR leave−one−out sensitivity analysis for
'Glucuronate levels' on 'Rheumatoid arthritis'

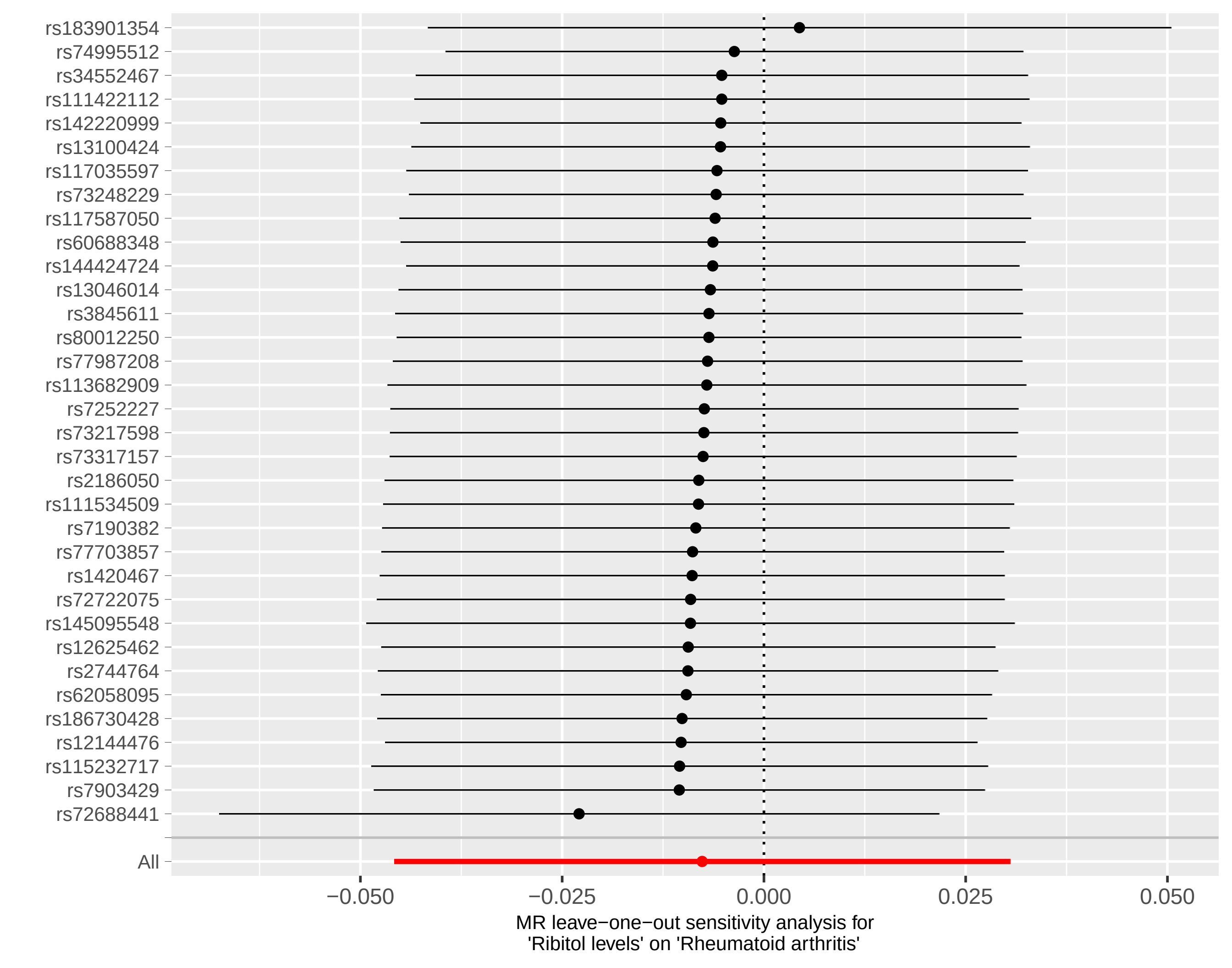
rs183901354
rs74995512
rs34552467
rs111422112
rs142220999
rs13100424
rs117035597
rs73248229
rs117587050
rs60688348
rs144424724
rs13046014
rs3845611
rs80012250
rs77987208
rs113682909
rs7252227
rs73217598
rs73317157
rs2186050
rs111534509
rs7190382
rs77703857
rs1420467
rs72722075
rs145095548
rs12625462
rs2744764
rs62058095
rs186730428
rs12144476
rs115232717
rs7903429
rs72688441
All
−0.050
−0.025
0.000
0.025
0.050
MR leave−one−out sensitivity analysis for
'Ribitol levels' on 'Rheumatoid arthritis'

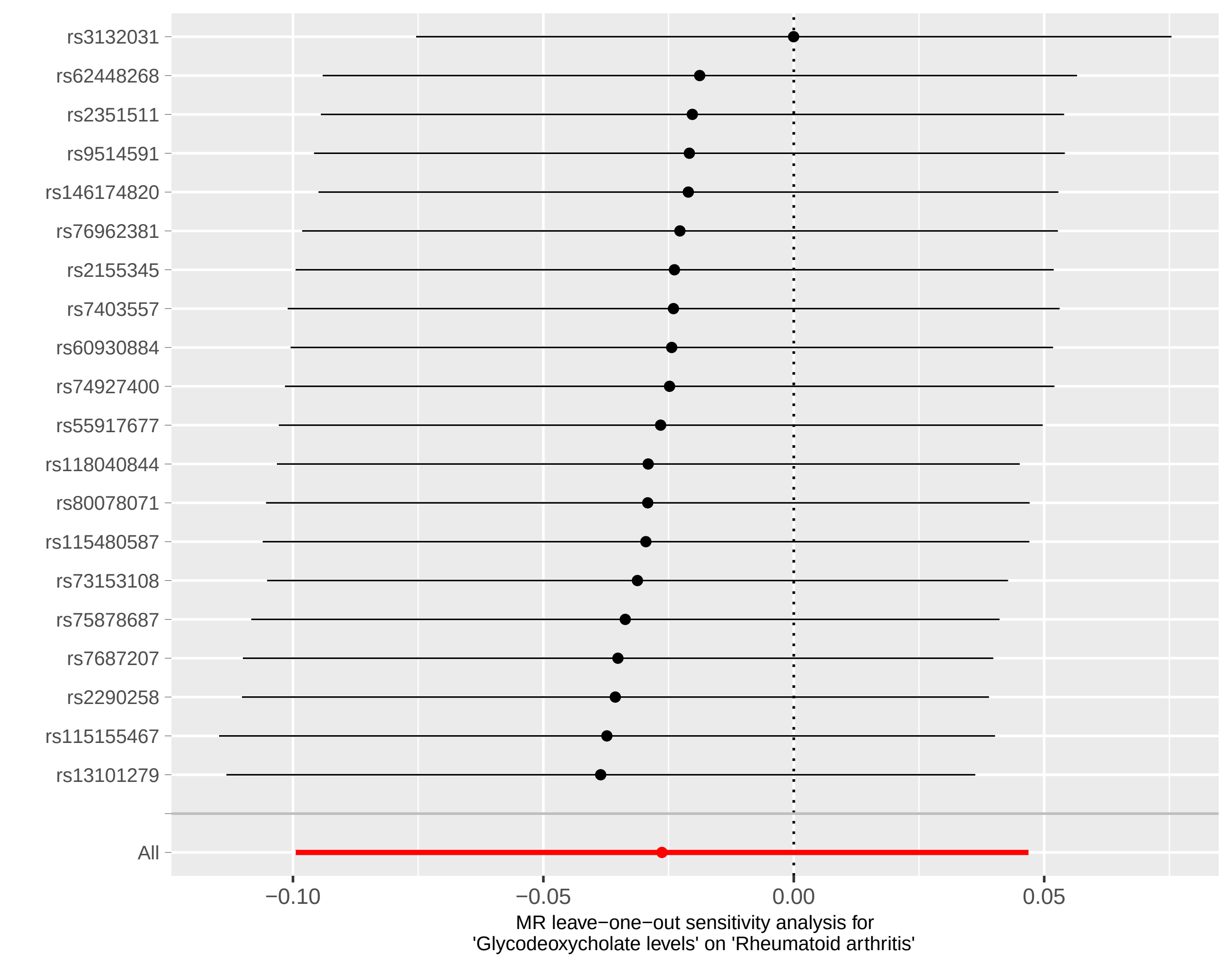
rs3132031
rs62448268
rs2351511
rs9514591
rs146174820
rs76962381
rs2155345
rs7403557
rs60930884
rs74927400
rs55917677
rs118040844
rs80078071
rs115480587
rs73153108
rs75878687
rs7687207
rs2290258
rs115155467
rs13101279
All
−0.10
−0.05
0.00
0.05
MR leave−one−out sensitivity analysis for
'Glycodeoxycholate levels' on 'Rheumatoid arthritis'

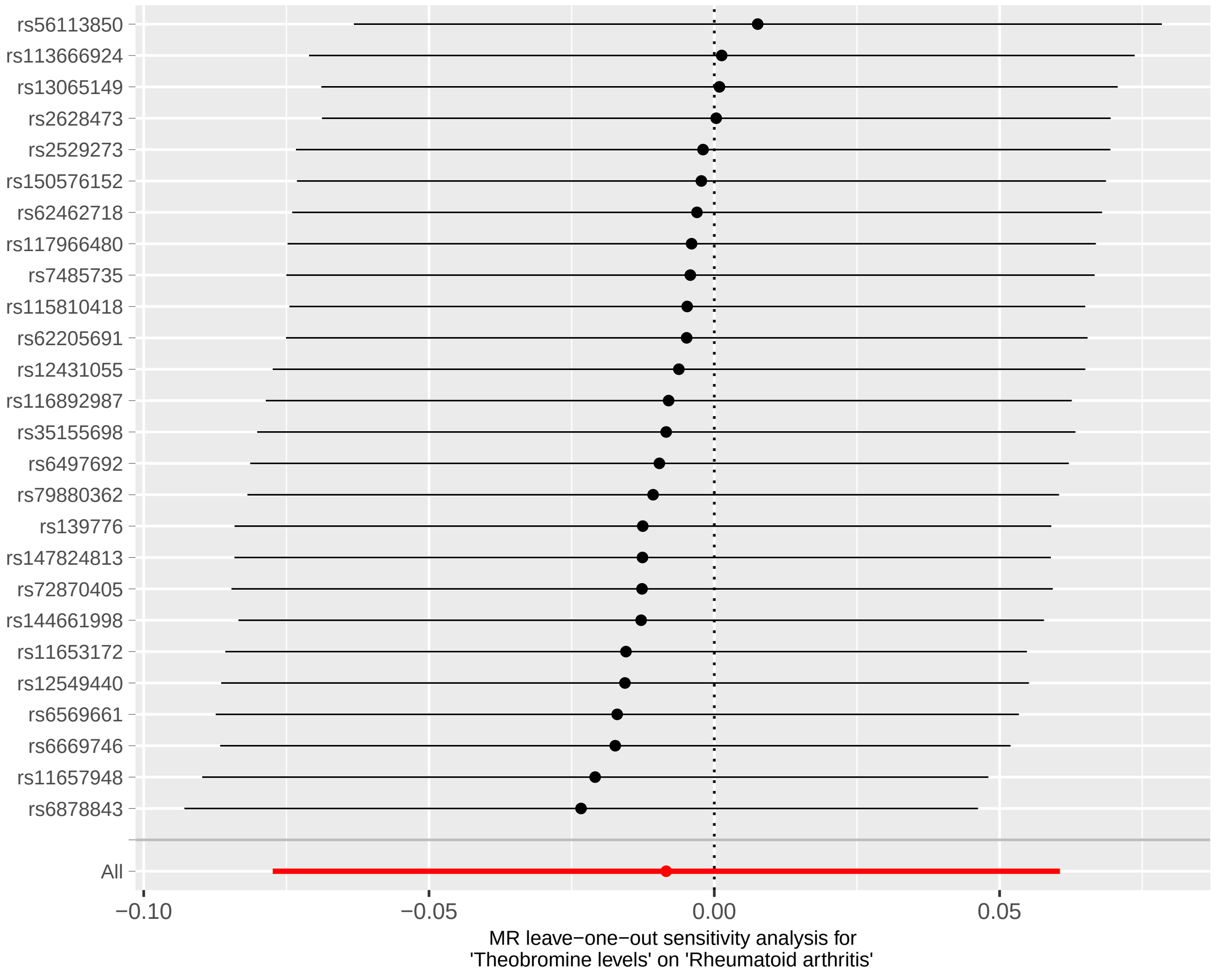

rs56113850
rs113666924
rs13065149
rs2628473
rs2529273
rs150576152
rs62462718
rs117966480
rs7485735
rs115810418
rs62205691
rs12431055
rs116892987
rs35155698
rs6497692
rs79880362
rs139776
rs147824813
rs72870405
rs144661998
rs11653172
rs12549440
rs6569661
rs6669746
rs11657948
rs6878843
All
−0.10
−0.05
0.00
0.05
MR leave−one−out sensitivity analysis for
'Theobromine levels' on 'Rheumatoid arthritis'

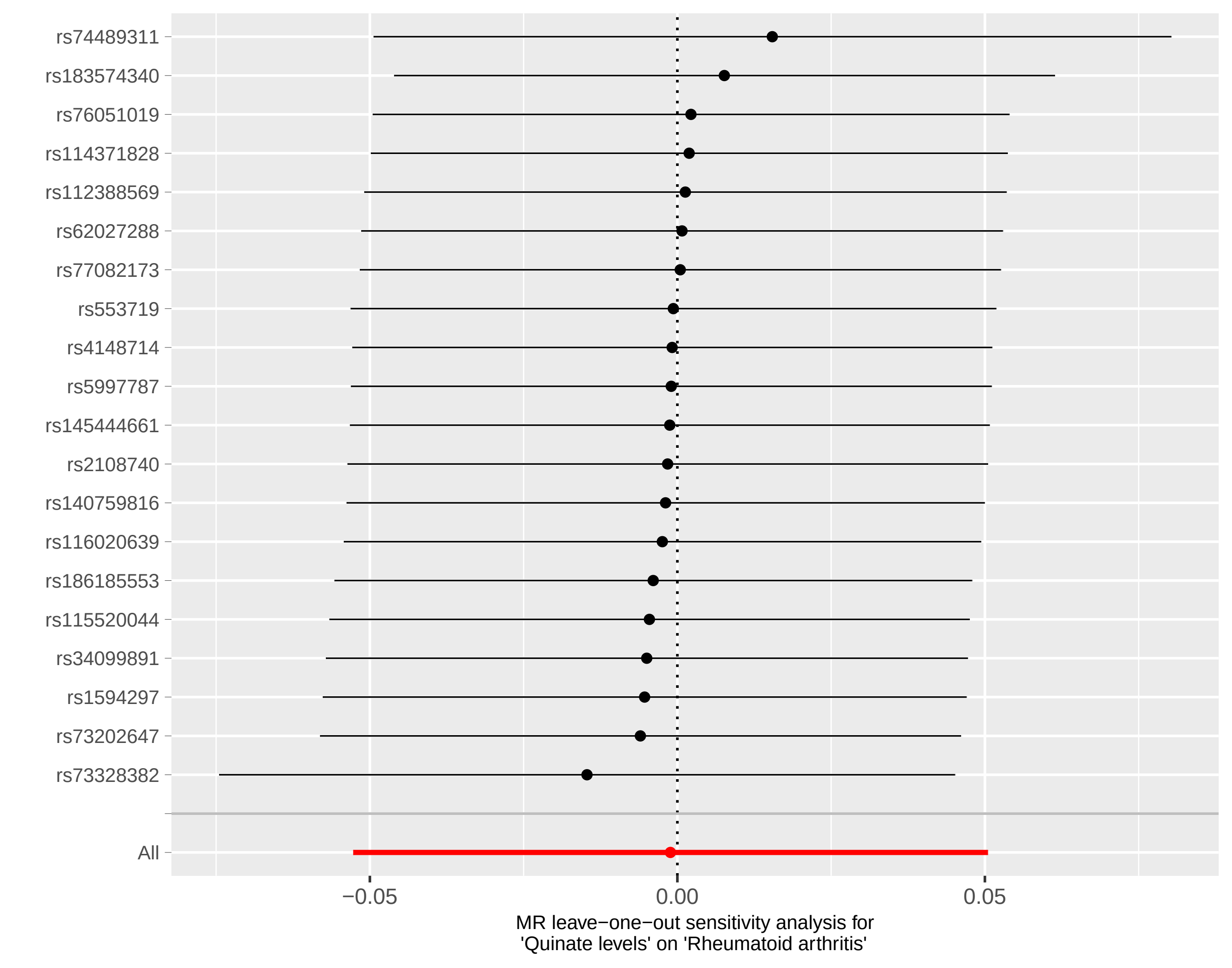
rs74489311
rs183574340
rs76051019
rs114371828
rs112388569
rs62027288
rs77082173
rs553719
rs4148714
rs5997787
rs145444661
rs2108740
rs140759816
rs116020639
rs186185553
rs115520044
rs34099891
rs1594297
rs73202647
rs73328382
All
−0.05
0.00
0.05
MR leave−one−out sensitivity analysis for
'Quinate levels' on 'Rheumatoid arthritis'

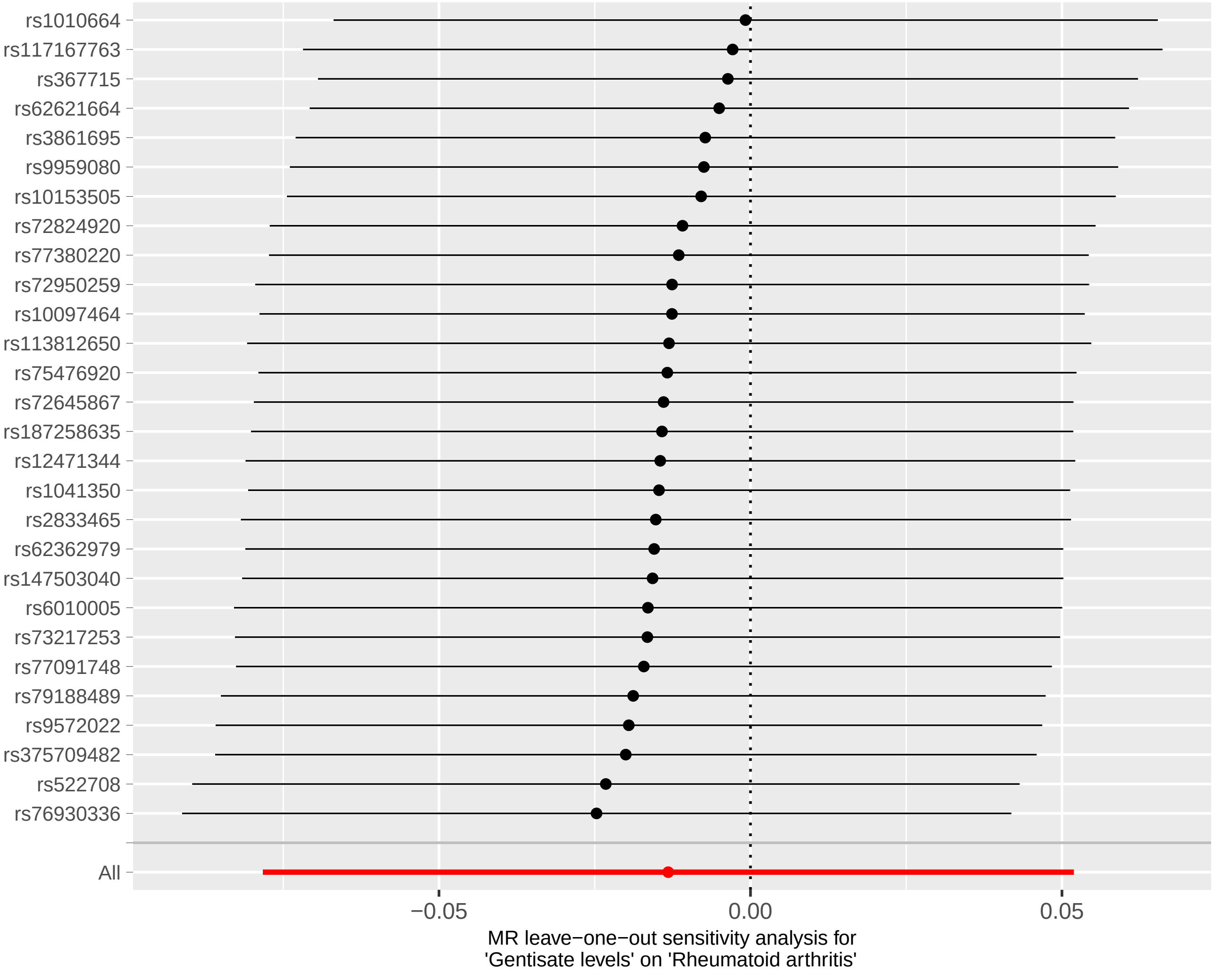
rs1010664
rs117167763
rs367715
rs62621664
rs3861695
rs9959080
rs10153505
rs72824920
rs77380220
rs72950259
rs10097464
rs113812650
rs75476920
rs72645867
rs187258635
rs12471344
rs1041350
rs2833465
rs62362979
rs147503040
rs6010005
rs73217253
rs77091748
rs79188489
rs9572022
rs375709482
rs522708
rs76930336
All
−0.05
0.00
0.05
MR leave−one−out sensitivity analysis for
'Gentisate levels' on 'Rheumatoid arthritis'

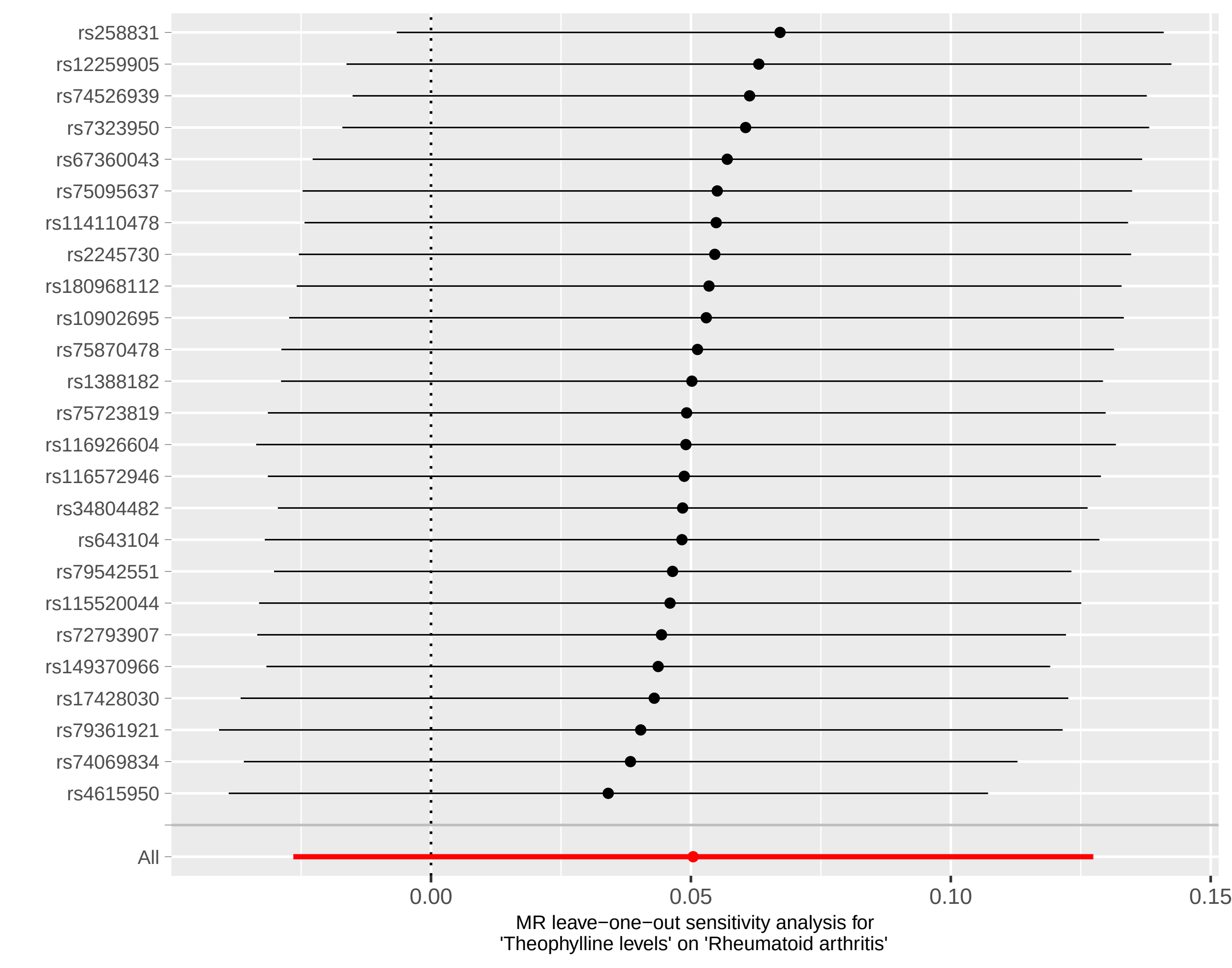

rs258831
rs12259905
rs74526939
rs7323950
rs67360043
rs75095637
rs114110478
rs2245730
rs180968112
rs10902695
rs75870478
rs1388182
rs75723819
rs116926604
rs116572946
rs34804482
rs643104
rs79542551
rs115520044
rs72793907
rs149370966
rs17428030
rs79361921
rs74069834
rs4615950
All
0.00
0.05
0.10
0.15
MR leave−one−out sensitivity analysis for
'Theophylline levels' on 'Rheumatoid arthritis'

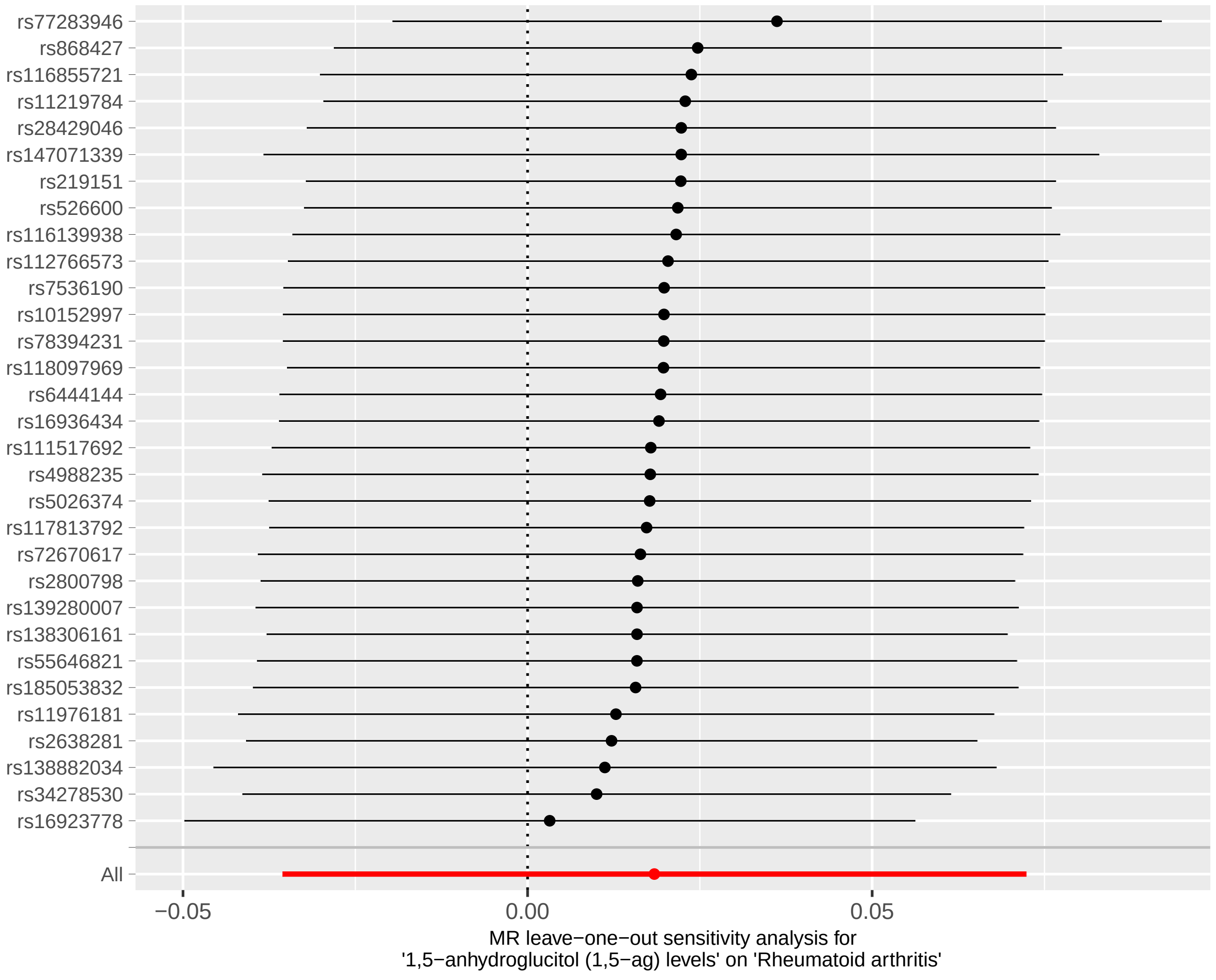
rs77283946
rs868427
rs116855721
rs11219784
rs28429046
rs147071339
rs219151
rs526600
rs116139938
rs112766573
rs7536190
rs10152997
rs78394231
rs118097969
rs6444144
rs16936434
rs111517692
rs4988235
rs5026374
rs117813792
rs72670617
rs2800798
rs139280007
rs138306161
rs55646821
rs185053832
rs11976181
rs2638281
rs138882034
rs34278530
rs16923778
All
−0.05
0.00
0.05
MR leave−one−out sensitivity analysis for
'1,5−anhydroglucitol (1,5−ag) levels' on 'Rheumatoid arthritis'

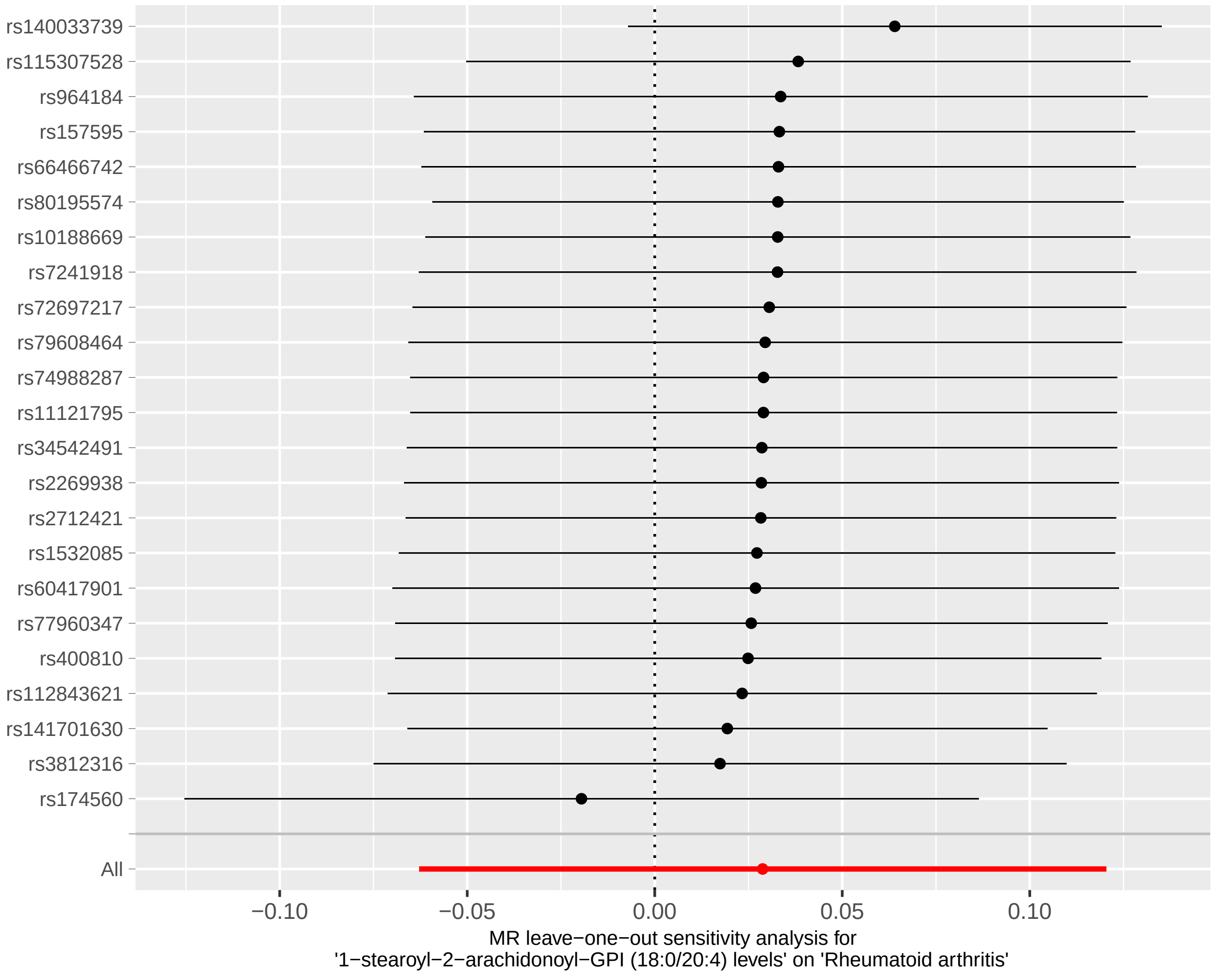

rs140033739
rs115307528
rs964184
rs157595
rs66466742
rs80195574
rs10188669
rs7241918
rs72697217
rs79608464
rs74988287
rs11121795
rs34542491
rs2269938
rs2712421
rs1532085
rs60417901
rs77960347
rs400810
rs112843621
rs141701630
rs3812316
rs174560
All
−0.10
−0.05
0.00
0.05
0.10
MR leave−one−out sensitivity analysis for
'1−stearoyl−2−arachidonoyl−GPI (18:0/20:4) levels' on 'Rheumatoid arthritis'

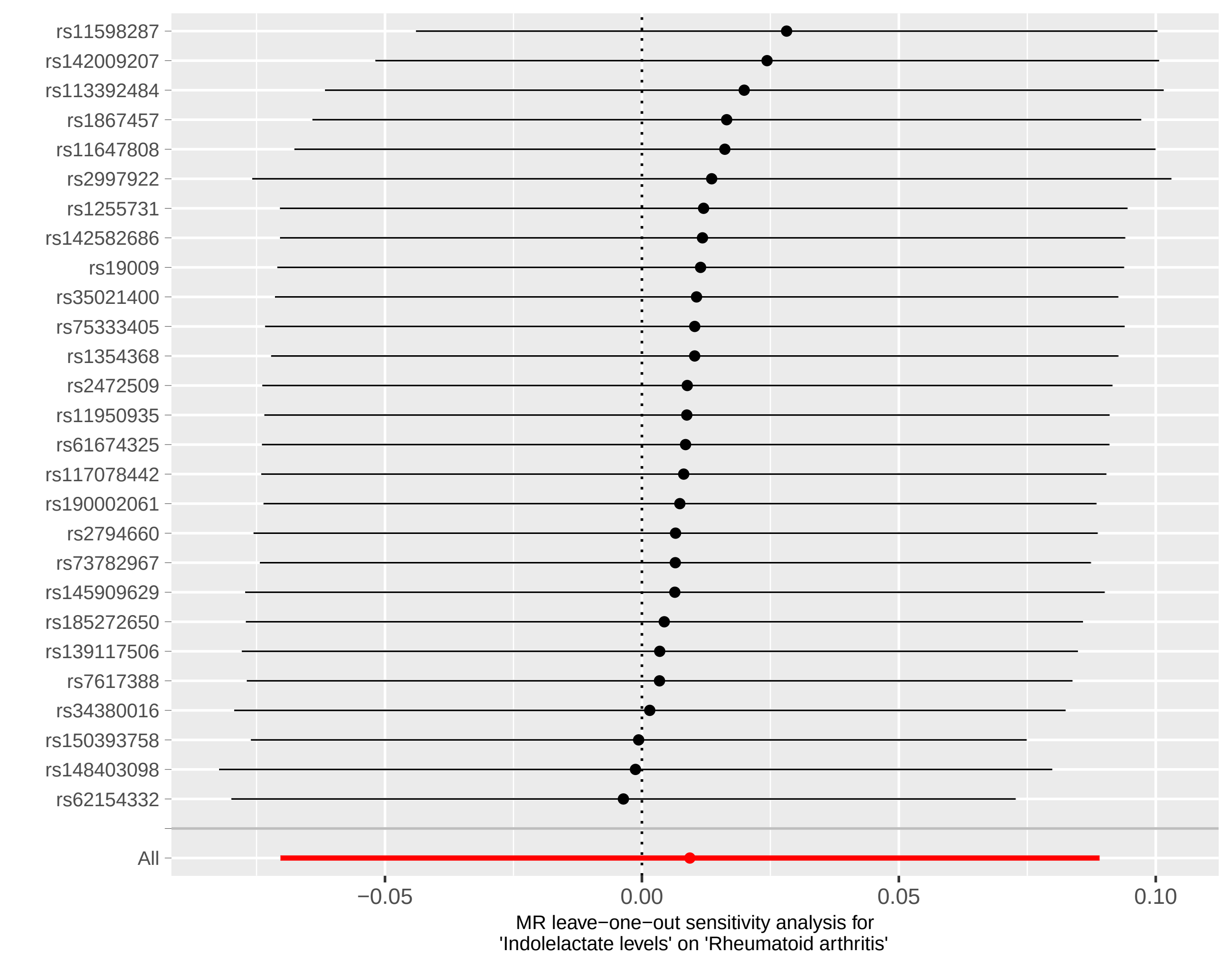
rs11598287
rs142009207
rs113392484
rs1867457
rs11647808
rs2997922
rs1255731
rs142582686
rs19009
rs35021400
rs75333405
rs1354368
rs2472509
rs11950935
rs61674325
rs117078442
rs190002061
rs2794660
rs73782967
rs145909629
rs185272650
rs139117506
rs7617388
rs34380016
rs150393758
rs148403098
rs62154332
All
−0.05
0.00
0.05
0.10
MR leave−one−out sensitivity analysis for
'Indolelactate levels' on 'Rheumatoid arthritis'

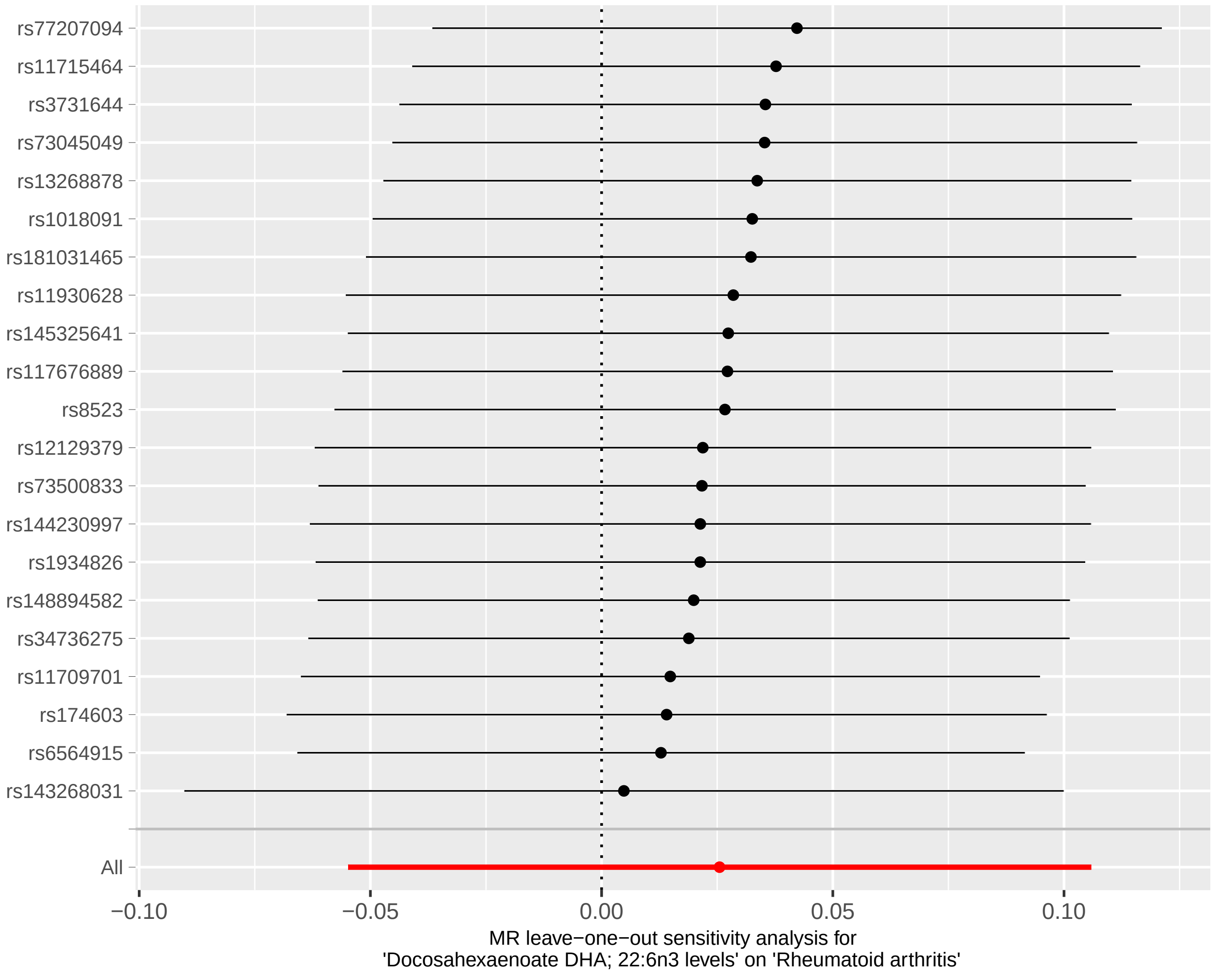
rs77207094
rs11715464
rs3731644
rs73045049
rs13268878
rs1018091
rs181031465
rs11930628
rs145325641
rs117676889
rs8523
rs12129379
rs73500833
rs144230997
rs1934826
rs148894582
rs34736275
rs11709701
rs174603
rs6564915
rs143268031
All
−0.10
−0.05
0.00
0.05
0.10
MR leave−one−out sensitivity analysis for
'Docosahexaenoate DHA; 22:6n3 levels' on 'Rheumatoid arthritis'

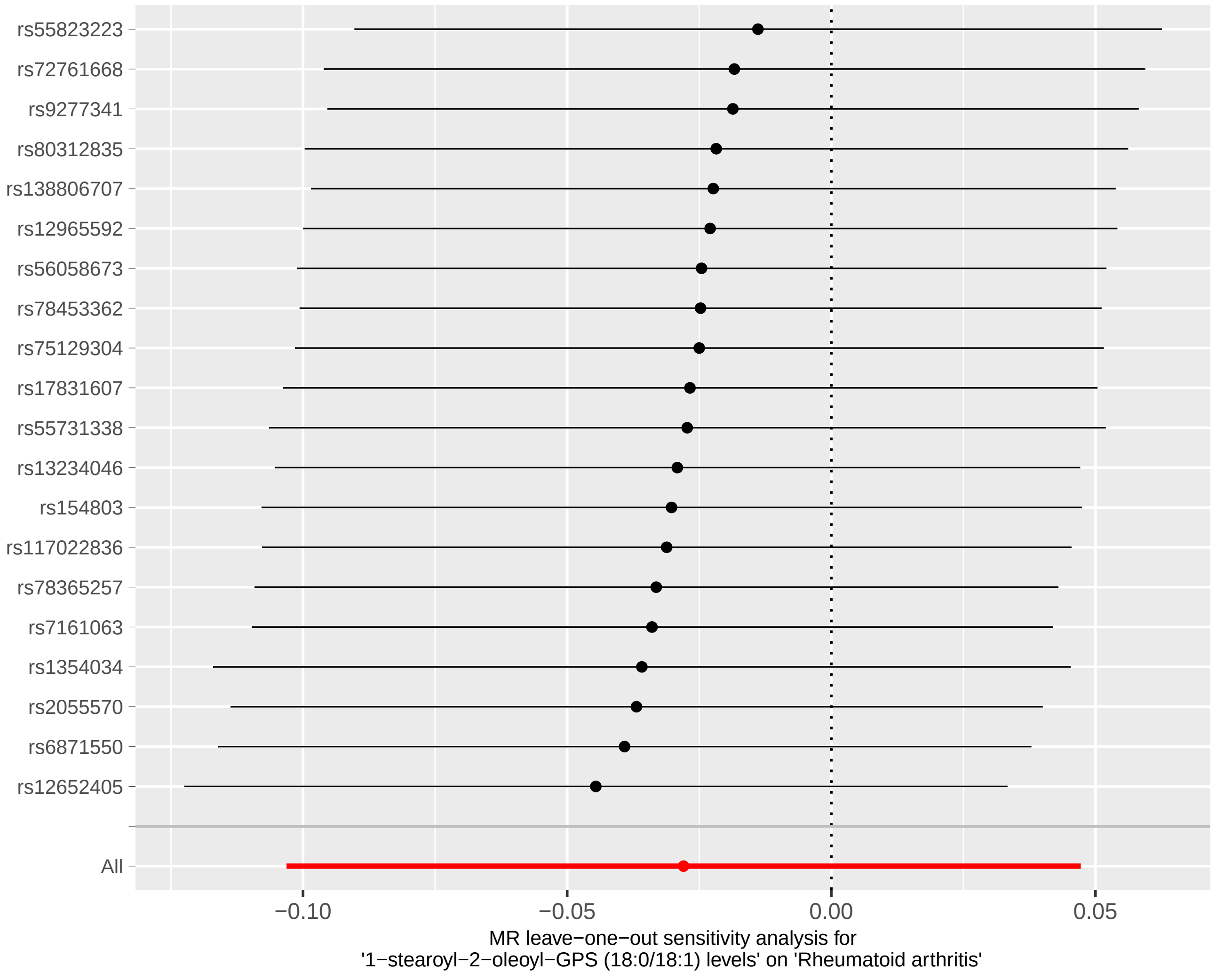
rs55823223
rs72761668
rs9277341
rs80312835
rs138806707
rs12965592
rs56058673
rs78453362
rs75129304
rs17831607
rs55731338
rs13234046
rs154803
rs117022836
rs78365257
rs7161063
rs1354034
rs2055570
rs6871550
rs12652405
All
−0.10
−0.05
0.00
0.05
MR leave−one−out sensitivity analysis for
'1−stearoyl−2−oleoyl−GPS (18:0/18:1) levels' on 'Rheumatoid arthritis'

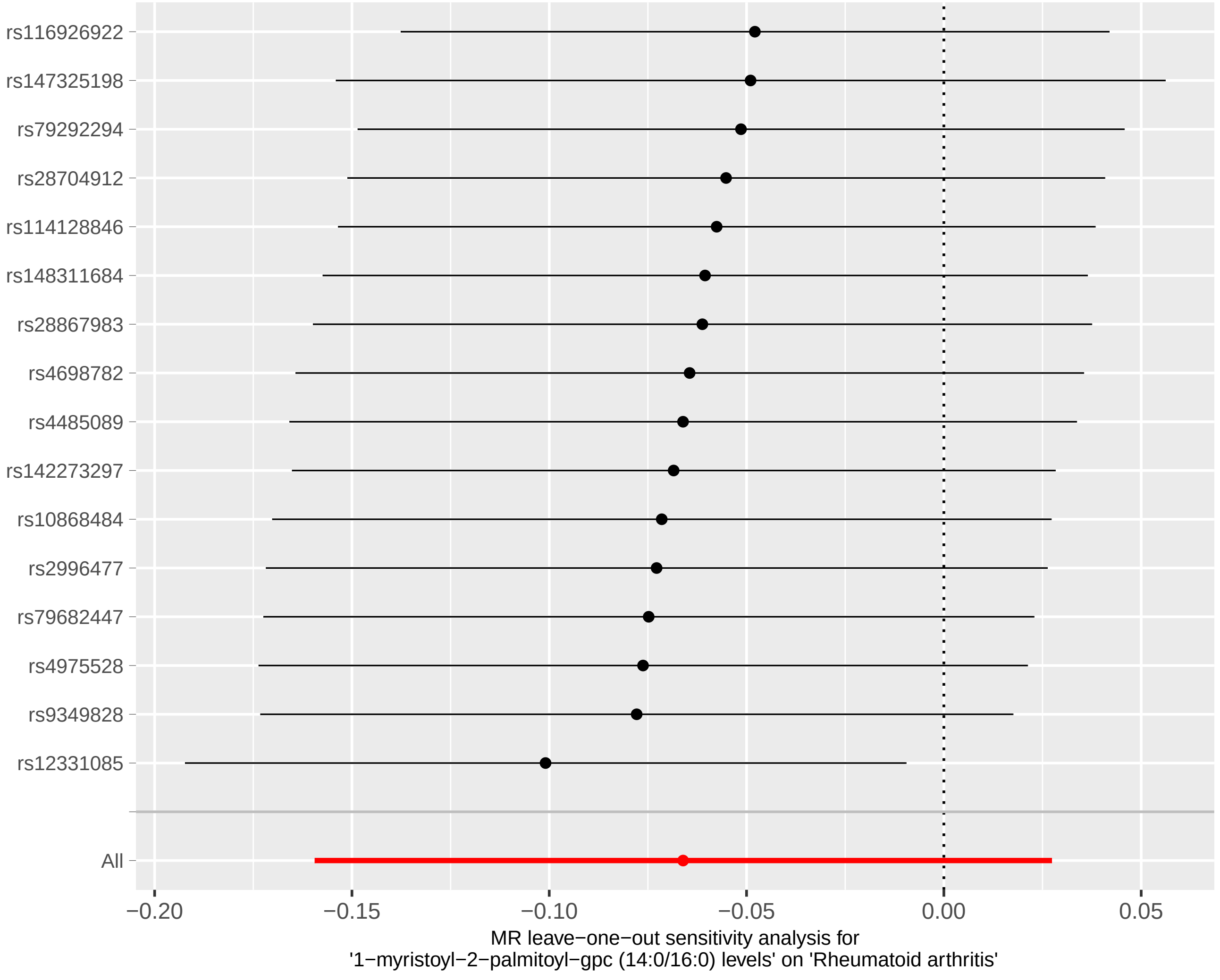
rs116926922
rs147325198
rs79292294
rs28704912
rs114128846
rs148311684
rs28867983
rs4698782
rs4485089
rs142273297
rs10868484
rs2996477
rs79682447
rs4975528
rs9349828
rs12331085
All
−0.20
−0.15
−0.10
−0.05
0.00
0.05
MR leave−one−out sensitivity analysis for
'1−myristoyl−2−palmitoyl−gpc (14:0/16:0) levels' on 'Rheumatoid arthritis'

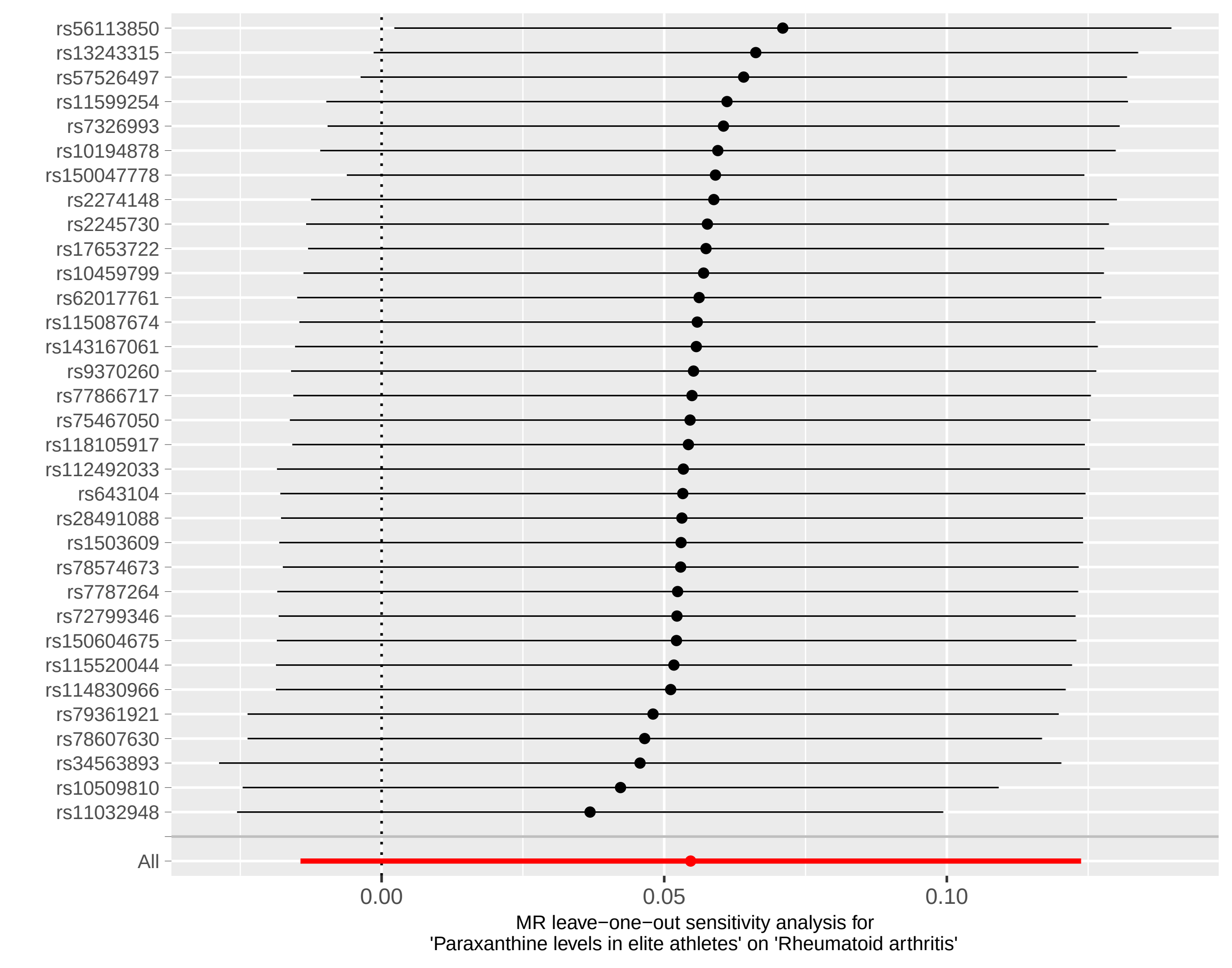
rs56113850
rs13243315
rs57526497
rs11599254
rs7326993
rs10194878
rs150047778
rs2274148
rs2245730
rs17653722
rs10459799
rs62017761
rs115087674
rs143167061
rs9370260
rs77866717
rs75467050
rs118105917
rs112492033
rs643104
rs28491088
rs1503609
rs78574673
rs7787264
rs72799346
rs150604675
rs115520044
rs114830966
rs79361921
rs78607630
rs34563893
rs10509810
rs11032948
All
0.00
0.05
0.10
MR leave−one−out sensitivity analysis for
'Paraxanthine levels in elite athletes' on 'Rheumatoid arthritis'

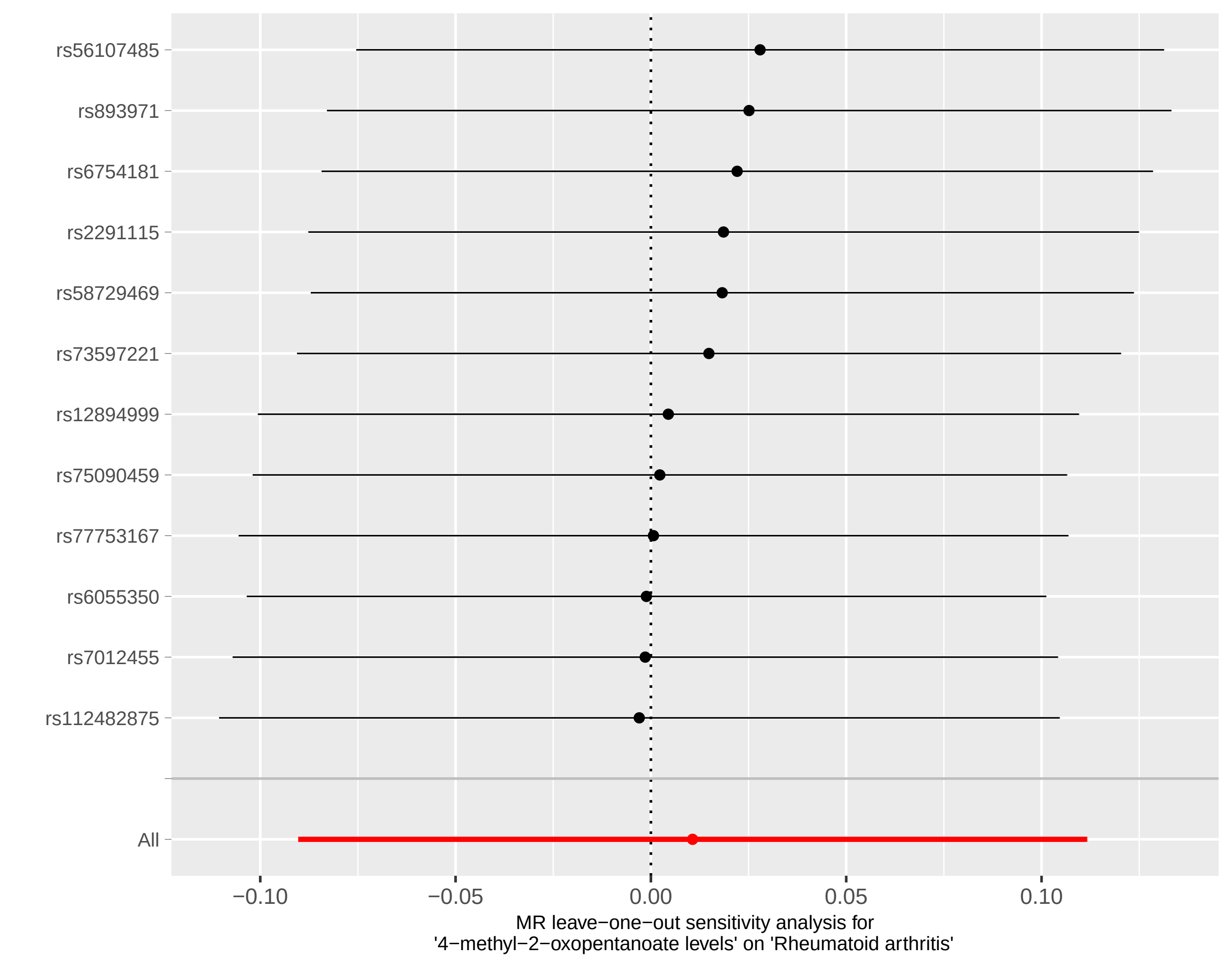
rs56107485
rs893971
rs6754181
rs2291115
rs58729469
rs73597221
rs12894999
rs75090459
rs77753167
rs6055350
rs7012455
rs112482875
All
−0.10
−0.05
0.00
0.05
0.10
MR leave−one−out sensitivity analysis for
'4−methyl−2−oxopentanoate levels' on 'Rheumatoid arthritis'

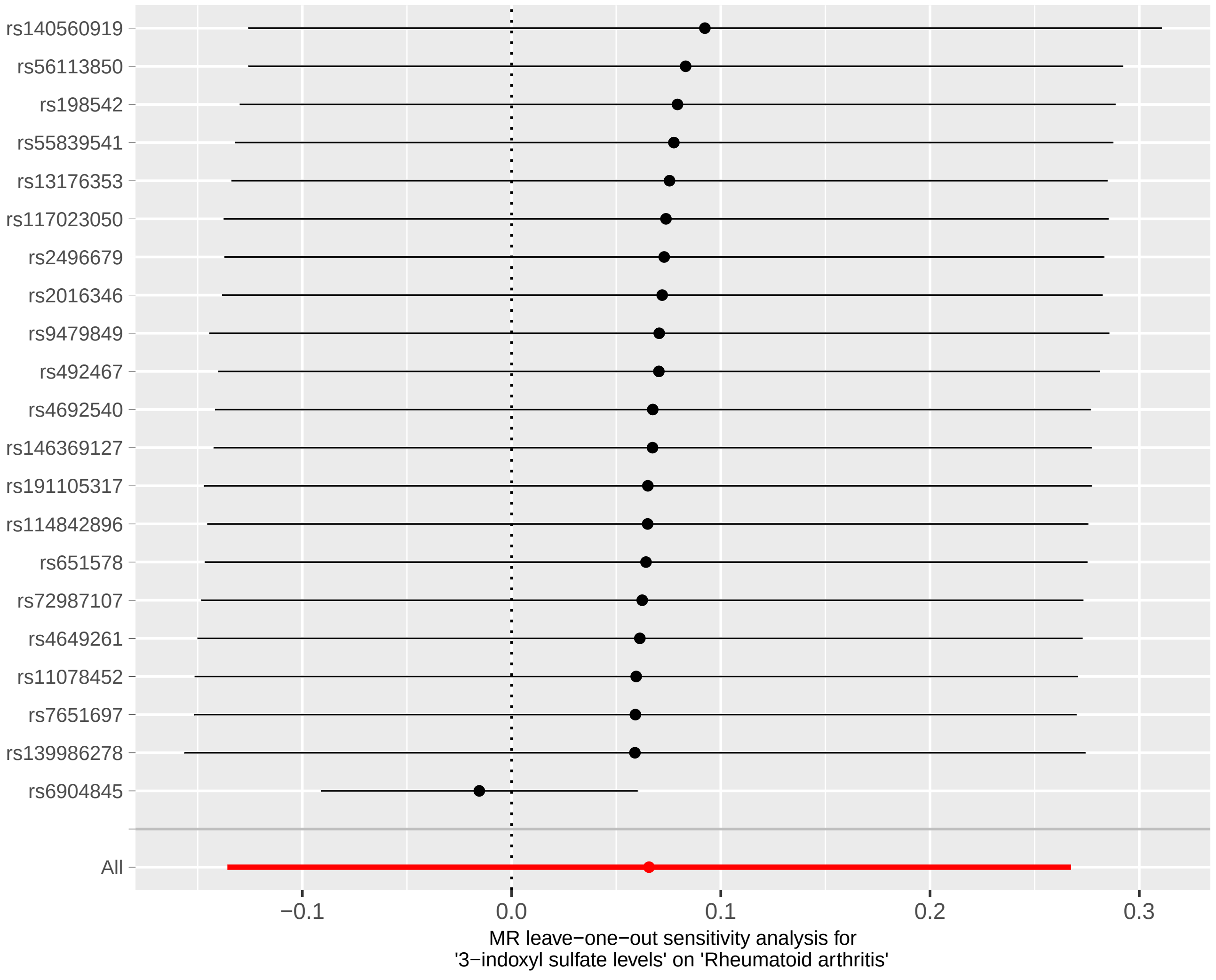

rs140560919
rs56113850
rs198542
rs55839541
rs13176353
rs117023050
rs2496679
rs2016346
rs9479849
rs492467
rs4692540
rs146369127
rs191105317
rs114842896
rs651578
rs72987107
rs4649261
rs11078452
rs7651697
rs139986278
rs6904845
All
−0.1
0.0
0.1
0.2
0.3
MR leave−one−out sensitivity analysis for
'3−indoxyl sulfate levels' on 'Rheumatoid arthritis'

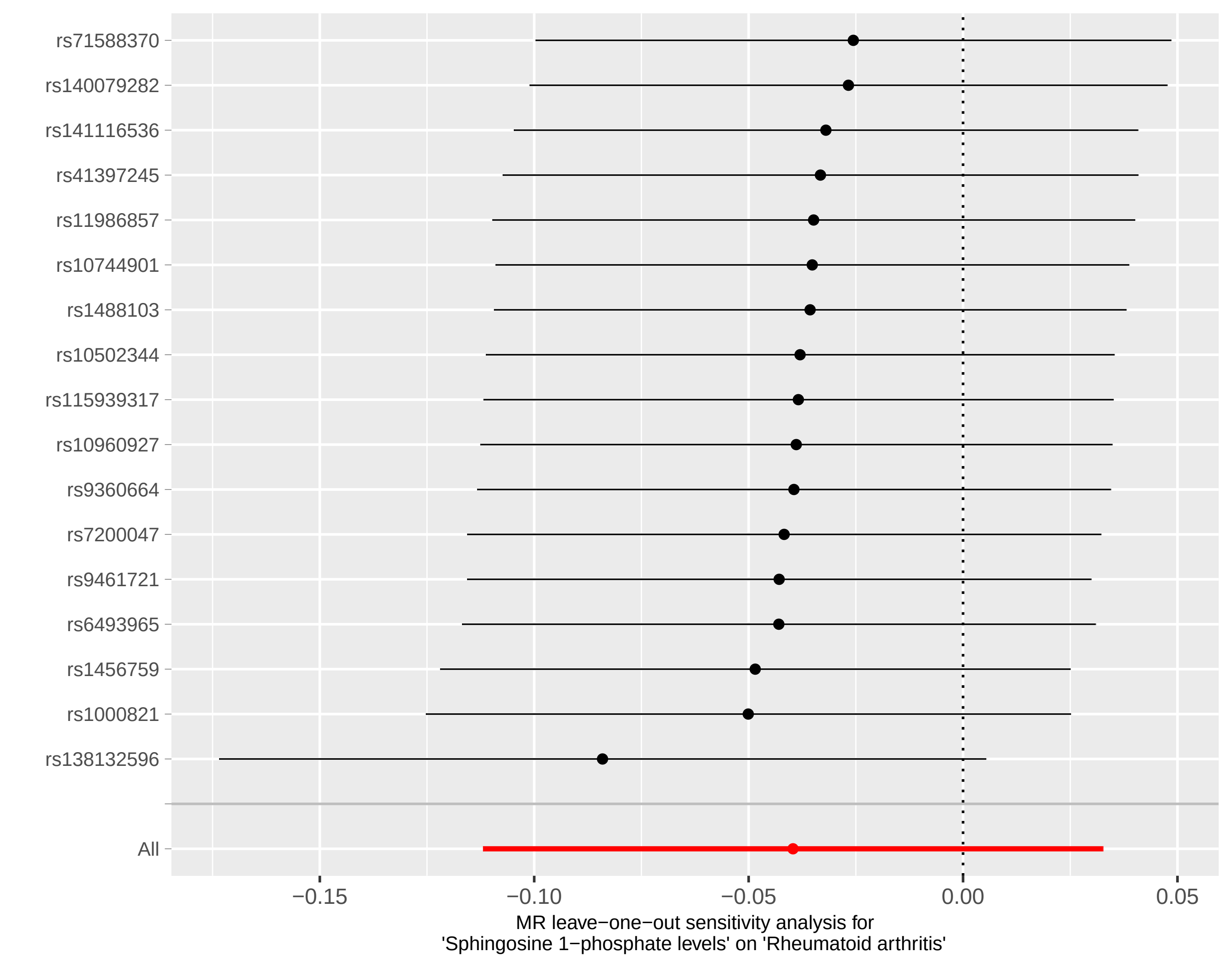
rs71588370
rs140079282
rs141116536
rs41397245
rs11986857
rs10744901
rs1488103
rs10502344
rs115939317
rs10960927
rs9360664
rs7200047
rs9461721
rs6493965
rs1456759
rs1000821
rs138132596
All
−0.15
−0.10
−0.05
0.00
0.05
MR leave−one−out sensitivity analysis for
'Sphingosine 1−phosphate levels' on 'Rheumatoid arthritis'

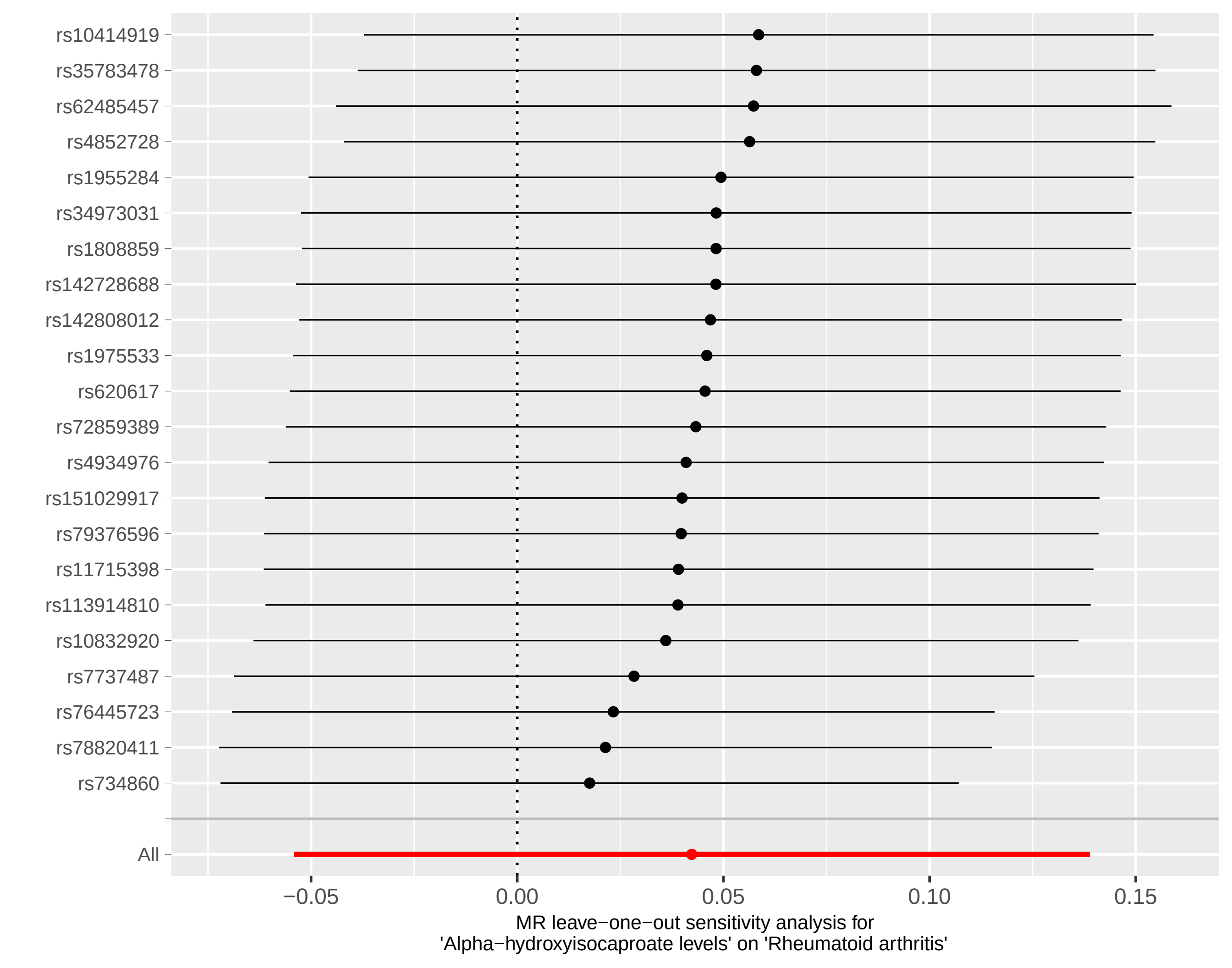
rs10414919
rs35783478
rs62485457
rs4852728
rs1955284
rs34973031
rs1808859
rs142728688
rs142808012
rs1975533
rs620617
rs72859389
rs4934976
rs151029917
rs79376596
rs11715398
rs113914810
rs10832920
rs7737487
rs76445723
rs78820411
rs734860
All
−0.05
0.00
0.05
0.10
0.15
MR leave−one−out sensitivity analysis for
'Alpha−hydroxyisocaproate levels' on 'Rheumatoid arthritis'

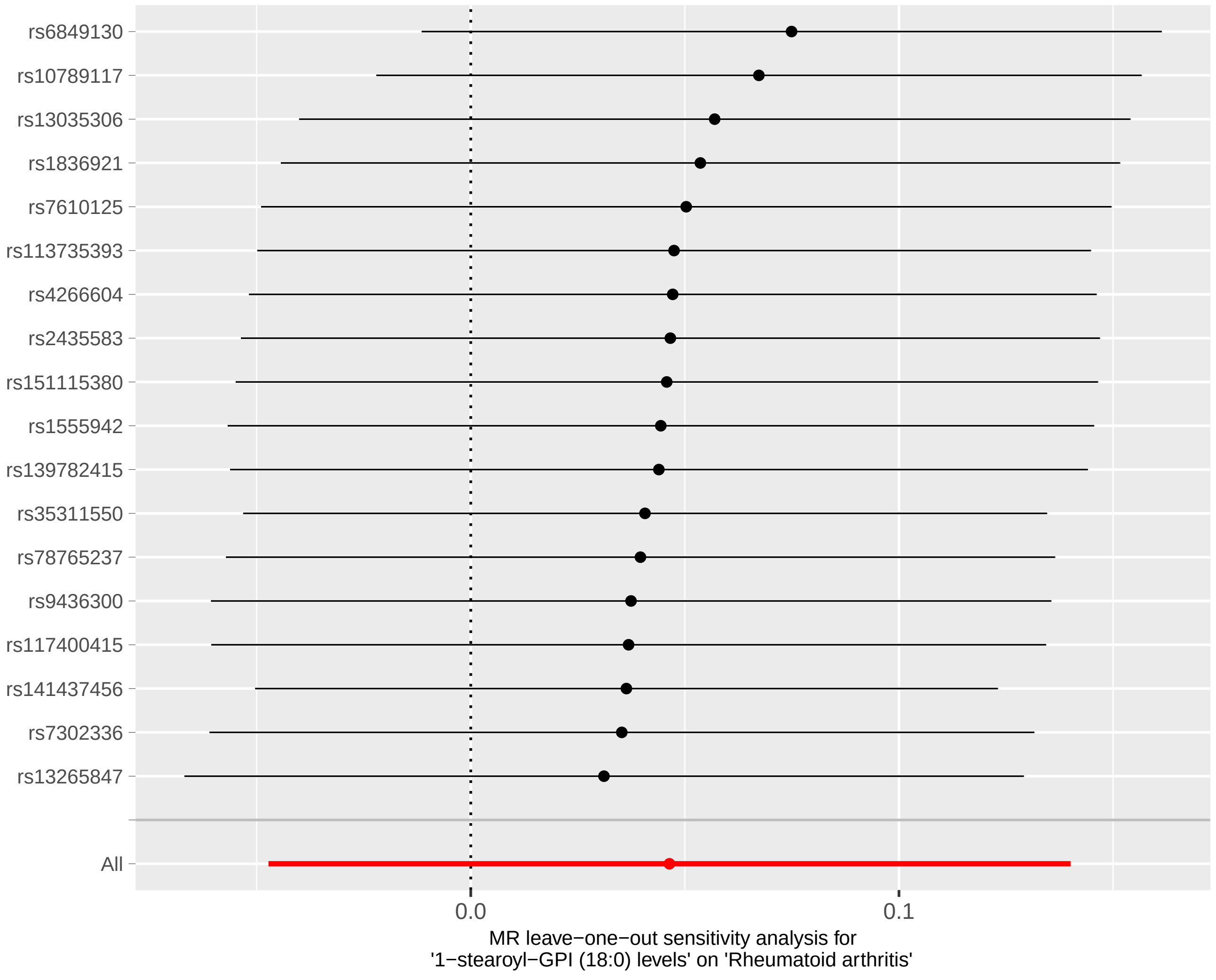

rs6849130
rs10789117
rs13035306
rs1836921
rs7610125
rs113735393
rs4266604
rs2435583
rs151115380
rs1555942
rs139782415
rs35311550
rs78765237
rs9436300
rs117400415
rs141437456
rs7302336
rs13265847
All
0.0
0.1
MR leave−one−out sensitivity analysis for
'1−stearoyl−GPI (18:0) levels' on 'Rheumatoid arthritis'

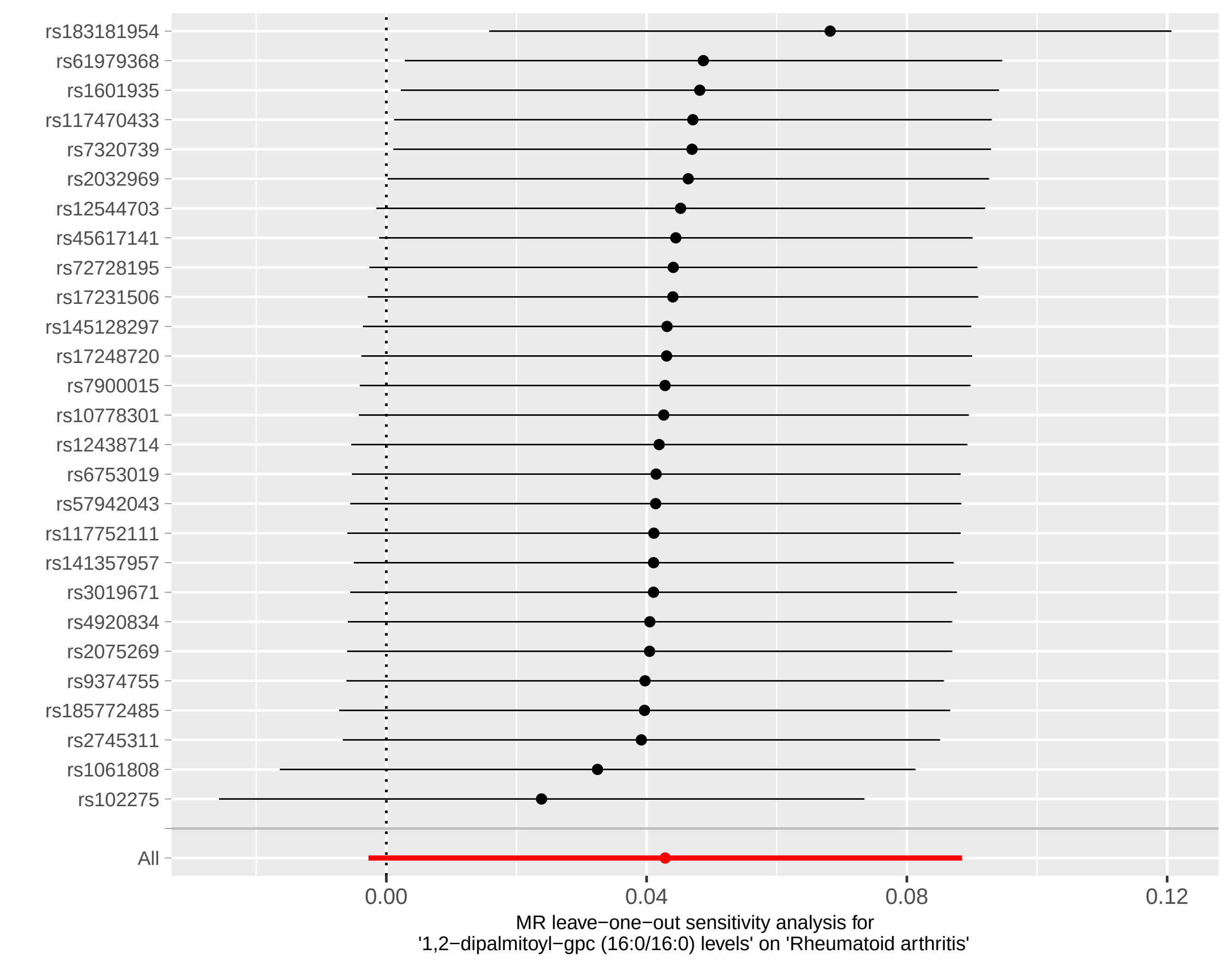
rs183181954
rs61979368
rs1601935
rs117470433
rs7320739
rs2032969
rs12544703
rs45617141
rs72728195
rs17231506
rs145128297
rs17248720
rs7900015
rs10778301
rs12438714
rs6753019
rs57942043
rs117752111
rs141357957
rs3019671
rs4920834
rs2075269
rs9374755
rs185772485
rs2745311
rs1061808
rs102275
All
0.00
0.04
0.08
0.12
MR leave−one−out sensitivity analysis for
'1,2−dipalmitoyl−gpc (16:0/16:0) levels' on 'Rheumatoid arthritis'

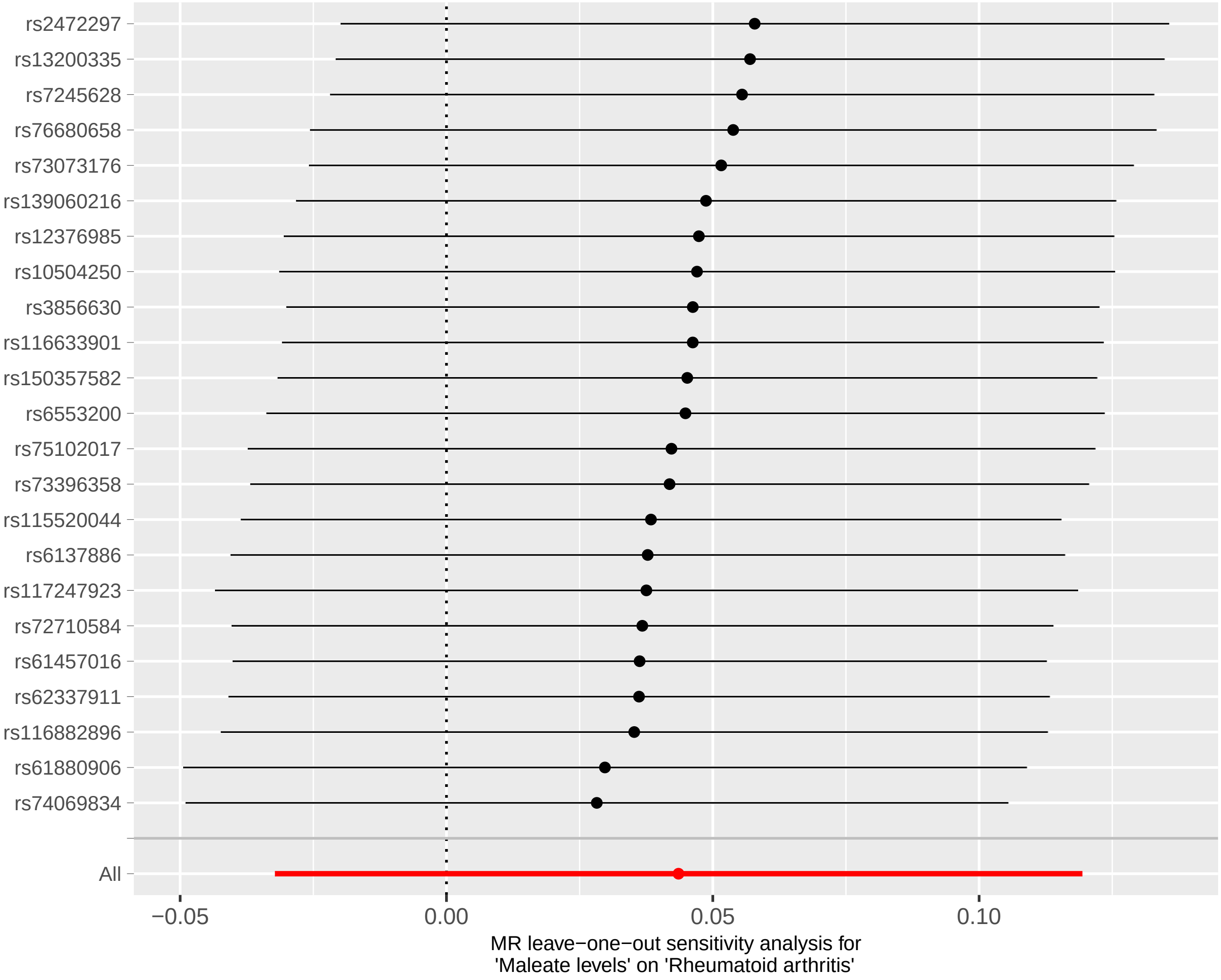
rs2472297
rs13200335
rs7245628
rs76680658
rs73073176
rs139060216
rs12376985
rs10504250
rs3856630
rs116633901
rs150357582
rs6553200
rs75102017
rs73396358
rs115520044
rs6137886
rs117247923
rs72710584
rs61457016
rs62337911
rs116882896
rs61880906
rs74069834
All
−0.05
0.00
0.05
0.10
MR leave−one−out sensitivity analysis for
'Maleate levels' on 'Rheumatoid arthritis'

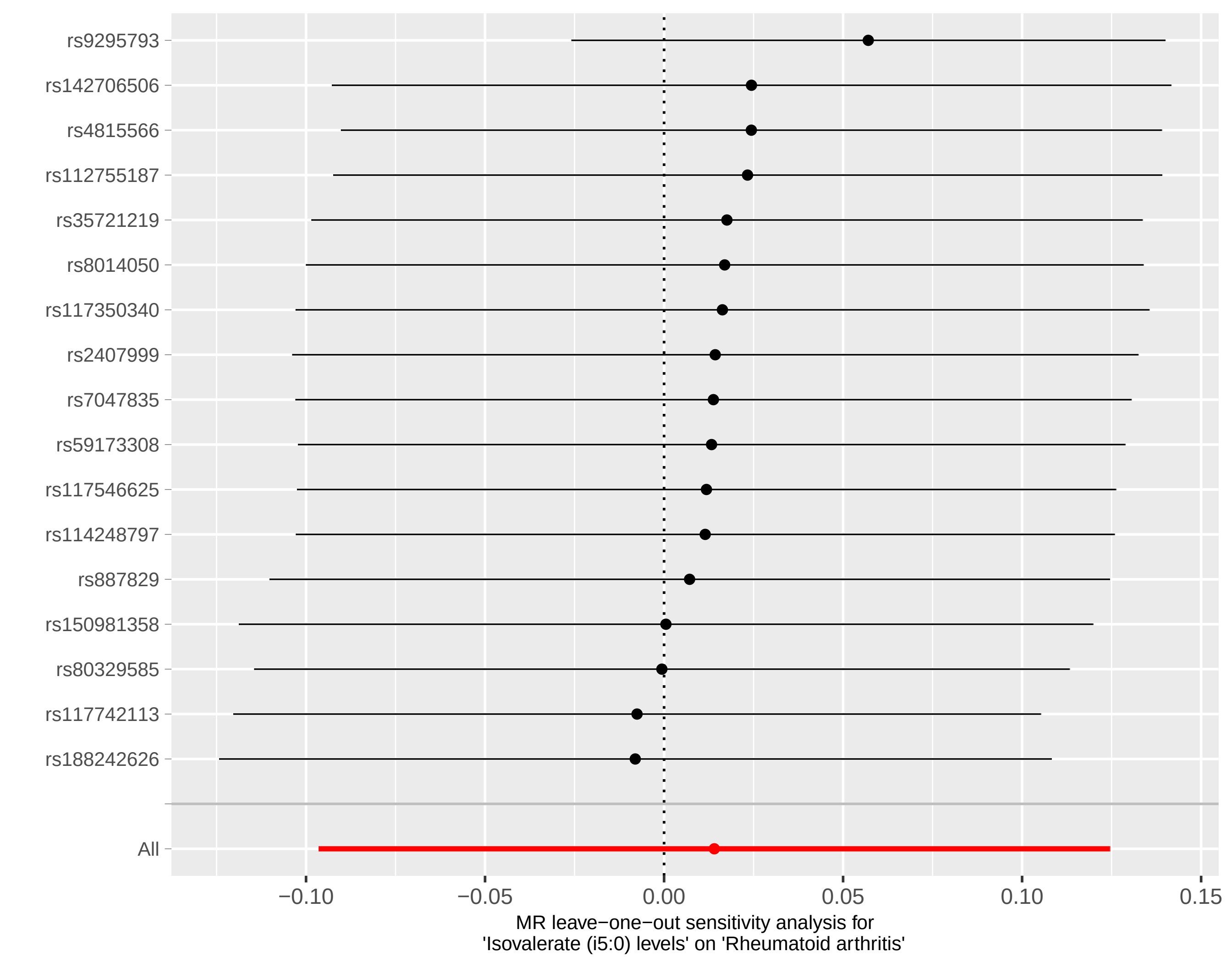
rs9295793
rs142706506
rs4815566
rs112755187
rs35721219
rs8014050
rs117350340
rs2407999
rs7047835
rs59173308
rs117546625
rs114248797
rs887829
rs150981358
rs80329585
rs117742113
rs188242626
All
−0.10
−0.05
0.00
0.05
0.10
0.15
MR leave−one−out sensitivity analysis for
'Isovalerate (i5:0) levels' on 'Rheumatoid arthritis'

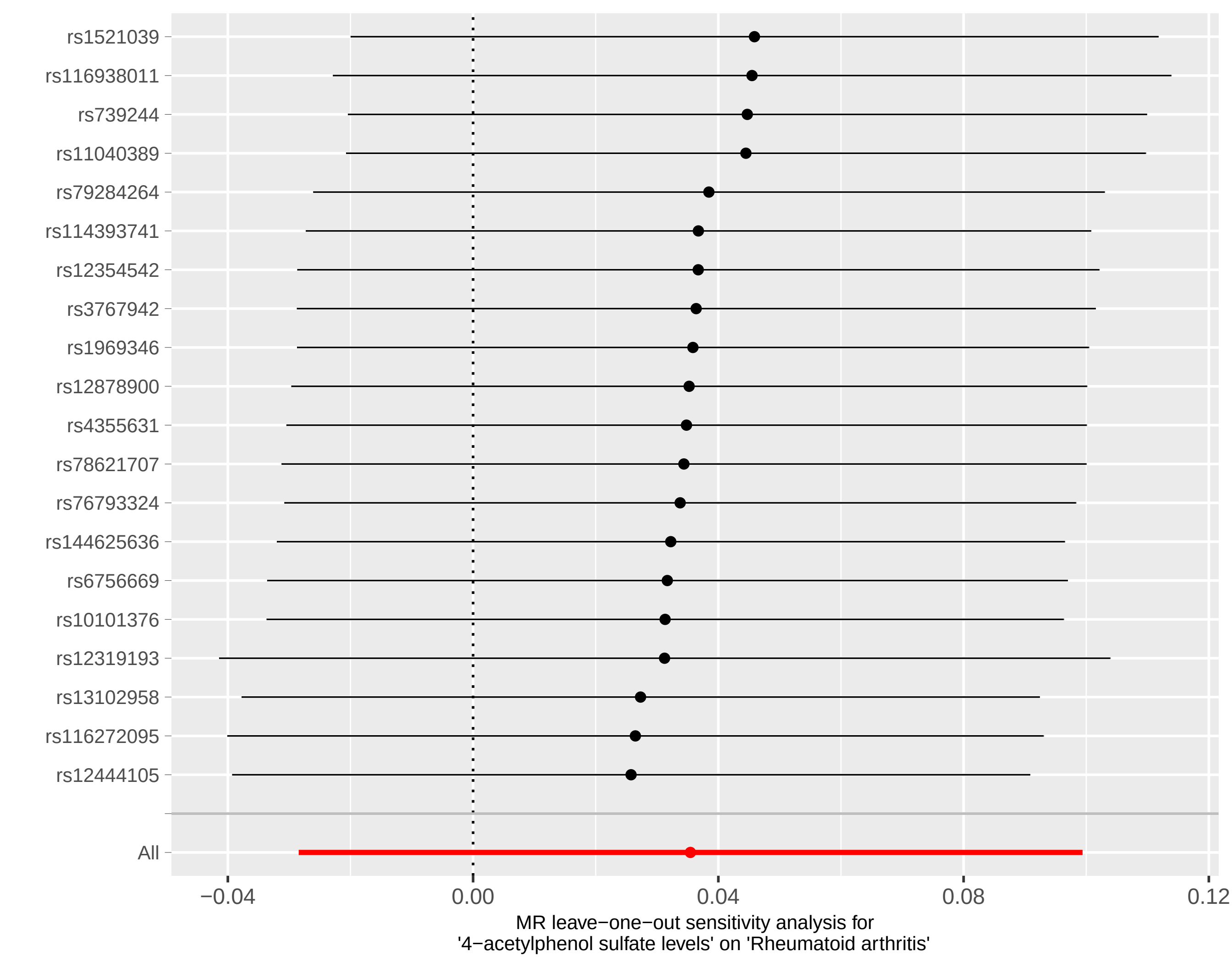

rs1521039
rs116938011
rs739244
rs11040389
rs79284264
rs114393741
rs12354542
rs3767942
rs1969346
rs12878900
rs4355631
rs78621707
rs76793324
rs144625636
rs6756669
rs10101376
rs12319193
rs13102958
rs116272095
rs12444105
All
−0.04
0.00
0.04
0.08
0.12
MR leave−one−out sensitivity analysis for
'4−acetylphenol sulfate levels' on 'Rheumatoid arthritis'

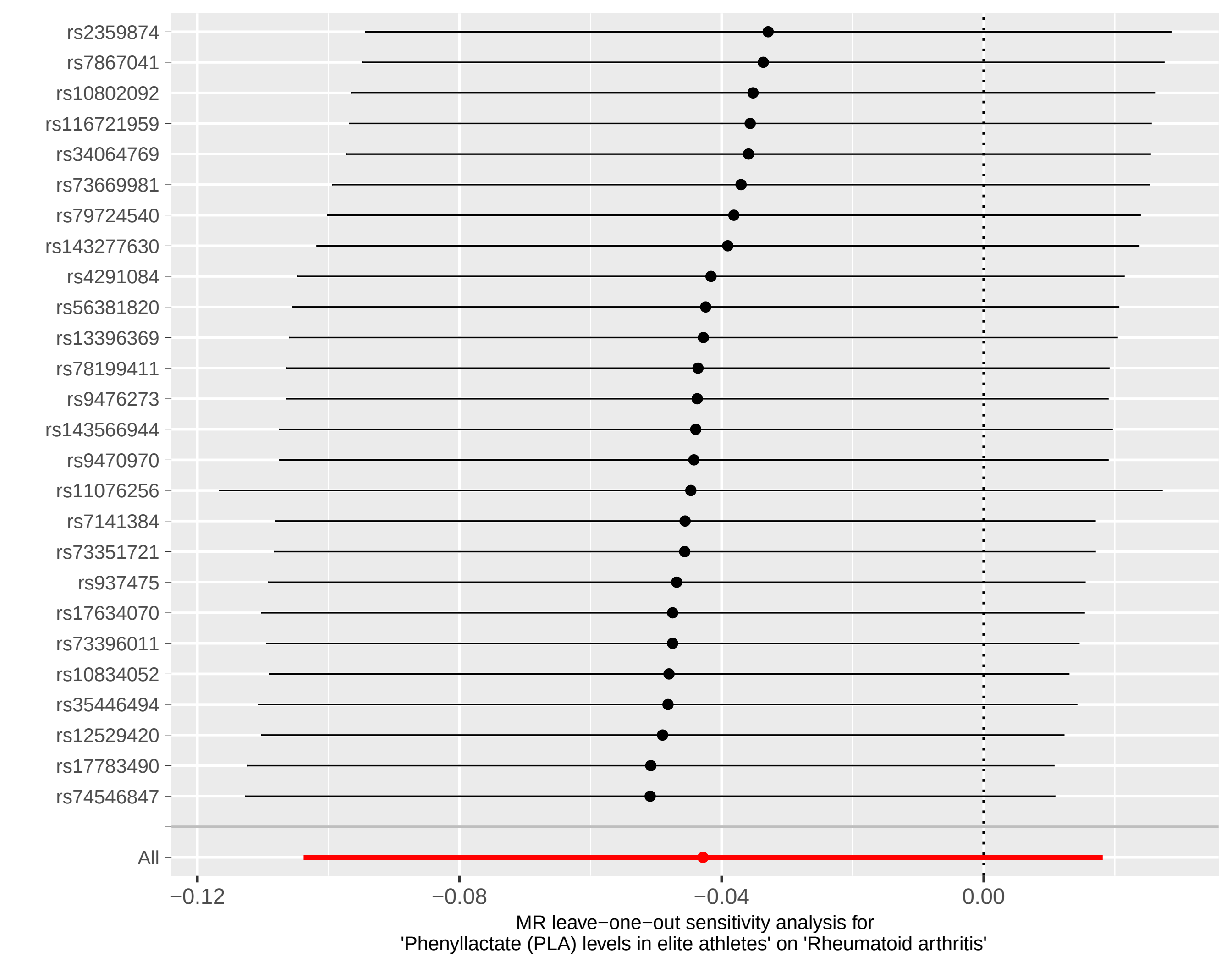
rs2359874
rs7867041
rs10802092
rs116721959
rs34064769
rs73669981
rs79724540
rs143277630
rs4291084
rs56381820
rs13396369
rs78199411
rs9476273
rs143566944
rs9470970
rs11076256
rs7141384
rs73351721
rs937475
rs17634070
rs73396011
rs10834052
rs35446494
rs12529420
rs17783490
rs74546847
All
−0.12
−0.08
−0.04
0.00
MR leave−one−out sensitivity analysis for
'Phenyllactate (PLA) levels in elite athletes' on 'Rheumatoid arthritis'

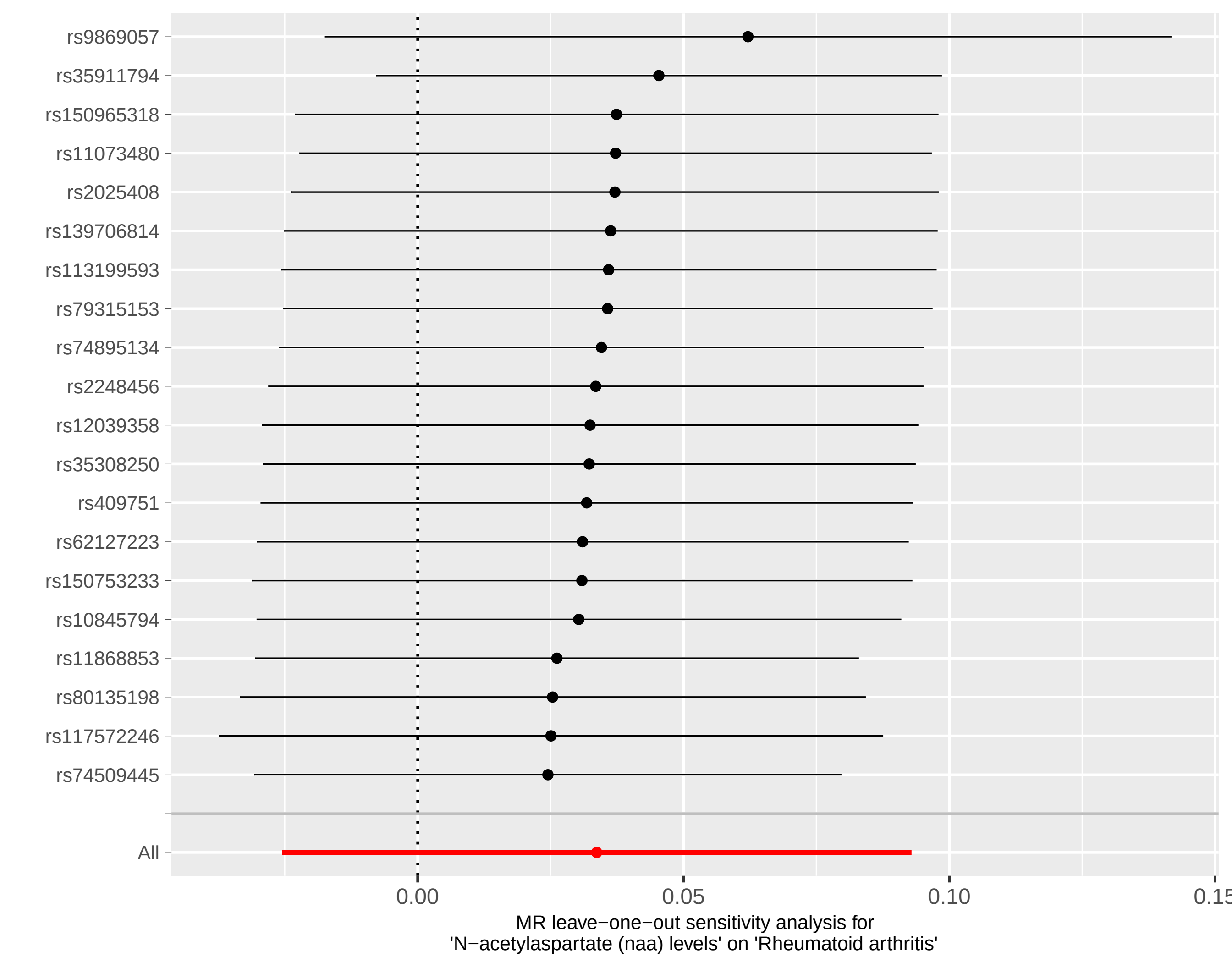
rs9869057
rs35911794
rs150965318
rs11073480
rs2025408
rs139706814
rs113199593
rs79315153
rs74895134
rs2248456
rs12039358
rs35308250
rs409751
rs62127223
rs150753233
rs10845794
rs11868853
rs80135198
rs117572246
rs74509445
All
0.00
0.05
0.10
MR leave−one−out sensitivity analysis for
'N−acetylaspartate (naa) levels' on 'Rheumatoid arthritis'

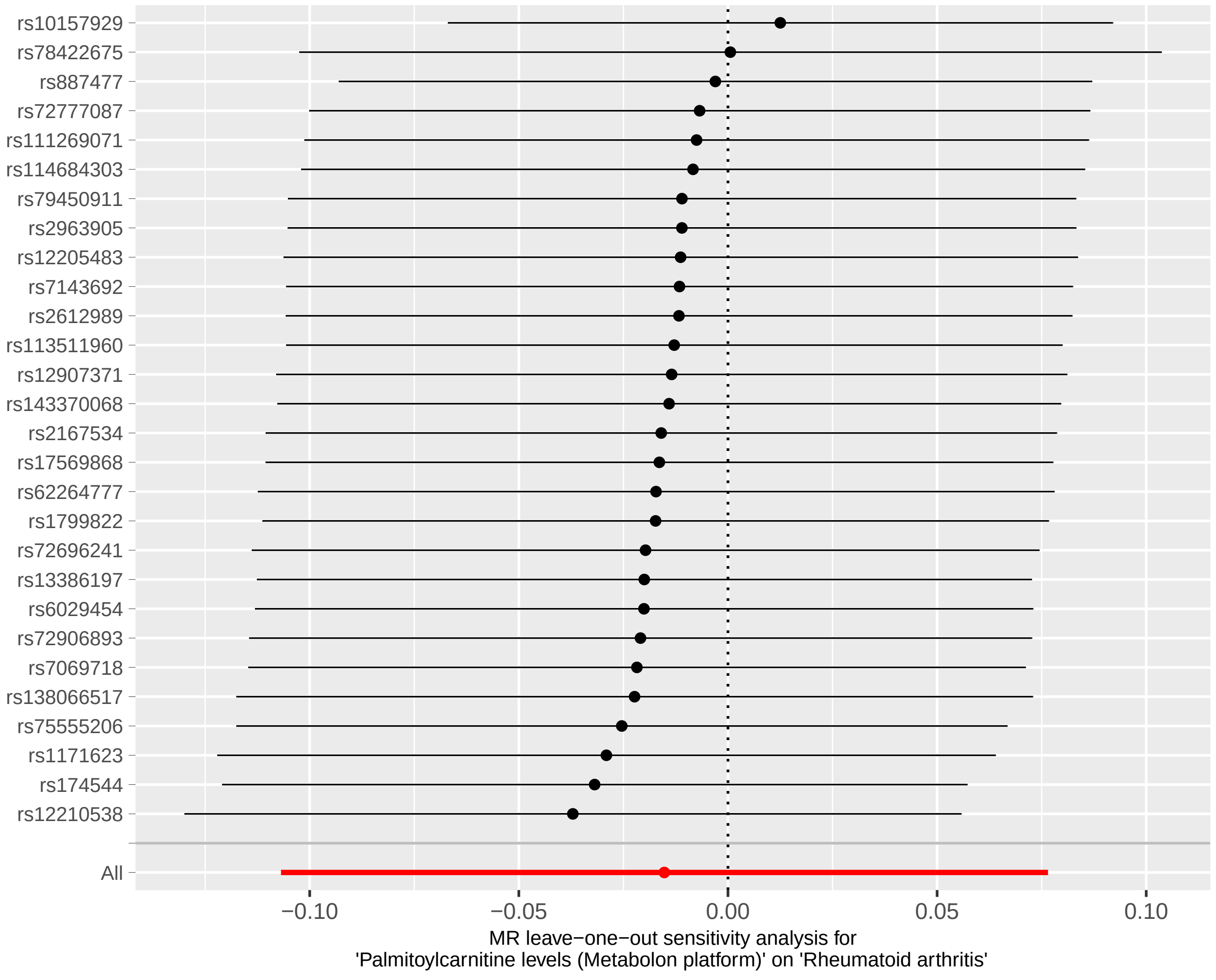
rs10157929
rs78422675
rs887477
rs72777087
rs111269071
rs114684303
rs79450911
rs2963905
rs12205483
rs7143692
rs2612989
rs113511960
rs12907371
rs143370068
rs2167534
rs17569868
rs62264777
rs1799822
rs72696241
rs13386197
rs6029454
rs72906893
rs7069718
rs138066517
rs75555206
rs1171623
rs174544
rs12210538
All
−0.10
−0.05
0.00
0.05
0.10
MR leave−one−out sensitivity analysis for
'Palmitoylcarnitine levels (Metabolon platform)' on 'Rheumatoid arthritis'

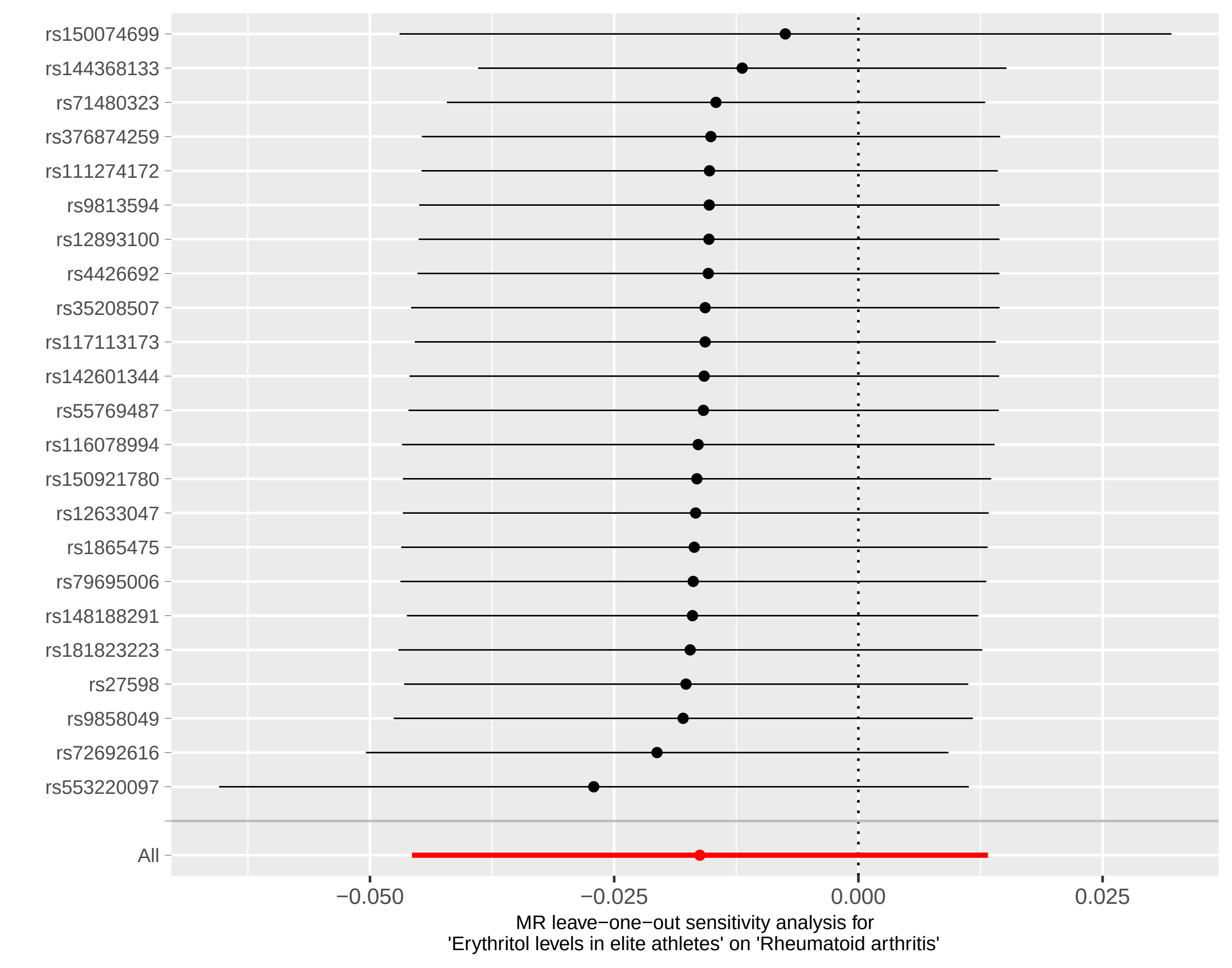
rs150074699
rs144368133
rs71480323
rs376874259
rs111274172
rs9813594
rs12893100
rs4426692
rs35208507
rs117113173
rs142601344
rs55769487
rs116078994
rs150921780
rs12633047
rs1865475
rs79695006
rs148188291
rs181823223
rs27598
rs9858049
rs72692616
rs553220097
All
−0.050
−0.025
0.000
0.025
MR leave−one−out sensitivity analysis for
'Erythritol levels in elite athletes' on 'Rheumatoid arthritis'

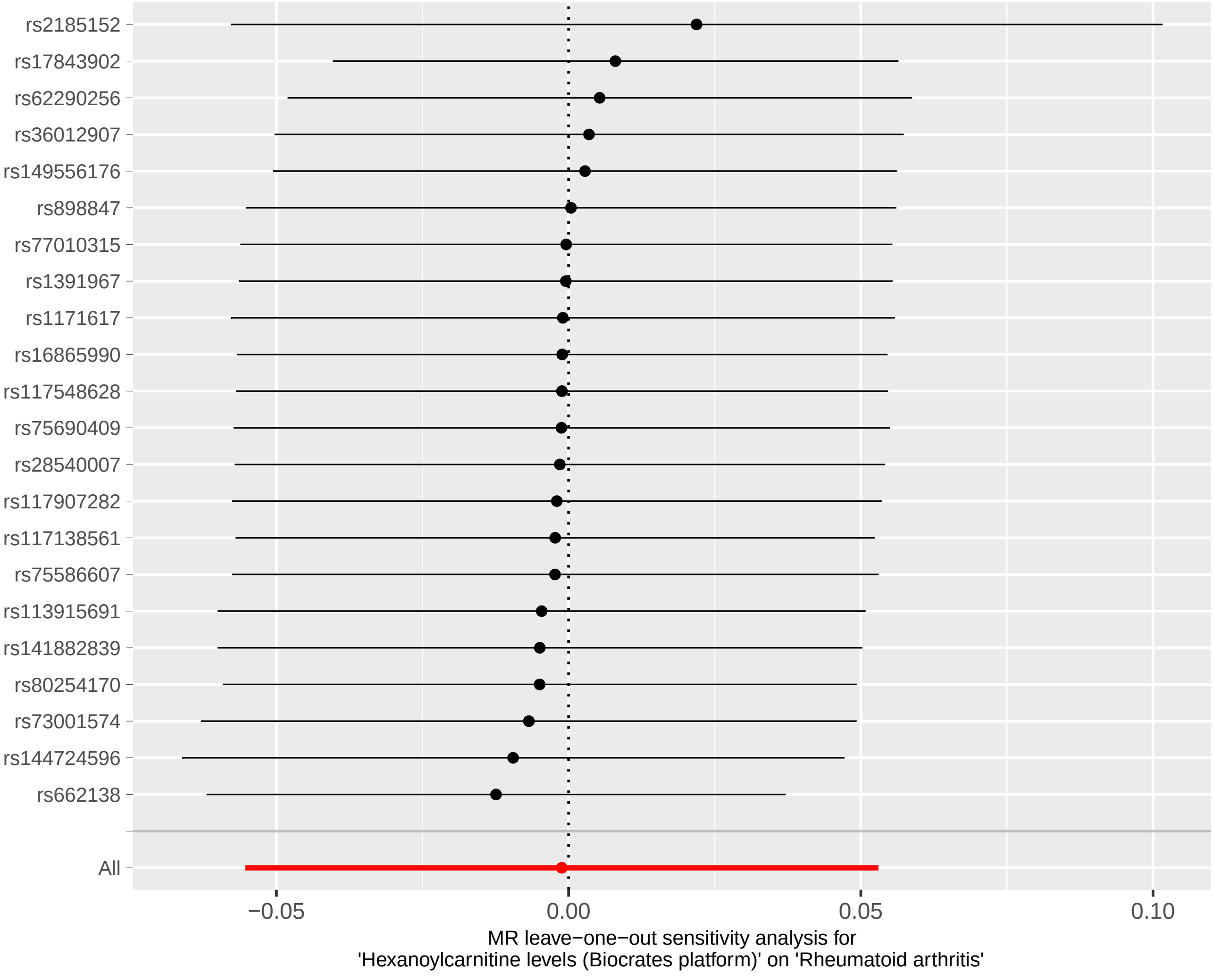
rs2185152
rs17843902
rs62290256
rs36012907
rs149556176
rs898847
rs77010315
rs1391967
rs1171617
rs16865990
rs117548628
rs75690409
rs28540007
rs117907282
rs117138561
rs75586607
rs113915691
rs141882839
rs80254170
rs73001574
rs144724596
rs662138
All
−0.05
0.00
0.05
0.10
MR leave−one−out sensitivity analysis for
'Hexanoylcarnitine levels (Biocrates platform)' on 'Rheumatoid arthritis'

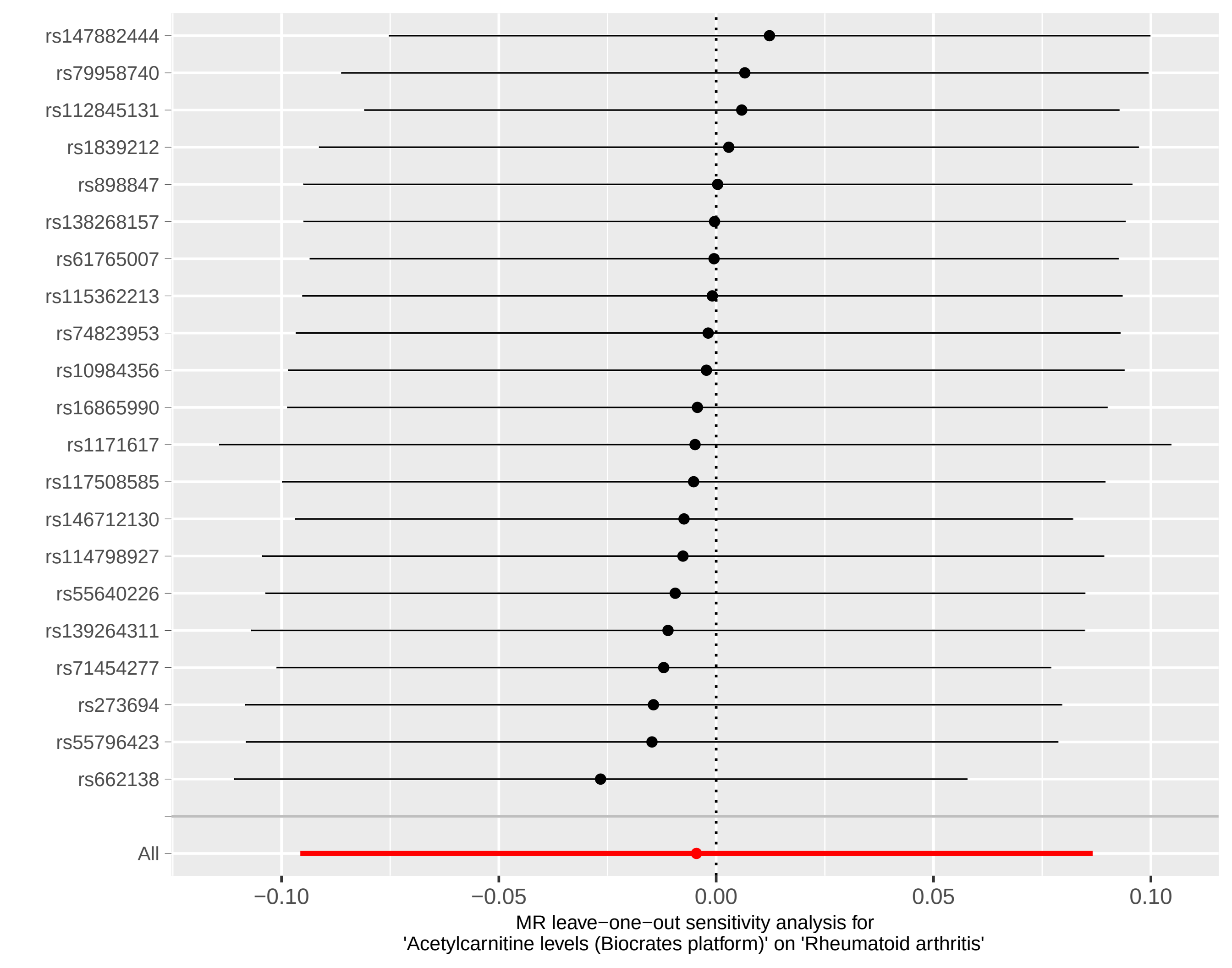
rs147882444
rs79958740
rs112845131
rs1839212
rs898847
rs138268157
rs61765007
rs115362213
rs74823953
rs10984356
rs16865990
rs1171617
rs117508585
rs146712130
rs114798927
rs55640226
rs139264311
rs71454277
rs273694
rs55796423
rs662138
All
−0.10
−0.05
0.00
0.05
0.10
MR leave−one−out sensitivity analysis for
'Acetylcarnitine levels (Biocrates platform)' on 'Rheumatoid arthritis'

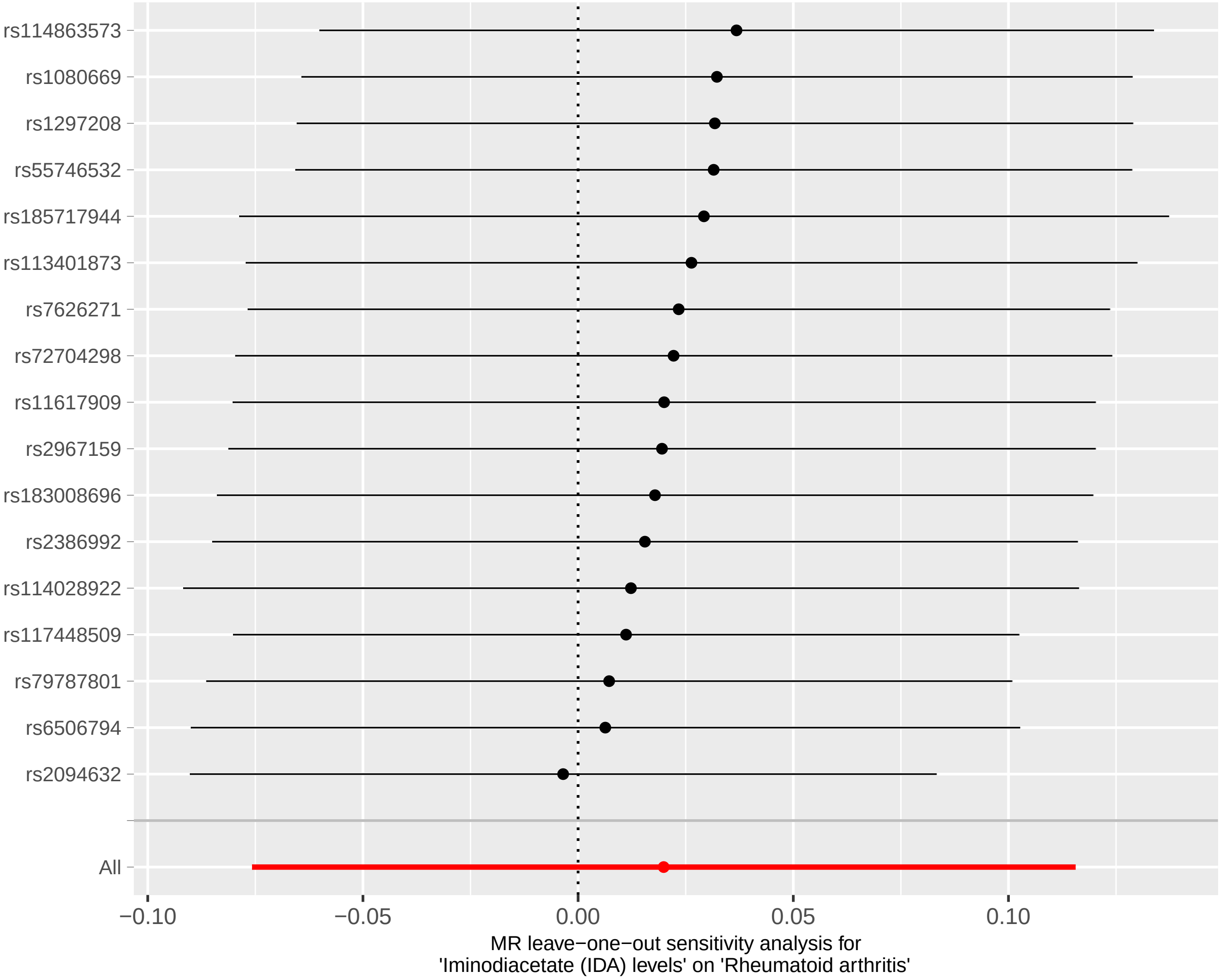

rs114863573
rs1080669
rs1297208
rs55746532
rs185717944
rs113401873
rs7626271
rs72704298
rs11617909
rs2967159
rs183008696
rs2386992
rs114028922
rs117448509
rs79787801
rs6506794
rs2094632
All
−0.10
−0.05
0.00
0.05
0.10
MR leave−one−out sensitivity analysis for
'Iminodiacetate (IDA) levels' on 'Rheumatoid arthritis'

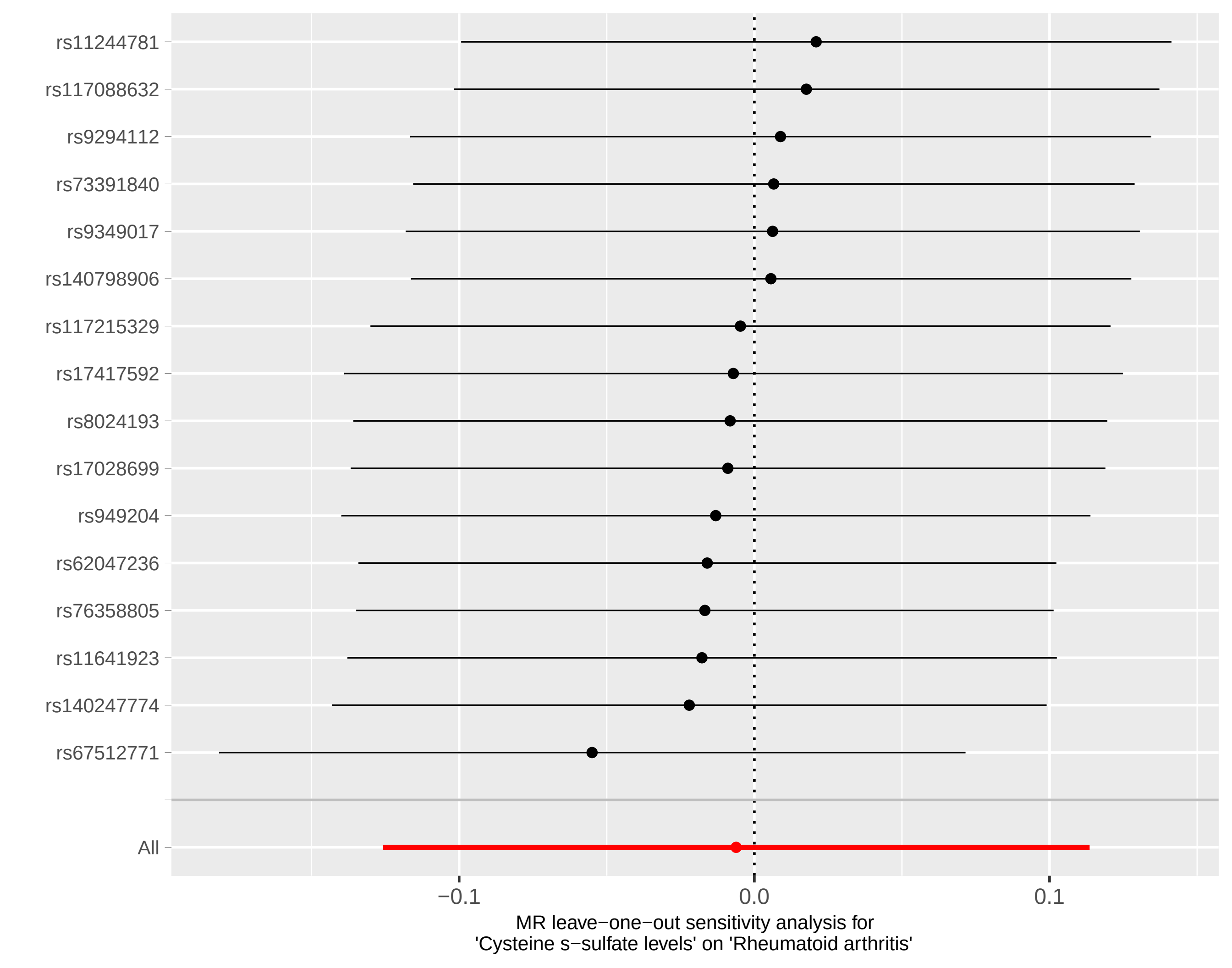

rs11244781
rs117088632
rs9294112
rs73391840
rs9349017
rs140798906
rs117215329
rs17417592
rs8024193
rs17028699
rs949204
rs62047236
rs76358805
rs11641923
rs140247774
rs67512771
All
−0.1
0.0
0.1
MR leave−one−out sensitivity analysis for
'Cysteine s−sulfate levels' on 'Rheumatoid arthritis'

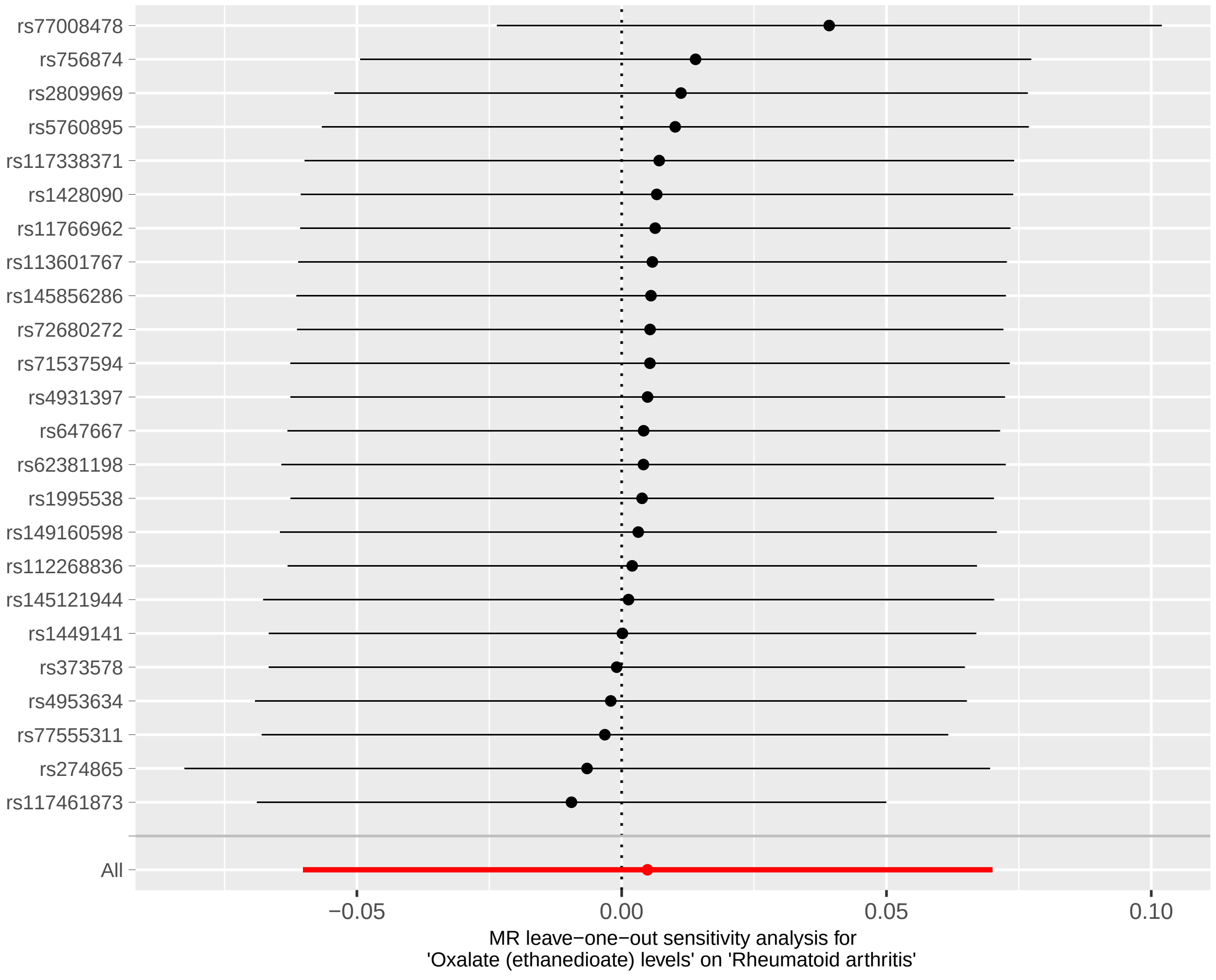
rs77008478
rs756874
rs2809969
rs5760895
rs117338371
rs1428090
rs11766962
rs113601767
rs145856286
rs72680272
rs71537594
rs4931397
rs647667
rs62381198
rs1995538
rs149160598
rs112268836
rs145121944
rs1449141
rs373578
rs4953634
rs77555311
rs274865
rs117461873
All
−0.05
0.00
0.05
0.10
MR leave−one−out sensitivity analysis for
'Oxalate (ethanedioate) levels' on 'Rheumatoid arthritis'

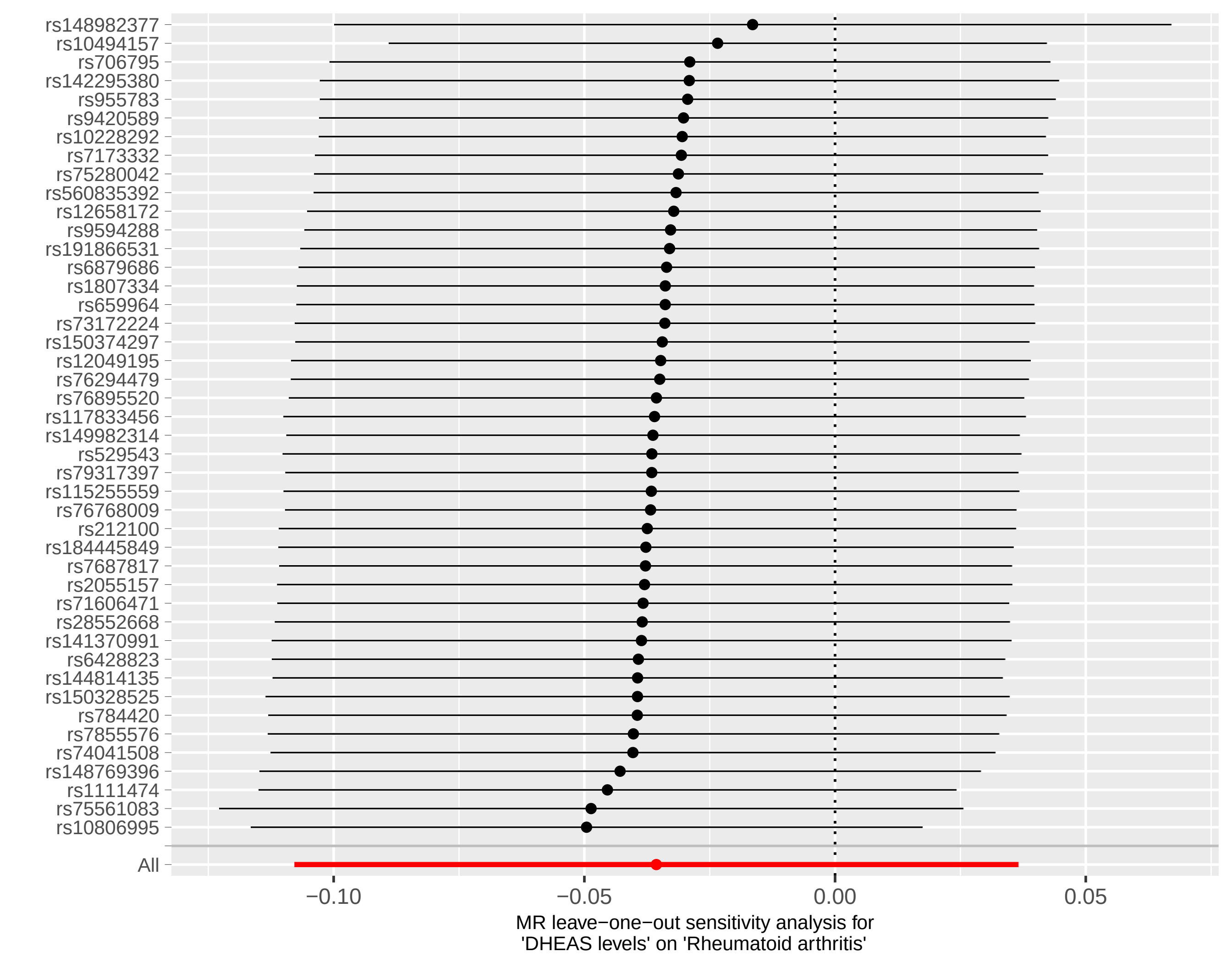
rs148982377
rs10494157
rs706795
rs142295380
rs955783
rs9420589
rs10228292
rs7173332
rs75280042
rs560835392
rs12658172
rs9594288
rs191866531
rs6879686
rs1807334
rs659964
rs73172224
rs150374297
rs12049195
rs76294479
rs76895520
rs117833456
rs149982314
rs529543
rs79317397
rs115255559
rs76768009
rs212100
rs184445849
rs7687817
rs2055157
rs71606471
rs28552668
rs141370991
rs6428823
rs144814135
rs150328525
rs784420
rs7855576
rs74041508
rs148769396
rs1111474
rs75561083
rs10806995
All
−0.10
−0.05
0.00
0.05
MR leave−one−out sensitivity analysis for
'DHEAS levels' on 'Rheumatoid arthritis'

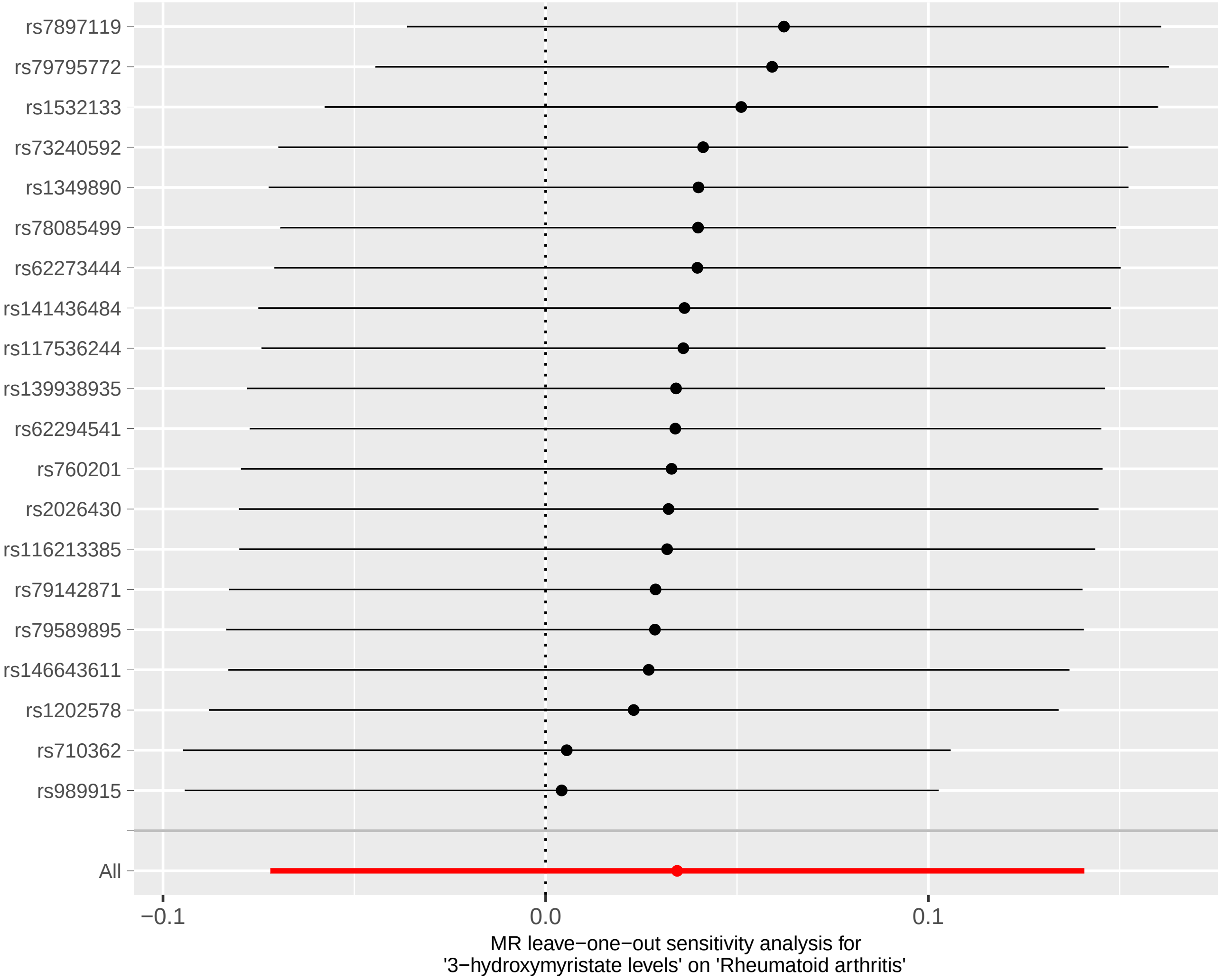
rs7897119
rs79795772
rs1532133
rs73240592
rs1349890
rs78085499
rs62273444
rs141436484
rs117536244
rs139938935
rs62294541
rs760201
rs2026430
rs116213385
rs79142871
rs79589895
rs146643611
rs1202578
rs710362
rs989915
All
−0.1
0.0
0.1
MR leave−one−out sensitivity analysis for
'3−hydroxymyristate levels' on 'Rheumatoid arthritis'

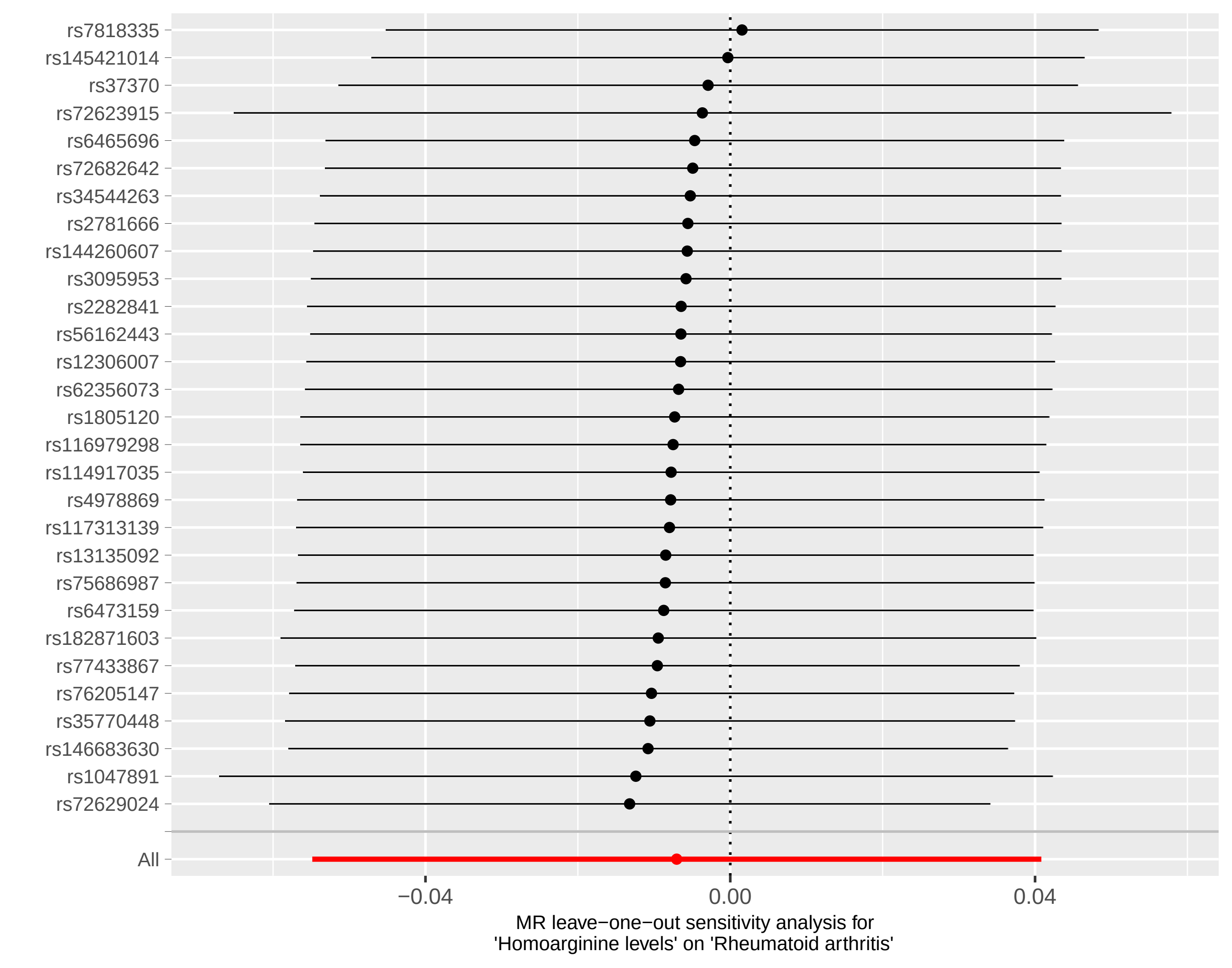
rs7818335
rs145421014
rs37370
rs72623915
rs6465696
rs72682642
rs34544263
rs2781666
rs144260607
rs3095953
rs2282841
rs56162443
rs12306007
rs62356073
rs1805120
rs116979298
rs114917035
rs4978869
rs117313139
rs13135092
rs75686987
rs6473159
rs182871603
rs77433867
rs76205147
rs35770448
rs146683630
rs1047891
rs72629024
All
−0.04
0.00
0.04
MR leave−one−out sensitivity analysis for
'Homoarginine levels' on 'Rheumatoid arthritis'

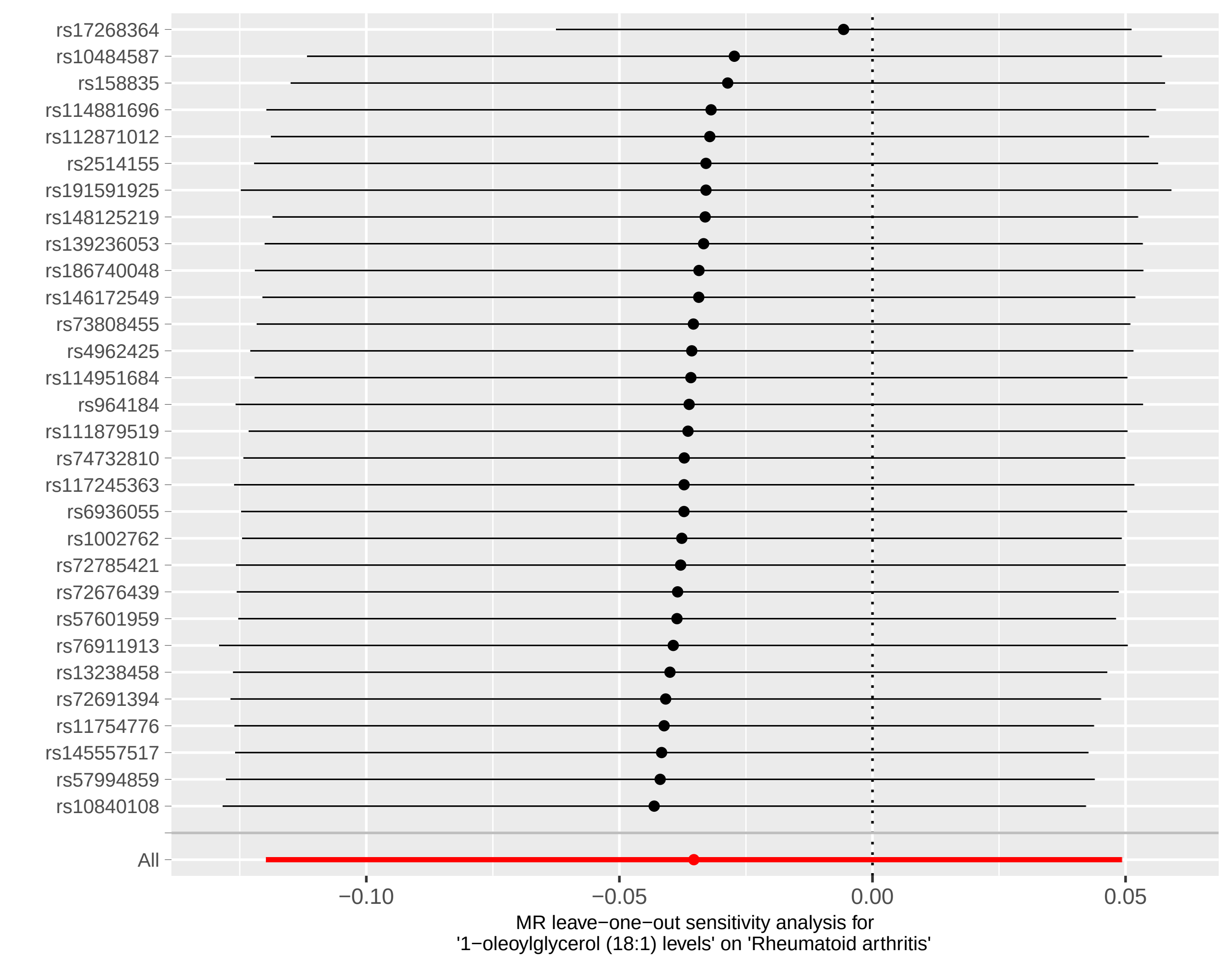

rs17268364
rs10484587
rs158835
rs114881696
rs112871012
rs2514155
rs191591925
rs148125219
rs139236053
rs186740048
rs146172549
rs73808455
rs4962425
rs114951684
rs964184
rs111879519
rs74732810
rs117245363
rs6936055
rs1002762
rs72785421
rs72676439
rs57601959
rs76911913
rs13238458
rs72691394
rs11754776
rs145557517
rs57994859
rs10840108
All
−0.10
−0.05
0.00
0.05
MR leave−one−out sensitivity analysis for
'1−oleoylglycerol (18:1) levels' on 'Rheumatoid arthritis'

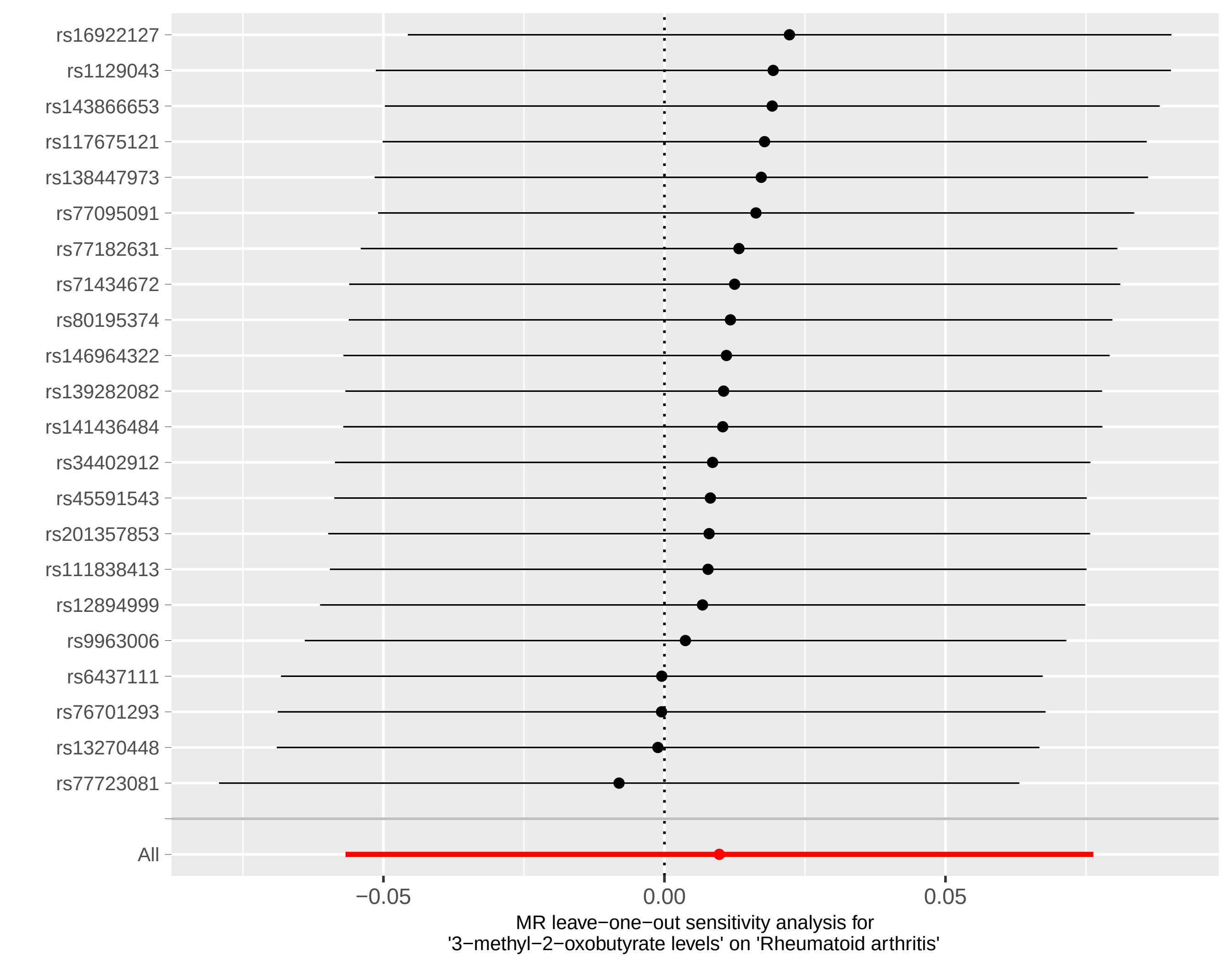
rs16922127
rs1129043
rs143866653
rs117675121
rs138447973
rs77095091
rs77182631
rs71434672
rs80195374
rs146964322
rs139282082
rs141436484
rs34402912
rs45591543
rs201357853
rs111838413
rs12894999
rs9963006
rs6437111
rs76701293
rs13270448
rs77723081
All
−0.05
0.00
0.05
MR leave−one−out sensitivity analysis for
'3−methyl−2−oxobutyrate levels' on 'Rheumatoid arthritis'

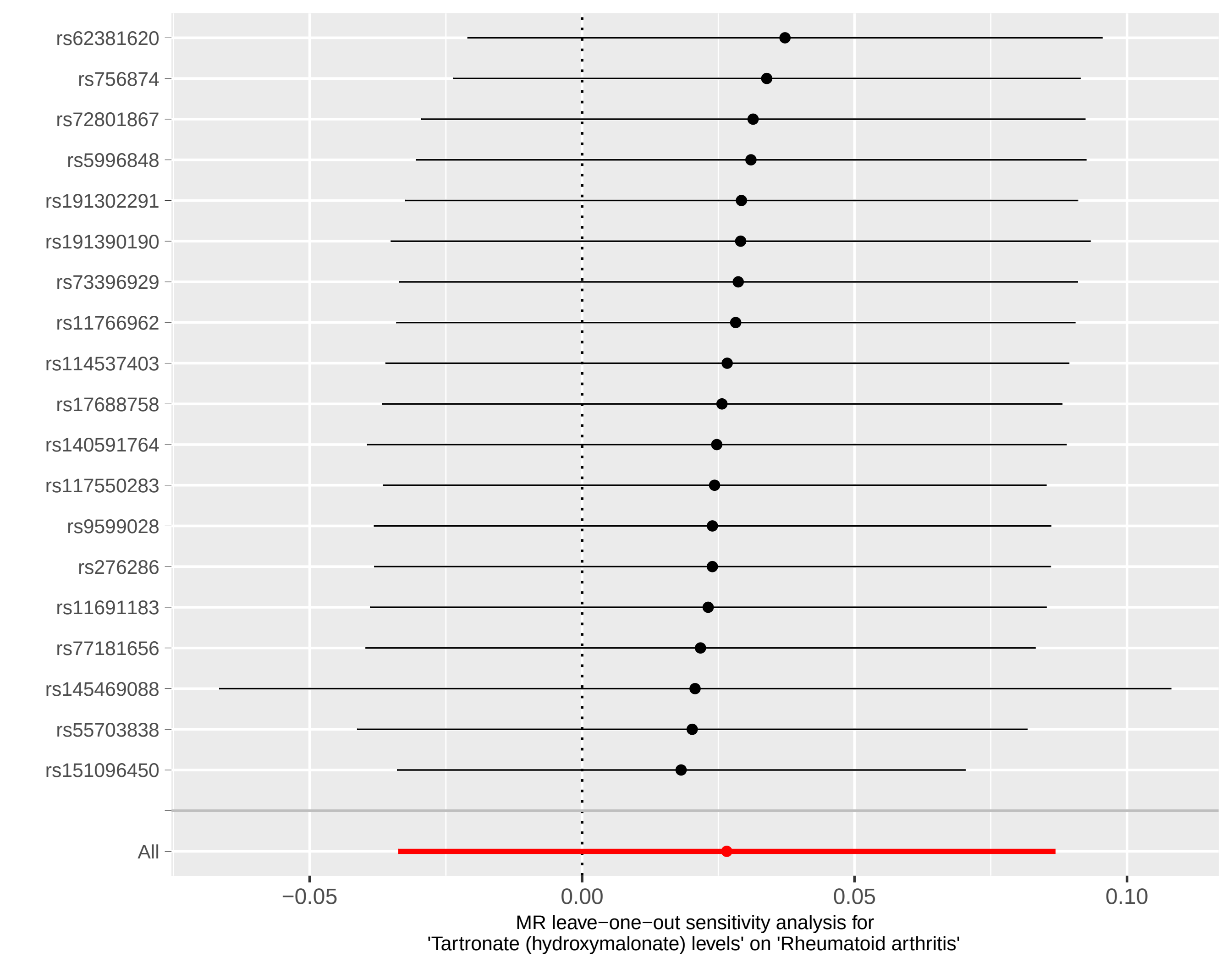
rs62381620
rs756874
rs72801867
rs5996848
rs191302291
rs191390190
rs73396929
rs11766962
rs114537403
rs17688758
rs140591764
rs117550283
rs9599028
rs276286
rs11691183
rs77181656
rs145469088
rs55703838
rs151096450
All
−0.05
0.00
0.05
0.10
MR leave−one−out sensitivity analysis for
'Tartronate (hydroxymalonate) levels' on 'Rheumatoid arthritis'

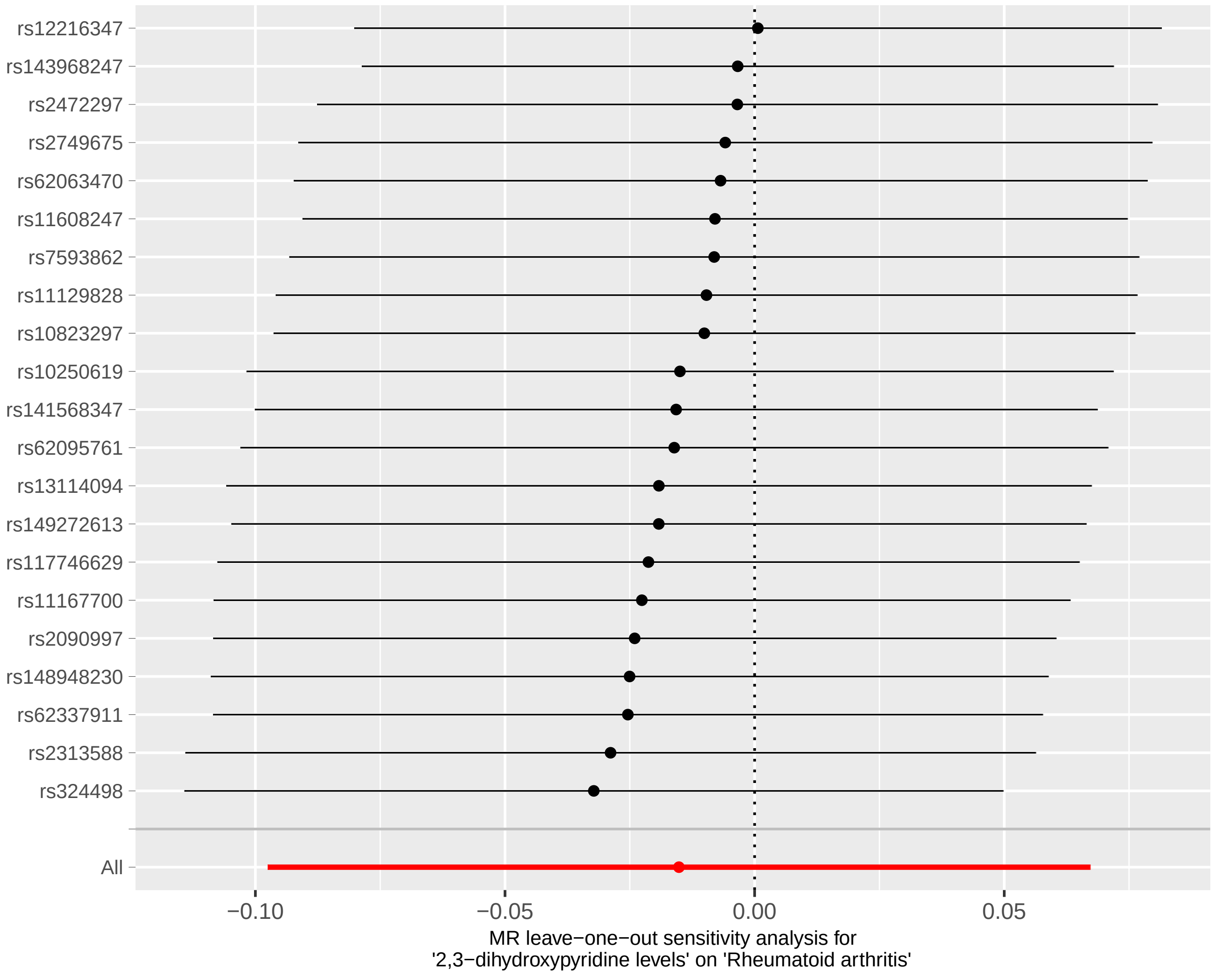

rs12216347
rs143968247
rs2472297
rs2749675
rs62063470
rs11608247
rs7593862
rs11129828
rs10823297
rs10250619
rs141568347
rs62095761
rs13114094
rs149272613
rs117746629
rs11167700
rs2090997
rs148948230
rs62337911
rs2313588
rs324498
All
−0.10
−0.05
0.00
0.05
MR leave−one−out sensitivity analysis for
'2,3−dihydroxypyridine levels' on 'Rheumatoid arthritis'

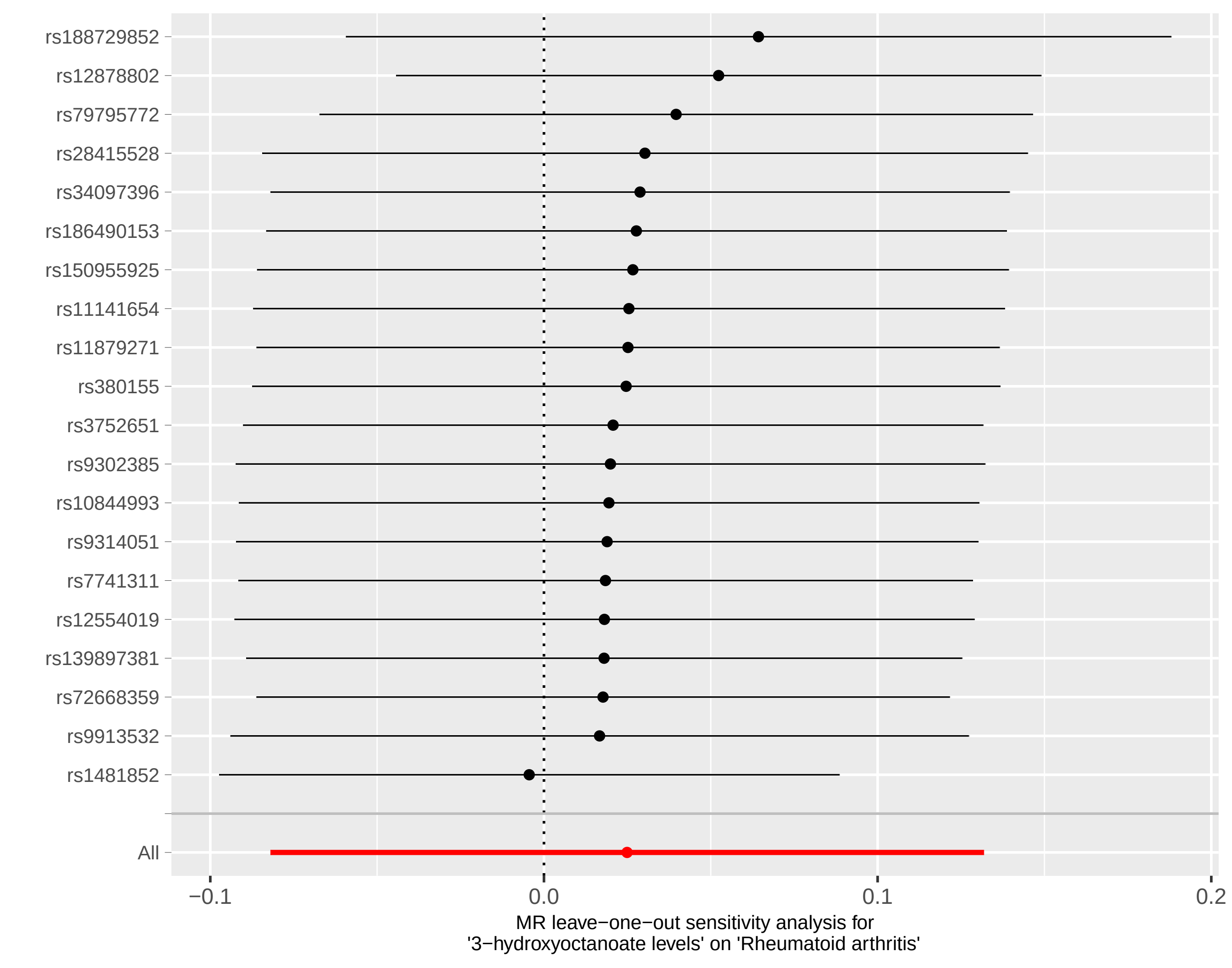
rs188729852
rs12878802
rs79795772
rs28415528
rs34097396
rs186490153
rs150955925
rs11141654
rs11879271
rs380155
rs3752651
rs9302385
rs10844993
rs9314051
rs7741311
rs12554019
rs139897381
rs72668359
rs9913532
rs1481852
All
−0.1
0.0
0.1
0.2
MR leave−one−out sensitivity analysis for
'3−hydroxyoctanoate levels' on 'Rheumatoid arthritis'

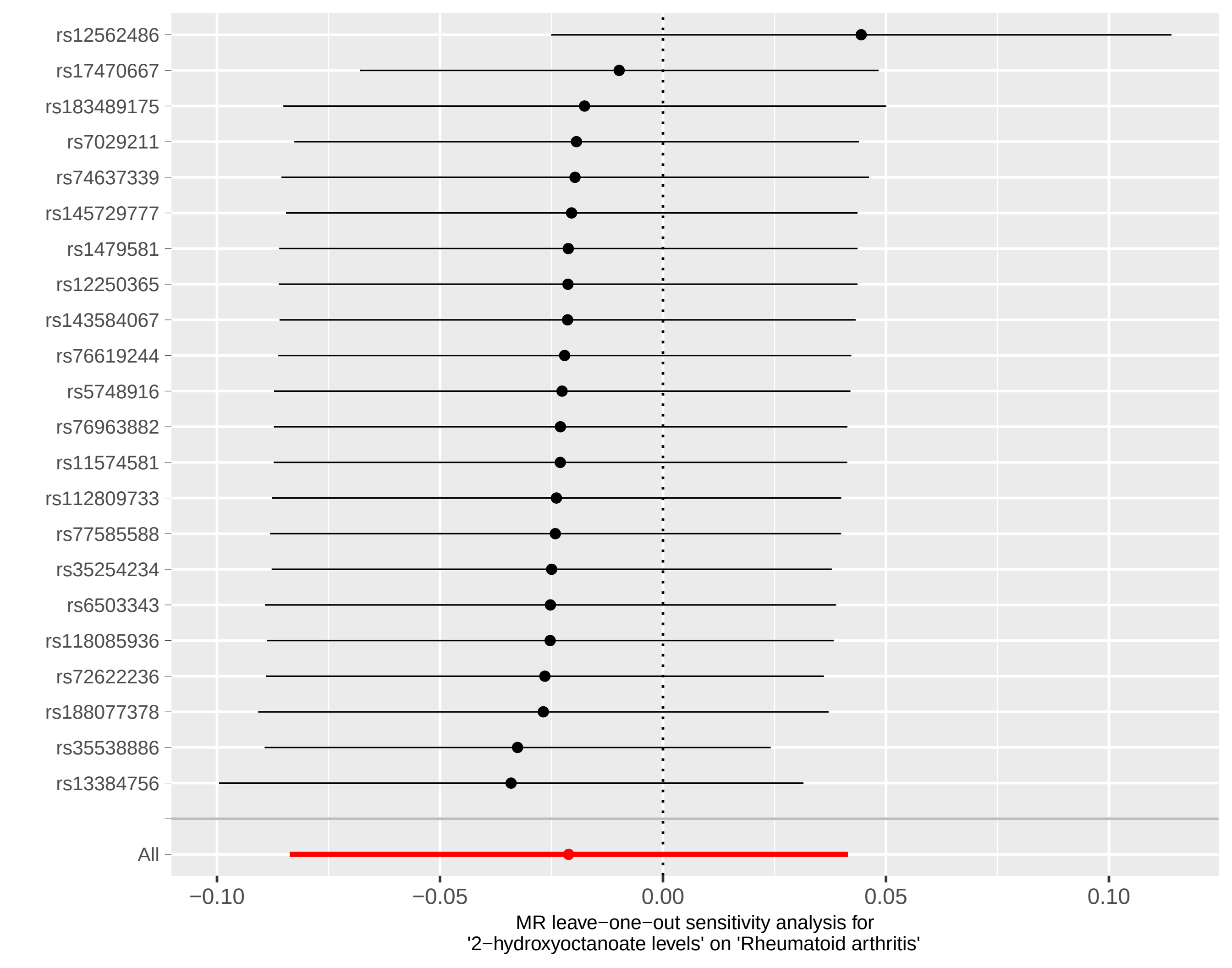

rs12562486
rs17470667
rs183489175
rs7029211
rs74637339
rs145729777
rs1479581
rs12250365
rs143584067
rs76619244
rs5748916
rs76963882
rs11574581
rs112809733
rs77585588
rs35254234
rs6503343
rs118085936
rs72622236
rs188077378
rs35538886
rs13384756
All
−0.10
−0.05
0.00
0.05
0.10
MR leave−one−out sensitivity analysis for
'2−hydroxyoctanoate levels' on 'Rheumatoid arthritis'

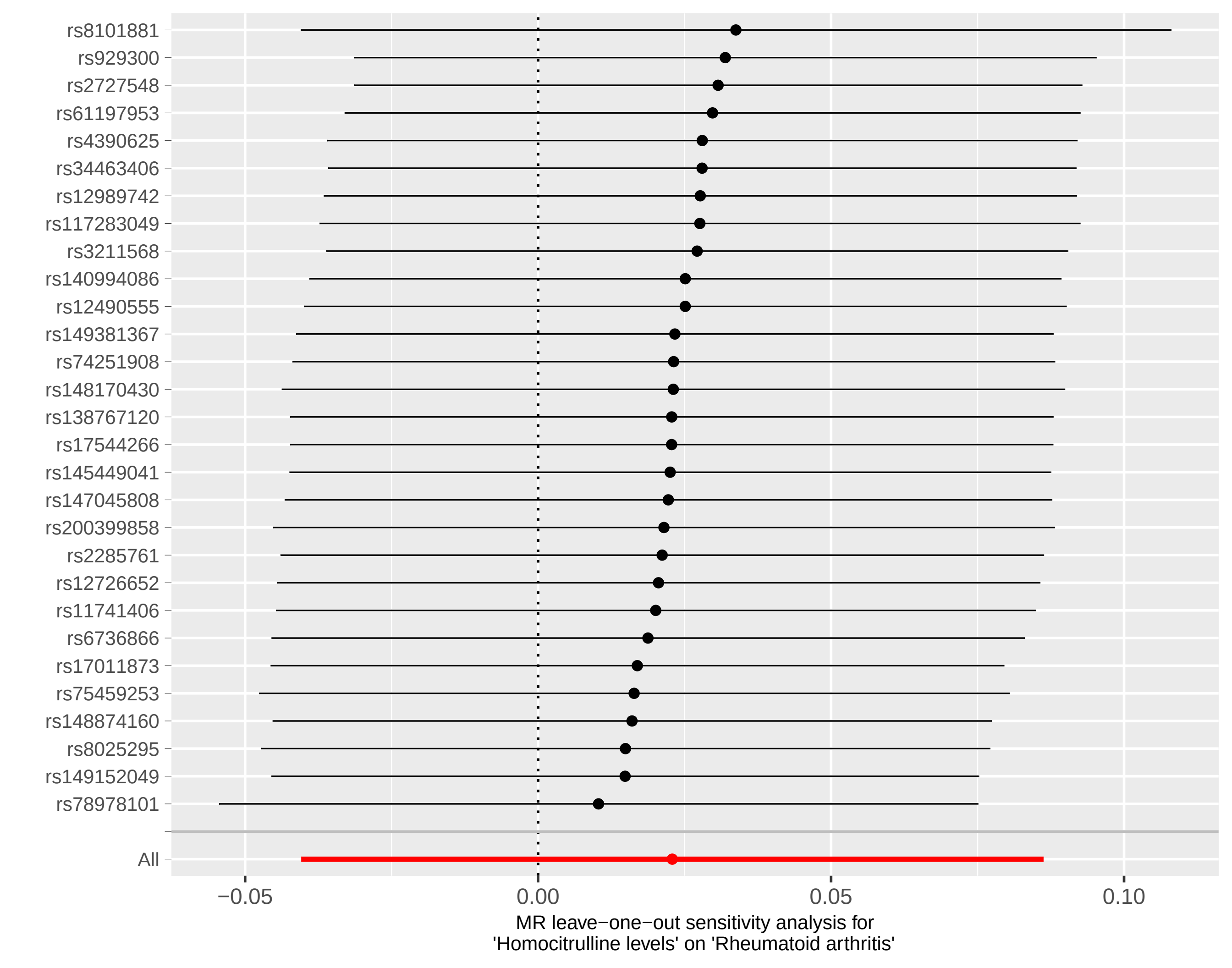
rs8101881
rs929300
rs2727548
rs61197953
rs4390625
rs34463406
rs12989742
rs117283049
rs3211568
rs140994086
rs12490555
rs149381367
rs74251908
rs148170430
rs138767120
rs17544266
rs145449041
rs147045808
rs200399858
rs2285761
rs12726652
rs11741406
rs6736866
rs17011873
rs75459253
rs148874160
rs8025295
rs149152049
rs78978101
All
−0.05
0.00
0.05
0.10
MR leave−one−out sensitivity analysis for
'Homocitrulline levels' on 'Rheumatoid arthritis'

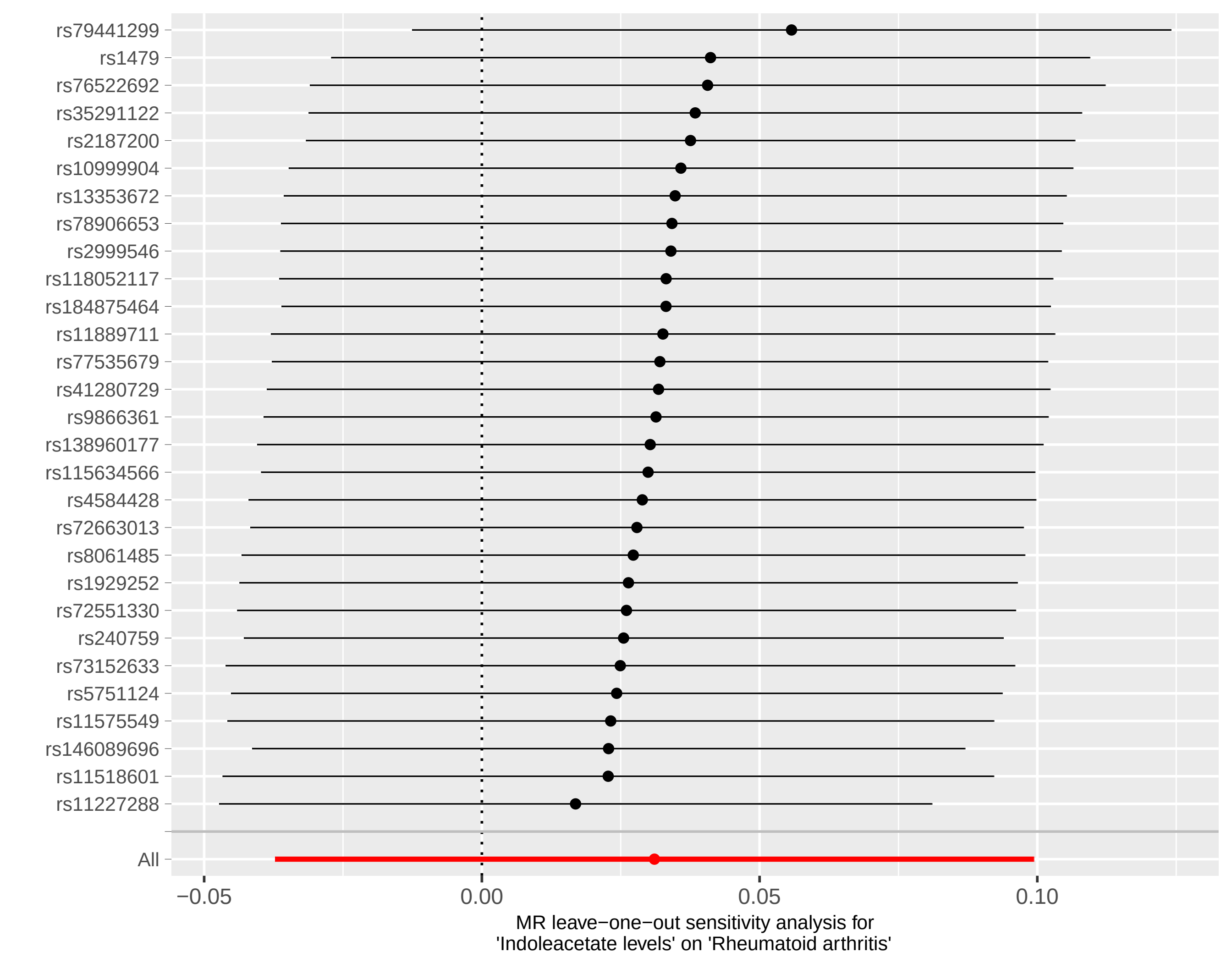
rs79441299
rs1479
rs76522692
rs35291122
rs2187200
rs10999904
rs13353672
rs78906653
rs2999546
rs118052117
rs184875464
rs11889711
rs77535679
rs41280729
rs9866361
rs138960177
rs115634566
rs4584428
rs72663013
rs8061485
rs1929252
rs72551330
rs240759
rs73152633
rs5751124
rs11575549
rs146089696
rs11518601
rs11227288
All
−0.05
0.00
0.05
0.10
MR leave−one−out sensitivity analysis for
'Indoleacetate levels' on 'Rheumatoid arthritis'

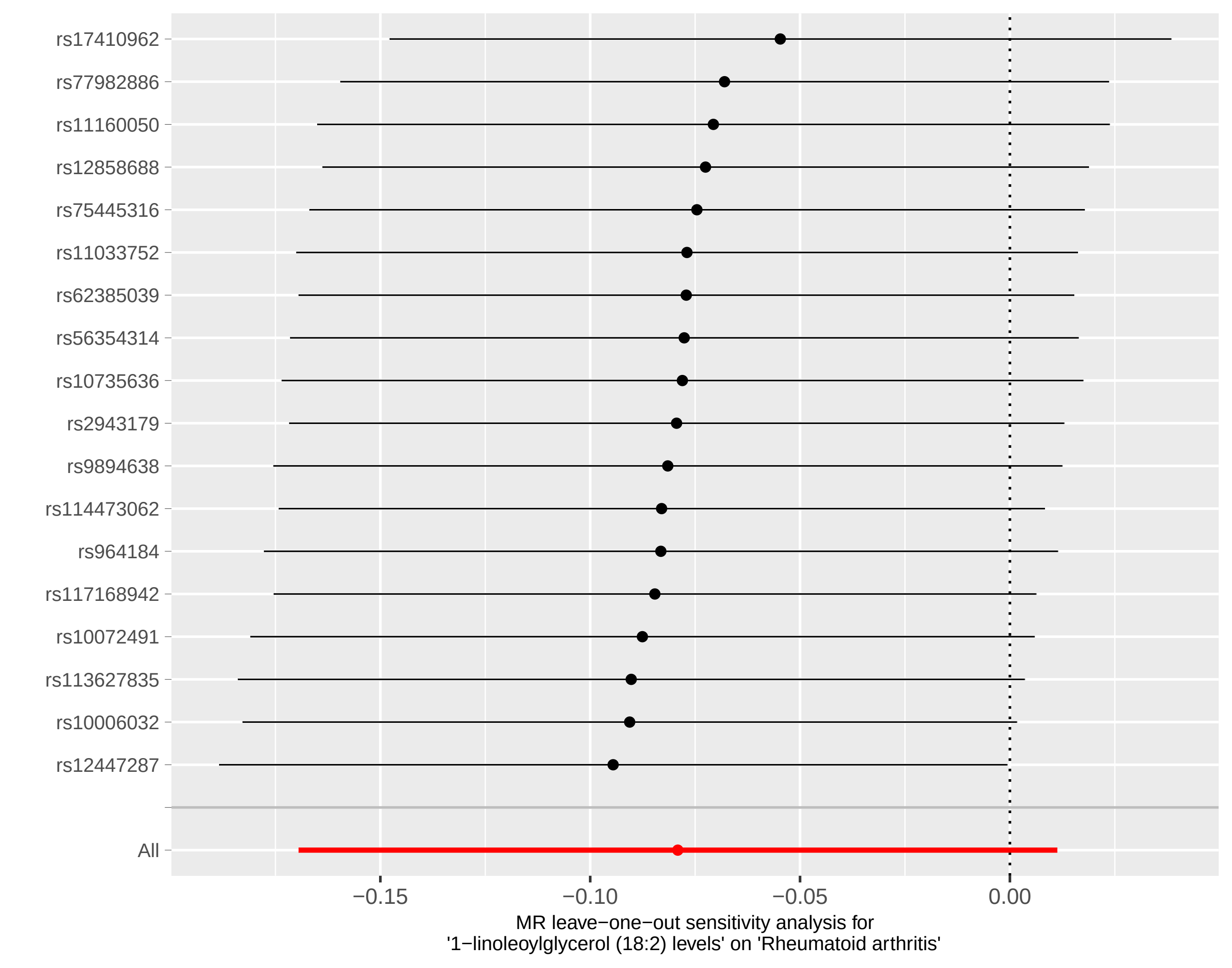
rs17410962
rs77982886
rs11160050
rs12858688
rs75445316
rs11033752
rs62385039
rs56354314
rs10735636
rs2943179
rs9894638
rs114473062
rs964184
rs117168942
rs10072491
rs113627835
rs10006032
rs12447287
All
−0.15
−0.10
−0.05
0.00
MR leave−one−out sensitivity analysis for
'1−linoleoylglycerol (18:2) levels' on 'Rheumatoid arthritis'

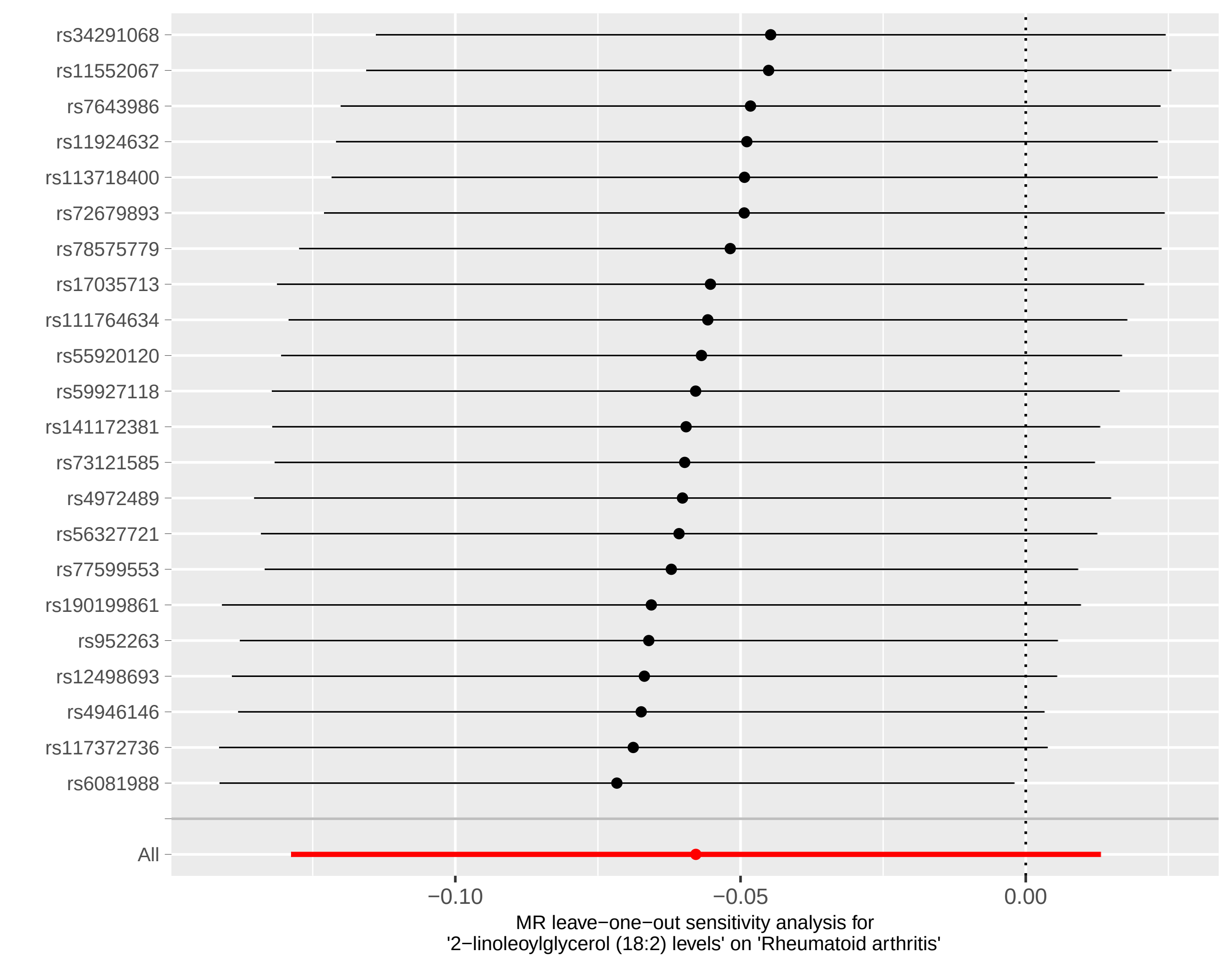
rs34291068
rs11552067
rs7643986
rs11924632
rs113718400
rs72679893
rs78575779
rs17035713
rs111764634
rs55920120
rs59927118
rs141172381
rs73121585
rs4972489
rs56327721
rs77599553
rs190199861
rs952263
rs12498693
rs4946146
rs117372736
rs6081988
All
−0.10
−0.05
0.00
MR leave−one−out sensitivity analysis for
'2−linoleoylglycerol (18:2) levels' on 'Rheumatoid arthritis'

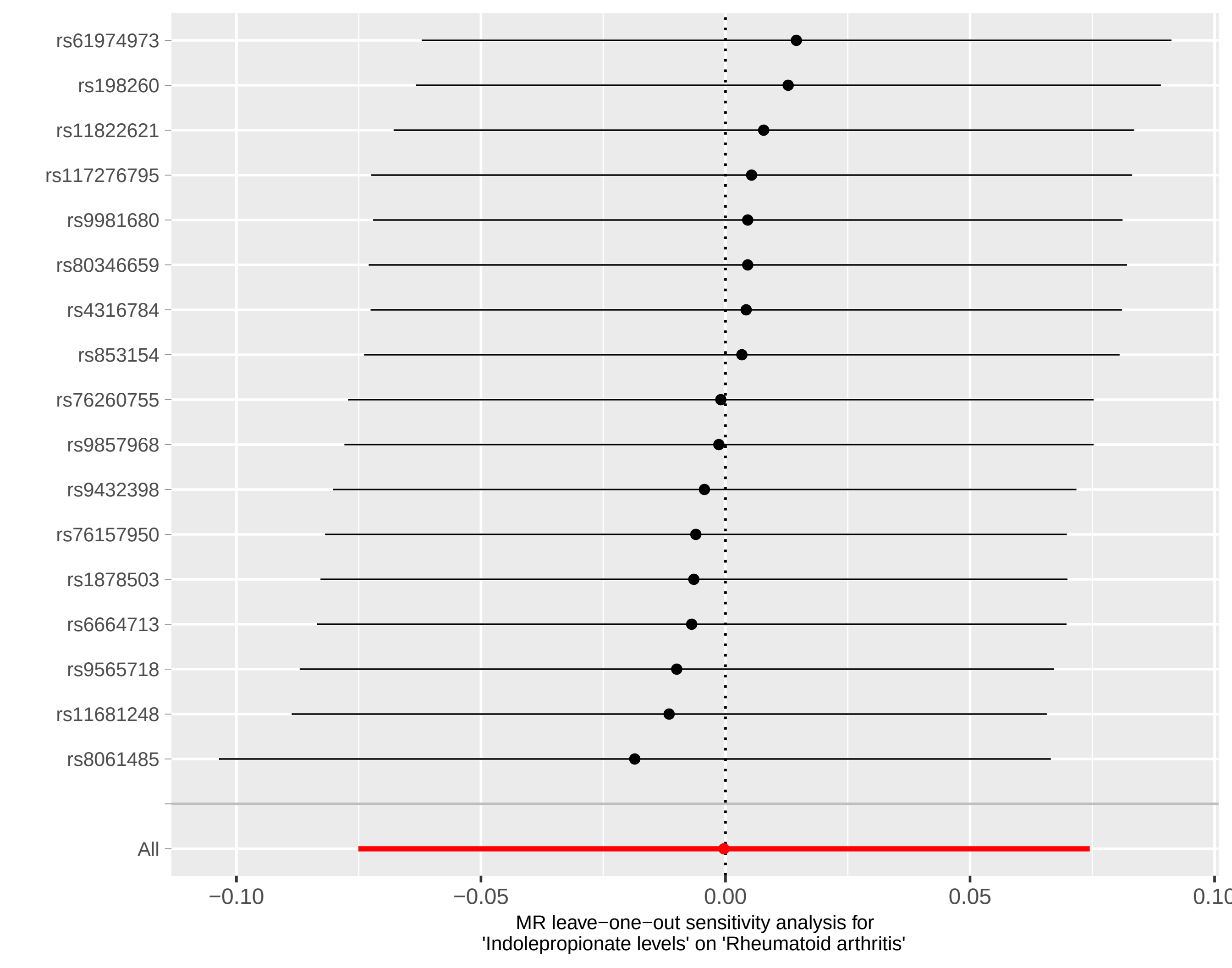
rs61974973
rs198260
rs11822621
rs117276795
rs9981680
rs80346659
rs4316784
rs853154
rs76260755
rs9857968
rs9432398
rs76157950
rs1878503
rs6664713
rs9565718
rs11681248
rs8061485
All
−0.10
−0.05
0.00
0.05
0.10
MR leave−one−out sensitivity analysis for
'Indolepropionate levels' on 'Rheumatoid arthritis'

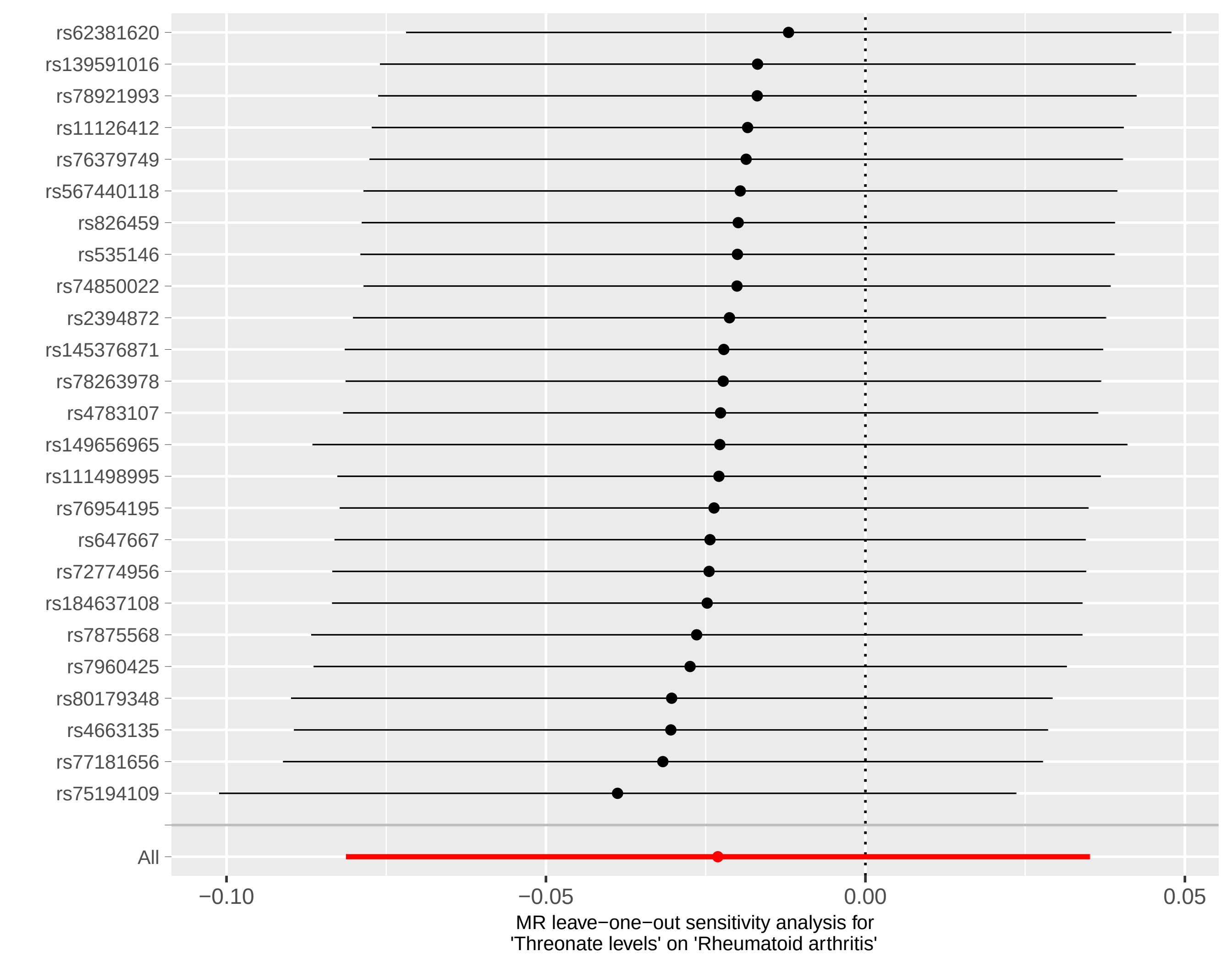
rs62381620
rs139591016
rs78921993
rs11126412
rs76379749
rs567440118
rs826459
rs535146
rs74850022
rs2394872
rs145376871
rs78263978
rs4783107
rs149656965
rs111498995
rs76954195
rs647667
rs72774956
rs184637108
rs7875568
rs7960425
rs80179348
rs4663135
rs77181656
rs75194109
All
−0.10
−0.05
0.00
0.05
MR leave−one−out sensitivity analysis for
'Threonate levels' on 'Rheumatoid arthritis'

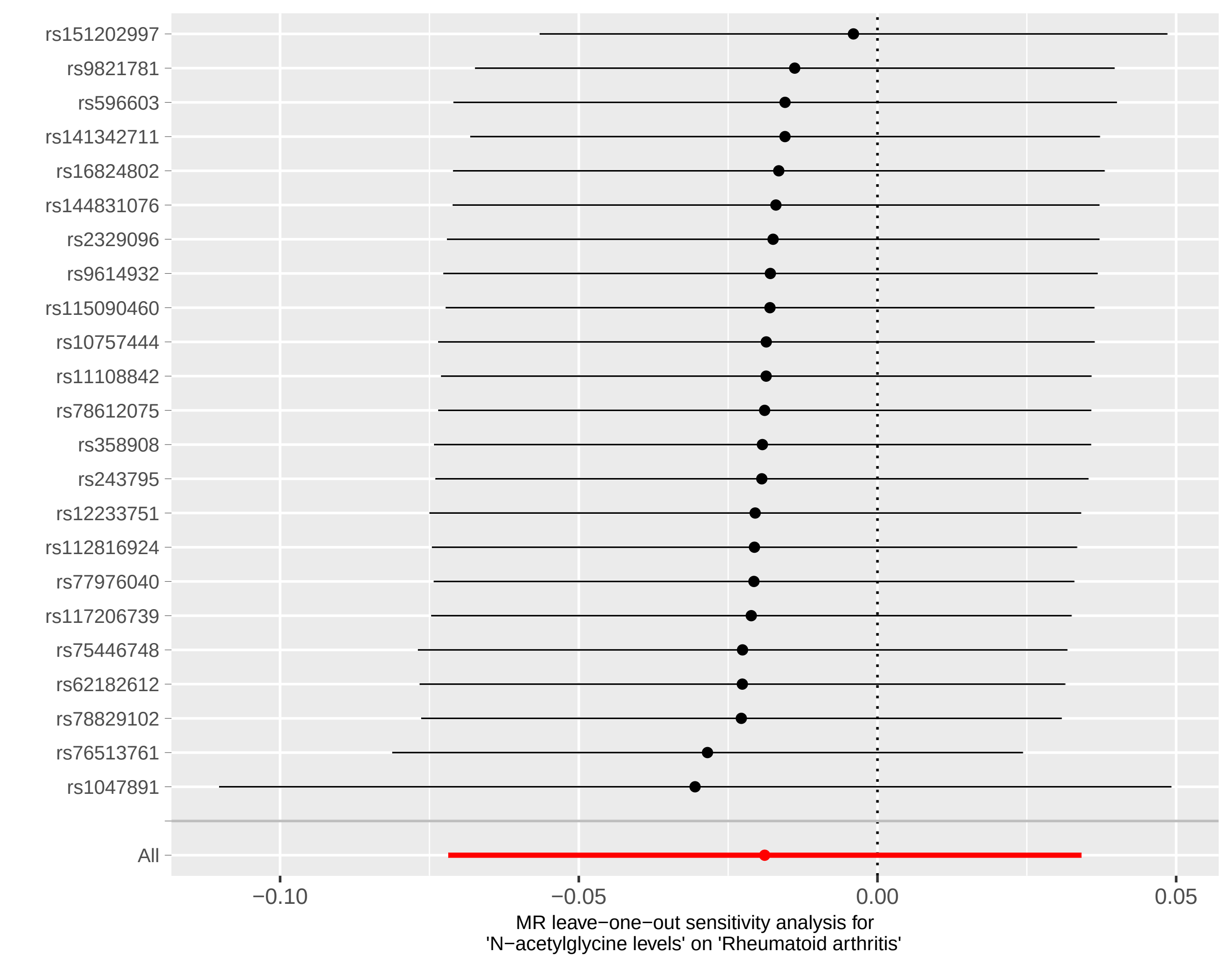
rs151202997
rs9821781
rs596603
rs141342711
rs16824802
rs144831076
rs2329096
rs9614932
rs115090460
rs10757444
rs11108842
rs78612075
rs358908
rs243795
rs12233751
rs112816924
rs77976040
rs117206739
rs75446748
rs62182612
rs78829102
rs76513761
rs1047891
All
−0.10
−0.05
0.00
0.05
MR leave−one−out sensitivity analysis for
'N−acetylglycine levels' on 'Rheumatoid arthritis'

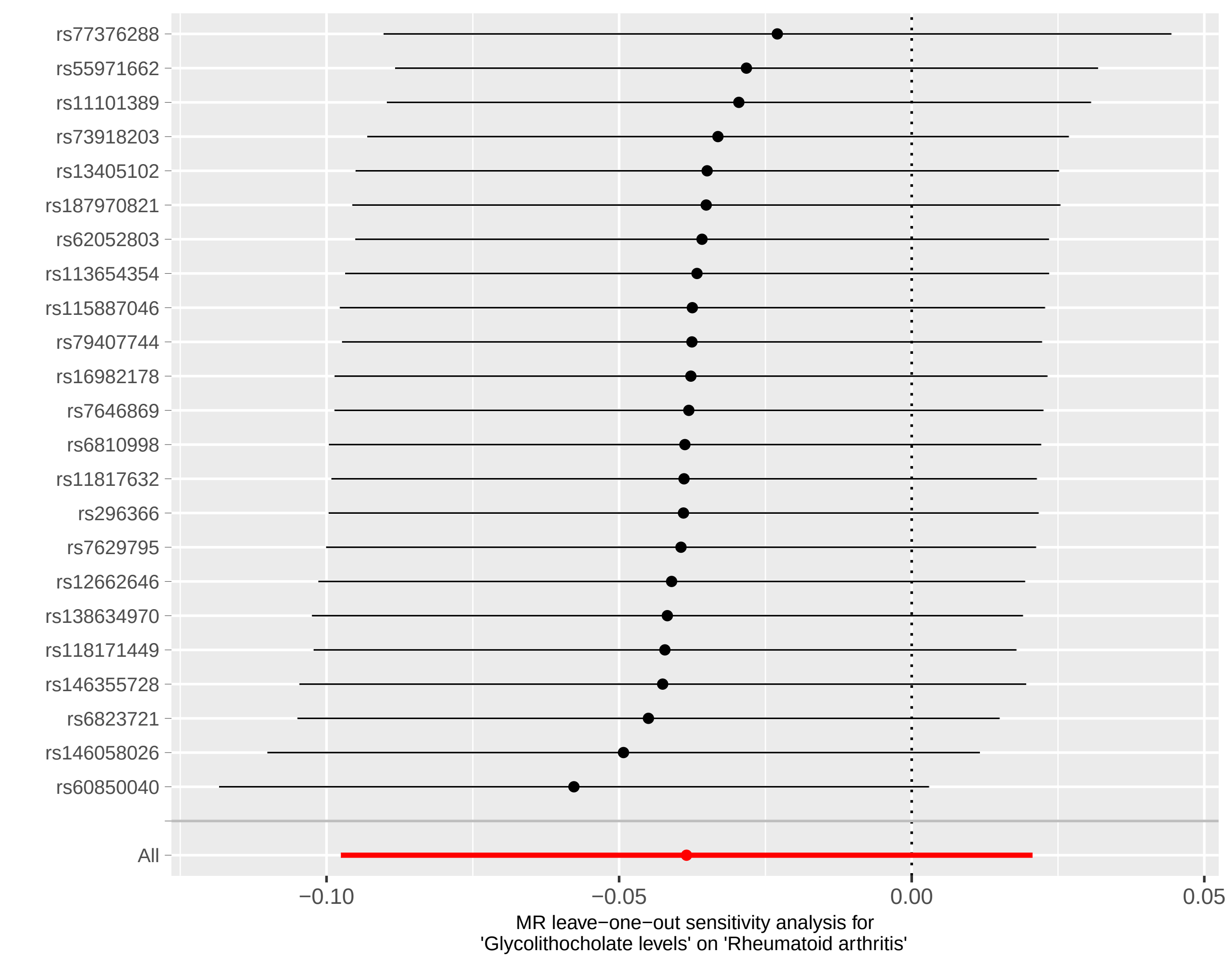
rs77376288
rs55971662
rs11101389
rs73918203
rs13405102
rs187970821
rs62052803
rs113654354
rs115887046
rs79407744
rs16982178
rs7646869
rs6810998
rs11817632
rs296366
rs7629795
rs12662646
rs138634970
rs118171449
rs146355728
rs6823721
rs146058026
rs60850040
All
−0.10
−0.05
0.00
0.05
MR leave−one−out sensitivity analysis for
'Glycolithocholate levels' on 'Rheumatoid arthritis'

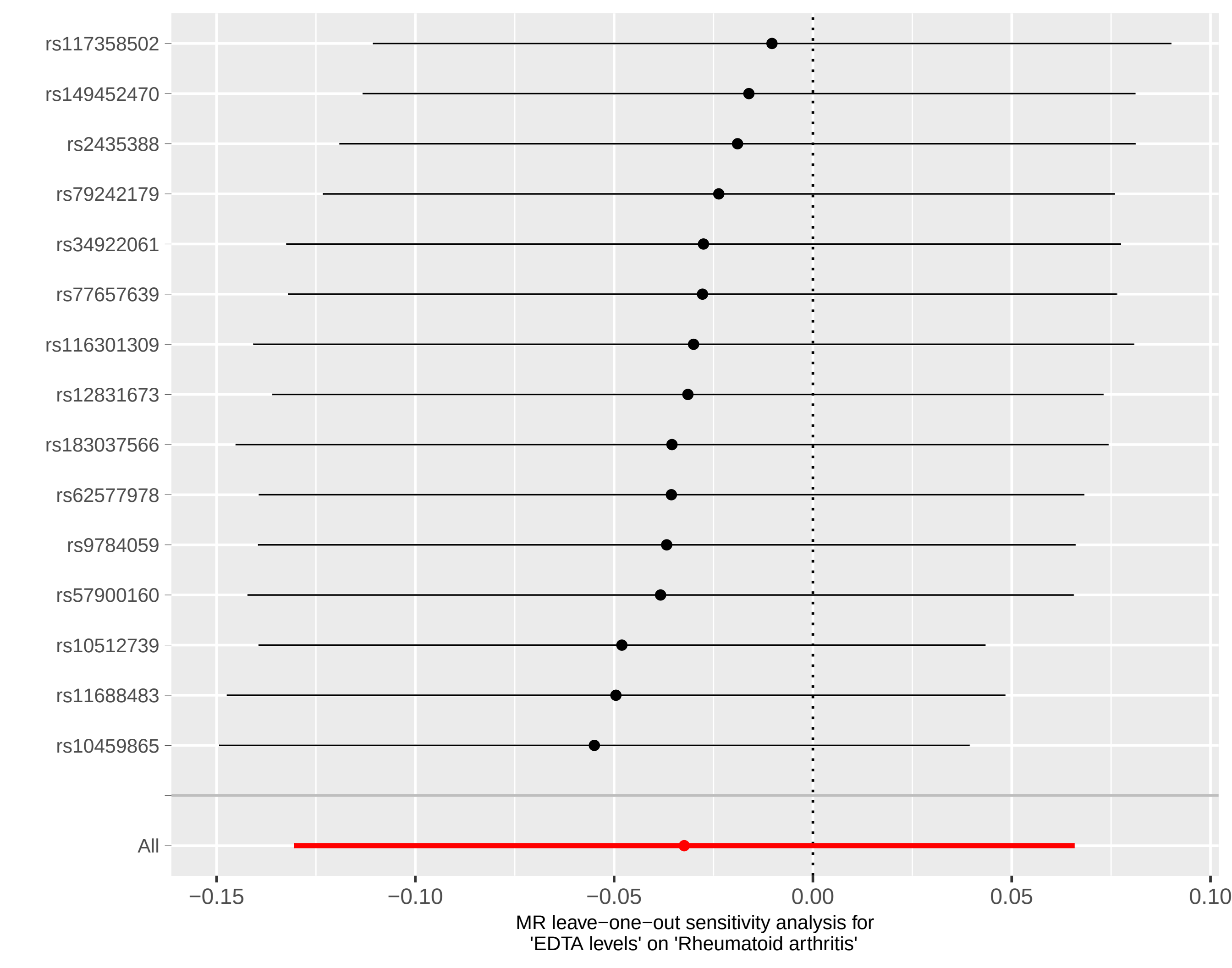
rs117358502
rs149452470
rs2435388
rs79242179
rs34922061
rs77657639
rs116301309
rs12831673
rs183037566
rs62577978
rs9784059
rs57900160
rs10512739
rs11688483
rs10459865
All
−0.15
−0.10
−0.05
0.00
0.05
0.10
MR leave−one−out sensitivity analysis for
'EDTA levels' on 'Rheumatoid arthritis'

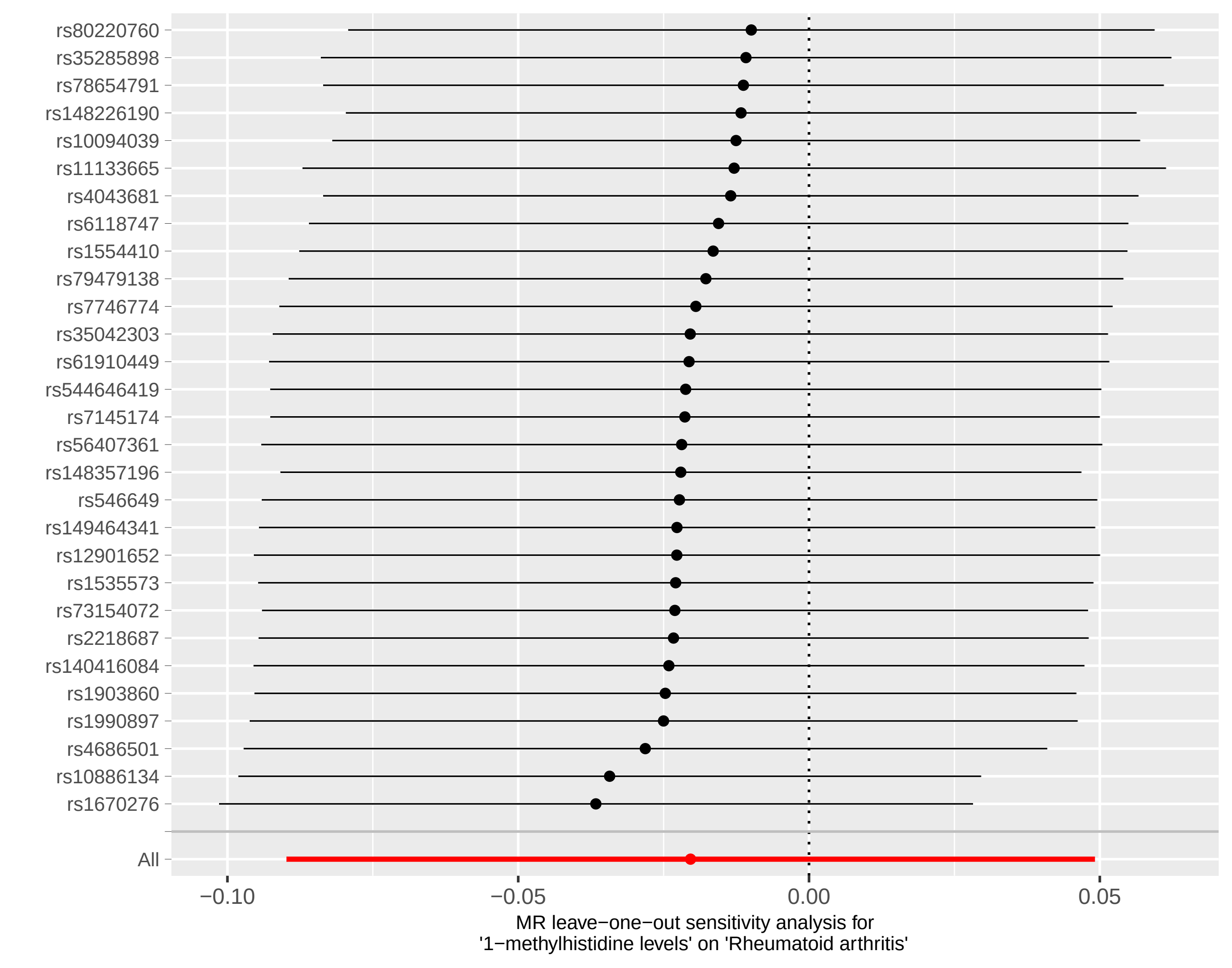

rs80220760
rs35285898
rs78654791
rs148226190
rs10094039
rs11133665
rs4043681
rs6118747
rs1554410
rs79479138
rs7746774
rs35042303
rs61910449
rs544646419
rs7145174
rs56407361
rs148357196
rs546649
rs149464341
rs12901652
rs1535573
rs73154072
rs2218687
rs140416084
rs1903860
rs1990897
rs4686501
rs10886134
rs1670276
All
−0.10
−0.05
0.00
0.05
MR leave−one−out sensitivity analysis for
'1−methylhistidine levels' on 'Rheumatoid arthritis'

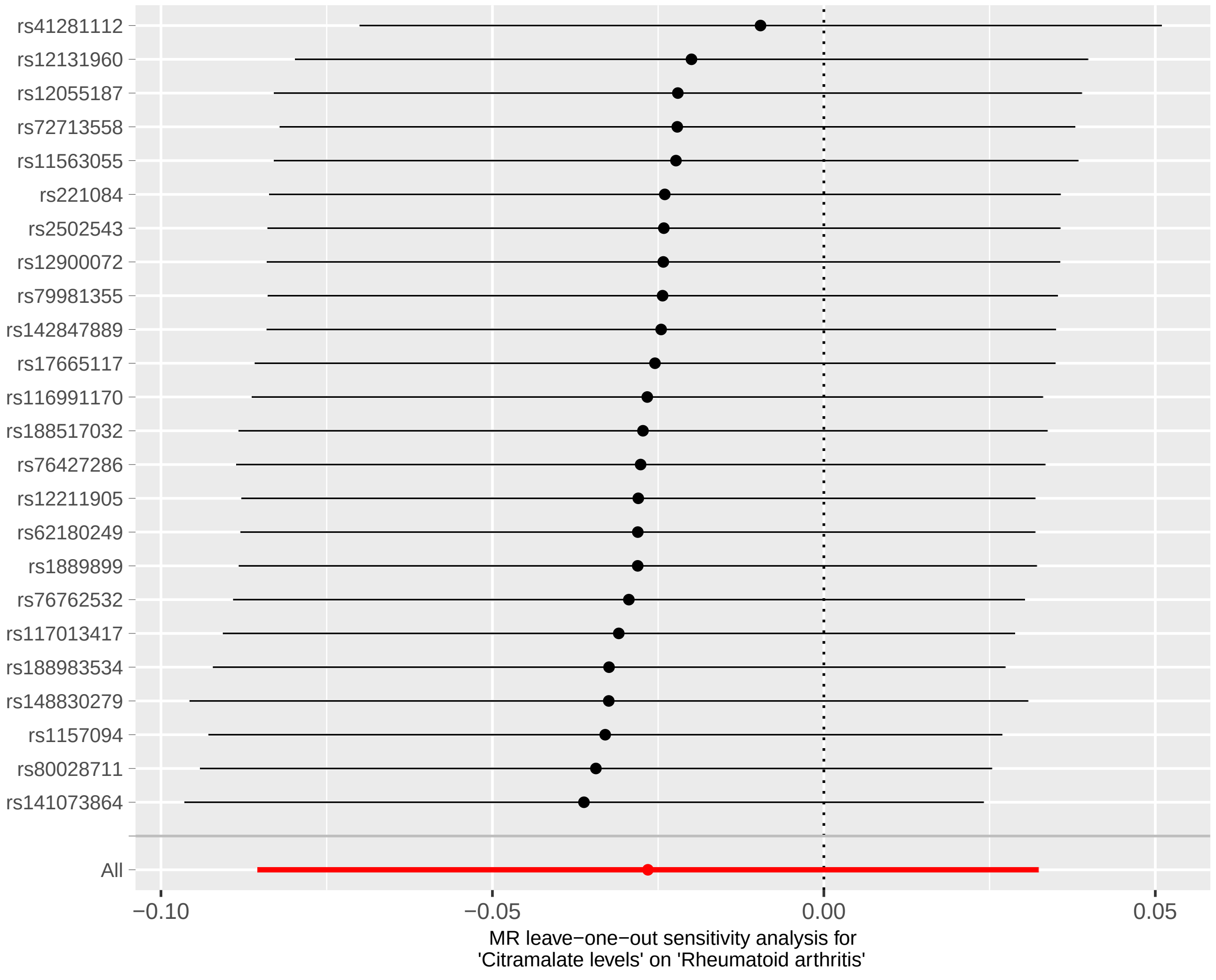

rs41281112
rs12131960
rs12055187
rs72713558
rs11563055
rs221084
rs2502543
rs12900072
rs79981355
rs142847889
rs17665117
rs116991170
rs188517032
rs76427286
rs12211905
rs62180249
rs1889899
rs76762532
rs117013417
rs188983534
rs148830279
rs1157094
rs80028711
rs141073864
All
−0.10
−0.05
0.00
0.05
MR leave−one−out sensitivity analysis for
'Citramalate levels' on 'Rheumatoid arthritis'

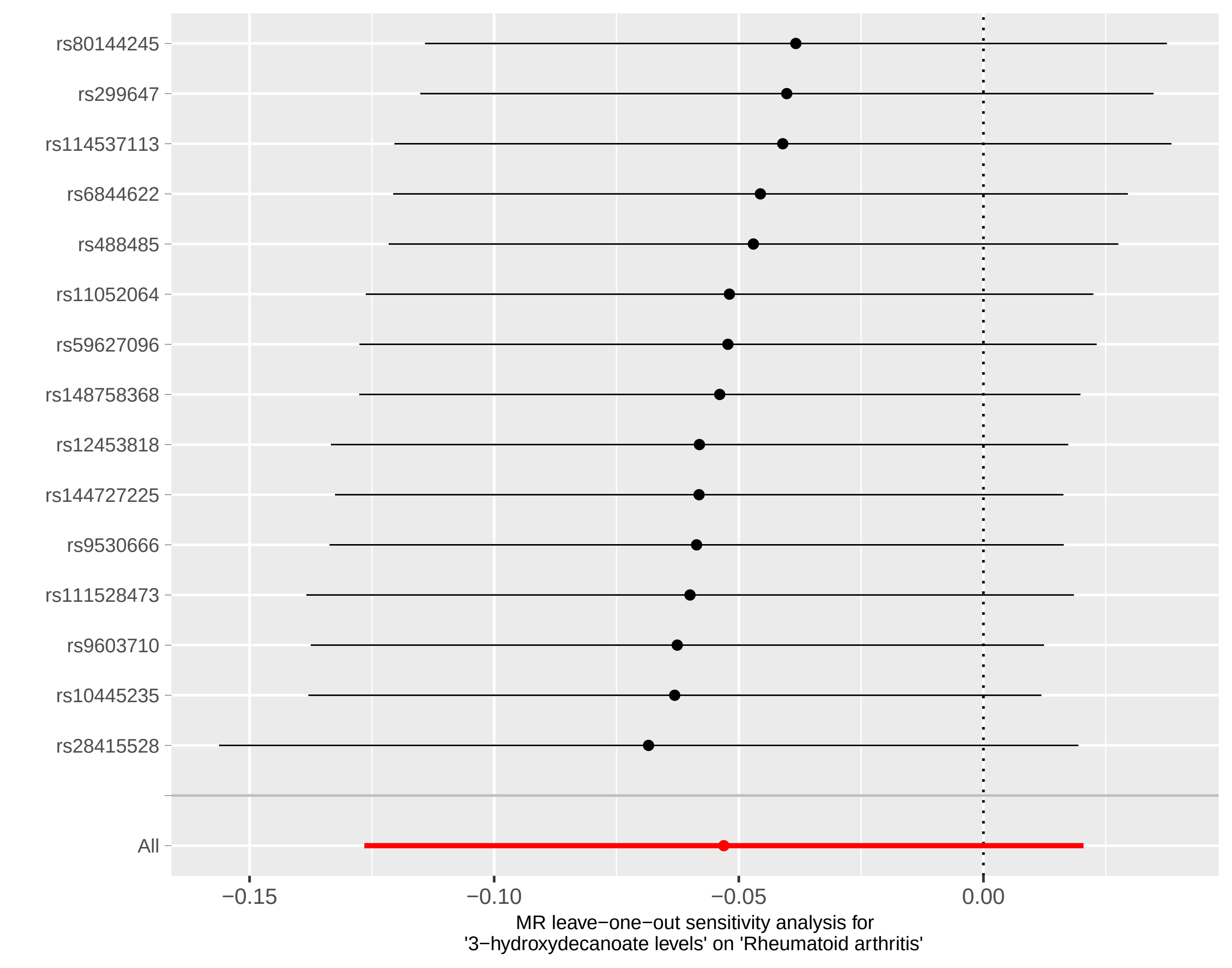

rs80144245
rs299647
rs114537113
rs6844622
rs488485
rs11052064
rs59627096
rs148758368
rs12453818
rs144727225
rs9530666
rs111528473
rs9603710
rs10445235
rs28415528
All
−0.15
−0.10
−0.05
0.00
MR leave−one−out sensitivity analysis for
'3−hydroxydecanoate levels' on 'Rheumatoid arthritis'

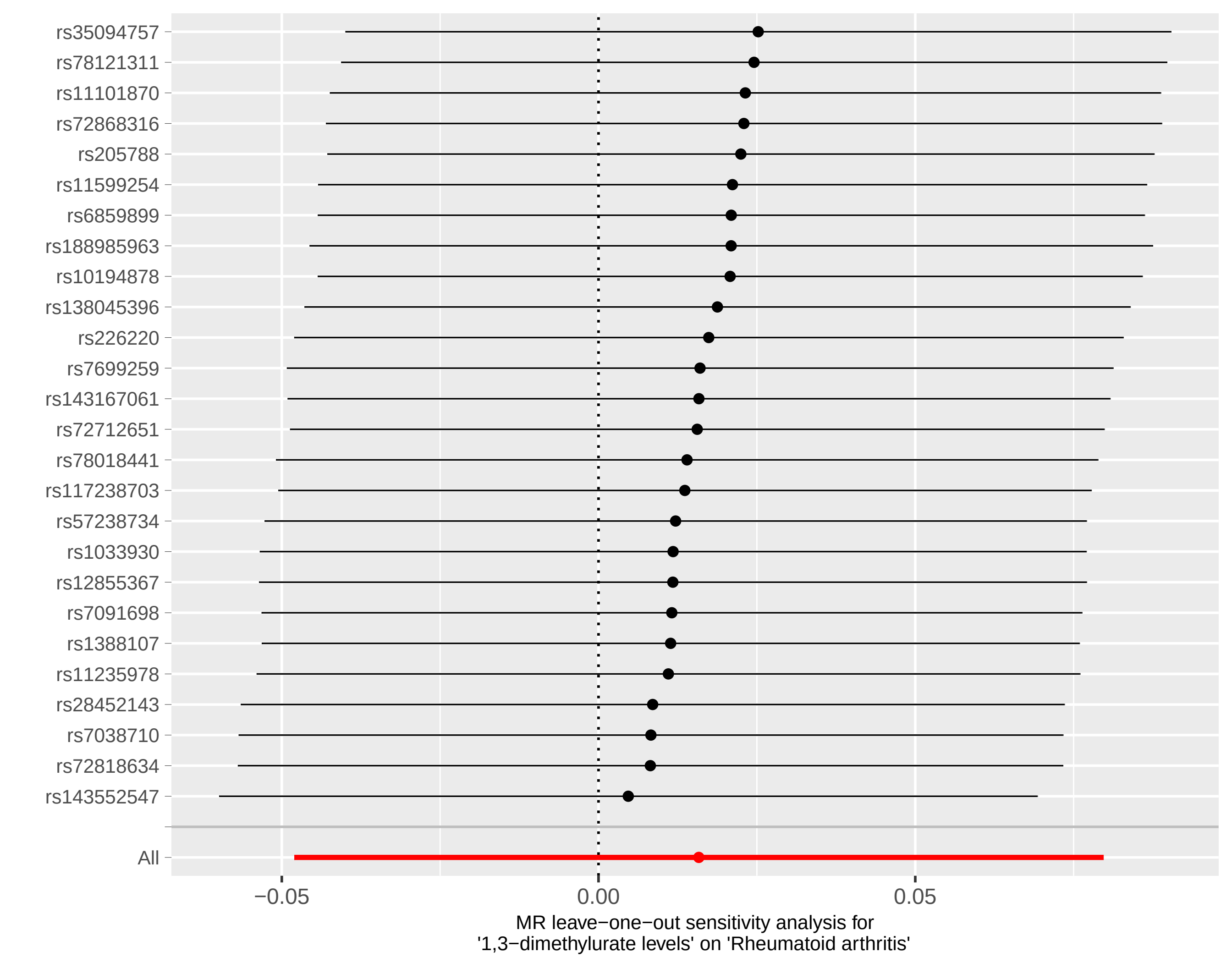

rs35094757
rs78121311
rs11101870
rs72868316
rs205788
rs11599254
rs6859899
rs188985963
rs10194878
rs138045396
rs226220
rs7699259
rs143167061
rs72712651
rs78018441
rs117238703
rs57238734
rs1033930
rs12855367
rs7091698
rs1388107
rs11235978
rs28452143
rs7038710
rs72818634
rs143552547
All
−0.05
0.00
0.05
MR leave−one−out sensitivity analysis for
'1,3−dimethylurate levels' on 'Rheumatoid arthritis'

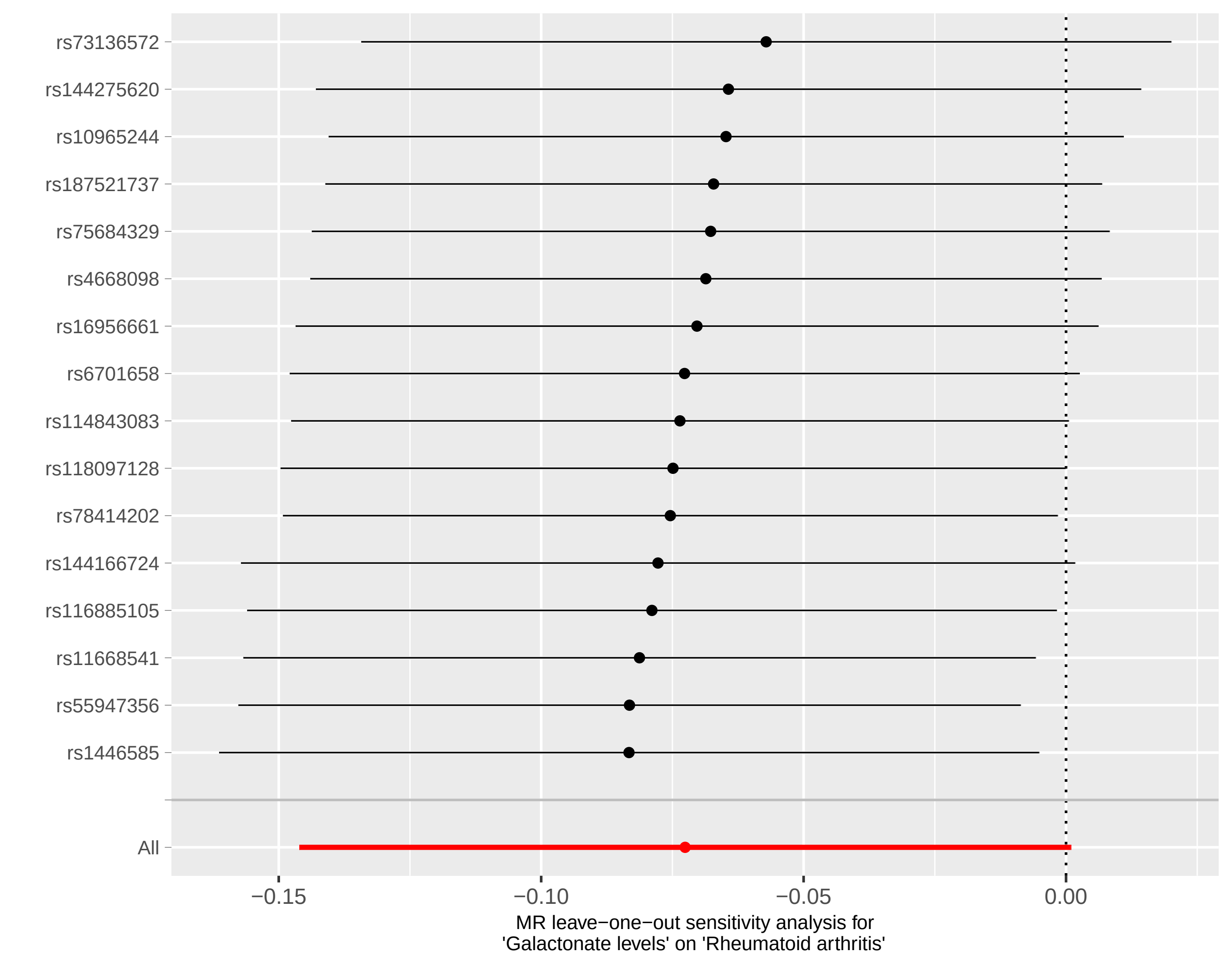
rs73136572
rs144275620
rs10965244
rs187521737
rs75684329
rs4668098
rs16956661
rs6701658
rs114843083
rs118097128
rs78414202
rs144166724
rs116885105
rs11668541
rs55947356
rs1446585
All
−0.15
−0.10
−0.05
0.00
MR leave−one−out sensitivity analysis for
'Galactonate levels' on 'Rheumatoid arthritis'

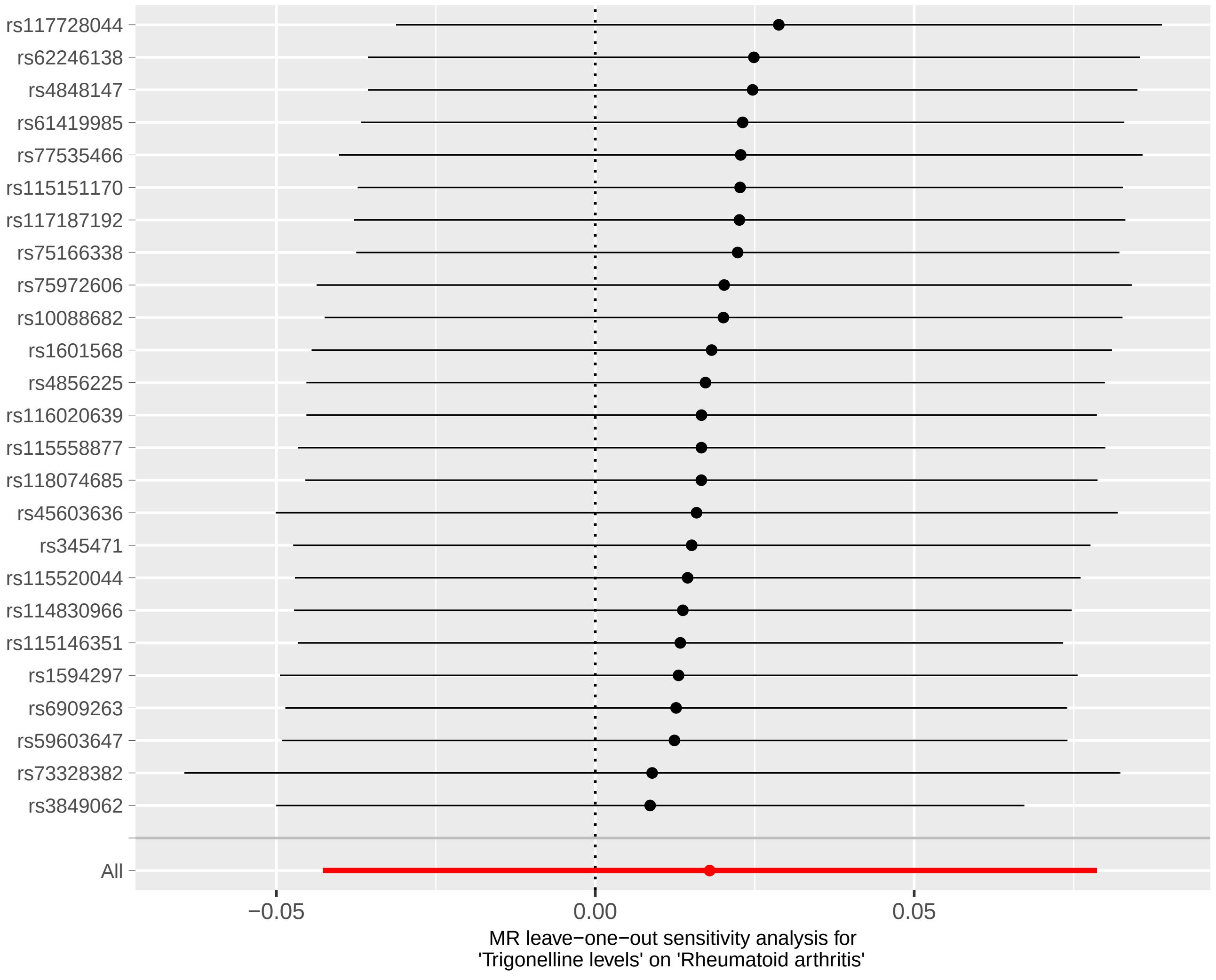
rs117728044
rs62246138
rs4848147
rs61419985
rs77535466
rs115151170
rs117187192
rs75166338
rs75972606
rs10088682
rs1601568
rs4856225
rs116020639
rs115558877
rs118074685
rs45603636
rs345471
rs115520044
rs114830966
rs115146351
rs1594297
rs6909263
rs59603647
rs73328382
rs3849062
All
−0.05
0.00
0.05
MR leave−one−out sensitivity analysis for
'Trigonelline levels' on 'Rheumatoid arthritis'

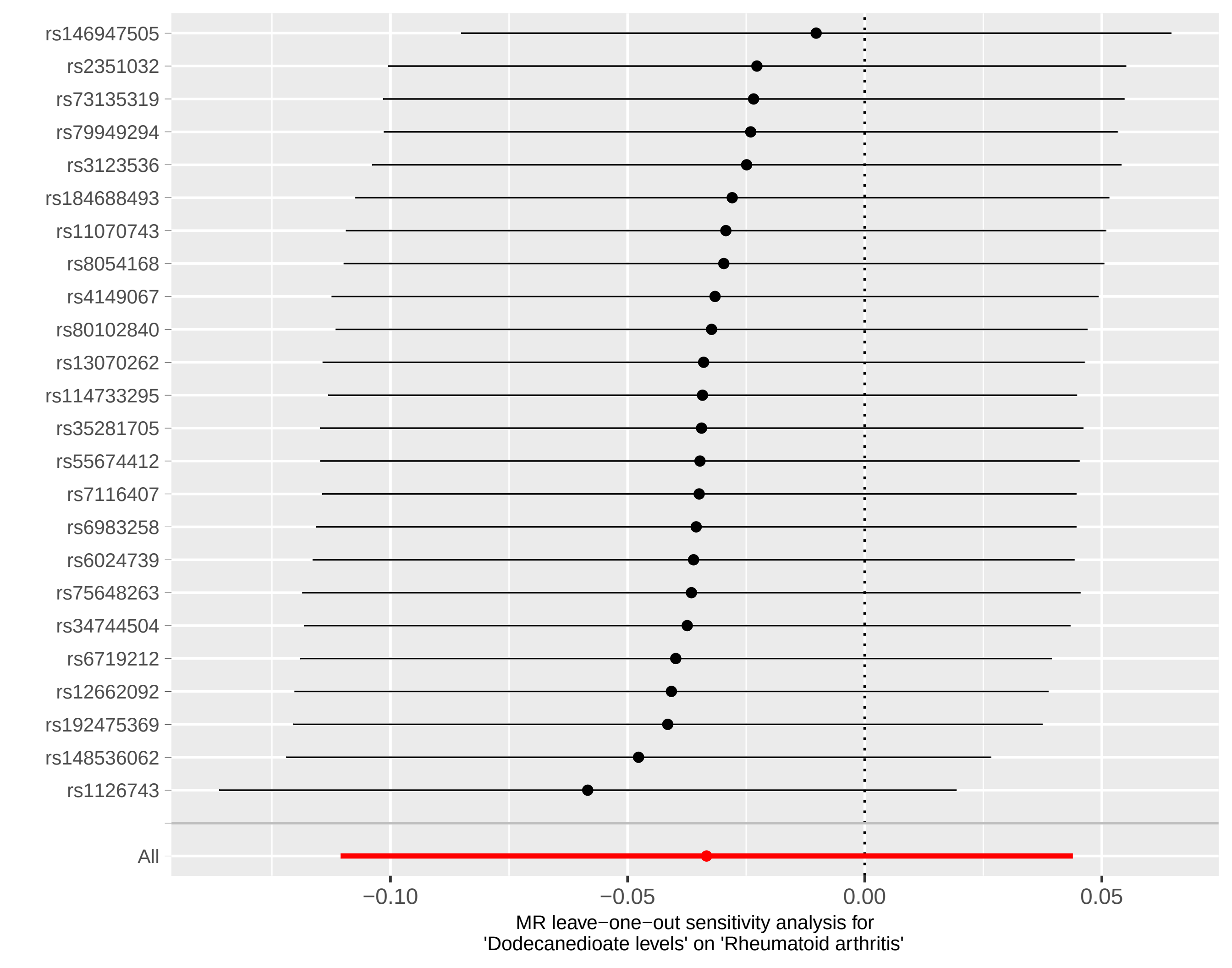

rs146947505
rs2351032
rs73135319
rs79949294
rs3123536
rs184688493
rs11070743
rs8054168
rs4149067
rs80102840
rs13070262
rs114733295
rs35281705
rs55674412
rs7116407
rs6983258
rs6024739
rs75648263
rs34744504
rs6719212
rs12662092
rs192475369
rs148536062
rs1126743
All
−0.10
−0.05
0.00
0.05
MR leave−one−out sensitivity analysis for
'Dodecanedioate levels' on 'Rheumatoid arthritis'

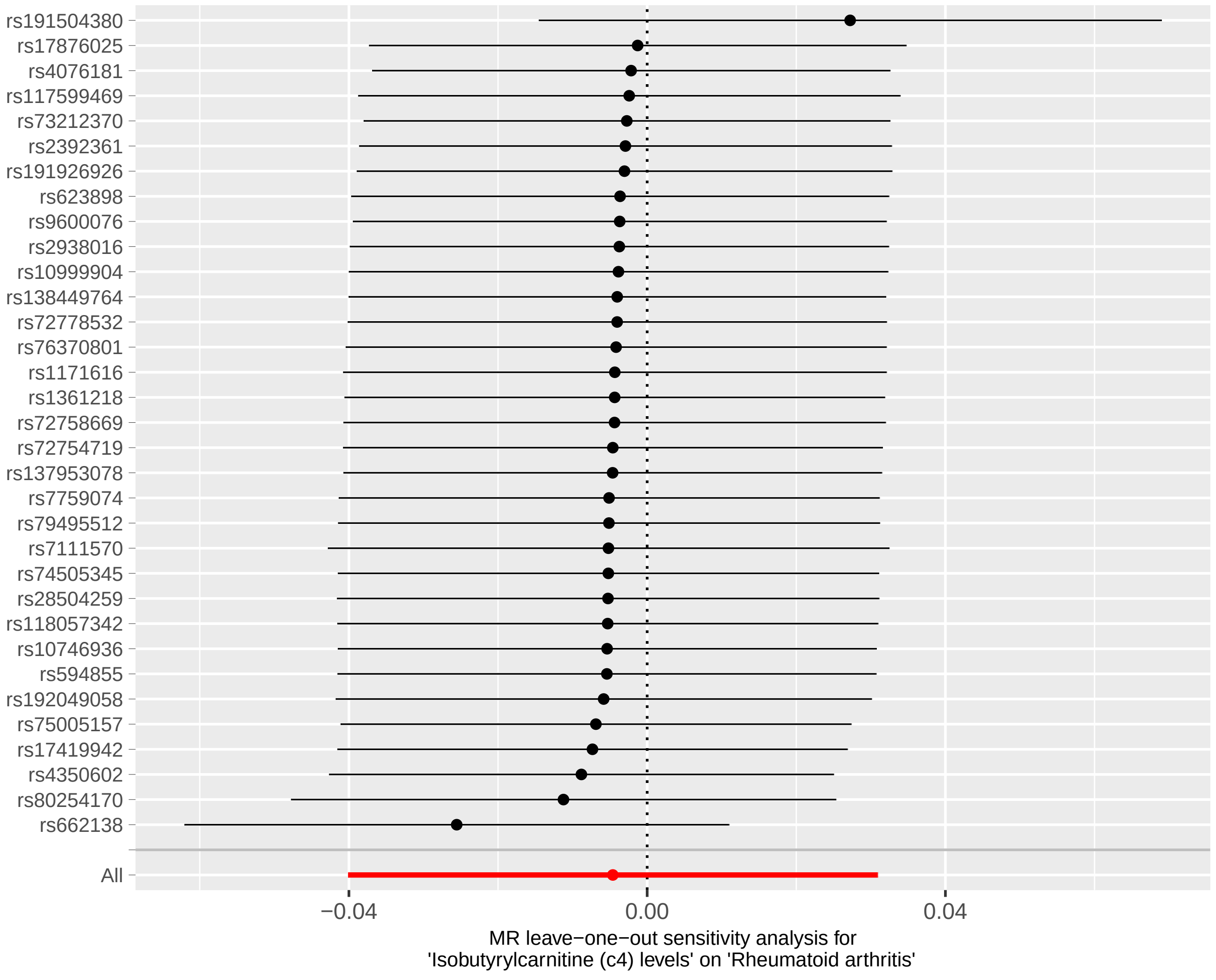
rs191504380
rs17876025
rs4076181
rs117599469
rs73212370
rs2392361
rs191926926
rs623898
rs9600076
rs2938016
rs10999904
rs138449764
rs72778532
rs76370801
rs1171616
rs1361218
rs72758669
rs72754719
rs137953078
rs7759074
rs79495512
rs7111570
rs74505345
rs28504259
rs118057342
rs10746936
rs594855
rs192049058
rs75005157
rs17419942
rs4350602
rs80254170
rs662138
All
−0.04
0.00
0.04
MR leave−one−out sensitivity analysis for
'Isobutyrylcarnitine (c4) levels' on 'Rheumatoid arthritis'

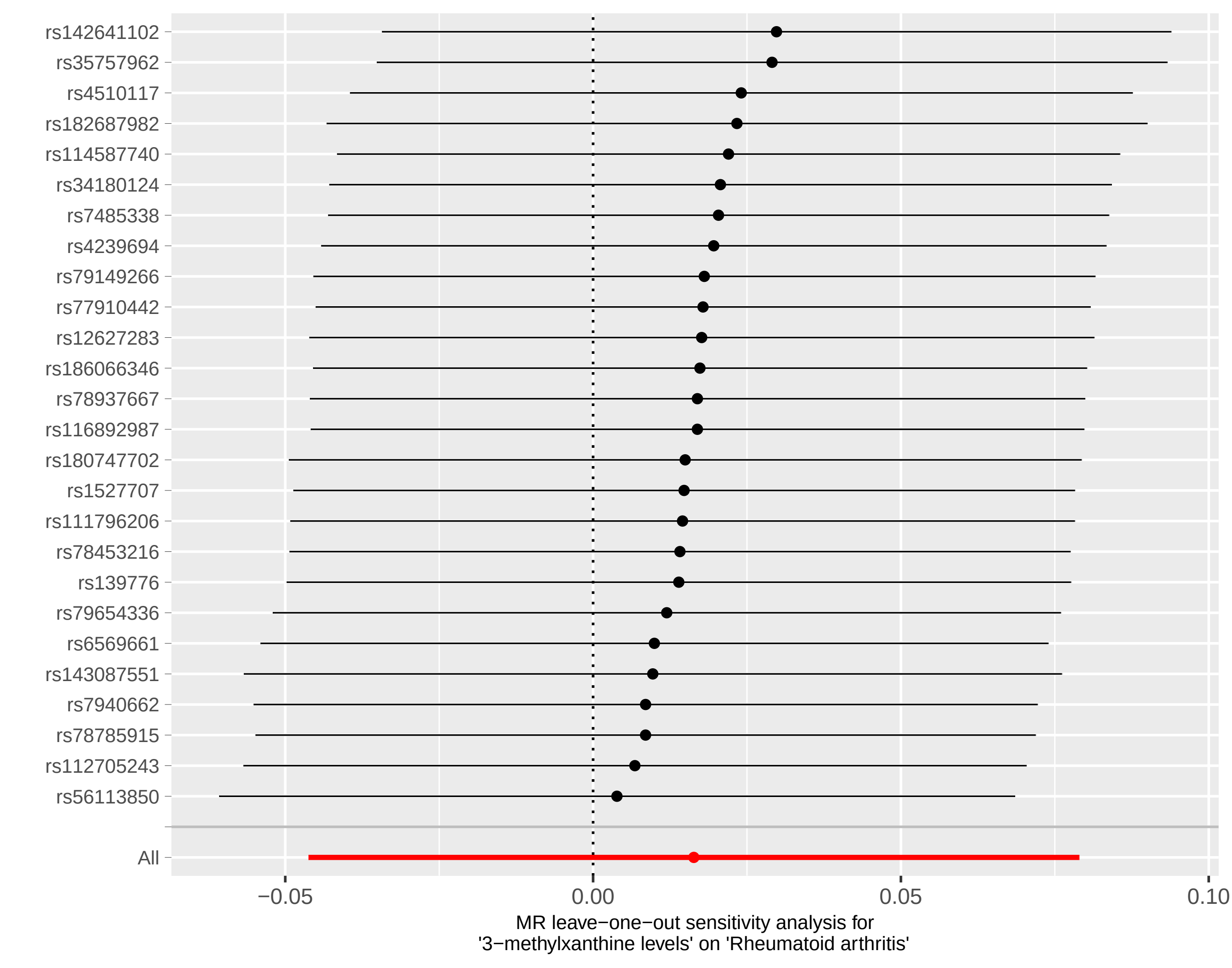
rs142641102
rs35757962
rs4510117
rs182687982
rs114587740
rs34180124
rs7485338
rs4239694
rs79149266
rs77910442
rs12627283
rs186066346
rs78937667
rs116892987
rs180747702
rs1527707
rs111796206
rs78453216
rs139776
rs79654336
rs6569661
rs143087551
rs7940662
rs78785915
rs112705243
rs56113850
All
−0.05
0.00
0.05
0.10
MR leave−one−out sensitivity analysis for
'3−methylxanthine levels' on 'Rheumatoid arthritis'

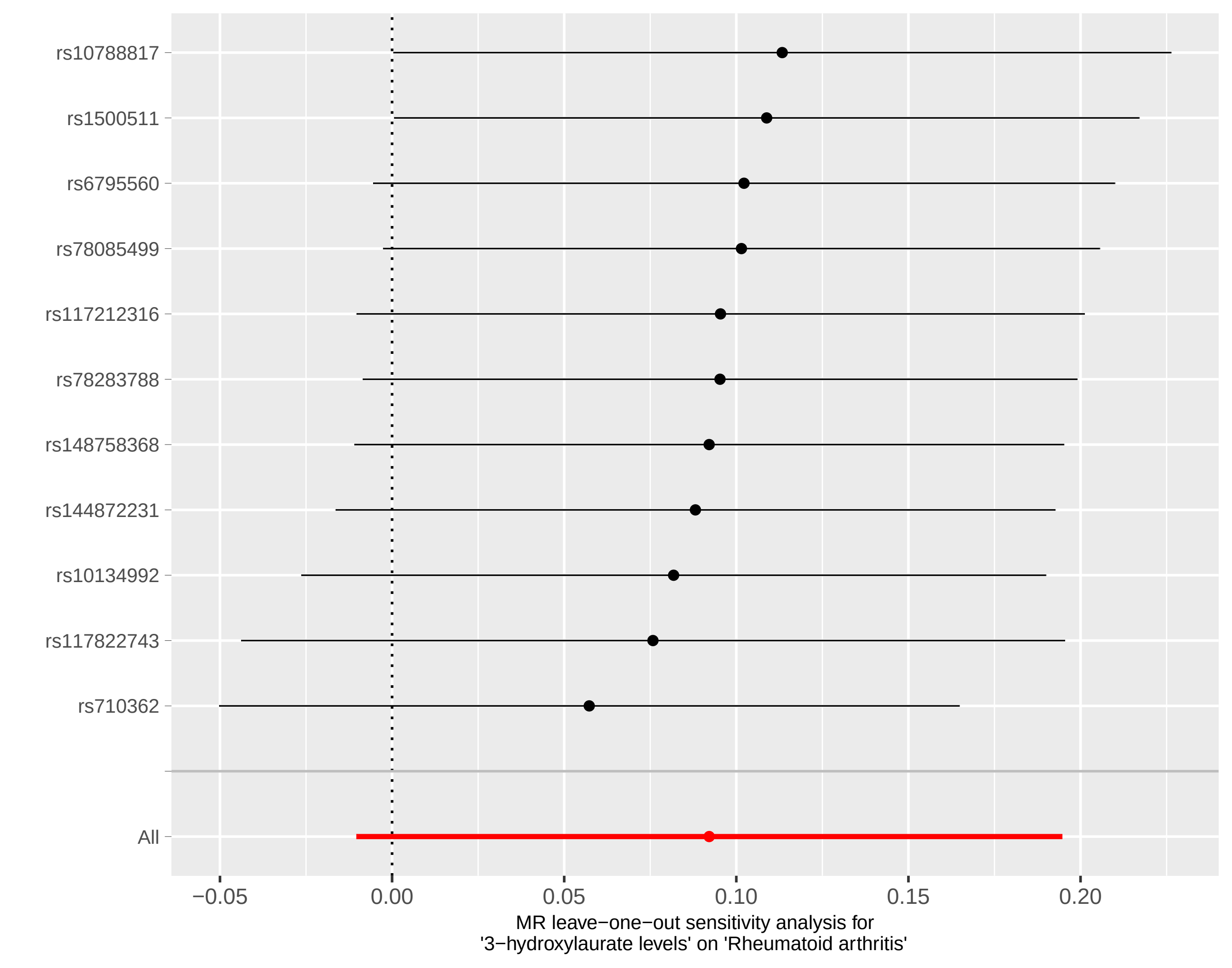
rs10788817
rs1500511
rs6795560
rs78085499
rs117212316
rs78283788
rs148758368
rs144872231
rs10134992
rs117822743
rs710362
All
−0.05
0.00
0.05
0.10
0.15
0.20
MR leave−one−out sensitivity analysis for
'3−hydroxylaurate levels' on 'Rheumatoid arthritis'

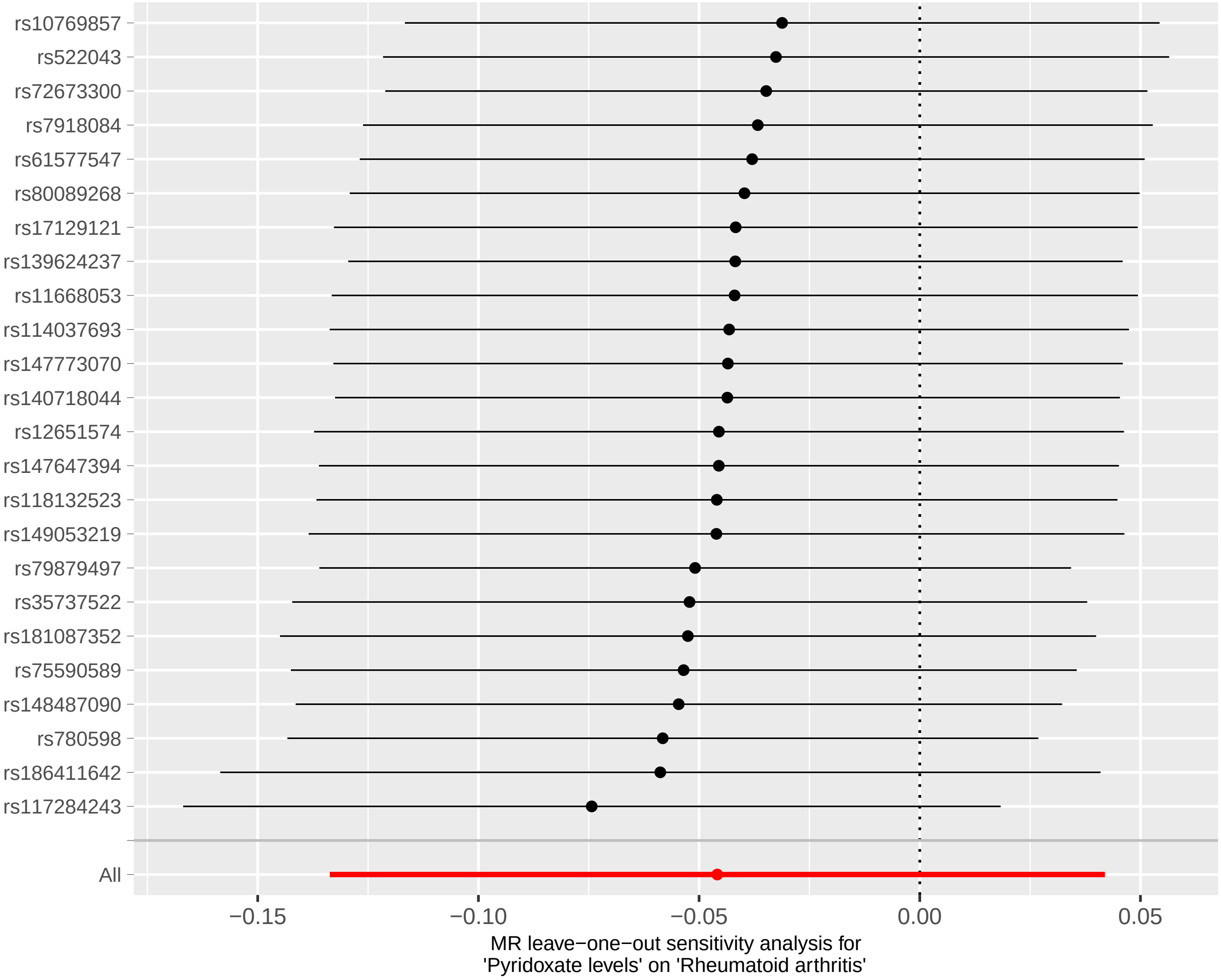
rs10769857
rs522043
rs72673300
rs7918084
rs61577547
rs80089268
rs17129121
rs139624237
rs11668053
rs114037693
rs147773070
rs140718044
rs12651574
rs147647394
rs118132523
rs149053219
rs79879497
rs35737522
rs181087352
rs75590589
rs148487090
rs780598
rs186411642
rs117284243
All
−0.15
−0.10
−0.05
0.00
0.05
MR leave−one−out sensitivity analysis for
'Pyridoxate levels' on 'Rheumatoid arthritis'

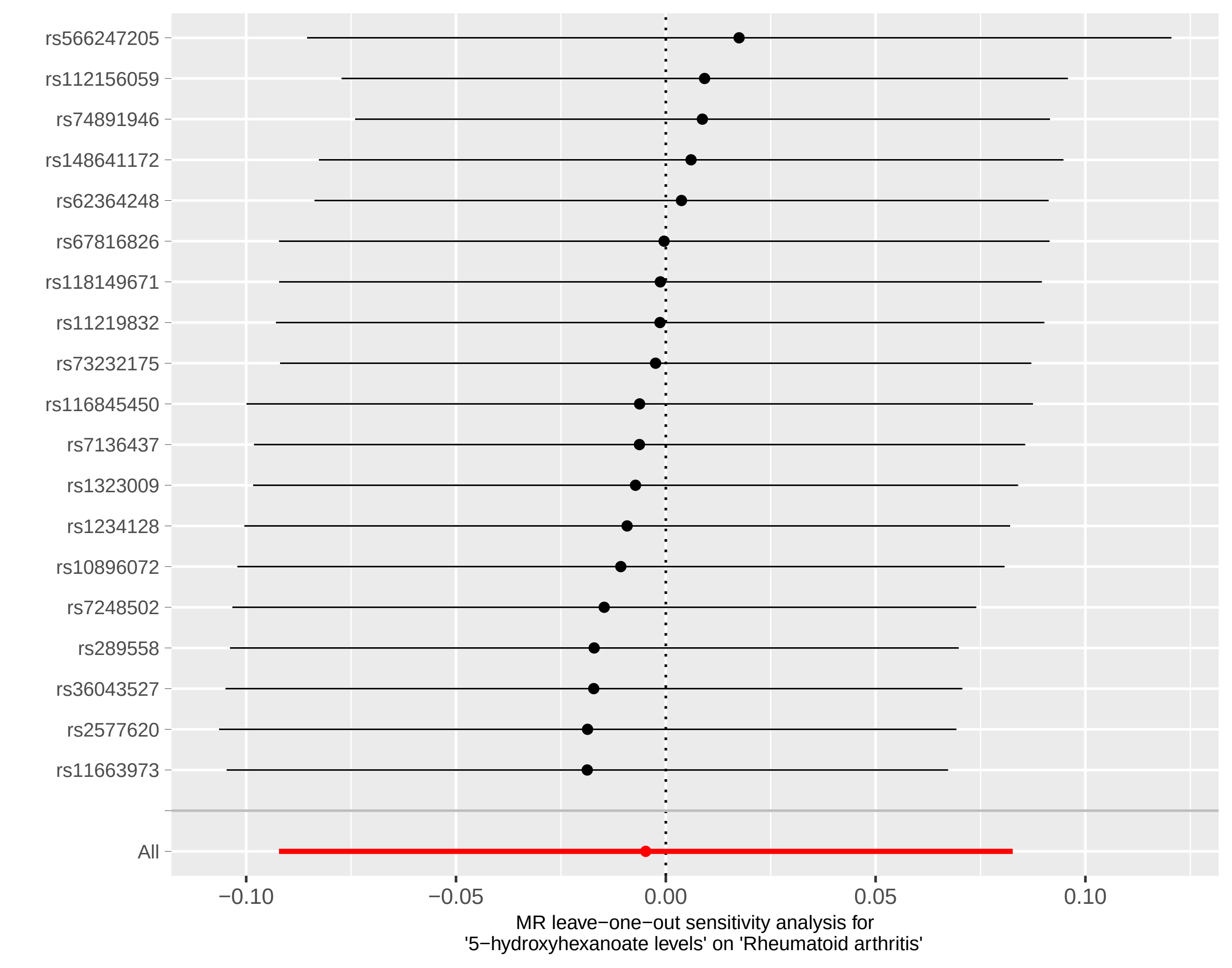

rs566247205
rs112156059
rs74891946
rs148641172
rs62364248
rs67816826
rs118149671
rs11219832
rs73232175
rs116845450
rs7136437
rs1323009
rs1234128
rs10896072
rs7248502
rs289558
rs36043527
rs2577620
rs11663973
All
−0.10
−0.05
0.00
0.05
0.10
MR leave−one−out sensitivity analysis for
'5−hydroxyhexanoate levels' on 'Rheumatoid arthritis'

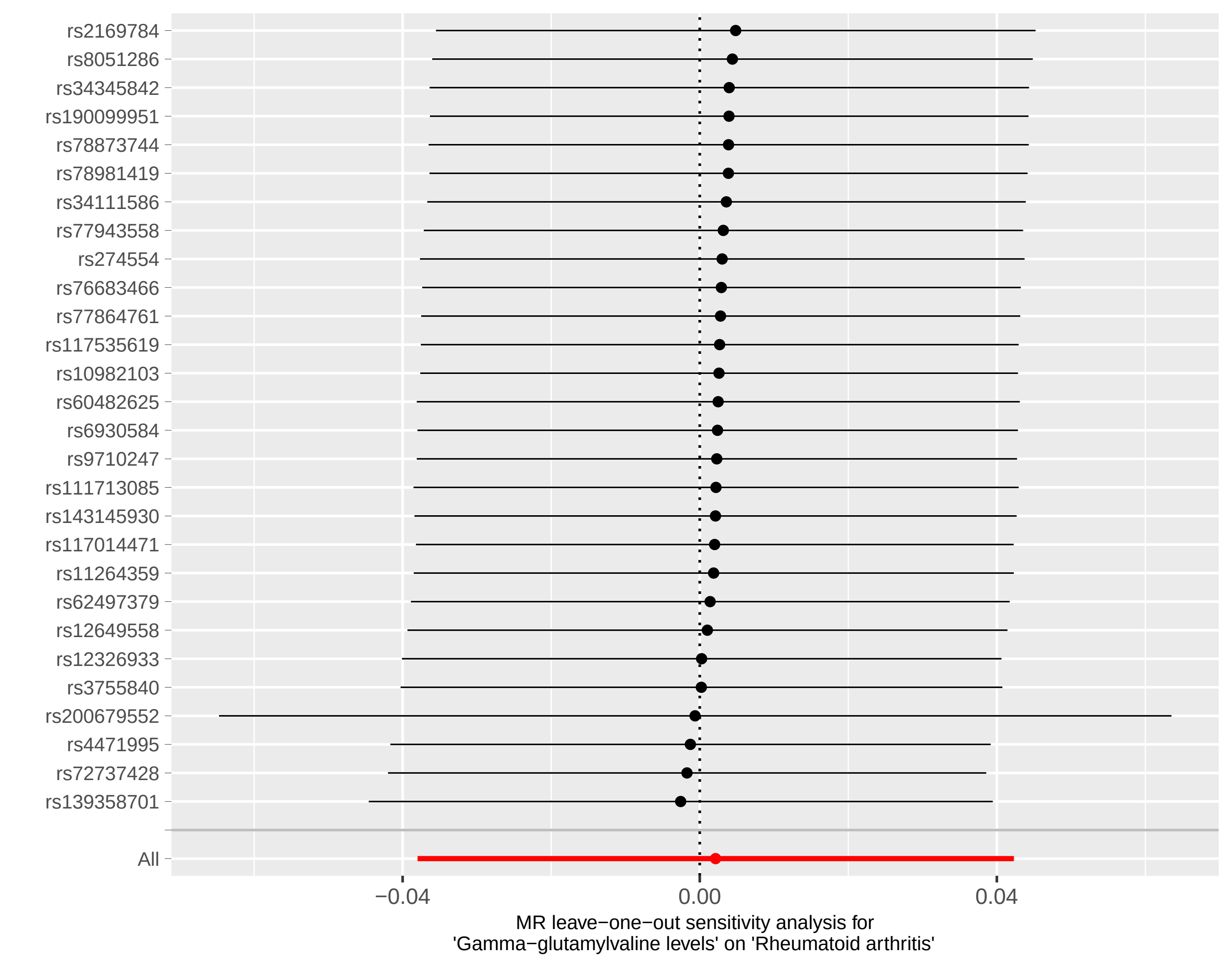

rs2169784
rs8051286
rs34345842
rs190099951
rs78873744
rs78981419
rs34111586
rs77943558
rs274554
rs76683466
rs77864761
rs117535619
rs10982103
rs60482625
rs6930584
rs9710247
rs111713085
rs143145930
rs117014471
rs11264359
rs62497379
rs12649558
rs12326933
rs3755840
rs200679552
rs4471995
rs72737428
rs139358701
All
−0.04
0.00
0.04
MR leave−one−out sensitivity analysis for
'Gamma−glutamylvaline levels' on 'Rheumatoid arthritis'

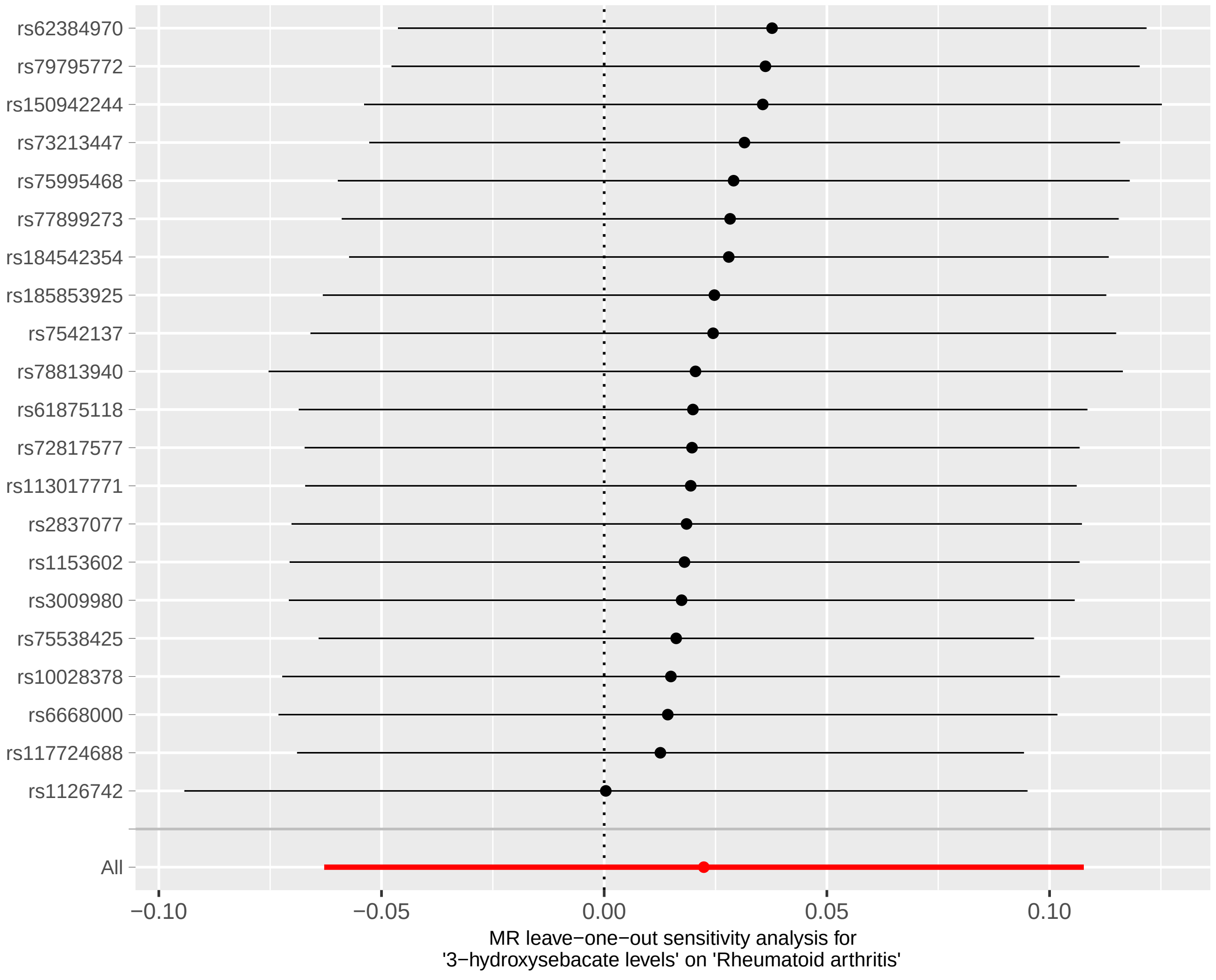
rs62384970
rs79795772
rs150942244
rs73213447
rs75995468
rs77899273
rs184542354
rs185853925
rs7542137
rs78813940
rs61875118
rs72817577
rs113017771
rs2837077
rs1153602
rs3009980
rs75538425
rs10028378
rs6668000
rs117724688
rs1126742
All
−0.10
−0.05
0.00
0.05
0.10
MR leave−one−out sensitivity analysis for
'3−hydroxysebacate levels' on 'Rheumatoid arthritis'

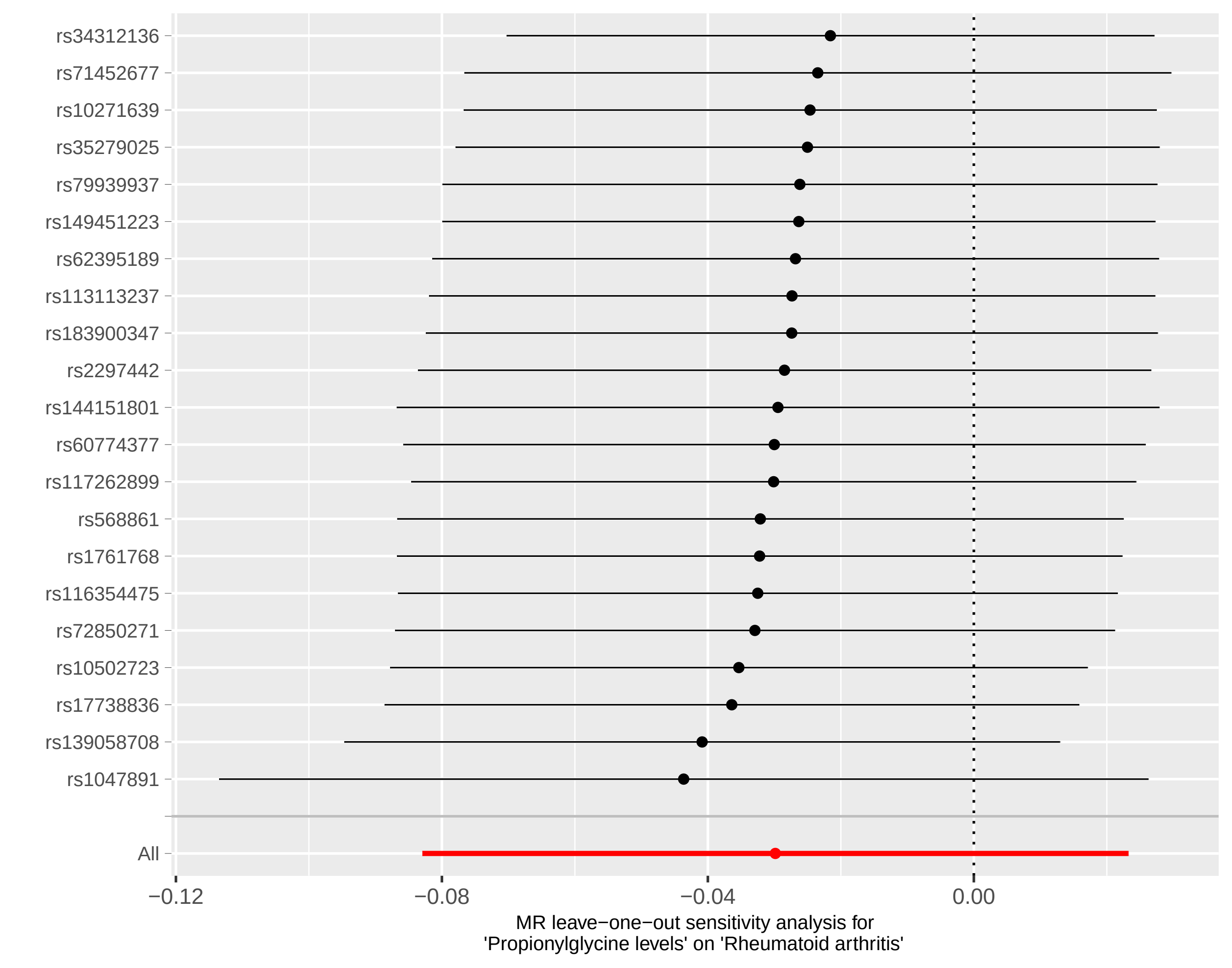

rs34312136
rs71452677
rs10271639
rs35279025
rs79939937
rs149451223
rs62395189
rs113113237
rs183900347
rs2297442
rs144151801
rs60774377
rs117262899
rs568861
rs1761768
rs116354475
rs72850271
rs10502723
rs17738836
rs139058708
rs1047891
All
−0.12
−0.08
−0.04
0.00
MR leave−one−out sensitivity analysis for
'Propionylglycine levels' on 'Rheumatoid arthritis'

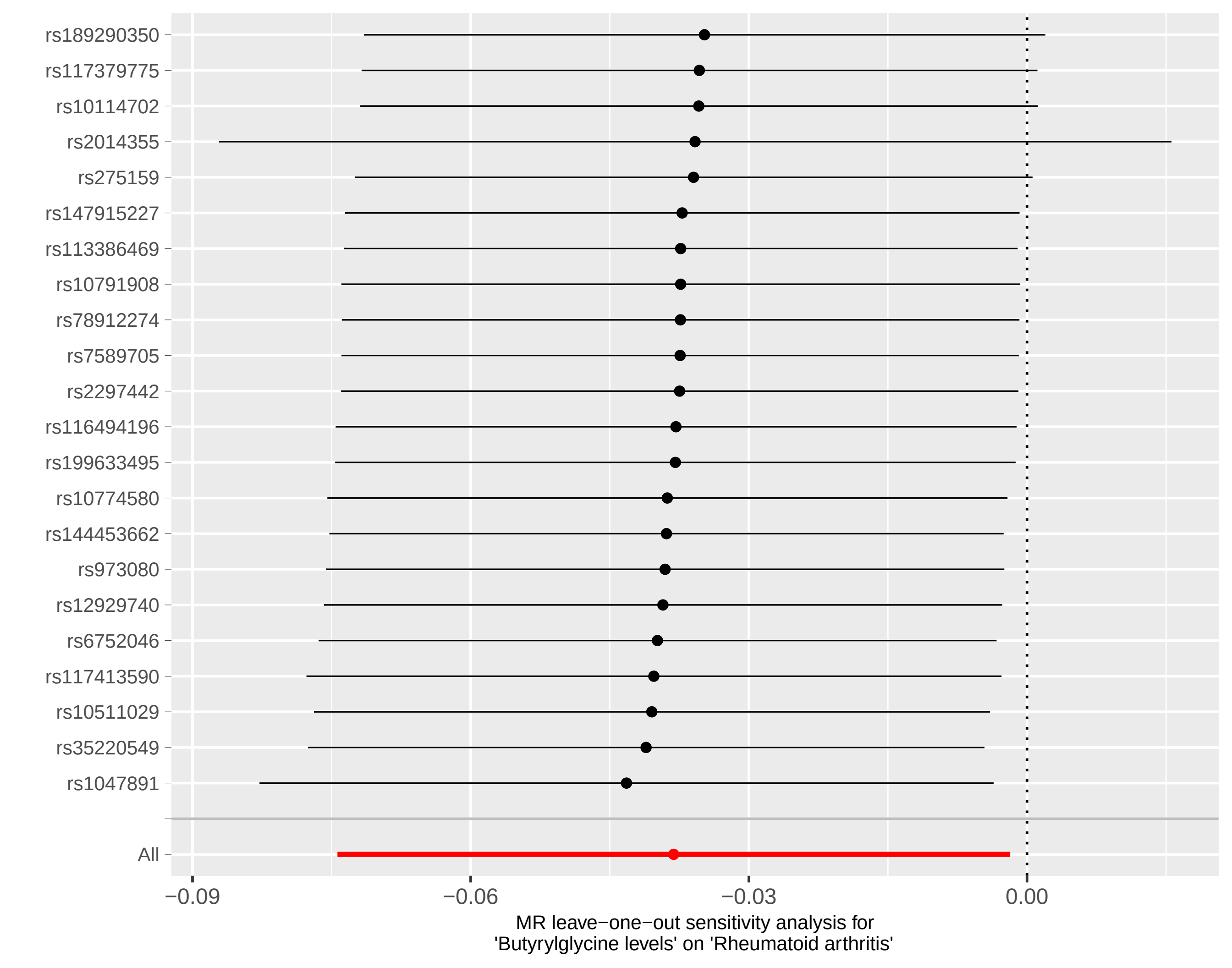
rs189290350
rs117379775
rs10114702
rs2014355
rs275159
rs147915227
rs113386469
rs10791908
rs78912274
rs7589705
rs2297442
rs116494196
rs199633495
rs10774580
rs144453662
rs973080
rs12929740
rs6752046
rs117413590
rs10511029
rs35220549
rs1047891
All
−0.09
−0.06
−0.03
0.00
MR leave−one−out sensitivity analysis for
'Butyrylglycine levels' on 'Rheumatoid arthritis'

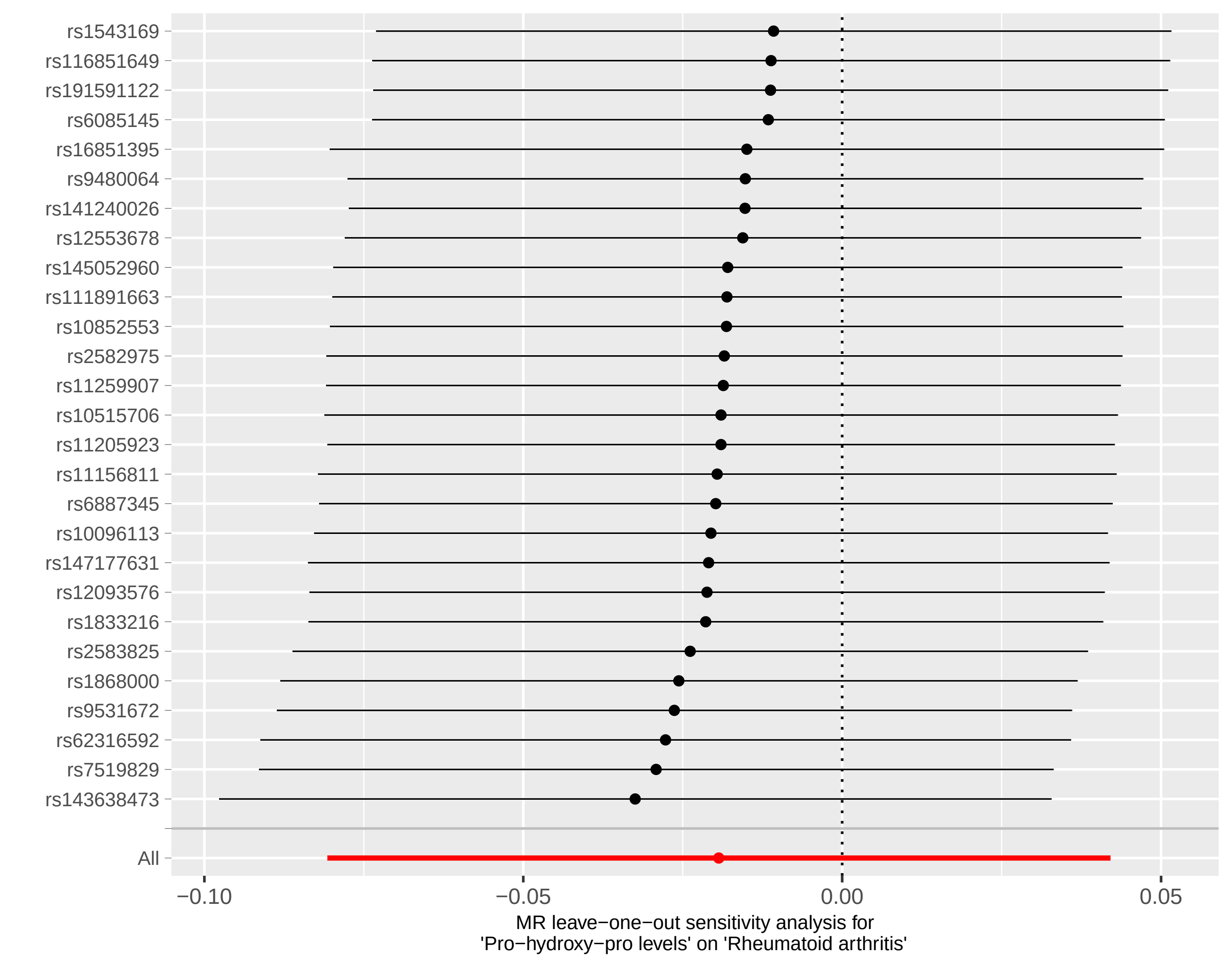
rs1543169
rs116851649
rs191591122
rs6085145
rs16851395
rs9480064
rs141240026
rs12553678
rs145052960
rs111891663
rs10852553
rs2582975
rs11259907
rs10515706
rs11205923
rs11156811
rs6887345
rs10096113
rs147177631
rs12093576
rs1833216
rs2583825
rs1868000
rs9531672
rs62316592
rs7519829
rs143638473
All
−0.10
−0.05
0.00
0.05
MR leave−one−out sensitivity analysis for
'Pro−hydroxy−pro levels' on 'Rheumatoid arthritis'

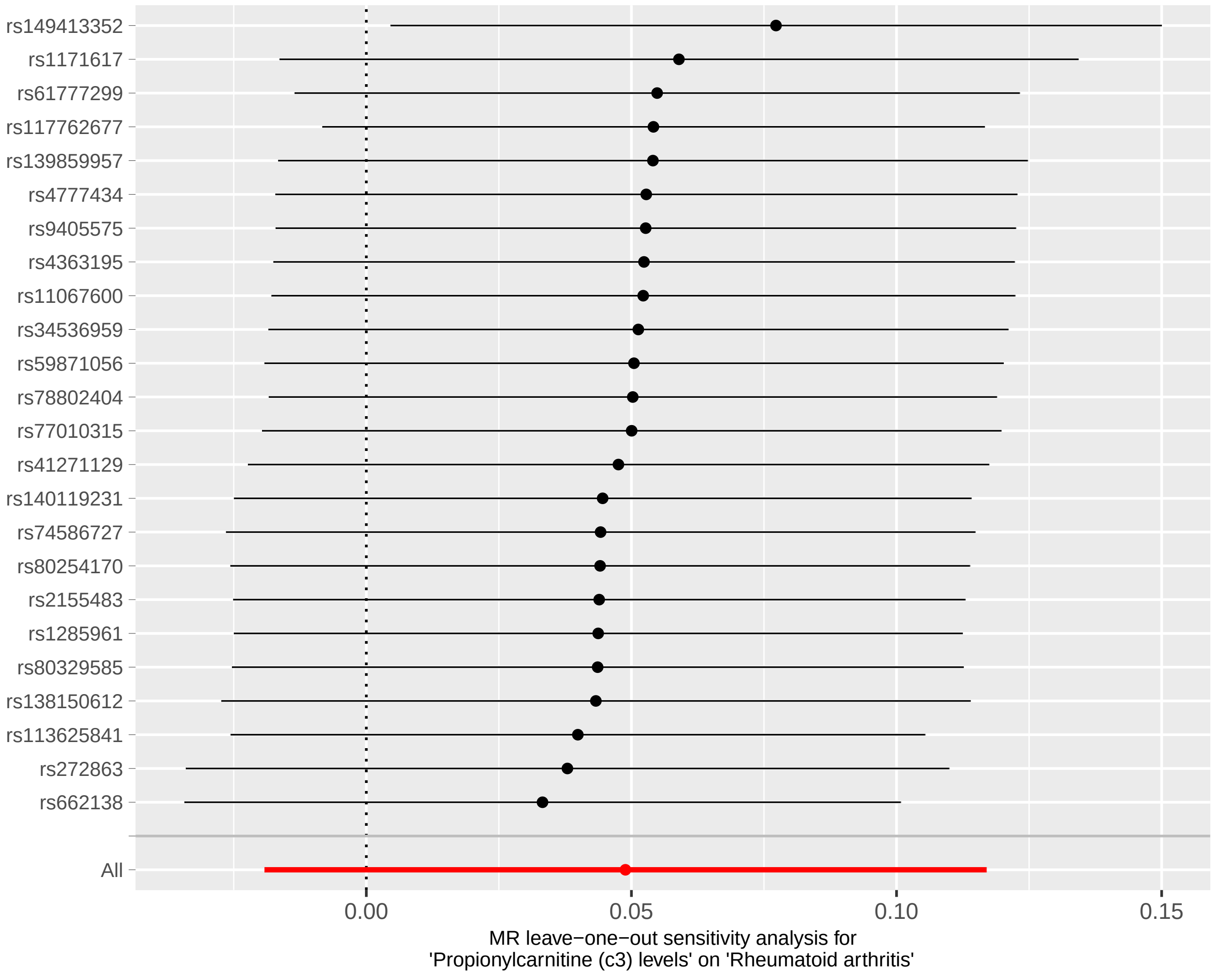
rs149413352
rs1171617
rs61777299
rs117762677
rs139859957
rs4777434
rs9405575
rs4363195
rs11067600
rs34536959
rs59871056
rs78802404
rs77010315
rs41271129
rs140119231
rs74586727
rs80254170
rs2155483
rs1285961
rs80329585
rs138150612
rs113625841
rs272863
rs662138
All
0.00
0.05
0.10
0.15
MR leave−one−out sensitivity analysis for
'Propionylcarnitine (c3) levels' on 'Rheumatoid arthritis'

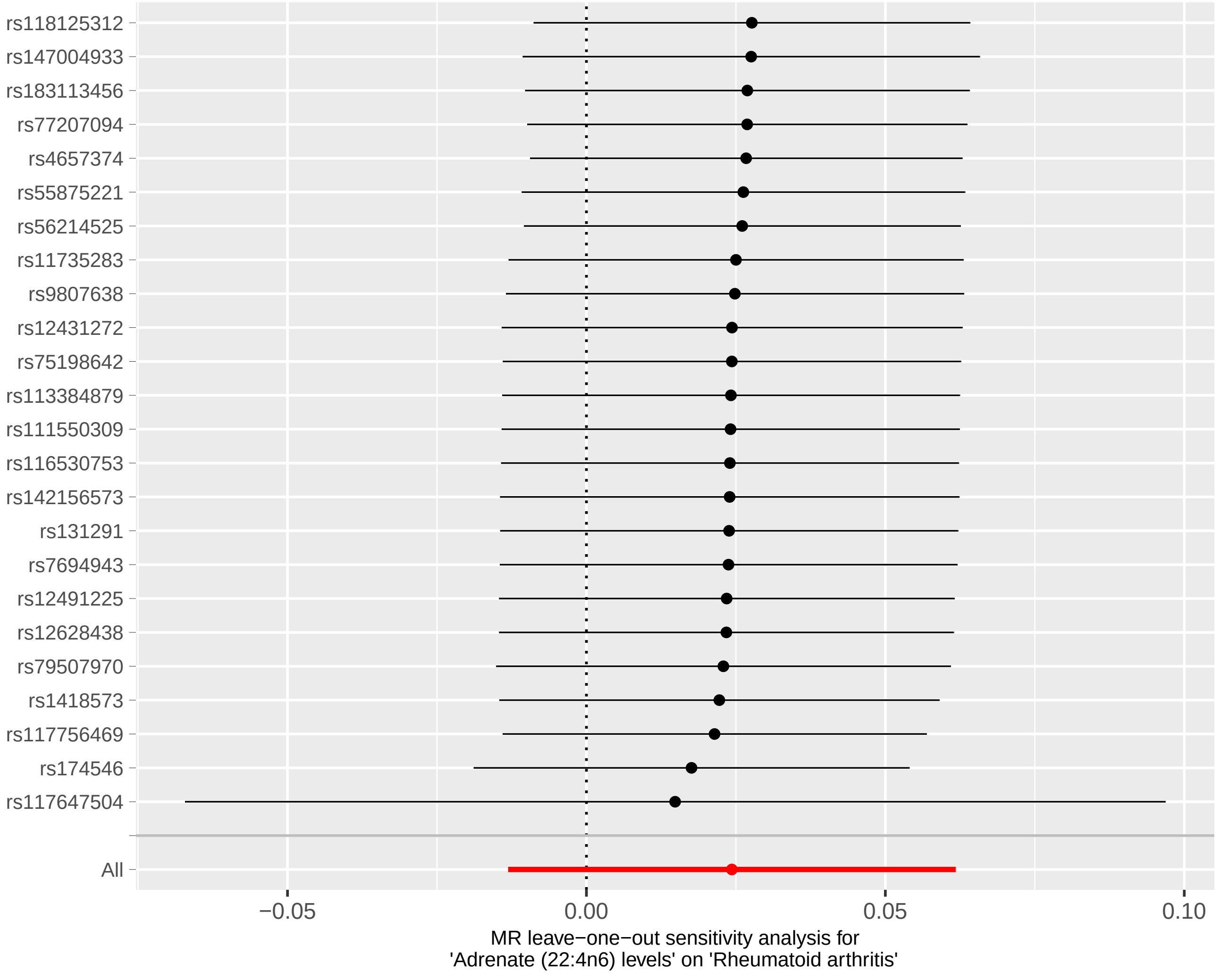
rs118125312
rs147004933
rs183113456
rs77207094
rs4657374
rs55875221
rs56214525
rs11735283
rs9807638
rs12431272
rs75198642
rs113384879
rs111550309
rs116530753
rs142156573
rs131291
rs7694943
rs12491225
rs12628438
rs79507970
rs1418573
rs117756469
rs174546
rs117647504
All
−0.05
0.00
0.05
0.10
MR leave−one−out sensitivity analysis for
'Adrenate (22:4n6) levels' on 'Rheumatoid arthritis'

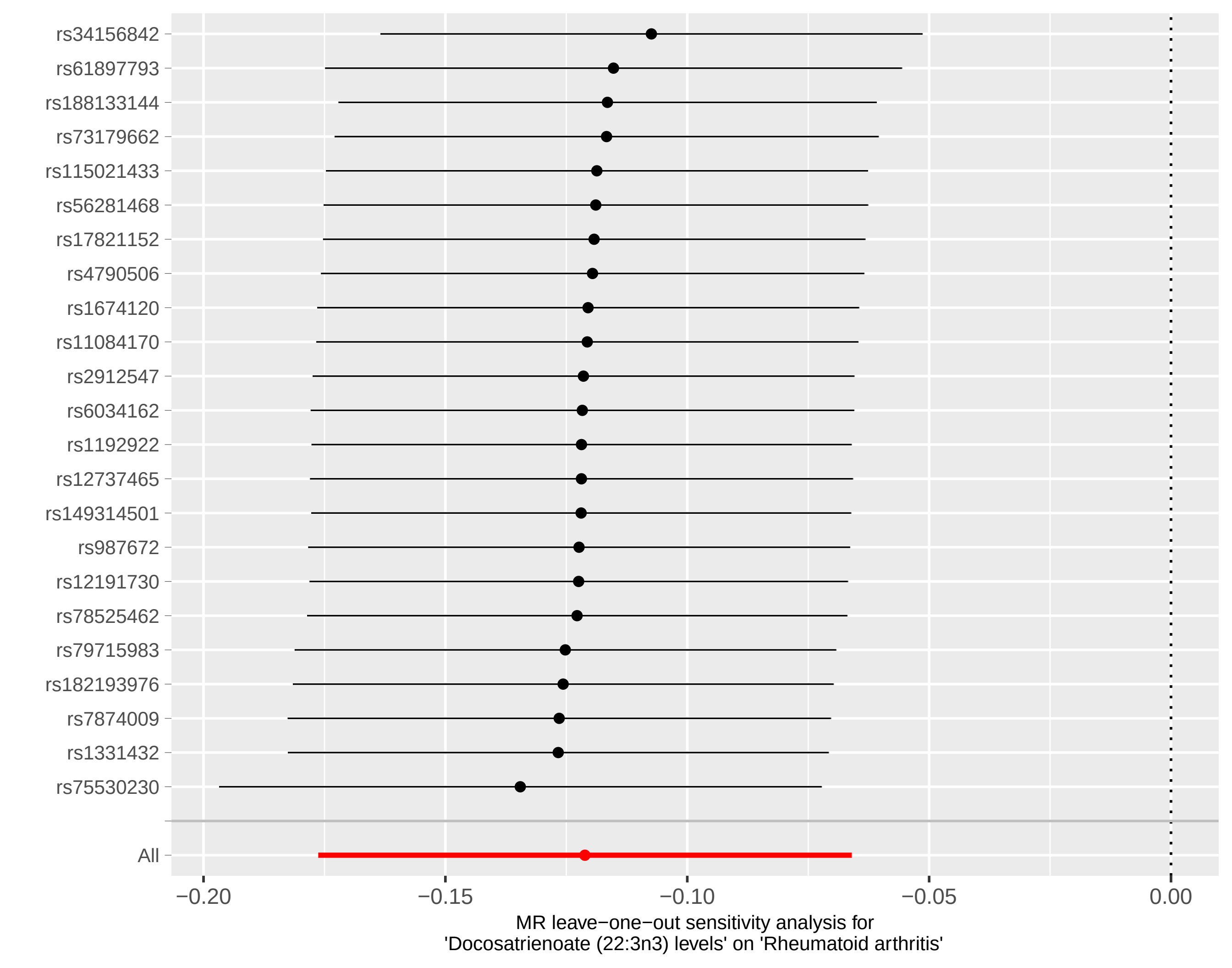
rs34156842
rs61897793
rs188133144
rs73179662
rs115021433
rs56281468
rs17821152
rs4790506
rs1674120
rs11084170
rs2912547
rs6034162
rs1192922
rs12737465
rs149314501
rs987672
rs12191730
rs78525462
rs79715983
rs182193976
rs7874009
rs1331432
rs75530230
All
−0.20
−0.15
−0.10
−0.05
0.00
MR leave−one−out sensitivity analysis for
'Docosatrienoate (22:3n3) levels' on 'Rheumatoid arthritis'

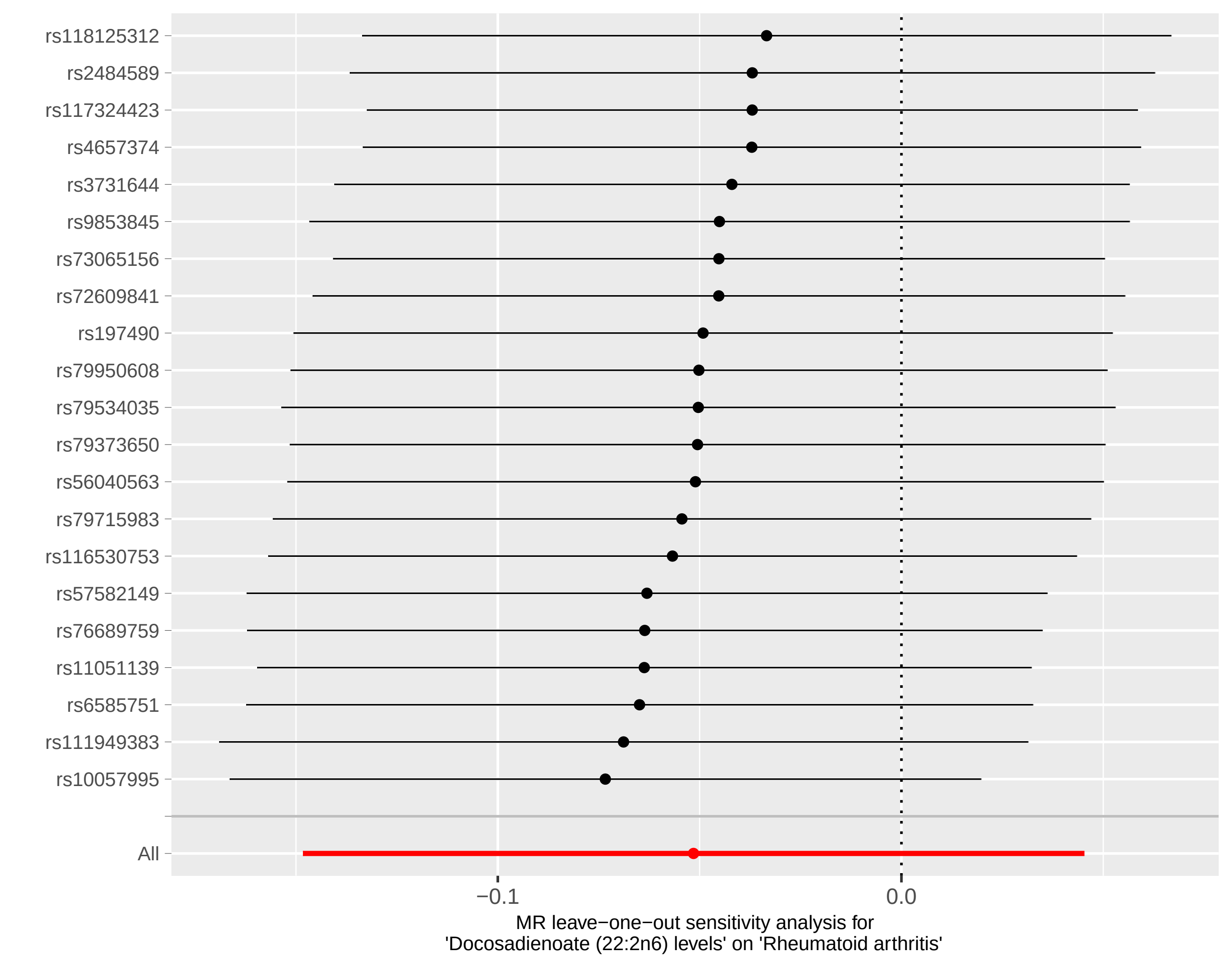
rs118125312
rs2484589
rs117324423
rs4657374
rs3731644
rs9853845
rs73065156
rs72609841
rs197490
rs79950608
rs79534035
rs79373650
rs56040563
rs79715983
rs116530753
rs57582149
rs76689759
rs11051139
rs6585751
rs111949383
rs10057995
All
−0.1
0.0
MR leave−one−out sensitivity analysis for
'Docosadienoate (22:2n6) levels' on 'Rheumatoid arthritis'

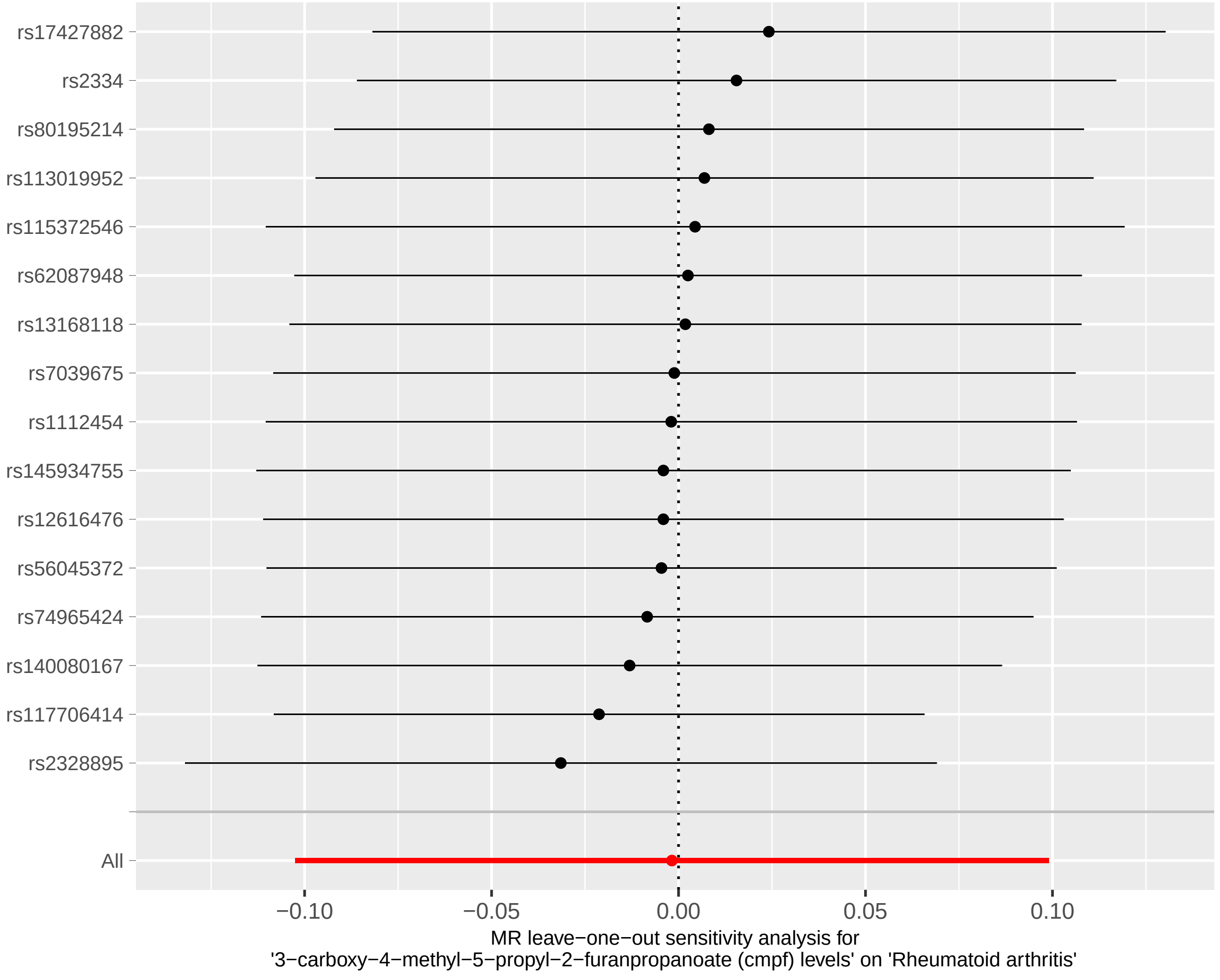
rs17427882
rs2334
rs80195214
rs113019952
rs115372546
rs62087948
rs13168118
rs7039675
rs1112454
rs145934755
rs12616476
rs56045372
rs74965424
rs140080167
rs117706414
rs2328895
All
−0.10
−0.05
0.00
0.05
0.10
MR leave−one−out sensitivity analysis for
'3−carboxy−4−methyl−5−propyl−2−furanpropanoate (cmpf) levels' on 'Rheumatoid arthritis'

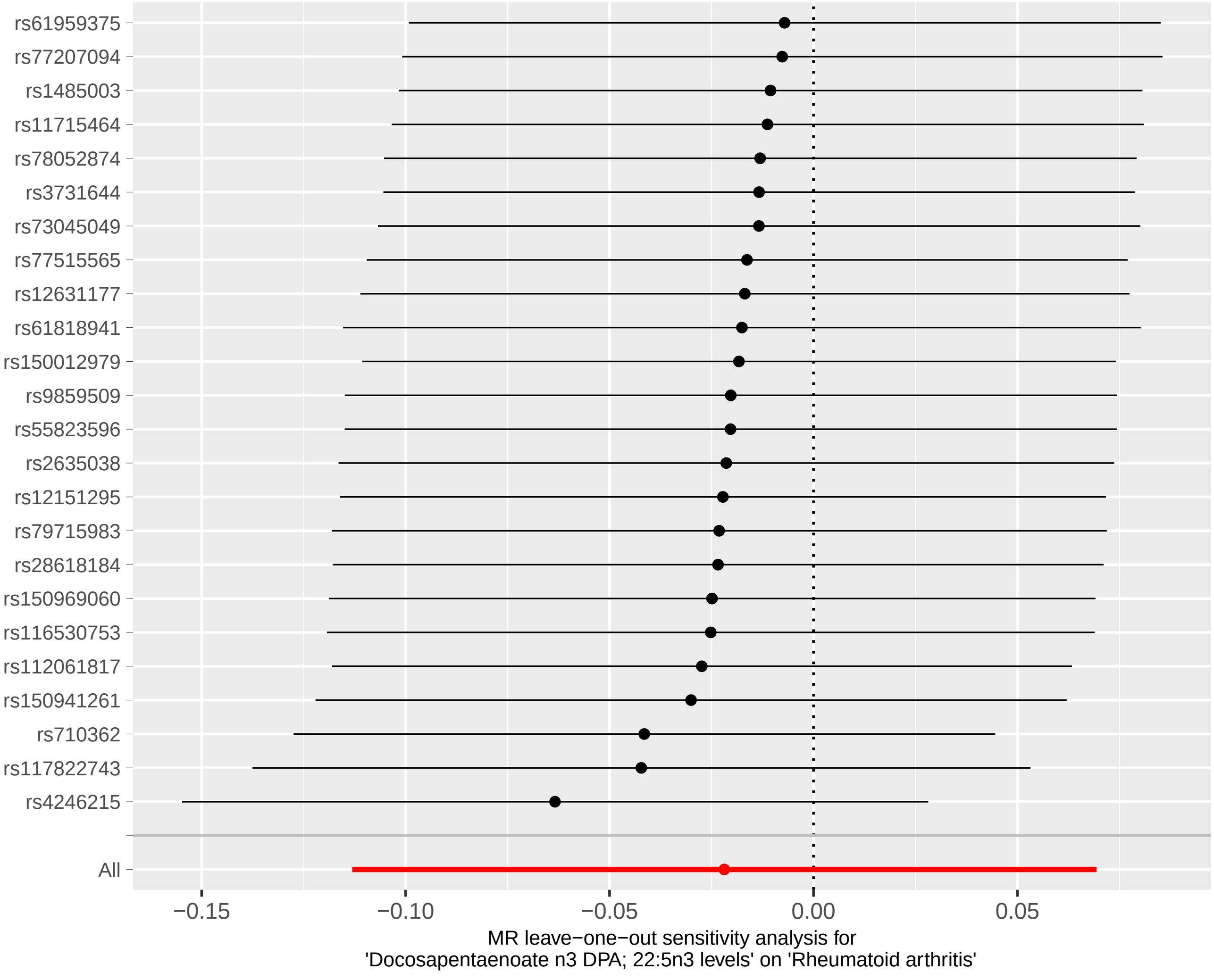

rs61959375
rs77207094
rs1485003
rs11715464
rs78052874
rs3731644
rs73045049
rs77515565
rs12631177
rs61818941
rs150012979
rs9859509
rs55823596
rs2635038
rs12151295
rs79715983
rs28618184
rs150969060
rs116530753
rs112061817
rs150941261
rs710362
rs117822743
rs4246215
All
−0.15
−0.10
−0.05
0.00
0.05
MR leave−one−out sensitivity analysis for
'Docosapentaenoate n3 DPA; 22:5n3 levels' on 'Rheumatoid arthritis'

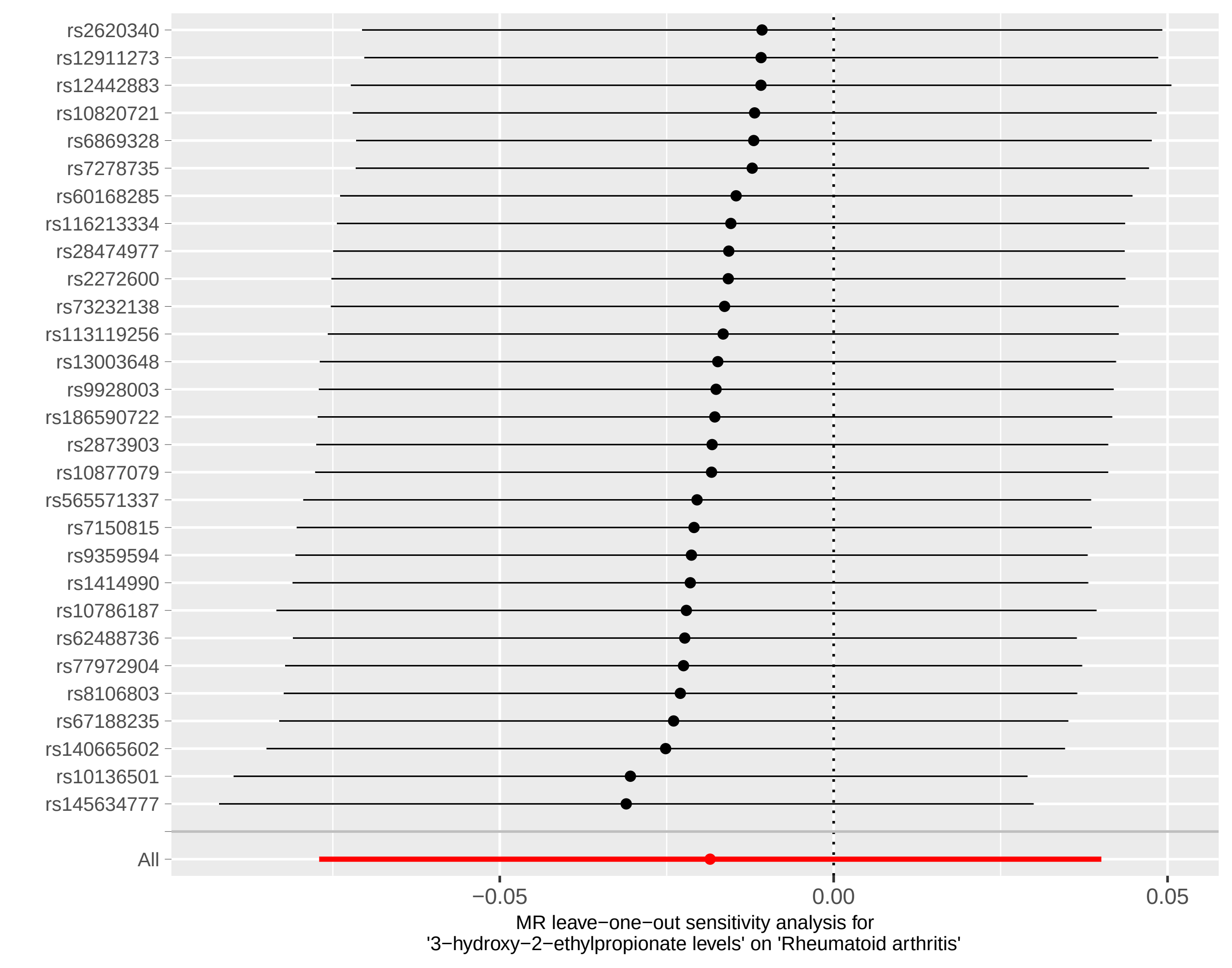

rs2620340
rs12911273
rs12442883
rs10820721
rs6869328
rs7278735
rs60168285
rs116213334
rs28474977
rs2272600
rs73232138
rs113119256
rs13003648
rs9928003
rs186590722
rs2873903
rs10877079
rs565571337
rs7150815
rs9359594
rs1414990
rs10786187
rs62488736
rs77972904
rs8106803
rs67188235
rs140665602
rs10136501
rs145634777
All
−0.05
0.00
0.05
MR leave−one−out sensitivity analysis for
'3−hydroxy−2−ethylpropionate levels' on 'Rheumatoid arthritis'

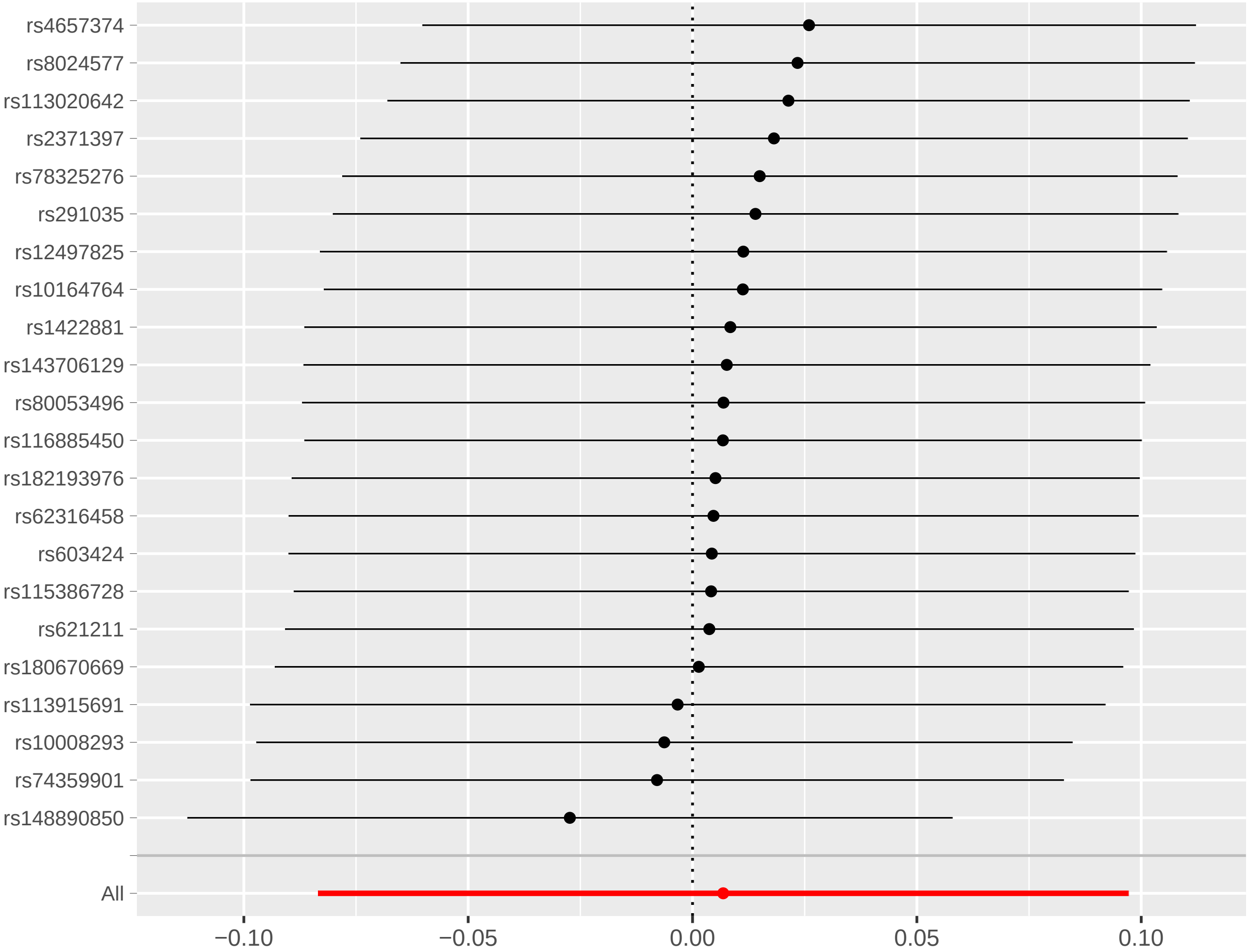

MR leave−one−out sensitivity analysis for
'Myristoleate (14:1n5) levels' on 'Rheumatoid arthritis'

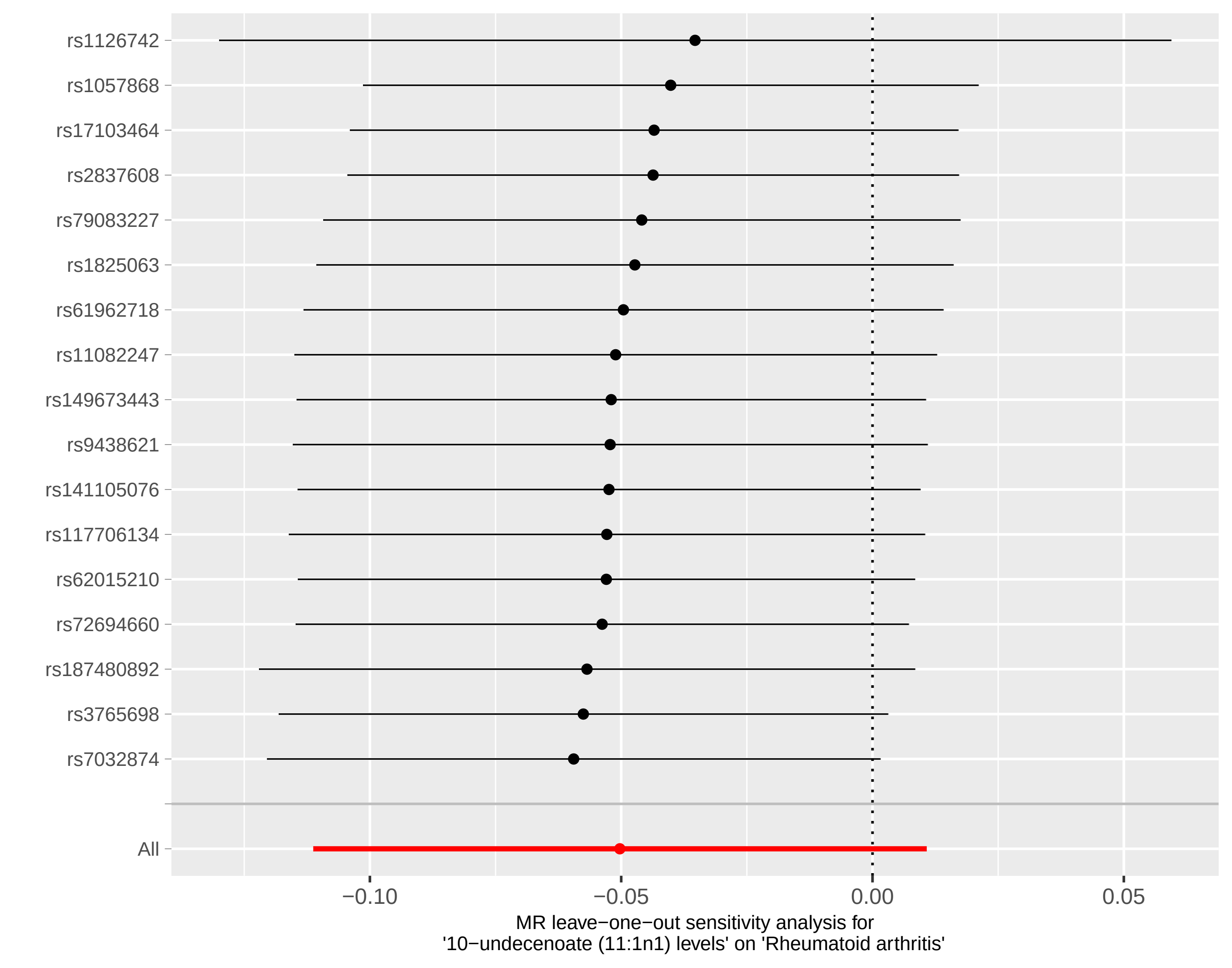
rs1126742
rs1057868
rs17103464
rs2837608
rs79083227
rs1825063
rs61962718
rs11082247
rs149673443
rs9438621
rs141105076
rs117706134
rs62015210
rs72694660
rs187480892
rs3765698
rs7032874
All
−0.10
−0.05
0.00
0.05
MR leave−one−out sensitivity analysis for
'10−undecenoate (11:1n1) levels' on 'Rheumatoid arthritis'

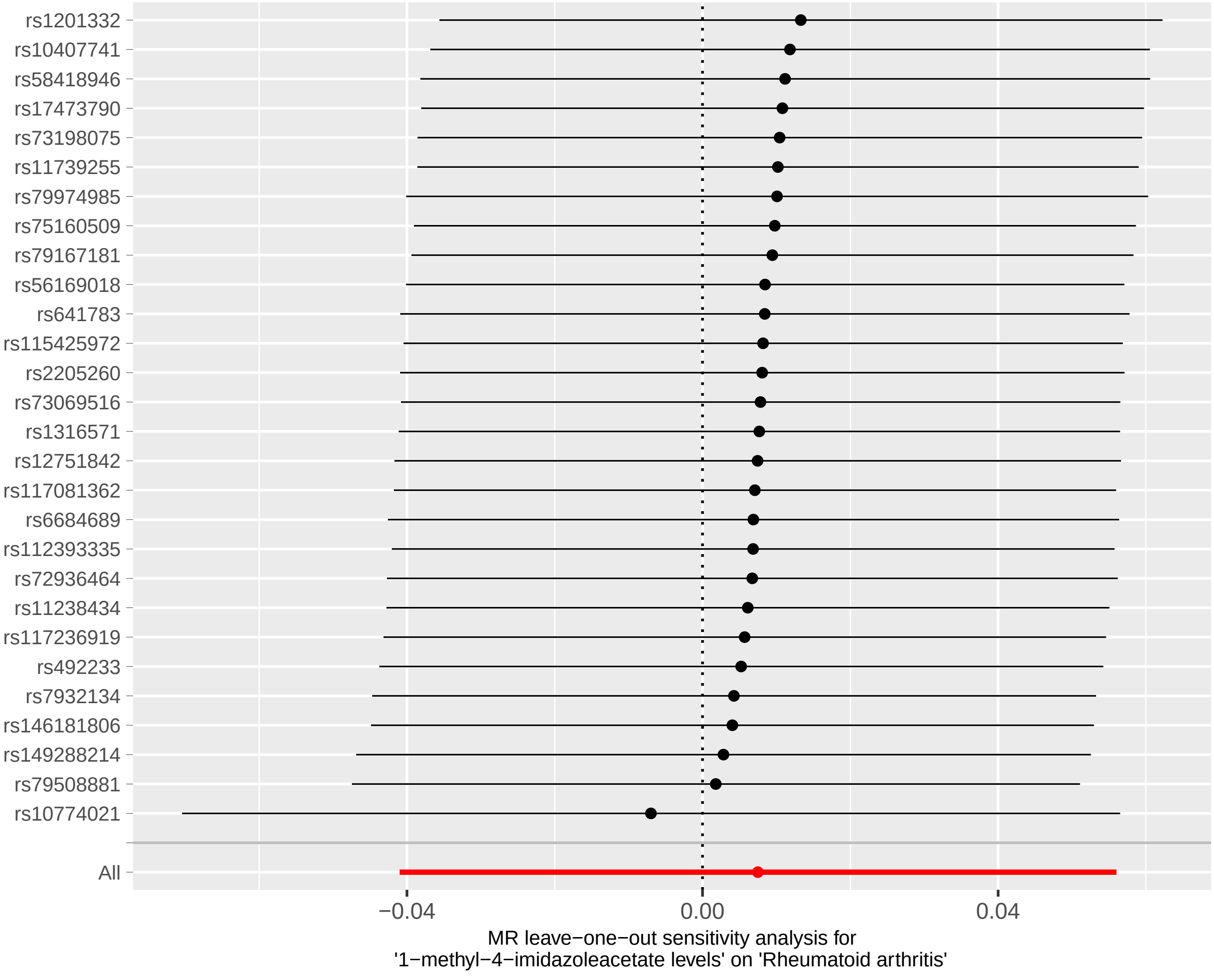
rs1201332
rs10407741
rs58418946
rs17473790
rs73198075
rs11739255
rs79974985
rs75160509
rs79167181
rs56169018
rs641783
rs115425972
rs2205260
rs73069516
rs1316571
rs12751842
rs117081362
rs6684689
rs112393335
rs72936464
rs11238434
rs117236919
rs492233
rs7932134
rs146181806
rs149288214
rs79508881
rs10774021
All
−0.04
0.00
0.04
MR leave−one−out sensitivity analysis for
'1−methyl−4−imidazoleacetate levels' on 'Rheumatoid arthritis'

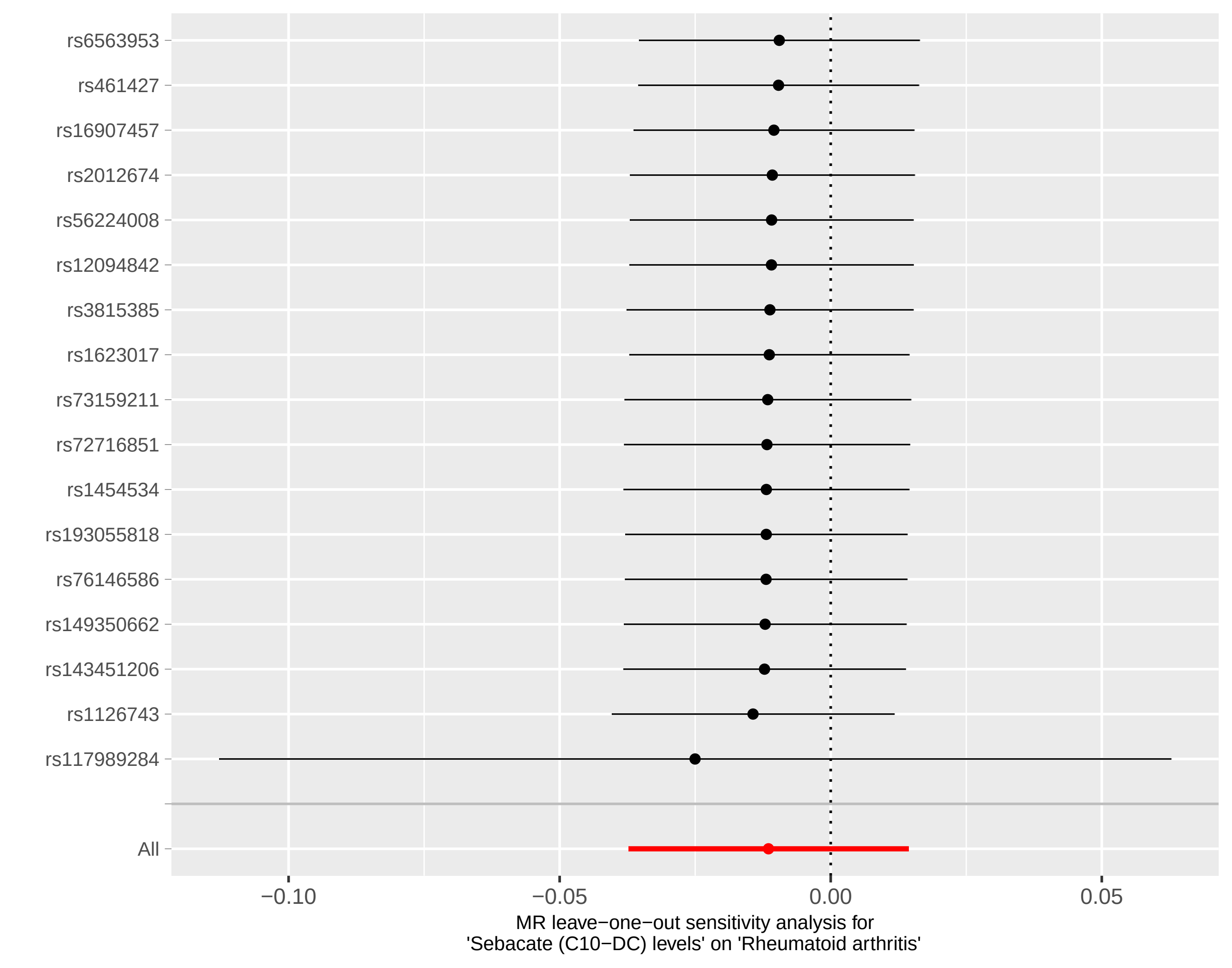
rs6563953
rs461427
rs16907457
rs2012674
rs56224008
rs12094842
rs3815385
rs1623017
rs73159211
rs72716851
rs1454534
rs193055818
rs76146586
rs149350662
rs143451206
rs1126743
rs117989284
All
−0.10
−0.05
0.00
0.05
MR leave−one−out sensitivity analysis for
'Sebacate (C10−DC) levels' on 'Rheumatoid arthritis'

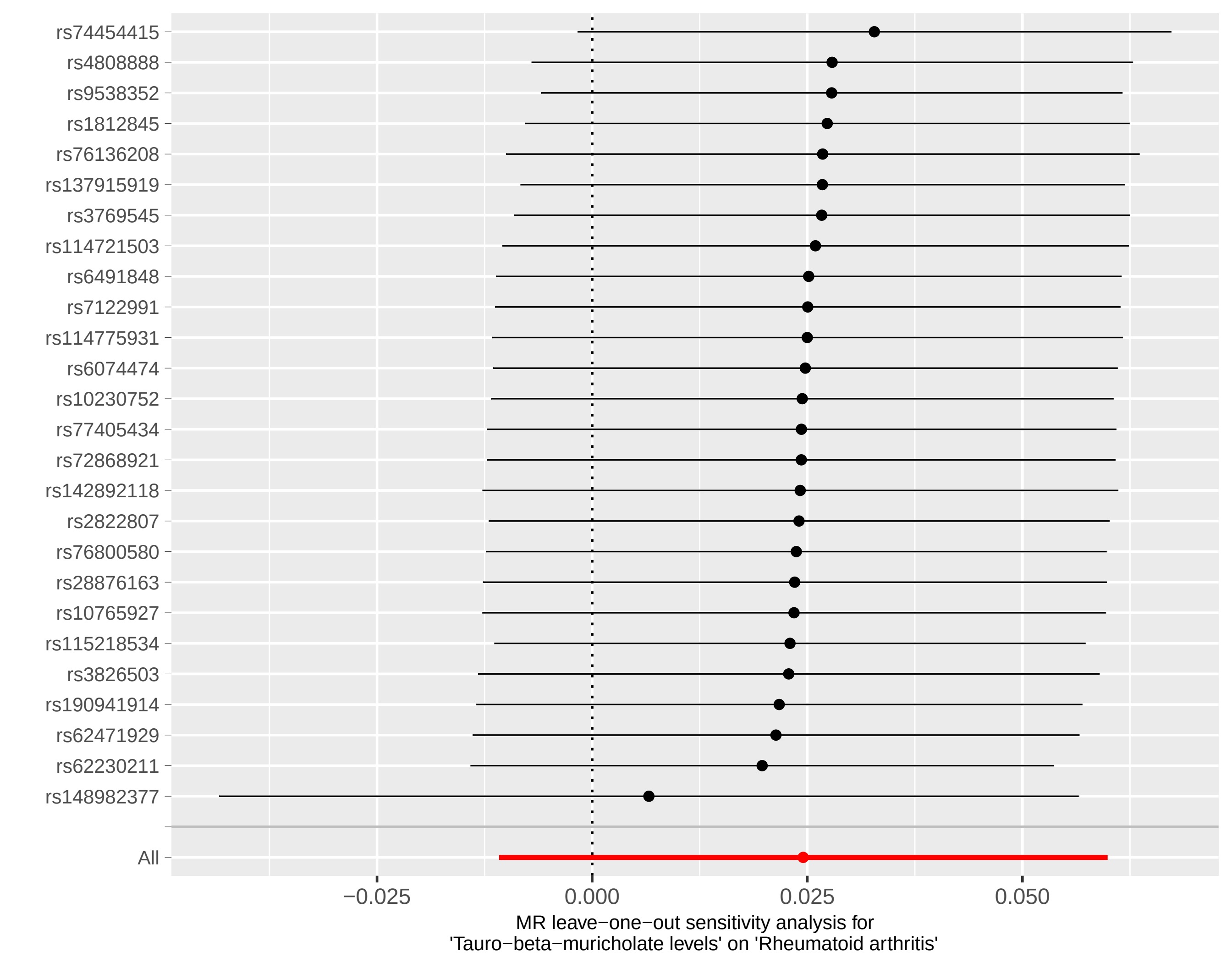

rs74454415
rs4808888
rs9538352
rs1812845
rs76136208
rs137915919
rs3769545
rs114721503
rs6491848
rs7122991
rs114775931
rs6074474
rs10230752
rs77405434
rs72868921
rs142892118
rs2822807
rs76800580
rs28876163
rs10765927
rs115218534
rs3826503
rs190941914
rs62471929
rs62230211
rs148982377
All
−0.025
0.000
0.025
0.050
MR leave−one−out sensitivity analysis for
'Tauro−beta−muricholate levels' on 'Rheumatoid arthritis'

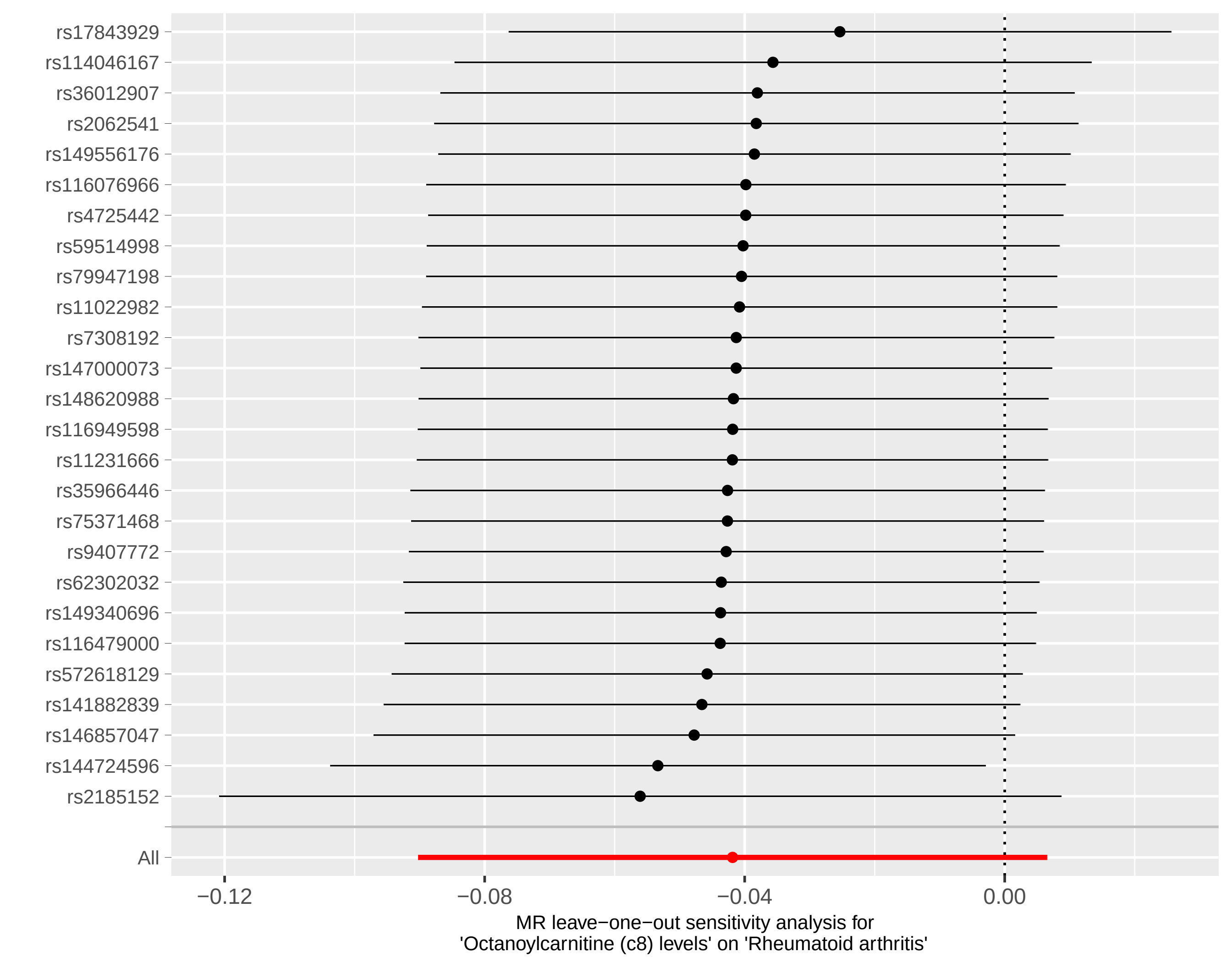
rs17843929
rs114046167
rs36012907
rs2062541
rs149556176
rs116076966
rs4725442
rs59514998
rs79947198
rs11022982
rs7308192
rs147000073
rs148620988
rs116949598
rs11231666
rs35966446
rs75371468
rs9407772
rs62302032
rs149340696
rs116479000
rs572618129
rs141882839
rs146857047
rs144724596
rs2185152
All
−0.12
−0.08
−0.04
0.00
MR leave−one−out sensitivity analysis for
'Octanoylcarnitine (c8) levels' on 'Rheumatoid arthritis'

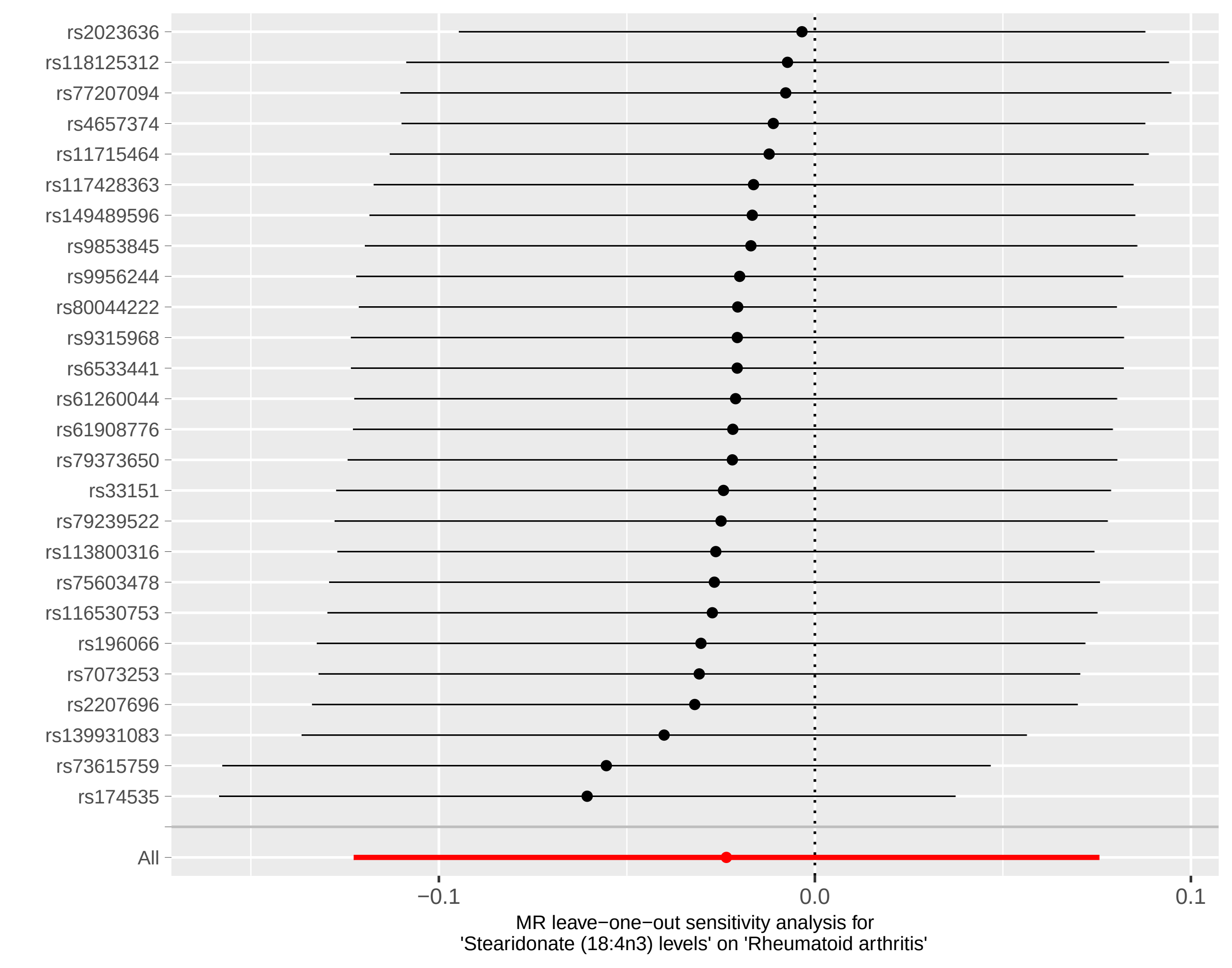
rs2023636
rs118125312
rs77207094
rs4657374
rs11715464
rs117428363
rs149489596
rs9853845
rs9956244
rs80044222
rs9315968
rs6533441
rs61260044
rs61908776
rs79373650
rs33151
rs79239522
rs113800316
rs75603478
rs116530753
rs196066
rs7073253
rs2207696
rs139931083
rs73615759
rs174535
All
−0.1
0.0
0.1
MR leave−one−out sensitivity analysis for
'Stearidonate (18:4n3) levels' on 'Rheumatoid arthritis'

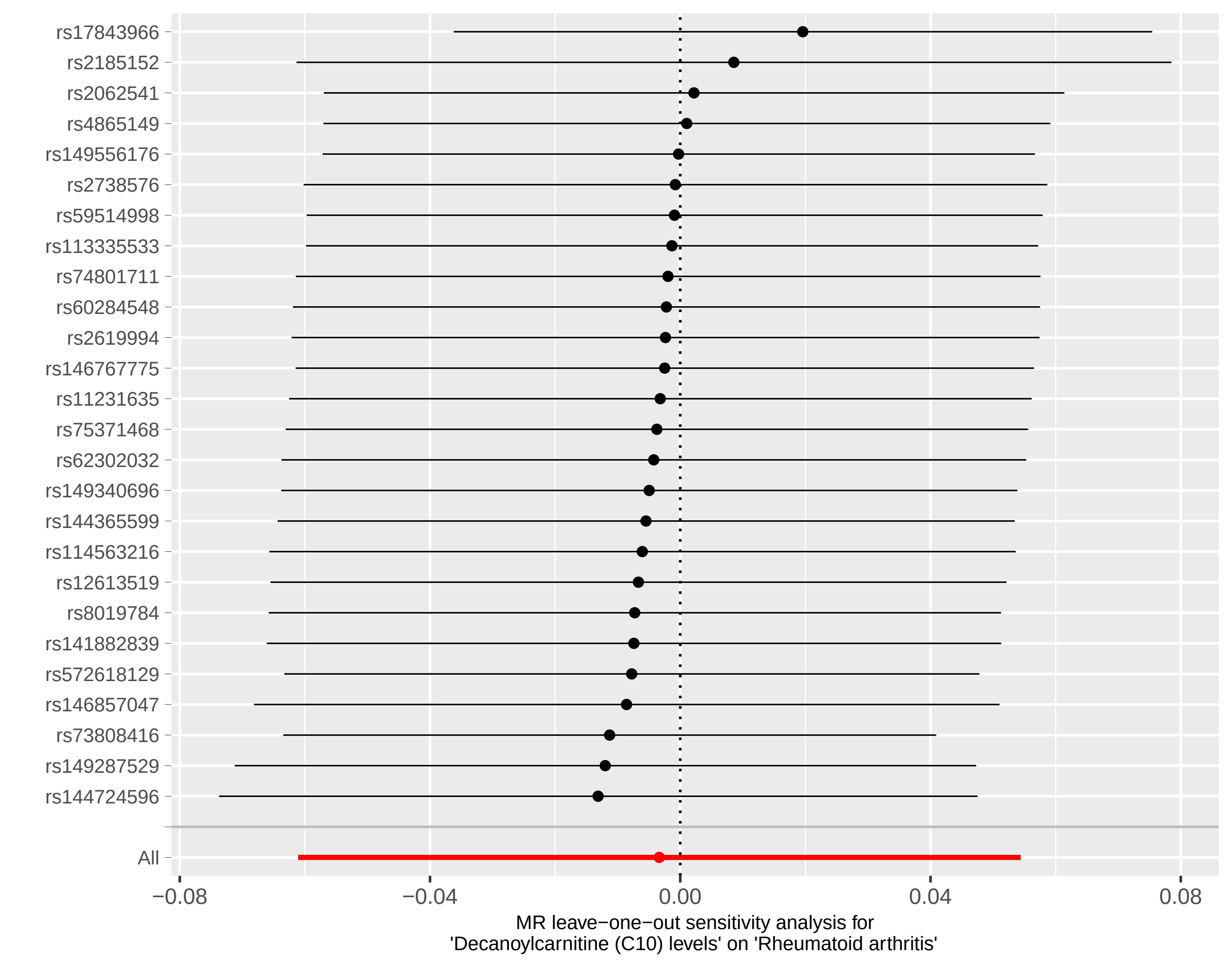

rs17843966
rs2185152
rs2062541
rs4865149
rs149556176
rs2738576
rs59514998
rs113335533
rs74801711
rs60284548
rs2619994
rs146767775
rs11231635
rs75371468
rs62302032
rs149340696
rs144365599
rs114563216
rs12613519
rs8019784
rs141882839
rs572618129
rs146857047
rs73808416
rs149287529
rs144724596
All
−0.08
−0.04
0.00
0.04
0.08
MR leave−one−out sensitivity analysis for
'Decanoylcarnitine (C10) levels' on 'Rheumatoid arthritis'

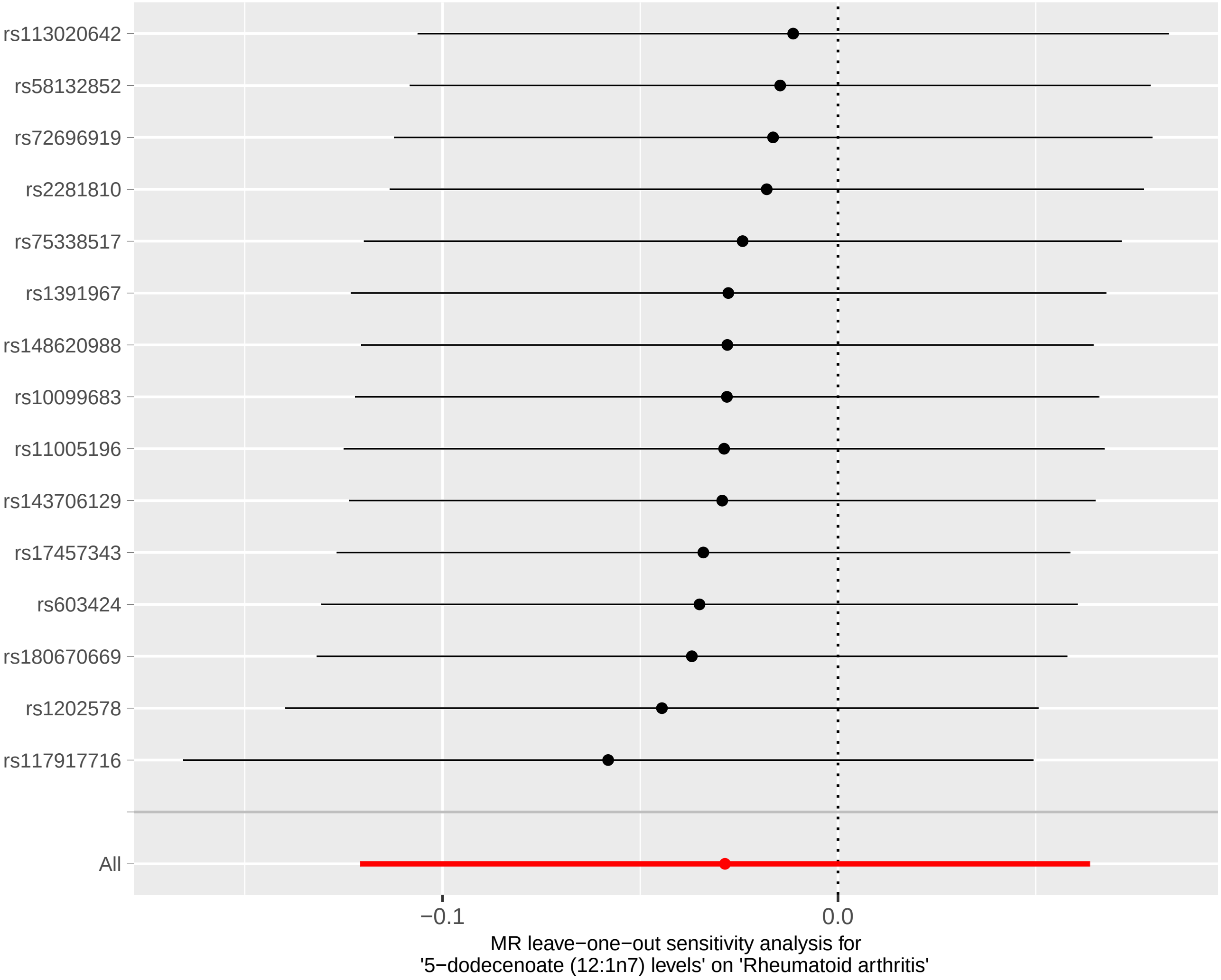
rs113020642
rs58132852
rs72696919
rs2281810
rs75338517
rs1391967
rs148620988
rs10099683
rs11005196
rs143706129
rs17457343
rs603424
rs180670669
rs1202578
rs117917716
All
−0.1
0.0
MR leave−one−out sensitivity analysis for
'5−dodecenoate (12:1n7) levels' on 'Rheumatoid arthritis'

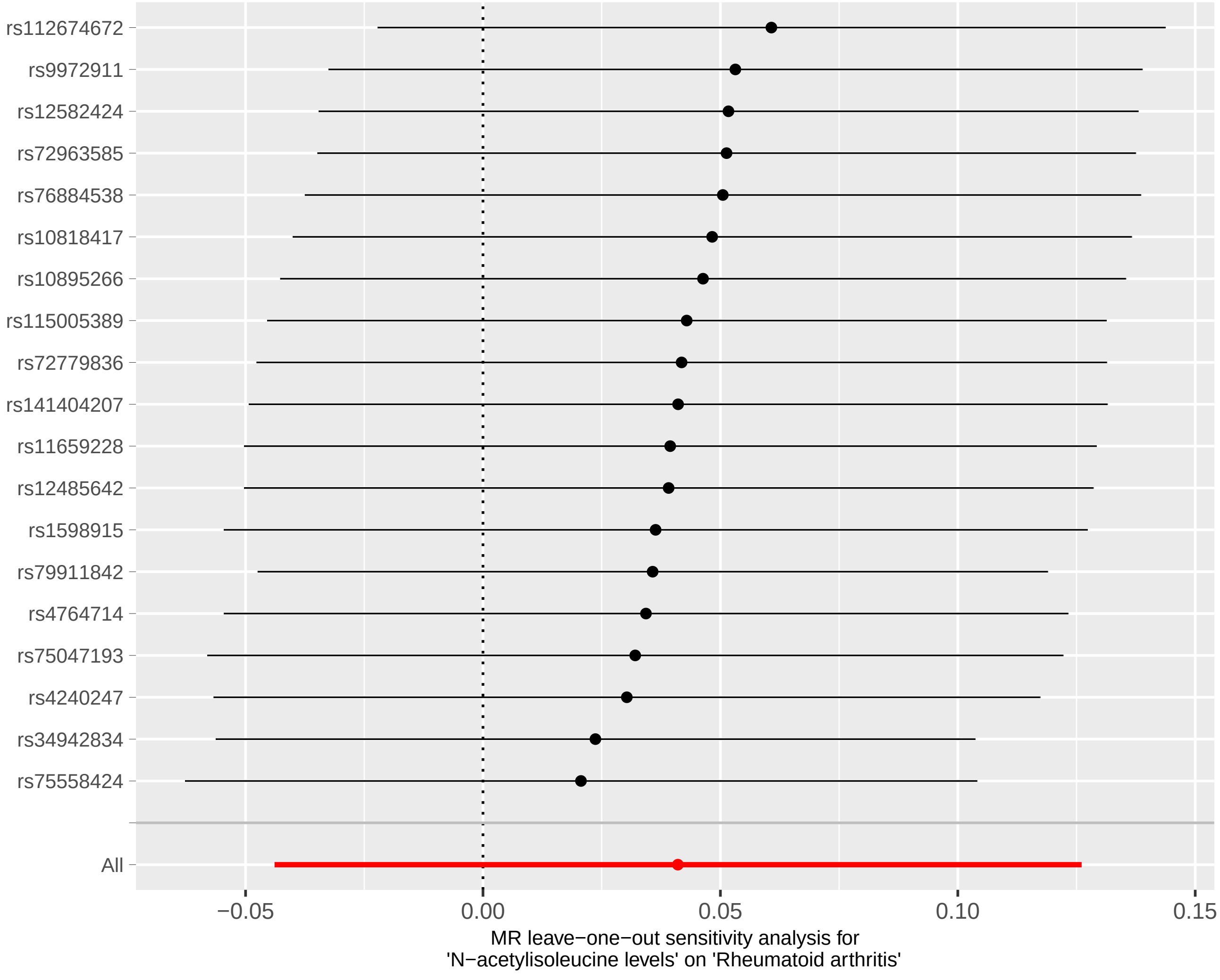

rs112674672
rs9972911
rs12582424
rs72963585
rs76884538
rs10818417
rs10895266
rs115005389
rs72779836
rs141404207
rs11659228
rs12485642
rs1598915
rs79911842
rs4764714
rs75047193
rs4240247
rs34942834
rs75558424
All
−0.05
0.00
0.05
0.10
0.15
MR leave−one−out sensitivity analysis for
'N−acetylisoleucine levels' on 'Rheumatoid arthritis'

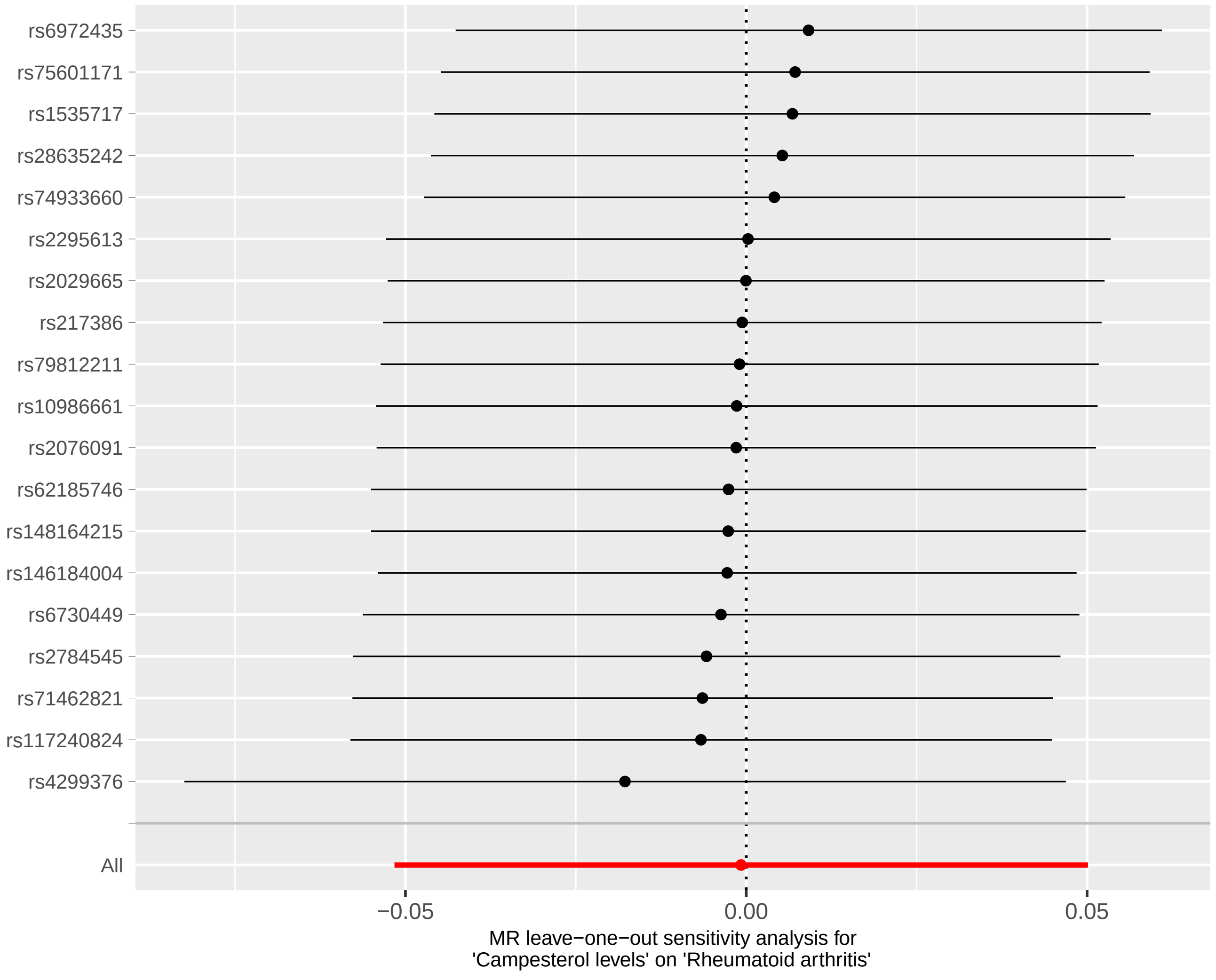

rs6972435
rs75601171
rs1535717
rs28635242
rs74933660
rs2295613
rs2029665
rs217386
rs79812211
rs10986661
rs2076091
rs62185746
rs148164215
rs146184004
rs6730449
rs2784545
rs71462821
rs117240824
rs4299376
All
−0.05
0.00
0.05
MR leave−one−out sensitivity analysis for
'Campesterol levels' on 'Rheumatoid arthritis'

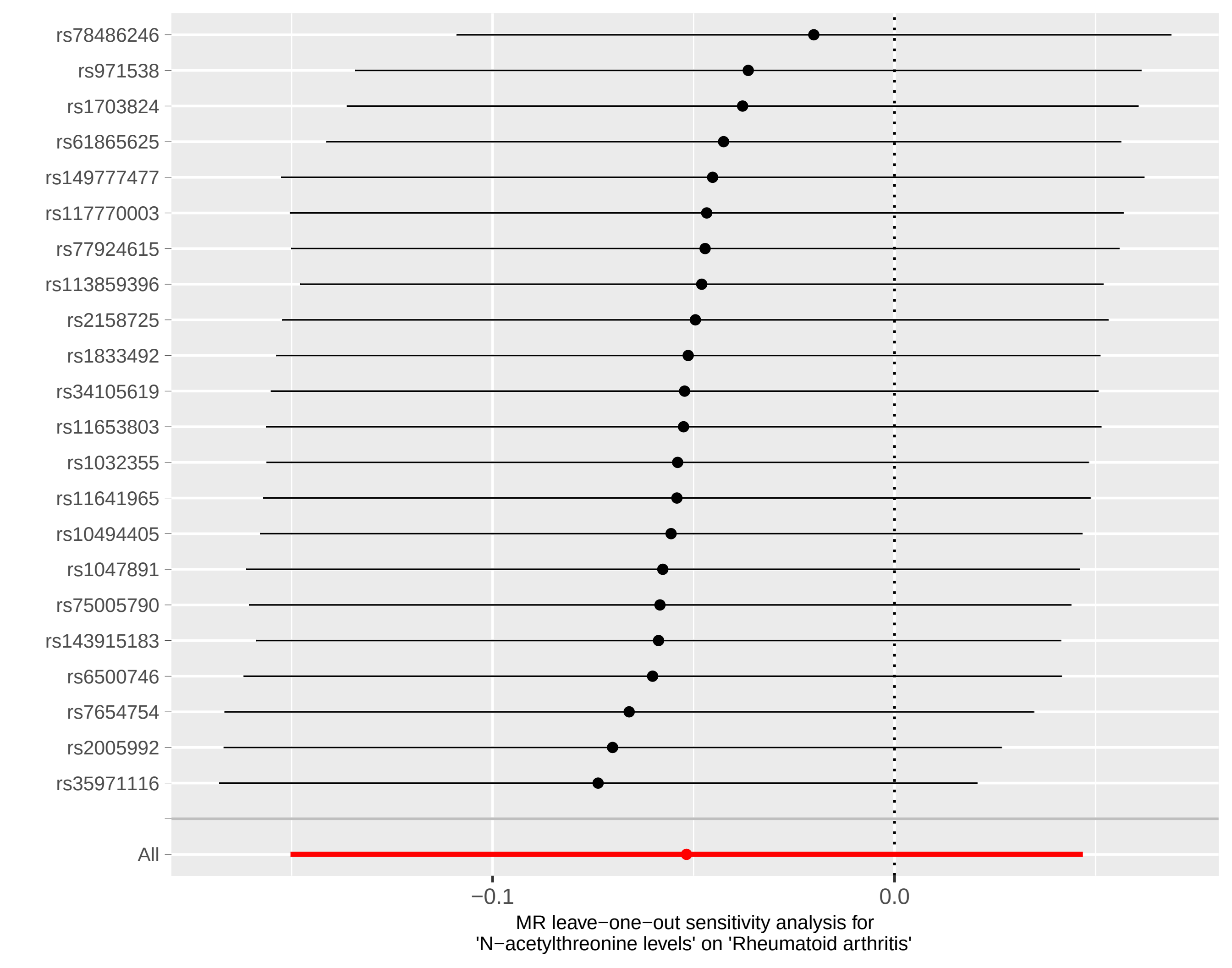
rs78486246
rs971538
rs1703824
rs61865625
rs149777477
rs117770003
rs77924615
rs113859396
rs2158725
rs1833492
rs34105619
rs11653803
rs1032355
rs11641965
rs10494405
rs1047891
rs75005790
rs143915183
rs6500746
rs7654754
rs2005992
rs35971116
All
−0.1
0.0
MR leave−one−out sensitivity analysis for
'N−acetylthreonine levels' on 'Rheumatoid arthritis'

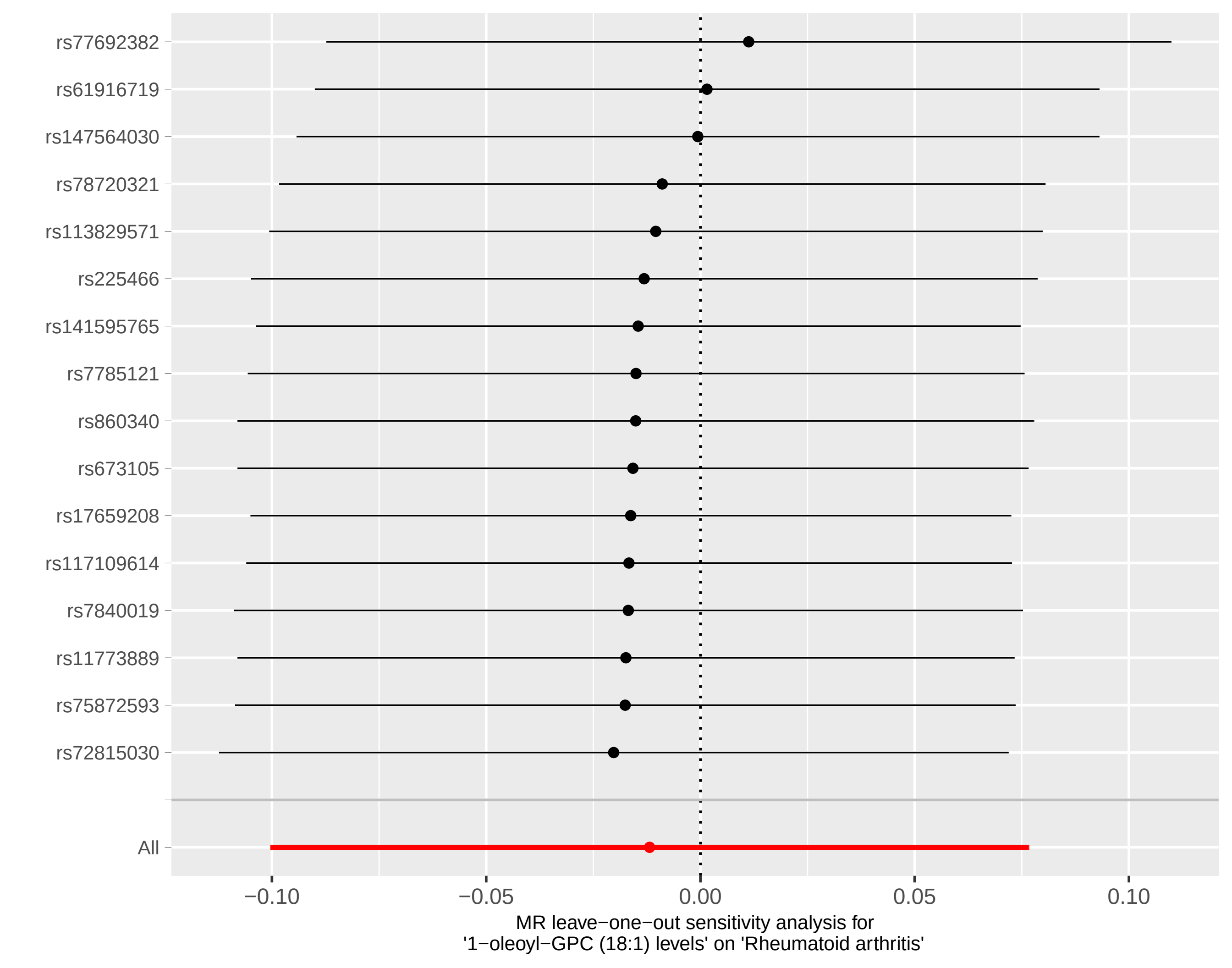
rs77692382
rs61916719
rs147564030
rs78720321
rs113829571
rs225466
rs141595765
rs7785121
rs860340
rs673105
rs17659208
rs117109614
rs7840019
rs11773889
rs75872593
rs72815030
All
−0.10
−0.05
0.00
0.05
0.10
MR leave−one−out sensitivity analysis for
'1−oleoyl−GPC (18:1) levels' on 'Rheumatoid arthritis'

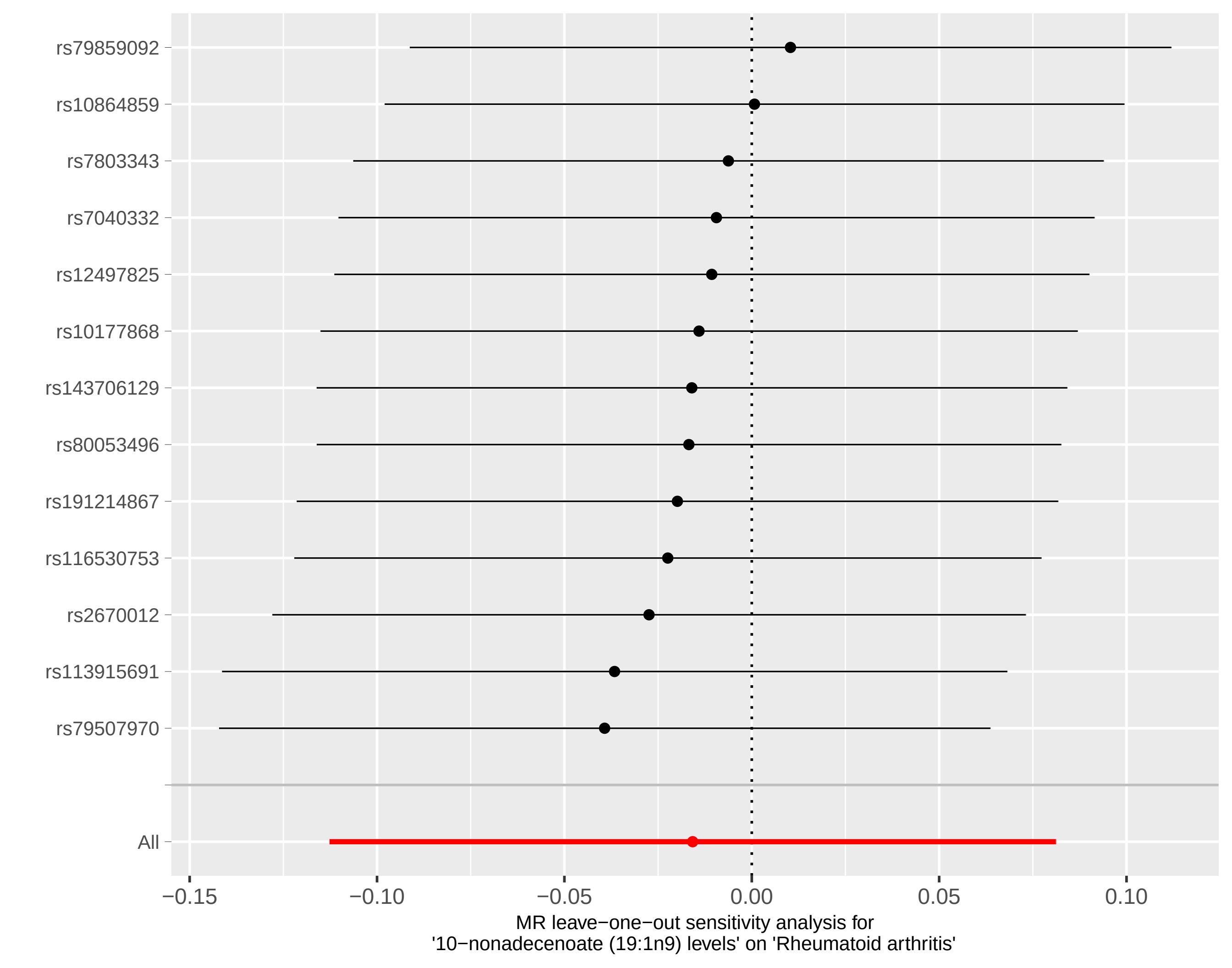

rs79859092
rs10864859
rs7803343
rs7040332
rs12497825
rs10177868
rs143706129
rs80053496
rs191214867
rs116530753
rs2670012
rs113915691
rs79507970
All
−0.15
−0.10
−0.05
0.00
0.05
0.10
MR leave−one−out sensitivity analysis for
'10−nonadecenoate (19:1n9) levels' on 'Rheumatoid arthritis'

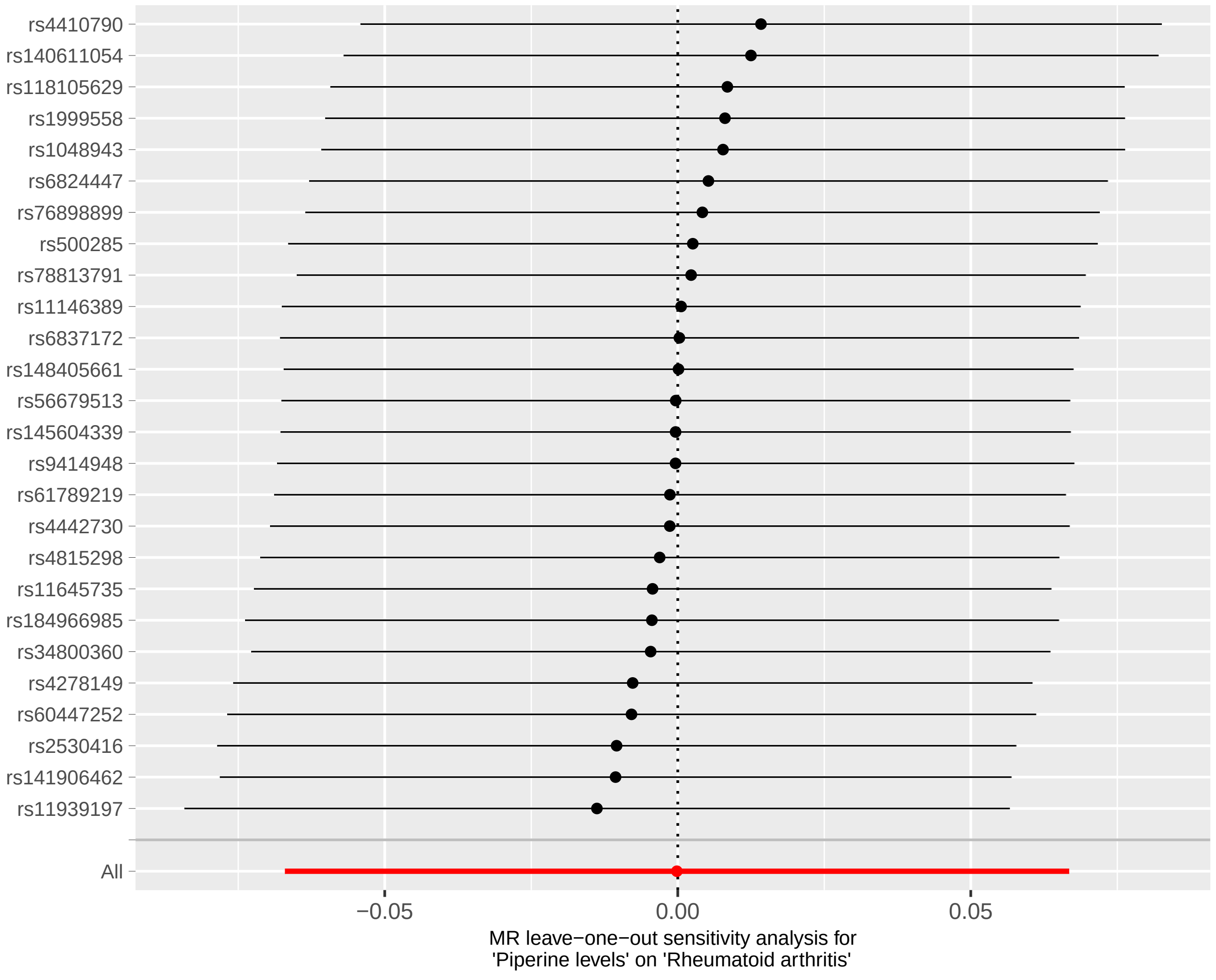
rs4410790
rs140611054
rs118105629
rs1999558
rs1048943
rs6824447
rs76898899
rs500285
rs78813791
rs11146389
rs6837172
rs148405661
rs56679513
rs145604339
rs9414948
rs61789219
rs4442730
rs4815298
rs11645735
rs184966985
rs34800360
rs4278149
rs60447252
rs2530416
rs141906462
rs11939197
All
−0.05
0.00
0.05
MR leave−one−out sensitivity analysis for
'Piperine levels' on 'Rheumatoid arthritis'

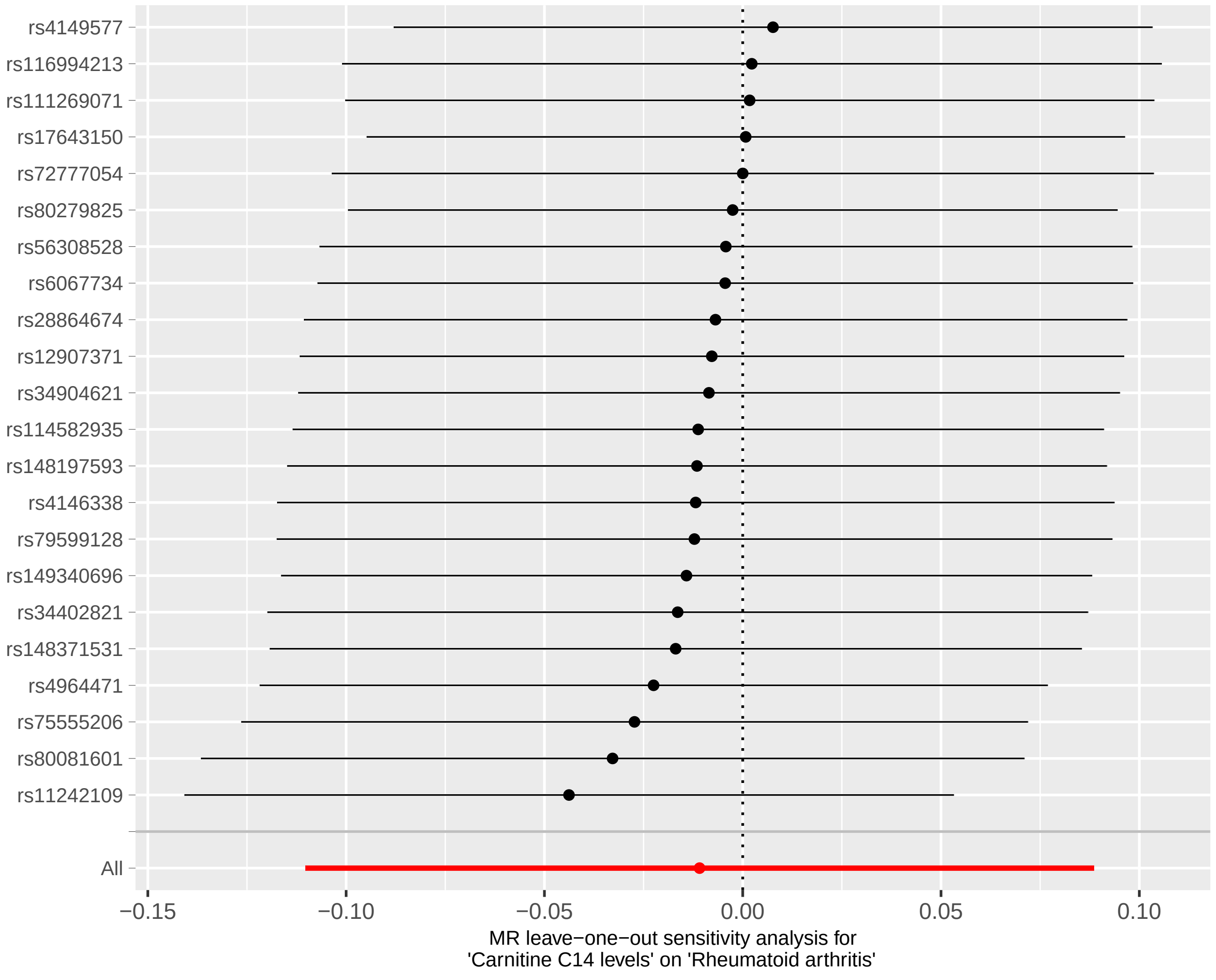
rs4149577
rs116994213
rs111269071
rs17643150
rs72777054
rs80279825
rs56308528
rs6067734
rs28864674
rs12907371
rs34904621
rs114582935
rs148197593
rs4146338
rs79599128
rs149340696
rs34402821
rs148371531
rs4964471
rs75555206
rs80081601
rs11242109
All
−0.15
−0.10
−0.05
0.00
0.05
0.10
MR leave−one−out sensitivity analysis for
'Carnitine C14 levels' on 'Rheumatoid arthritis'

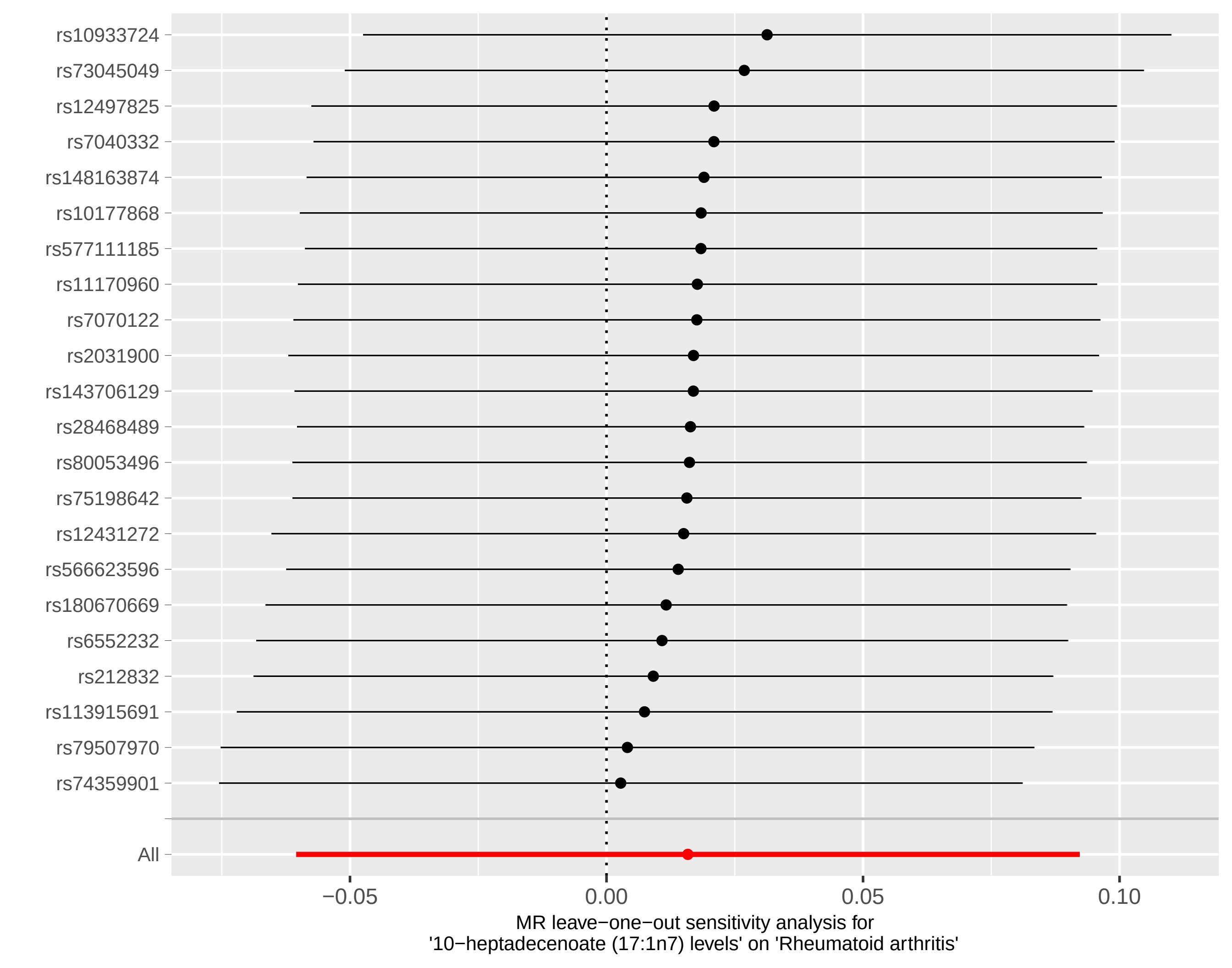
rs10933724
rs73045049
rs12497825
rs7040332
rs148163874
rs10177868
rs577111185
rs11170960
rs7070122
rs2031900
rs143706129
rs28468489
rs80053496
rs75198642
rs12431272
rs566623596
rs180670669
rs6552232
rs212832
rs113915691
rs79507970
rs74359901
All
−0.05
0.00
0.05
0.10
MR leave−one−out sensitivity analysis for
'10−heptadecenoate (17:1n7) levels' on 'Rheumatoid arthritis'

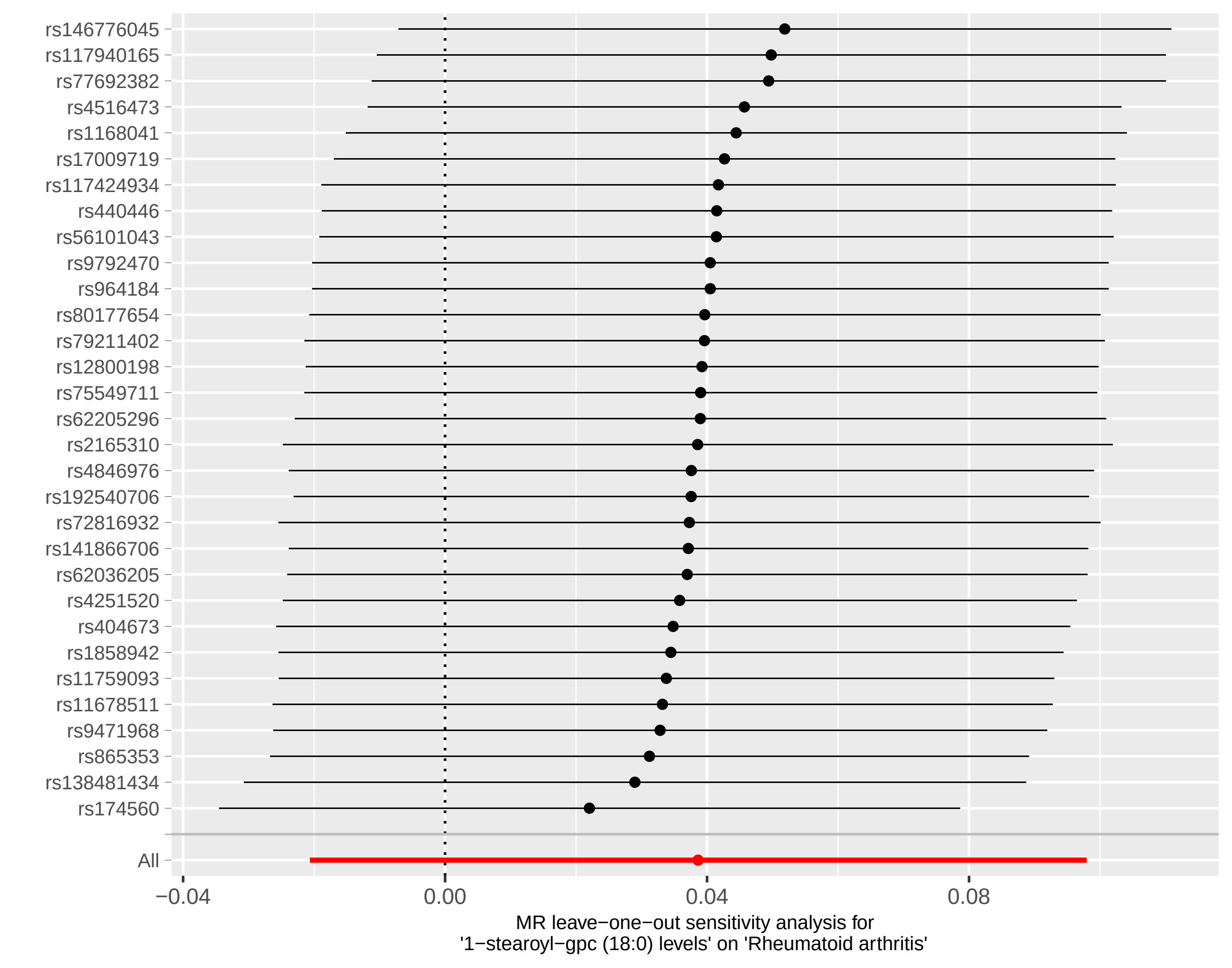
rs146776045
rs117940165
rs77692382
rs4516473
rs1168041
rs17009719
rs117424934
rs440446
rs56101043
rs9792470
rs964184
rs80177654
rs79211402
rs12800198
rs75549711
rs62205296
rs2165310
rs4846976
rs192540706
rs72816932
rs141866706
rs62036205
rs4251520
rs404673
rs1858942
rs11759093
rs11678511
rs9471968
rs865353
rs138481434
rs174560
All
−0.04
0.00
0.04
0.08
MR leave−one−out sensitivity analysis for
'1−stearoyl−gpc (18:0) levels' on 'Rheumatoid arthritis'

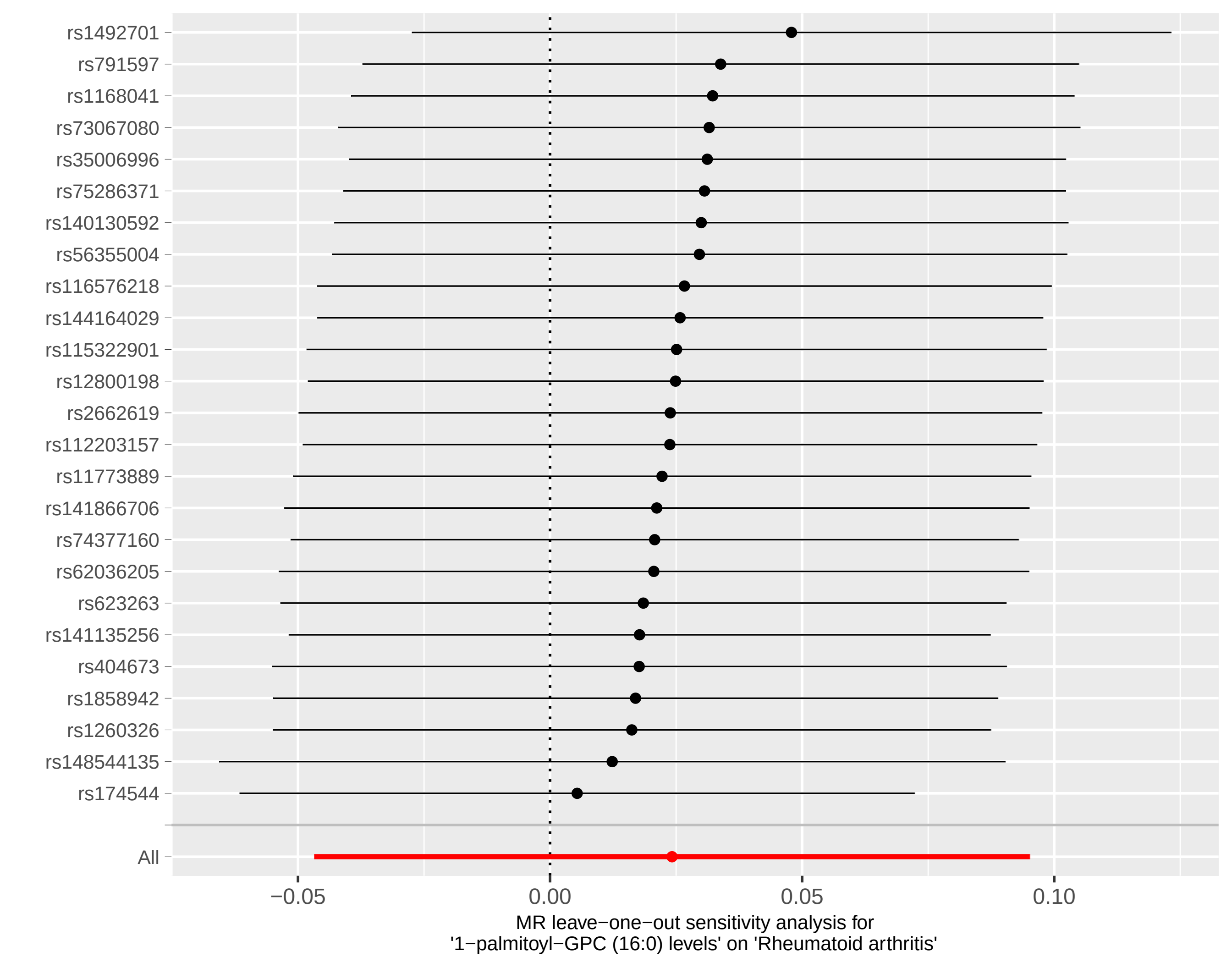

rs1492701
rs791597
rs1168041
rs73067080
rs35006996
rs75286371
rs140130592
rs56355004
rs116576218
rs144164029
rs115322901
rs12800198
rs2662619
rs112203157
rs11773889
rs141866706
rs74377160
rs62036205
rs623263
rs141135256
rs404673
rs1858942
rs1260326
rs148544135
rs174544
All
−0.05
0.00
0.05
0.10
MR leave−one−out sensitivity analysis for
'1−palmitoyl−GPC (16:0) levels' on 'Rheumatoid arthritis'

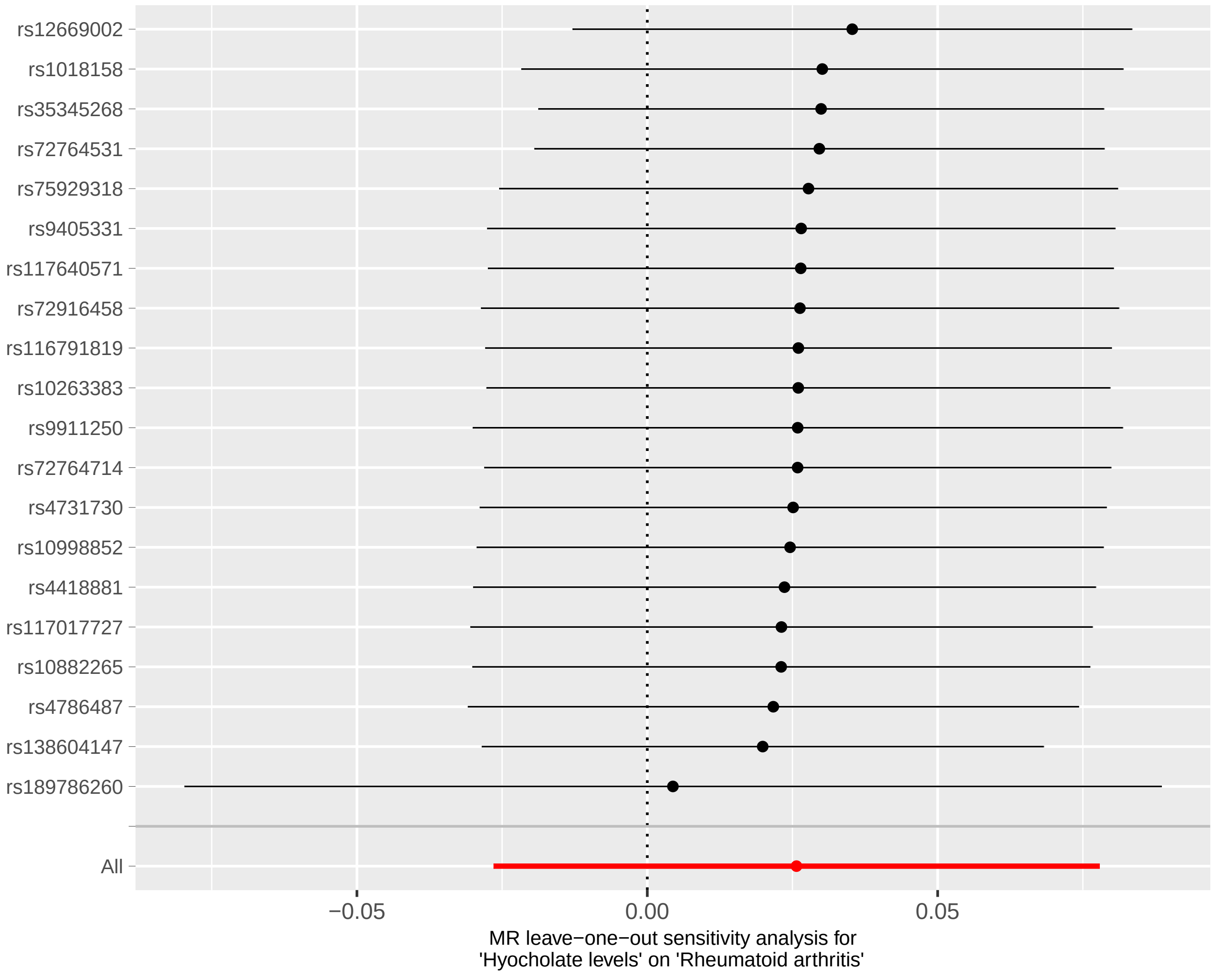
rs12669002
rs1018158
rs35345268
rs72764531
rs75929318
rs9405331
rs117640571
rs72916458
rs116791819
rs10263383
rs9911250
rs72764714
rs4731730
rs10998852
rs4418881
rs117017727
rs10882265
rs4786487
rs138604147
rs189786260
All
−0.05
0.00
0.05
MR leave−one−out sensitivity analysis for
'Hyocholate levels' on 'Rheumatoid arthritis'

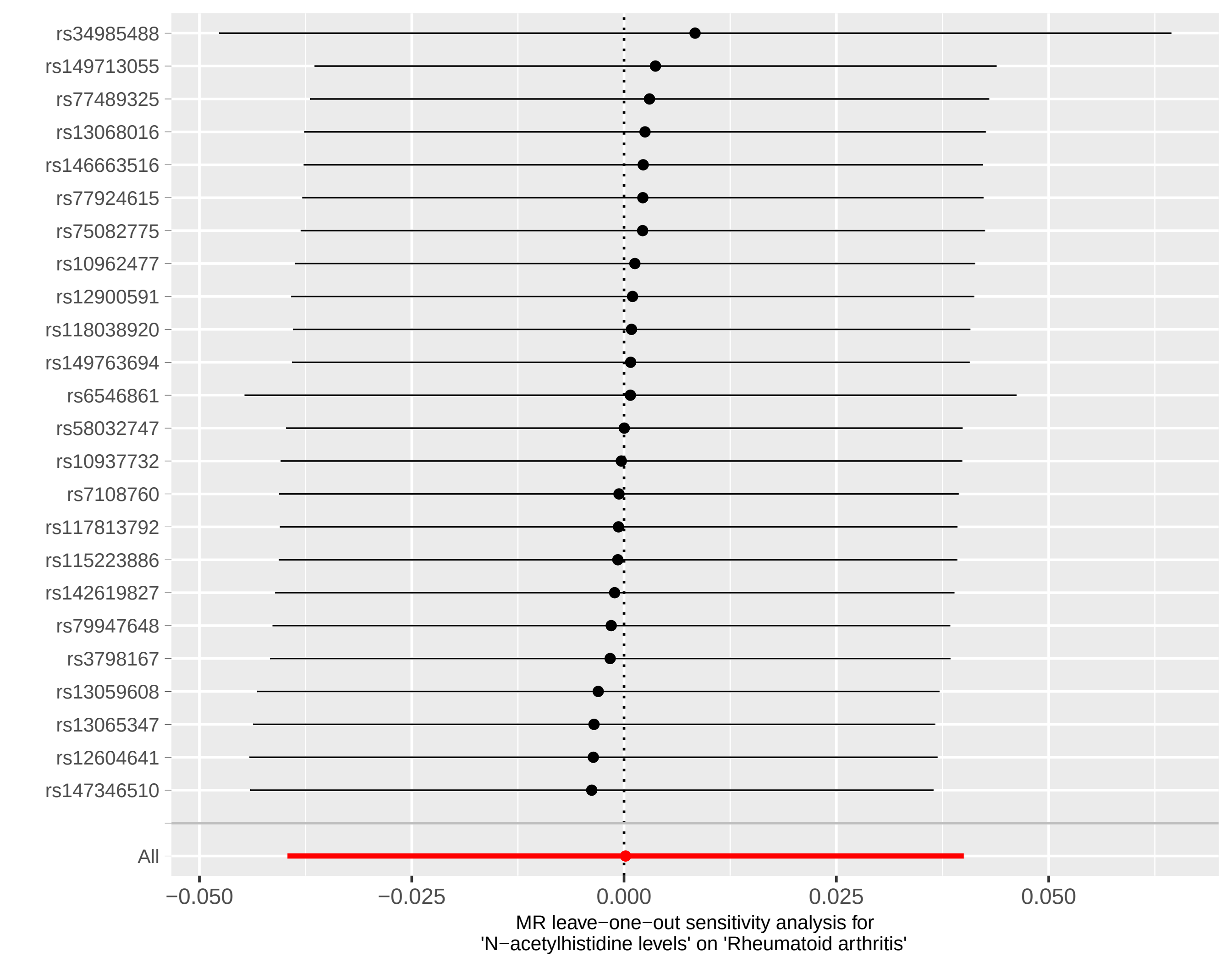
rs34985488
rs149713055
rs77489325
rs13068016
rs146663516
rs77924615
rs75082775
rs10962477
rs12900591
rs118038920
rs149763694
rs6546861
rs58032747
rs10937732
rs7108760
rs117813792
rs115223886
rs142619827
rs79947648
rs3798167
rs13059608
rs13065347
rs12604641
rs147346510
All
−0.050
−0.025
0.000
0.025
0.050
MR leave−one−out sensitivity analysis for
'N−acetylhistidine levels' on 'Rheumatoid arthritis'

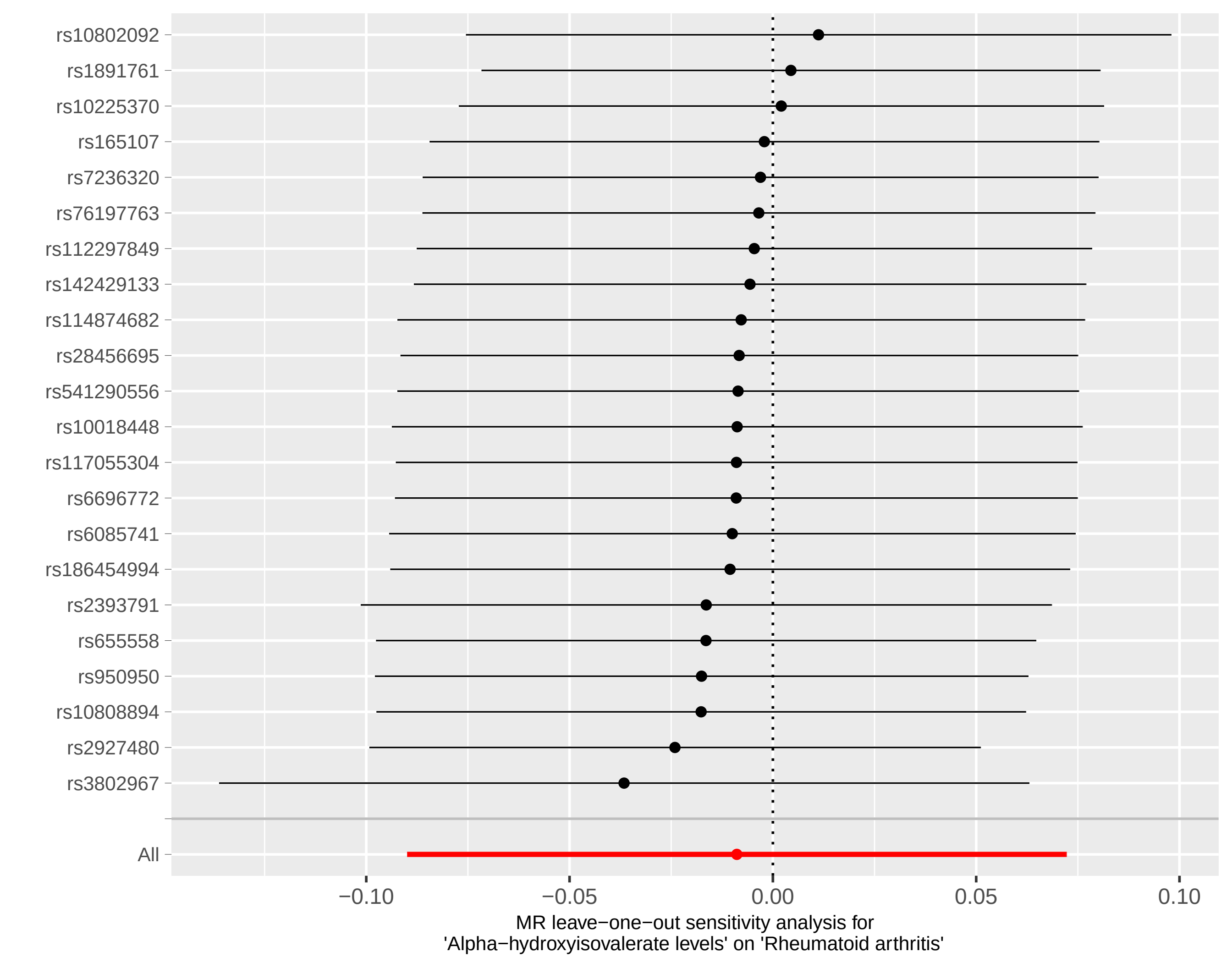
rs10802092
rs1891761
rs10225370
rs165107
rs7236320
rs76197763
rs112297849
rs142429133
rs114874682
rs28456695
rs541290556
rs10018448
rs117055304
rs6696772
rs6085741
rs186454994
rs2393791
rs655558
rs950950
rs10808894
rs2927480
rs3802967
All
−0.10
−0.05
0.00
0.05
0.10
MR leave−one−out sensitivity analysis for
'Alpha−hydroxyisovalerate levels' on 'Rheumatoid arthritis'

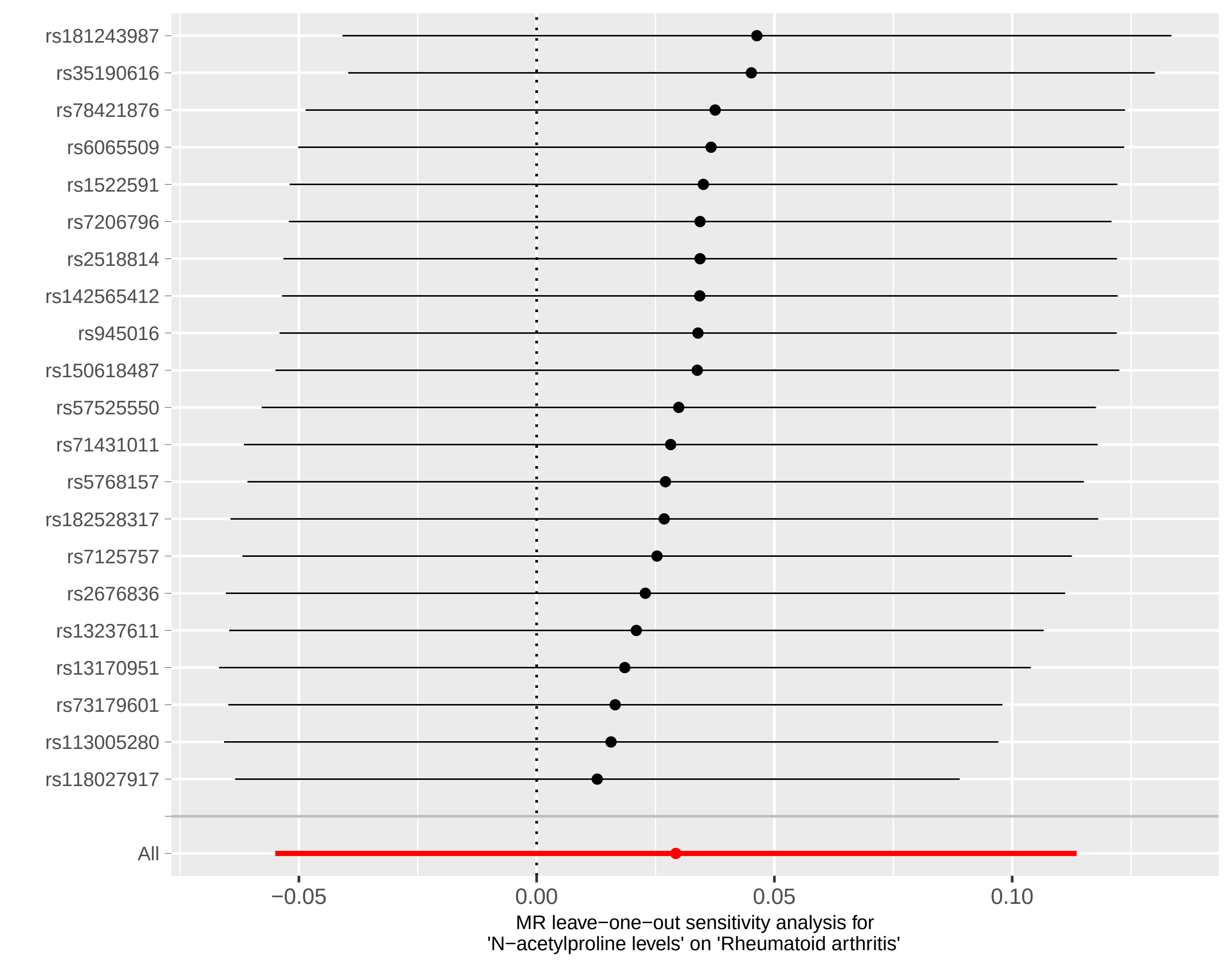

rs181243987
rs35190616
rs78421876
rs6065509
rs1522591
rs7206796
rs2518814
rs142565412
rs945016
rs150618487
rs57525550
rs71431011
rs5768157
rs182528317
rs7125757
rs2676836
rs13237611
rs13170951
rs73179601
rs113005280
rs118027917
All
−0.05
0.00
0.05
0.10
MR leave−one−out sensitivity analysis for
'N−acetylproline levels' on 'Rheumatoid arthritis'

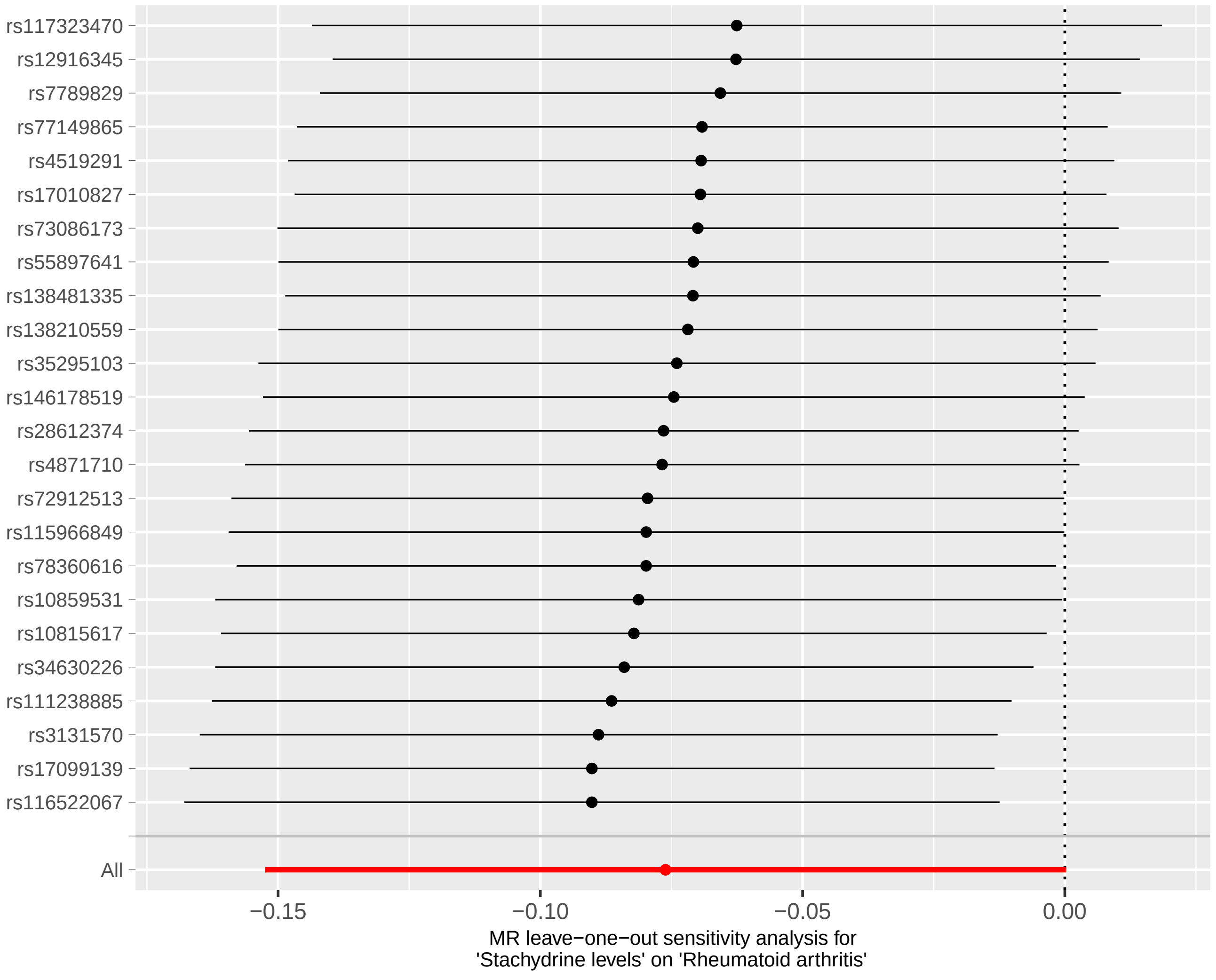
rs117323470
rs12916345
rs7789829
rs77149865
rs4519291
rs17010827
rs73086173
rs55897641
rs138481335
rs138210559
rs35295103
rs146178519
rs28612374
rs4871710
rs72912513
rs115966849
rs78360616
rs10859531
rs10815617
rs34630226
rs111238885
rs3131570
rs17099139
rs116522067
All
−0.15
−0.10
−0.05
0.00
MR leave−one−out sensitivity analysis for
'Stachydrine levels' on 'Rheumatoid arthritis'

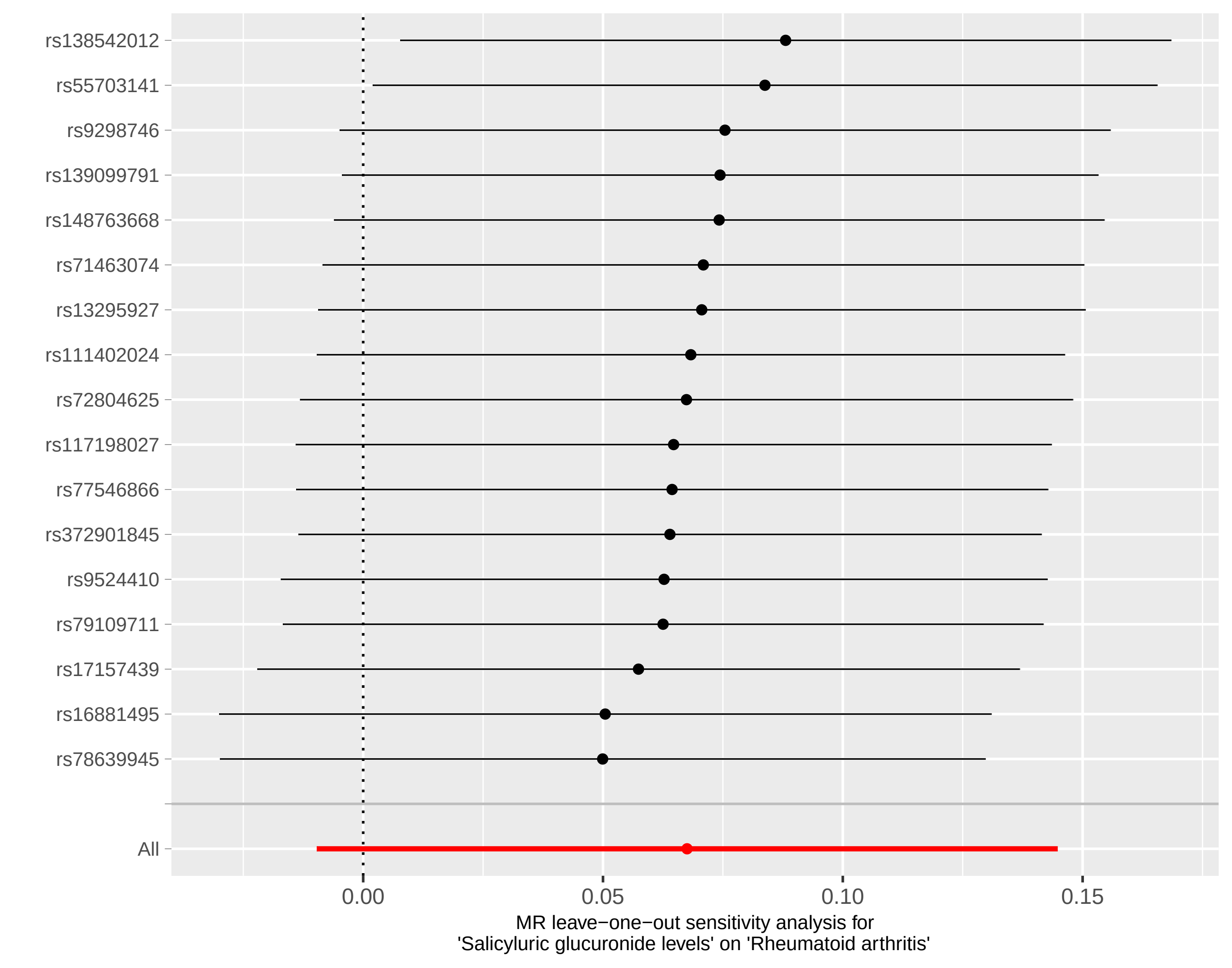
rs138542012
rs55703141
rs9298746
rs139099791
rs148763668
rs71463074
rs13295927
rs111402024
rs72804625
rs117198027
rs77546866
rs372901845
rs9524410
rs79109711
rs17157439
rs16881495
rs78639945
All
0.00
0.05
0.10
0.15
MR leave−one−out sensitivity analysis for
'Salicyluric glucuronide levels' on 'Rheumatoid arthritis'

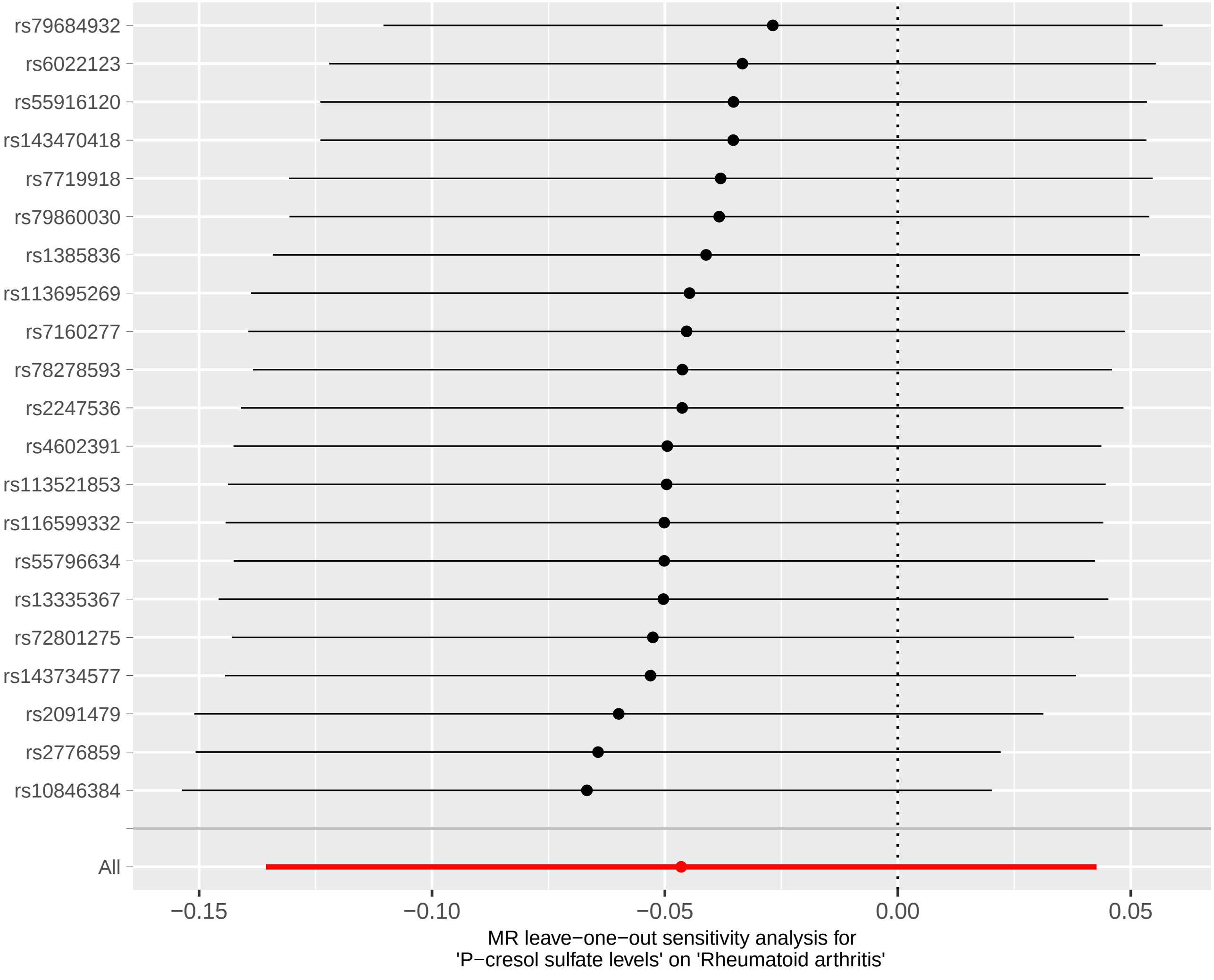
rs79684932
rs6022123
rs55916120
rs143470418
rs7719918
rs79860030
rs1385836
rs113695269
rs7160277
rs78278593
rs2247536
rs4602391
rs113521853
rs116599332
rs55796634
rs13335367
rs72801275
rs143734577
rs2091479
rs2776859
rs10846384
All
−0.15
−0.10
−0.05
0.00
0.05
MR leave−one−out sensitivity analysis for
'P−cresol sulfate levels' on 'Rheumatoid arthritis'

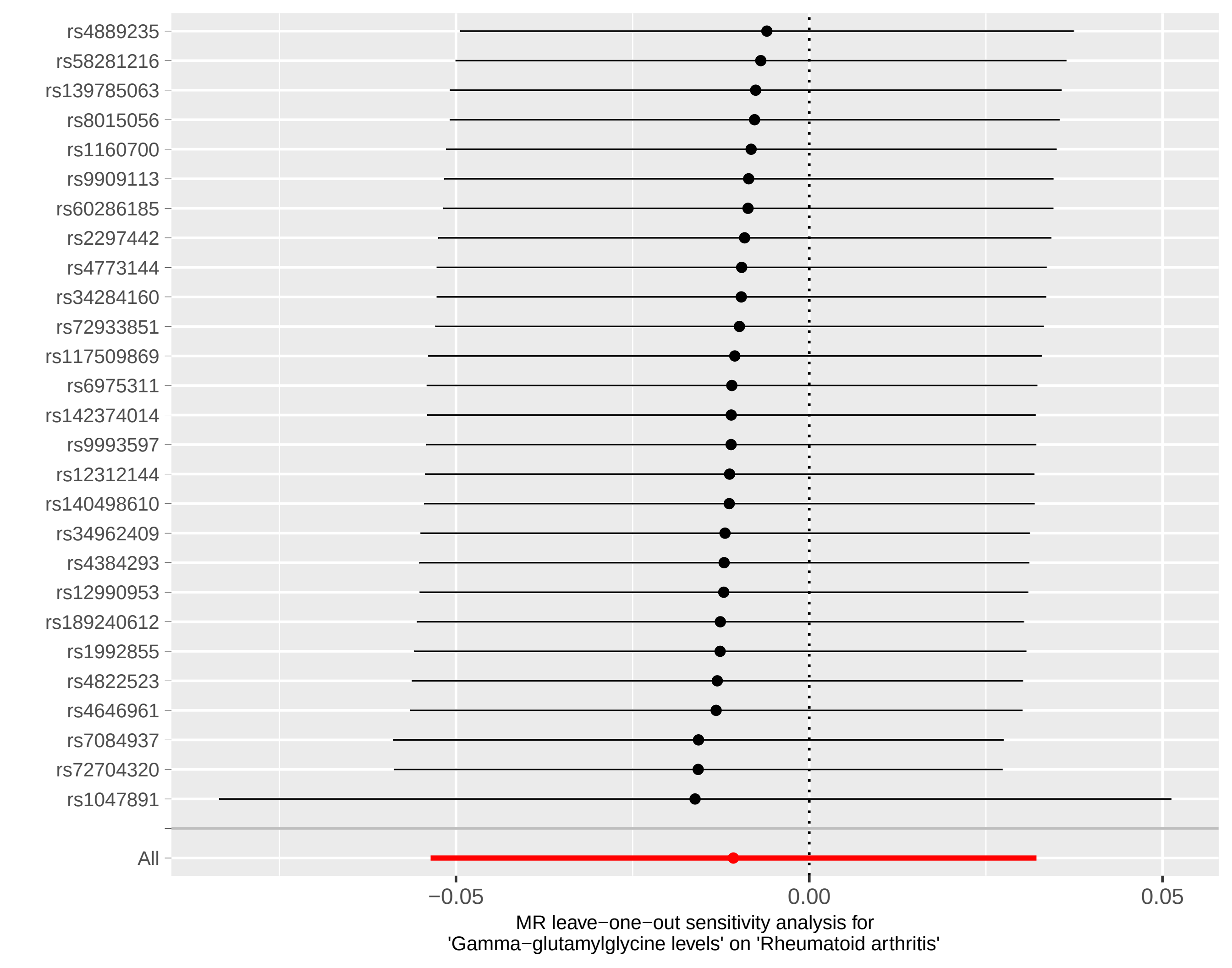

rs4889235
rs58281216
rs139785063
rs8015056
rs1160700
rs9909113
rs60286185
rs2297442
rs4773144
rs34284160
rs72933851
rs117509869
rs6975311
rs142374014
rs9993597
rs12312144
rs140498610
rs34962409
rs4384293
rs12990953
rs189240612
rs1992855
rs4822523
rs4646961
rs7084937
rs72704320
rs1047891
All
−0.05
0.00
0.05
MR leave−one−out sensitivity analysis for
'Gamma−glutamylglycine levels' on 'Rheumatoid arthritis'

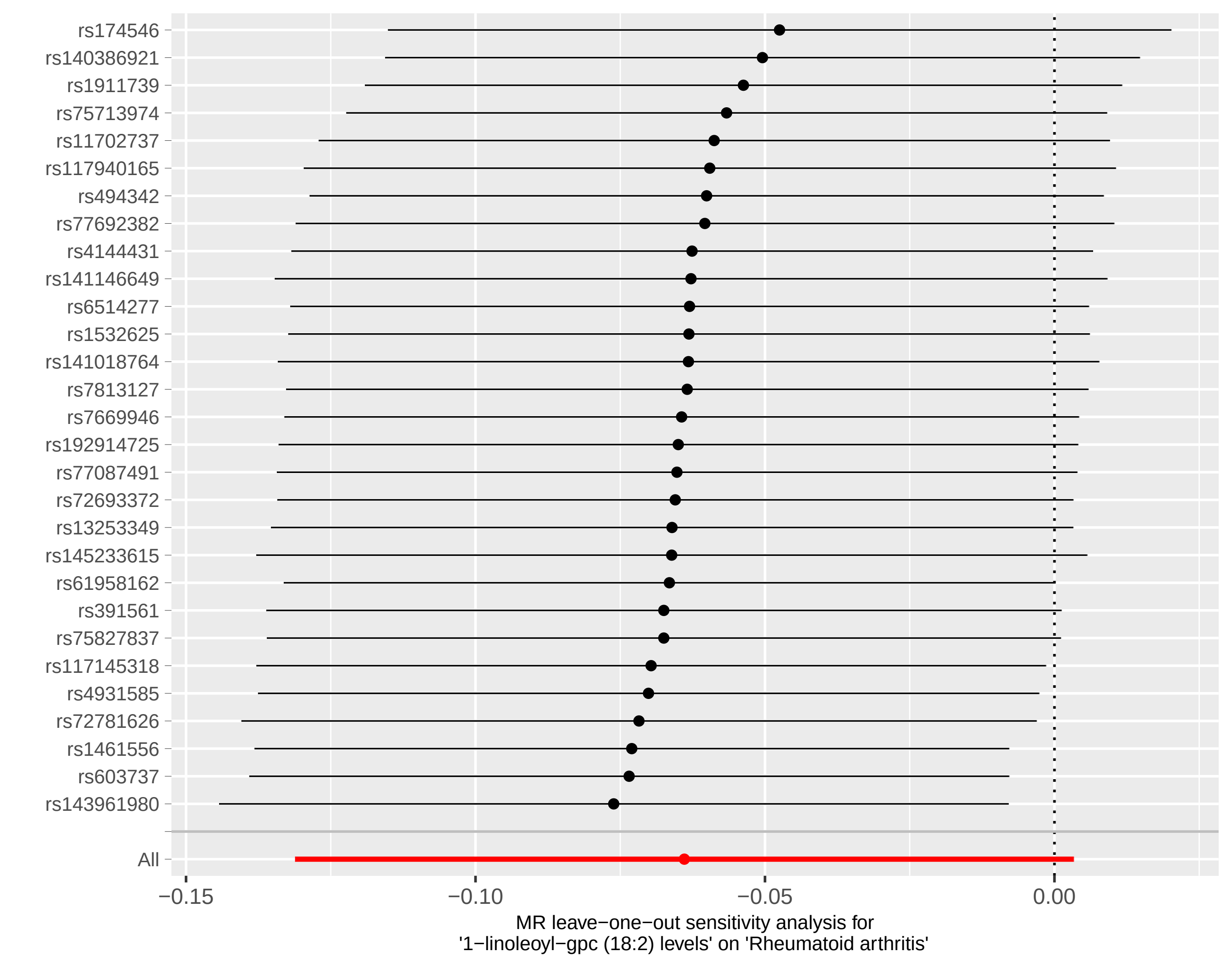

rs174546
rs140386921
rs1911739
rs75713974
rs11702737
rs117940165
rs494342
rs77692382
rs4144431
rs141146649
rs6514277
rs1532625
rs141018764
rs7813127
rs7669946
rs192914725
rs77087491
rs72693372
rs13253349
rs145233615
rs61958162
rs391561
rs75827837
rs117145318
rs4931585
rs72781626
rs1461556
rs603737
rs143961980
All
−0.15
−0.10
−0.05
0.00
MR leave−one−out sensitivity analysis for
'1−linoleoyl−gpc (18:2) levels' on 'Rheumatoid arthritis'

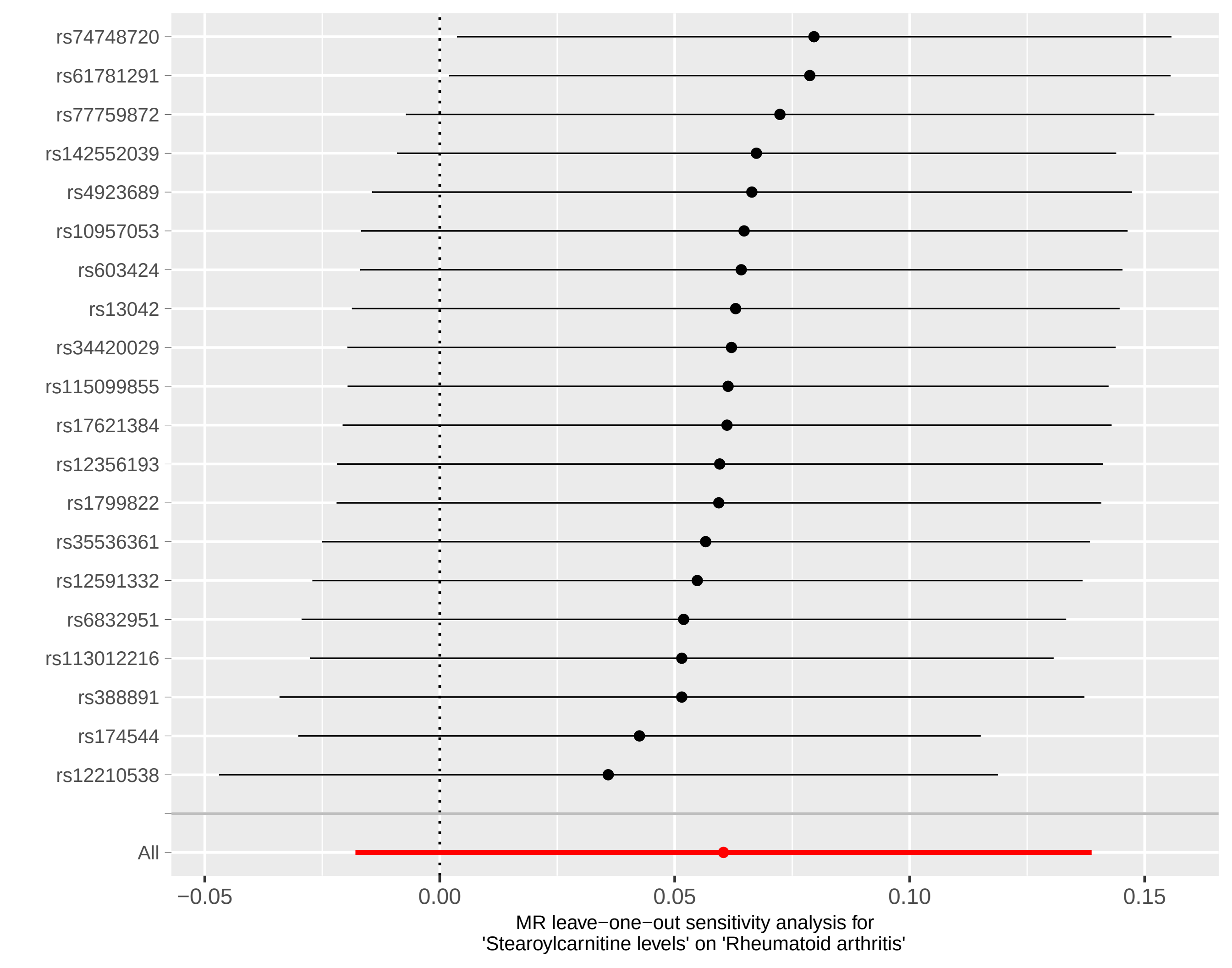
rs74748720
rs61781291
rs77759872
rs142552039
rs4923689
rs10957053
rs603424
rs13042
rs34420029
rs115099855
rs17621384
rs12356193
rs1799822
rs35536361
rs12591332
rs6832951
rs113012216
rs388891
rs174544
rs12210538
All
−0.05
0.00
0.05
0.10
0.15
MR leave−one−out sensitivity analysis for
'Stearoylcarnitine levels' on 'Rheumatoid arthritis'

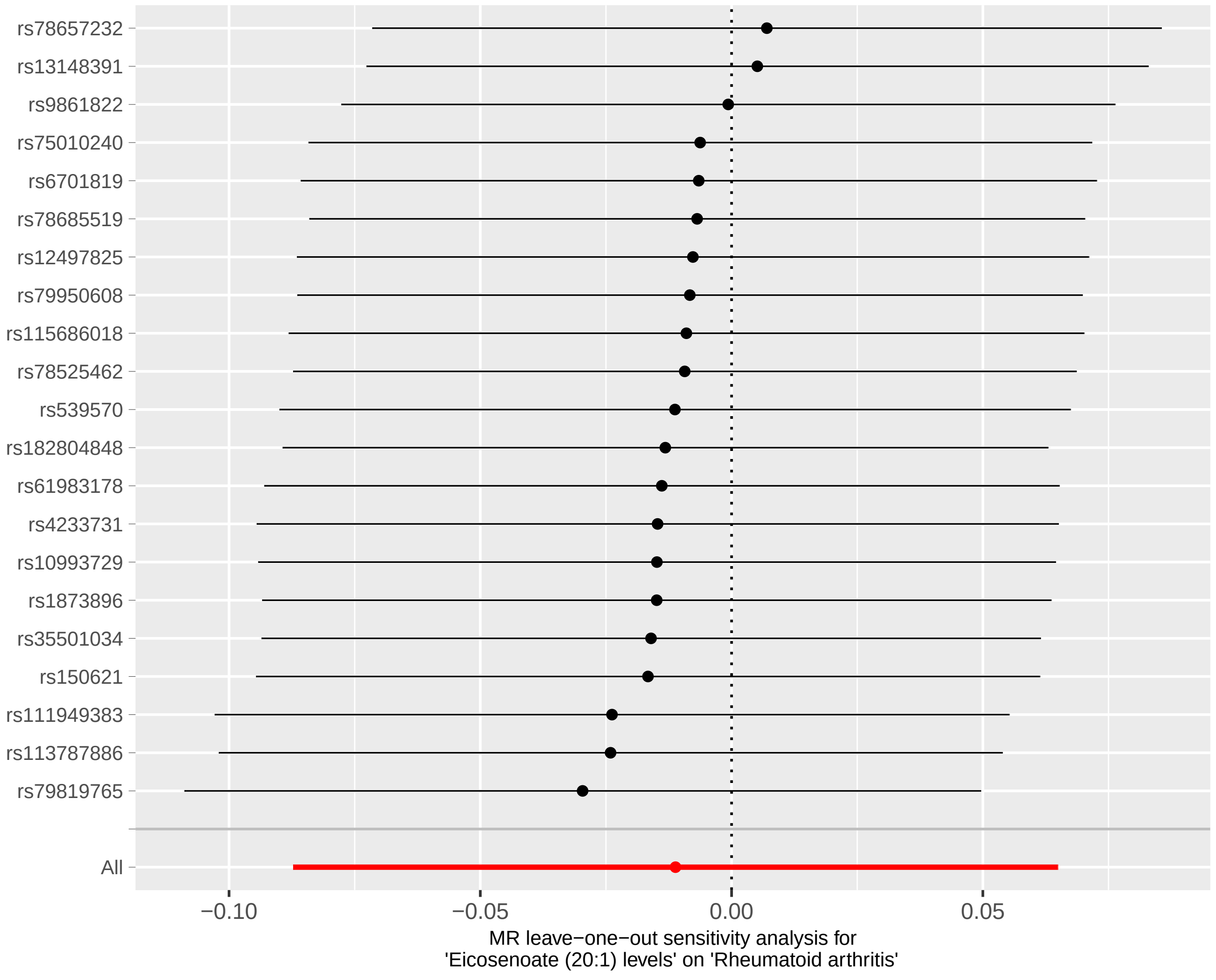
rs78657232
rs13148391
rs9861822
rs75010240
rs6701819
rs78685519
rs12497825
rs79950608
rs115686018
rs78525462
rs539570
rs182804848
rs61983178
rs4233731
rs10993729
rs1873896
rs35501034
rs150621
rs111949383
rs113787886
rs79819765
All
−0.10
−0.05
0.00
0.05
MR leave−one−out sensitivity analysis for
'Eicosenoate (20:1) levels' on 'Rheumatoid arthritis'

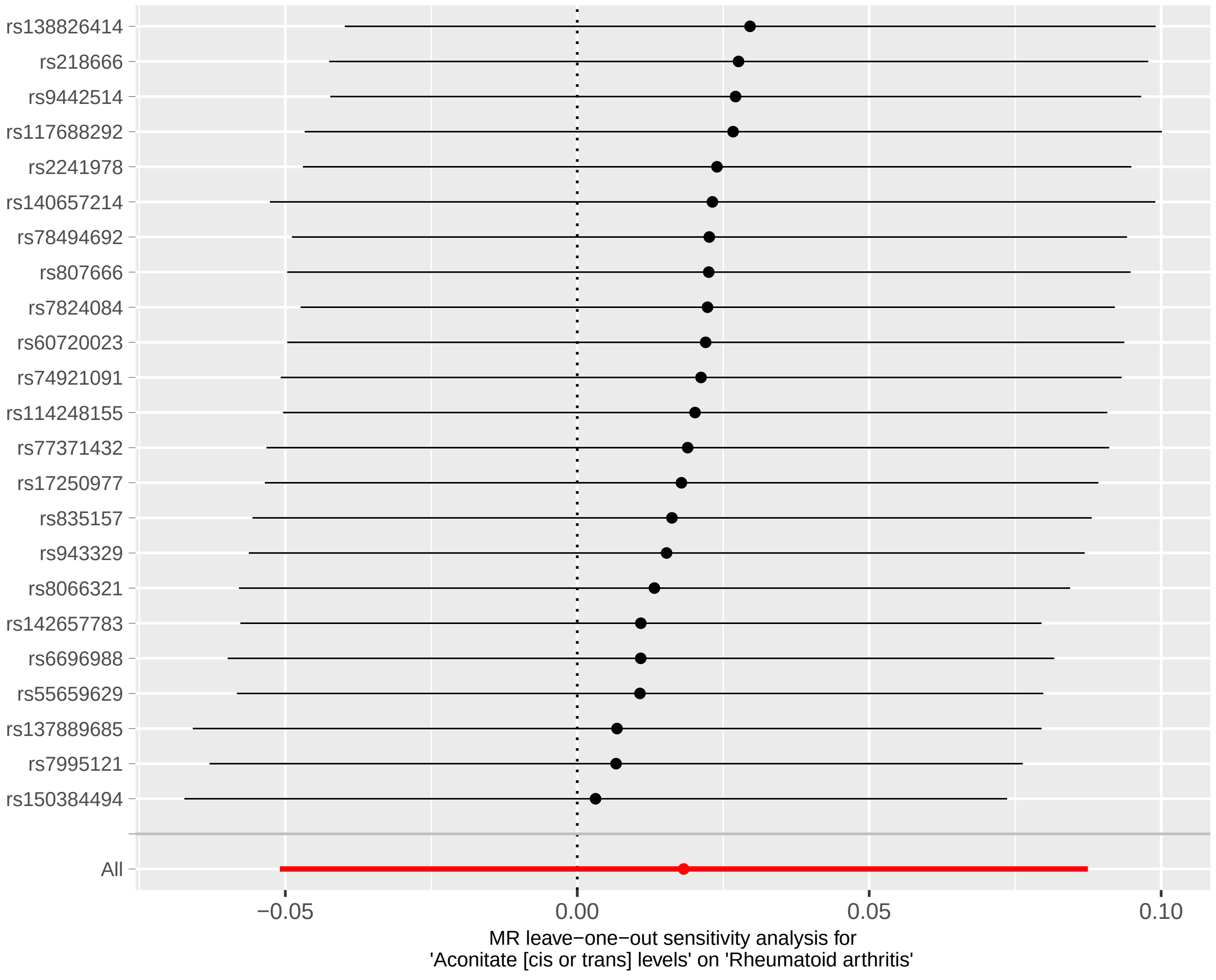
rs138826414
rs218666
rs9442514
rs117688292
rs2241978
rs140657214
rs78494692
rs807666
rs7824084
rs60720023
rs74921091
rs114248155
rs77371432
rs17250977
rs835157
rs943329
rs8066321
rs142657783
rs6696988
rs55659629
rs137889685
rs7995121
rs150384494
All
−0.05
0.00
0.05
0.10
MR leave−one−out sensitivity analysis for
'Aconitate [cis or trans] levels' on 'Rheumatoid arthritis'

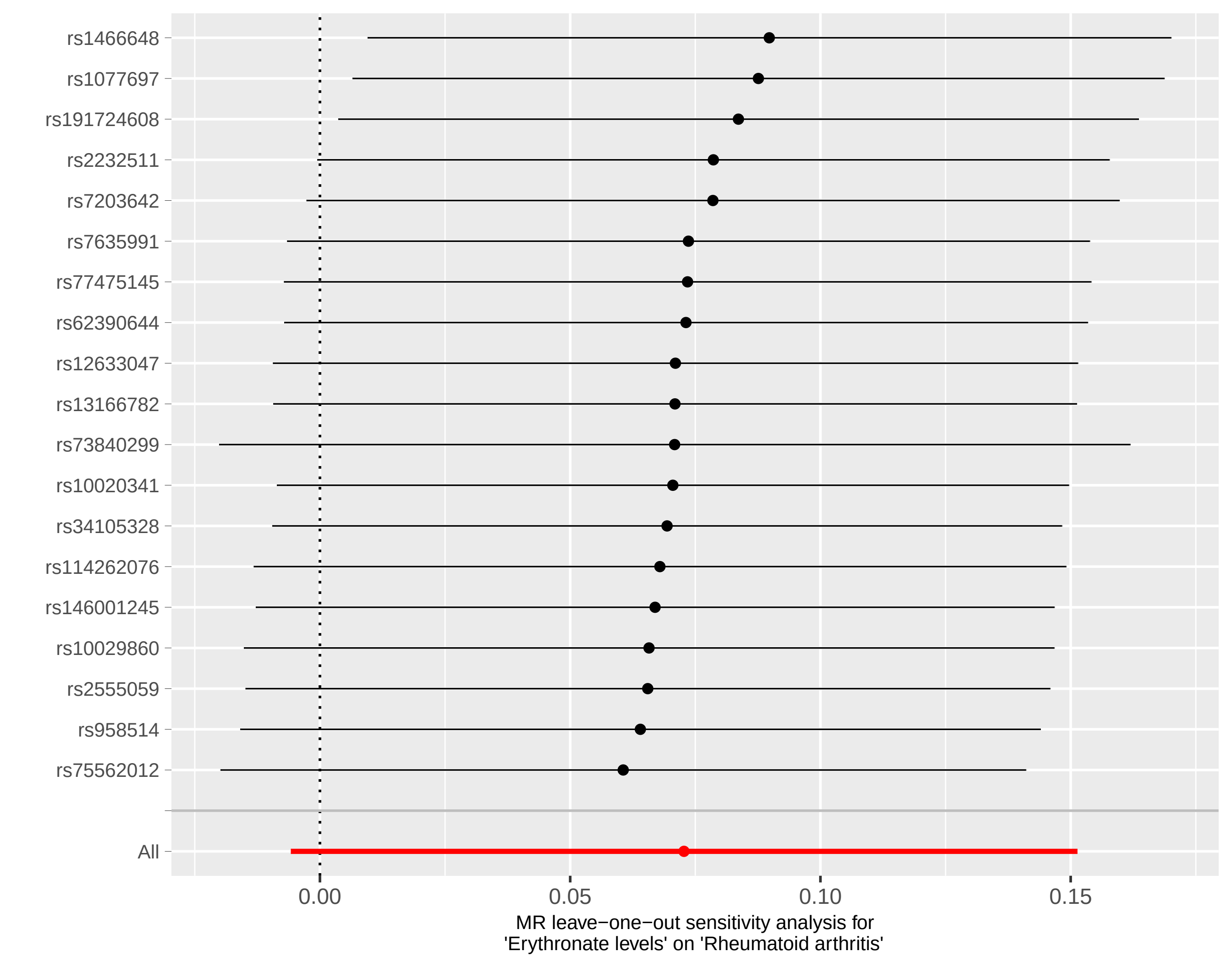

rs1466648
rs1077697
rs191724608
rs2232511
rs7203642
rs7635991
rs77475145
rs62390644
rs12633047
rs13166782
rs73840299
rs10020341
rs34105328
rs114262076
rs146001245
rs10029860
rs2555059
rs958514
rs75562012
All
0.00
0.05
0.10
0.15
MR leave−one−out sensitivity analysis for
'Erythronate levels' on 'Rheumatoid arthritis'

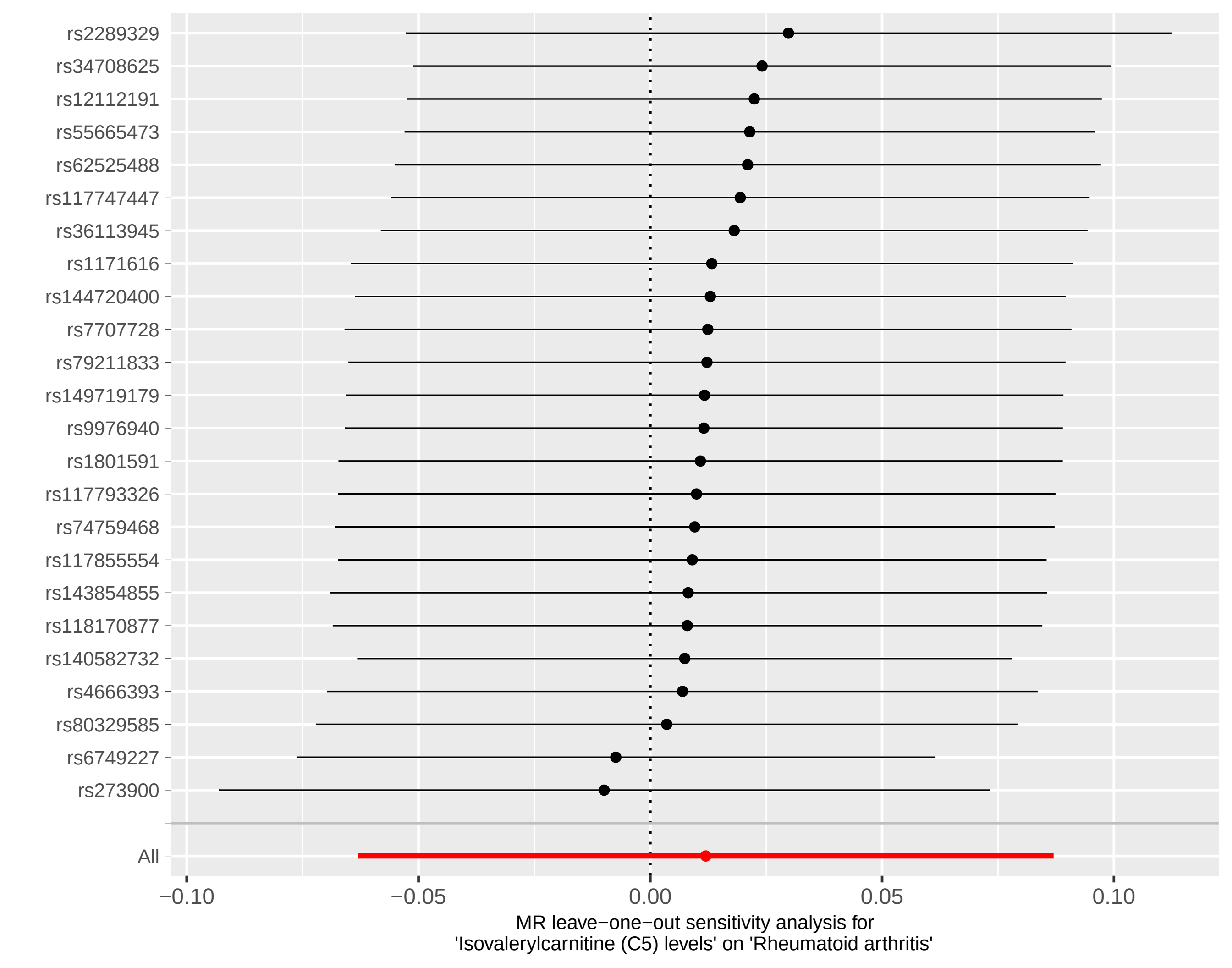
rs2289329
rs34708625
rs12112191
rs55665473
rs62525488
rs117747447
rs36113945
rs1171616
rs144720400
rs7707728
rs79211833
rs149719179
rs9976940
rs1801591
rs117793326
rs74759468
rs117855554
rs143854855
rs118170877
rs140582732
rs4666393
rs80329585
rs6749227
rs273900
All
−0.10
−0.05
0.00
0.05
0.10
MR leave−one−out sensitivity analysis for
'Isovalerylcarnitine (C5) levels' on 'Rheumatoid arthritis'

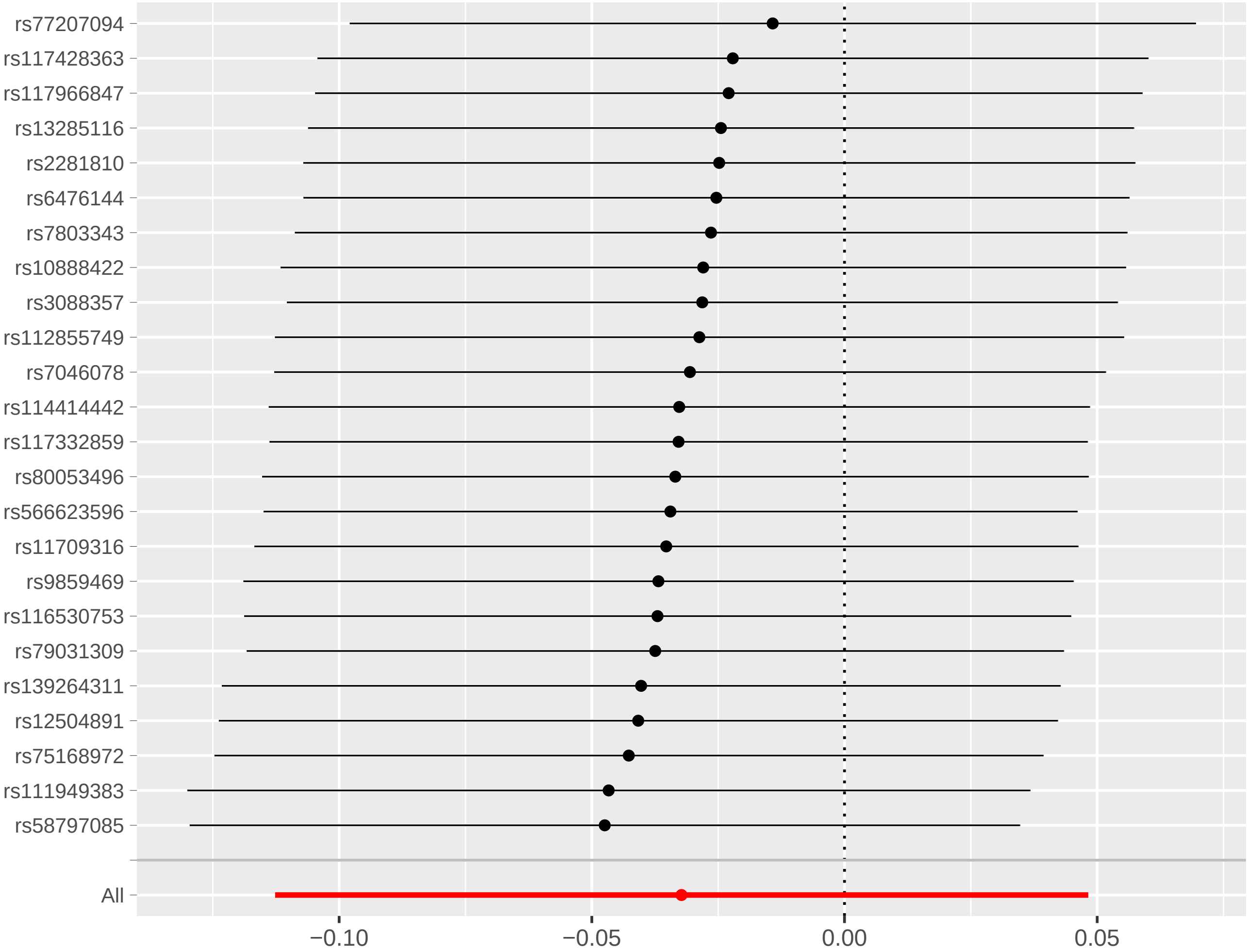

MR leave−one−out sensitivity analysis for
'Linolenate [alpha or gamma; (18:3n3 or 6)] levels' on 'Rheumatoid arthritis'

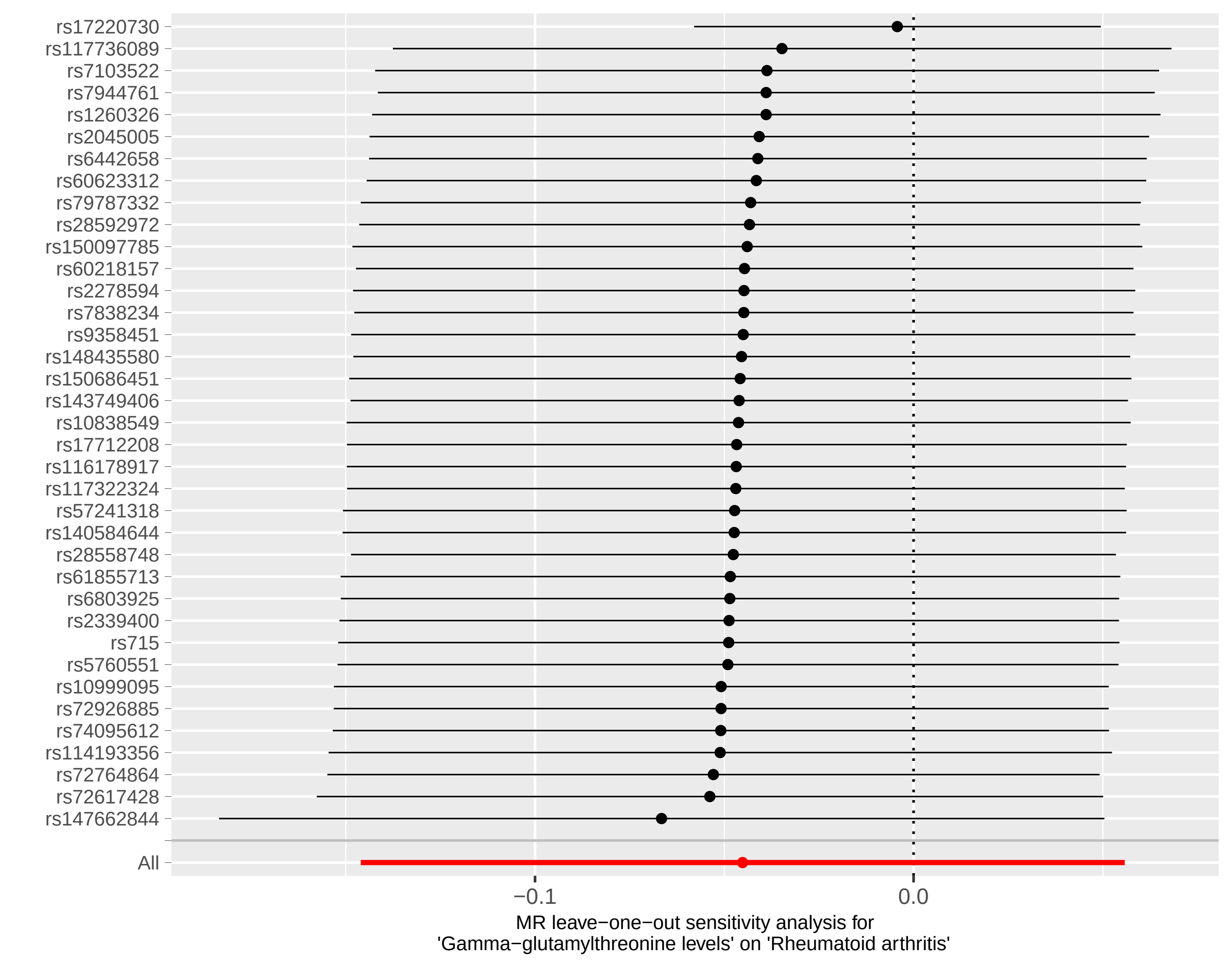
rs17220730
rs117736089
rs7103522
rs7944761
rs1260326
rs2045005
rs6442658
rs60623312
rs79787332
rs28592972
rs150097785
rs60218157
rs2278594
rs7838234
rs9358451
rs148435580
rs150686451
rs143749406
rs10838549
rs17712208
rs116178917
rs117322324
rs57241318
rs140584644
rs28558748
rs61855713
rs6803925
rs2339400
rs715
rs5760551
rs10999095
rs72926885
rs74095612
rs114193356
rs72764864
rs72617428
rs147662844
All
−0.1
0.0
MR leave−one−out sensitivity analysis for
'Gamma−glutamylthreonine levels' on 'Rheumatoid arthritis'

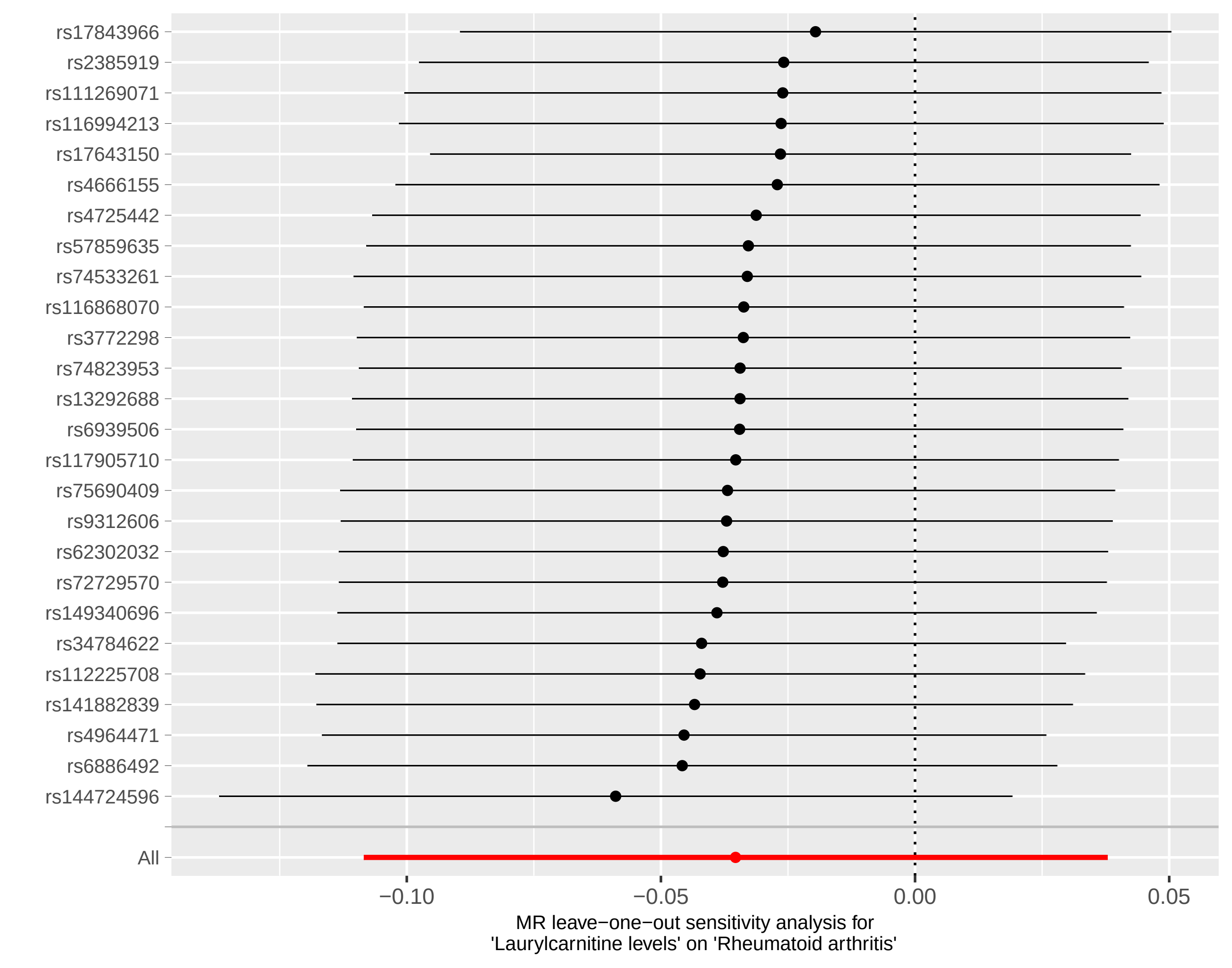
rs17843966
rs2385919
rs111269071
rs116994213
rs17643150
rs4666155
rs4725442
rs57859635
rs74533261
rs116868070
rs3772298
rs74823953
rs13292688
rs6939506
rs117905710
rs75690409
rs9312606
rs62302032
rs72729570
rs149340696
rs34784622
rs112225708
rs141882839
rs4964471
rs6886492
rs144724596
All
−0.10
−0.05
0.00
0.05
MR leave−one−out sensitivity analysis for
'Laurylcarnitine levels' on 'Rheumatoid arthritis'

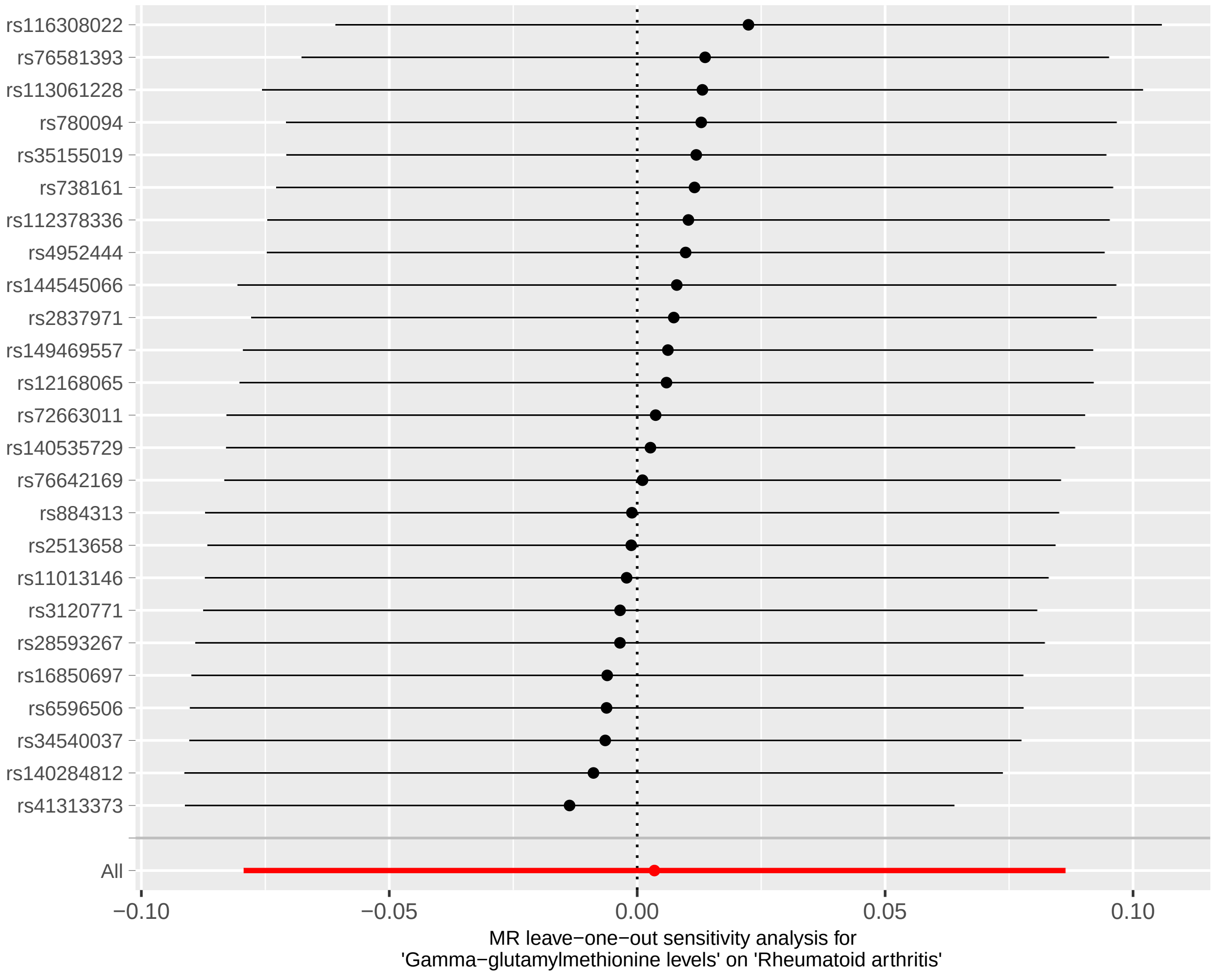
rs116308022
rs76581393
rs113061228
rs780094
rs35155019
rs738161
rs112378336
rs4952444
rs144545066
rs2837971
rs149469557
rs12168065
rs72663011
rs140535729
rs76642169
rs884313
rs2513658
rs11013146
rs3120771
rs28593267
rs16850697
rs6596506
rs34540037
rs140284812
rs41313373
All
−0.10
−0.05
0.00
0.05
0.10
MR leave−one−out sensitivity analysis for
'Gamma−glutamylmethionine levels' on 'Rheumatoid arthritis'

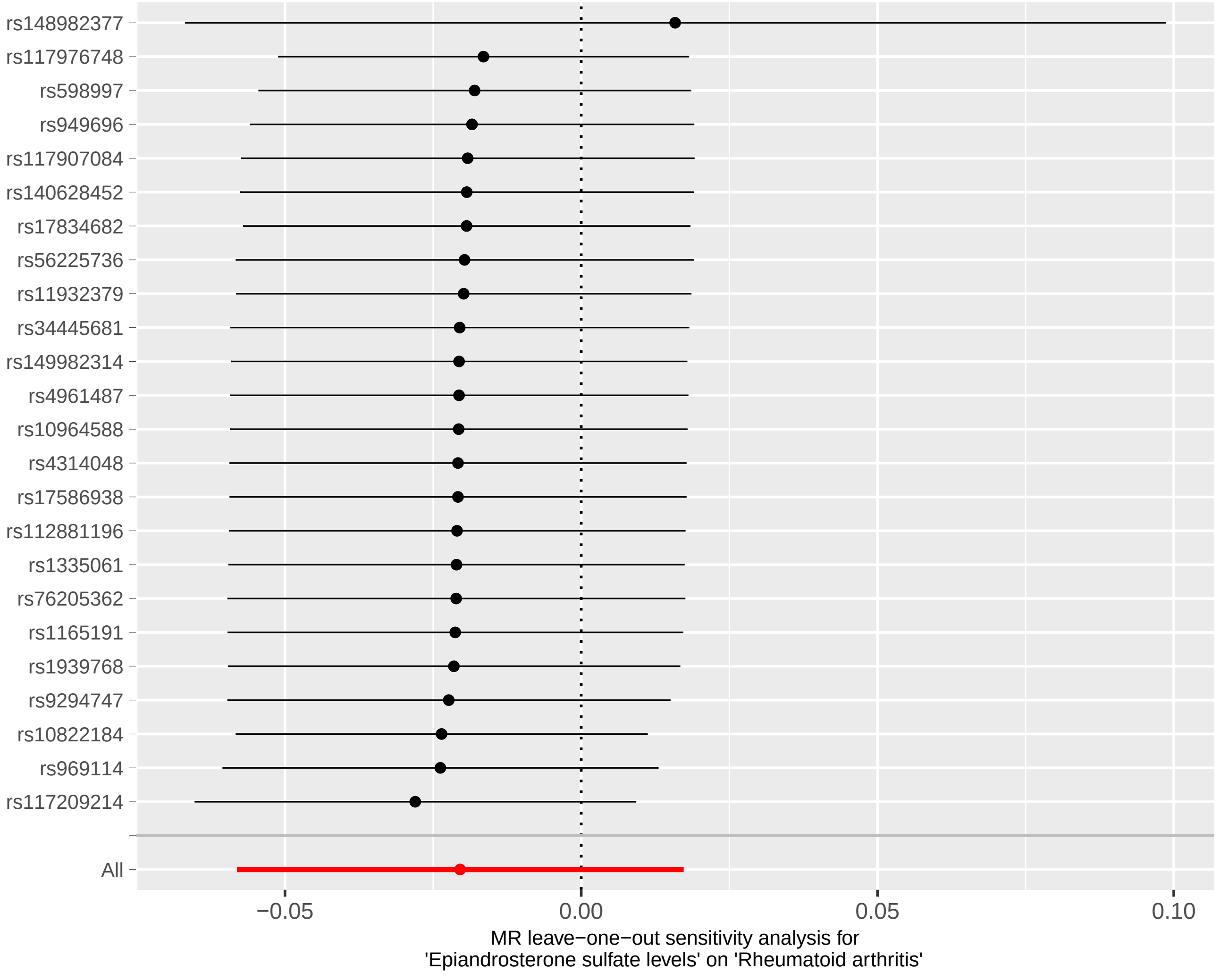
rs148982377
rs117976748
rs598997
rs949696
rs117907084
rs140628452
rs17834682
rs56225736
rs11932379
rs34445681
rs149982314
rs4961487
rs10964588
rs4314048
rs17586938
rs112881196
rs1335061
rs76205362
rs1165191
rs1939768
rs9294747
rs10822184
rs969114
rs117209214
All
−0.05
0.00
0.05
0.10
MR leave−one−out sensitivity analysis for
'Epiandrosterone sulfate levels' on 'Rheumatoid arthritis'

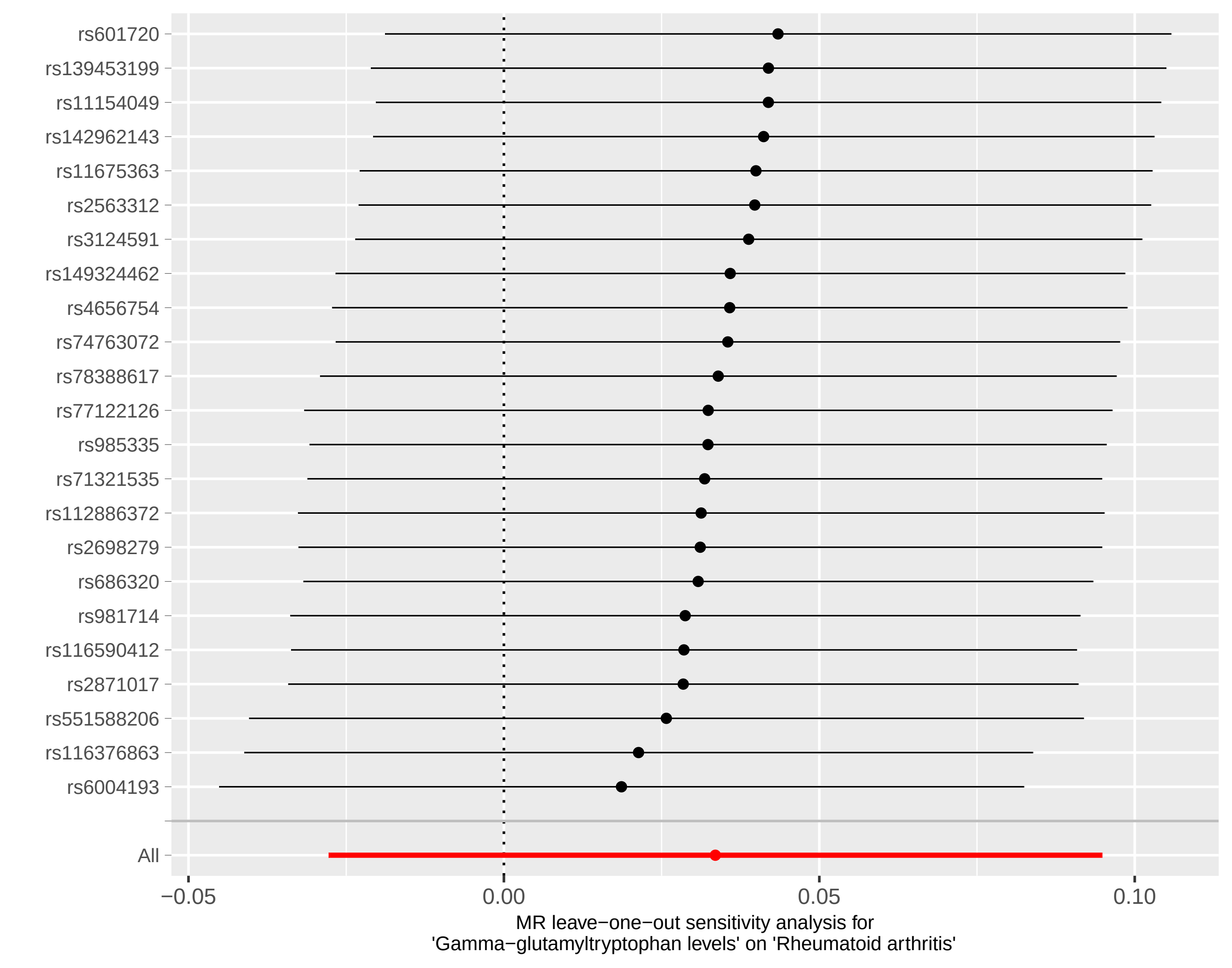
rs601720
rs139453199
rs11154049
rs142962143
rs11675363
rs2563312
rs3124591
rs149324462
rs4656754
rs74763072
rs78388617
rs77122126
rs985335
rs71321535
rs112886372
rs2698279
rs686320
rs981714
rs116590412
rs2871017
rs551588206
rs116376863
rs6004193
All
−0.05
0.00
0.05
0.10
MR leave−one−out sensitivity analysis for
'Gamma−glutamyltryptophan levels' on 'Rheumatoid arthritis'

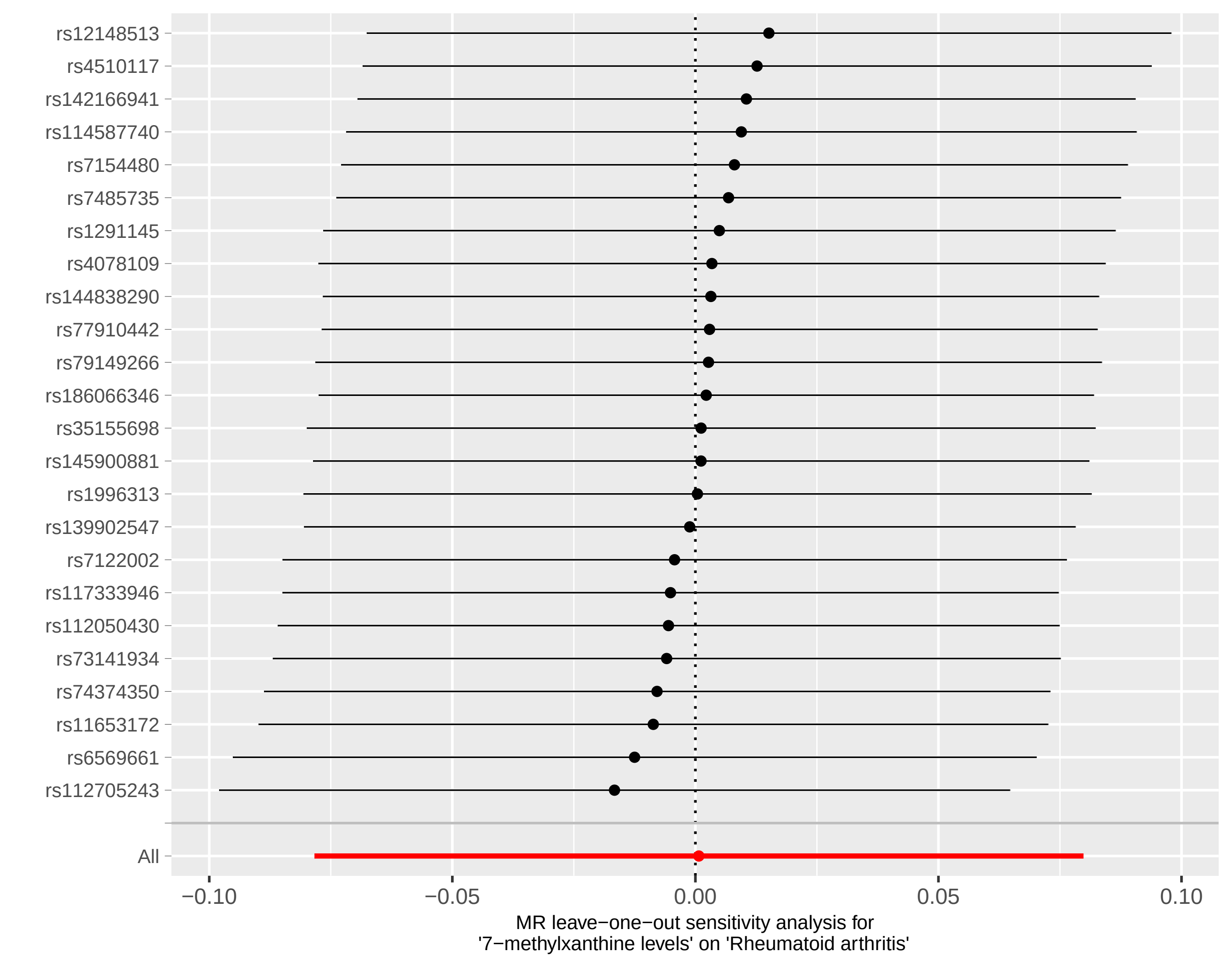
rs12148513
rs4510117
rs142166941
rs114587740
rs7154480
rs7485735
rs1291145
rs4078109
rs144838290
rs77910442
rs79149266
rs186066346
rs35155698
rs145900881
rs1996313
rs139902547
rs7122002
rs117333946
rs112050430
rs73141934
rs74374350
rs11653172
rs6569661
rs112705243
All
−0.10
−0.05
0.00
0.05
0.10
MR leave−one−out sensitivity analysis for
'7−methylxanthine levels' on 'Rheumatoid arthritis'

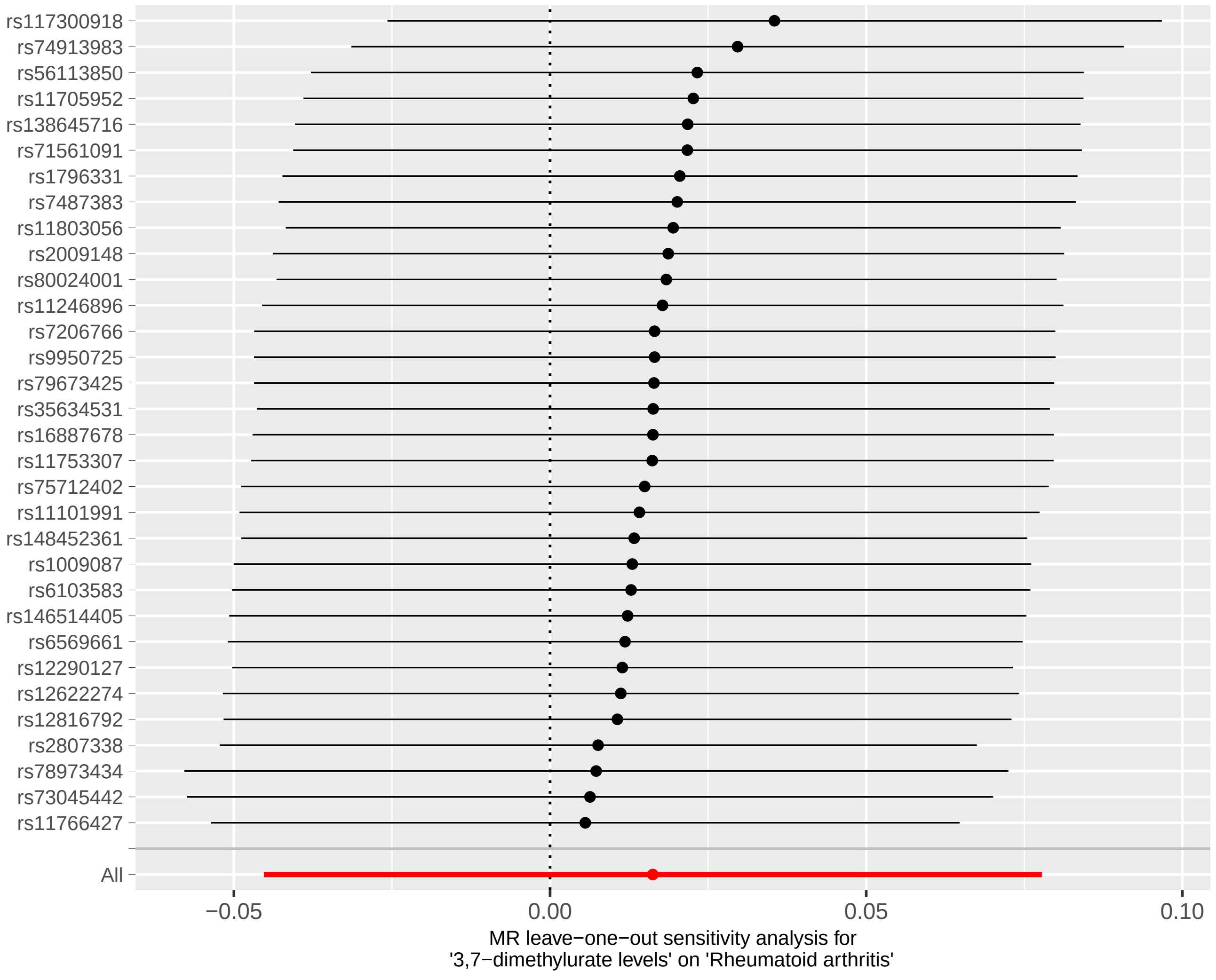

rs117300918
rs74913983
rs56113850
rs11705952
rs138645716
rs71561091
rs1796331
rs7487383
rs11803056
rs2009148
rs80024001
rs11246896
rs7206766
rs9950725
rs79673425
rs35634531
rs16887678
rs11753307
rs75712402
rs11101991
rs148452361
rs1009087
rs6103583
rs146514405
rs6569661
rs12290127
rs12622274
rs12816792
rs2807338
rs78973434
rs73045442
rs11766427
All
−0.05
0.00
0.05
0.10
MR leave−one−out sensitivity analysis for
'3,7−dimethylurate levels' on 'Rheumatoid arthritis'

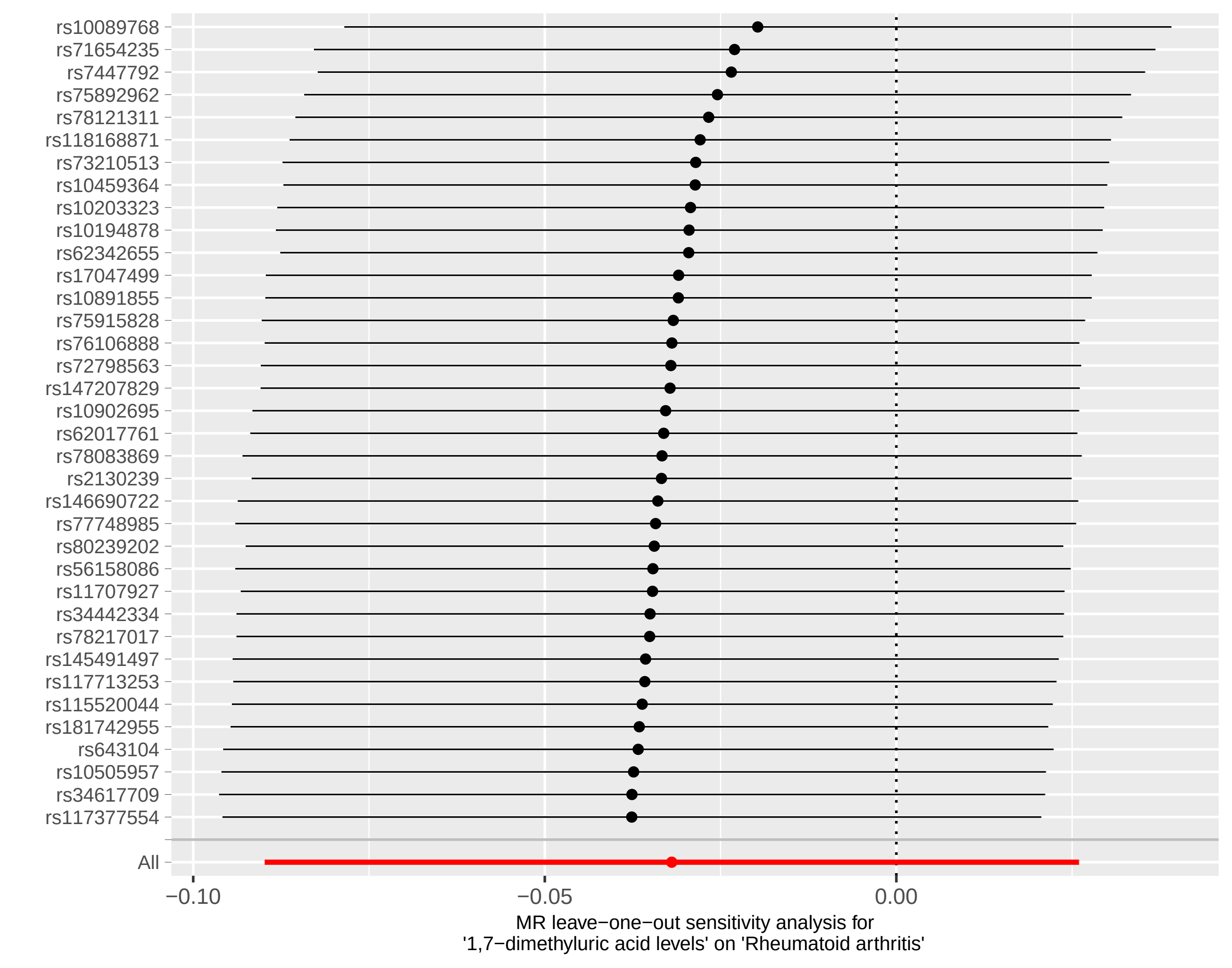
rs10089768
rs71654235
rs7447792
rs75892962
rs78121311
rs118168871
rs73210513
rs10459364
rs10203323
rs10194878
rs62342655
rs17047499
rs10891855
rs75915828
rs76106888
rs72798563
rs147207829
rs10902695
rs62017761
rs78083869
rs2130239
rs146690722
rs77748985
rs80239202
rs56158086
rs11707927
rs34442334
rs78217017
rs145491497
rs117713253
rs115520044
rs181742955
rs643104
rs10505957
rs34617709
rs117377554
All
−0.10
−0.05
0.00
MR leave−one−out sensitivity analysis for
'1,7−dimethyluric acid levels' on 'Rheumatoid arthritis'

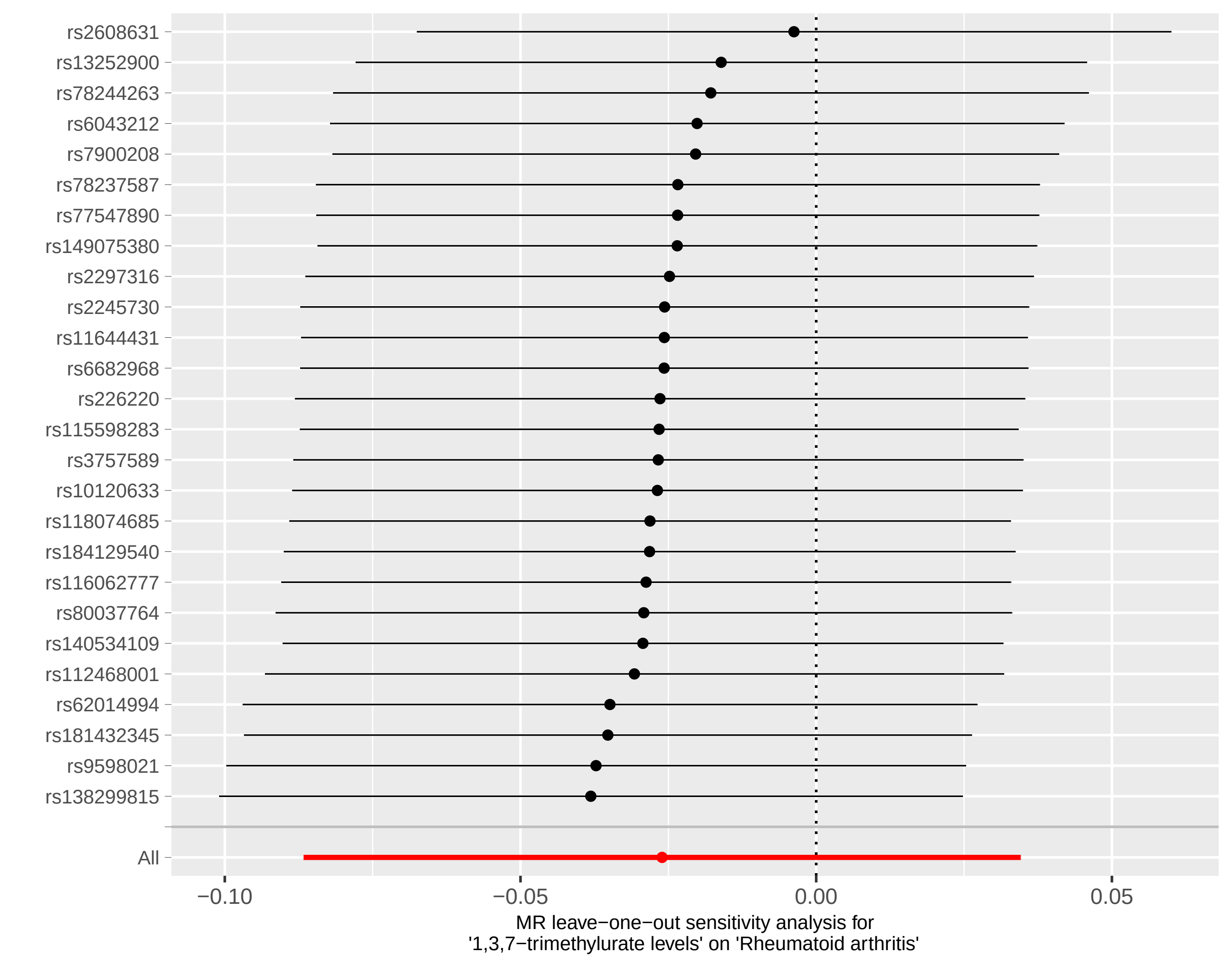
rs2608631
rs13252900
rs78244263
rs6043212
rs7900208
rs78237587
rs77547890
rs149075380
rs2297316
rs2245730
rs11644431
rs6682968
rs226220
rs115598283
rs3757589
rs10120633
rs118074685
rs184129540
rs116062777
rs80037764
rs140534109
rs112468001
rs62014994
rs181432345
rs9598021
rs138299815
All
−0.10
−0.05
0.00
0.05
MR leave−one−out sensitivity analysis for
'1,3,7−trimethylurate levels' on 'Rheumatoid arthritis'

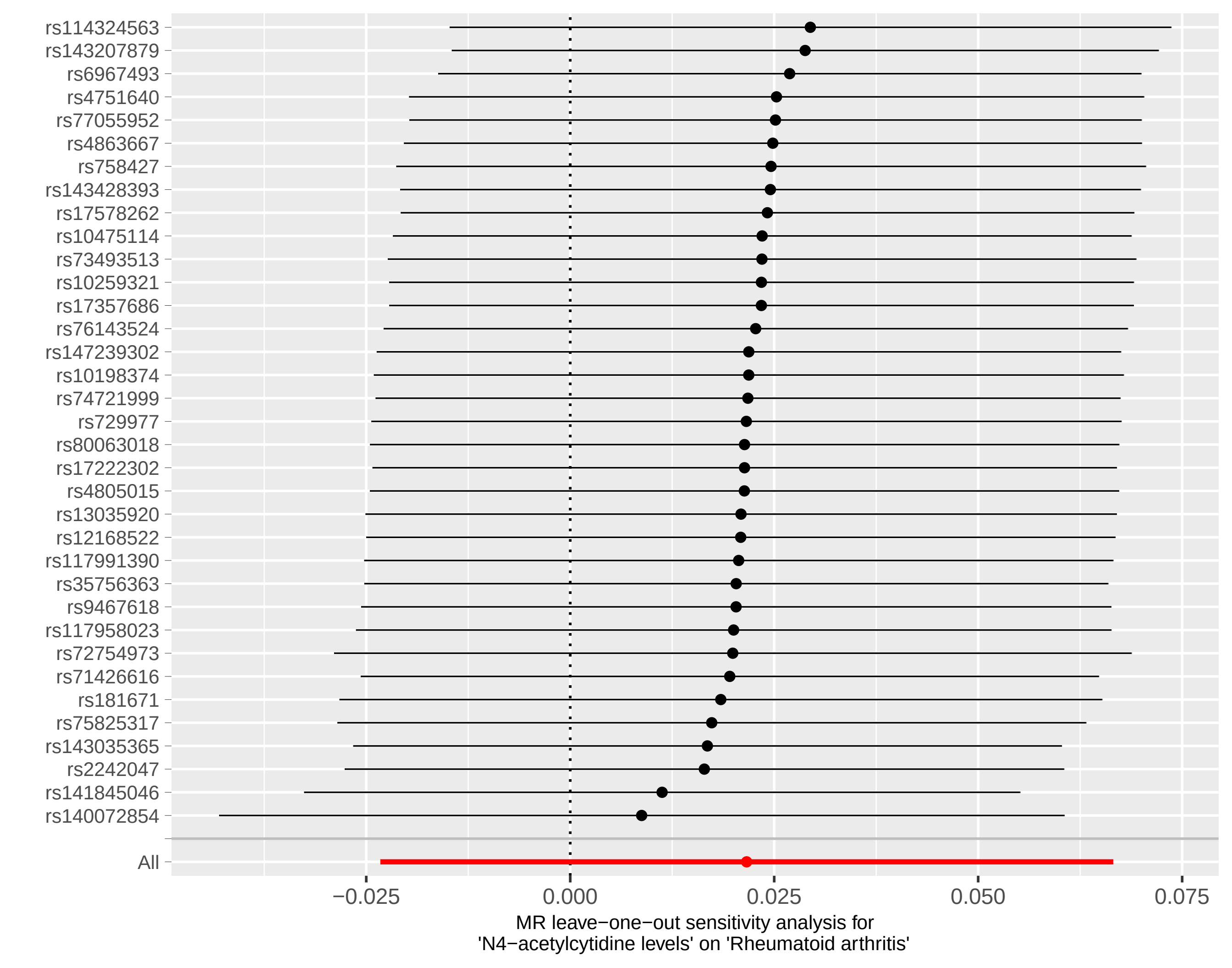

rs114324563
rs143207879
rs6967493
rs4751640
rs77055952
rs4863667
rs758427
rs143428393
rs17578262
rs10475114
rs73493513
rs10259321
rs17357686
rs76143524
rs147239302
rs10198374
rs74721999
rs729977
rs80063018
rs17222302
rs4805015
rs13035920
rs12168522
rs117991390
rs35756363
rs9467618
rs117958023
rs72754973
rs71426616
rs181671
rs75825317
rs143035365
rs2242047
rs141845046
rs140072854
All
−0.025
0.000
0.025
0.050
0.075
MR leave−one−out sensitivity analysis for
'N4−acetylcytidine levels' on 'Rheumatoid arthritis'

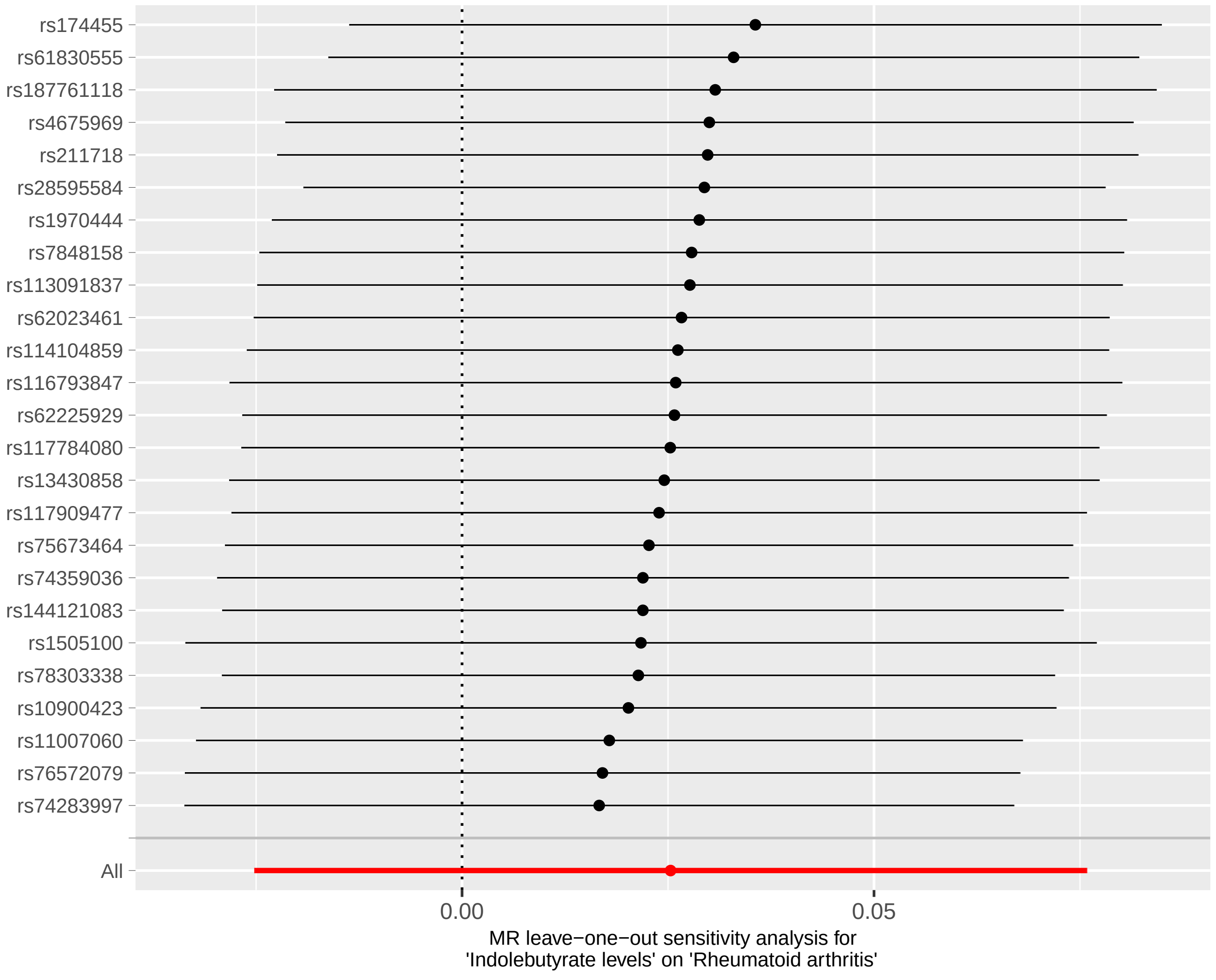
rs174455
rs61830555
rs187761118
rs4675969
rs211718
rs28595584
rs1970444
rs7848158
rs113091837
rs62023461
rs114104859
rs116793847
rs62225929
rs117784080
rs13430858
rs117909477
rs75673464
rs74359036
rs144121083
rs1505100
rs78303338
rs10900423
rs11007060
rs76572079
rs74283997
All
0.00
0.05
MR leave−one−out sensitivity analysis for
'Indolebutyrate levels' on 'Rheumatoid arthritis'

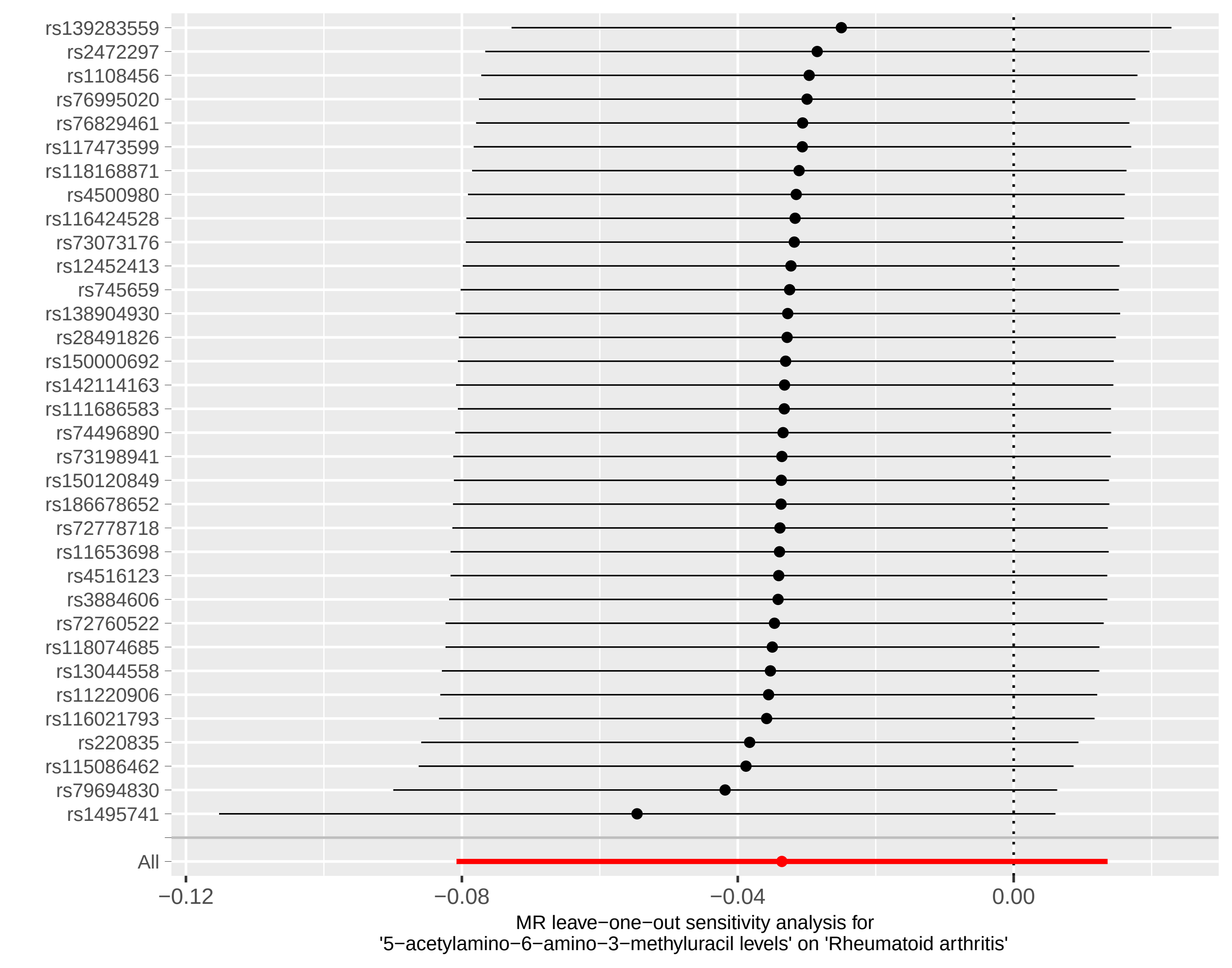

rs139283559
rs2472297
rs1108456
rs76995020
rs76829461
rs117473599
rs118168871
rs4500980
rs116424528
rs73073176
rs12452413
rs745659
rs138904930
rs28491826
rs150000692
rs142114163
rs111686583
rs74496890
rs73198941
rs150120849
rs186678652
rs72778718
rs11653698
rs4516123
rs3884606
rs72760522
rs118074685
rs13044558
rs11220906
rs116021793
rs220835
rs115086462
rs79694830
rs1495741
All
−0.12
−0.08
−0.04
0.00
MR leave−one−out sensitivity analysis for
'5−acetylamino−6−amino−3−methyluracil levels' on 'Rheumatoid arthritis'

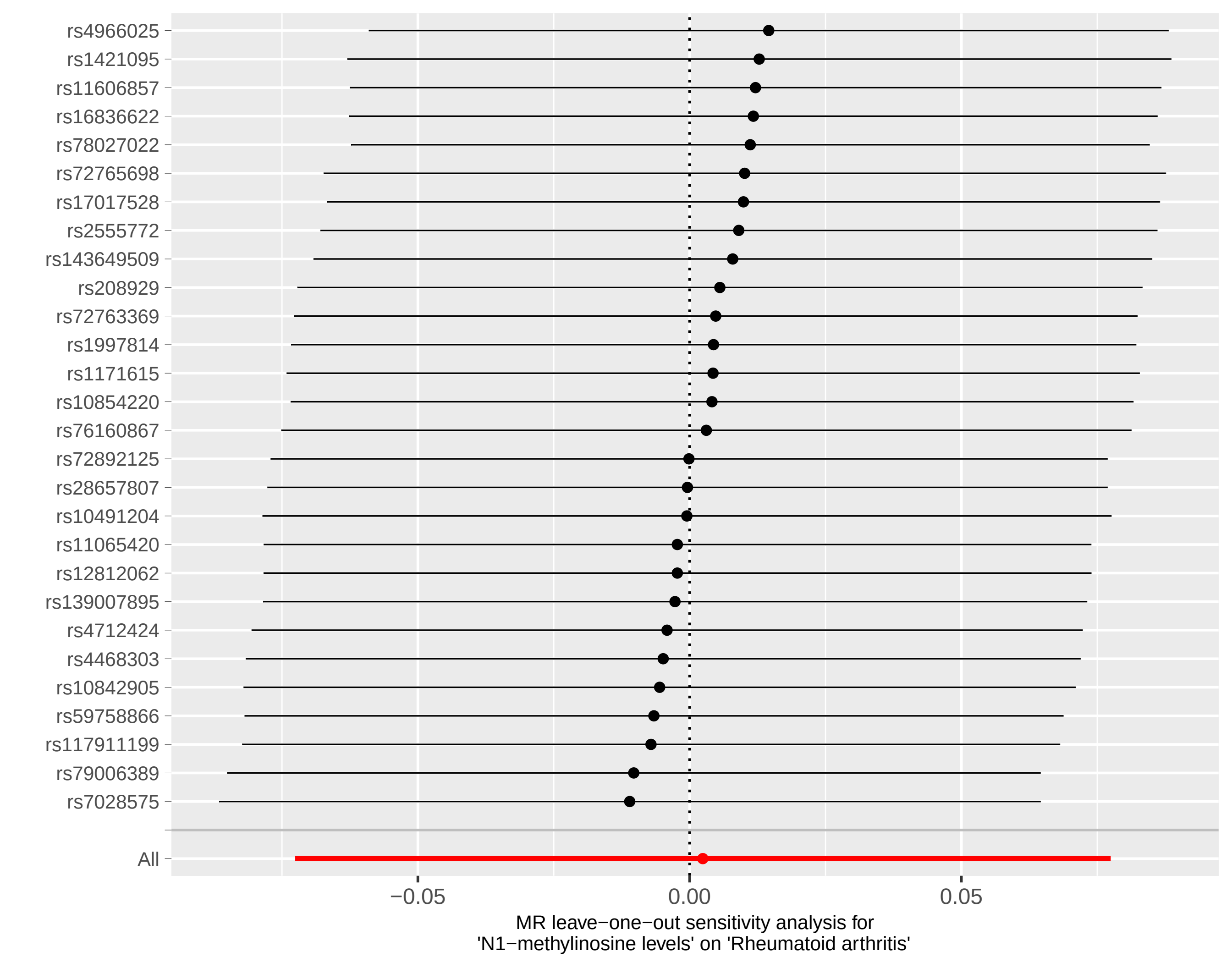
rs4966025
rs1421095
rs11606857
rs16836622
rs78027022
rs72765698
rs17017528
rs2555772
rs143649509
rs208929
rs72763369
rs1997814
rs1171615
rs10854220
rs76160867
rs72892125
rs28657807
rs10491204
rs11065420
rs12812062
rs139007895
rs4712424
rs4468303
rs10842905
rs59758866
rs117911199
rs79006389
rs7028575
All
−0.05
0.00
0.05
MR leave−one−out sensitivity analysis for
'N1−methylinosine levels' on 'Rheumatoid arthritis'

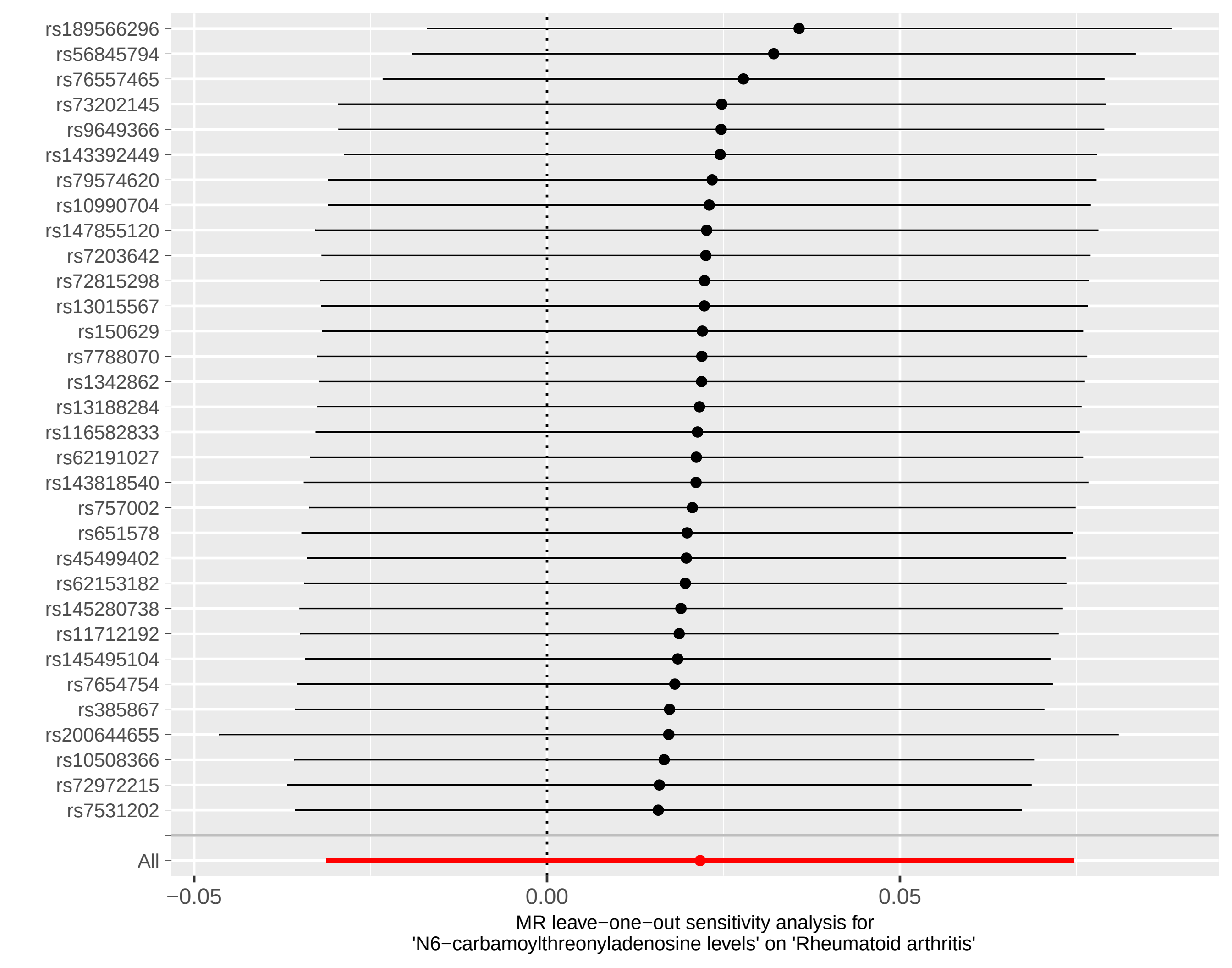
rs189566296
rs56845794
rs76557465
rs73202145
rs9649366
rs143392449
rs79574620
rs10990704
rs147855120
rs7203642
rs72815298
rs13015567
rs150629
rs7788070
rs1342862
rs13188284
rs116582833
rs62191027
rs143818540
rs757002
rs651578
rs45499402
rs62153182
rs145280738
rs11712192
rs145495104
rs7654754
rs385867
rs200644655
rs10508366
rs72972215
rs7531202
All
−0.05
0.00
0.05
MR leave−one−out sensitivity analysis for
'N6−carbamoylthreonyladenosine levels' on 'Rheumatoid arthritis'

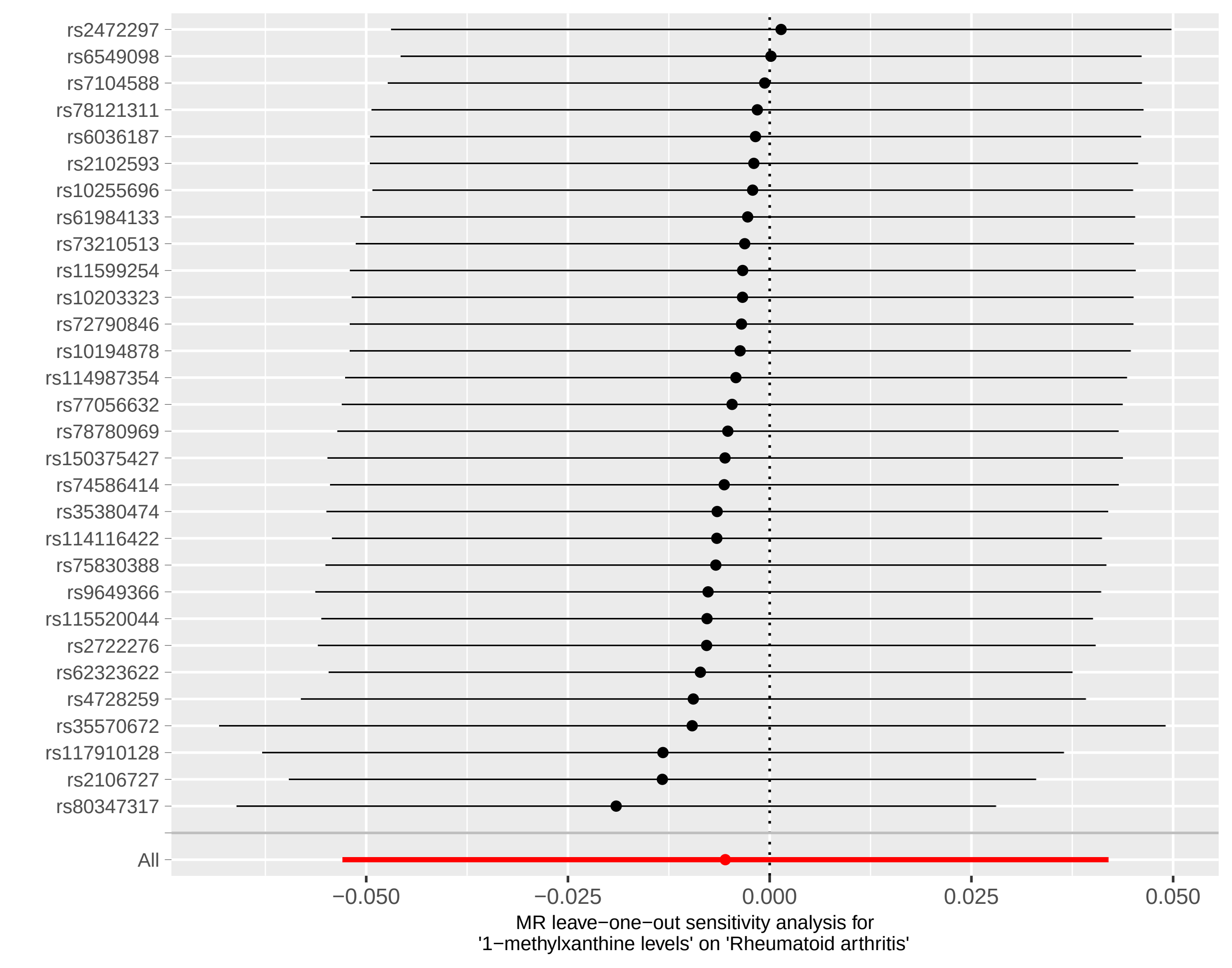
rs2472297
rs6549098
rs7104588
rs78121311
rs6036187
rs2102593
rs10255696
rs61984133
rs73210513
rs11599254
rs10203323
rs72790846
rs10194878
rs114987354
rs77056632
rs78780969
rs150375427
rs74586414
rs35380474
rs114116422
rs75830388
rs9649366
rs115520044
rs2722276
rs62323622
rs4728259
rs35570672
rs117910128
rs2106727
rs80347317
All
−0.050
−0.025
0.000
0.025
0.050
MR leave−one−out sensitivity analysis for
'1−methylxanthine levels' on 'Rheumatoid arthritis'

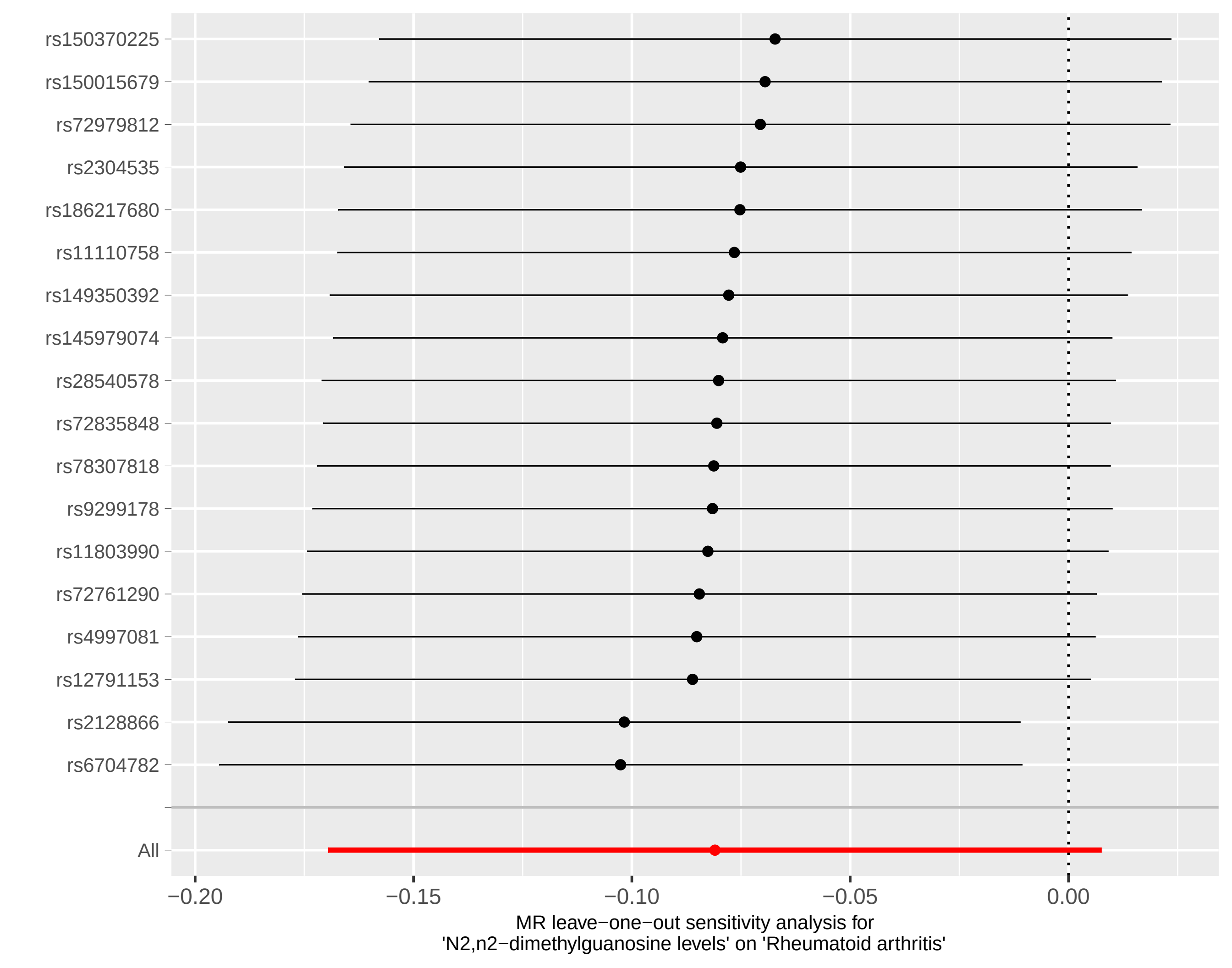
rs150370225
rs150015679
rs72979812
rs2304535
rs186217680
rs11110758
rs149350392
rs145979074
rs28540578
rs72835848
rs78307818
rs9299178
rs11803990
rs72761290
rs4997081
rs12791153
rs2128866
rs6704782
All
−0.20
−0.15
−0.10
−0.05
0.00
MR leave−one−out sensitivity analysis for
'N2,n2−dimethylguanosine levels' on 'Rheumatoid arthritis'

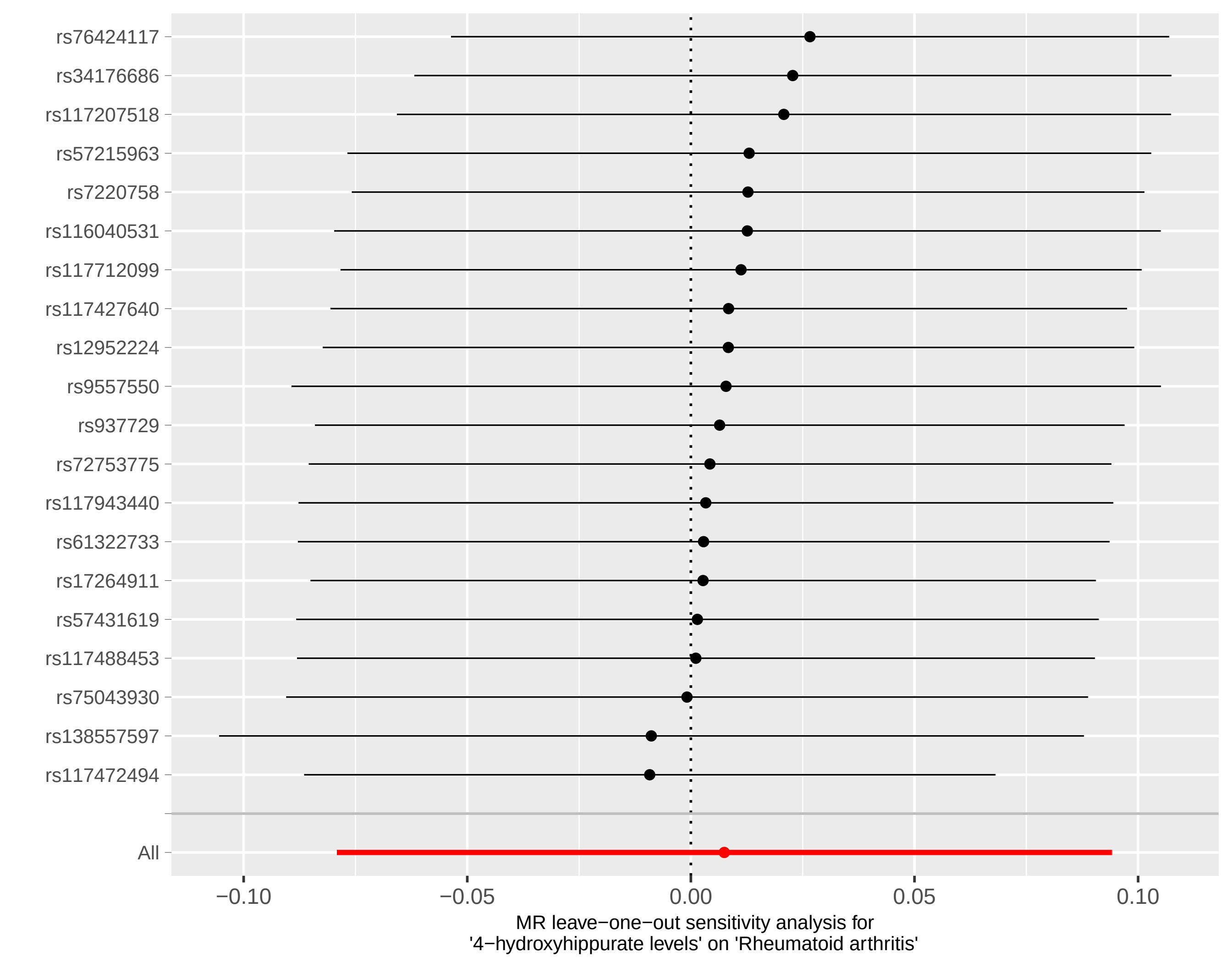

rs76424117
rs34176686
rs117207518
rs57215963
rs7220758
rs116040531
rs117712099
rs117427640
rs12952224
rs9557550
rs937729
rs72753775
rs117943440
rs61322733
rs17264911
rs57431619
rs117488453
rs75043930
rs138557597
rs117472494
All
−0.10
−0.05
0.00
0.05
0.10
MR leave−one−out sensitivity analysis for
'4−hydroxyhippurate levels' on 'Rheumatoid arthritis'

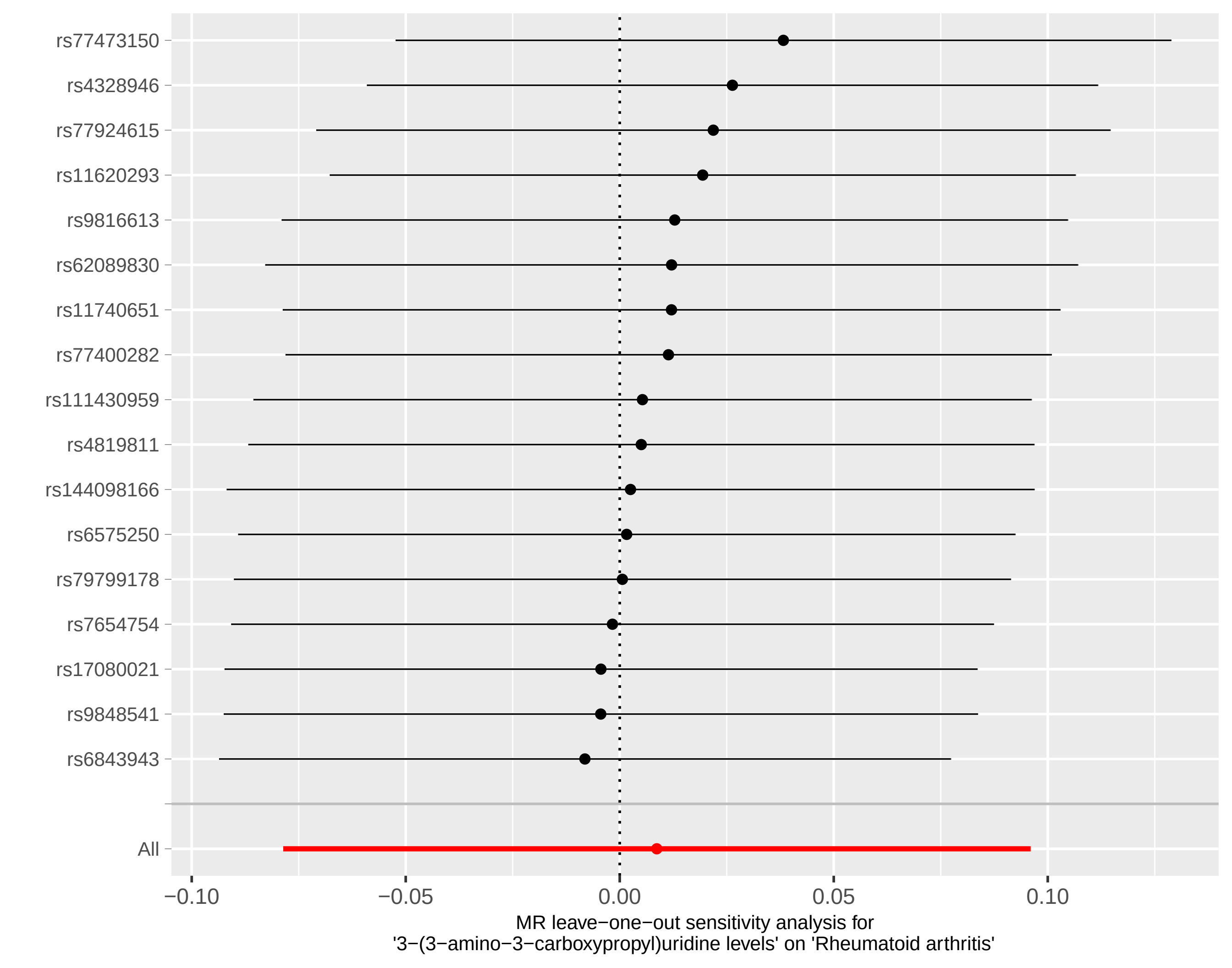
rs77473150
rs4328946
rs77924615
rs11620293
rs9816613
rs62089830
rs11740651
rs77400282
rs111430959
rs4819811
rs144098166
rs6575250
rs79799178
rs7654754
rs17080021
rs9848541
rs6843943
All
−0.10
−0.05
0.00
0.05
0.10
MR leave−one−out sensitivity analysis for
'3−(3−amino−3−carboxypropyl)uridine levels' on 'Rheumatoid arthritis'

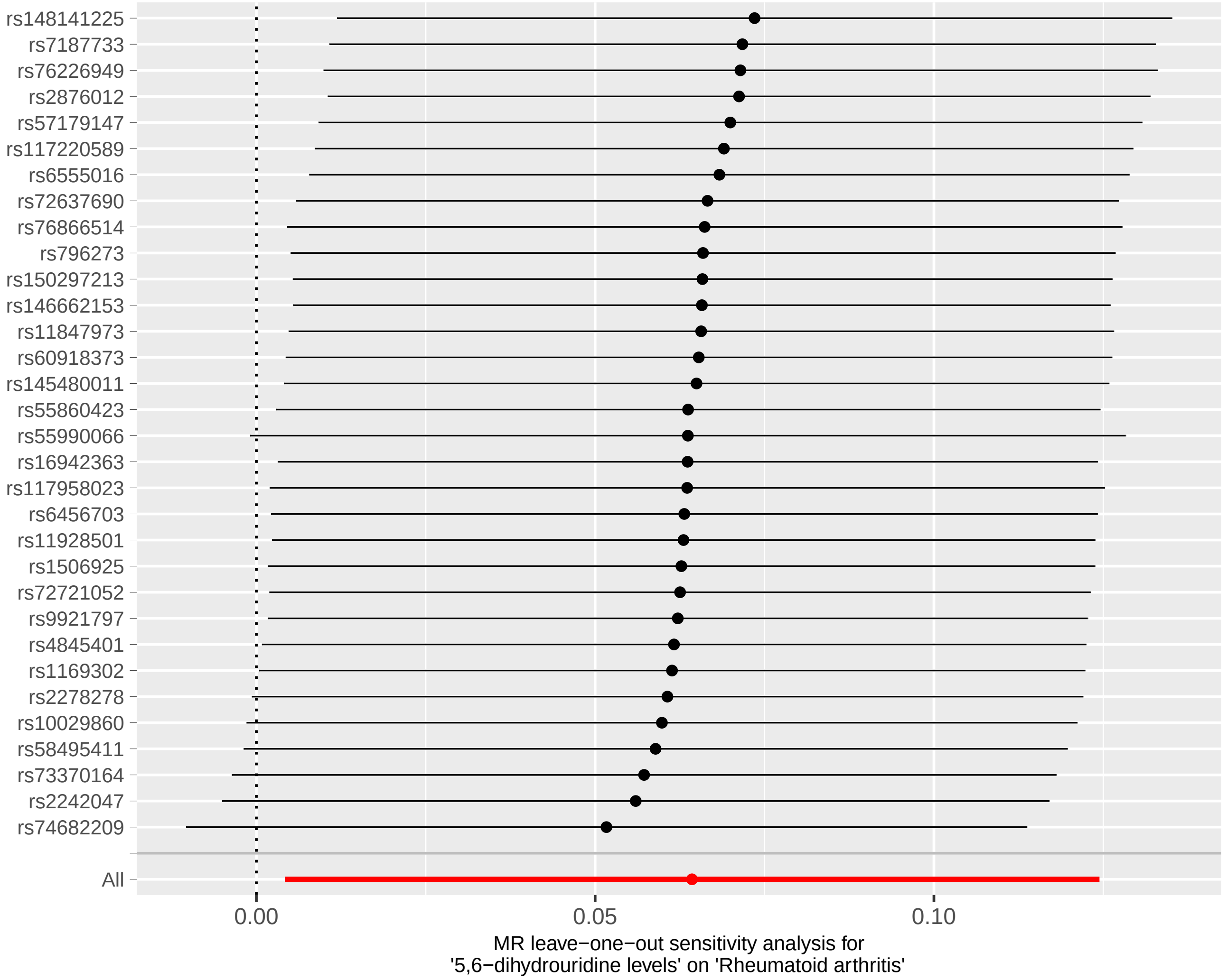
rs148141225
rs7187733
rs76226949
rs2876012
rs57179147
rs117220589
rs6555016
rs72637690
rs76866514
rs796273
rs150297213
rs146662153
rs11847973
rs60918373
rs145480011
rs55860423
rs55990066
rs16942363
rs117958023
rs6456703
rs11928501
rs1506925
rs72721052
rs9921797
rs4845401
rs1169302
rs2278278
rs10029860
rs58495411
rs73370164
rs2242047
rs74682209
All
0.00
0.05
0.10
MR leave−one−out sensitivity analysis for
'5,6−dihydrouridine levels' on 'Rheumatoid arthritis'

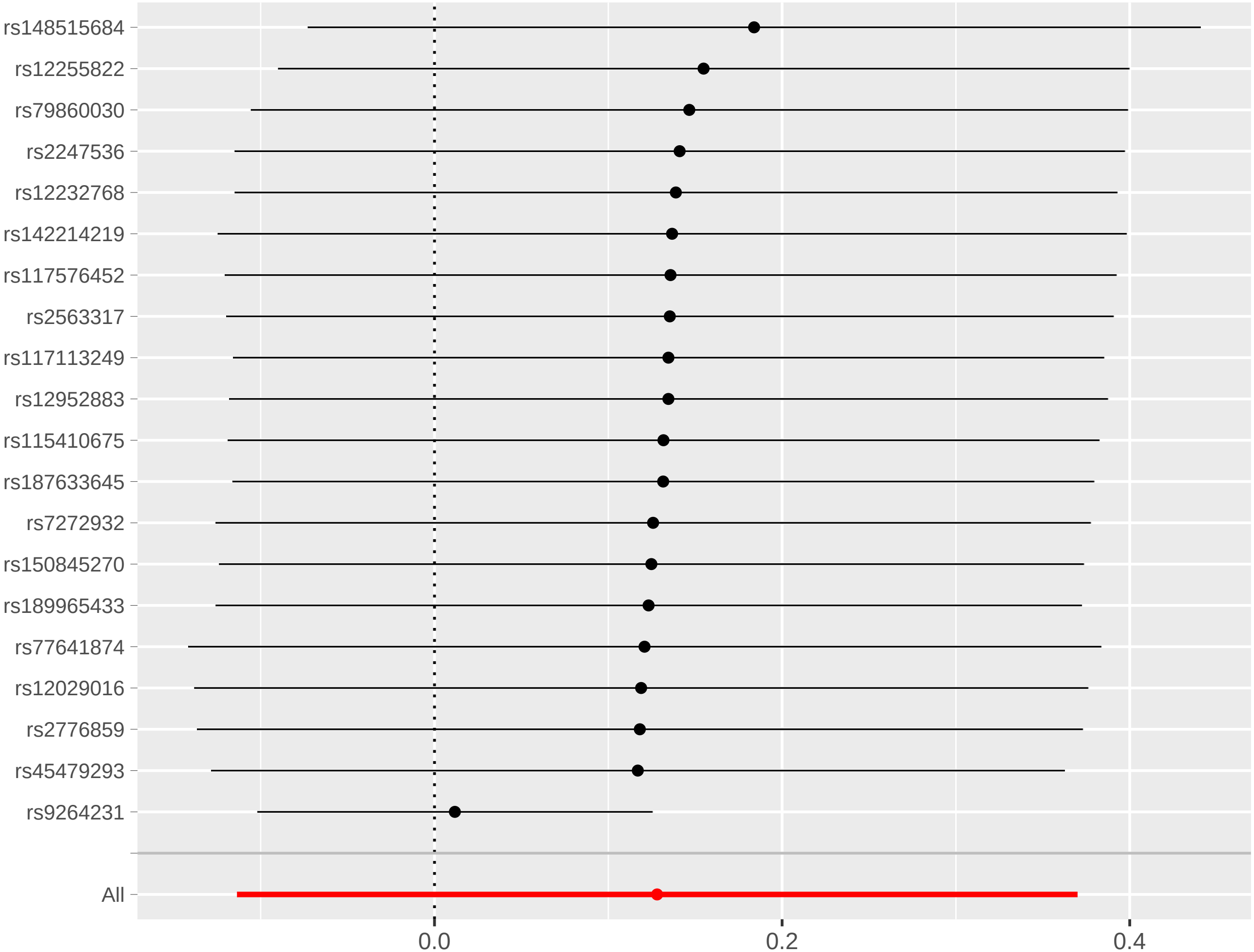

MR leave−one−out sensitivity analysis for
'Phenylacetylglutamine levels' on 'Rheumatoid arthritis'

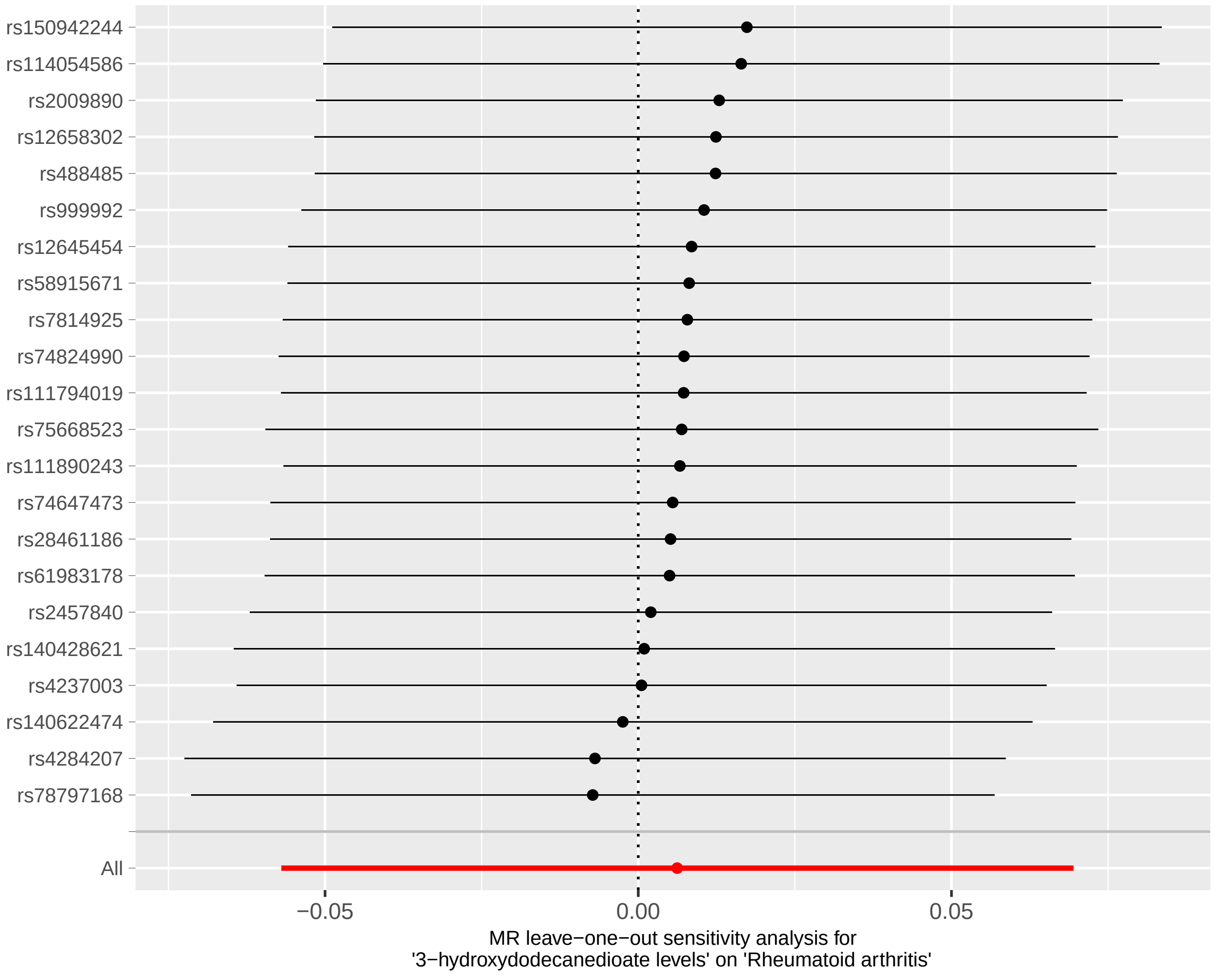

rs150942244
rs114054586
rs2009890
rs12658302
rs488485
rs999992
rs12645454
rs58915671
rs7814925
rs74824990
rs111794019
rs75668523
rs111890243
rs74647473
rs28461186
rs61983178
rs2457840
rs140428621
rs4237003
rs140622474
rs4284207
rs78797168
All
−0.05
0.00
0.05
MR leave−one−out sensitivity analysis for
'3−hydroxydodecanedioate levels' on 'Rheumatoid arthritis'

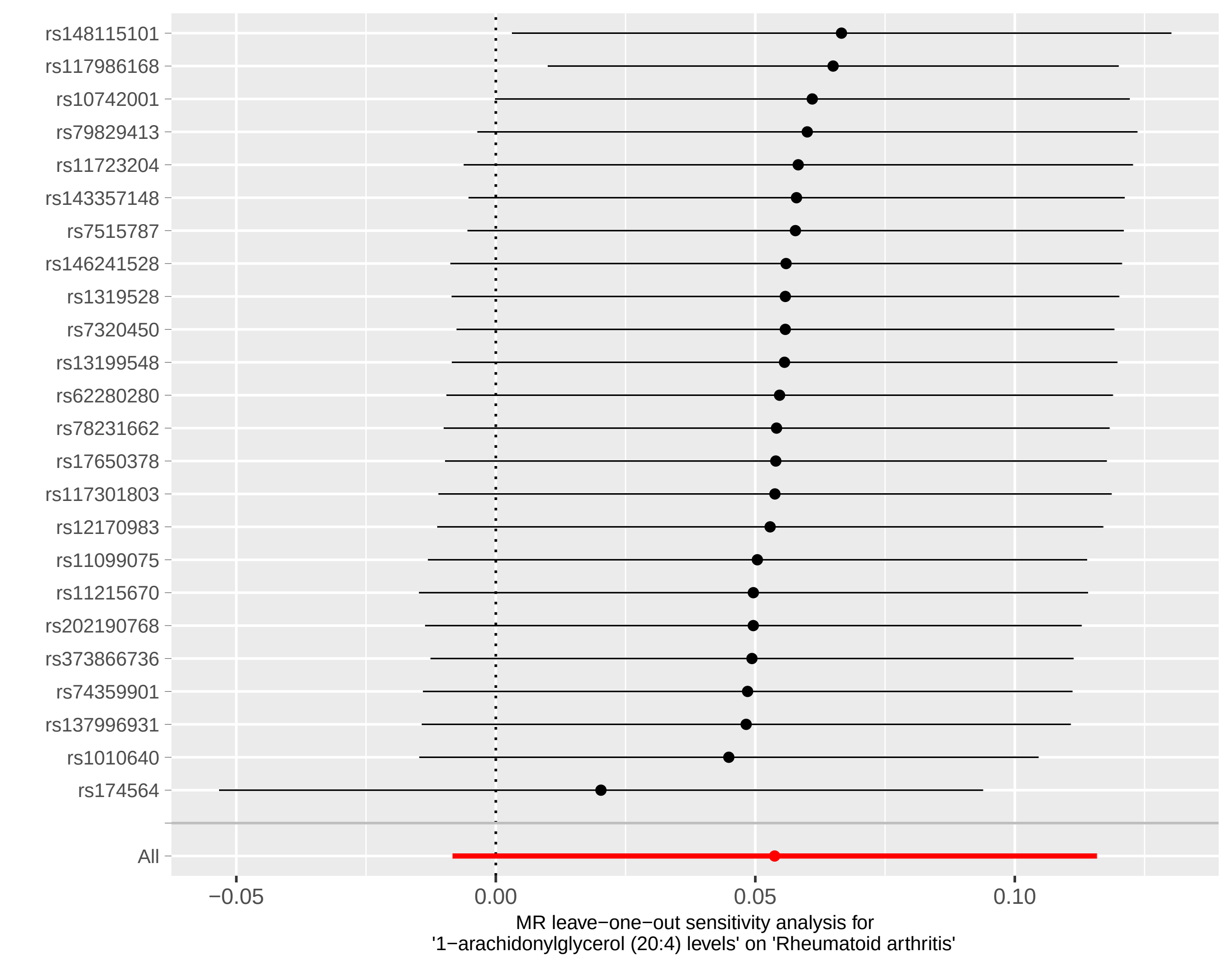

rs148115101
rs117986168
rs10742001
rs79829413
rs11723204
rs143357148
rs7515787
rs146241528
rs1319528
rs7320450
rs13199548
rs62280280
rs78231662
rs17650378
rs117301803
rs12170983
rs11099075
rs11215670
rs202190768
rs373866736
rs74359901
rs137996931
rs1010640
rs174564
All
−0.05
0.00
0.05
0.10
MR leave−one−out sensitivity analysis for
'1−arachidonylglycerol (20:4) levels' on 'Rheumatoid arthritis'

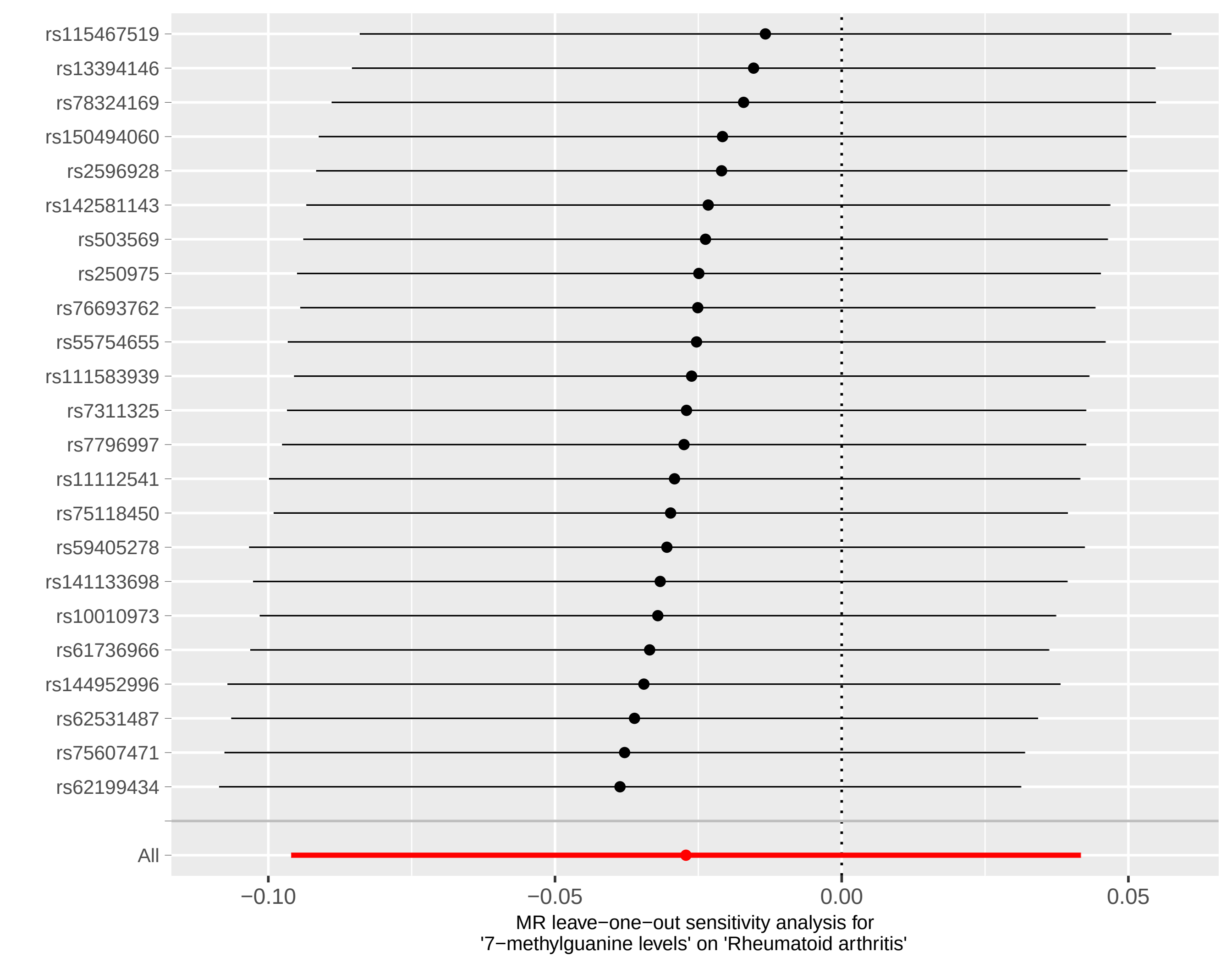
rs115467519
rs13394146
rs78324169
rs150494060
rs2596928
rs142581143
rs503569
rs250975
rs76693762
rs55754655
rs111583939
rs7311325
rs7796997
rs11112541
rs75118450
rs59405278
rs141133698
rs10010973
rs61736966
rs144952996
rs62531487
rs75607471
rs62199434
All
−0.10
−0.05
0.00
0.05
MR leave−one−out sensitivity analysis for
'7−methylguanine levels' on 'Rheumatoid arthritis'

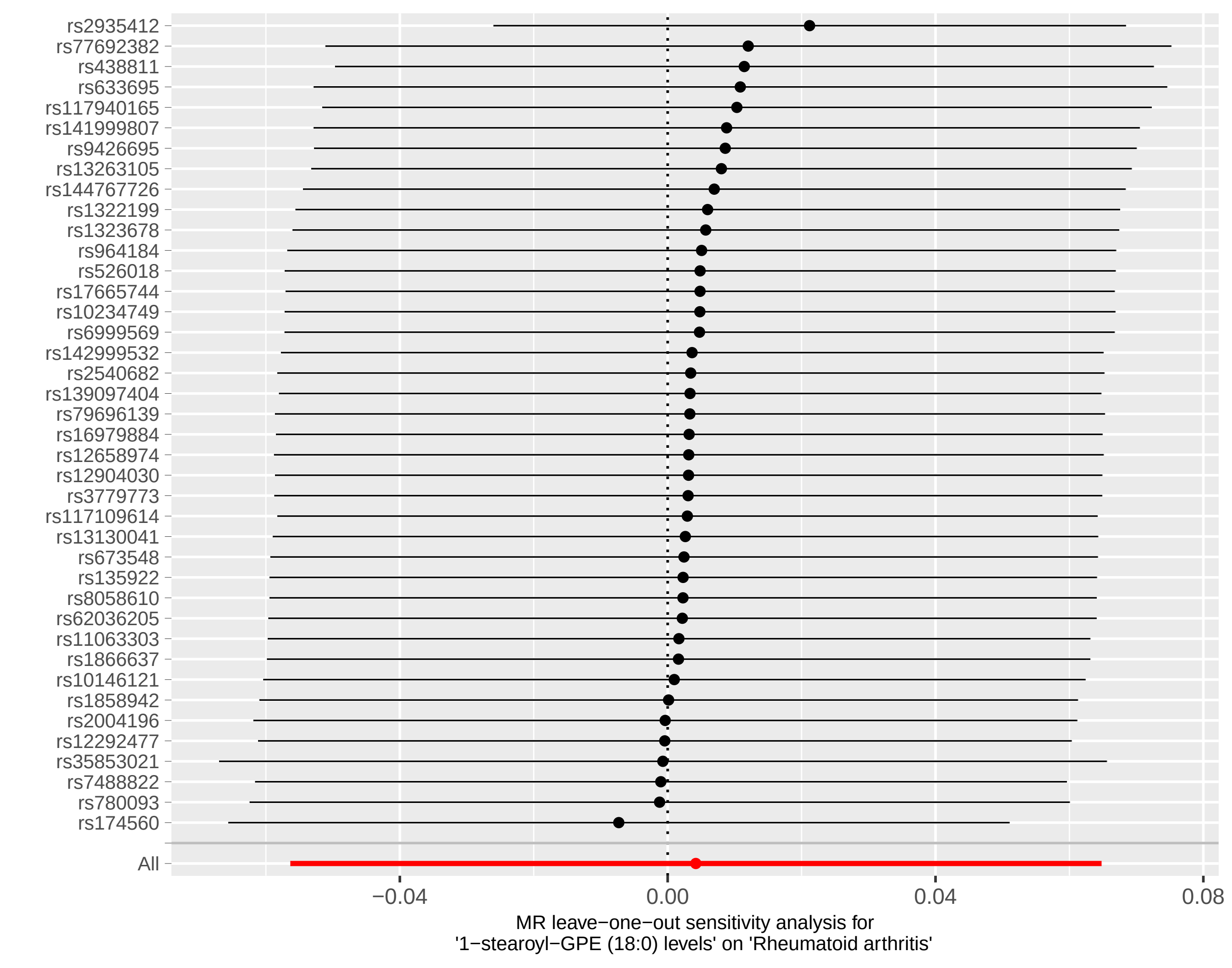
rs2935412
rs77692382
rs438811
rs633695
rs117940165
rs141999807
rs9426695
rs13263105
rs144767726
rs1322199
rs1323678
rs964184
rs526018
rs17665744
rs10234749
rs6999569
rs142999532
rs2540682
rs139097404
rs79696139
rs16979884
rs12658974
rs12904030
rs3779773
rs117109614
rs13130041
rs673548
rs135922
rs8058610
rs62036205
rs11063303
rs1866637
rs10146121
rs1858942
rs2004196
rs12292477
rs35853021
rs7488822
rs780093
rs174560
All
−0.04
0.00
0.04
0.08
MR leave−one−out sensitivity analysis for
'1−stearoyl−GPE (18:0) levels' on 'Rheumatoid arthritis'

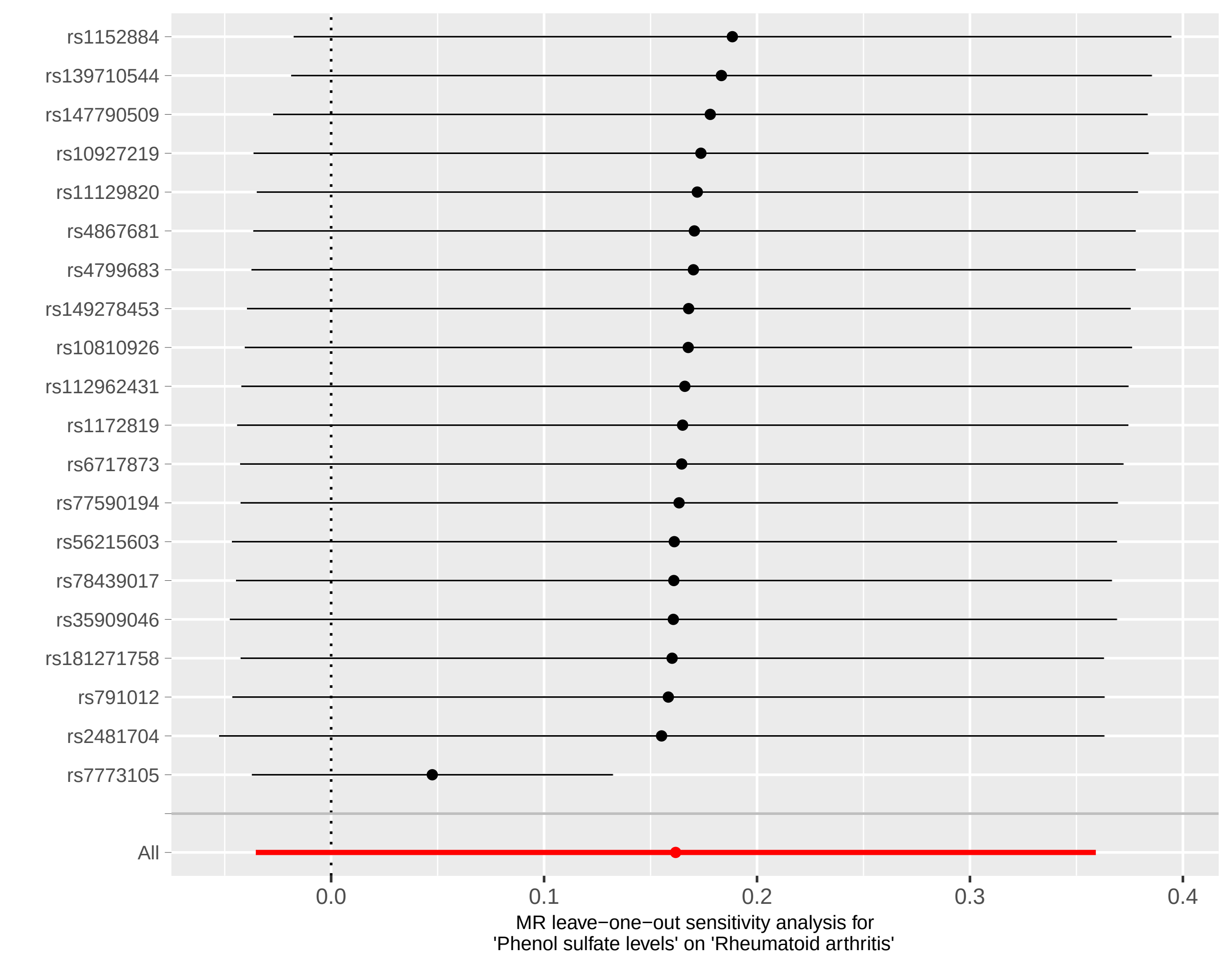
rs1152884
rs139710544
rs147790509
rs10927219
rs11129820
rs4867681
rs4799683
rs149278453
rs10810926
rs112962431
rs1172819
rs6717873
rs77590194
rs56215603
rs78439017
rs35909046
rs181271758
rs791012
rs2481704
rs7773105
All
0.0
0.1
0.2
0.3
0.4
MR leave−one−out sensitivity analysis for
'Phenol sulfate levels' on 'Rheumatoid arthritis'

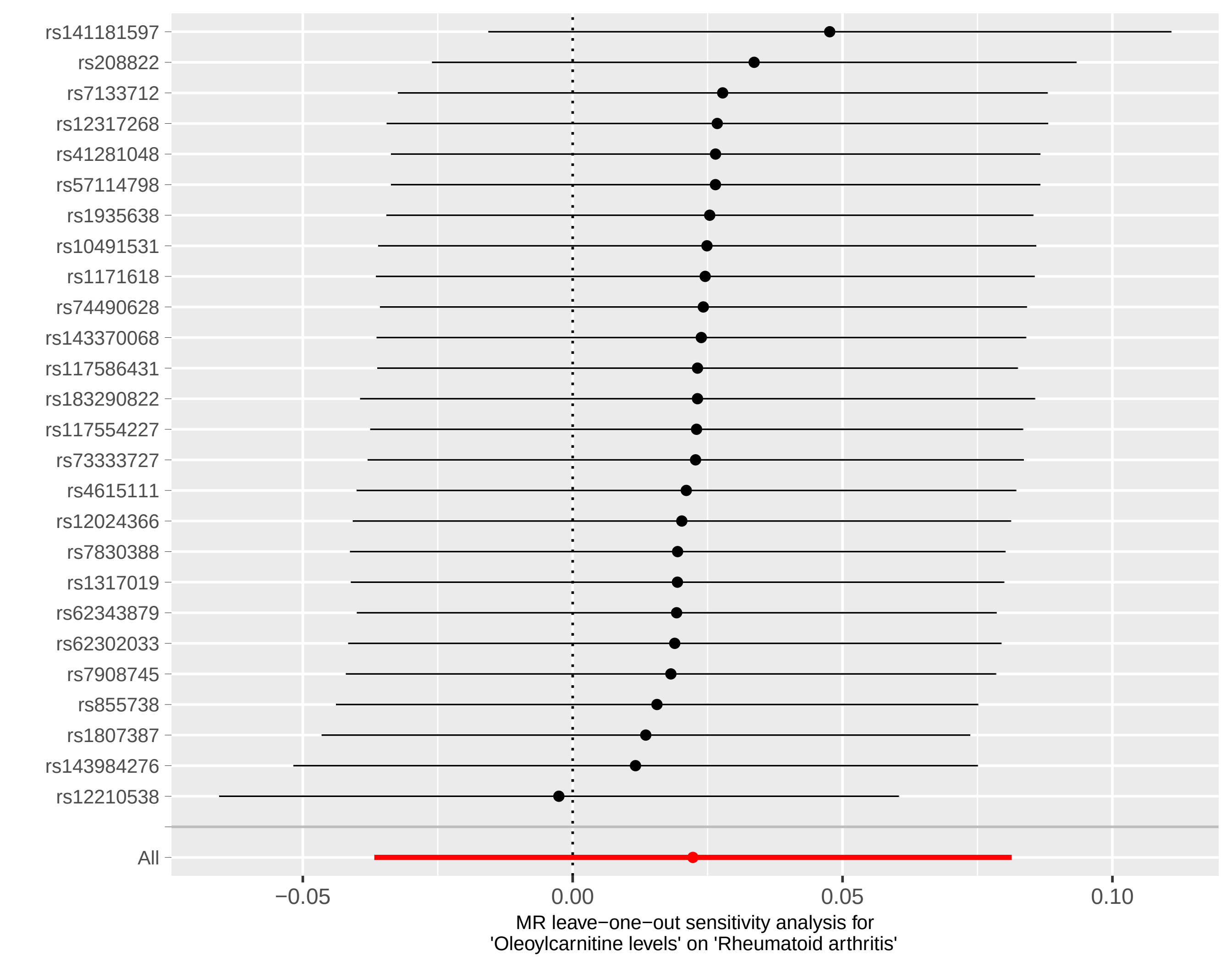
rs141181597
rs208822
rs7133712
rs12317268
rs41281048
rs57114798
rs1935638
rs10491531
rs1171618
rs74490628
rs143370068
rs117586431
rs183290822
rs117554227
rs73333727
rs4615111
rs12024366
rs7830388
rs1317019
rs62343879
rs62302033
rs7908745
rs855738
rs1807387
rs143984276
rs12210538
All
−0.05
0.00
0.05
0.10
MR leave−one−out sensitivity analysis for
'Oleoylcarnitine levels' on 'Rheumatoid arthritis'

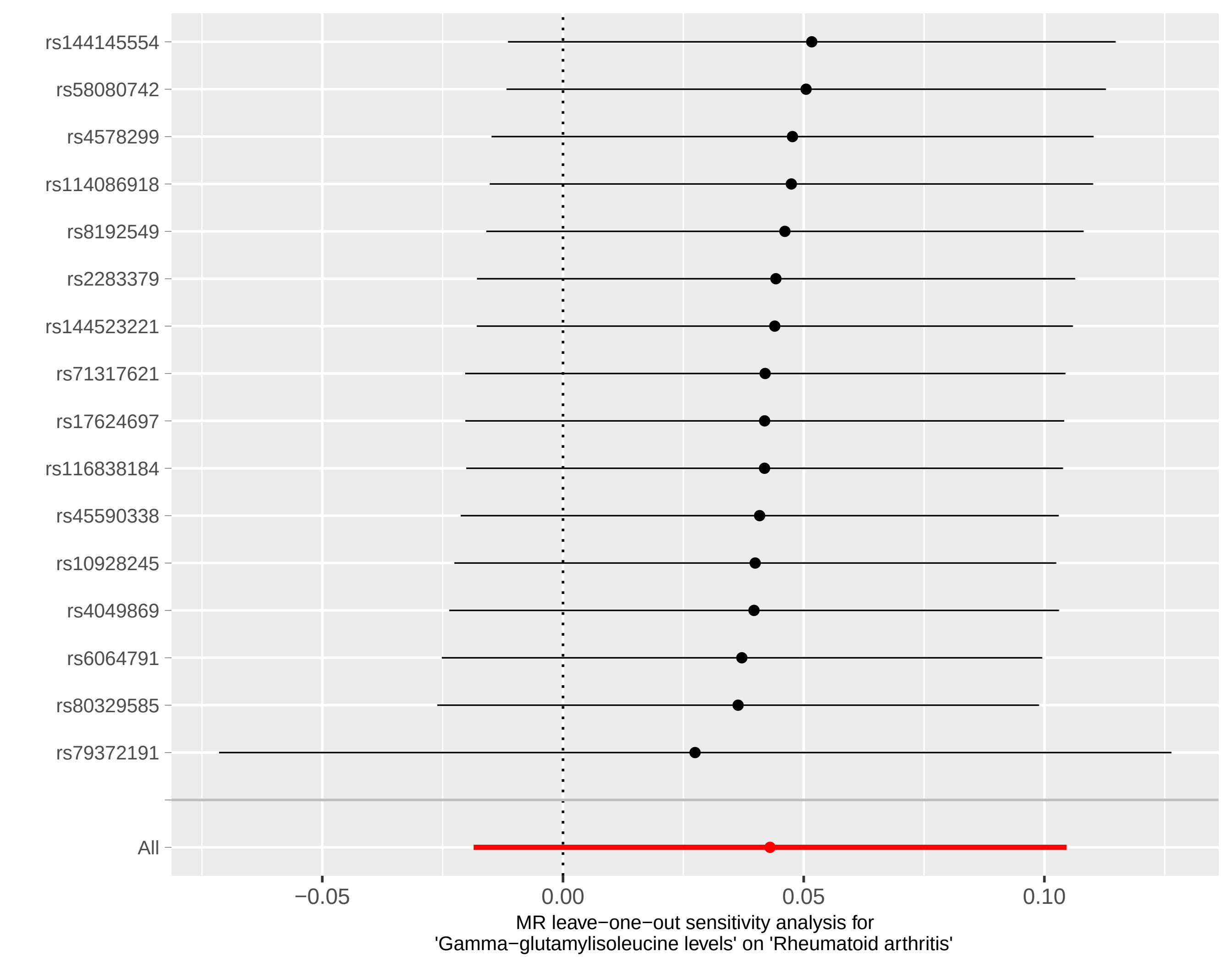
rs144145554
rs58080742
rs4578299
rs114086918
rs8192549
rs2283379
rs144523221
rs71317621
rs17624697
rs116838184
rs45590338
rs10928245
rs4049869
rs6064791
rs80329585
rs79372191
All
−0.05
0.00
0.05
0.10
MR leave−one−out sensitivity analysis for
'Gamma−glutamylisoleucine levels' on 'Rheumatoid arthritis'

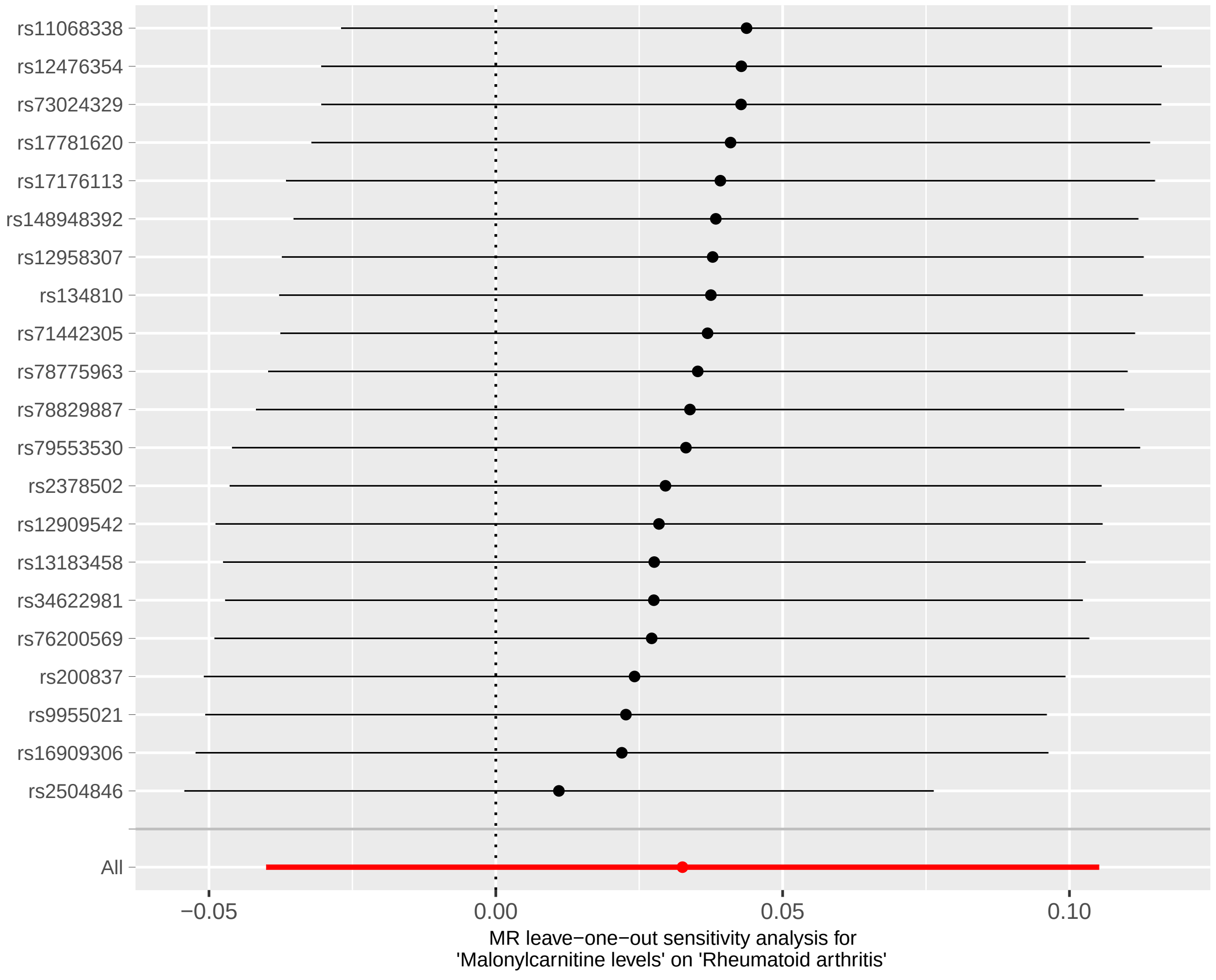
rs11068338
rs12476354
rs73024329
rs17781620
rs17176113
rs148948392
rs12958307
rs134810
rs71442305
rs78775963
rs78829887
rs79553530
rs2378502
rs12909542
rs13183458
rs34622981
rs76200569
rs200837
rs9955021
rs16909306
rs2504846
All
−0.05
0.00
0.05
0.10
MR leave−one−out sensitivity analysis for
'Malonylcarnitine levels' on 'Rheumatoid arthritis'

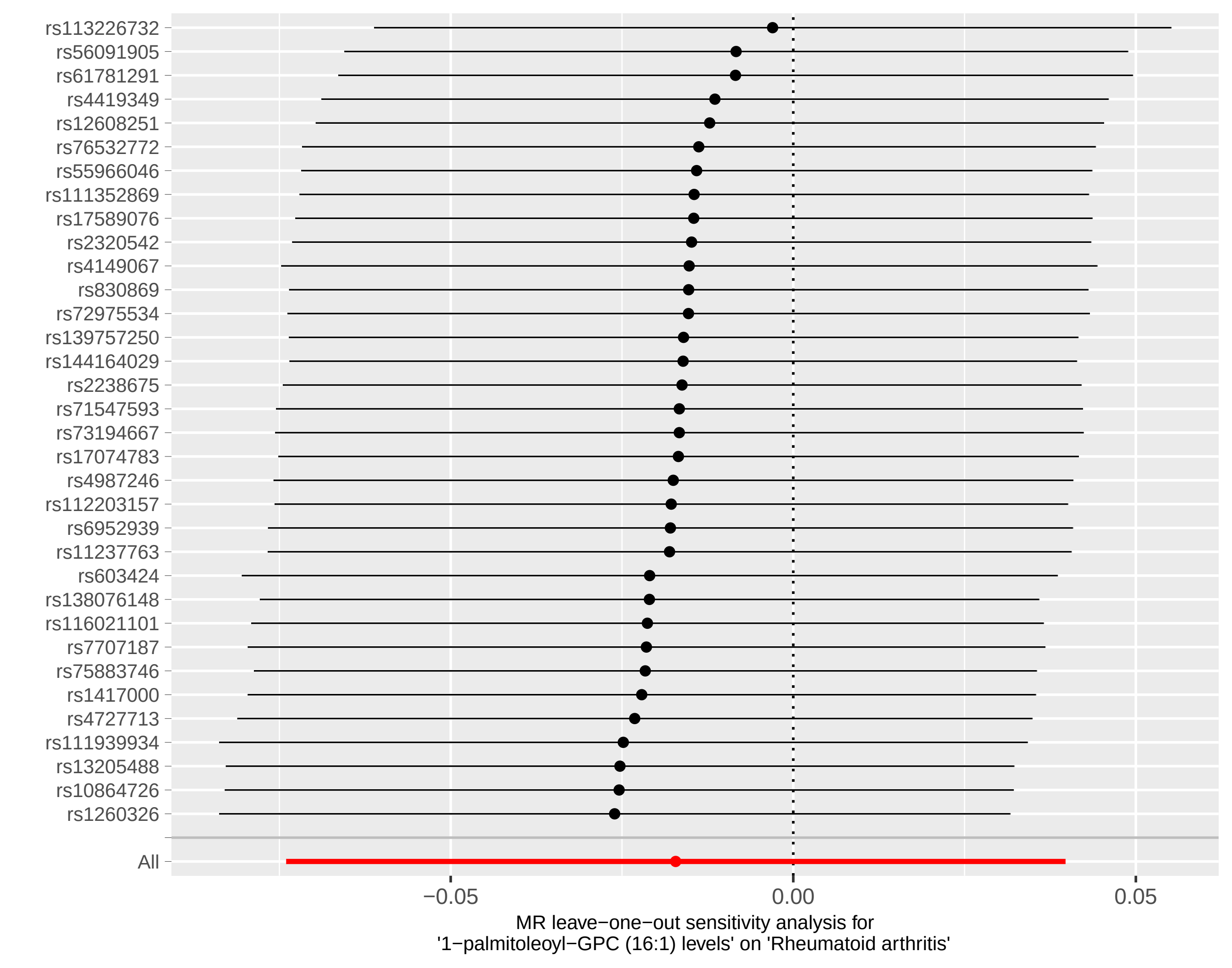

rs113226732
rs56091905
rs61781291
rs4419349
rs12608251
rs76532772
rs55966046
rs111352869
rs17589076
rs2320542
rs4149067
rs830869
rs72975534
rs139757250
rs144164029
rs2238675
rs71547593
rs73194667
rs17074783
rs4987246
rs112203157
rs6952939
rs11237763
rs603424
rs138076148
rs116021101
rs7707187
rs75883746
rs1417000
rs4727713
rs111939934
rs13205488
rs10864726
rs1260326
All
−0.05
0.00
0.05
MR leave−one−out sensitivity analysis for
'1−palmitoleoyl−GPC (16:1) levels' on 'Rheumatoid arthritis'

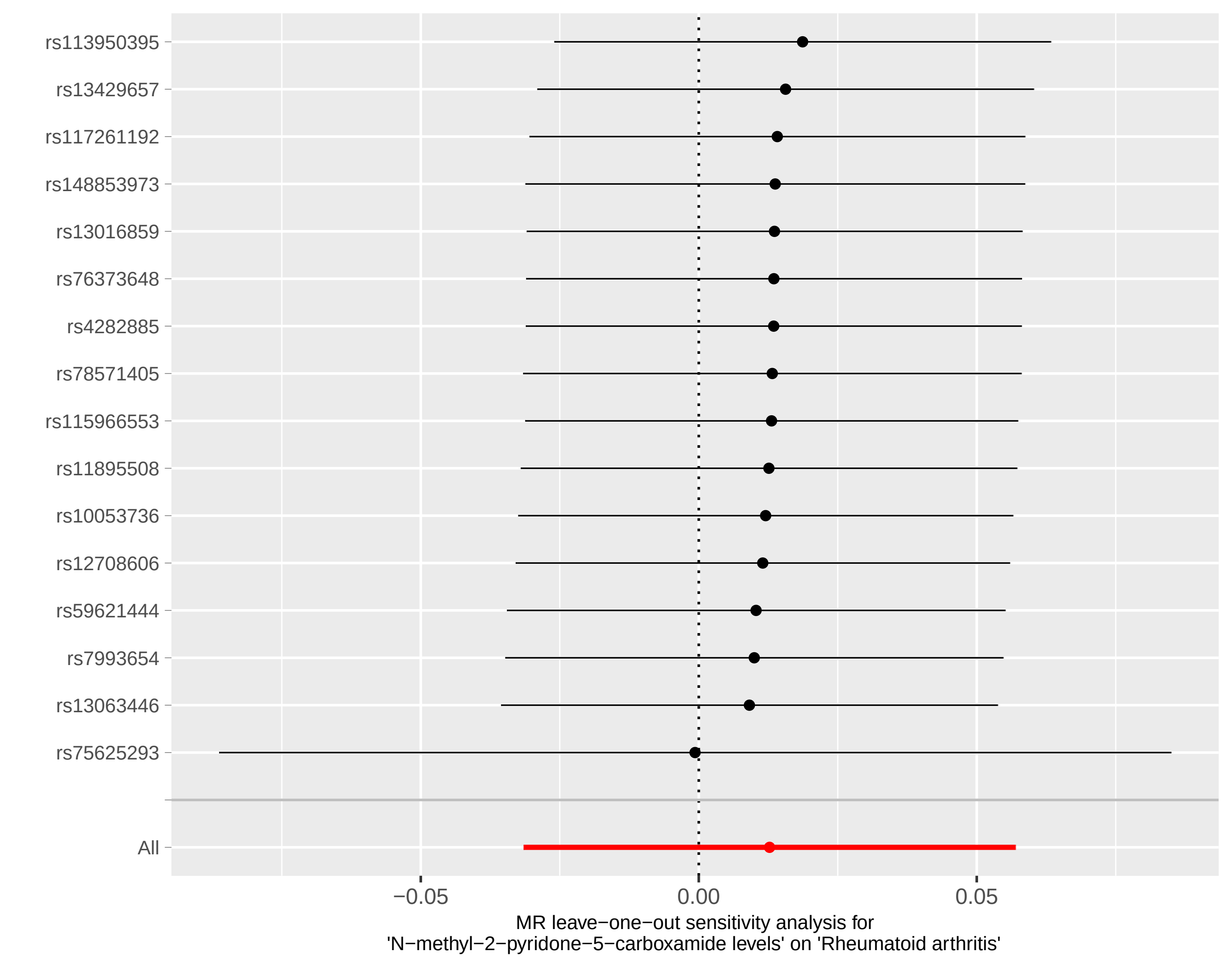
rs113950395
rs13429657
rs117261192
rs148853973
rs13016859
rs76373648
rs4282885
rs78571405
rs115966553
rs11895508
rs10053736
rs12708606
rs59621444
rs7993654
rs13063446
rs75625293
All
−0.05
0.00
0.05
MR leave−one−out sensitivity analysis for
'N−methyl−2−pyridone−5−carboxamide levels' on 'Rheumatoid arthritis'

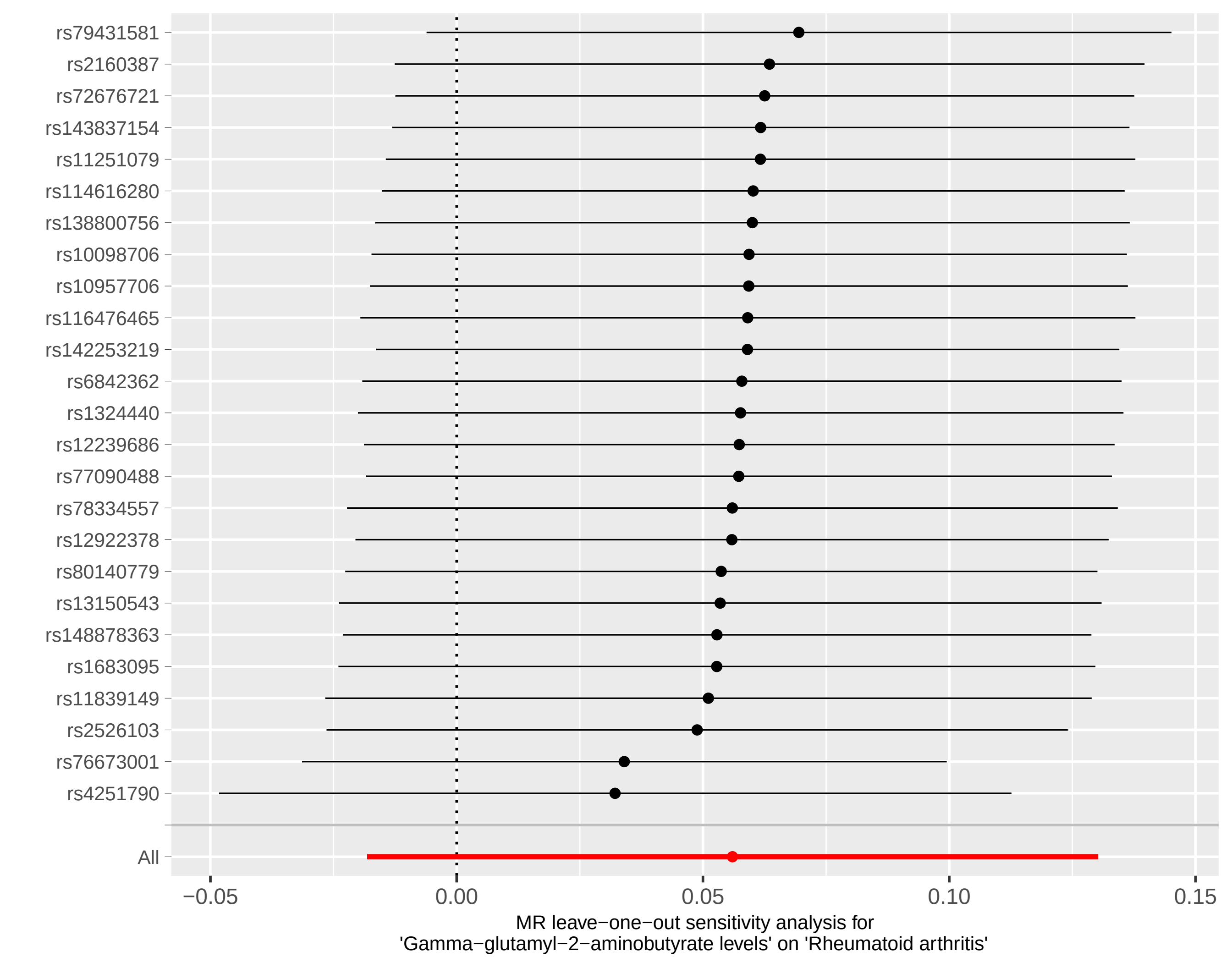
rs79431581
rs2160387
rs72676721
rs143837154
rs11251079
rs114616280
rs138800756
rs10098706
rs10957706
rs116476465
rs142253219
rs6842362
rs1324440
rs12239686
rs77090488
rs78334557
rs12922378
rs80140779
rs13150543
rs148878363
rs1683095
rs11839149
rs2526103
rs76673001
rs4251790
All
−0.05
0.00
0.05
0.10
0.15
MR leave−one−out sensitivity analysis for
'Gamma−glutamyl−2−aminobutyrate levels' on 'Rheumatoid arthritis'

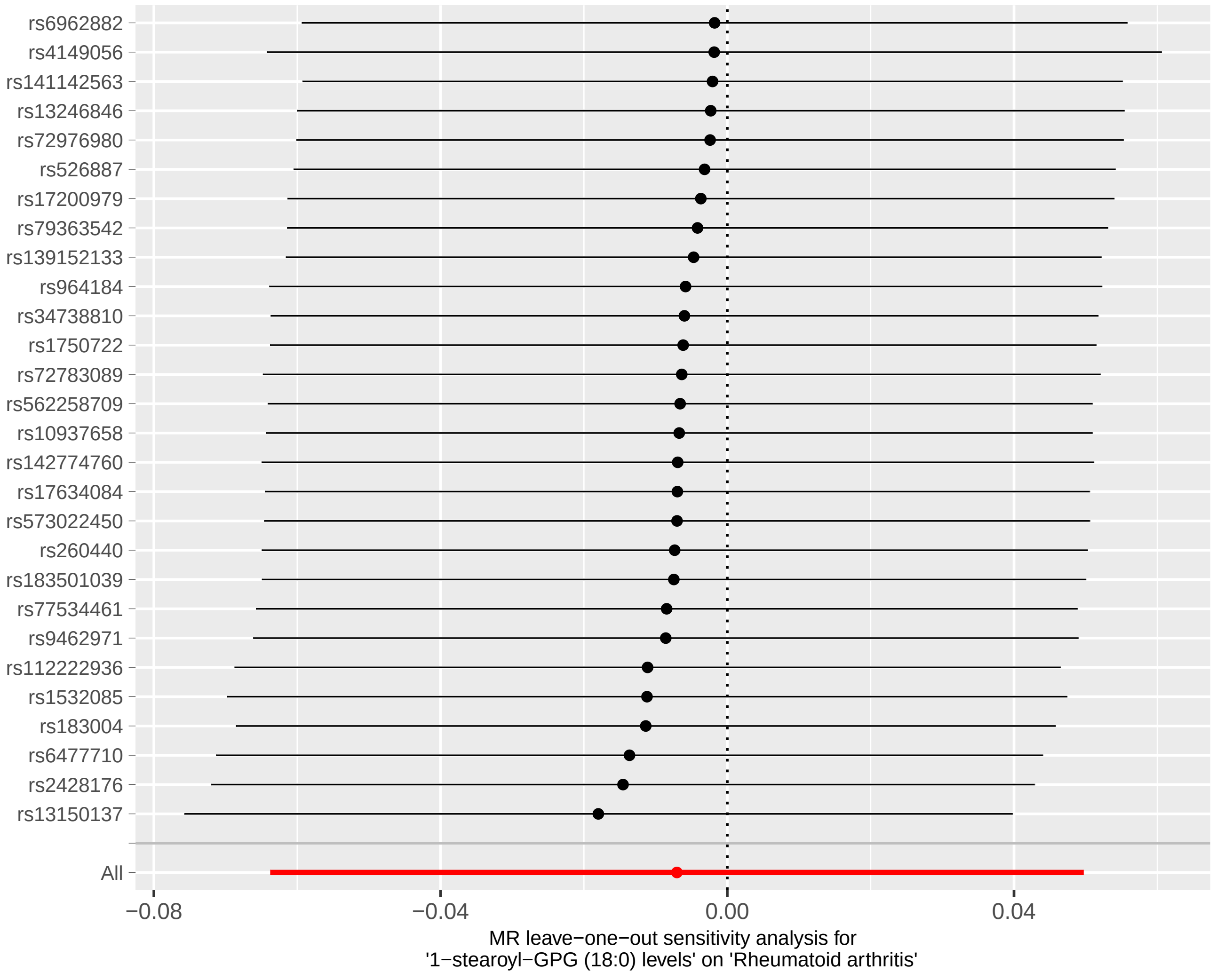
rs6962882
rs4149056
rs141142563
rs13246846
rs72976980
rs526887
rs17200979
rs79363542
rs139152133
rs964184
rs34738810
rs1750722
rs72783089
rs562258709
rs10937658
rs142774760
rs17634084
rs573022450
rs260440
rs183501039
rs77534461
rs9462971
rs112222936
rs1532085
rs183004
rs6477710
rs2428176
rs13150137
All
−0.08
−0.04
0.00
0.04
MR leave−one−out sensitivity analysis for
'1−stearoyl−GPG (18:0) levels' on 'Rheumatoid arthritis'

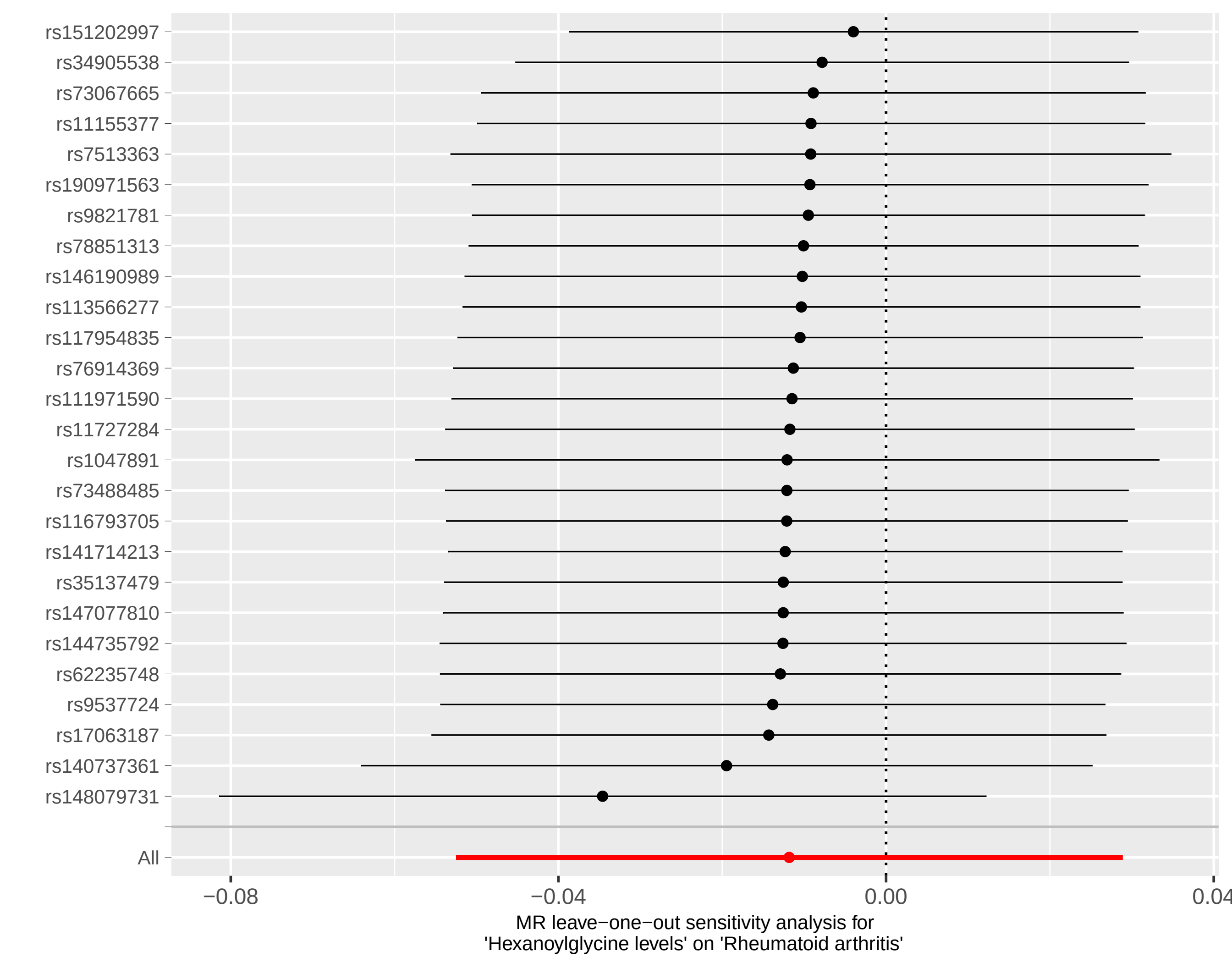
rs151202997
rs34905538
rs73067665
rs11155377
rs7513363
rs190971563
rs9821781
rs78851313
rs146190989
rs113566277
rs117954835
rs76914369
rs111971590
rs11727284
rs1047891
rs73488485
rs116793705
rs141714213
rs35137479
rs147077810
rs144735792
rs62235748
rs9537724
rs17063187
rs140737361
rs148079731
All
−0.08
−0.04
0.00
0.04
MR leave−one−out sensitivity analysis for
'Hexanoylglycine levels' on 'Rheumatoid arthritis'

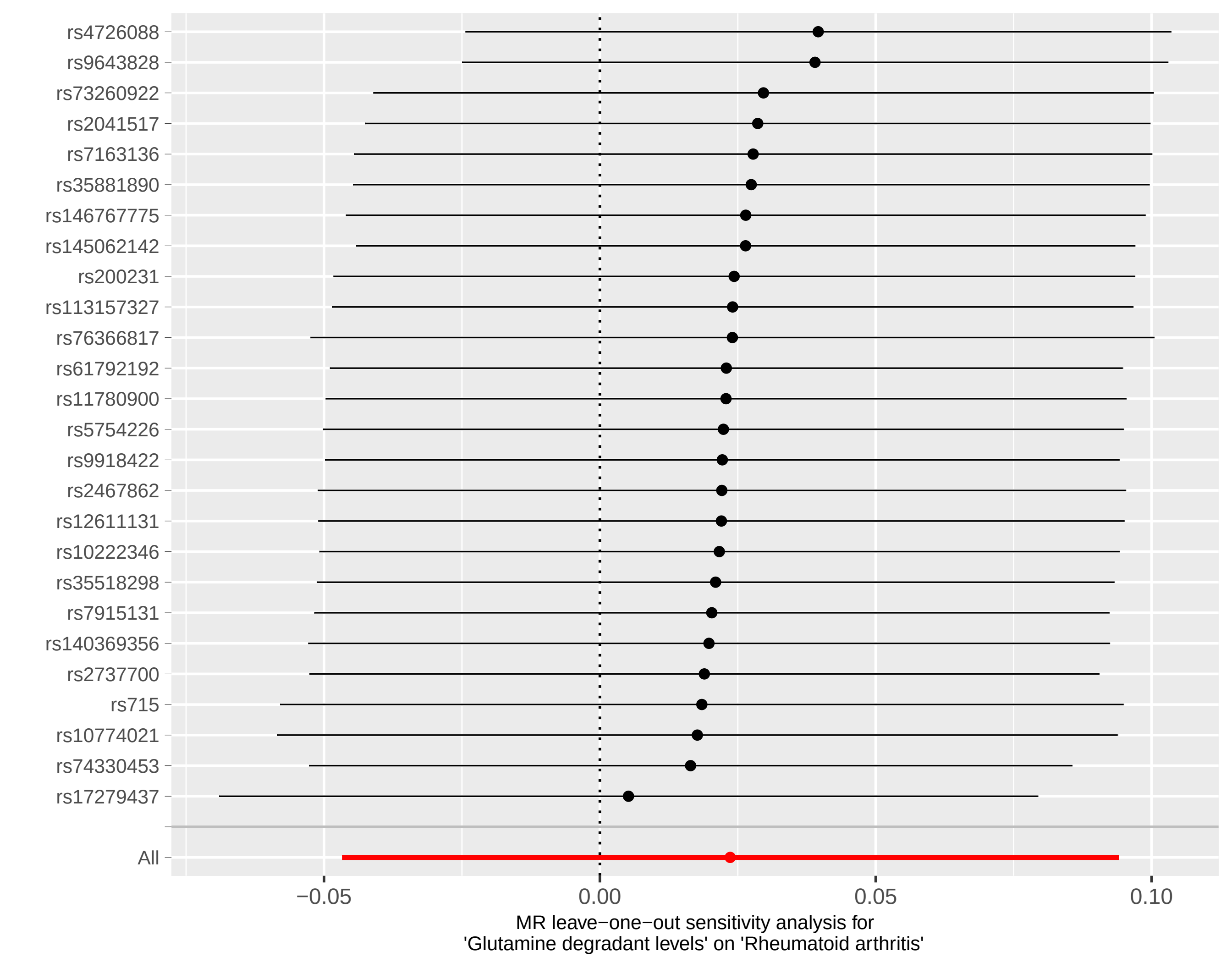
rs4726088
rs9643828
rs73260922
rs2041517
rs7163136
rs35881890
rs146767775
rs145062142
rs200231
rs113157327
rs76366817
rs61792192
rs11780900
rs5754226
rs9918422
rs2467862
rs12611131
rs10222346
rs35518298
rs7915131
rs140369356
rs2737700
rs715
rs10774021
rs74330453
rs17279437
All
−0.05
0.00
0.05
0.10
MR leave−one−out sensitivity analysis for
'Glutamine degradant levels' on 'Rheumatoid arthritis'

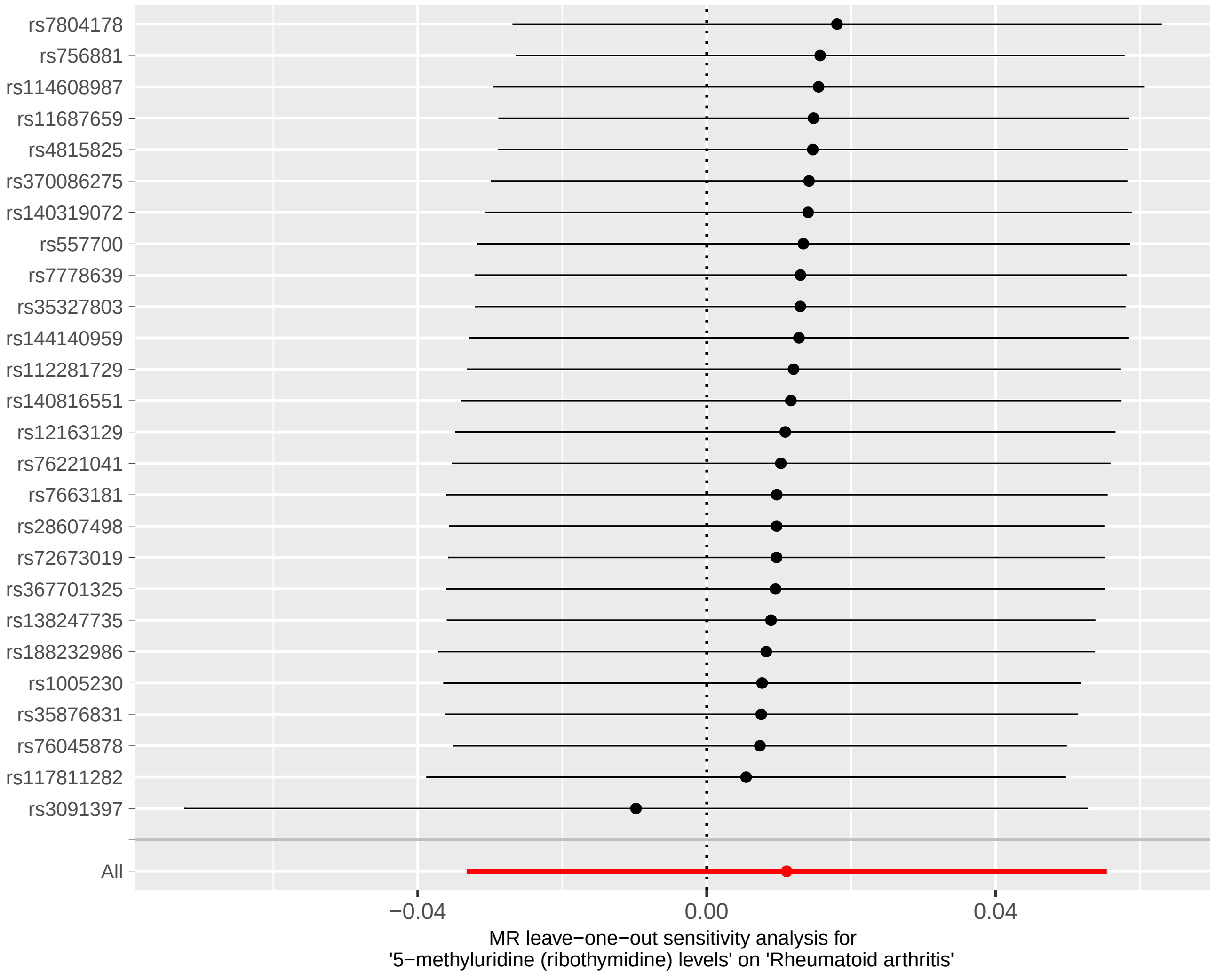

rs7804178
rs756881
rs114608987
rs11687659
rs4815825
rs370086275
rs140319072
rs557700
rs7778639
rs35327803
rs144140959
rs112281729
rs140816551
rs12163129
rs76221041
rs7663181
rs28607498
rs72673019
rs367701325
rs138247735
rs188232986
rs1005230
rs35876831
rs76045878
rs117811282
rs3091397
All
−0.04
0.00
0.04
MR leave−one−out sensitivity analysis for
'5−methyluridine (ribothymidine) levels' on 'Rheumatoid arthritis'

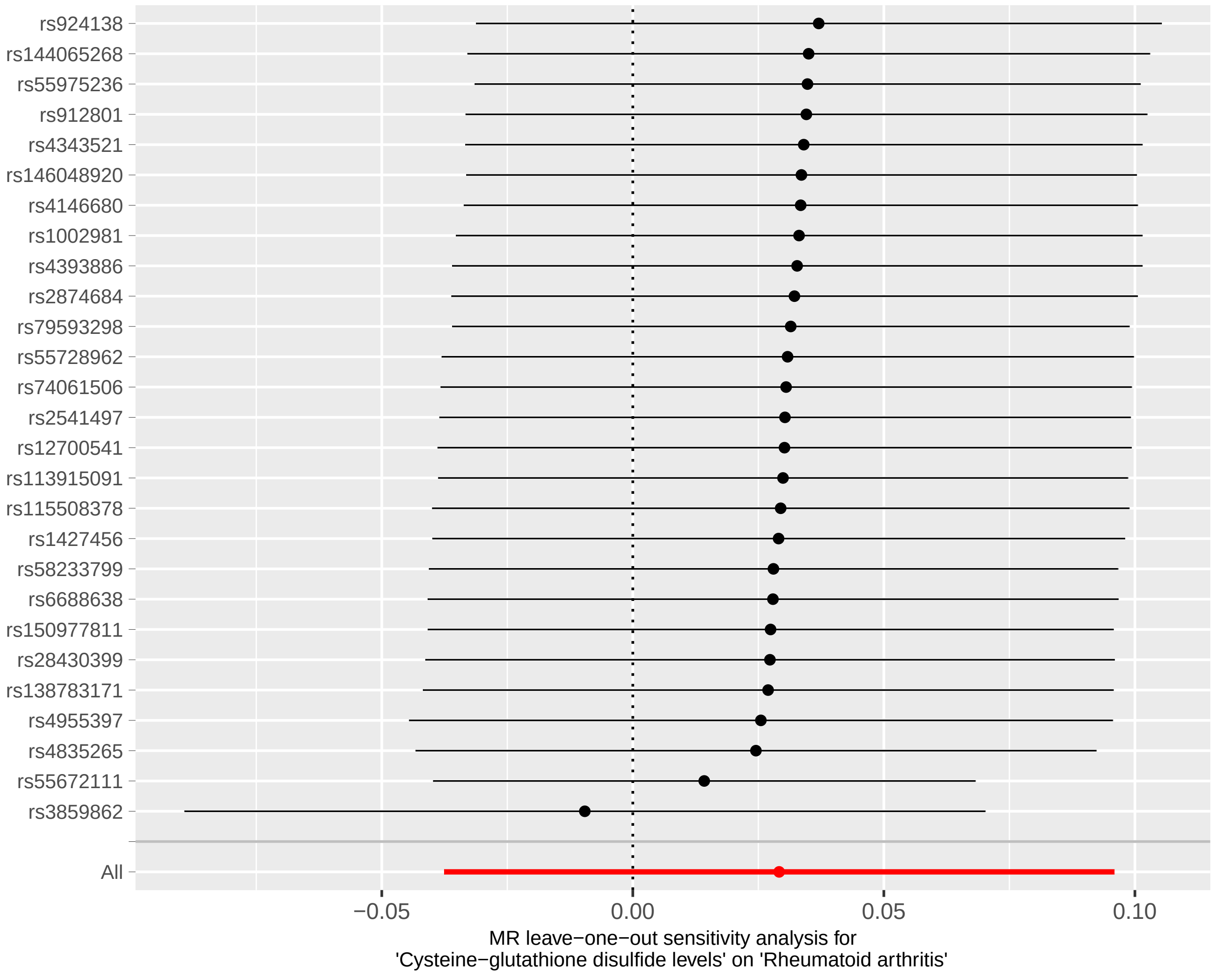
rs924138
rs144065268
rs55975236
rs912801
rs4343521
rs146048920
rs4146680
rs1002981
rs4393886
rs2874684
rs79593298
rs55728962
rs74061506
rs2541497
rs12700541
rs113915091
rs115508378
rs1427456
rs58233799
rs6688638
rs150977811
rs28430399
rs138783171
rs4955397
rs4835265
rs55672111
rs3859862
All
−0.05
0.00
0.05
0.10
MR leave−one−out sensitivity analysis for
'Cysteine−glutathione disulfide levels' on 'Rheumatoid arthritis'

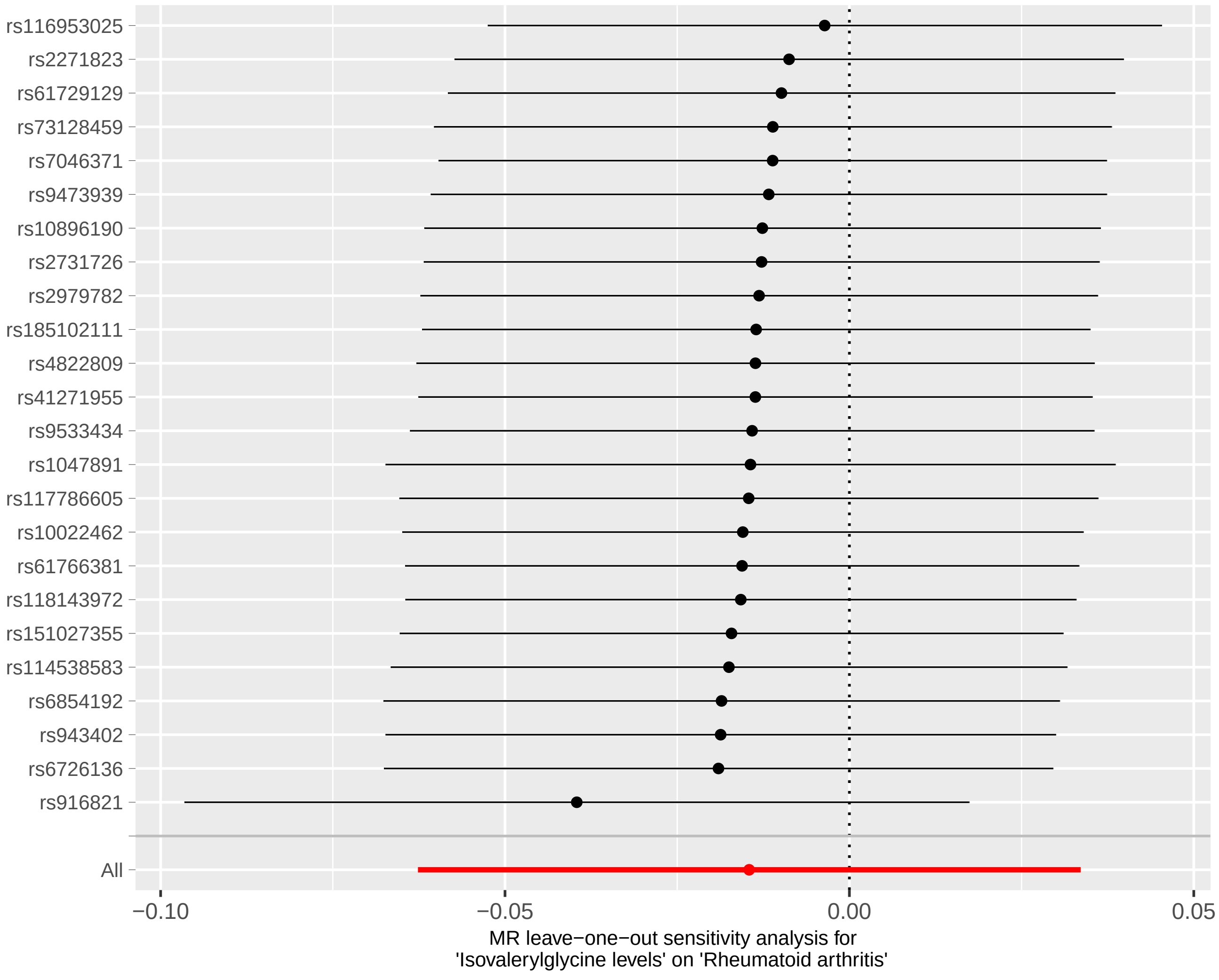
rs116953025
rs2271823
rs61729129
rs73128459
rs7046371
rs9473939
rs10896190
rs2731726
rs2979782
rs185102111
rs4822809
rs41271955
rs9533434
rs1047891
rs117786605
rs10022462
rs61766381
rs118143972
rs151027355
rs114538583
rs6854192
rs943402
rs6726136
rs916821
All
−0.10
−0.05
0.00
0.05
MR leave−one−out sensitivity analysis for
'Isovalerylglycine levels' on 'Rheumatoid arthritis'

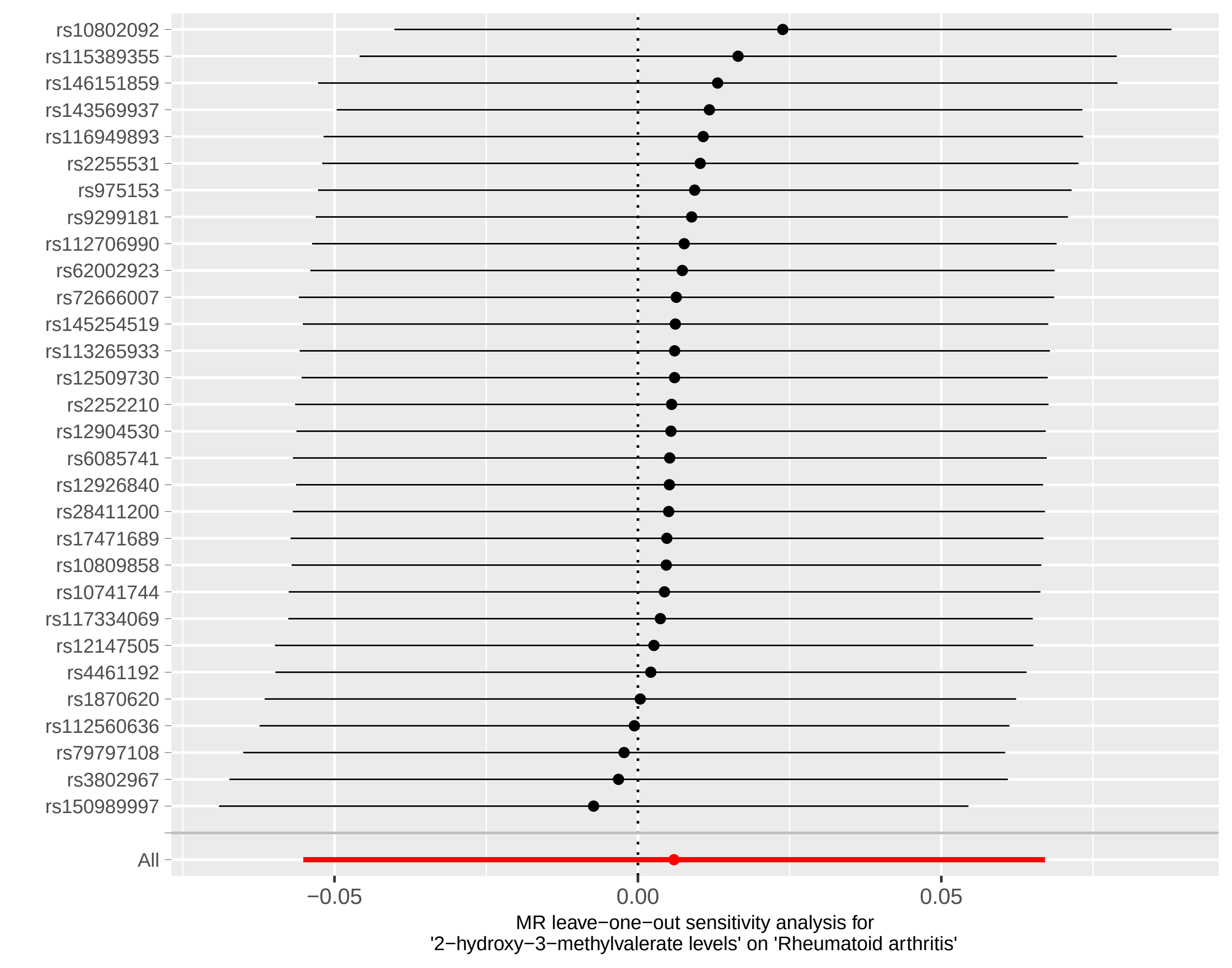
rs10802092
rs115389355
rs146151859
rs143569937
rs116949893
rs2255531
rs975153
rs9299181
rs112706990
rs62002923
rs72666007
rs145254519
rs113265933
rs12509730
rs2252210
rs12904530
rs6085741
rs12926840
rs28411200
rs17471689
rs10809858
rs10741744
rs117334069
rs12147505
rs4461192
rs1870620
rs112560636
rs79797108
rs3802967
rs150989997
All
−0.05
0.00
0.05
MR leave−one−out sensitivity analysis for
'2−hydroxy−3−methylvalerate levels' on 'Rheumatoid arthritis'

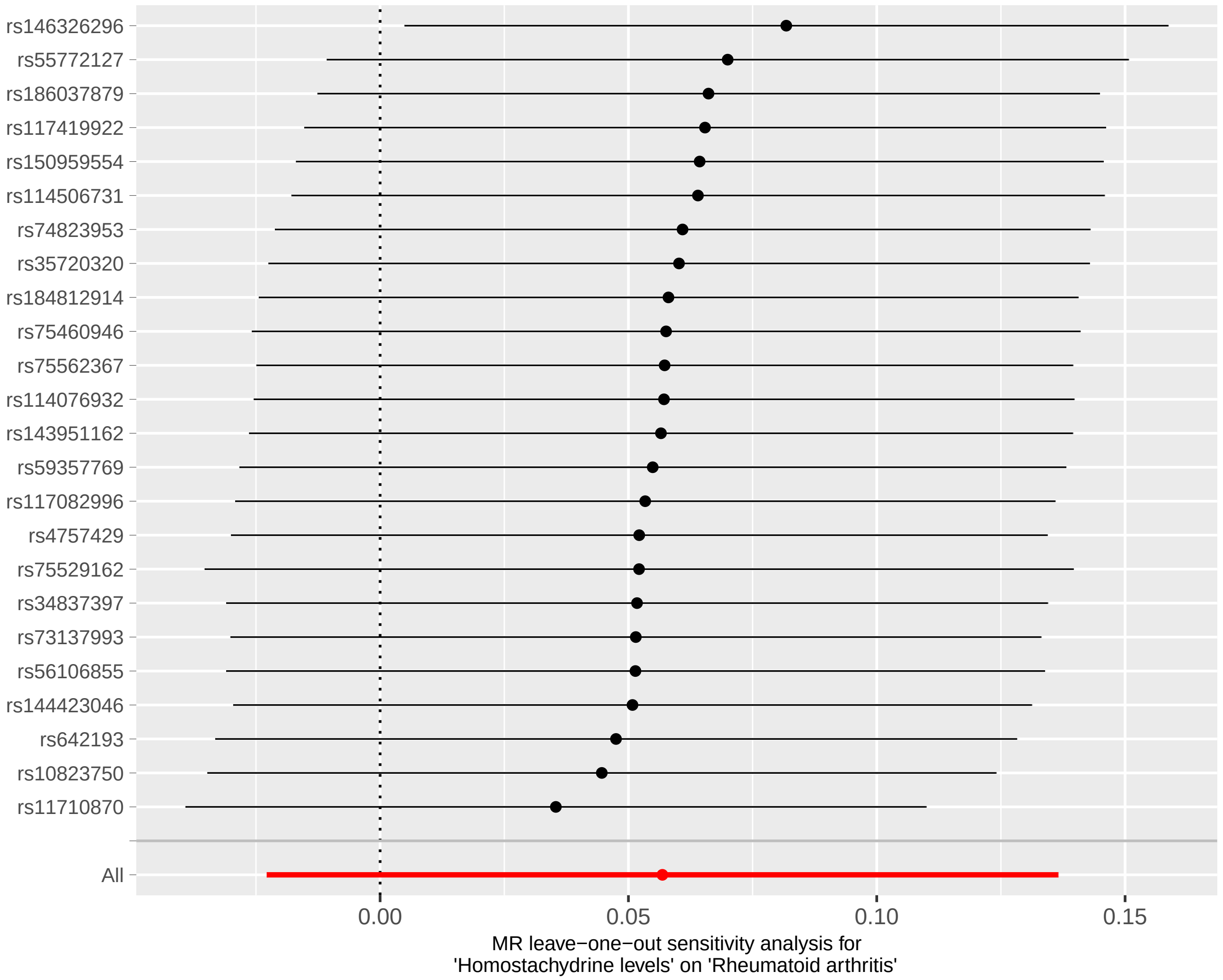
rs146326296
rs55772127
rs186037879
rs117419922
rs150959554
rs114506731
rs74823953
rs35720320
rs184812914
rs75460946
rs75562367
rs114076932
rs143951162
rs59357769
rs117082996
rs4757429
rs75529162
rs34837397
rs73137993
rs56106855
rs144423046
rs642193
rs10823750
rs11710870
All
0.00
0.05
0.10
0.15
MR leave−one−out sensitivity analysis for
'Homostachydrine levels' on 'Rheumatoid arthritis'

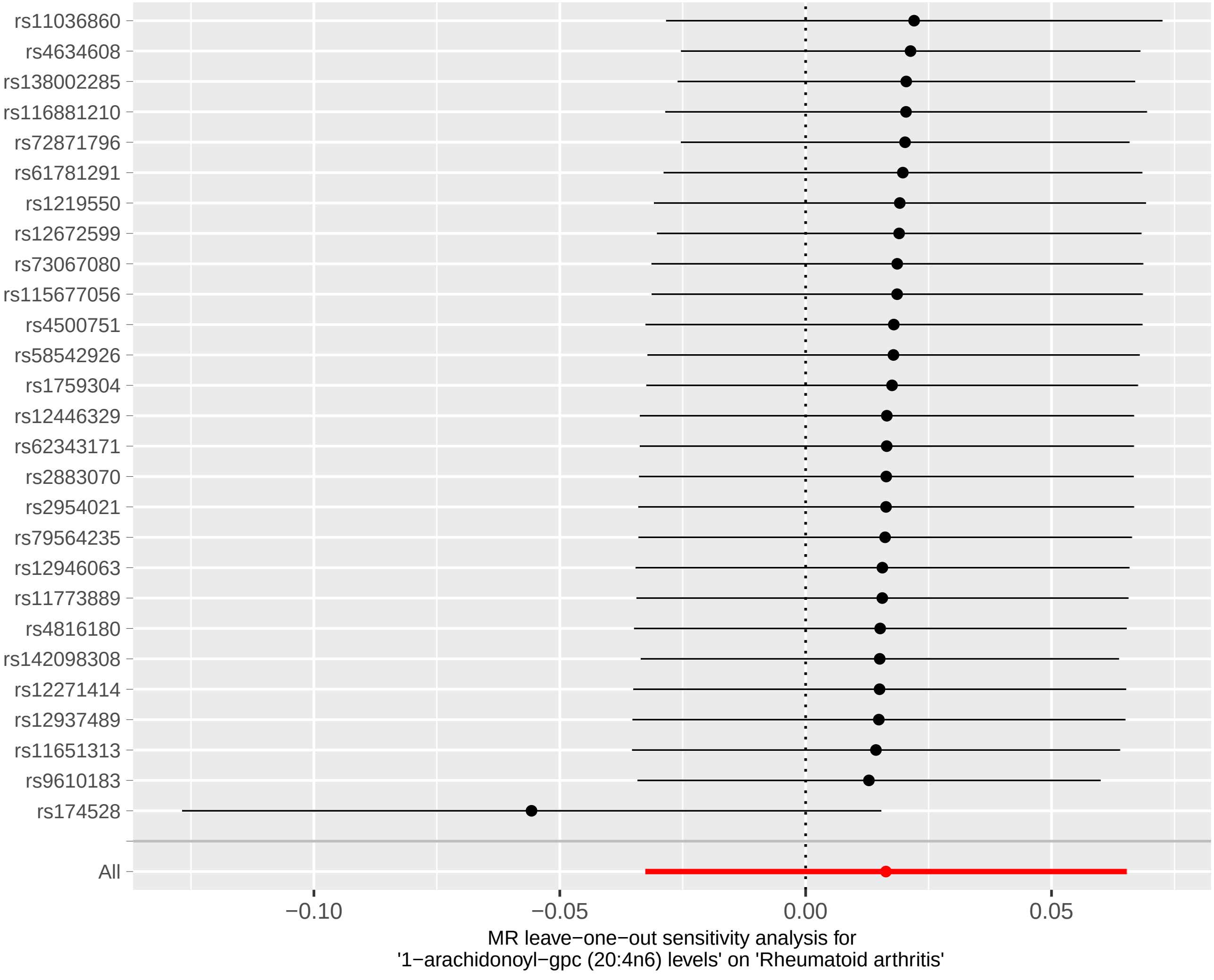
rs11036860
rs4634608
rs138002285
rs116881210
rs72871796
rs61781291
rs1219550
rs12672599
rs73067080
rs115677056
rs4500751
rs58542926
rs1759304
rs12446329
rs62343171
rs2883070
rs2954021
rs79564235
rs12946063
rs11773889
rs4816180
rs142098308
rs12271414
rs12937489
rs11651313
rs9610183
rs174528
All
−0.10
−0.05
0.00
0.05
MR leave−one−out sensitivity analysis for
'1−arachidonoyl−gpc (20:4n6) levels' on 'Rheumatoid arthritis'

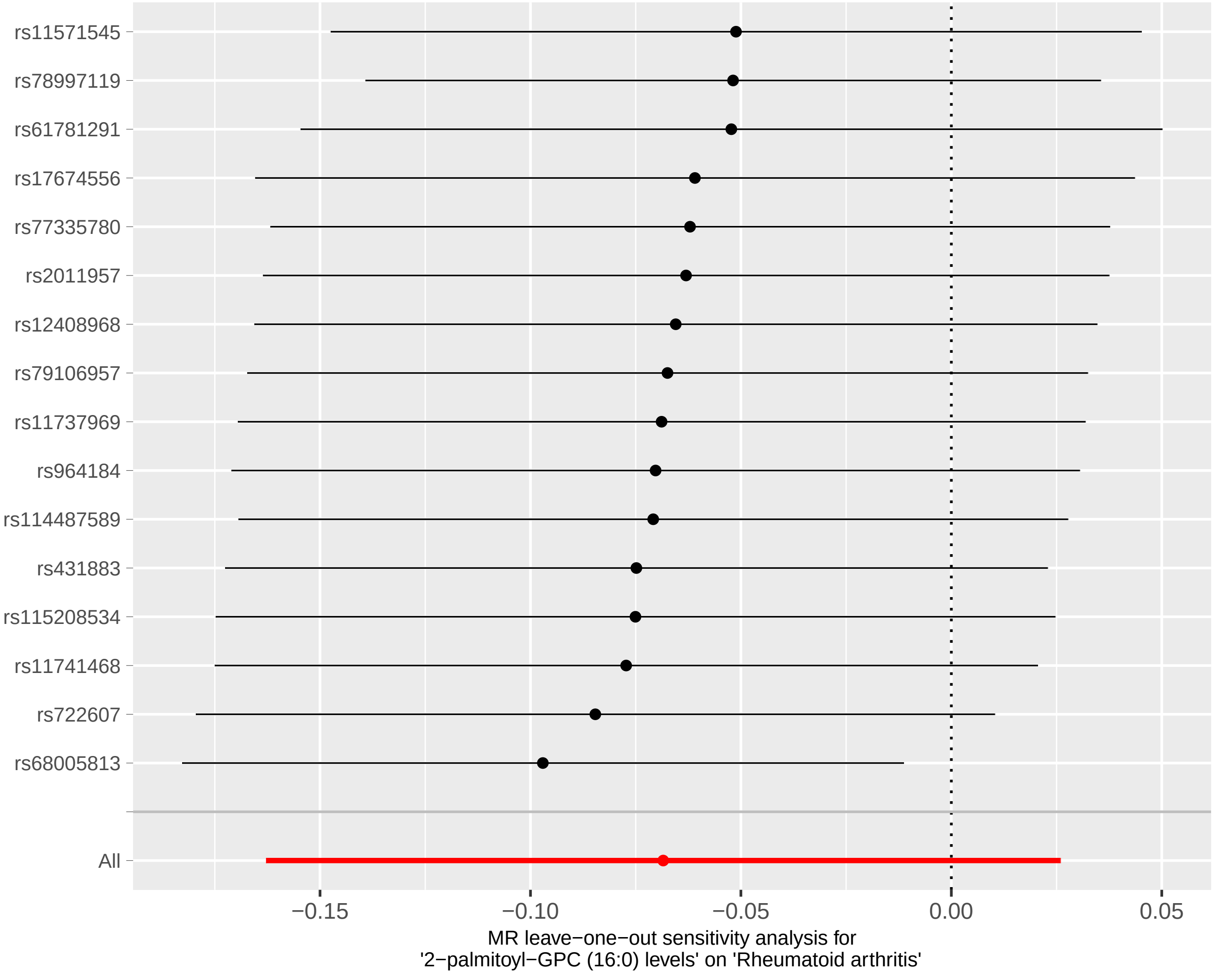
rs11571545
rs78997119
rs61781291
rs17674556
rs77335780
rs2011957
rs12408968
rs79106957
rs11737969
rs964184
rs114487589
rs431883
rs115208534
rs11741468
rs722607
rs68005813
All
−0.15
−0.10
−0.05
0.00
0.05
MR leave−one−out sensitivity analysis for
'2−palmitoyl−GPC (16:0) levels' on 'Rheumatoid arthritis'

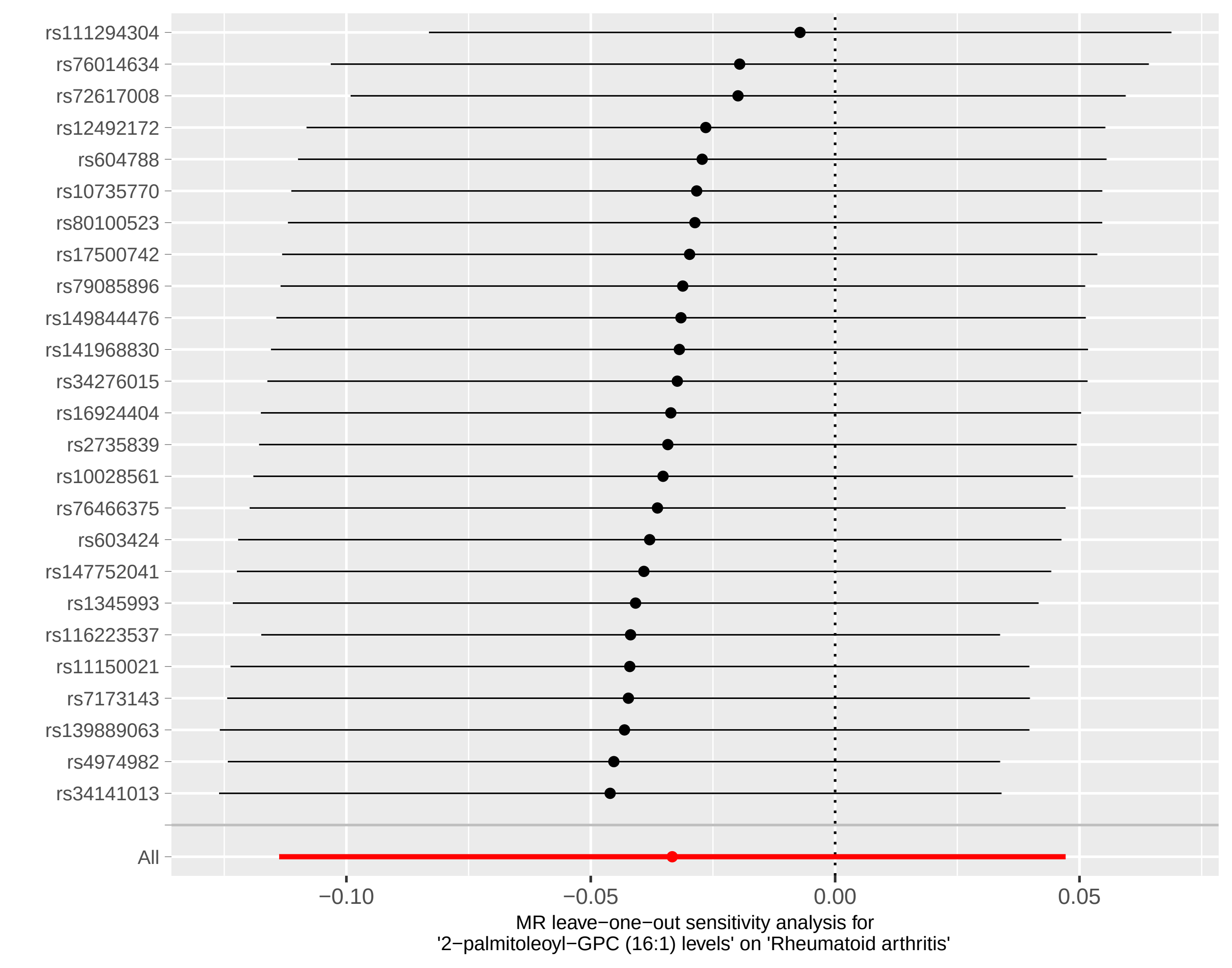
rs111294304
rs76014634
rs72617008
rs12492172
rs604788
rs10735770
rs80100523
rs17500742
rs79085896
rs149844476
rs141968830
rs34276015
rs16924404
rs2735839
rs10028561
rs76466375
rs603424
rs147752041
rs1345993
rs116223537
rs11150021
rs7173143
rs139889063
rs4974982
rs34141013
All
−0.10
−0.05
0.00
0.05
MR leave−one−out sensitivity analysis for
'2−palmitoleoyl−GPC (16:1) levels' on 'Rheumatoid arthritis'

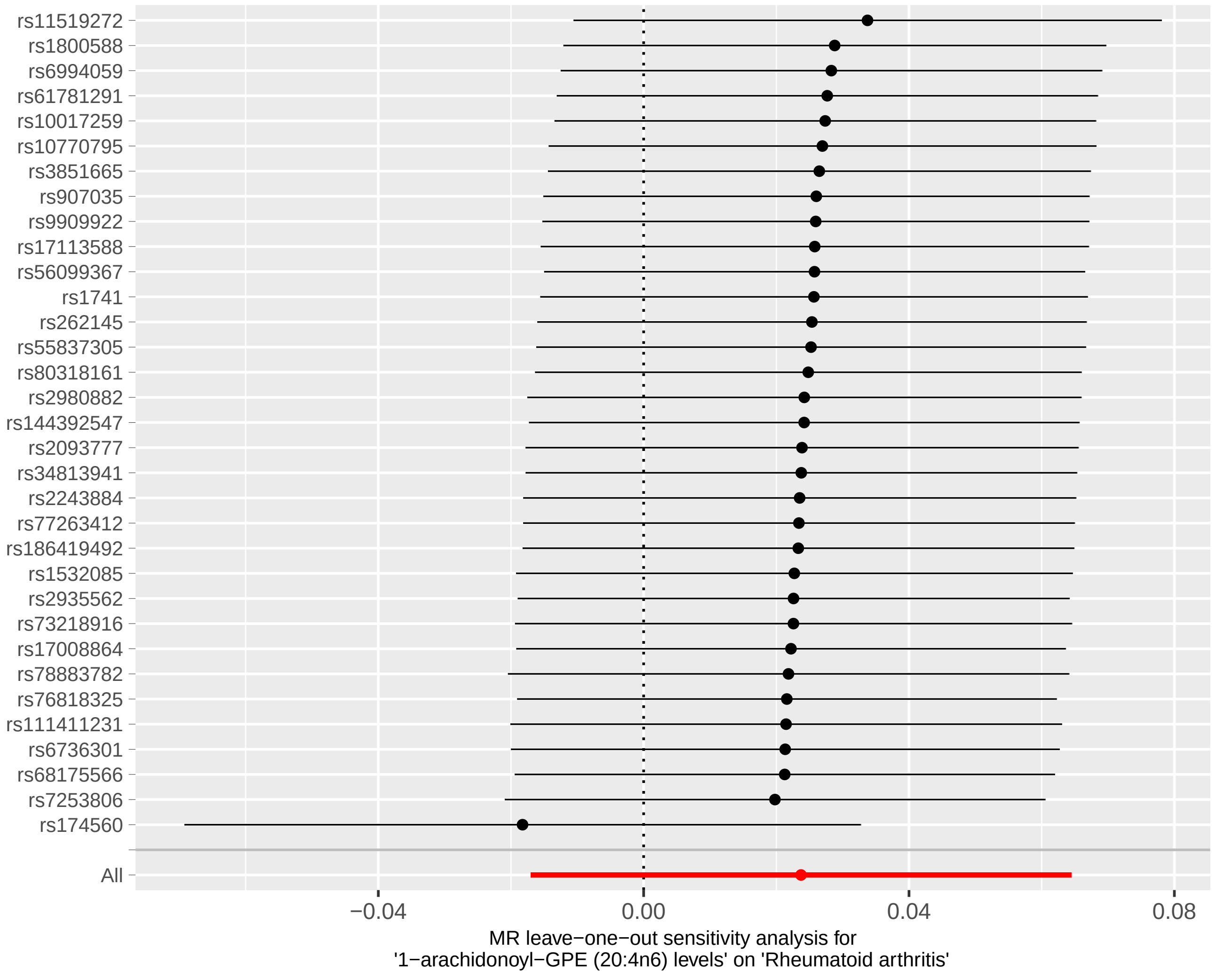
rs11519272
rs1800588
rs6994059
rs61781291
rs10017259
rs10770795
rs3851665
rs907035
rs9909922
rs17113588
rs56099367
rs1741
rs262145
rs55837305
rs80318161
rs2980882
rs144392547
rs2093777
rs34813941
rs2243884
rs77263412
rs186419492
rs1532085
rs2935562
rs73218916
rs17008864
rs78883782
rs76818325
rs111411231
rs6736301
rs68175566
rs7253806
rs174560
All
−0.04
0.00
0.04
0.08
MR leave−one−out sensitivity analysis for
'1−arachidonoyl−GPE (20:4n6) levels' on 'Rheumatoid arthritis'

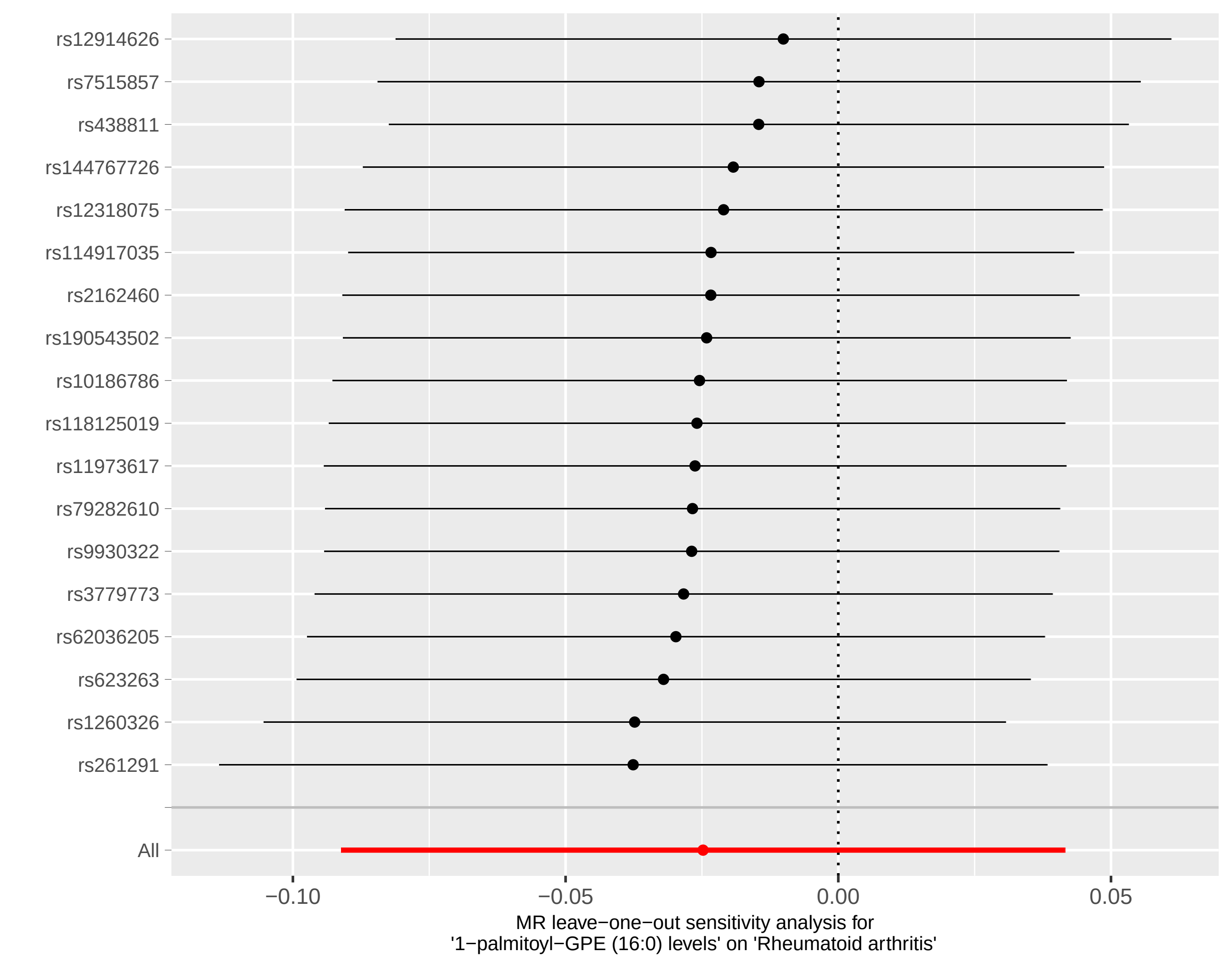
rs12914626
rs7515857
rs438811
rs144767726
rs12318075
rs114917035
rs2162460
rs190543502
rs10186786
rs118125019
rs11973617
rs79282610
rs9930322
rs3779773
rs62036205
rs623263
rs1260326
rs261291
All
−0.10
−0.05
0.00
0.05
MR leave−one−out sensitivity analysis for
'1−palmitoyl−GPE (16:0) levels' on 'Rheumatoid arthritis'

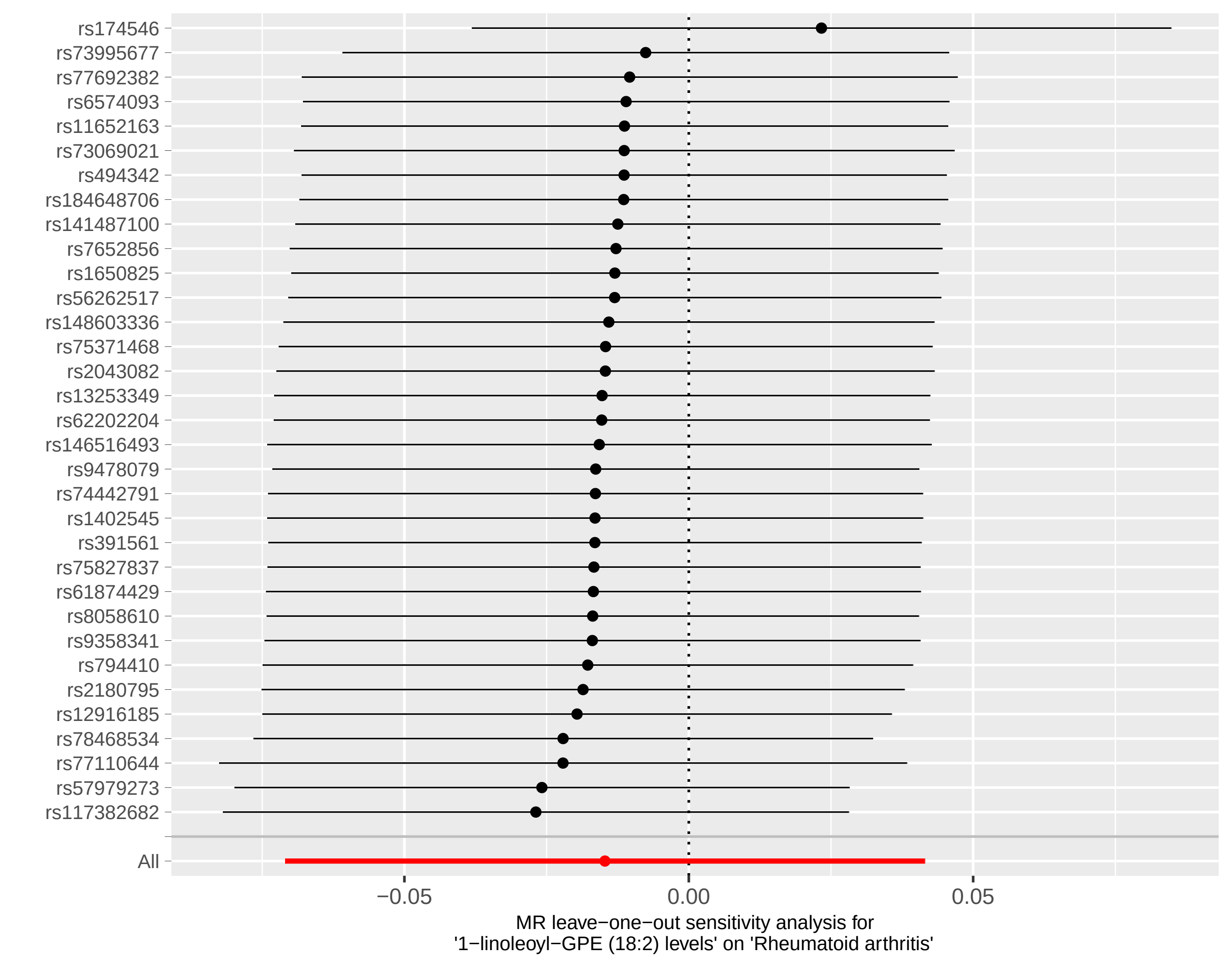

rs174546
rs73995677
rs77692382
rs6574093
rs11652163
rs73069021
rs494342
rs184648706
rs141487100
rs7652856
rs1650825
rs56262517
rs148603336
rs75371468
rs2043082
rs13253349
rs62202204
rs146516493
rs9478079
rs74442791
rs1402545
rs391561
rs75827837
rs61874429
rs8058610
rs9358341
rs794410
rs2180795
rs12916185
rs78468534
rs77110644
rs57979273
rs117382682
All
−0.05
0.00
0.05
MR leave−one−out sensitivity analysis for
'1−linoleoyl−GPE (18:2) levels' on 'Rheumatoid arthritis'

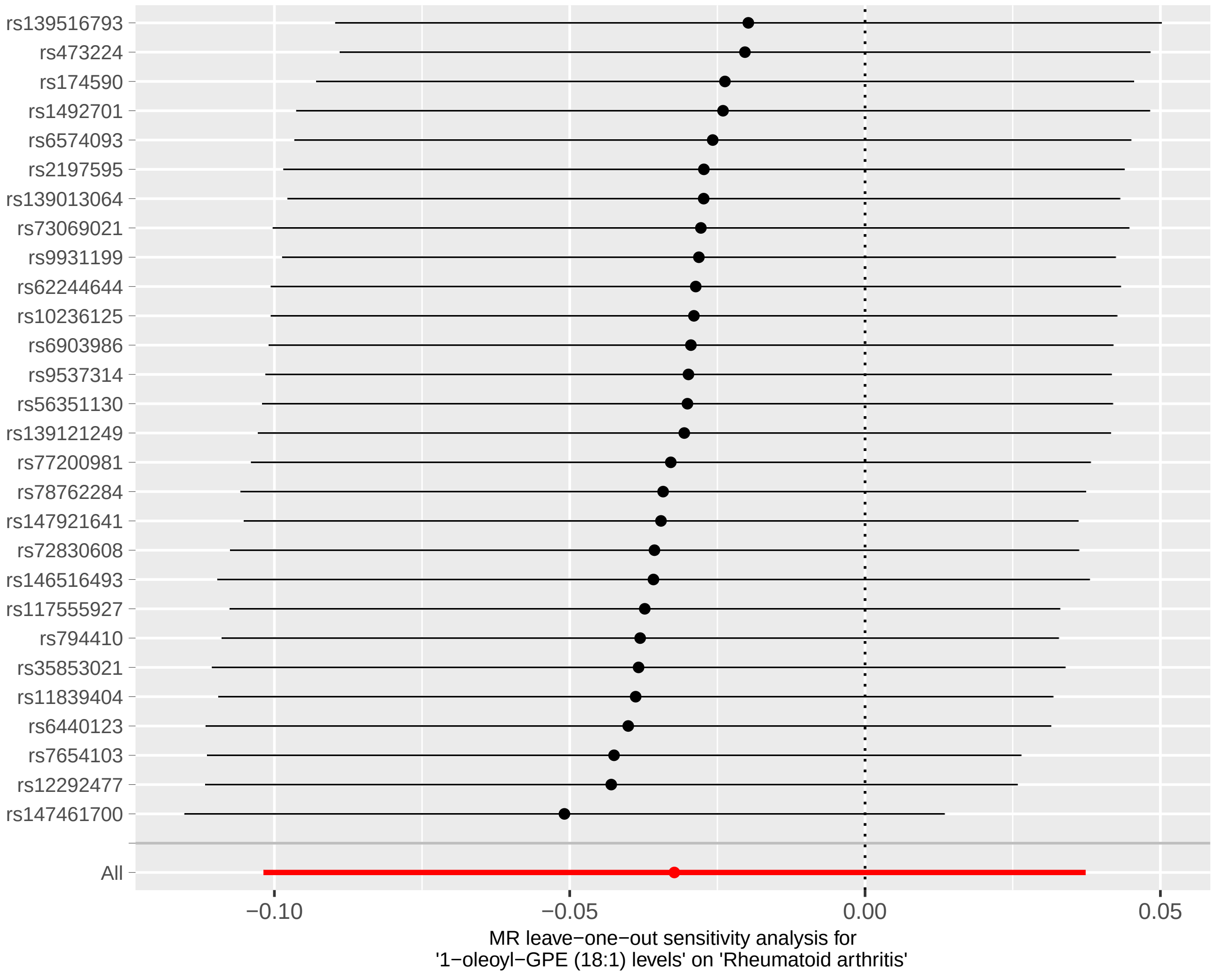

rs139516793
rs473224
rs174590
rs1492701
rs6574093
rs2197595
rs139013064
rs73069021
rs9931199
rs62244644
rs10236125
rs6903986
rs9537314
rs56351130
rs139121249
rs77200981
rs78762284
rs147921641
rs72830608
rs146516493
rs117555927
rs794410
rs35853021
rs11839404
rs6440123
rs7654103
rs12292477
rs147461700
All
−0.10
−0.05
0.00
0.05
MR leave−one−out sensitivity analysis for
'1−oleoyl−GPE (18:1) levels' on 'Rheumatoid arthritis'

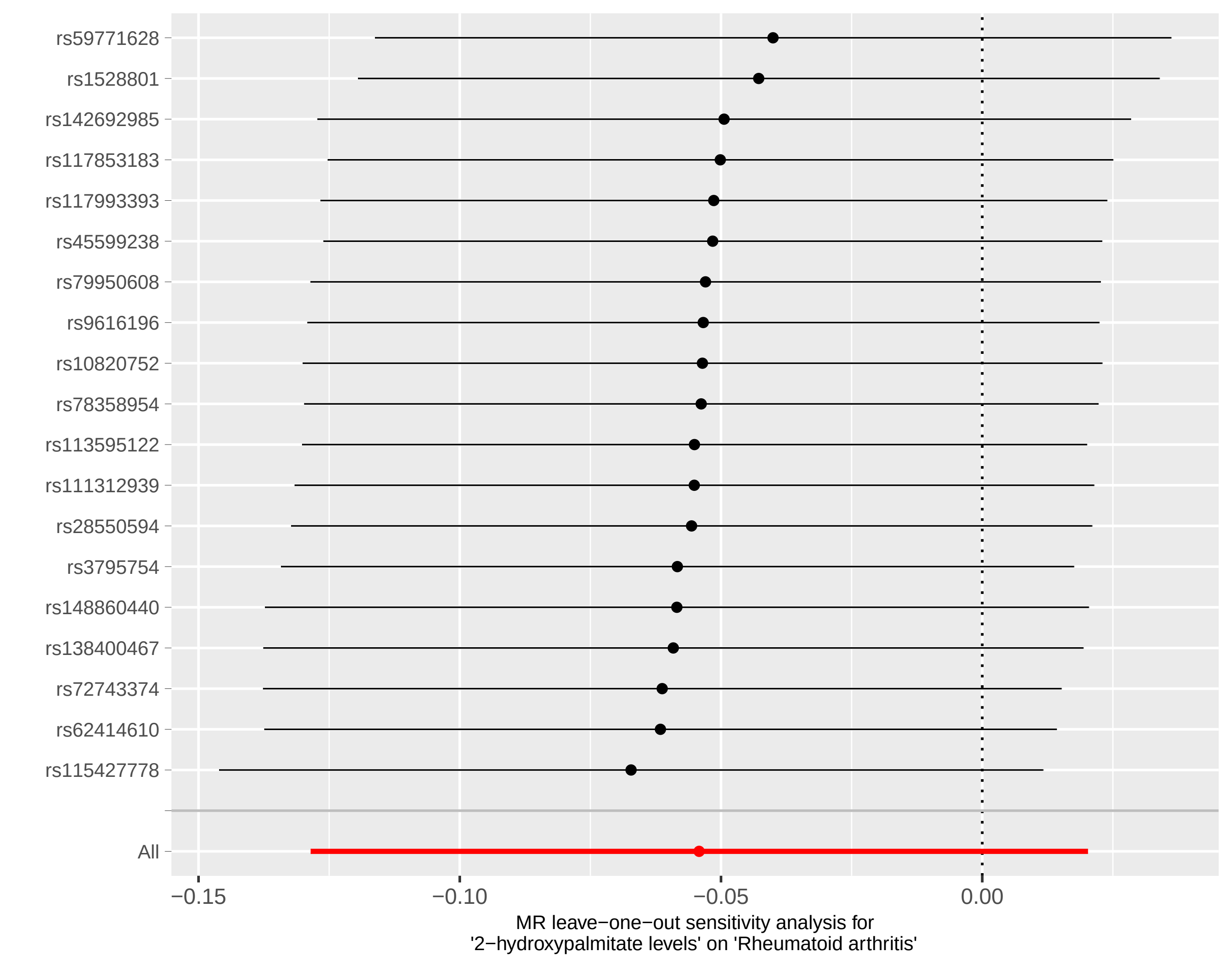

rs59771628
rs1528801
rs142692985
rs117853183
rs117993393
rs45599238
rs79950608
rs9616196
rs10820752
rs78358954
rs113595122
rs111312939
rs28550594
rs3795754
rs148860440
rs138400467
rs72743374
rs62414610
rs115427778
All
−0.15
−0.10
−0.05
0.00
MR leave−one−out sensitivity analysis for
'2−hydroxypalmitate levels' on 'Rheumatoid arthritis'

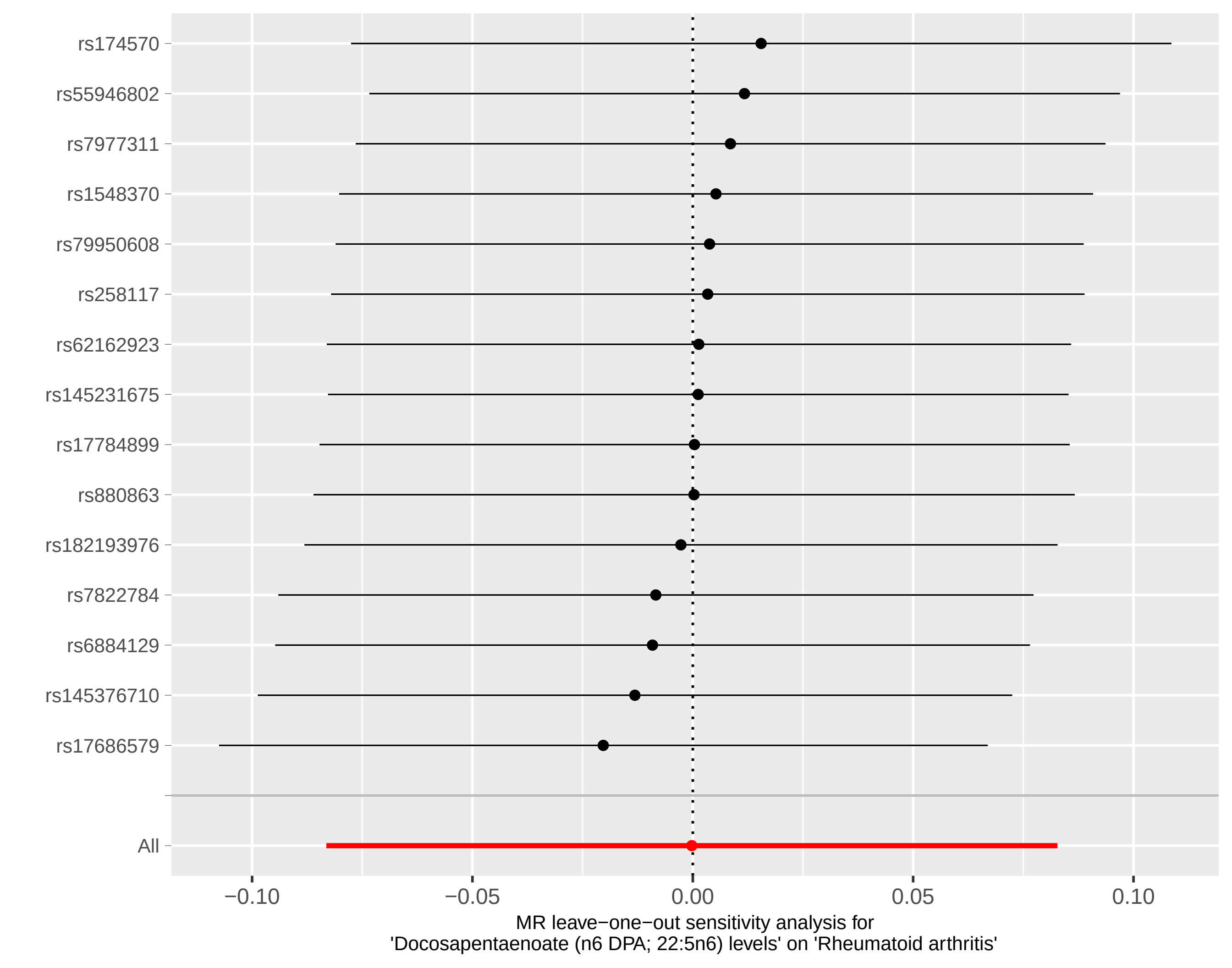

rs174570
rs55946802
rs7977311
rs1548370
rs79950608
rs258117
rs62162923
rs145231675
rs17784899
rs880863
rs182193976
rs7822784
rs6884129
rs145376710
rs17686579
All
−0.10
−0.05
0.00
0.05
0.10
MR leave−one−out sensitivity analysis for
'Docosapentaenoate (n6 DPA; 22:5n6) levels' on 'Rheumatoid arthritis'

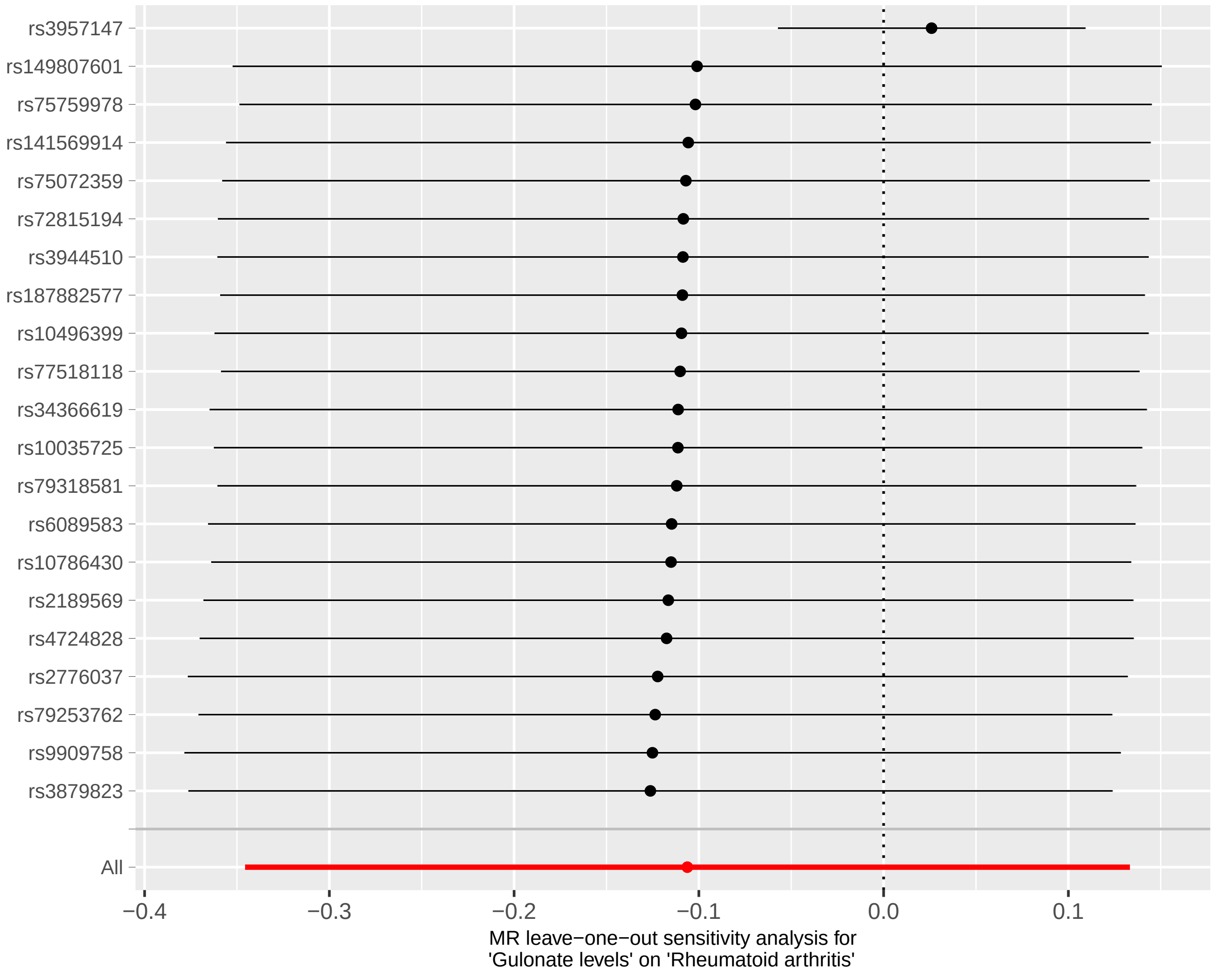

rs3957147
rs149807601
rs75759978
rs141569914
rs75072359
rs72815194
rs3944510
rs187882577
rs10496399
rs77518118
rs34366619
rs10035725
rs79318581
rs6089583
rs10786430
rs2189569
rs4724828
rs2776037
rs79253762
rs9909758
rs3879823
All
−0.4
−0.3
−0.2
−0.1
0.0
0.1
MR leave−one−out sensitivity analysis for
'Gulonate levels' on 'Rheumatoid arthritis'

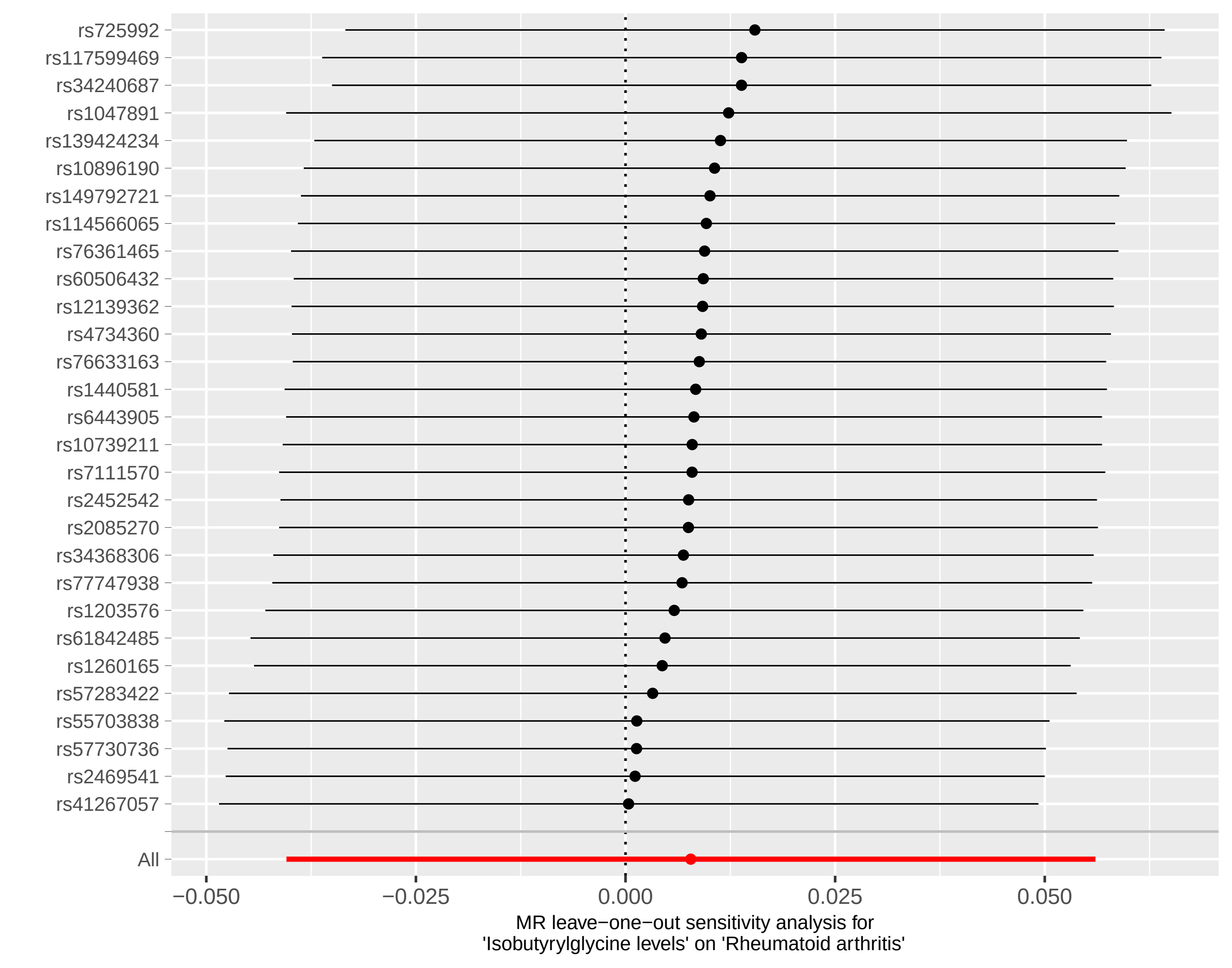

rs725992
rs117599469
rs34240687
rs1047891
rs139424234
rs10896190
rs149792721
rs114566065
rs76361465
rs60506432
rs12139362
rs4734360
rs76633163
rs1440581
rs6443905
rs10739211
rs7111570
rs2452542
rs2085270
rs34368306
rs77747938
rs1203576
rs61842485
rs1260165
rs57283422
rs55703838
rs57730736
rs2469541
rs41267057
All
−0.050
−0.025
0.000
0.025
0.050
MR leave−one−out sensitivity analysis for
'Isobutyrylglycine levels' on 'Rheumatoid arthritis'

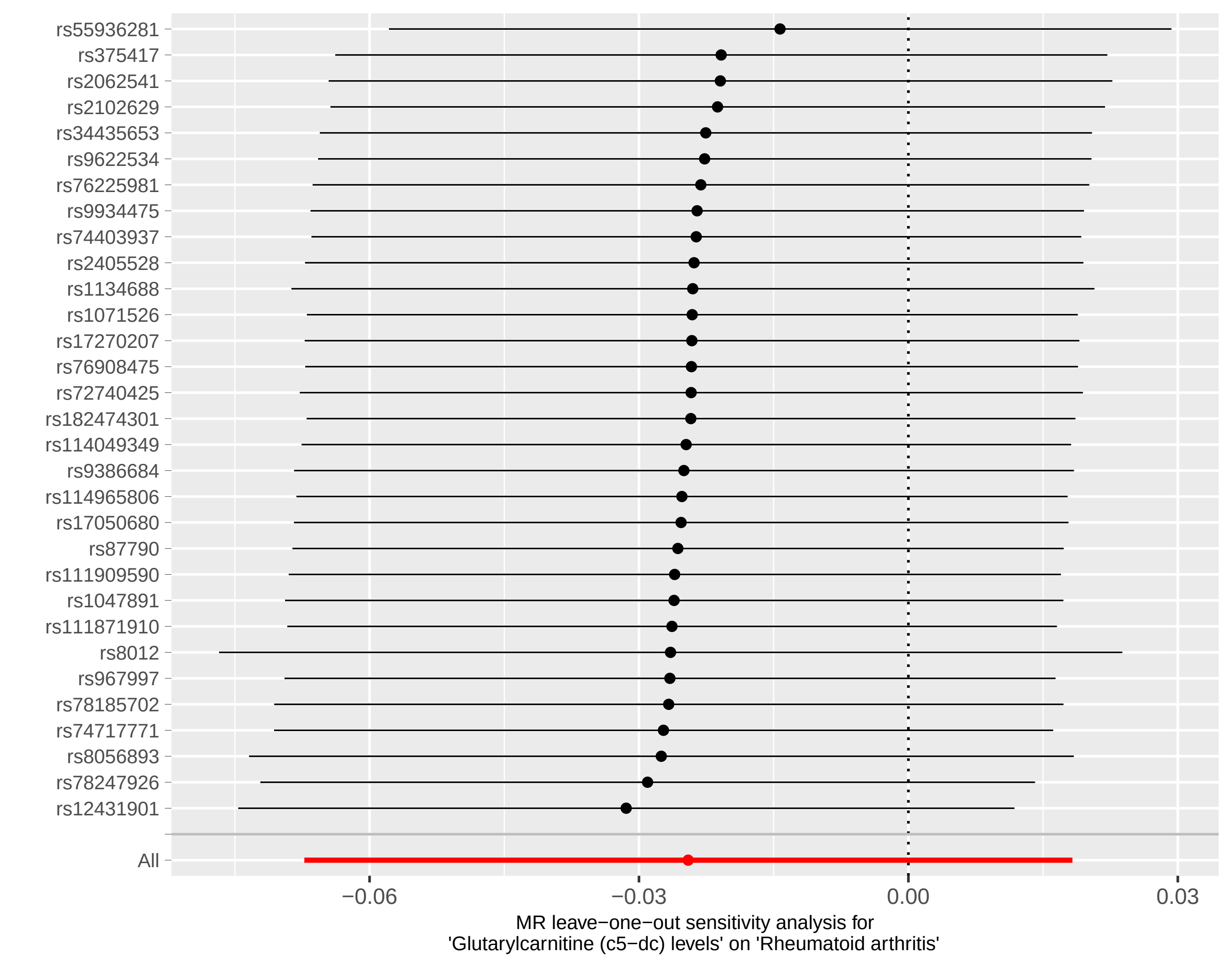
rs55936281
rs375417
rs2062541
rs2102629
rs34435653
rs9622534
rs76225981
rs9934475
rs74403937
rs2405528
rs1134688
rs1071526
rs17270207
rs76908475
rs72740425
rs182474301
rs114049349
rs9386684
rs114965806
rs17050680
rs87790
rs111909590
rs1047891
rs111871910
rs8012
rs967997
rs78185702
rs74717771
rs8056893
rs78247926
rs12431901
All
−0.06
−0.03
0.00
0.03
MR leave−one−out sensitivity analysis for
'Glutarylcarnitine (c5−dc) levels' on 'Rheumatoid arthritis'

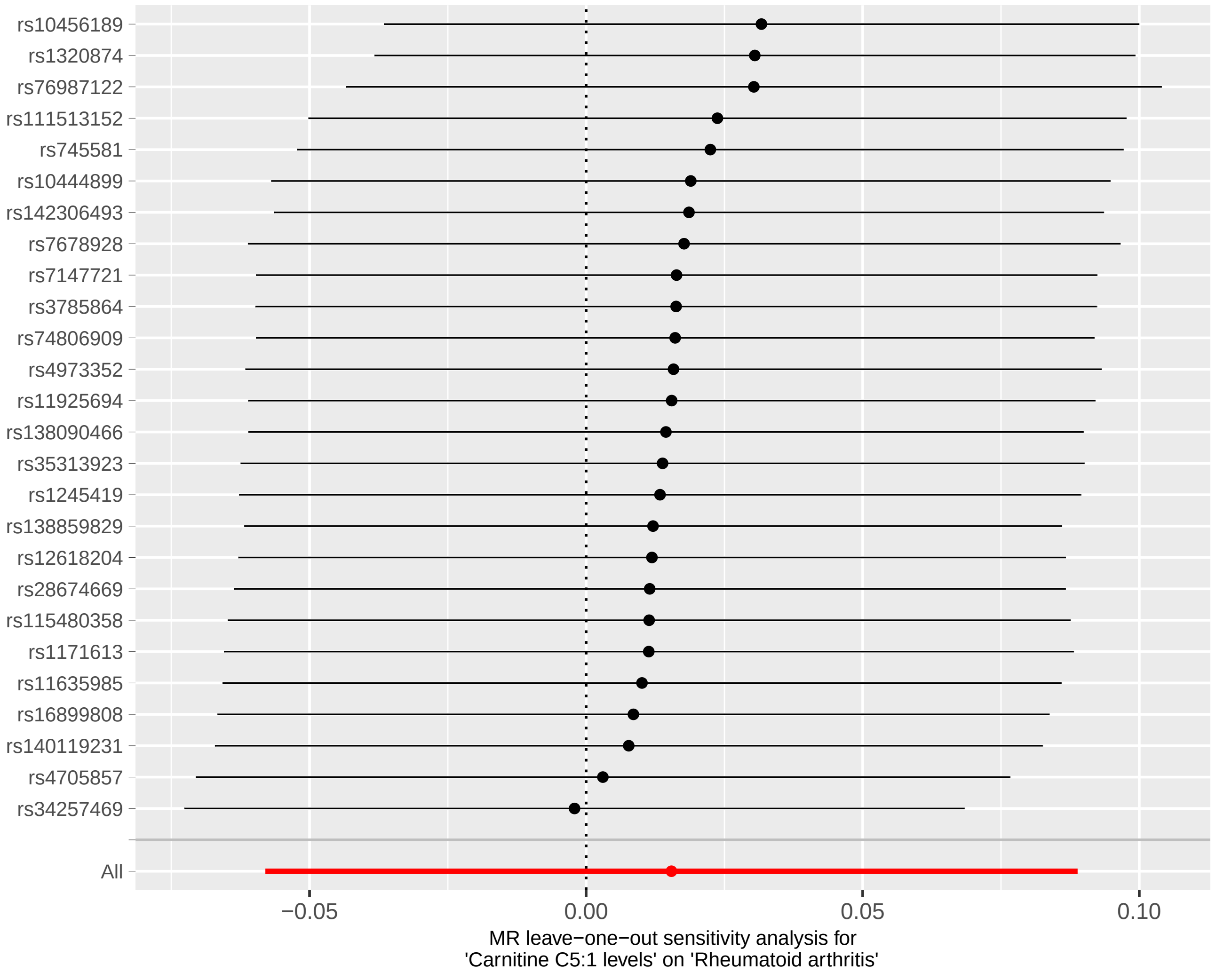

rs10456189
rs1320874
rs76987122
rs111513152
rs745581
rs10444899
rs142306493
rs7678928
rs7147721
rs3785864
rs74806909
rs4973352
rs11925694
rs138090466
rs35313923
rs1245419
rs138859829
rs12618204
rs28674669
rs115480358
rs1171613
rs11635985
rs16899808
rs140119231
rs4705857
rs34257469
All
−0.05
0.00
0.05
0.10
MR leave−one−out sensitivity analysis for
'Carnitine C5:1 levels' on 'Rheumatoid arthritis'

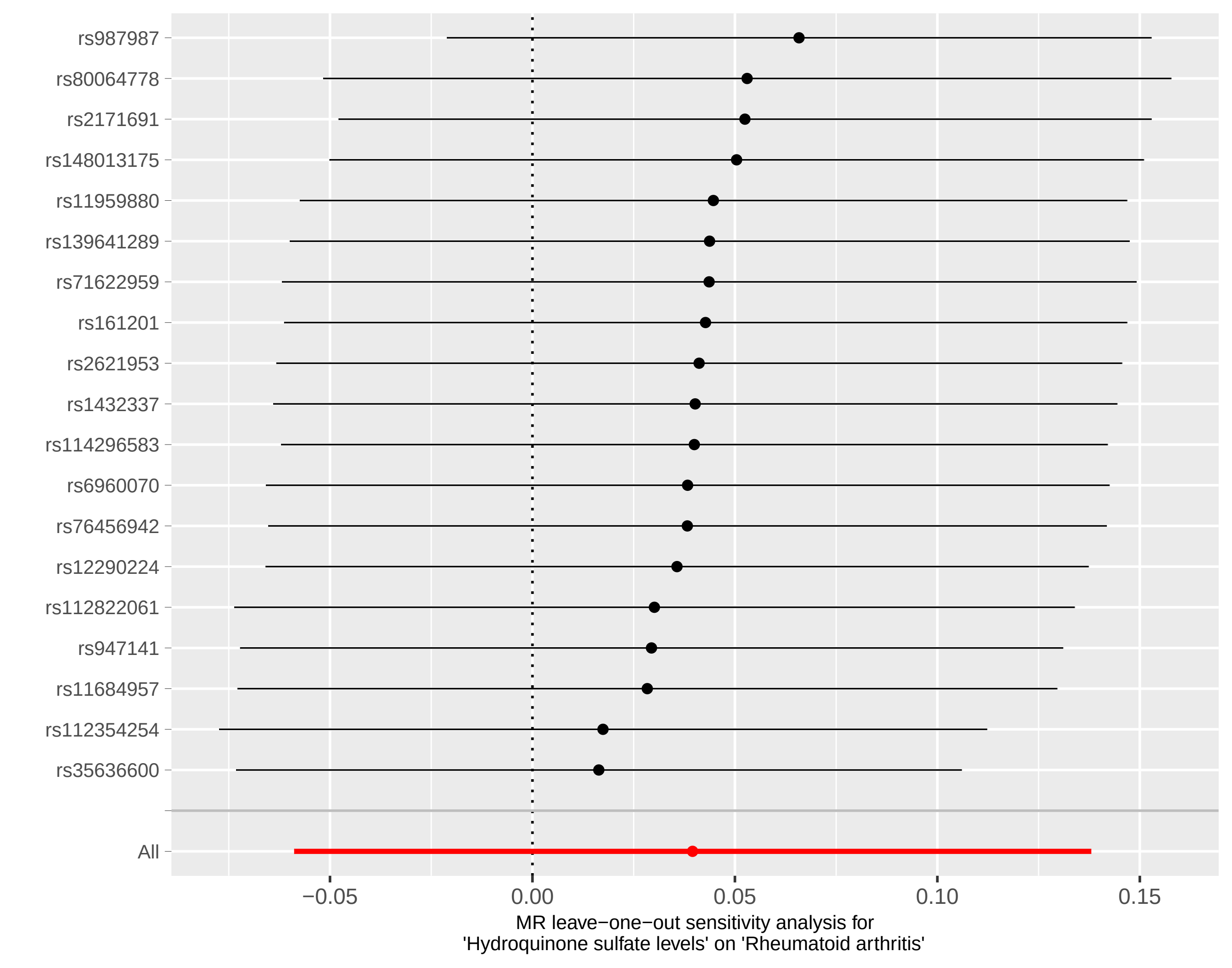
rs987987
rs80064778
rs2171691
rs148013175
rs11959880
rs139641289
rs71622959
rs161201
rs2621953
rs1432337
rs114296583
rs6960070
rs76456942
rs12290224
rs112822061
rs947141
rs11684957
rs112354254
rs35636600
All
−0.05
0.00
0.05
0.10
0.15
MR leave−one−out sensitivity analysis for
'Hydroquinone sulfate levels' on 'Rheumatoid arthritis'

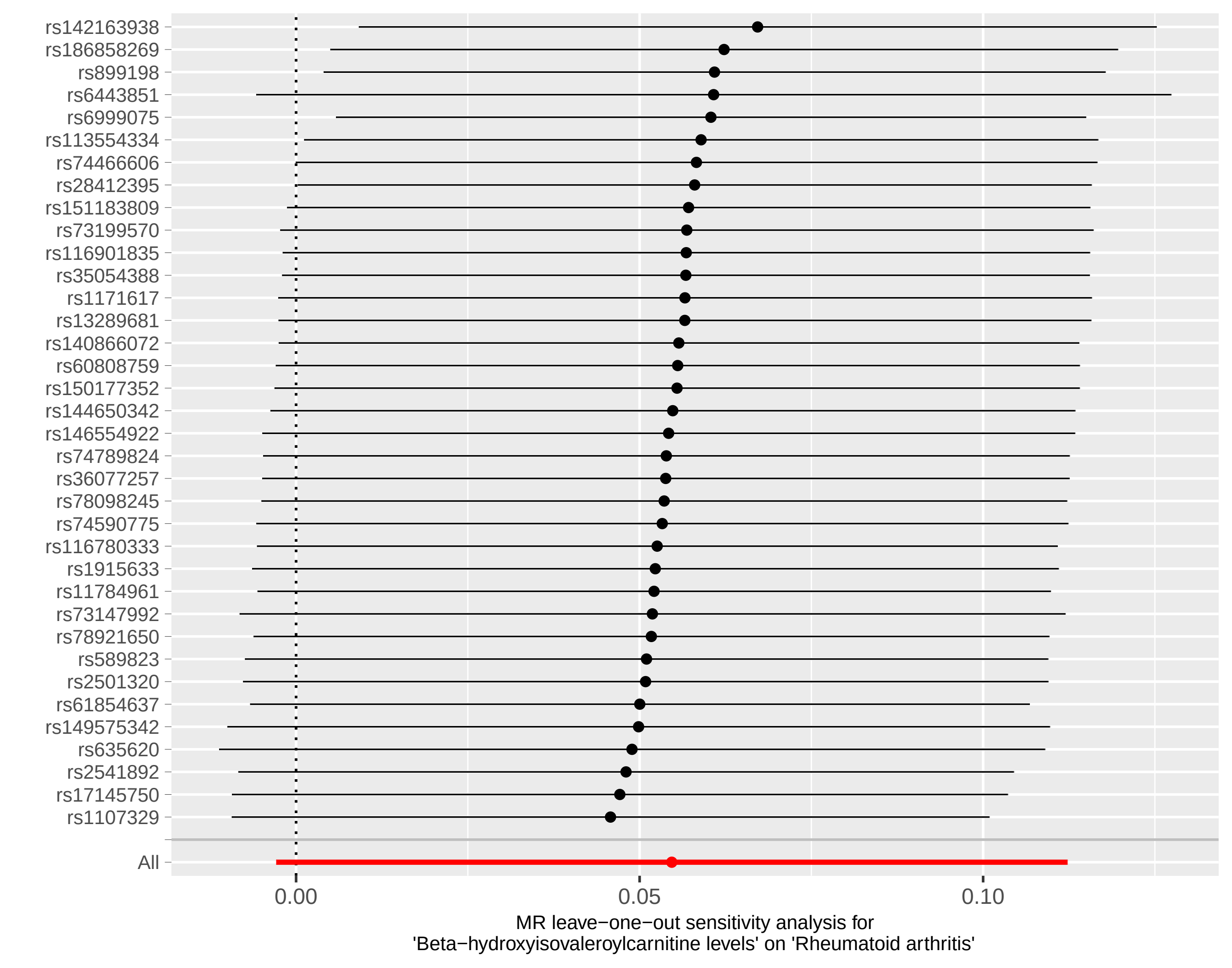

rs142163938
rs186858269
rs899198
rs6443851
rs6999075
rs113554334
rs74466606
rs28412395
rs151183809
rs73199570
rs116901835
rs35054388
rs1171617
rs13289681
rs140866072
rs60808759
rs150177352
rs144650342
rs146554922
rs74789824
rs36077257
rs78098245
rs74590775
rs116780333
rs1915633
rs11784961
rs73147992
rs78921650
rs589823
rs2501320
rs61854637
rs149575342
rs635620
rs2541892
rs17145750
rs1107329
All
0.00
0.05
0.10
MR leave−one−out sensitivity analysis for
'Beta−hydroxyisovaleroylcarnitine levels' on 'Rheumatoid arthritis'

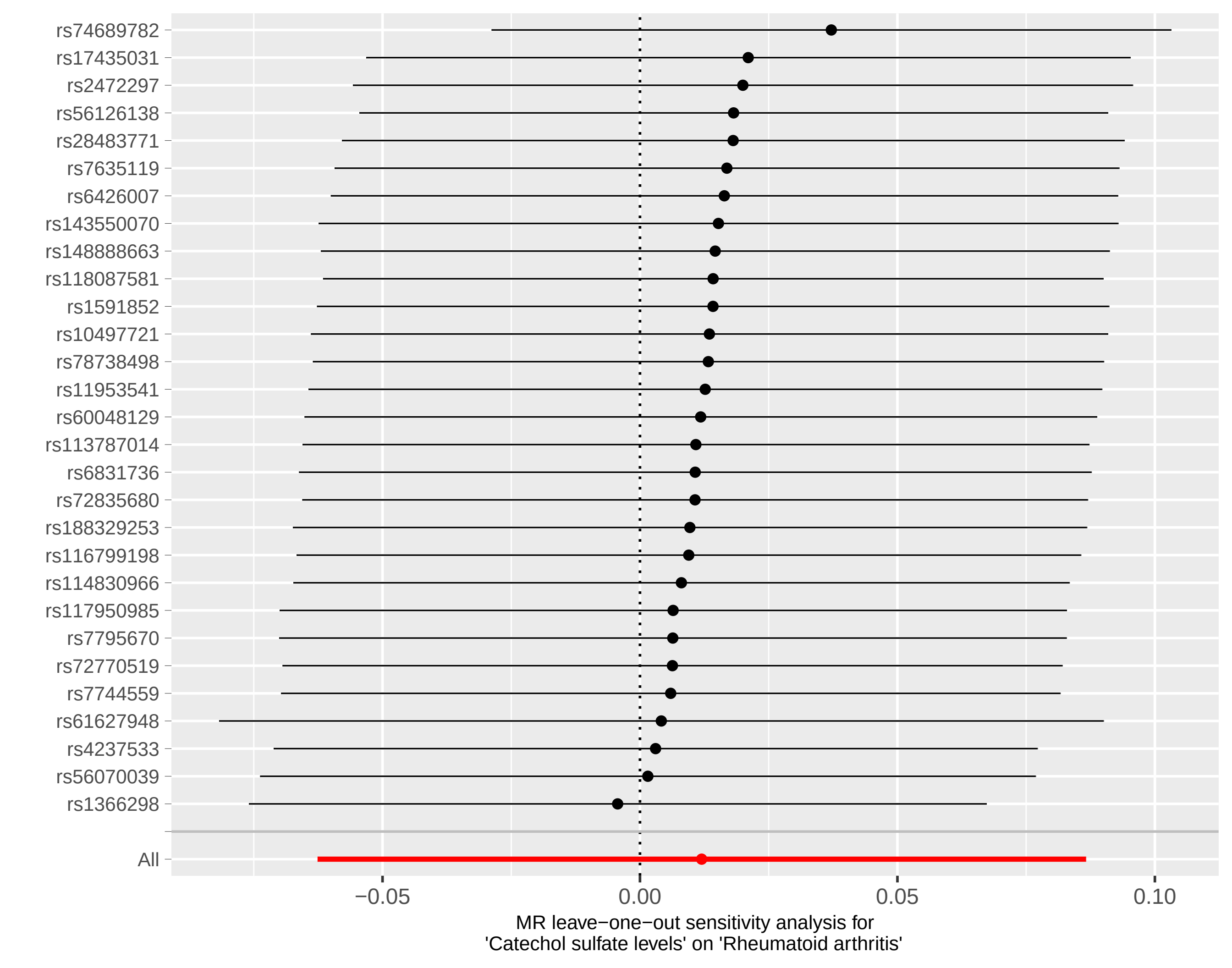
rs74689782
rs17435031
rs2472297
rs56126138
rs28483771
rs7635119
rs6426007
rs143550070
rs148888663
rs118087581
rs1591852
rs10497721
rs78738498
rs11953541
rs60048129
rs113787014
rs6831736
rs72835680
rs188329253
rs116799198
rs114830966
rs117950985
rs7795670
rs72770519
rs7744559
rs61627948
rs4237533
rs56070039
rs1366298
All
−0.05
0.00
0.05
0.10
MR leave−one−out sensitivity analysis for
'Catechol sulfate levels' on 'Rheumatoid arthritis'

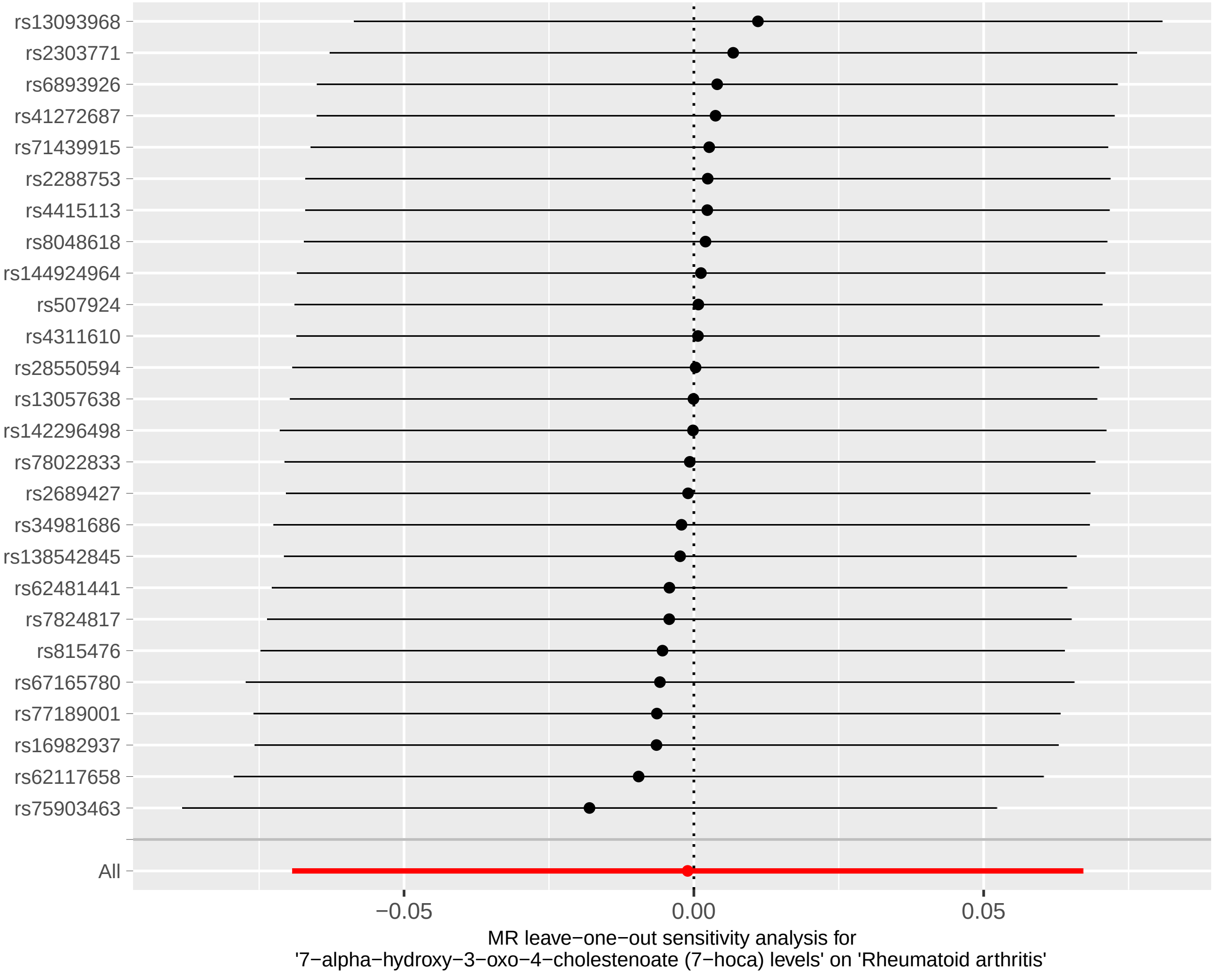
rs13093968
rs2303771
rs6893926
rs41272687
rs71439915
rs2288753
rs4415113
rs8048618
rs144924964
rs507924
rs4311610
rs28550594
rs13057638
rs142296498
rs78022833
rs2689427
rs34981686
rs138542845
rs62481441
rs7824817
rs815476
rs67165780
rs77189001
rs16982937
rs62117658
rs75903463
All
−0.05
0.00
0.05
MR leave−one−out sensitivity analysis for
'7−alpha−hydroxy−3−oxo−4−cholestenoate (7−hoca) levels' on 'Rheumatoid arthritis'

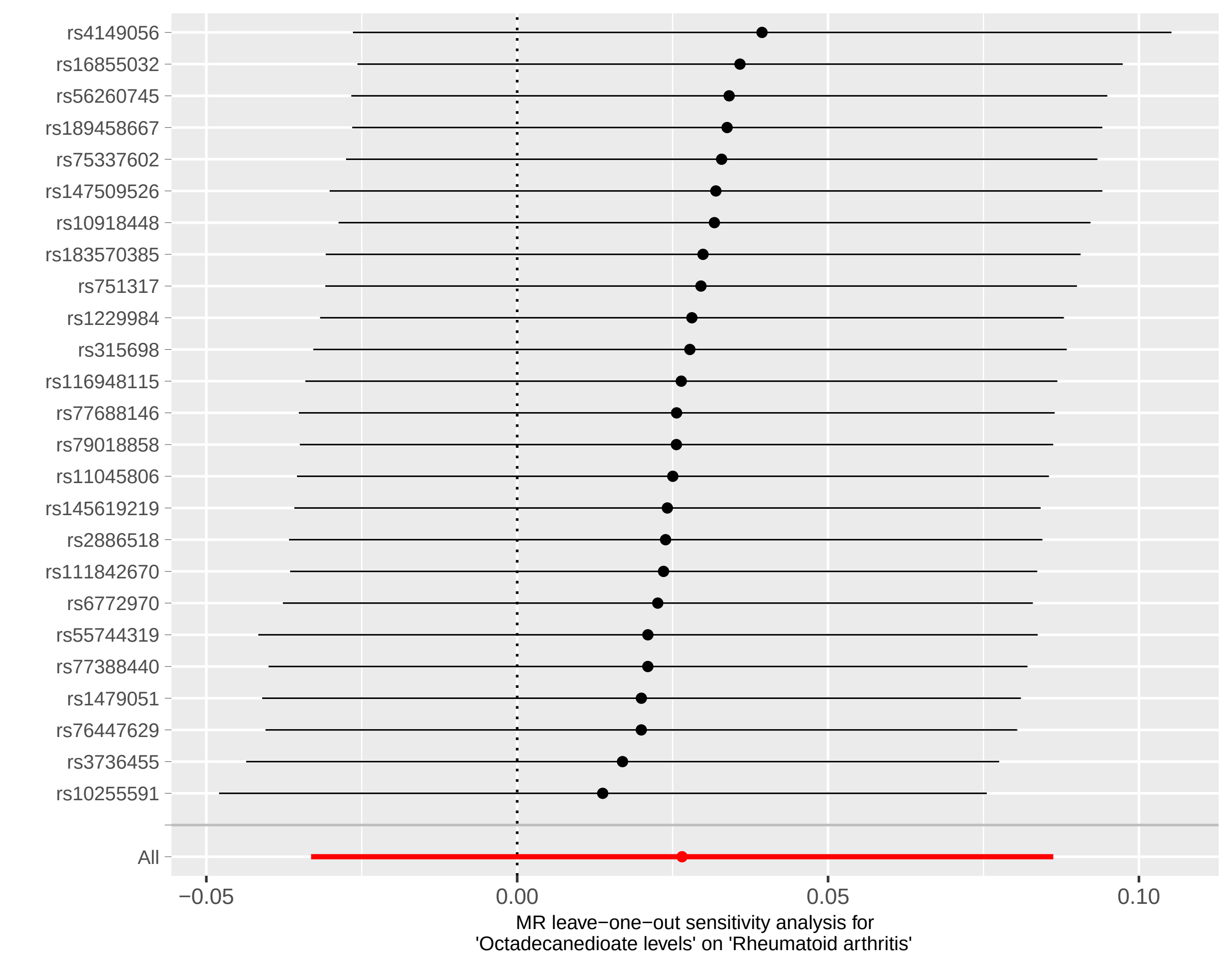
rs4149056
rs16855032
rs56260745
rs189458667
rs75337602
rs147509526
rs10918448
rs183570385
rs751317
rs1229984
rs315698
rs116948115
rs77688146
rs79018858
rs11045806
rs145619219
rs2886518
rs111842670
rs6772970
rs55744319
rs77388440
rs1479051
rs76447629
rs3736455
rs10255591
All
−0.05
0.00
0.05
0.10
MR leave−one−out sensitivity analysis for
'Octadecanedioate levels' on 'Rheumatoid arthritis'

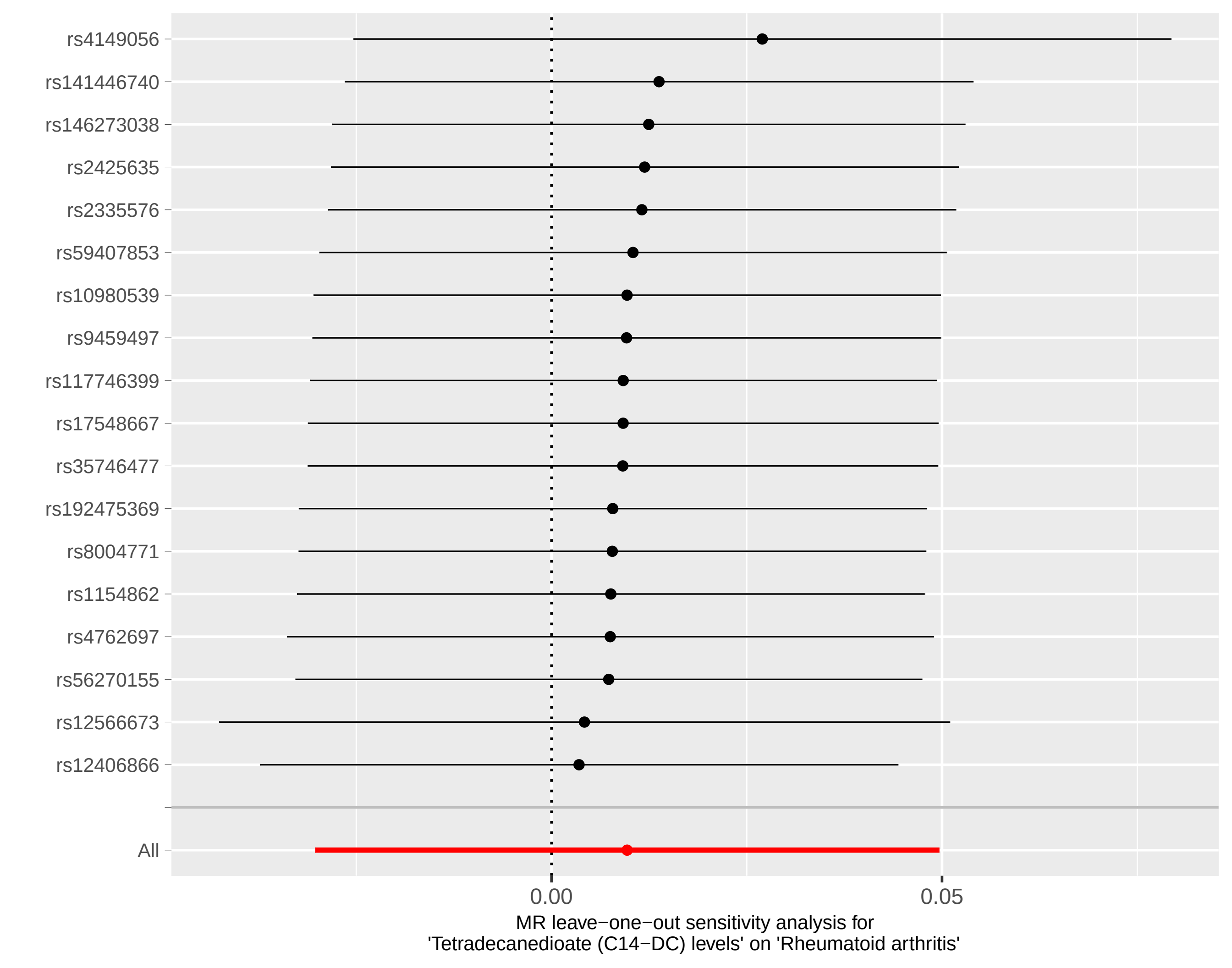
rs4149056
rs141446740
rs146273038
rs2425635
rs2335576
rs59407853
rs10980539
rs9459497
rs117746399
rs17548667
rs35746477
rs192475369
rs8004771
rs1154862
rs4762697
rs56270155
rs12566673
rs12406866
All
0.00
0.05
MR leave−one−out sensitivity analysis for
'Tetradecanedioate (C14−DC) levels' on 'Rheumatoid arthritis'

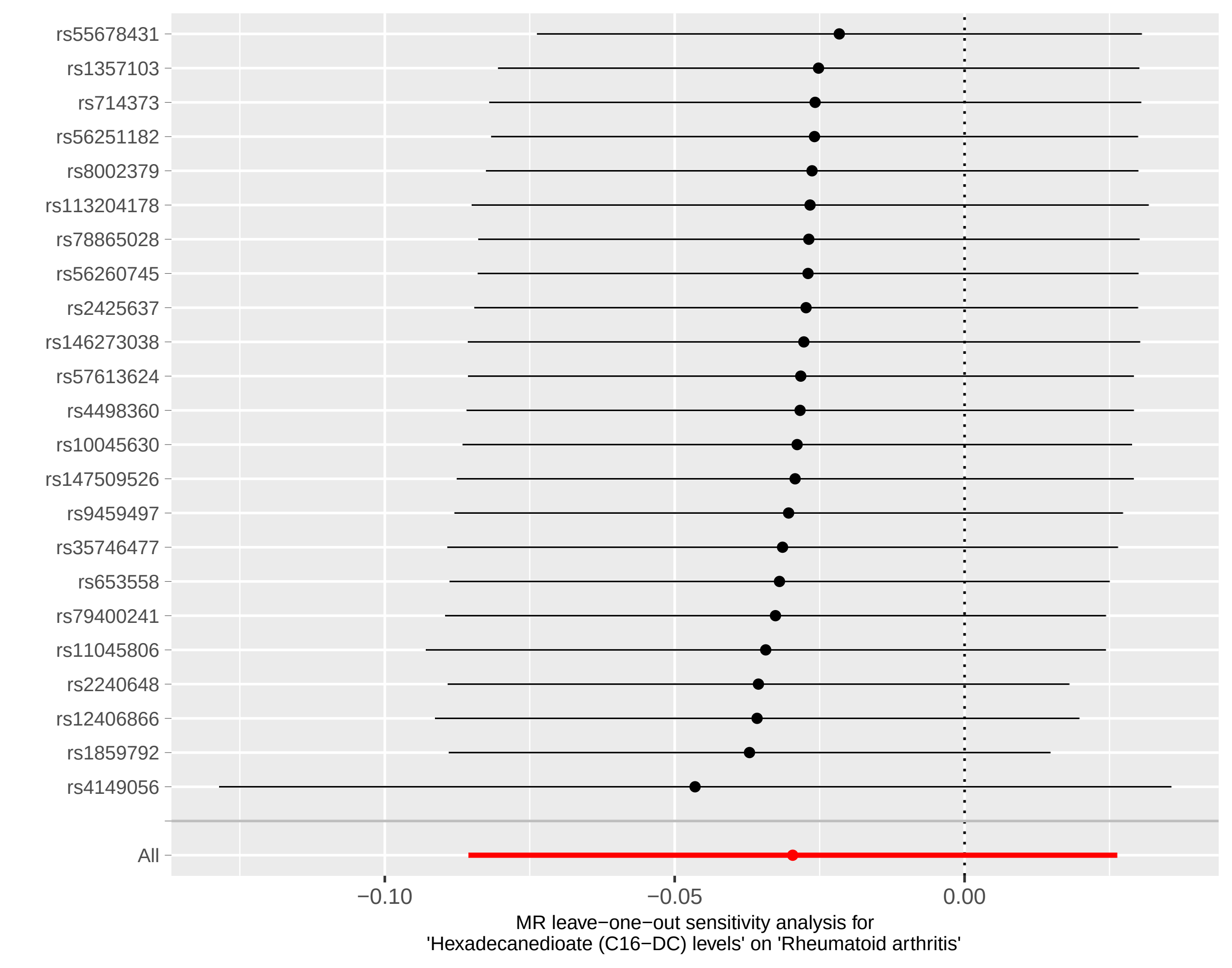
rs55678431
rs1357103
rs714373
rs56251182
rs8002379
rs113204178
rs78865028
rs56260745
rs2425637
rs146273038
rs57613624
rs4498360
rs10045630
rs147509526
rs9459497
rs35746477
rs653558
rs79400241
rs11045806
rs2240648
rs12406866
rs1859792
rs4149056
All
−0.10
−0.05
0.00
MR leave−one−out sensitivity analysis for
'Hexadecanedioate (C16−DC) levels' on 'Rheumatoid arthritis'

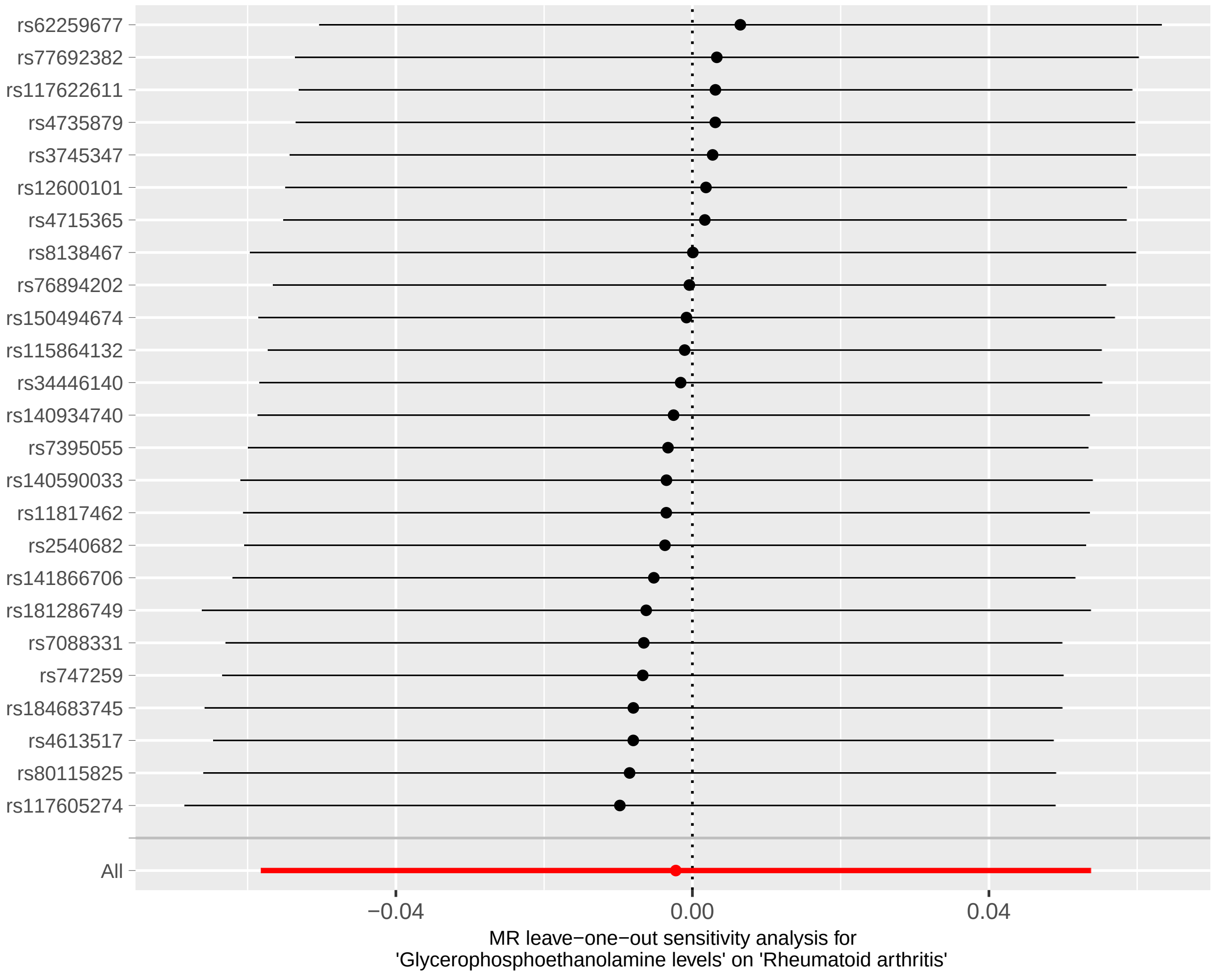
rs62259677
rs77692382
rs117622611
rs4735879
rs3745347
rs12600101
rs4715365
rs8138467
rs76894202
rs150494674
rs115864132
rs34446140
rs140934740
rs7395055
rs140590033
rs11817462
rs2540682
rs141866706
rs181286749
rs7088331
rs747259
rs184683745
rs4613517
rs80115825
rs117605274
All
−0.04
0.00
0.04
MR leave−one−out sensitivity analysis for
'Glycerophosphoethanolamine levels' on 'Rheumatoid arthritis'

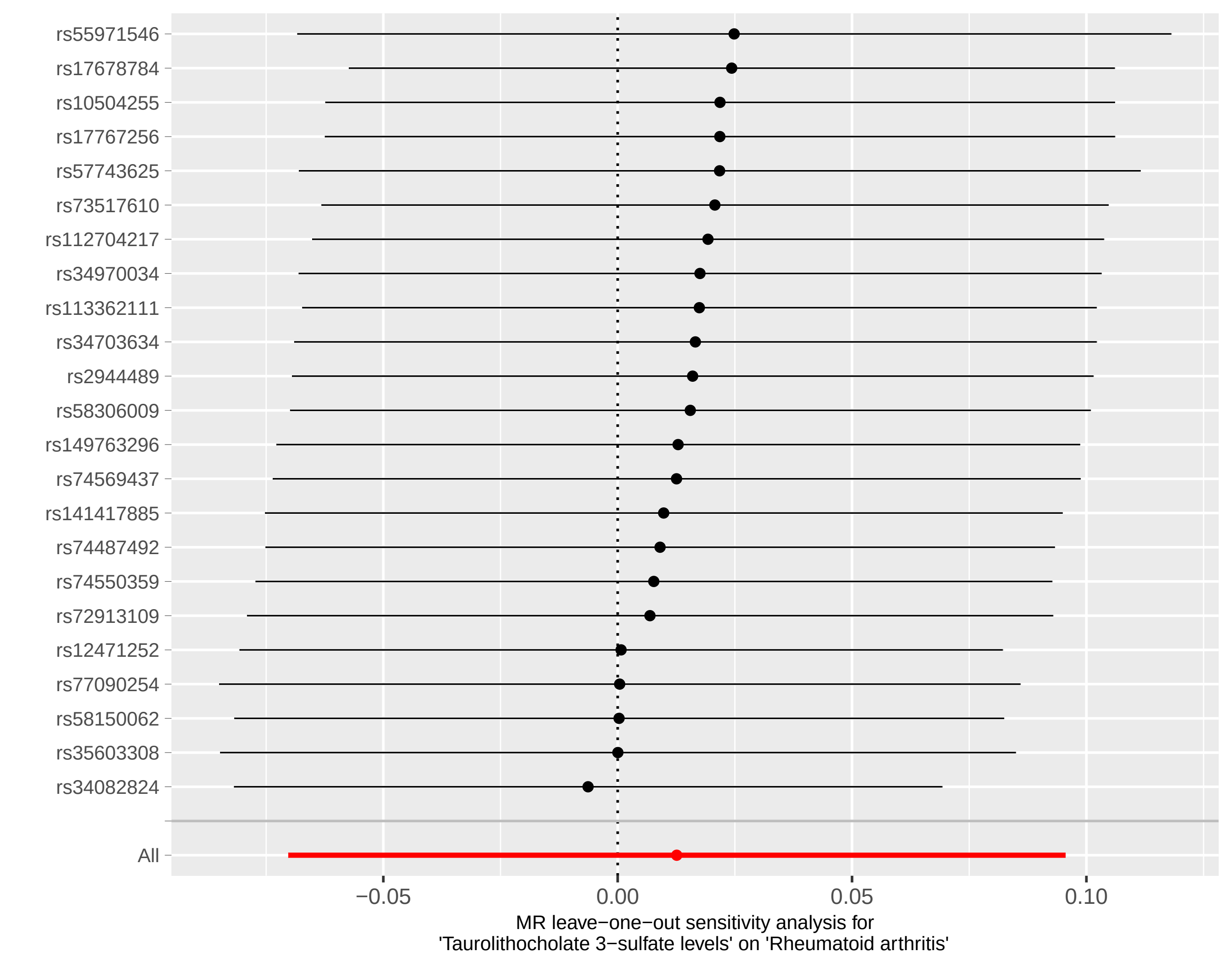
rs55971546
rs17678784
rs10504255
rs17767256
rs57743625
rs73517610
rs112704217
rs34970034
rs113362111
rs34703634
rs2944489
rs58306009
rs149763296
rs74569437
rs141417885
rs74487492
rs74550359
rs72913109
rs12471252
rs77090254
rs58150062
rs35603308
rs34082824
All
−0.05
0.00
0.05
0.10
MR leave−one−out sensitivity analysis for
'Taurolithocholate 3−sulfate levels' on 'Rheumatoid arthritis'

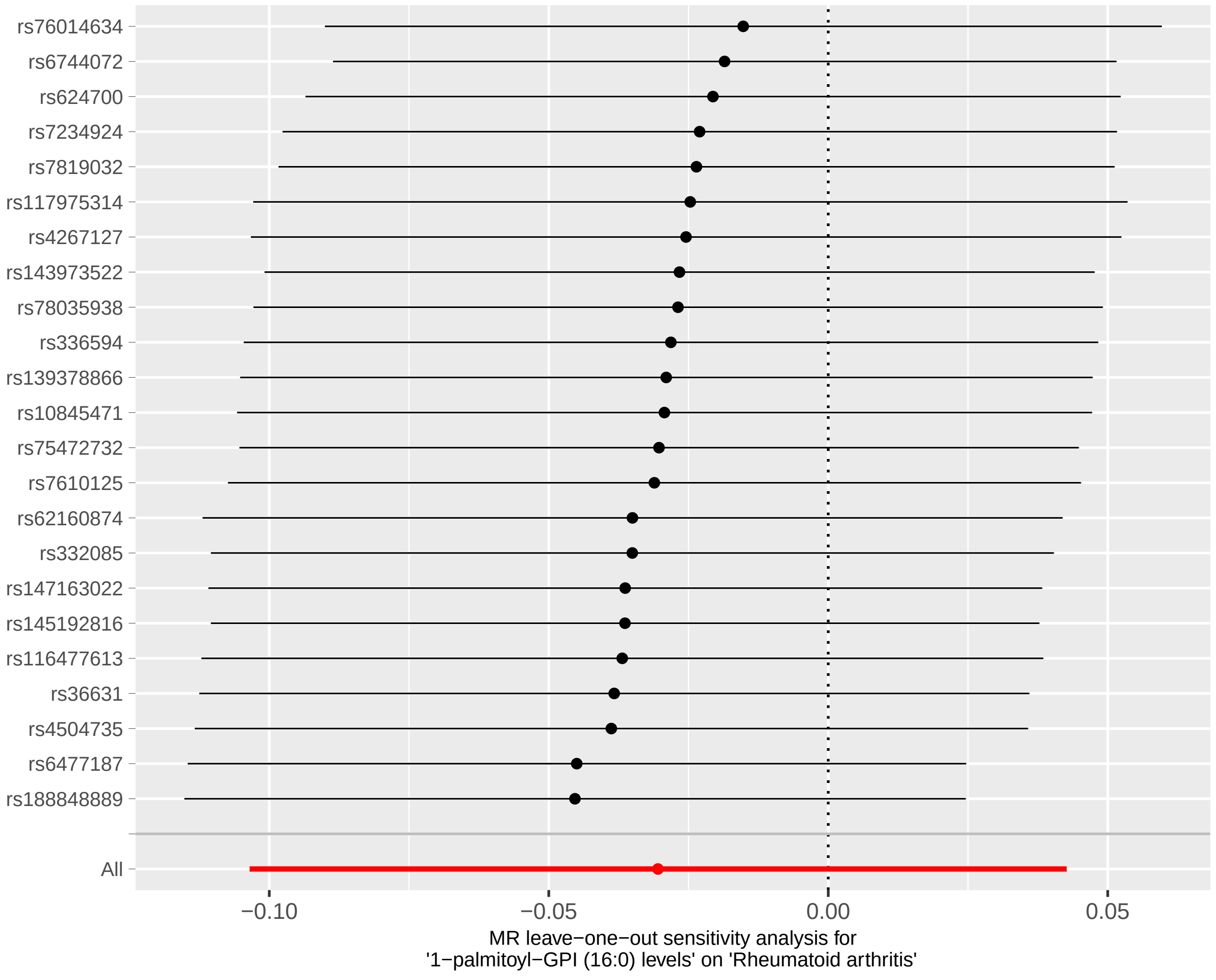

rs76014634
rs6744072
rs624700
rs7234924
rs7819032
rs117975314
rs4267127
rs143973522
rs78035938
rs336594
rs139378866
rs10845471
rs75472732
rs7610125
rs62160874
rs332085
rs147163022
rs145192816
rs116477613
rs36631
rs4504735
rs6477187
rs188848889
All
−0.10
−0.05
0.00
0.05
MR leave−one−out sensitivity analysis for
'1−palmitoyl−GPI (16:0) levels' on 'Rheumatoid arthritis'

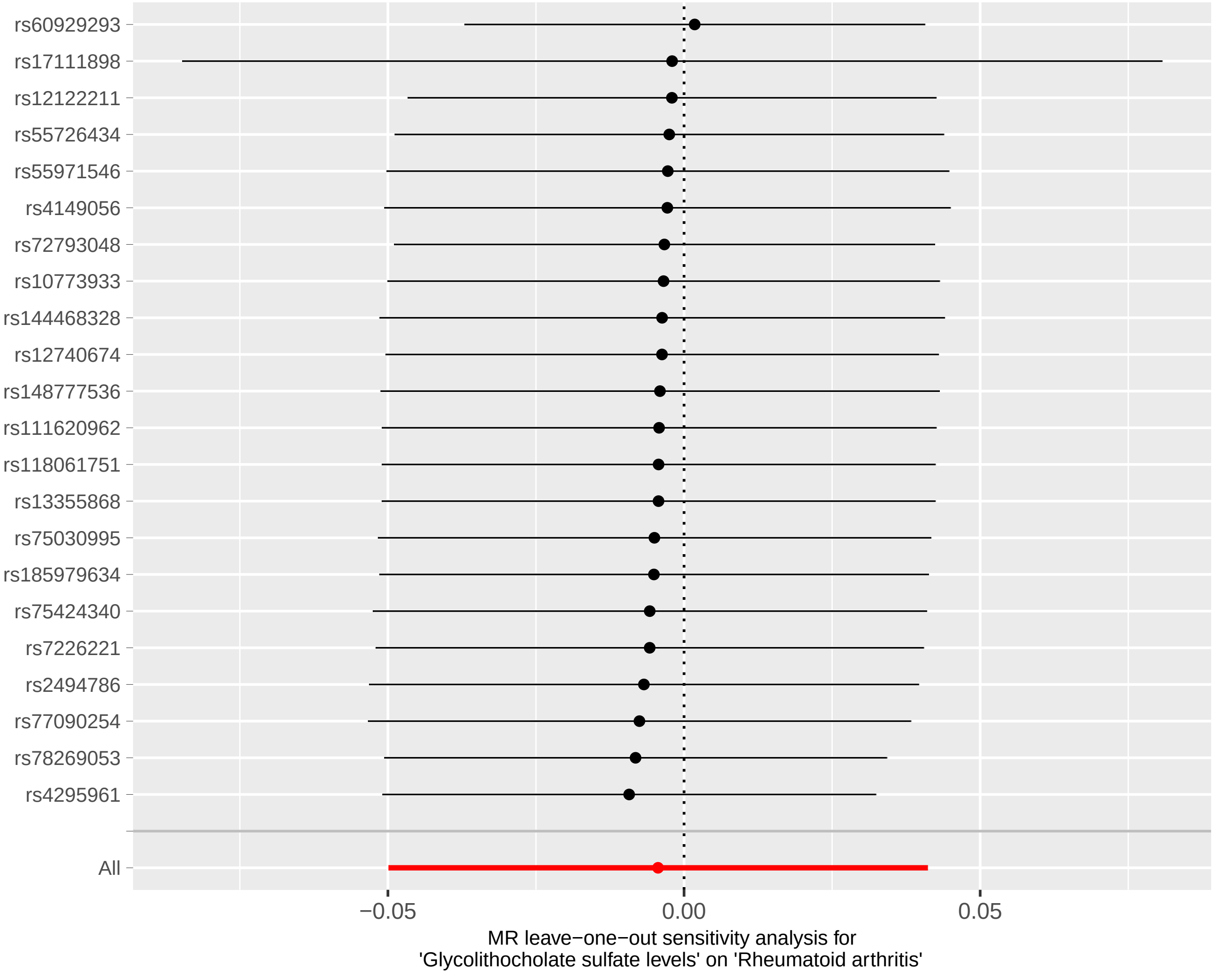
rs60929293
rs17111898
rs12122211
rs55726434
rs55971546
rs4149056
rs72793048
rs10773933
rs144468328
rs12740674
rs148777536
rs111620962
rs118061751
rs13355868
rs75030995
rs185979634
rs75424340
rs7226221
rs2494786
rs77090254
rs78269053
rs4295961
All
−0.05
0.00
0.05
MR leave−one−out sensitivity analysis for
'Glycolithocholate sulfate levels' on 'Rheumatoid arthritis'

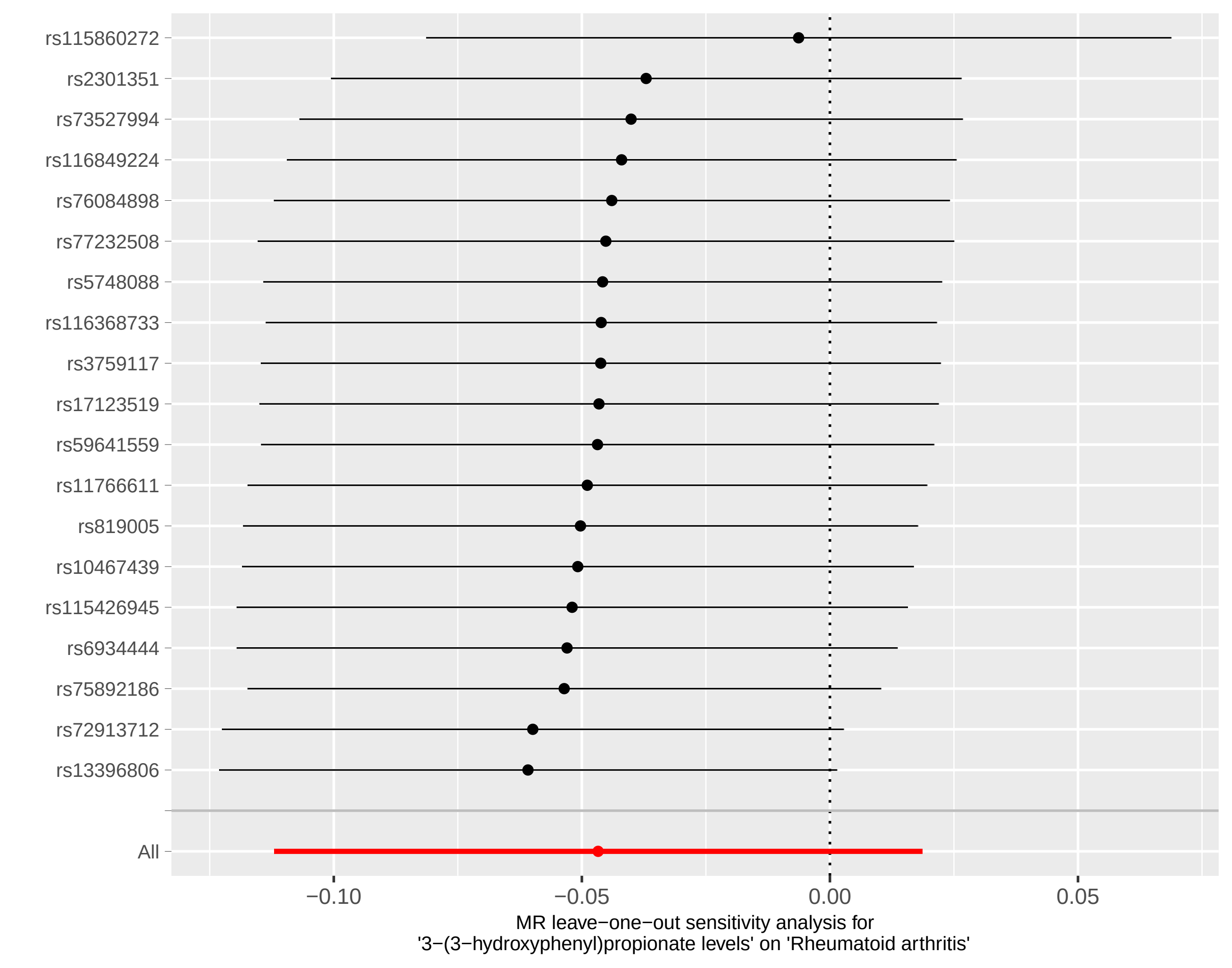
rs115860272
rs2301351
rs73527994
rs116849224
rs76084898
rs77232508
rs5748088
rs116368733
rs3759117
rs17123519
rs59641559
rs11766611
rs819005
rs10467439
rs115426945
rs6934444
rs75892186
rs72913712
rs13396806
All
−0.10
−0.05
0.00
0.05
MR leave−one−out sensitivity analysis for
'3−(3−hydroxyphenyl)propionate levels' on 'Rheumatoid arthritis'

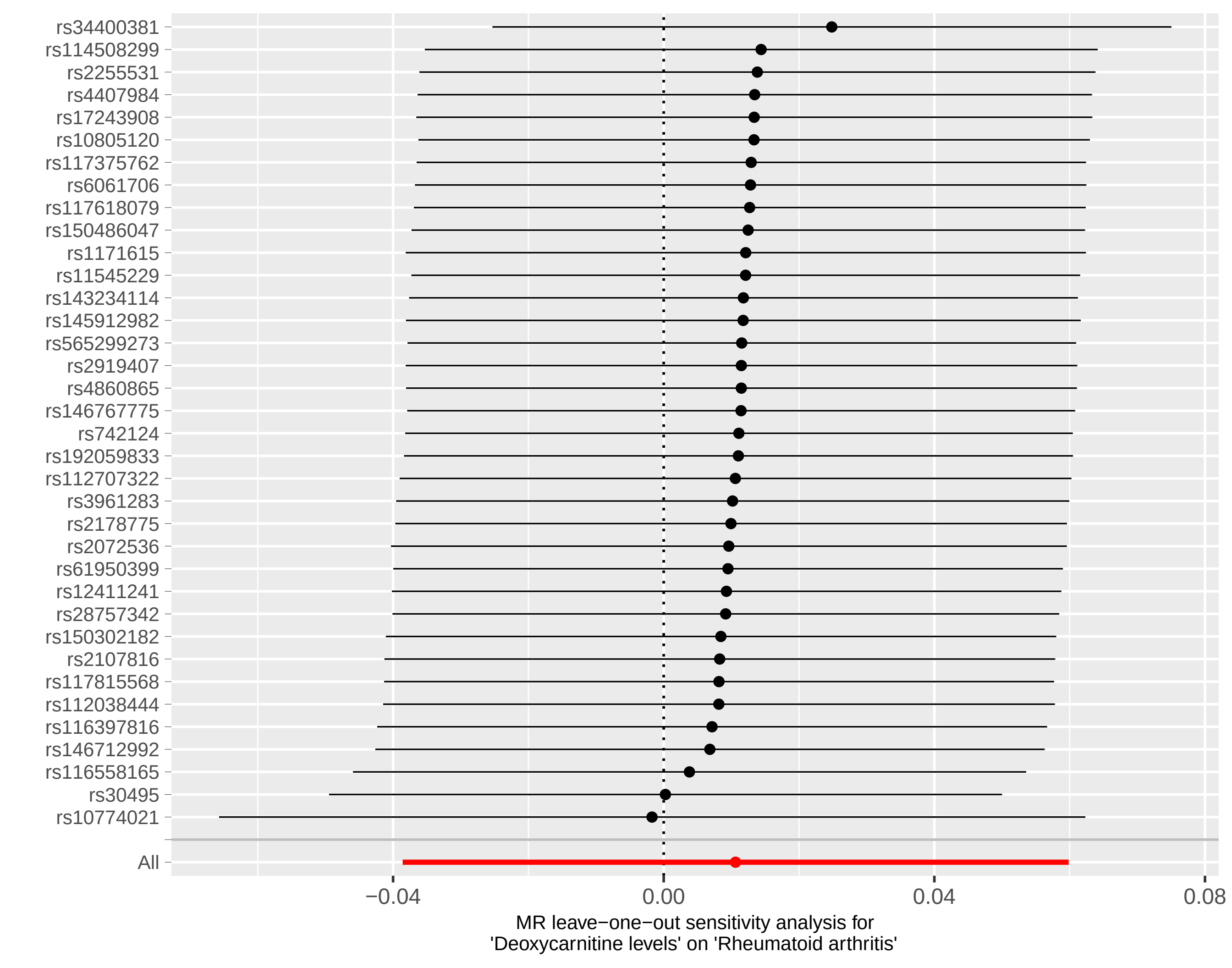

rs34400381
rs114508299
rs2255531
rs4407984
rs17243908
rs10805120
rs117375762
rs6061706
rs117618079
rs150486047
rs1171615
rs11545229
rs143234114
rs145912982
rs565299273
rs2919407
rs4860865
rs146767775
rs742124
rs192059833
rs112707322
rs3961283
rs2178775
rs2072536
rs61950399
rs12411241
rs28757342
rs150302182
rs2107816
rs117815568
rs112038444
rs116397816
rs146712992
rs116558165
rs30495
rs10774021
All
−0.04
0.00
0.04
0.08
MR leave−one−out sensitivity analysis for
'Deoxycarnitine levels' on 'Rheumatoid arthritis'

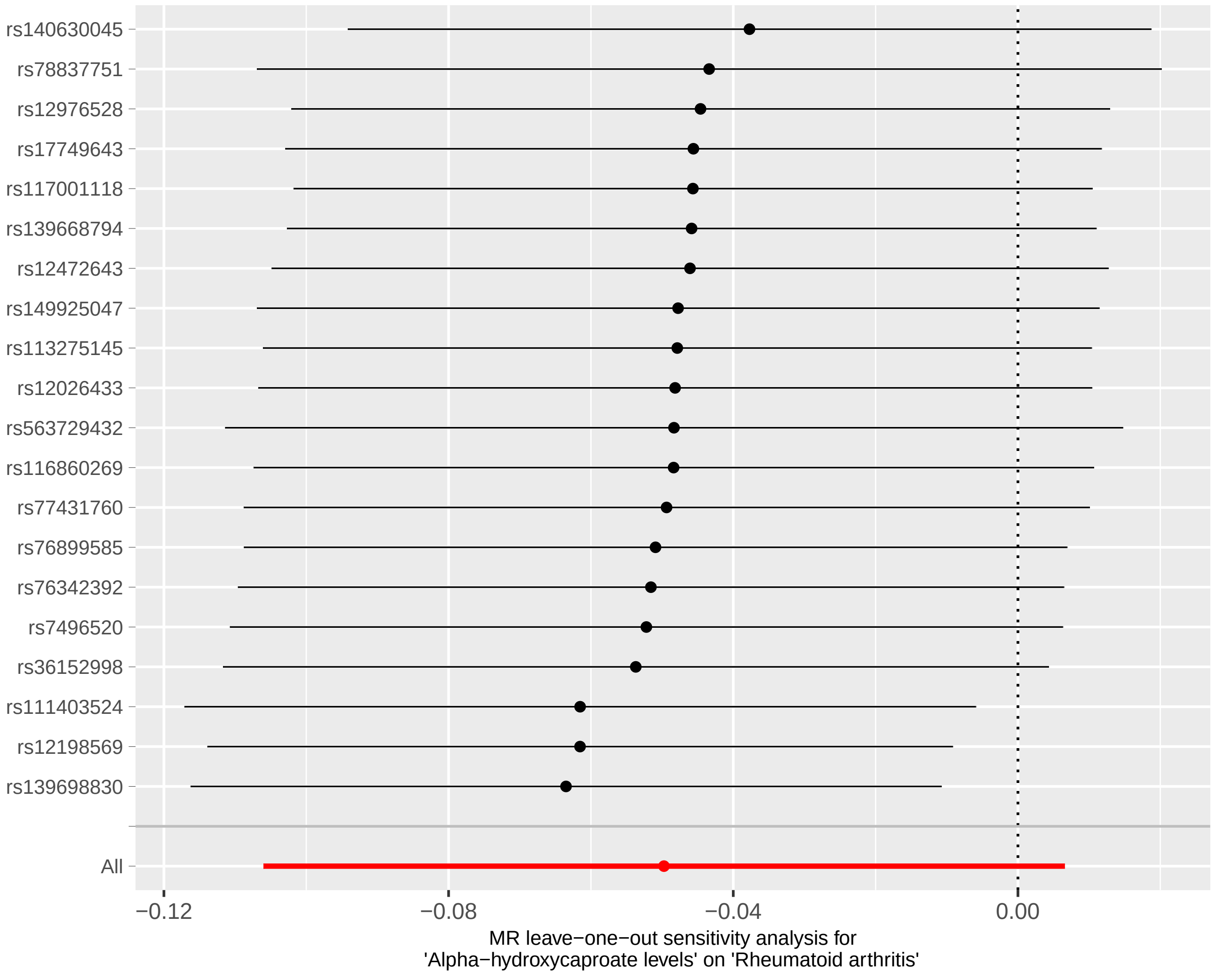

rs140630045
rs78837751
rs12976528
rs17749643
rs117001118
rs139668794
rs12472643
rs149925047
rs113275145
rs12026433
rs563729432
rs116860269
rs77431760
rs76899585
rs76342392
rs7496520
rs36152998
rs111403524
rs12198569
rs139698830
All
−0.12
−0.08
−0.04
0.00
MR leave−one−out sensitivity analysis for
'Alpha−hydroxycaproate levels' on 'Rheumatoid arthritis'

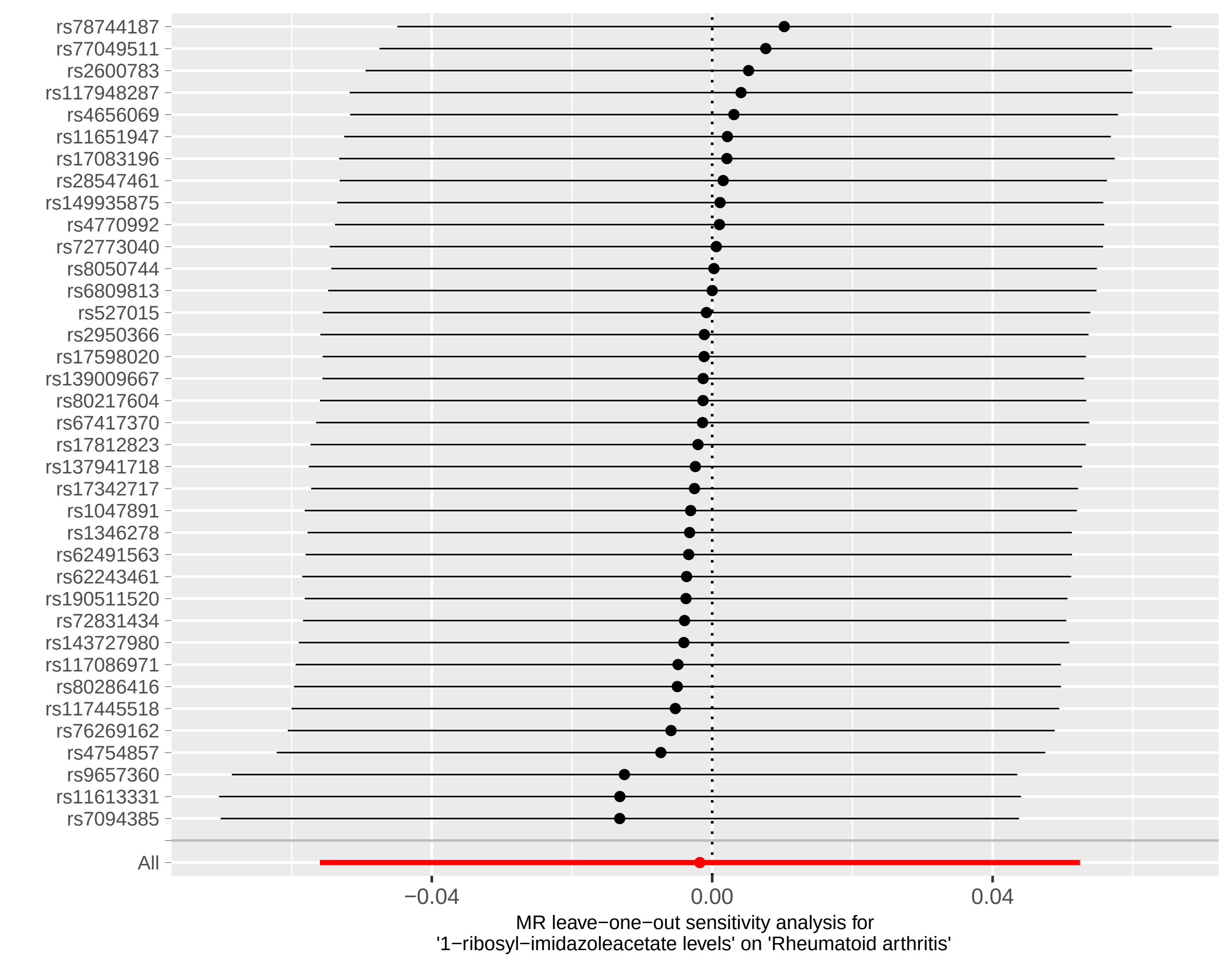
rs78744187
rs77049511
rs2600783
rs117948287
rs4656069
rs11651947
rs17083196
rs28547461
rs149935875
rs4770992
rs72773040
rs8050744
rs6809813
rs527015
rs2950366
rs17598020
rs139009667
rs80217604
rs67417370
rs17812823
rs137941718
rs17342717
rs1047891
rs1346278
rs62491563
rs62243461
rs190511520
rs72831434
rs143727980
rs117086971
rs80286416
rs117445518
rs76269162
rs4754857
rs9657360
rs11613331
rs7094385
All
−0.04
0.00
0.04
MR leave−one−out sensitivity analysis for
'1−ribosyl−imidazoleacetate levels' on 'Rheumatoid arthritis'

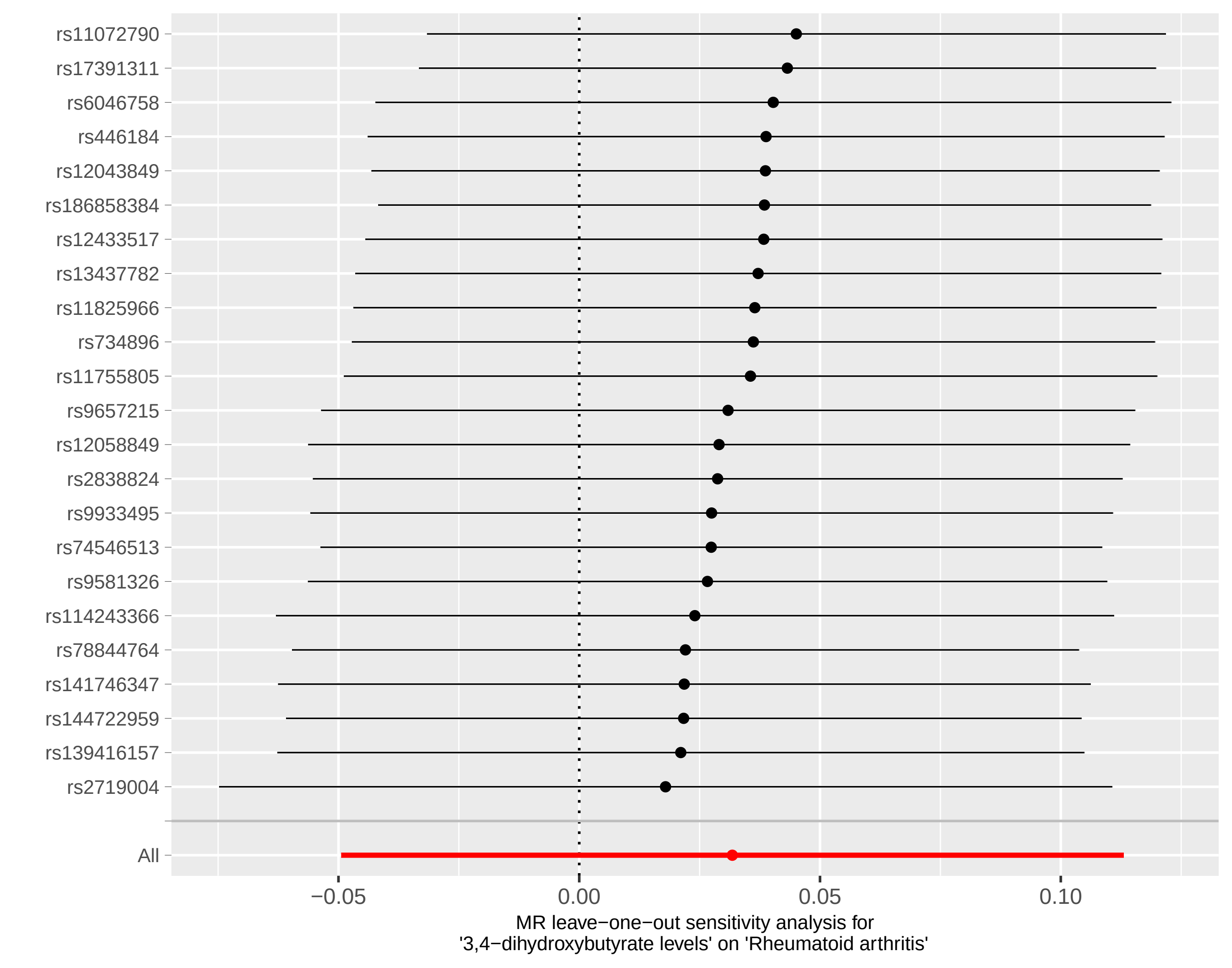
rs11072790
rs17391311
rs6046758
rs446184
rs12043849
rs186858384
rs12433517
rs13437782
rs11825966
rs734896
rs11755805
rs9657215
rs12058849
rs2838824
rs9933495
rs74546513
rs9581326
rs114243366
rs78844764
rs141746347
rs144722959
rs139416157
rs2719004
All
−0.05
0.00
0.05
0.10
MR leave−one−out sensitivity analysis for
'3,4−dihydroxybutyrate levels' on 'Rheumatoid arthritis'

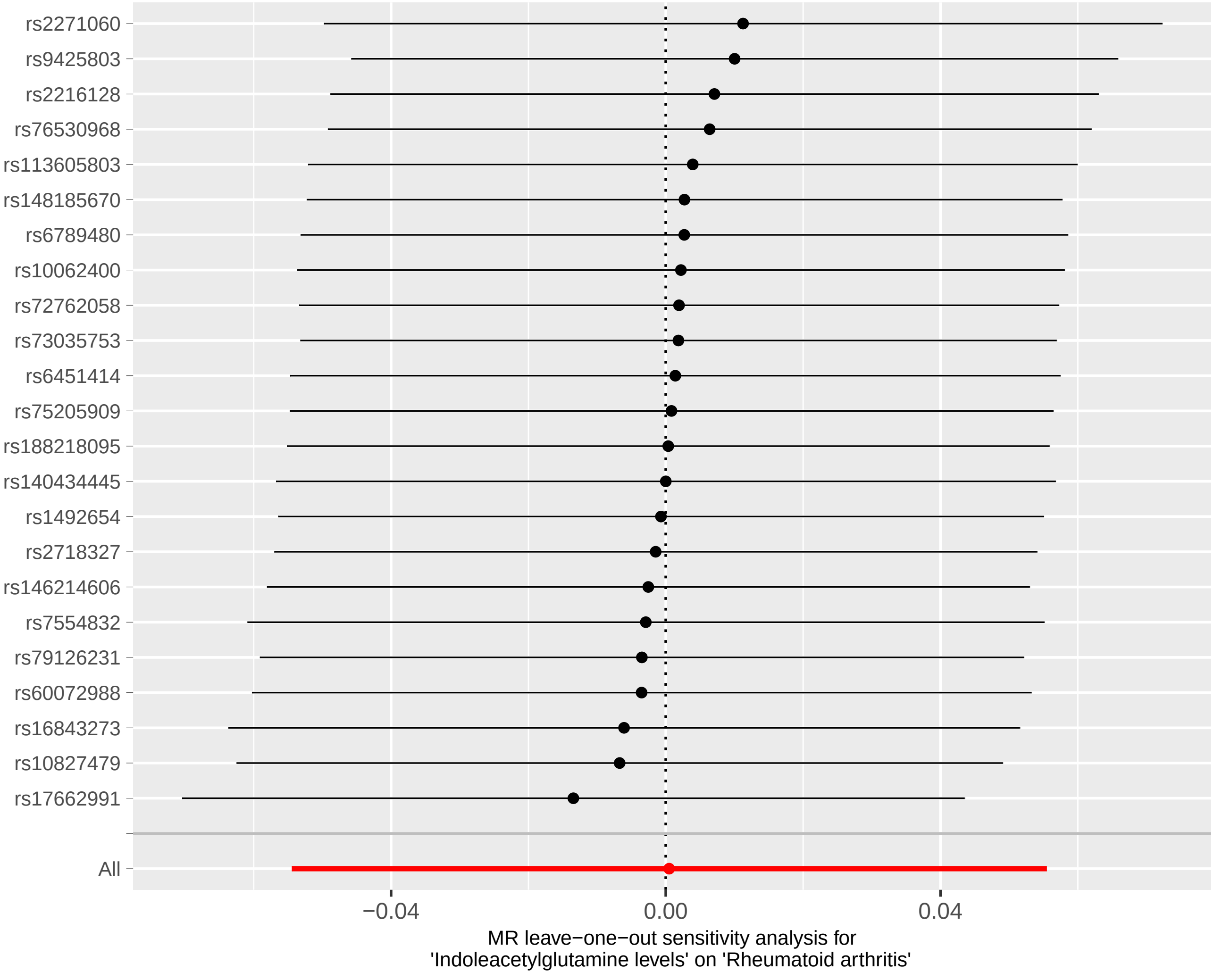
rs2271060
rs9425803
rs2216128
rs76530968
rs113605803
rs148185670
rs6789480
rs10062400
rs72762058
rs73035753
rs6451414
rs75205909
rs188218095
rs140434445
rs1492654
rs2718327
rs146214606
rs7554832
rs79126231
rs60072988
rs16843273
rs10827479
rs17662991
All
−0.04
0.00
0.04
MR leave−one−out sensitivity analysis for
'Indoleacetylglutamine levels' on 'Rheumatoid arthritis'

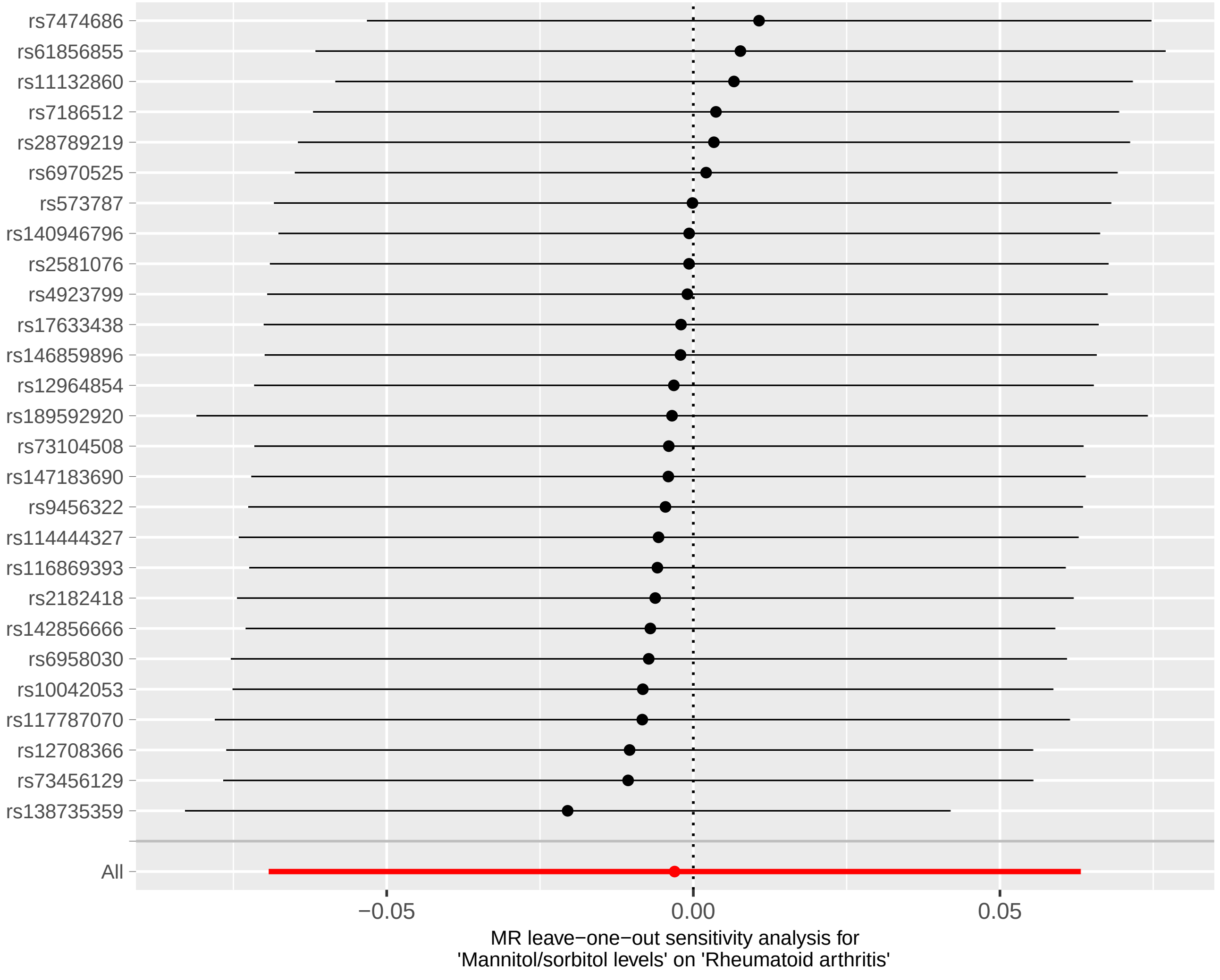

rs7474686
rs61856855
rs11132860
rs7186512
rs28789219
rs6970525
rs573787
rs140946796
rs2581076
rs4923799
rs17633438
rs146859896
rs12964854
rs189592920
rs73104508
rs147183690
rs9456322
rs114444327
rs116869393
rs2182418
rs142856666
rs6958030
rs10042053
rs117787070
rs12708366
rs73456129
rs138735359
All
−0.05
0.00
0.05
MR leave−one−out sensitivity analysis for
'Mannitol/sorbitol levels' on 'Rheumatoid arthritis'

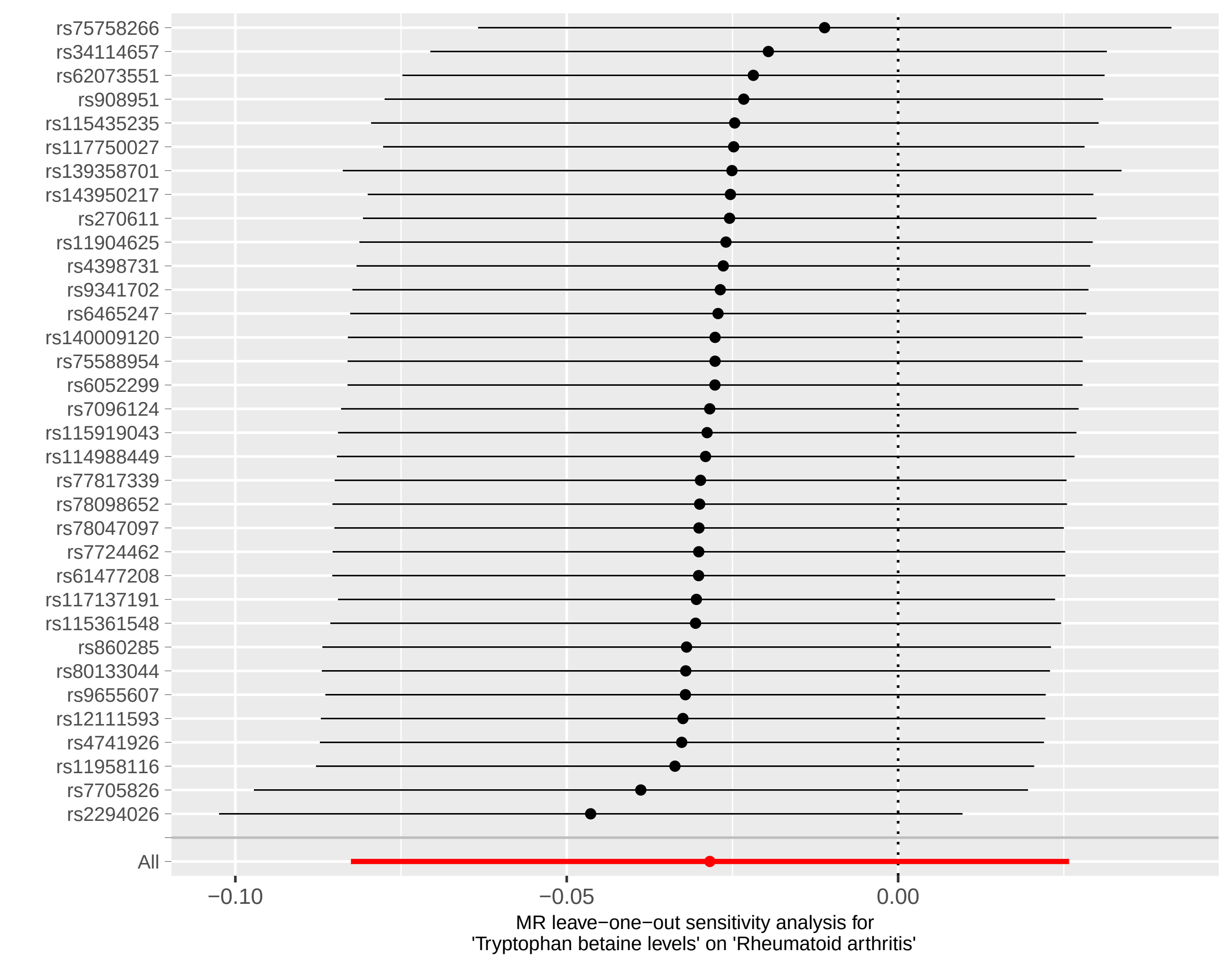
rs75758266
rs34114657
rs62073551
rs908951
rs115435235
rs117750027
rs139358701
rs143950217
rs270611
rs11904625
rs4398731
rs9341702
rs6465247
rs140009120
rs75588954
rs6052299
rs7096124
rs115919043
rs114988449
rs77817339
rs78098652
rs78047097
rs7724462
rs61477208
rs117137191
rs115361548
rs860285
rs80133044
rs9655607
rs12111593
rs4741926
rs11958116
rs7705826
rs2294026
All
−0.10
−0.05
0.00
MR leave−one−out sensitivity analysis for
'Tryptophan betaine levels' on 'Rheumatoid arthritis'

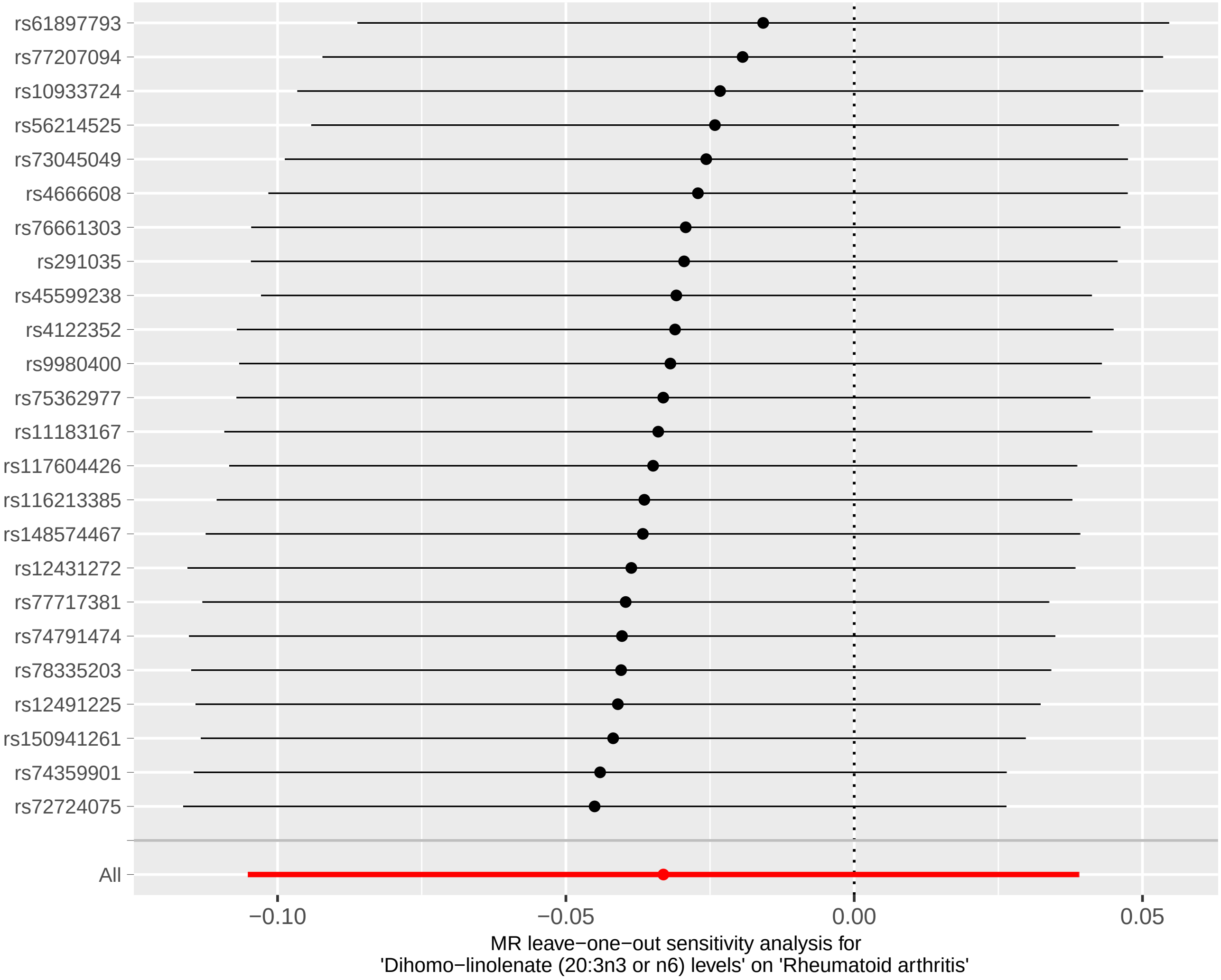
rs61897793
rs77207094
rs10933724
rs56214525
rs73045049
rs4666608
rs76661303
rs291035
rs45599238
rs4122352
rs9980400
rs75362977
rs11183167
rs117604426
rs116213385
rs148574467
rs12431272
rs77717381
rs74791474
rs78335203
rs12491225
rs150941261
rs74359901
rs72724075
All
−0.10
−0.05
0.00
0.05
MR leave−one−out sensitivity analysis for
'Dihomo−linolenate (20:3n3 or n6) levels' on 'Rheumatoid arthritis'

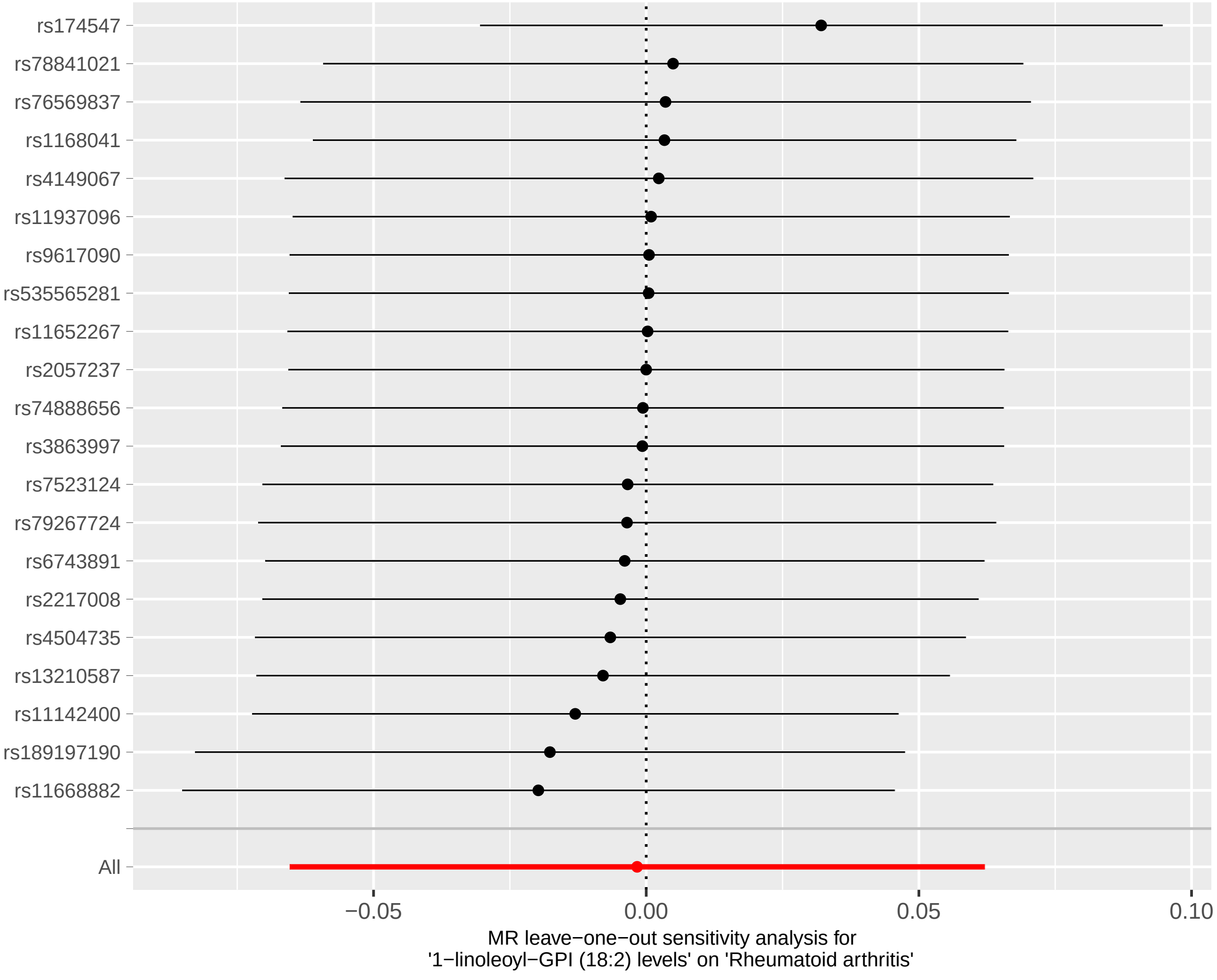

rs174547
rs78841021
rs76569837
rs1168041
rs4149067
rs11937096
rs9617090
rs535565281
rs11652267
rs2057237
rs74888656
rs3863997
rs7523124
rs79267724
rs6743891
rs2217008
rs4504735
rs13210587
rs11142400
rs189197190
rs11668882
All
−0.05
0.00
0.05
0.10
MR leave−one−out sensitivity analysis for
'1−linoleoyl−GPI (18:2) levels' on 'Rheumatoid arthritis'

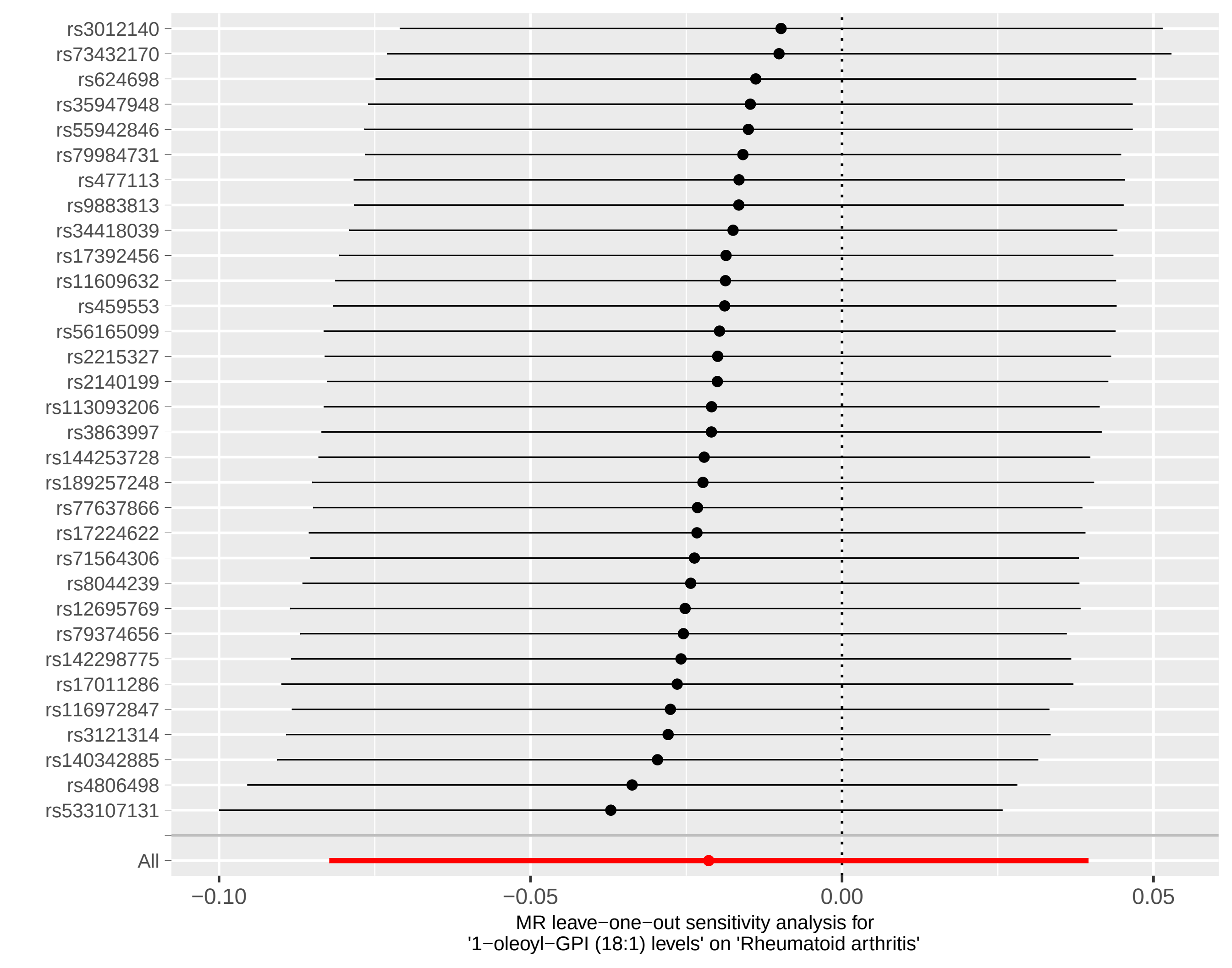
rs3012140
rs73432170
rs624698
rs35947948
rs55942846
rs79984731
rs477113
rs9883813
rs34418039
rs17392456
rs11609632
rs459553
rs56165099
rs2215327
rs2140199
rs113093206
rs3863997
rs144253728
rs189257248
rs77637866
rs17224622
rs71564306
rs8044239
rs12695769
rs79374656
rs142298775
rs17011286
rs116972847
rs3121314
rs140342885
rs4806498
rs533107131
All
−0.10
−0.05
0.00
0.05
MR leave−one−out sensitivity analysis for
'1−oleoyl−GPI (18:1) levels' on 'Rheumatoid arthritis'

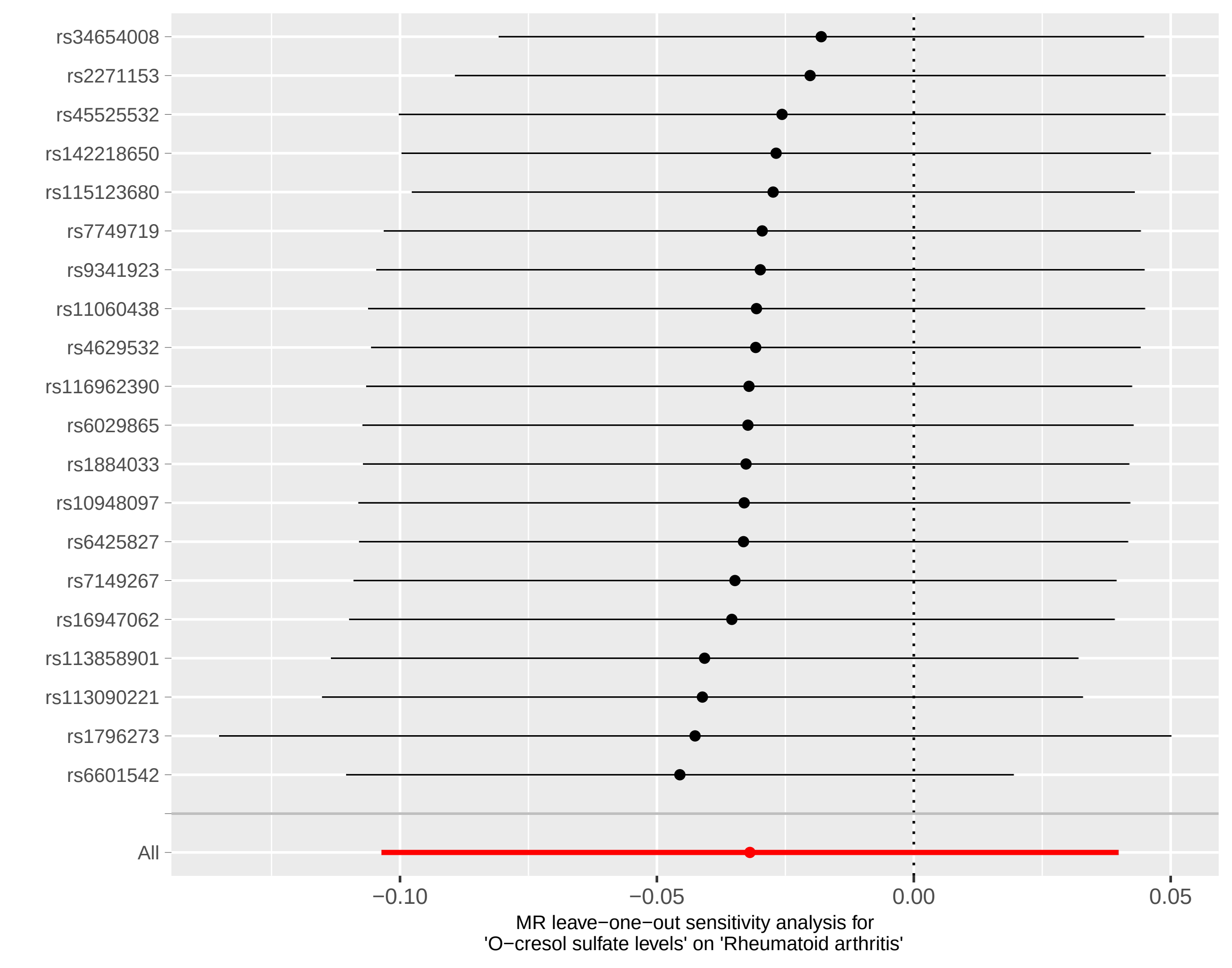
rs34654008
rs2271153
rs45525532
rs142218650
rs115123680
rs7749719
rs9341923
rs11060438
rs4629532
rs116962390
rs6029865
rs1884033
rs10948097
rs6425827
rs7149267
rs16947062
rs113858901
rs113090221
rs1796273
rs6601542
All
−0.10
−0.05
0.00
0.05
MR leave−one−out sensitivity analysis for
'O−cresol sulfate levels' on 'Rheumatoid arthritis'

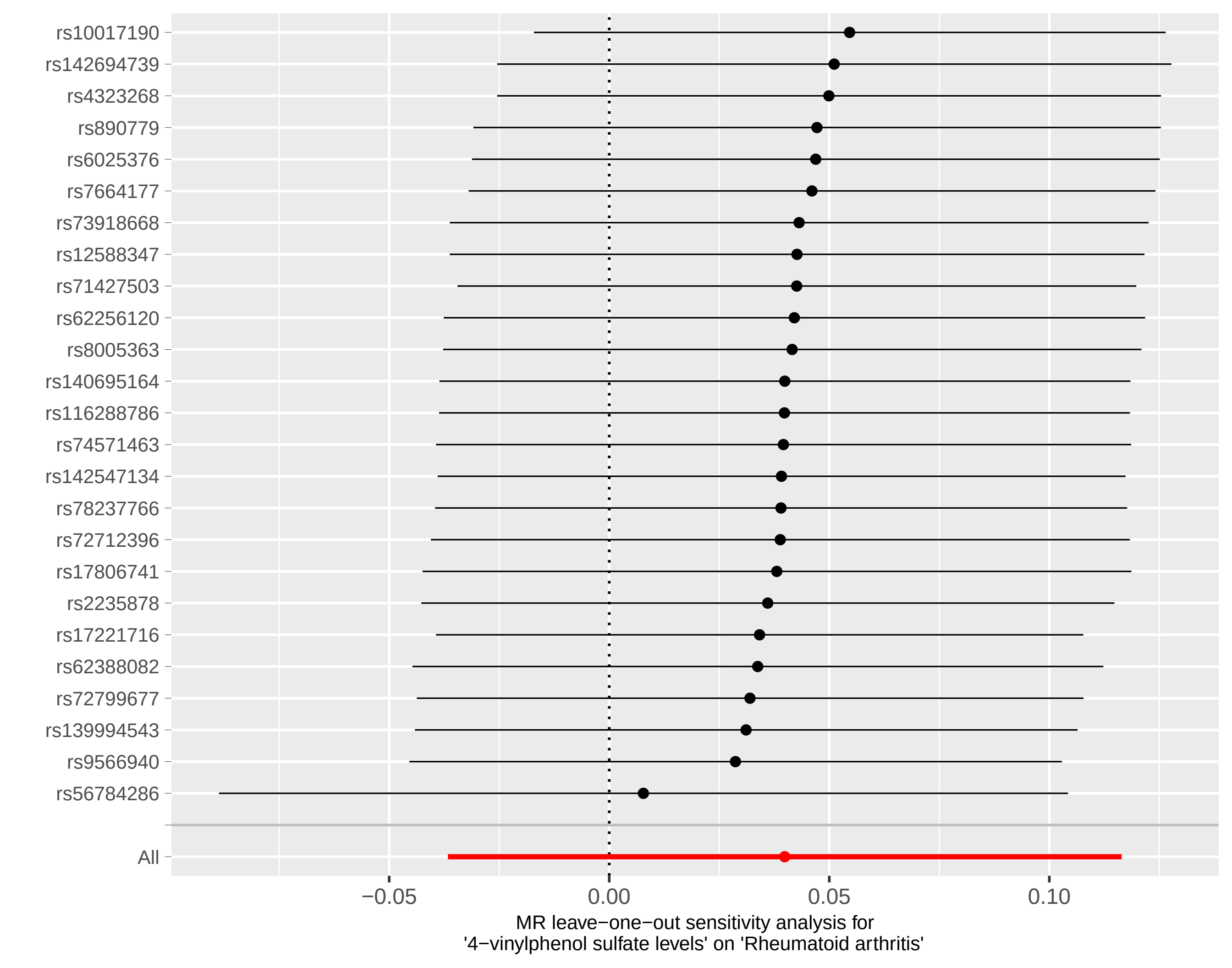
rs10017190
rs142694739
rs4323268
rs890779
rs6025376
rs7664177
rs73918668
rs12588347
rs71427503
rs62256120
rs8005363
rs140695164
rs116288786
rs74571463
rs142547134
rs78237766
rs72712396
rs17806741
rs2235878
rs17221716
rs62388082
rs72799677
rs139994543
rs9566940
rs56784286
All
−0.05
0.00
0.05
0.10
MR leave−one−out sensitivity analysis for
'4−vinylphenol sulfate levels' on 'Rheumatoid arthritis'

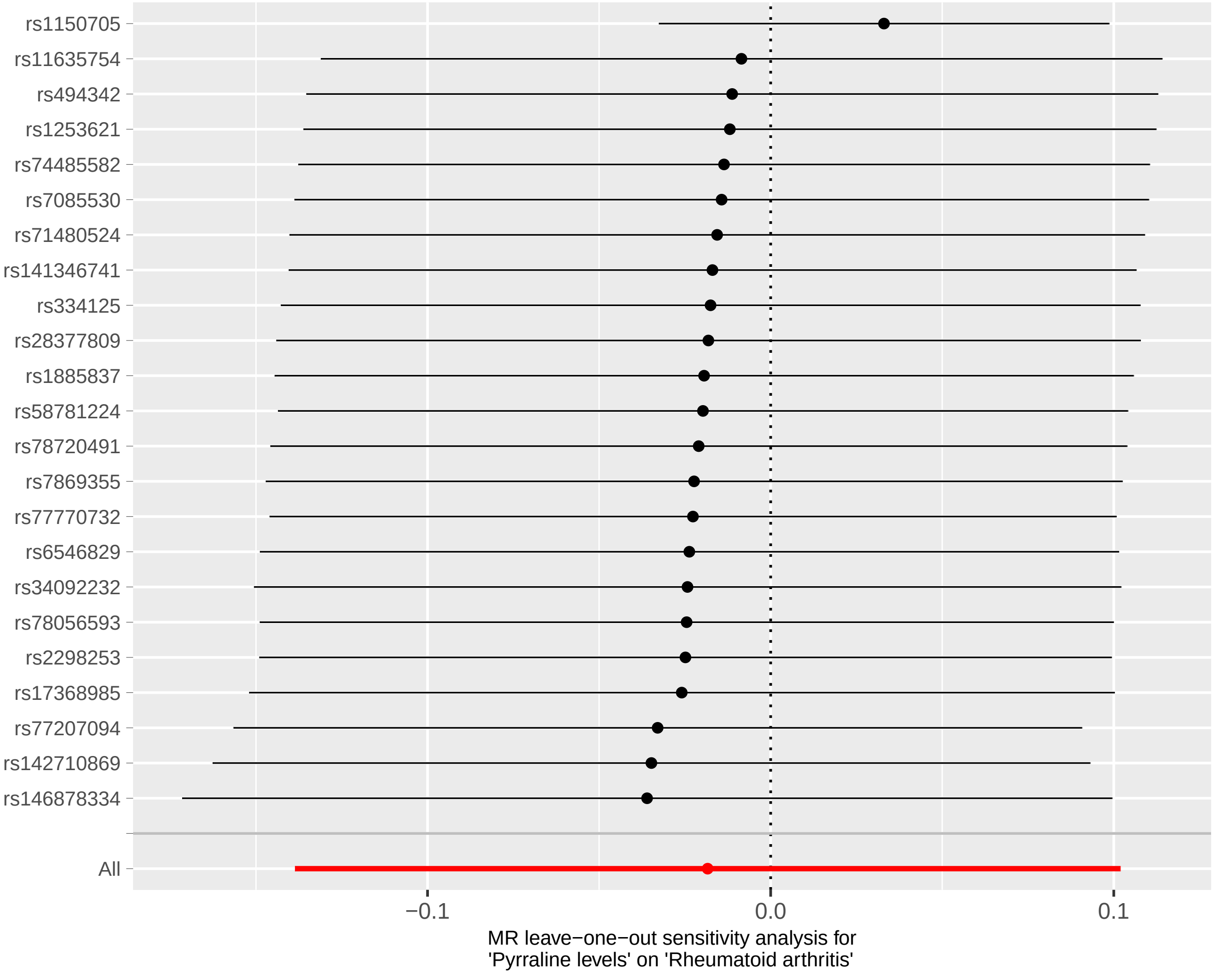
rs1150705
rs11635754
rs494342
rs1253621
rs74485582
rs7085530
rs71480524
rs141346741
rs334125
rs28377809
rs1885837
rs58781224
rs78720491
rs7869355
rs77770732
rs6546829
rs34092232
rs78056593
rs2298253
rs17368985
rs77207094
rs142710869
rs146878334
All
−0.1
0.0
0.1
MR leave−one−out sensitivity analysis for
'Pyrraline levels' on 'Rheumatoid arthritis'

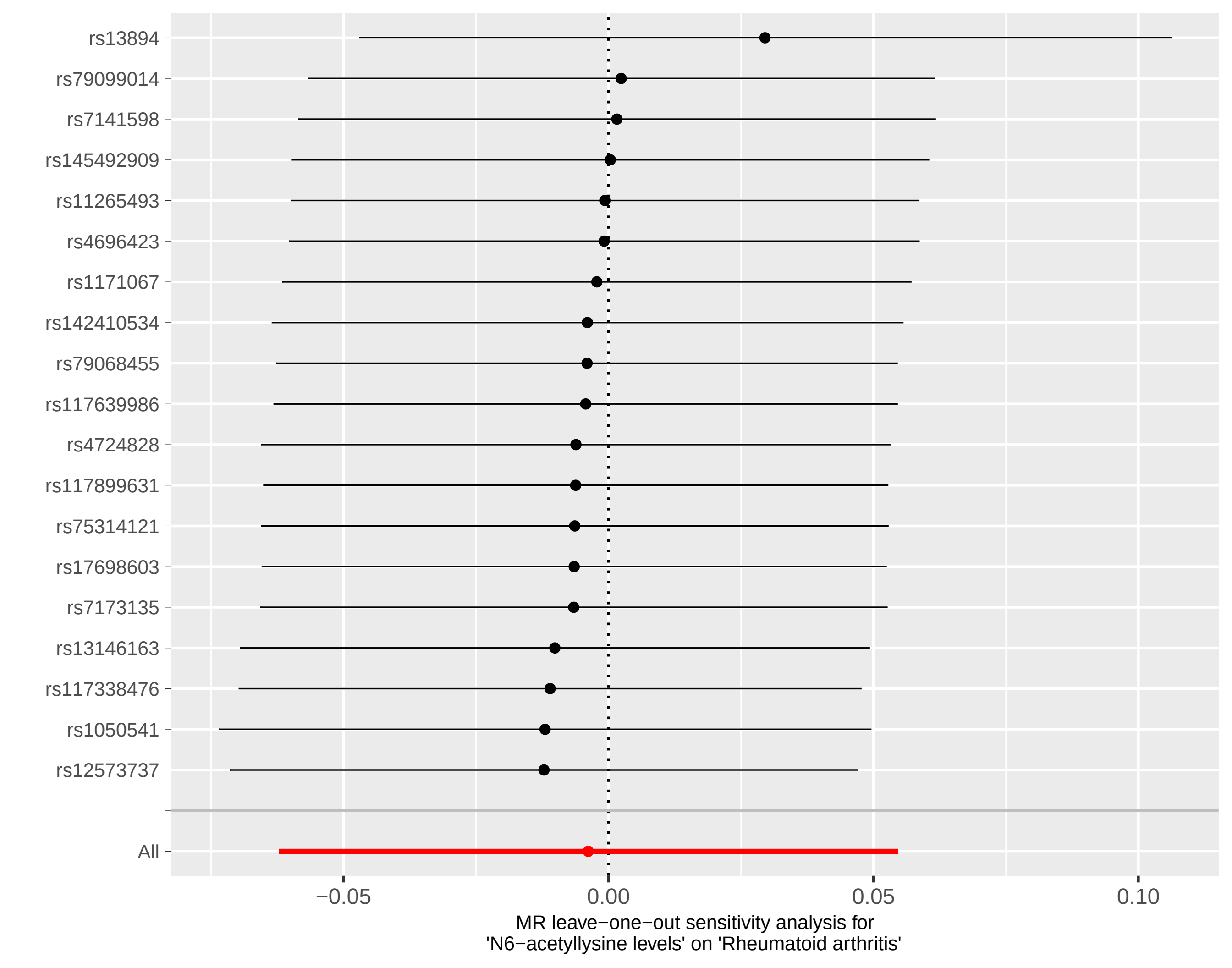
rs13894
rs79099014
rs7141598
rs145492909
rs11265493
rs4696423
rs1171067
rs142410534
rs79068455
rs117639986
rs4724828
rs117899631
rs75314121
rs17698603
rs7173135
rs13146163
rs117338476
rs1050541
rs12573737
All
−0.05
0.00
0.05
0.10
MR leave−one−out sensitivity analysis for
'N6−acetyllysine levels' on 'Rheumatoid arthritis'

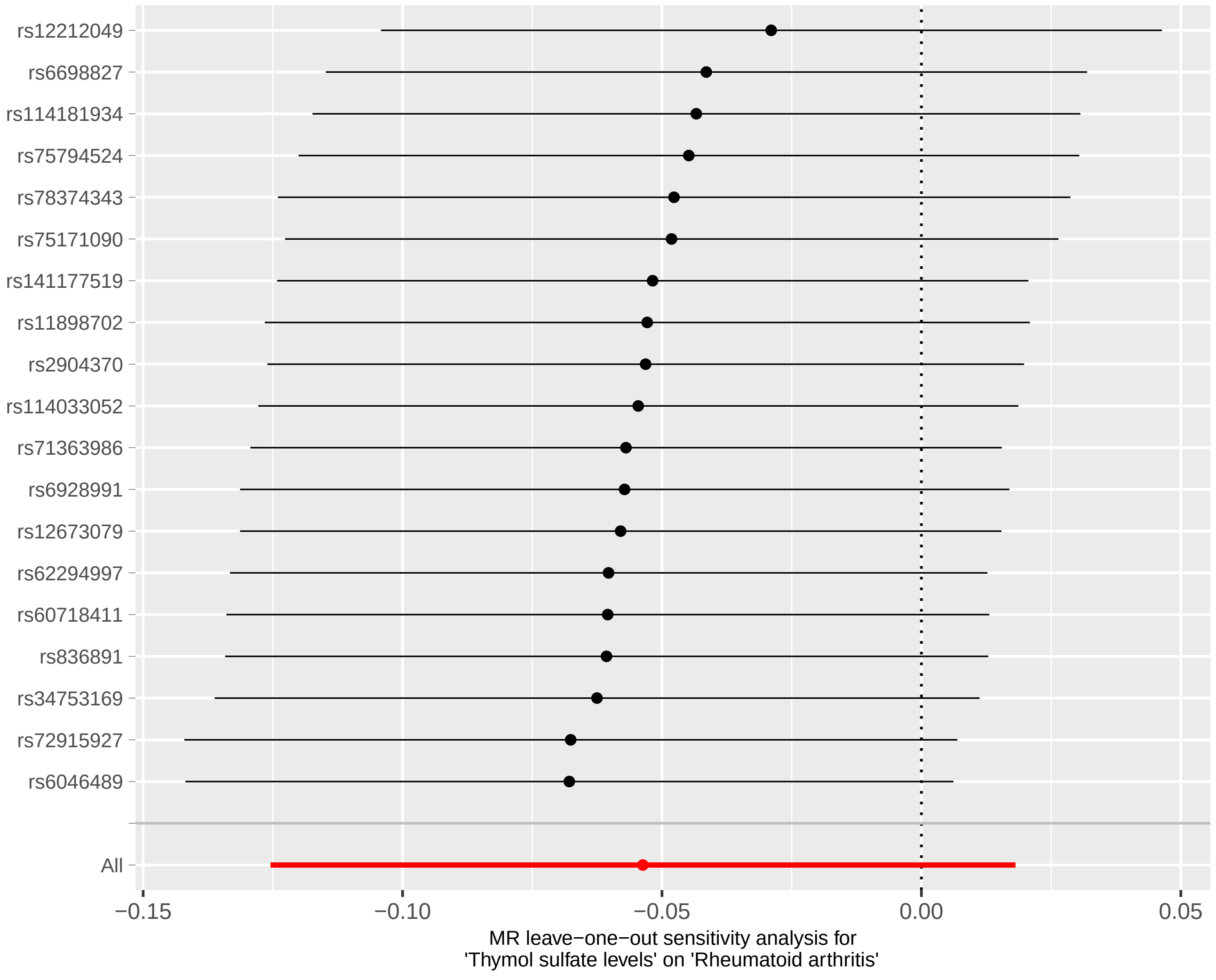
rs12212049
rs6698827
rs114181934
rs75794524
rs78374343
rs75171090
rs141177519
rs11898702
rs2904370
rs114033052
rs71363986
rs6928991
rs12673079
rs62294997
rs60718411
rs836891
rs34753169
rs72915927
rs6046489
All
−0.15
−0.10
−0.05
0.00
0.05
MR leave−one−out sensitivity analysis for
'Thymol sulfate levels' on 'Rheumatoid arthritis'

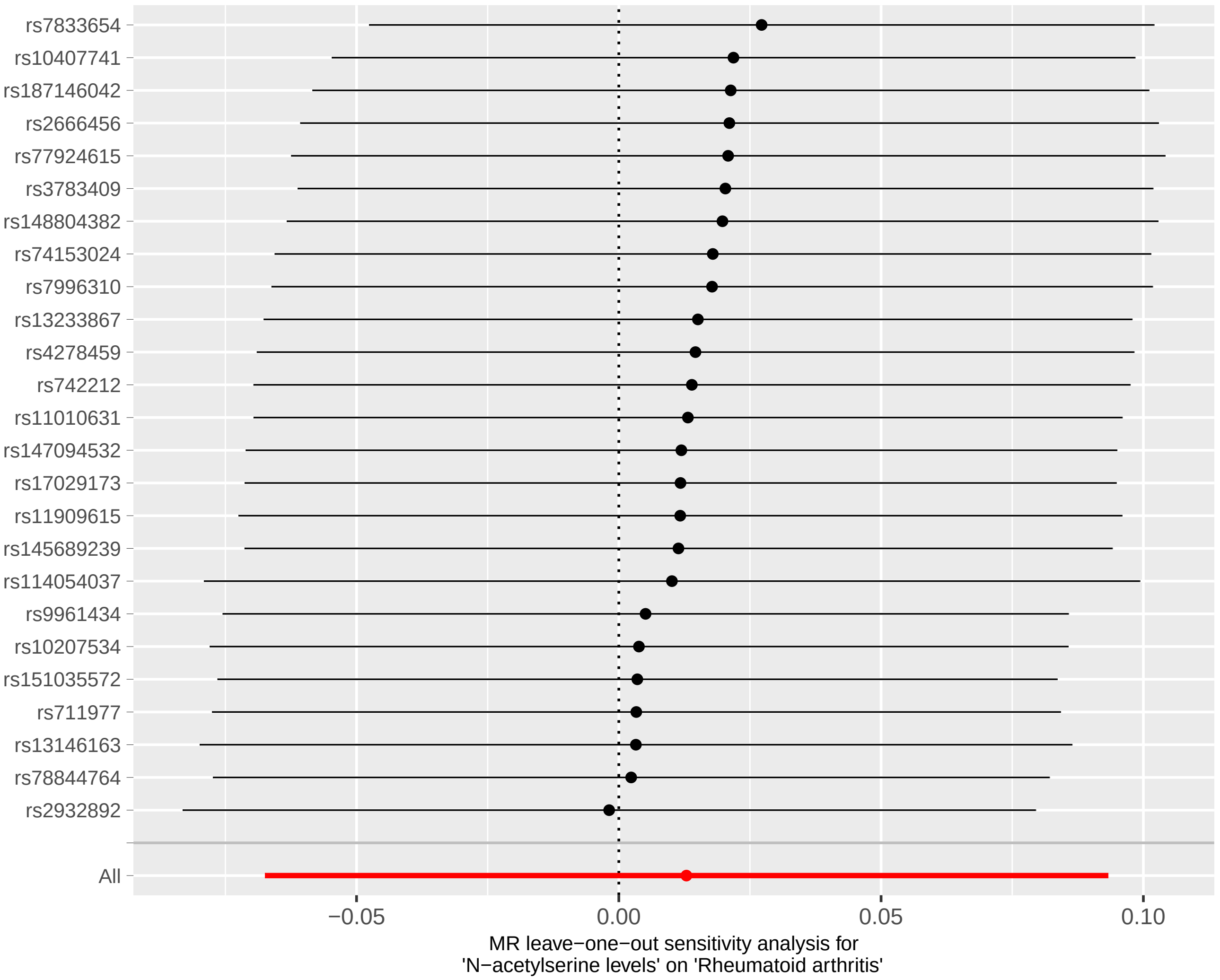
rs7833654
rs10407741
rs187146042
rs2666456
rs77924615
rs3783409
rs148804382
rs74153024
rs7996310
rs13233867
rs4278459
rs742212
rs11010631
rs147094532
rs17029173
rs11909615
rs145689239
rs114054037
rs9961434
rs10207534
rs151035572
rs711977
rs13146163
rs78844764
rs2932892
All
−0.05
0.00
0.05
0.10
MR leave−one−out sensitivity analysis for
'N−acetylserine levels' on 'Rheumatoid arthritis'

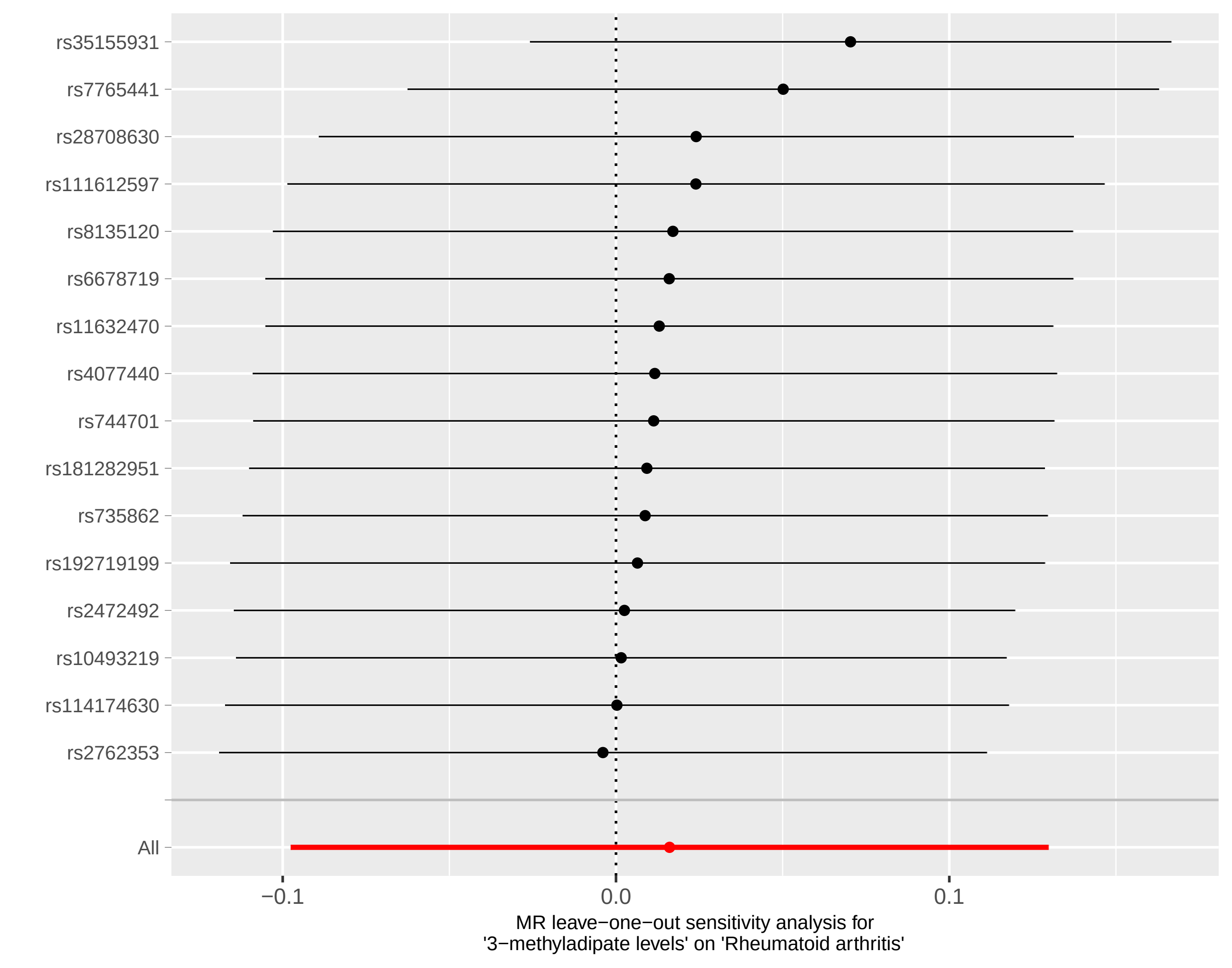

rs35155931
rs7765441
rs28708630
rs111612597
rs8135120
rs6678719
rs11632470
rs4077440
rs744701
rs181282951
rs735862
rs192719199
rs2472492
rs10493219
rs114174630
rs2762353
All
−0.1
0.0
0.1
MR leave−one−out sensitivity analysis for
'3−methyladipate levels' on 'Rheumatoid arthritis'

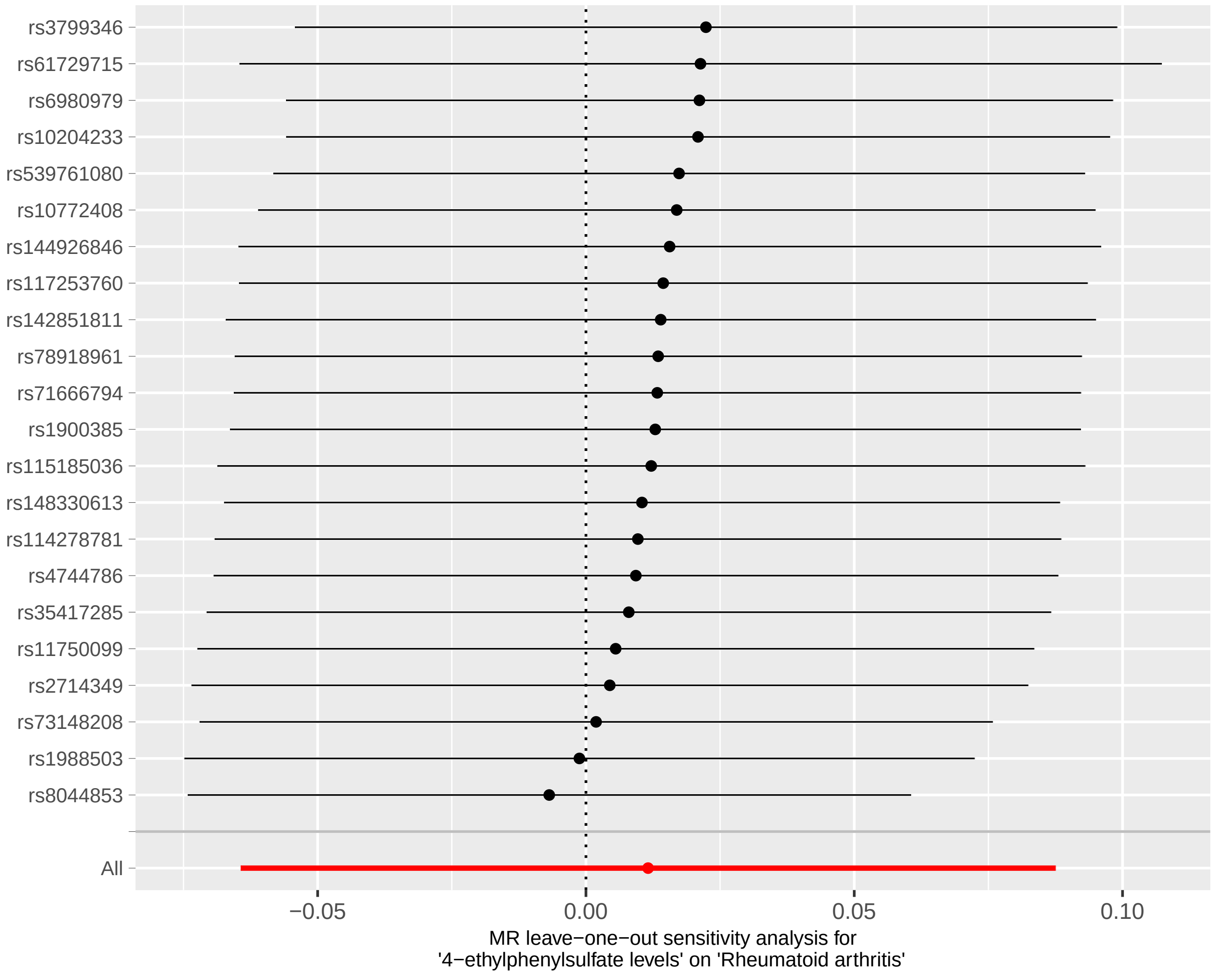

rs3799346
rs61729715
rs6980979
rs10204233
rs539761080
rs10772408
rs144926846
rs117253760
rs142851811
rs78918961
rs71666794
rs1900385
rs115185036
rs148330613
rs114278781
rs4744786
rs35417285
rs11750099
rs2714349
rs73148208
rs1988503
rs8044853
All
−0.05
0.00
0.05
0.10
MR leave−one−out sensitivity analysis for
'4−ethylphenylsulfate levels' on 'Rheumatoid arthritis'

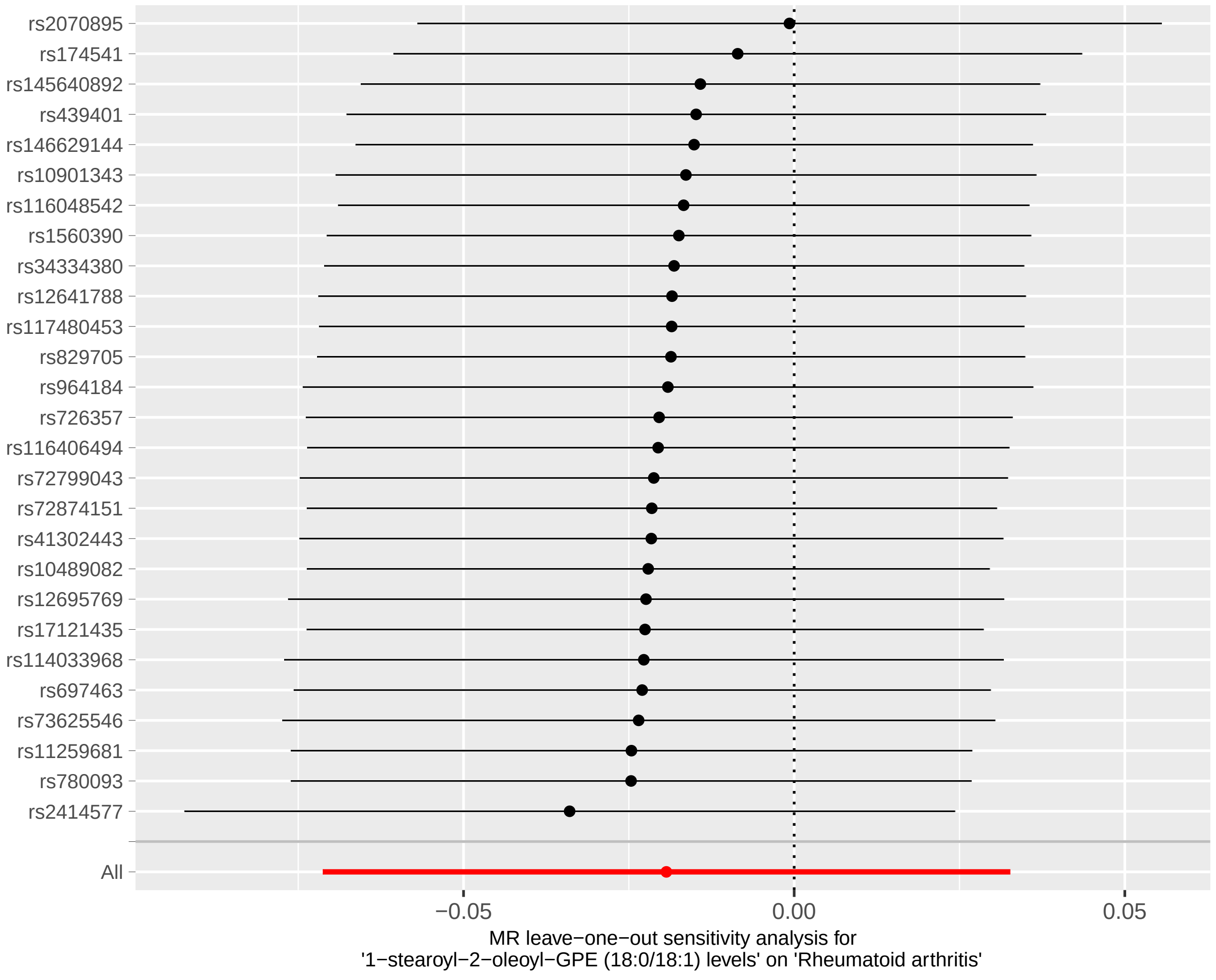

rs2070895
rs174541
rs145640892
rs439401
rs146629144
rs10901343
rs116048542
rs1560390
rs34334380
rs12641788
rs117480453
rs829705
rs964184
rs726357
rs116406494
rs72799043
rs72874151
rs41302443
rs10489082
rs12695769
rs17121435
rs114033968
rs697463
rs73625546
rs11259681
rs780093
rs2414577
All
−0.05
0.00
0.05
MR leave−one−out sensitivity analysis for
'1−stearoyl−2−oleoyl−GPE (18:0/18:1) levels' on 'Rheumatoid arthritis'

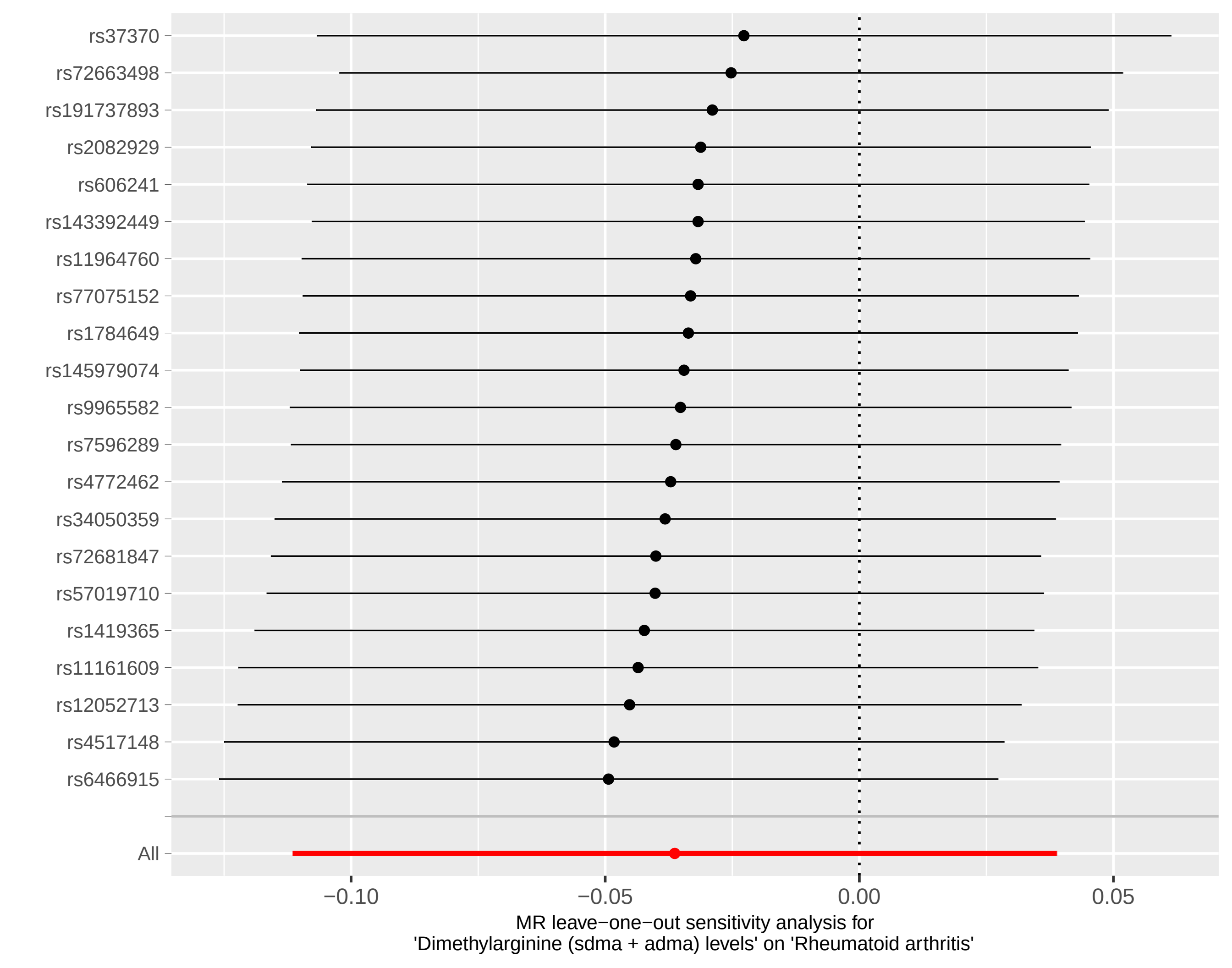
rs37370
rs72663498
rs191737893
rs2082929
rs606241
rs143392449
rs11964760
rs77075152
rs1784649
rs145979074
rs9965582
rs7596289
rs4772462
rs34050359
rs72681847
rs57019710
rs1419365
rs11161609
rs12052713
rs4517148
rs6466915
All
−0.10
−0.05
0.00
0.05
MR leave−one−out sensitivity analysis for
'Dimethylarginine (sdma + adma) levels' on 'Rheumatoid arthritis'

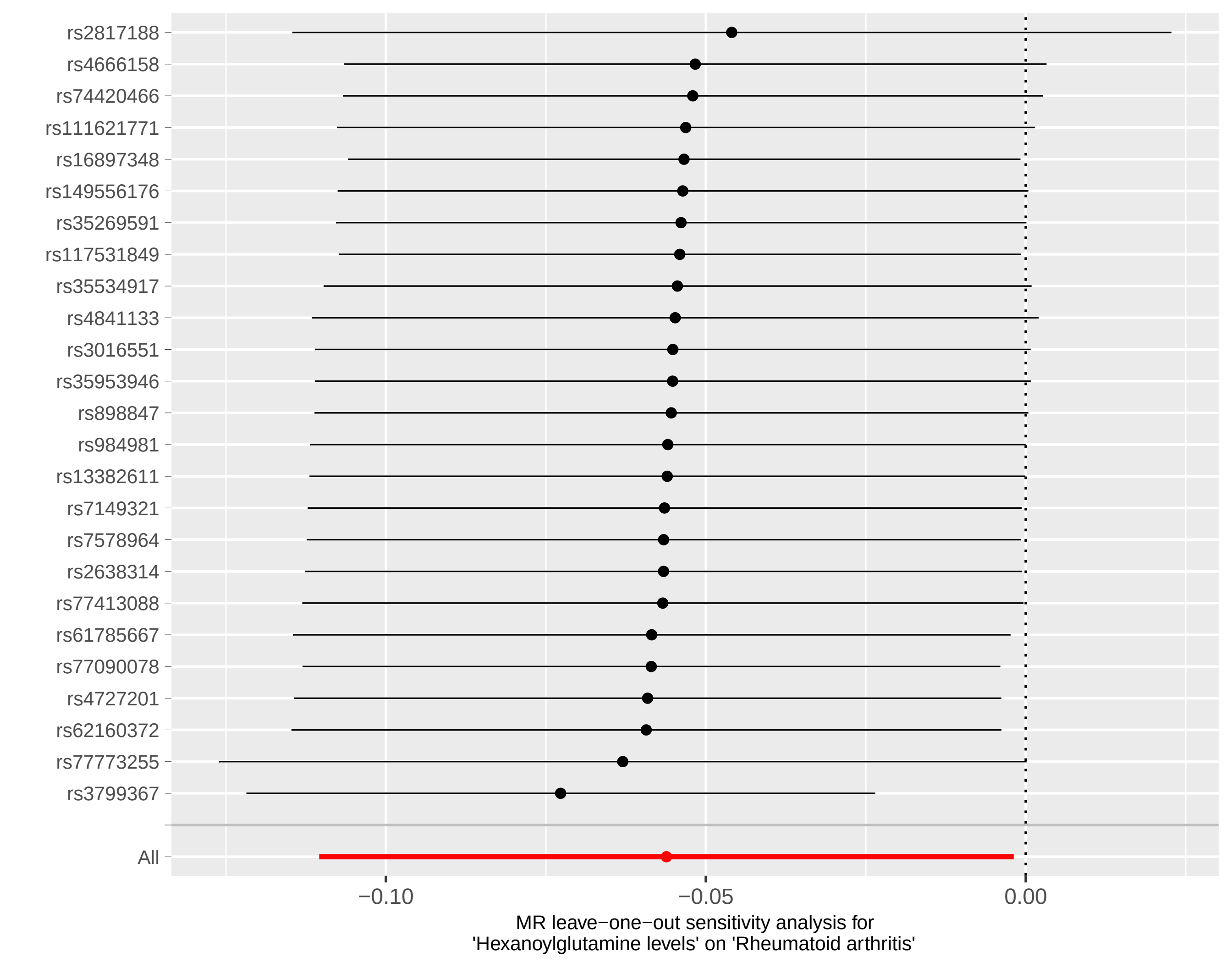

rs2817188
rs4666158
rs74420466
rs111621771
rs16897348
rs149556176
rs35269591
rs117531849
rs35534917
rs4841133
rs3016551
rs35953946
rs898847
rs984981
rs13382611
rs7149321
rs7578964
rs2638314
rs77413088
rs61785667
rs77090078
rs4727201
rs62160372
rs77773255
rs3799367
All
−0.10
−0.05
0.00
MR leave−one−out sensitivity analysis for
'Hexanoylglutamine levels' on 'Rheumatoid arthritis'

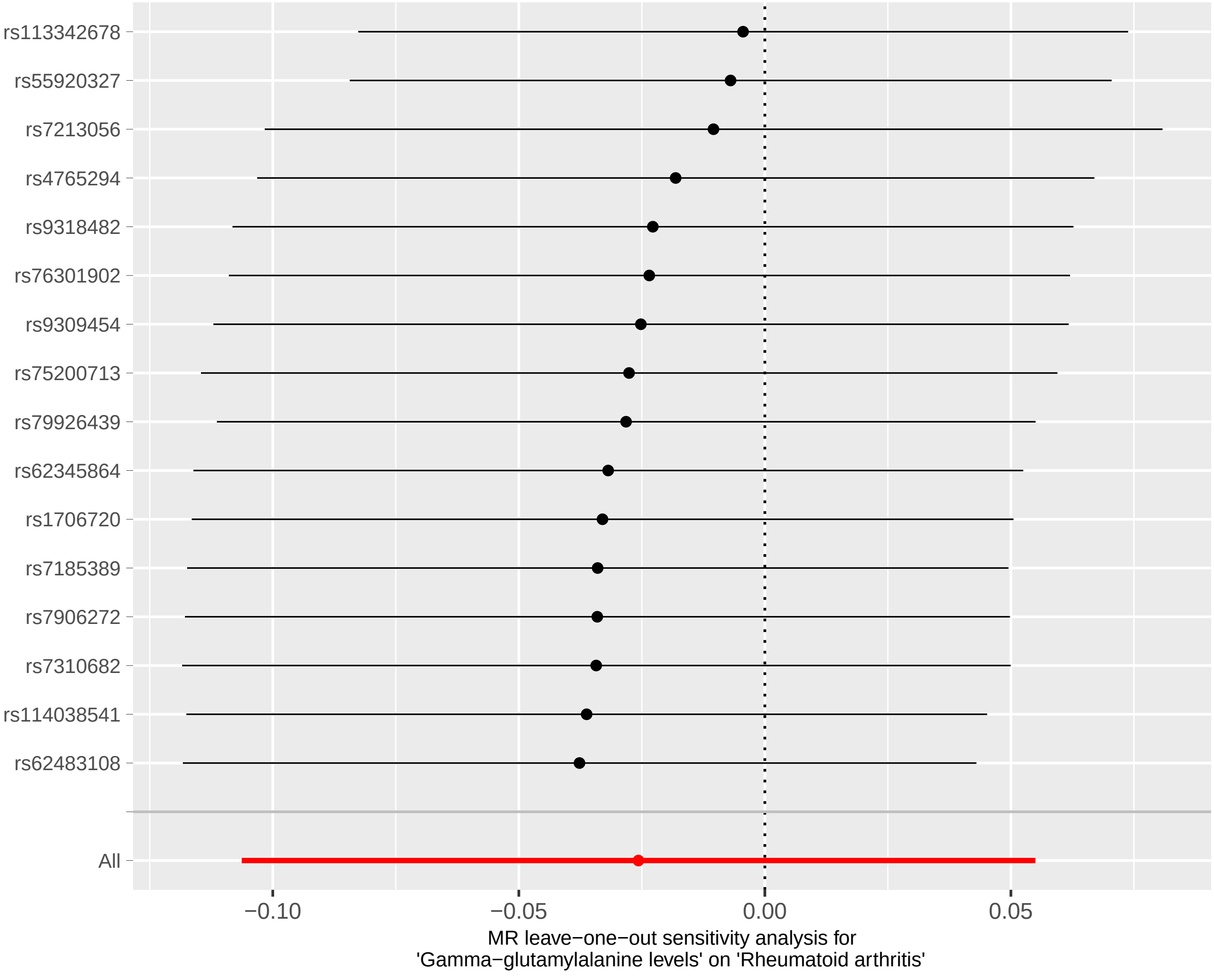
rs113342678
rs55920327
rs7213056
rs4765294
rs9318482
rs76301902
rs9309454
rs75200713
rs79926439
rs62345864
rs1706720
rs7185389
rs7906272
rs7310682
rs114038541
rs62483108
All
−0.10
−0.05
0.00
0.05
MR leave−one−out sensitivity analysis for
'Gamma−glutamylalanine levels' on 'Rheumatoid arthritis'

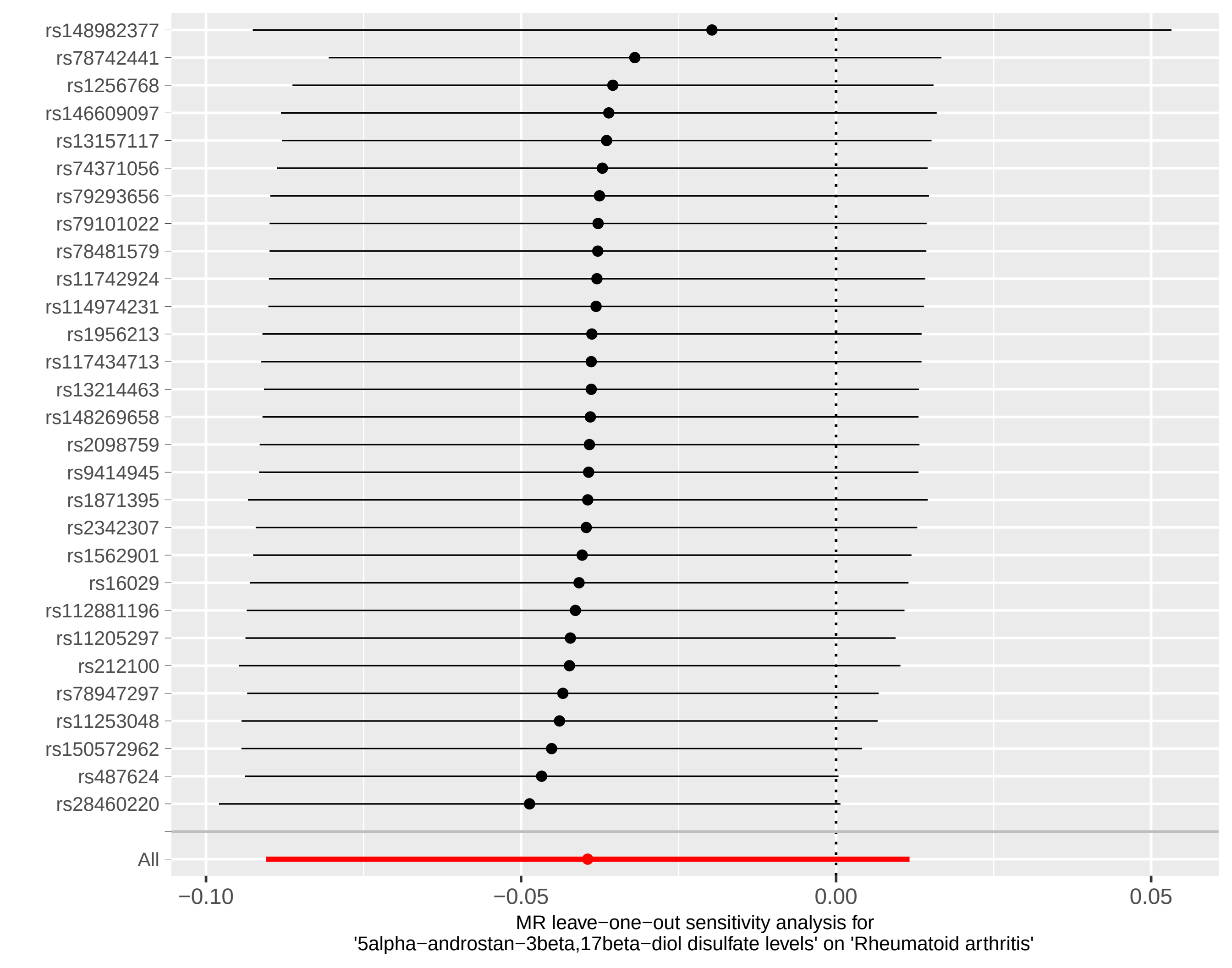
rs148982377
rs78742441
rs1256768
rs146609097
rs13157117
rs74371056
rs79293656
rs79101022
rs78481579
rs11742924
rs114974231
rs1956213
rs117434713
rs13214463
rs148269658
rs2098759
rs9414945
rs1871395
rs2342307
rs1562901
rs16029
rs112881196
rs11205297
rs212100
rs78947297
rs11253048
rs150572962
rs487624
rs28460220
All
−0.10
−0.05
0.00
0.05
MR leave−one−out sensitivity analysis for
'5alpha−androstan−3beta,17beta−diol disulfate levels' on 'Rheumatoid arthritis'

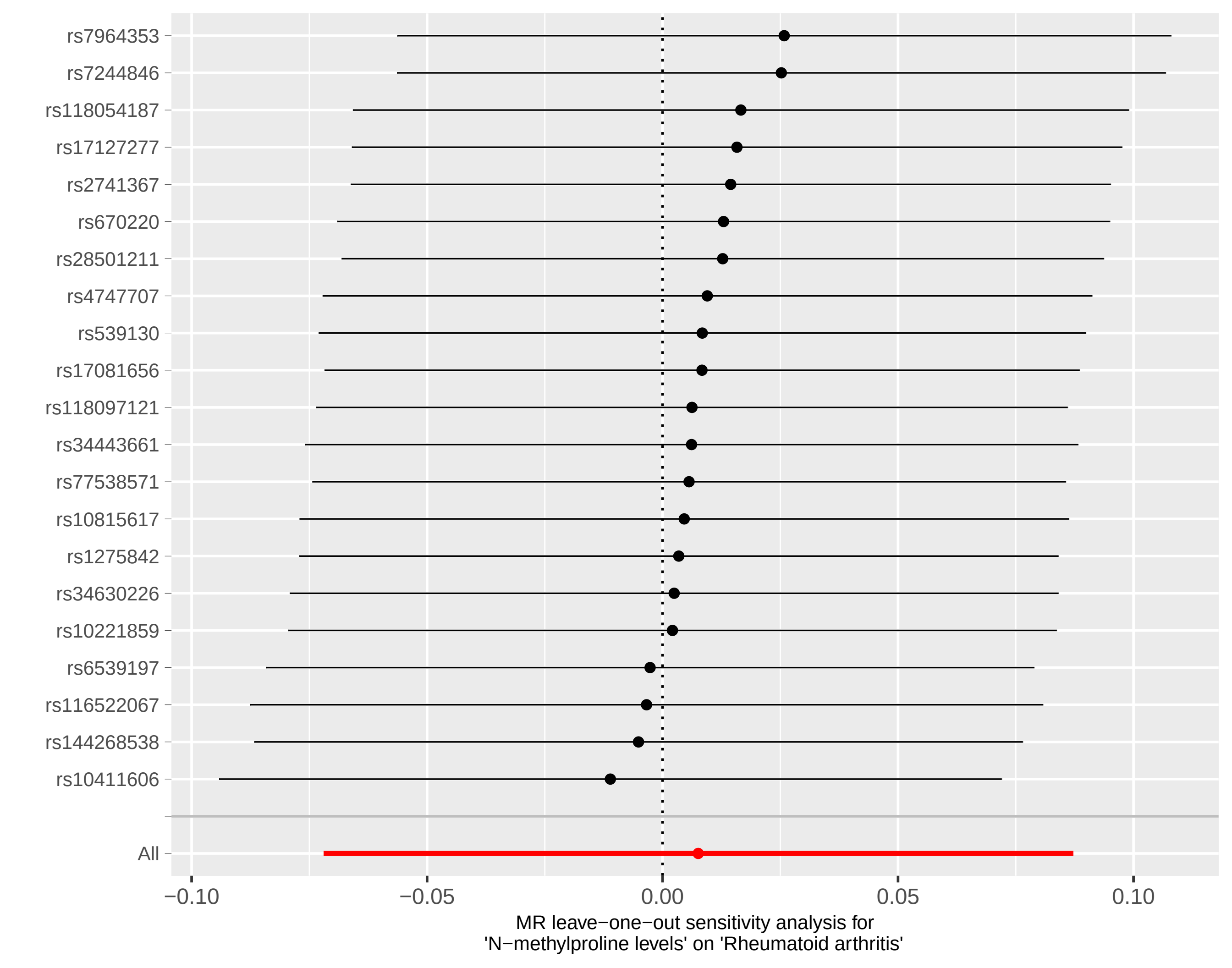
rs7964353
rs7244846
rs118054187
rs17127277
rs2741367
rs670220
rs28501211
rs4747707
rs539130
rs17081656
rs118097121
rs34443661
rs77538571
rs10815617
rs1275842
rs34630226
rs10221859
rs6539197
rs116522067
rs144268538
rs10411606
All
−0.10
−0.05
0.00
0.05
0.10
MR leave−one−out sensitivity analysis for
'N−methylproline levels' on 'Rheumatoid arthritis'

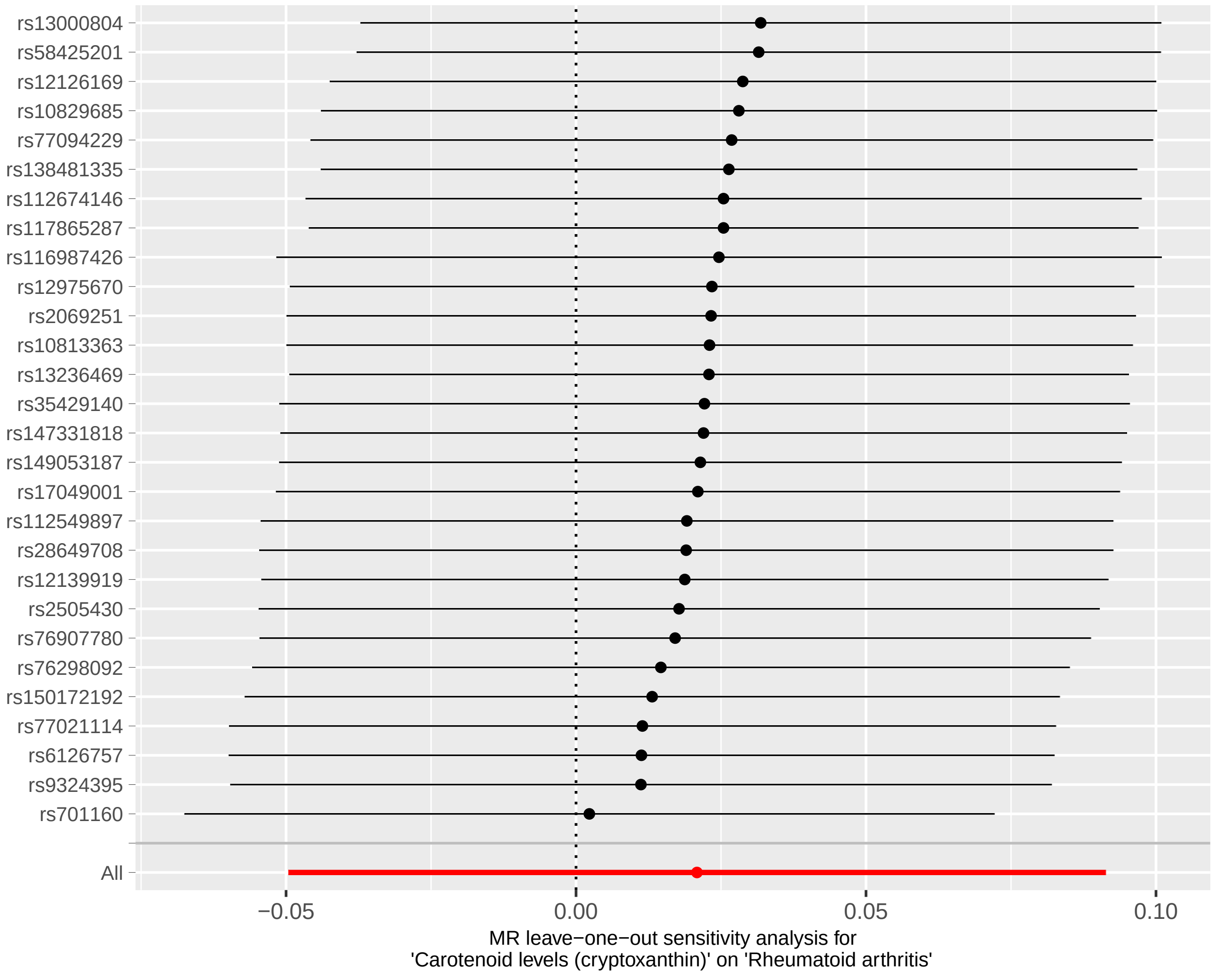
rs13000804
rs58425201
rs12126169
rs10829685
rs77094229
rs138481335
rs112674146
rs117865287
rs116987426
rs12975670
rs2069251
rs10813363
rs13236469
rs35429140
rs147331818
rs149053187
rs17049001
rs112549897
rs28649708
rs12139919
rs2505430
rs76907780
rs76298092
rs150172192
rs77021114
rs6126757
rs9324395
rs701160
All
−0.05
0.00
0.05
0.10
MR leave−one−out sensitivity analysis for
'Carotenoid levels (cryptoxanthin)' on 'Rheumatoid arthritis'

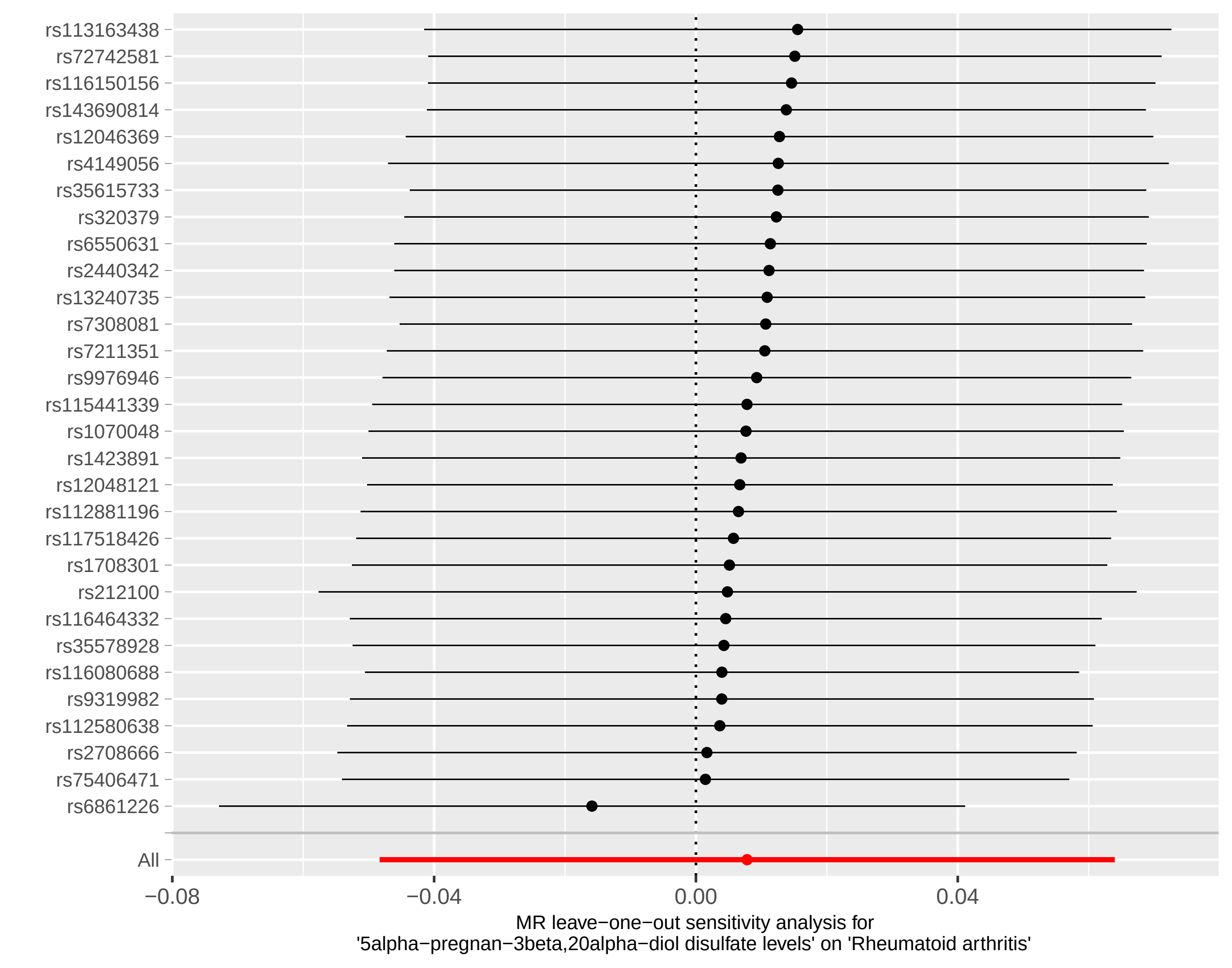
rs113163438
rs72742581
rs116150156
rs143690814
rs12046369
rs4149056
rs35615733
rs320379
rs6550631
rs2440342
rs13240735
rs7308081
rs7211351
rs9976946
rs115441339
rs1070048
rs1423891
rs12048121
rs112881196
rs117518426
rs1708301
rs212100
rs116464332
rs35578928
rs116080688
rs9319982
rs112580638
rs2708666
rs75406471
rs6861226
All
−0.08
−0.04
0.00
0.04
MR leave−one−out sensitivity analysis for
'5alpha−pregnan−3beta,20alpha−diol disulfate levels' on 'Rheumatoid arthritis'

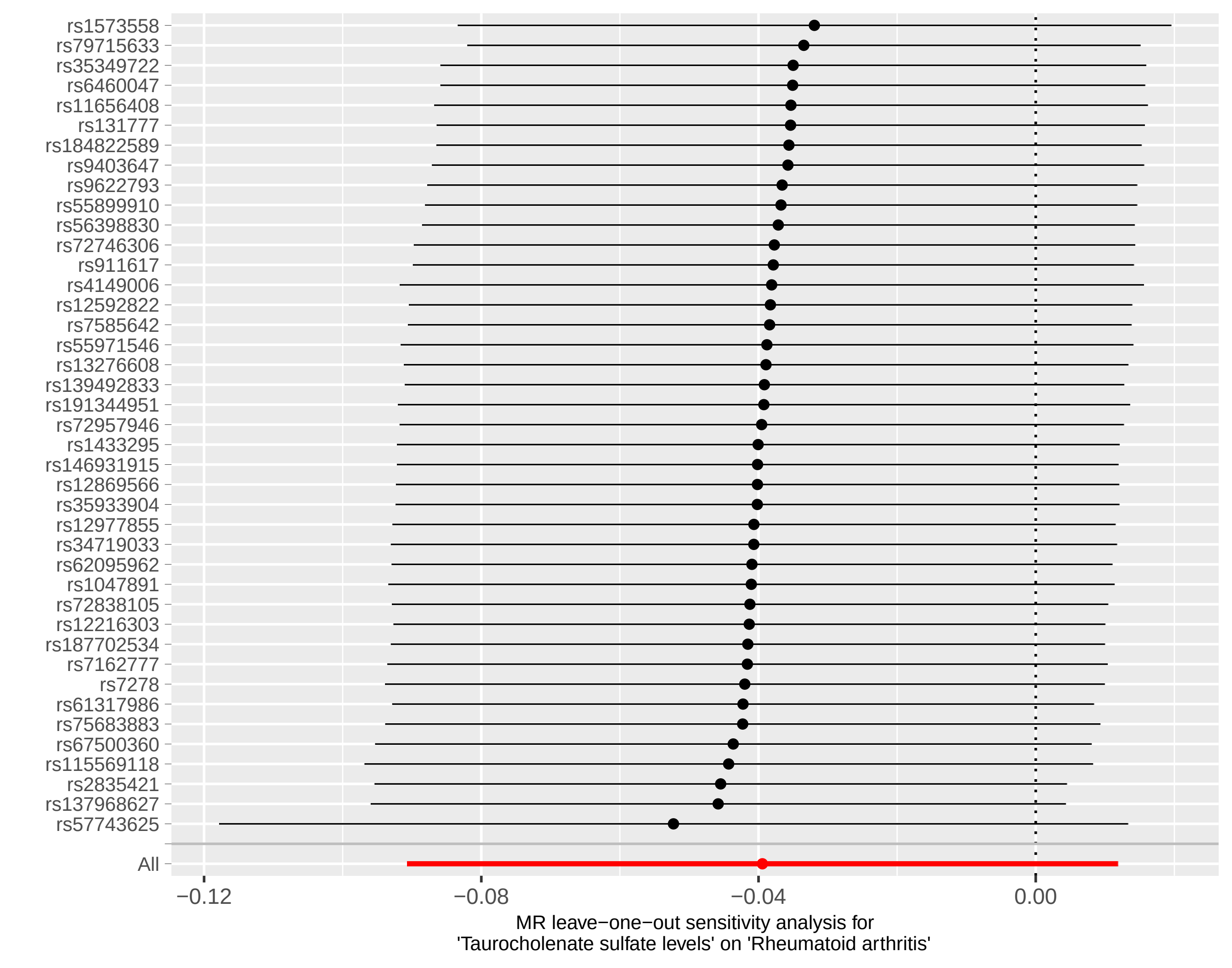

rs1573558
rs79715633
rs35349722
rs6460047
rs11656408
rs131777
rs184822589
rs9403647
rs9622793
rs55899910
rs56398830
rs72746306
rs911617
rs4149006
rs12592822
rs7585642
rs55971546
rs13276608
rs139492833
rs191344951
rs72957946
rs1433295
rs146931915
rs12869566
rs35933904
rs12977855
rs34719033
rs62095962
rs1047891
rs72838105
rs12216303
rs187702534
rs7162777
rs7278
rs61317986
rs75683883
rs67500360
rs115569118
rs2835421
rs137968627
rs57743625
All
−0.12
−0.08
−0.04
0.00
MR leave−one−out sensitivity analysis for
'Taurocholenate sulfate levels' on 'Rheumatoid arthritis'

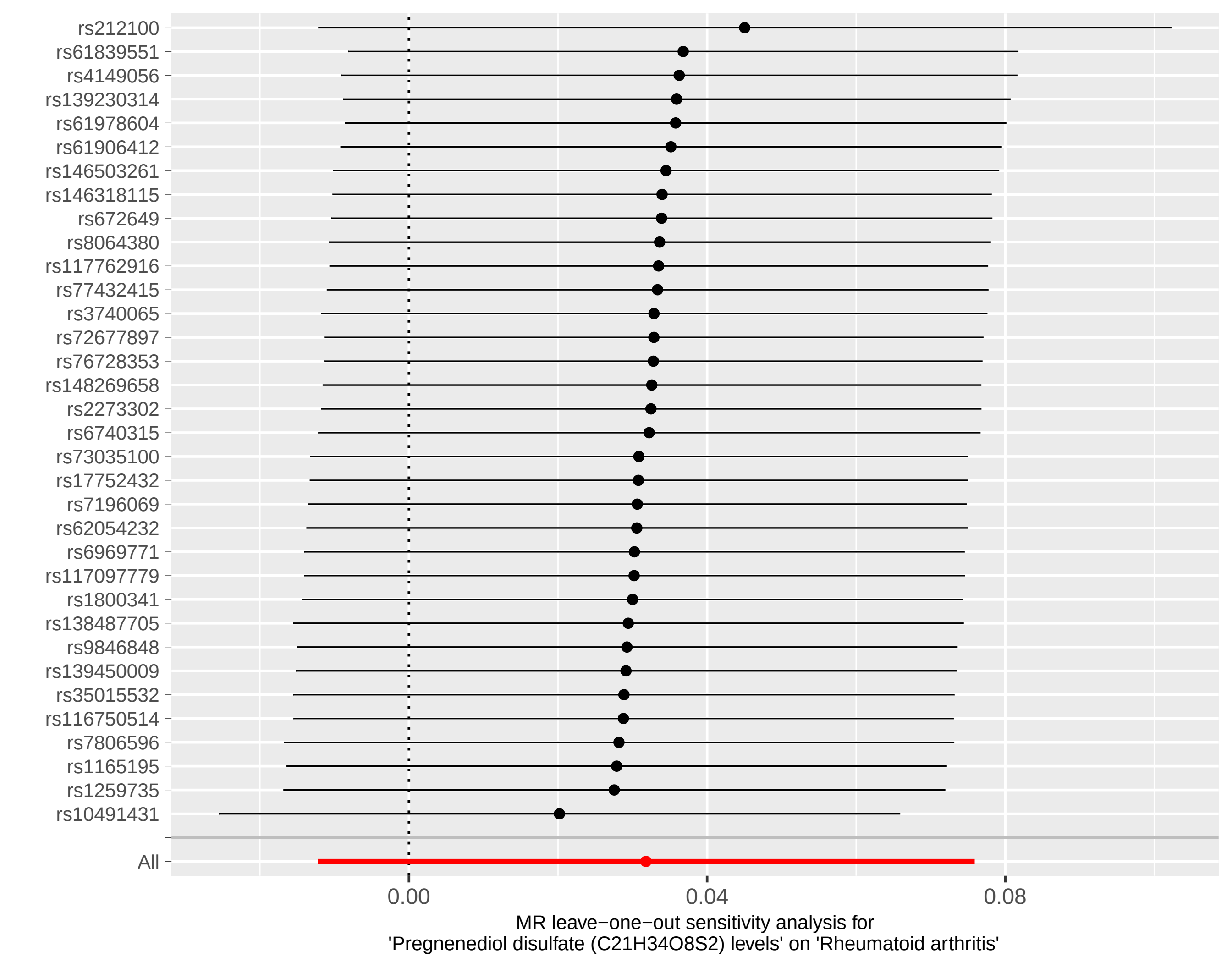

rs212100
rs61839551
rs4149056
rs139230314
rs61978604
rs61906412
rs146503261
rs146318115
rs672649
rs8064380
rs117762916
rs77432415
rs3740065
rs72677897
rs76728353
rs148269658
rs2273302
rs6740315
rs73035100
rs17752432
rs7196069
rs62054232
rs6969771
rs117097779
rs1800341
rs138487705
rs9846848
rs139450009
rs35015532
rs116750514
rs7806596
rs1165195
rs1259735
rs10491431
All
0.00
0.04
0.08
MR leave−one−out sensitivity analysis for
'Pregnenediol disulfate (C21H34O8S2) levels' on 'Rheumatoid arthritis'

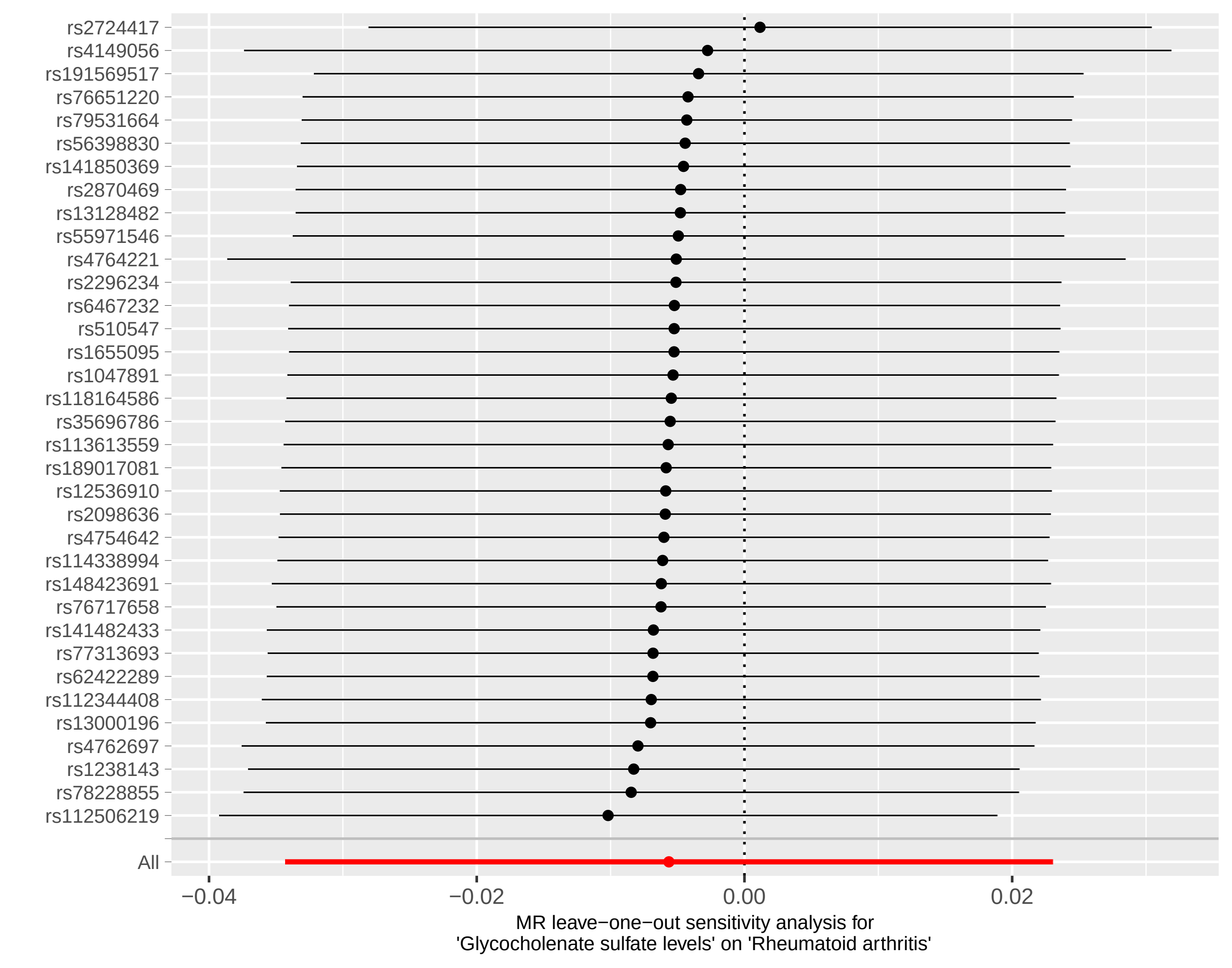

rs2724417
rs4149056
rs191569517
rs76651220
rs79531664
rs56398830
rs141850369
rs2870469
rs13128482
rs55971546
rs4764221
rs2296234
rs6467232
rs510547
rs1655095
rs1047891
rs118164586
rs35696786
rs113613559
rs189017081
rs12536910
rs2098636
rs4754642
rs114338994
rs148423691
rs76717658
rs141482433
rs77313693
rs62422289
rs112344408
rs13000196
rs4762697
rs1238143
rs78228855
rs112506219
All
−0.04
−0.02
0.00
0.02
MR leave−one−out sensitivity analysis for
'Glycocholenate sulfate levels' on 'Rheumatoid arthritis'

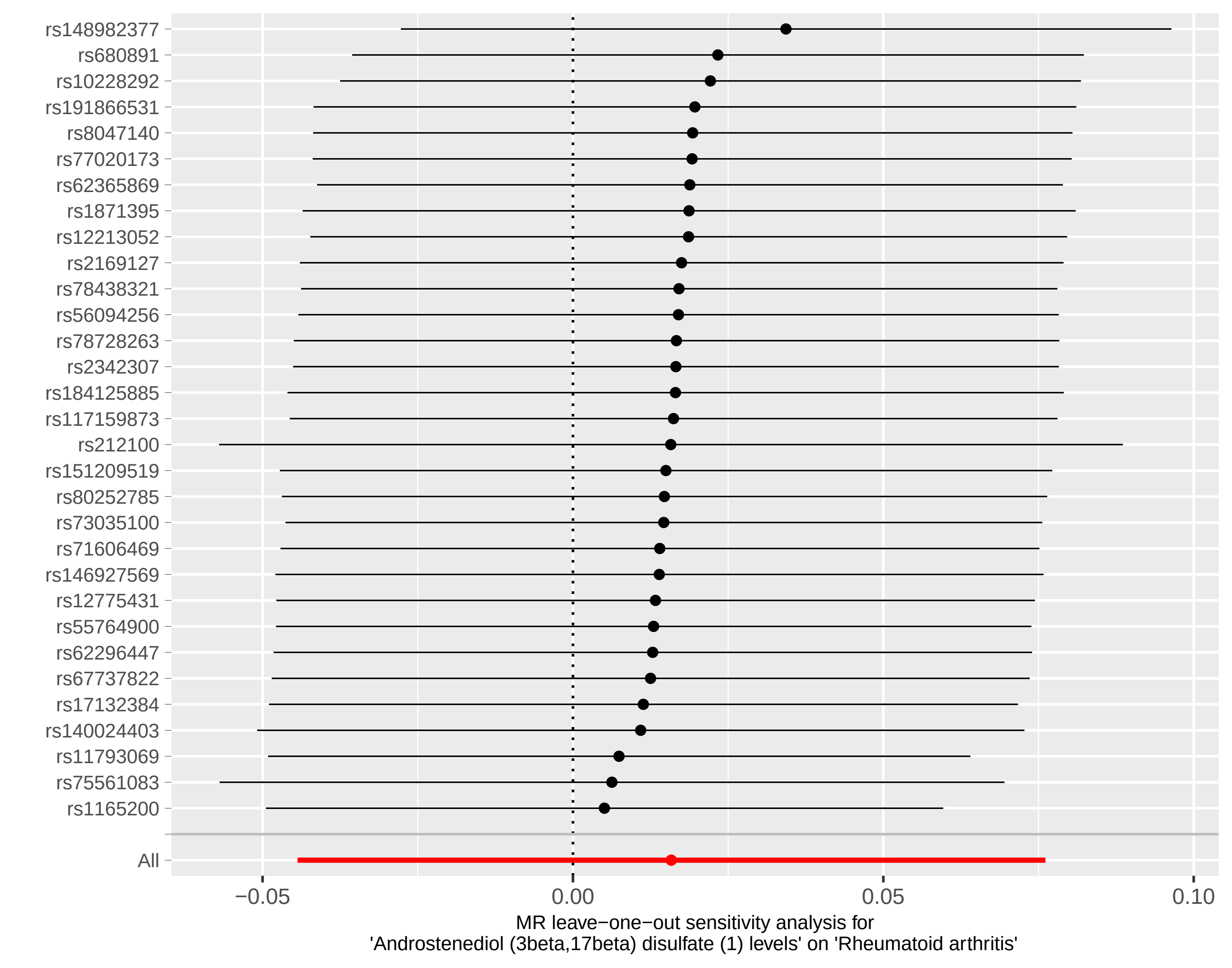
rs148982377
rs680891
rs10228292
rs191866531
rs8047140
rs77020173
rs62365869
rs1871395
rs12213052
rs2169127
rs78438321
rs56094256
rs78728263
rs2342307
rs184125885
rs117159873
rs212100
rs151209519
rs80252785
rs73035100
rs71606469
rs146927569
rs12775431
rs55764900
rs62296447
rs67737822
rs17132384
rs140024403
rs11793069
rs75561083
rs1165200
All
−0.05
0.00
0.05
0.10
MR leave−one−out sensitivity analysis for
'Androstenediol (3beta,17beta) disulfate (1) levels' on 'Rheumatoid arthritis'

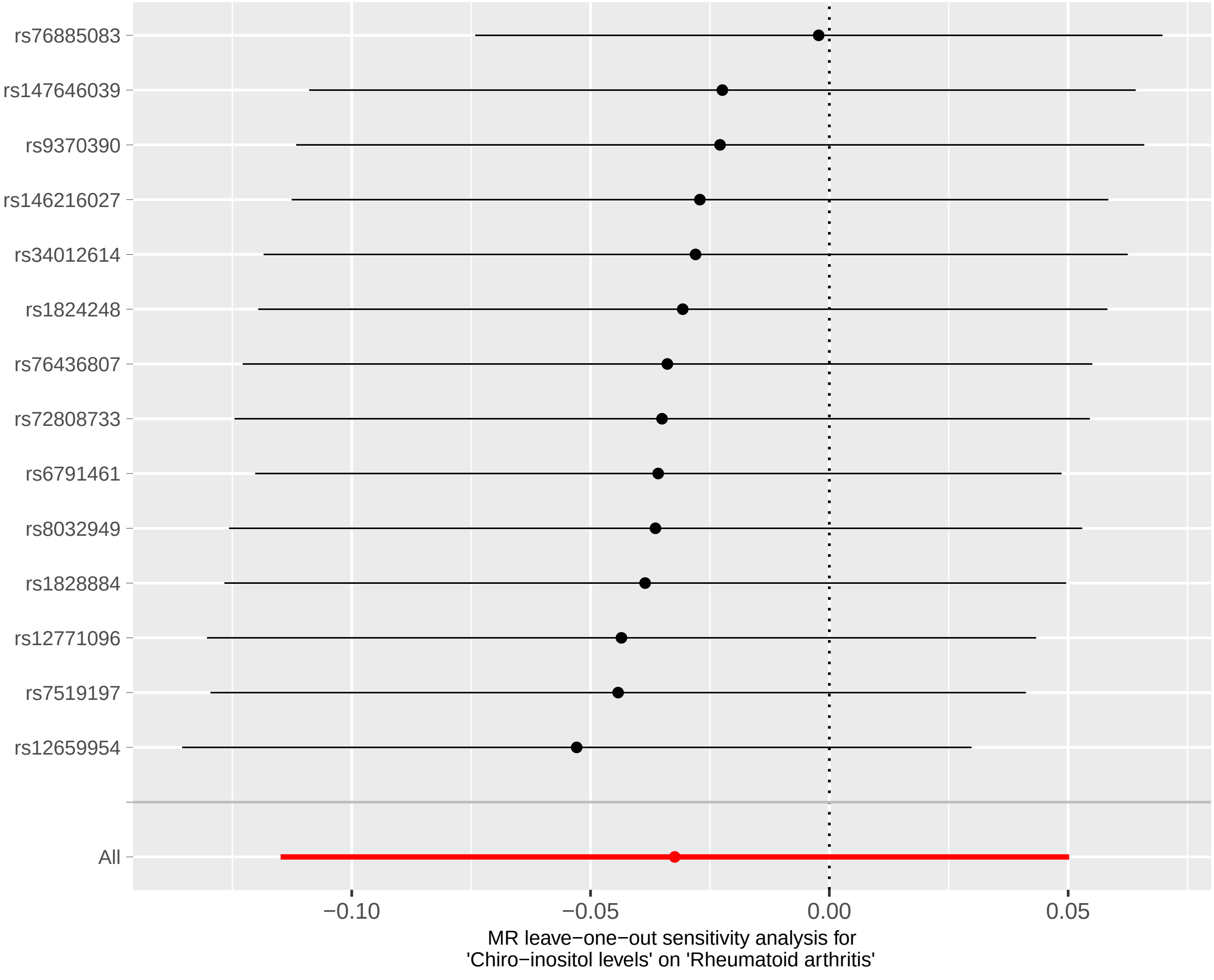

rs76885083
rs147646039
rs9370390
rs146216027
rs34012614
rs1824248
rs76436807
rs72808733
rs6791461
rs8032949
rs1828884
rs12771096
rs7519197
rs12659954
All
−0.10
−0.05
0.00
0.05
MR leave−one−out sensitivity analysis for
'Chiro−inositol levels' on 'Rheumatoid arthritis'

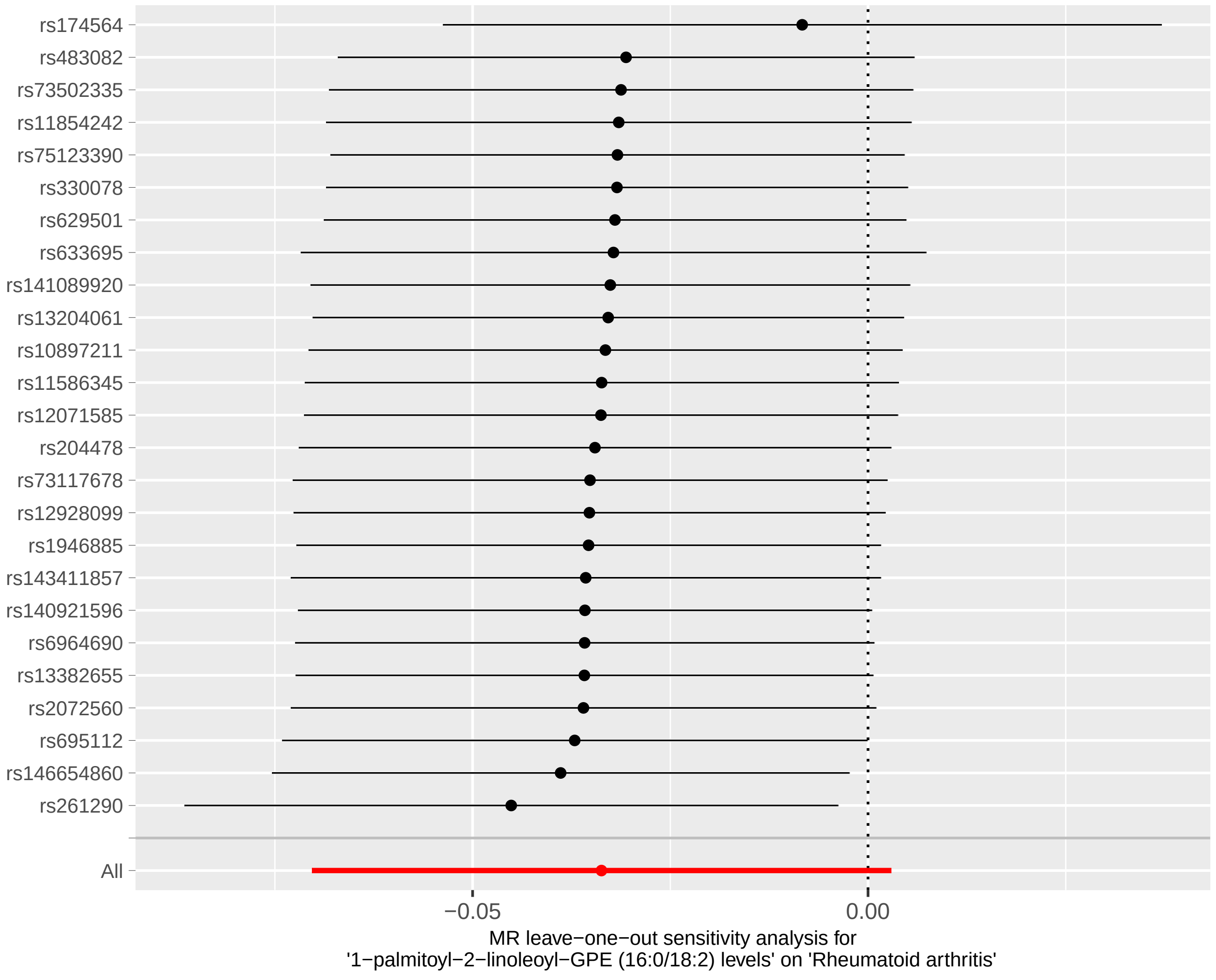
rs174564
rs483082
rs73502335
rs11854242
rs75123390
rs330078
rs629501
rs633695
rs141089920
rs13204061
rs10897211
rs11586345
rs12071585
rs204478
rs73117678
rs12928099
rs1946885
rs143411857
rs140921596
rs6964690
rs13382655
rs2072560
rs695112
rs146654860
rs261290
All
−0.05
0.00
MR leave−one−out sensitivity analysis for
'1−palmitoyl−2−linoleoyl−GPE (16:0/18:2) levels' on 'Rheumatoid arthritis'

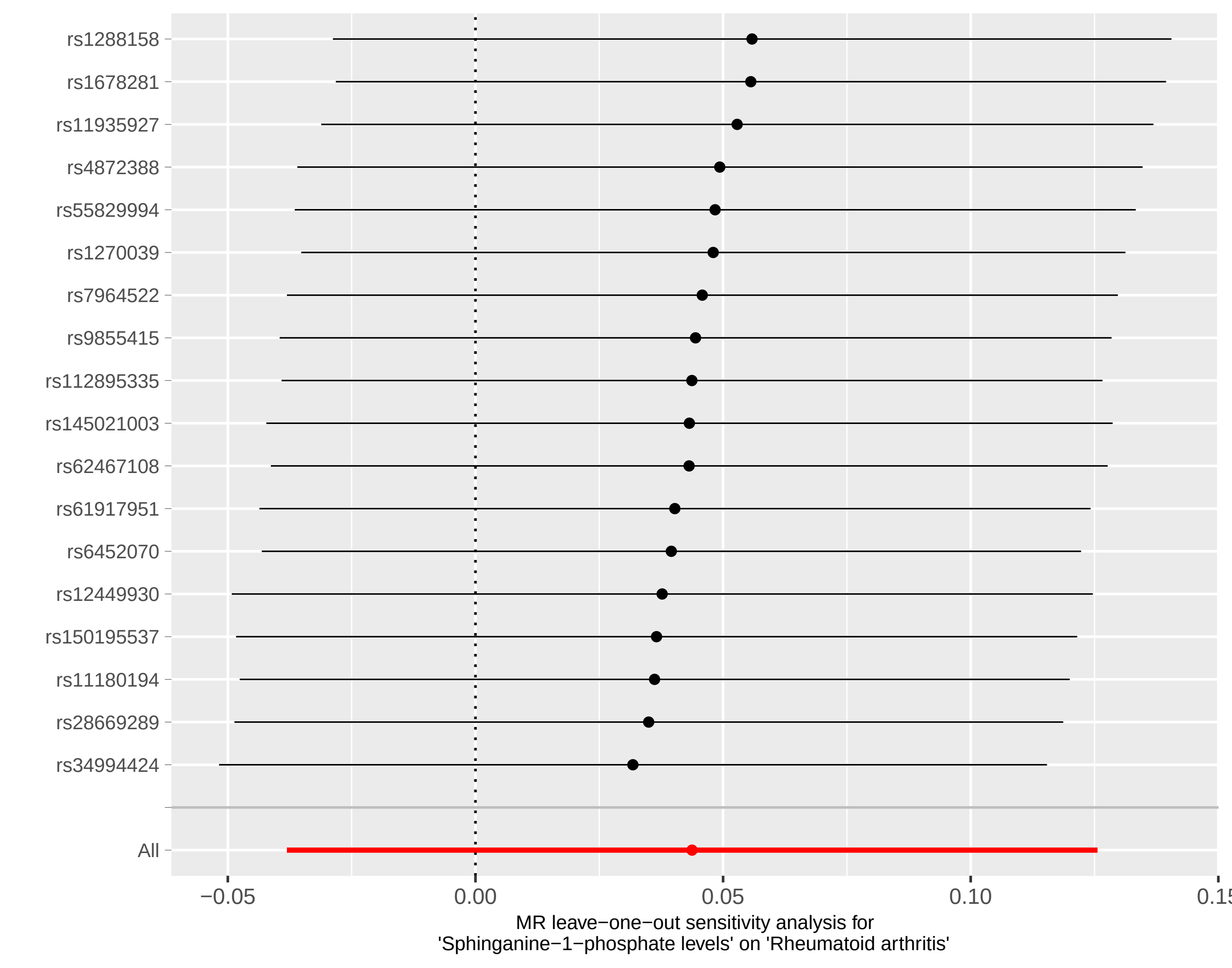
rs1288158
rs1678281
rs11935927
rs4872388
rs55829994
rs1270039
rs7964522
rs9855415
rs112895335
rs145021003
rs62467108
rs61917951
rs6452070
rs12449930
rs150195537
rs11180194
rs28669289
rs34994424
All
−0.05
0.00
0.05
0.10
MR leave−one−out sensitivity analysis for
'Sphinganine−1−phosphate levels' on 'Rheumatoid arthritis'

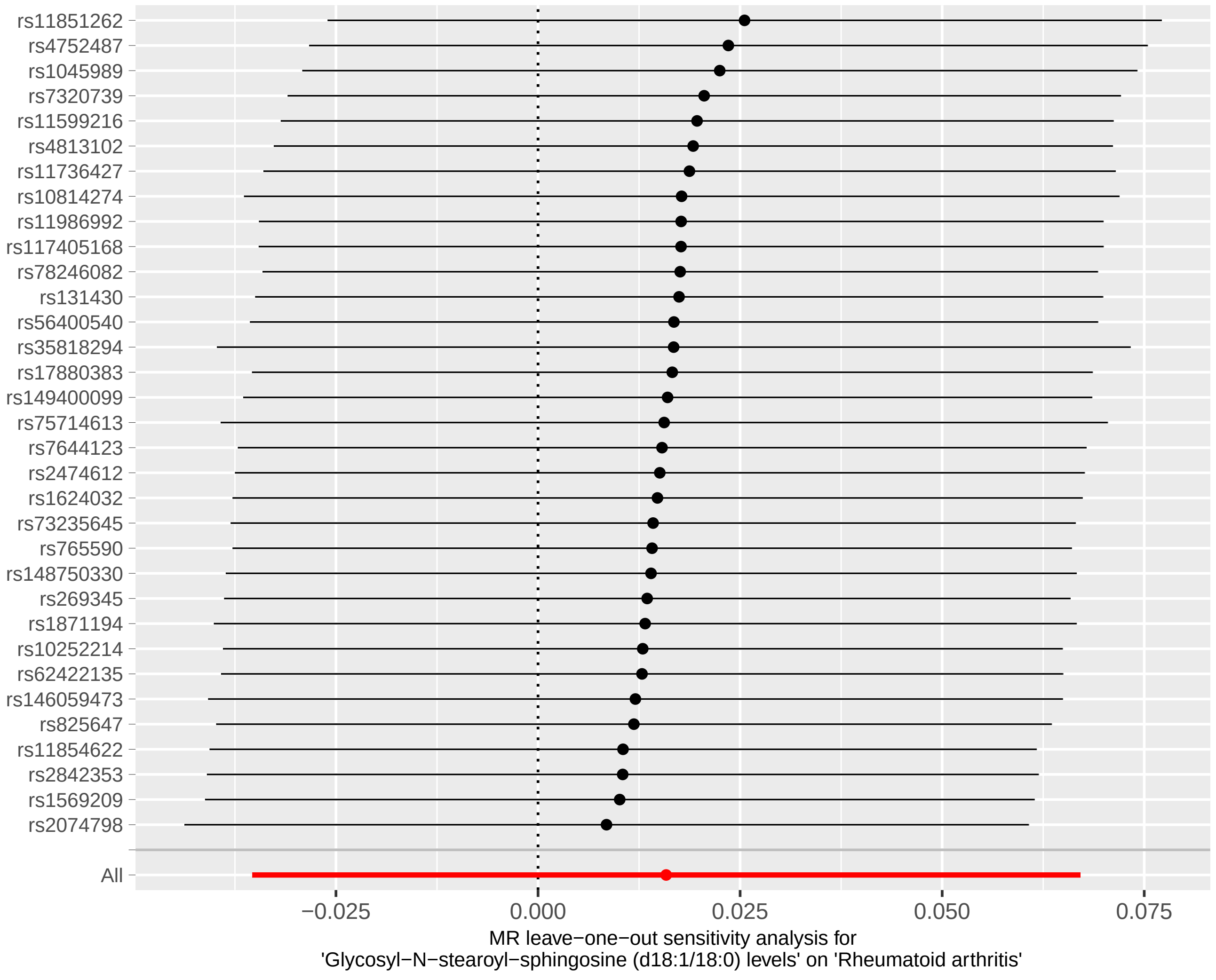
rs11851262
rs4752487
rs1045989
rs7320739
rs11599216
rs4813102
rs11736427
rs10814274
rs11986992
rs117405168
rs78246082
rs131430
rs56400540
rs35818294
rs17880383
rs149400099
rs75714613
rs7644123
rs2474612
rs1624032
rs73235645
rs765590
rs148750330
rs269345
rs1871194
rs10252214
rs62422135
rs146059473
rs825647
rs11854622
rs2842353
rs1569209
rs2074798
All
−0.025
0.000
0.025
0.050
0.075
MR leave−one−out sensitivity analysis for
'Glycosyl−N−stearoyl−sphingosine (d18:1/18:0) levels' on 'Rheumatoid arthritis'

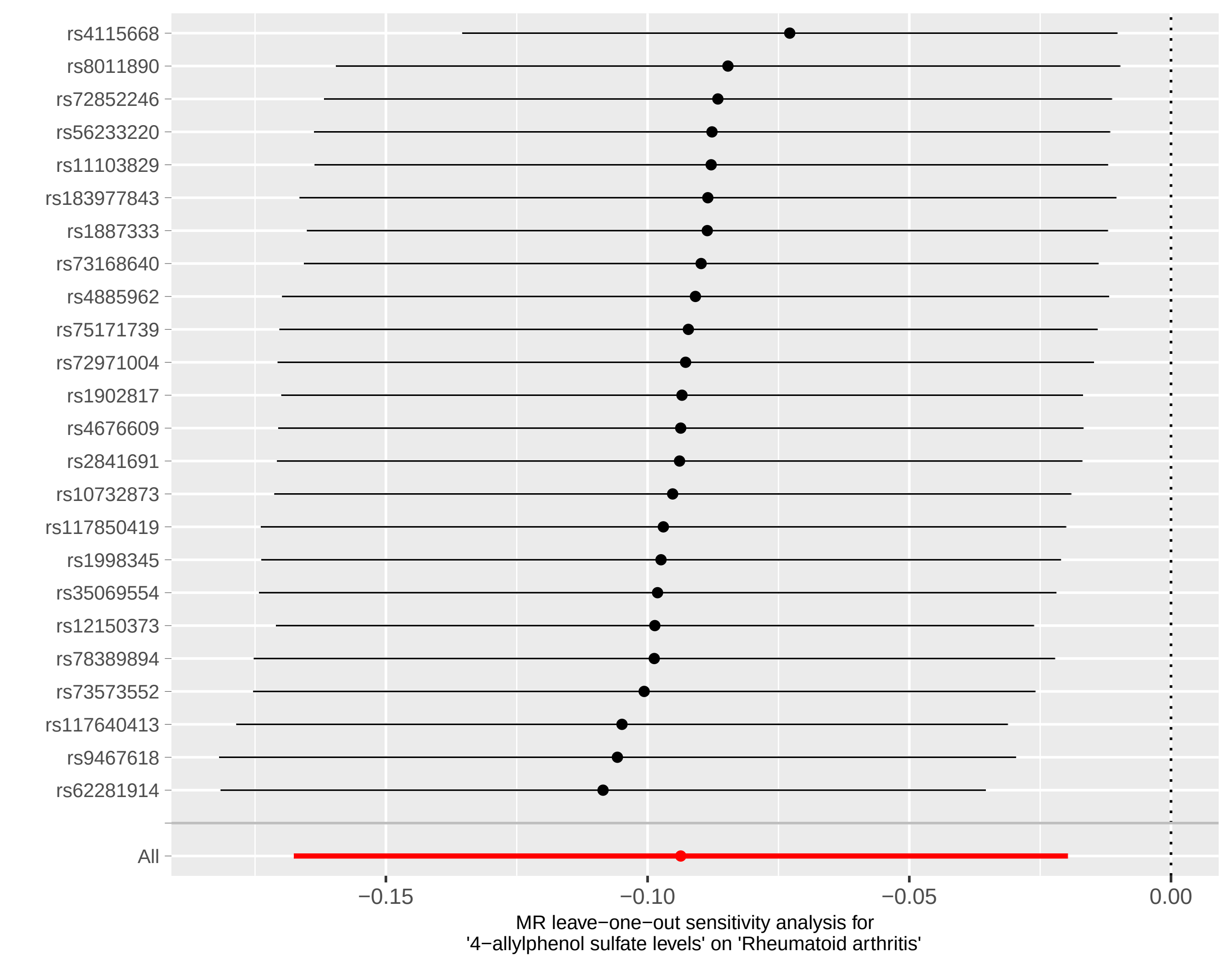

rs4115668
rs8011890
rs72852246
rs56233220
rs11103829
rs183977843
rs1887333
rs73168640
rs4885962
rs75171739
rs72971004
rs1902817
rs4676609
rs2841691
rs10732873
rs117850419
rs1998345
rs35069554
rs12150373
rs78389894
rs73573552
rs117640413
rs9467618
rs62281914
All
−0.15
−0.10
−0.05
0.00
MR leave−one−out sensitivity analysis for
'4−allylphenol sulfate levels' on 'Rheumatoid arthritis'

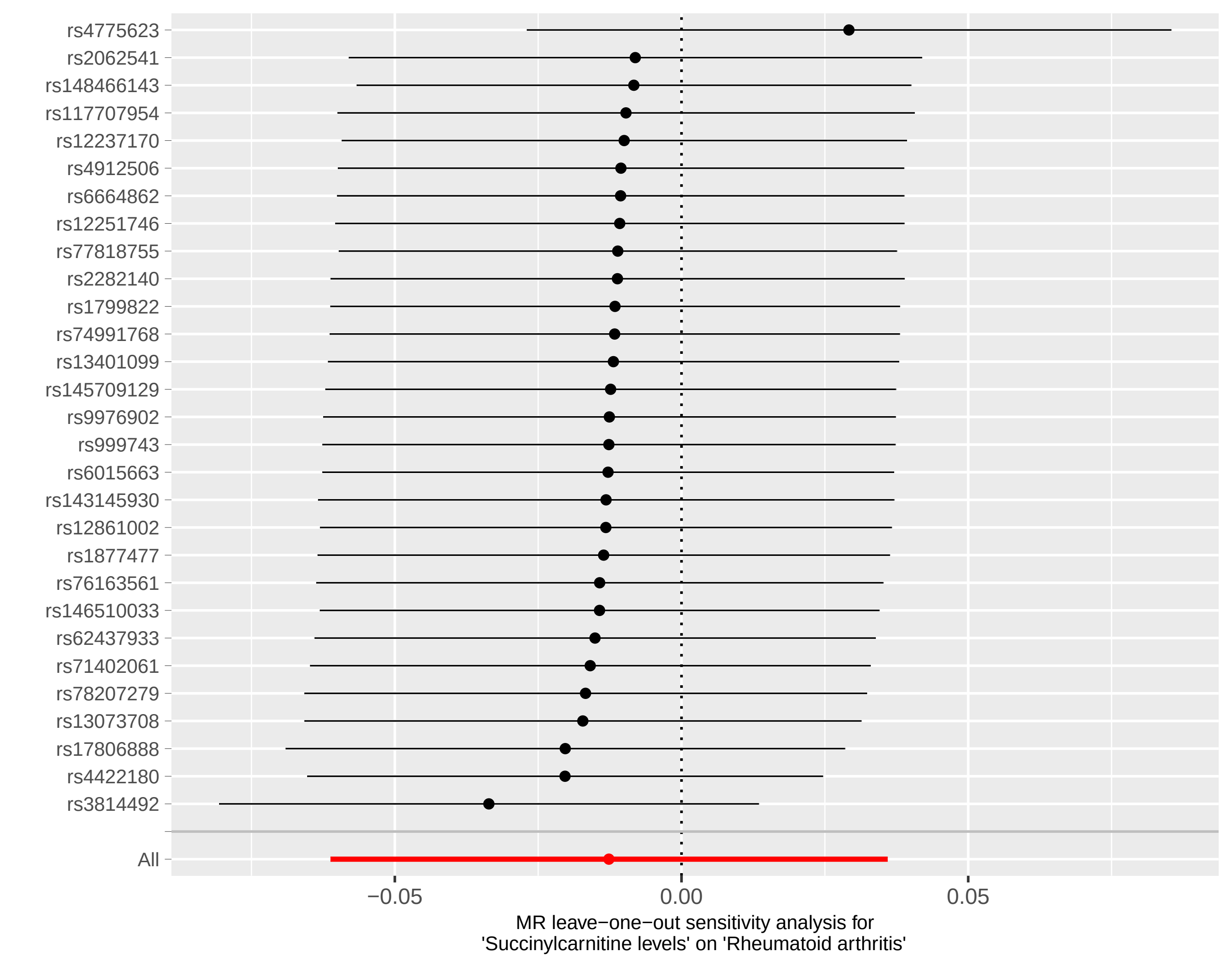

rs4775623
rs2062541
rs148466143
rs117707954
rs12237170
rs4912506
rs6664862
rs12251746
rs77818755
rs2282140
rs1799822
rs74991768
rs13401099
rs145709129
rs9976902
rs999743
rs6015663
rs143145930
rs12861002
rs1877477
rs76163561
rs146510033
rs62437933
rs71402061
rs78207279
rs13073708
rs17806888
rs4422180
rs3814492
All
−0.05
0.00
0.05
MR leave−one−out sensitivity analysis for
'Succinylcarnitine levels' on 'Rheumatoid arthritis'

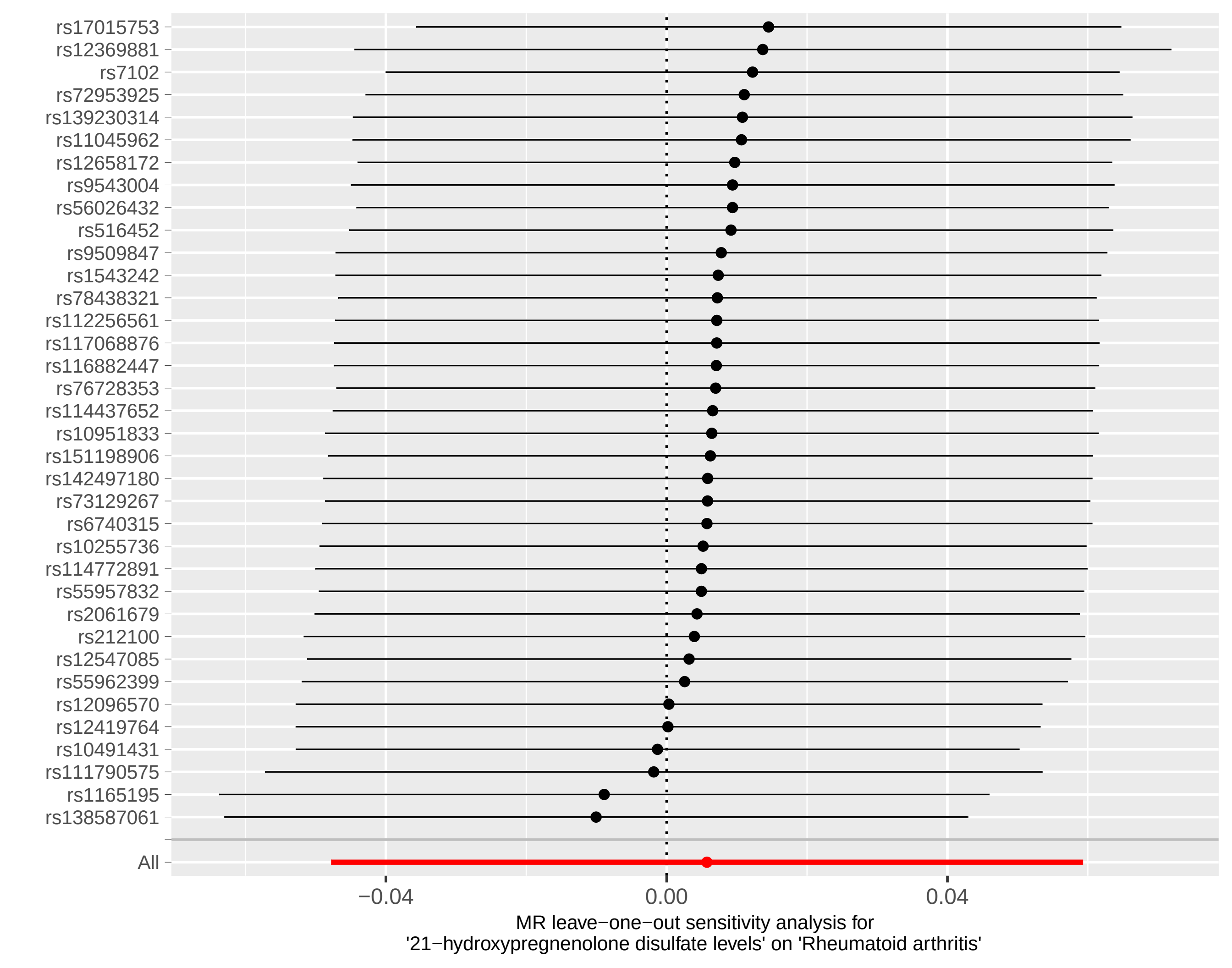

rs17015753
rs12369881
rs7102
rs72953925
rs139230314
rs11045962
rs12658172
rs9543004
rs56026432
rs516452
rs9509847
rs1543242
rs78438321
rs112256561
rs117068876
rs116882447
rs76728353
rs114437652
rs10951833
rs151198906
rs142497180
rs73129267
rs6740315
rs10255736
rs114772891
rs55957832
rs2061679
rs212100
rs12547085
rs55962399
rs12096570
rs12419764
rs10491431
rs111790575
rs1165195
rs138587061
All
−0.04
0.00
0.04
MR leave−one−out sensitivity analysis for
'21−hydroxypregnenolone disulfate levels' on 'Rheumatoid arthritis'

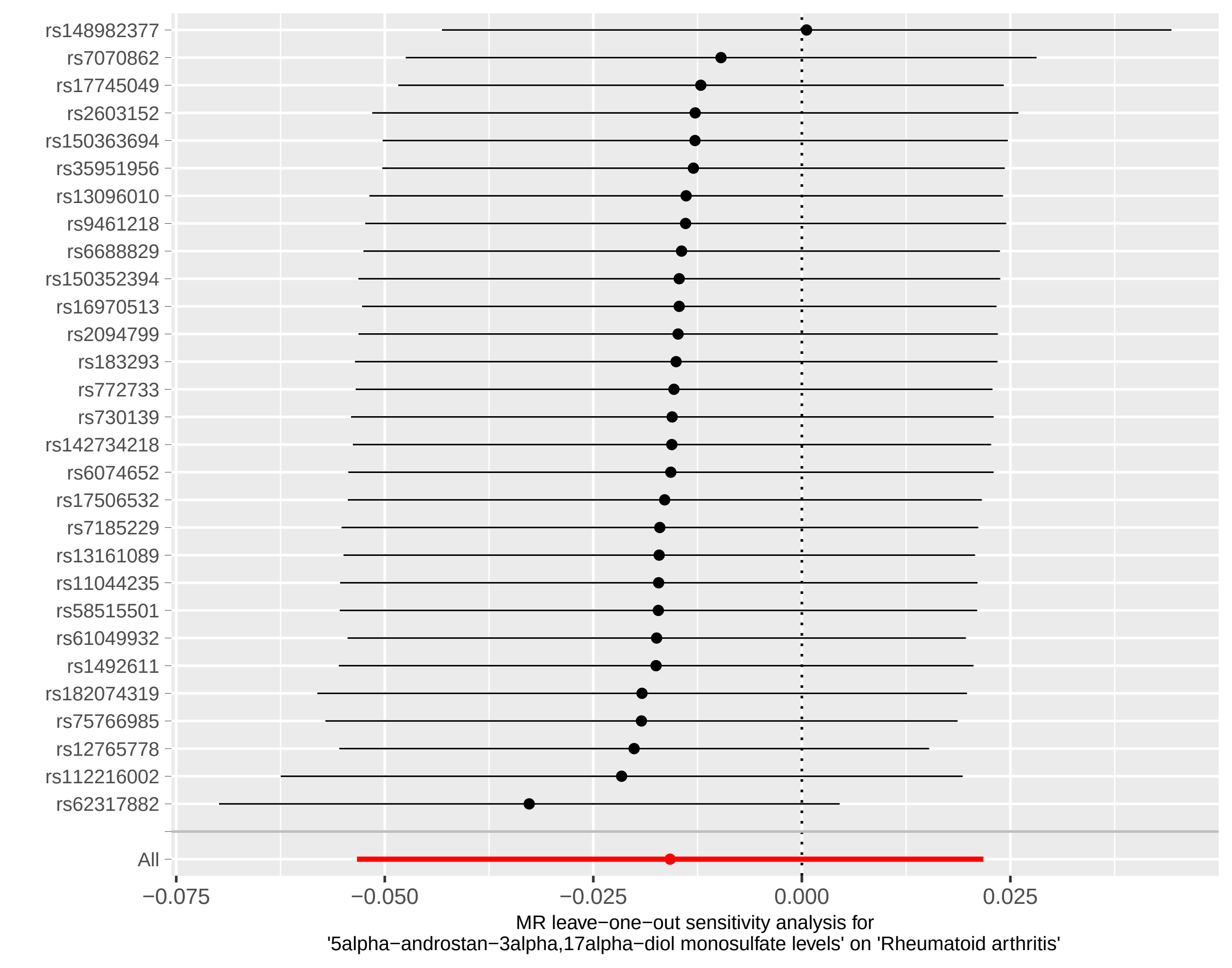
rs148982377
rs7070862
rs17745049
rs2603152
rs150363694
rs35951956
rs13096010
rs9461218
rs6688829
rs150352394
rs16970513
rs2094799
rs183293
rs772733
rs730139
rs142734218
rs6074652
rs17506532
rs7185229
rs13161089
rs11044235
rs58515501
rs61049932
rs1492611
rs182074319
rs75766985
rs12765778
rs112216002
rs62317882
All
−0.075
−0.050
−0.025
0.000
0.025
MR leave−one−out sensitivity analysis for
'5alpha−androstan−3alpha,17alpha−diol monosulfate levels' on 'Rheumatoid arthritis'

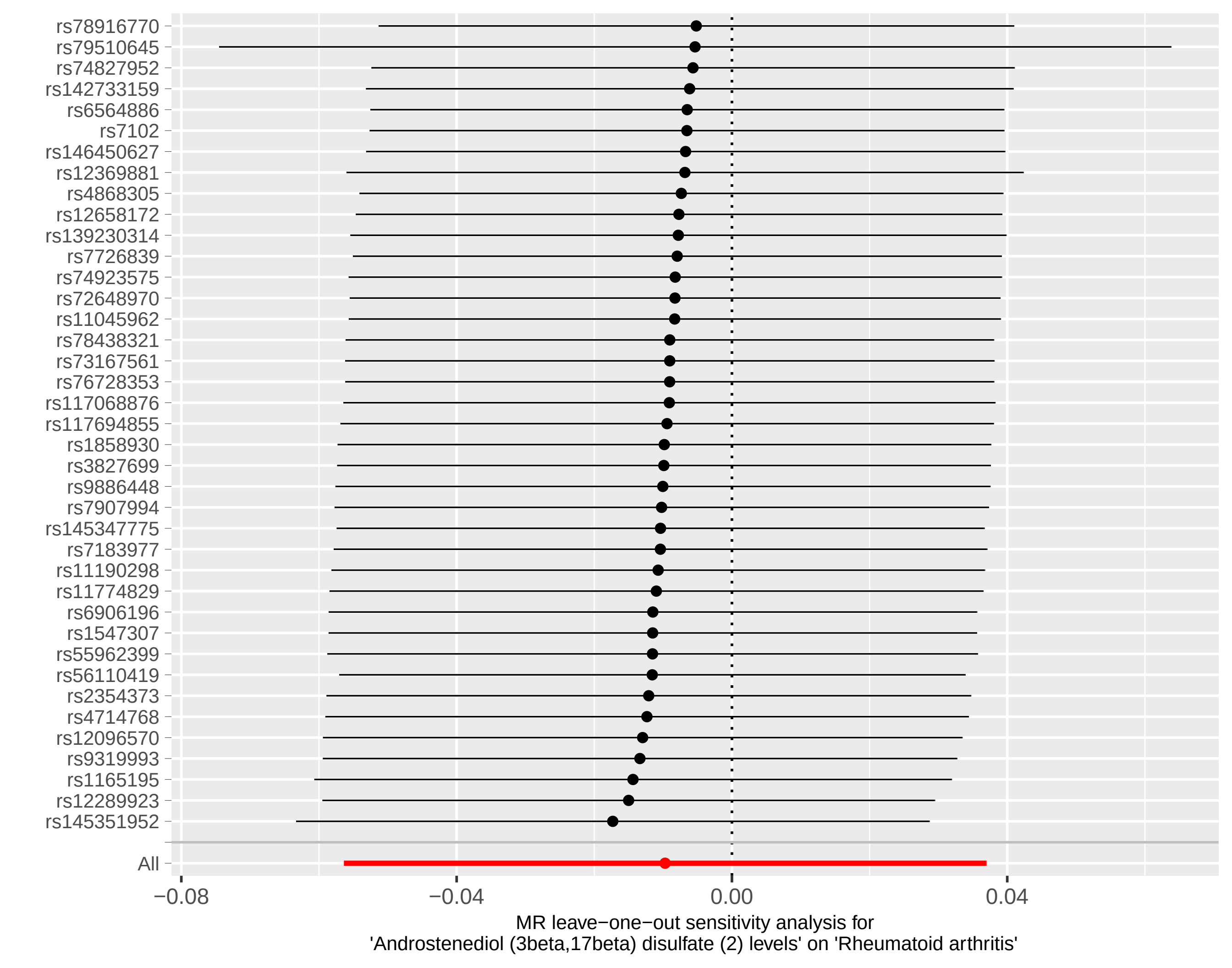

rs78916770
rs79510645
rs74827952
rs142733159
rs6564886
rs7102
rs146450627
rs12369881
rs4868305
rs12658172
rs139230314
rs7726839
rs74923575
rs72648970
rs11045962
rs78438321
rs73167561
rs76728353
rs117068876
rs117694855
rs1858930
rs3827699
rs9886448
rs7907994
rs145347775
rs7183977
rs11190298
rs11774829
rs6906196
rs1547307
rs55962399
rs56110419
rs2354373
rs4714768
rs12096570
rs9319993
rs1165195
rs12289923
rs145351952
All
−0.08
−0.04
0.00
0.04
MR leave−one−out sensitivity analysis for
'Androstenediol (3beta,17beta) disulfate (2) levels' on 'Rheumatoid arthritis'

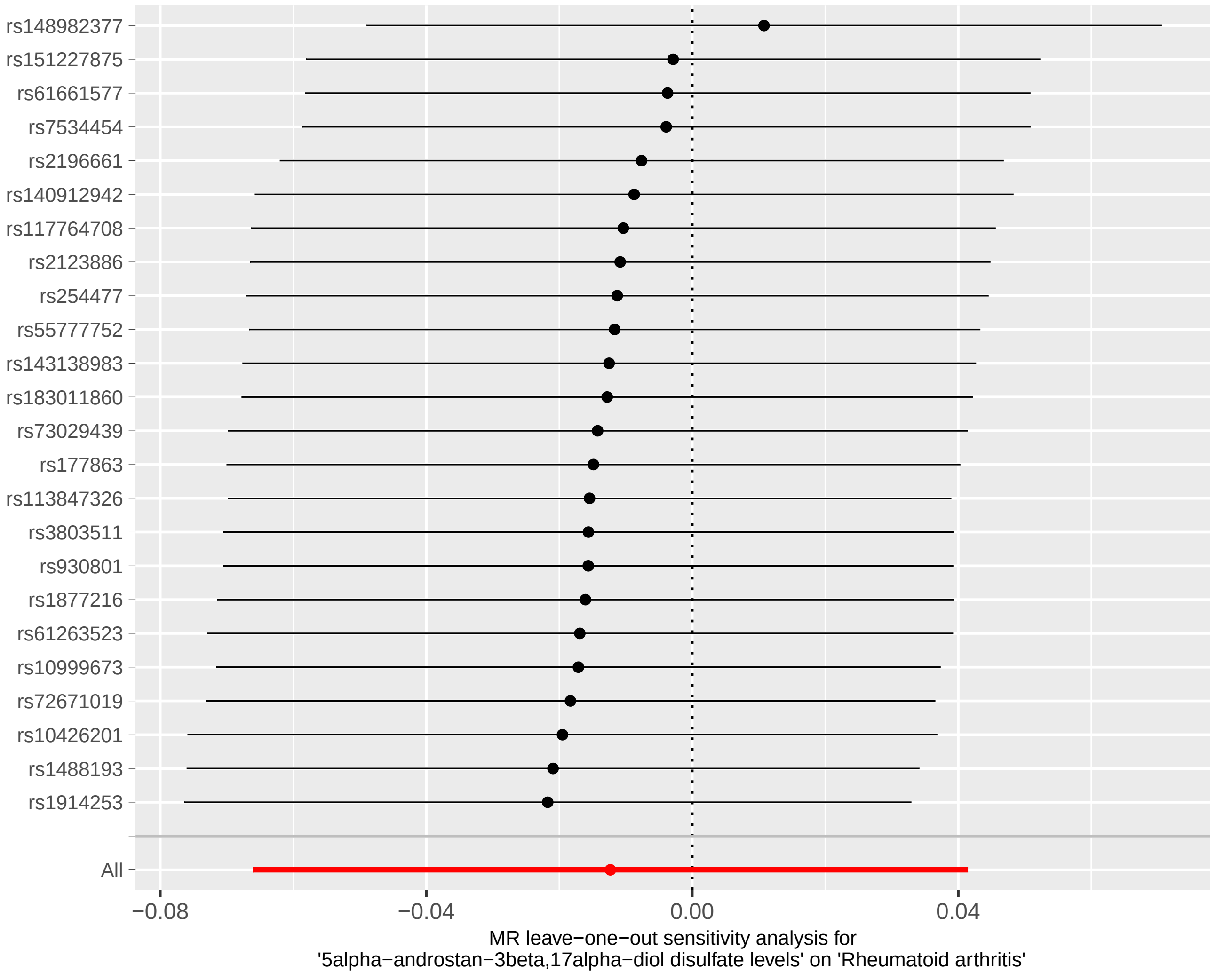
rs148982377
rs151227875
rs61661577
rs7534454
rs2196661
rs140912942
rs117764708
rs2123886
rs254477
rs55777752
rs143138983
rs183011860
rs73029439
rs177863
rs113847326
rs3803511
rs930801
rs1877216
rs61263523
rs10999673
rs72671019
rs10426201
rs1488193
rs1914253
All
−0.08
−0.04
0.00
0.04
MR leave−one−out sensitivity analysis for
'5alpha−androstan−3beta,17alpha−diol disulfate levels' on 'Rheumatoid arthritis'

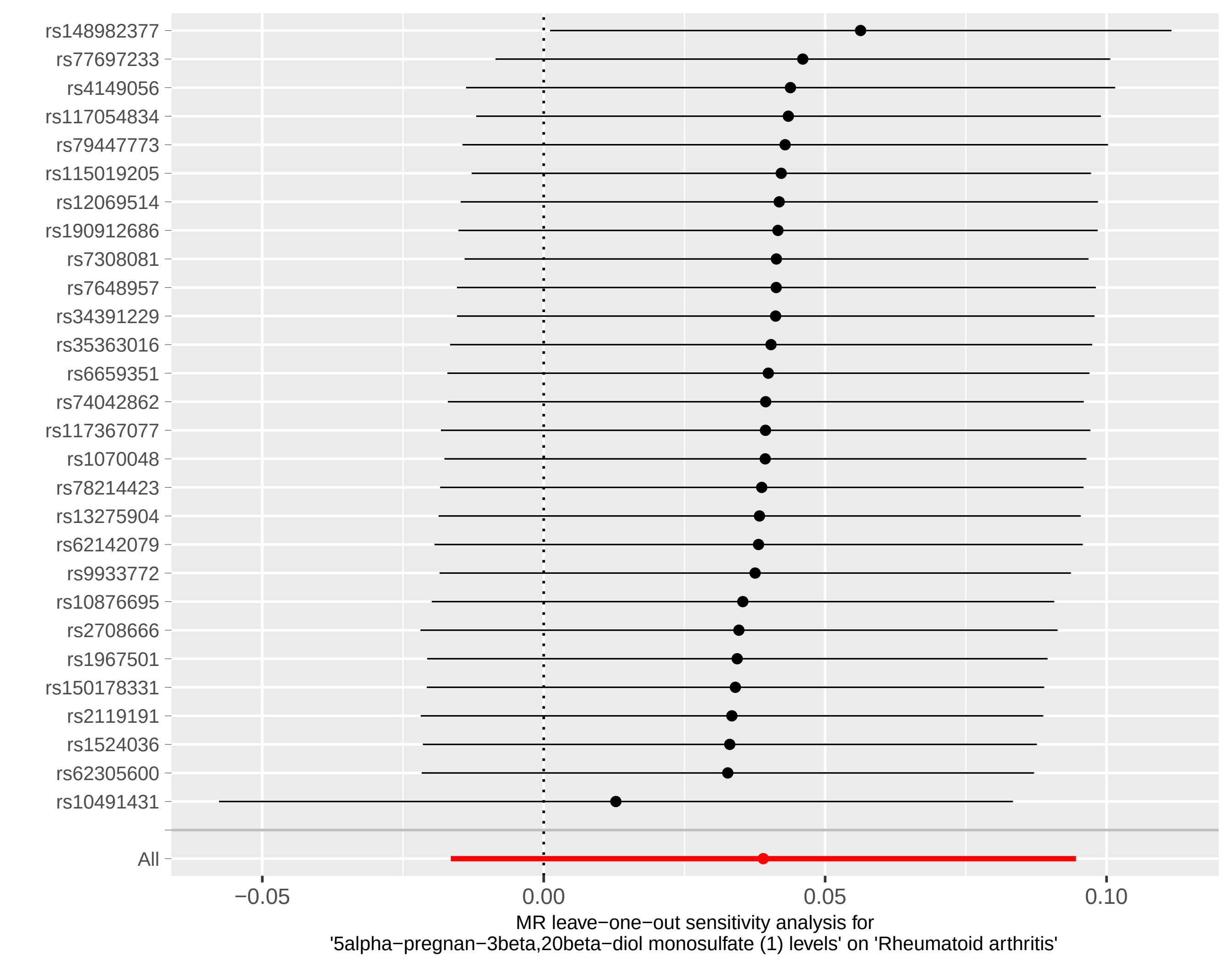
rs148982377
rs77697233
rs4149056
rs117054834
rs79447773
rs115019205
rs12069514
rs190912686
rs7308081
rs7648957
rs34391229
rs35363016
rs6659351
rs74042862
rs117367077
rs1070048
rs78214423
rs13275904
rs62142079
rs9933772
rs10876695
rs2708666
rs1967501
rs150178331
rs2119191
rs1524036
rs62305600
rs10491431
All
−0.05
0.00
0.05
0.10
MR leave−one−out sensitivity analysis for
'5alpha−pregnan−3beta,20beta−diol monosulfate (1) levels' on 'Rheumatoid arthritis'

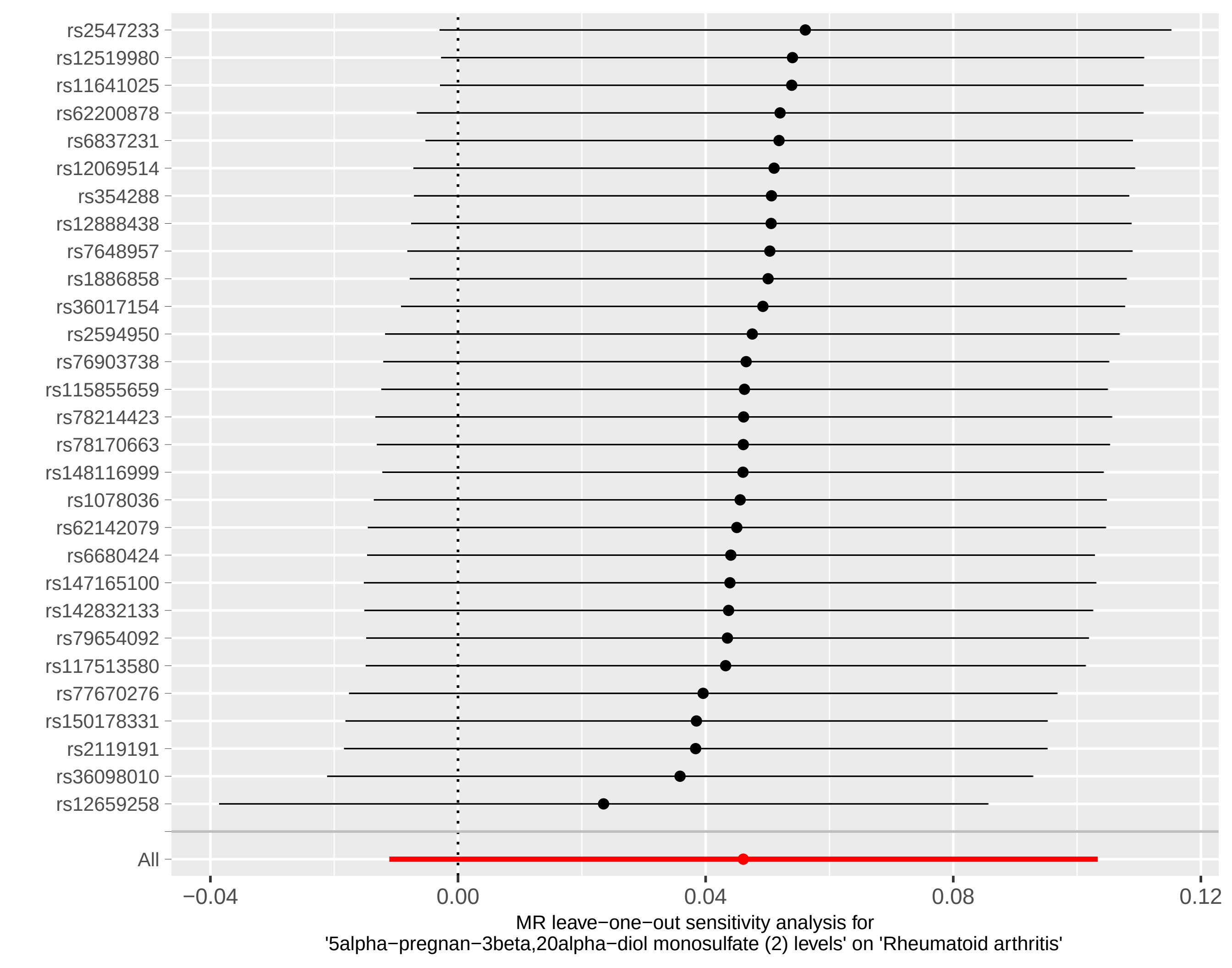
rs2547233
rs12519980
rs11641025
rs62200878
rs6837231
rs12069514
rs354288
rs12888438
rs7648957
rs1886858
rs36017154
rs2594950
rs76903738
rs115855659
rs78214423
rs78170663
rs148116999
rs1078036
rs62142079
rs6680424
rs147165100
rs142832133
rs79654092
rs117513580
rs77670276
rs150178331
rs2119191
rs36098010
rs12659258
All
−0.04
0.00
0.04
0.08
0.12
MR leave−one−out sensitivity analysis for
'5alpha−pregnan−3beta,20alpha−diol monosulfate (2) levels' on 'Rheumatoid arthritis'

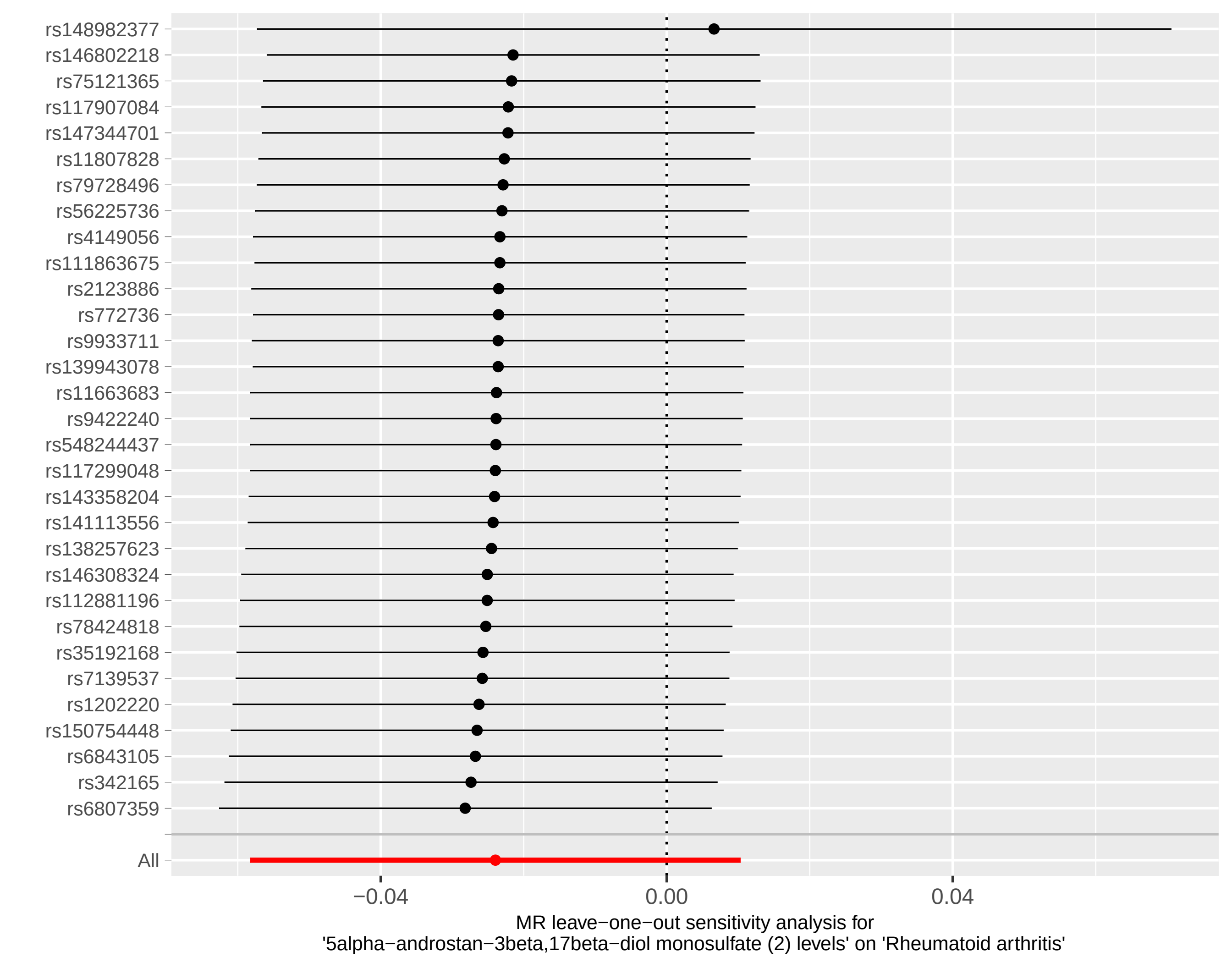
rs148982377
rs146802218
rs75121365
rs117907084
rs147344701
rs11807828
rs79728496
rs56225736
rs4149056
rs111863675
rs2123886
rs772736
rs9933711
rs139943078
rs11663683
rs9422240
rs548244437
rs117299048
rs143358204
rs141113556
rs138257623
rs146308324
rs112881196
rs78424818
rs35192168
rs7139537
rs1202220
rs150754448
rs6843105
rs342165
rs6807359
All
−0.04
0.00
0.04
MR leave−one−out sensitivity analysis for
'5alpha−androstan−3beta,17beta−diol monosulfate (2) levels' on 'Rheumatoid arthritis'

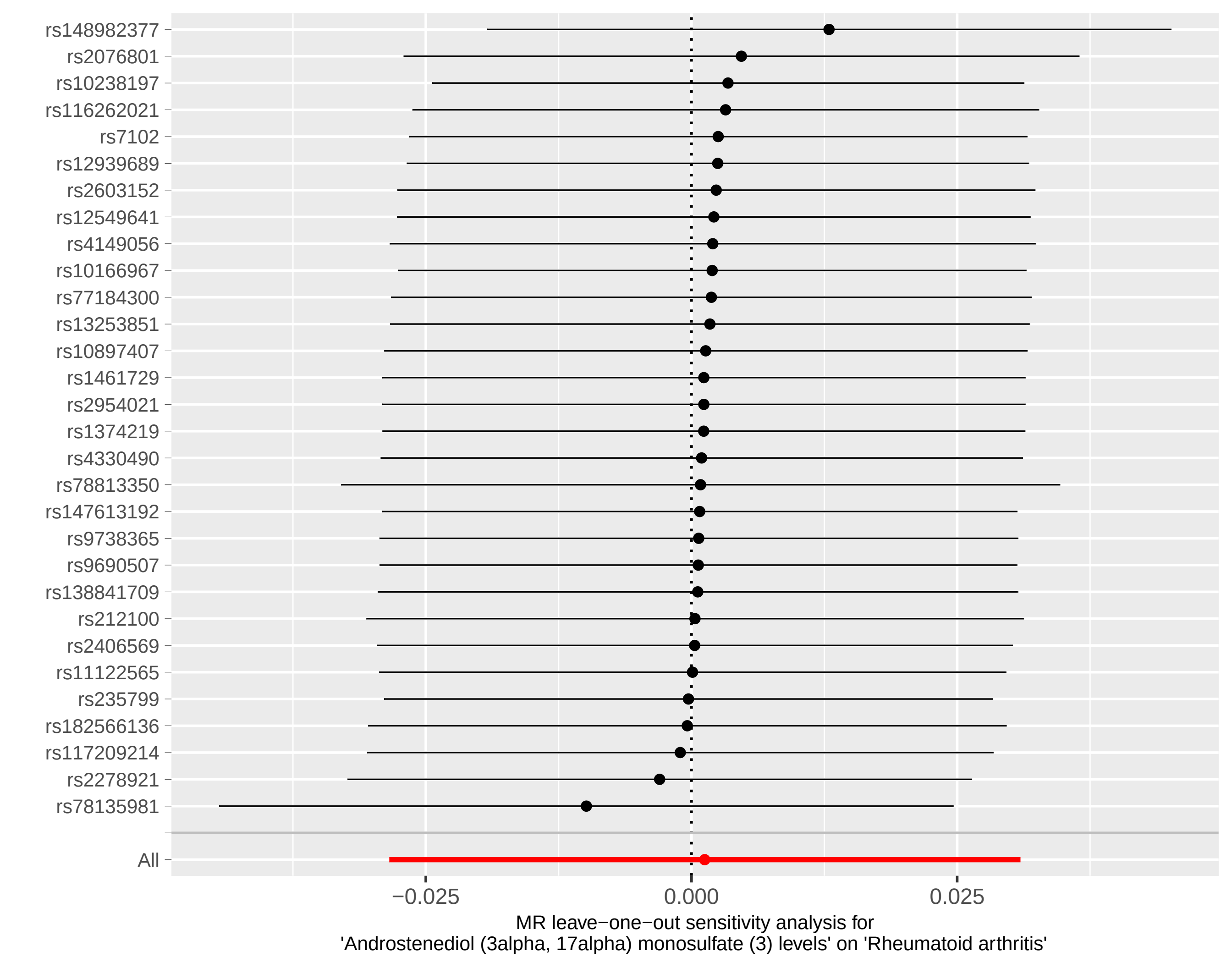
rs148982377
rs2076801
rs10238197
rs116262021
rs7102
rs12939689
rs2603152
rs12549641
rs4149056
rs10166967
rs77184300
rs13253851
rs10897407
rs1461729
rs2954021
rs1374219
rs4330490
rs78813350
rs147613192
rs9738365
rs9690507
rs138841709
rs212100
rs2406569
rs11122565
rs235799
rs182566136
rs117209214
rs2278921
rs78135981
All
−0.025
0.000
0.025
MR leave−one−out sensitivity analysis for
'Androstenediol (3alpha, 17alpha) monosulfate (3) levels' on 'Rheumatoid arthritis'

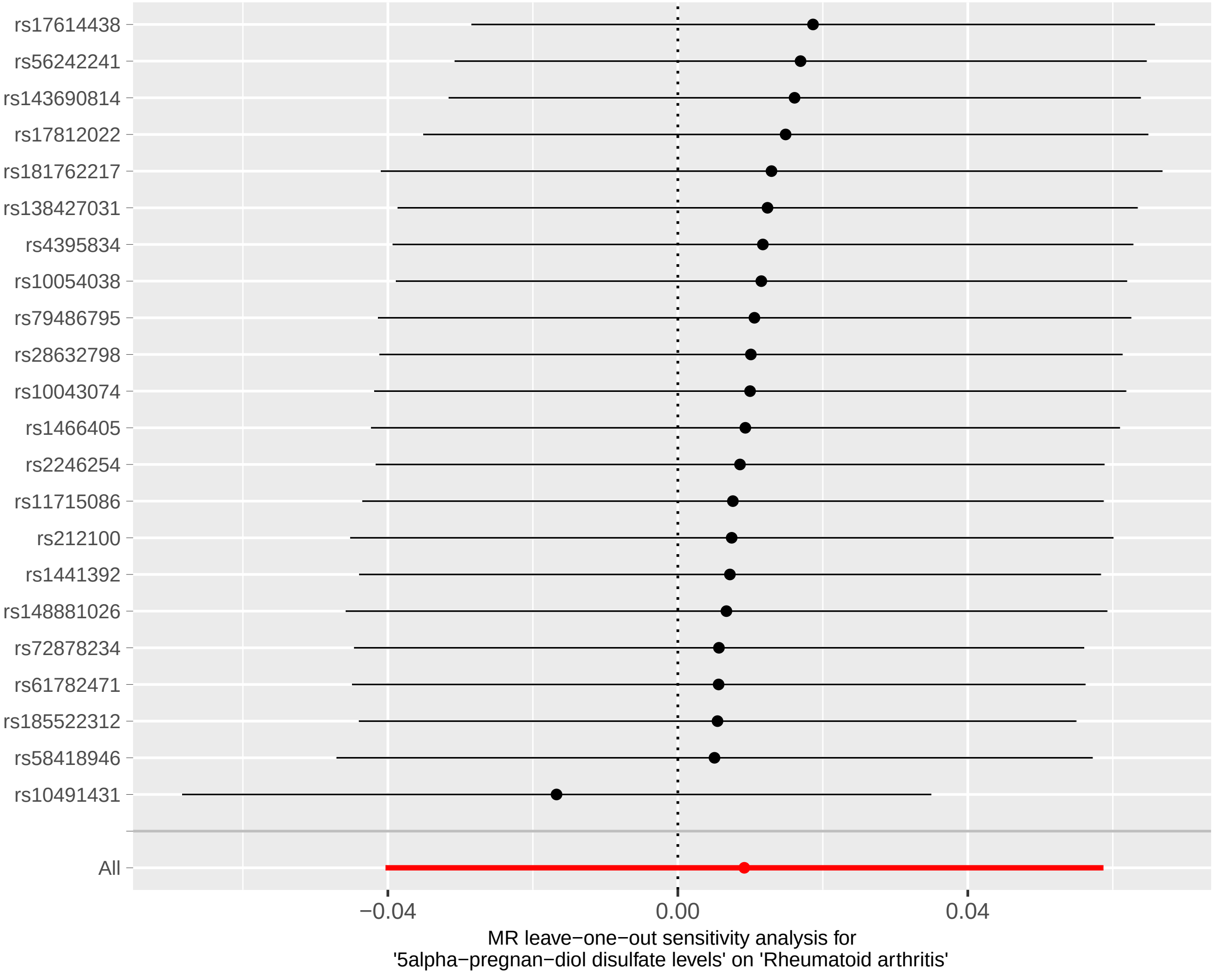

rs17614438
rs56242241
rs143690814
rs17812022
rs181762217
rs138427031
rs4395834
rs10054038
rs79486795
rs28632798
rs10043074
rs1466405
rs2246254
rs11715086
rs212100
rs1441392
rs148881026
rs72878234
rs61782471
rs185522312
rs58418946
rs10491431
All
−0.04
0.00
0.04
MR leave−one−out sensitivity analysis for
'5alpha−pregnan−diol disulfate levels' on 'Rheumatoid arthritis'

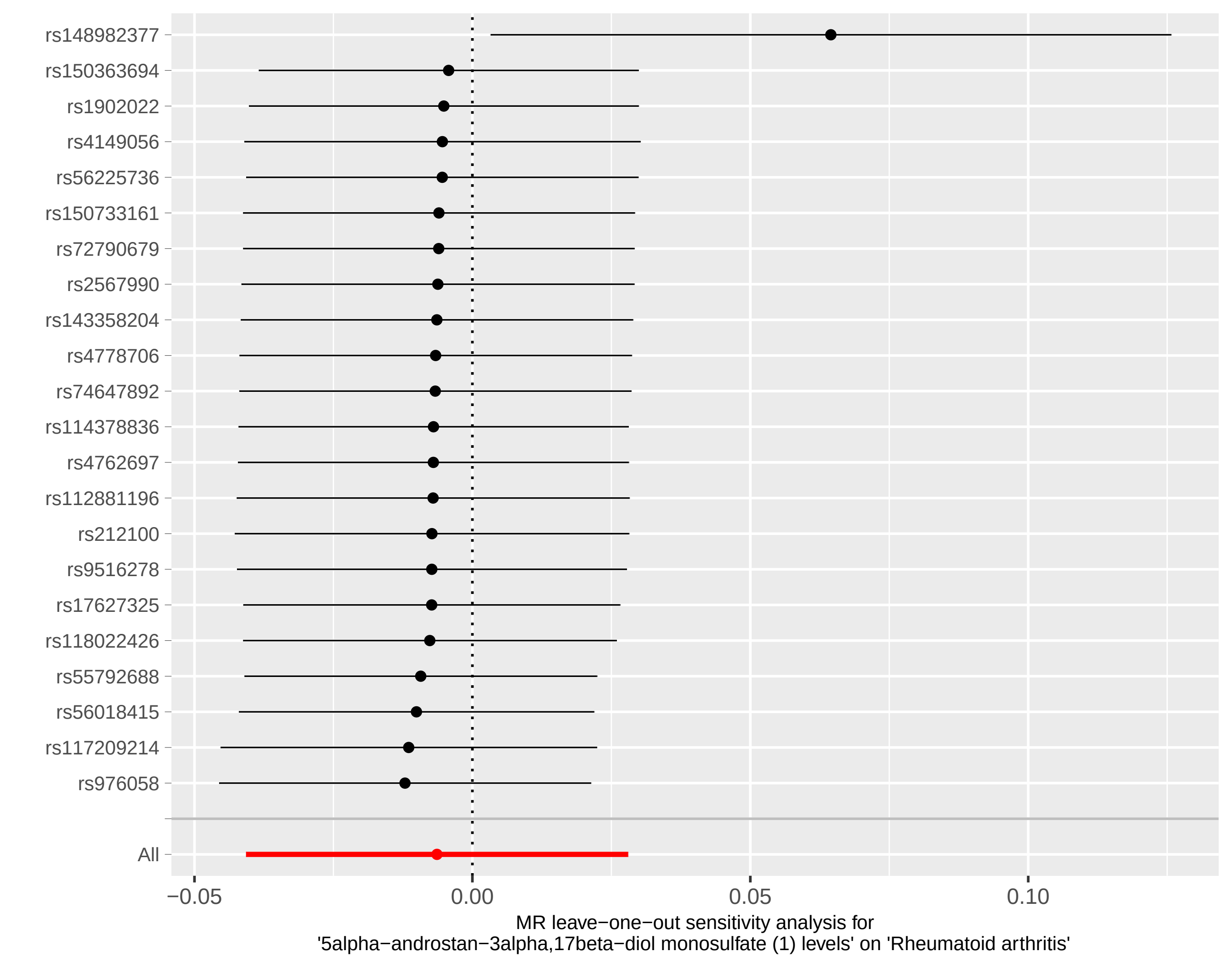

rs148982377
rs150363694
rs1902022
rs4149056
rs56225736
rs150733161
rs72790679
rs2567990
rs143358204
rs4778706
rs74647892
rs114378836
rs4762697
rs112881196
rs212100
rs9516278
rs17627325
rs118022426
rs55792688
rs56018415
rs117209214
rs976058
All
−0.05
0.00
0.05
0.10
MR leave−one−out sensitivity analysis for
'5alpha−androstan−3alpha,17beta−diol monosulfate (1) levels' on 'Rheumatoid arthritis'

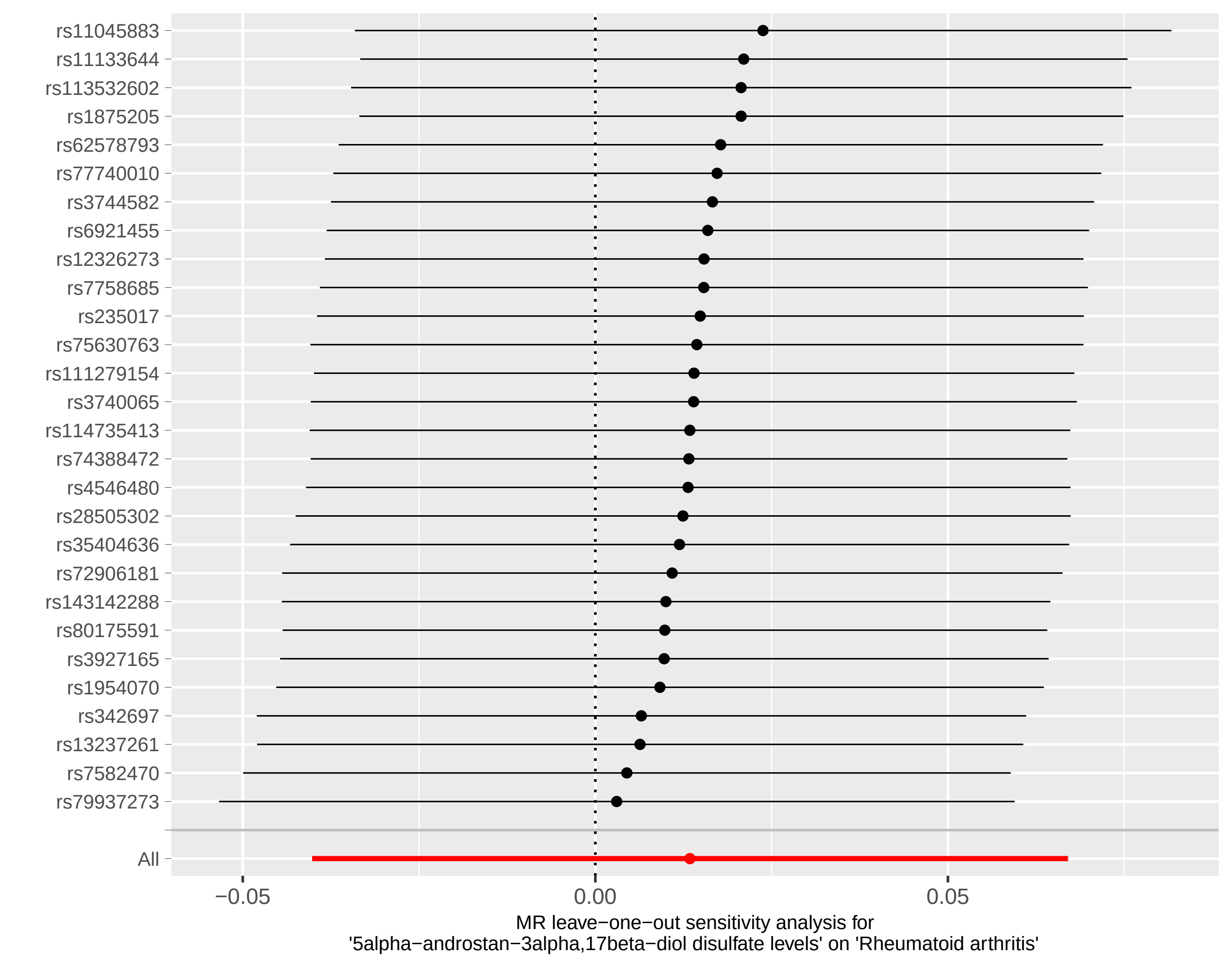

rs11045883
rs11133644
rs113532602
rs1875205
rs62578793
rs77740010
rs3744582
rs6921455
rs12326273
rs7758685
rs235017
rs75630763
rs111279154
rs3740065
rs114735413
rs74388472
rs4546480
rs28505302
rs35404636
rs72906181
rs143142288
rs80175591
rs3927165
rs1954070
rs342697
rs13237261
rs7582470
rs79937273
All
−0.05
0.00
0.05
MR leave−one−out sensitivity analysis for
'5alpha−androstan−3alpha,17beta−diol disulfate levels' on 'Rheumatoid arthritis'

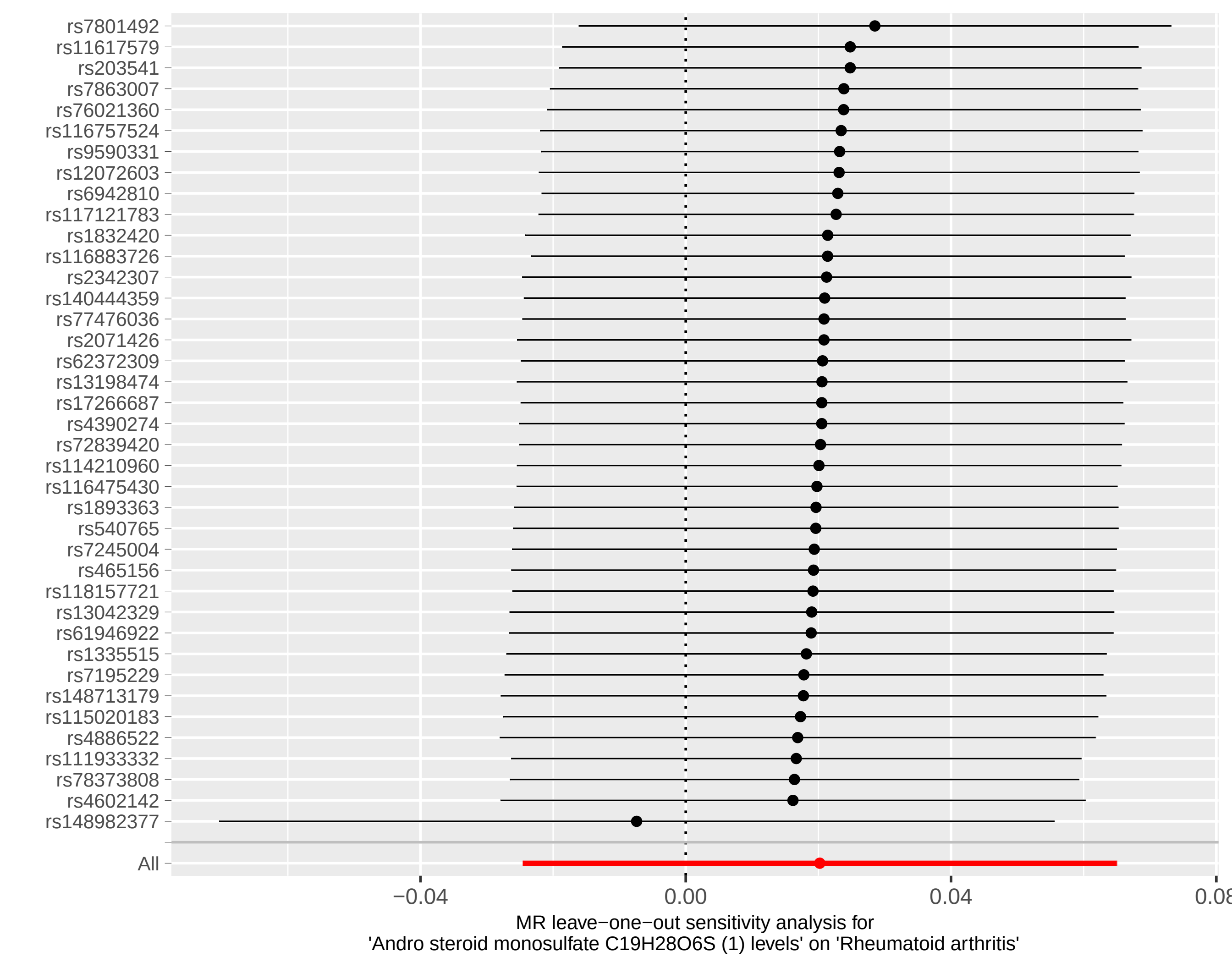
rs7801492
rs11617579
rs203541
rs7863007
rs76021360
rs116757524
rs9590331
rs12072603
rs6942810
rs117121783
rs1832420
rs116883726
rs2342307
rs140444359
rs77476036
rs2071426
rs62372309
rs13198474
rs17266687
rs4390274
rs72839420
rs114210960
rs116475430
rs1893363
rs540765
rs7245004
rs465156
rs118157721
rs13042329
rs61946922
rs1335515
rs7195229
rs148713179
rs115020183
rs4886522
rs111933332
rs78373808
rs4602142
rs148982377
All
−0.04
0.00
0.04
MR leave−one−out sensitivity analysis for
'Andro steroid monosulfate C19H28O6S (1) levels' on 'Rheumatoid arthritis'

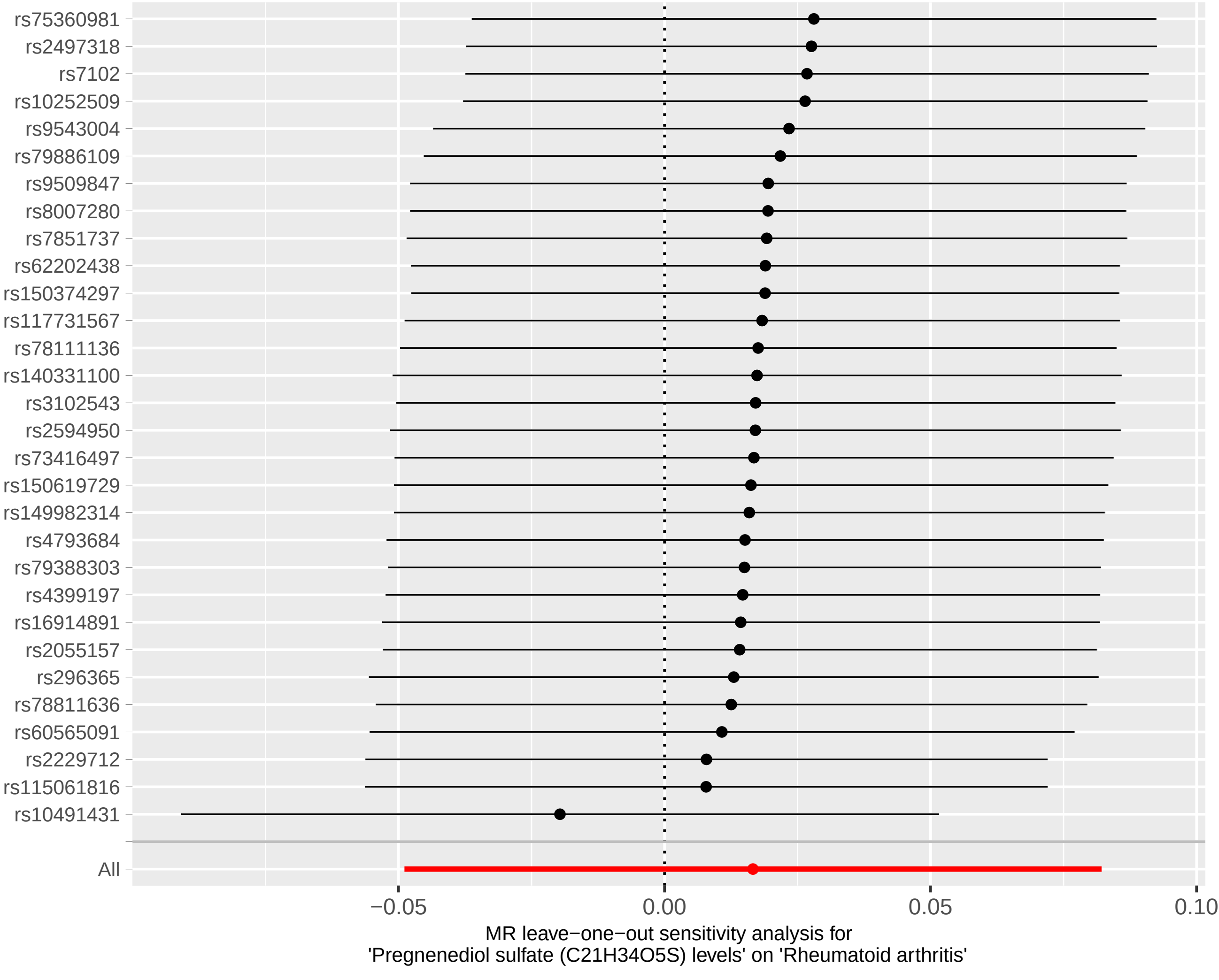
rs75360981
rs2497318
rs7102
rs10252509
rs9543004
rs79886109
rs9509847
rs8007280
rs7851737
rs62202438
rs150374297
rs117731567
rs78111136
rs140331100
rs3102543
rs2594950
rs73416497
rs150619729
rs149982314
rs4793684
rs79388303
rs4399197
rs16914891
rs2055157
rs296365
rs78811636
rs60565091
rs2229712
rs115061816
rs10491431
All
−0.05
0.00
0.05
0.10
MR leave−one−out sensitivity analysis for
'Pregnenediol sulfate (C21H34O5S) levels' on 'Rheumatoid arthritis'

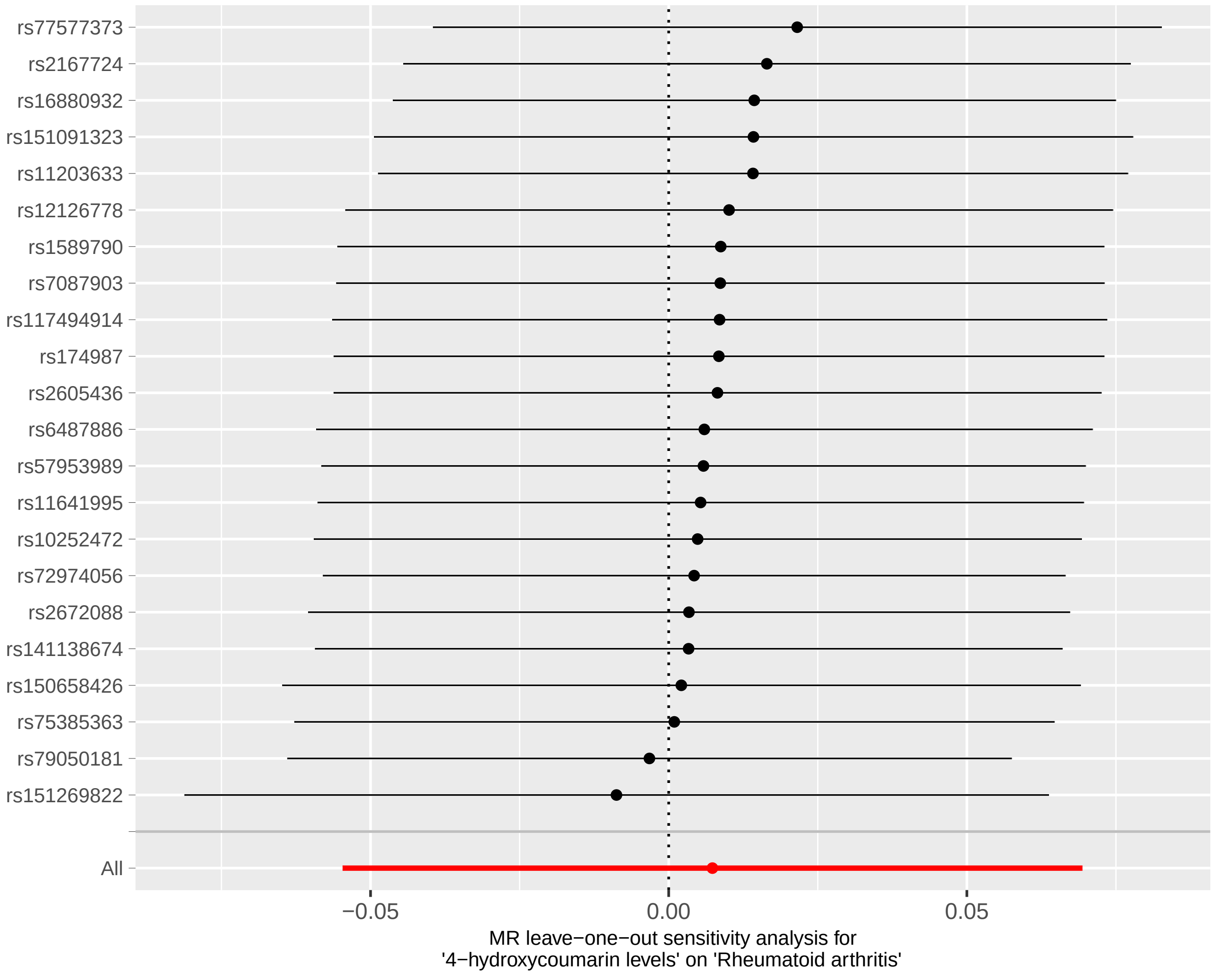
rs77577373
rs2167724
rs16880932
rs151091323
rs11203633
rs12126778
rs1589790
rs7087903
rs117494914
rs174987
rs2605436
rs6487886
rs57953989
rs11641995
rs10252472
rs72974056
rs2672088
rs141138674
rs150658426
rs75385363
rs79050181
rs151269822
All
−0.05
0.00
0.05
MR leave−one−out sensitivity analysis for
'4−hydroxycoumarin levels' on 'Rheumatoid arthritis'

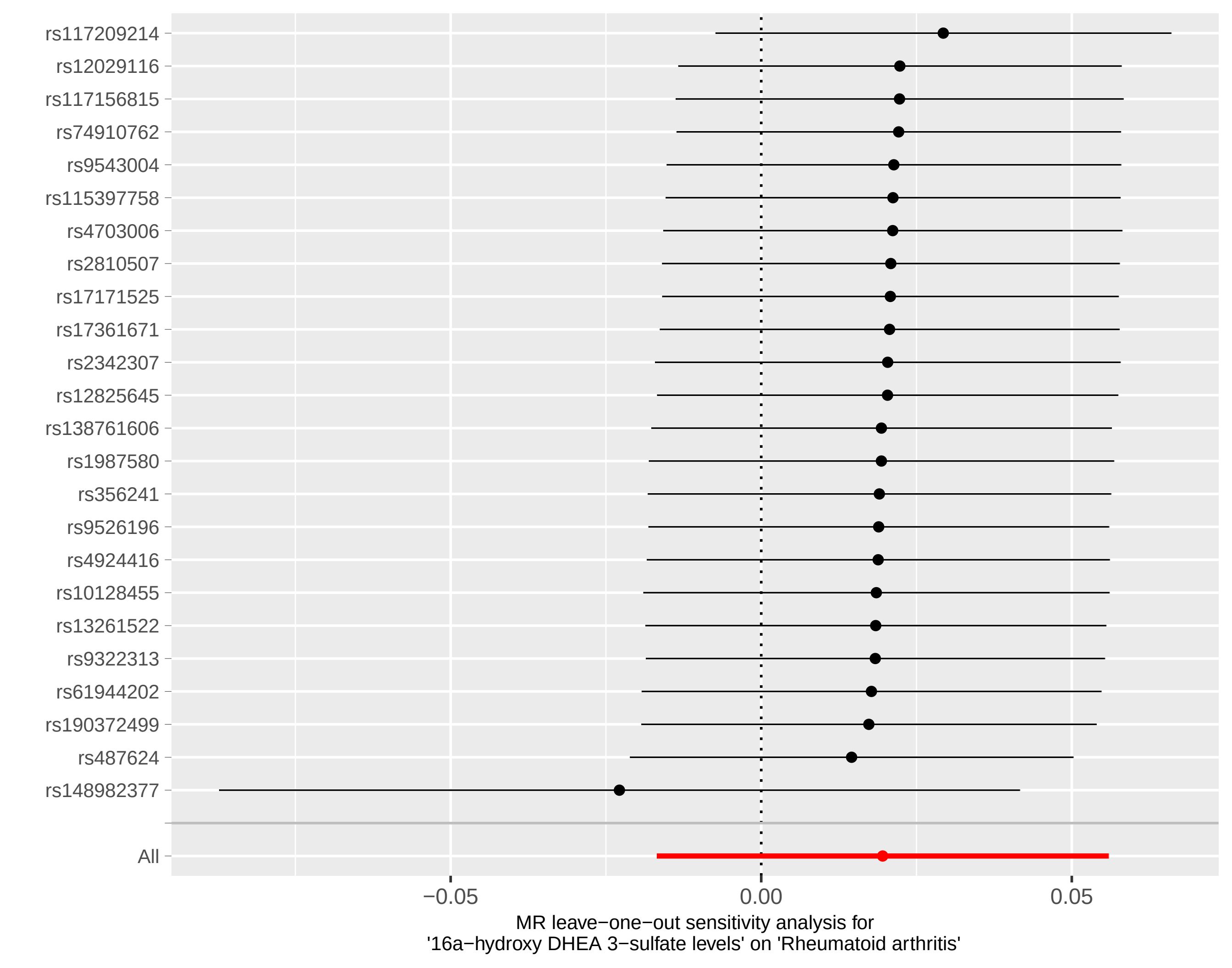

rs117209214
rs12029116
rs117156815
rs74910762
rs9543004
rs115397758
rs4703006
rs2810507
rs17171525
rs17361671
rs2342307
rs12825645
rs138761606
rs1987580
rs356241
rs9526196
rs4924416
rs10128455
rs13261522
rs9322313
rs61944202
rs190372499
rs487624
rs148982377
All
−0.05
0.00
0.05
MR leave−one−out sensitivity analysis for
'16a−hydroxy DHEA 3−sulfate levels' on 'Rheumatoid arthritis'

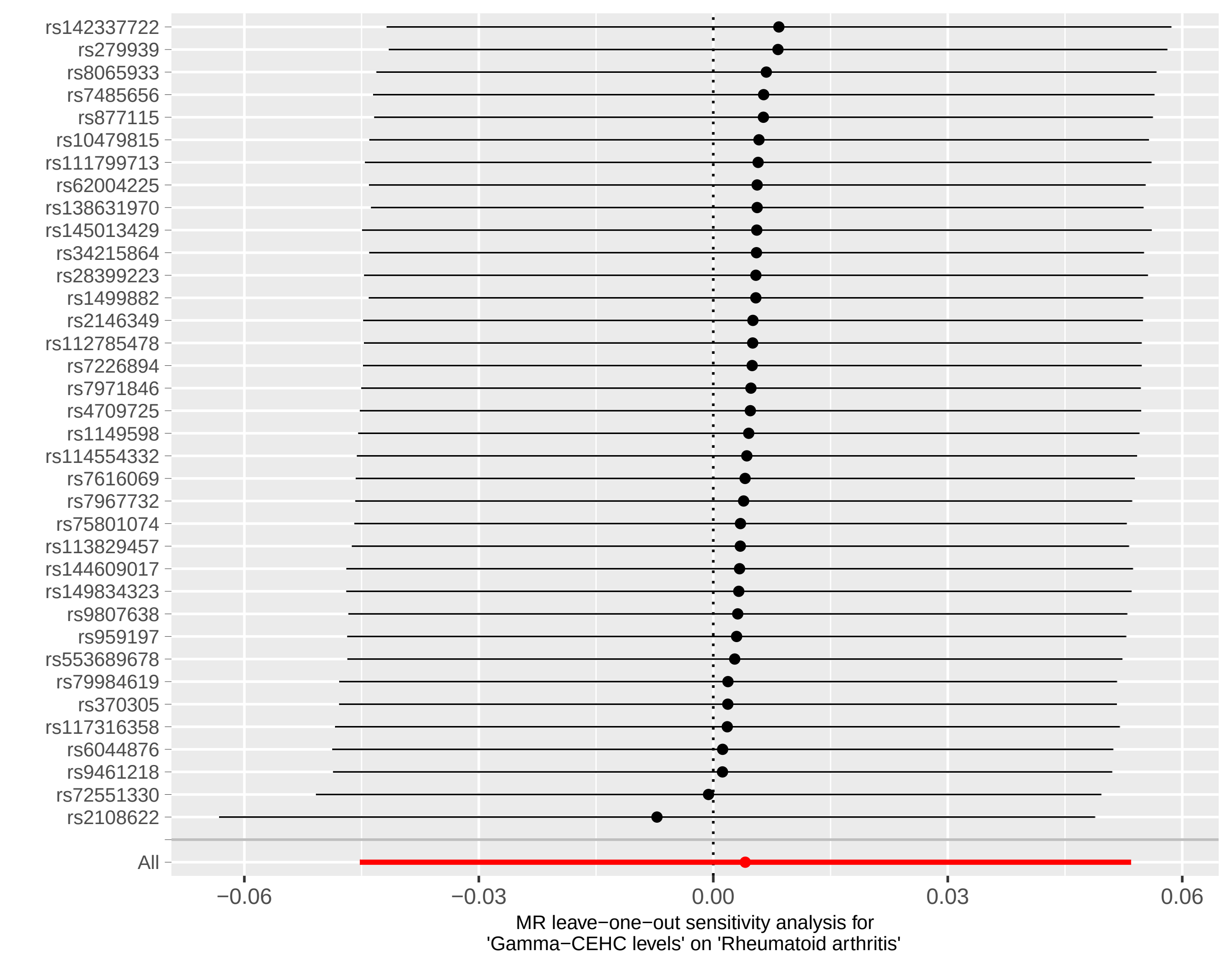
rs142337722
rs279939
rs8065933
rs7485656
rs877115
rs10479815
rs111799713
rs62004225
rs138631970
rs145013429
rs34215864
rs28399223
rs1499882
rs2146349
rs112785478
rs7226894
rs7971846
rs4709725
rs1149598
rs114554332
rs7616069
rs7967732
rs75801074
rs113829457
rs144609017
rs149834323
rs9807638
rs959197
rs553689678
rs79984619
rs370305
rs117316358
rs6044876
rs9461218
rs72551330
rs2108622
All
−0.06
−0.03
0.00
0.03
0.06
MR leave−one−out sensitivity analysis for
'Gamma−CEHC levels' on 'Rheumatoid arthritis'

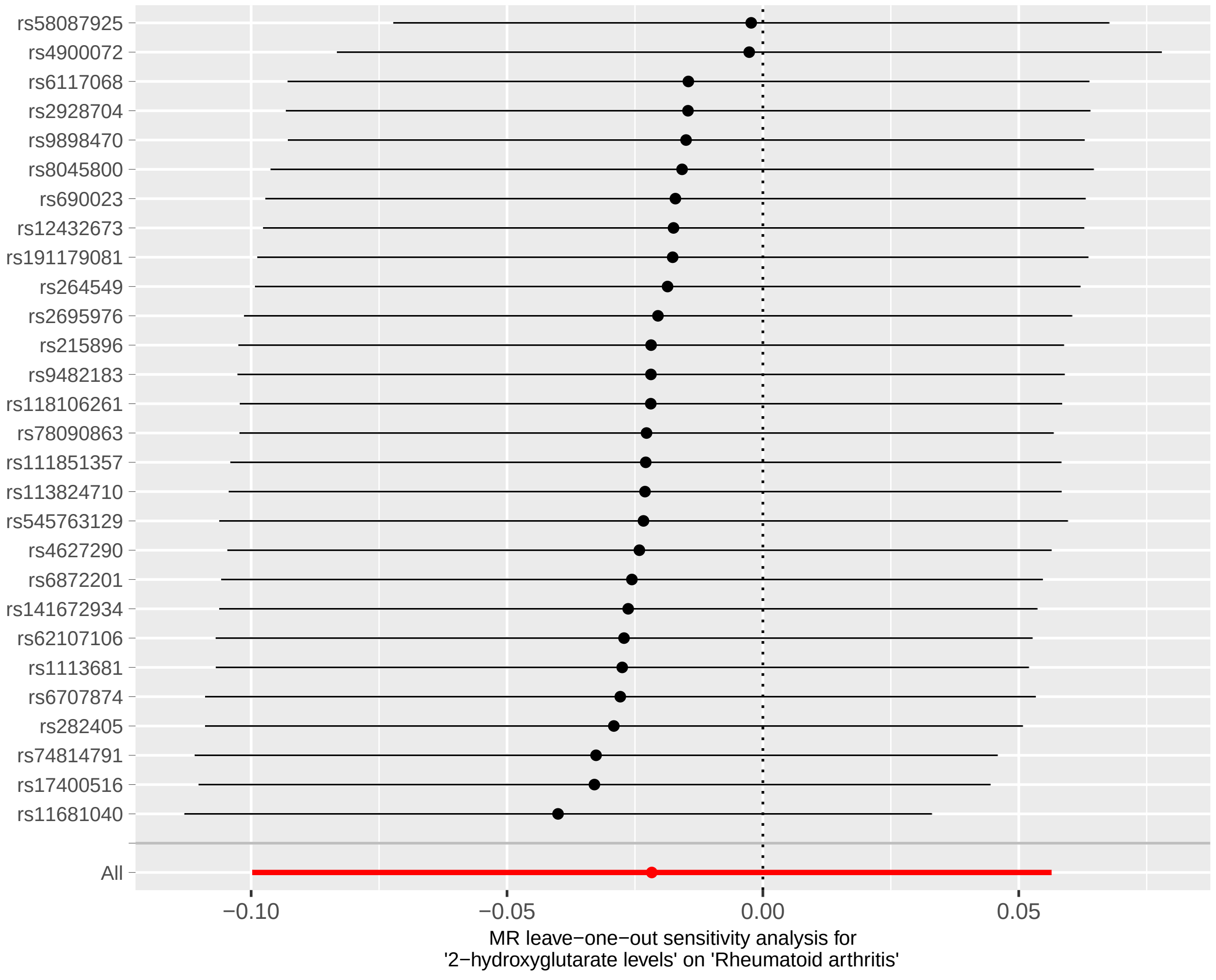
rs58087925
rs4900072
rs6117068
rs2928704
rs9898470
rs8045800
rs690023
rs12432673
rs191179081
rs264549
rs2695976
rs215896
rs9482183
rs118106261
rs78090863
rs111851357
rs113824710
rs545763129
rs4627290
rs6872201
rs141672934
rs62107106
rs1113681
rs6707874
rs282405
rs74814791
rs17400516
rs11681040
All
−0.10
−0.05
0.00
0.05
MR leave−one−out sensitivity analysis for
'2−hydroxyglutarate levels' on 'Rheumatoid arthritis'

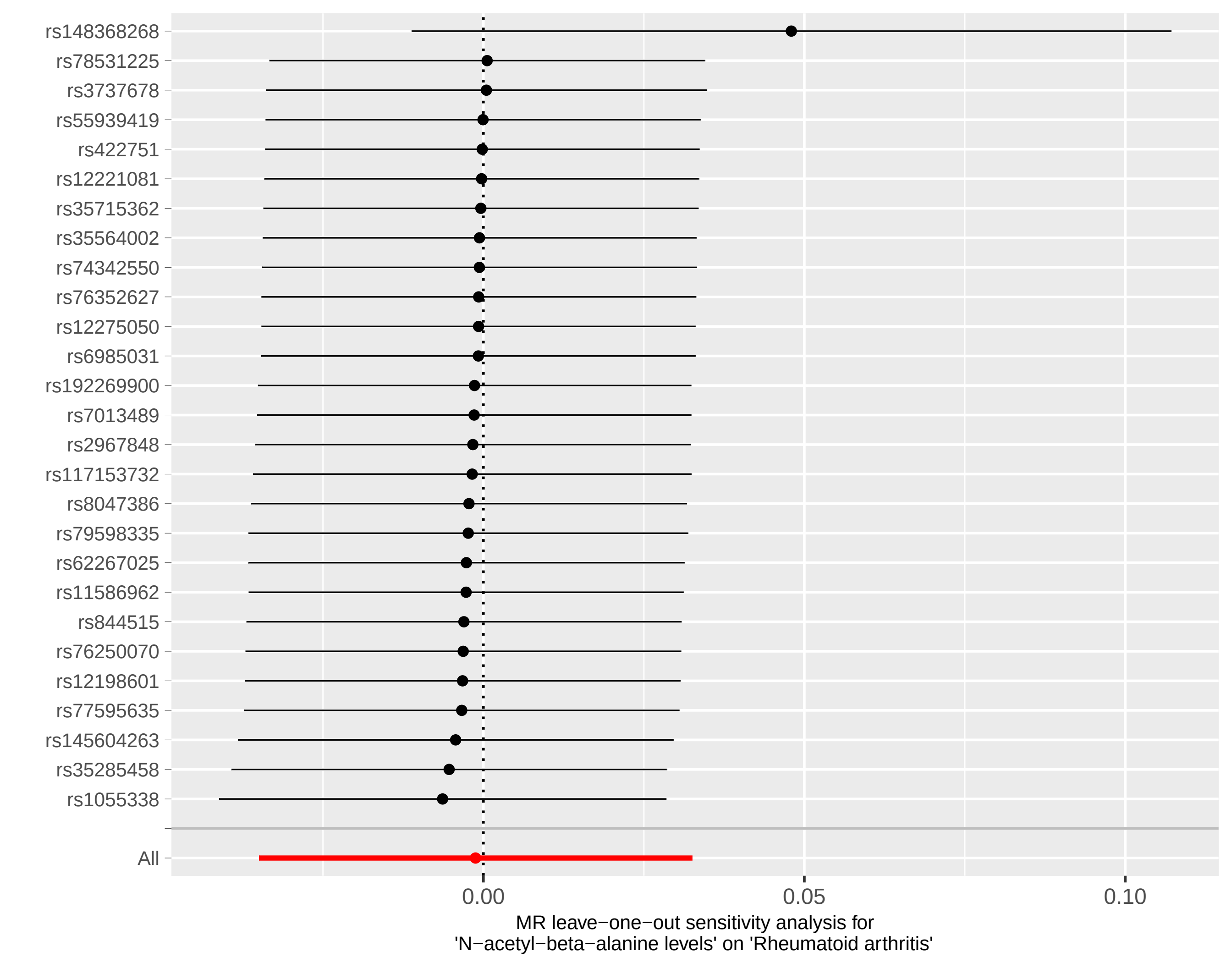

rs148368268
rs78531225
rs3737678
rs55939419
rs422751
rs12221081
rs35715362
rs35564002
rs74342550
rs76352627
rs12275050
rs6985031
rs192269900
rs7013489
rs2967848
rs117153732
rs8047386
rs79598335
rs62267025
rs11586962
rs844515
rs76250070
rs12198601
rs77595635
rs145604263
rs35285458
rs1055338
All
0.00
0.05
0.10
MR leave−one−out sensitivity analysis for
'N−acetyl−beta−alanine levels' on 'Rheumatoid arthritis'

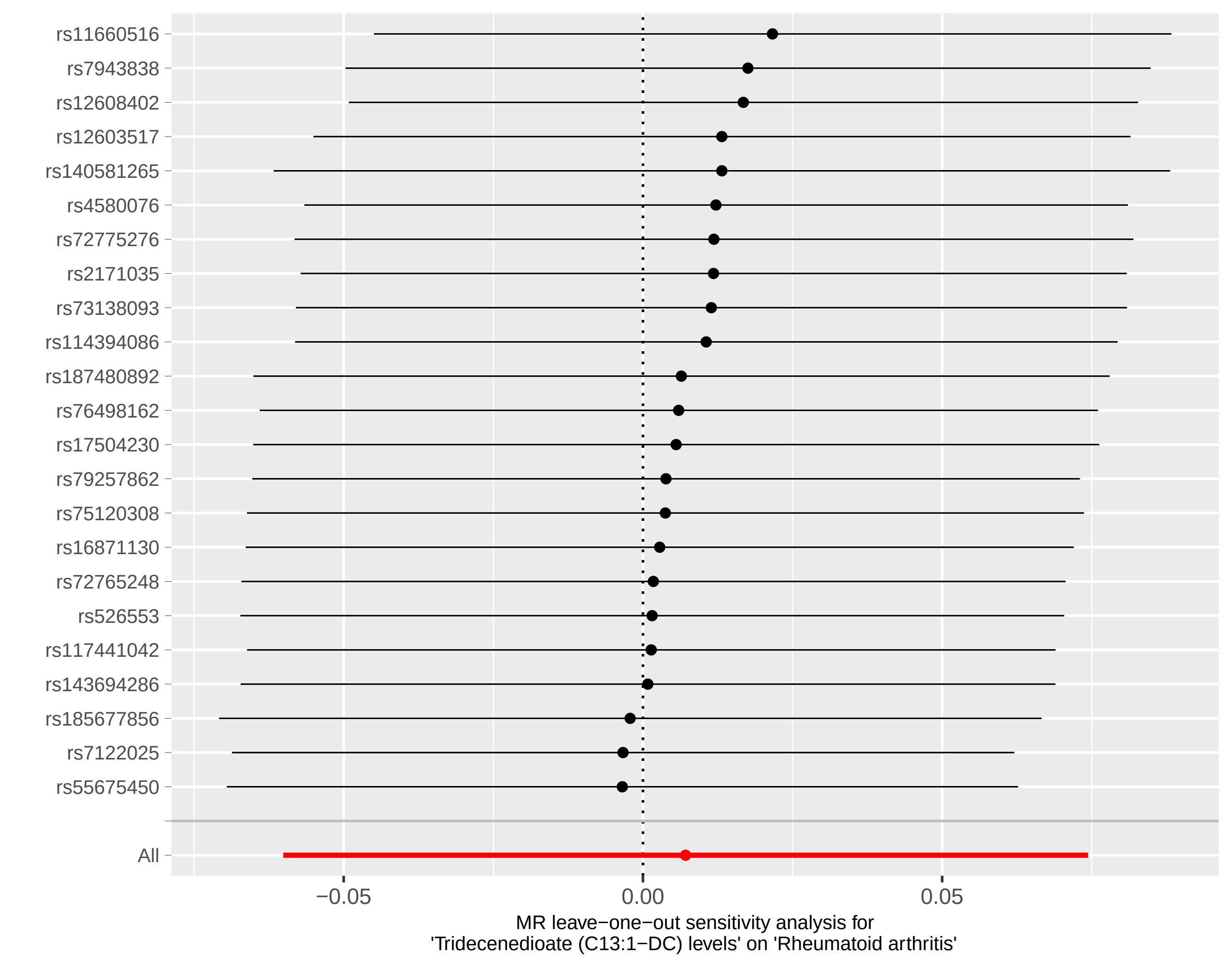

rs11660516
rs7943838
rs12608402
rs12603517
rs140581265
rs4580076
rs72775276
rs2171035
rs73138093
rs114394086
rs187480892
rs76498162
rs17504230
rs79257862
rs75120308
rs16871130
rs72765248
rs526553
rs117441042
rs143694286
rs185677856
rs7122025
rs55675450
All
−0.05
0.00
0.05
MR leave−one−out sensitivity analysis for
'Tridecenedioate (C13:1−DC) levels' on 'Rheumatoid arthritis'

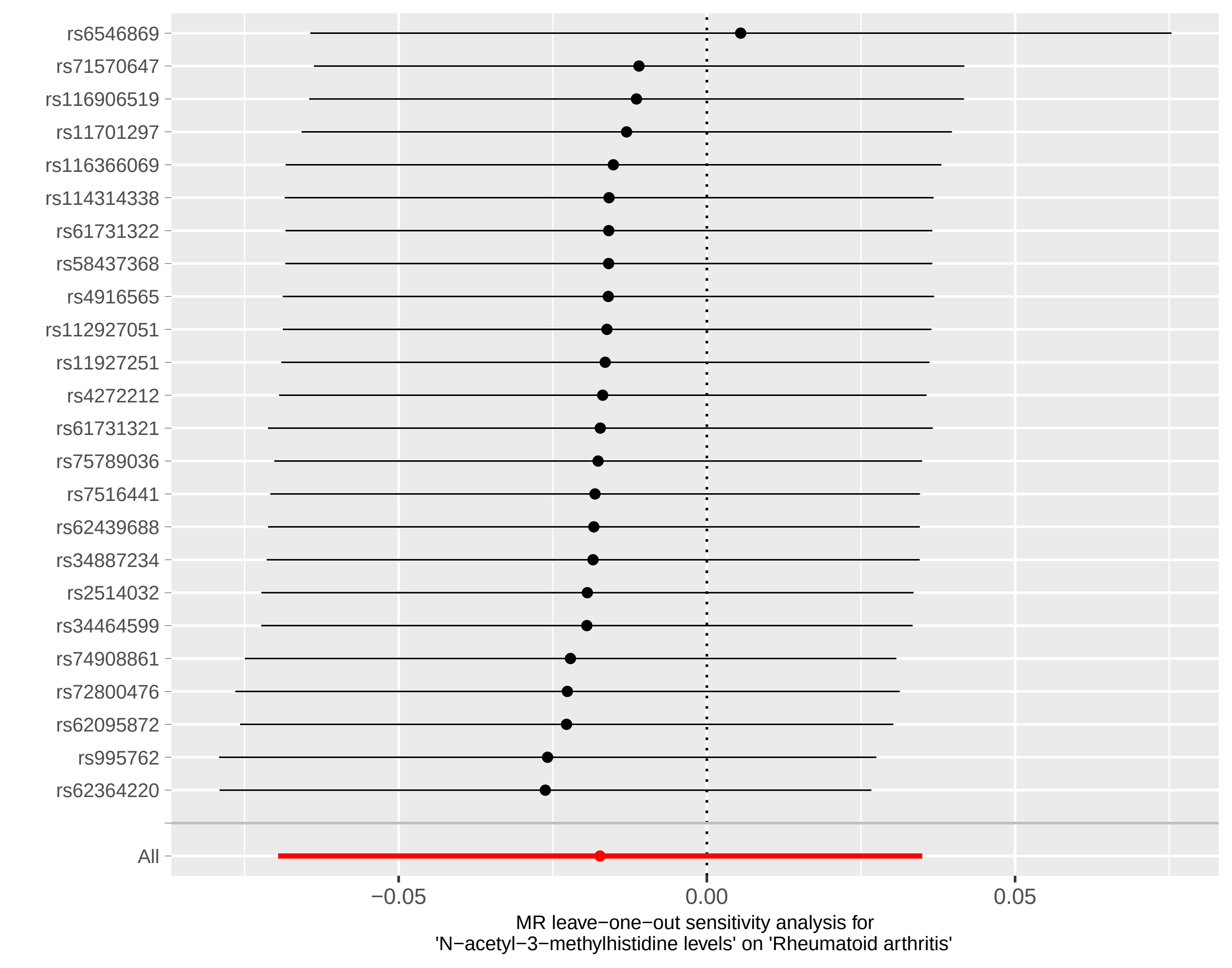
rs6546869
rs71570647
rs116906519
rs11701297
rs116366069
rs114314338
rs61731322
rs58437368
rs4916565
rs112927051
rs11927251
rs4272212
rs61731321
rs75789036
rs7516441
rs62439688
rs34887234
rs2514032
rs34464599
rs74908861
rs72800476
rs62095872
rs995762
rs62364220
All
−0.05
0.00
0.05
MR leave−one−out sensitivity analysis for
'N−acetyl−3−methylhistidine levels' on 'Rheumatoid arthritis'

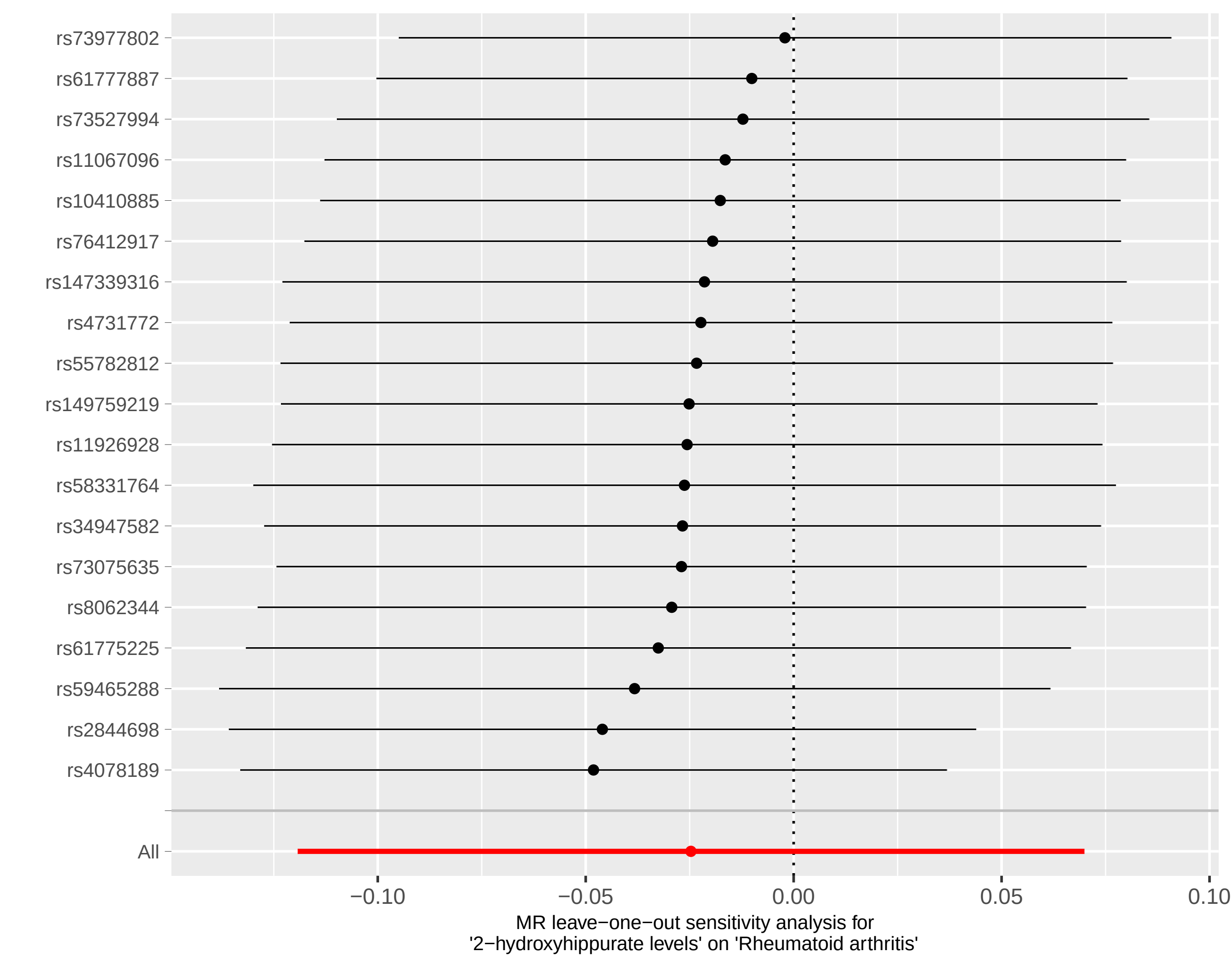
rs73977802
rs61777887
rs73527994
rs11067096
rs10410885
rs76412917
rs147339316
rs4731772
rs55782812
rs149759219
rs11926928
rs58331764
rs34947582
rs73075635
rs8062344
rs61775225
rs59465288
rs2844698
rs4078189
All
−0.10
−0.05
0.00
0.05
0.10
MR leave−one−out sensitivity analysis for
'2−hydroxyhippurate levels' on 'Rheumatoid arthritis'

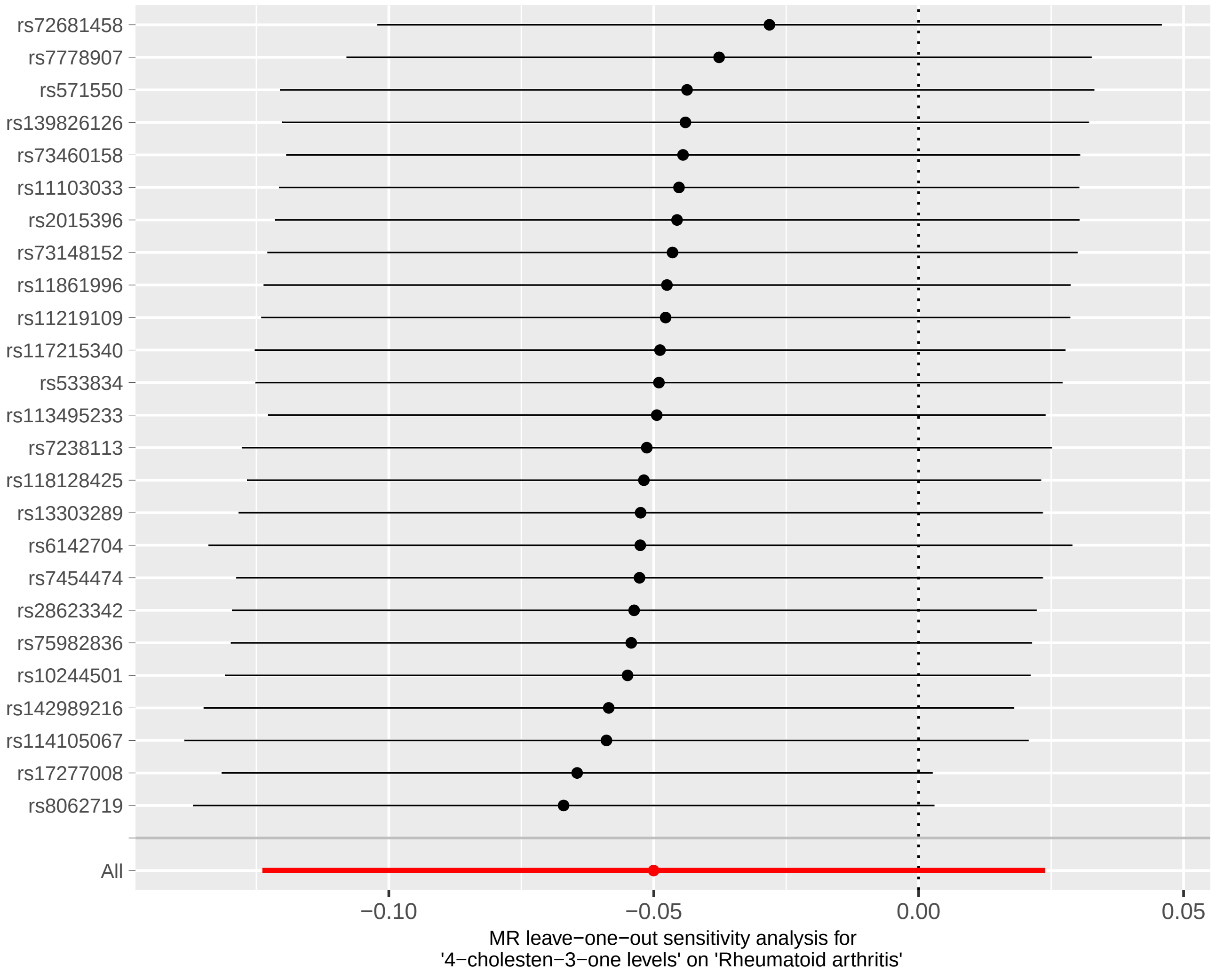
rs72681458
rs7778907
rs571550
rs139826126
rs73460158
rs11103033
rs2015396
rs73148152
rs11861996
rs11219109
rs117215340
rs533834
rs113495233
rs7238113
rs118128425
rs13303289
rs6142704
rs7454474
rs28623342
rs75982836
rs10244501
rs142989216
rs114105067
rs17277008
rs8062719
All
−0.10
−0.05
0.00
0.05
MR leave−one−out sensitivity analysis for
'4−cholesten−3−one levels' on 'Rheumatoid arthritis'

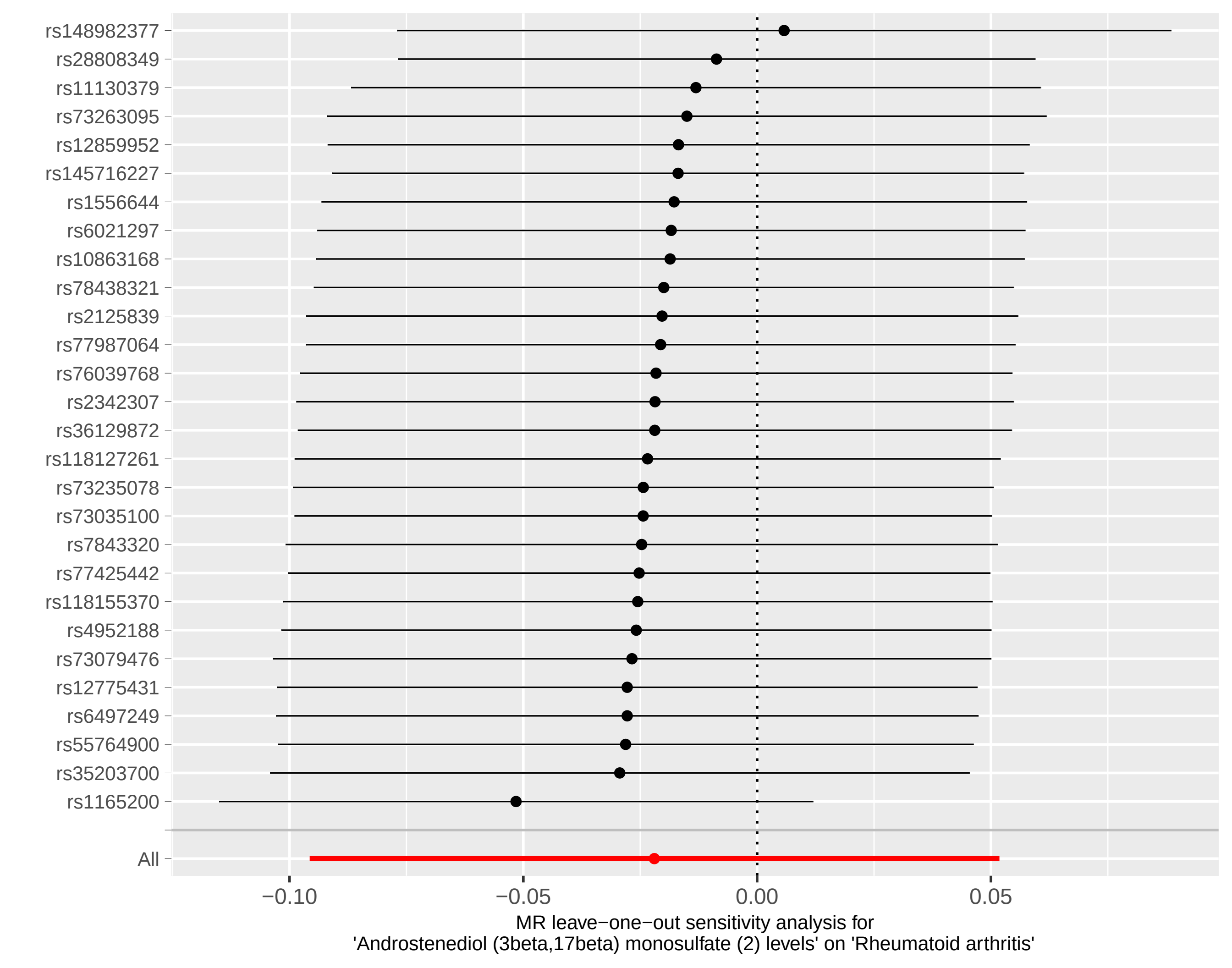

rs148982377
rs28808349
rs11130379
rs73263095
rs12859952
rs145716227
rs1556644
rs6021297
rs10863168
rs78438321
rs2125839
rs77987064
rs76039768
rs2342307
rs36129872
rs118127261
rs73235078
rs73035100
rs7843320
rs77425442
rs118155370
rs4952188
rs73079476
rs12775431
rs6497249
rs55764900
rs35203700
rs1165200
All
−0.10
−0.05
0.00
0.05
MR leave−one−out sensitivity analysis for
'Androstenediol (3beta,17beta) monosulfate (2) levels' on 'Rheumatoid arthritis'

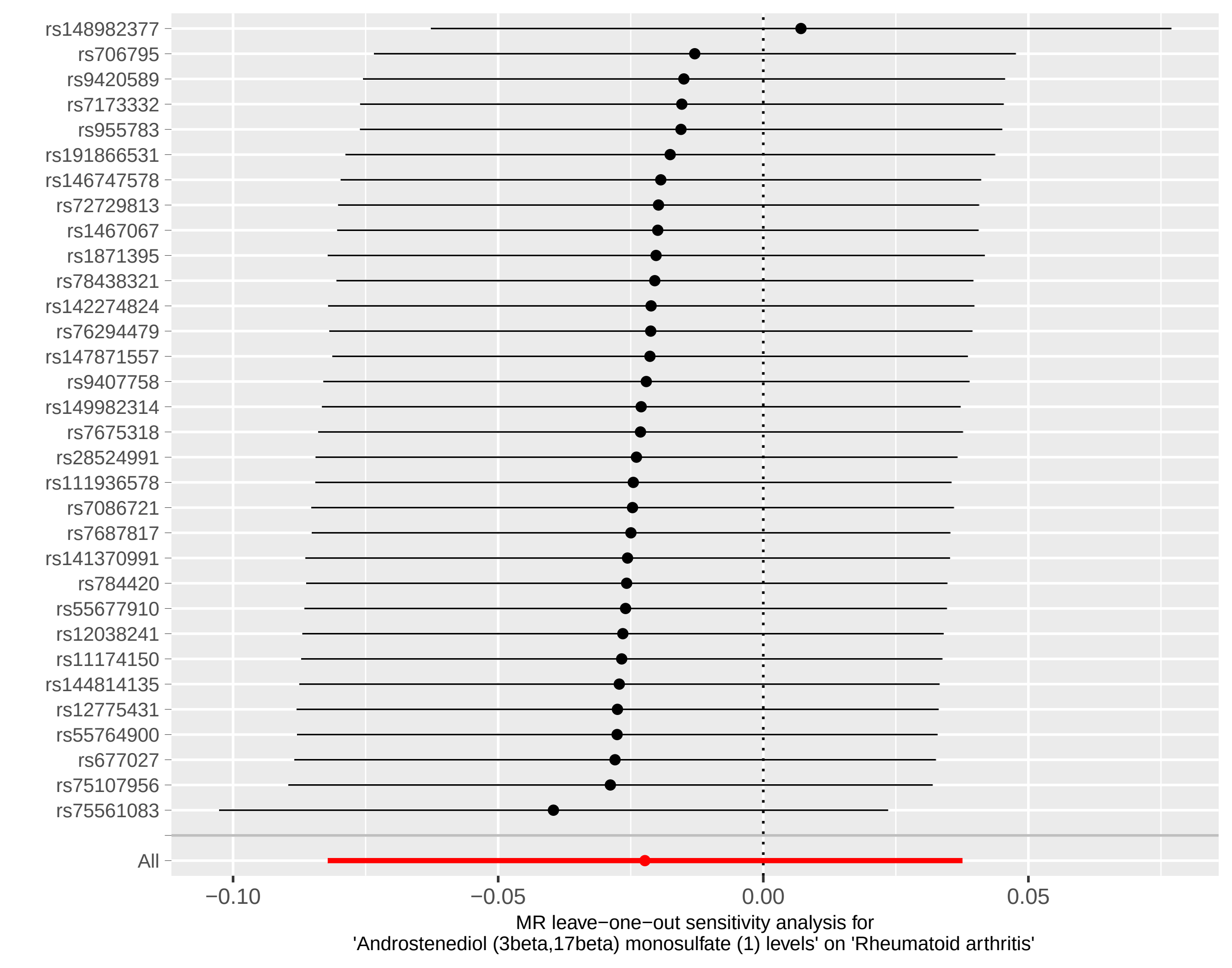
rs148982377
rs706795
rs9420589
rs7173332
rs955783
rs191866531
rs146747578
rs72729813
rs1467067
rs1871395
rs78438321
rs142274824
rs76294479
rs147871557
rs9407758
rs149982314
rs7675318
rs28524991
rs111936578
rs7086721
rs7687817
rs141370991
rs784420
rs55677910
rs12038241
rs11174150
rs144814135
rs12775431
rs55764900
rs677027
rs75107956
rs75561083
All
−0.10
−0.05
0.00
0.05
MR leave−one−out sensitivity analysis for
'Androstenediol (3beta,17beta) monosulfate (1) levels' on 'Rheumatoid arthritis'

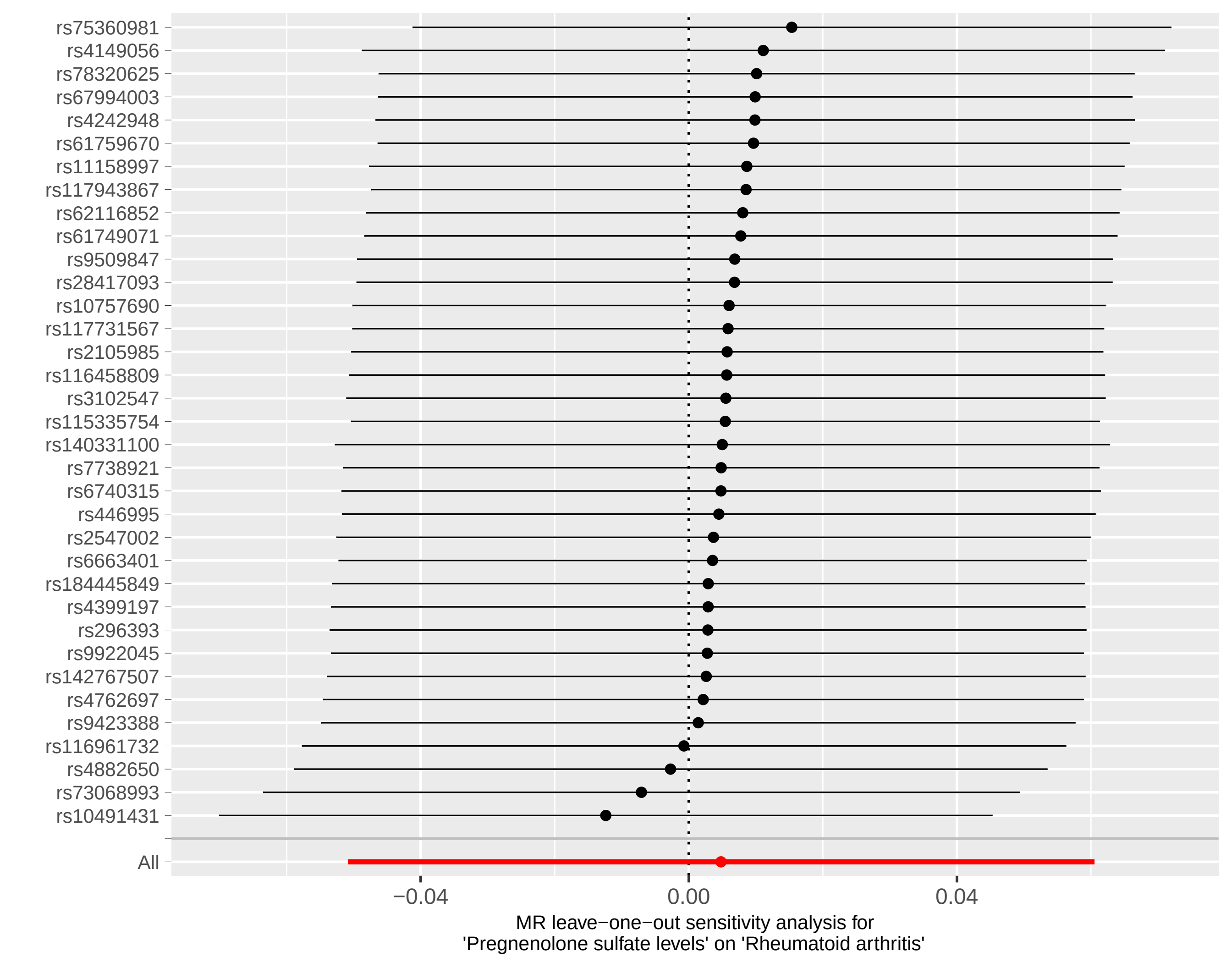
rs75360981
rs4149056
rs78320625
rs67994003
rs4242948
rs61759670
rs11158997
rs117943867
rs62116852
rs61749071
rs9509847
rs28417093
rs10757690
rs117731567
rs2105985
rs116458809
rs3102547
rs115335754
rs140331100
rs7738921
rs6740315
rs446995
rs2547002
rs6663401
rs184445849
rs4399197
rs296393
rs9922045
rs142767507
rs4762697
rs9423388
rs116961732
rs4882650
rs73068993
rs10491431
All
−0.04
0.00
0.04
MR leave−one−out sensitivity analysis for
'Pregnenolone sulfate levels' on 'Rheumatoid arthritis'

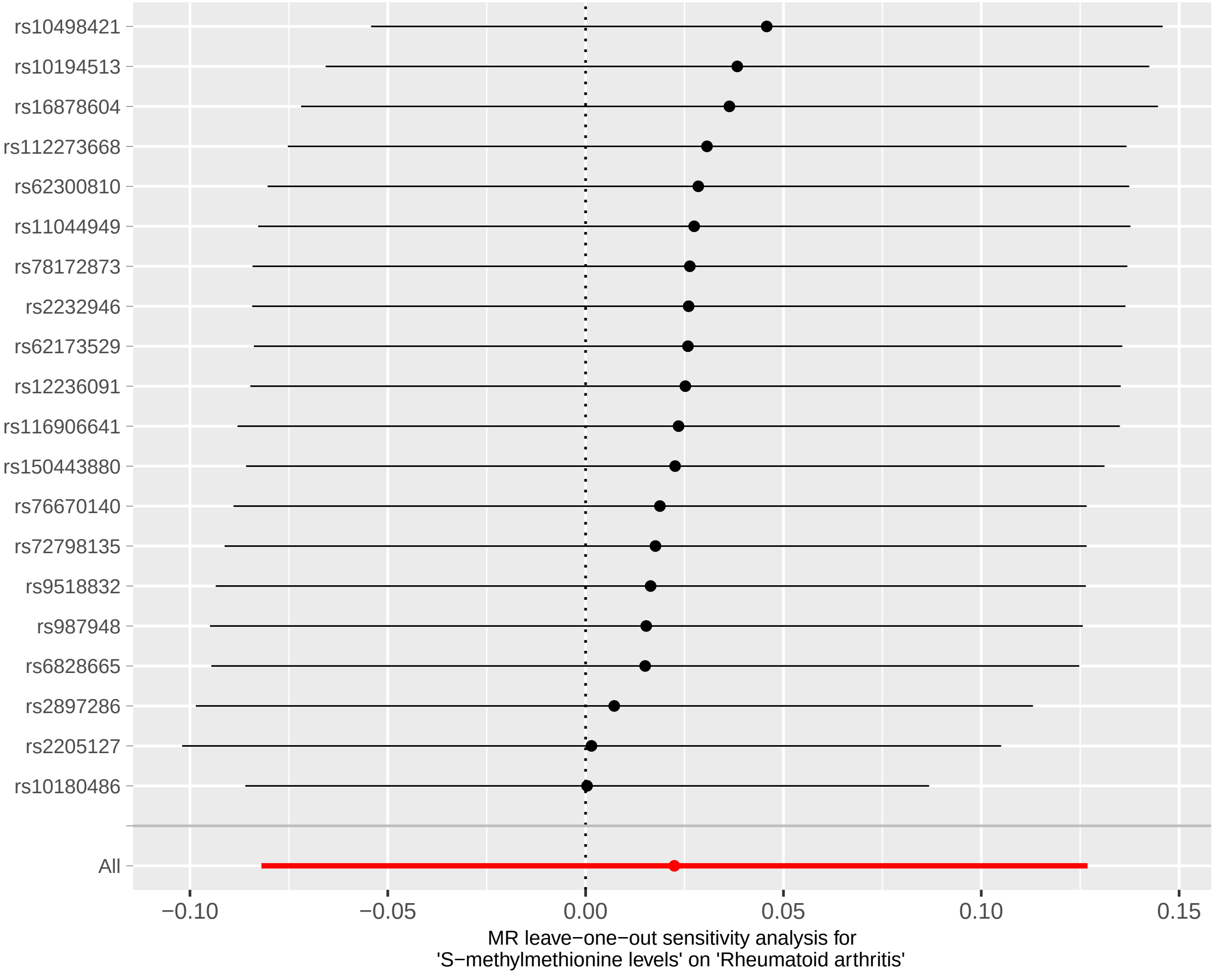
rs10498421
rs10194513
rs16878604
rs112273668
rs62300810
rs11044949
rs78172873
rs2232946
rs62173529
rs12236091
rs116906641
rs150443880
rs76670140
rs72798135
rs9518832
rs987948
rs6828665
rs2897286
rs2205127
rs10180486
All
−0.10
−0.05
0.00
0.05
0.10
0.15
MR leave−one−out sensitivity analysis for
'S−methylmethionine levels' on 'Rheumatoid arthritis'

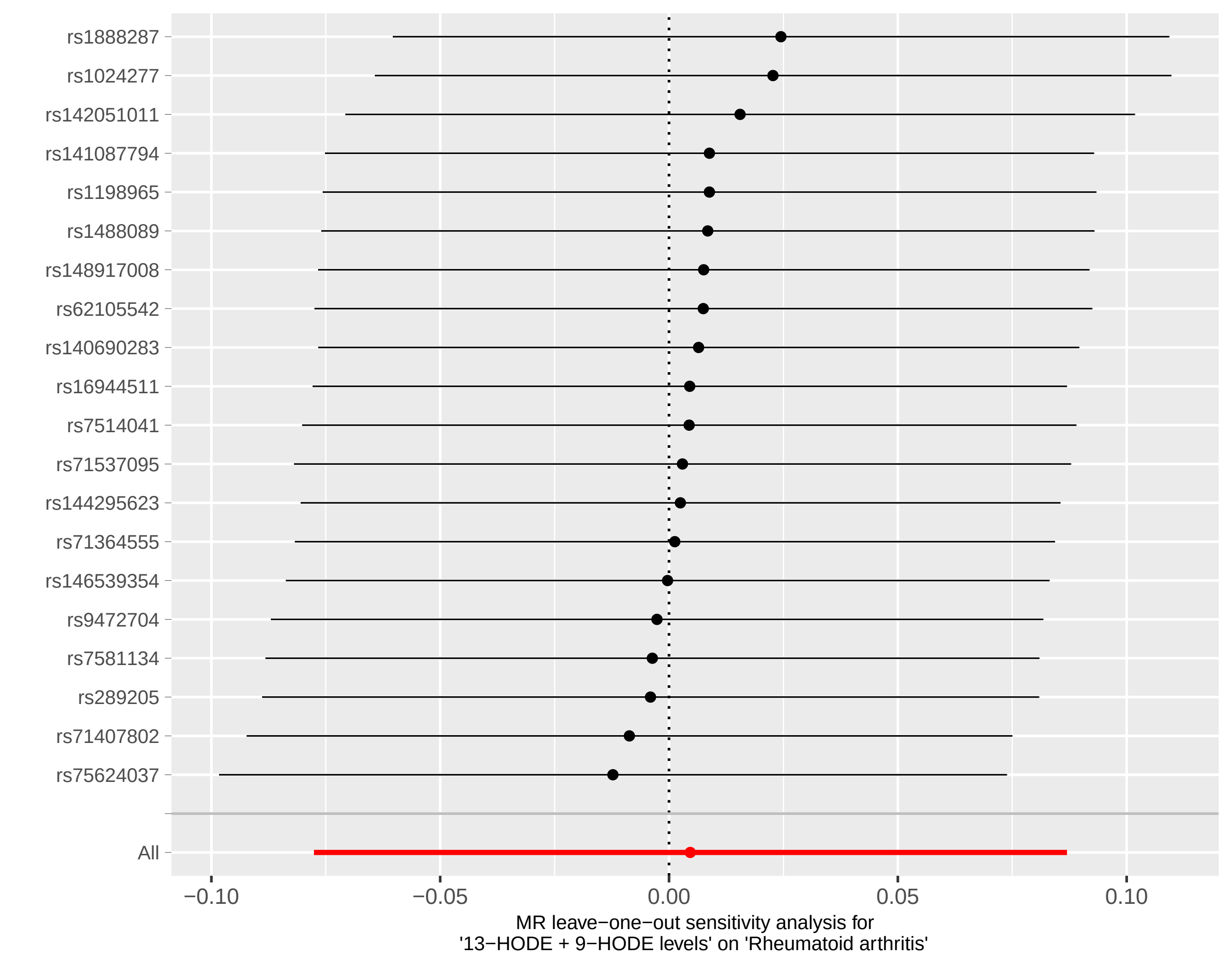

rs1888287
rs1024277
rs142051011
rs141087794
rs1198965
rs1488089
rs148917008
rs62105542
rs140690283
rs16944511
rs7514041
rs71537095
rs144295623
rs71364555
rs146539354
rs9472704
rs7581134
rs289205
rs71407802
rs75624037
All
−0.10
−0.05
0.00
0.05
0.10
MR leave−one−out sensitivity analysis for
'13−HODE + 9−HODE levels' on 'Rheumatoid arthritis'

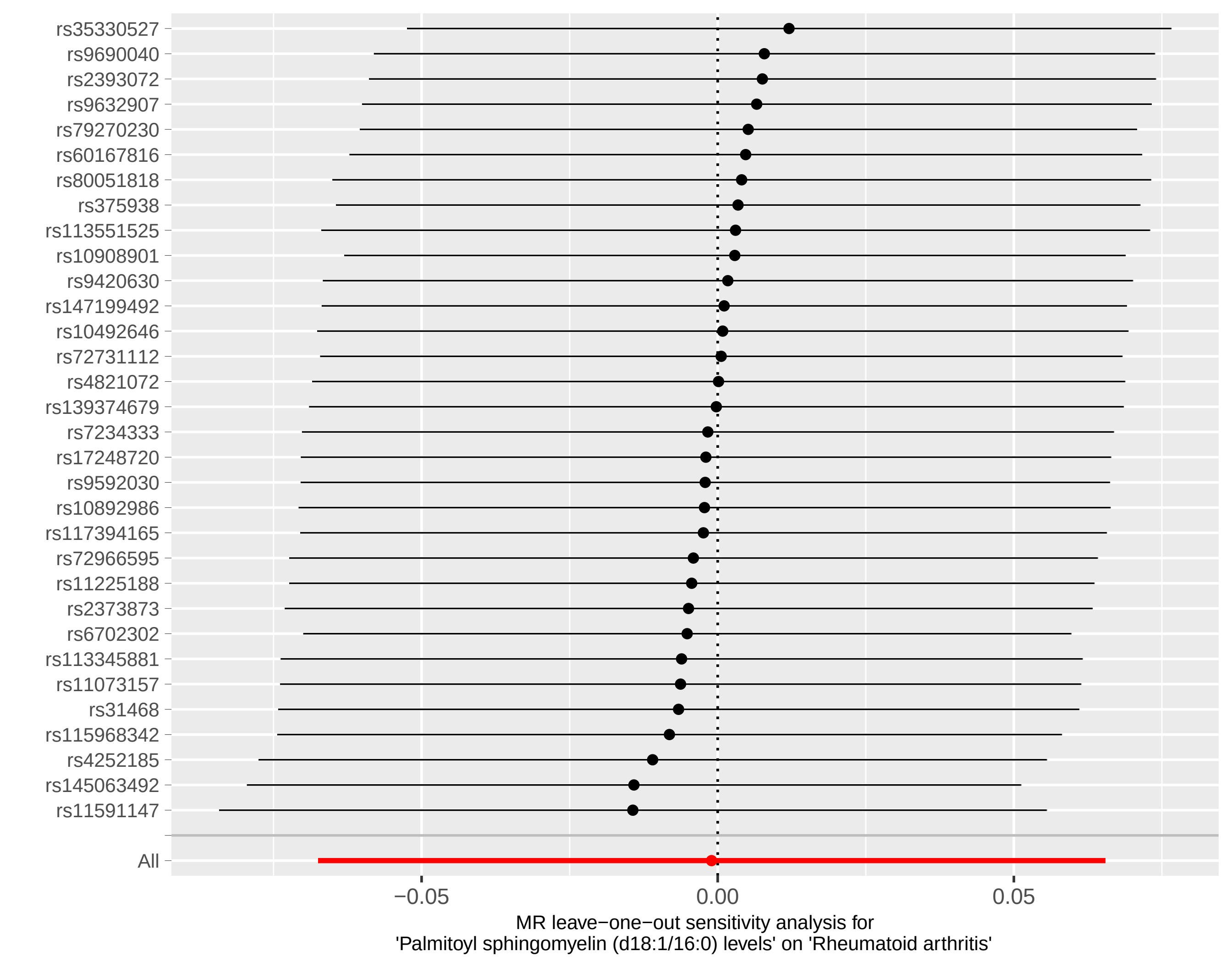
rs35330527
rs9690040
rs2393072
rs9632907
rs79270230
rs60167816
rs80051818
rs375938
rs113551525
rs10908901
rs9420630
rs147199492
rs10492646
rs72731112
rs4821072
rs139374679
rs7234333
rs17248720
rs9592030
rs10892986
rs117394165
rs72966595
rs11225188
rs2373873
rs6702302
rs113345881
rs11073157
rs31468
rs115968342
rs4252185
rs145063492
rs11591147
All
−0.05
0.00
0.05
MR leave−one−out sensitivity analysis for
'Palmitoyl sphingomyelin (d18:1/16:0) levels' on 'Rheumatoid arthritis'

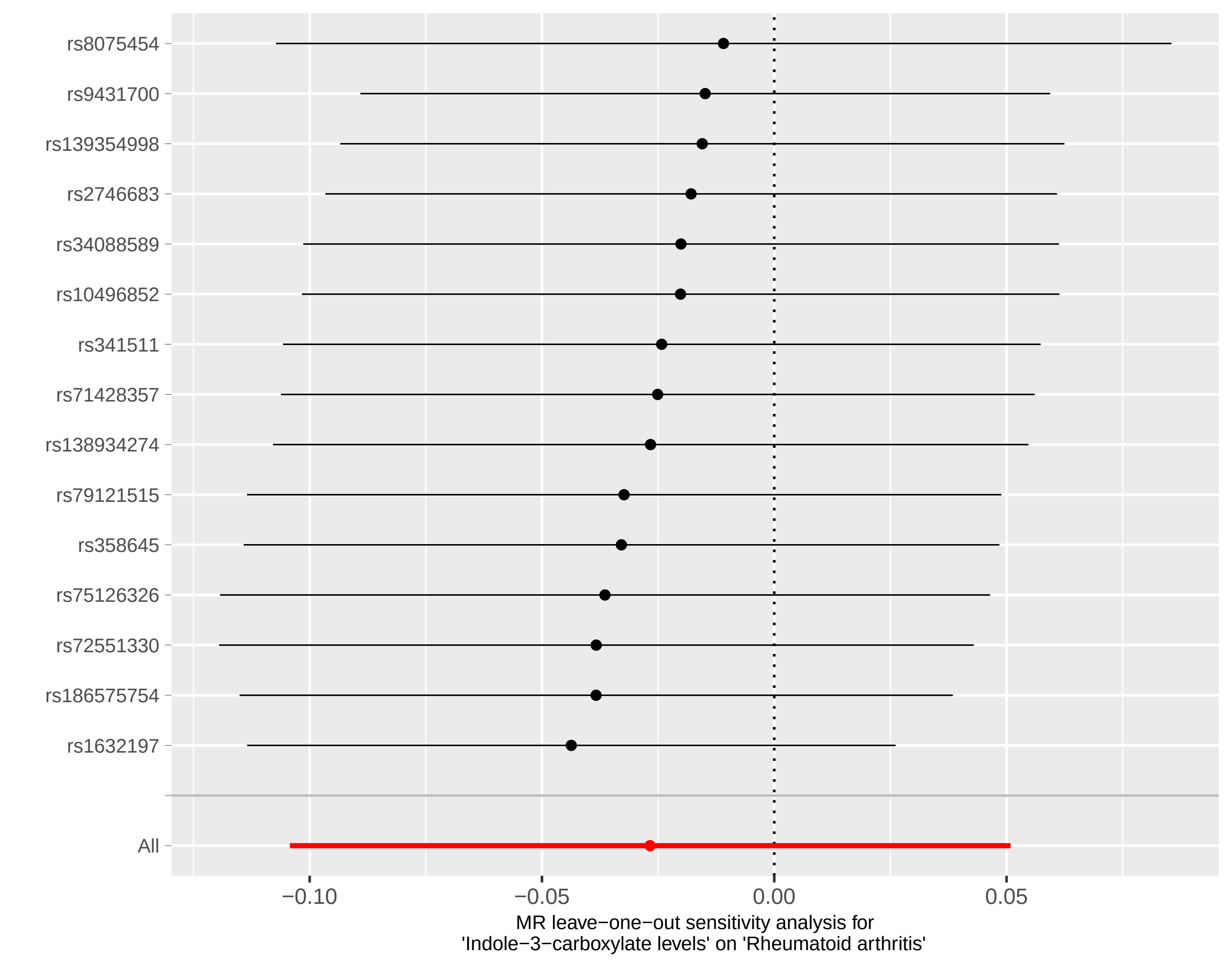
rs8075454
rs9431700
rs139354998
rs2746683
rs34088589
rs10496852
rs341511
rs71428357
rs138934274
rs79121515
rs358645
rs75126326
rs72551330
rs186575754
rs1632197
All
−0.10
−0.05
0.00
0.05
MR leave−one−out sensitivity analysis for
'Indole−3−carboxylate levels' on 'Rheumatoid arthritis'

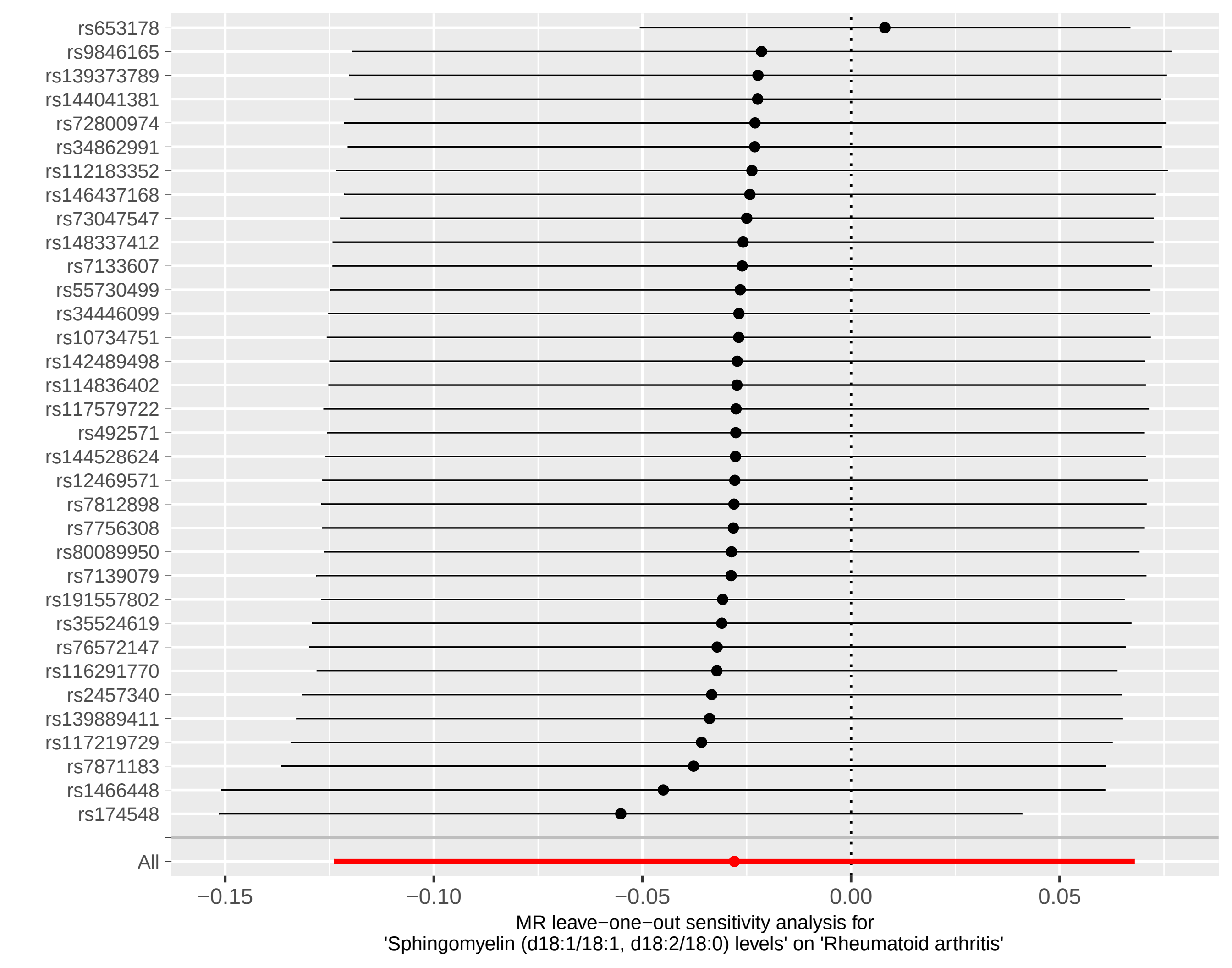

rs653178
rs9846165
rs139373789
rs144041381
rs72800974
rs34862991
rs112183352
rs146437168
rs73047547
rs148337412
rs7133607
rs55730499
rs34446099
rs10734751
rs142489498
rs114836402
rs117579722
rs492571
rs144528624
rs12469571
rs7812898
rs7756308
rs80089950
rs7139079
rs191557802
rs35524619
rs76572147
rs116291770
rs2457340
rs139889411
rs117219729
rs7871183
rs1466448
rs174548
All
−0.15
−0.10
−0.05
0.00
0.05
MR leave−one−out sensitivity analysis for
'Sphingomyelin (d18:1/18:1, d18:2/18:0) levels' on 'Rheumatoid arthritis'

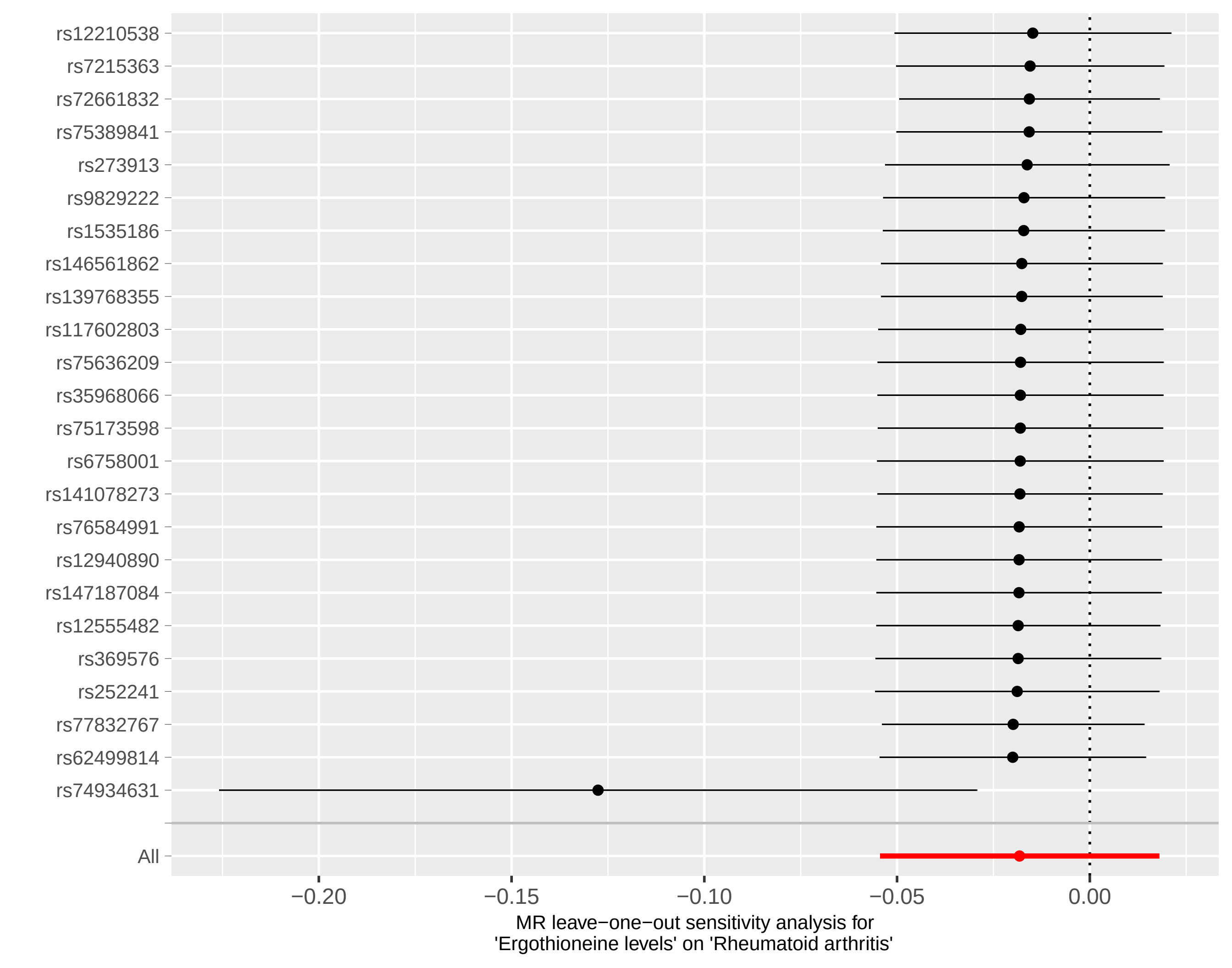

rs12210538
rs7215363
rs72661832
rs75389841
rs273913
rs9829222
rs1535186
rs146561862
rs139768355
rs117602803
rs75636209
rs35968066
rs75173598
rs6758001
rs141078273
rs76584991
rs12940890
rs147187084
rs12555482
rs369576
rs252241
rs77832767
rs62499814
rs74934631
All
−0.20
−0.15
−0.10
−0.05
0.00
MR leave−one−out sensitivity analysis for
'Ergothioneine levels' on 'Rheumatoid arthritis'

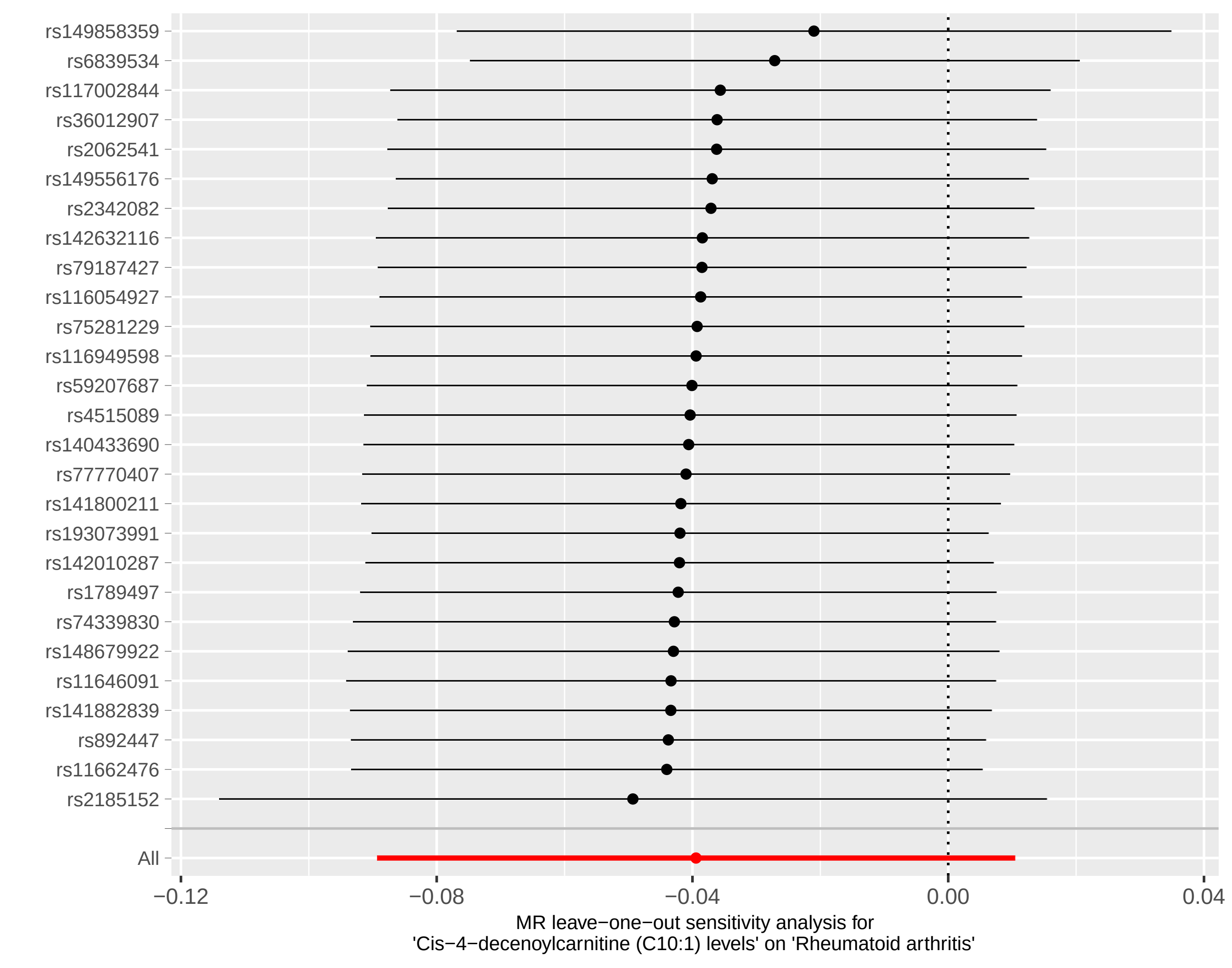

rs149858359
rs6839534
rs117002844
rs36012907
rs2062541
rs149556176
rs2342082
rs142632116
rs79187427
rs116054927
rs75281229
rs116949598
rs59207687
rs4515089
rs140433690
rs77770407
rs141800211
rs193073991
rs142010287
rs1789497
rs74339830
rs148679922
rs11646091
rs141882839
rs892447
rs11662476
rs2185152
All
−0.12
−0.08
−0.04
0.00
0.04
MR leave−one−out sensitivity analysis for
'Cis−4−decenoylcarnitine (C10:1) levels' on 'Rheumatoid arthritis'

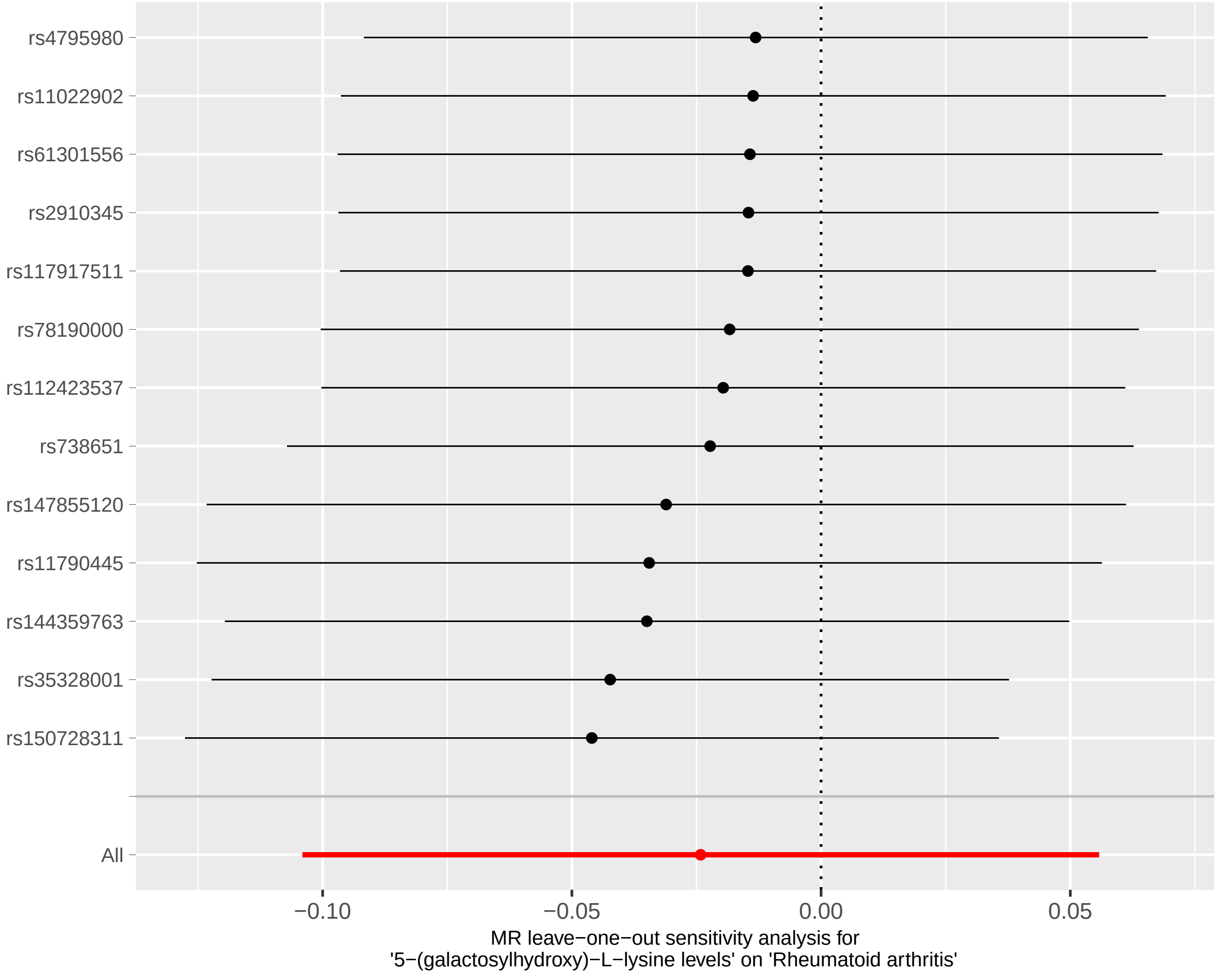
rs4795980
rs11022902
rs61301556
rs2910345
rs117917511
rs78190000
rs112423537
rs738651
rs147855120
rs11790445
rs144359763
rs35328001
rs150728311
All
−0.10
−0.05
0.00
0.05
MR leave−one−out sensitivity analysis for
'5−(galactosylhydroxy)−L−lysine levels' on 'Rheumatoid arthritis'

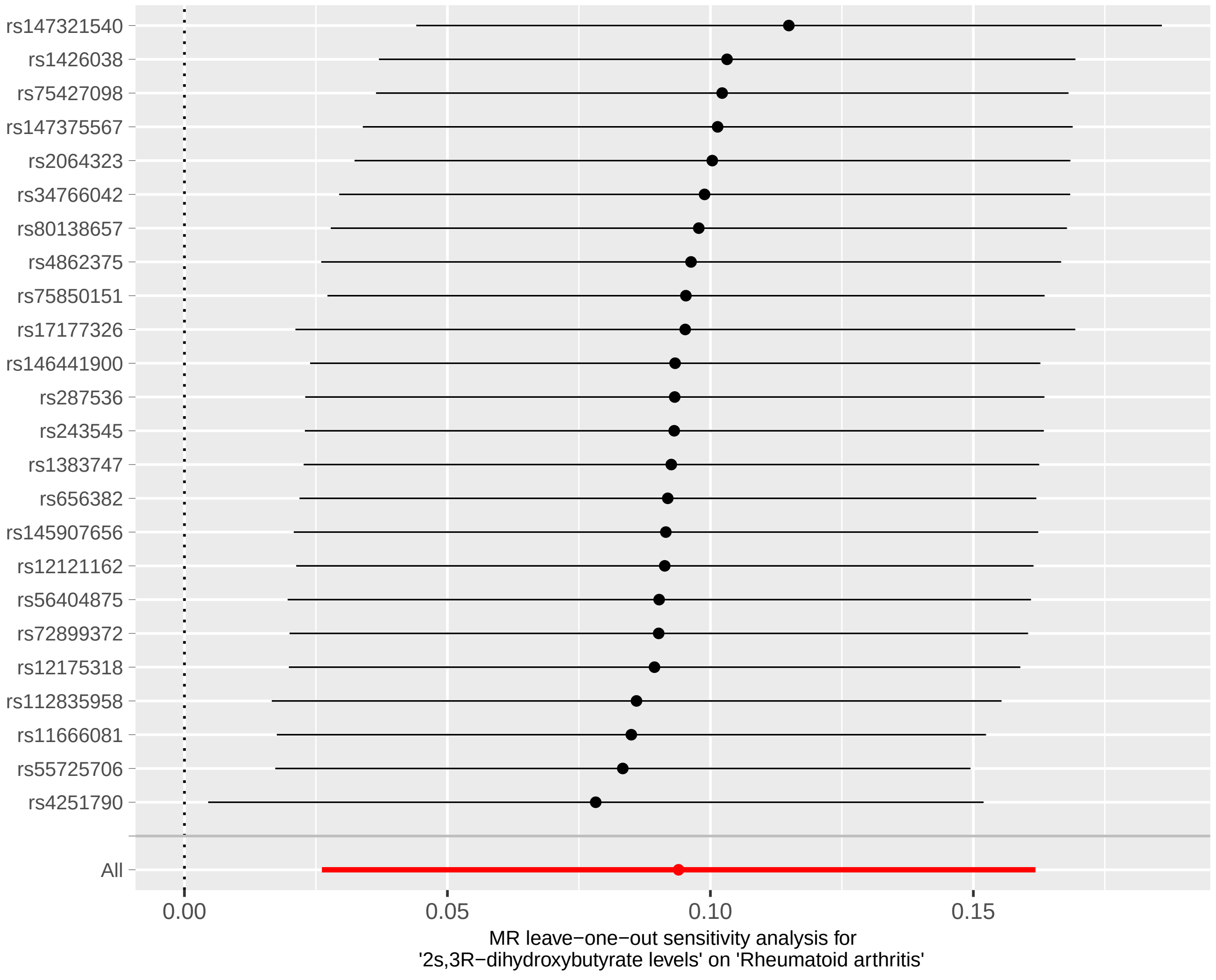
rs147321540
rs1426038
rs75427098
rs147375567
rs2064323
rs34766042
rs80138657
rs4862375
rs75850151
rs17177326
rs146441900
rs287536
rs243545
rs1383747
rs656382
rs145907656
rs12121162
rs56404875
rs72899372
rs12175318
rs112835958
rs11666081
rs55725706
rs4251790
All
0.00
0.05
0.10
0.15
MR leave−one−out sensitivity analysis for
'2s,3R−dihydroxybutyrate levels' on 'Rheumatoid arthritis'

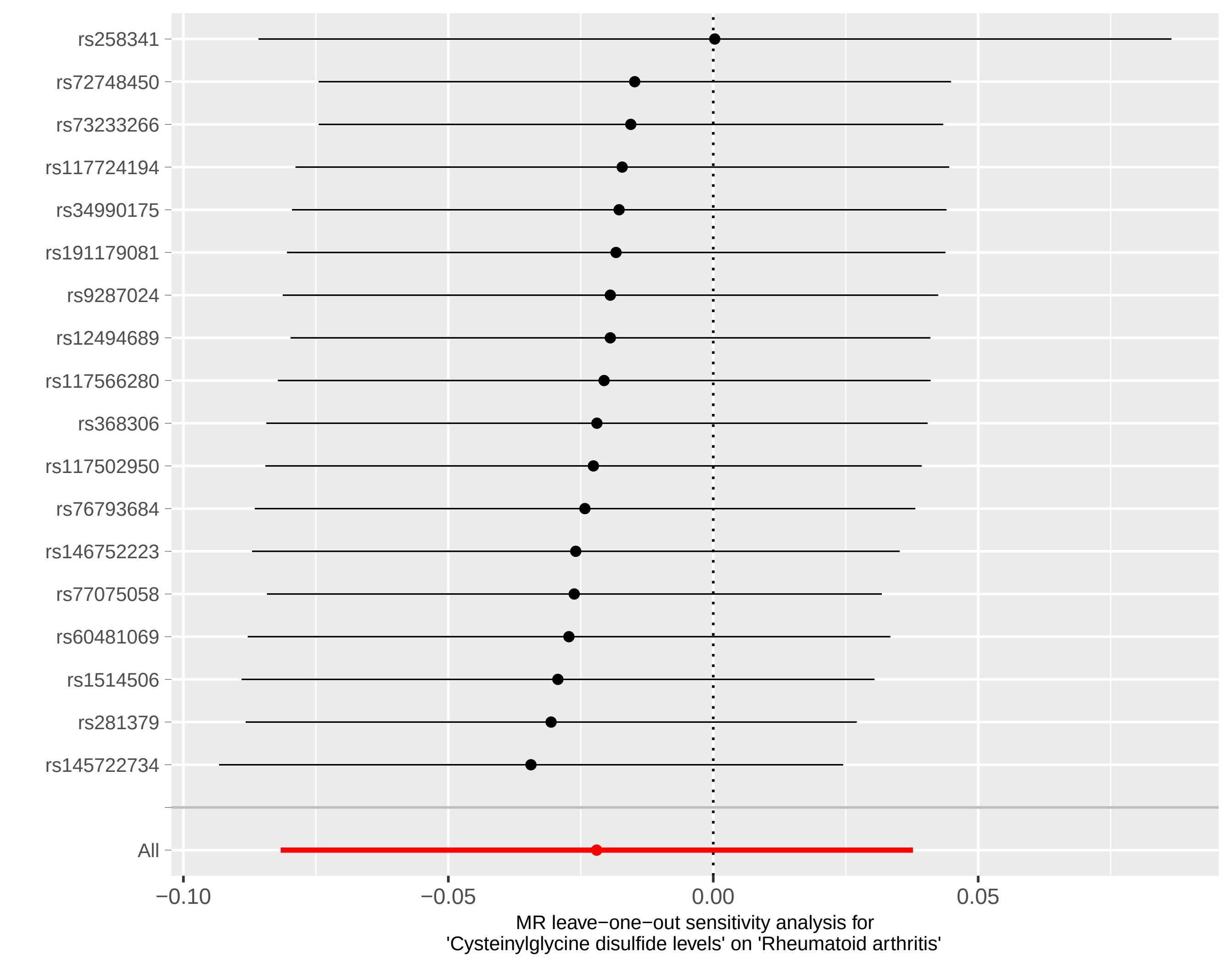
rs258341
rs72748450
rs73233266
rs117724194
rs34990175
rs191179081
rs9287024
rs12494689
rs117566280
rs368306
rs117502950
rs76793684
rs146752223
rs77075058
rs60481069
rs1514506
rs281379
rs145722734
All
−0.10
−0.05
0.00
0.05
MR leave−one−out sensitivity analysis for
'Cysteinylglycine disulfide levels' on 'Rheumatoid arthritis'

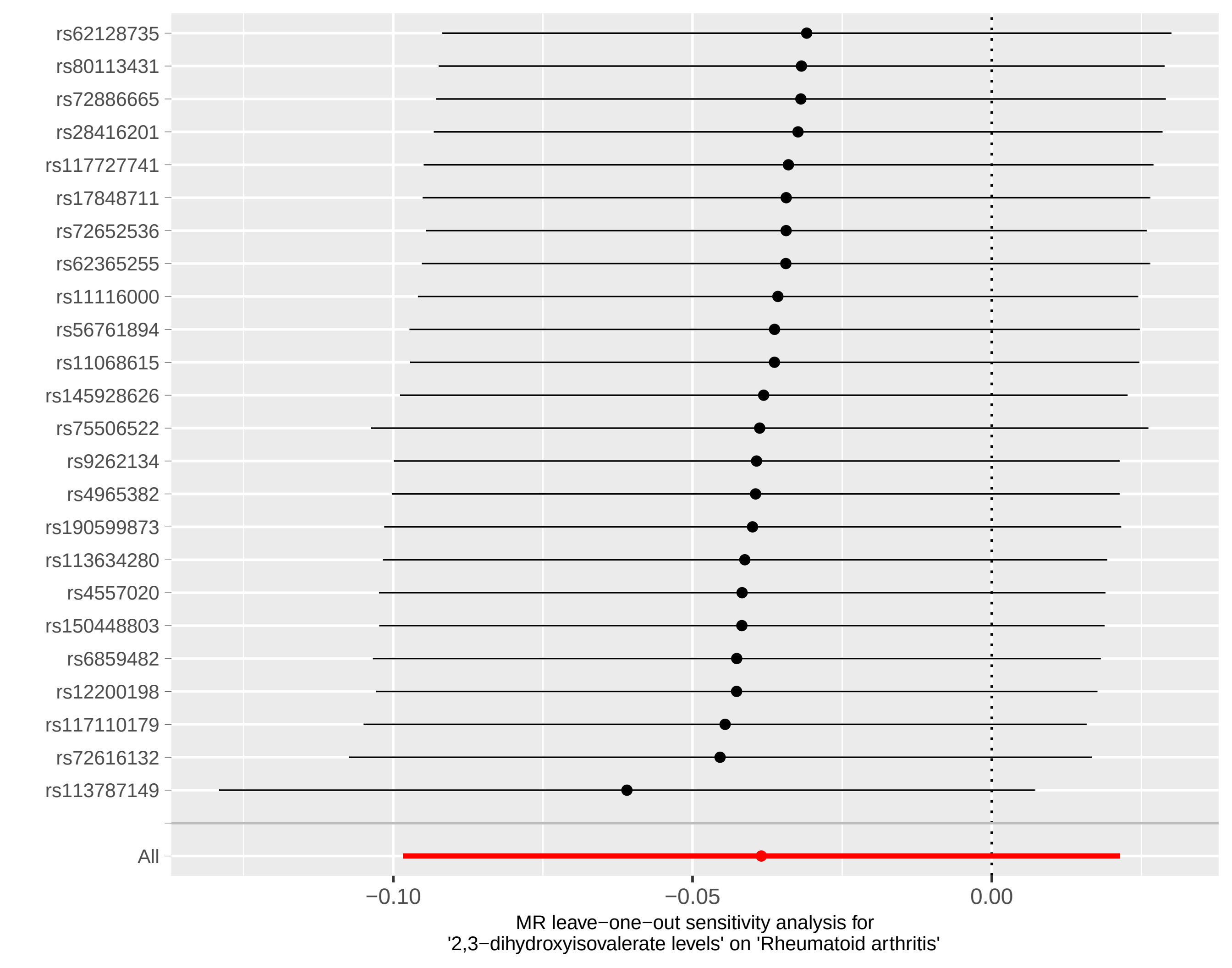
rs62128735
rs80113431
rs72886665
rs28416201
rs117727741
rs17848711
rs72652536
rs62365255
rs11116000
rs56761894
rs11068615
rs145928626
rs75506522
rs9262134
rs4965382
rs190599873
rs113634280
rs4557020
rs150448803
rs6859482
rs12200198
rs117110179
rs72616132
rs113787149
All
−0.10
−0.05
0.00
MR leave−one−out sensitivity analysis for
'2,3−dihydroxyisovalerate levels' on 'Rheumatoid arthritis'

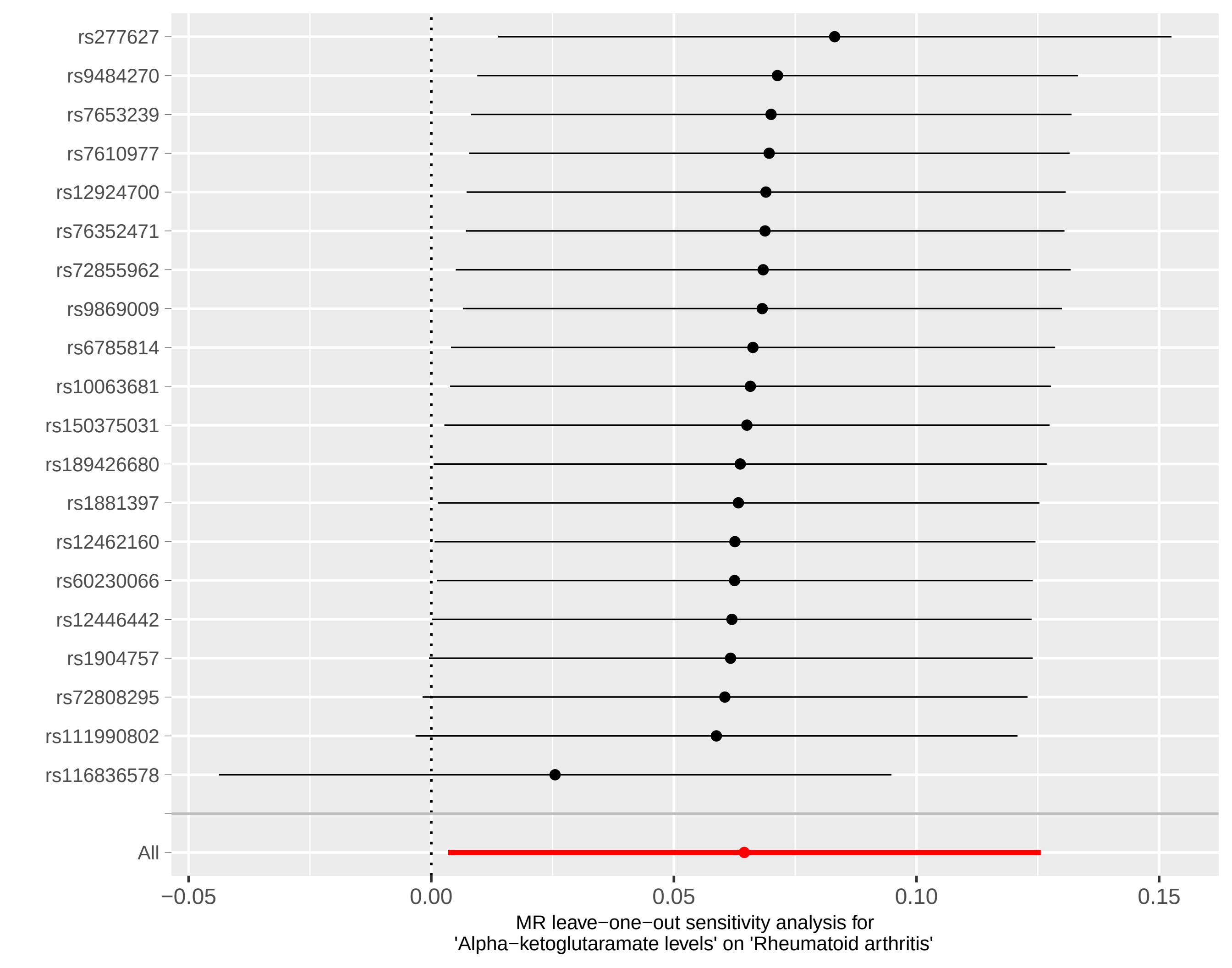
rs277627
rs9484270
rs7653239
rs7610977
rs12924700
rs76352471
rs72855962
rs9869009
rs6785814
rs10063681
rs150375031
rs189426680
rs1881397
rs12462160
rs60230066
rs12446442
rs1904757
rs72808295
rs111990802
rs116836578
All
−0.05
0.00
0.05
0.10
0.15
MR leave−one−out sensitivity analysis for
'Alpha−ketoglutaramate levels' on 'Rheumatoid arthritis'

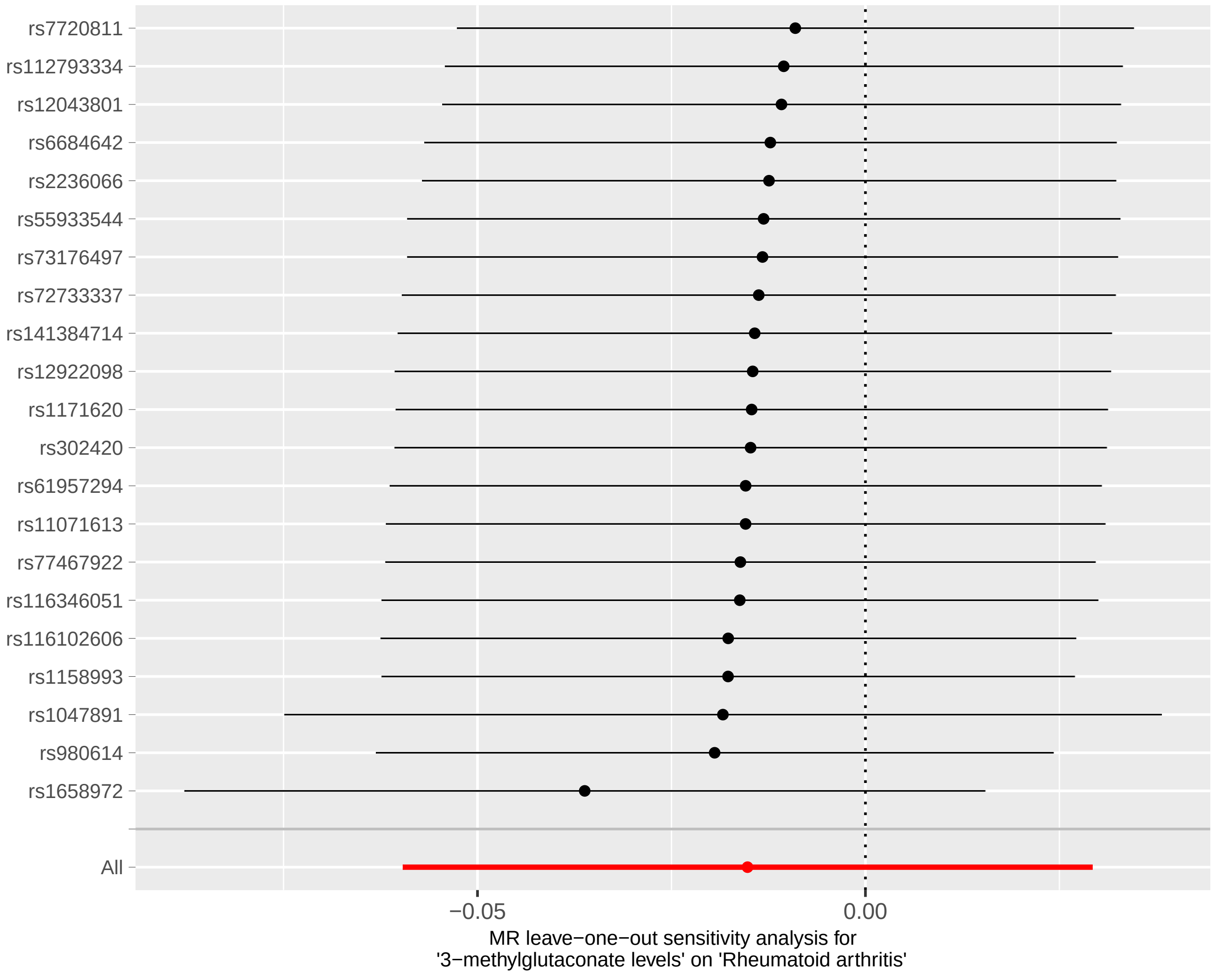
rs7720811
rs112793334
rs12043801
rs6684642
rs2236066
rs55933544
rs73176497
rs72733337
rs141384714
rs12922098
rs1171620
rs302420
rs61957294
rs11071613
rs77467922
rs116346051
rs116102606
rs1158993
rs1047891
rs980614
rs1658972
All
−0.05
0.00
MR leave−one−out sensitivity analysis for
'3−methylglutaconate levels' on 'Rheumatoid arthritis'

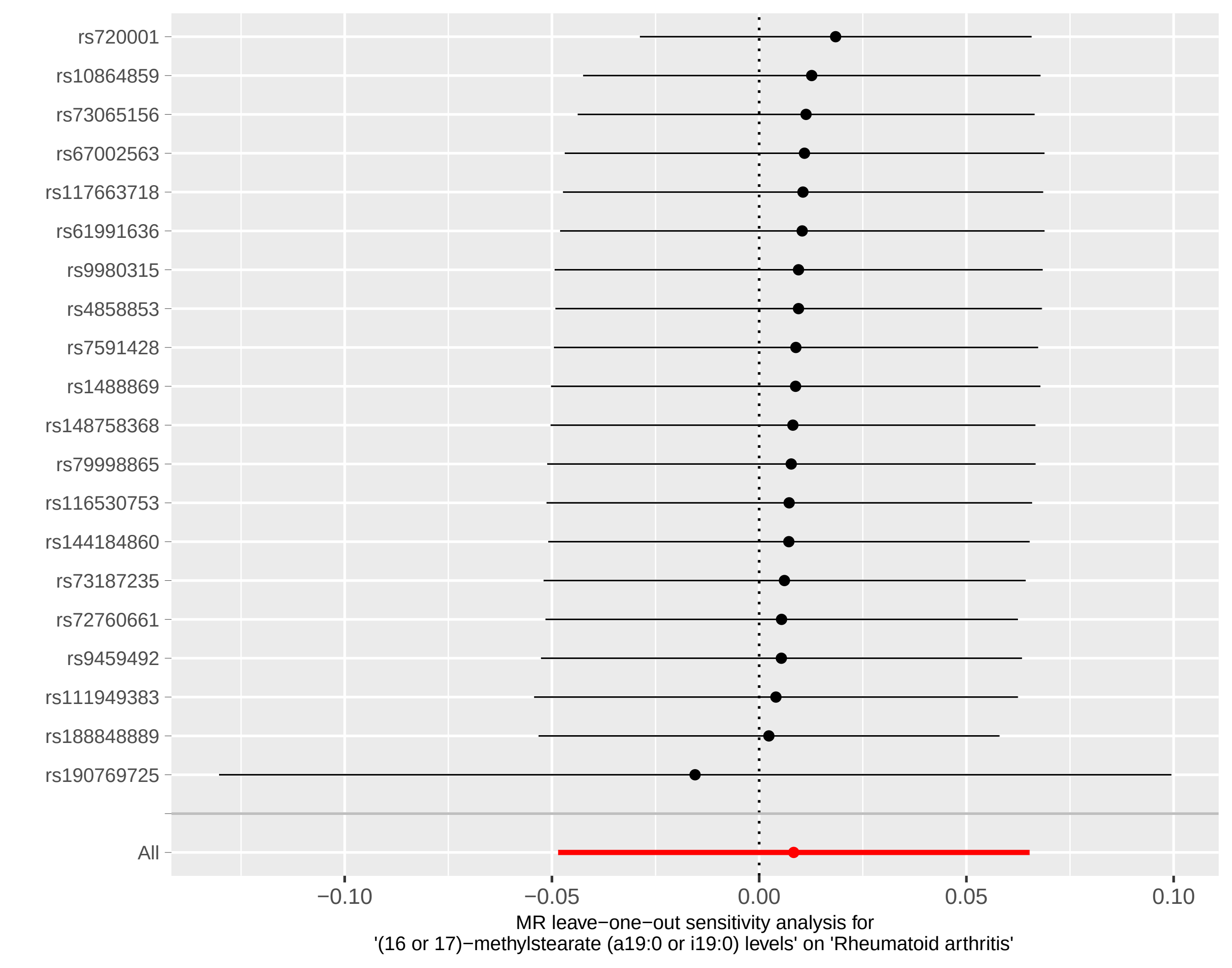
rs720001
rs10864859
rs73065156
rs67002563
rs117663718
rs61991636
rs9980315
rs4858853
rs7591428
rs1488869
rs148758368
rs79998865
rs116530753
rs144184860
rs73187235
rs72760661
rs9459492
rs111949383
rs188848889
rs190769725
All
−0.10
−0.05
0.00
0.05
0.10
MR leave−one−out sensitivity analysis for
'(16 or 17)−methylstearate (a19:0 or i19:0) levels' on 'Rheumatoid arthritis'

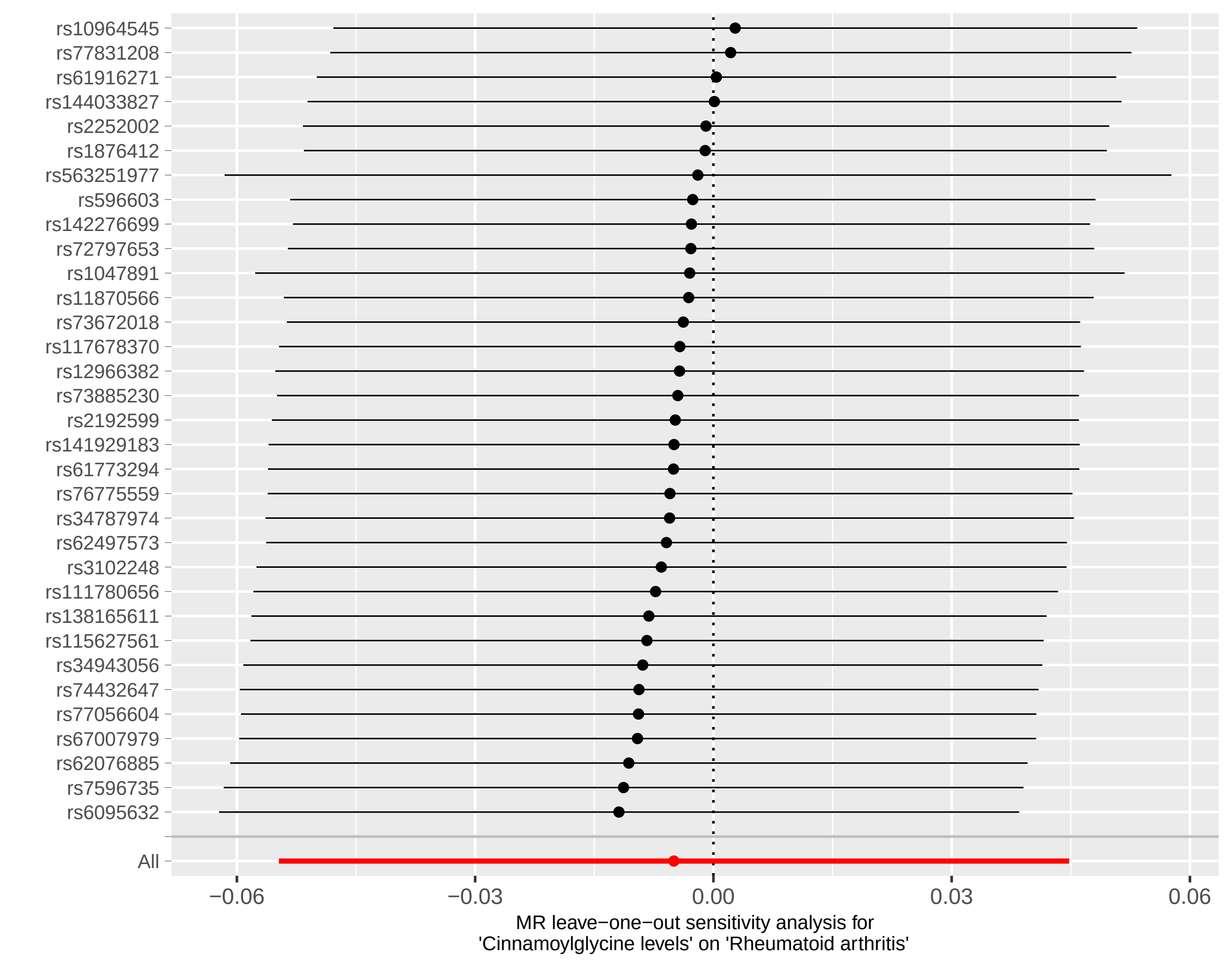
rs10964545
rs77831208
rs61916271
rs144033827
rs2252002
rs1876412
rs563251977
rs596603
rs142276699
rs72797653
rs1047891
rs11870566
rs73672018
rs117678370
rs12966382
rs73885230
rs2192599
rs141929183
rs61773294
rs76775559
rs34787974
rs62497573
rs3102248
rs111780656
rs138165611
rs115627561
rs34943056
rs74432647
rs77056604
rs67007979
rs62076885
rs7596735
rs6095632
All
−0.06
−0.03
0.00
0.03
0.06
MR leave−one−out sensitivity analysis for
'Cinnamoylglycine levels' on 'Rheumatoid arthritis'

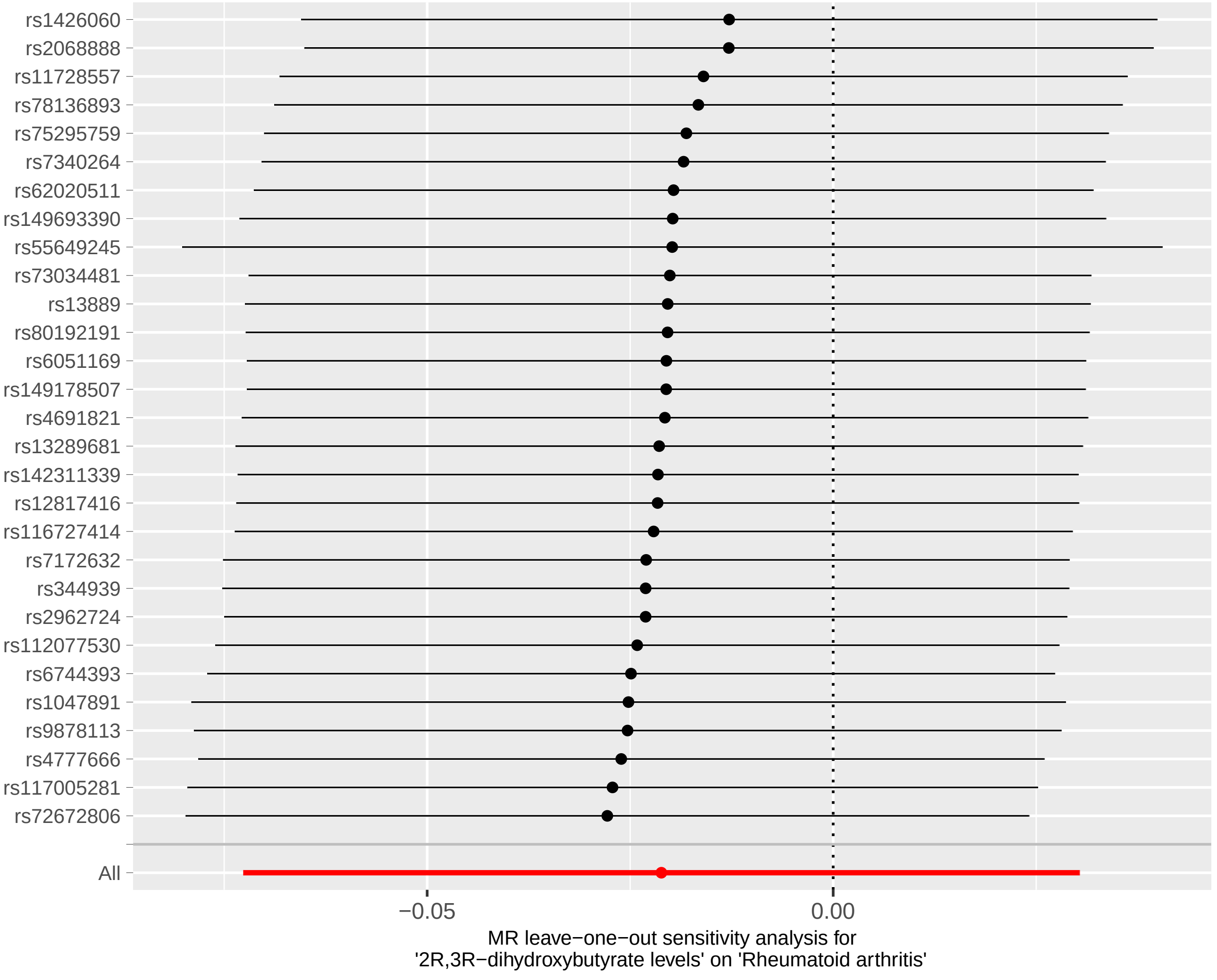
rs1426060
rs2068888
rs11728557
rs78136893
rs75295759
rs7340264
rs62020511
rs149693390
rs55649245
rs73034481
rs13889
rs80192191
rs6051169
rs149178507
rs4691821
rs13289681
rs142311339
rs12817416
rs116727414
rs7172632
rs344939
rs2962724
rs112077530
rs6744393
rs1047891
rs9878113
rs4777666
rs117005281
rs72672806
All
−0.05
0.00
MR leave−one−out sensitivity analysis for
'2R,3R−dihydroxybutyrate levels' on 'Rheumatoid arthritis'

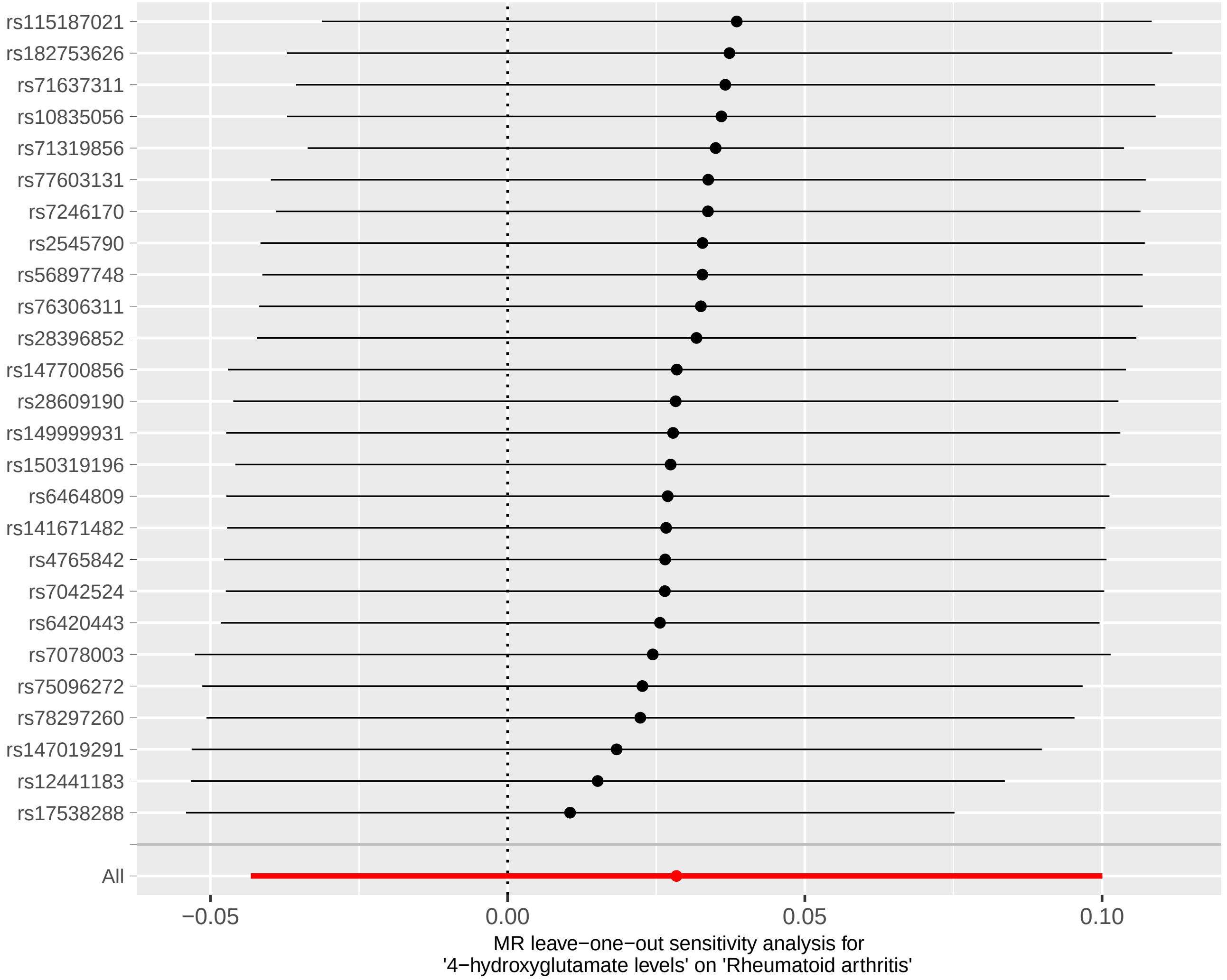

rs115187021
rs182753626
rs71637311
rs10835056
rs71319856
rs77603131
rs7246170
rs2545790
rs56897748
rs76306311
rs28396852
rs147700856
rs28609190
rs149999931
rs150319196
rs6464809
rs141671482
rs4765842
rs7042524
rs6420443
rs7078003
rs75096272
rs78297260
rs147019291
rs12441183
rs17538288
All
−0.05
0.00
0.05
0.10
MR leave−one−out sensitivity analysis for
'4−hydroxyglutamate levels' on 'Rheumatoid arthritis'

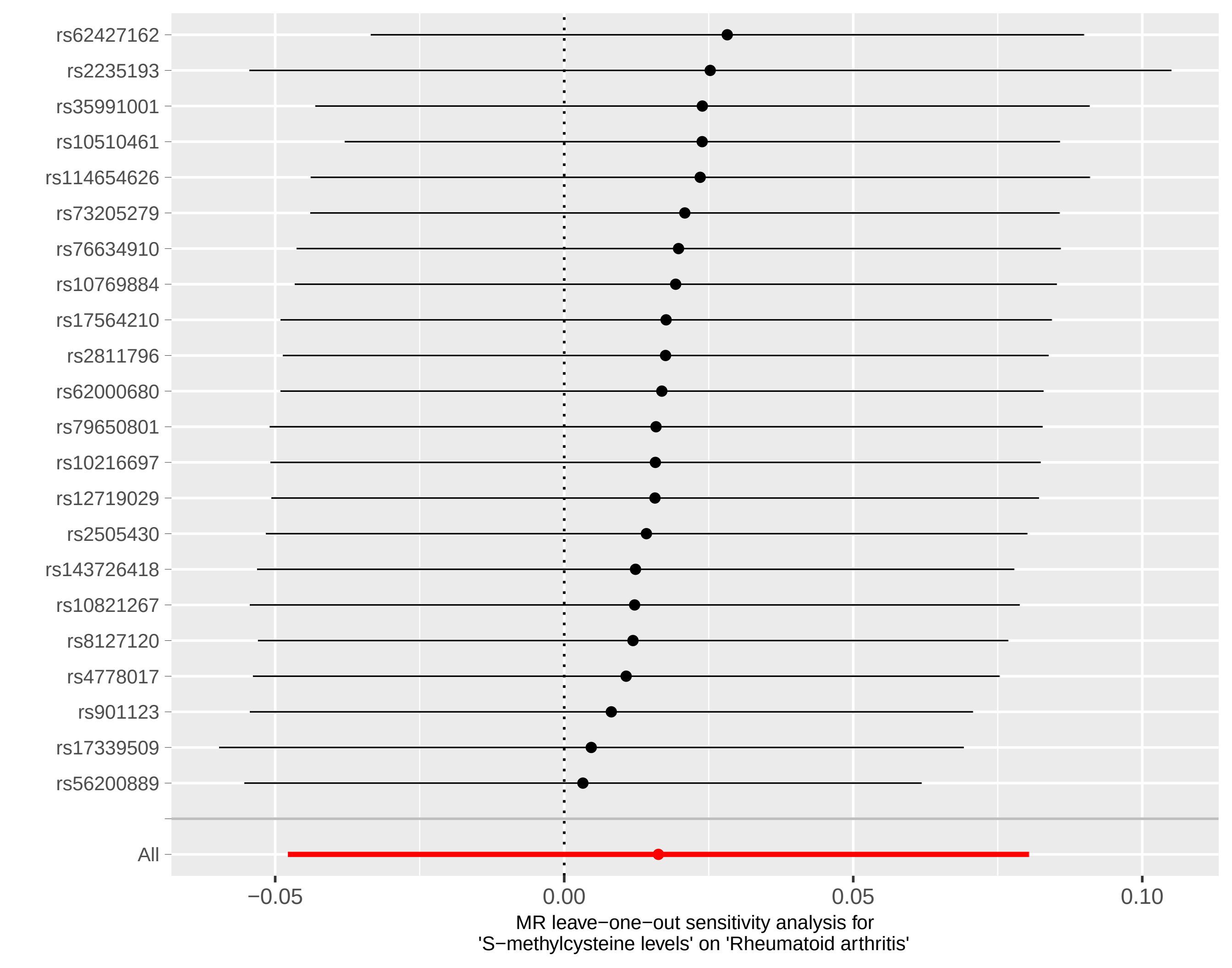
rs62427162
rs2235193
rs35991001
rs10510461
rs114654626
rs73205279
rs76634910
rs10769884
rs17564210
rs2811796
rs62000680
rs79650801
rs10216697
rs12719029
rs2505430
rs143726418
rs10821267
rs8127120
rs4778017
rs901123
rs17339509
rs56200889
All
−0.05
0.00
0.05
0.10
MR leave−one−out sensitivity analysis for
'S−methylcysteine levels' on 'Rheumatoid arthritis'

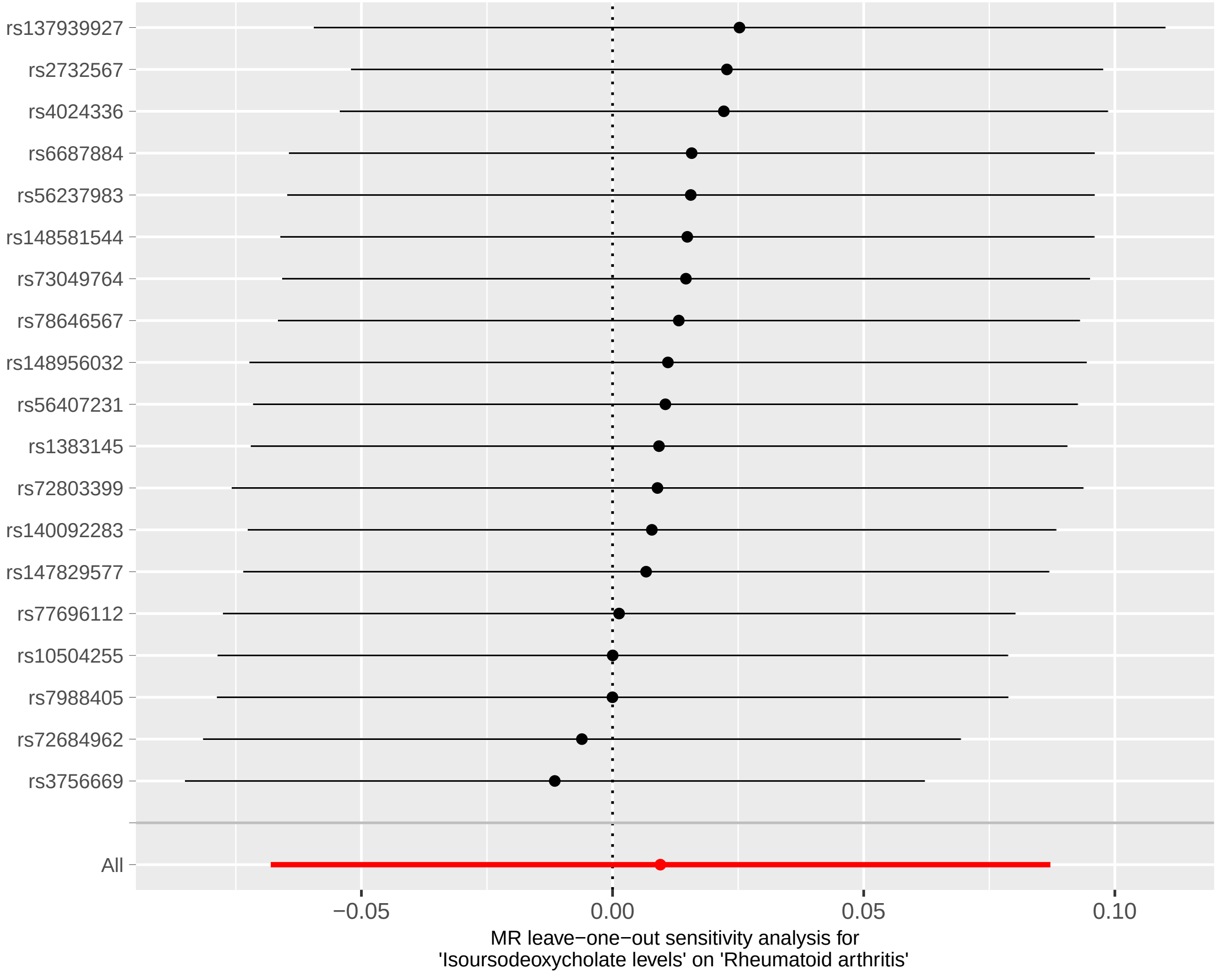

rs137939927
rs2732567
rs4024336
rs6687884
rs56237983
rs148581544
rs73049764
rs78646567
rs148956032
rs56407231
rs1383145
rs72803399
rs140092283
rs147829577
rs77696112
rs10504255
rs7988405
rs72684962
rs3756669
All
−0.05
0.00
0.05
0.10
MR leave−one−out sensitivity analysis for
'Isoursodeoxycholate levels' on 'Rheumatoid arthritis'

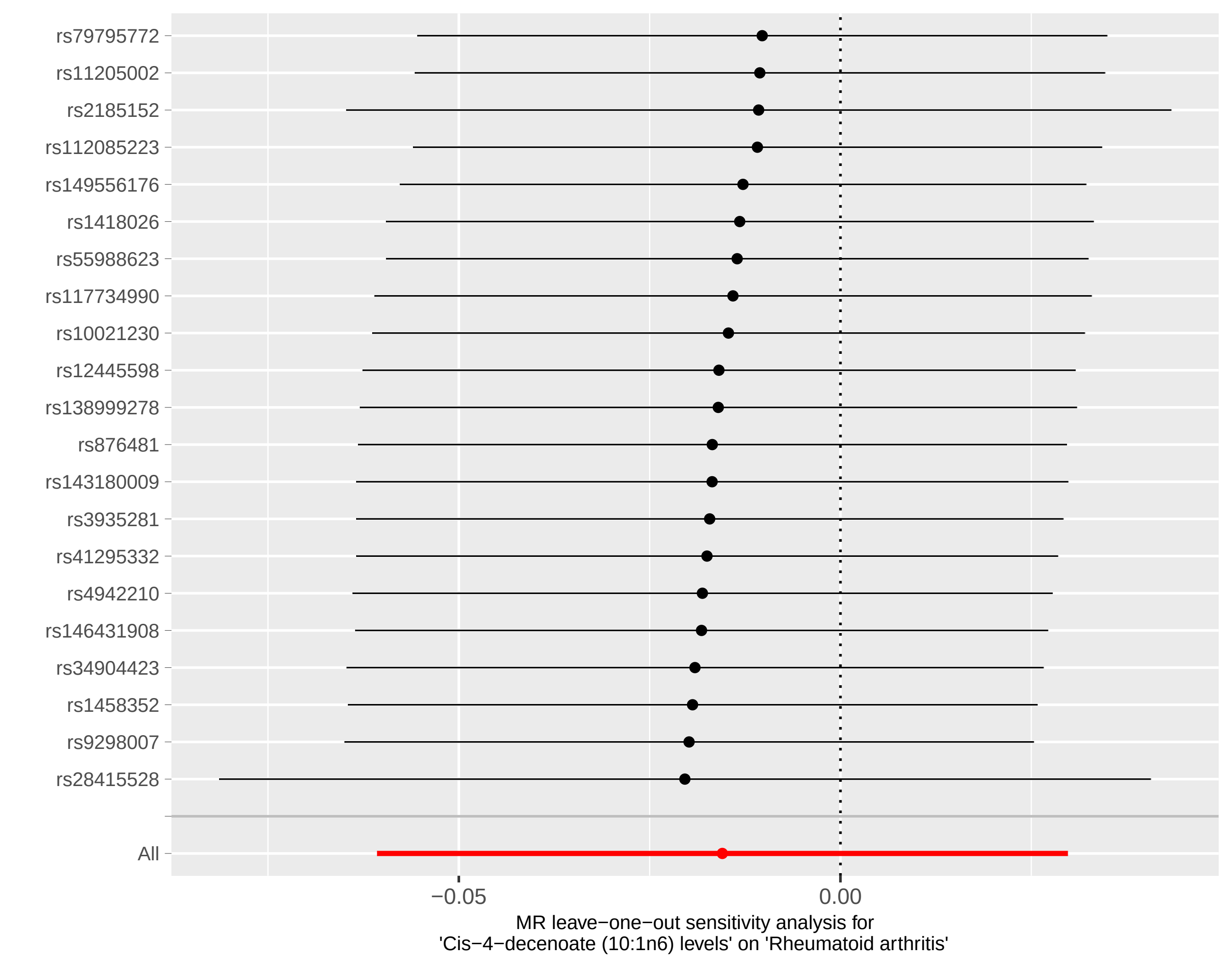

rs79795772
rs11205002
rs2185152
rs112085223
rs149556176
rs1418026
rs55988623
rs117734990
rs10021230
rs12445598
rs138999278
rs876481
rs143180009
rs3935281
rs41295332
rs4942210
rs146431908
rs34904423
rs1458352
rs9298007
rs28415528
All
−0.05
0.00
MR leave−one−out sensitivity analysis for
'Cis−4−decenoate (10:1n6) levels' on 'Rheumatoid arthritis'

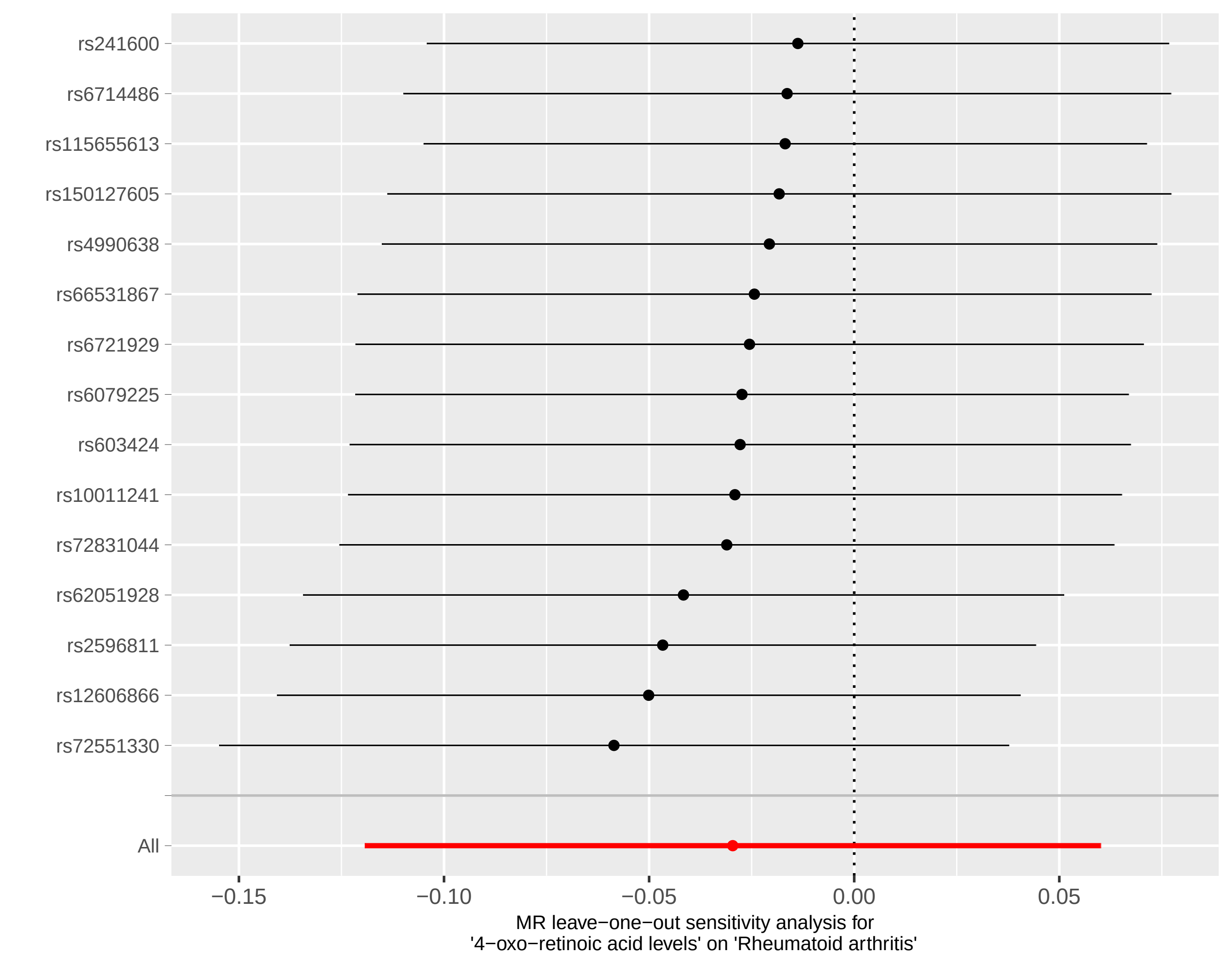
rs241600
rs6714486
rs115655613
rs150127605
rs4990638
rs66531867
rs6721929
rs6079225
rs603424
rs10011241
rs72831044
rs62051928
rs2596811
rs12606866
rs72551330
All
−0.15
−0.10
−0.05
0.00
0.05
MR leave−one−out sensitivity analysis for
'4−oxo−retinoic acid levels' on 'Rheumatoid arthritis'

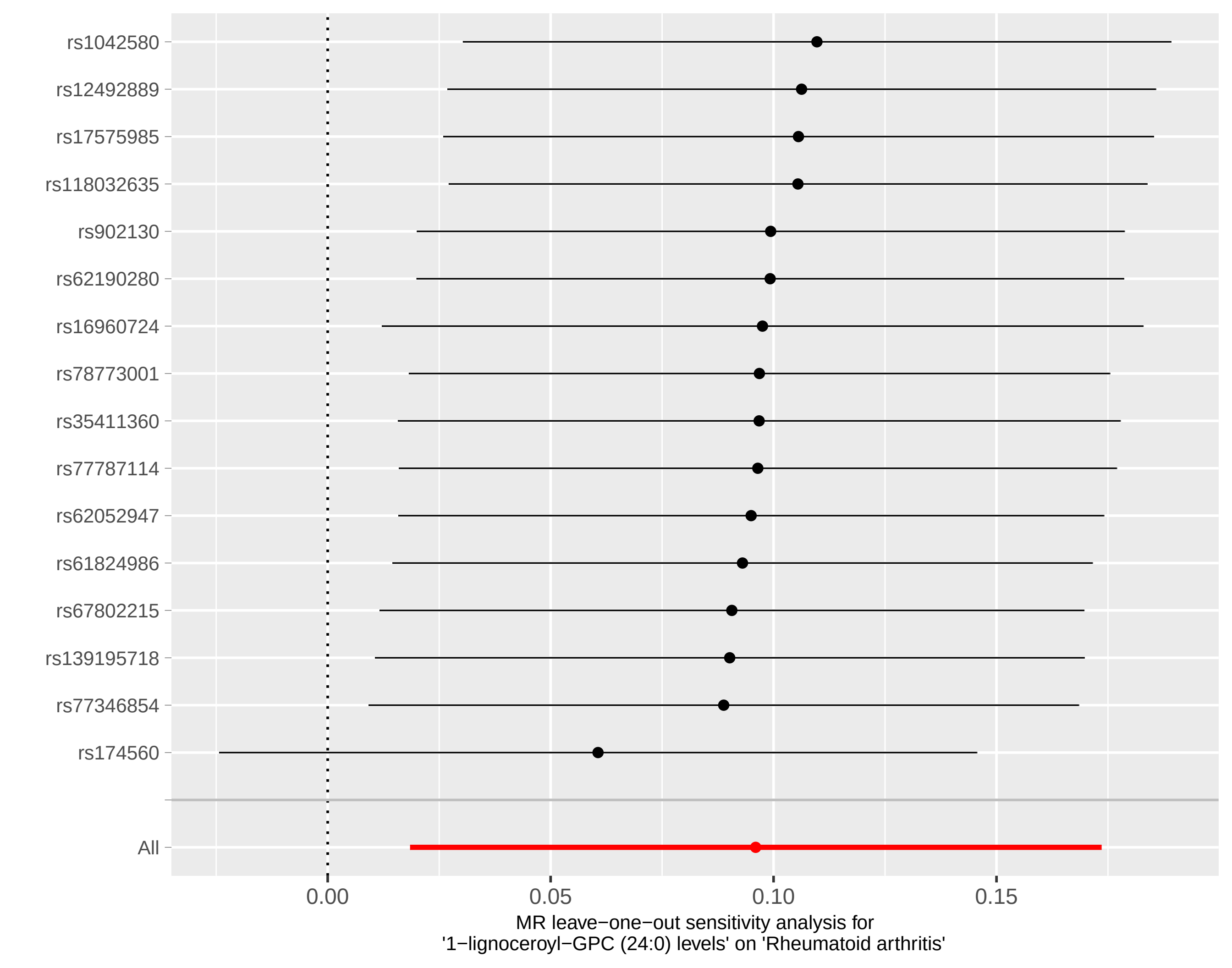

rs1042580
rs12492889
rs17575985
rs118032635
rs902130
rs62190280
rs16960724
rs78773001
rs35411360
rs77787114
rs62052947
rs61824986
rs67802215
rs139195718
rs77346854
rs174560
All
0.00
0.05
0.10
0.15
MR leave−one−out sensitivity analysis for
'1−lignoceroyl−GPC (24:0) levels' on 'Rheumatoid arthritis'

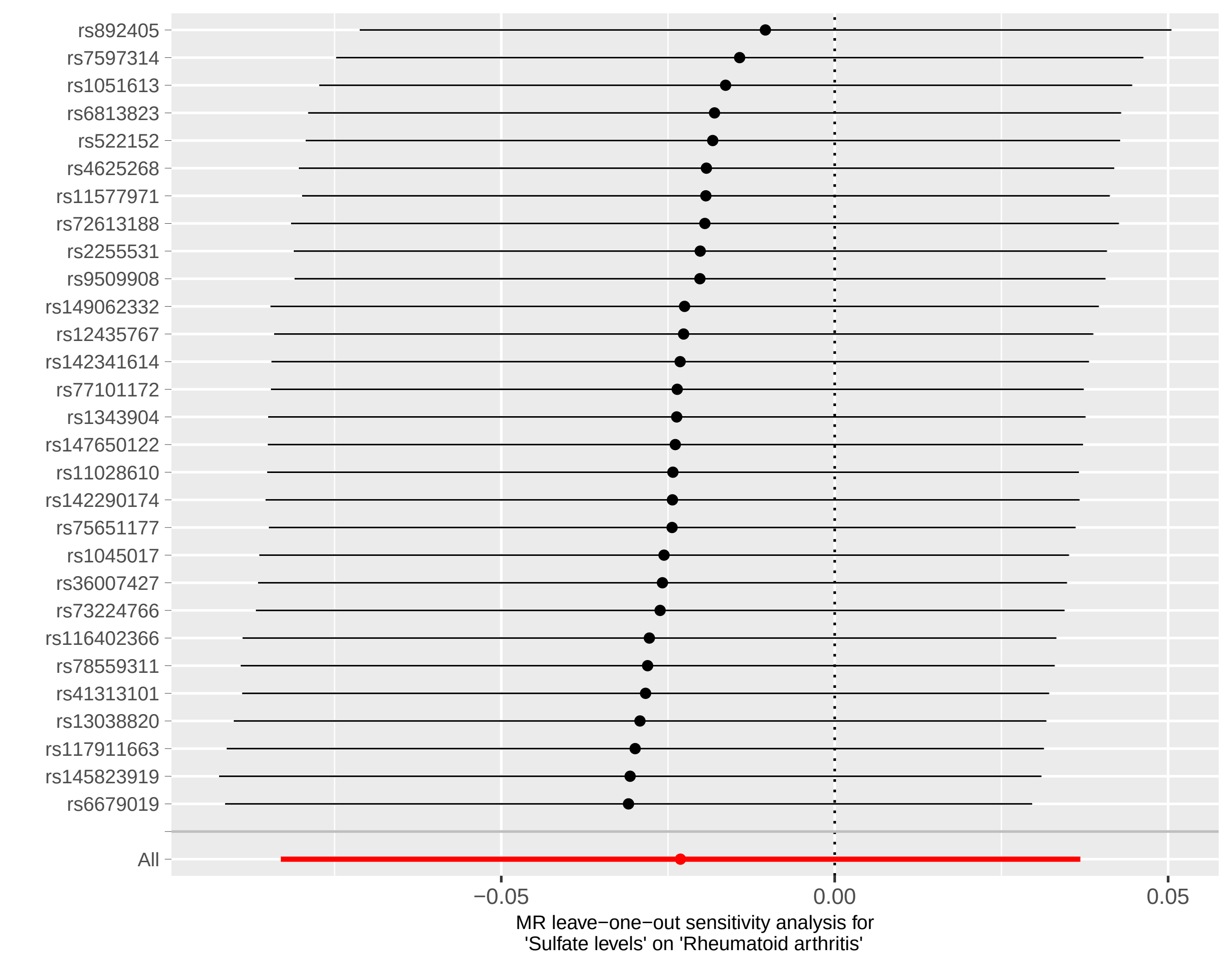

rs892405
rs7597314
rs1051613
rs6813823
rs522152
rs4625268
rs11577971
rs72613188
rs2255531
rs9509908
rs149062332
rs12435767
rs142341614
rs77101172
rs1343904
rs147650122
rs11028610
rs142290174
rs75651177
rs1045017
rs36007427
rs73224766
rs116402366
rs78559311
rs41313101
rs13038820
rs117911663
rs145823919
rs6679019
All
−0.05
0.00
0.05
MR leave−one−out sensitivity analysis for
'Sulfate levels' on 'Rheumatoid arthritis'

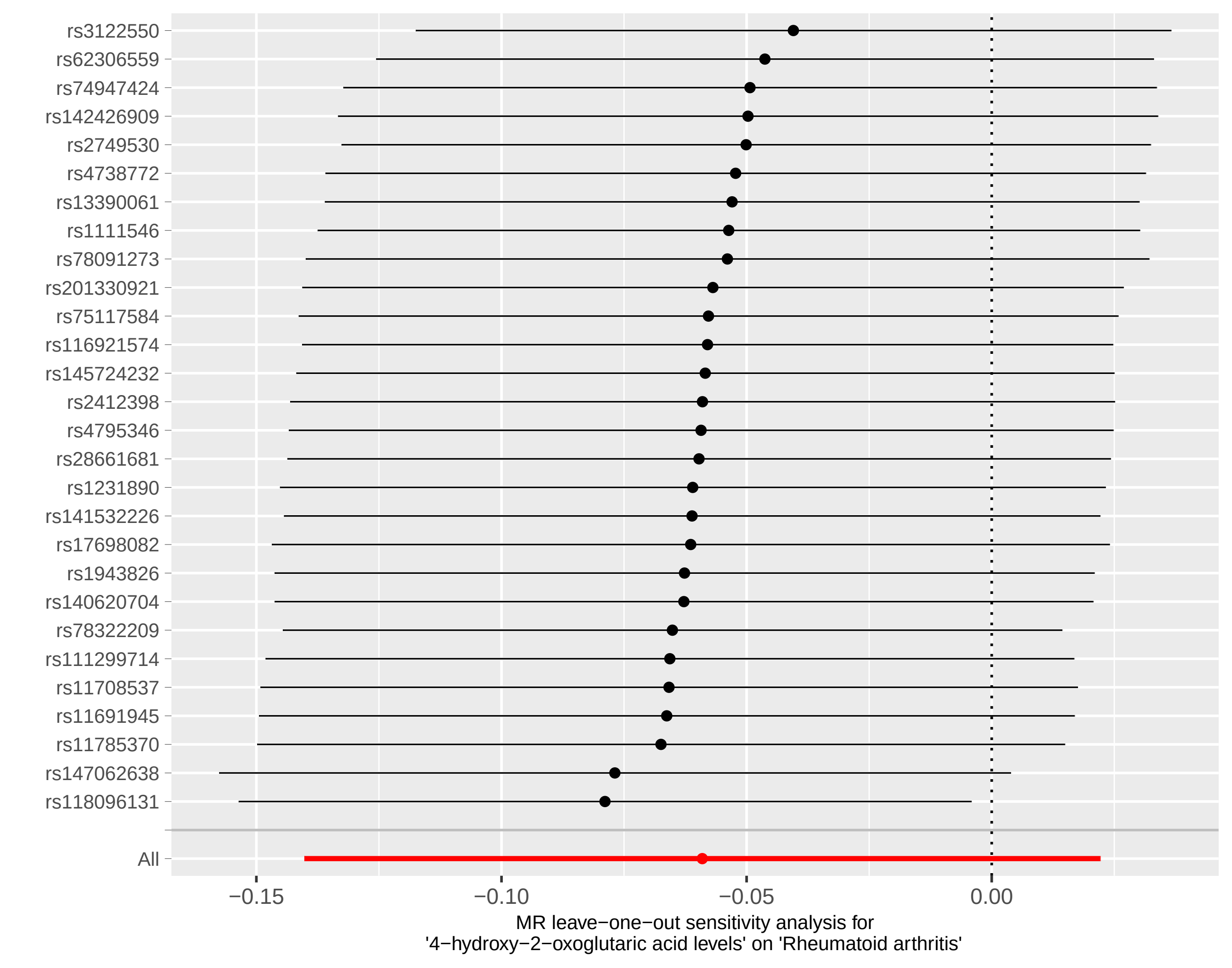
rs3122550
rs62306559
rs74947424
rs142426909
rs2749530
rs4738772
rs13390061
rs1111546
rs78091273
rs201330921
rs75117584
rs116921574
rs145724232
rs2412398
rs4795346
rs28661681
rs1231890
rs141532226
rs17698082
rs1943826
rs140620704
rs78322209
rs111299714
rs11708537
rs11691945
rs11785370
rs147062638
rs118096131
All
−0.15
−0.10
−0.05
0.00
MR leave−one−out sensitivity analysis for
'4−hydroxy−2−oxoglutaric acid levels' on 'Rheumatoid arthritis'

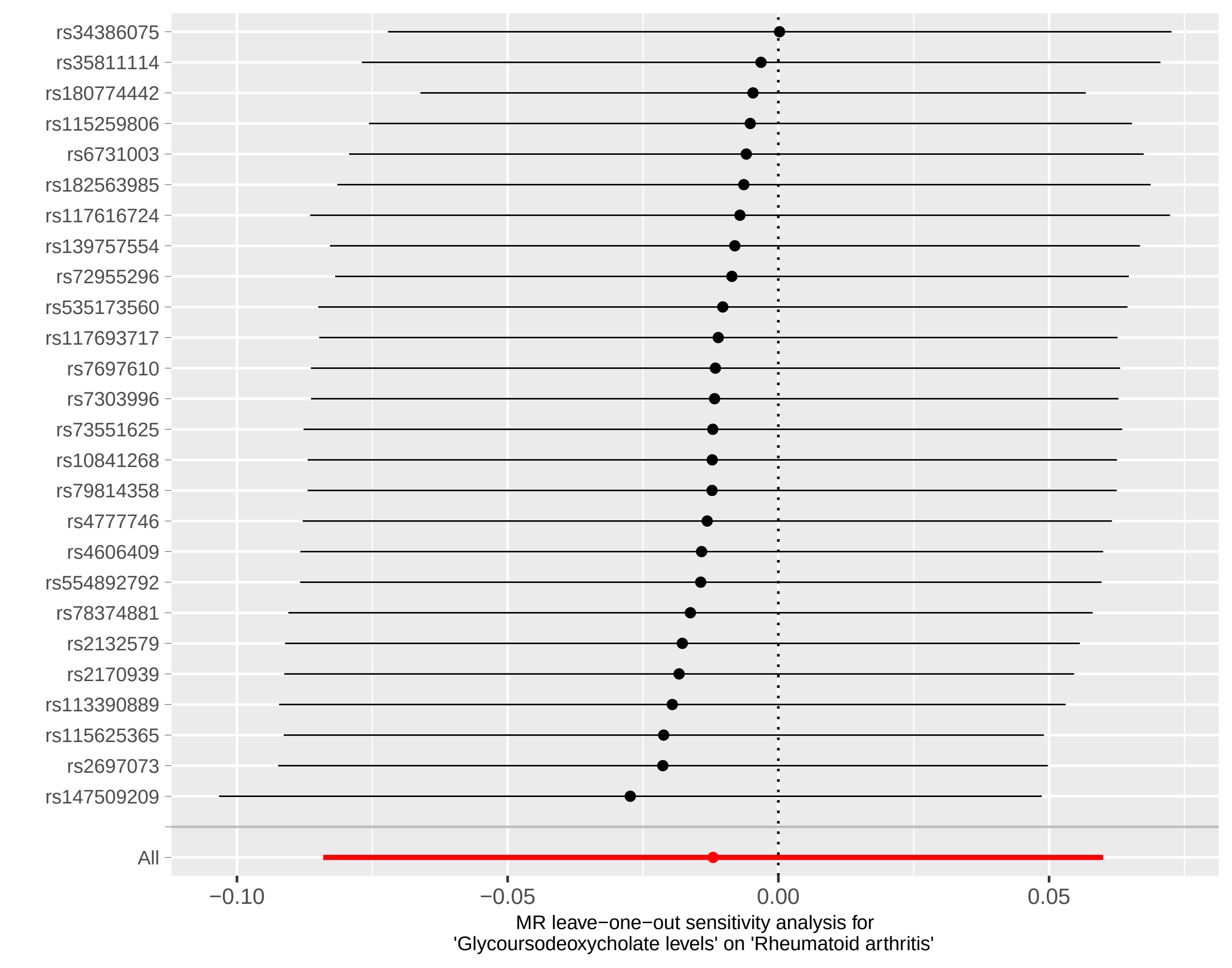
rs34386075
rs35811114
rs180774442
rs115259806
rs6731003
rs182563985
rs117616724
rs139757554
rs72955296
rs535173560
rs117693717
rs7697610
rs7303996
rs73551625
rs10841268
rs79814358
rs4777746
rs4606409
rs554892792
rs78374881
rs2132579
rs2170939
rs113390889
rs115625365
rs2697073
rs147509209
All
−0.10
−0.05
0.00
0.05
MR leave−one−out sensitivity analysis for
'Glycoursodeoxycholate levels' on 'Rheumatoid arthritis'

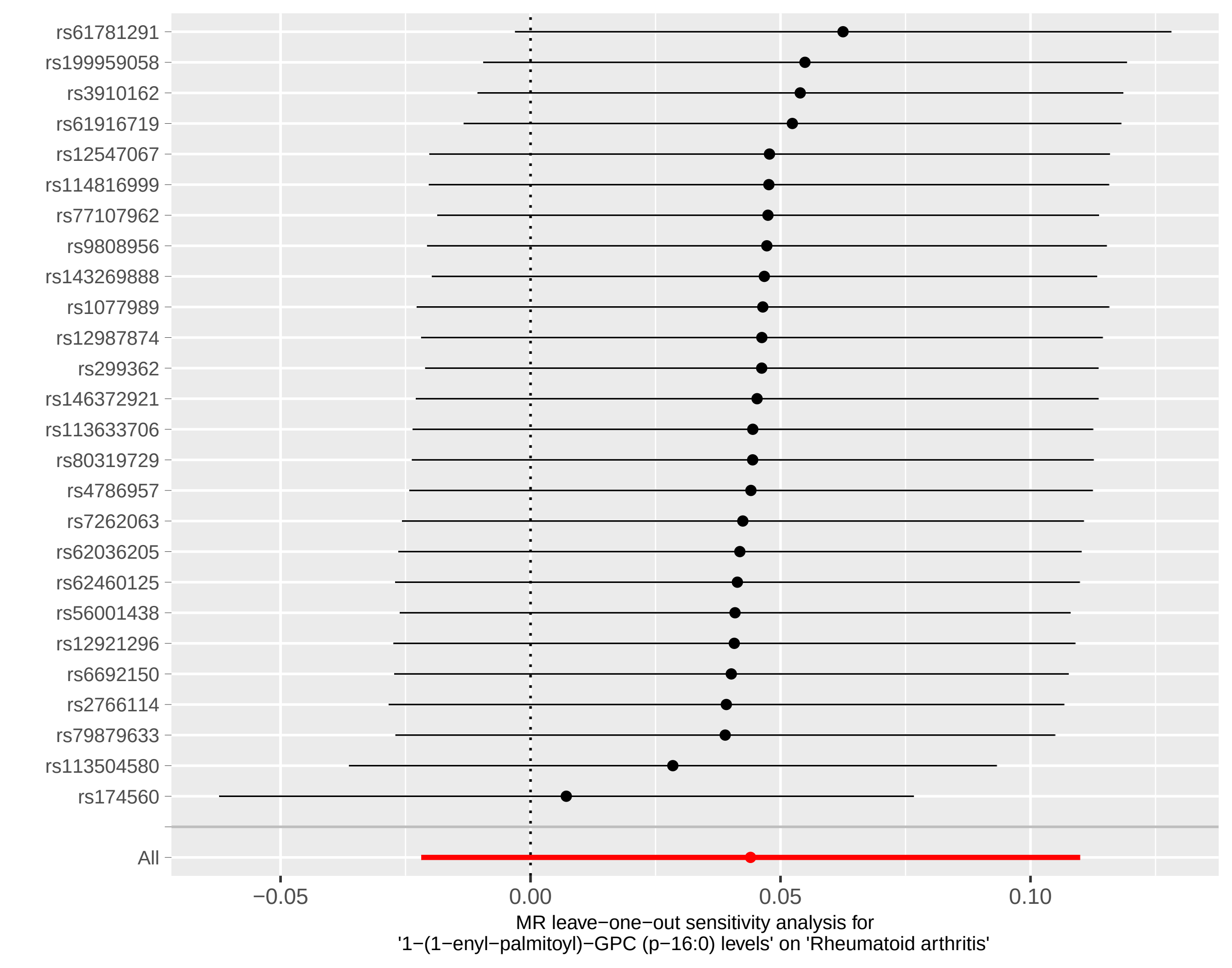

rs61781291
rs199959058
rs3910162
rs61916719
rs12547067
rs114816999
rs77107962
rs9808956
rs143269888
rs1077989
rs12987874
rs299362
rs146372921
rs113633706
rs80319729
rs4786957
rs7262063
rs62036205
rs62460125
rs56001438
rs12921296
rs6692150
rs2766114
rs79879633
rs113504580
rs174560
All
−0.05
0.00
0.05
0.10
MR leave−one−out sensitivity analysis for
'1−(1−enyl−palmitoyl)−GPC (p−16:0) levels' on 'Rheumatoid arthritis'

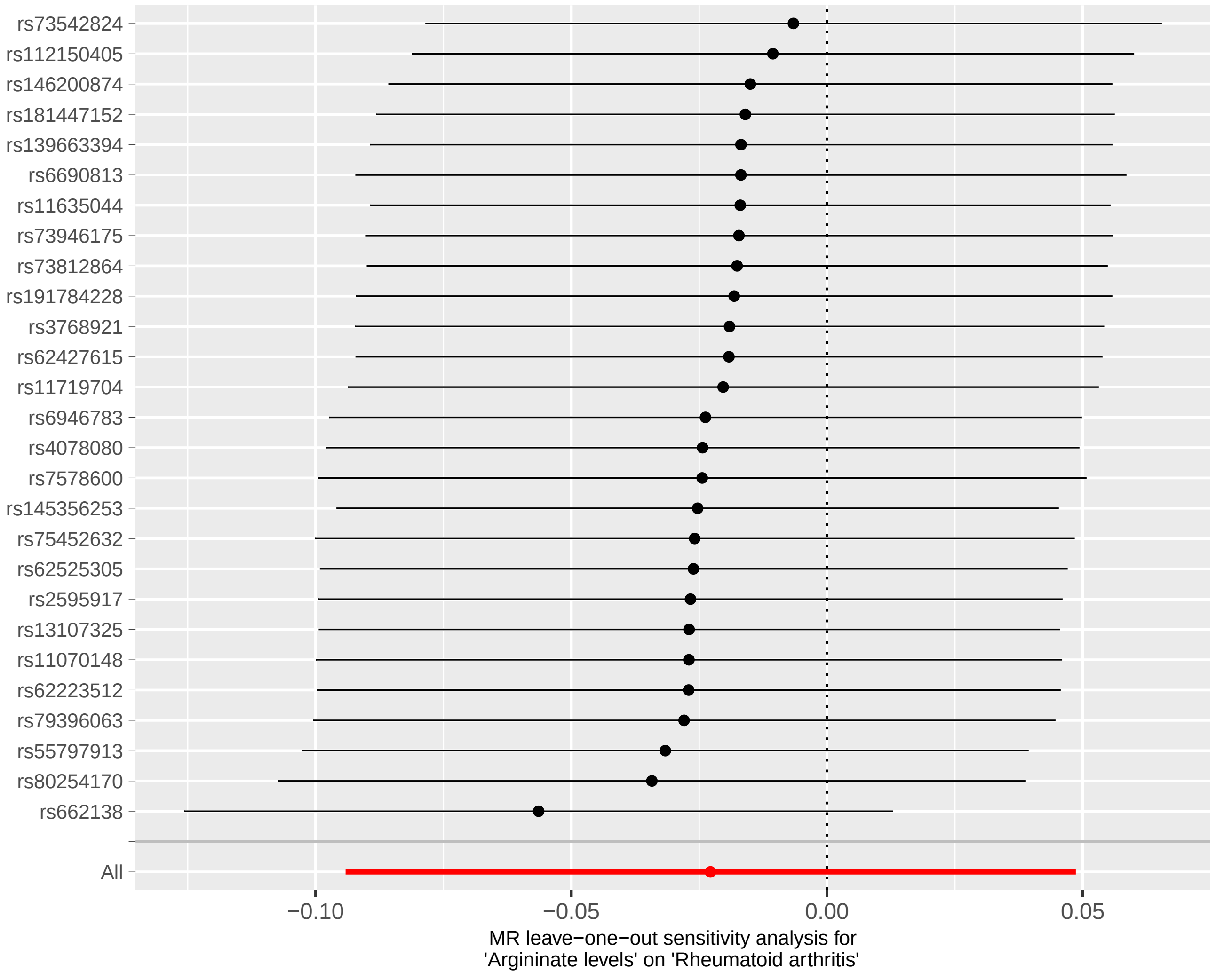

rs73542824
rs112150405
rs146200874
rs181447152
rs139663394
rs6690813
rs11635044
rs73946175
rs73812864
rs191784228
rs3768921
rs62427615
rs11719704
rs6946783
rs4078080
rs7578600
rs145356253
rs75452632
rs62525305
rs2595917
rs13107325
rs11070148
rs62223512
rs79396063
rs55797913
rs80254170
rs662138
All
−0.10
−0.05
0.00
0.05
MR leave−one−out sensitivity analysis for
'Argininate levels' on 'Rheumatoid arthritis'

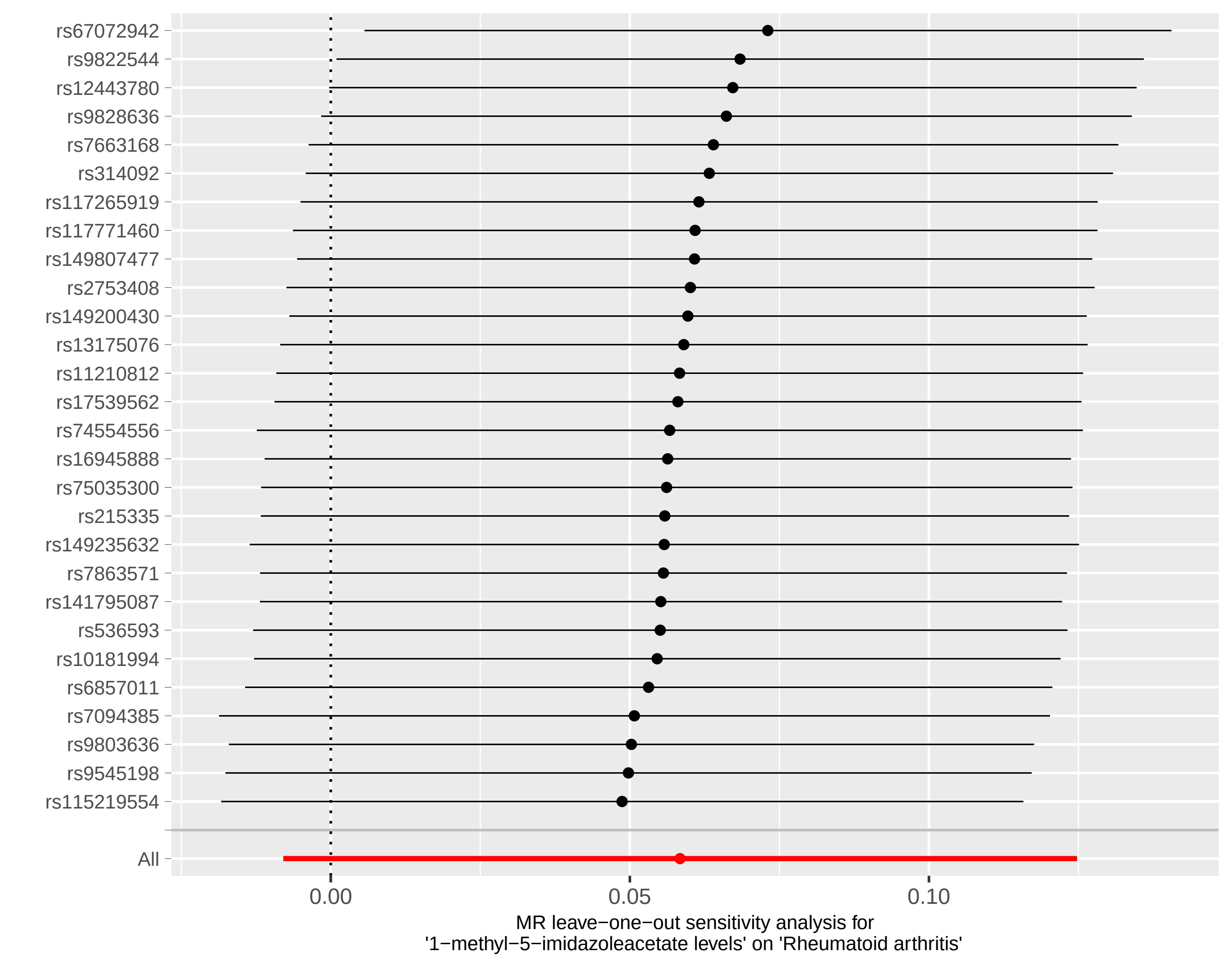
rs67072942
rs9822544
rs12443780
rs9828636
rs7663168
rs314092
rs117265919
rs117771460
rs149807477
rs2753408
rs149200430
rs13175076
rs11210812
rs17539562
rs74554556
rs16945888
rs75035300
rs215335
rs149235632
rs7863571
rs141795087
rs536593
rs10181994
rs6857011
rs7094385
rs9803636
rs9545198
rs115219554
All
0.00
0.05
0.10
MR leave−one−out sensitivity analysis for
'1−methyl−5−imidazoleacetate levels' on 'Rheumatoid arthritis'

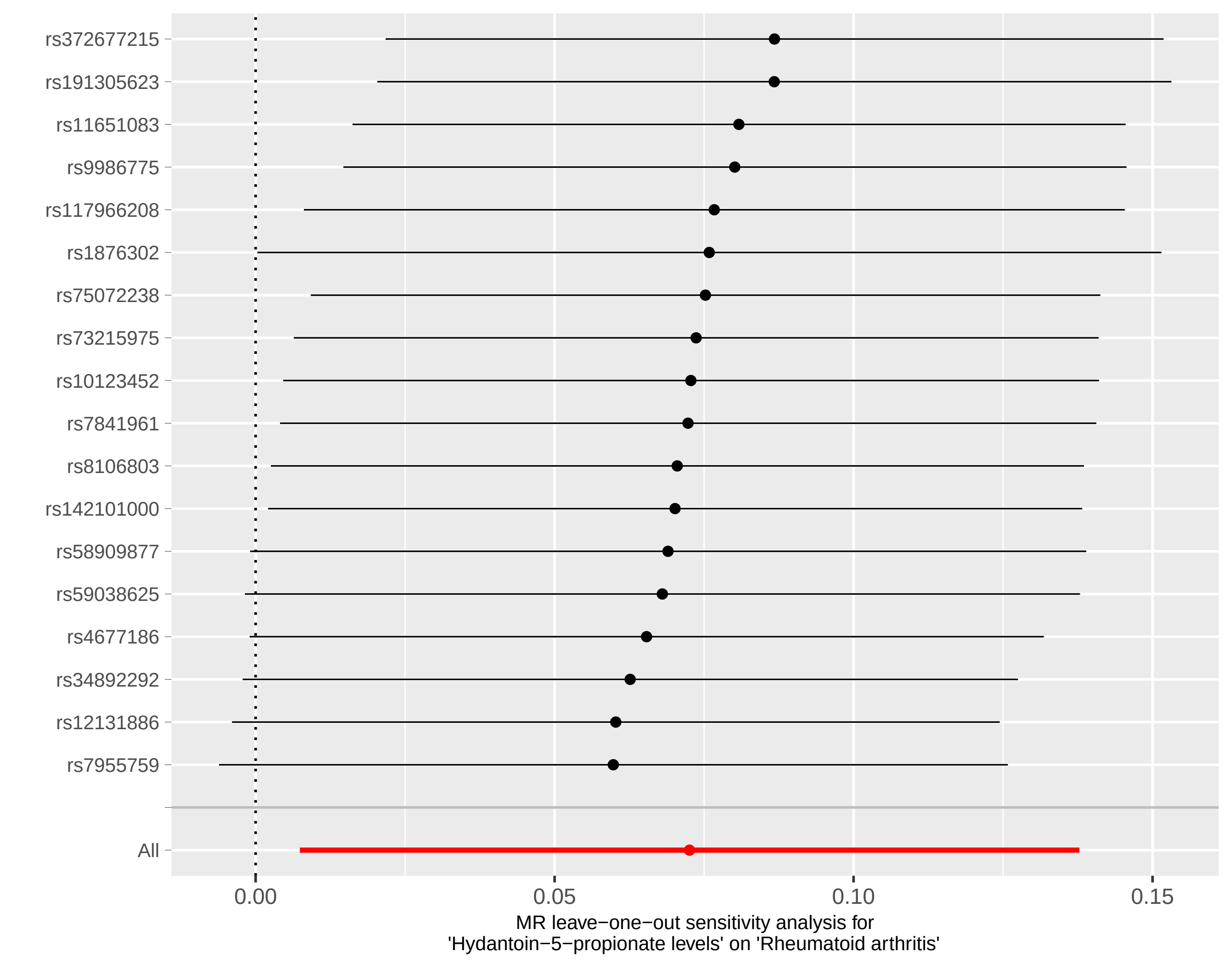

rs372677215
rs191305623
rs11651083
rs9986775
rs117966208
rs1876302
rs75072238
rs73215975
rs10123452
rs7841961
rs8106803
rs142101000
rs58909877
rs59038625
rs4677186
rs34892292
rs12131886
rs7955759
All
0.00
0.05
0.10
0.15
MR leave−one−out sensitivity analysis for
'Hydantoin−5−propionate levels' on 'Rheumatoid arthritis'

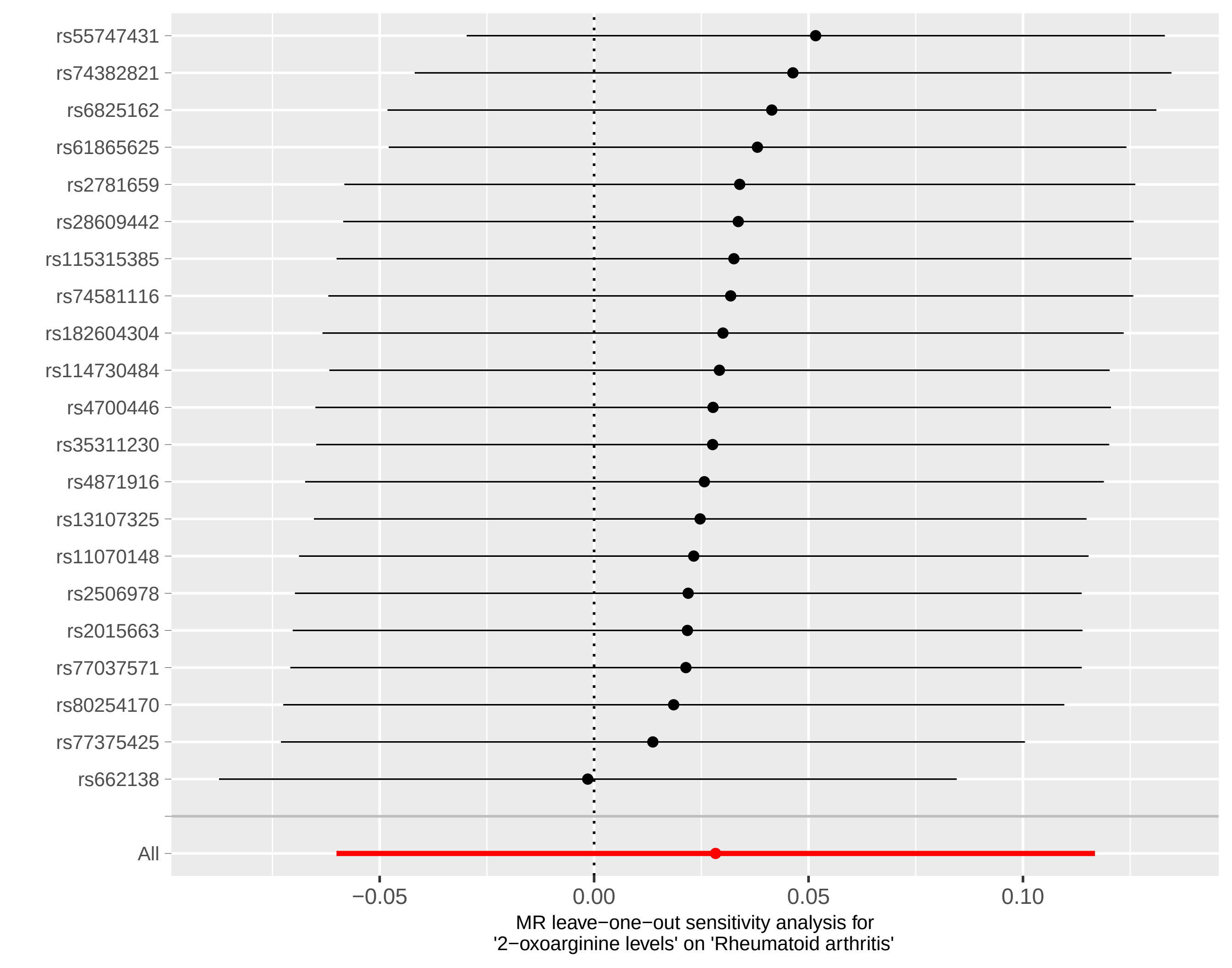
rs55747431
rs74382821
rs6825162
rs61865625
rs2781659
rs28609442
rs115315385
rs74581116
rs182604304
rs114730484
rs4700446
rs35311230
rs4871916
rs13107325
rs11070148
rs2506978
rs2015663
rs77037571
rs80254170
rs77375425
rs662138
All
−0.05
0.00
0.05
0.10
MR leave−one−out sensitivity analysis for
'2−oxoarginine levels' on 'Rheumatoid arthritis'

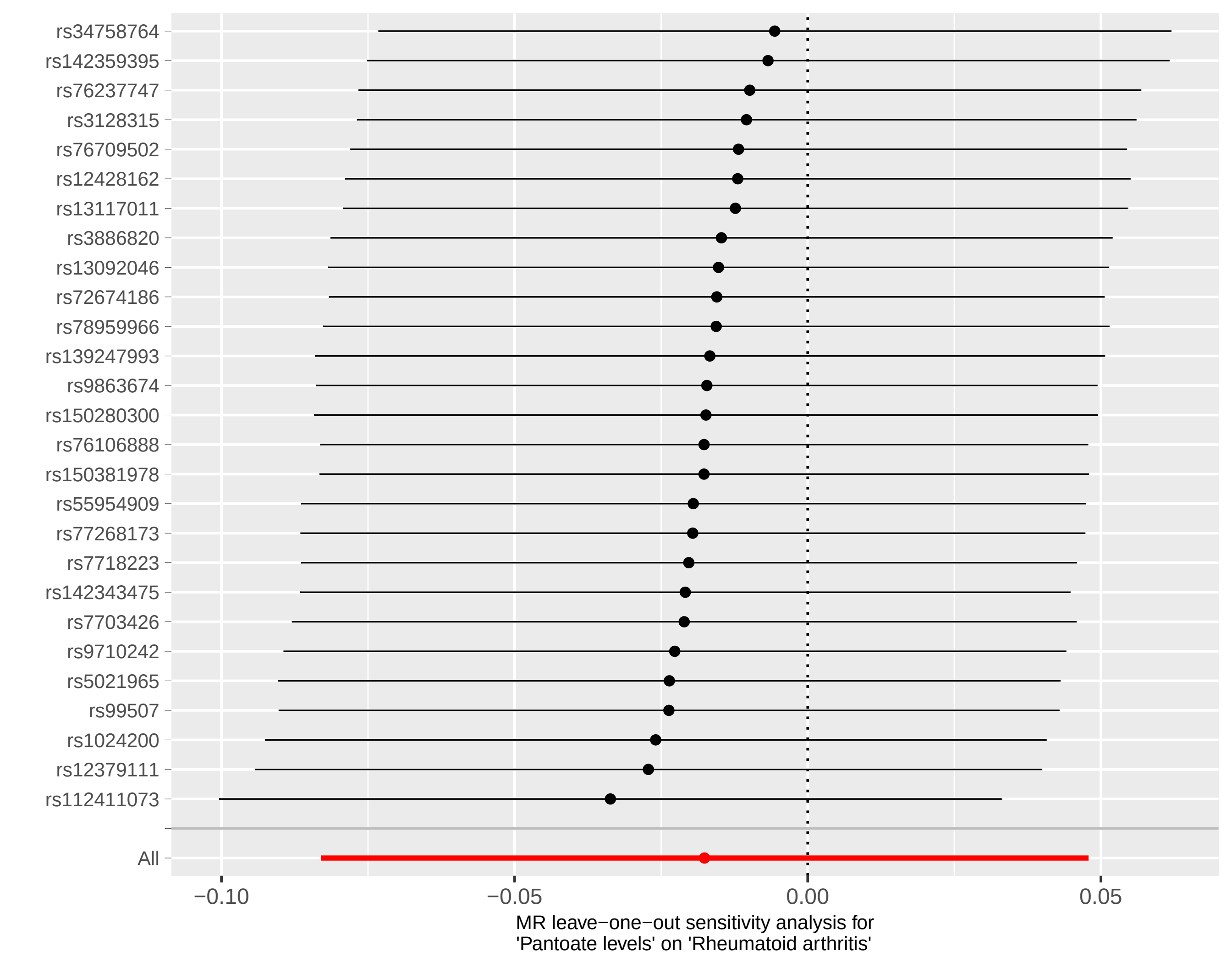

rs34758764
rs142359395
rs76237747
rs3128315
rs76709502
rs12428162
rs13117011
rs3886820
rs13092046
rs72674186
rs78959966
rs139247993
rs9863674
rs150280300
rs76106888
rs150381978
rs55954909
rs77268173
rs7718223
rs142343475
rs7703426
rs9710242
rs5021965
rs99507
rs1024200
rs12379111
rs112411073
All
−0.10
−0.05
0.00
0.05
MR leave−one−out sensitivity analysis for
'Pantoate levels' on 'Rheumatoid arthritis'

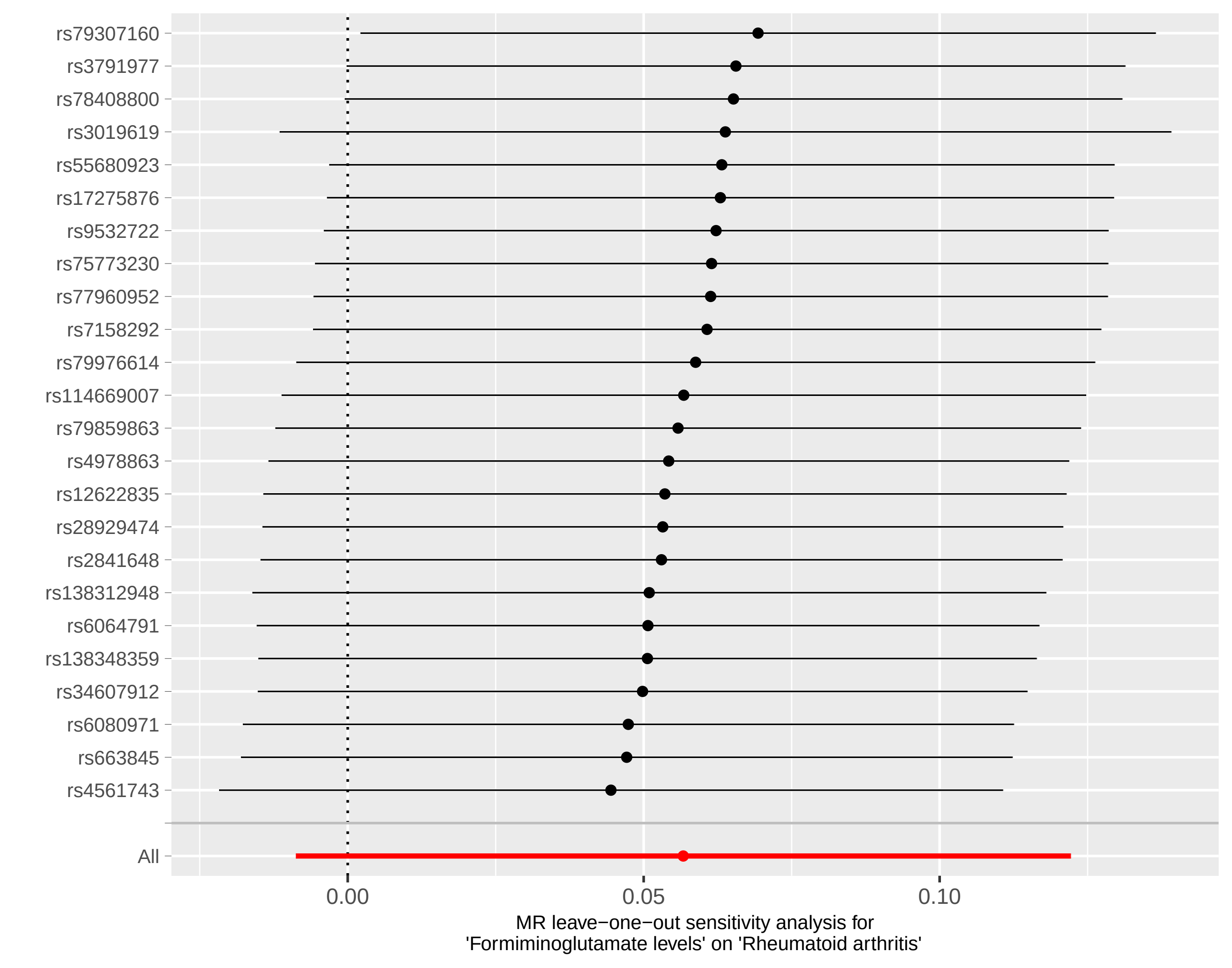

rs79307160
rs3791977
rs78408800
rs3019619
rs55680923
rs17275876
rs9532722
rs75773230
rs77960952
rs7158292
rs79976614
rs114669007
rs79859863
rs4978863
rs12622835
rs28929474
rs2841648
rs138312948
rs6064791
rs138348359
rs34607912
rs6080971
rs663845
rs4561743
All
0.00
0.05
0.10
MR leave−one−out sensitivity analysis for
'Formiminoglutamate levels' on 'Rheumatoid arthritis'

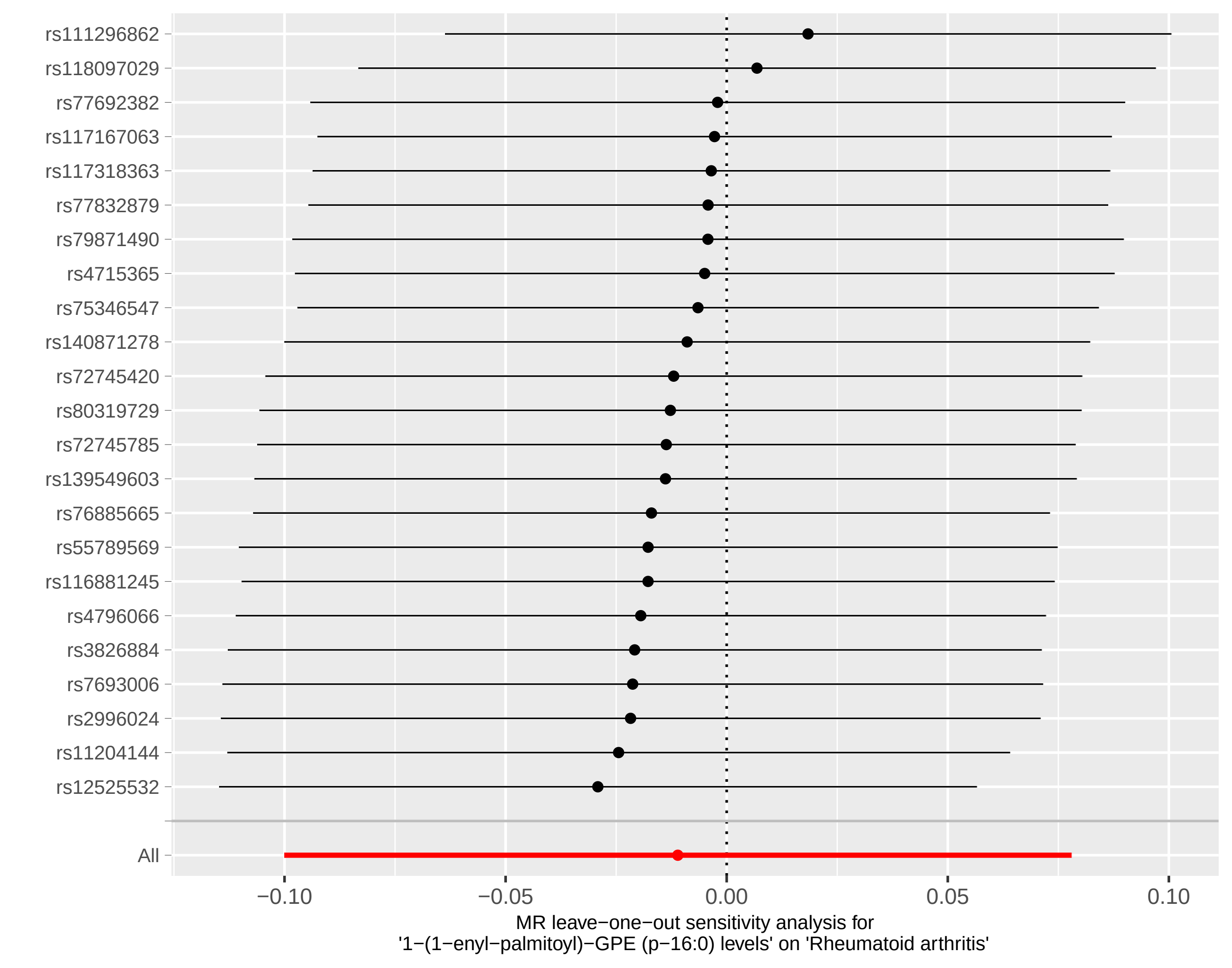
rs111296862
rs118097029
rs77692382
rs117167063
rs117318363
rs77832879
rs79871490
rs4715365
rs75346547
rs140871278
rs72745420
rs80319729
rs72745785
rs139549603
rs76885665
rs55789569
rs116881245
rs4796066
rs3826884
rs7693006
rs2996024
rs11204144
rs12525532
All
−0.10
−0.05
0.00
0.05
0.10
MR leave−one−out sensitivity analysis for
'1−(1−enyl−palmitoyl)−GPE (p−16:0) levels' on 'Rheumatoid arthritis'

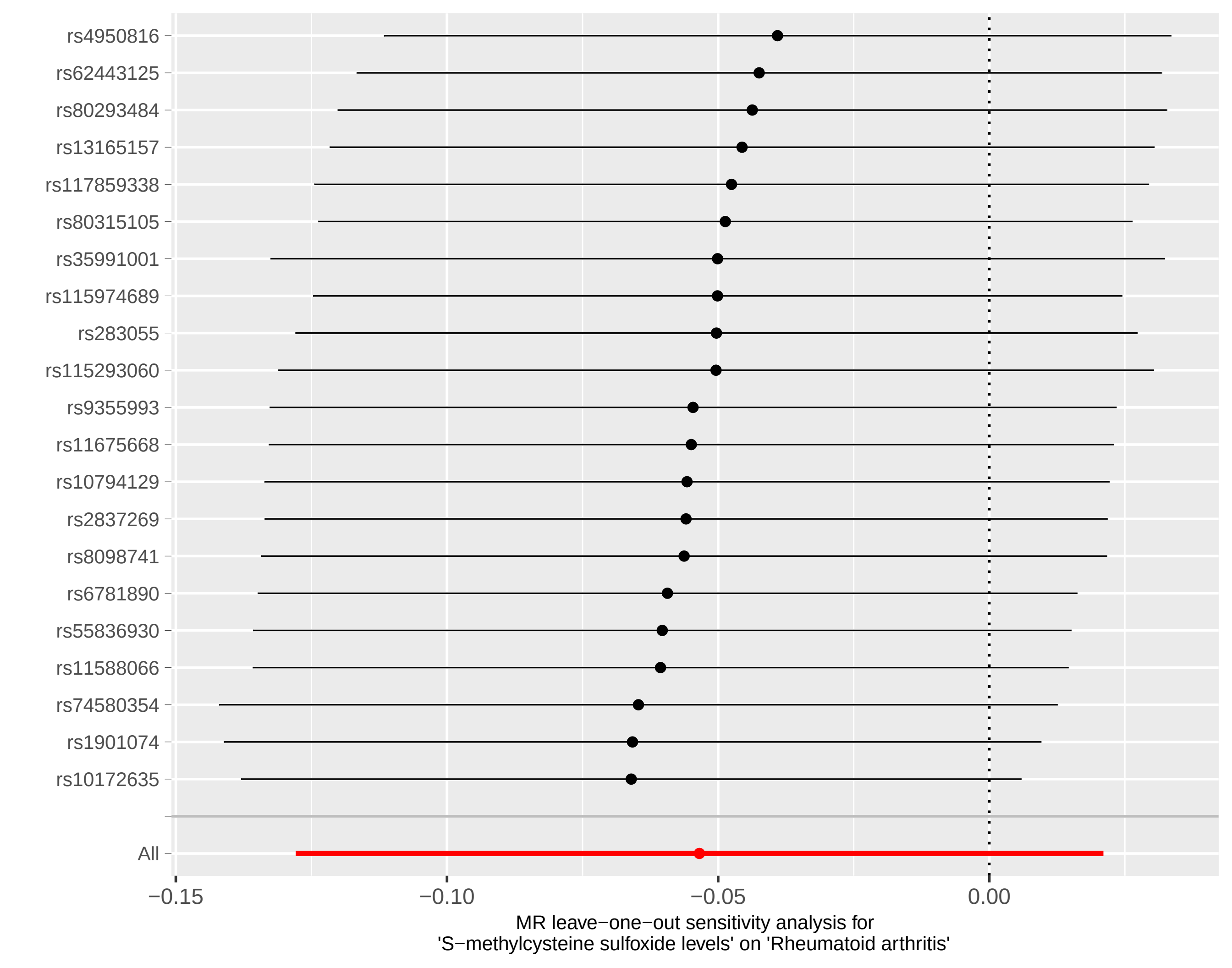
rs4950816
rs62443125
rs80293484
rs13165157
rs117859338
rs80315105
rs35991001
rs115974689
rs283055
rs115293060
rs9355993
rs11675668
rs10794129
rs2837269
rs8098741
rs6781890
rs55836930
rs11588066
rs74580354
rs1901074
rs10172635
All
−0.15
−0.10
−0.05
0.00
MR leave−one−out sensitivity analysis for
'S−methylcysteine sulfoxide levels' on 'Rheumatoid arthritis'

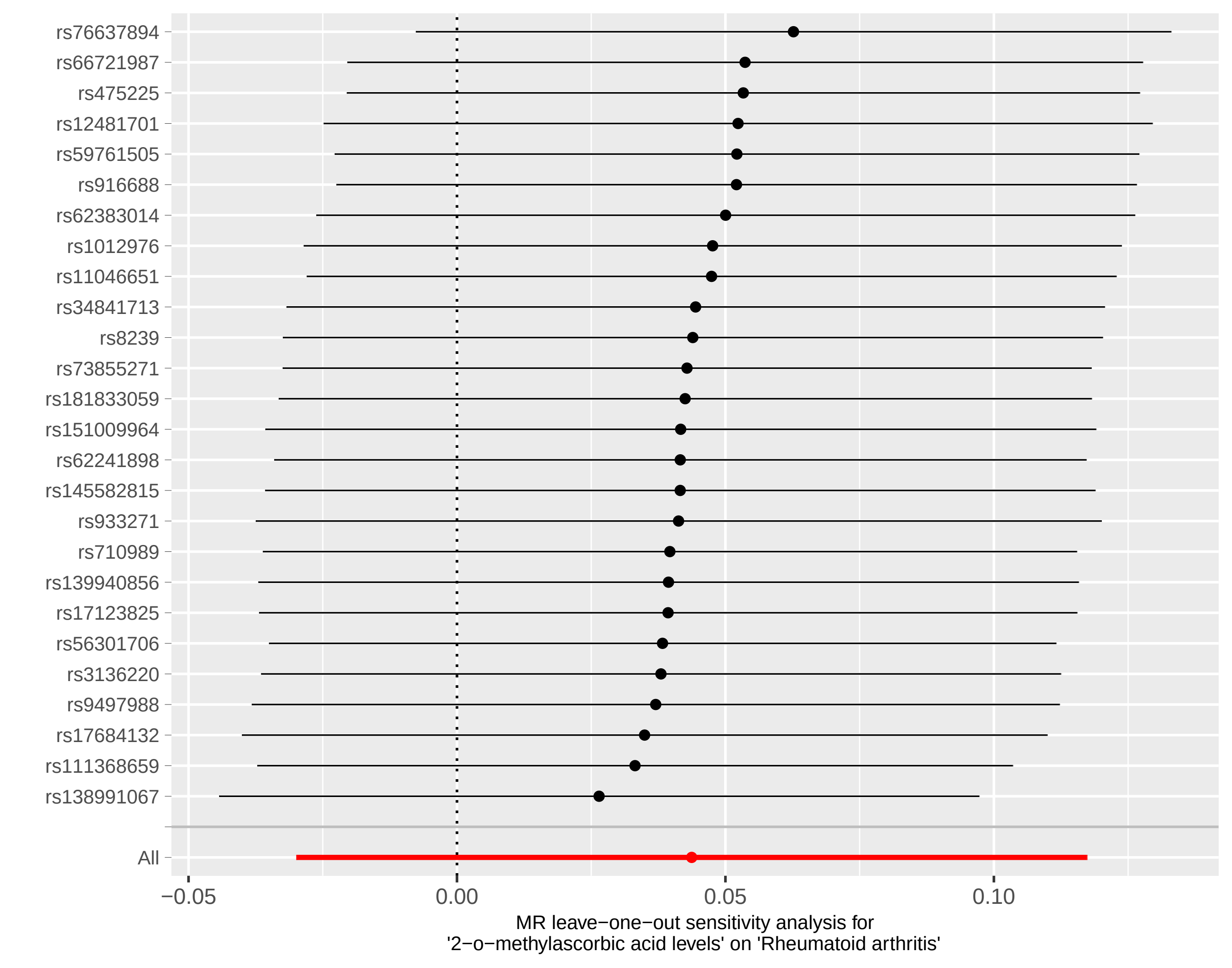
rs76637894
rs66721987
rs475225
rs12481701
rs59761505
rs916688
rs62383014
rs1012976
rs11046651
rs34841713
rs8239
rs73855271
rs181833059
rs151009964
rs62241898
rs145582815
rs933271
rs710989
rs139940856
rs17123825
rs56301706
rs3136220
rs9497988
rs17684132
rs111368659
rs138991067
All
−0.05
0.00
0.05
0.10
MR leave−one−out sensitivity analysis for
'2−o−methylascorbic acid levels' on 'Rheumatoid arthritis'

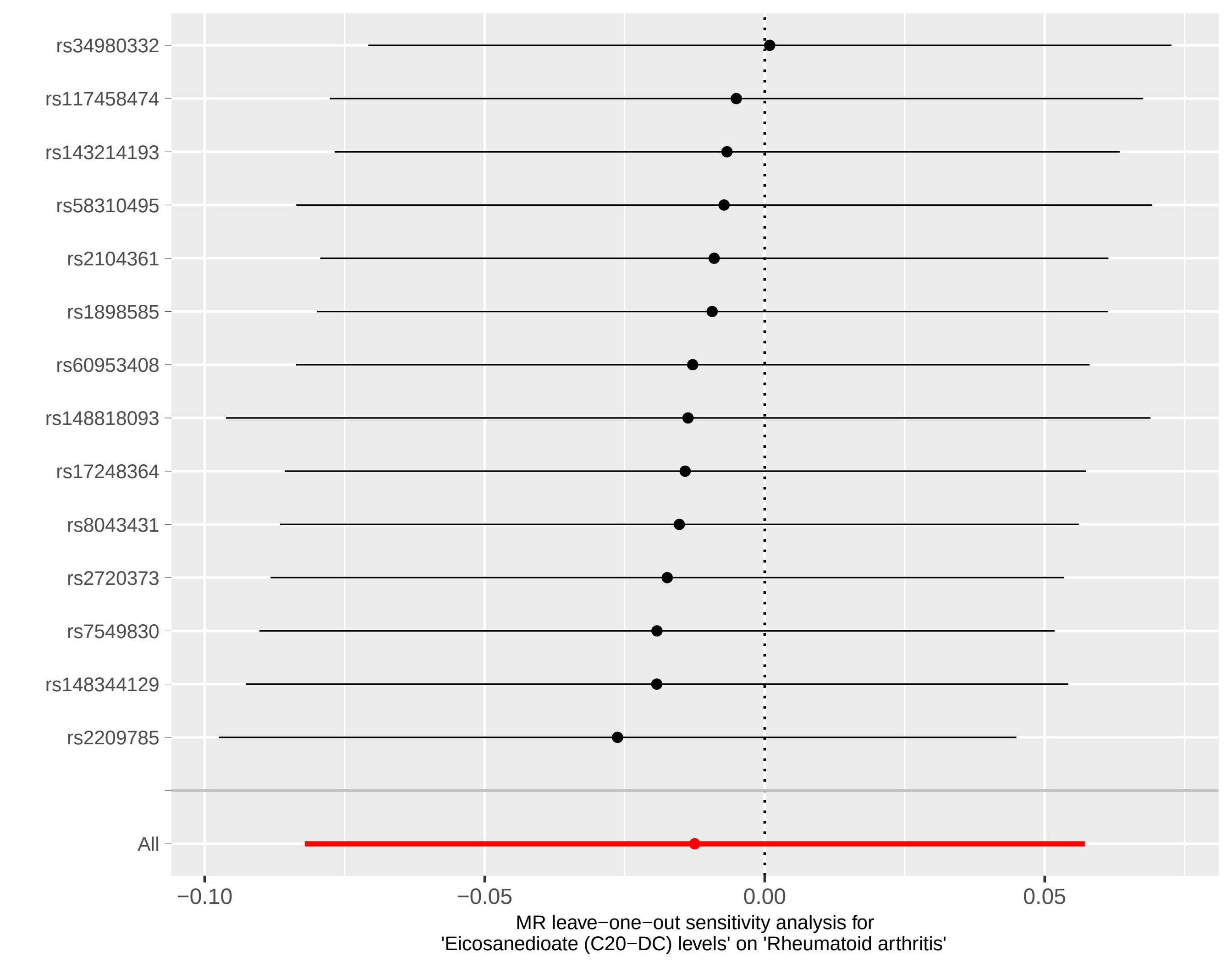

rs34980332
rs117458474
rs143214193
rs58310495
rs2104361
rs1898585
rs60953408
rs148818093
rs17248364
rs8043431
rs2720373
rs7549830
rs148344129
rs2209785
All
−0.10
−0.05
0.00
0.05
MR leave−one−out sensitivity analysis for
'Eicosanedioate (C20−DC) levels' on 'Rheumatoid arthritis'

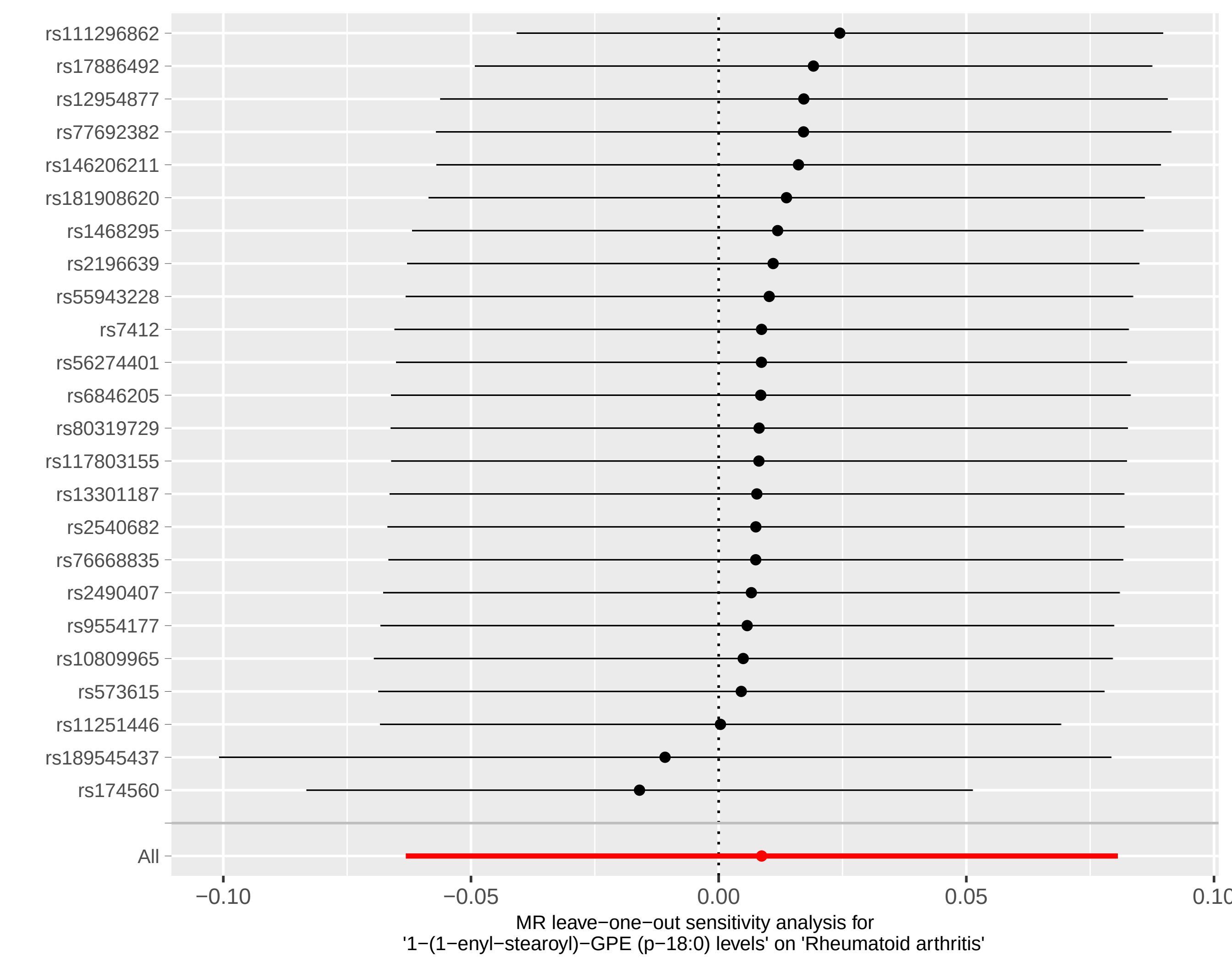
rs111296862
rs17886492
rs12954877
rs77692382
rs146206211
rs181908620
rs1468295
rs2196639
rs55943228
rs7412
rs56274401
rs6846205
rs80319729
rs117803155
rs13301187
rs2540682
rs76668835
rs2490407
rs9554177
rs10809965
rs573615
rs11251446
rs189545437
rs174560
All
−0.10
−0.05
0.00
0.05
0.10
MR leave−one−out sensitivity analysis for
'1−(1−enyl−stearoyl)−GPE (p−18:0) levels' on 'Rheumatoid arthritis'

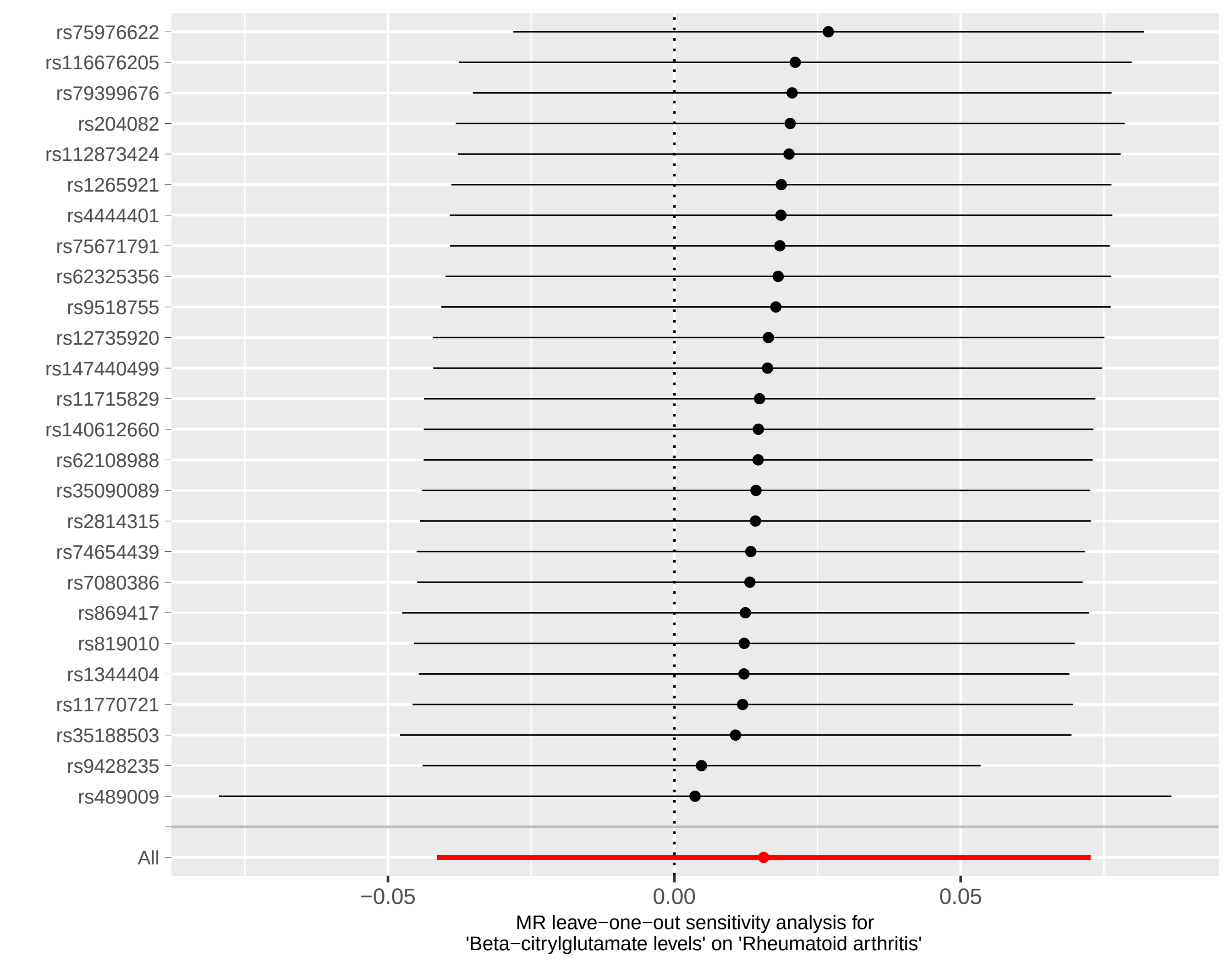

rs75976622
rs116676205
rs79399676
rs204082
rs112873424
rs1265921
rs4444401
rs75671791
rs62325356
rs9518755
rs12735920
rs147440499
rs11715829
rs140612660
rs62108988
rs35090089
rs2814315
rs74654439
rs7080386
rs869417
rs819010
rs1344404
rs11770721
rs35188503
rs9428235
rs489009
All
−0.05
0.00
0.05
MR leave−one−out sensitivity analysis for
'Beta−citrylglutamate levels' on 'Rheumatoid arthritis'

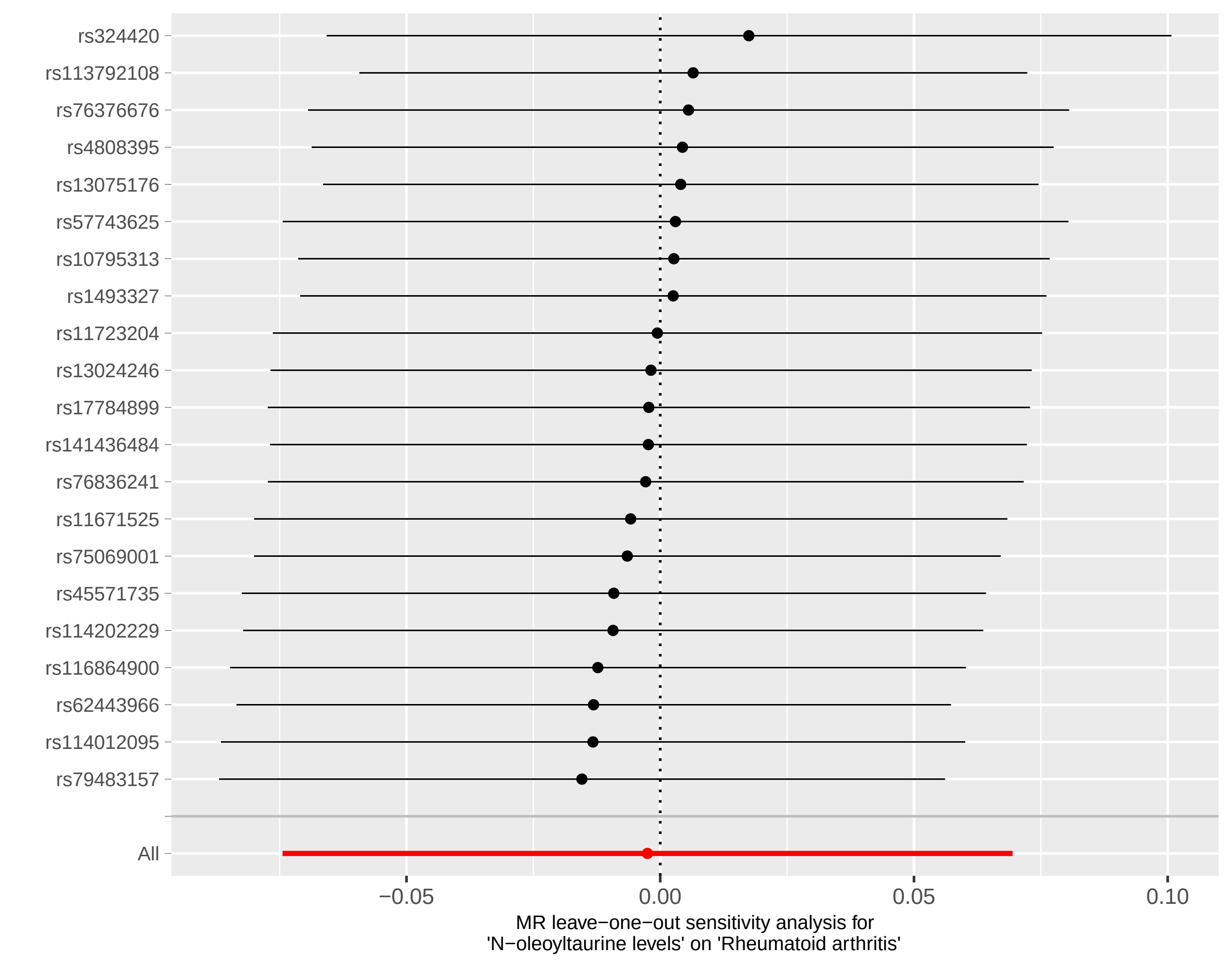

rs324420
rs113792108
rs76376676
rs4808395
rs13075176
rs57743625
rs10795313
rs1493327
rs11723204
rs13024246
rs17784899
rs141436484
rs76836241
rs11671525
rs75069001
rs45571735
rs114202229
rs116864900
rs62443966
rs114012095
rs79483157
All
−0.05
0.00
0.05
0.10
MR leave−one−out sensitivity analysis for
'N−oleoyltaurine levels' on 'Rheumatoid arthritis'

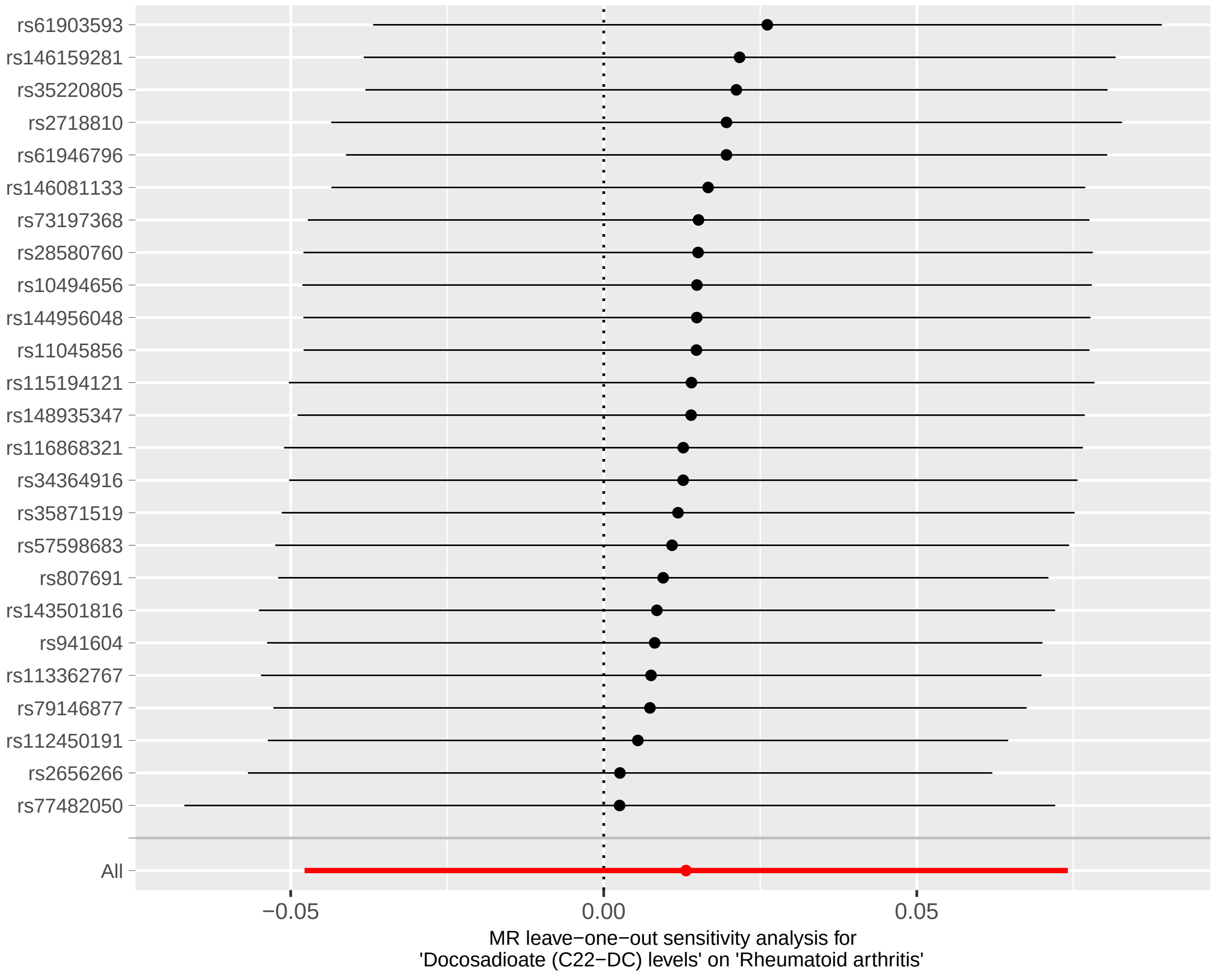
rs61903593
rs146159281
rs35220805
rs2718810
rs61946796
rs146081133
rs73197368
rs28580760
rs10494656
rs144956048
rs11045856
rs115194121
rs148935347
rs116868321
rs34364916
rs35871519
rs57598683
rs807691
rs143501816
rs941604
rs113362767
rs79146877
rs112450191
rs2656266
rs77482050
All
−0.05
0.00
0.05
MR leave−one−out sensitivity analysis for
'Docosadioate (C22−DC) levels' on 'Rheumatoid arthritis'

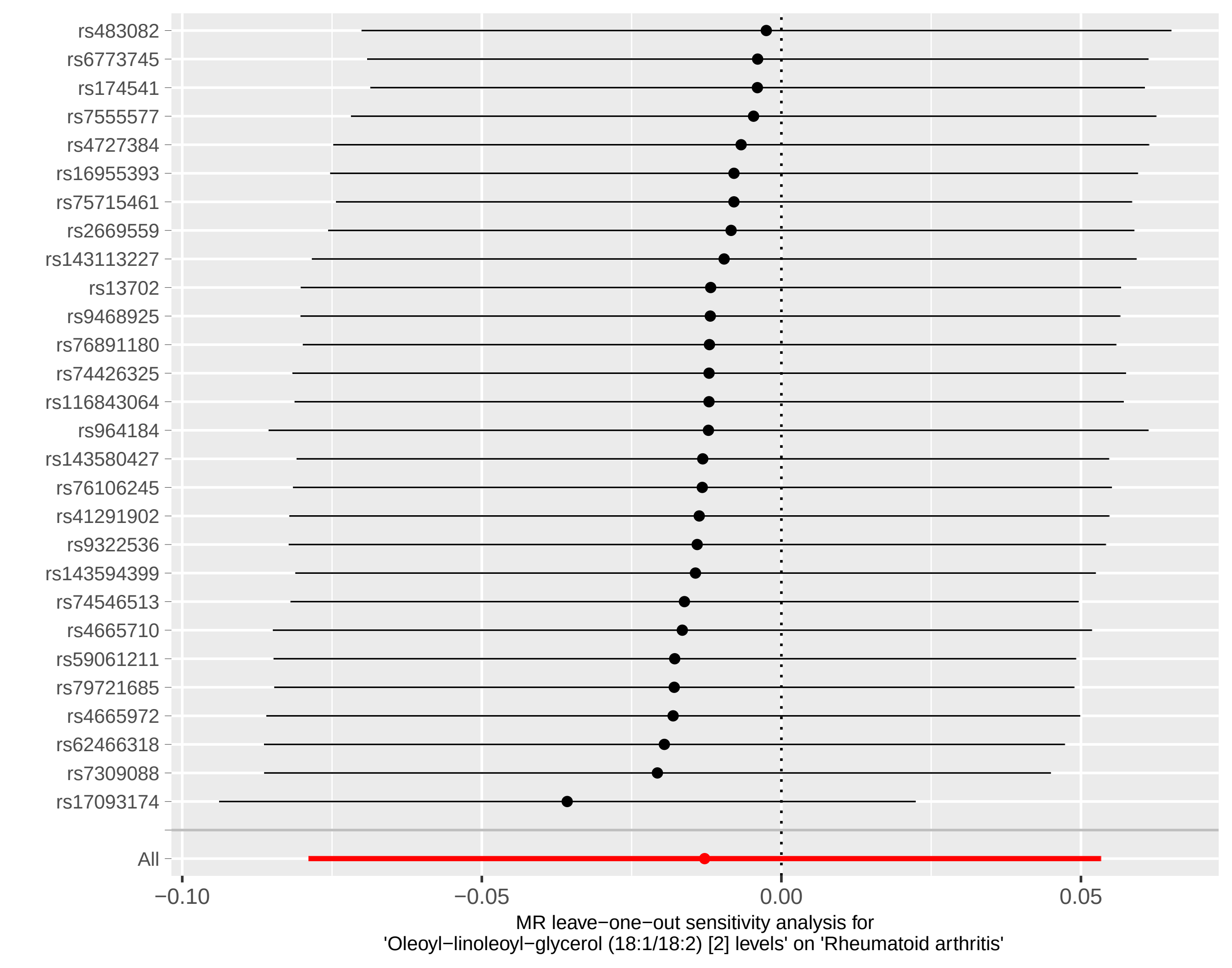
rs483082
rs6773745
rs174541
rs7555577
rs4727384
rs16955393
rs75715461
rs2669559
rs143113227
rs13702
rs9468925
rs76891180
rs74426325
rs116843064
rs964184
rs143580427
rs76106245
rs41291902
rs9322536
rs143594399
rs74546513
rs4665710
rs59061211
rs79721685
rs4665972
rs62466318
rs7309088
rs17093174
All
−0.10
−0.05
0.00
0.05
MR leave−one−out sensitivity analysis for
'Oleoyl−linoleoyl−glycerol (18:1/18:2) [2] levels' on 'Rheumatoid arthritis'

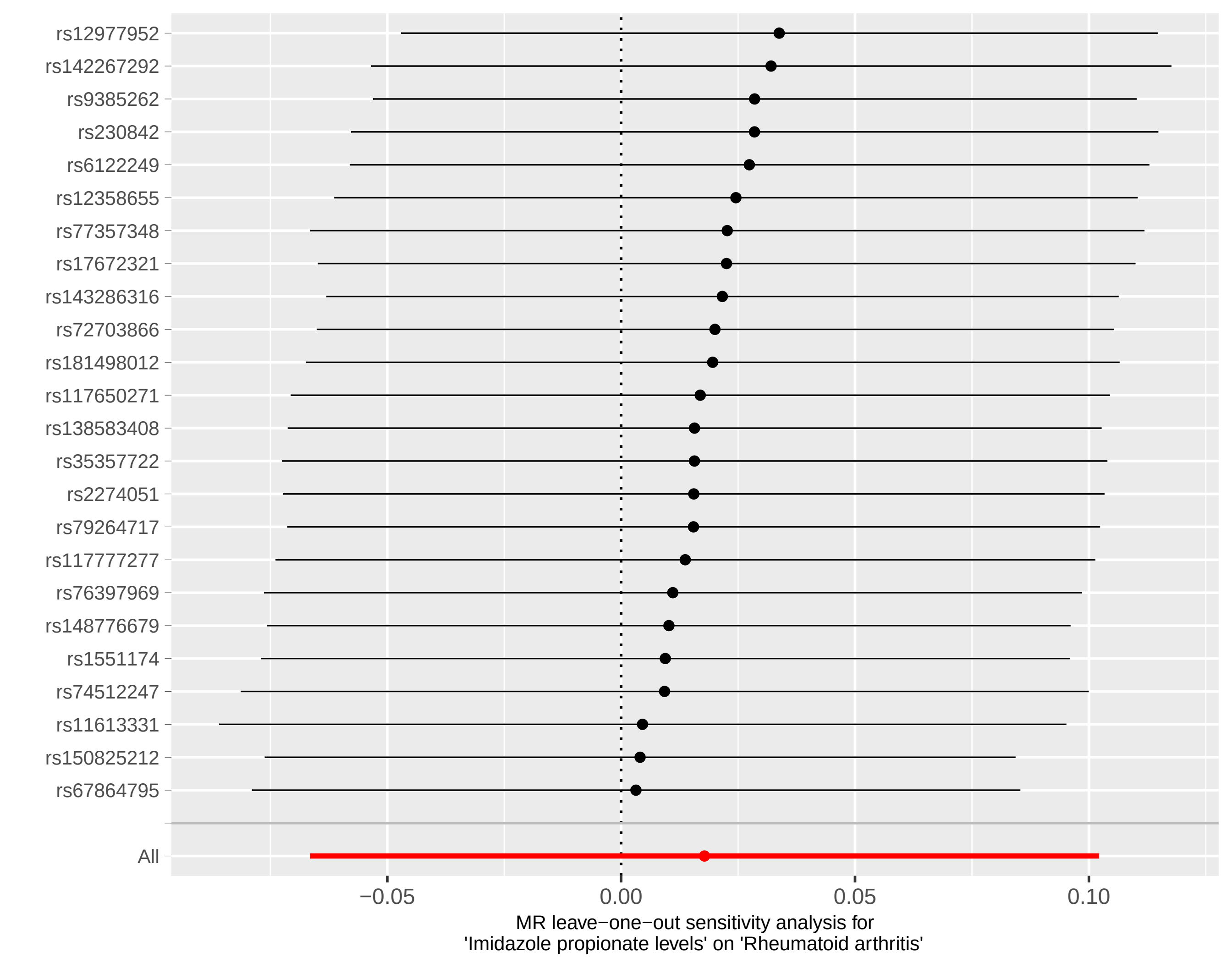
rs12977952
rs142267292
rs9385262
rs230842
rs6122249
rs12358655
rs77357348
rs17672321
rs143286316
rs72703866
rs181498012
rs117650271
rs138583408
rs35357722
rs2274051
rs79264717
rs117777277
rs76397969
rs148776679
rs1551174
rs74512247
rs11613331
rs150825212
rs67864795
All
−0.05
0.00
0.05
0.10
MR leave−one−out sensitivity analysis for
'Imidazole propionate levels' on 'Rheumatoid arthritis'

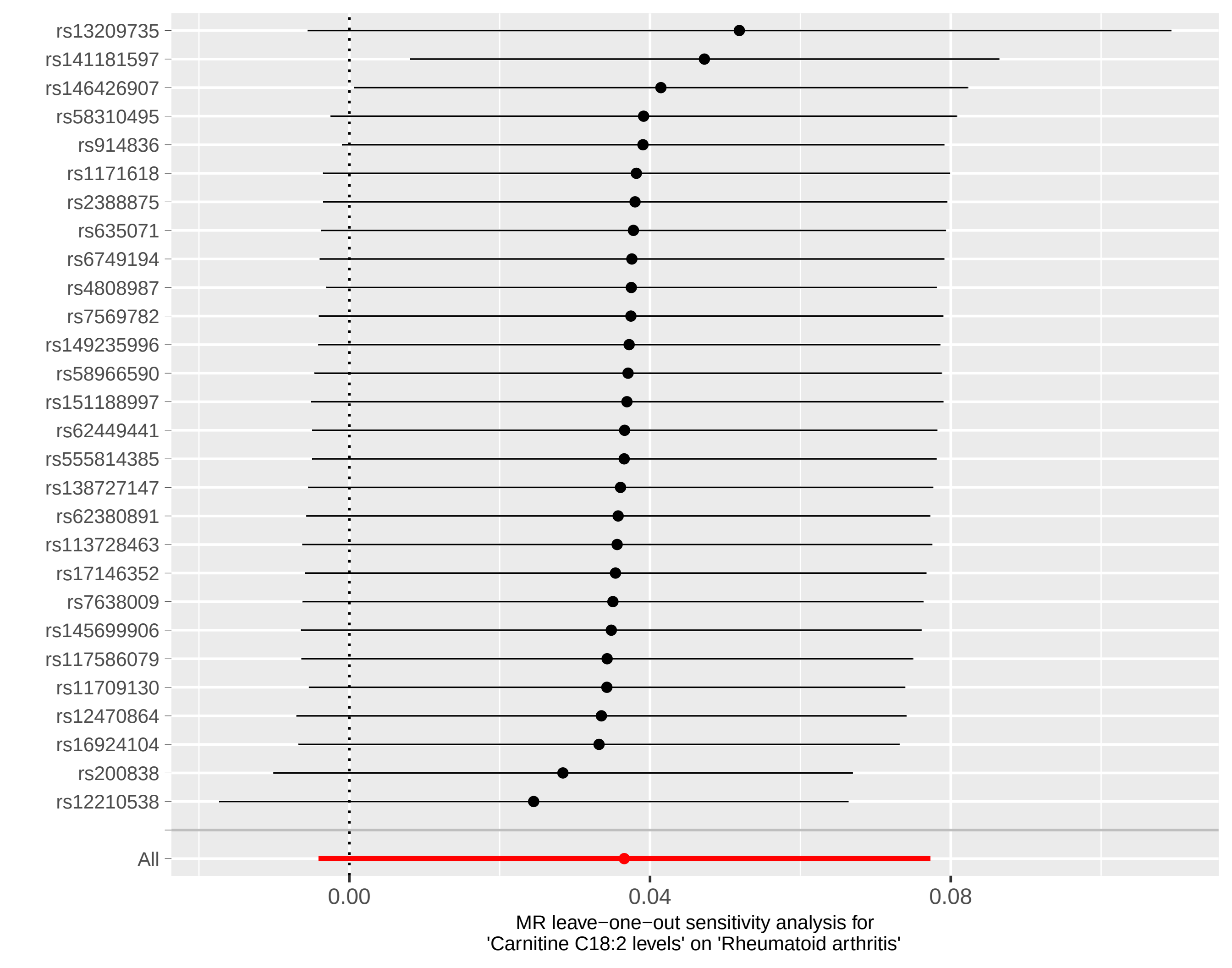

rs13209735
rs141181597
rs146426907
rs58310495
rs914836
rs1171618
rs2388875
rs635071
rs6749194
rs4808987
rs7569782
rs149235996
rs58966590
rs151188997
rs62449441
rs555814385
rs138727147
rs62380891
rs113728463
rs17146352
rs7638009
rs145699906
rs117586079
rs11709130
rs12470864
rs16924104
rs200838
rs12210538
All
0.00
0.04
0.08
MR leave−one−out sensitivity analysis for
'Carnitine C18:2 levels' on 'Rheumatoid arthritis'

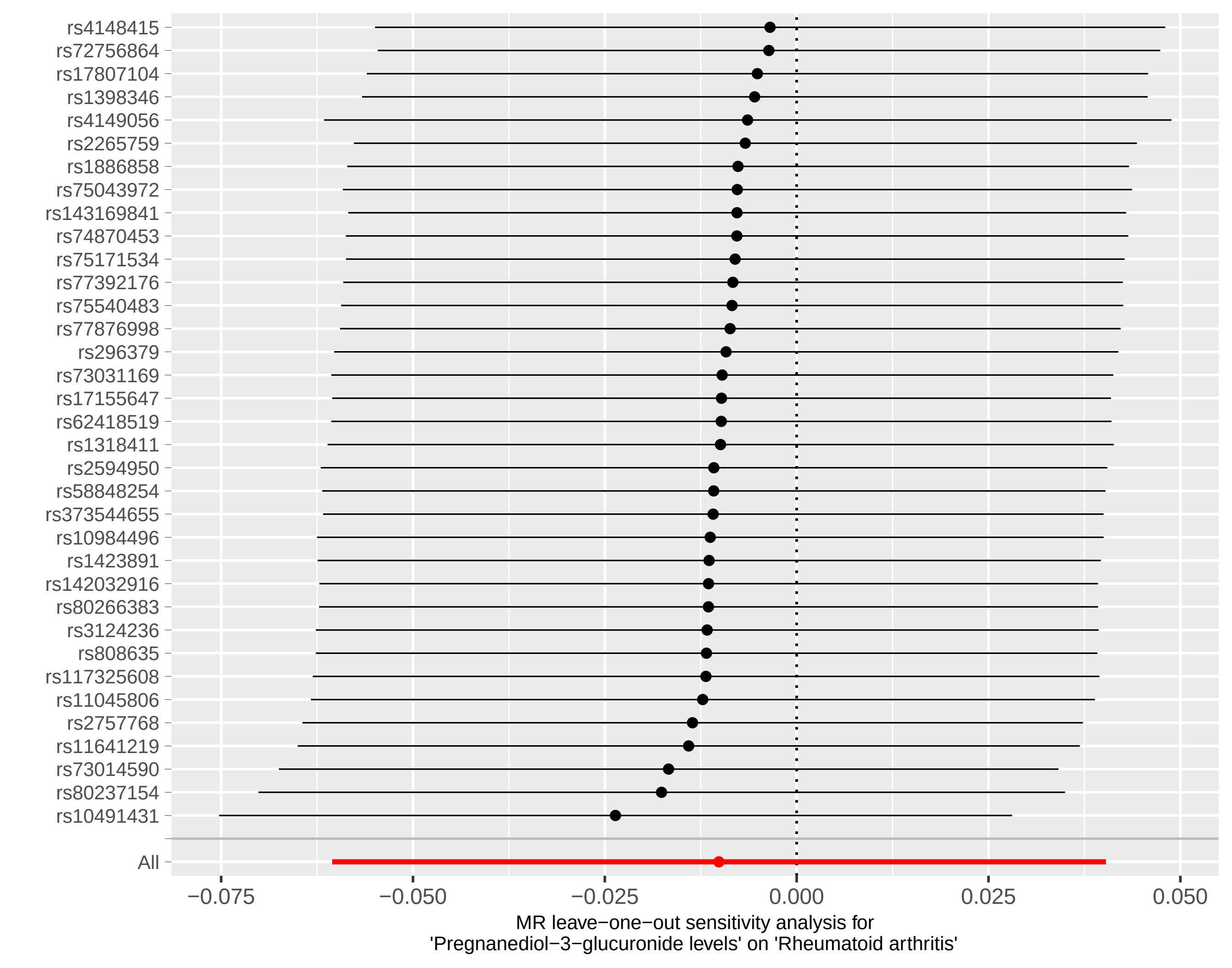
rs4148415
rs72756864
rs17807104
rs1398346
rs4149056
rs2265759
rs1886858
rs75043972
rs143169841
rs74870453
rs75171534
rs77392176
rs75540483
rs77876998
rs296379
rs73031169
rs17155647
rs62418519
rs1318411
rs2594950
rs58848254
rs373544655
rs10984496
rs1423891
rs142032916
rs80266383
rs3124236
rs808635
rs117325608
rs11045806
rs2757768
rs11641219
rs73014590
rs80237154
rs10491431
All
−0.075
−0.050
−0.025
0.000
0.025
0.050
MR leave−one−out sensitivity analysis for
'Pregnanediol−3−glucuronide levels' on 'Rheumatoid arthritis'

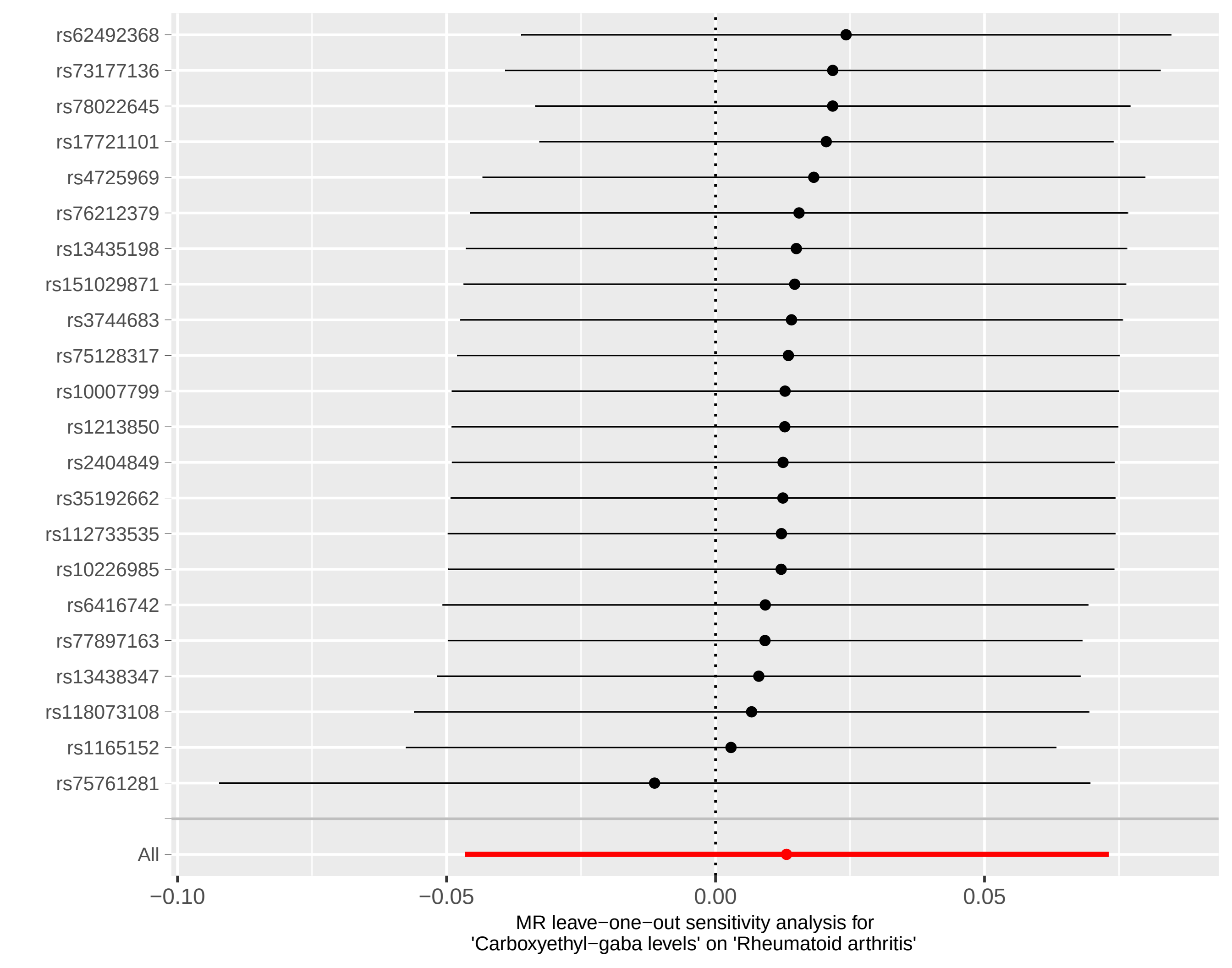
rs62492368
rs73177136
rs78022645
rs17721101
rs4725969
rs76212379
rs13435198
rs151029871
rs3744683
rs75128317
rs10007799
rs1213850
rs2404849
rs35192662
rs112733535
rs10226985
rs6416742
rs77897163
rs13438347
rs118073108
rs1165152
rs75761281
All
−0.10
−0.05
0.00
0.05
MR leave−one−out sensitivity analysis for
'Carboxyethyl−gaba levels' on 'Rheumatoid arthritis'

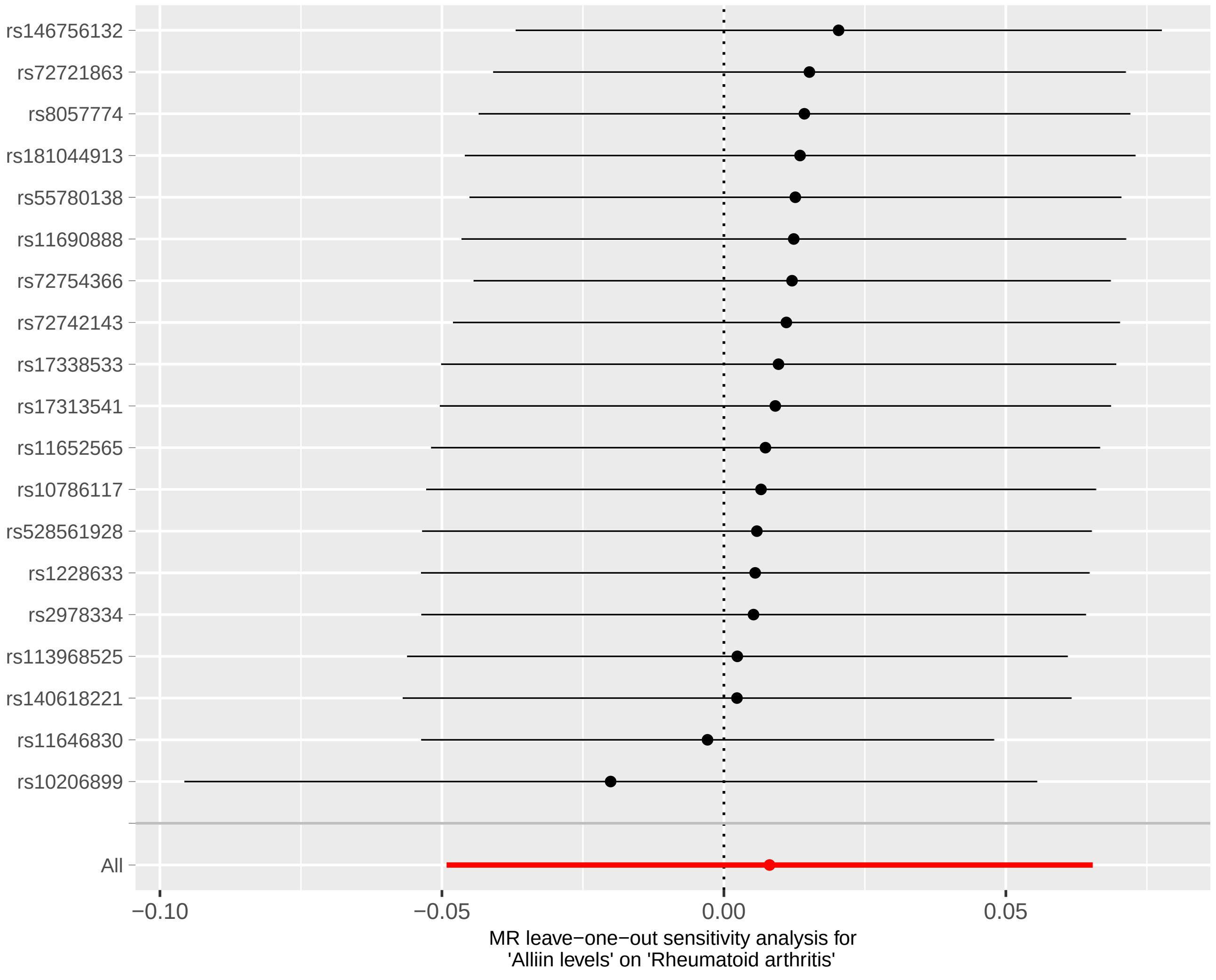
rs146756132
rs72721863
rs8057774
rs181044913
rs55780138
rs11690888
rs72754366
rs72742143
rs17338533
rs17313541
rs11652565
rs10786117
rs528561928
rs1228633
rs2978334
rs113968525
rs140618221
rs11646830
rs10206899
All
−0.10
−0.05
0.00
0.05
MR leave−one−out sensitivity analysis for
'Alliin levels' on 'Rheumatoid arthritis'

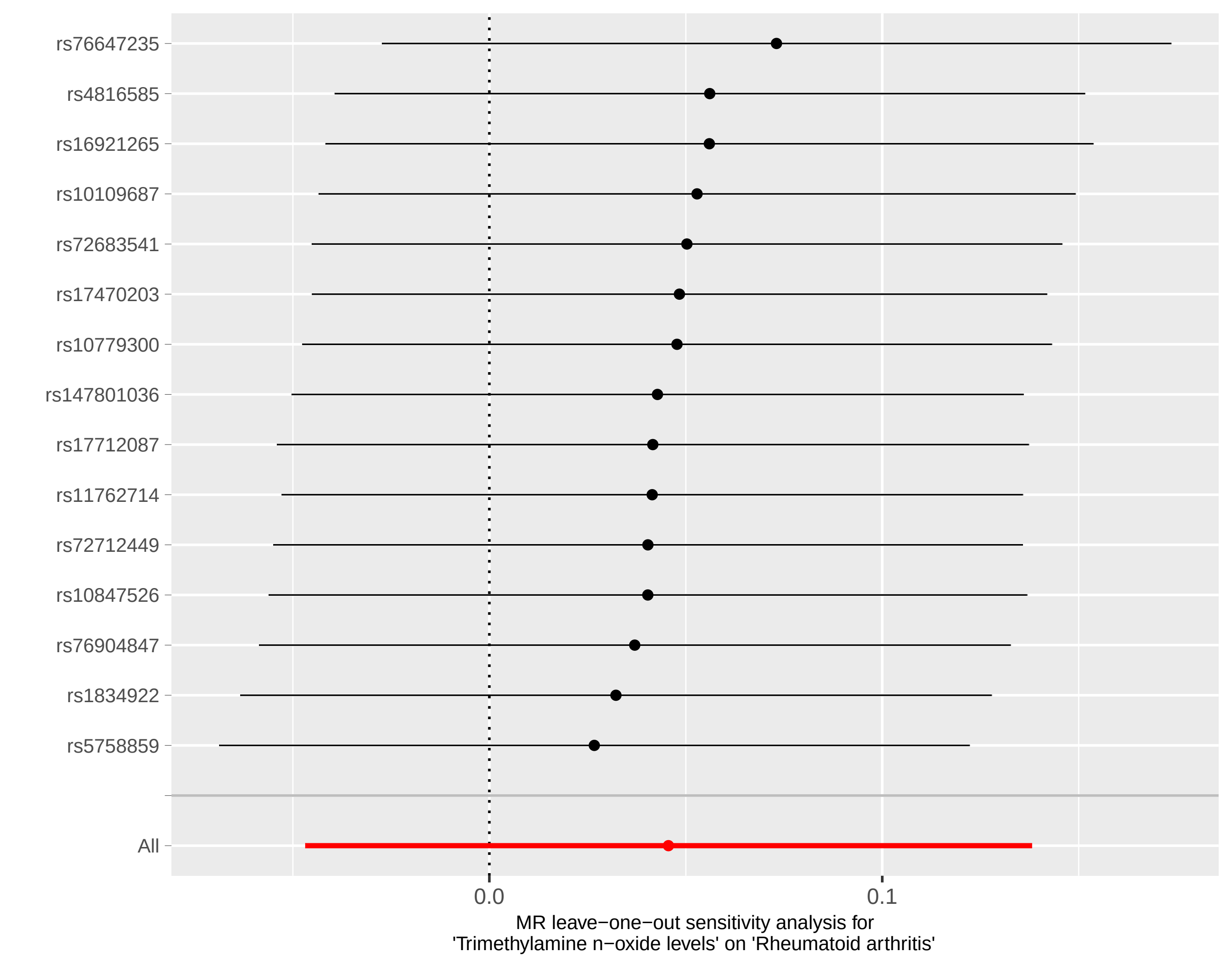
rs76647235
rs4816585
rs16921265
rs10109687
rs72683541
rs17470203
rs10779300
rs147801036
rs17712087
rs11762714
rs72712449
rs10847526
rs76904847
rs1834922
rs5758859
All
0.0
0.1
MR leave−one−out sensitivity analysis for
'Trimethylamine n−oxide levels' on 'Rheumatoid arthritis'

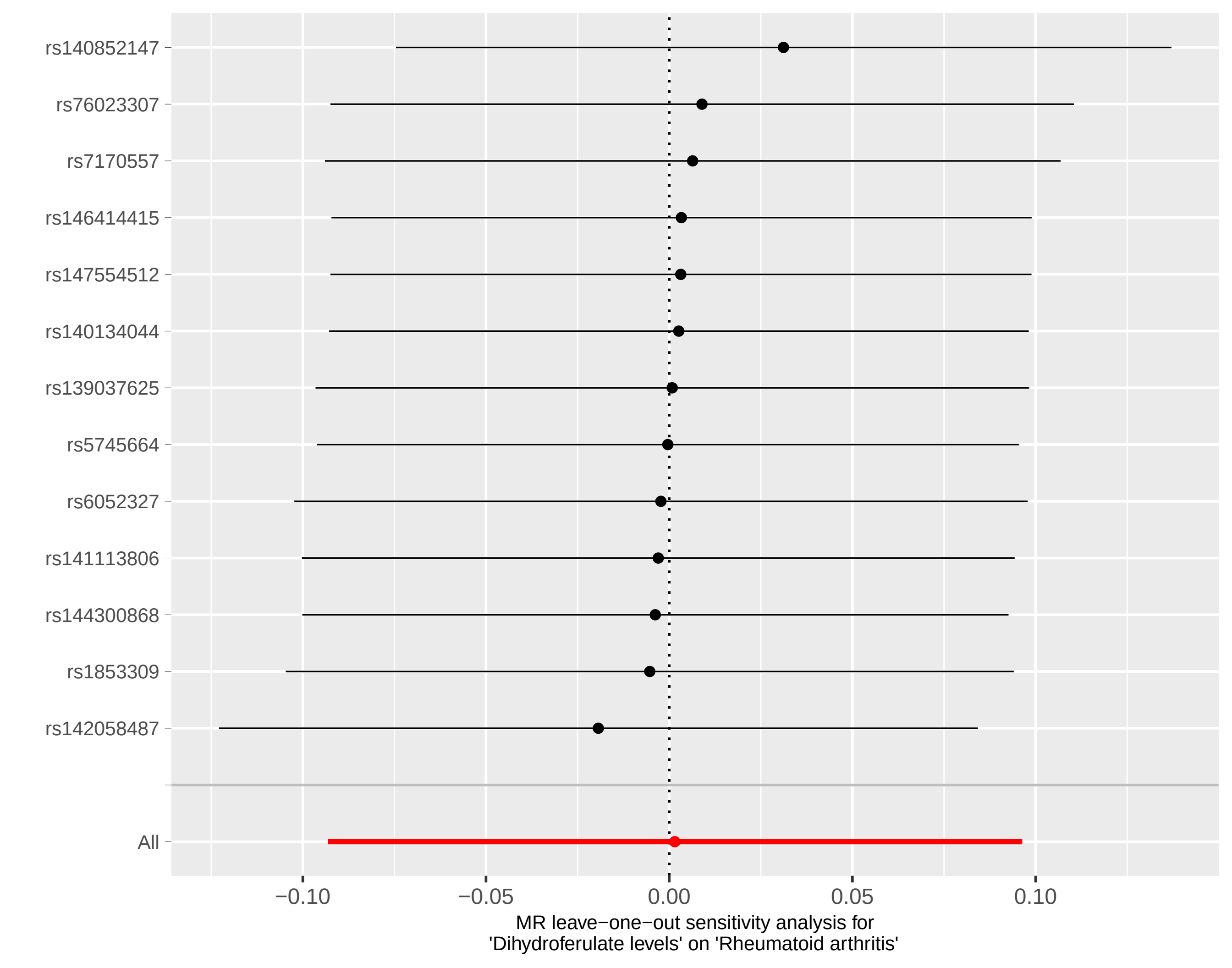
rs140852147
rs76023307
rs7170557
rs146414415
rs147554512
rs140134044
rs139037625
rs5745664
rs6052327
rs141113806
rs144300868
rs1853309
rs142058487
All
−0.10
−0.05
0.00
0.05
0.10
MR leave−one−out sensitivity analysis for
'Dihydroferulate levels' on 'Rheumatoid arthritis'

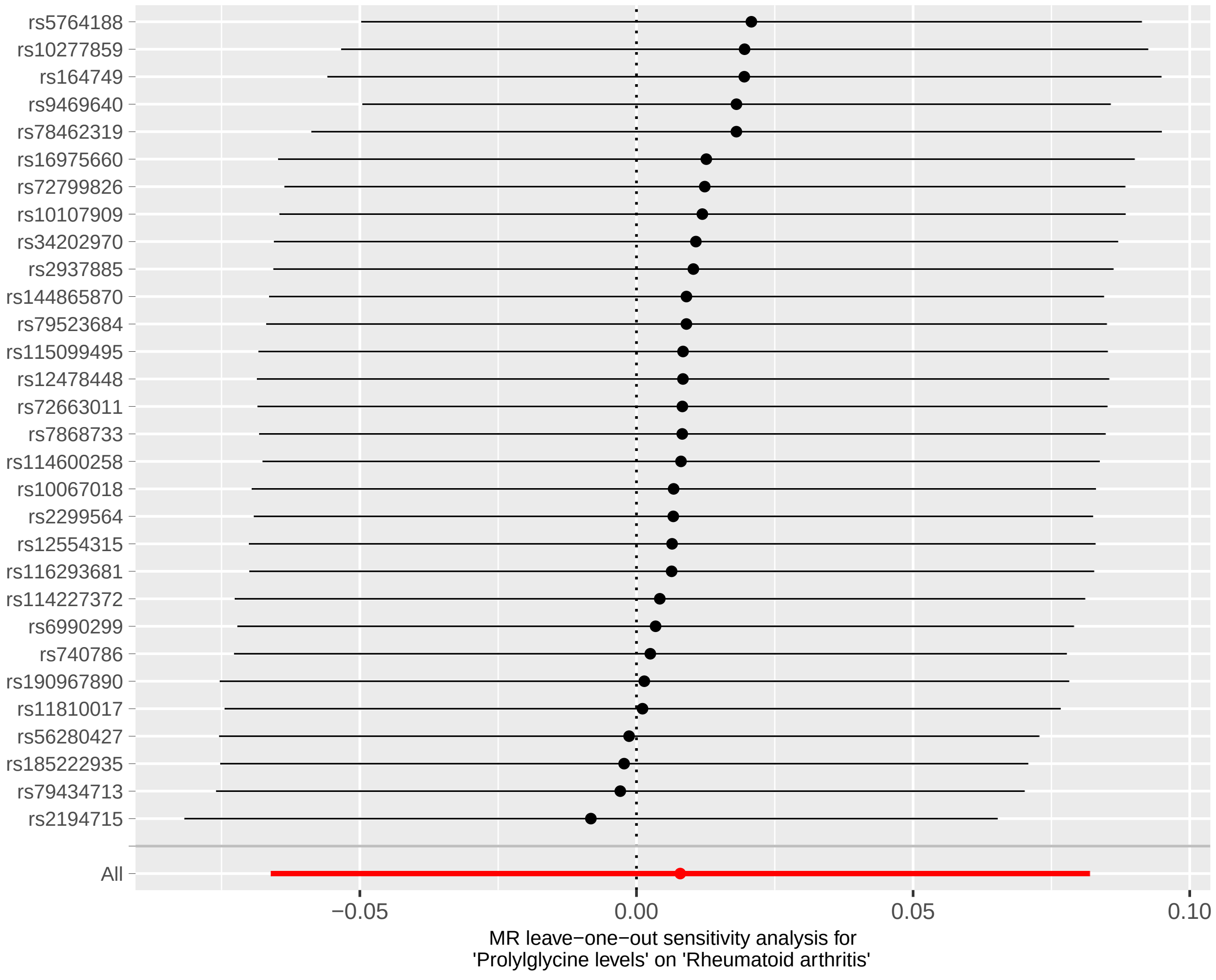

rs5764188
rs10277859
rs164749
rs9469640
rs78462319
rs16975660
rs72799826
rs10107909
rs34202970
rs2937885
rs144865870
rs79523684
rs115099495
rs12478448
rs72663011
rs7868733
rs114600258
rs10067018
rs2299564
rs12554315
rs116293681
rs114227372
rs6990299
rs740786
rs190967890
rs11810017
rs56280427
rs185222935
rs79434713
rs2194715
All
−0.05
0.00
0.05
0.10
MR leave−one−out sensitivity analysis for
'Prolylglycine levels' on 'Rheumatoid arthritis'

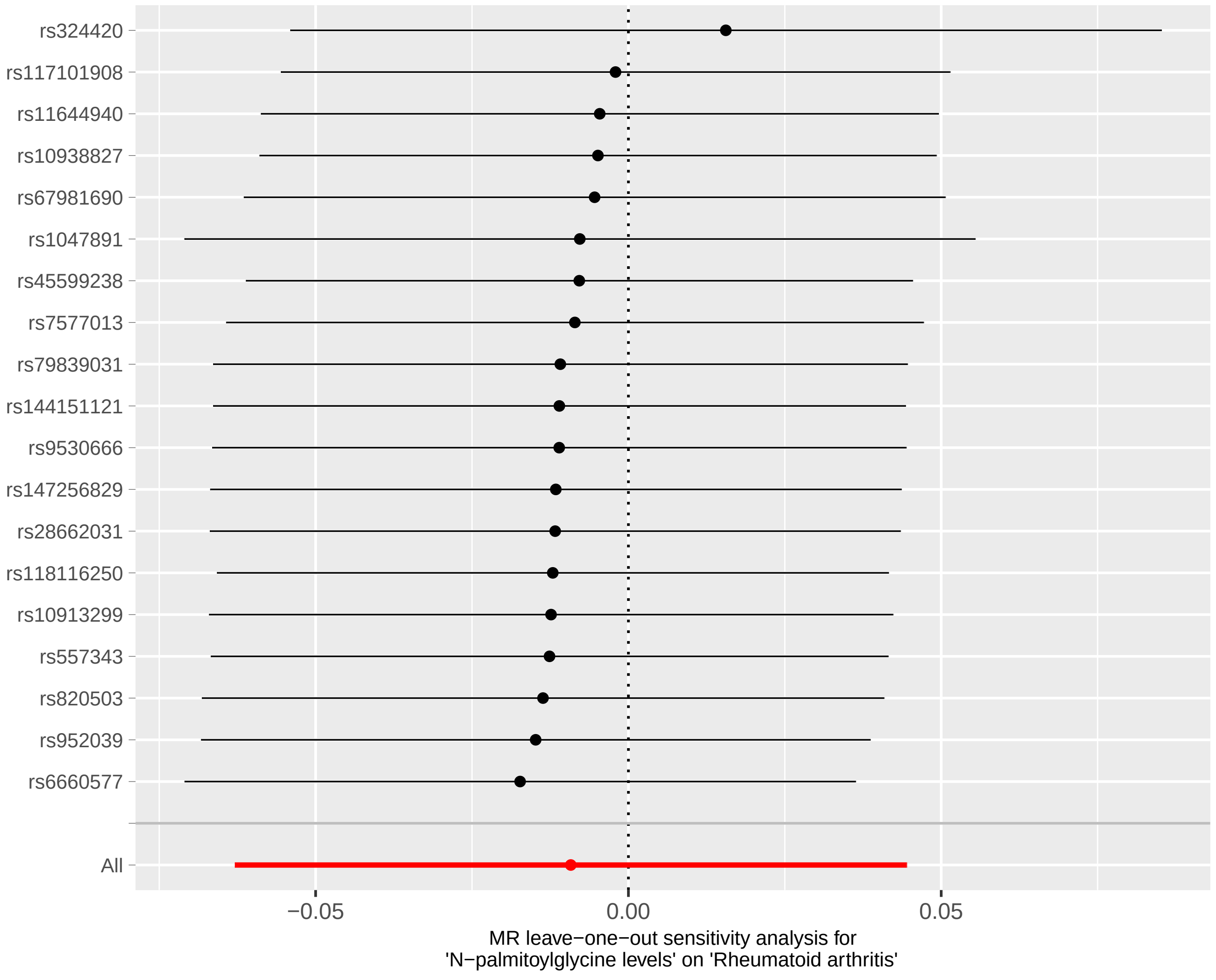
rs324420
rs117101908
rs11644940
rs10938827
rs67981690
rs1047891
rs45599238
rs7577013
rs79839031
rs144151121
rs9530666
rs147256829
rs28662031
rs118116250
rs10913299
rs557343
rs820503
rs952039
rs6660577
All
−0.05
0.00
0.05
MR leave−one−out sensitivity analysis for
'N−palmitoylglycine levels' on 'Rheumatoid arthritis'

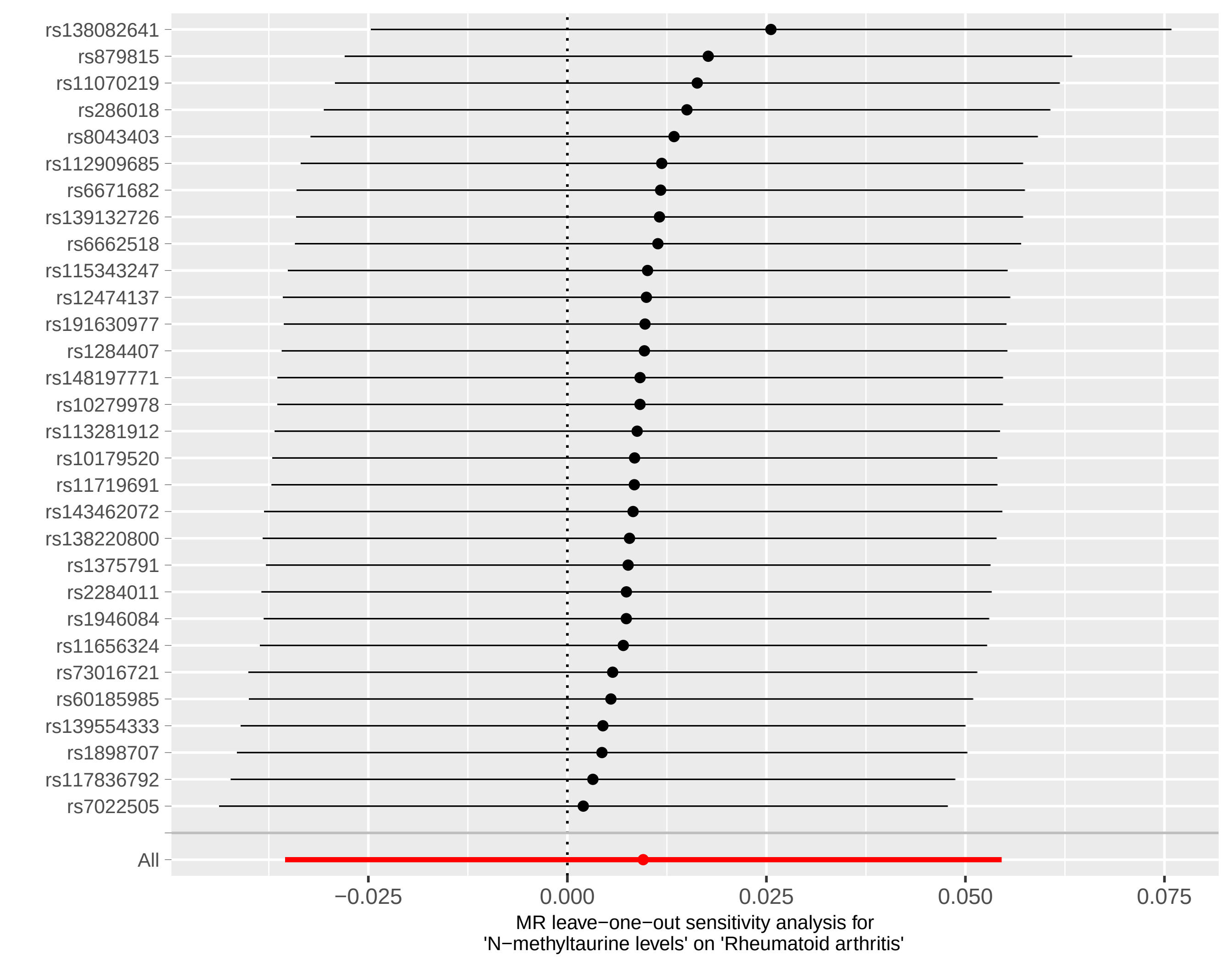
rs138082641
rs879815
rs11070219
rs286018
rs8043403
rs112909685
rs6671682
rs139132726
rs6662518
rs115343247
rs12474137
rs191630977
rs1284407
rs148197771
rs10279978
rs113281912
rs10179520
rs11719691
rs143462072
rs138220800
rs1375791
rs2284011
rs1946084
rs11656324
rs73016721
rs60185985
rs139554333
rs1898707
rs117836792
rs7022505
All
−0.025
0.000
0.025
0.050
0.075
MR leave−one−out sensitivity analysis for
'N−methyltaurine levels' on 'Rheumatoid arthritis'

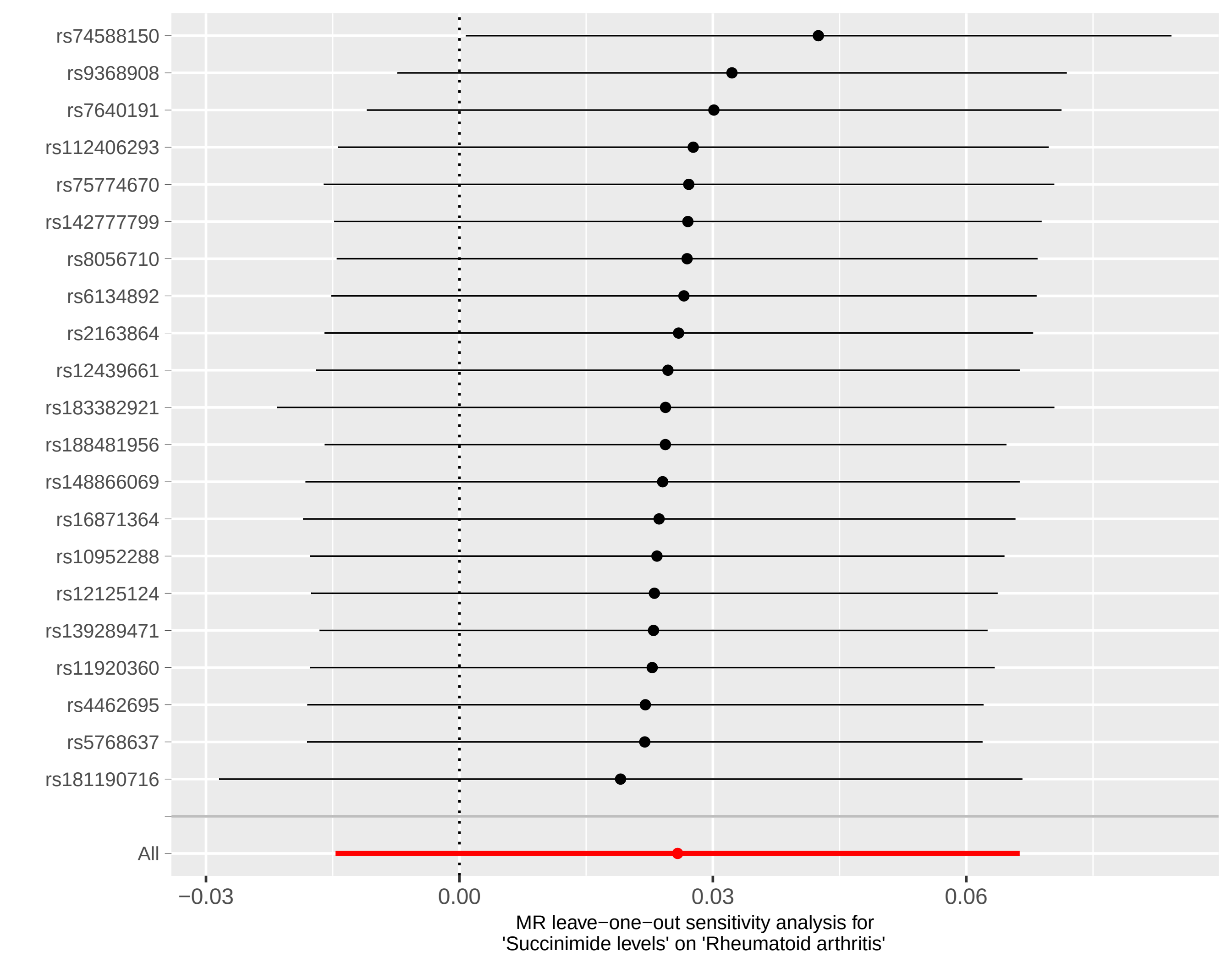
rs74588150
rs9368908
rs7640191
rs112406293
rs75774670
rs142777799
rs8056710
rs6134892
rs2163864
rs12439661
rs183382921
rs188481956
rs148866069
rs16871364
rs10952288
rs12125124
rs139289471
rs11920360
rs4462695
rs5768637
rs181190716
All
−0.03
0.00
0.03
0.06
MR leave−one−out sensitivity analysis for
'Succinimide levels' on 'Rheumatoid arthritis'

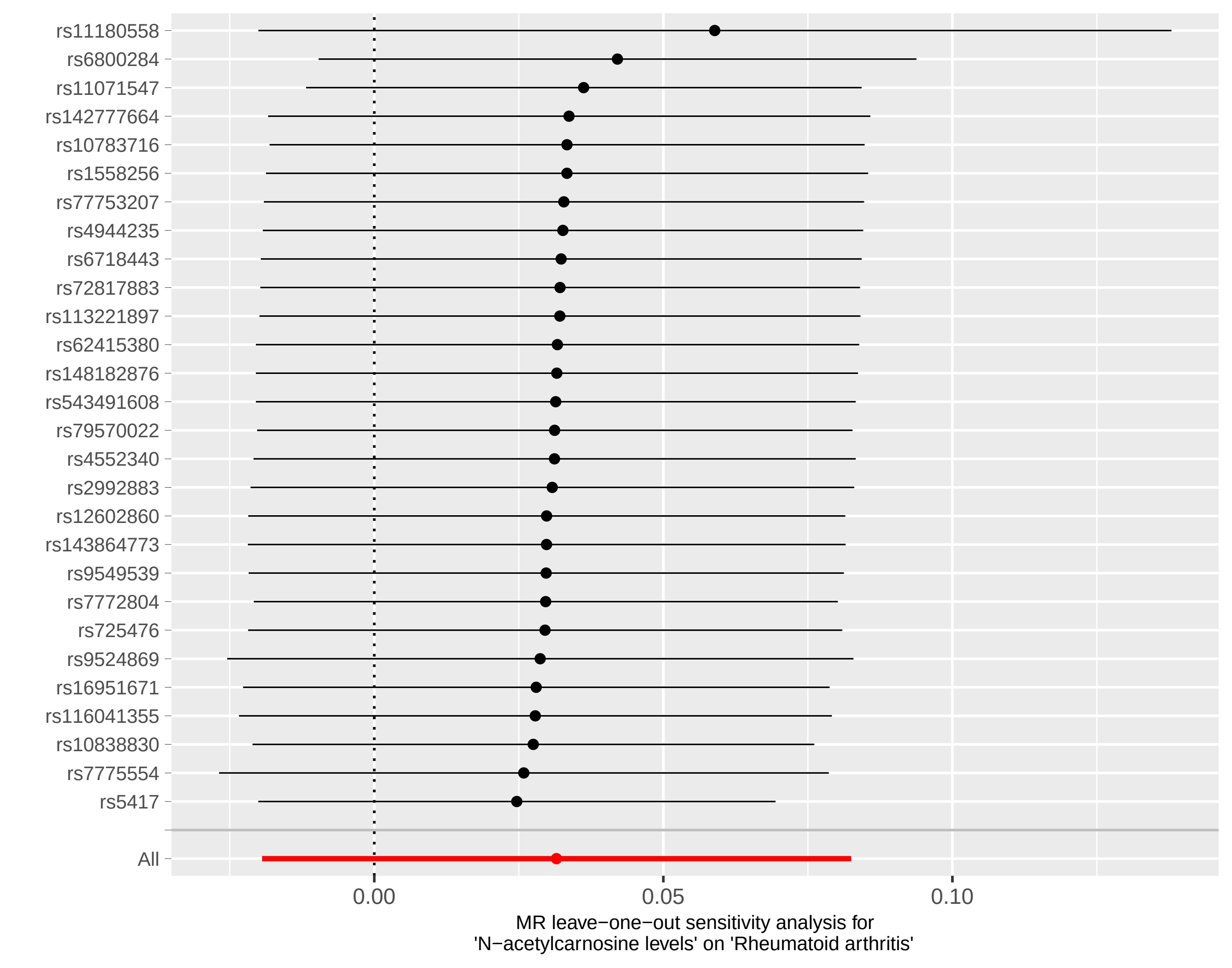
rs11180558
rs6800284
rs11071547
rs142777664
rs10783716
rs1558256
rs77753207
rs4944235
rs6718443
rs72817883
rs113221897
rs62415380
rs148182876
rs543491608
rs79570022
rs4552340
rs2992883
rs12602860
rs143864773
rs9549539
rs7772804
rs725476
rs9524869
rs16951671
rs116041355
rs10838830
rs7775554
rs5417
All
0.00
0.05
0.10
MR leave−one−out sensitivity analysis for
'N−acetylcarnosine levels' on 'Rheumatoid arthritis'

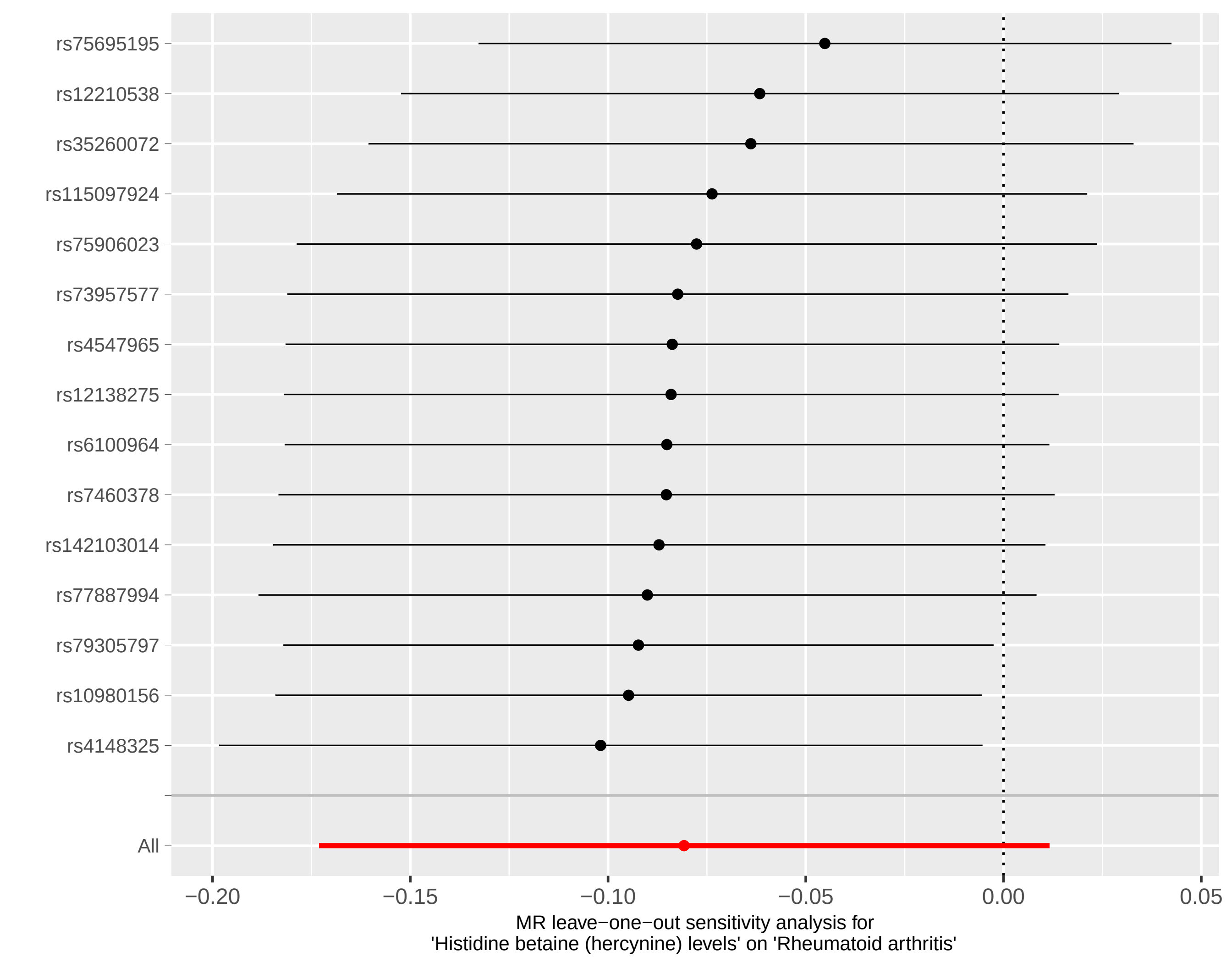
rs75695195
rs12210538
rs35260072
rs115097924
rs75906023
rs73957577
rs4547965
rs12138275
rs6100964
rs7460378
rs142103014
rs77887994
rs79305797
rs10980156
rs4148325
All
−0.20
−0.15
−0.10
−0.05
0.00
0.05
MR leave−one−out sensitivity analysis for
'Histidine betaine (hercynine) levels' on 'Rheumatoid arthritis'

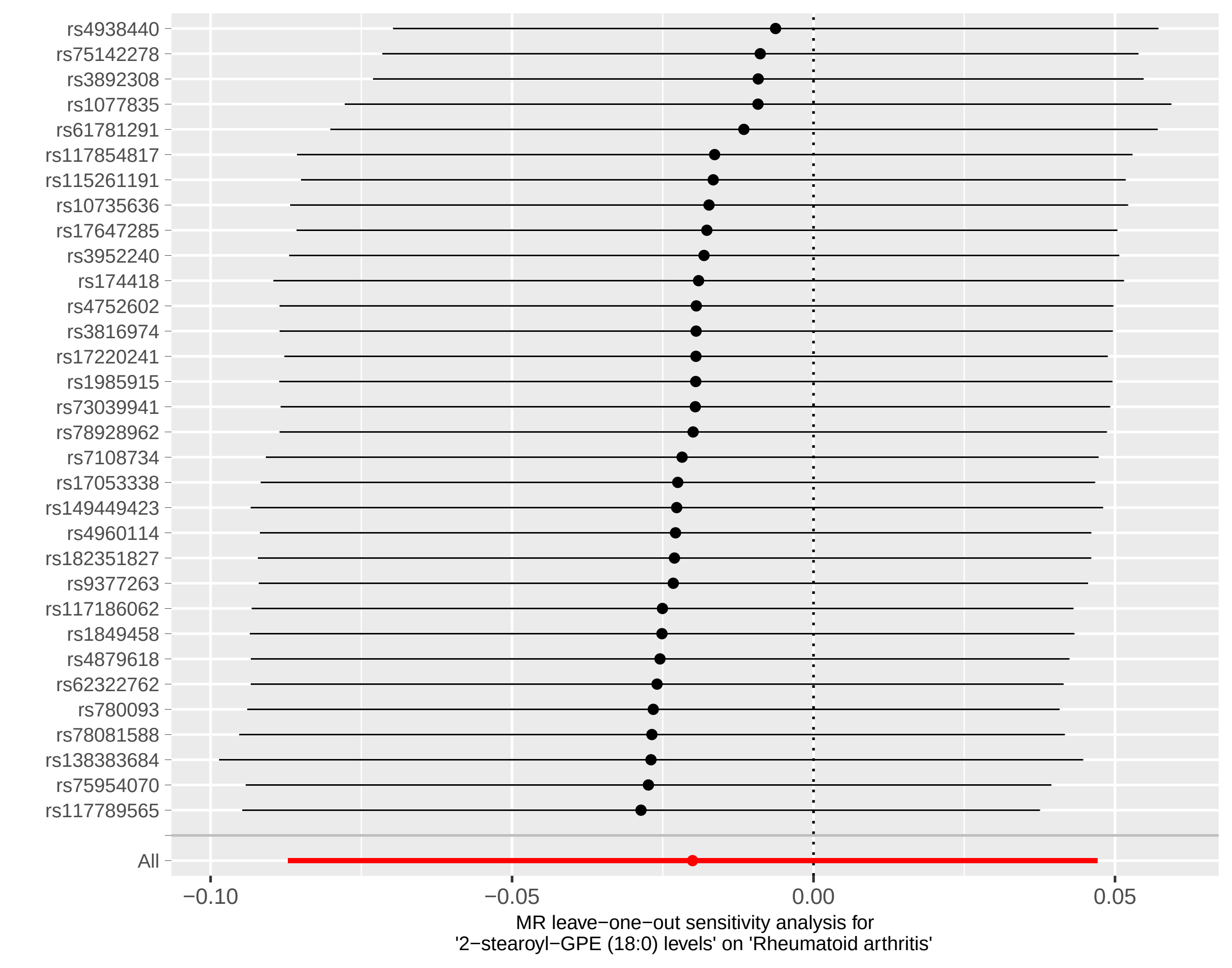

rs4938440
rs75142278
rs3892308
rs1077835
rs61781291
rs117854817
rs115261191
rs10735636
rs17647285
rs3952240
rs174418
rs4752602
rs3816974
rs17220241
rs1985915
rs73039941
rs78928962
rs7108734
rs17053338
rs149449423
rs4960114
rs182351827
rs9377263
rs117186062
rs1849458
rs4879618
rs62322762
rs780093
rs78081588
rs138383684
rs75954070
rs117789565
All
−0.10
−0.05
0.00
0.05
MR leave−one−out sensitivity analysis for
'2−stearoyl−GPE (18:0) levels' on 'Rheumatoid arthritis'

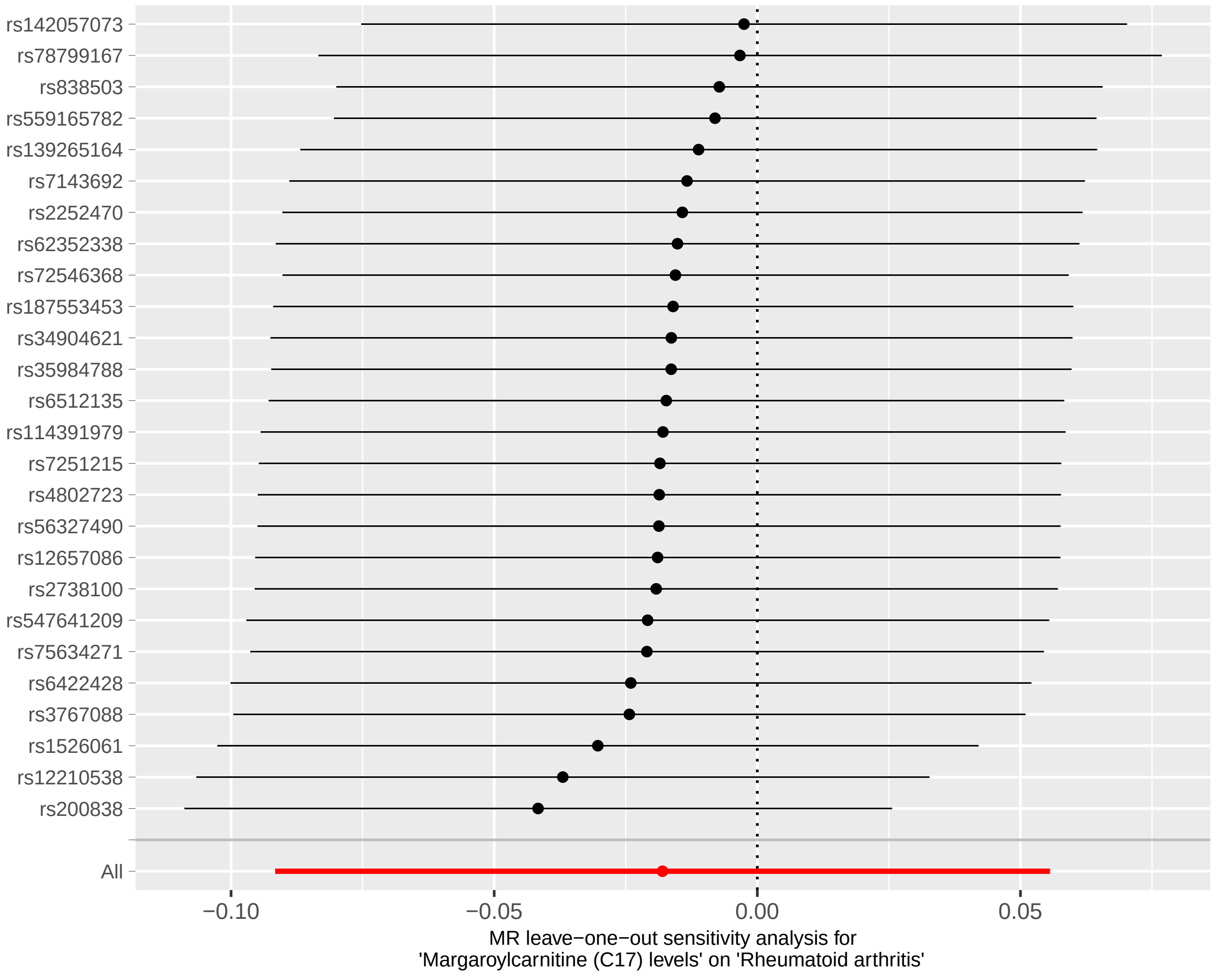
rs142057073
rs78799167
rs838503
rs559165782
rs139265164
rs7143692
rs2252470
rs62352338
rs72546368
rs187553453
rs34904621
rs35984788
rs6512135
rs114391979
rs7251215
rs4802723
rs56327490
rs12657086
rs2738100
rs547641209
rs75634271
rs6422428
rs3767088
rs1526061
rs12210538
rs200838
All
−0.10
−0.05
0.00
0.05
MR leave−one−out sensitivity analysis for
'Margaroylcarnitine (C17) levels' on 'Rheumatoid arthritis'

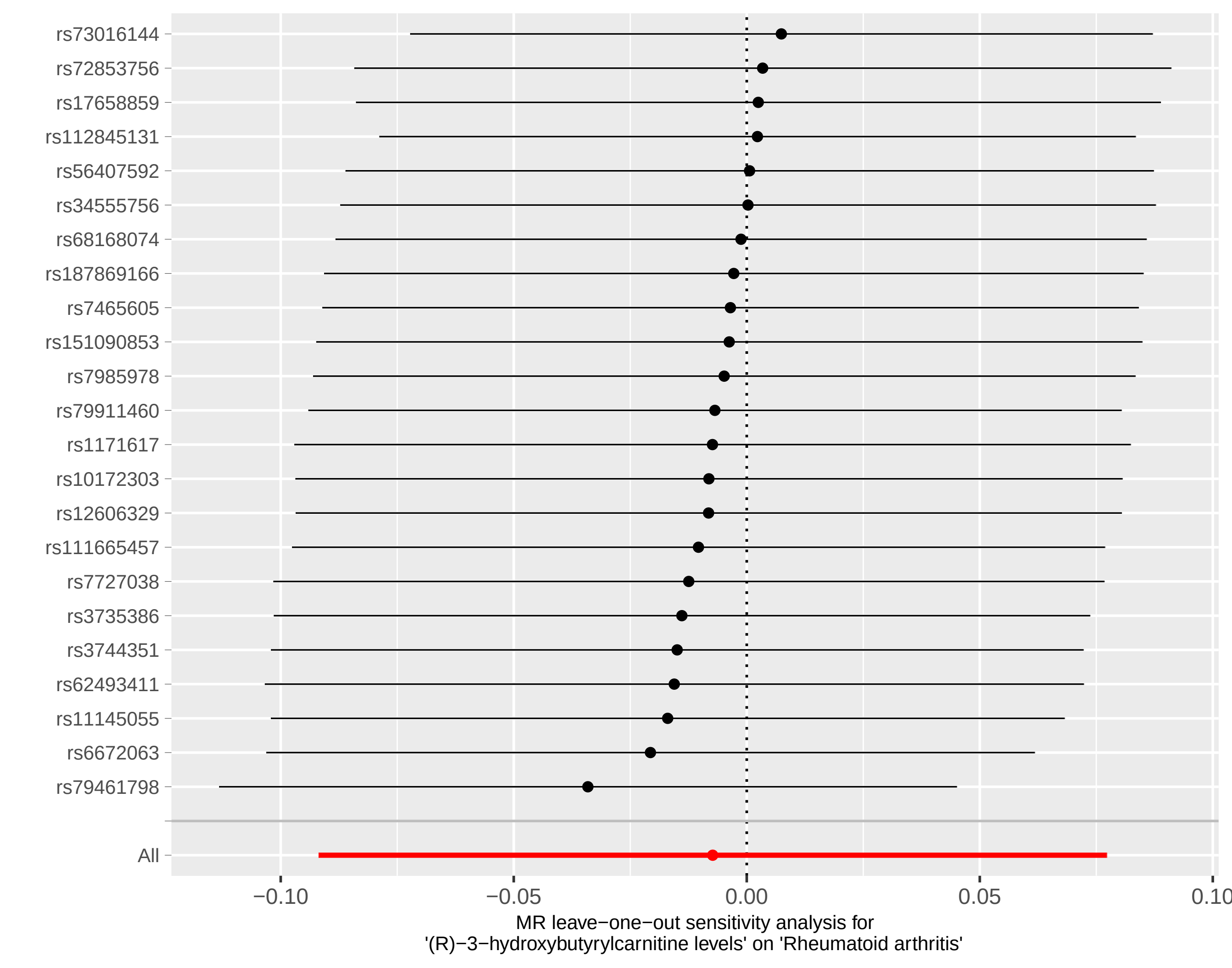

rs73016144
rs72853756
rs17658859
rs112845131
rs56407592
rs34555756
rs68168074
rs187869166
rs7465605
rs151090853
rs7985978
rs79911460
rs1171617
rs10172303
rs12606329
rs111665457
rs7727038
rs3735386
rs3744351
rs62493411
rs11145055
rs6672063
rs79461798
All
−0.10
−0.05
0.00
0.05
0.10
MR leave−one−out sensitivity analysis for
'(R)−3−hydroxybutyrylcarnitine levels' on 'Rheumatoid arthritis'

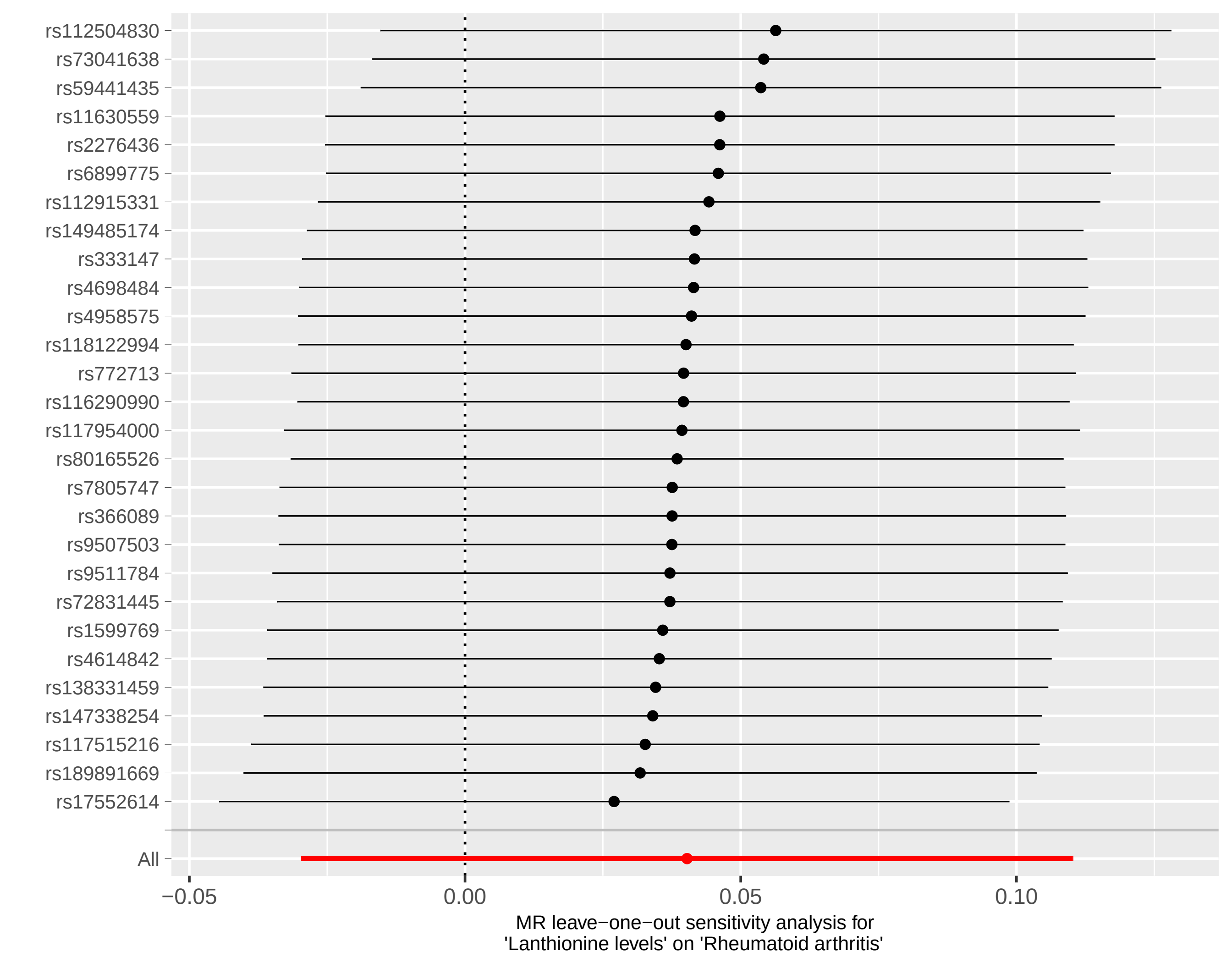
rs112504830
rs73041638
rs59441435
rs11630559
rs2276436
rs6899775
rs112915331
rs149485174
rs333147
rs4698484
rs4958575
rs118122994
rs772713
rs116290990
rs117954000
rs80165526
rs7805747
rs366089
rs9507503
rs9511784
rs72831445
rs1599769
rs4614842
rs138331459
rs147338254
rs117515216
rs189891669
rs17552614
All
−0.05
0.00
0.05
0.10
MR leave−one−out sensitivity analysis for
'Lanthionine levels' on 'Rheumatoid arthritis'

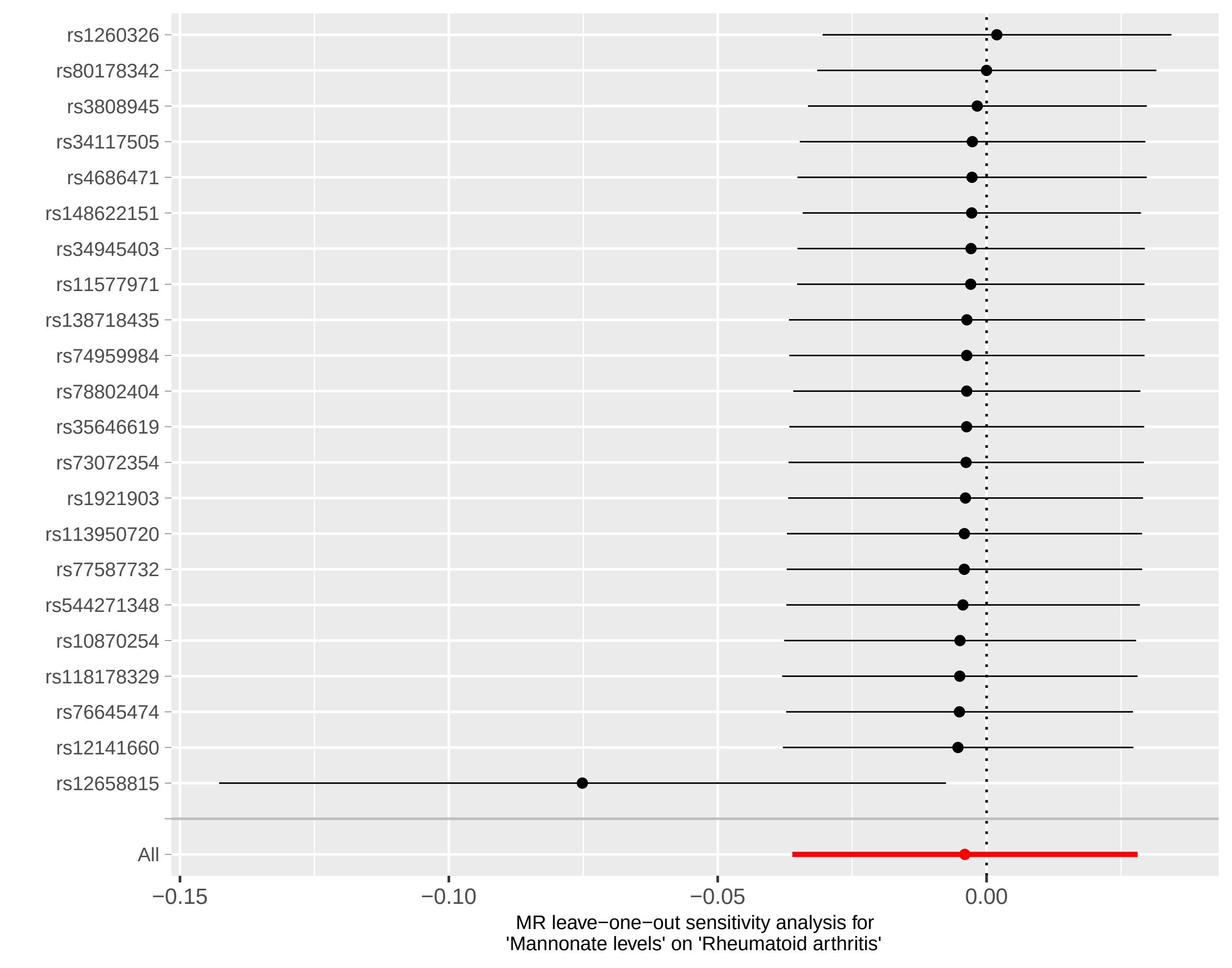

rs1260326
rs80178342
rs3808945
rs34117505
rs4686471
rs148622151
rs34945403
rs11577971
rs138718435
rs74959984
rs78802404
rs35646619
rs73072354
rs1921903
rs113950720
rs77587732
rs544271348
rs10870254
rs118178329
rs76645474
rs12141660
rs12658815
All
−0.15
−0.10
−0.05
0.00
MR leave−one−out sensitivity analysis for
'Mannonate levels' on 'Rheumatoid arthritis'

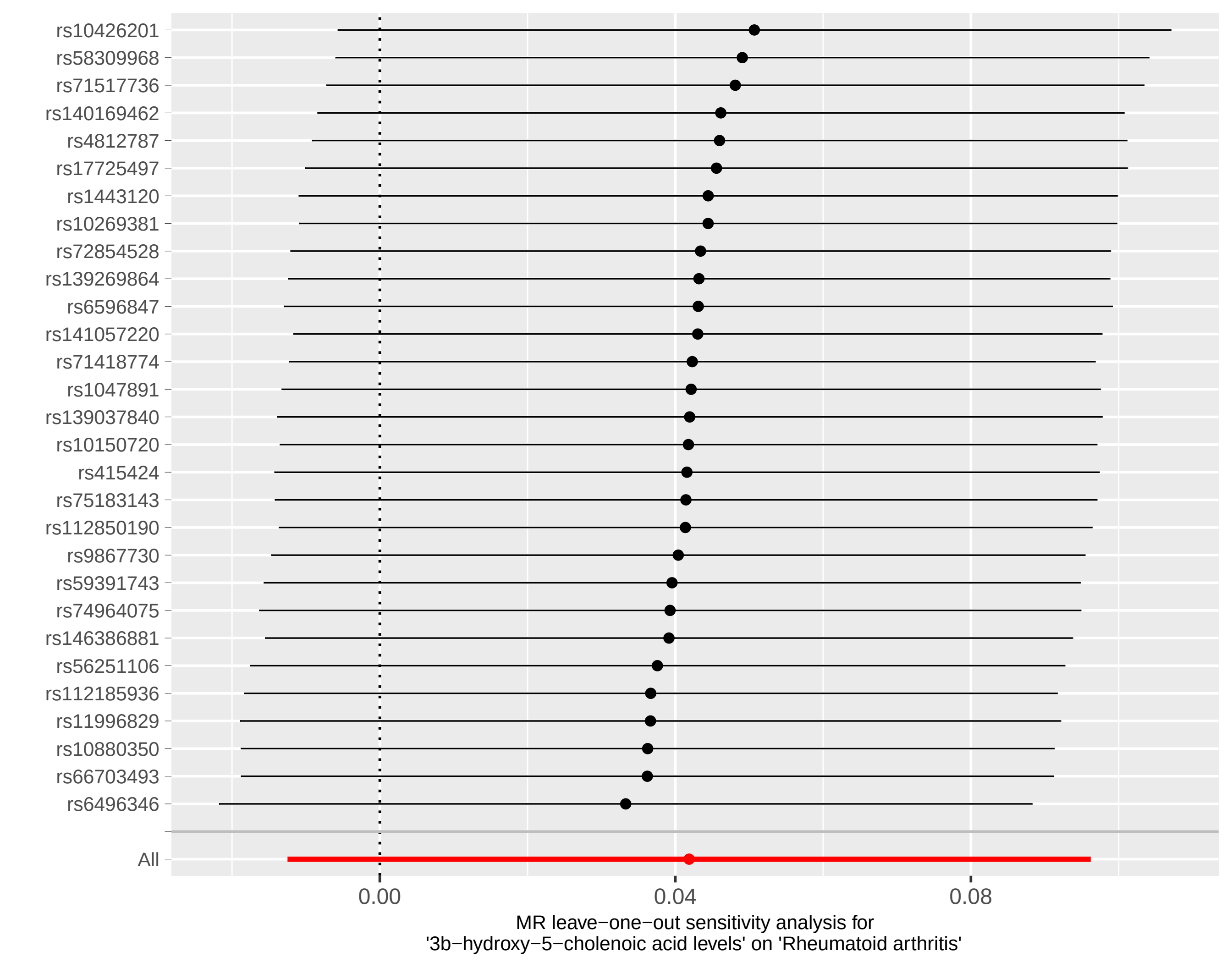
rs10426201
rs58309968
rs71517736
rs140169462
rs4812787
rs17725497
rs1443120
rs10269381
rs72854528
rs139269864
rs6596847
rs141057220
rs71418774
rs1047891
rs139037840
rs10150720
rs415424
rs75183143
rs112850190
rs9867730
rs59391743
rs74964075
rs146386881
rs56251106
rs112185936
rs11996829
rs10880350
rs66703493
rs6496346
All
0.00
0.04
0.08
MR leave−one−out sensitivity analysis for
'3b−hydroxy−5−cholenoic acid levels' on 'Rheumatoid arthritis'

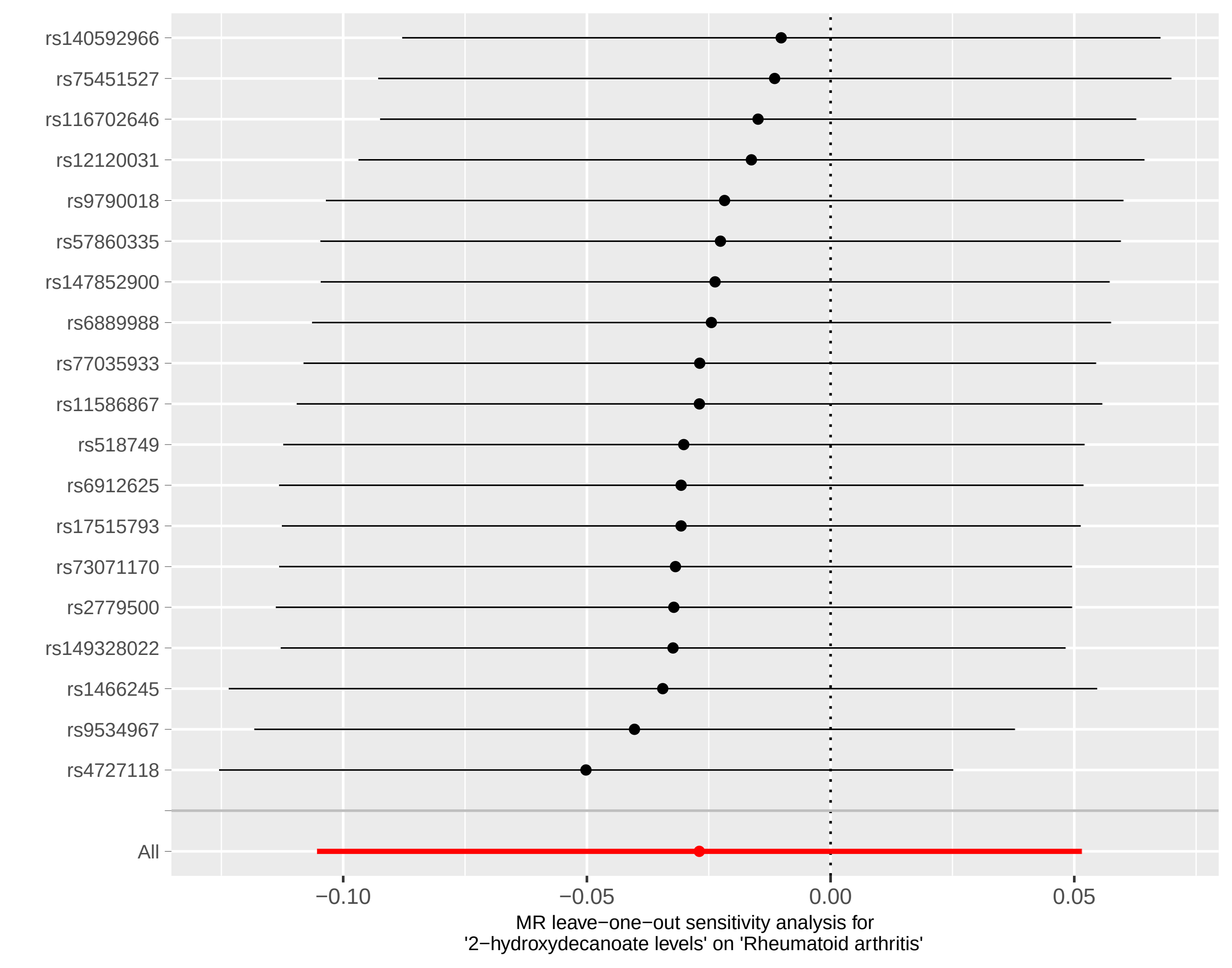
rs140592966
rs75451527
rs116702646
rs12120031
rs9790018
rs57860335
rs147852900
rs6889988
rs77035933
rs11586867
rs518749
rs6912625
rs17515793
rs73071170
rs2779500
rs149328022
rs1466245
rs9534967
rs4727118
All
−0.10
−0.05
0.00
0.05
MR leave−one−out sensitivity analysis for
'2−hydroxydecanoate levels' on 'Rheumatoid arthritis'

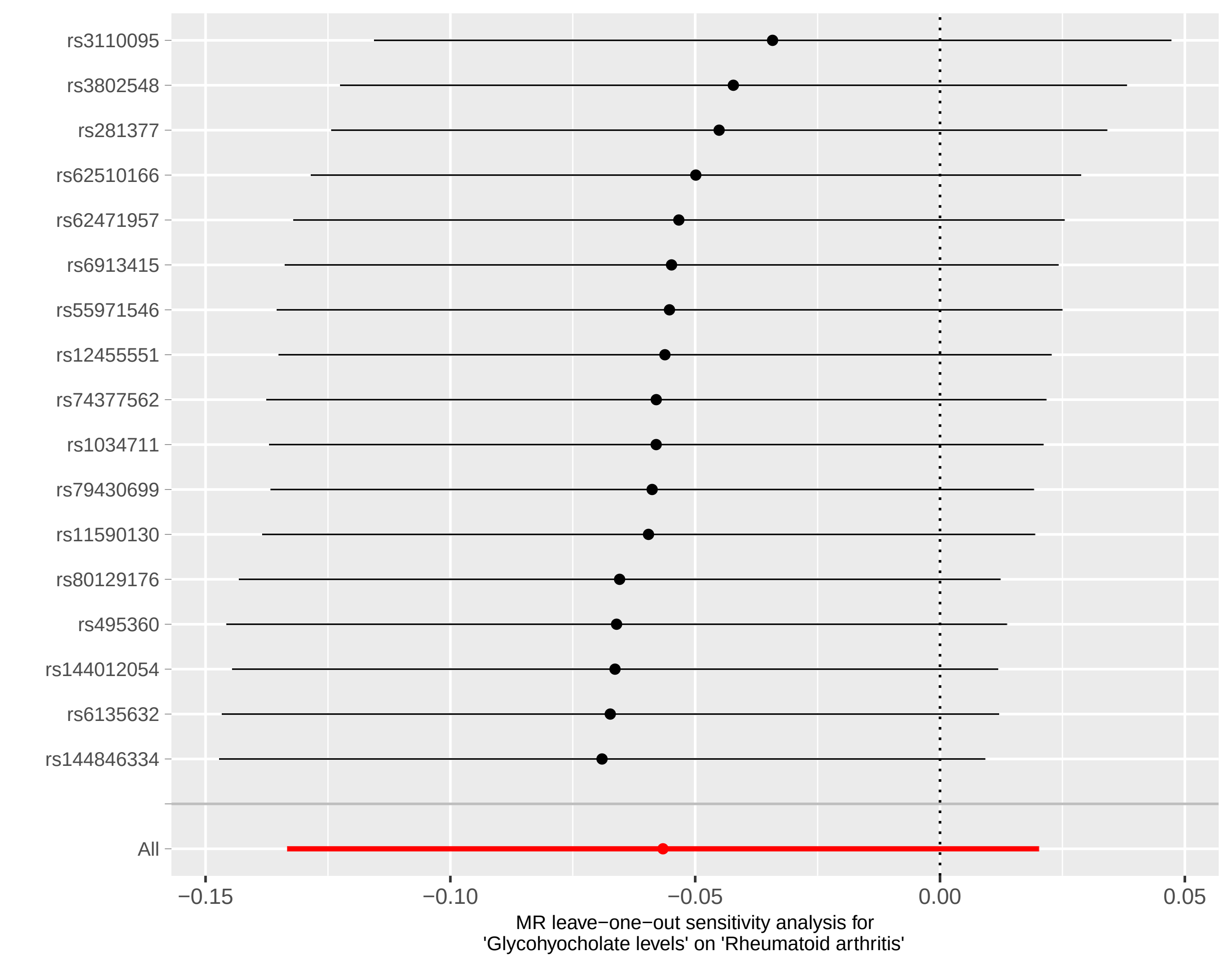
rs3110095
rs3802548
rs281377
rs62510166
rs62471957
rs6913415
rs55971546
rs12455551
rs74377562
rs1034711
rs79430699
rs11590130
rs80129176
rs495360
rs144012054
rs6135632
rs144846334
All
−0.15
−0.10
−0.05
0.00
0.05
MR leave−one−out sensitivity analysis for
'Glycohyocholate levels' on 'Rheumatoid arthritis'

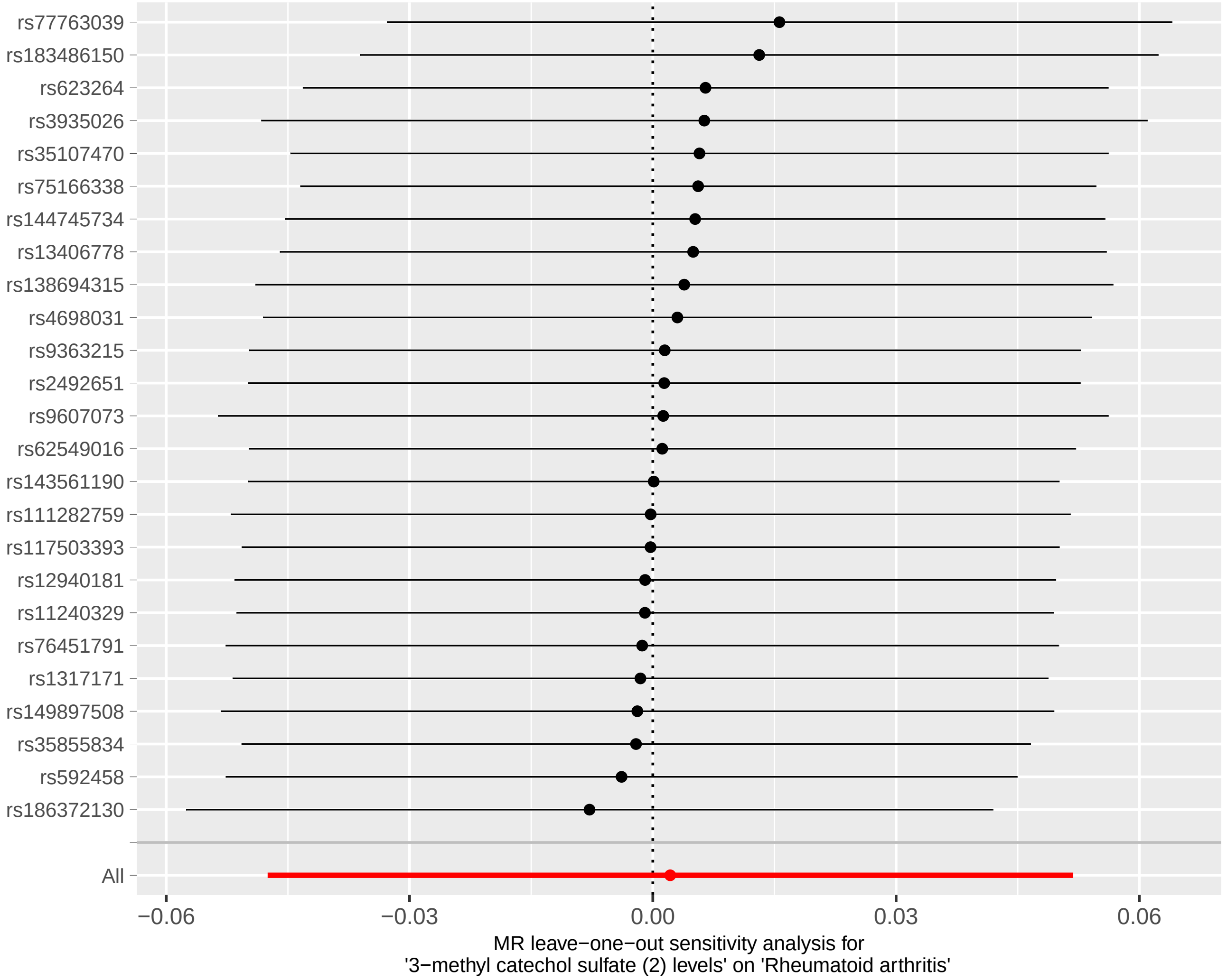
rs77763039
rs183486150
rs623264
rs3935026
rs35107470
rs75166338
rs144745734
rs13406778
rs138694315
rs4698031
rs9363215
rs2492651
rs9607073
rs62549016
rs143561190
rs111282759
rs117503393
rs12940181
rs11240329
rs76451791
rs1317171
rs149897508
rs35855834
rs592458
rs186372130
All
−0.06
−0.03
0.00
0.03
0.06
MR leave−one−out sensitivity analysis for
'3−methyl catechol sulfate (2) levels' on 'Rheumatoid arthritis'

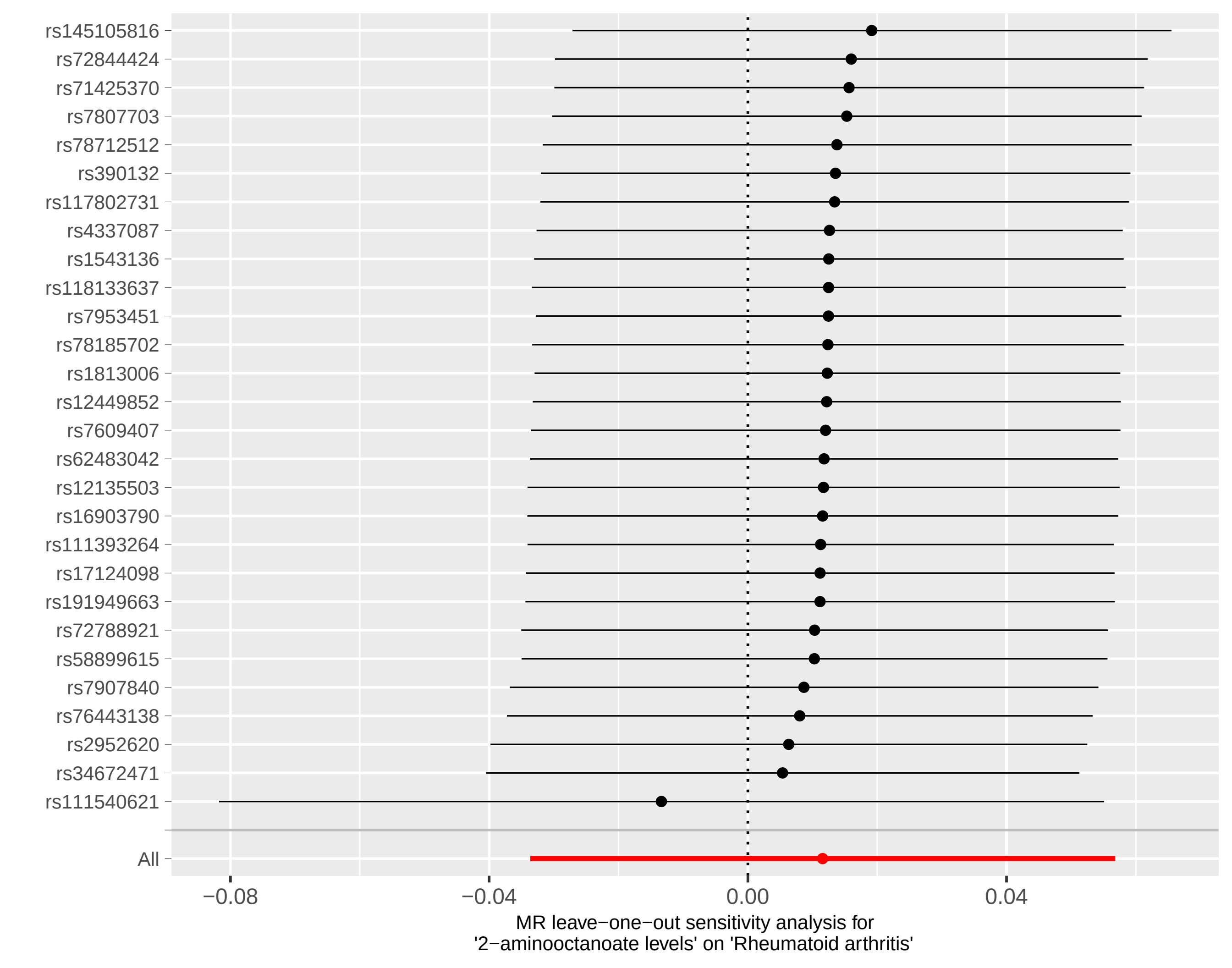

rs145105816
rs72844424
rs71425370
rs7807703
rs78712512
rs390132
rs117802731
rs4337087
rs1543136
rs118133637
rs7953451
rs78185702
rs1813006
rs12449852
rs7609407
rs62483042
rs12135503
rs16903790
rs111393264
rs17124098
rs191949663
rs72788921
rs58899615
rs7907840
rs76443138
rs2952620
rs34672471
rs111540621
All
−0.08
−0.04
0.00
0.04
MR leave−one−out sensitivity analysis for
'2−aminooctanoate levels' on 'Rheumatoid arthritis'

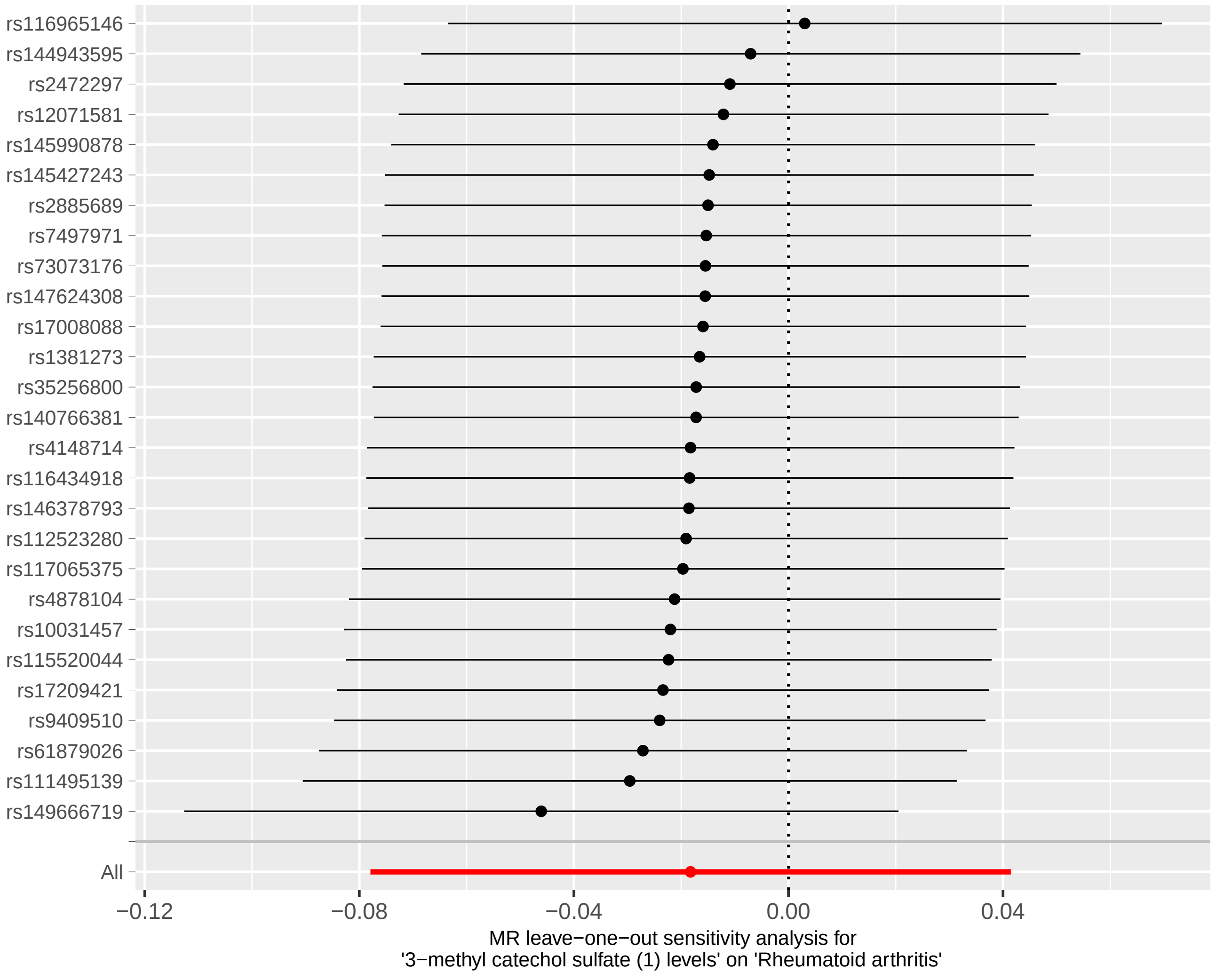

rs116965146
rs144943595
rs2472297
rs12071581
rs145990878
rs145427243
rs2885689
rs7497971
rs73073176
rs147624308
rs17008088
rs1381273
rs35256800
rs140766381
rs4148714
rs116434918
rs146378793
rs112523280
rs117065375
rs4878104
rs10031457
rs115520044
rs17209421
rs9409510
rs61879026
rs111495139
rs149666719
All
−0.12
−0.08
−0.04
0.00
0.04
MR leave−one−out sensitivity analysis for
'3−methyl catechol sulfate (1) levels' on 'Rheumatoid arthritis'

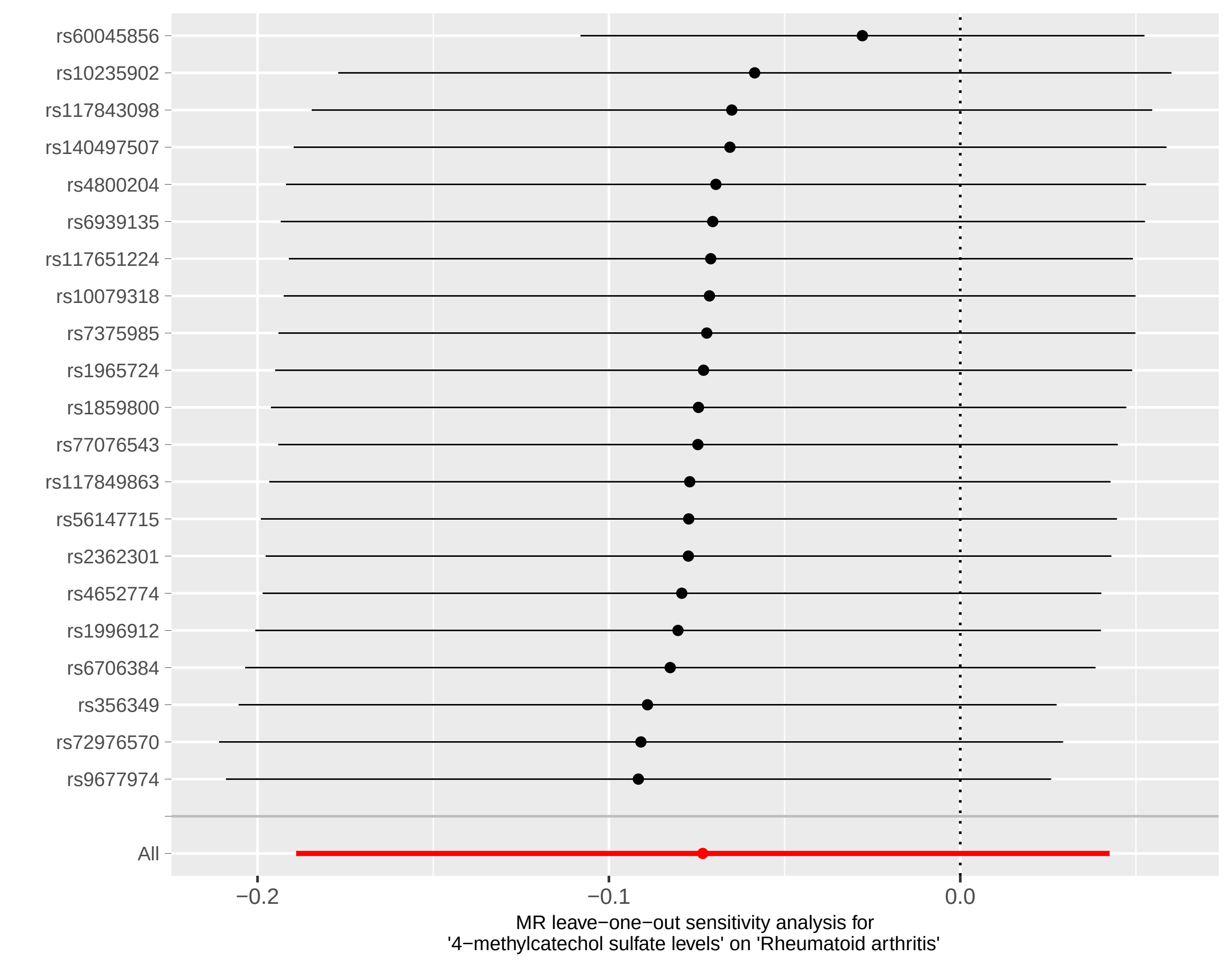
rs60045856
rs10235902
rs117843098
rs140497507
rs4800204
rs6939135
rs117651224
rs10079318
rs7375985
rs1965724
rs1859800
rs77076543
rs117849863
rs56147715
rs2362301
rs4652774
rs1996912
rs6706384
rs356349
rs72976570
rs9677974
All
−0.2
−0.1
0.0
MR leave−one−out sensitivity analysis for
'4−methylcatechol sulfate levels' on 'Rheumatoid arthritis'

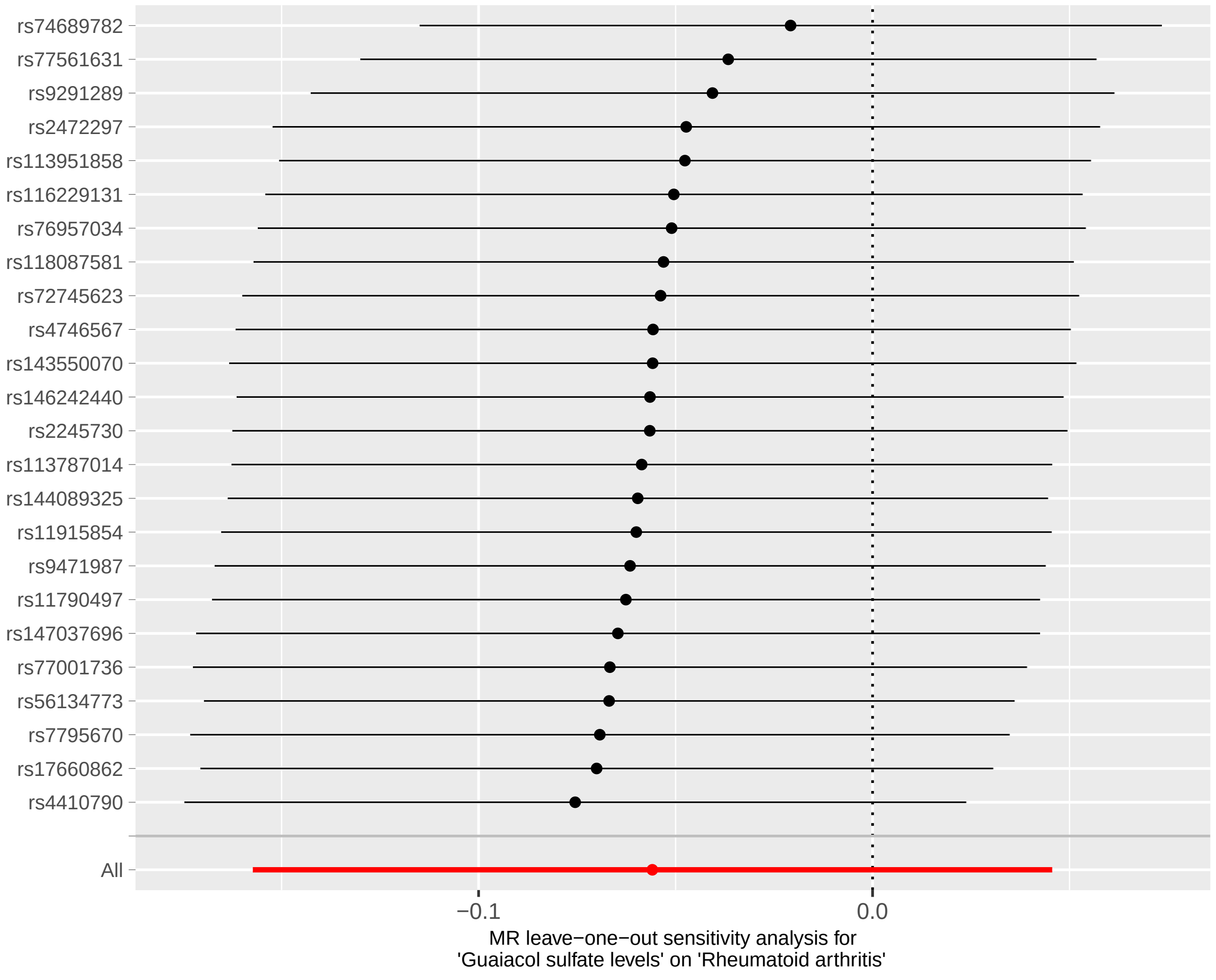
rs74689782
rs77561631
rs9291289
rs2472297
rs113951858
rs116229131
rs76957034
rs118087581
rs72745623
rs4746567
rs143550070
rs146242440
rs2245730
rs113787014
rs144089325
rs11915854
rs9471987
rs11790497
rs147037696
rs77001736
rs56134773
rs7795670
rs17660862
rs4410790
All
−0.1
0.0
MR leave−one−out sensitivity analysis for
'Guaiacol sulfate levels' on 'Rheumatoid arthritis'

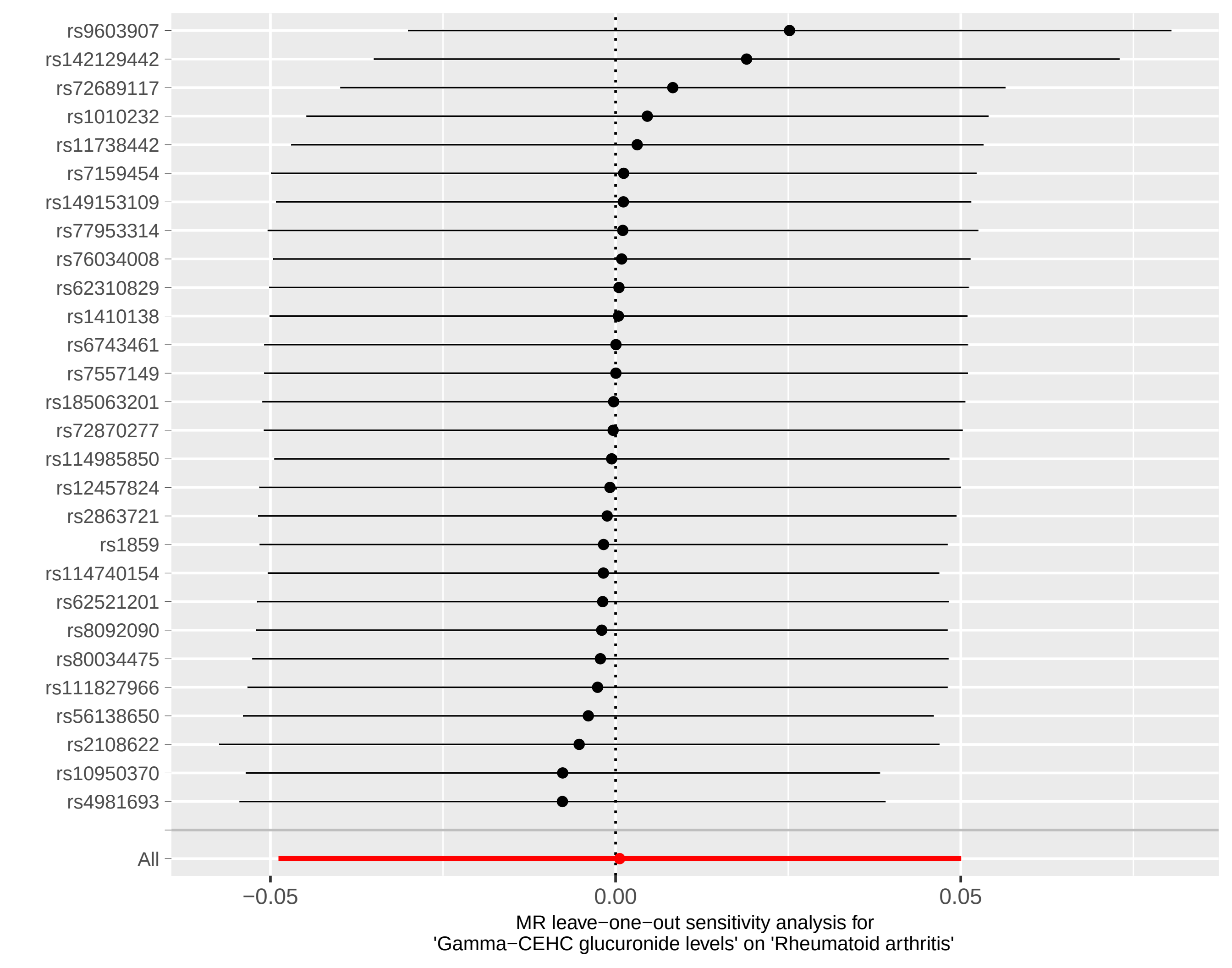

rs9603907
rs142129442
rs72689117
rs1010232
rs11738442
rs7159454
rs149153109
rs77953314
rs76034008
rs62310829
rs1410138
rs6743461
rs7557149
rs185063201
rs72870277
rs114985850
rs12457824
rs2863721
rs1859
rs114740154
rs62521201
rs8092090
rs80034475
rs111827966
rs56138650
rs2108622
rs10950370
rs4981693
All
−0.05
0.00
0.05
MR leave−one−out sensitivity analysis for
'Gamma−CEHC glucuronide levels' on 'Rheumatoid arthritis'

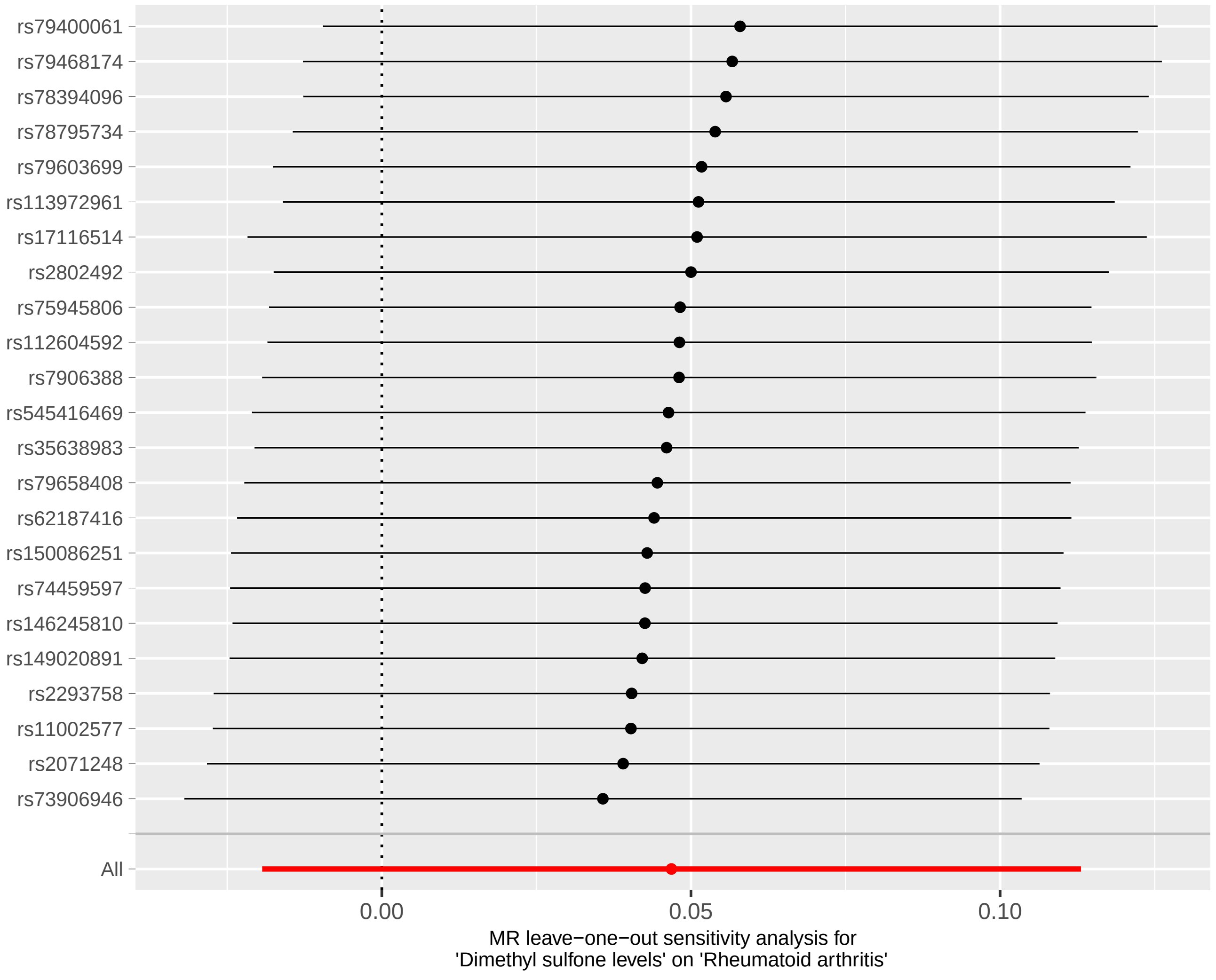

rs79400061
rs79468174
rs78394096
rs78795734
rs79603699
rs113972961
rs17116514
rs2802492
rs75945806
rs112604592
rs7906388
rs545416469
rs35638983
rs79658408
rs62187416
rs150086251
rs74459597
rs146245810
rs149020891
rs2293758
rs11002577
rs2071248
rs73906946
All
0.00
0.05
0.10
MR leave−one−out sensitivity analysis for
'Dimethyl sulfone levels' on 'Rheumatoid arthritis'

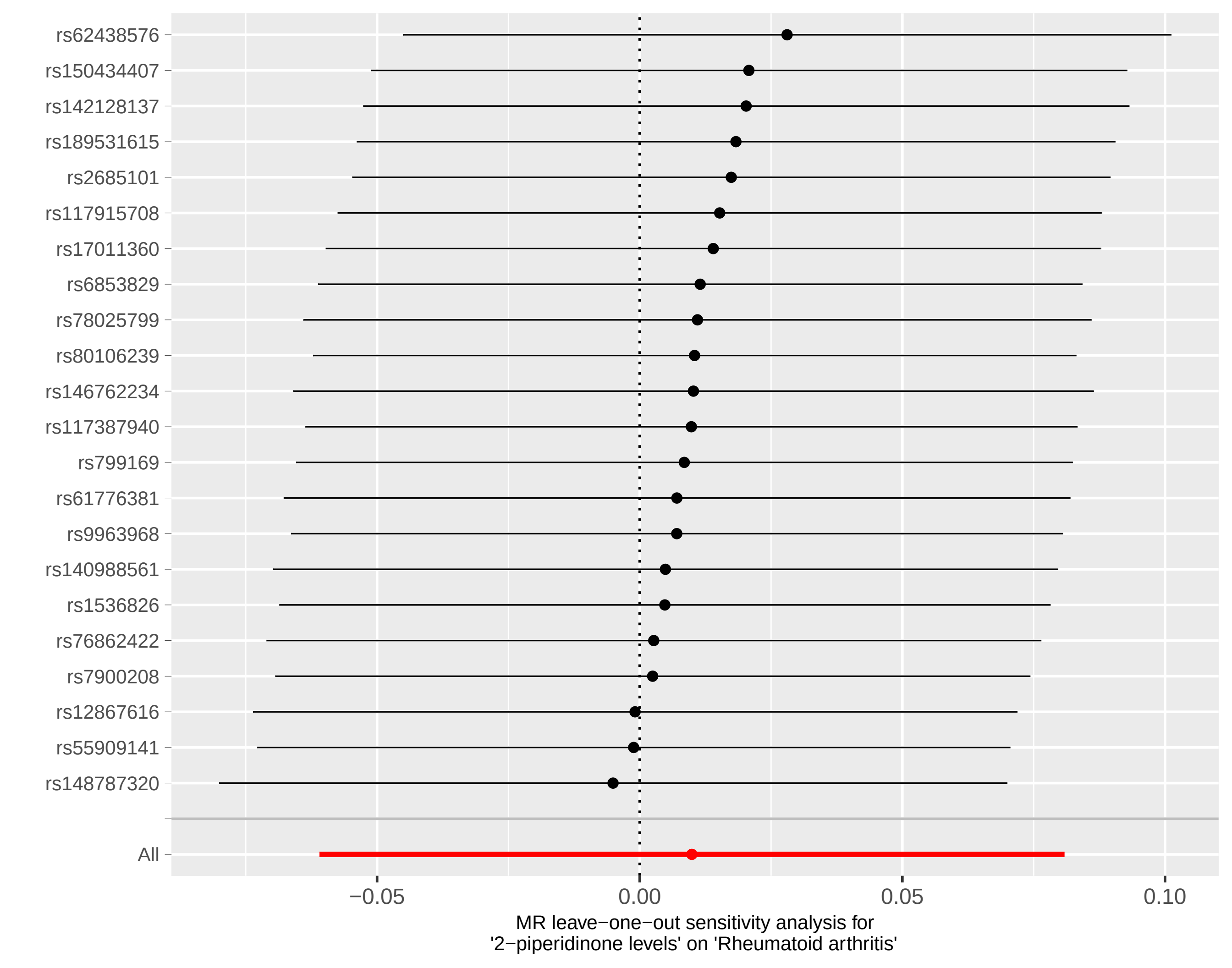
rs62438576
rs150434407
rs142128137
rs189531615
rs2685101
rs117915708
rs17011360
rs6853829
rs78025799
rs80106239
rs146762234
rs117387940
rs799169
rs61776381
rs9963968
rs140988561
rs1536826
rs76862422
rs7900208
rs12867616
rs55909141
rs148787320
All
−0.05
0.00
0.05
0.10
MR leave−one−out sensitivity analysis for
'2−piperidinone levels' on 'Rheumatoid arthritis'

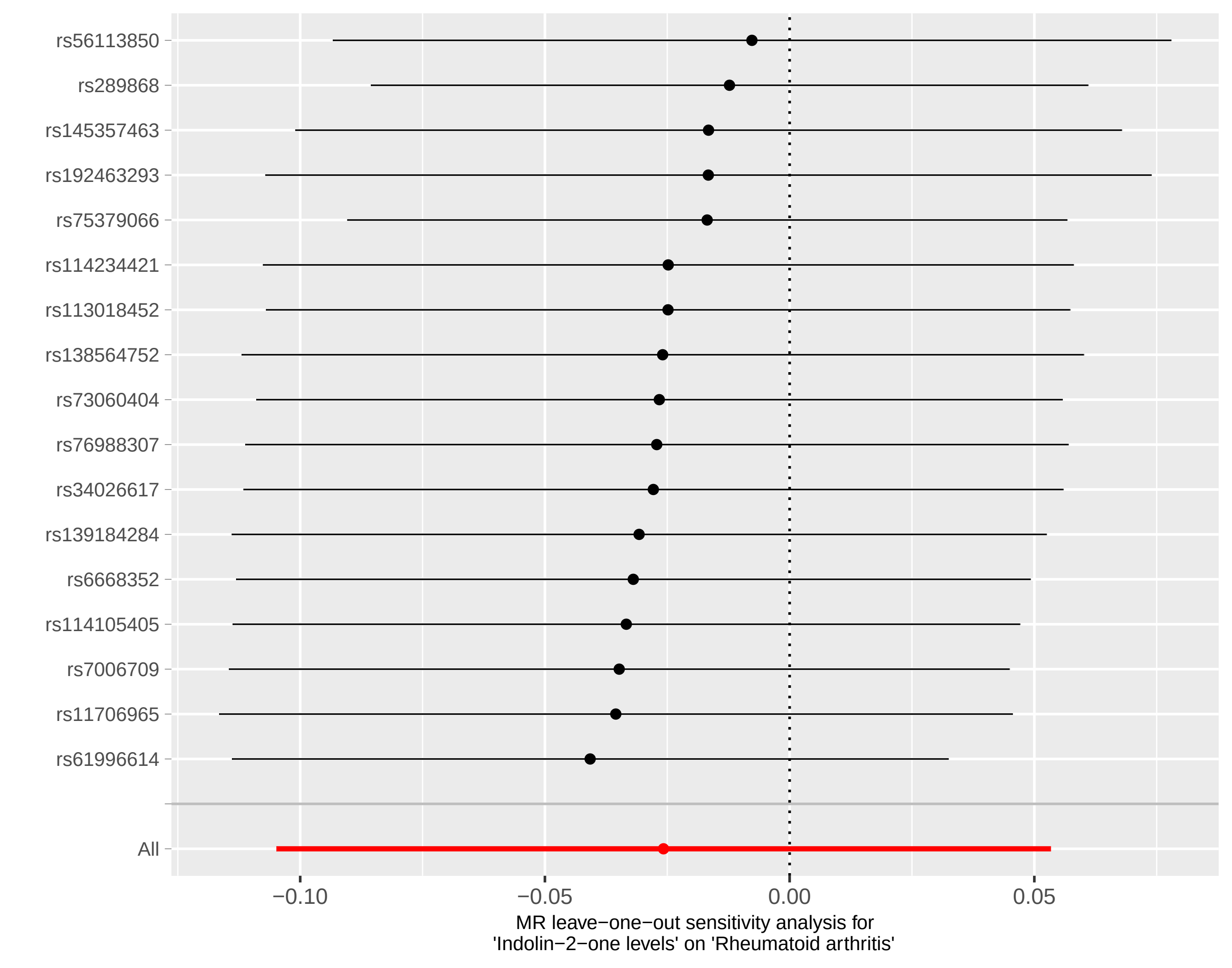

rs56113850
rs289868
rs145357463
rs192463293
rs75379066
rs114234421
rs113018452
rs138564752
rs73060404
rs76988307
rs34026617
rs139184284
rs6668352
rs114105405
rs7006709
rs11706965
rs61996614
All
−0.10
−0.05
0.00
0.05
MR leave−one−out sensitivity analysis for
'Indolin−2−one levels' on 'Rheumatoid arthritis'

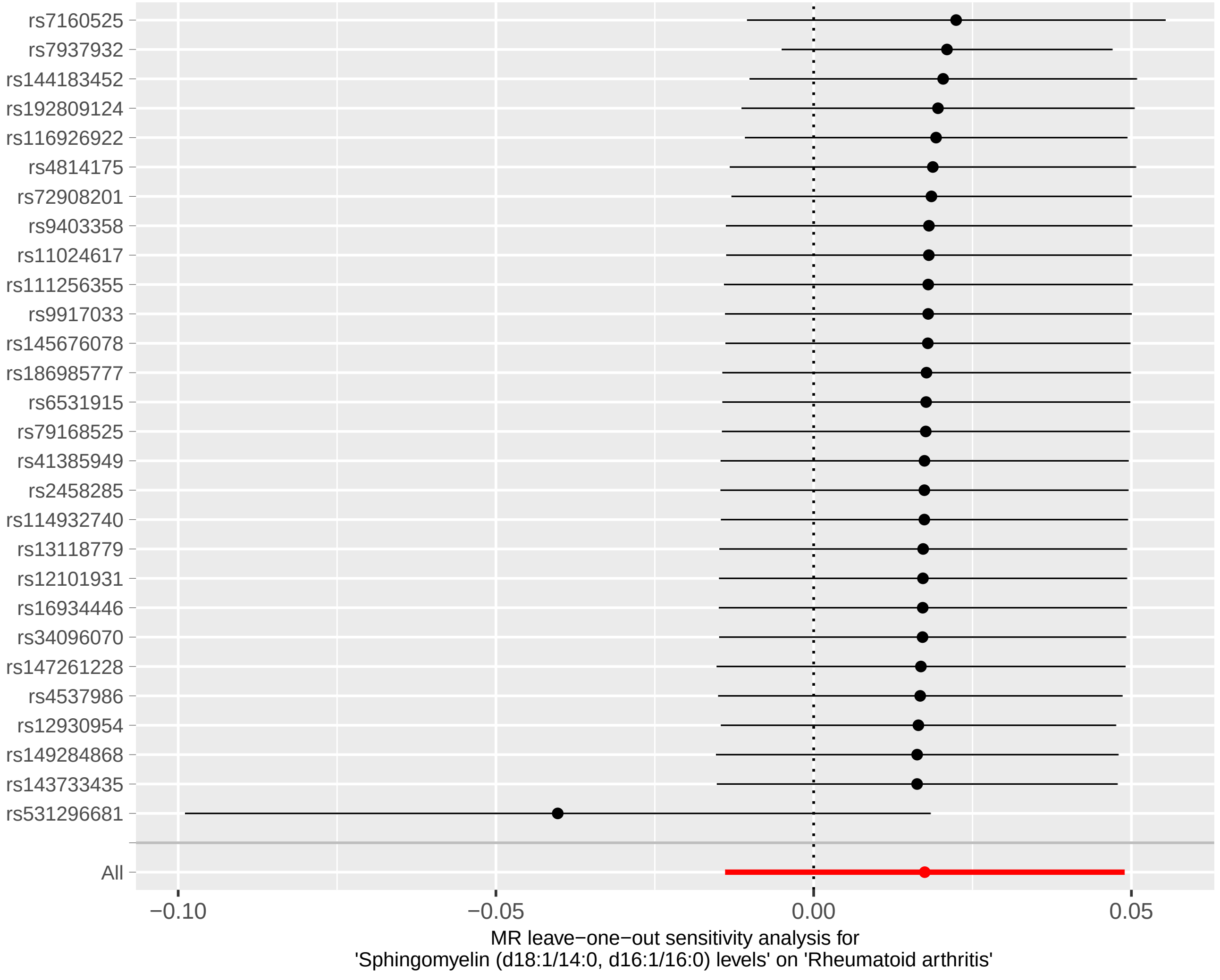

rs7160525
rs7937932
rs144183452
rs192809124
rs116926922
rs4814175
rs72908201
rs9403358
rs11024617
rs111256355
rs9917033
rs145676078
rs186985777
rs6531915
rs79168525
rs41385949
rs2458285
rs114932740
rs13118779
rs12101931
rs16934446
rs34096070
rs147261228
rs4537986
rs12930954
rs149284868
rs143733435
rs531296681
All
−0.10
−0.05
0.00
0.05
MR leave−one−out sensitivity analysis for
'Sphingomyelin (d18:1/14:0, d16:1/16:0) levels' on 'Rheumatoid arthritis'

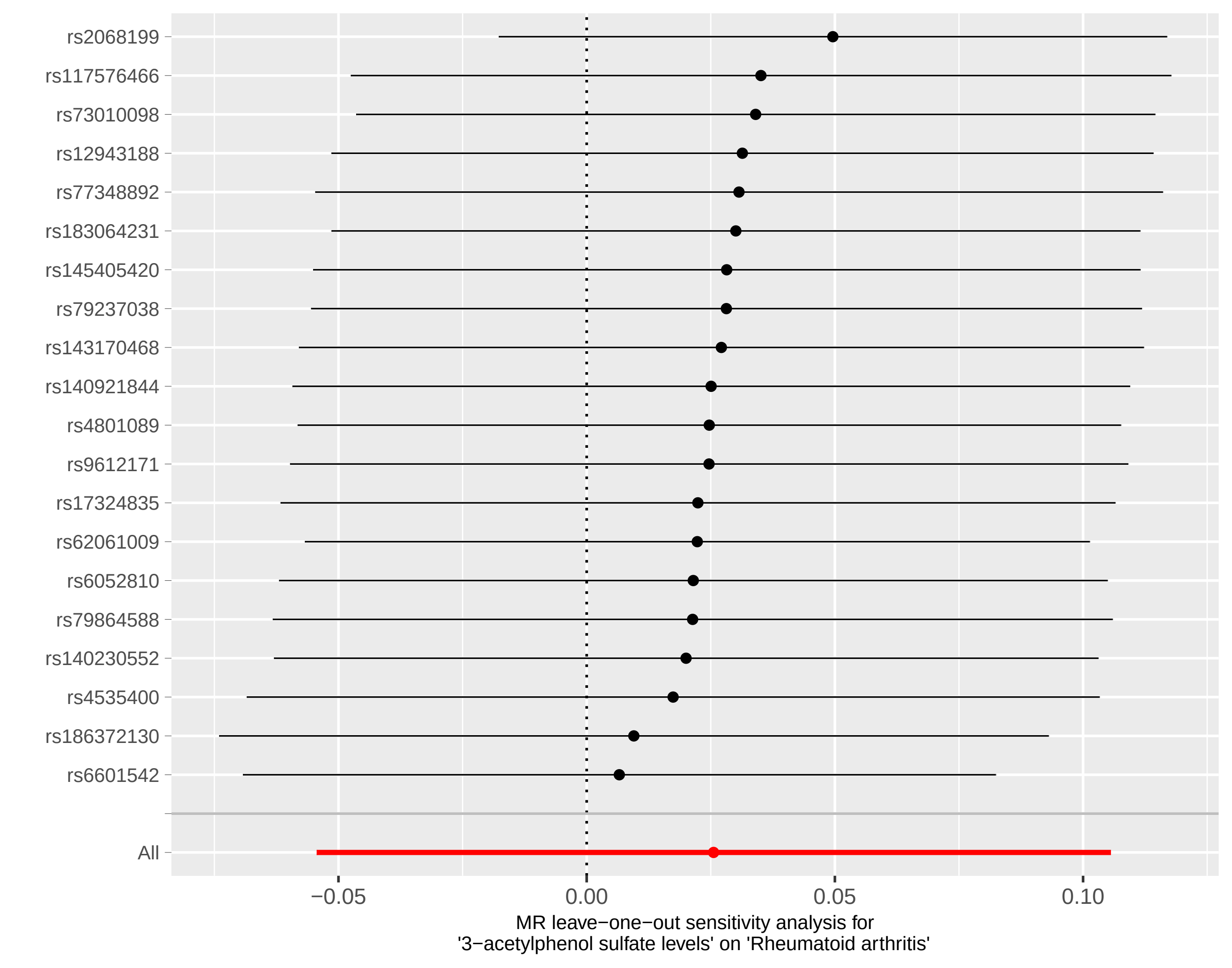

rs2068199
rs117576466
rs73010098
rs12943188
rs77348892
rs183064231
rs145405420
rs79237038
rs143170468
rs140921844
rs4801089
rs9612171
rs17324835
rs62061009
rs6052810
rs79864588
rs140230552
rs4535400
rs186372130
rs6601542
All
−0.05
0.00
0.05
0.10
MR leave−one−out sensitivity analysis for
'3−acetylphenol sulfate levels' on 'Rheumatoid arthritis'

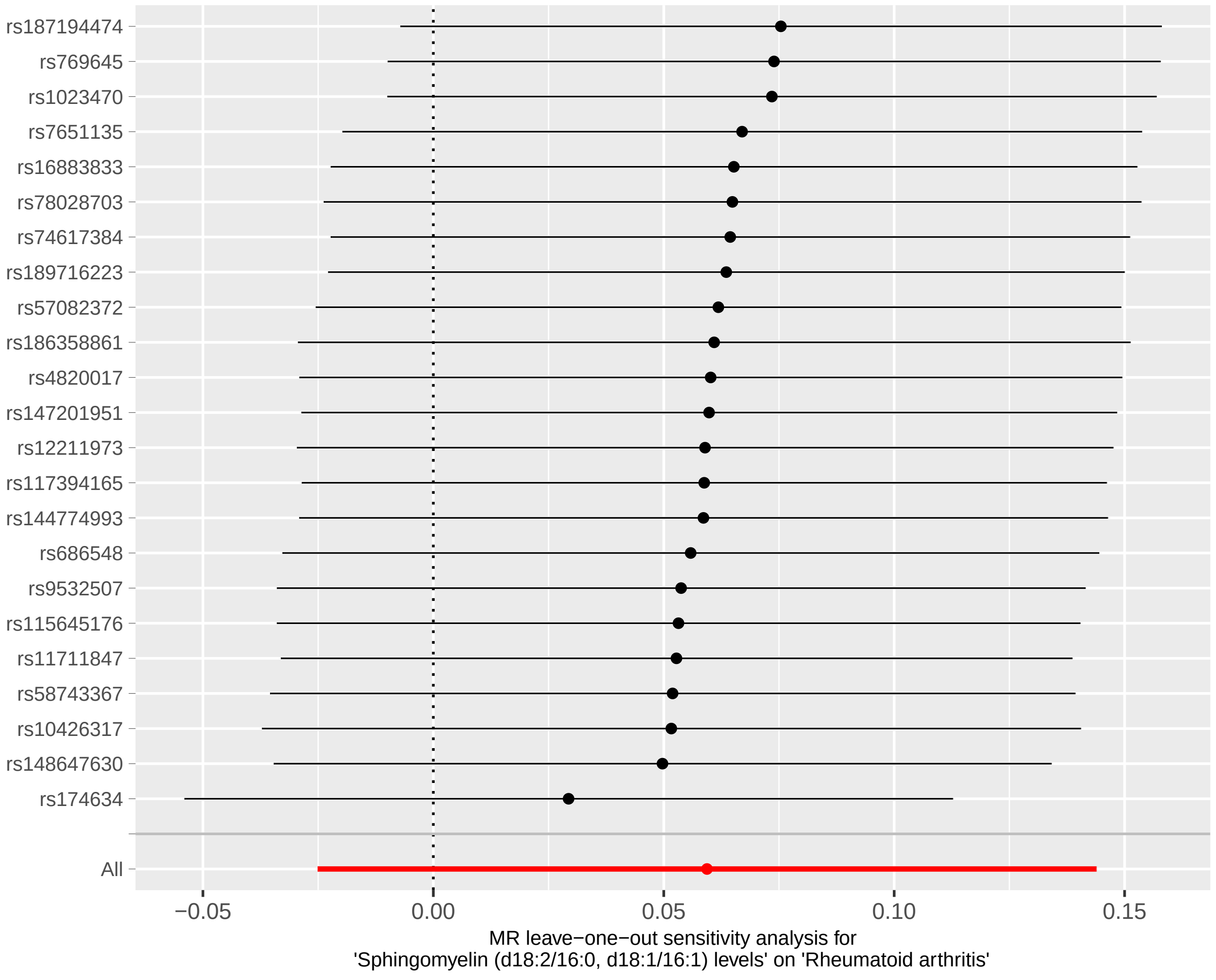

rs187194474
rs769645
rs1023470
rs7651135
rs16883833
rs78028703
rs74617384
rs189716223
rs57082372
rs186358861
rs4820017
rs147201951
rs12211973
rs117394165
rs144774993
rs686548
rs9532507
rs115645176
rs11711847
rs58743367
rs10426317
rs148647630
rs174634
All
−0.05
0.00
0.05
0.10
0.15
MR leave−one−out sensitivity analysis for
'Sphingomyelin (d18:2/16:0, d18:1/16:1) levels' on 'Rheumatoid arthritis'

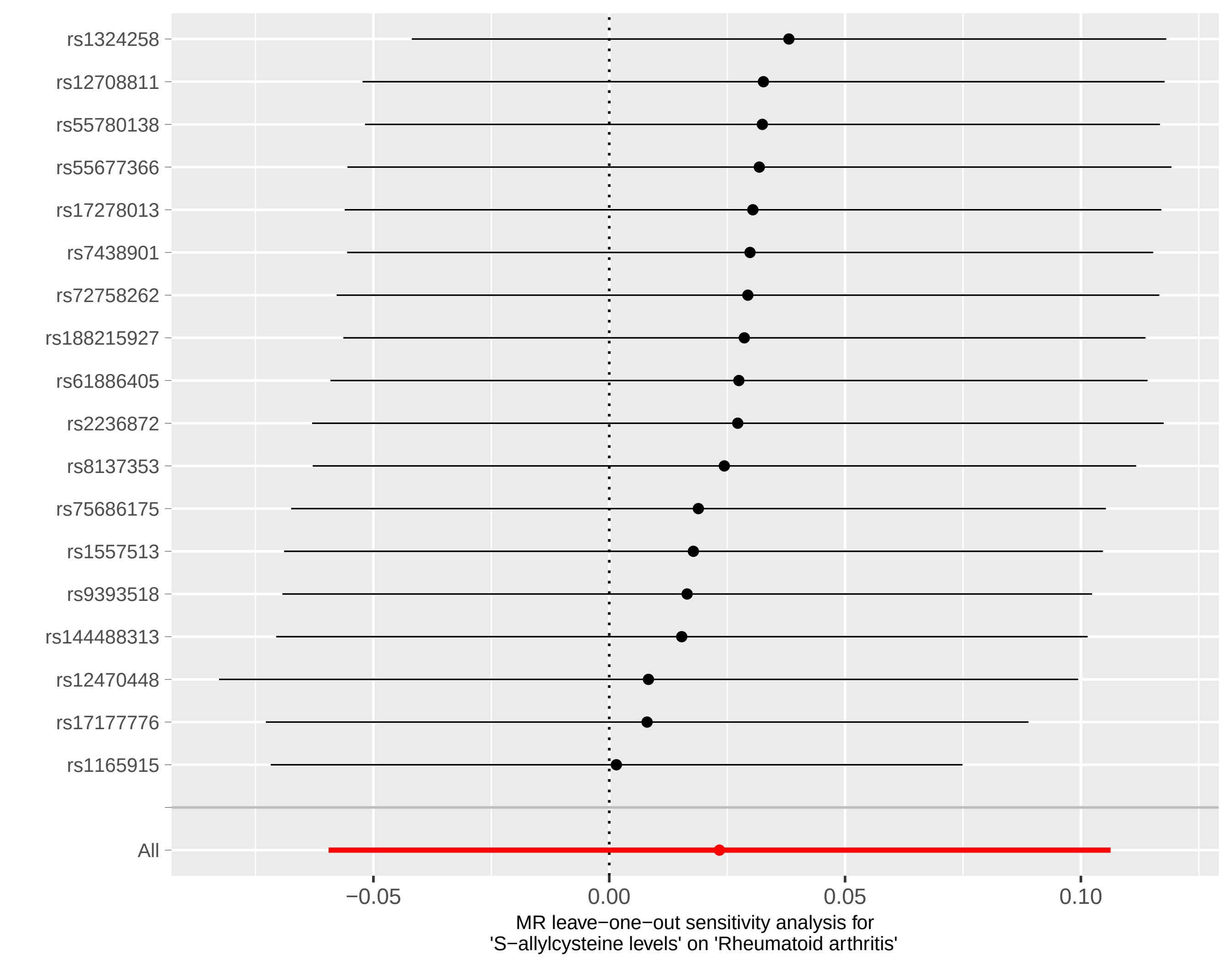
rs1324258
rs12708811
rs55780138
rs55677366
rs17278013
rs7438901
rs72758262
rs188215927
rs61886405
rs2236872
rs8137353
rs75686175
rs1557513
rs9393518
rs144488313
rs12470448
rs17177776
rs1165915
All
−0.05
0.00
0.05
0.10
MR leave−one−out sensitivity analysis for
'S−allylcysteine levels' on 'Rheumatoid arthritis'

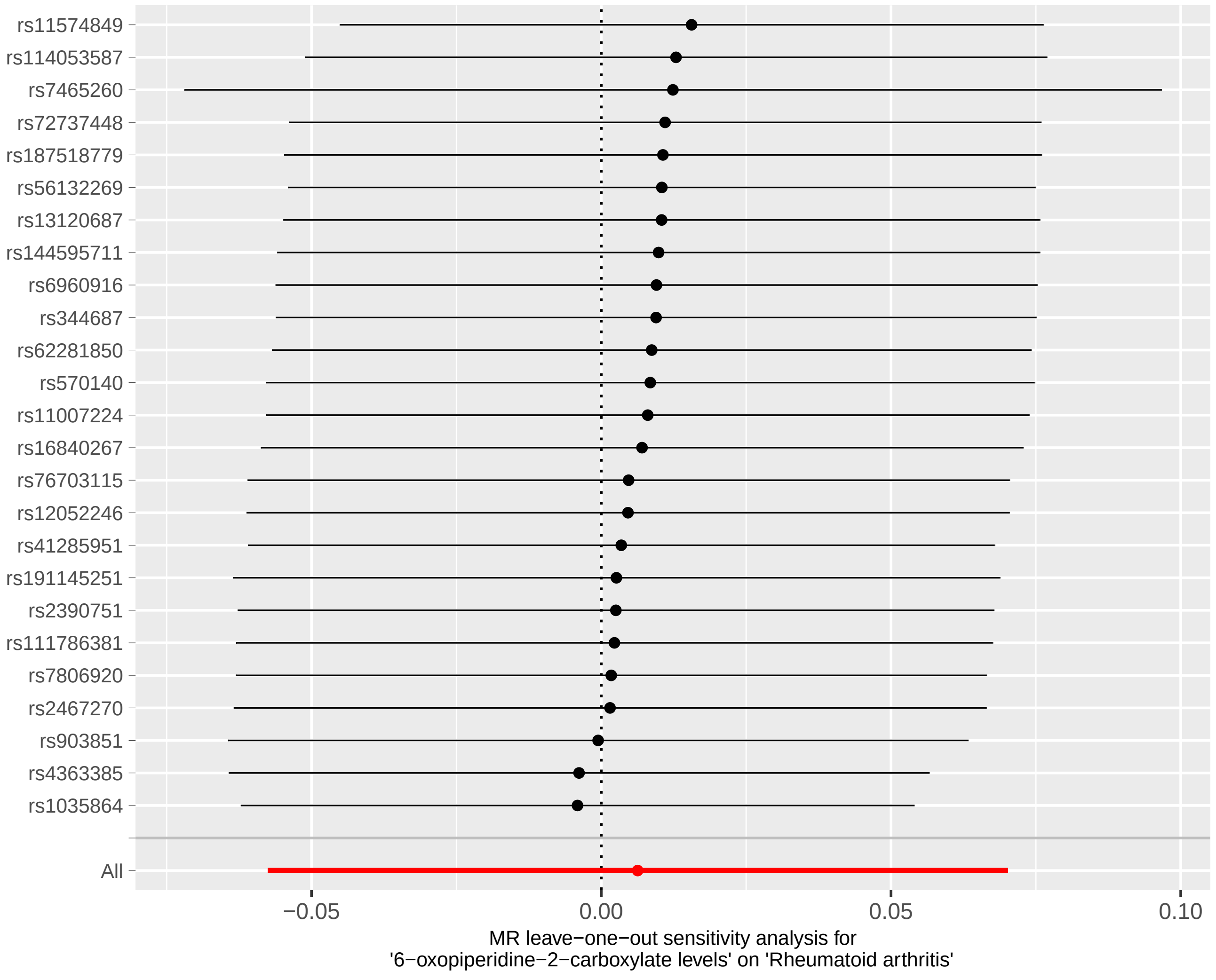
rs11574849
rs114053587
rs7465260
rs72737448
rs187518779
rs56132269
rs13120687
rs144595711
rs6960916
rs344687
rs62281850
rs570140
rs11007224
rs16840267
rs76703115
rs12052246
rs41285951
rs191145251
rs2390751
rs111786381
rs7806920
rs2467270
rs903851
rs4363385
rs1035864
All
−0.05
0.00
0.05
0.10
MR leave−one−out sensitivity analysis for
'6−oxopiperidine−2−carboxylate levels' on 'Rheumatoid arthritis'

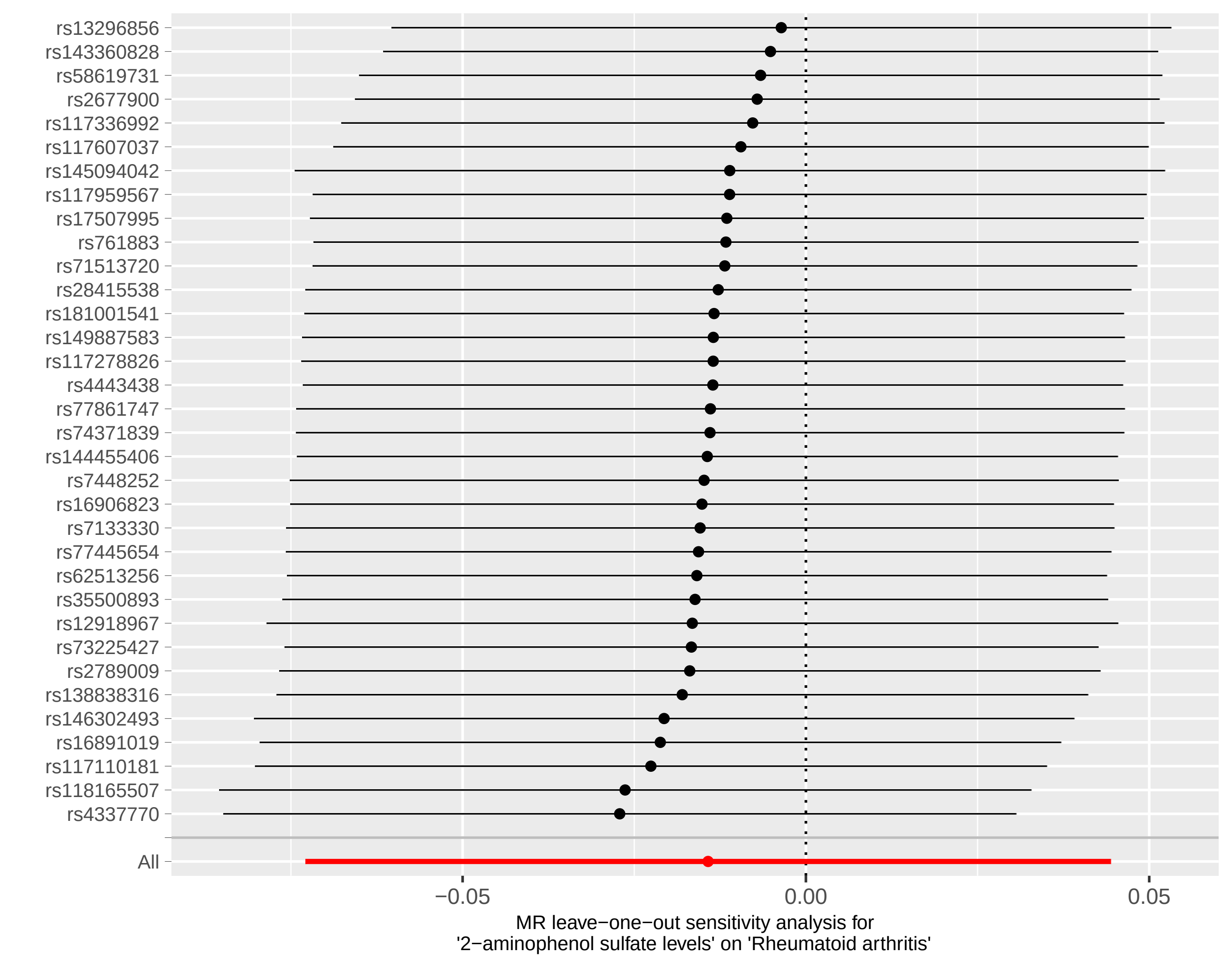
rs13296856
rs143360828
rs58619731
rs2677900
rs117336992
rs117607037
rs145094042
rs117959567
rs17507995
rs761883
rs71513720
rs28415538
rs181001541
rs149887583
rs117278826
rs4443438
rs77861747
rs74371839
rs144455406
rs7448252
rs16906823
rs7133330
rs77445654
rs62513256
rs35500893
rs12918967
rs73225427
rs2789009
rs138838316
rs146302493
rs16891019
rs117110181
rs118165507
rs4337770
All
−0.05
0.00
0.05
MR leave−one−out sensitivity analysis for
'2−aminophenol sulfate levels' on 'Rheumatoid arthritis'

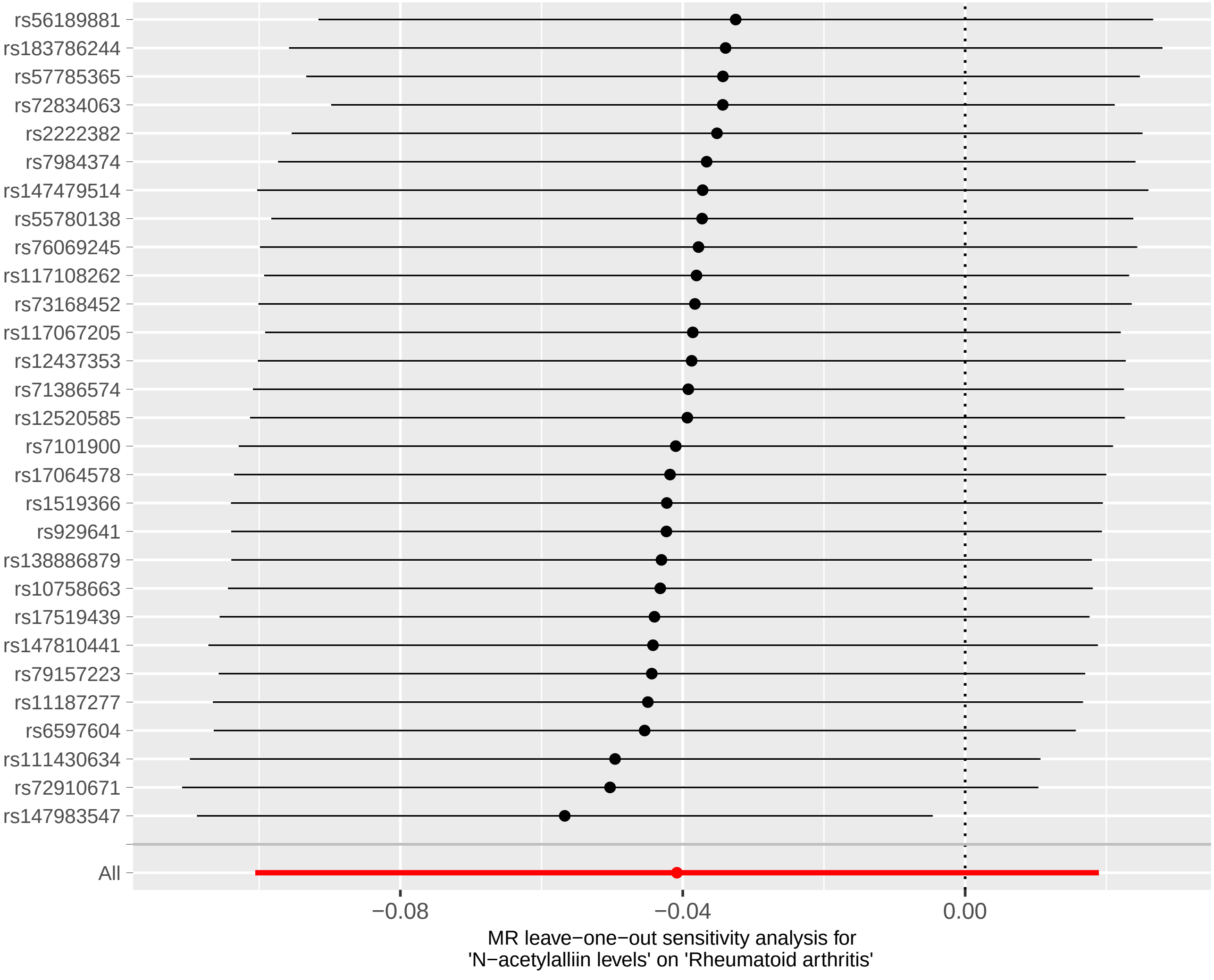

rs56189881
rs183786244
rs57785365
rs72834063
rs2222382
rs7984374
rs147479514
rs55780138
rs76069245
rs117108262
rs73168452
rs117067205
rs12437353
rs71386574
rs12520585
rs7101900
rs17064578
rs1519366
rs929641
rs138886879
rs10758663
rs17519439
rs147810441
rs79157223
rs11187277
rs6597604
rs111430634
rs72910671
rs147983547
All
−0.08
−0.04
0.00
MR leave−one−out sensitivity analysis for
'N−acetylalliin levels' on 'Rheumatoid arthritis'

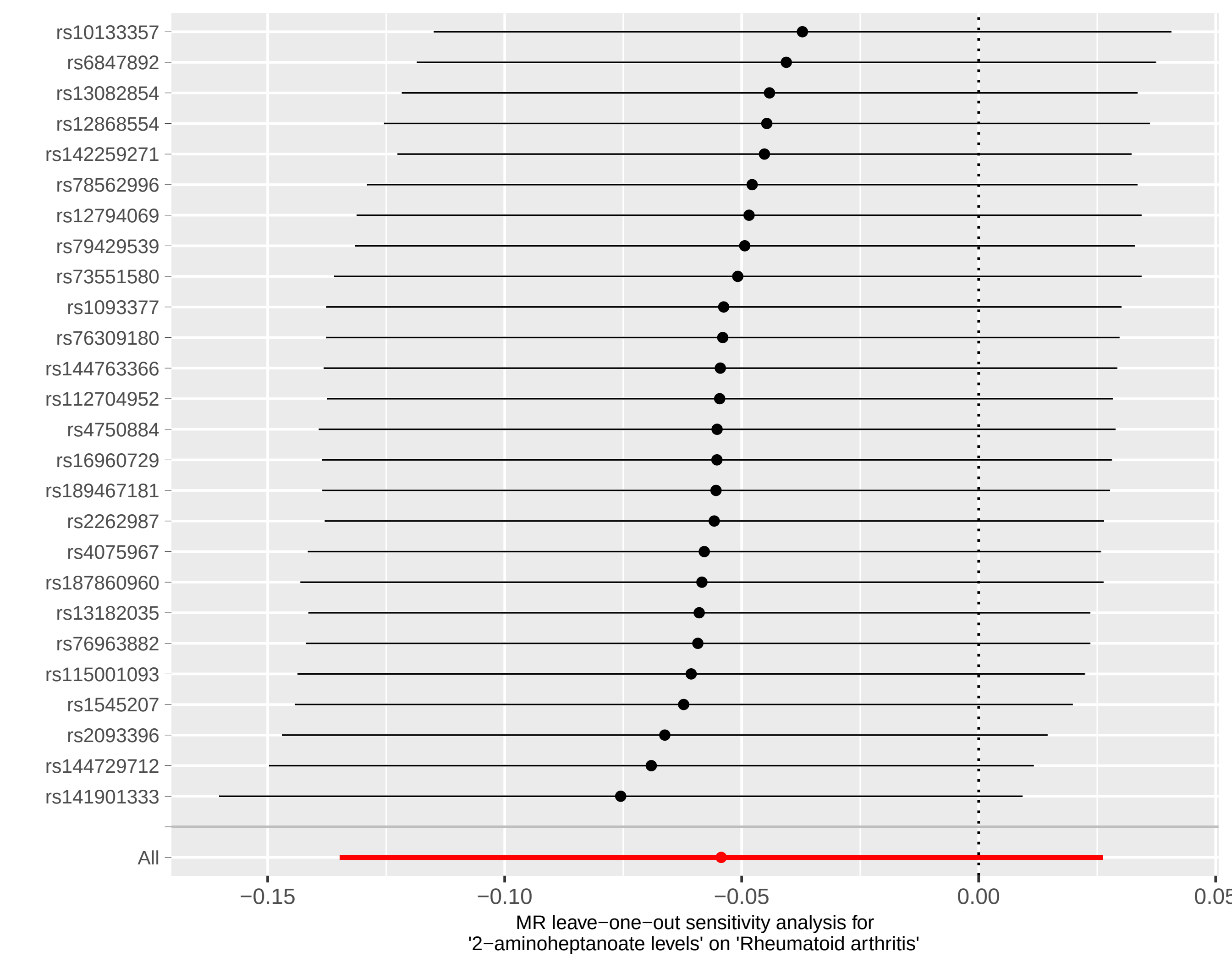
rs10133357
rs6847892
rs13082854
rs12868554
rs142259271
rs78562996
rs12794069
rs79429539
rs73551580
rs1093377
rs76309180
rs144763366
rs112704952
rs4750884
rs16960729
rs189467181
rs2262987
rs4075967
rs187860960
rs13182035
rs76963882
rs115001093
rs1545207
rs2093396
rs144729712
rs141901333
All
−0.15
−0.10
−0.05
0.00
MR leave−one−out sensitivity analysis for
'2−aminoheptanoate levels' on 'Rheumatoid arthritis'

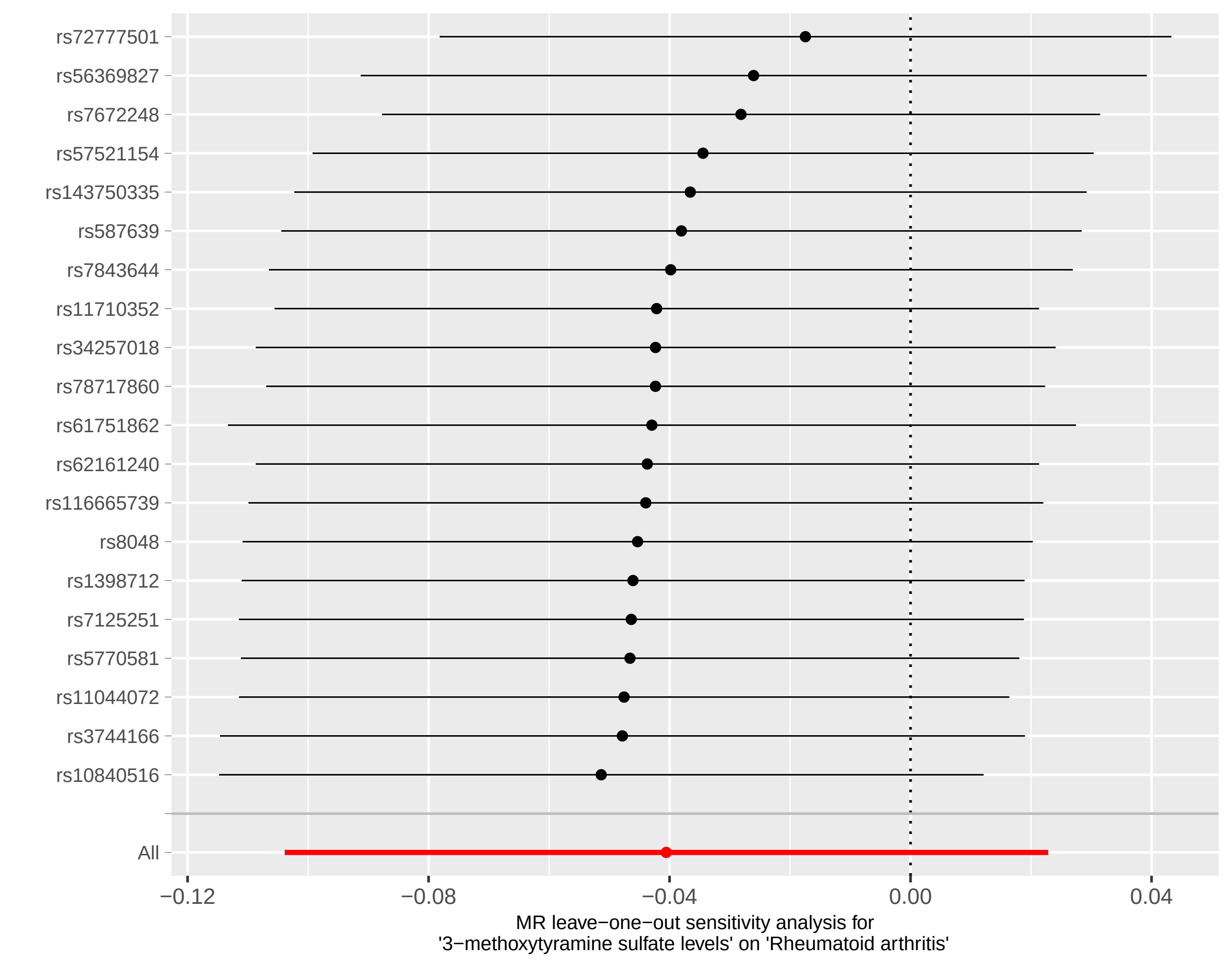

rs72777501
rs56369827
rs7672248
rs57521154
rs143750335
rs587639
rs7843644
rs11710352
rs34257018
rs78717860
rs61751862
rs62161240
rs116665739
rs8048
rs1398712
rs7125251
rs5770581
rs11044072
rs3744166
rs10840516
All
−0.12
−0.08
−0.04
0.00
0.04
MR leave−one−out sensitivity analysis for
'3−methoxytyramine sulfate levels' on 'Rheumatoid arthritis'

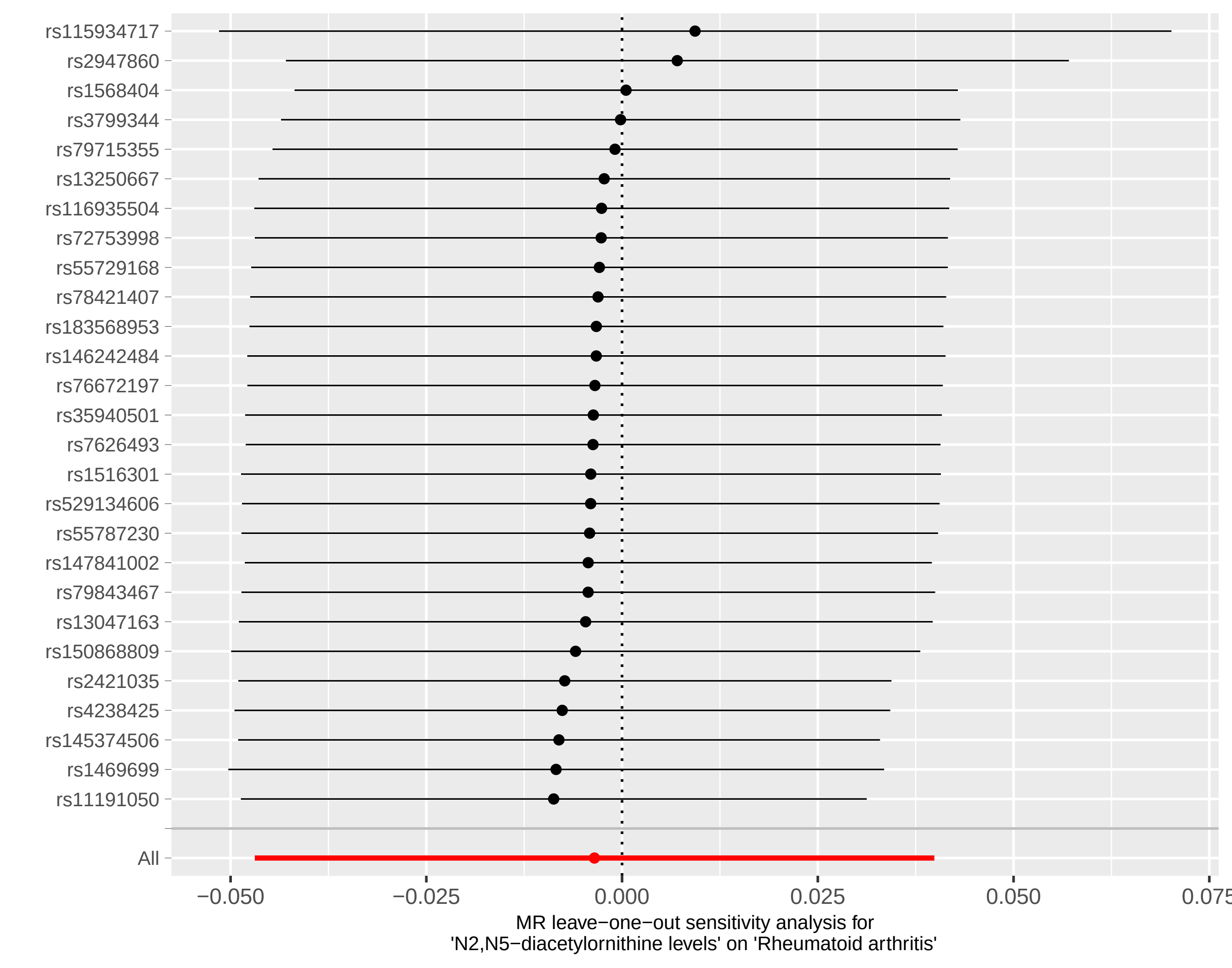
rs115934717
rs2947860
rs1568404
rs3799344
rs79715355
rs13250667
rs116935504
rs72753998
rs55729168
rs78421407
rs183568953
rs146242484
rs76672197
rs35940501
rs7626493
rs1516301
rs529134606
rs55787230
rs147841002
rs79843467
rs13047163
rs150868809
rs2421035
rs4238425
rs145374506
rs1469699
rs11191050
All
−0.050
−0.025
0.000
0.025
0.050
MR leave−one−out sensitivity analysis for
'N2,N5−diacetylornithine levels' on 'Rheumatoid arthritis'

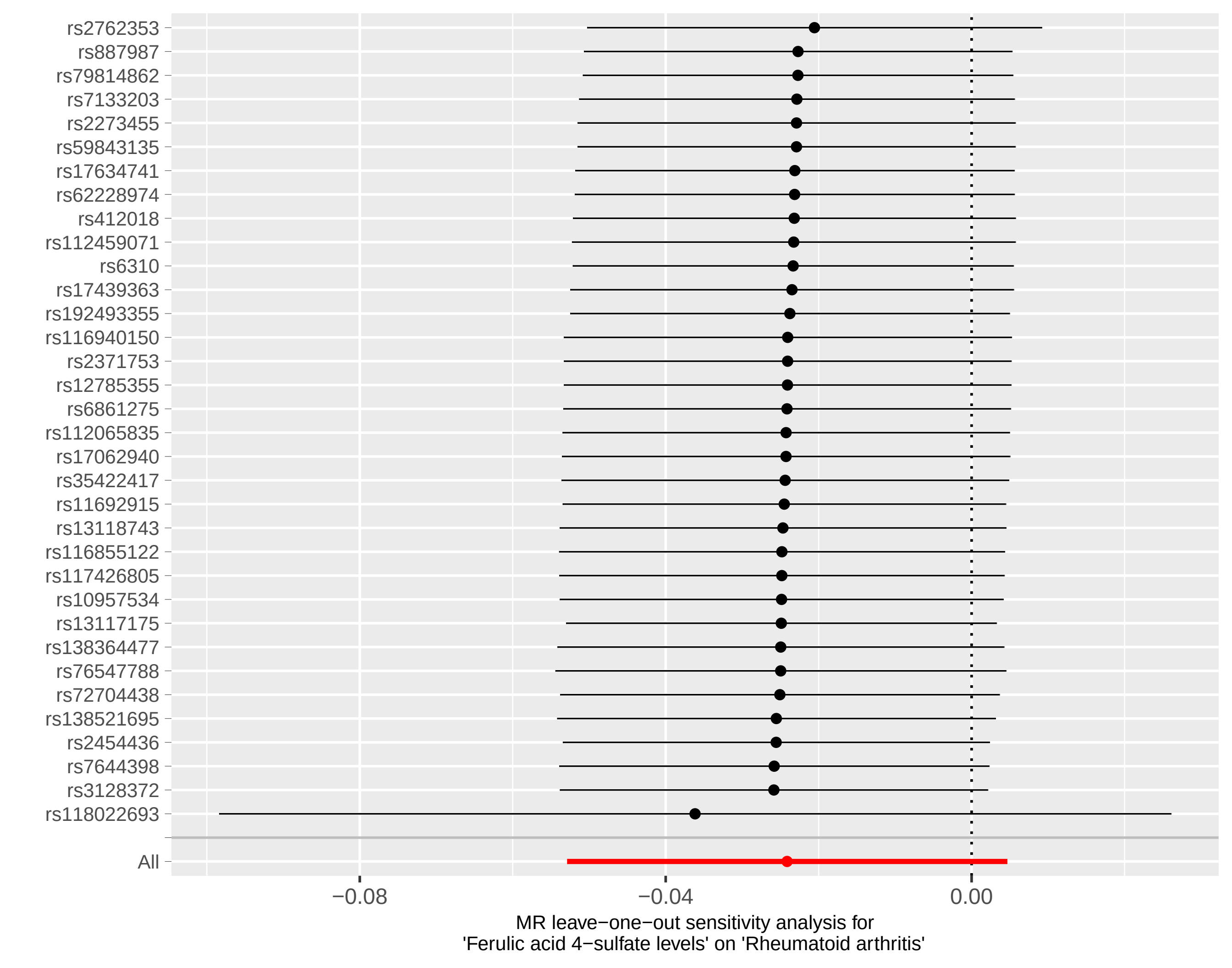
rs2762353
rs887987
rs79814862
rs7133203
rs2273455
rs59843135
rs17634741
rs62228974
rs412018
rs112459071
rs6310
rs17439363
rs192493355
rs116940150
rs2371753
rs12785355
rs6861275
rs112065835
rs17062940
rs35422417
rs11692915
rs13118743
rs116855122
rs117426805
rs10957534
rs13117175
rs138364477
rs76547788
rs72704438
rs138521695
rs2454436
rs7644398
rs3128372
rs118022693
All
−0.08
−0.04
0.00
MR leave−one−out sensitivity analysis for
'Ferulic acid 4−sulfate levels' on 'Rheumatoid arthritis'

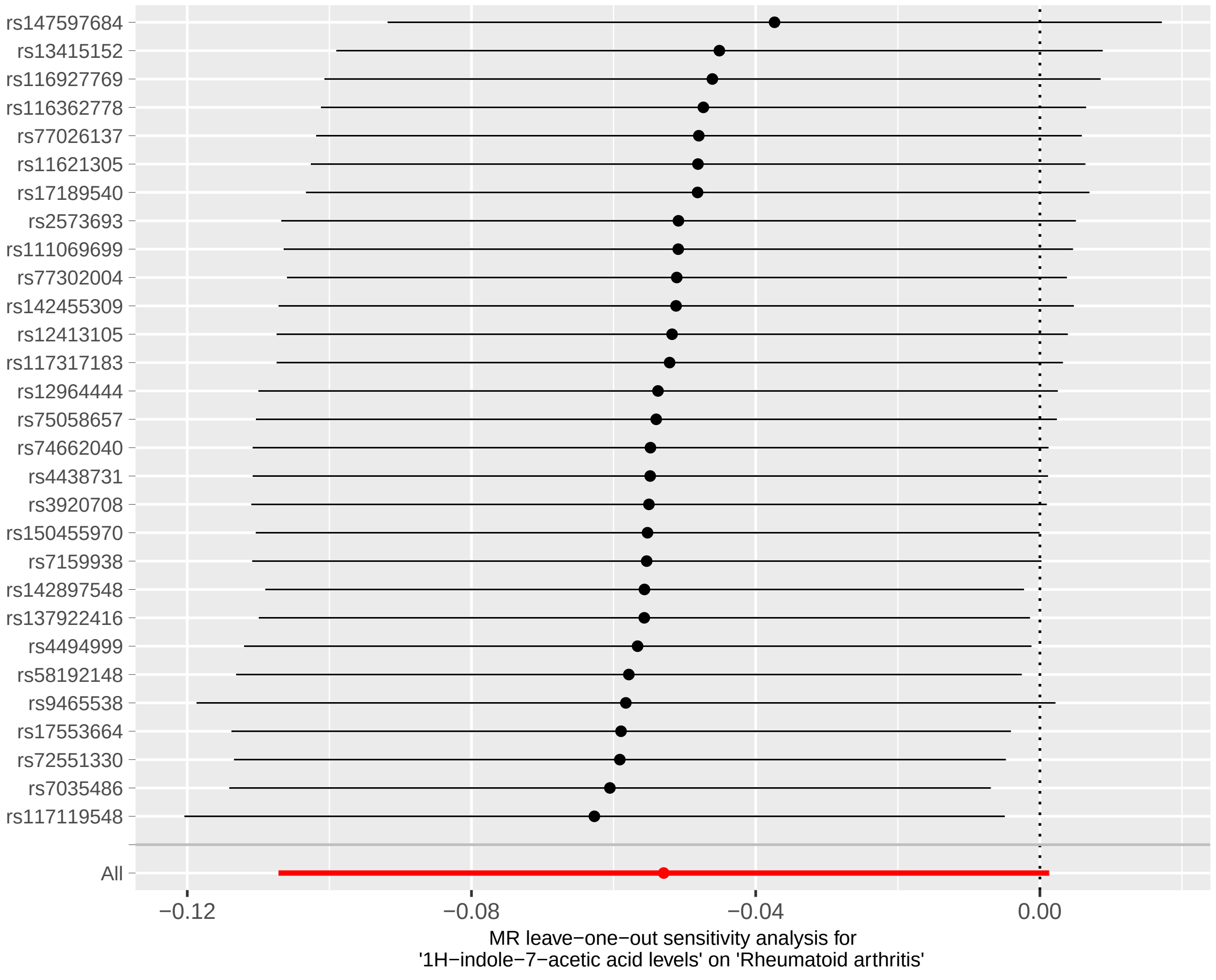

rs147597684
rs13415152
rs116927769
rs116362778
rs77026137
rs11621305
rs17189540
rs2573693
rs111069699
rs77302004
rs142455309
rs12413105
rs117317183
rs12964444
rs75058657
rs74662040
rs4438731
rs3920708
rs150455970
rs7159938
rs142897548
rs137922416
rs4494999
rs58192148
rs9465538
rs17553664
rs72551330
rs7035486
rs117119548
All
−0.12
−0.08
−0.04
0.00
MR leave−one−out sensitivity analysis for
'1H−indole−7−acetic acid levels' on 'Rheumatoid arthritis'

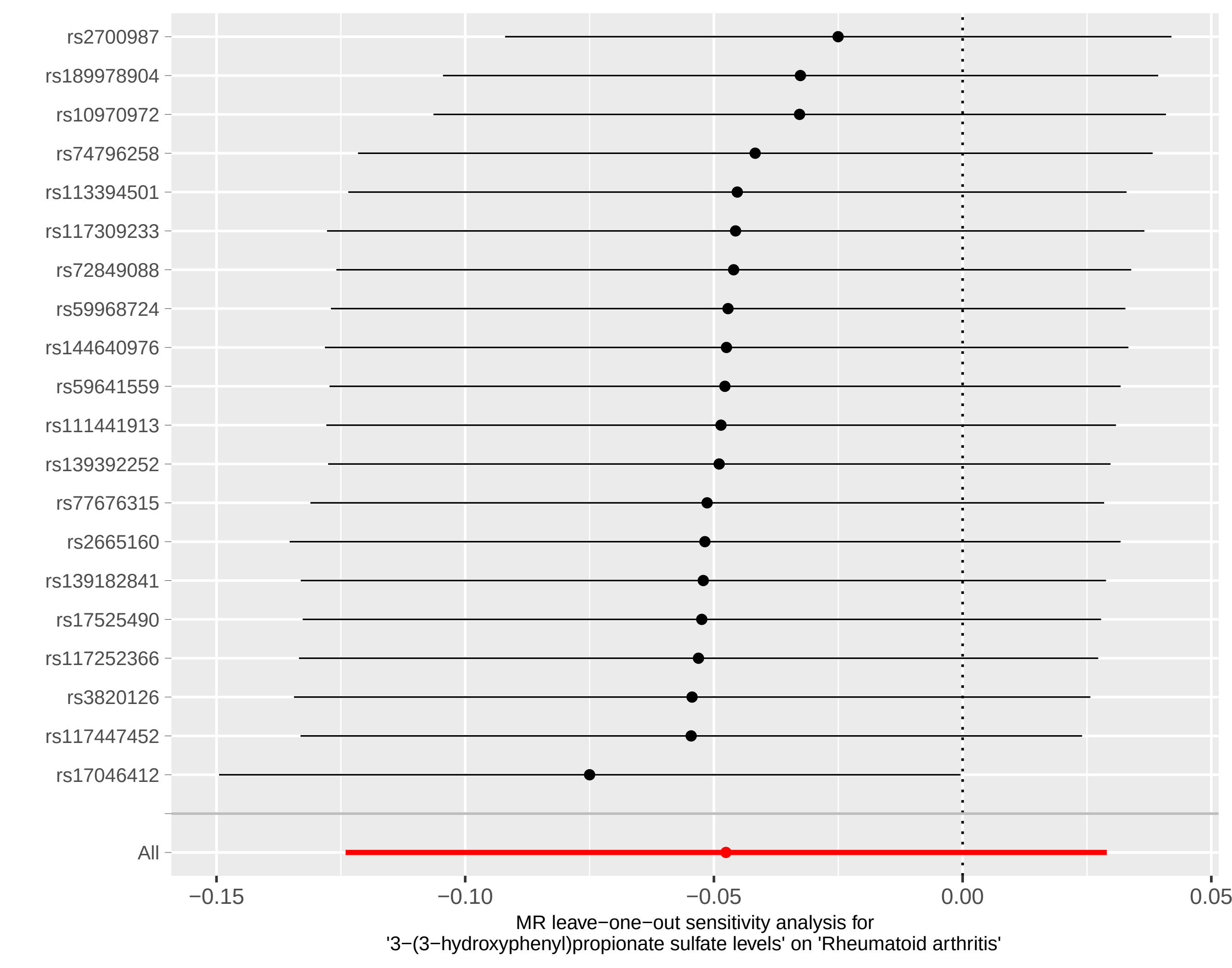
rs2700987
rs189978904
rs10970972
rs74796258
rs113394501
rs117309233
rs72849088
rs59968724
rs144640976
rs59641559
rs111441913
rs139392252
rs77676315
rs2665160
rs139182841
rs17525490
rs117252366
rs3820126
rs117447452
rs17046412
All
−0.15
−0.10
−0.05
0.00
0.05
MR leave−one−out sensitivity analysis for
'3−(3−hydroxyphenyl)propionate sulfate levels' on 'Rheumatoid arthritis'

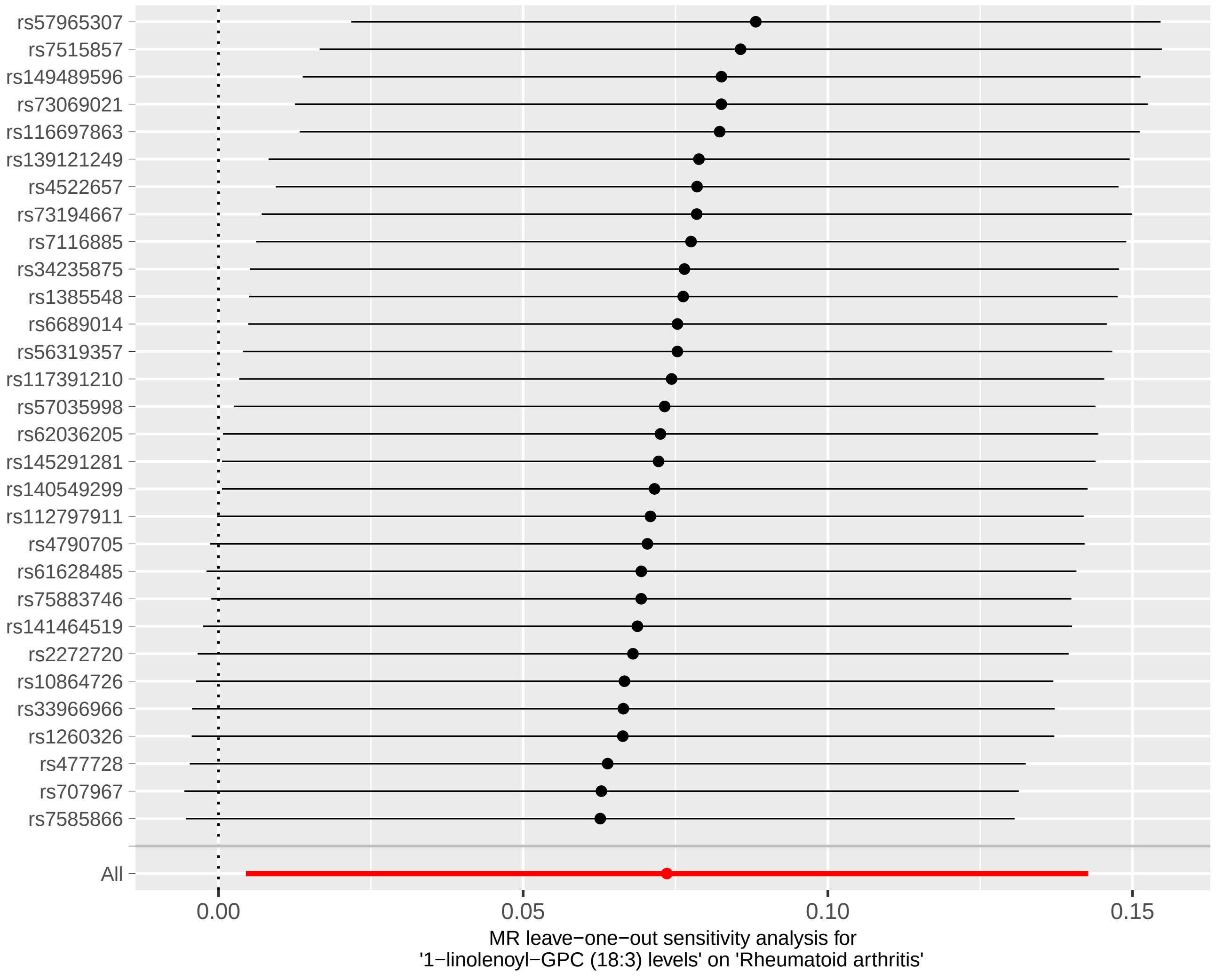
rs57965307
rs7515857
rs149489596
rs73069021
rs116697863
rs139121249
rs4522657
rs73194667
rs7116885
rs34235875
rs1385548
rs6689014
rs56319357
rs117391210
rs57035998
rs62036205
rs145291281
rs140549299
rs112797911
rs4790705
rs61628485
rs75883746
rs141464519
rs2272720
rs10864726
rs33966966
rs1260326
rs477728
rs707967
rs7585866
All
0.00
0.05
0.10
0.15
MR leave−one−out sensitivity analysis for
'1−linolenoyl−GPC (18:3) levels' on 'Rheumatoid arthritis'

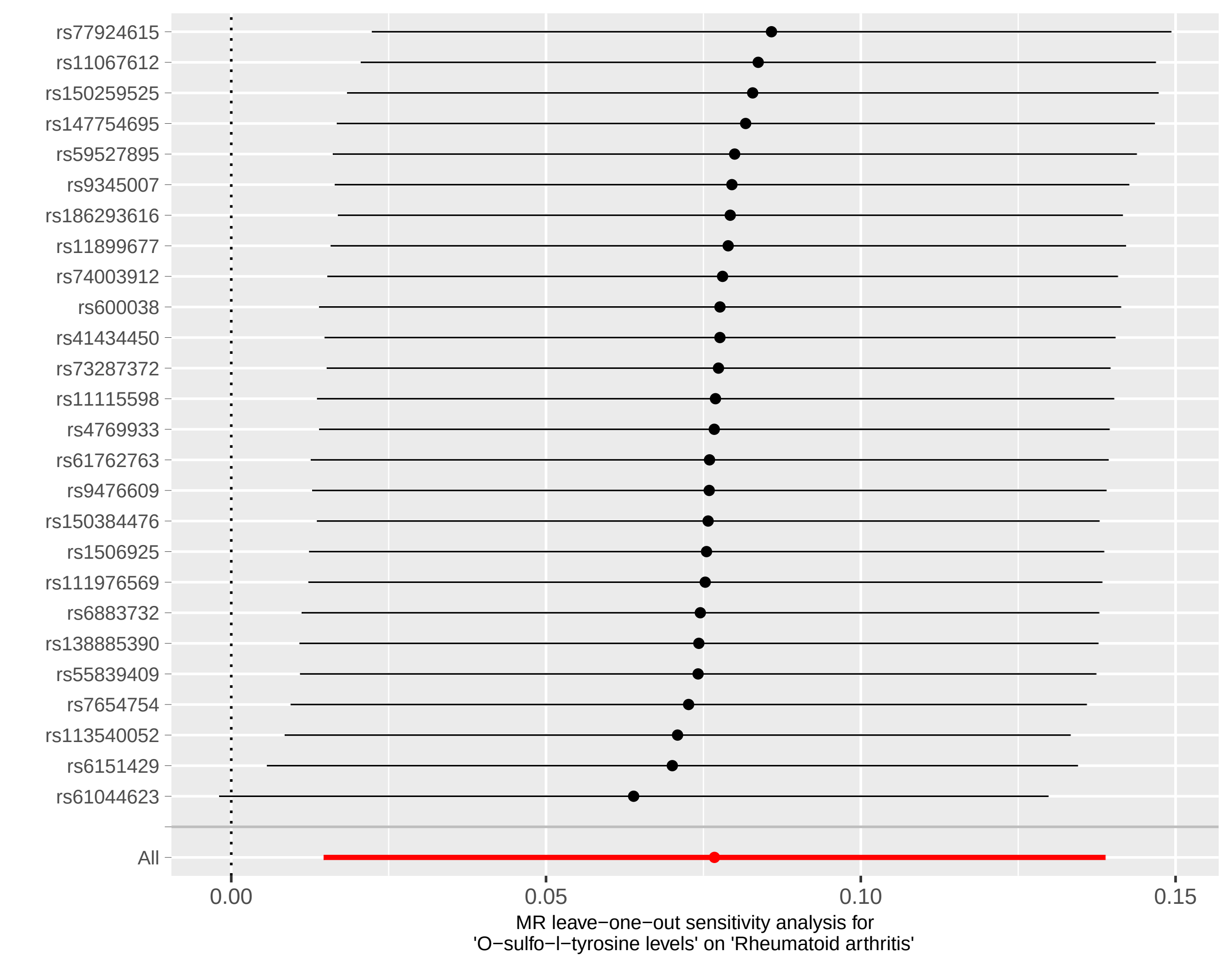

rs77924615
rs11067612
rs150259525
rs147754695
rs59527895
rs9345007
rs186293616
rs11899677
rs74003912
rs600038
rs41434450
rs73287372
rs11115598
rs4769933
rs61762763
rs9476609
rs150384476
rs1506925
rs111976569
rs6883732
rs138885390
rs55839409
rs7654754
rs113540052
rs6151429
rs61044623
All
0.00
0.05
0.10
0.15
MR leave−one−out sensitivity analysis for
'O−sulfo−l−tyrosine levels' on 'Rheumatoid arthritis'

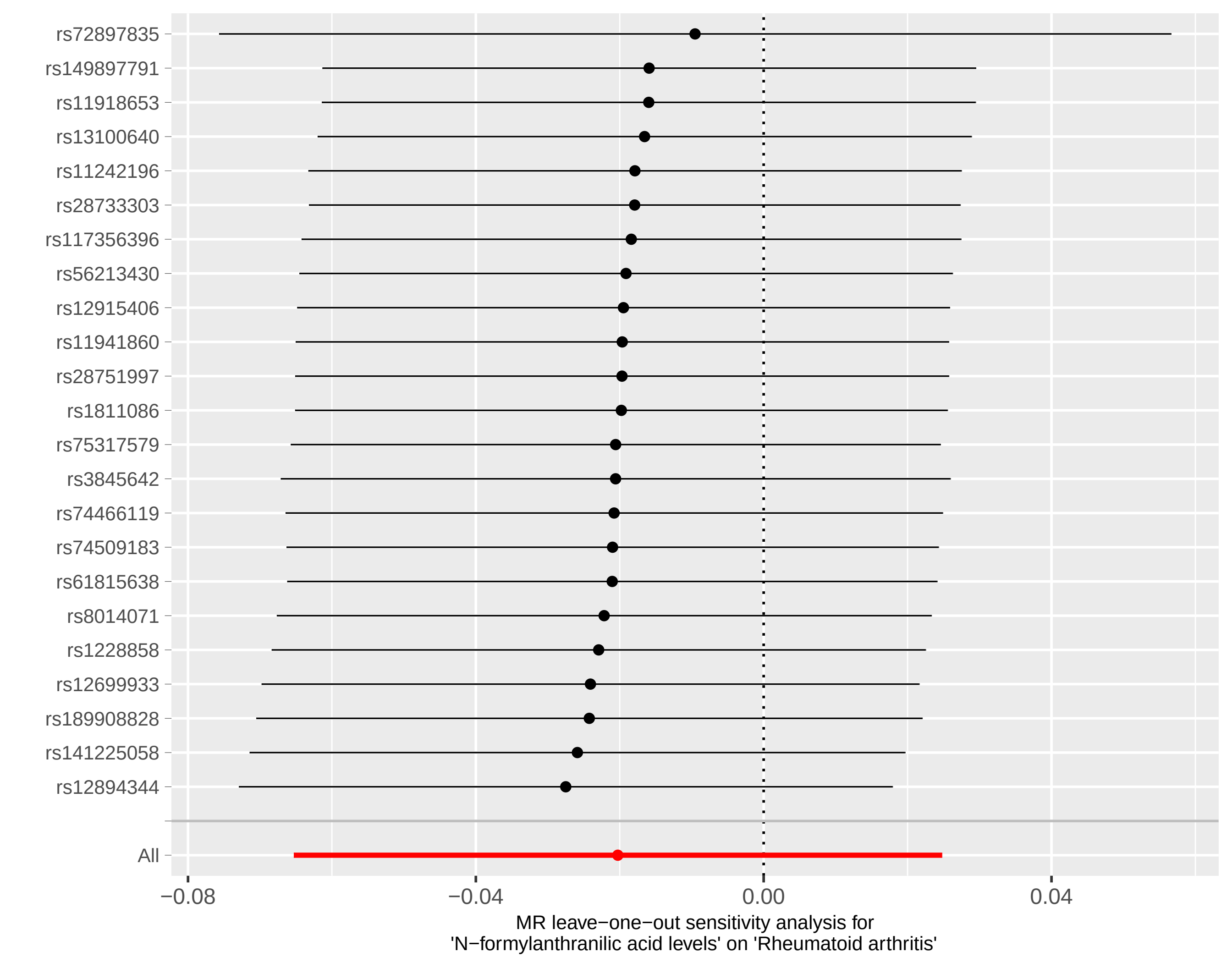
rs72897835
rs149897791
rs11918653
rs13100640
rs11242196
rs28733303
rs117356396
rs56213430
rs12915406
rs11941860
rs28751997
rs1811086
rs75317579
rs3845642
rs74466119
rs74509183
rs61815638
rs8014071
rs1228858
rs12699933
rs189908828
rs141225058
rs12894344
All
−0.08
−0.04
0.00
0.04
MR leave−one−out sensitivity analysis for
'N−formylanthranilic acid levels' on 'Rheumatoid arthritis'

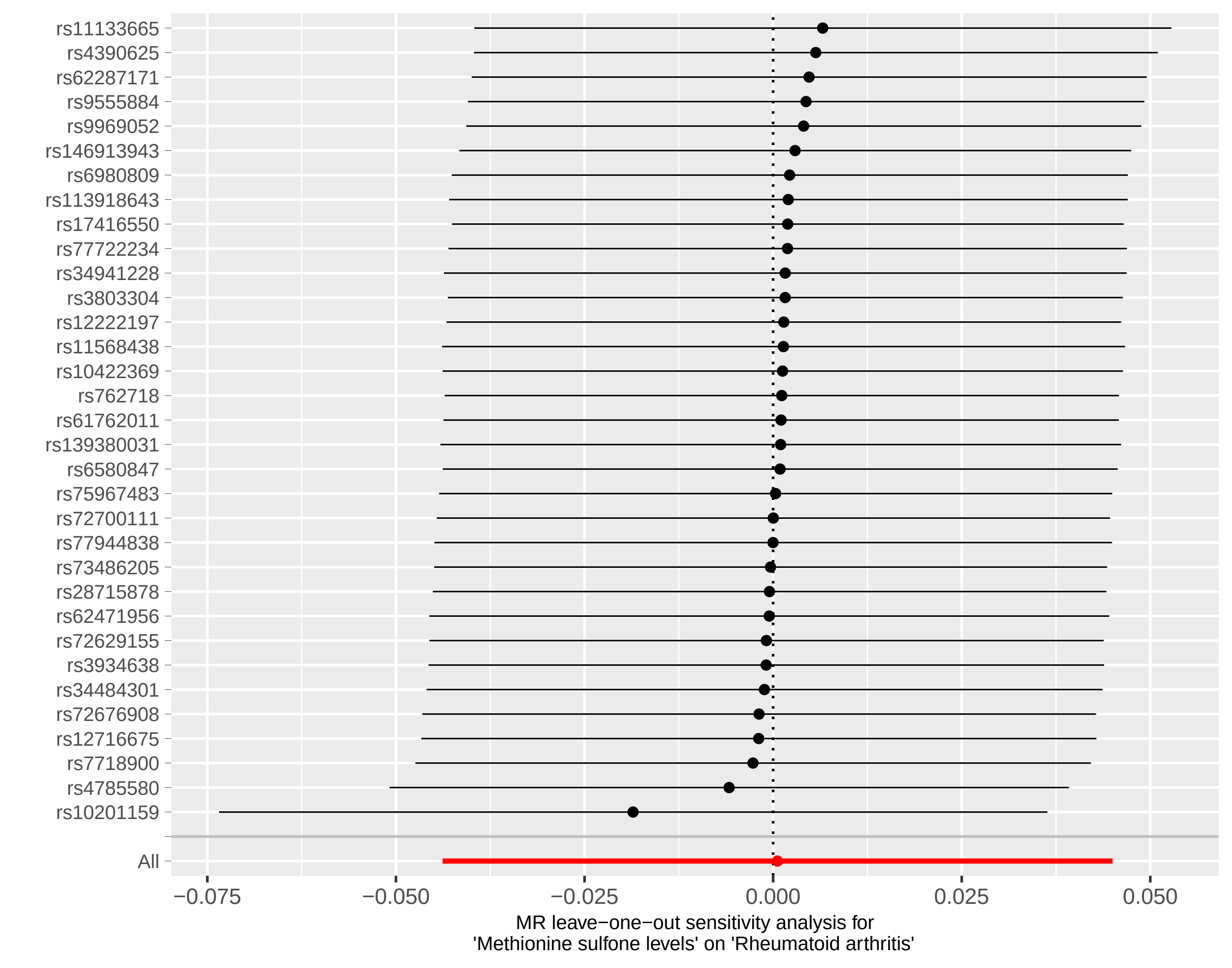

rs11133665
rs4390625
rs62287171
rs9555884
rs9969052
rs146913943
rs6980809
rs113918643
rs17416550
rs77722234
rs34941228
rs3803304
rs12222197
rs11568438
rs10422369
rs762718
rs61762011
rs139380031
rs6580847
rs75967483
rs72700111
rs77944838
rs73486205
rs28715878
rs62471956
rs72629155
rs3934638
rs34484301
rs72676908
rs12716675
rs7718900
rs4785580
rs10201159
All
−0.075
−0.050
−0.025
0.000
0.025
0.050
MR leave−one−out sensitivity analysis for
'Methionine sulfone levels' on 'Rheumatoid arthritis'

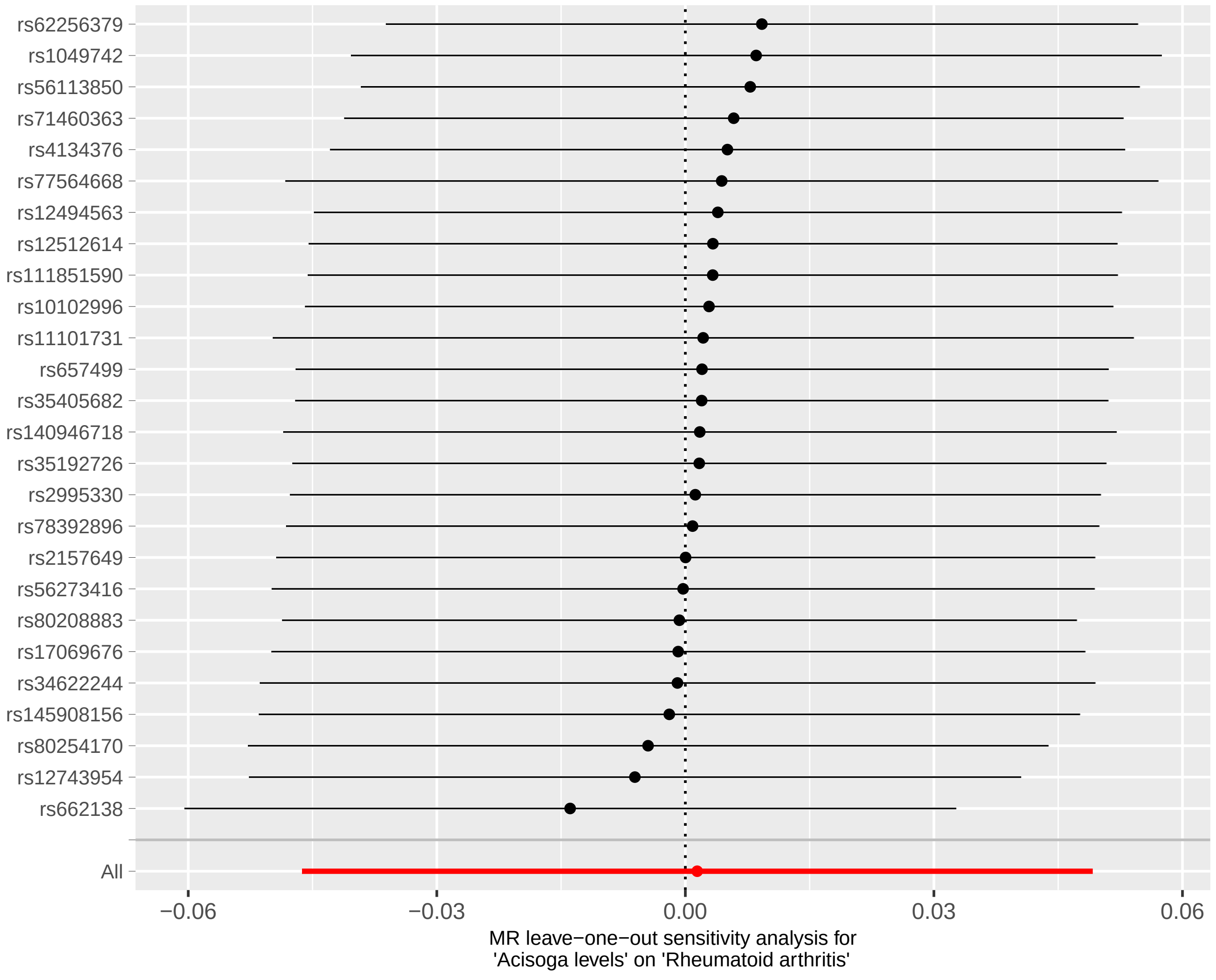
rs62256379
rs1049742
rs56113850
rs71460363
rs4134376
rs77564668
rs12494563
rs12512614
rs111851590
rs10102996
rs11101731
rs657499
rs35405682
rs140946718
rs35192726
rs2995330
rs78392896
rs2157649
rs56273416
rs80208883
rs17069676
rs34622244
rs145908156
rs80254170
rs12743954
rs662138
All
−0.06
−0.03
0.00
0.03
0.06
MR leave−one−out sensitivity analysis for
'Acisoga levels' on 'Rheumatoid arthritis'

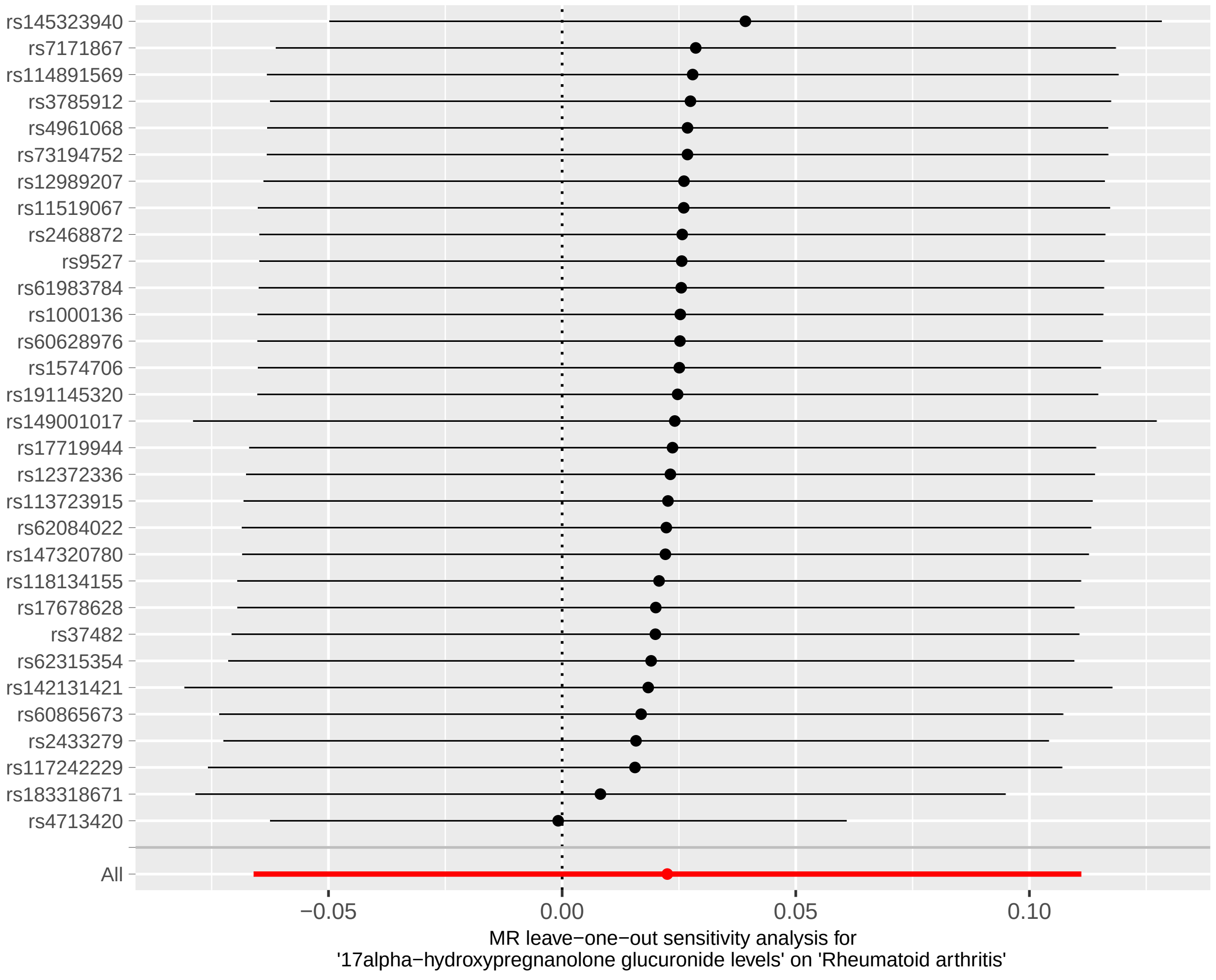
rs145323940
rs7171867
rs114891569
rs3785912
rs4961068
rs73194752
rs12989207
rs11519067
rs2468872
rs9527
rs61983784
rs1000136
rs60628976
rs1574706
rs191145320
rs149001017
rs17719944
rs12372336
rs113723915
rs62084022
rs147320780
rs118134155
rs17678628
rs37482
rs62315354
rs142131421
rs60865673
rs2433279
rs117242229
rs183318671
rs4713420
All
−0.05
0.00
0.05
0.10
MR leave−one−out sensitivity analysis for
'17alpha−hydroxypregnanolone glucuronide levels' on 'Rheumatoid arthritis'

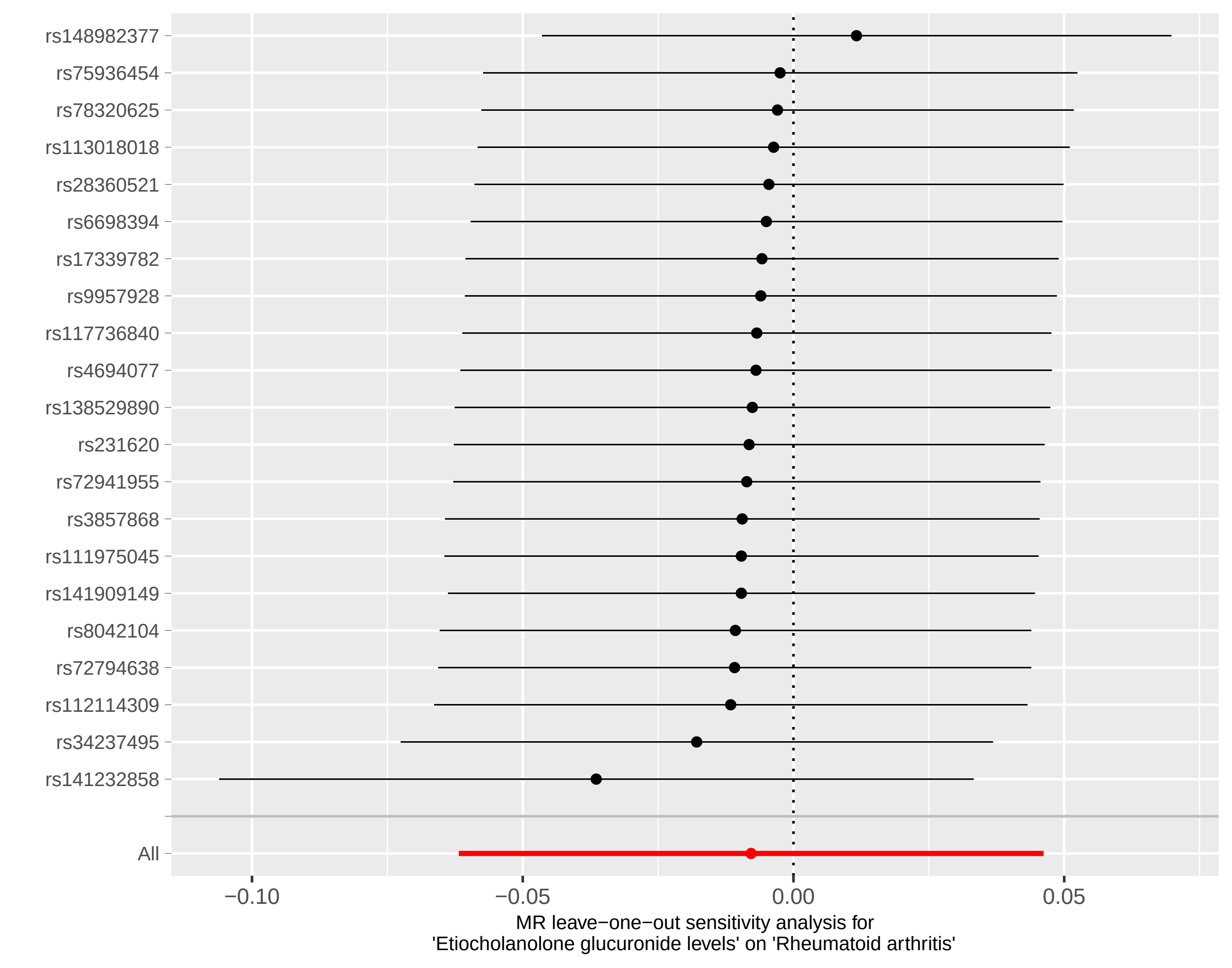

rs148982377
rs75936454
rs78320625
rs113018018
rs28360521
rs6698394
rs17339782
rs9957928
rs117736840
rs4694077
rs138529890
rs231620
rs72941955
rs3857868
rs111975045
rs141909149
rs8042104
rs72794638
rs112114309
rs34237495
rs141232858
All
−0.10
−0.05
0.00
0.05
MR leave−one−out sensitivity analysis for
'Etiocholanolone glucuronide levels' on 'Rheumatoid arthritis'

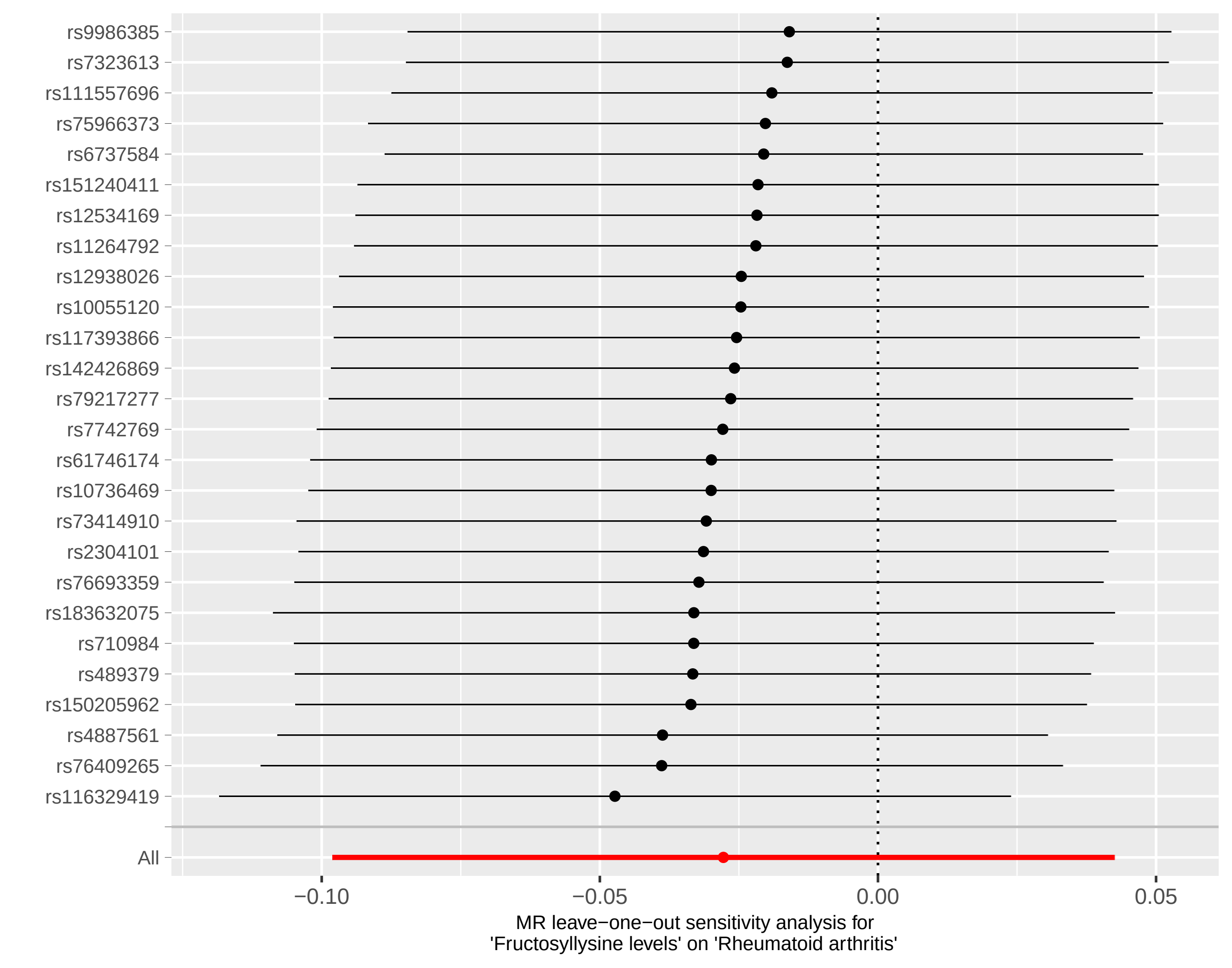
rs9986385
rs7323613
rs111557696
rs75966373
rs6737584
rs151240411
rs12534169
rs11264792
rs12938026
rs10055120
rs117393866
rs142426869
rs79217277
rs7742769
rs61746174
rs10736469
rs73414910
rs2304101
rs76693359
rs183632075
rs710984
rs489379
rs150205962
rs4887561
rs76409265
rs116329419
All
−0.10
−0.05
0.00
0.05
MR leave−one−out sensitivity analysis for
'Fructosyllysine levels' on 'Rheumatoid arthritis'

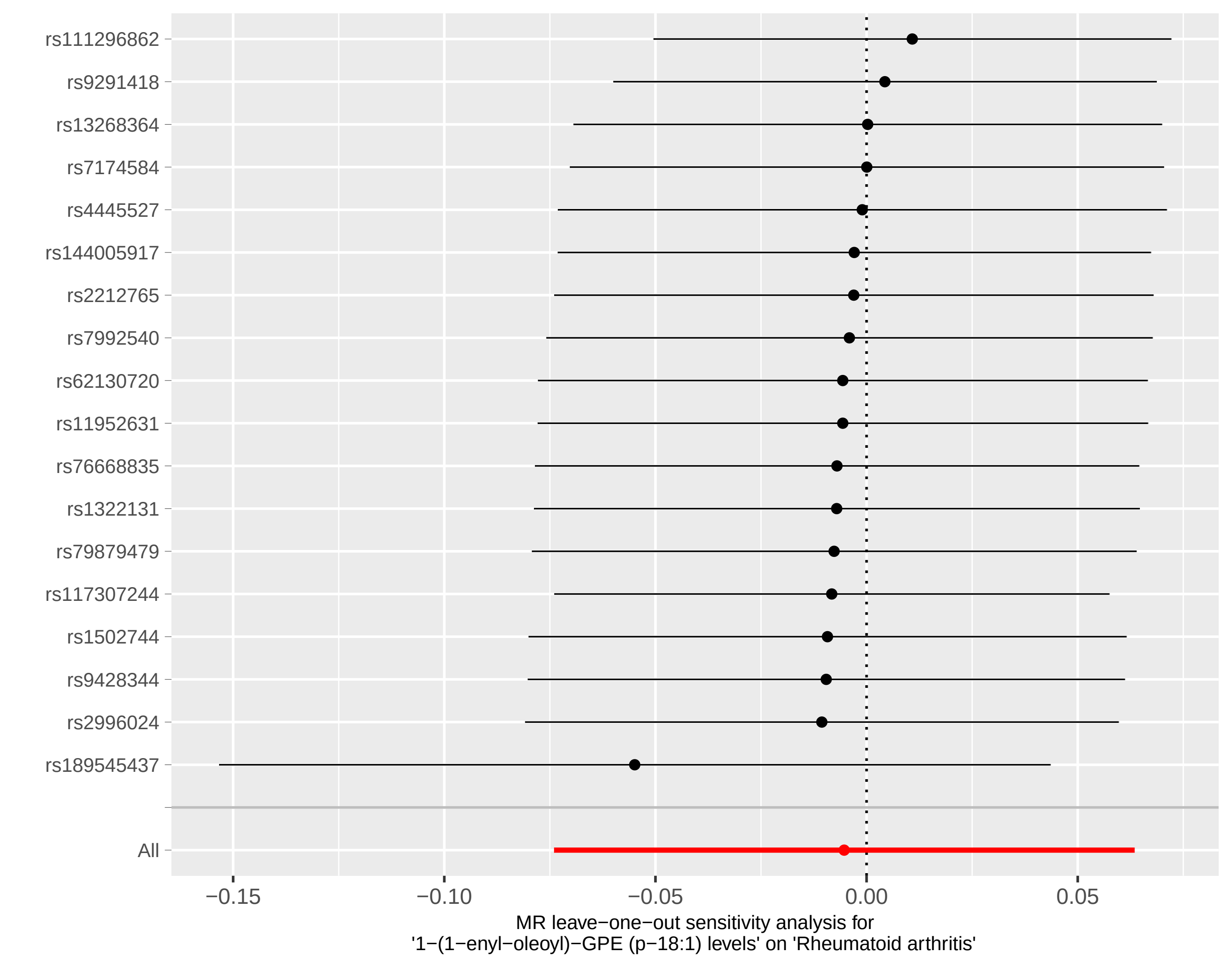
rs111296862
rs9291418
rs13268364
rs7174584
rs4445527
rs144005917
rs2212765
rs7992540
rs62130720
rs11952631
rs76668835
rs1322131
rs79879479
rs117307244
rs1502744
rs9428344
rs2996024
rs189545437
All
−0.15
−0.10
−0.05
0.00
0.05
MR leave−one−out sensitivity analysis for
'1−(1−enyl−oleoyl)−GPE (p−18:1) levels' on 'Rheumatoid arthritis'

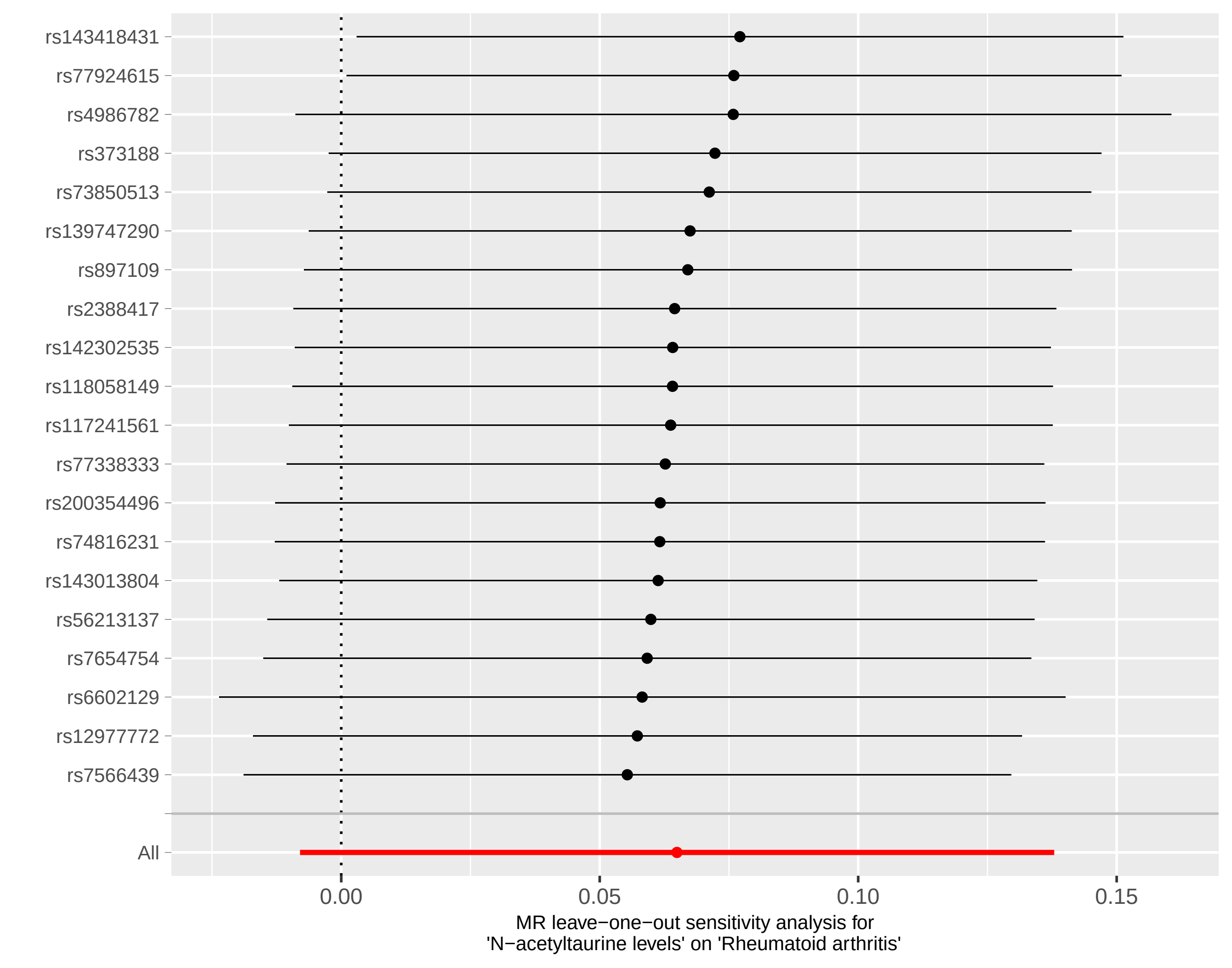
rs143418431
rs77924615
rs4986782
rs373188
rs73850513
rs139747290
rs897109
rs2388417
rs142302535
rs118058149
rs117241561
rs77338333
rs200354496
rs74816231
rs143013804
rs56213137
rs7654754
rs6602129
rs12977772
rs7566439
All
0.00
0.05
0.10
0.15
MR leave−one−out sensitivity analysis for
'N−acetyltaurine levels' on 'Rheumatoid arthritis'

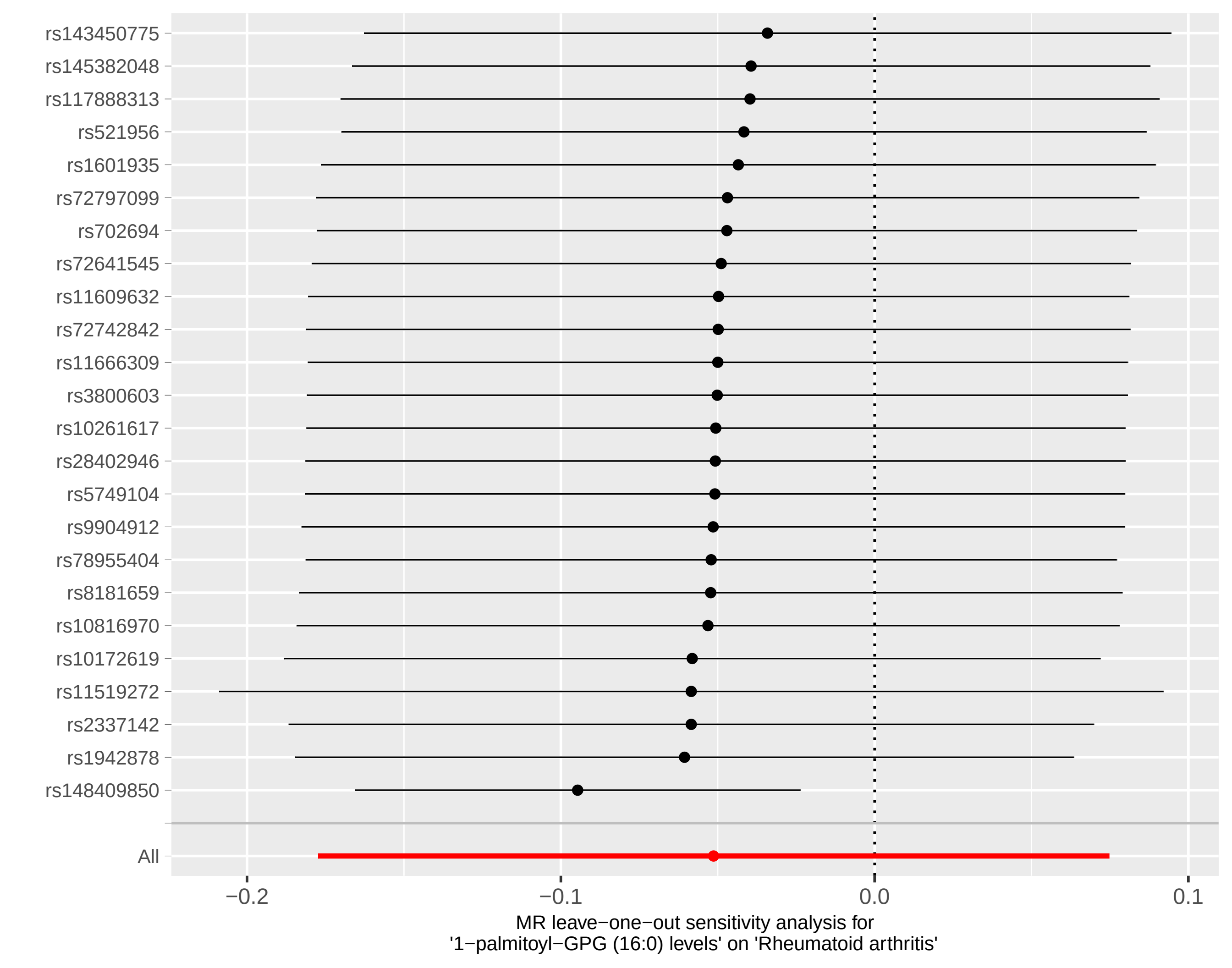

rs143450775
rs145382048
rs117888313
rs521956
rs1601935
rs72797099
rs702694
rs72641545
rs11609632
rs72742842
rs11666309
rs3800603
rs10261617
rs28402946
rs5749104
rs9904912
rs78955404
rs8181659
rs10816970
rs10172619
rs11519272
rs2337142
rs1942878
rs148409850
All
−0.2
−0.1
0.0
0.1
MR leave−one−out sensitivity analysis for
'1−palmitoyl−GPG (16:0) levels' on 'Rheumatoid arthritis'

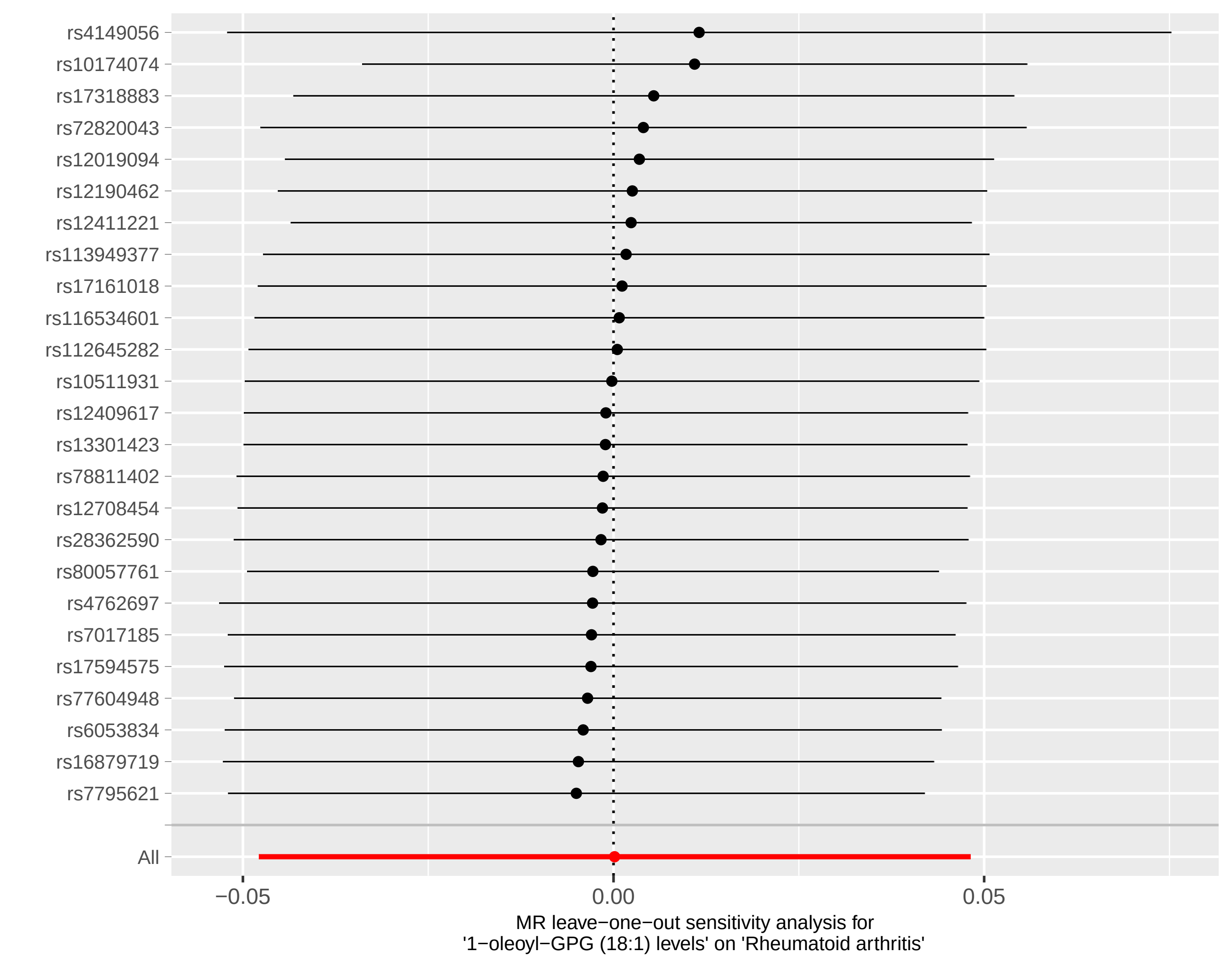

rs4149056
rs10174074
rs17318883
rs72820043
rs12019094
rs12190462
rs12411221
rs113949377
rs17161018
rs116534601
rs112645282
rs10511931
rs12409617
rs13301423
rs78811402
rs12708454
rs28362590
rs80057761
rs4762697
rs7017185
rs17594575
rs77604948
rs6053834
rs16879719
rs7795621
All
−0.05
0.00
0.05
MR leave−one−out sensitivity analysis for
'1−oleoyl−GPG (18:1) levels' on 'Rheumatoid arthritis'

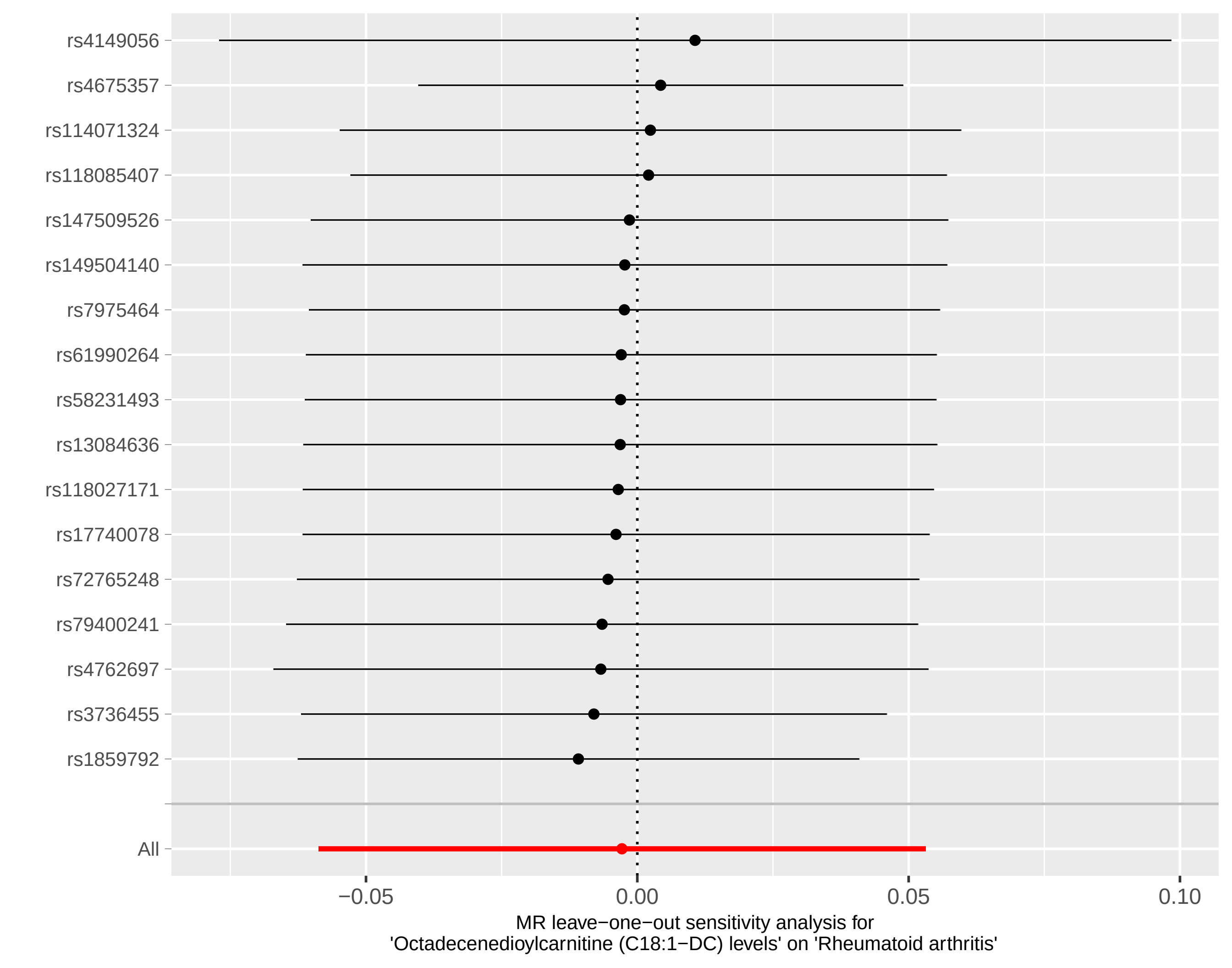
rs4149056
rs4675357
rs114071324
rs118085407
rs147509526
rs149504140
rs7975464
rs61990264
rs58231493
rs13084636
rs118027171
rs17740078
rs72765248
rs79400241
rs4762697
rs3736455
rs1859792
All
−0.05
0.00
0.05
0.10
MR leave−one−out sensitivity analysis for
'Octadecenedioylcarnitine (C18:1−DC) levels' on 'Rheumatoid arthritis'

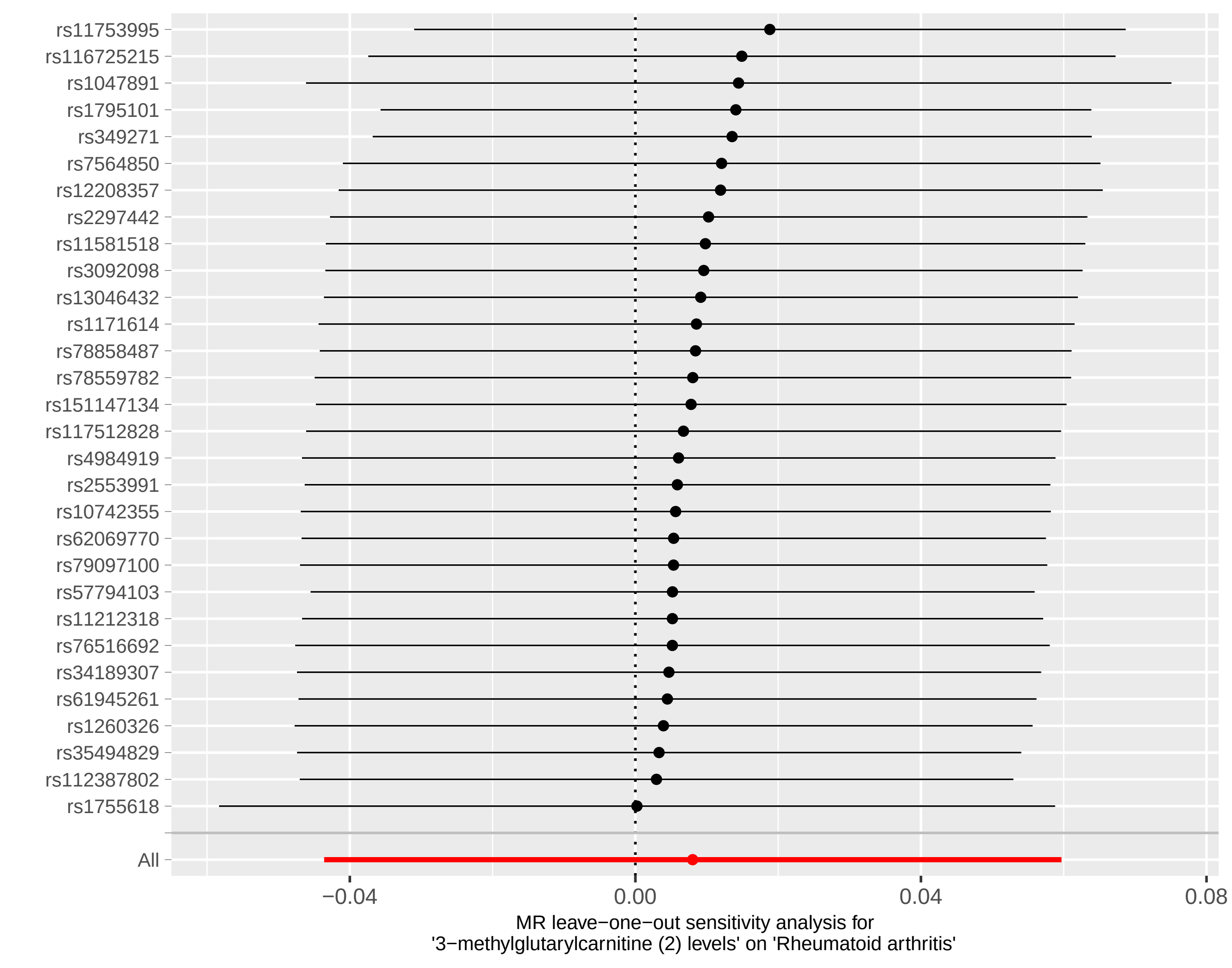
rs11753995
rs116725215
rs1047891
rs1795101
rs349271
rs7564850
rs12208357
rs2297442
rs11581518
rs3092098
rs13046432
rs1171614
rs78858487
rs78559782
rs151147134
rs117512828
rs4984919
rs2553991
rs10742355
rs62069770
rs79097100
rs57794103
rs11212318
rs76516692
rs34189307
rs61945261
rs1260326
rs35494829
rs112387802
rs1755618
All
−0.04
0.00
0.04
0.08
MR leave−one−out sensitivity analysis for
'3−methylglutarylcarnitine (2) levels' on 'Rheumatoid arthritis'

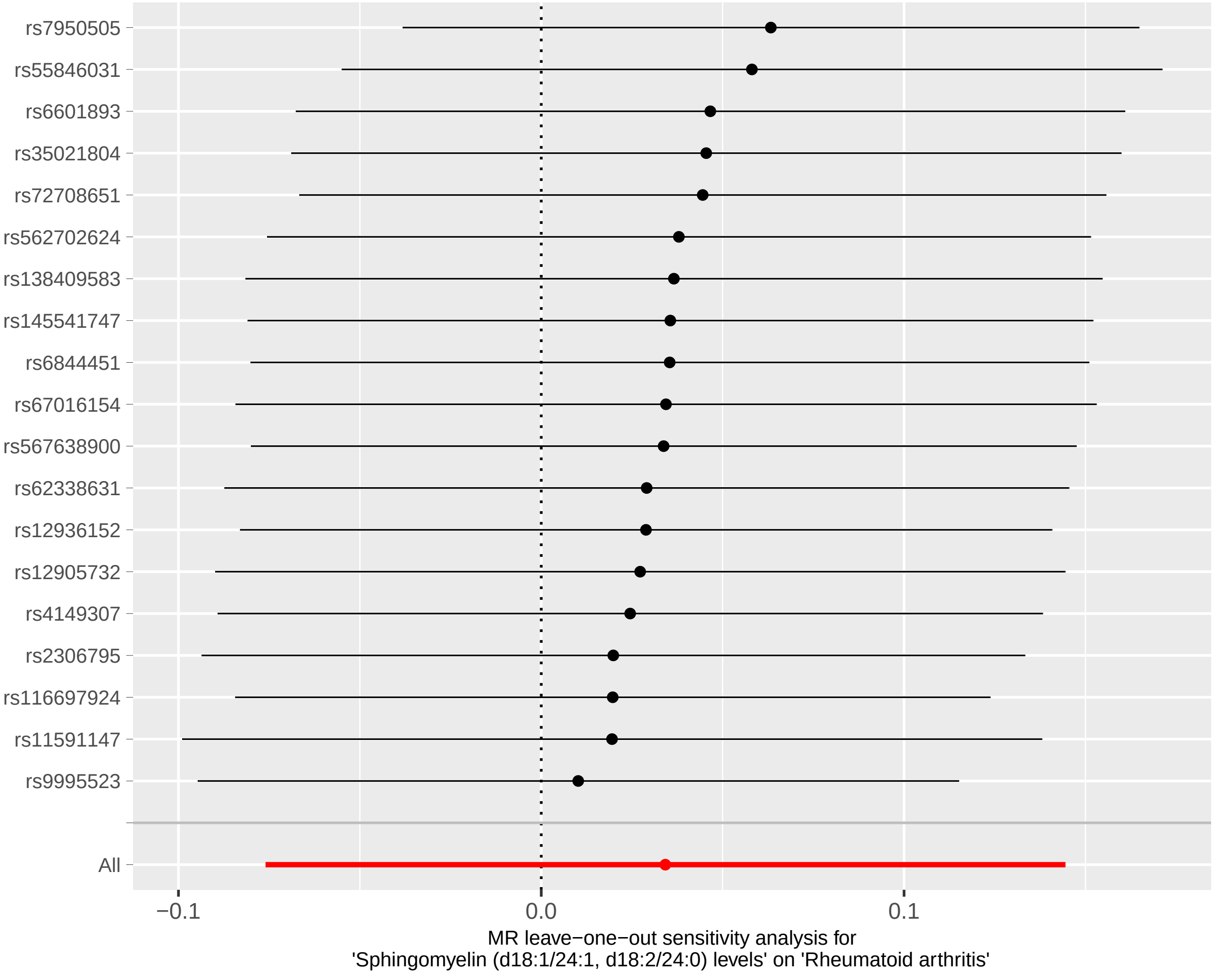
rs7950505
rs55846031
rs6601893
rs35021804
rs72708651
rs562702624
rs138409583
rs145541747
rs6844451
rs67016154
rs567638900
rs62338631
rs12936152
rs12905732
rs4149307
rs2306795
rs116697924
rs11591147
rs9995523
All
−0.1
0.0
0.1
MR leave−one−out sensitivity analysis for
'Sphingomyelin (d18:1/24:1, d18:2/24:0) levels' on 'Rheumatoid arthritis'

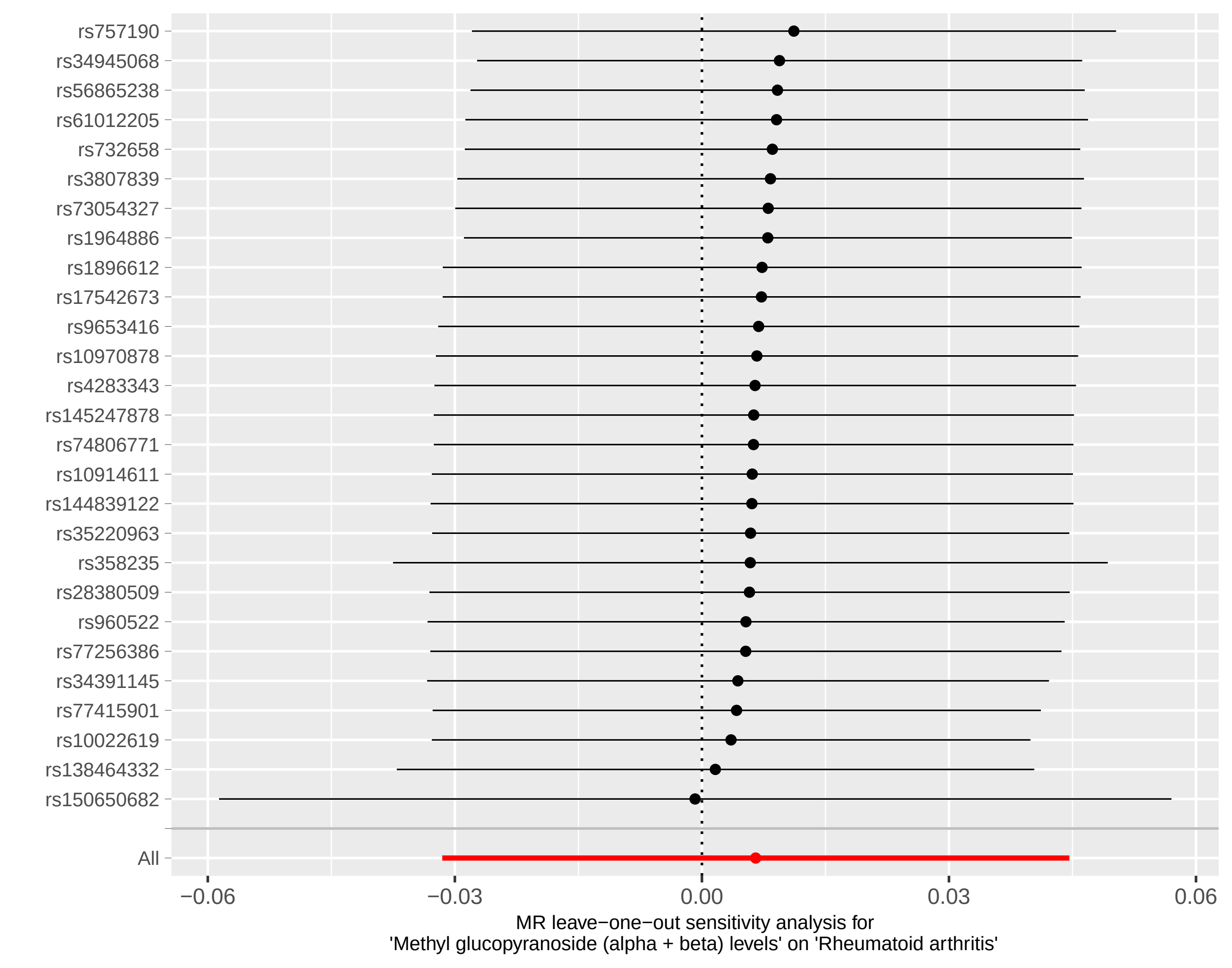

rs757190
rs34945068
rs56865238
rs61012205
rs732658
rs3807839
rs73054327
rs1964886
rs1896612
rs17542673
rs9653416
rs10970878
rs4283343
rs145247878
rs74806771
rs10914611
rs144839122
rs35220963
rs358235
rs28380509
rs960522
rs77256386
rs34391145
rs77415901
rs10022619
rs138464332
rs150650682
All
−0.06
−0.03
0.00
0.03
0.06
MR leave−one−out sensitivity analysis for
'Methyl glucopyranoside (alpha + beta) levels' on 'Rheumatoid arthritis'

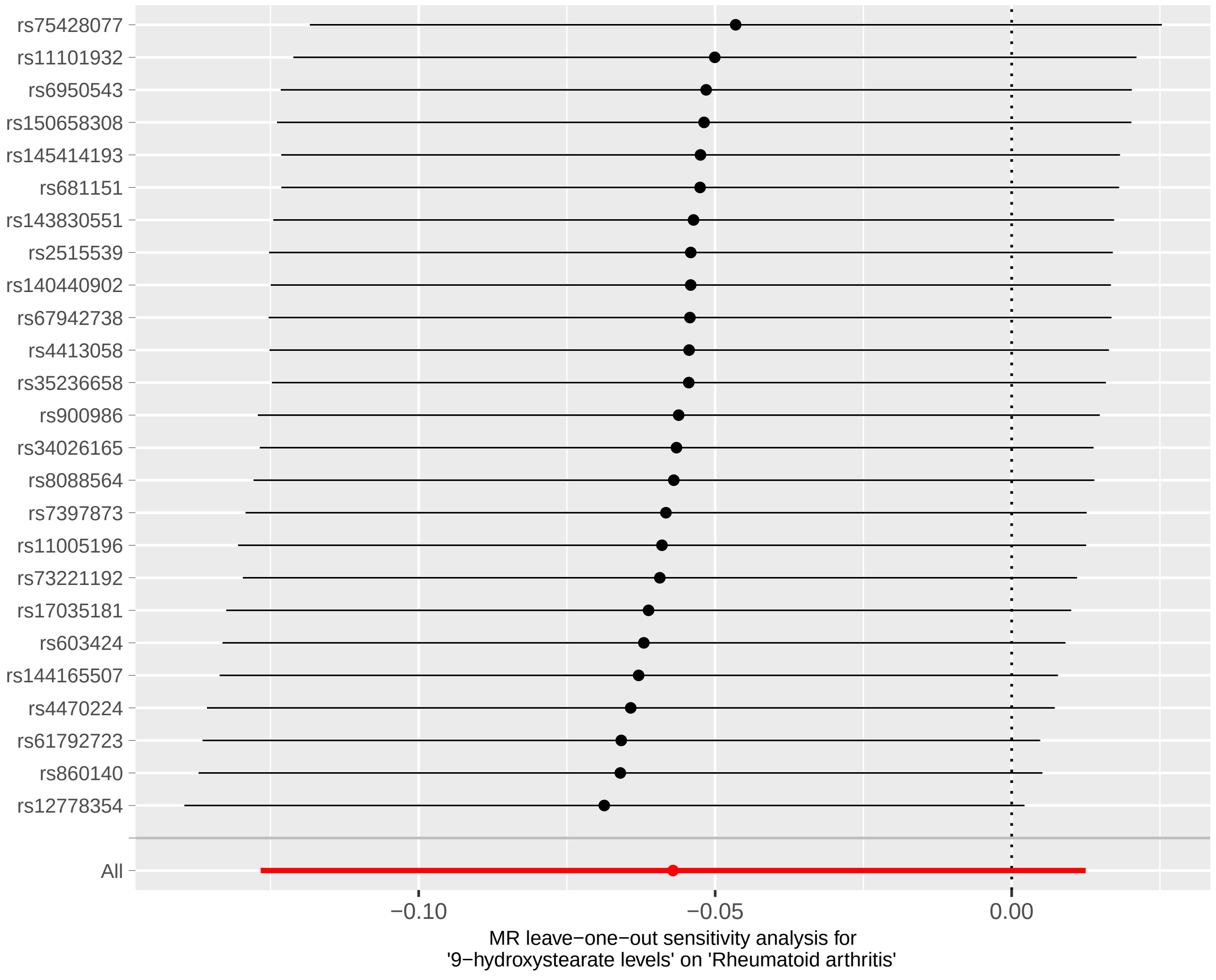
rs75428077
rs11101932
rs6950543
rs150658308
rs145414193
rs681151
rs143830551
rs2515539
rs140440902
rs67942738
rs4413058
rs35236658
rs900986
rs34026165
rs8088564
rs7397873
rs11005196
rs73221192
rs17035181
rs603424
rs144165507
rs4470224
rs61792723
rs860140
rs12778354
All
−0.10
−0.05
0.00
MR leave−one−out sensitivity analysis for
'9−hydroxystearate levels' on 'Rheumatoid arthritis'

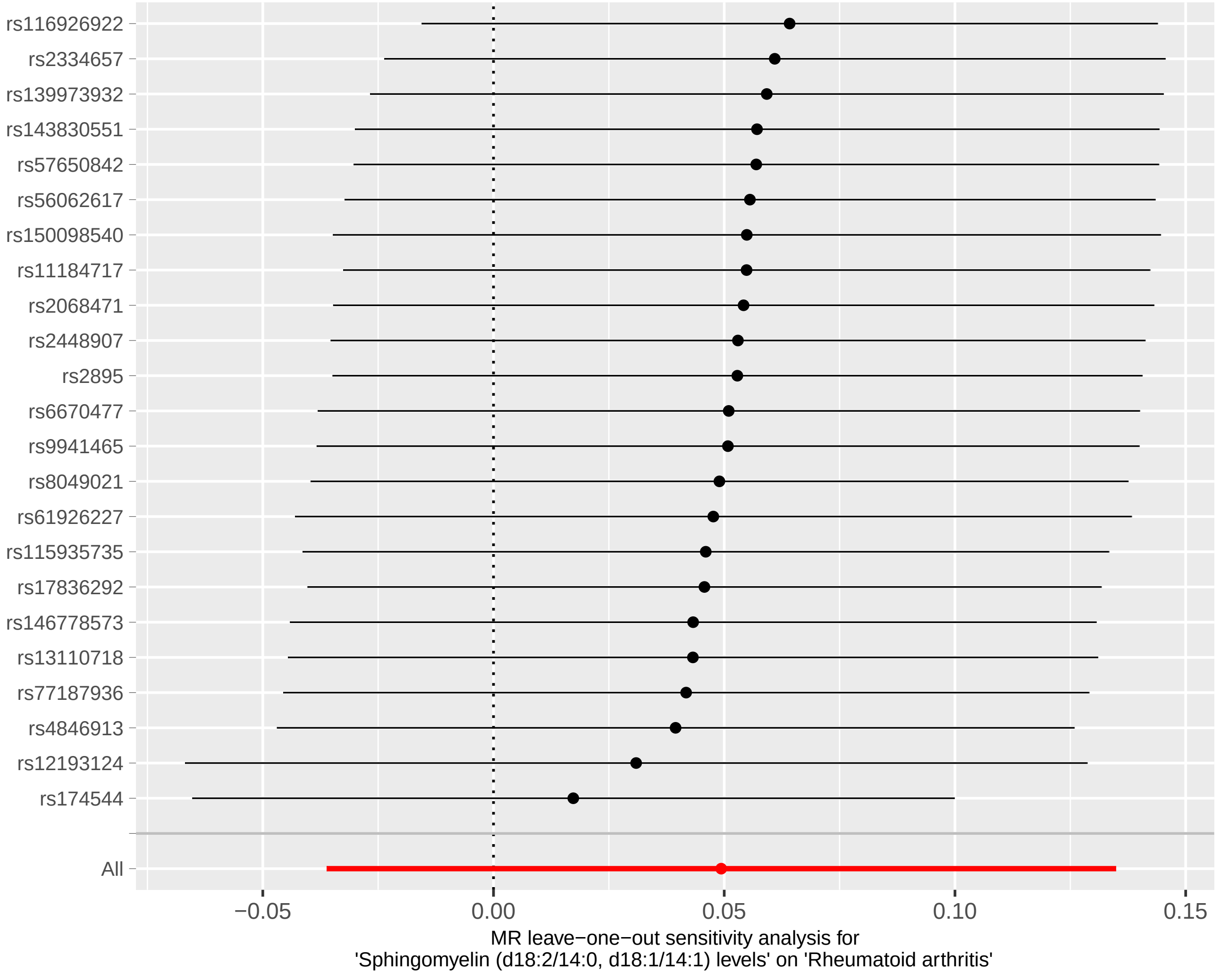
rs116926922
rs2334657
rs139973932
rs143830551
rs57650842
rs56062617
rs150098540
rs11184717
rs2068471
rs2448907
rs2895
rs6670477
rs9941465
rs8049021
rs61926227
rs115935735
rs17836292
rs146778573
rs13110718
rs77187936
rs4846913
rs12193124
rs174544
All
−0.05
0.00
0.05
0.10
0.15
MR leave−one−out sensitivity analysis for
'Sphingomyelin (d18:2/14:0, d18:1/14:1) levels' on 'Rheumatoid arthritis'

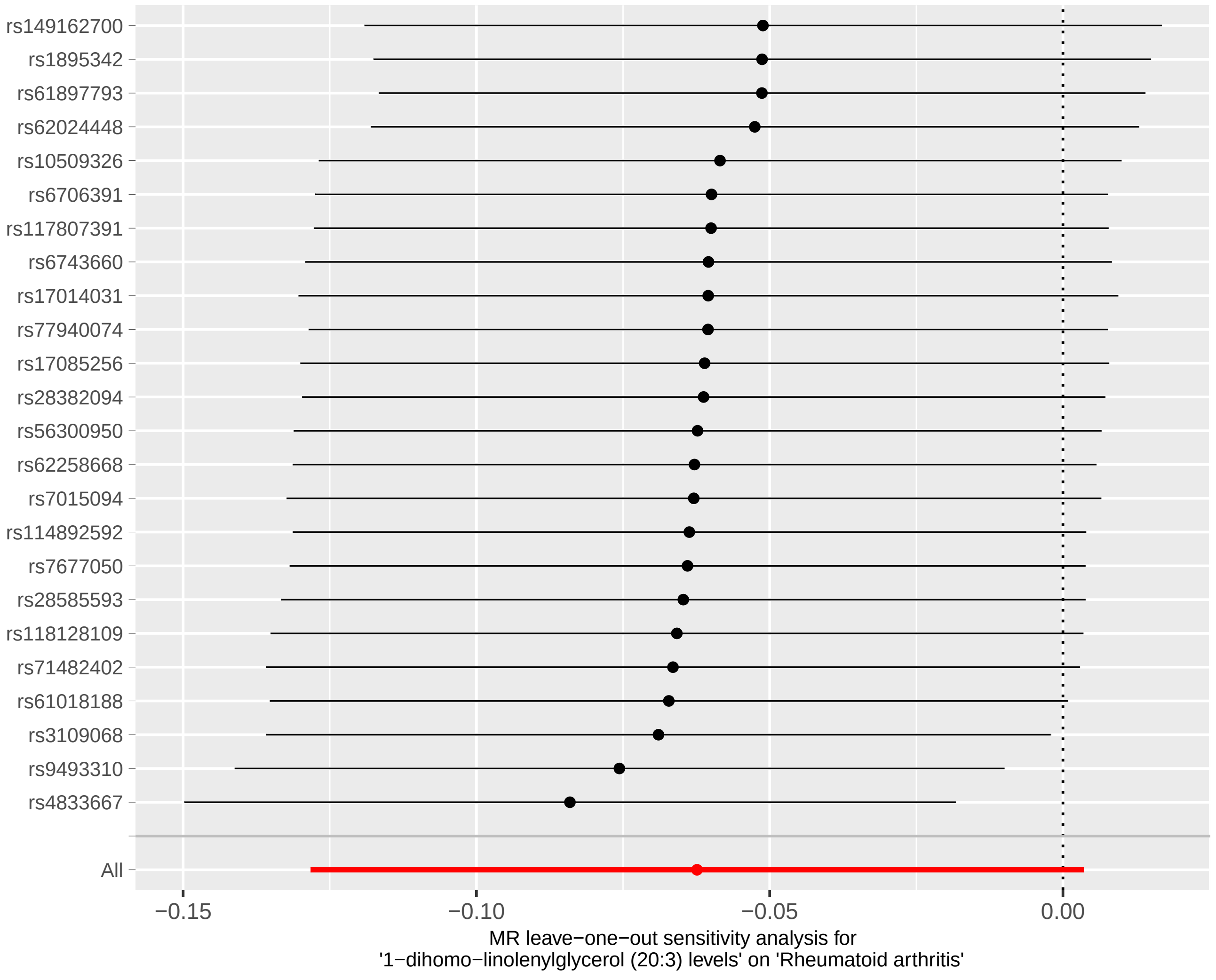
rs149162700
rs1895342
rs61897793
rs62024448
rs10509326
rs6706391
rs117807391
rs6743660
rs17014031
rs77940074
rs17085256
rs28382094
rs56300950
rs62258668
rs7015094
rs114892592
rs7677050
rs28585593
rs118128109
rs71482402
rs61018188
rs3109068
rs9493310
rs4833667
All
−0.15
−0.10
−0.05
0.00
MR leave−one−out sensitivity analysis for
'1−dihomo−linolenylglycerol (20:3) levels' on 'Rheumatoid arthritis'

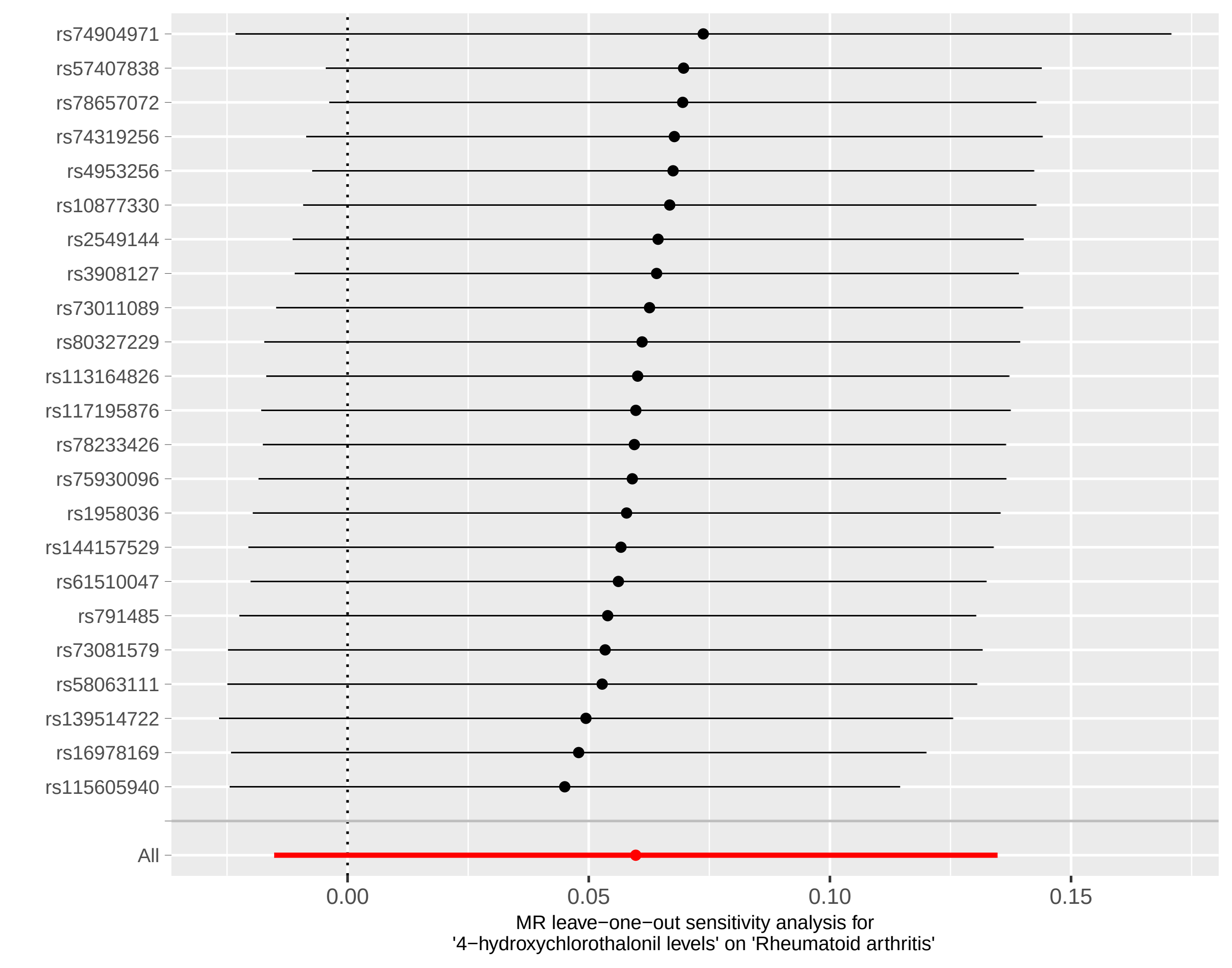
rs74904971
rs57407838
rs78657072
rs74319256
rs4953256
rs10877330
rs2549144
rs3908127
rs73011089
rs80327229
rs113164826
rs117195876
rs78233426
rs75930096
rs1958036
rs144157529
rs61510047
rs791485
rs73081579
rs58063111
rs139514722
rs16978169
rs115605940
All
0.00
0.05
0.10
0.15
MR leave−one−out sensitivity analysis for
'4−hydroxychlorothalonil levels' on 'Rheumatoid arthritis'

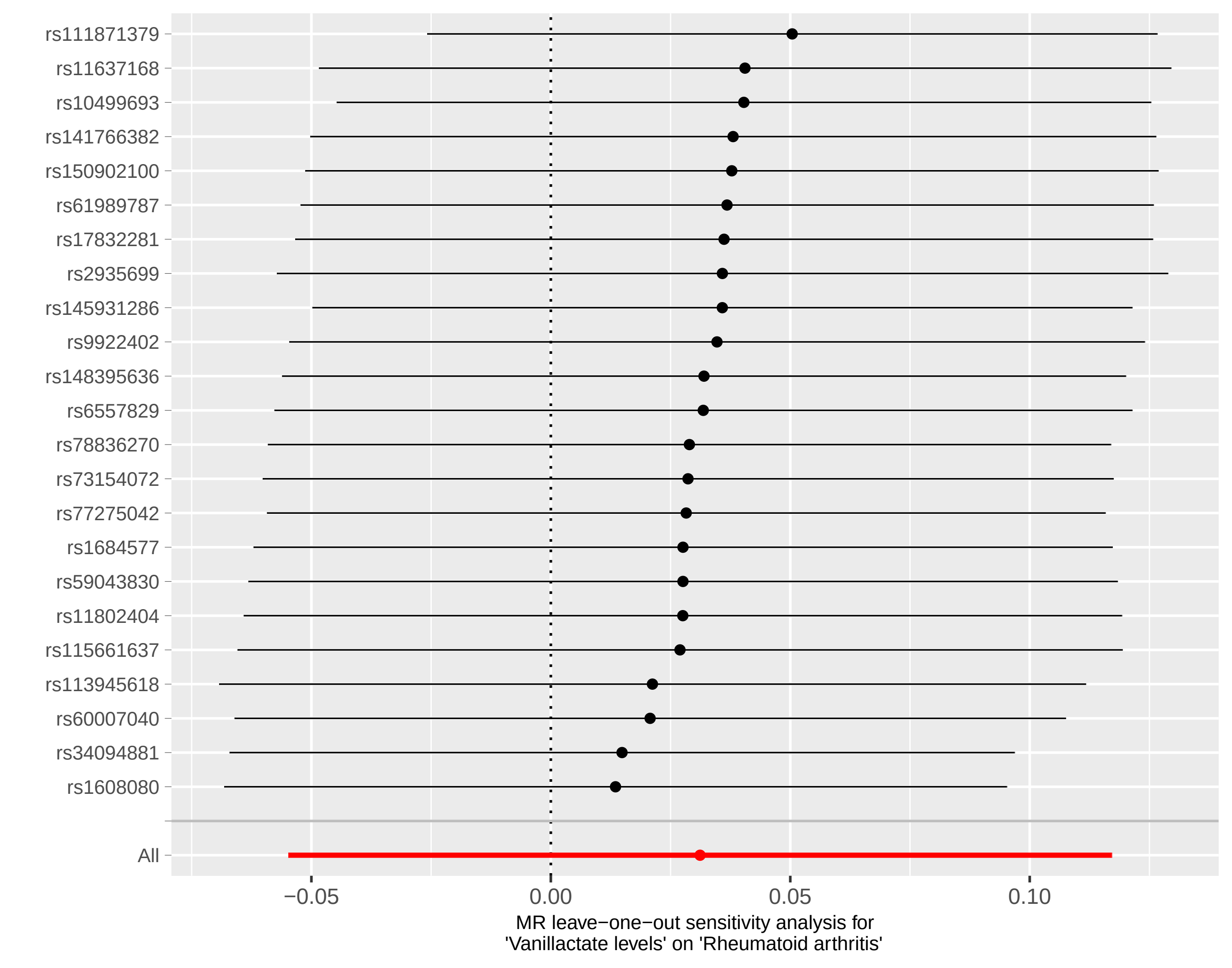
rs111871379
rs11637168
rs10499693
rs141766382
rs150902100
rs61989787
rs17832281
rs2935699
rs145931286
rs9922402
rs148395636
rs6557829
rs78836270
rs73154072
rs77275042
rs1684577
rs59043830
rs11802404
rs115661637
rs113945618
rs60007040
rs34094881
rs1608080
All
−0.05
0.00
0.05
0.10
MR leave−one−out sensitivity analysis for
'Vanillactate levels' on 'Rheumatoid arthritis'

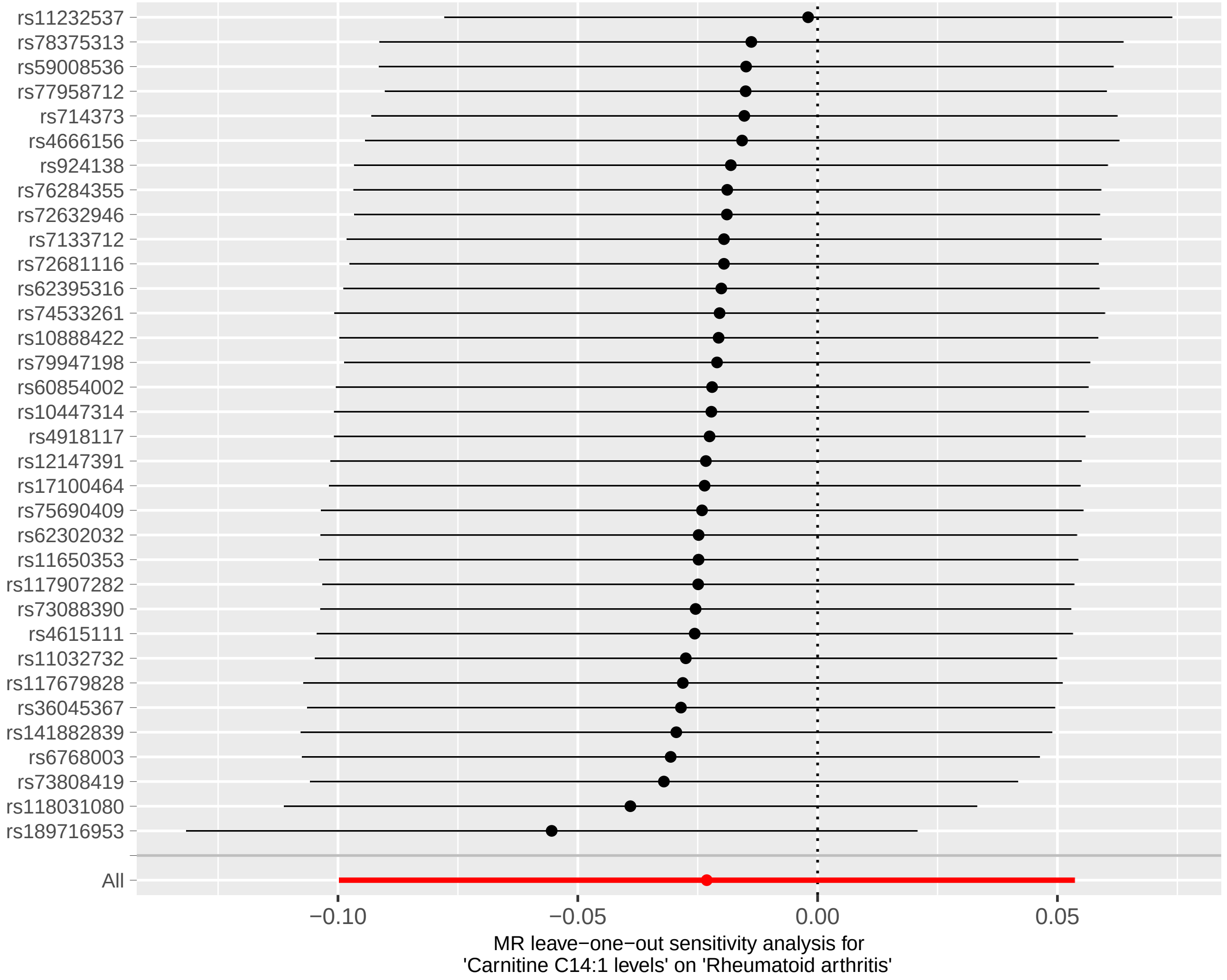
rs11232537
rs78375313
rs59008536
rs77958712
rs714373
rs4666156
rs924138
rs76284355
rs72632946
rs7133712
rs72681116
rs62395316
rs74533261
rs10888422
rs79947198
rs60854002
rs10447314
rs4918117
rs12147391
rs17100464
rs75690409
rs62302032
rs11650353
rs117907282
rs73088390
rs4615111
rs11032732
rs117679828
rs36045367
rs141882839
rs6768003
rs73808419
rs118031080
rs189716953
All
−0.10
−0.05
0.00
0.05
MR leave−one−out sensitivity analysis for
'Carnitine C14:1 levels' on 'Rheumatoid arthritis'

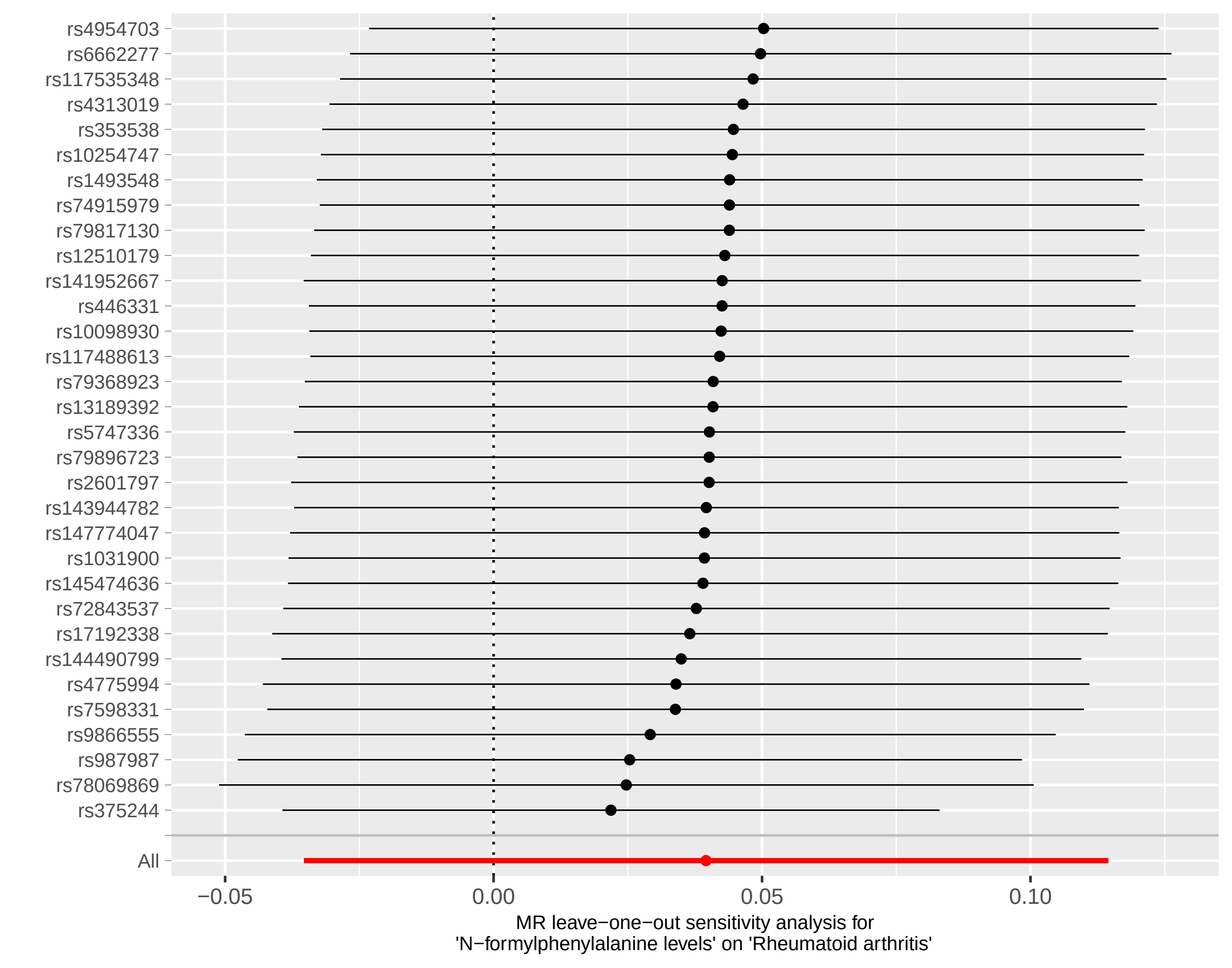

rs4954703
rs6662277
rs117535348
rs4313019
rs353538
rs10254747
rs1493548
rs74915979
rs79817130
rs12510179
rs141952667
rs446331
rs10098930
rs117488613
rs79368923
rs13189392
rs5747336
rs79896723
rs2601797
rs143944782
rs147774047
rs1031900
rs145474636
rs72843537
rs17192338
rs144490799
rs4775994
rs7598331
rs9866555
rs987987
rs78069869
rs375244
All
−0.05
0.00
0.05
0.10
MR leave−one−out sensitivity analysis for
'N−formylphenylalanine levels' on 'Rheumatoid arthritis'

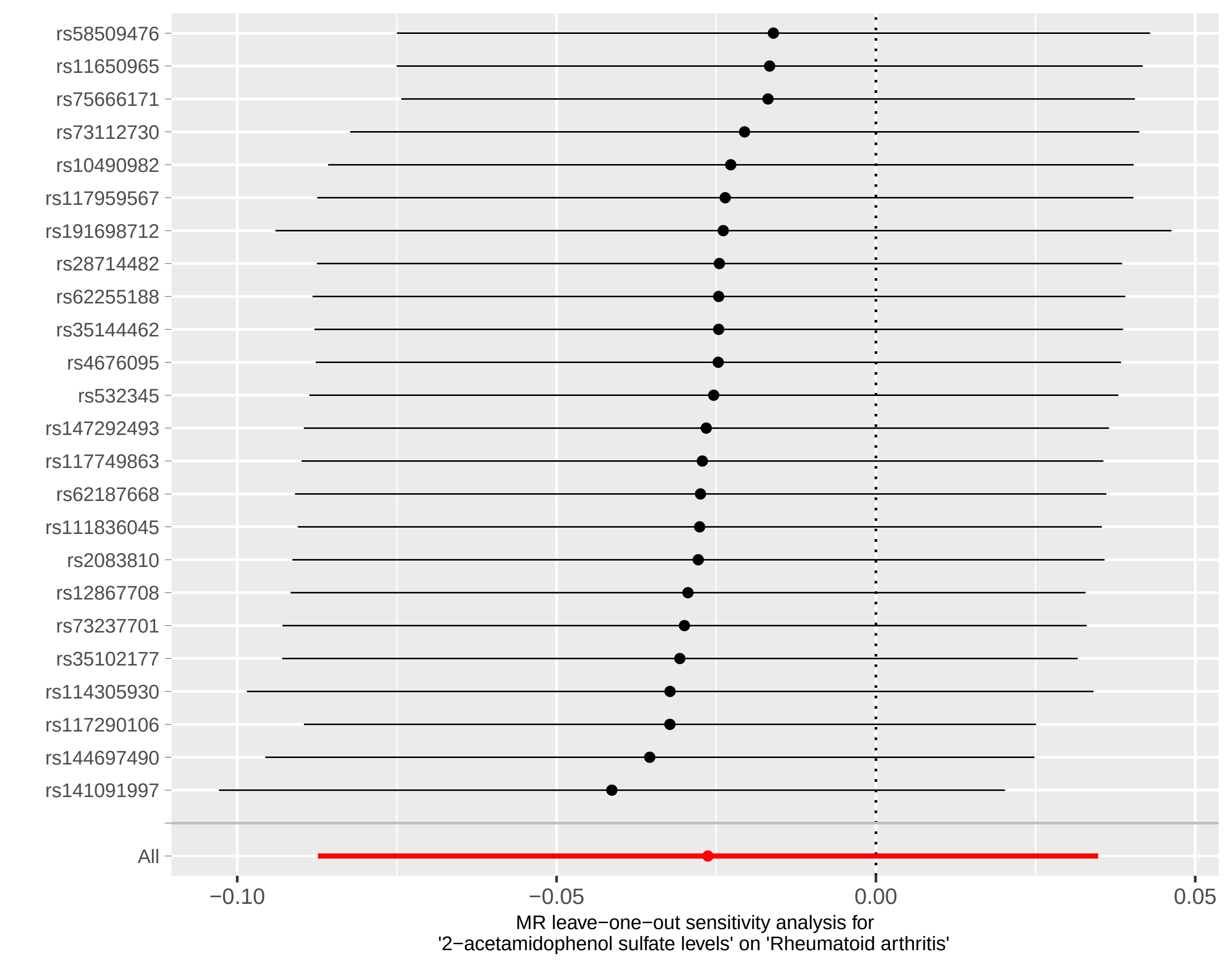
rs58509476
rs11650965
rs75666171
rs73112730
rs10490982
rs117959567
rs191698712
rs28714482
rs62255188
rs35144462
rs4676095
rs532345
rs147292493
rs117749863
rs62187668
rs111836045
rs2083810
rs12867708
rs73237701
rs35102177
rs114305930
rs117290106
rs144697490
rs141091997
All
−0.10
−0.05
0.00
0.05
MR leave−one−out sensitivity analysis for
'2−acetamidophenol sulfate levels' on 'Rheumatoid arthritis'

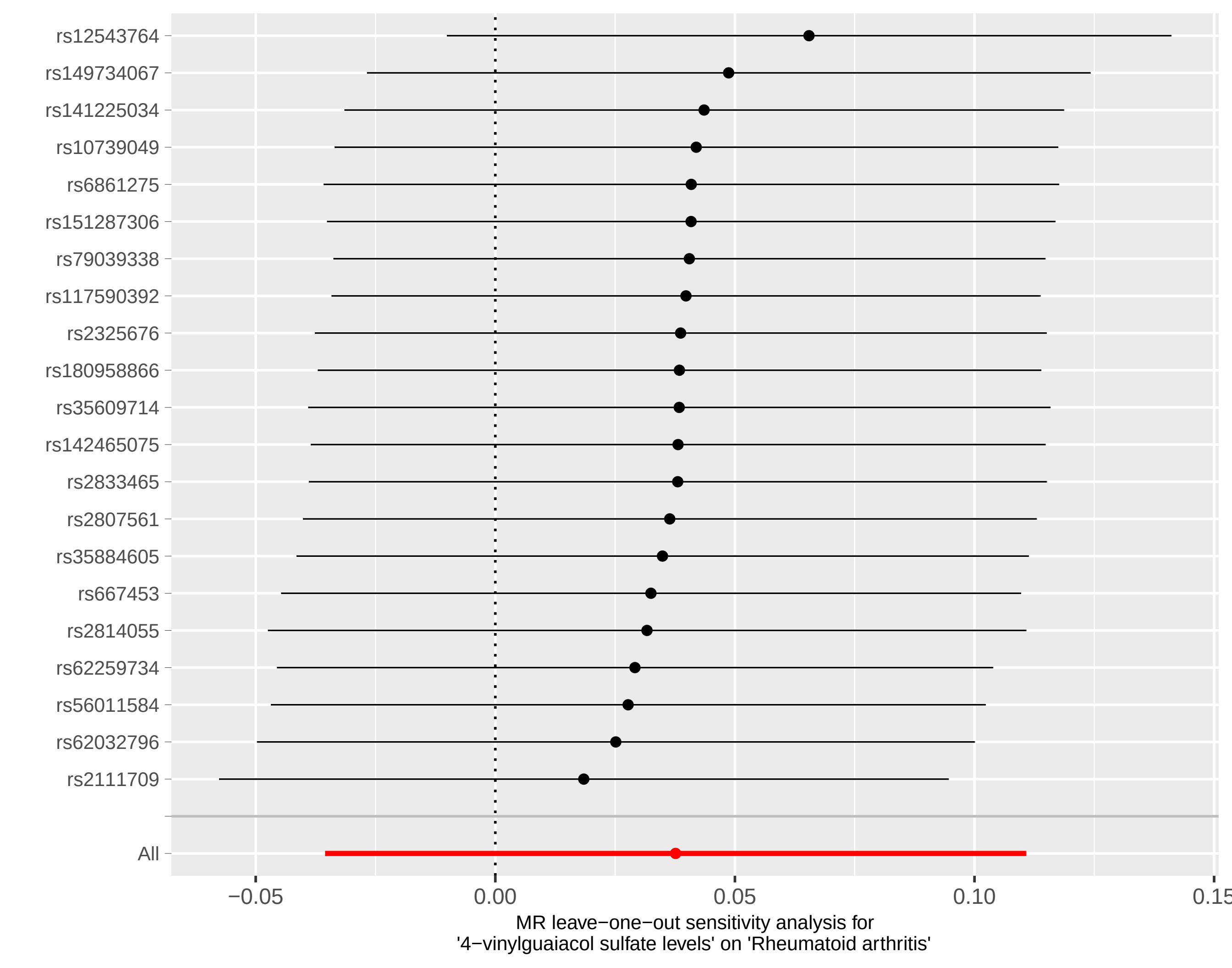

rs12543764
rs149734067
rs141225034
rs10739049
rs6861275
rs151287306
rs79039338
rs117590392
rs2325676
rs180958866
rs35609714
rs142465075
rs2833465
rs2807561
rs35884605
rs667453
rs2814055
rs62259734
rs56011584
rs62032796
rs2111709
All
−0.05
0.00
0.05
0.10
0.15
MR leave−one−out sensitivity analysis for
'4−vinylguaiacol sulfate levels' on 'Rheumatoid arthritis'

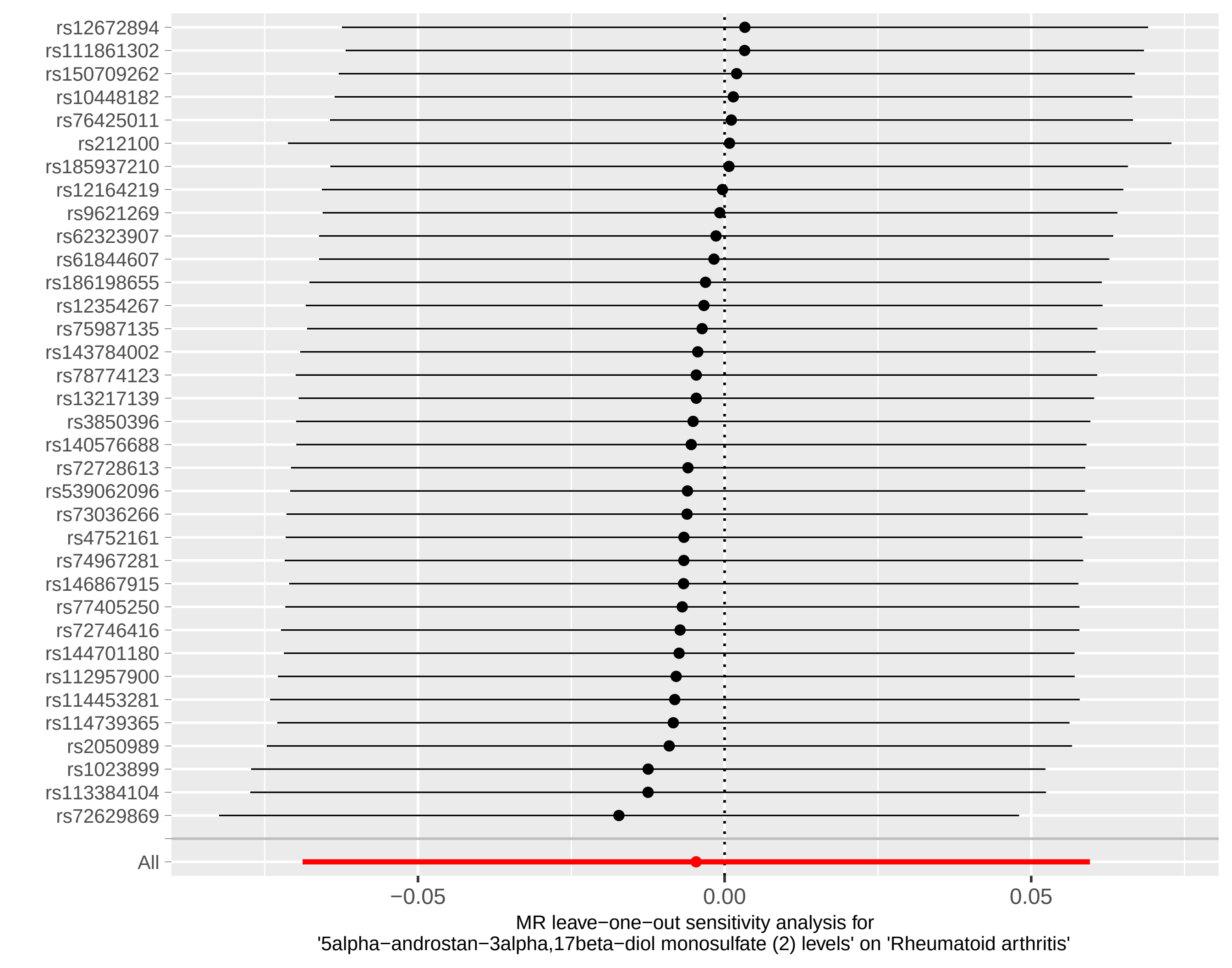

rs12672894
rs111861302
rs150709262
rs10448182
rs76425011
rs212100
rs185937210
rs12164219
rs9621269
rs62323907
rs61844607
rs186198655
rs12354267
rs75987135
rs143784002
rs78774123
rs13217139
rs3850396
rs140576688
rs72728613
rs539062096
rs73036266
rs4752161
rs74967281
rs146867915
rs77405250
rs72746416
rs144701180
rs112957900
rs114453281
rs114739365
rs2050989
rs1023899
rs113384104
rs72629869
All
−0.05
0.00
0.05
MR leave−one−out sensitivity analysis for
'5alpha−androstan−3alpha,17beta−diol monosulfate (2) levels' on 'Rheumatoid arthritis'

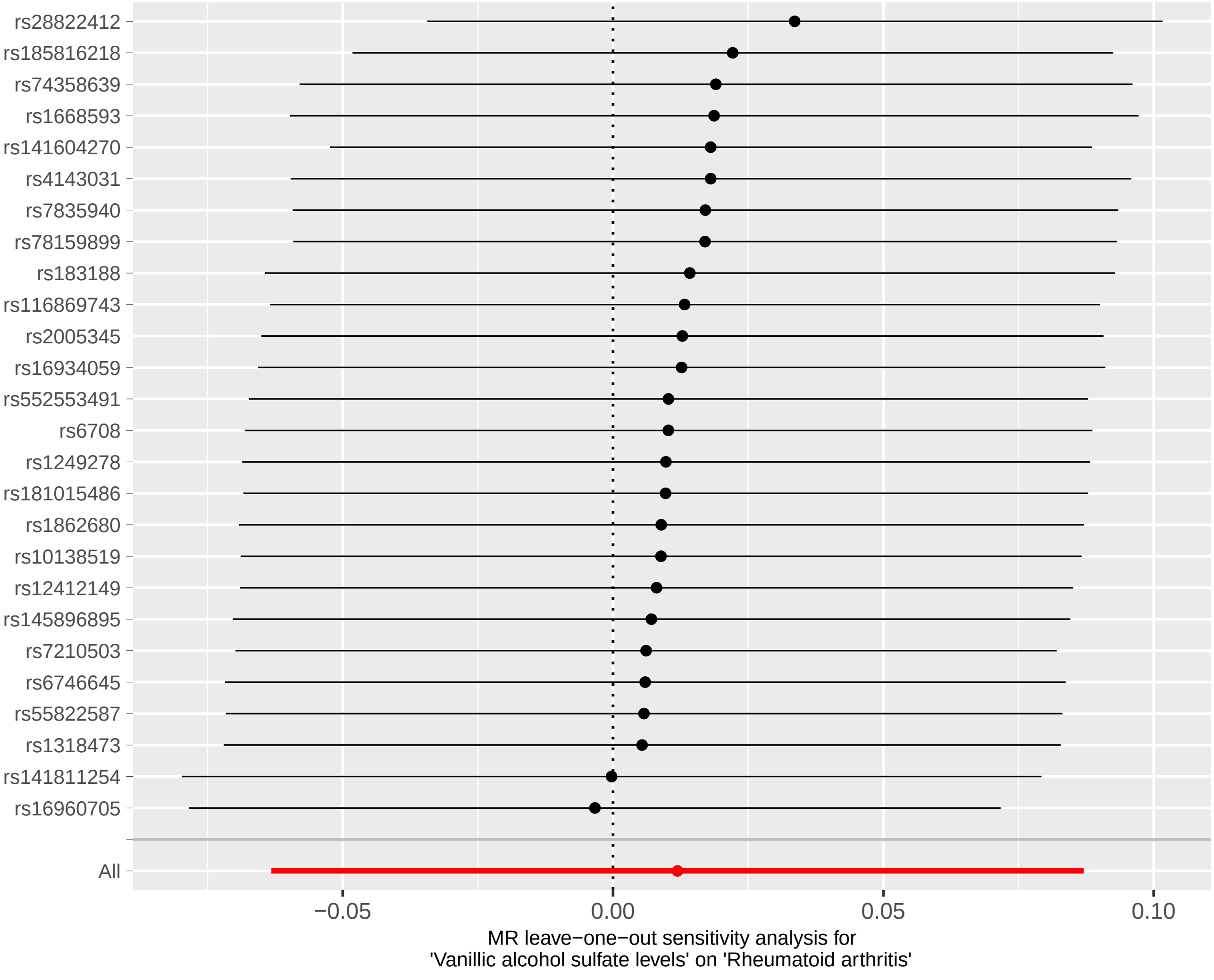
rs28822412
rs185816218
rs74358639
rs1668593
rs141604270
rs4143031
rs7835940
rs78159899
rs183188
rs116869743
rs2005345
rs16934059
rs552553491
rs6708
rs1249278
rs181015486
rs1862680
rs10138519
rs12412149
rs145896895
rs7210503
rs6746645
rs55822587
rs1318473
rs141811254
rs16960705
All
−0.05
0.00
0.05
0.10
MR leave−one−out sensitivity analysis for
'Vanillic alcohol sulfate levels' on 'Rheumatoid arthritis'

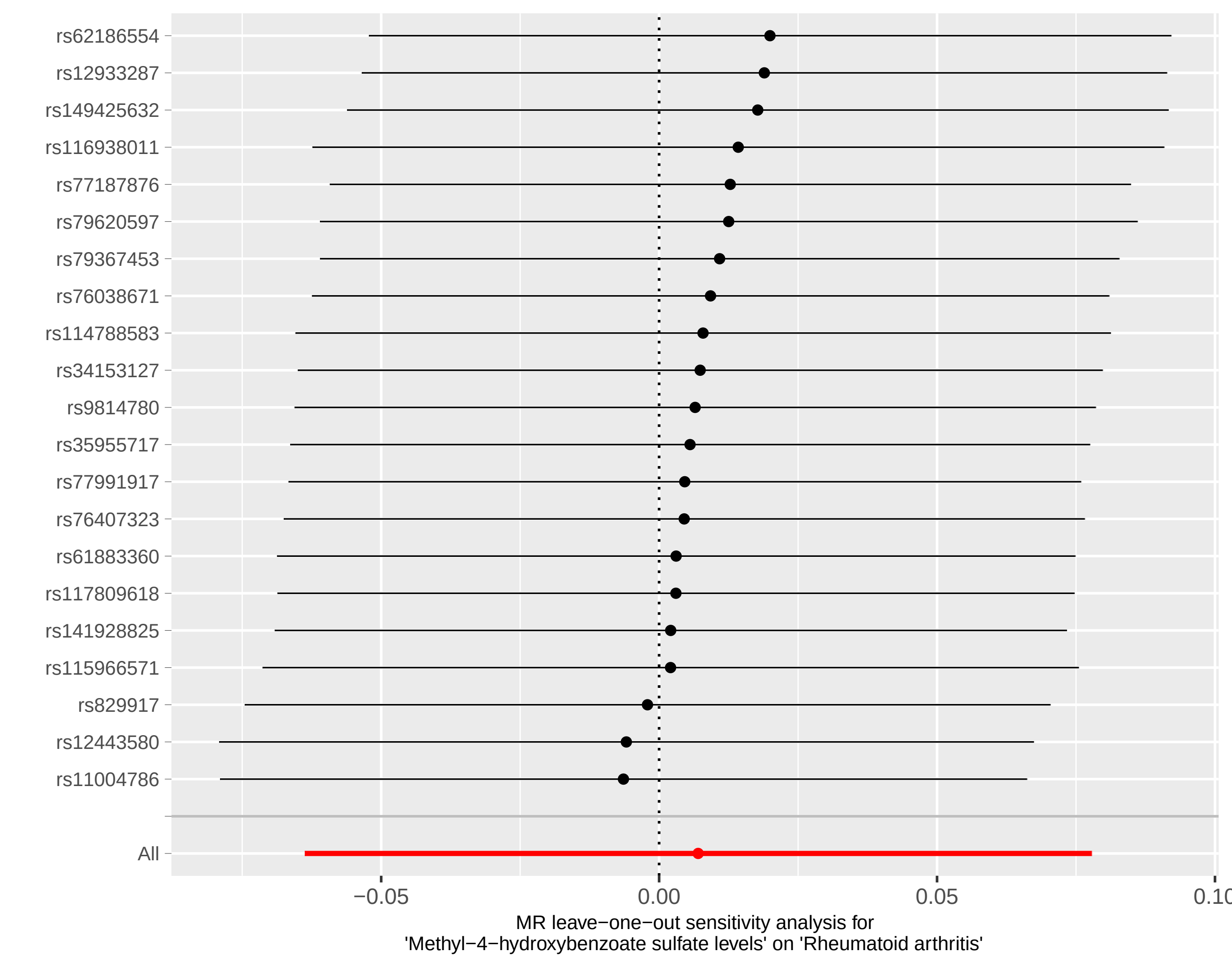
rs62186554
rs12933287
rs149425632
rs116938011
rs77187876
rs79620597
rs79367453
rs76038671
rs114788583
rs34153127
rs9814780
rs35955717
rs77991917
rs76407323
rs61883360
rs117809618
rs141928825
rs115966571
rs829917
rs12443580
rs11004786
All
−0.05
0.00
0.05
0.10
MR leave−one−out sensitivity analysis for
'Methyl−4−hydroxybenzoate sulfate levels' on 'Rheumatoid arthritis'

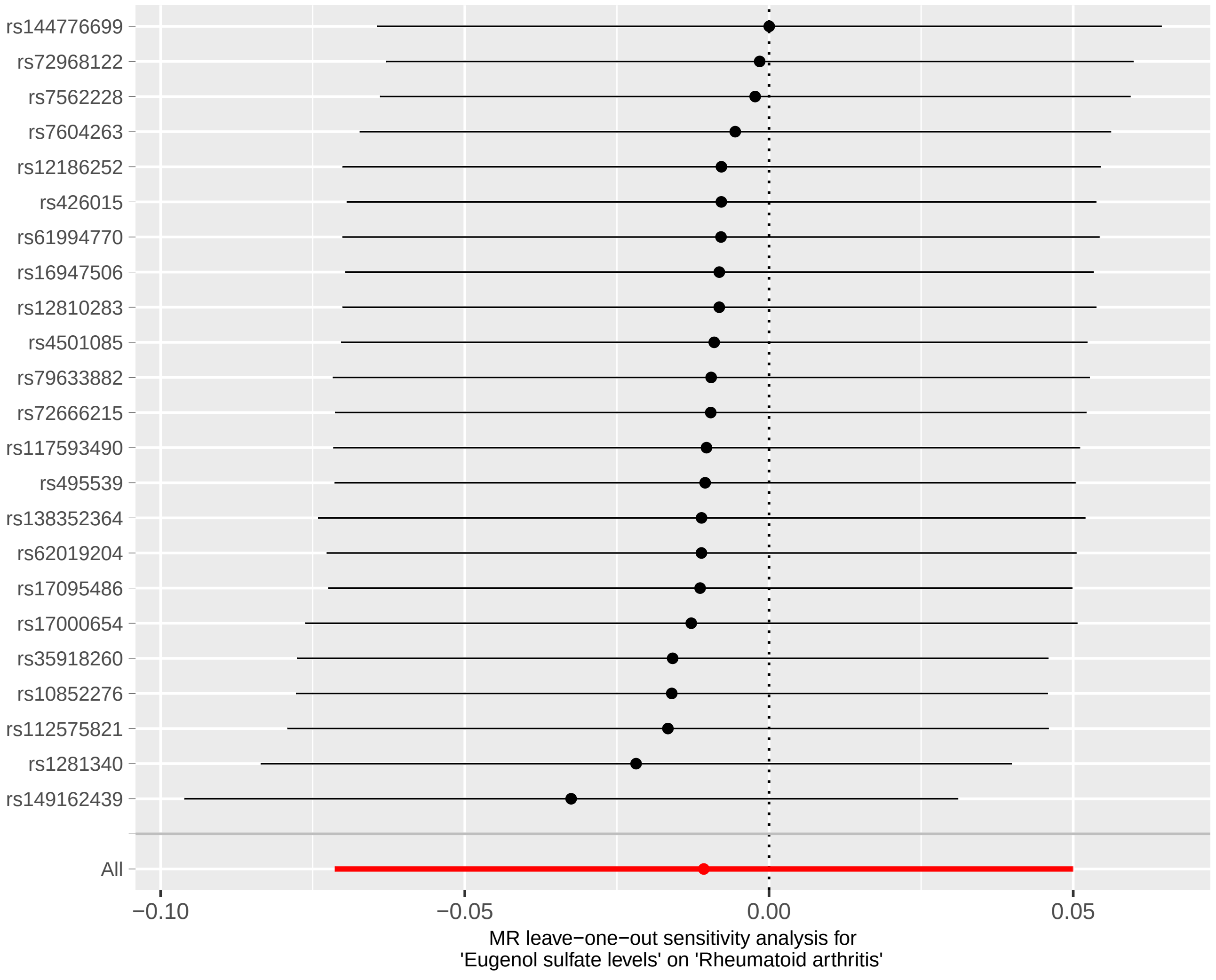
rs144776699
rs72968122
rs7562228
rs7604263
rs12186252
rs426015
rs61994770
rs16947506
rs12810283
rs4501085
rs79633882
rs72666215
rs117593490
rs495539
rs138352364
rs62019204
rs17095486
rs17000654
rs35918260
rs10852276
rs112575821
rs1281340
rs149162439
All
−0.10
−0.05
0.00
0.05
MR leave−one−out sensitivity analysis for
'Eugenol sulfate levels' on 'Rheumatoid arthritis'

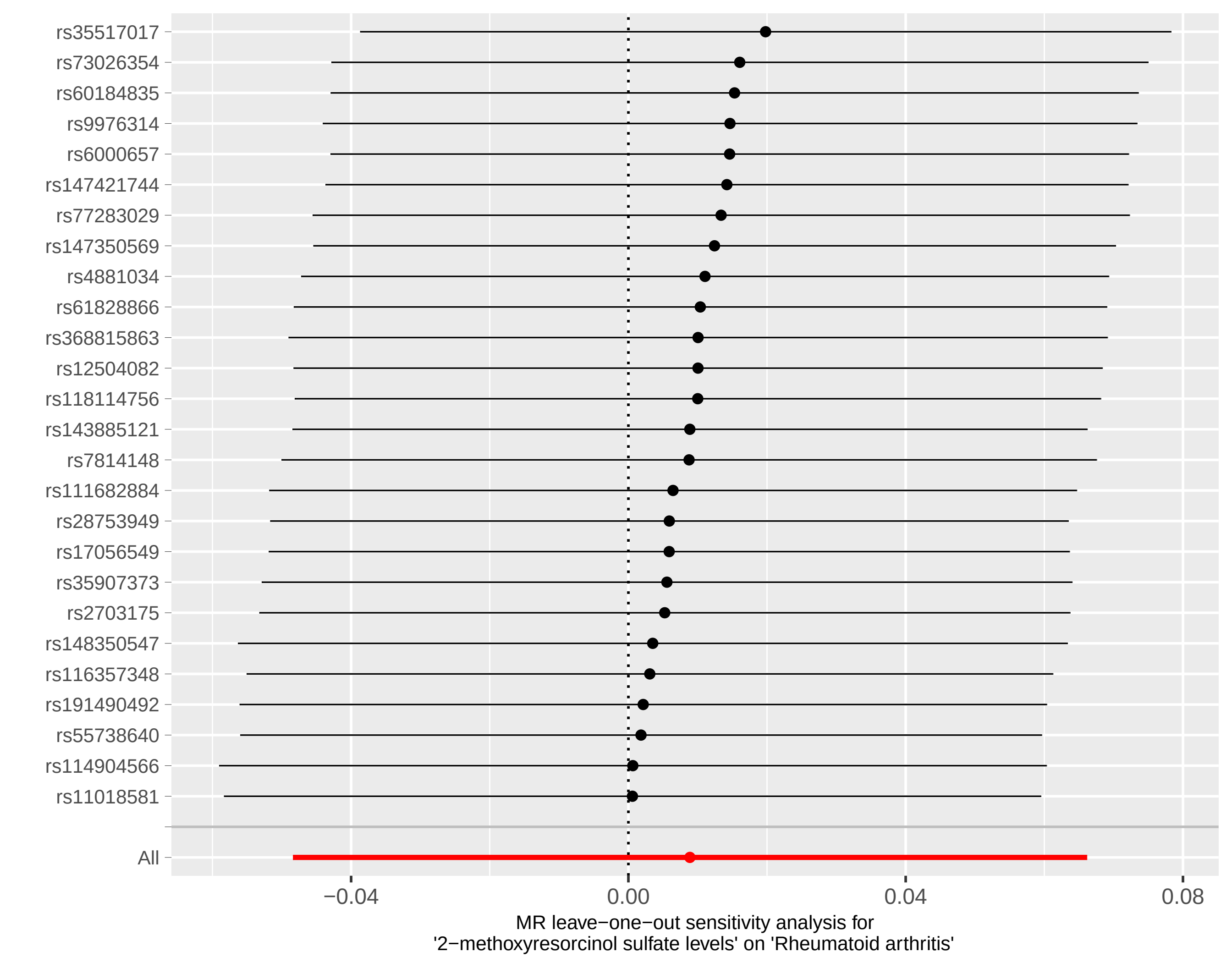
rs35517017
rs73026354
rs60184835
rs9976314
rs6000657
rs147421744
rs77283029
rs147350569
rs4881034
rs61828866
rs368815863
rs12504082
rs118114756
rs143885121
rs7814148
rs111682884
rs28753949
rs17056549
rs35907373
rs2703175
rs148350547
rs116357348
rs191490492
rs55738640
rs114904566
rs11018581
All
−0.04
0.00
0.04
0.08
MR leave−one−out sensitivity analysis for
'2−methoxyresorcinol sulfate levels' on 'Rheumatoid arthritis'

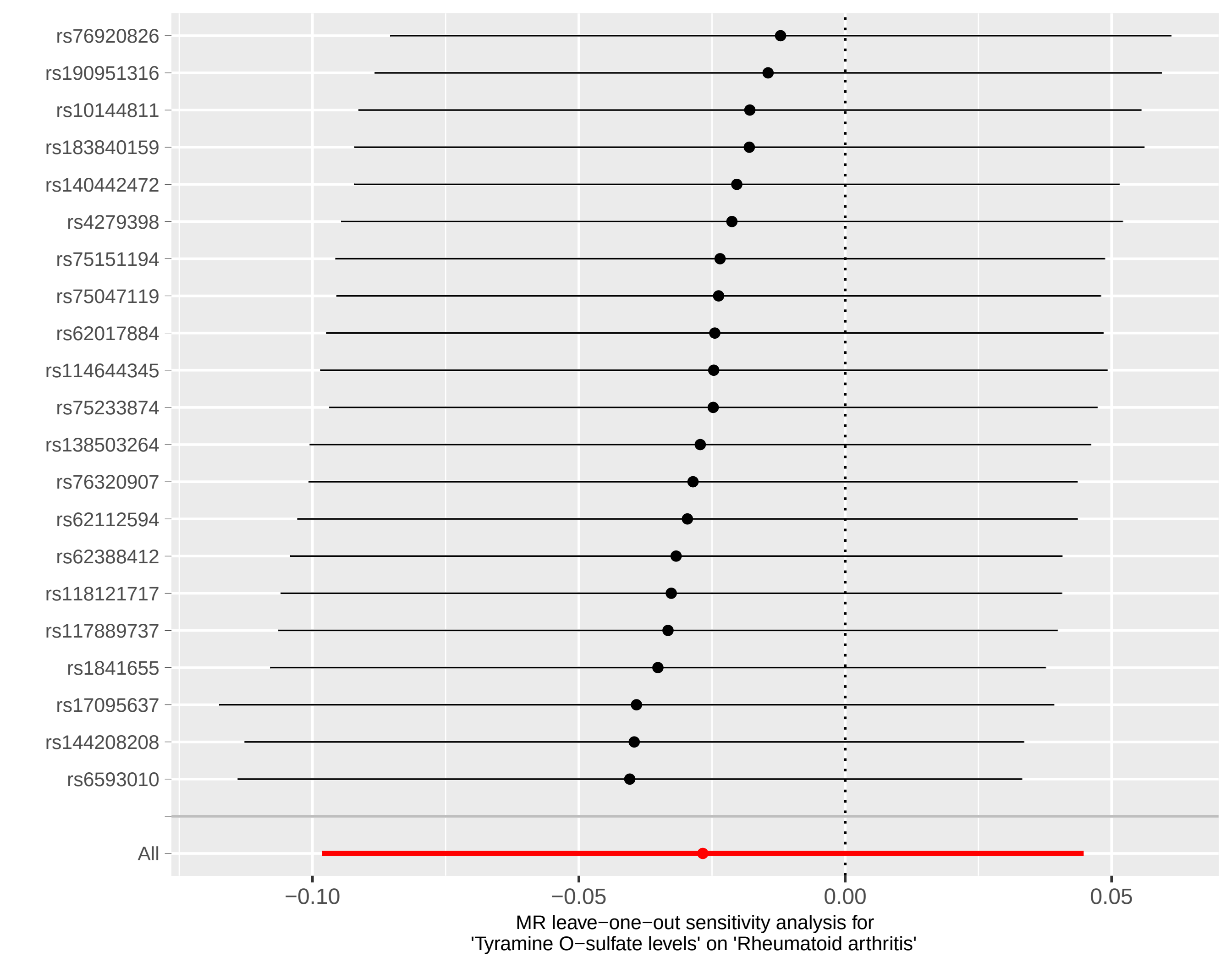
rs76920826
rs190951316
rs10144811
rs183840159
rs140442472
rs4279398
rs75151194
rs75047119
rs62017884
rs114644345
rs75233874
rs138503264
rs76320907
rs62112594
rs62388412
rs118121717
rs117889737
rs1841655
rs17095637
rs144208208
rs6593010
All
−0.10
−0.05
0.00
0.05
MR leave−one−out sensitivity analysis for
'Tyramine O−sulfate levels' on 'Rheumatoid arthritis'

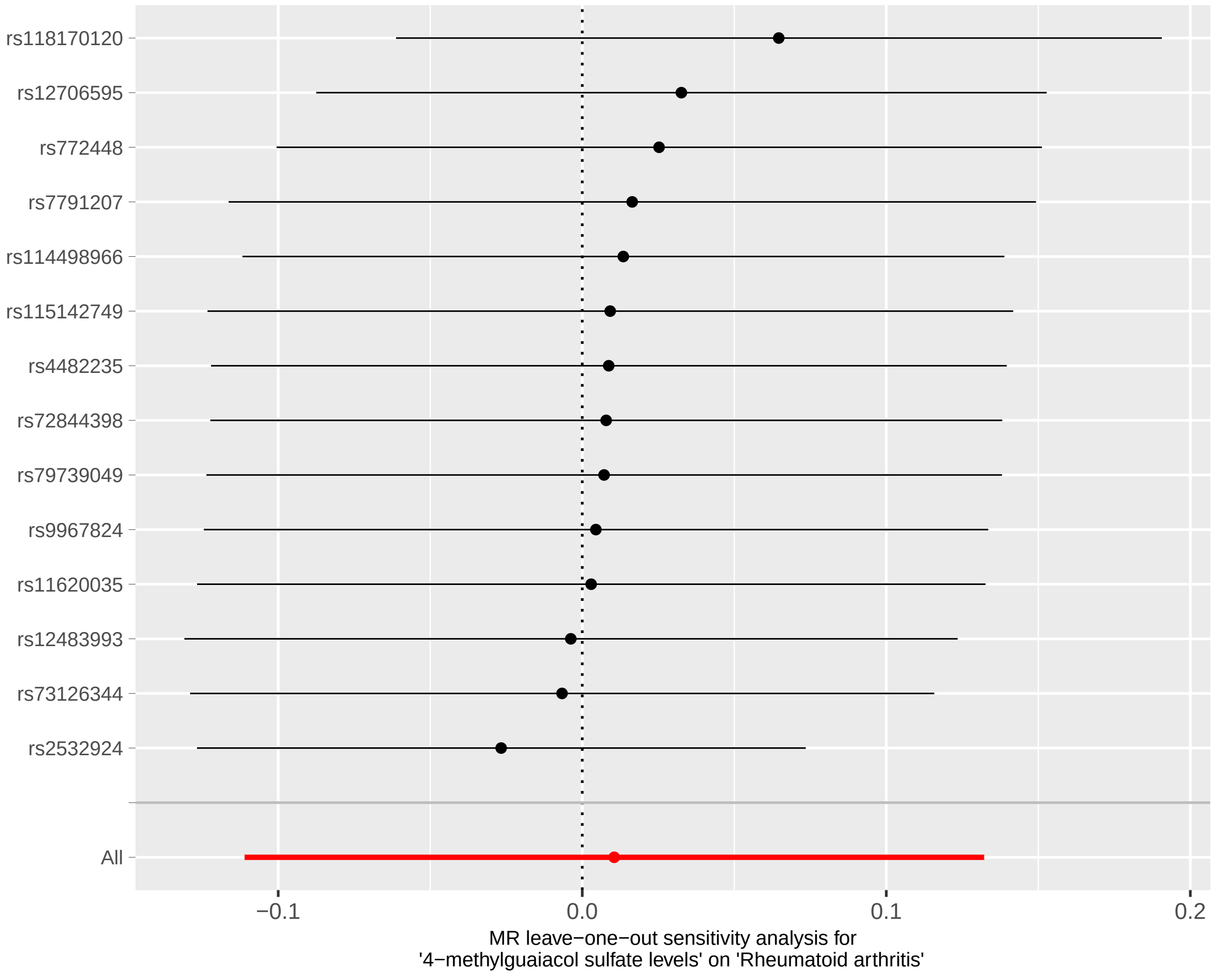
rs118170120
rs12706595
rs772448
rs7791207
rs114498966
rs115142749
rs4482235
rs72844398
rs79739049
rs9967824
rs11620035
rs12483993
rs73126344
rs2532924
All
−0.1
0.0
0.1
0.2
MR leave−one−out sensitivity analysis for
'4−methylguaiacol sulfate levels' on 'Rheumatoid arthritis'

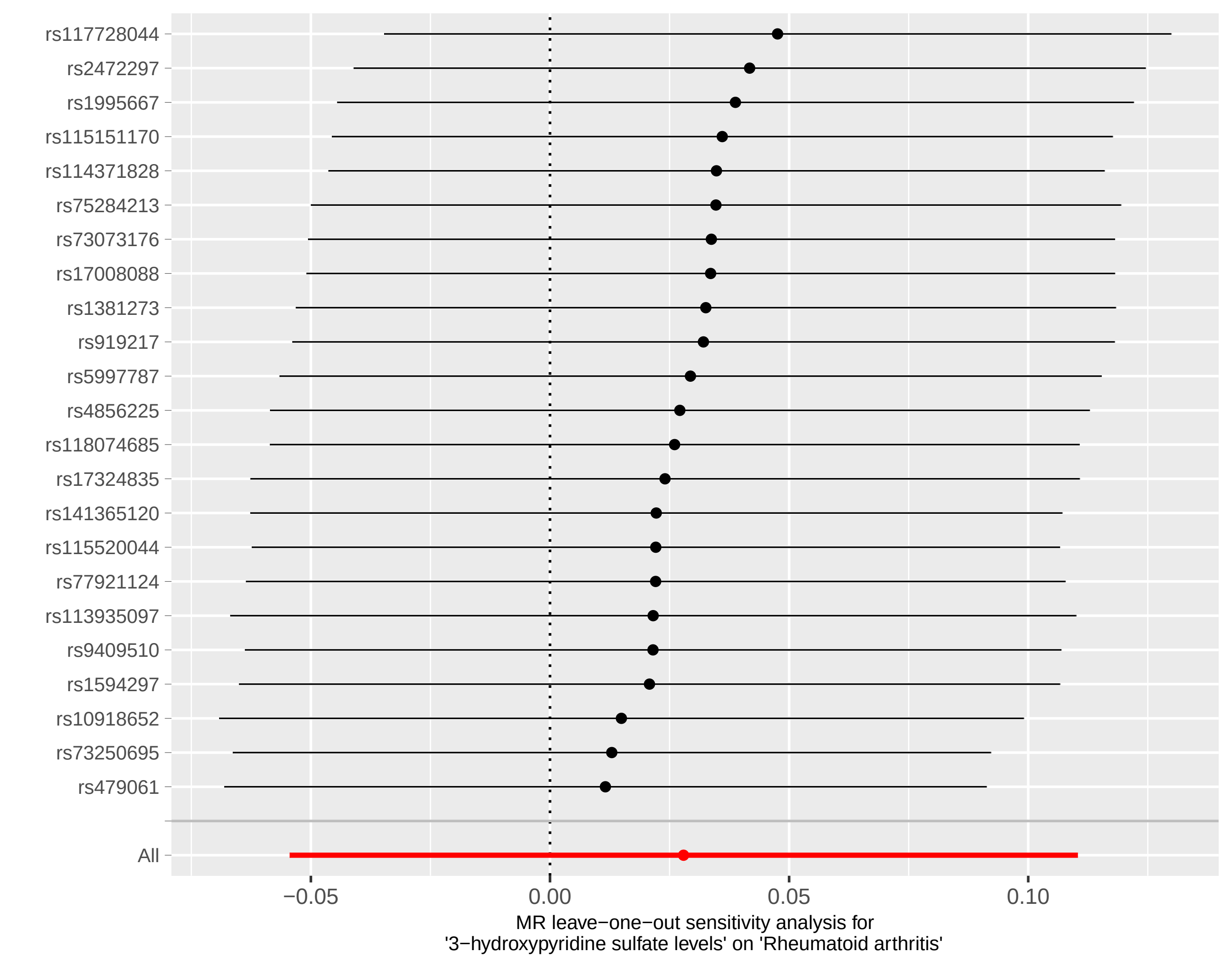
rs117728044
rs2472297
rs1995667
rs115151170
rs114371828
rs75284213
rs73073176
rs17008088
rs1381273
rs919217
rs5997787
rs4856225
rs118074685
rs17324835
rs141365120
rs115520044
rs77921124
rs113935097
rs9409510
rs1594297
rs10918652
rs73250695
rs479061
All
−0.05
0.00
0.05
0.10
MR leave−one−out sensitivity analysis for
'3−hydroxypyridine sulfate levels' on 'Rheumatoid arthritis'

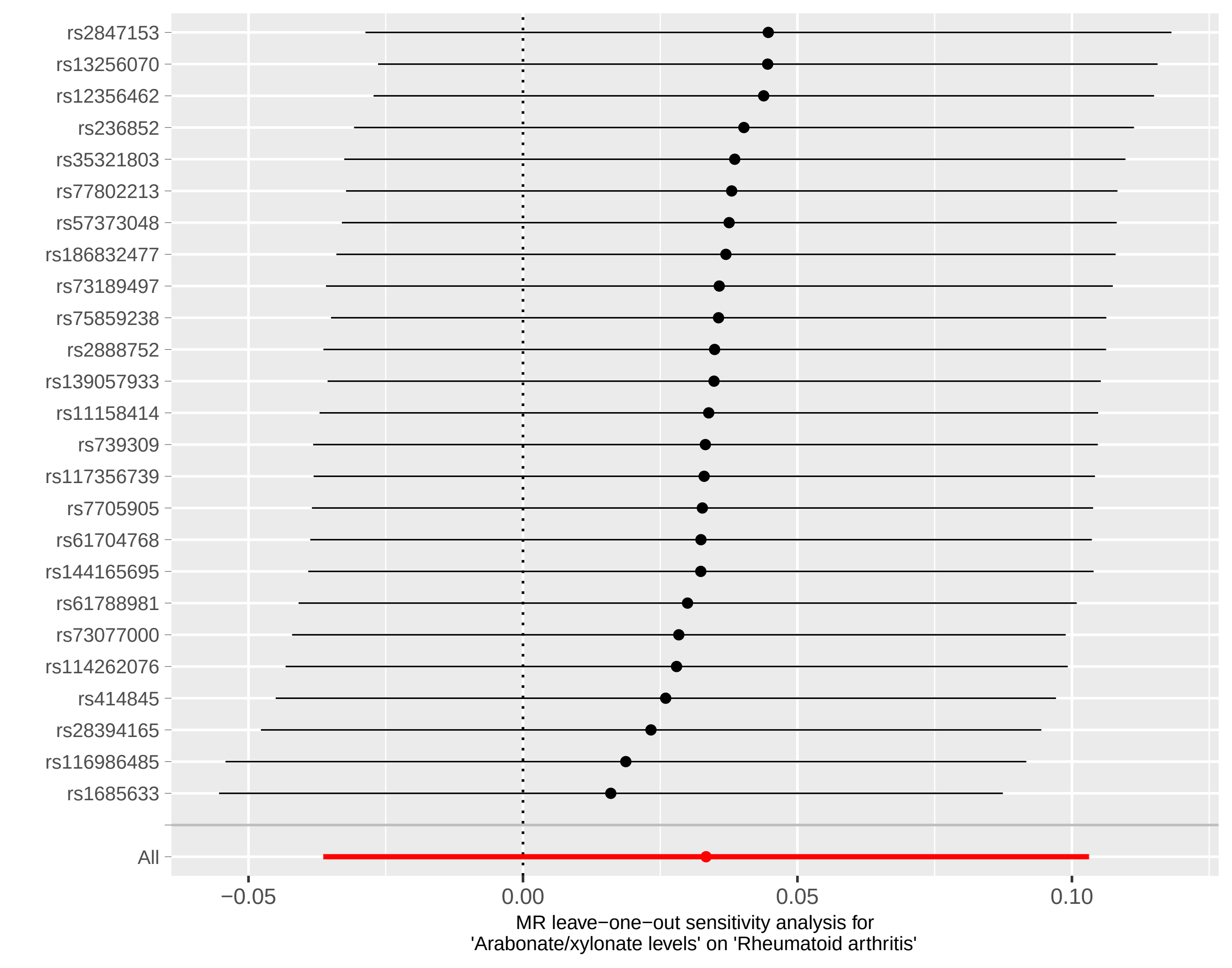
rs2847153
rs13256070
rs12356462
rs236852
rs35321803
rs77802213
rs57373048
rs186832477
rs73189497
rs75859238
rs2888752
rs139057933
rs11158414
rs739309
rs117356739
rs7705905
rs61704768
rs144165695
rs61788981
rs73077000
rs114262076
rs414845
rs28394165
rs116986485
rs1685633
All
−0.05
0.00
0.05
0.10
MR leave−one−out sensitivity analysis for
'Arabonate/xylonate levels' on 'Rheumatoid arthritis'

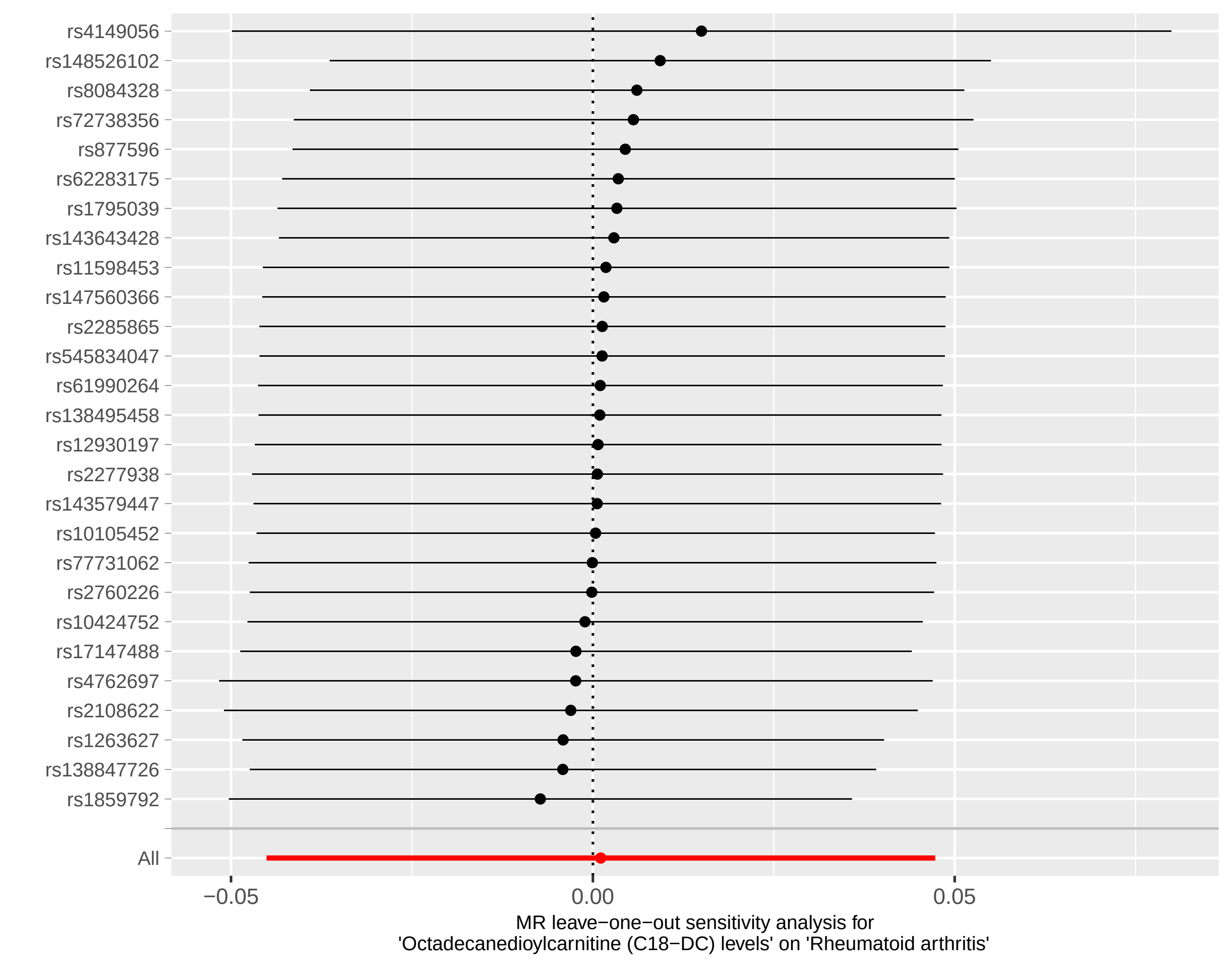

rs4149056
rs148526102
rs8084328
rs72738356
rs877596
rs62283175
rs1795039
rs143643428
rs11598453
rs147560366
rs2285865
rs545834047
rs61990264
rs138495458
rs12930197
rs2277938
rs143579447
rs10105452
rs77731062
rs2760226
rs10424752
rs17147488
rs4762697
rs2108622
rs1263627
rs138847726
rs1859792
All
−0.05
0.00
0.05
MR leave−one−out sensitivity analysis for
'Octadecanedioylcarnitine (C18−DC) levels' on 'Rheumatoid arthritis'

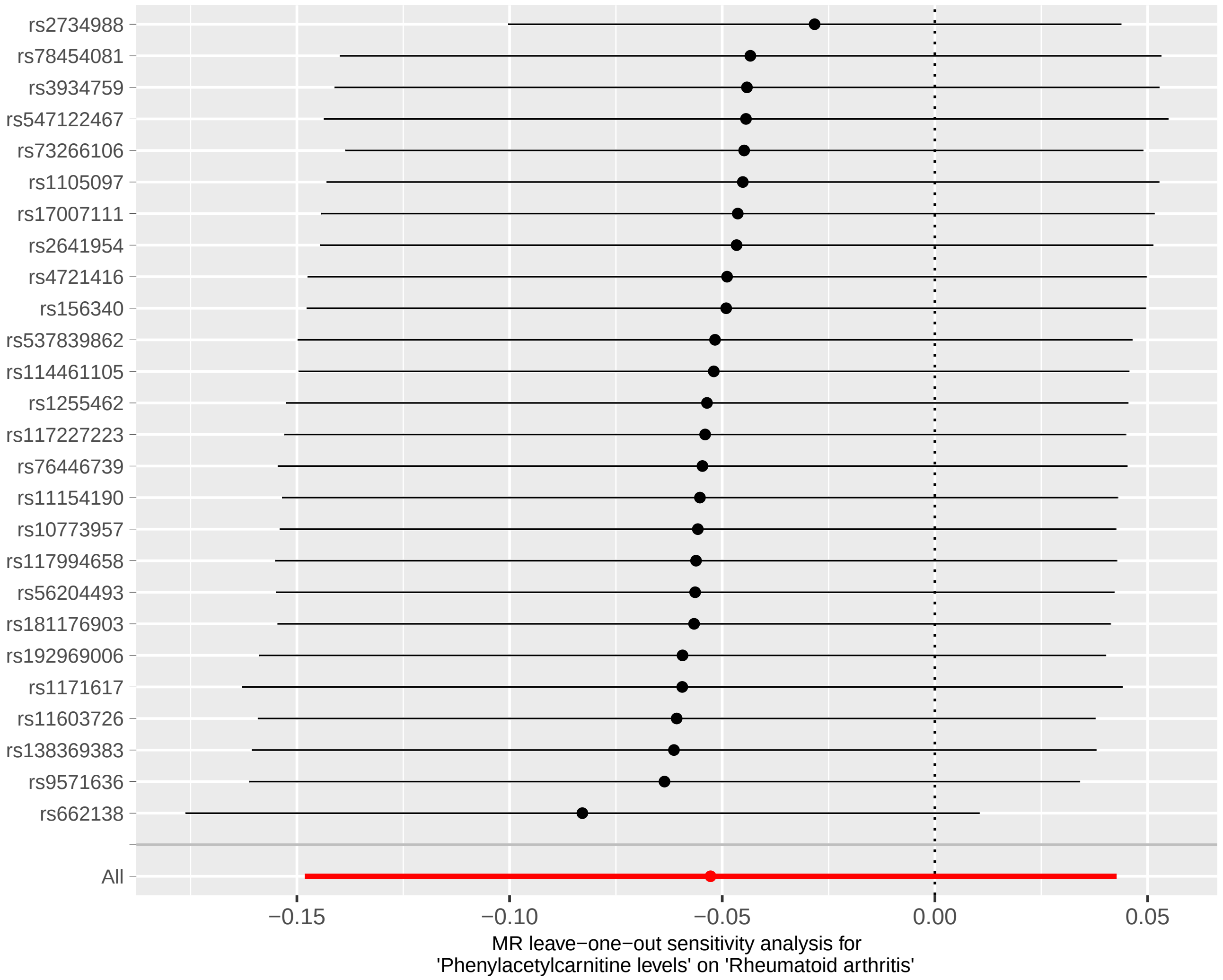
rs2734988
rs78454081
rs3934759
rs547122467
rs73266106
rs1105097
rs17007111
rs2641954
rs4721416
rs156340
rs537839862
rs114461105
rs1255462
rs117227223
rs76446739
rs11154190
rs10773957
rs117994658
rs56204493
rs181176903
rs192969006
rs1171617
rs11603726
rs138369383
rs9571636
rs662138
All
−0.15
−0.10
−0.05
0.00
0.05
MR leave−one−out sensitivity analysis for
'Phenylacetylcarnitine levels' on 'Rheumatoid arthritis'

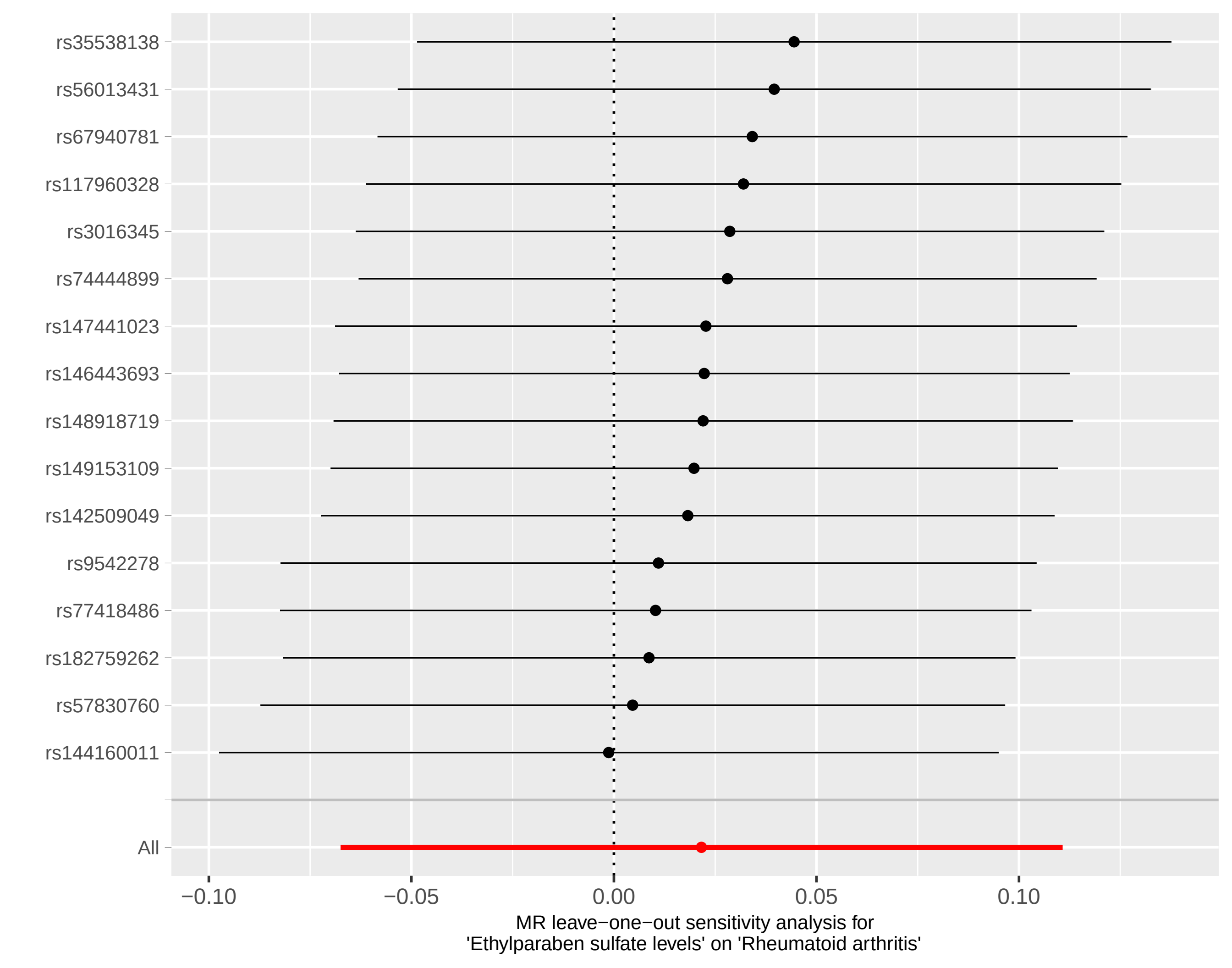

rs35538138
rs56013431
rs67940781
rs117960328
rs3016345
rs74444899
rs147441023
rs146443693
rs148918719
rs149153109
rs142509049
rs9542278
rs77418486
rs182759262
rs57830760
rs144160011
All
−0.10
−0.05
0.00
0.05
0.10
MR leave−one−out sensitivity analysis for
'Ethylparaben sulfate levels' on 'Rheumatoid arthritis'

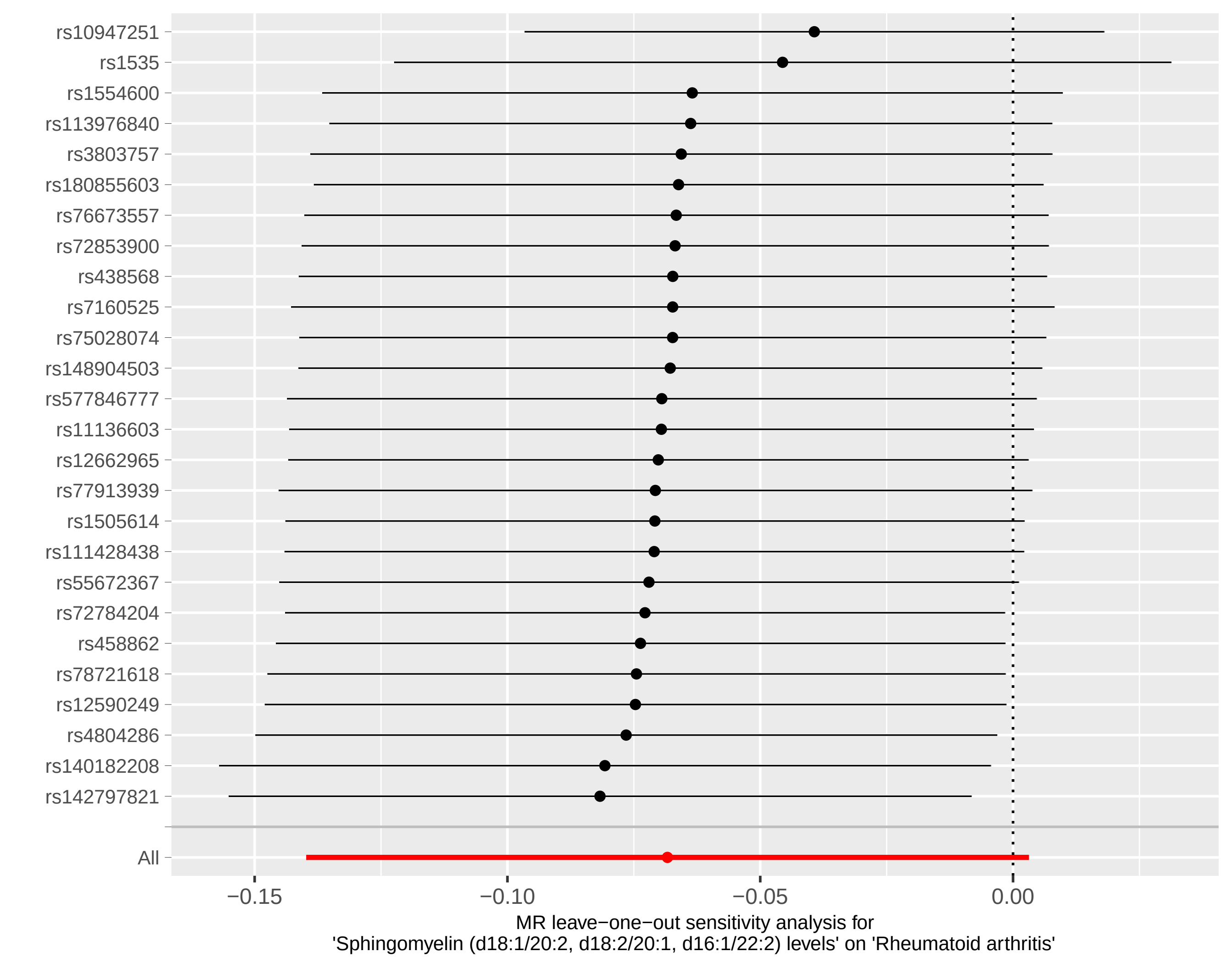
rs10947251
rs1535
rs1554600
rs113976840
rs3803757
rs180855603
rs76673557
rs72853900
rs438568
rs7160525
rs75028074
rs148904503
rs577846777
rs11136603
rs12662965
rs77913939
rs1505614
rs111428438
rs55672367
rs72784204
rs458862
rs78721618
rs12590249
rs4804286
rs140182208
rs142797821
All
−0.15
−0.10
−0.05
0.00
MR leave−one−out sensitivity analysis for
'Sphingomyelin (d18:1/20:2, d18:2/20:1, d16:1/22:2) levels' on 'Rheumatoid arthritis'

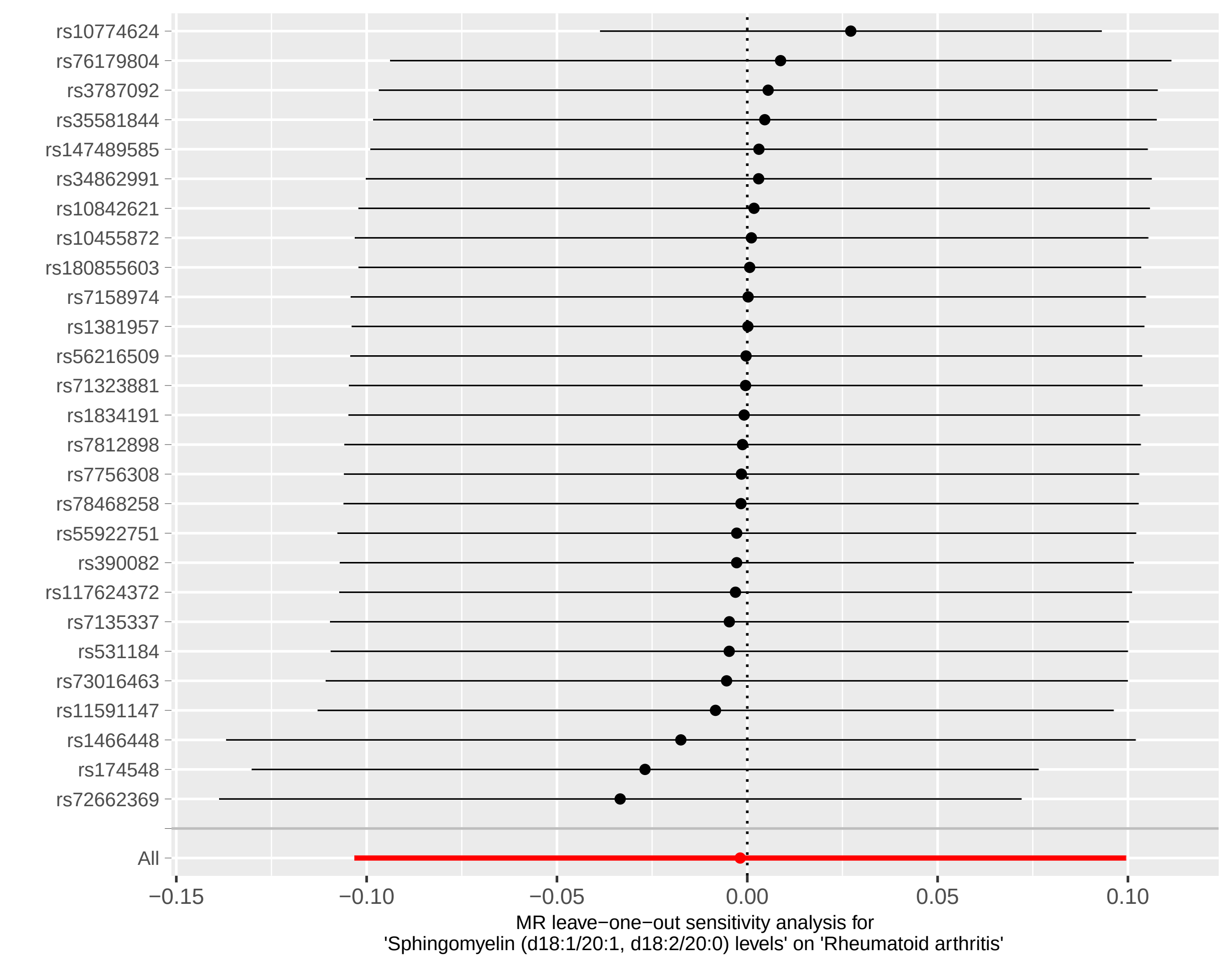
rs10774624
rs76179804
rs3787092
rs35581844
rs147489585
rs34862991
rs10842621
rs10455872
rs180855603
rs7158974
rs1381957
rs56216509
rs71323881
rs1834191
rs7812898
rs7756308
rs78468258
rs55922751
rs390082
rs117624372
rs7135337
rs531184
rs73016463
rs11591147
rs1466448
rs174548
rs72662369
All
−0.15
−0.10
−0.05
0.00
0.05
0.10
MR leave−one−out sensitivity analysis for
'Sphingomyelin (d18:1/20:1, d18:2/20:0) levels' on 'Rheumatoid arthritis'

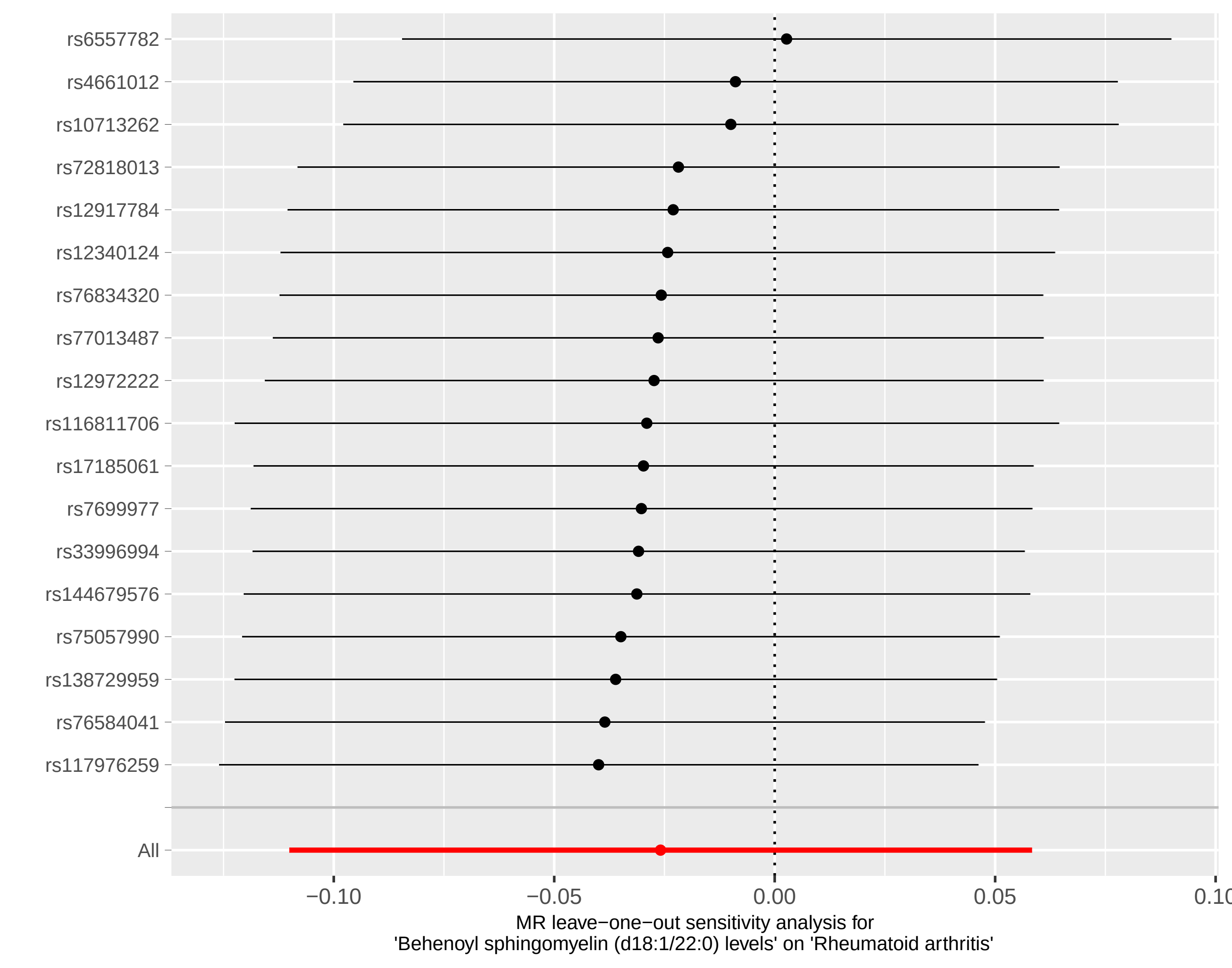
rs6557782
rs4661012
rs10713262
rs72818013
rs12917784
rs12340124
rs76834320
rs77013487
rs12972222
rs116811706
rs17185061
rs7699977
rs33996994
rs144679576
rs75057990
rs138729959
rs76584041
rs117976259
All
−0.10
−0.05
0.00
0.05
0.10
MR leave−one−out sensitivity analysis for
'Behenoyl sphingomyelin (d18:1/22:0) levels' on 'Rheumatoid arthritis'

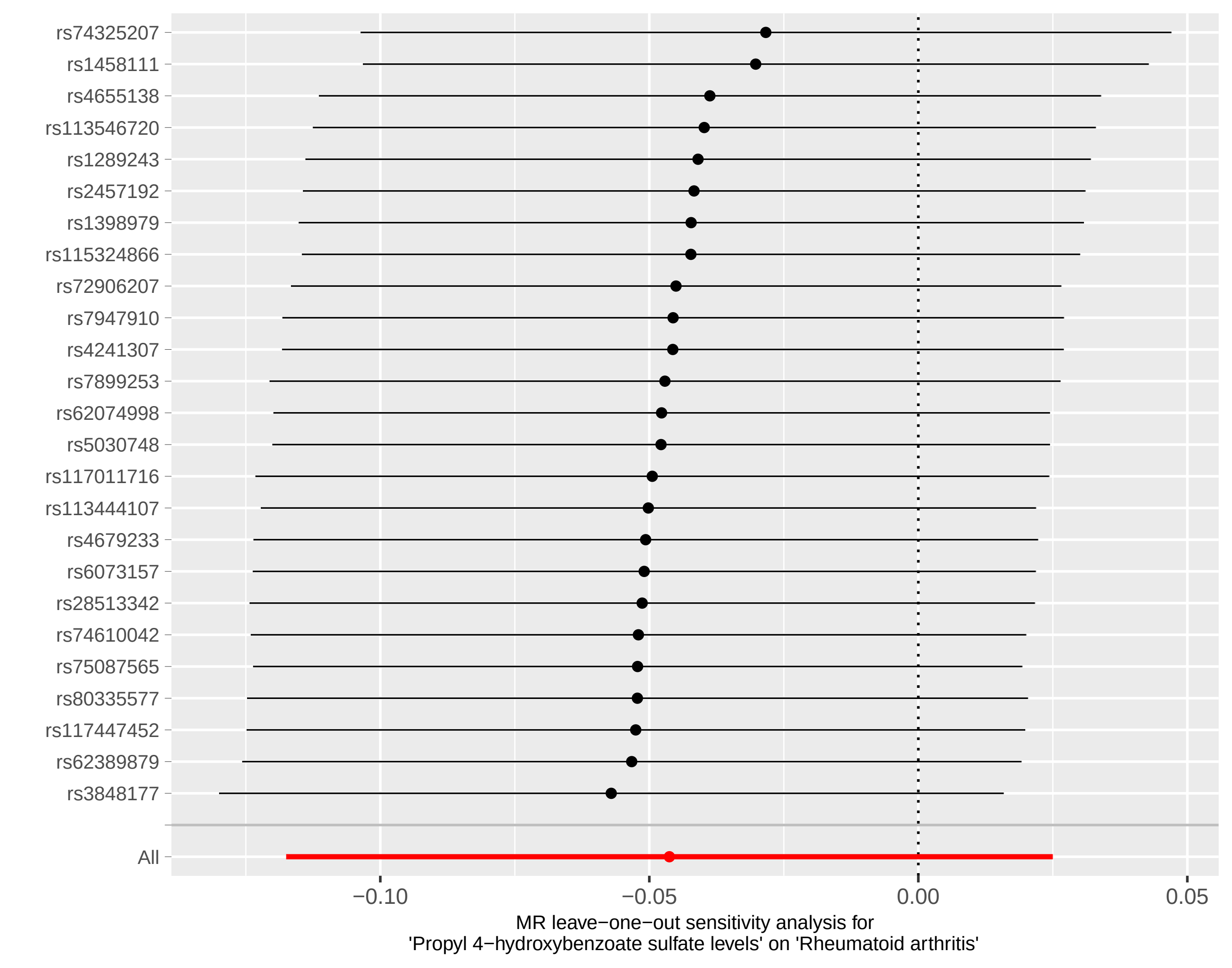
rs74325207
rs1458111
rs4655138
rs113546720
rs1289243
rs2457192
rs1398979
rs115324866
rs72906207
rs7947910
rs4241307
rs7899253
rs62074998
rs5030748
rs117011716
rs113444107
rs4679233
rs6073157
rs28513342
rs74610042
rs75087565
rs80335577
rs117447452
rs62389879
rs3848177
All
−0.10
−0.05
0.00
0.05
MR leave−one−out sensitivity analysis for
'Propyl 4−hydroxybenzoate sulfate levels' on 'Rheumatoid arthritis'

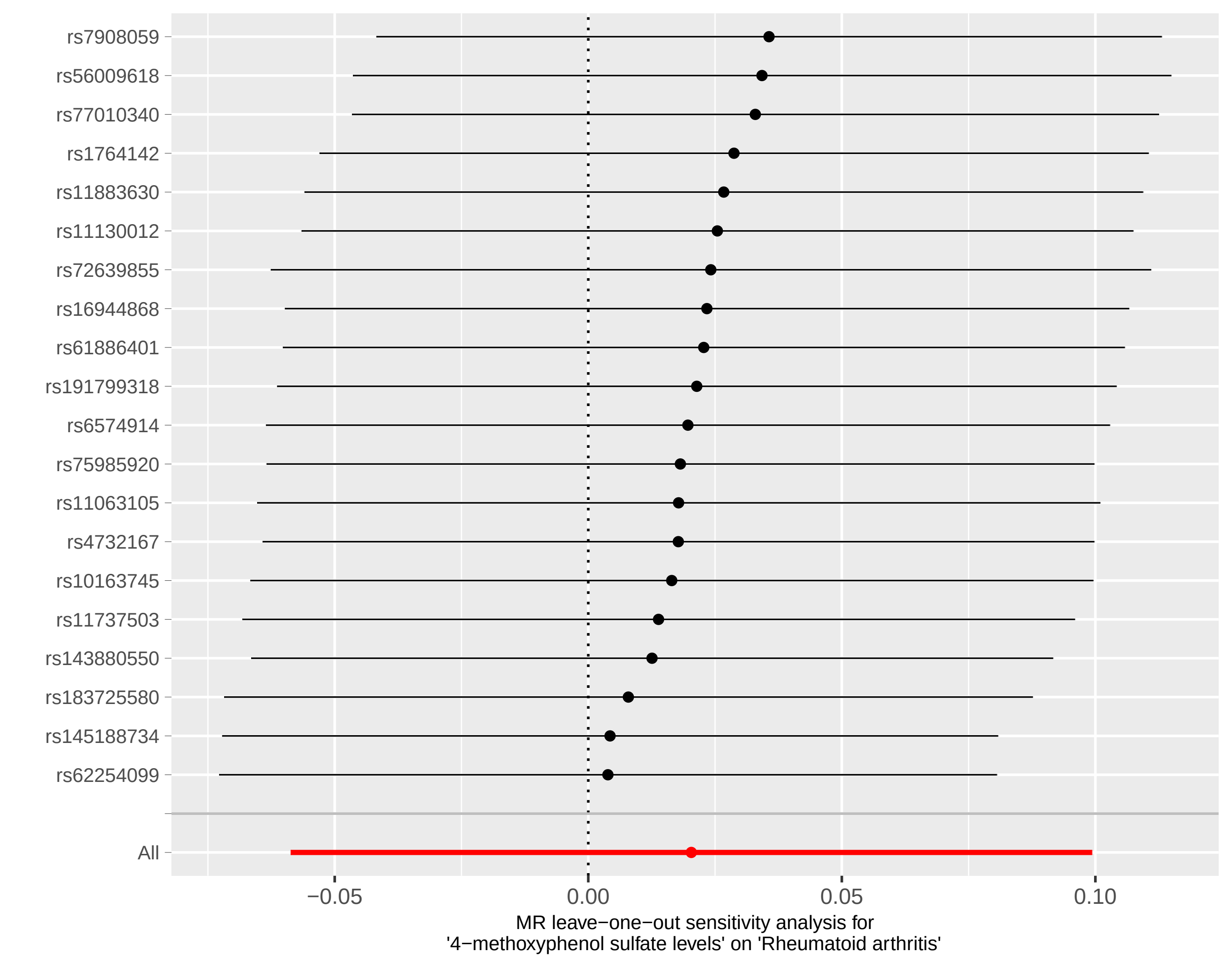
rs7908059
rs56009618
rs77010340
rs1764142
rs11883630
rs11130012
rs72639855
rs16944868
rs61886401
rs191799318
rs6574914
rs75985920
rs11063105
rs4732167
rs10163745
rs11737503
rs143880550
rs183725580
rs145188734
rs62254099
All
−0.05
0.00
0.05
0.10
MR leave−one−out sensitivity analysis for
'4−methoxyphenol sulfate levels' on 'Rheumatoid arthritis'

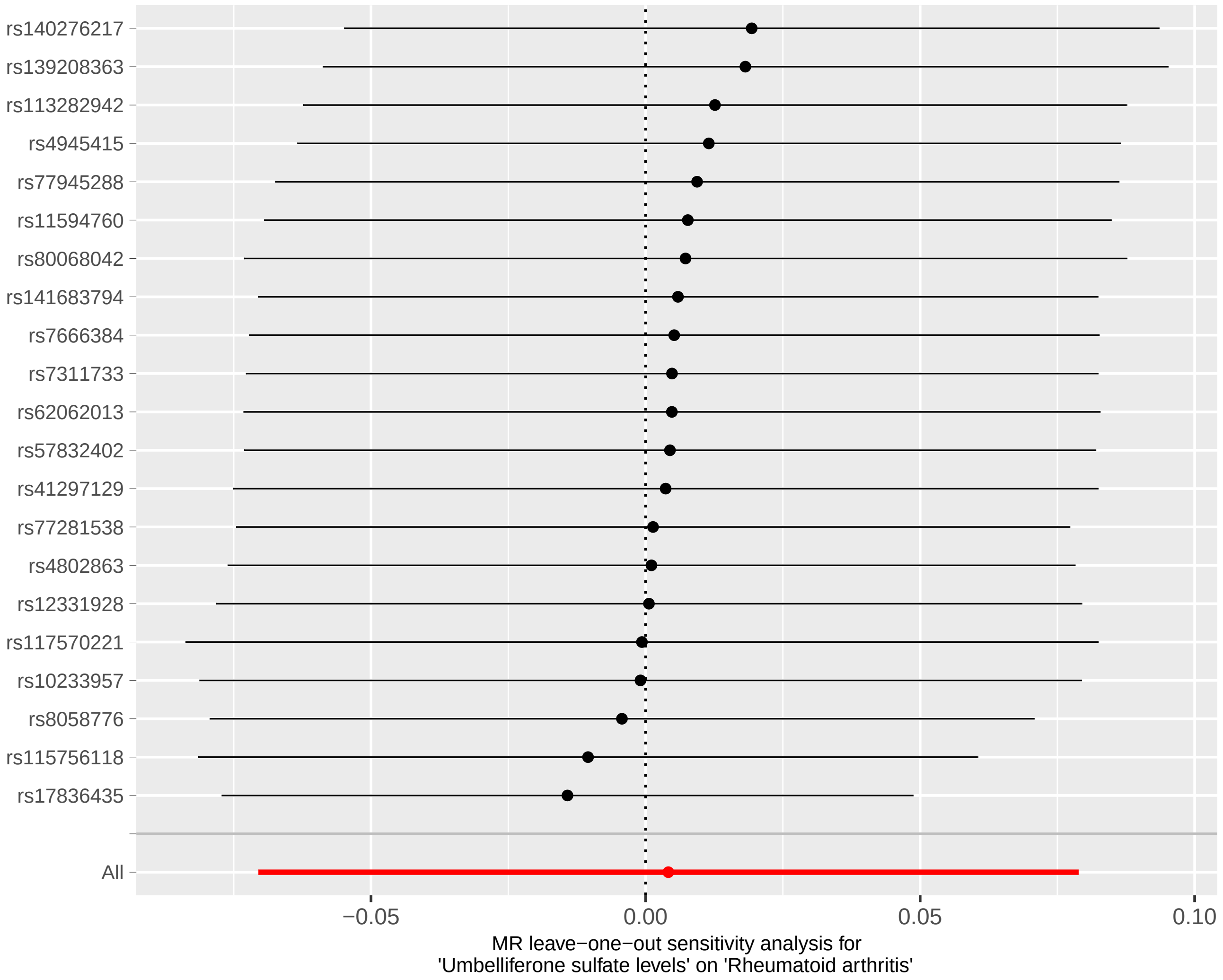

rs140276217
rs139208363
rs113282942
rs4945415
rs77945288
rs11594760
rs80068042
rs141683794
rs7666384
rs7311733
rs62062013
rs57832402
rs41297129
rs77281538
rs4802863
rs12331928
rs117570221
rs10233957
rs8058776
rs115756118
rs17836435
All
−0.05
0.00
0.05
0.10
MR leave−one−out sensitivity analysis for
'Umbelliferone sulfate levels' on 'Rheumatoid arthritis'

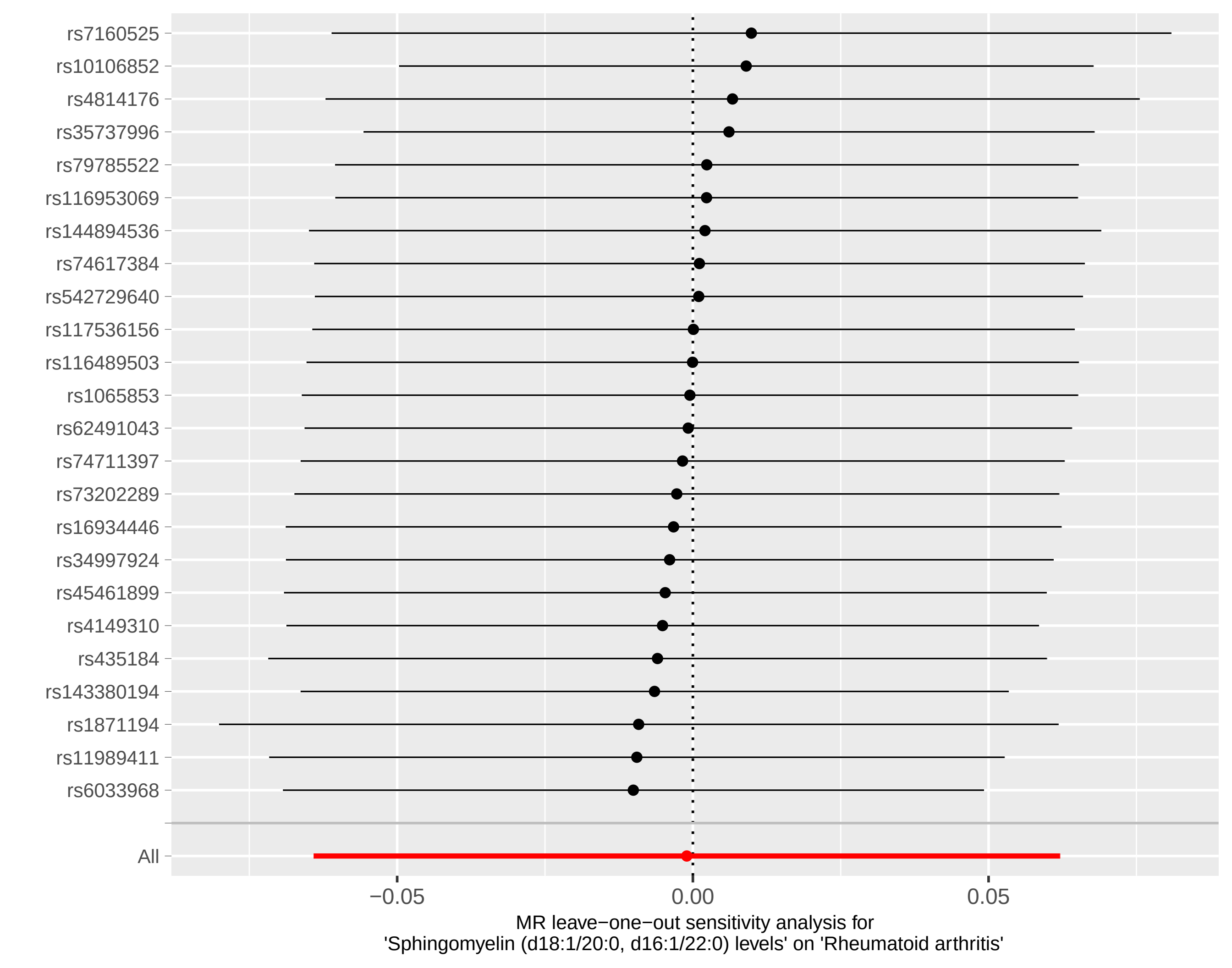
rs7160525
rs10106852
rs4814176
rs35737996
rs79785522
rs116953069
rs144894536
rs74617384
rs542729640
rs117536156
rs116489503
rs1065853
rs62491043
rs74711397
rs73202289
rs16934446
rs34997924
rs45461899
rs4149310
rs435184
rs143380194
rs1871194
rs11989411
rs6033968
All
−0.05
0.00
0.05
MR leave−one−out sensitivity analysis for
'Sphingomyelin (d18:1/20:0, d16:1/22:0) levels' on 'Rheumatoid arthritis'

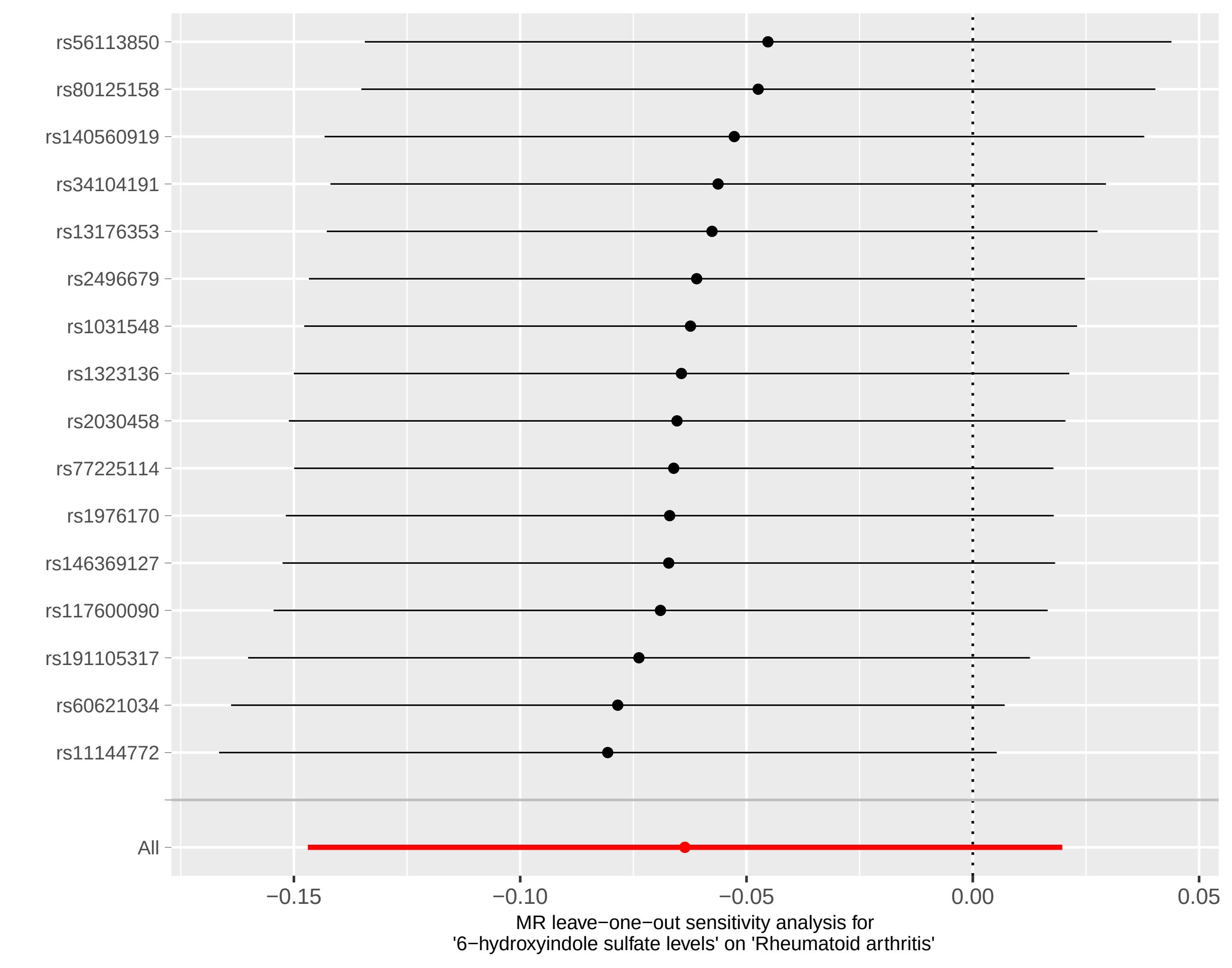
rs56113850
rs80125158
rs140560919
rs34104191
rs13176353
rs2496679
rs1031548
rs1323136
rs2030458
rs77225114
rs1976170
rs146369127
rs117600090
rs191105317
rs60621034
rs11144772
All
−0.15
−0.10
−0.05
0.00
0.05
MR leave−one−out sensitivity analysis for
'6−hydroxyindole sulfate levels' on 'Rheumatoid arthritis'

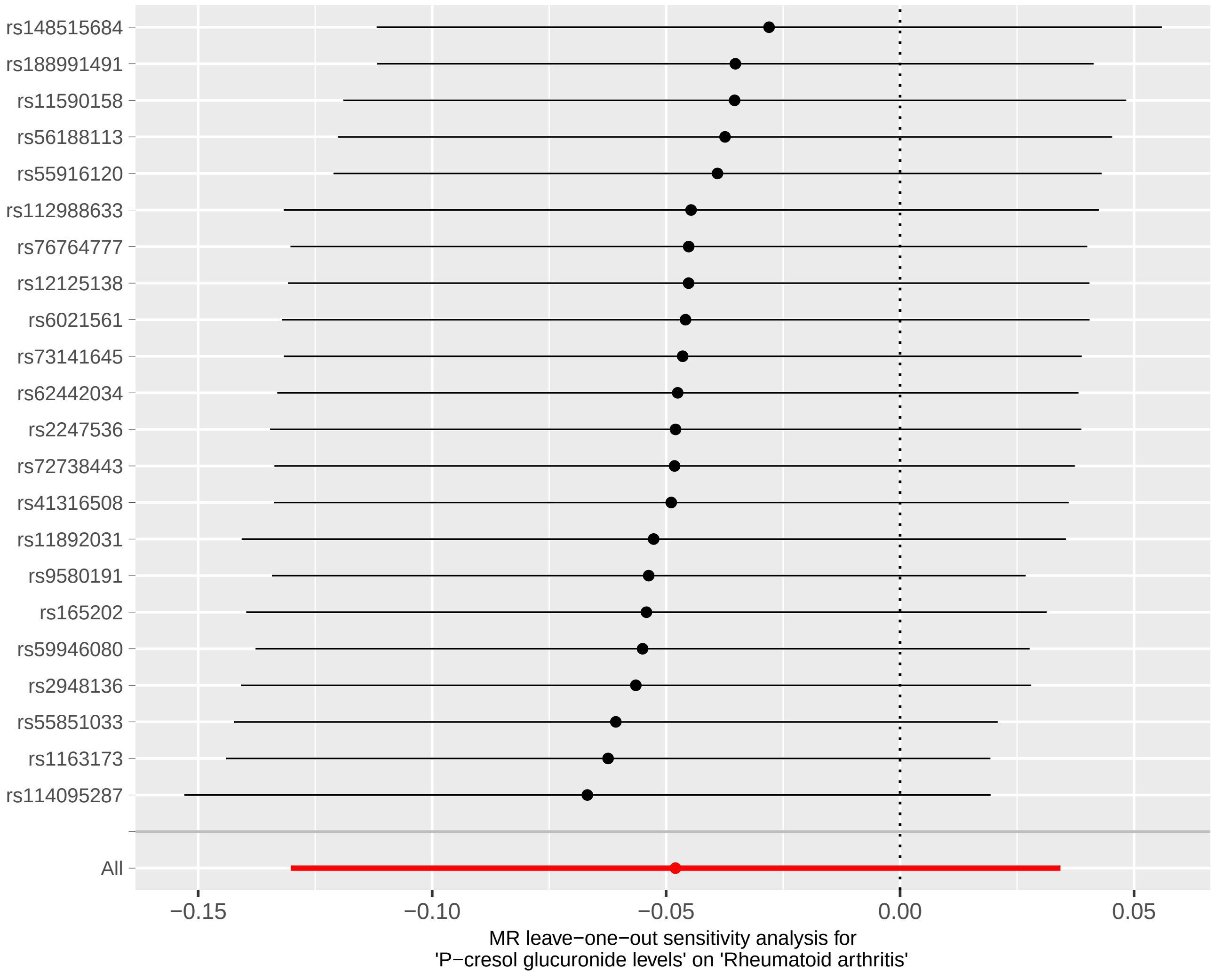
rs148515684
rs188991491
rs11590158
rs56188113
rs55916120
rs112988633
rs76764777
rs12125138
rs6021561
rs73141645
rs62442034
rs2247536
rs72738443
rs41316508
rs11892031
rs9580191
rs165202
rs59946080
rs2948136
rs55851033
rs1163173
rs114095287
All
−0.15
−0.10
−0.05
0.00
0.05
MR leave−one−out sensitivity analysis for
'P−cresol glucuronide levels' on 'Rheumatoid arthritis'

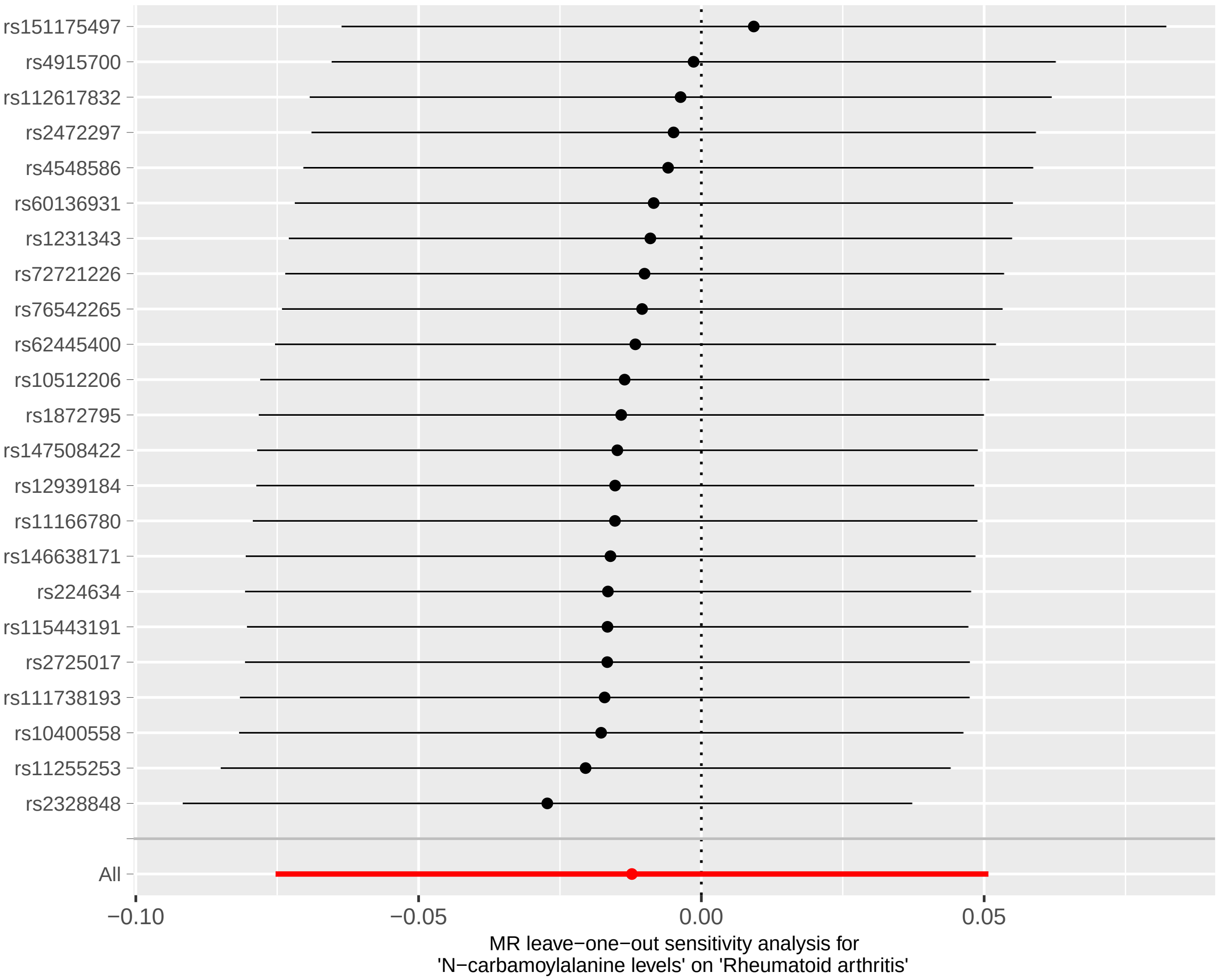
rs151175497
rs4915700
rs112617832
rs2472297
rs4548586
rs60136931
rs1231343
rs72721226
rs76542265
rs62445400
rs10512206
rs1872795
rs147508422
rs12939184
rs11166780
rs146638171
rs224634
rs115443191
rs2725017
rs111738193
rs10400558
rs11255253
rs2328848
All
−0.10
−0.05
0.00
0.05
MR leave−one−out sensitivity analysis for
'N−carbamoylalanine levels' on 'Rheumatoid arthritis'

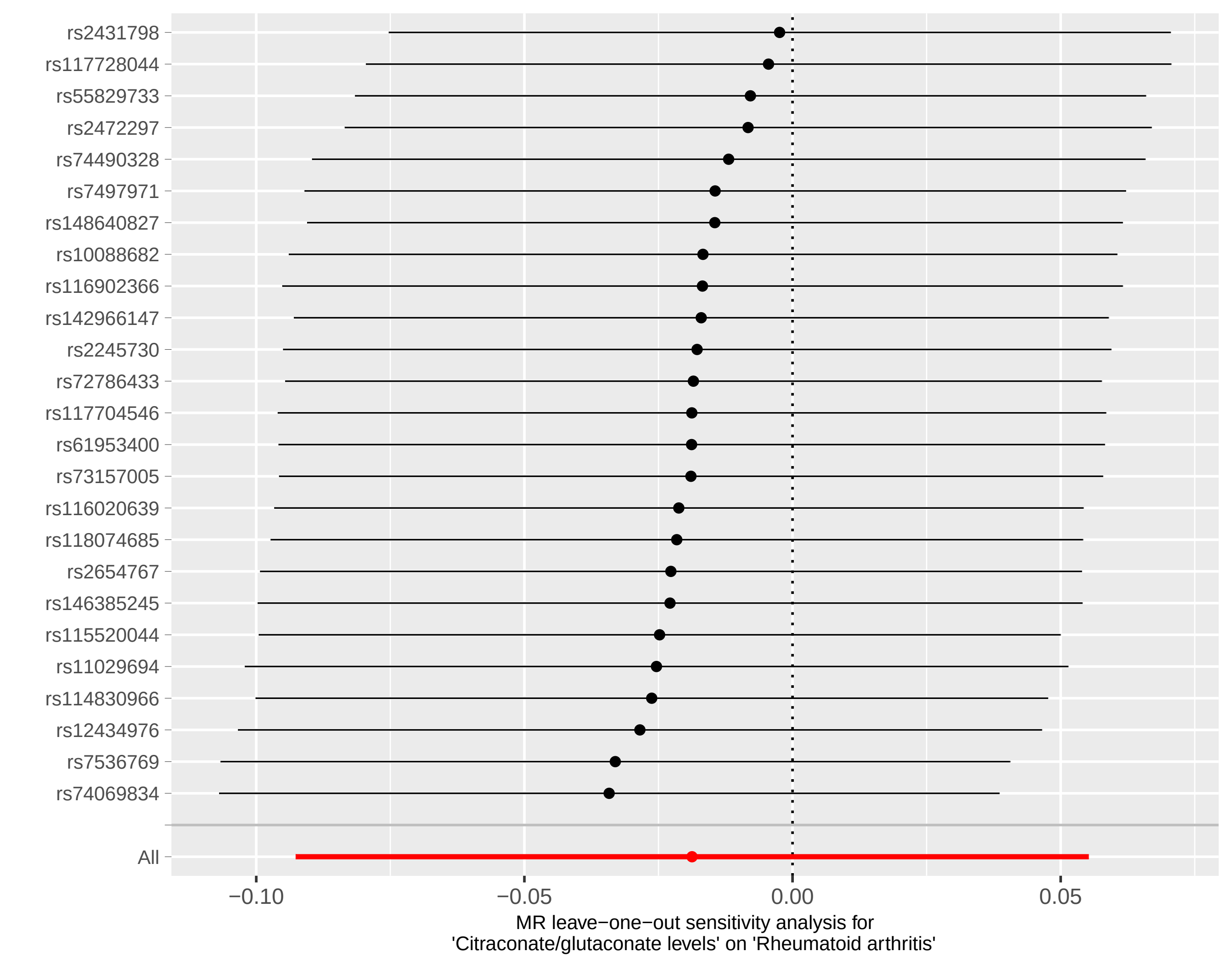

rs2431798
rs117728044
rs55829733
rs2472297
rs74490328
rs7497971
rs148640827
rs10088682
rs116902366
rs142966147
rs2245730
rs72786433
rs117704546
rs61953400
rs73157005
rs116020639
rs118074685
rs2654767
rs146385245
rs115520044
rs11029694
rs114830966
rs12434976
rs7536769
rs74069834
All
−0.10
−0.05
0.00
0.05
MR leave−one−out sensitivity analysis for
'Citraconate/glutaconate levels' on 'Rheumatoid arthritis'

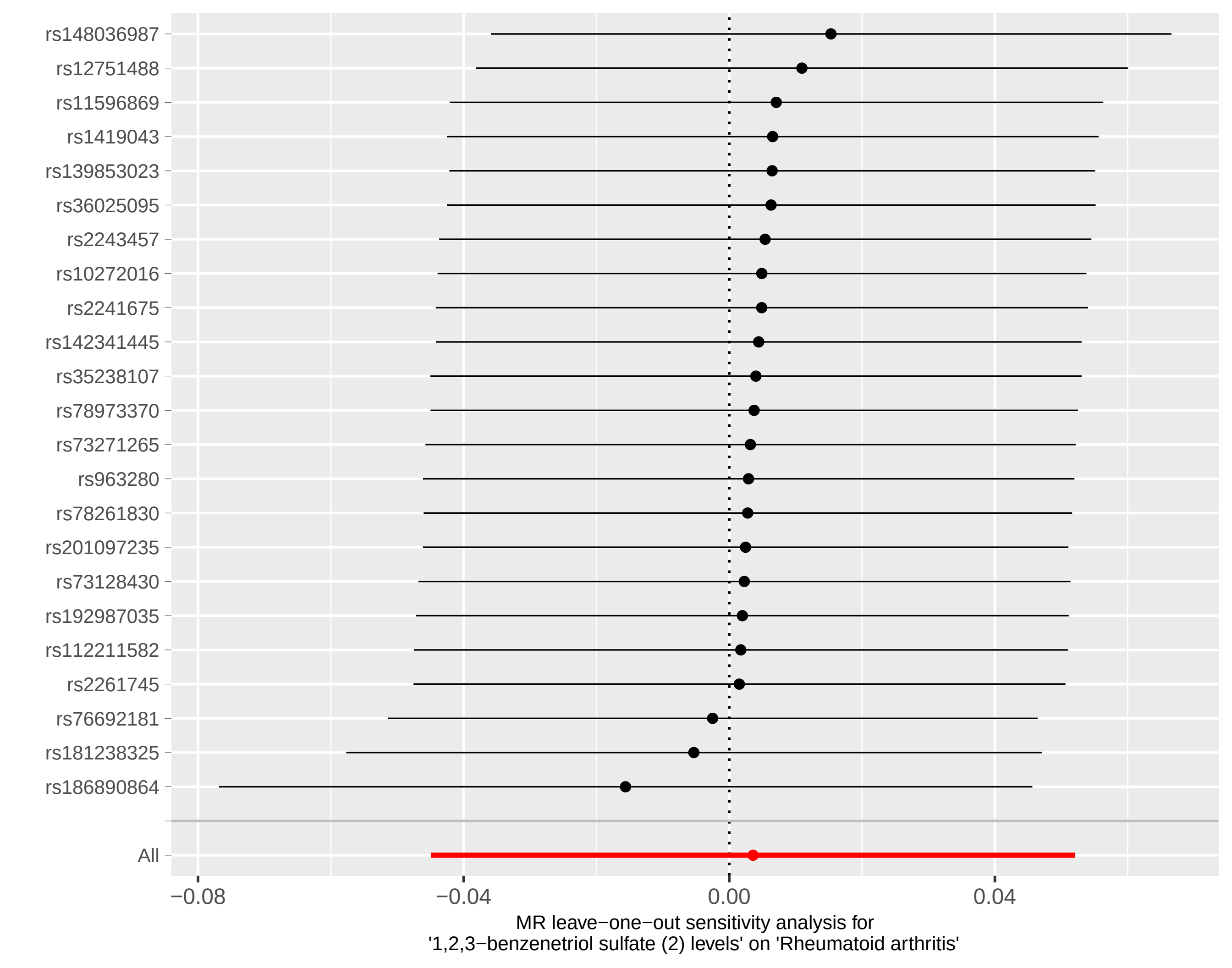
rs148036987
rs12751488
rs11596869
rs1419043
rs139853023
rs36025095
rs2243457
rs10272016
rs2241675
rs142341445
rs35238107
rs78973370
rs73271265
rs963280
rs78261830
rs201097235
rs73128430
rs192987035
rs112211582
rs2261745
rs76692181
rs181238325
rs186890864
All
−0.08
−0.04
0.00
0.04
MR leave−one−out sensitivity analysis for
'1,2,3−benzenetriol sulfate (2) levels' on 'Rheumatoid arthritis'

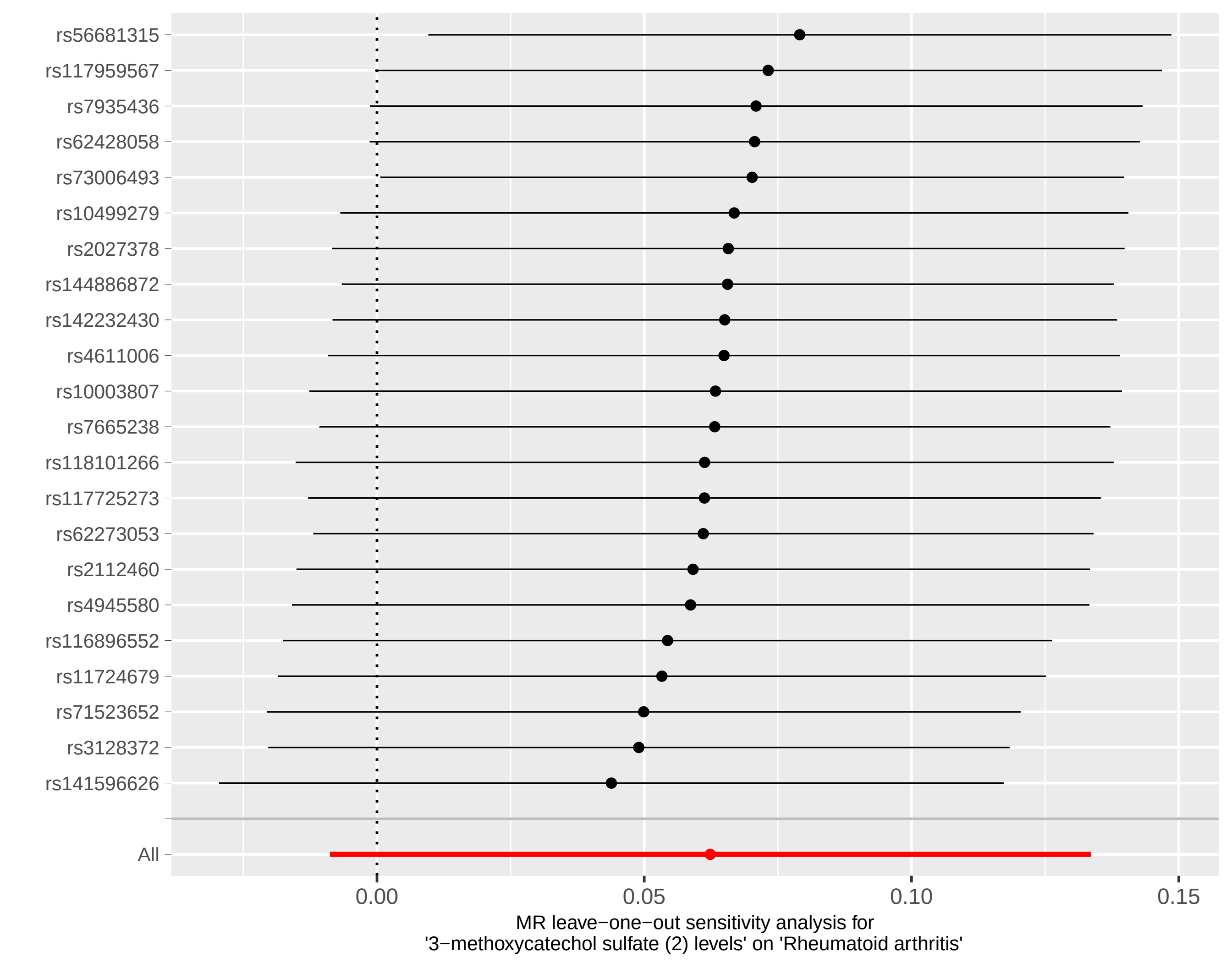
rs56681315
rs117959567
rs7935436
rs62428058
rs73006493
rs10499279
rs2027378
rs144886872
rs142232430
rs4611006
rs10003807
rs7665238
rs118101266
rs117725273
rs62273053
rs2112460
rs4945580
rs116896552
rs11724679
rs71523652
rs3128372
rs141596626
All
0.00
0.05
0.10
0.15
MR leave−one−out sensitivity analysis for
'3−methoxycatechol sulfate (2) levels' on 'Rheumatoid arthritis'

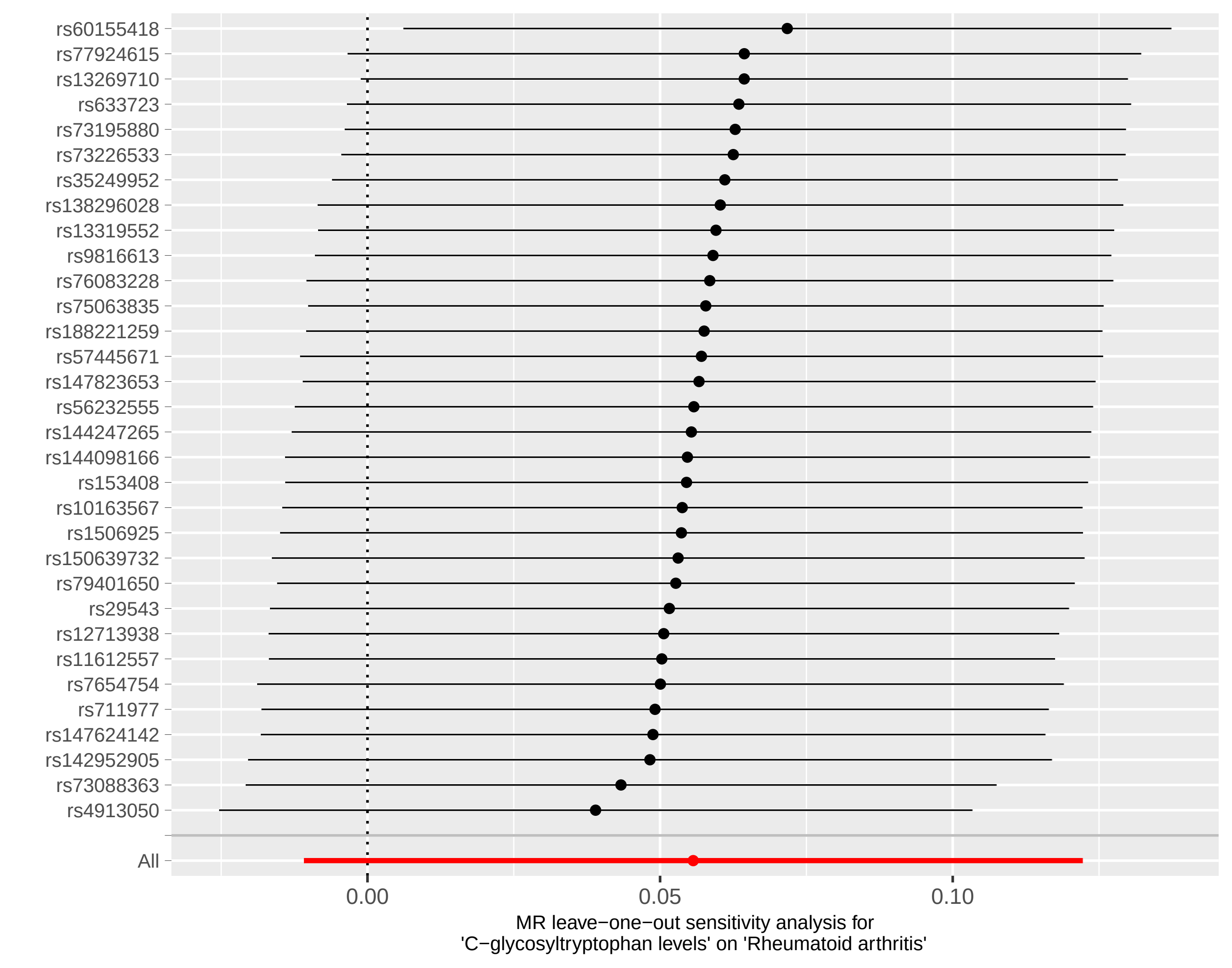
rs60155418
rs77924615
rs13269710
rs633723
rs73195880
rs73226533
rs35249952
rs138296028
rs13319552
rs9816613
rs76083228
rs75063835
rs188221259
rs57445671
rs147823653
rs56232555
rs144247265
rs144098166
rs153408
rs10163567
rs1506925
rs150639732
rs79401650
rs29543
rs12713938
rs11612557
rs7654754
rs711977
rs147624142
rs142952905
rs73088363
rs4913050
All
0.00
0.05
0.10
MR leave−one−out sensitivity analysis for
'C−glycosyltryptophan levels' on 'Rheumatoid arthritis'

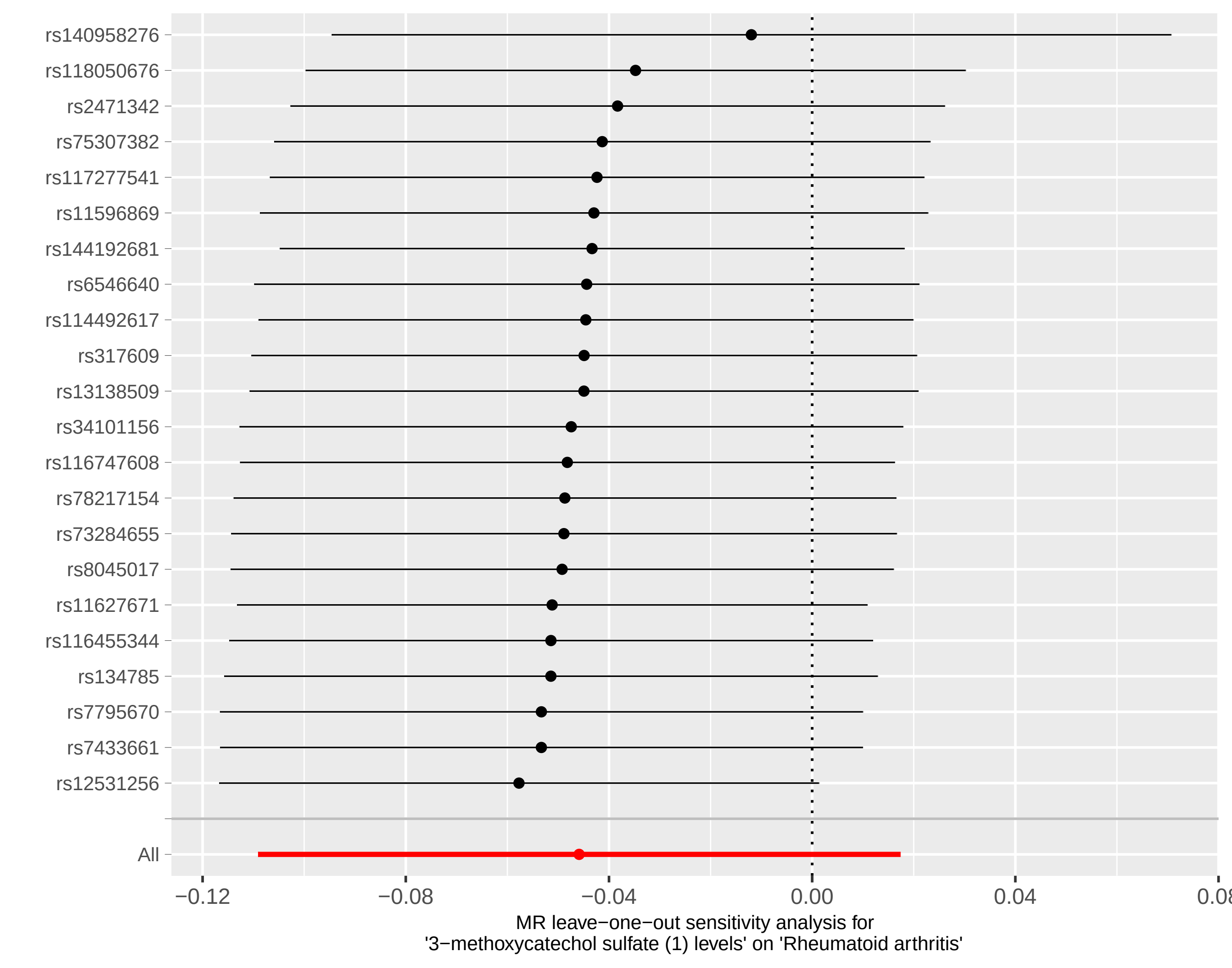
rs140958276
rs118050676
rs2471342
rs75307382
rs117277541
rs11596869
rs144192681
rs6546640
rs114492617
rs317609
rs13138509
rs34101156
rs116747608
rs78217154
rs73284655
rs8045017
rs11627671
rs116455344
rs134785
rs7795670
rs7433661
rs12531256
All
−0.12
−0.08
−0.04
0.00
0.04
0.08
MR leave−one−out sensitivity analysis for
'3−methoxycatechol sulfate (1) levels' on 'Rheumatoid arthritis'

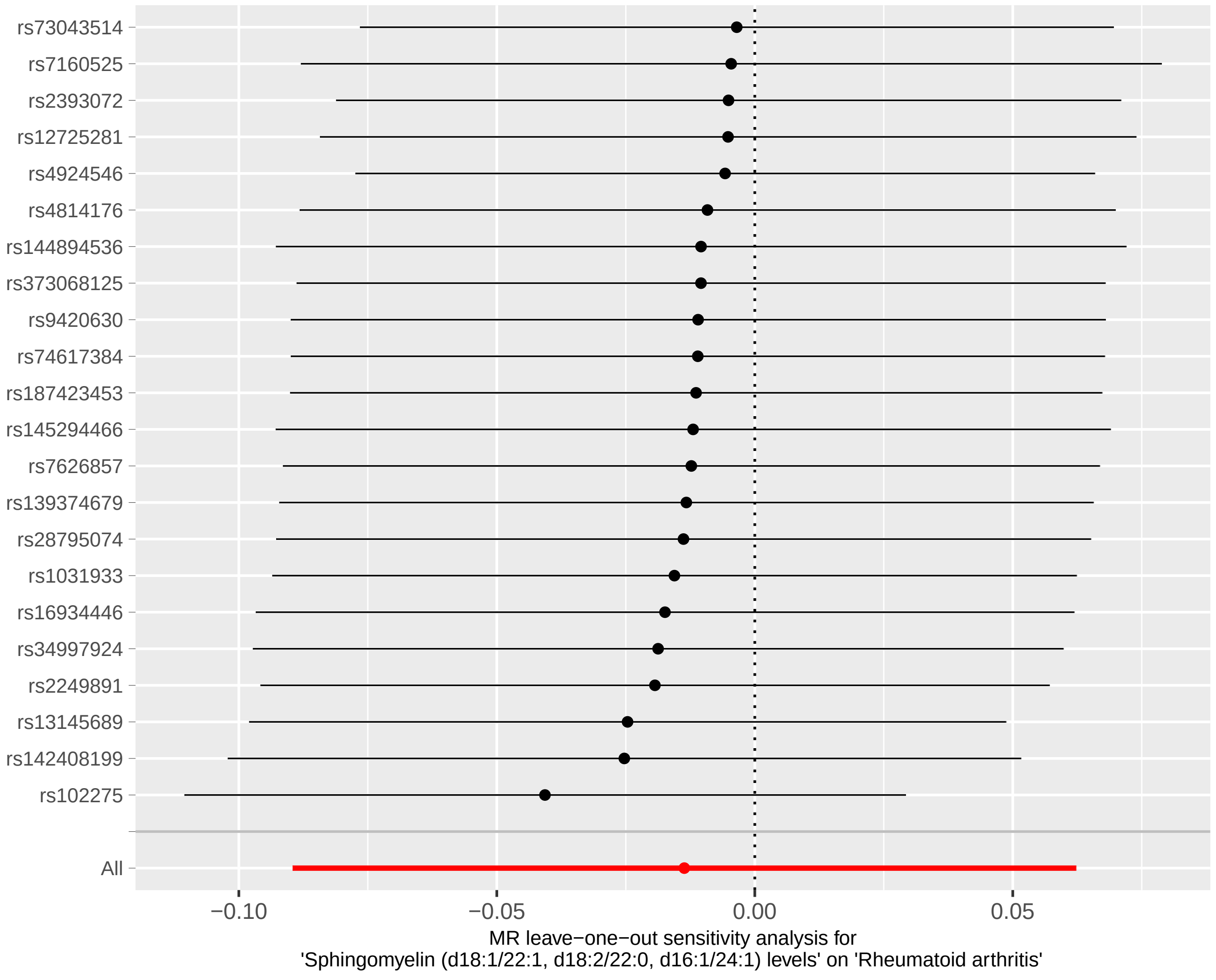

rs73043514
rs7160525
rs2393072
rs12725281
rs4924546
rs4814176
rs144894536
rs373068125
rs9420630
rs74617384
rs187423453
rs145294466
rs7626857
rs139374679
rs28795074
rs1031933
rs16934446
rs34997924
rs2249891
rs13145689
rs142408199
rs102275
All
−0.10
−0.05
0.00
0.05
MR leave−one−out sensitivity analysis for
'Sphingomyelin (d18:1/22:1, d18:2/22:0, d16:1/24:1) levels' on 'Rheumatoid arthritis'

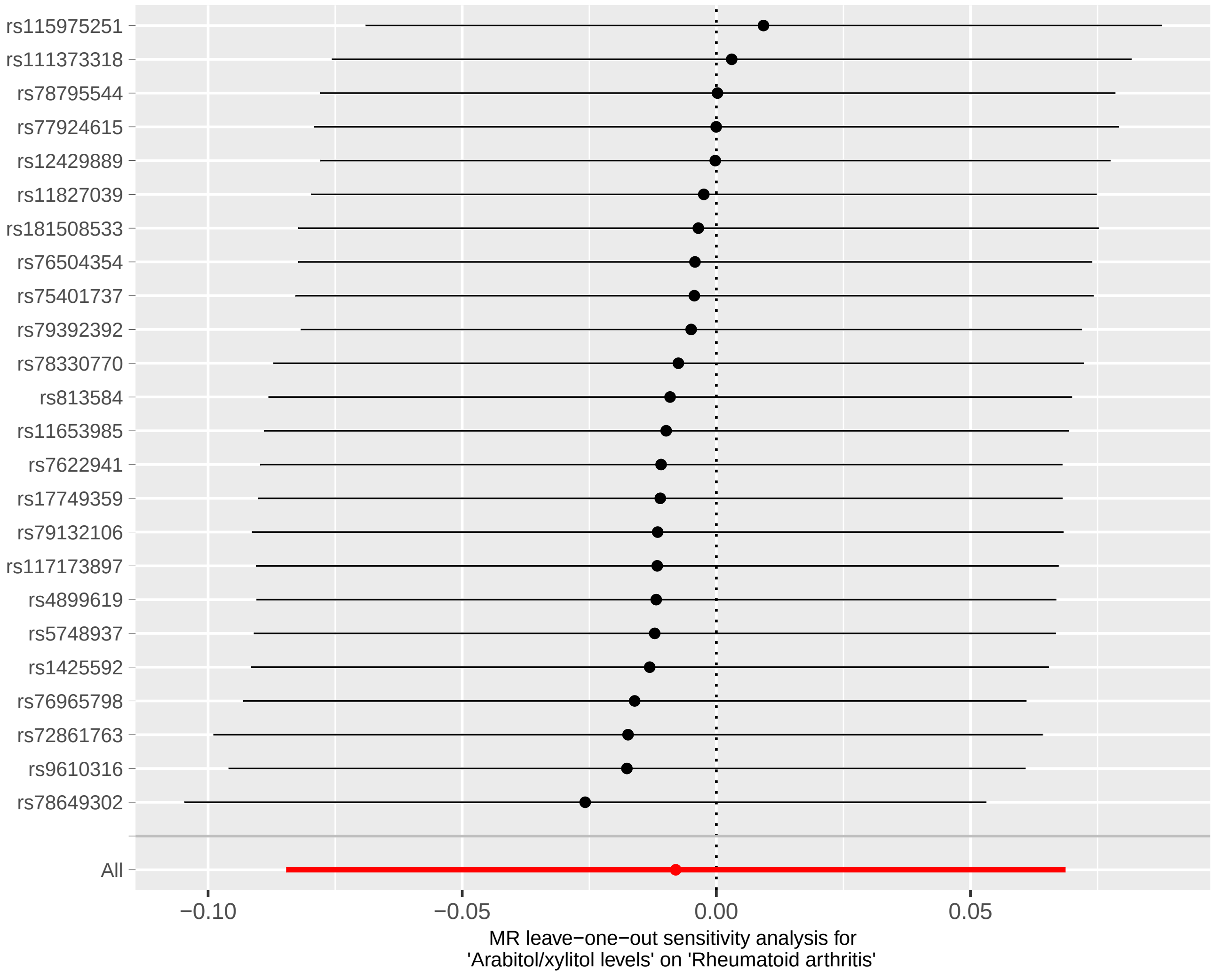

rs115975251
rs111373318
rs78795544
rs77924615
rs12429889
rs11827039
rs181508533
rs76504354
rs75401737
rs79392392
rs78330770
rs813584
rs11653985
rs7622941
rs17749359
rs79132106
rs117173897
rs4899619
rs5748937
rs1425592
rs76965798
rs72861763
rs9610316
rs78649302
All
−0.10
−0.05
0.00
0.05
MR leave−one−out sensitivity analysis for
'Arabitol/xylitol levels' on 'Rheumatoid arthritis'

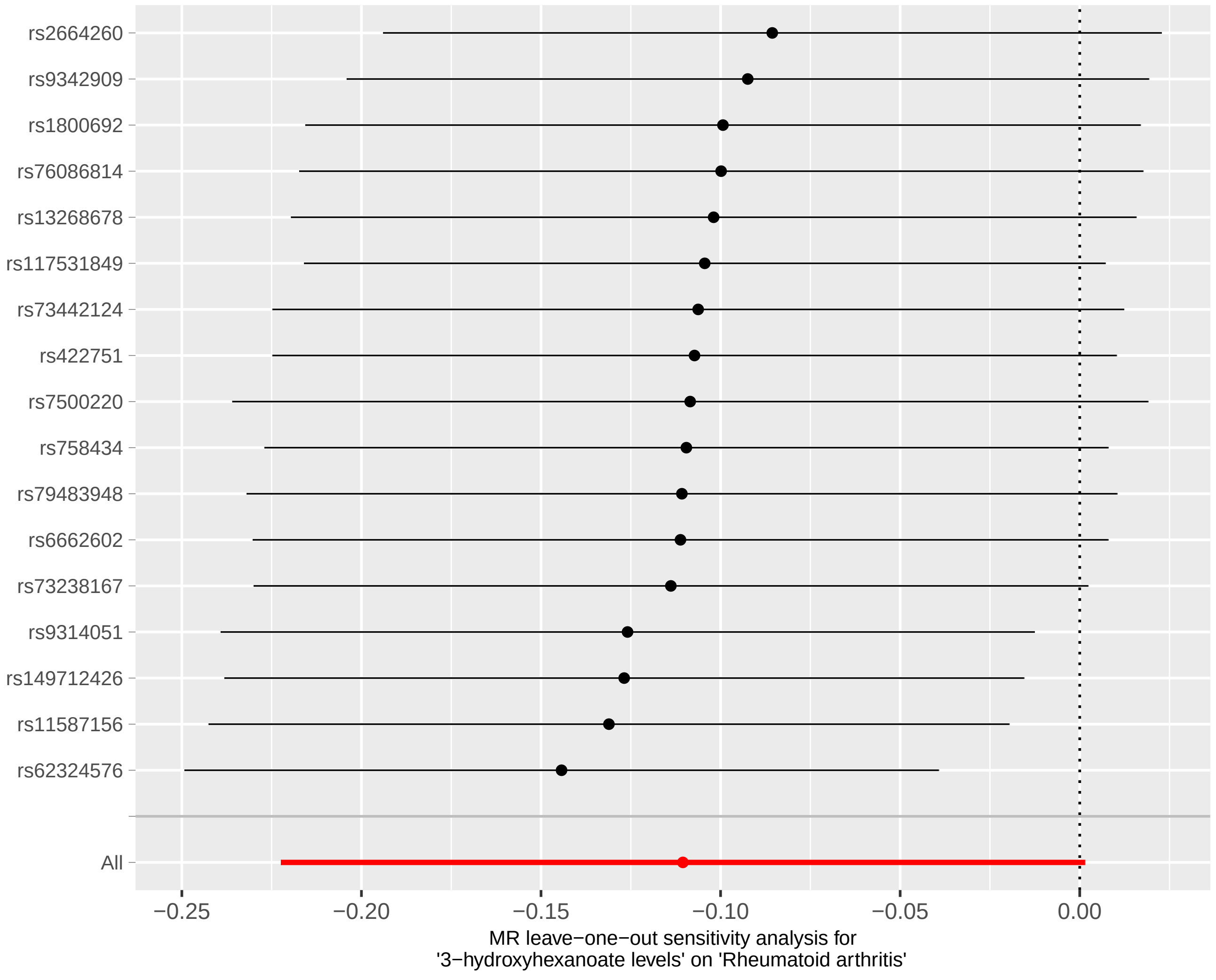

rs2664260
rs9342909
rs1800692
rs76086814
rs13268678
rs117531849
rs73442124
rs422751
rs7500220
rs758434
rs79483948
rs6662602
rs73238167
rs9314051
rs149712426
rs11587156
rs62324576
All
−0.25
−0.20
−0.15
−0.10
−0.05
0.00
MR leave−one−out sensitivity analysis for
'3−hydroxyhexanoate levels' on 'Rheumatoid arthritis'

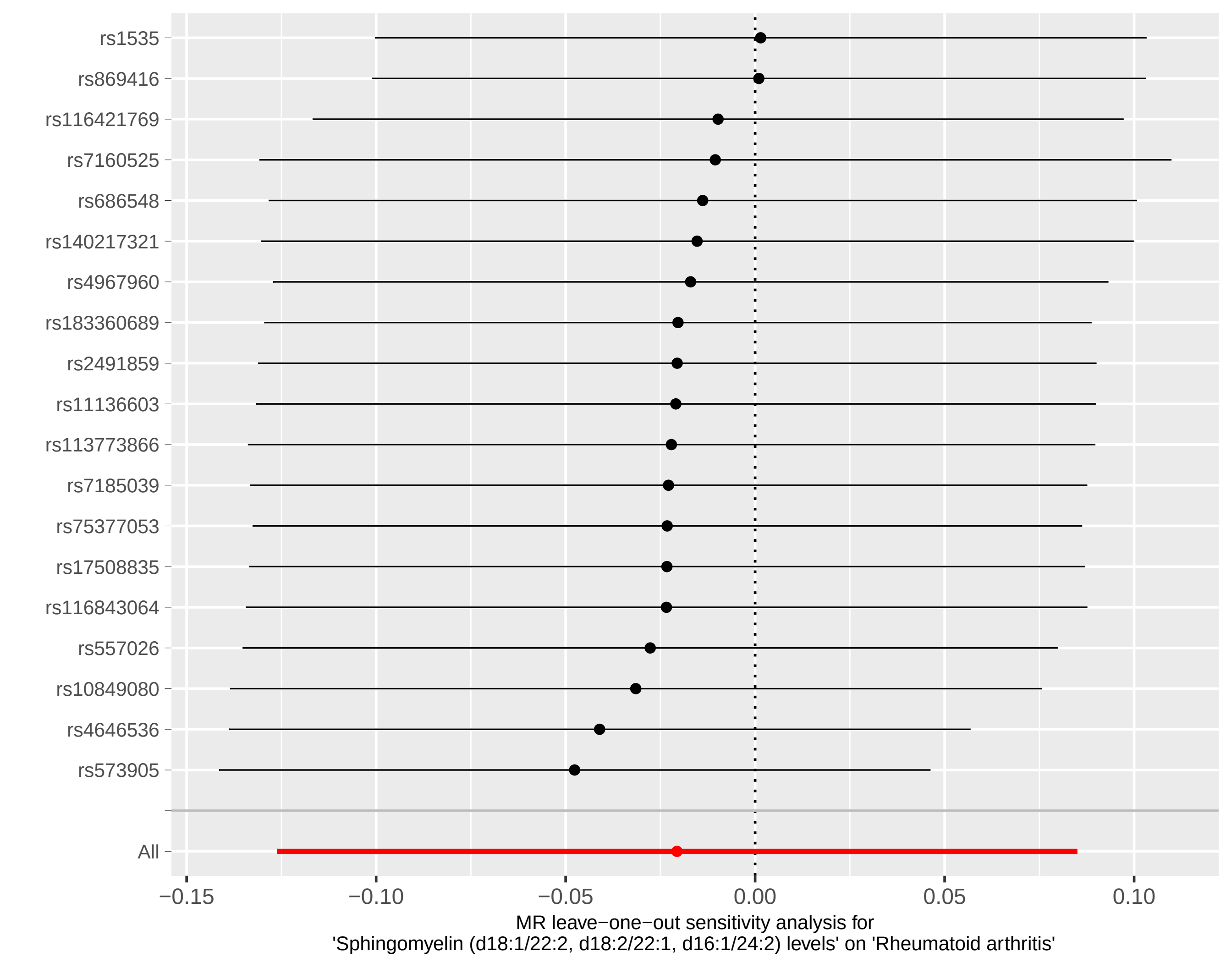

rs1535
rs869416
rs116421769
rs7160525
rs686548
rs140217321
rs4967960
rs183360689
rs2491859
rs11136603
rs113773866
rs7185039
rs75377053
rs17508835
rs116843064
rs557026
rs10849080
rs4646536
rs573905
All
−0.15
−0.10
−0.05
0.00
0.05
0.10
MR leave−one−out sensitivity analysis for
'Sphingomyelin (d18:1/22:2, d18:2/22:1, d16:1/24:2) levels' on 'Rheumatoid arthritis'

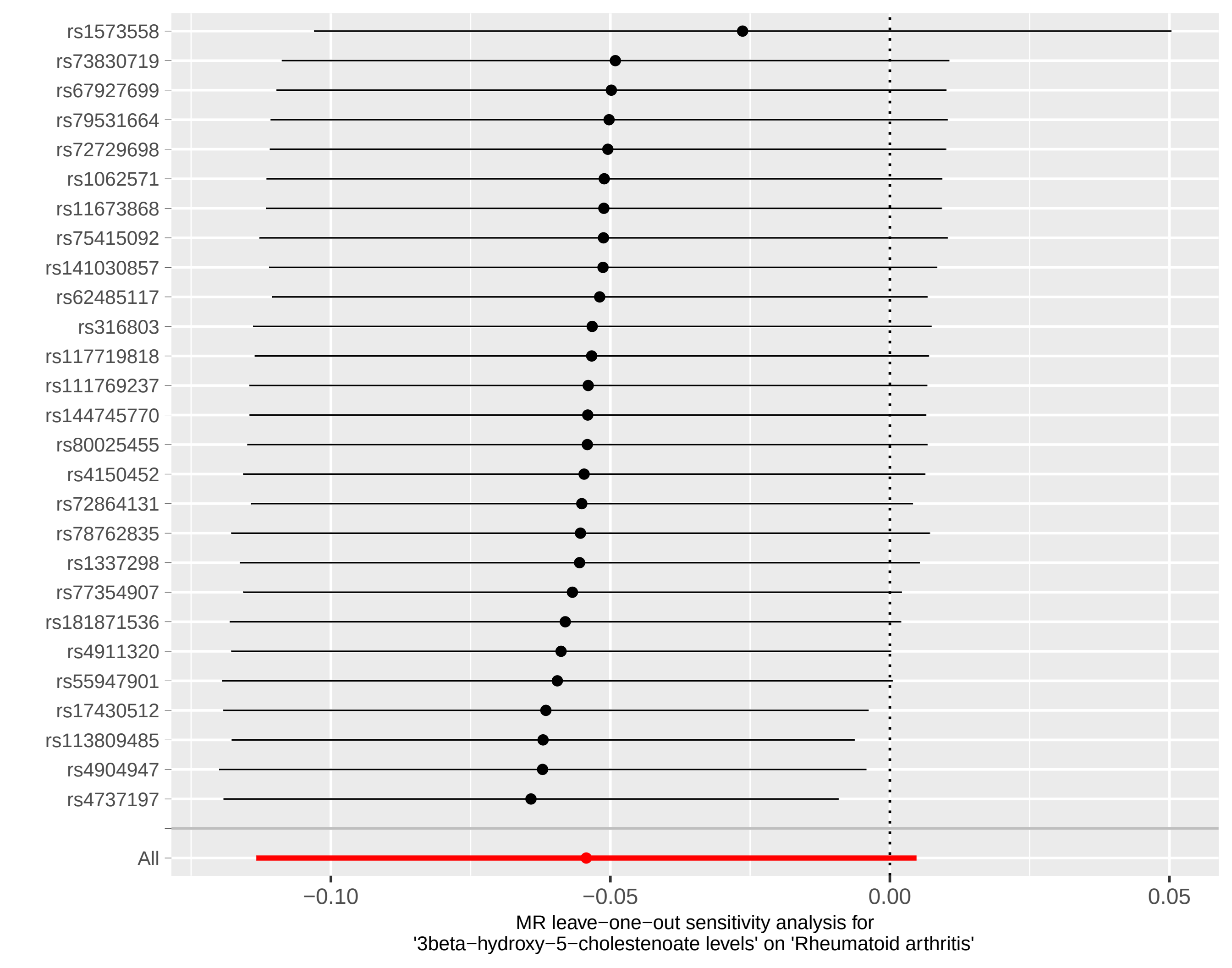

rs1573558
rs73830719
rs67927699
rs79531664
rs72729698
rs1062571
rs11673868
rs75415092
rs141030857
rs62485117
rs316803
rs117719818
rs111769237
rs144745770
rs80025455
rs4150452
rs72864131
rs78762835
rs1337298
rs77354907
rs181871536
rs4911320
rs55947901
rs17430512
rs113809485
rs4904947
rs4737197
All
−0.10
−0.05
0.00
0.05
MR leave−one−out sensitivity analysis for
'3beta−hydroxy−5−cholestenoate levels' on 'Rheumatoid arthritis'

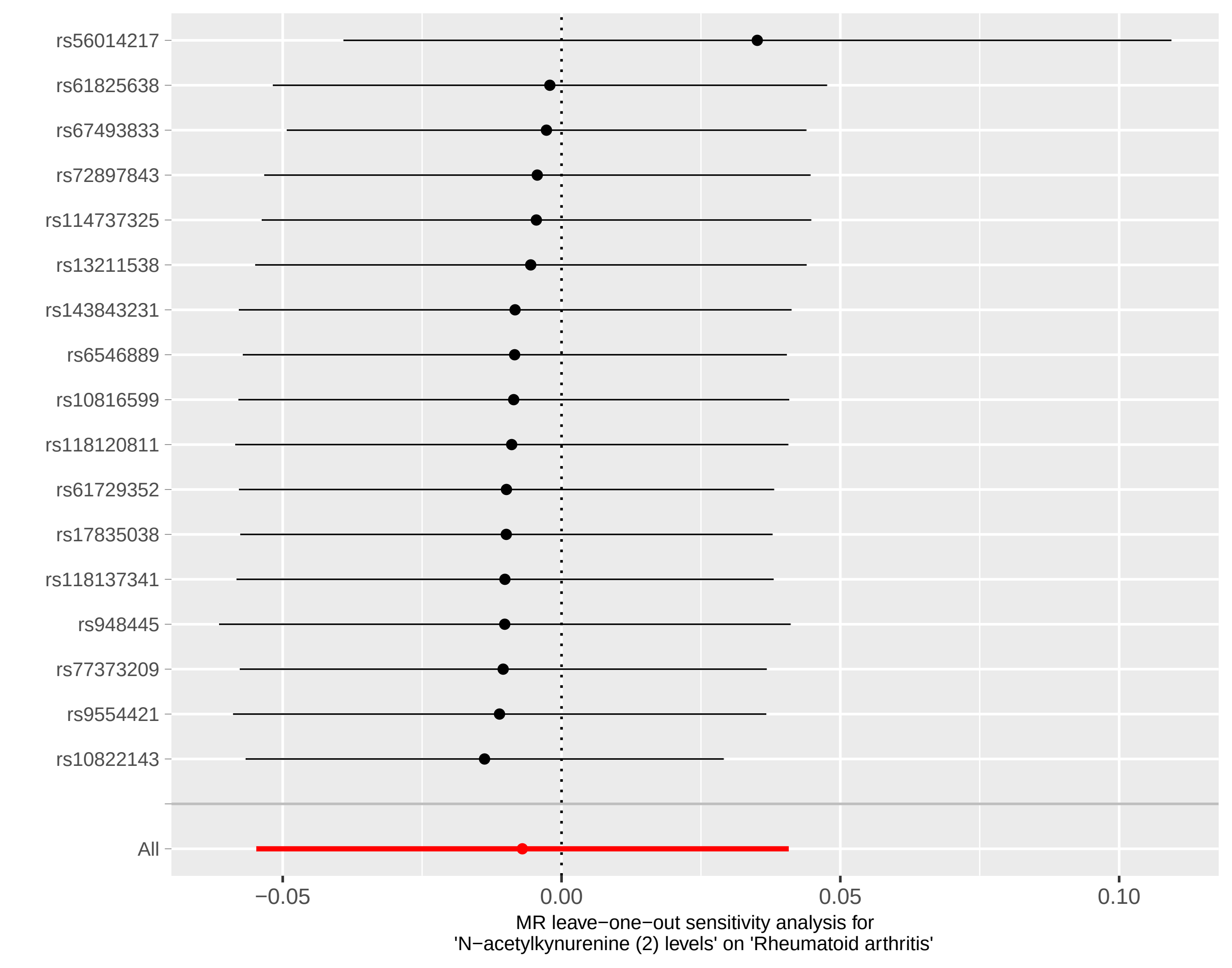
rs56014217
rs61825638
rs67493833
rs72897843
rs114737325
rs13211538
rs143843231
rs6546889
rs10816599
rs118120811
rs61729352
rs17835038
rs118137341
rs948445
rs77373209
rs9554421
rs10822143
All
−0.05
0.00
0.05
0.10
MR leave−one−out sensitivity analysis for
'N−acetylkynurenine (2) levels' on 'Rheumatoid arthritis'

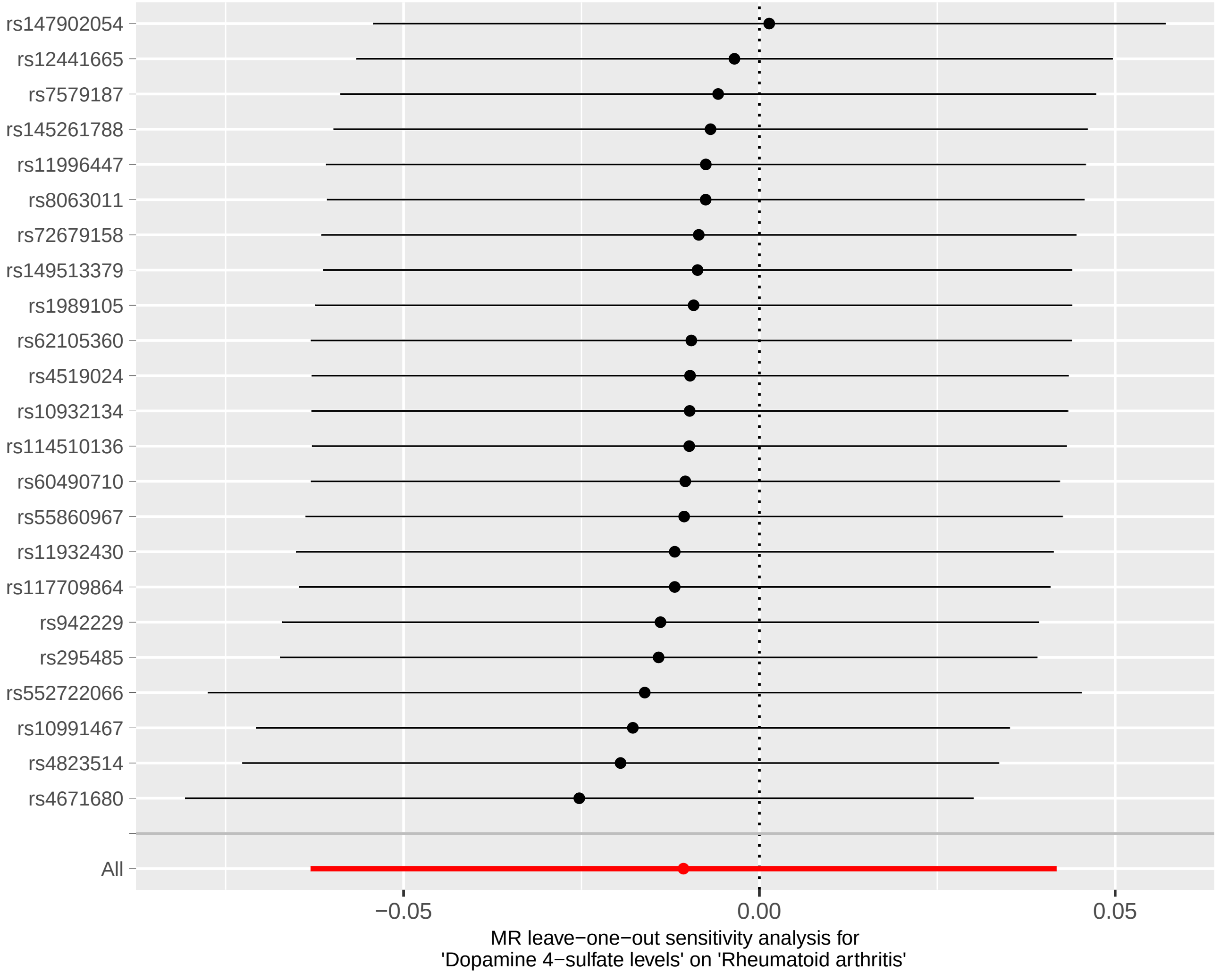
rs147902054
rs12441665
rs7579187
rs145261788
rs11996447
rs8063011
rs72679158
rs149513379
rs1989105
rs62105360
rs4519024
rs10932134
rs114510136
rs60490710
rs55860967
rs11932430
rs117709864
rs942229
rs295485
rs552722066
rs10991467
rs4823514
rs4671680
All
−0.05
0.00
0.05
MR leave−one−out sensitivity analysis for
'Dopamine 4−sulfate levels' on 'Rheumatoid arthritis'

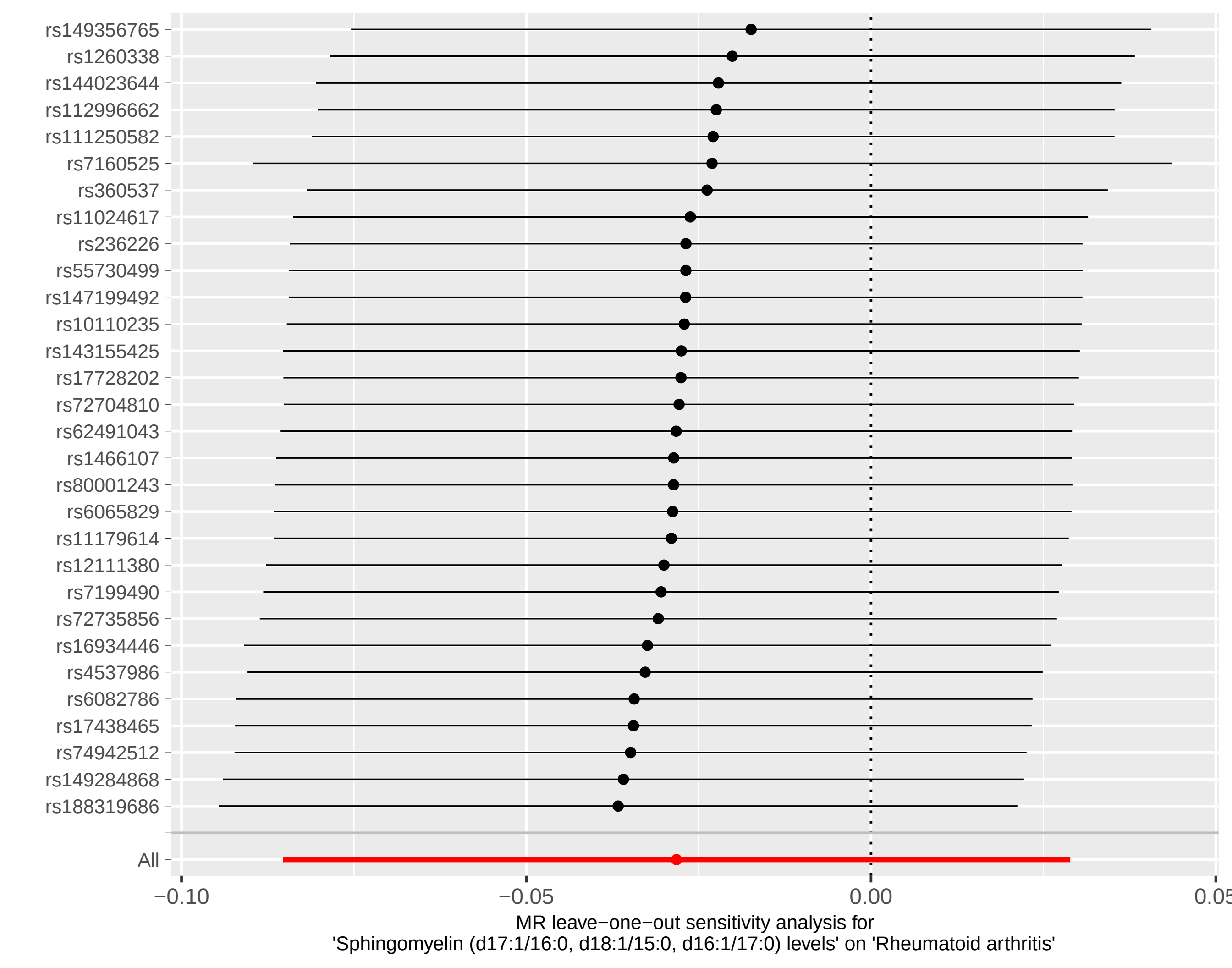

rs149356765
rs1260338
rs144023644
rs112996662
rs111250582
rs7160525
rs360537
rs11024617
rs236226
rs55730499
rs147199492
rs10110235
rs143155425
rs17728202
rs72704810
rs62491043
rs1466107
rs80001243
rs6065829
rs11179614
rs12111380
rs7199490
rs72735856
rs16934446
rs4537986
rs6082786
rs17438465
rs74942512
rs149284868
rs188319686
All
−0.10
−0.05
0.00
0.05
MR leave−one−out sensitivity analysis for
'Sphingomyelin (d17:1/16:0, d18:1/15:0, d16:1/17:0) levels' on 'Rheumatoid arthritis'

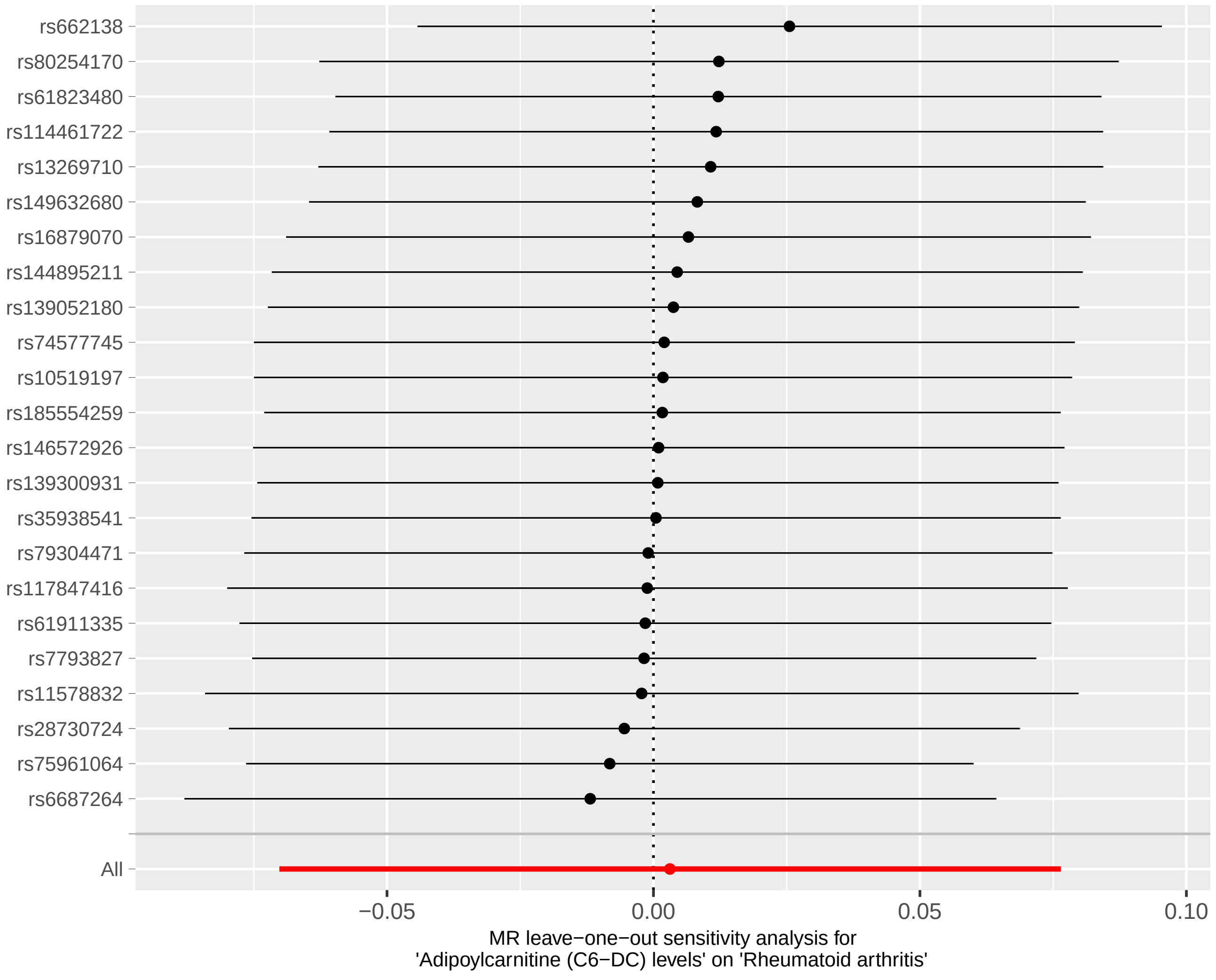
rs662138
rs80254170
rs61823480
rs114461722
rs13269710
rs149632680
rs16879070
rs144895211
rs139052180
rs74577745
rs10519197
rs185554259
rs146572926
rs139300931
rs35938541
rs79304471
rs117847416
rs61911335
rs7793827
rs11578832
rs28730724
rs75961064
rs6687264
All
−0.05
0.00
0.05
0.10
MR leave−one−out sensitivity analysis for
'Adipoylcarnitine (C6−DC) levels' on 'Rheumatoid arthritis'

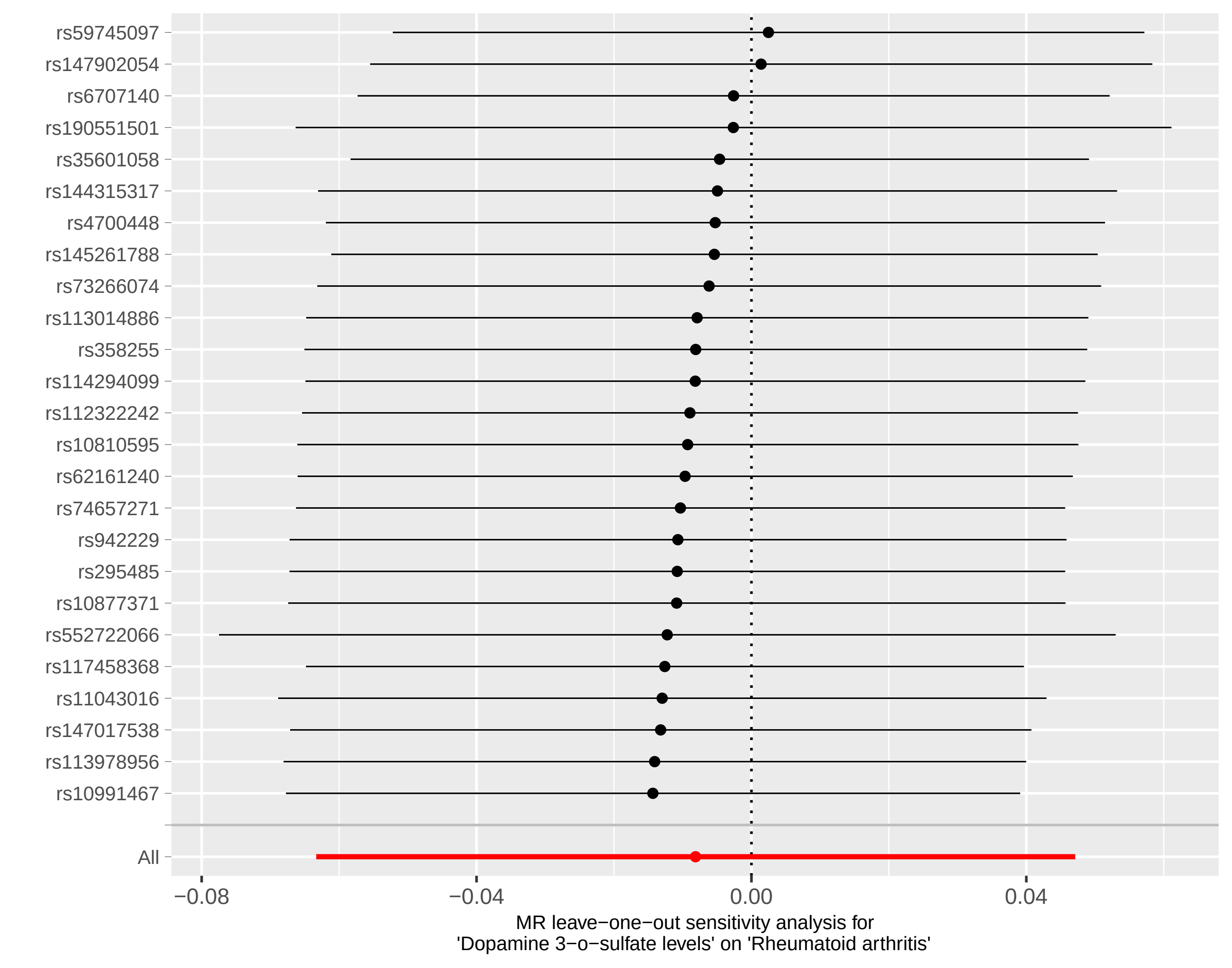
rs59745097
rs147902054
rs6707140
rs190551501
rs35601058
rs144315317
rs4700448
rs145261788
rs73266074
rs113014886
rs358255
rs114294099
rs112322242
rs10810595
rs62161240
rs74657271
rs942229
rs295485
rs10877371
rs552722066
rs117458368
rs11043016
rs147017538
rs113978956
rs10991467
All
−0.08
−0.04
0.00
0.04
MR leave−one−out sensitivity analysis for
'Dopamine 3−o−sulfate levels' on 'Rheumatoid arthritis'

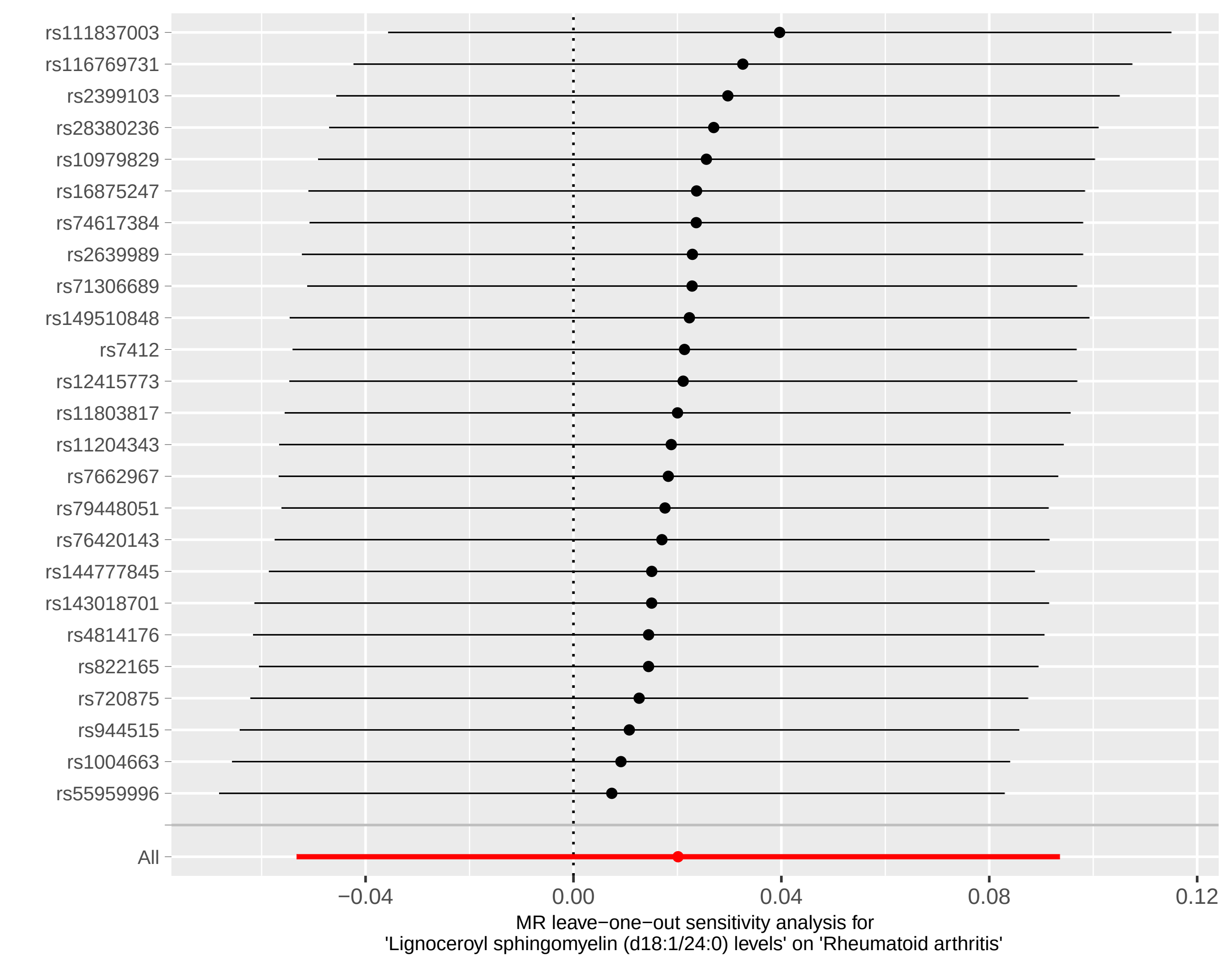
rs111837003
rs116769731
rs2399103
rs28380236
rs10979829
rs16875247
rs74617384
rs2639989
rs71306689
rs149510848
rs7412
rs12415773
rs11803817
rs11204343
rs7662967
rs79448051
rs76420143
rs144777845
rs143018701
rs4814176
rs822165
rs720875
rs944515
rs1004663
rs55959996
All
−0.04
0.00
0.04
0.08
0.12
MR leave−one−out sensitivity analysis for
'Lignoceroyl sphingomyelin (d18:1/24:0) levels' on 'Rheumatoid arthritis'

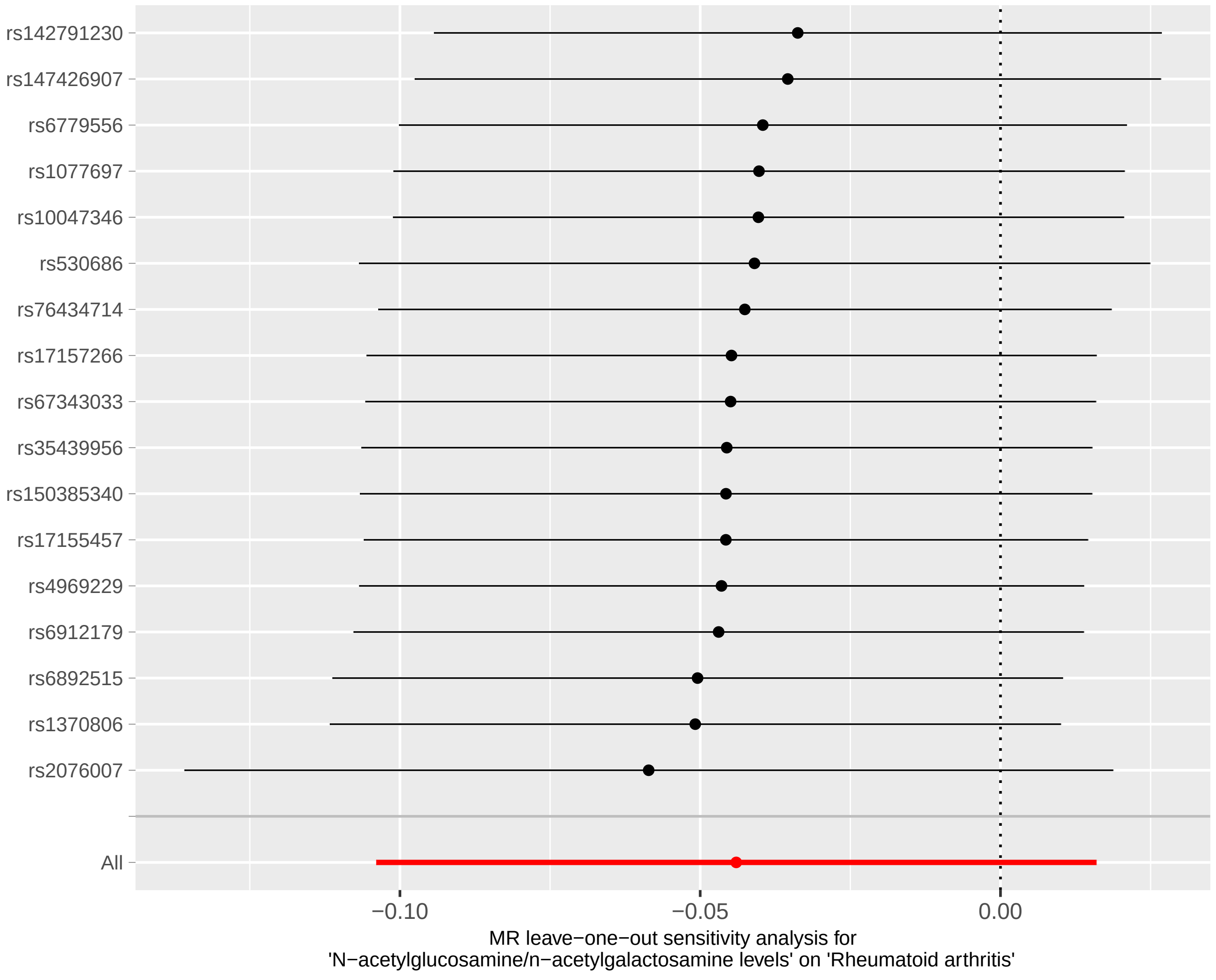
rs142791230
rs147426907
rs6779556
rs1077697
rs10047346
rs530686
rs76434714
rs17157266
rs67343033
rs35439956
rs150385340
rs17155457
rs4969229
rs6912179
rs6892515
rs1370806
rs2076007
All
−0.10
−0.05
0.00
MR leave−one−out sensitivity analysis for
'N−acetylglucosamine/n−acetylgalactosamine levels' on 'Rheumatoid arthritis'

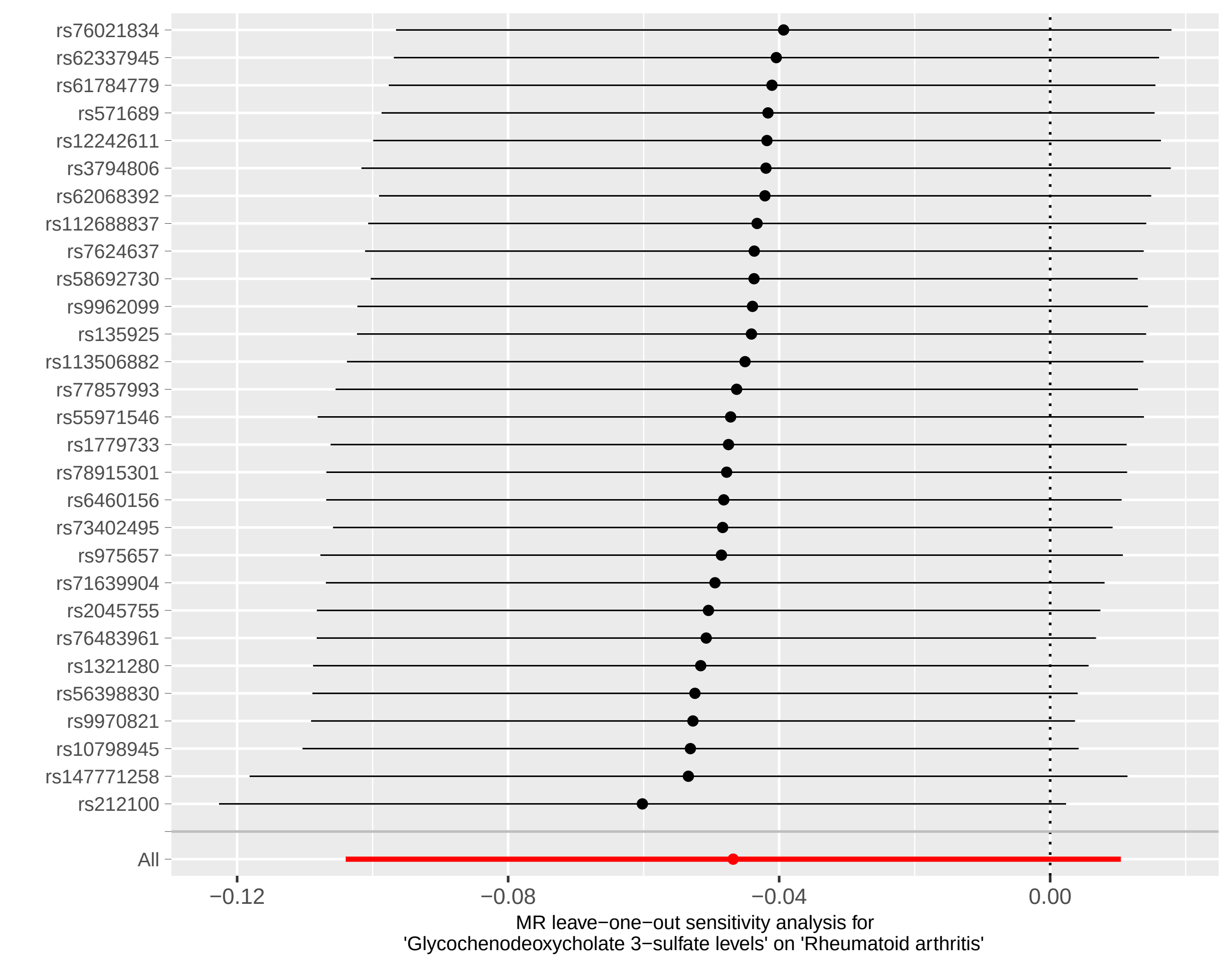
rs76021834
rs62337945
rs61784779
rs571689
rs12242611
rs3794806
rs62068392
rs112688837
rs7624637
rs58692730
rs9962099
rs135925
rs113506882
rs77857993
rs55971546
rs1779733
rs78915301
rs6460156
rs73402495
rs975657
rs71639904
rs2045755
rs76483961
rs1321280
rs56398830
rs9970821
rs10798945
rs147771258
rs212100
All
−0.12
−0.08
−0.04
0.00
MR leave−one−out sensitivity analysis for
'Glycochenodeoxycholate 3−sulfate levels' on 'Rheumatoid arthritis'

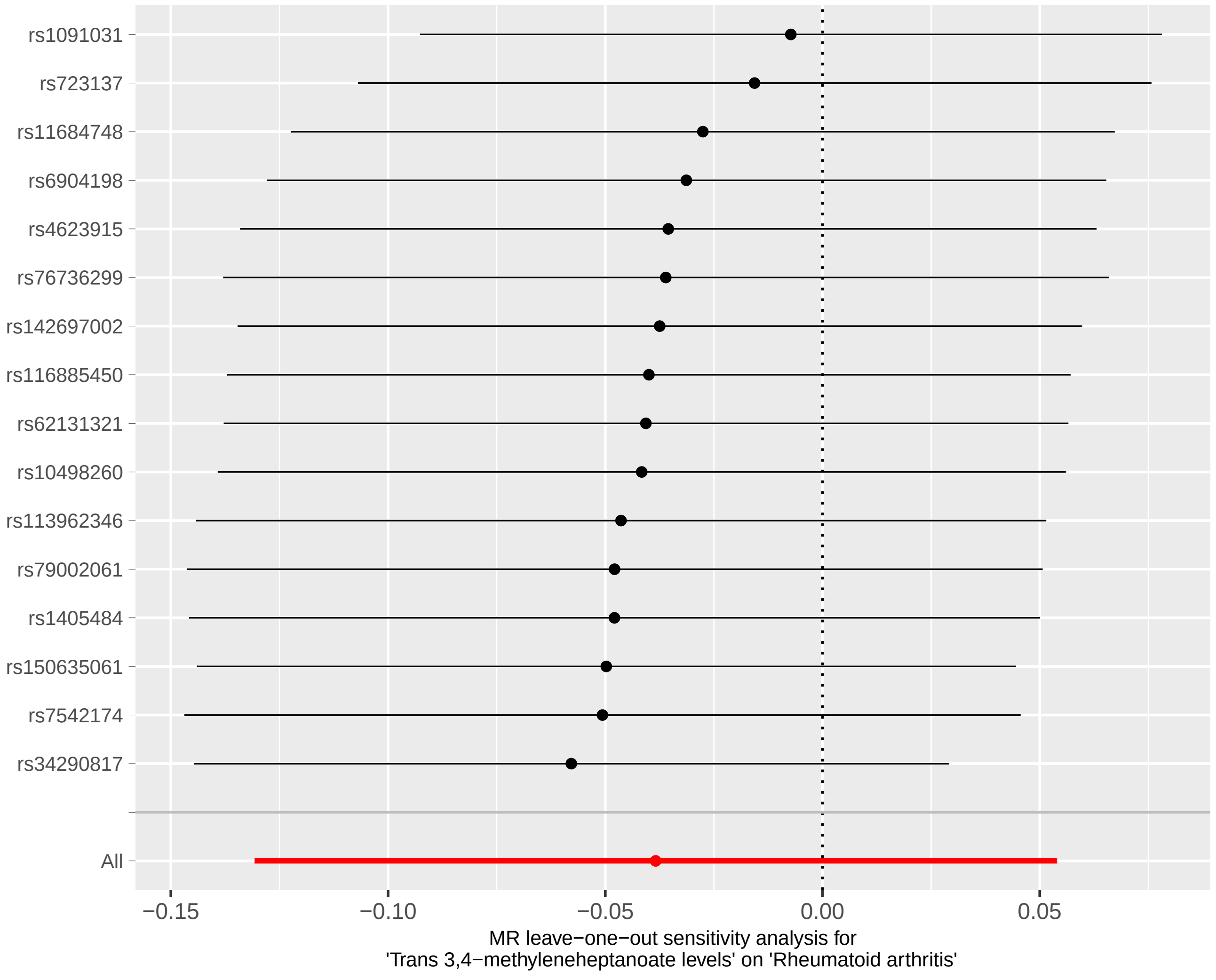
rs1091031
rs723137
rs11684748
rs6904198
rs4623915
rs76736299
rs142697002
rs116885450
rs62131321
rs10498260
rs113962346
rs79002061
rs1405484
rs150635061
rs7542174
rs34290817
All
−0.15
−0.10
−0.05
0.00
0.05
MR leave−one−out sensitivity analysis for
'Trans 3,4−methyleneheptanoate levels' on 'Rheumatoid arthritis'

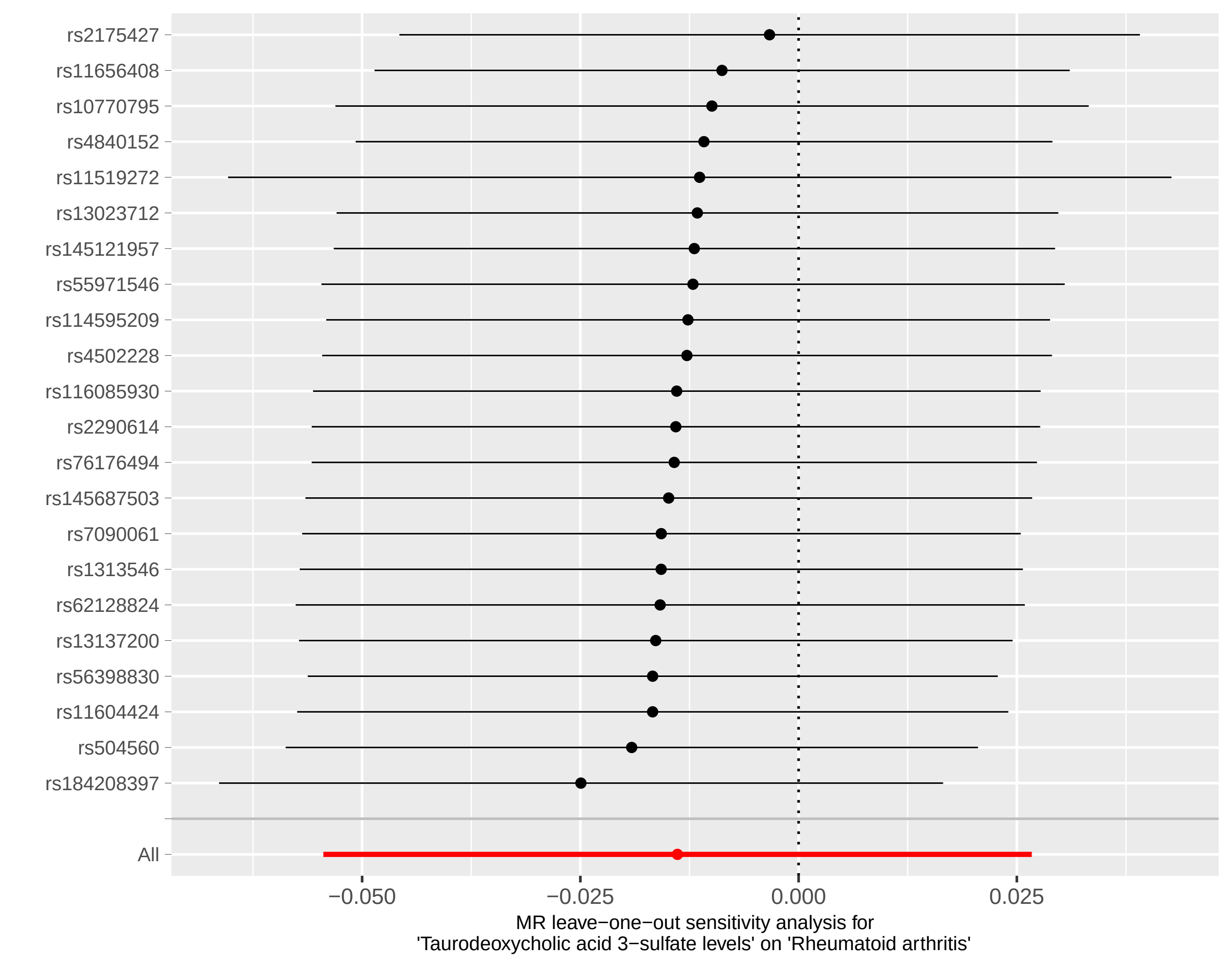
rs2175427
rs11656408
rs10770795
rs4840152
rs11519272
rs13023712
rs145121957
rs55971546
rs114595209
rs4502228
rs116085930
rs2290614
rs76176494
rs145687503
rs7090061
rs1313546
rs62128824
rs13137200
rs56398830
rs11604424
rs504560
rs184208397
All
−0.050
−0.025
0.000
0.025
MR leave−one−out sensitivity analysis for
'Taurodeoxycholic acid 3−sulfate levels' on 'Rheumatoid arthritis'

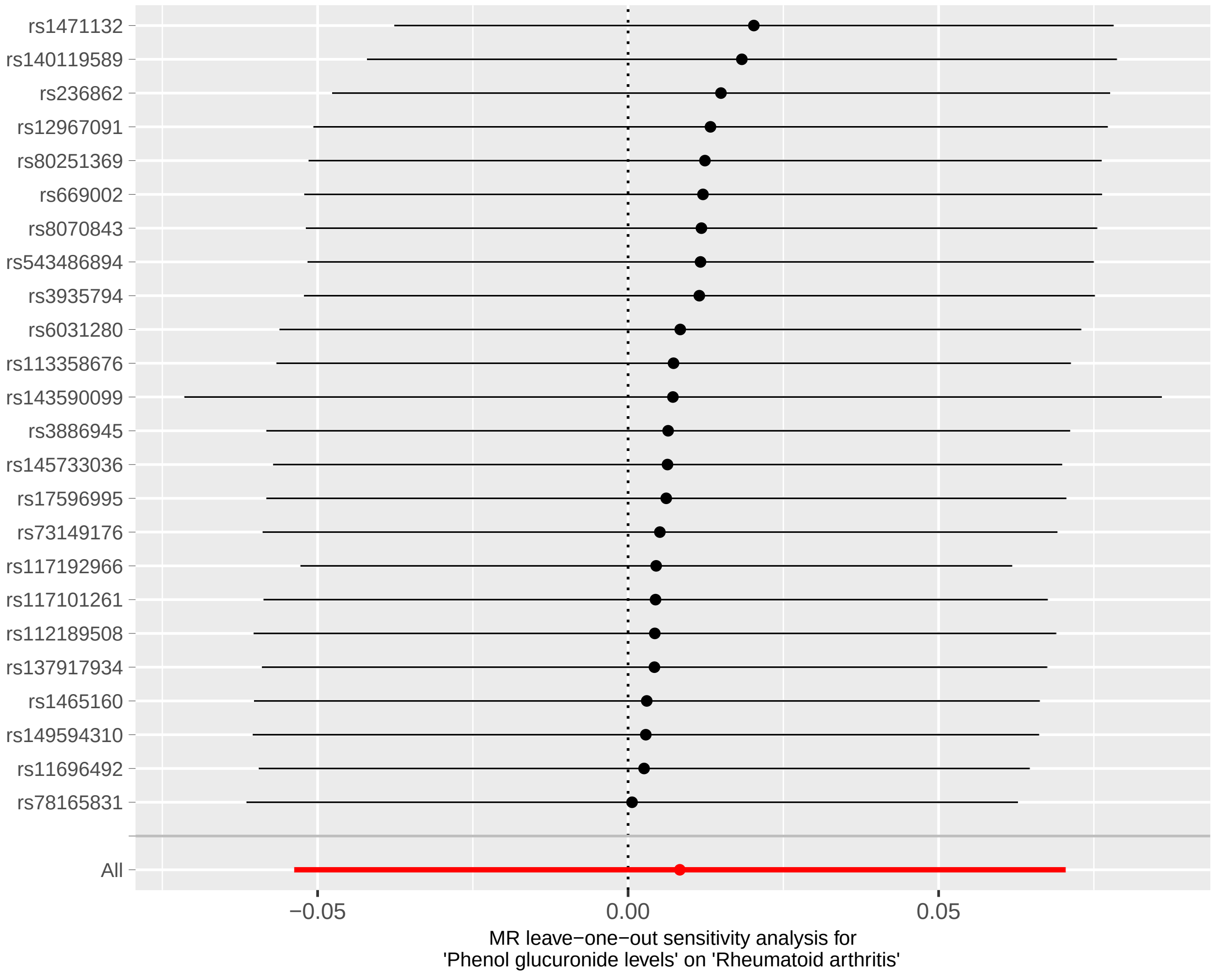

rs1471132
rs140119589
rs236862
rs12967091
rs80251369
rs669002
rs8070843
rs543486894
rs3935794
rs6031280
rs113358676
rs143590099
rs3886945
rs145733036
rs17596995
rs73149176
rs117192966
rs117101261
rs112189508
rs137917934
rs1465160
rs149594310
rs11696492
rs78165831
All
−0.05
0.00
0.05
MR leave−one−out sensitivity analysis for
'Phenol glucuronide levels' on 'Rheumatoid arthritis'

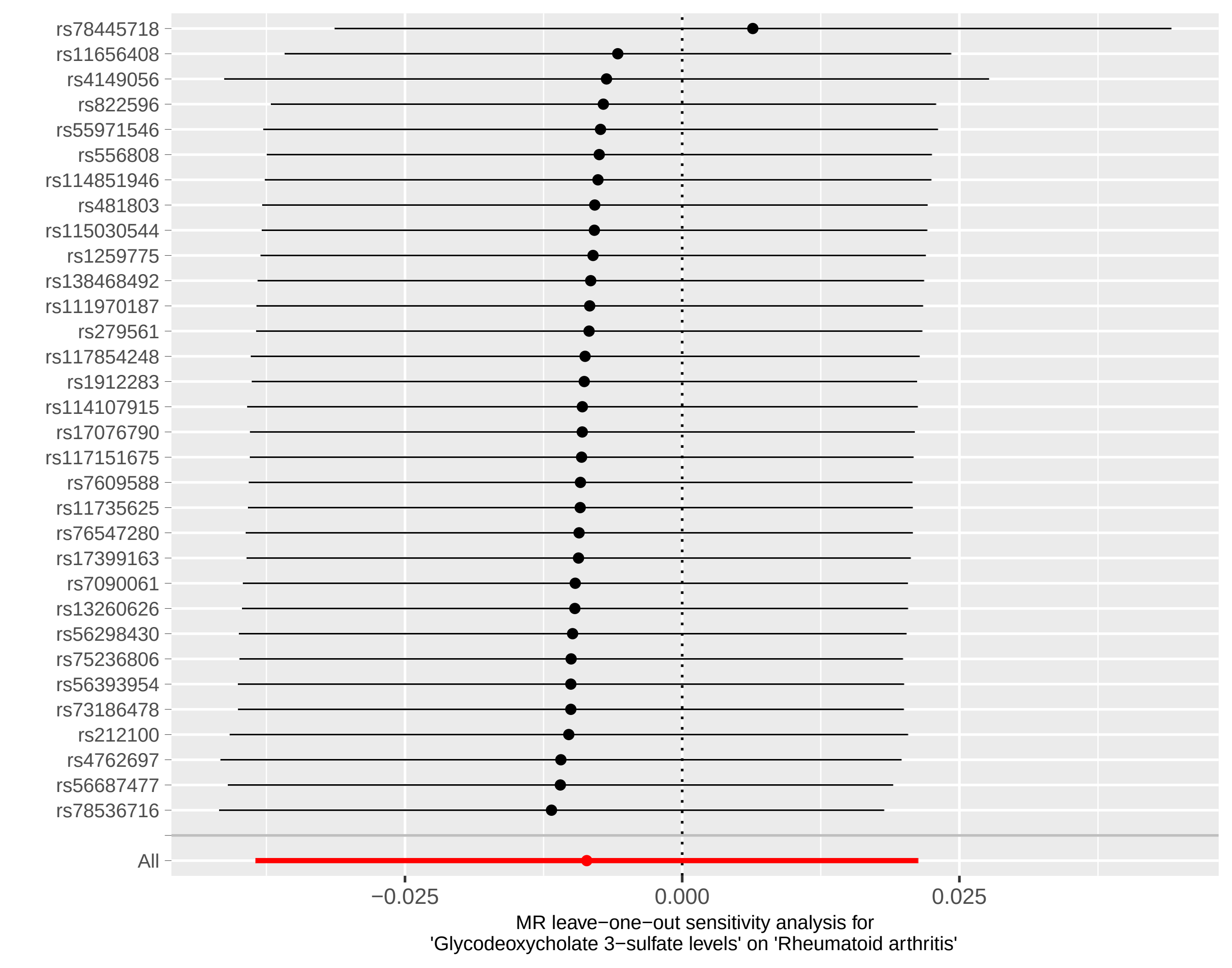

rs78445718
rs11656408
rs4149056
rs822596
rs55971546
rs556808
rs114851946
rs481803
rs115030544
rs1259775
rs138468492
rs111970187
rs279561
rs117854248
rs1912283
rs114107915
rs17076790
rs117151675
rs7609588
rs11735625
rs76547280
rs17399163
rs7090061
rs13260626
rs56298430
rs75236806
rs56393954
rs73186478
rs212100
rs4762697
rs56687477
rs78536716
All
−0.025
0.000
0.025
MR leave−one−out sensitivity analysis for
'Glycodeoxycholate 3−sulfate levels' on 'Rheumatoid arthritis'

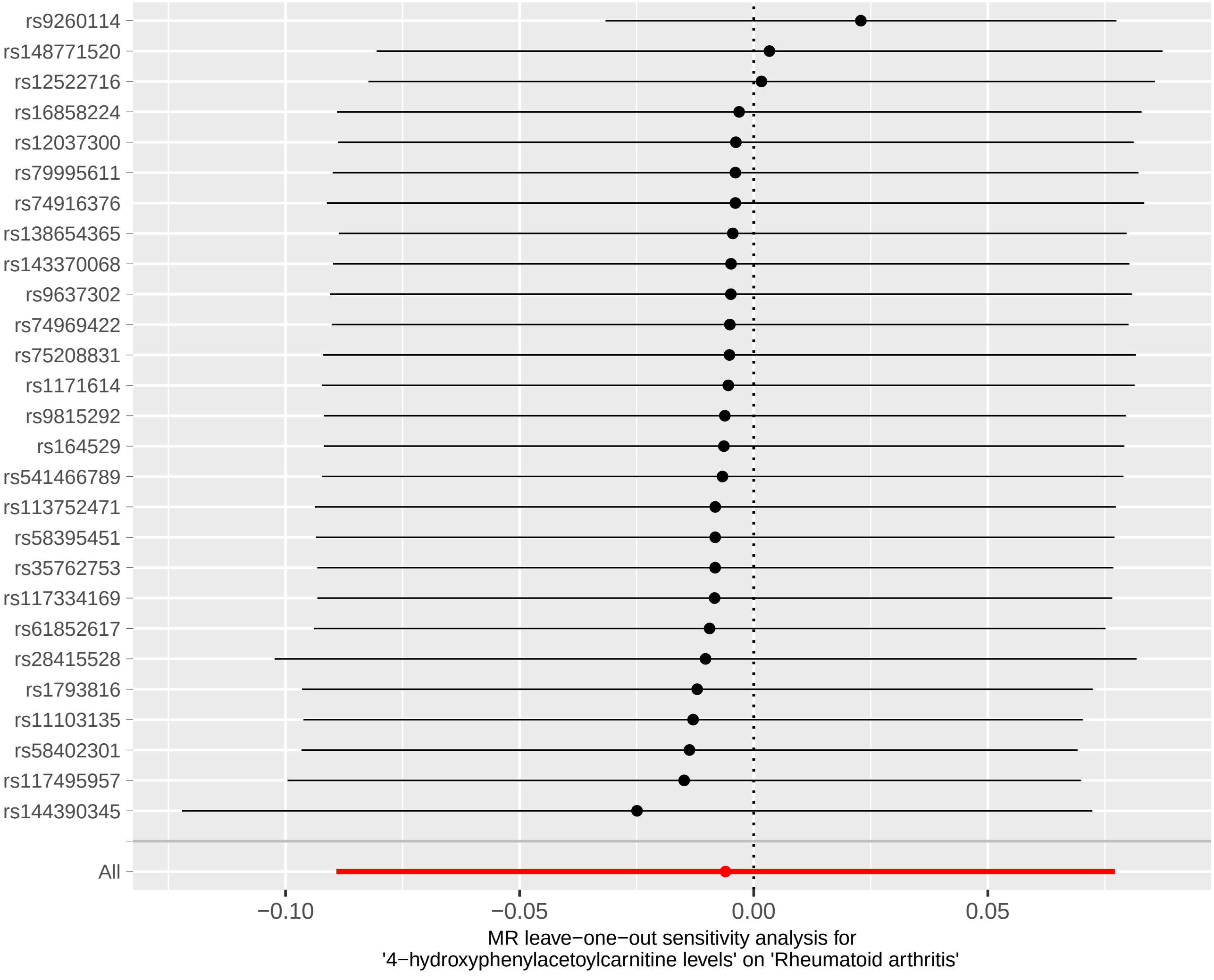
rs9260114
rs148771520
rs12522716
rs16858224
rs12037300
rs79995611
rs74916376
rs138654365
rs143370068
rs9637302
rs74969422
rs75208831
rs1171614
rs9815292
rs164529
rs541466789
rs113752471
rs58395451
rs35762753
rs117334169
rs61852617
rs28415528
rs1793816
rs11103135
rs58402301
rs117495957
rs144390345
All
−0.10
−0.05
0.00
0.05
MR leave−one−out sensitivity analysis for
'4−hydroxyphenylacetoylcarnitine levels' on 'Rheumatoid arthritis'

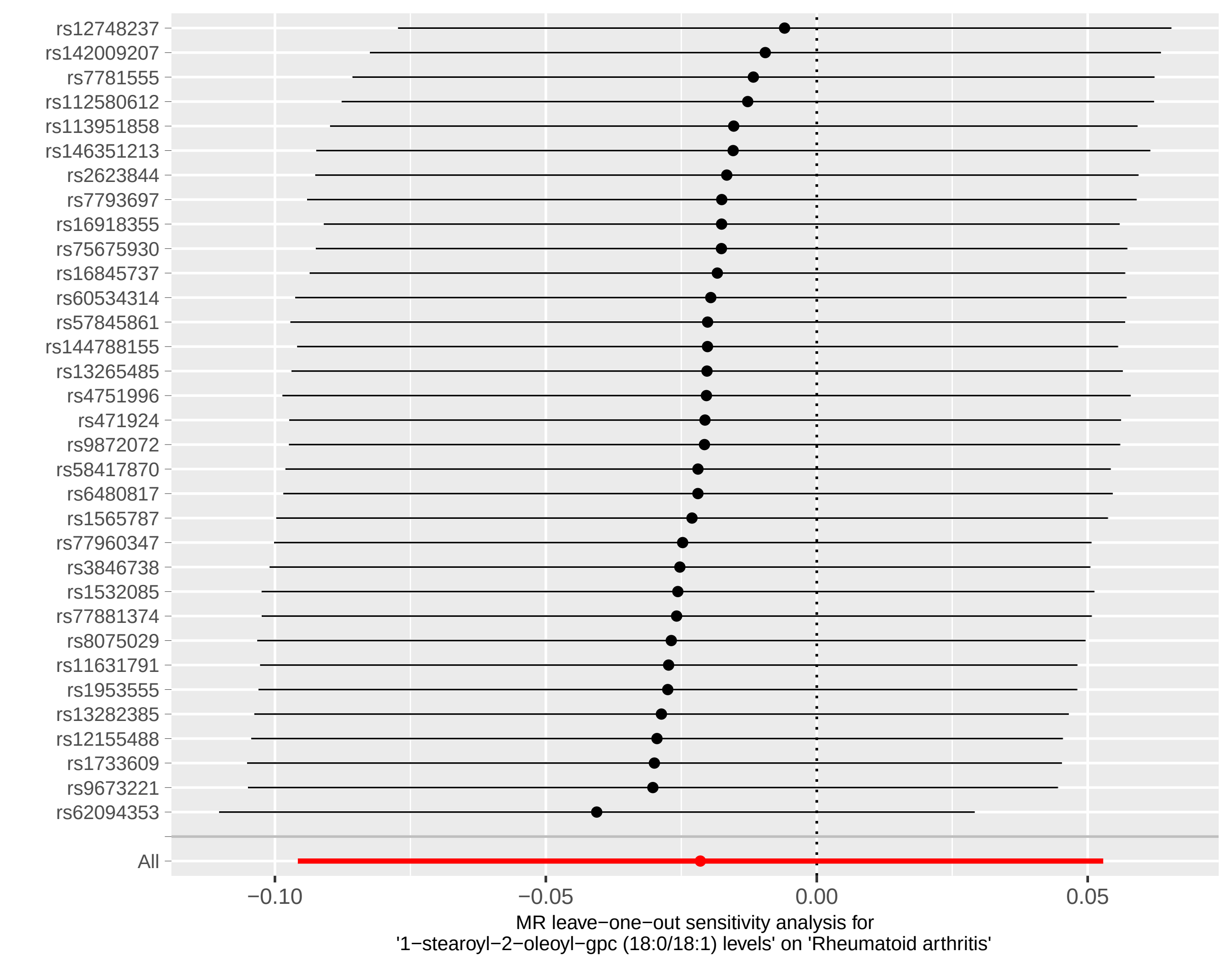
rs12748237
rs142009207
rs7781555
rs112580612
rs113951858
rs146351213
rs2623844
rs7793697
rs16918355
rs75675930
rs16845737
rs60534314
rs57845861
rs144788155
rs13265485
rs4751996
rs471924
rs9872072
rs58417870
rs6480817
rs1565787
rs77960347
rs3846738
rs1532085
rs77881374
rs8075029
rs11631791
rs1953555
rs13282385
rs12155488
rs1733609
rs9673221
rs62094353
All
−0.10
−0.05
0.00
0.05
MR leave−one−out sensitivity analysis for
'1−stearoyl−2−oleoyl−gpc (18:0/18:1) levels' on 'Rheumatoid arthritis'

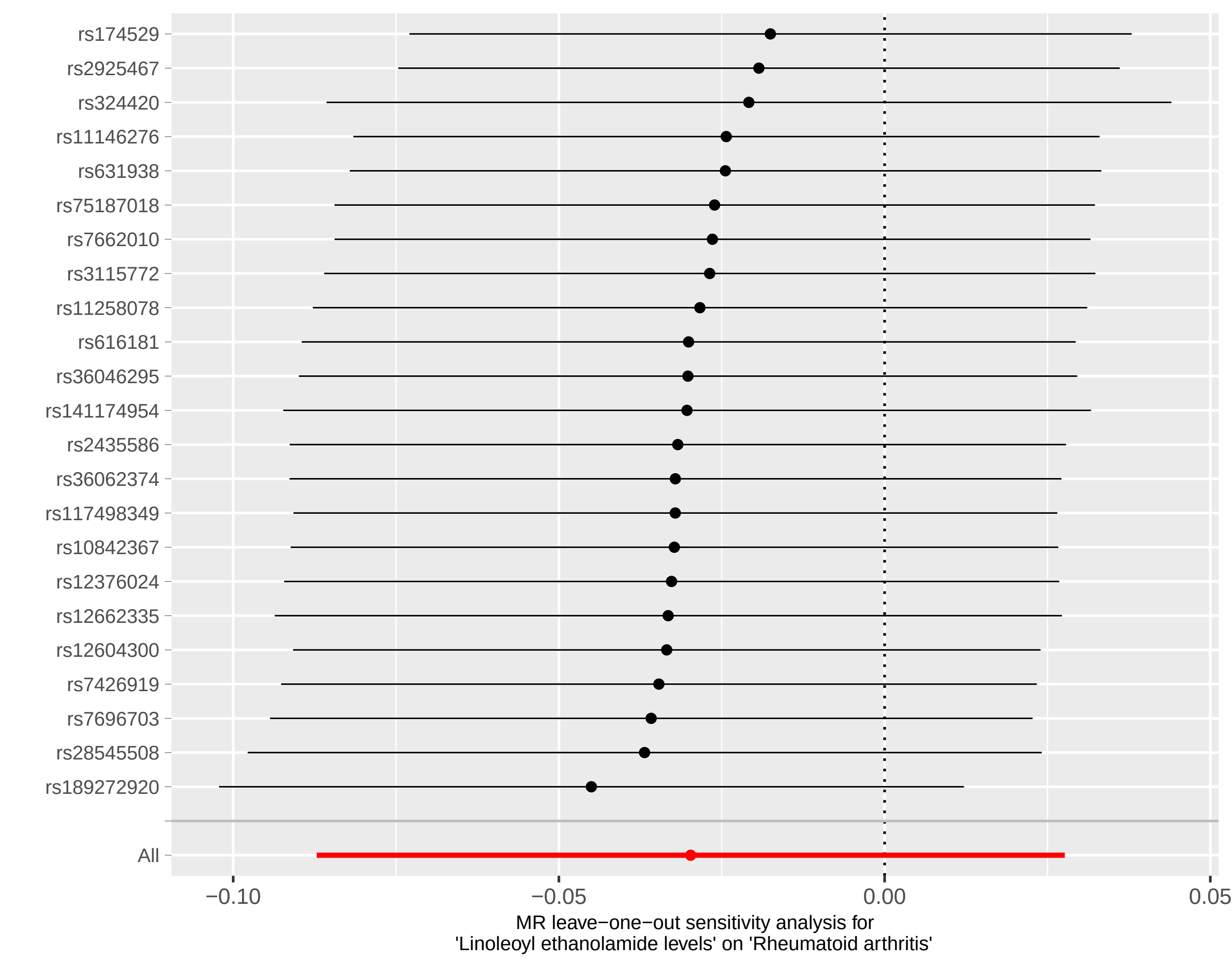

rs174529
rs2925467
rs324420
rs11146276
rs631938
rs75187018
rs7662010
rs3115772
rs11258078
rs616181
rs36046295
rs141174954
rs2435586
rs36062374
rs117498349
rs10842367
rs12376024
rs12662335
rs12604300
rs7426919
rs7696703
rs28545508
rs189272920
All
−0.10
−0.05
0.00
0.05
MR leave−one−out sensitivity analysis for
'Linoleoyl ethanolamide levels' on 'Rheumatoid arthritis'

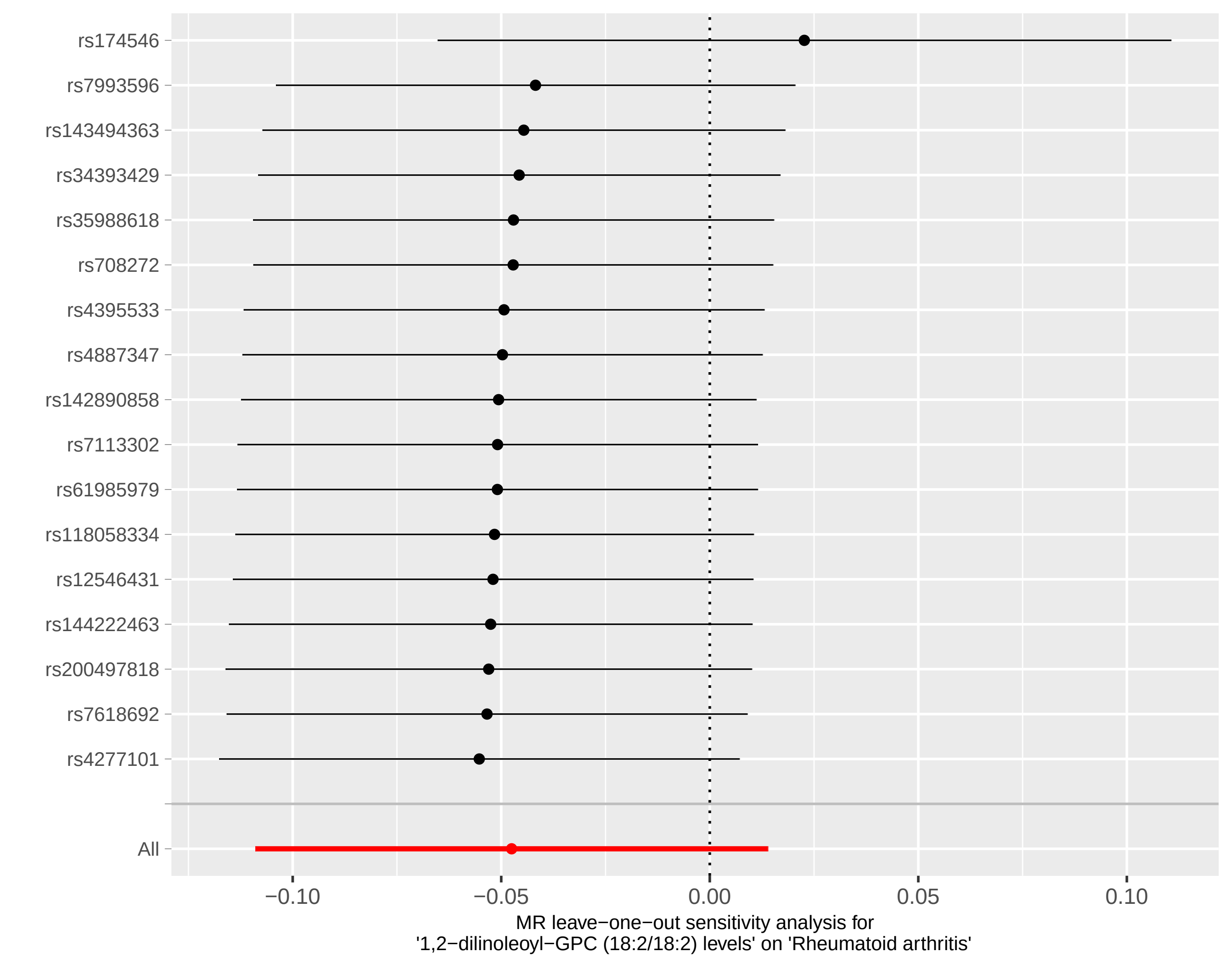

rs174546
rs7993596
rs143494363
rs34393429
rs35988618
rs708272
rs4395533
rs4887347
rs142890858
rs7113302
rs61985979
rs118058334
rs12546431
rs144222463
rs200497818
rs7618692
rs4277101
All
−0.10
−0.05
0.00
0.05
0.10
MR leave−one−out sensitivity analysis for
'1,2−dilinoleoyl−GPC (18:2/18:2) levels' on 'Rheumatoid arthritis'

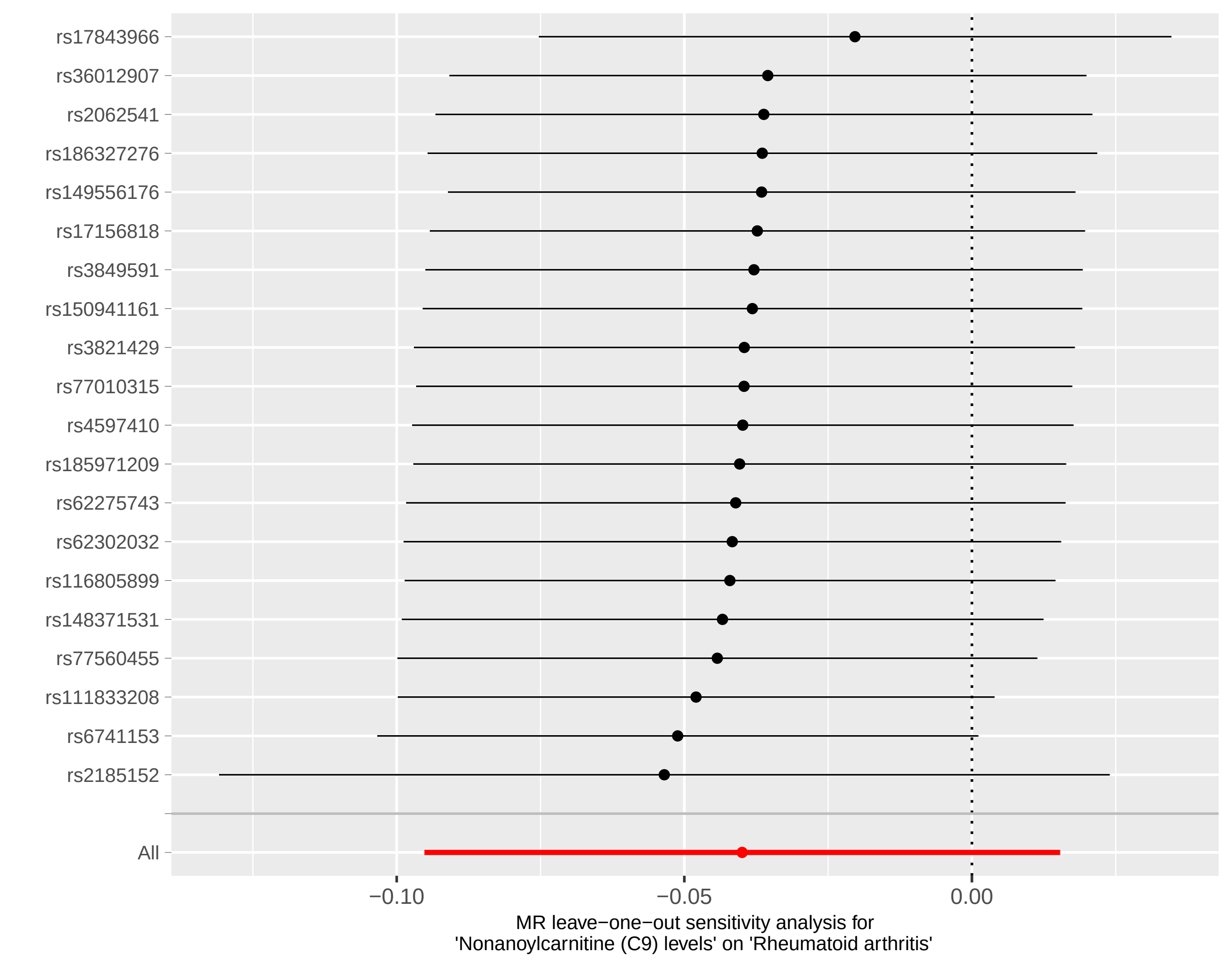
rs17843966
rs36012907
rs2062541
rs186327276
rs149556176
rs17156818
rs3849591
rs150941161
rs3821429
rs77010315
rs4597410
rs185971209
rs62275743
rs62302032
rs116805899
rs148371531
rs77560455
rs111833208
rs6741153
rs2185152
All
−0.10
−0.05
0.00
MR leave−one−out sensitivity analysis for
'Nonanoylcarnitine (C9) levels' on 'Rheumatoid arthritis'

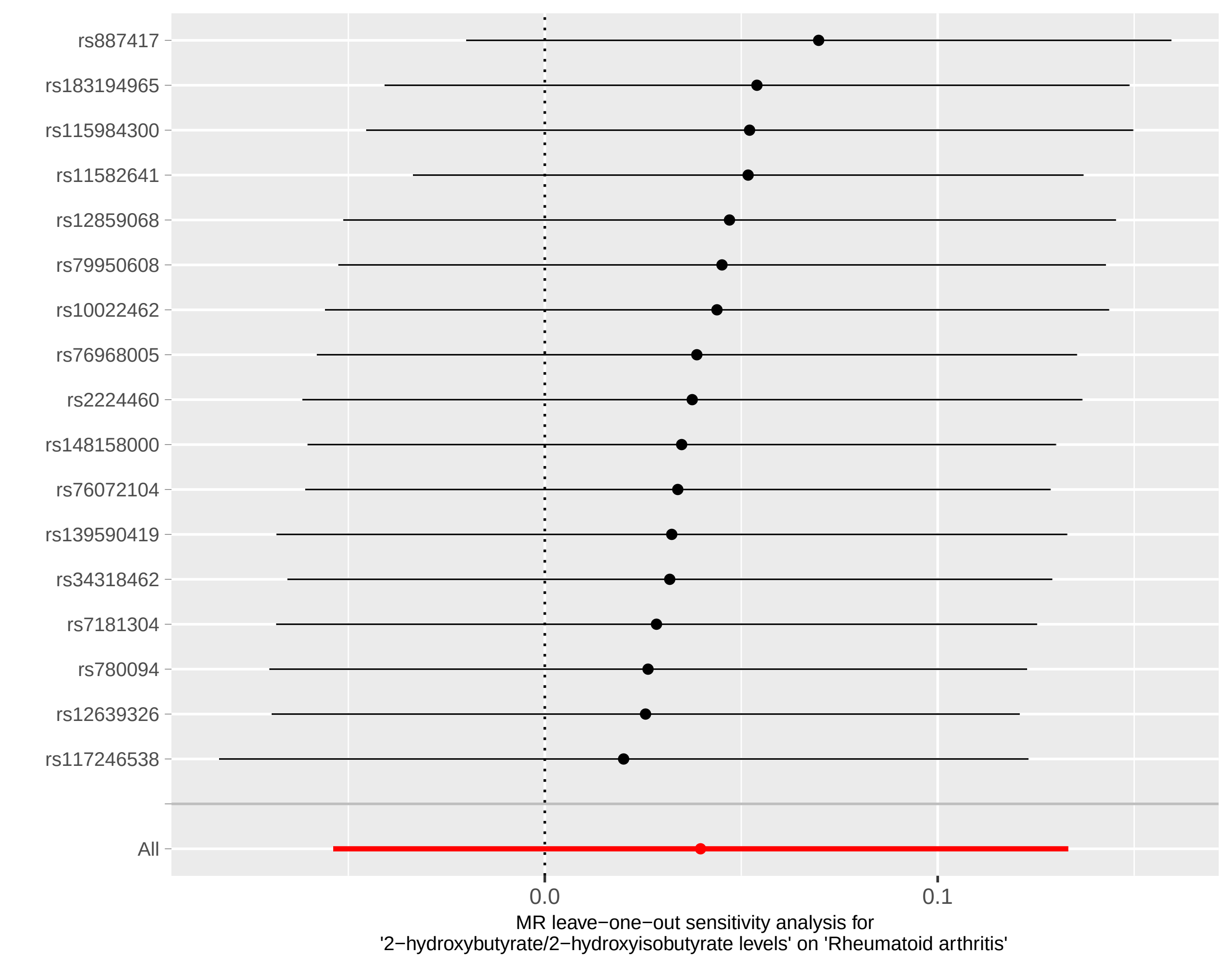
rs887417
rs183194965
rs115984300
rs11582641
rs12859068
rs79950608
rs10022462
rs76968005
rs2224460
rs148158000
rs76072104
rs139590419
rs34318462
rs7181304
rs780094
rs12639326
rs117246538
All
0.0
0.1
MR leave−one−out sensitivity analysis for
'2−hydroxybutyrate/2−hydroxyisobutyrate levels' on 'Rheumatoid arthritis'

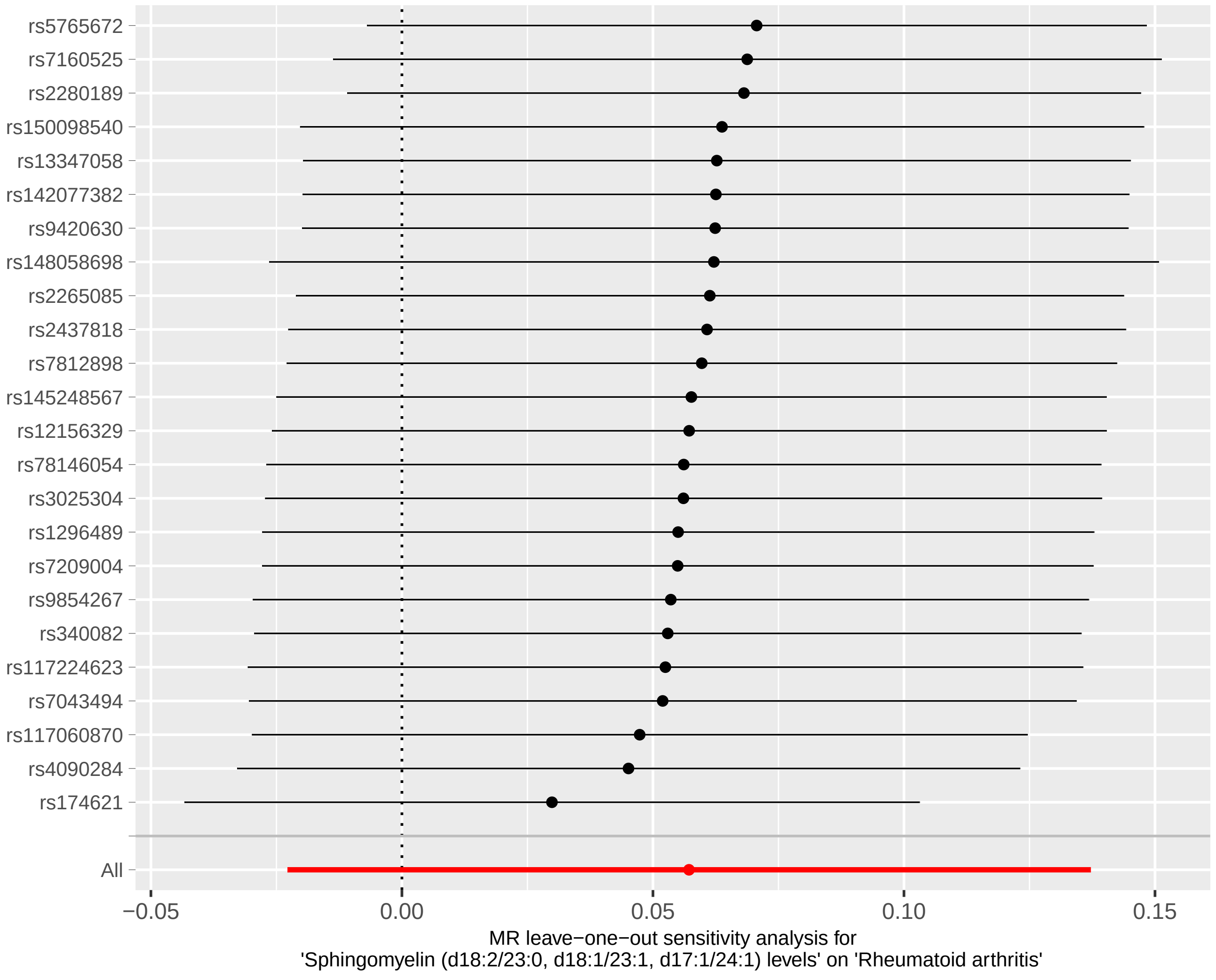
rs5765672
rs7160525
rs2280189
rs150098540
rs13347058
rs142077382
rs9420630
rs148058698
rs2265085
rs2437818
rs7812898
rs145248567
rs12156329
rs78146054
rs3025304
rs1296489
rs7209004
rs9854267
rs340082
rs117224623
rs7043494
rs117060870
rs4090284
rs174621
All
−0.05
0.00
0.05
0.10
0.15
MR leave−one−out sensitivity analysis for
'Sphingomyelin (d18:2/23:0, d18:1/23:1, d17:1/24:1) levels' on 'Rheumatoid arthritis'

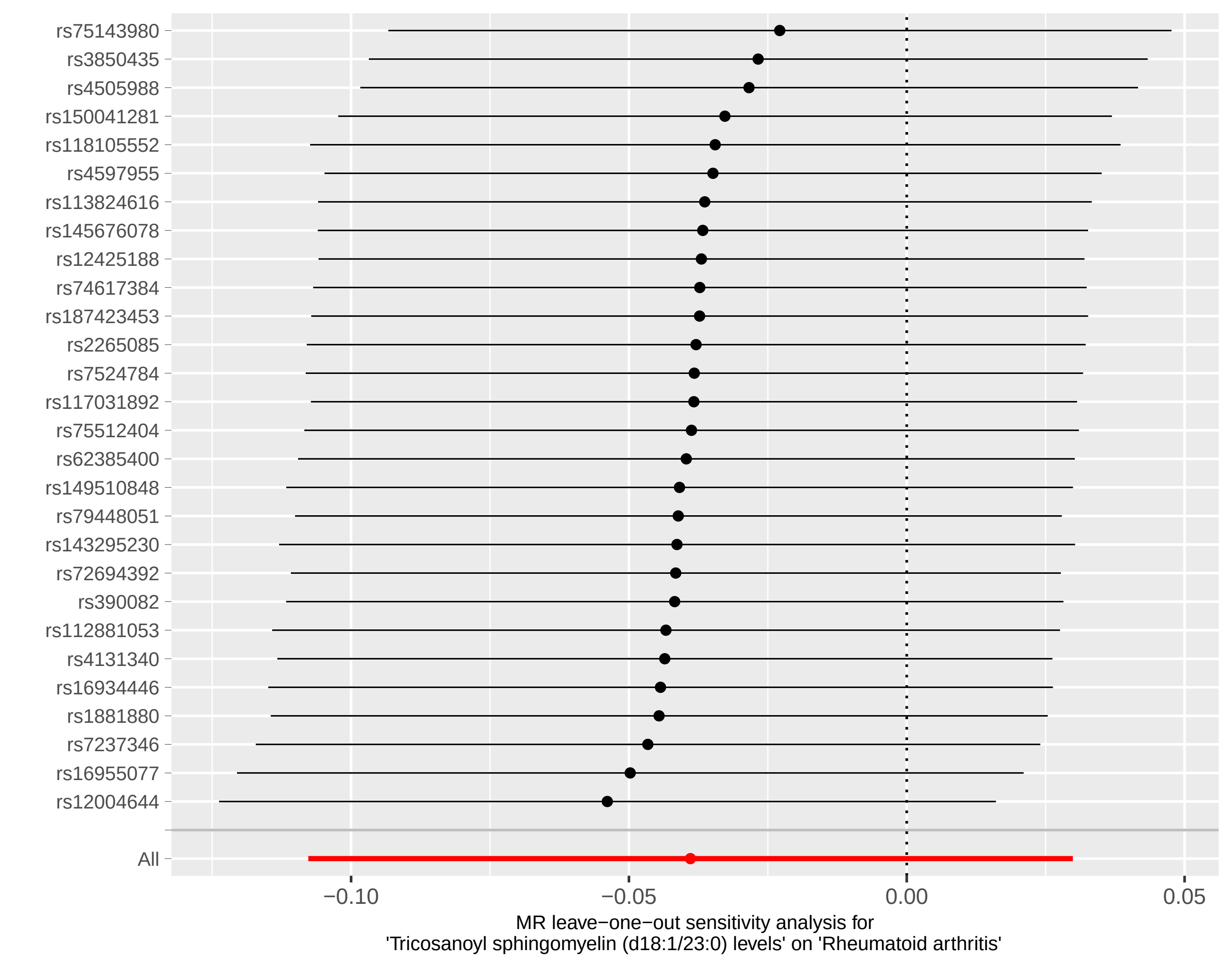
rs75143980
rs3850435
rs4505988
rs150041281
rs118105552
rs4597955
rs113824616
rs145676078
rs12425188
rs74617384
rs187423453
rs2265085
rs7524784
rs117031892
rs75512404
rs62385400
rs149510848
rs79448051
rs143295230
rs72694392
rs390082
rs112881053
rs4131340
rs16934446
rs1881880
rs7237346
rs16955077
rs12004644
All
−0.10
−0.05
0.00
0.05
MR leave−one−out sensitivity analysis for
'Tricosanoyl sphingomyelin (d18:1/23:0) levels' on 'Rheumatoid arthritis'

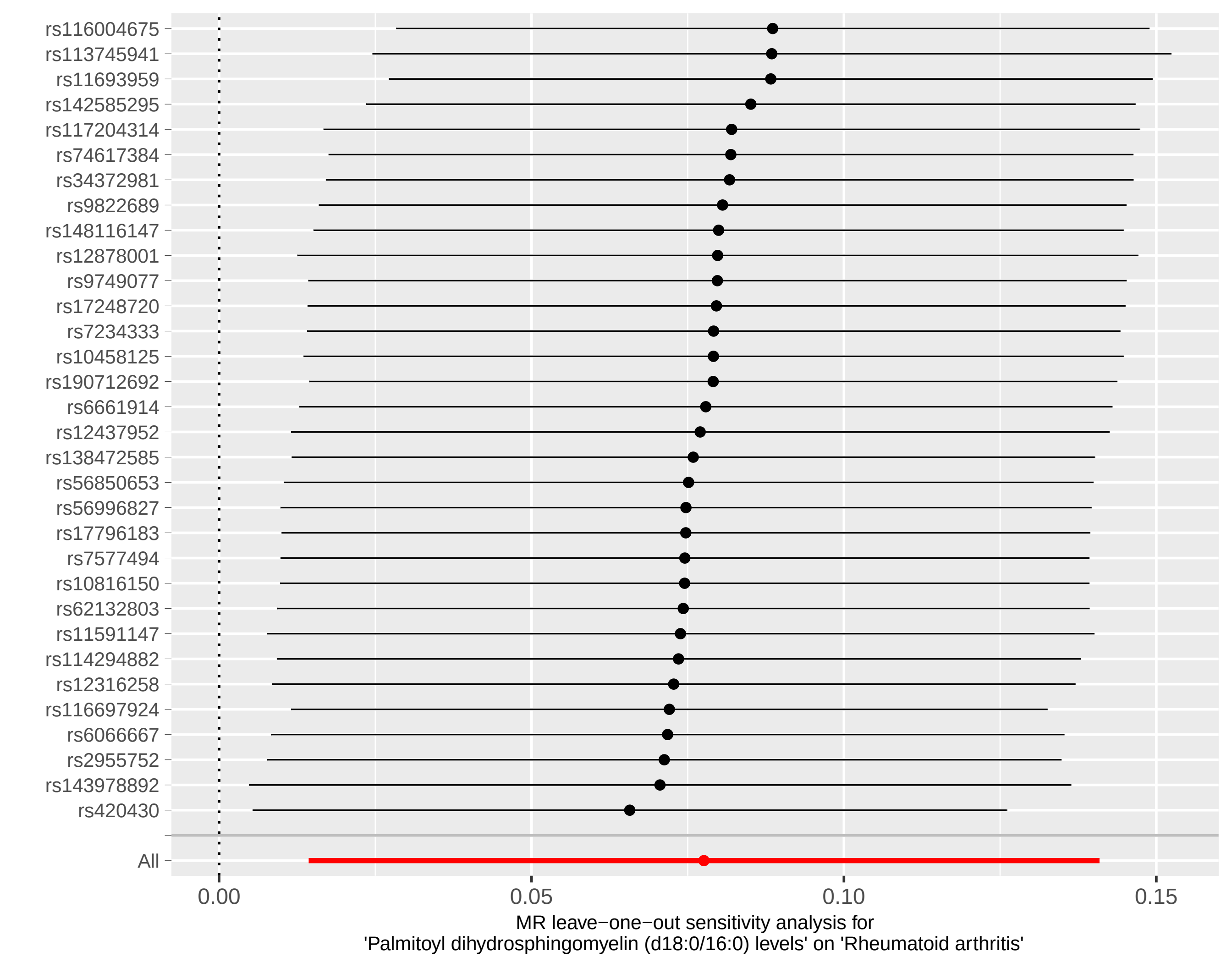
rs116004675
rs113745941
rs11693959
rs142585295
rs117204314
rs74617384
rs34372981
rs9822689
rs148116147
rs12878001
rs9749077
rs17248720
rs7234333
rs10458125
rs190712692
rs6661914
rs12437952
rs138472585
rs56850653
rs56996827
rs17796183
rs7577494
rs10816150
rs62132803
rs11591147
rs114294882
rs12316258
rs116697924
rs6066667
rs2955752
rs143978892
rs420430
All
0.00
0.05
0.10
0.15
MR leave−one−out sensitivity analysis for
'Palmitoyl dihydrosphingomyelin (d18:0/16:0) levels' on 'Rheumatoid arthritis'

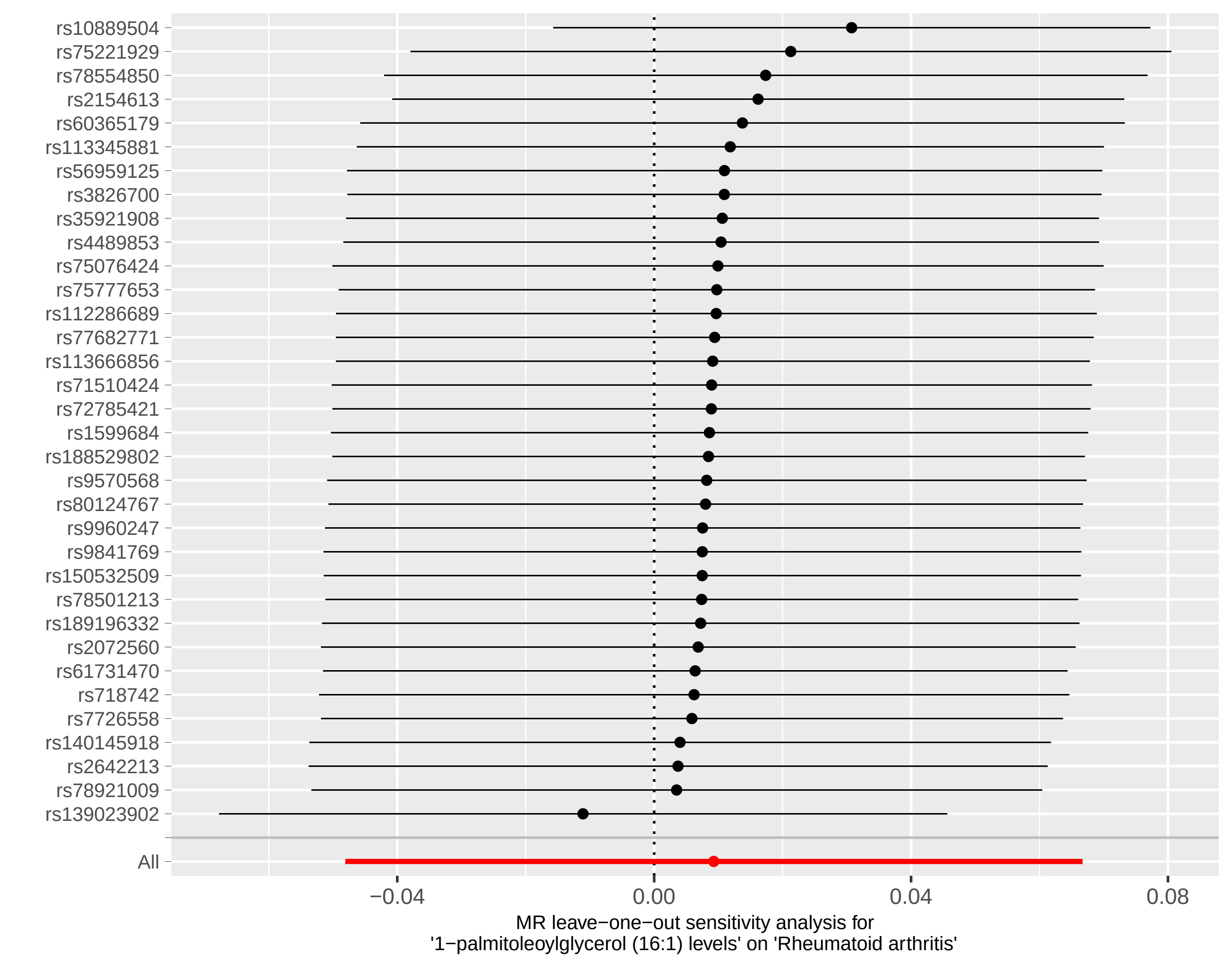
rs10889504
rs75221929
rs78554850
rs2154613
rs60365179
rs113345881
rs56959125
rs3826700
rs35921908
rs4489853
rs75076424
rs75777653
rs112286689
rs77682771
rs113666856
rs71510424
rs72785421
rs1599684
rs188529802
rs9570568
rs80124767
rs9960247
rs9841769
rs150532509
rs78501213
rs189196332
rs2072560
rs61731470
rs718742
rs7726558
rs140145918
rs2642213
rs78921009
rs139023902
All
−0.04
0.00
0.04
0.08
MR leave−one−out sensitivity analysis for
'1−palmitoleoylglycerol (16:1) levels' on 'Rheumatoid arthritis'

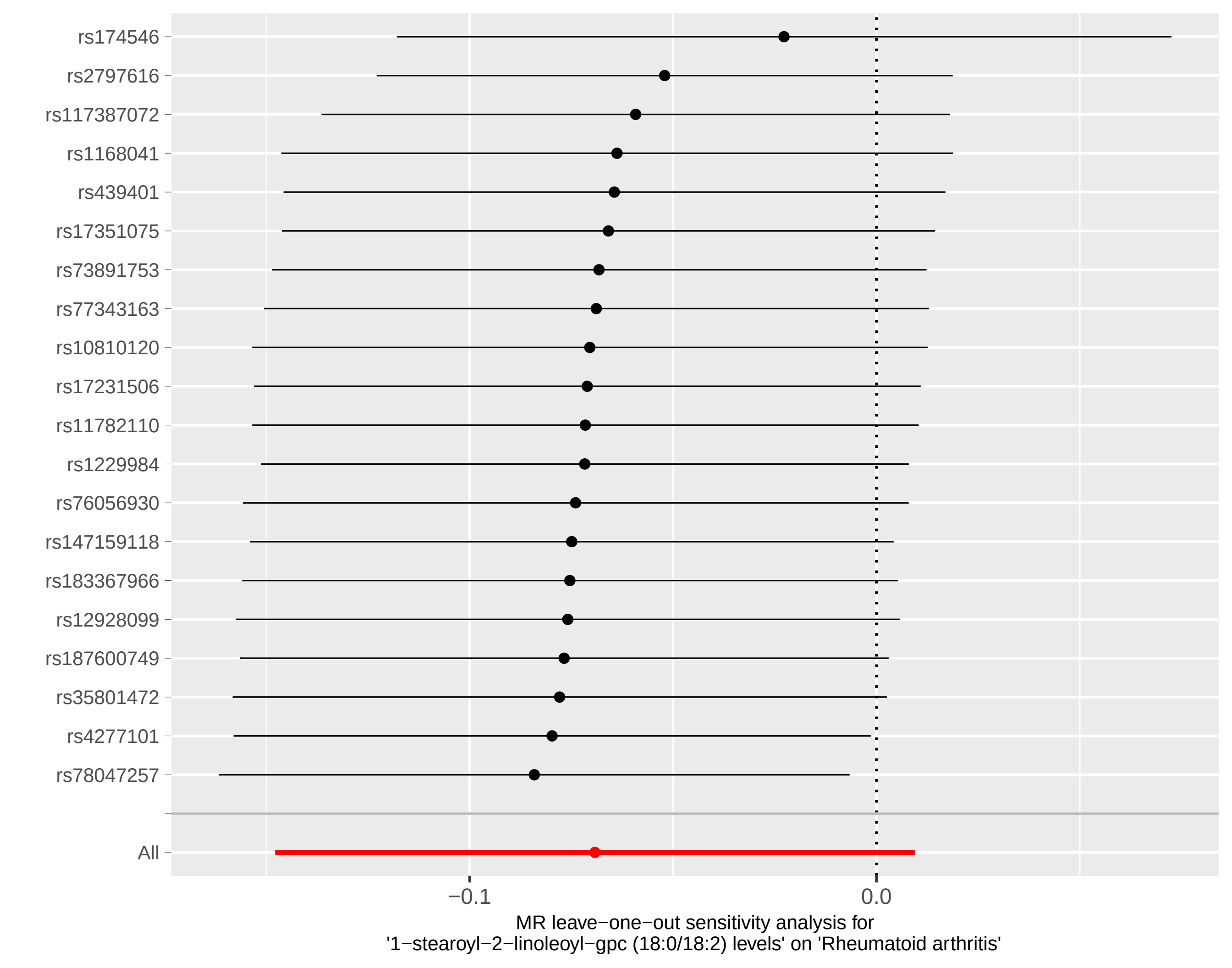

rs174546
rs2797616
rs117387072
rs1168041
rs439401
rs17351075
rs73891753
rs77343163
rs10810120
rs17231506
rs11782110
rs1229984
rs76056930
rs147159118
rs183367966
rs12928099
rs187600749
rs35801472
rs4277101
rs78047257
All
−0.1
0.0
MR leave−one−out sensitivity analysis for
'1−stearoyl−2−linoleoyl−gpc (18:0/18:2) levels' on 'Rheumatoid arthritis'

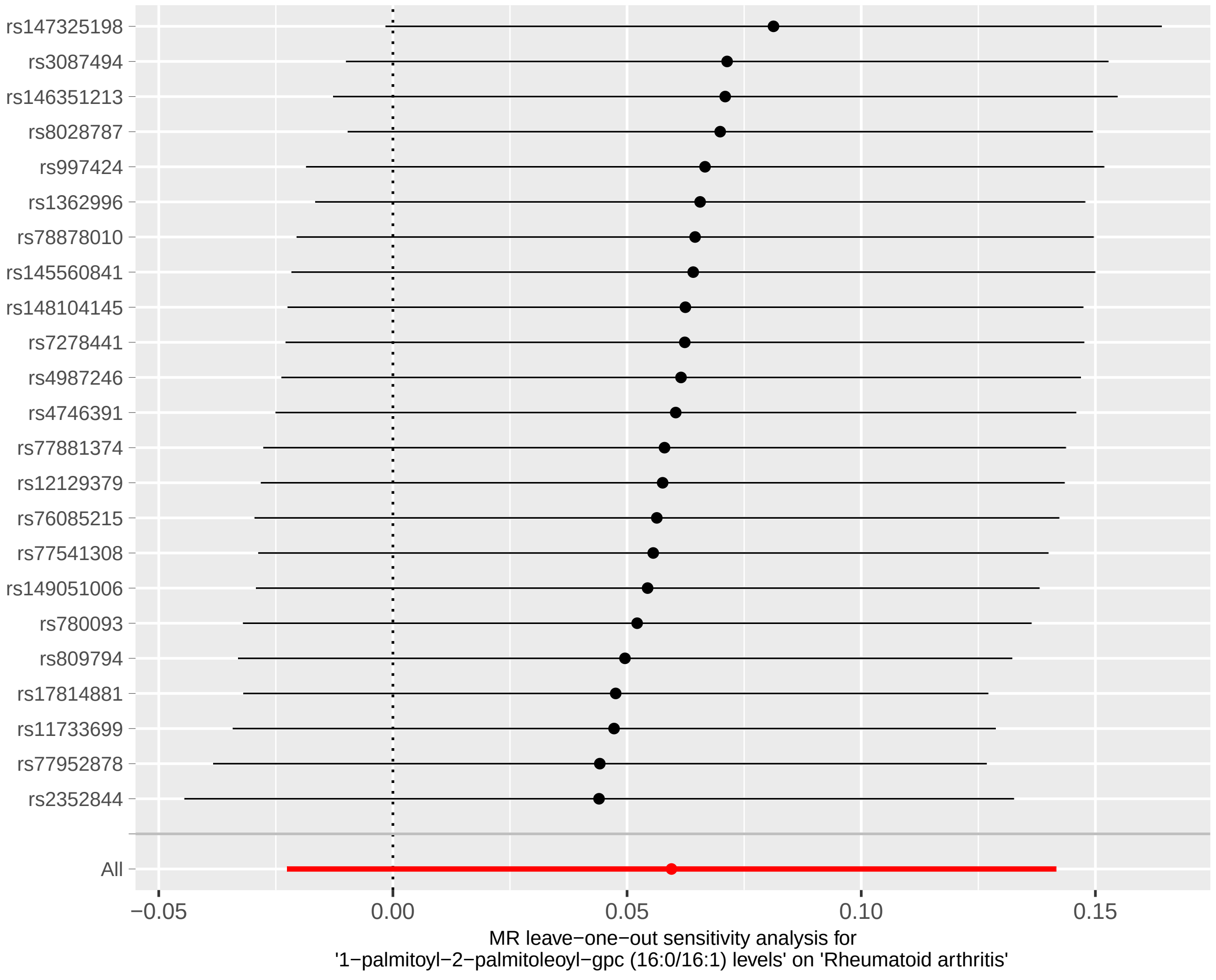

rs147325198
rs3087494
rs146351213
rs8028787
rs997424
rs1362996
rs78878010
rs145560841
rs148104145
rs7278441
rs4987246
rs4746391
rs77881374
rs12129379
rs76085215
rs77541308
rs149051006
rs780093
rs809794
rs17814881
rs11733699
rs77952878
rs2352844
All
−0.05
0.00
0.05
0.10
0.15
MR leave−one−out sensitivity analysis for
'1−palmitoyl−2−palmitoleoyl−gpc (16:0/16:1) levels' on 'Rheumatoid arthritis'

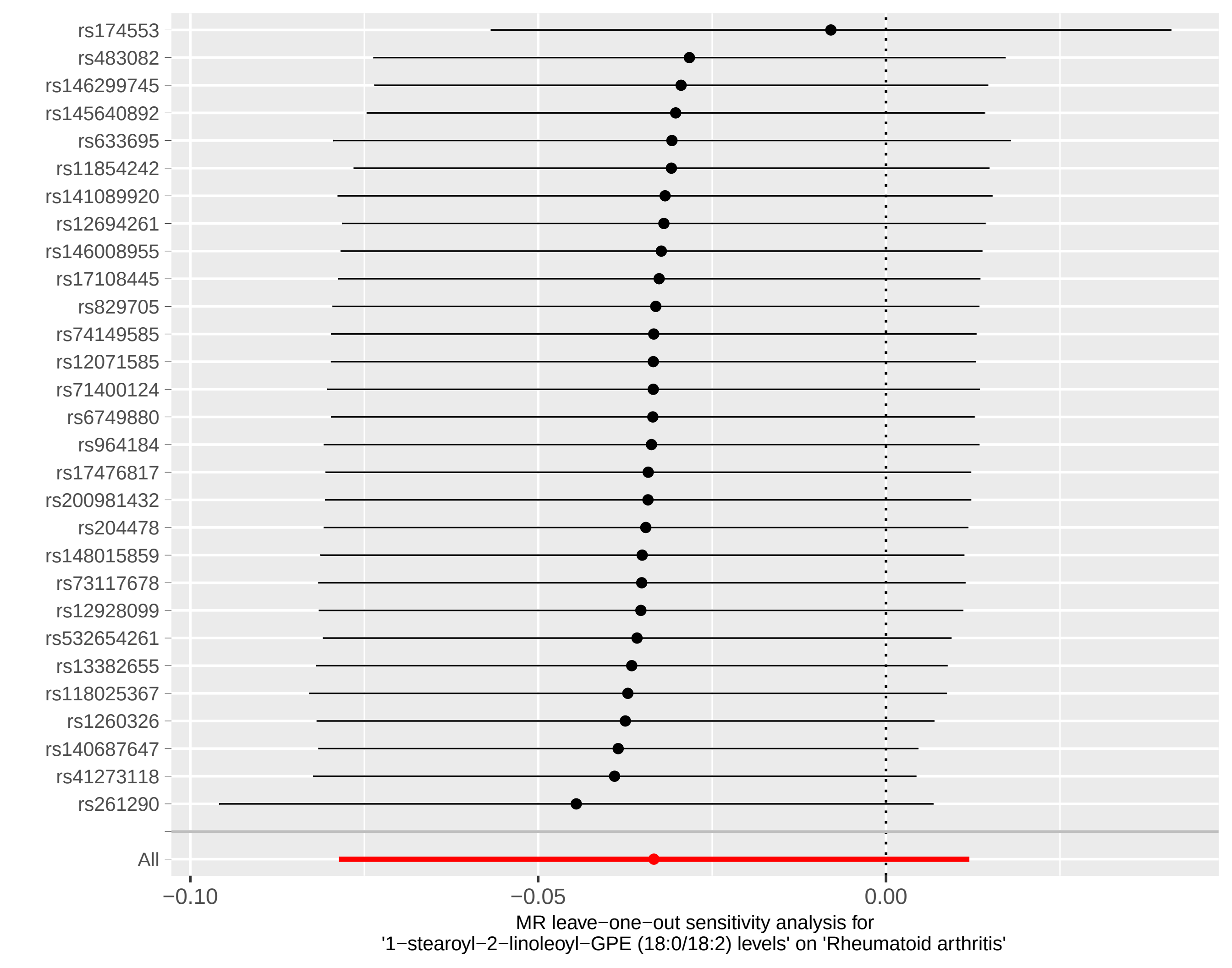

rs174553
rs483082
rs146299745
rs145640892
rs633695
rs11854242
rs141089920
rs12694261
rs146008955
rs17108445
rs829705
rs74149585
rs12071585
rs71400124
rs6749880
rs964184
rs17476817
rs200981432
rs204478
rs148015859
rs73117678
rs12928099
rs532654261
rs13382655
rs118025367
rs1260326
rs140687647
rs41273118
rs261290
All
−0.10
−0.05
0.00
MR leave−one−out sensitivity analysis for
'1−stearoyl−2−linoleoyl−GPE (18:0/18:2) levels' on 'Rheumatoid arthritis'

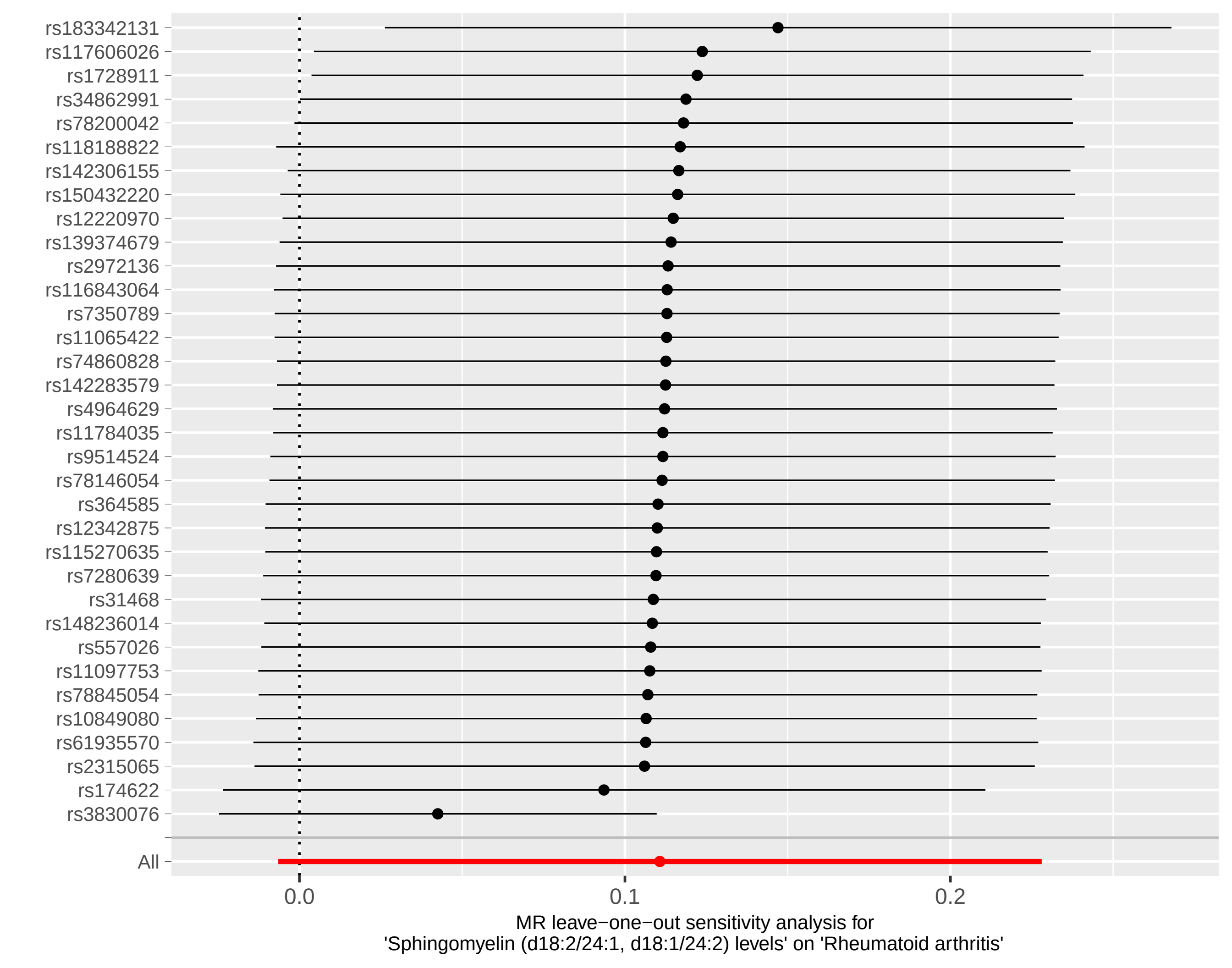

rs183342131
rs117606026
rs1728911
rs34862991
rs78200042
rs118188822
rs142306155
rs150432220
rs12220970
rs139374679
rs2972136
rs116843064
rs7350789
rs11065422
rs74860828
rs142283579
rs4964629
rs11784035
rs9514524
rs78146054
rs364585
rs12342875
rs115270635
rs7280639
rs31468
rs148236014
rs557026
rs11097753
rs78845054
rs10849080
rs61935570
rs2315065
rs174622
rs3830076
All
0.0
0.1
0.2
MR leave−one−out sensitivity analysis for
'Sphingomyelin (d18:2/24:1, d18:1/24:2) levels' on 'Rheumatoid arthritis'

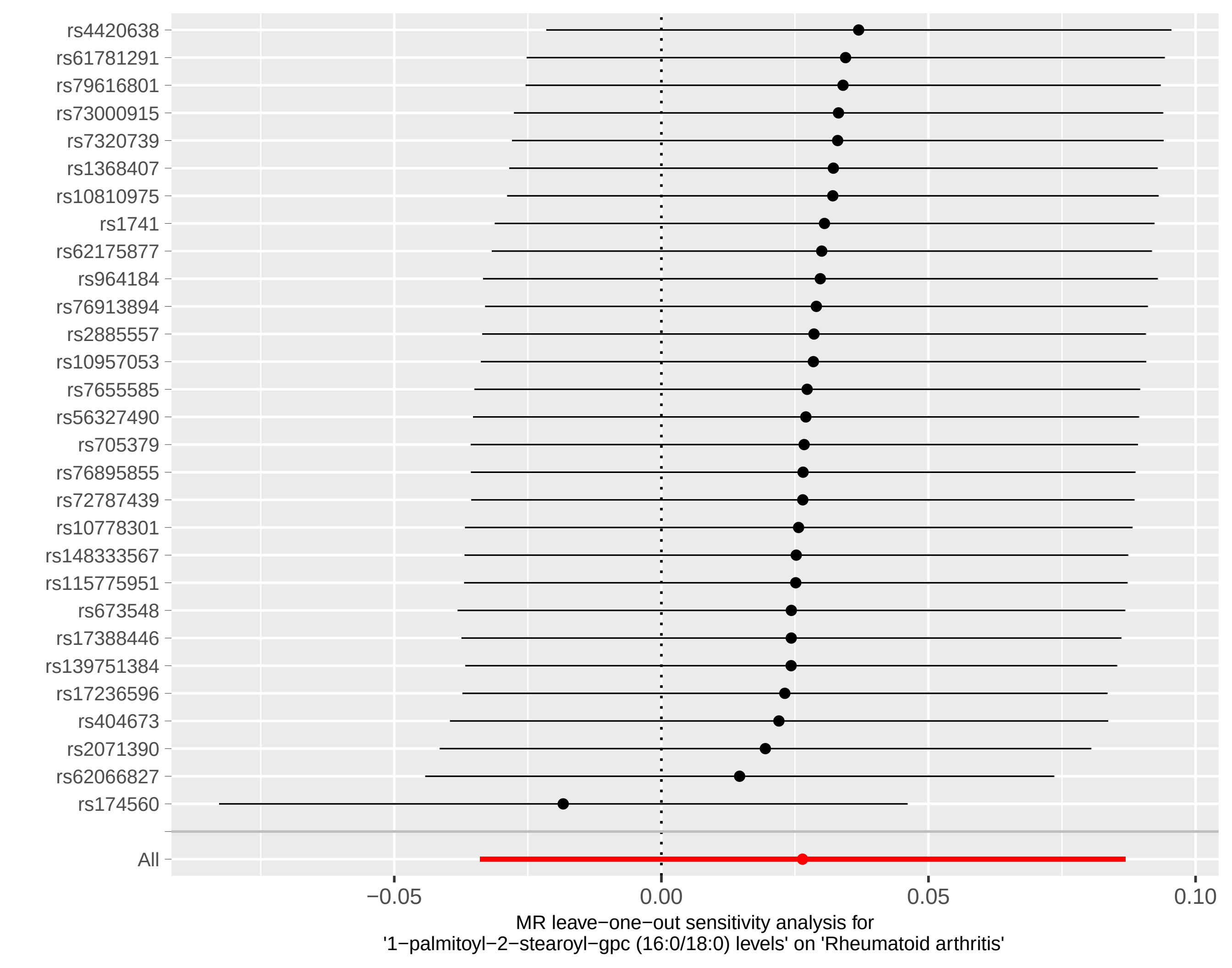
rs4420638
rs61781291
rs79616801
rs73000915
rs7320739
rs1368407
rs10810975
rs1741
rs62175877
rs964184
rs76913894
rs2885557
rs10957053
rs7655585
rs56327490
rs705379
rs76895855
rs72787439
rs10778301
rs148333567
rs115775951
rs673548
rs17388446
rs139751384
rs17236596
rs404673
rs2071390
rs62066827
rs174560
All
−0.05
0.00
0.05
0.10
MR leave−one−out sensitivity analysis for
'1−palmitoyl−2−stearoyl−gpc (16:0/18:0) levels' on 'Rheumatoid arthritis'

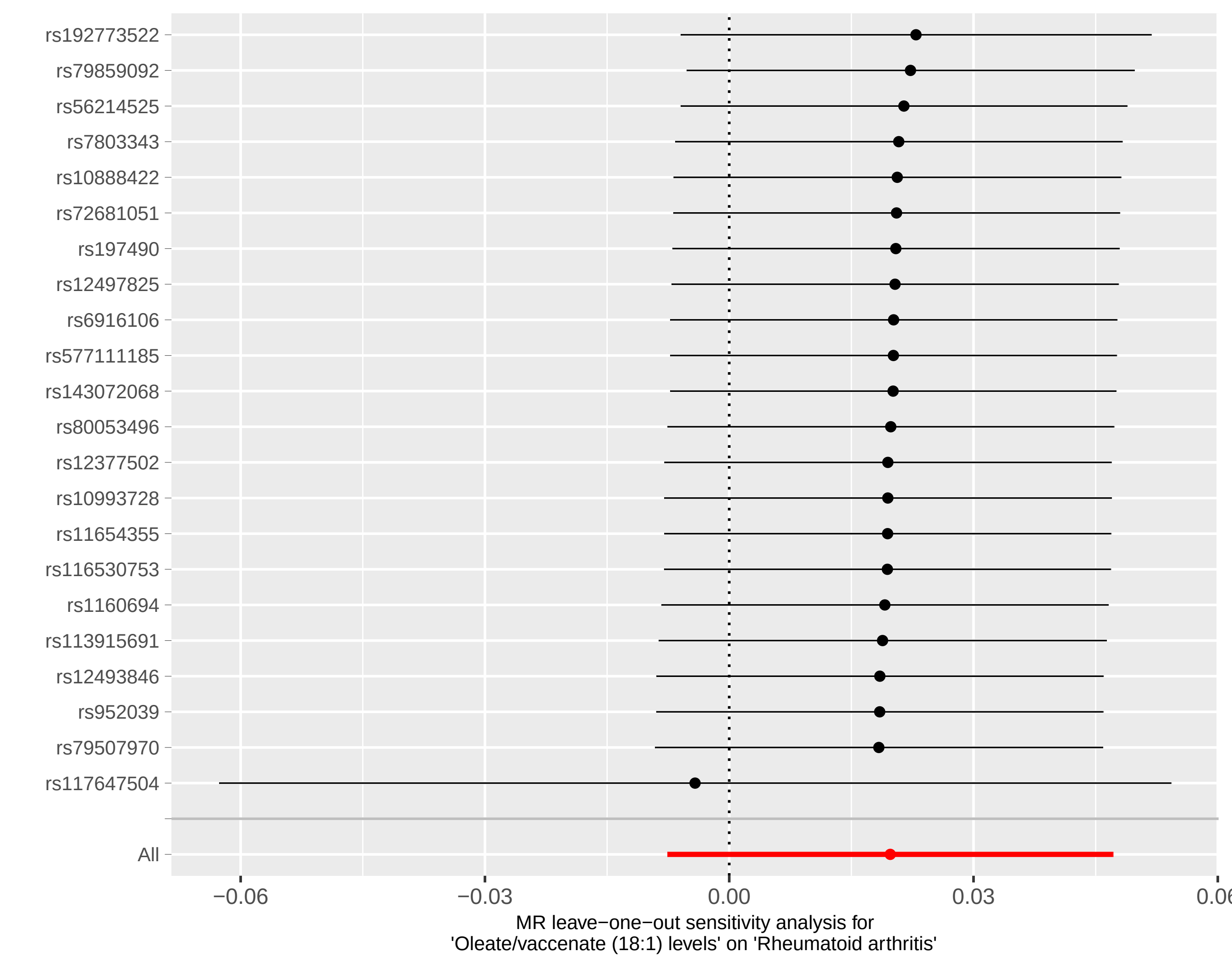
rs192773522
rs79859092
rs56214525
rs7803343
rs10888422
rs72681051
rs197490
rs12497825
rs6916106
rs577111185
rs143072068
rs80053496
rs12377502
rs10993728
rs11654355
rs116530753
rs1160694
rs113915691
rs12493846
rs952039
rs79507970
rs117647504
All
−0.06
−0.03
0.00
0.03
MR leave−one−out sensitivity analysis for
'Oleate/vaccenate (18:1) levels' on 'Rheumatoid arthritis'

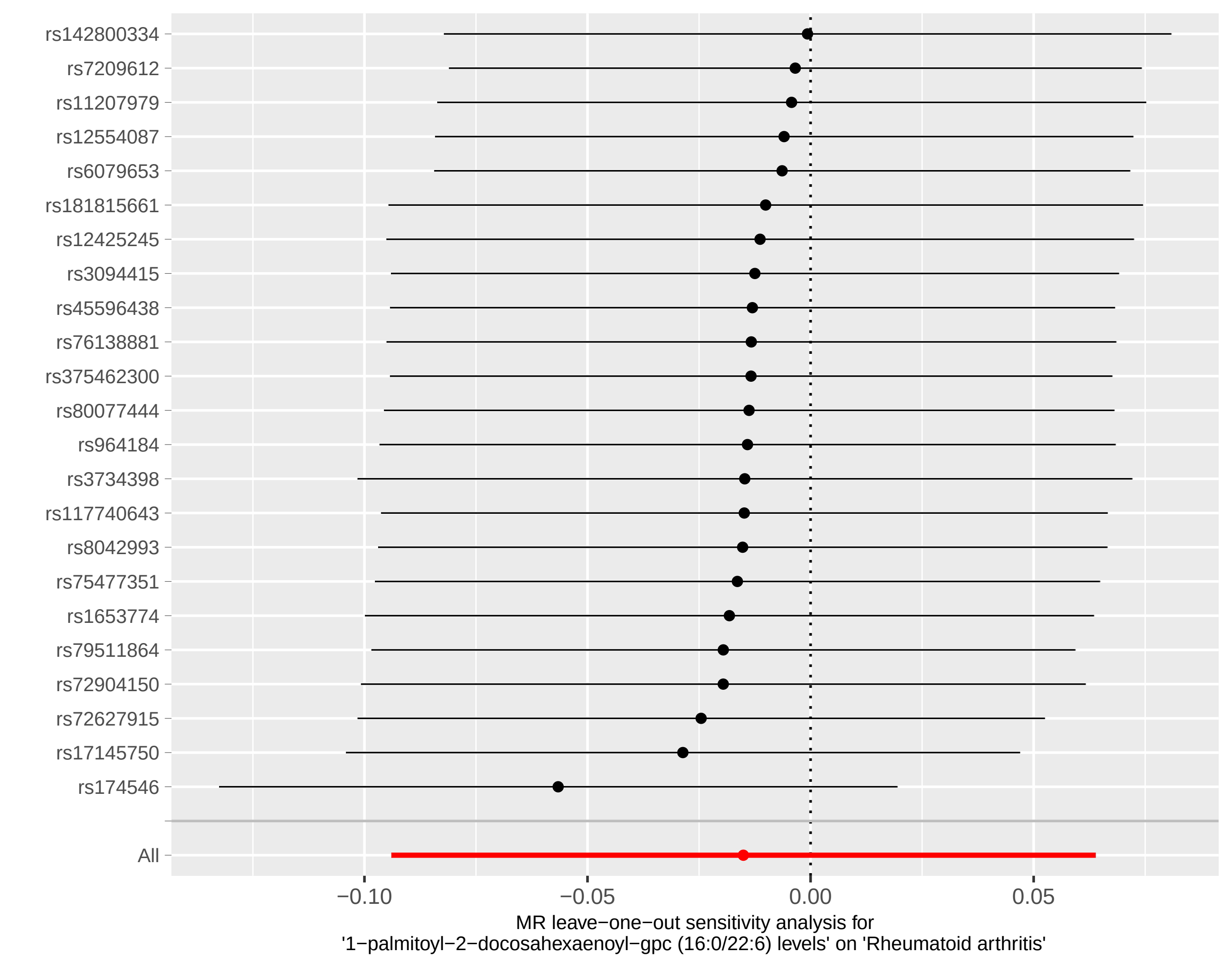
rs142800334
rs7209612
rs11207979
rs12554087
rs6079653
rs181815661
rs12425245
rs3094415
rs45596438
rs76138881
rs375462300
rs80077444
rs964184
rs3734398
rs117740643
rs8042993
rs75477351
rs1653774
rs79511864
rs72904150
rs72627915
rs17145750
rs174546
All
−0.10
−0.05
0.00
0.05
MR leave−one−out sensitivity analysis for
'1−palmitoyl−2−docosahexaenoyl−gpc (16:0/22:6) levels' on 'Rheumatoid arthritis'

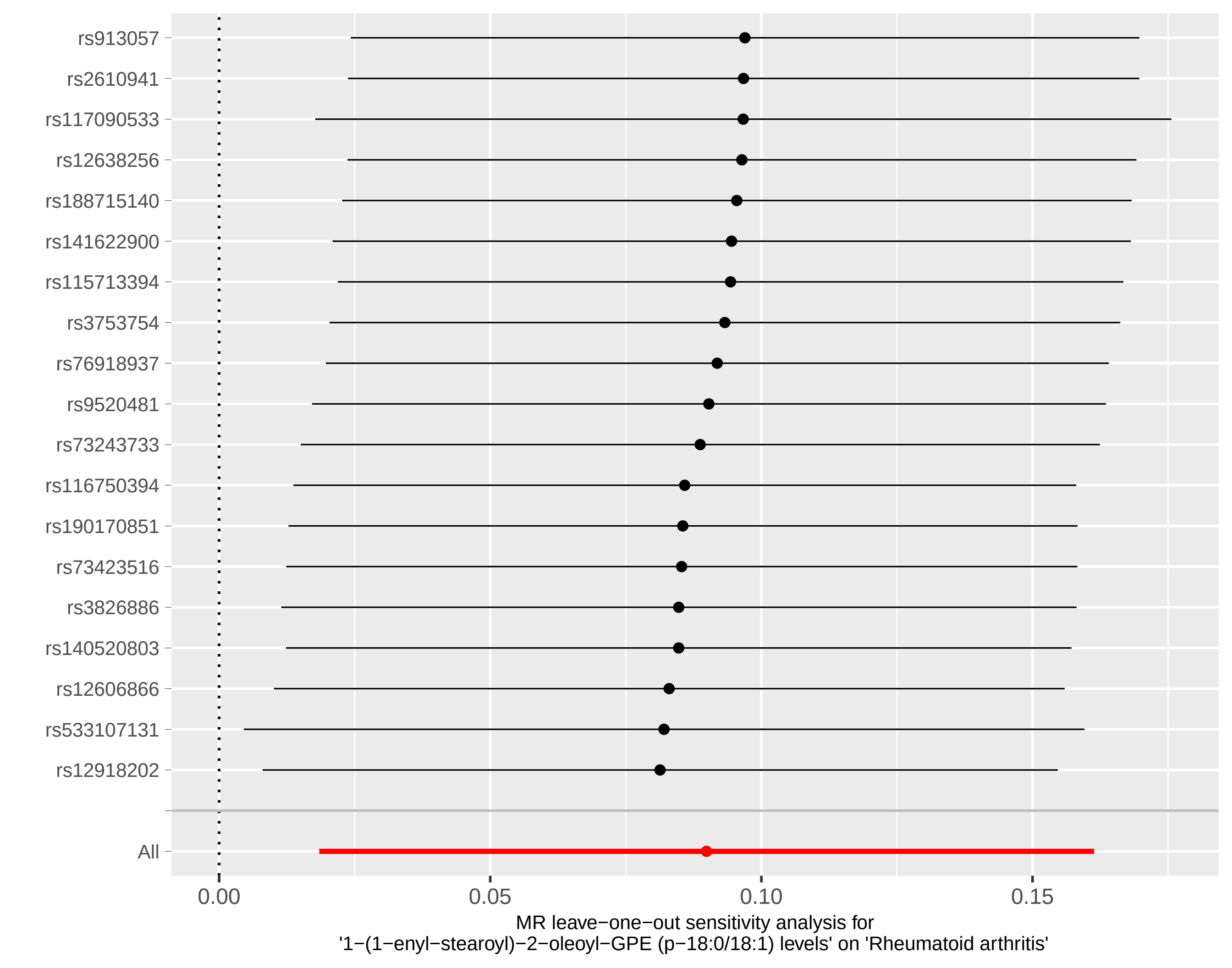
rs913057
rs2610941
rs117090533
rs12638256
rs188715140
rs141622900
rs115713394
rs3753754
rs76918937
rs9520481
rs73243733
rs116750394
rs190170851
rs73423516
rs3826886
rs140520803
rs12606866
rs533107131
rs12918202
All
0.00
0.05
0.10
0.15
MR leave−one−out sensitivity analysis for
'1−(1−enyl−stearoyl)−2−oleoyl−GPE (p−18:0/18:1) levels' on 'Rheumatoid arthritis'

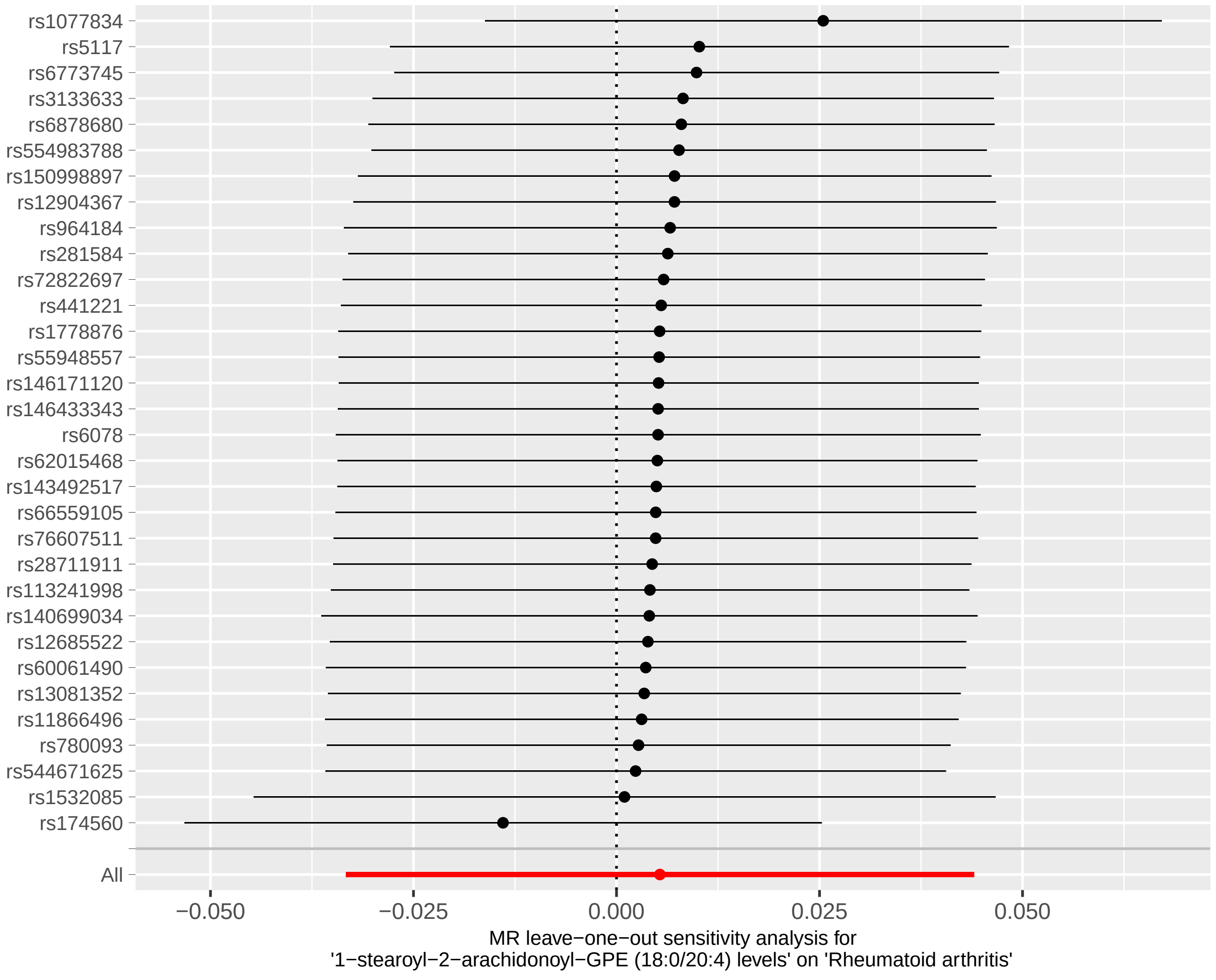
rs1077834
rs5117
rs6773745
rs3133633
rs6878680
rs554983788
rs150998897
rs12904367
rs964184
rs281584
rs72822697
rs441221
rs1778876
rs55948557
rs146171120
rs146433343
rs6078
rs62015468
rs143492517
rs66559105
rs76607511
rs28711911
rs113241998
rs140699034
rs12685522
rs60061490
rs13081352
rs11866496
rs780093
rs544671625
rs1532085
rs174560
All
−0.050
−0.025
0.000
0.025
0.050
MR leave−one−out sensitivity analysis for
'1−stearoyl−2−arachidonoyl−GPE (18:0/20:4) levels' on 'Rheumatoid arthritis'

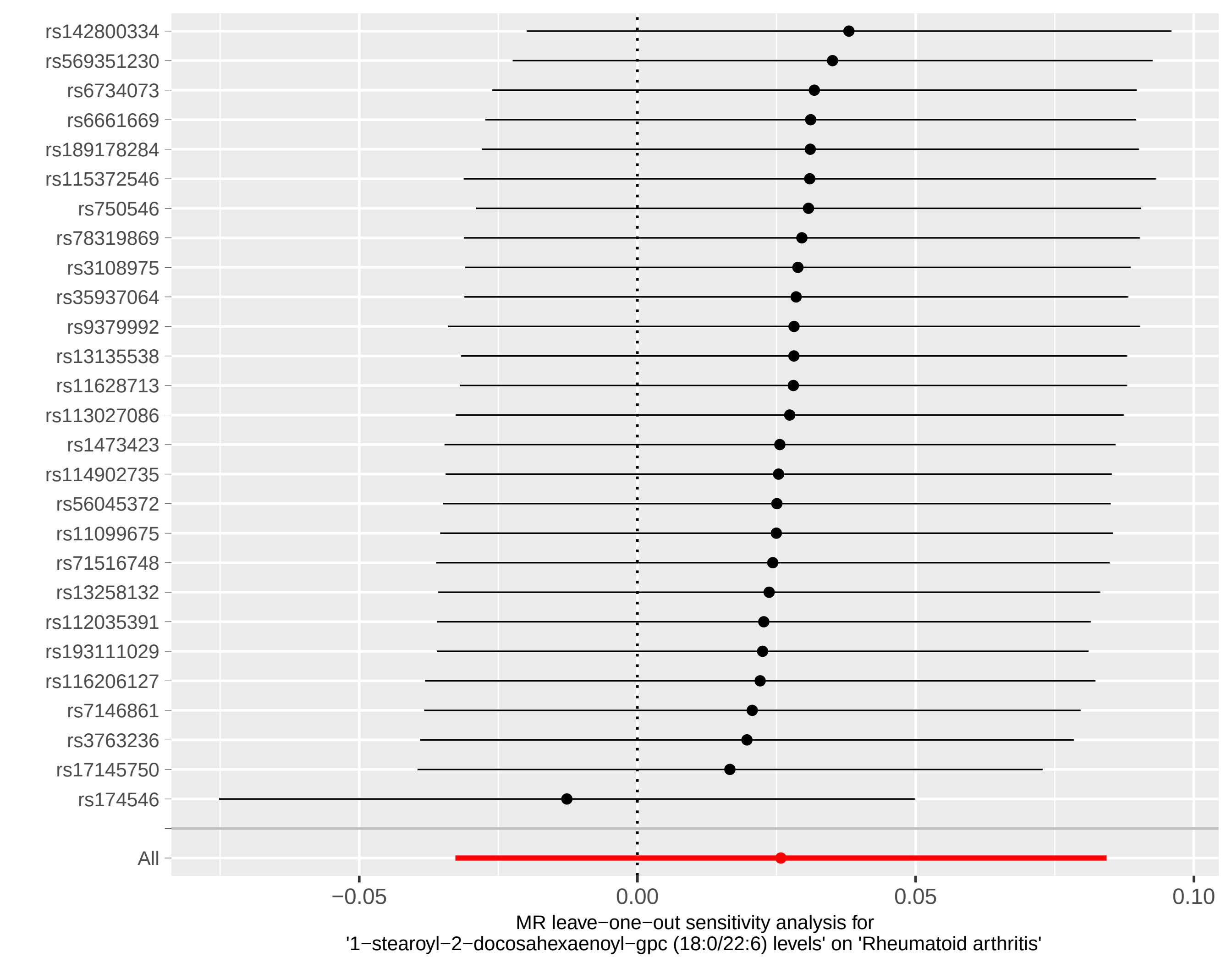
rs142800334
rs569351230
rs6734073
rs6661669
rs189178284
rs115372546
rs750546
rs78319869
rs3108975
rs35937064
rs9379992
rs13135538
rs11628713
rs113027086
rs1473423
rs114902735
rs56045372
rs11099675
rs71516748
rs13258132
rs112035391
rs193111029
rs116206127
rs7146861
rs3763236
rs17145750
rs174546
All
−0.05
0.00
0.05
0.10
MR leave−one−out sensitivity analysis for
'1−stearoyl−2−docosahexaenoyl−gpc (18:0/22:6) levels' on 'Rheumatoid arthritis'

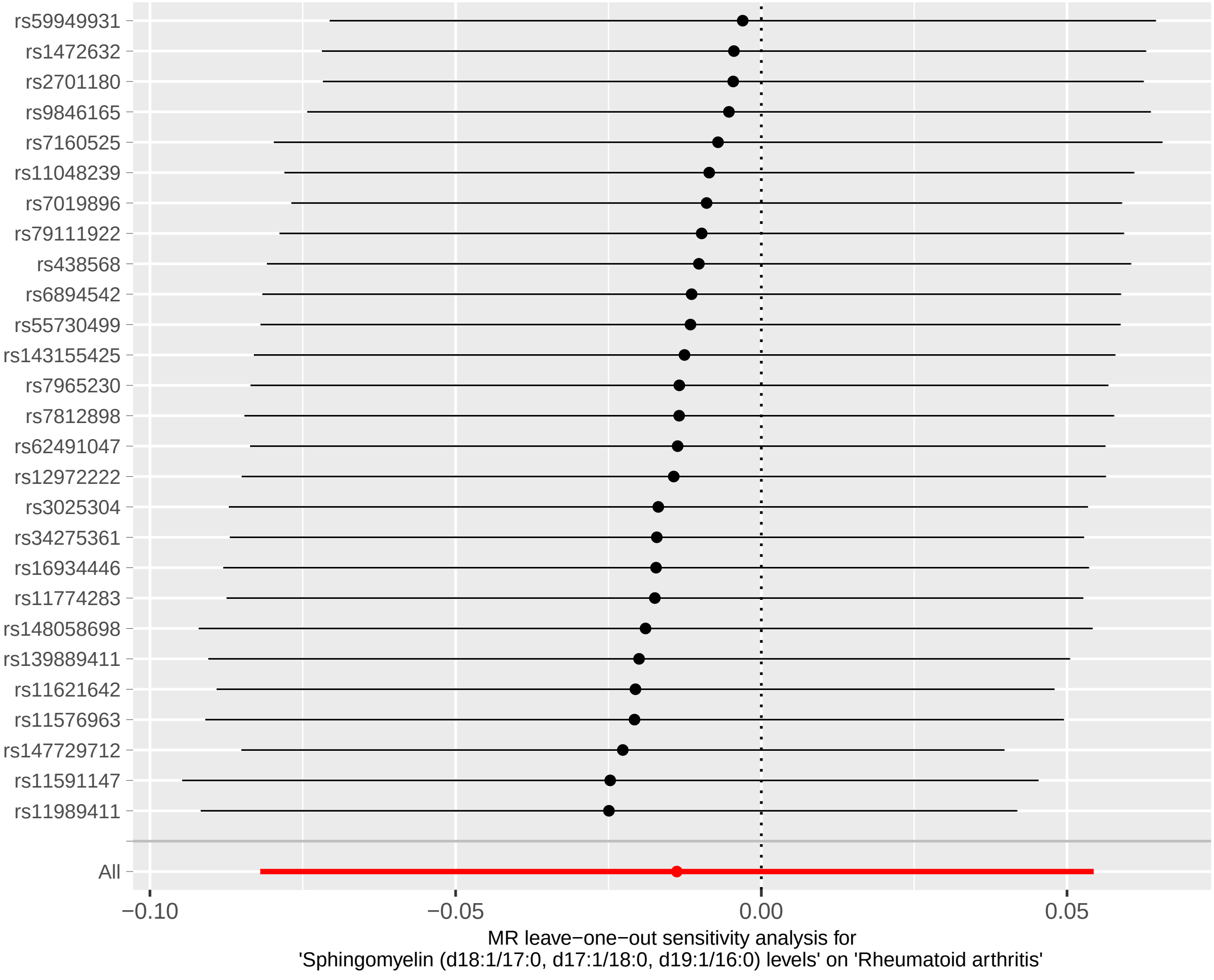

rs59949931
rs1472632
rs2701180
rs9846165
rs7160525
rs11048239
rs7019896
rs79111922
rs438568
rs6894542
rs55730499
rs143155425
rs7965230
rs7812898
rs62491047
rs12972222
rs3025304
rs34275361
rs16934446
rs11774283
rs148058698
rs139889411
rs11621642
rs11576963
rs147729712
rs11591147
rs11989411
All
−0.10
−0.05
0.00
0.05
MR leave−one−out sensitivity analysis for
'Sphingomyelin (d18:1/17:0, d17:1/18:0, d19:1/16:0) levels' on 'Rheumatoid arthritis'

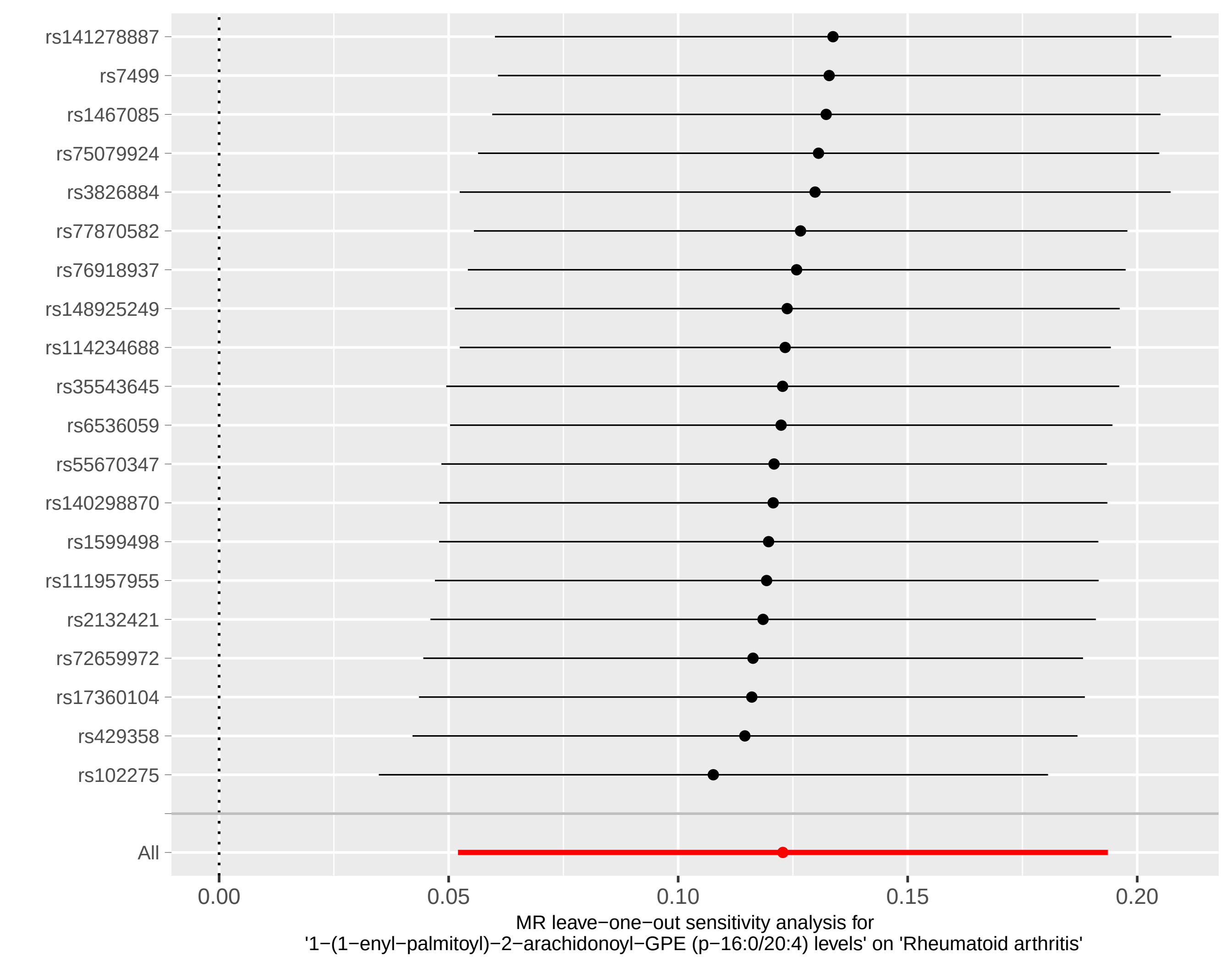
rs141278887
rs7499
rs1467085
rs75079924
rs3826884
rs77870582
rs76918937
rs148925249
rs114234688
rs35543645
rs6536059
rs55670347
rs140298870
rs1599498
rs111957955
rs2132421
rs72659972
rs17360104
rs429358
rs102275
All
0.00
0.05
0.10
0.15
0.20
MR leave−one−out sensitivity analysis for
'1−(1−enyl−palmitoyl)−2−arachidonoyl−GPE (p−16:0/20:4) levels' on 'Rheumatoid arthritis'

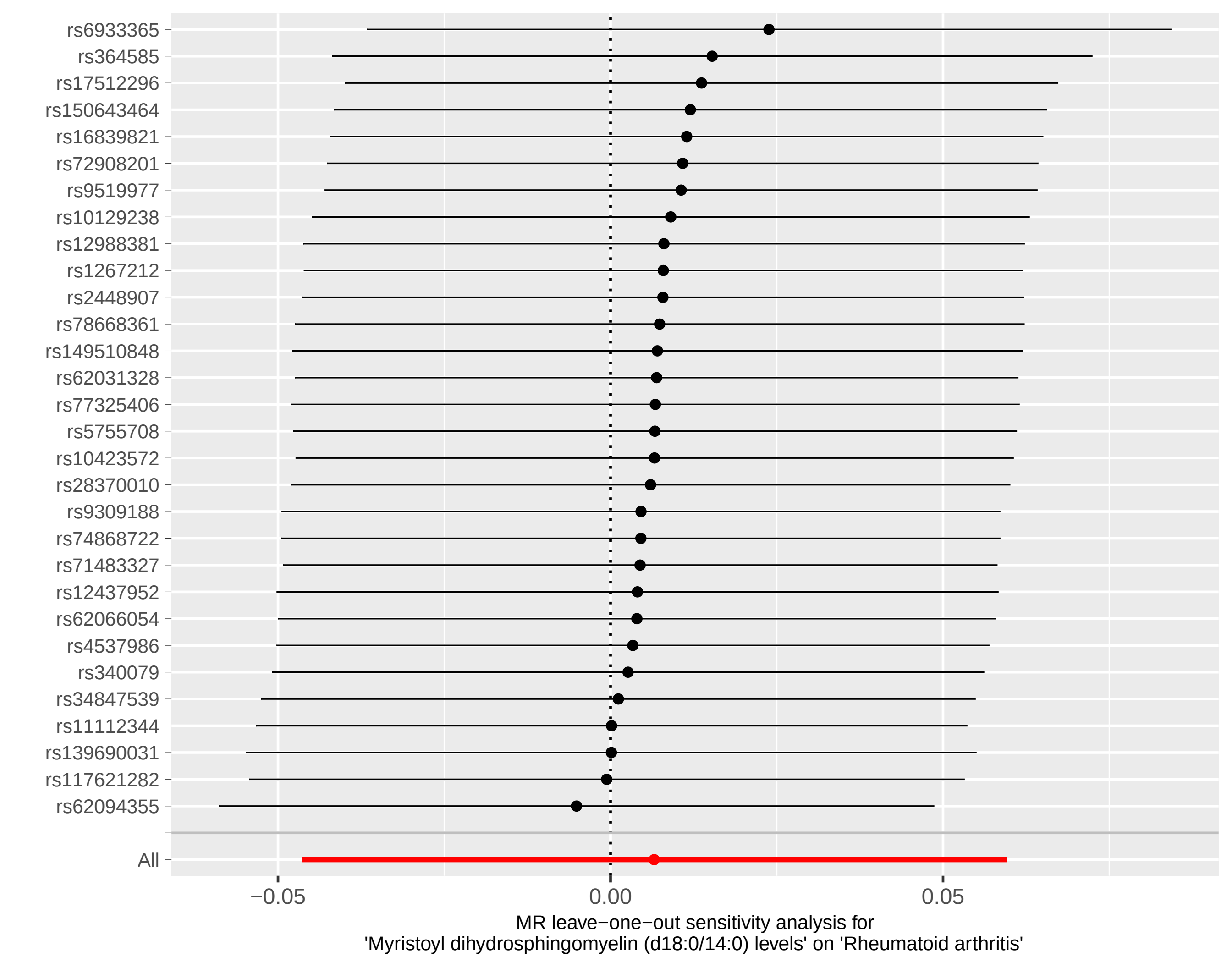
rs6933365
rs364585
rs17512296
rs150643464
rs16839821
rs72908201
rs9519977
rs10129238
rs12988381
rs1267212
rs2448907
rs78668361
rs149510848
rs62031328
rs77325406
rs5755708
rs10423572
rs28370010
rs9309188
rs74868722
rs71483327
rs12437952
rs62066054
rs4537986
rs340079
rs34847539
rs11112344
rs139690031
rs117621282
rs62094355
All
−0.05
0.00
0.05
MR leave−one−out sensitivity analysis for
'Myristoyl dihydrosphingomyelin (d18:0/14:0) levels' on 'Rheumatoid arthritis'

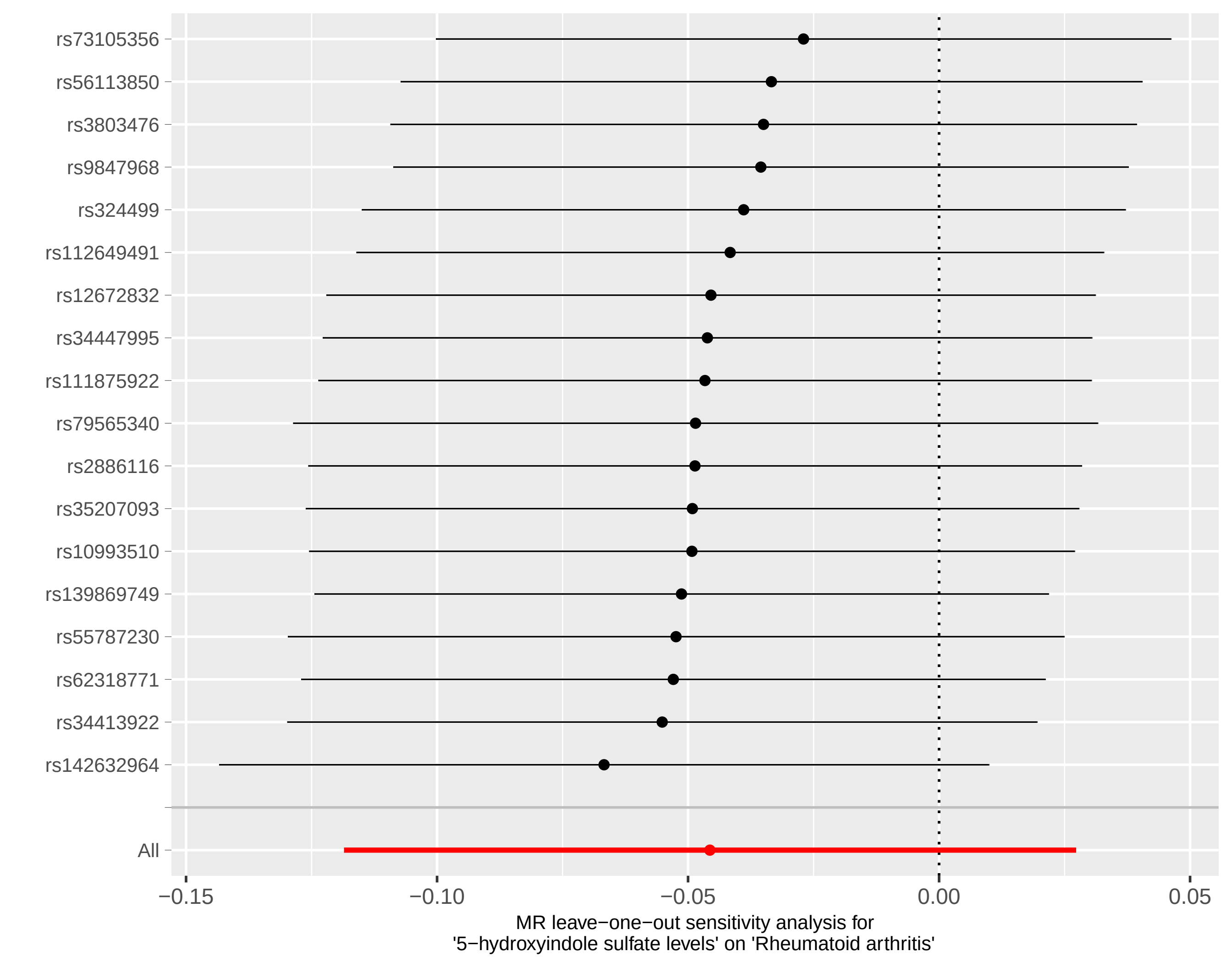
rs73105356
rs56113850
rs3803476
rs9847968
rs324499
rs112649491
rs12672832
rs34447995
rs111875922
rs79565340
rs2886116
rs35207093
rs10993510
rs139869749
rs55787230
rs62318771
rs34413922
rs142632964
All
−0.15
−0.10
−0.05
0.00
0.05
MR leave−one−out sensitivity analysis for
'5−hydroxyindole sulfate levels' on 'Rheumatoid arthritis'

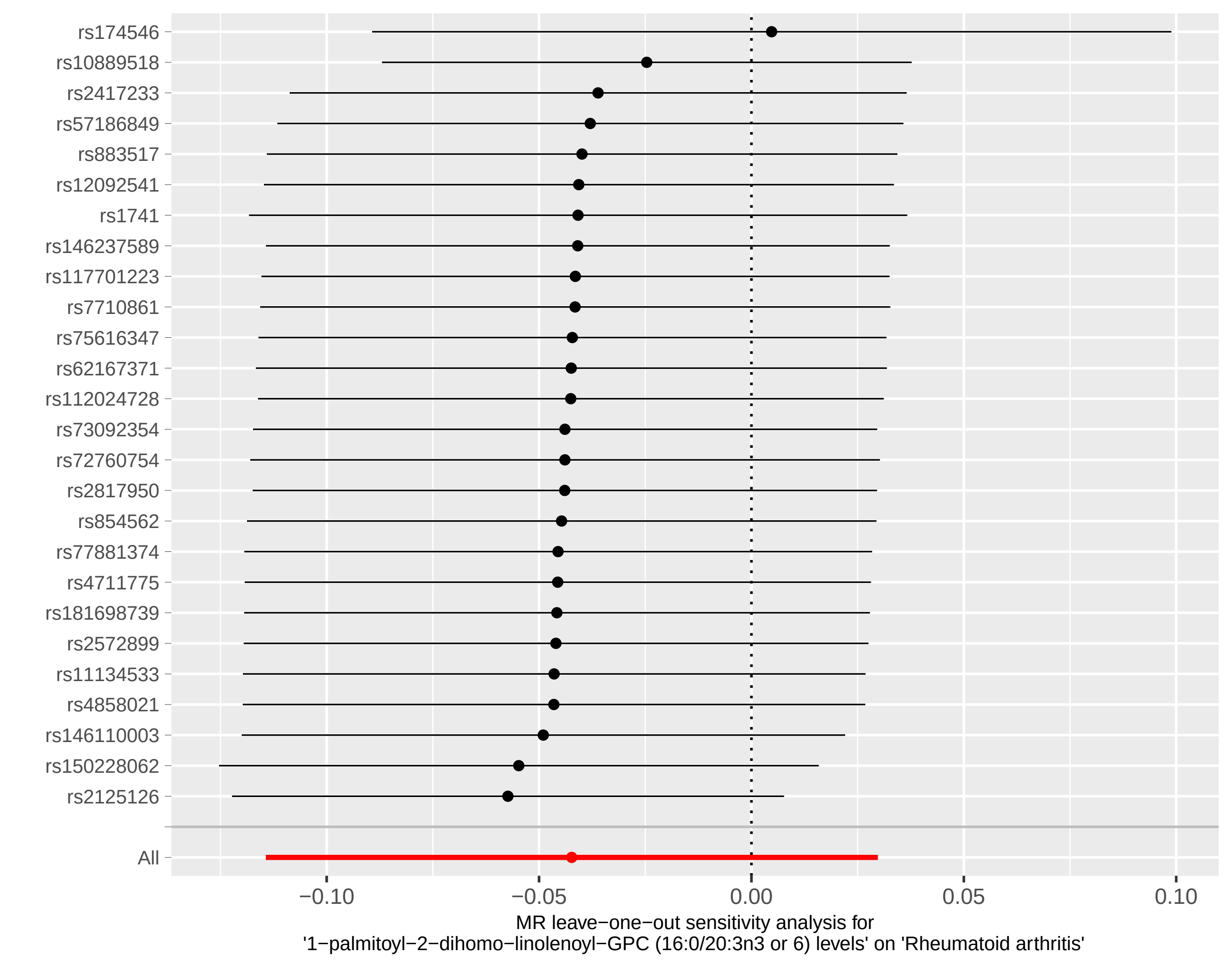
rs174546
rs10889518
rs2417233
rs57186849
rs883517
rs12092541
rs1741
rs146237589
rs117701223
rs7710861
rs75616347
rs62167371
rs112024728
rs73092354
rs72760754
rs2817950
rs854562
rs77881374
rs4711775
rs181698739
rs2572899
rs11134533
rs4858021
rs146110003
rs150228062
rs2125126
All
−0.10
−0.05
0.00
0.05
0.10
MR leave−one−out sensitivity analysis for
'1−palmitoyl−2−dihomo−linolenoyl−GPC (16:0/20:3n3 or 6) levels' on 'Rheumatoid arthritis'

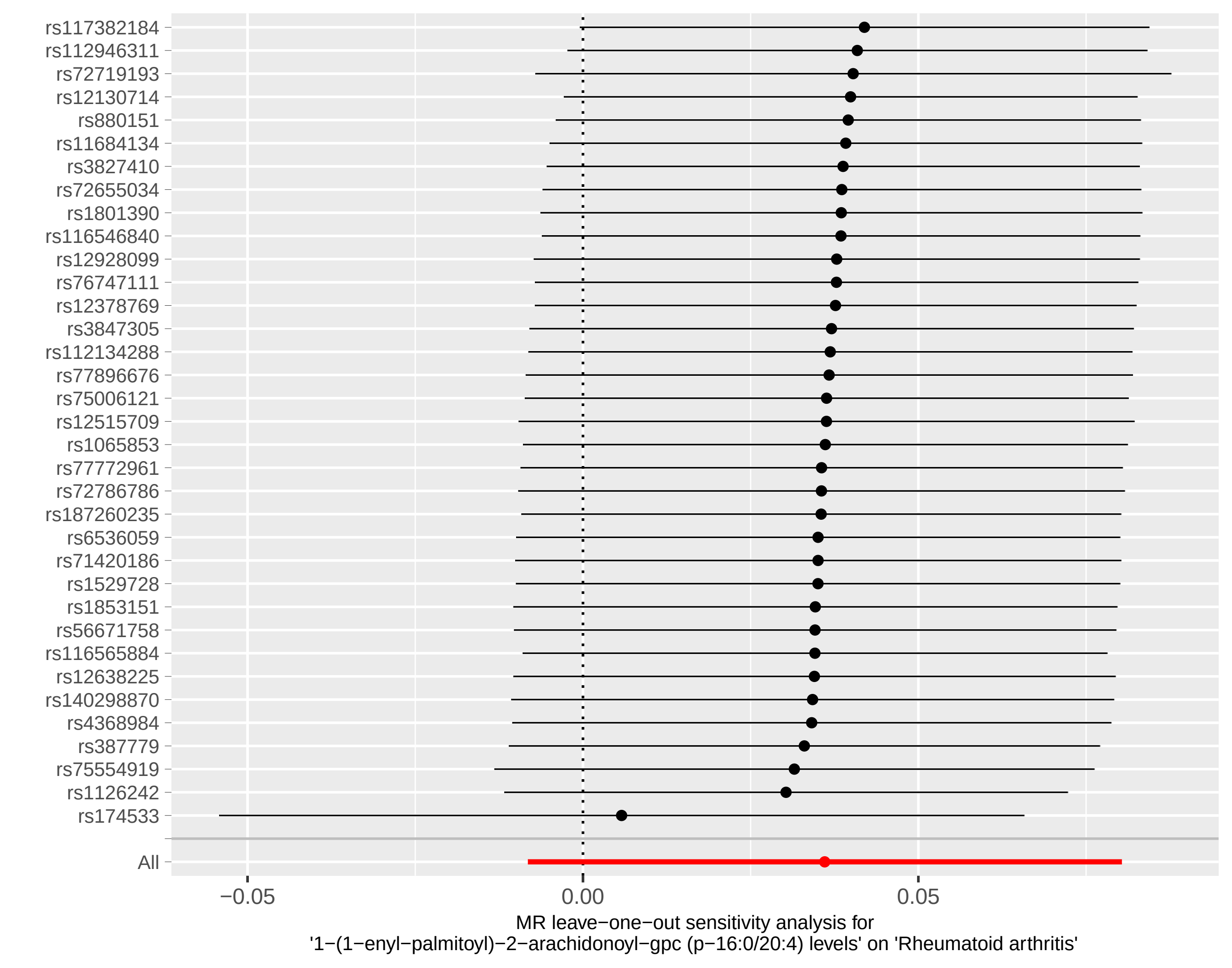
rs117382184
rs112946311
rs72719193
rs12130714
rs880151
rs11684134
rs3827410
rs72655034
rs1801390
rs116546840
rs12928099
rs76747111
rs12378769
rs3847305
rs112134288
rs77896676
rs75006121
rs12515709
rs1065853
rs77772961
rs72786786
rs187260235
rs6536059
rs71420186
rs1529728
rs1853151
rs56671758
rs116565884
rs12638225
rs140298870
rs4368984
rs387779
rs75554919
rs1126242
rs174533
All
−0.05
0.00
0.05
MR leave−one−out sensitivity analysis for
'1−(1−enyl−palmitoyl)−2−arachidonoyl−gpc (p−16:0/20:4) levels' on 'Rheumatoid arthritis'

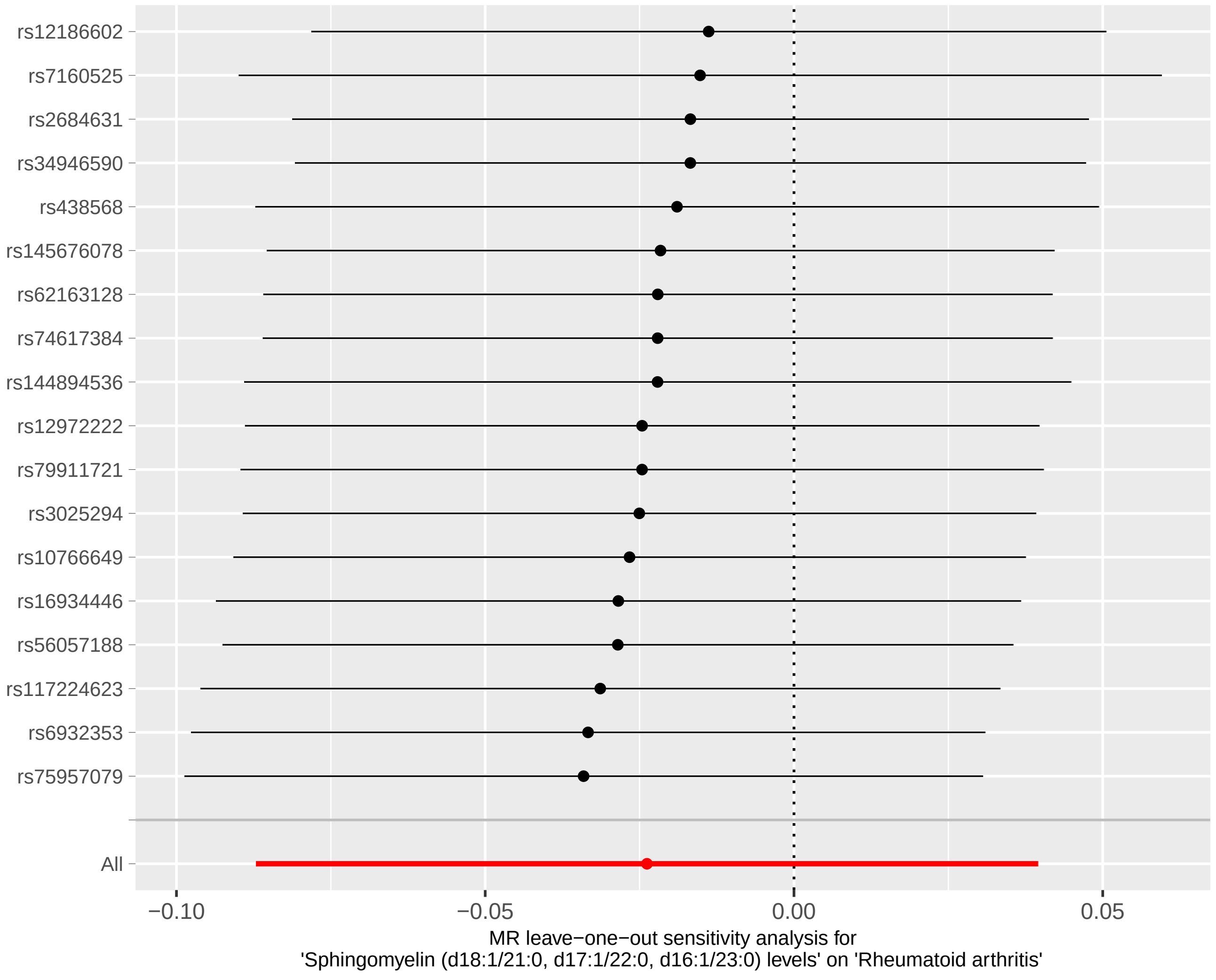
rs12186602
rs7160525
rs2684631
rs34946590
rs438568
rs145676078
rs62163128
rs74617384
rs144894536
rs12972222
rs79911721
rs3025294
rs10766649
rs16934446
rs56057188
rs117224623
rs6932353
rs75957079
All
−0.10
−0.05
0.00
0.05
MR leave−one−out sensitivity analysis for
'Sphingomyelin (d18:1/21:0, d17:1/22:0, d16:1/23:0) levels' on 'Rheumatoid arthritis'

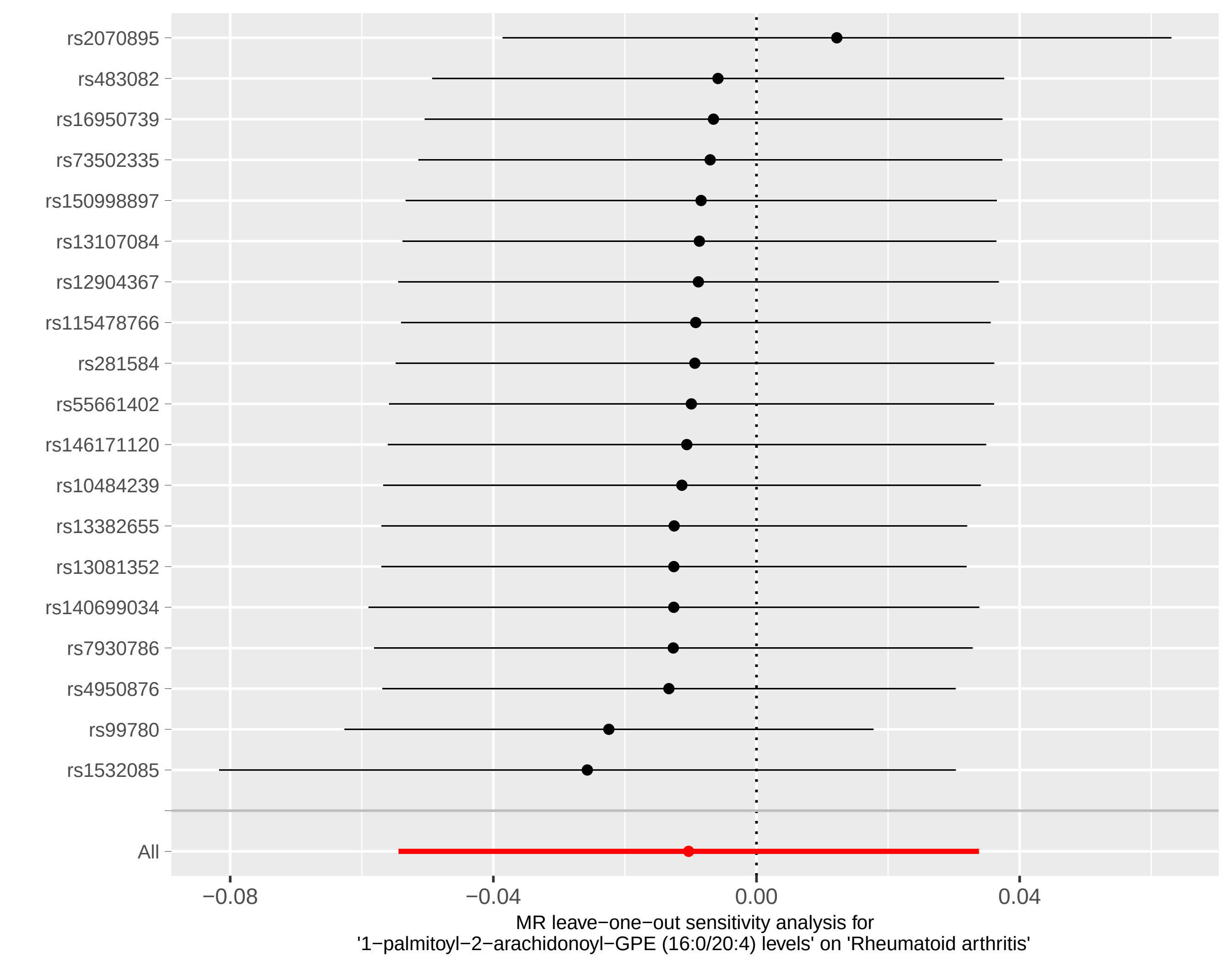
rs2070895
rs483082
rs16950739
rs73502335
rs150998897
rs13107084
rs12904367
rs115478766
rs281584
rs55661402
rs146171120
rs10484239
rs13382655
rs13081352
rs140699034
rs7930786
rs4950876
rs99780
rs1532085
All
−0.08
−0.04
0.00
0.04
MR leave−one−out sensitivity analysis for
'1−palmitoyl−2−arachidonoyl−GPE (16:0/20:4) levels' on 'Rheumatoid arthritis'

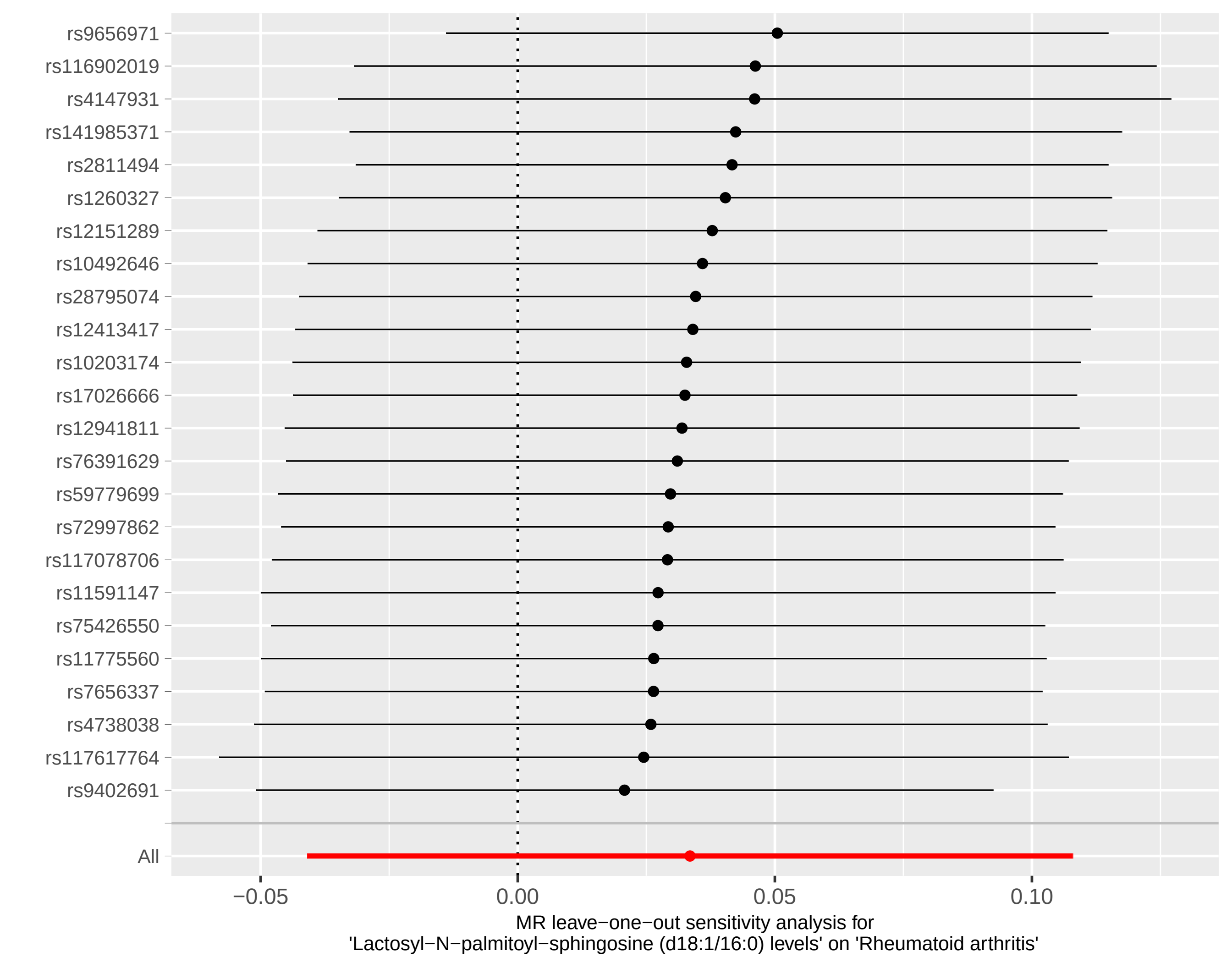

rs9656971
rs116902019
rs4147931
rs141985371
rs2811494
rs1260327
rs12151289
rs10492646
rs28795074
rs12413417
rs10203174
rs17026666
rs12941811
rs76391629
rs59779699
rs72997862
rs117078706
rs11591147
rs75426550
rs11775560
rs7656337
rs4738038
rs117617764
rs9402691
All
−0.05
0.00
0.05
0.10
MR leave−one−out sensitivity analysis for
'Lactosyl−N−palmitoyl−sphingosine (d18:1/16:0) levels' on 'Rheumatoid arthritis'

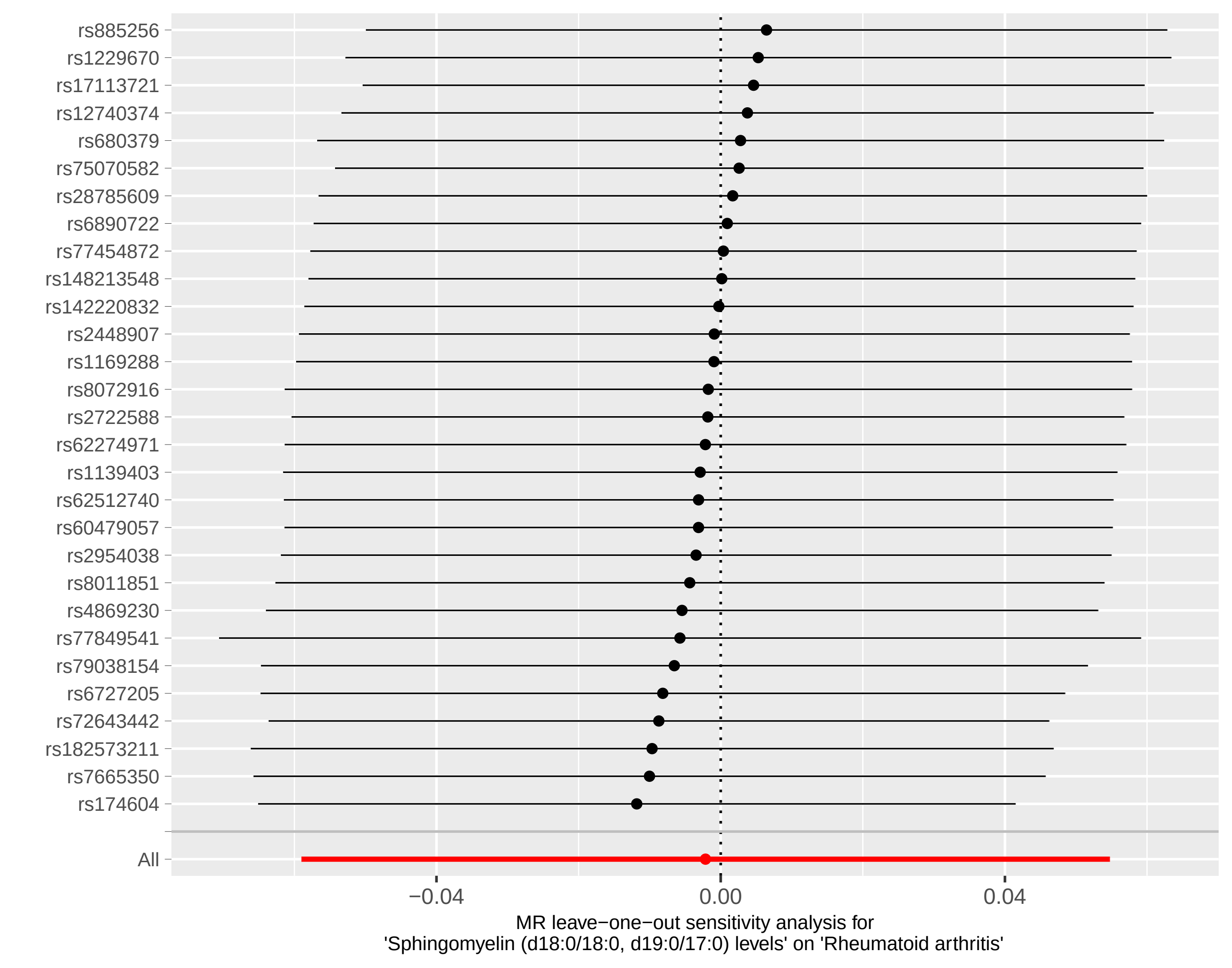
rs885256
rs1229670
rs17113721
rs12740374
rs680379
rs75070582
rs28785609
rs6890722
rs77454872
rs148213548
rs142220832
rs2448907
rs1169288
rs8072916
rs2722588
rs62274971
rs1139403
rs62512740
rs60479057
rs2954038
rs8011851
rs4869230
rs77849541
rs79038154
rs6727205
rs72643442
rs182573211
rs7665350
rs174604
All
−0.04
0.00
0.04
MR leave−one−out sensitivity analysis for
'Sphingomyelin (d18:0/18:0, d19:0/17:0) levels' on 'Rheumatoid arthritis'

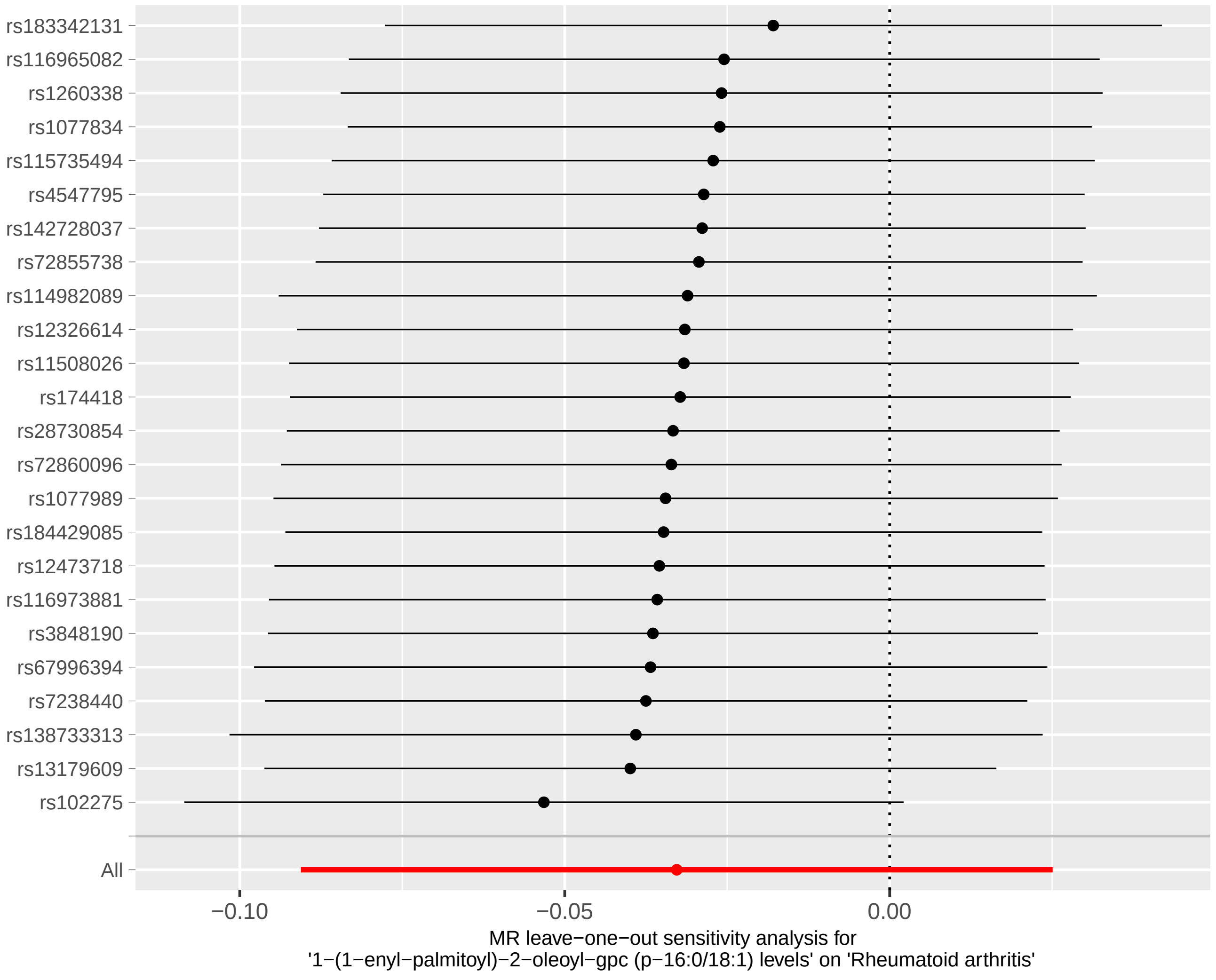
rs183342131
rs116965082
rs1260338
rs1077834
rs115735494
rs4547795
rs142728037
rs72855738
rs114982089
rs12326614
rs11508026
rs174418
rs28730854
rs72860096
rs1077989
rs184429085
rs12473718
rs116973881
rs3848190
rs67996394
rs7238440
rs138733313
rs13179609
rs102275
All
−0.10
−0.05
0.00
MR leave−one−out sensitivity analysis for
'1−(1−enyl−palmitoyl)−2−oleoyl−gpc (p−16:0/18:1) levels' on 'Rheumatoid arthritis'

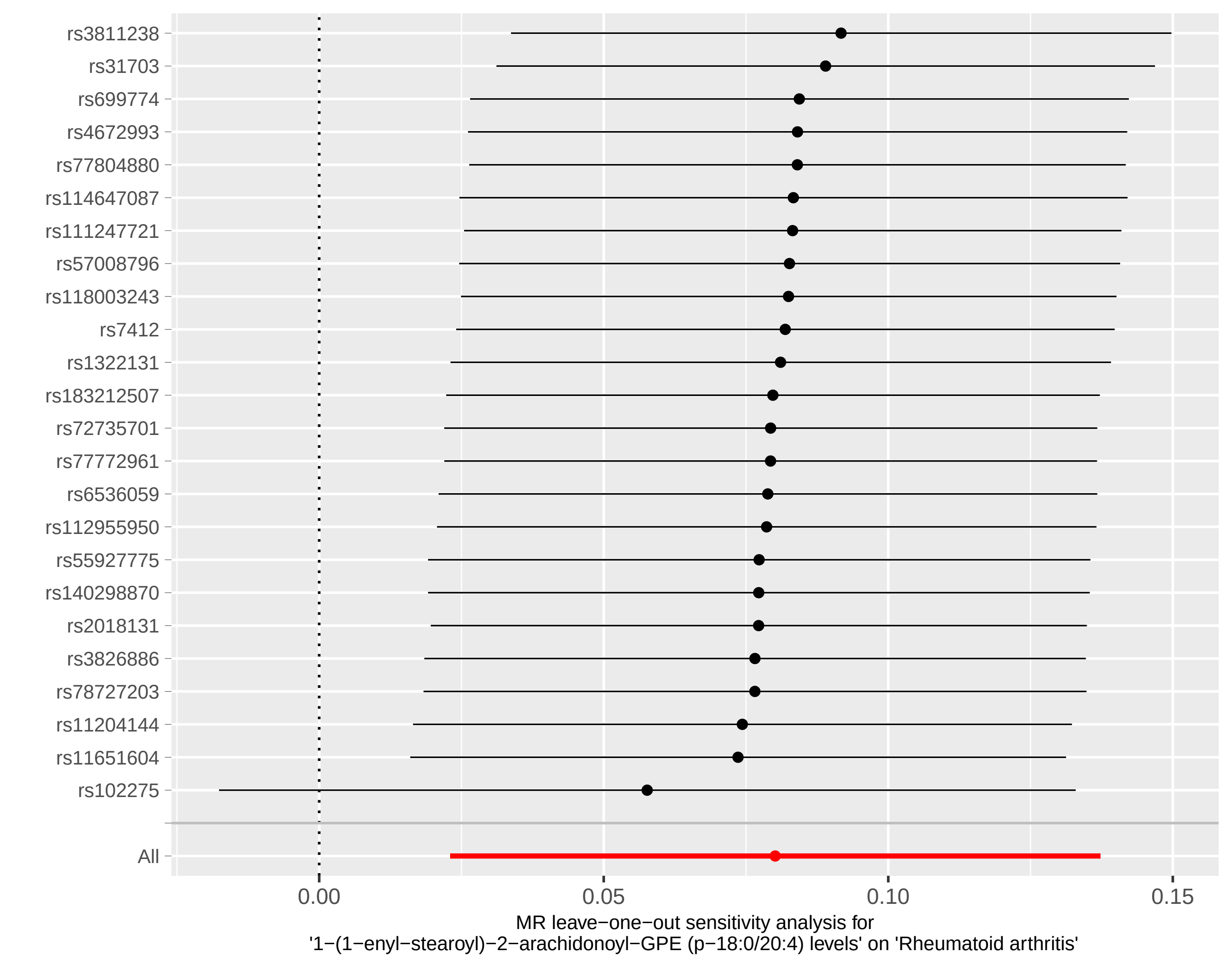
rs3811238
rs31703
rs699774
rs4672993
rs77804880
rs114647087
rs111247721
rs57008796
rs118003243
rs7412
rs1322131
rs183212507
rs72735701
rs77772961
rs6536059
rs112955950
rs55927775
rs140298870
rs2018131
rs3826886
rs78727203
rs11204144
rs11651604
rs102275
All
0.00
0.05
0.10
0.15
MR leave−one−out sensitivity analysis for
'1−(1−enyl−stearoyl)−2−arachidonoyl−GPE (p−18:0/20:4) levels' on 'Rheumatoid arthritis'

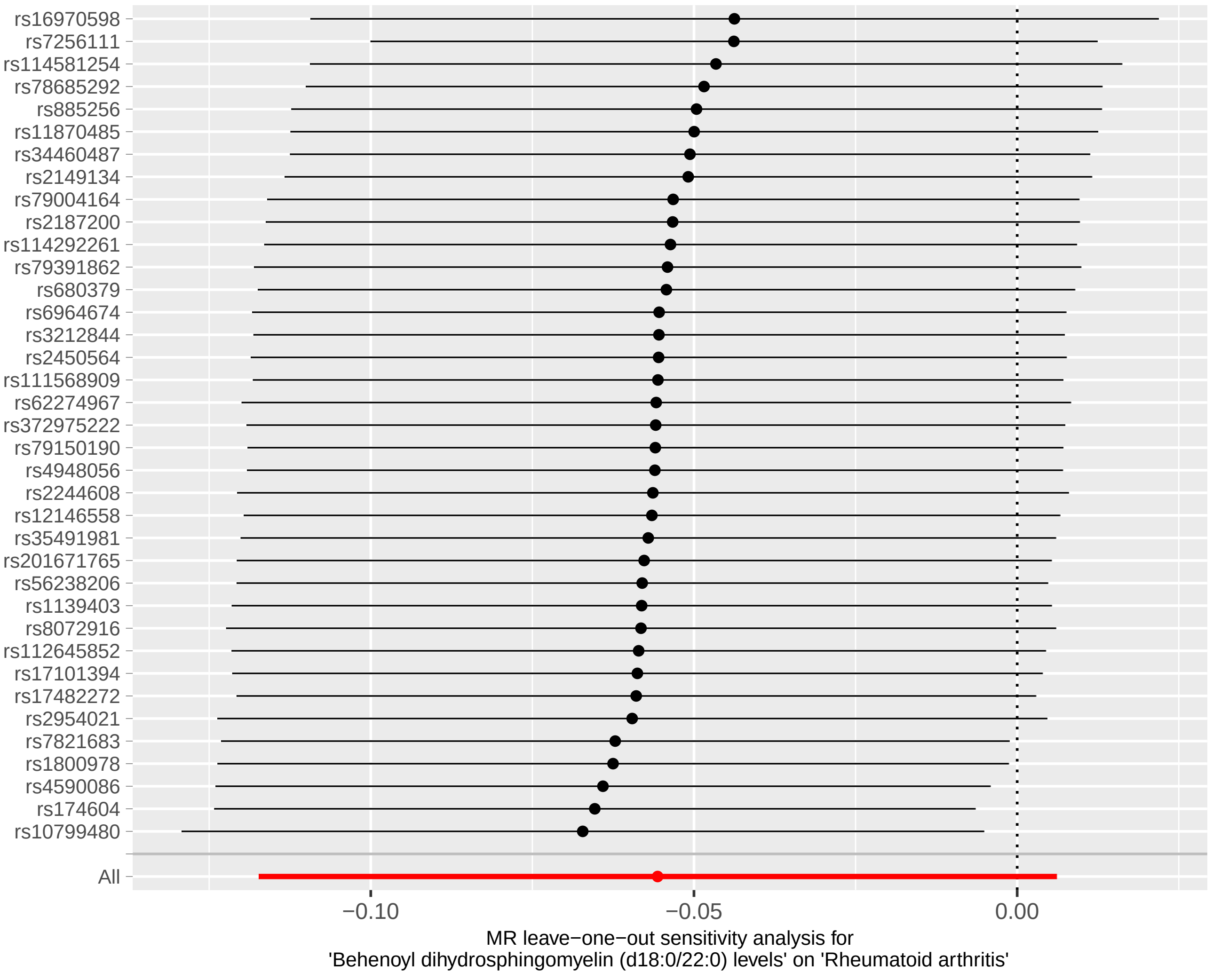
rs16970598
rs7256111
rs114581254
rs78685292
rs885256
rs11870485
rs34460487
rs2149134
rs79004164
rs2187200
rs114292261
rs79391862
rs680379
rs6964674
rs3212844
rs2450564
rs111568909
rs62274967
rs372975222
rs79150190
rs4948056
rs2244608
rs12146558
rs35491981
rs201671765
rs56238206
rs1139403
rs8072916
rs112645852
rs17101394
rs17482272
rs2954021
rs7821683
rs1800978
rs4590086
rs174604
rs10799480
All
−0.10
−0.05
0.00
MR leave−one−out sensitivity analysis for
'Behenoyl dihydrosphingomyelin (d18:0/22:0) levels' on 'Rheumatoid arthritis'

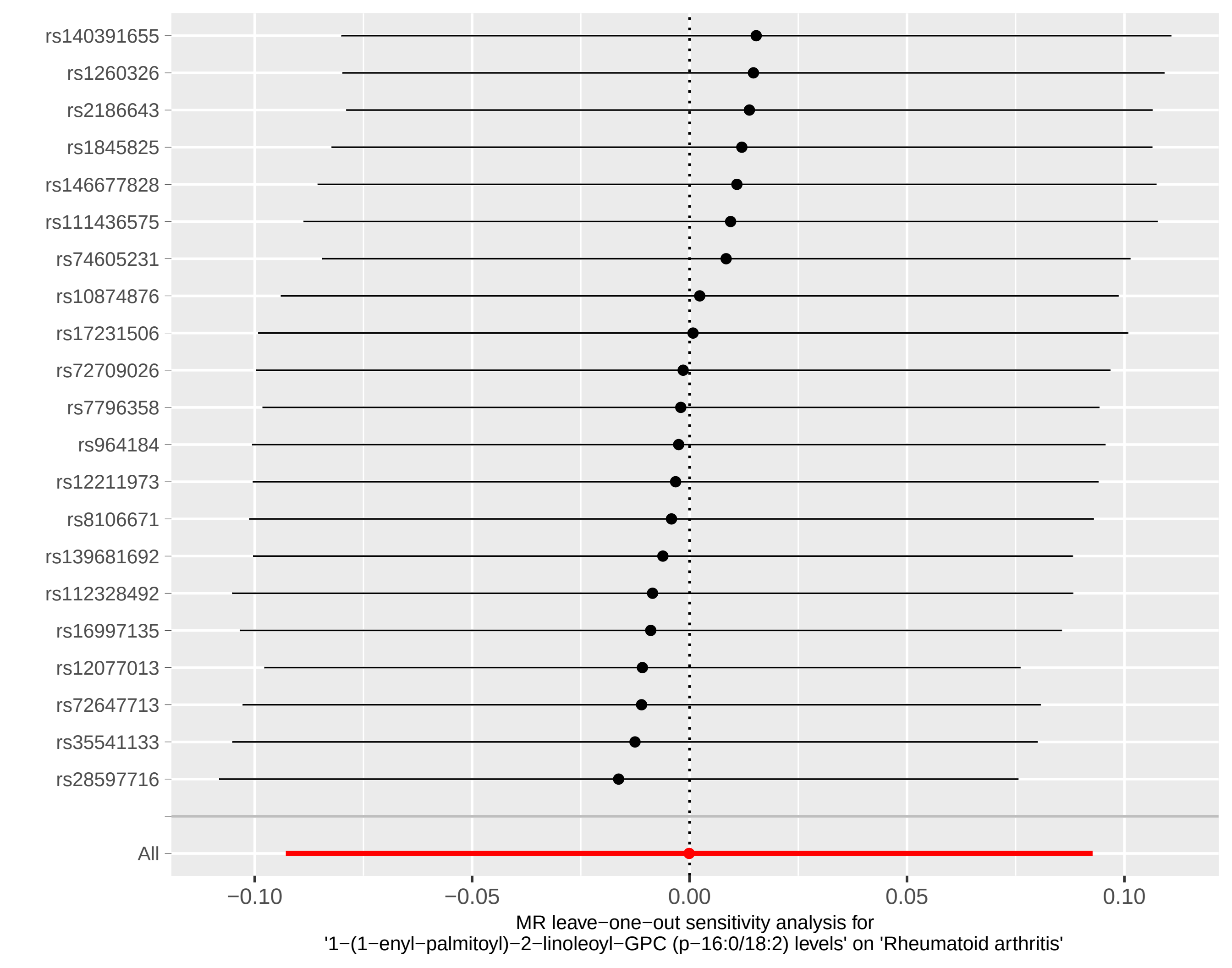
rs140391655
rs1260326
rs2186643
rs1845825
rs146677828
rs111436575
rs74605231
rs10874876
rs17231506
rs72709026
rs7796358
rs964184
rs12211973
rs8106671
rs139681692
rs112328492
rs16997135
rs12077013
rs72647713
rs35541133
rs28597716
All
−0.10
−0.05
0.00
0.05
0.10
MR leave−one−out sensitivity analysis for
'1−(1−enyl−palmitoyl)−2−linoleoyl−GPC (p−16:0/18:2) levels' on 'Rheumatoid arthritis'

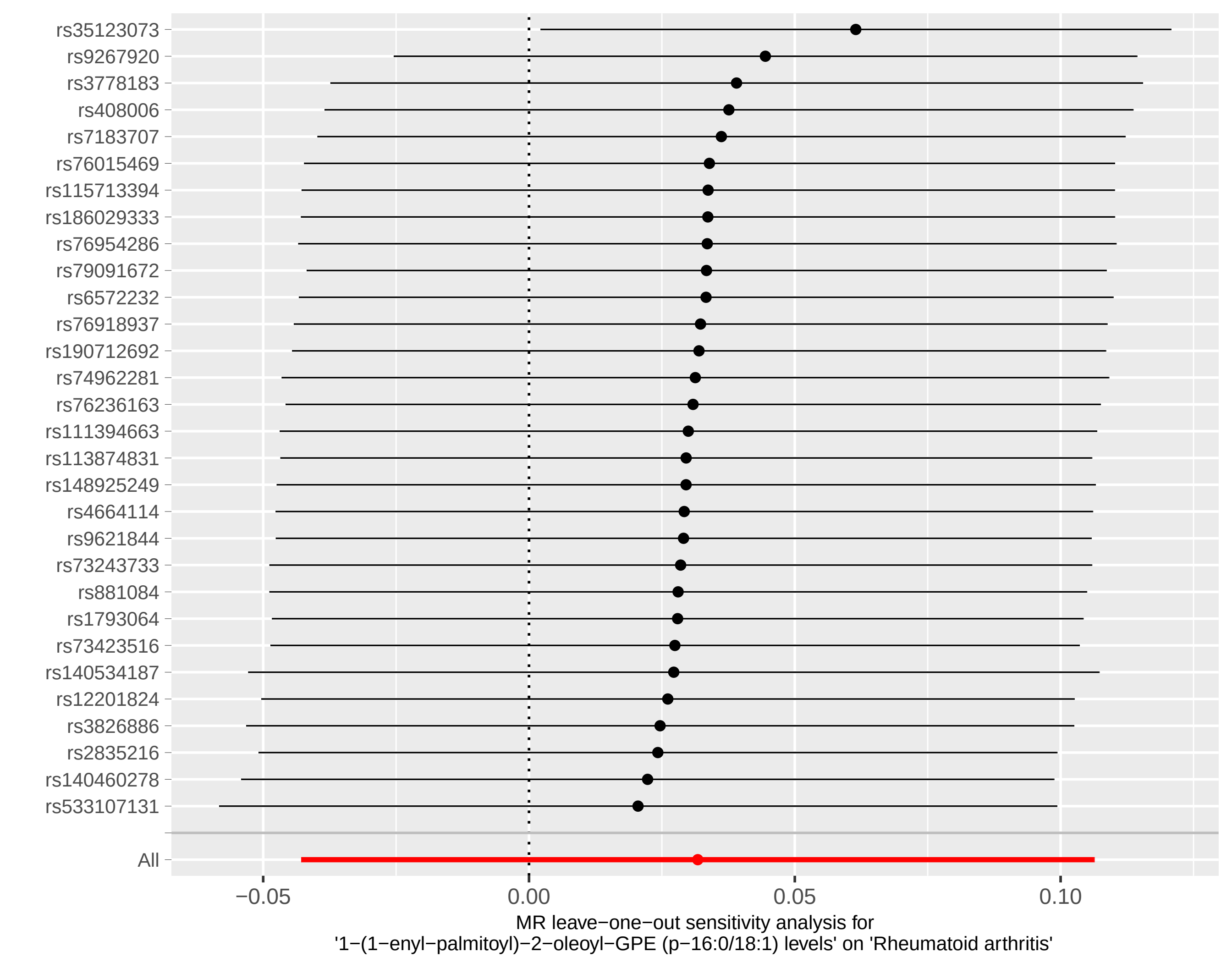
rs35123073
rs9267920
rs3778183
rs408006
rs7183707
rs76015469
rs115713394
rs186029333
rs76954286
rs79091672
rs6572232
rs76918937
rs190712692
rs74962281
rs76236163
rs111394663
rs113874831
rs148925249
rs4664114
rs9621844
rs73243733
rs881084
rs1793064
rs73423516
rs140534187
rs12201824
rs3826886
rs2835216
rs140460278
rs533107131
All
−0.05
0.00
0.05
0.10
MR leave−one−out sensitivity analysis for
'1−(1−enyl−palmitoyl)−2−oleoyl−GPE (p−16:0/18:1) levels' on 'Rheumatoid arthritis'

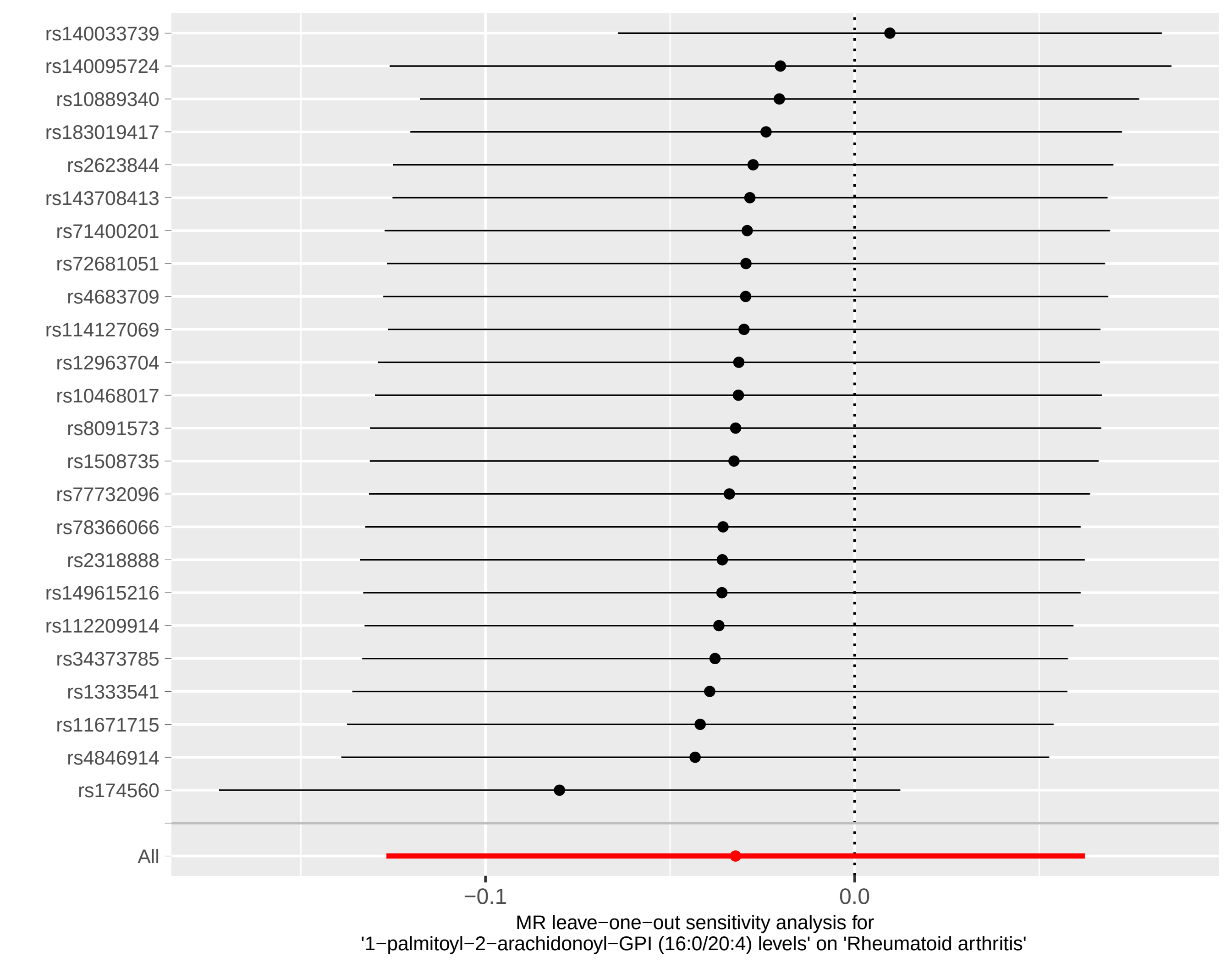
rs140033739
rs140095724
rs10889340
rs183019417
rs2623844
rs143708413
rs71400201
rs72681051
rs4683709
rs114127069
rs12963704
rs10468017
rs8091573
rs1508735
rs77732096
rs78366066
rs2318888
rs149615216
rs112209914
rs34373785
rs1333541
rs11671715
rs4846914
rs174560
All
−0.1
0.0
MR leave−one−out sensitivity analysis for
'1−palmitoyl−2−arachidonoyl−GPI (16:0/20:4) levels' on 'Rheumatoid arthritis'

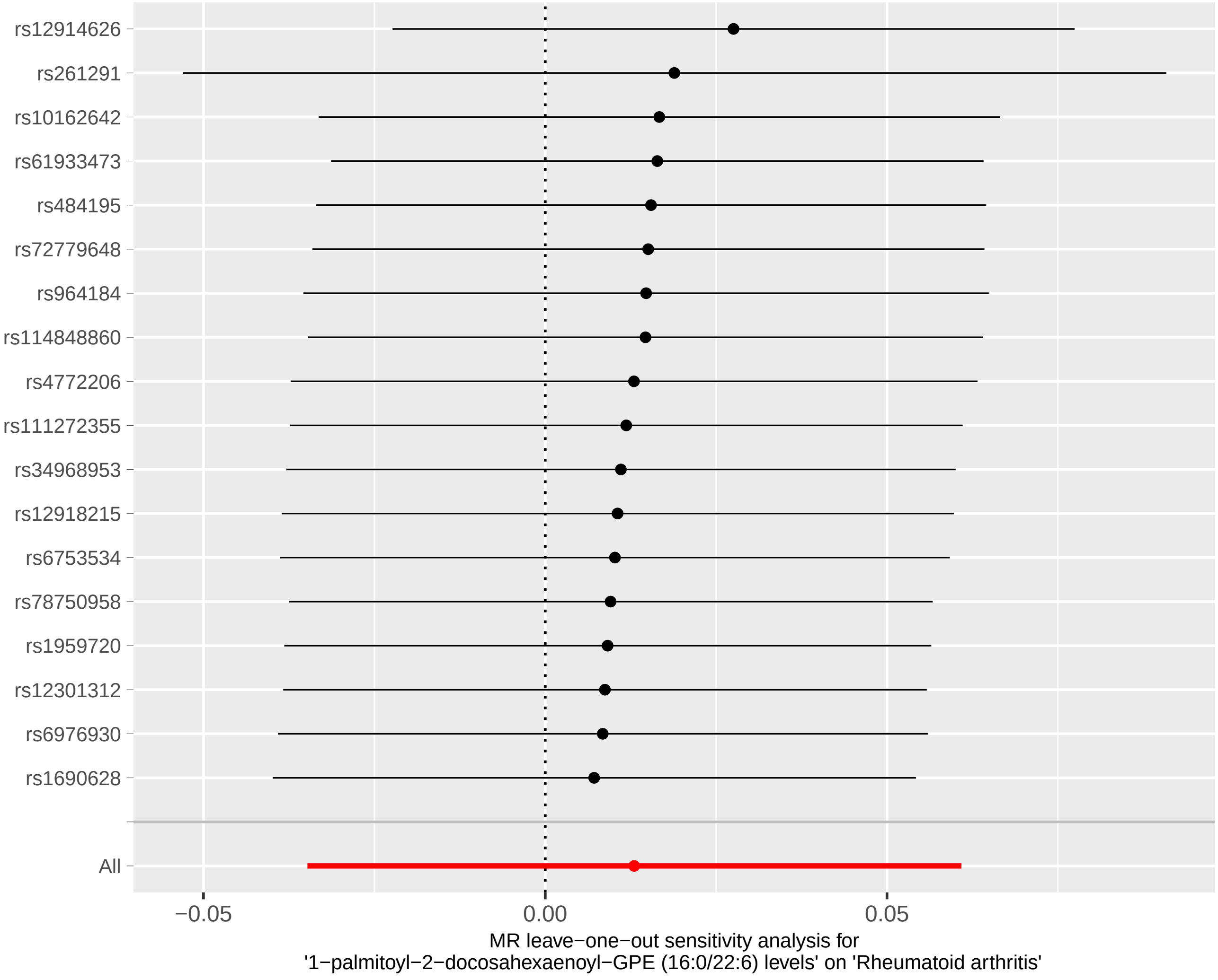

rs12914626
rs261291
rs10162642
rs61933473
rs484195
rs72779648
rs964184
rs114848860
rs4772206
rs111272355
rs34968953
rs12918215
rs6753534
rs78750958
rs1959720
rs12301312
rs6976930
rs1690628
All
−0.05
0.00
0.05
MR leave−one−out sensitivity analysis for
'1−palmitoyl−2−docosahexaenoyl−GPE (16:0/22:6) levels' on 'Rheumatoid arthritis'

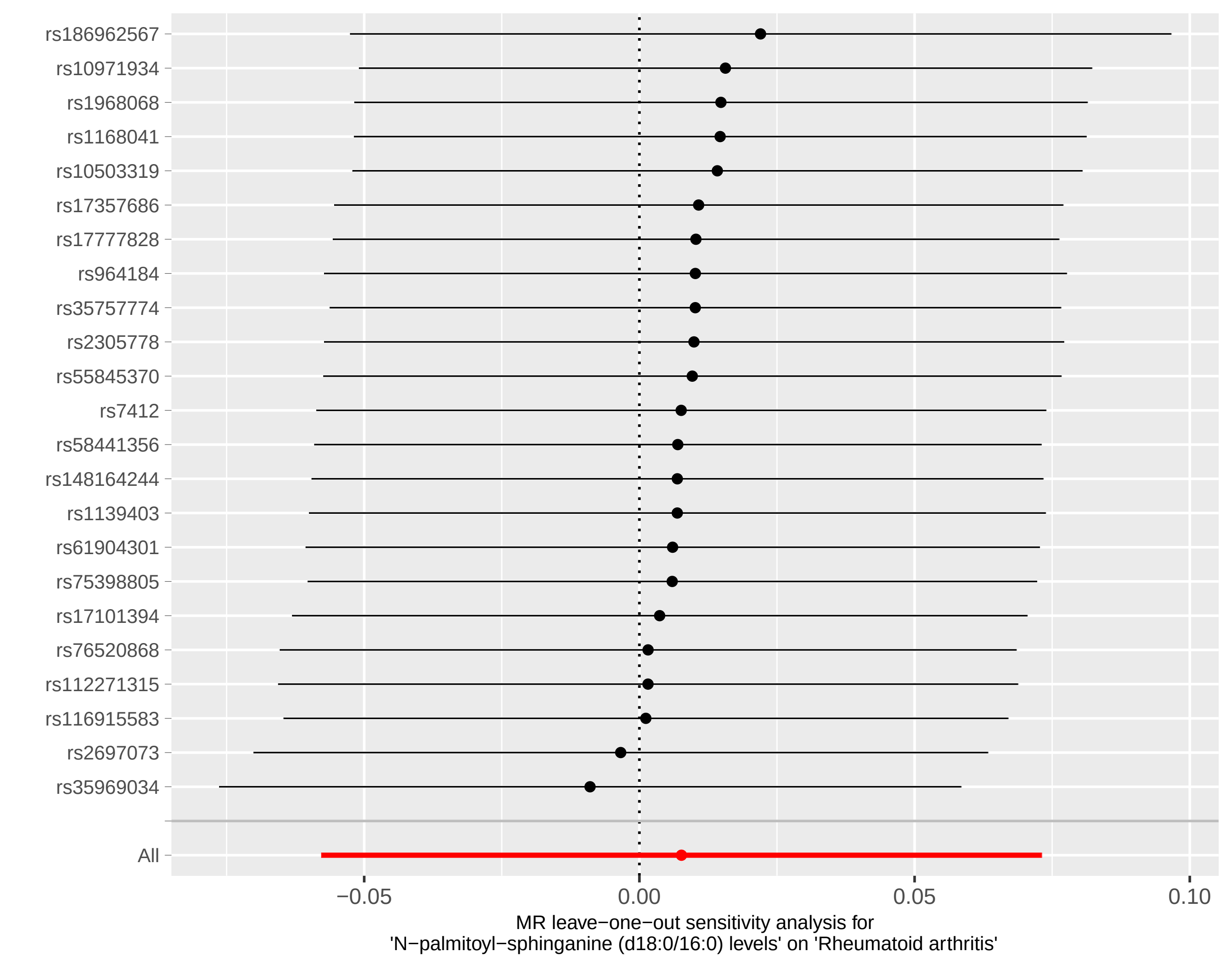
rs186962567
rs10971934
rs1968068
rs1168041
rs10503319
rs17357686
rs17777828
rs964184
rs35757774
rs2305778
rs55845370
rs7412
rs58441356
rs148164244
rs1139403
rs61904301
rs75398805
rs17101394
rs76520868
rs112271315
rs116915583
rs2697073
rs35969034
All
−0.05
0.00
0.05
0.10
MR leave−one−out sensitivity analysis for
'N−palmitoyl−sphinganine (d18:0/16:0) levels' on 'Rheumatoid arthritis'

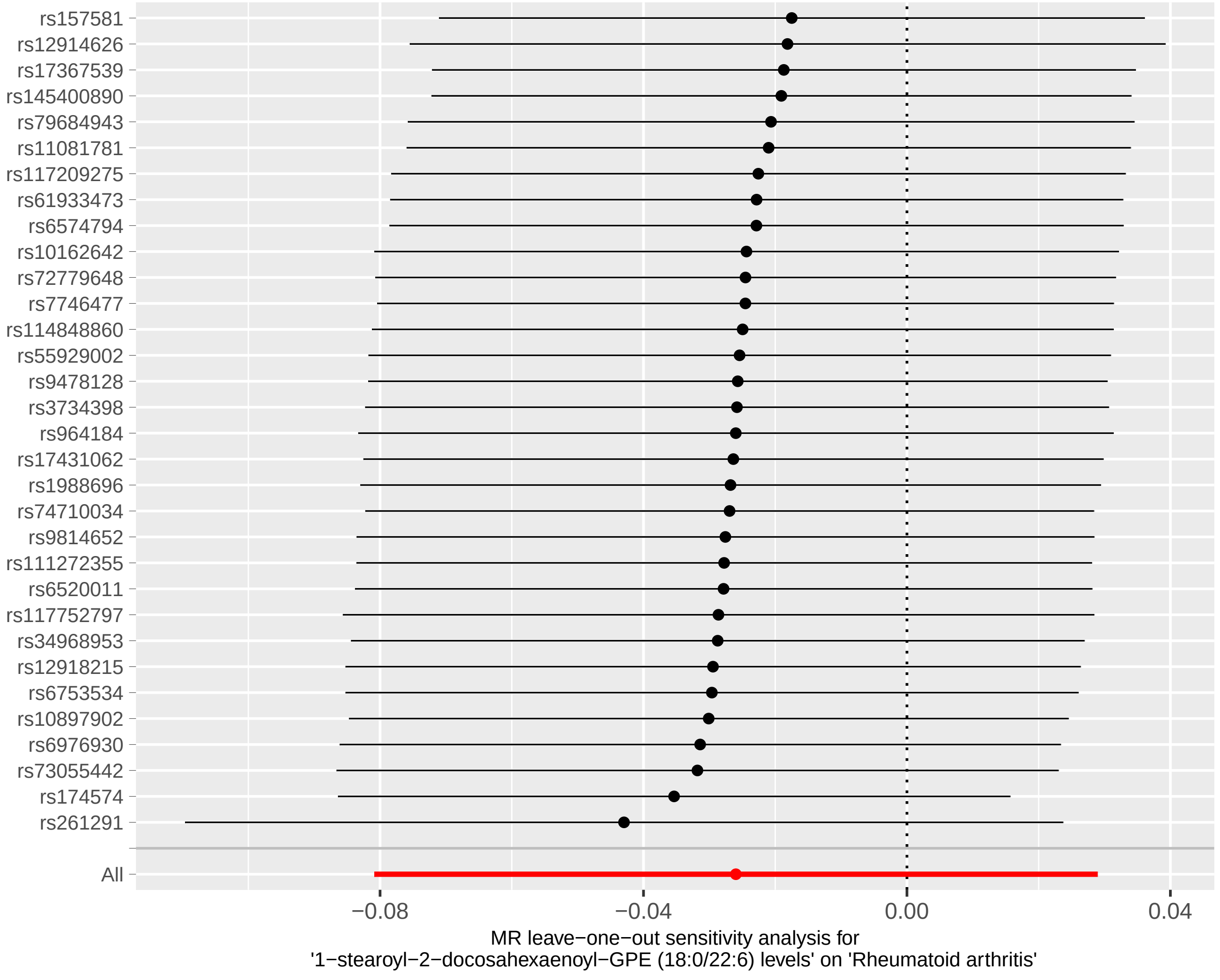
rs157581
rs12914626
rs17367539
rs145400890
rs79684943
rs11081781
rs117209275
rs61933473
rs6574794
rs10162642
rs72779648
rs7746477
rs114848860
rs55929002
rs9478128
rs3734398
rs964184
rs17431062
rs1988696
rs74710034
rs9814652
rs111272355
rs6520011
rs117752797
rs34968953
rs12918215
rs6753534
rs10897902
rs6976930
rs73055442
rs174574
rs261291
All
−0.08
−0.04
0.00
0.04
MR leave−one−out sensitivity analysis for
'1−stearoyl−2−docosahexaenoyl−GPE (18:0/22:6) levels' on 'Rheumatoid arthritis'

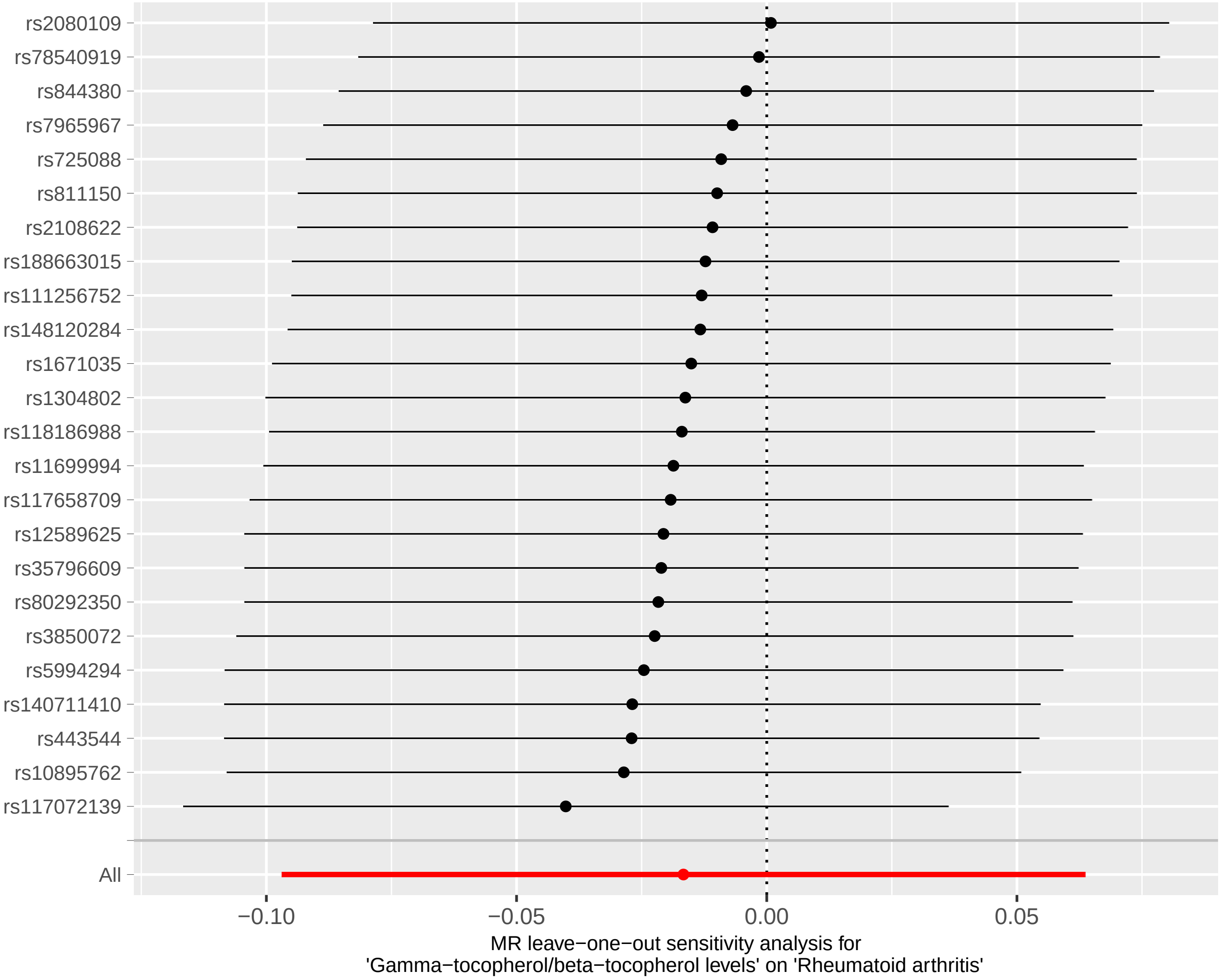
rs2080109
rs78540919
rs844380
rs7965967
rs725088
rs811150
rs2108622
rs188663015
rs111256752
rs148120284
rs1671035
rs1304802
rs118186988
rs11699994
rs117658709
rs12589625
rs35796609
rs80292350
rs3850072
rs5994294
rs140711410
rs443544
rs10895762
rs117072139
All
−0.10
−0.05
0.00
0.05
MR leave−one−out sensitivity analysis for
'Gamma−tocopherol/beta−tocopherol levels' on 'Rheumatoid arthritis'

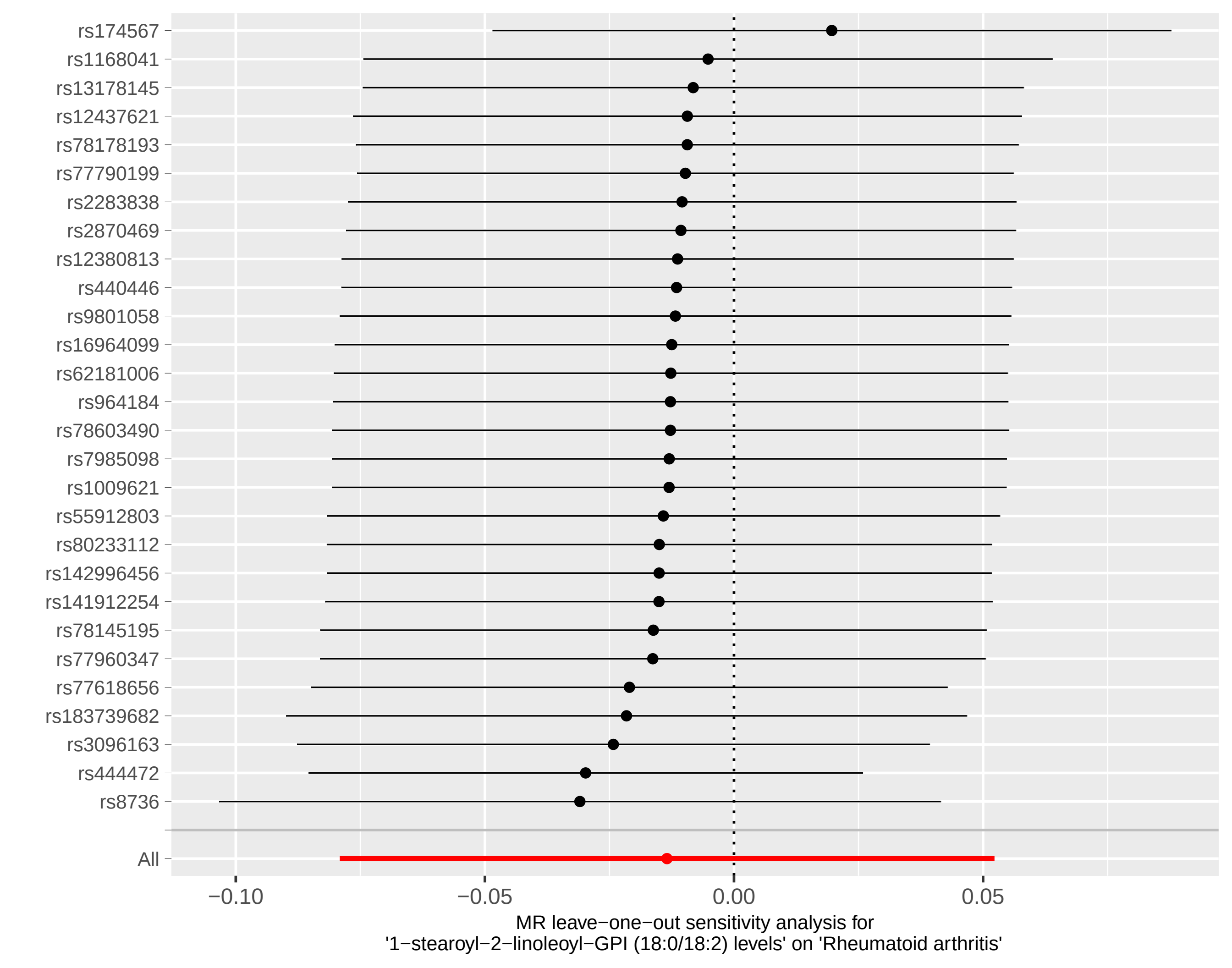
rs174567
rs1168041
rs13178145
rs12437621
rs78178193
rs77790199
rs2283838
rs2870469
rs12380813
rs440446
rs9801058
rs16964099
rs62181006
rs964184
rs78603490
rs7985098
rs1009621
rs55912803
rs80233112
rs142996456
rs141912254
rs78145195
rs77960347
rs77618656
rs183739682
rs3096163
rs444472
rs8736
All
−0.10
−0.05
0.00
0.05
MR leave−one−out sensitivity analysis for
'1−stearoyl−2−linoleoyl−GPI (18:0/18:2) levels' on 'Rheumatoid arthritis'

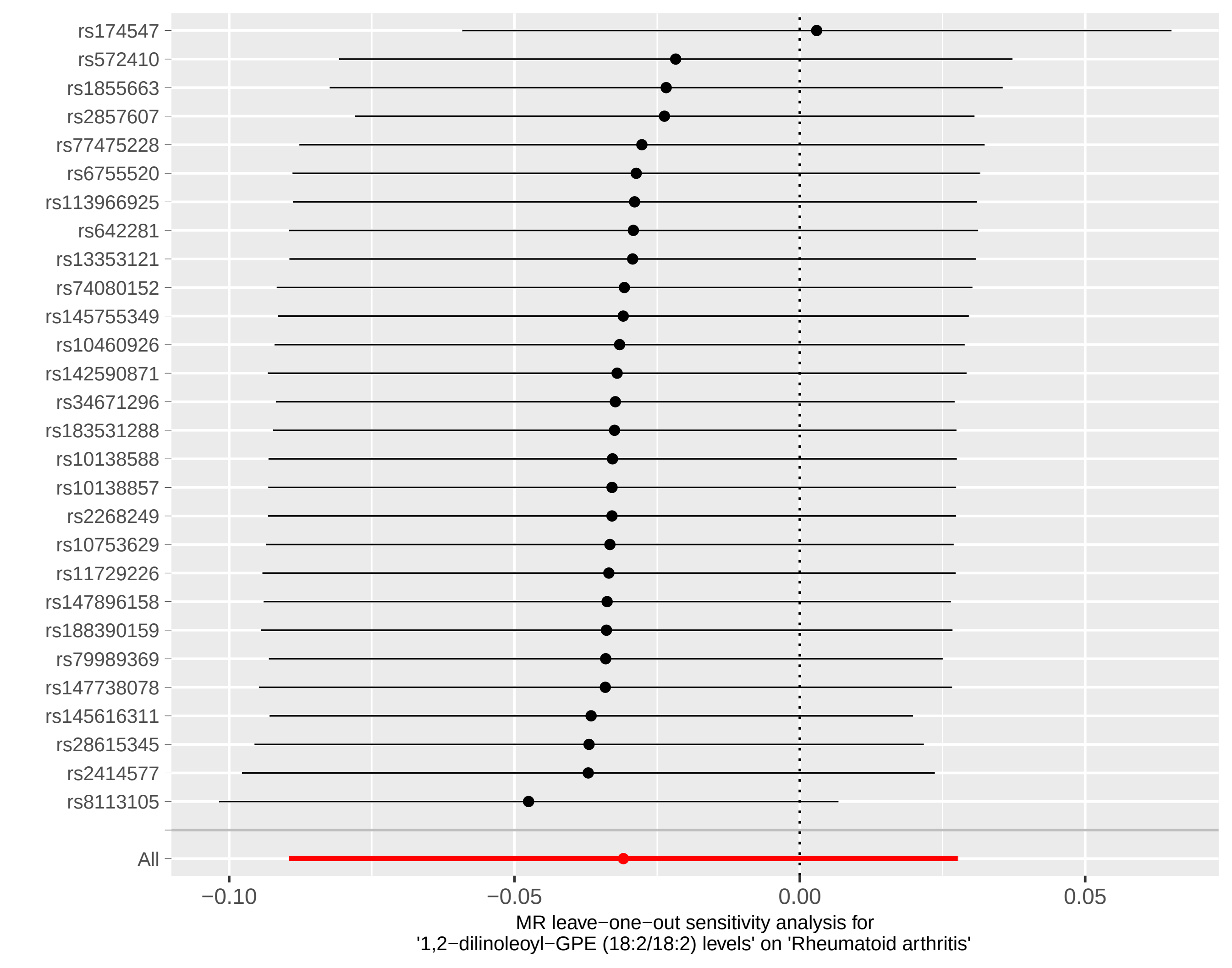

rs174547
rs572410
rs1855663
rs2857607
rs77475228
rs6755520
rs113966925
rs642281
rs13353121
rs74080152
rs145755349
rs10460926
rs142590871
rs34671296
rs183531288
rs10138588
rs10138857
rs2268249
rs10753629
rs11729226
rs147896158
rs188390159
rs79989369
rs147738078
rs145616311
rs28615345
rs2414577
rs8113105
All
−0.10
−0.05
0.00
0.05
MR leave−one−out sensitivity analysis for
'1,2−dilinoleoyl−GPE (18:2/18:2) levels' on 'Rheumatoid arthritis'

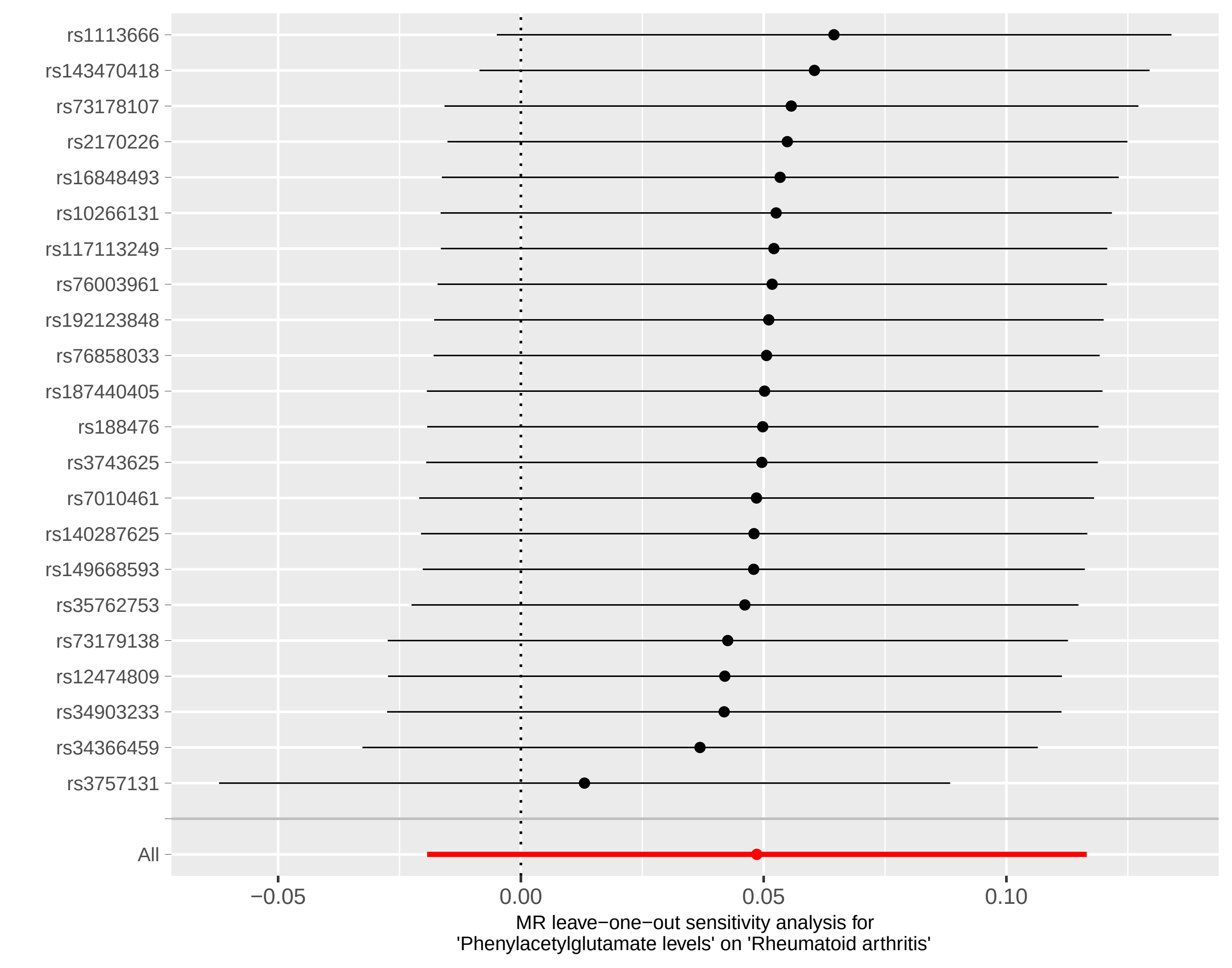
rs1113666
rs143470418
rs73178107
rs2170226
rs16848493
rs10266131
rs117113249
rs76003961
rs192123848
rs76858033
rs187440405
rs188476
rs3743625
rs7010461
rs140287625
rs149668593
rs35762753
rs73179138
rs12474809
rs34903233
rs34366459
rs3757131
All
−0.05
0.00
0.05
0.10
MR leave−one−out sensitivity analysis for
'Phenylacetylglutamate levels' on 'Rheumatoid arthritis'

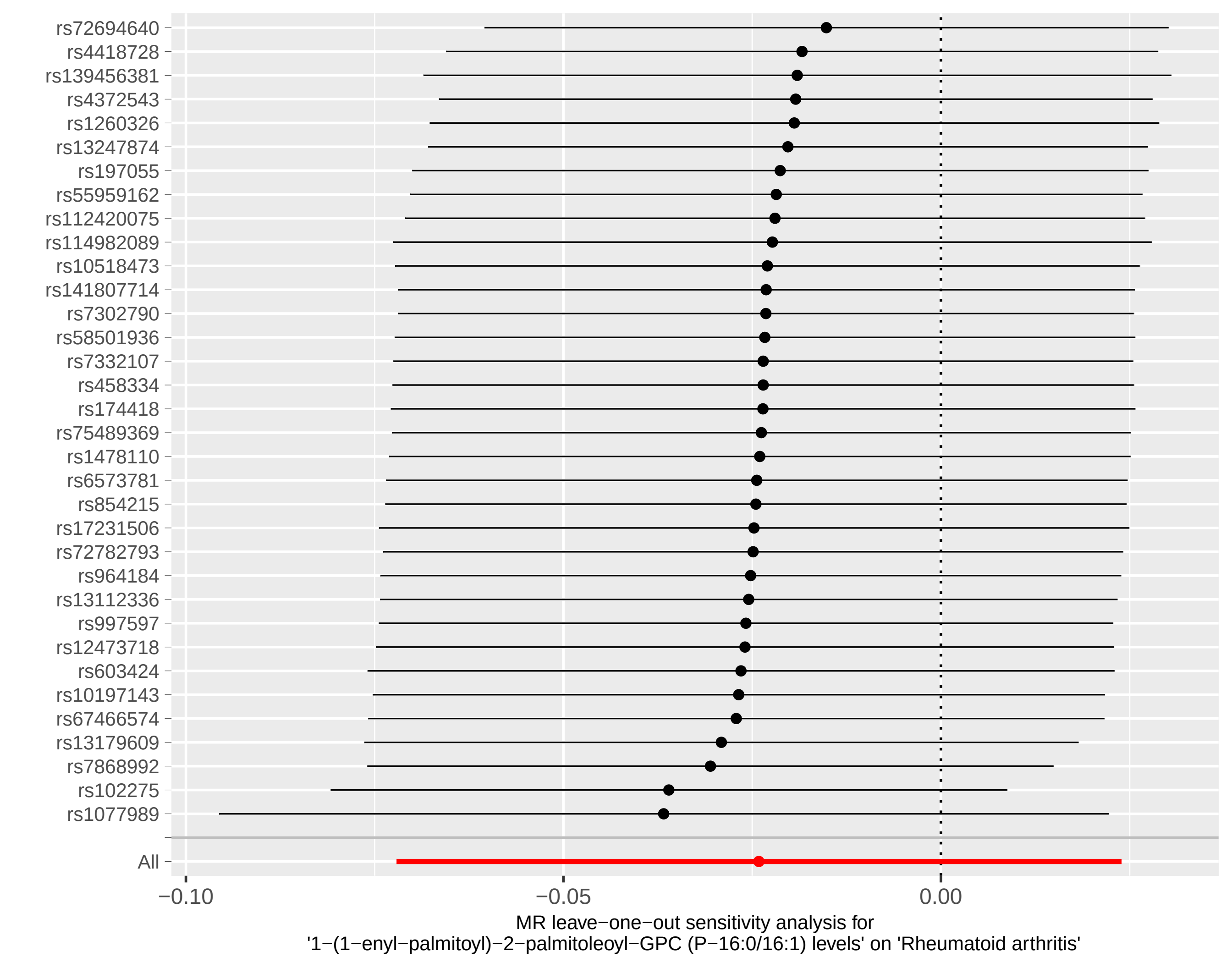

rs72694640
rs4418728
rs139456381
rs4372543
rs1260326
rs13247874
rs197055
rs55959162
rs112420075
rs114982089
rs10518473
rs141807714
rs7302790
rs58501936
rs7332107
rs458334
rs174418
rs75489369
rs1478110
rs6573781
rs854215
rs17231506
rs72782793
rs964184
rs13112336
rs997597
rs12473718
rs603424
rs10197143
rs67466574
rs13179609
rs7868992
rs102275
rs1077989
All
−0.10
−0.05
0.00
MR leave−one−out sensitivity analysis for
'1−(1−enyl−palmitoyl)−2−palmitoleoyl−GPC (P−16:0/16:1) levels' on 'Rheumatoid arthritis'

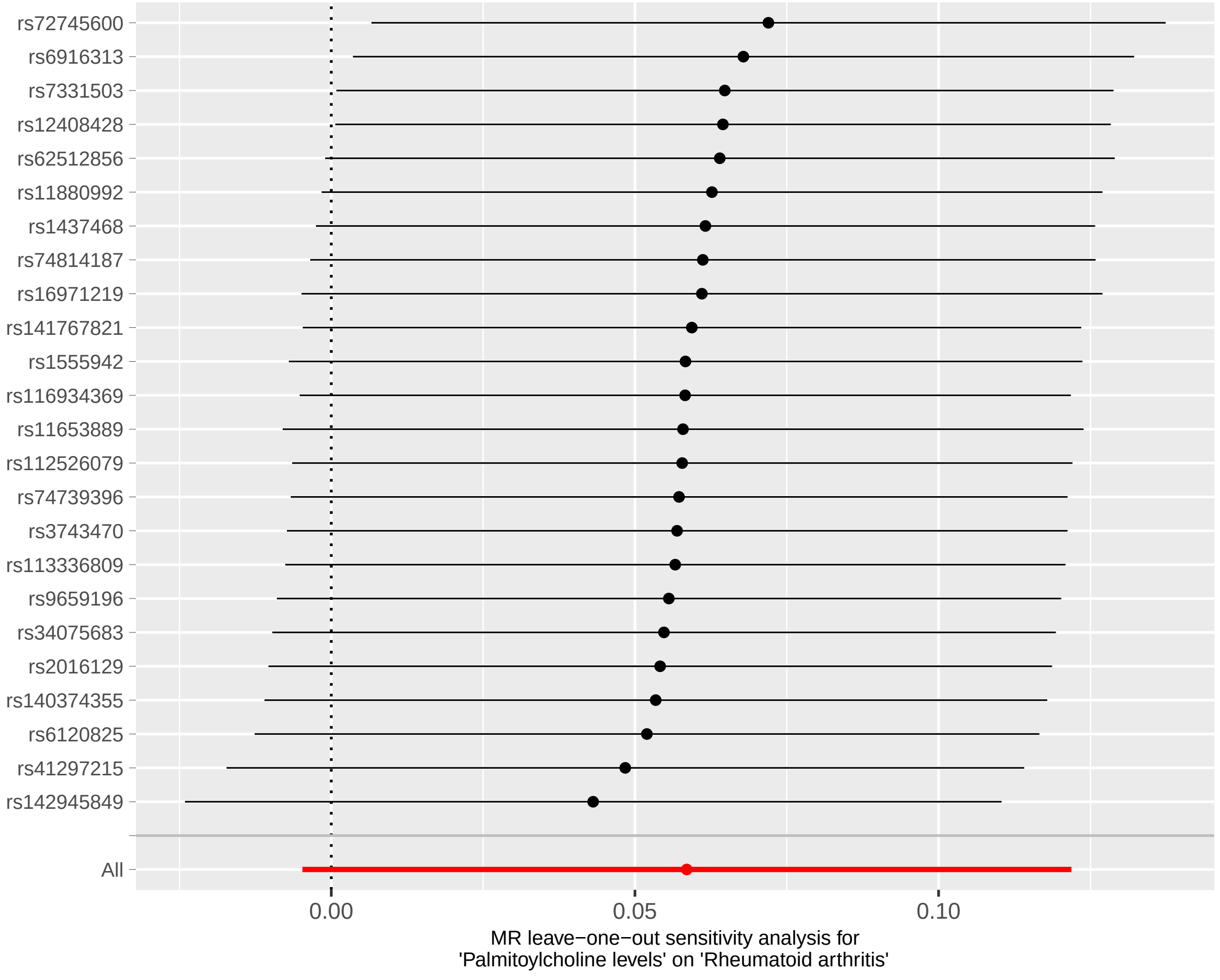
rs72745600
rs6916313
rs7331503
rs12408428
rs62512856
rs11880992
rs1437468
rs74814187
rs16971219
rs141767821
rs1555942
rs116934369
rs11653889
rs112526079
rs74739396
rs3743470
rs113336809
rs9659196
rs34075683
rs2016129
rs140374355
rs6120825
rs41297215
rs142945849
All
0.00
0.05
0.10
MR leave−one−out sensitivity analysis for
'Palmitoylcholine levels' on 'Rheumatoid arthritis'

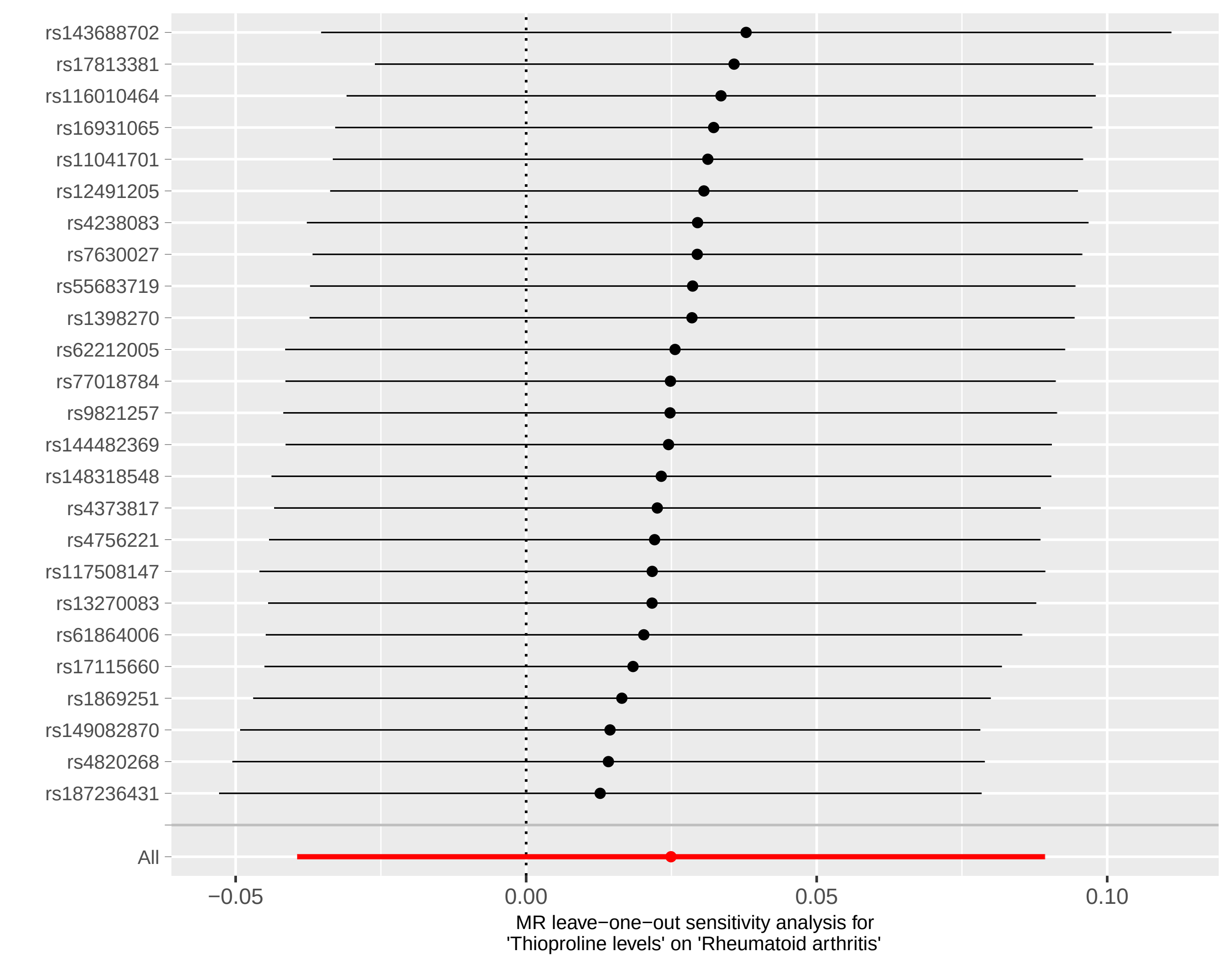

rs143688702
rs17813381
rs116010464
rs16931065
rs11041701
rs12491205
rs4238083
rs7630027
rs55683719
rs1398270
rs62212005
rs77018784
rs9821257
rs144482369
rs148318548
rs4373817
rs4756221
rs117508147
rs13270083
rs61864006
rs17115660
rs1869251
rs149082870
rs4820268
rs187236431
All
−0.05
0.00
0.05
0.10
MR leave−one−out sensitivity analysis for
'Thioproline levels' on 'Rheumatoid arthritis'

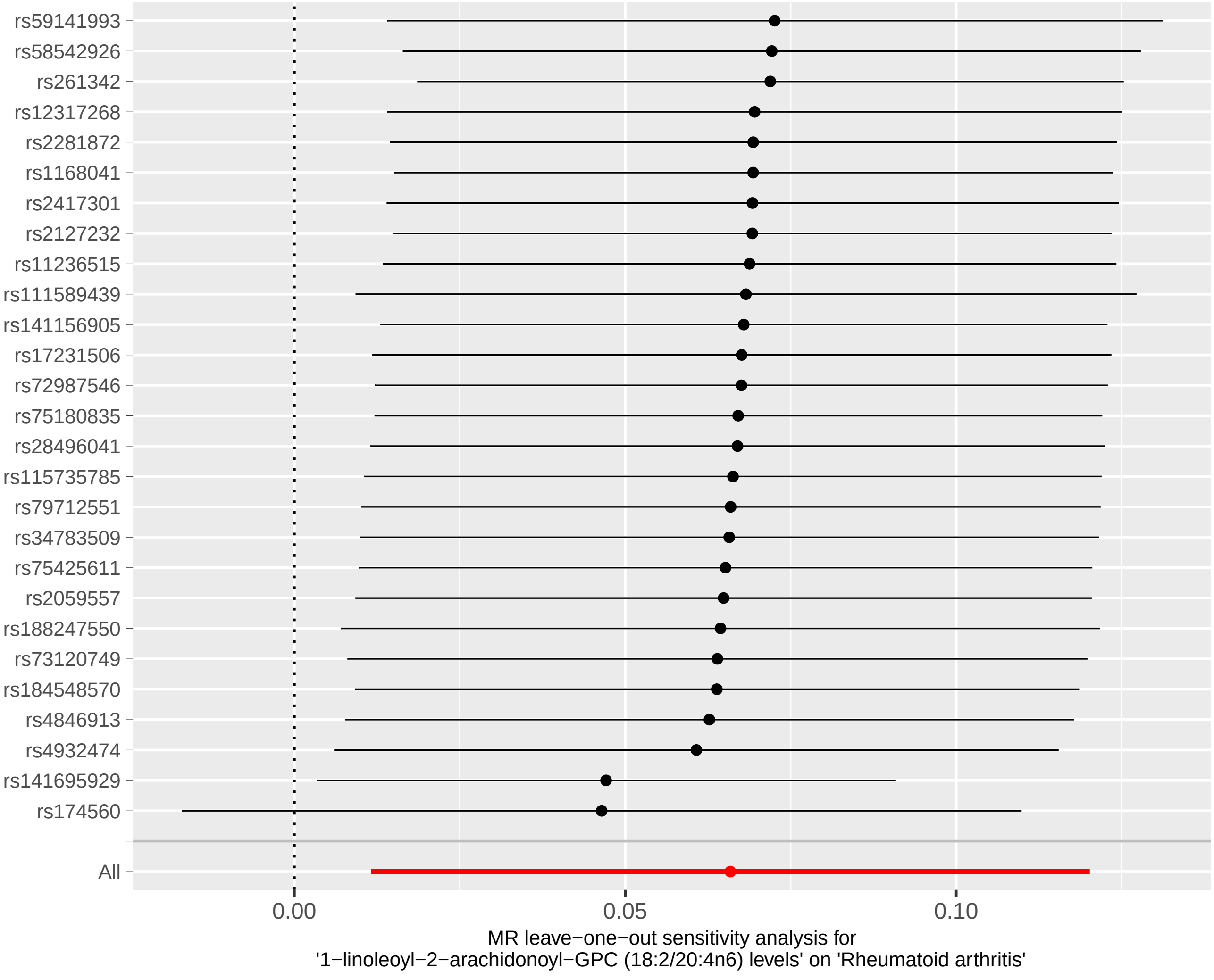
rs59141993
rs58542926
rs261342
rs12317268
rs2281872
rs1168041
rs2417301
rs2127232
rs11236515
rs111589439
rs141156905
rs17231506
rs72987546
rs75180835
rs28496041
rs115735785
rs79712551
rs34783509
rs75425611
rs2059557
rs188247550
rs73120749
rs184548570
rs4846913
rs4932474
rs141695929
rs174560
All
0.00
0.05
0.10
MR leave−one−out sensitivity analysis for
'1−linoleoyl−2−arachidonoyl−GPC (18:2/20:4n6) levels' on 'Rheumatoid arthritis'

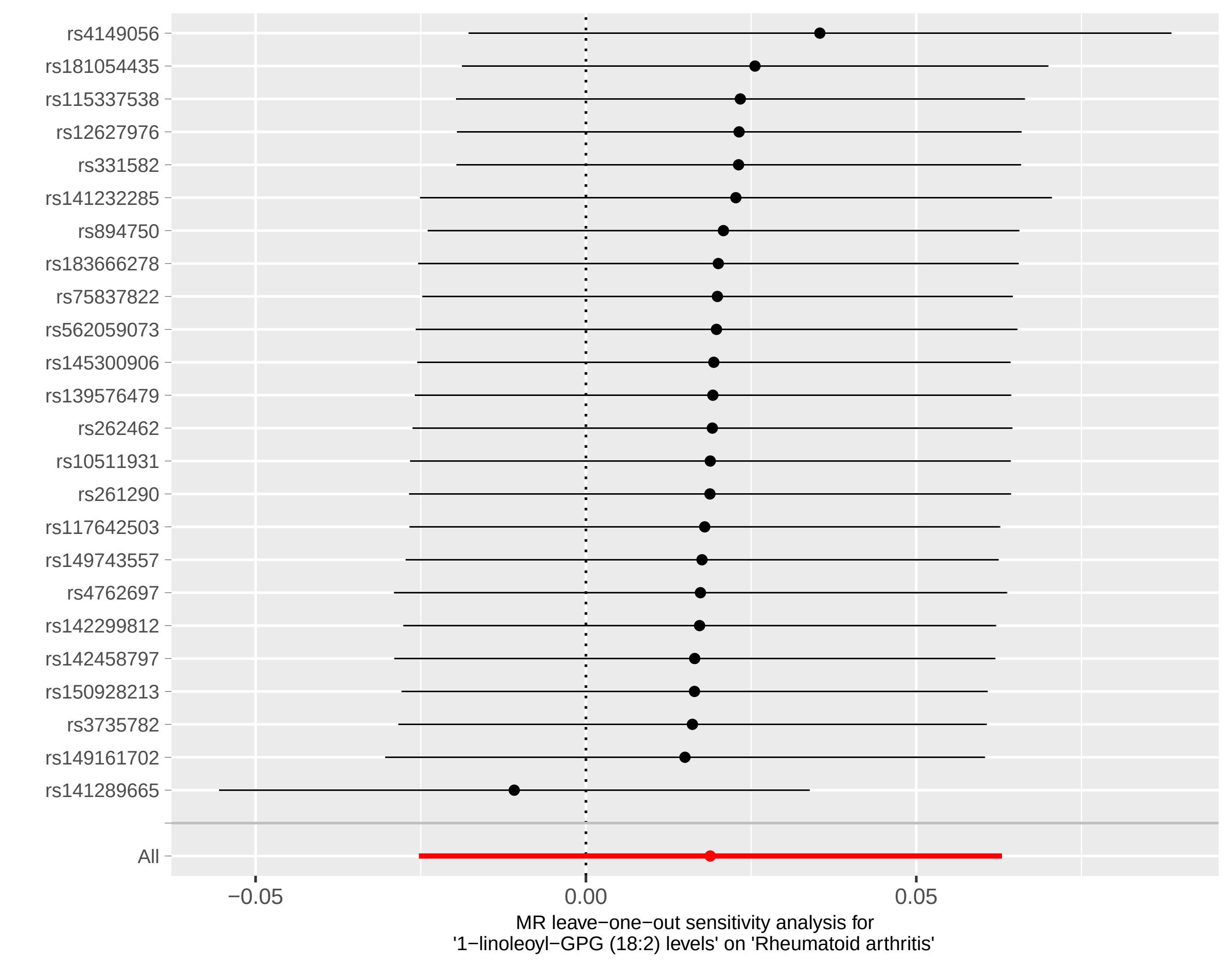
rs4149056
rs181054435
rs115337538
rs12627976
rs331582
rs141232285
rs894750
rs183666278
rs75837822
rs562059073
rs145300906
rs139576479
rs262462
rs10511931
rs261290
rs117642503
rs149743557
rs4762697
rs142299812
rs142458797
rs150928213
rs3735782
rs149161702
rs141289665
All
−0.05
0.00
0.05
MR leave−one−out sensitivity analysis for
'1−linoleoyl−GPG (18:2) levels' on 'Rheumatoid arthritis'

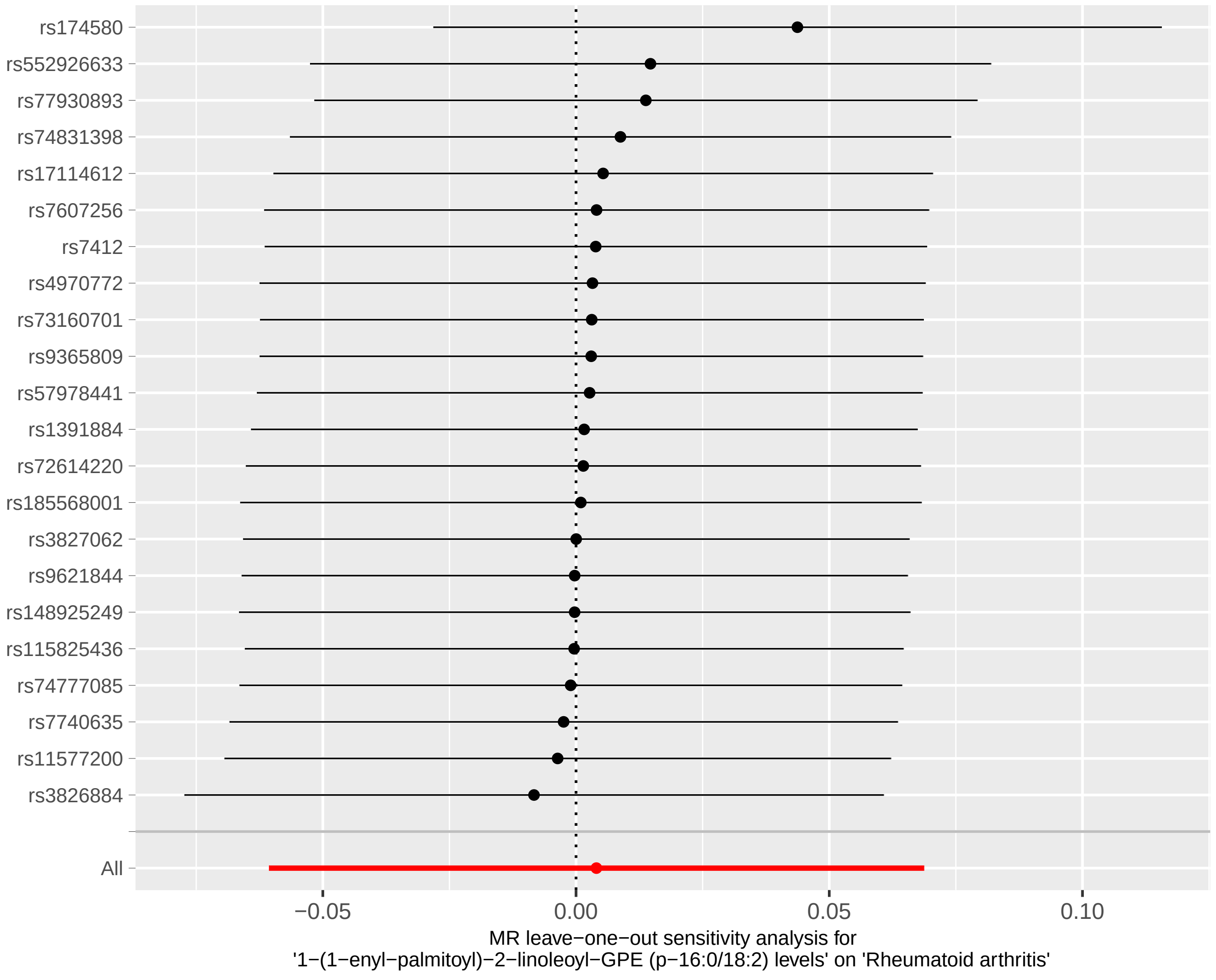
rs174580
rs552926633
rs77930893
rs74831398
rs17114612
rs7607256
rs7412
rs4970772
rs73160701
rs9365809
rs57978441
rs1391884
rs72614220
rs185568001
rs3827062
rs9621844
rs148925249
rs115825436
rs74777085
rs7740635
rs11577200
rs3826884
All
−0.05
0.00
0.05
0.10
MR leave−one−out sensitivity analysis for
'1−(1−enyl−palmitoyl)−2−linoleoyl−GPE (p−16:0/18:2) levels' on 'Rheumatoid arthritis'

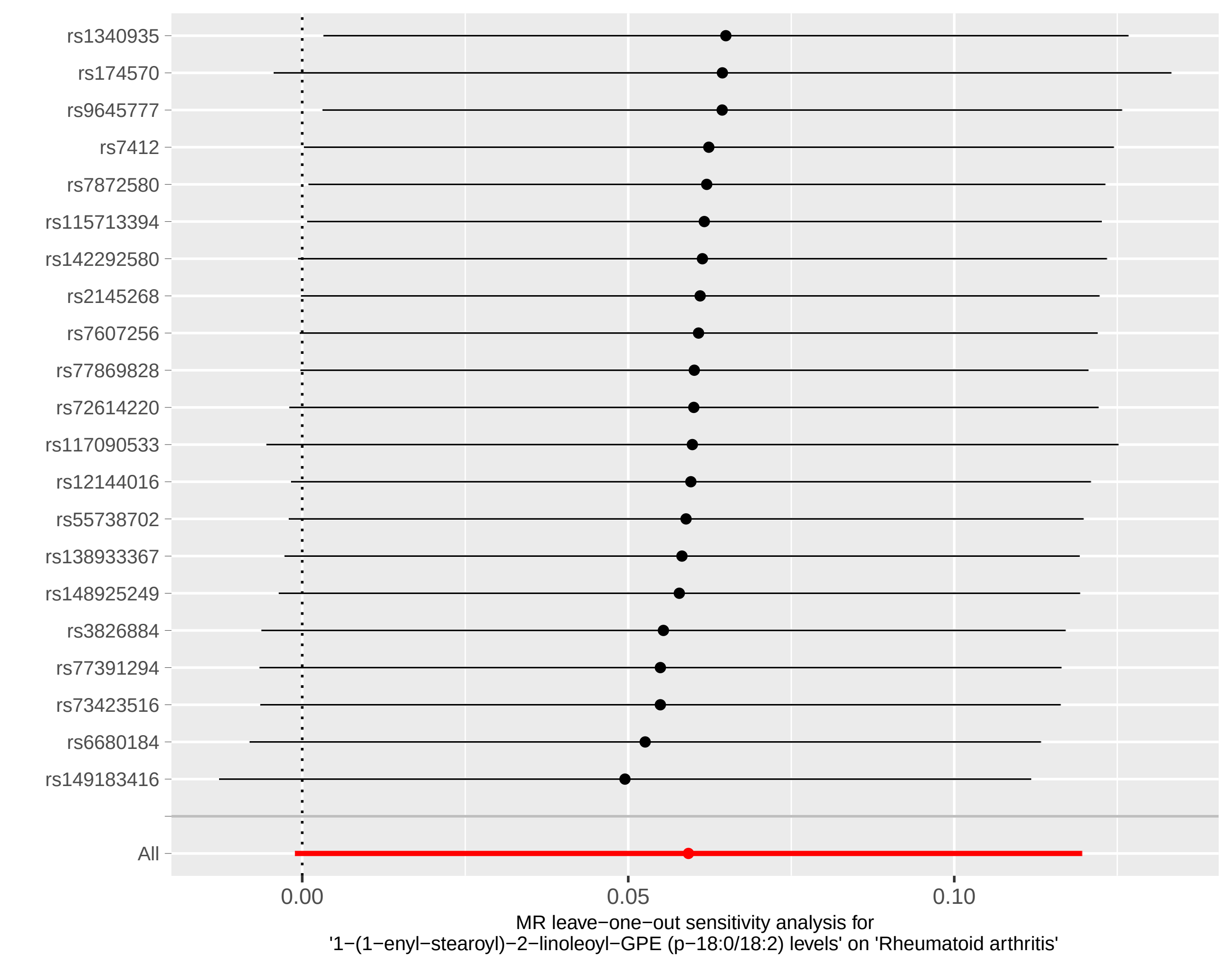

rs1340935
rs174570
rs9645777
rs7412
rs7872580
rs115713394
rs142292580
rs2145268
rs7607256
rs77869828
rs72614220
rs117090533
rs12144016
rs55738702
rs138933367
rs148925249
rs3826884
rs77391294
rs73423516
rs6680184
rs149183416
All
0.00
0.05
0.10
MR leave−one−out sensitivity analysis for
'1−(1−enyl−stearoyl)−2−linoleoyl−GPE (p−18:0/18:2) levels' on 'Rheumatoid arthritis'

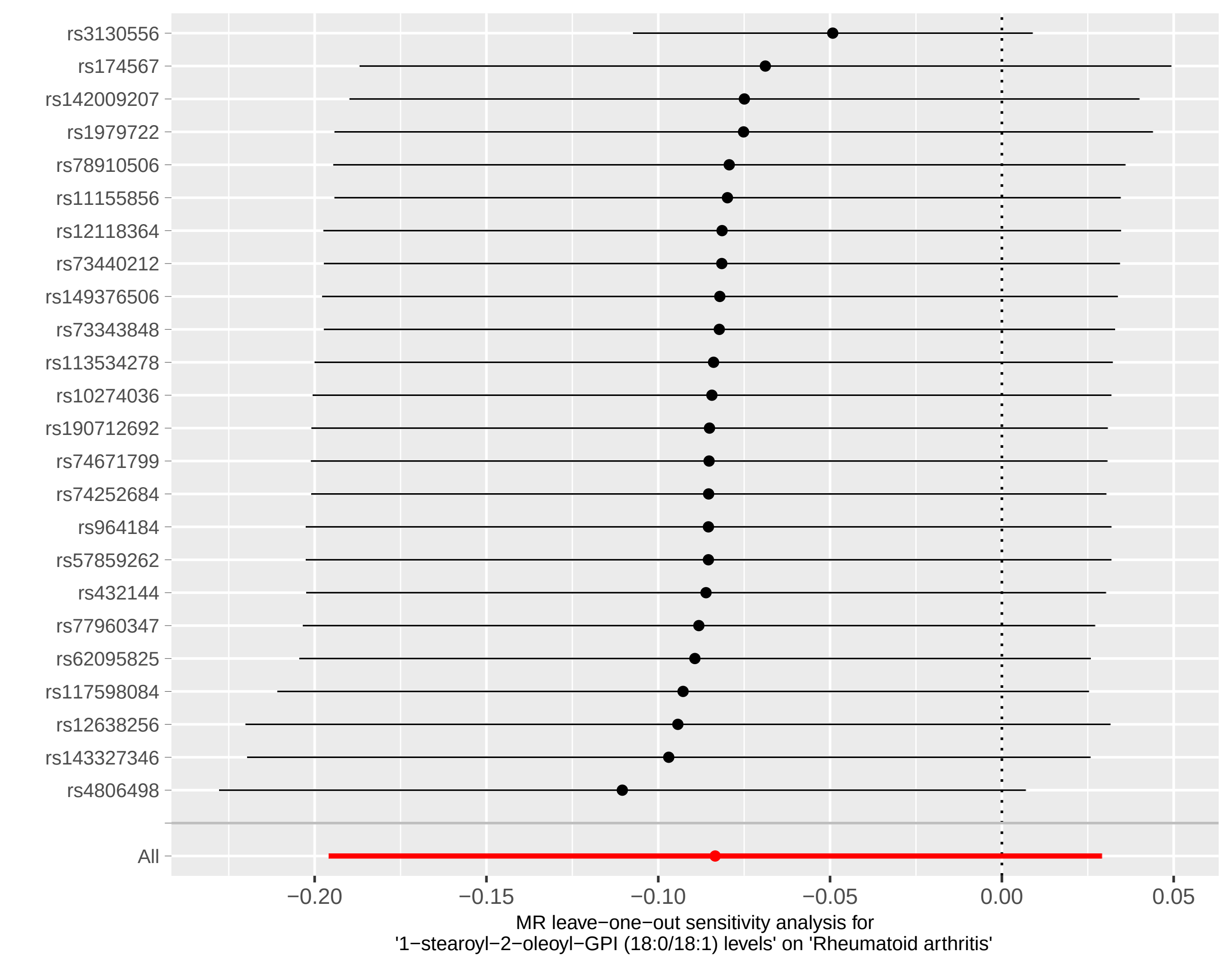
rs3130556
rs174567
rs142009207
rs1979722
rs78910506
rs11155856
rs12118364
rs73440212
rs149376506
rs73343848
rs113534278
rs10274036
rs190712692
rs74671799
rs74252684
rs964184
rs57859262
rs432144
rs77960347
rs62095825
rs117598084
rs12638256
rs143327346
rs4806498
All
−0.20
−0.15
−0.10
−0.05
0.00
0.05
MR leave−one−out sensitivity analysis for
'1−stearoyl−2−oleoyl−GPI (18:0/18:1) levels' on 'Rheumatoid arthritis'

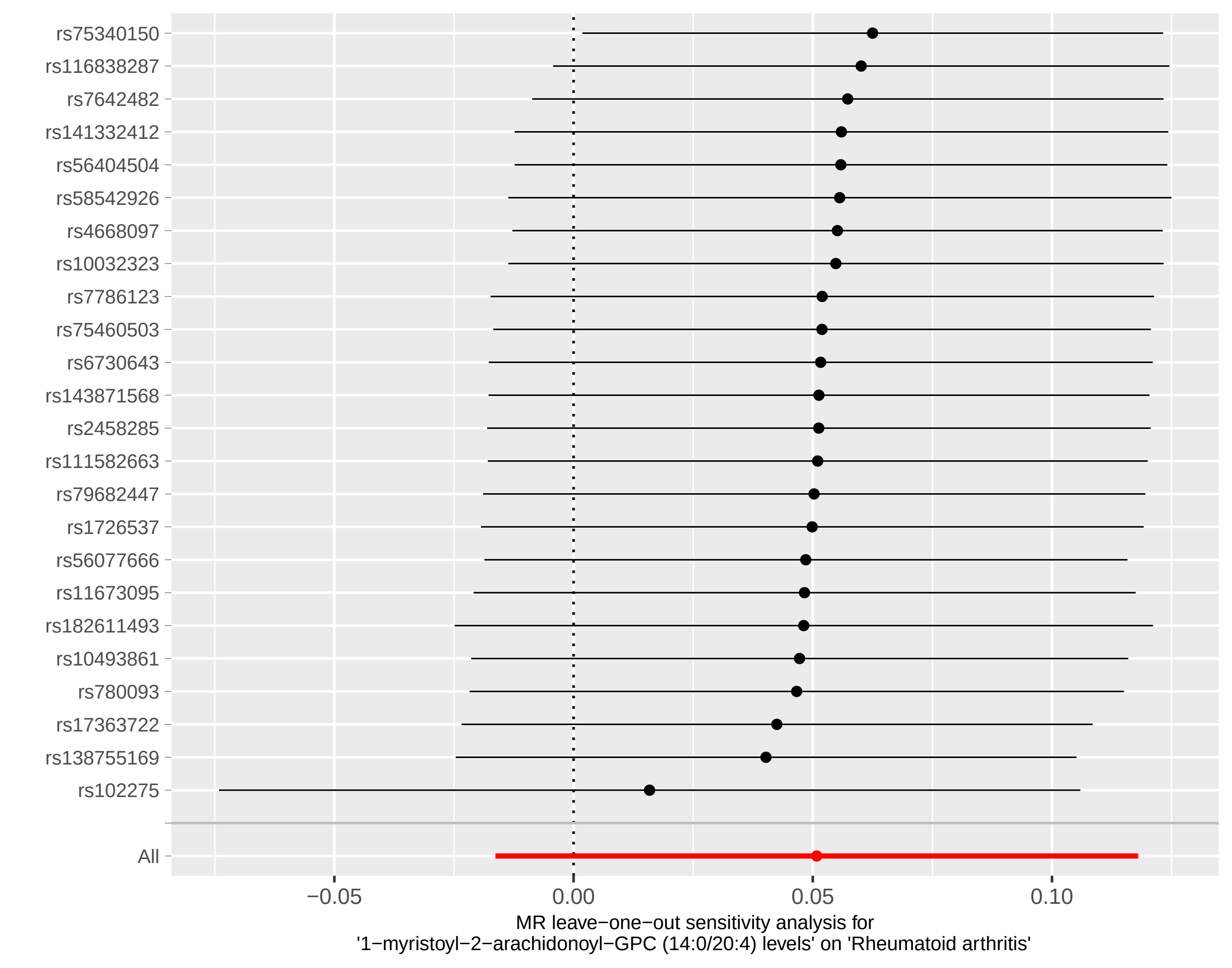
rs75340150
rs116838287
rs7642482
rs141332412
rs56404504
rs58542926
rs4668097
rs10032323
rs7786123
rs75460503
rs6730643
rs143871568
rs2458285
rs111582663
rs79682447
rs1726537
rs56077666
rs11673095
rs182611493
rs10493861
rs780093
rs17363722
rs138755169
rs102275
All
−0.05
0.00
0.05
0.10
MR leave−one−out sensitivity analysis for
'1−myristoyl−2−arachidonoyl−GPC (14:0/20:4) levels' on 'Rheumatoid arthritis'

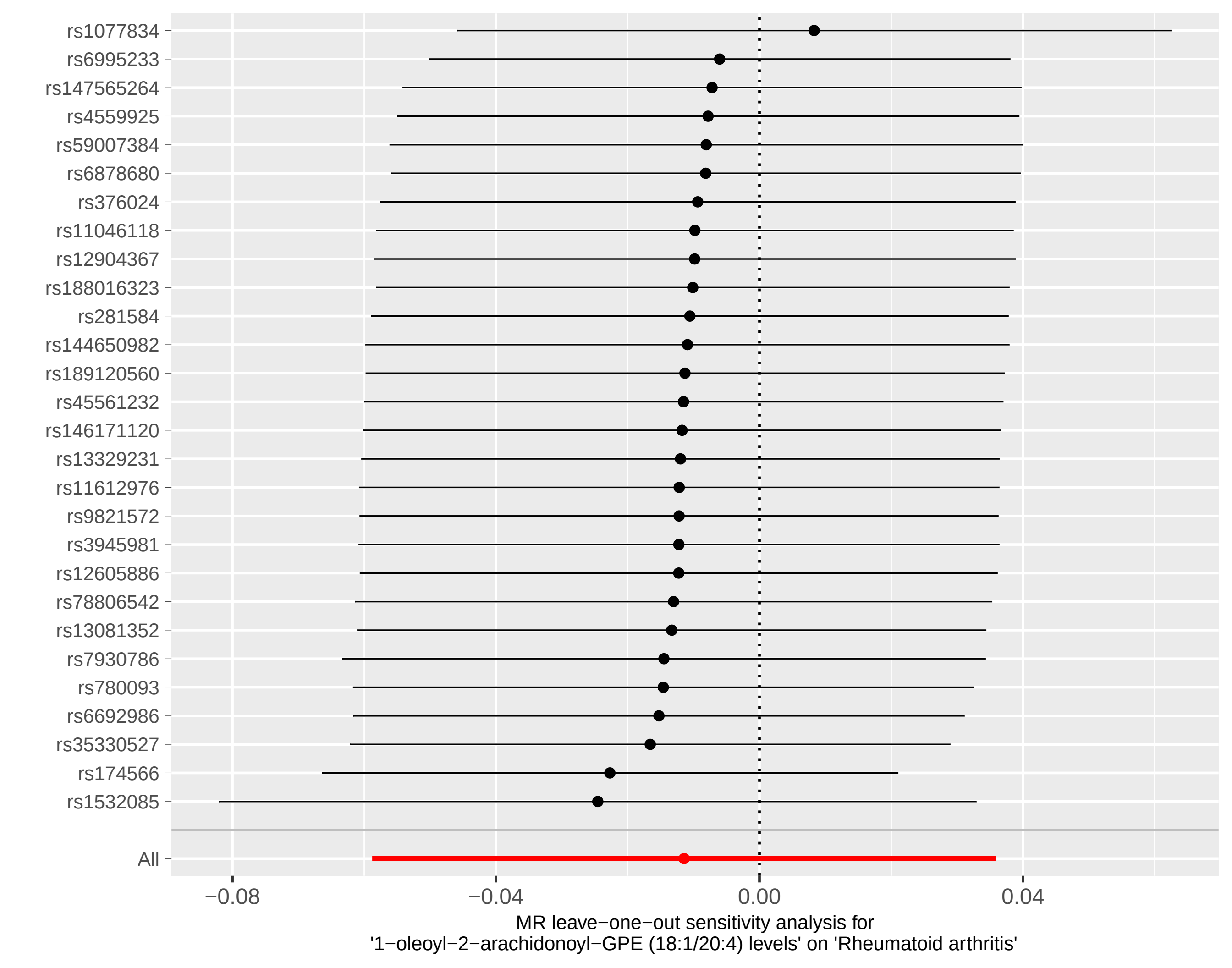
rs1077834
rs6995233
rs147565264
rs4559925
rs59007384
rs6878680
rs376024
rs11046118
rs12904367
rs188016323
rs281584
rs144650982
rs189120560
rs45561232
rs146171120
rs13329231
rs11612976
rs9821572
rs3945981
rs12605886
rs78806542
rs13081352
rs7930786
rs780093
rs6692986
rs35330527
rs174566
rs1532085
All
−0.08
−0.04
0.00
0.04
MR leave−one−out sensitivity analysis for
'1−oleoyl−2−arachidonoyl−GPE (18:1/20:4) levels' on 'Rheumatoid arthritis'

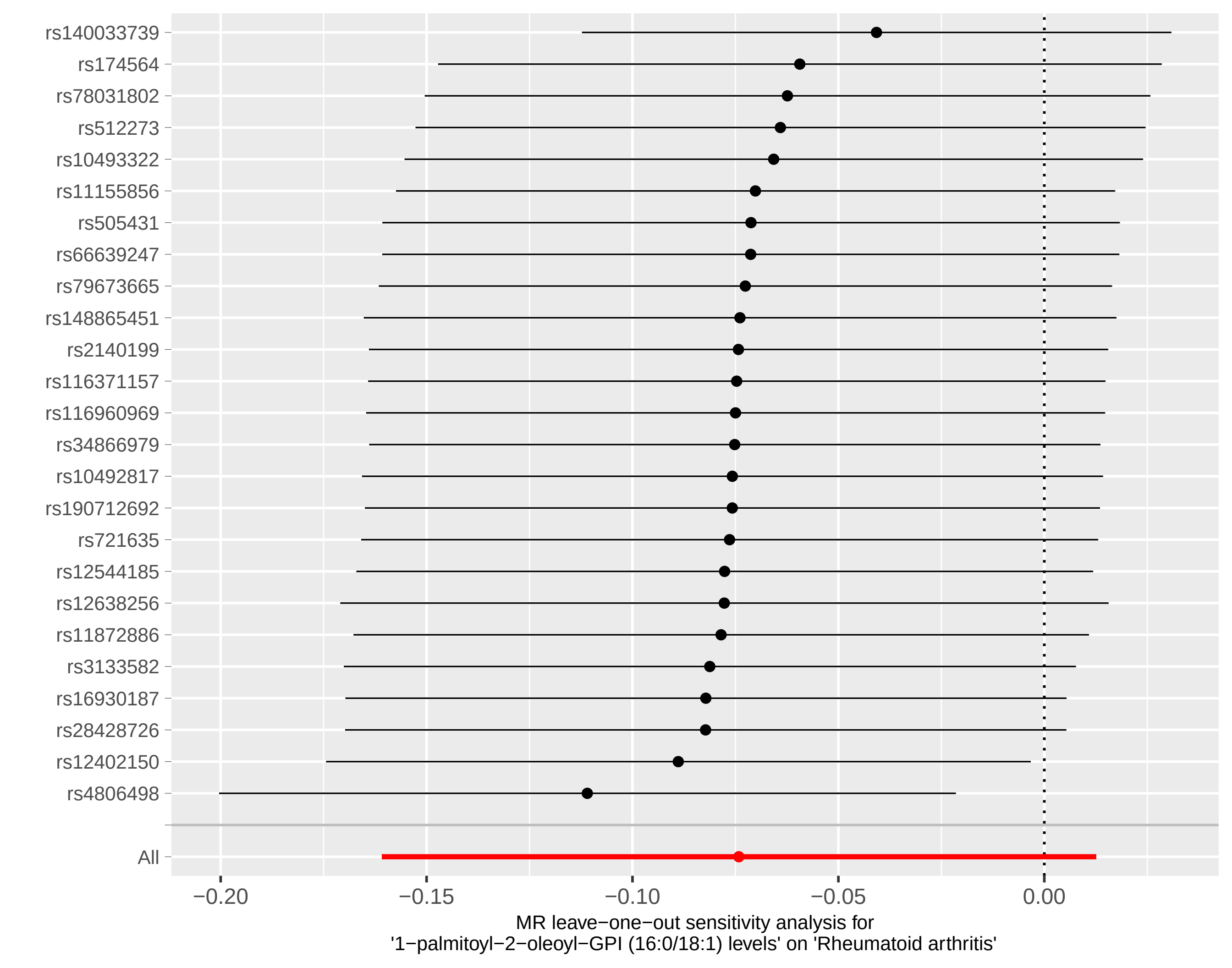

rs140033739
rs174564
rs78031802
rs512273
rs10493322
rs11155856
rs505431
rs66639247
rs79673665
rs148865451
rs2140199
rs116371157
rs116960969
rs34866979
rs10492817
rs190712692
rs721635
rs12544185
rs12638256
rs11872886
rs3133582
rs16930187
rs28428726
rs12402150
rs4806498
All
−0.20
−0.15
−0.10
−0.05
0.00
MR leave−one−out sensitivity analysis for
'1−palmitoyl−2−oleoyl−GPI (16:0/18:1) levels' on 'Rheumatoid arthritis'

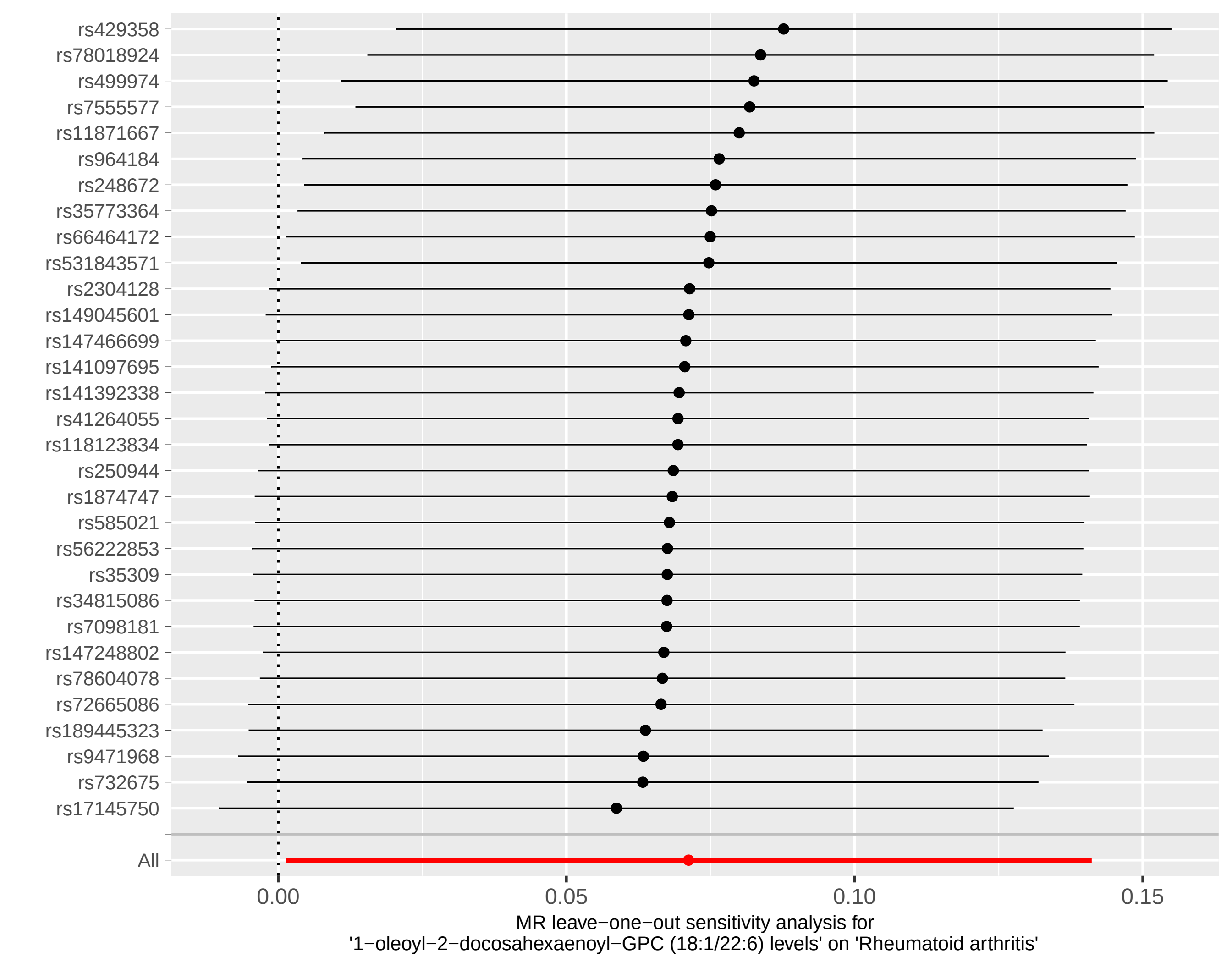
rs429358
rs78018924
rs499974
rs7555577
rs11871667
rs964184
rs248672
rs35773364
rs66464172
rs531843571
rs2304128
rs149045601
rs147466699
rs141097695
rs141392338
rs41264055
rs118123834
rs250944
rs1874747
rs585021
rs56222853
rs35309
rs34815086
rs7098181
rs147248802
rs78604078
rs72665086
rs189445323
rs9471968
rs732675
rs17145750
All
0.00
0.05
0.10
0.15
MR leave−one−out sensitivity analysis for
'1−oleoyl−2−docosahexaenoyl−GPC (18:1/22:6) levels' on 'Rheumatoid arthritis'

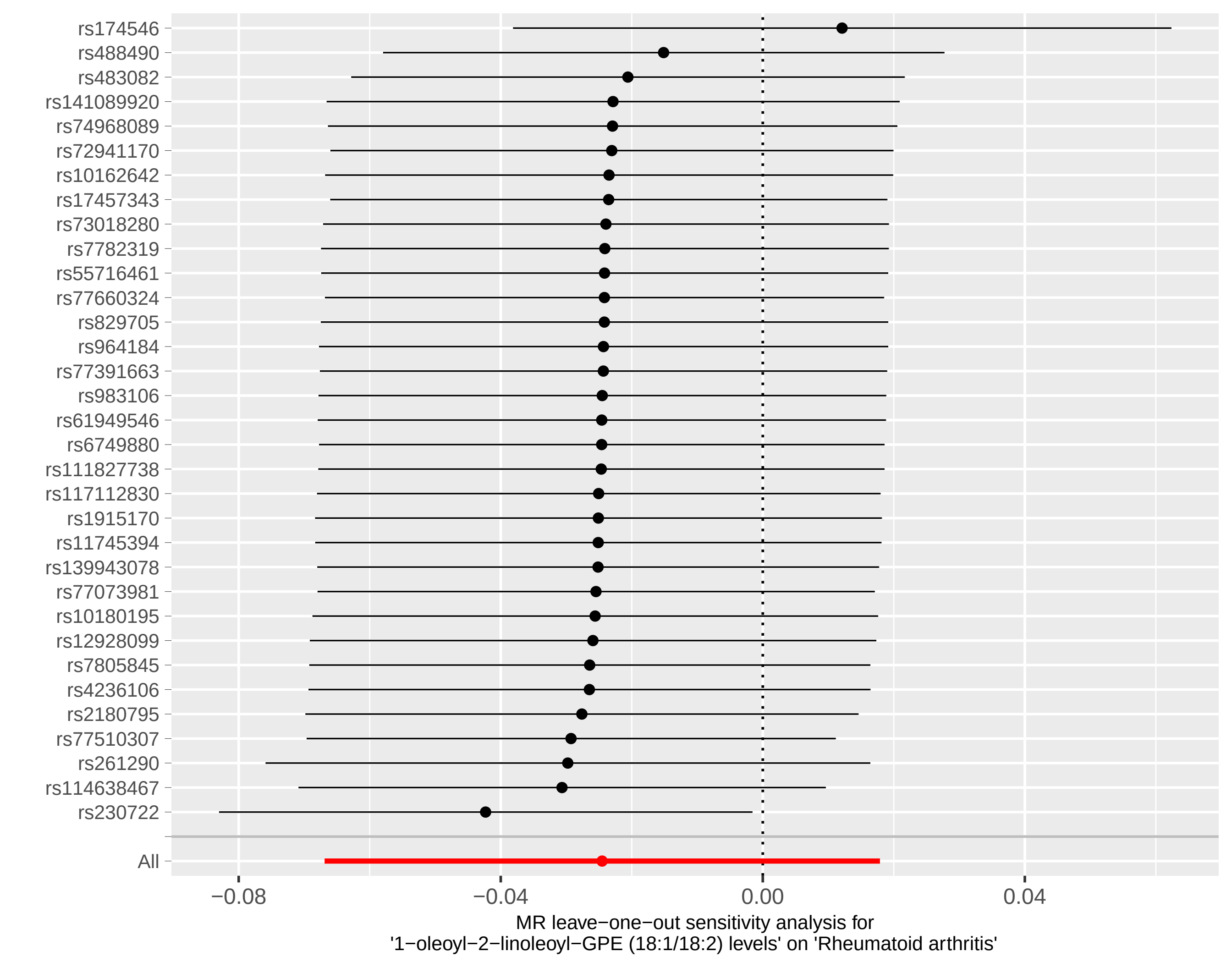
rs174546
rs488490
rs483082
rs141089920
rs74968089
rs72941170
rs10162642
rs17457343
rs73018280
rs7782319
rs55716461
rs77660324
rs829705
rs964184
rs77391663
rs983106
rs61949546
rs6749880
rs111827738
rs117112830
rs1915170
rs11745394
rs139943078
rs77073981
rs10180195
rs12928099
rs7805845
rs4236106
rs2180795
rs77510307
rs261290
rs114638467
rs230722
All
−0.08
−0.04
0.00
0.04
MR leave−one−out sensitivity analysis for
'1−oleoyl−2−linoleoyl−GPE (18:1/18:2) levels' on 'Rheumatoid arthritis'

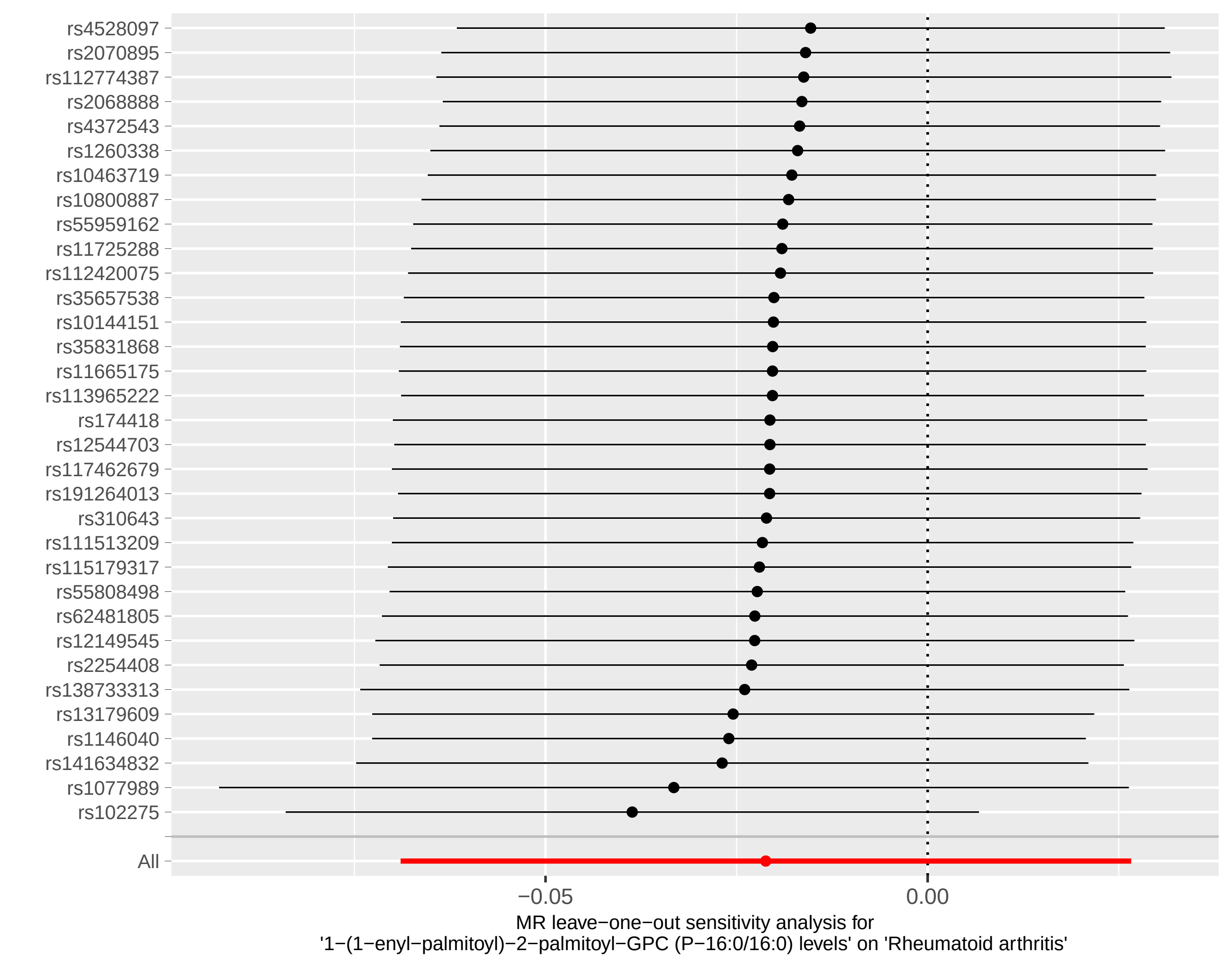
rs4528097
rs2070895
rs112774387
rs2068888
rs4372543
rs1260338
rs10463719
rs10800887
rs55959162
rs11725288
rs112420075
rs35657538
rs10144151
rs35831868
rs11665175
rs113965222
rs174418
rs12544703
rs117462679
rs191264013
rs310643
rs111513209
rs115179317
rs55808498
rs62481805
rs12149545
rs2254408
rs138733313
rs13179609
rs1146040
rs141634832
rs1077989
rs102275
All
−0.05
0.00
MR leave−one−out sensitivity analysis for
'1−(1−enyl−palmitoyl)−2−palmitoyl−GPC (P−16:0/16:0) levels' on 'Rheumatoid arthritis'

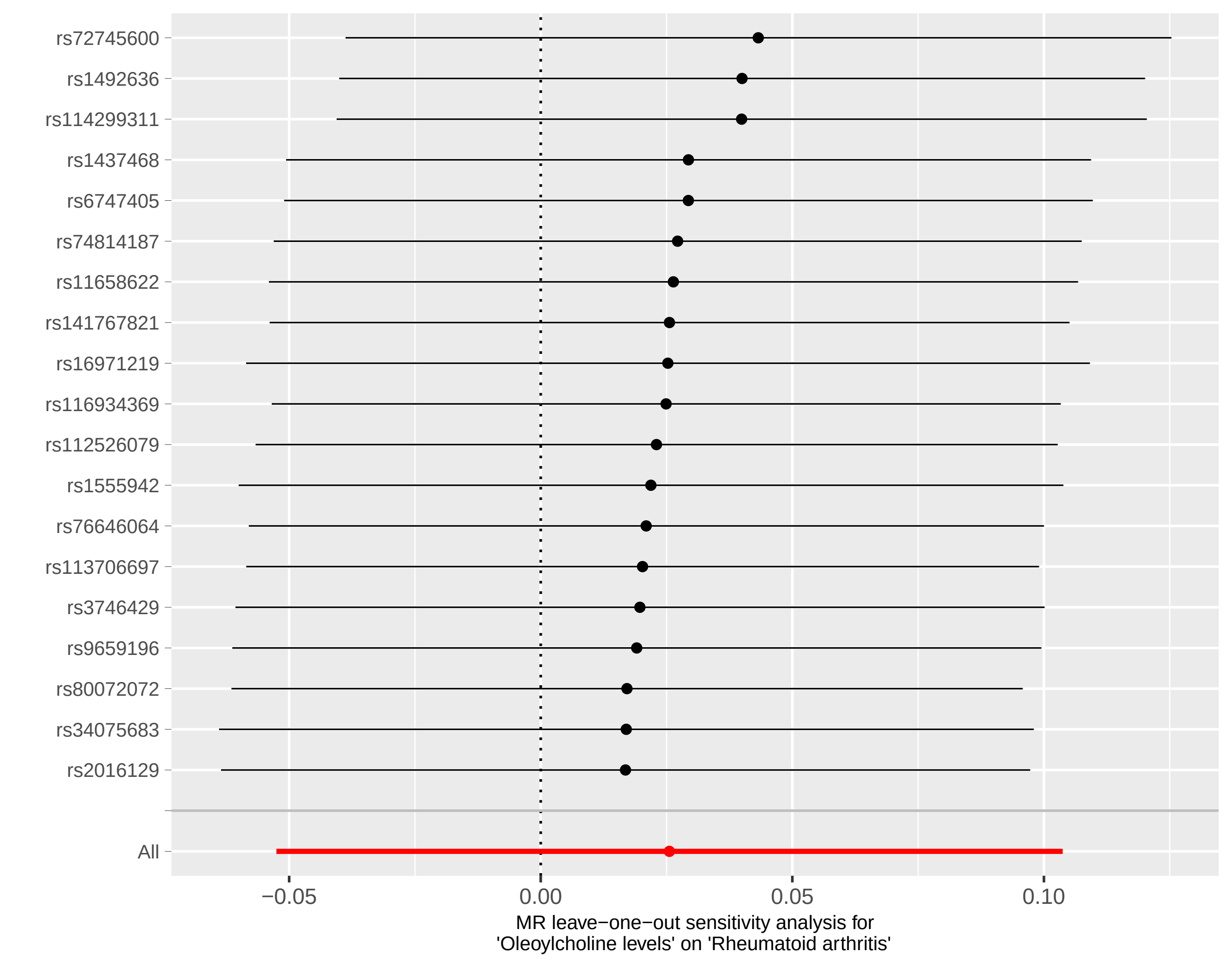
rs72745600
rs1492636
rs114299311
rs1437468
rs6747405
rs74814187
rs11658622
rs141767821
rs16971219
rs116934369
rs112526079
rs1555942
rs76646064
rs113706697
rs3746429
rs9659196
rs80072072
rs34075683
rs2016129
All
−0.05
0.00
0.05
0.10
MR leave−one−out sensitivity analysis for
'Oleoylcholine levels' on 'Rheumatoid arthritis'

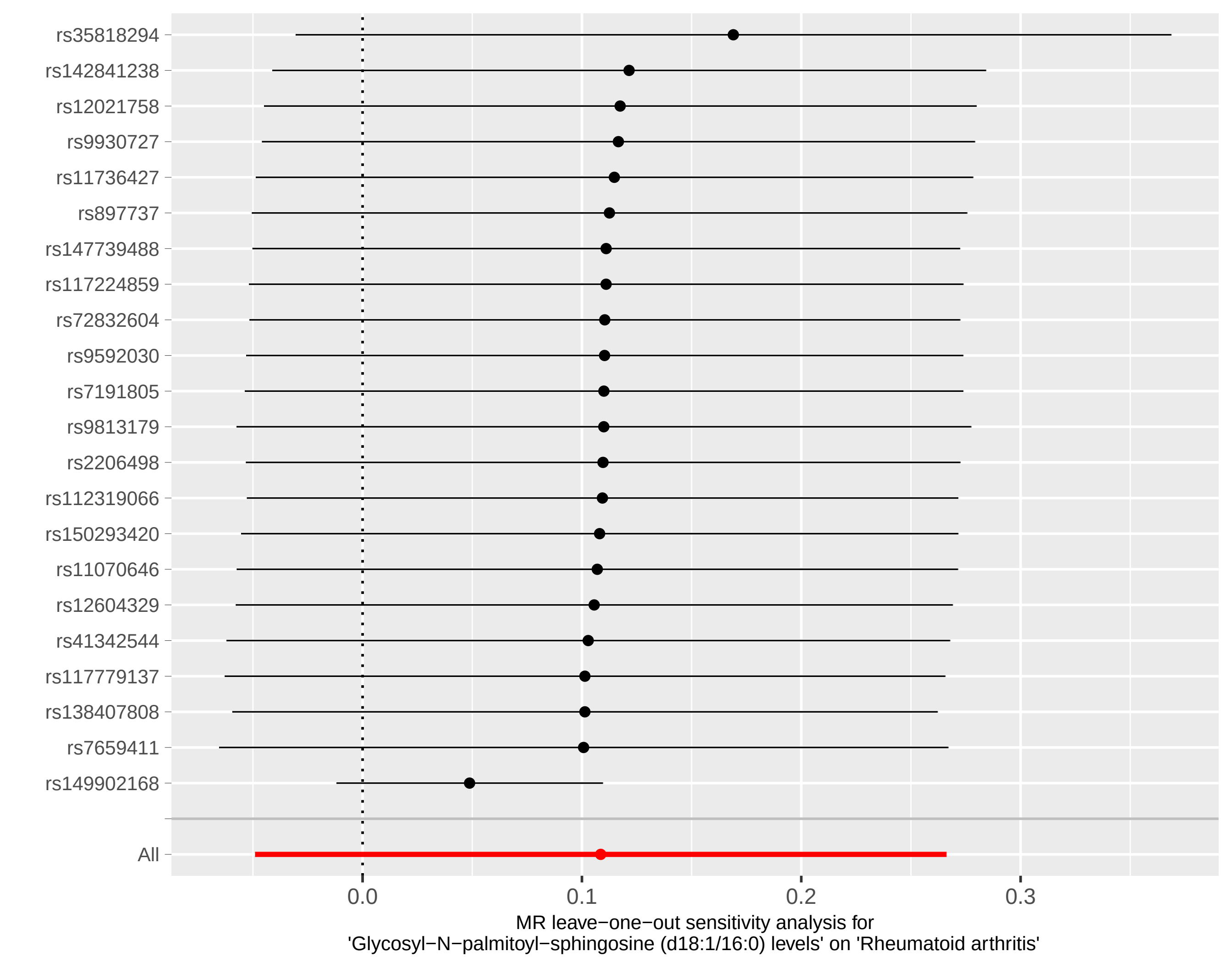

rs35818294
rs142841238
rs12021758
rs9930727
rs11736427
rs897737
rs147739488
rs117224859
rs72832604
rs9592030
rs7191805
rs9813179
rs2206498
rs112319066
rs150293420
rs11070646
rs12604329
rs41342544
rs117779137
rs138407808
rs7659411
rs149902168
All
0.0
0.1
0.2
0.3
MR leave−one−out sensitivity analysis for
'Glycosyl−N−palmitoyl−sphingosine (d18:1/16:0) levels' on 'Rheumatoid arthritis'

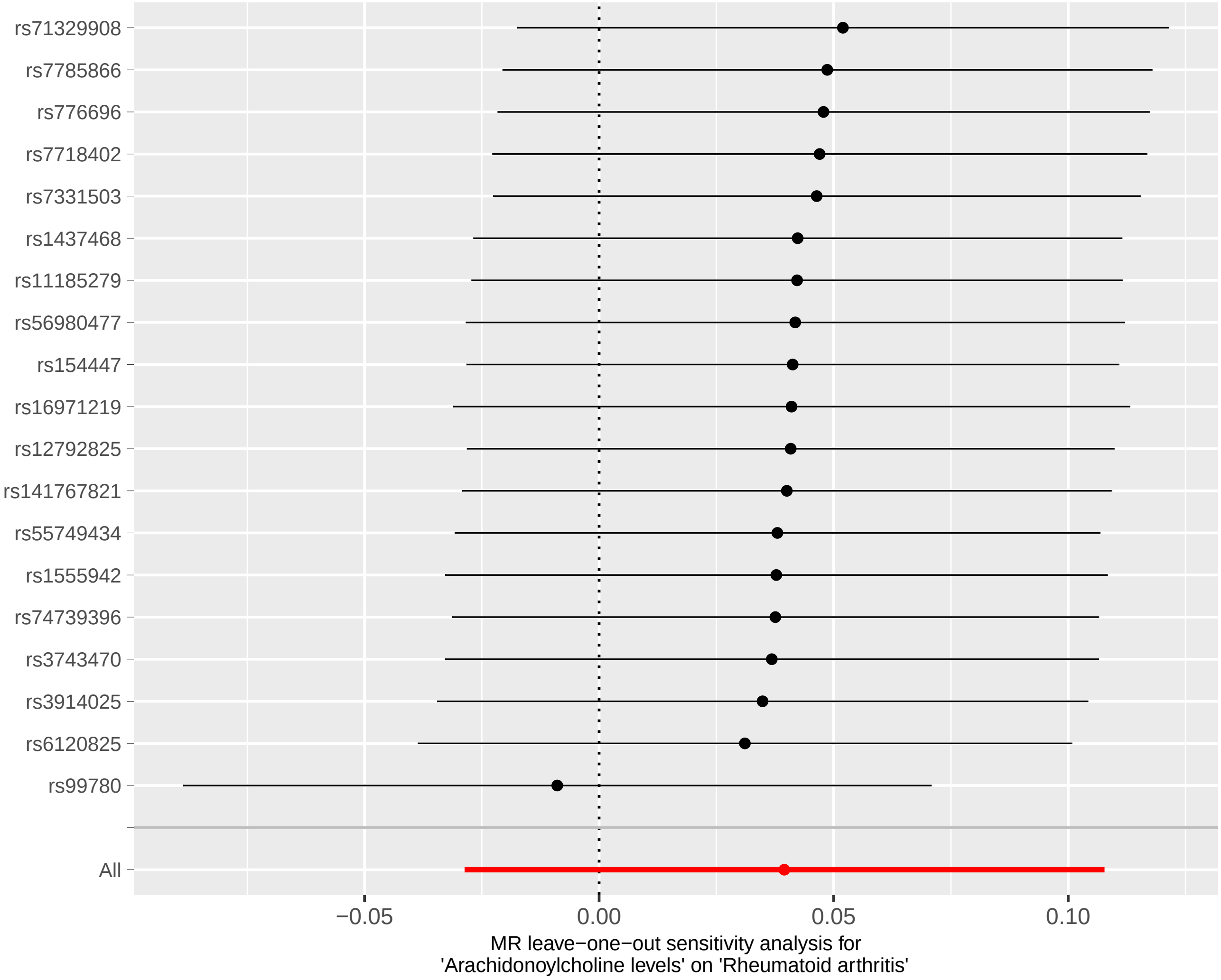
rs71329908
rs7785866
rs776696
rs7718402
rs7331503
rs1437468
rs11185279
rs56980477
rs154447
rs16971219
rs12792825
rs141767821
rs55749434
rs1555942
rs74739396
rs3743470
rs3914025
rs6120825
rs99780
All
−0.05
0.00
0.05
0.10
MR leave−one−out sensitivity analysis for
'Arachidonoylcholine levels' on 'Rheumatoid arthritis'

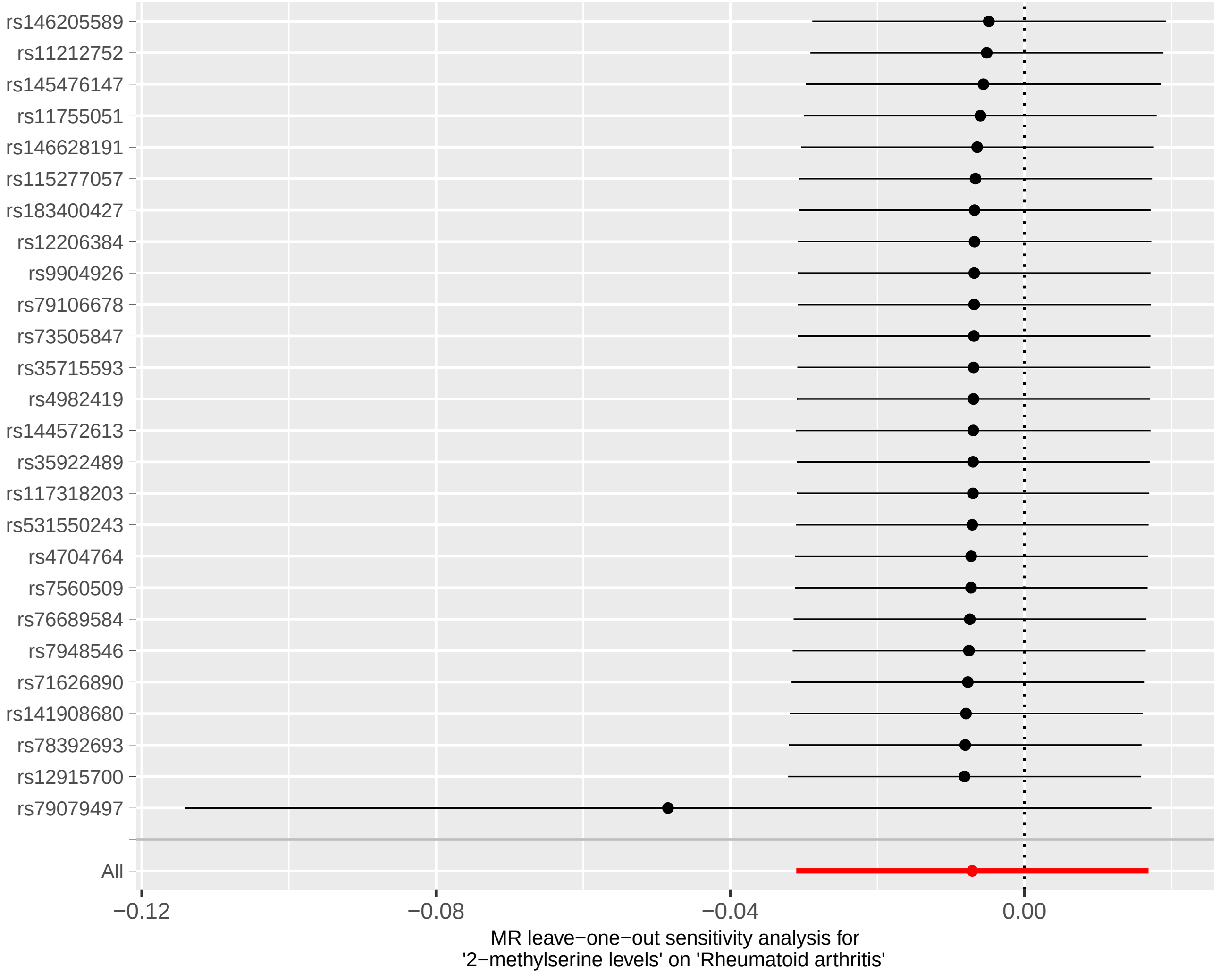

rs146205589
rs11212752
rs145476147
rs11755051
rs146628191
rs115277057
rs183400427
rs12206384
rs9904926
rs79106678
rs73505847
rs35715593
rs4982419
rs144572613
rs35922489
rs117318203
rs531550243
rs4704764
rs7560509
rs76689584
rs7948546
rs71626890
rs141908680
rs78392693
rs12915700
rs79079497
All
−0.12
−0.08
−0.04
0.00
MR leave−one−out sensitivity analysis for
'2−methylserine levels' on 'Rheumatoid arthritis'

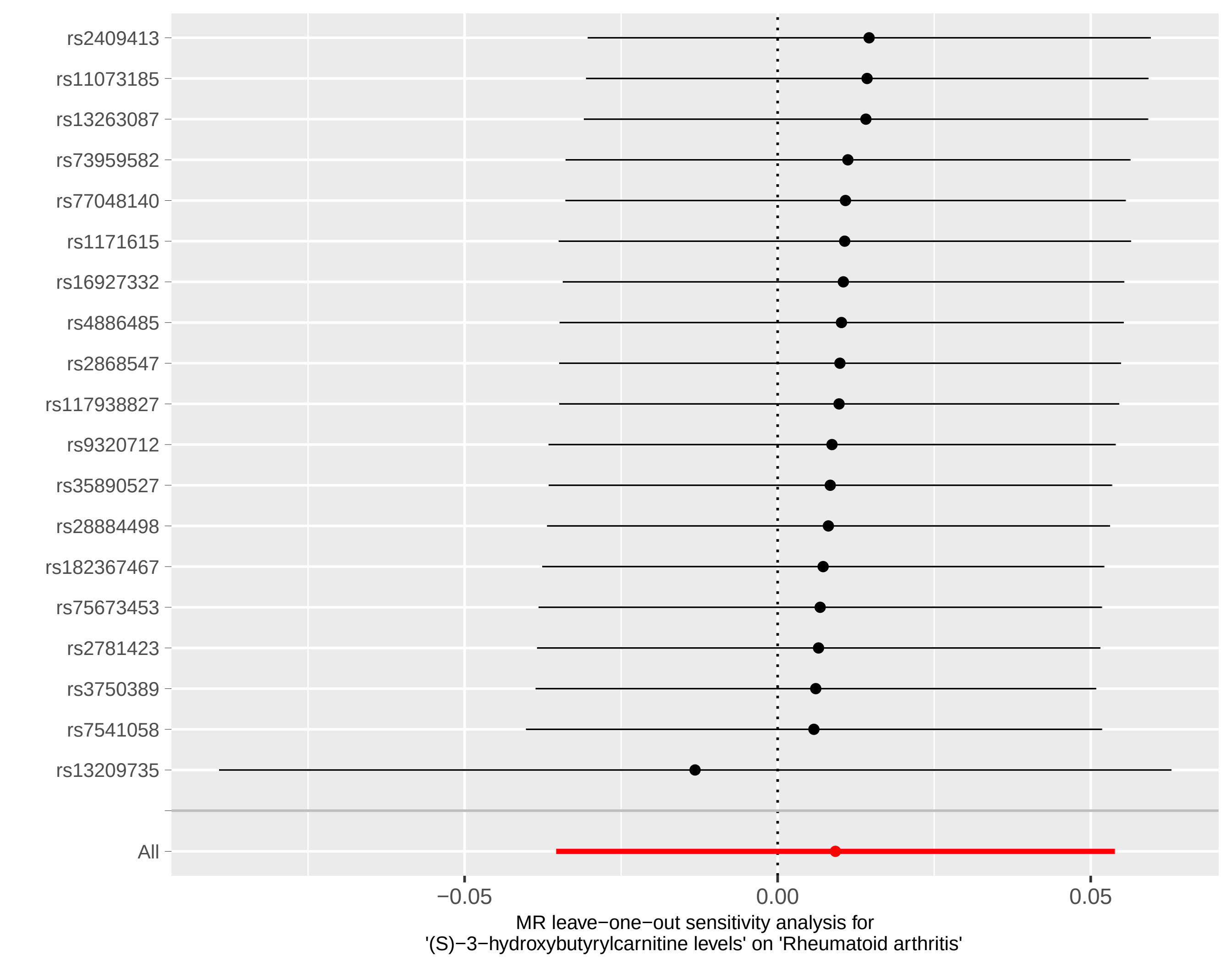
rs2409413
rs11073185
rs13263087
rs73959582
rs77048140
rs1171615
rs16927332
rs4886485
rs2868547
rs117938827
rs9320712
rs35890527
rs28884498
rs182367467
rs75673453
rs2781423
rs3750389
rs7541058
rs13209735
All
−0.05
0.00
0.05
MR leave−one−out sensitivity analysis for
'(S)−3−hydroxybutyrylcarnitine levels' on 'Rheumatoid arthritis'

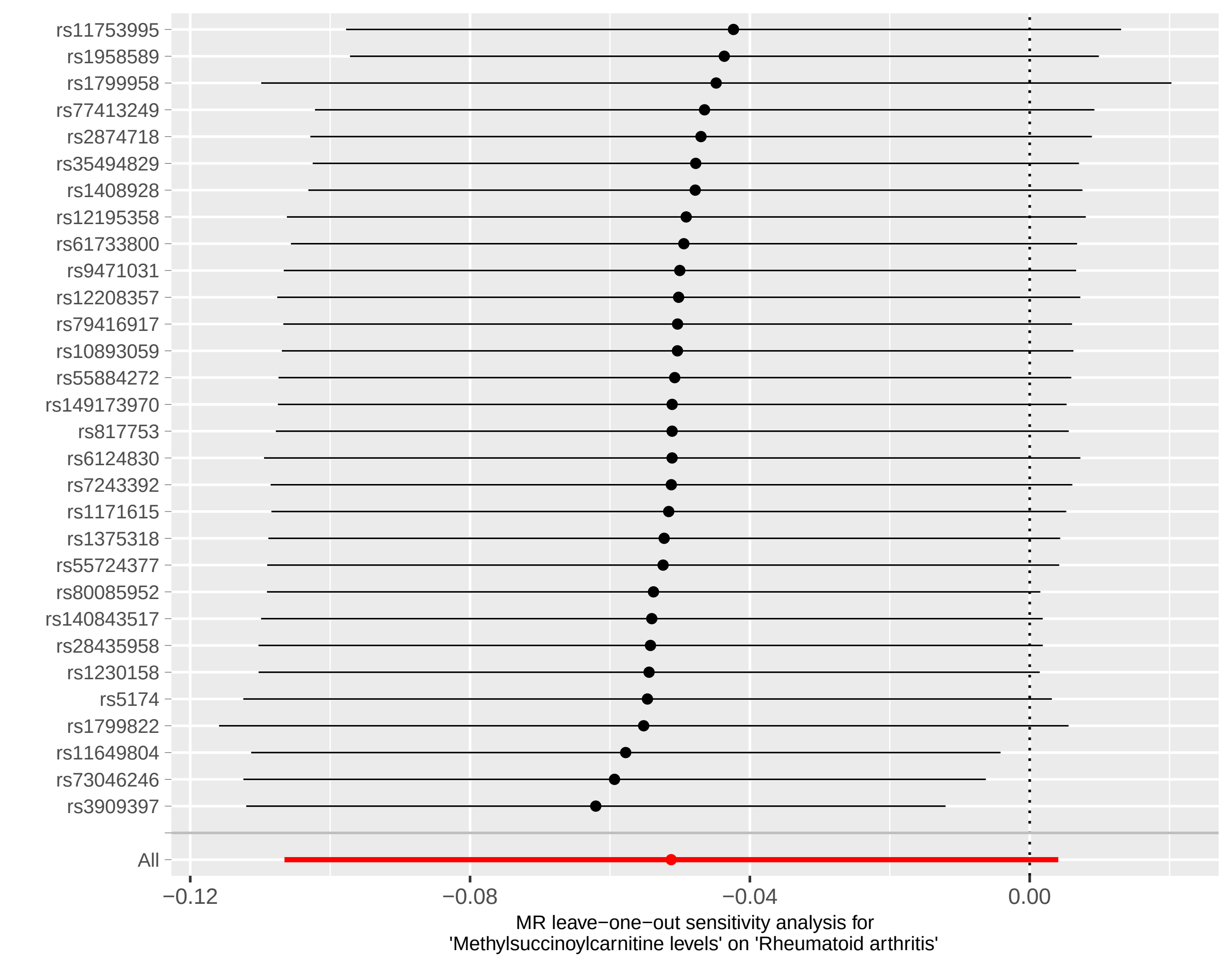
rs11753995
rs1958589
rs1799958
rs77413249
rs2874718
rs35494829
rs1408928
rs12195358
rs61733800
rs9471031
rs12208357
rs79416917
rs10893059
rs55884272
rs149173970
rs817753
rs6124830
rs7243392
rs1171615
rs1375318
rs55724377
rs80085952
rs140843517
rs28435958
rs1230158
rs5174
rs1799822
rs11649804
rs73046246
rs3909397
All
−0.12
−0.08
−0.04
0.00
MR leave−one−out sensitivity analysis for
'Methylsuccinoylcarnitine levels' on 'Rheumatoid arthritis'

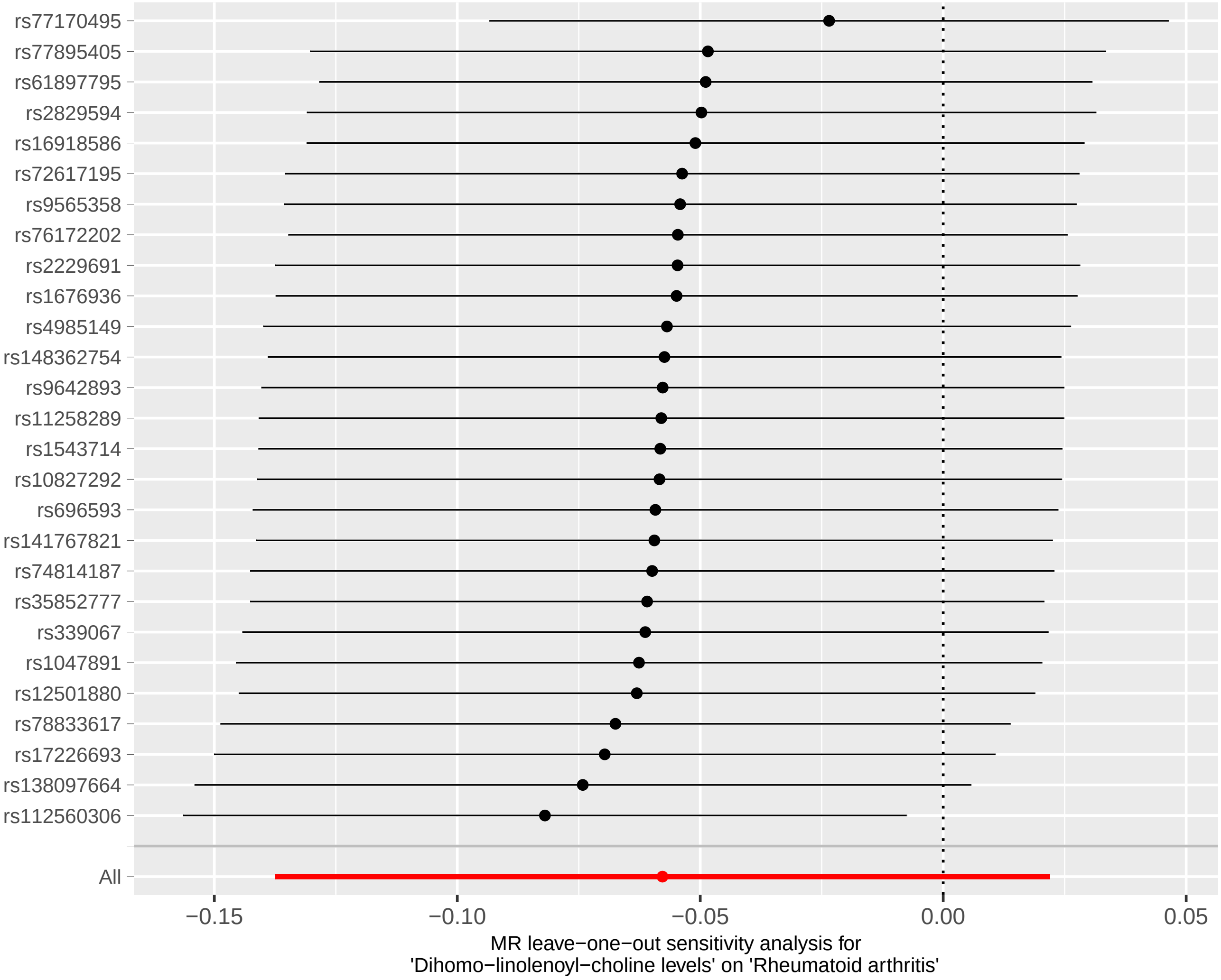
rs77170495
rs77895405
rs61897795
rs2829594
rs16918586
rs72617195
rs9565358
rs76172202
rs2229691
rs1676936
rs4985149
rs148362754
rs9642893
rs11258289
rs1543714
rs10827292
rs696593
rs141767821
rs74814187
rs35852777
rs339067
rs1047891
rs12501880
rs78833617
rs17226693
rs138097664
rs112560306
All
−0.15
−0.10
−0.05
0.00
0.05
MR leave−one−out sensitivity analysis for
'Dihomo−linolenoyl−choline levels' on 'Rheumatoid arthritis'

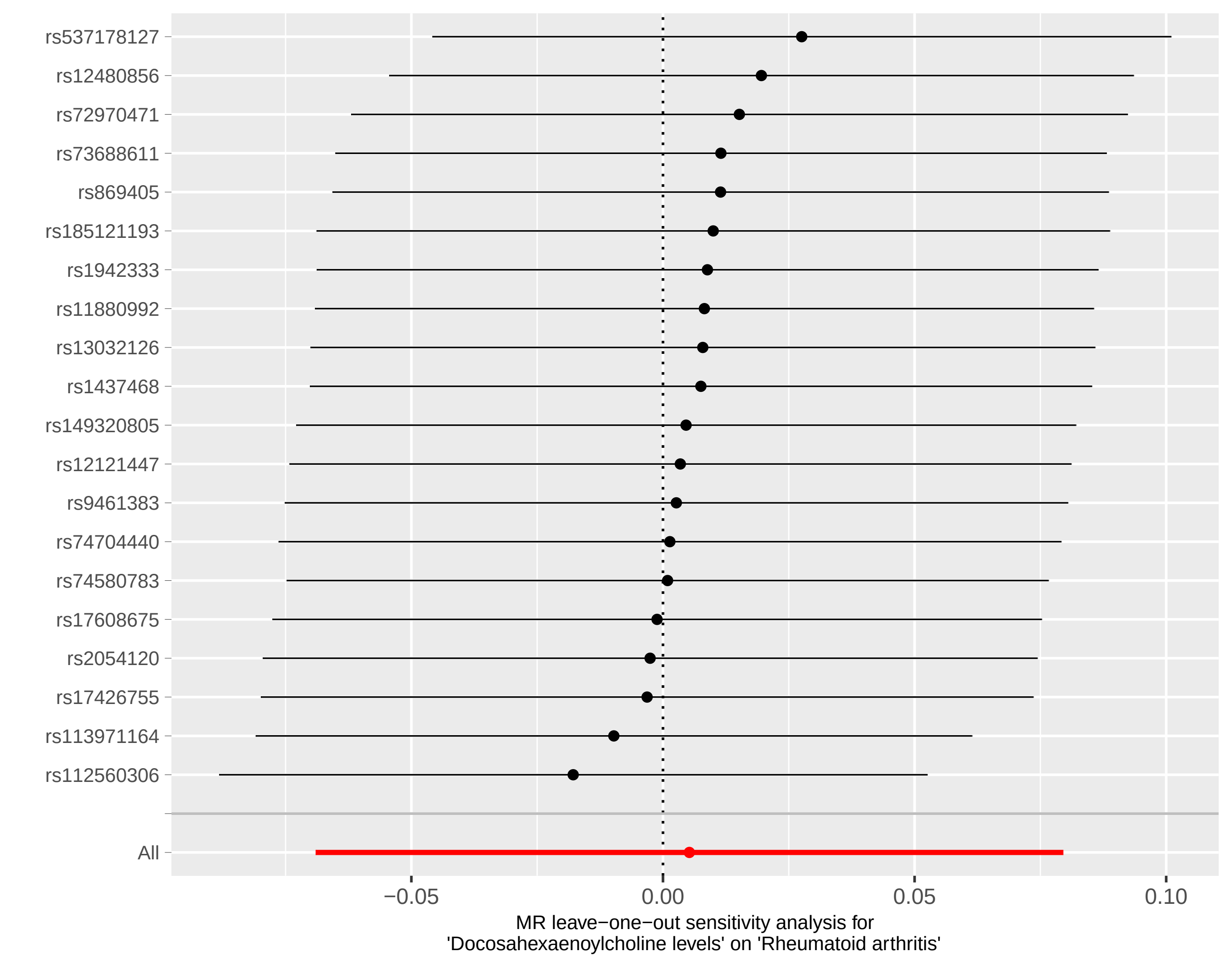
rs537178127
rs12480856
rs72970471
rs73688611
rs869405
rs185121193
rs1942333
rs11880992
rs13032126
rs1437468
rs149320805
rs12121447
rs9461383
rs74704440
rs74580783
rs17608675
rs2054120
rs17426755
rs113971164
rs112560306
All
−0.05
0.00
0.05
0.10
MR leave−one−out sensitivity analysis for
'Docosahexaenoylcholine levels' on 'Rheumatoid arthritis'

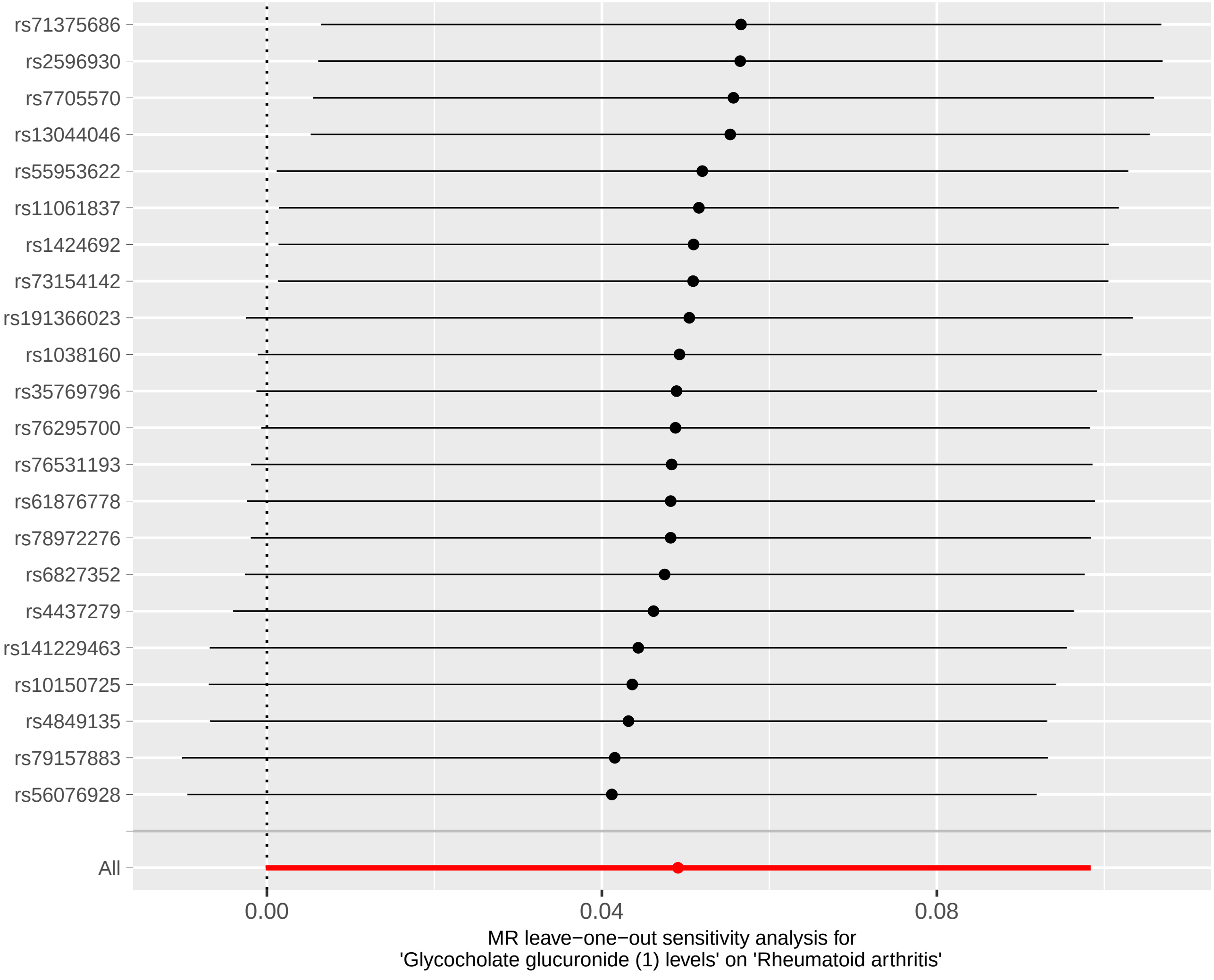
rs71375686
rs2596930
rs7705570
rs13044046
rs55953622
rs11061837
rs1424692
rs73154142
rs191366023
rs1038160
rs35769796
rs76295700
rs76531193
rs61876778
rs78972276
rs6827352
rs4437279
rs141229463
rs10150725
rs4849135
rs79157883
rs56076928
All
0.00
0.04
0.08
MR leave−one−out sensitivity analysis for
'Glycocholate glucuronide (1) levels' on 'Rheumatoid arthritis'

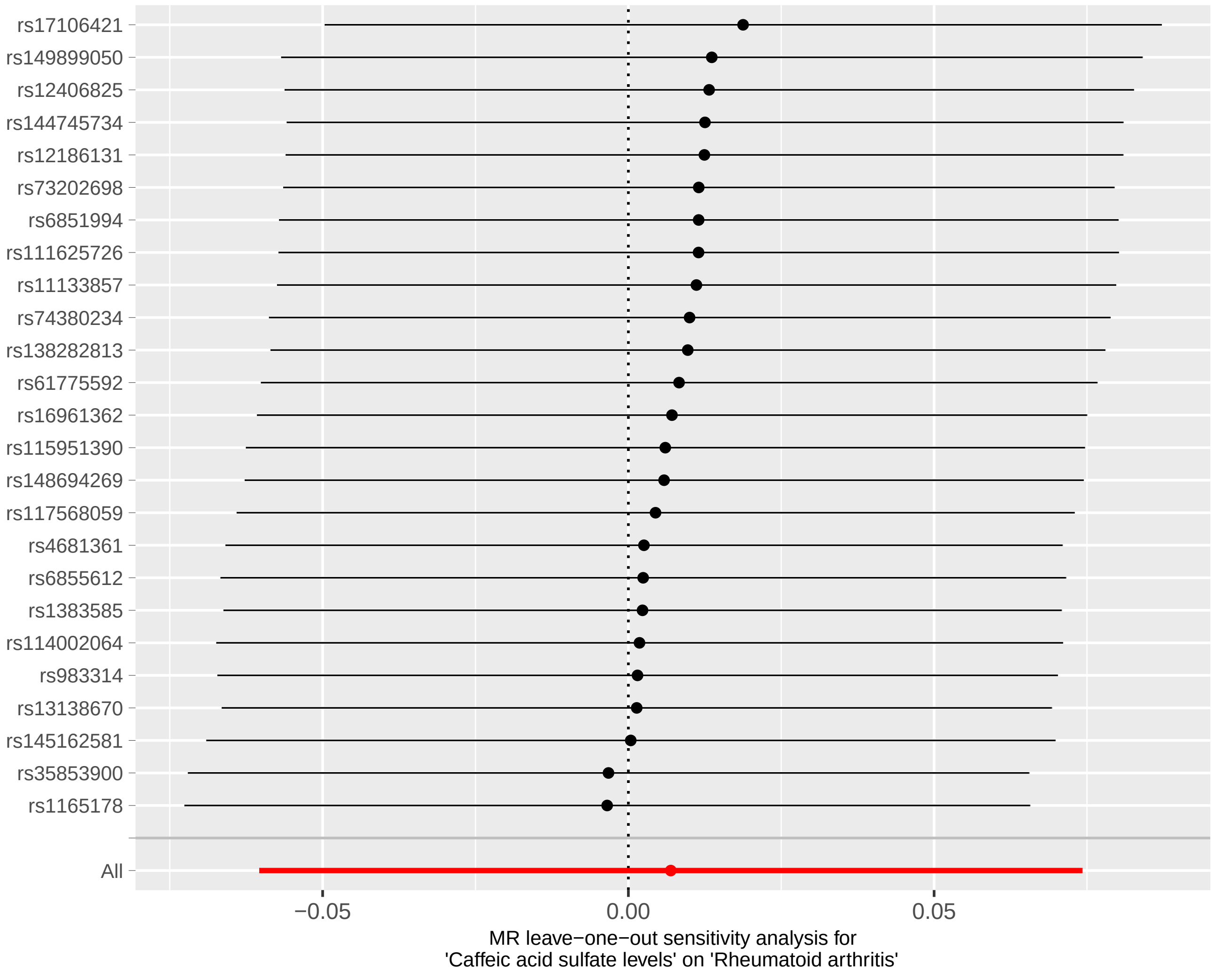
rs17106421
rs149899050
rs12406825
rs144745734
rs12186131
rs73202698
rs6851994
rs111625726
rs11133857
rs74380234
rs138282813
rs61775592
rs16961362
rs115951390
rs148694269
rs117568059
rs4681361
rs6855612
rs1383585
rs114002064
rs983314
rs13138670
rs145162581
rs35853900
rs1165178
All
−0.05
0.00
0.05
MR leave−one−out sensitivity analysis for
'Caffeic acid sulfate levels' on 'Rheumatoid arthritis'

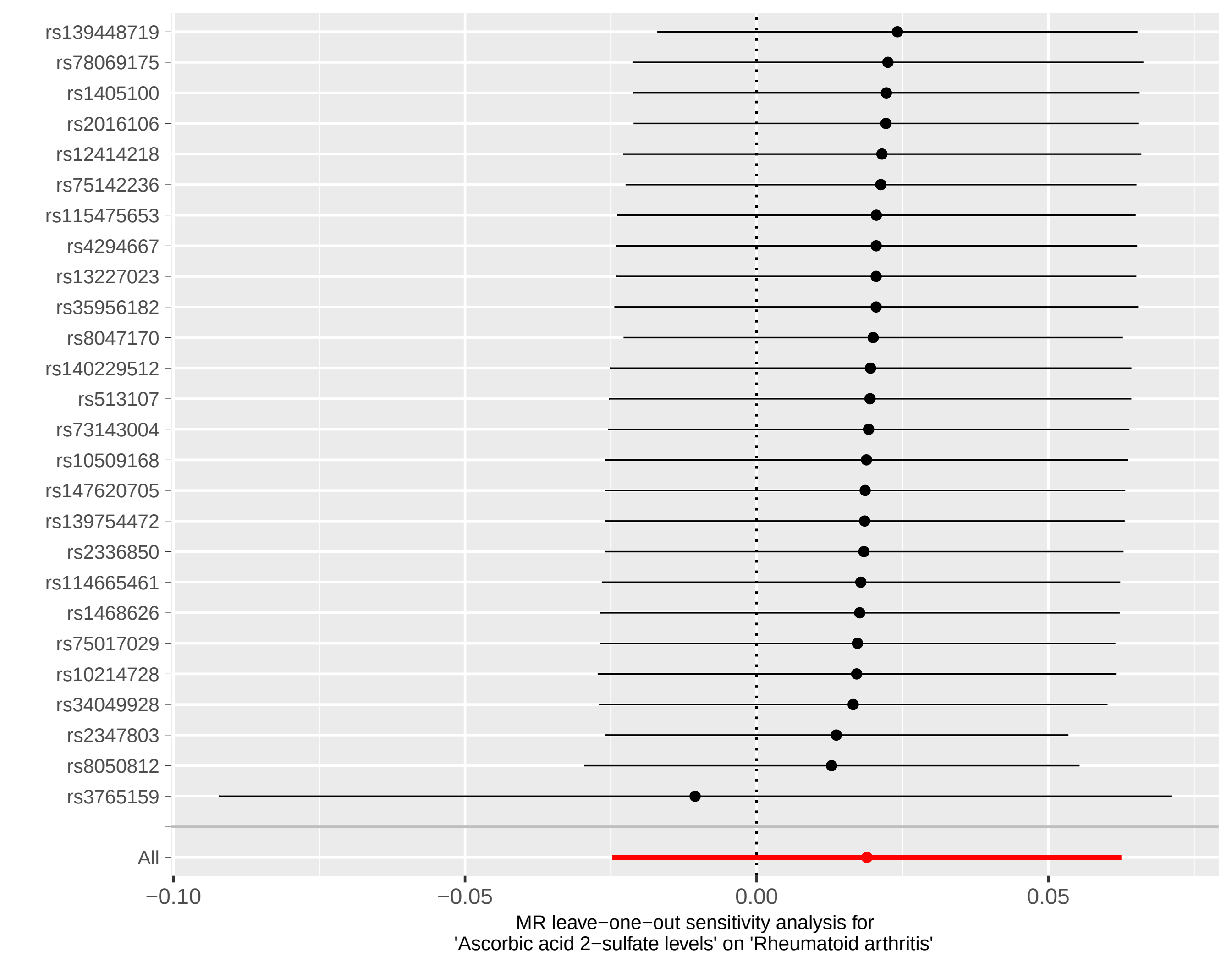
rs139448719
rs78069175
rs1405100
rs2016106
rs12414218
rs75142236
rs115475653
rs4294667
rs13227023
rs35956182
rs8047170
rs140229512
rs513107
rs73143004
rs10509168
rs147620705
rs139754472
rs2336850
rs114665461
rs1468626
rs75017029
rs10214728
rs34049928
rs2347803
rs8050812
rs3765159
All
−0.10
−0.05
0.00
0.05
MR leave−one−out sensitivity analysis for
'Ascorbic acid 2−sulfate levels' on 'Rheumatoid arthritis'

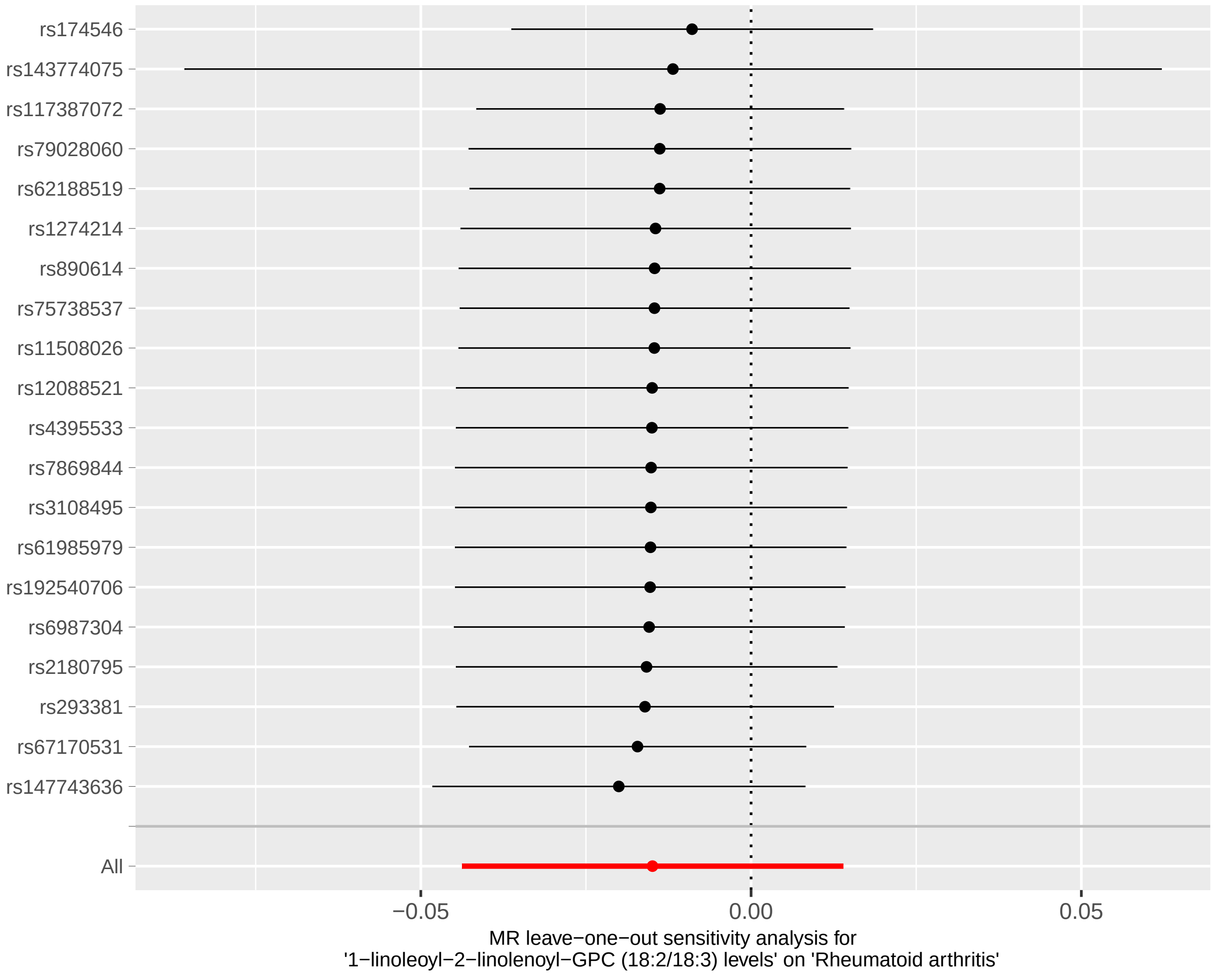

rs174546
rs143774075
rs117387072
rs79028060
rs62188519
rs1274214
rs890614
rs75738537
rs11508026
rs12088521
rs4395533
rs7869844
rs3108495
rs61985979
rs192540706
rs6987304
rs2180795
rs293381
rs67170531
rs147743636
All
−0.05
0.00
0.05
MR leave−one−out sensitivity analysis for
'1−linoleoyl−2−linolenoyl−GPC (18:2/18:3) levels' on 'Rheumatoid arthritis'

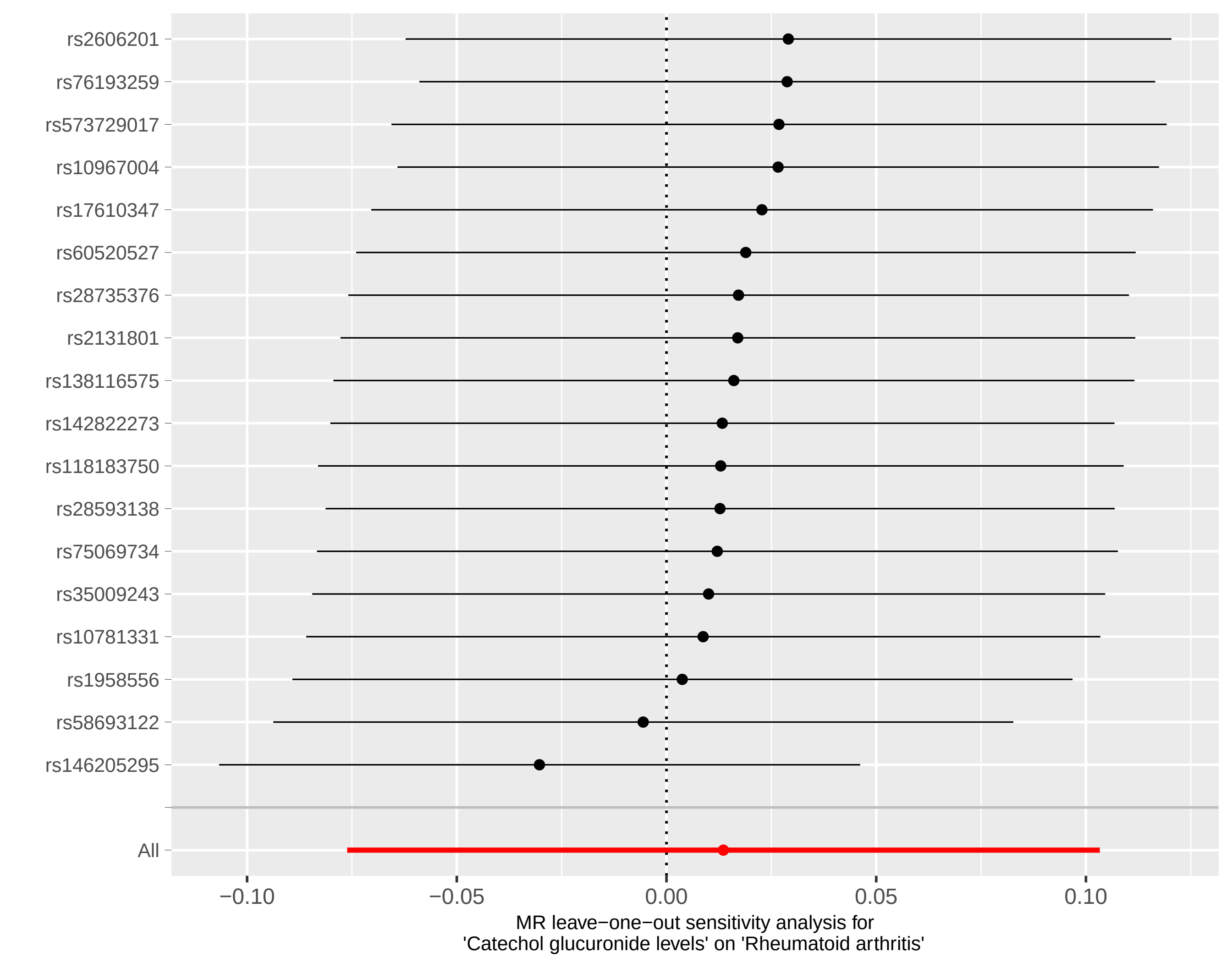
rs2606201
rs76193259
rs573729017
rs10967004
rs17610347
rs60520527
rs28735376
rs2131801
rs138116575
rs142822273
rs118183750
rs28593138
rs75069734
rs35009243
rs10781331
rs1958556
rs58693122
rs146205295
All
−0.10
−0.05
0.00
0.05
0.10
MR leave−one−out sensitivity analysis for
'Catechol glucuronide levels' on 'Rheumatoid arthritis'

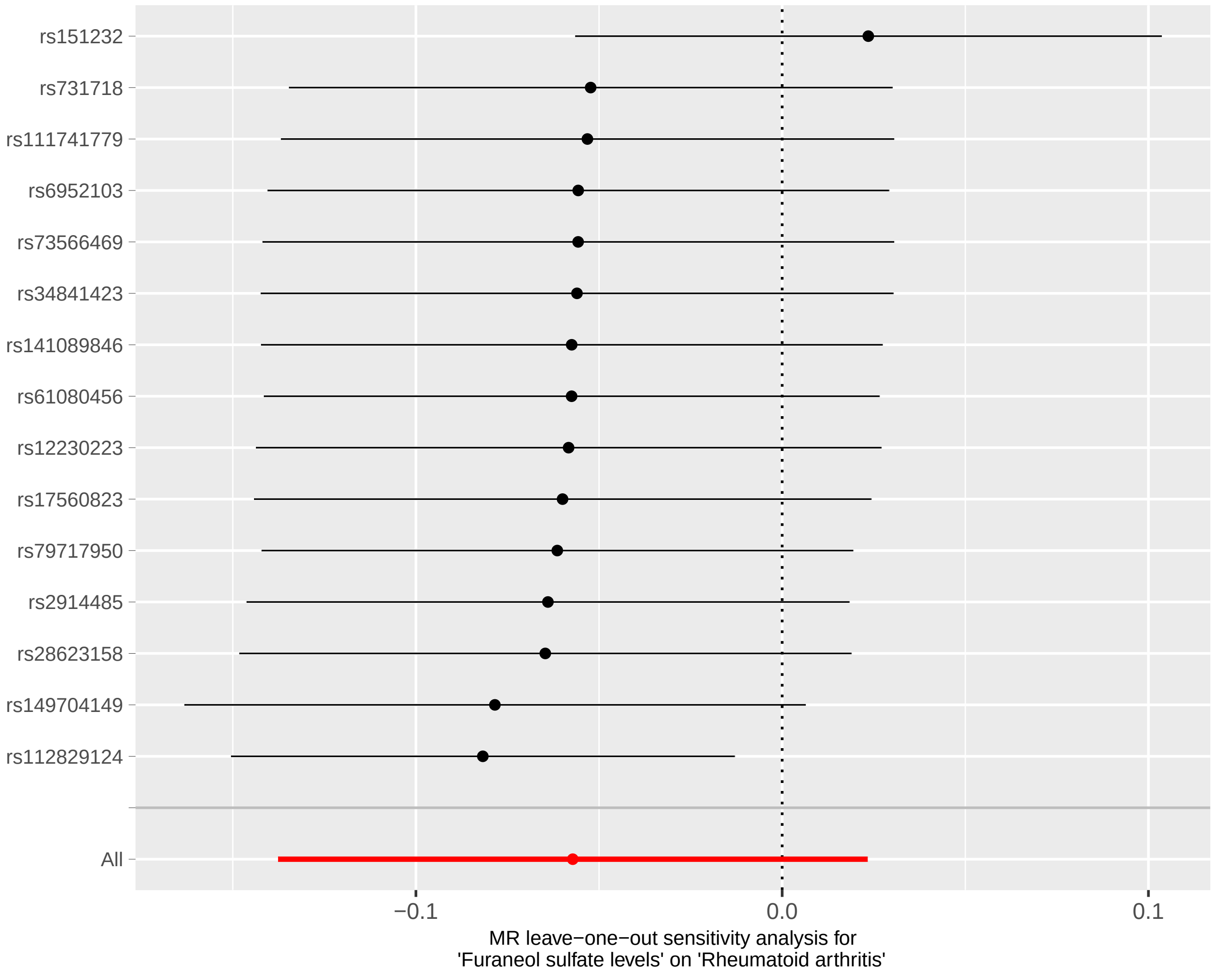
rs151232
rs731718
rs111741779
rs6952103
rs73566469
rs34841423
rs141089846
rs61080456
rs12230223
rs17560823
rs79717950
rs2914485
rs28623158
rs149704149
rs112829124
All
−0.1
0.0
0.1
MR leave−one−out sensitivity analysis for
'Furaneol sulfate levels' on 'Rheumatoid arthritis'

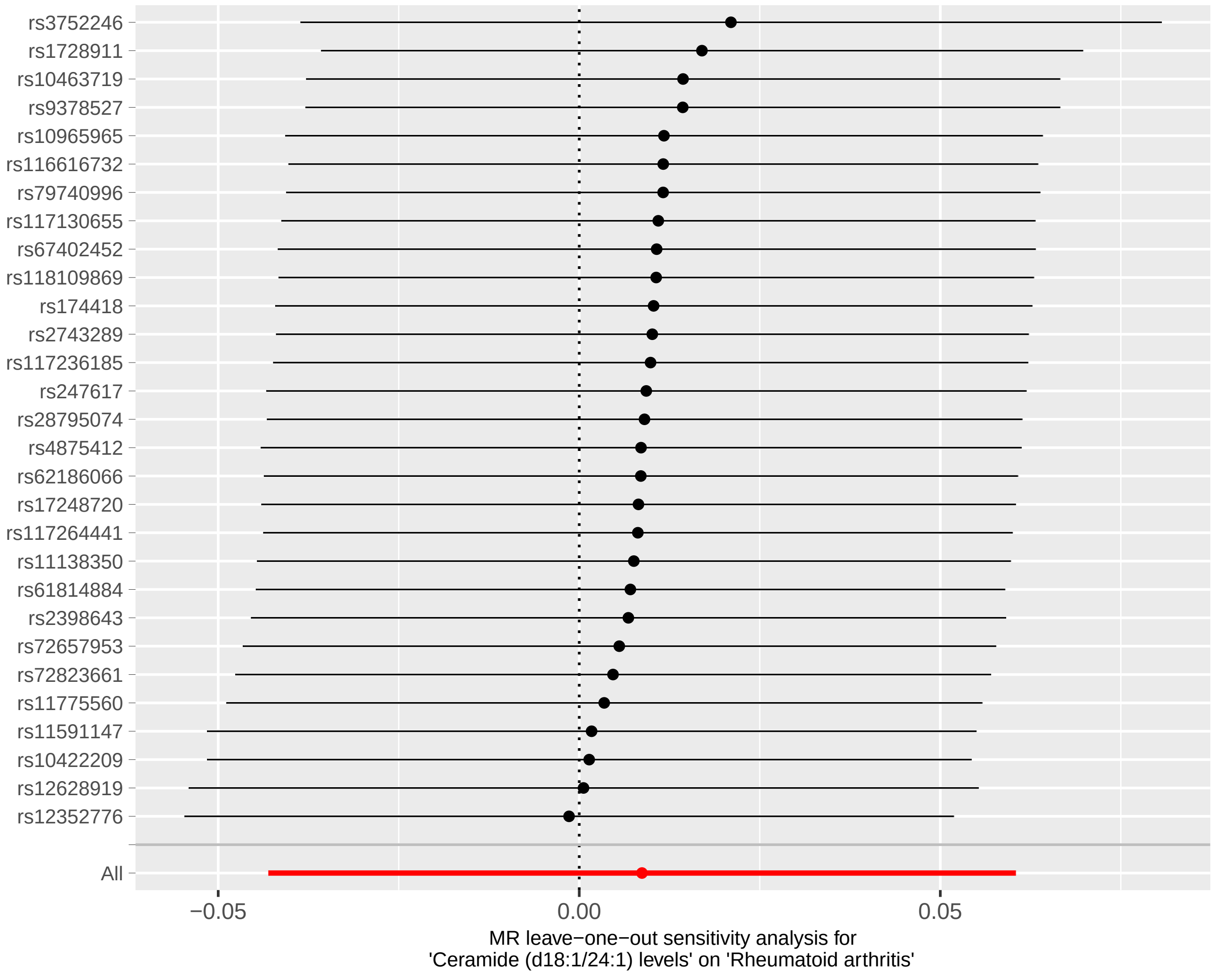
rs3752246
rs1728911
rs10463719
rs9378527
rs10965965
rs116616732
rs79740996
rs117130655
rs67402452
rs118109869
rs174418
rs2743289
rs117236185
rs247617
rs28795074
rs4875412
rs62186066
rs17248720
rs117264441
rs11138350
rs61814884
rs2398643
rs72657953
rs72823661
rs11775560
rs11591147
rs10422209
rs12628919
rs12352776
All
−0.05
0.00
0.05
MR leave−one−out sensitivity analysis for
'Ceramide (d18:1/24:1) levels' on 'Rheumatoid arthritis'

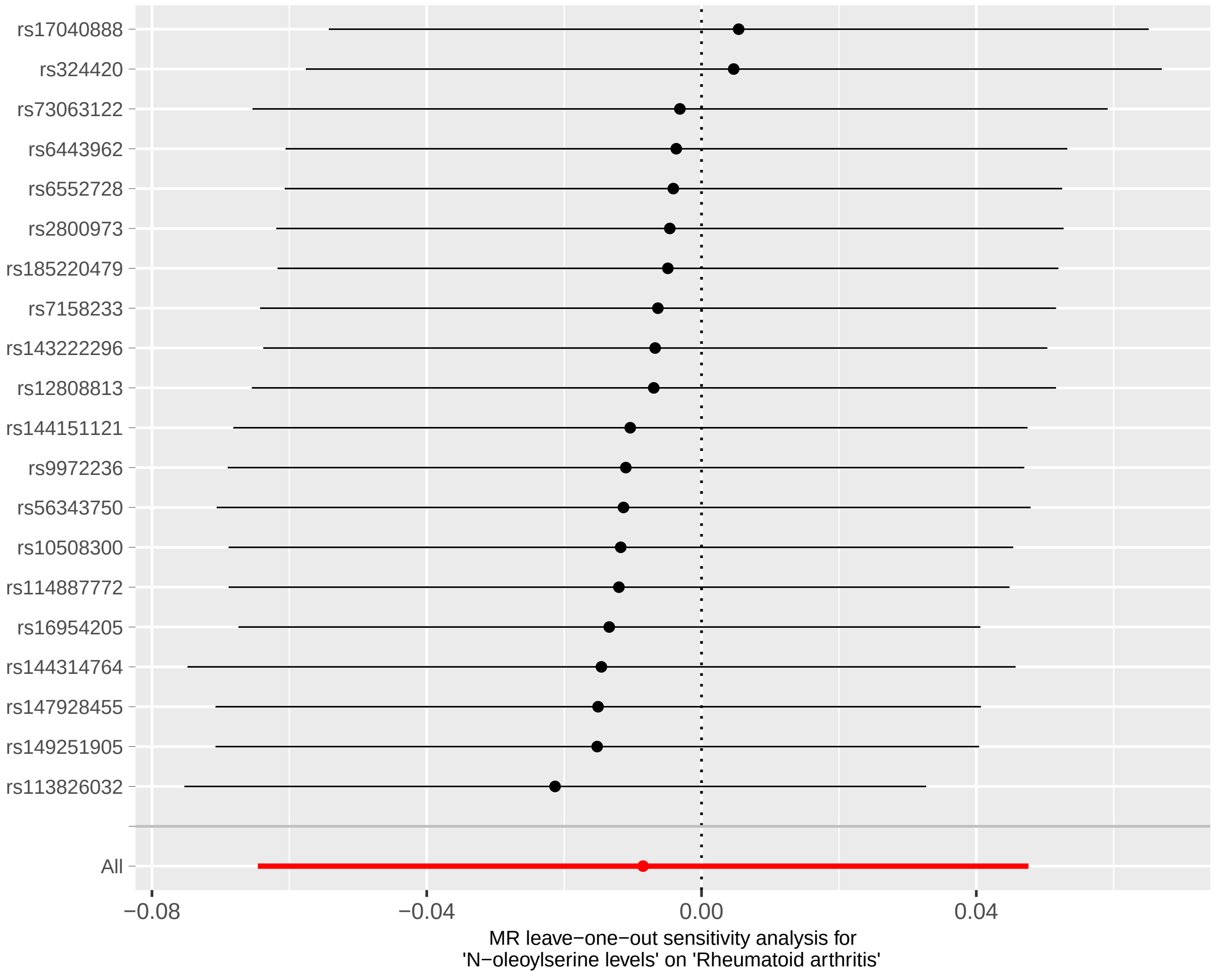

rs17040888
rs324420
rs73063122
rs6443962
rs6552728
rs2800973
rs185220479
rs7158233
rs143222296
rs12808813
rs144151121
rs9972236
rs56343750
rs10508300
rs114887772
rs16954205
rs144314764
rs147928455
rs149251905
rs113826032
All
−0.08
−0.04
0.00
0.04
MR leave−one−out sensitivity analysis for
'N−oleoylserine levels' on 'Rheumatoid arthritis'

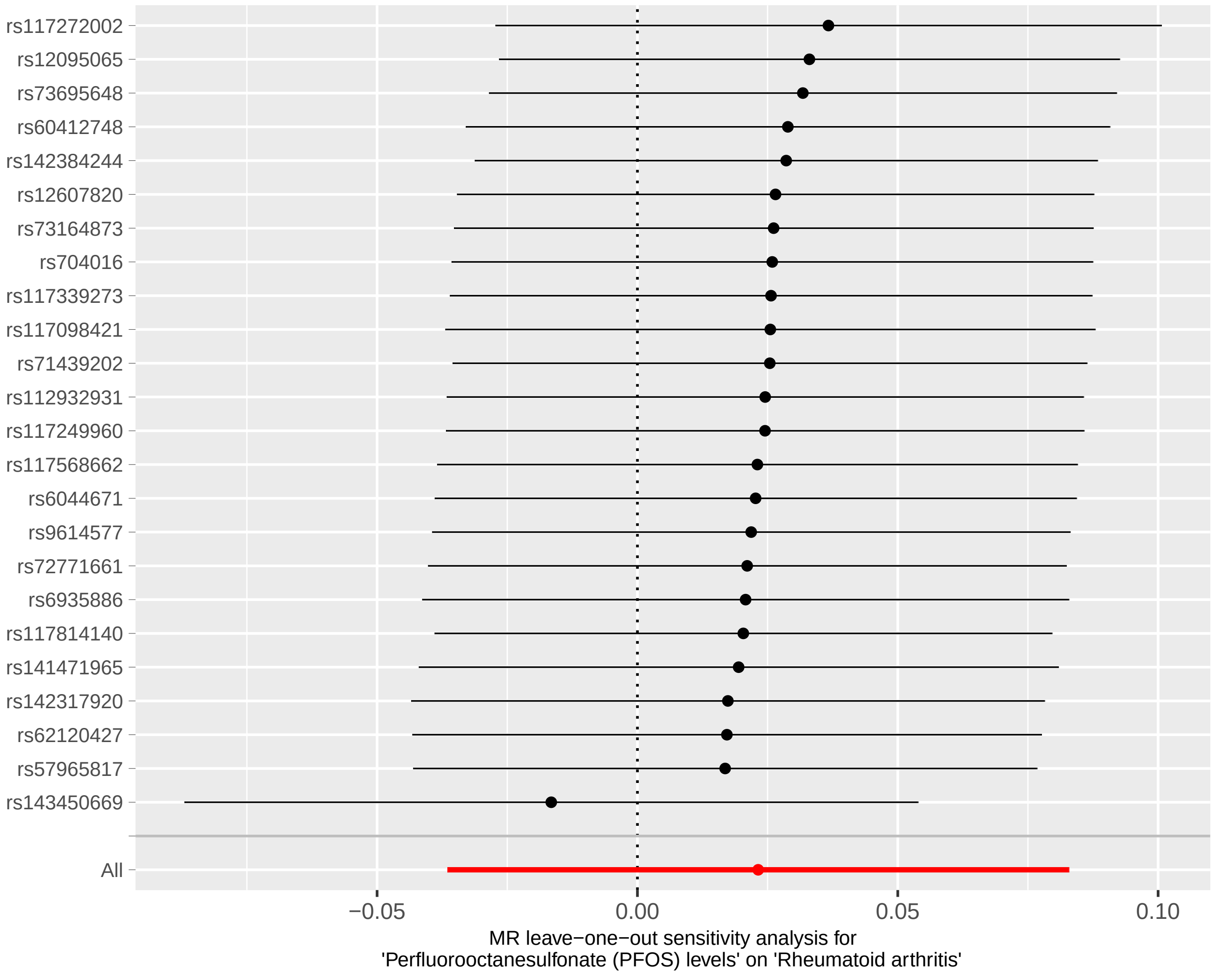

rs117272002
rs12095065
rs73695648
rs60412748
rs142384244
rs12607820
rs73164873
rs704016
rs117339273
rs117098421
rs71439202
rs112932931
rs117249960
rs117568662
rs6044671
rs9614577
rs72771661
rs6935886
rs117814140
rs141471965
rs142317920
rs62120427
rs57965817
rs143450669
All
−0.05
0.00
0.05
0.10
MR leave−one−out sensitivity analysis for
'Perfluorooctanesulfonate (PFOS) levels' on 'Rheumatoid arthritis'

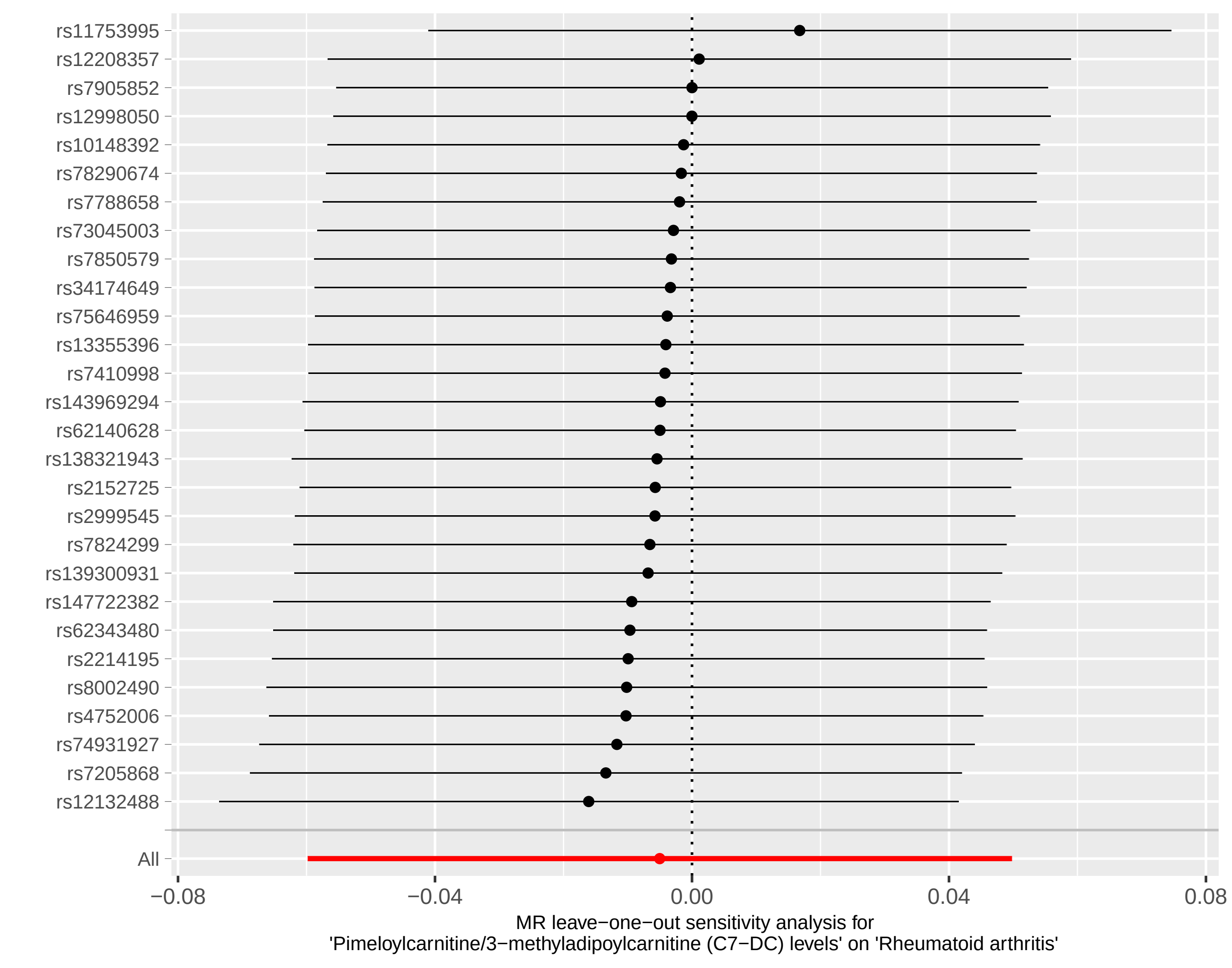
rs11753995
rs12208357
rs7905852
rs12998050
rs10148392
rs78290674
rs7788658
rs73045003
rs7850579
rs34174649
rs75646959
rs13355396
rs7410998
rs143969294
rs62140628
rs138321943
rs2152725
rs2999545
rs7824299
rs139300931
rs147722382
rs62343480
rs2214195
rs8002490
rs4752006
rs74931927
rs7205868
rs12132488
All
−0.08
−0.04
0.00
0.04
0.08
MR leave−one−out sensitivity analysis for
'Pimeloylcarnitine/3−methyladipoylcarnitine (C7−DC) levels' on 'Rheumatoid arthritis'

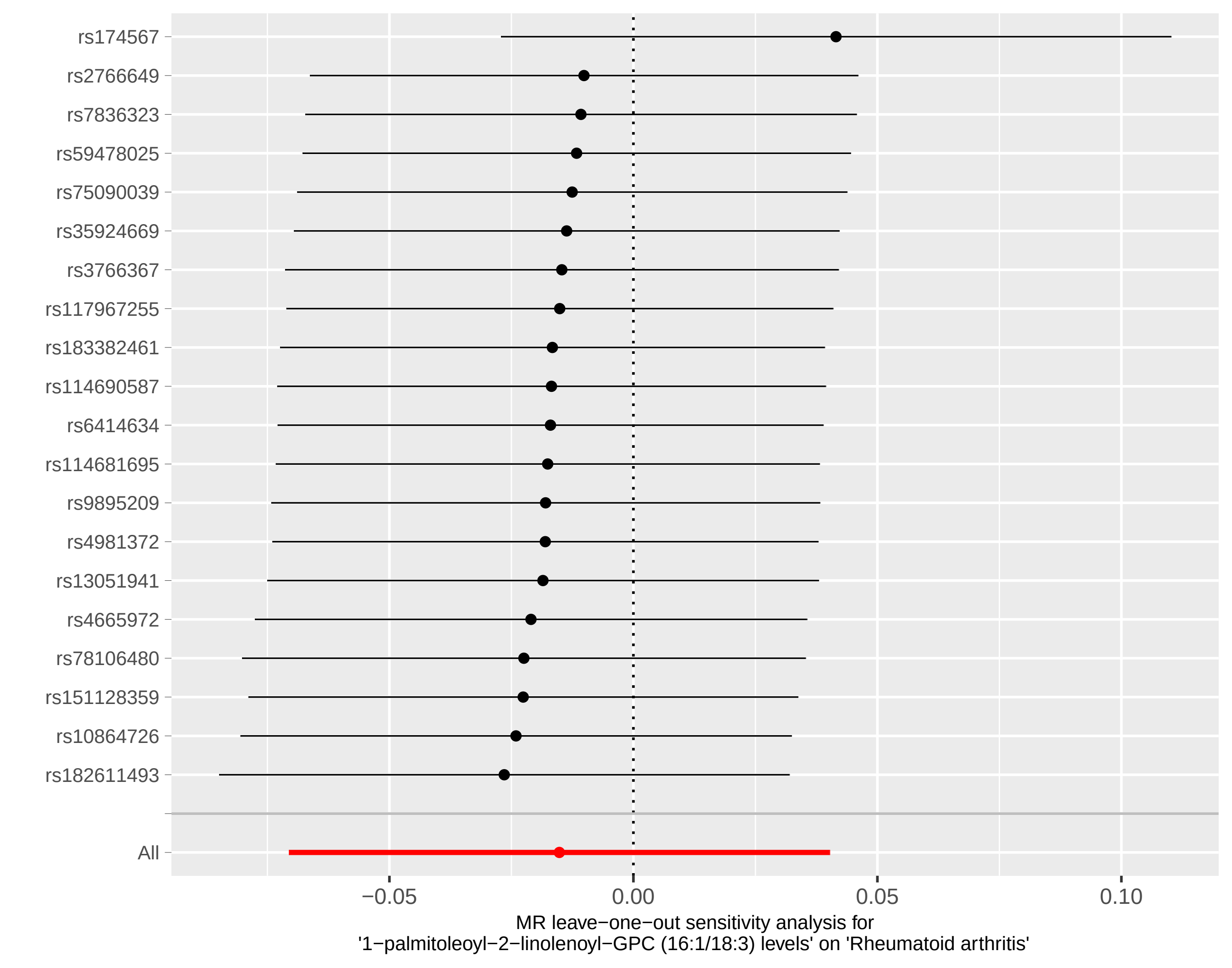
rs174567
rs2766649
rs7836323
rs59478025
rs75090039
rs35924669
rs3766367
rs117967255
rs183382461
rs114690587
rs6414634
rs114681695
rs9895209
rs4981372
rs13051941
rs4665972
rs78106480
rs151128359
rs10864726
rs182611493
All
−0.05
0.00
0.05
0.10
MR leave−one−out sensitivity analysis for
'1−palmitoleoyl−2−linolenoyl−GPC (16:1/18:3) levels' on 'Rheumatoid arthritis'

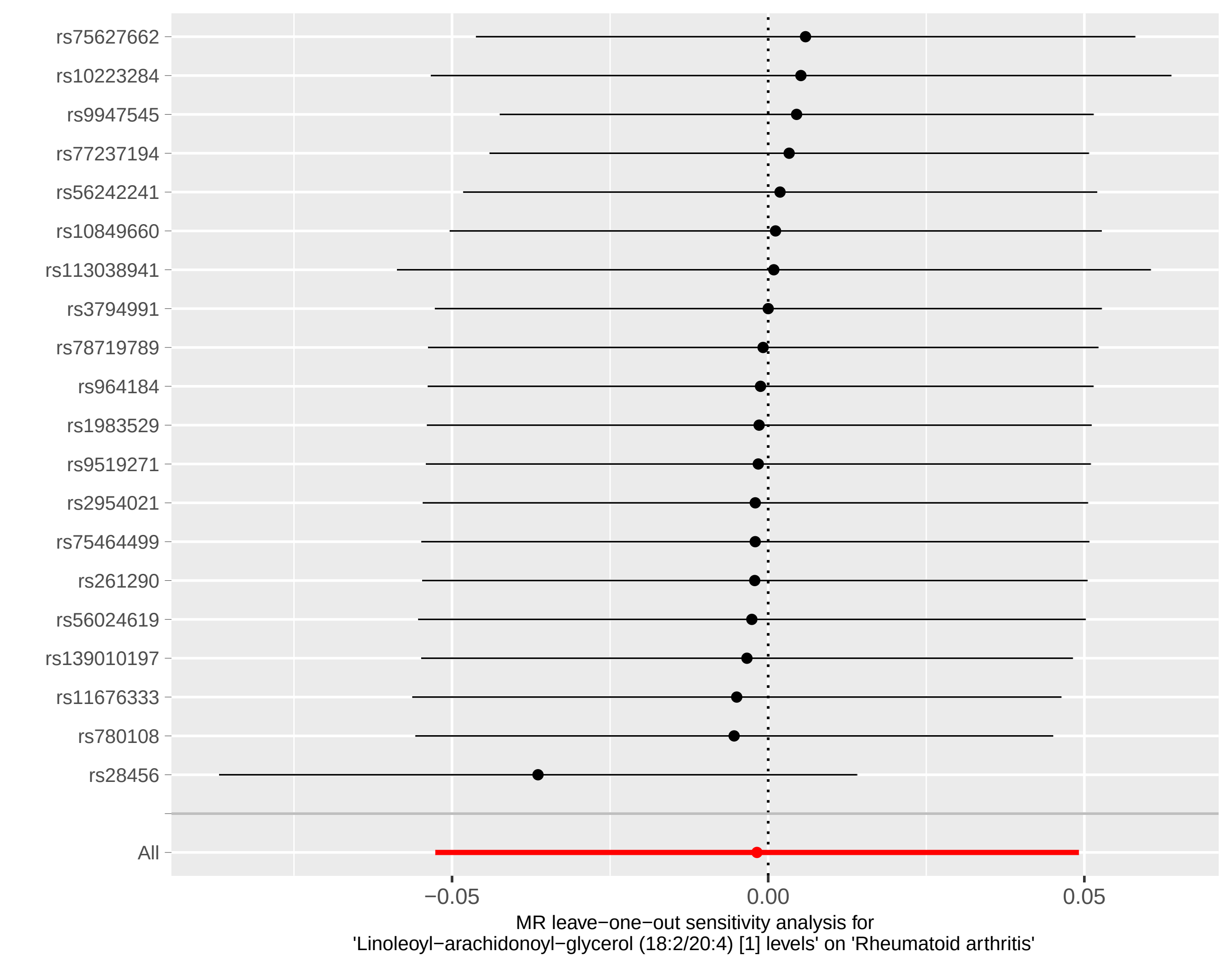

rs75627662
rs10223284
rs9947545
rs77237194
rs56242241
rs10849660
rs113038941
rs3794991
rs78719789
rs964184
rs1983529
rs9519271
rs2954021
rs75464499
rs261290
rs56024619
rs139010197
rs11676333
rs780108
rs28456
All
−0.05
0.00
0.05
MR leave−one−out sensitivity analysis for
'Linoleoyl−arachidonoyl−glycerol (18:2/20:4) [1] levels' on 'Rheumatoid arthritis'

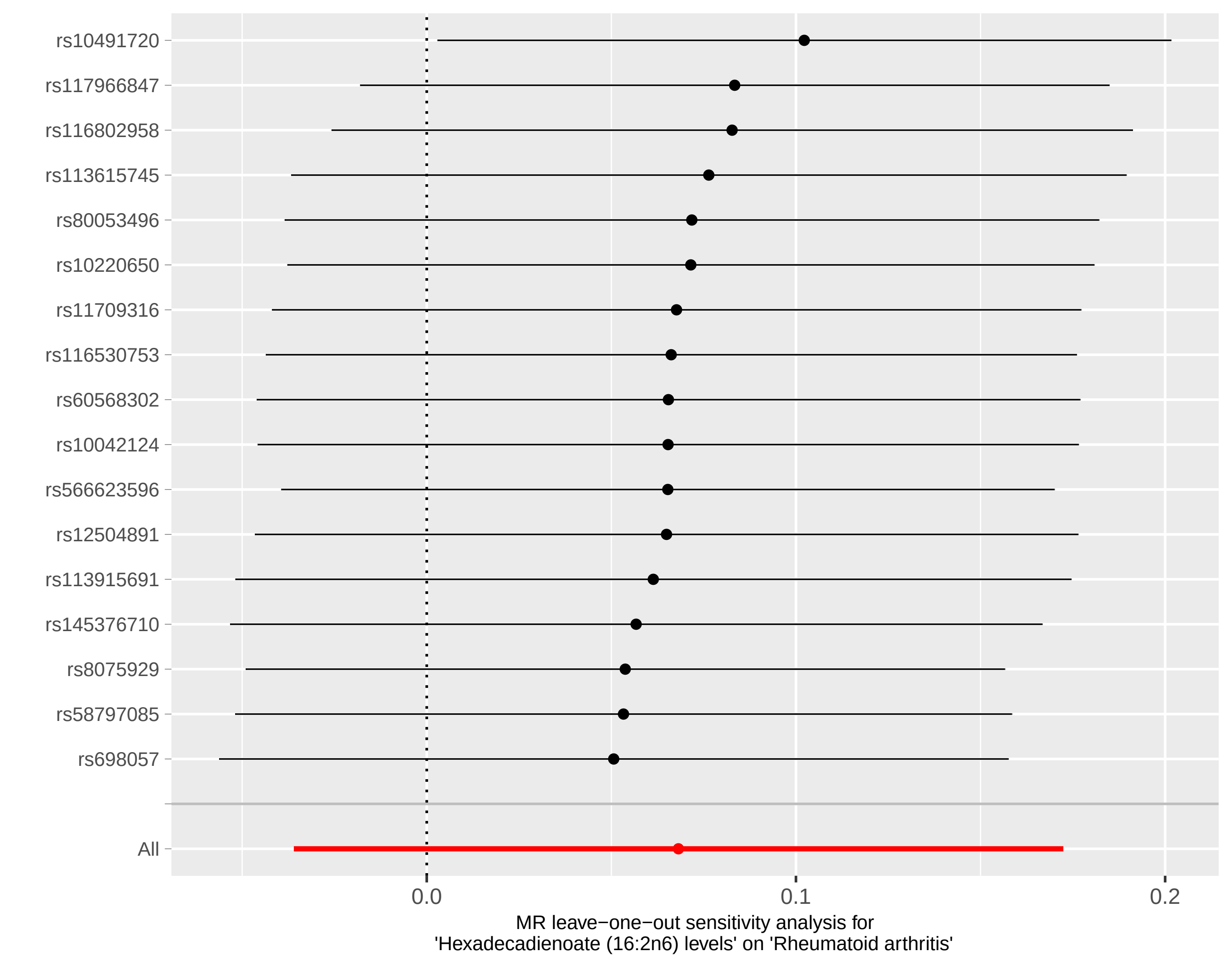

rs10491720
rs117966847
rs116802958
rs113615745
rs80053496
rs10220650
rs11709316
rs116530753
rs60568302
rs10042124
rs566623596
rs12504891
rs113915691
rs145376710
rs8075929
rs58797085
rs698057
All
0.0
0.1
0.2
MR leave−one−out sensitivity analysis for
'Hexadecadienoate (16:2n6) levels' on 'Rheumatoid arthritis'

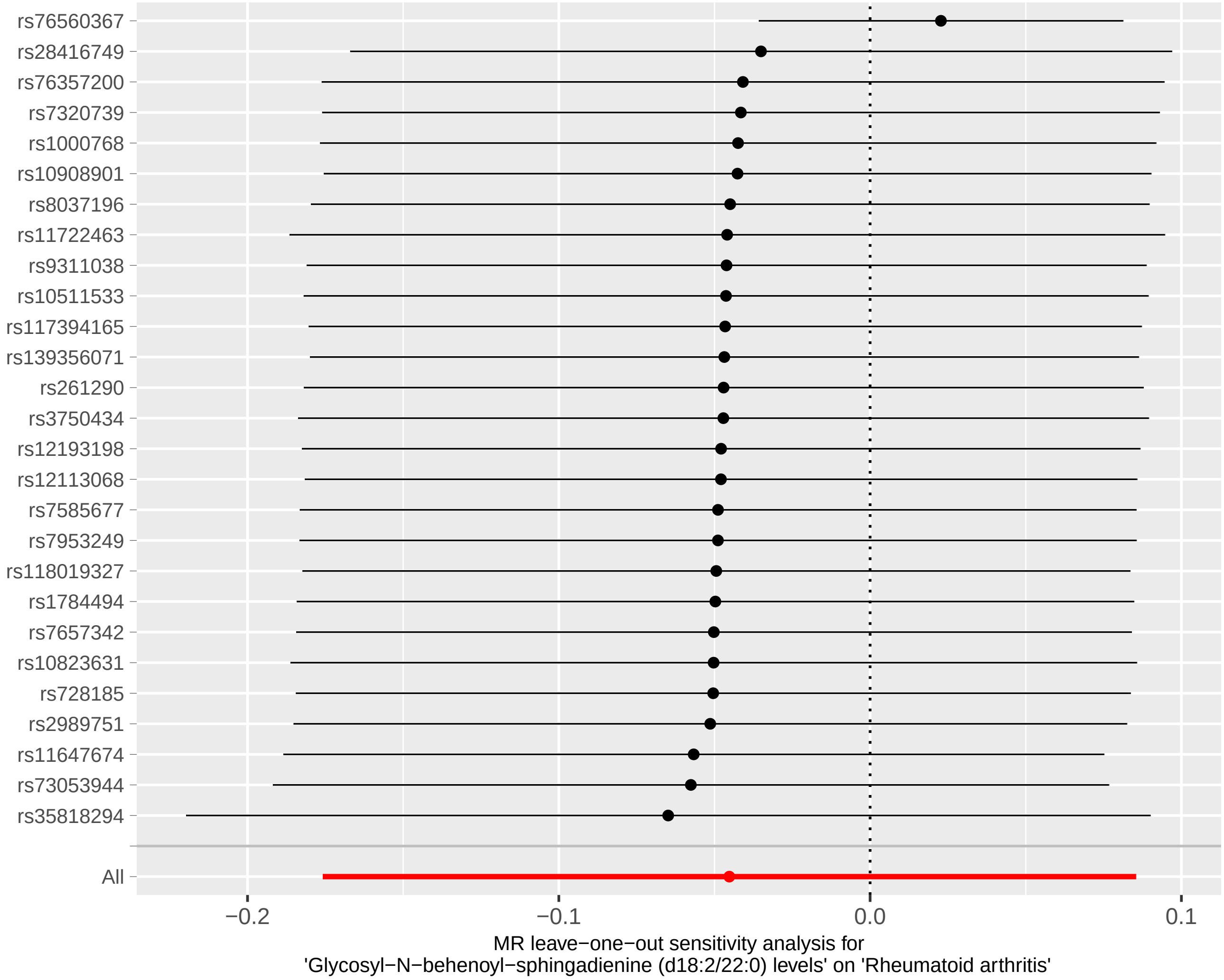

rs76560367
rs28416749
rs76357200
rs7320739
rs1000768
rs10908901
rs8037196
rs11722463
rs9311038
rs10511533
rs117394165
rs139356071
rs261290
rs3750434
rs12193198
rs12113068
rs7585677
rs7953249
rs118019327
rs1784494
rs7657342
rs10823631
rs728185
rs2989751
rs11647674
rs73053944
rs35818294
All
−0.2
−0.1
0.0
0.1
MR leave−one−out sensitivity analysis for
'Glycosyl−N−behenoyl−sphingadienine (d18:2/22:0) levels' on 'Rheumatoid arthritis'

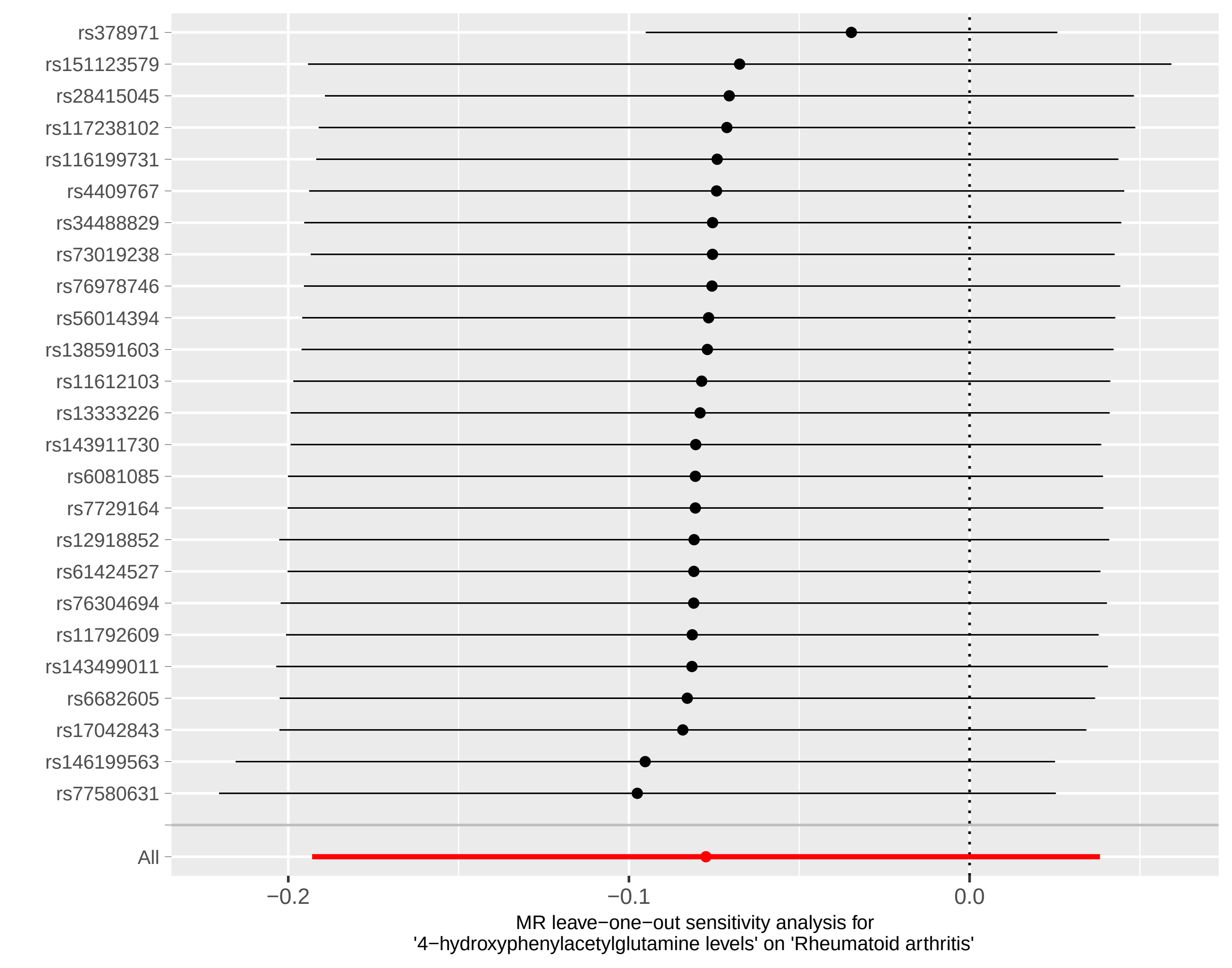

rs378971
rs151123579
rs28415045
rs117238102
rs116199731
rs4409767
rs34488829
rs73019238
rs76978746
rs56014394
rs138591603
rs11612103
rs13333226
rs143911730
rs6081085
rs7729164
rs12918852
rs61424527
rs76304694
rs11792609
rs143499011
rs6682605
rs17042843
rs146199563
rs77580631
All
−0.2
−0.1
0.0
MR leave−one−out sensitivity analysis for
'4−hydroxyphenylacetylglutamine levels' on 'Rheumatoid arthritis'

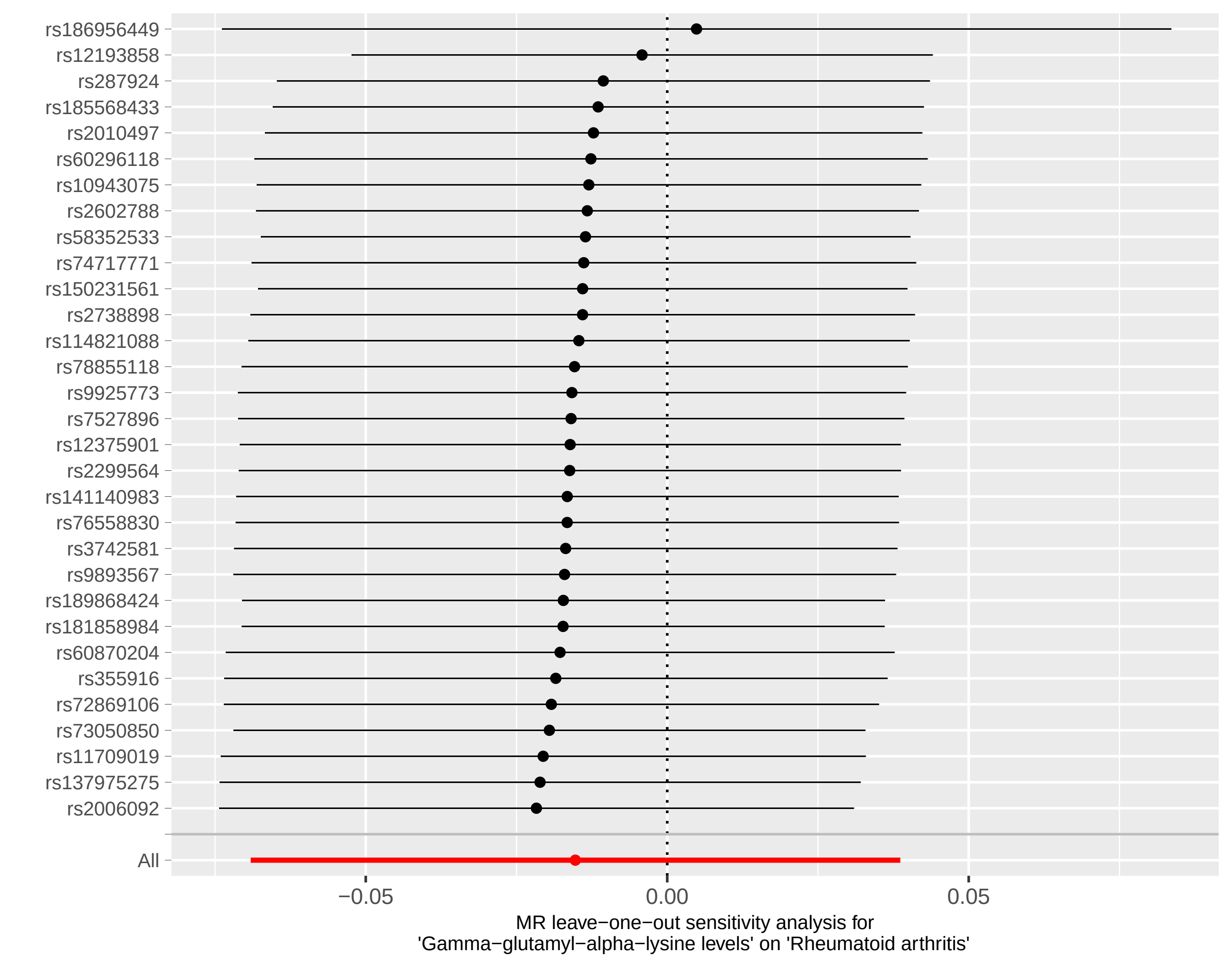
rs186956449
rs12193858
rs287924
rs185568433
rs2010497
rs60296118
rs10943075
rs2602788
rs58352533
rs74717771
rs150231561
rs2738898
rs114821088
rs78855118
rs9925773
rs7527896
rs12375901
rs2299564
rs141140983
rs76558830
rs3742581
rs9893567
rs189868424
rs181858984
rs60870204
rs355916
rs72869106
rs73050850
rs11709019
rs137975275
rs2006092
All
−0.05
0.00
0.05
MR leave−one−out sensitivity analysis for
'Gamma−glutamyl−alpha−lysine levels' on 'Rheumatoid arthritis'

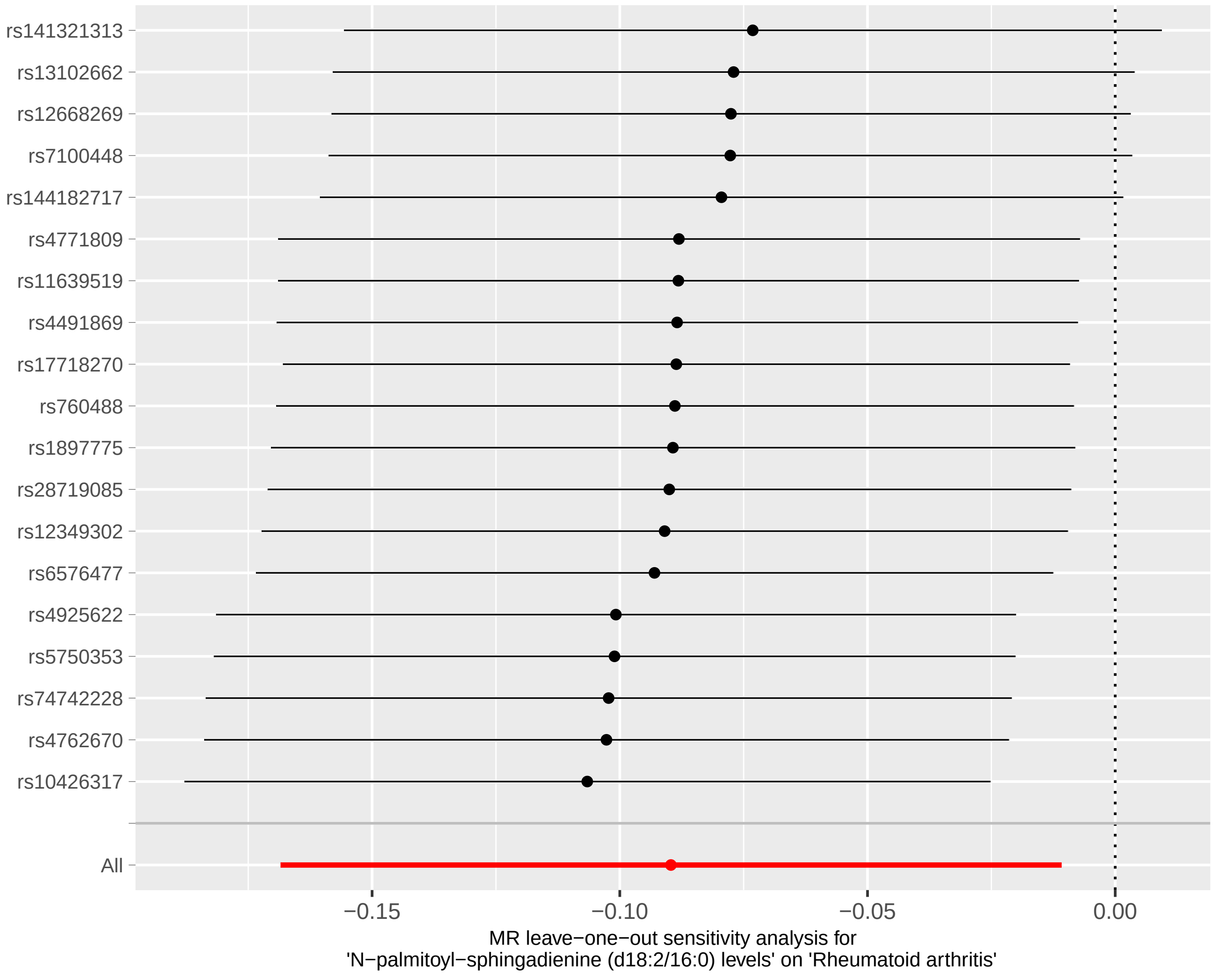
rs141321313
rs13102662
rs12668269
rs7100448
rs144182717
rs4771809
rs11639519
rs4491869
rs17718270
rs760488
rs1897775
rs28719085
rs12349302
rs6576477
rs4925622
rs5750353
rs74742228
rs4762670
rs10426317
All
−0.15
−0.10
−0.05
0.00
MR leave−one−out sensitivity analysis for
'N−palmitoyl−sphingadienine (d18:2/16:0) levels' on 'Rheumatoid arthritis'

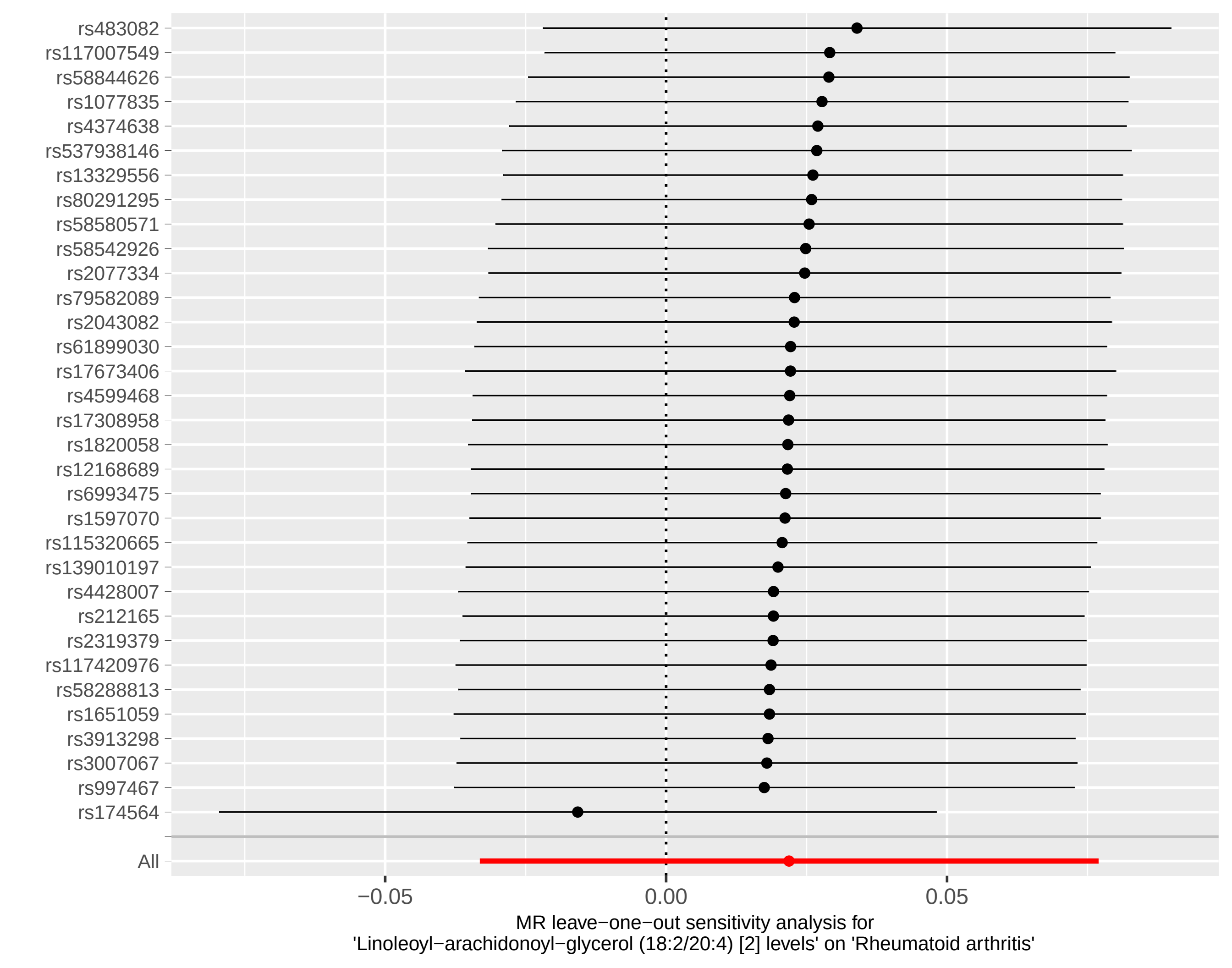
rs483082
rs117007549
rs58844626
rs1077835
rs4374638
rs537938146
rs13329556
rs80291295
rs58580571
rs58542926
rs2077334
rs79582089
rs2043082
rs61899030
rs17673406
rs4599468
rs17308958
rs1820058
rs12168689
rs6993475
rs1597070
rs115320665
rs139010197
rs4428007
rs212165
rs2319379
rs117420976
rs58288813
rs1651059
rs3913298
rs3007067
rs997467
rs174564
All
−0.05
0.00
0.05
MR leave−one−out sensitivity analysis for
'Linoleoyl−arachidonoyl−glycerol (18:2/20:4) [2] levels' on 'Rheumatoid arthritis'

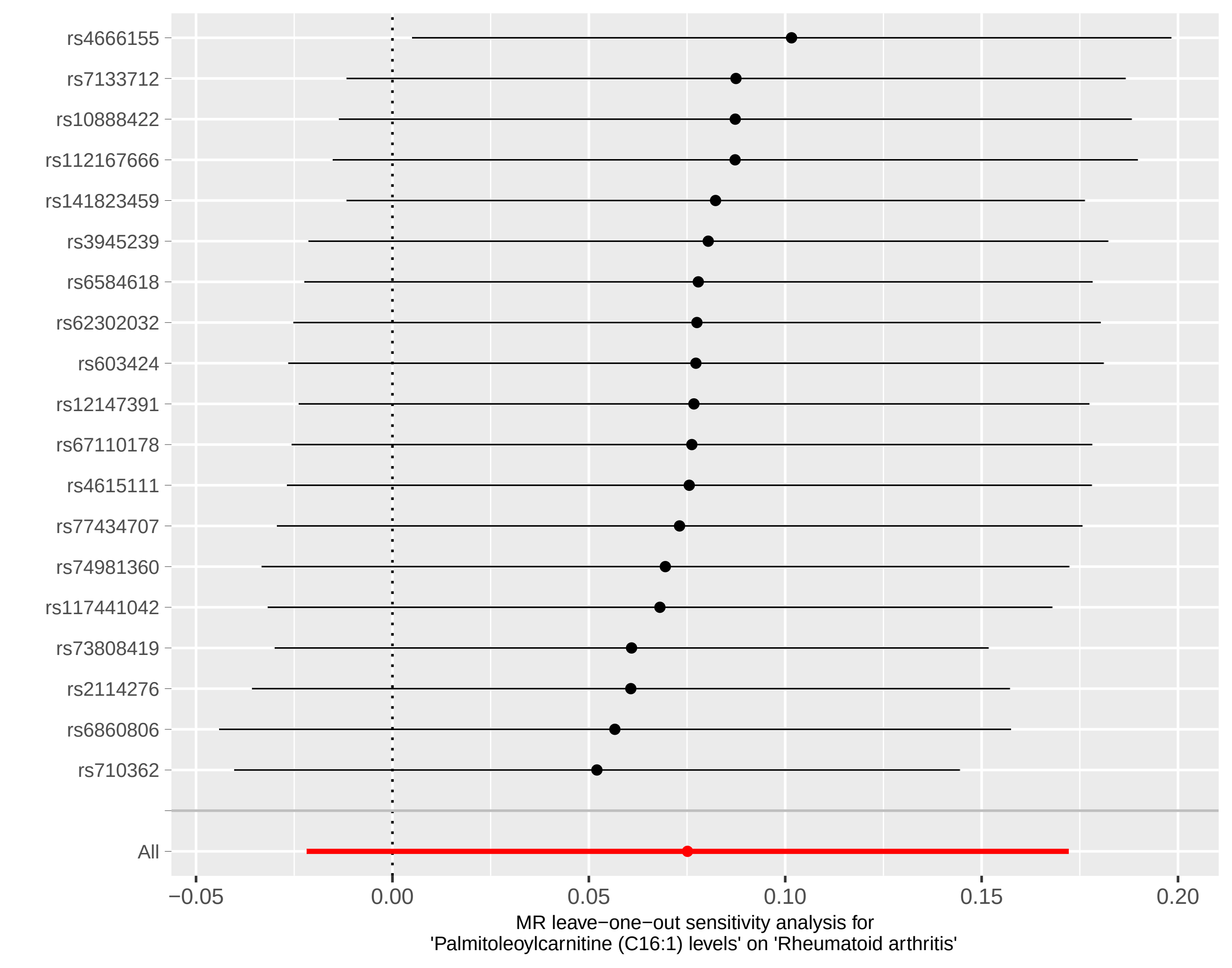

rs4666155
rs7133712
rs10888422
rs112167666
rs141823459
rs3945239
rs6584618
rs62302032
rs603424
rs12147391
rs67110178
rs4615111
rs77434707
rs74981360
rs117441042
rs73808419
rs2114276
rs6860806
rs710362
All
−0.05
0.00
0.05
0.10
0.15
0.20
MR leave−one−out sensitivity analysis for
'Palmitoleoylcarnitine (C16:1) levels' on 'Rheumatoid arthritis'

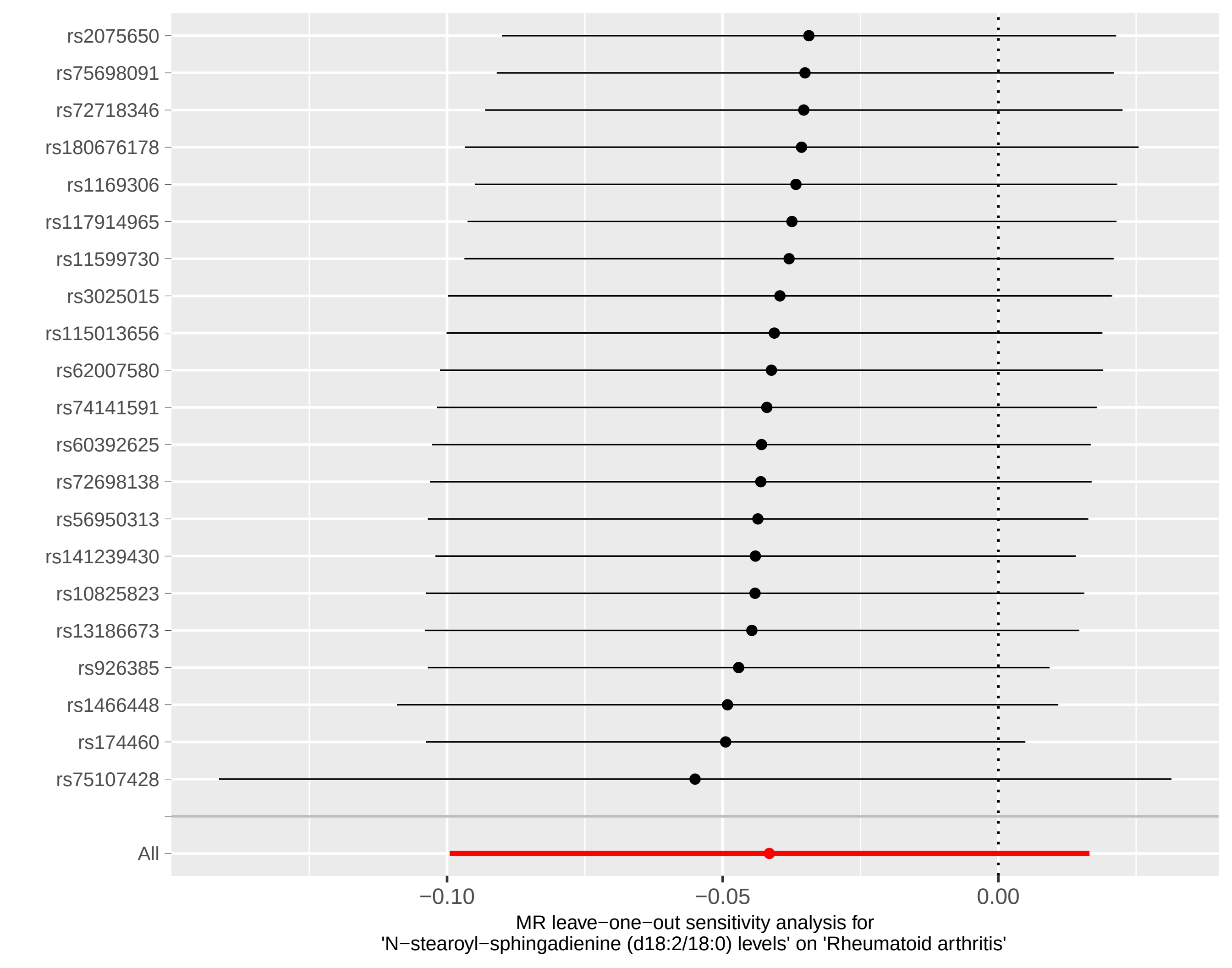
rs2075650
rs75698091
rs72718346
rs180676178
rs1169306
rs117914965
rs11599730
rs3025015
rs115013656
rs62007580
rs74141591
rs60392625
rs72698138
rs56950313
rs141239430
rs10825823
rs13186673
rs926385
rs1466448
rs174460
rs75107428
All
−0.10
−0.05
0.00
MR leave−one−out sensitivity analysis for
'N−stearoyl−sphingadienine (d18:2/18:0) levels' on 'Rheumatoid arthritis'

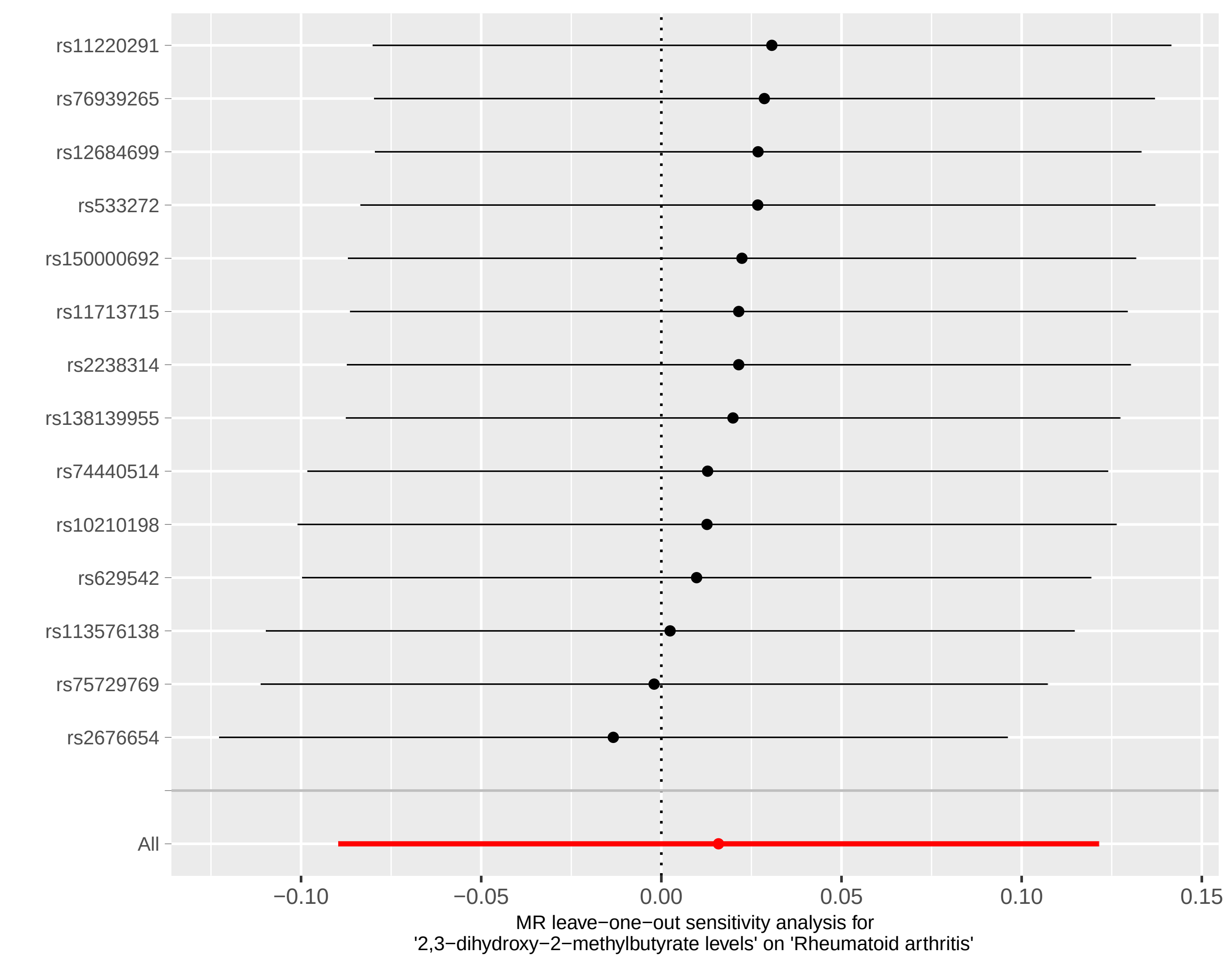
rs11220291
rs76939265
rs12684699
rs533272
rs150000692
rs11713715
rs2238314
rs138139955
rs74440514
rs10210198
rs629542
rs113576138
rs75729769
rs2676654
All
−0.10
−0.05
0.00
0.05
0.10
0.15
MR leave−one−out sensitivity analysis for
'2,3−dihydroxy−2−methylbutyrate levels' on 'Rheumatoid arthritis'

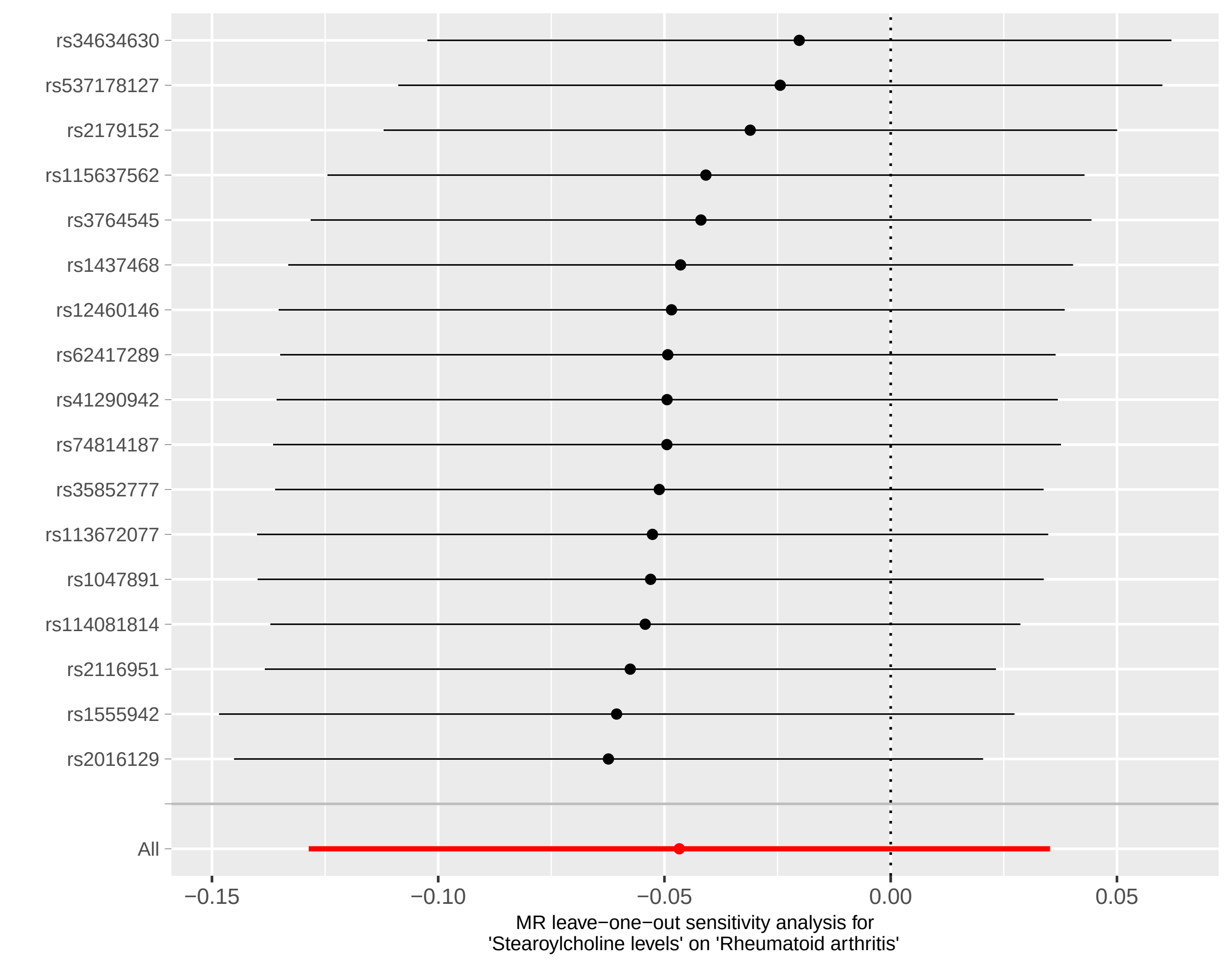
rs34634630
rs537178127
rs2179152
rs115637562
rs3764545
rs1437468
rs12460146
rs62417289
rs41290942
rs74814187
rs35852777
rs113672077
rs1047891
rs114081814
rs2116951
rs1555942
rs2016129
All
−0.15
−0.10
−0.05
0.00
0.05
MR leave−one−out sensitivity analysis for
'Stearoylcholine levels' on 'Rheumatoid arthritis'

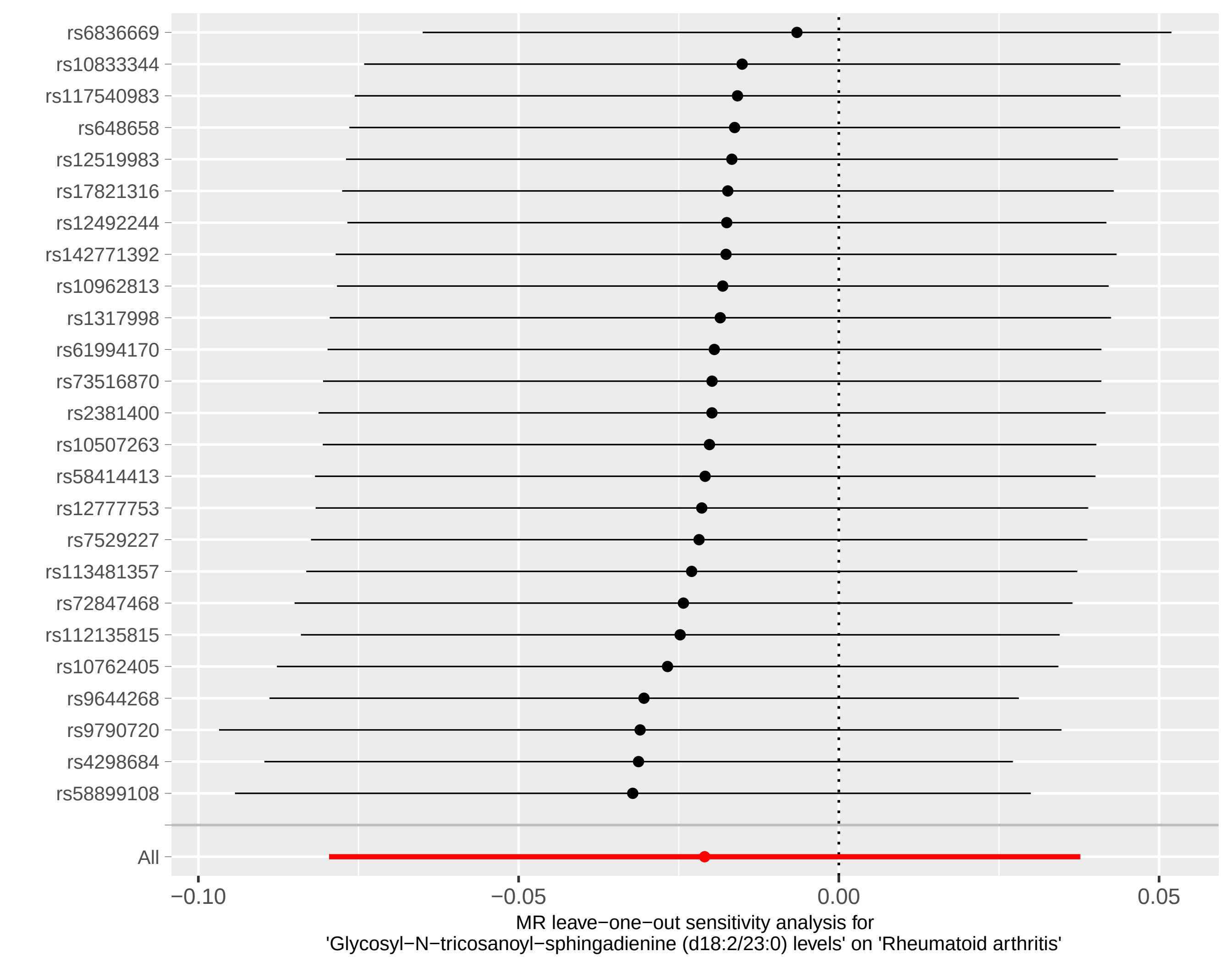

rs6836669
rs10833344
rs117540983
rs648658
rs12519983
rs17821316
rs12492244
rs142771392
rs10962813
rs1317998
rs61994170
rs73516870
rs2381400
rs10507263
rs58414413
rs12777753
rs7529227
rs113481357
rs72847468
rs112135815
rs10762405
rs9644268
rs9790720
rs4298684
rs58899108
All
−0.10
−0.05
0.00
0.05
MR leave−one−out sensitivity analysis for
'Glycosyl−N−tricosanoyl−sphingadienine (d18:2/23:0) levels' on 'Rheumatoid arthritis'

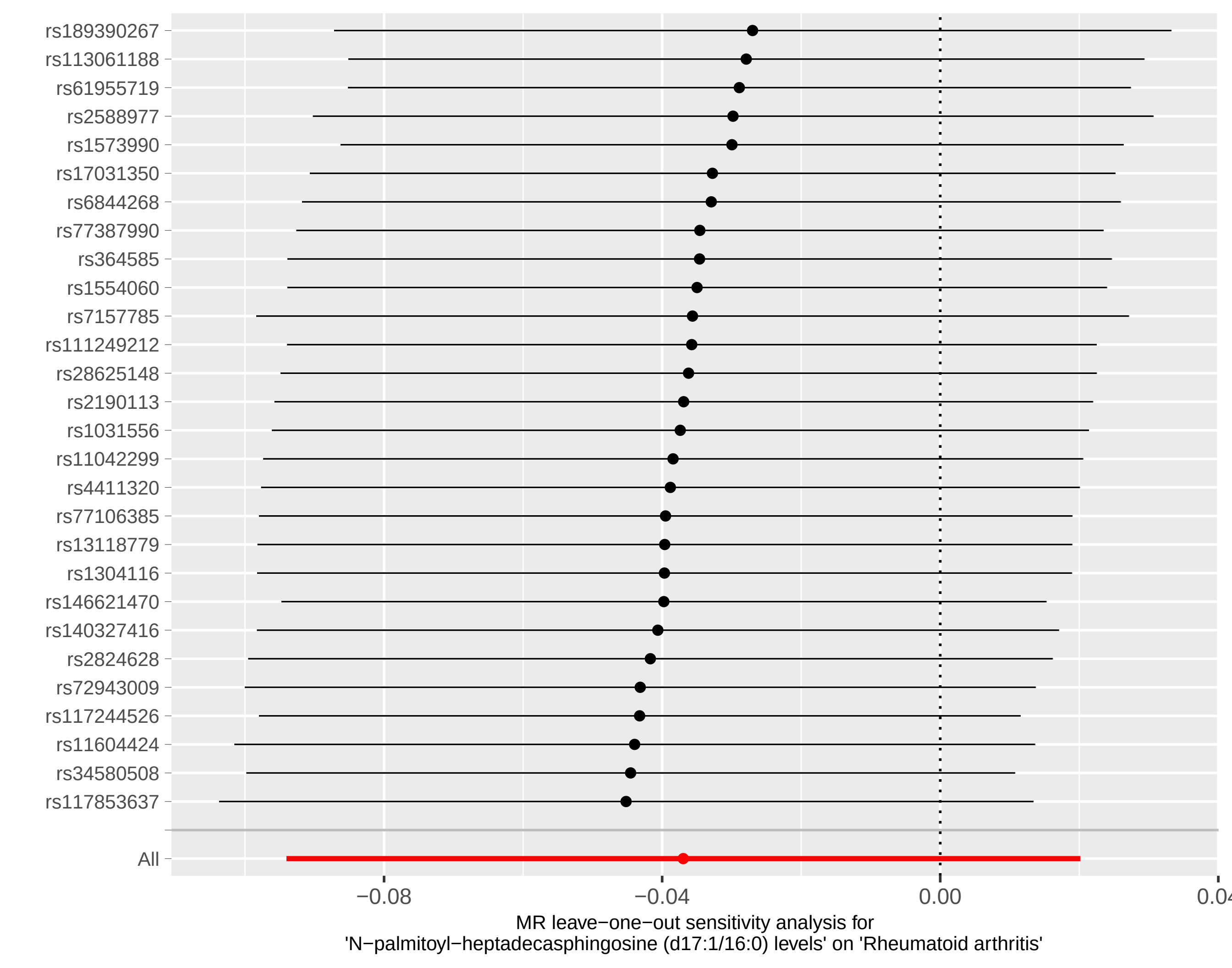
rs189390267
rs113061188
rs61955719
rs2588977
rs1573990
rs17031350
rs6844268
rs77387990
rs364585
rs1554060
rs7157785
rs111249212
rs28625148
rs2190113
rs1031556
rs11042299
rs4411320
rs77106385
rs13118779
rs1304116
rs146621470
rs140327416
rs2824628
rs72943009
rs117244526
rs11604424
rs34580508
rs117853637
All
−0.08
−0.04
0.00
MR leave−one−out sensitivity analysis for
'N−palmitoyl−heptadecasphingosine (d17:1/16:0) levels' on 'Rheumatoid arthritis'

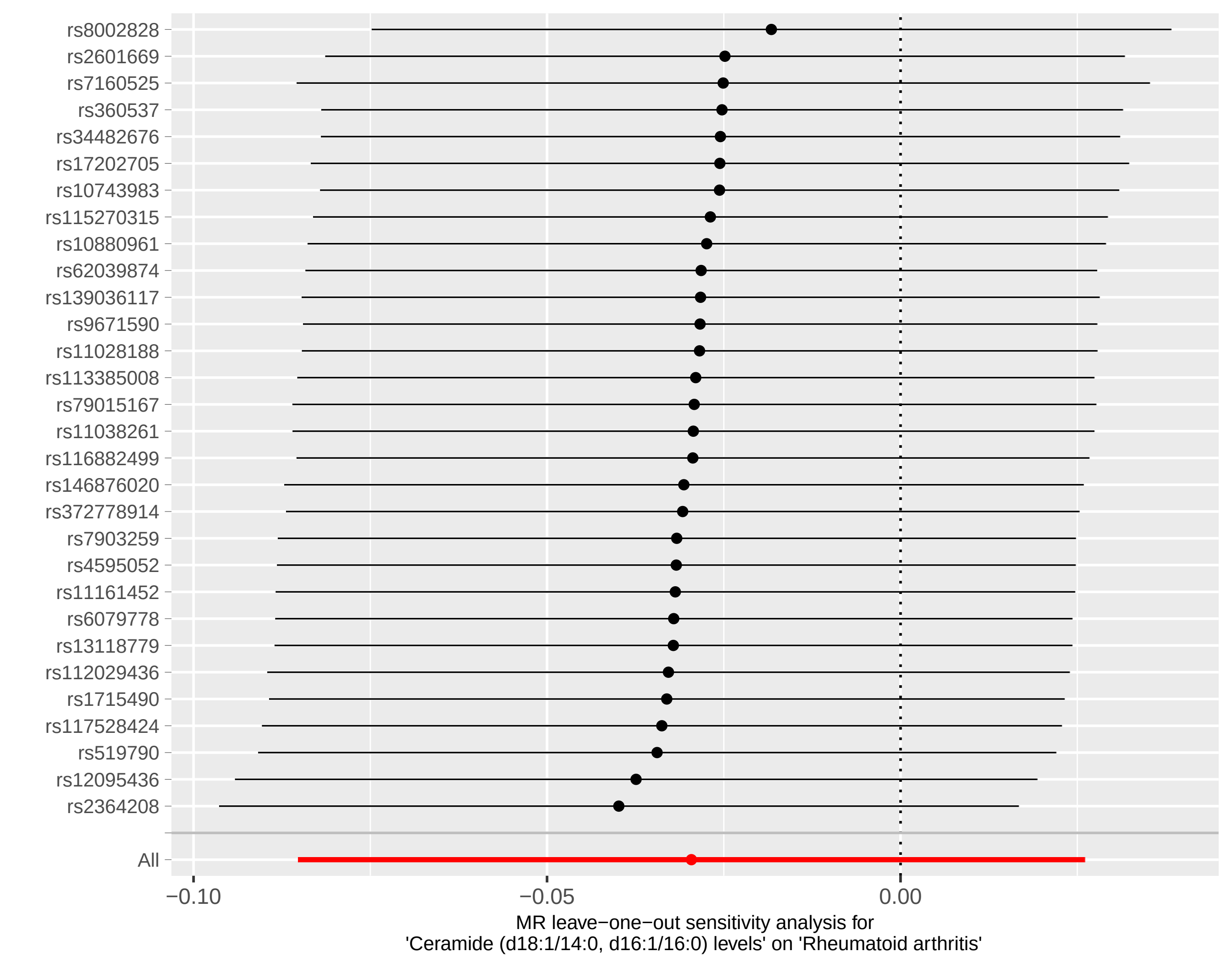

rs8002828
rs2601669
rs7160525
rs360537
rs34482676
rs17202705
rs10743983
rs115270315
rs10880961
rs62039874
rs139036117
rs9671590
rs11028188
rs113385008
rs79015167
rs11038261
rs116882499
rs146876020
rs372778914
rs7903259
rs4595052
rs11161452
rs6079778
rs13118779
rs112029436
rs1715490
rs117528424
rs519790
rs12095436
rs2364208
All
−0.10
−0.05
0.00
MR leave−one−out sensitivity analysis for
'Ceramide (d18:1/14:0, d16:1/16:0) levels' on 'Rheumatoid arthritis'

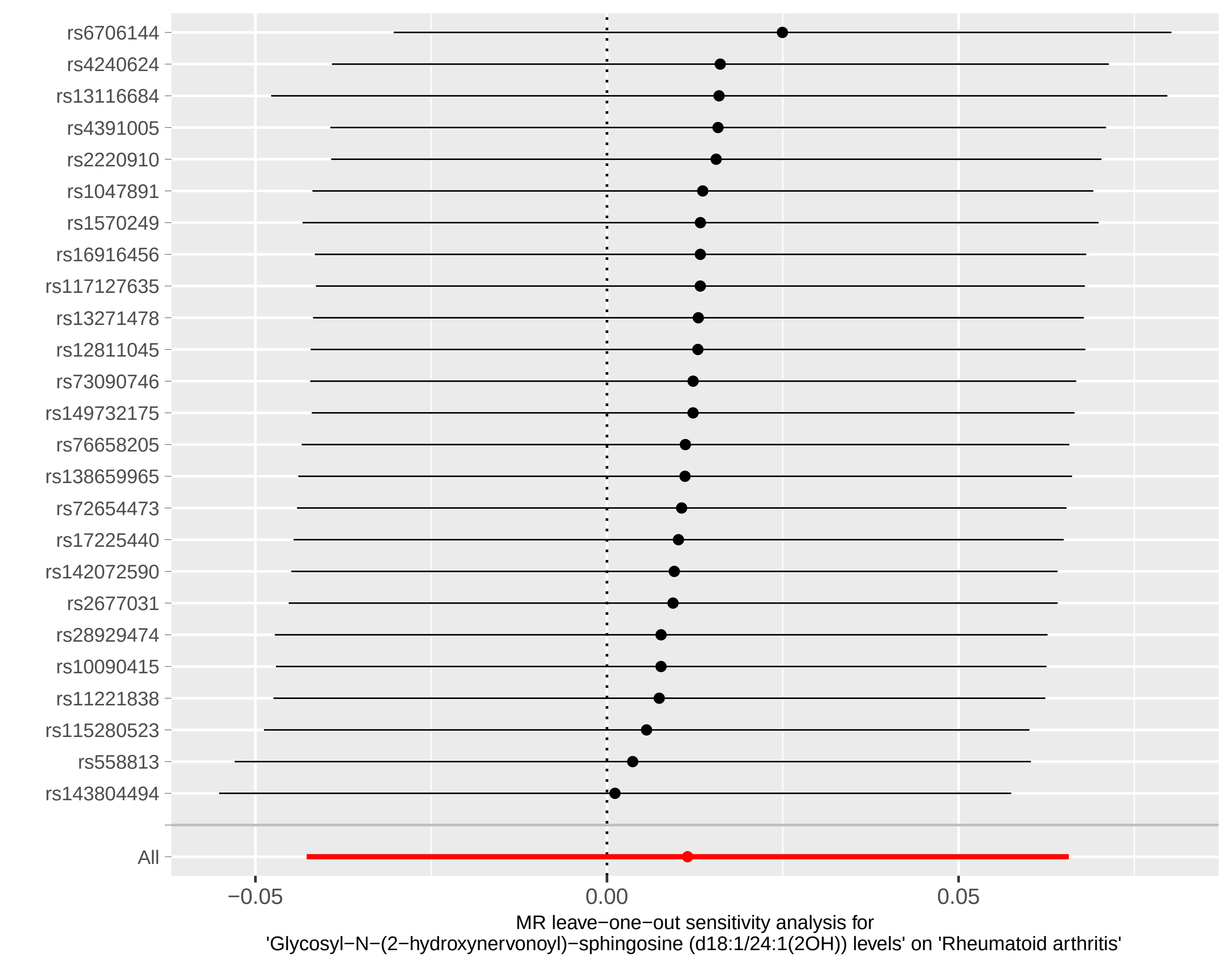
rs6706144
rs4240624
rs13116684
rs4391005
rs2220910
rs1047891
rs1570249
rs16916456
rs117127635
rs13271478
rs12811045
rs73090746
rs149732175
rs76658205
rs138659965
rs72654473
rs17225440
rs142072590
rs2677031
rs28929474
rs10090415
rs11221838
rs115280523
rs558813
rs143804494
All
−0.05
0.00
0.05
MR leave−one−out sensitivity analysis for
'Glycosyl−N−(2−hydroxynervonoyl)−sphingosine (d18:1/24:1(2OH)) levels' on 'Rheumatoid arthritis'

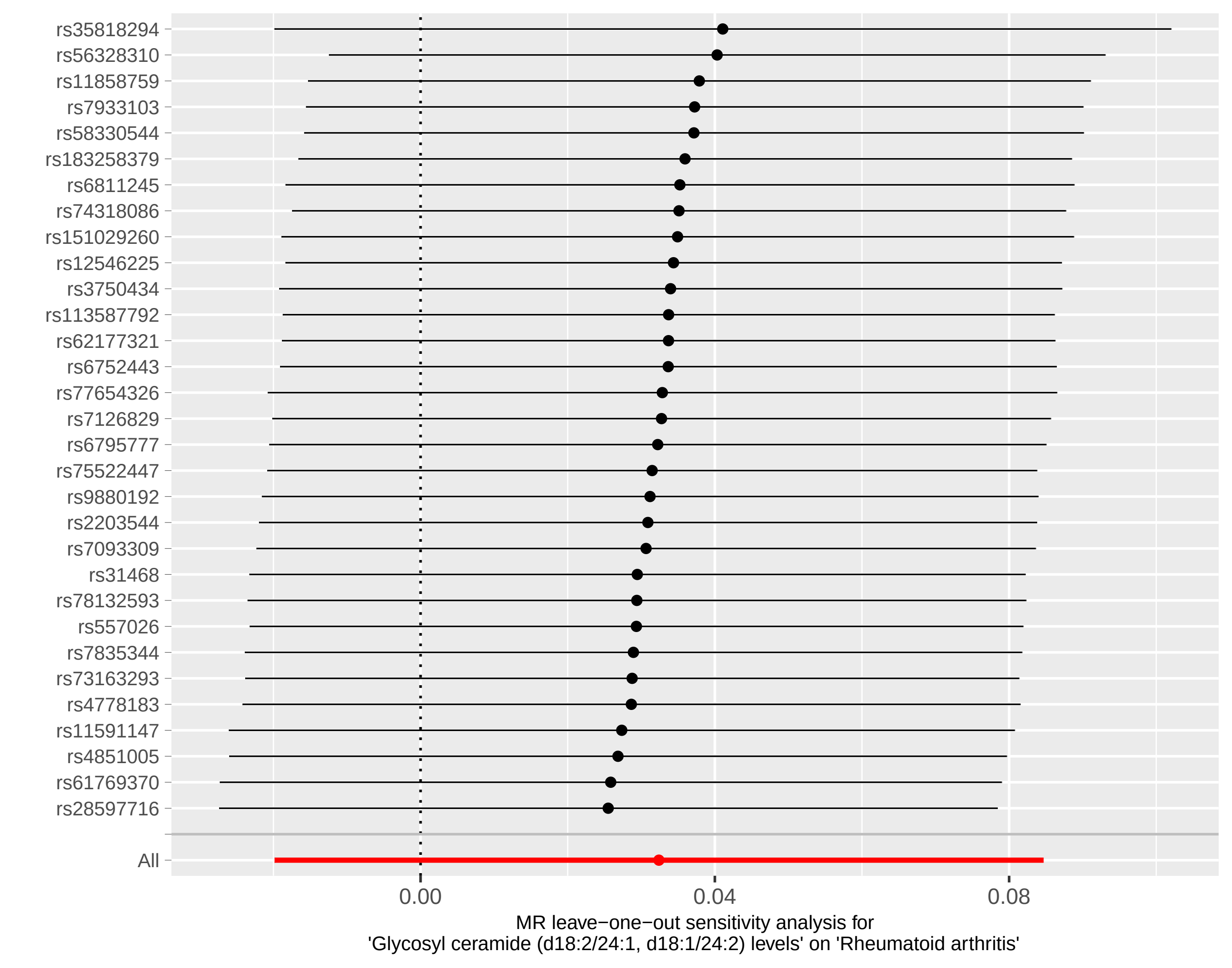

rs35818294
rs56328310
rs11858759
rs7933103
rs58330544
rs183258379
rs6811245
rs74318086
rs151029260
rs12546225
rs3750434
rs113587792
rs62177321
rs6752443
rs77654326
rs7126829
rs6795777
rs75522447
rs9880192
rs2203544
rs7093309
rs31468
rs78132593
rs557026
rs7835344
rs73163293
rs4778183
rs11591147
rs4851005
rs61769370
rs28597716
All
0.00
0.04
0.08
MR leave−one−out sensitivity analysis for
'Glycosyl ceramide (d18:2/24:1, d18:1/24:2) levels' on 'Rheumatoid arthritis'

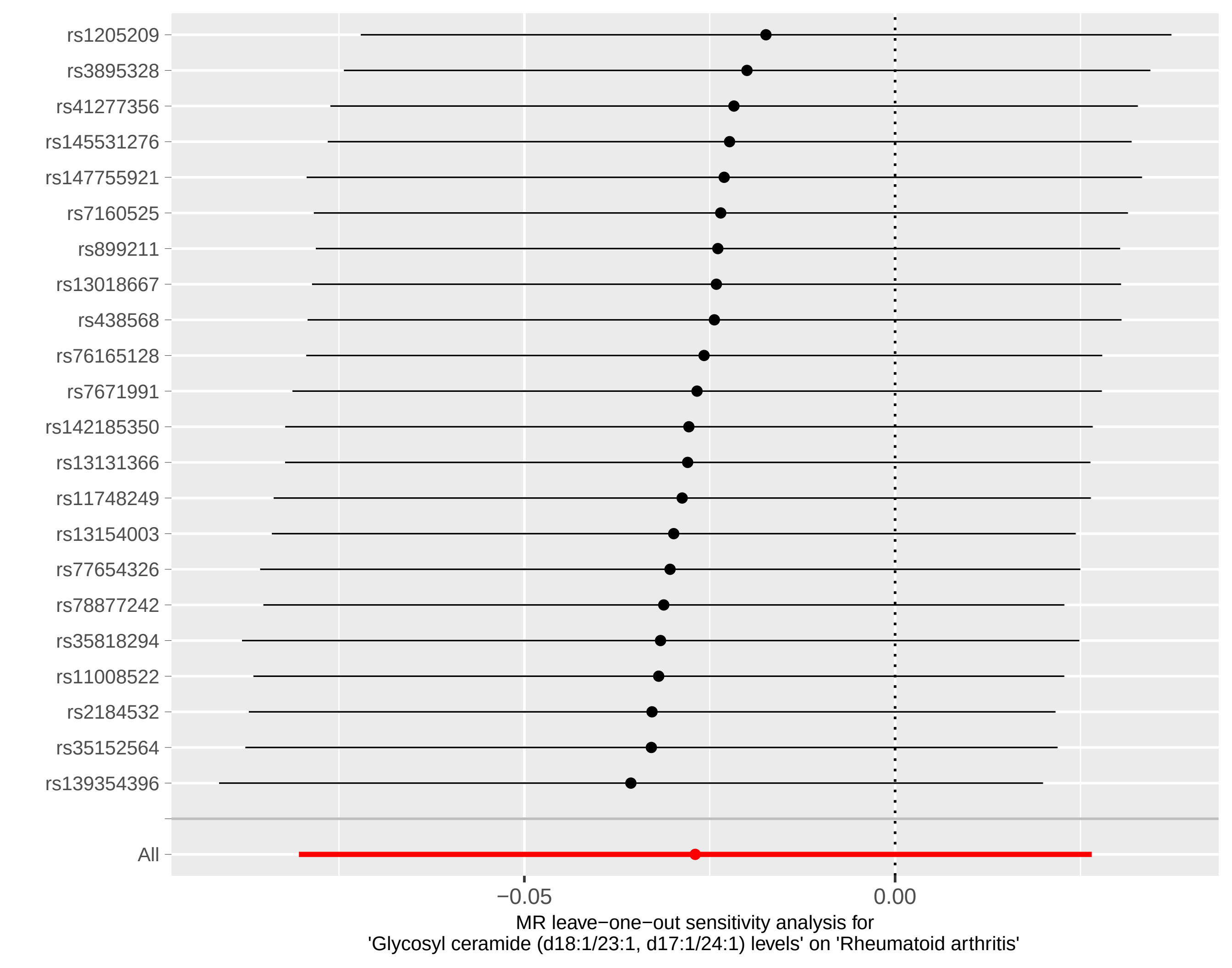
rs1205209
rs3895328
rs41277356
rs145531276
rs147755921
rs7160525
rs899211
rs13018667
rs438568
rs76165128
rs7671991
rs142185350
rs13131366
rs11748249
rs13154003
rs77654326
rs78877242
rs35818294
rs11008522
rs2184532
rs35152564
rs139354396
All
−0.05
0.00
MR leave−one−out sensitivity analysis for
'Glycosyl ceramide (d18:1/23:1, d17:1/24:1) levels' on 'Rheumatoid arthritis'

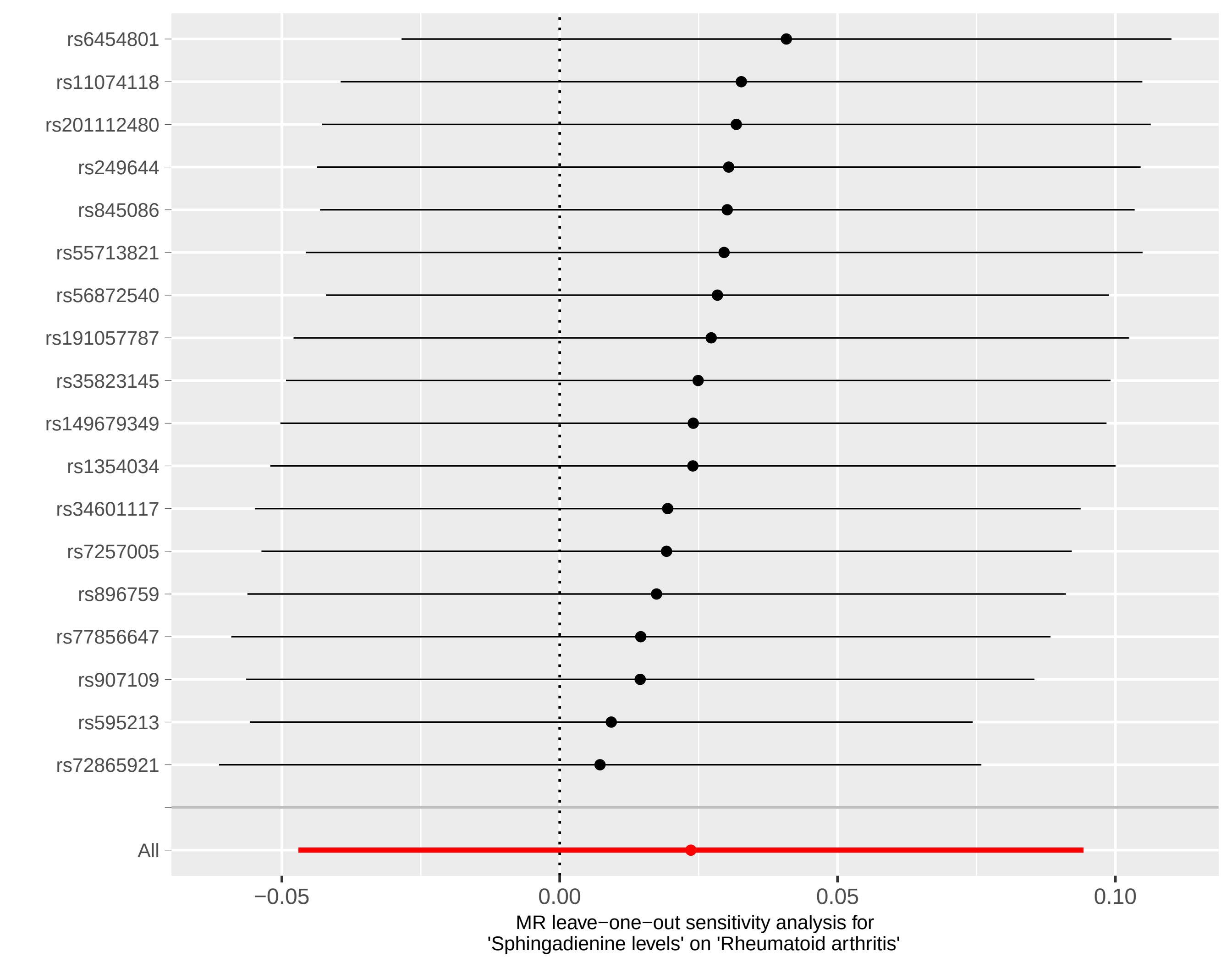
rs6454801
rs11074118
rs201112480
rs249644
rs845086
rs55713821
rs56872540
rs191057787
rs35823145
rs149679349
rs1354034
rs34601117
rs7257005
rs896759
rs77856647
rs907109
rs595213
rs72865921
All
−0.05
0.00
0.05
0.10
MR leave−one−out sensitivity analysis for
'Sphingadienine levels' on 'Rheumatoid arthritis'

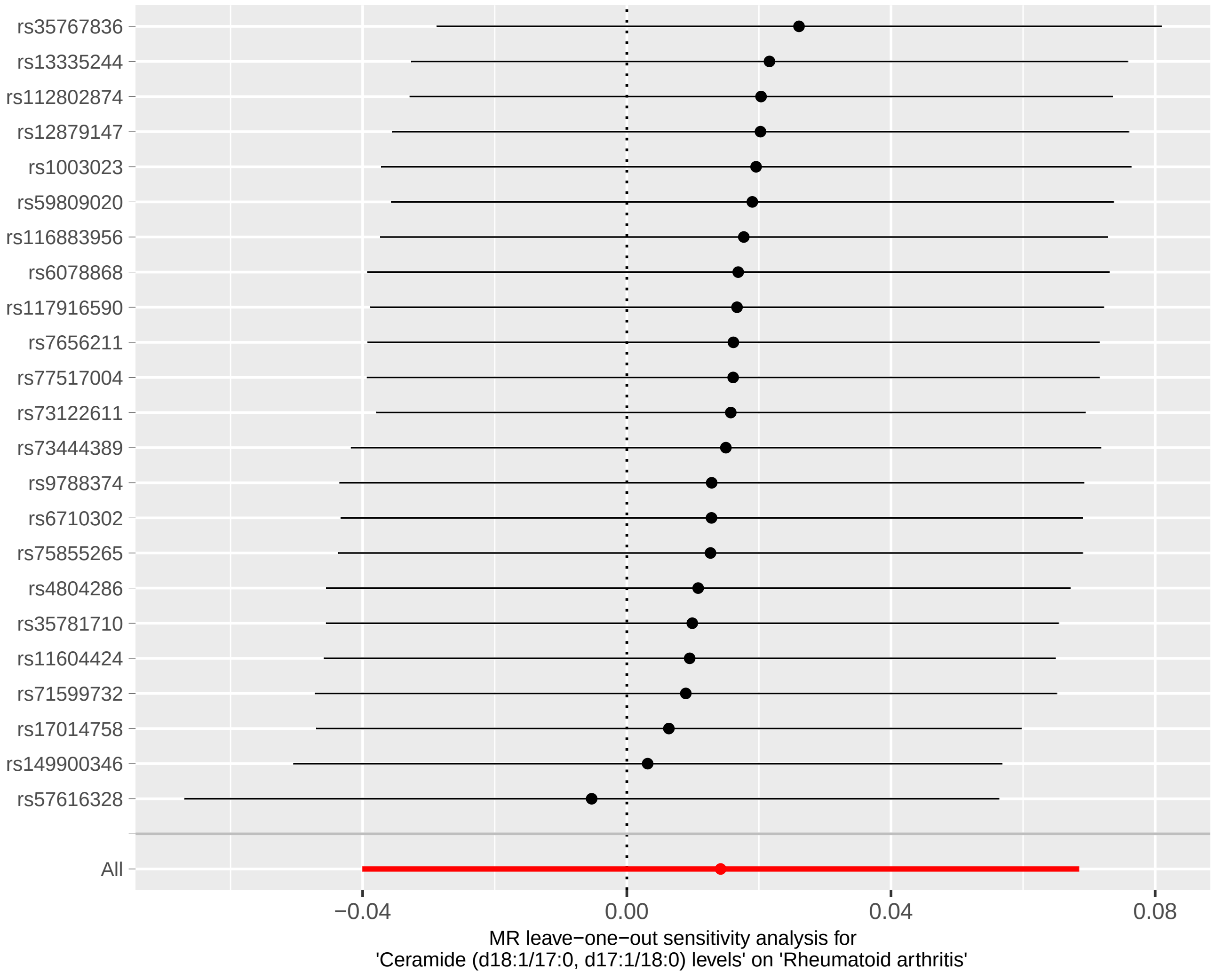
rs35767836
rs13335244
rs112802874
rs12879147
rs1003023
rs59809020
rs116883956
rs6078868
rs117916590
rs7656211
rs77517004
rs73122611
rs73444389
rs9788374
rs6710302
rs75855265
rs4804286
rs35781710
rs11604424
rs71599732
rs17014758
rs149900346
rs57616328
All
−0.04
0.00
0.04
0.08
MR leave−one−out sensitivity analysis for
'Ceramide (d18:1/17:0, d17:1/18:0) levels' on 'Rheumatoid arthritis'

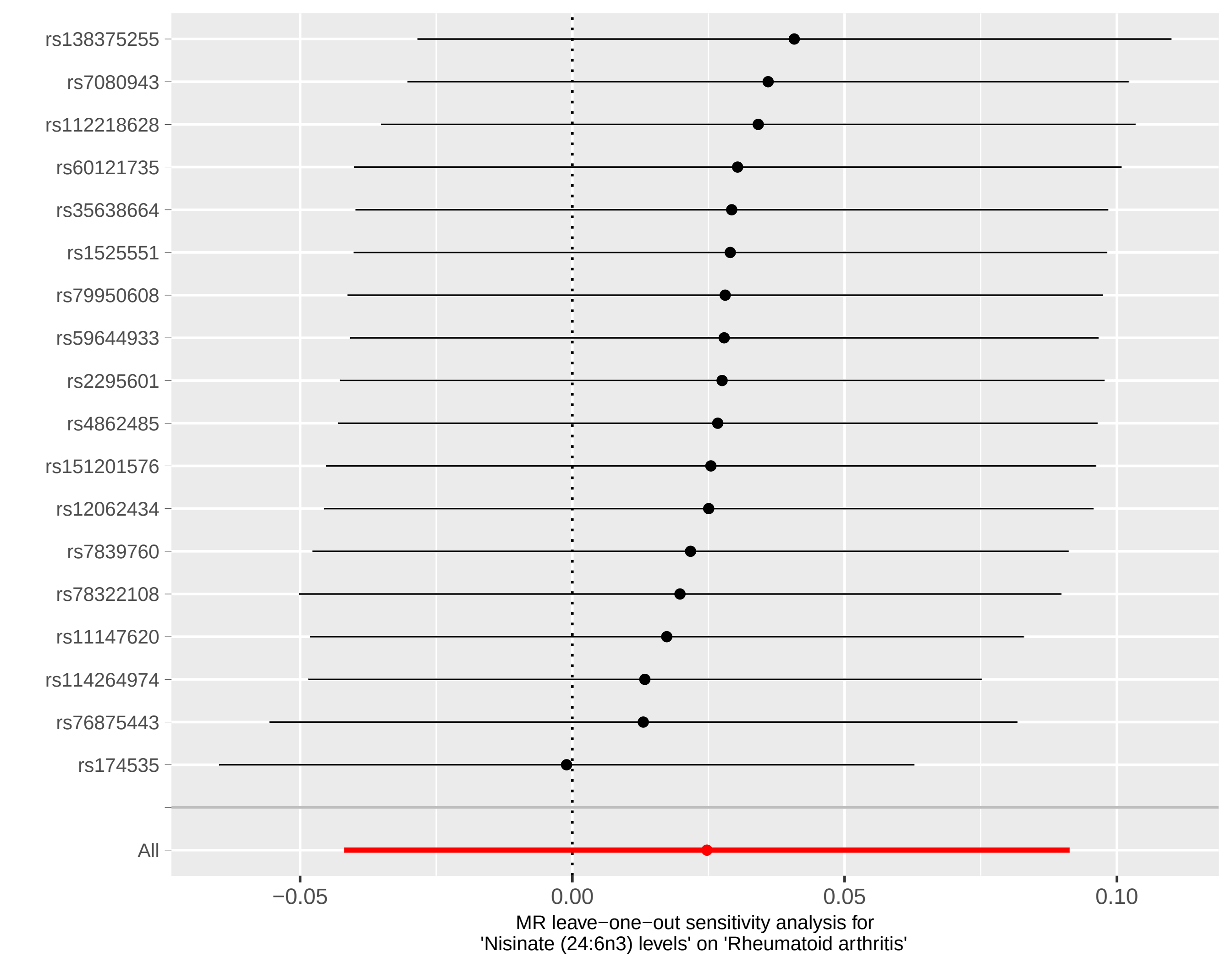
rs138375255
rs7080943
rs112218628
rs60121735
rs35638664
rs1525551
rs79950608
rs59644933
rs2295601
rs4862485
rs151201576
rs12062434
rs7839760
rs78322108
rs11147620
rs114264974
rs76875443
rs174535
All
−0.05
0.00
0.05
0.10
MR leave−one−out sensitivity analysis for
'Nisinate (24:6n3) levels' on 'Rheumatoid arthritis'

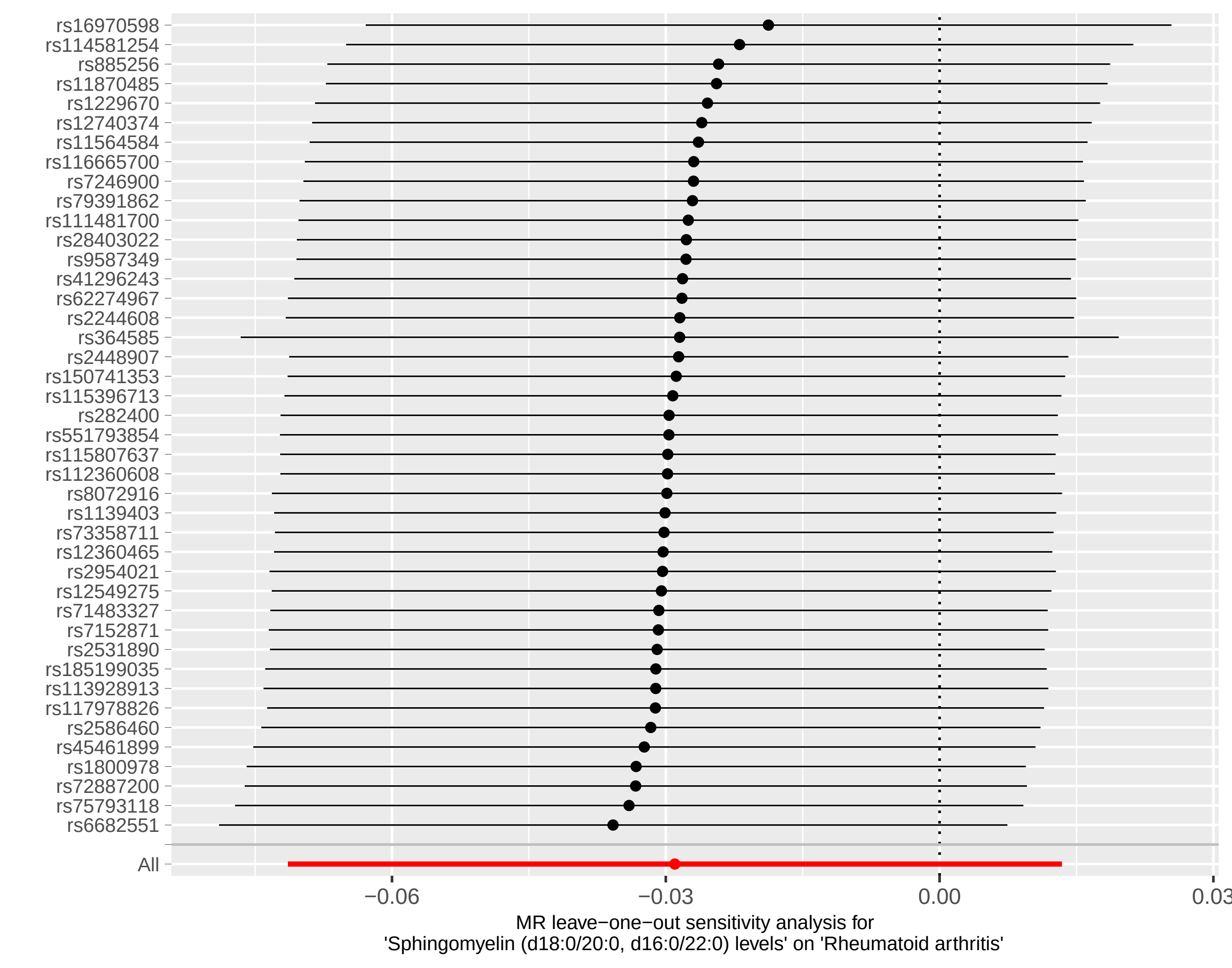

rs16970598
rs114581254
rs885256
rs11870485
rs1229670
rs12740374
rs11564584
rs116665700
rs7246900
rs79391862
rs111481700
rs28403022
rs9587349
rs41296243
rs62274967
rs2244608
rs364585
rs2448907
rs150741353
rs115396713
rs282400
rs551793854
rs115807637
rs112360608
rs8072916
rs1139403
rs73358711
rs12360465
rs2954021
rs12549275
rs71483327
rs7152871
rs2531890
rs185199035
rs113928913
rs117978826
rs2586460
rs45461899
rs1800978
rs72887200
rs75793118
rs6682551
All
−0.06
−0.03
0.00
0.03
MR leave−one−out sensitivity analysis for
'Sphingomyelin (d18:0/20:0, d16:0/22:0) levels' on 'Rheumatoid arthritis'

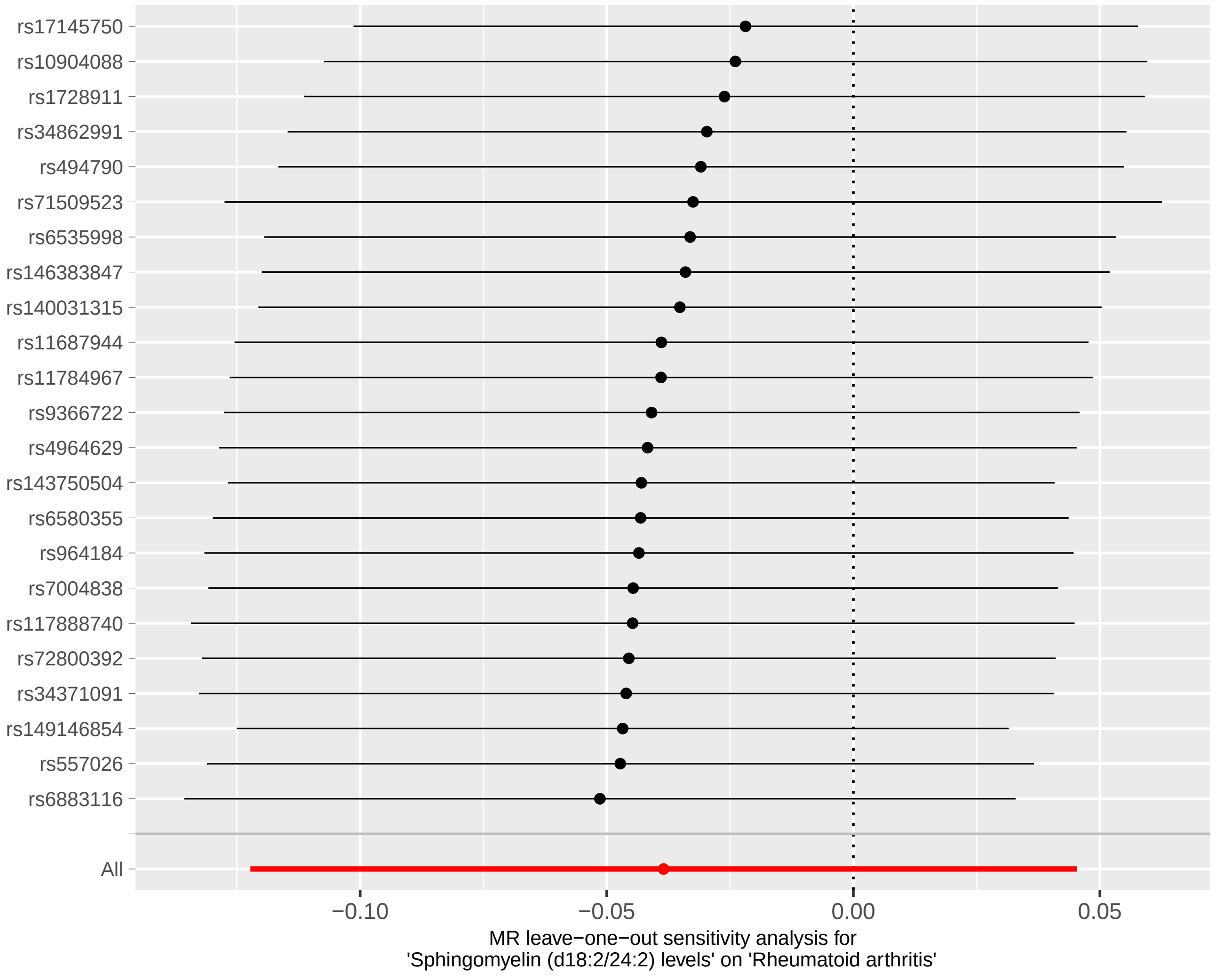
rs17145750
rs10904088
rs1728911
rs34862991
rs494790
rs71509523
rs6535998
rs146383847
rs140031315
rs11687944
rs11784967
rs9366722
rs4964629
rs143750504
rs6580355
rs964184
rs7004838
rs117888740
rs72800392
rs34371091
rs149146854
rs557026
rs6883116
All
−0.10
−0.05
0.00
0.05
MR leave−one−out sensitivity analysis for
'Sphingomyelin (d18:2/24:2) levels' on 'Rheumatoid arthritis'

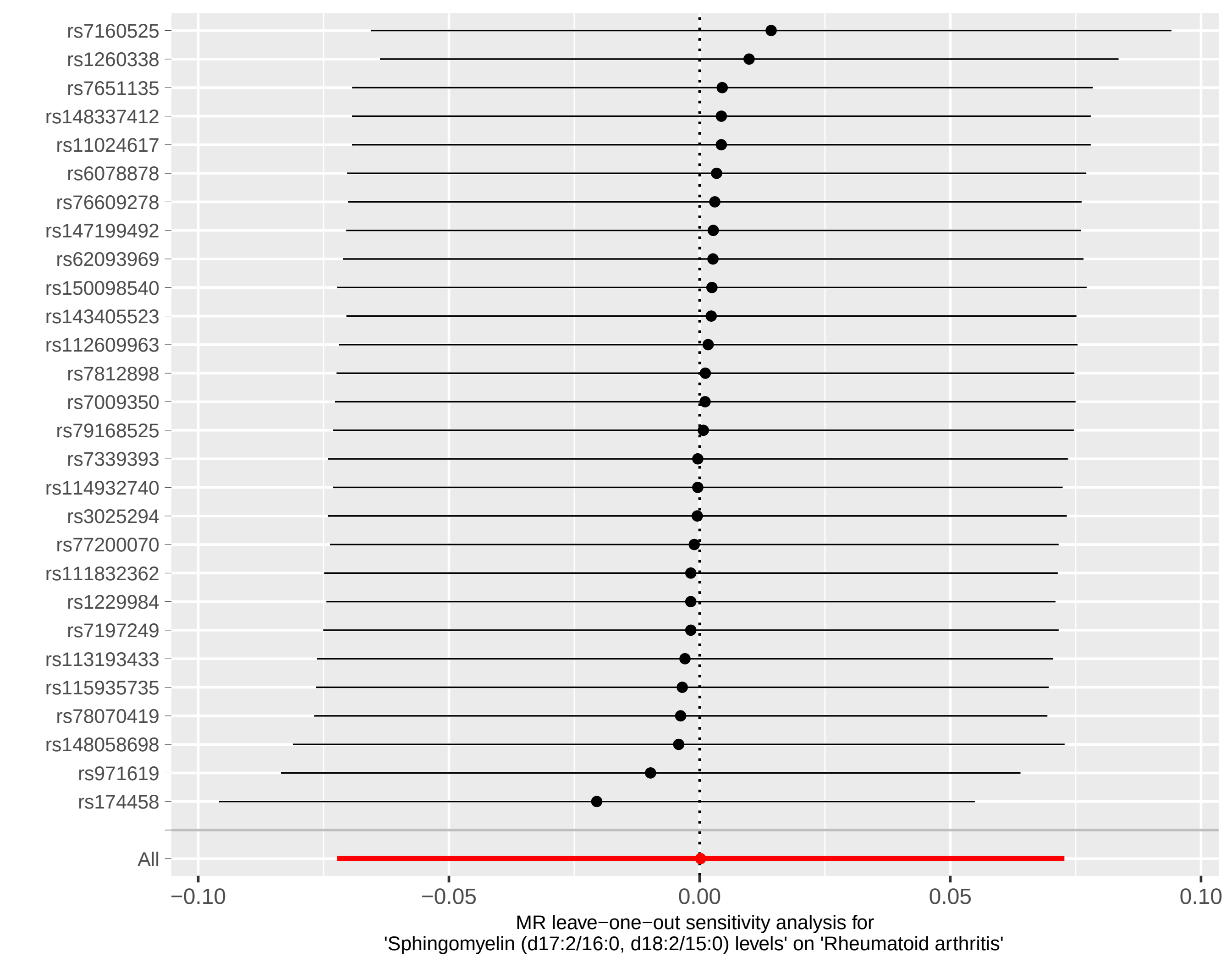
rs7160525
rs1260338
rs7651135
rs148337412
rs11024617
rs6078878
rs76609278
rs147199492
rs62093969
rs150098540
rs143405523
rs112609963
rs7812898
rs7009350
rs79168525
rs7339393
rs114932740
rs3025294
rs77200070
rs111832362
rs1229984
rs7197249
rs113193433
rs115935735
rs78070419
rs148058698
rs971619
rs174458
All
−0.10
−0.05
0.00
0.05
0.10
MR leave−one−out sensitivity analysis for
'Sphingomyelin (d17:2/16:0, d18:2/15:0) levels' on 'Rheumatoid arthritis'

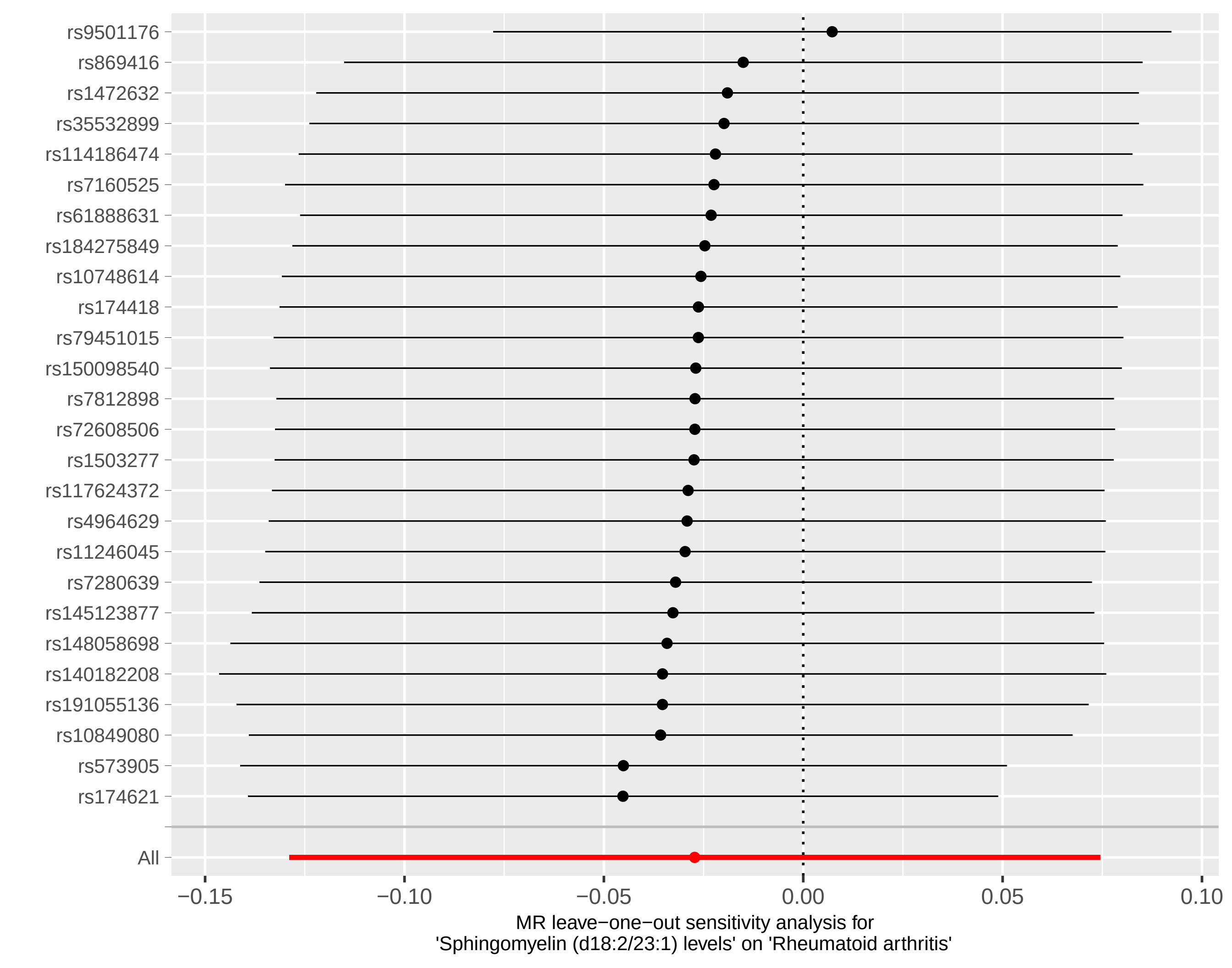

rs9501176
rs869416
rs1472632
rs35532899
rs114186474
rs7160525
rs61888631
rs184275849
rs10748614
rs174418
rs79451015
rs150098540
rs7812898
rs72608506
rs1503277
rs117624372
rs4964629
rs11246045
rs7280639
rs145123877
rs148058698
rs140182208
rs191055136
rs10849080
rs573905
rs174621
All
−0.15
−0.10
−0.05
0.00
0.05
0.10
MR leave−one−out sensitivity analysis for
'Sphingomyelin (d18:2/23:1) levels' on 'Rheumatoid arthritis'

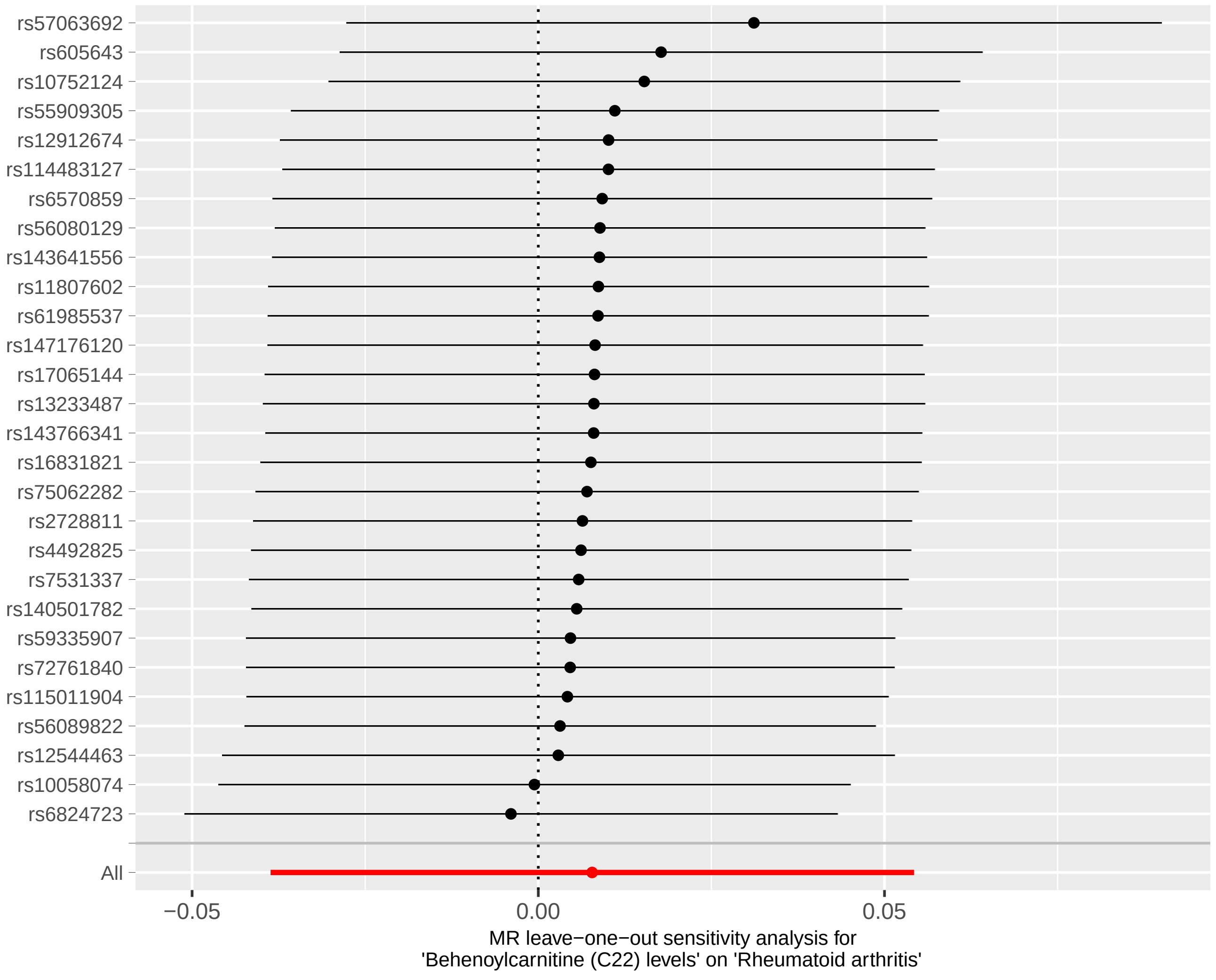
rs57063692
rs605643
rs10752124
rs55909305
rs12912674
rs114483127
rs6570859
rs56080129
rs143641556
rs11807602
rs61985537
rs147176120
rs17065144
rs13233487
rs143766341
rs16831821
rs75062282
rs2728811
rs4492825
rs7531337
rs140501782
rs59335907
rs72761840
rs115011904
rs56089822
rs12544463
rs10058074
rs6824723
All
−0.05
0.00
0.05
MR leave−one−out sensitivity analysis for
'Behenoylcarnitine (C22) levels' on 'Rheumatoid arthritis'

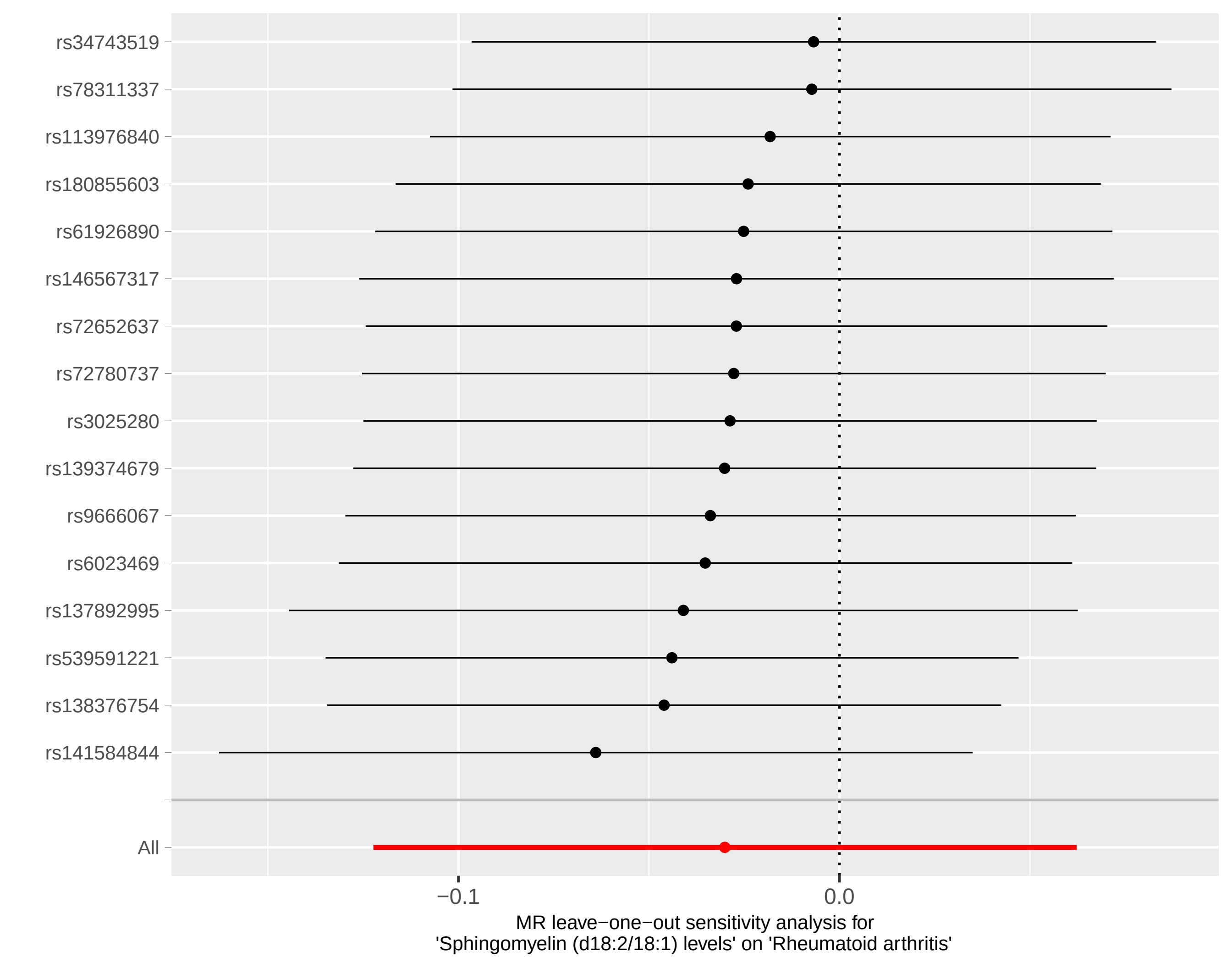
rs34743519
rs78311337
rs113976840
rs180855603
rs61926890
rs146567317
rs72652637
rs72780737
rs3025280
rs139374679
rs9666067
rs6023469
rs137892995
rs539591221
rs138376754
rs141584844
All
−0.1
0.0
MR leave−one−out sensitivity analysis for
'Sphingomyelin (d18:2/18:1) levels' on 'Rheumatoid arthritis'

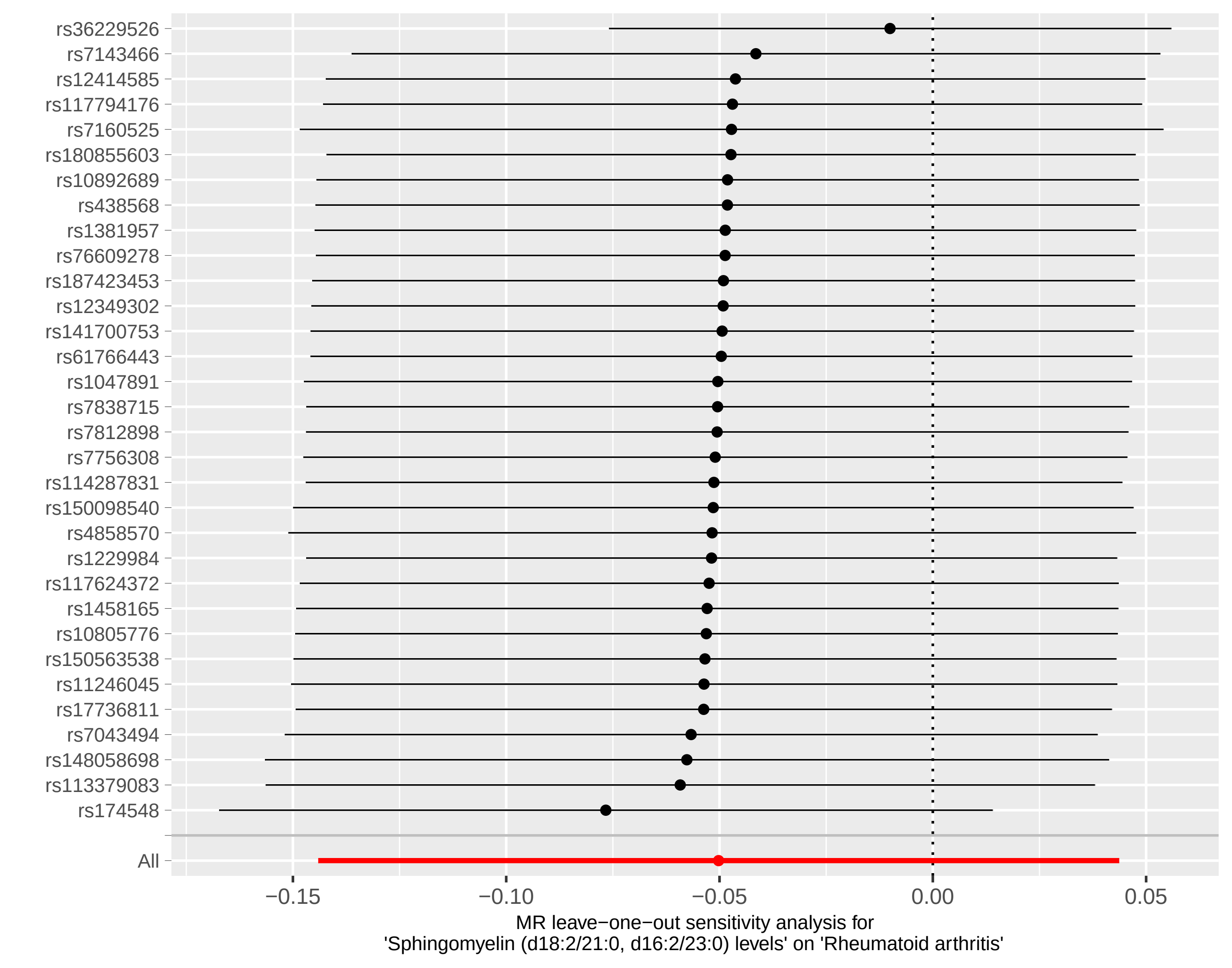
rs36229526
rs7143466
rs12414585
rs117794176
rs7160525
rs180855603
rs10892689
rs438568
rs1381957
rs76609278
rs187423453
rs12349302
rs141700753
rs61766443
rs1047891
rs7838715
rs7812898
rs7756308
rs114287831
rs150098540
rs4858570
rs1229984
rs117624372
rs1458165
rs10805776
rs150563538
rs11246045
rs17736811
rs7043494
rs148058698
rs113379083
rs174548
All
−0.15
−0.10
−0.05
0.00
0.05
MR leave−one−out sensitivity analysis for
'Sphingomyelin (d18:2/21:0, d16:2/23:0) levels' on 'Rheumatoid arthritis'

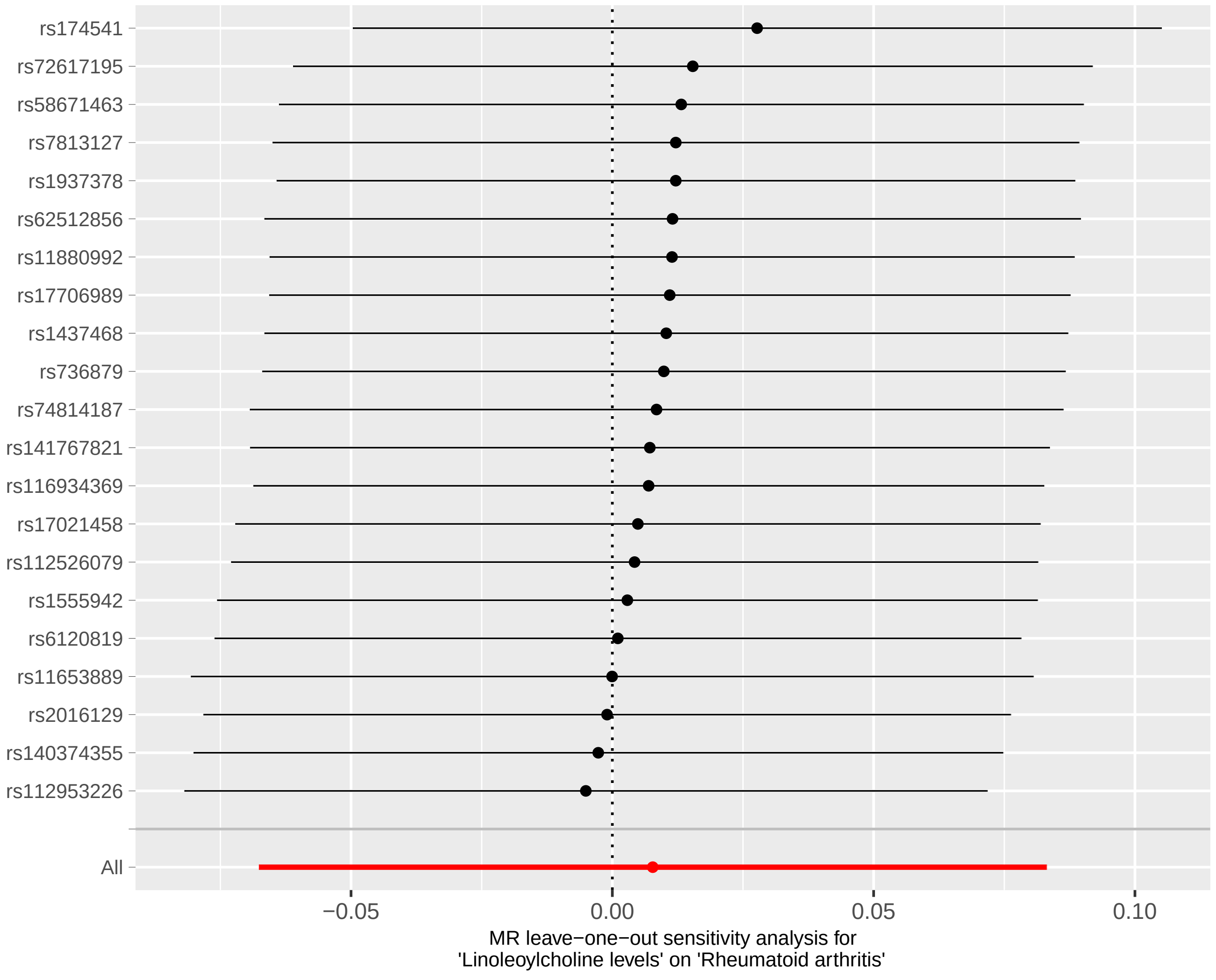
rs174541
rs72617195
rs58671463
rs7813127
rs1937378
rs62512856
rs11880992
rs17706989
rs1437468
rs736879
rs74814187
rs141767821
rs116934369
rs17021458
rs112526079
rs1555942
rs6120819
rs11653889
rs2016129
rs140374355
rs112953226
All
−0.05
0.00
0.05
0.10
MR leave−one−out sensitivity analysis for
'Linoleoylcholine levels' on 'Rheumatoid arthritis'

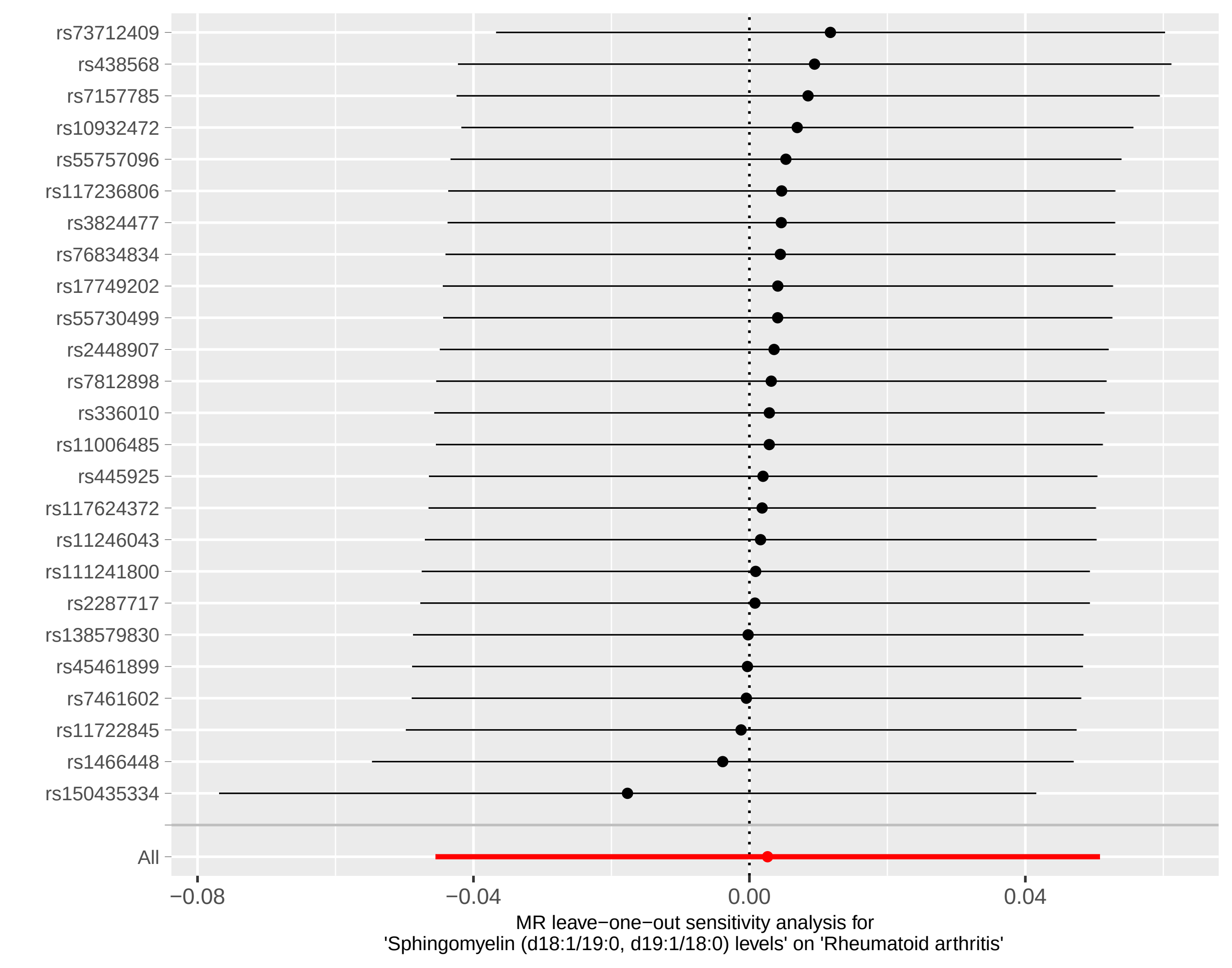
rs73712409
rs438568
rs7157785
rs10932472
rs55757096
rs117236806
rs3824477
rs76834834
rs17749202
rs55730499
rs2448907
rs7812898
rs336010
rs11006485
rs445925
rs117624372
rs11246043
rs111241800
rs2287717
rs138579830
rs45461899
rs7461602
rs11722845
rs1466448
rs150435334
All
−0.08
−0.04
0.00
0.04
MR leave−one−out sensitivity analysis for
'Sphingomyelin (d18:1/19:0, d19:1/18:0) levels' on 'Rheumatoid arthritis'

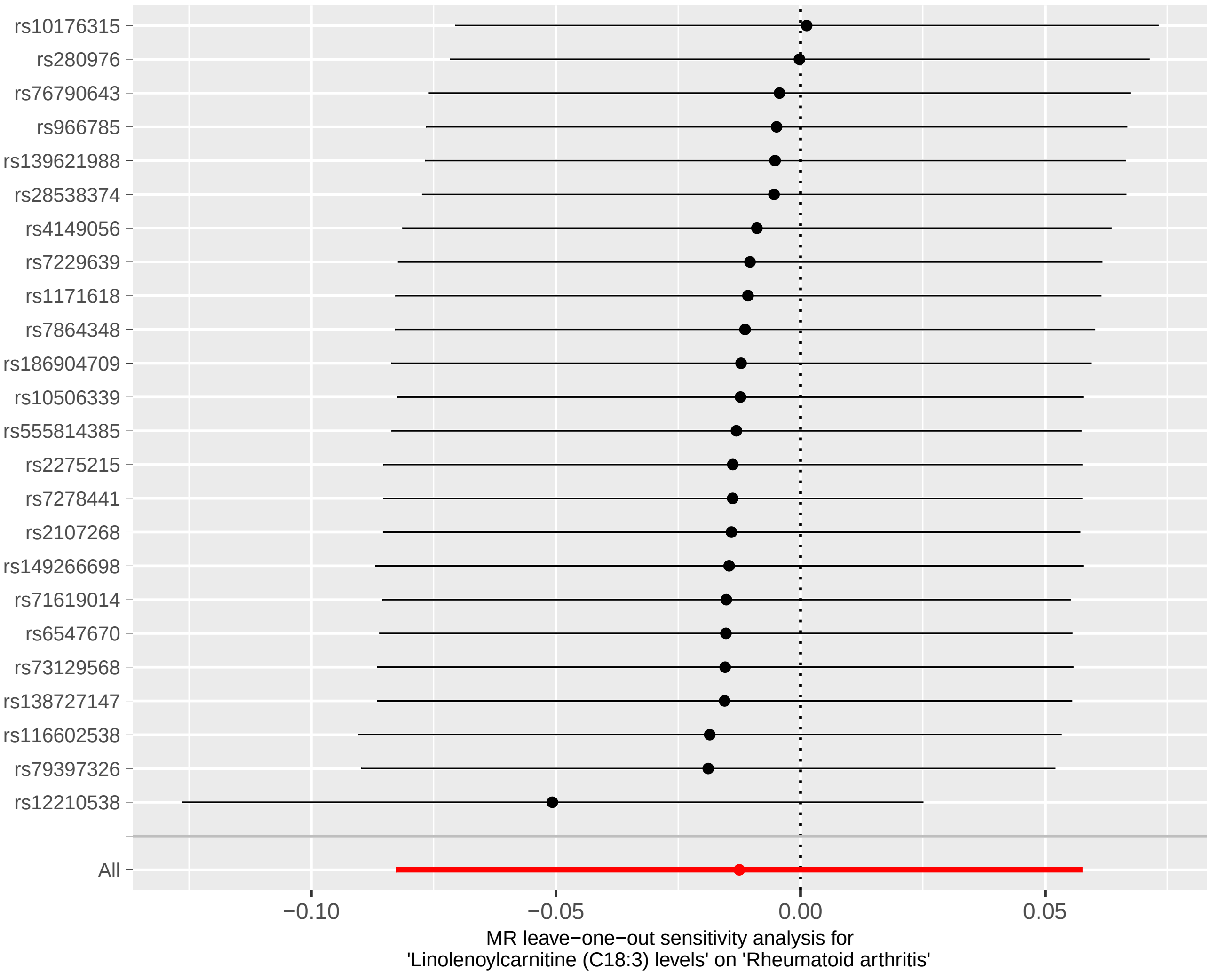
rs10176315
rs280976
rs76790643
rs966785
rs139621988
rs28538374
rs4149056
rs7229639
rs1171618
rs7864348
rs186904709
rs10506339
rs555814385
rs2275215
rs7278441
rs2107268
rs149266698
rs71619014
rs6547670
rs73129568
rs138727147
rs116602538
rs79397326
rs12210538
All
−0.10
−0.05
0.00
0.05
MR leave−one−out sensitivity analysis for
'Linolenoylcarnitine (C18:3) levels' on 'Rheumatoid arthritis'

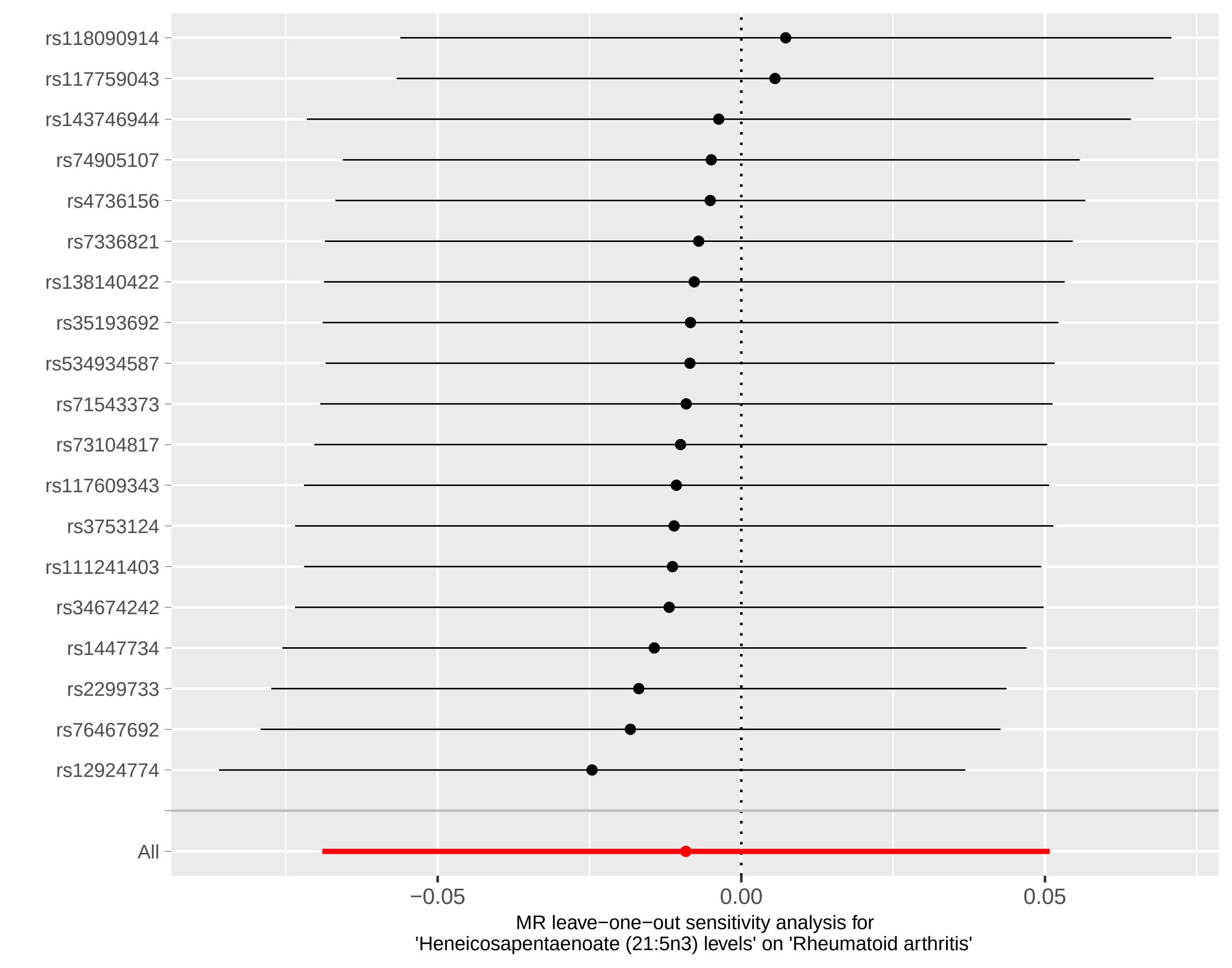
rs118090914
rs117759043
rs143746944
rs74905107
rs4736156
rs7336821
rs138140422
rs35193692
rs534934587
rs71543373
rs73104817
rs117609343
rs3753124
rs111241403
rs34674242
rs1447734
rs2299733
rs76467692
rs12924774
All
−0.05
0.00
0.05
MR leave−one−out sensitivity analysis for
'Heneicosapentaenoate (21:5n3) levels' on 'Rheumatoid arthritis'

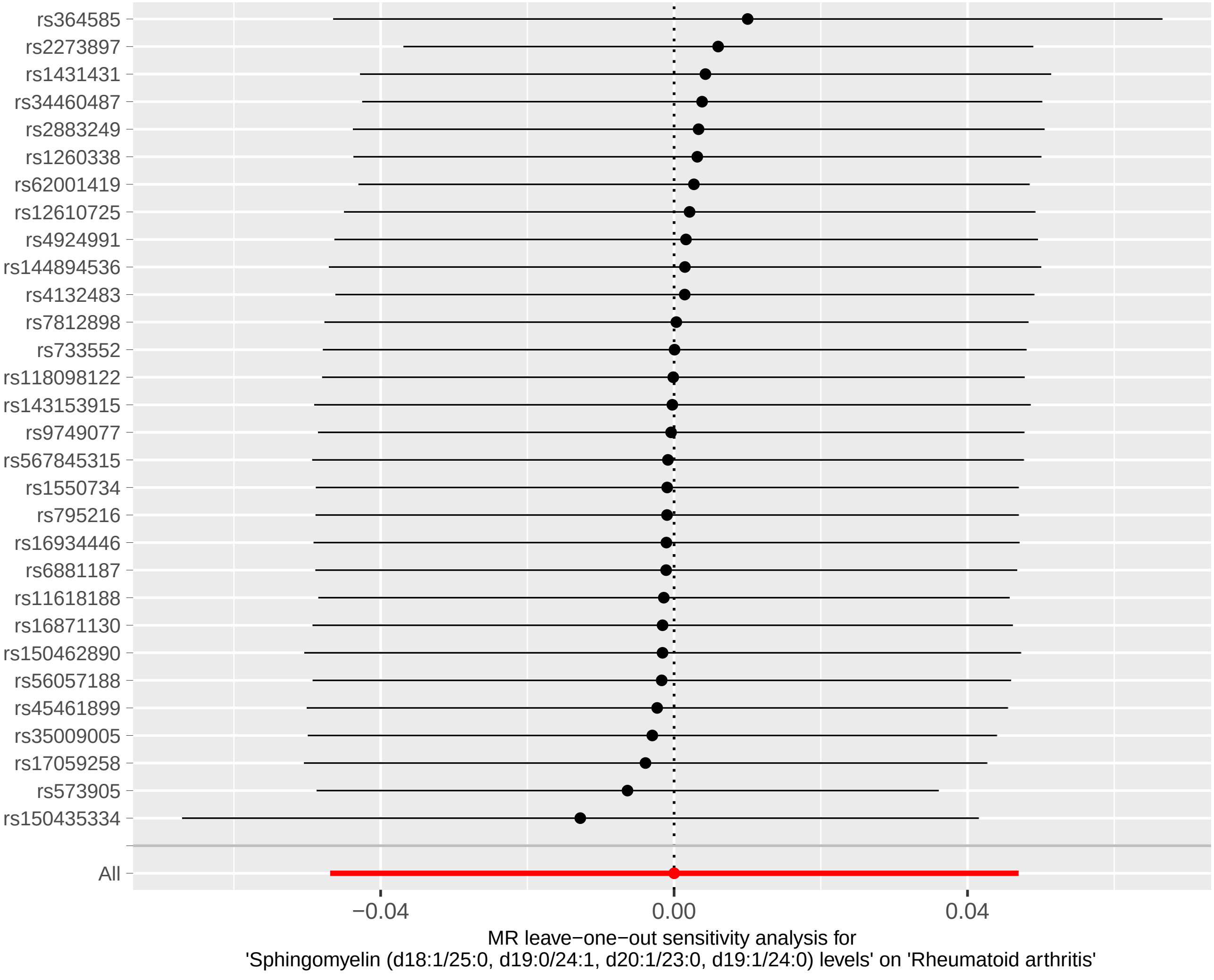
rs364585
rs2273897
rs1431431
rs34460487
rs2883249
rs1260338
rs62001419
rs12610725
rs4924991
rs144894536
rs4132483
rs7812898
rs733552
rs118098122
rs143153915
rs9749077
rs567845315
rs1550734
rs795216
rs16934446
rs6881187
rs11618188
rs16871130
rs150462890
rs56057188
rs45461899
rs35009005
rs17059258
rs573905
rs150435334
All
−0.04
0.00
0.04
MR leave−one−out sensitivity analysis for
'Sphingomyelin (d18:1/25:0, d19:0/24:1, d20:1/23:0, d19:1/24:0) levels' on 'Rheumatoid arthritis'

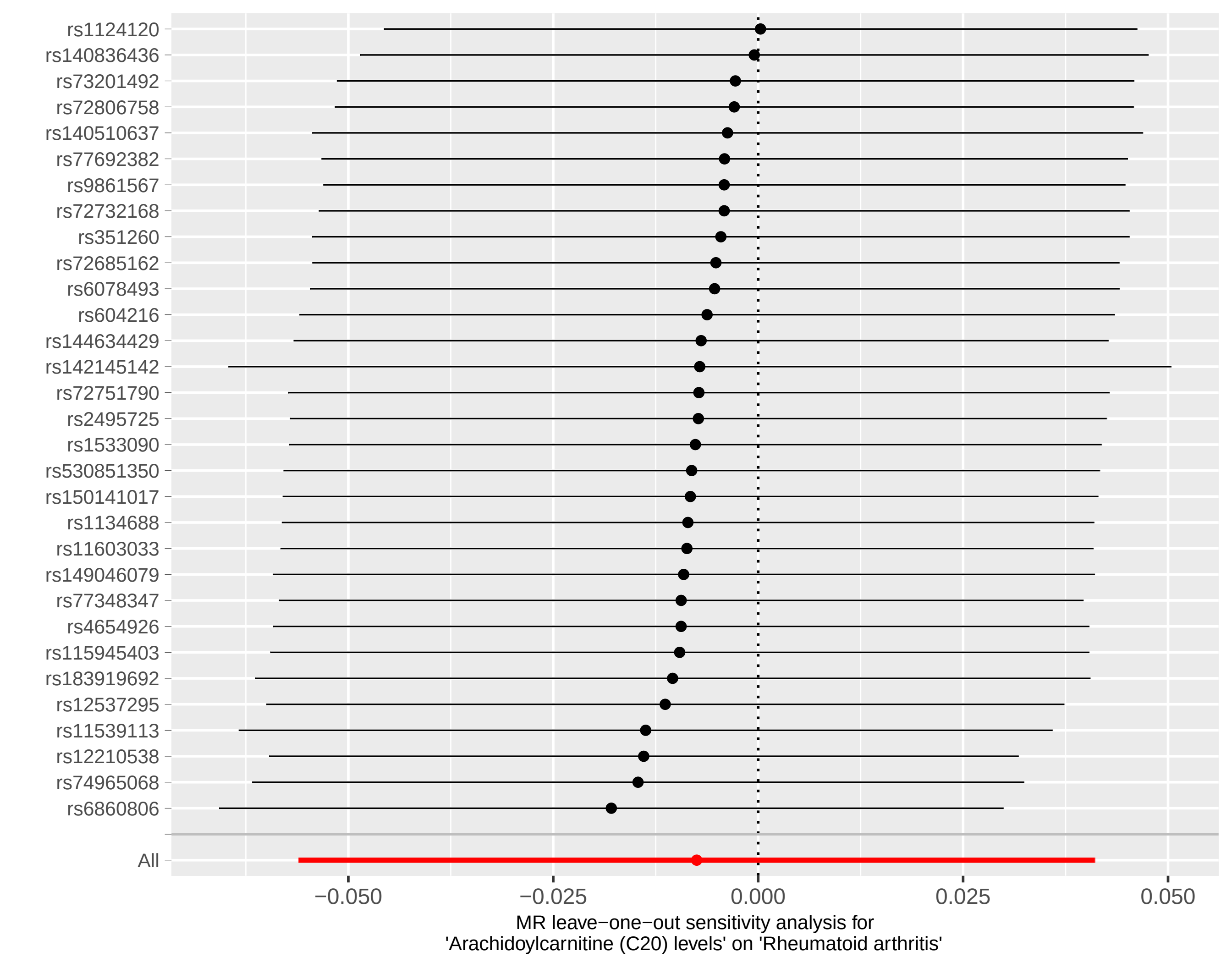
rs1124120
rs140836436
rs73201492
rs72806758
rs140510637
rs77692382
rs9861567
rs72732168
rs351260
rs72685162
rs6078493
rs604216
rs144634429
rs142145142
rs72751790
rs2495725
rs1533090
rs530851350
rs150141017
rs1134688
rs11603033
rs149046079
rs77348347
rs4654926
rs115945403
rs183919692
rs12537295
rs11539113
rs12210538
rs74965068
rs6860806
All
−0.050
−0.025
0.000
0.025
0.050
MR leave−one−out sensitivity analysis for
'Arachidoylcarnitine (C20) levels' on 'Rheumatoid arthritis'

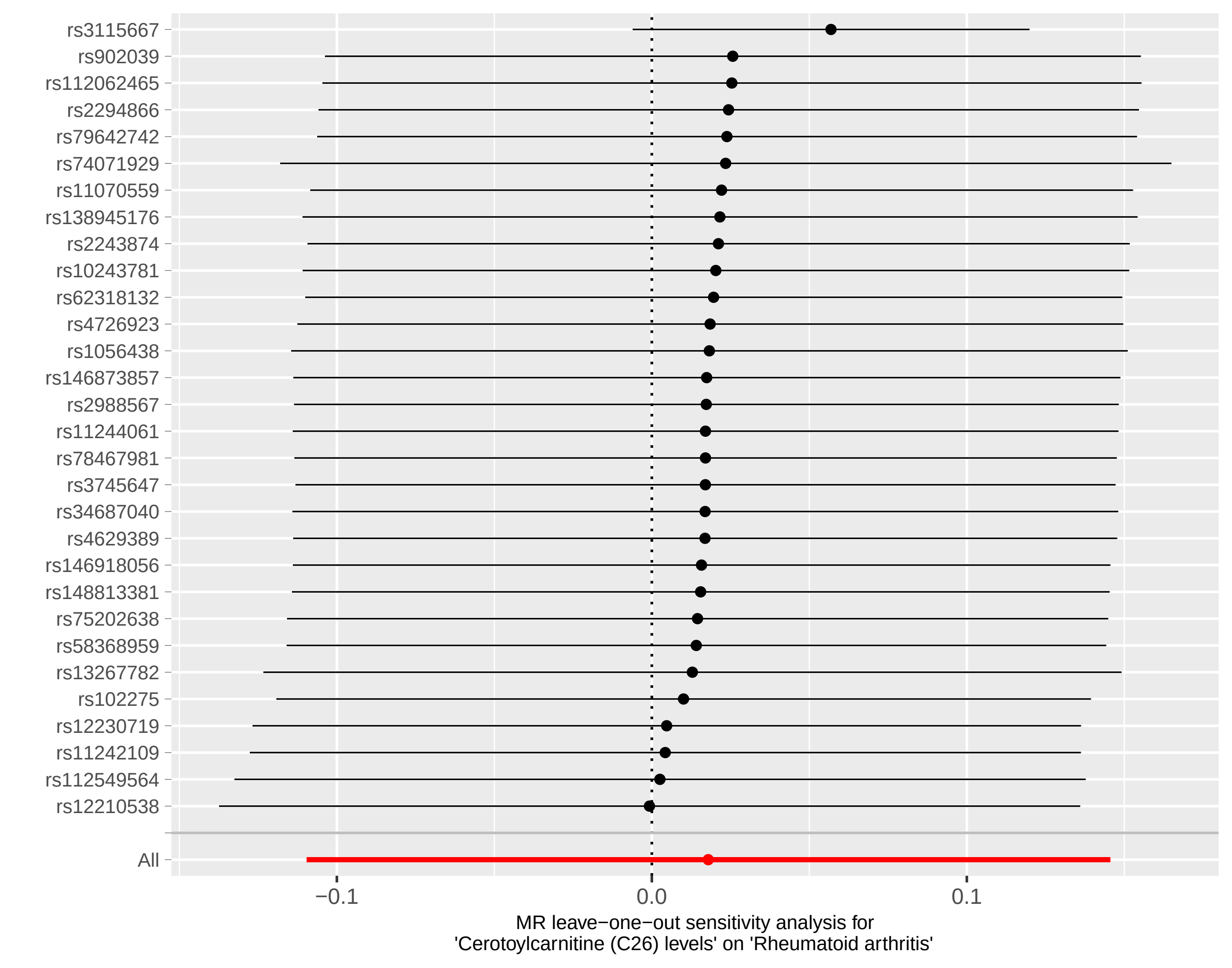

rs3115667
rs902039
rs112062465
rs2294866
rs79642742
rs74071929
rs11070559
rs138945176
rs2243874
rs10243781
rs62318132
rs4726923
rs1056438
rs146873857
rs2988567
rs11244061
rs78467981
rs3745647
rs34687040
rs4629389
rs146918056
rs148813381
rs75202638
rs58368959
rs13267782
rs102275
rs12230719
rs11242109
rs112549564
rs12210538
All
−0.1
0.0
0.1
MR leave−one−out sensitivity analysis for
'Cerotoylcarnitine (C26) levels' on 'Rheumatoid arthritis'

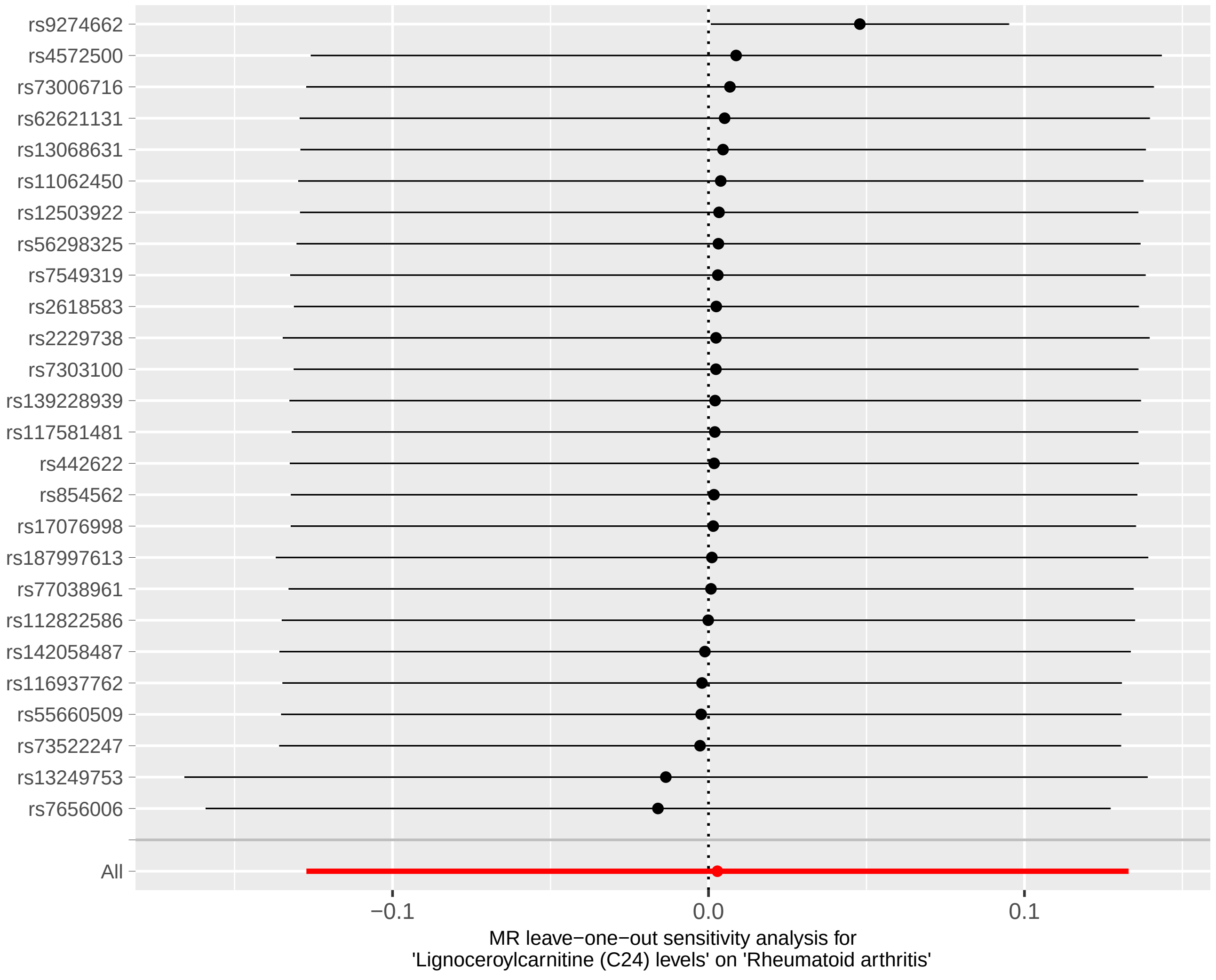
rs9274662
rs4572500
rs73006716
rs62621131
rs13068631
rs11062450
rs12503922
rs56298325
rs7549319
rs2618583
rs2229738
rs7303100
rs139228939
rs117581481
rs442622
rs854562
rs17076998
rs187997613
rs77038961
rs112822586
rs142058487
rs116937762
rs55660509
rs73522247
rs13249753
rs7656006
All
−0.1
0.0
0.1
MR leave−one−out sensitivity analysis for
'Lignoceroylcarnitine (C24) levels' on 'Rheumatoid arthritis'

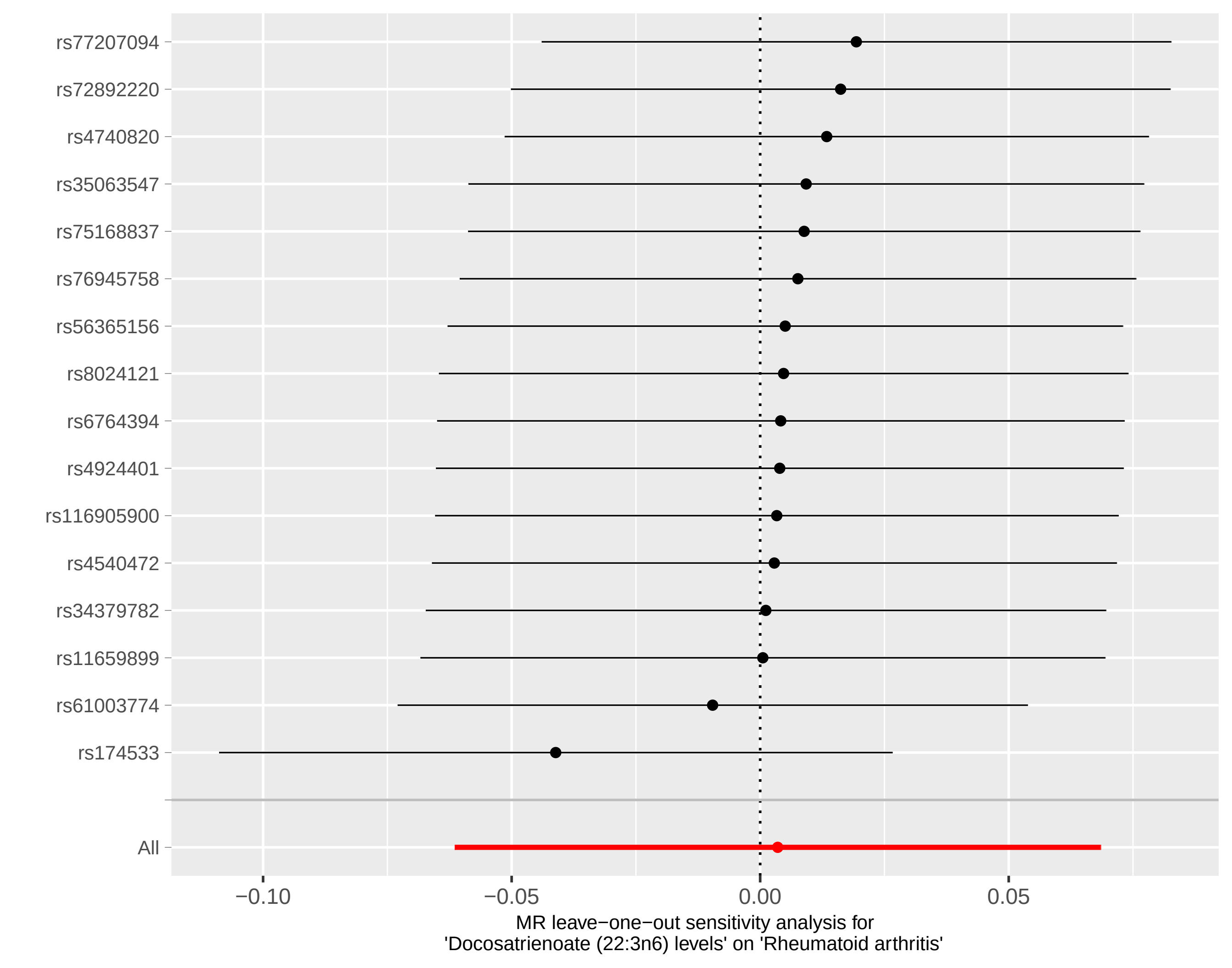
rs77207094
rs72892220
rs4740820
rs35063547
rs75168837
rs76945758
rs56365156
rs8024121
rs6764394
rs4924401
rs116905900
rs4540472
rs34379782
rs11659899
rs61003774
rs174533
All
−0.10
−0.05
0.00
0.05
MR leave−one−out sensitivity analysis for
'Docosatrienoate (22:3n6) levels' on 'Rheumatoid arthritis'

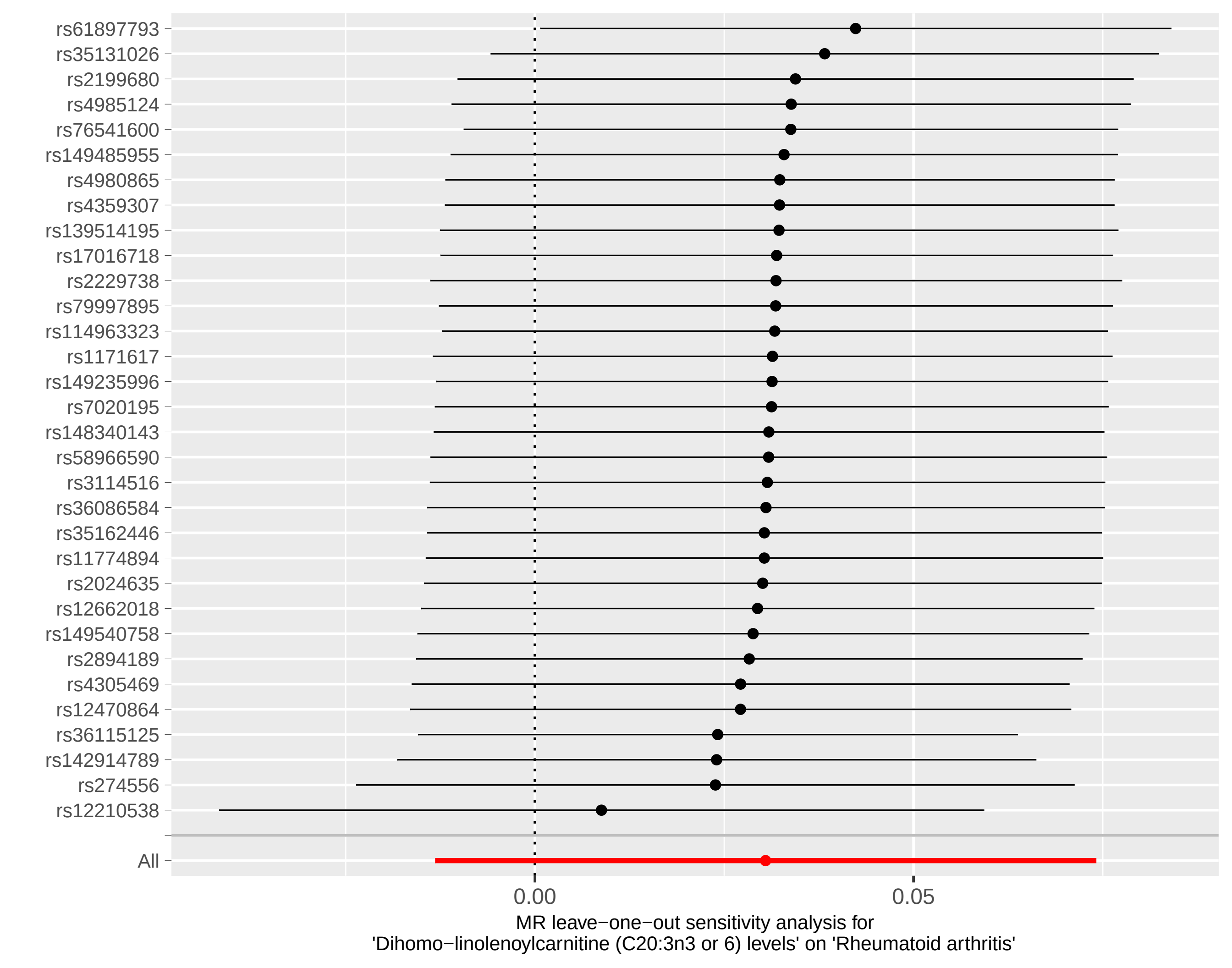
rs61897793
rs35131026
rs2199680
rs4985124
rs76541600
rs149485955
rs4980865
rs4359307
rs139514195
rs17016718
rs2229738
rs79997895
rs114963323
rs1171617
rs149235996
rs7020195
rs148340143
rs58966590
rs3114516
rs36086584
rs35162446
rs11774894
rs2024635
rs12662018
rs149540758
rs2894189
rs4305469
rs12470864
rs36115125
rs142914789
rs274556
rs12210538
All
0.00
0.05
MR leave−one−out sensitivity analysis for
'Dihomo−linolenoylcarnitine (C20:3n3 or 6) levels' on 'Rheumatoid arthritis'

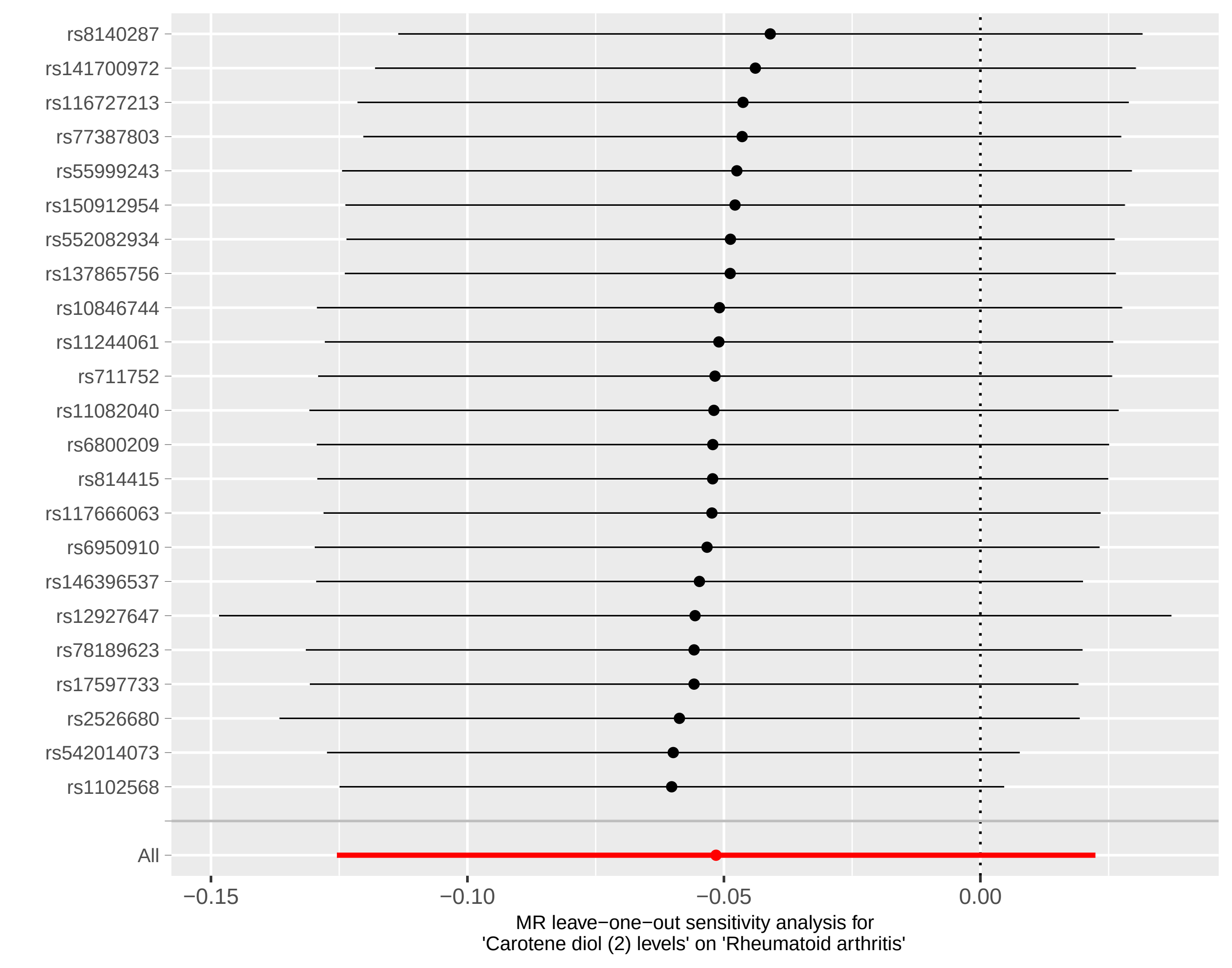
rs8140287
rs141700972
rs116727213
rs77387803
rs55999243
rs150912954
rs552082934
rs137865756
rs10846744
rs11244061
rs711752
rs11082040
rs6800209
rs814415
rs117666063
rs6950910
rs146396537
rs12927647
rs78189623
rs17597733
rs2526680
rs542014073
rs1102568
All
−0.15
−0.10
−0.05
0.00
MR leave−one−out sensitivity analysis for
'Carotene diol (2) levels' on 'Rheumatoid arthritis'

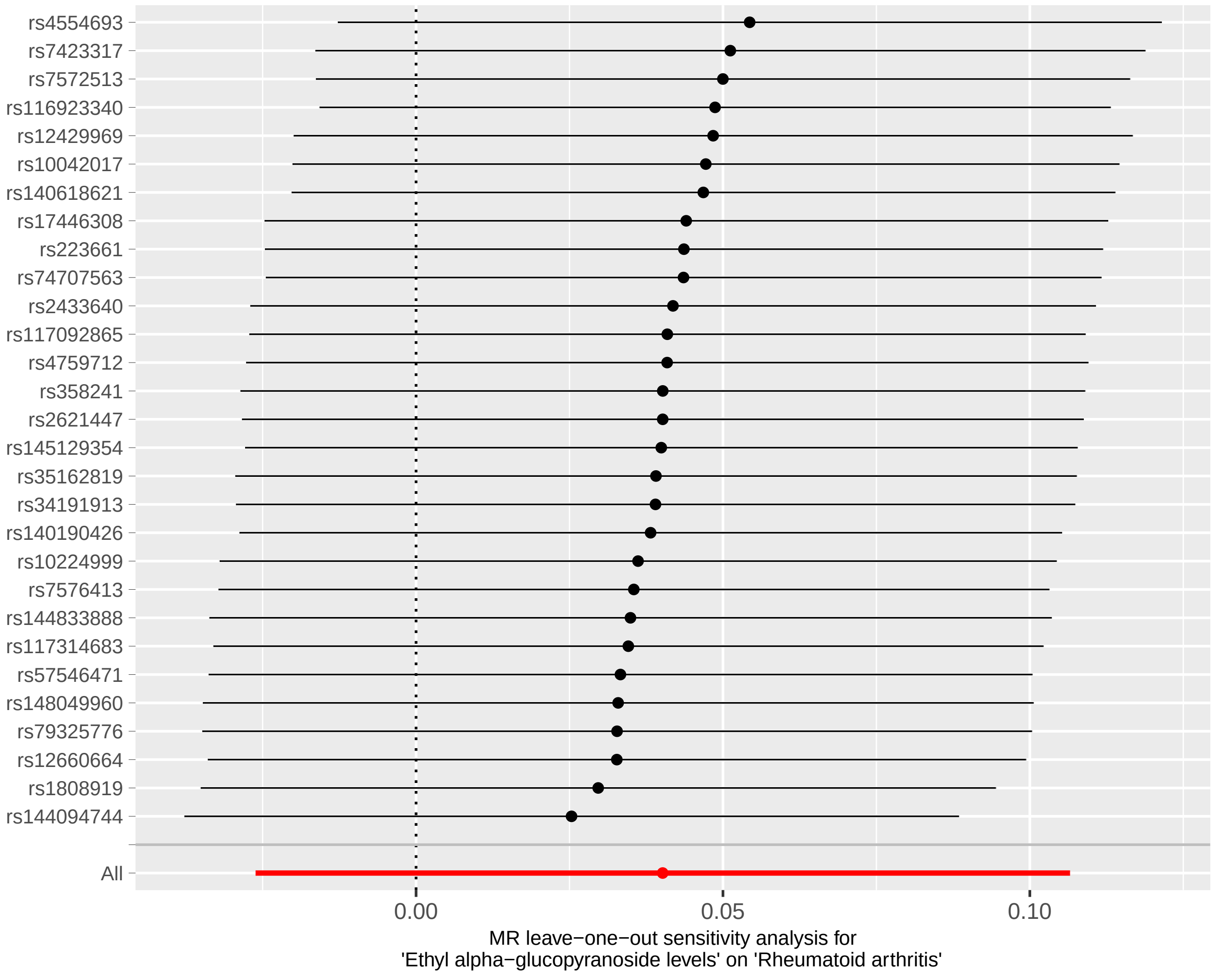
rs4554693
rs7423317
rs7572513
rs116923340
rs12429969
rs10042017
rs140618621
rs17446308
rs223661
rs74707563
rs2433640
rs117092865
rs4759712
rs358241
rs2621447
rs145129354
rs35162819
rs34191913
rs140190426
rs10224999
rs7576413
rs144833888
rs117314683
rs57546471
rs148049960
rs79325776
rs12660664
rs1808919
rs144094744
All
0.00
0.05
0.10
MR leave−one−out sensitivity analysis for
'Ethyl alpha−glucopyranoside levels' on 'Rheumatoid arthritis'

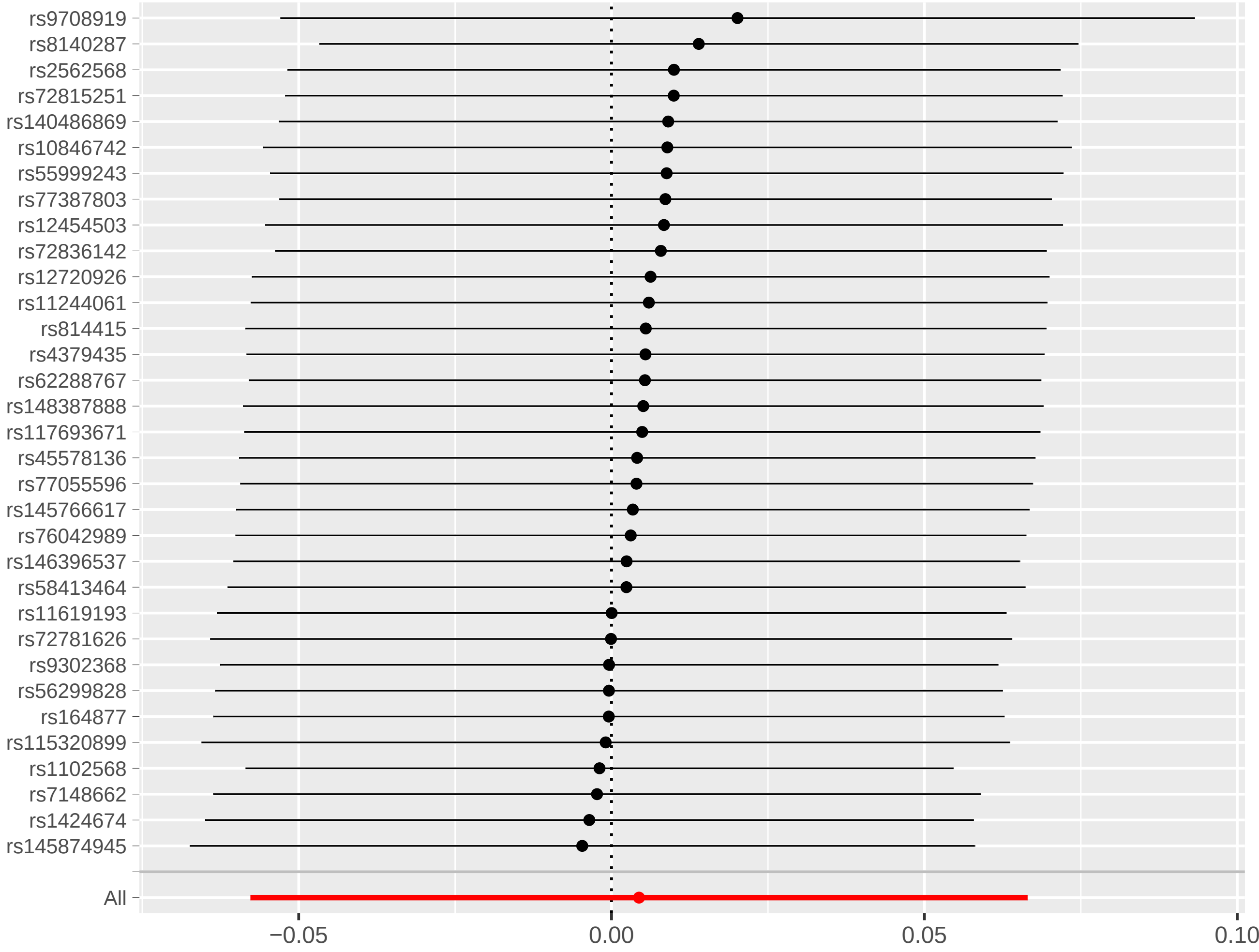

MR leave−one−out sensitivity analysis for
'Carotene diol (1) levels' on 'Rheumatoid arthritis'

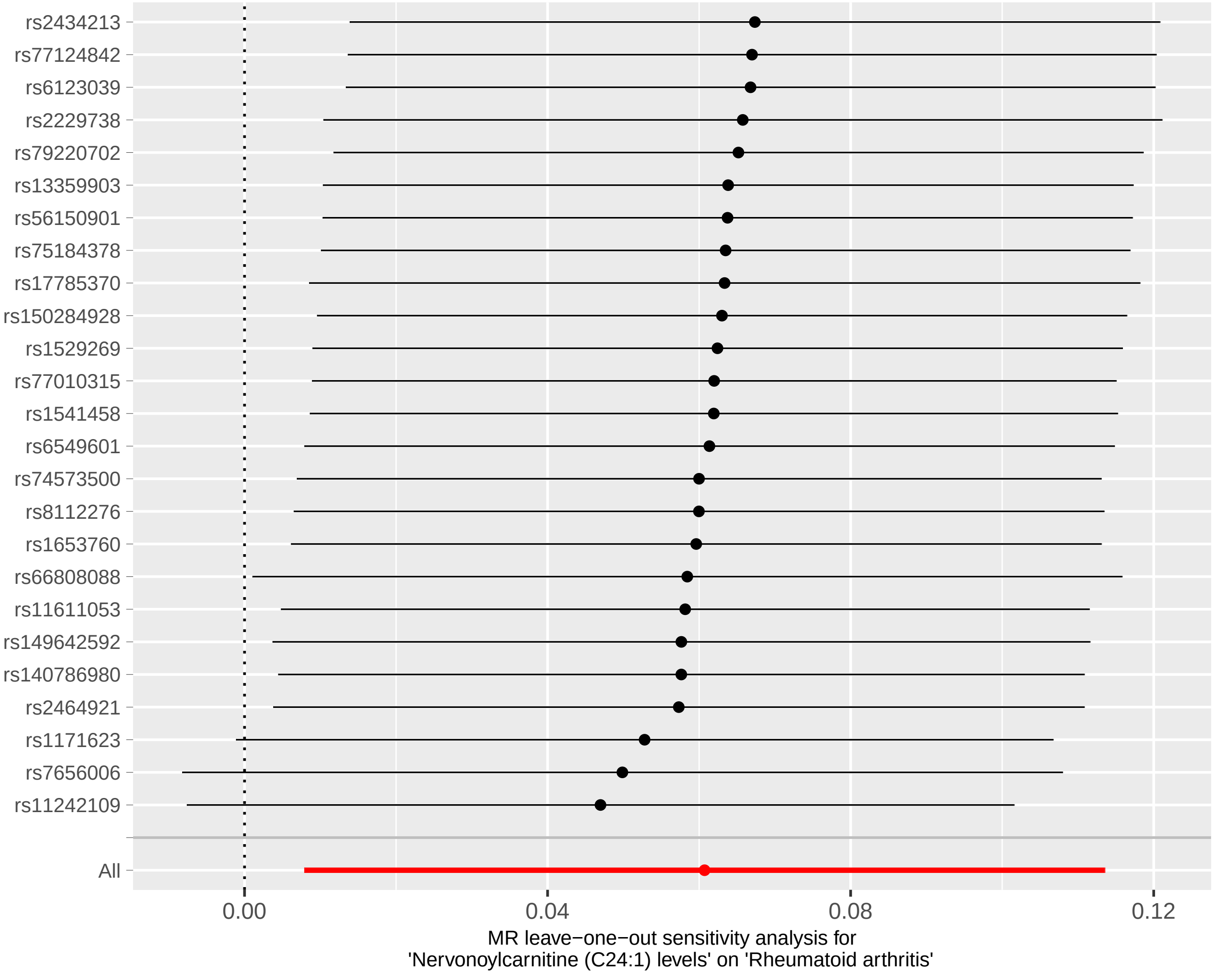
rs2434213
rs77124842
rs6123039
rs2229738
rs79220702
rs13359903
rs56150901
rs75184378
rs17785370
rs150284928
rs1529269
rs77010315
rs1541458
rs6549601
rs74573500
rs8112276
rs1653760
rs66808088
rs11611053
rs149642592
rs140786980
rs2464921
rs1171623
rs7656006
rs11242109
All
0.00
0.04
0.08
0.12
MR leave−one−out sensitivity analysis for
'Nervonoylcarnitine (C24:1) levels' on 'Rheumatoid arthritis'

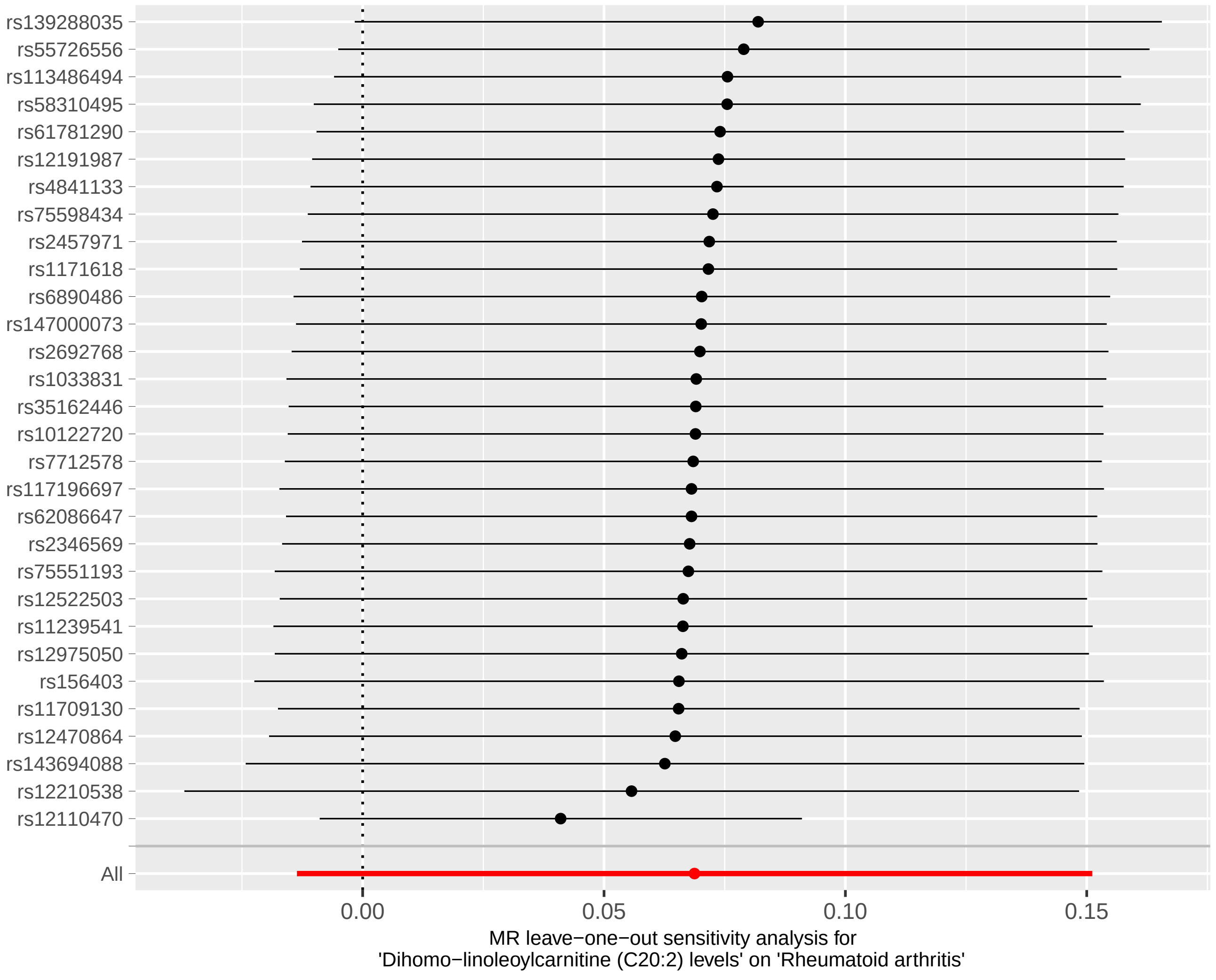

rs139288035
rs55726556
rs113486494
rs58310495
rs61781290
rs12191987
rs4841133
rs75598434
rs2457971
rs1171618
rs6890486
rs147000073
rs2692768
rs1033831
rs35162446
rs10122720
rs7712578
rs117196697
rs62086647
rs2346569
rs75551193
rs12522503
rs11239541
rs12975050
rs156403
rs11709130
rs12470864
rs143694088
rs12210538
rs12110470
All
0.00
0.05
0.10
0.15
MR leave−one−out sensitivity analysis for
'Dihomo−linoleoylcarnitine (C20:2) levels' on 'Rheumatoid arthritis'

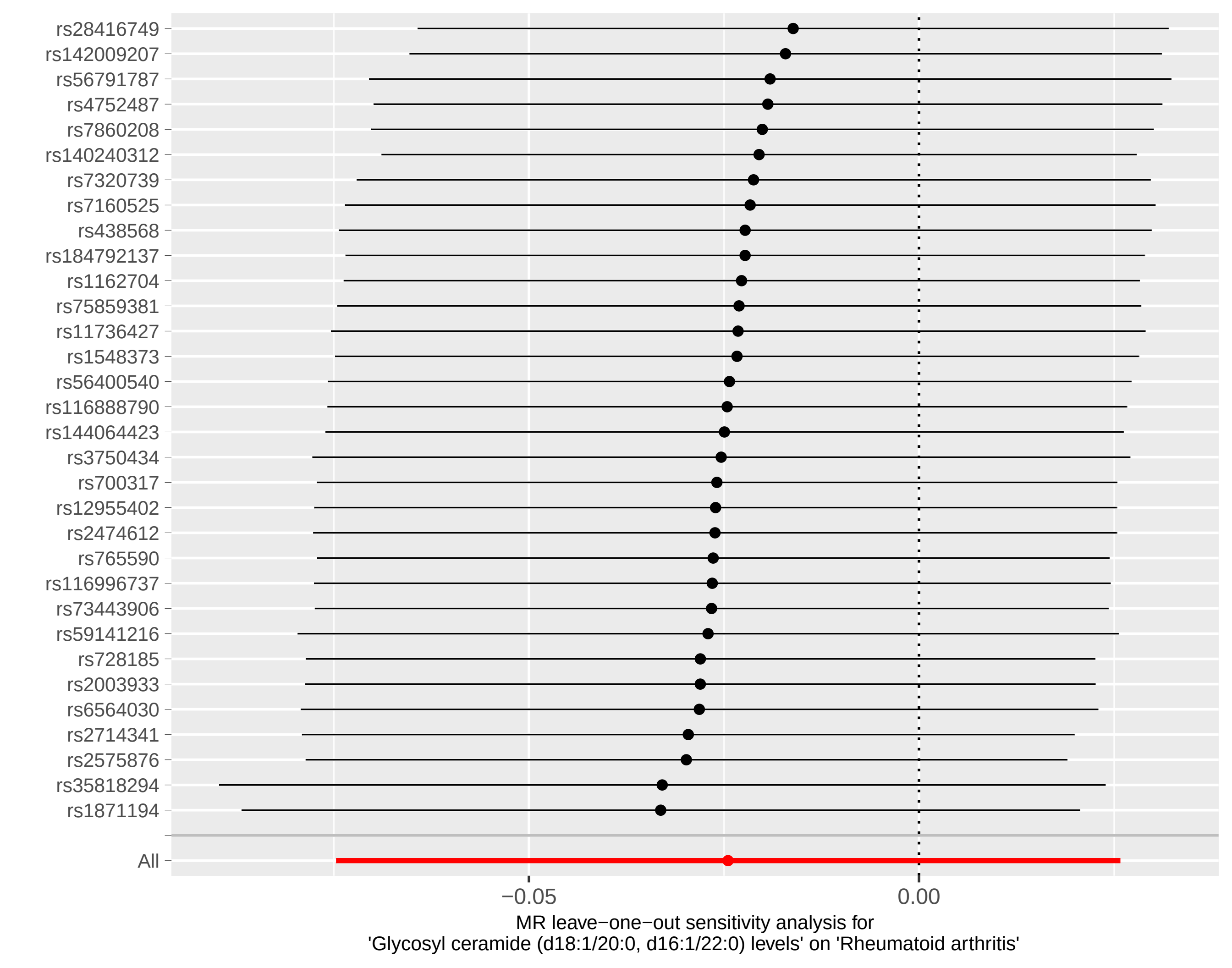
rs28416749
rs142009207
rs56791787
rs4752487
rs7860208
rs140240312
rs7320739
rs7160525
rs438568
rs184792137
rs1162704
rs75859381
rs11736427
rs1548373
rs56400540
rs116888790
rs144064423
rs3750434
rs700317
rs12955402
rs2474612
rs765590
rs116996737
rs73443906
rs59141216
rs728185
rs2003933
rs6564030
rs2714341
rs2575876
rs35818294
rs1871194
All
−0.05
0.00
MR leave−one−out sensitivity analysis for
'Glycosyl ceramide (d18:1/20:0, d16:1/22:0) levels' on 'Rheumatoid arthritis'

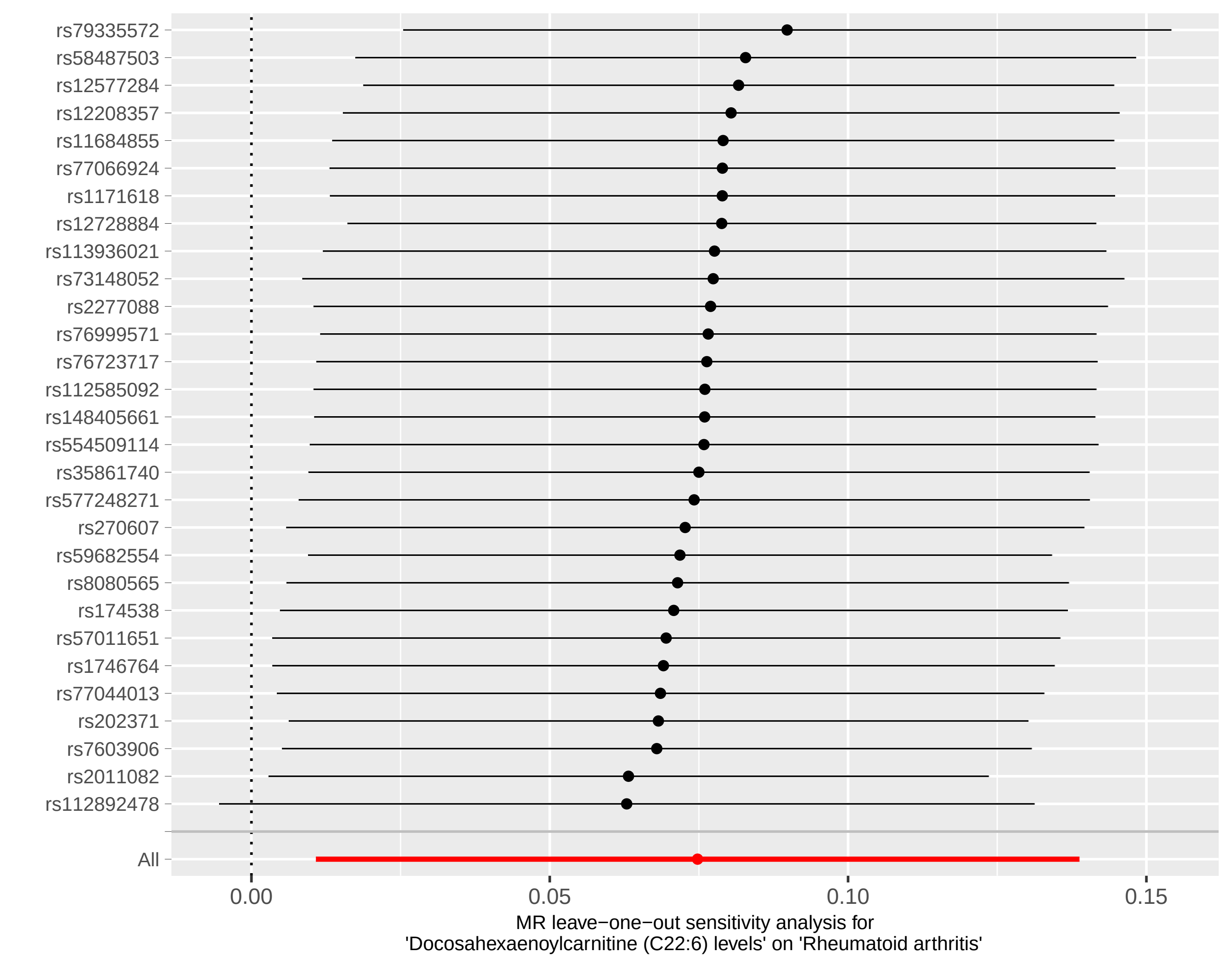

rs79335572
rs58487503
rs12577284
rs12208357
rs11684855
rs77066924
rs1171618
rs12728884
rs113936021
rs73148052
rs2277088
rs76999571
rs76723717
rs112585092
rs148405661
rs554509114
rs35861740
rs577248271
rs270607
rs59682554
rs8080565
rs174538
rs57011651
rs1746764
rs77044013
rs202371
rs7603906
rs2011082
rs112892478
All
0.00
0.05
0.10
0.15
MR leave−one−out sensitivity analysis for
'Docosahexaenoylcarnitine (C22:6) levels' on 'Rheumatoid arthritis'

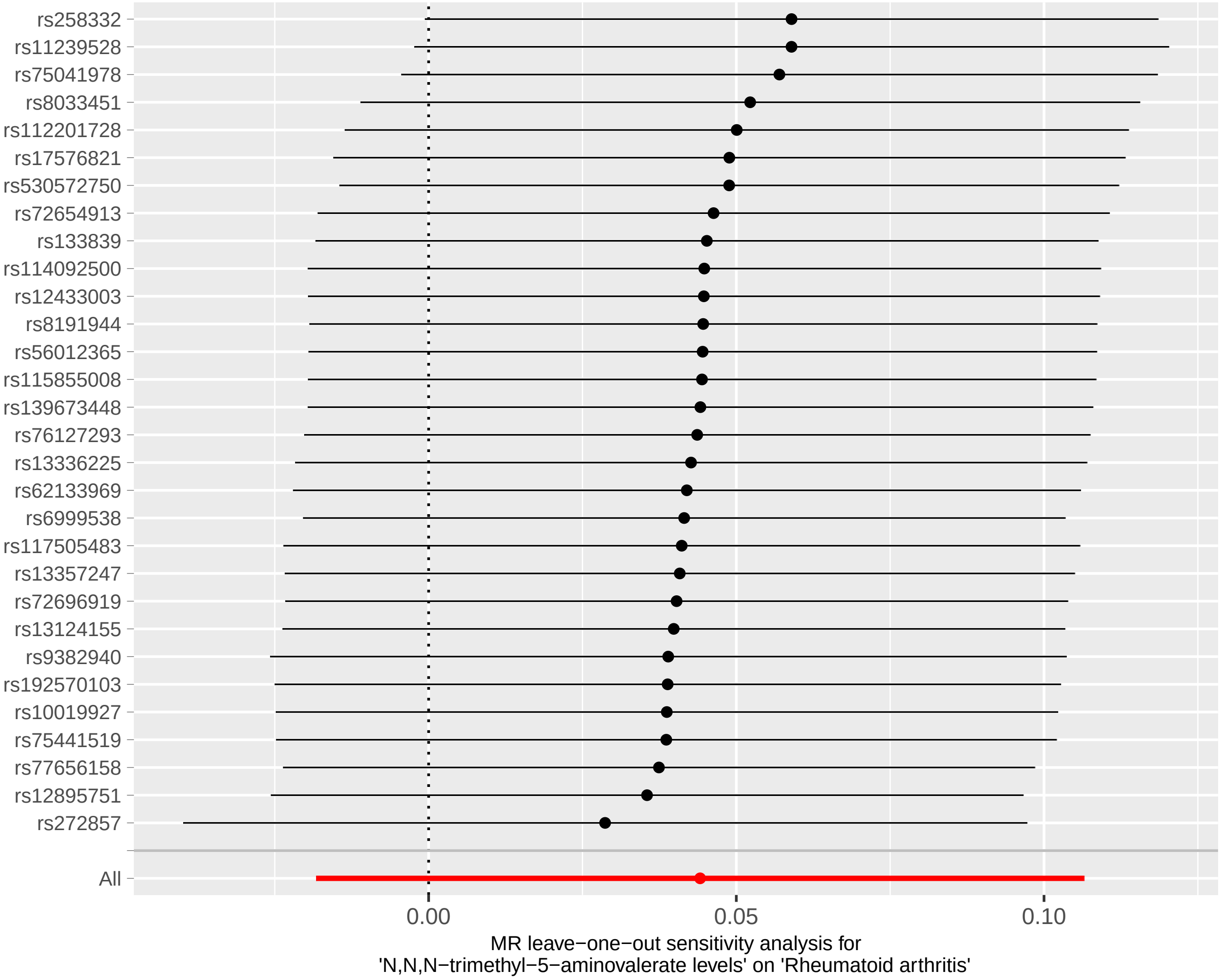
rs258332
rs11239528
rs75041978
rs8033451
rs112201728
rs17576821
rs530572750
rs72654913
rs133839
rs114092500
rs12433003
rs8191944
rs56012365
rs115855008
rs139673448
rs76127293
rs13336225
rs62133969
rs6999538
rs117505483
rs13357247
rs72696919
rs13124155
rs9382940
rs192570103
rs10019927
rs75441519
rs77656158
rs12895751
rs272857
All
0.00
0.05
0.10
MR leave−one−out sensitivity analysis for
'N,N,N−trimethyl−5−aminovalerate levels' on 'Rheumatoid arthritis'

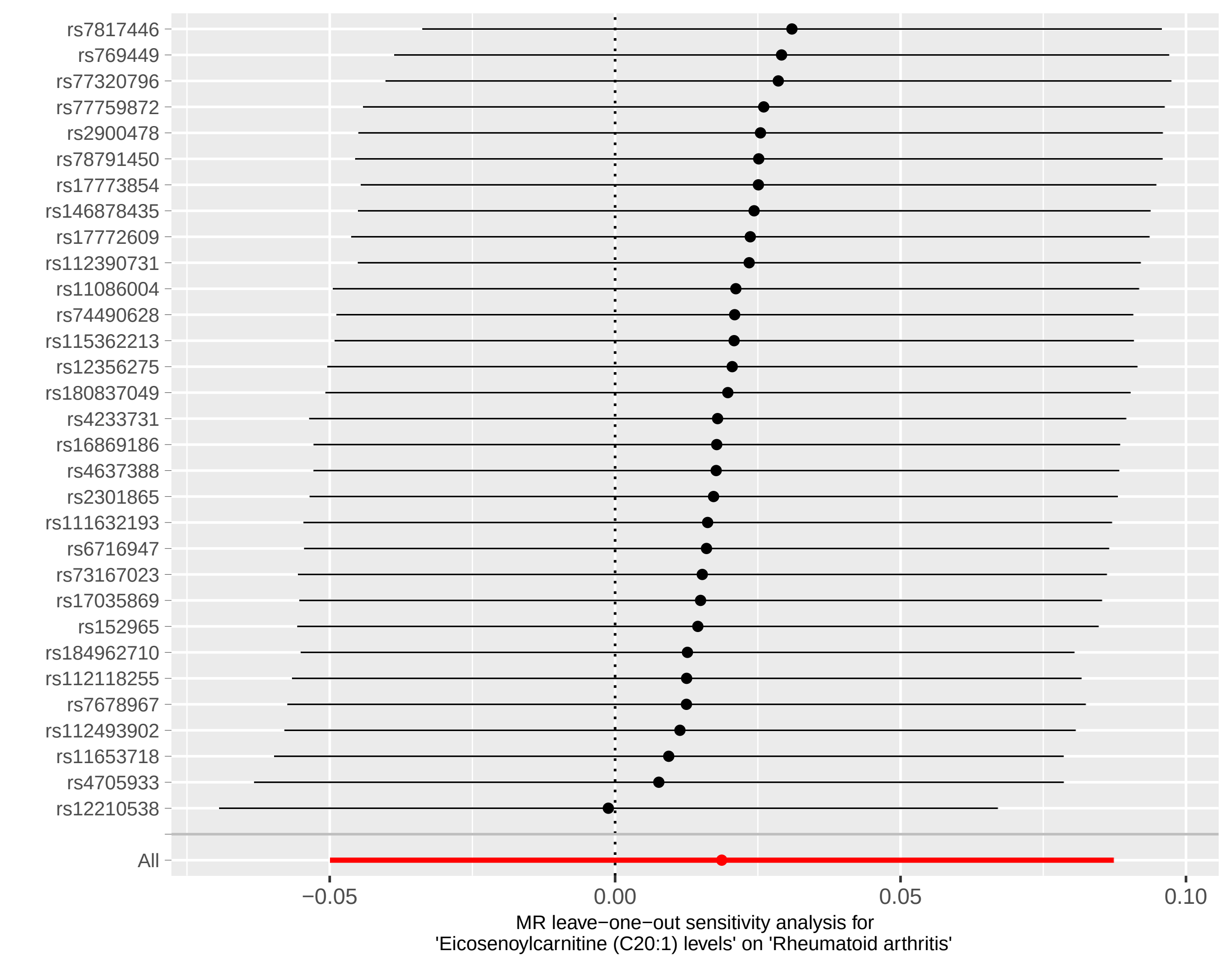
rs7817446
rs769449
rs77320796
rs77759872
rs2900478
rs78791450
rs17773854
rs146878435
rs17772609
rs112390731
rs11086004
rs74490628
rs115362213
rs12356275
rs180837049
rs4233731
rs16869186
rs4637388
rs2301865
rs111632193
rs6716947
rs73167023
rs17035869
rs152965
rs184962710
rs112118255
rs7678967
rs112493902
rs11653718
rs4705933
rs12210538
All
−0.05
0.00
0.05
0.10
MR leave−one−out sensitivity analysis for
'Eicosenoylcarnitine (C20:1) levels' on 'Rheumatoid arthritis'

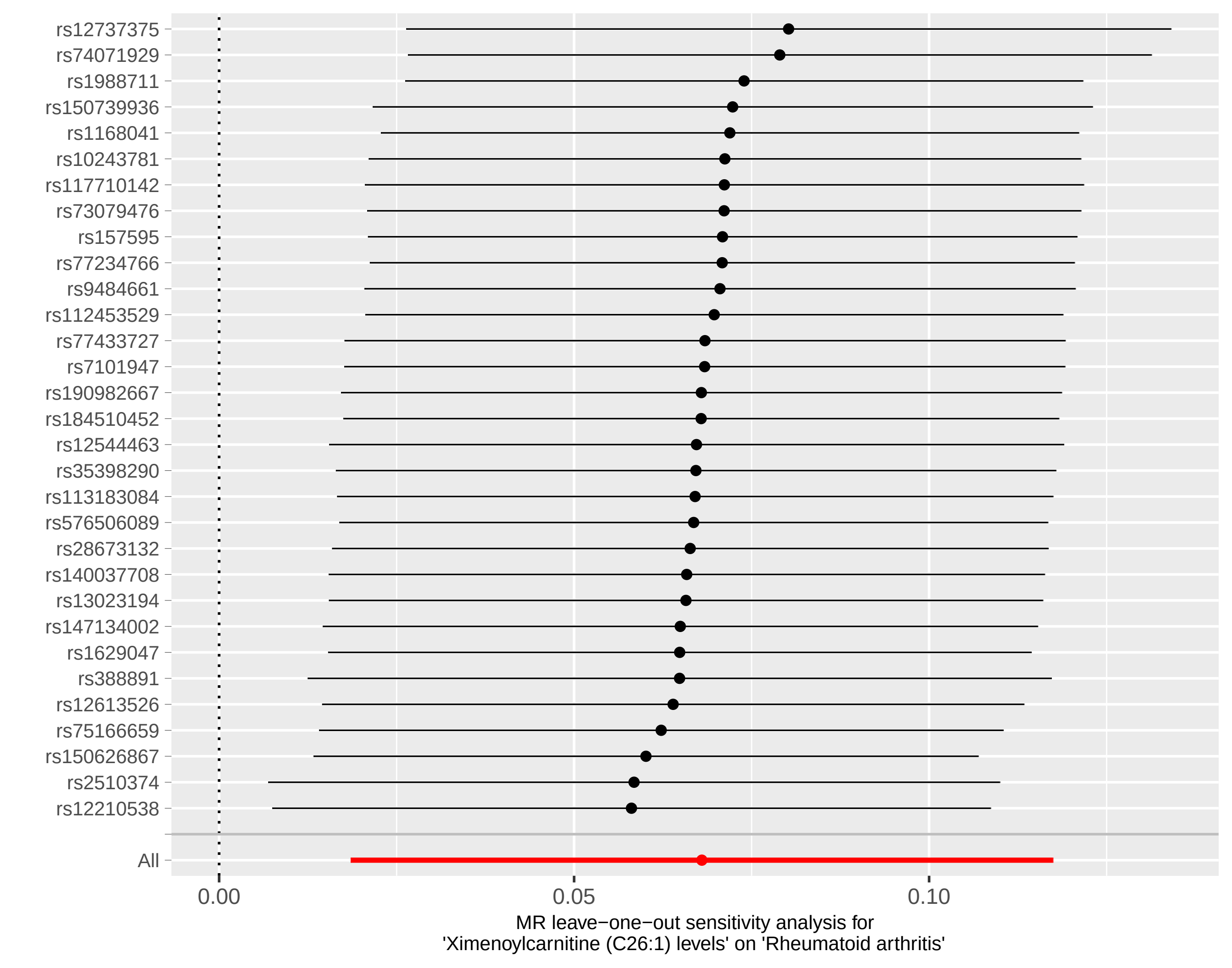
rs12737375
rs74071929
rs1988711
rs150739936
rs1168041
rs10243781
rs117710142
rs73079476
rs157595
rs77234766
rs9484661
rs112453529
rs77433727
rs7101947
rs190982667
rs184510452
rs12544463
rs35398290
rs113183084
rs576506089
rs28673132
rs140037708
rs13023194
rs147134002
rs1629047
rs388891
rs12613526
rs75166659
rs150626867
rs2510374
rs12210538
All
0.00
0.05
0.10
MR leave−one−out sensitivity analysis for
'Ximenoylcarnitine (C26:1) levels' on 'Rheumatoid arthritis'

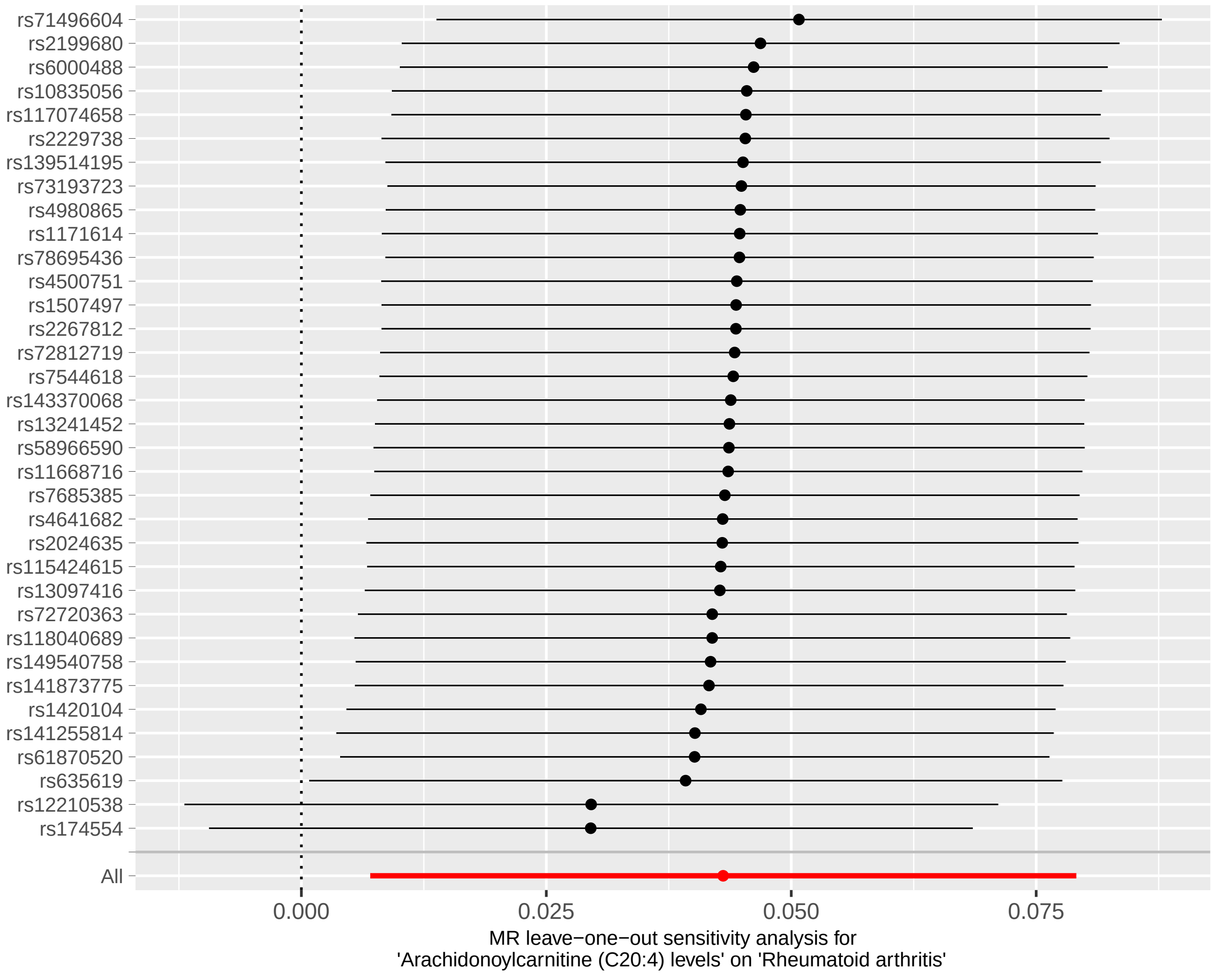
rs71496604
rs2199680
rs6000488
rs10835056
rs117074658
rs2229738
rs139514195
rs73193723
rs4980865
rs1171614
rs78695436
rs4500751
rs1507497
rs2267812
rs72812719
rs7544618
rs143370068
rs13241452
rs58966590
rs11668716
rs7685385
rs4641682
rs2024635
rs115424615
rs13097416
rs72720363
rs118040689
rs149540758
rs141873775
rs1420104
rs141255814
rs61870520
rs635619
rs12210538
rs174554
All
0.000
0.025
0.050
0.075
MR leave−one−out sensitivity analysis for
'Arachidonoylcarnitine (C20:4) levels' on 'Rheumatoid arthritis'

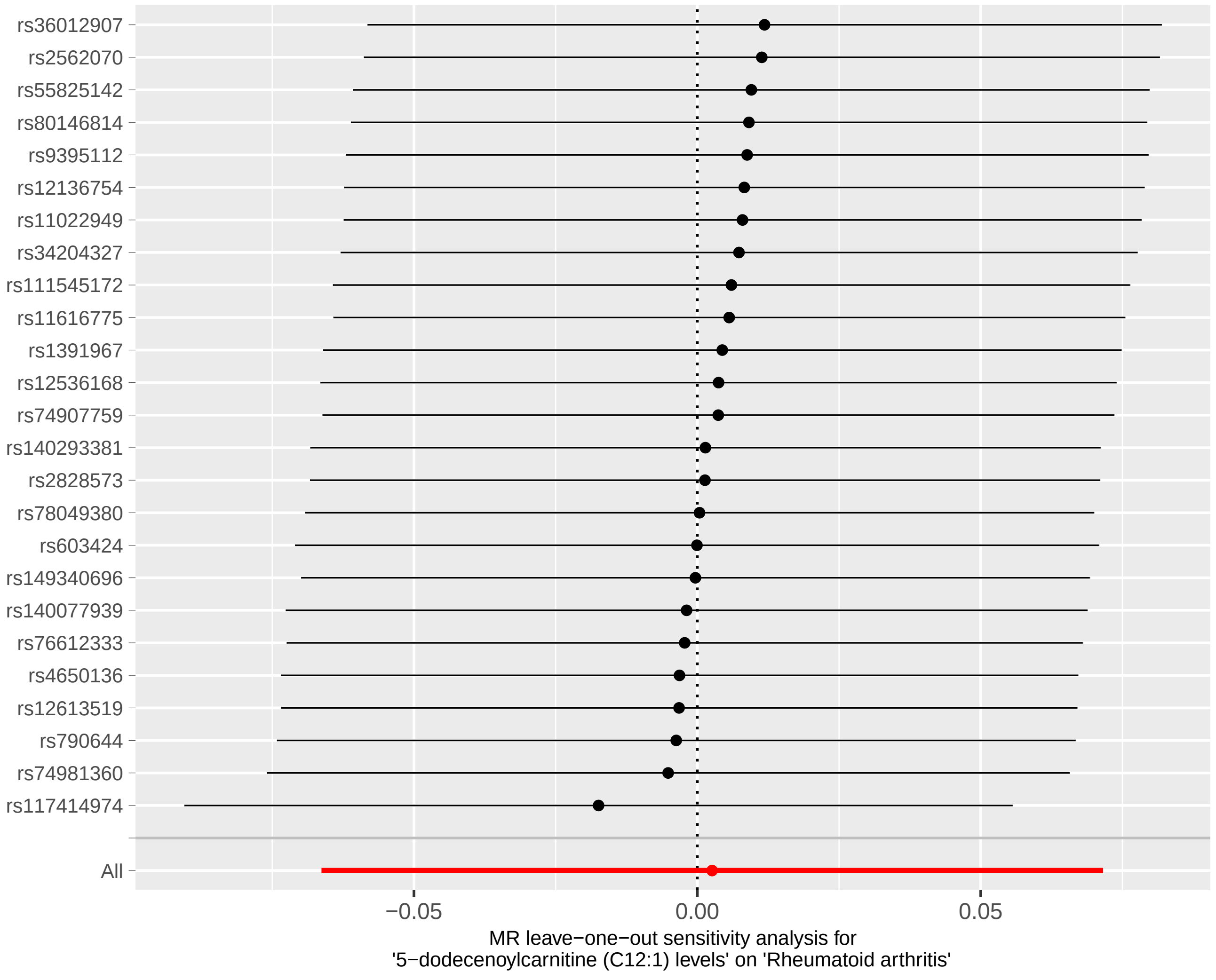
rs36012907
rs2562070
rs55825142
rs80146814
rs9395112
rs12136754
rs11022949
rs34204327
rs111545172
rs11616775
rs1391967
rs12536168
rs74907759
rs140293381
rs2828573
rs78049380
rs603424
rs149340696
rs140077939
rs76612333
rs4650136
rs12613519
rs790644
rs74981360
rs117414974
All
−0.05
0.00
0.05
MR leave−one−out sensitivity analysis for
'5−dodecenoylcarnitine (C12:1) levels' on 'Rheumatoid arthritis'

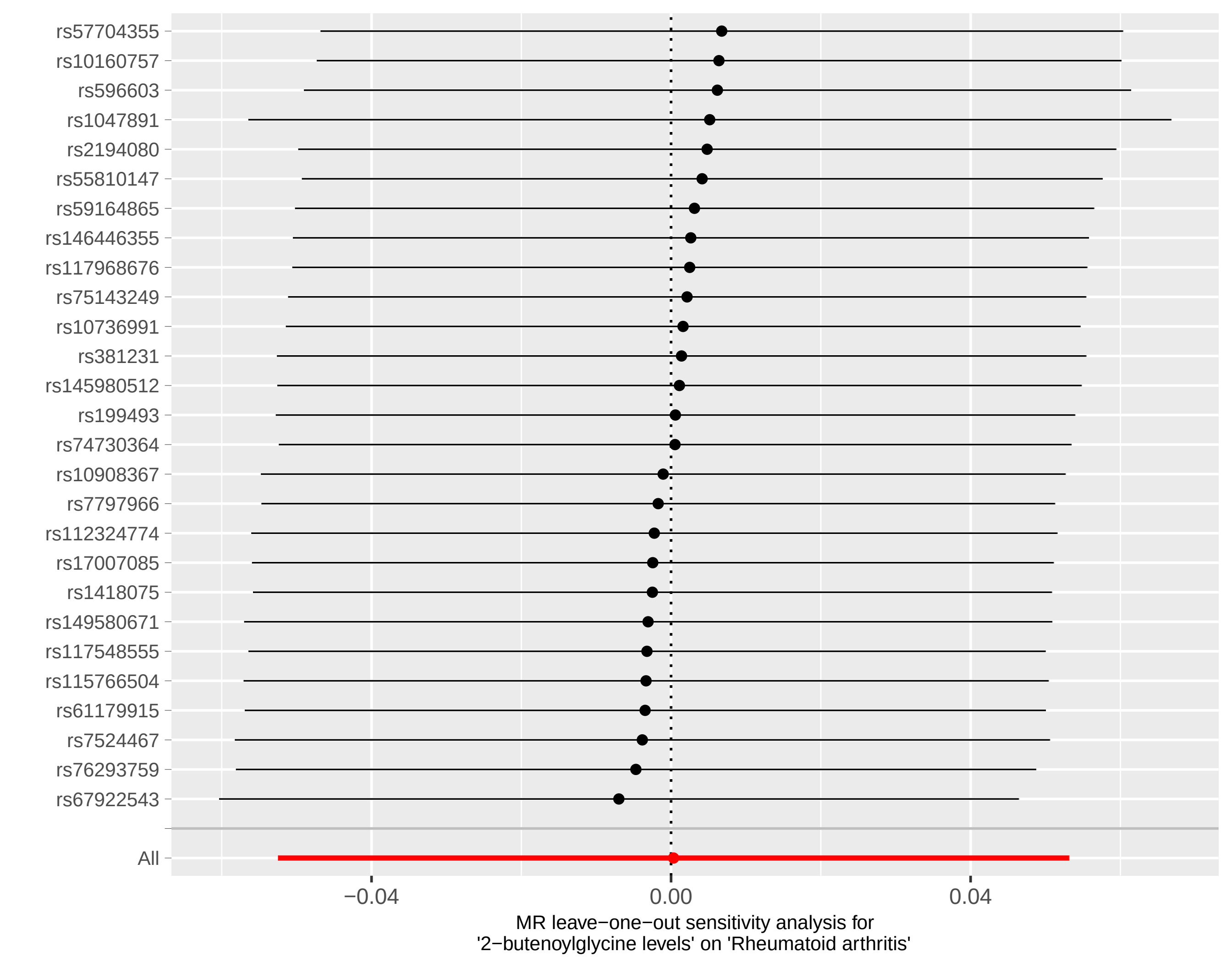
rs57704355
rs10160757
rs596603
rs1047891
rs2194080
rs55810147
rs59164865
rs146446355
rs117968676
rs75143249
rs10736991
rs381231
rs145980512
rs199493
rs74730364
rs10908367
rs7797966
rs112324774
rs17007085
rs1418075
rs149580671
rs117548555
rs115766504
rs61179915
rs7524467
rs76293759
rs67922543
All
−0.04
0.00
0.04
MR leave−one−out sensitivity analysis for
'2−butenoylglycine levels' on 'Rheumatoid arthritis'

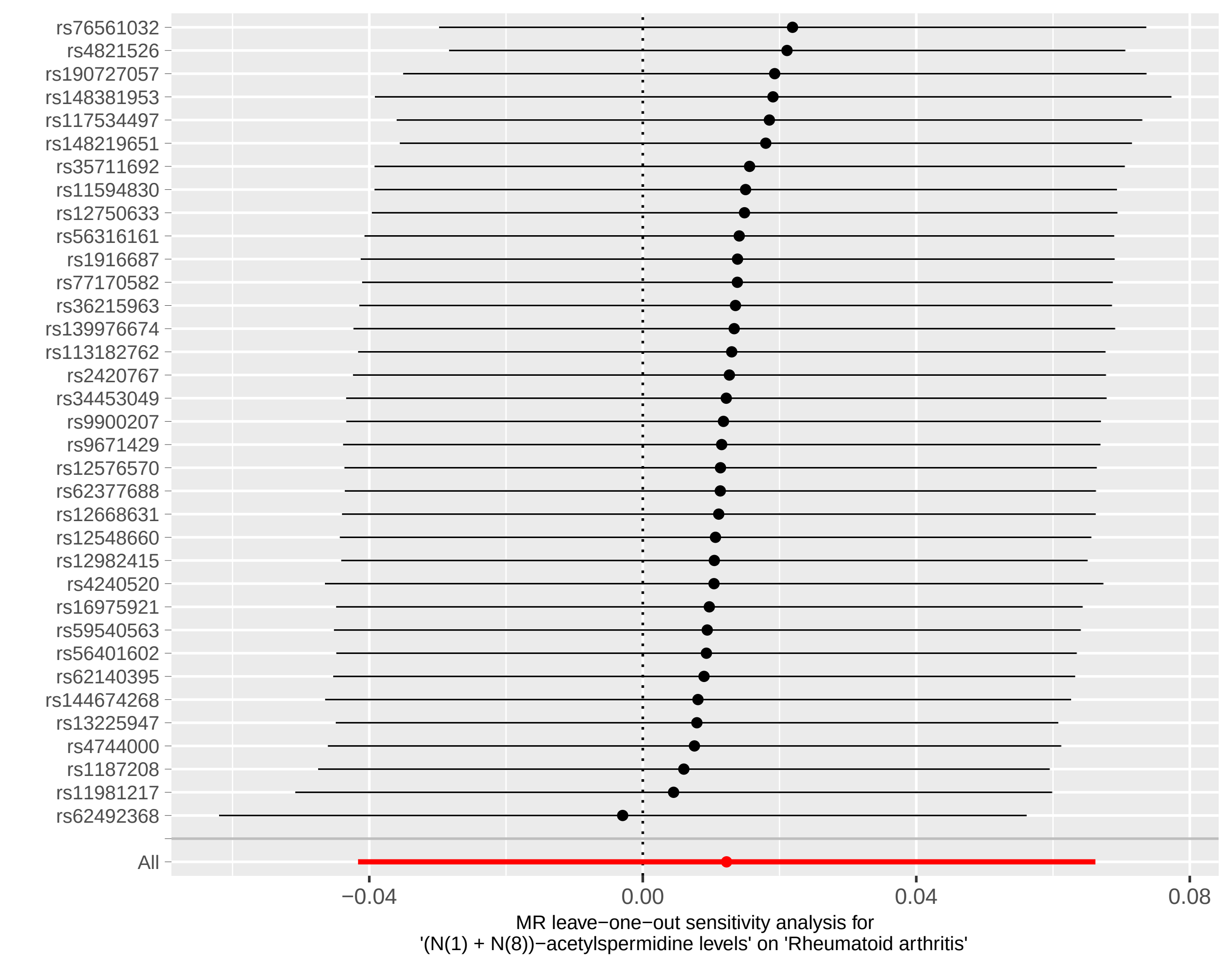

rs76561032
rs4821526
rs190727057
rs148381953
rs117534497
rs148219651
rs35711692
rs11594830
rs12750633
rs56316161
rs1916687
rs77170582
rs36215963
rs139976674
rs113182762
rs2420767
rs34453049
rs9900207
rs9671429
rs12576570
rs62377688
rs12668631
rs12548660
rs12982415
rs4240520
rs16975921
rs59540563
rs56401602
rs62140395
rs144674268
rs13225947
rs4744000
rs1187208
rs11981217
rs62492368
All
−0.04
0.00
0.04
0.08
MR leave−one−out sensitivity analysis for
'(N(1) + N(8))−acetylspermidine levels' on 'Rheumatoid arthritis'

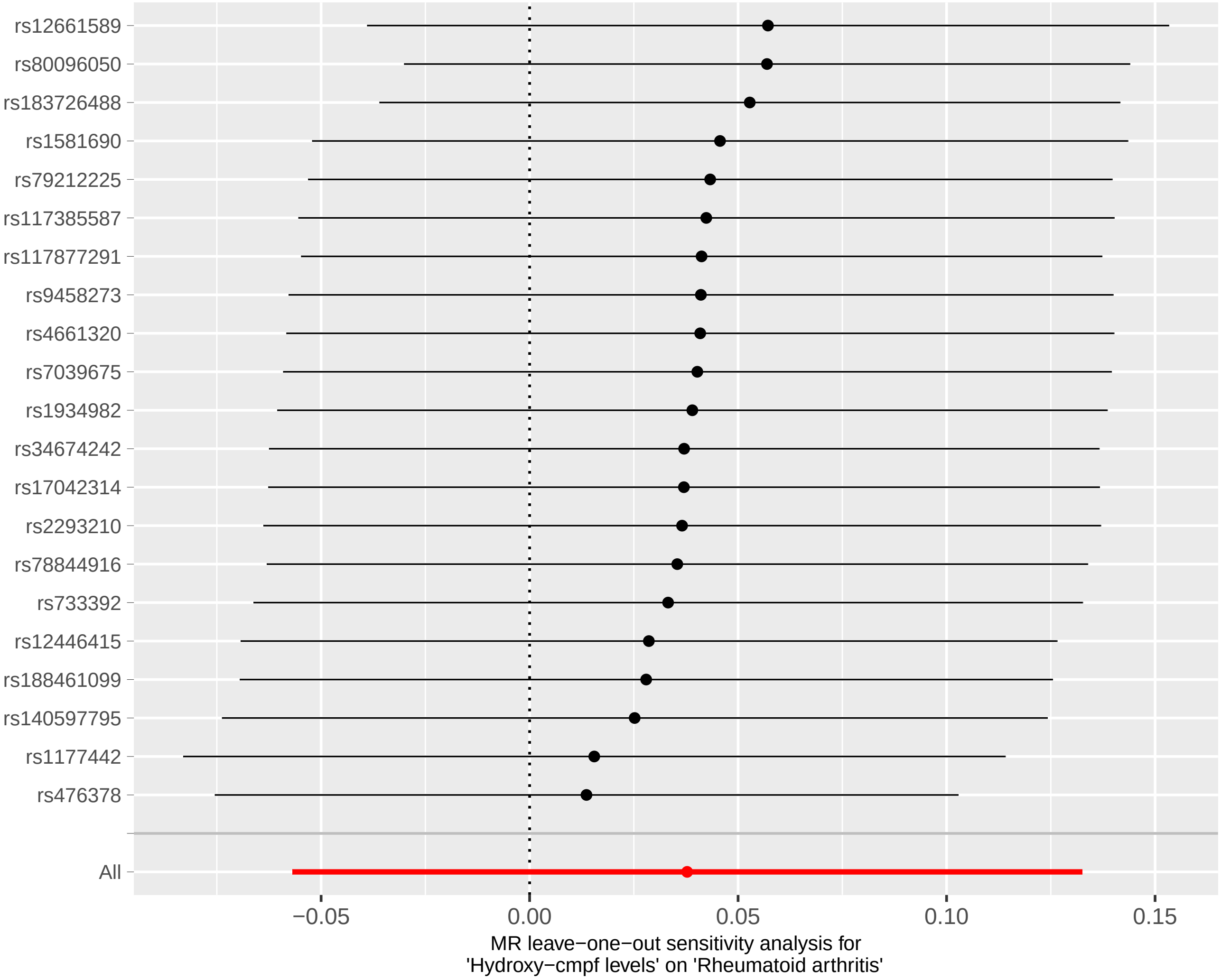

rs12661589
rs80096050
rs183726488
rs1581690
rs79212225
rs117385587
rs117877291
rs9458273
rs4661320
rs7039675
rs1934982
rs34674242
rs17042314
rs2293210
rs78844916
rs733392
rs12446415
rs188461099
rs140597795
rs1177442
rs476378
All
−0.05
0.00
0.05
0.10
0.15
MR leave−one−out sensitivity analysis for
'Hydroxy−cmpf levels' on 'Rheumatoid arthritis'

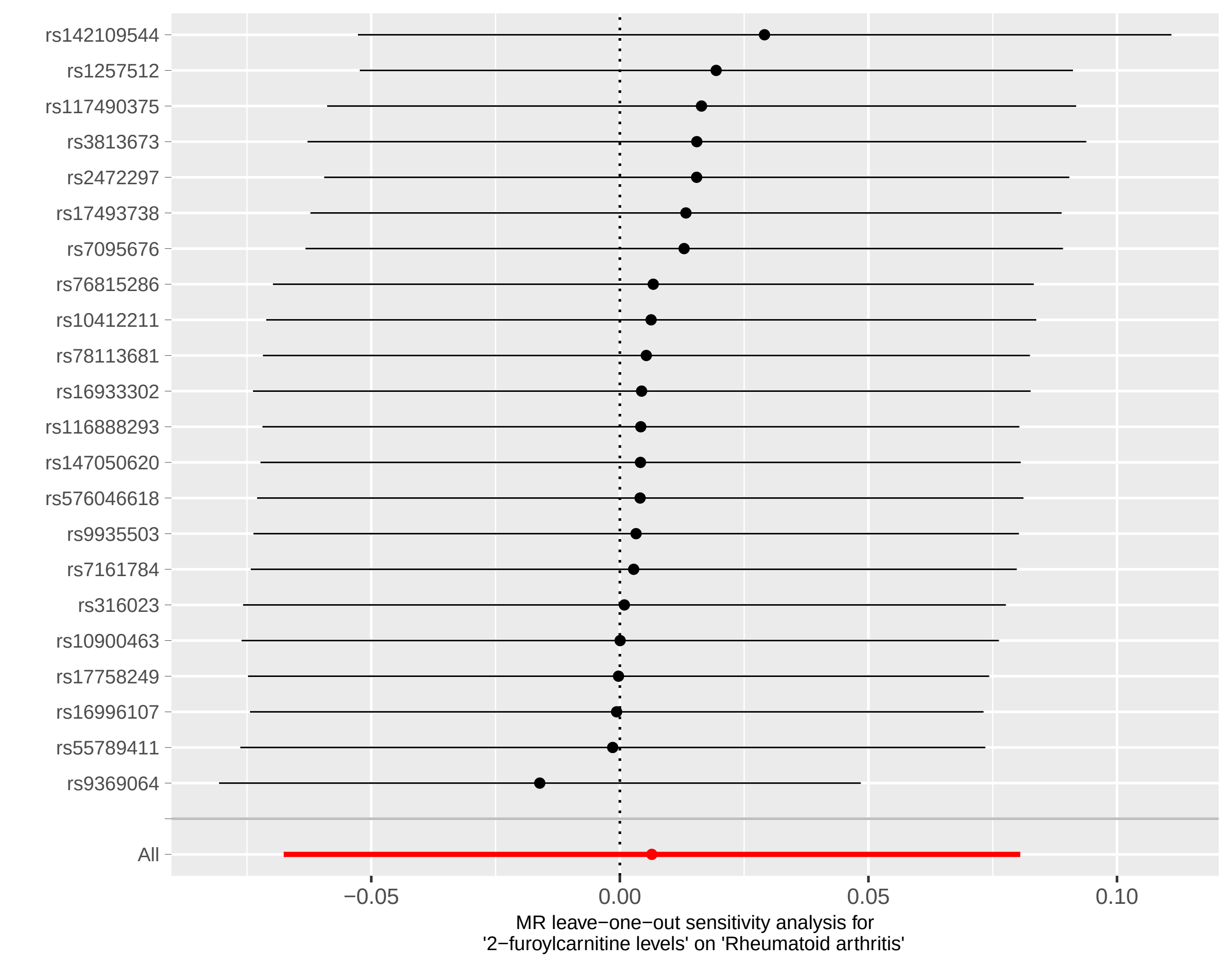
rs142109544
rs1257512
rs117490375
rs3813673
rs2472297
rs17493738
rs7095676
rs76815286
rs10412211
rs78113681
rs16933302
rs116888293
rs147050620
rs576046618
rs9935503
rs7161784
rs316023
rs10900463
rs17758249
rs16996107
rs55789411
rs9369064
All
−0.05
0.00
0.05
0.10
MR leave−one−out sensitivity analysis for
'2−furoylcarnitine levels' on 'Rheumatoid arthritis'

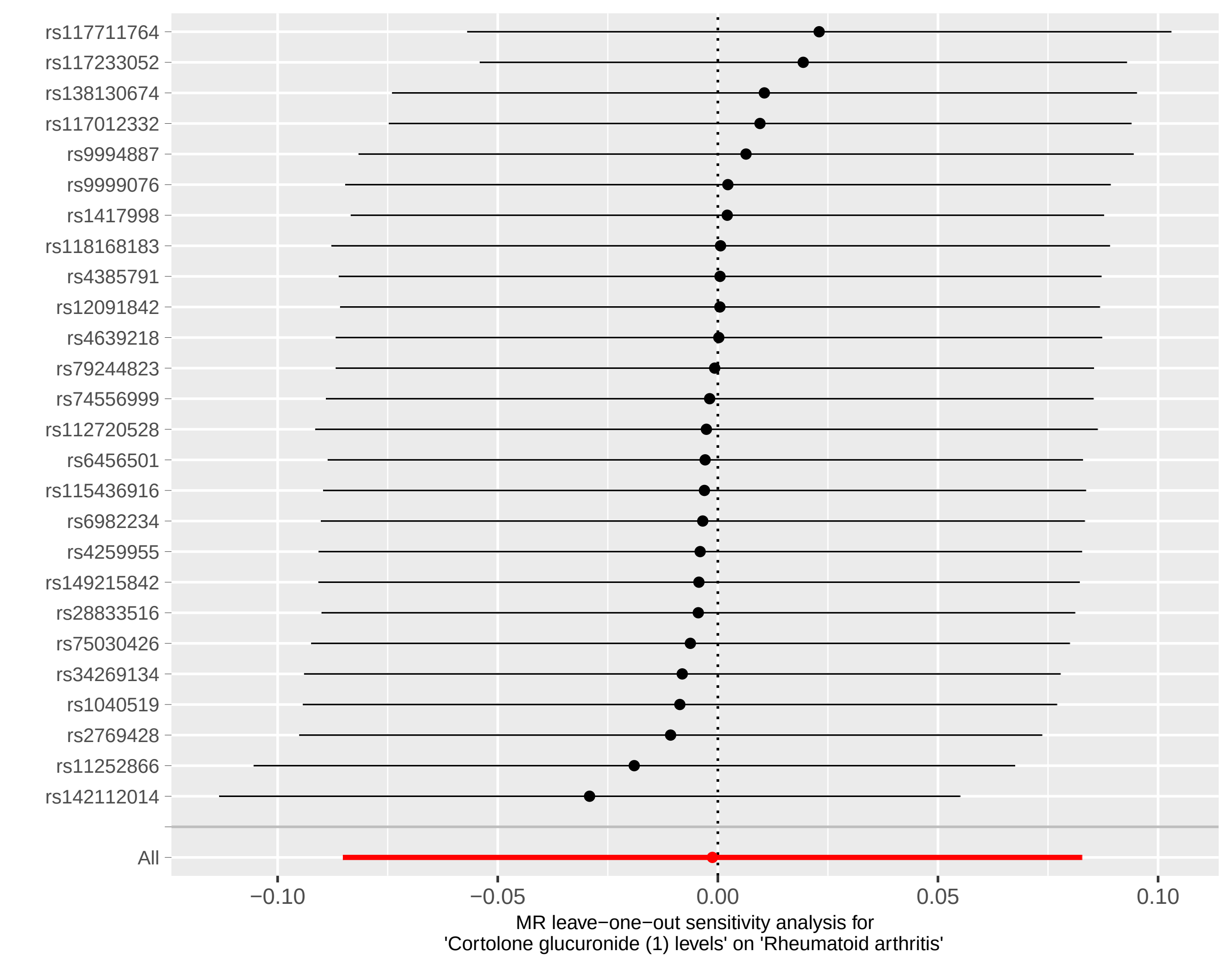
rs117711764
rs117233052
rs138130674
rs117012332
rs9994887
rs9999076
rs1417998
rs118168183
rs4385791
rs12091842
rs4639218
rs79244823
rs74556999
rs112720528
rs6456501
rs115436916
rs6982234
rs4259955
rs149215842
rs28833516
rs75030426
rs34269134
rs1040519
rs2769428
rs11252866
rs142112014
All
−0.10
−0.05
0.00
0.05
0.10
MR leave−one−out sensitivity analysis for
'Cortolone glucuronide (1) levels' on 'Rheumatoid arthritis'

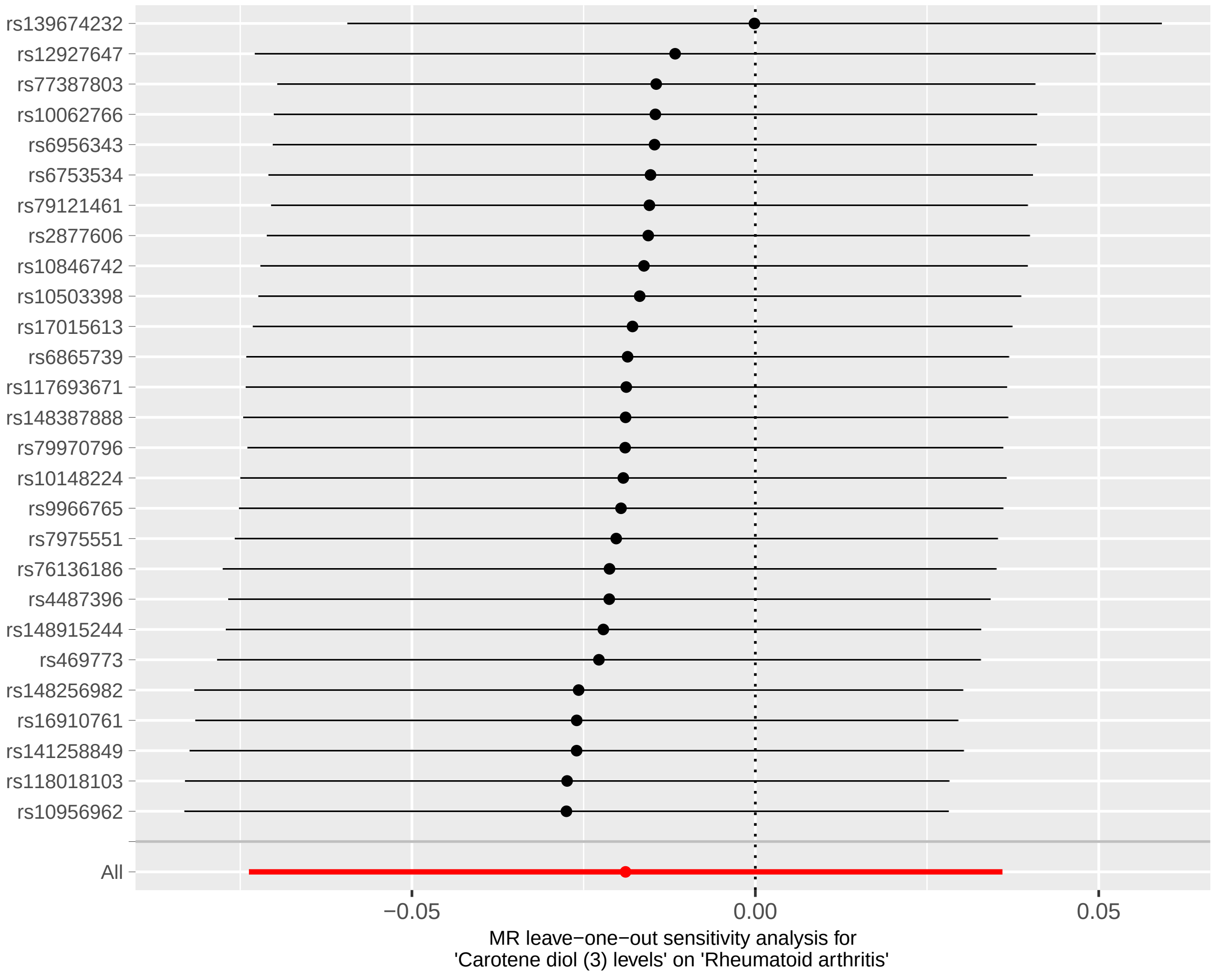

rs139674232
rs12927647
rs77387803
rs10062766
rs6956343
rs6753534
rs79121461
rs2877606
rs10846742
rs10503398
rs17015613
rs6865739
rs117693671
rs148387888
rs79970796
rs10148224
rs9966765
rs7975551
rs76136186
rs4487396
rs148915244
rs469773
rs148256982
rs16910761
rs141258849
rs118018103
rs10956962
All
−0.05
0.00
0.05
MR leave−one−out sensitivity analysis for
'Carotene diol (3) levels' on 'Rheumatoid arthritis'

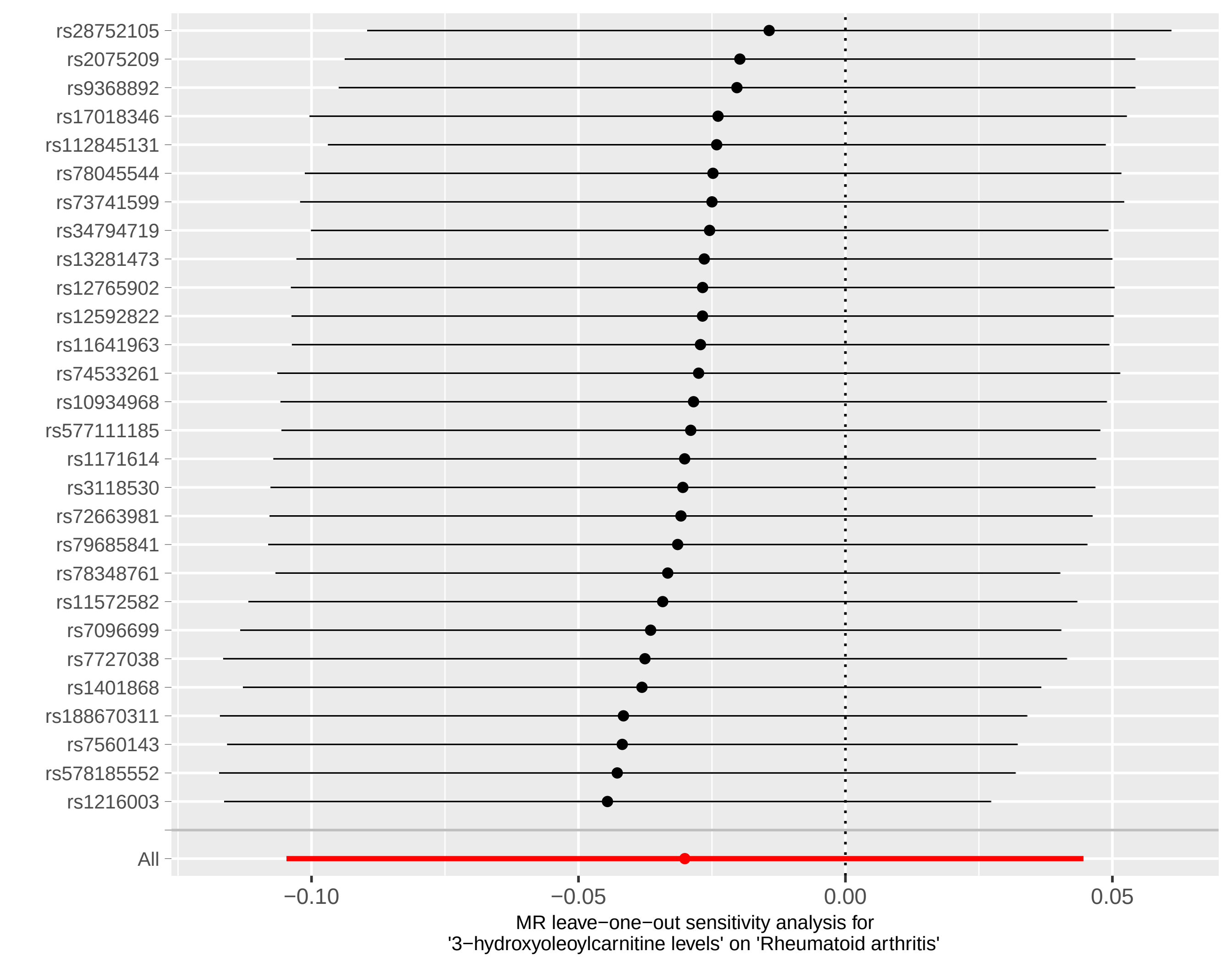
rs28752105
rs2075209
rs9368892
rs17018346
rs112845131
rs78045544
rs73741599
rs34794719
rs13281473
rs12765902
rs12592822
rs11641963
rs74533261
rs10934968
rs577111185
rs1171614
rs3118530
rs72663981
rs79685841
rs78348761
rs11572582
rs7096699
rs7727038
rs1401868
rs188670311
rs7560143
rs578185552
rs1216003
All
−0.10
−0.05
0.00
0.05
MR leave−one−out sensitivity analysis for
'3−hydroxyoleoylcarnitine levels' on 'Rheumatoid arthritis'

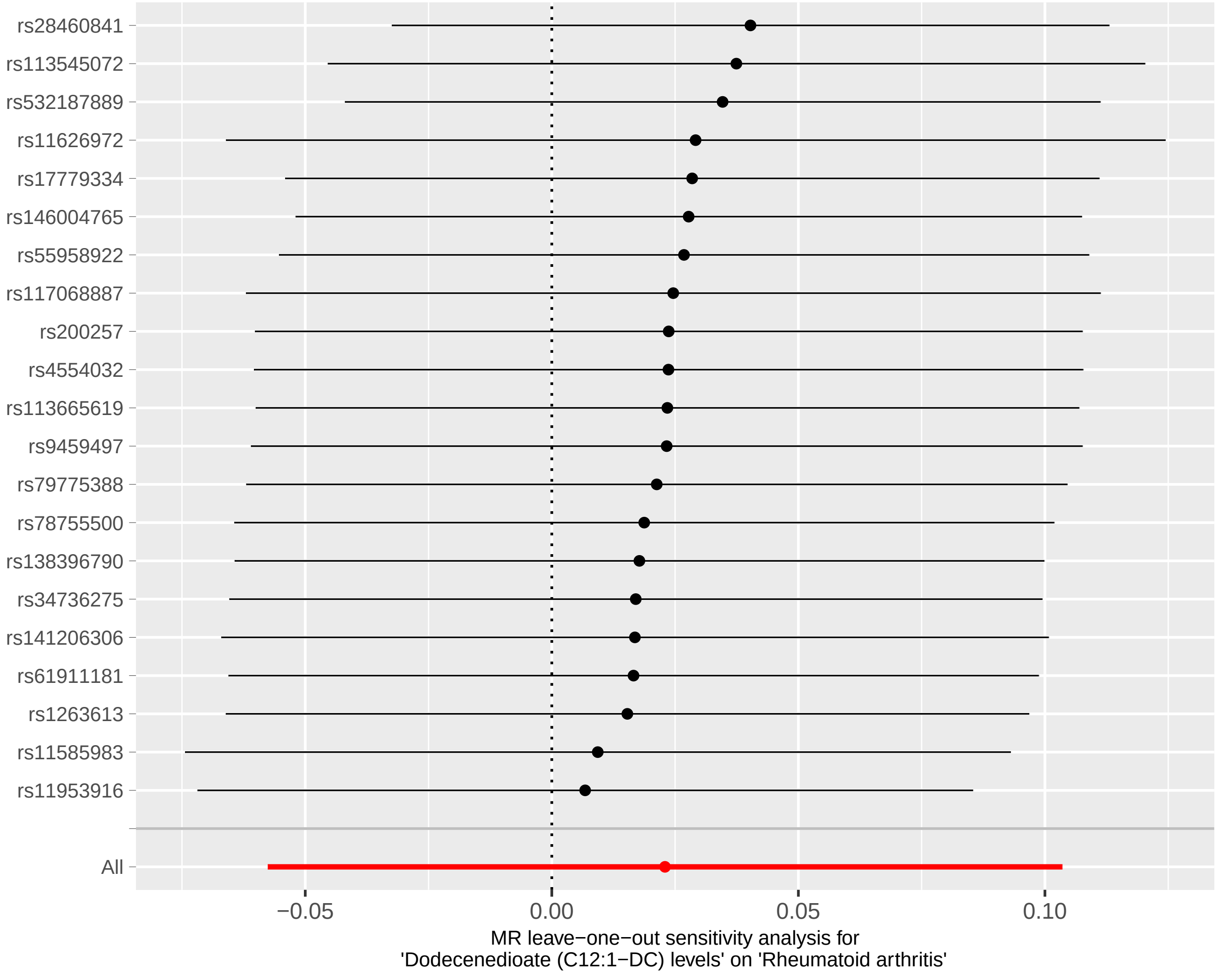
rs28460841
rs113545072
rs532187889
rs11626972
rs17779334
rs146004765
rs55958922
rs117068887
rs200257
rs4554032
rs113665619
rs9459497
rs79775388
rs78755500
rs138396790
rs34736275
rs141206306
rs61911181
rs1263613
rs11585983
rs11953916
All
−0.05
0.00
0.05
0.10
MR leave−one−out sensitivity analysis for
'Dodecenedioate (C12:1−DC) levels' on 'Rheumatoid arthritis'

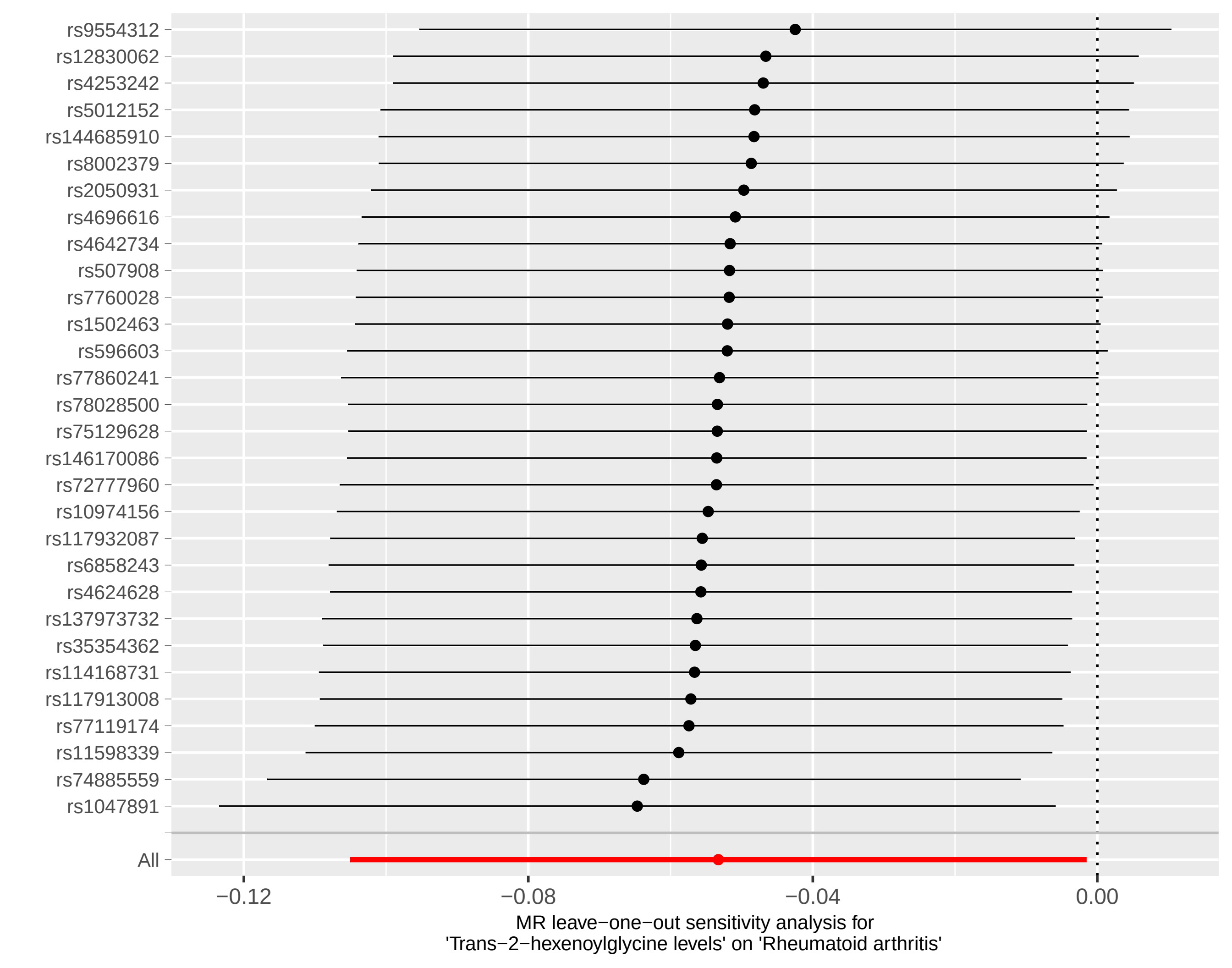
rs9554312
rs12830062
rs4253242
rs5012152
rs144685910
rs8002379
rs2050931
rs4696616
rs4642734
rs507908
rs7760028
rs1502463
rs596603
rs77860241
rs78028500
rs75129628
rs146170086
rs72777960
rs10974156
rs117932087
rs6858243
rs4624628
rs137973732
rs35354362
rs114168731
rs117913008
rs77119174
rs11598339
rs74885559
rs1047891
All
−0.12
−0.08
−0.04
0.00
MR leave−one−out sensitivity analysis for
'Trans−2−hexenoylglycine levels' on 'Rheumatoid arthritis'

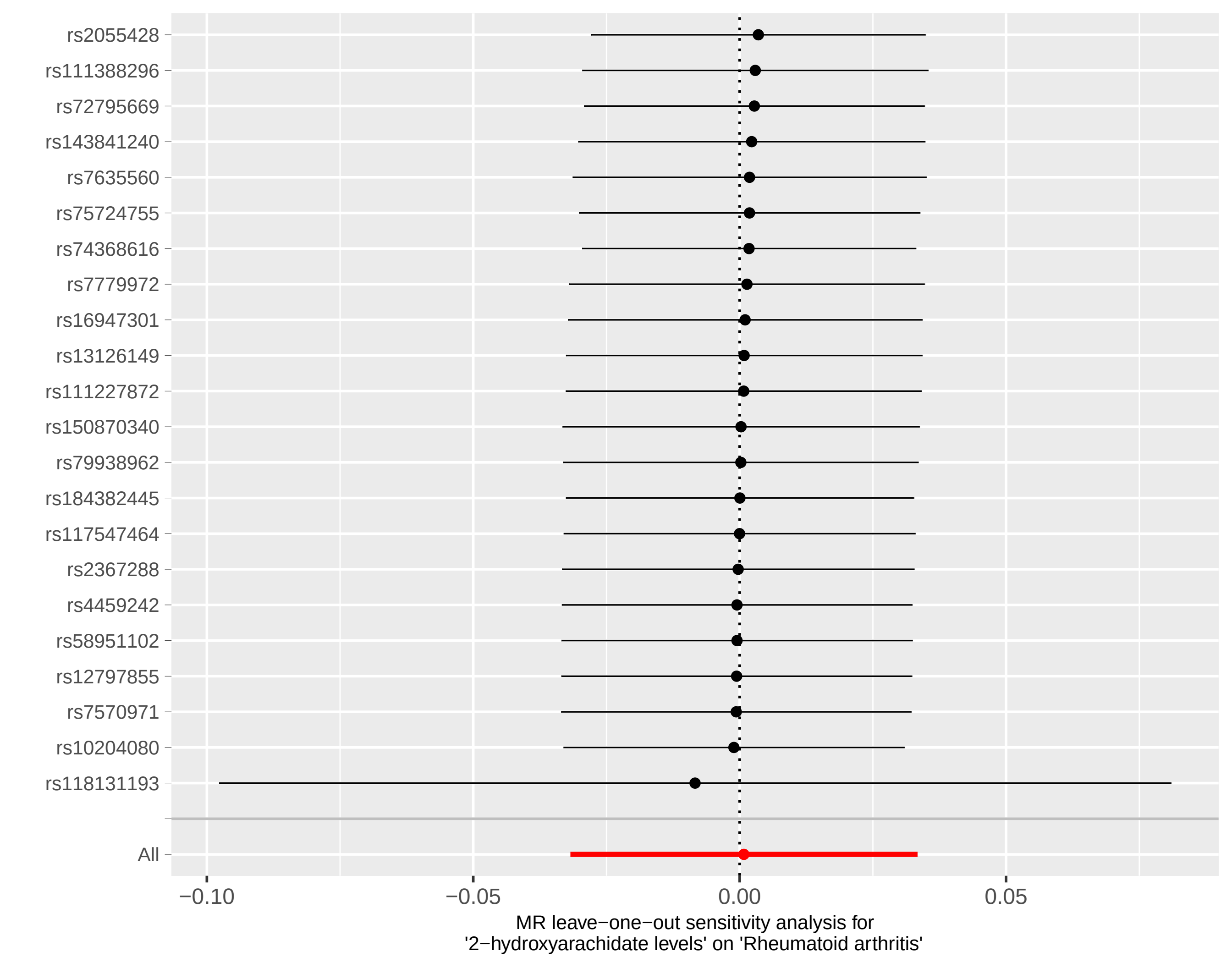

rs2055428
rs111388296
rs72795669
rs143841240
rs7635560
rs75724755
rs74368616
rs7779972
rs16947301
rs13126149
rs111227872
rs150870340
rs79938962
rs184382445
rs117547464
rs2367288
rs4459242
rs58951102
rs12797855
rs7570971
rs10204080
rs118131193
All
−0.10
−0.05
0.00
0.05
MR leave−one−out sensitivity analysis for
'2−hydroxyarachidate levels' on 'Rheumatoid arthritis'

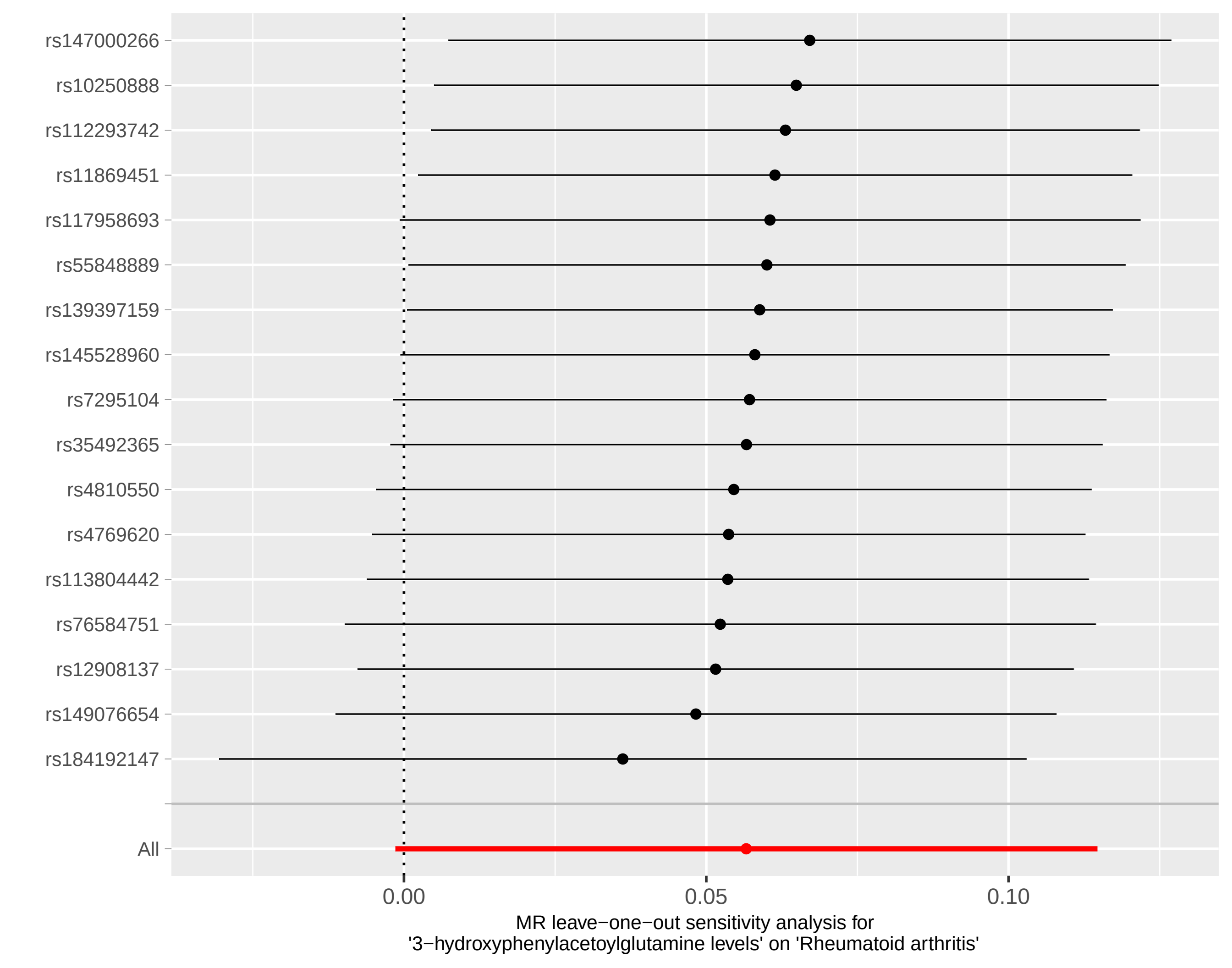
rs147000266
rs10250888
rs112293742
rs11869451
rs117958693
rs55848889
rs139397159
rs145528960
rs7295104
rs35492365
rs4810550
rs4769620
rs113804442
rs76584751
rs12908137
rs149076654
rs184192147
All
0.00
0.05
0.10
MR leave−one−out sensitivity analysis for
'3−hydroxyphenylacetoylglutamine levels' on 'Rheumatoid arthritis'

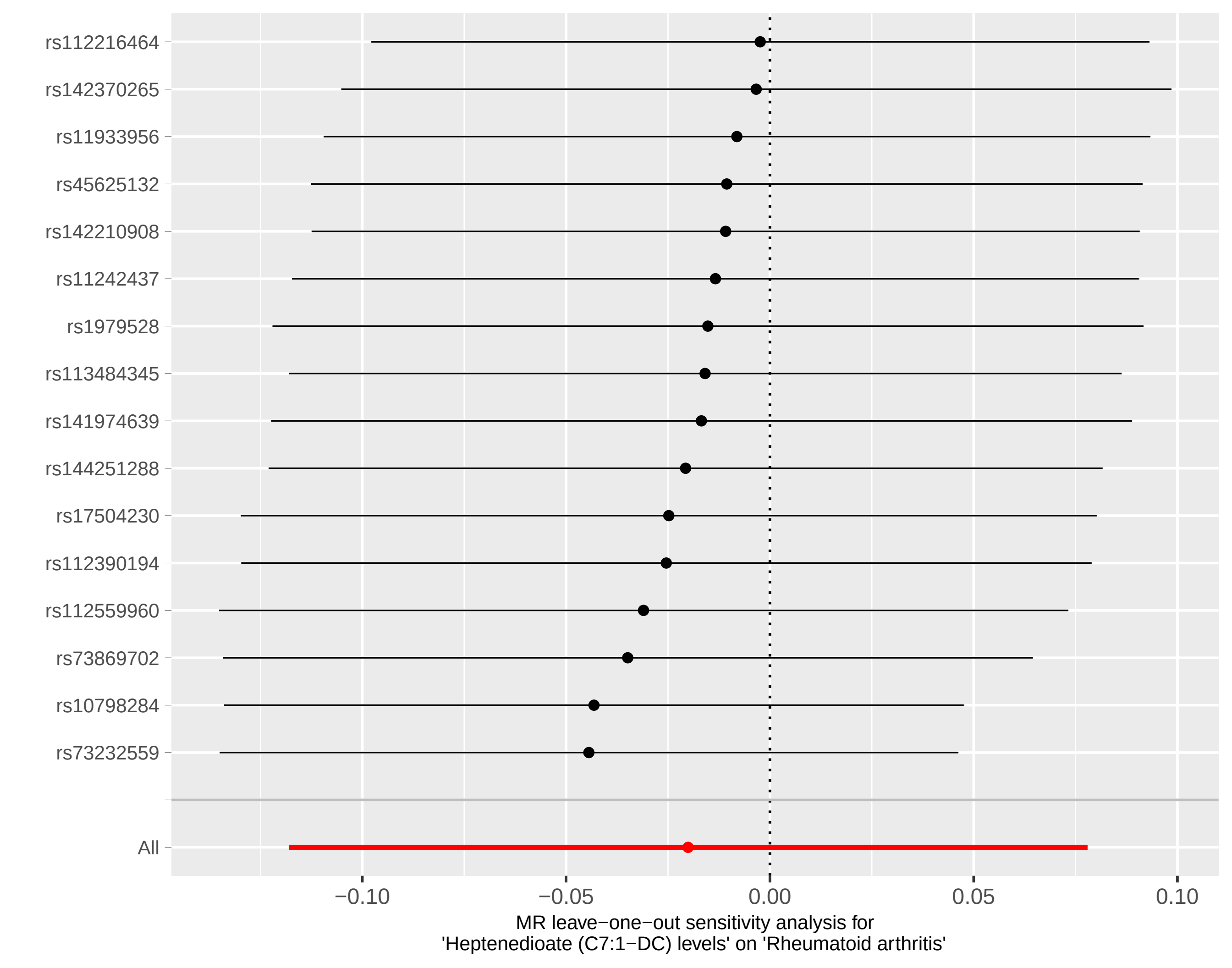

rs112216464
rs142370265
rs11933956
rs45625132
rs142210908
rs11242437
rs1979528
rs113484345
rs141974639
rs144251288
rs17504230
rs112390194
rs112559960
rs73869702
rs10798284
rs73232559
All
−0.10
−0.05
0.00
0.05
0.10
MR leave−one−out sensitivity analysis for
'Heptenedioate (C7:1−DC) levels' on 'Rheumatoid arthritis'

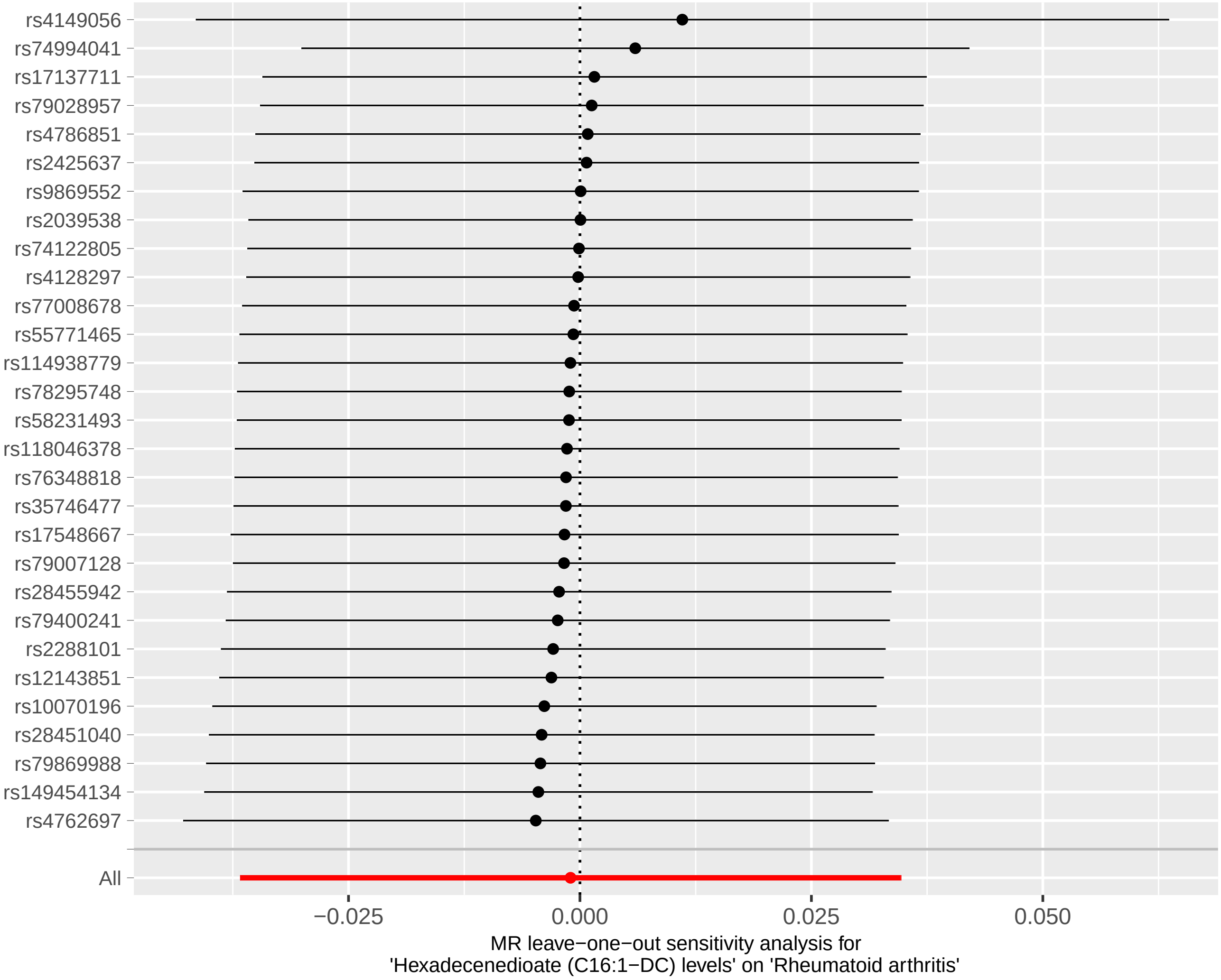
rs4149056
rs74994041
rs17137711
rs79028957
rs4786851
rs2425637
rs9869552
rs2039538
rs74122805
rs4128297
rs77008678
rs55771465
rs114938779
rs78295748
rs58231493
rs118046378
rs76348818
rs35746477
rs17548667
rs79007128
rs28455942
rs79400241
rs2288101
rs12143851
rs10070196
rs28451040
rs79869988
rs149454134
rs4762697
All
−0.025
0.000
0.025
0.050
MR leave−one−out sensitivity analysis for
'Hexadecenedioate (C16:1−DC) levels' on 'Rheumatoid arthritis'

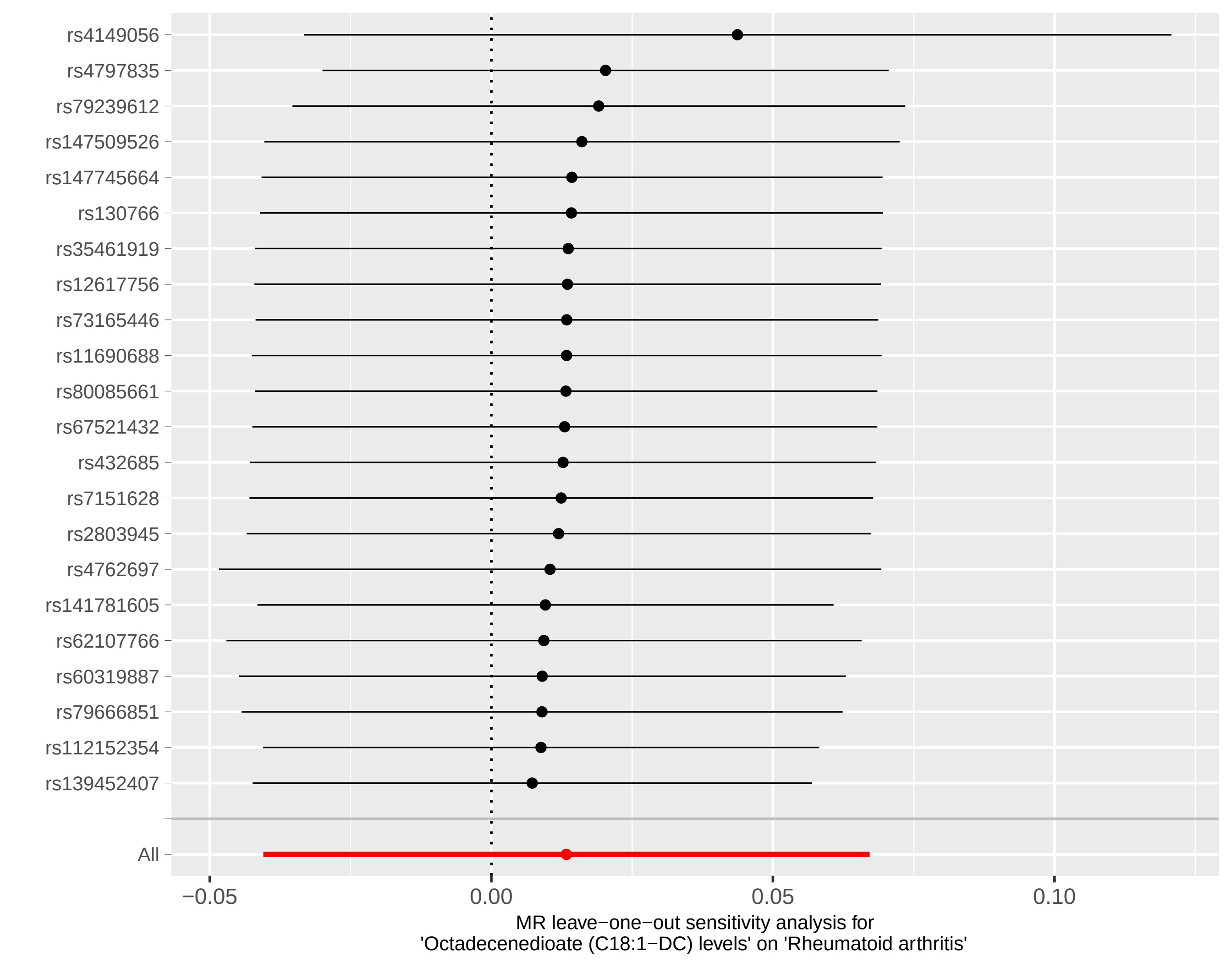
rs4149056
rs4797835
rs79239612
rs147509526
rs147745664
rs130766
rs35461919
rs12617756
rs73165446
rs11690688
rs80085661
rs67521432
rs432685
rs7151628
rs2803945
rs4762697
rs141781605
rs62107766
rs60319887
rs79666851
rs112152354
rs139452407
All
−0.05
0.00
0.05
0.10
MR leave−one−out sensitivity analysis for
'Octadecenedioate (C18:1−DC) levels' on 'Rheumatoid arthritis'

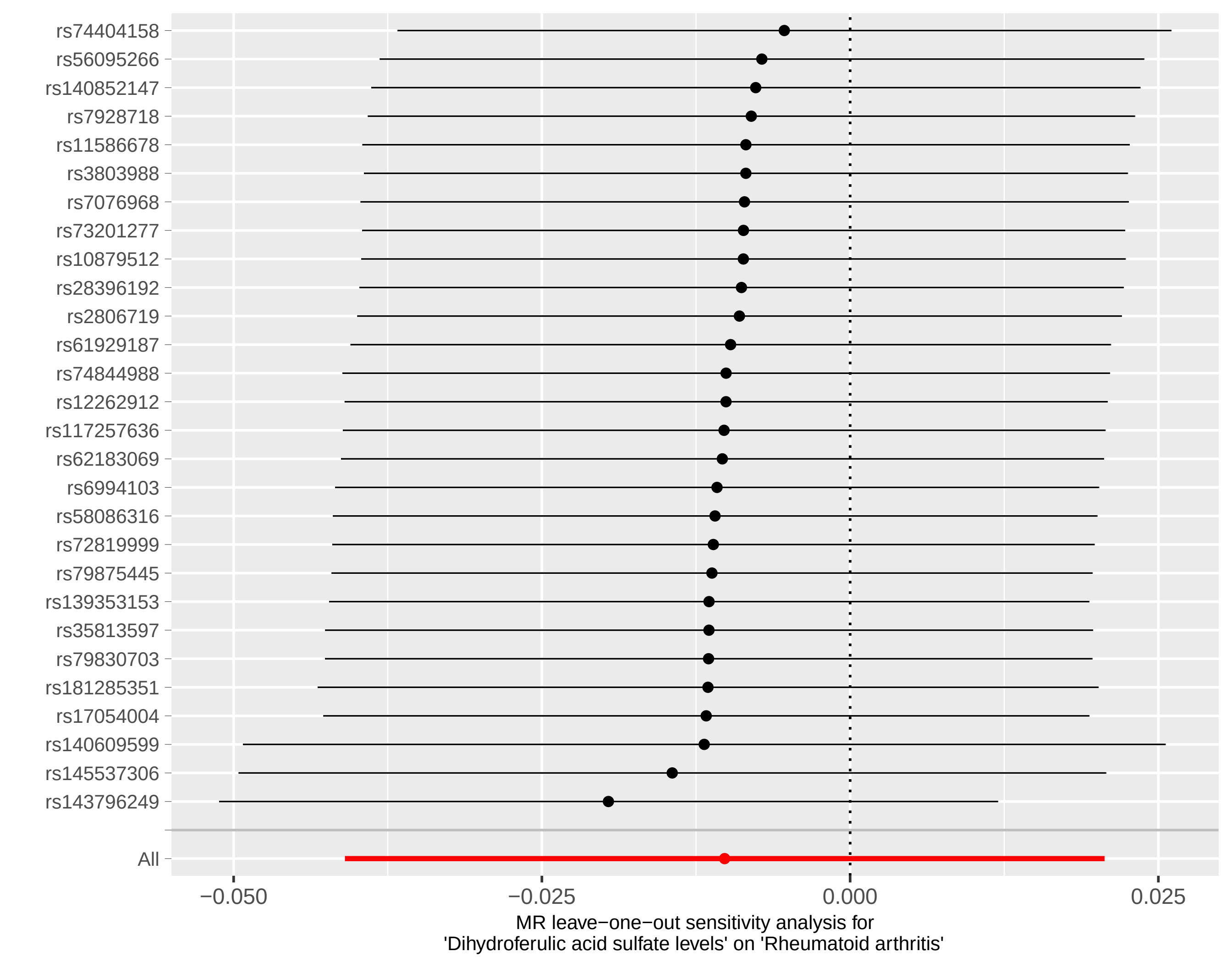
rs74404158
rs56095266
rs140852147
rs7928718
rs11586678
rs3803988
rs7076968
rs73201277
rs10879512
rs28396192
rs2806719
rs61929187
rs74844988
rs12262912
rs117257636
rs62183069
rs6994103
rs58086316
rs72819999
rs79875445
rs139353153
rs35813597
rs79830703
rs181285351
rs17054004
rs140609599
rs145537306
rs143796249
All
−0.050
−0.025
0.000
0.025
MR leave−one−out sensitivity analysis for
'Dihydroferulic acid sulfate levels' on 'Rheumatoid arthritis'

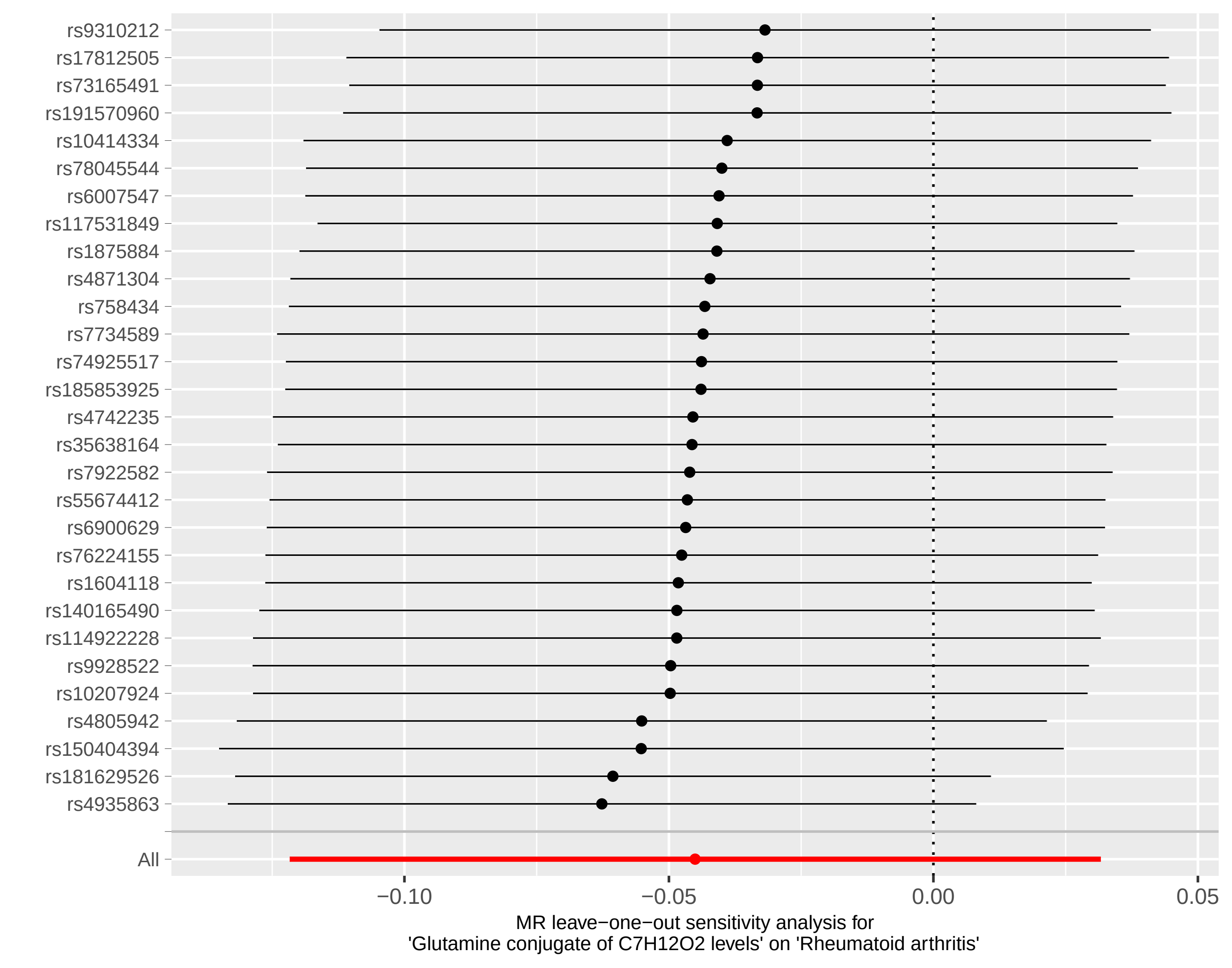
rs9310212
rs17812505
rs73165491
rs191570960
rs10414334
rs78045544
rs6007547
rs117531849
rs1875884
rs4871304
rs758434
rs7734589
rs74925517
rs185853925
rs4742235
rs35638164
rs7922582
rs55674412
rs6900629
rs76224155
rs1604118
rs140165490
rs114922228
rs9928522
rs10207924
rs4805942
rs150404394
rs181629526
rs4935863
All
−0.10
−0.05
0.00
0.05
MR leave−one−out sensitivity analysis for
'Glutamine conjugate of C7H12O2 levels' on 'Rheumatoid arthritis'

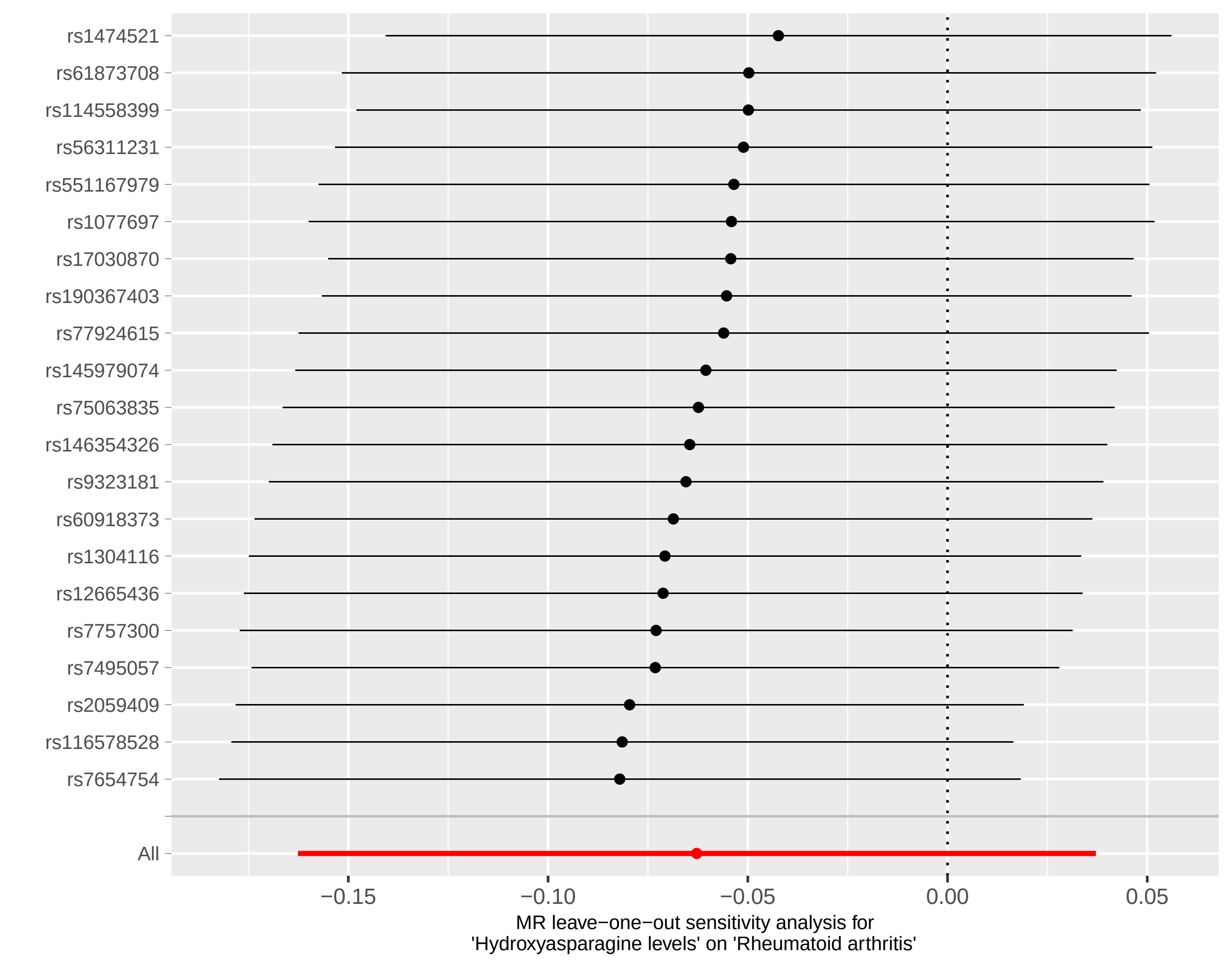
rs1474521
rs61873708
rs114558399
rs56311231
rs551167979
rs1077697
rs17030870
rs190367403
rs77924615
rs145979074
rs75063835
rs146354326
rs9323181
rs60918373
rs1304116
rs12665436
rs7757300
rs7495057
rs2059409
rs116578528
rs7654754
All
−0.15
−0.10
−0.05
0.00
0.05
MR leave−one−out sensitivity analysis for
'Hydroxyasparagine levels' on 'Rheumatoid arthritis'

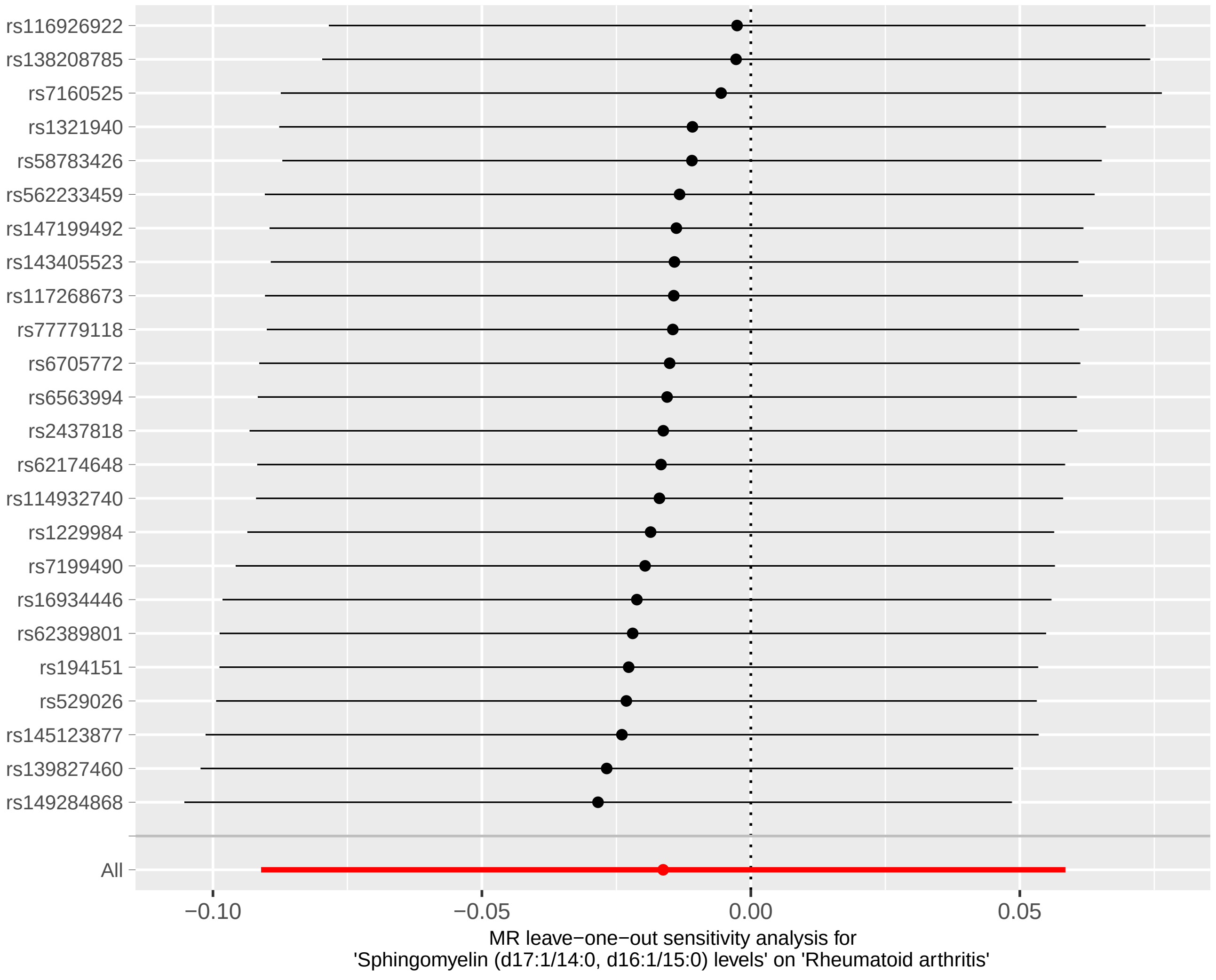
rs116926922
rs138208785
rs7160525
rs1321940
rs58783426
rs562233459
rs147199492
rs143405523
rs117268673
rs77779118
rs6705772
rs6563994
rs2437818
rs62174648
rs114932740
rs1229984
rs7199490
rs16934446
rs62389801
rs194151
rs529026
rs145123877
rs139827460
rs149284868
All
−0.10
−0.05
0.00
0.05
MR leave−one−out sensitivity analysis for
'Sphingomyelin (d17:1/14:0, d16:1/15:0) levels' on 'Rheumatoid arthritis'

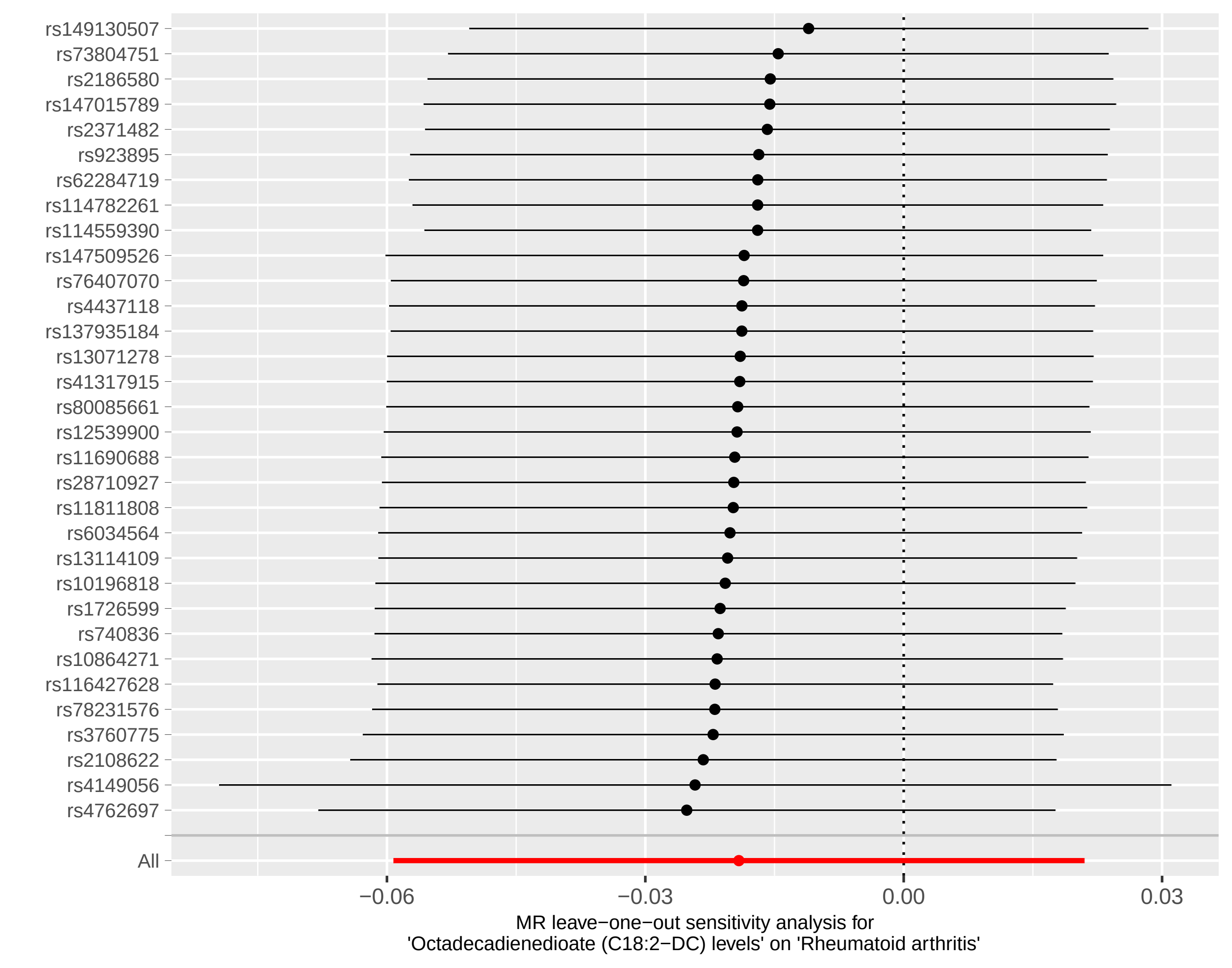
rs149130507
rs73804751
rs2186580
rs147015789
rs2371482
rs923895
rs62284719
rs114782261
rs114559390
rs147509526
rs76407070
rs4437118
rs137935184
rs13071278
rs41317915
rs80085661
rs12539900
rs11690688
rs28710927
rs11811808
rs6034564
rs13114109
rs10196818
rs1726599
rs740836
rs10864271
rs116427628
rs78231576
rs3760775
rs2108622
rs4149056
rs4762697
All
−0.06
−0.03
0.00
0.03
MR leave−one−out sensitivity analysis for
'Octadecadienedioate (C18:2−DC) levels' on 'Rheumatoid arthritis'

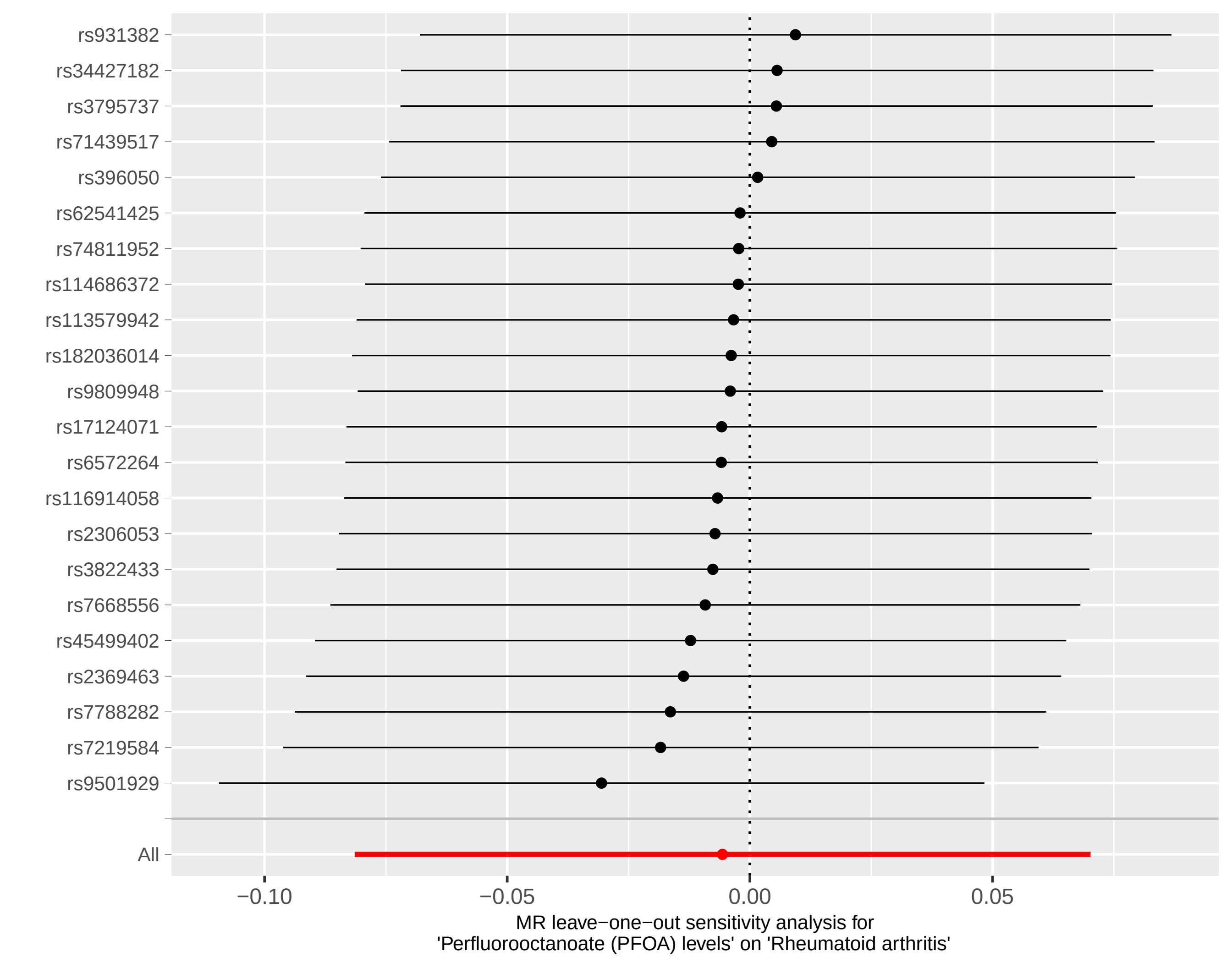

rs931382
rs34427182
rs3795737
rs71439517
rs396050
rs62541425
rs74811952
rs114686372
rs113579942
rs182036014
rs9809948
rs17124071
rs6572264
rs116914058
rs2306053
rs3822433
rs7668556
rs45499402
rs2369463
rs7788282
rs7219584
rs9501929
All
−0.10
−0.05
0.00
0.05
MR leave−one−out sensitivity analysis for
'Perfluorooctanoate (PFOA) levels' on 'Rheumatoid arthritis'

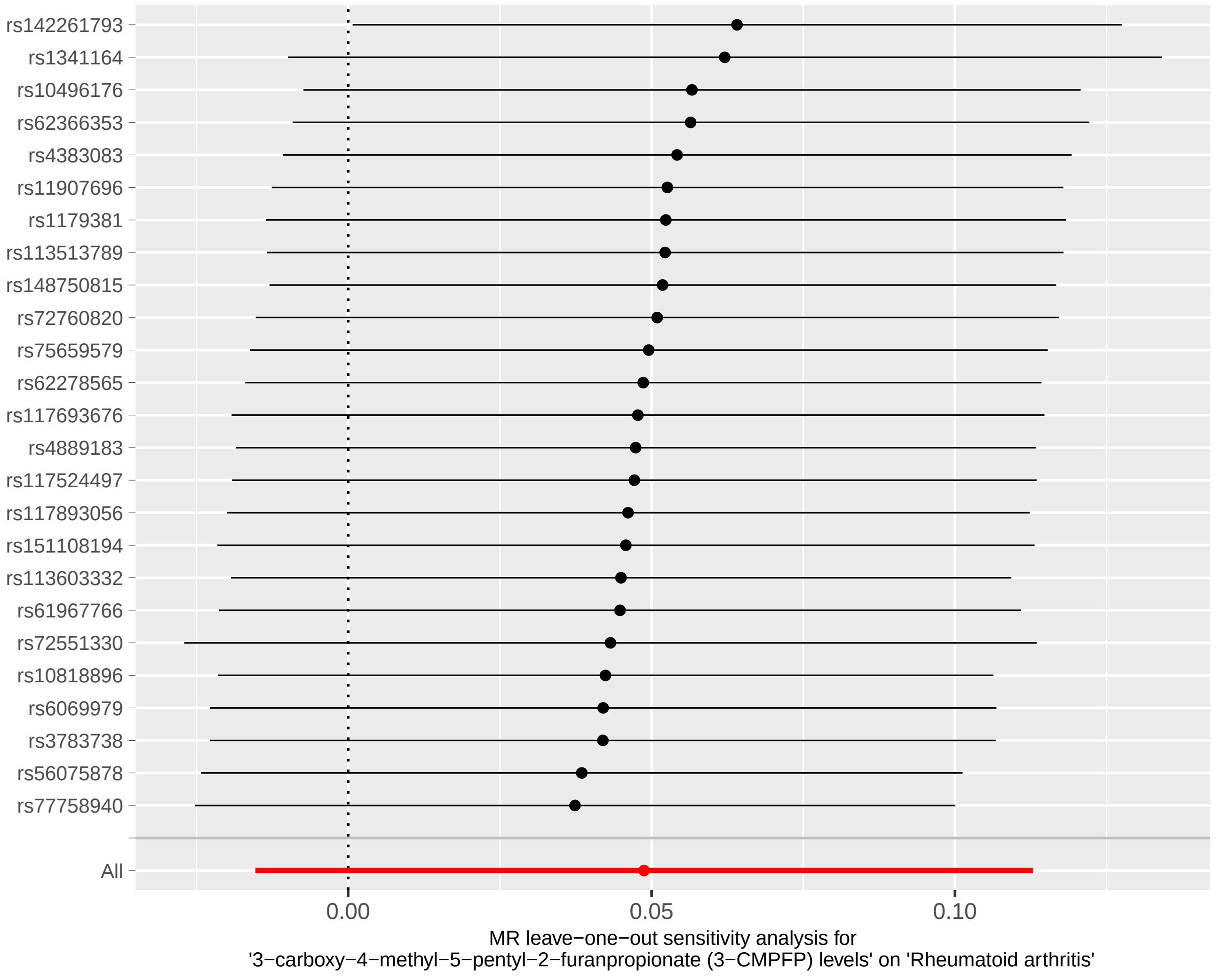
rs142261793
rs1341164
rs10496176
rs62366353
rs4383083
rs11907696
rs1179381
rs113513789
rs148750815
rs72760820
rs75659579
rs62278565
rs117693676
rs4889183
rs117524497
rs117893056
rs151108194
rs113603332
rs61967766
rs72551330
rs10818896
rs6069979
rs3783738
rs56075878
rs77758940
All
0.00
0.05
0.10
MR leave−one−out sensitivity analysis for
'3−carboxy−4−methyl−5−pentyl−2−furanpropionate (3−CMPFP) levels' on 'Rheumatoid arthritis'

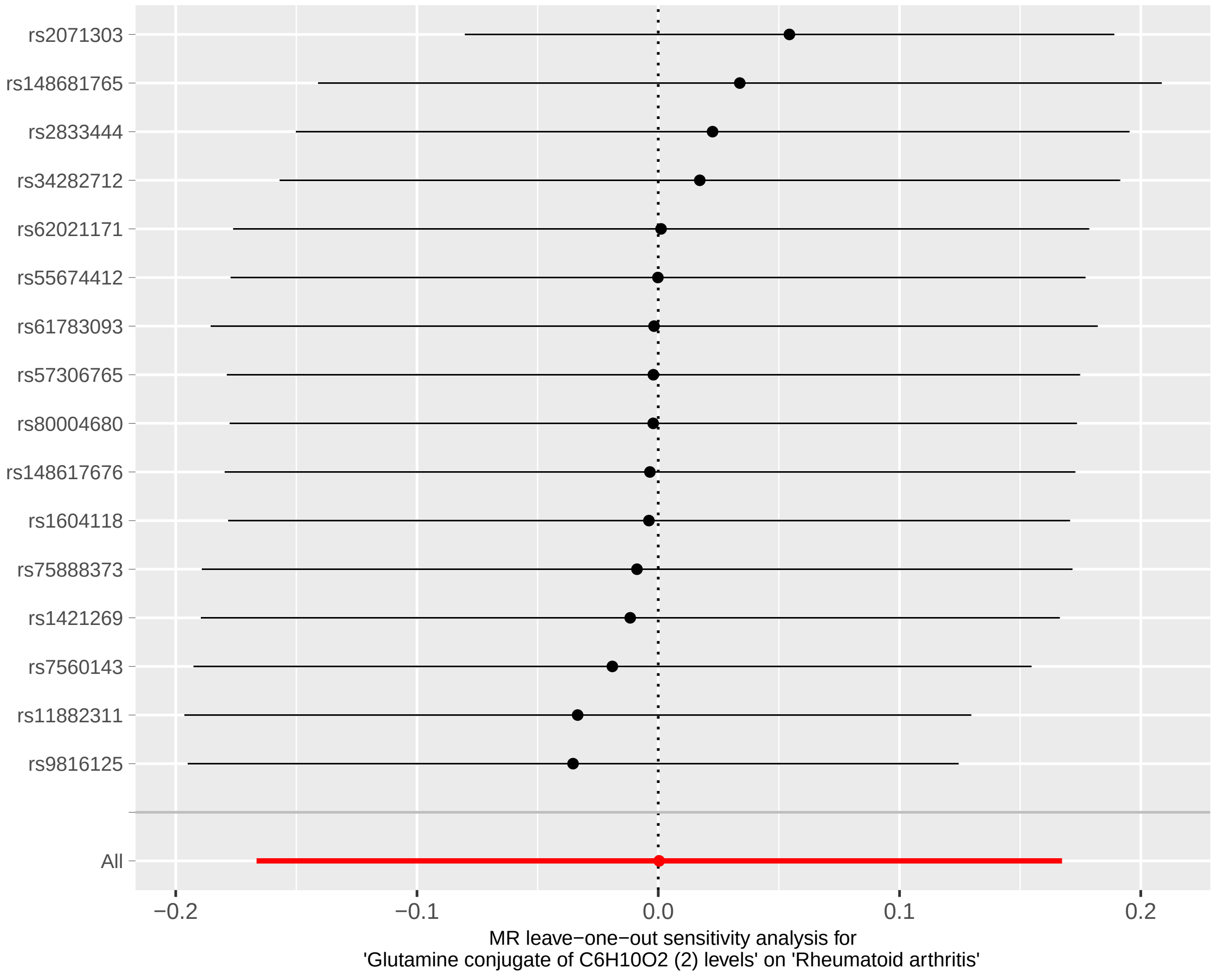
rs2071303
rs148681765
rs2833444
rs34282712
rs62021171
rs55674412
rs61783093
rs57306765
rs80004680
rs148617676
rs1604118
rs75888373
rs1421269
rs7560143
rs11882311
rs9816125
All
−0.2
−0.1
0.0
0.1
0.2
MR leave−one−out sensitivity analysis for
'Glutamine conjugate of C6H10O2 (2) levels' on 'Rheumatoid arthritis'

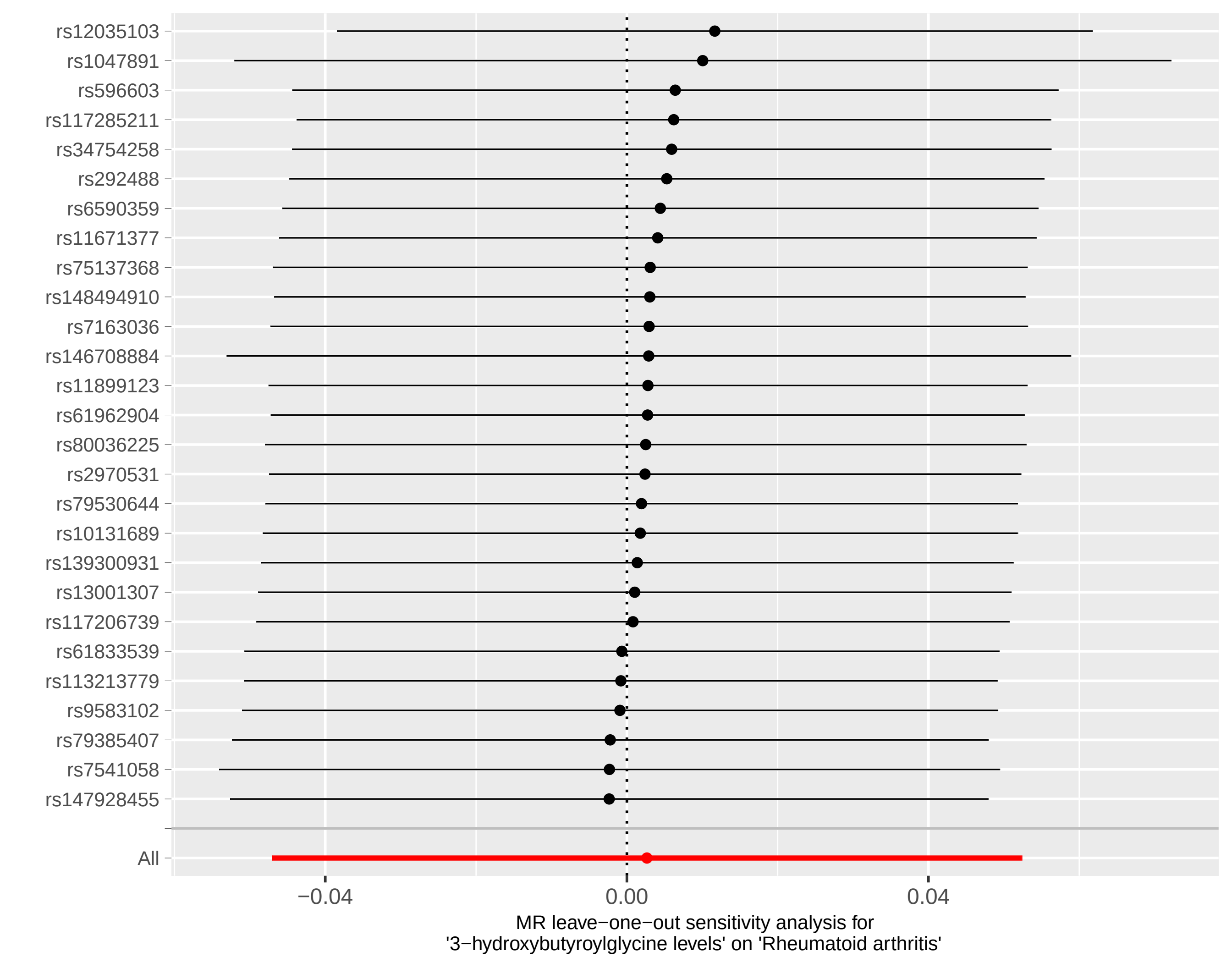

rs12035103
rs1047891
rs596603
rs117285211
rs34754258
rs292488
rs6590359
rs11671377
rs75137368
rs148494910
rs7163036
rs146708884
rs11899123
rs61962904
rs80036225
rs2970531
rs79530644
rs10131689
rs139300931
rs13001307
rs117206739
rs61833539
rs113213779
rs9583102
rs79385407
rs7541058
rs147928455
All
−0.04
0.00
0.04
MR leave−one−out sensitivity analysis for
'3−hydroxybutyroylglycine levels' on 'Rheumatoid arthritis'

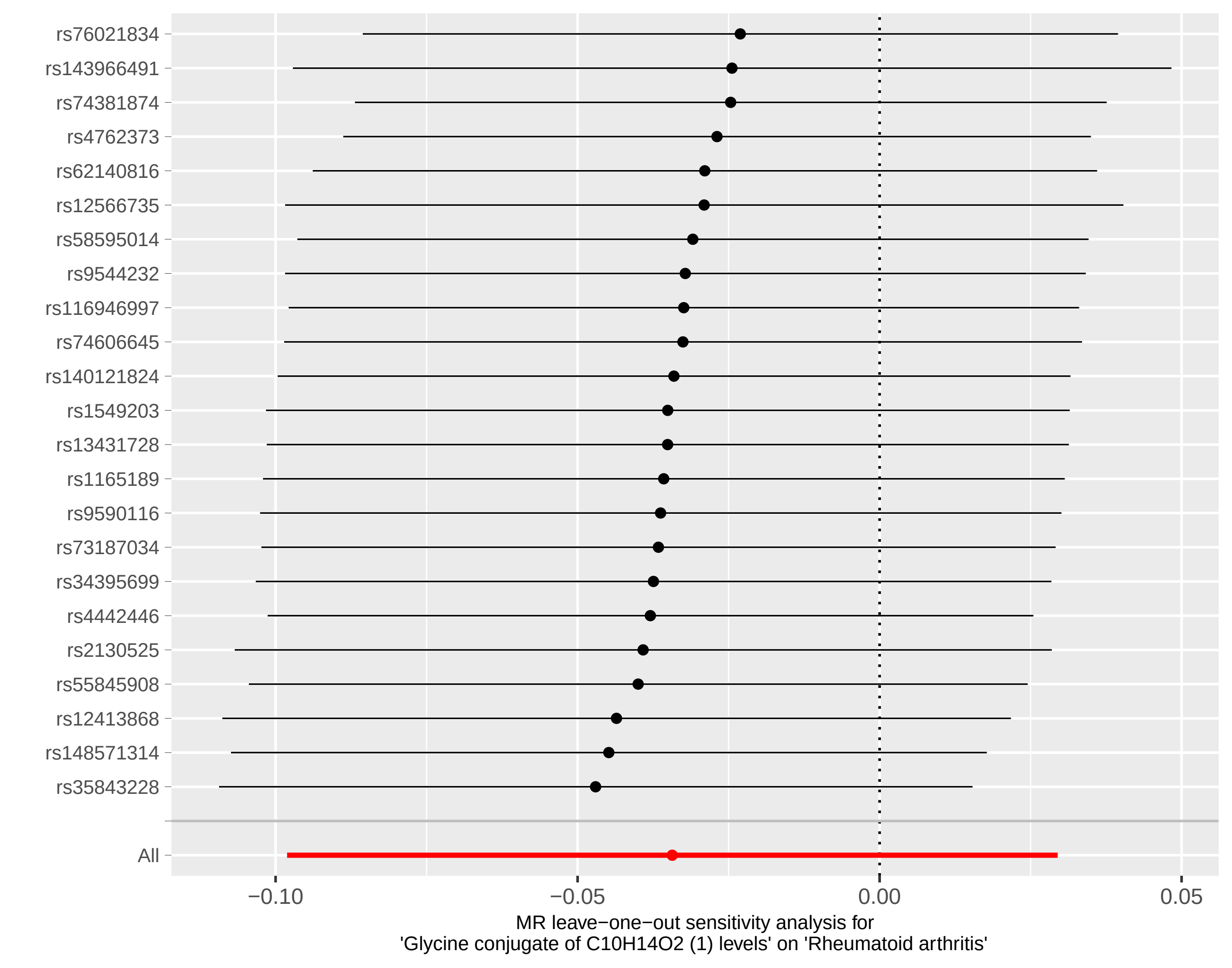

rs76021834
rs143966491
rs74381874
rs4762373
rs62140816
rs12566735
rs58595014
rs9544232
rs116946997
rs74606645
rs140121824
rs1549203
rs13431728
rs1165189
rs9590116
rs73187034
rs34395699
rs4442446
rs2130525
rs55845908
rs12413868
rs148571314
rs35843228
All
−0.10
−0.05
0.00
0.05
MR leave−one−out sensitivity analysis for
'Glycine conjugate of C10H14O2 (1) levels' on 'Rheumatoid arthritis'

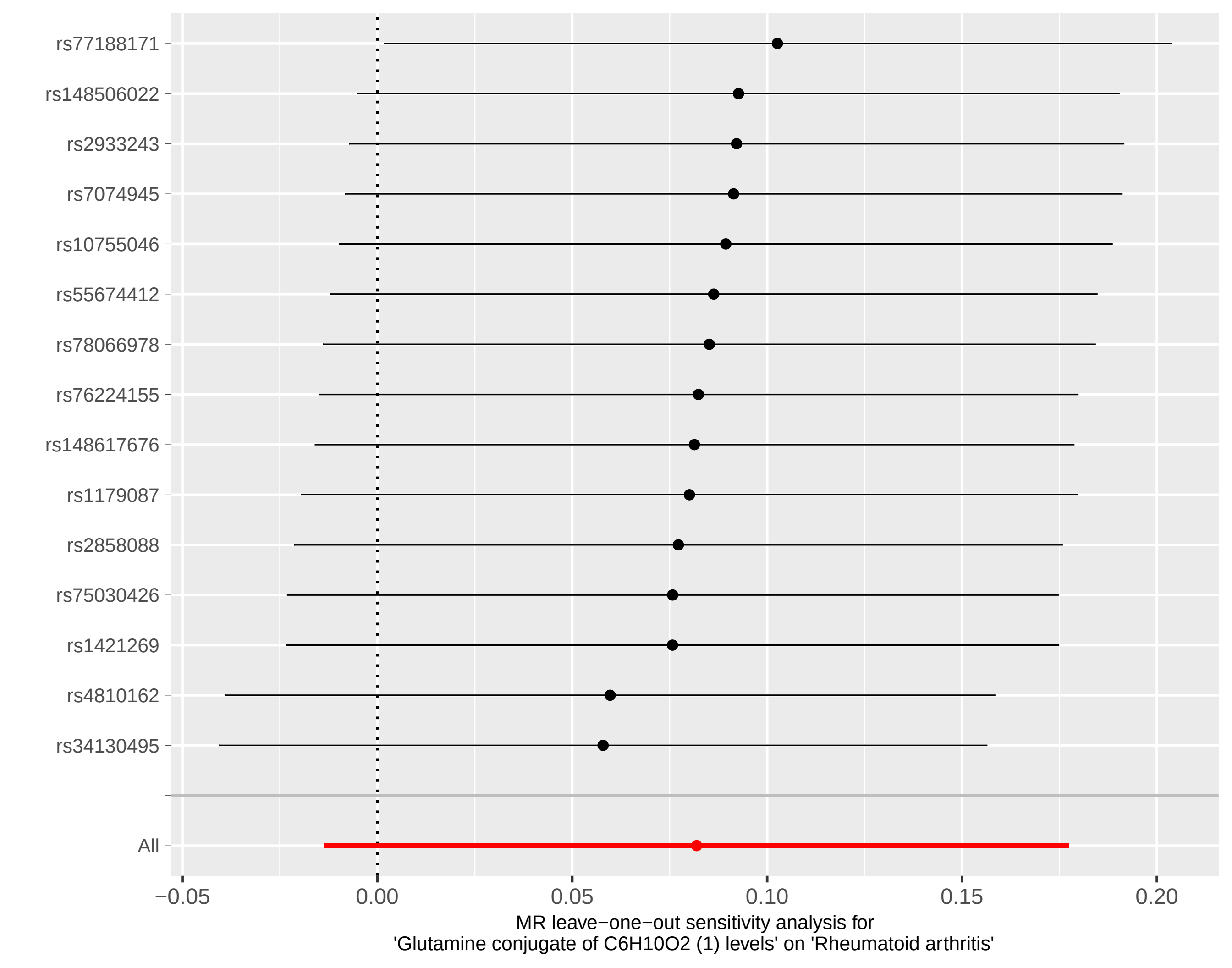
rs77188171
rs148506022
rs2933243
rs7074945
rs10755046
rs55674412
rs78066978
rs76224155
rs148617676
rs1179087
rs2858088
rs75030426
rs1421269
rs4810162
rs34130495
All
−0.05
0.00
0.05
0.10
0.15
0.20
MR leave−one−out sensitivity analysis for
'Glutamine conjugate of C6H10O2 (1) levels' on 'Rheumatoid arthritis'

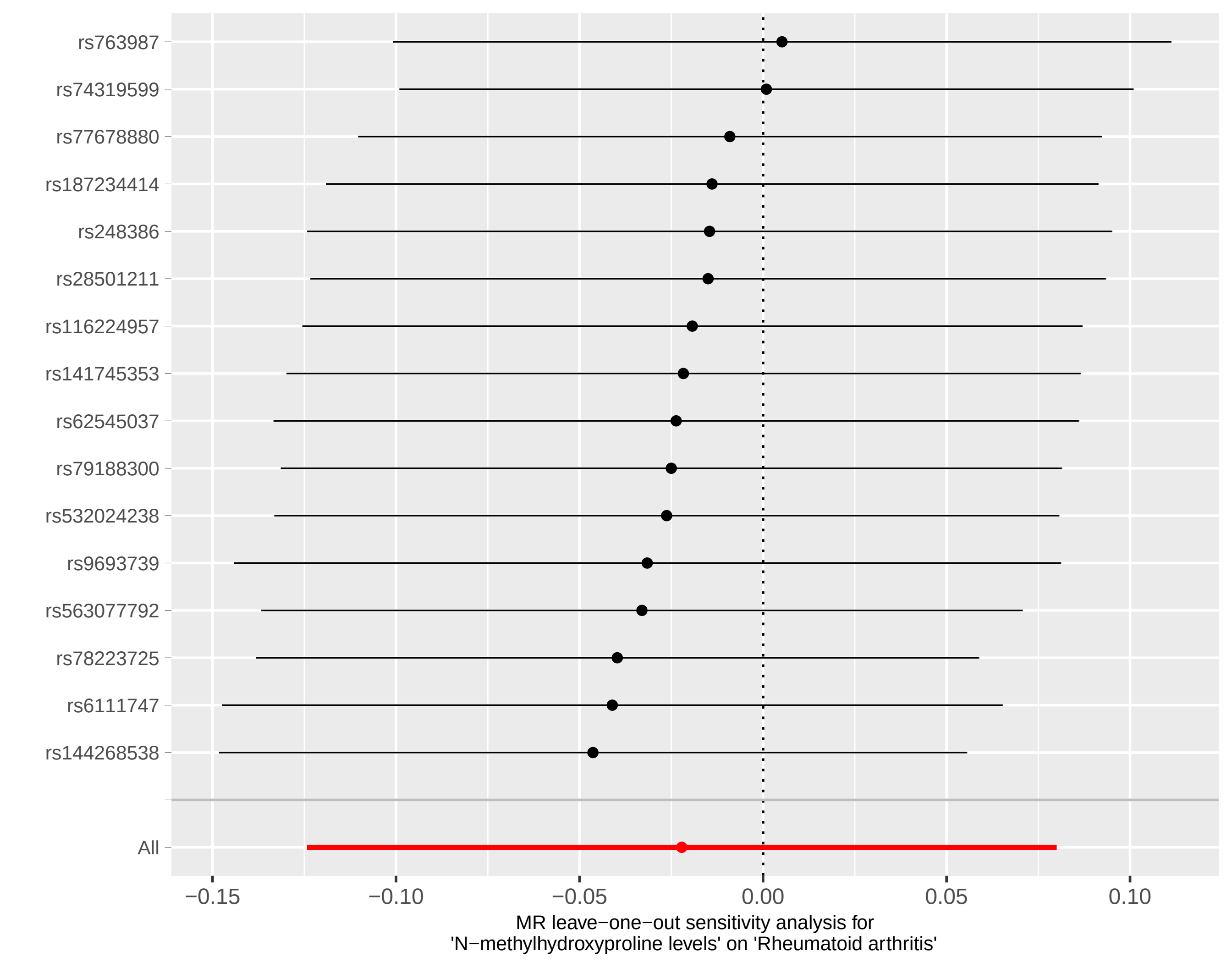
rs763987
rs74319599
rs77678880
rs187234414
rs248386
rs28501211
rs116224957
rs141745353
rs62545037
rs79188300
rs532024238
rs9693739
rs563077792
rs78223725
rs6111747
rs144268538
All
−0.15
−0.10
−0.05
0.00
0.05
0.10
MR leave−one−out sensitivity analysis for
'N−methylhydroxyproline levels' on 'Rheumatoid arthritis'

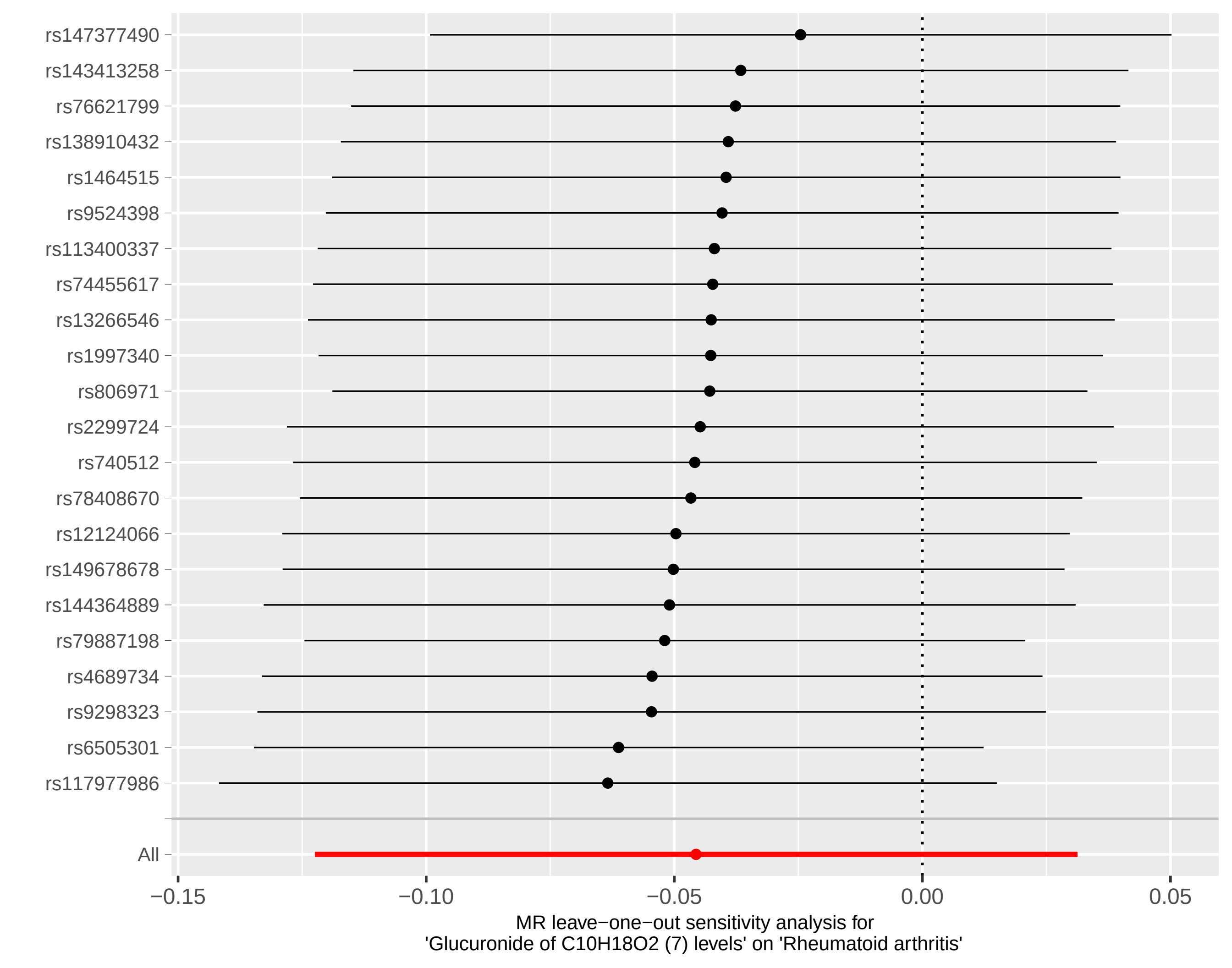
rs147377490
rs143413258
rs76621799
rs138910432
rs1464515
rs9524398
rs113400337
rs74455617
rs13266546
rs1997340
rs806971
rs2299724
rs740512
rs78408670
rs12124066
rs149678678
rs144364889
rs79887198
rs4689734
rs9298323
rs6505301
rs117977986
All
−0.15
−0.10
−0.05
0.00
0.05
MR leave−one−out sensitivity analysis for
'Glucuronide of C10H18O2 (7) levels' on 'Rheumatoid arthritis'

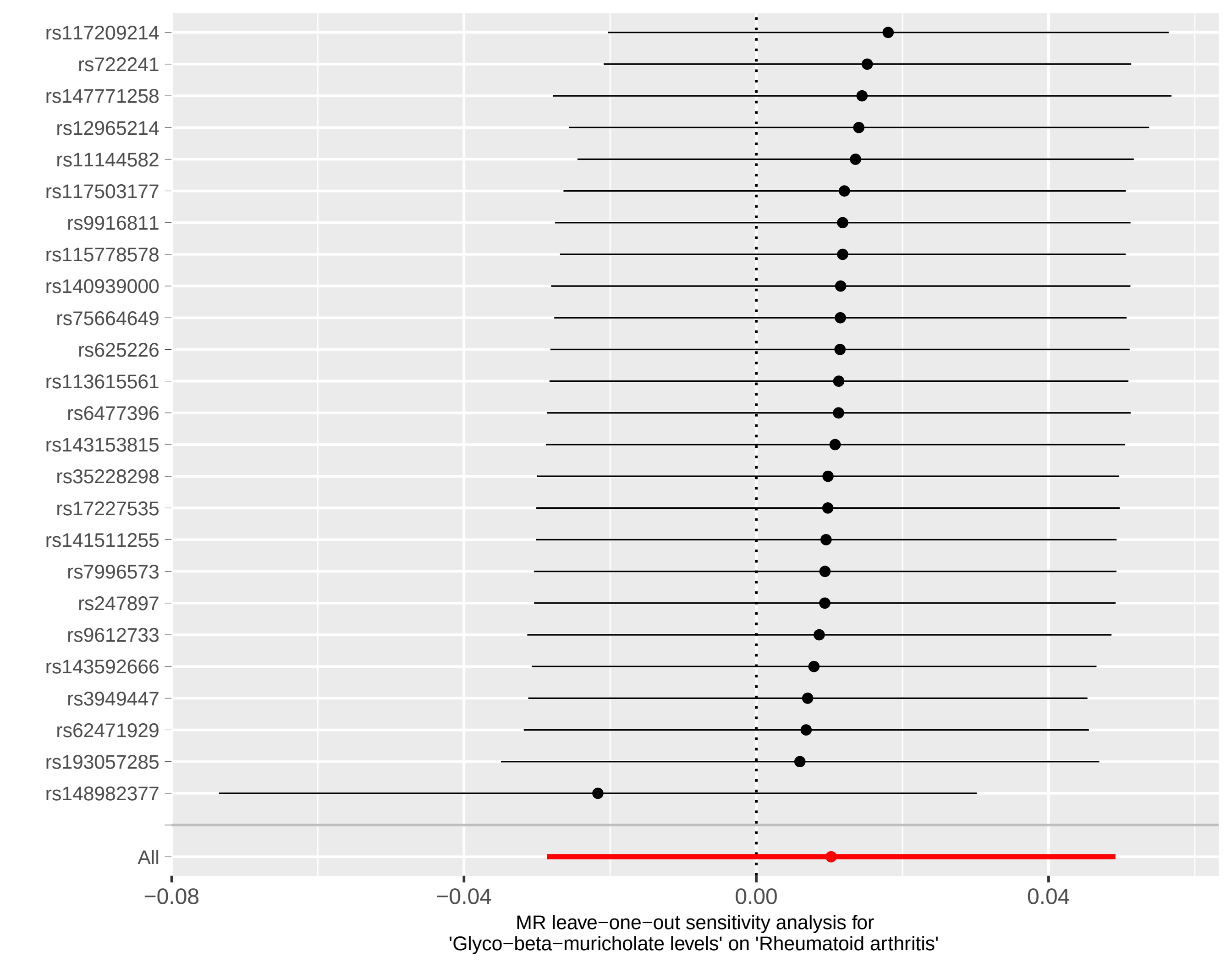
rs117209214
rs722241
rs147771258
rs12965214
rs11144582
rs117503177
rs9916811
rs115778578
rs140939000
rs75664649
rs625226
rs113615561
rs6477396
rs143153815
rs35228298
rs17227535
rs141511255
rs7996573
rs247897
rs9612733
rs143592666
rs3949447
rs62471929
rs193057285
rs148982377
All
−0.08
−0.04
0.00
0.04
MR leave−one−out sensitivity analysis for
'Glyco−beta−muricholate levels' on 'Rheumatoid arthritis'

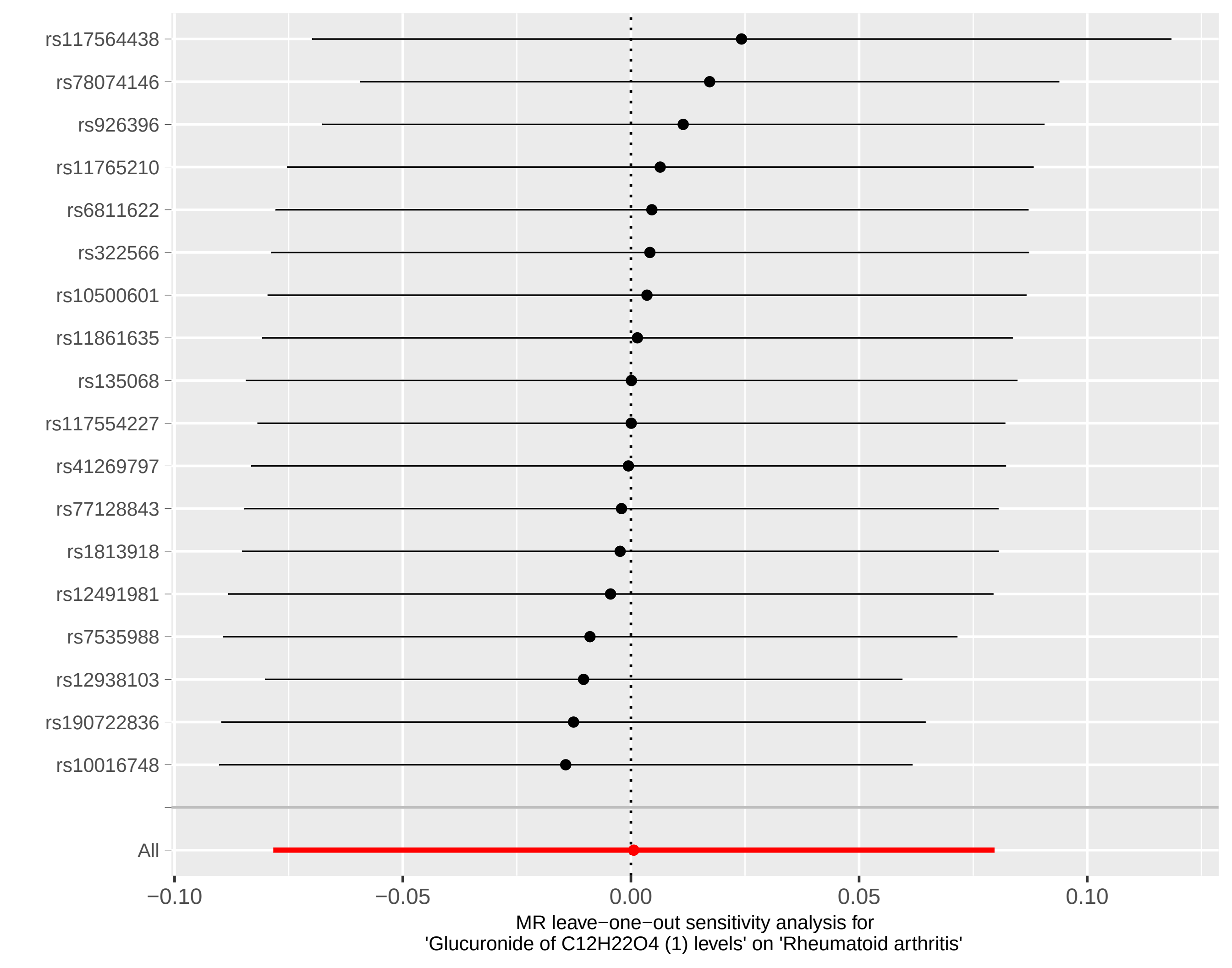

rs117564438
rs78074146
rs926396
rs11765210
rs6811622
rs322566
rs10500601
rs11861635
rs135068
rs117554227
rs41269797
rs77128843
rs1813918
rs12491981
rs7535988
rs12938103
rs190722836
rs10016748
All
−0.10
−0.05
0.00
0.05
0.10
MR leave−one−out sensitivity analysis for
'Glucuronide of C12H22O4 (1) levels' on 'Rheumatoid arthritis'

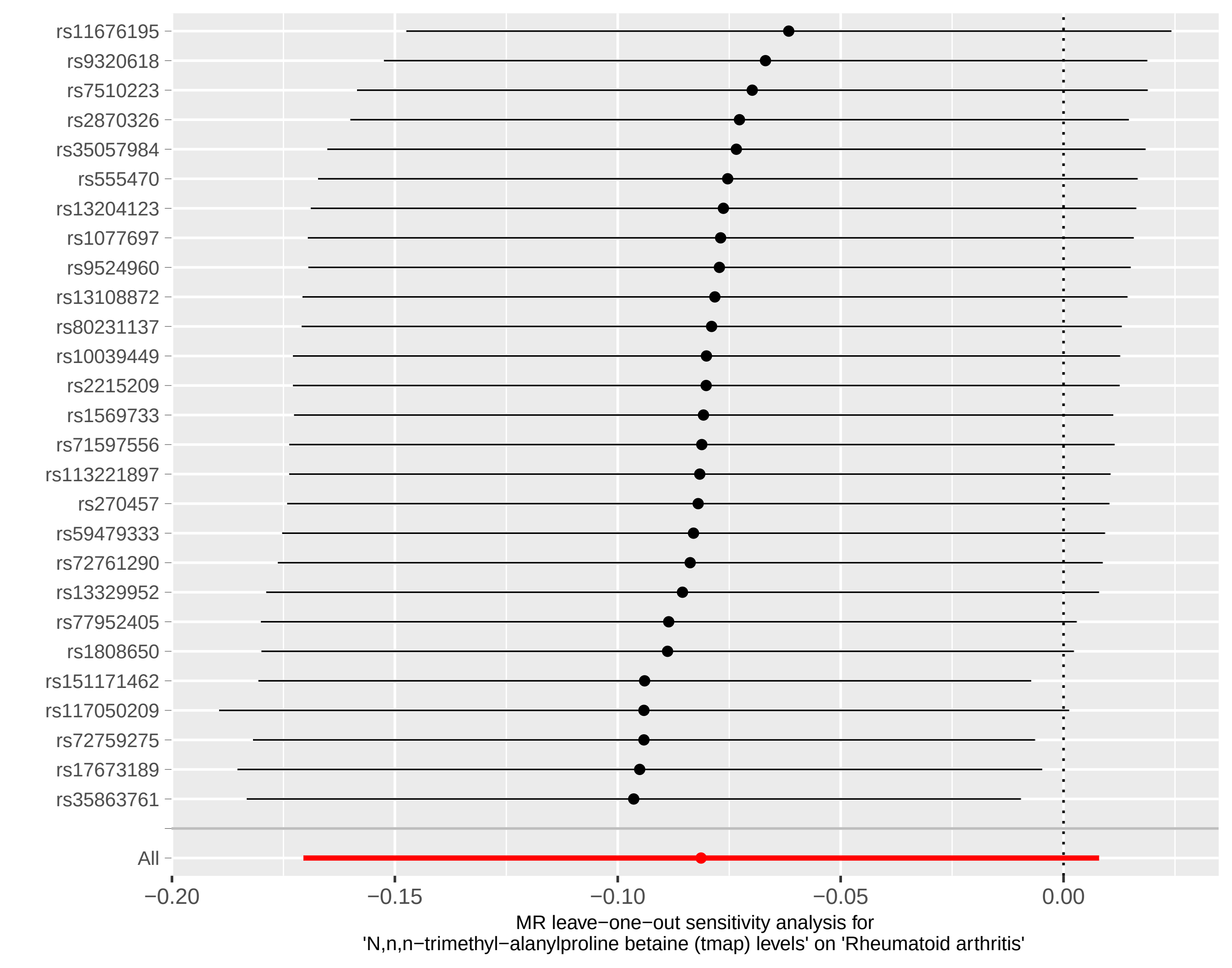

rs11676195
rs9320618
rs7510223
rs2870326
rs35057984
rs555470
rs13204123
rs1077697
rs9524960
rs13108872
rs80231137
rs10039449
rs2215209
rs1569733
rs71597556
rs113221897
rs270457
rs59479333
rs72761290
rs13329952
rs77952405
rs1808650
rs151171462
rs117050209
rs72759275
rs17673189
rs35863761
All
−0.20
−0.15
−0.10
−0.05
0.00
MR leave−one−out sensitivity analysis for
'N,n,n−trimethyl−alanylproline betaine (tmap) levels' on 'Rheumatoid arthritis'

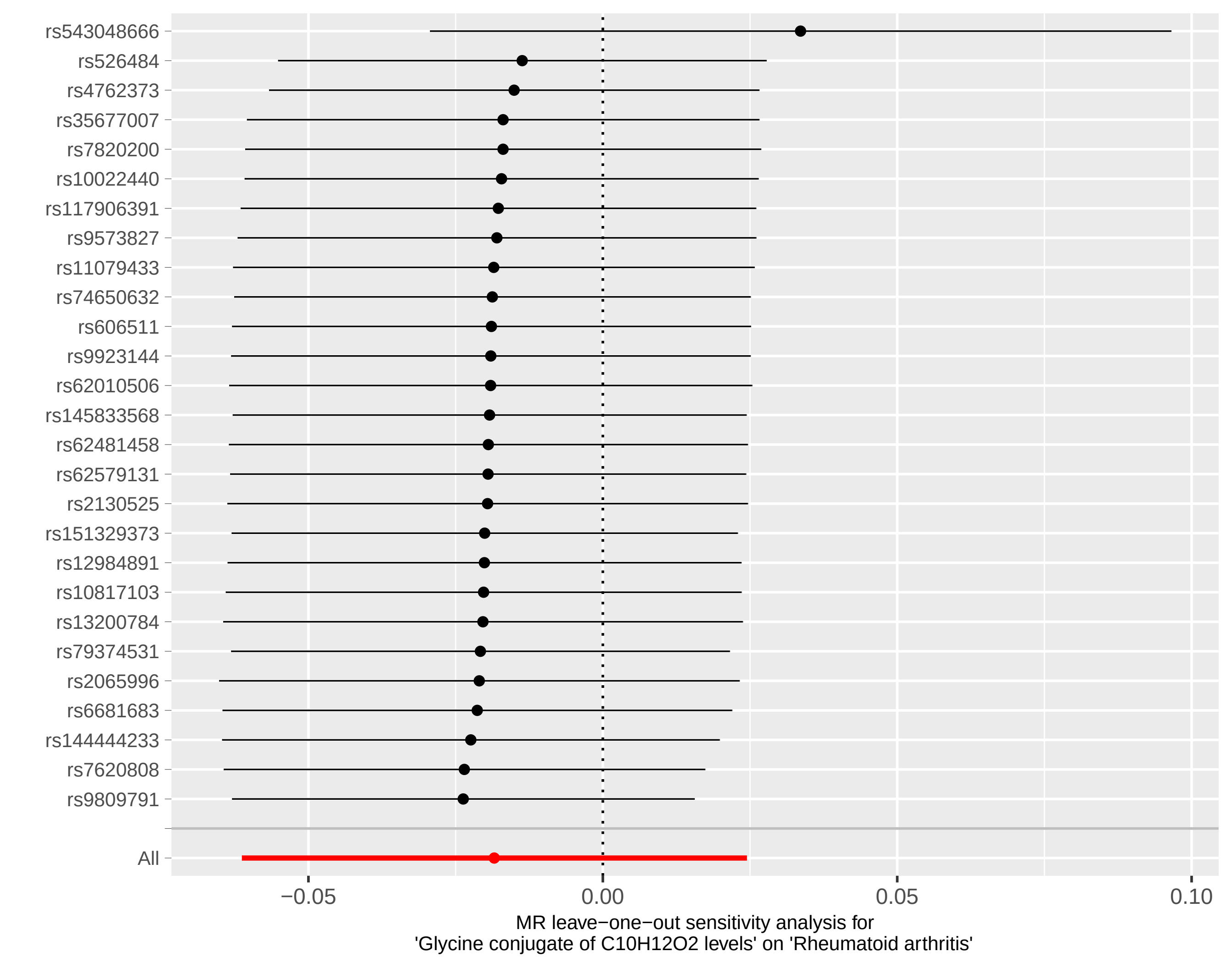

rs543048666
rs526484
rs4762373
rs35677007
rs7820200
rs10022440
rs117906391
rs9573827
rs11079433
rs74650632
rs606511
rs9923144
rs62010506
rs145833568
rs62481458
rs62579131
rs2130525
rs151329373
rs12984891
rs10817103
rs13200784
rs79374531
rs2065996
rs6681683
rs144444233
rs7620808
rs9809791
All
−0.05
0.00
0.05
0.10
MR leave−one−out sensitivity analysis for
'Glycine conjugate of C10H12O2 levels' on 'Rheumatoid arthritis'

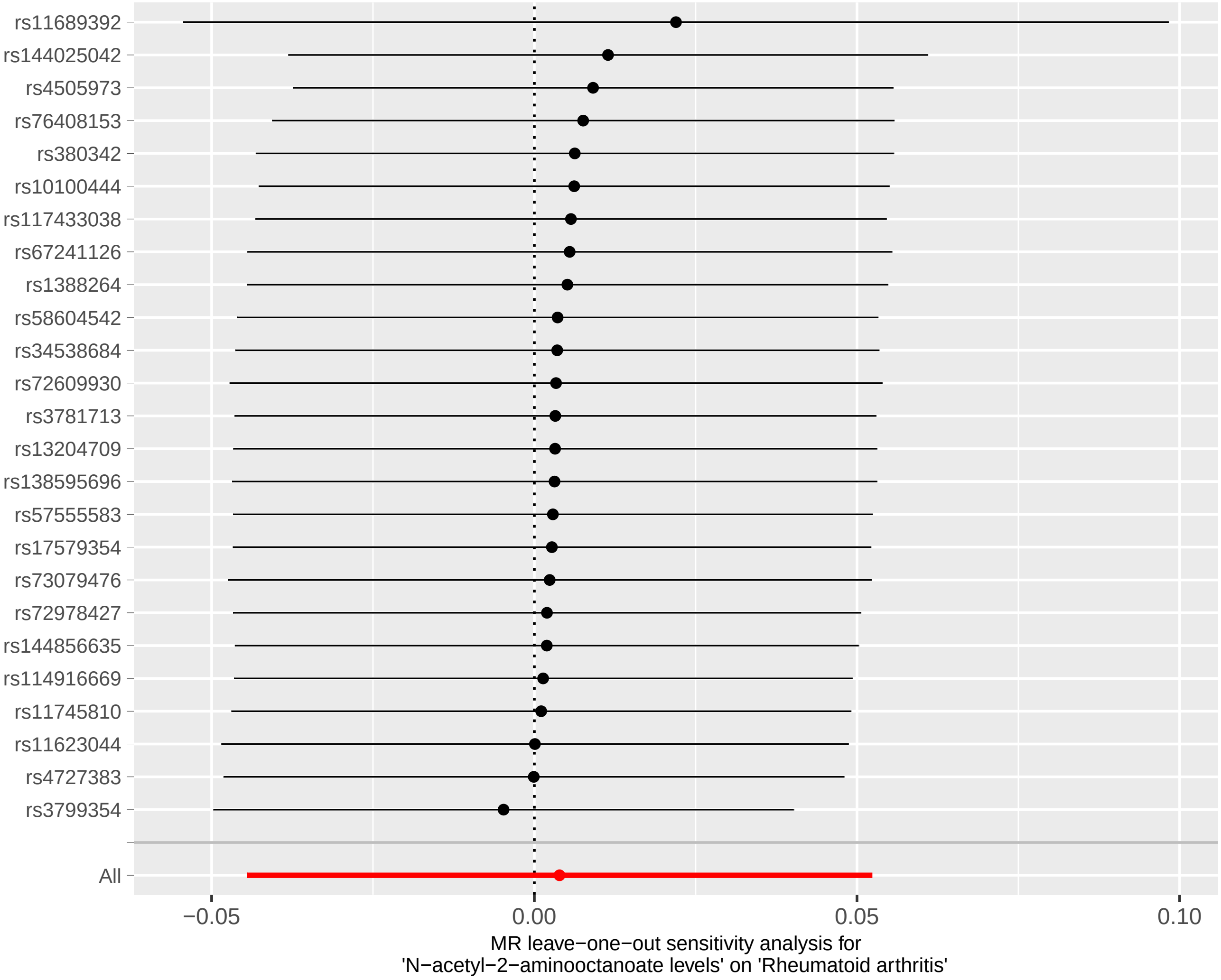
rs11689392
rs144025042
rs4505973
rs76408153
rs380342
rs10100444
rs117433038
rs67241126
rs1388264
rs58604542
rs34538684
rs72609930
rs3781713
rs13204709
rs138595696
rs57555583
rs17579354
rs73079476
rs72978427
rs144856635
rs114916669
rs11745810
rs11623044
rs4727383
rs3799354
All
−0.05
0.00
0.05
0.10
MR leave−one−out sensitivity analysis for
'N−acetyl−2−aminooctanoate levels' on 'Rheumatoid arthritis'

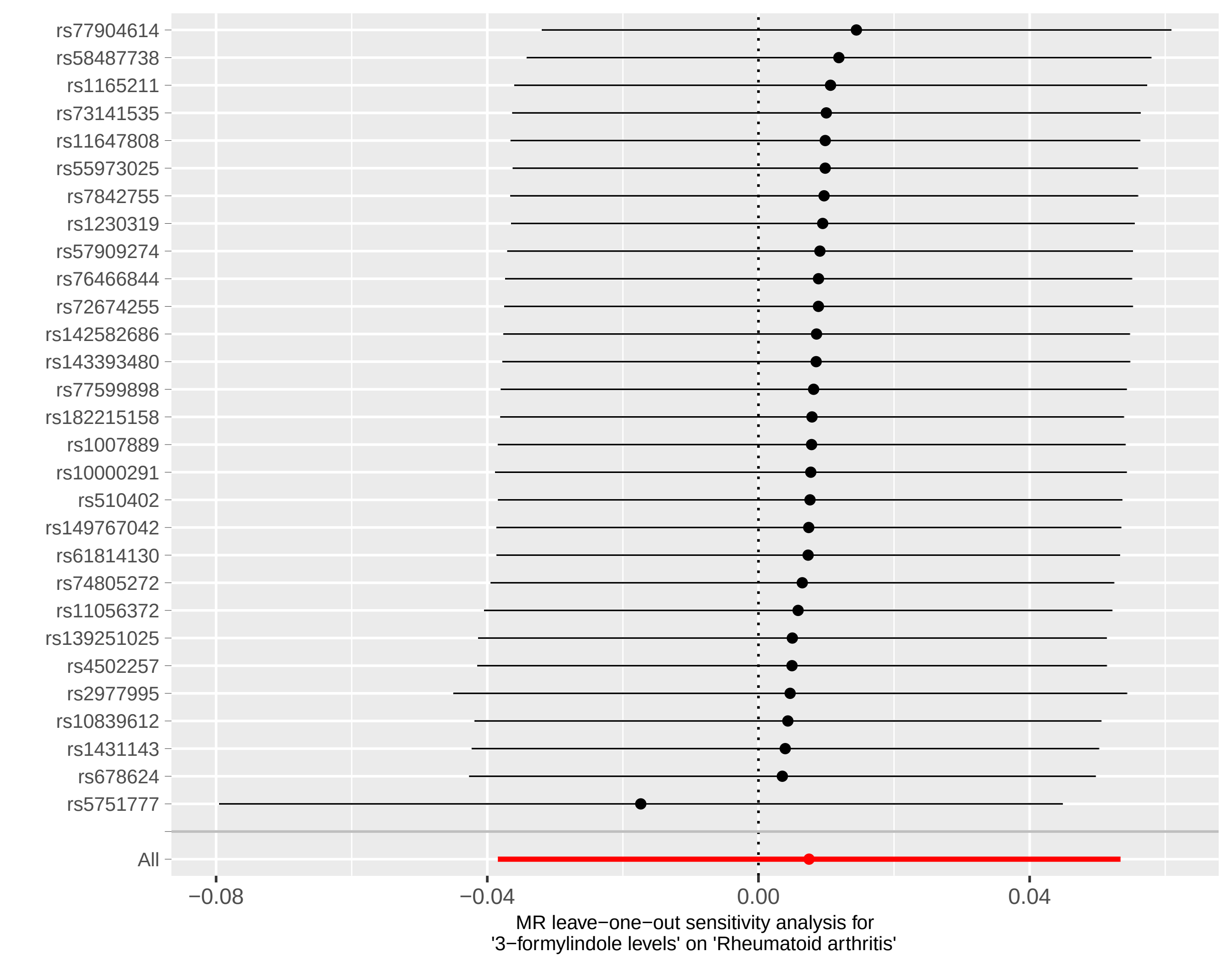

rs77904614
rs58487738
rs1165211
rs73141535
rs11647808
rs55973025
rs7842755
rs1230319
rs57909274
rs76466844
rs72674255
rs142582686
rs143393480
rs77599898
rs182215158
rs1007889
rs10000291
rs510402
rs149767042
rs61814130
rs74805272
rs11056372
rs139251025
rs4502257
rs2977995
rs10839612
rs1431143
rs678624
rs5751777
All
−0.08
−0.04
0.00
0.04
MR leave−one−out sensitivity analysis for
'3−formylindole levels' on 'Rheumatoid arthritis'

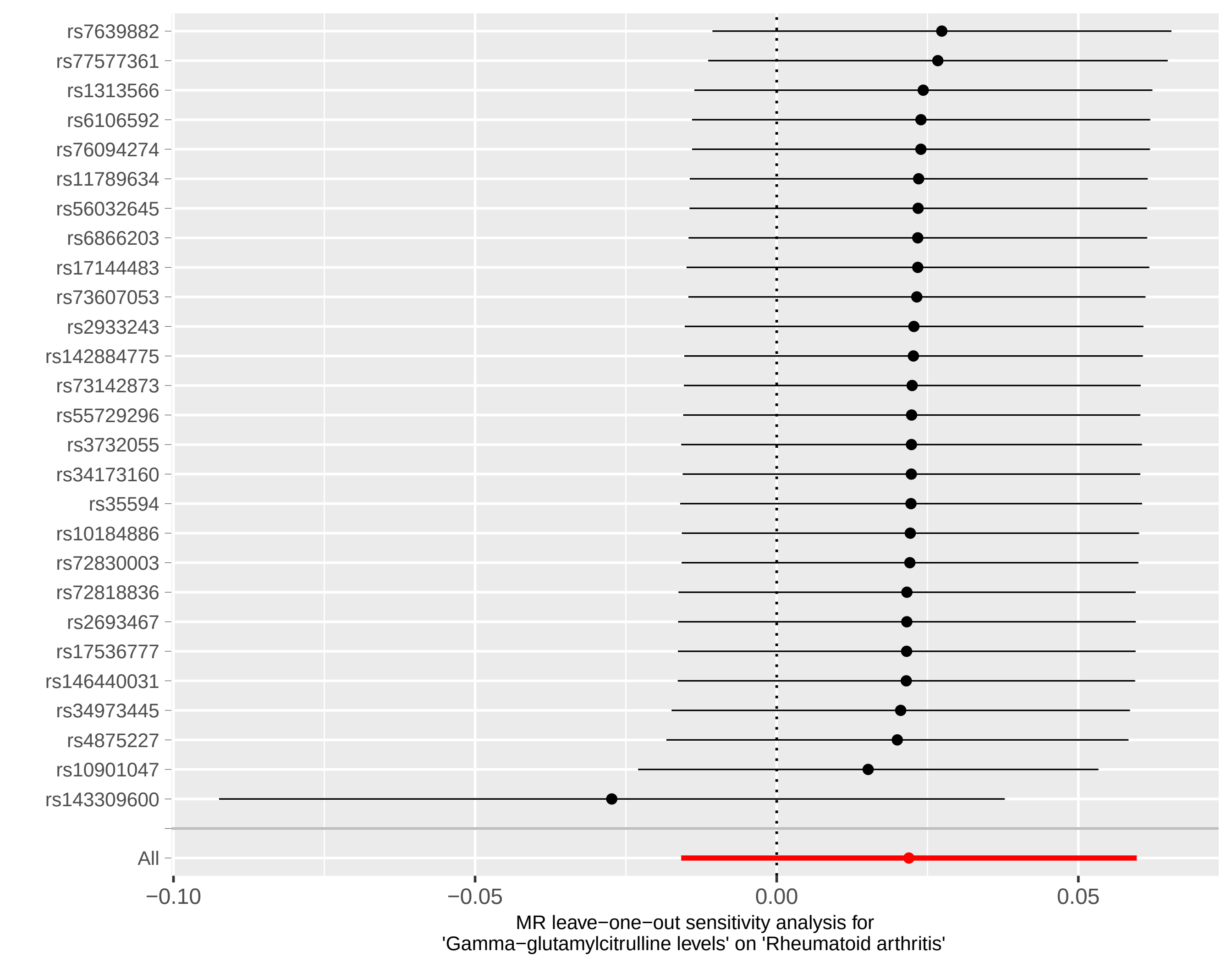
rs7639882
rs77577361
rs1313566
rs6106592
rs76094274
rs11789634
rs56032645
rs6866203
rs17144483
rs73607053
rs2933243
rs142884775
rs73142873
rs55729296
rs3732055
rs34173160
rs35594
rs10184886
rs72830003
rs72818836
rs2693467
rs17536777
rs146440031
rs34973445
rs4875227
rs10901047
rs143309600
All
−0.10
−0.05
0.00
0.05
MR leave−one−out sensitivity analysis for
'Gamma−glutamylcitrulline levels' on 'Rheumatoid arthritis'

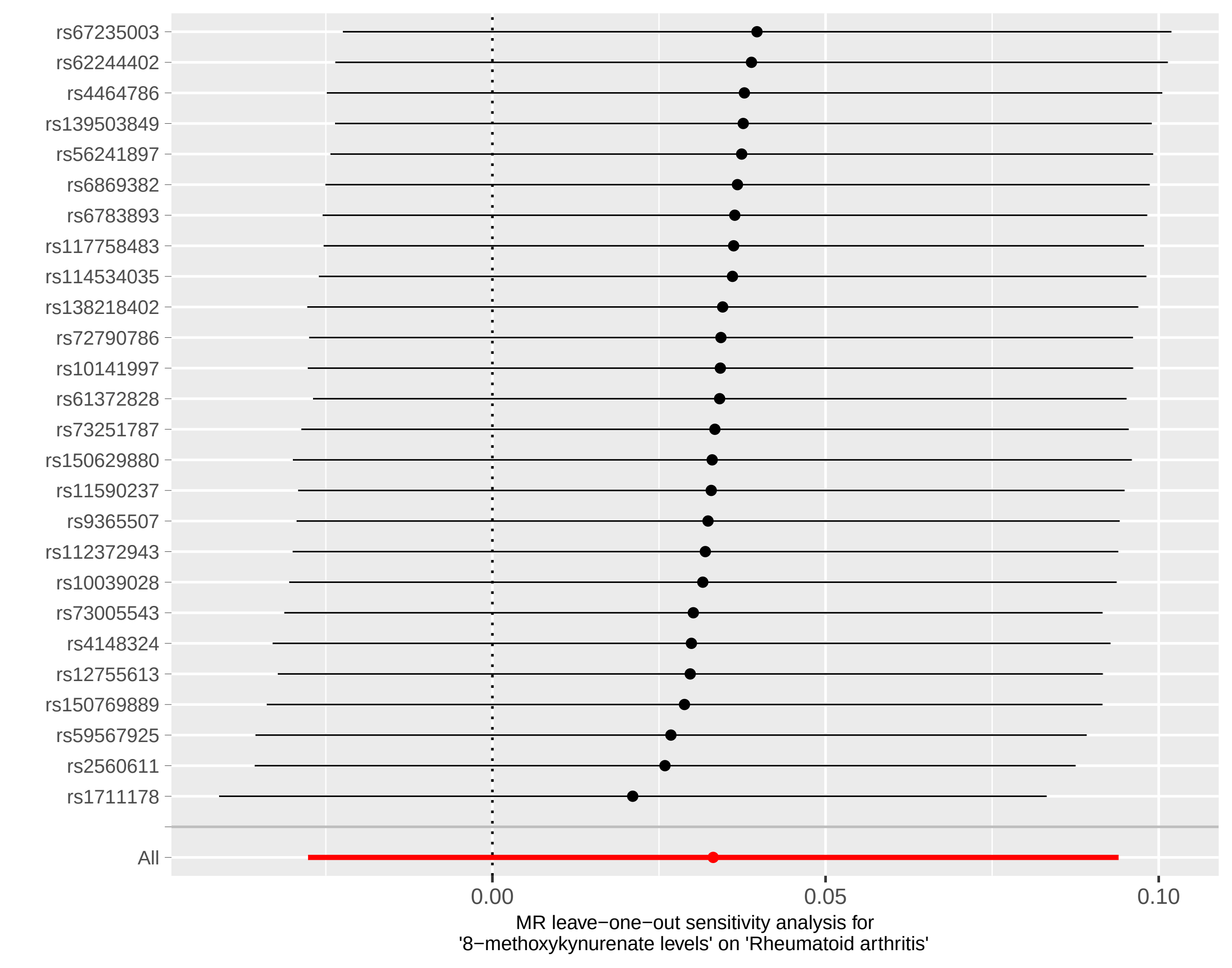

rs67235003
rs62244402
rs4464786
rs139503849
rs56241897
rs6869382
rs6783893
rs117758483
rs114534035
rs138218402
rs72790786
rs10141997
rs61372828
rs73251787
rs150629880
rs11590237
rs9365507
rs112372943
rs10039028
rs73005543
rs4148324
rs12755613
rs150769889
rs59567925
rs2560611
rs1711178
All
0.00
0.05
0.10
MR leave−one−out sensitivity analysis for
'8−methoxykynurenate levels' on 'Rheumatoid arthritis'

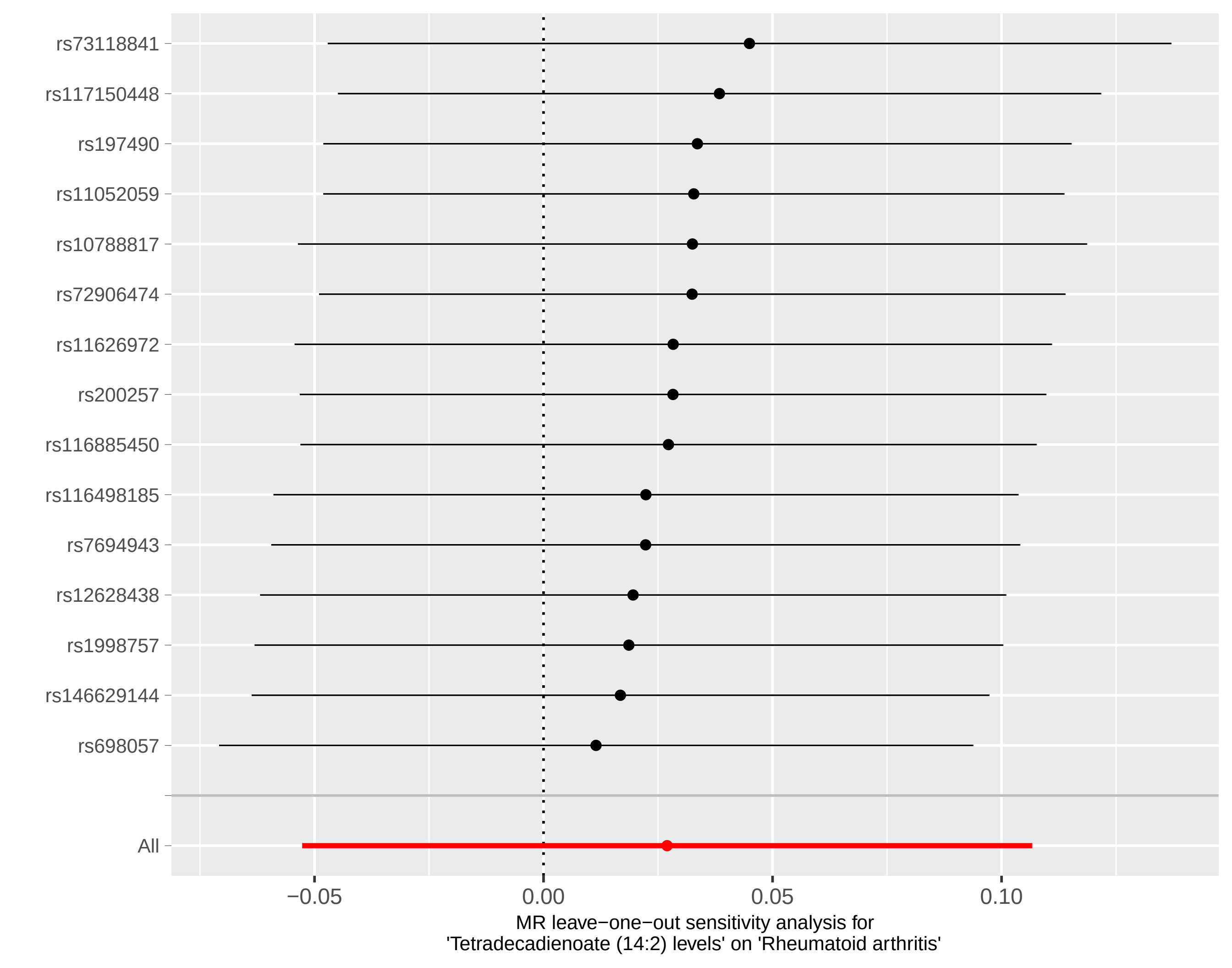
rs73118841
rs117150448
rs197490
rs11052059
rs10788817
rs72906474
rs11626972
rs200257
rs116885450
rs116498185
rs7694943
rs12628438
rs1998757
rs146629144
rs698057
All
−0.05
0.00
0.05
0.10
MR leave−one−out sensitivity analysis for
'Tetradecadienoate (14:2) levels' on 'Rheumatoid arthritis'

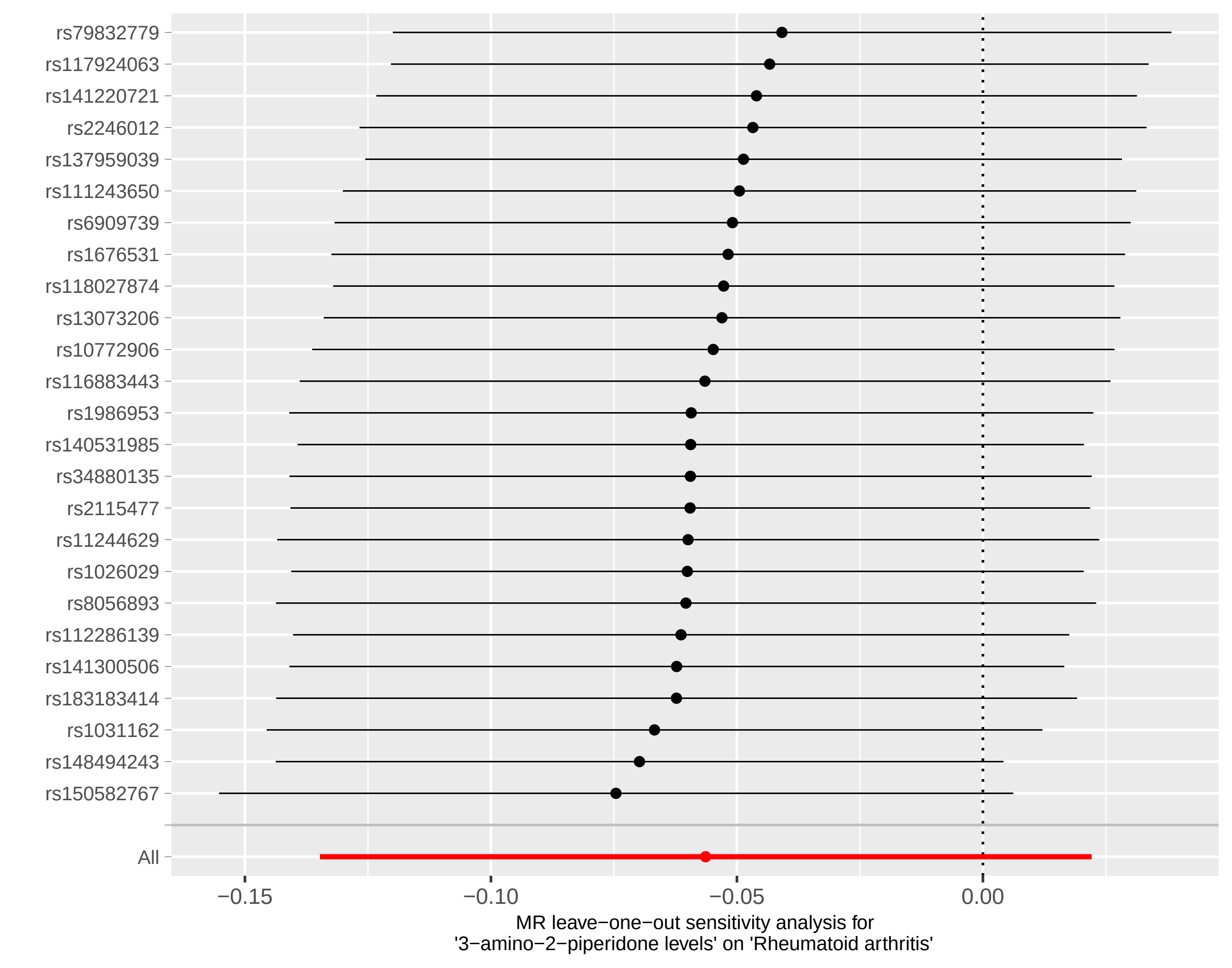
rs79832779
rs117924063
rs141220721
rs2246012
rs137959039
rs111243650
rs6909739
rs1676531
rs118027874
rs13073206
rs10772906
rs116883443
rs1986953
rs140531985
rs34880135
rs2115477
rs11244629
rs1026029
rs8056893
rs112286139
rs141300506
rs183183414
rs1031162
rs148494243
rs150582767
All
−0.15
−0.10
−0.05
0.00
MR leave−one−out sensitivity analysis for
'3−amino−2−piperidone levels' on 'Rheumatoid arthritis'

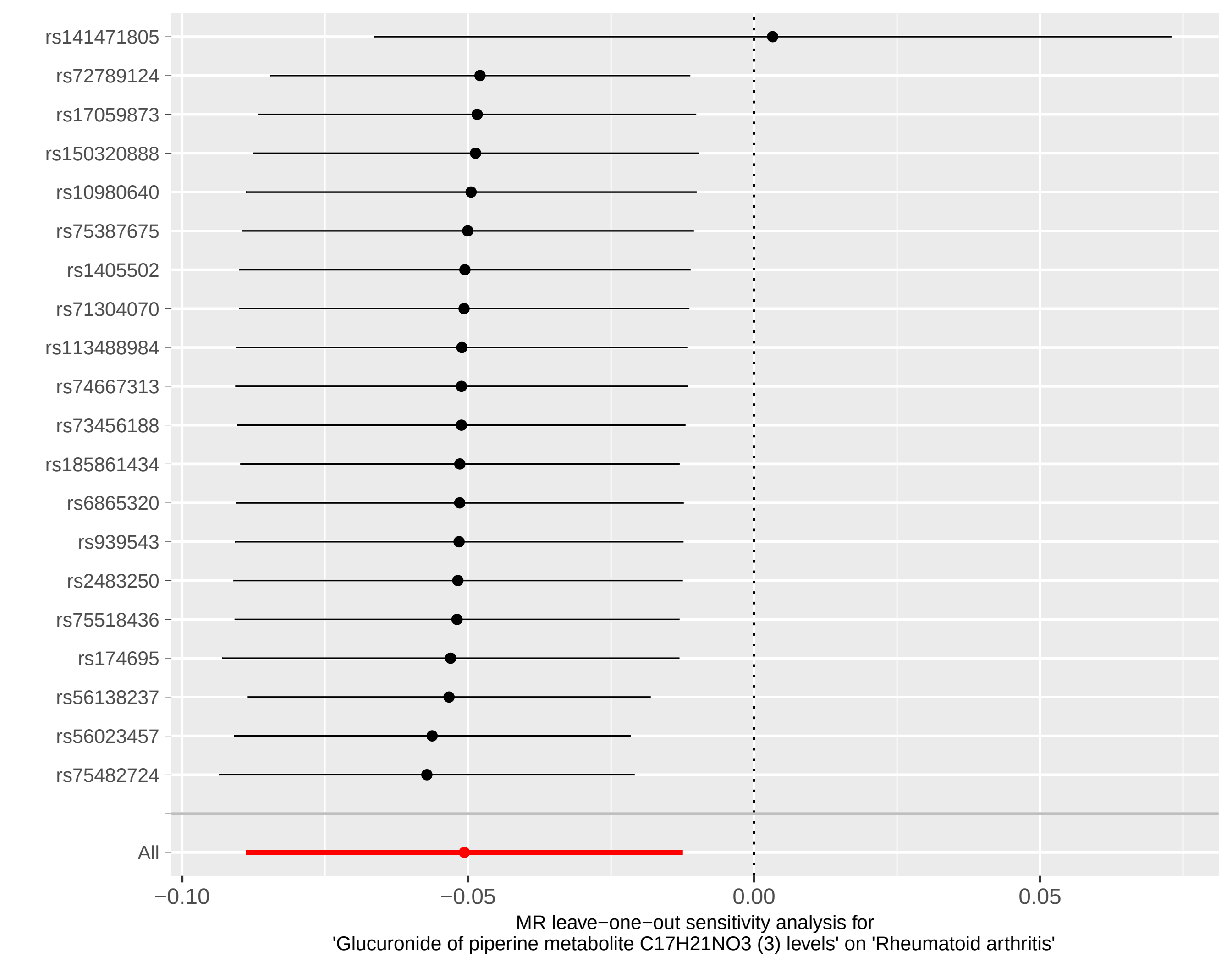
rs141471805
rs72789124
rs17059873
rs150320888
rs10980640
rs75387675
rs1405502
rs71304070
rs113488984
rs74667313
rs73456188
rs185861434
rs6865320
rs939543
rs2483250
rs75518436
rs174695
rs56138237
rs56023457
rs75482724
All
−0.10
−0.05
0.00
0.05
MR leave−one−out sensitivity analysis for
'Glucuronide of piperine metabolite C17H21NO3 (3) levels' on 'Rheumatoid arthritis'

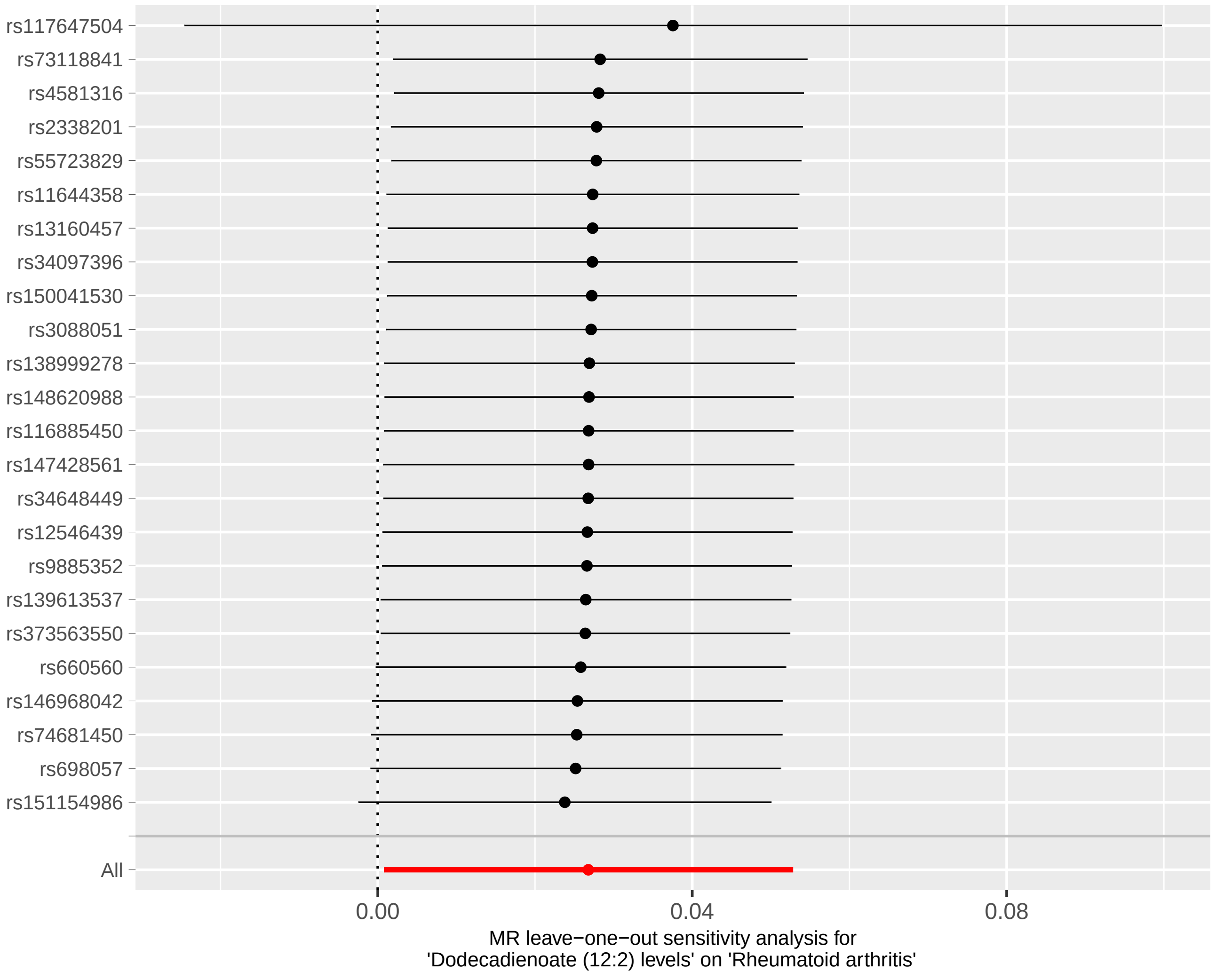

rs117647504
rs73118841
rs4581316
rs2338201
rs55723829
rs11644358
rs13160457
rs34097396
rs150041530
rs3088051
rs138999278
rs148620988
rs116885450
rs147428561
rs34648449
rs12546439
rs9885352
rs139613537
rs373563550
rs660560
rs146968042
rs74681450
rs698057
rs151154986
All
0.00
0.04
0.08
MR leave−one−out sensitivity analysis for
'Dodecadienoate (12:2) levels' on 'Rheumatoid arthritis'

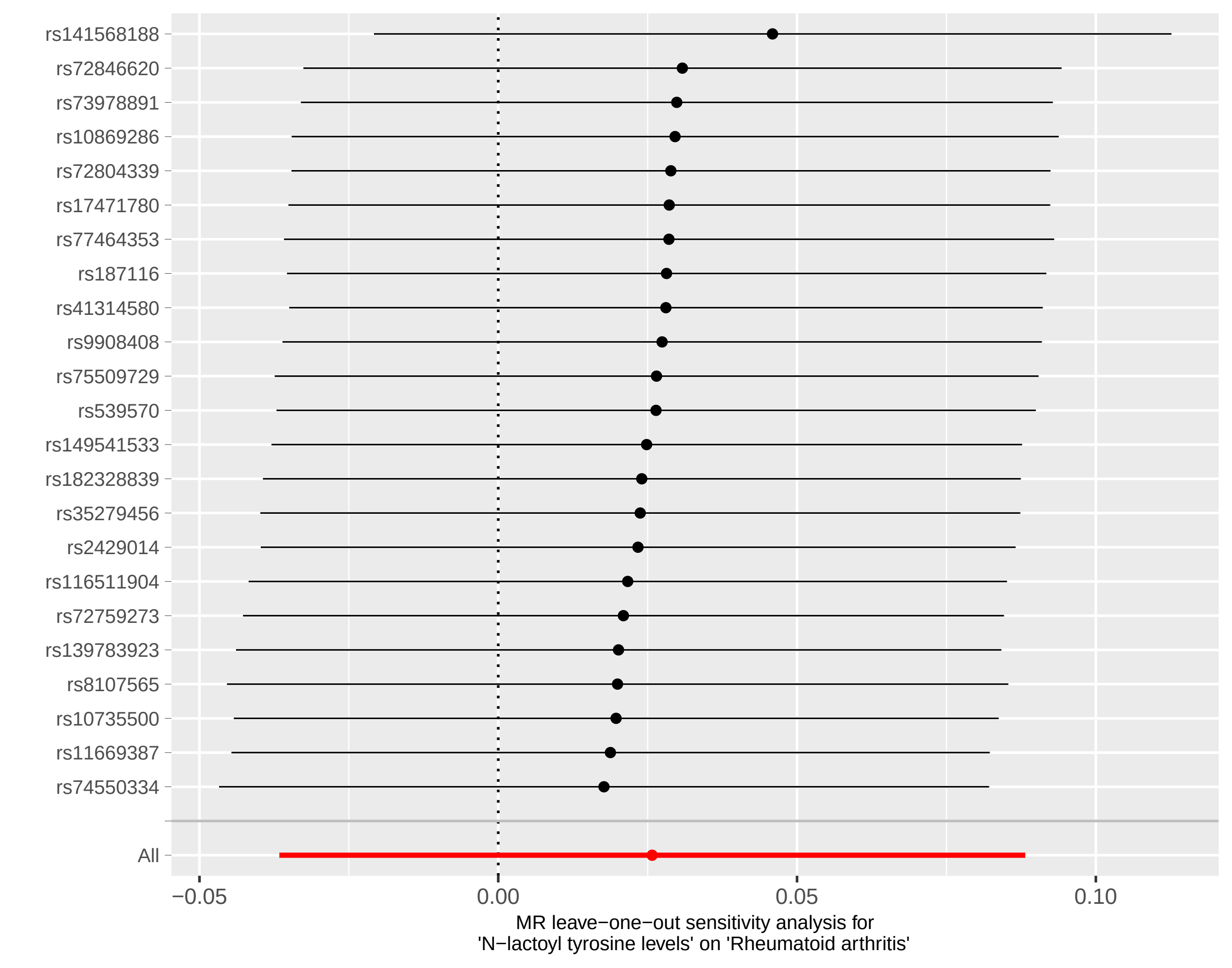
rs141568188
rs72846620
rs73978891
rs10869286
rs72804339
rs17471780
rs77464353
rs187116
rs41314580
rs9908408
rs75509729
rs539570
rs149541533
rs182328839
rs35279456
rs2429014
rs116511904
rs72759273
rs139783923
rs8107565
rs10735500
rs11669387
rs74550334
All
−0.05
0.00
0.05
0.10
MR leave−one−out sensitivity analysis for
'N−lactoyl tyrosine levels' on 'Rheumatoid arthritis'

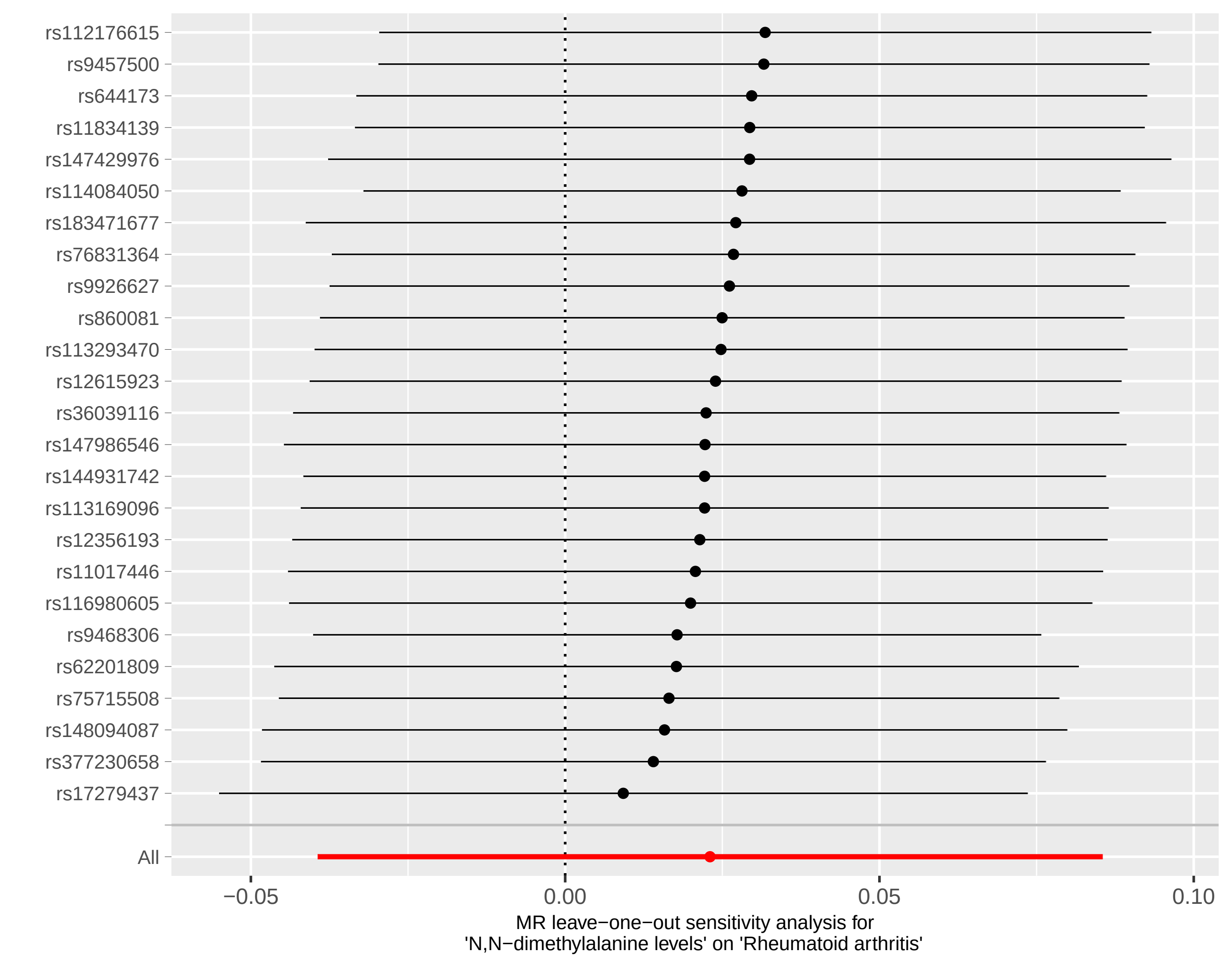
rs112176615
rs9457500
rs644173
rs11834139
rs147429976
rs114084050
rs183471677
rs76831364
rs9926627
rs860081
rs113293470
rs12615923
rs36039116
rs147986546
rs144931742
rs113169096
rs12356193
rs11017446
rs116980605
rs9468306
rs62201809
rs75715508
rs148094087
rs377230658
rs17279437
All
−0.05
0.00
0.05
0.10
MR leave−one−out sensitivity analysis for
'N,N−dimethylalanine levels' on 'Rheumatoid arthritis'

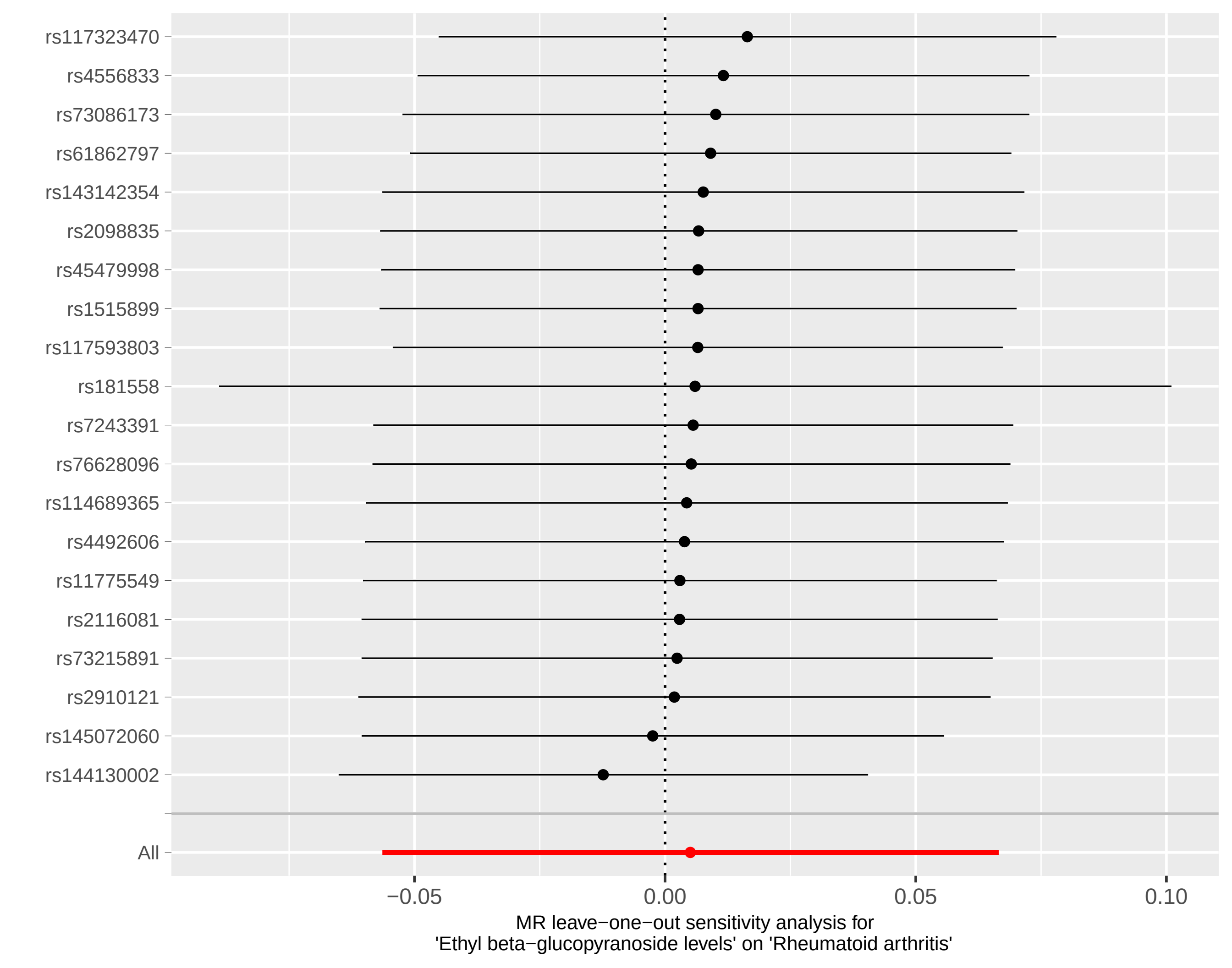
rs117323470
rs4556833
rs73086173
rs61862797
rs143142354
rs2098835
rs45479998
rs1515899
rs117593803
rs181558
rs7243391
rs76628096
rs114689365
rs4492606
rs11775549
rs2116081
rs73215891
rs2910121
rs145072060
rs144130002
All
−0.05
0.00
0.05
0.10
MR leave−one−out sensitivity analysis for
'Ethyl beta−glucopyranoside levels' on 'Rheumatoid arthritis'

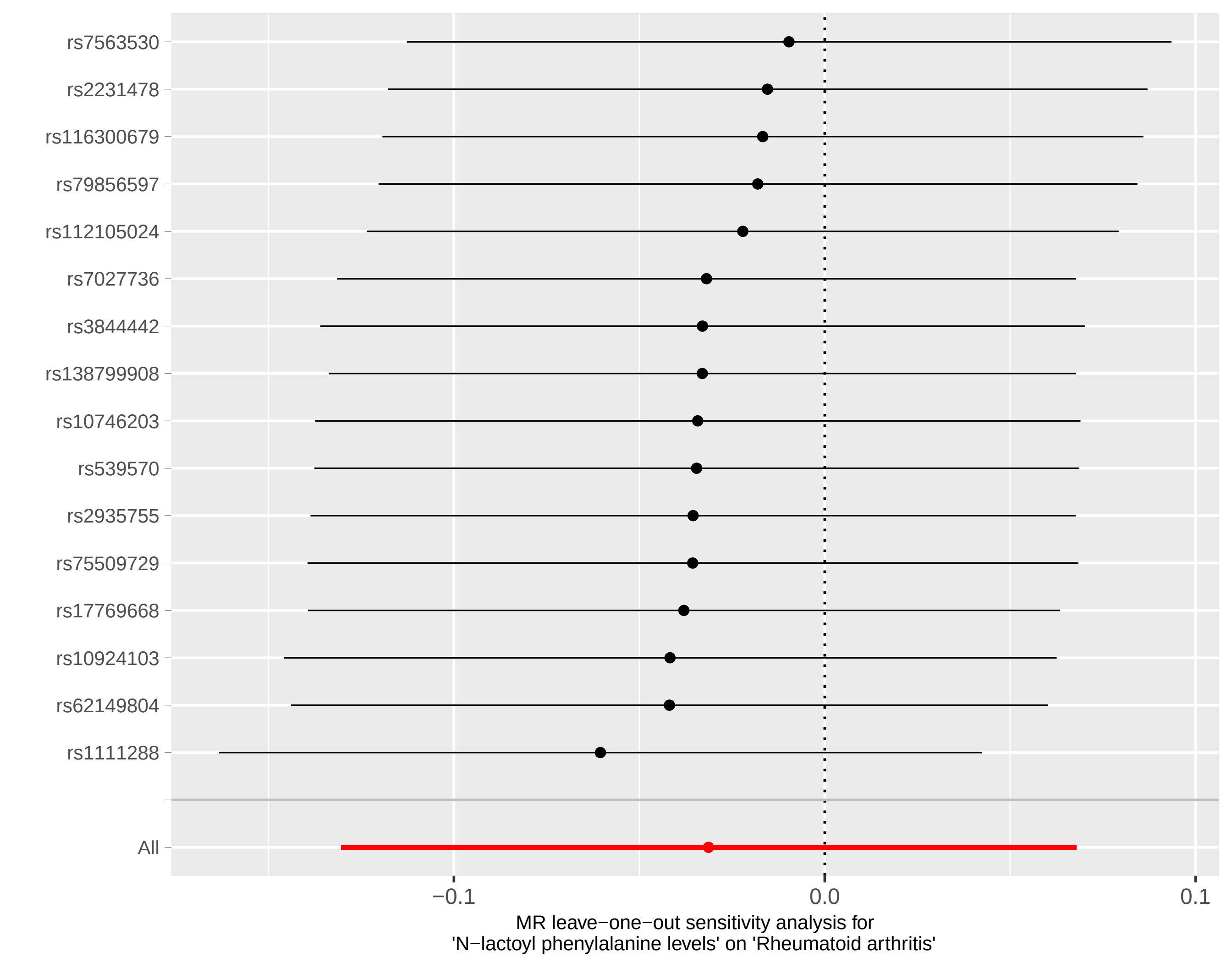

rs7563530
rs2231478
rs116300679
rs79856597
rs112105024
rs7027736
rs3844442
rs138799908
rs10746203
rs539570
rs2935755
rs75509729
rs17769668
rs10924103
rs62149804
rs1111288
All
−0.1
0.0
0.1
MR leave−one−out sensitivity analysis for
'N−lactoyl phenylalanine levels' on 'Rheumatoid arthritis'

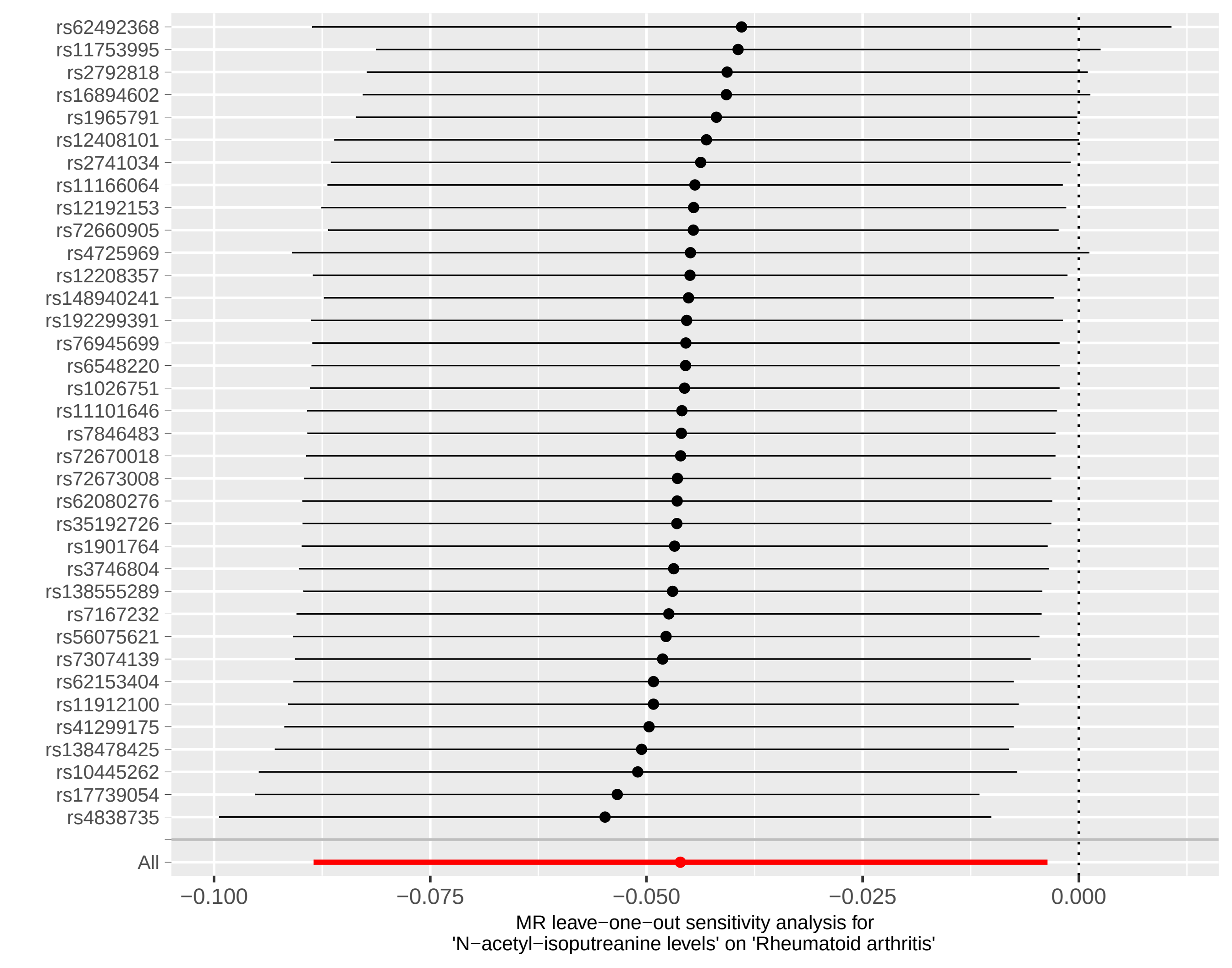

rs62492368
rs11753995
rs2792818
rs16894602
rs1965791
rs12408101
rs2741034
rs11166064
rs12192153
rs72660905
rs4725969
rs12208357
rs148940241
rs192299391
rs76945699
rs6548220
rs1026751
rs11101646
rs7846483
rs72670018
rs72673008
rs62080276
rs35192726
rs1901764
rs3746804
rs138555289
rs7167232
rs56075621
rs73074139
rs62153404
rs11912100
rs41299175
rs138478425
rs10445262
rs17739054
rs4838735
All
−0.100
−0.075
−0.050
−0.025
0.000
MR leave−one−out sensitivity analysis for
'N−acetyl−isoputreanine levels' on 'Rheumatoid arthritis'

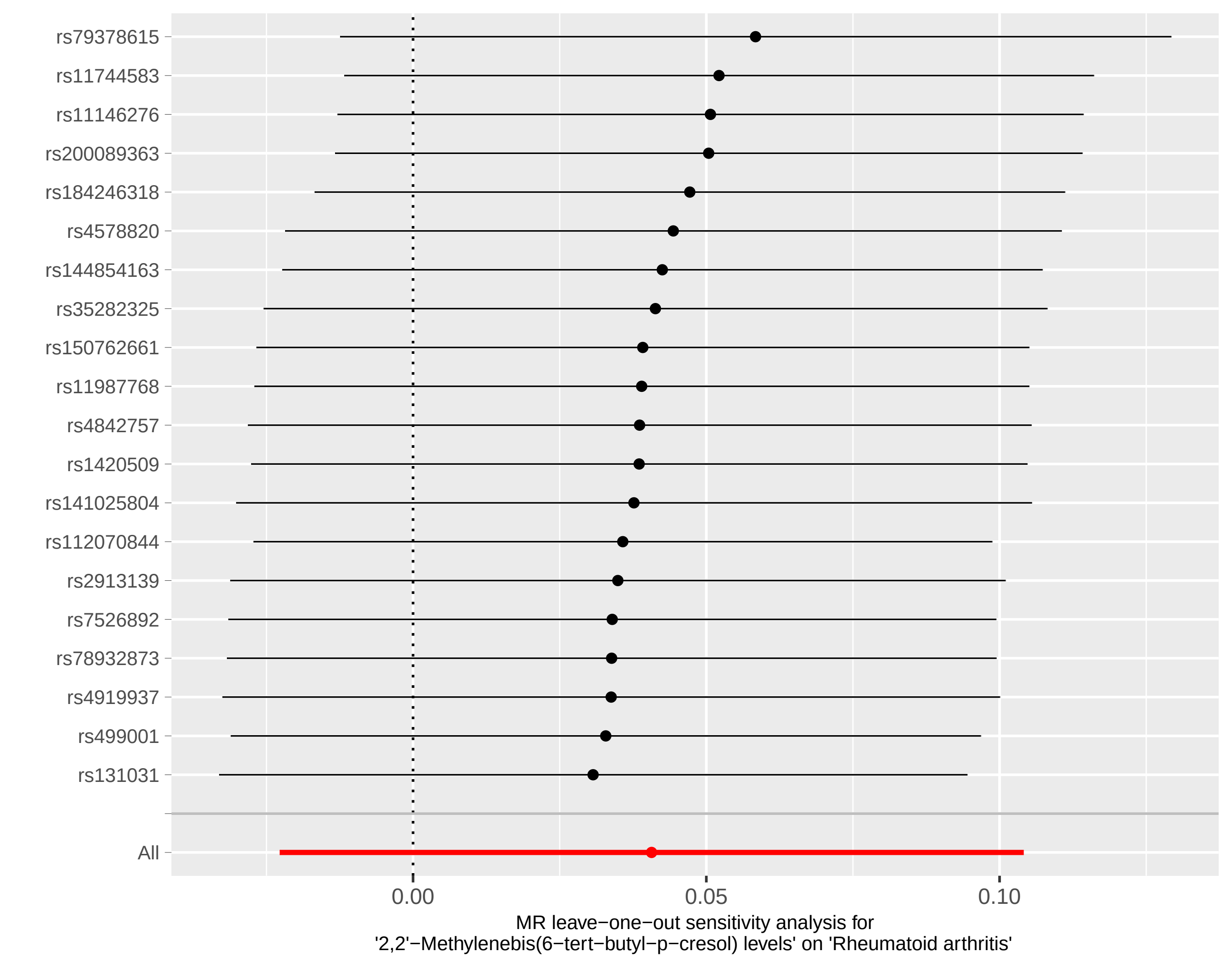
rs79378615
rs11744583
rs11146276
rs200089363
rs184246318
rs4578820
rs144854163
rs35282325
rs150762661
rs11987768
rs4842757
rs1420509
rs141025804
rs112070844
rs2913139
rs7526892
rs78932873
rs4919937
rs499001
rs131031
All
0.00
0.05
0.10
MR leave−one−out sensitivity analysis for
'2,2'−Methylenebis(6−tert−butyl−p−cresol) levels' on 'Rheumatoid arthritis'

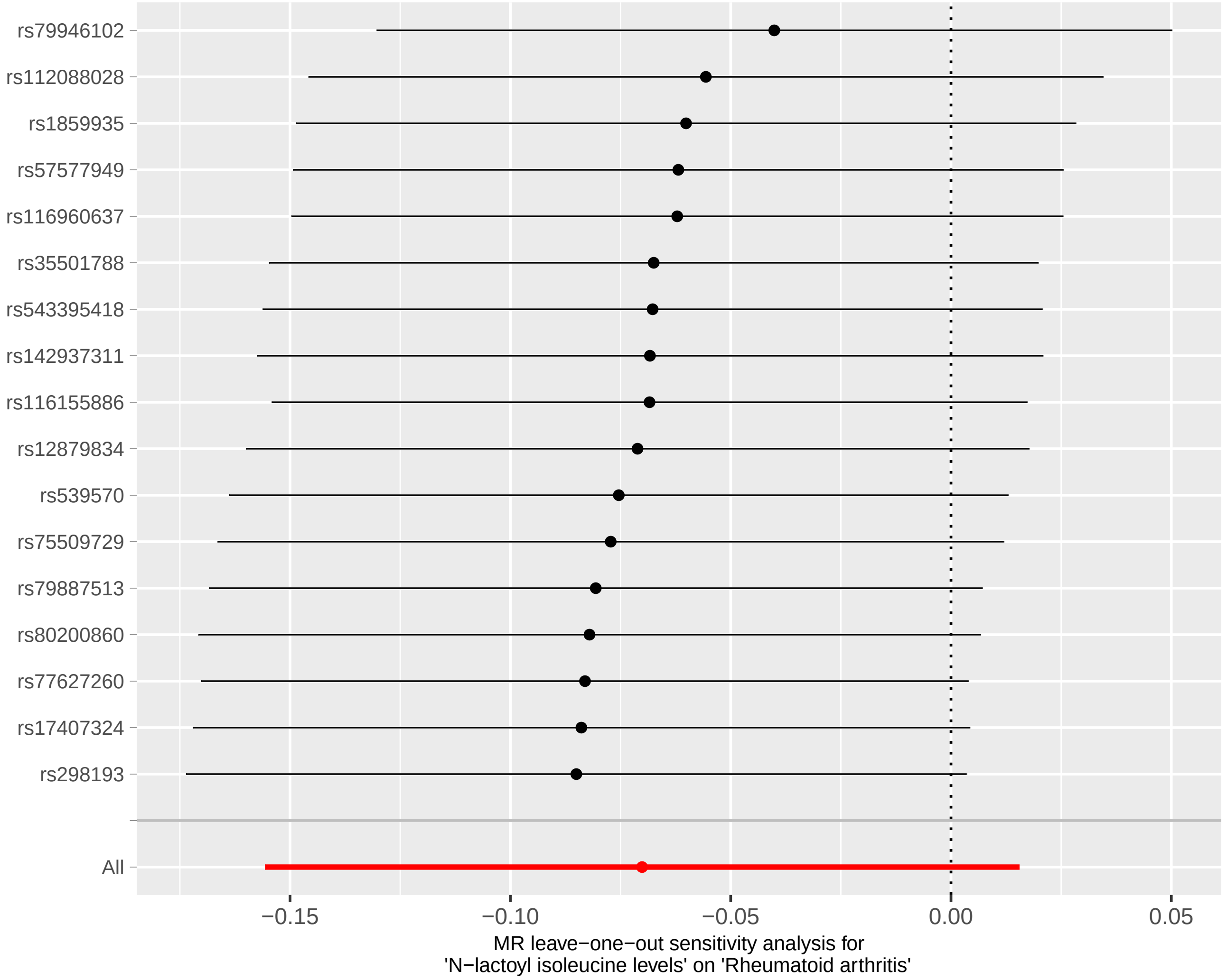
rs79946102
rs112088028
rs1859935
rs57577949
rs116960637
rs35501788
rs543395418
rs142937311
rs116155886
rs12879834
rs539570
rs75509729
rs79887513
rs80200860
rs77627260
rs17407324
rs298193
All
−0.15
−0.10
−0.05
0.00
0.05
MR leave−one−out sensitivity analysis for
'N−lactoyl isoleucine levels' on 'Rheumatoid arthritis'

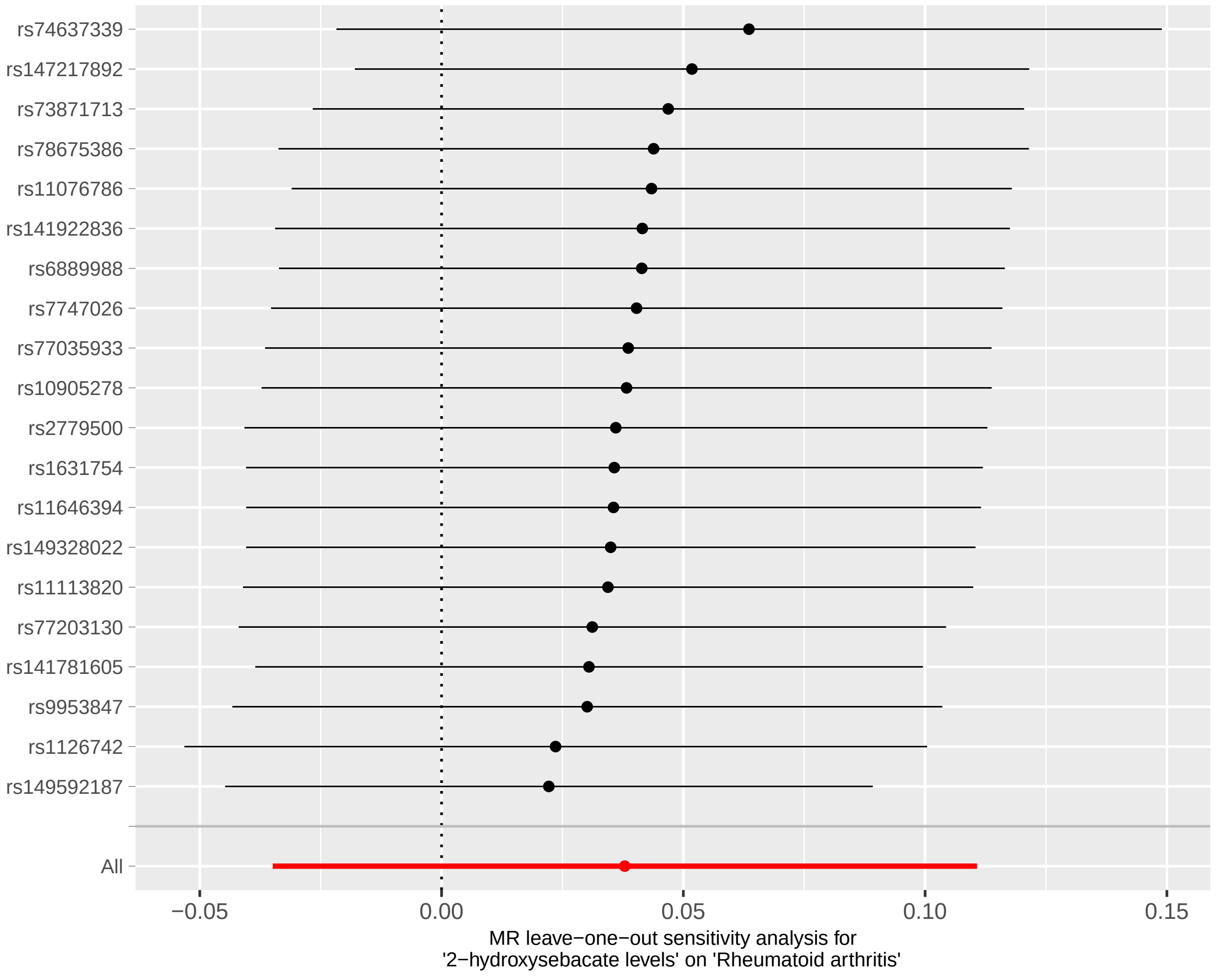
rs74637339
rs147217892
rs73871713
rs78675386
rs11076786
rs141922836
rs6889988
rs7747026
rs77035933
rs10905278
rs2779500
rs1631754
rs11646394
rs149328022
rs11113820
rs77203130
rs141781605
rs9953847
rs1126742
rs149592187
All
−0.05
0.00
0.05
0.10
0.15
MR leave−one−out sensitivity analysis for
'2−hydroxysebacate levels' on 'Rheumatoid arthritis'

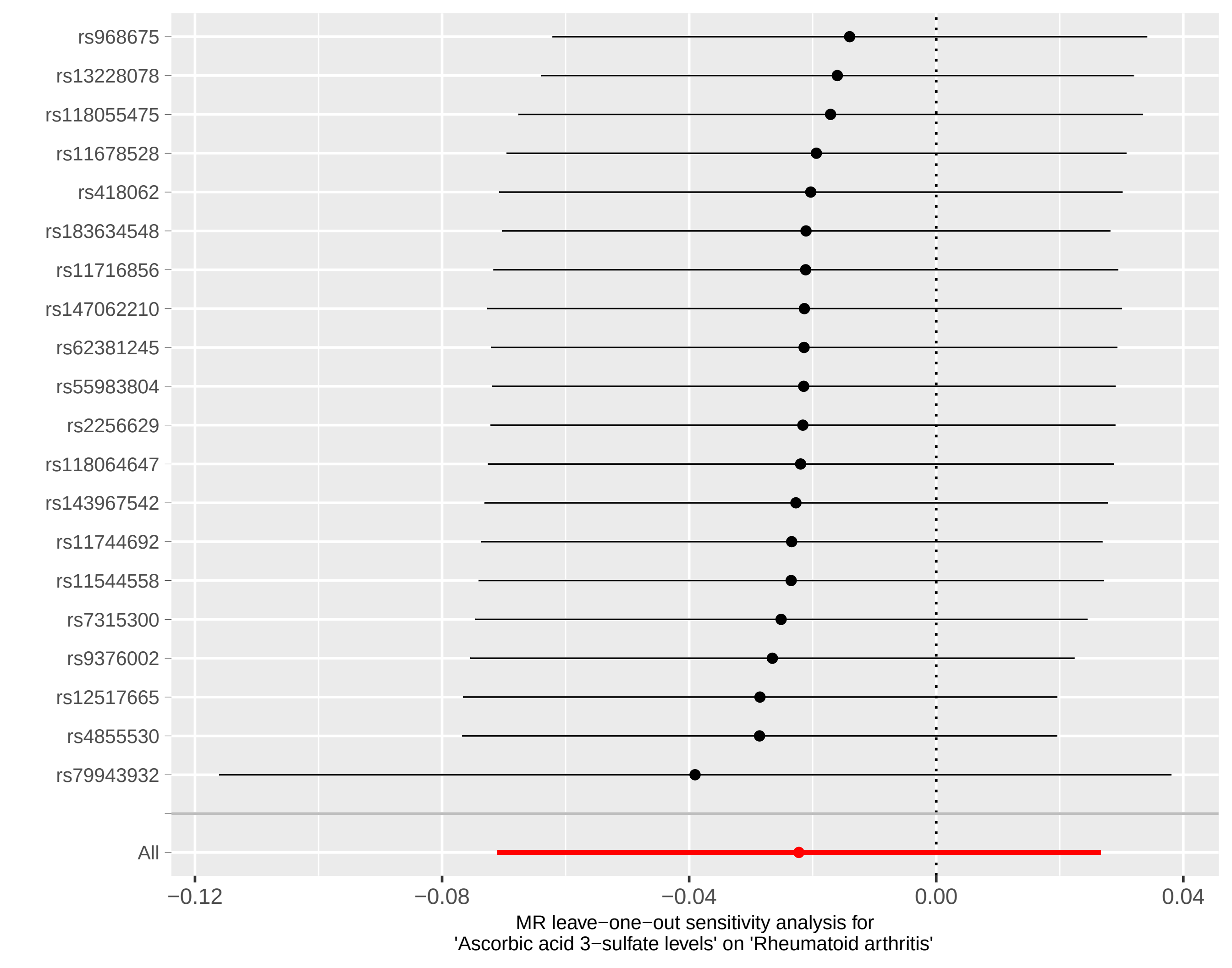
rs968675
rs13228078
rs118055475
rs11678528
rs418062
rs183634548
rs11716856
rs147062210
rs62381245
rs55983804
rs2256629
rs118064647
rs143967542
rs11744692
rs11544558
rs7315300
rs9376002
rs12517665
rs4855530
rs79943932
All
−0.12
−0.08
−0.04
0.00
0.04
MR leave−one−out sensitivity analysis for
'Ascorbic acid 3−sulfate levels' on 'Rheumatoid arthritis'

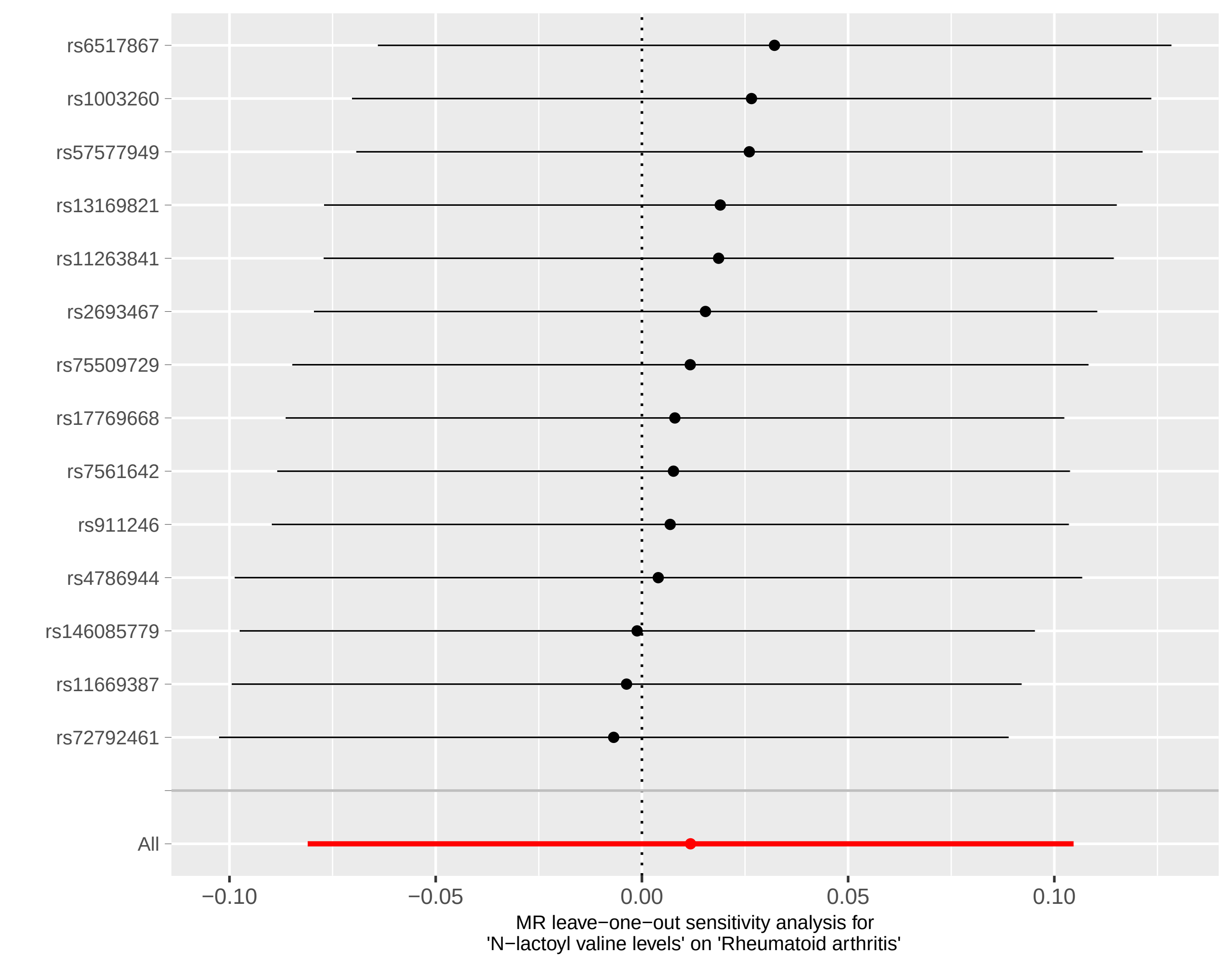
rs6517867
rs1003260
rs57577949
rs13169821
rs11263841
rs2693467
rs75509729
rs17769668
rs7561642
rs911246
rs4786944
rs146085779
rs11669387
rs72792461
All
−0.10
−0.05
0.00
0.05
0.10
MR leave−one−out sensitivity analysis for
'N−lactoyl valine levels' on 'Rheumatoid arthritis'

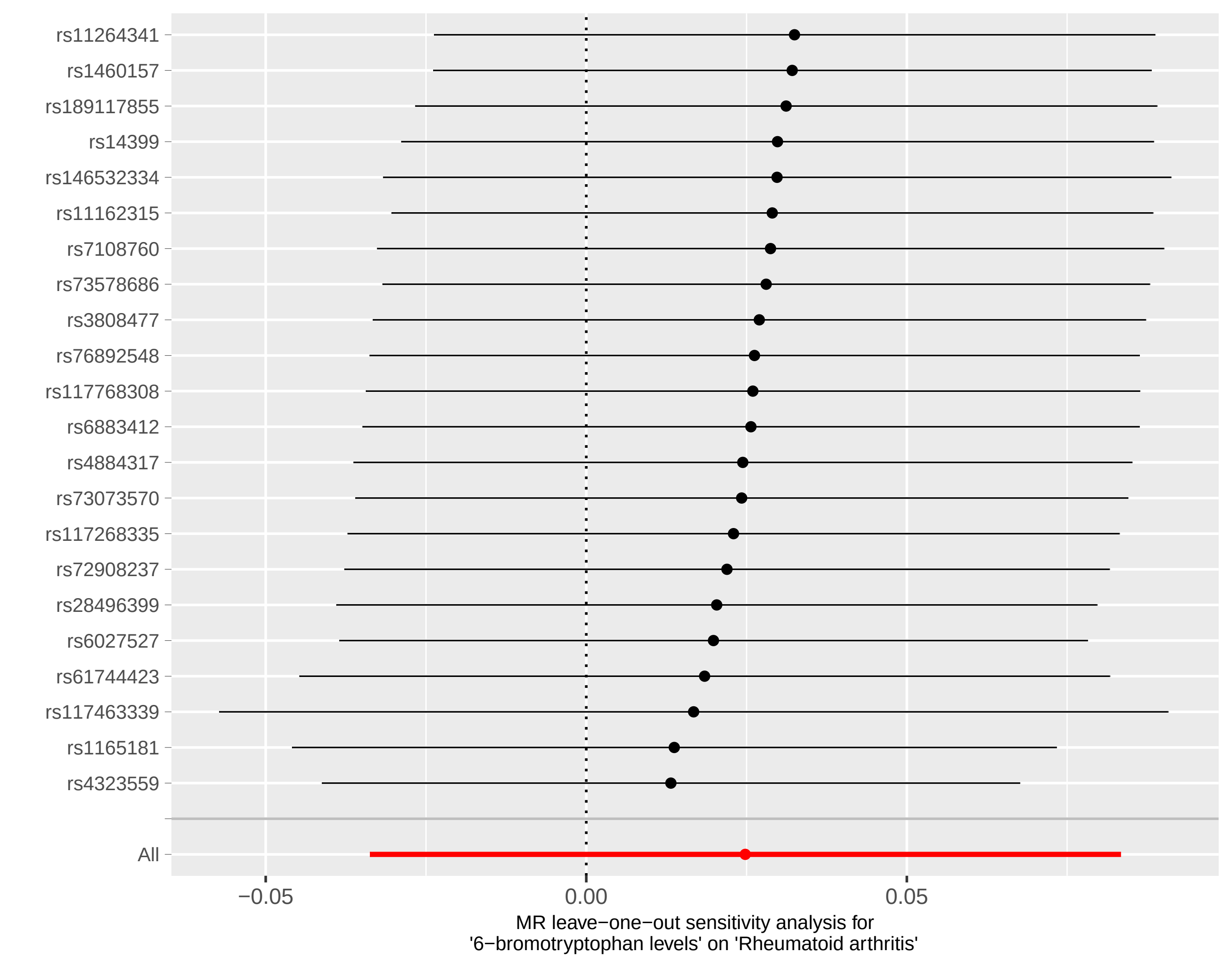
rs11264341
rs1460157
rs189117855
rs14399
rs146532334
rs11162315
rs7108760
rs73578686
rs3808477
rs76892548
rs117768308
rs6883412
rs4884317
rs73073570
rs117268335
rs72908237
rs28496399
rs6027527
rs61744423
rs117463339
rs1165181
rs4323559
All
−0.05
0.00
0.05
MR leave−one−out sensitivity analysis for
'6−bromotryptophan levels' on 'Rheumatoid arthritis'

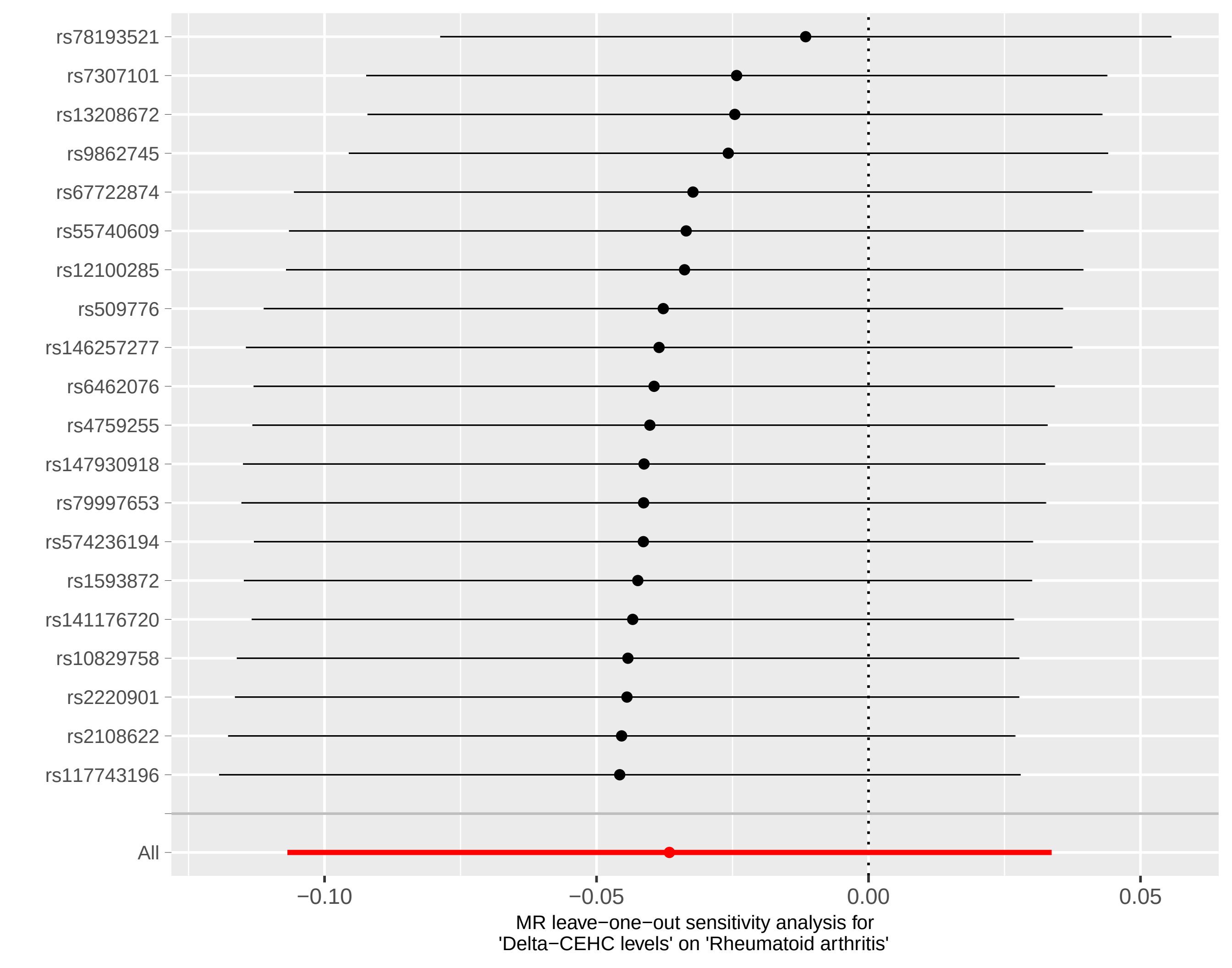
rs78193521
rs7307101
rs13208672
rs9862745
rs67722874
rs55740609
rs12100285
rs509776
rs146257277
rs6462076
rs4759255
rs147930918
rs79997653
rs574236194
rs1593872
rs141176720
rs10829758
rs2220901
rs2108622
rs117743196
All
−0.10
−0.05
0.00
0.05
MR leave−one−out sensitivity analysis for
'Delta−CEHC levels' on 'Rheumatoid arthritis'

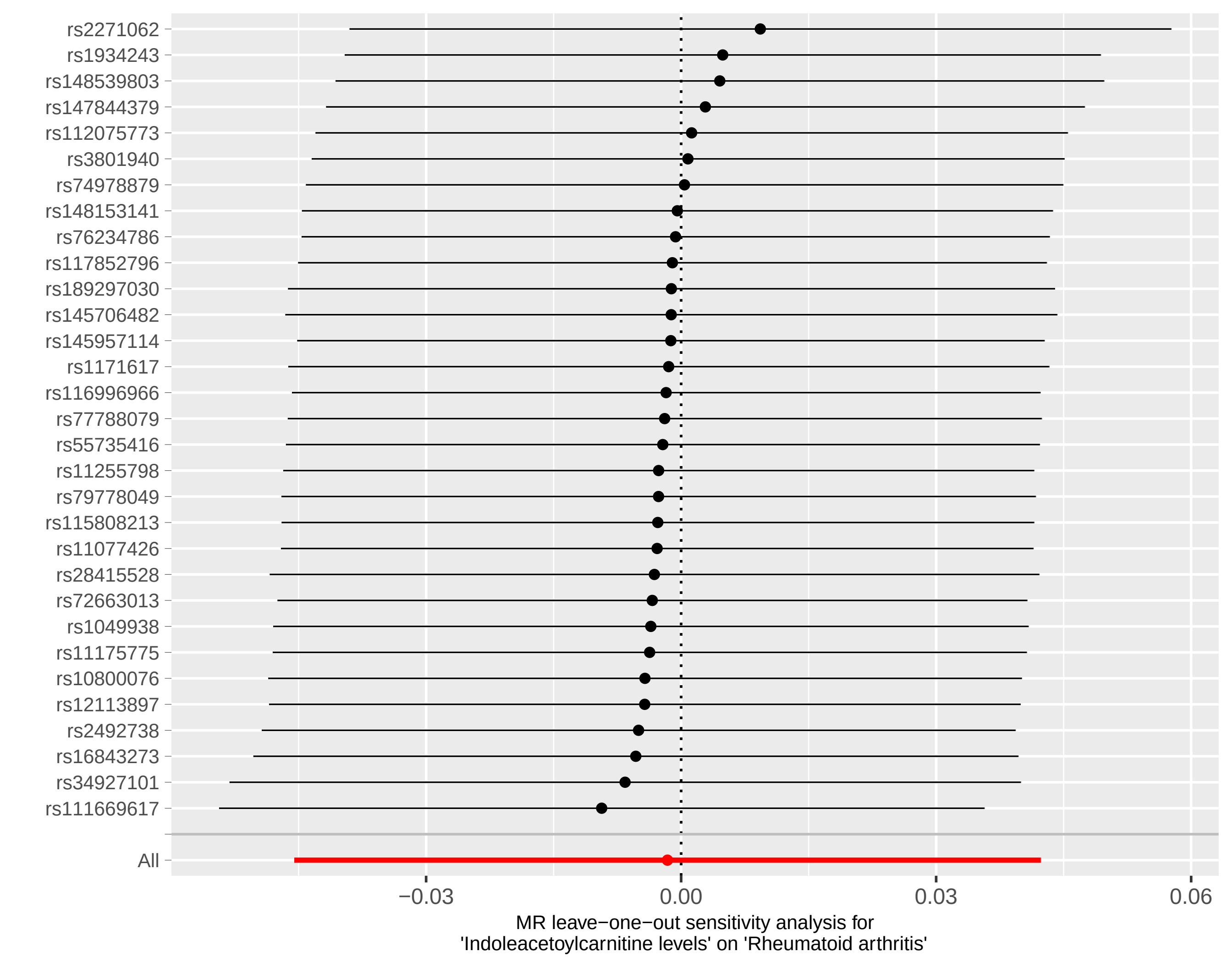

rs2271062
rs1934243
rs148539803
rs147844379
rs112075773
rs3801940
rs74978879
rs148153141
rs76234786
rs117852796
rs189297030
rs145706482
rs145957114
rs1171617
rs116996966
rs77788079
rs55735416
rs11255798
rs79778049
rs115808213
rs11077426
rs28415528
rs72663013
rs1049938
rs11175775
rs10800076
rs12113897
rs2492738
rs16843273
rs34927101
rs111669617
All
−0.03
0.00
0.03
0.06
MR leave−one−out sensitivity analysis for
'Indoleacetoylcarnitine levels' on 'Rheumatoid arthritis'

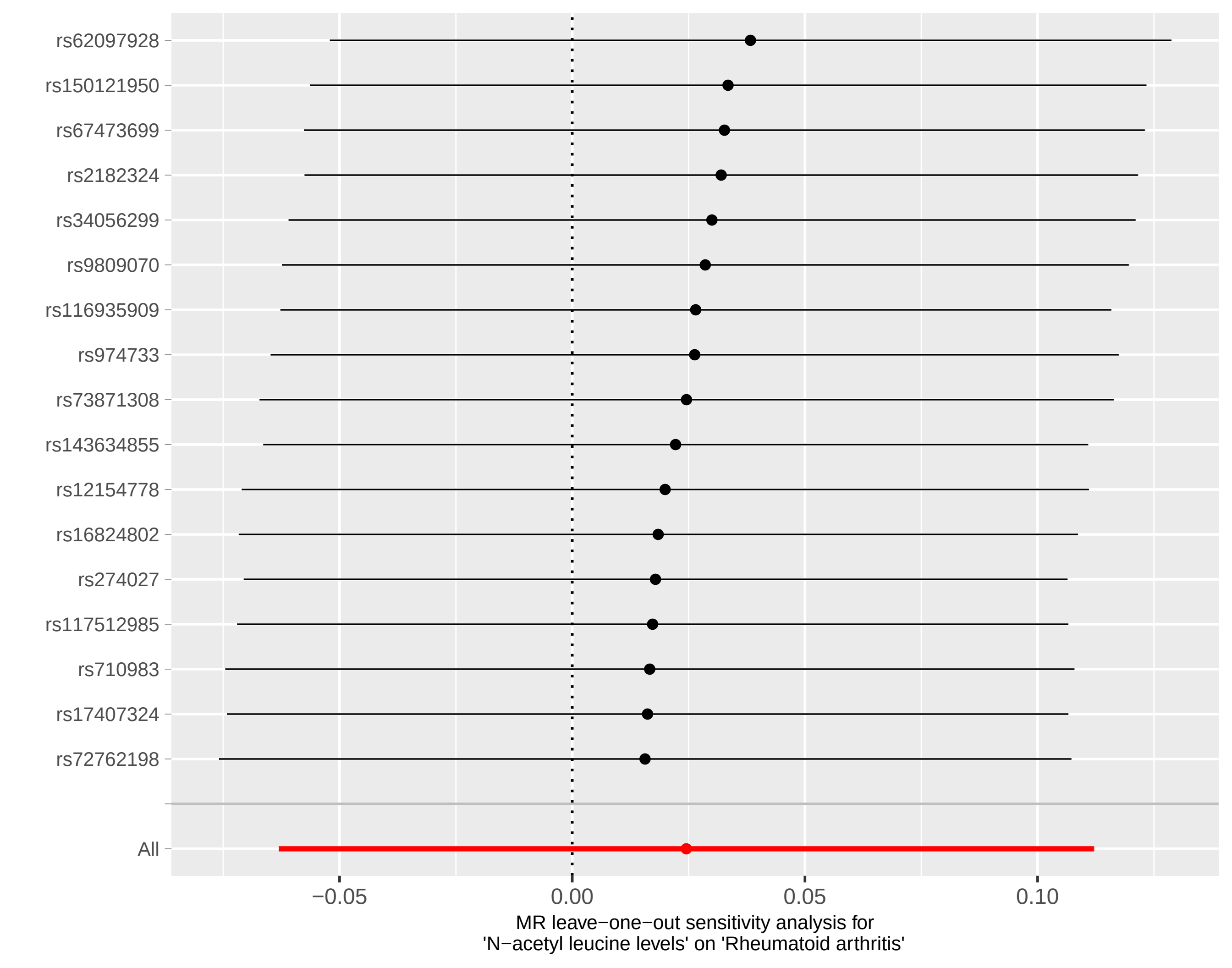

rs62097928
rs150121950
rs67473699
rs2182324
rs34056299
rs9809070
rs116935909
rs974733
rs73871308
rs143634855
rs12154778
rs16824802
rs274027
rs117512985
rs710983
rs17407324
rs72762198
All
−0.05
0.00
0.05
0.10
MR leave−one−out sensitivity analysis for
'N−acetyl leucine levels' on 'Rheumatoid arthritis'

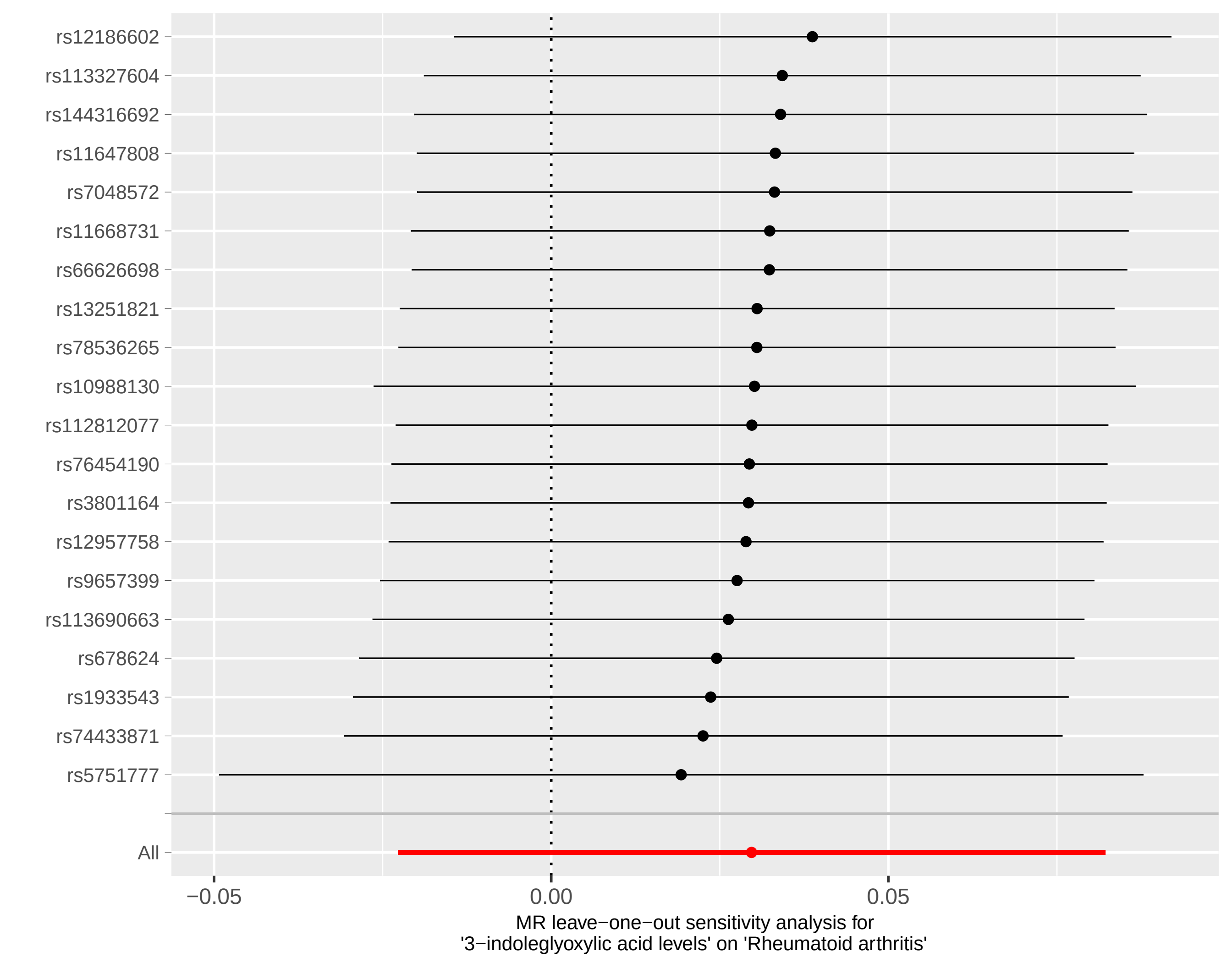
rs12186602
rs113327604
rs144316692
rs11647808
rs7048572
rs11668731
rs66626698
rs13251821
rs78536265
rs10988130
rs112812077
rs76454190
rs3801164
rs12957758
rs9657399
rs113690663
rs678624
rs1933543
rs74433871
rs5751777
All
−0.05
0.00
0.05
MR leave−one−out sensitivity analysis for
'3−indoleglyoxylic acid levels' on 'Rheumatoid arthritis'

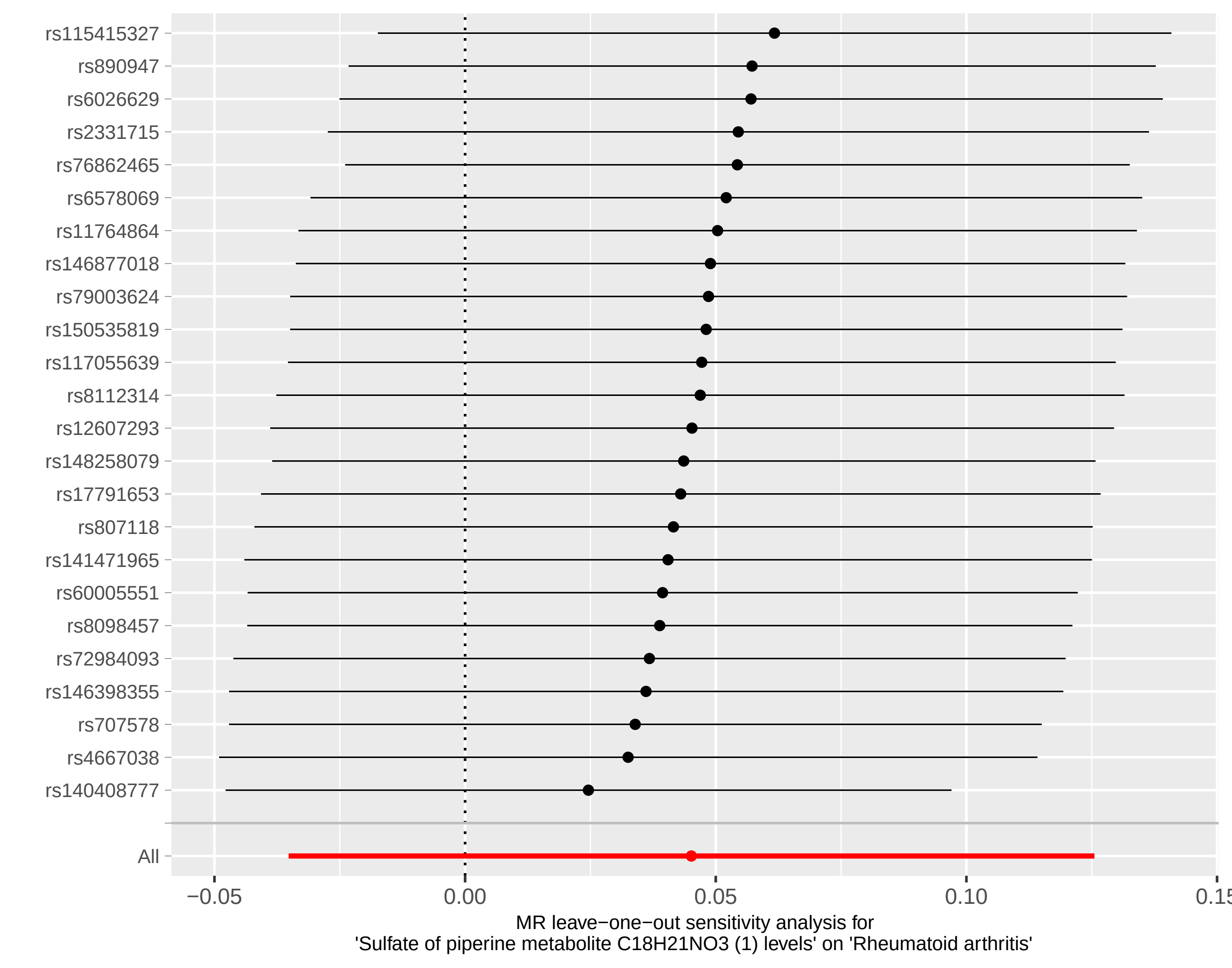

rs115415327
rs890947
rs6026629
rs2331715
rs76862465
rs6578069
rs11764864
rs146877018
rs79003624
rs150535819
rs117055639
rs8112314
rs12607293
rs148258079
rs17791653
rs807118
rs141471965
rs60005551
rs8098457
rs72984093
rs146398355
rs707578
rs4667038
rs140408777
All
−0.05
0.00
0.05
0.10
0.15
MR leave−one−out sensitivity analysis for
'Sulfate of piperine metabolite C18H21NO3 (1) levels' on 'Rheumatoid arthritis'

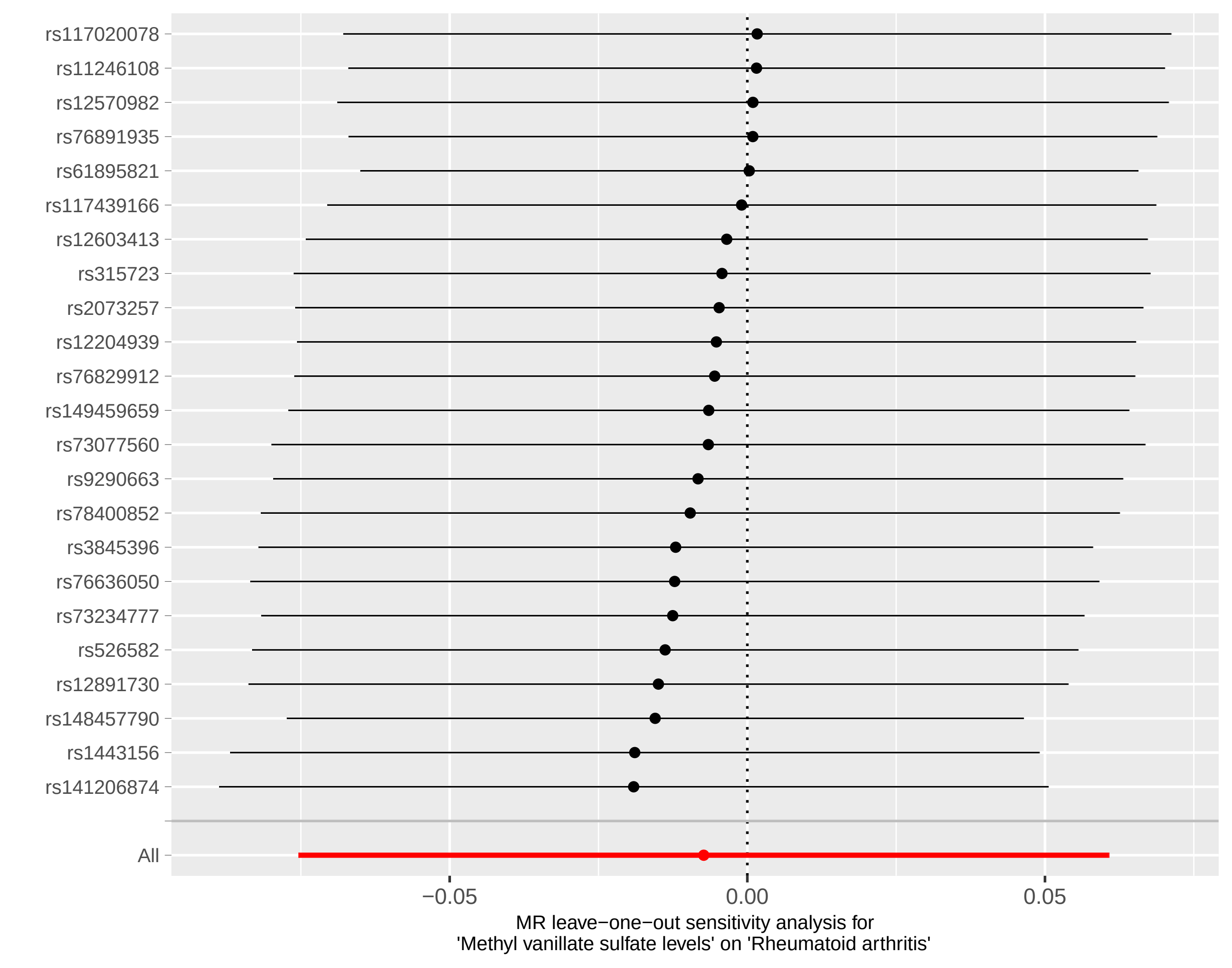
rs117020078
rs11246108
rs12570982
rs76891935
rs61895821
rs117439166
rs12603413
rs315723
rs2073257
rs12204939
rs76829912
rs149459659
rs73077560
rs9290663
rs78400852
rs3845396
rs76636050
rs73234777
rs526582
rs12891730
rs148457790
rs1443156
rs141206874
All
−0.05
0.00
0.05
MR leave−one−out sensitivity analysis for
'Methyl vanillate sulfate levels' on 'Rheumatoid arthritis'

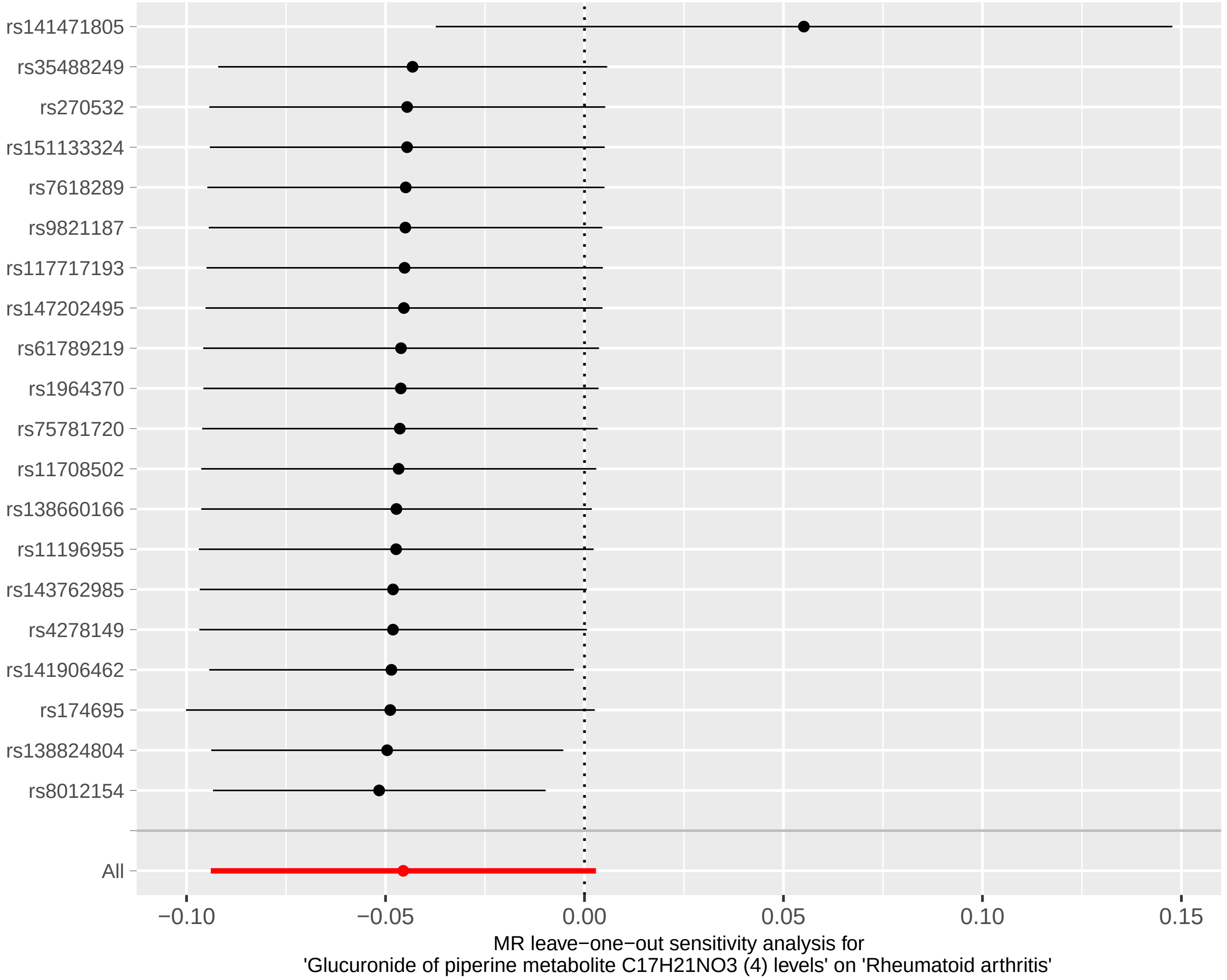
rs141471805
rs35488249
rs270532
rs151133324
rs7618289
rs9821187
rs117717193
rs147202495
rs61789219
rs1964370
rs75781720
rs11708502
rs138660166
rs11196955
rs143762985
rs4278149
rs141906462
rs174695
rs138824804
rs8012154
All
−0.10
−0.05
0.00
0.05
0.10
0.15
MR leave−one−out sensitivity analysis for
'Glucuronide of piperine metabolite C17H21NO3 (4) levels' on 'Rheumatoid arthritis'

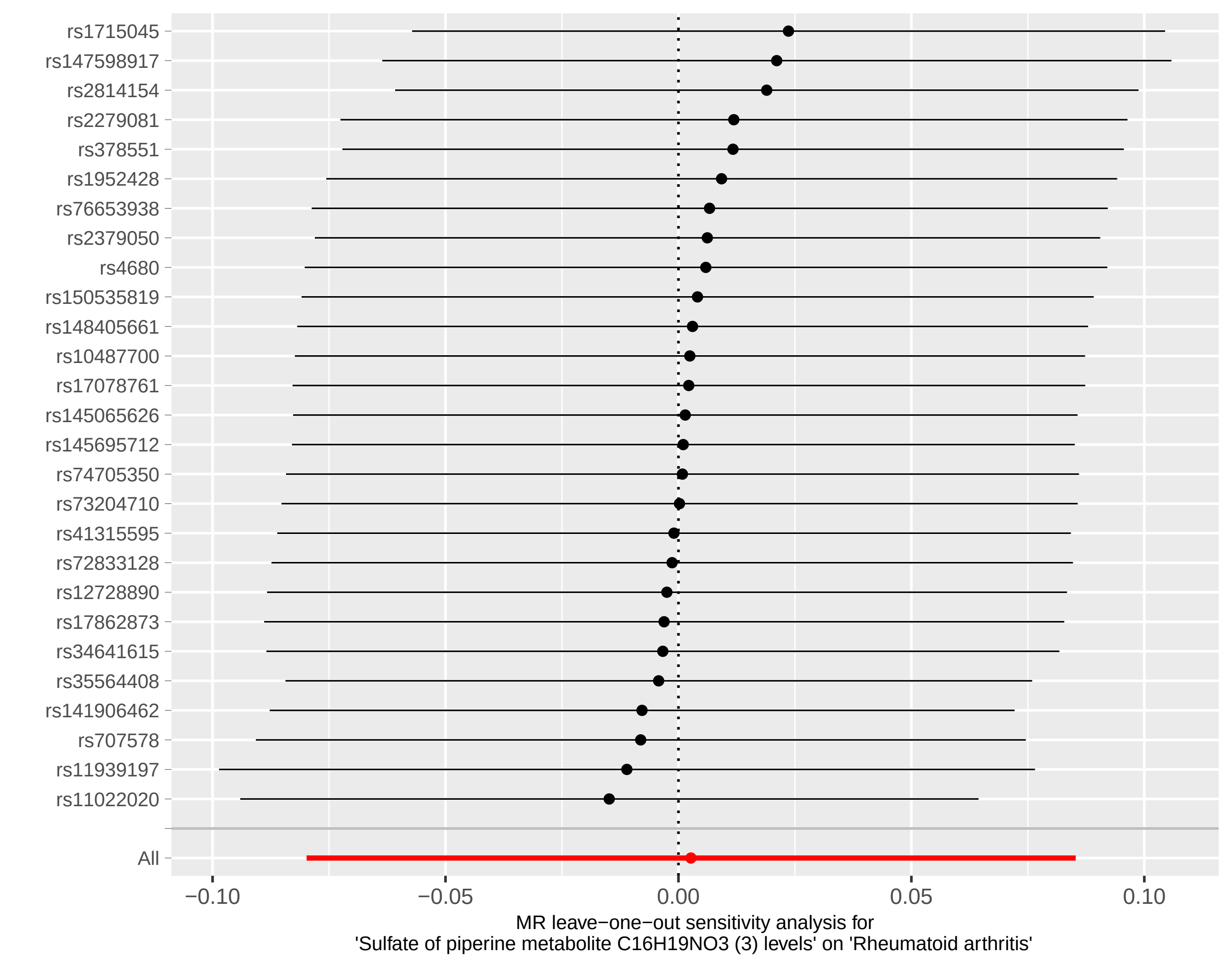
rs1715045
rs147598917
rs2814154
rs2279081
rs378551
rs1952428
rs76653938
rs2379050
rs4680
rs150535819
rs148405661
rs10487700
rs17078761
rs145065626
rs145695712
rs74705350
rs73204710
rs41315595
rs72833128
rs12728890
rs17862873
rs34641615
rs35564408
rs141906462
rs707578
rs11939197
rs11022020
All
−0.10
−0.05
0.00
0.05
0.10
MR leave−one−out sensitivity analysis for
'Sulfate of piperine metabolite C16H19NO3 (3) levels' on 'Rheumatoid arthritis'

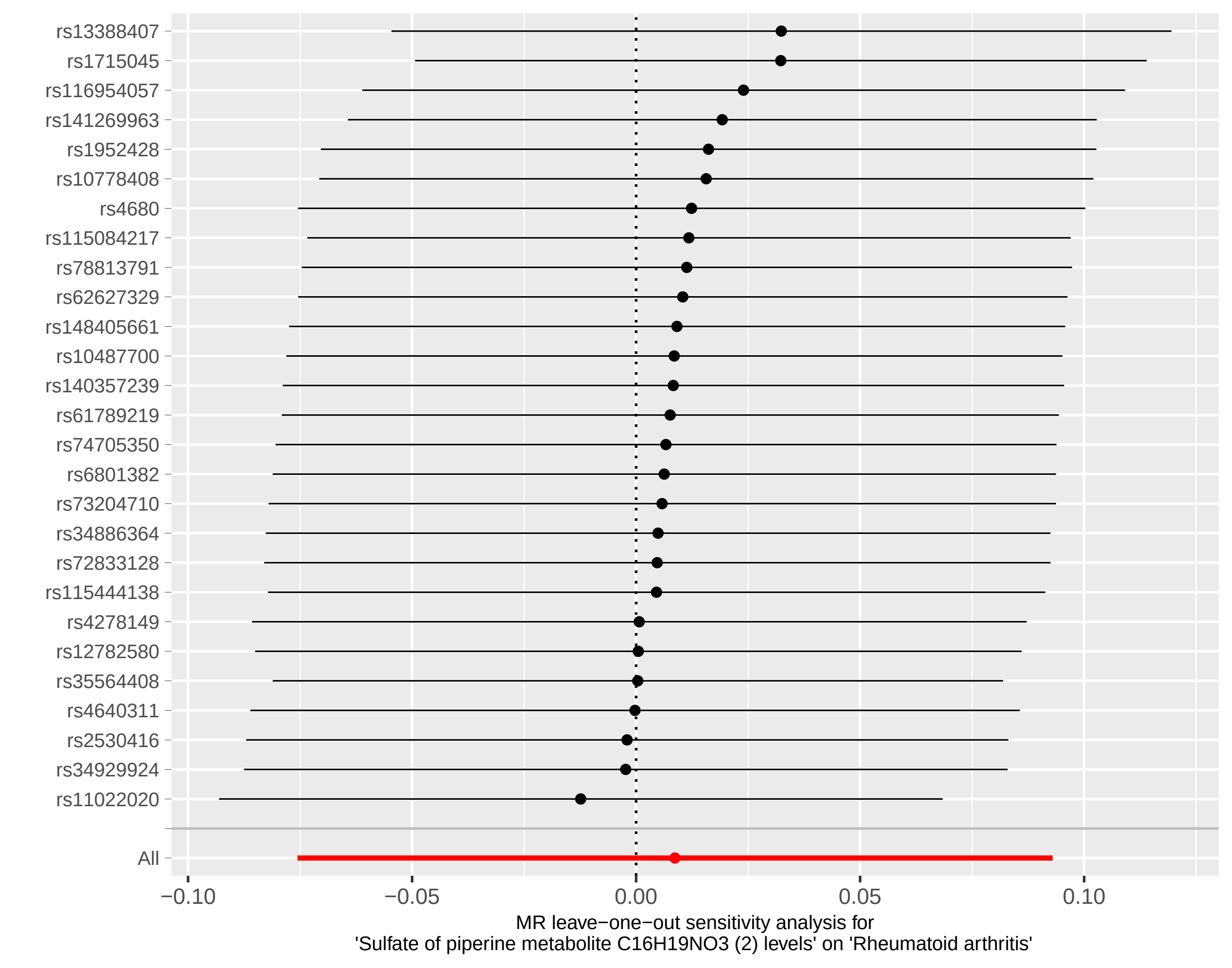
rs13388407
rs1715045
rs116954057
rs141269963
rs1952428
rs10778408
rs4680
rs115084217
rs78813791
rs62627329
rs148405661
rs10487700
rs140357239
rs61789219
rs74705350
rs6801382
rs73204710
rs34886364
rs72833128
rs115444138
rs4278149
rs12782580
rs35564408
rs4640311
rs2530416
rs34929924
rs11022020
All
−0.10
−0.05
0.00
0.05
0.10
MR leave−one−out sensitivity analysis for
'Sulfate of piperine metabolite C16H19NO3 (2) levels' on 'Rheumatoid arthritis'

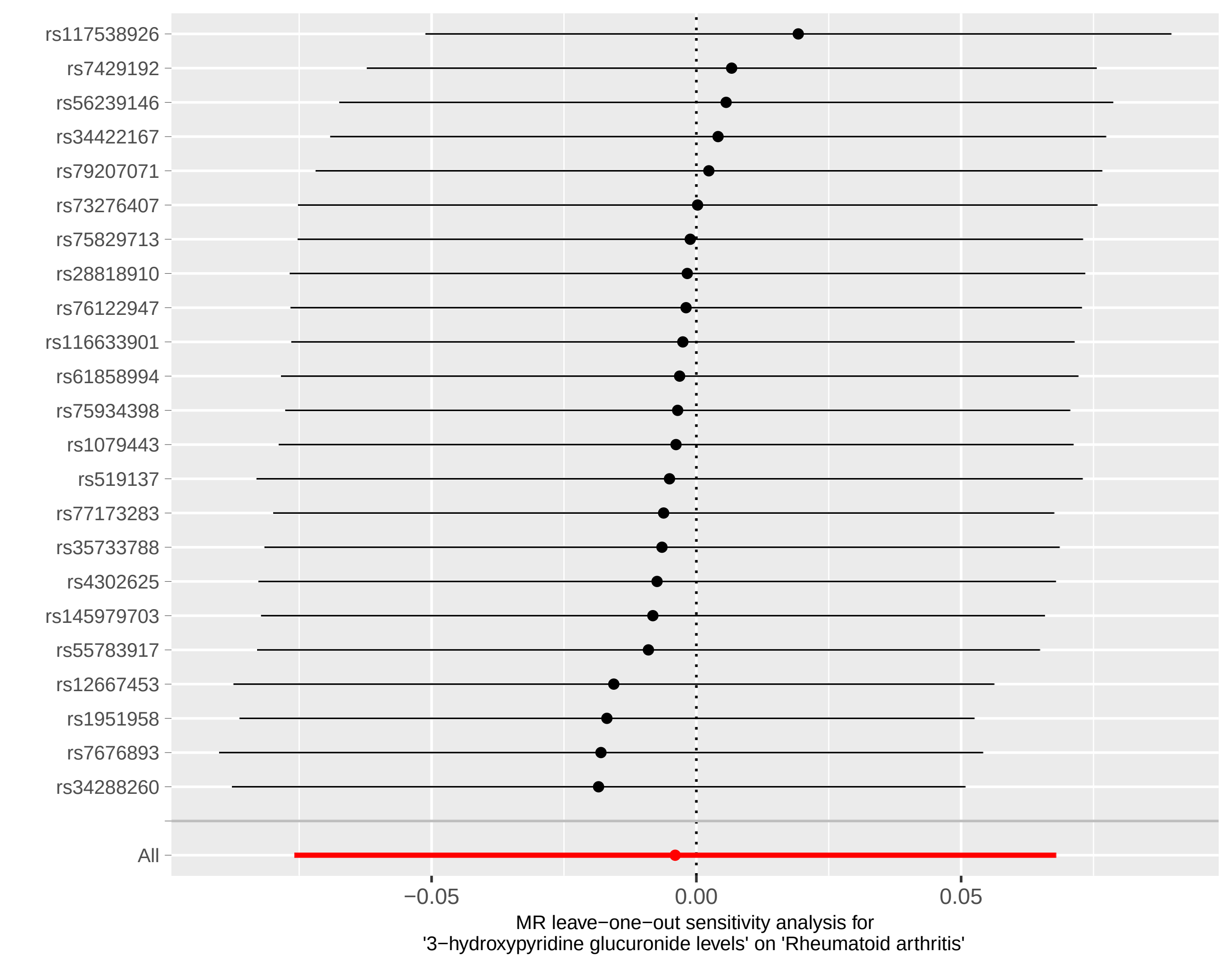
rs117538926
rs7429192
rs56239146
rs34422167
rs79207071
rs73276407
rs75829713
rs28818910
rs76122947
rs116633901
rs61858994
rs75934398
rs1079443
rs519137
rs77173283
rs35733788
rs4302625
rs145979703
rs55783917
rs12667453
rs1951958
rs7676893
rs34288260
All
−0.05
0.00
0.05
MR leave−one−out sensitivity analysis for
'3−hydroxypyridine glucuronide levels' on 'Rheumatoid arthritis'

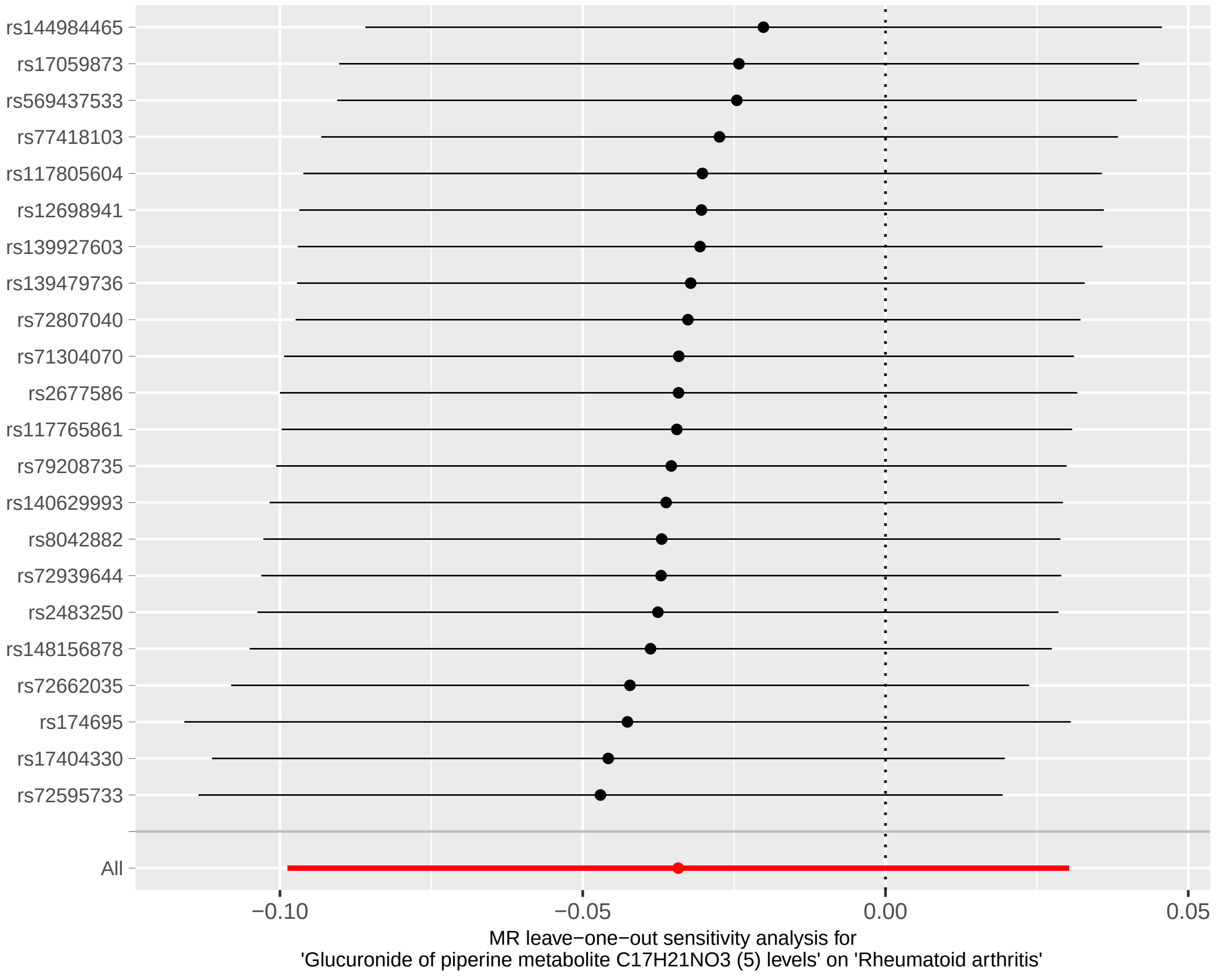
rs144984465
rs17059873
rs569437533
rs77418103
rs117805604
rs12698941
rs139927603
rs139479736
rs72807040
rs71304070
rs2677586
rs117765861
rs79208735
rs140629993
rs8042882
rs72939644
rs2483250
rs148156878
rs72662035
rs174695
rs17404330
rs72595733
All
−0.10
−0.05
0.00
0.05
MR leave−one−out sensitivity analysis for
'Glucuronide of piperine metabolite C17H21NO3 (5) levels' on 'Rheumatoid arthritis'

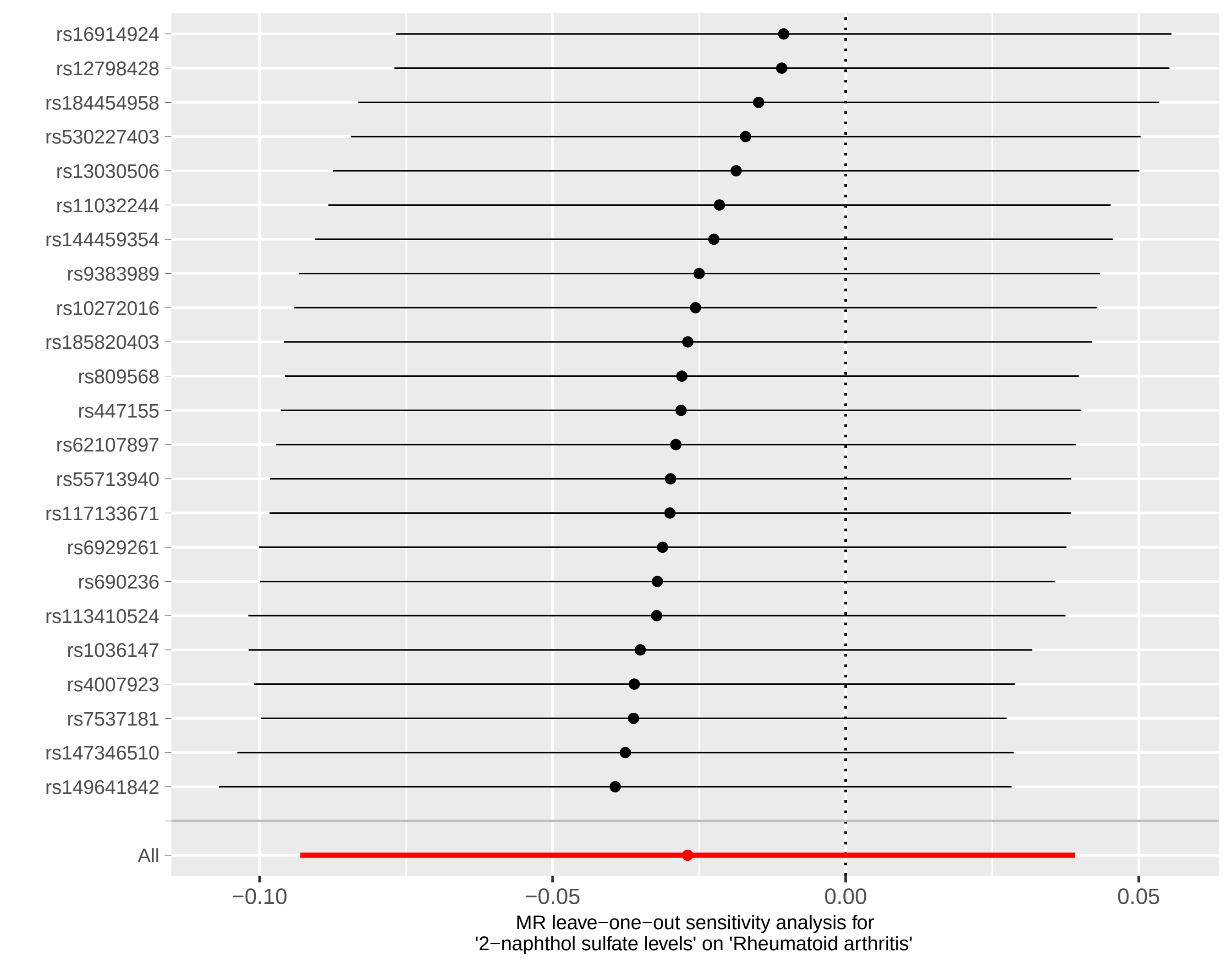
rs16914924
rs12798428
rs184454958
rs530227403
rs13030506
rs11032244
rs144459354
rs9383989
rs10272016
rs185820403
rs809568
rs447155
rs62107897
rs55713940
rs117133671
rs6929261
rs690236
rs113410524
rs1036147
rs4007923
rs7537181
rs147346510
rs149641842
All
−0.10
−0.05
0.00
0.05
MR leave−one−out sensitivity analysis for
'2−naphthol sulfate levels' on 'Rheumatoid arthritis'

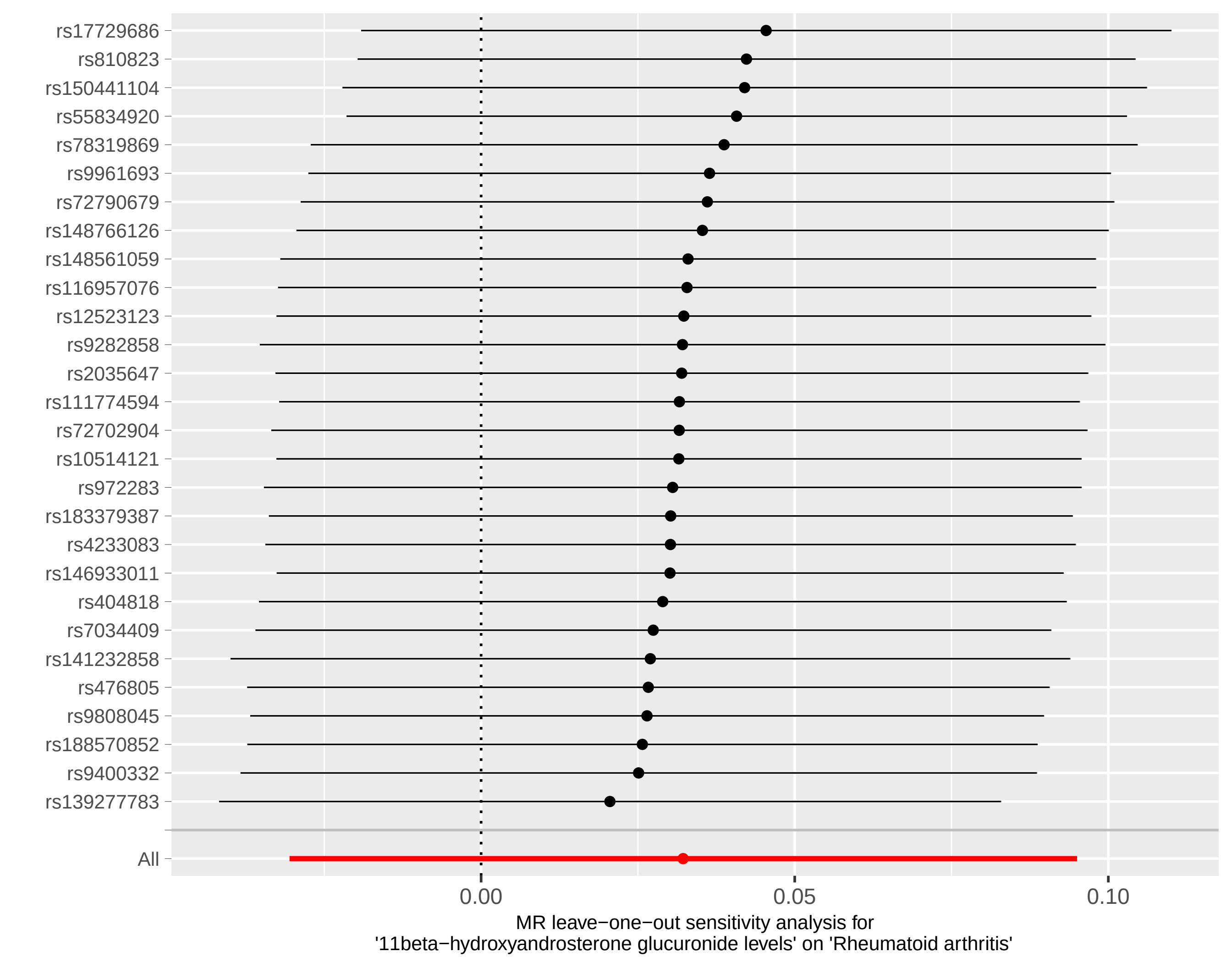
rs17729686
rs810823
rs150441104
rs55834920
rs78319869
rs9961693
rs72790679
rs148766126
rs148561059
rs116957076
rs12523123
rs9282858
rs2035647
rs111774594
rs72702904
rs10514121
rs972283
rs183379387
rs4233083
rs146933011
rs404818
rs7034409
rs141232858
rs476805
rs9808045
rs188570852
rs9400332
rs139277783
All
0.00
0.05
0.10
MR leave−one−out sensitivity analysis for
'11beta−hydroxyandrosterone glucuronide levels' on 'Rheumatoid arthritis'

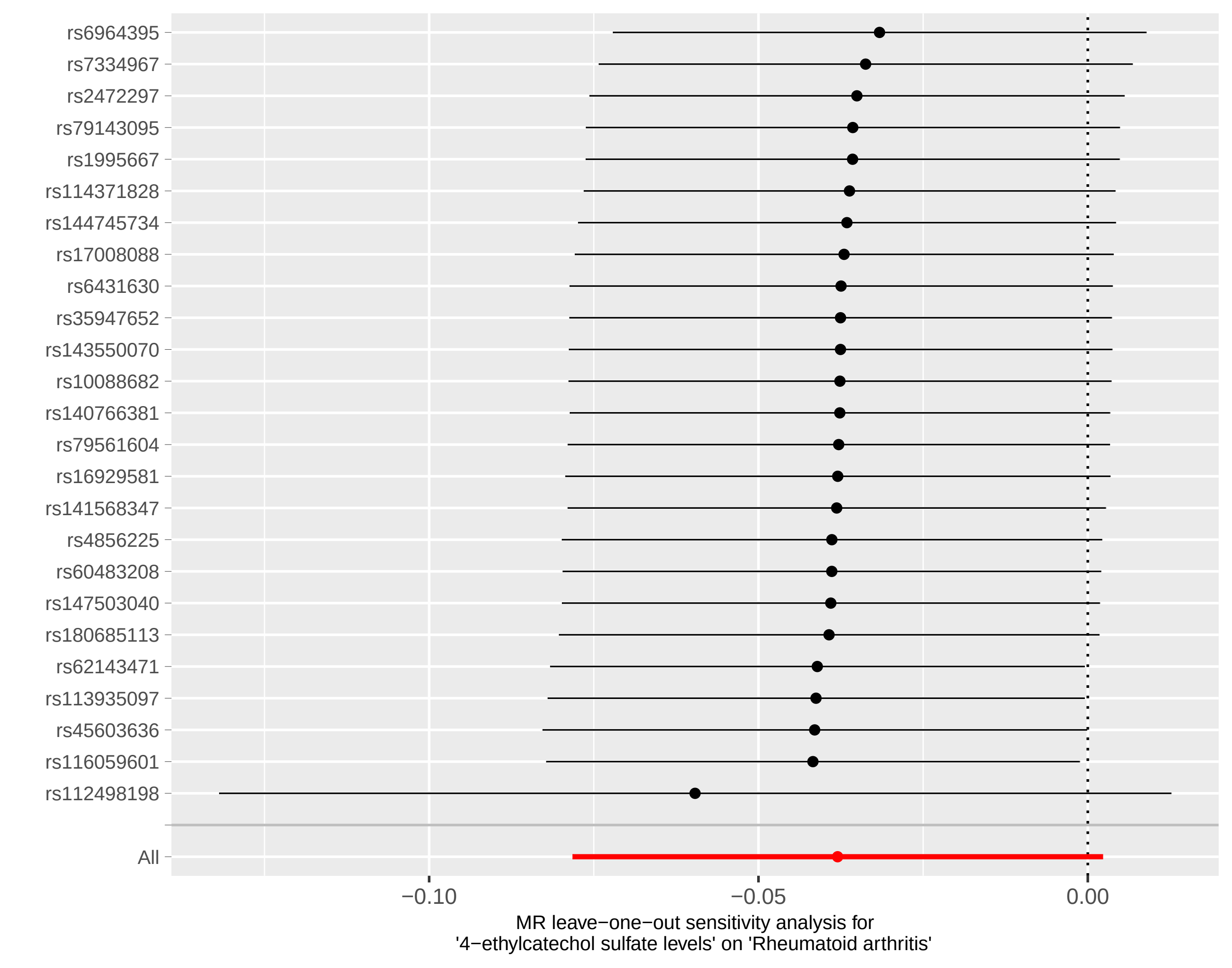

rs6964395
rs7334967
rs2472297
rs79143095
rs1995667
rs114371828
rs144745734
rs17008088
rs6431630
rs35947652
rs143550070
rs10088682
rs140766381
rs79561604
rs16929581
rs141568347
rs4856225
rs60483208
rs147503040
rs180685113
rs62143471
rs113935097
rs45603636
rs116059601
rs112498198
All
−0.10
−0.05
0.00
MR leave−one−out sensitivity analysis for
'4−ethylcatechol sulfate levels' on 'Rheumatoid arthritis'

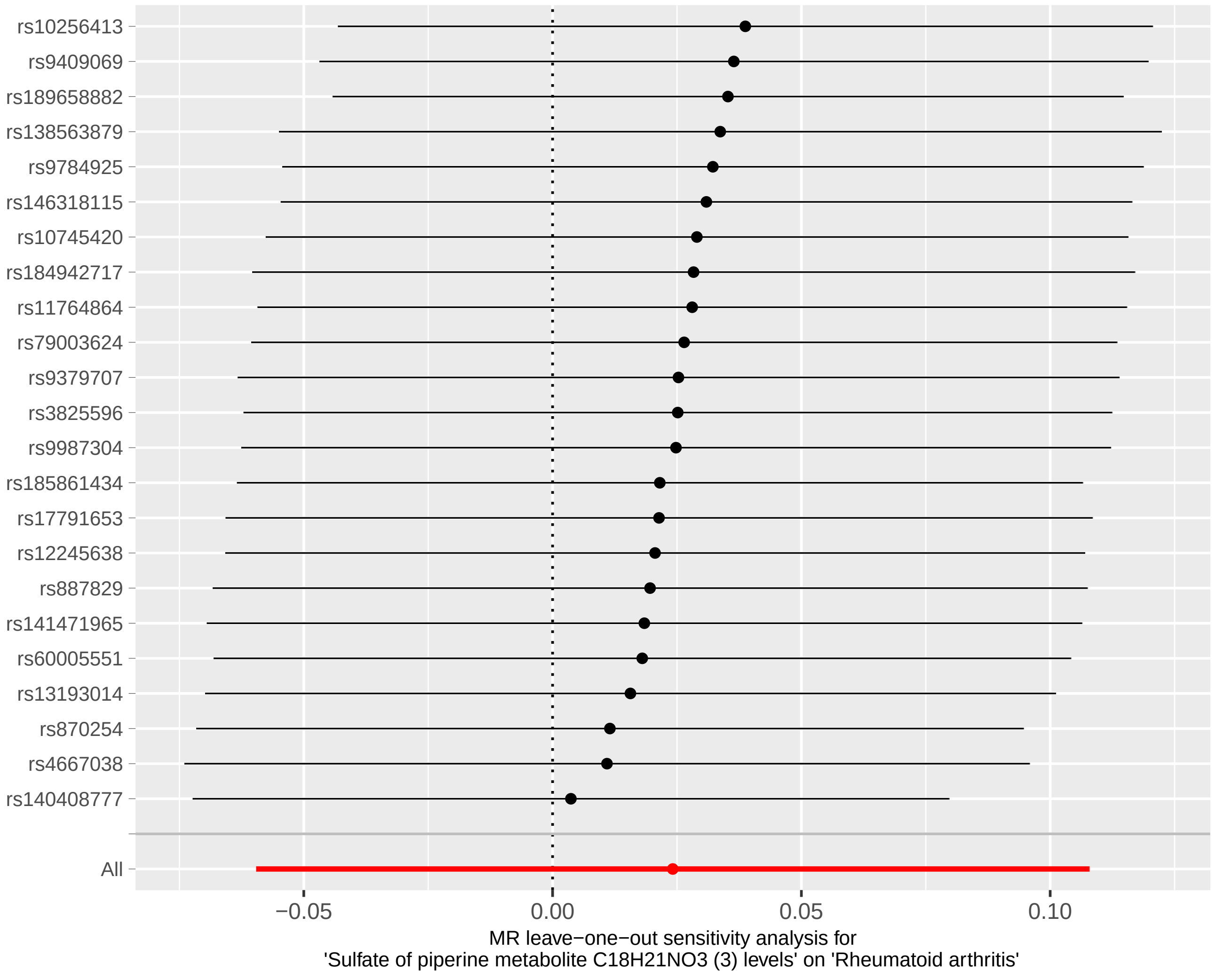
rs10256413
rs9409069
rs189658882
rs138563879
rs9784925
rs146318115
rs10745420
rs184942717
rs11764864
rs79003624
rs9379707
rs3825596
rs9987304
rs185861434
rs17791653
rs12245638
rs887829
rs141471965
rs60005551
rs13193014
rs870254
rs4667038
rs140408777
All
−0.05
0.00
0.05
0.10
MR leave−one−out sensitivity analysis for
'Sulfate of piperine metabolite C18H21NO3 (3) levels' on 'Rheumatoid arthritis'

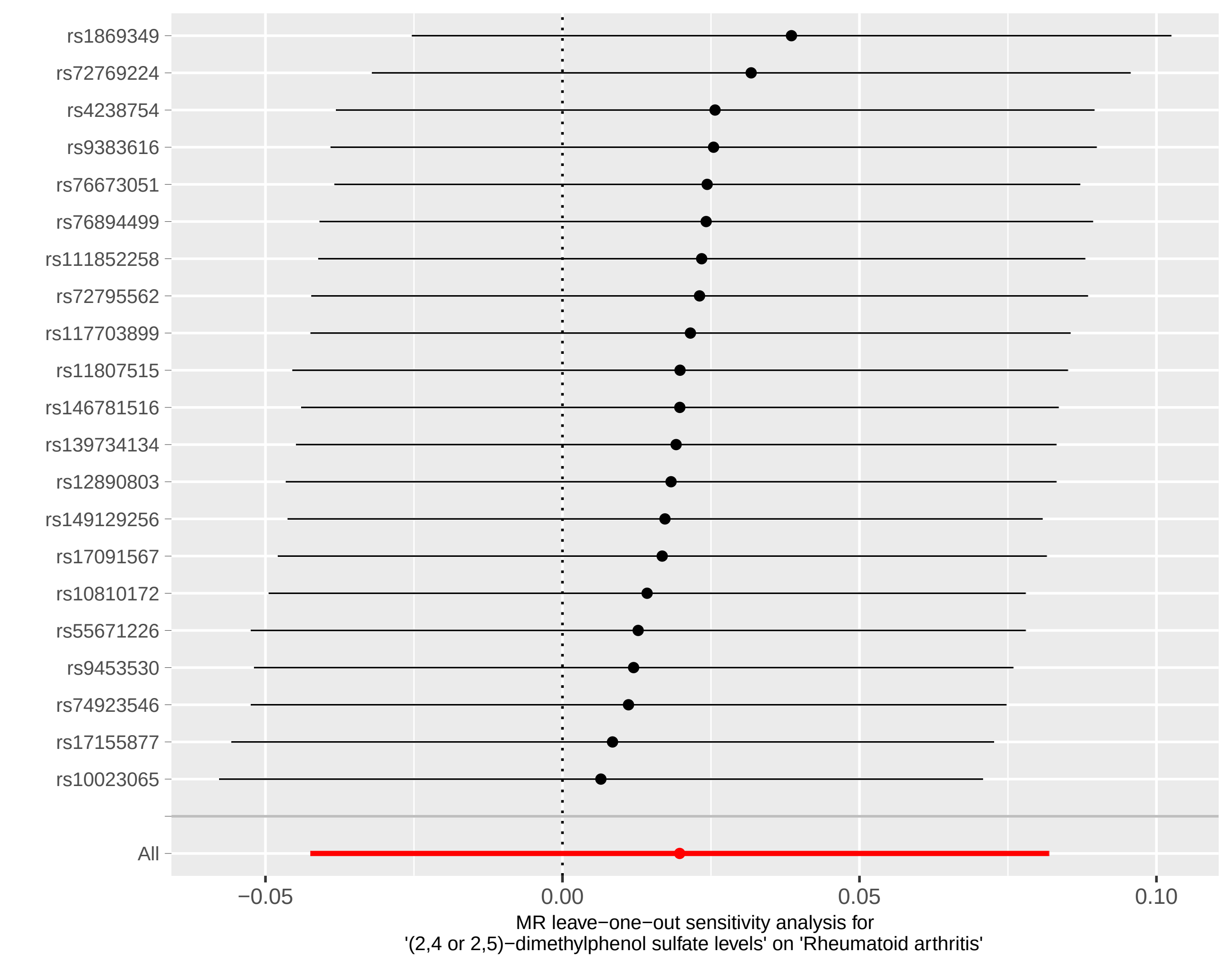
rs1869349
rs72769224
rs4238754
rs9383616
rs76673051
rs76894499
rs111852258
rs72795562
rs117703899
rs11807515
rs146781516
rs139734134
rs12890803
rs149129256
rs17091567
rs10810172
rs55671226
rs9453530
rs74923546
rs17155877
rs10023065
All
−0.05
0.00
0.05
0.10
MR leave−one−out sensitivity analysis for
'(2,4 or 2,5)−dimethylphenol sulfate levels' on 'Rheumatoid arthritis'

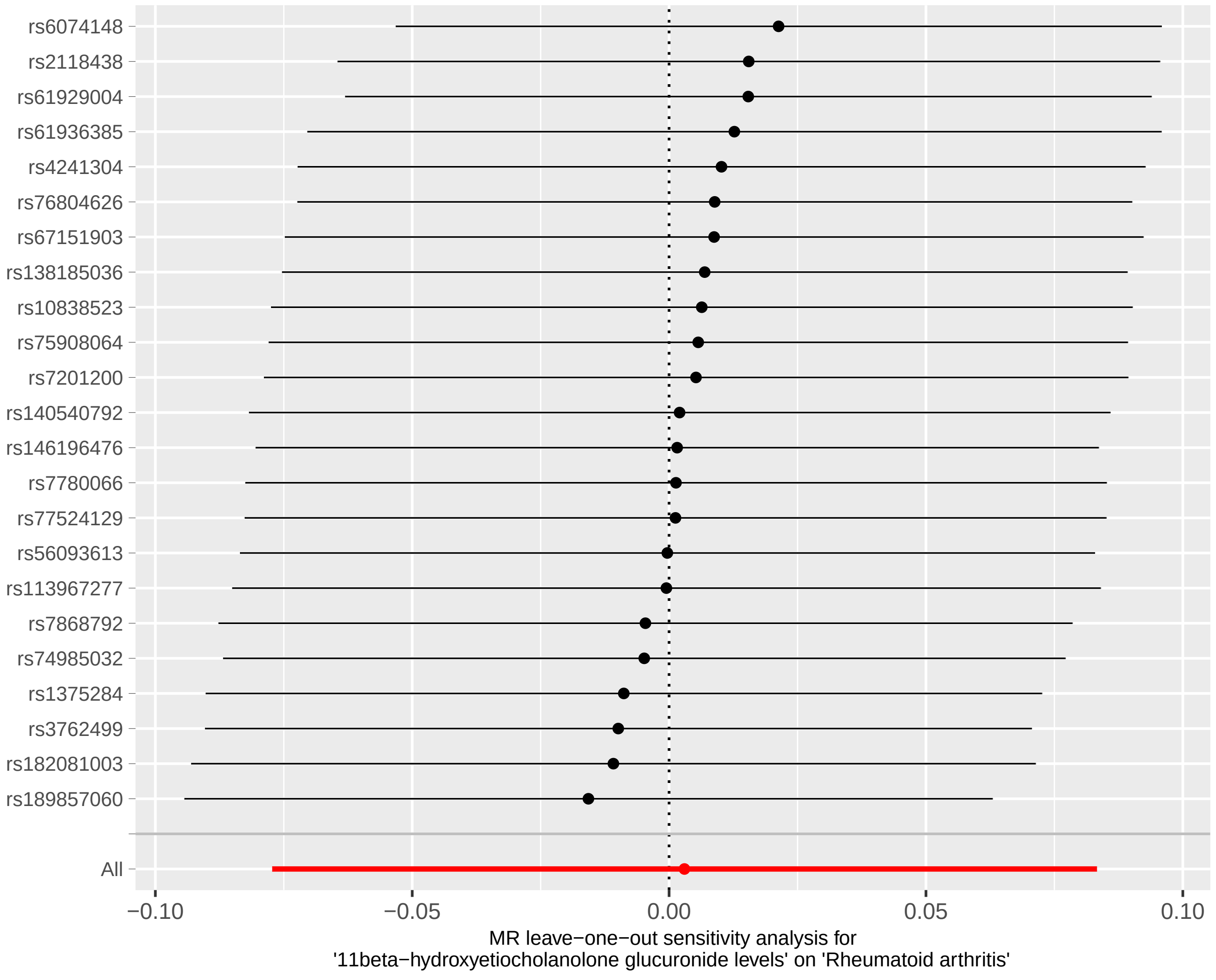
rs6074148
rs2118438
rs61929004
rs61936385
rs4241304
rs76804626
rs67151903
rs138185036
rs10838523
rs75908064
rs7201200
rs140540792
rs146196476
rs7780066
rs77524129
rs56093613
rs113967277
rs7868792
rs74985032
rs1375284
rs3762499
rs182081003
rs189857060
All
−0.10
−0.05
0.00
0.05
0.10
MR leave−one−out sensitivity analysis for
'11beta−hydroxyetiocholanolone glucuronide levels' on 'Rheumatoid arthritis'

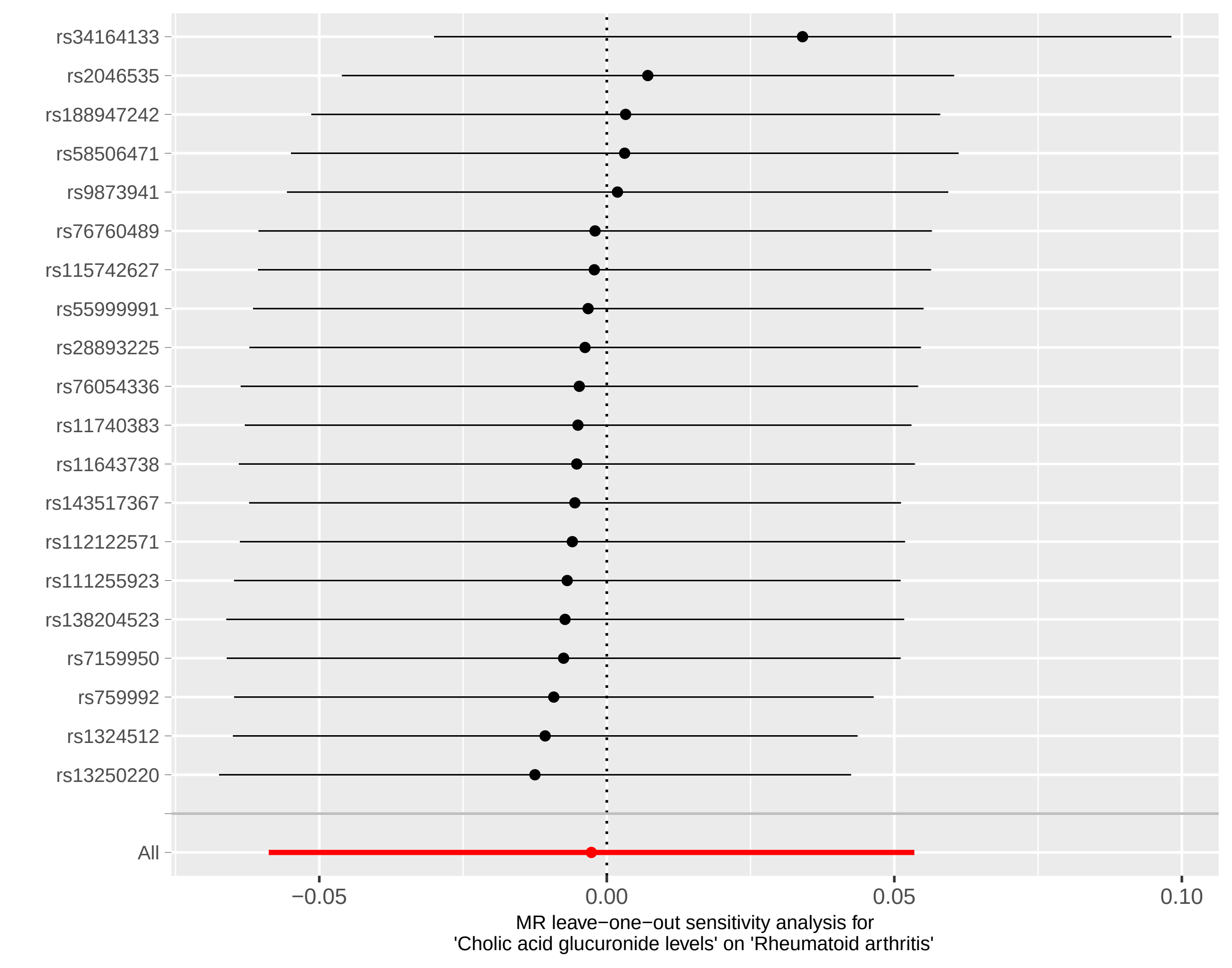
rs34164133
rs2046535
rs188947242
rs58506471
rs9873941
rs76760489
rs115742627
rs55999991
rs28893225
rs76054336
rs11740383
rs11643738
rs143517367
rs112122571
rs111255923
rs138204523
rs7159950
rs759992
rs1324512
rs13250220
All
−0.05
0.00
0.05
0.10
MR leave−one−out sensitivity analysis for
'Cholic acid glucuronide levels' on 'Rheumatoid arthritis'

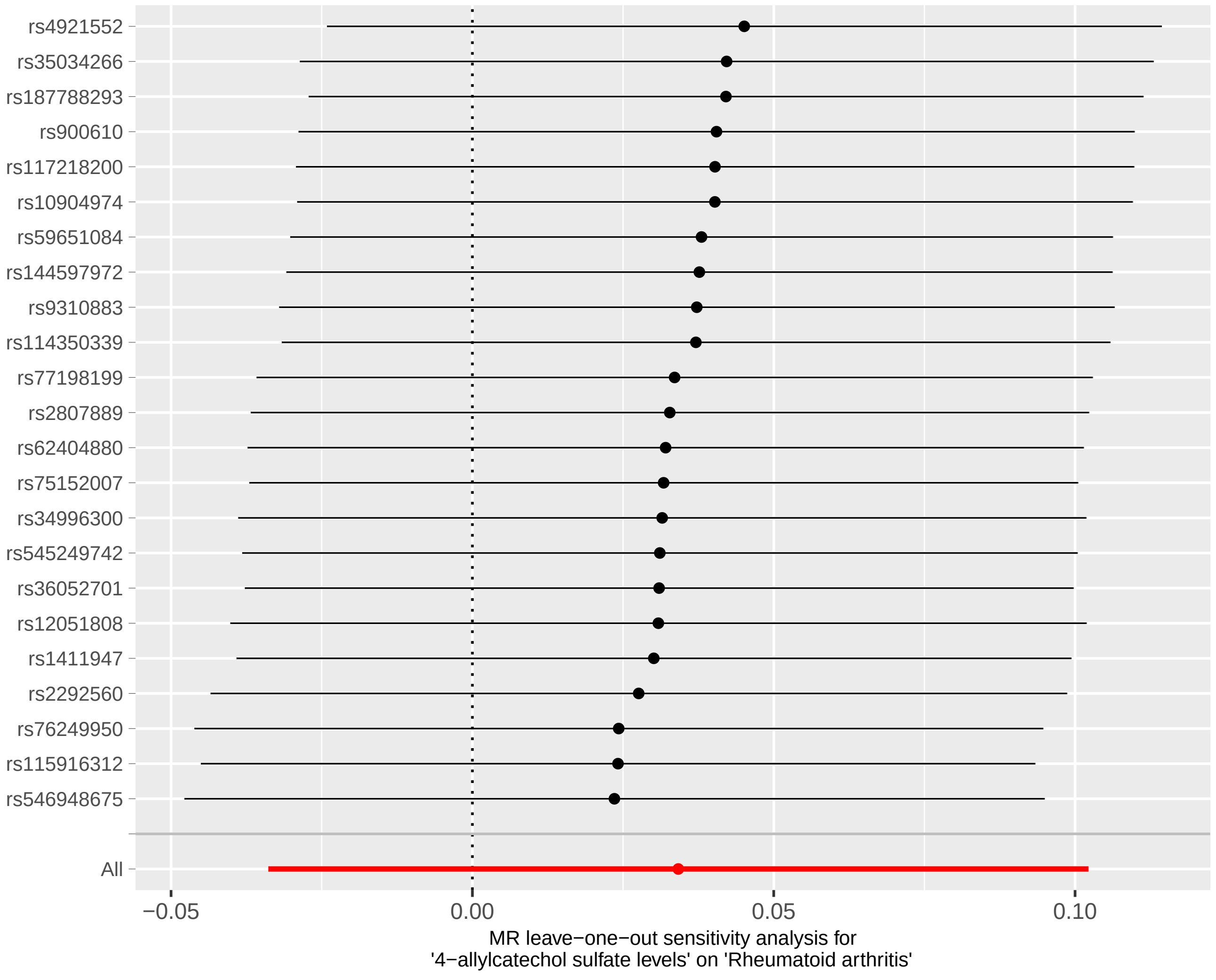
rs4921552
rs35034266
rs187788293
rs900610
rs117218200
rs10904974
rs59651084
rs144597972
rs9310883
rs114350339
rs77198199
rs2807889
rs62404880
rs75152007
rs34996300
rs545249742
rs36052701
rs12051808
rs1411947
rs2292560
rs76249950
rs115916312
rs546948675
All
−0.05
0.00
0.05
0.10
MR leave−one−out sensitivity analysis for
'4−allylcatechol sulfate levels' on 'Rheumatoid arthritis'

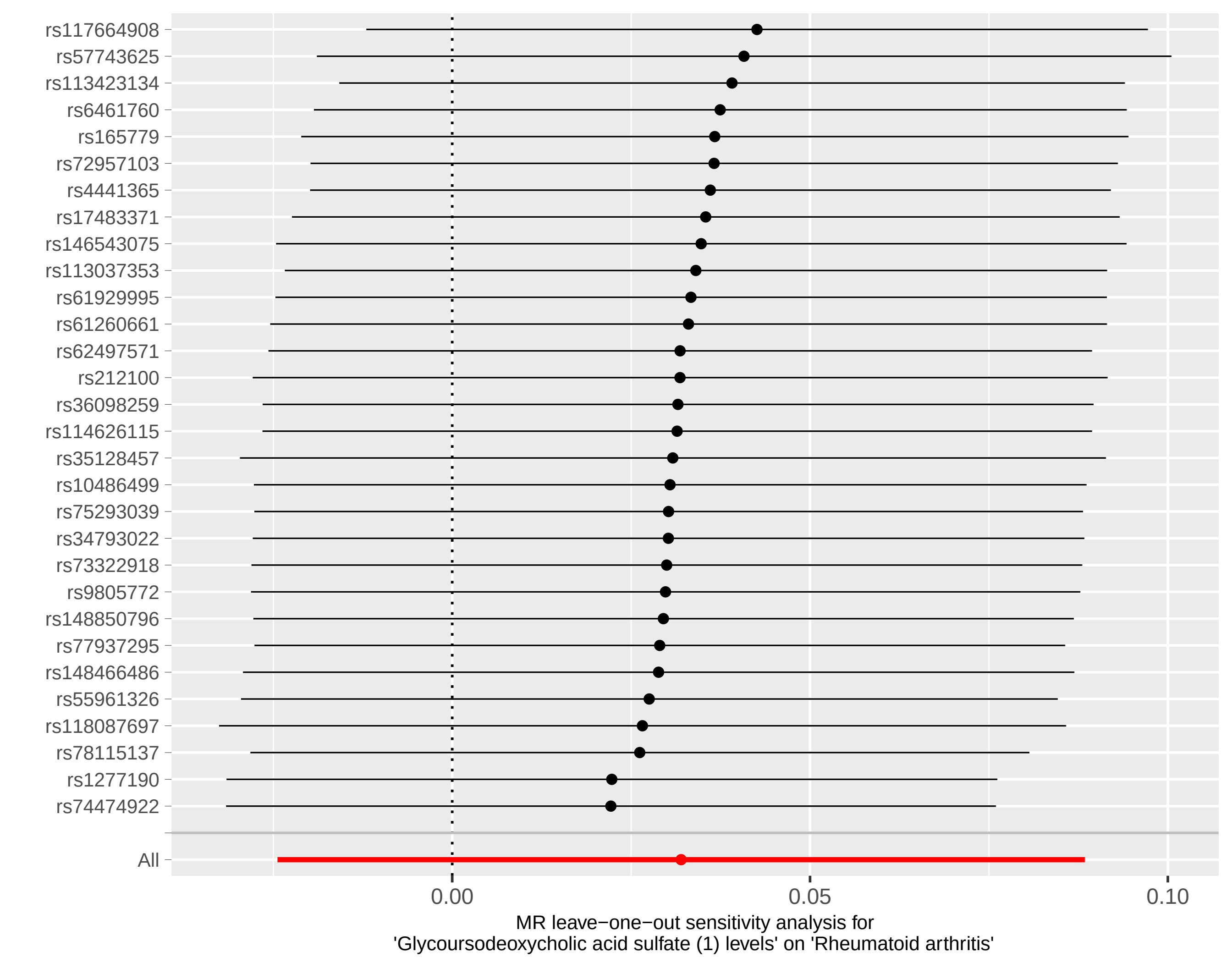
rs117664908
rs57743625
rs113423134
rs6461760
rs165779
rs72957103
rs4441365
rs17483371
rs146543075
rs113037353
rs61929995
rs61260661
rs62497571
rs212100
rs36098259
rs114626115
rs35128457
rs10486499
rs75293039
rs34793022
rs73322918
rs9805772
rs148850796
rs77937295
rs148466486
rs55961326
rs118087697
rs78115137
rs1277190
rs74474922
All
0.00
0.05
0.10
MR leave−one−out sensitivity analysis for
'Glycoursodeoxycholic acid sulfate (1) levels' on 'Rheumatoid arthritis'

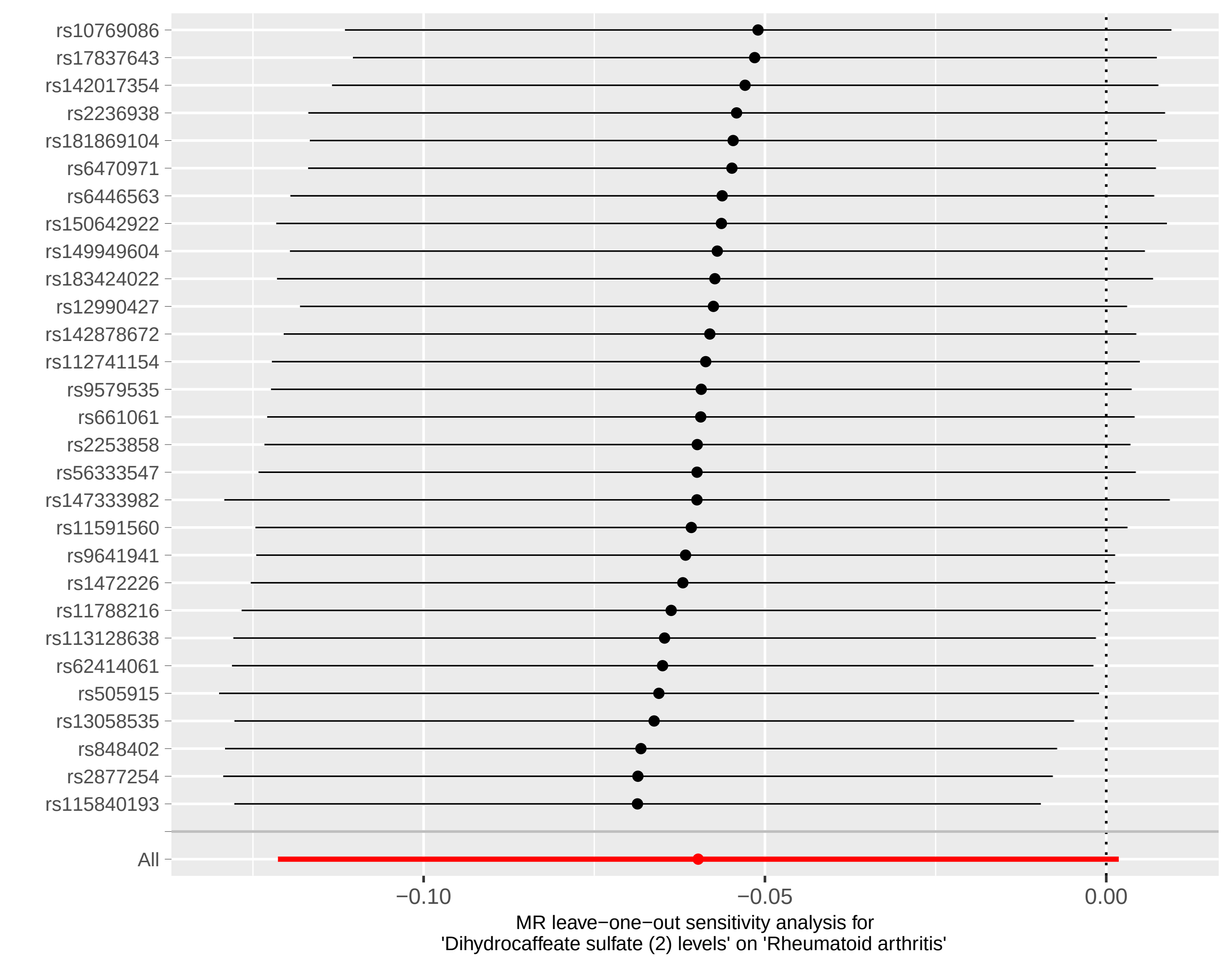
rs10769086
rs17837643
rs142017354
rs2236938
rs181869104
rs6470971
rs6446563
rs150642922
rs149949604
rs183424022
rs12990427
rs142878672
rs112741154
rs9579535
rs661061
rs2253858
rs56333547
rs147333982
rs11591560
rs9641941
rs1472226
rs11788216
rs113128638
rs62414061
rs505915
rs13058535
rs848402
rs2877254
rs115840193
All
−0.10
−0.05
0.00
MR leave−one−out sensitivity analysis for
'Dihydrocaffeate sulfate (2) levels' on 'Rheumatoid arthritis'

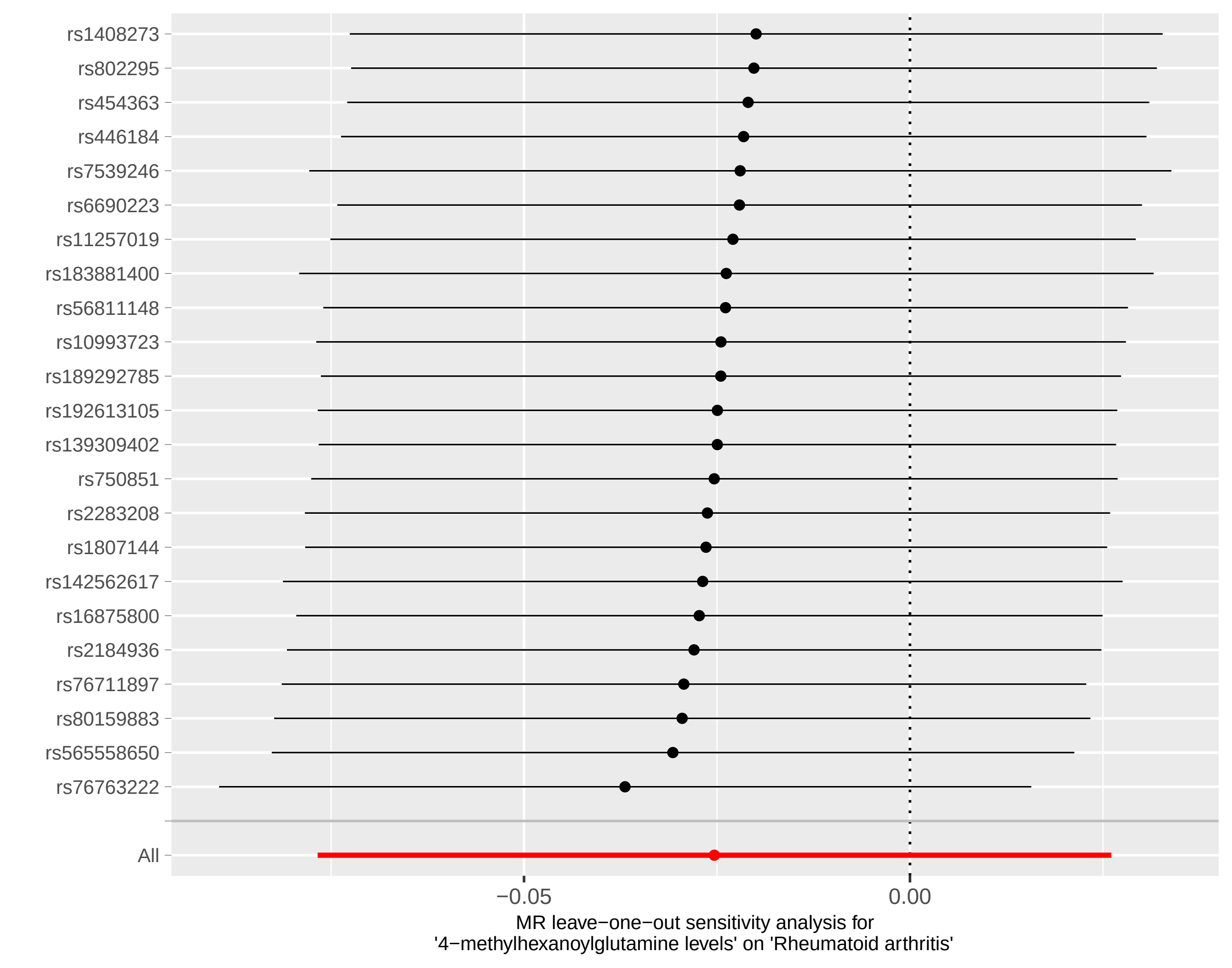

rs1408273
rs802295
rs454363
rs446184
rs7539246
rs6690223
rs11257019
rs183881400
rs56811148
rs10993723
rs189292785
rs192613105
rs139309402
rs750851
rs2283208
rs1807144
rs142562617
rs16875800
rs2184936
rs76711897
rs80159883
rs565558650
rs76763222
All
−0.05
0.00
MR leave−one−out sensitivity analysis for
'4−methylhexanoylglutamine levels' on 'Rheumatoid arthritis'

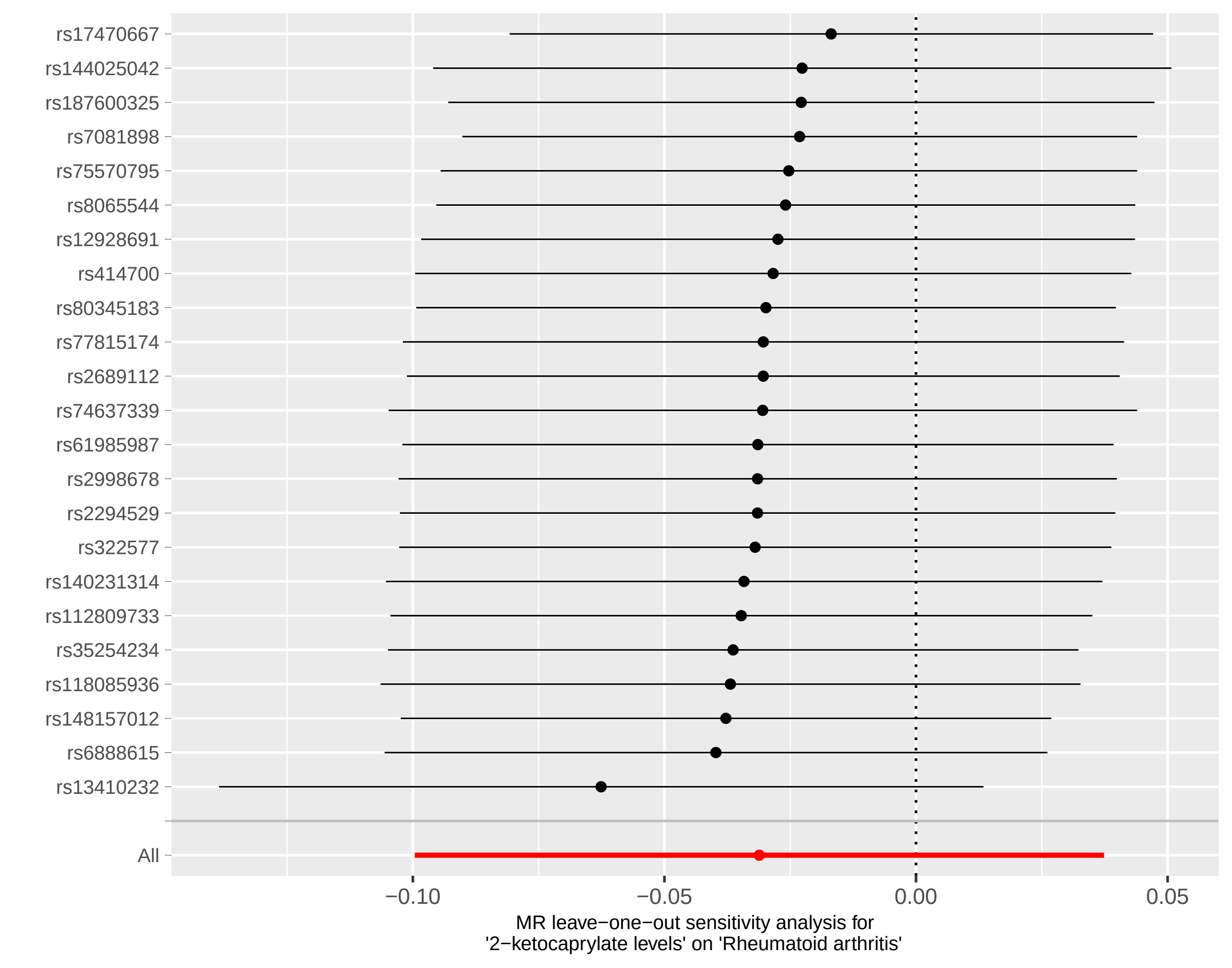

rs17470667
rs144025042
rs187600325
rs7081898
rs75570795
rs8065544
rs12928691
rs414700
rs80345183
rs77815174
rs2689112
rs74637339
rs61985987
rs2998678
rs2294529
rs322577
rs140231314
rs112809733
rs35254234
rs118085936
rs148157012
rs6888615
rs13410232
All
−0.10
−0.05
0.00
0.05
MR leave−one−out sensitivity analysis for
'2−ketocaprylate levels' on 'Rheumatoid arthritis'

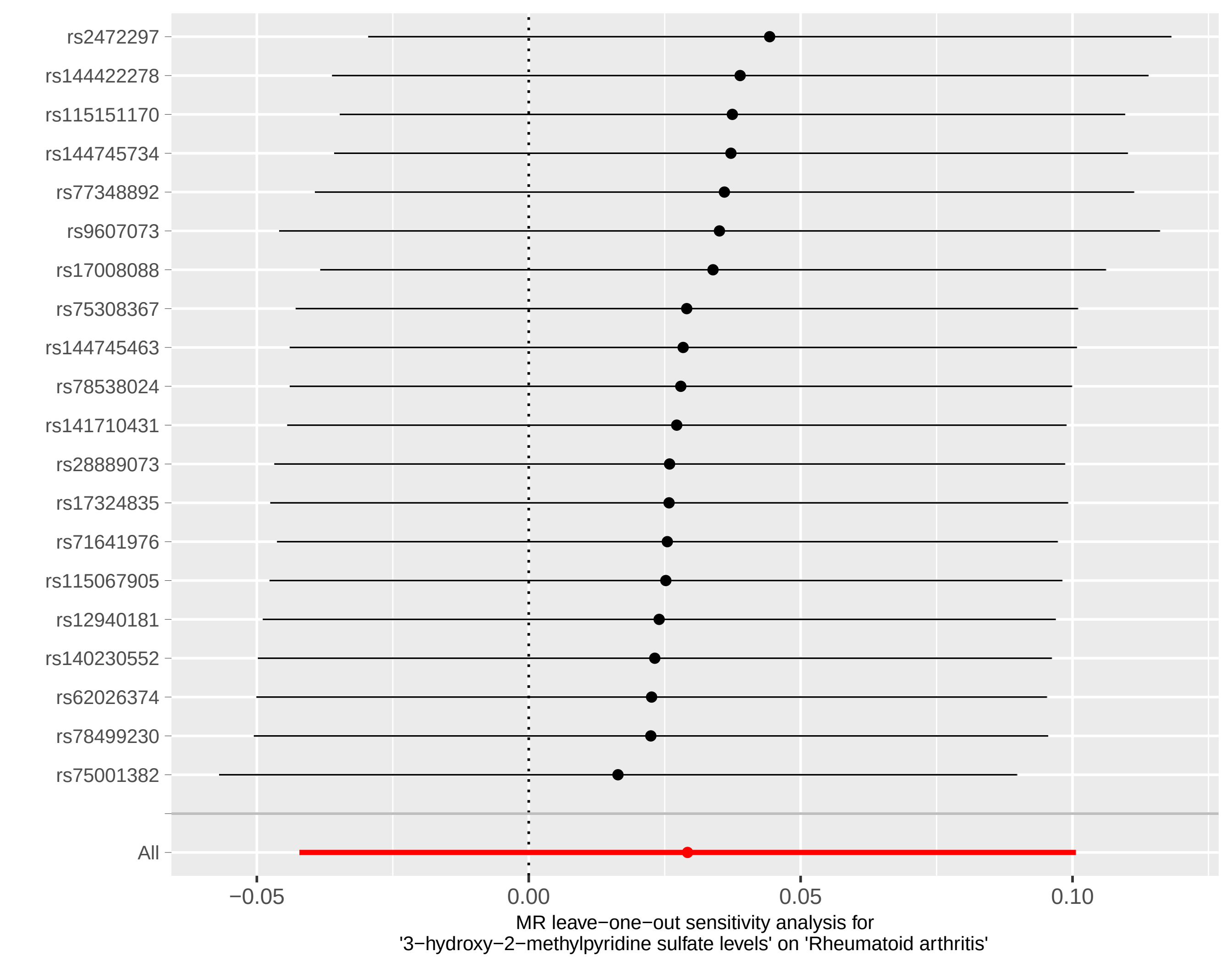
rs2472297
rs144422278
rs115151170
rs144745734
rs77348892
rs9607073
rs17008088
rs75308367
rs144745463
rs78538024
rs141710431
rs28889073
rs17324835
rs71641976
rs115067905
rs12940181
rs140230552
rs62026374
rs78499230
rs75001382
All
−0.05
0.00
0.05
0.10
MR leave−one−out sensitivity analysis for
'3−hydroxy−2−methylpyridine sulfate levels' on 'Rheumatoid arthritis'

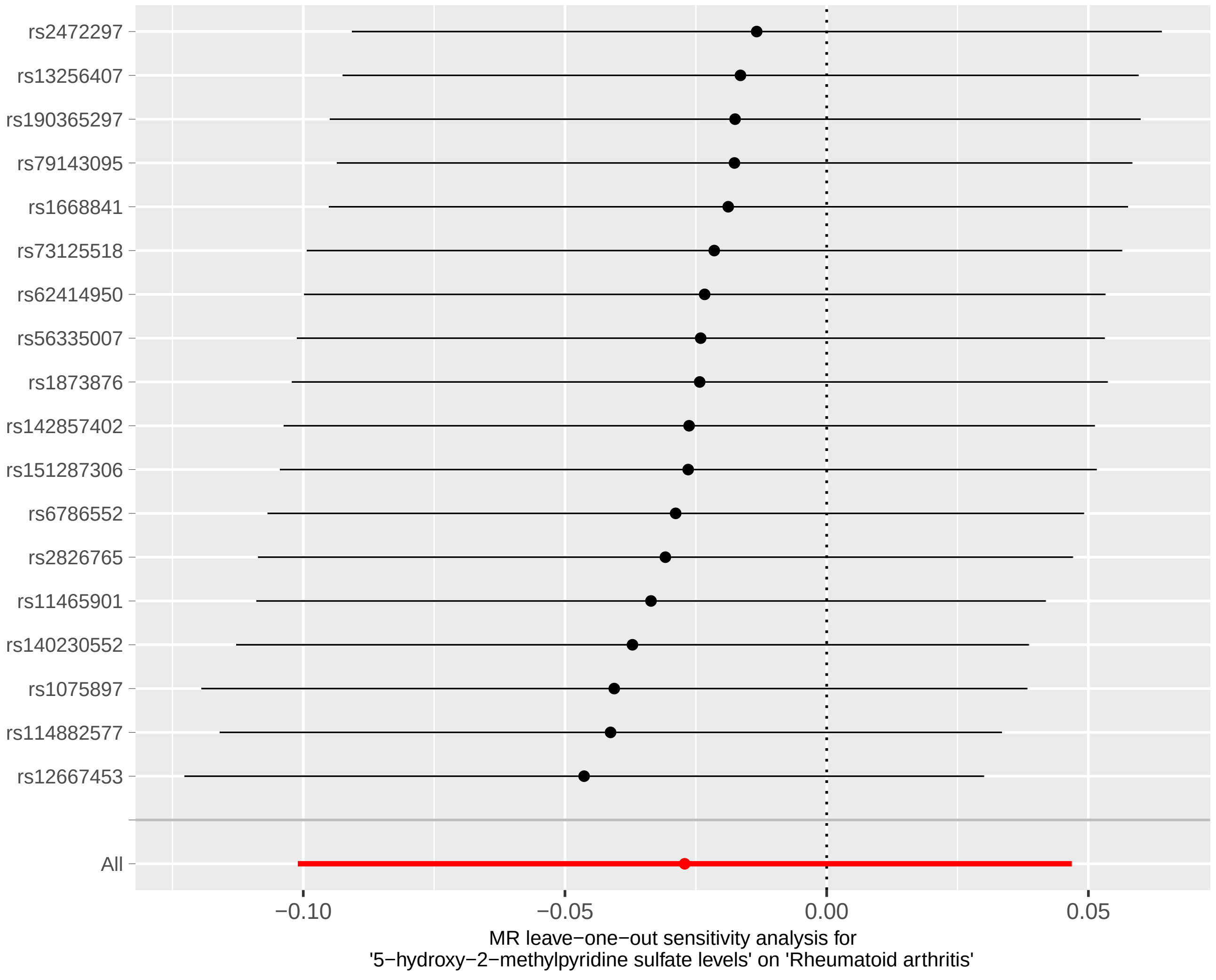
rs2472297
rs13256407
rs190365297
rs79143095
rs1668841
rs73125518
rs62414950
rs56335007
rs1873876
rs142857402
rs151287306
rs6786552
rs2826765
rs11465901
rs140230552
rs1075897
rs114882577
rs12667453
All
−0.10
−0.05
0.00
0.05
MR leave−one−out sensitivity analysis for
'5−hydroxy−2−methylpyridine sulfate levels' on 'Rheumatoid arthritis'

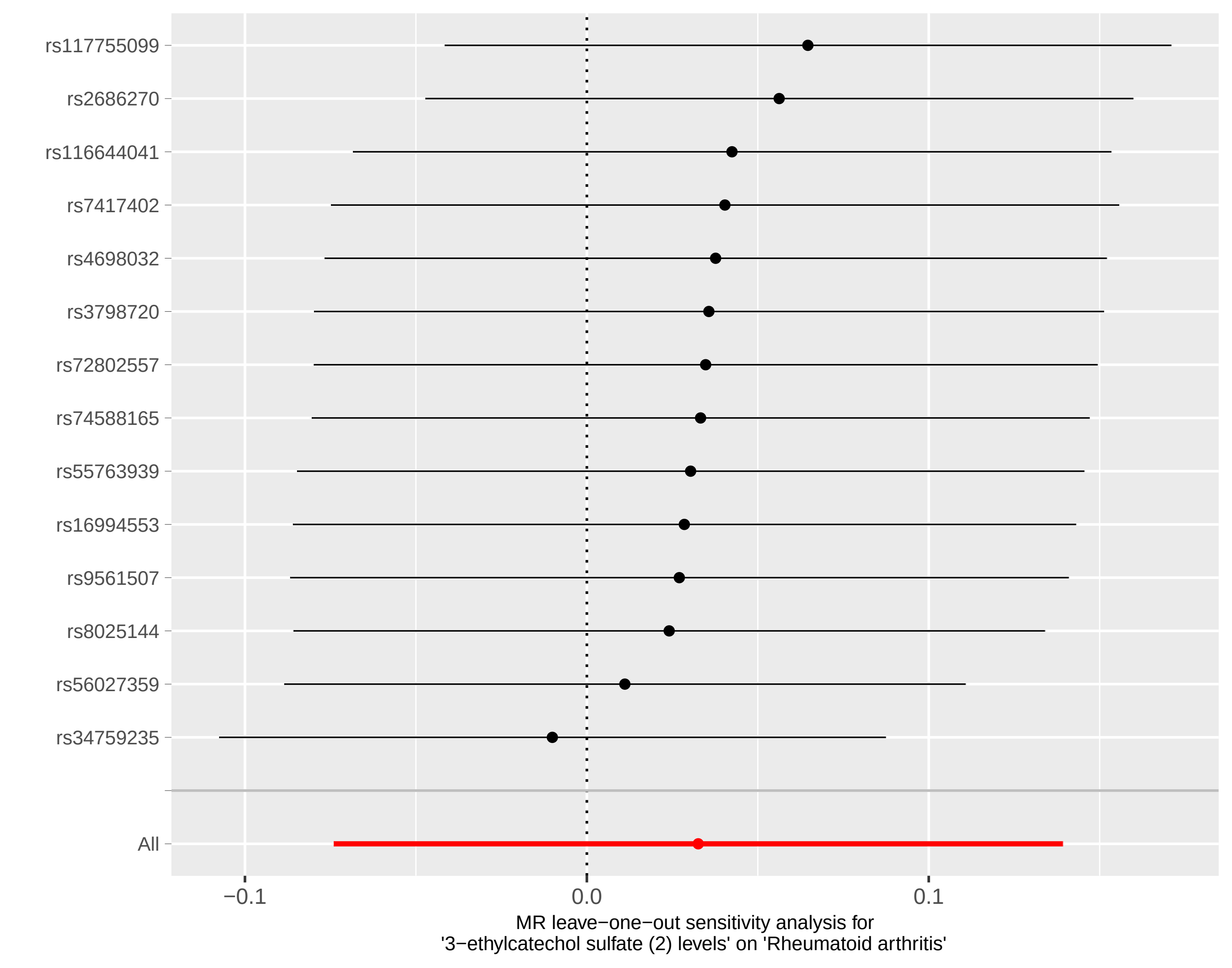
rs117755099
rs2686270
rs116644041
rs7417402
rs4698032
rs3798720
rs72802557
rs74588165
rs55763939
rs16994553
rs9561507
rs8025144
rs56027359
rs34759235
All
−0.1
0.0
0.1
MR leave−one−out sensitivity analysis for
'3−ethylcatechol sulfate (2) levels' on 'Rheumatoid arthritis'

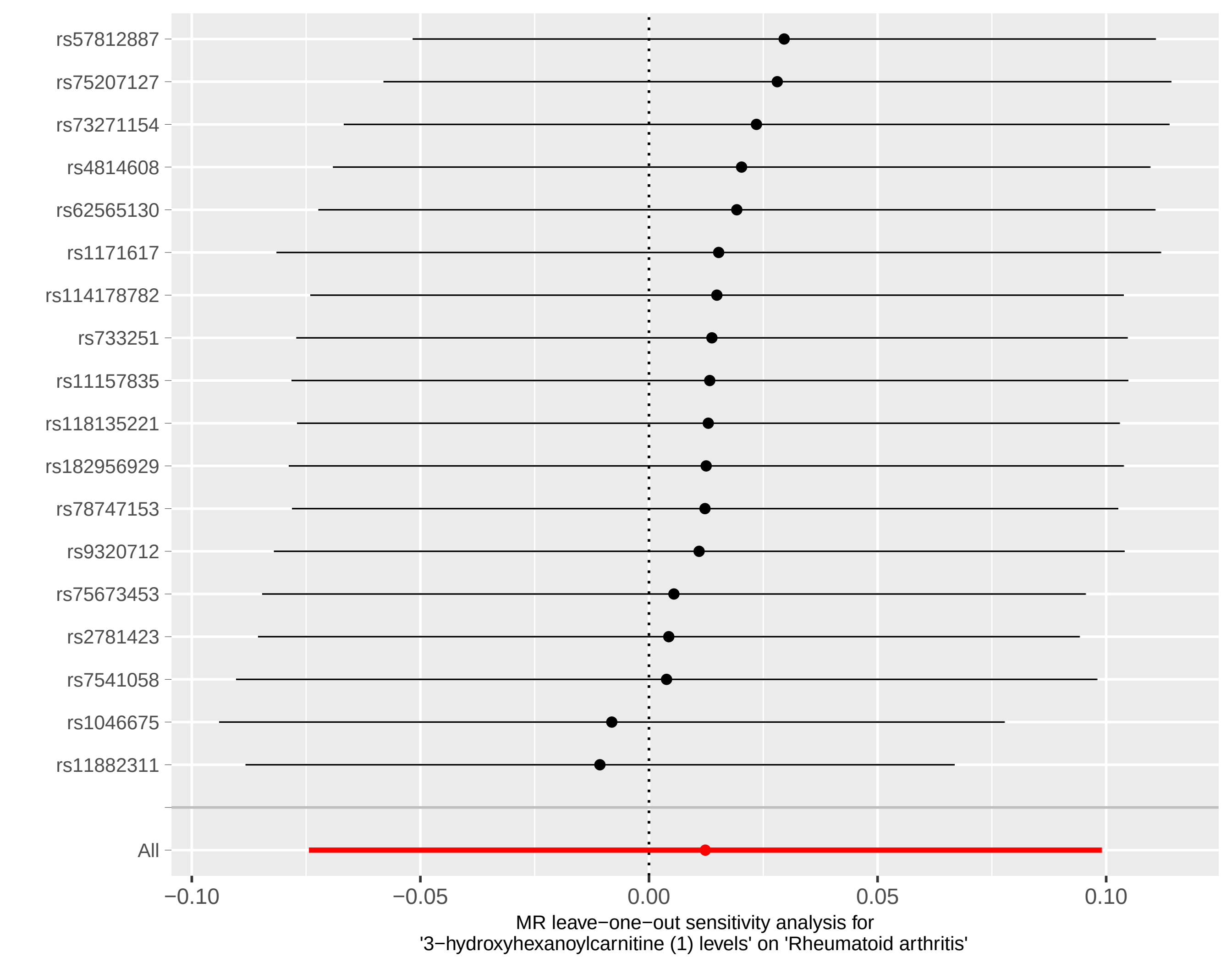

rs57812887
rs75207127
rs73271154
rs4814608
rs62565130
rs1171617
rs114178782
rs733251
rs11157835
rs118135221
rs182956929
rs78747153
rs9320712
rs75673453
rs2781423
rs7541058
rs1046675
rs11882311
All
−0.10
−0.05
0.00
0.05
0.10
MR leave−one−out sensitivity analysis for
'3−hydroxyhexanoylcarnitine (1) levels' on 'Rheumatoid arthritis'

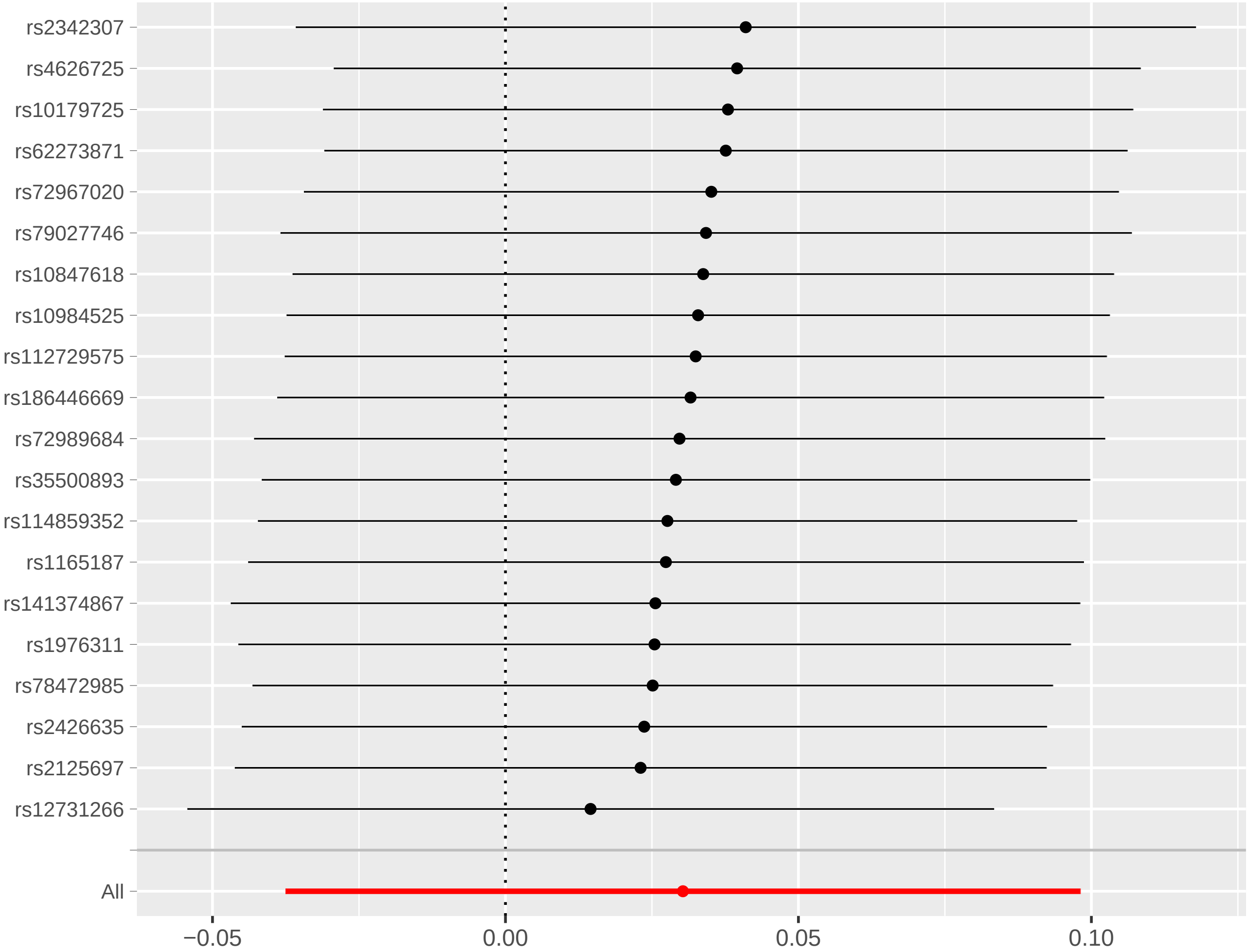

MR leave−one−out sensitivity analysis for
'2,6−dihydroxybenzoic acid levels' on 'Rheumatoid arthritis'

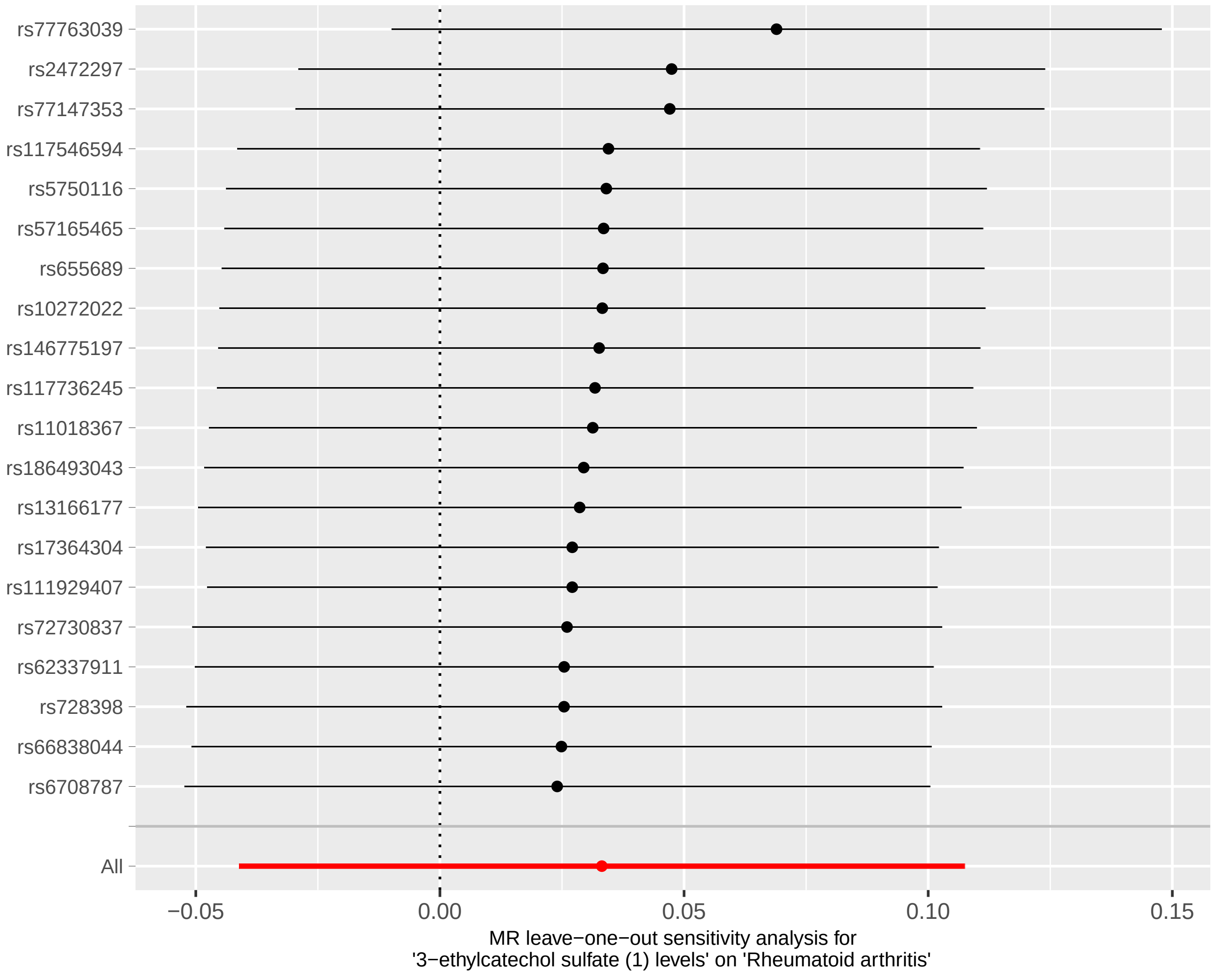

rs77763039
rs2472297
rs77147353
rs117546594
rs5750116
rs57165465
rs655689
rs10272022
rs146775197
rs117736245
rs11018367
rs186493043
rs13166177
rs17364304
rs111929407
rs72730837
rs62337911
rs728398
rs66838044
rs6708787
All
−0.05
0.00
0.05
0.10
0.15
MR leave−one−out sensitivity analysis for
'3−ethylcatechol sulfate (1) levels' on 'Rheumatoid arthritis'

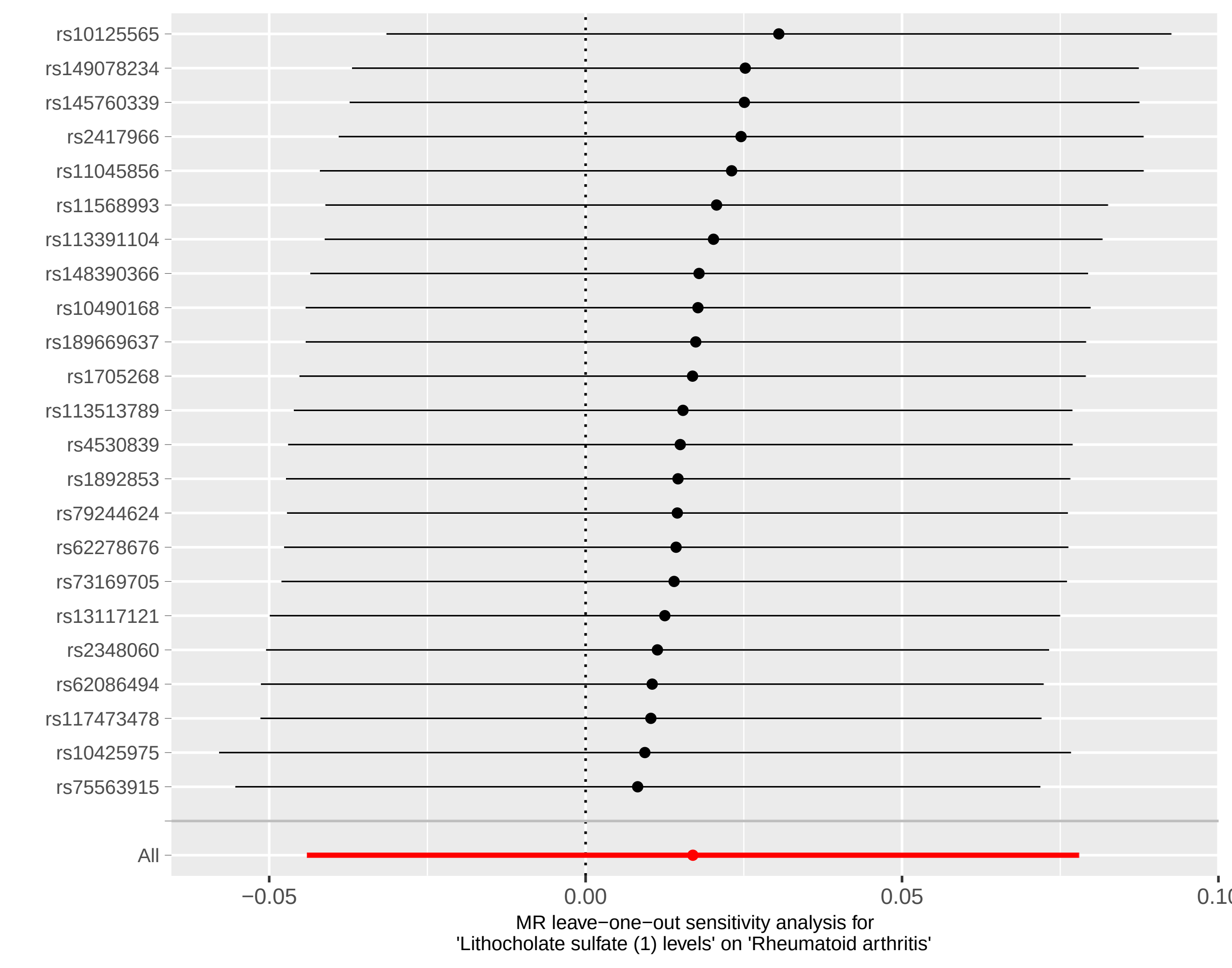

rs10125565
rs149078234
rs145760339
rs2417966
rs11045856
rs11568993
rs113391104
rs148390366
rs10490168
rs189669637
rs1705268
rs113513789
rs4530839
rs1892853
rs79244624
rs62278676
rs73169705
rs13117121
rs2348060
rs62086494
rs117473478
rs10425975
rs75563915
All
−0.05
0.00
0.05
0.10
MR leave−one−out sensitivity analysis for
'Lithocholate sulfate (1) levels' on 'Rheumatoid arthritis'

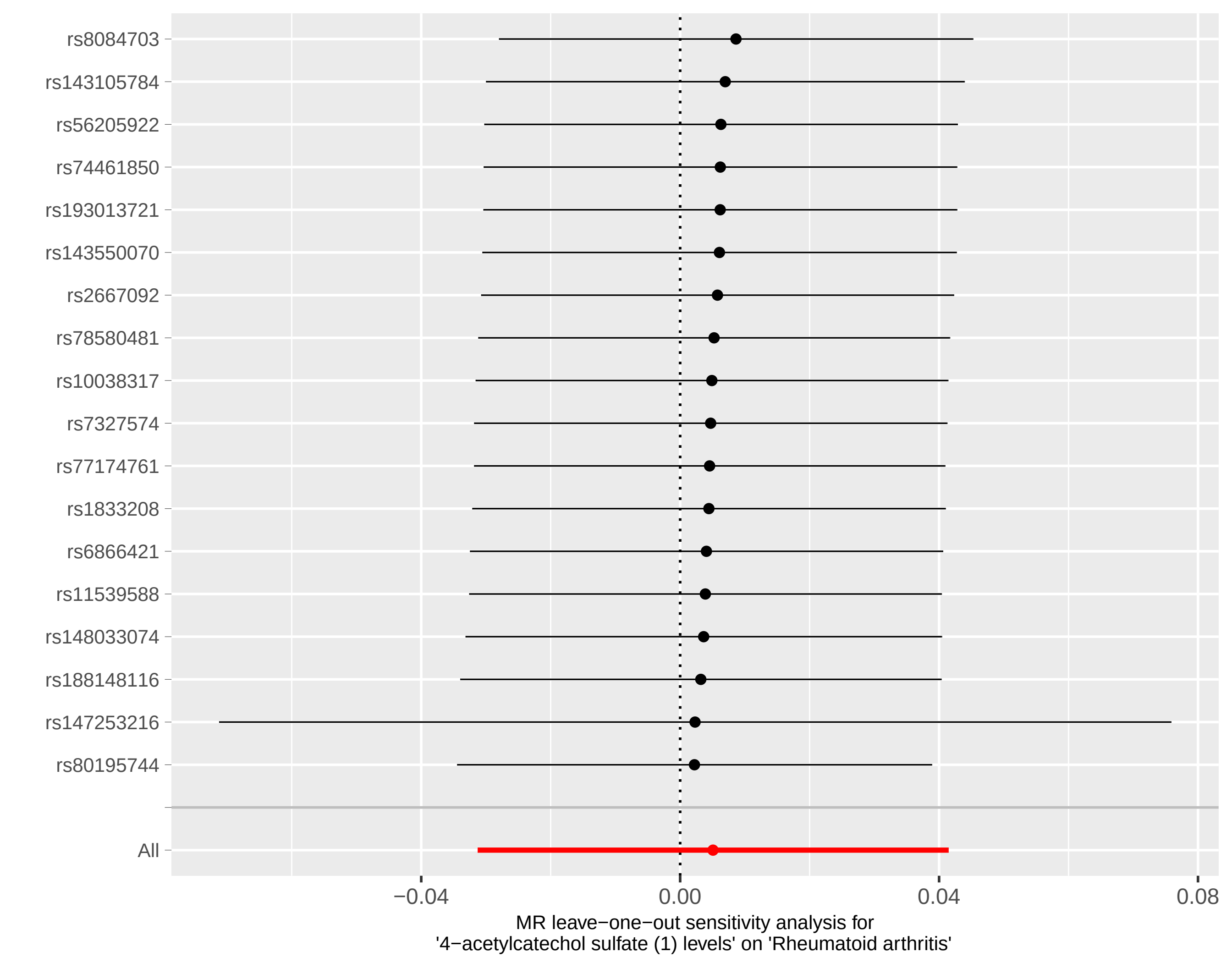
rs8084703
rs143105784
rs56205922
rs74461850
rs193013721
rs143550070
rs2667092
rs78580481
rs10038317
rs7327574
rs77174761
rs1833208
rs6866421
rs11539588
rs148033074
rs188148116
rs147253216
rs80195744
All
−0.04
0.00
0.04
0.08
MR leave−one−out sensitivity analysis for
'4−acetylcatechol sulfate (1) levels' on 'Rheumatoid arthritis'

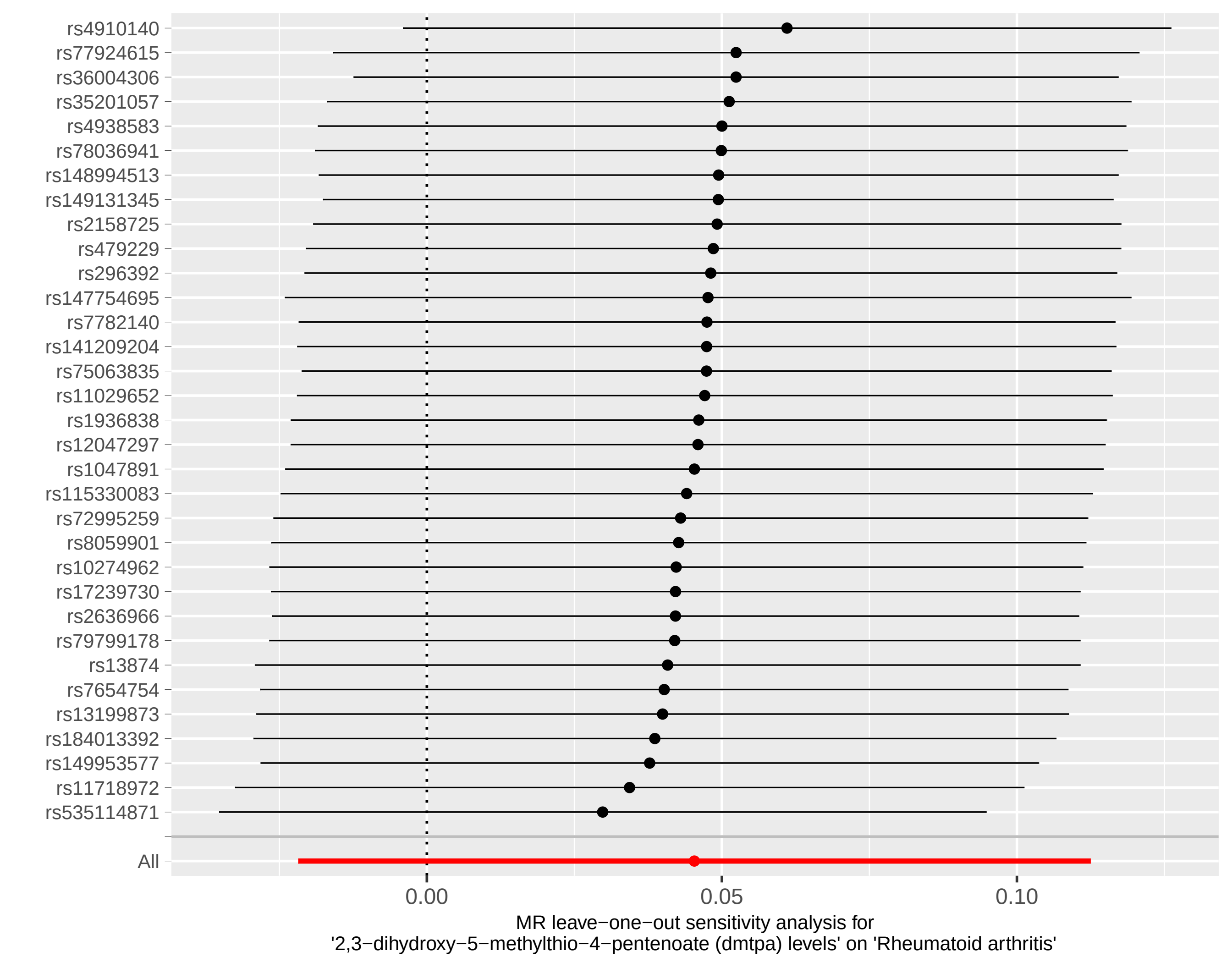
rs4910140
rs77924615
rs36004306
rs35201057
rs4938583
rs78036941
rs148994513
rs149131345
rs2158725
rs479229
rs296392
rs147754695
rs7782140
rs141209204
rs75063835
rs11029652
rs1936838
rs12047297
rs1047891
rs115330083
rs72995259
rs8059901
rs10274962
rs17239730
rs2636966
rs79799178
rs13874
rs7654754
rs13199873
rs184013392
rs149953577
rs11718972
rs535114871
All
0.00
0.05
0.10
MR leave−one−out sensitivity analysis for
'2,3−dihydroxy−5−methylthio−4−pentenoate (dmtpa) levels' on 'Rheumatoid arthritis'

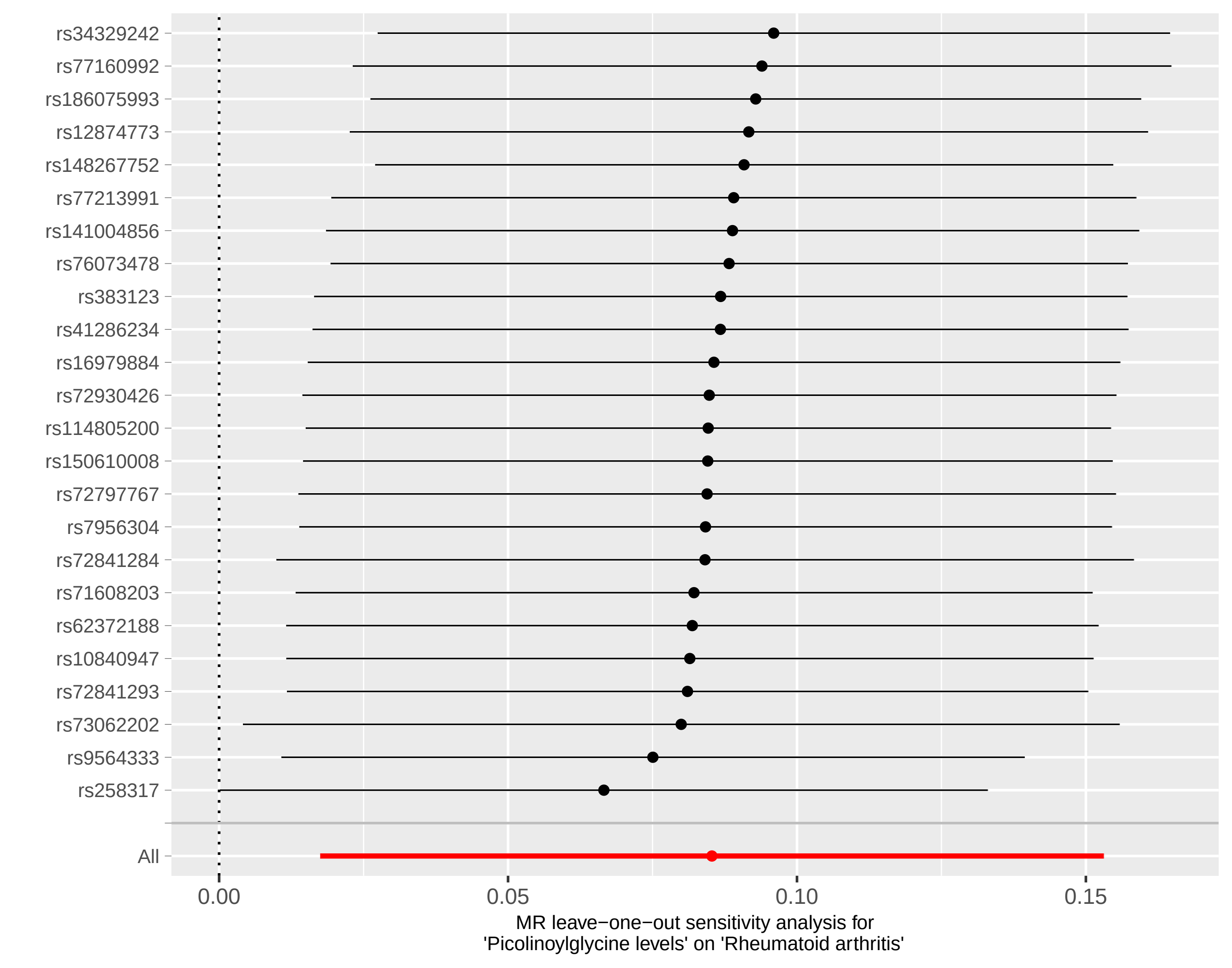
rs34329242
rs77160992
rs186075993
rs12874773
rs148267752
rs77213991
rs141004856
rs76073478
rs383123
rs41286234
rs16979884
rs72930426
rs114805200
rs150610008
rs72797767
rs7956304
rs72841284
rs71608203
rs62372188
rs10840947
rs72841293
rs73062202
rs9564333
rs258317
All
0.00
0.05
0.10
0.15
MR leave−one−out sensitivity analysis for
'Picolinoylglycine levels' on 'Rheumatoid arthritis'

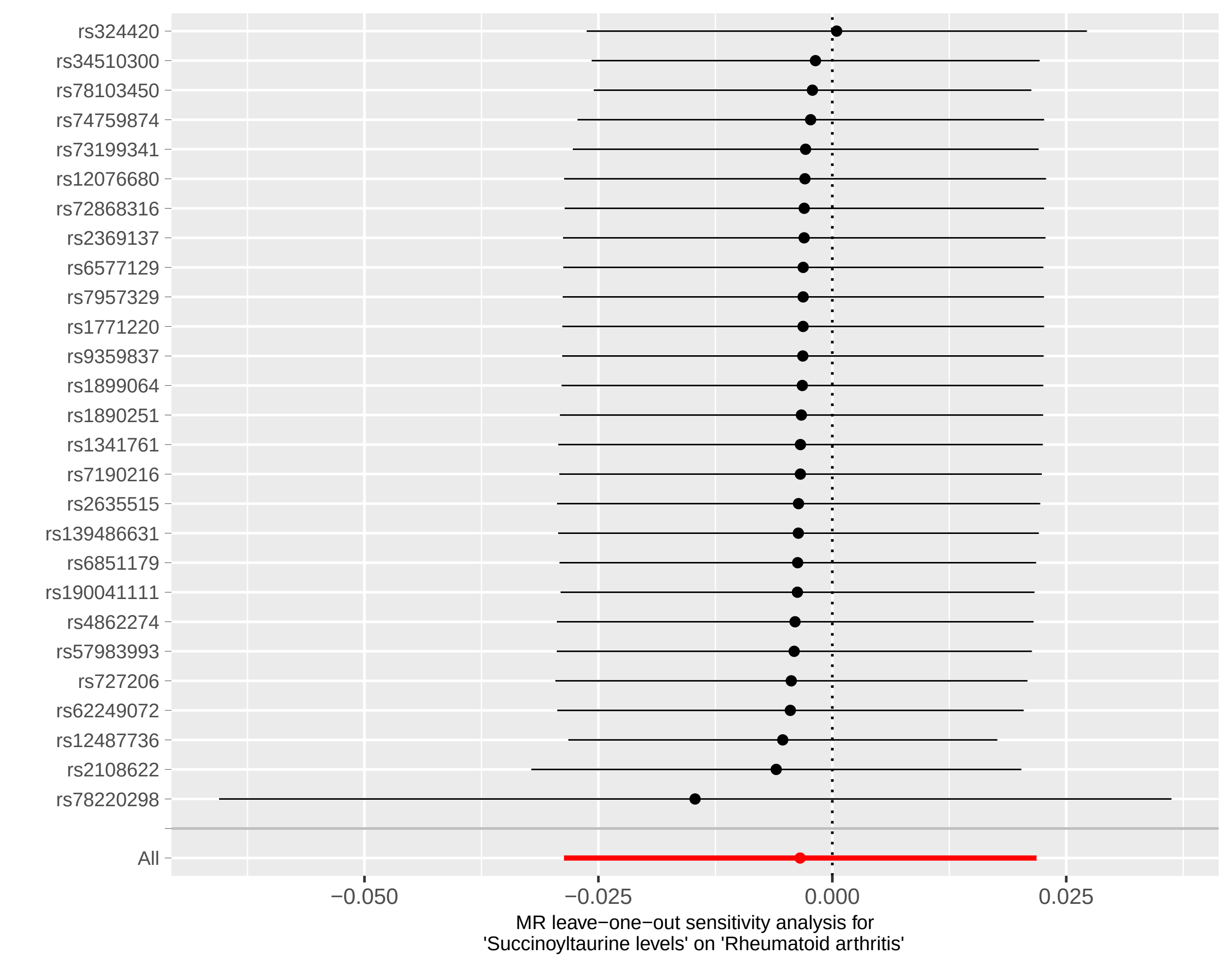

rs324420
rs34510300
rs78103450
rs74759874
rs73199341
rs12076680
rs72868316
rs2369137
rs6577129
rs7957329
rs1771220
rs9359837
rs1899064
rs1890251
rs1341761
rs7190216
rs2635515
rs139486631
rs6851179
rs190041111
rs4862274
rs57983993
rs727206
rs62249072
rs12487736
rs2108622
rs78220298
All
−0.050
−0.025
0.000
0.025
MR leave−one−out sensitivity analysis for
'Succinoyltaurine levels' on 'Rheumatoid arthritis'

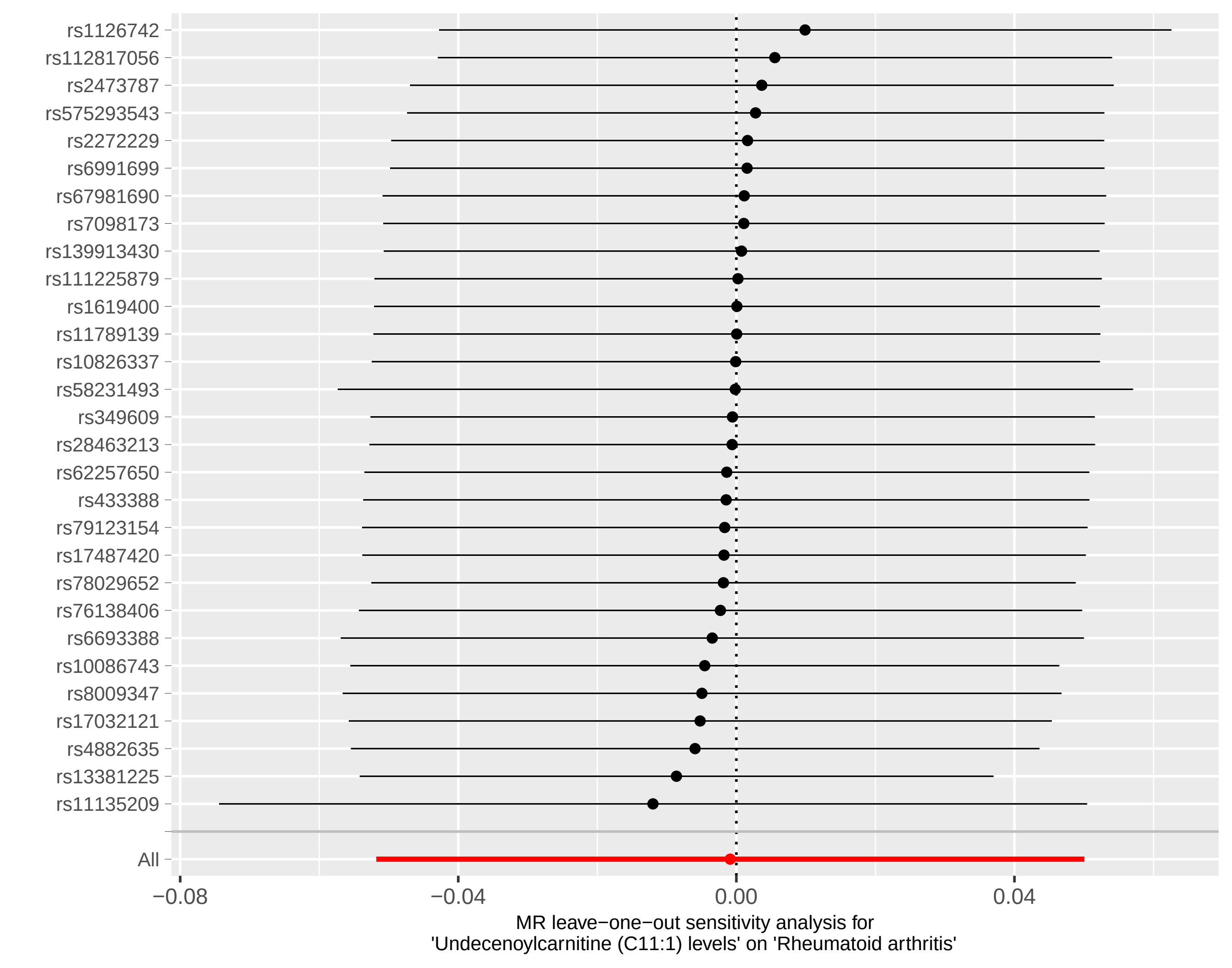
rs1126742
rs112817056
rs2473787
rs575293543
rs2272229
rs6991699
rs67981690
rs7098173
rs139913430
rs111225879
rs1619400
rs11789139
rs10826337
rs58231493
rs349609
rs28463213
rs62257650
rs433388
rs79123154
rs17487420
rs78029652
rs76138406
rs6693388
rs10086743
rs8009347
rs17032121
rs4882635
rs13381225
rs11135209
All
−0.08
−0.04
0.00
0.04
MR leave−one−out sensitivity analysis for
'Undecenoylcarnitine (C11:1) levels' on 'Rheumatoid arthritis'

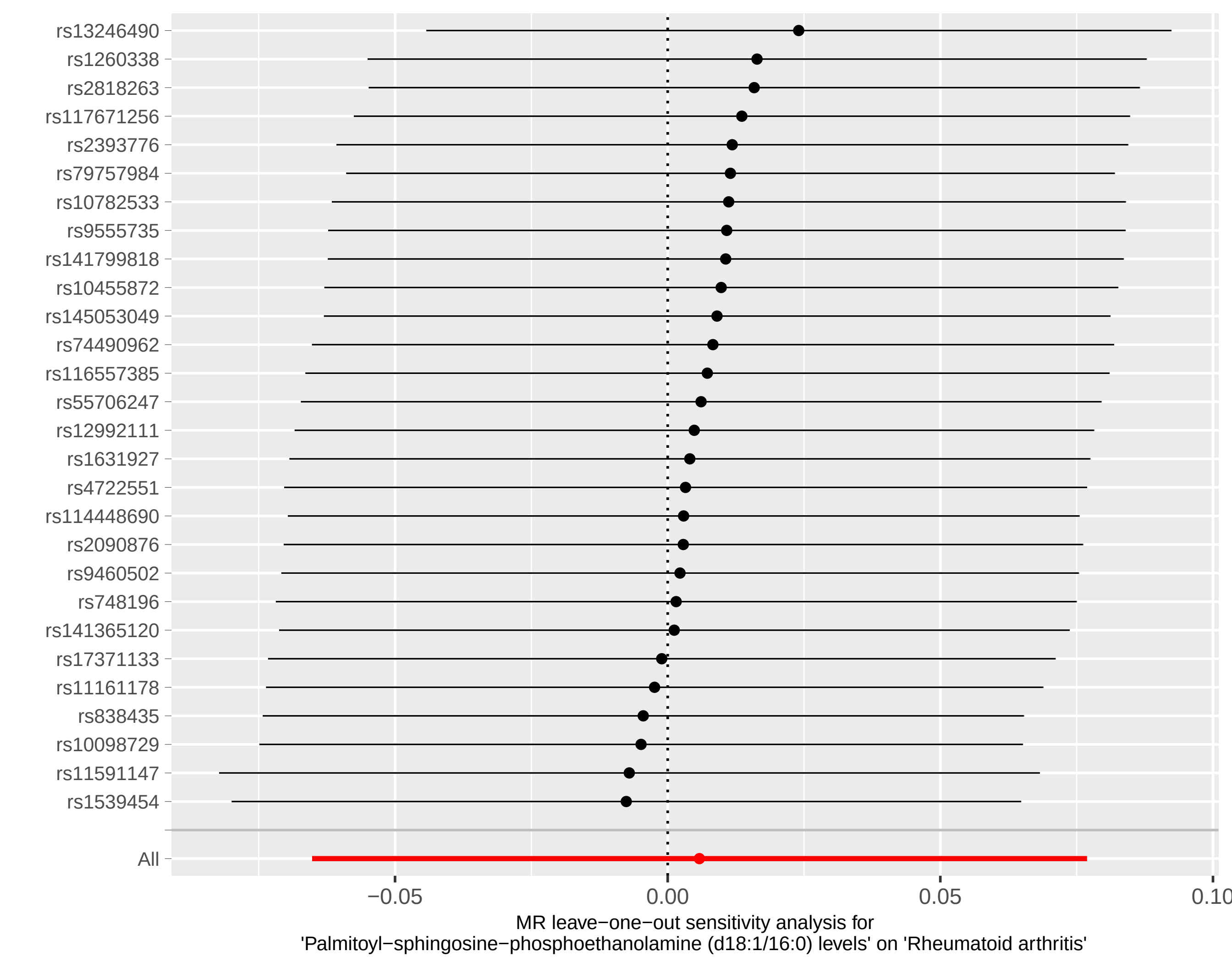
rs13246490
rs1260338
rs2818263
rs117671256
rs2393776
rs79757984
rs10782533
rs9555735
rs141799818
rs10455872
rs145053049
rs74490962
rs116557385
rs55706247
rs12992111
rs1631927
rs4722551
rs114448690
rs2090876
rs9460502
rs748196
rs141365120
rs17371133
rs11161178
rs838435
rs10098729
rs11591147
rs1539454
All
−0.05
0.00
0.05
0.10
MR leave−one−out sensitivity analysis for
'Palmitoyl−sphingosine−phosphoethanolamine (d18:1/16:0) levels' on 'Rheumatoid arthritis'

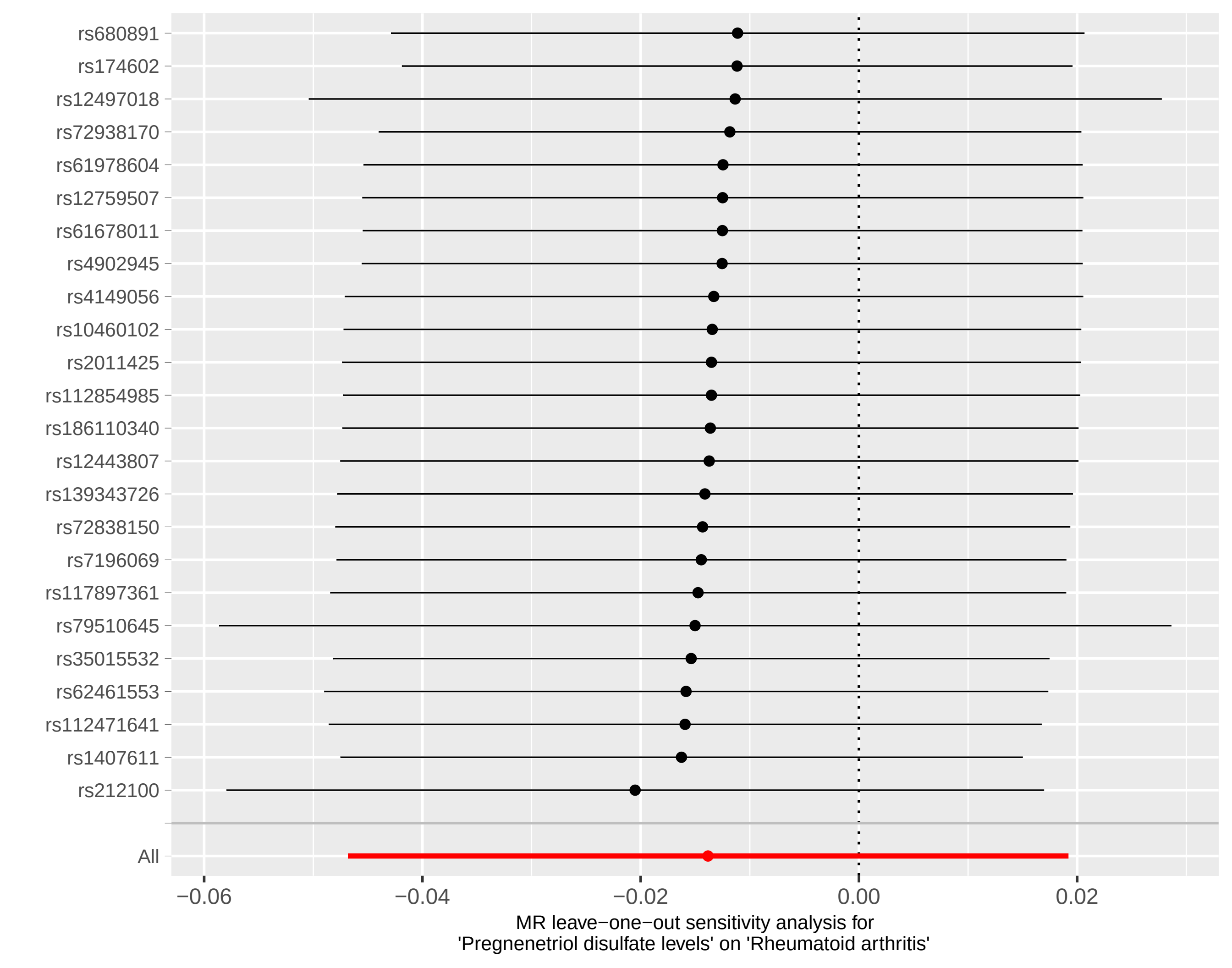
rs680891
rs174602
rs12497018
rs72938170
rs61978604
rs12759507
rs61678011
rs4902945
rs4149056
rs10460102
rs2011425
rs112854985
rs186110340
rs12443807
rs139343726
rs72838150
rs7196069
rs117897361
rs79510645
rs35015532
rs62461553
rs112471641
rs1407611
rs212100
All
−0.06
−0.04
−0.02
0.00
0.02
MR leave−one−out sensitivity analysis for
'Pregnenetriol disulfate levels' on 'Rheumatoid arthritis'

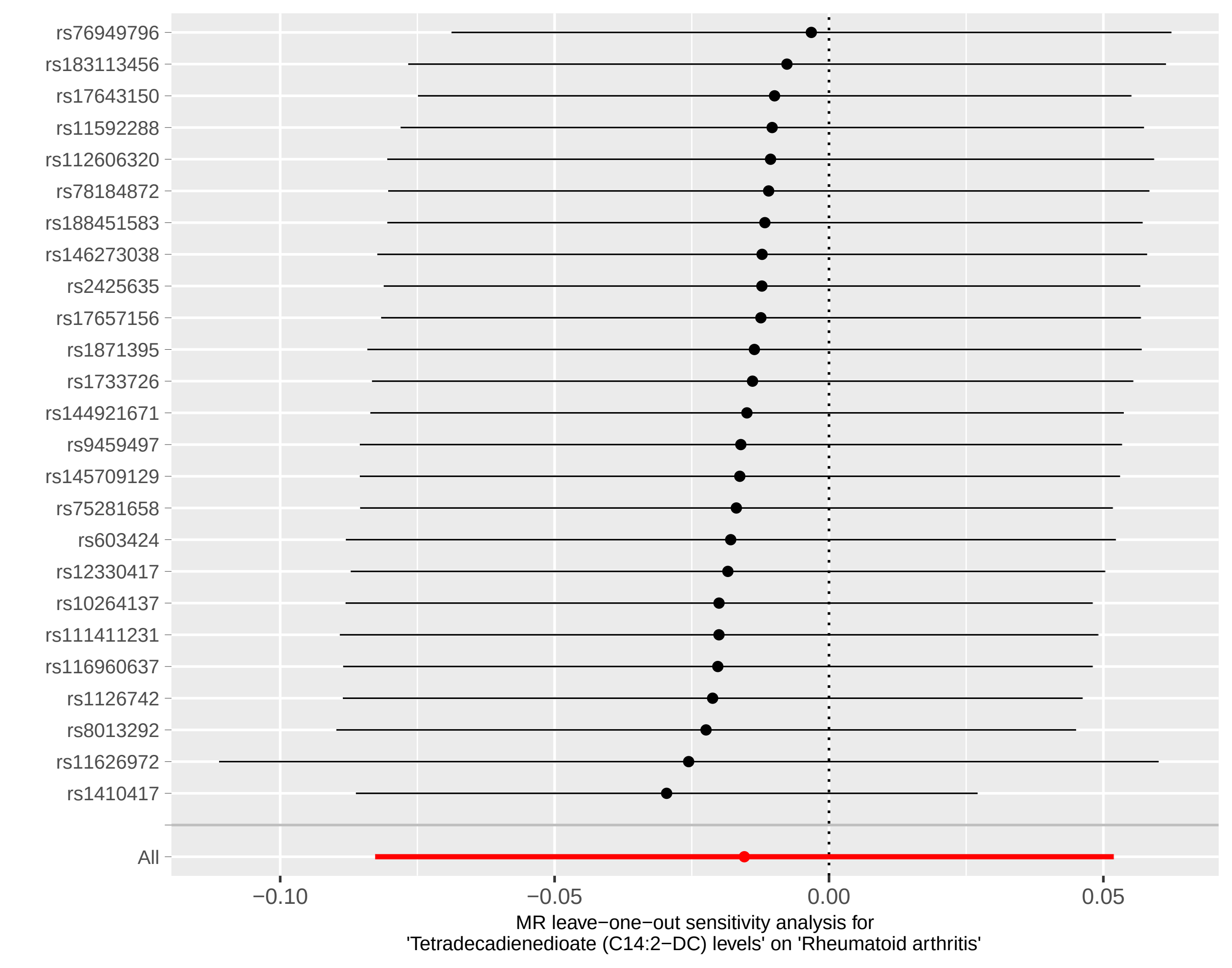
rs76949796
rs183113456
rs17643150
rs11592288
rs112606320
rs78184872
rs188451583
rs146273038
rs2425635
rs17657156
rs1871395
rs1733726
rs144921671
rs9459497
rs145709129
rs75281658
rs603424
rs12330417
rs10264137
rs111411231
rs116960637
rs1126742
rs8013292
rs11626972
rs1410417
All
−0.10
−0.05
0.00
0.05
MR leave−one−out sensitivity analysis for
'Tetradecadienedioate (C14:2−DC) levels' on 'Rheumatoid arthritis'

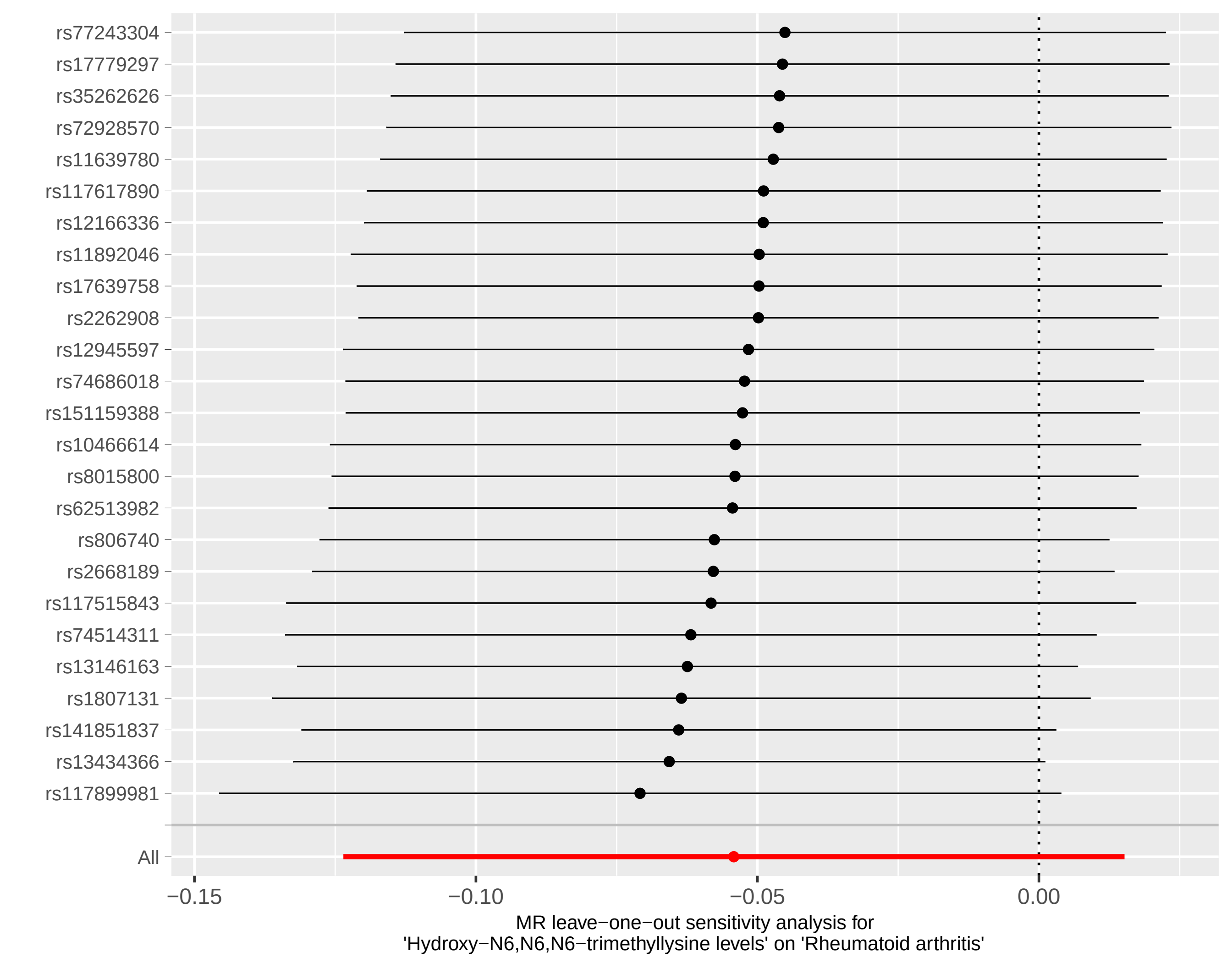
rs77243304
rs17779297
rs35262626
rs72928570
rs11639780
rs117617890
rs12166336
rs11892046
rs17639758
rs2262908
rs12945597
rs74686018
rs151159388
rs10466614
rs8015800
rs62513982
rs806740
rs2668189
rs117515843
rs74514311
rs13146163
rs1807131
rs141851837
rs13434366
rs117899981
All
−0.15
−0.10
−0.05
0.00
MR leave−one−out sensitivity analysis for
'Hydroxy−N6,N6,N6−trimethyllysine levels' on 'Rheumatoid arthritis'

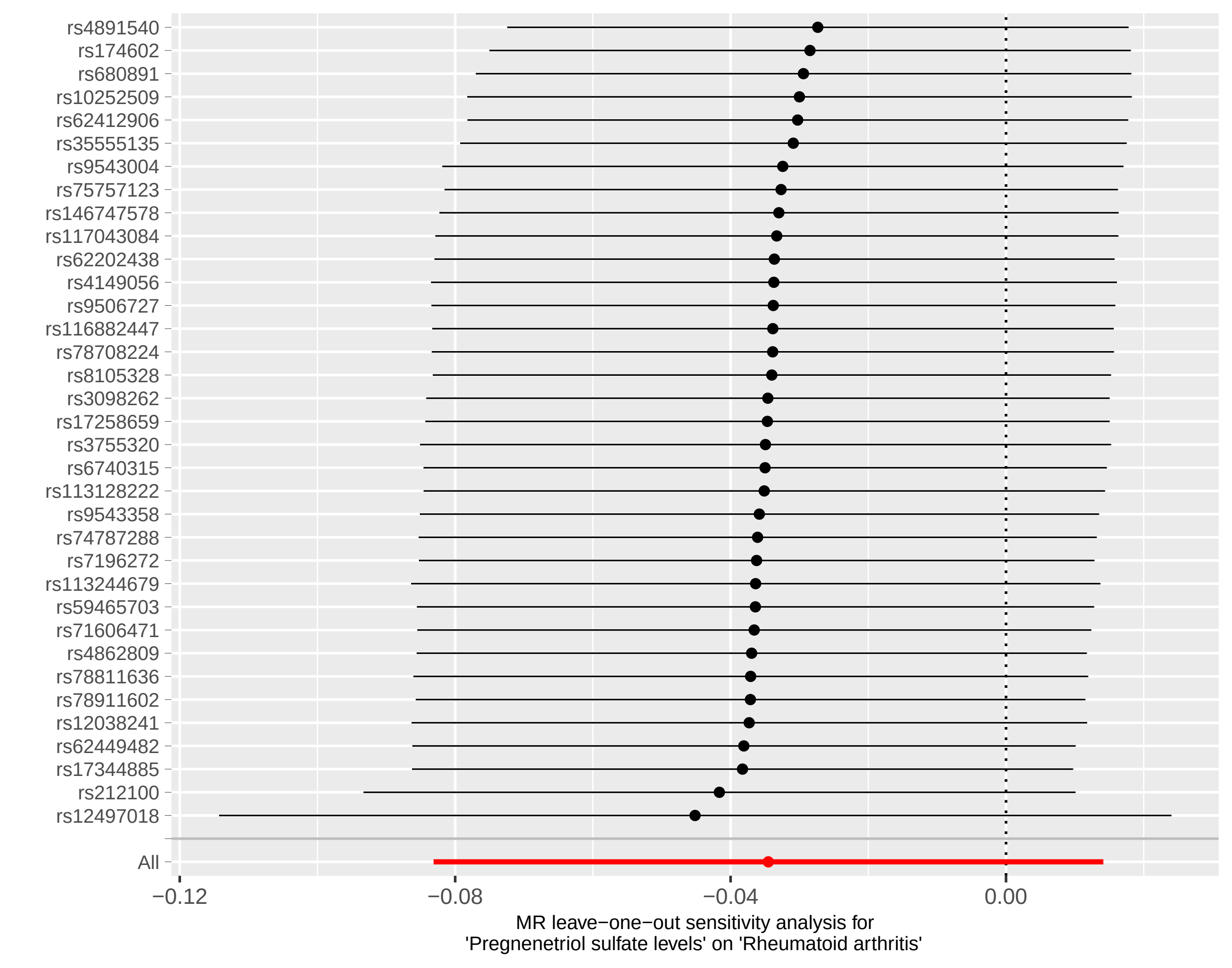
rs4891540
rs174602
rs680891
rs10252509
rs62412906
rs35555135
rs9543004
rs75757123
rs146747578
rs117043084
rs62202438
rs4149056
rs9506727
rs116882447
rs78708224
rs8105328
rs3098262
rs17258659
rs3755320
rs6740315
rs113128222
rs9543358
rs74787288
rs7196272
rs113244679
rs59465703
rs71606471
rs4862809
rs78811636
rs78911602
rs12038241
rs62449482
rs17344885
rs212100
rs12497018
All
−0.12
−0.08
−0.04
0.00
MR leave−one−out sensitivity analysis for
'Pregnenetriol sulfate levels' on 'Rheumatoid arthritis'

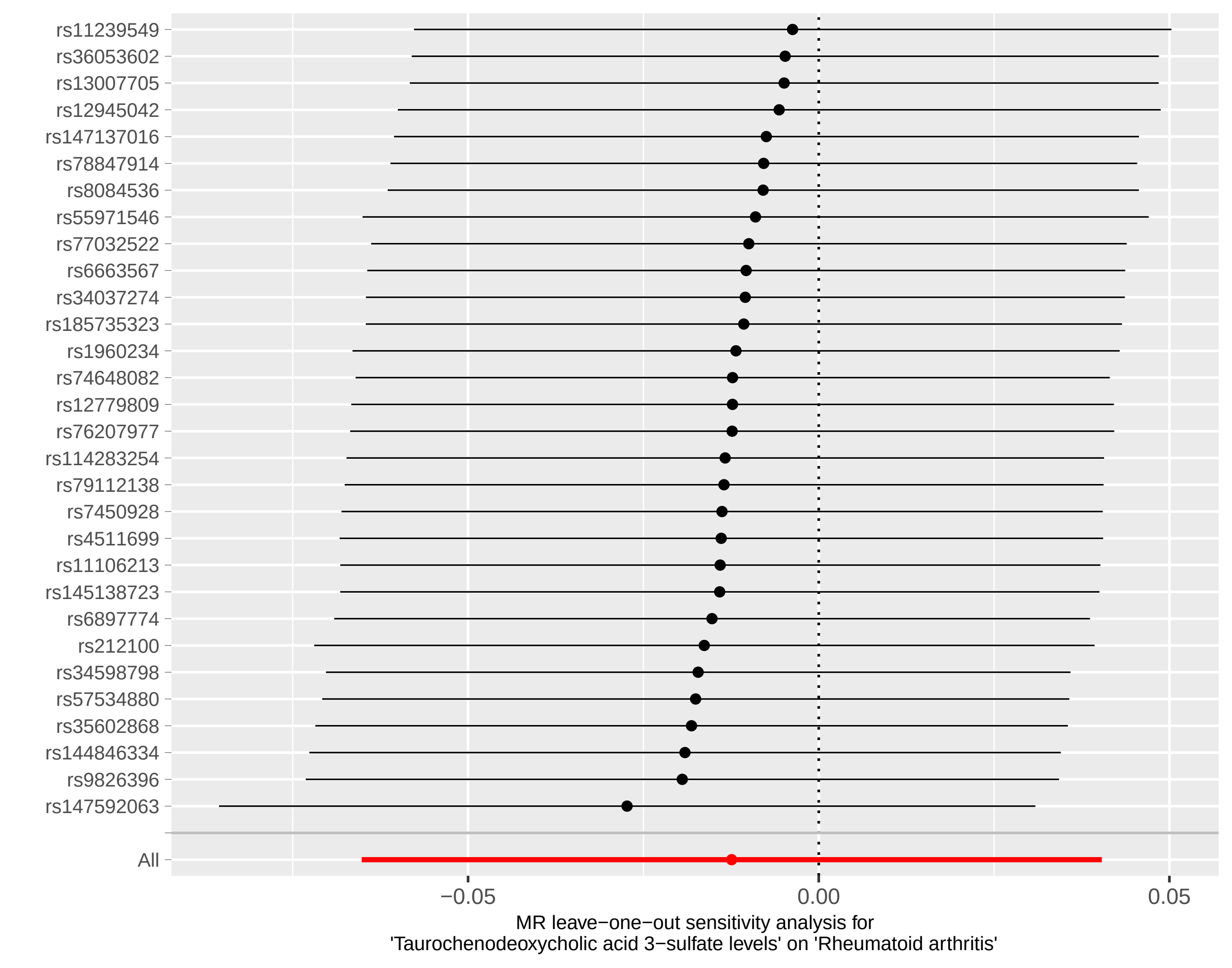
rs11239549
rs36053602
rs13007705
rs12945042
rs147137016
rs78847914
rs8084536
rs55971546
rs77032522
rs6663567
rs34037274
rs185735323
rs1960234
rs74648082
rs12779809
rs76207977
rs114283254
rs79112138
rs7450928
rs4511699
rs11106213
rs145138723
rs6897774
rs212100
rs34598798
rs57534880
rs35602868
rs144846334
rs9826396
rs147592063
All
−0.05
0.00
0.05
MR leave−one−out sensitivity analysis for
'Taurochenodeoxycholic acid 3−sulfate levels' on 'Rheumatoid arthritis'

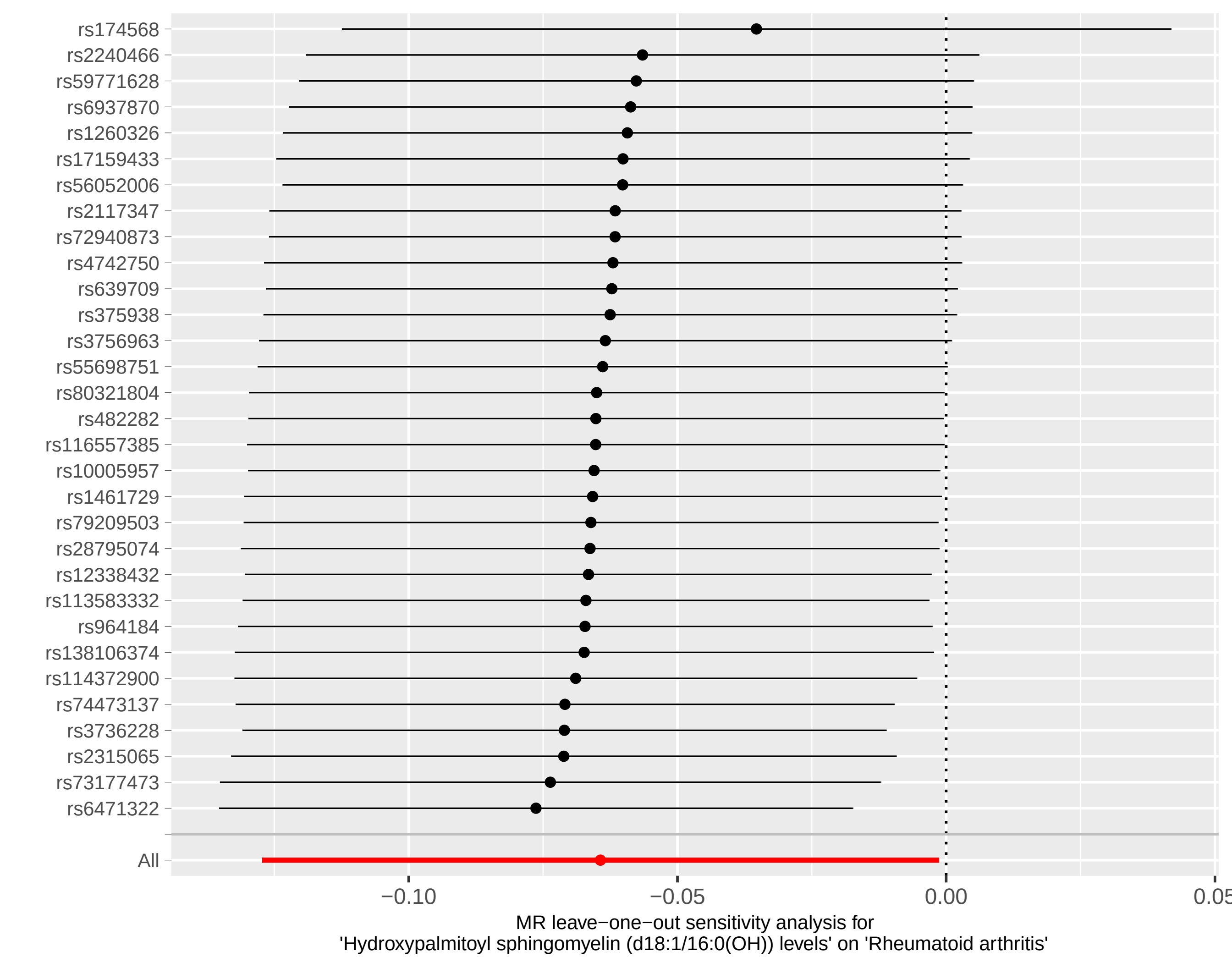
rs174568
rs2240466
rs59771628
rs6937870
rs1260326
rs17159433
rs56052006
rs2117347
rs72940873
rs4742750
rs639709
rs375938
rs3756963
rs55698751
rs80321804
rs482282
rs116557385
rs10005957
rs1461729
rs79209503
rs28795074
rs12338432
rs113583332
rs964184
rs138106374
rs114372900
rs74473137
rs3736228
rs2315065
rs73177473
rs6471322
All
−0.10
−0.05
0.00
0.05
MR leave−one−out sensitivity analysis for
'Hydroxypalmitoyl sphingomyelin (d18:1/16:0(OH)) levels' on 'Rheumatoid arthritis'

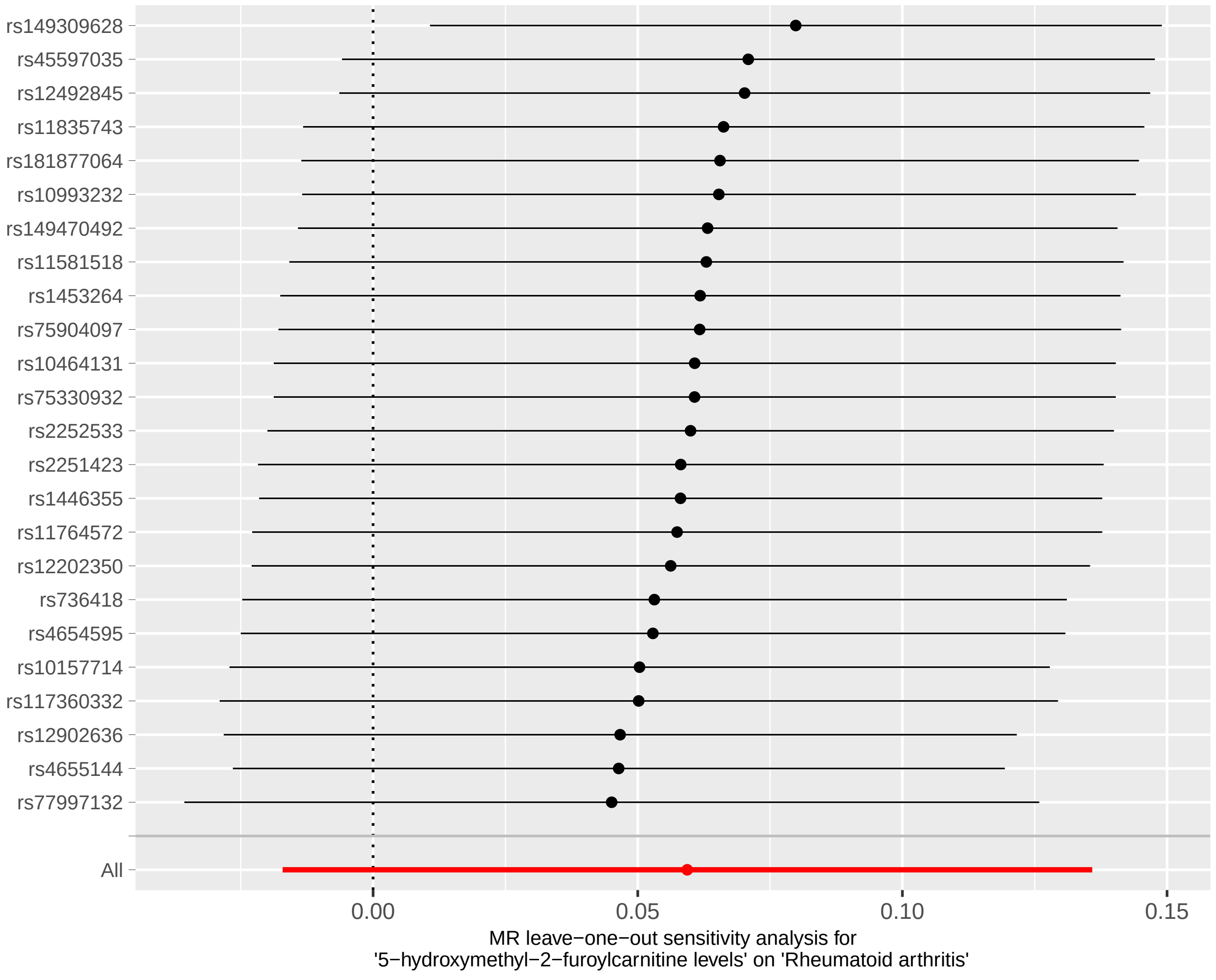
rs149309628
rs45597035
rs12492845
rs11835743
rs181877064
rs10993232
rs149470492
rs11581518
rs1453264
rs75904097
rs10464131
rs75330932
rs2252533
rs2251423
rs1446355
rs11764572
rs12202350
rs736418
rs4654595
rs10157714
rs117360332
rs12902636
rs4655144
rs77997132
All
0.00
0.05
0.10
0.15
MR leave−one−out sensitivity analysis for
'5−hydroxymethyl−2−furoylcarnitine levels' on 'Rheumatoid arthritis'

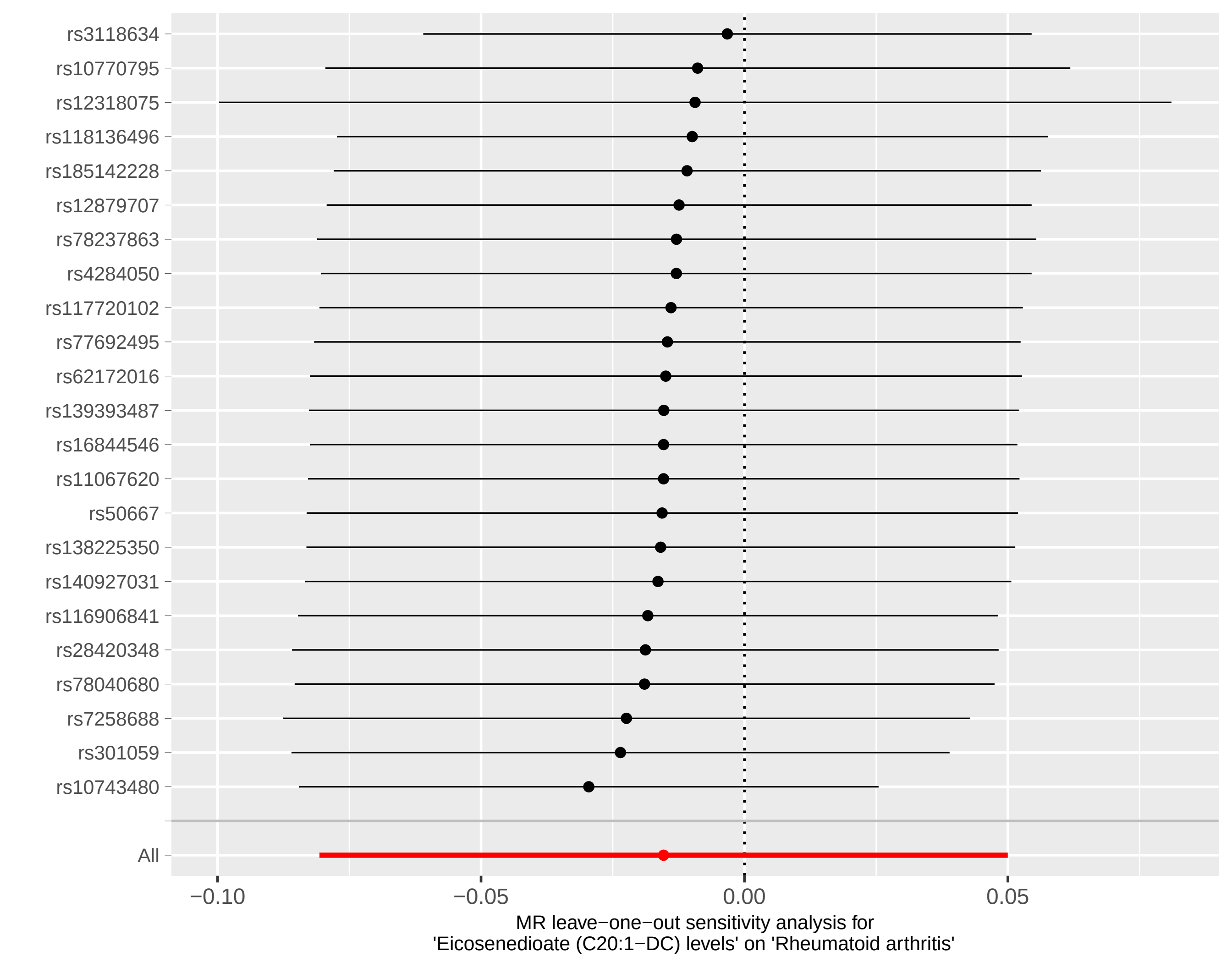

rs3118634
rs10770795
rs12318075
rs118136496
rs185142228
rs12879707
rs78237863
rs4284050
rs117720102
rs77692495
rs62172016
rs139393487
rs16844546
rs11067620
rs50667
rs138225350
rs140927031
rs116906841
rs28420348
rs78040680
rs7258688
rs301059
rs10743480
All
−0.10
−0.05
0.00
0.05
MR leave−one−out sensitivity analysis for
'Eicosenedioate (C20:1−DC) levels' on 'Rheumatoid arthritis'

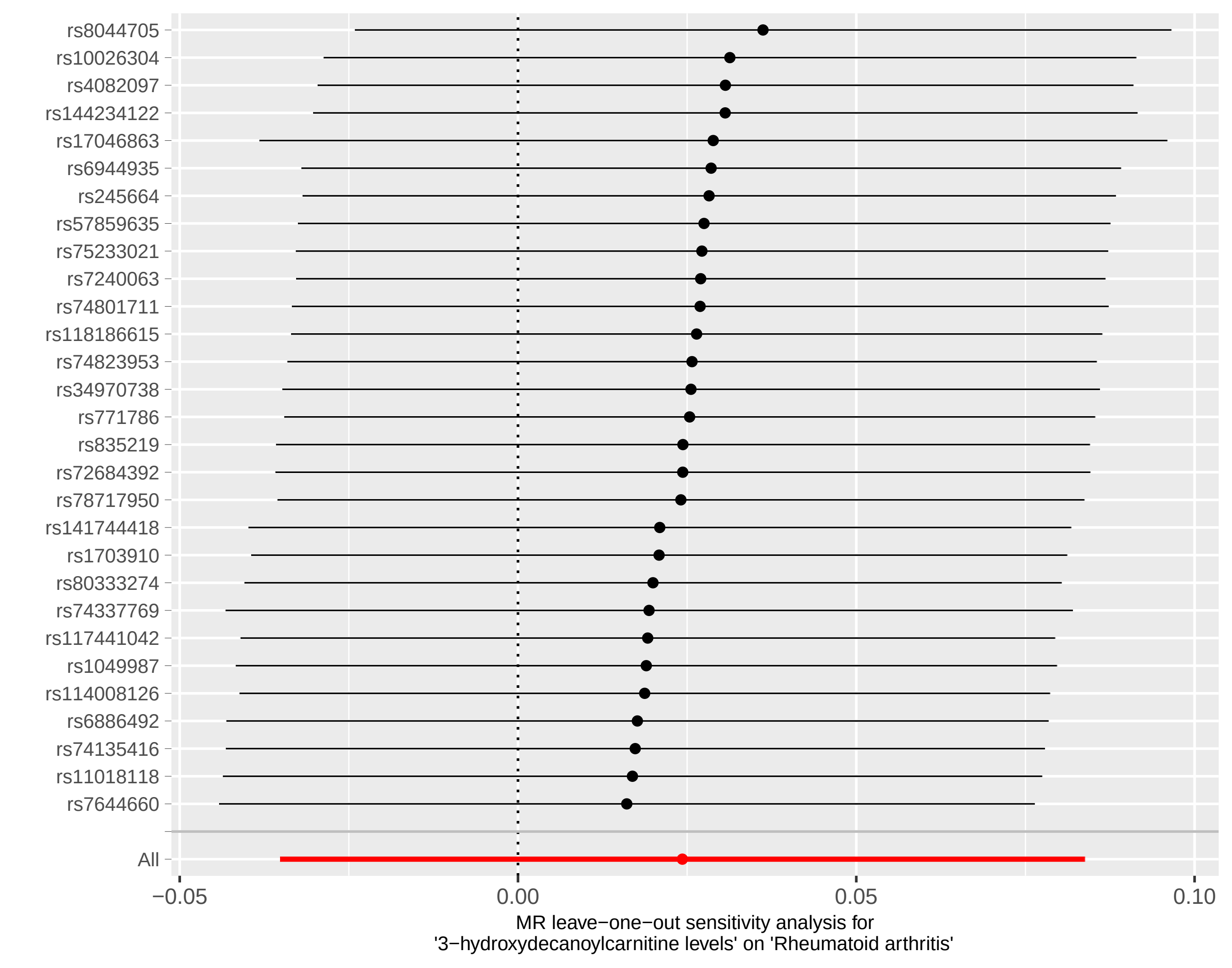
rs8044705
rs10026304
rs4082097
rs144234122
rs17046863
rs6944935
rs245664
rs57859635
rs75233021
rs7240063
rs74801711
rs118186615
rs74823953
rs34970738
rs771786
rs835219
rs72684392
rs78717950
rs141744418
rs1703910
rs80333274
rs74337769
rs117441042
rs1049987
rs114008126
rs6886492
rs74135416
rs11018118
rs7644660
All
−0.05
0.00
0.05
0.10
MR leave−one−out sensitivity analysis for
'3−hydroxydecanoylcarnitine levels' on 'Rheumatoid arthritis'

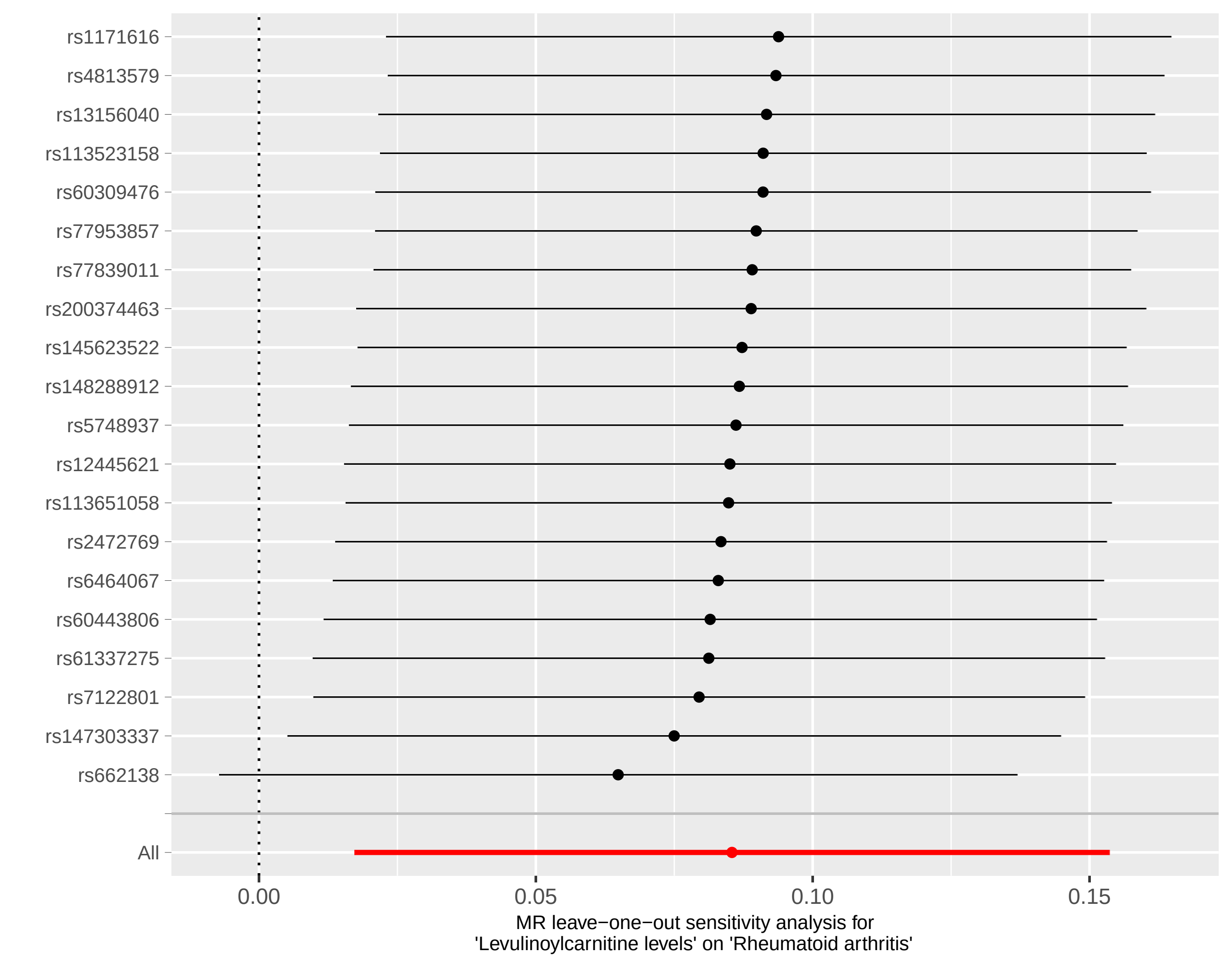
rs1171616
rs4813579
rs13156040
rs113523158
rs60309476
rs77953857
rs77839011
rs200374463
rs145623522
rs148288912
rs5748937
rs12445621
rs113651058
rs2472769
rs6464067
rs60443806
rs61337275
rs7122801
rs147303337
rs662138
All
0.00
0.05
0.10
0.15
MR leave−one−out sensitivity analysis for
'Levulinoylcarnitine levels' on 'Rheumatoid arthritis'

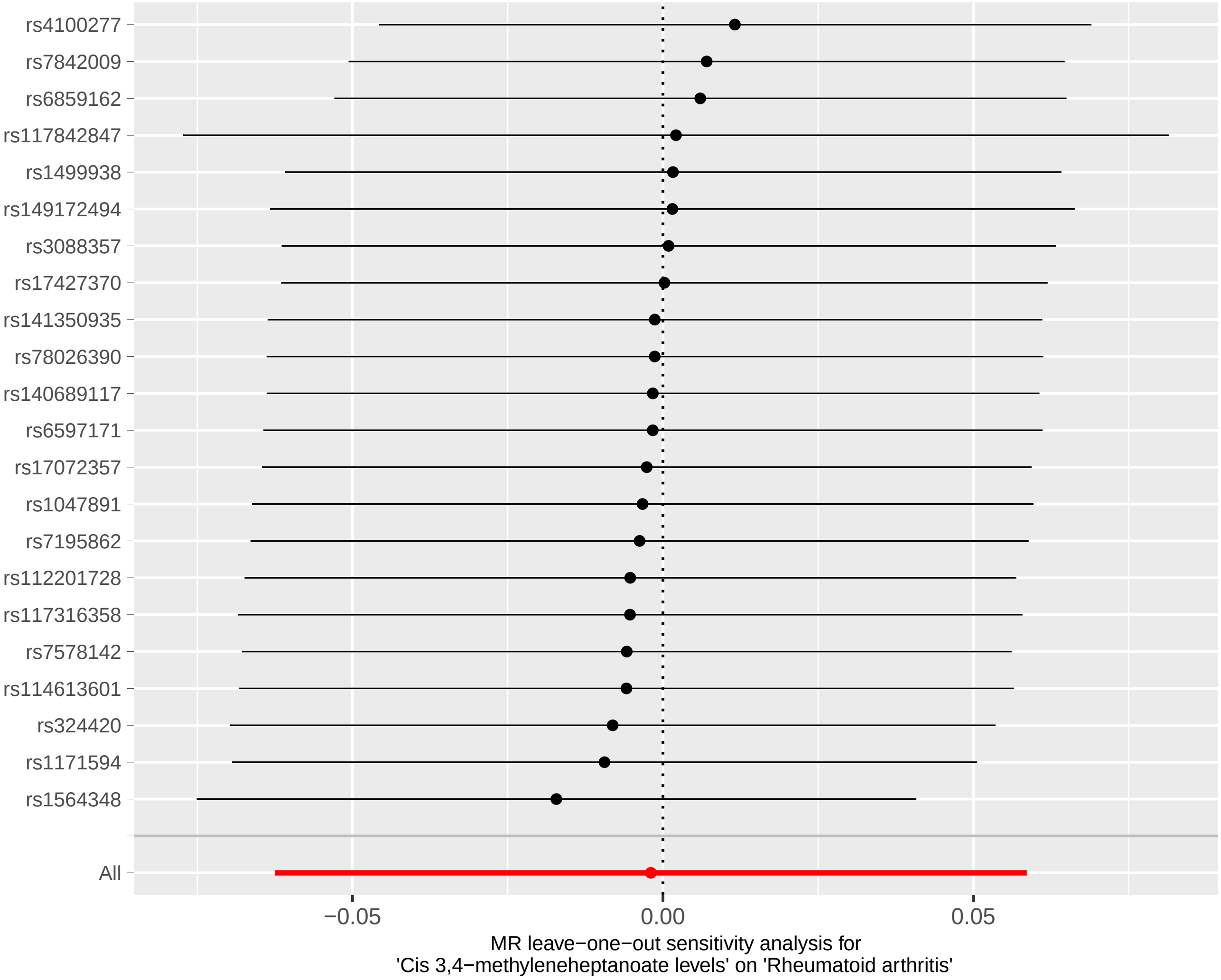
rs4100277
rs7842009
rs6859162
rs117842847
rs1499938
rs149172494
rs3088357
rs17427370
rs141350935
rs78026390
rs140689117
rs6597171
rs17072357
rs1047891
rs7195862
rs112201728
rs117316358
rs7578142
rs114613601
rs324420
rs1171594
rs1564348
All
−0.05
0.00
0.05
MR leave−one−out sensitivity analysis for
'Cis 3,4−methyleneheptanoate levels' on 'Rheumatoid arthritis'

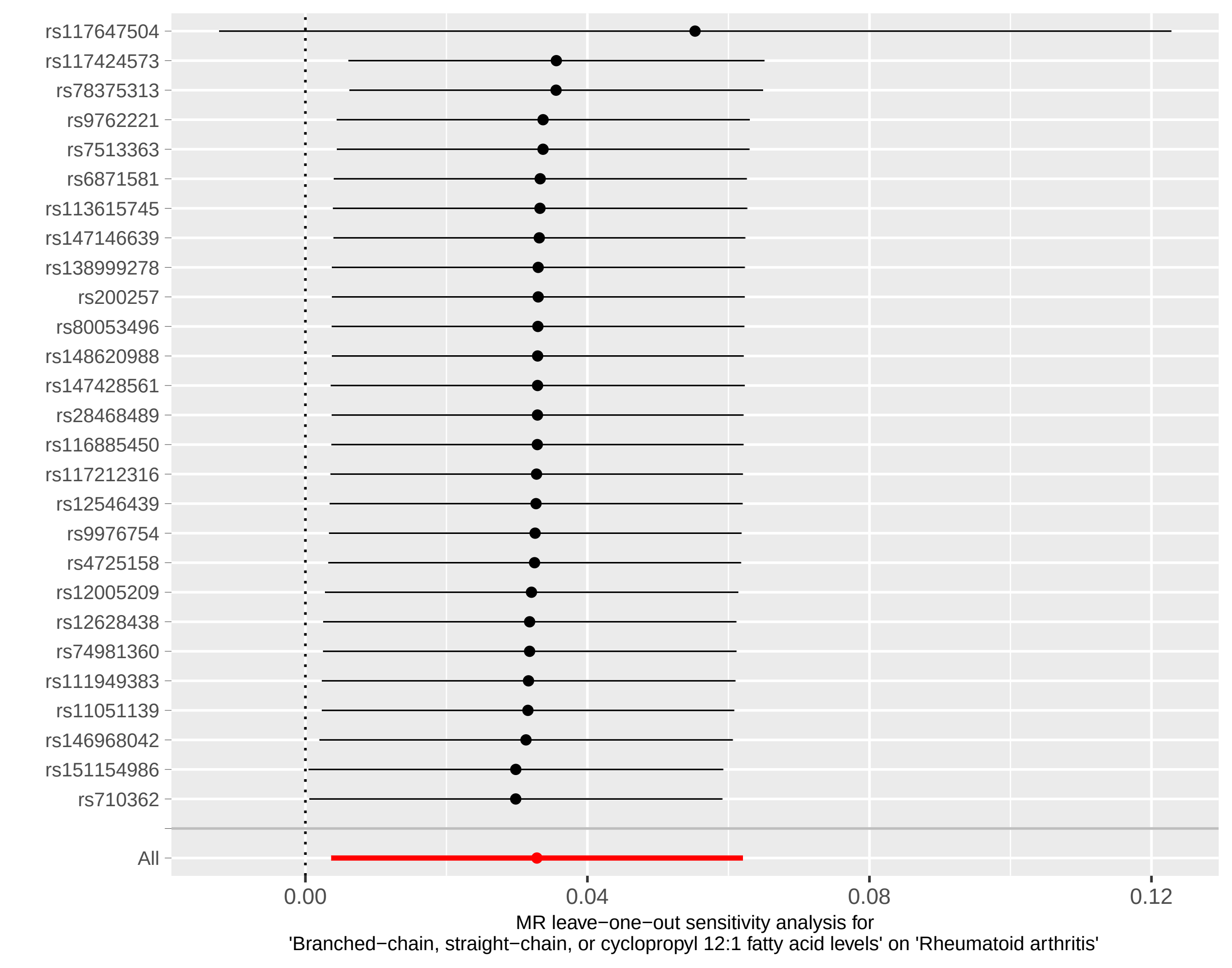
rs117647504
rs117424573
rs78375313
rs9762221
rs7513363
rs6871581
rs113615745
rs147146639
rs138999278
rs200257
rs80053496
rs148620988
rs147428561
rs28468489
rs116885450
rs117212316
rs12546439
rs9976754
rs4725158
rs12005209
rs12628438
rs74981360
rs111949383
rs11051139
rs146968042
rs151154986
rs710362
All
0.00
0.04
0.08
0.12
MR leave−one−out sensitivity analysis for
'Branched−chain, straight−chain, or cyclopropyl 12:1 fatty acid levels' on 'Rheumatoid arthritis'

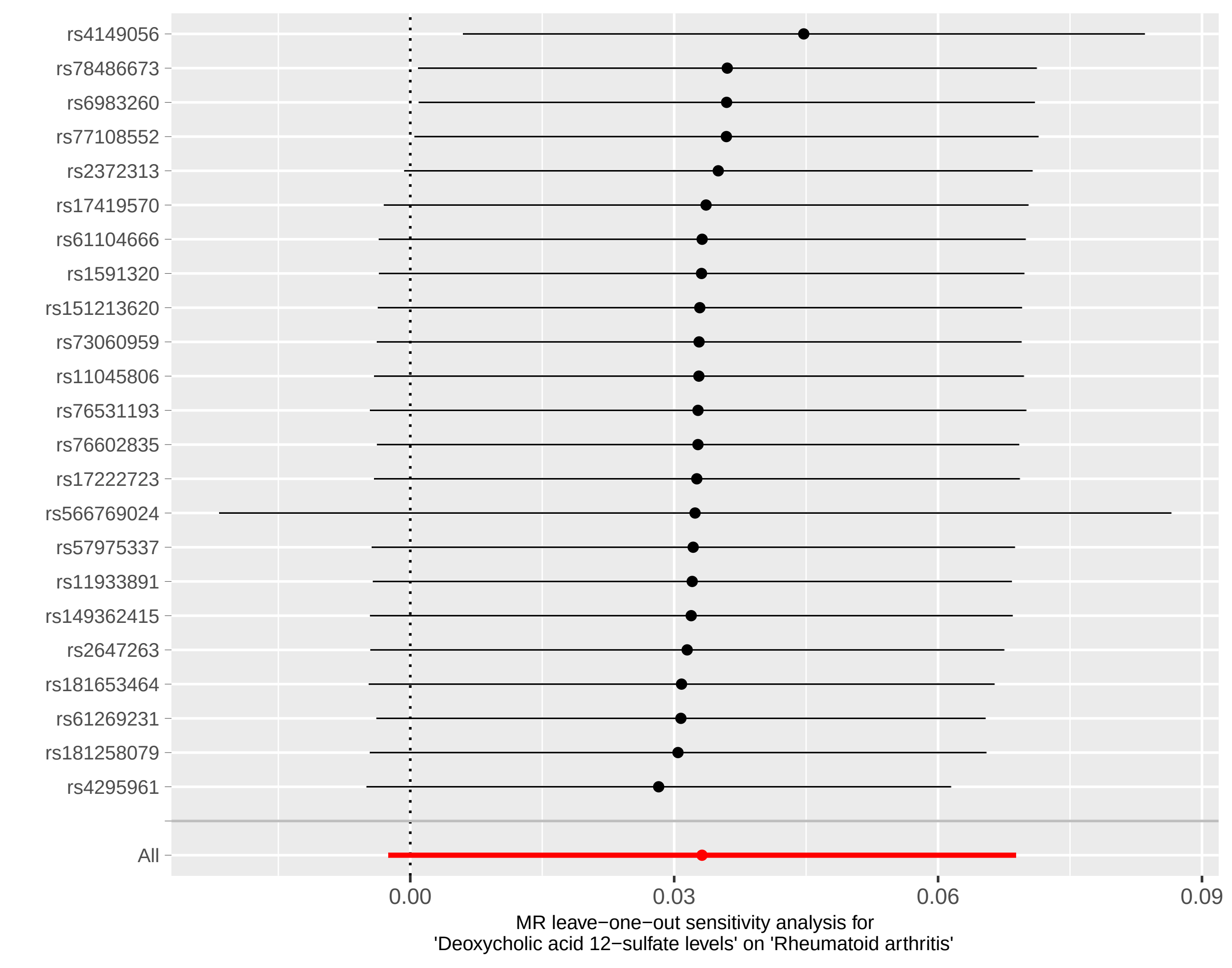

rs4149056
rs78486673
rs6983260
rs77108552
rs2372313
rs17419570
rs61104666
rs1591320
rs151213620
rs73060959
rs11045806
rs76531193
rs76602835
rs17222723
rs566769024
rs57975337
rs11933891
rs149362415
rs2647263
rs181653464
rs61269231
rs181258079
rs4295961
All
0.00
0.03
0.06
0.09
MR leave−one−out sensitivity analysis for
'Deoxycholic acid 12−sulfate levels' on 'Rheumatoid arthritis'

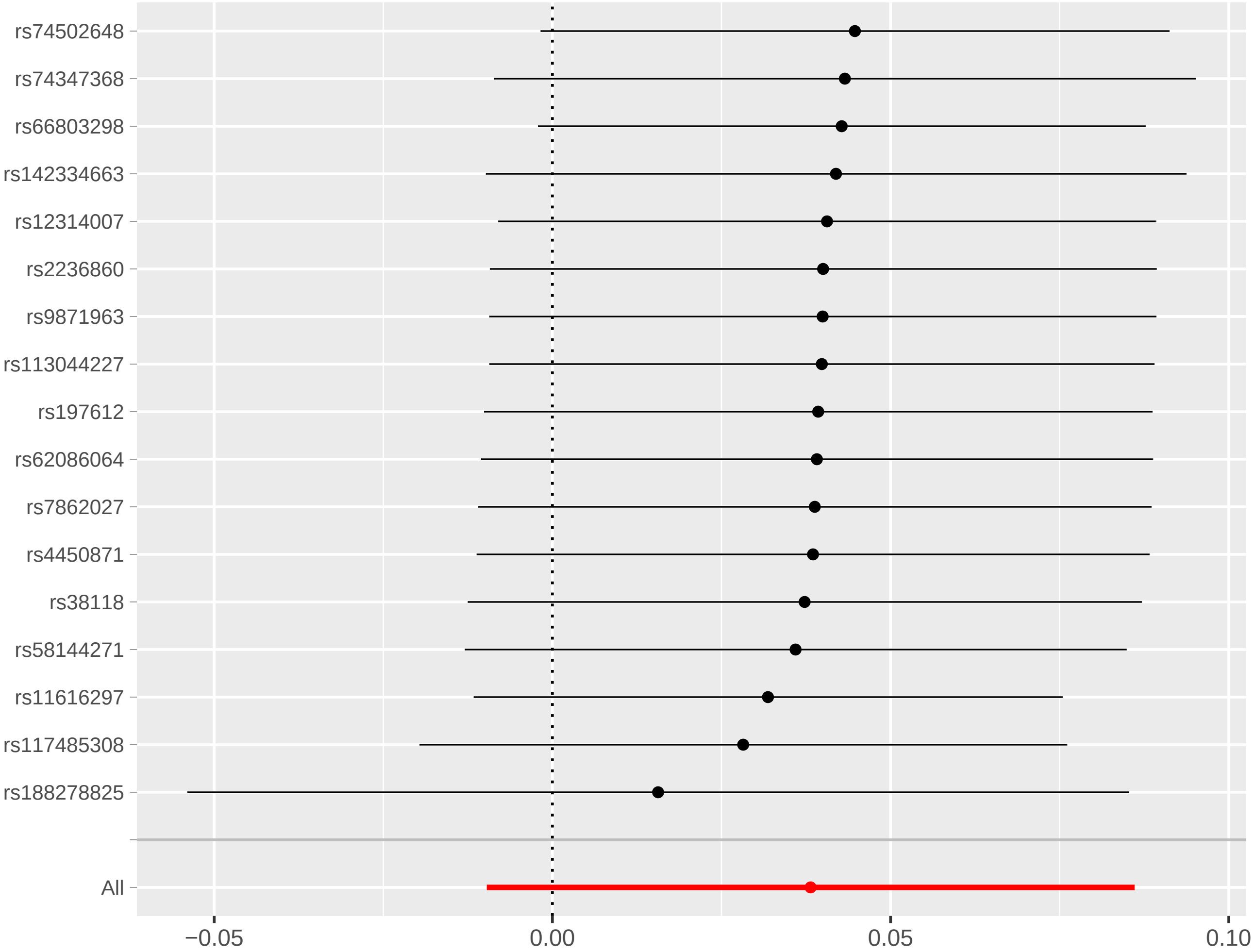

MR leave−one−out sensitivity analysis for
'Branched−chain, straight−chain, or cyclopropyl 10:1 fatty acid (1) levels' on 'Rheumatoid arthritis'

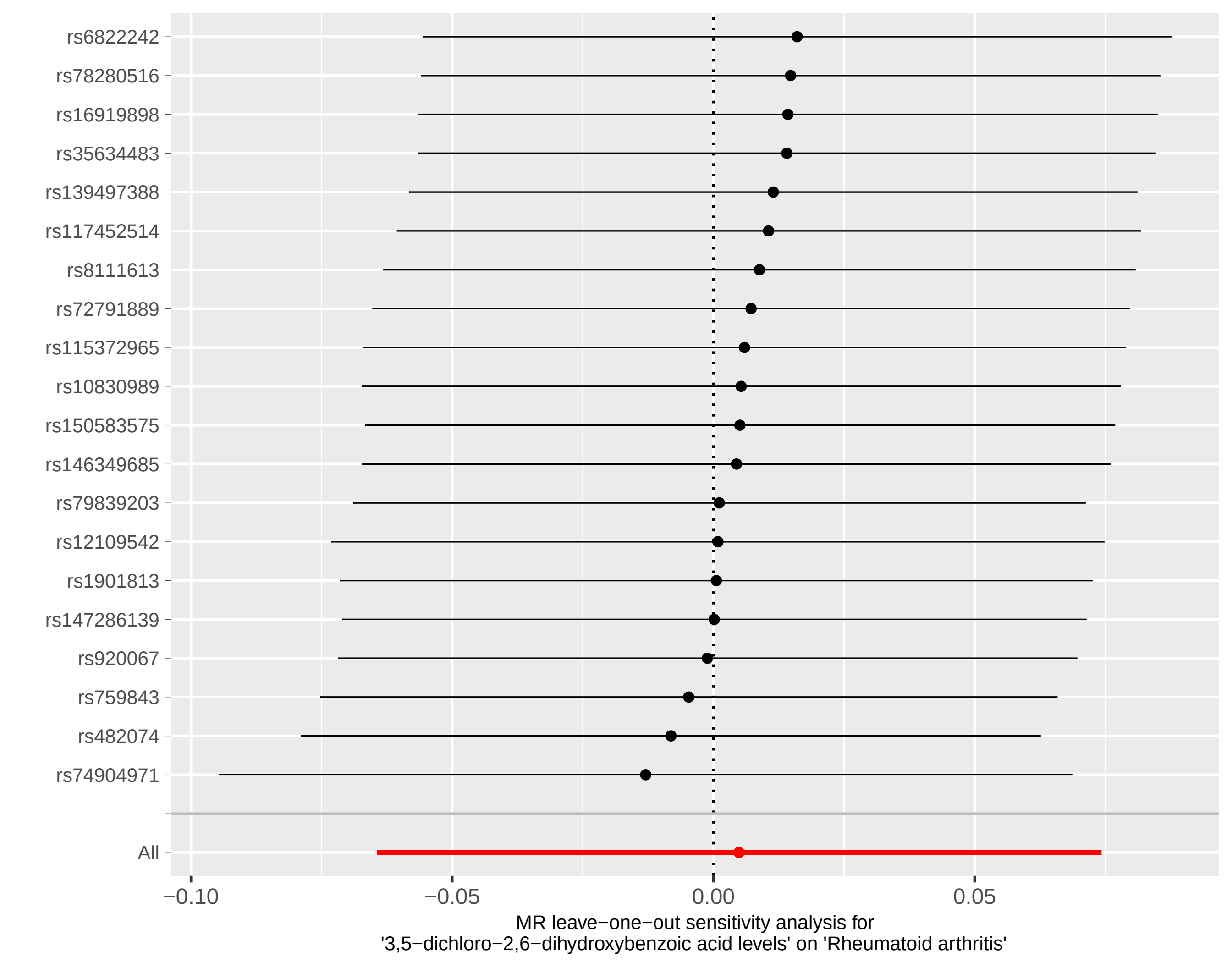
rs6822242
rs78280516
rs16919898
rs35634483
rs139497388
rs117452514
rs8111613
rs72791889
rs115372965
rs10830989
rs150583575
rs146349685
rs79839203
rs12109542
rs1901813
rs147286139
rs920067
rs759843
rs482074
rs74904971
All
−0.10
−0.05
0.00
0.05
MR leave−one−out sensitivity analysis for
'3,5−dichloro−2,6−dihydroxybenzoic acid levels' on 'Rheumatoid arthritis'

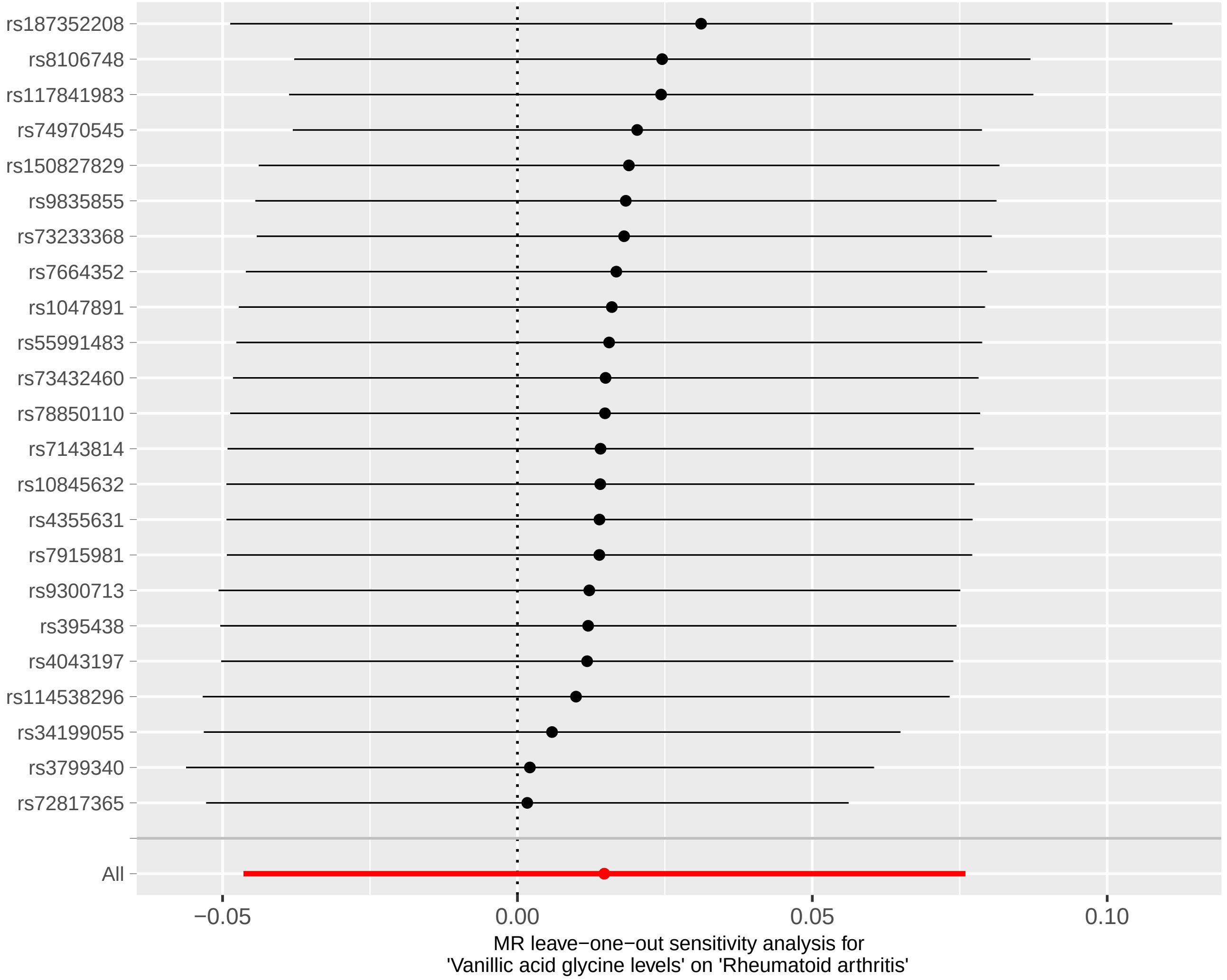
rs187352208
rs8106748
rs117841983
rs74970545
rs150827829
rs9835855
rs73233368
rs7664352
rs1047891
rs55991483
rs73432460
rs78850110
rs7143814
rs10845632
rs4355631
rs7915981
rs9300713
rs395438
rs4043197
rs114538296
rs34199055
rs3799340
rs72817365
All
−0.05
0.00
0.05
0.10
MR leave−one−out sensitivity analysis for
'Vanillic acid glycine levels' on 'Rheumatoid arthritis'

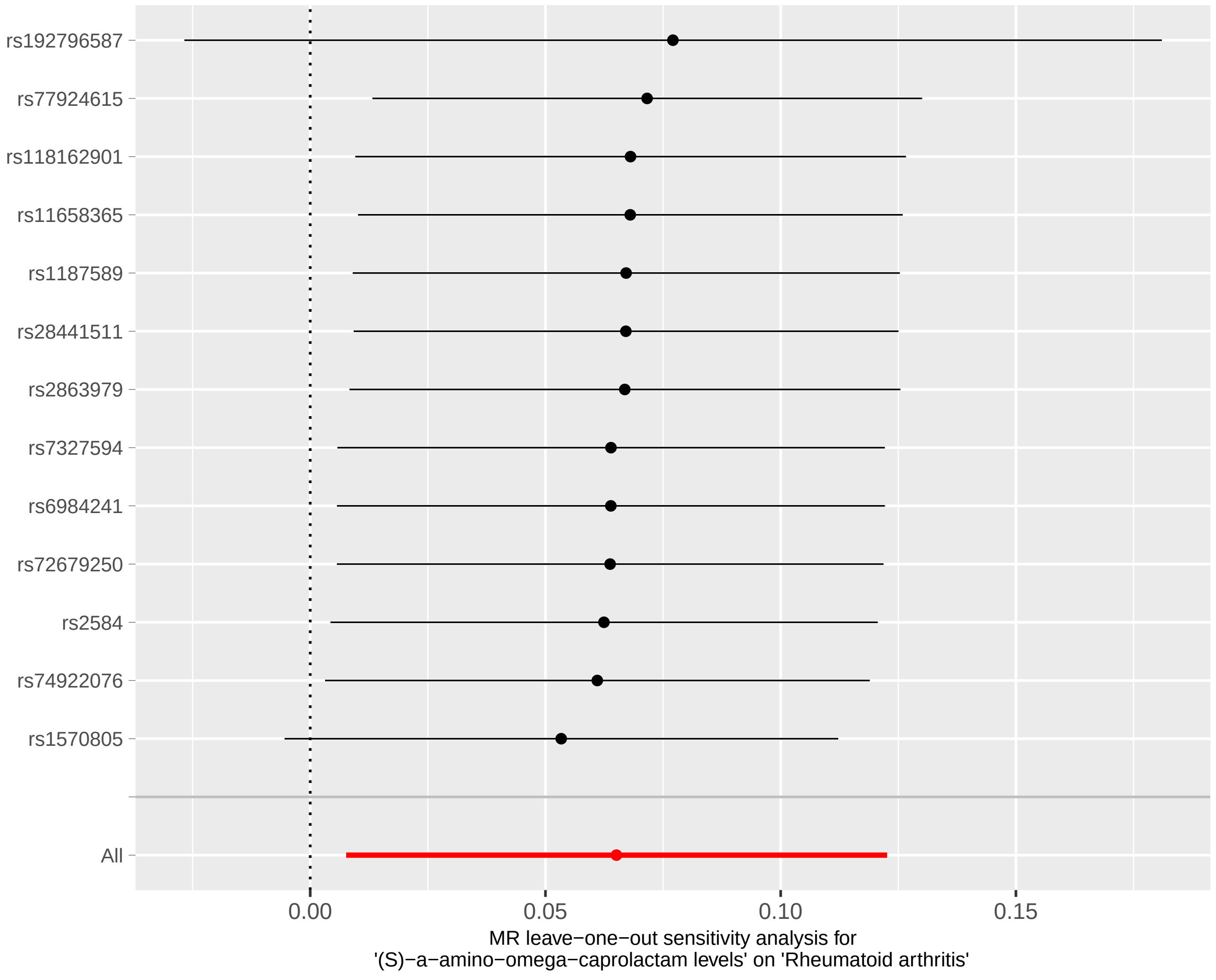
rs192796587
rs77924615
rs118162901
rs11658365
rs1187589
rs28441511
rs2863979
rs7327594
rs6984241
rs72679250
rs2584
rs74922076
rs1570805
All
0.00
0.05
0.10
0.15
MR leave−one−out sensitivity analysis for
'(S)−a−amino−omega−caprolactam levels' on 'Rheumatoid arthritis'

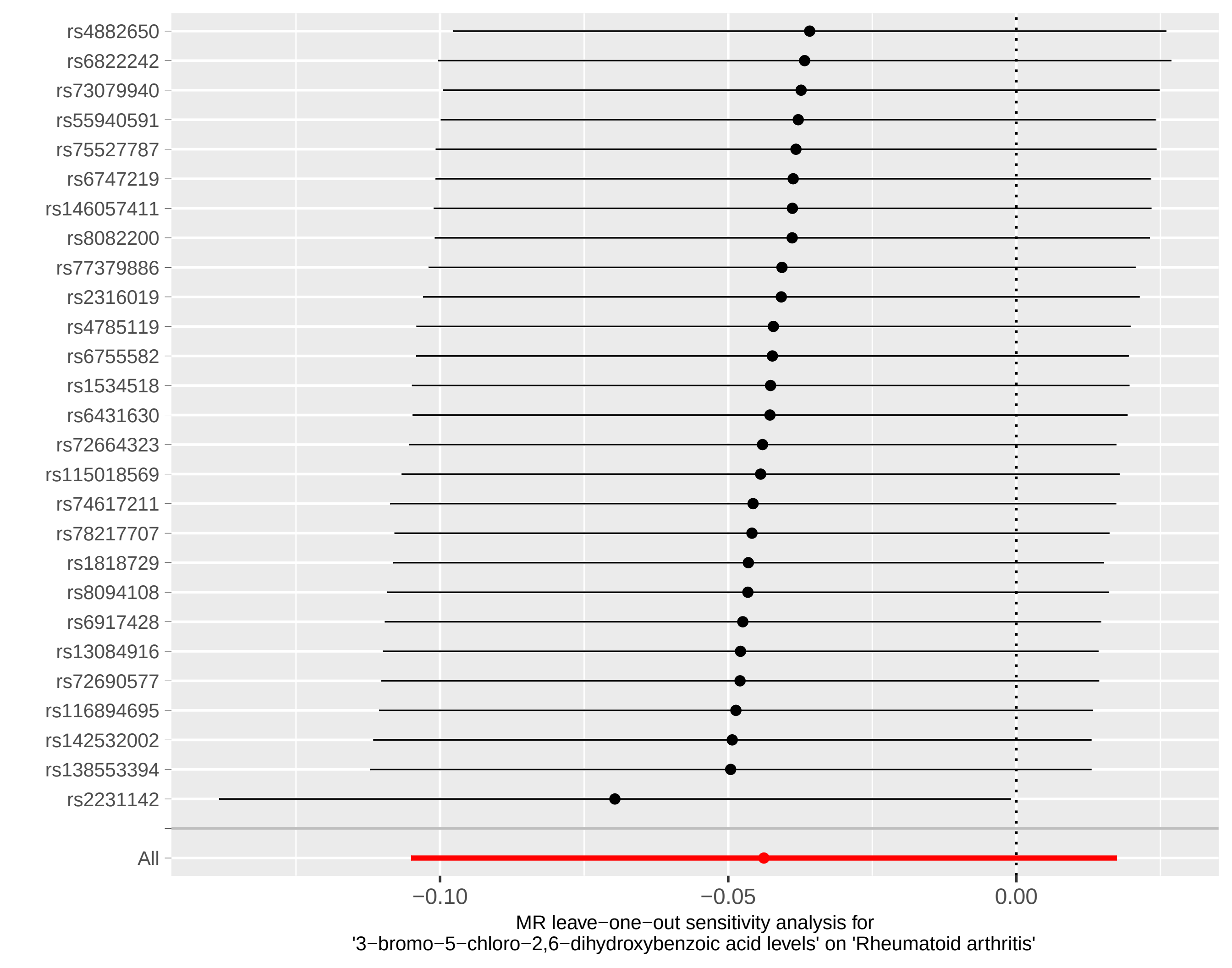
rs4882650
rs6822242
rs73079940
rs55940591
rs75527787
rs6747219
rs146057411
rs8082200
rs77379886
rs2316019
rs4785119
rs6755582
rs1534518
rs6431630
rs72664323
rs115018569
rs74617211
rs78217707
rs1818729
rs8094108
rs6917428
rs13084916
rs72690577
rs116894695
rs142532002
rs138553394
rs2231142
All
−0.10
−0.05
0.00
MR leave−one−out sensitivity analysis for
'3−bromo−5−chloro−2,6−dihydroxybenzoic acid levels' on 'Rheumatoid arthritis'

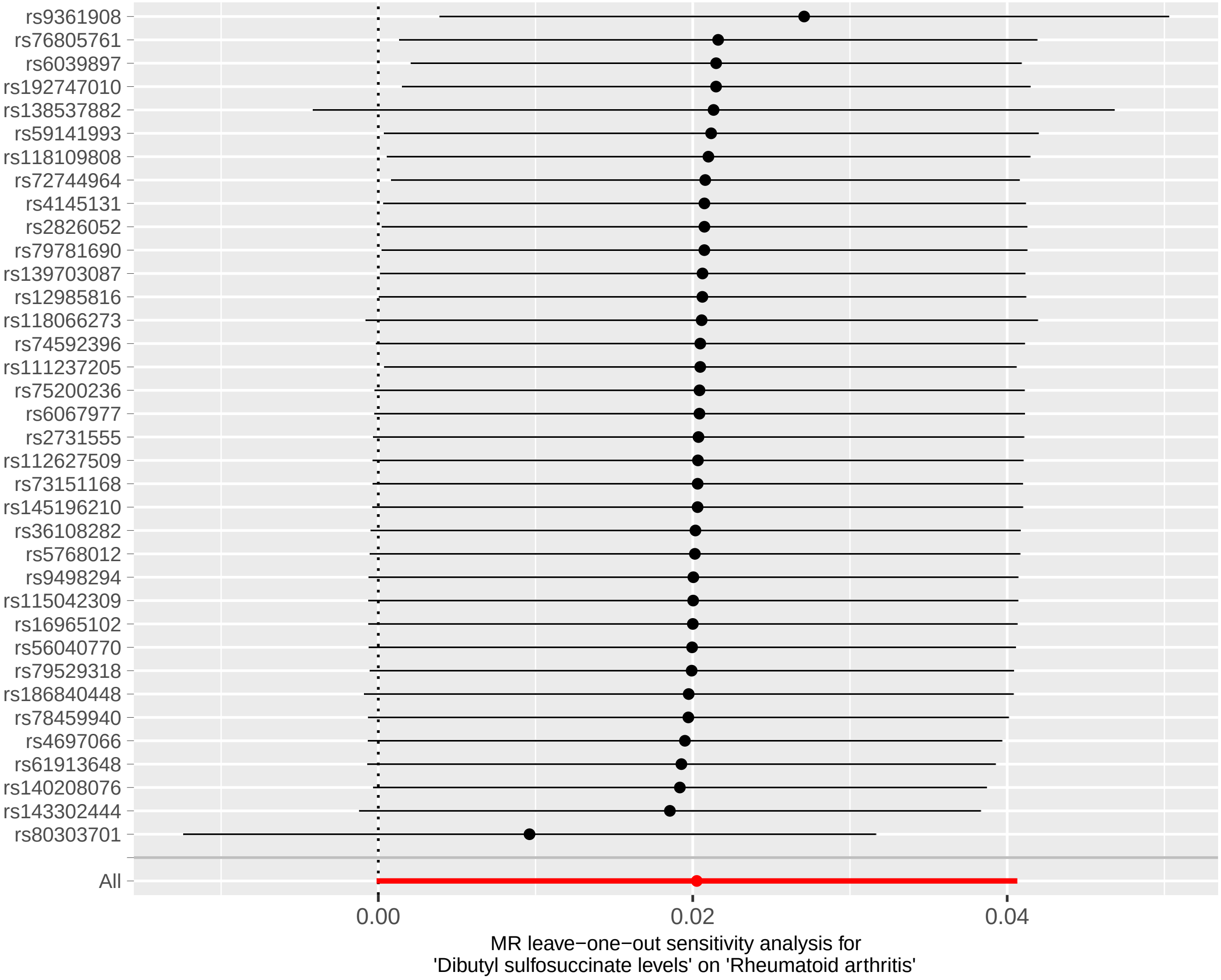
rs9361908
rs76805761
rs6039897
rs192747010
rs138537882
rs59141993
rs118109808
rs72744964
rs4145131
rs2826052
rs79781690
rs139703087
rs12985816
rs118066273
rs74592396
rs111237205
rs75200236
rs6067977
rs2731555
rs112627509
rs73151168
rs145196210
rs36108282
rs5768012
rs9498294
rs115042309
rs16965102
rs56040770
rs79529318
rs186840448
rs78459940
rs4697066
rs61913648
rs140208076
rs143302444
rs80303701
All
0.00
0.02
0.04
MR leave−one−out sensitivity analysis for
'Dibutyl sulfosuccinate levels' on 'Rheumatoid arthritis'

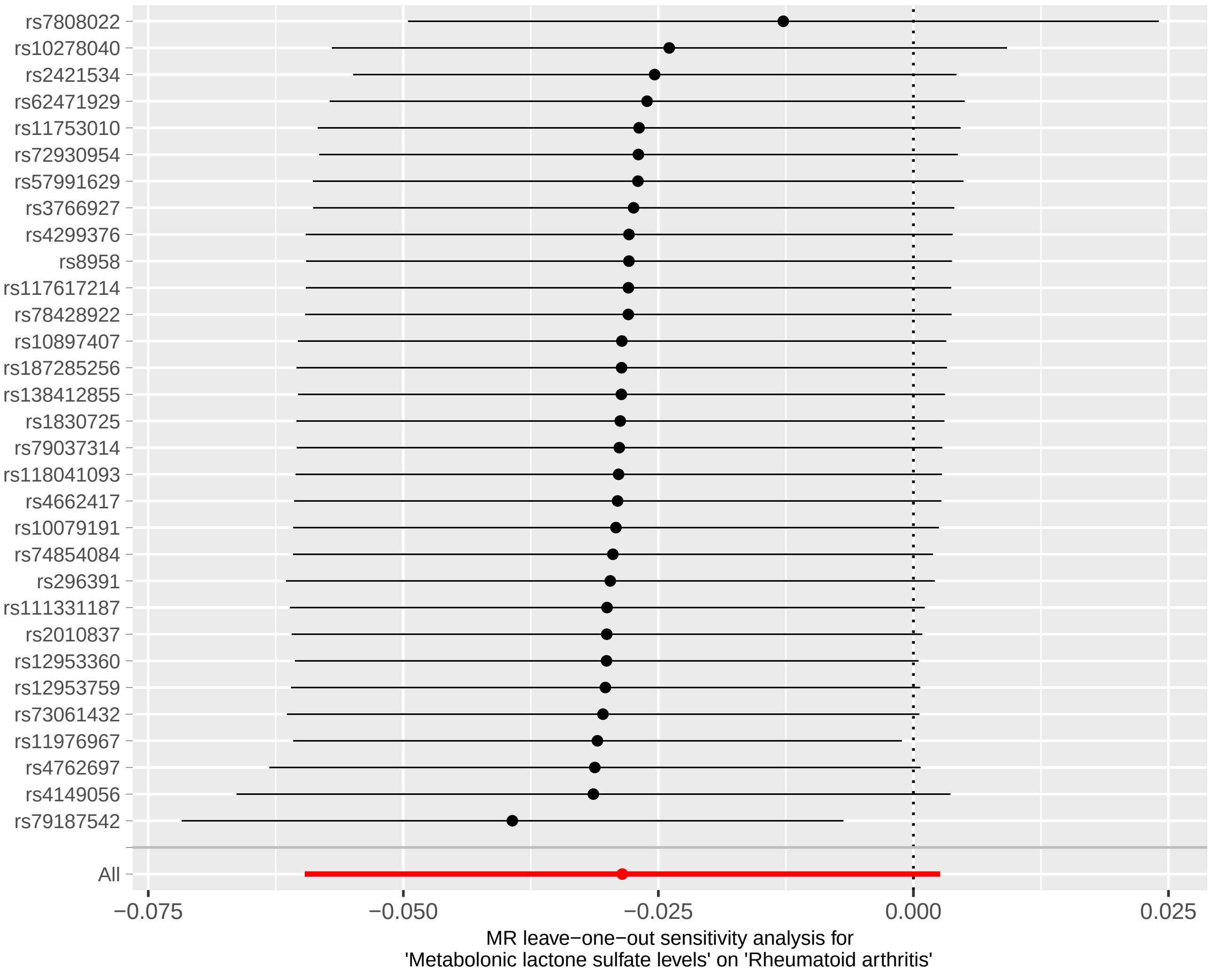
rs7808022
rs10278040
rs2421534
rs62471929
rs11753010
rs72930954
rs57991629
rs3766927
rs4299376
rs8958
rs117617214
rs78428922
rs10897407
rs187285256
rs138412855
rs1830725
rs79037314
rs118041093
rs4662417
rs10079191
rs74854084
rs296391
rs111331187
rs2010837
rs12953360
rs12953759
rs73061432
rs11976967
rs4762697
rs4149056
rs79187542
All
−0.075
−0.050
−0.025
0.000
0.025
MR leave−one−out sensitivity analysis for
'Metabolonic lactone sulfate levels' on 'Rheumatoid arthritis'

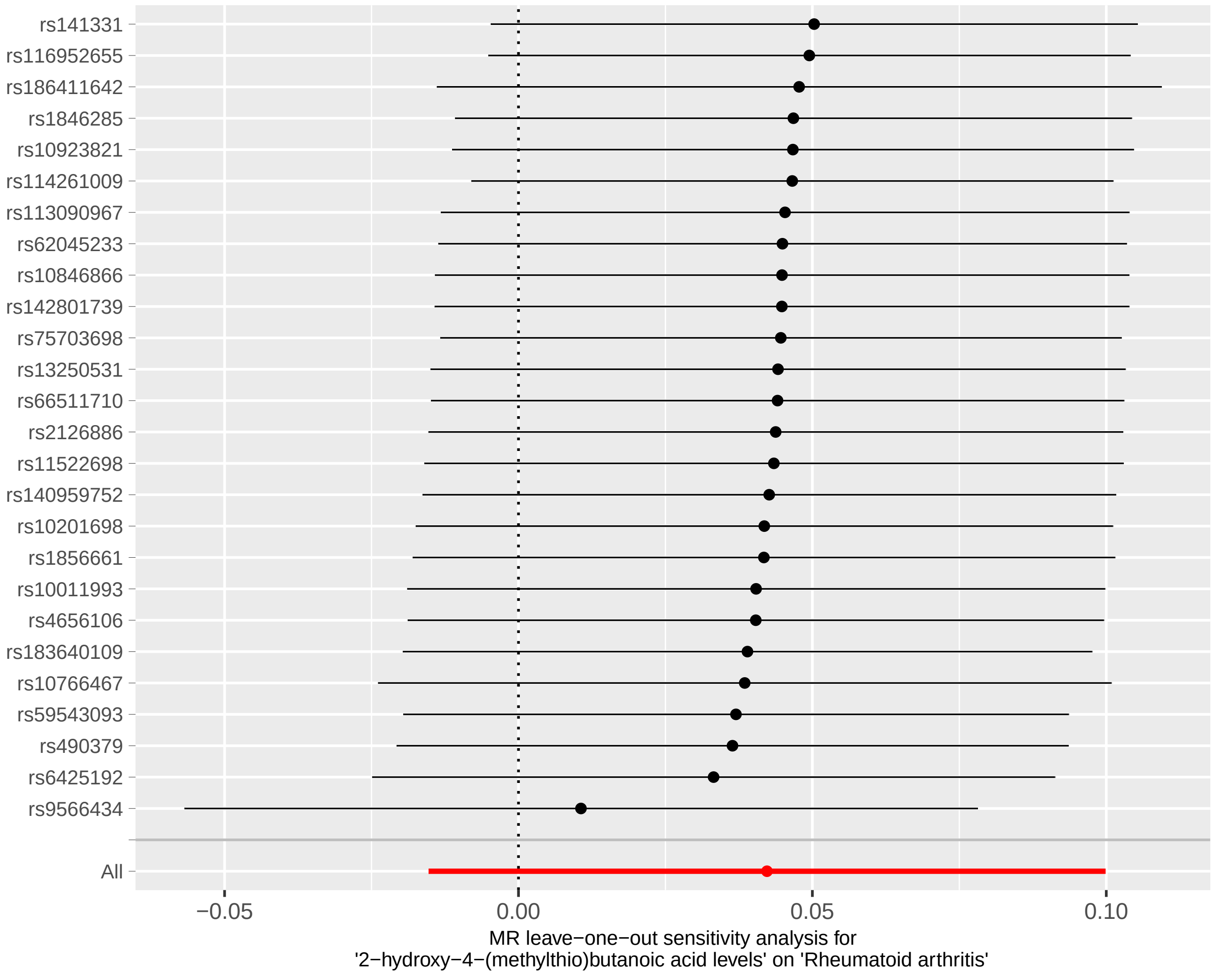
rs141331
rs116952655
rs186411642
rs1846285
rs10923821
rs114261009
rs113090967
rs62045233
rs10846866
rs142801739
rs75703698
rs13250531
rs66511710
rs2126886
rs11522698
rs140959752
rs10201698
rs1856661
rs10011993
rs4656106
rs183640109
rs10766467
rs59543093
rs490379
rs6425192
rs9566434
All
−0.05
0.00
0.05
0.10
MR leave−one−out sensitivity analysis for
'2−hydroxy−4−(methylthio)butanoic acid levels' on 'Rheumatoid arthritis'

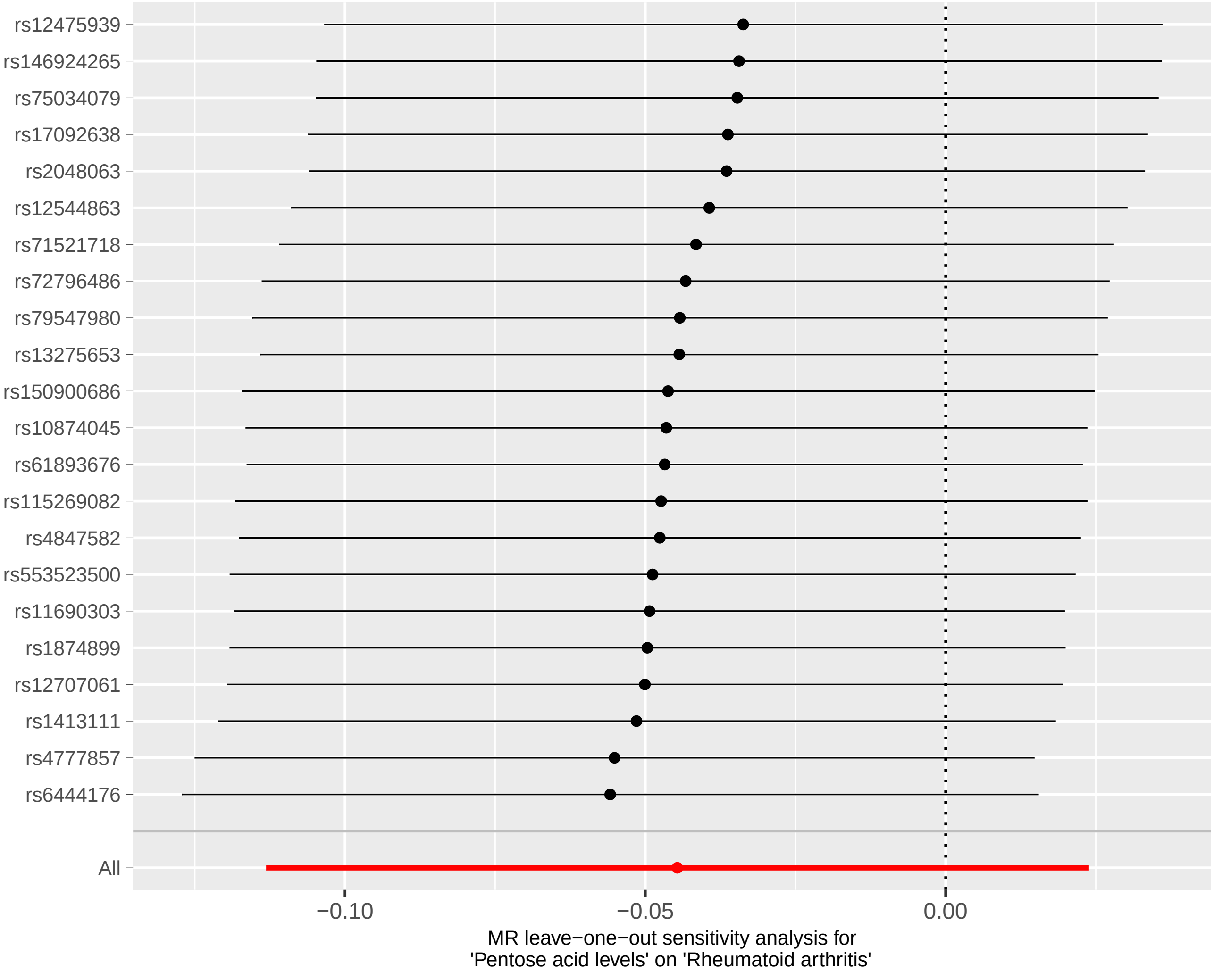
rs12475939
rs146924265
rs75034079
rs17092638
rs2048063
rs12544863
rs71521718
rs72796486
rs79547980
rs13275653
rs150900686
rs10874045
rs61893676
rs115269082
rs4847582
rs553523500
rs11690303
rs1874899
rs12707061
rs1413111
rs4777857
rs6444176
All
−0.10
−0.05
0.00
MR leave−one−out sensitivity analysis for
'Pentose acid levels' on 'Rheumatoid arthritis'

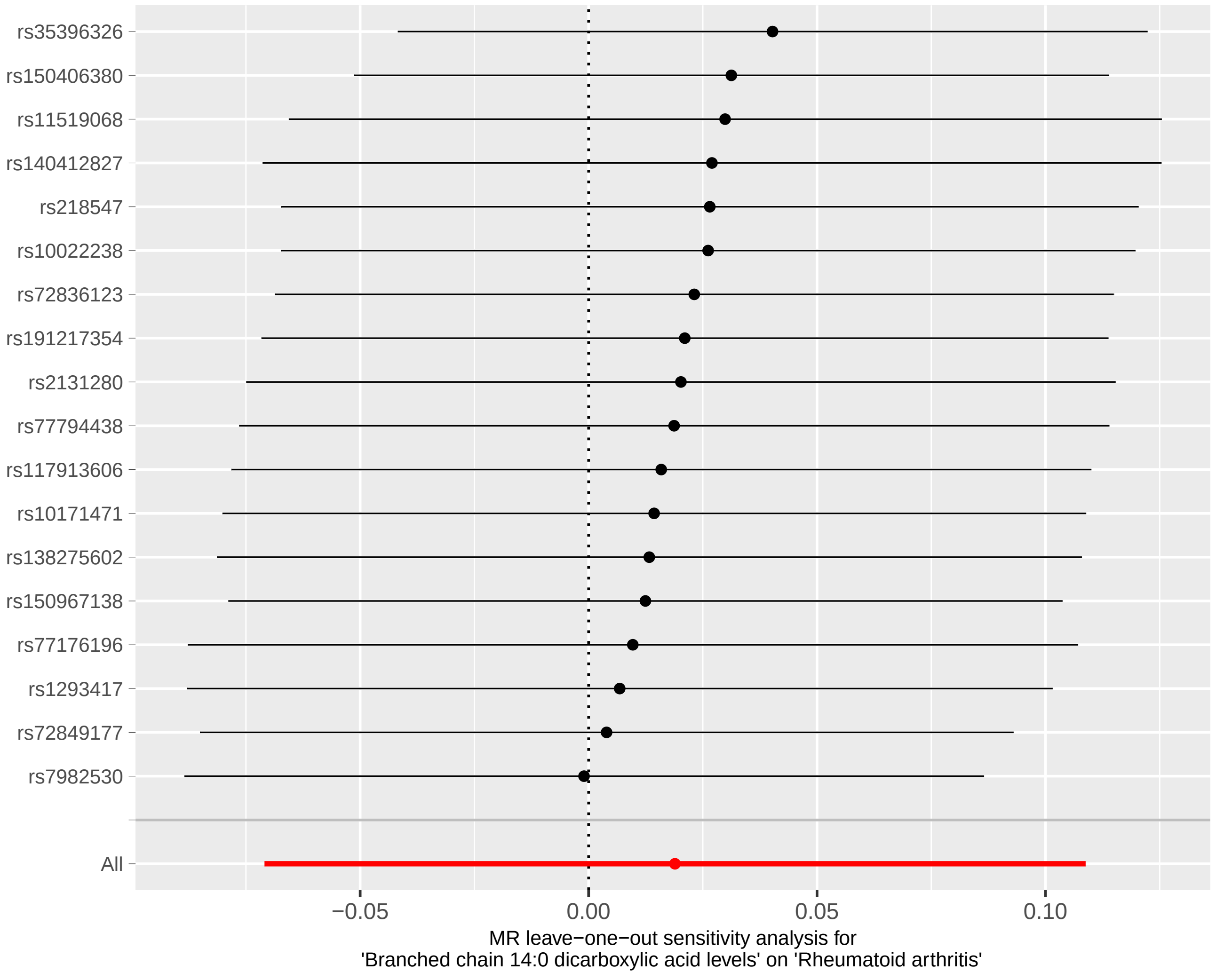

rs35396326
rs150406380
rs11519068
rs140412827
rs218547
rs10022238
rs72836123
rs191217354
rs2131280
rs77794438
rs117913606
rs10171471
rs138275602
rs150967138
rs77176196
rs1293417
rs72849177
rs7982530
All
−0.05
0.00
0.05
0.10
MR leave−one−out sensitivity analysis for
'Branched chain 14:0 dicarboxylic acid levels' on 'Rheumatoid arthritis'

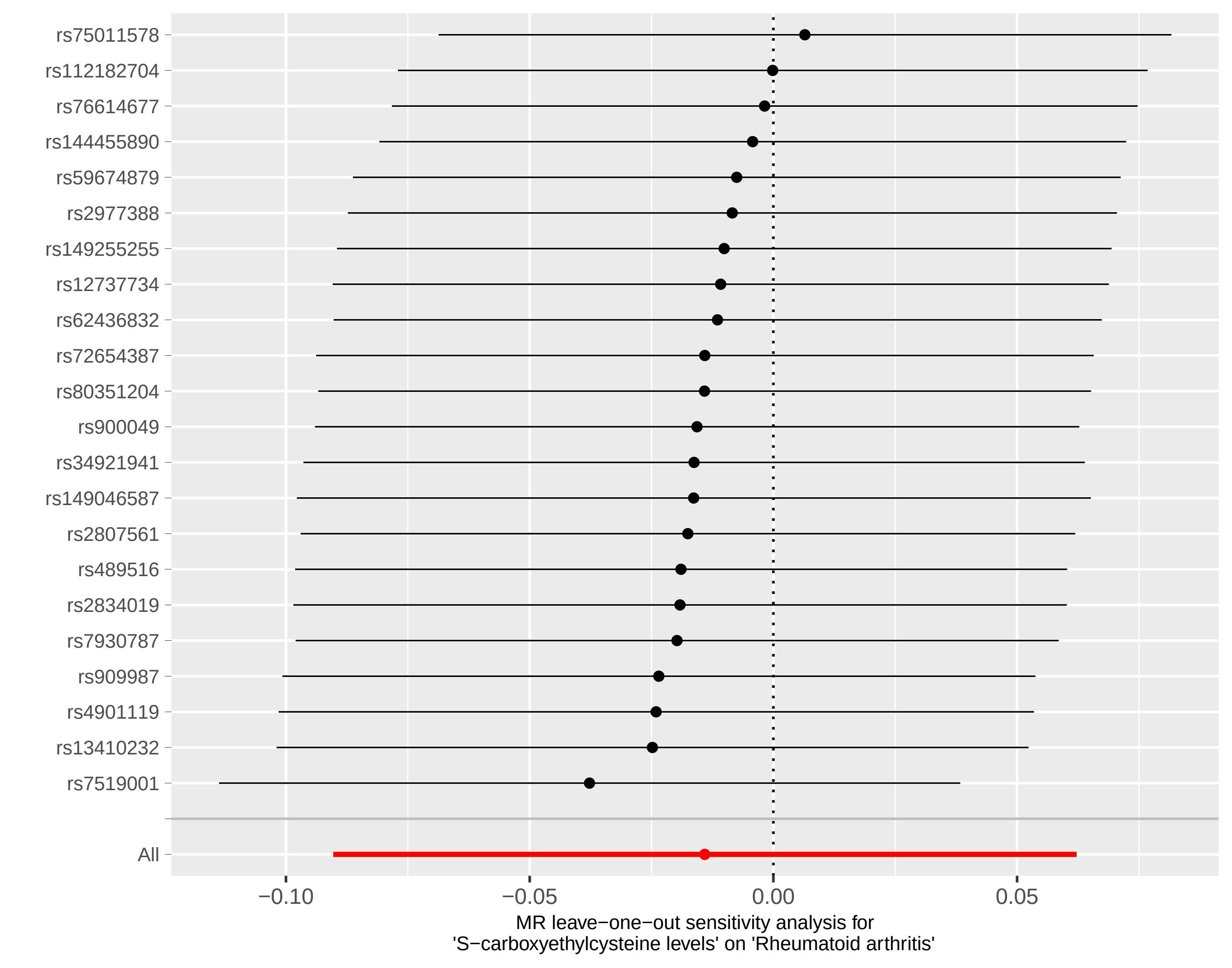
rs75011578
rs112182704
rs76614677
rs144455890
rs59674879
rs2977388
rs149255255
rs12737734
rs62436832
rs72654387
rs80351204
rs900049
rs34921941
rs149046587
rs2807561
rs489516
rs2834019
rs7930787
rs909987
rs4901119
rs13410232
rs7519001
All
−0.10
−0.05
0.00
0.05
MR leave−one−out sensitivity analysis for
'S−carboxyethylcysteine levels' on 'Rheumatoid arthritis'

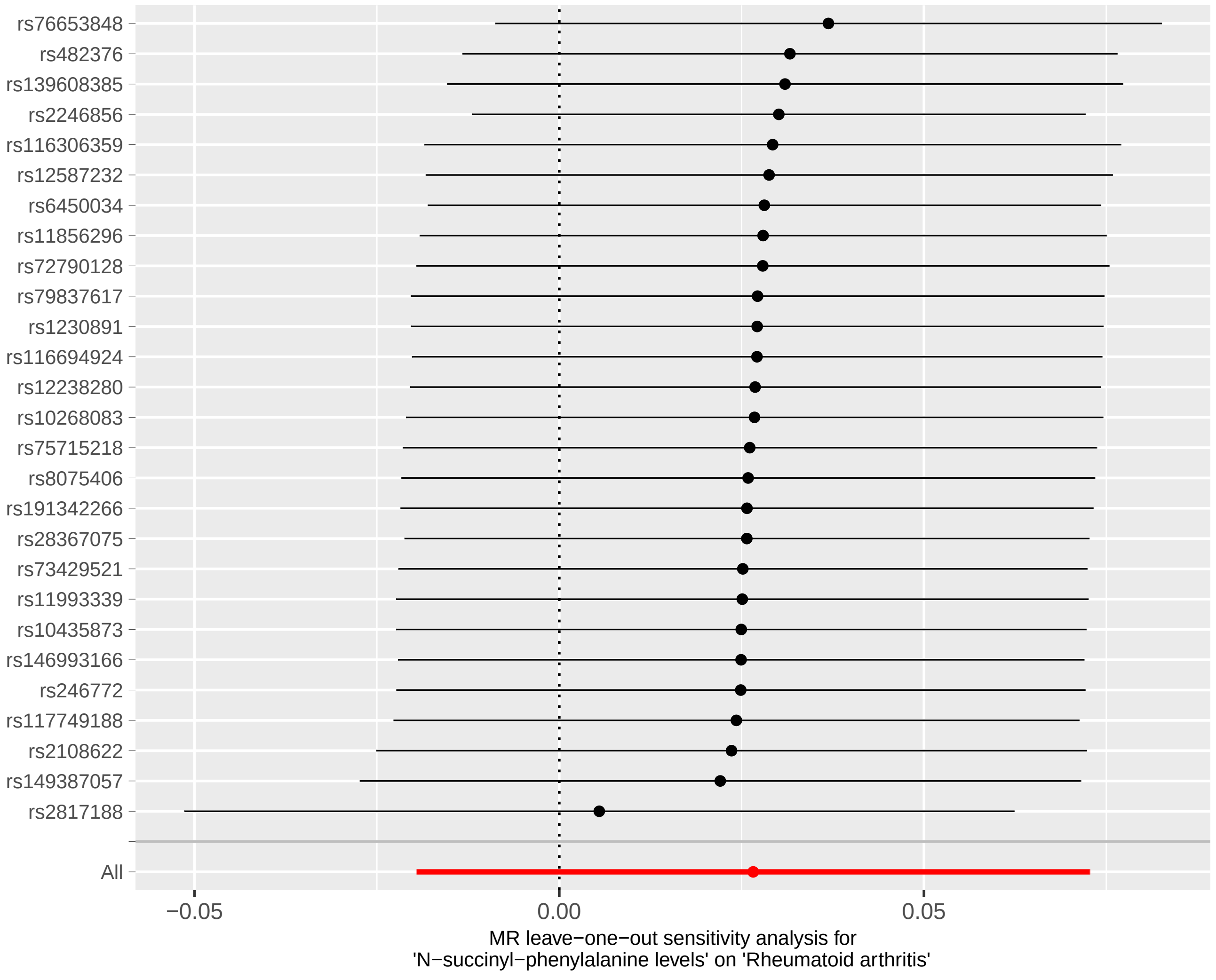

rs76653848
rs482376
rs139608385
rs2246856
rs116306359
rs12587232
rs6450034
rs11856296
rs72790128
rs79837617
rs1230891
rs116694924
rs12238280
rs10268083
rs75715218
rs8075406
rs191342266
rs28367075
rs73429521
rs11993339
rs10435873
rs146993166
rs246772
rs117749188
rs2108622
rs149387057
rs2817188
All
−0.05
0.00
0.05
MR leave−one−out sensitivity analysis for
'N−succinyl−phenylalanine levels' on 'Rheumatoid arthritis'

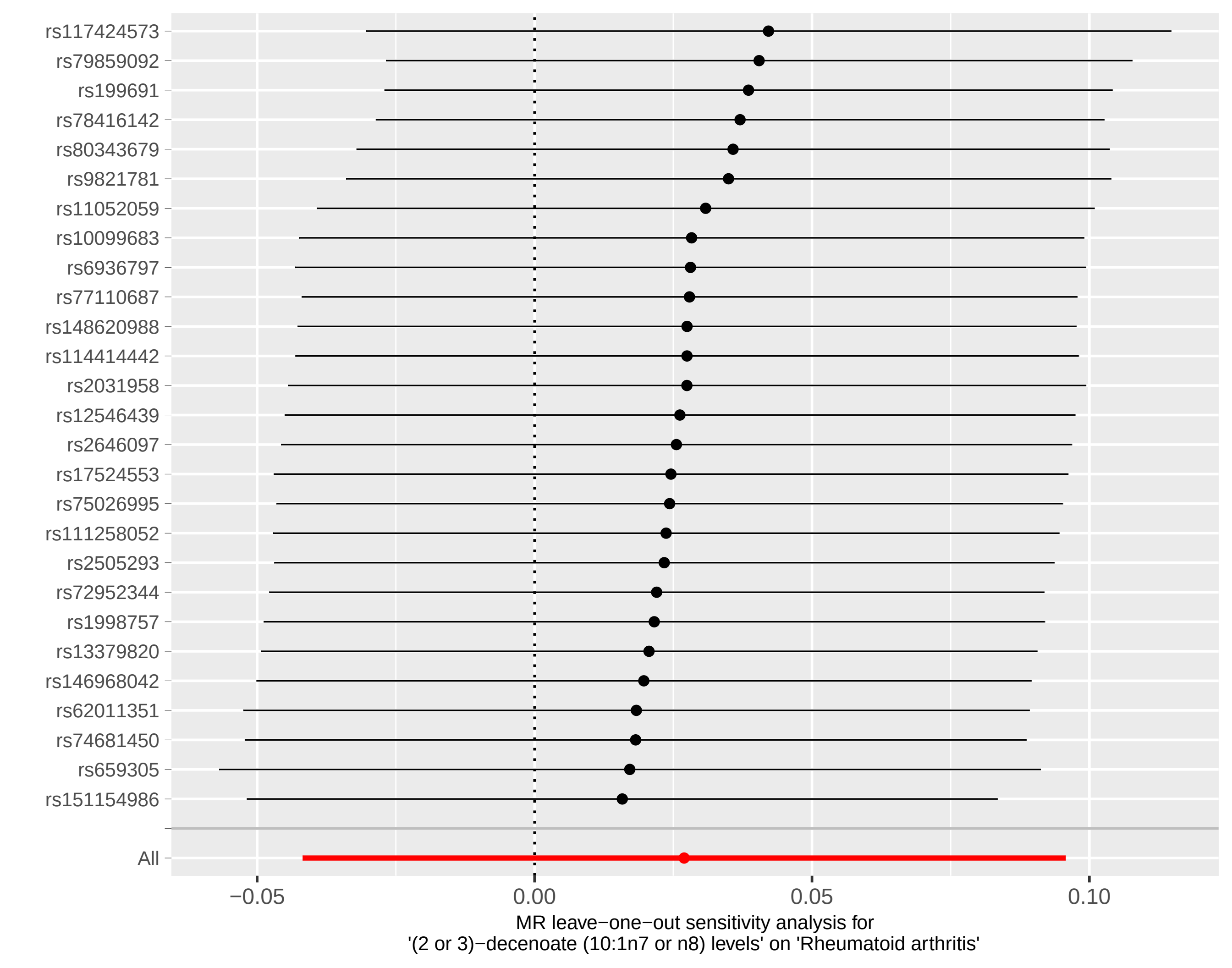

rs117424573
rs79859092
rs199691
rs78416142
rs80343679
rs9821781
rs11052059
rs10099683
rs6936797
rs77110687
rs148620988
rs114414442
rs2031958
rs12546439
rs2646097
rs17524553
rs75026995
rs111258052
rs2505293
rs72952344
rs1998757
rs13379820
rs146968042
rs62011351
rs74681450
rs659305
rs151154986
All
−0.05
0.00
0.05
0.10
MR leave−one−out sensitivity analysis for
'(2 or 3)−decenoate (10:1n7 or n8) levels' on 'Rheumatoid arthritis'

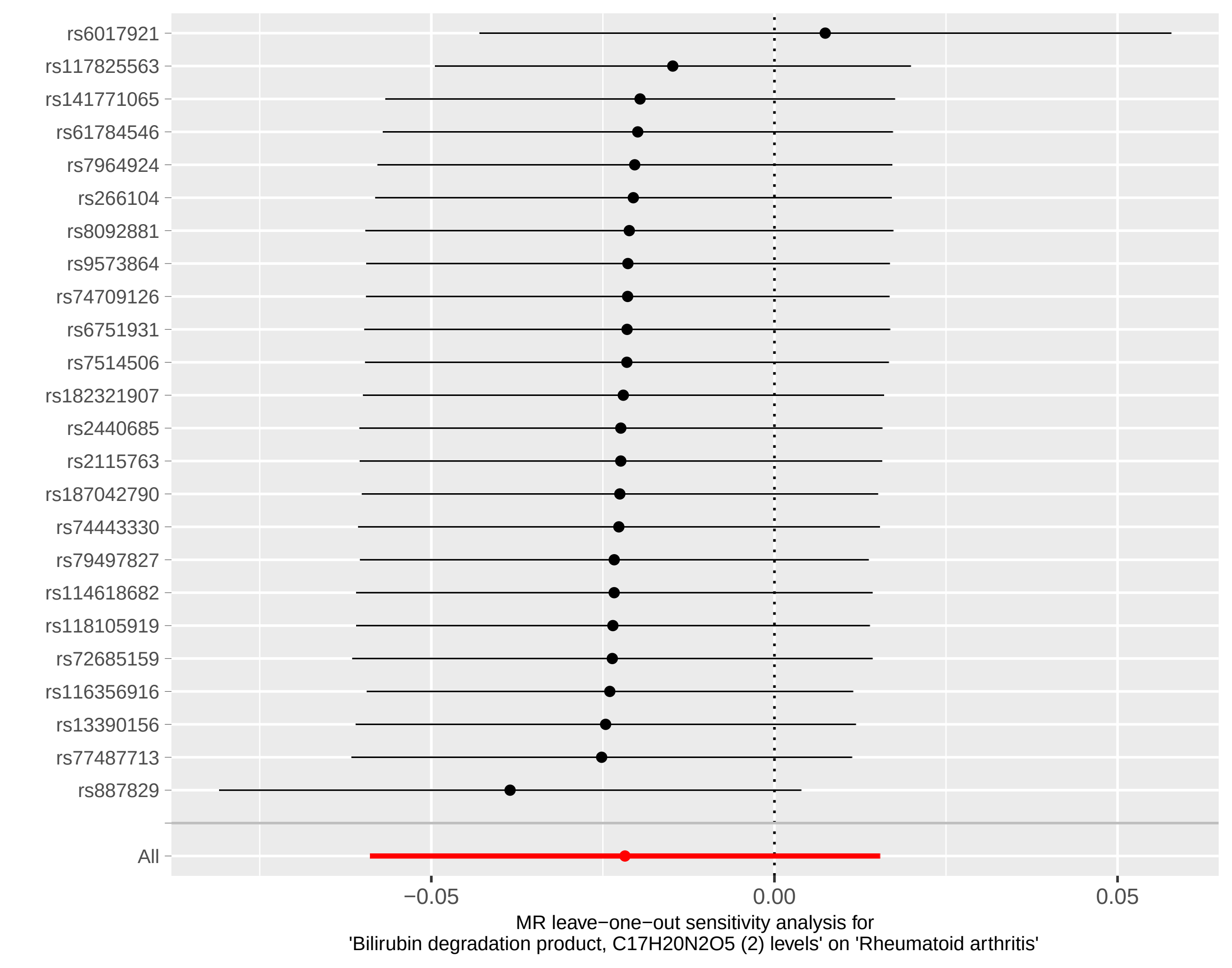
rs6017921
rs117825563
rs141771065
rs61784546
rs7964924
rs266104
rs8092881
rs9573864
rs74709126
rs6751931
rs7514506
rs182321907
rs2440685
rs2115763
rs187042790
rs74443330
rs79497827
rs114618682
rs118105919
rs72685159
rs116356916
rs13390156
rs77487713
rs887829
All
−0.05
0.00
0.05
MR leave−one−out sensitivity analysis for
'Bilirubin degradation product, C17H20N2O5 (2) levels' on 'Rheumatoid arthritis'

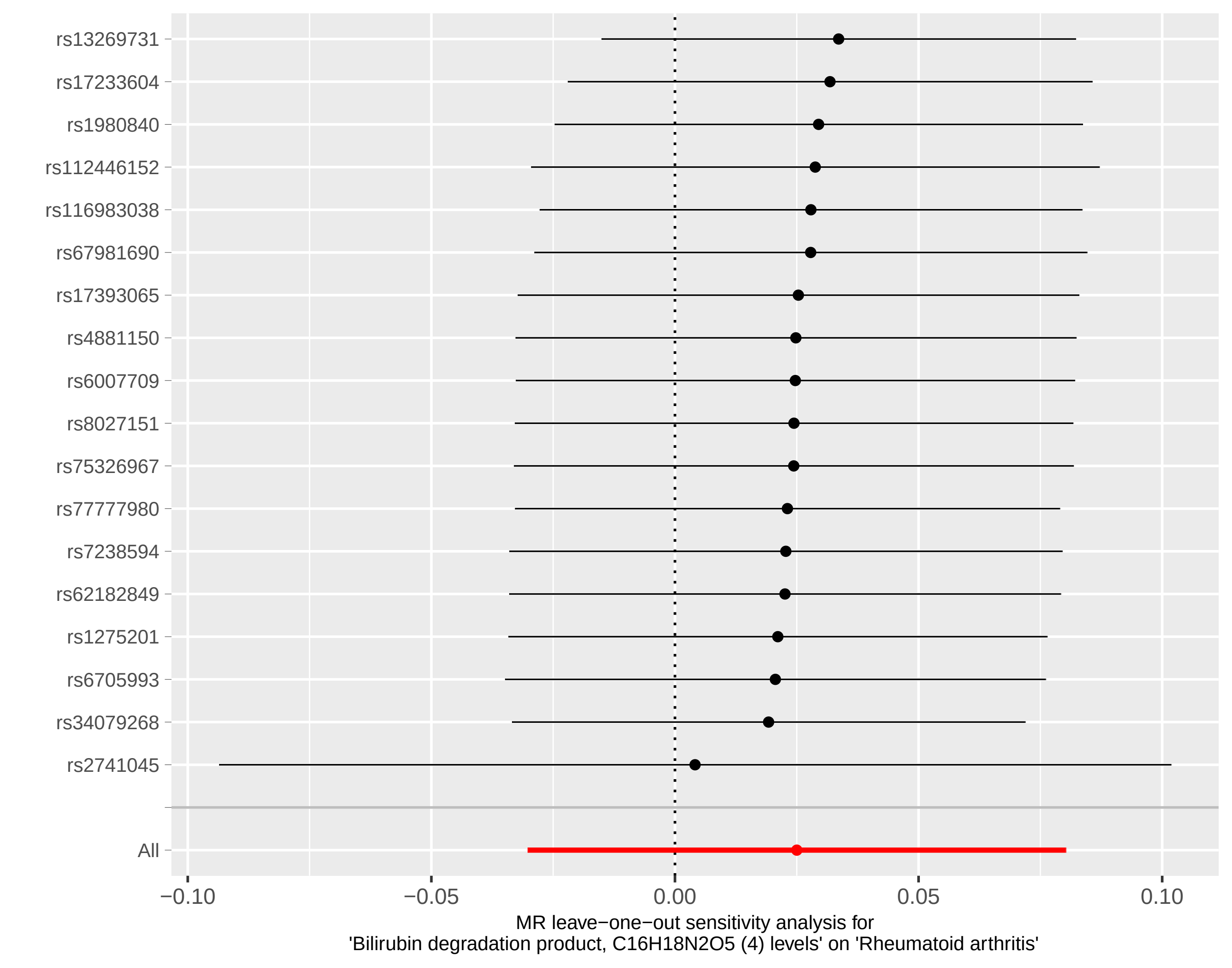

rs13269731
rs17233604
rs1980840
rs112446152
rs116983038
rs67981690
rs17393065
rs4881150
rs6007709
rs8027151
rs75326967
rs77777980
rs7238594
rs62182849
rs1275201
rs6705993
rs34079268
rs2741045
All
−0.10
−0.05
0.00
0.05
0.10
MR leave−one−out sensitivity analysis for
'Bilirubin degradation product, C16H18N2O5 (4) levels' on 'Rheumatoid arthritis'

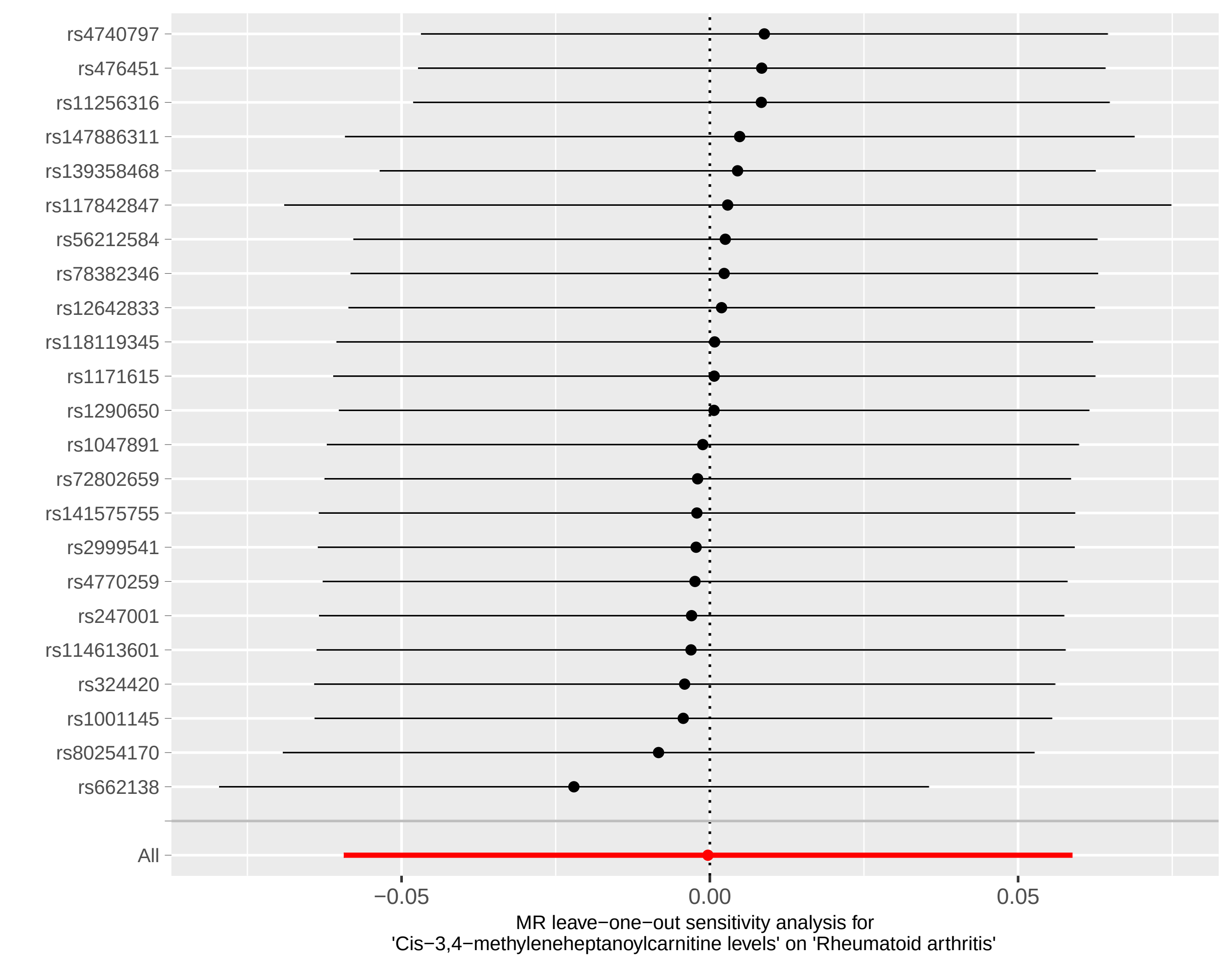
rs4740797
rs476451
rs11256316
rs147886311
rs139358468
rs117842847
rs56212584
rs78382346
rs12642833
rs118119345
rs1171615
rs1290650
rs1047891
rs72802659
rs141575755
rs2999541
rs4770259
rs247001
rs114613601
rs324420
rs1001145
rs80254170
rs662138
All
−0.05
0.00
0.05
MR leave−one−out sensitivity analysis for
'Cis−3,4−methyleneheptanoylcarnitine levels' on 'Rheumatoid arthritis'

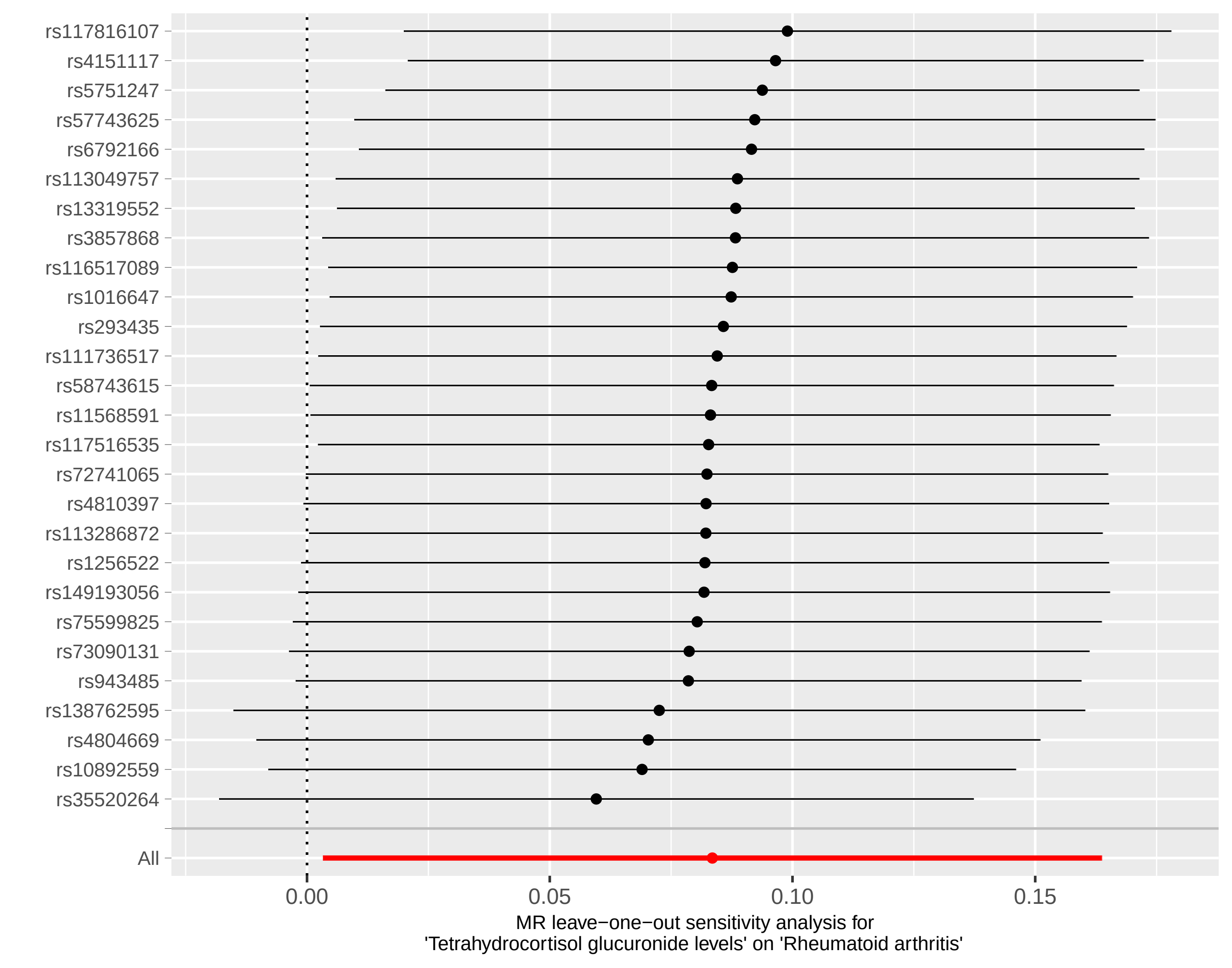
rs117816107
rs4151117
rs5751247
rs57743625
rs6792166
rs113049757
rs13319552
rs3857868
rs116517089
rs1016647
rs293435
rs111736517
rs58743615
rs11568591
rs117516535
rs72741065
rs4810397
rs113286872
rs1256522
rs149193056
rs75599825
rs73090131
rs943485
rs138762595
rs4804669
rs10892559
rs35520264
All
0.00
0.05
0.10
0.15
MR leave−one−out sensitivity analysis for
'Tetrahydrocortisol glucuronide levels' on 'Rheumatoid arthritis'

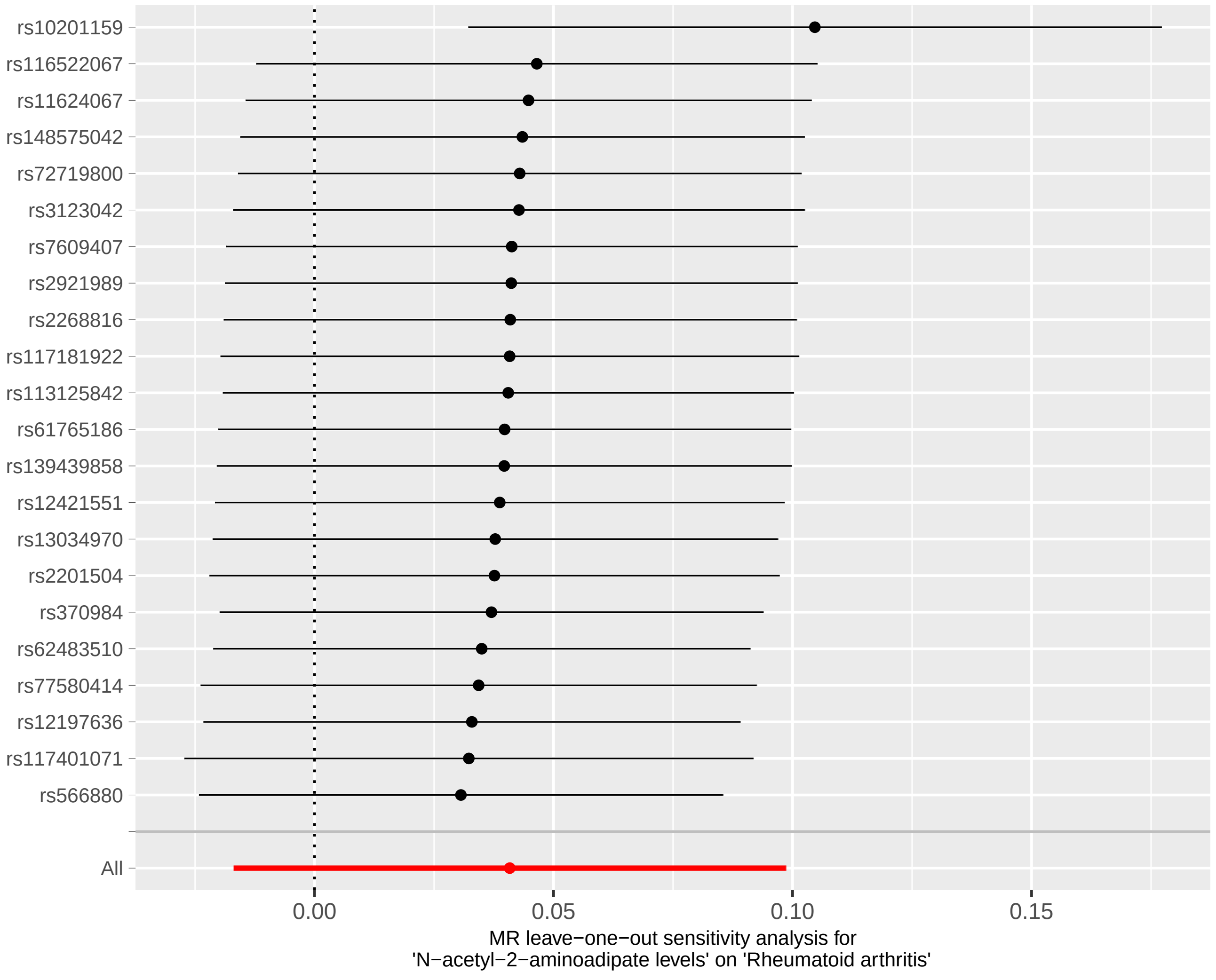
rs10201159
rs116522067
rs11624067
rs148575042
rs72719800
rs3123042
rs7609407
rs2921989
rs2268816
rs117181922
rs113125842
rs61765186
rs139439858
rs12421551
rs13034970
rs2201504
rs370984
rs62483510
rs77580414
rs12197636
rs117401071
rs566880
All
0.00
0.05
0.10
0.15
MR leave−one−out sensitivity analysis for
'N−acetyl−2−aminoadipate levels' on 'Rheumatoid arthritis'

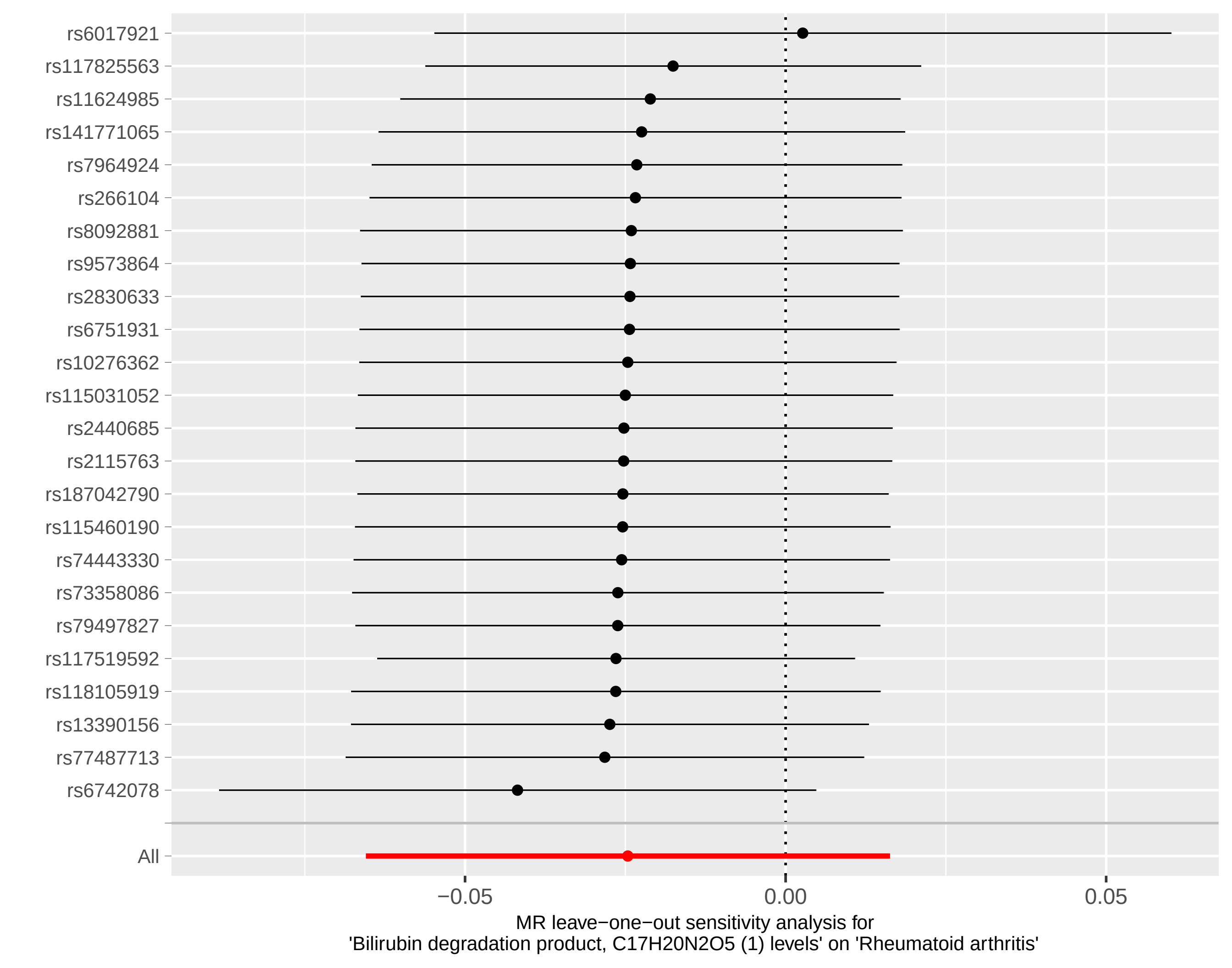
rs6017921
rs117825563
rs11624985
rs141771065
rs7964924
rs266104
rs8092881
rs9573864
rs2830633
rs6751931
rs10276362
rs115031052
rs2440685
rs2115763
rs187042790
rs115460190
rs74443330
rs73358086
rs79497827
rs117519592
rs118105919
rs13390156
rs77487713
rs6742078
All
−0.05
0.00
0.05
MR leave−one−out sensitivity analysis for
'Bilirubin degradation product, C17H20N2O5 (1) levels' on 'Rheumatoid arthritis'

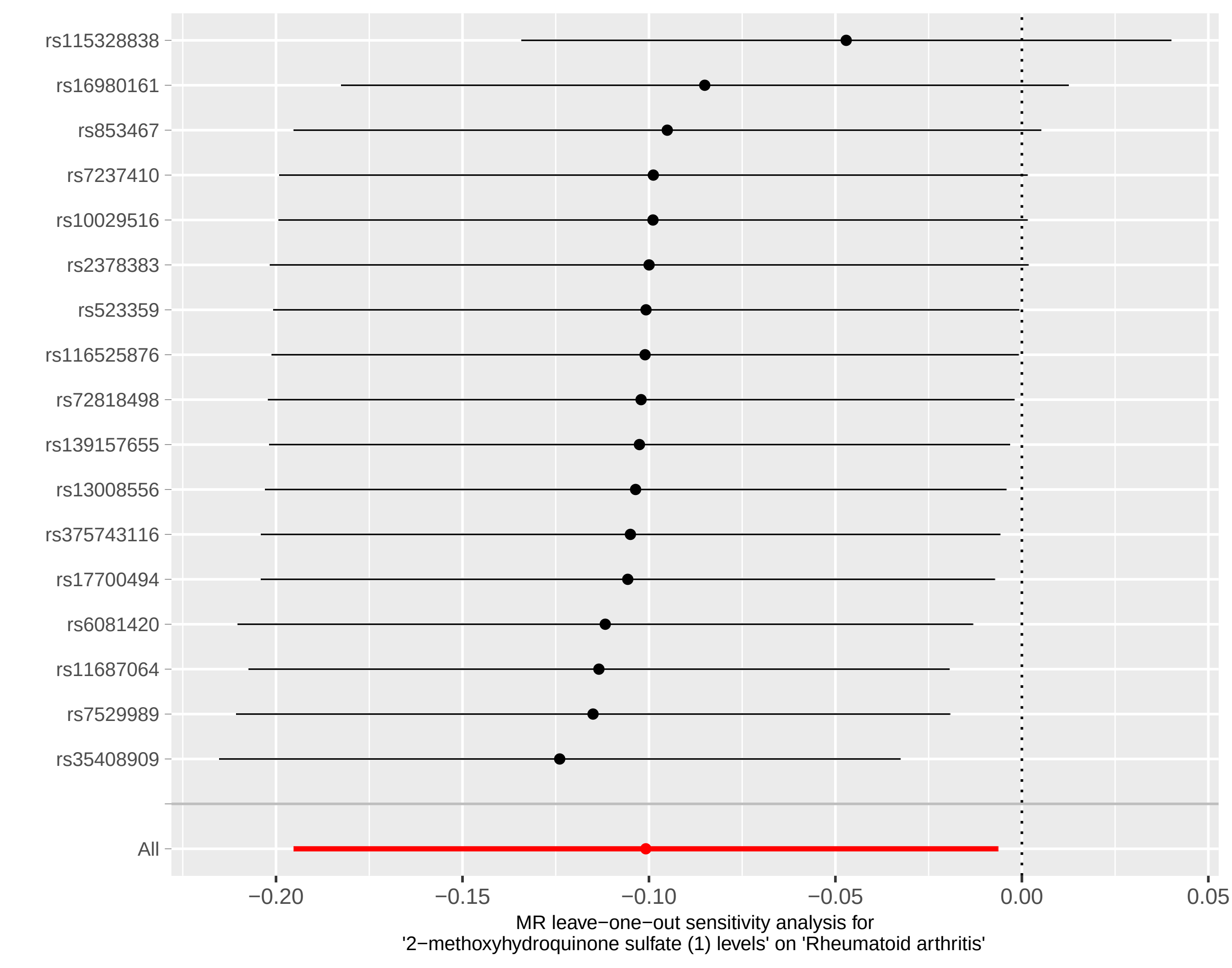

rs115328838
rs16980161
rs853467
rs7237410
rs10029516
rs2378383
rs523359
rs116525876
rs72818498
rs139157655
rs13008556
rs375743116
rs17700494
rs6081420
rs11687064
rs7529989
rs35408909
All
−0.20
−0.15
−0.10
−0.05
0.00
0.05
MR leave−one−out sensitivity analysis for
'2−methoxyhydroquinone sulfate (1) levels' on 'Rheumatoid arthritis'

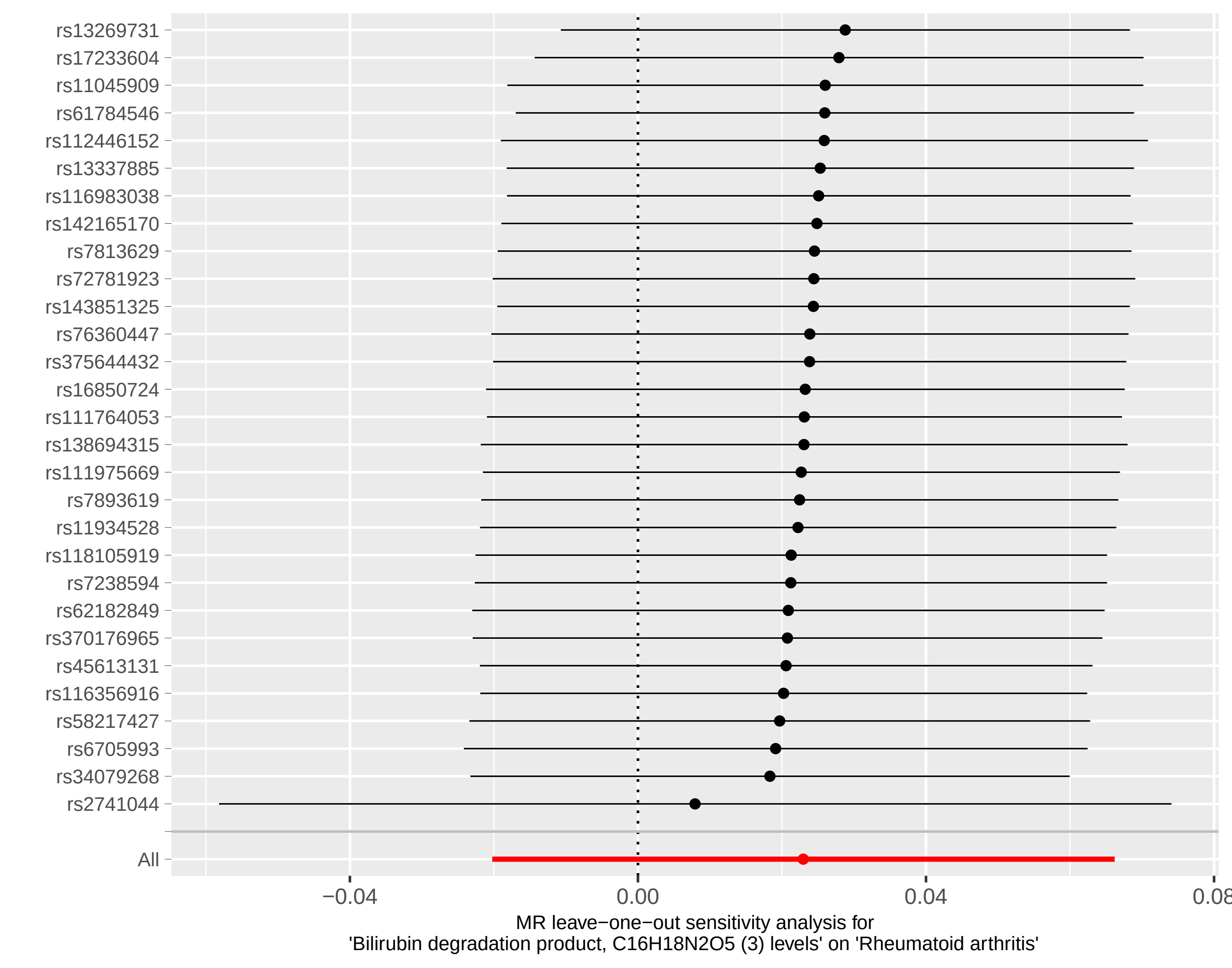

rs13269731
rs17233604
rs11045909
rs61784546
rs112446152
rs13337885
rs116983038
rs142165170
rs7813629
rs72781923
rs143851325
rs76360447
rs375644432
rs16850724
rs111764053
rs138694315
rs111975669
rs7893619
rs11934528
rs118105919
rs7238594
rs62182849
rs370176965
rs45613131
rs116356916
rs58217427
rs6705993
rs34079268
rs2741044
All
−0.04
0.00
0.04
0.08
MR leave−one−out sensitivity analysis for
'Bilirubin degradation product, C16H18N2O5 (3) levels' on 'Rheumatoid arthritis'

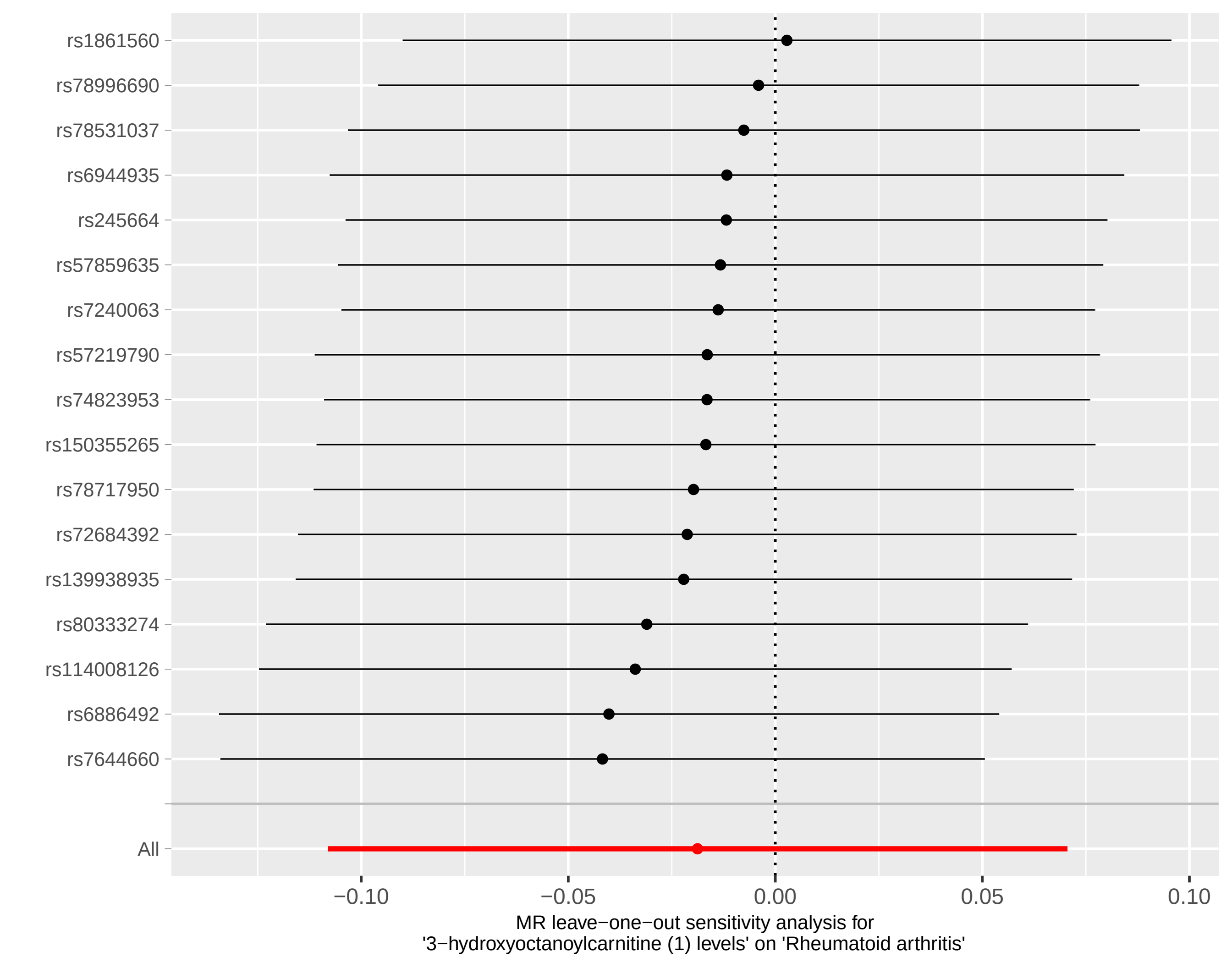

rs1861560
rs78996690
rs78531037
rs6944935
rs245664
rs57859635
rs7240063
rs57219790
rs74823953
rs150355265
rs78717950
rs72684392
rs139938935
rs80333274
rs114008126
rs6886492
rs7644660
All
−0.10
−0.05
0.00
0.05
0.10
MR leave−one−out sensitivity analysis for
'3−hydroxyoctanoylcarnitine (1) levels' on 'Rheumatoid arthritis'

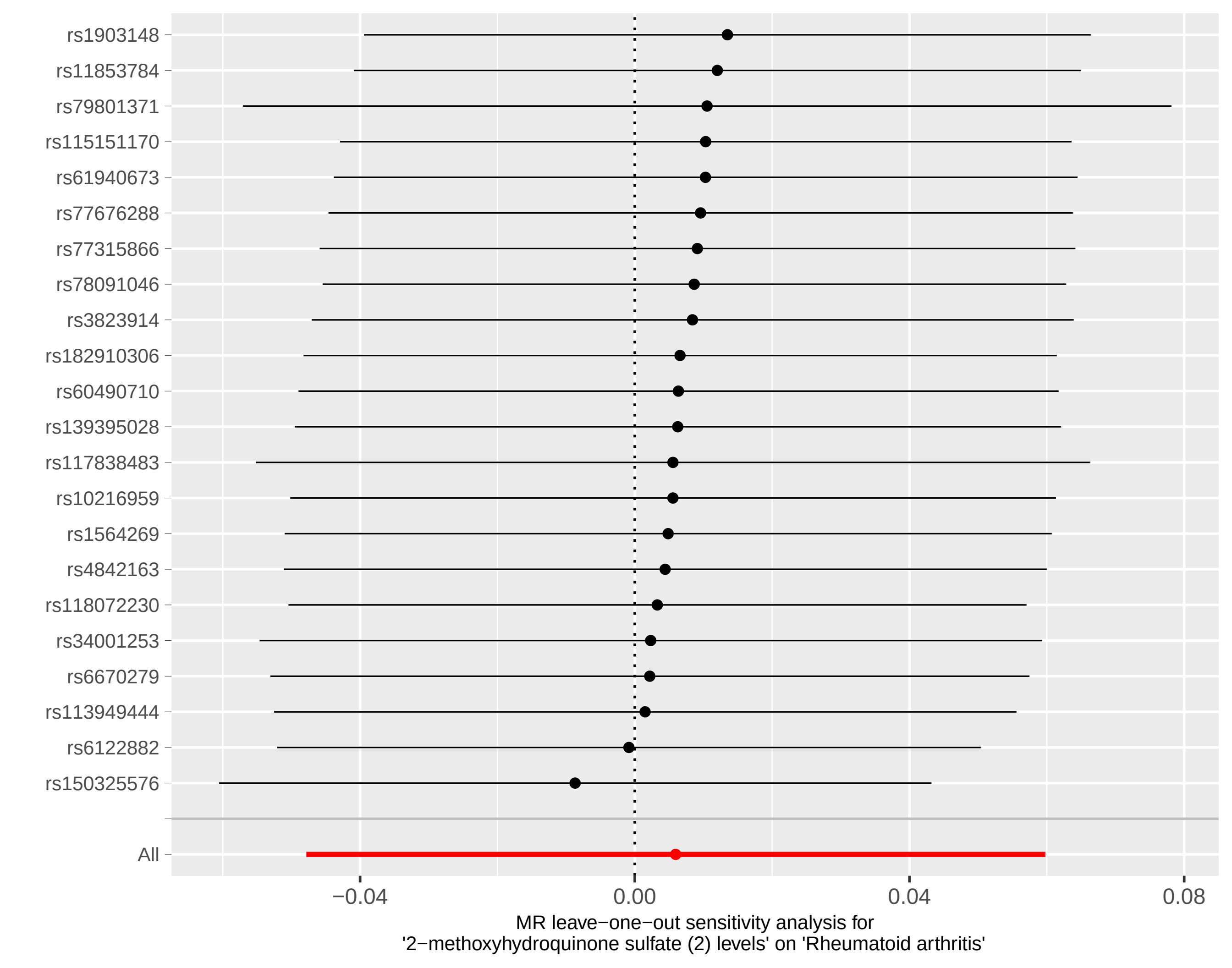
rs1903148
rs11853784
rs79801371
rs115151170
rs61940673
rs77676288
rs77315866
rs78091046
rs3823914
rs182910306
rs60490710
rs139395028
rs117838483
rs10216959
rs1564269
rs4842163
rs118072230
rs34001253
rs6670279
rs113949444
rs6122882
rs150325576
All
−0.04
0.00
0.04
0.08
MR leave−one−out sensitivity analysis for
'2−methoxyhydroquinone sulfate (2) levels' on 'Rheumatoid arthritis'

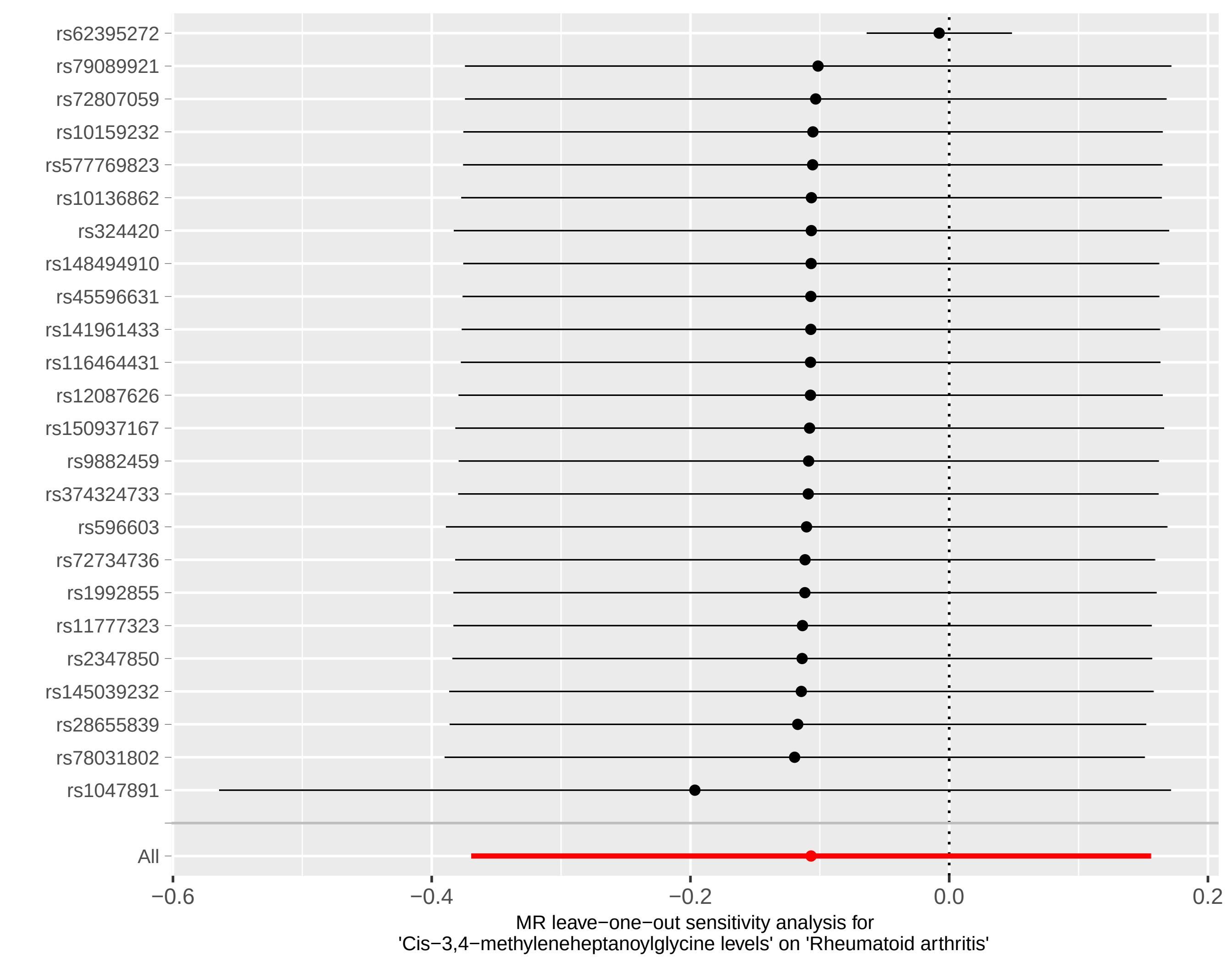
rs62395272
rs79089921
rs72807059
rs10159232
rs577769823
rs10136862
rs324420
rs148494910
rs45596631
rs141961433
rs116464431
rs12087626
rs150937167
rs9882459
rs374324733
rs596603
rs72734736
rs1992855
rs11777323
rs2347850
rs145039232
rs28655839
rs78031802
rs1047891
All
−0.6
−0.4
−0.2
0.0
0.2
MR leave−one−out sensitivity analysis for
'Cis−3,4−methyleneheptanoylglycine levels' on 'Rheumatoid arthritis'

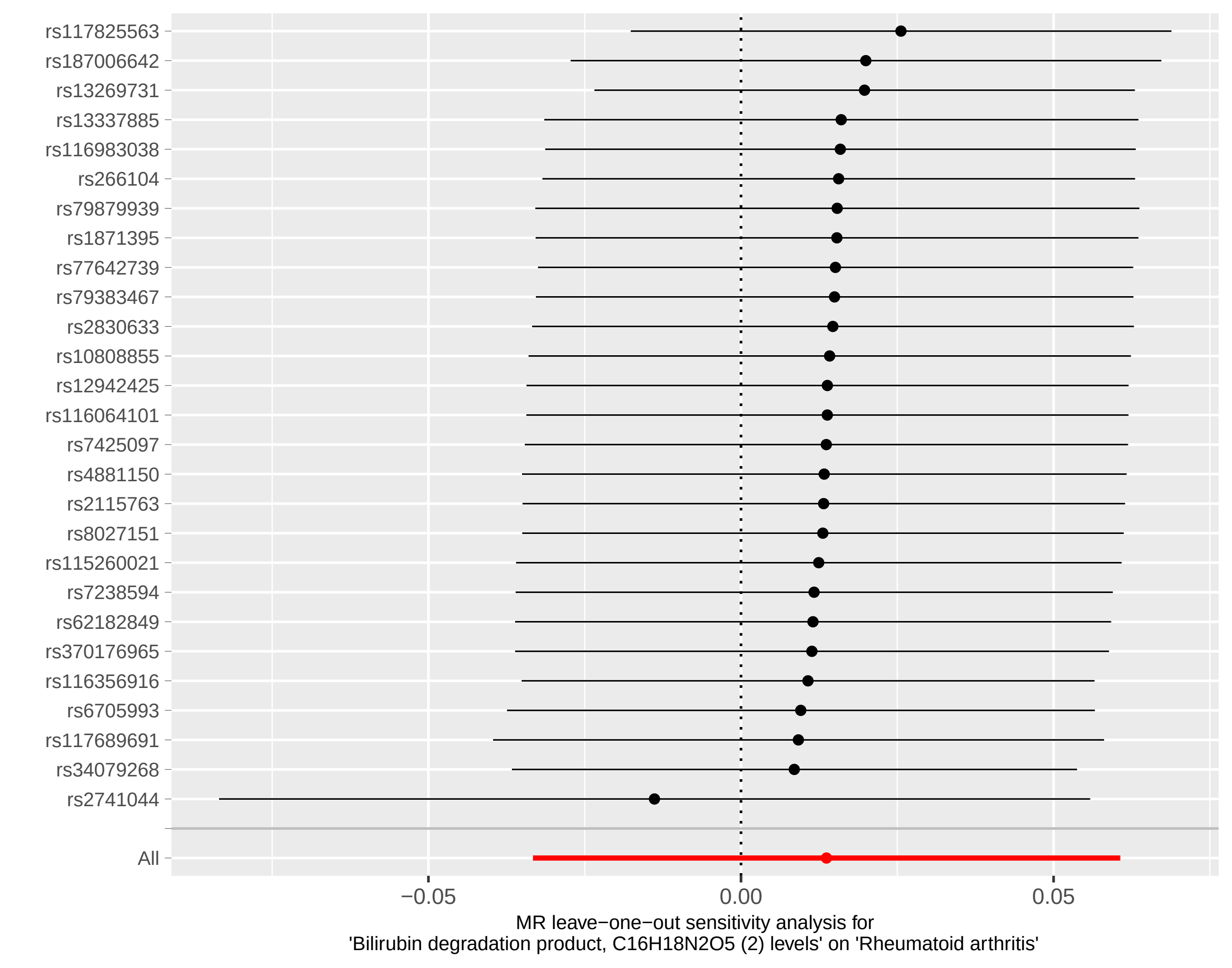
rs117825563
rs187006642
rs13269731
rs13337885
rs116983038
rs266104
rs79879939
rs1871395
rs77642739
rs79383467
rs2830633
rs10808855
rs12942425
rs116064101
rs7425097
rs4881150
rs2115763
rs8027151
rs115260021
rs7238594
rs62182849
rs370176965
rs116356916
rs6705993
rs117689691
rs34079268
rs2741044
All
−0.05
0.00
0.05
MR leave−one−out sensitivity analysis for
'Bilirubin degradation product, C16H18N2O5 (2) levels' on 'Rheumatoid arthritis'

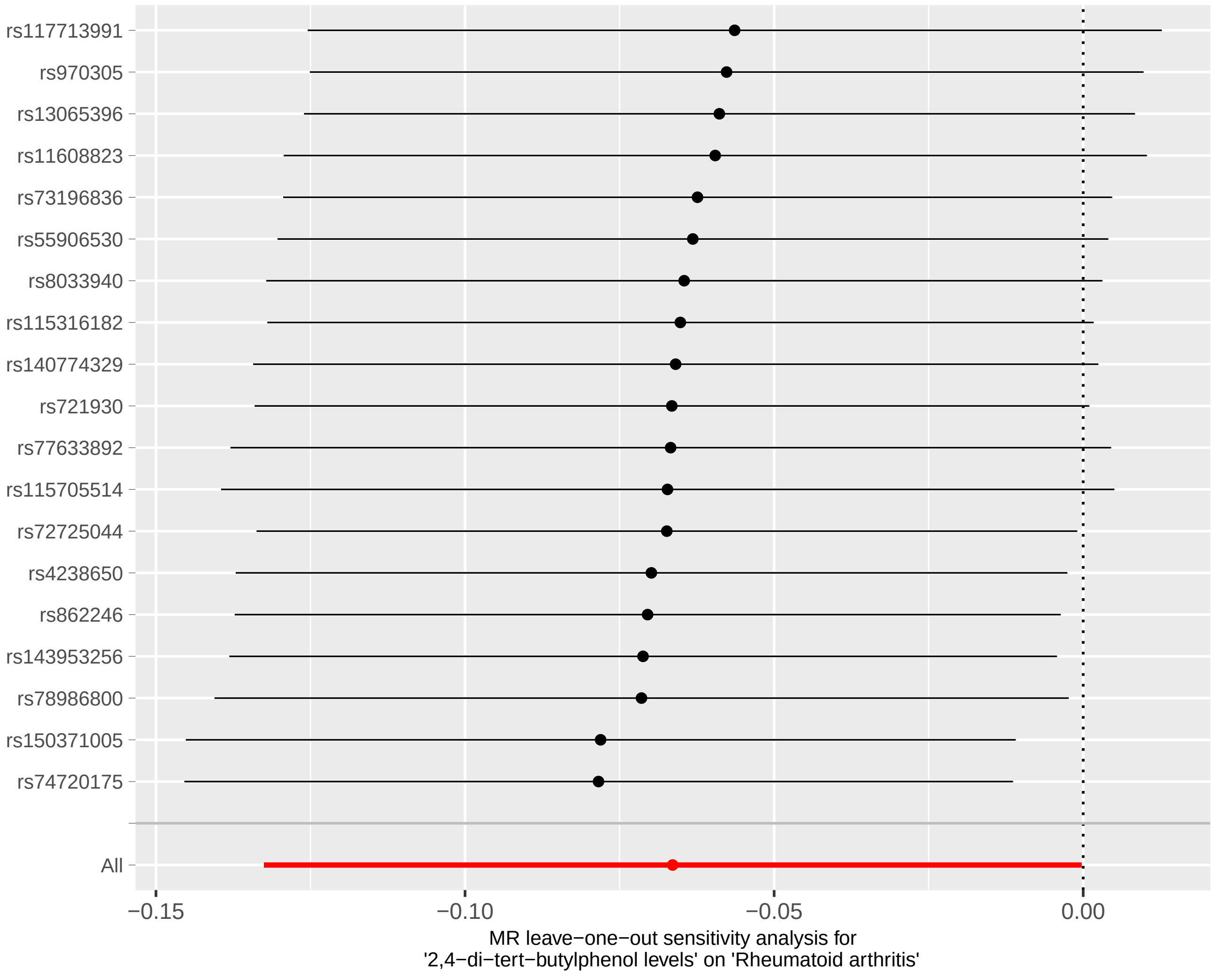
rs117713991
rs970305
rs13065396
rs11608823
rs73196836
rs55906530
rs8033940
rs115316182
rs140774329
rs721930
rs77633892
rs115705514
rs72725044
rs4238650
rs862246
rs143953256
rs78986800
rs150371005
rs74720175
All
−0.15
−0.10
−0.05
0.00
MR leave−one−out sensitivity analysis for
'2,4−di−tert−butylphenol levels' on 'Rheumatoid arthritis'

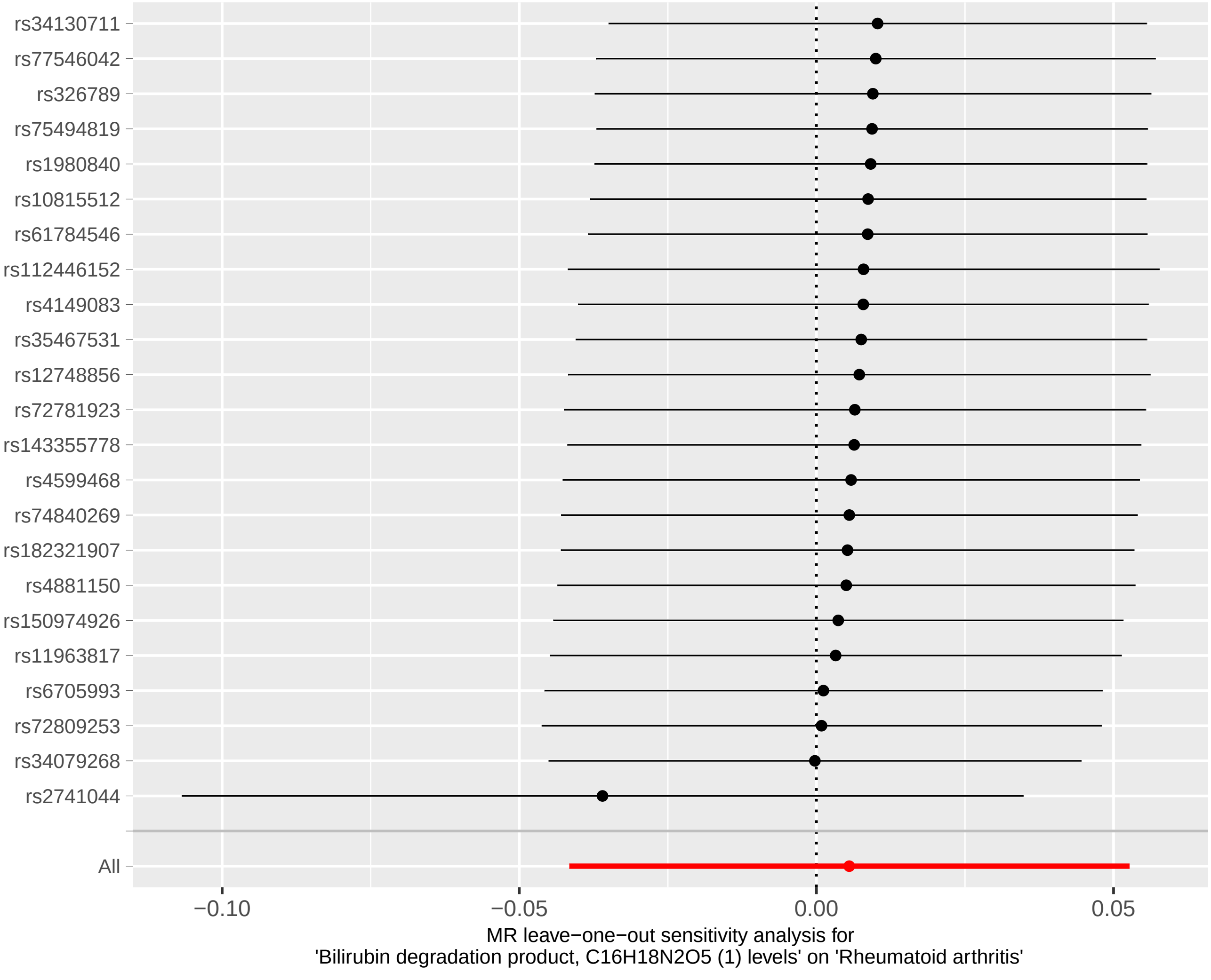

rs34130711
rs77546042
rs326789
rs75494819
rs1980840
rs10815512
rs61784546
rs112446152
rs4149083
rs35467531
rs12748856
rs72781923
rs143355778
rs4599468
rs74840269
rs182321907
rs4881150
rs150974926
rs11963817
rs6705993
rs72809253
rs34079268
rs2741044
All
−0.10
−0.05
0.00
0.05
MR leave−one−out sensitivity analysis for
'Bilirubin degradation product, C16H18N2O5 (1) levels' on 'Rheumatoid arthritis'

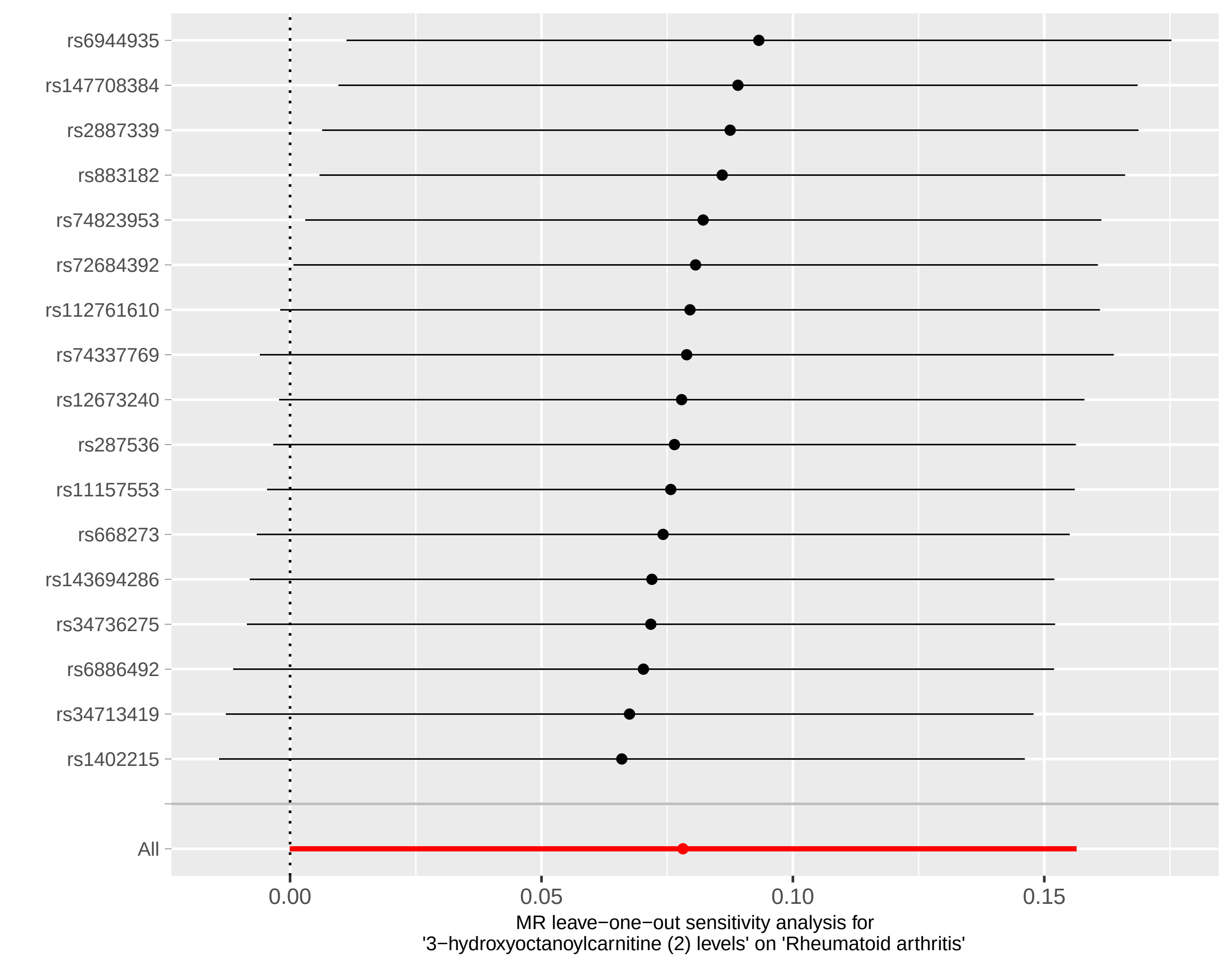
rs6944935
rs147708384
rs2887339
rs883182
rs74823953
rs72684392
rs112761610
rs74337769
rs12673240
rs287536
rs11157553
rs668273
rs143694286
rs34736275
rs6886492
rs34713419
rs1402215
All
0.00
0.05
0.10
0.15
MR leave−one−out sensitivity analysis for
'3−hydroxyoctanoylcarnitine (2) levels' on 'Rheumatoid arthritis'

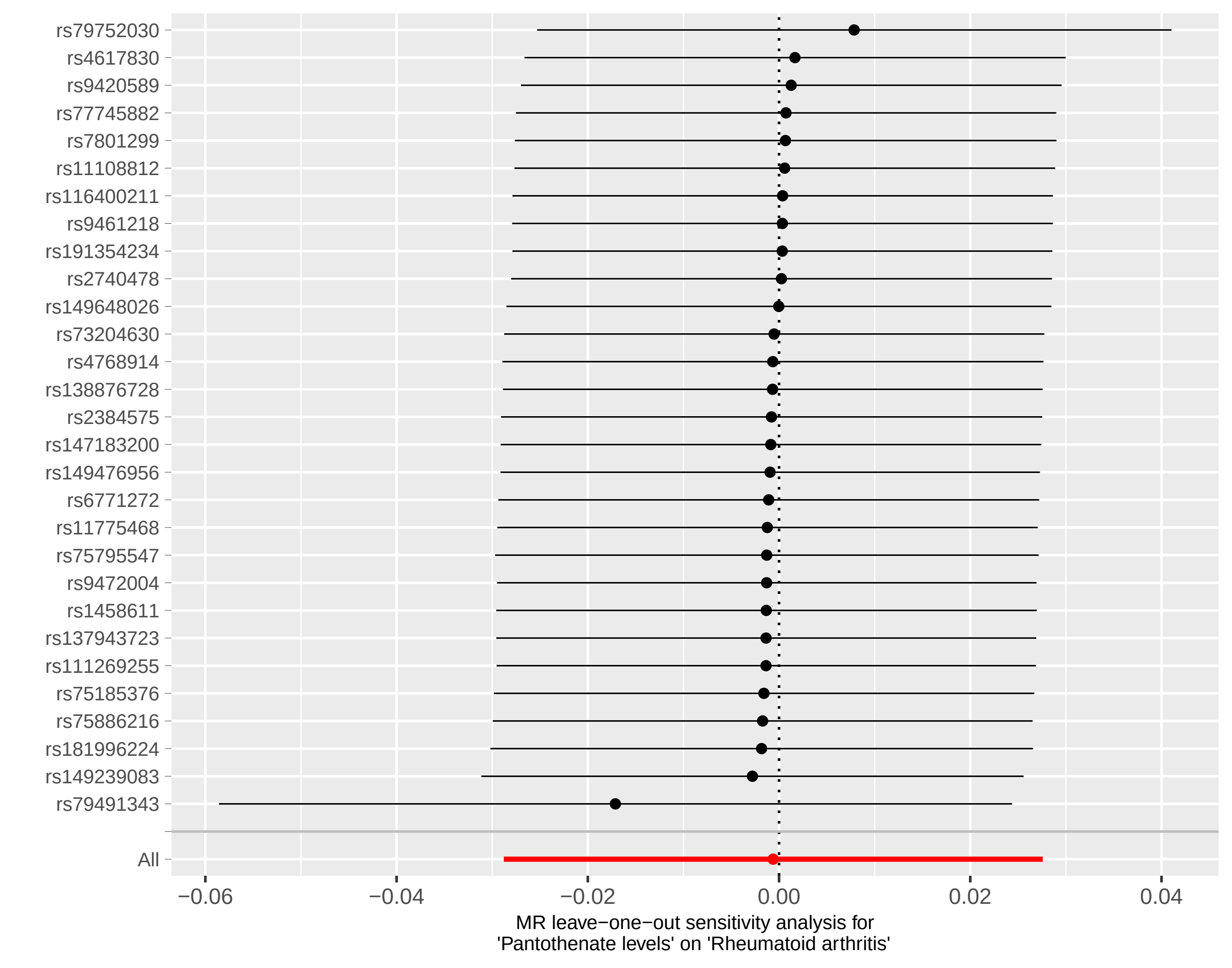

rs79752030
rs4617830
rs9420589
rs77745882
rs7801299
rs11108812
rs116400211
rs9461218
rs191354234
rs2740478
rs149648026
rs73204630
rs4768914
rs138876728
rs2384575
rs147183200
rs149476956
rs6771272
rs11775468
rs75795547
rs9472004
rs1458611
rs137943723
rs111269255
rs75185376
rs75886216
rs181996224
rs149239083
rs79491343
All
−0.06
−0.04
−0.02
0.00
0.02
0.04
MR leave−one−out sensitivity analysis for
'Pantothenate levels' on 'Rheumatoid arthritis'

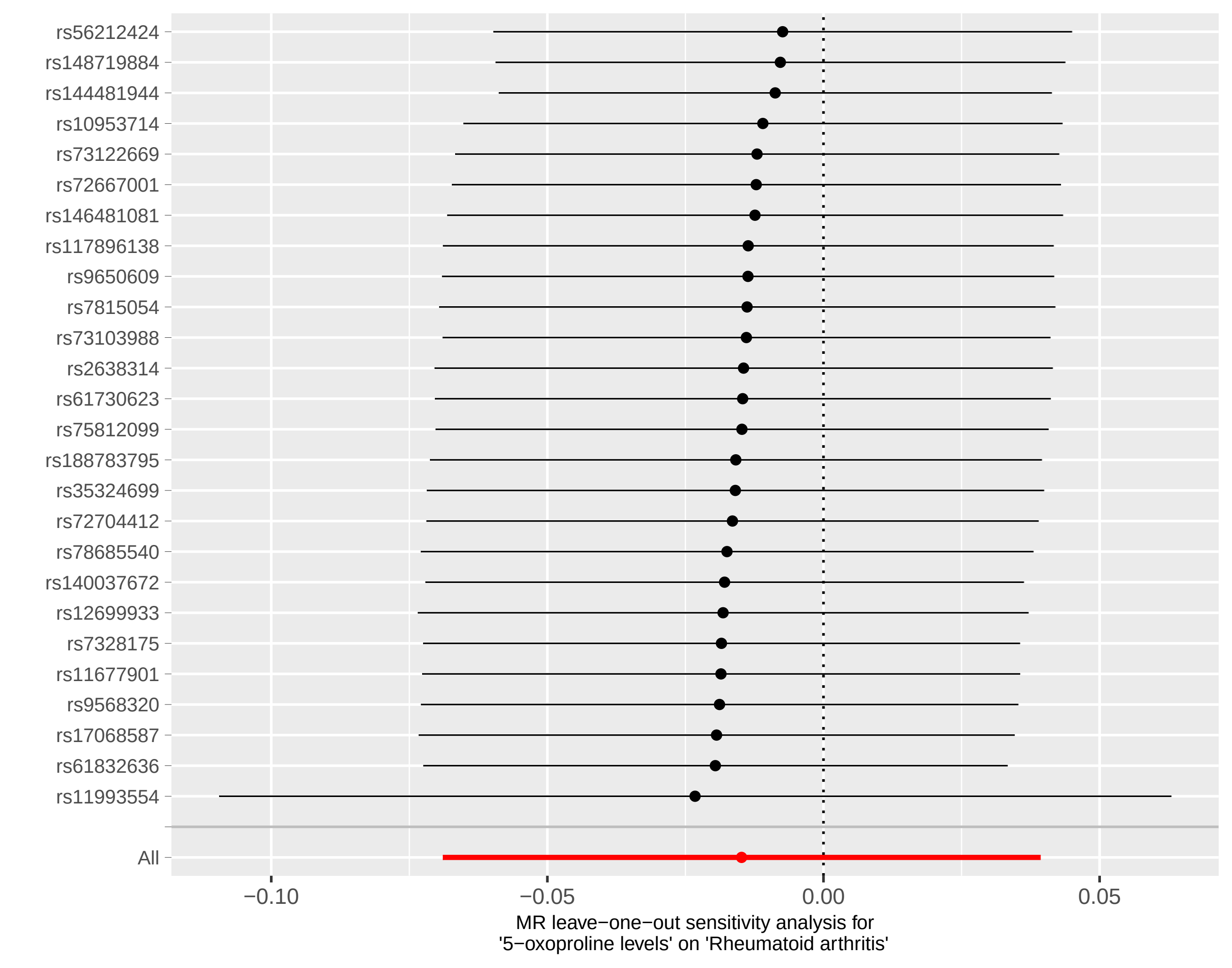
rs56212424
rs148719884
rs144481944
rs10953714
rs73122669
rs72667001
rs146481081
rs117896138
rs9650609
rs7815054
rs73103988
rs2638314
rs61730623
rs75812099
rs188783795
rs35324699
rs72704412
rs78685540
rs140037672
rs12699933
rs7328175
rs11677901
rs9568320
rs17068587
rs61832636
rs11993554
All
−0.10
−0.05
0.00
0.05
MR leave−one−out sensitivity analysis for
'5−oxoproline levels' on 'Rheumatoid arthritis'

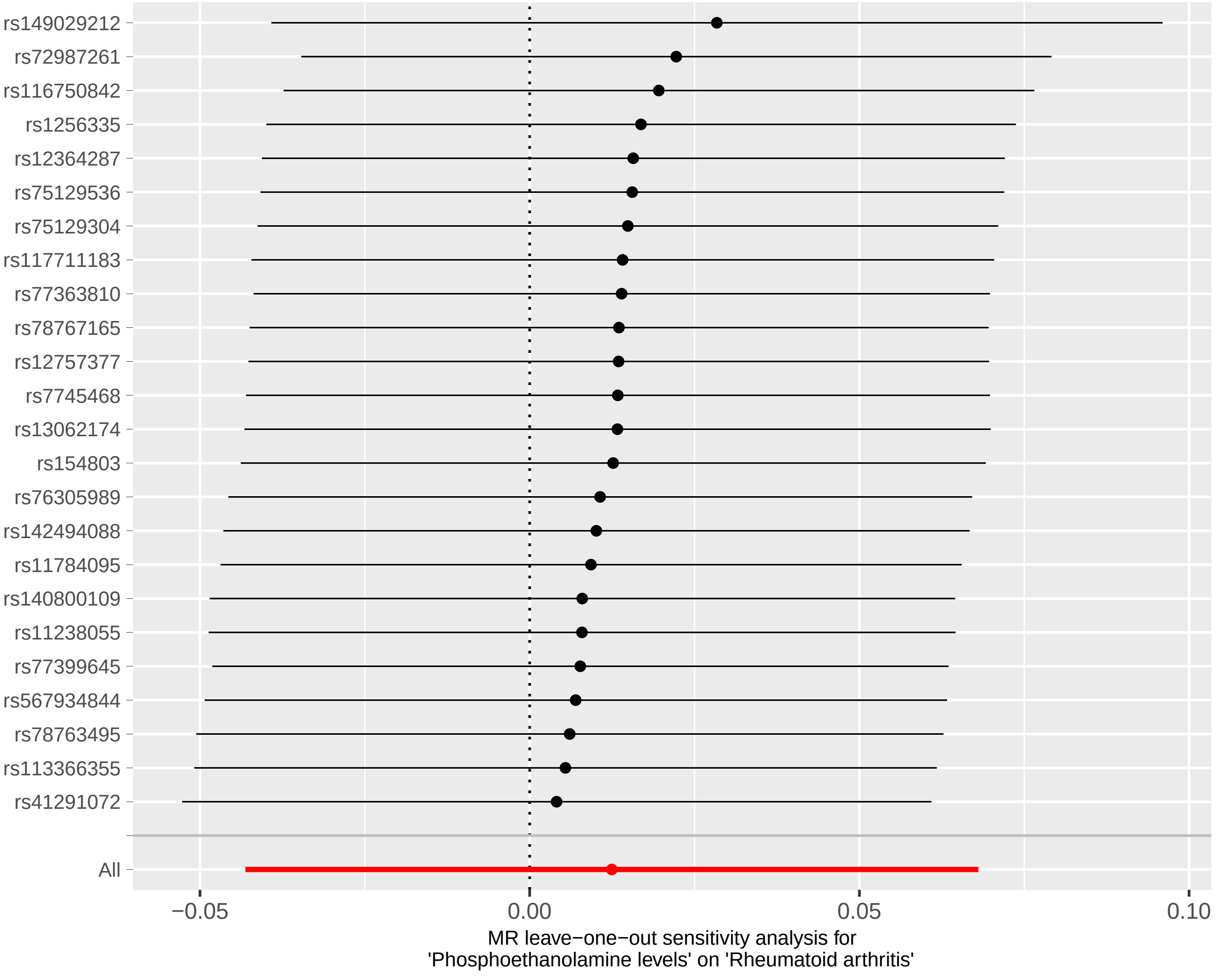

rs149029212
rs72987261
rs116750842
rs1256335
rs12364287
rs75129536
rs75129304
rs117711183
rs77363810
rs78767165
rs12757377
rs7745468
rs13062174
rs154803
rs76305989
rs142494088
rs11784095
rs140800109
rs11238055
rs77399645
rs567934844
rs78763495
rs113366355
rs41291072
All
−0.05
0.00
0.05
0.10
MR leave−one−out sensitivity analysis for
'Phosphoethanolamine levels' on 'Rheumatoid arthritis'

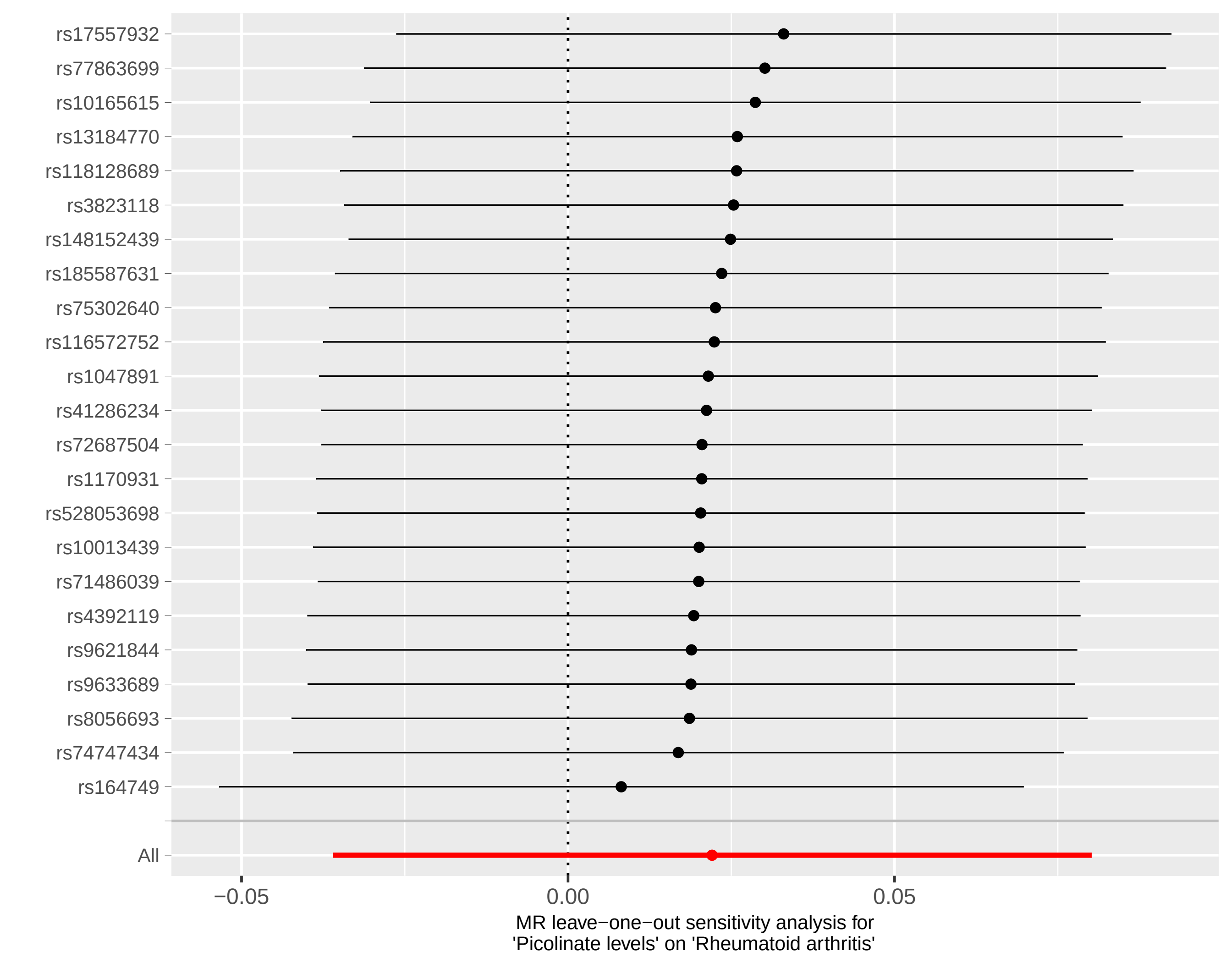

rs17557932
rs77863699
rs10165615
rs13184770
rs118128689
rs3823118
rs148152439
rs185587631
rs75302640
rs116572752
rs1047891
rs41286234
rs72687504
rs1170931
rs528053698
rs10013439
rs71486039
rs4392119
rs9621844
rs9633689
rs8056693
rs74747434
rs164749
All
−0.05
0.00
0.05
MR leave−one−out sensitivity analysis for
'Picolinate levels' on 'Rheumatoid arthritis'

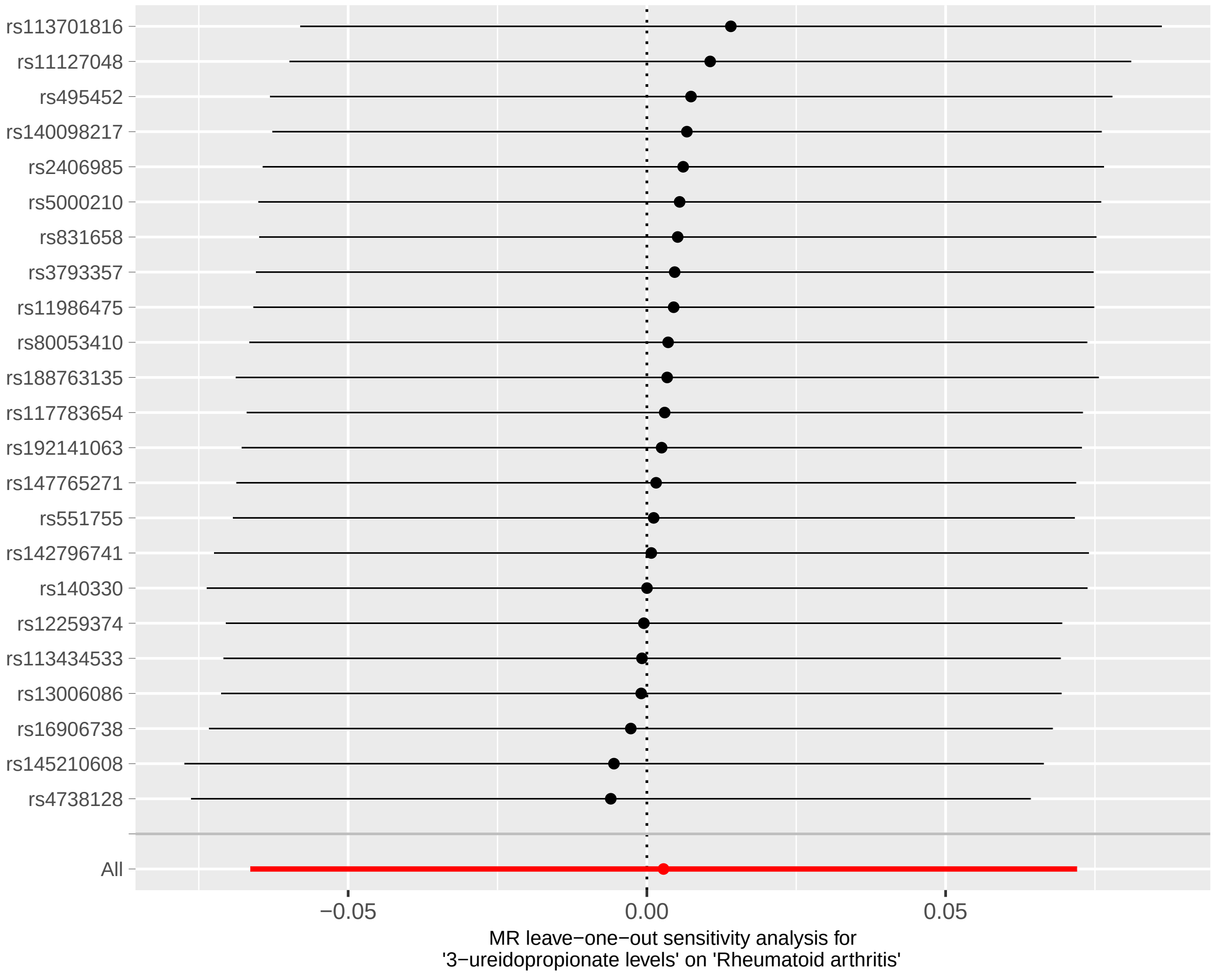
rs113701816
rs11127048
rs495452
rs140098217
rs2406985
rs5000210
rs831658
rs3793357
rs11986475
rs80053410
rs188763135
rs117783654
rs192141063
rs147765271
rs551755
rs142796741
rs140330
rs12259374
rs113434533
rs13006086
rs16906738
rs145210608
rs4738128
All
−0.05
0.00
0.05
MR leave−one−out sensitivity analysis for
'3−ureidopropionate levels' on 'Rheumatoid arthritis'

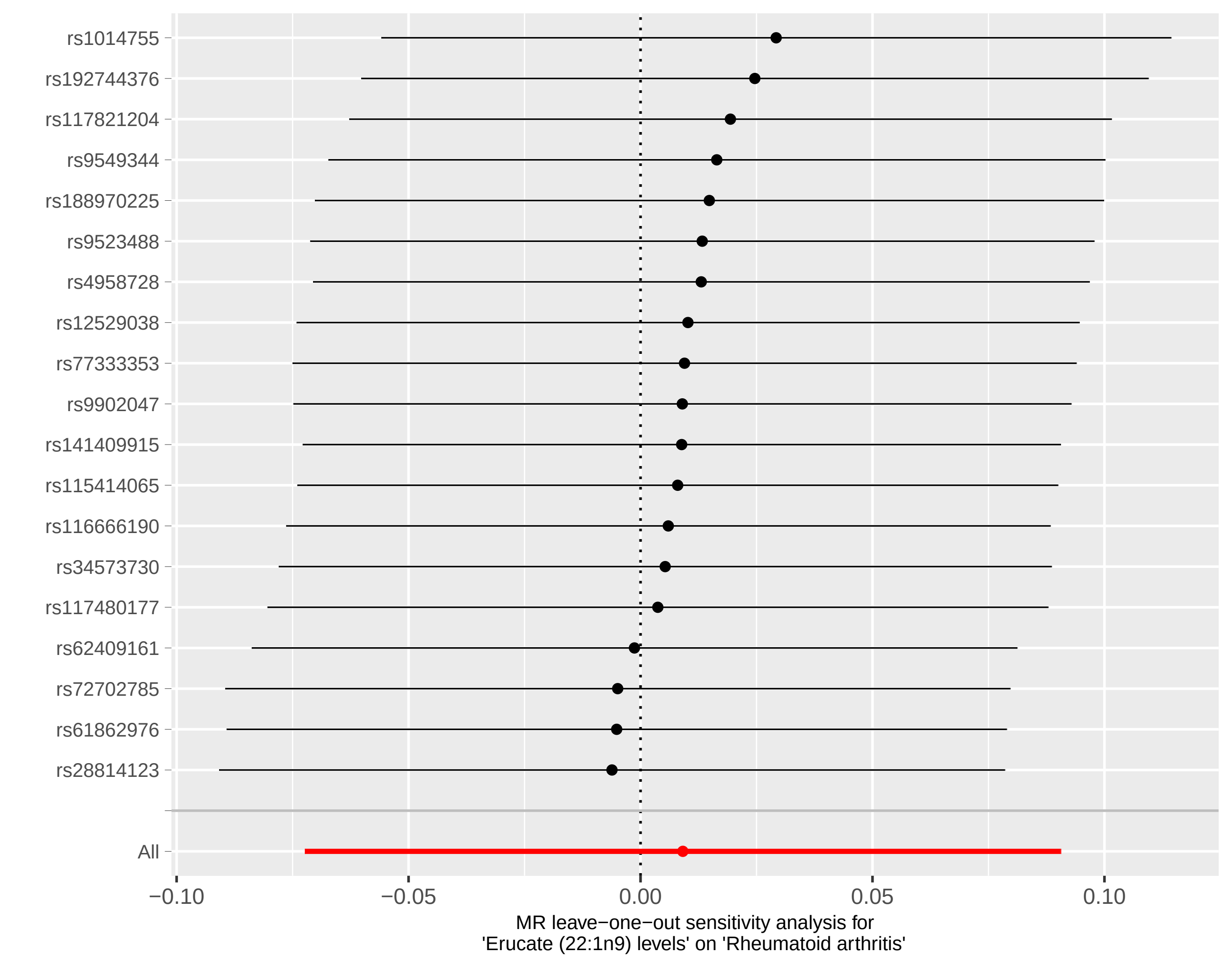
rs1014755
rs192744376
rs117821204
rs9549344
rs188970225
rs9523488
rs4958728
rs12529038
rs77333353
rs9902047
rs141409915
rs115414065
rs116666190
rs34573730
rs117480177
rs62409161
rs72702785
rs61862976
rs28814123
All
−0.10
−0.05
0.00
0.05
0.10
MR leave−one−out sensitivity analysis for
'Erucate (22:1n9) levels' on 'Rheumatoid arthritis'

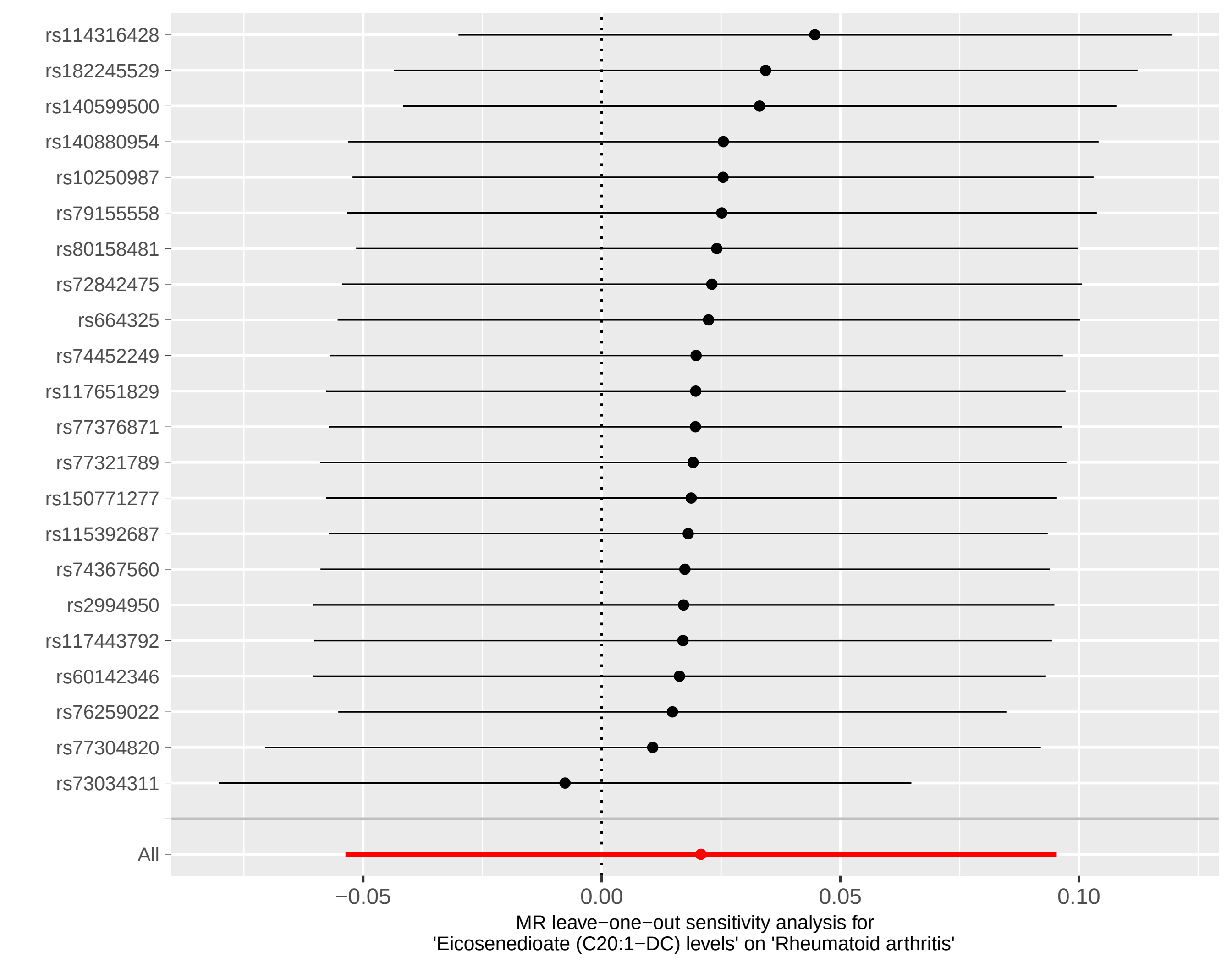
rs114316428
rs182245529
rs140599500
rs140880954
rs10250987
rs79155558
rs80158481
rs72842475
rs664325
rs74452249
rs117651829
rs77376871
rs77321789
rs150771277
rs115392687
rs74367560
rs2994950
rs117443792
rs60142346
rs76259022
rs77304820
rs73034311
All
−0.05
0.00
0.05
0.10
MR leave−one−out sensitivity analysis for
'Eicosenedioate (C20:1−DC) levels' on 'Rheumatoid arthritis'

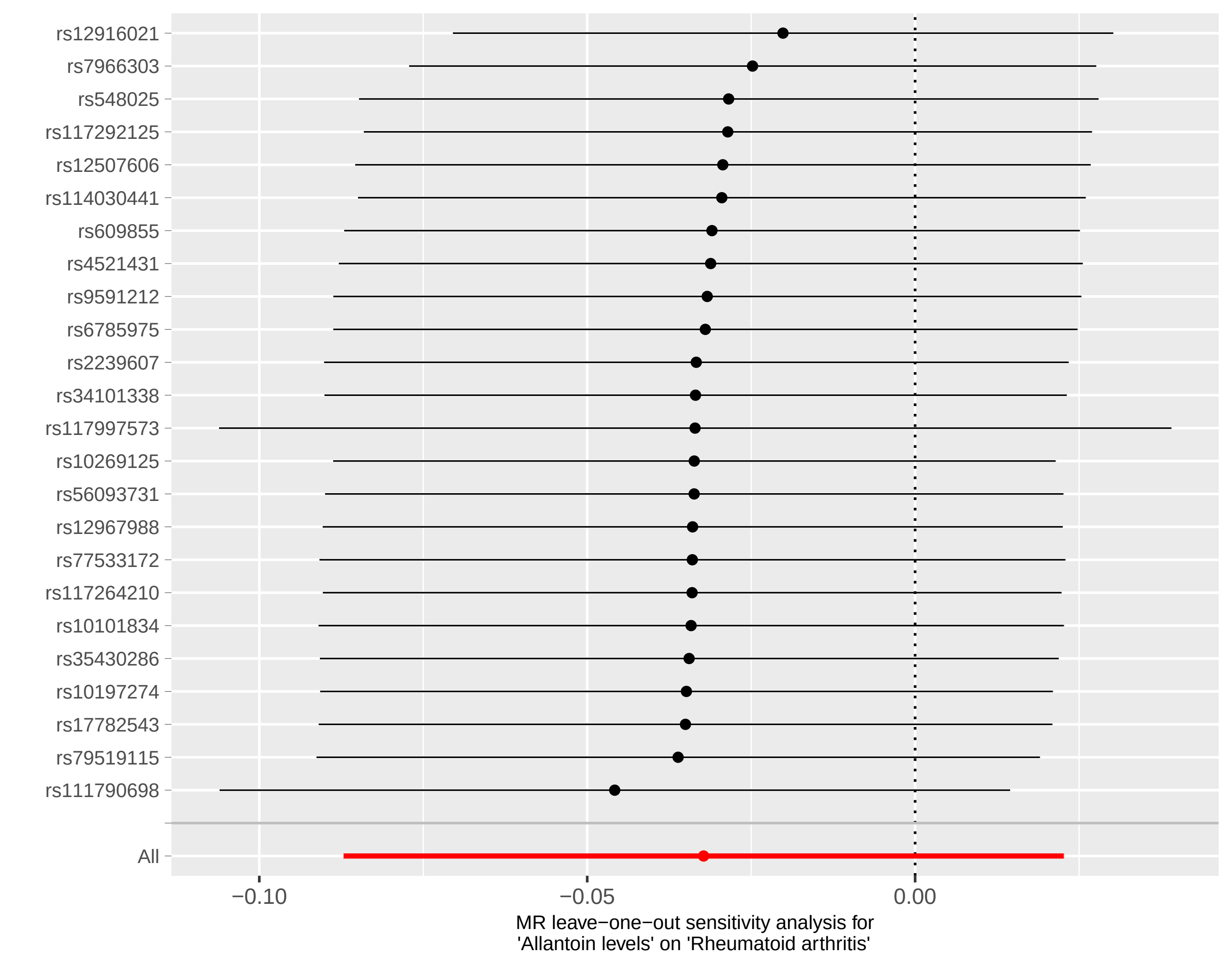
rs12916021
rs7966303
rs548025
rs117292125
rs12507606
rs114030441
rs609855
rs4521431
rs9591212
rs6785975
rs2239607
rs34101338
rs117997573
rs10269125
rs56093731
rs12967988
rs77533172
rs117264210
rs10101834
rs35430286
rs10197274
rs17782543
rs79519115
rs111790698
All
−0.10
−0.05
0.00
MR leave−one−out sensitivity analysis for
'Allantoin levels' on 'Rheumatoid arthritis'

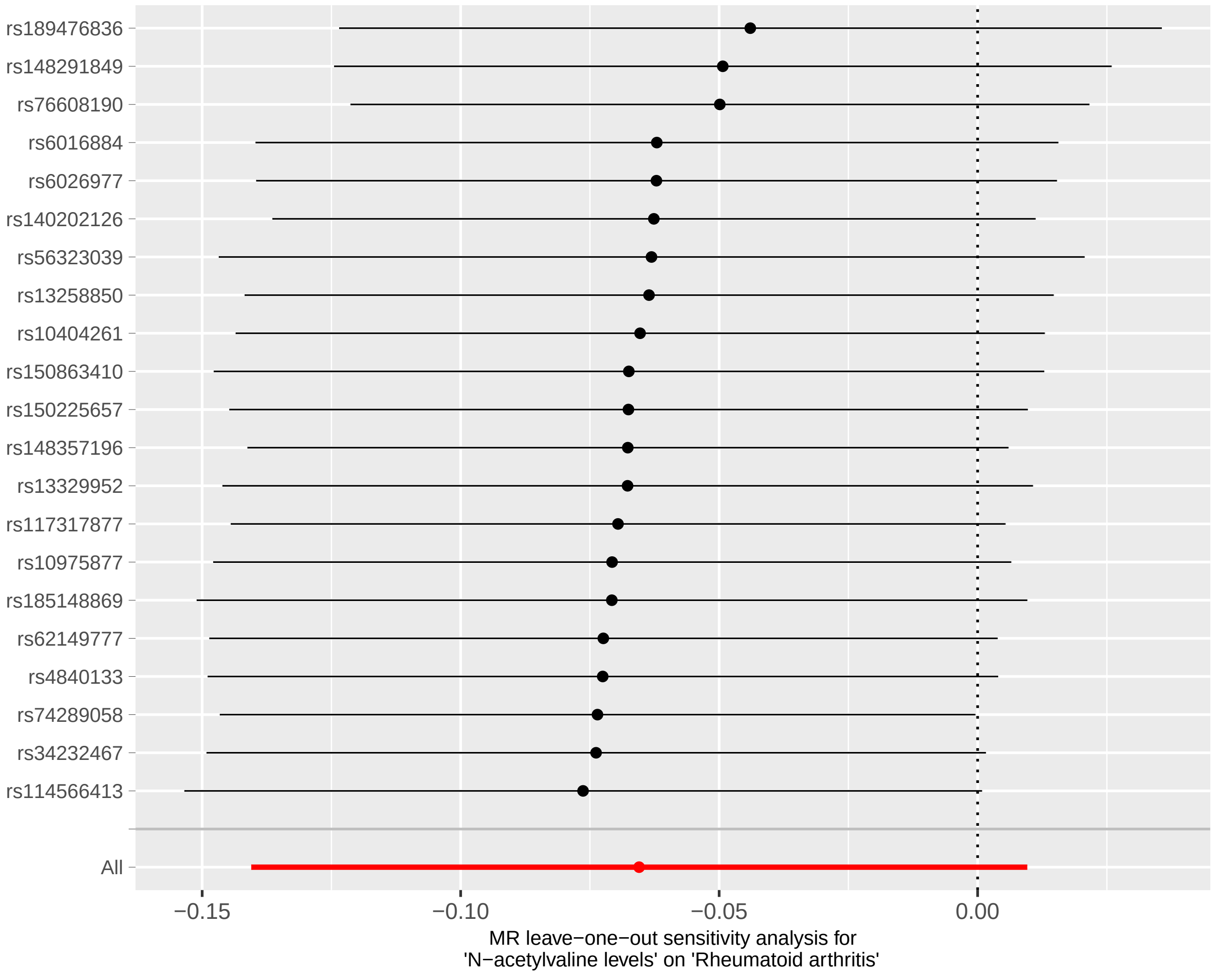
rs189476836
rs148291849
rs76608190
rs6016884
rs6026977
rs140202126
rs56323039
rs13258850
rs10404261
rs150863410
rs150225657
rs148357196
rs13329952
rs117317877
rs10975877
rs185148869
rs62149777
rs4840133
rs74289058
rs34232467
rs114566413
All
−0.15
−0.10
−0.05
0.00
MR leave−one−out sensitivity analysis for
'N−acetylvaline levels' on 'Rheumatoid arthritis'

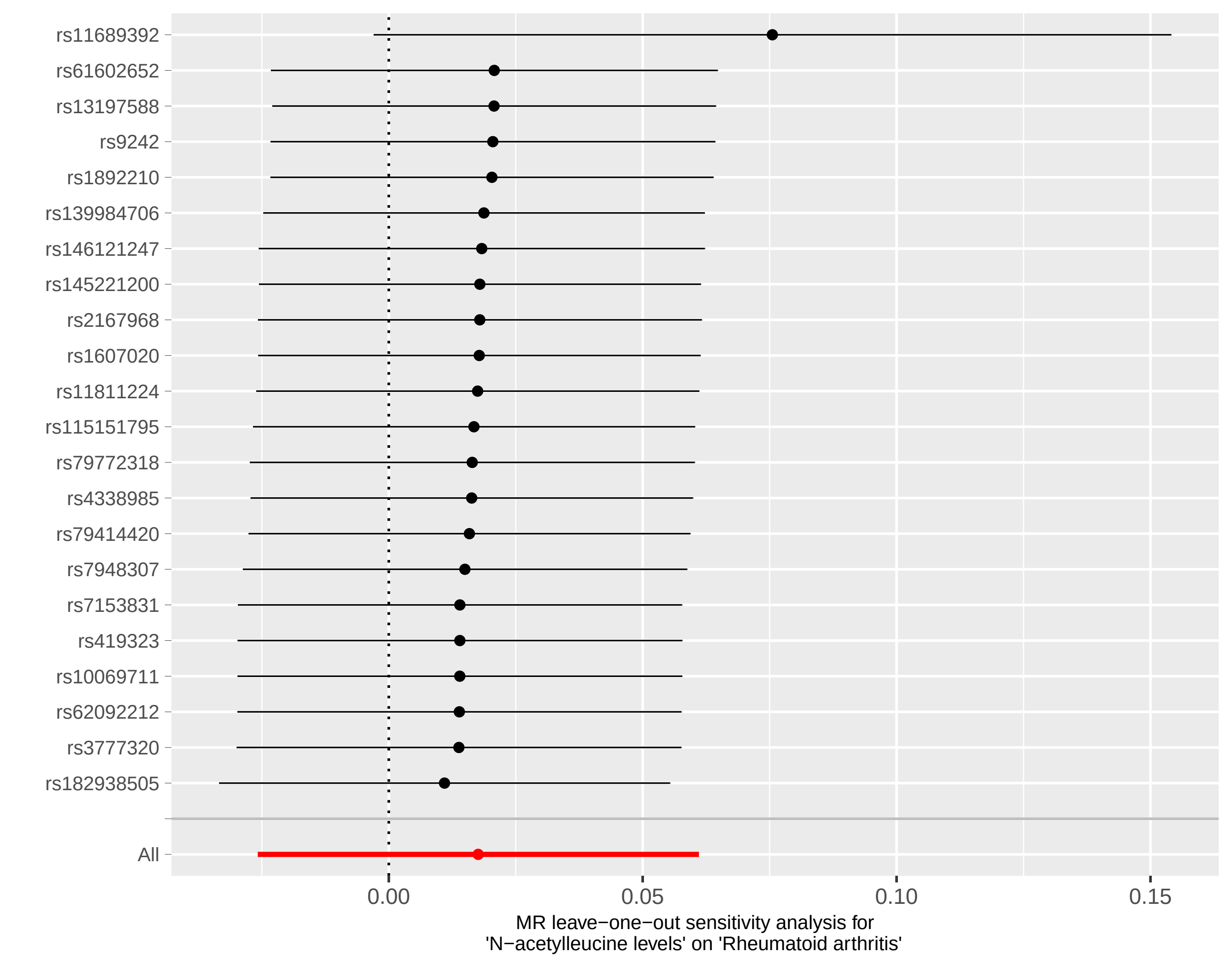

rs11689392
rs61602652
rs13197588
rs9242
rs1892210
rs139984706
rs146121247
rs145221200
rs2167968
rs1607020
rs11811224
rs115151795
rs79772318
rs4338985
rs79414420
rs7948307
rs7153831
rs419323
rs10069711
rs62092212
rs3777320
rs182938505
All
0.00
0.05
0.10
0.15
MR leave−one−out sensitivity analysis for
'N−acetylleucine levels' on 'Rheumatoid arthritis'

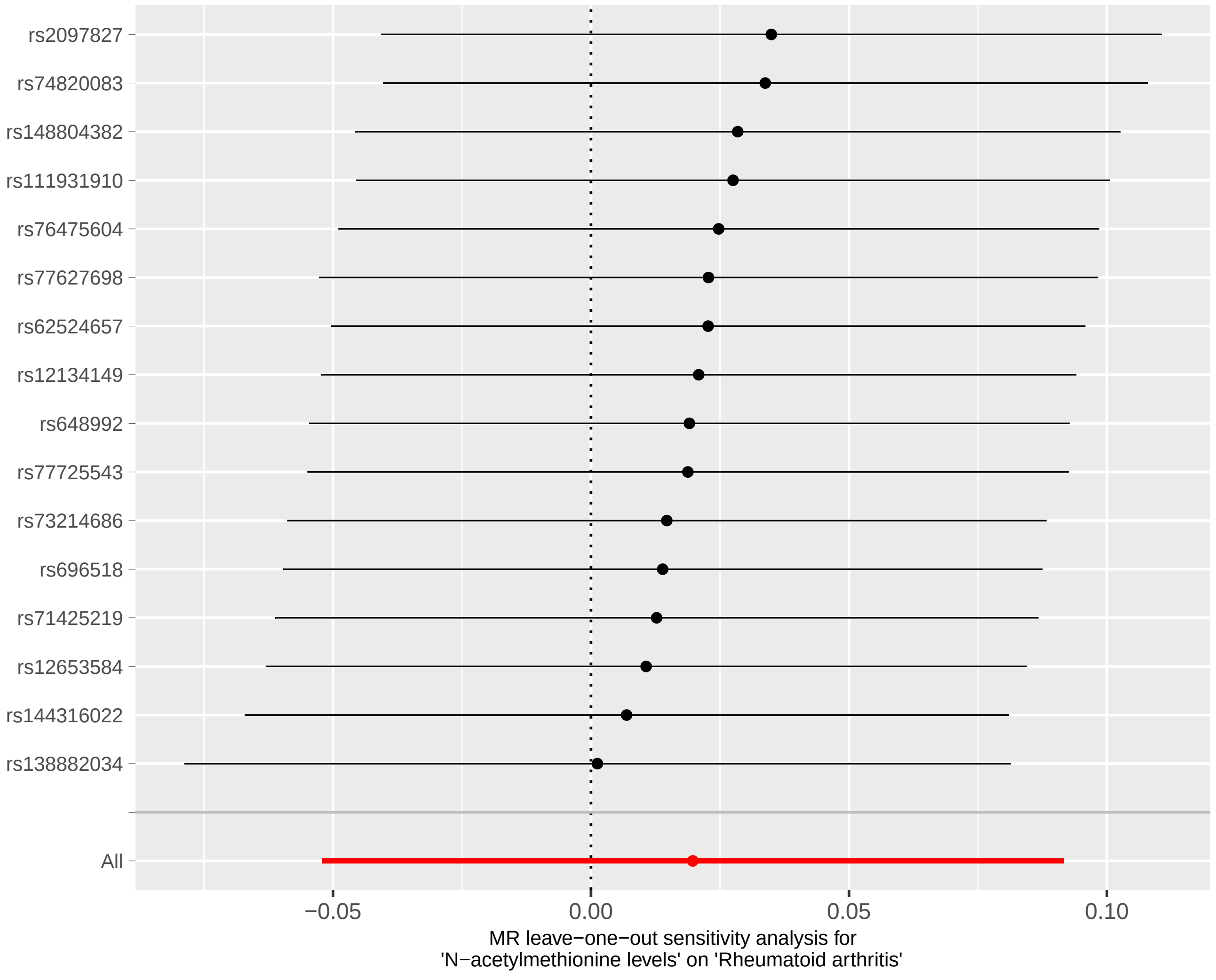
rs2097827
rs74820083
rs148804382
rs111931910
rs76475604
rs77627698
rs62524657
rs12134149
rs648992
rs77725543
rs73214686
rs696518
rs71425219
rs12653584
rs144316022
rs138882034
All
−0.05
0.00
0.05
0.10
MR leave−one−out sensitivity analysis for
'N−acetylmethionine levels' on 'Rheumatoid arthritis'

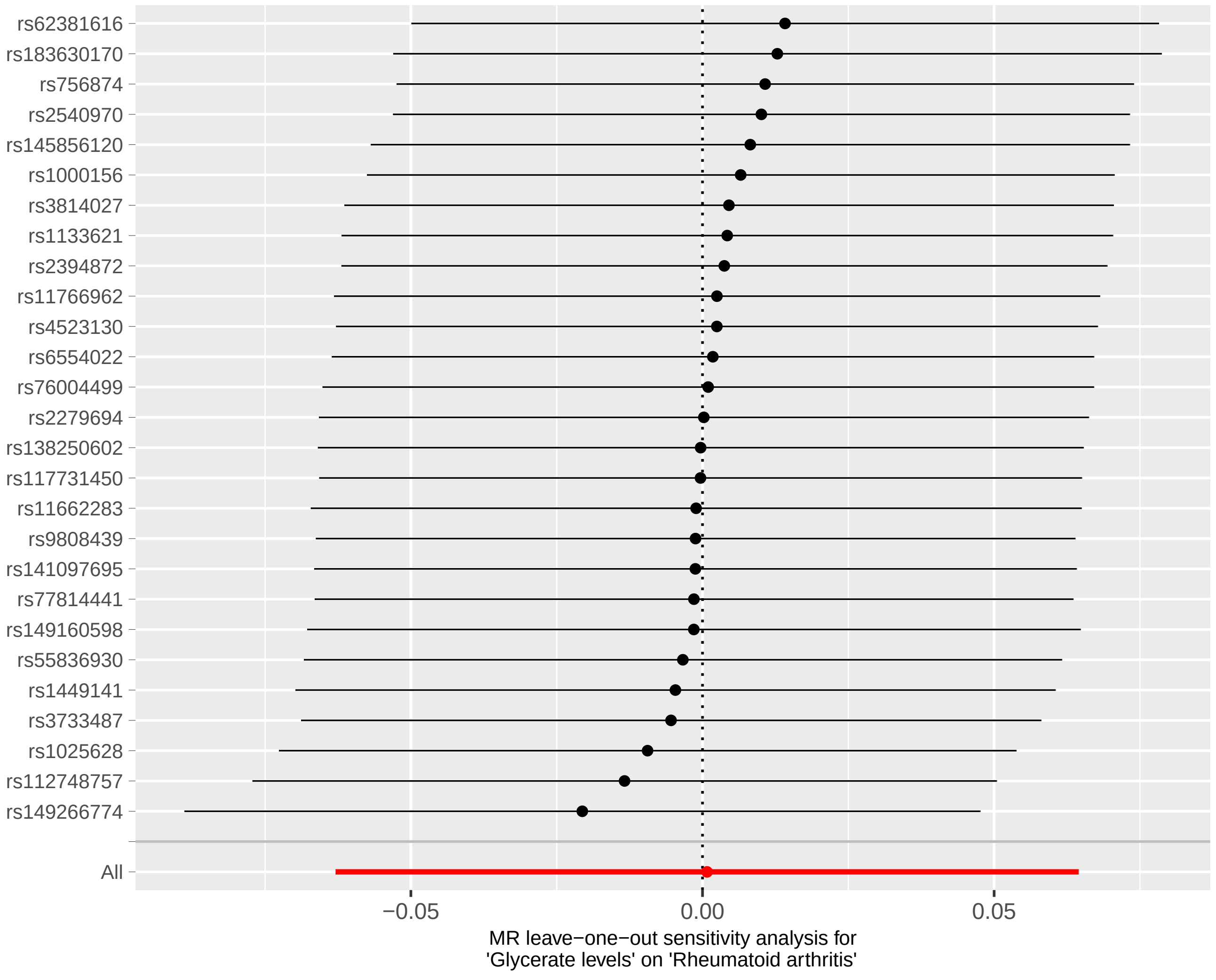
rs62381616
rs183630170
rs756874
rs2540970
rs145856120
rs1000156
rs3814027
rs1133621
rs2394872
rs11766962
rs4523130
rs6554022
rs76004499
rs2279694
rs138250602
rs117731450
rs11662283
rs9808439
rs141097695
rs77814441
rs149160598
rs55836930
rs1449141
rs3733487
rs1025628
rs112748757
rs149266774
All
−0.05
0.00
0.05
MR leave−one−out sensitivity analysis for
'Glycerate levels' on 'Rheumatoid arthritis'

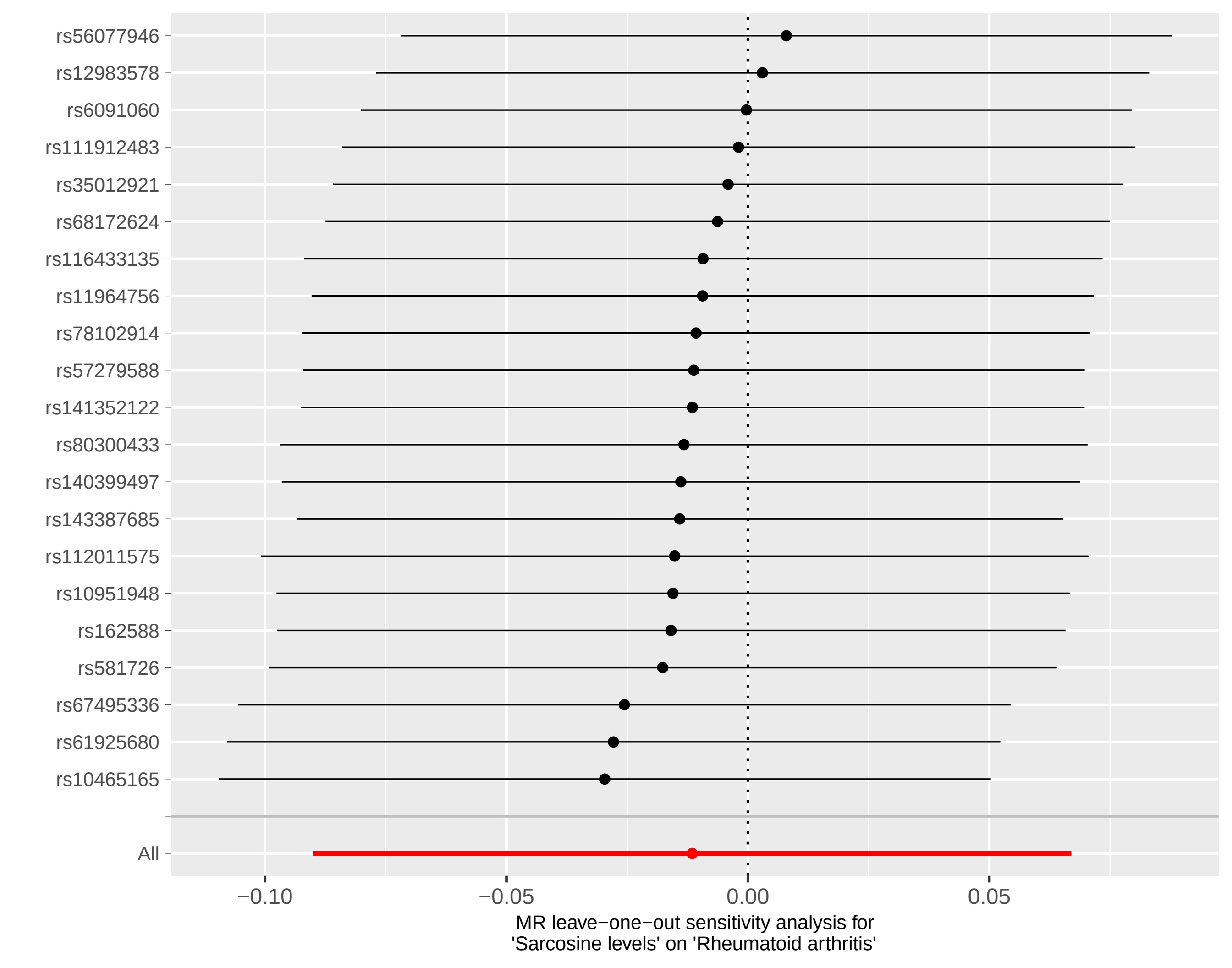
rs56077946
rs12983578
rs6091060
rs111912483
rs35012921
rs68172624
rs116433135
rs11964756
rs78102914
rs57279588
rs141352122
rs80300433
rs140399497
rs143387685
rs112011575
rs10951948
rs162588
rs581726
rs67495336
rs61925680
rs10465165
All
−0.10
−0.05
0.00
0.05
MR leave−one−out sensitivity analysis for
'Sarcosine levels' on 'Rheumatoid arthritis'

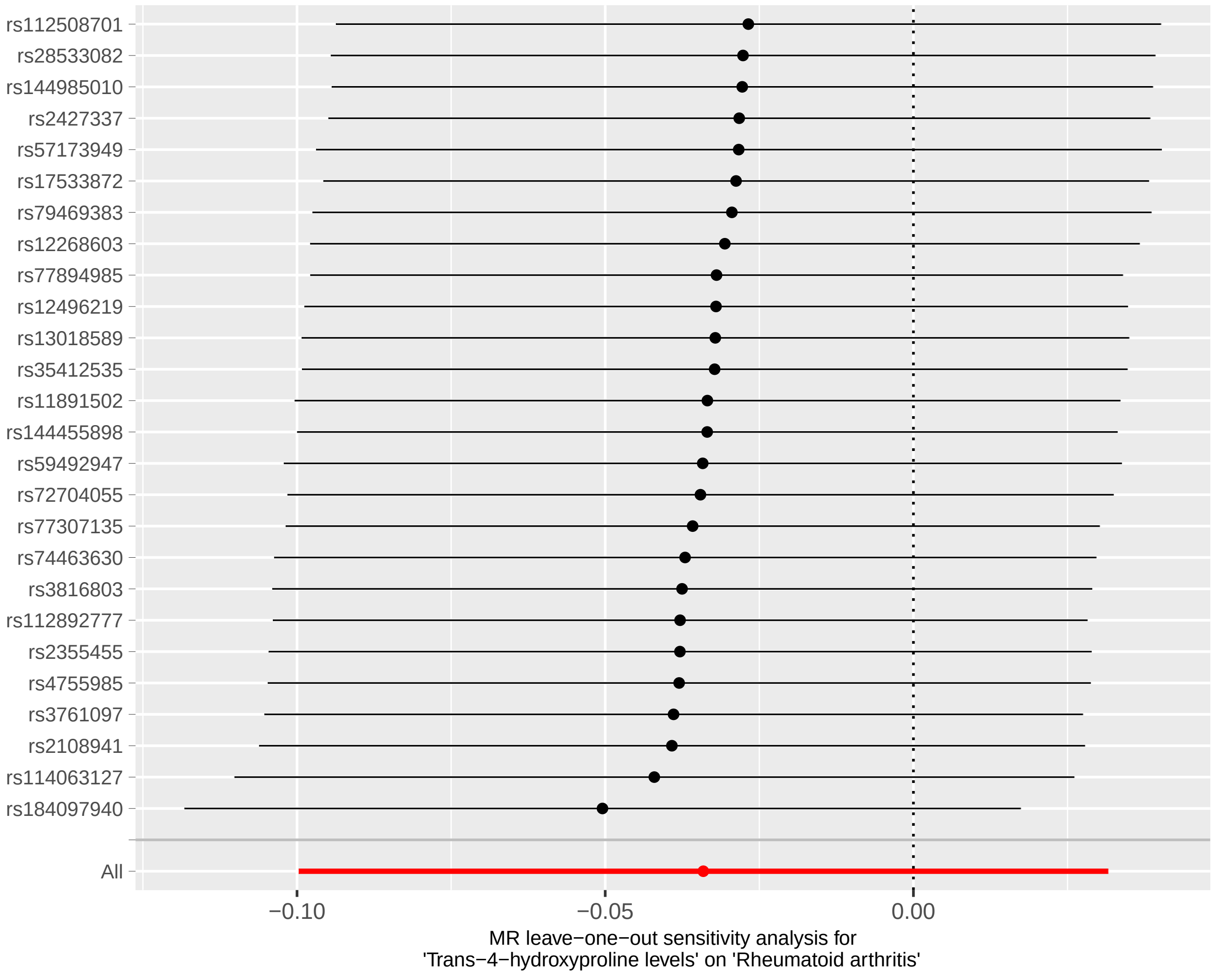
rs112508701
rs28533082
rs144985010
rs2427337
rs57173949
rs17533872
rs79469383
rs12268603
rs77894985
rs12496219
rs13018589
rs35412535
rs11891502
rs144455898
rs59492947
rs72704055
rs77307135
rs74463630
rs3816803
rs112892777
rs2355455
rs4755985
rs3761097
rs2108941
rs114063127
rs184097940
All
−0.10
−0.05
0.00
MR leave−one−out sensitivity analysis for
'Trans−4−hydroxyproline levels' on 'Rheumatoid arthritis'

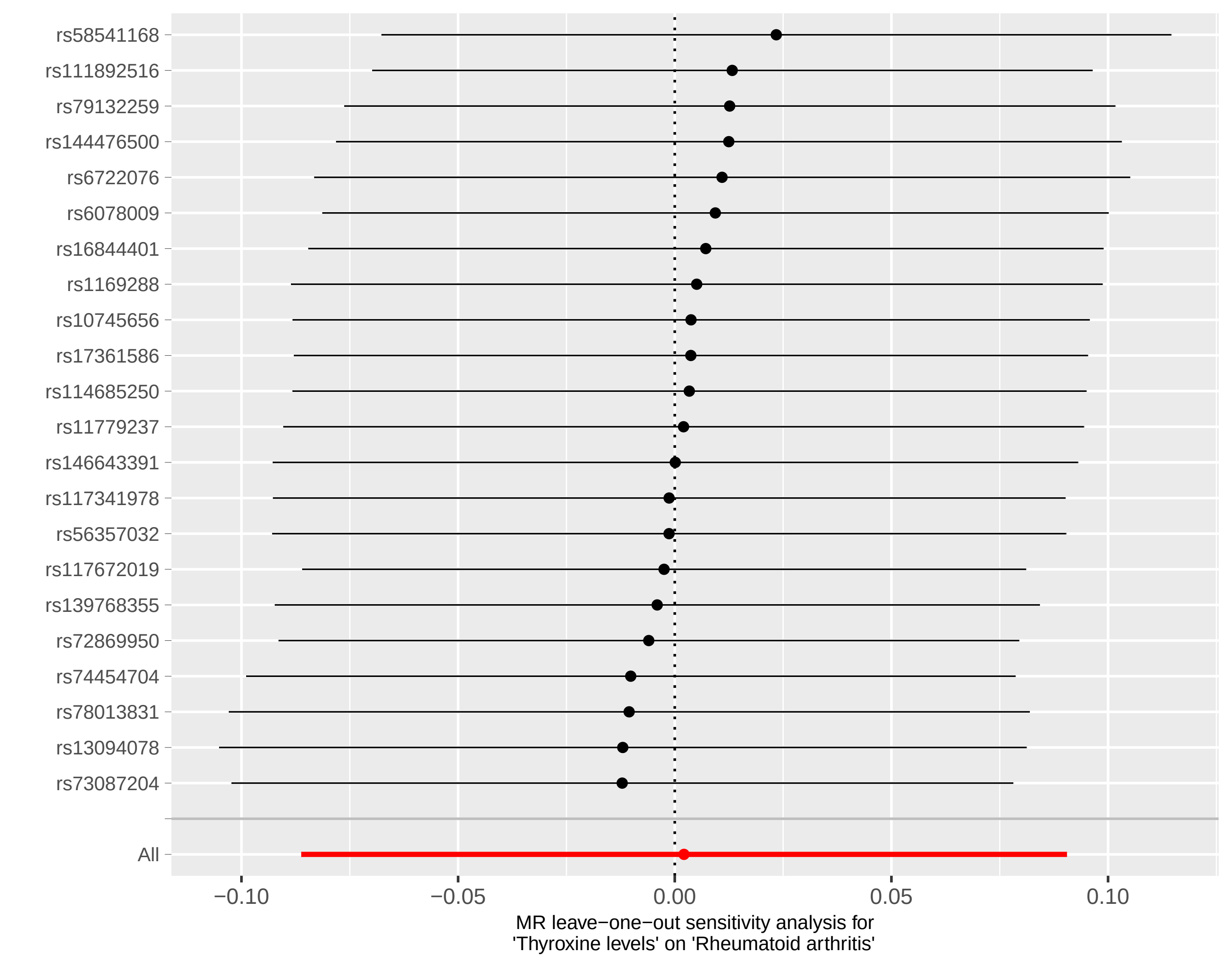
rs58541168
rs111892516
rs79132259
rs144476500
rs6722076
rs6078009
rs16844401
rs1169288
rs10745656
rs17361586
rs114685250
rs11779237
rs146643391
rs117341978
rs56357032
rs117672019
rs139768355
rs72869950
rs74454704
rs78013831
rs13094078
rs73087204
All
−0.10
−0.05
0.00
0.05
0.10
MR leave−one−out sensitivity analysis for
'Thyroxine levels' on 'Rheumatoid arthritis'

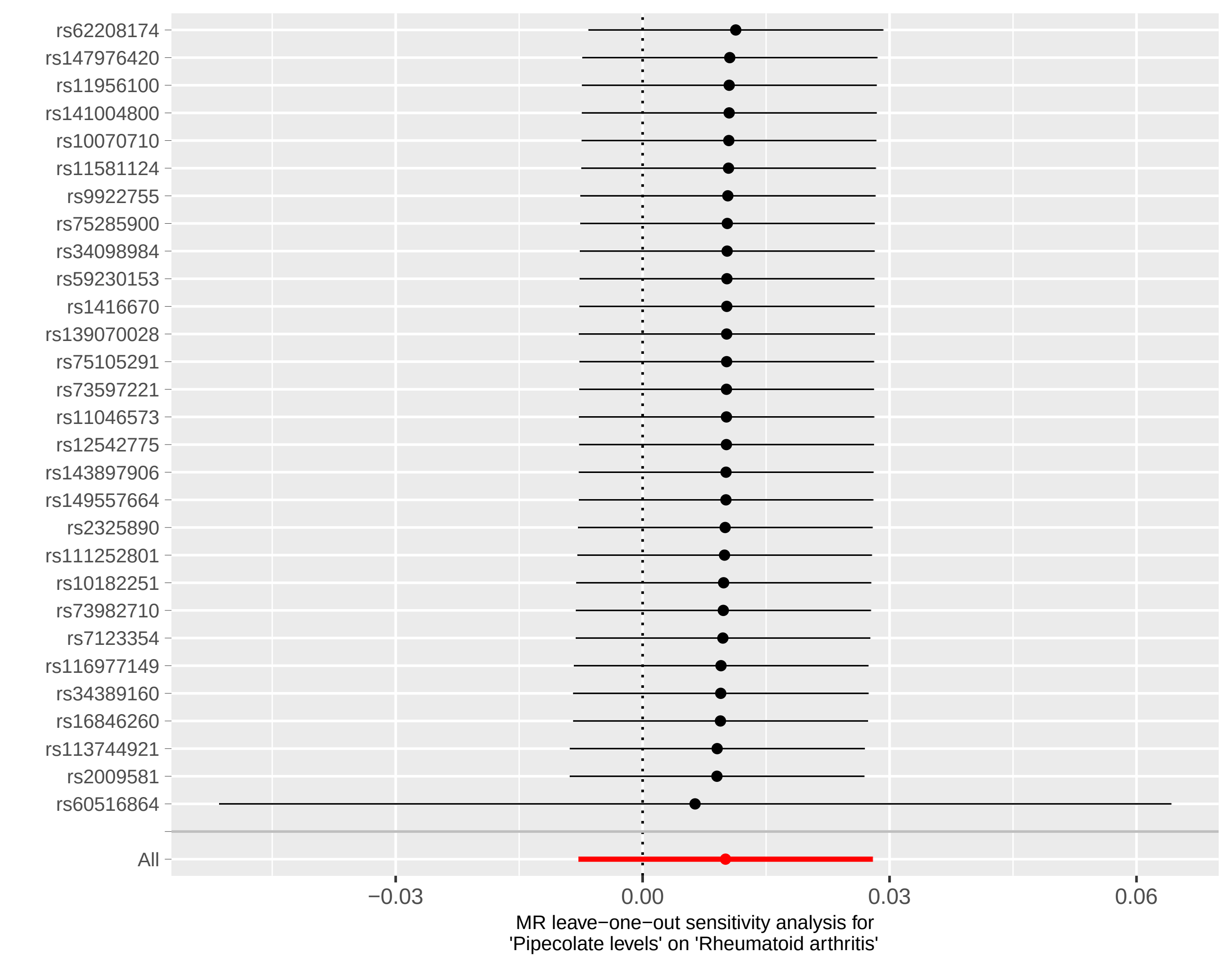
rs62208174
rs147976420
rs11956100
rs141004800
rs10070710
rs11581124
rs9922755
rs75285900
rs34098984
rs59230153
rs1416670
rs139070028
rs75105291
rs73597221
rs11046573
rs12542775
rs143897906
rs149557664
rs2325890
rs111252801
rs10182251
rs73982710
rs7123354
rs116977149
rs34389160
rs16846260
rs113744921
rs2009581
rs60516864
All
−0.03
0.00
0.03
0.06
MR leave−one−out sensitivity analysis for
'Pipecolate levels' on 'Rheumatoid arthritis'

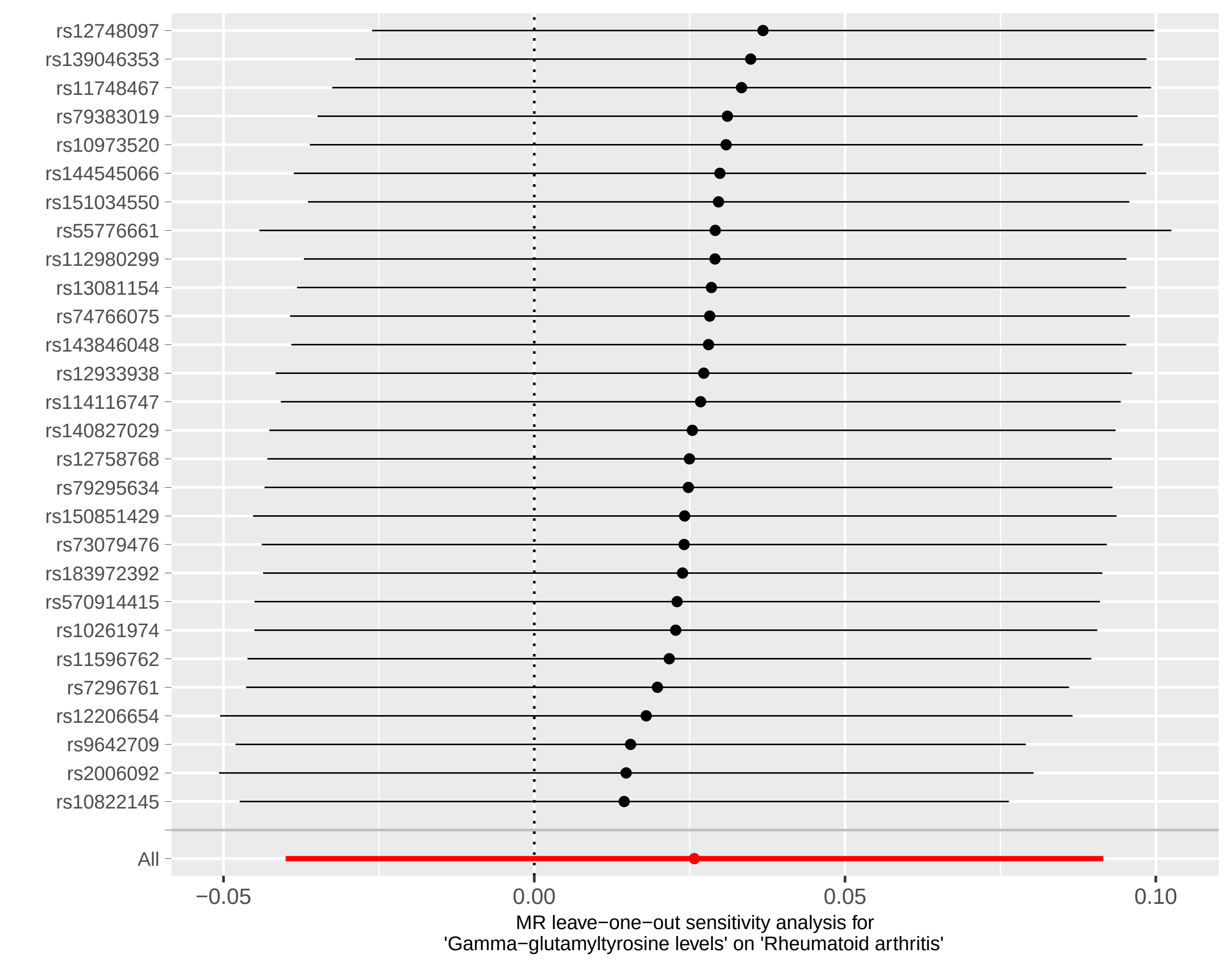
rs12748097
rs139046353
rs11748467
rs79383019
rs10973520
rs144545066
rs151034550
rs55776661
rs112980299
rs13081154
rs74766075
rs143846048
rs12933938
rs114116747
rs140827029
rs12758768
rs79295634
rs150851429
rs73079476
rs183972392
rs570914415
rs10261974
rs11596762
rs7296761
rs12206654
rs9642709
rs2006092
rs10822145
All
−0.05
0.00
0.05
0.10
MR leave−one−out sensitivity analysis for
'Gamma−glutamyltyrosine levels' on 'Rheumatoid arthritis'

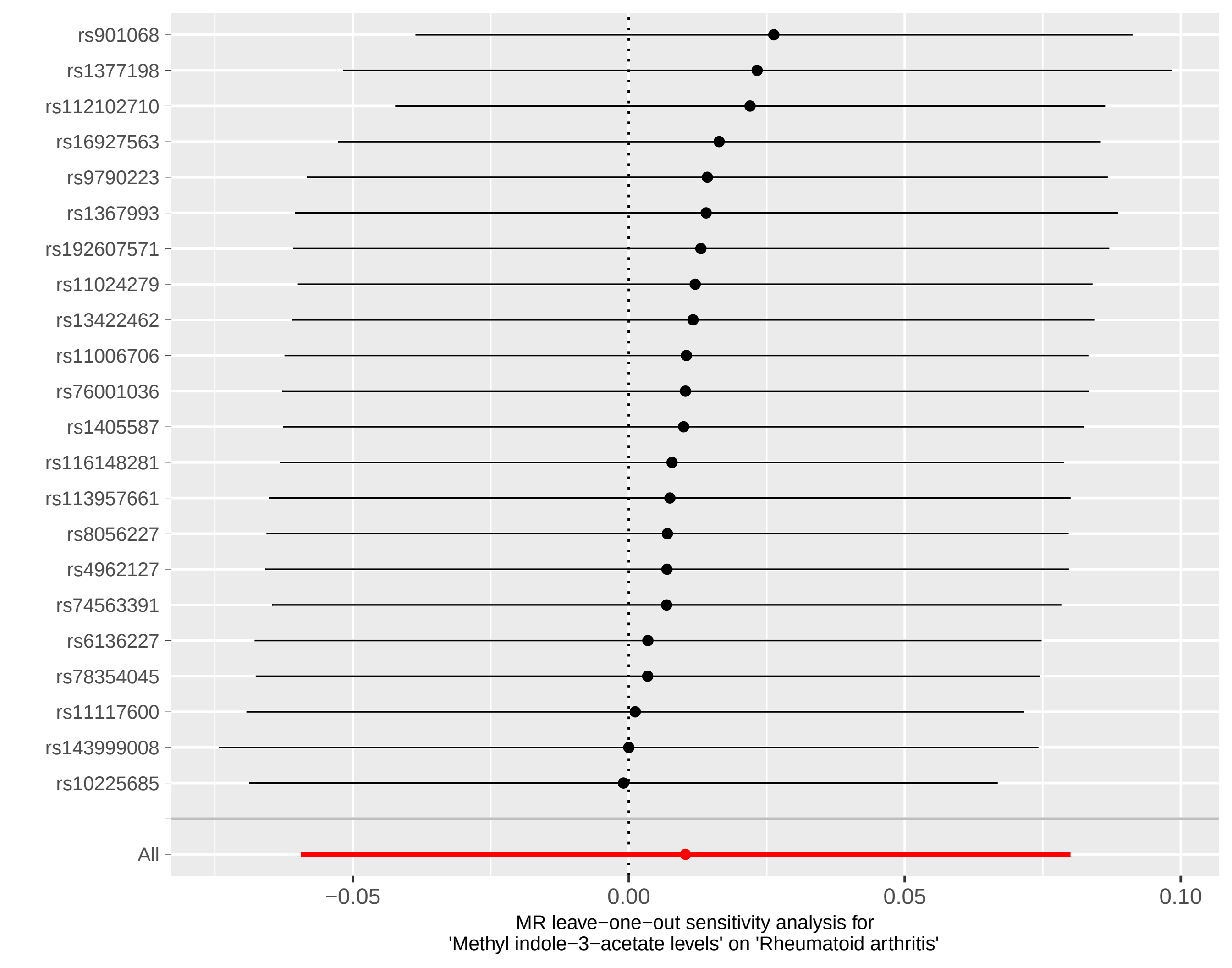

rs901068
rs1377198
rs112102710
rs16927563
rs9790223
rs1367993
rs192607571
rs11024279
rs13422462
rs11006706
rs76001036
rs1405587
rs116148281
rs113957661
rs8056227
rs4962127
rs74563391
rs6136227
rs78354045
rs11117600
rs143999008
rs10225685
All
−0.05
0.00
0.05
0.10
MR leave−one−out sensitivity analysis for
'Methyl indole−3−acetate levels' on 'Rheumatoid arthritis'

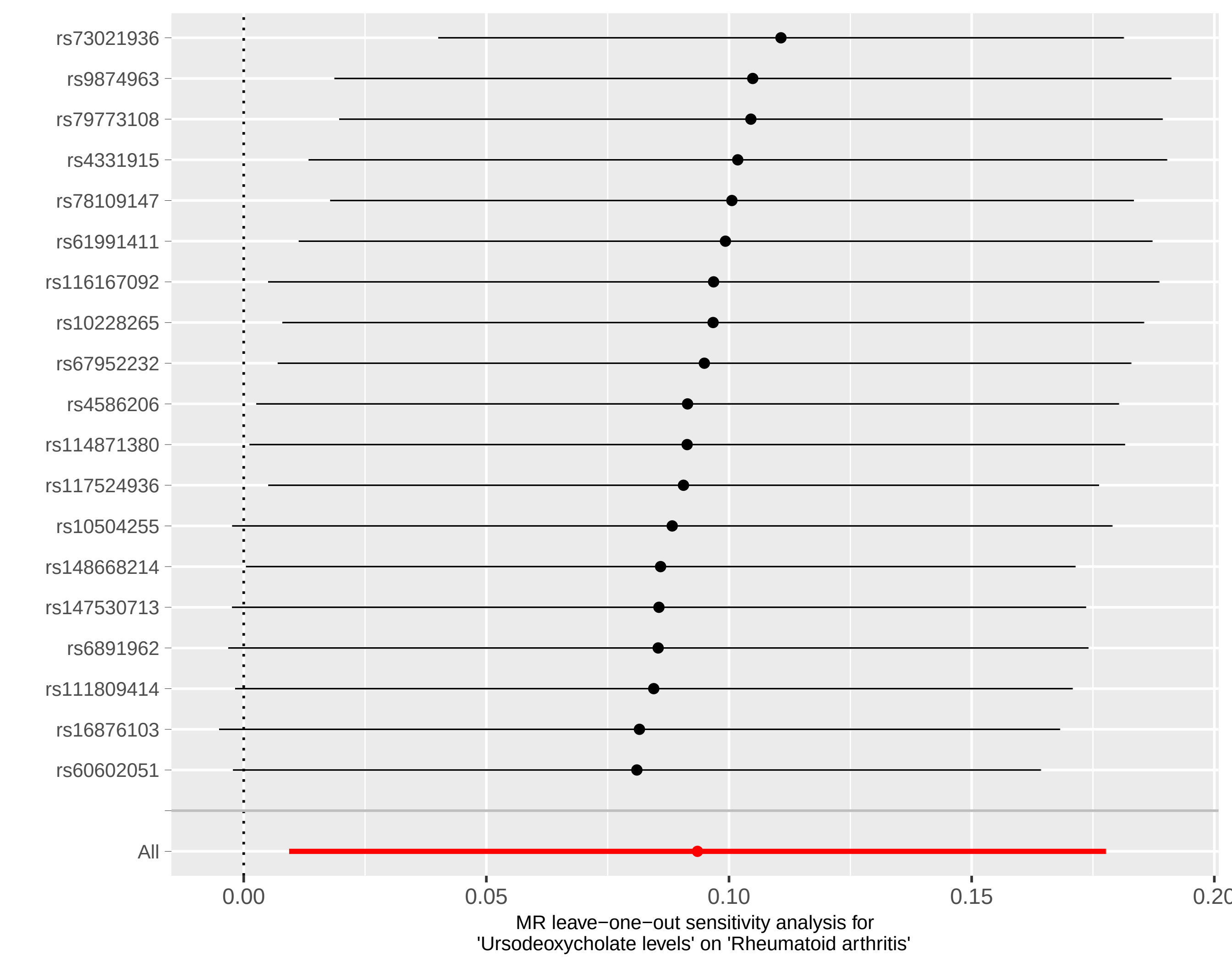
rs73021936
rs9874963
rs79773108
rs4331915
rs78109147
rs61991411
rs116167092
rs10228265
rs67952232
rs4586206
rs114871380
rs117524936
rs10504255
rs148668214
rs147530713
rs6891962
rs111809414
rs16876103
rs60602051
All
0.00
0.05
0.10
0.15
0.20
MR leave−one−out sensitivity analysis for
'Ursodeoxycholate levels' on 'Rheumatoid arthritis'

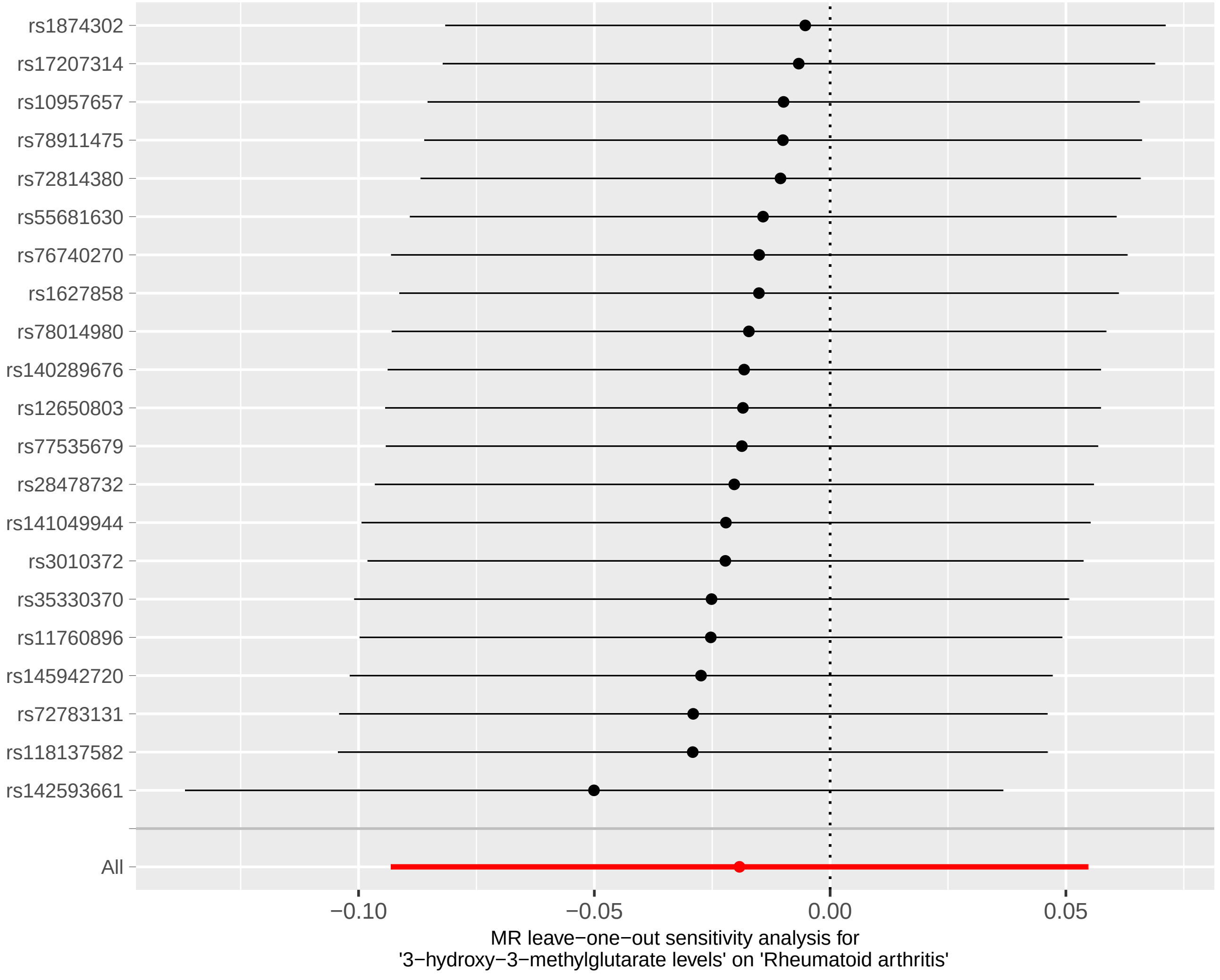
rs1874302
rs17207314
rs10957657
rs78911475
rs72814380
rs55681630
rs76740270
rs1627858
rs78014980
rs140289676
rs12650803
rs77535679
rs28478732
rs141049944
rs3010372
rs35330370
rs11760896
rs145942720
rs72783131
rs118137582
rs142593661
All
−0.10
−0.05
0.00
0.05
MR leave−one−out sensitivity analysis for
'3−hydroxy−3−methylglutarate levels' on 'Rheumatoid arthritis'

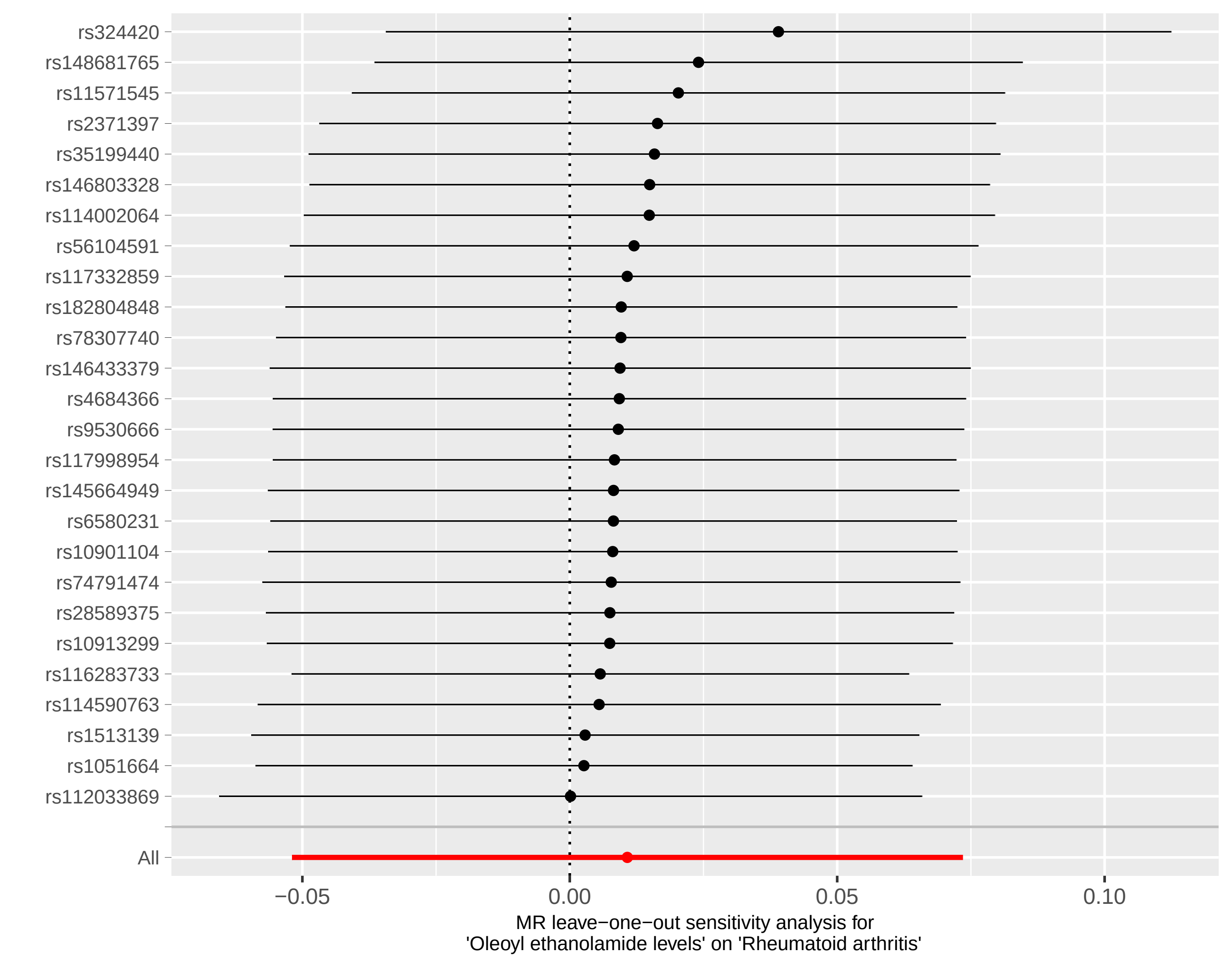

rs324420
rs148681765
rs11571545
rs2371397
rs35199440
rs146803328
rs114002064
rs56104591
rs117332859
rs182804848
rs78307740
rs146433379
rs4684366
rs9530666
rs117998954
rs145664949
rs6580231
rs10901104
rs74791474
rs28589375
rs10913299
rs116283733
rs114590763
rs1513139
rs1051664
rs112033869
All
−0.05
0.00
0.05
0.10
MR leave−one−out sensitivity analysis for
'Oleoyl ethanolamide levels' on 'Rheumatoid arthritis'

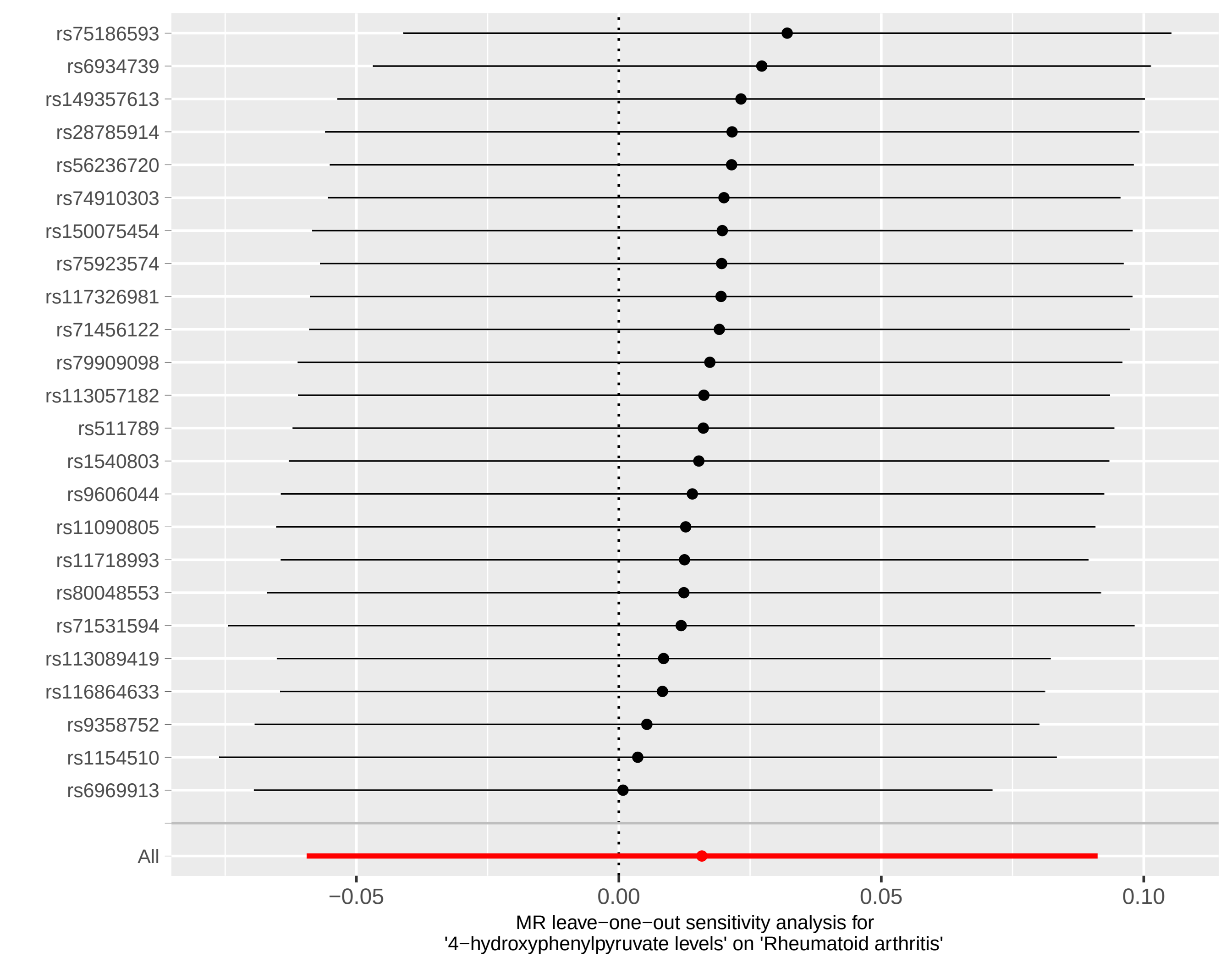
rs75186593
rs6934739
rs149357613
rs28785914
rs56236720
rs74910303
rs150075454
rs75923574
rs117326981
rs71456122
rs79909098
rs113057182
rs511789
rs1540803
rs9606044
rs11090805
rs11718993
rs80048553
rs71531594
rs113089419
rs116864633
rs9358752
rs1154510
rs6969913
All
−0.05
0.00
0.05
0.10
MR leave−one−out sensitivity analysis for
'4−hydroxyphenylpyruvate levels' on 'Rheumatoid arthritis'

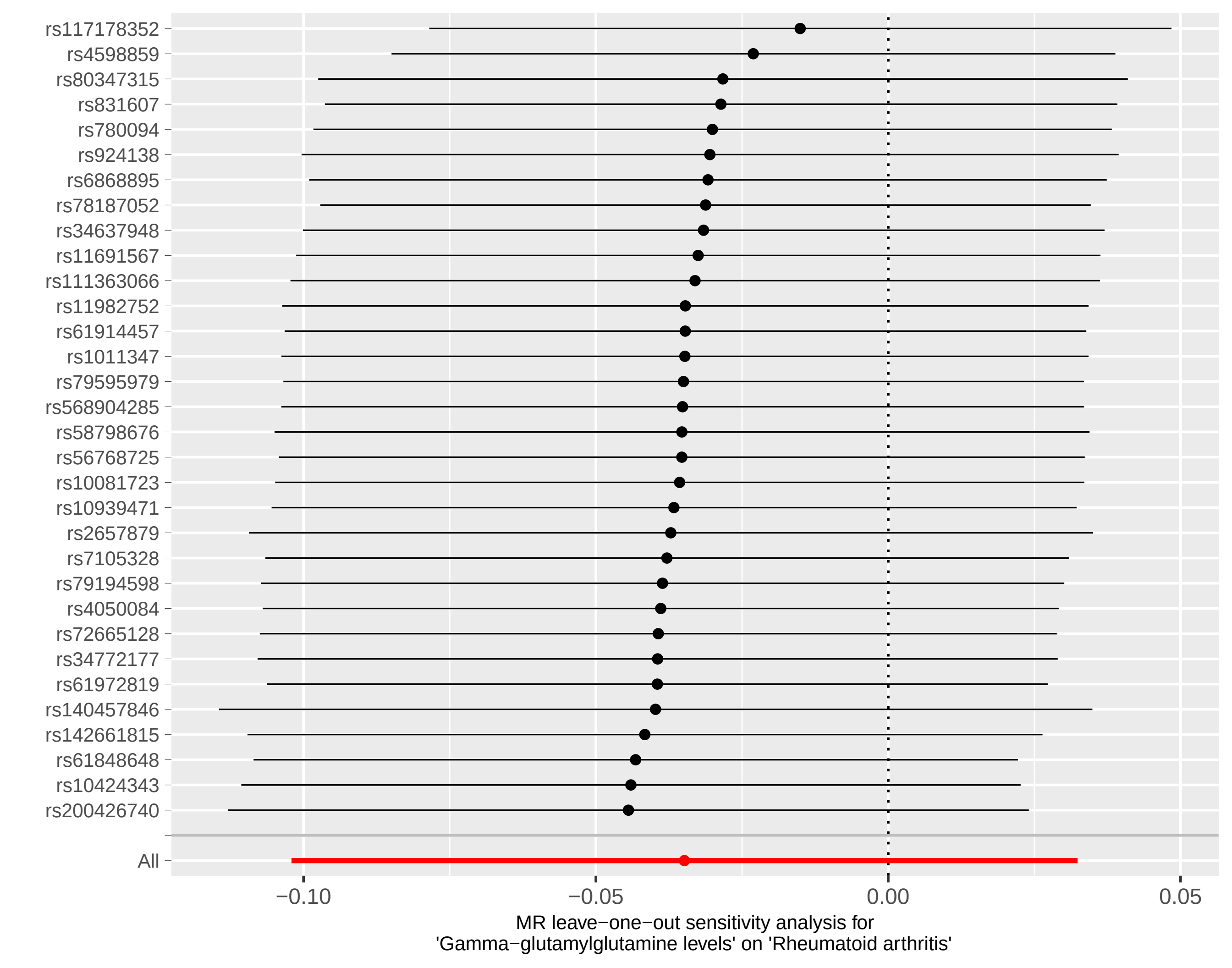
rs117178352
rs4598859
rs80347315
rs831607
rs780094
rs924138
rs6868895
rs78187052
rs34637948
rs11691567
rs111363066
rs11982752
rs61914457
rs1011347
rs79595979
rs568904285
rs58798676
rs56768725
rs10081723
rs10939471
rs2657879
rs7105328
rs79194598
rs4050084
rs72665128
rs34772177
rs61972819
rs140457846
rs142661815
rs61848648
rs10424343
rs200426740
All
−0.10
−0.05
0.00
0.05
MR leave−one−out sensitivity analysis for
'Gamma−glutamylglutamine levels' on 'Rheumatoid arthritis'

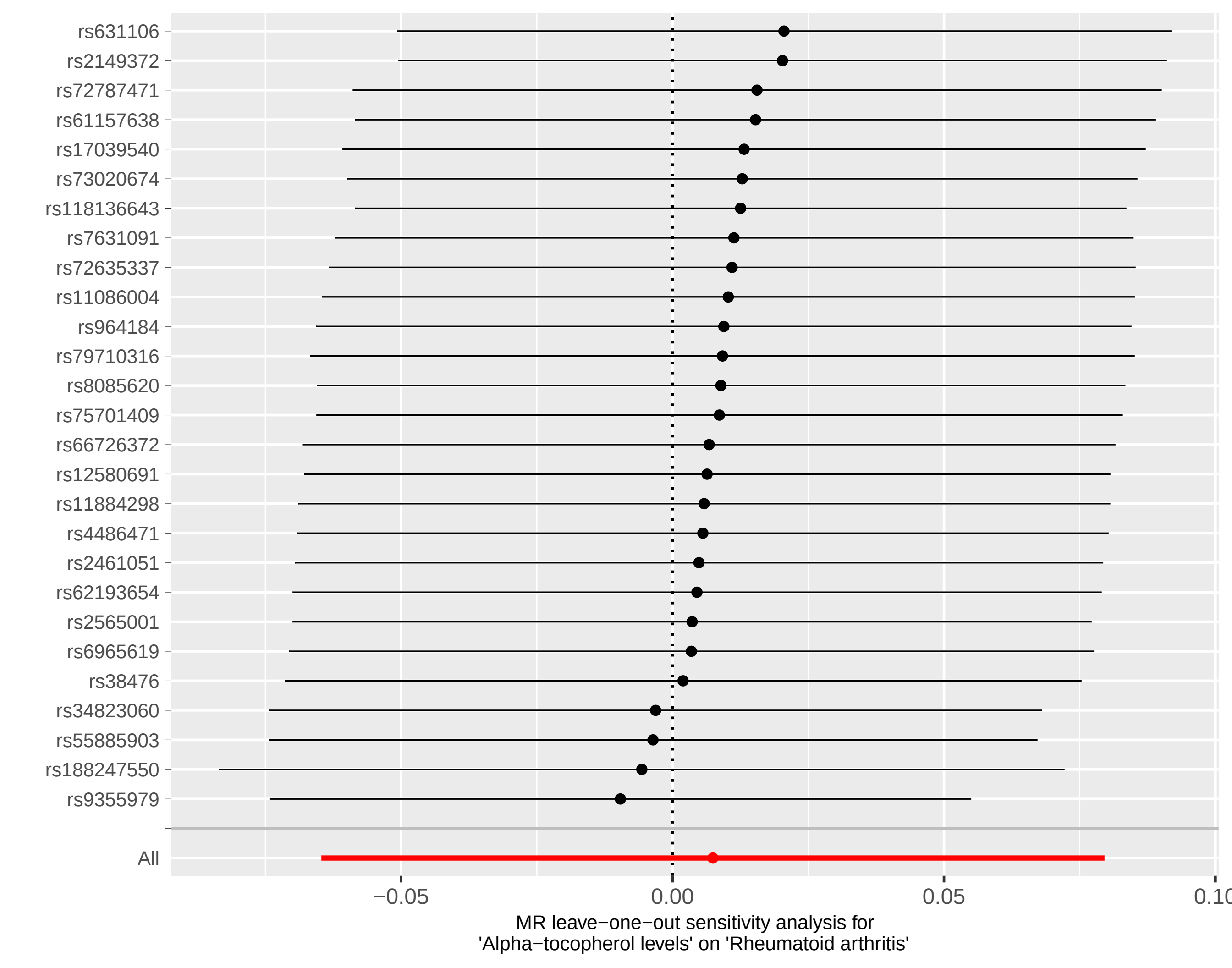
rs631106
rs2149372
rs72787471
rs61157638
rs17039540
rs73020674
rs118136643
rs7631091
rs72635337
rs11086004
rs964184
rs79710316
rs8085620
rs75701409
rs66726372
rs12580691
rs11884298
rs4486471
rs2461051
rs62193654
rs2565001
rs6965619
rs38476
rs34823060
rs55885903
rs188247550
rs9355979
All
−0.05
0.00
0.05
0.10
MR leave−one−out sensitivity analysis for
'Alpha−tocopherol levels' on 'Rheumatoid arthritis'

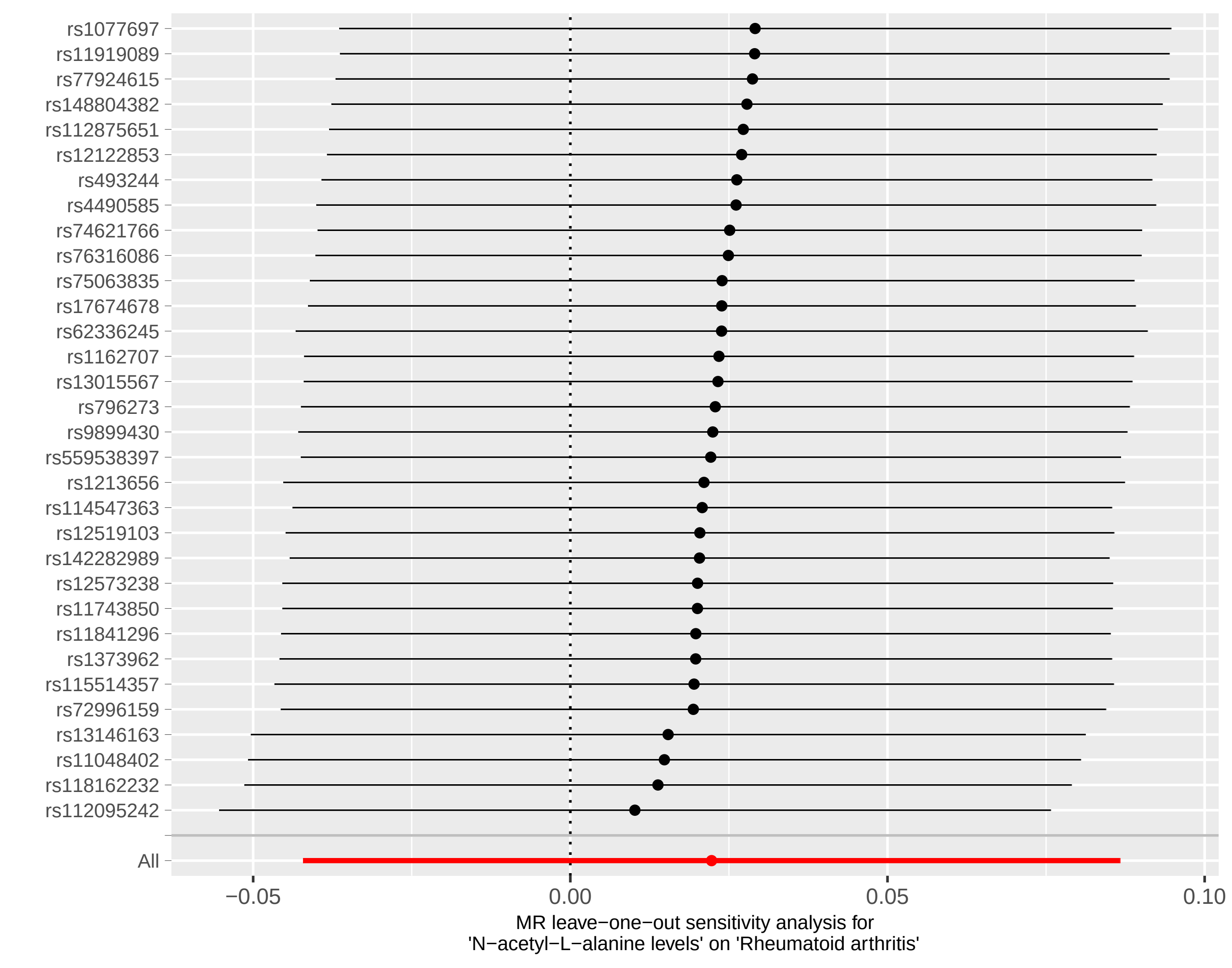
rs1077697
rs11919089
rs77924615
rs148804382
rs112875651
rs12122853
rs493244
rs4490585
rs74621766
rs76316086
rs75063835
rs17674678
rs62336245
rs1162707
rs13015567
rs796273
rs9899430
rs559538397
rs1213656
rs114547363
rs12519103
rs142282989
rs12573238
rs11743850
rs11841296
rs1373962
rs115514357
rs72996159
rs13146163
rs11048402
rs118162232
rs112095242
All
−0.05
0.00
0.05
0.10
MR leave−one−out sensitivity analysis for
'N−acetyl−L−alanine levels' on 'Rheumatoid arthritis'

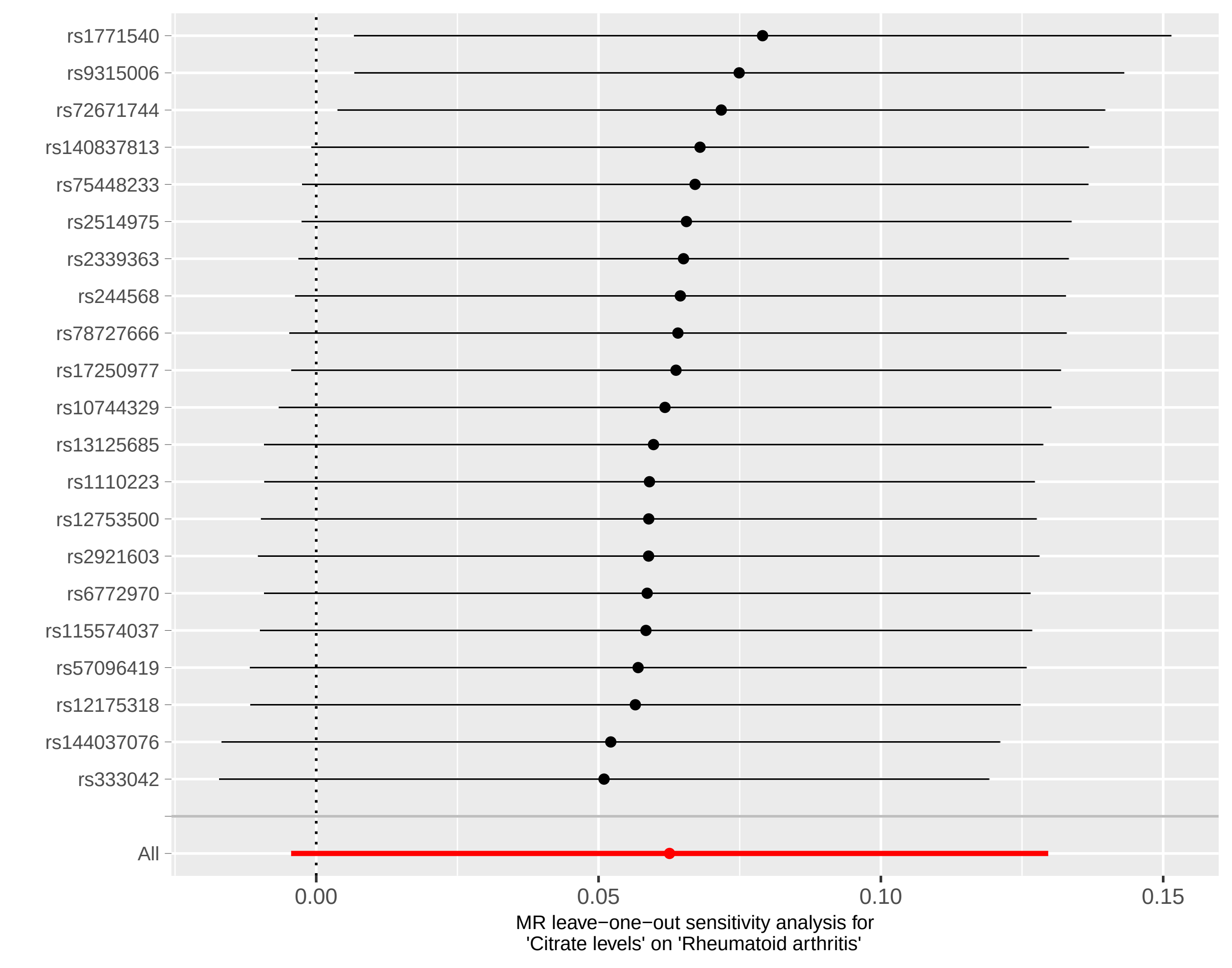

rs1771540
rs9315006
rs72671744
rs140837813
rs75448233
rs2514975
rs2339363
rs244568
rs78727666
rs17250977
rs10744329
rs13125685
rs1110223
rs12753500
rs2921603
rs6772970
rs115574037
rs57096419
rs12175318
rs144037076
rs333042
All
0.00
0.05
0.10
0.15
MR leave−one−out sensitivity analysis for
'Citrate levels' on 'Rheumatoid arthritis'

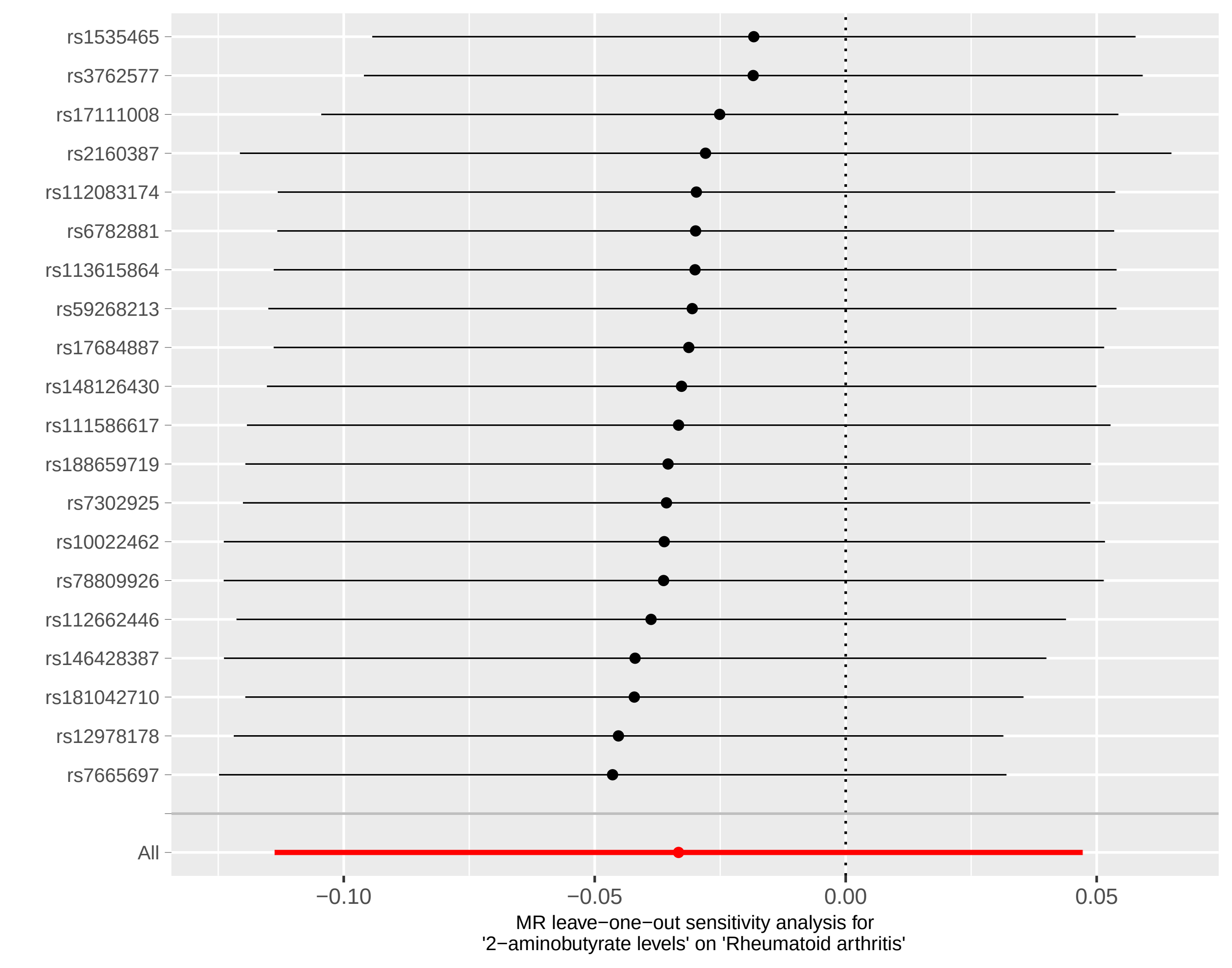
rs1535465
rs3762577
rs17111008
rs2160387
rs112083174
rs6782881
rs113615864
rs59268213
rs17684887
rs148126430
rs111586617
rs188659719
rs7302925
rs10022462
rs78809926
rs112662446
rs146428387
rs181042710
rs12978178
rs7665697
All
−0.10
−0.05
0.00
0.05
MR leave−one−out sensitivity analysis for
'2−aminobutyrate levels' on 'Rheumatoid arthritis'

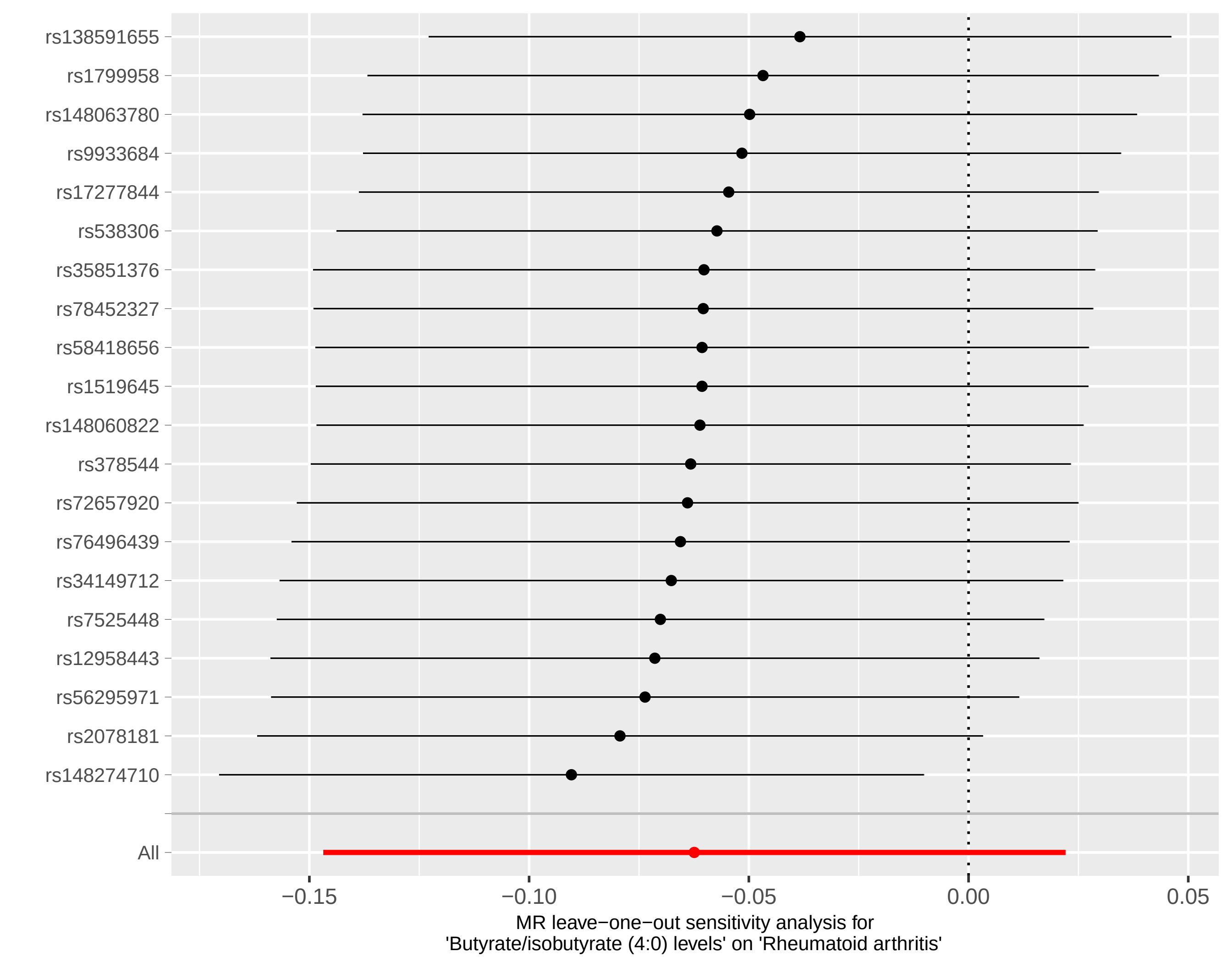
rs138591655
rs1799958
rs148063780
rs9933684
rs17277844
rs538306
rs35851376
rs78452327
rs58418656
rs1519645
rs148060822
rs378544
rs72657920
rs76496439
rs34149712
rs7525448
rs12958443
rs56295971
rs2078181
rs148274710
All
−0.15
−0.10
−0.05
0.00
0.05
MR leave−one−out sensitivity analysis for
'Butyrate/isobutyrate (4:0) levels' on 'Rheumatoid arthritis'

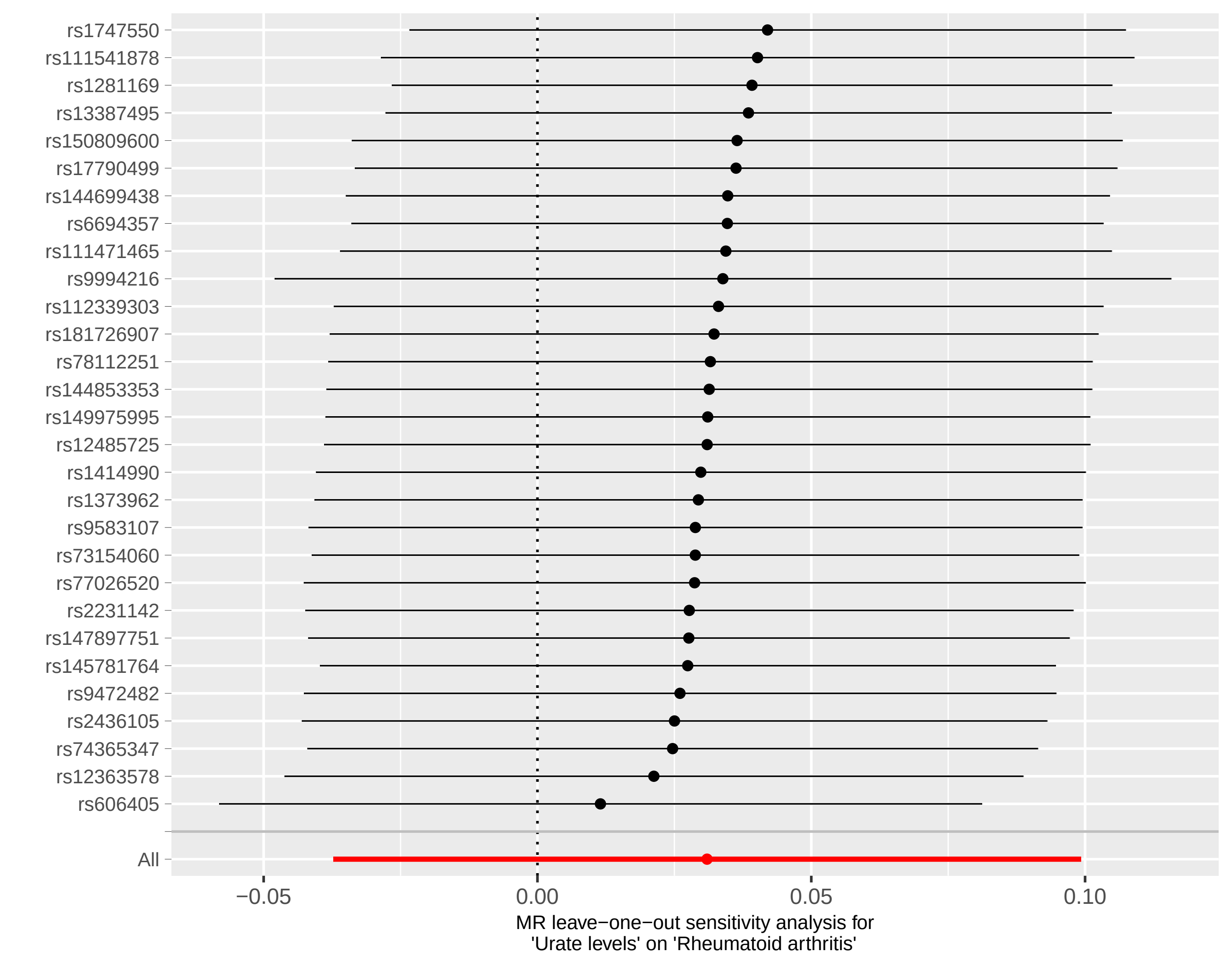

rs1747550
rs111541878
rs1281169
rs13387495
rs150809600
rs17790499
rs144699438
rs6694357
rs111471465
rs9994216
rs112339303
rs181726907
rs78112251
rs144853353
rs149975995
rs12485725
rs1414990
rs1373962
rs9583107
rs73154060
rs77026520
rs2231142
rs147897751
rs145781764
rs9472482
rs2436105
rs74365347
rs12363578
rs606405
All
−0.05
0.00
0.05
0.10
MR leave−one−out sensitivity analysis for
'Urate levels' on 'Rheumatoid arthritis'

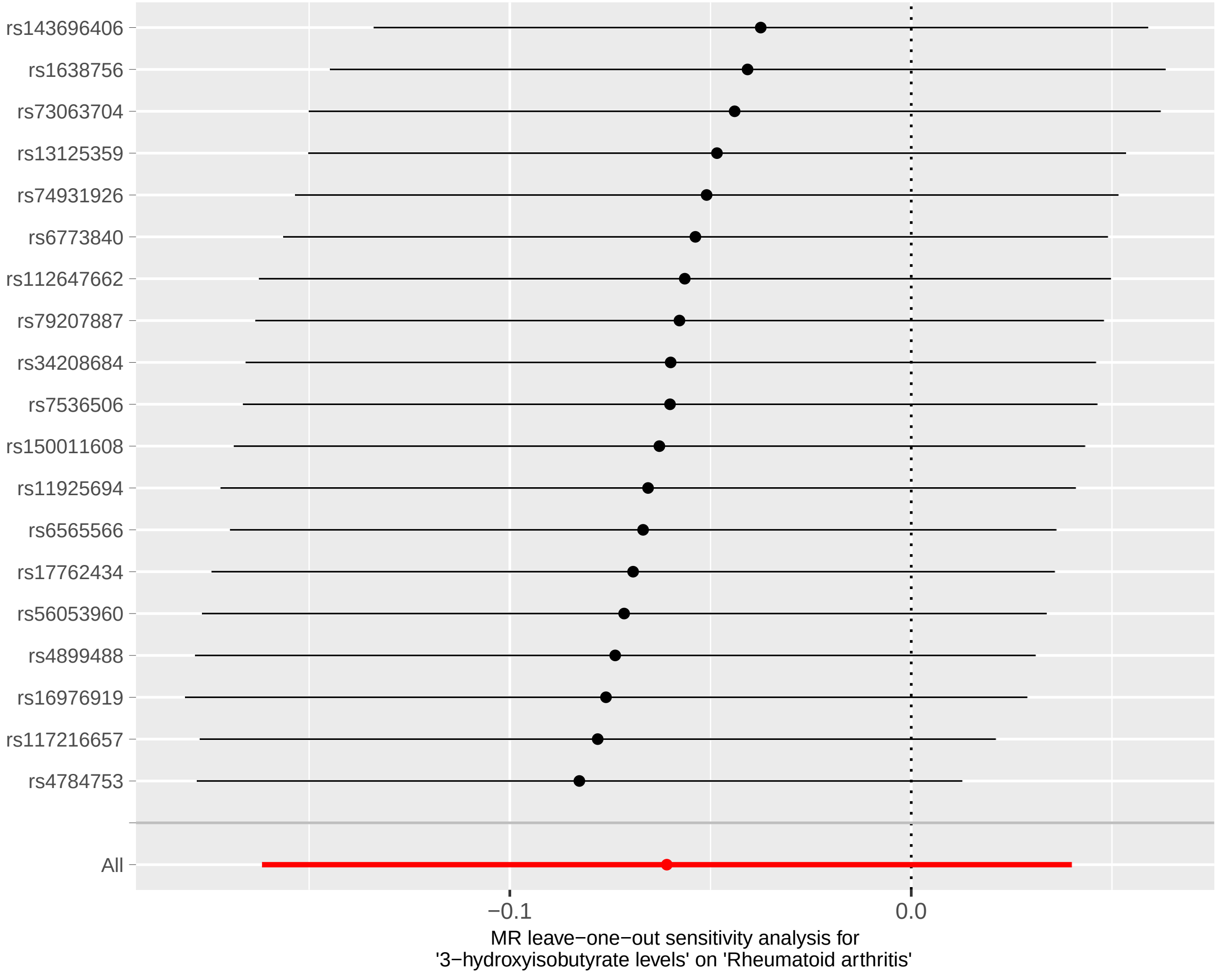
rs143696406
rs1638756
rs73063704
rs13125359
rs74931926
rs6773840
rs112647662
rs79207887
rs34208684
rs7536506
rs150011608
rs11925694
rs6565566
rs17762434
rs56053960
rs4899488
rs16976919
rs117216657
rs4784753
All
−0.1
0.0
MR leave−one−out sensitivity analysis for
'3−hydroxyisobutyrate levels' on 'Rheumatoid arthritis'

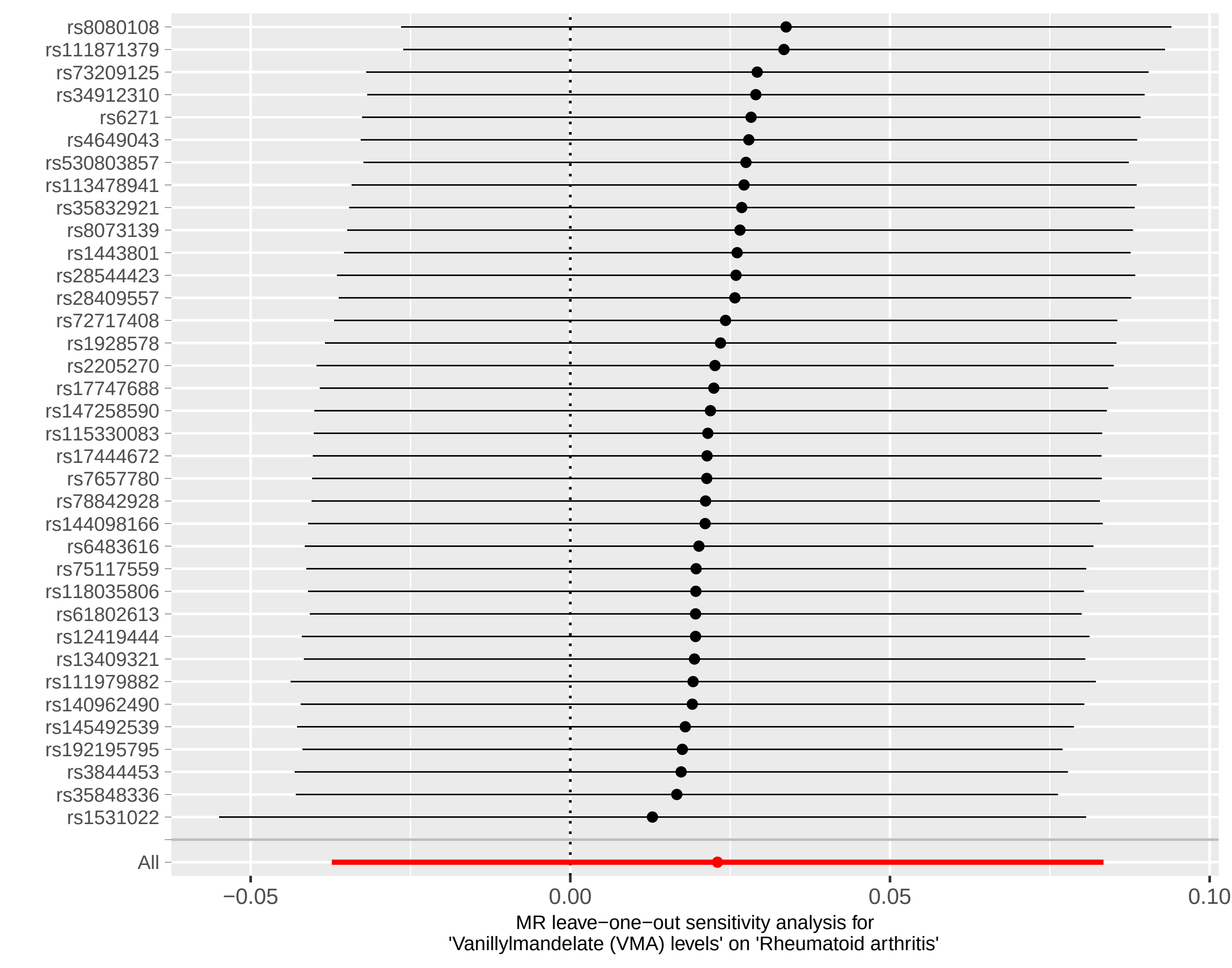
rs8080108
rs111871379
rs73209125
rs34912310
rs6271
rs4649043
rs530803857
rs113478941
rs35832921
rs8073139
rs1443801
rs28544423
rs28409557
rs72717408
rs1928578
rs2205270
rs17747688
rs147258590
rs115330083
rs17444672
rs7657780
rs78842928
rs144098166
rs6483616
rs75117559
rs118035806
rs61802613
rs12419444
rs13409321
rs111979882
rs140962490
rs145492539
rs192195795
rs3844453
rs35848336
rs1531022
All
−0.05
0.00
0.05
0.10
MR leave−one−out sensitivity analysis for
'Vanillylmandelate (VMA) levels' on 'Rheumatoid arthritis'

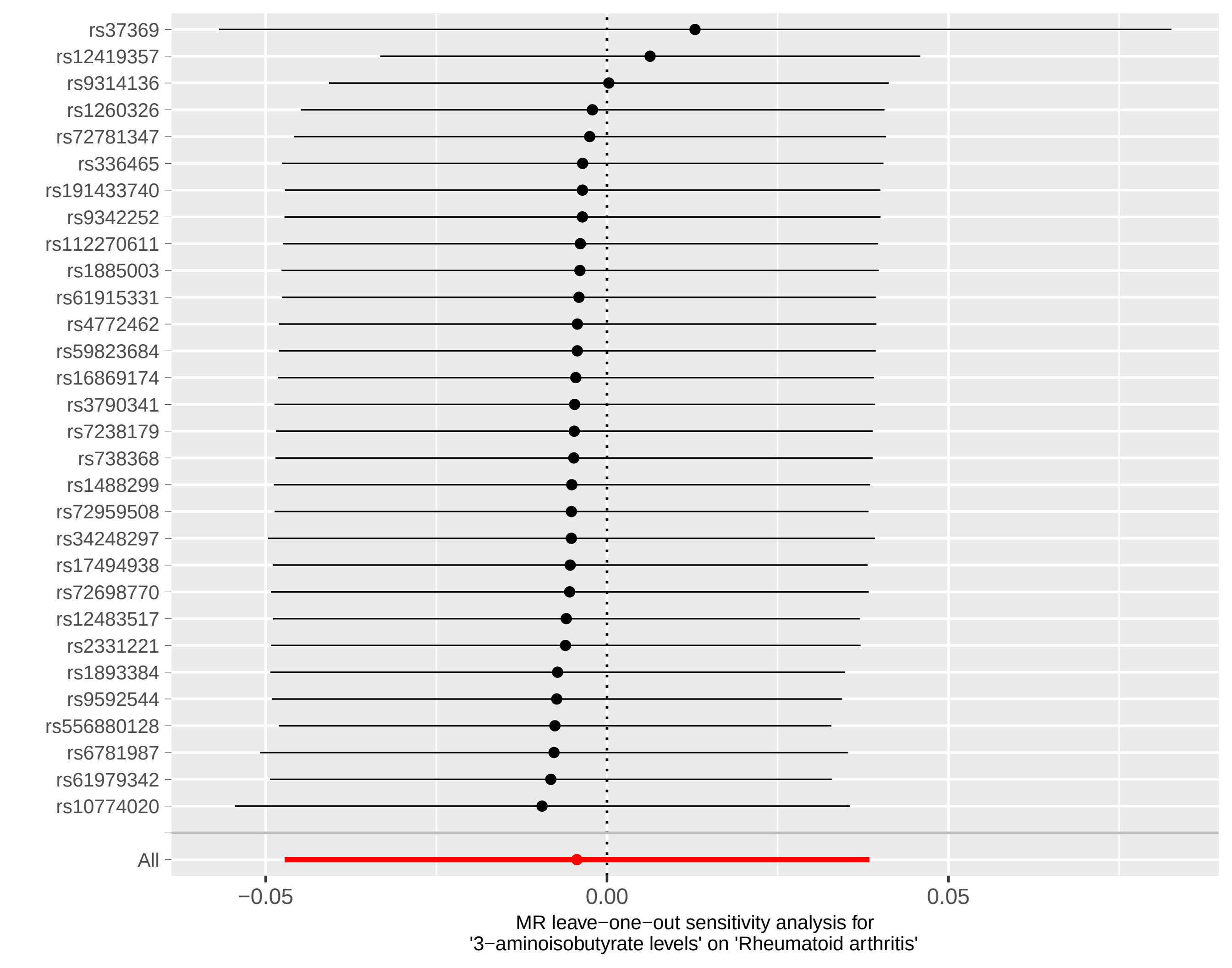
rs37369
rs12419357
rs9314136
rs1260326
rs72781347
rs336465
rs191433740
rs9342252
rs112270611
rs1885003
rs61915331
rs4772462
rs59823684
rs16869174
rs3790341
rs7238179
rs738368
rs1488299
rs72959508
rs34248297
rs17494938
rs72698770
rs12483517
rs2331221
rs1893384
rs9592544
rs556880128
rs6781987
rs61979342
rs10774020
All
−0.05
0.00
0.05
MR leave−one−out sensitivity analysis for
'3−aminoisobutyrate levels' on 'Rheumatoid arthritis'

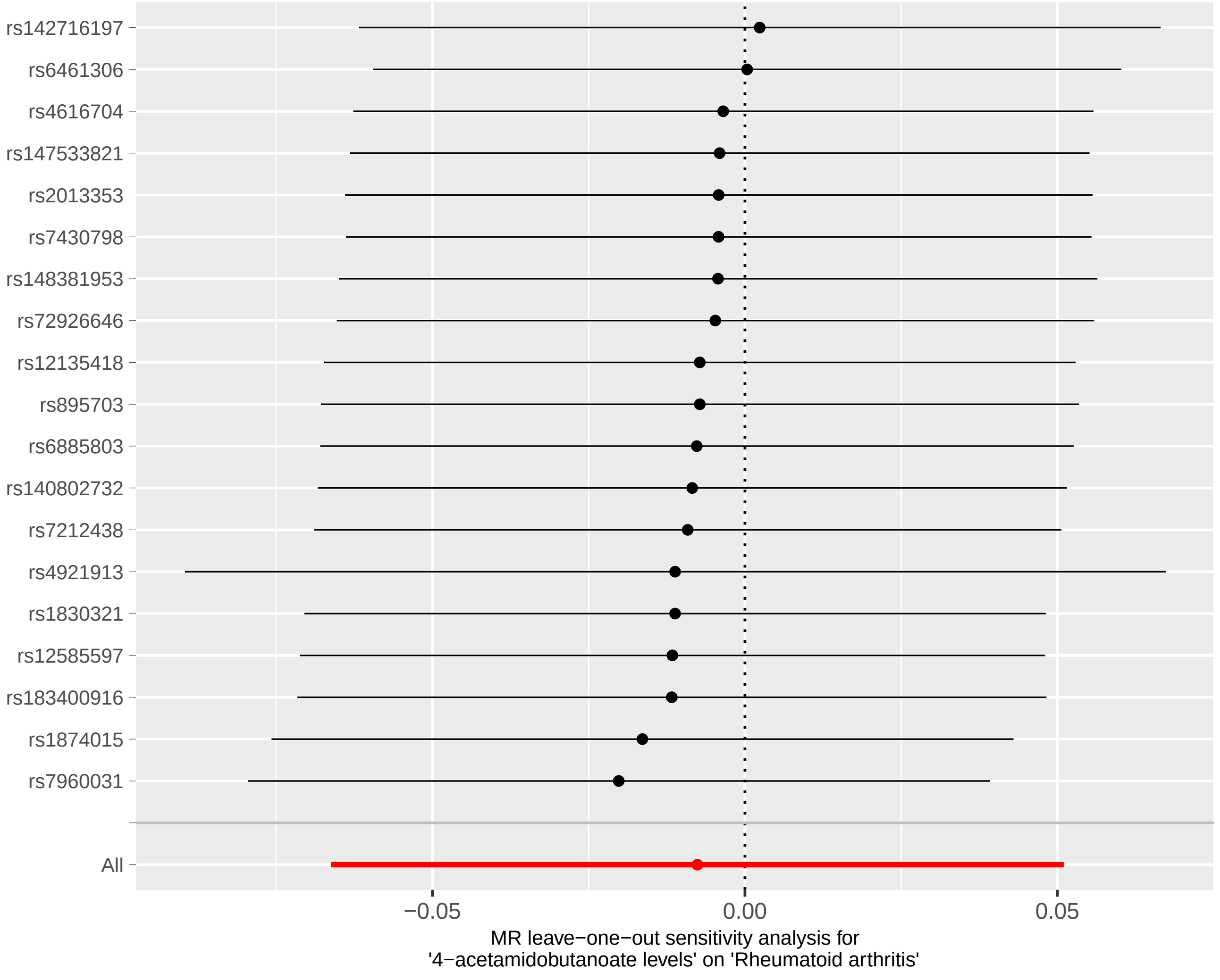

rs142716197
rs6461306
rs4616704
rs147533821
rs2013353
rs7430798
rs148381953
rs72926646
rs12135418
rs895703
rs6885803
rs140802732
rs7212438
rs4921913
rs1830321
rs12585597
rs183400916
rs1874015
rs7960031
All
−0.05
0.00
0.05
MR leave−one−out sensitivity analysis for
'4−acetamidobutanoate levels' on 'Rheumatoid arthritis'

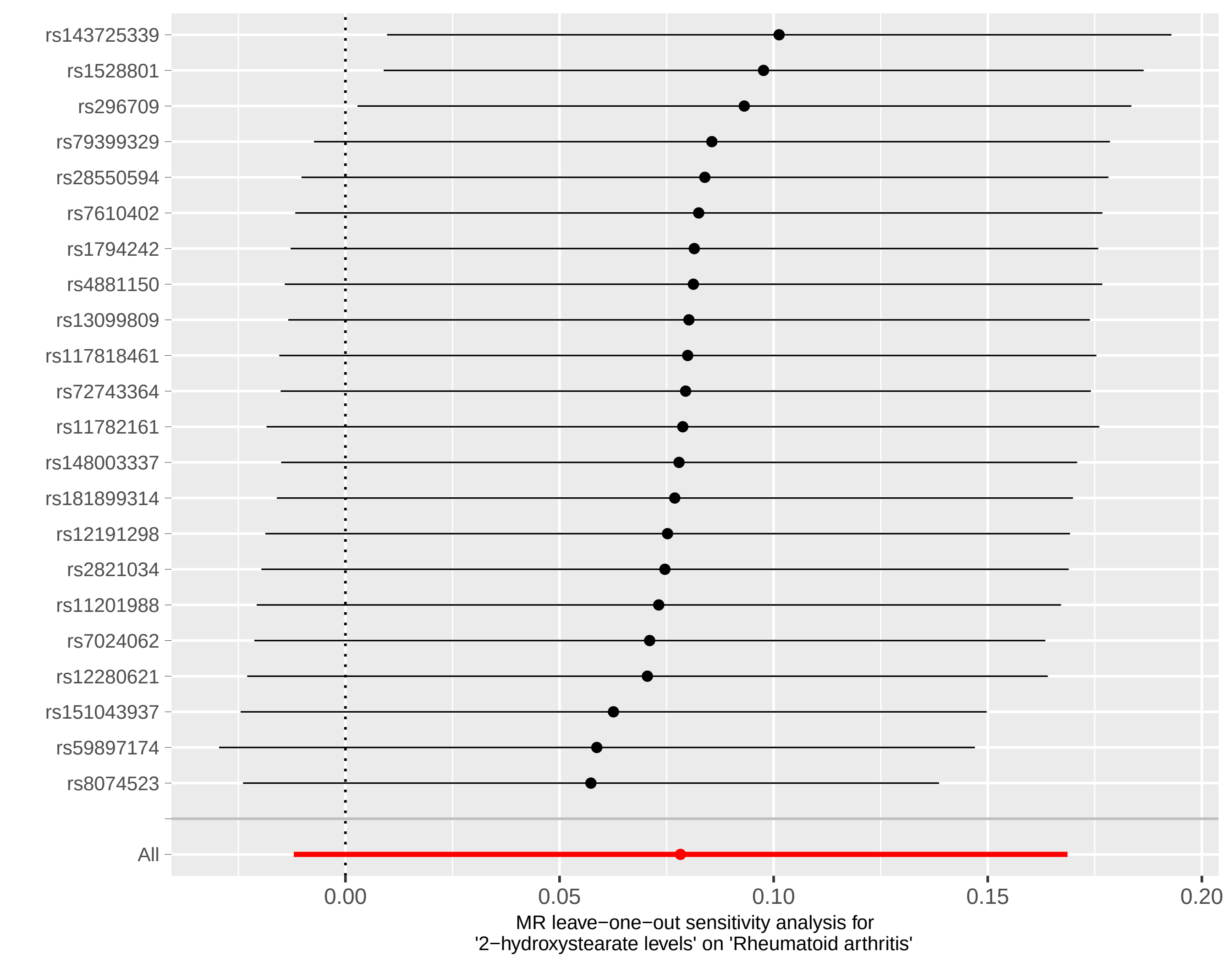
rs143725339
rs1528801
rs296709
rs79399329
rs28550594
rs7610402
rs1794242
rs4881150
rs13099809
rs117818461
rs72743364
rs11782161
rs148003337
rs181899314
rs12191298
rs2821034
rs11201988
rs7024062
rs12280621
rs151043937
rs59897174
rs8074523
All
0.00
0.05
0.10
0.15
0.20
MR leave−one−out sensitivity analysis for
'2−hydroxystearate levels' on 'Rheumatoid arthritis'

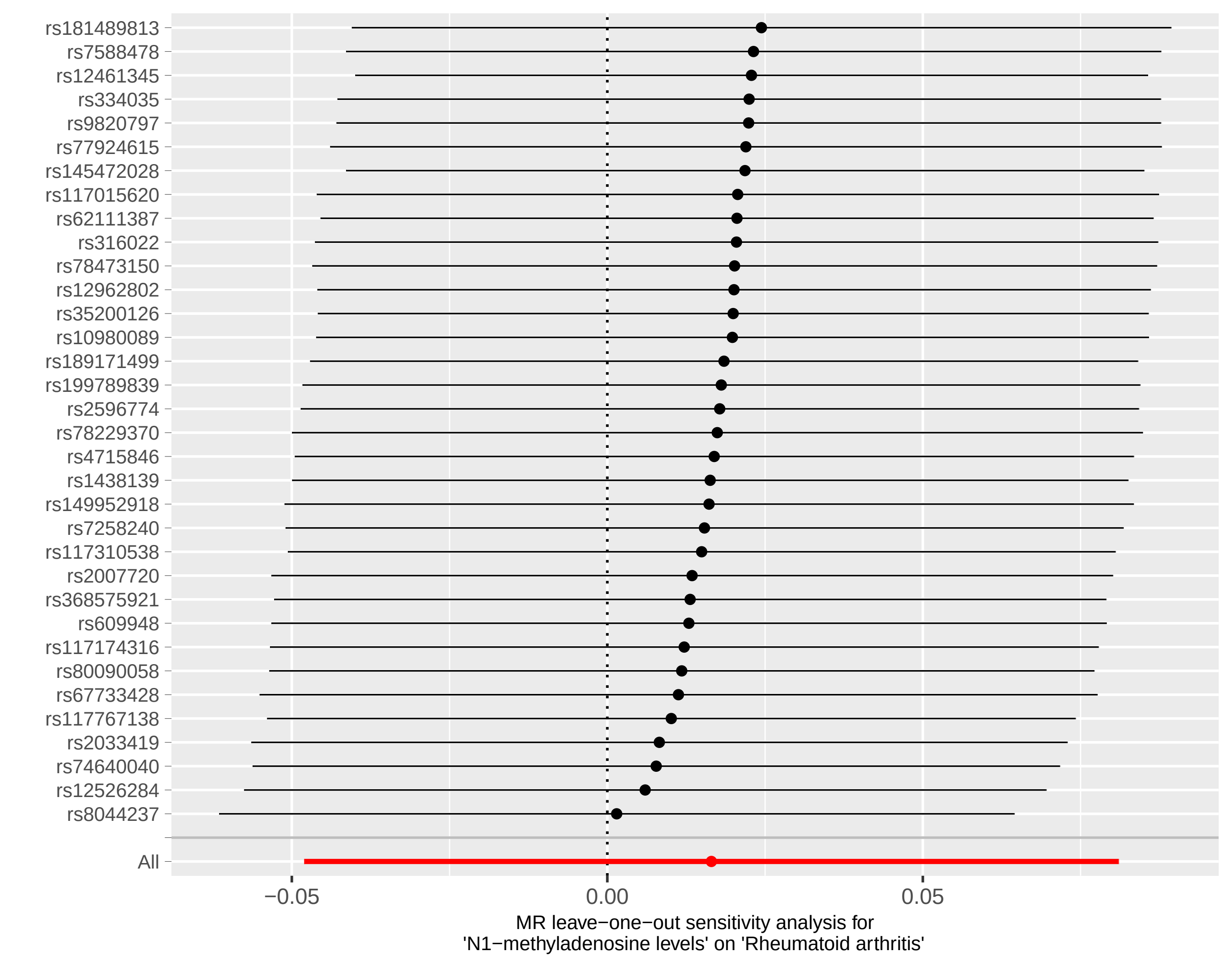

rs181489813
rs7588478
rs12461345
rs334035
rs9820797
rs77924615
rs145472028
rs117015620
rs62111387
rs316022
rs78473150
rs12962802
rs35200126
rs10980089
rs189171499
rs199789839
rs2596774
rs78229370
rs4715846
rs1438139
rs149952918
rs7258240
rs117310538
rs2007720
rs368575921
rs609948
rs117174316
rs80090058
rs67733428
rs117767138
rs2033419
rs74640040
rs12526284
rs8044237
All
−0.05
0.00
0.05
MR leave−one−out sensitivity analysis for
'N1−methyladenosine levels' on 'Rheumatoid arthritis'

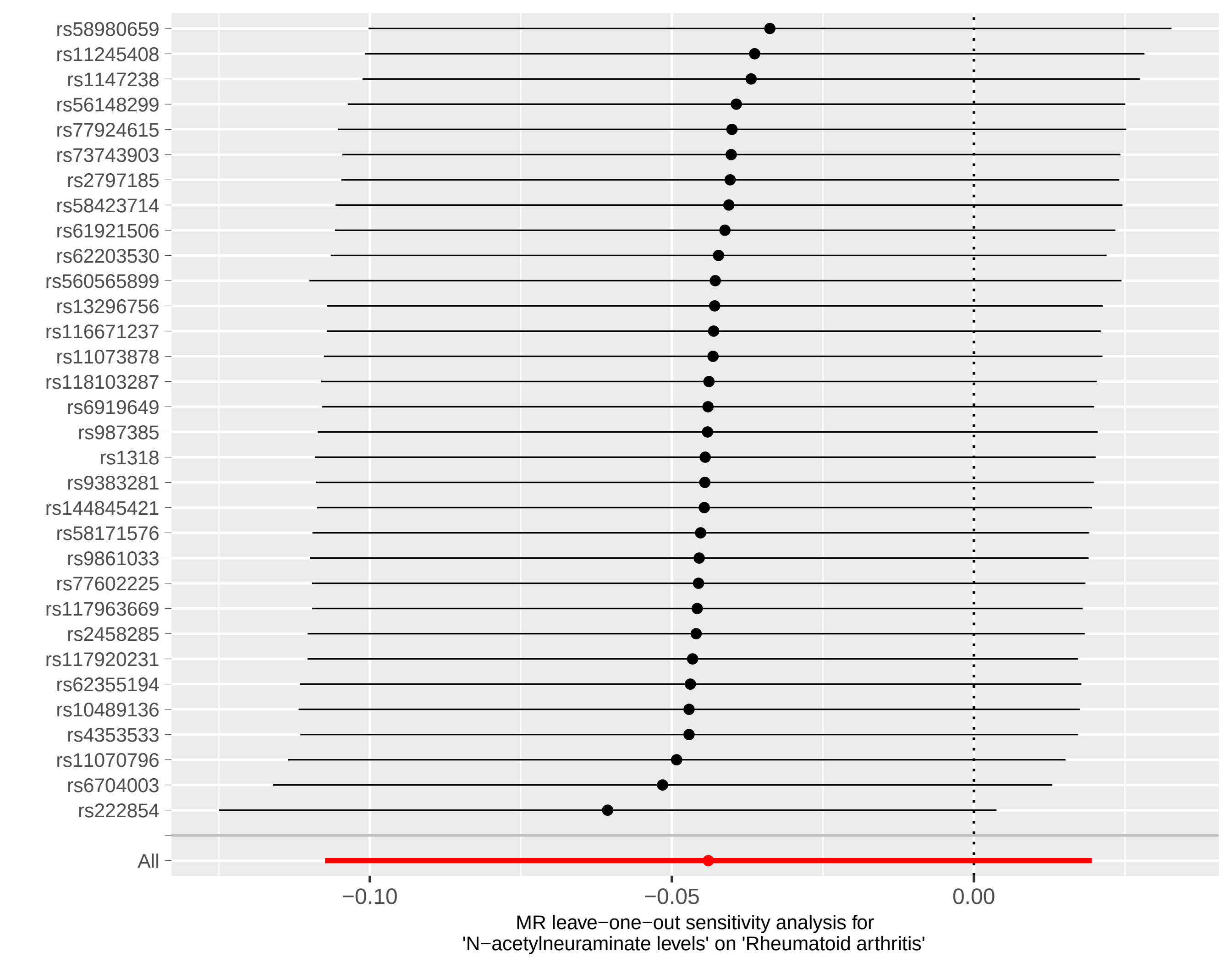
rs58980659
rs11245408
rs1147238
rs56148299
rs77924615
rs73743903
rs2797185
rs58423714
rs61921506
rs62203530
rs560565899
rs13296756
rs116671237
rs11073878
rs118103287
rs6919649
rs987385
rs1318
rs9383281
rs144845421
rs58171576
rs9861033
rs77602225
rs117963669
rs2458285
rs117920231
rs62355194
rs10489136
rs4353533
rs11070796
rs6704003
rs222854
All
−0.10
−0.05
0.00
MR leave−one−out sensitivity analysis for
'N−acetylneuraminate levels' on 'Rheumatoid arthritis'

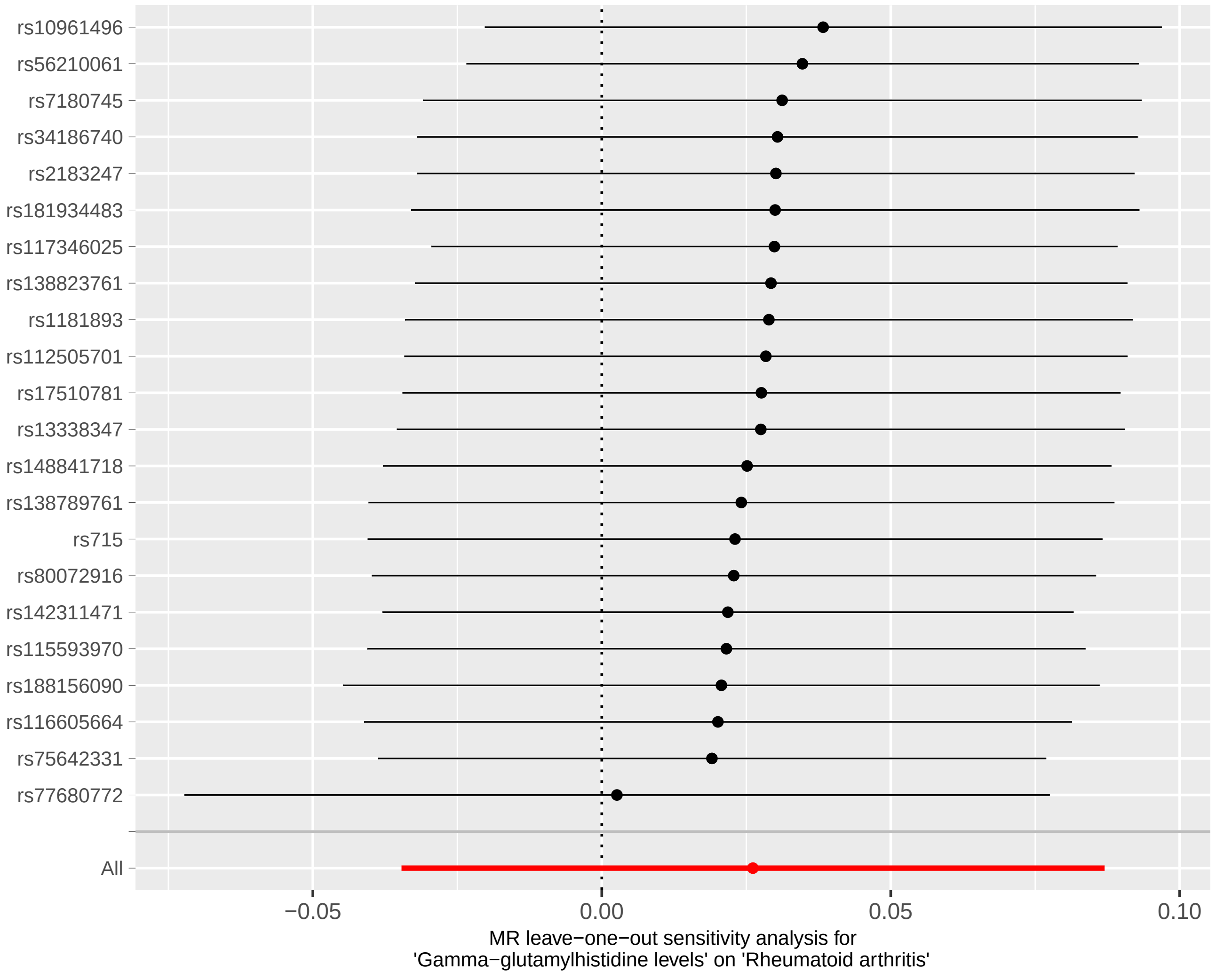
rs10961496
rs56210061
rs7180745
rs34186740
rs2183247
rs181934483
rs117346025
rs138823761
rs1181893
rs112505701
rs17510781
rs13338347
rs148841718
rs138789761
rs715
rs80072916
rs142311471
rs115593970
rs188156090
rs116605664
rs75642331
rs77680772
All
−0.05
0.00
0.05
0.10
MR leave−one−out sensitivity analysis for
'Gamma−glutamylhistidine levels' on 'Rheumatoid arthritis'

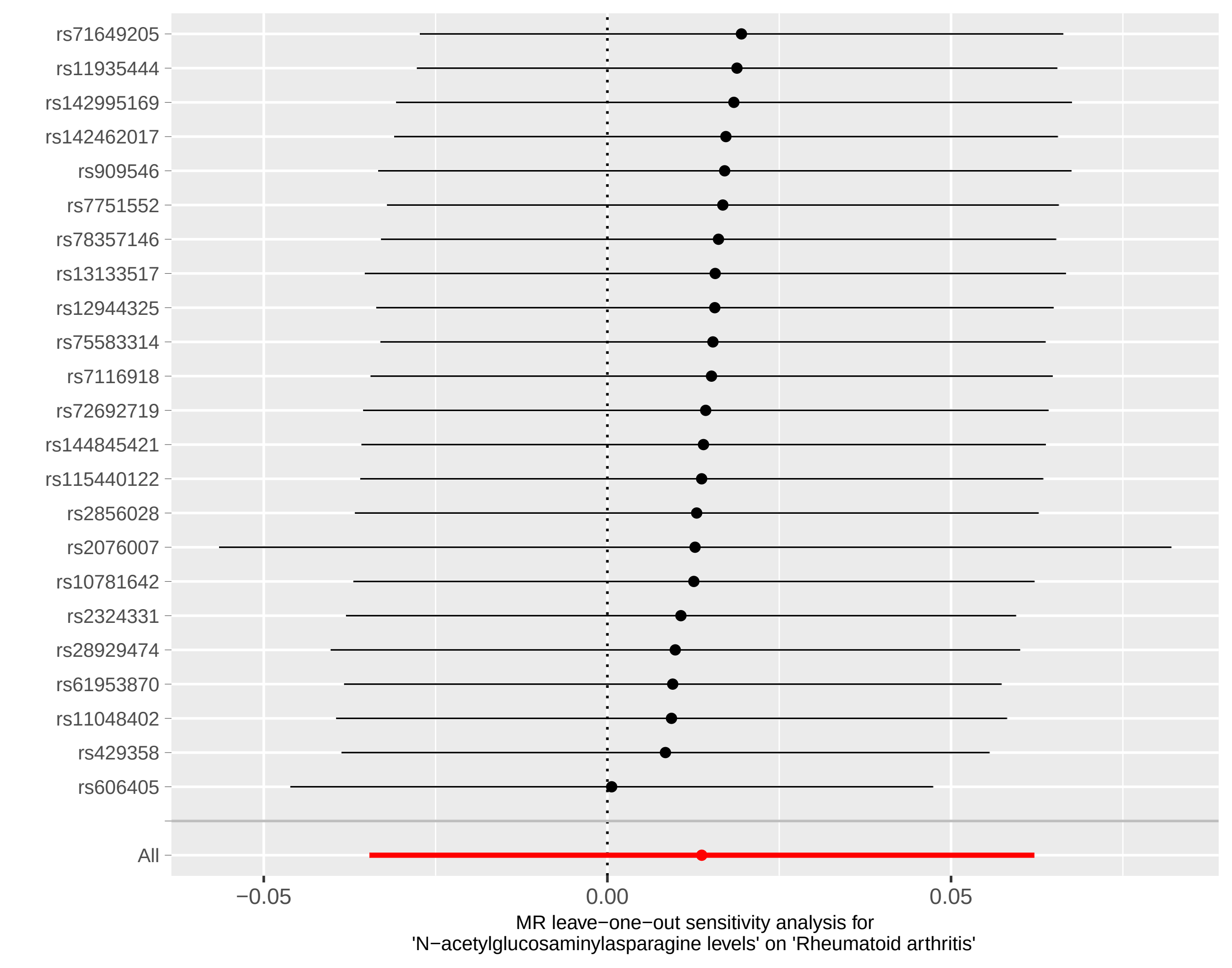
rs71649205
rs11935444
rs142995169
rs142462017
rs909546
rs7751552
rs78357146
rs13133517
rs12944325
rs75583314
rs7116918
rs72692719
rs144845421
rs115440122
rs2856028
rs2076007
rs10781642
rs2324331
rs28929474
rs61953870
rs11048402
rs429358
rs606405
All
−0.05
0.00
0.05
MR leave−one−out sensitivity analysis for
'N−acetylglucosaminylasparagine levels' on 'Rheumatoid arthritis'

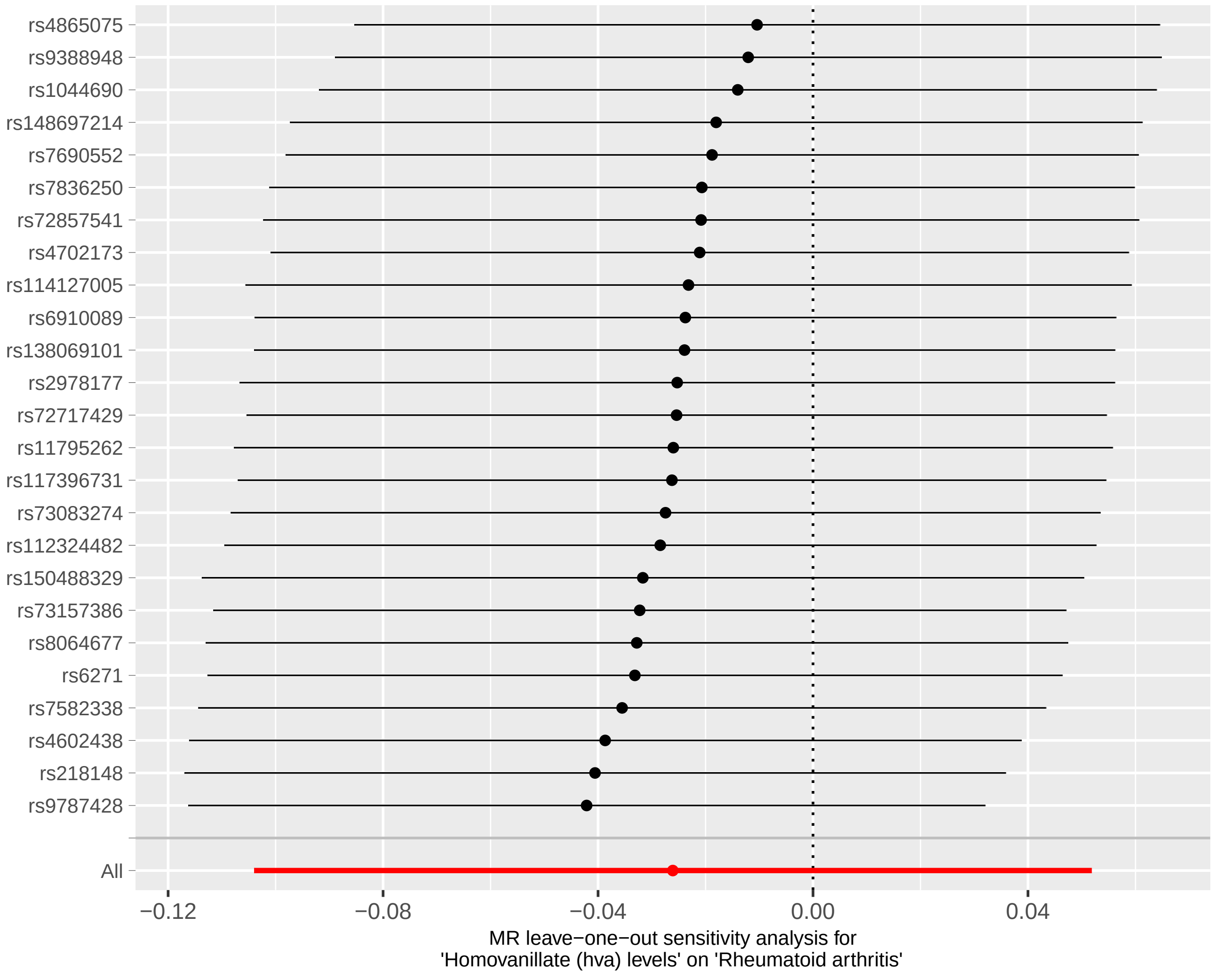
rs4865075
rs9388948
rs1044690
rs148697214
rs7690552
rs7836250
rs72857541
rs4702173
rs114127005
rs6910089
rs138069101
rs2978177
rs72717429
rs11795262
rs117396731
rs73083274
rs112324482
rs150488329
rs73157386
rs8064677
rs6271
rs7582338
rs4602438
rs218148
rs9787428
All
−0.12
−0.08
−0.04
0.00
0.04
MR leave−one−out sensitivity analysis for
'Homovanillate (hva) levels' on 'Rheumatoid arthritis'

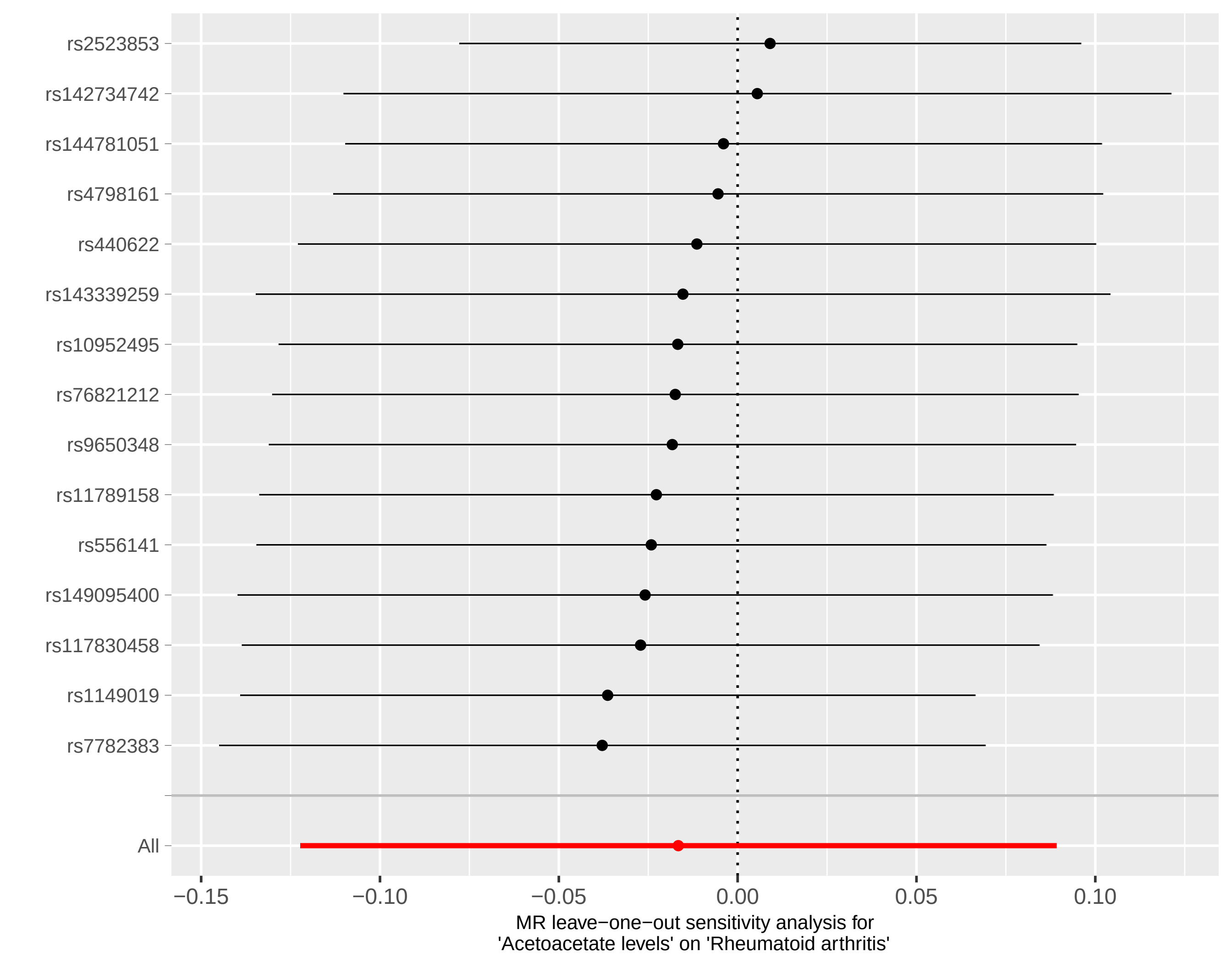
rs2523853
rs142734742
rs144781051
rs4798161
rs440622
rs143339259
rs10952495
rs76821212
rs9650348
rs11789158
rs556141
rs149095400
rs117830458
rs1149019
rs7782383
All
−0.15
−0.10
−0.05
0.00
0.05
0.10
MR leave−one−out sensitivity analysis for
'Acetoacetate levels' on 'Rheumatoid arthritis'

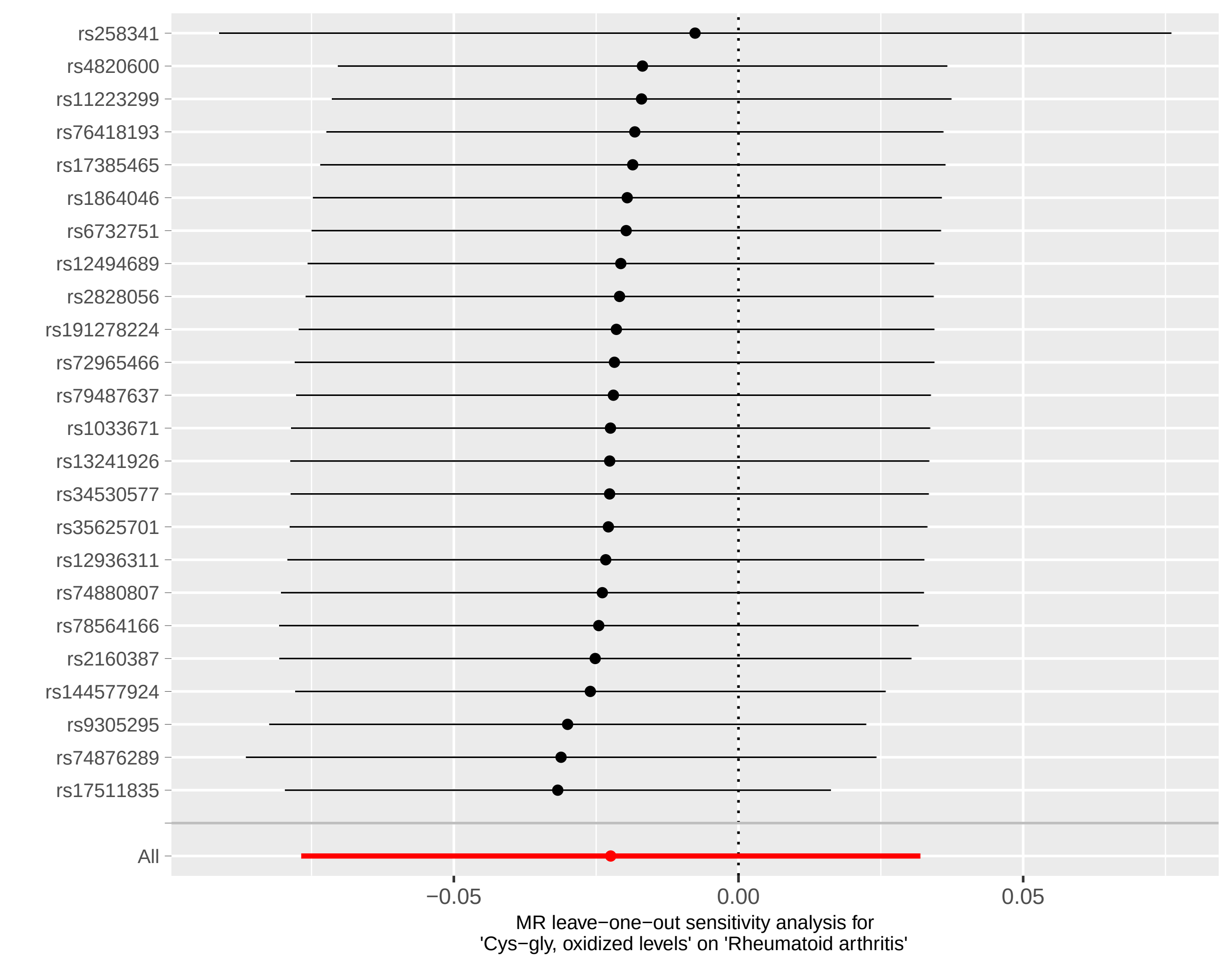
rs258341
rs4820600
rs11223299
rs76418193
rs17385465
rs1864046
rs6732751
rs12494689
rs2828056
rs191278224
rs72965466
rs79487637
rs1033671
rs13241926
rs34530577
rs35625701
rs12936311
rs74880807
rs78564166
rs2160387
rs144577924
rs9305295
rs74876289
rs17511835
All
−0.05
0.00
0.05
MR leave−one−out sensitivity analysis for
'Cys−gly, oxidized levels' on 'Rheumatoid arthritis'

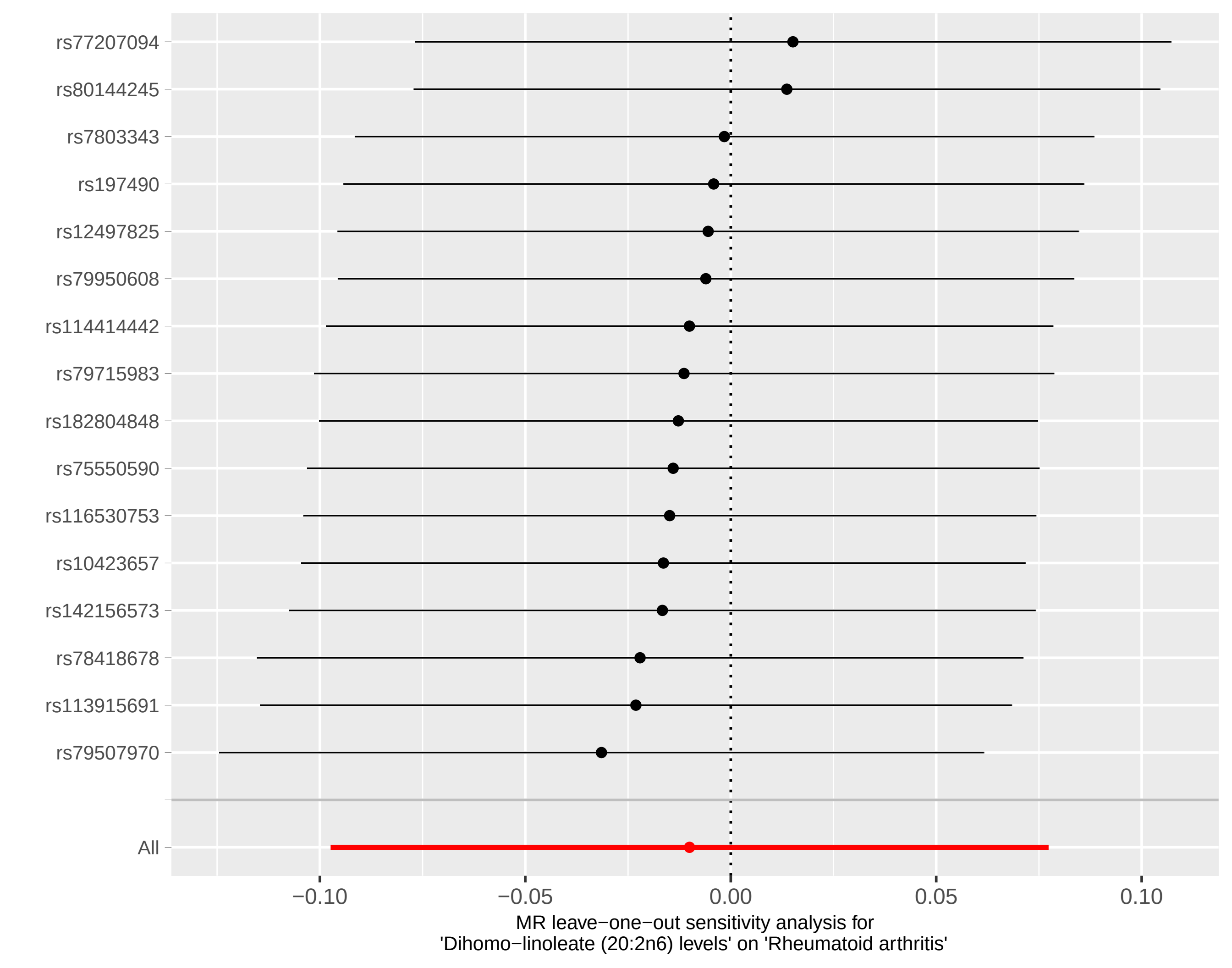

rs77207094
rs80144245
rs7803343
rs197490
rs12497825
rs79950608
rs114414442
rs79715983
rs182804848
rs75550590
rs116530753
rs10423657
rs142156573
rs78418678
rs113915691
rs79507970
All
−0.10
−0.05
0.00
0.05
0.10
MR leave−one−out sensitivity analysis for
'Dihomo−linoleate (20:2n6) levels' on 'Rheumatoid arthritis'

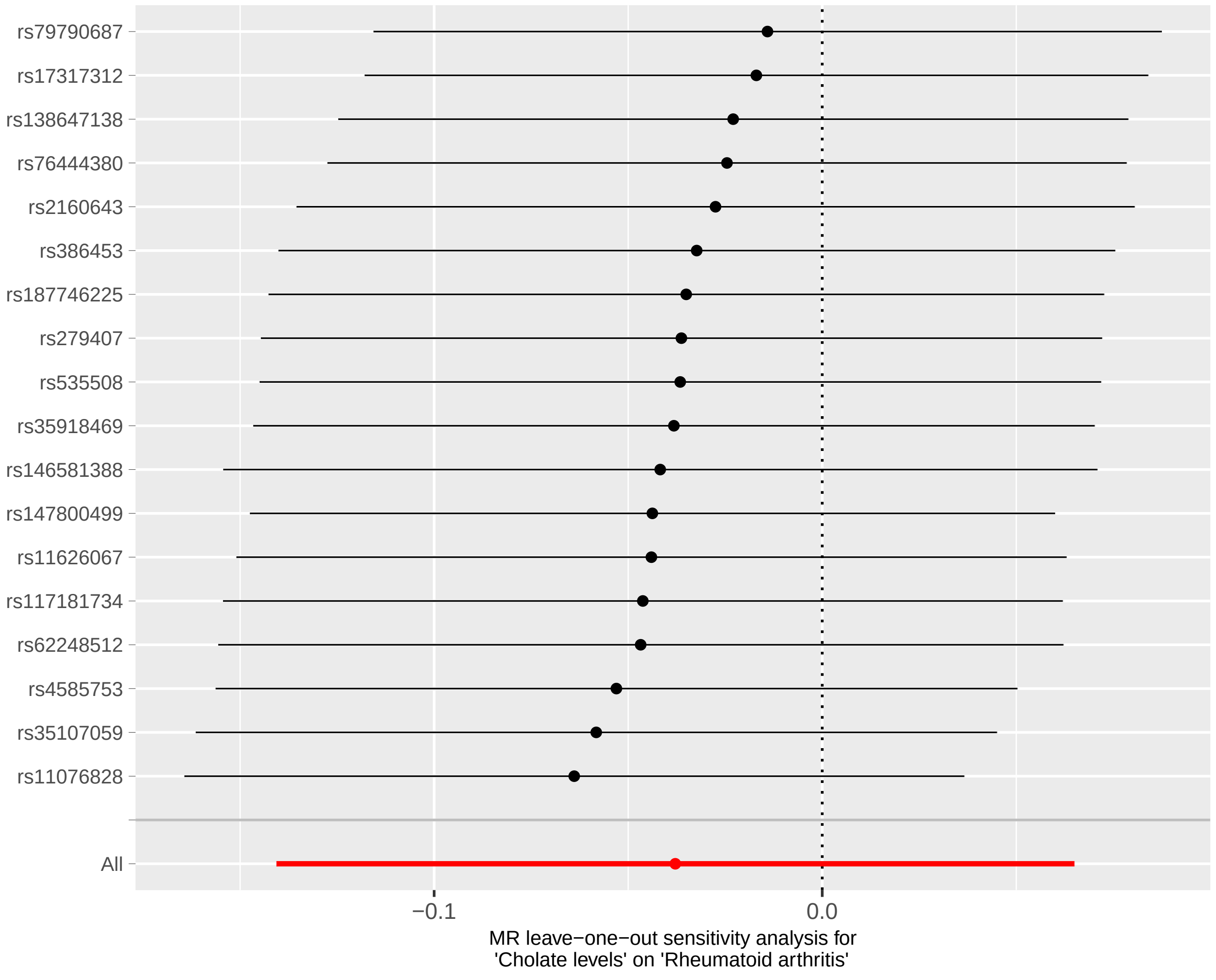
rs79790687
rs17317312
rs138647138
rs76444380
rs2160643
rs386453
rs187746225
rs279407
rs535508
rs35918469
rs146581388
rs147800499
rs11626067
rs117181734
rs62248512
rs4585753
rs35107059
rs11076828
All
−0.1
0.0
MR leave−one−out sensitivity analysis for
'Cholate levels' on 'Rheumatoid arthritis'

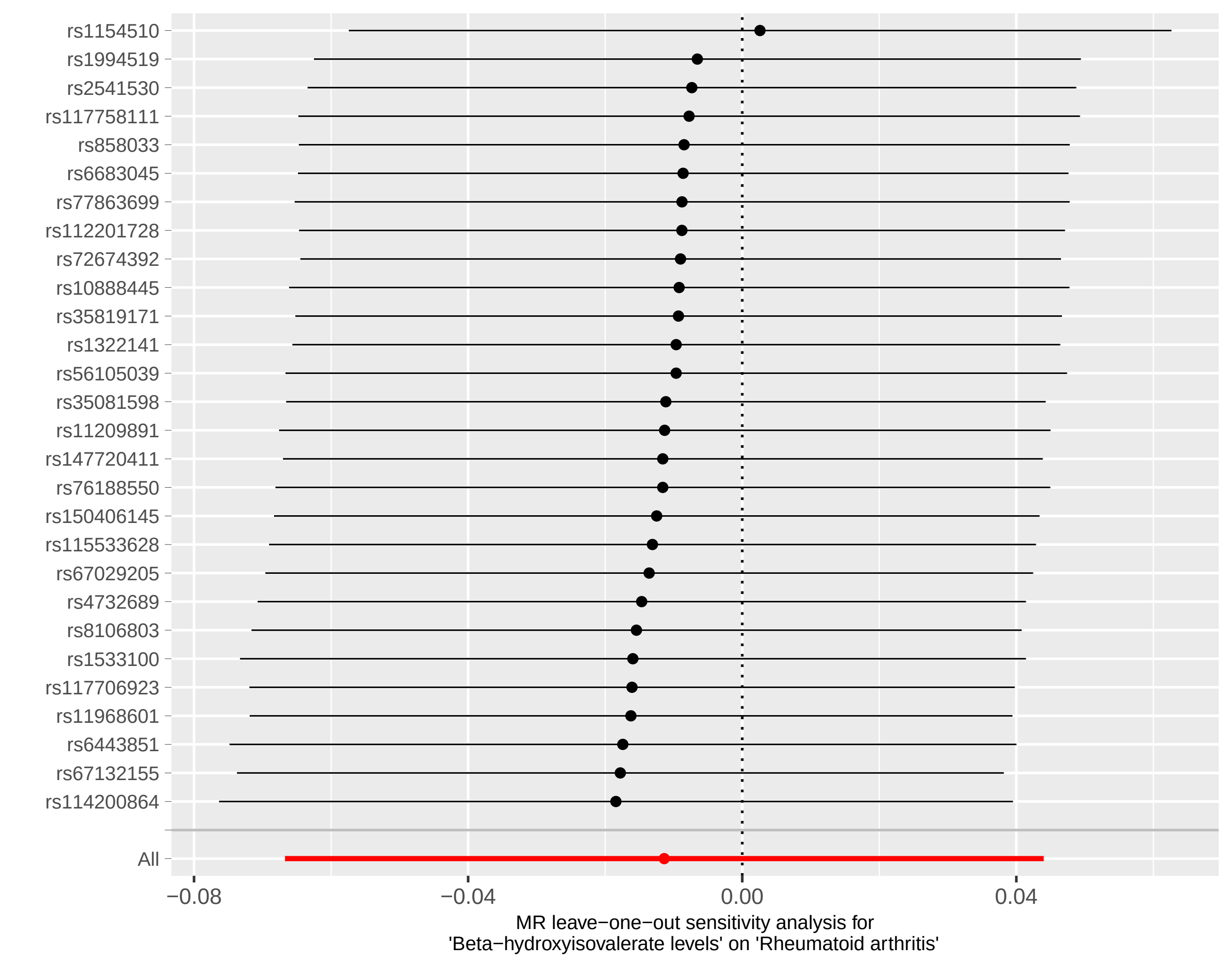

rs1154510
rs1994519
rs2541530
rs117758111
rs858033
rs6683045
rs77863699
rs112201728
rs72674392
rs10888445
rs35819171
rs1322141
rs56105039
rs35081598
rs11209891
rs147720411
rs76188550
rs150406145
rs115533628
rs67029205
rs4732689
rs8106803
rs1533100
rs117706923
rs11968601
rs6443851
rs67132155
rs114200864
All
−0.08
−0.04
0.00
0.04
MR leave−one−out sensitivity analysis for
'Beta−hydroxyisovalerate levels' on 'Rheumatoid arthritis'

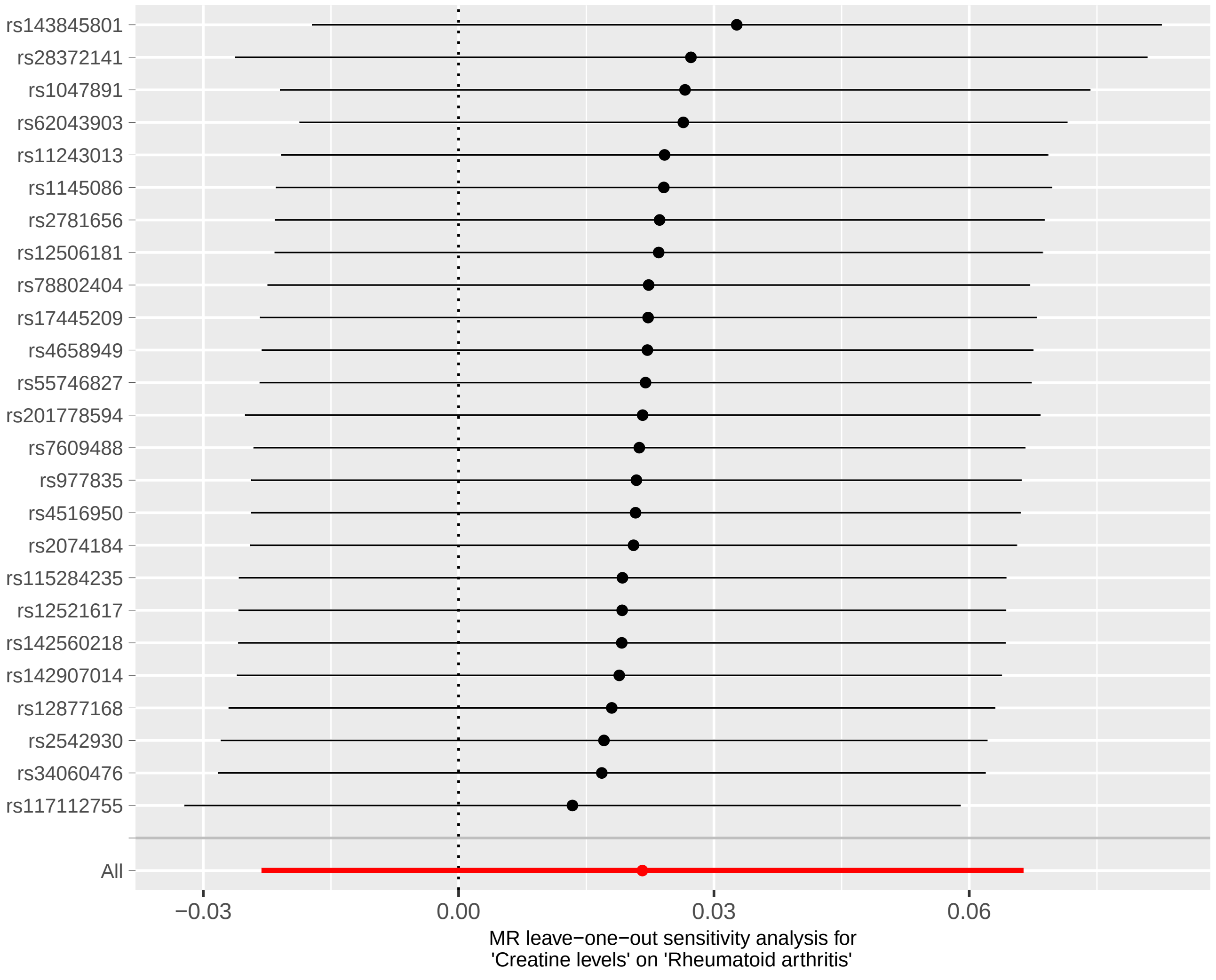
rs143845801
rs28372141
rs1047891
rs62043903
rs11243013
rs1145086
rs2781656
rs12506181
rs78802404
rs17445209
rs4658949
rs55746827
rs201778594
rs7609488
rs977835
rs4516950
rs2074184
rs115284235
rs12521617
rs142560218
rs142907014
rs12877168
rs2542930
rs34060476
rs117112755
All
−0.03
0.00
0.03
0.06
MR leave−one−out sensitivity analysis for
'Creatine levels' on 'Rheumatoid arthritis'

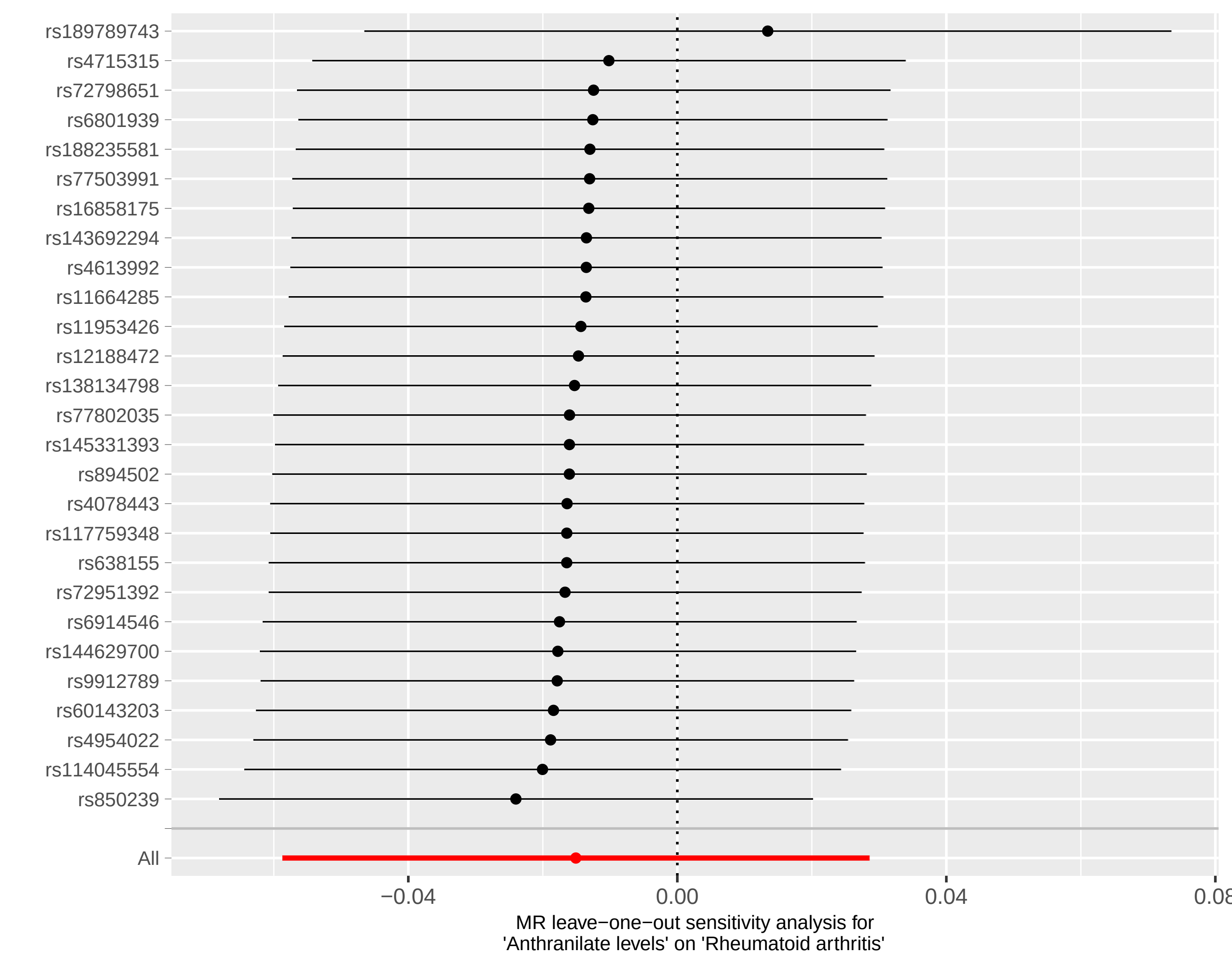
rs189789743
rs4715315
rs72798651
rs6801939
rs188235581
rs77503991
rs16858175
rs143692294
rs4613992
rs11664285
rs11953426
rs12188472
rs138134798
rs77802035
rs145331393
rs894502
rs4078443
rs117759348
rs638155
rs72951392
rs6914546
rs144629700
rs9912789
rs60143203
rs4954022
rs114045554
rs850239
All
−0.04
0.00
0.04
0.08
MR leave−one−out sensitivity analysis for
'Anthranilate levels' on 'Rheumatoid arthritis'

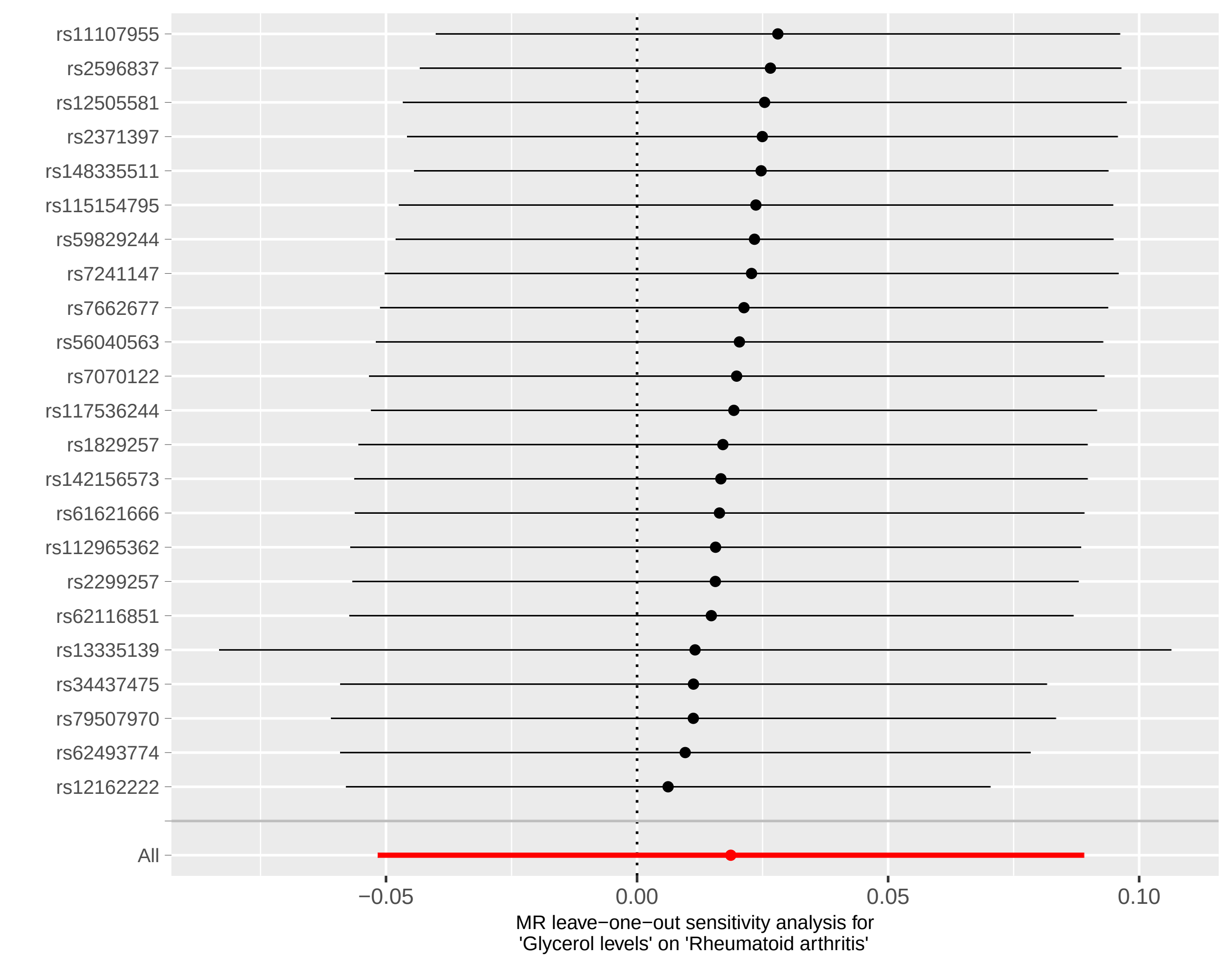
rs11107955
rs2596837
rs12505581
rs2371397
rs148335511
rs115154795
rs59829244
rs7241147
rs7662677
rs56040563
rs7070122
rs117536244
rs1829257
rs142156573
rs61621666
rs112965362
rs2299257
rs62116851
rs13335139
rs34437475
rs79507970
rs62493774
rs12162222
All
−0.05
0.00
0.05
0.10
MR leave−one−out sensitivity analysis for
'Glycerol levels' on 'Rheumatoid arthritis'

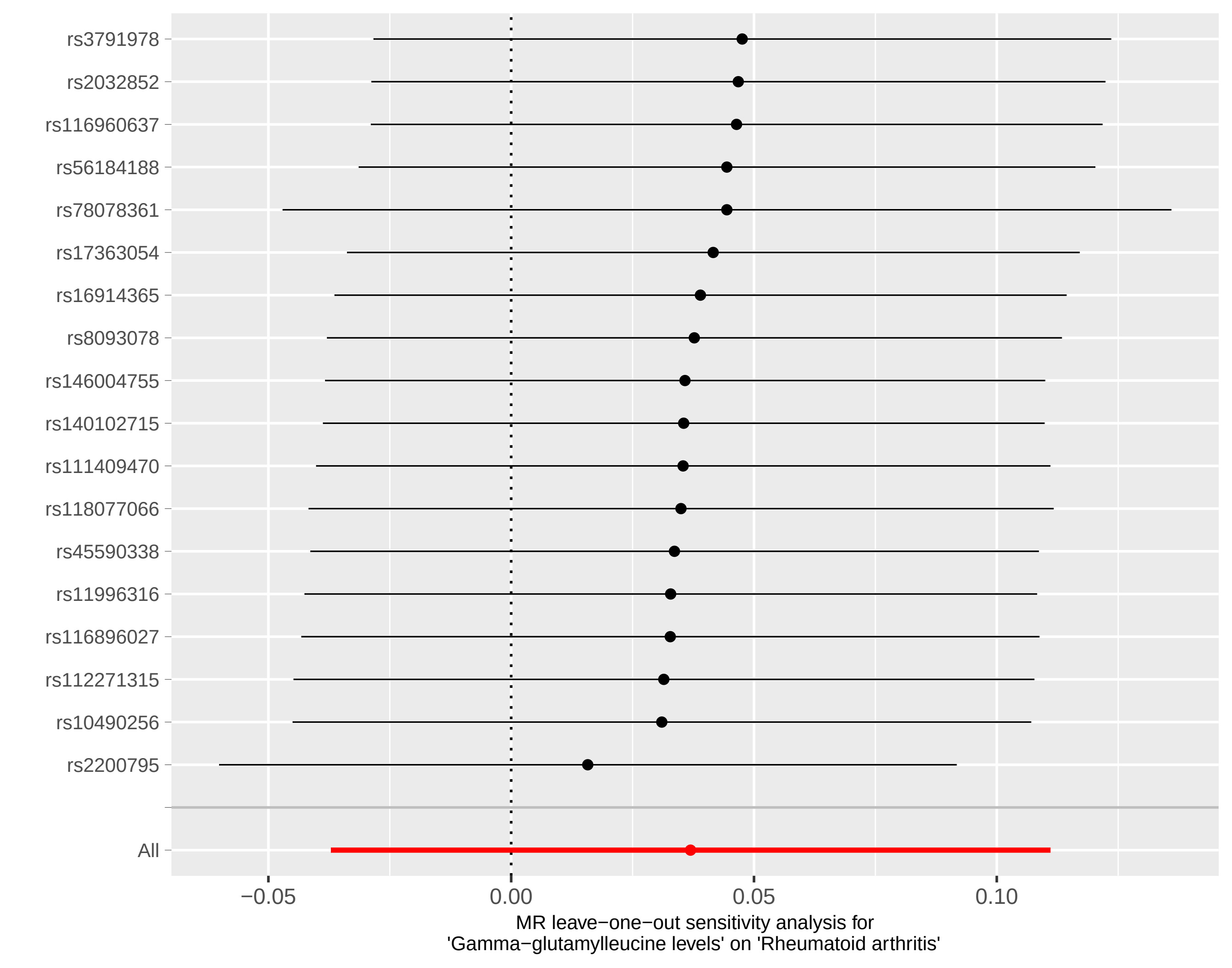
rs3791978
rs2032852
rs116960637
rs56184188
rs78078361
rs17363054
rs16914365
rs8093078
rs146004755
rs140102715
rs111409470
rs118077066
rs45590338
rs11996316
rs116896027
rs112271315
rs10490256
rs2200795
All
−0.05
0.00
0.05
0.10
MR leave−one−out sensitivity analysis for
'Gamma−glutamylleucine levels' on 'Rheumatoid arthritis'

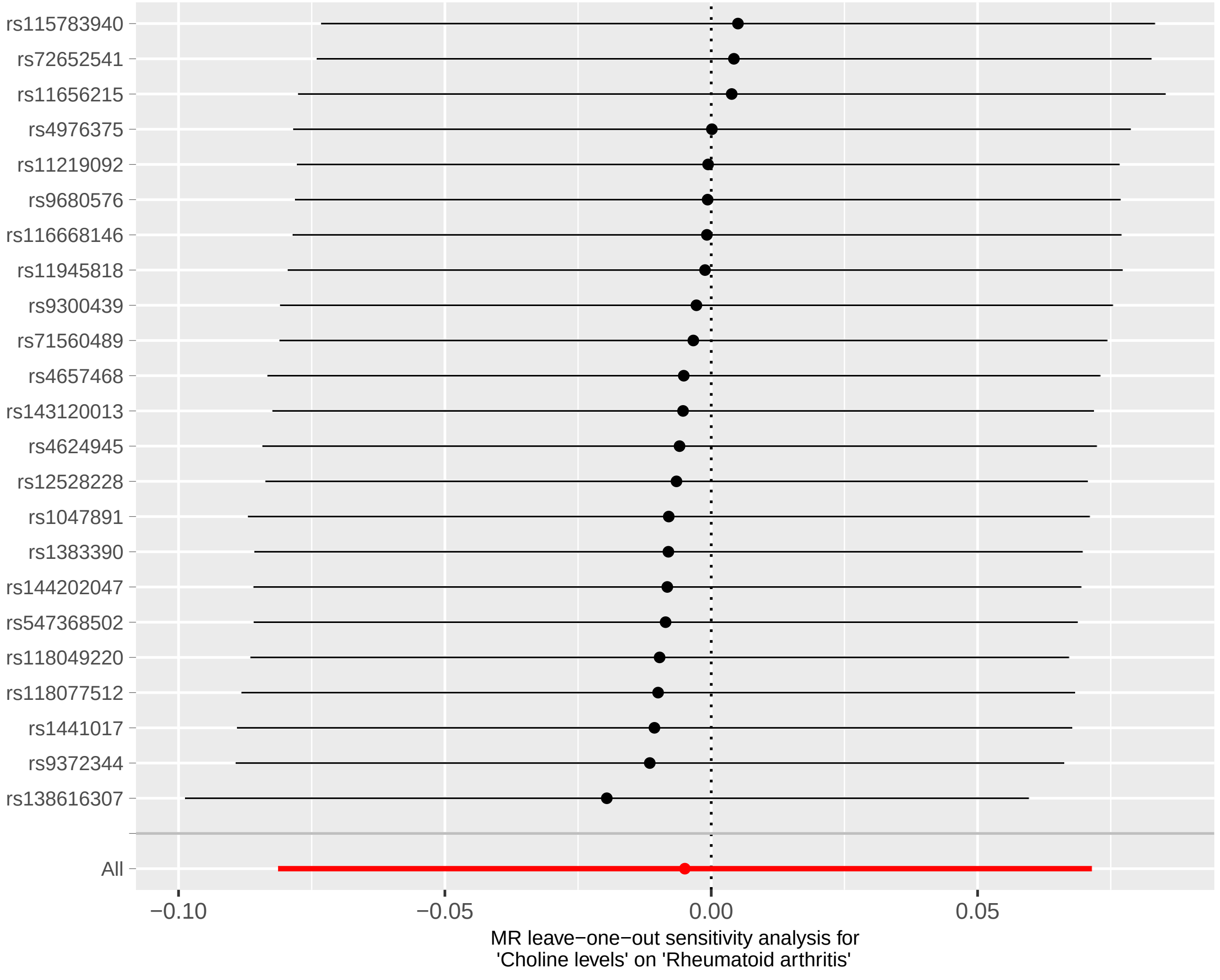
rs115783940
rs72652541
rs11656215
rs4976375
rs11219092
rs9680576
rs116668146
rs11945818
rs9300439
rs71560489
rs4657468
rs143120013
rs4624945
rs12528228
rs1047891
rs1383390
rs144202047
rs547368502
rs118049220
rs118077512
rs1441017
rs9372344
rs138616307
All
−0.10
−0.05
0.00
0.05
MR leave−one−out sensitivity analysis for
'Choline levels' on 'Rheumatoid arthritis'

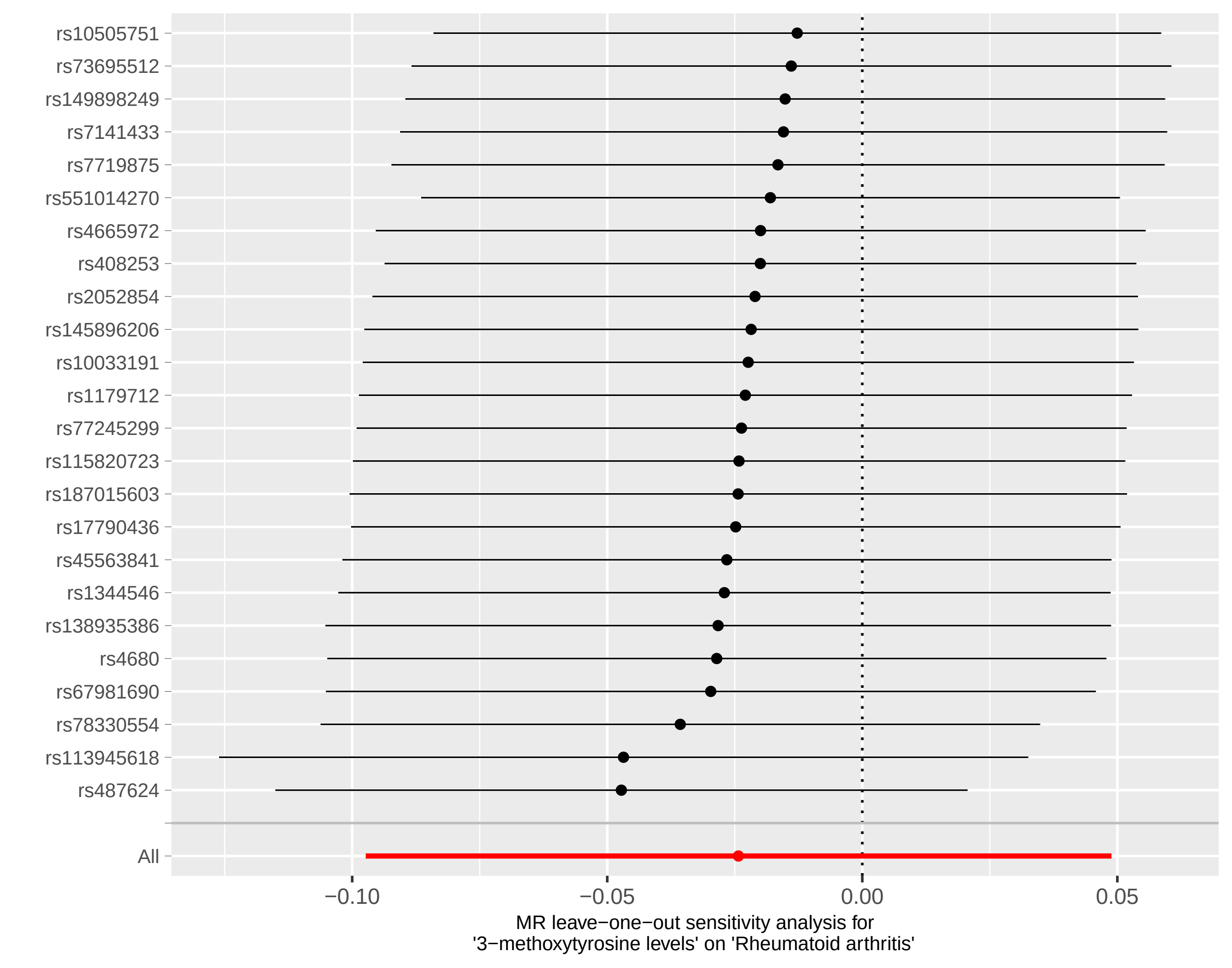
rs10505751
rs73695512
rs149898249
rs7141433
rs7719875
rs551014270
rs4665972
rs408253
rs2052854
rs145896206
rs10033191
rs1179712
rs77245299
rs115820723
rs187015603
rs17790436
rs45563841
rs1344546
rs138935386
rs4680
rs67981690
rs78330554
rs113945618
rs487624
All
−0.10
−0.05
0.00
0.05
MR leave−one−out sensitivity analysis for
'3−methoxytyrosine levels' on 'Rheumatoid arthritis'

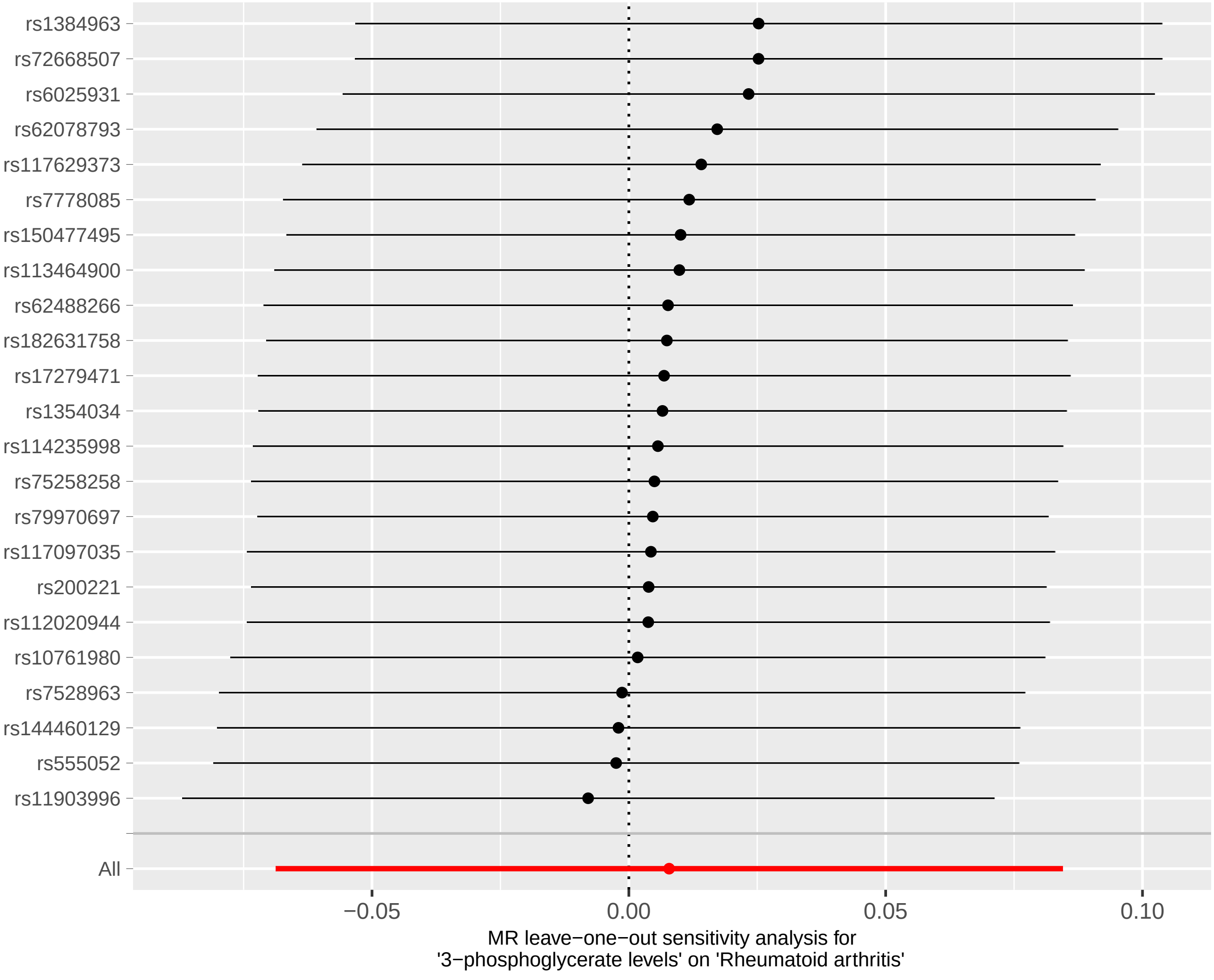
rs1384963
rs72668507
rs6025931
rs62078793
rs117629373
rs7778085
rs150477495
rs113464900
rs62488266
rs182631758
rs17279471
rs1354034
rs114235998
rs75258258
rs79970697
rs117097035
rs200221
rs112020944
rs10761980
rs7528963
rs144460129
rs555052
rs11903996
All
−0.05
0.00
0.05
0.10
MR leave−one−out sensitivity analysis for
'3−phosphoglycerate levels' on 'Rheumatoid arthritis'

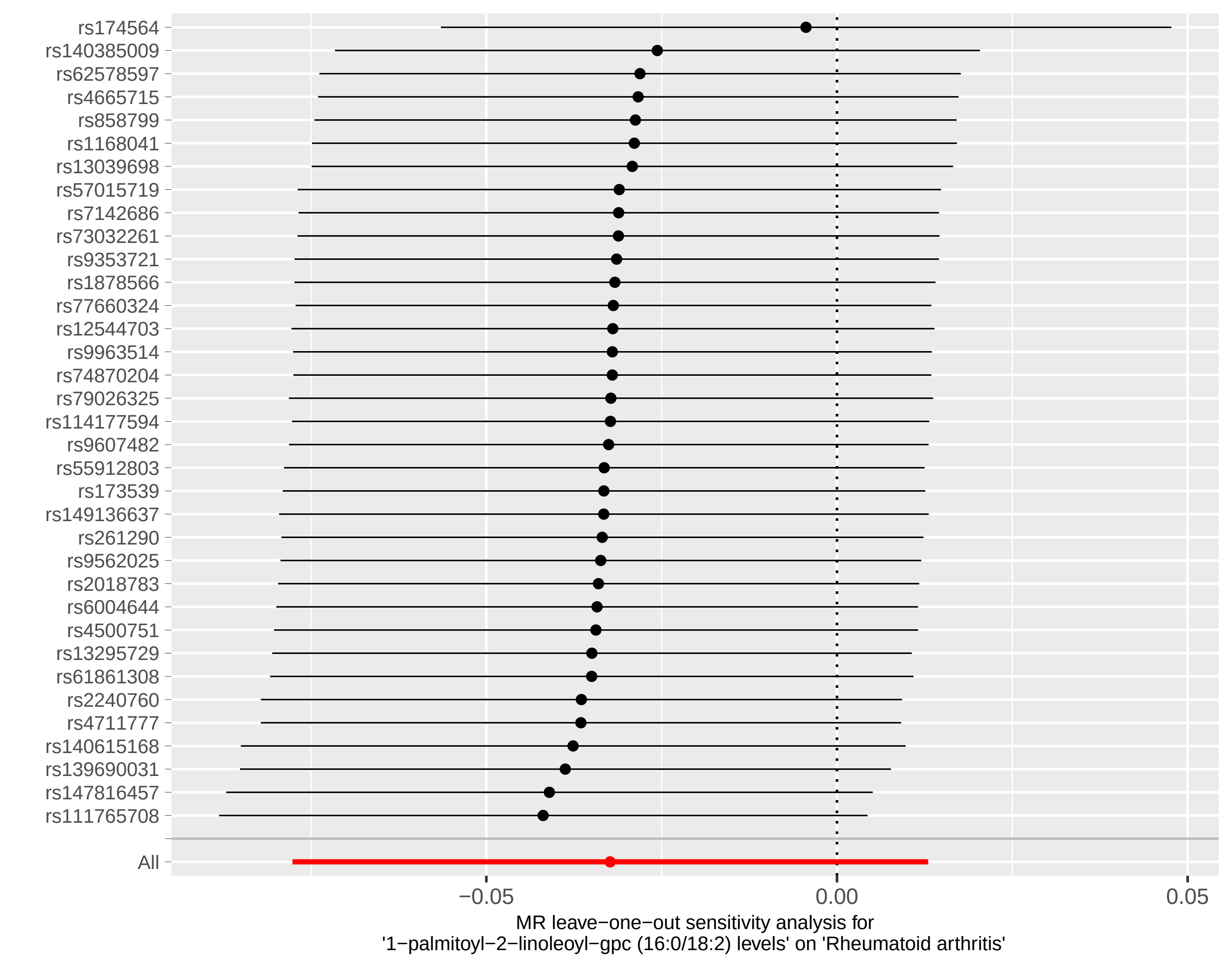
rs174564
rs140385009
rs62578597
rs4665715
rs858799
rs1168041
rs13039698
rs57015719
rs7142686
rs73032261
rs9353721
rs1878566
rs77660324
rs12544703
rs9963514
rs74870204
rs79026325
rs114177594
rs9607482
rs55912803
rs173539
rs149136637
rs261290
rs9562025
rs2018783
rs6004644
rs4500751
rs13295729
rs61861308
rs2240760
rs4711777
rs140615168
rs139690031
rs147816457
rs111765708
All
−0.05
0.00
0.05
MR leave−one−out sensitivity analysis for
'1−palmitoyl−2−linoleoyl−gpc (16:0/18:2) levels' on 'Rheumatoid arthritis'

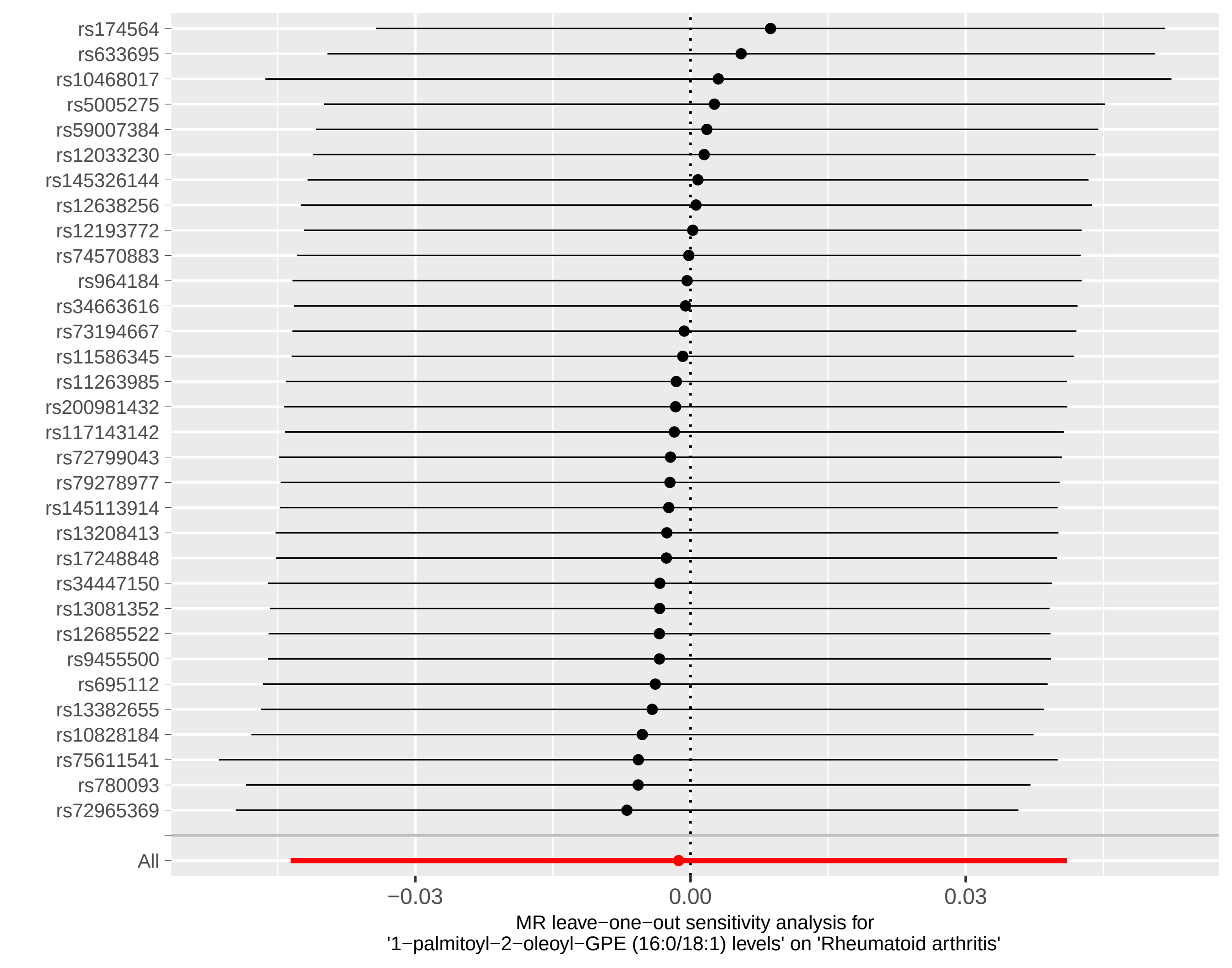
rs174564
rs633695
rs10468017
rs5005275
rs59007384
rs12033230
rs145326144
rs12638256
rs12193772
rs74570883
rs964184
rs34663616
rs73194667
rs11586345
rs11263985
rs200981432
rs117143142
rs72799043
rs79278977
rs145113914
rs13208413
rs17248848
rs34447150
rs13081352
rs12685522
rs9455500
rs695112
rs13382655
rs10828184
rs75611541
rs780093
rs72965369
All
−0.03
0.00
0.03
MR leave−one−out sensitivity analysis for
'1−palmitoyl−2−oleoyl−GPE (16:0/18:1) levels' on 'Rheumatoid arthritis'

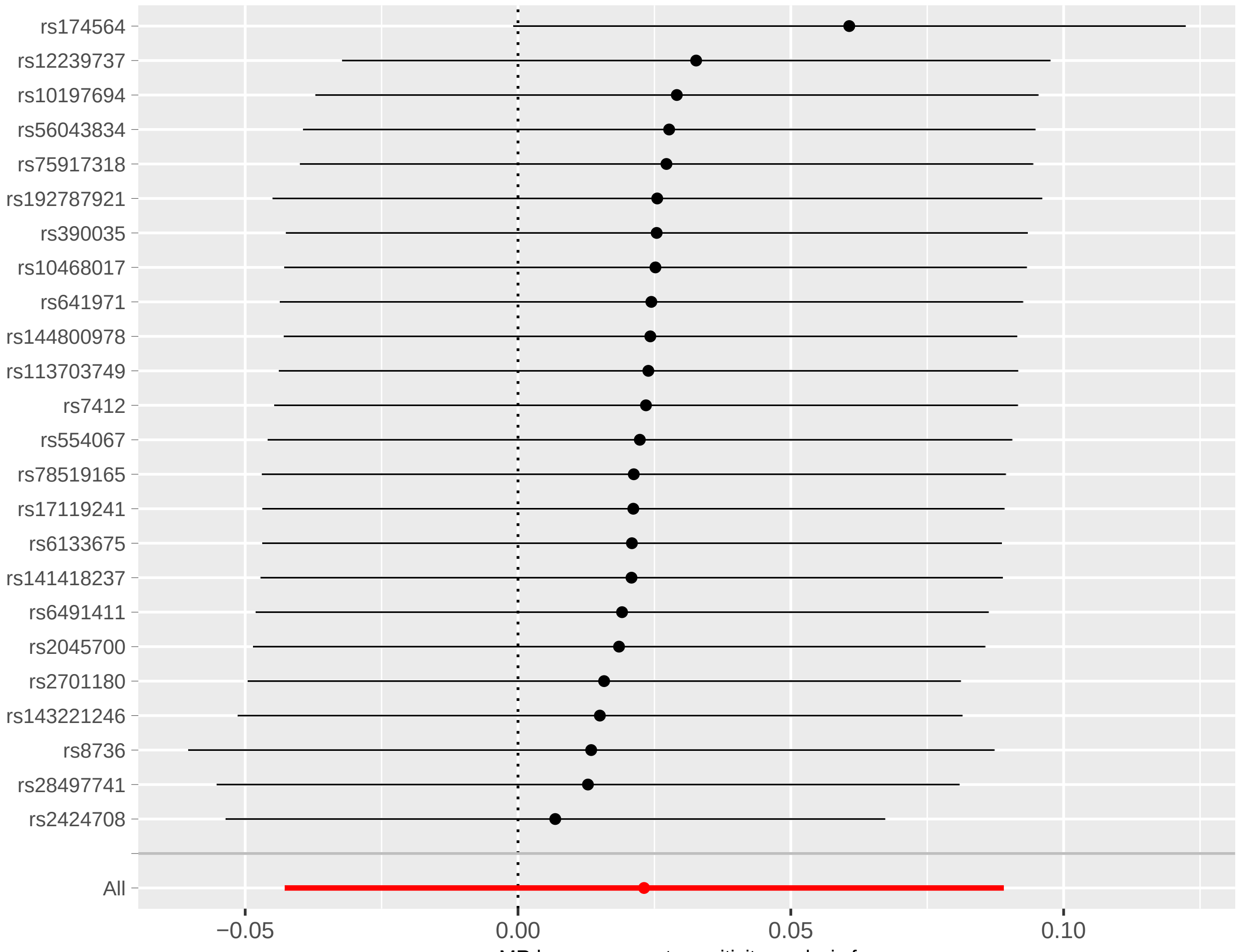

MR leave−one−out sensitivity analysis for
'1−palmitoyl−2−linoleoyl−GPI (16:0/18:2) levels' on 'Rheumatoid arthritis'

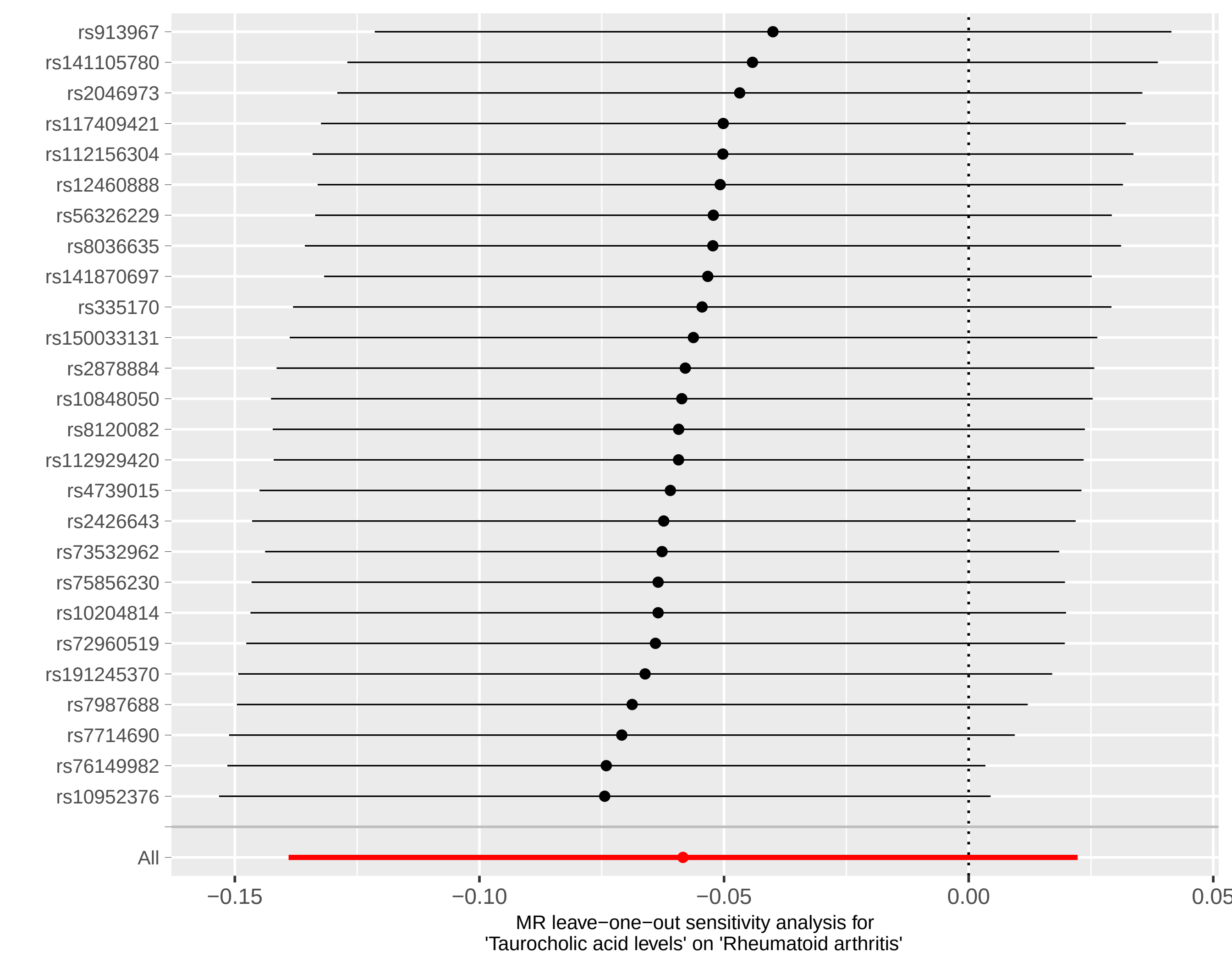
rs913967
rs141105780
rs2046973
rs117409421
rs112156304
rs12460888
rs56326229
rs8036635
rs141870697
rs335170
rs150033131
rs2878884
rs10848050
rs8120082
rs112929420
rs4739015
rs2426643
rs73532962
rs75856230
rs10204814
rs72960519
rs191245370
rs7987688
rs7714690
rs76149982
rs10952376
All
−0.15
−0.10
−0.05
0.00
0.05
MR leave−one−out sensitivity analysis for
'Taurocholic acid levels' on 'Rheumatoid arthritis'

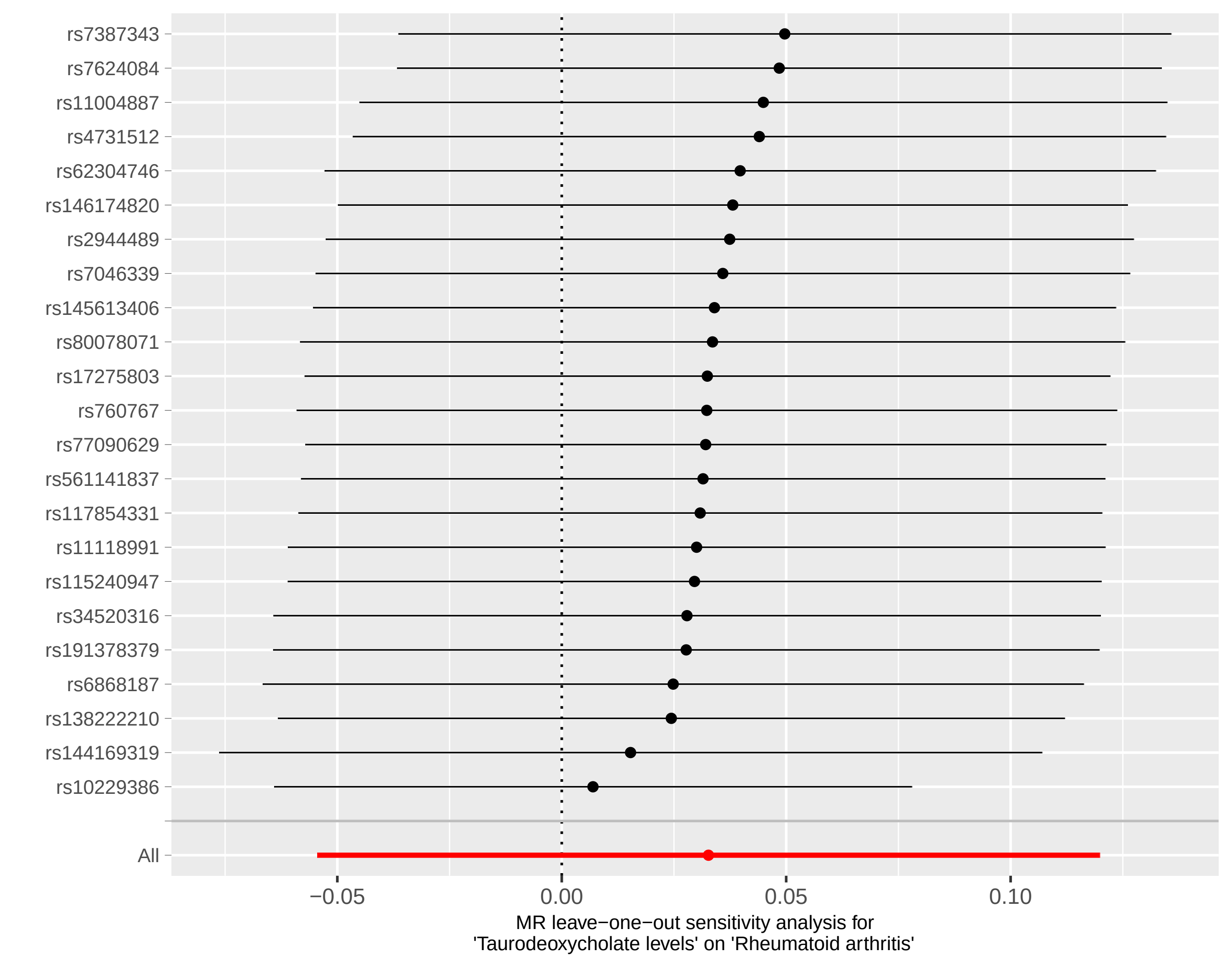
rs7387343
rs7624084
rs11004887
rs4731512
rs62304746
rs146174820
rs2944489
rs7046339
rs145613406
rs80078071
rs17275803
rs760767
rs77090629
rs561141837
rs117854331
rs11118991
rs115240947
rs34520316
rs191378379
rs6868187
rs138222210
rs144169319
rs10229386
All
−0.05
0.00
0.05
0.10
MR leave−one−out sensitivity analysis for
'Taurodeoxycholate levels' on 'Rheumatoid arthritis'

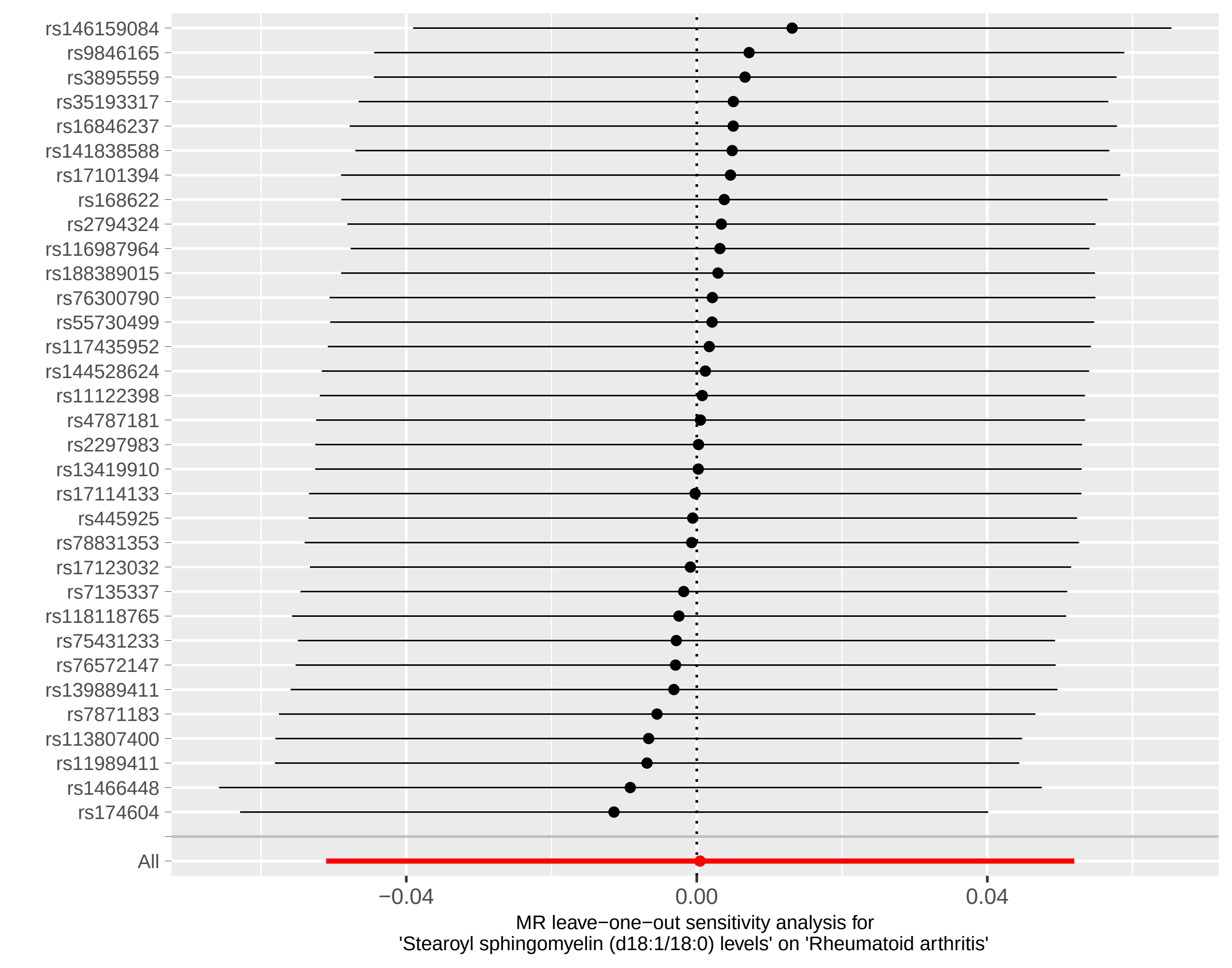
rs146159084
rs9846165
rs3895559
rs35193317
rs16846237
rs141838588
rs17101394
rs168622
rs2794324
rs116987964
rs188389015
rs76300790
rs55730499
rs117435952
rs144528624
rs11122398
rs4787181
rs2297983
rs13419910
rs17114133
rs445925
rs78831353
rs17123032
rs7135337
rs118118765
rs75431233
rs76572147
rs139889411
rs7871183
rs113807400
rs11989411
rs1466448
rs174604
All
−0.04
0.00
0.04
MR leave−one−out sensitivity analysis for
'Stearoyl sphingomyelin (d18:1/18:0) levels' on 'Rheumatoid arthritis'

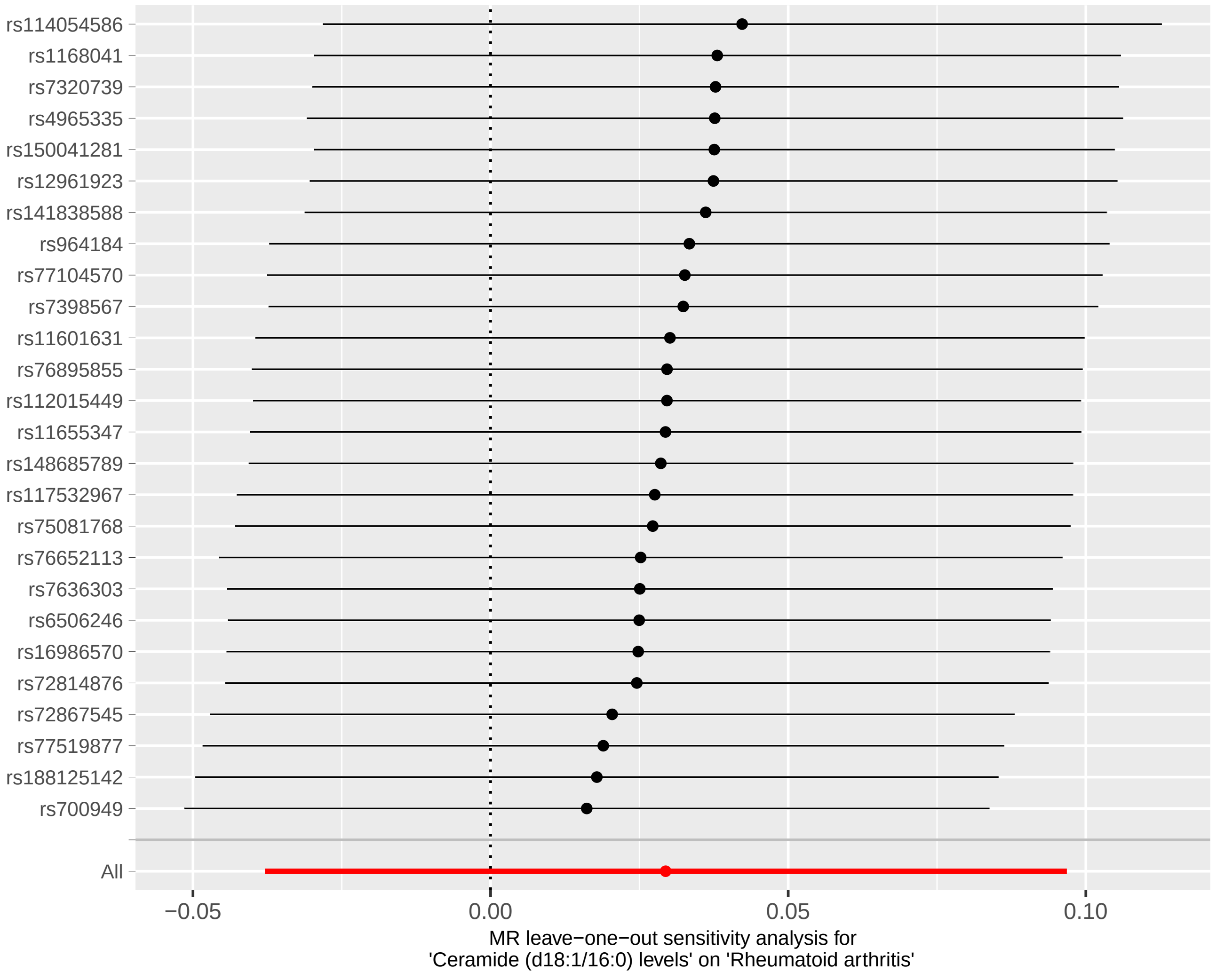

rs114054586
rs1168041
rs7320739
rs4965335
rs150041281
rs12961923
rs141838588
rs964184
rs77104570
rs7398567
rs11601631
rs76895855
rs112015449
rs11655347
rs148685789
rs117532967
rs75081768
rs76652113
rs7636303
rs6506246
rs16986570
rs72814876
rs72867545
rs77519877
rs188125142
rs700949
All
−0.05
0.00
0.05
0.10
MR leave−one−out sensitivity analysis for
'Ceramide (d18:1/16:0) levels' on 'Rheumatoid arthritis'

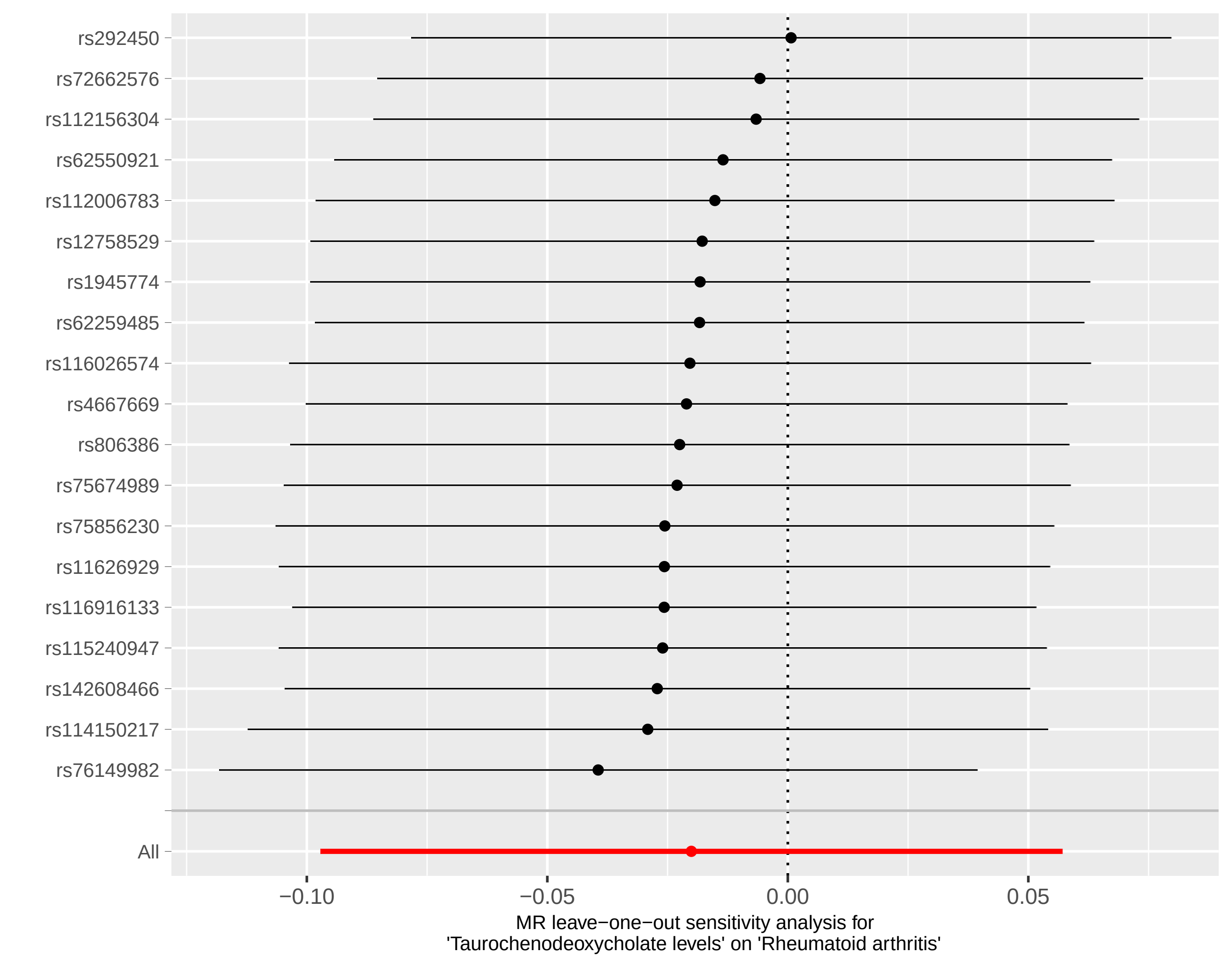
rs292450
rs72662576
rs112156304
rs62550921
rs112006783
rs12758529
rs1945774
rs62259485
rs116026574
rs4667669
rs806386
rs75674989
rs75856230
rs11626929
rs116916133
rs115240947
rs142608466
rs114150217
rs76149982
All
−0.10
−0.05
0.00
0.05
MR leave−one−out sensitivity analysis for
'Taurochenodeoxycholate levels' on 'Rheumatoid arthritis'

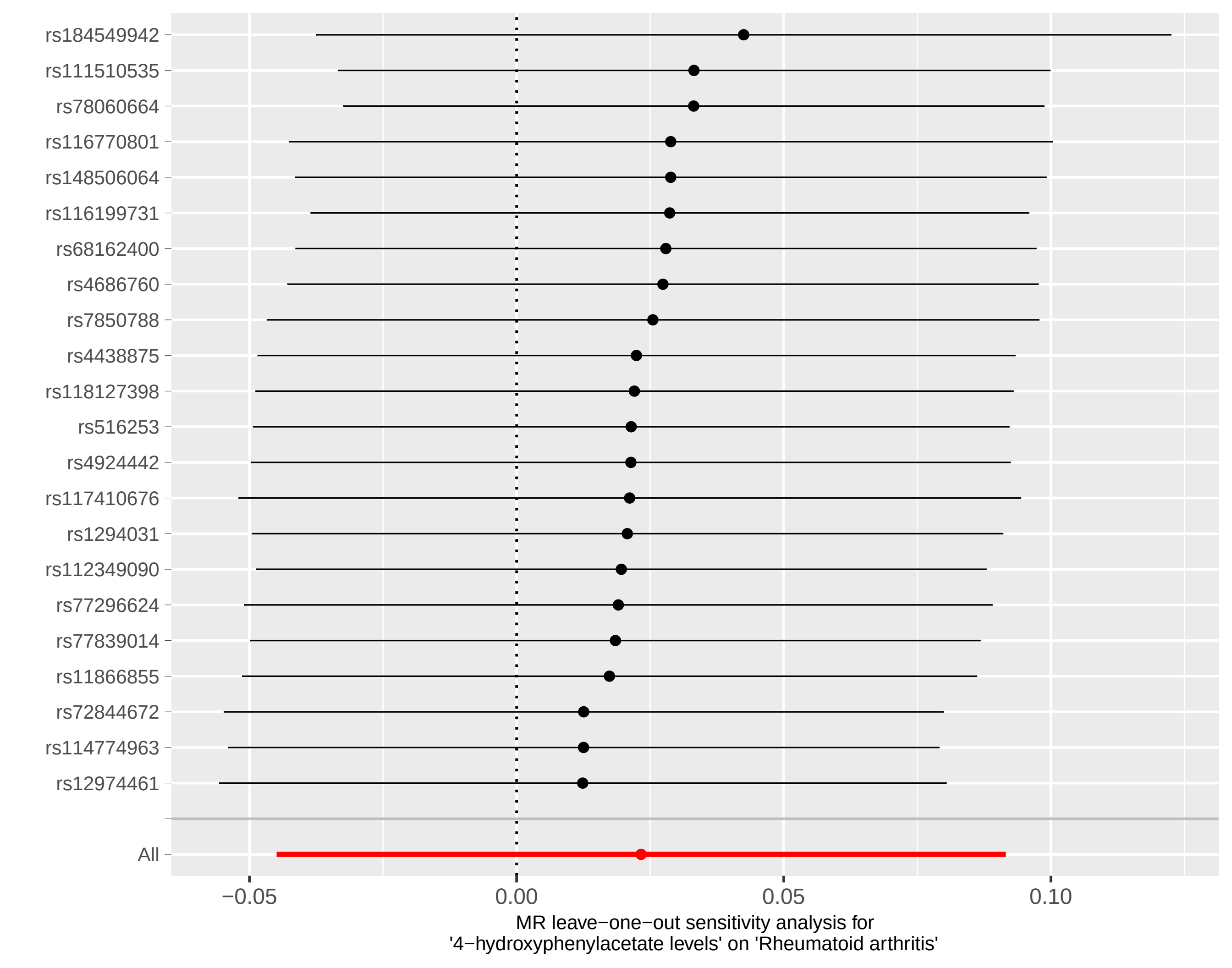

rs184549942
rs111510535
rs78060664
rs116770801
rs148506064
rs116199731
rs68162400
rs4686760
rs7850788
rs4438875
rs118127398
rs516253
rs4924442
rs117410676
rs1294031
rs112349090
rs77296624
rs77839014
rs11866855
rs72844672
rs114774963
rs12974461
All
−0.05
0.00
0.05
0.10
MR leave−one−out sensitivity analysis for
'4−hydroxyphenylacetate levels' on 'Rheumatoid arthritis'

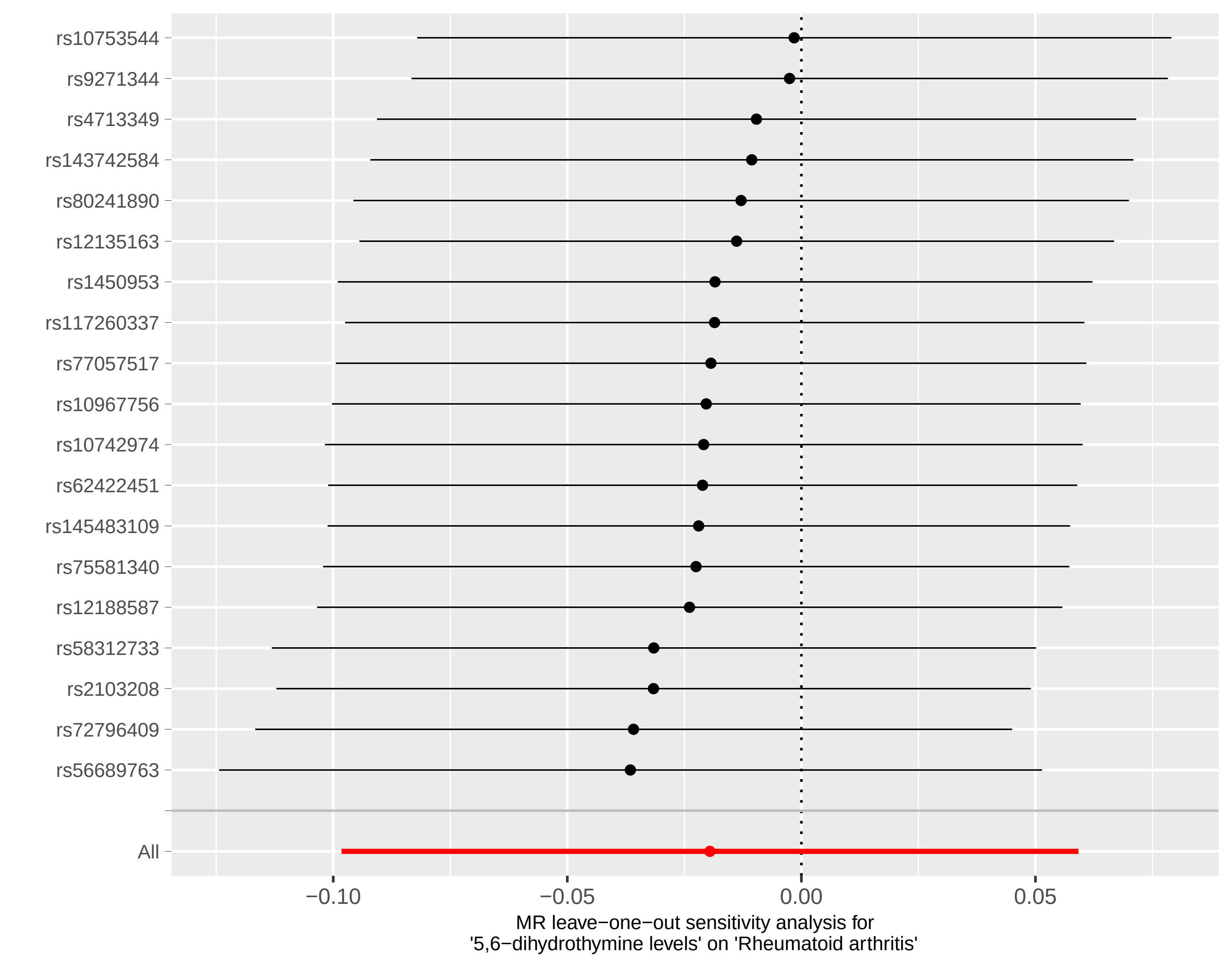
rs10753544
rs9271344
rs4713349
rs143742584
rs80241890
rs12135163
rs1450953
rs117260337
rs77057517
rs10967756
rs10742974
rs62422451
rs145483109
rs75581340
rs12188587
rs58312733
rs2103208
rs72796409
rs56689763
All
−0.10
−0.05
0.00
0.05
MR leave−one−out sensitivity analysis for
'5,6−dihydrothymine levels' on 'Rheumatoid arthritis'

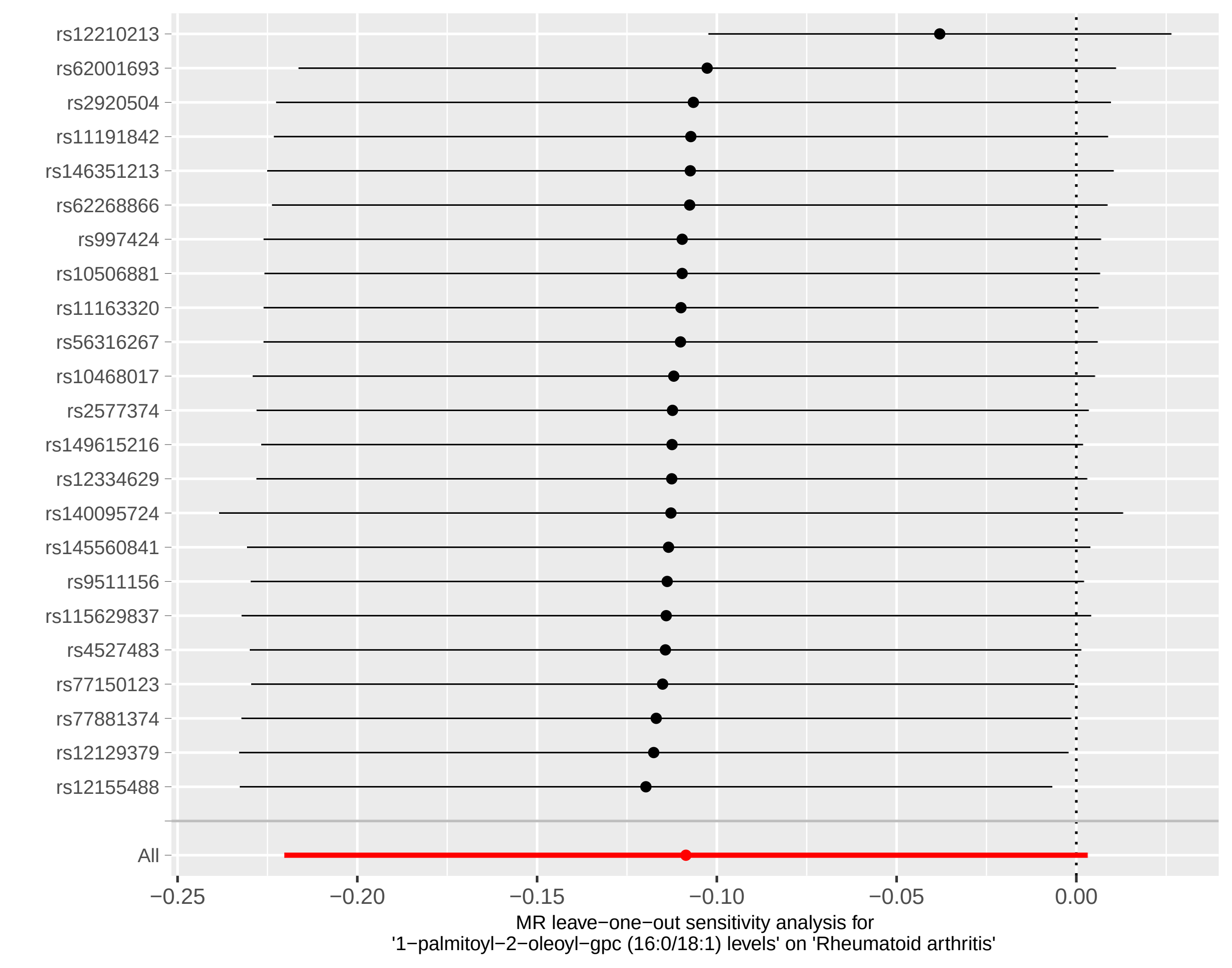

rs12210213
rs62001693
rs2920504
rs11191842
rs146351213
rs62268866
rs997424
rs10506881
rs11163320
rs56316267
rs10468017
rs2577374
rs149615216
rs12334629
rs140095724
rs145560841
rs9511156
rs115629837
rs4527483
rs77150123
rs77881374
rs12129379
rs12155488
All
−0.25
−0.20
−0.15
−0.10
−0.05
0.00
MR leave−one−out sensitivity analysis for
'1−palmitoyl−2−oleoyl−gpc (16:0/18:1) levels' on 'Rheumatoid arthritis'

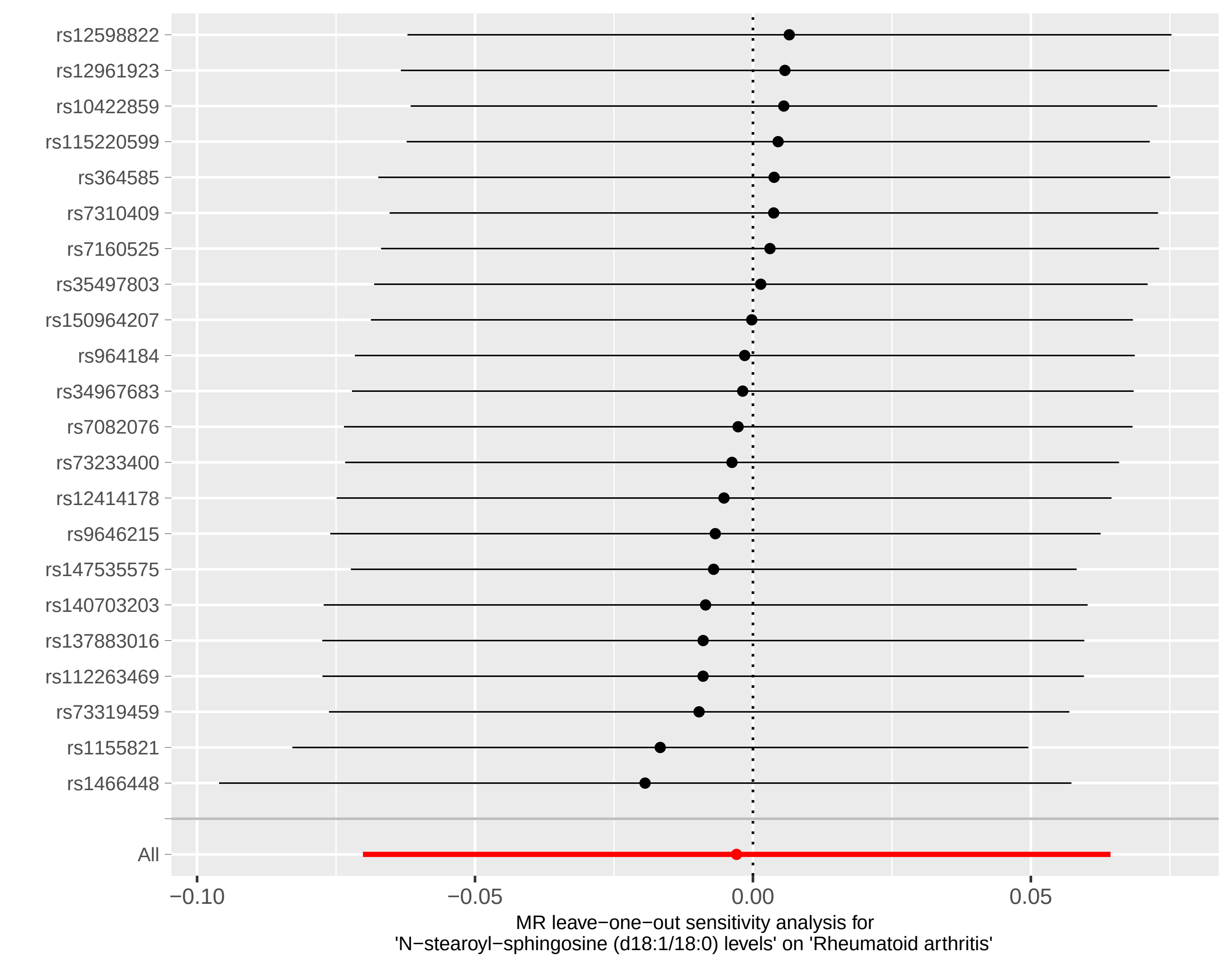
rs12598822
rs12961923
rs10422859
rs115220599
rs364585
rs7310409
rs7160525
rs35497803
rs150964207
rs964184
rs34967683
rs7082076
rs73233400
rs12414178
rs9646215
rs147535575
rs140703203
rs137883016
rs112263469
rs73319459
rs1155821
rs1466448
All
−0.10
−0.05
0.00
0.05
MR leave−one−out sensitivity analysis for
'N−stearoyl−sphingosine (d18:1/18:0) levels' on 'Rheumatoid arthritis'

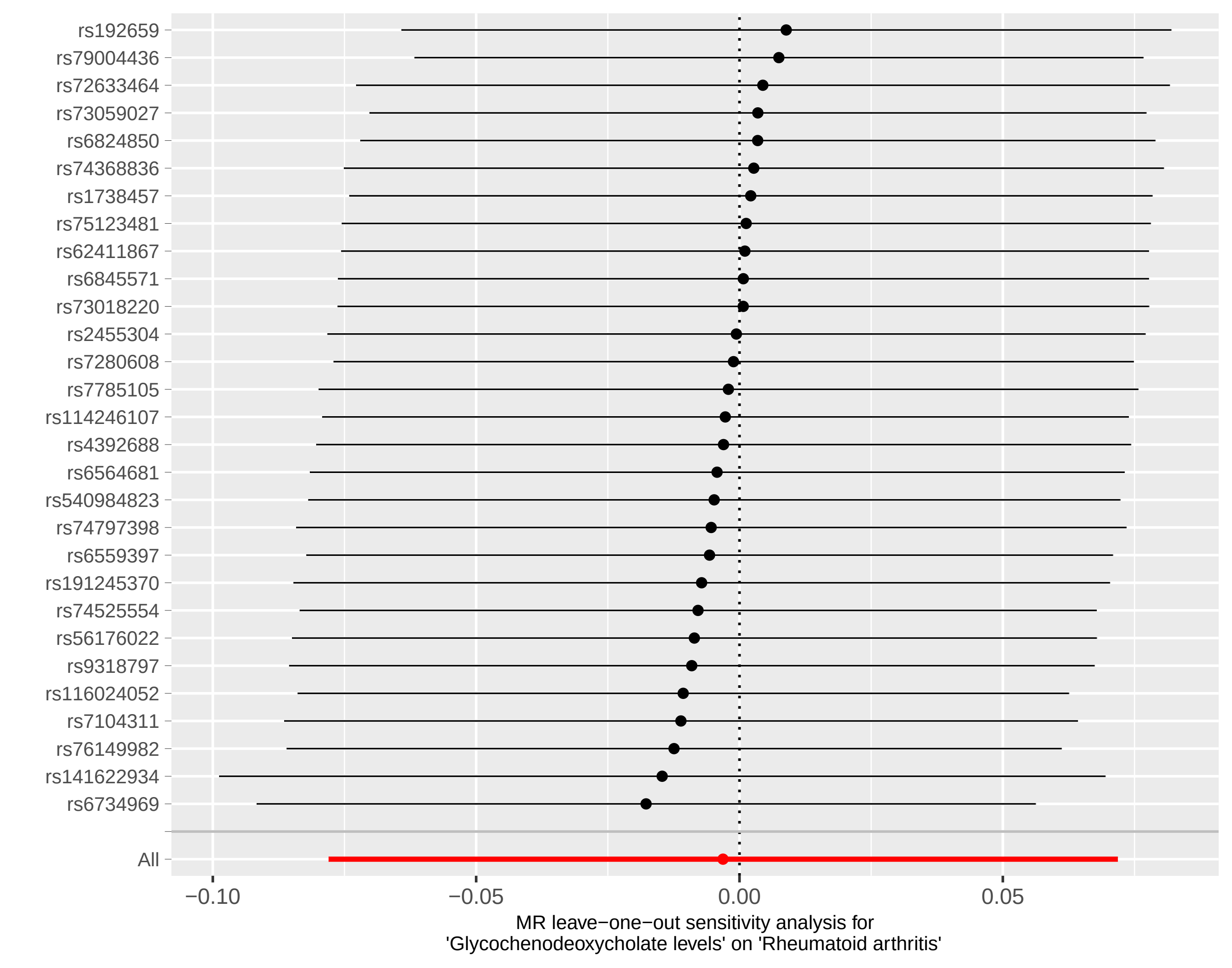

rs192659
rs79004436
rs72633464
rs73059027
rs6824850
rs74368836
rs1738457
rs75123481
rs62411867
rs6845571
rs73018220
rs2455304
rs7280608
rs7785105
rs114246107
rs4392688
rs6564681
rs540984823
rs74797398
rs6559397
rs191245370
rs74525554
rs56176022
rs9318797
rs116024052
rs7104311
rs76149982
rs141622934
rs6734969
All
−0.10
−0.05
0.00
0.05
MR leave−one−out sensitivity analysis for
'Glycochenodeoxycholate levels' on 'Rheumatoid arthritis'

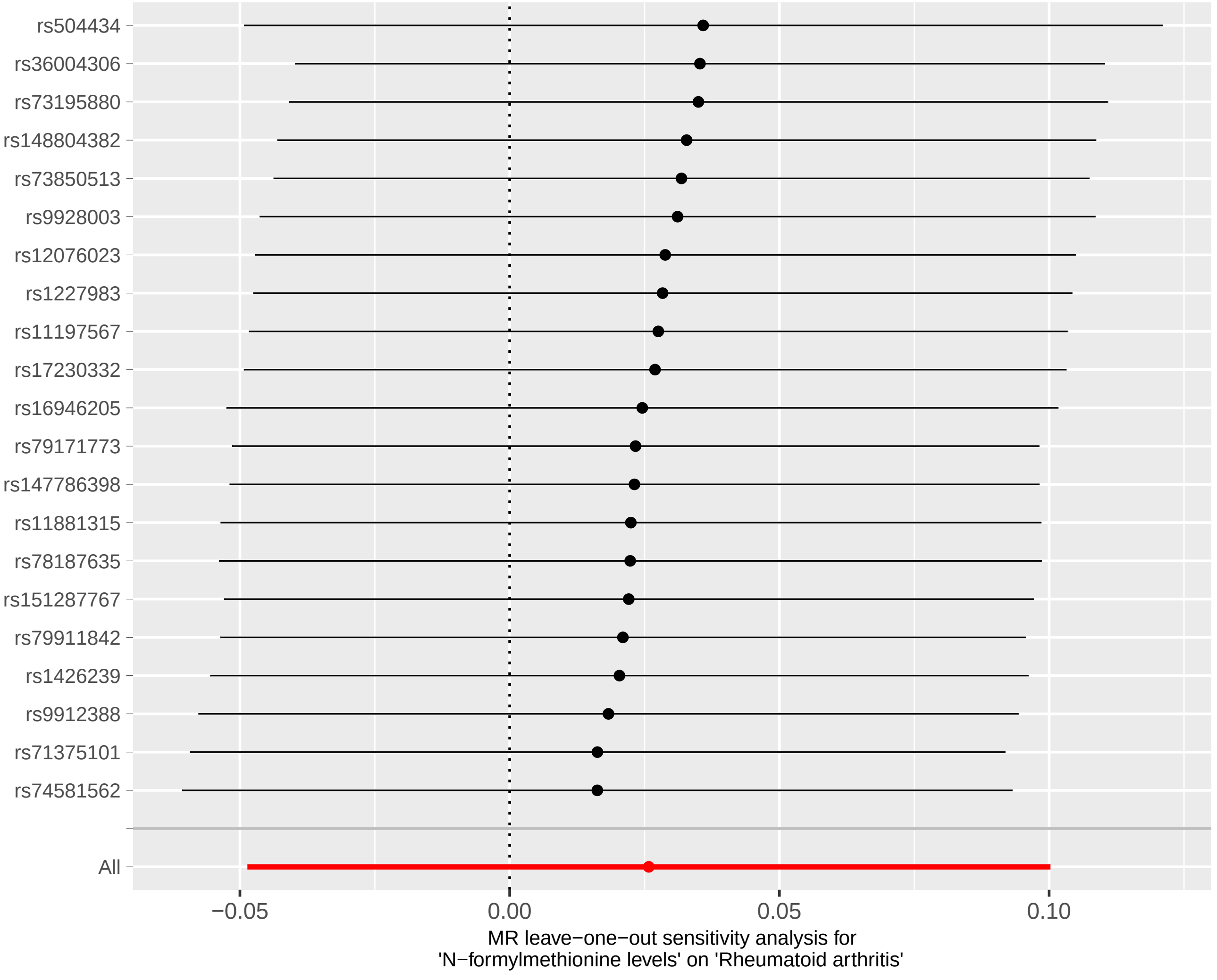
rs504434
rs36004306
rs73195880
rs148804382
rs73850513
rs9928003
rs12076023
rs1227983
rs11197567
rs17230332
rs16946205
rs79171773
rs147786398
rs11881315
rs78187635
rs151287767
rs79911842
rs1426239
rs9912388
rs71375101
rs74581562
All
−0.05
0.00
0.05
0.10
MR leave−one−out sensitivity analysis for
'N−formylmethionine levels' on 'Rheumatoid arthritis'

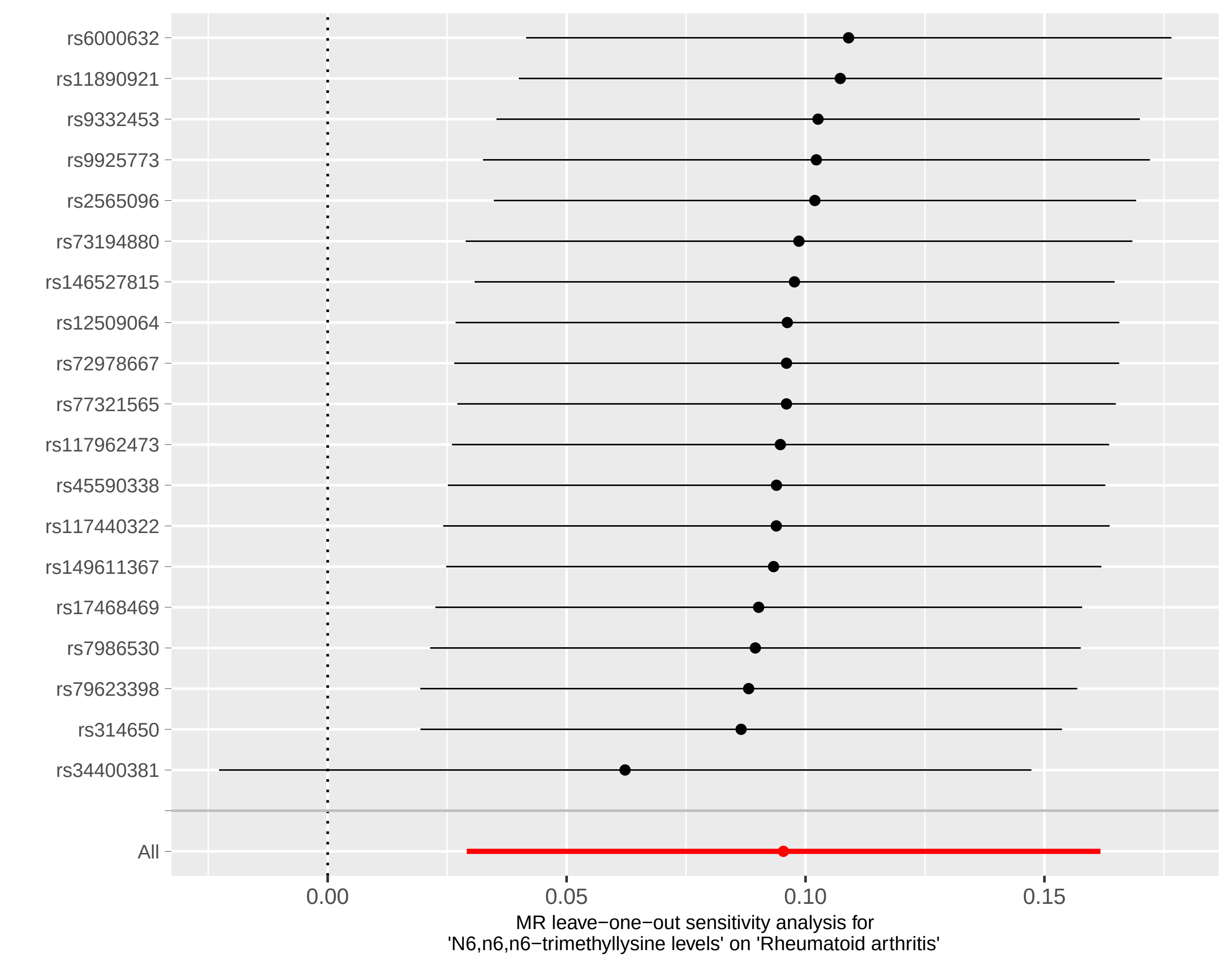
rs6000632
rs11890921
rs9332453
rs9925773
rs2565096
rs73194880
rs146527815
rs12509064
rs72978667
rs77321565
rs117962473
rs45590338
rs117440322
rs149611367
rs17468469
rs7986530
rs79623398
rs314650
rs34400381
All
0.00
0.05
0.10
0.15
MR leave−one−out sensitivity analysis for
'N6,n6,n6−trimethyllysine levels' on 'Rheumatoid arthritis'

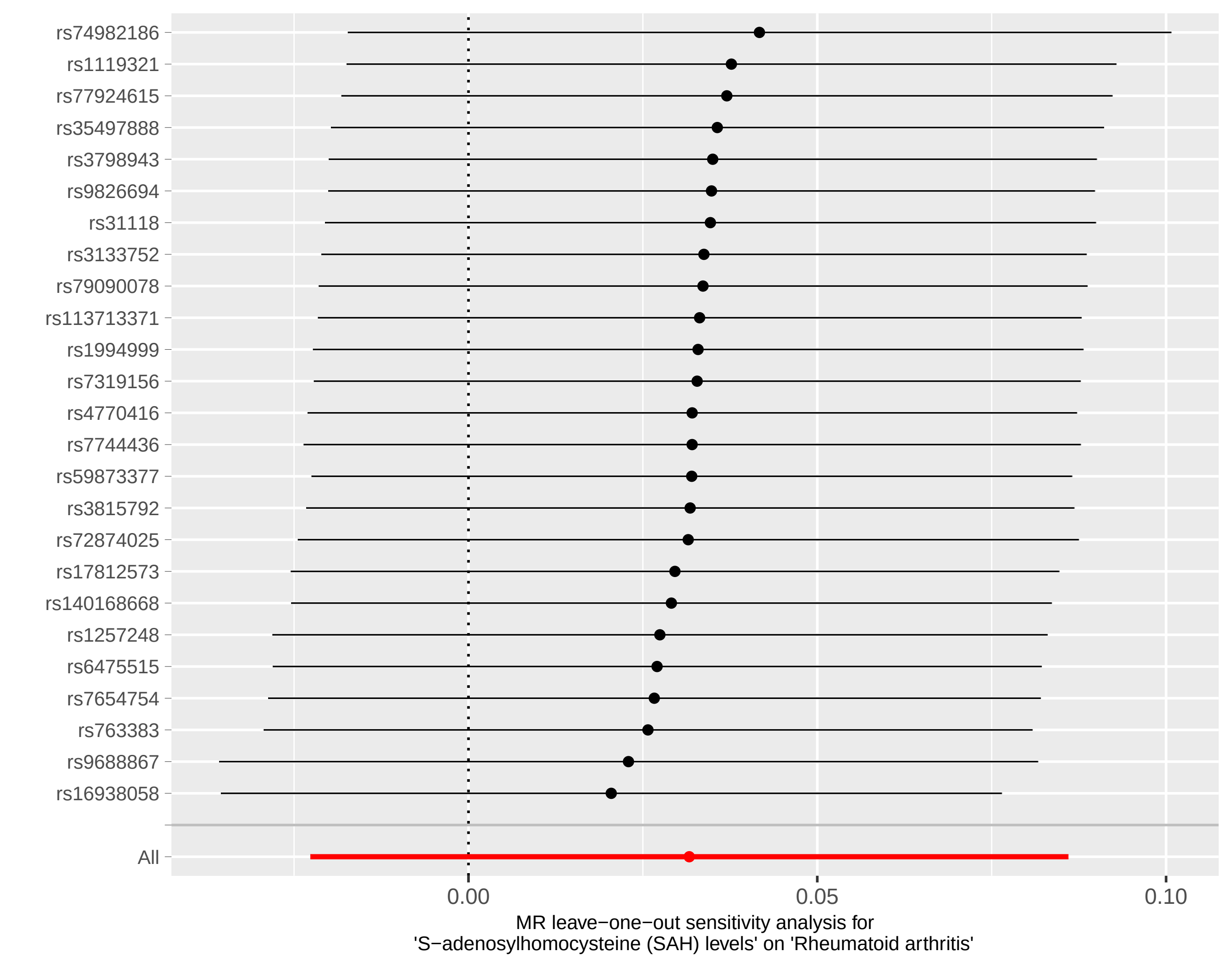
rs74982186
rs1119321
rs77924615
rs35497888
rs3798943
rs9826694
rs31118
rs3133752
rs79090078
rs113713371
rs1994999
rs7319156
rs4770416
rs7744436
rs59873377
rs3815792
rs72874025
rs17812573
rs140168668
rs1257248
rs6475515
rs7654754
rs763383
rs9688867
rs16938058
All
0.00
0.05
0.10
MR leave−one−out sensitivity analysis for
'S−adenosylhomocysteine (SAH) levels' on 'Rheumatoid arthritis'

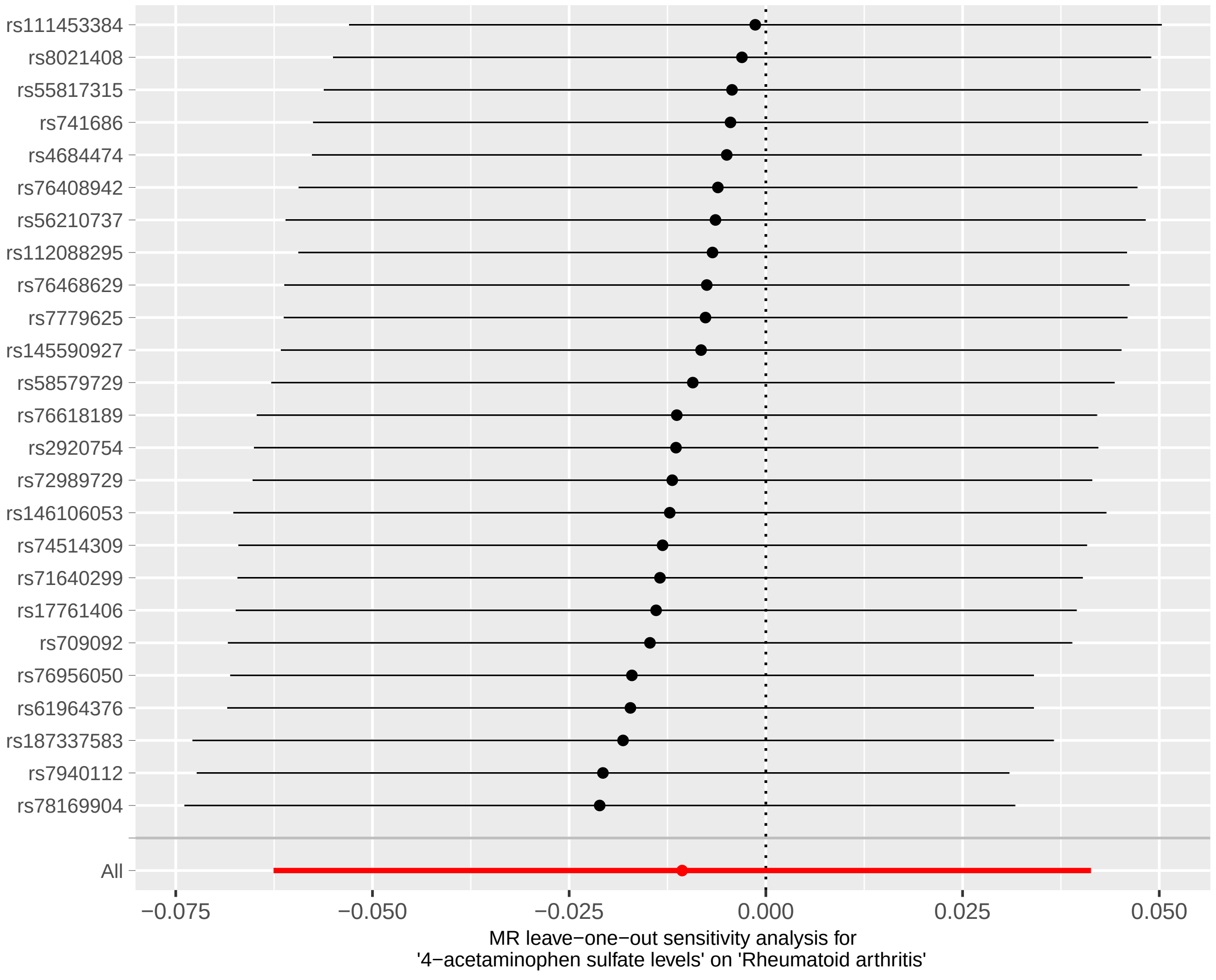

rs111453384
rs8021408
rs55817315
rs741686
rs4684474
rs76408942
rs56210737
rs112088295
rs76468629
rs7779625
rs145590927
rs58579729
rs76618189
rs2920754
rs72989729
rs146106053
rs74514309
rs71640299
rs17761406
rs709092
rs76956050
rs61964376
rs187337583
rs7940112
rs78169904
All
−0.075
−0.050
−0.025
0.000
0.025
0.050
MR leave−one−out sensitivity analysis for
'4−acetaminophen sulfate levels' on 'Rheumatoid arthritis'

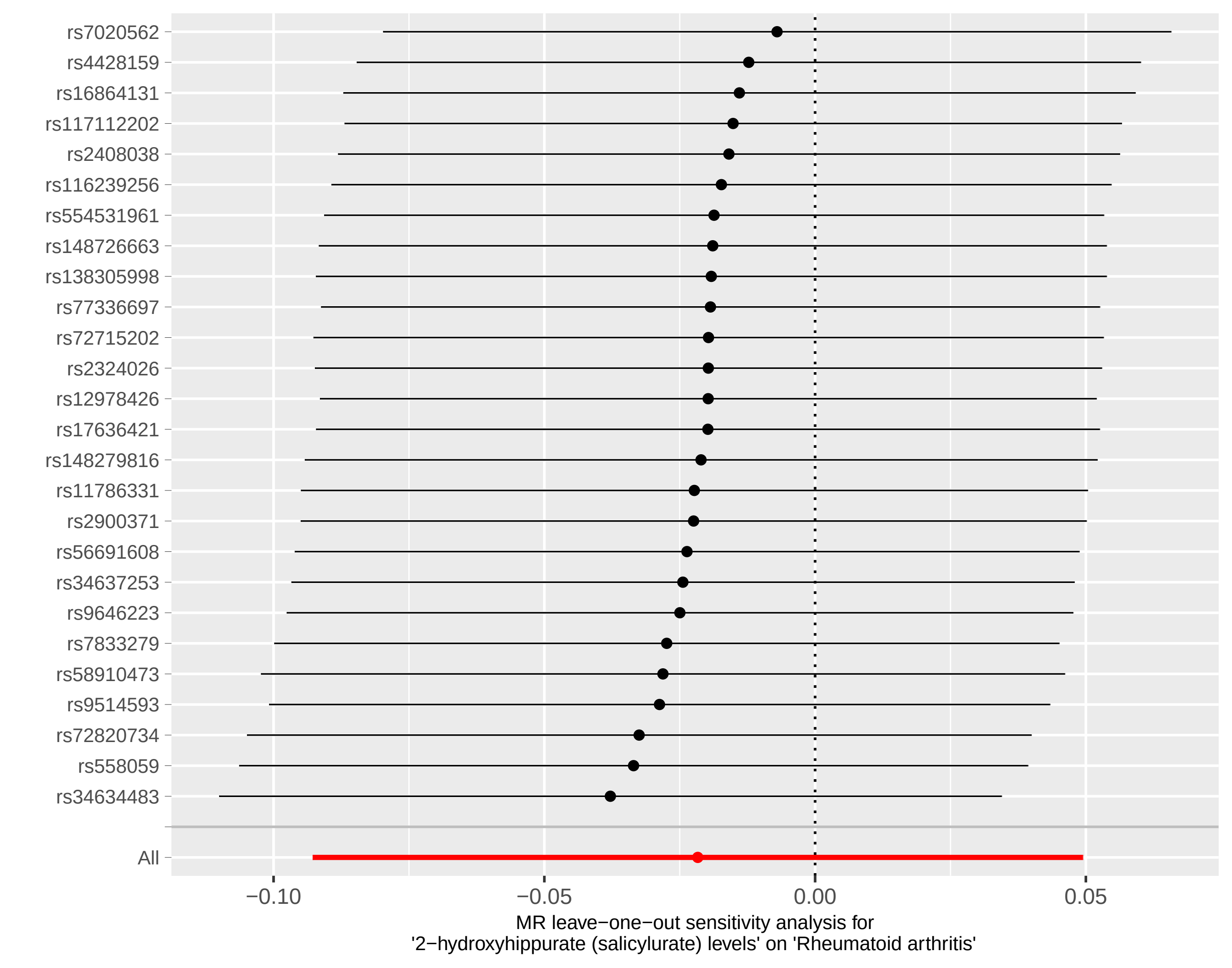
rs7020562
rs4428159
rs16864131
rs117112202
rs2408038
rs116239256
rs554531961
rs148726663
rs138305998
rs77336697
rs72715202
rs2324026
rs12978426
rs17636421
rs148279816
rs11786331
rs2900371
rs56691608
rs34637253
rs9646223
rs7833279
rs58910473
rs9514593
rs72820734
rs558059
rs34634483
All
−0.10
−0.05
0.00
0.05
MR leave−one−out sensitivity analysis for
'2−hydroxyhippurate (salicylurate) levels' on 'Rheumatoid arthritis'

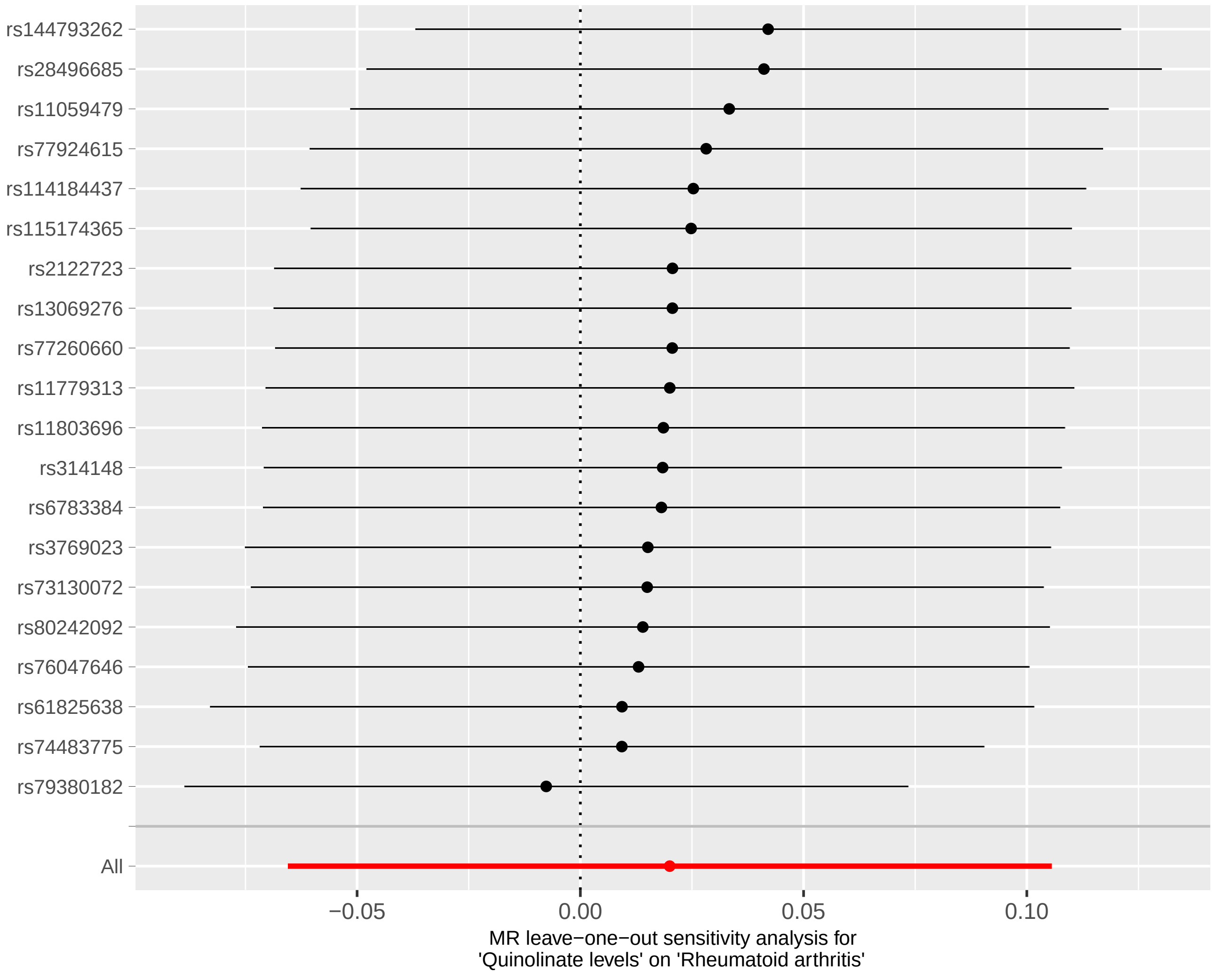
rs144793262
rs28496685
rs11059479
rs77924615
rs114184437
rs115174365
rs2122723
rs13069276
rs77260660
rs11779313
rs11803696
rs314148
rs6783384
rs3769023
rs73130072
rs80242092
rs76047646
rs61825638
rs74483775
rs79380182
All
−0.05
0.00
0.05
0.10
MR leave−one−out sensitivity analysis for
'Quinolinate levels' on 'Rheumatoid arthritis'

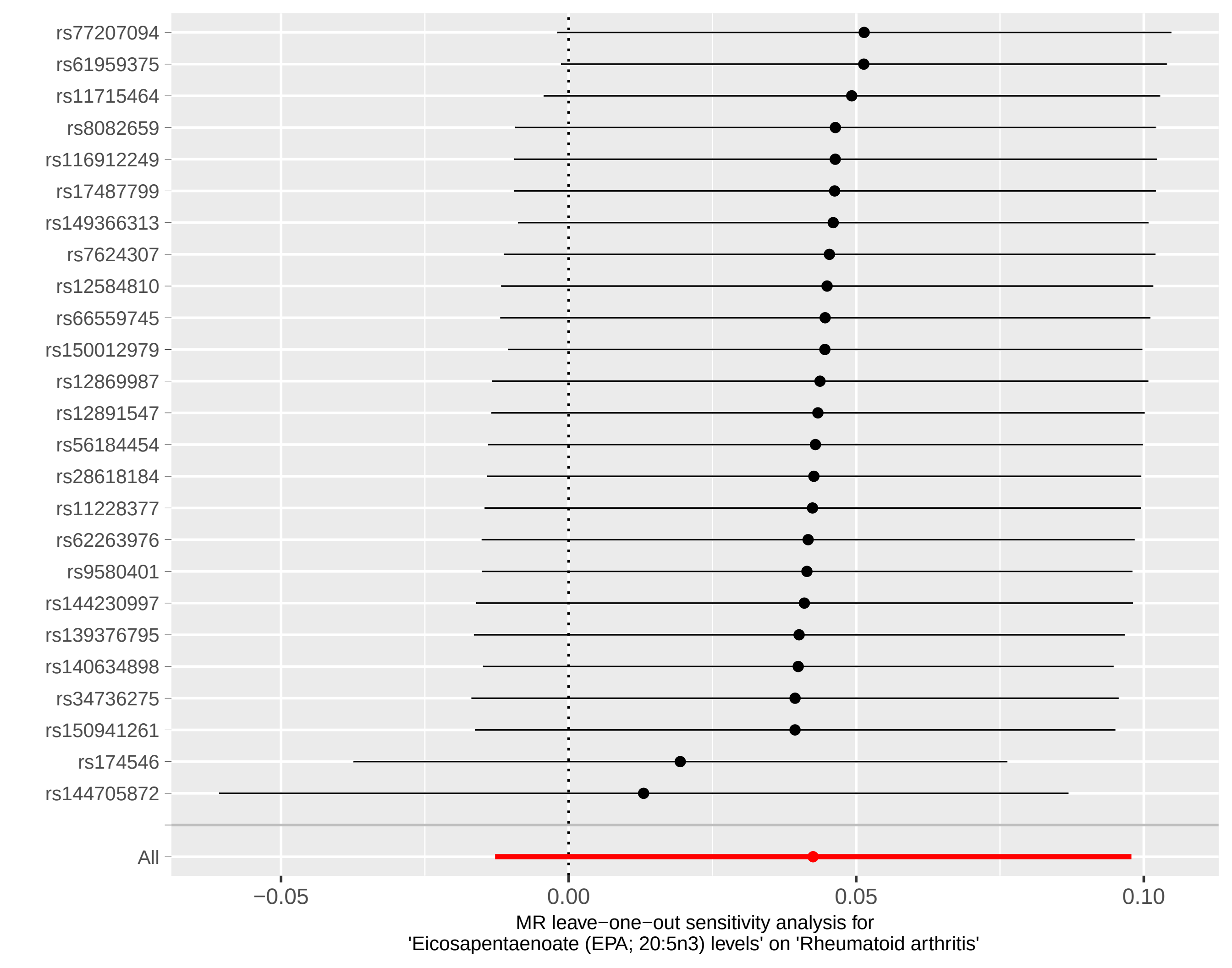
rs77207094
rs61959375
rs11715464
rs8082659
rs116912249
rs17487799
rs149366313
rs7624307
rs12584810
rs66559745
rs150012979
rs12869987
rs12891547
rs56184454
rs28618184
rs11228377
rs62263976
rs9580401
rs144230997
rs139376795
rs140634898
rs34736275
rs150941261
rs174546
rs144705872
All
−0.05
0.00
0.05
0.10
MR leave−one−out sensitivity analysis for
'Eicosapentaenoate (EPA; 20:5n3) levels' on 'Rheumatoid arthritis'

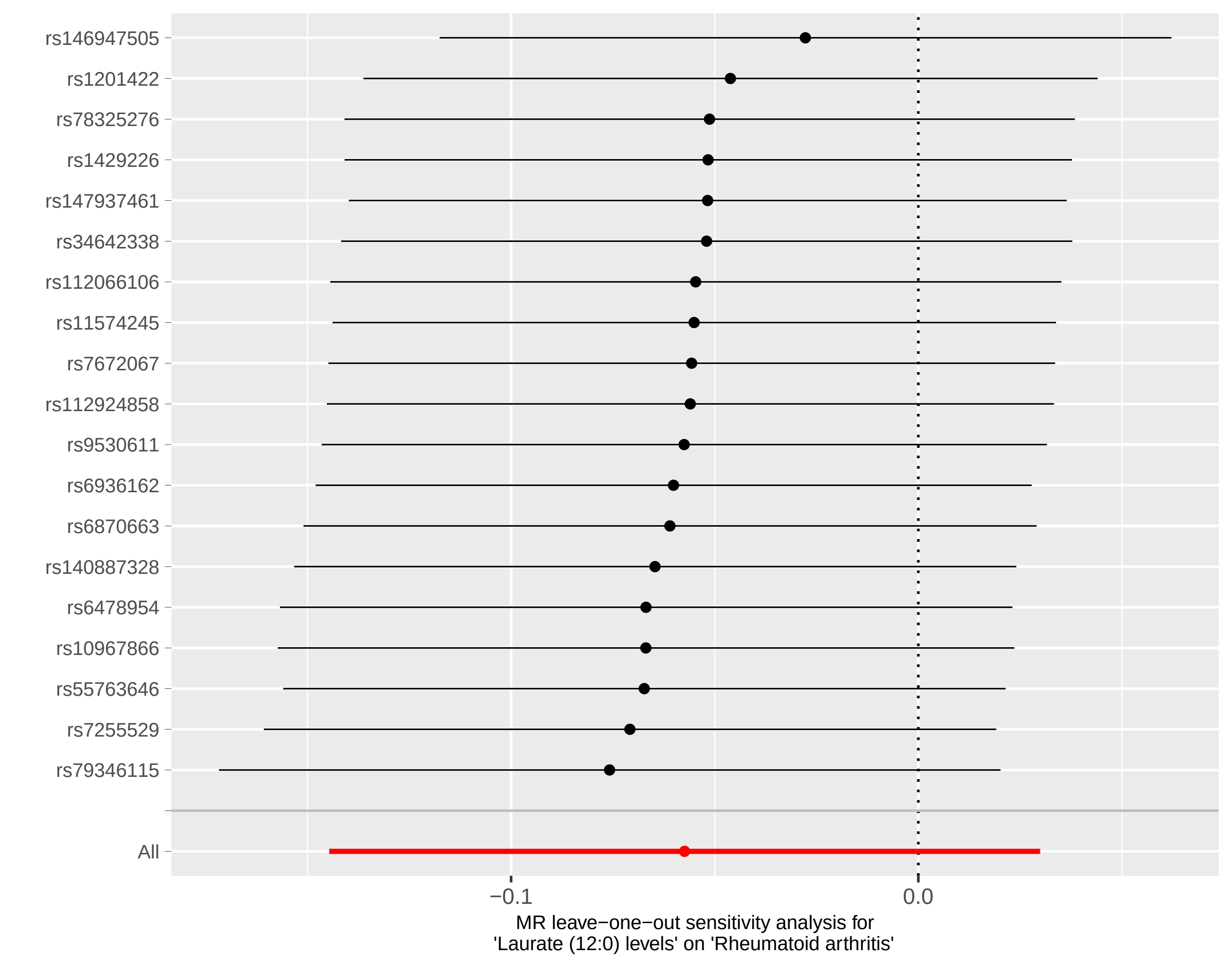
rs146947505
rs1201422
rs78325276
rs1429226
rs147937461
rs34642338
rs112066106
rs11574245
rs7672067
rs112924858
rs9530611
rs6936162
rs6870663
rs140887328
rs6478954
rs10967866
rs55763646
rs7255529
rs79346115
All
−0.1
0.0
MR leave−one−out sensitivity analysis for
'Laurate (12:0) levels' on 'Rheumatoid arthritis'

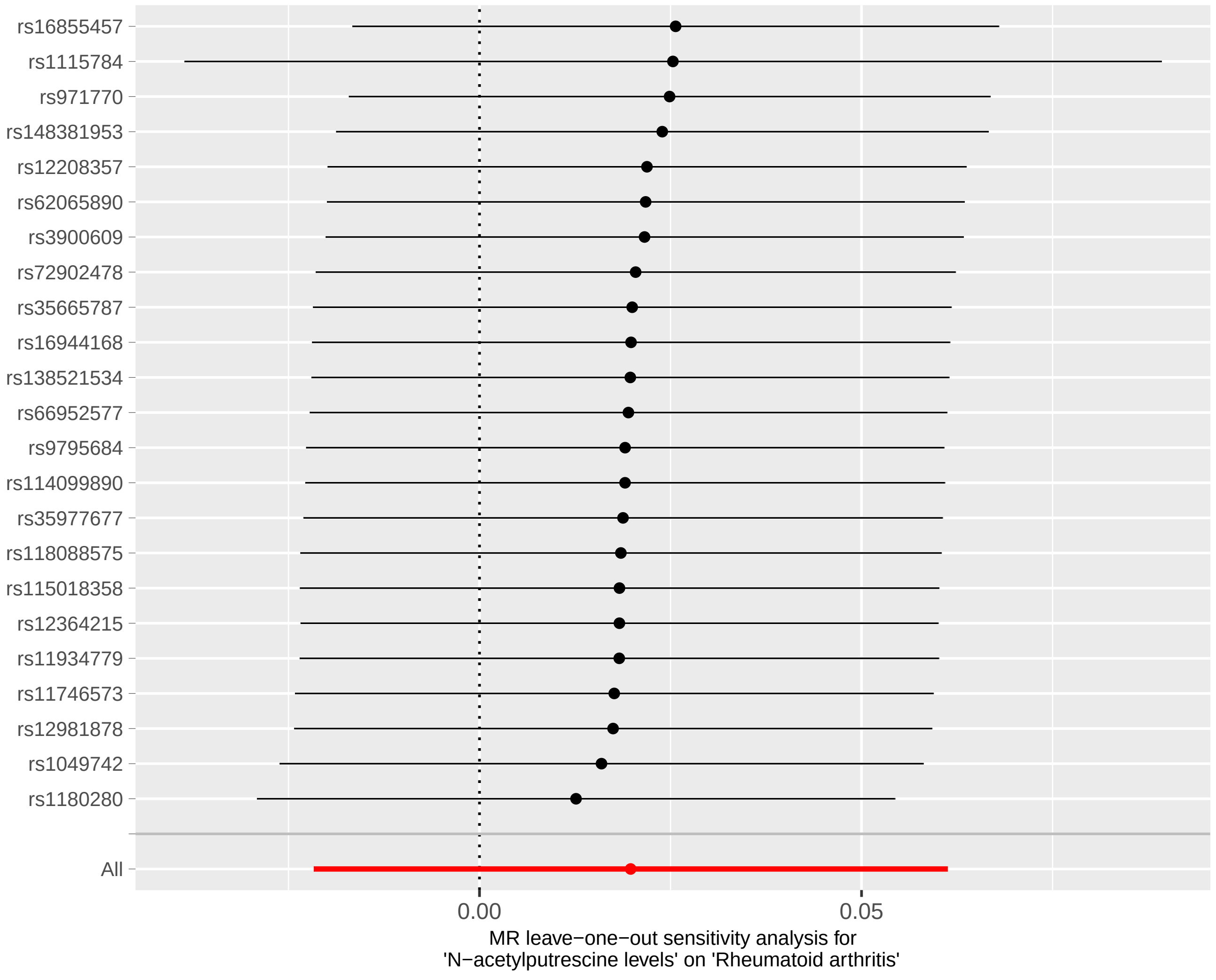
rs16855457
rs1115784
rs971770
rs148381953
rs12208357
rs62065890
rs3900609
rs72902478
rs35665787
rs16944168
rs138521534
rs66952577
rs9795684
rs114099890
rs35977677
rs118088575
rs115018358
rs12364215
rs11934779
rs11746573
rs12981878
rs1049742
rs1180280
All
0.00
0.05
MR leave−one−out sensitivity analysis for
'N−acetylputrescine levels' on 'Rheumatoid arthritis'

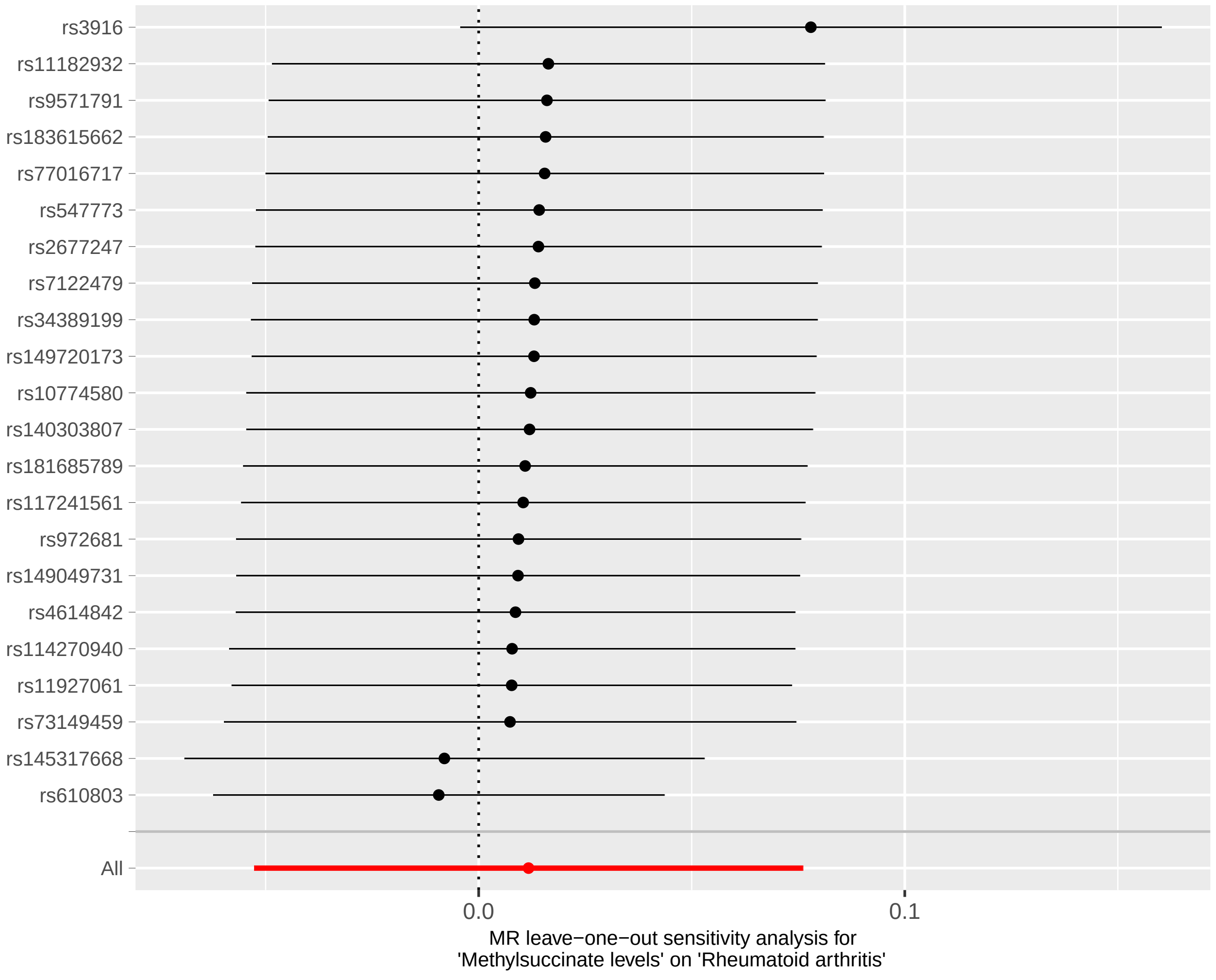
rs3916
rs11182932
rs9571791
rs183615662
rs77016717
rs547773
rs2677247
rs7122479
rs34389199
rs149720173
rs10774580
rs140303807
rs181685789
rs117241561
rs972681
rs149049731
rs4614842
rs114270940
rs11927061
rs73149459
rs145317668
rs610803
All
0.0
0.1
MR leave−one−out sensitivity analysis for
'Methylsuccinate levels' on 'Rheumatoid arthritis'

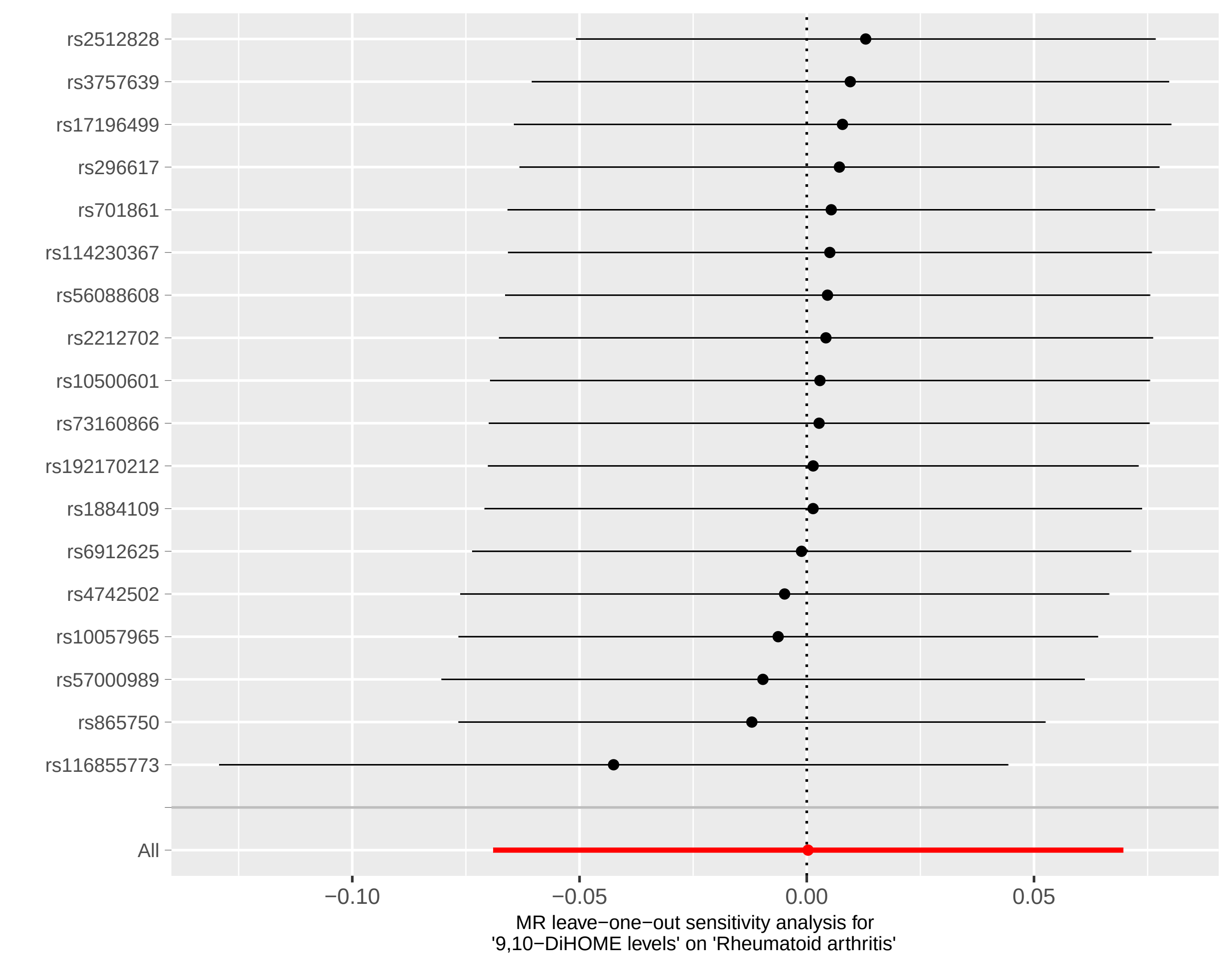
rs2512828
rs3757639
rs17196499
rs296617
rs701861
rs114230367
rs56088608
rs2212702
rs10500601
rs73160866
rs192170212
rs1884109
rs6912625
rs4742502
rs10057965
rs57000989
rs865750
rs116855773
All
−0.10
−0.05
0.00
0.05
MR leave−one−out sensitivity analysis for
'9,10−DiHOME levels' on 'Rheumatoid arthritis'

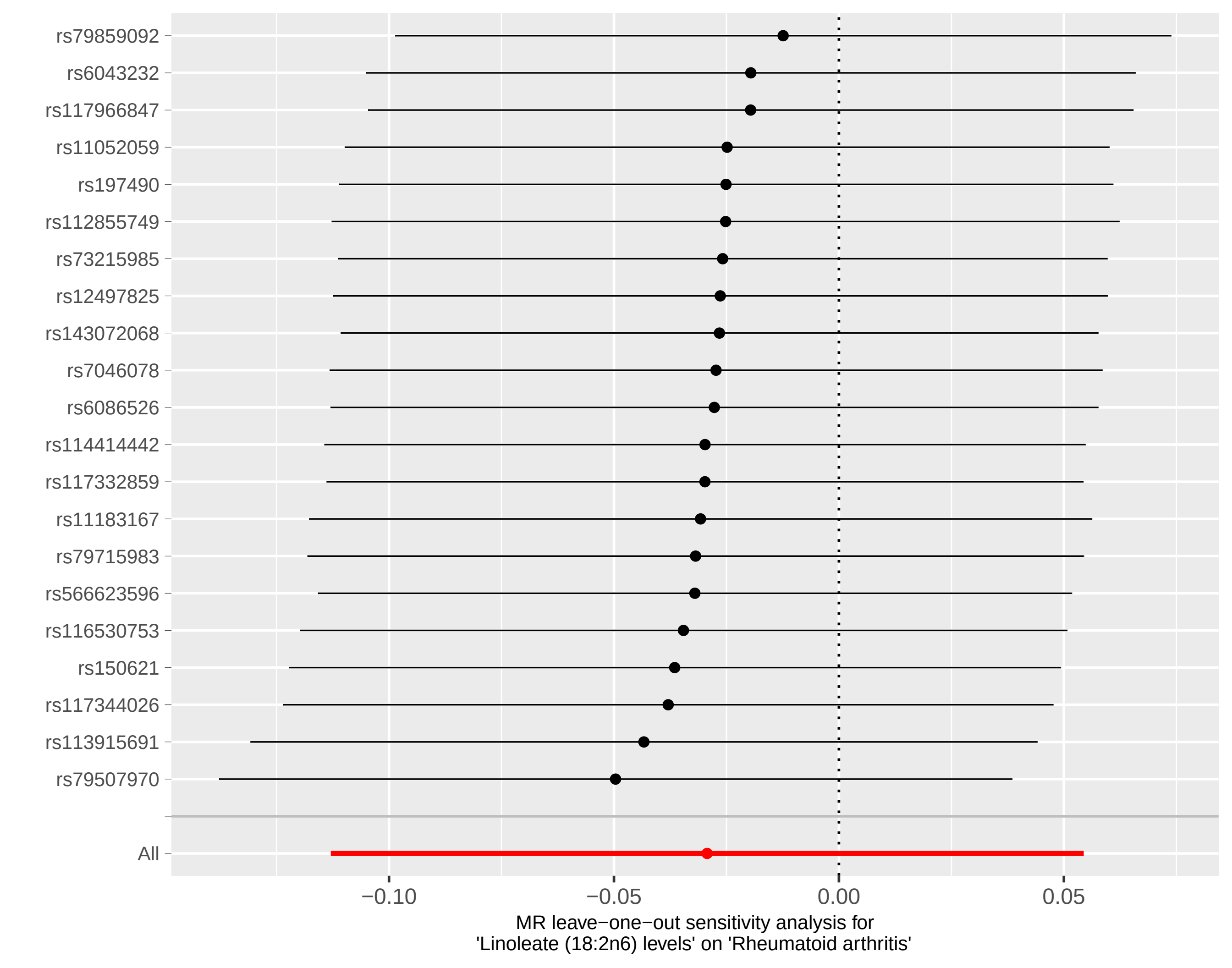
rs79859092
rs6043232
rs117966847
rs11052059
rs197490
rs112855749
rs73215985
rs12497825
rs143072068
rs7046078
rs6086526
rs114414442
rs117332859
rs11183167
rs79715983
rs566623596
rs116530753
rs150621
rs117344026
rs113915691
rs79507970
All
−0.10
−0.05
0.00
0.05
MR leave−one−out sensitivity analysis for
'Linoleate (18:2n6) levels' on 'Rheumatoid arthritis'

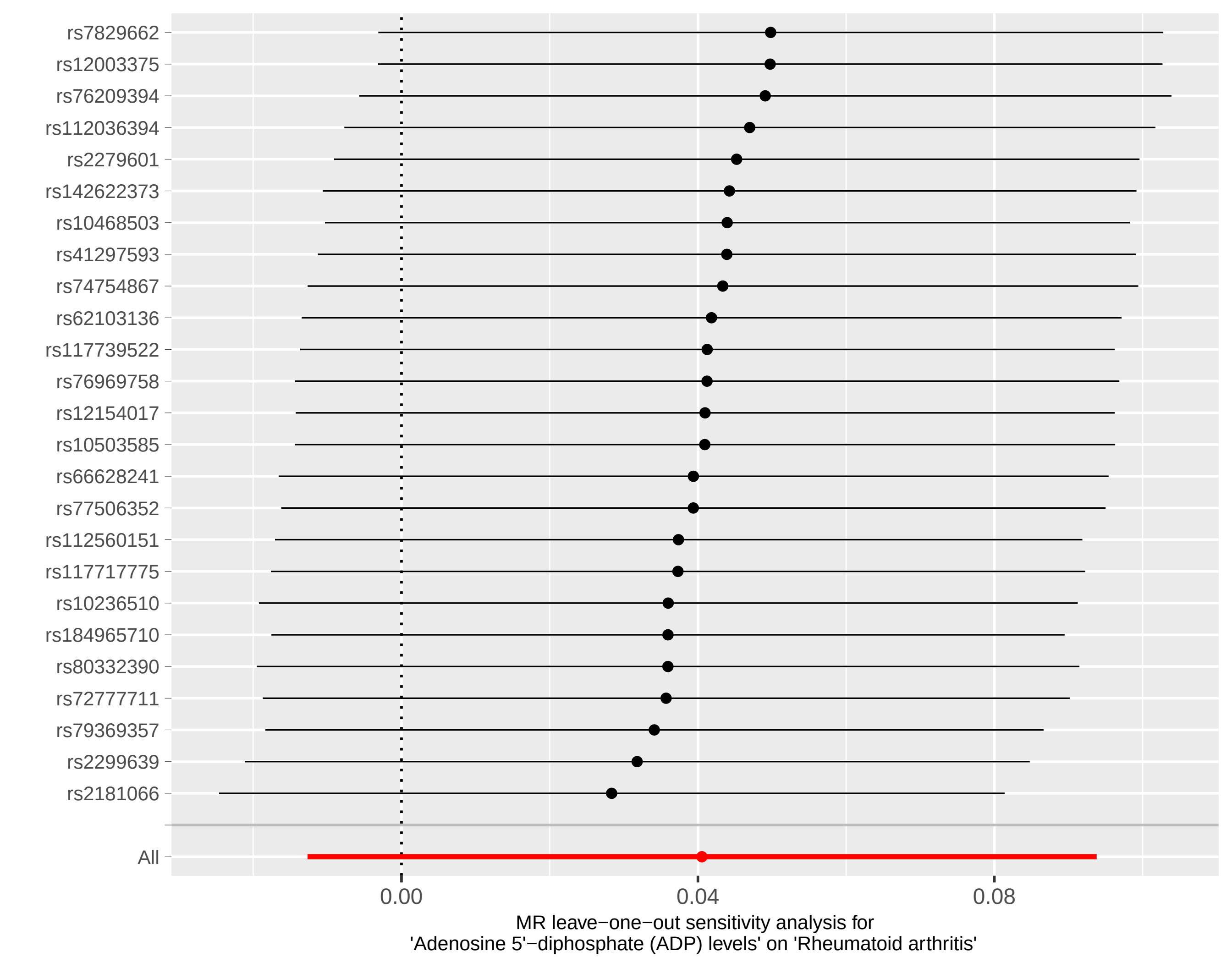
rs7829662
rs12003375
rs76209394
rs112036394
rs2279601
rs142622373
rs10468503
rs41297593
rs74754867
rs62103136
rs117739522
rs76969758
rs12154017
rs10503585
rs66628241
rs77506352
rs112560151
rs117717775
rs10236510
rs184965710
rs80332390
rs72777711
rs79369357
rs2299639
rs2181066
All
0.00
0.04
0.08
MR leave−one−out sensitivity analysis for
'Adenosine 5'−diphosphate (ADP) levels' on 'Rheumatoid arthritis'

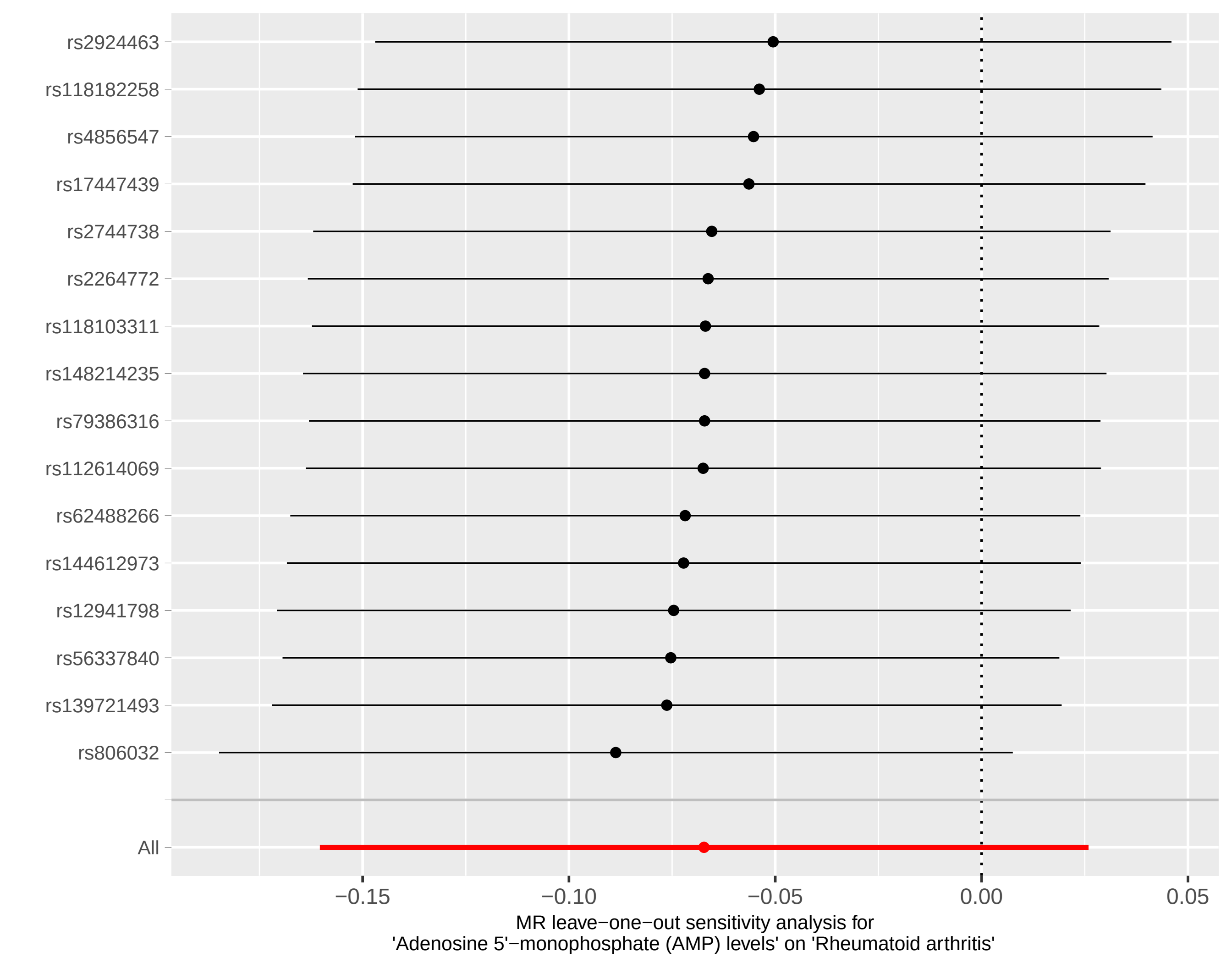
rs2924463
rs118182258
rs4856547
rs17447439
rs2744738
rs2264772
rs118103311
rs148214235
rs79386316
rs112614069
rs62488266
rs144612973
rs12941798
rs56337840
rs139721493
rs806032
All
−0.15
−0.10
−0.05
0.00
0.05
MR leave−one−out sensitivity analysis for
'Adenosine 5'−monophosphate (AMP) levels' on 'Rheumatoid arthritis'

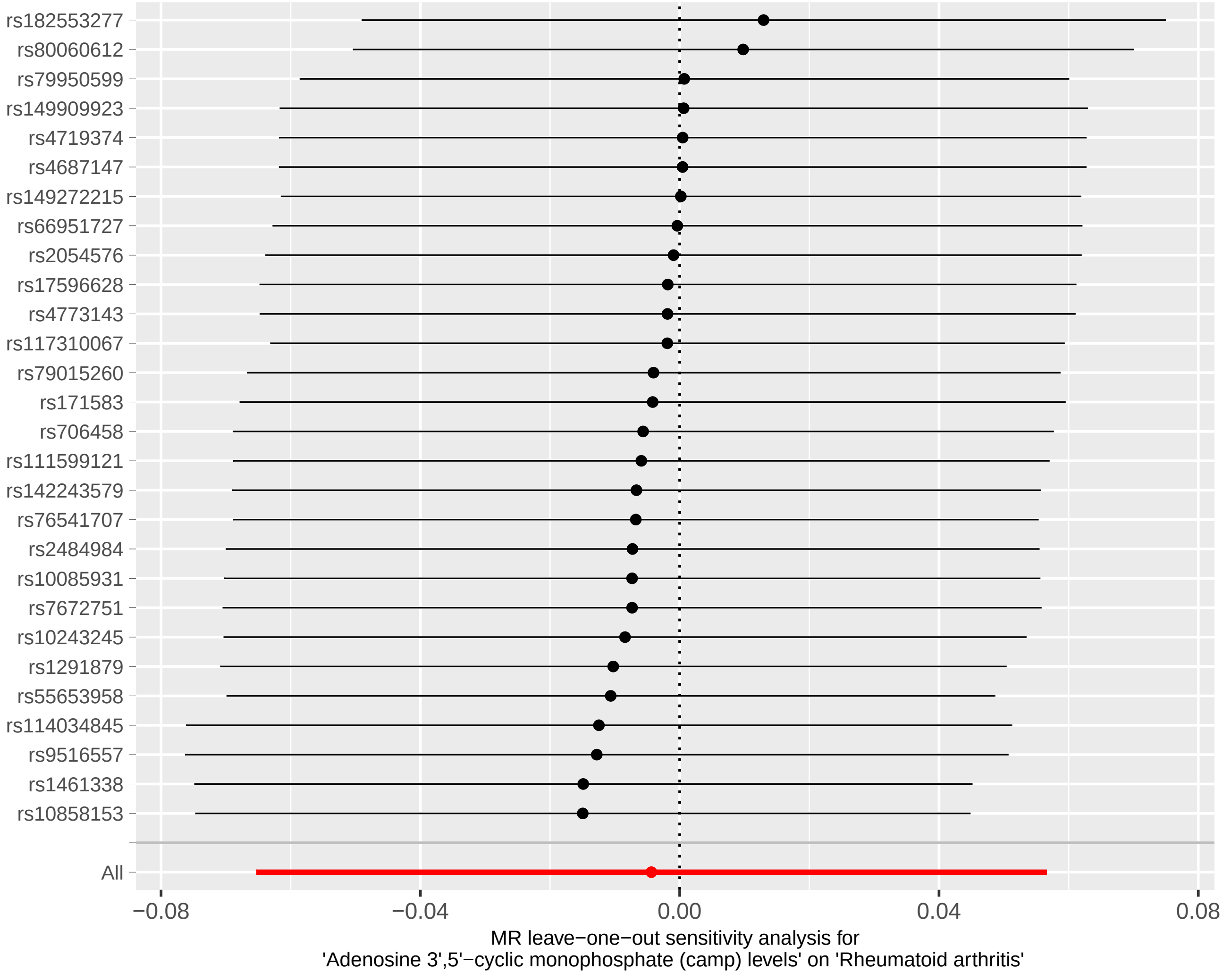
rs182553277
rs80060612
rs79950599
rs149909923
rs4719374
rs4687147
rs149272215
rs66951727
rs2054576
rs17596628
rs4773143
rs117310067
rs79015260
rs171583
rs706458
rs111599121
rs142243579
rs76541707
rs2484984
rs10085931
rs7672751
rs10243245
rs1291879
rs55653958
rs114034845
rs9516557
rs1461338
rs10858153
All
−0.08
−0.04
0.00
0.04
0.08
MR leave−one−out sensitivity analysis for
'Adenosine 3',5'−cyclic monophosphate (camp) levels' on 'Rheumatoid arthritis'

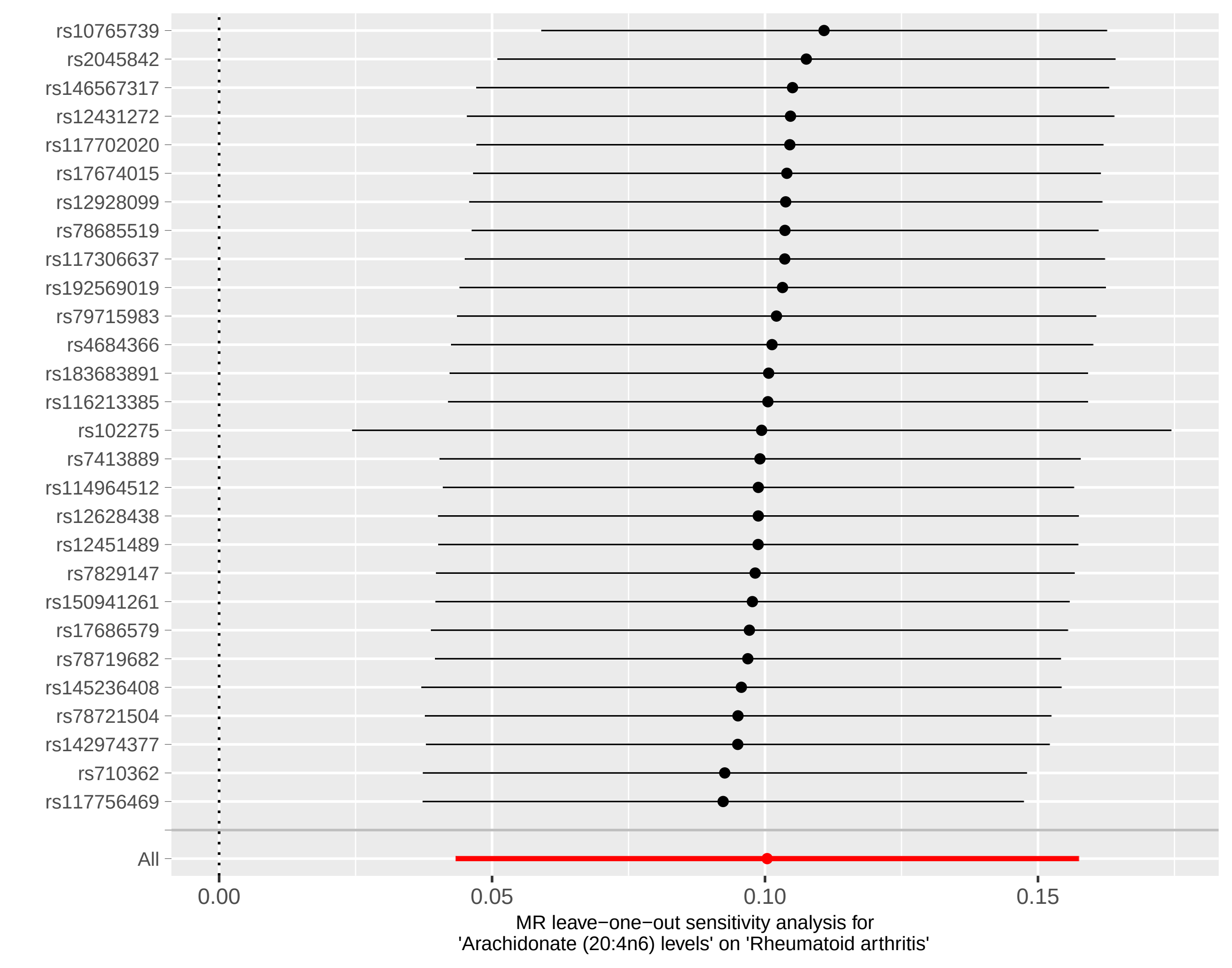
rs10765739
rs2045842
rs146567317
rs12431272
rs117702020
rs17674015
rs12928099
rs78685519
rs117306637
rs192569019
rs79715983
rs4684366
rs183683891
rs116213385
rs102275
rs7413889
rs114964512
rs12628438
rs12451489
rs7829147
rs150941261
rs17686579
rs78719682
rs145236408
rs78721504
rs142974377
rs710362
rs117756469
All
0.00
0.05
0.10
0.15
MR leave−one−out sensitivity analysis for
'Arachidonate (20:4n6) levels' on 'Rheumatoid arthritis'

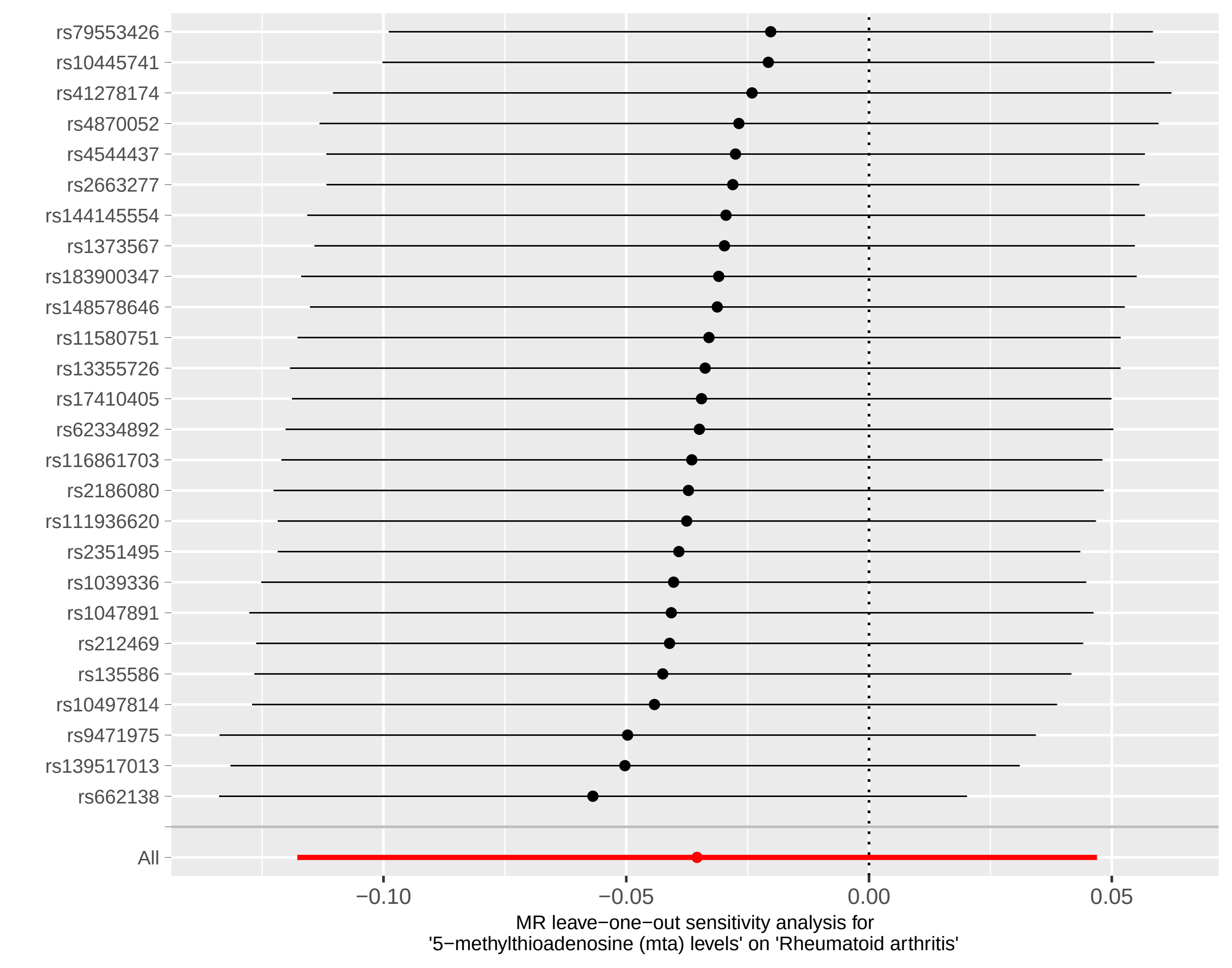
rs79553426
rs10445741
rs41278174
rs4870052
rs4544437
rs2663277
rs144145554
rs1373567
rs183900347
rs148578646
rs11580751
rs13355726
rs17410405
rs62334892
rs116861703
rs2186080
rs111936620
rs2351495
rs1039336
rs1047891
rs212469
rs135586
rs10497814
rs9471975
rs139517013
rs662138
All
−0.10
−0.05
0.00
0.05
MR leave−one−out sensitivity analysis for
'5−methylthioadenosine (mta) levels' on 'Rheumatoid arthritis'

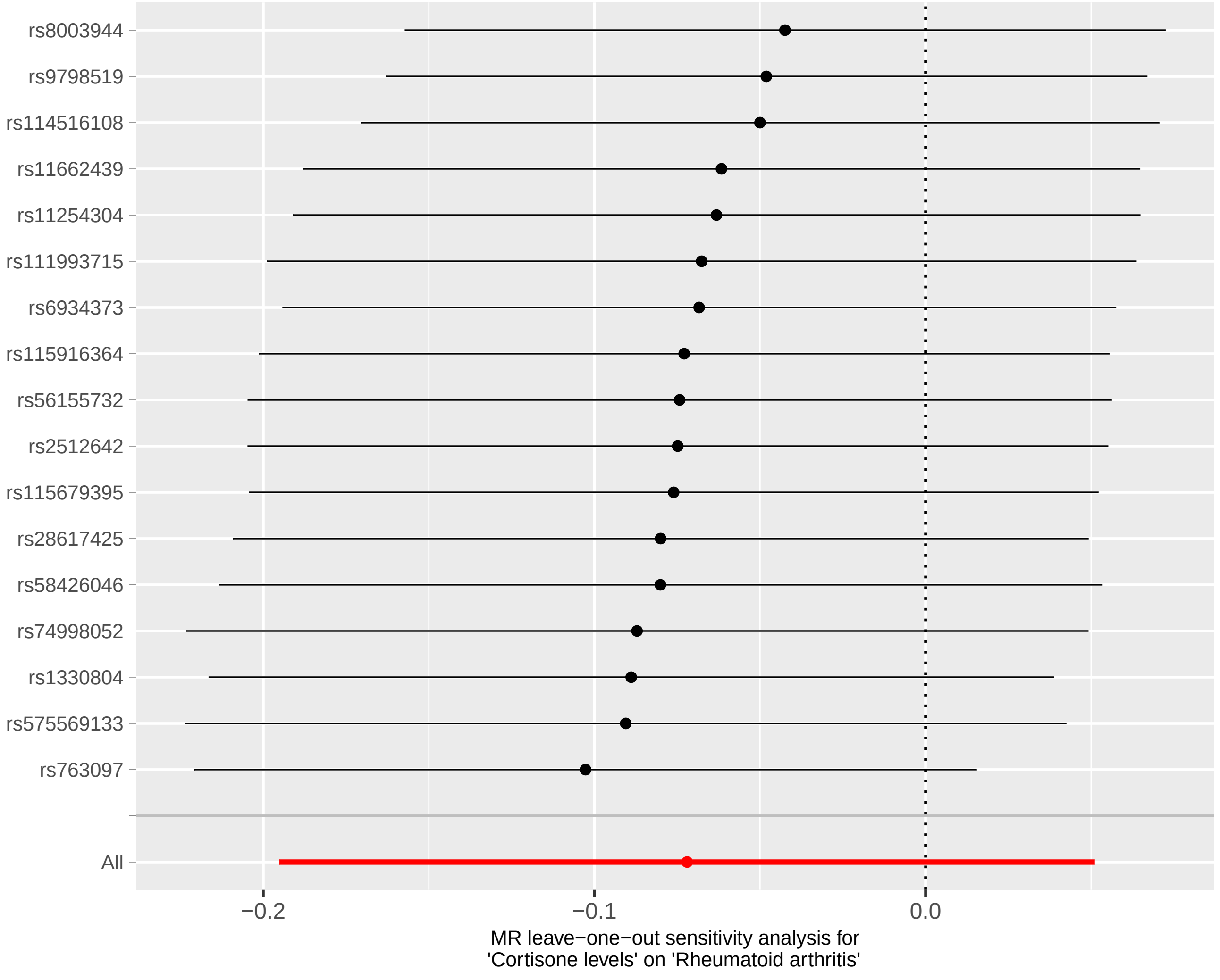
rs8003944
rs9798519
rs114516108
rs11662439
rs11254304
rs111993715
rs6934373
rs115916364
rs56155732
rs2512642
rs115679395
rs28617425
rs58426046
rs74998052
rs1330804
rs575569133
rs763097
All
−0.2
−0.1
0.0
MR leave−one−out sensitivity analysis for
'Cortisone levels' on 'Rheumatoid arthritis'

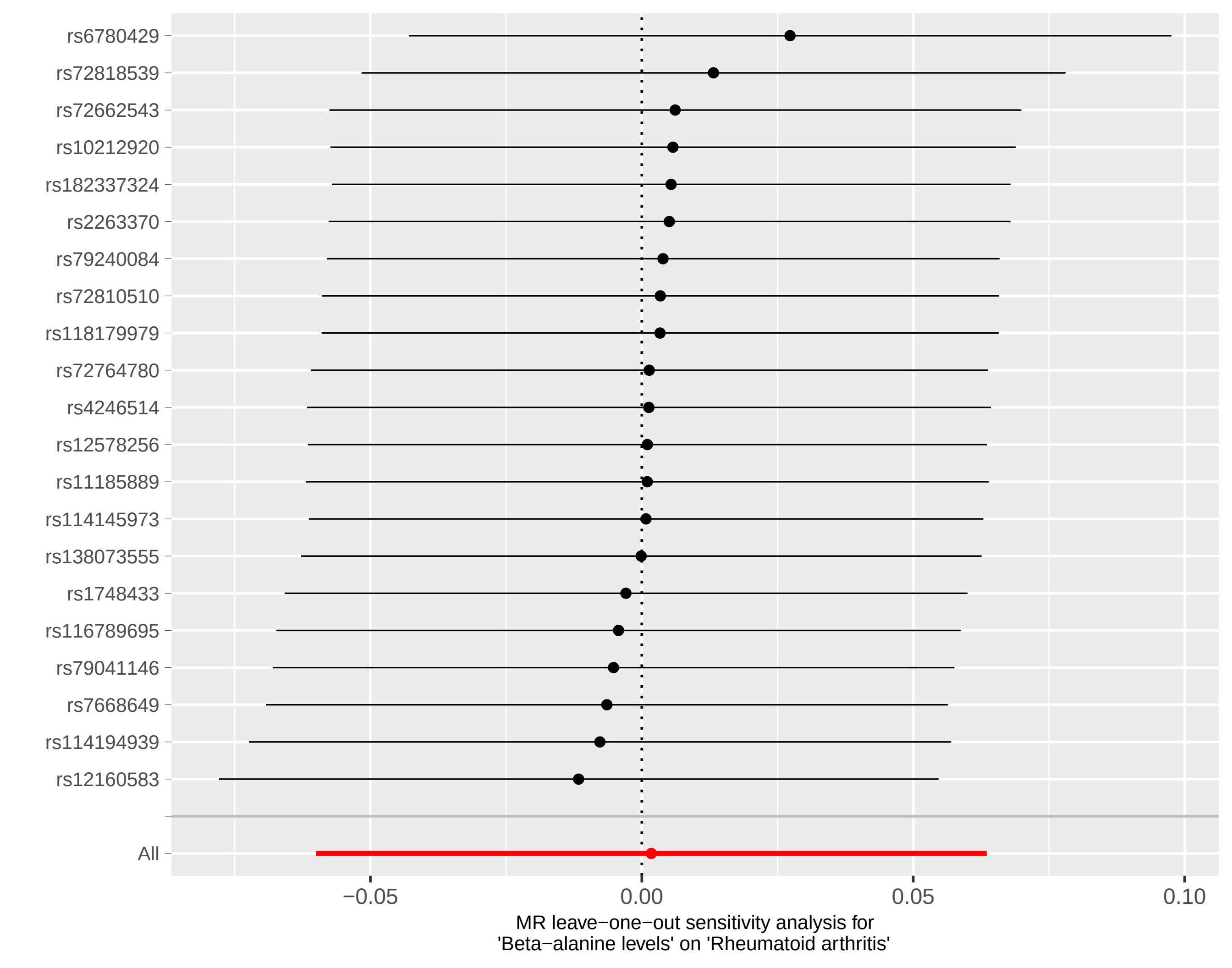
rs6780429
rs72818539
rs72662543
rs10212920
rs182337324
rs2263370
rs79240084
rs72810510
rs118179979
rs72764780
rs4246514
rs12578256
rs11185889
rs114145973
rs138073555
rs1748433
rs116789695
rs79041146
rs7668649
rs114194939
rs12160583
All
−0.05
0.00
0.05
0.10
MR leave−one−out sensitivity analysis for
'Beta−alanine levels' on 'Rheumatoid arthritis'

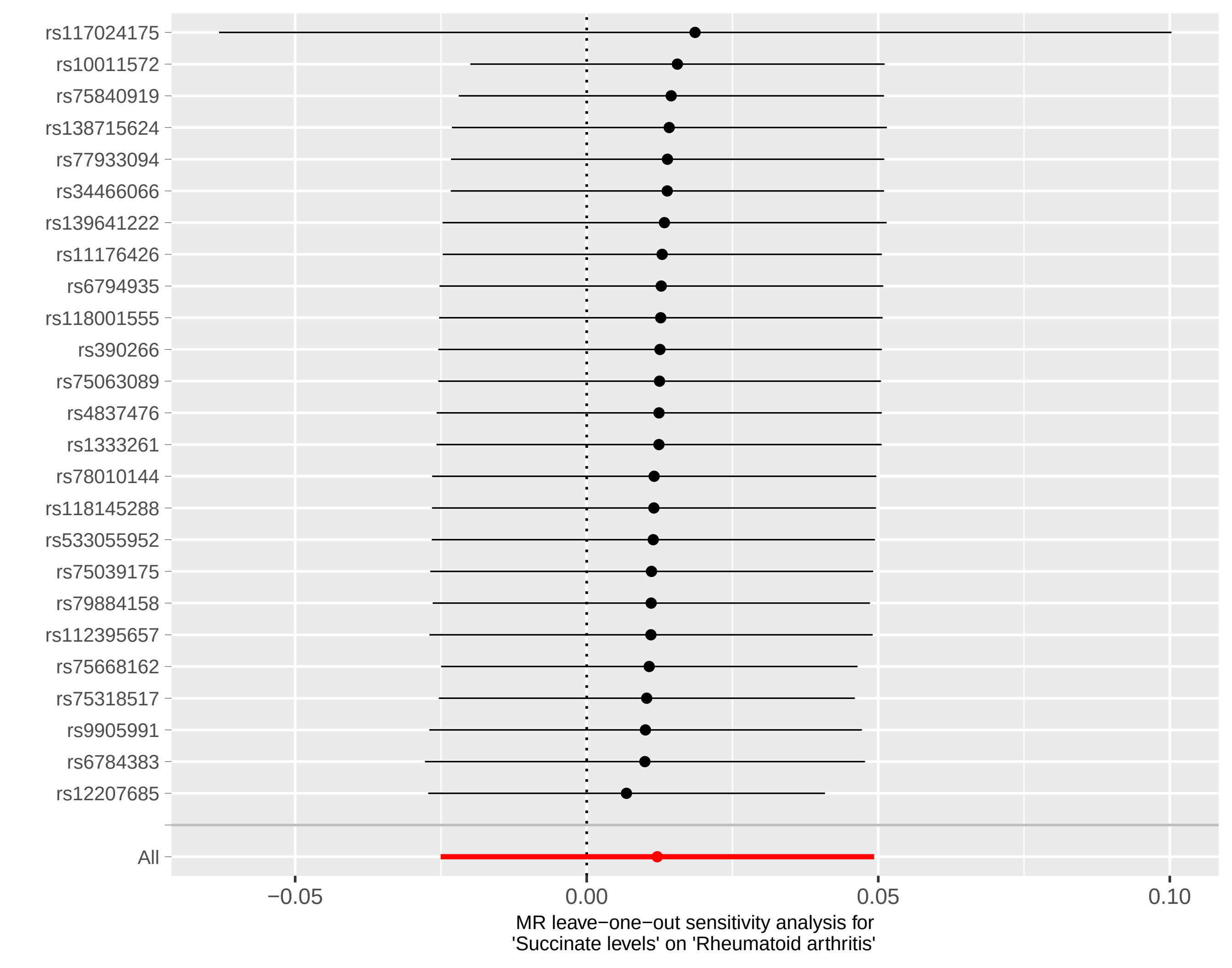
rs117024175
rs10011572
rs75840919
rs138715624
rs77933094
rs34466066
rs139641222
rs11176426
rs6794935
rs118001555
rs390266
rs75063089
rs4837476
rs1333261
rs78010144
rs118145288
rs533055952
rs75039175
rs79884158
rs112395657
rs75668162
rs75318517
rs9905991
rs6784383
rs12207685
All
−0.05
0.00
0.05
0.10
MR leave−one−out sensitivity analysis for
'Succinate levels' on 'Rheumatoid arthritis'

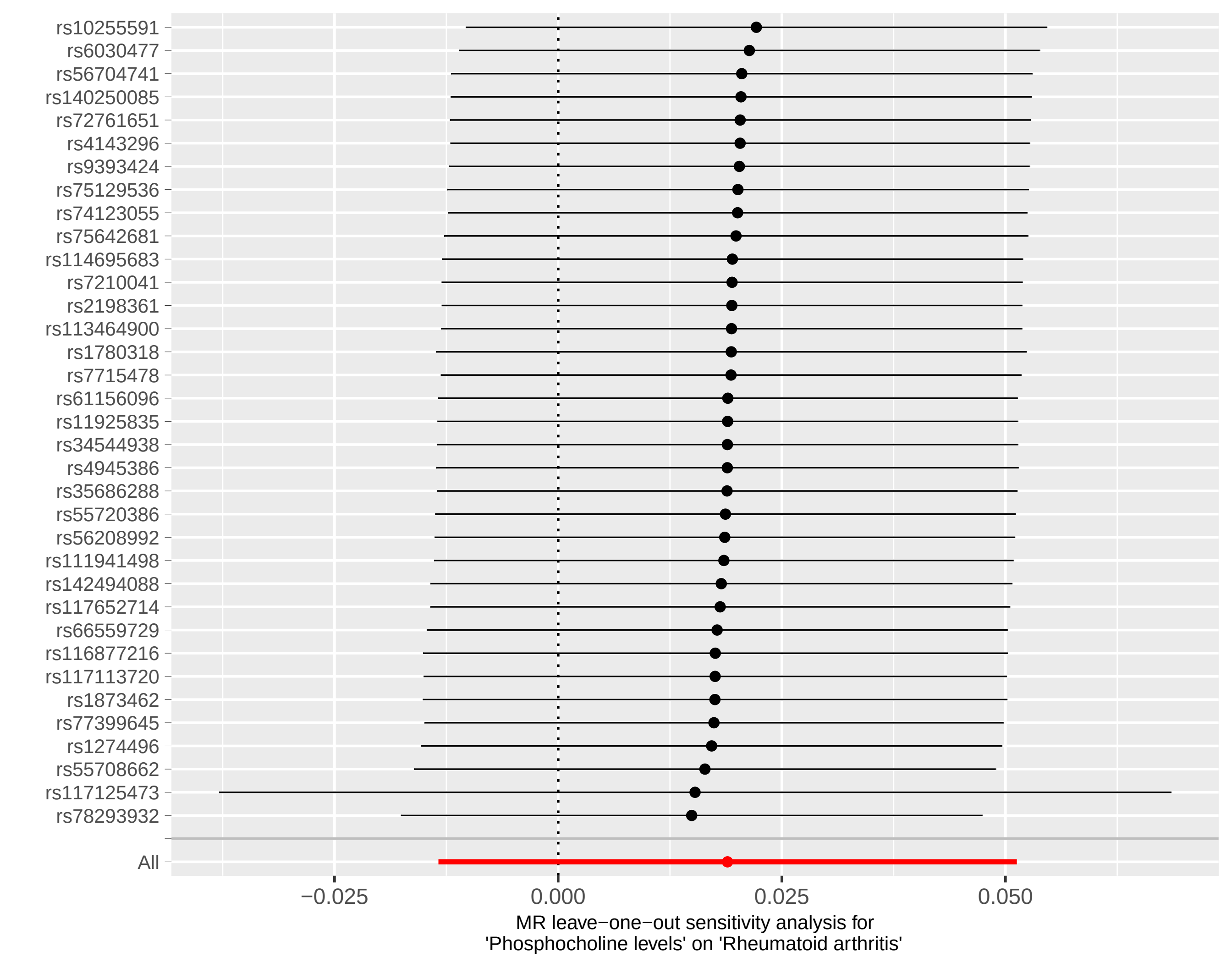

rs10255591
rs6030477
rs56704741
rs140250085
rs72761651
rs4143296
rs9393424
rs75129536
rs74123055
rs75642681
rs114695683
rs7210041
rs2198361
rs113464900
rs1780318
rs7715478
rs61156096
rs11925835
rs34544938
rs4945386
rs35686288
rs55720386
rs56208992
rs111941498
rs142494088
rs117652714
rs66559729
rs116877216
rs117113720
rs1873462
rs77399645
rs1274496
rs55708662
rs117125473
rs78293932
All
−0.025
0.000
0.025
0.050
MR leave−one−out sensitivity analysis for
'Phosphocholine levels' on 'Rheumatoid arthritis'

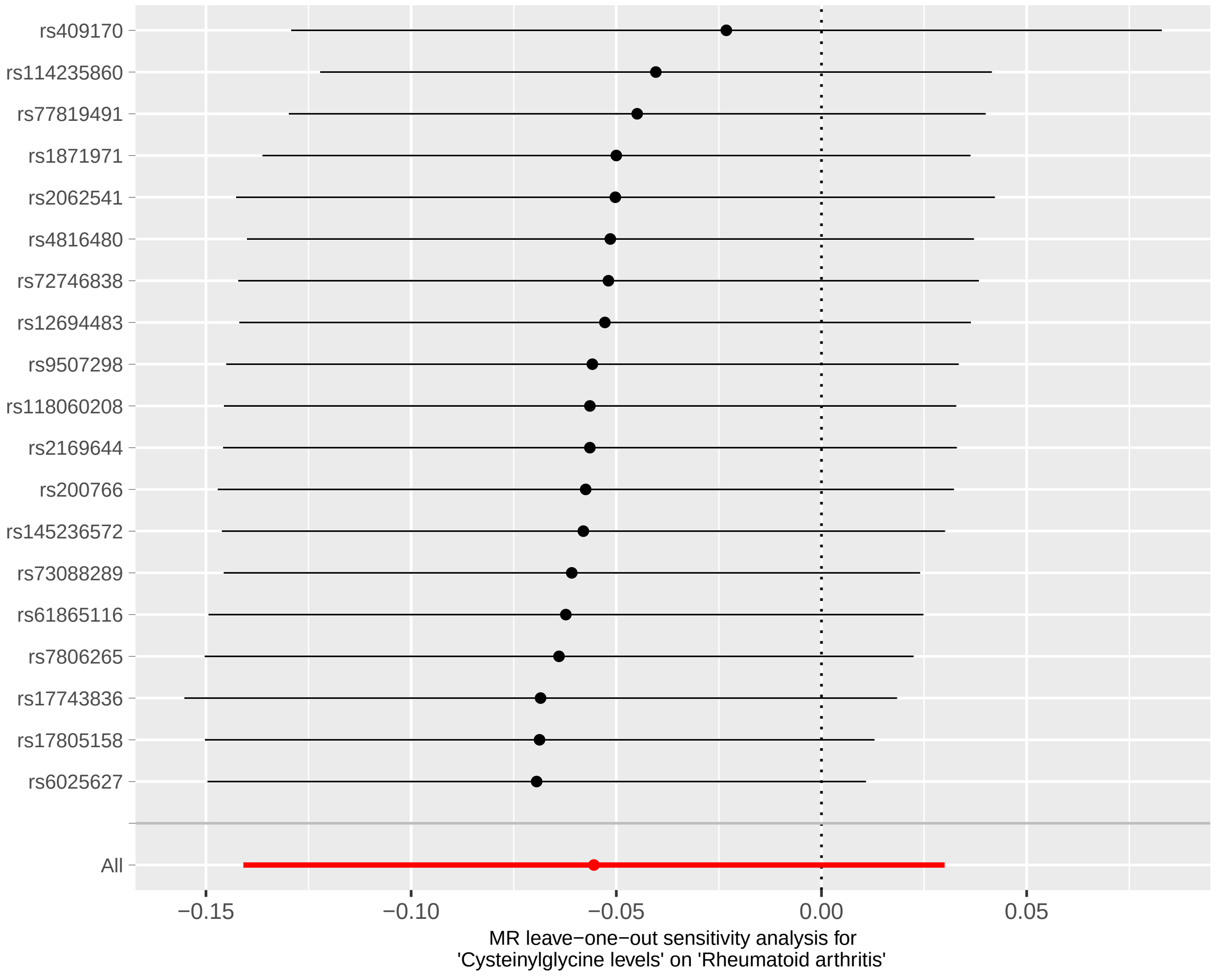
rs409170
rs114235860
rs77819491
rs1871971
rs2062541
rs4816480
rs72746838
rs12694483
rs9507298
rs118060208
rs2169644
rs200766
rs145236572
rs73088289
rs61865116
rs7806265
rs17743836
rs17805158
rs6025627
All
−0.15
−0.10
−0.05
0.00
0.05
MR leave−one−out sensitivity analysis for
'Cysteinylglycine levels' on 'Rheumatoid arthritis'

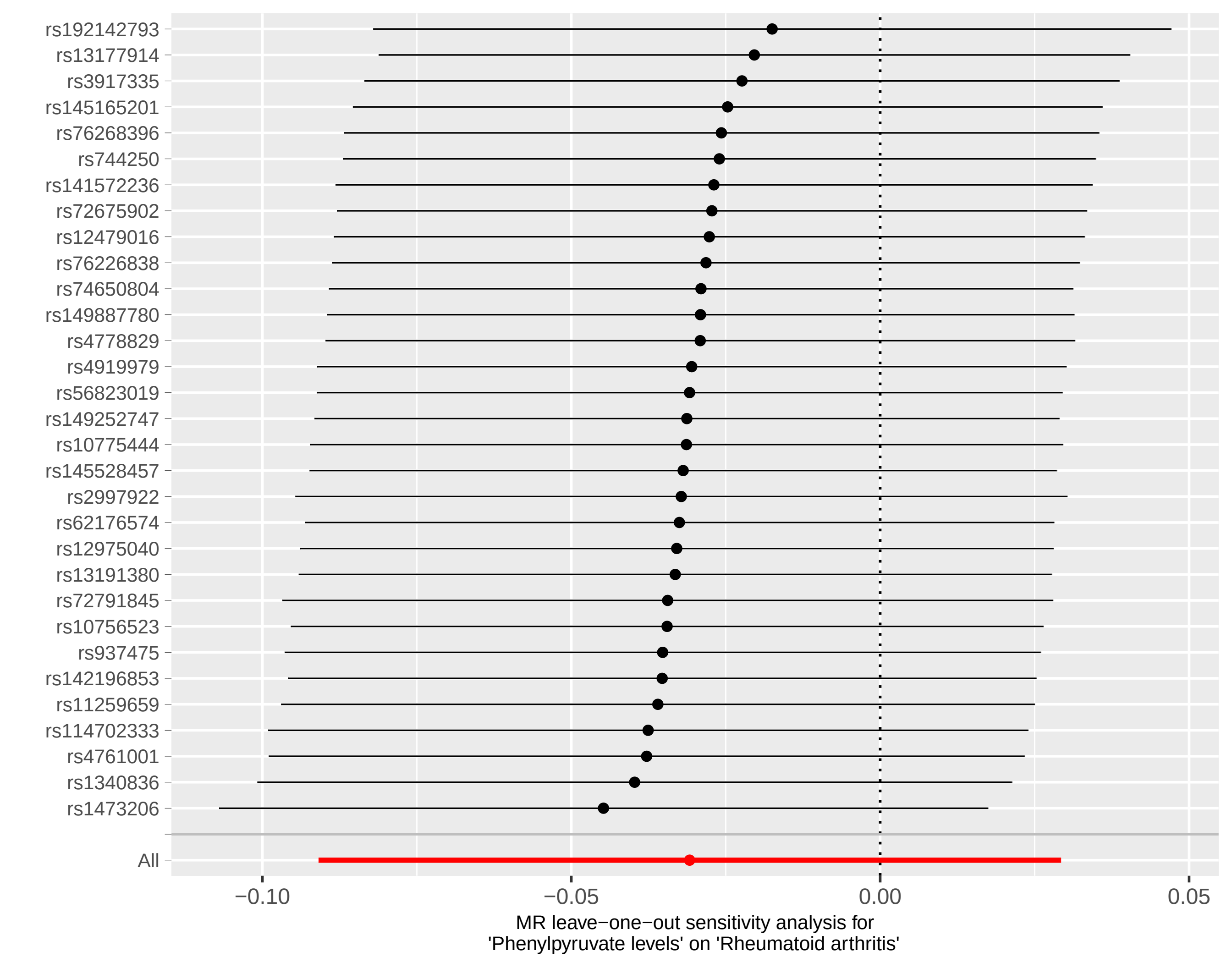
rs192142793
rs13177914
rs3917335
rs145165201
rs76268396
rs744250
rs141572236
rs72675902
rs12479016
rs76226838
rs74650804
rs149887780
rs4778829
rs4919979
rs56823019
rs149252747
rs10775444
rs145528457
rs2997922
rs62176574
rs12975040
rs13191380
rs72791845
rs10756523
rs937475
rs142196853
rs11259659
rs114702333
rs4761001
rs1340836
rs1473206
All
−0.10
−0.05
0.00
0.05
MR leave−one−out sensitivity analysis for
'Phenylpyruvate levels' on 'Rheumatoid arthritis'

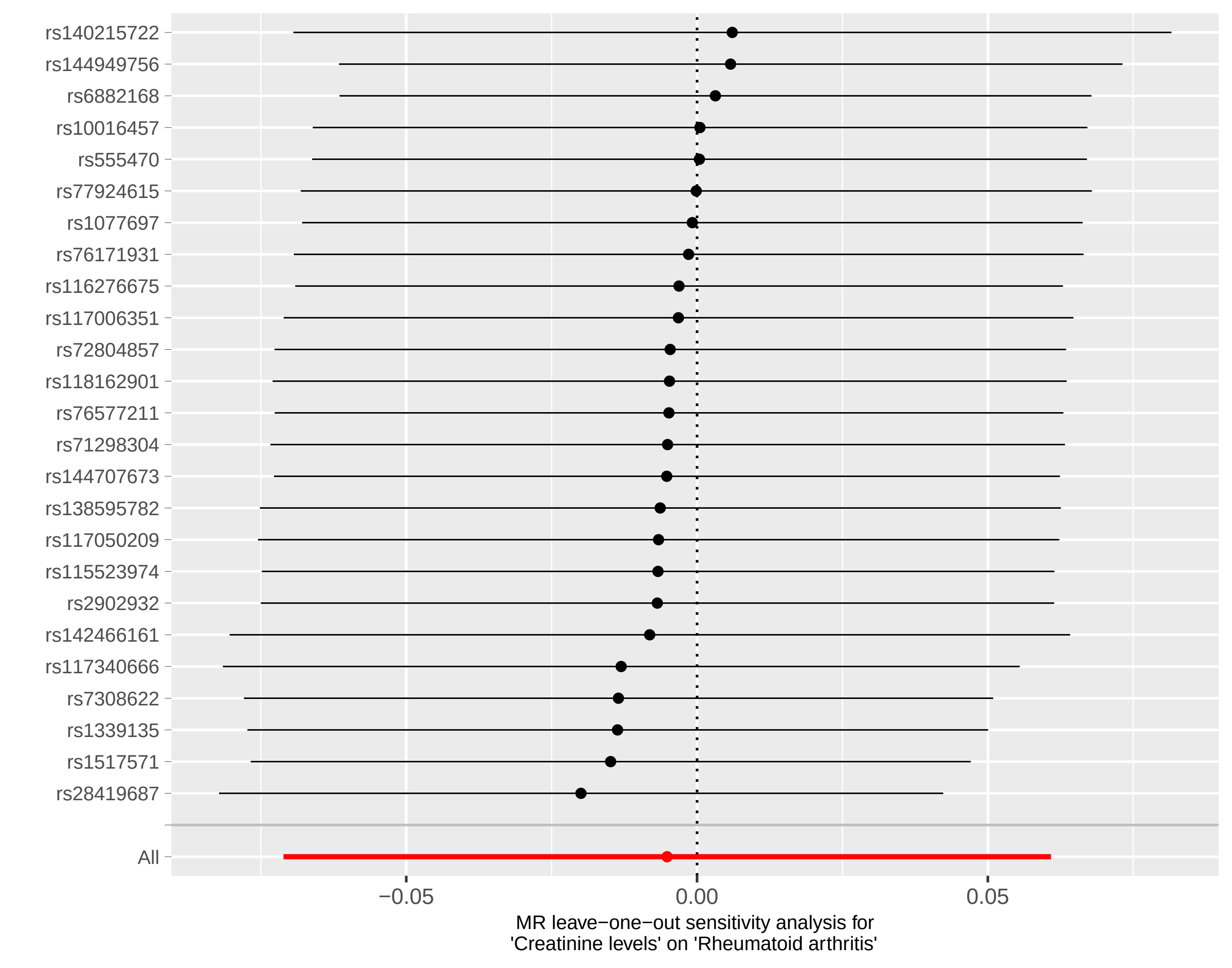

rs140215722
rs144949756
rs6882168
rs10016457
rs555470
rs77924615
rs1077697
rs76171931
rs116276675
rs117006351
rs72804857
rs118162901
rs76577211
rs71298304
rs144707673
rs138595782
rs117050209
rs115523974
rs2902932
rs142466161
rs117340666
rs7308622
rs1339135
rs1517571
rs28419687
All
−0.05
0.00
0.05
MR leave−one−out sensitivity analysis for
'Creatinine levels' on 'Rheumatoid arthritis'

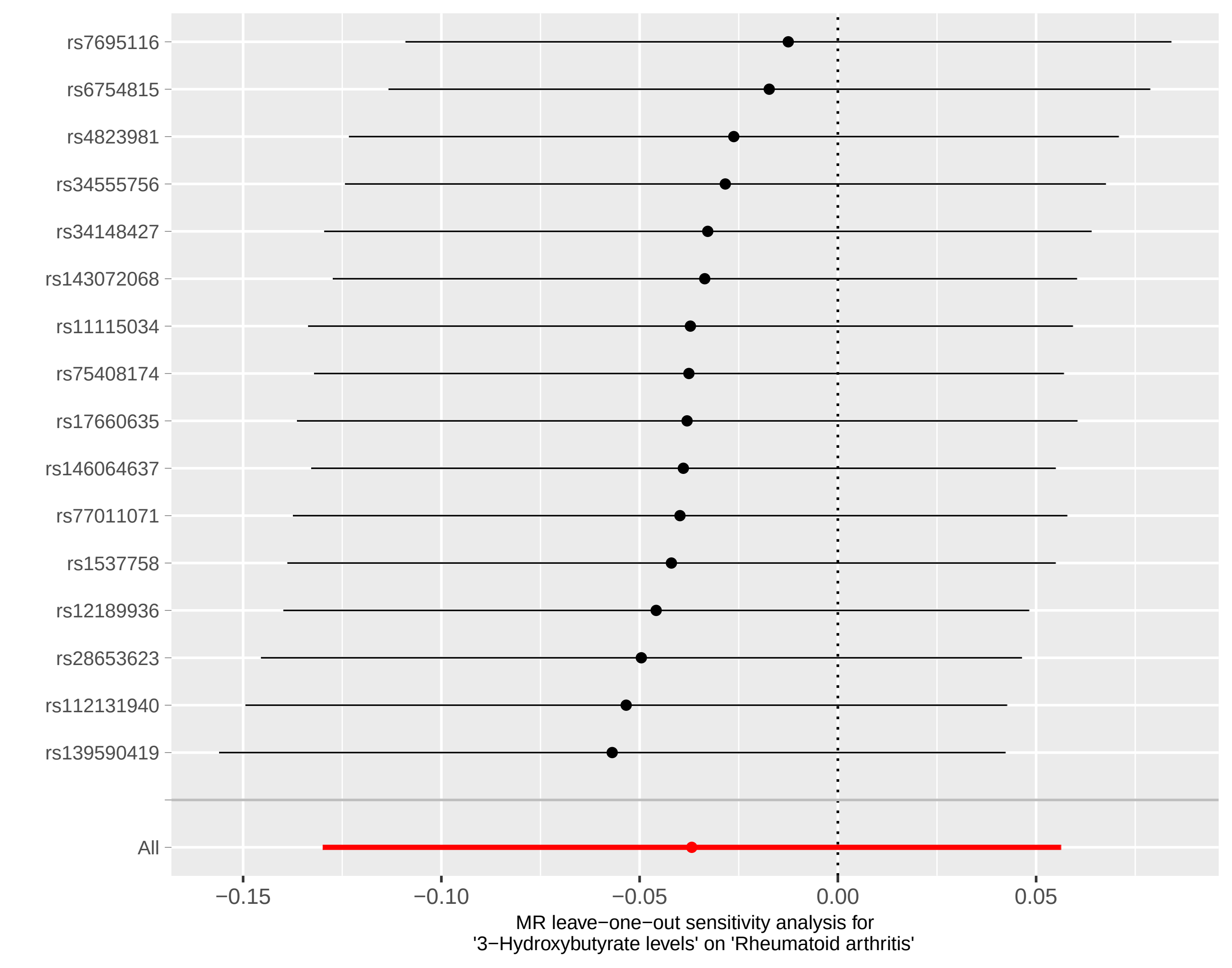
rs7695116
rs6754815
rs4823981
rs34555756
rs34148427
rs143072068
rs11115034
rs75408174
rs17660635
rs146064637
rs77011071
rs1537758
rs12189936
rs28653623
rs112131940
rs139590419
All
−0.15
−0.10
−0.05
0.00
0.05
MR leave−one−out sensitivity analysis for
'3−Hydroxybutyrate levels' on 'Rheumatoid arthritis'

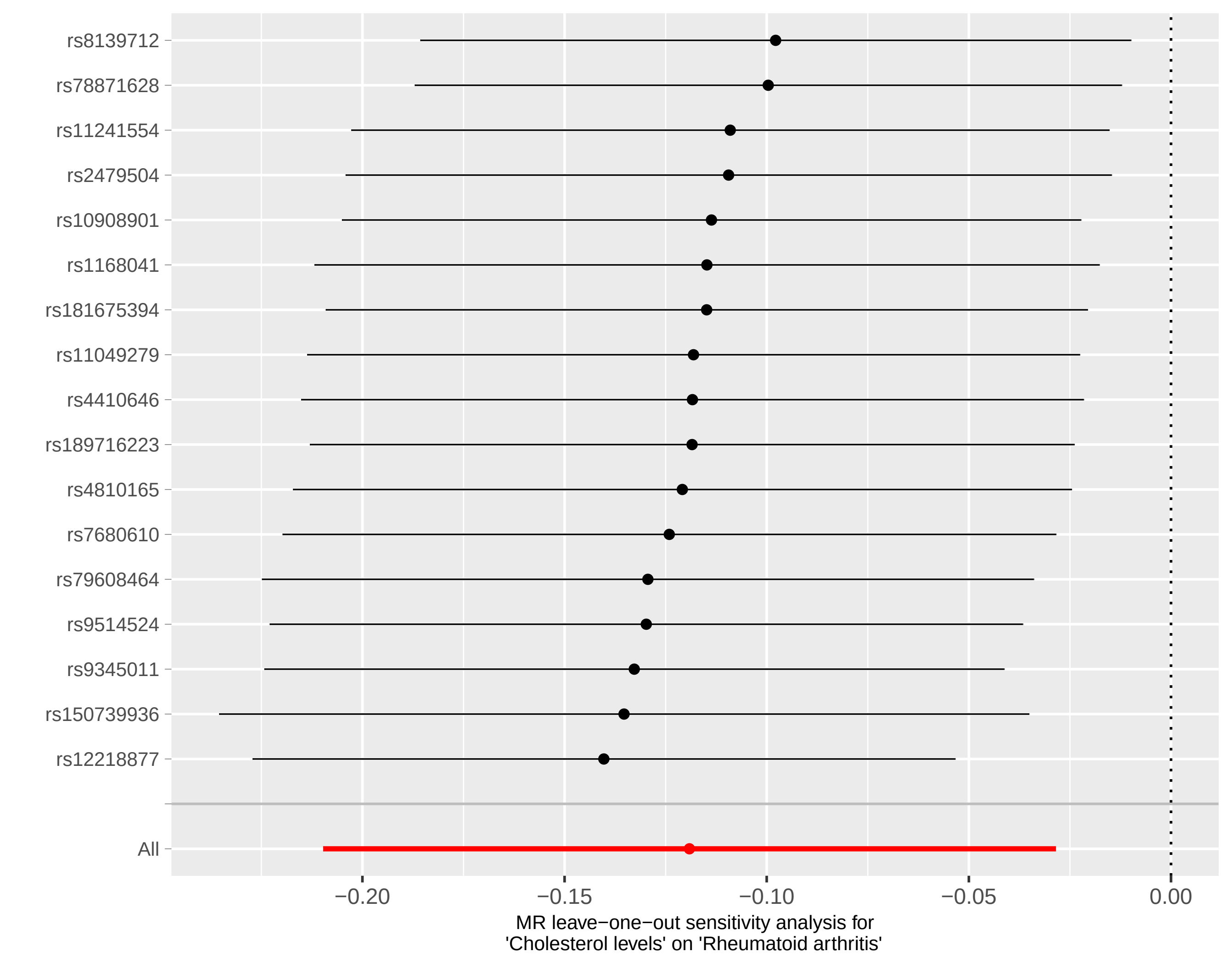
rs8139712
rs78871628
rs11241554
rs2479504
rs10908901
rs1168041
rs181675394
rs11049279
rs4410646
rs189716223
rs4810165
rs7680610
rs79608464
rs9514524
rs9345011
rs150739936
rs12218877
All
−0.20
−0.15
−0.10
−0.05
0.00
MR leave−one−out sensitivity analysis for
'Cholesterol levels' on 'Rheumatoid arthritis'

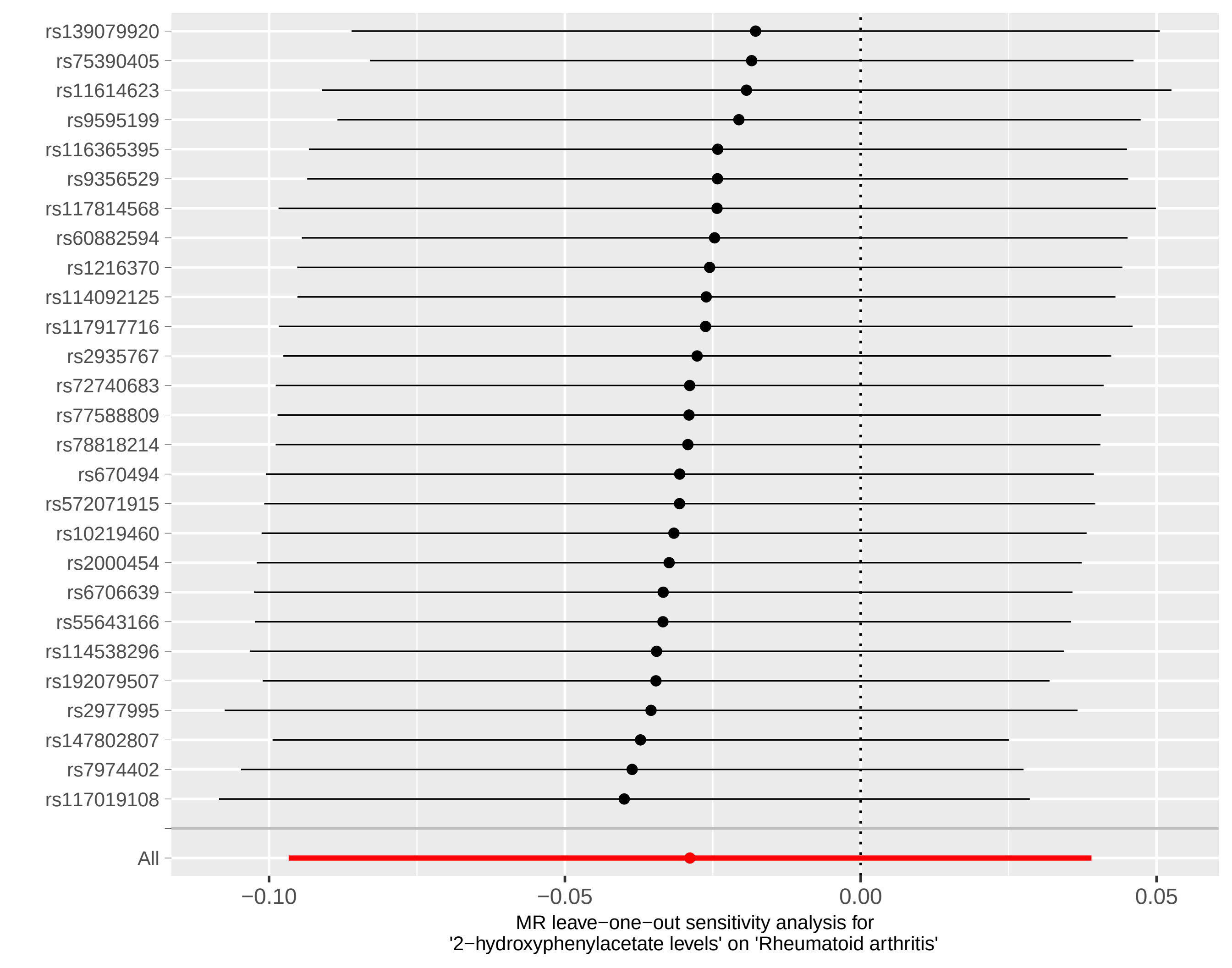

rs139079920
rs75390405
rs11614623
rs9595199
rs116365395
rs9356529
rs117814568
rs60882594
rs1216370
rs114092125
rs117917716
rs2935767
rs72740683
rs77588809
rs78818214
rs670494
rs572071915
rs10219460
rs2000454
rs6706639
rs55643166
rs114538296
rs192079507
rs2977995
rs147802807
rs7974402
rs117019108
All
−0.10
−0.05
0.00
0.05
MR leave−one−out sensitivity analysis for
'2−hydroxyphenylacetate levels' on 'Rheumatoid arthritis'

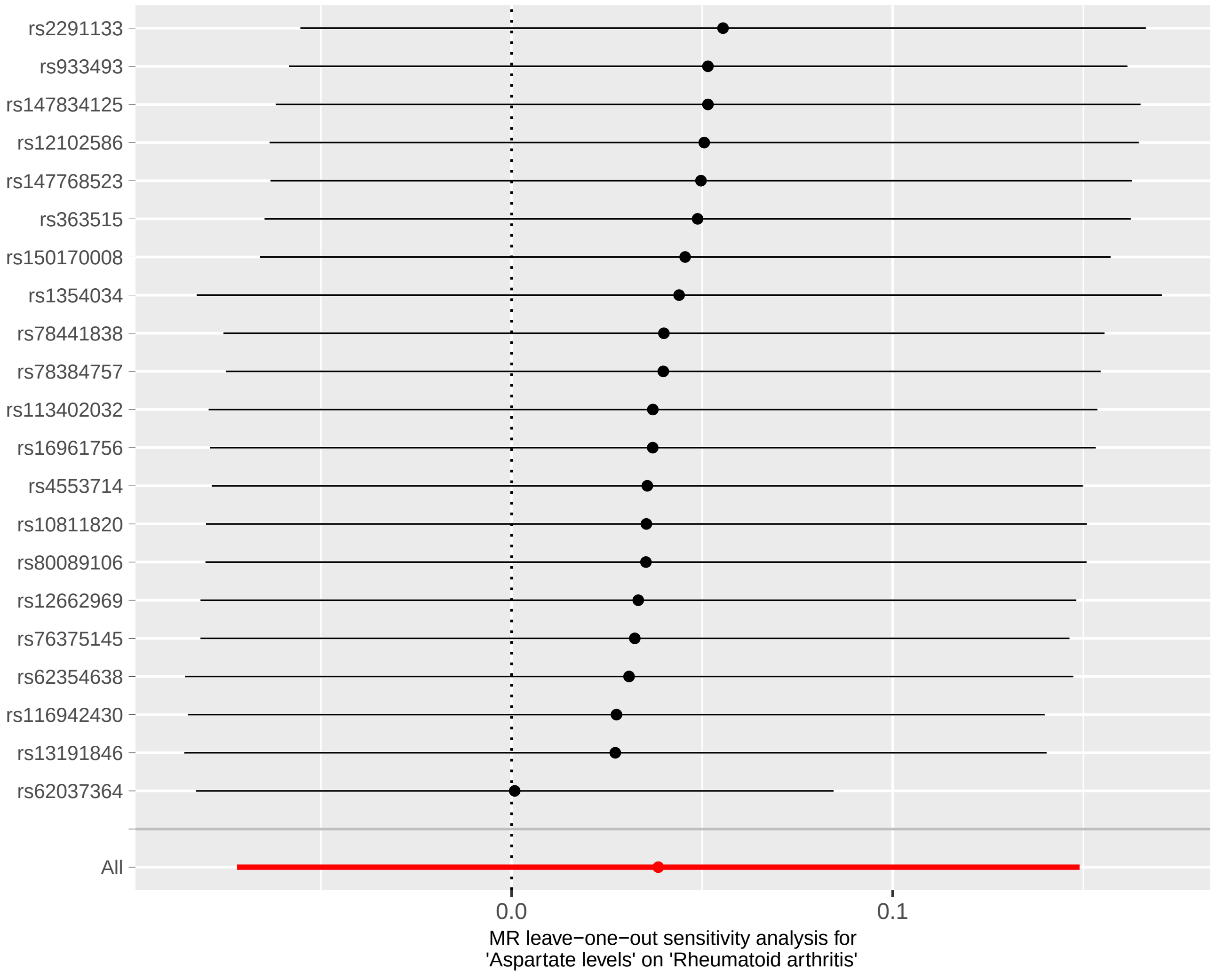

rs2291133
rs933493
rs147834125
rs12102586
rs147768523
rs363515
rs150170008
rs1354034
rs78441838
rs78384757
rs113402032
rs16961756
rs4553714
rs10811820
rs80089106
rs12662969
rs76375145
rs62354638
rs116942430
rs13191846
rs62037364
All
0.0
0.1
MR leave−one−out sensitivity analysis for
'Aspartate levels' on 'Rheumatoid arthritis'

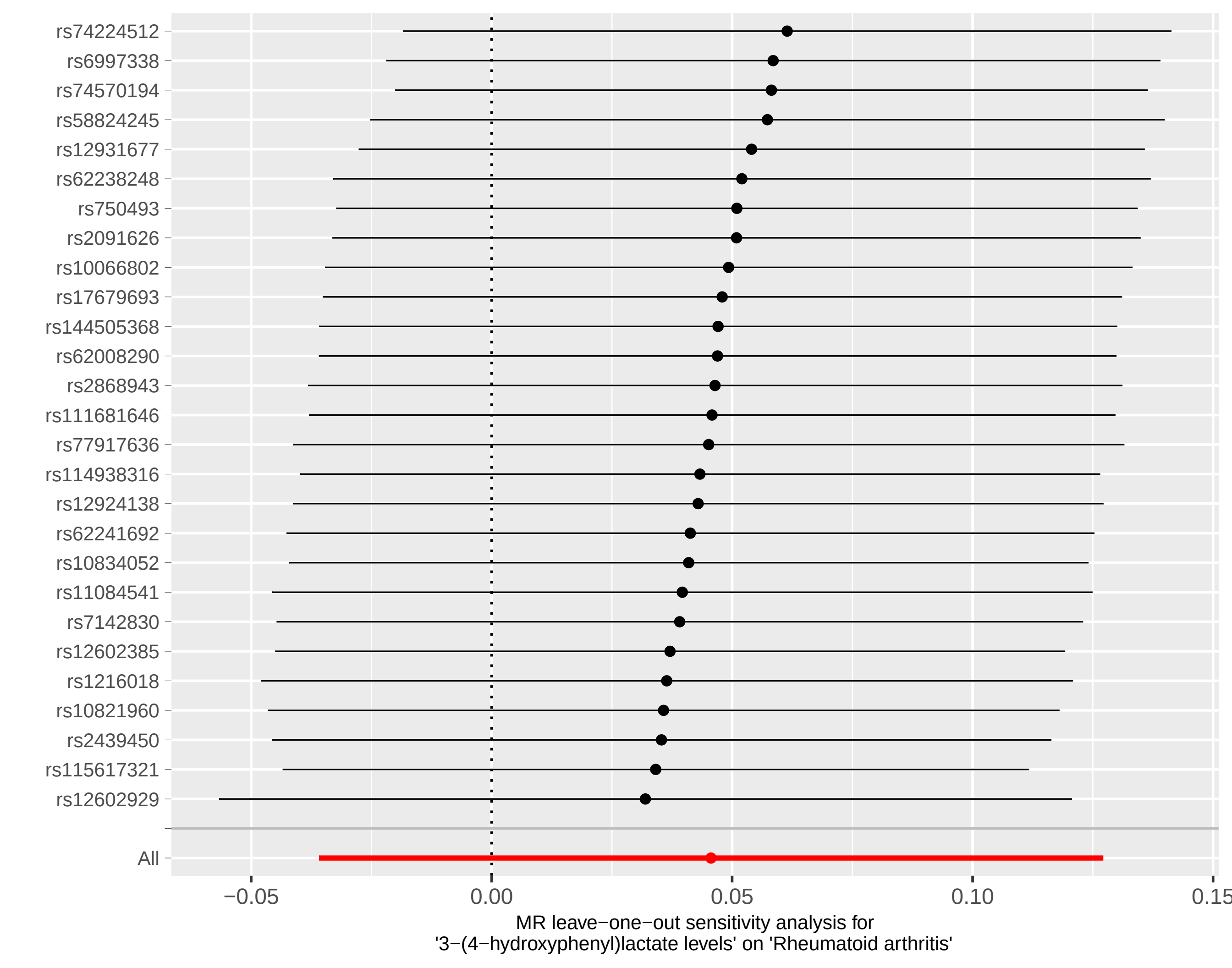
rs74224512
rs6997338
rs74570194
rs58824245
rs12931677
rs62238248
rs750493
rs2091626
rs10066802
rs17679693
rs144505368
rs62008290
rs2868943
rs111681646
rs77917636
rs114938316
rs12924138
rs62241692
rs10834052
rs11084541
rs7142830
rs12602385
rs1216018
rs10821960
rs2439450
rs115617321
rs12602929
All
−0.05
0.00
0.05
0.10
0.15
MR leave−one−out sensitivity analysis for
'3−(4−hydroxyphenyl)lactate levels' on 'Rheumatoid arthritis'

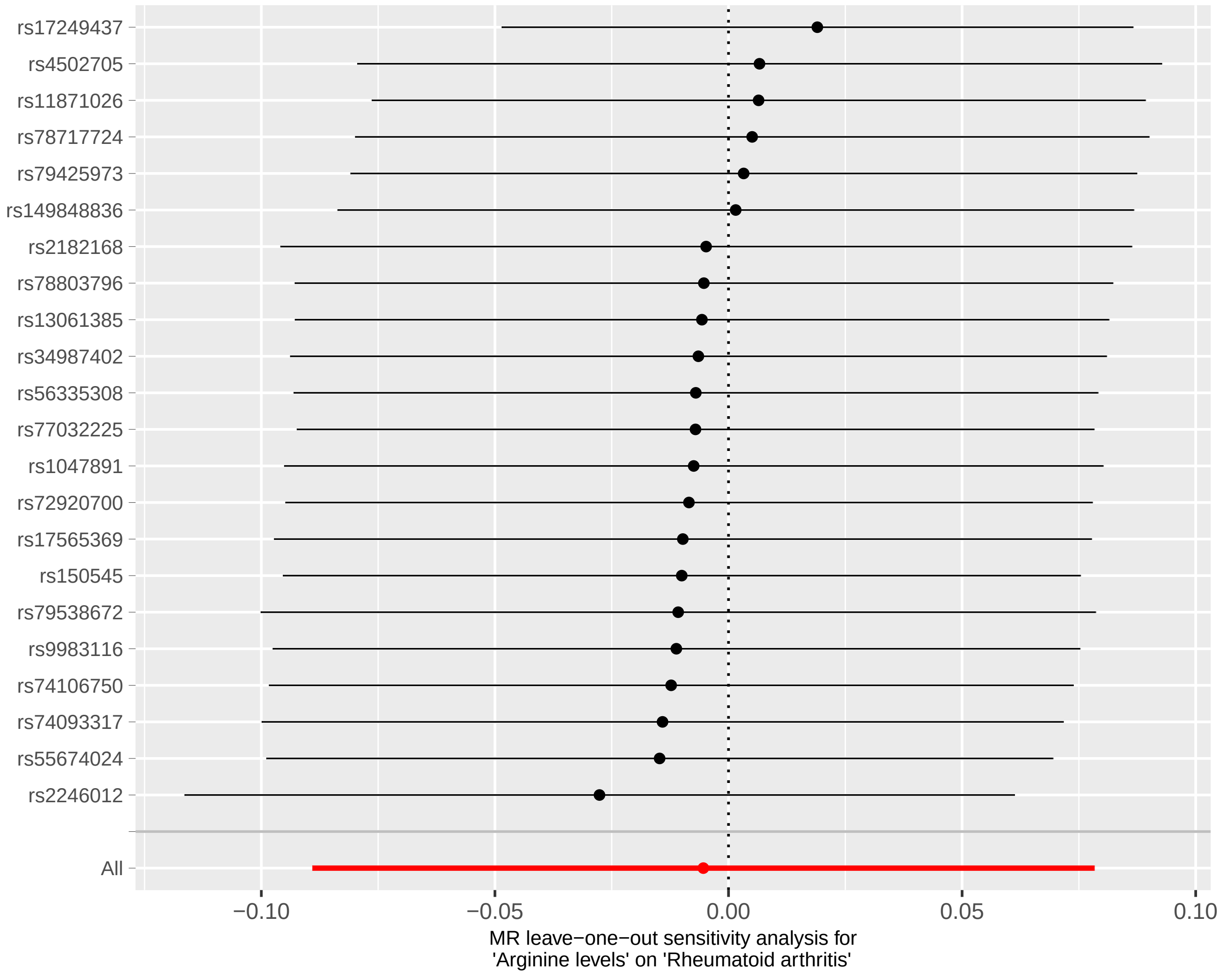
rs17249437
rs4502705
rs11871026
rs78717724
rs79425973
rs149848836
rs2182168
rs78803796
rs13061385
rs34987402
rs56335308
rs77032225
rs1047891
rs72920700
rs17565369
rs150545
rs79538672
rs9983116
rs74106750
rs74093317
rs55674024
rs2246012
All
−0.10
−0.05
0.00
0.05
0.10
MR leave−one−out sensitivity analysis for
'Arginine levels' on 'Rheumatoid arthritis'

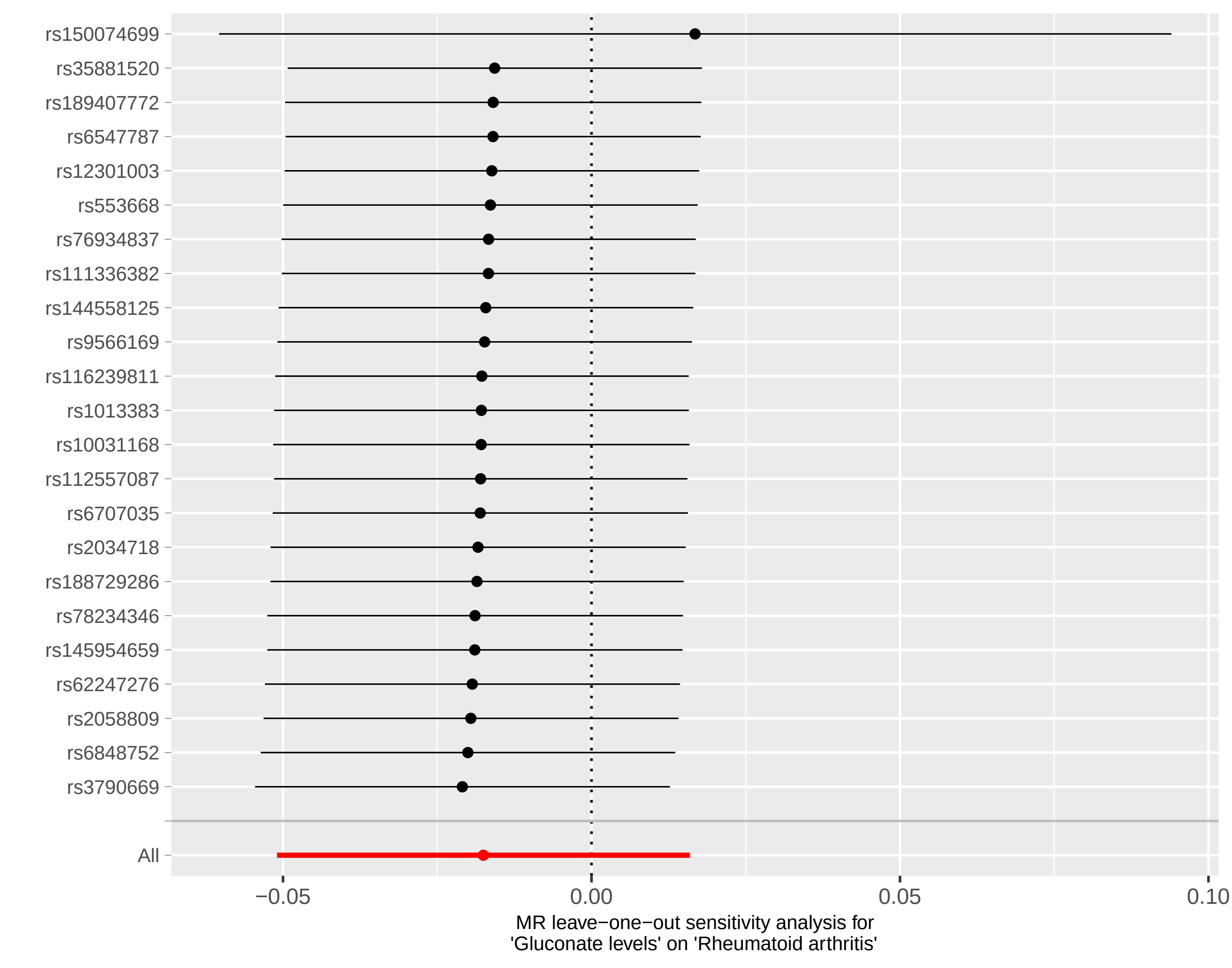

rs150074699
rs35881520
rs189407772
rs6547787
rs12301003
rs553668
rs76934837
rs111336382
rs144558125
rs9566169
rs116239811
rs1013383
rs10031168
rs112557087
rs6707035
rs2034718
rs188729286
rs78234346
rs145954659
rs62247276
rs2058809
rs6848752
rs3790669
All
−0.05
0.00
0.05
0.10
MR leave−one−out sensitivity analysis for
'Gluconate levels' on 'Rheumatoid arthritis'

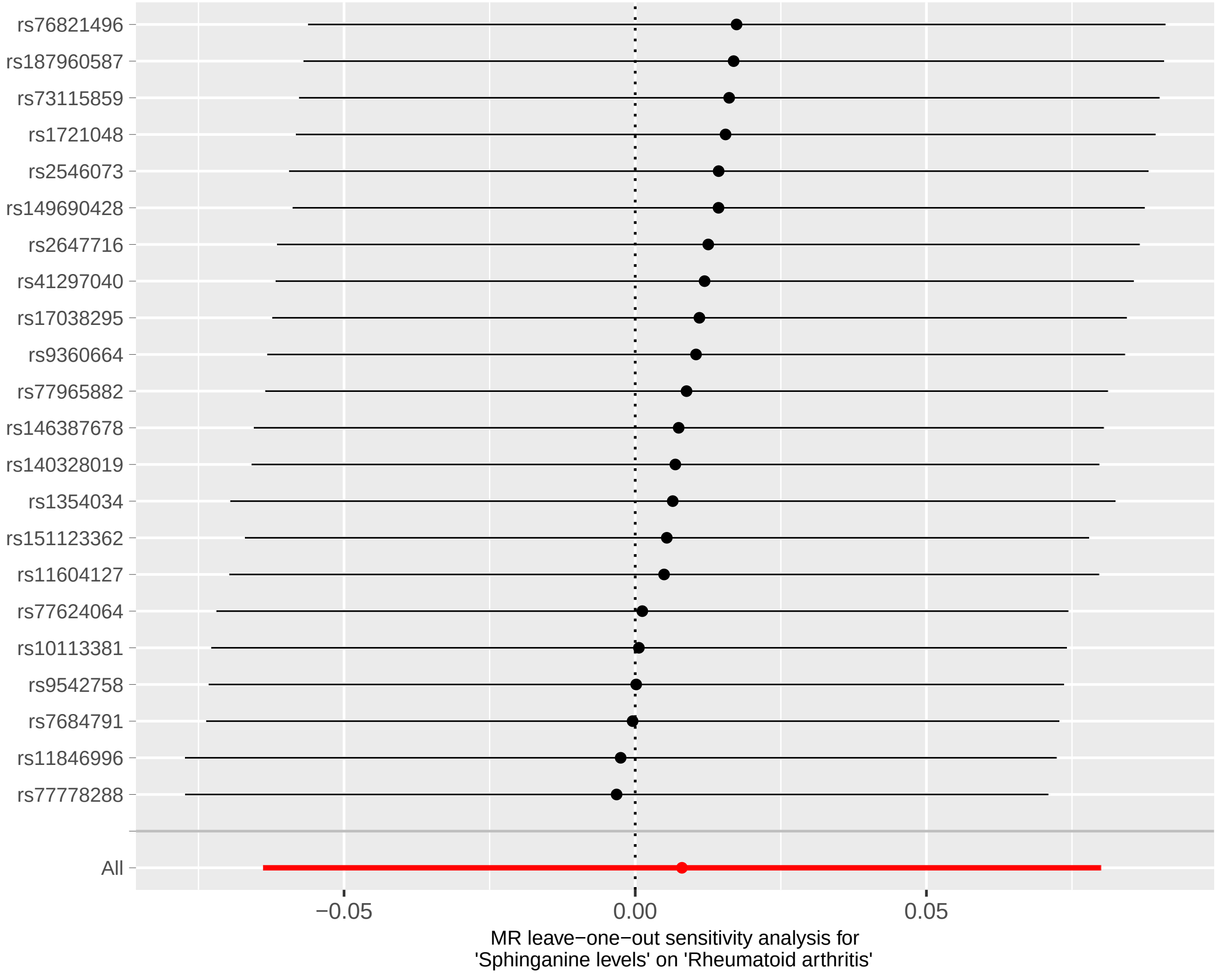

rs76821496
rs187960587
rs73115859
rs1721048
rs2546073
rs149690428
rs2647716
rs41297040
rs17038295
rs9360664
rs77965882
rs146387678
rs140328019
rs1354034
rs151123362
rs11604127
rs77624064
rs10113381
rs9542758
rs7684791
rs11846996
rs77778288
All
−0.05
0.00
0.05
MR leave−one−out sensitivity analysis for
'Sphinganine levels' on 'Rheumatoid arthritis'

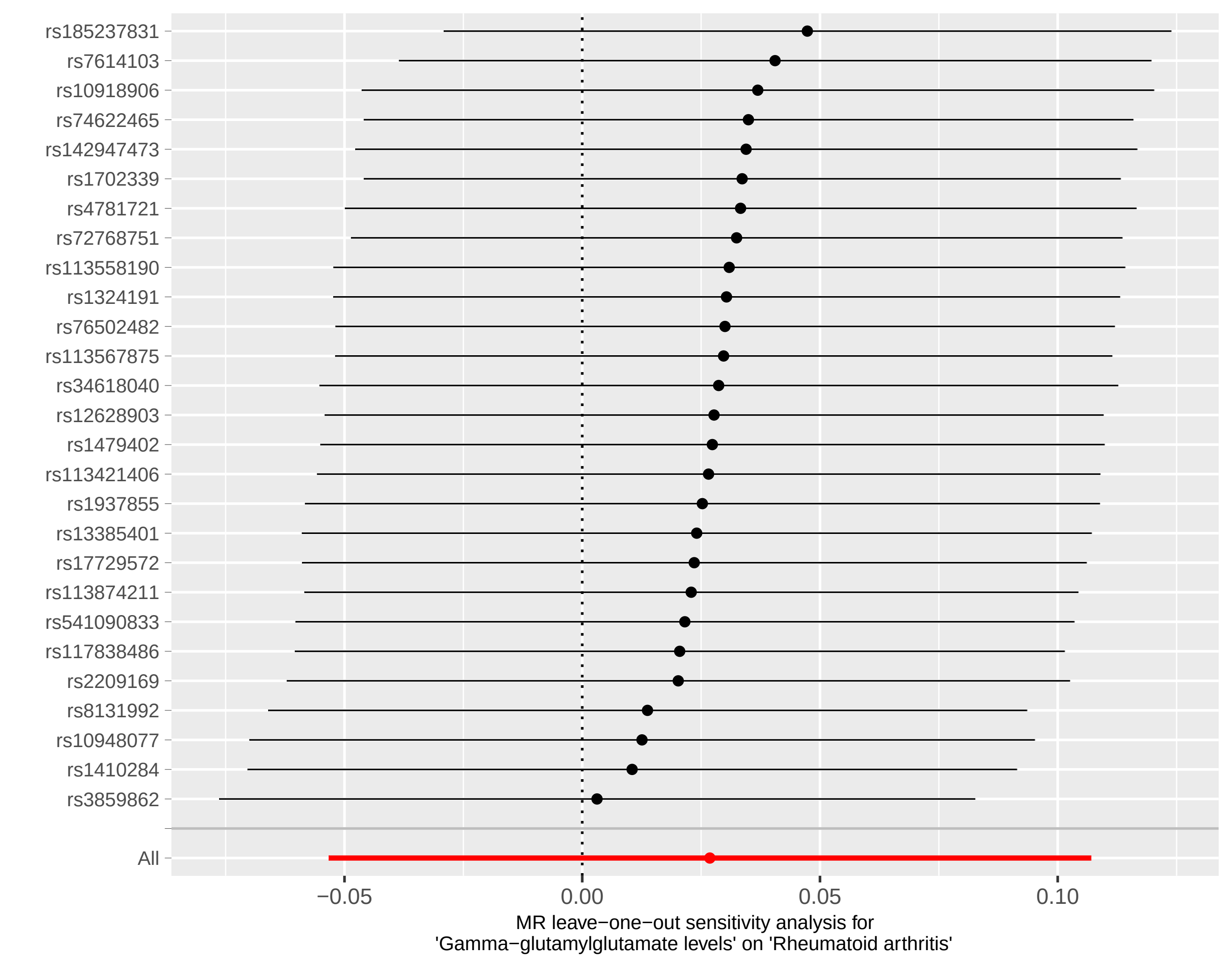

rs185237831
rs7614103
rs10918906
rs74622465
rs142947473
rs1702339
rs4781721
rs72768751
rs113558190
rs1324191
rs76502482
rs113567875
rs34618040
rs12628903
rs1479402
rs113421406
rs1937855
rs13385401
rs17729572
rs113874211
rs541090833
rs117838486
rs2209169
rs8131992
rs10948077
rs1410284
rs3859862
All
−0.05
0.00
0.05
0.10
MR leave−one−out sensitivity analysis for
'Gamma−glutamylglutamate levels' on 'Rheumatoid arthritis'

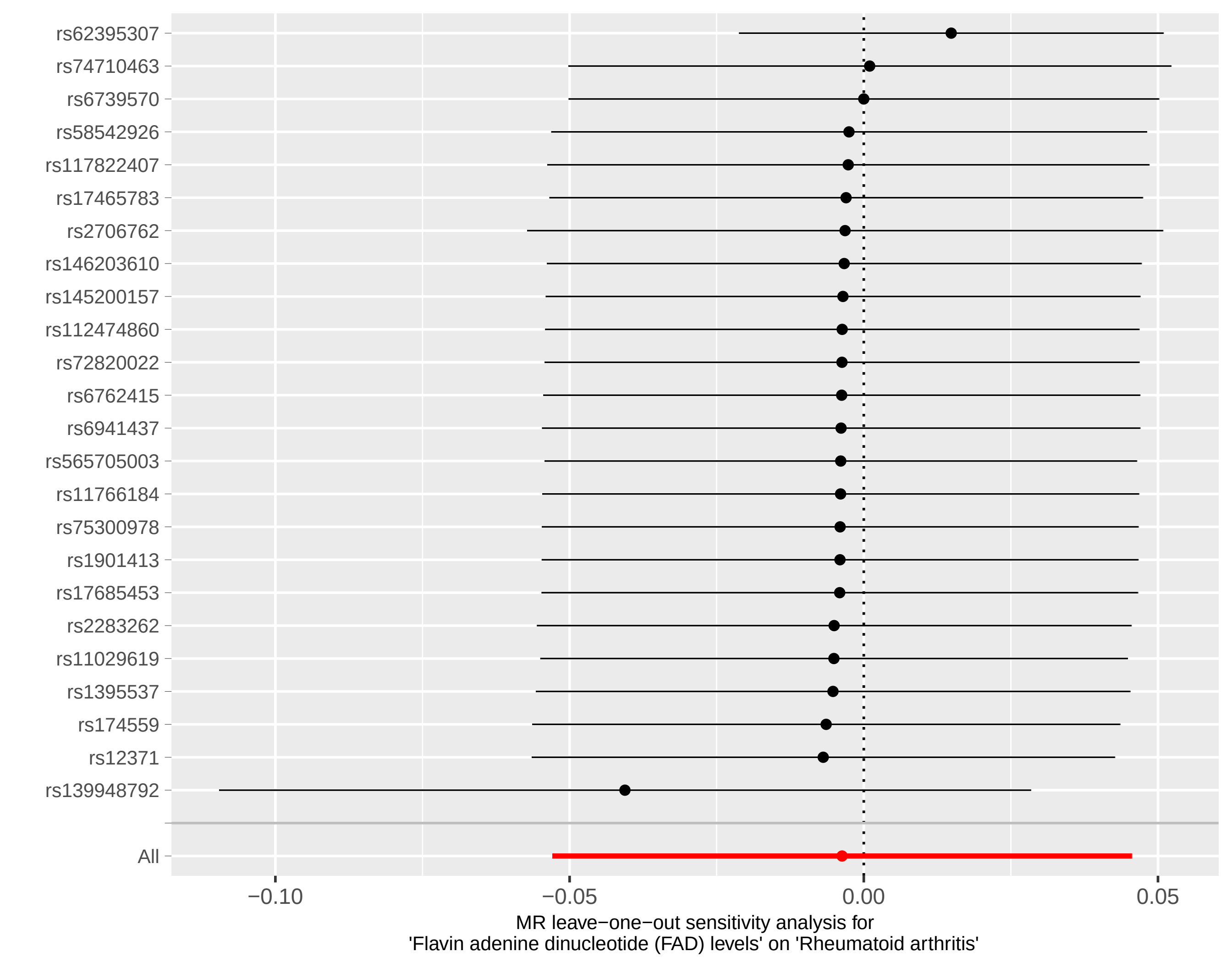
rs62395307
rs74710463
rs6739570
rs58542926
rs117822407
rs17465783
rs2706762
rs146203610
rs145200157
rs112474860
rs72820022
rs6762415
rs6941437
rs565705003
rs11766184
rs75300978
rs1901413
rs17685453
rs2283262
rs11029619
rs1395537
rs174559
rs12371
rs139948792
All
−0.10
−0.05
0.00
0.05
MR leave−one−out sensitivity analysis for
'Flavin adenine dinucleotide (FAD) levels' on 'Rheumatoid arthritis'

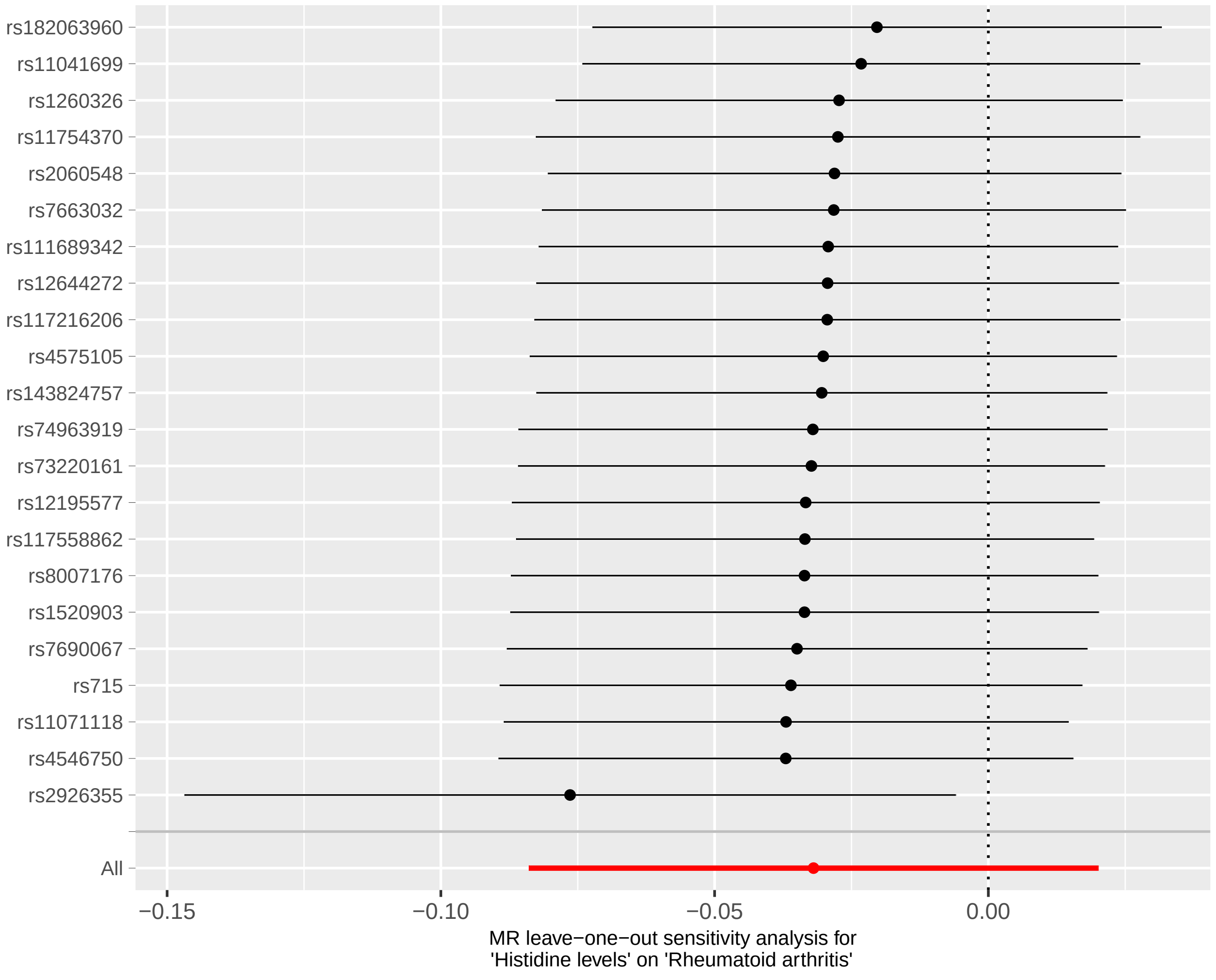
rs182063960
rs11041699
rs1260326
rs11754370
rs2060548
rs7663032
rs111689342
rs12644272
rs117216206
rs4575105
rs143824757
rs74963919
rs73220161
rs12195577
rs117558862
rs8007176
rs1520903
rs7690067
rs715
rs11071118
rs4546750
rs2926355
All
−0.15
−0.10
−0.05
0.00
MR leave−one−out sensitivity analysis for
'Histidine levels' on 'Rheumatoid arthritis'

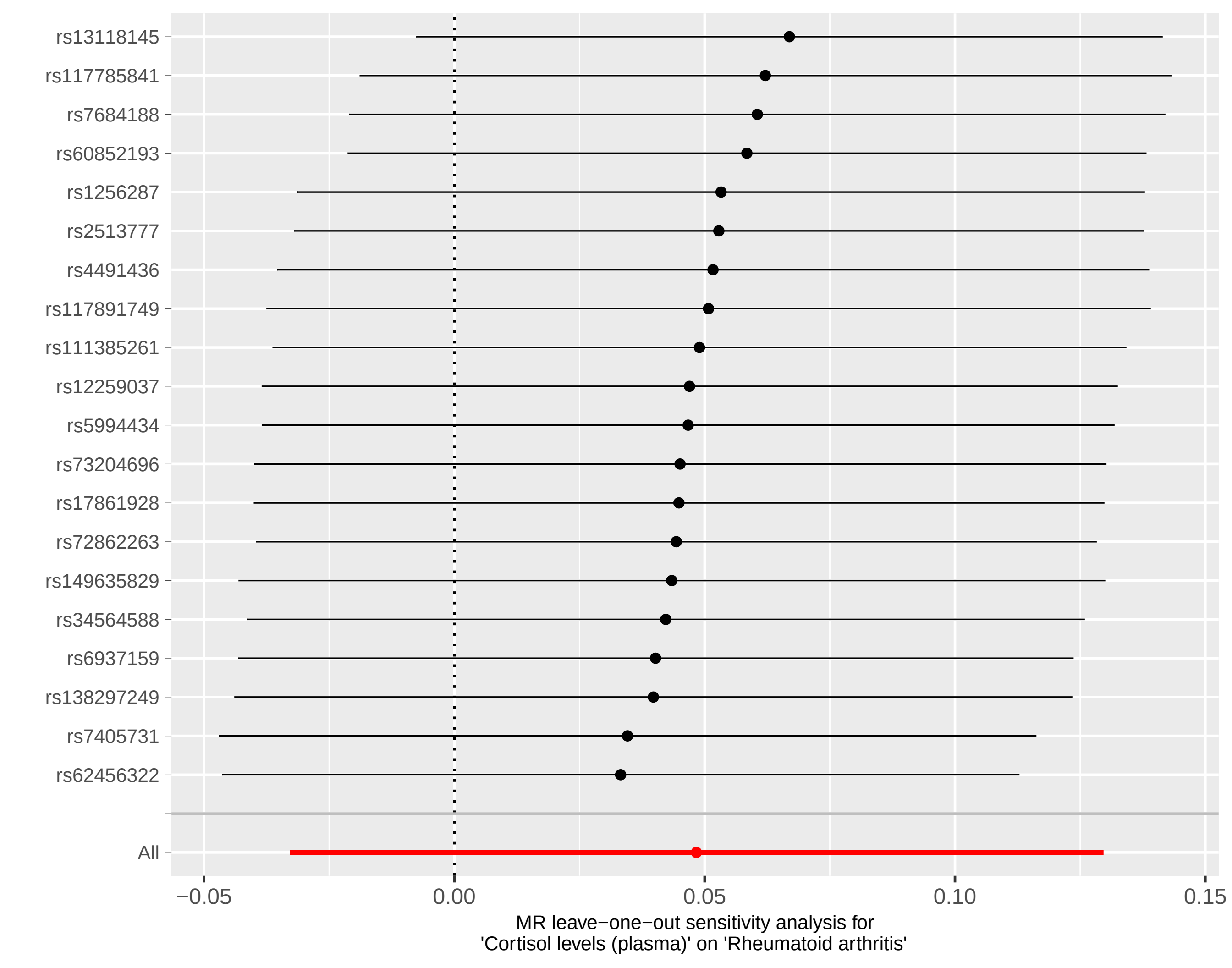
rs13118145
rs117785841
rs7684188
rs60852193
rs1256287
rs2513777
rs4491436
rs117891749
rs111385261
rs12259037
rs5994434
rs73204696
rs17861928
rs72862263
rs149635829
rs34564588
rs6937159
rs138297249
rs7405731
rs62456322
All
−0.05
0.00
0.05
0.10
0.15
MR leave−one−out sensitivity analysis for
'Cortisol levels (plasma)' on 'Rheumatoid arthritis'

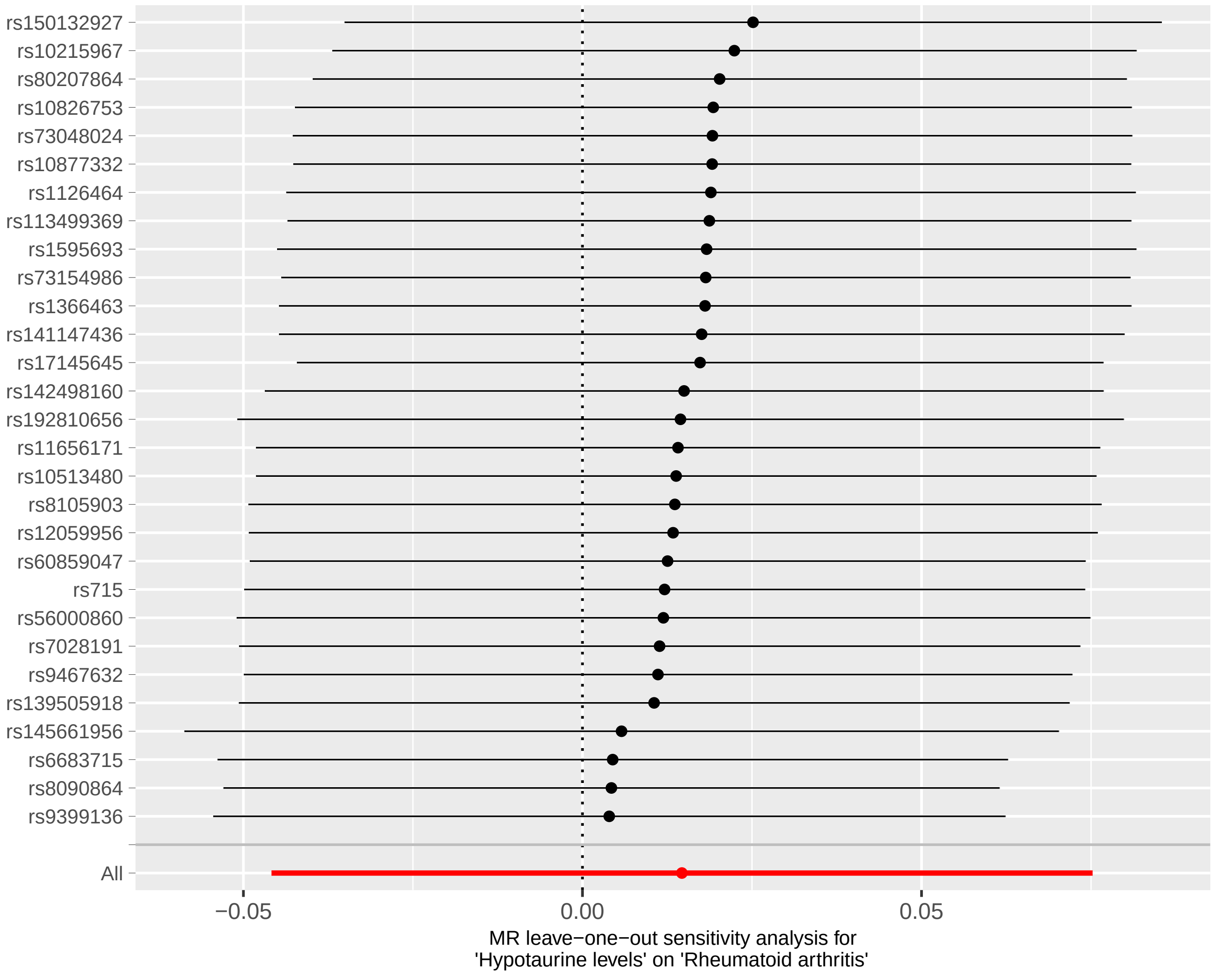

rs150132927
rs10215967
rs80207864
rs10826753
rs73048024
rs10877332
rs1126464
rs113499369
rs1595693
rs73154986
rs1366463
rs141147436
rs17145645
rs142498160
rs192810656
rs11656171
rs10513480
rs8105903
rs12059956
rs60859047
rs715
rs56000860
rs7028191
rs9467632
rs139505918
rs145661956
rs6683715
rs8090864
rs9399136
All
−0.05
0.00
0.05
MR leave−one−out sensitivity analysis for
'Hypotaurine levels' on 'Rheumatoid arthritis'

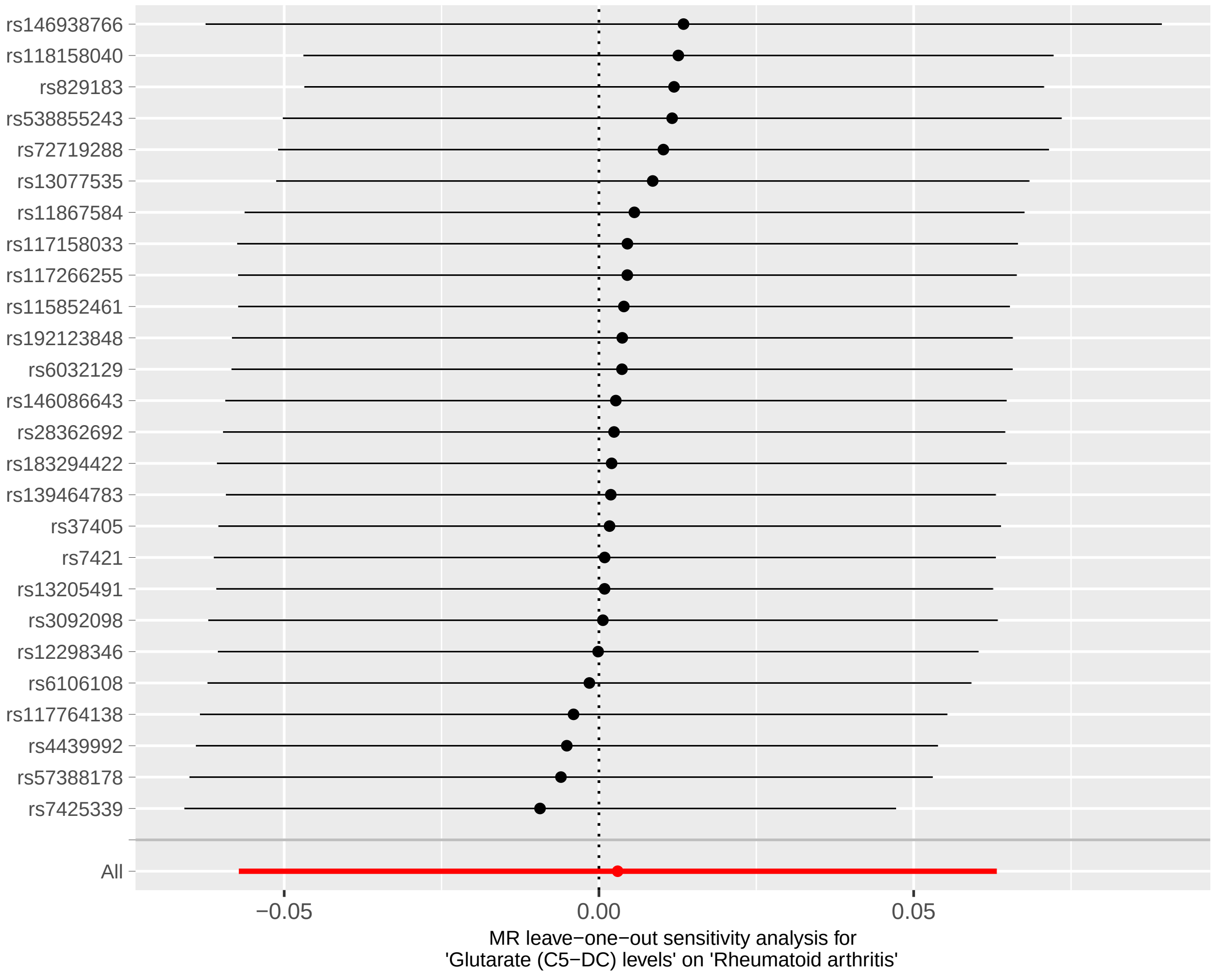
rs146938766
rs118158040
rs829183
rs538855243
rs72719288
rs13077535
rs11867584
rs117158033
rs117266255
rs115852461
rs192123848
rs6032129
rs146086643
rs28362692
rs183294422
rs139464783
rs37405
rs7421
rs13205491
rs3092098
rs12298346
rs6106108
rs117764138
rs4439992
rs57388178
rs7425339
All
−0.05
0.00
0.05
MR leave−one−out sensitivity analysis for
'Glutarate (C5−DC) levels' on 'Rheumatoid arthritis'

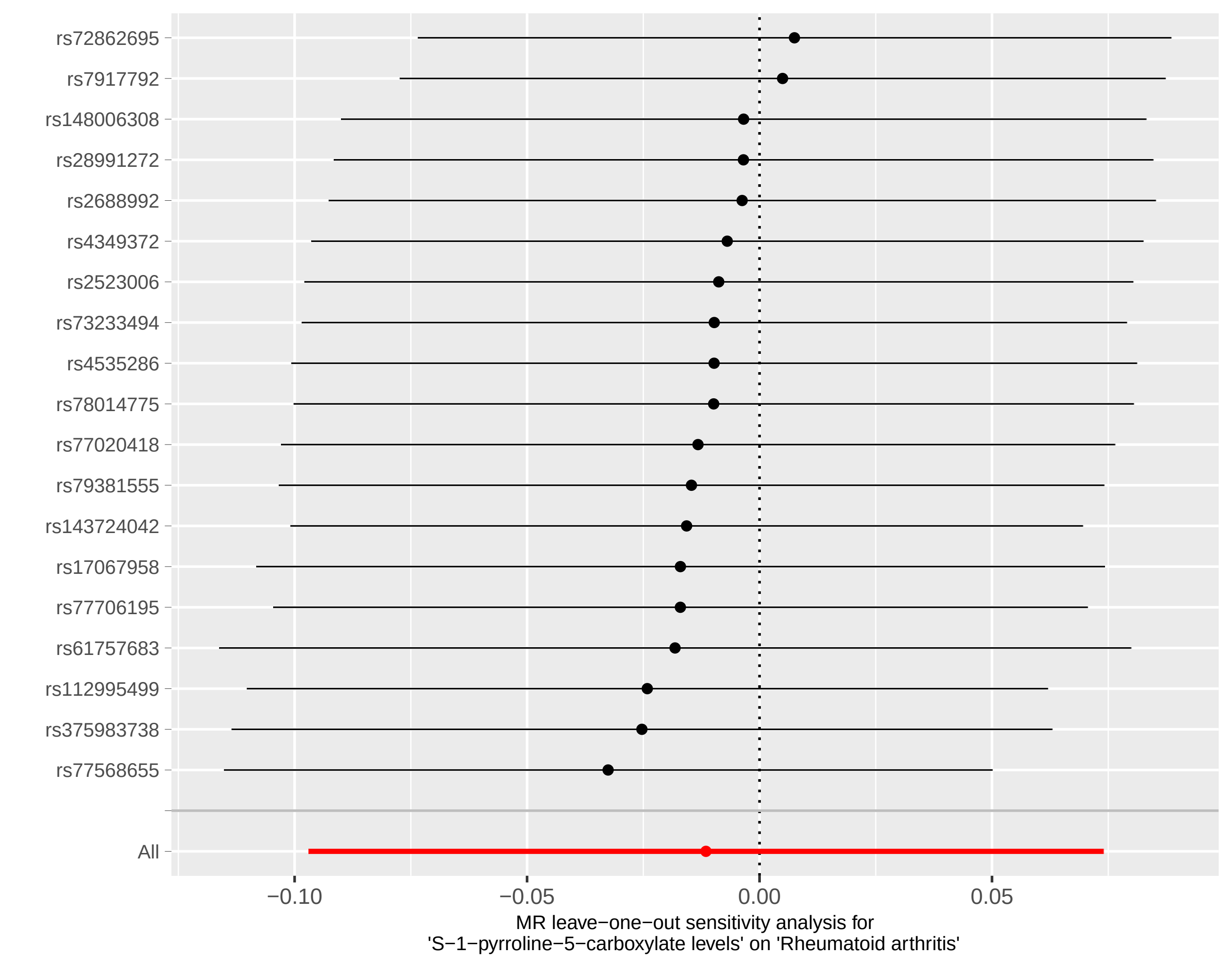
rs72862695
rs7917792
rs148006308
rs28991272
rs2688992
rs4349372
rs2523006
rs73233494
rs4535286
rs78014775
rs77020418
rs79381555
rs143724042
rs17067958
rs77706195
rs61757683
rs112995499
rs375983738
rs77568655
All
−0.10
−0.05
0.00
0.05
MR leave−one−out sensitivity analysis for
'S−1−pyrroline−5−carboxylate levels' on 'Rheumatoid arthritis'

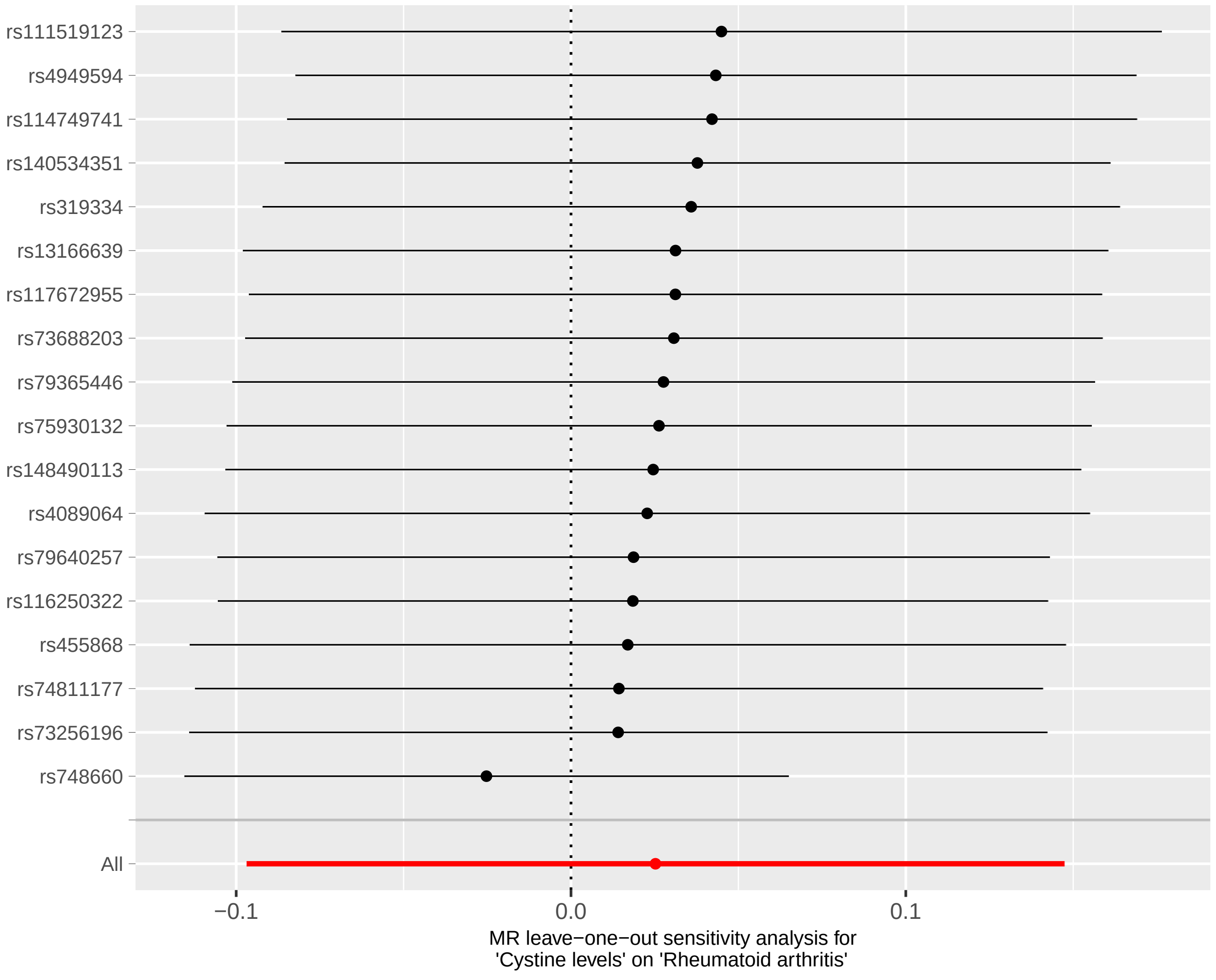
rs111519123
rs4949594
rs114749741
rs140534351
rs319334
rs13166639
rs117672955
rs73688203
rs79365446
rs75930132
rs148490113
rs4089064
rs79640257
rs116250322
rs455868
rs74811177
rs73256196
rs748660
All
−0.1
0.0
0.1
MR leave−one−out sensitivity analysis for
'Cystine levels' on 'Rheumatoid arthritis'

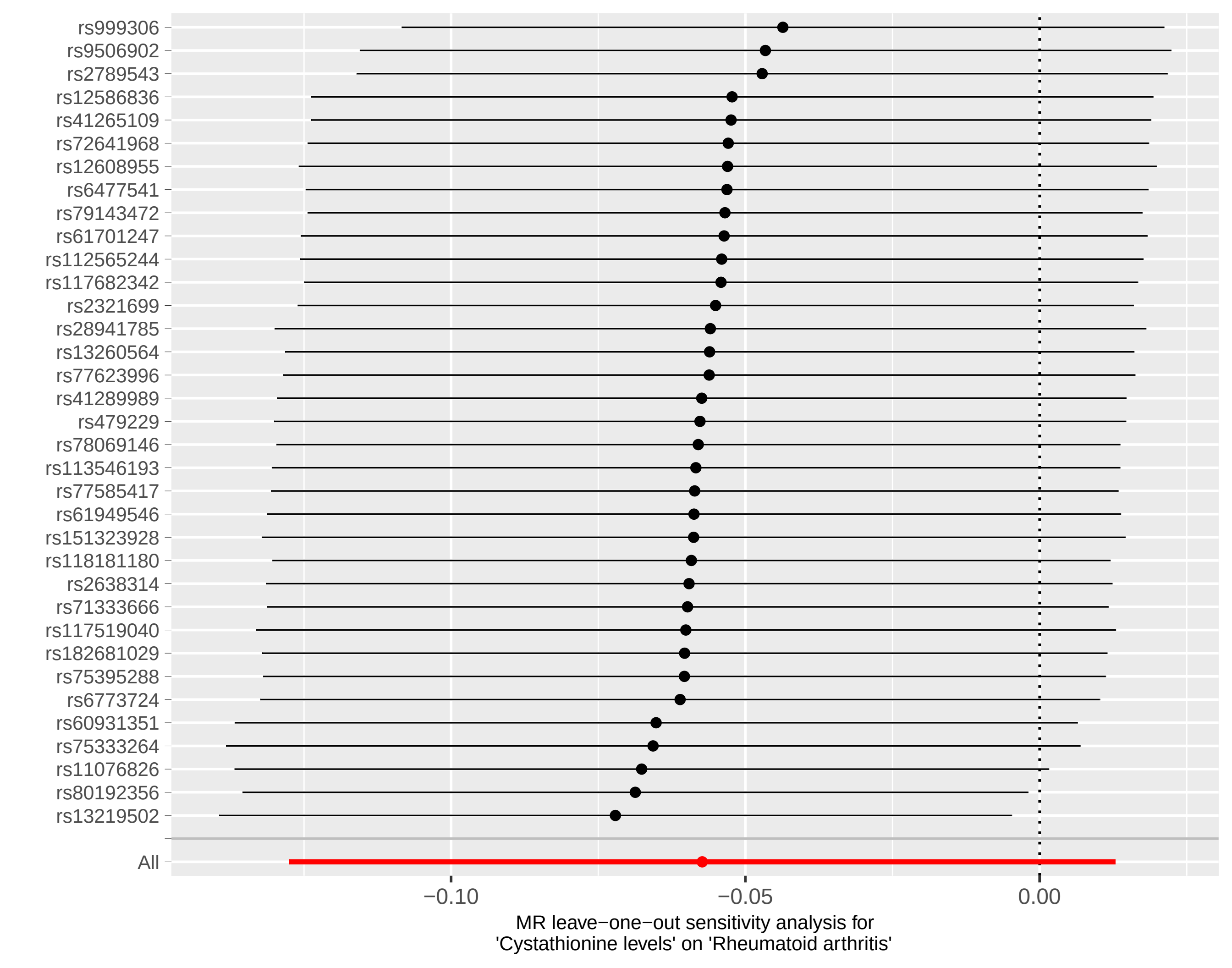

rs999306
rs9506902
rs2789543
rs12586836
rs41265109
rs72641968
rs12608955
rs6477541
rs79143472
rs61701247
rs112565244
rs117682342
rs2321699
rs28941785
rs13260564
rs77623996
rs41289989
rs479229
rs78069146
rs113546193
rs77585417
rs61949546
rs151323928
rs118181180
rs2638314
rs71333666
rs117519040
rs182681029
rs75395288
rs6773724
rs60931351
rs75333264
rs11076826
rs80192356
rs13219502
All
−0.10
−0.05
0.00
MR leave−one−out sensitivity analysis for
'Cystathionine levels' on 'Rheumatoid arthritis'

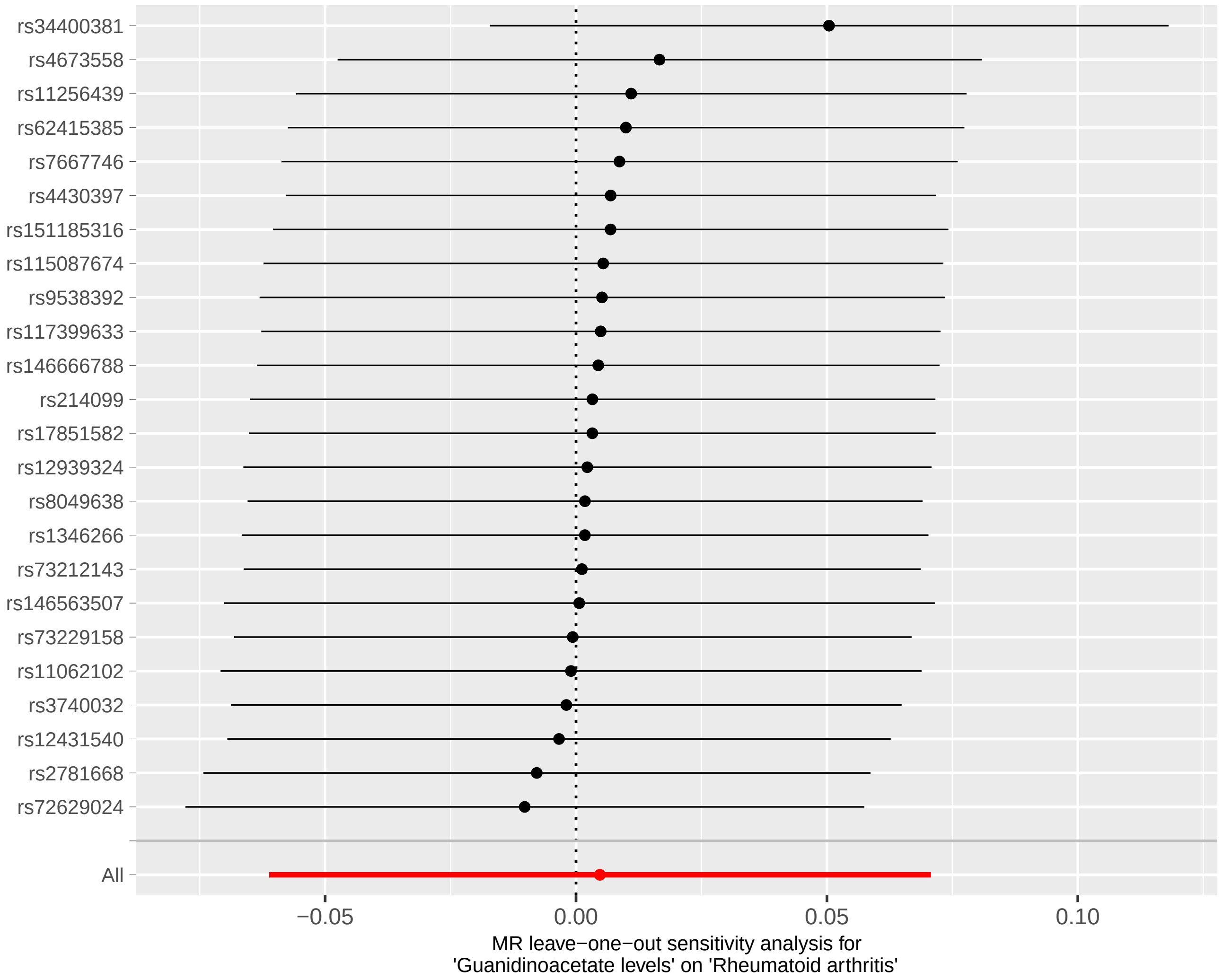
rs34400381
rs4673558
rs11256439
rs62415385
rs7667746
rs4430397
rs151185316
rs115087674
rs9538392
rs117399633
rs146666788
rs214099
rs17851582
rs12939324
rs8049638
rs1346266
rs73212143
rs146563507
rs73229158
rs11062102
rs3740032
rs12431540
rs2781668
rs72629024
All
−0.05
0.00
0.05
0.10
MR leave−one−out sensitivity analysis for
'Guanidinoacetate levels' on 'Rheumatoid arthritis'

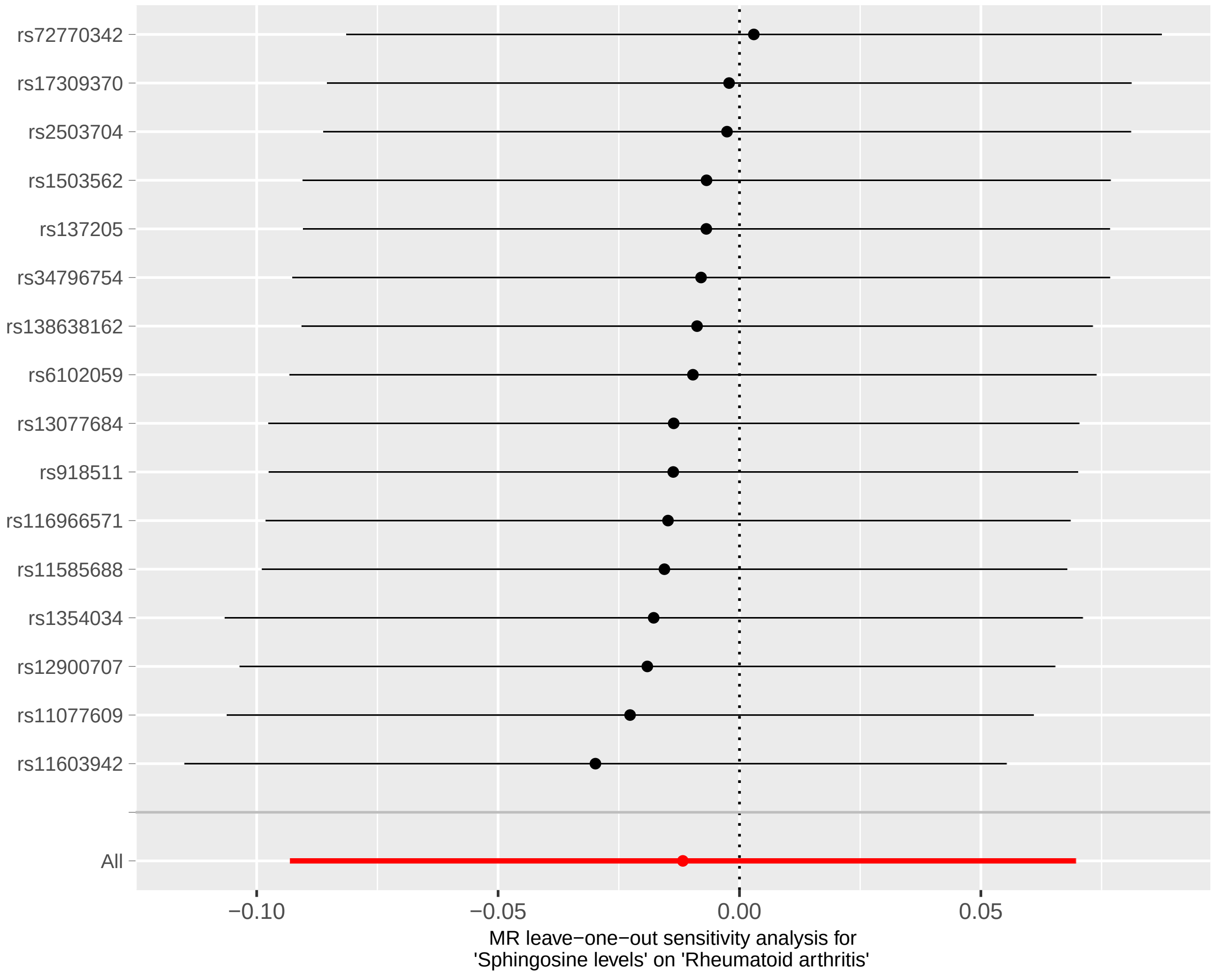
rs72770342
rs17309370
rs2503704
rs1503562
rs137205
rs34796754
rs138638162
rs6102059
rs13077684
rs918511
rs116966571
rs11585688
rs1354034
rs12900707
rs11077609
rs11603942
All
−0.10
−0.05
0.00
0.05
MR leave−one−out sensitivity analysis for
'Sphingosine levels' on 'Rheumatoid arthritis'

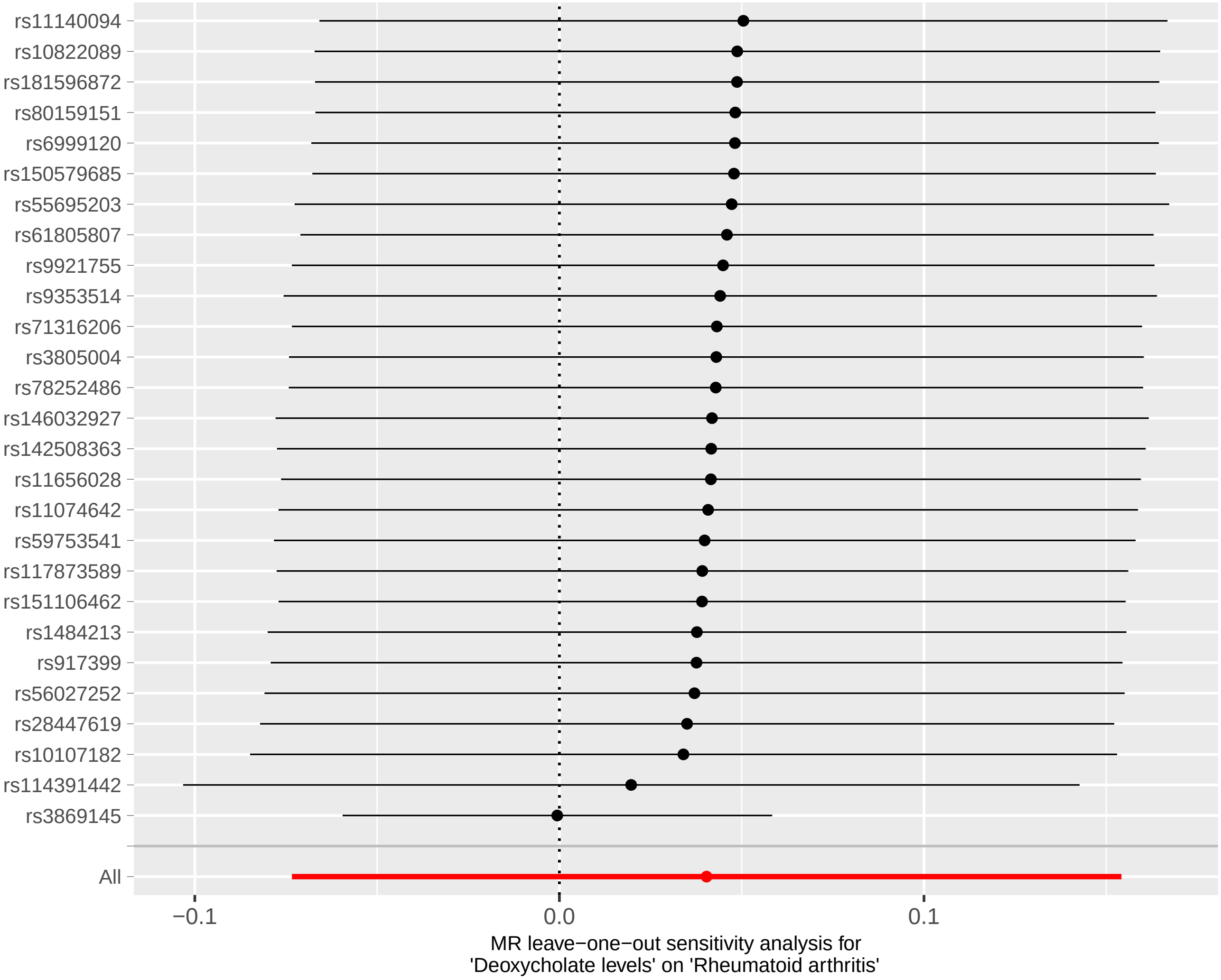
rs11140094
rs10822089
rs181596872
rs80159151
rs6999120
rs150579685
rs55695203
rs61805807
rs9921755
rs9353514
rs71316206
rs3805004
rs78252486
rs146032927
rs142508363
rs11656028
rs11074642
rs59753541
rs117873589
rs151106462
rs1484213
rs917399
rs56027252
rs28447619
rs10107182
rs114391442
rs3869145
All
−0.1
0.0
0.1
MR leave−one−out sensitivity analysis for
'Deoxycholate levels' on 'Rheumatoid arthritis'

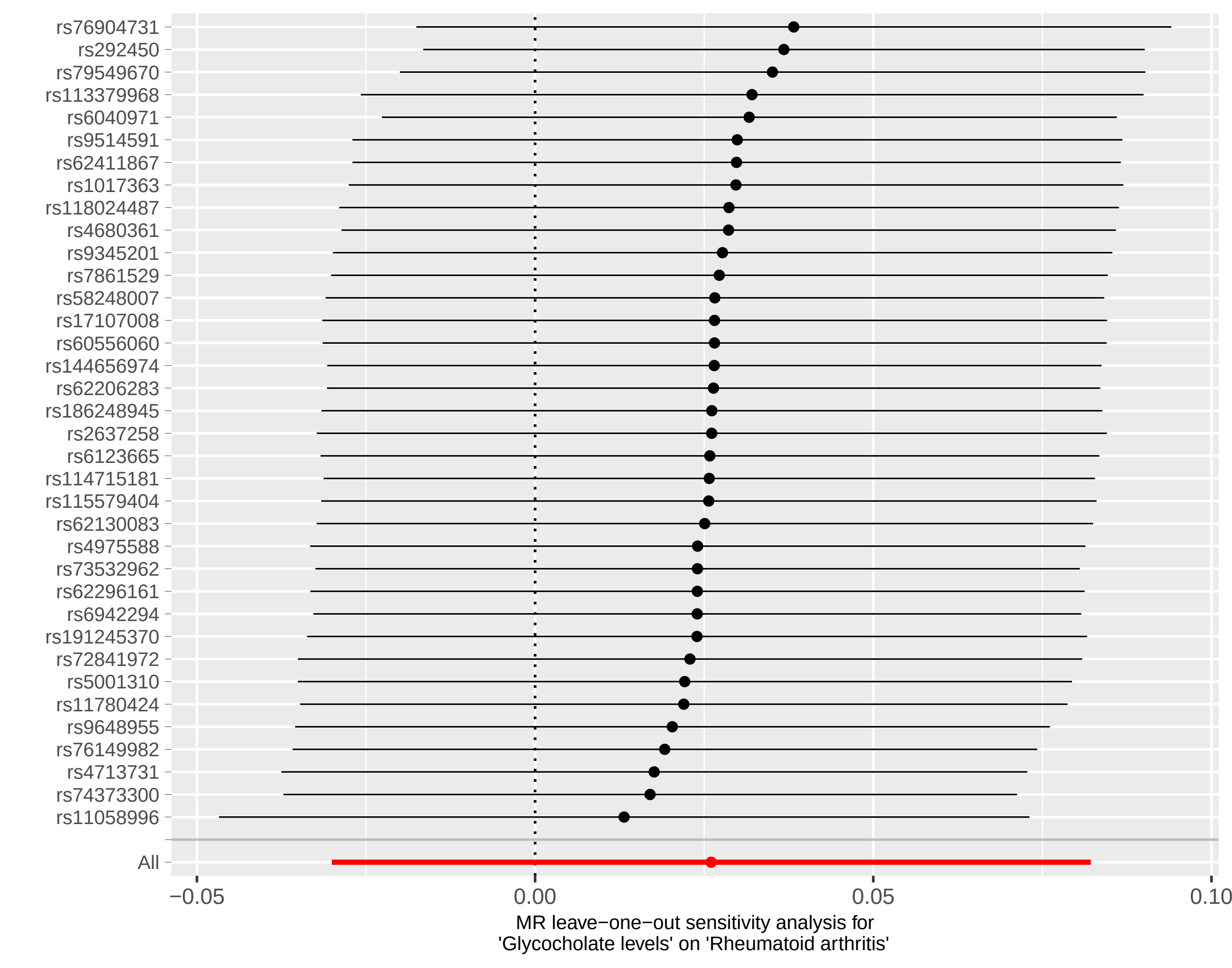
rs76904731
rs292450
rs79549670
rs113379968
rs6040971
rs9514591
rs62411867
rs1017363
rs118024487
rs4680361
rs9345201
rs7861529
rs58248007
rs17107008
rs60556060
rs144656974
rs62206283
rs186248945
rs2637258
rs6123665
rs114715181
rs115579404
rs62130083
rs4975588
rs73532962
rs62296161
rs6942294
rs191245370
rs72841972
rs5001310
rs11780424
rs9648955
rs76149982
rs4713731
rs74373300
rs11058996
All
−0.05
0.00
0.05
0.10
MR leave−one−out sensitivity analysis for
'Glycocholate levels' on 'Rheumatoid arthritis'

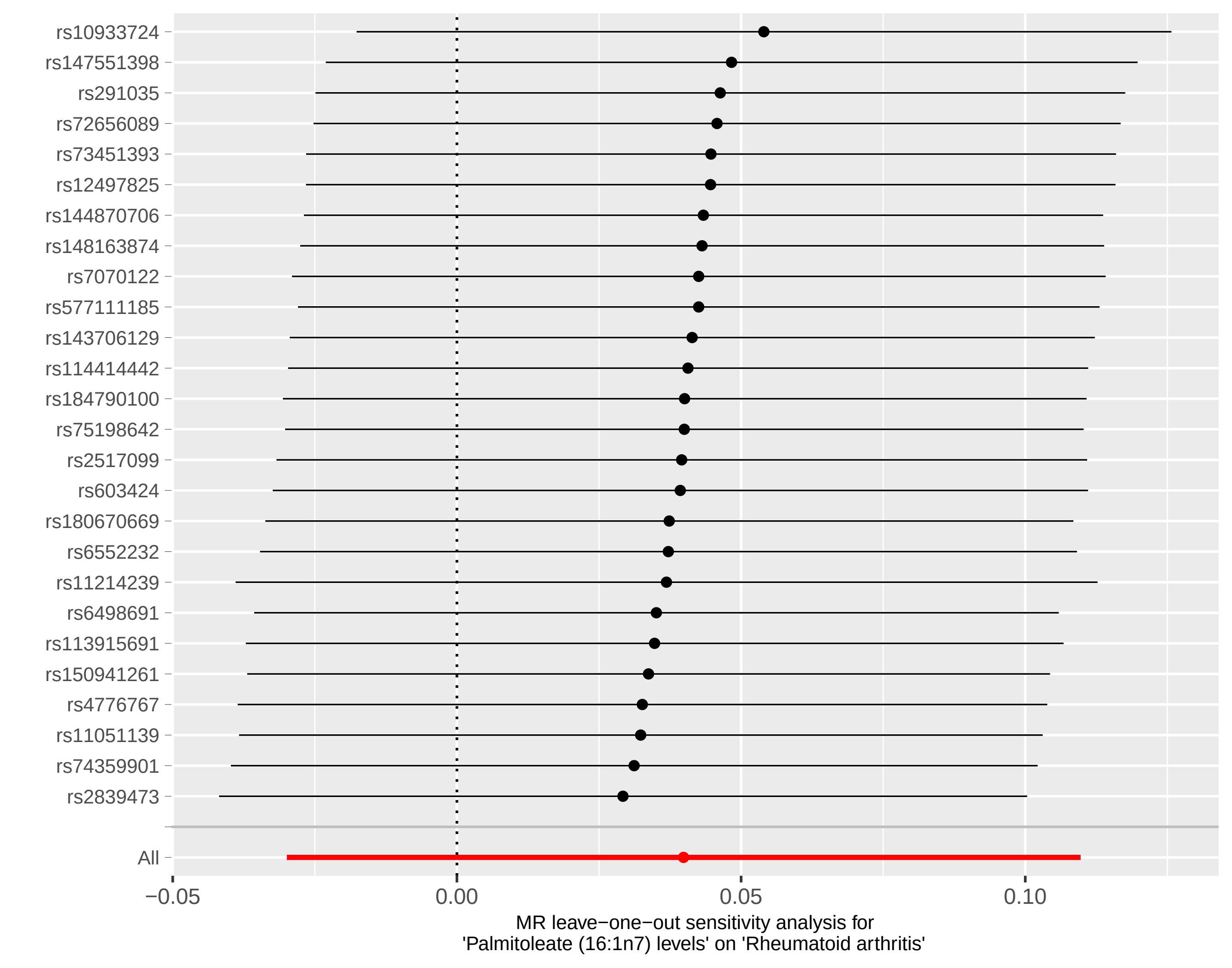

rs10933724
rs147551398
rs291035
rs72656089
rs73451393
rs12497825
rs144870706
rs148163874
rs7070122
rs577111185
rs143706129
rs114414442
rs184790100
rs75198642
rs2517099
rs603424
rs180670669
rs6552232
rs11214239
rs6498691
rs113915691
rs150941261
rs4776767
rs11051139
rs74359901
rs2839473
All
−0.05
0.00
0.05
0.10
MR leave−one−out sensitivity analysis for
'Palmitoleate (16:1n7) levels' on 'Rheumatoid arthritis'

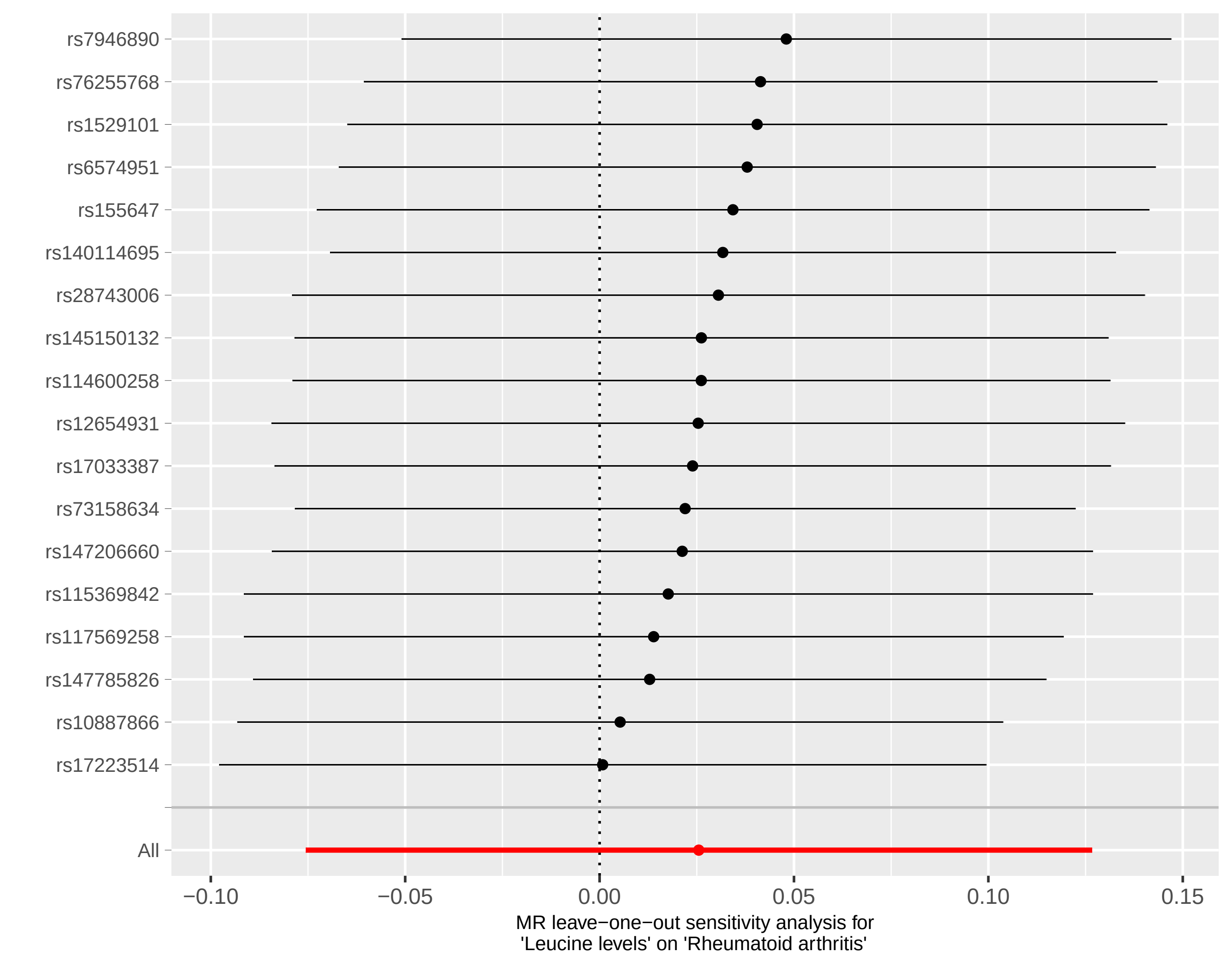
rs7946890
rs76255768
rs1529101
rs6574951
rs155647
rs140114695
rs28743006
rs145150132
rs114600258
rs12654931
rs17033387
rs73158634
rs147206660
rs115369842
rs117569258
rs147785826
rs10887866
rs17223514
All
−0.10
−0.05
0.00
0.05
0.10
0.15
MR leave−one−out sensitivity analysis for
'Leucine levels' on 'Rheumatoid arthritis'

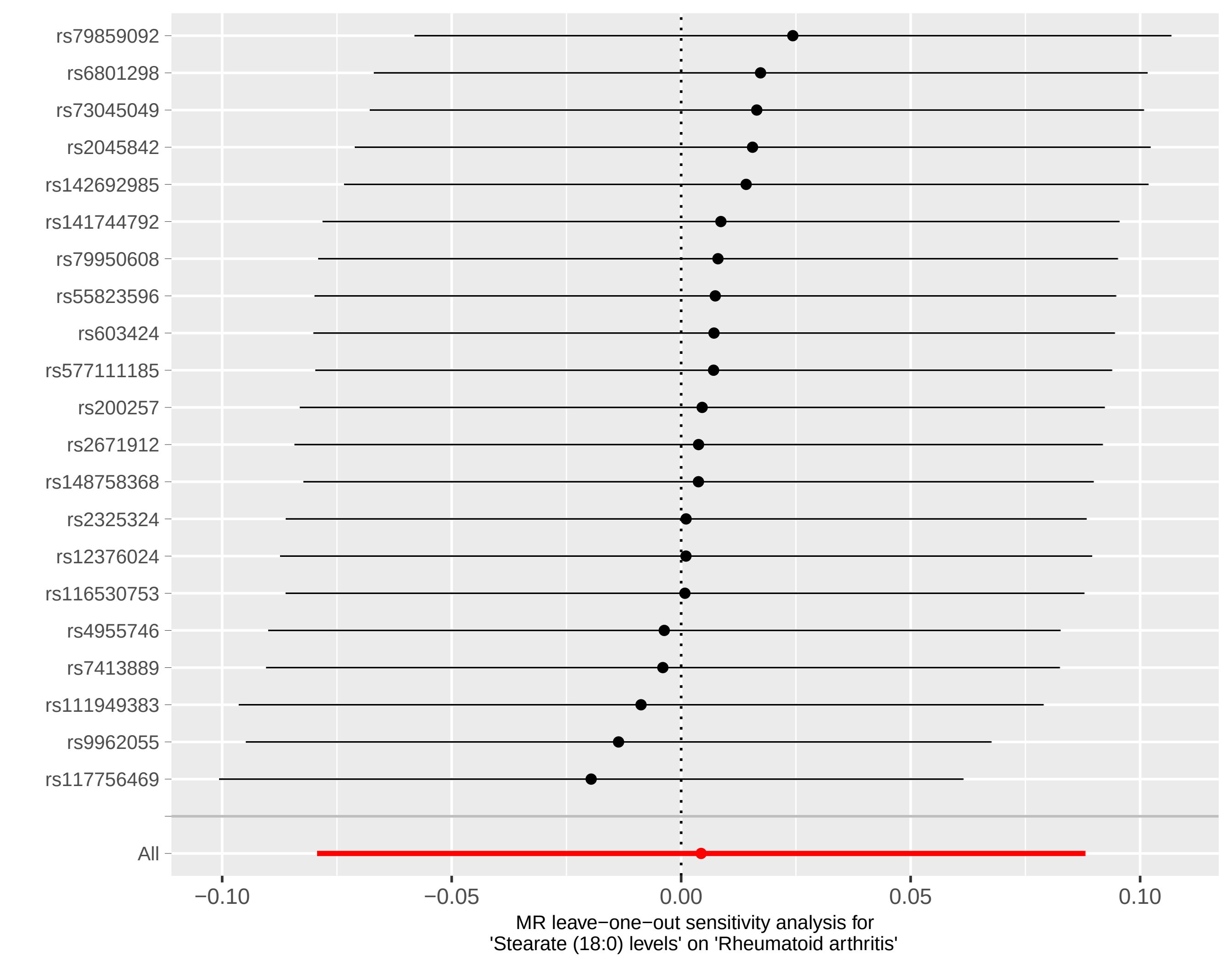
rs79859092
rs6801298
rs73045049
rs2045842
rs142692985
rs141744792
rs79950608
rs55823596
rs603424
rs577111185
rs200257
rs2671912
rs148758368
rs2325324
rs12376024
rs116530753
rs4955746
rs7413889
rs111949383
rs9962055
rs117756469
All
−0.10
−0.05
0.00
0.05
0.10
MR leave−one−out sensitivity analysis for
'Stearate (18:0) levels' on 'Rheumatoid arthritis'

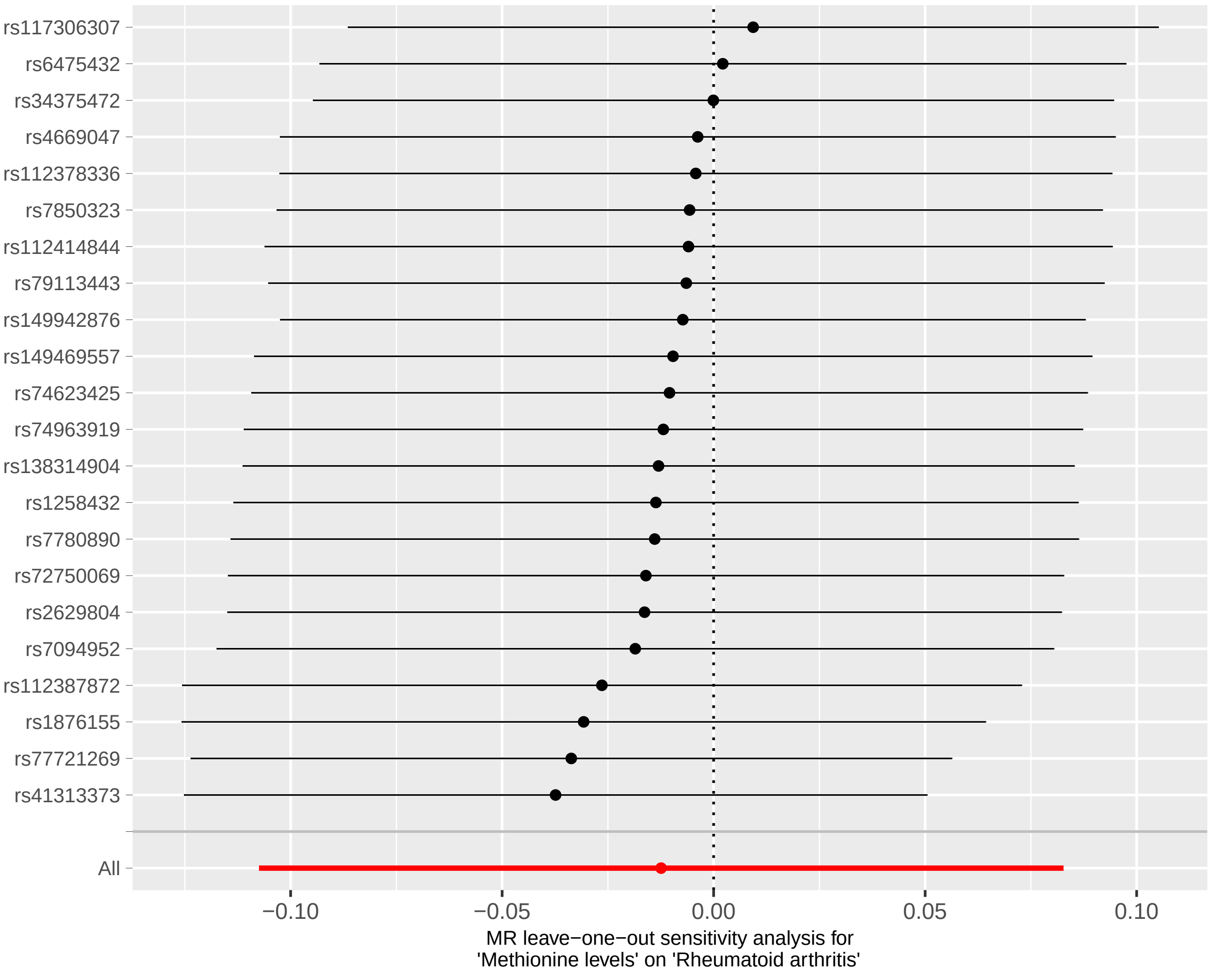
rs117306307
rs6475432
rs34375472
rs4669047
rs112378336
rs7850323
rs112414844
rs79113443
rs149942876
rs149469557
rs74623425
rs74963919
rs138314904
rs1258432
rs7780890
rs72750069
rs2629804
rs7094952
rs112387872
rs1876155
rs77721269
rs41313373
All
−0.10
−0.05
0.00
0.05
0.10
MR leave−one−out sensitivity analysis for
'Methionine levels' on 'Rheumatoid arthritis'

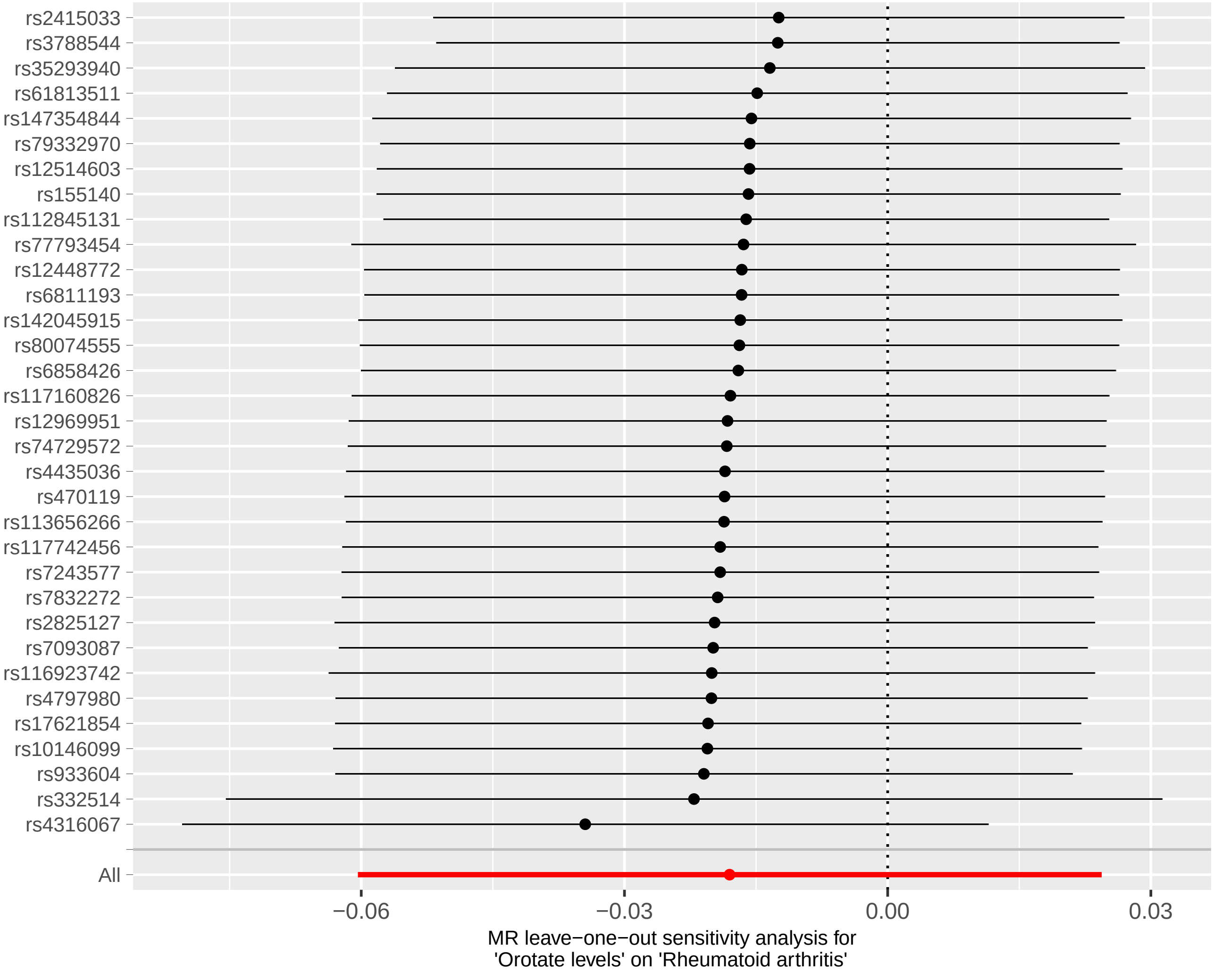
rs2415033
rs3788544
rs35293940
rs61813511
rs147354844
rs79332970
rs12514603
rs155140
rs112845131
rs77793454
rs12448772
rs6811193
rs142045915
rs80074555
rs6858426
rs117160826
rs12969951
rs74729572
rs4435036
rs470119
rs113656266
rs117742456
rs7243577
rs7832272
rs2825127
rs7093087
rs116923742
rs4797980
rs17621854
rs10146099
rs933604
rs332514
rs4316067
All
−0.06
−0.03
0.00
0.03
MR leave−one−out sensitivity analysis for
'Orotate levels' on 'Rheumatoid arthritis'

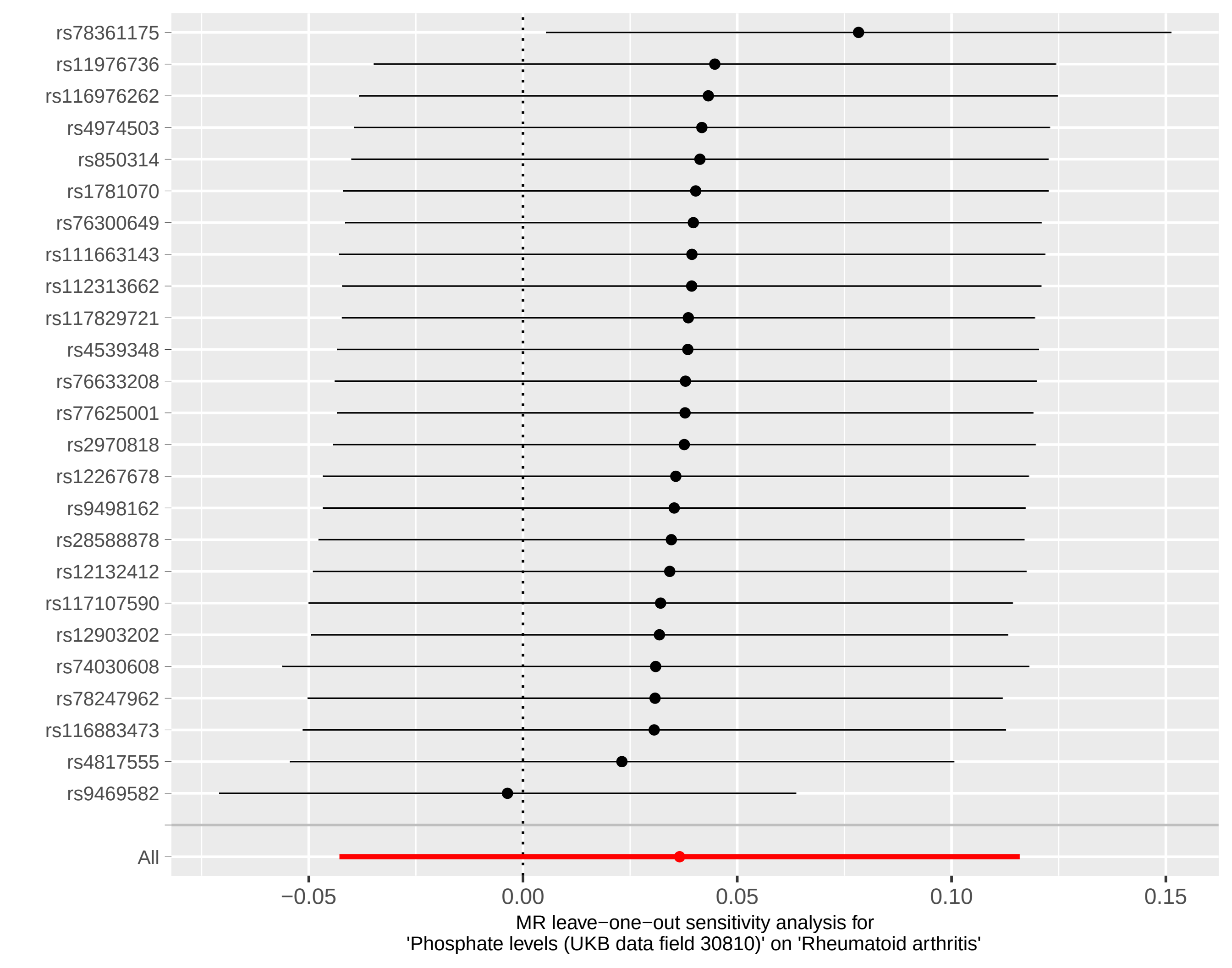
rs78361175
rs11976736
rs116976262
rs4974503
rs850314
rs1781070
rs76300649
rs111663143
rs112313662
rs117829721
rs4539348
rs76633208
rs77625001
rs2970818
rs12267678
rs9498162
rs28588878
rs12132412
rs117107590
rs12903202
rs74030608
rs78247962
rs116883473
rs4817555
rs9469582
All
−0.05
0.00
0.05
0.10
0.15
MR leave−one−out sensitivity analysis for
'Phosphate levels (UKB data field 30810)' on 'Rheumatoid arthritis'

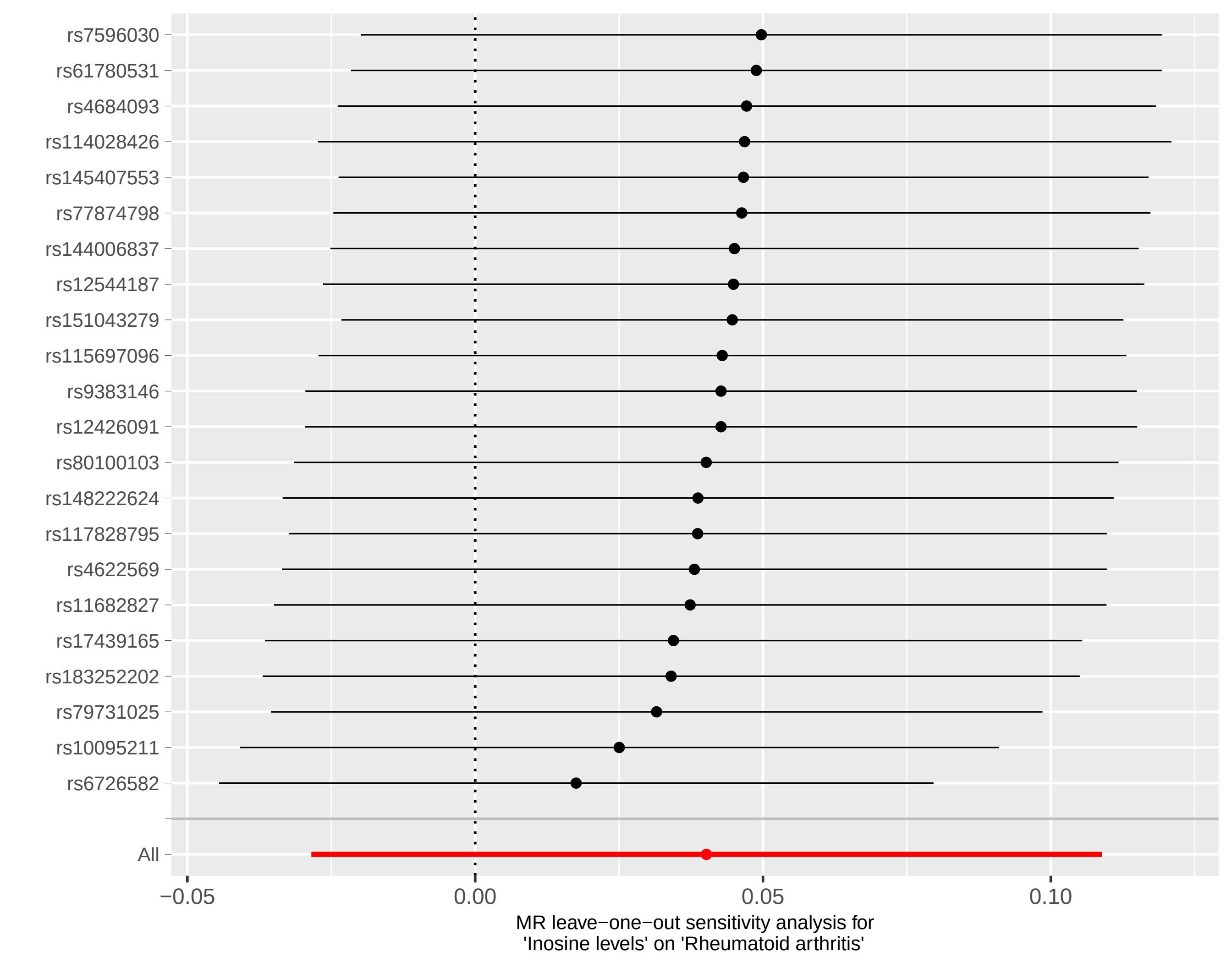
rs7596030
rs61780531
rs4684093
rs114028426
rs145407553
rs77874798
rs144006837
rs12544187
rs151043279
rs115697096
rs9383146
rs12426091
rs80100103
rs148222624
rs117828795
rs4622569
rs11682827
rs17439165
rs183252202
rs79731025
rs10095211
rs6726582
All
−0.05
0.00
0.05
0.10
MR leave−one−out sensitivity analysis for
'Inosine levels' on 'Rheumatoid arthritis'

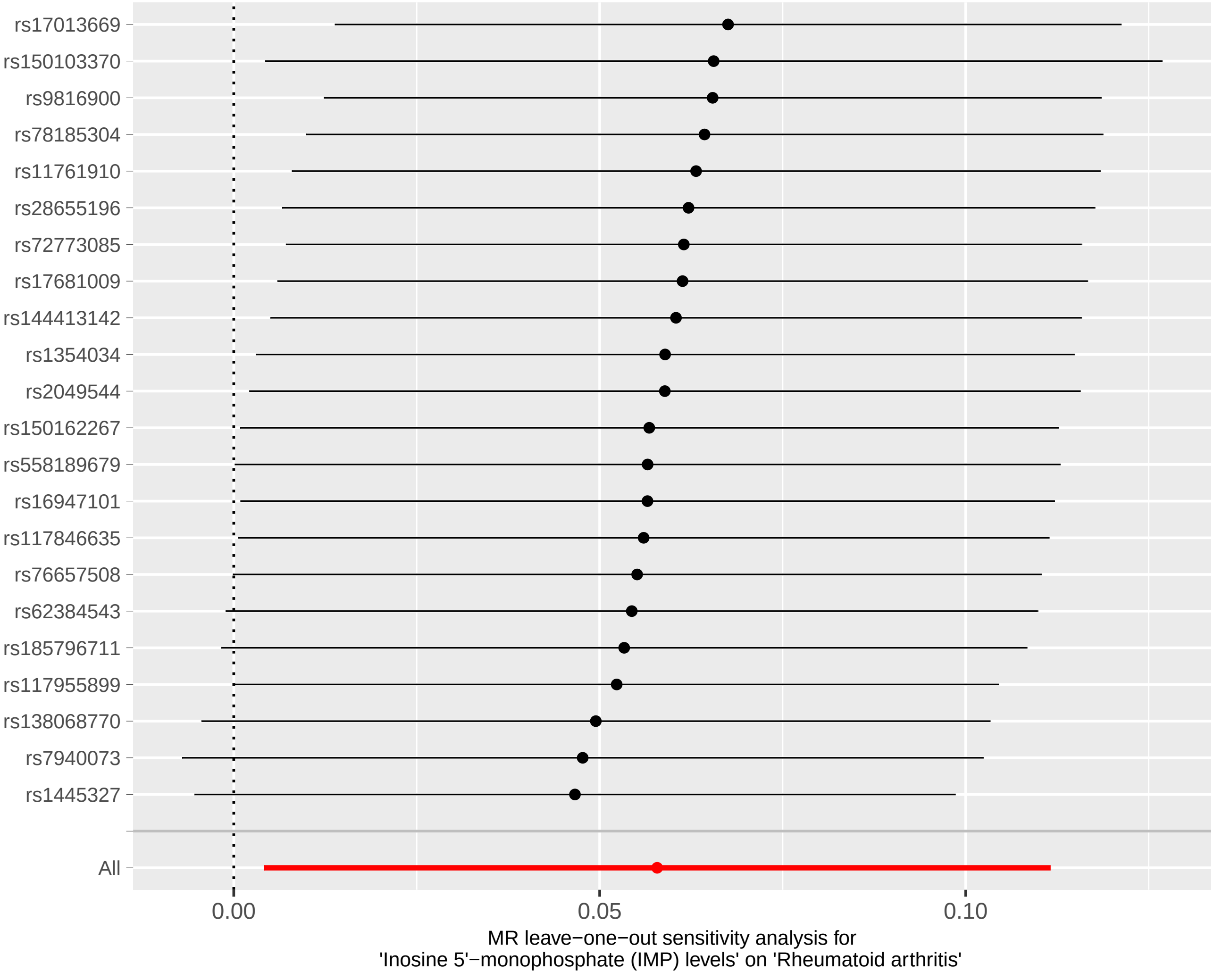

rs17013669
rs150103370
rs9816900
rs78185304
rs11761910
rs28655196
rs72773085
rs17681009
rs144413142
rs1354034
rs2049544
rs150162267
rs558189679
rs16947101
rs117846635
rs76657508
rs62384543
rs185796711
rs117955899
rs138068770
rs7940073
rs1445327
All
0.00
0.05
0.10
MR leave−one−out sensitivity analysis for
'Inosine 5'−monophosphate (IMP) levels' on 'Rheumatoid arthritis'

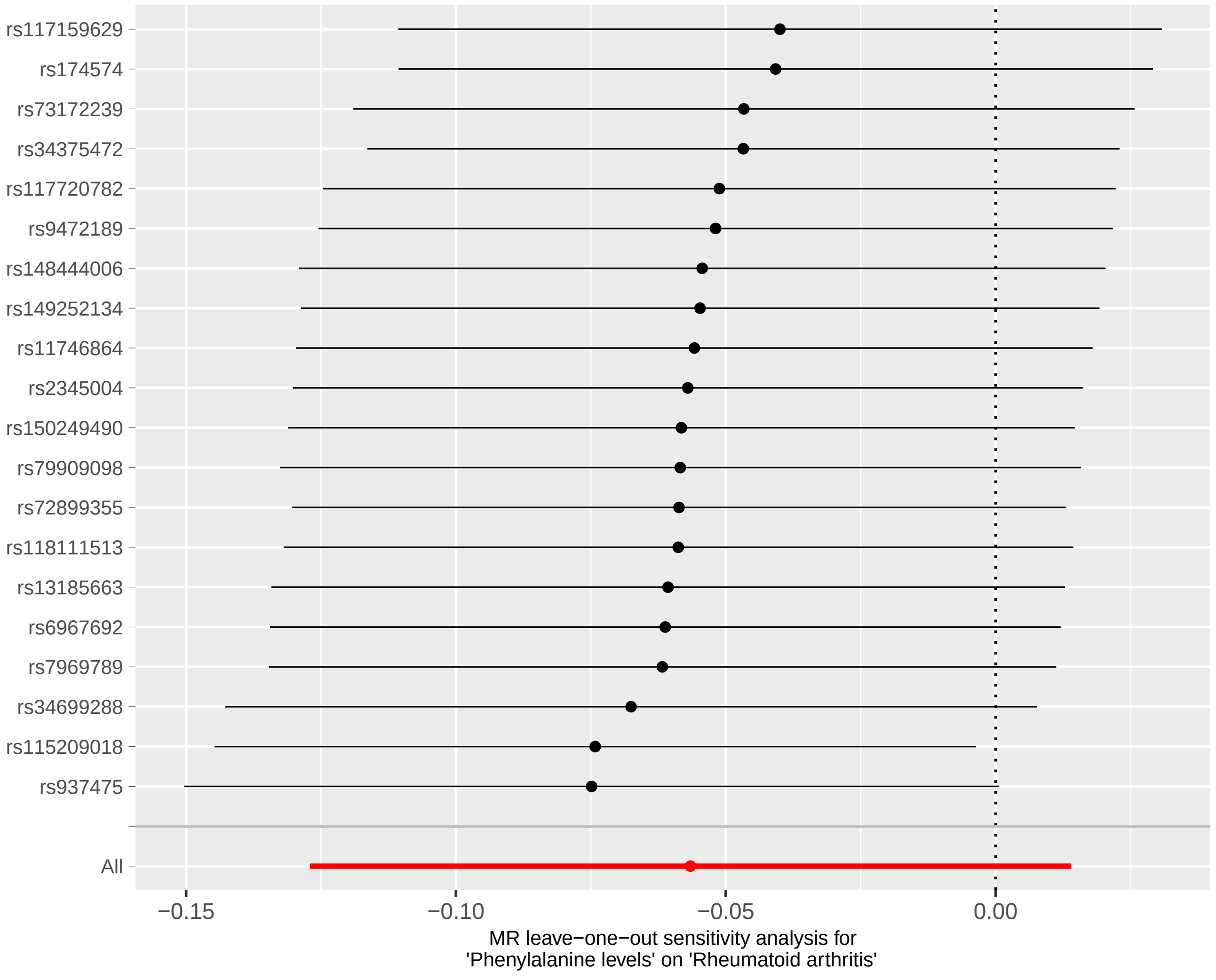
rs117159629
rs174574
rs73172239
rs34375472
rs117720782
rs9472189
rs148444006
rs149252134
rs11746864
rs2345004
rs150249490
rs79909098
rs72899355
rs118111513
rs13185663
rs6967692
rs7969789
rs34699288
rs115209018
rs937475
All
−0.15
−0.10
−0.05
0.00
MR leave−one−out sensitivity analysis for
'Phenylalanine levels' on 'Rheumatoid arthritis'

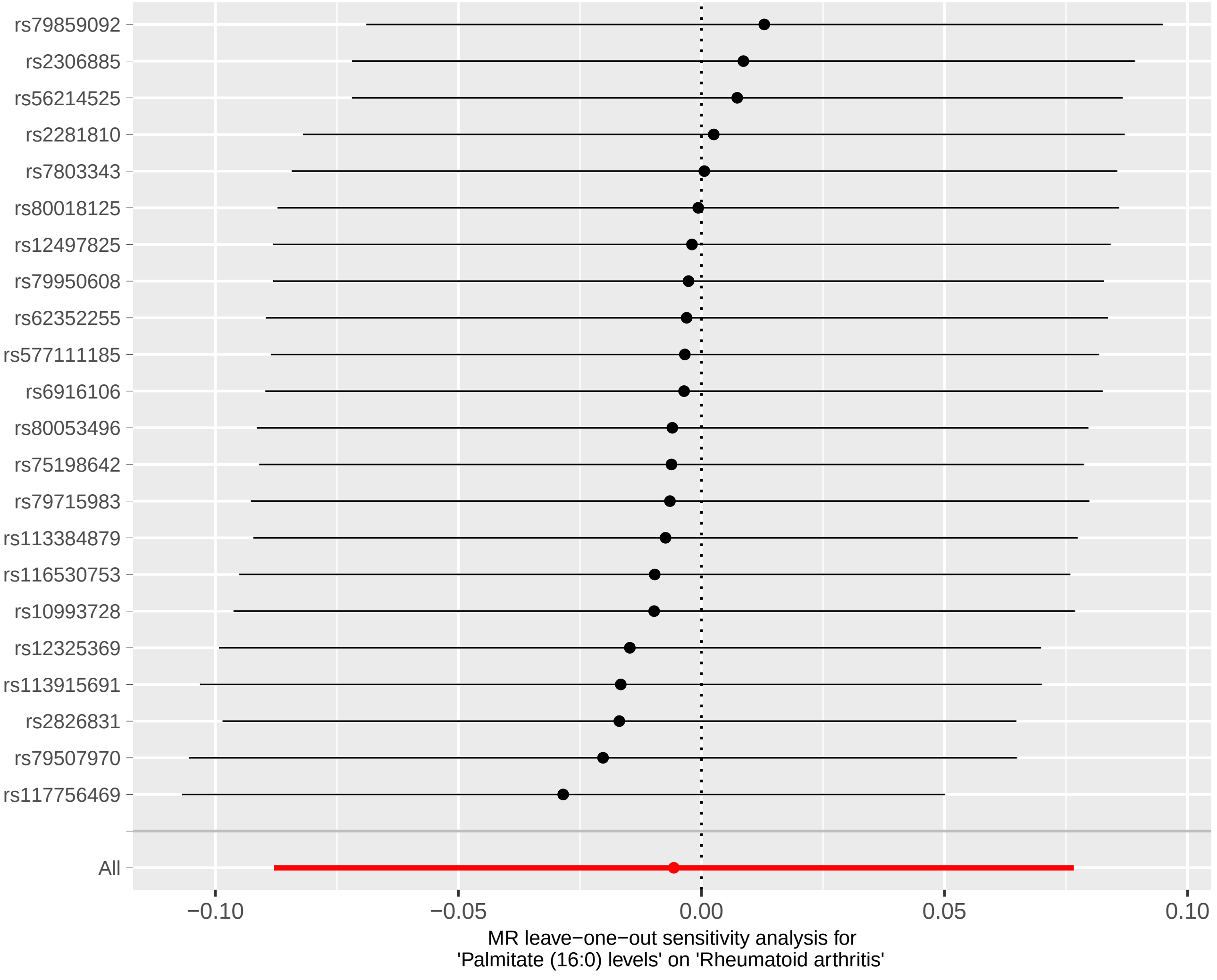

rs79859092
rs2306885
rs56214525
rs2281810
rs7803343
rs80018125
rs12497825
rs79950608
rs62352255
rs577111185
rs6916106
rs80053496
rs75198642
rs79715983
rs113384879
rs116530753
rs10993728
rs12325369
rs113915691
rs2826831
rs79507970
rs117756469
All
−0.10
−0.05
0.00
0.05
0.10
MR leave−one−out sensitivity analysis for
'Palmitate (16:0) levels' on 'Rheumatoid arthritis'

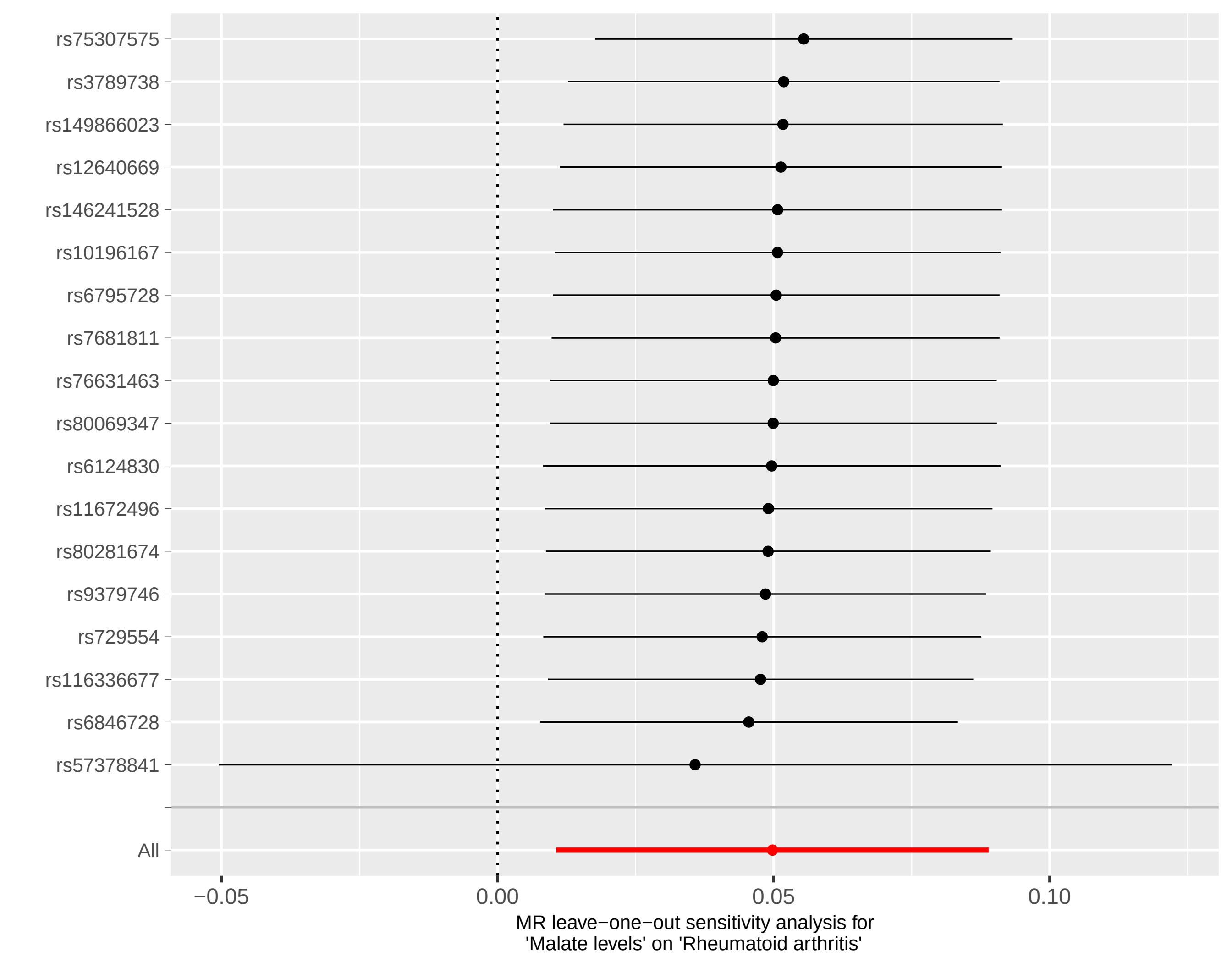
rs75307575
rs3789738
rs149866023
rs12640669
rs146241528
rs10196167
rs6795728
rs7681811
rs76631463
rs80069347
rs6124830
rs11672496
rs80281674
rs9379746
rs729554
rs116336677
rs6846728
rs57378841
All
−0.05
0.00
0.05
0.10
MR leave−one−out sensitivity analysis for
'Malate levels' on 'Rheumatoid arthritis'

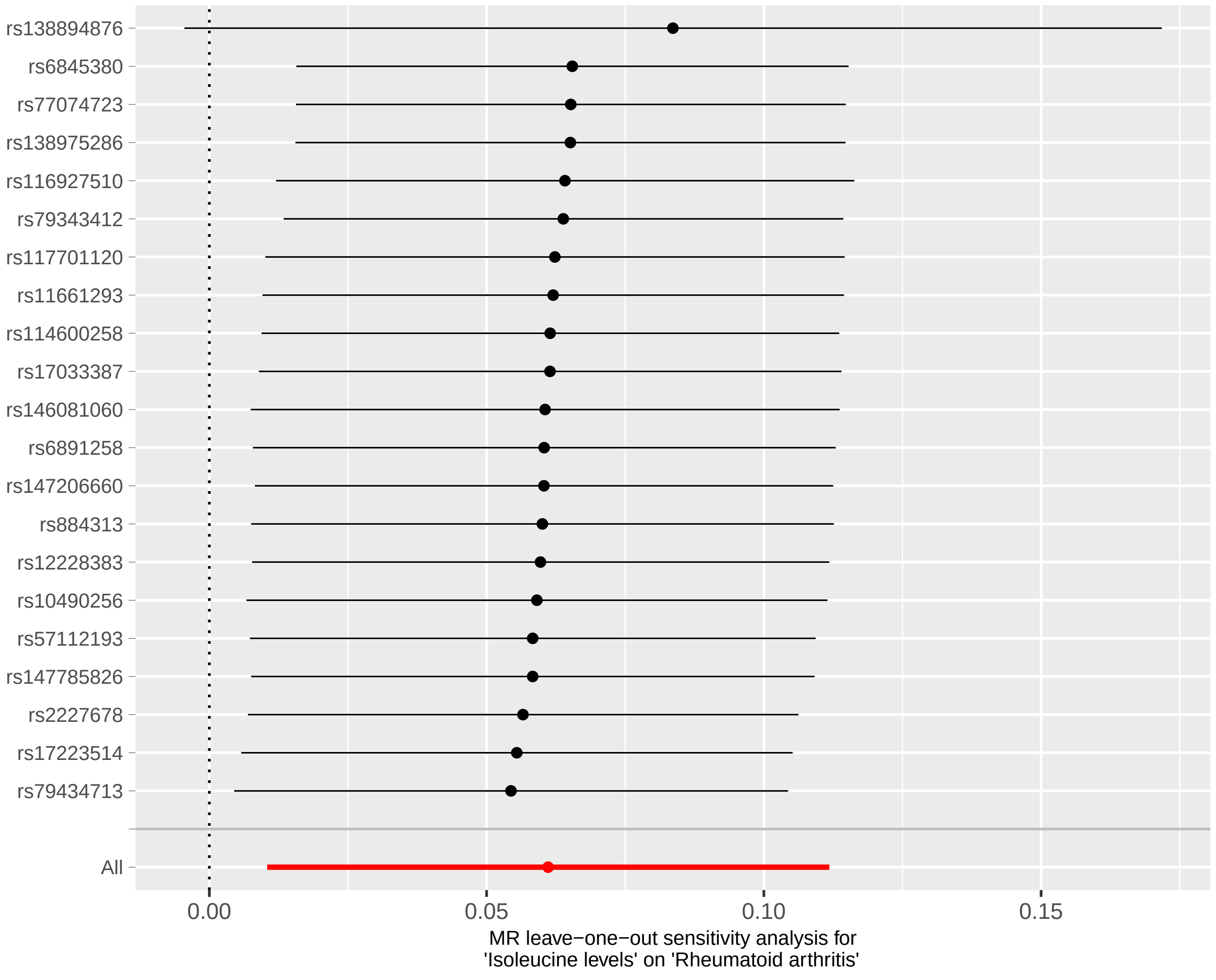
rs138894876
rs6845380
rs77074723
rs138975286
rs116927510
rs79343412
rs117701120
rs11661293
rs114600258
rs17033387
rs146081060
rs6891258
rs147206660
rs884313
rs12228383
rs10490256
rs57112193
rs147785826
rs2227678
rs17223514
rs79434713
All
0.00
0.05
0.10
0.15
MR leave−one−out sensitivity analysis for
'Isoleucine levels' on 'Rheumatoid arthritis'

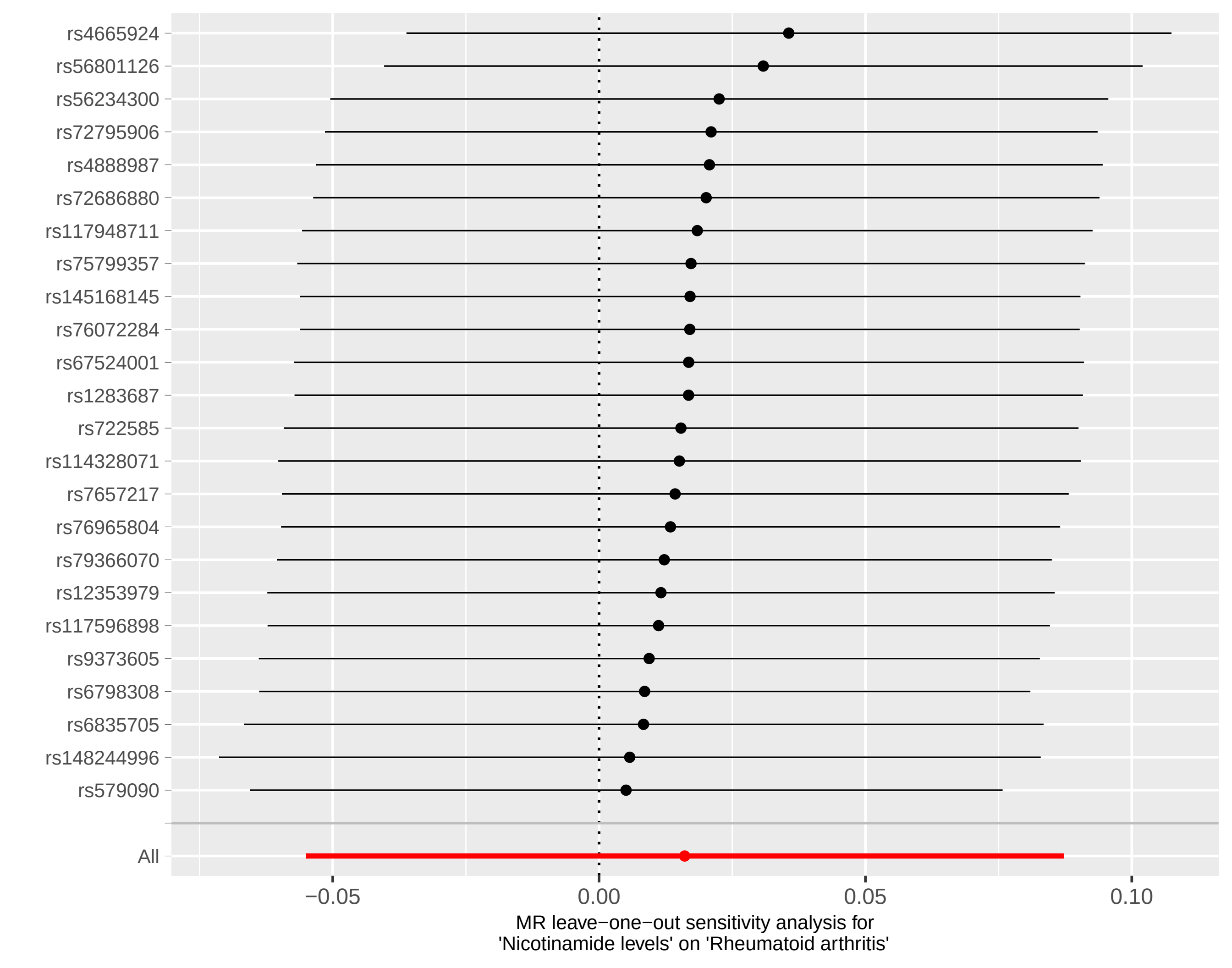
rs4665924
rs56801126
rs56234300
rs72795906
rs4888987
rs72686880
rs117948711
rs75799357
rs145168145
rs76072284
rs67524001
rs1283687
rs722585
rs114328071
rs7657217
rs76965804
rs79366070
rs12353979
rs117596898
rs9373605
rs6798308
rs6835705
rs148244996
rs579090
All
−0.05
0.00
0.05
0.10
MR leave−one−out sensitivity analysis for
'Nicotinamide levels' on 'Rheumatoid arthritis'

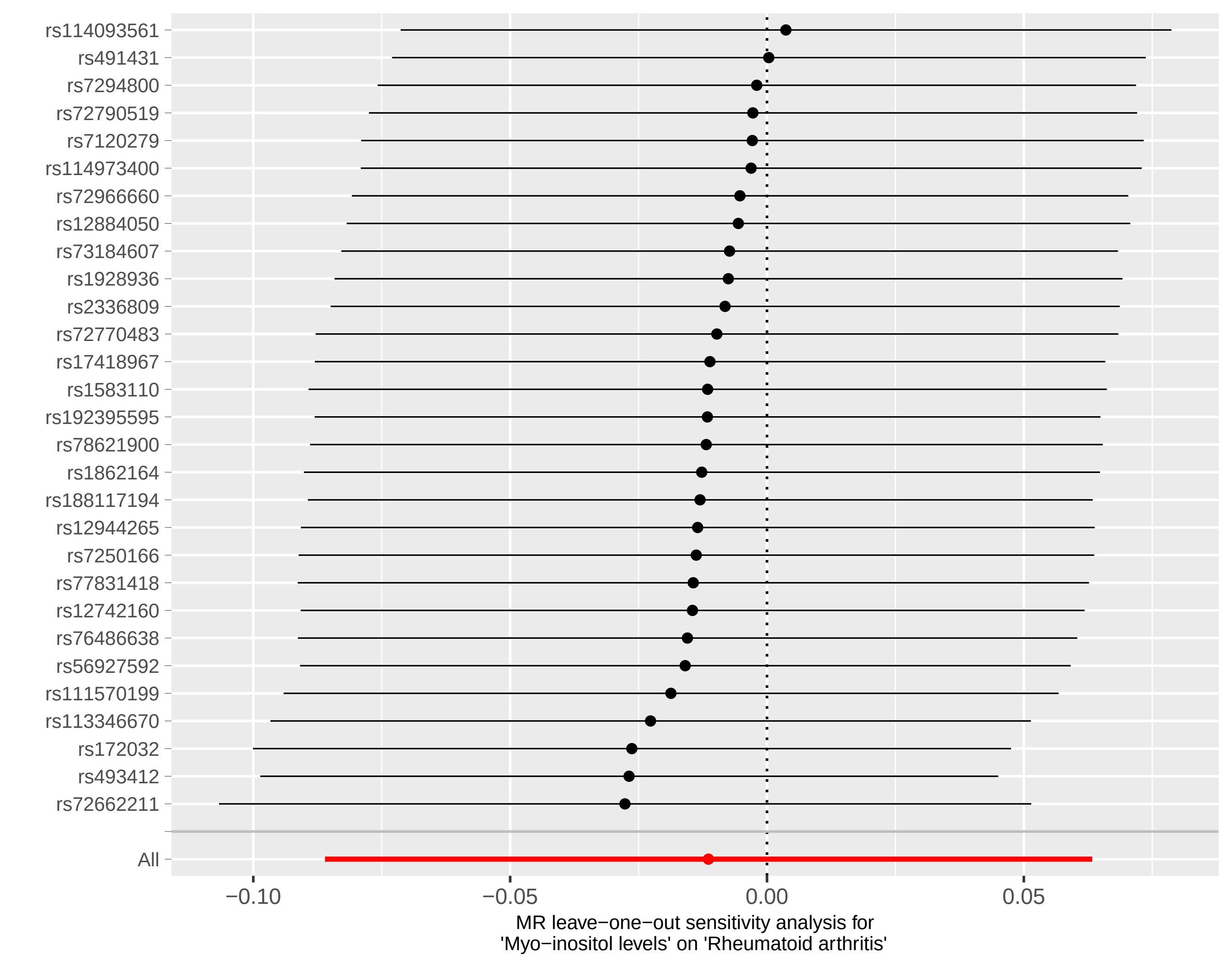

rs114093561
rs491431
rs7294800
rs72790519
rs7120279
rs114973400
rs72966660
rs12884050
rs73184607
rs1928936
rs2336809
rs72770483
rs17418967
rs1583110
rs192395595
rs78621900
rs1862164
rs188117194
rs12944265
rs7250166
rs77831418
rs12742160
rs76486638
rs56927592
rs111570199
rs113346670
rs172032
rs493412
rs72662211
All
−0.10
−0.05
0.00
0.05
MR leave−one−out sensitivity analysis for
'Myo−inositol levels' on 'Rheumatoid arthritis'

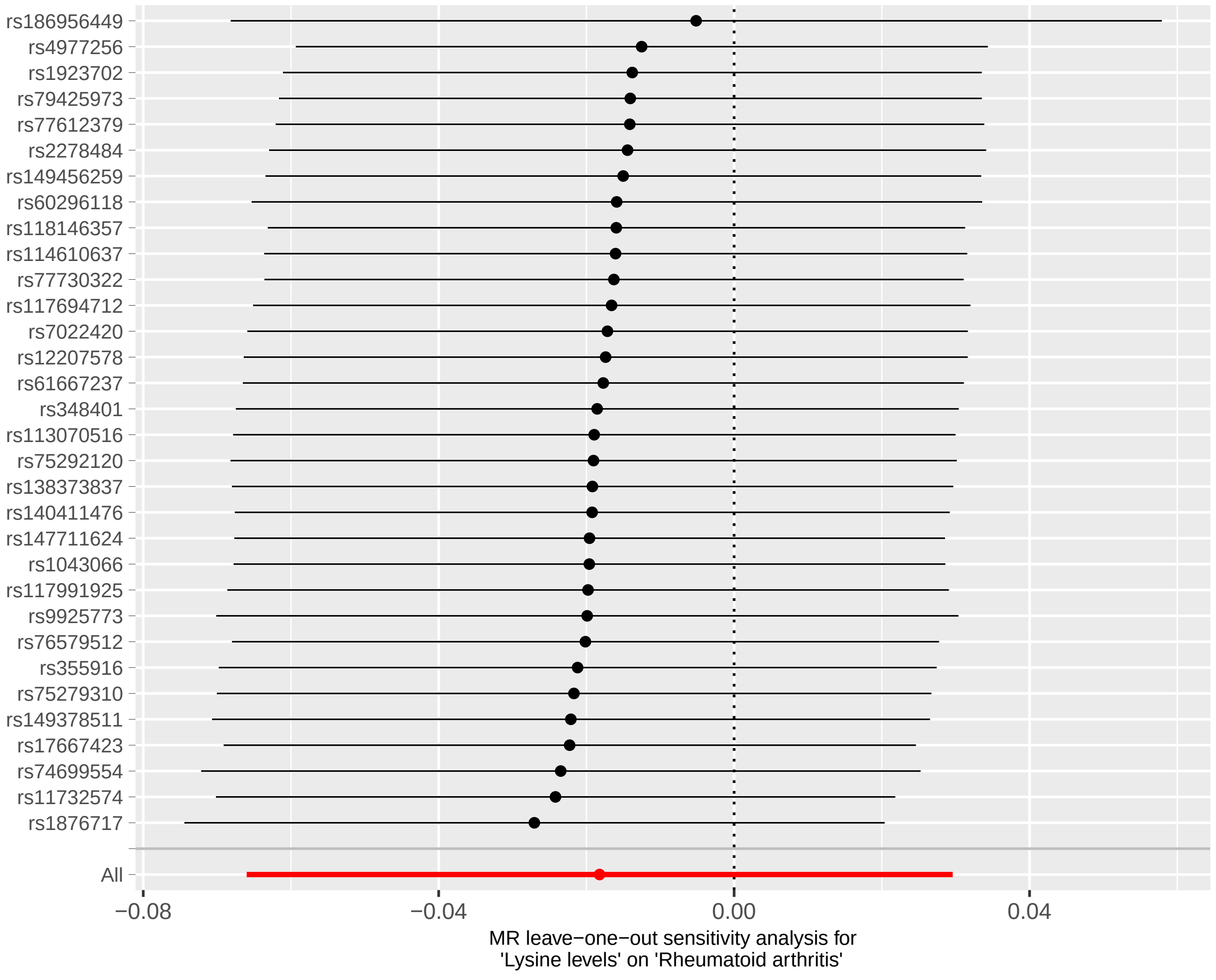
rs186956449
rs4977256
rs1923702
rs79425973
rs77612379
rs2278484
rs149456259
rs60296118
rs118146357
rs114610637
rs77730322
rs117694712
rs7022420
rs12207578
rs61667237
rs348401
rs113070516
rs75292120
rs138373837
rs140411476
rs147711624
rs1043066
rs117991925
rs9925773
rs76579512
rs355916
rs75279310
rs149378511
rs17667423
rs74699554
rs11732574
rs1876717
All
−0.08
−0.04
0.00
0.04
MR leave−one−out sensitivity analysis for
'Lysine levels' on 'Rheumatoid arthritis'

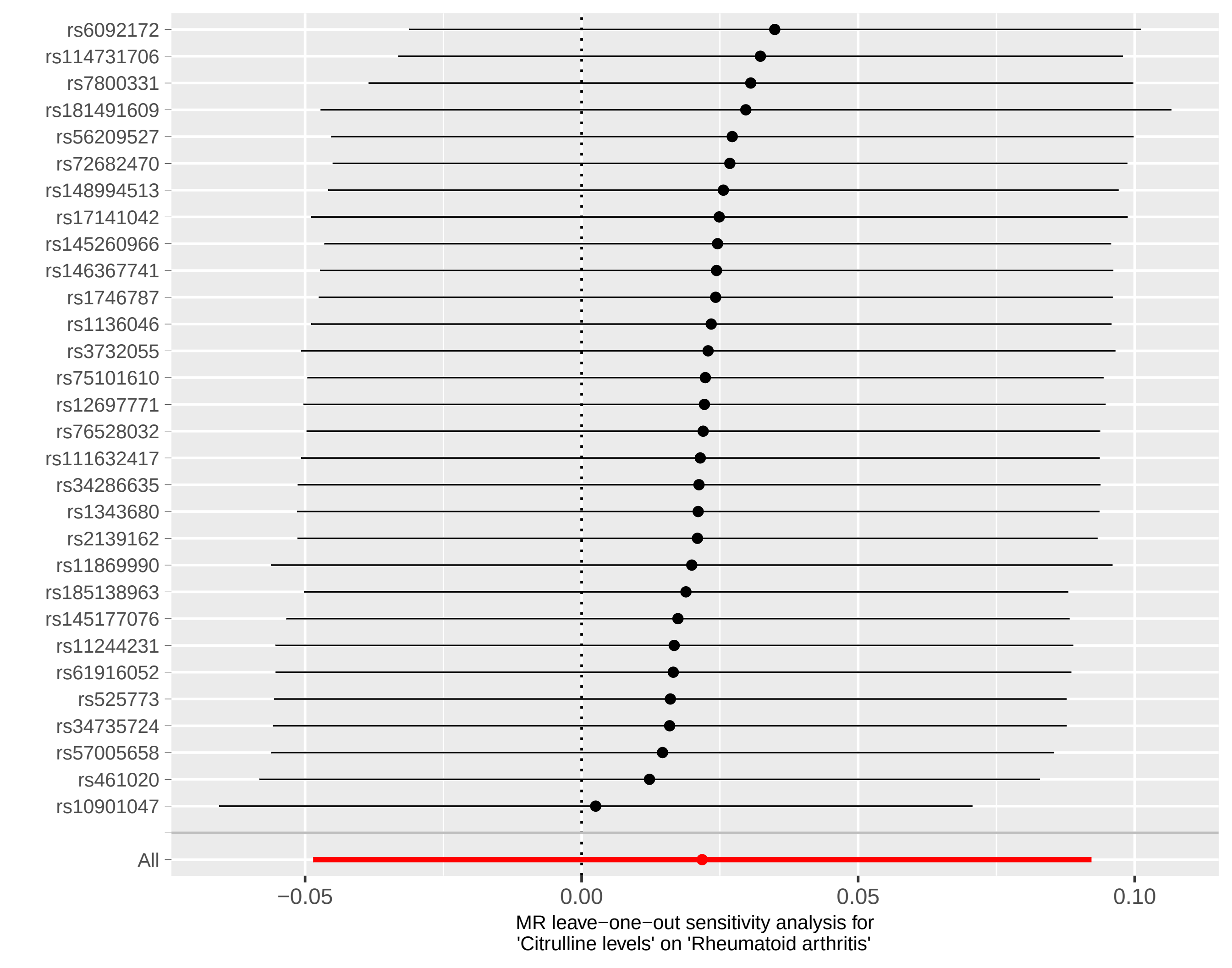

rs6092172
rs114731706
rs7800331
rs181491609
rs56209527
rs72682470
rs148994513
rs17141042
rs145260966
rs146367741
rs1746787
rs1136046
rs3732055
rs75101610
rs12697771
rs76528032
rs111632417
rs34286635
rs1343680
rs2139162
rs11869990
rs185138963
rs145177076
rs11244231
rs61916052
rs525773
rs34735724
rs57005658
rs461020
rs10901047
All
−0.05
0.00
0.05
0.10
MR leave−one−out sensitivity analysis for
'Citrulline levels' on 'Rheumatoid arthritis'

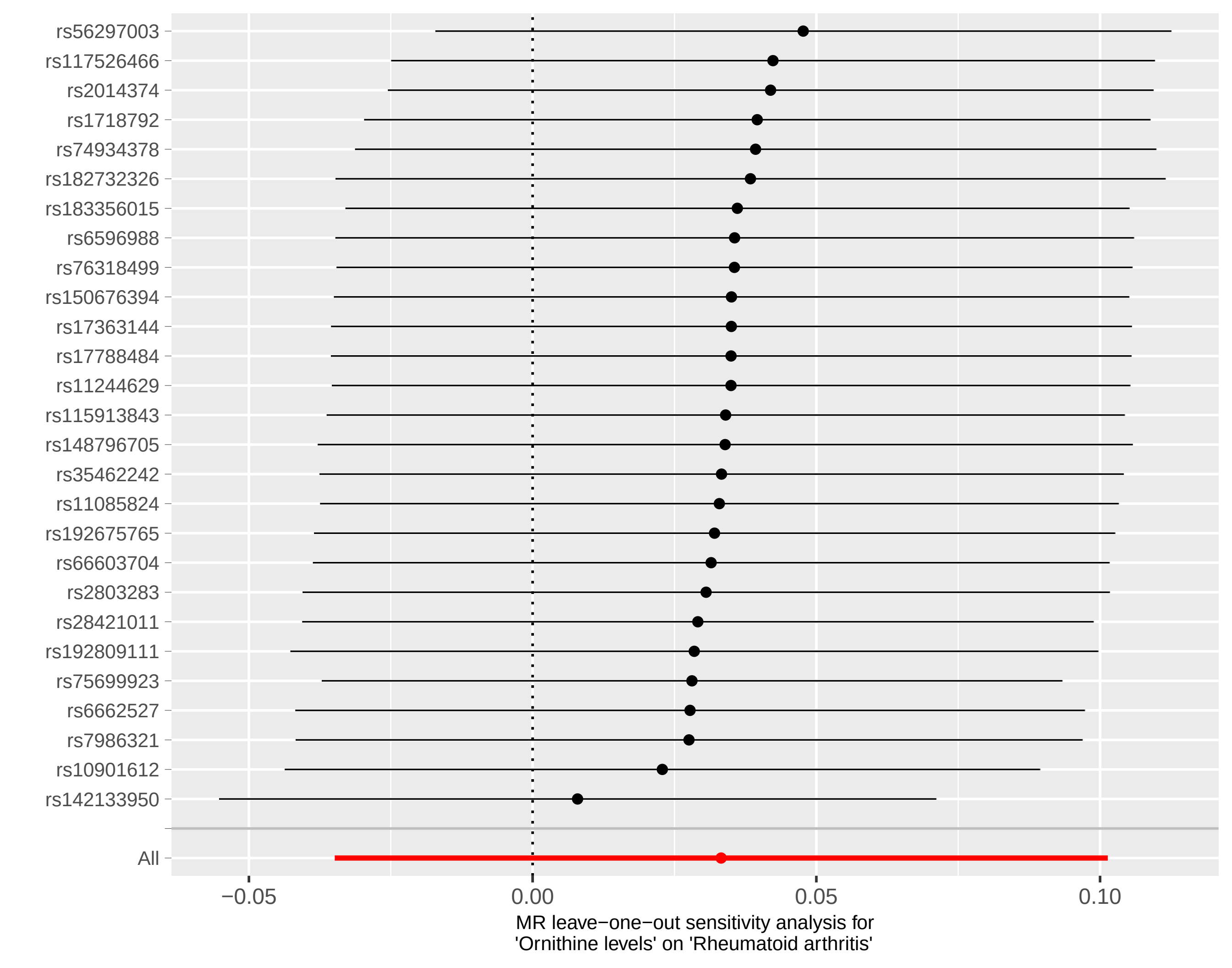
rs56297003
rs117526466
rs2014374
rs1718792
rs74934378
rs182732326
rs183356015
rs6596988
rs76318499
rs150676394
rs17363144
rs17788484
rs11244629
rs115913843
rs148796705
rs35462242
rs11085824
rs192675765
rs66603704
rs2803283
rs28421011
rs192809111
rs75699923
rs6662527
rs7986321
rs10901612
rs142133950
All
−0.05
0.00
0.05
0.10
MR leave−one−out sensitivity analysis for
'Ornithine levels' on 'Rheumatoid arthritis'

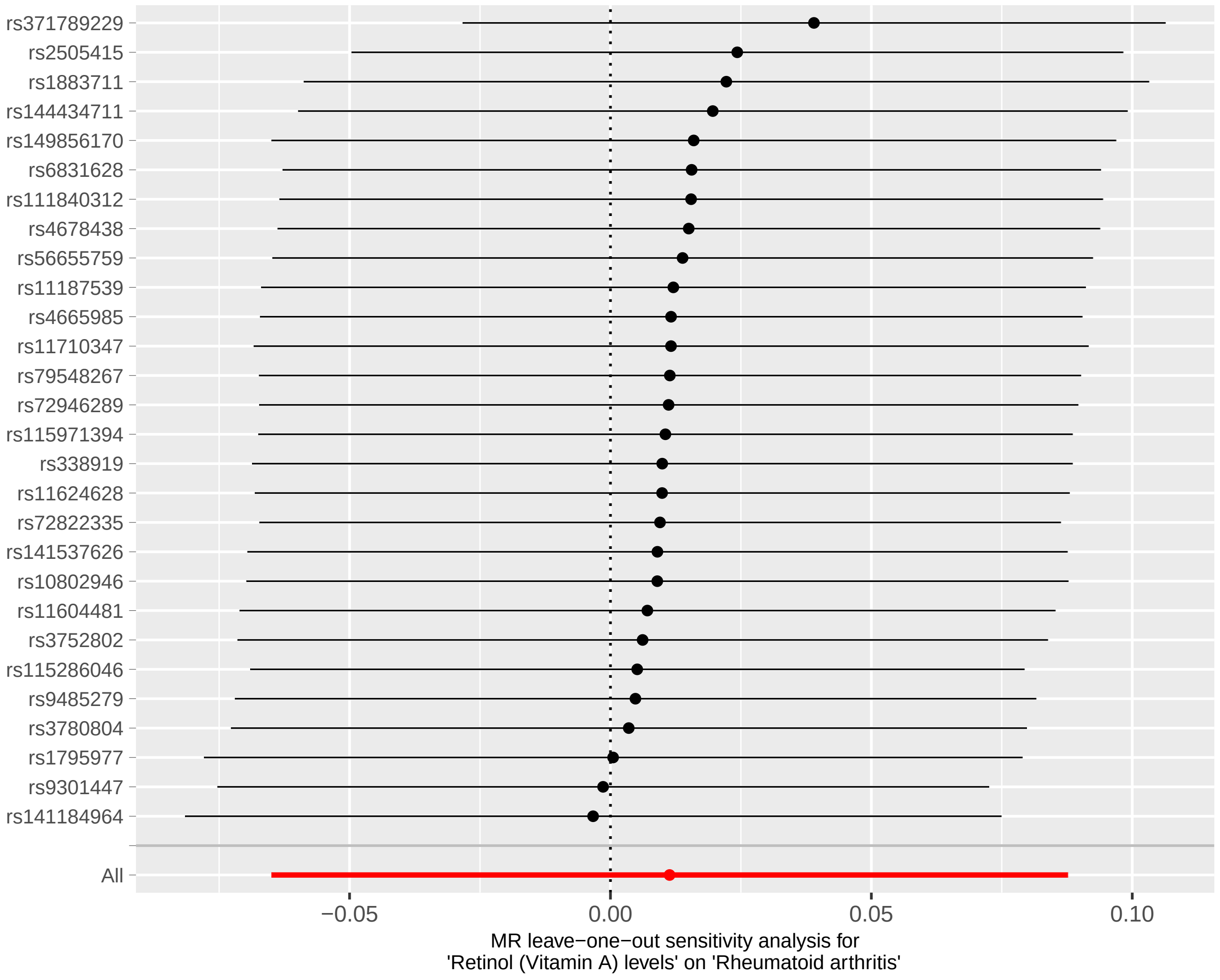
rs371789229
rs2505415
rs1883711
rs144434711
rs149856170
rs6831628
rs111840312
rs4678438
rs56655759
rs11187539
rs4665985
rs11710347
rs79548267
rs72946289
rs115971394
rs338919
rs11624628
rs72822335
rs141537626
rs10802946
rs11604481
rs3752802
rs115286046
rs9485279
rs3780804
rs1795977
rs9301447
rs141184964
All
−0.05
0.00
0.05
0.10
MR leave−one−out sensitivity analysis for
'Retinol (Vitamin A) levels' on 'Rheumatoid arthritis'

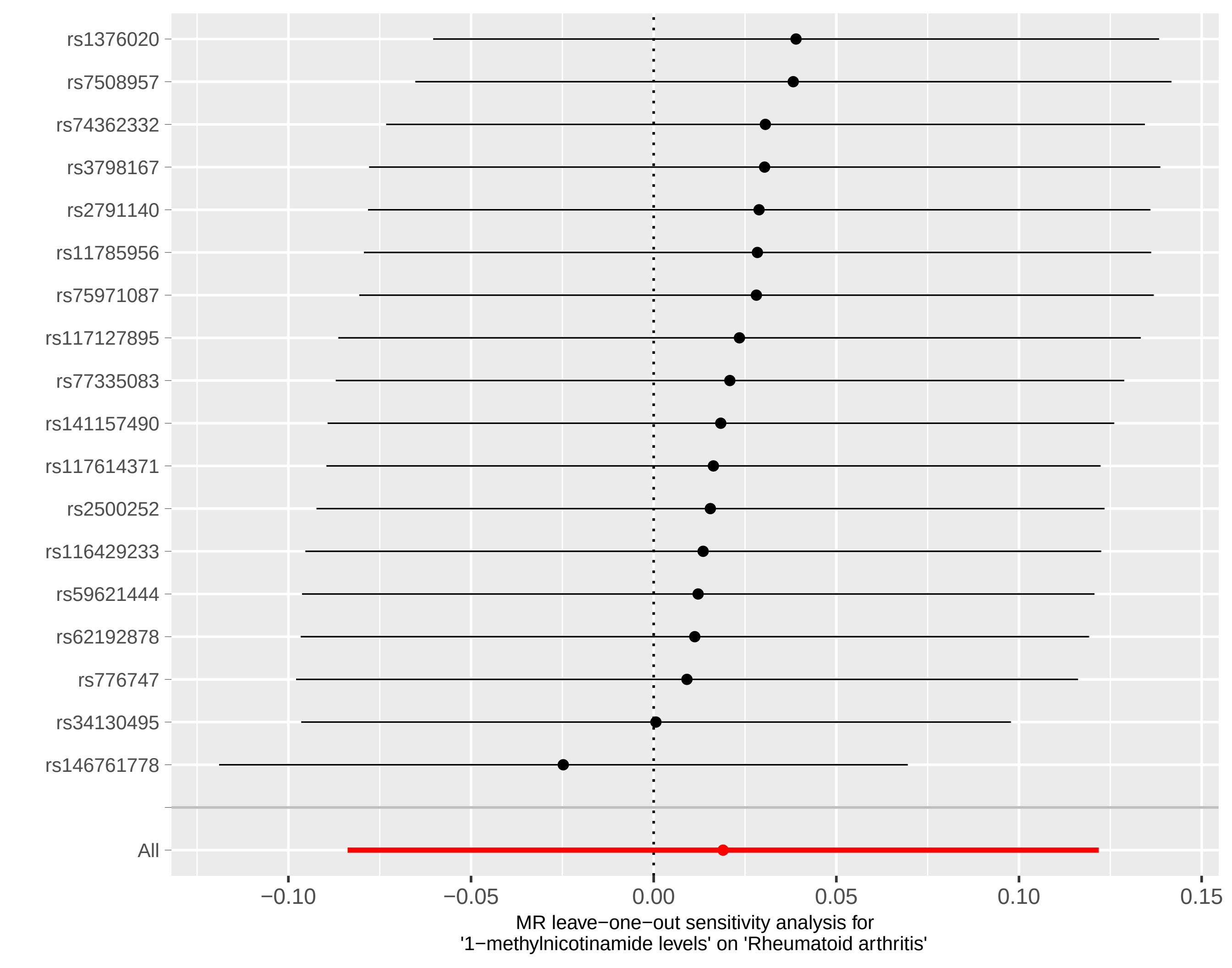
rs1376020
rs7508957
rs74362332
rs3798167
rs2791140
rs11785956
rs75971087
rs117127895
rs77335083
rs141157490
rs117614371
rs2500252
rs116429233
rs59621444
rs62192878
rs776747
rs34130495
rs146761778
All
−0.10
−0.05
0.00
0.05
0.10
0.15
MR leave−one−out sensitivity analysis for
'1−methylnicotinamide levels' on 'Rheumatoid arthritis'

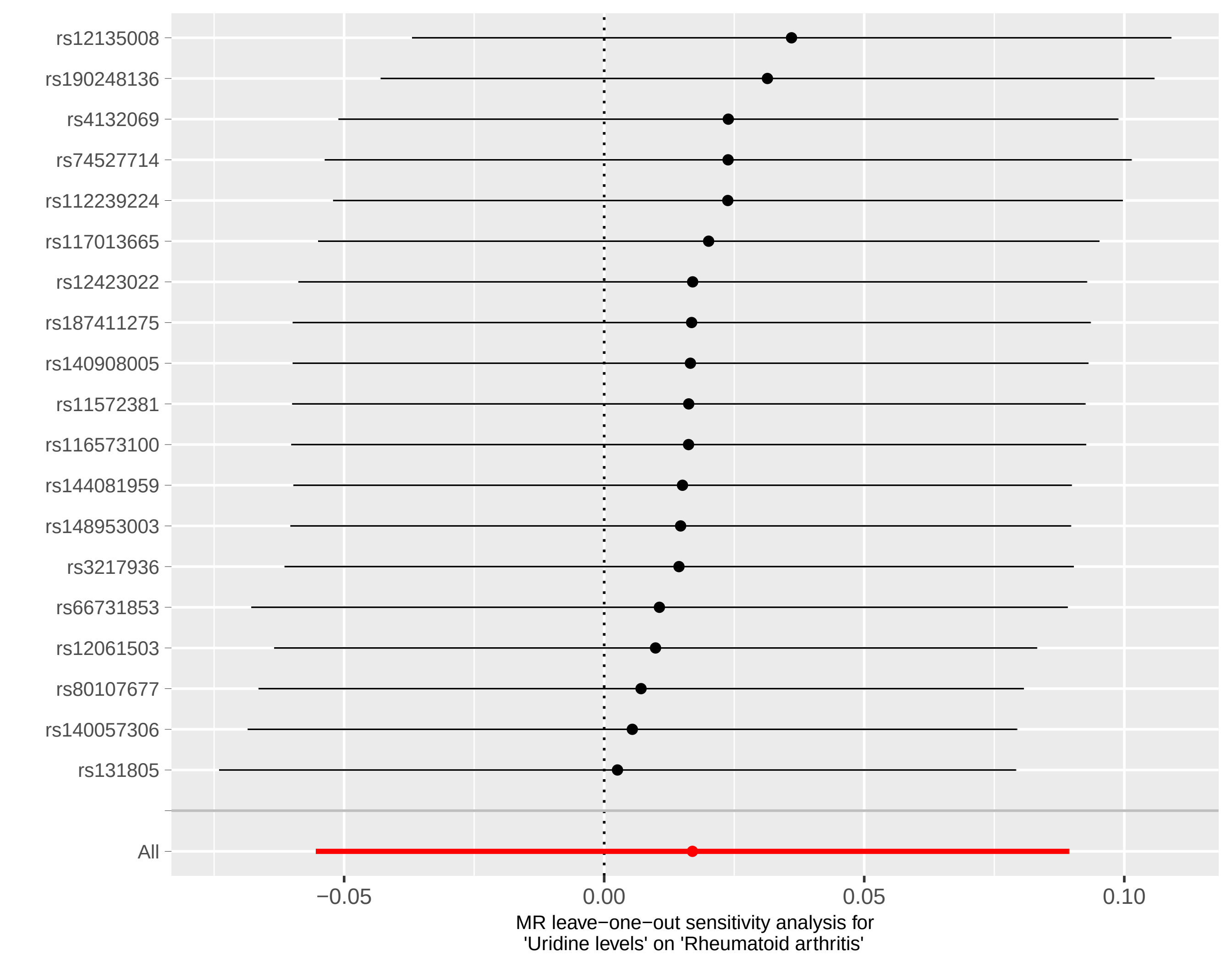
rs12135008
rs190248136
rs4132069
rs74527714
rs112239224
rs117013665
rs12423022
rs187411275
rs140908005
rs11572381
rs116573100
rs144081959
rs148953003
rs3217936
rs66731853
rs12061503
rs80107677
rs140057306
rs131805
All
−0.05
0.00
0.05
0.10
MR leave−one−out sensitivity analysis for
'Uridine levels' on 'Rheumatoid arthritis'

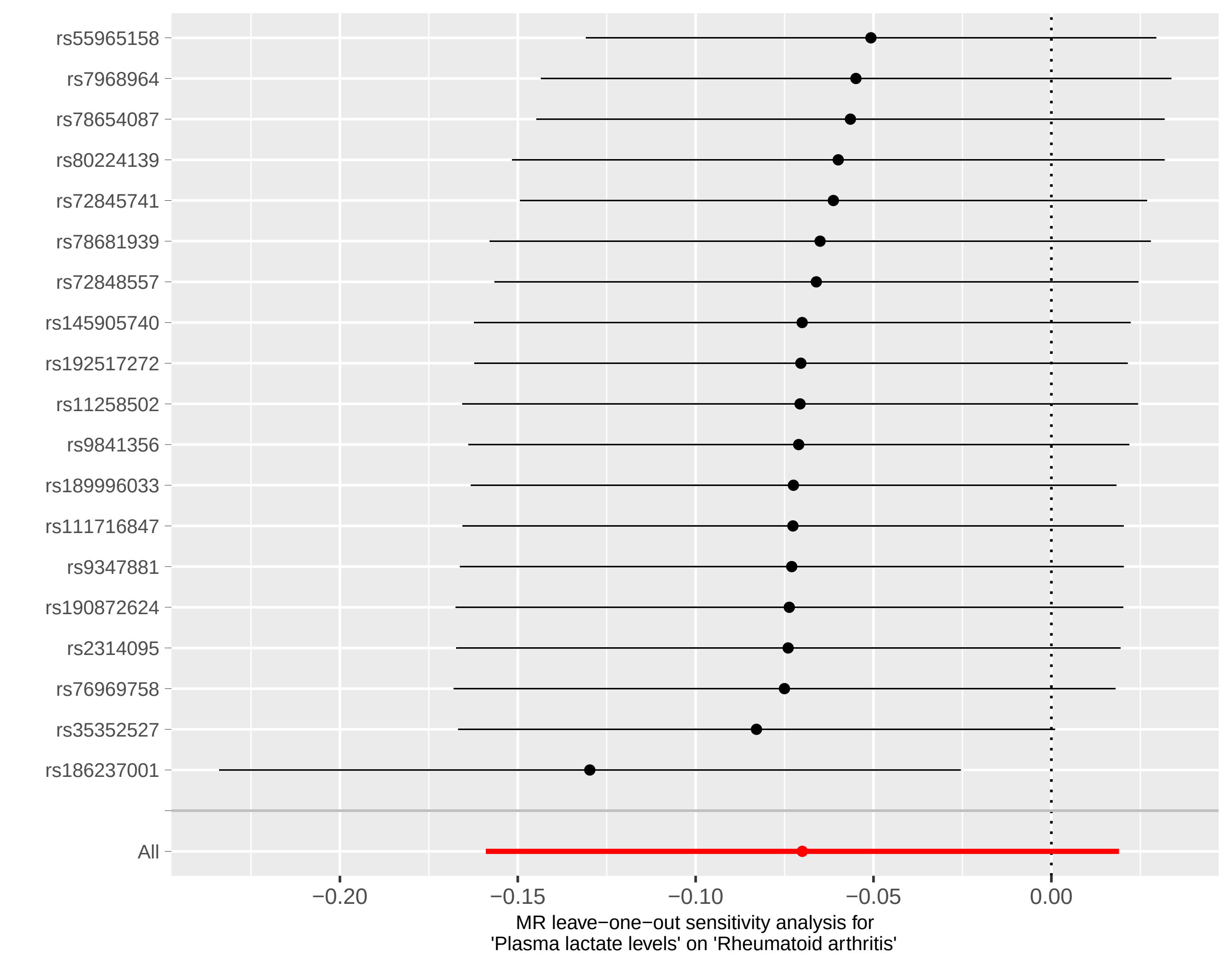
rs55965158
rs7968964
rs78654087
rs80224139
rs72845741
rs78681939
rs72848557
rs145905740
rs192517272
rs11258502
rs9841356
rs189996033
rs111716847
rs9347881
rs190872624
rs2314095
rs76969758
rs35352527
rs186237001
All
−0.20
−0.15
−0.10
−0.05
0.00
MR leave−one−out sensitivity analysis for
'Plasma lactate levels' on 'Rheumatoid arthritis'

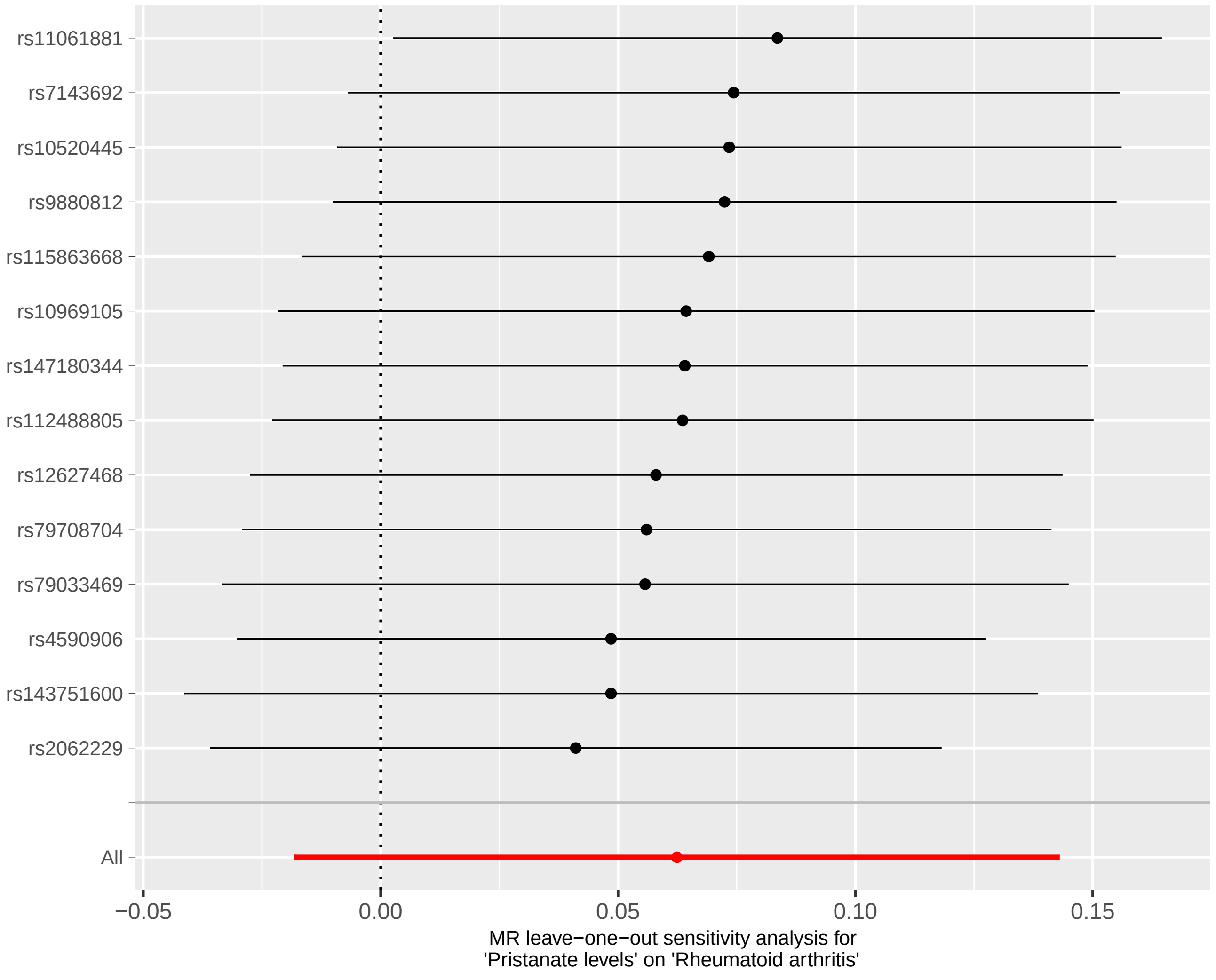

rs11061881
rs7143692
rs10520445
rs9880812
rs115863668
rs10969105
rs147180344
rs112488805
rs12627468
rs79708704
rs79033469
rs4590906
rs143751600
rs2062229
All
−0.05
0.00
0.05
0.10
0.15
MR leave−one−out sensitivity analysis for
'Pristanate levels' on 'Rheumatoid arthritis'

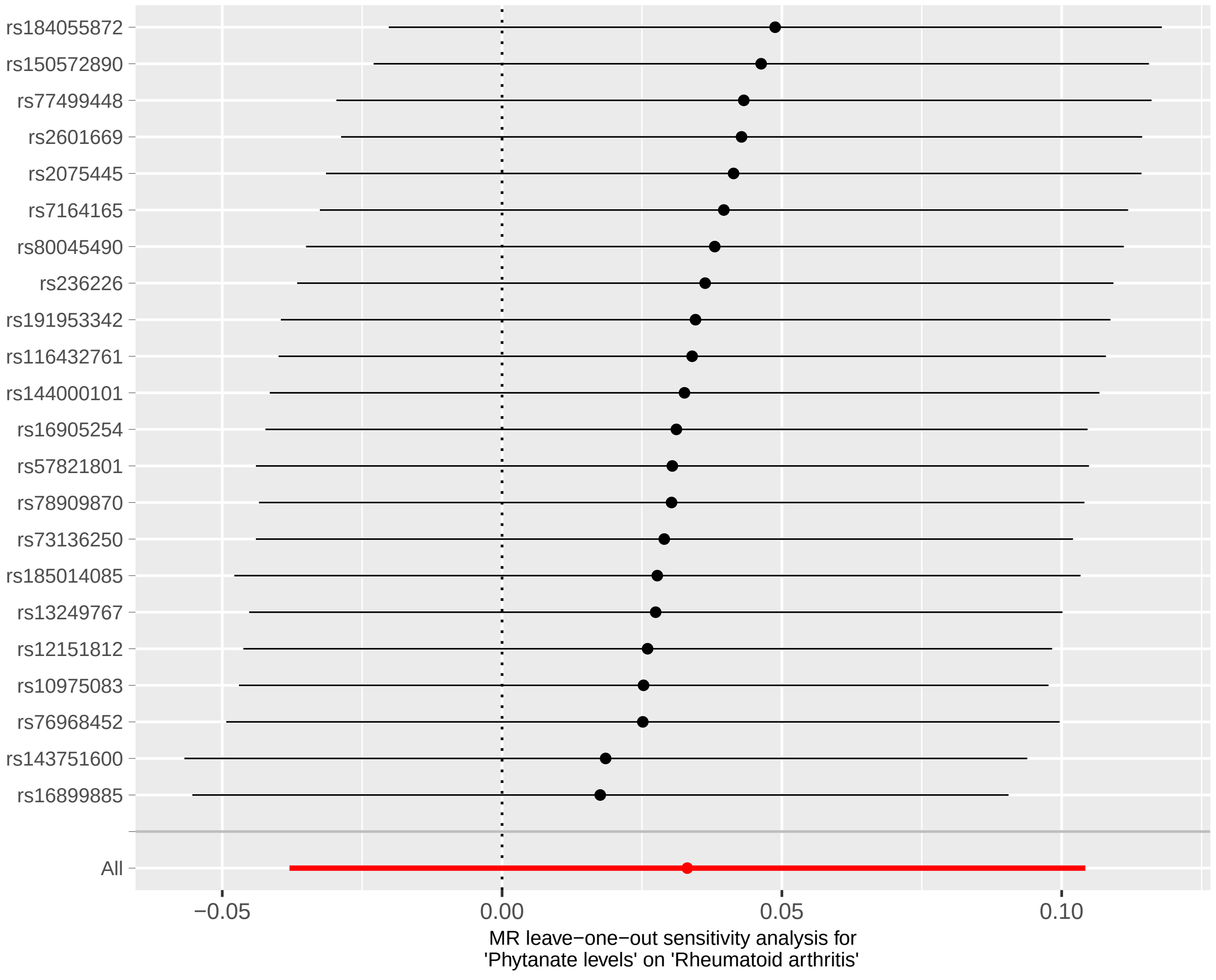

rs184055872
rs150572890
rs77499448
rs2601669
rs2075445
rs7164165
rs80045490
rs236226
rs191953342
rs116432761
rs144000101
rs16905254
rs57821801
rs78909870
rs73136250
rs185014085
rs13249767
rs12151812
rs10975083
rs76968452
rs143751600
rs16899885
All
−0.05
0.00
0.05
0.10
MR leave−one−out sensitivity analysis for
'Phytanate levels' on 'Rheumatoid arthritis'

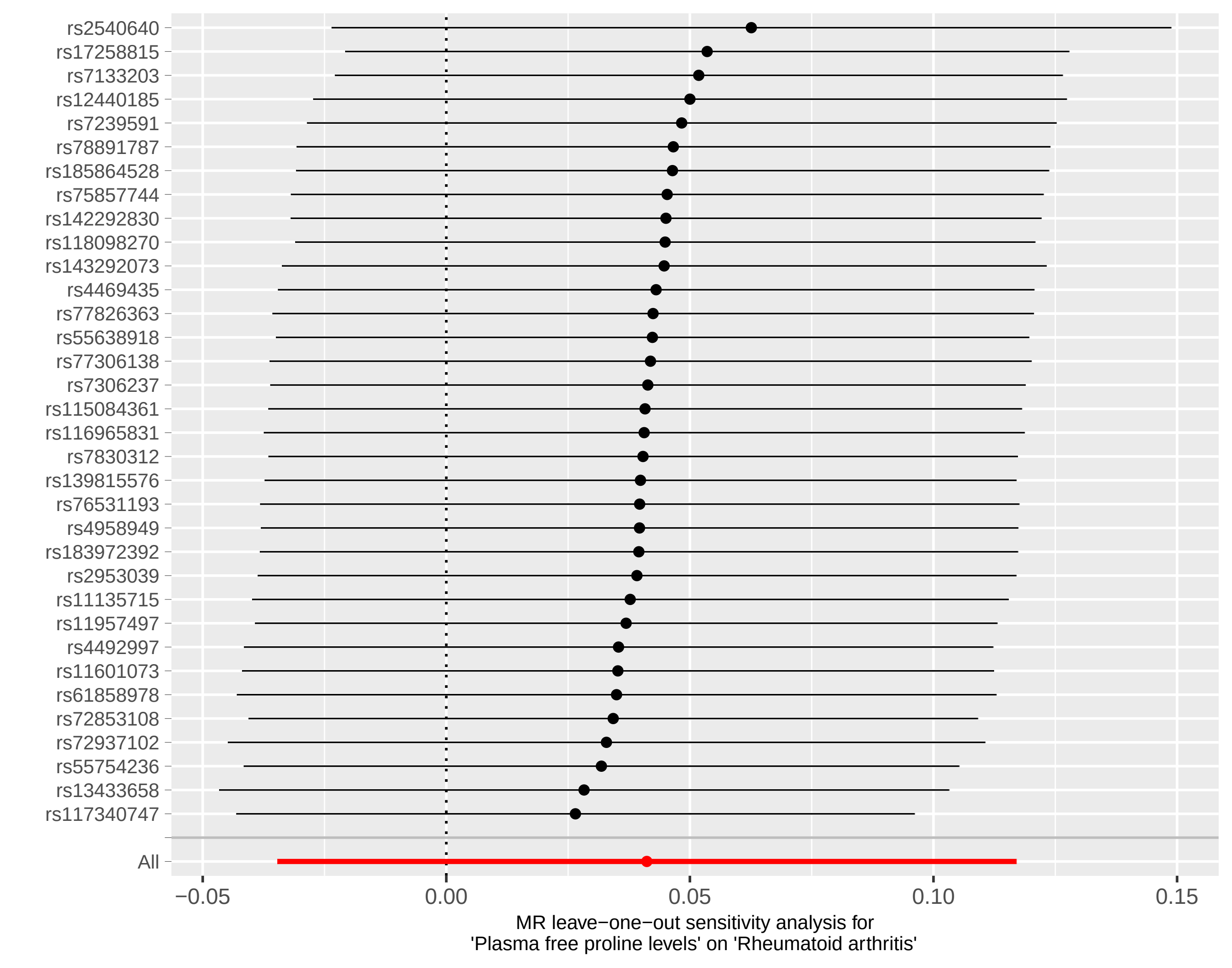
rs2540640
rs17258815
rs7133203
rs12440185
rs7239591
rs78891787
rs185864528
rs75857744
rs142292830
rs118098270
rs143292073
rs4469435
rs77826363
rs55638918
rs77306138
rs7306237
rs115084361
rs116965831
rs7830312
rs139815576
rs76531193
rs4958949
rs183972392
rs2953039
rs11135715
rs11957497
rs4492997
rs11601073
rs61858978
rs72853108
rs72937102
rs55754236
rs13433658
rs117340747
All
−0.05
0.00
0.05
0.10
0.15
MR leave−one−out sensitivity analysis for
'Plasma free proline levels' on 'Rheumatoid arthritis'

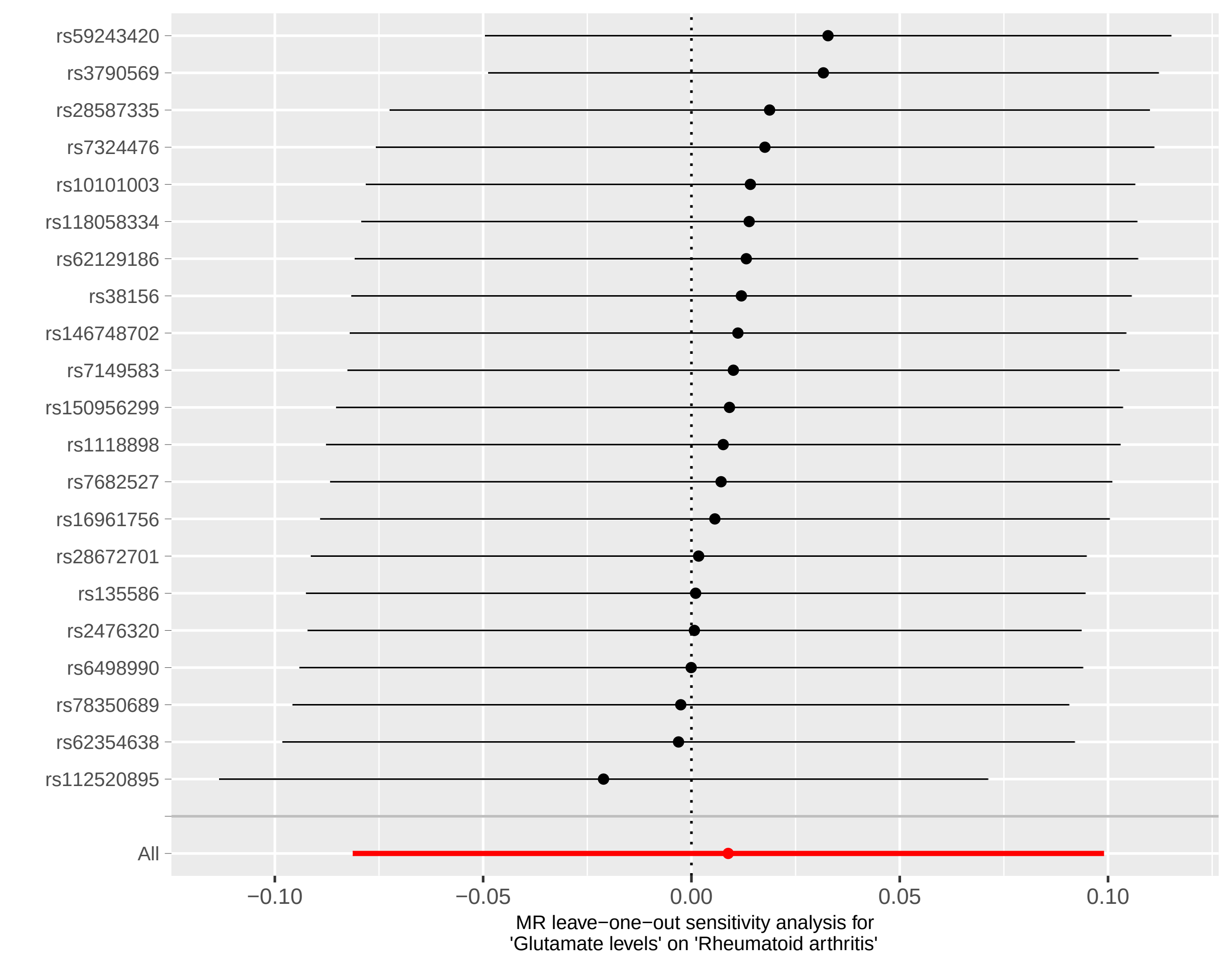
rs59243420
rs3790569
rs28587335
rs7324476
rs10101003
rs118058334
rs62129186
rs38156
rs146748702
rs7149583
rs150956299
rs1118898
rs7682527
rs16961756
rs28672701
rs135586
rs2476320
rs6498990
rs78350689
rs62354638
rs112520895
All
−0.10
−0.05
0.00
0.05
0.10
MR leave−one−out sensitivity analysis for
'Glutamate levels' on 'Rheumatoid arthritis'

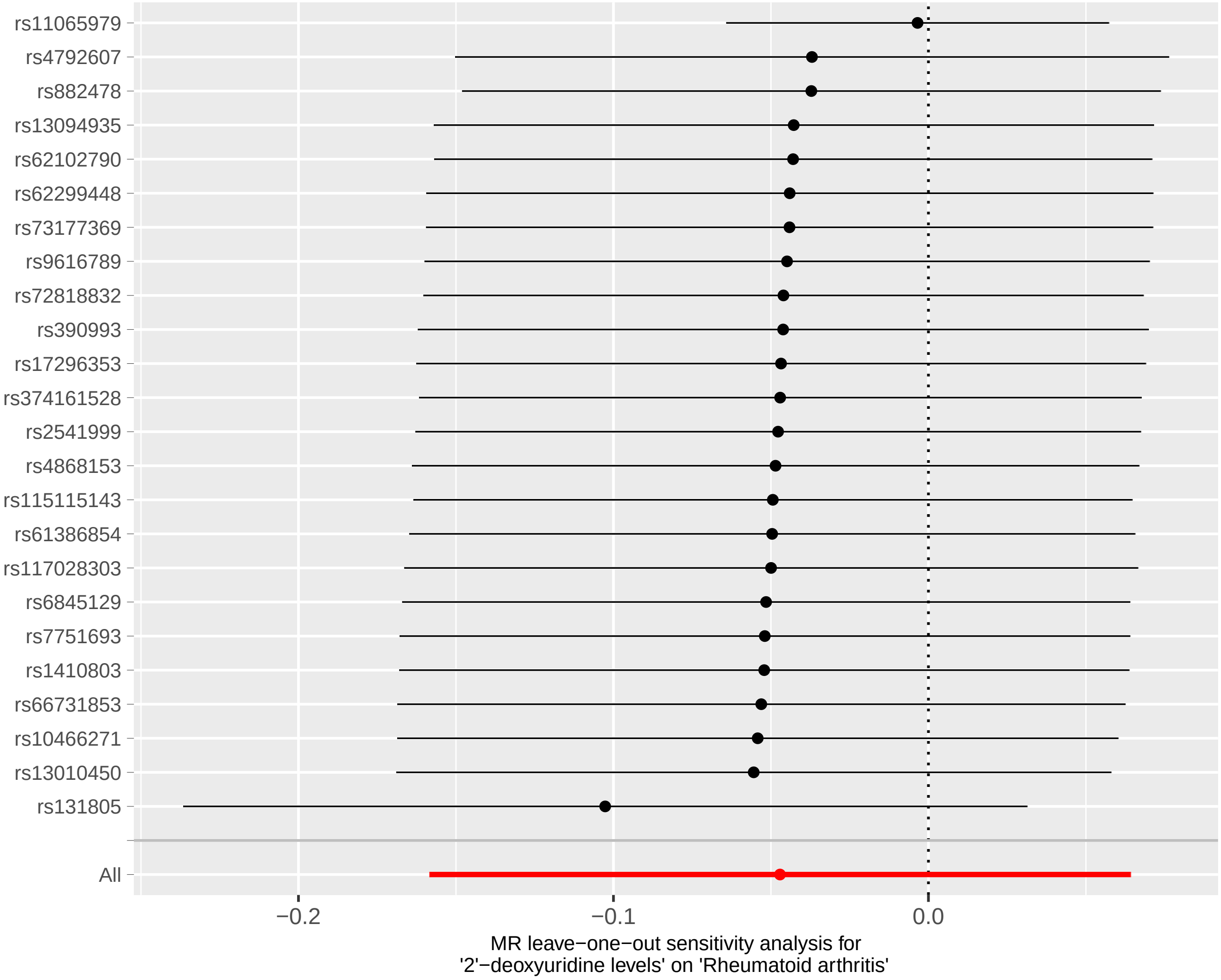

rs11065979
rs4792607
rs882478
rs13094935
rs62102790
rs62299448
rs73177369
rs9616789
rs72818832
rs390993
rs17296353
rs374161528
rs2541999
rs4868153
rs115115143
rs61386854
rs117028303
rs6845129
rs7751693
rs1410803
rs66731853
rs10466271
rs13010450
rs131805
All
−0.2
−0.1
0.0
MR leave−one−out sensitivity analysis for
'2'−deoxyuridine levels' on 'Rheumatoid arthritis'

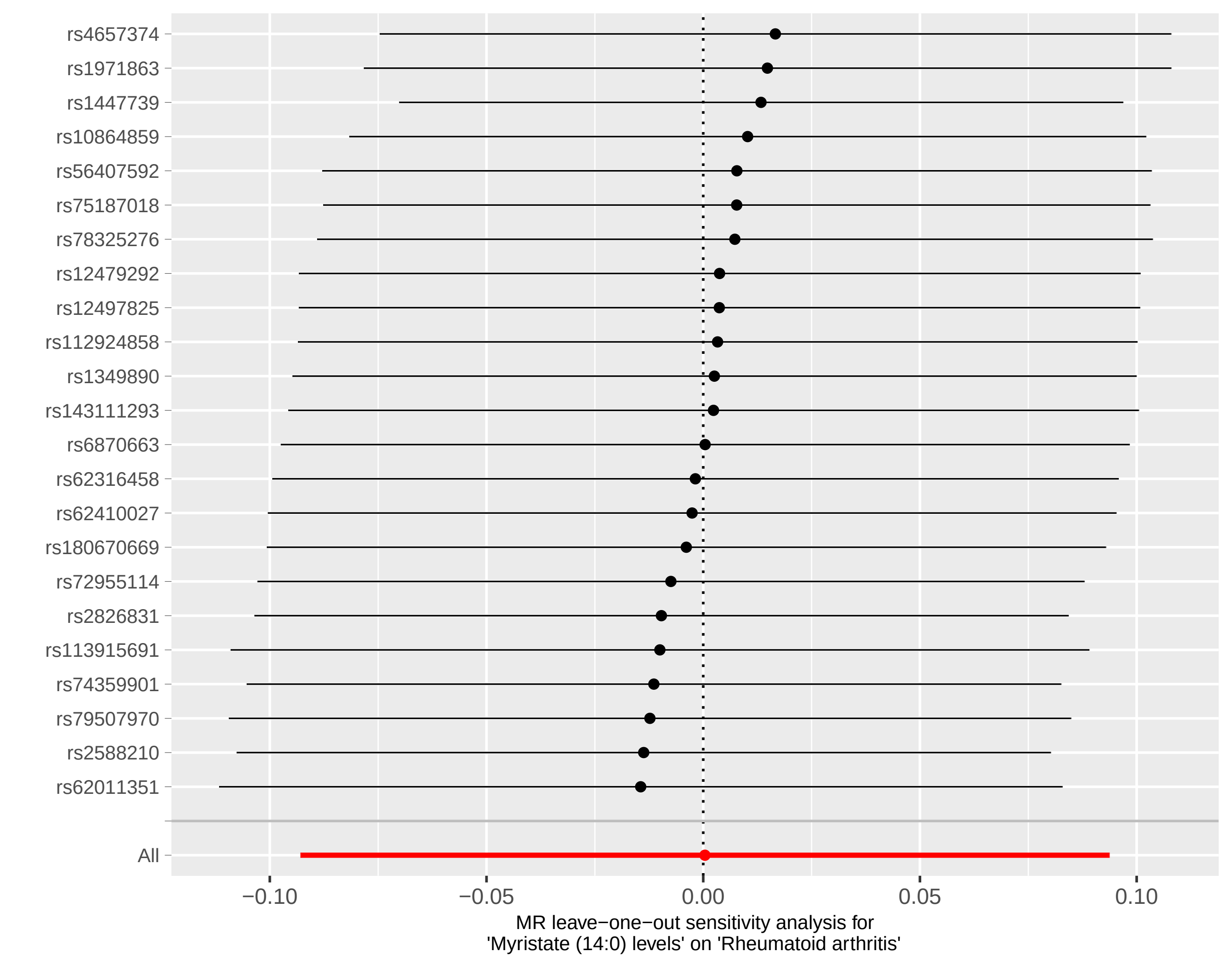
rs4657374
rs1971863
rs1447739
rs10864859
rs56407592
rs75187018
rs78325276
rs12479292
rs12497825
rs112924858
rs1349890
rs143111293
rs6870663
rs62316458
rs62410027
rs180670669
rs72955114
rs2826831
rs113915691
rs74359901
rs79507970
rs2588210
rs62011351
All
−0.10
−0.05
0.00
0.05
0.10
MR leave−one−out sensitivity analysis for
'Myristate (14:0) levels' on 'Rheumatoid arthritis'

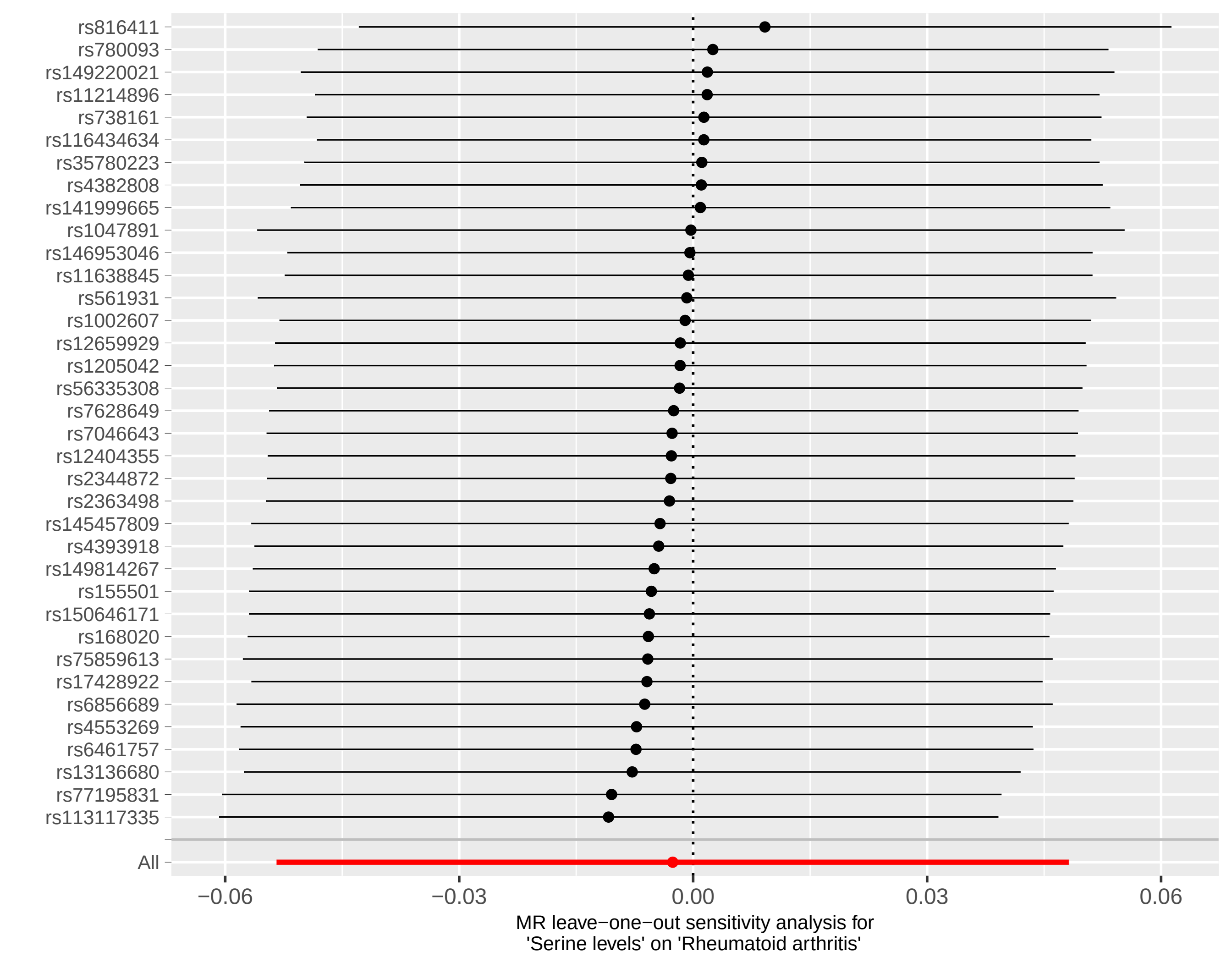
rs816411
rs780093
rs149220021
rs11214896
rs738161
rs116434634
rs35780223
rs4382808
rs141999665
rs1047891
rs146953046
rs11638845
rs561931
rs1002607
rs12659929
rs1205042
rs56335308
rs7628649
rs7046643
rs12404355
rs2344872
rs2363498
rs145457809
rs4393918
rs149814267
rs155501
rs150646171
rs168020
rs75859613
rs17428922
rs6856689
rs4553269
rs6461757
rs13136680
rs77195831
rs113117335
All
−0.06
−0.03
0.00
0.03
0.06
MR leave−one−out sensitivity analysis for
'Serine levels' on 'Rheumatoid arthritis'

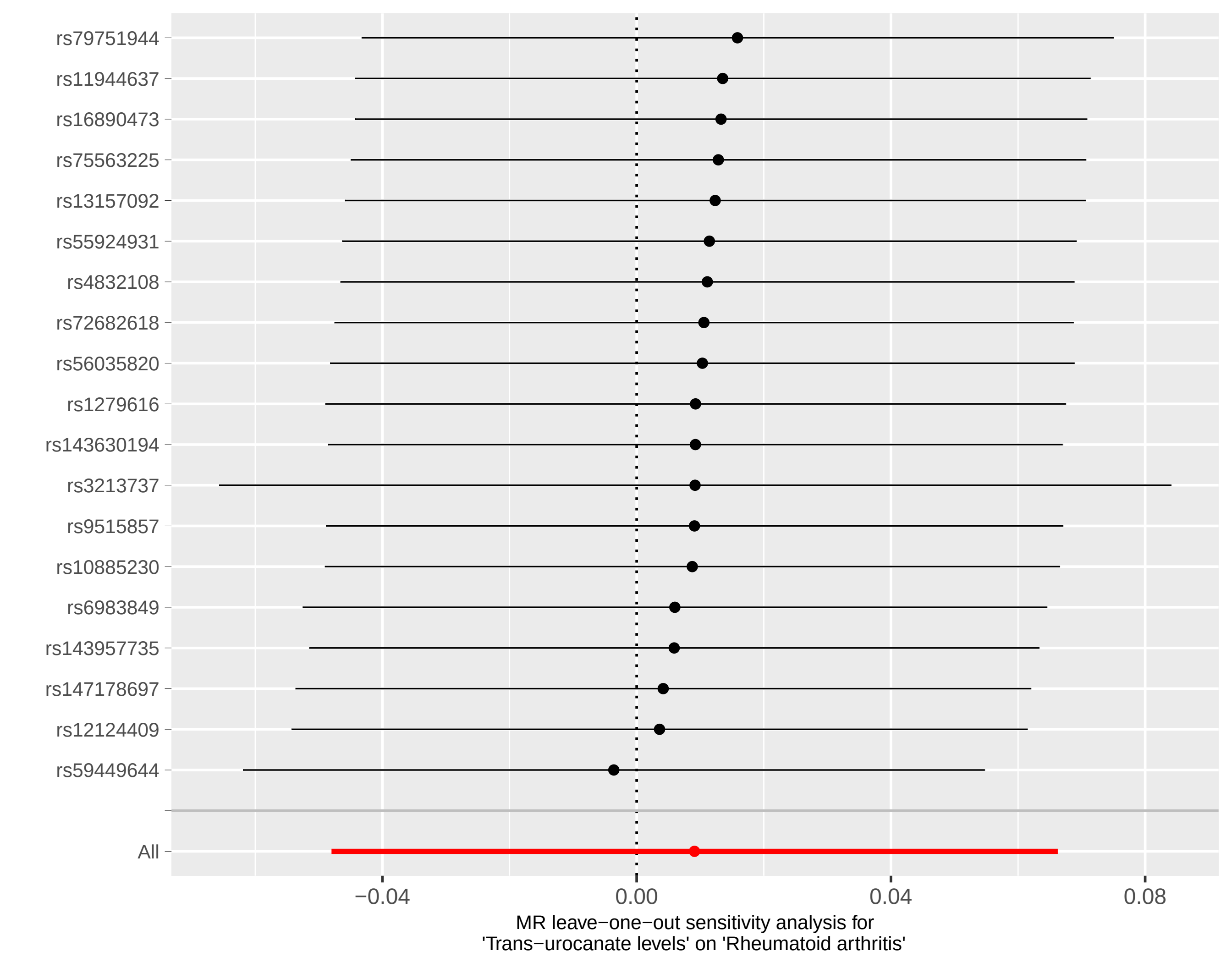

rs79751944
rs11944637
rs16890473
rs75563225
rs13157092
rs55924931
rs4832108
rs72682618
rs56035820
rs1279616
rs143630194
rs3213737
rs9515857
rs10885230
rs6983849
rs143957735
rs147178697
rs12124409
rs59449644
All
−0.04
0.00
0.04
0.08
MR leave−one−out sensitivity analysis for
'Trans−urocanate levels' on 'Rheumatoid arthritis'

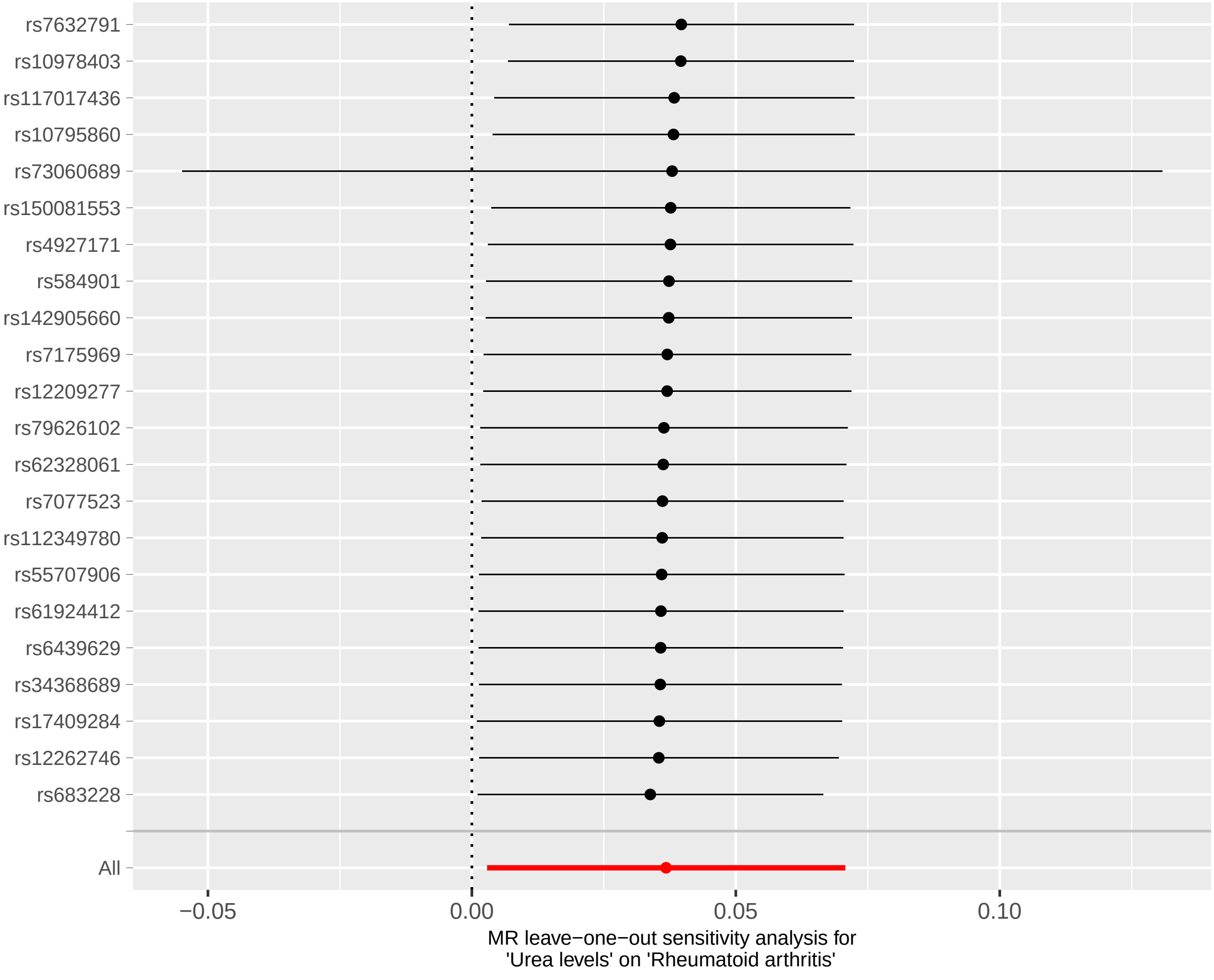
rs7632791
rs10978403
rs117017436
rs10795860
rs73060689
rs150081553
rs4927171
rs584901
rs142905660
rs7175969
rs12209277
rs79626102
rs62328061
rs7077523
rs112349780
rs55707906
rs61924412
rs6439629
rs34368689
rs17409284
rs12262746
rs683228
All
−0.05
0.00
0.05
0.10
MR leave−one−out sensitivity analysis for
'Urea levels' on 'Rheumatoid arthritis'

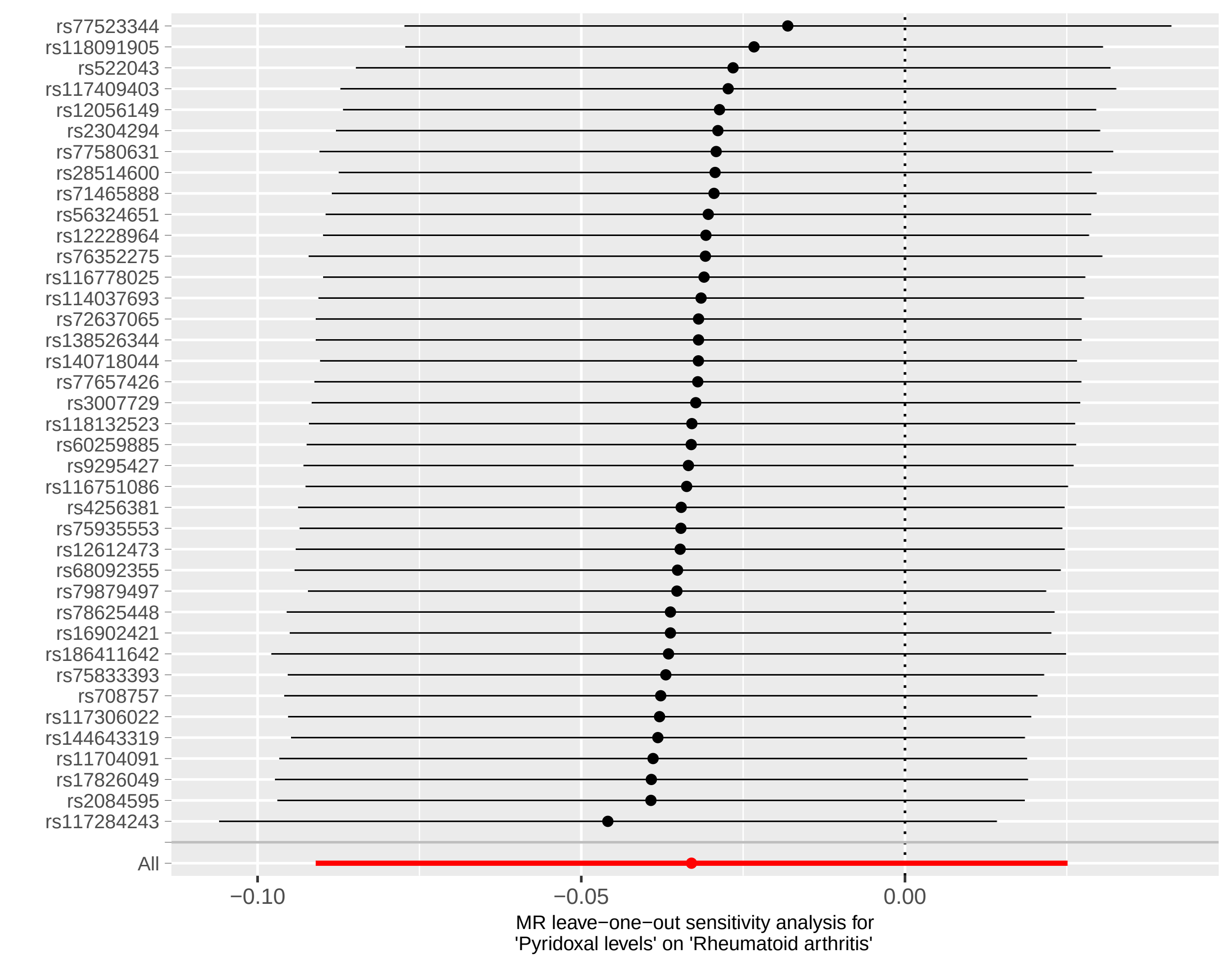

rs77523344
rs118091905
rs522043
rs117409403
rs12056149
rs2304294
rs77580631
rs28514600
rs71465888
rs56324651
rs12228964
rs76352275
rs116778025
rs114037693
rs72637065
rs138526344
rs140718044
rs77657426
rs3007729
rs118132523
rs60259885
rs9295427
rs116751086
rs4256381
rs75935553
rs12612473
rs68092355
rs79879497
rs78625448
rs16902421
rs186411642
rs75833393
rs708757
rs117306022
rs144643319
rs11704091
rs17826049
rs2084595
rs117284243
All
−0.10
−0.05
0.00
MR leave−one−out sensitivity analysis for
'Pyridoxal levels' on 'Rheumatoid arthritis'

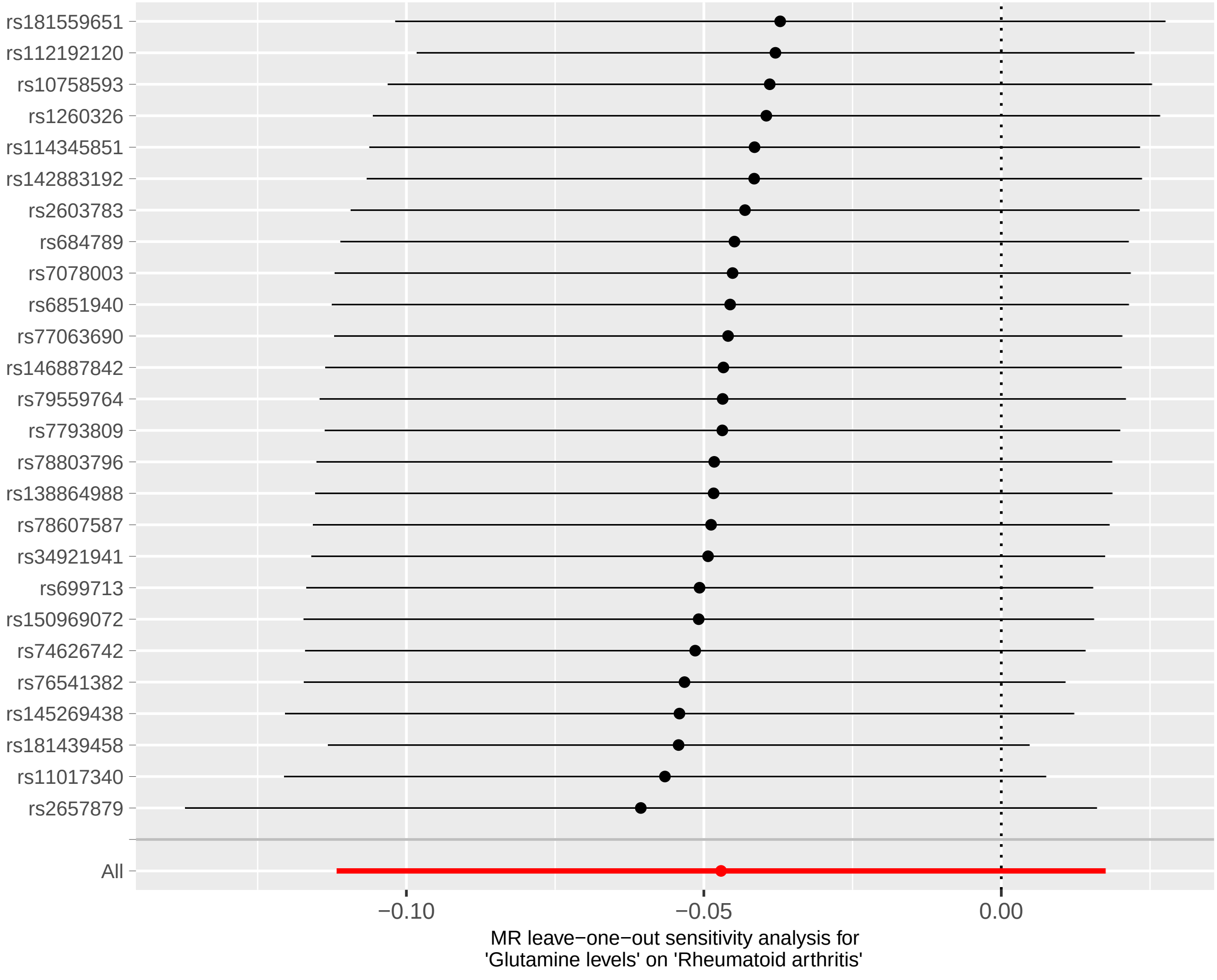
rs181559651
rs112192120
rs10758593
rs1260326
rs114345851
rs142883192
rs2603783
rs684789
rs7078003
rs6851940
rs77063690
rs146887842
rs79559764
rs7793809
rs78803796
rs138864988
rs78607587
rs34921941
rs699713
rs150969072
rs74626742
rs76541382
rs145269438
rs181439458
rs11017340
rs2657879
All
−0.10
−0.05
0.00
MR leave−one−out sensitivity analysis for
'Glutamine levels' on 'Rheumatoid arthritis'

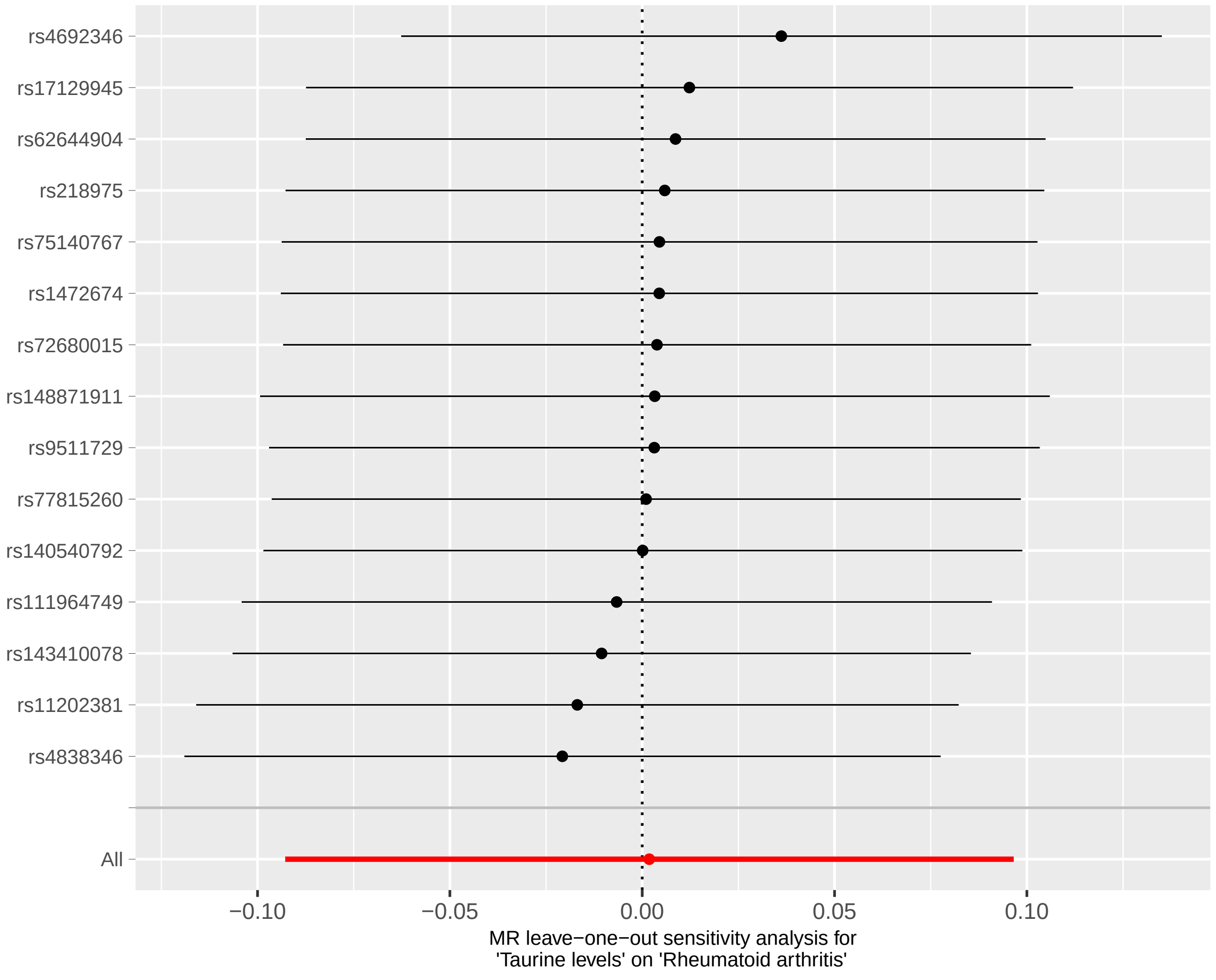
rs4692346
rs17129945
rs62644904
rs218975
rs75140767
rs1472674
rs72680015
rs148871911
rs9511729
rs77815260
rs140540792
rs111964749
rs143410078
rs11202381
rs4838346
All
−0.10
−0.05
0.00
0.05
0.10
MR leave−one−out sensitivity analysis for
'Taurine levels' on 'Rheumatoid arthritis'

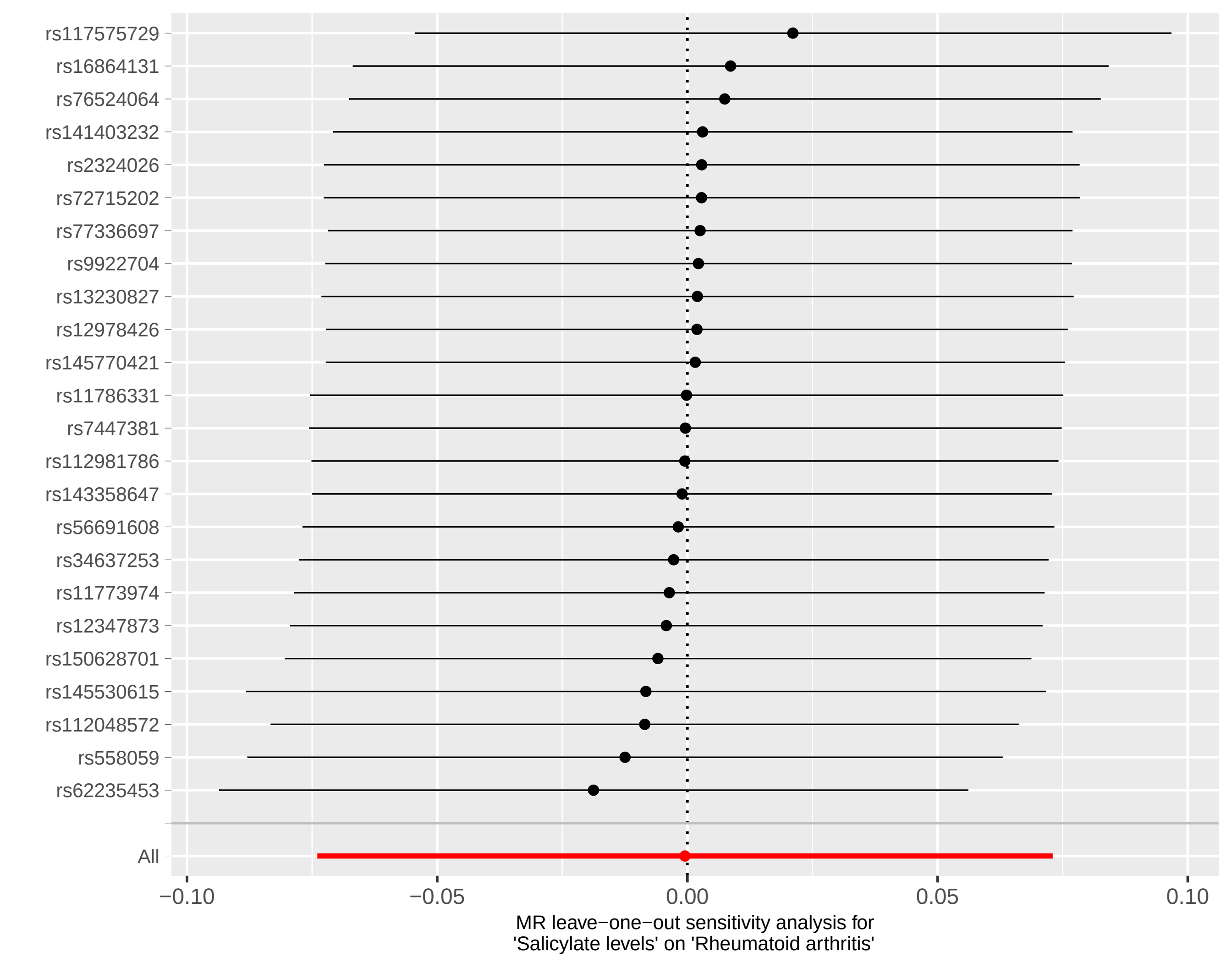
rs117575729
rs16864131
rs76524064
rs141403232
rs2324026
rs72715202
rs77336697
rs9922704
rs13230827
rs12978426
rs145770421
rs11786331
rs7447381
rs112981786
rs143358647
rs56691608
rs34637253
rs11773974
rs12347873
rs150628701
rs145530615
rs112048572
rs558059
rs62235453
All
−0.10
−0.05
0.00
0.05
0.10
MR leave−one−out sensitivity analysis for
'Salicylate levels' on 'Rheumatoid arthritis'

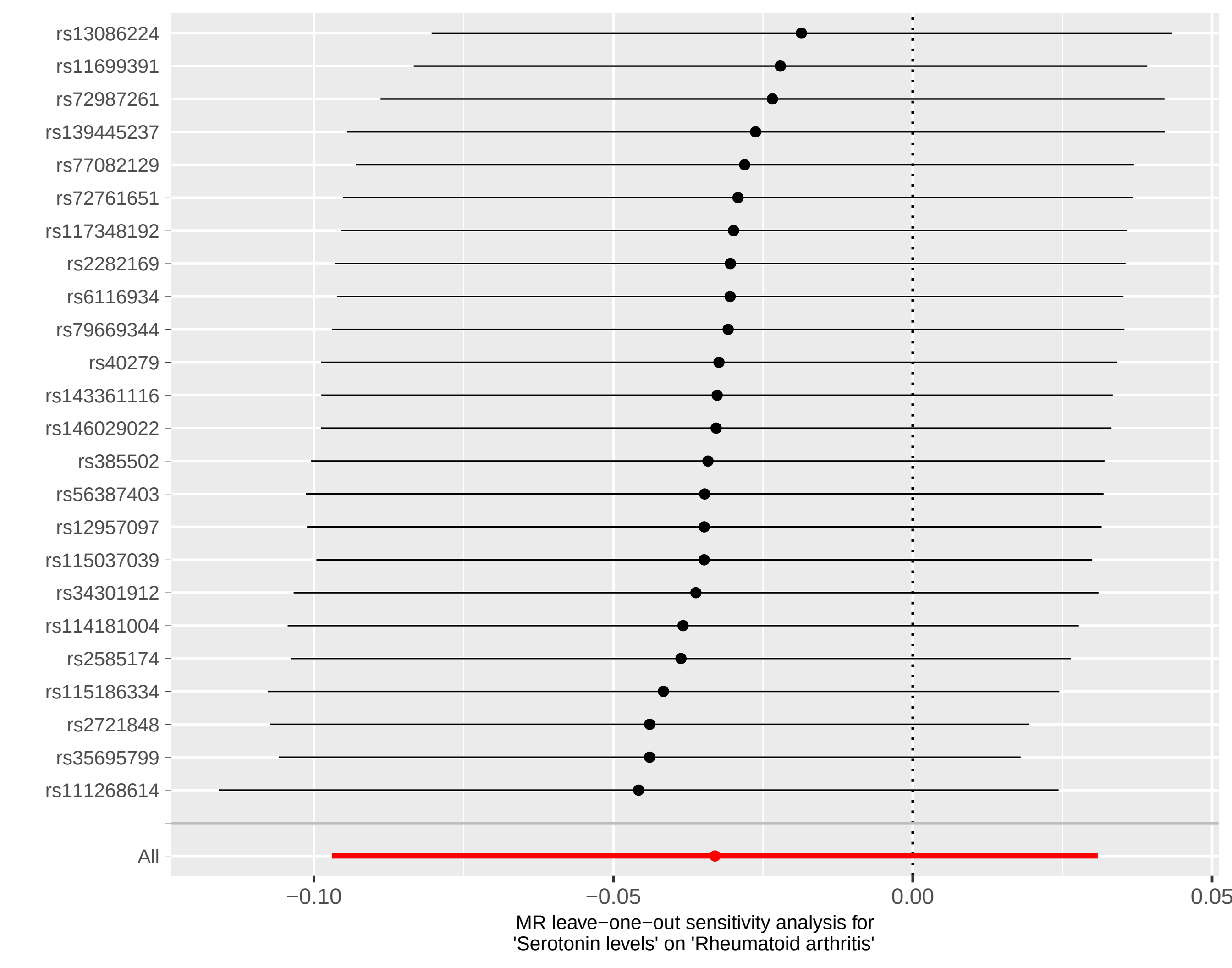

rs13086224
rs11699391
rs72987261
rs139445237
rs77082129
rs72761651
rs117348192
rs2282169
rs6116934
rs79669344
rs40279
rs143361116
rs146029022
rs385502
rs56387403
rs12957097
rs115037039
rs34301912
rs114181004
rs2585174
rs115186334
rs2721848
rs35695799
rs111268614
All
−0.10
−0.05
0.00
0.05
MR leave−one−out sensitivity analysis for
'Serotonin levels' on 'Rheumatoid arthritis'

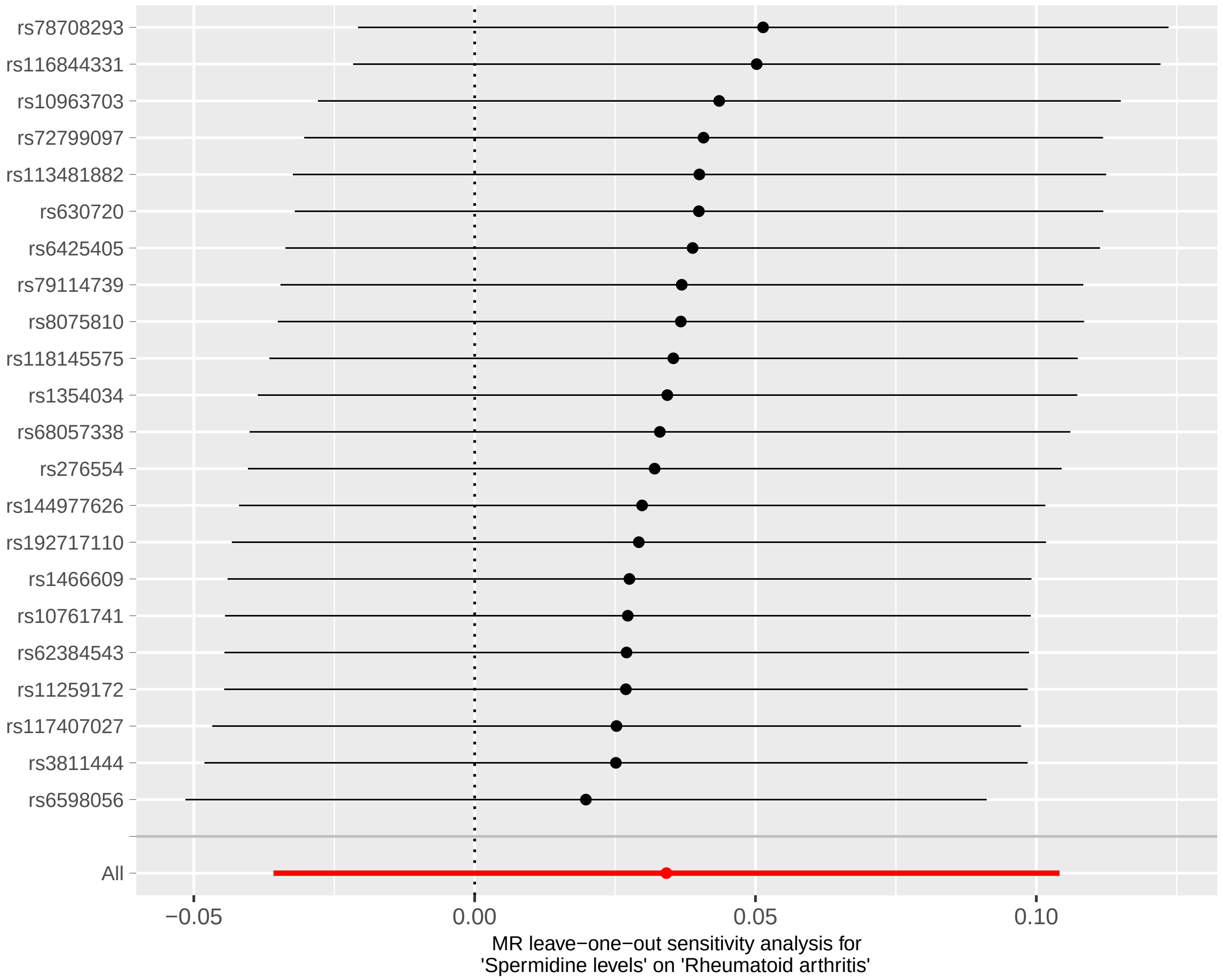
rs78708293
rs116844331
rs10963703
rs72799097
rs113481882
rs630720
rs6425405
rs79114739
rs8075810
rs118145575
rs1354034
rs68057338
rs276554
rs144977626
rs192717110
rs1466609
rs10761741
rs62384543
rs11259172
rs117407027
rs3811444
rs6598056
All
−0.05
0.00
0.05
0.10
MR leave−one−out sensitivity analysis for
'Spermidine levels' on 'Rheumatoid arthritis'

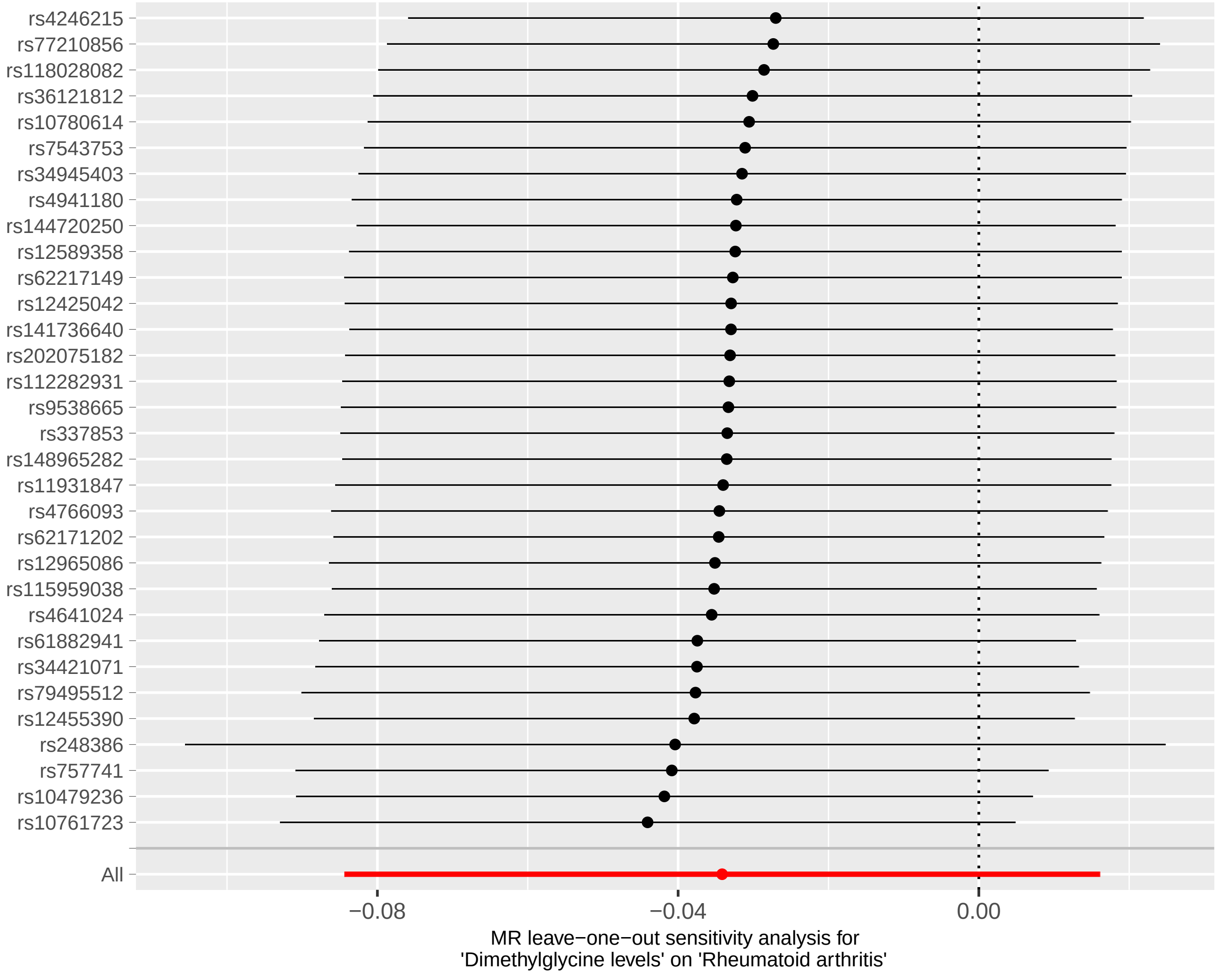
rs4246215
rs77210856
rs118028082
rs36121812
rs10780614
rs7543753
rs34945403
rs4941180
rs144720250
rs12589358
rs62217149
rs12425042
rs141736640
rs202075182
rs112282931
rs9538665
rs337853
rs148965282
rs11931847
rs4766093
rs62171202
rs12965086
rs115959038
rs4641024
rs61882941
rs34421071
rs79495512
rs12455390
rs248386
rs757741
rs10479236
rs10761723
All
−0.08
−0.04
0.00
MR leave−one−out sensitivity analysis for
'Dimethylglycine levels' on 'Rheumatoid arthritis'

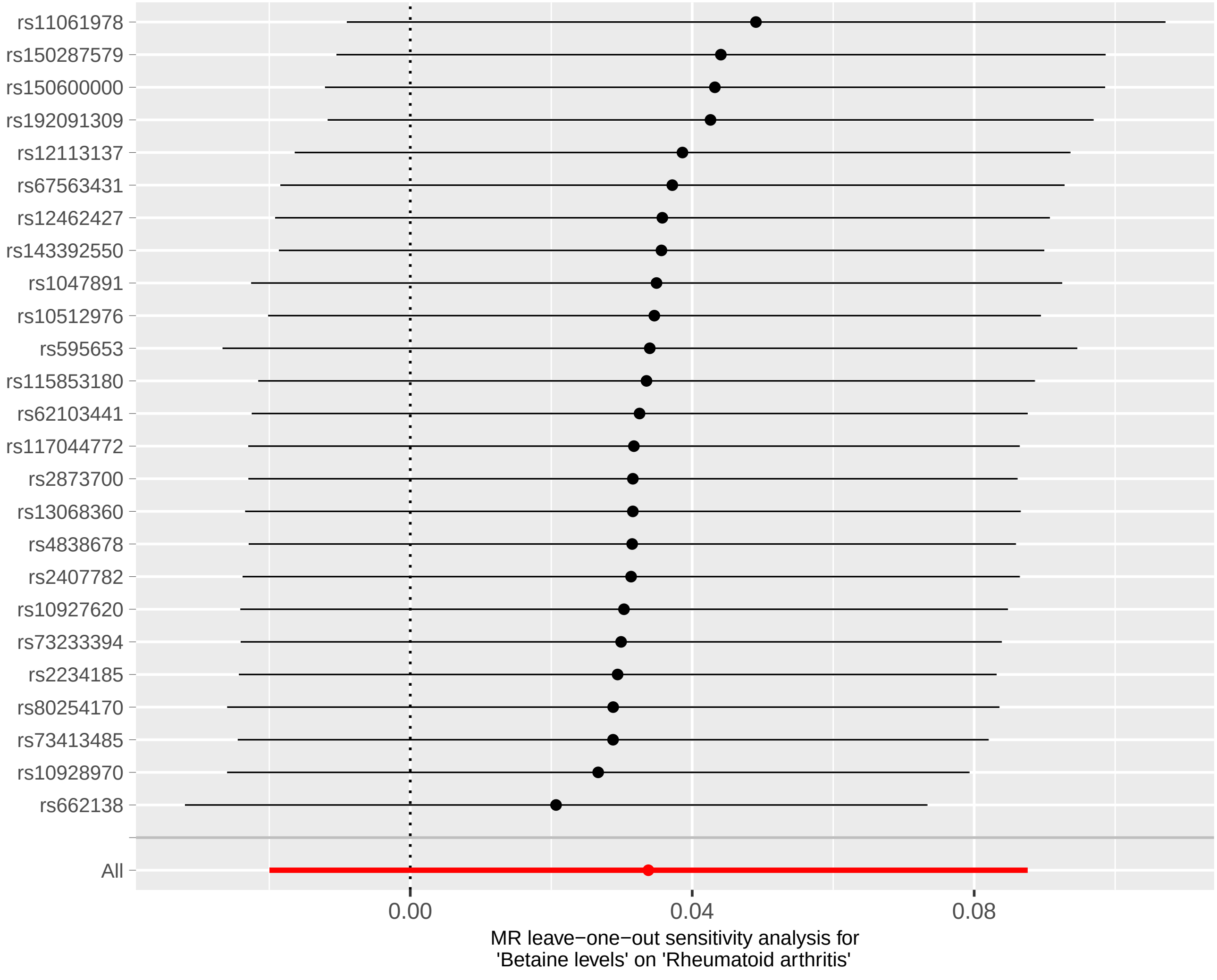
rs11061978
rs150287579
rs150600000
rs192091309
rs12113137
rs67563431
rs12462427
rs143392550
rs1047891
rs10512976
rs595653
rs115853180
rs62103441
rs117044772
rs2873700
rs13068360
rs4838678
rs2407782
rs10927620
rs73233394
rs2234185
rs80254170
rs73413485
rs10928970
rs662138
All
0.00
0.04
0.08
MR leave−one−out sensitivity analysis for
'Betaine levels' on 'Rheumatoid arthritis'

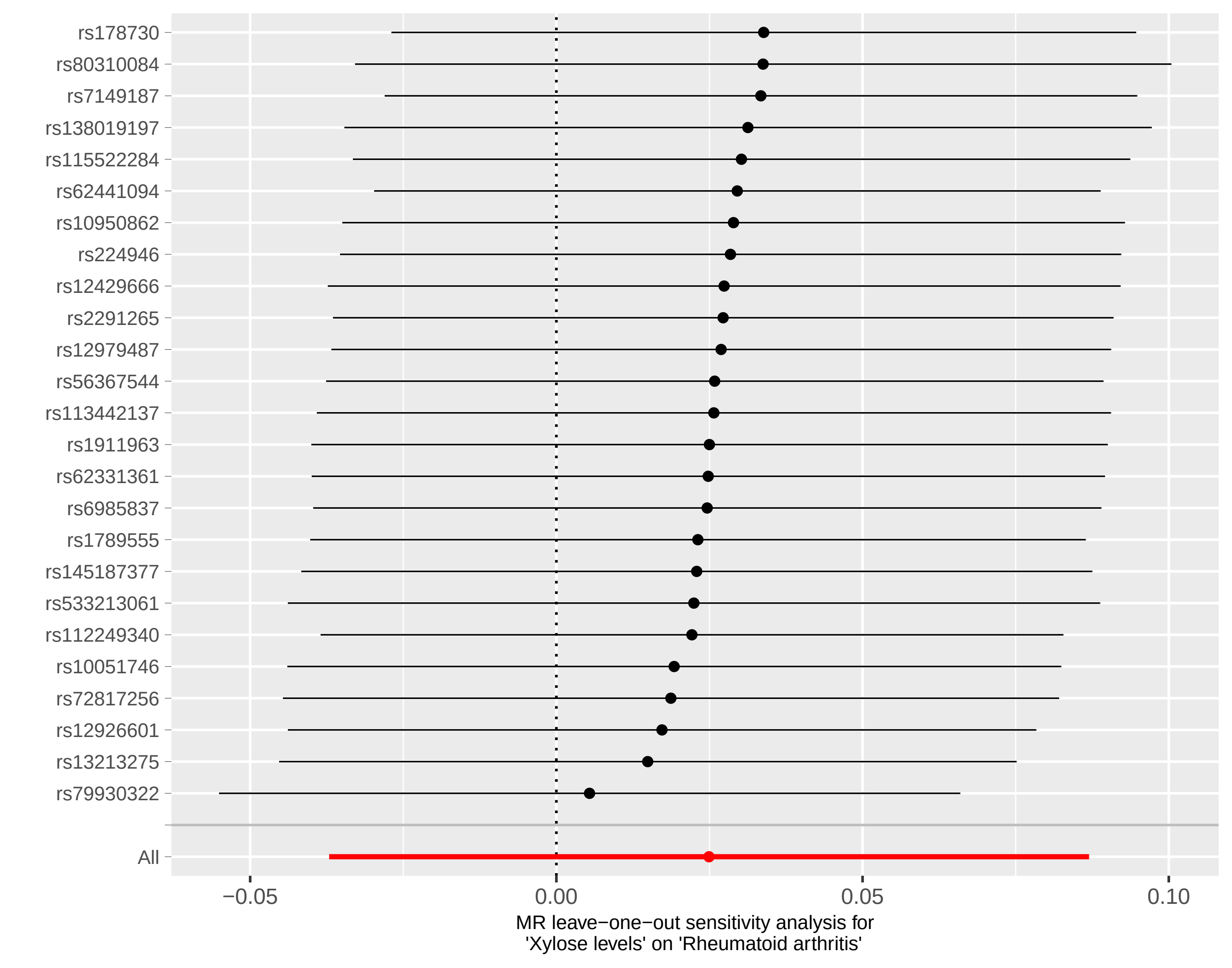
rs178730
rs80310084
rs7149187
rs138019197
rs115522284
rs62441094
rs10950862
rs224946
rs12429666
rs2291265
rs12979487
rs56367544
rs113442137
rs1911963
rs62331361
rs6985837
rs1789555
rs145187377
rs533213061
rs112249340
rs10051746
rs72817256
rs12926601
rs13213275
rs79930322
All
−0.05
0.00
0.05
0.10
MR leave−one−out sensitivity analysis for
'Xylose levels' on 'Rheumatoid arthritis'

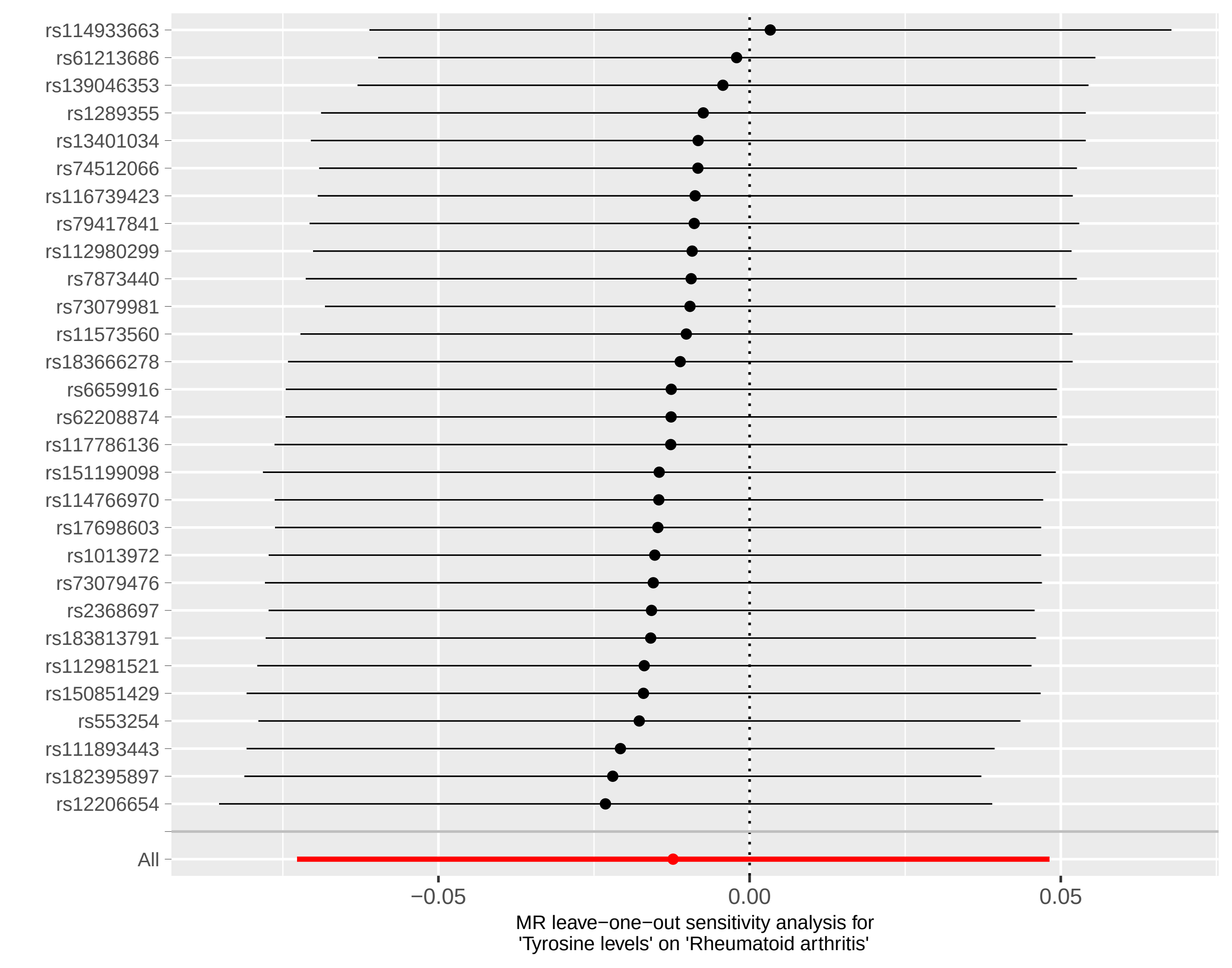
rs114933663
rs61213686
rs139046353
rs1289355
rs13401034
rs74512066
rs116739423
rs79417841
rs112980299
rs7873440
rs73079981
rs11573560
rs183666278
rs6659916
rs62208874
rs117786136
rs151199098
rs114766970
rs17698603
rs1013972
rs73079476
rs2368697
rs183813791
rs112981521
rs150851429
rs553254
rs111893443
rs182395897
rs12206654
All
−0.05
0.00
0.05
MR leave−one−out sensitivity analysis for
'Tyrosine levels' on 'Rheumatoid arthritis'

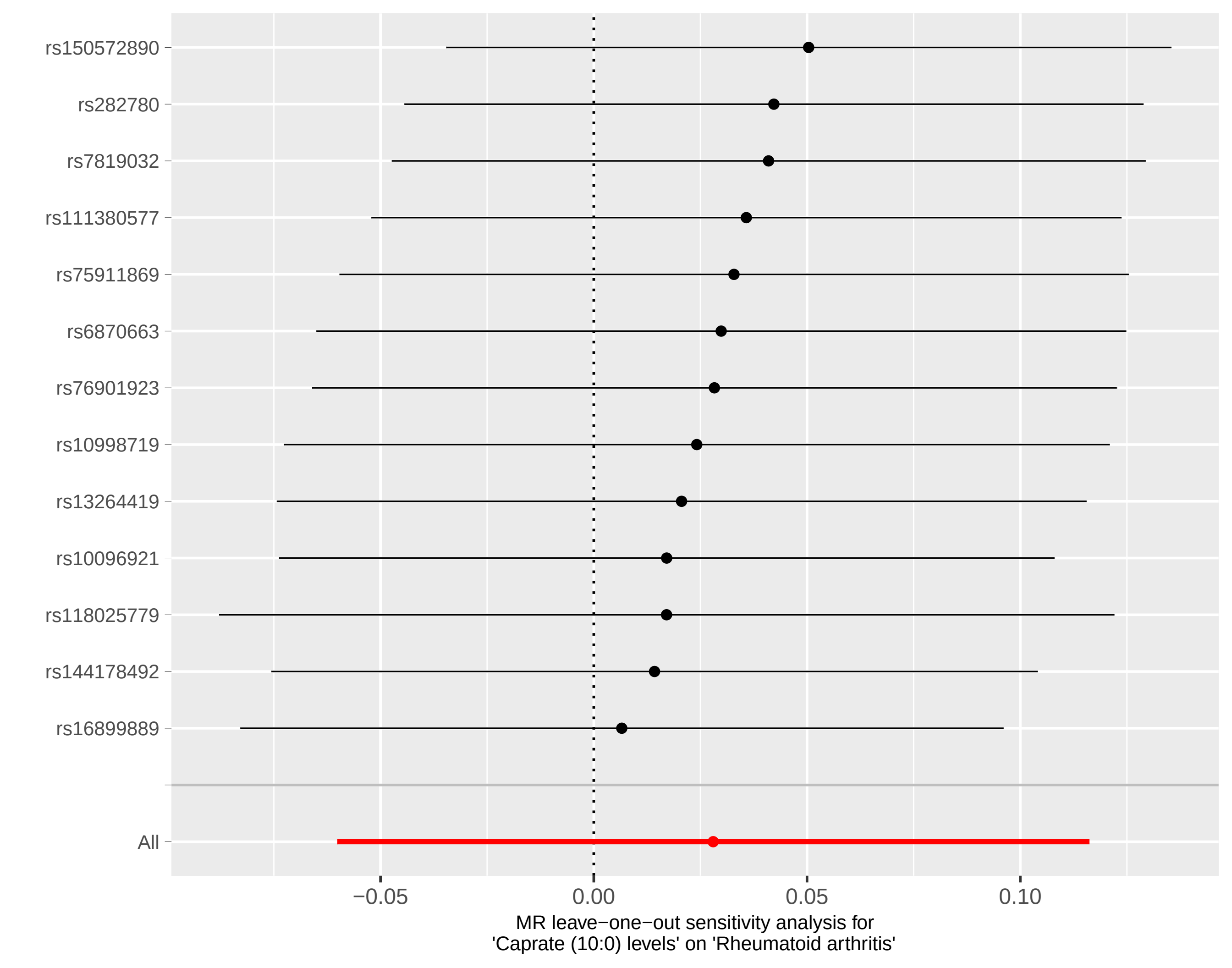
rs150572890
rs282780
rs7819032
rs111380577
rs75911869
rs6870663
rs76901923
rs10998719
rs13264419
rs10096921
rs118025779
rs144178492
rs16899889
All
−0.05
0.00
0.05
0.10
MR leave−one−out sensitivity analysis for
'Caprate (10:0) levels' on 'Rheumatoid arthritis'

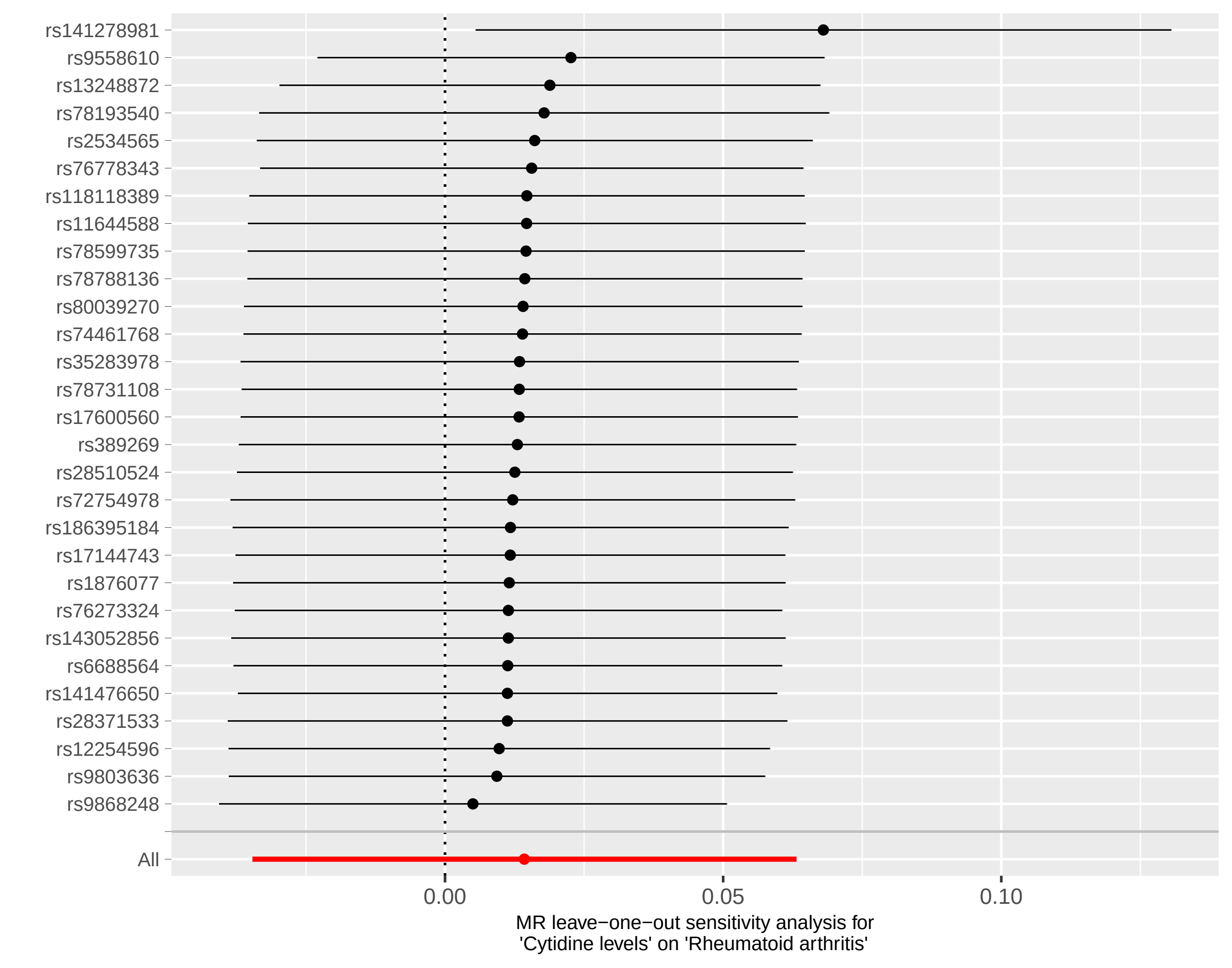

rs141278981
rs9558610
rs13248872
rs78193540
rs2534565
rs76778343
rs118118389
rs11644588
rs78599735
rs78788136
rs80039270
rs74461768
rs35283978
rs78731108
rs17600560
rs389269
rs28510524
rs72754978
rs186395184
rs17144743
rs1876077
rs76273324
rs143052856
rs6688564
rs141476650
rs28371533
rs12254596
rs9803636
rs9868248
All
0.00
0.05
0.10
MR leave−one−out sensitivity analysis for
'Cytidine levels' on 'Rheumatoid arthritis'

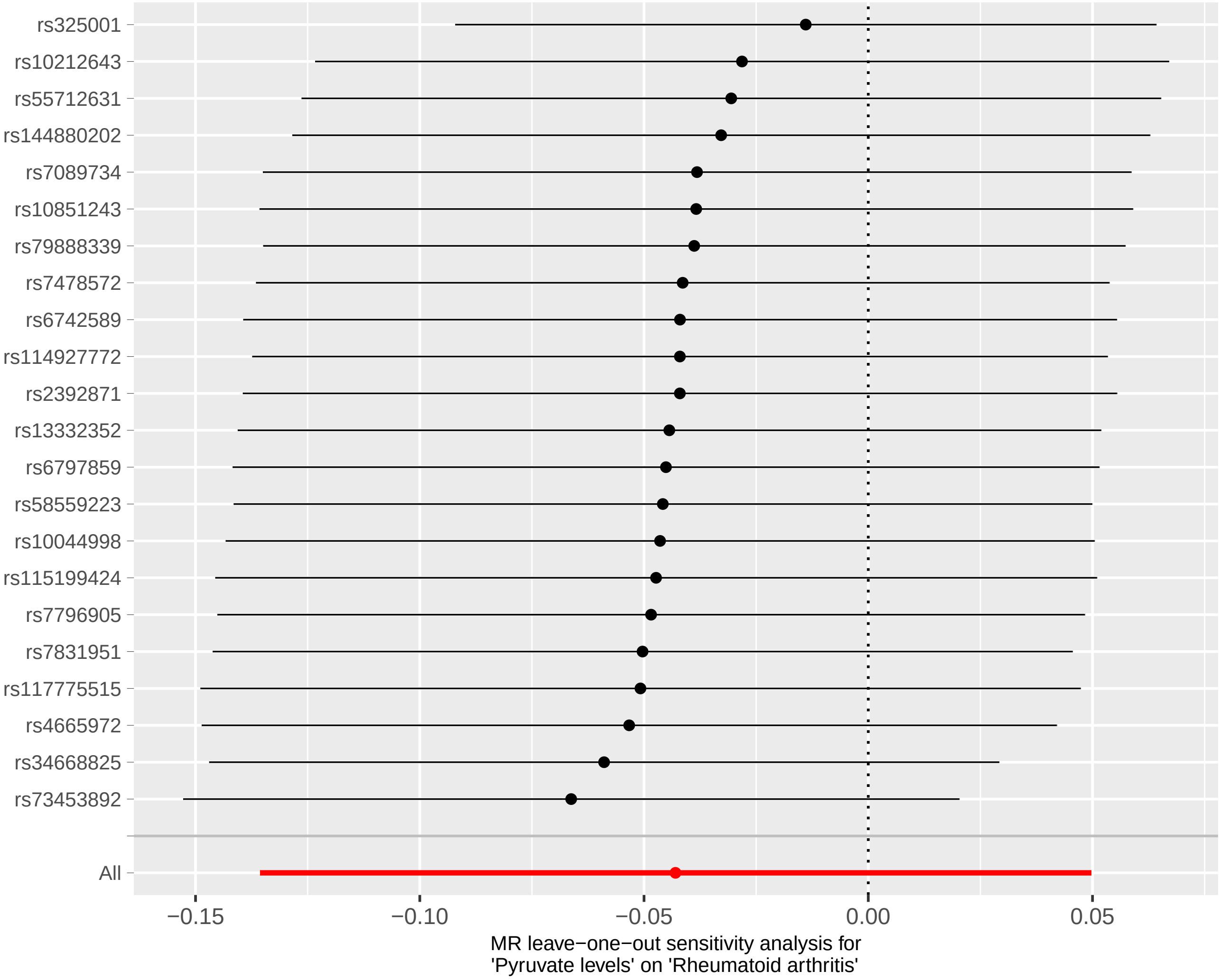
rs325001
rs10212643
rs55712631
rs144880202
rs7089734
rs10851243
rs79888339
rs7478572
rs6742589
rs114927772
rs2392871
rs13332352
rs6797859
rs58559223
rs10044998
rs115199424
rs7796905
rs7831951
rs117775515
rs4665972
rs34668825
rs73453892
All
−0.15
−0.10
−0.05
0.00
0.05
MR leave−one−out sensitivity analysis for
'Pyruvate levels' on 'Rheumatoid arthritis'

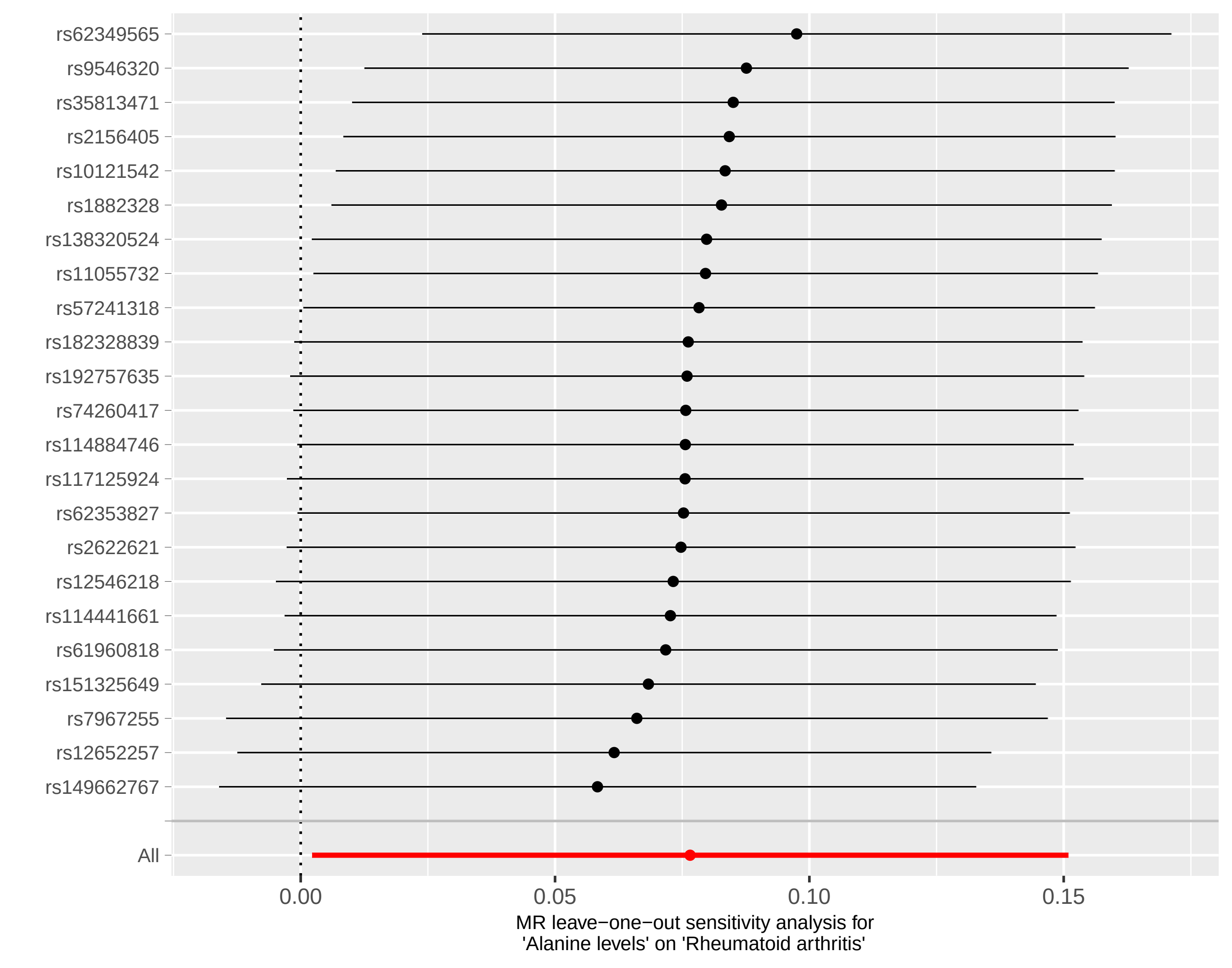

rs62349565
rs9546320
rs35813471
rs2156405
rs10121542
rs1882328
rs138320524
rs11055732
rs57241318
rs182328839
rs192757635
rs74260417
rs114884746
rs117125924
rs62353827
rs2622621
rs12546218
rs114441661
rs61960818
rs151325649
rs7967255
rs12652257
rs149662767
All
0.00
0.05
0.10
0.15
MR leave−one−out sensitivity analysis for
'Alanine levels' on 'Rheumatoid arthritis'

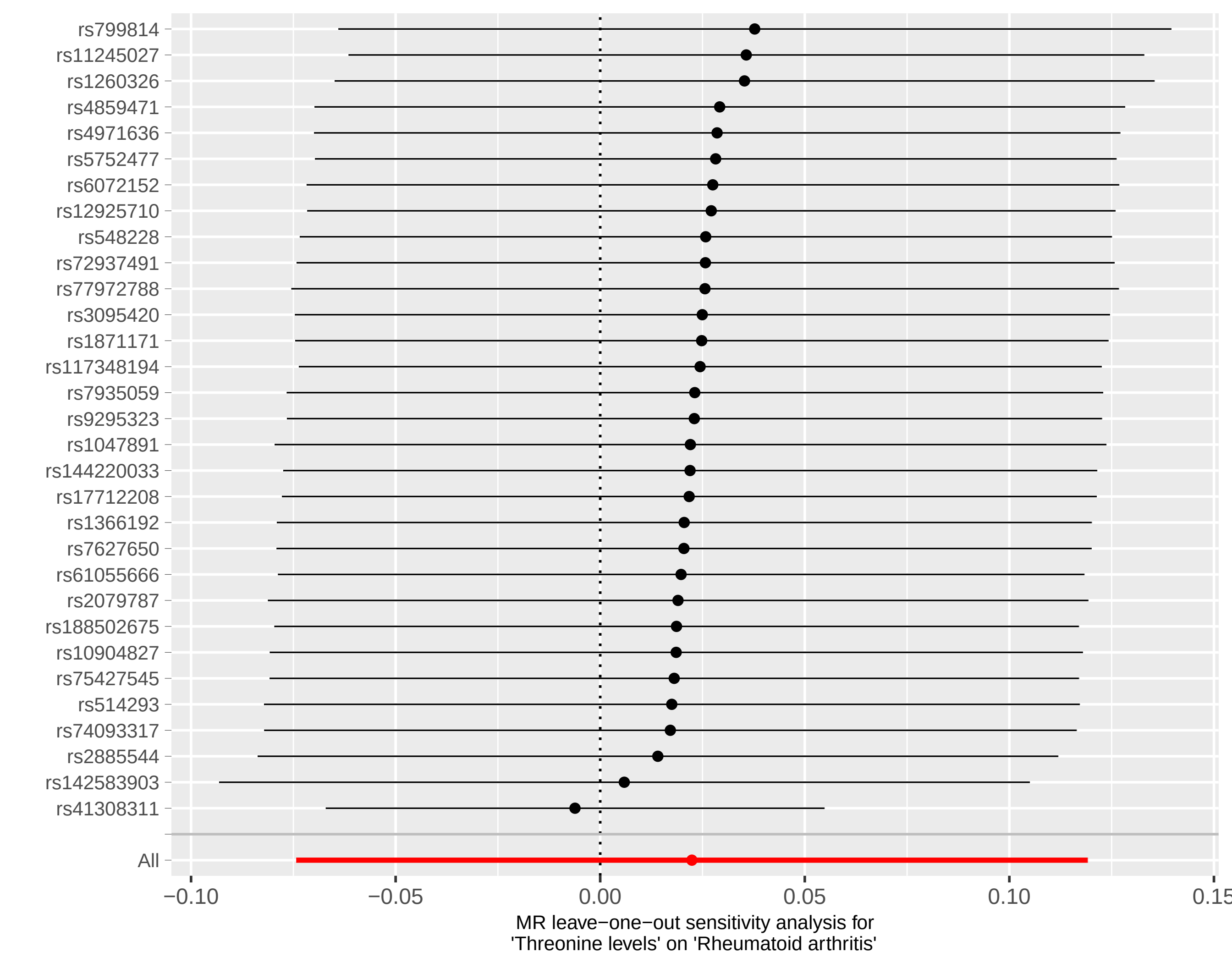

rs799814
rs11245027
rs1260326
rs4859471
rs4971636
rs5752477
rs6072152
rs12925710
rs548228
rs72937491
rs77972788
rs3095420
rs1871171
rs117348194
rs7935059
rs9295323
rs1047891
rs144220033
rs17712208
rs1366192
rs7627650
rs61055666
rs2079787
rs188502675
rs10904827
rs75427545
rs514293
rs74093317
rs2885544
rs142583903
rs41308311
All
−0.10
−0.05
0.00
0.05
0.10
0.15
MR leave−one−out sensitivity analysis for
'Threonine levels' on 'Rheumatoid arthritis'

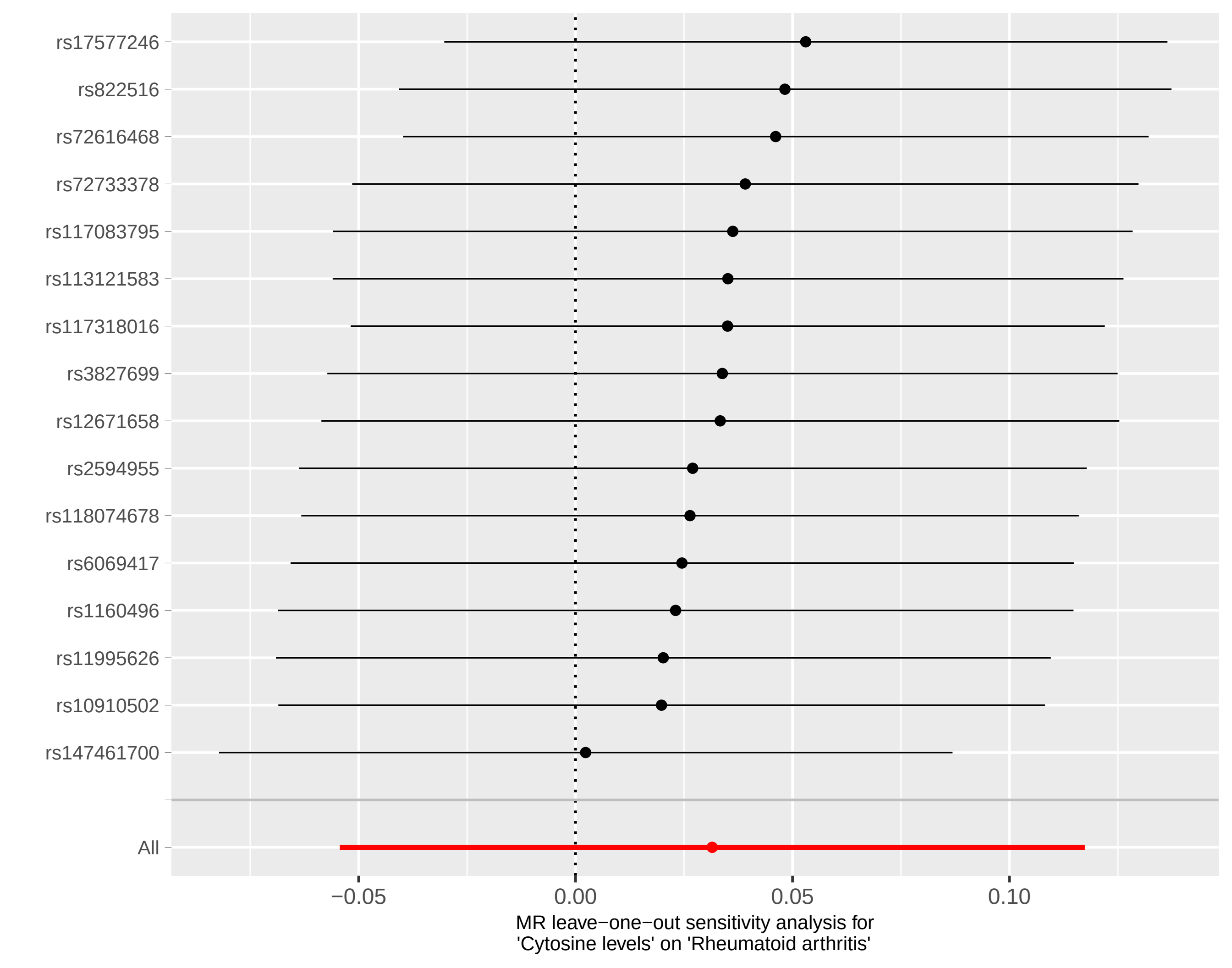
rs17577246
rs822516
rs72616468
rs72733378
rs117083795
rs113121583
rs117318016
rs3827699
rs12671658
rs2594955
rs118074678
rs6069417
rs1160496
rs11995626
rs10910502
rs147461700
All
−0.05
0.00
0.05
0.10
MR leave−one−out sensitivity analysis for
'Cytosine levels' on 'Rheumatoid arthritis'

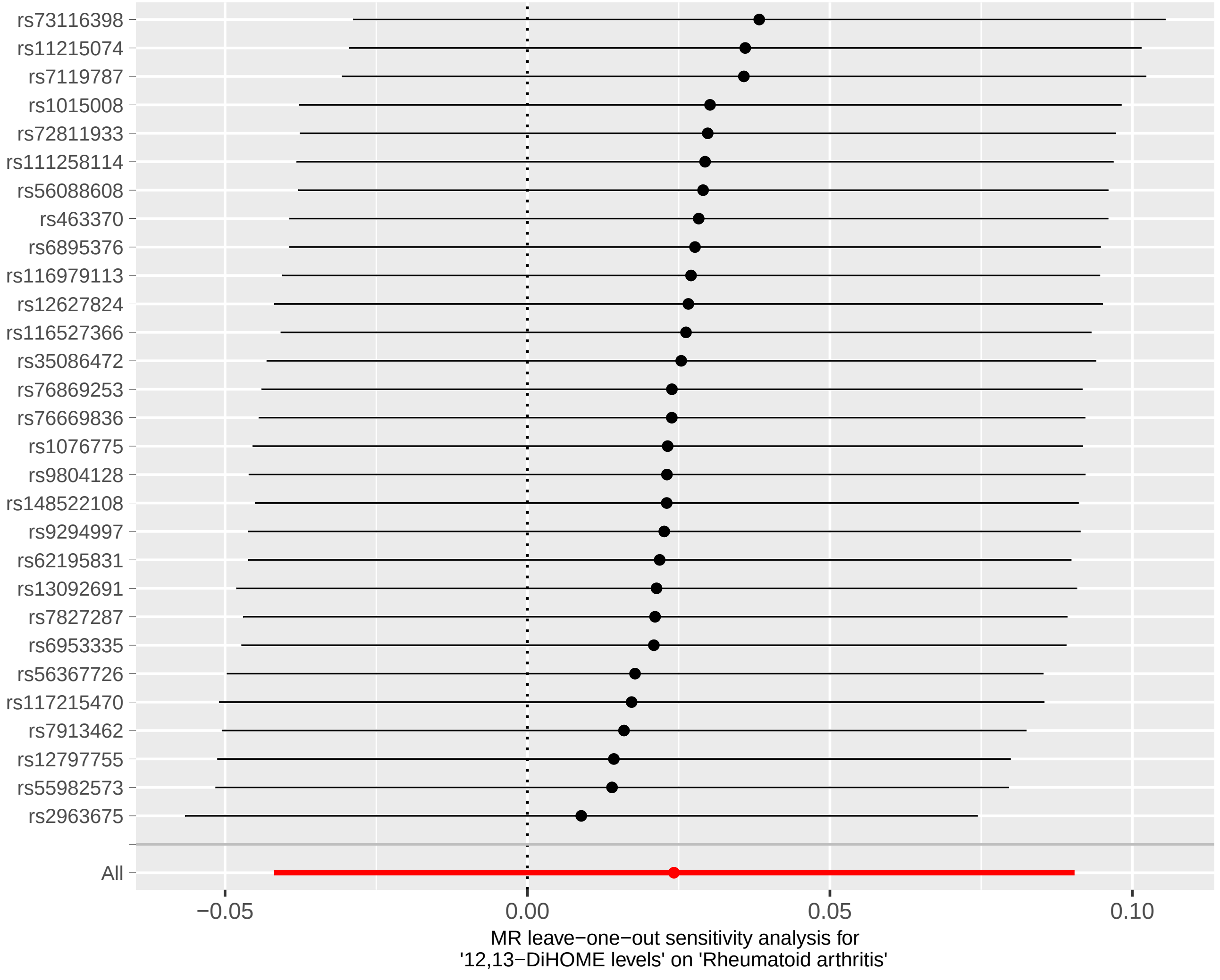

rs73116398
rs11215074
rs7119787
rs1015008
rs72811933
rs111258114
rs56088608
rs463370
rs6895376
rs116979113
rs12627824
rs116527366
rs35086472
rs76869253
rs76669836
rs1076775
rs9804128
rs148522108
rs9294997
rs62195831
rs13092691
rs7827287
rs6953335
rs56367726
rs117215470
rs7913462
rs12797755
rs55982573
rs2963675
All
−0.05
0.00
0.05
0.10
MR leave−one−out sensitivity analysis for
'12,13−DiHOME levels' on 'Rheumatoid arthritis'

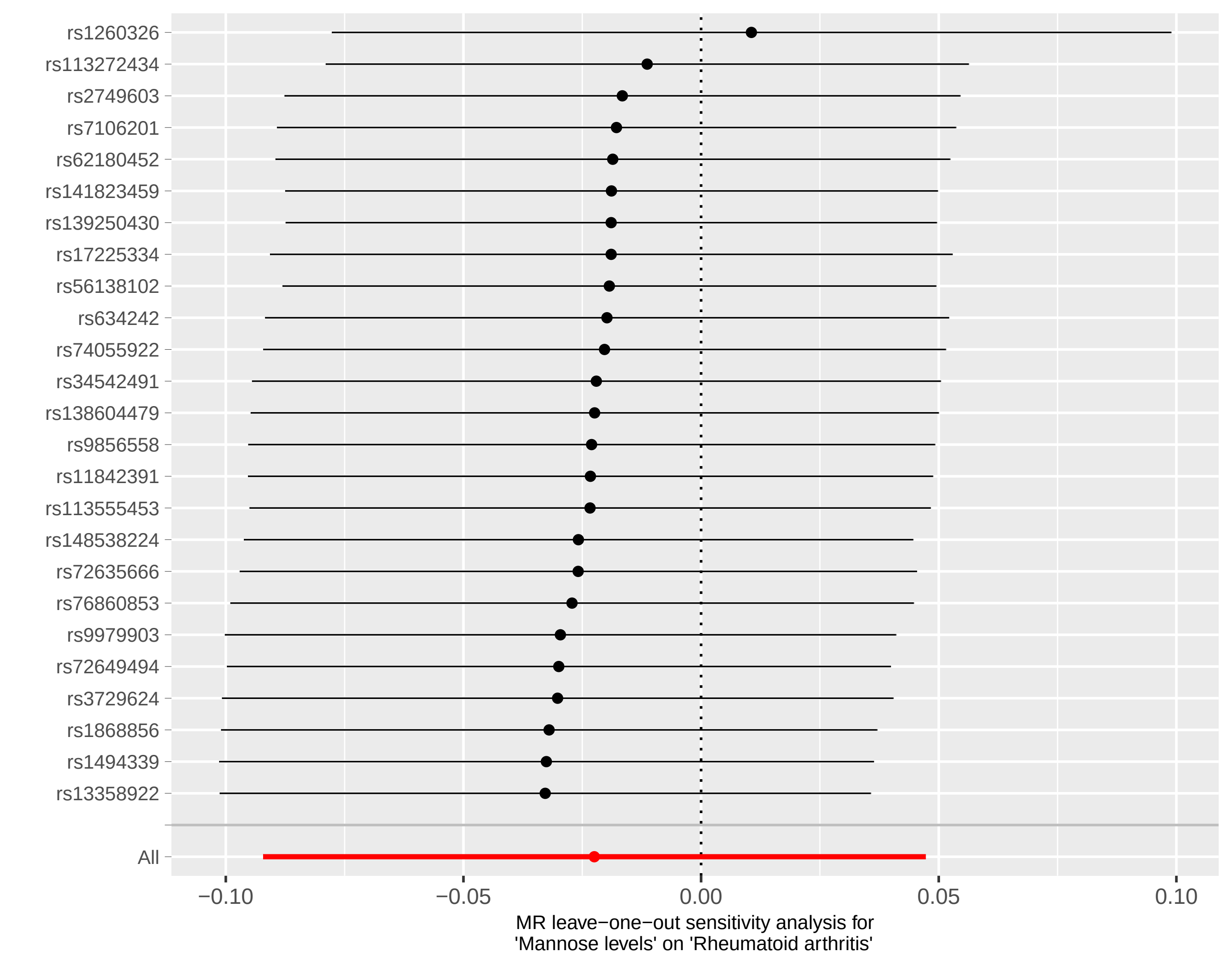
rs1260326
rs113272434
rs2749603
rs7106201
rs62180452
rs141823459
rs139250430
rs17225334
rs56138102
rs634242
rs74055922
rs34542491
rs138604479
rs9856558
rs11842391
rs113555453
rs148538224
rs72635666
rs76860853
rs9979903
rs72649494
rs3729624
rs1868856
rs1494339
rs13358922
All
−0.10
−0.05
0.00
0.05
0.10
MR leave−one−out sensitivity analysis for
'Mannose levels' on 'Rheumatoid arthritis'

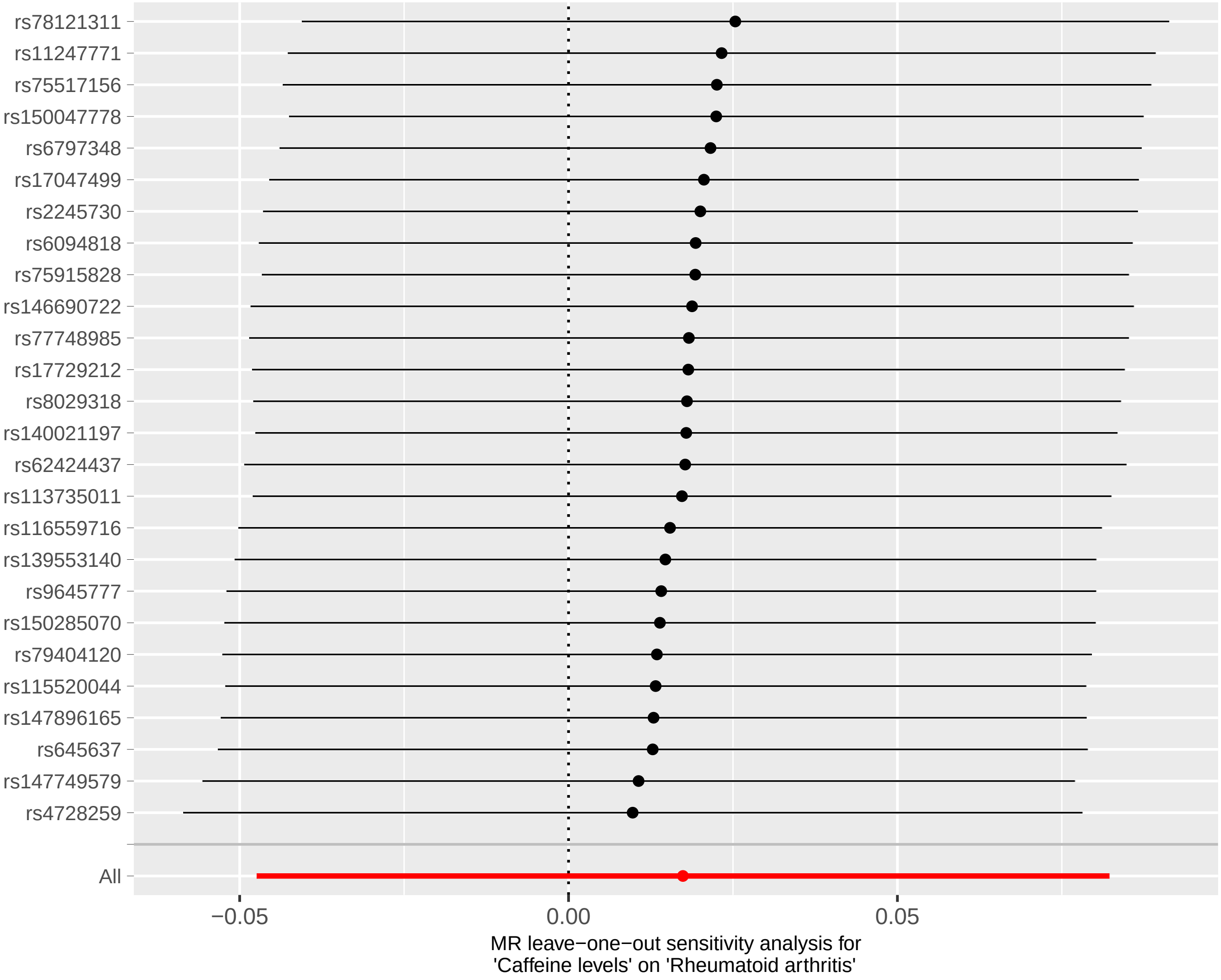
rs78121311
rs11247771
rs75517156
rs150047778
rs6797348
rs17047499
rs2245730
rs6094818
rs75915828
rs146690722
rs77748985
rs17729212
rs8029318
rs140021197
rs62424437
rs113735011
rs116559716
rs139553140
rs9645777
rs150285070
rs79404120
rs115520044
rs147896165
rs645637
rs147749579
rs4728259
All
−0.05
0.00
0.05
MR leave−one−out sensitivity analysis for
'Caffeine levels' on 'Rheumatoid arthritis'

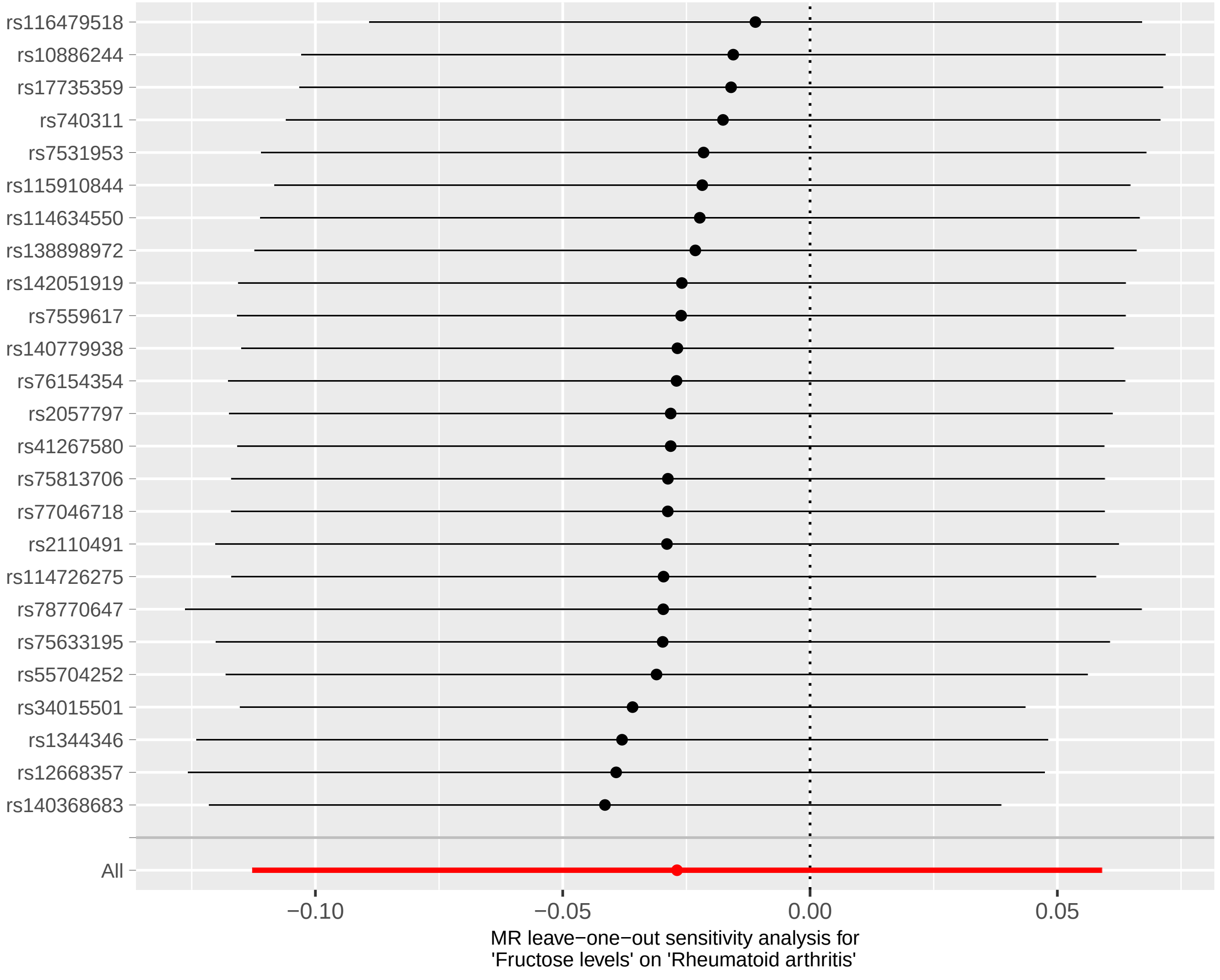
rs116479518
rs10886244
rs17735359
rs740311
rs7531953
rs115910844
rs114634550
rs138898972
rs142051919
rs7559617
rs140779938
rs76154354
rs2057797
rs41267580
rs75813706
rs77046718
rs2110491
rs114726275
rs78770647
rs75633195
rs55704252
rs34015501
rs1344346
rs12668357
rs140368683
All
−0.10
−0.05
0.00
0.05
MR leave−one−out sensitivity analysis for
'Fructose levels' on 'Rheumatoid arthritis'

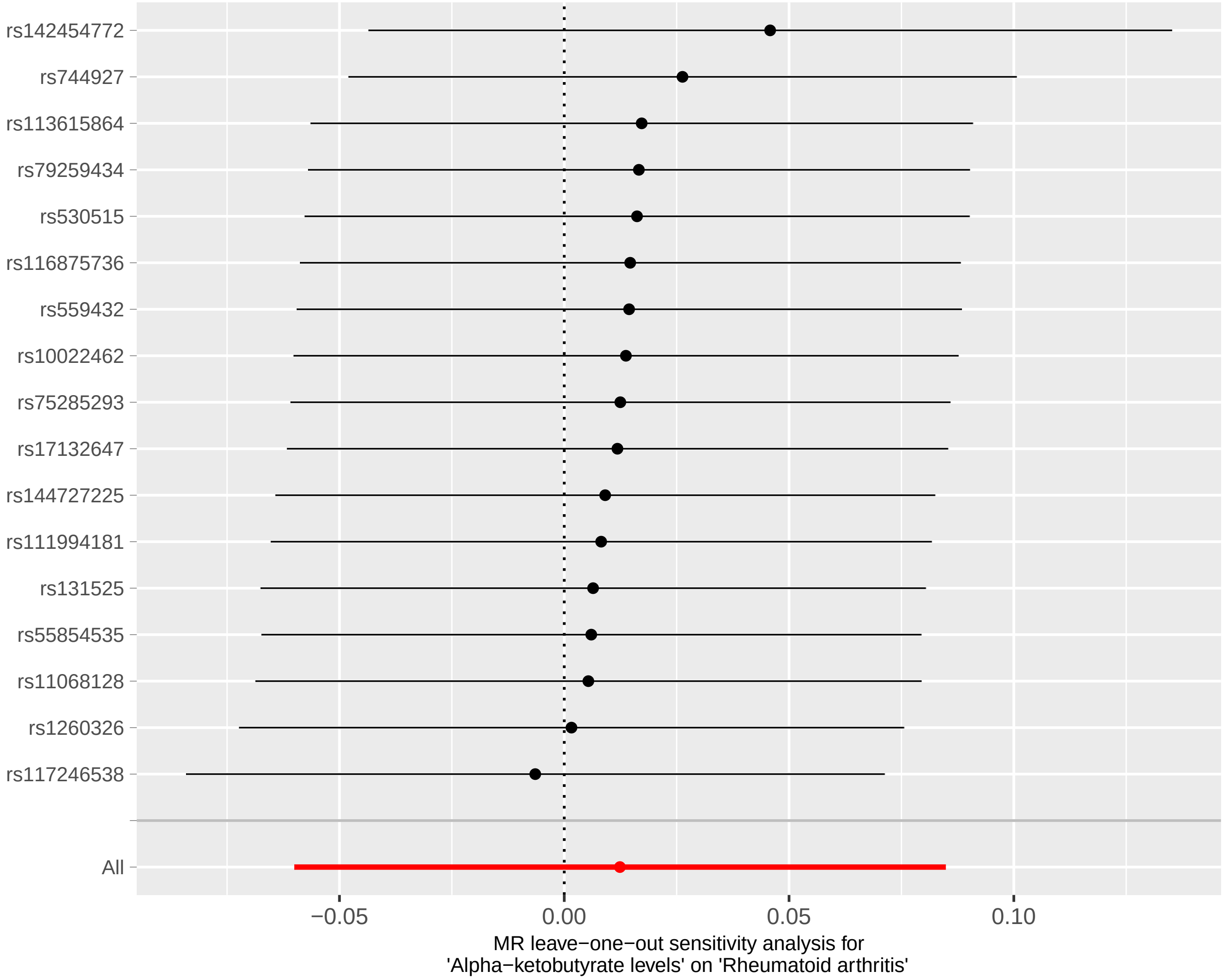
rs142454772
rs744927
rs113615864
rs79259434
rs530515
rs116875736
rs559432
rs10022462
rs75285293
rs17132647
rs144727225
rs111994181
rs131525
rs55854535
rs11068128
rs1260326
rs117246538
All
−0.05
0.00
0.05
0.10
MR leave−one−out sensitivity analysis for
'Alpha−ketobutyrate levels' on 'Rheumatoid arthritis'

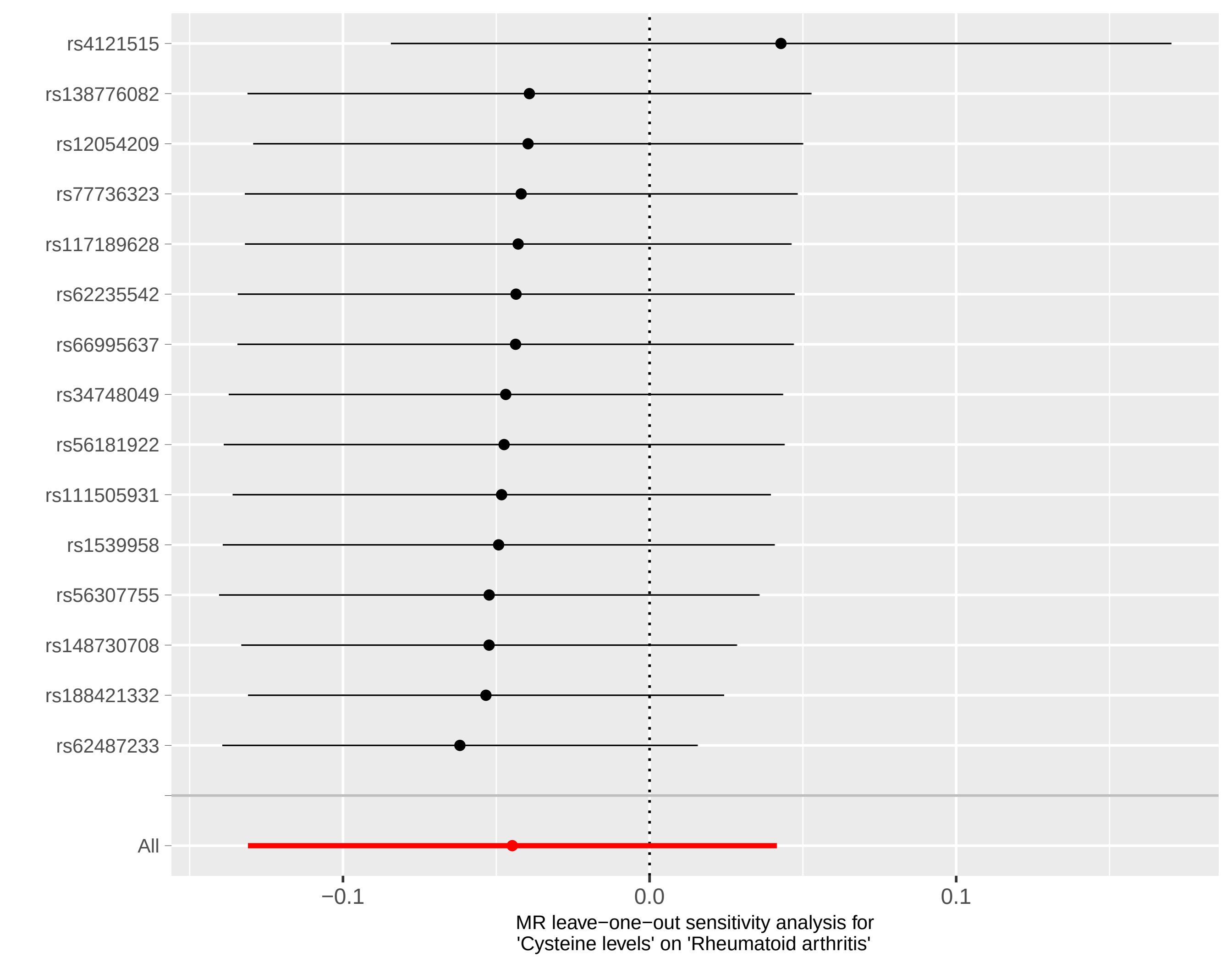
rs4121515
rs138776082
rs12054209
rs77736323
rs117189628
rs62235542
rs66995637
rs34748049
rs56181922
rs111505931
rs1539958
rs56307755
rs148730708
rs188421332
rs62487233
All
−0.1
0.0
0.1
MR leave−one−out sensitivity analysis for
'Cysteine levels' on 'Rheumatoid arthritis'

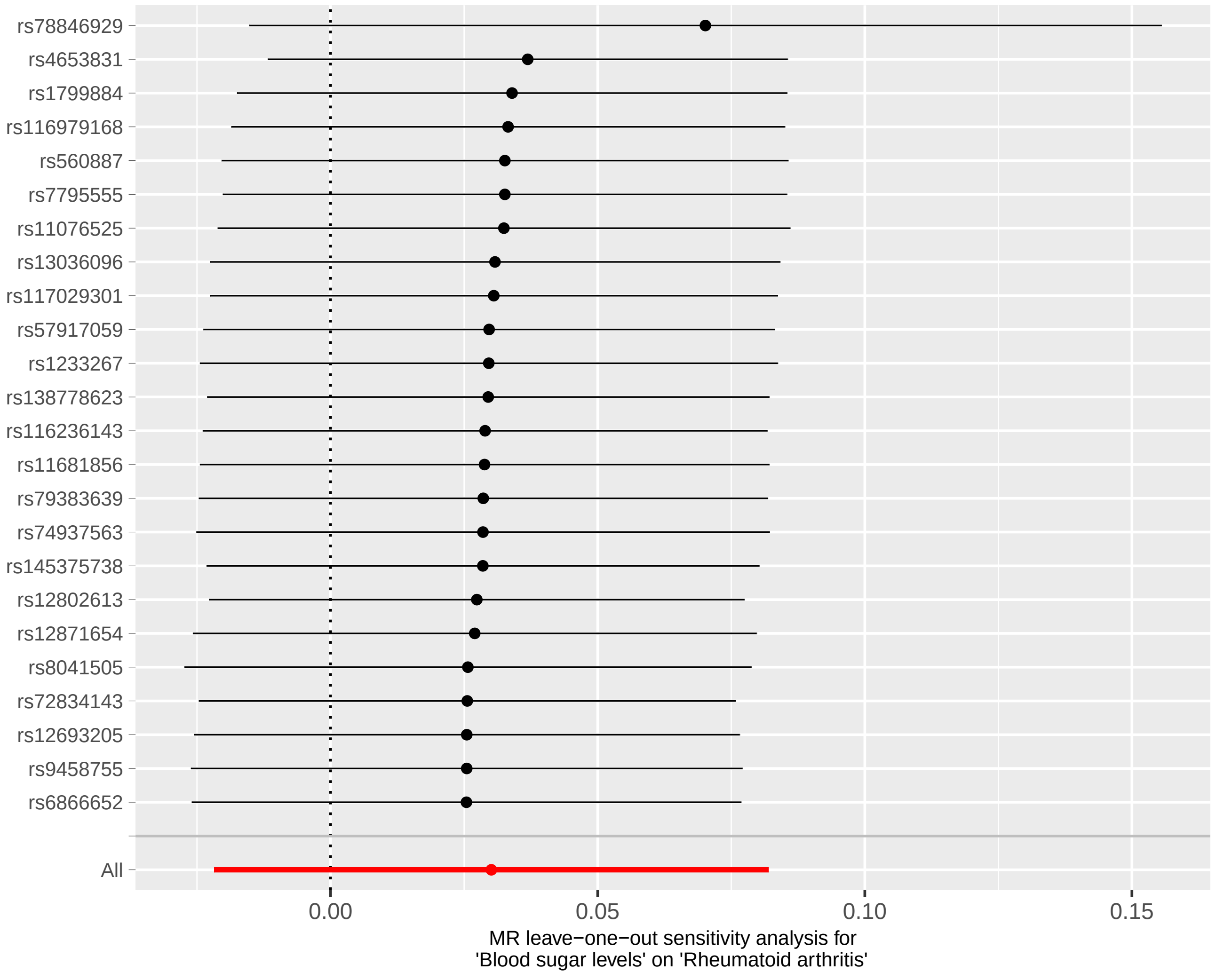
rs78846929
rs4653831
rs1799884
rs116979168
rs560887
rs7795555
rs11076525
rs13036096
rs117029301
rs57917059
rs1233267
rs138778623
rs116236143
rs11681856
rs79383639
rs74937563
rs145375738
rs12802613
rs12871654
rs8041505
rs72834143
rs12693205
rs9458755
rs6866652
All
0.00
0.05
0.10
0.15
MR leave−one−out sensitivity analysis for
'Blood sugar levels' on 'Rheumatoid arthritis'

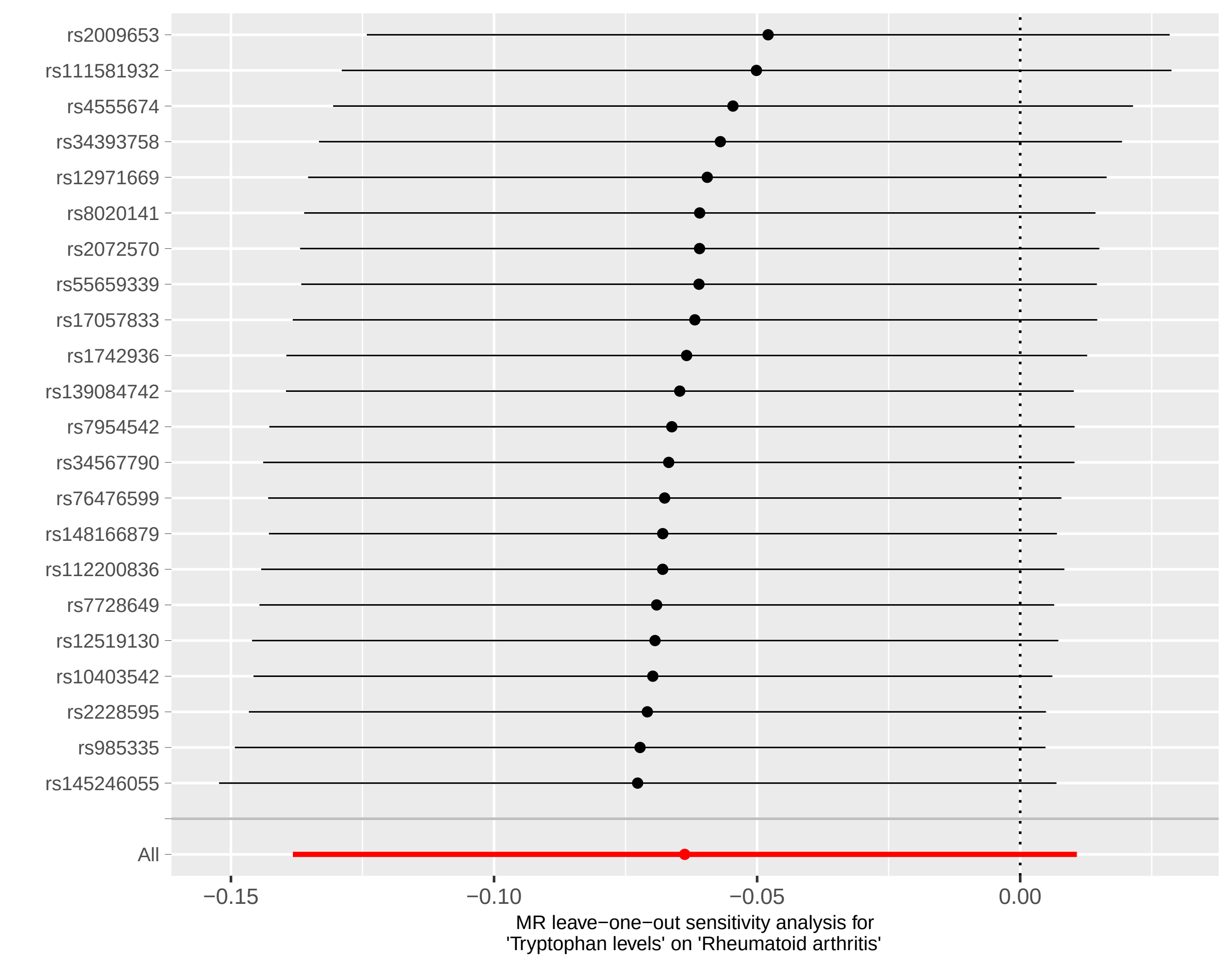
rs2009653
rs111581932
rs4555674
rs34393758
rs12971669
rs8020141
rs2072570
rs55659339
rs17057833
rs1742936
rs139084742
rs7954542
rs34567790
rs76476599
rs148166879
rs112200836
rs7728649
rs12519130
rs10403542
rs2228595
rs985335
rs145246055
All
−0.15
−0.10
−0.05
0.00
MR leave−one−out sensitivity analysis for
'Tryptophan levels' on 'Rheumatoid arthritis'

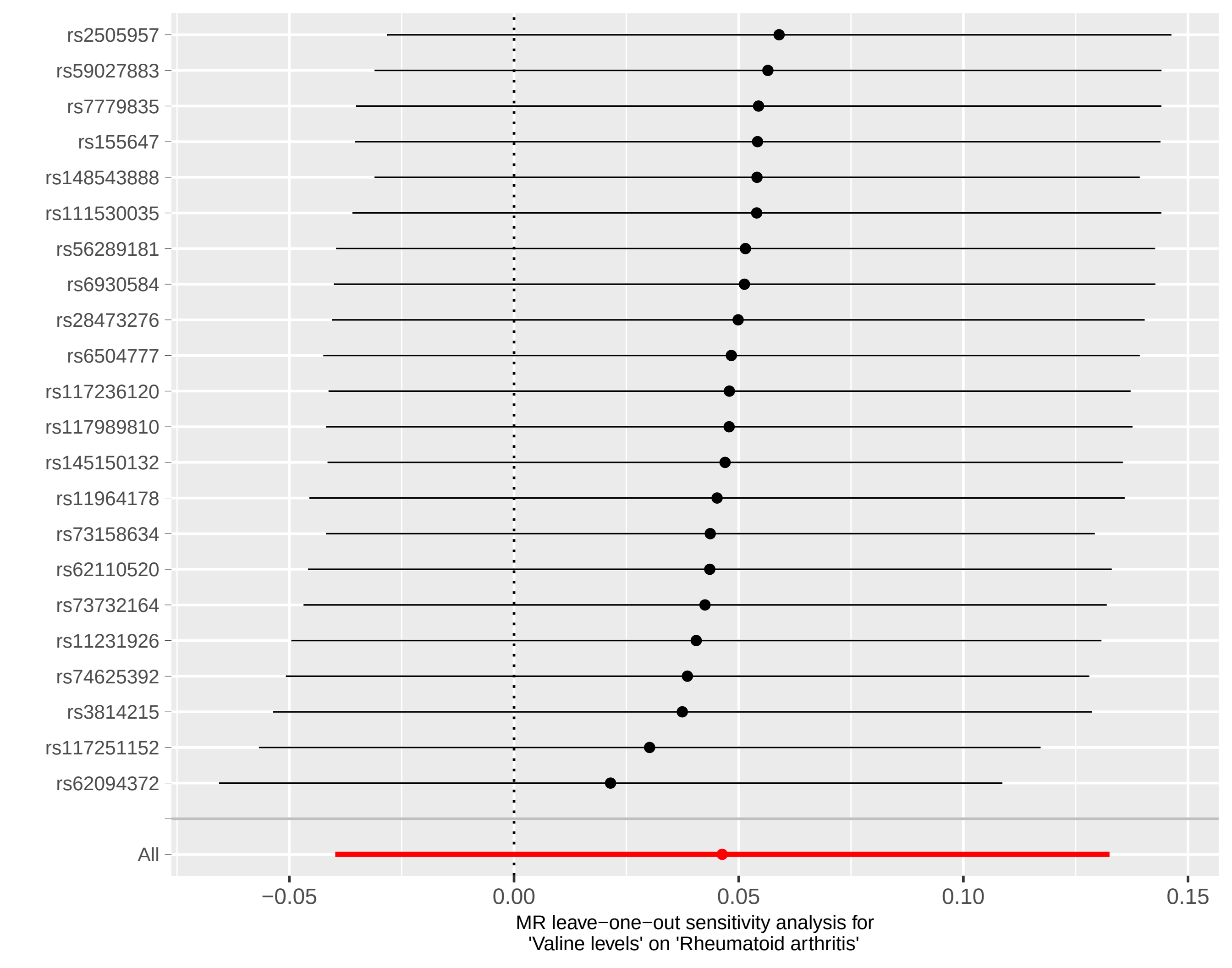

rs2505957
rs59027883
rs7779835
rs155647
rs148543888
rs111530035
rs56289181
rs6930584
rs28473276
rs6504777
rs117236120
rs117989810
rs145150132
rs11964178
rs73158634
rs62110520
rs73732164
rs11231926
rs74625392
rs3814215
rs117251152
rs62094372
All
−0.05
0.00
0.05
0.10
0.15
MR leave−one−out sensitivity analysis for
'Valine levels' on 'Rheumatoid arthritis'

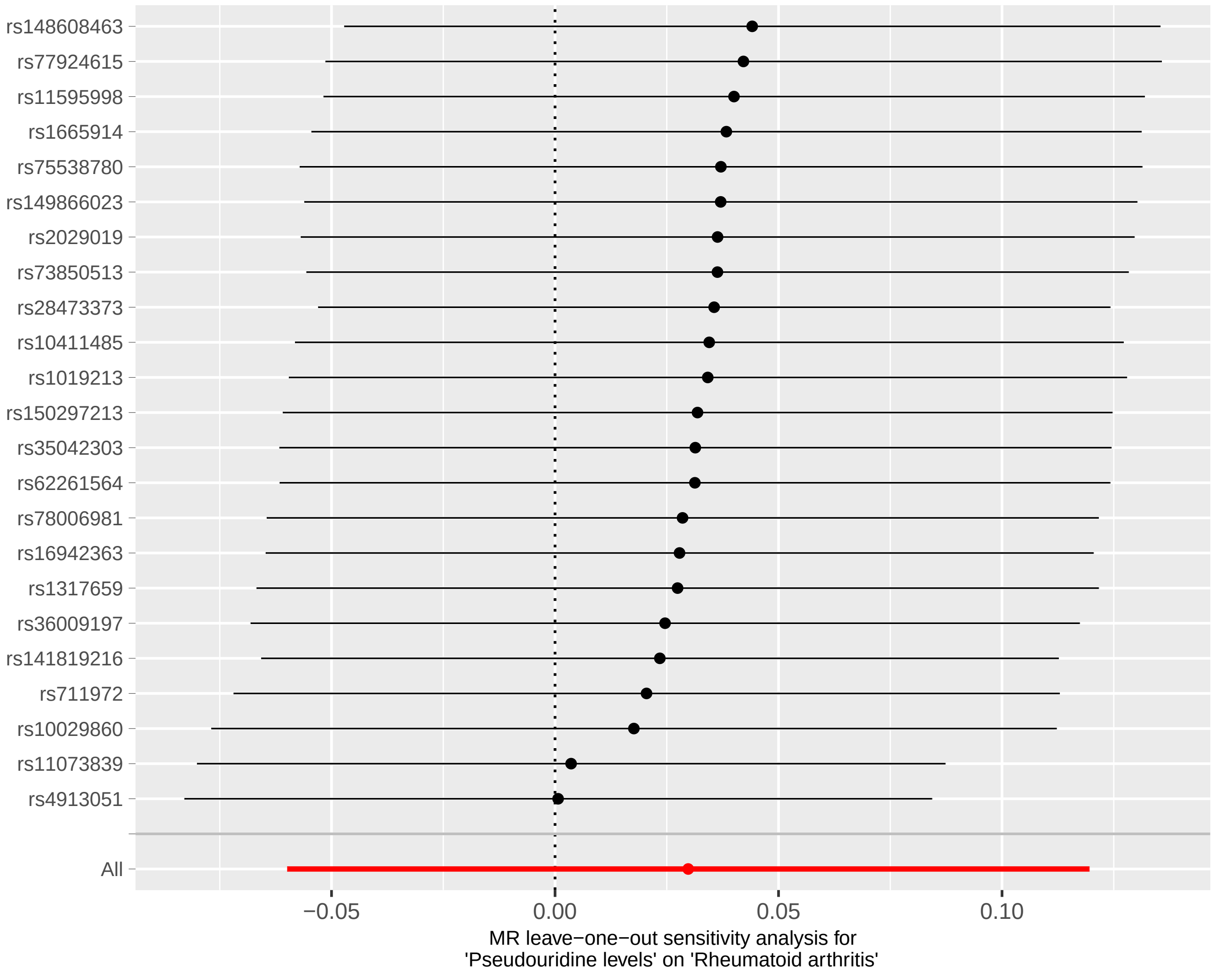

rs148608463
rs77924615
rs11595998
rs1665914
rs75538780
rs149866023
rs2029019
rs73850513
rs28473373
rs10411485
rs1019213
rs150297213
rs35042303
rs62261564
rs78006981
rs16942363
rs1317659
rs36009197
rs141819216
rs711972
rs10029860
rs11073839
rs4913051
All
−0.05
0.00
0.05
0.10
MR leave−one−out sensitivity analysis for
'Pseudouridine levels' on 'Rheumatoid arthritis'

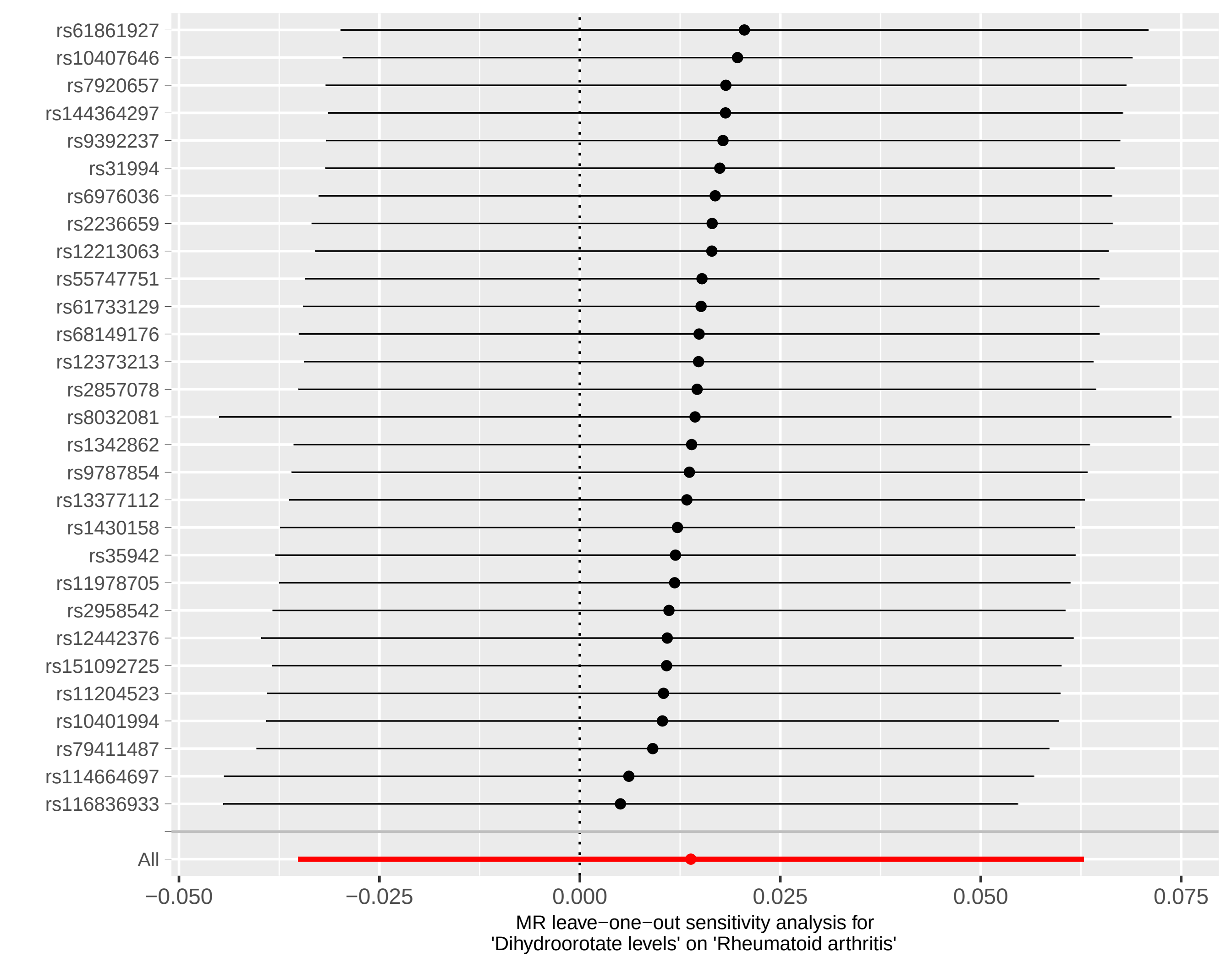

rs61861927
rs10407646
rs7920657
rs144364297
rs9392237
rs31994
rs6976036
rs2236659
rs12213063
rs55747751
rs61733129
rs68149176
rs12373213
rs2857078
rs8032081
rs1342862
rs9787854
rs13377112
rs1430158
rs35942
rs11978705
rs2958542
rs12442376
rs151092725
rs11204523
rs10401994
rs79411487
rs114664697
rs116836933
All
−0.050
−0.025
0.000
0.025
0.050
0.075
MR leave−one−out sensitivity analysis for
'Dihydroorotate levels' on 'Rheumatoid arthritis'

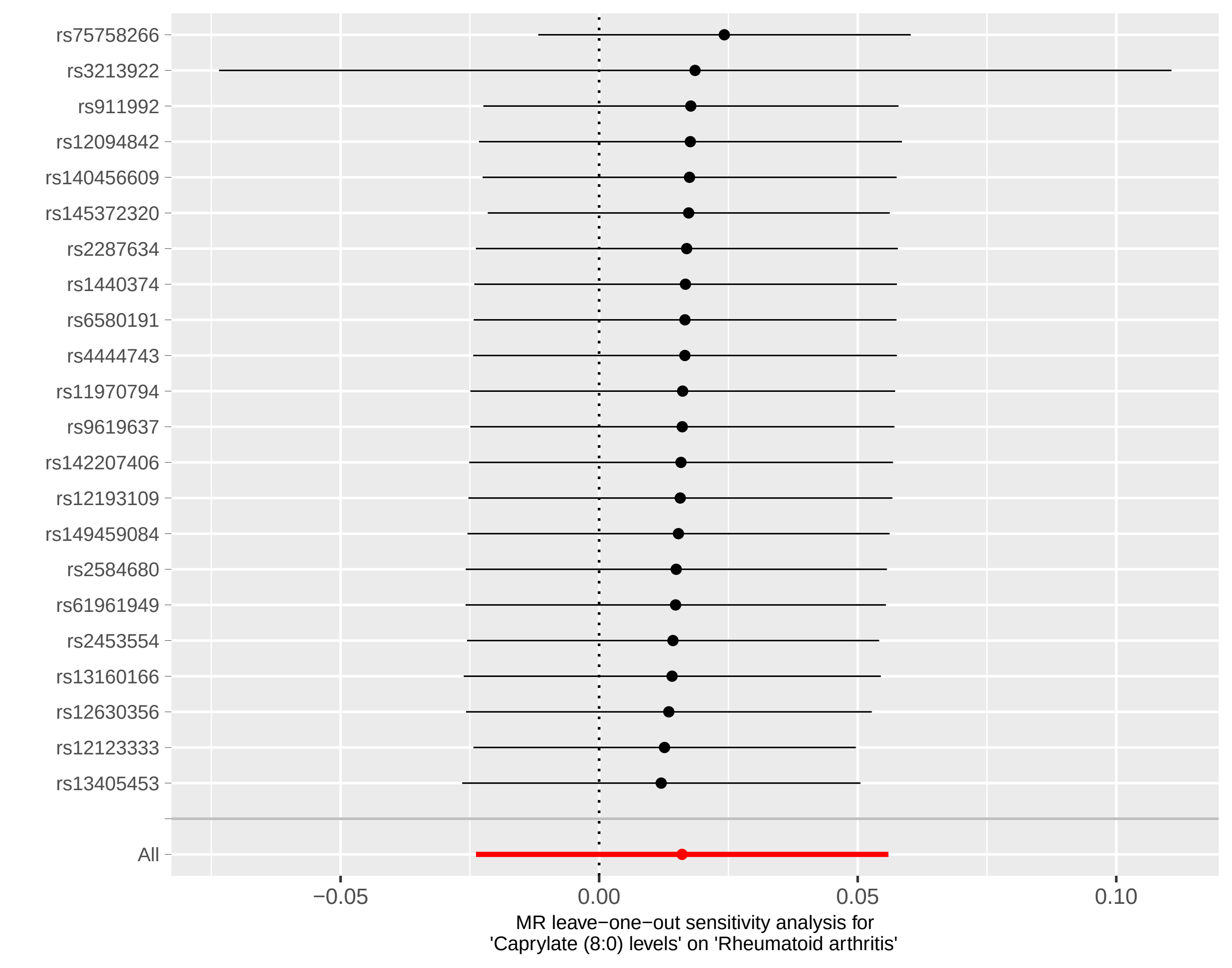
rs75758266
rs3213922
rs911992
rs12094842
rs140456609
rs145372320
rs2287634
rs1440374
rs6580191
rs4444743
rs11970794
rs9619637
rs142207406
rs12193109
rs149459084
rs2584680
rs61961949
rs2453554
rs13160166
rs12630356
rs12123333
rs13405453
All
−0.05
0.00
0.05
0.10
MR leave−one−out sensitivity analysis for
'Caprylate (8:0) levels' on 'Rheumatoid arthritis'

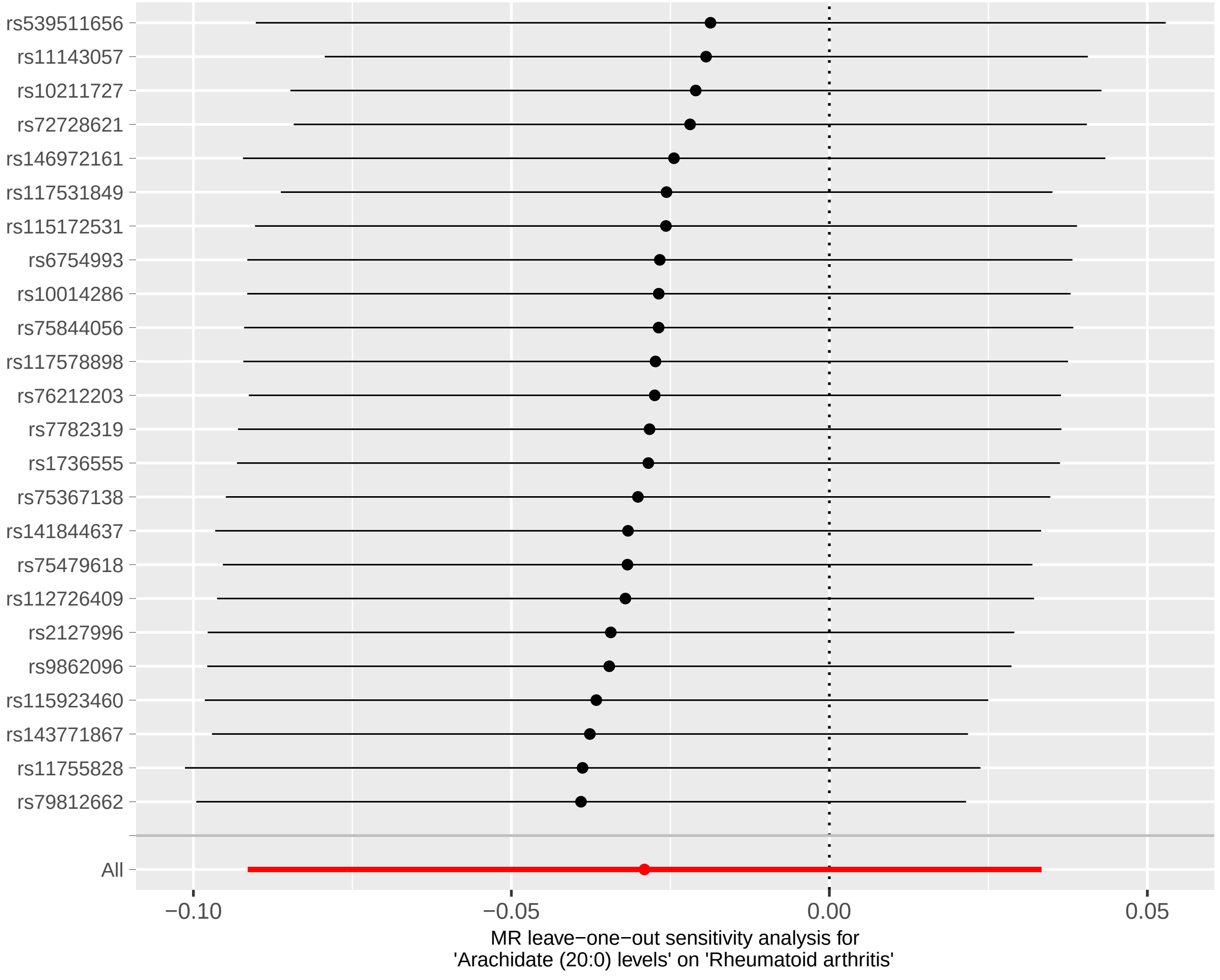
rs539511656
rs11143057
rs10211727
rs72728621
rs146972161
rs117531849
rs115172531
rs6754993
rs10014286
rs75844056
rs117578898
rs76212203
rs7782319
rs1736555
rs75367138
rs141844637
rs75479618
rs112726409
rs2127996
rs9862096
rs115923460
rs143771867
rs11755828
rs79812662
All
−0.10
−0.05
0.00
0.05
MR leave−one−out sensitivity analysis for
'Arachidate (20:0) levels' on 'Rheumatoid arthritis'

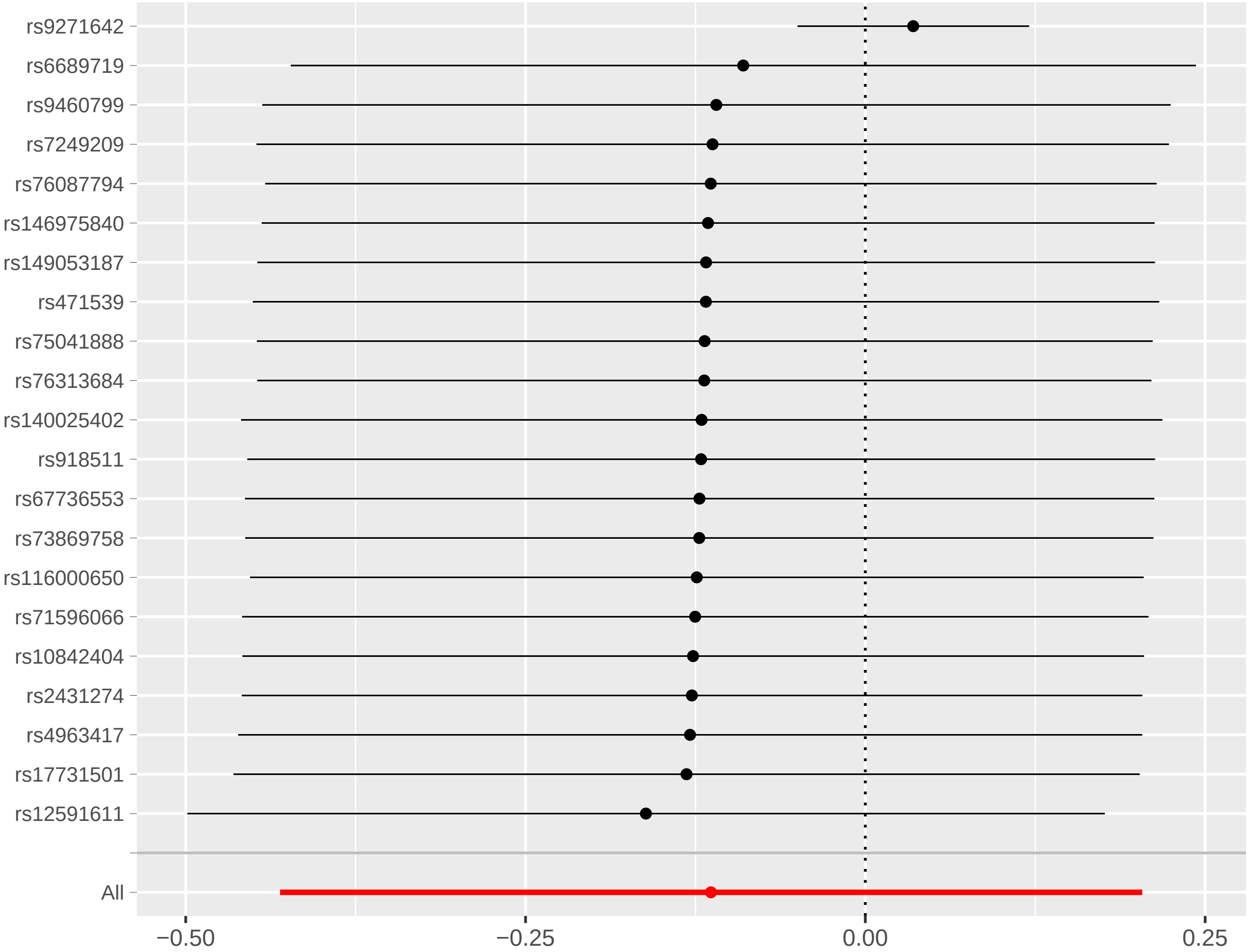

MR leave−one−out sensitivity analysis for
'Maltose levels in coronary artery disease' on 'Rheumatoid arthritis'

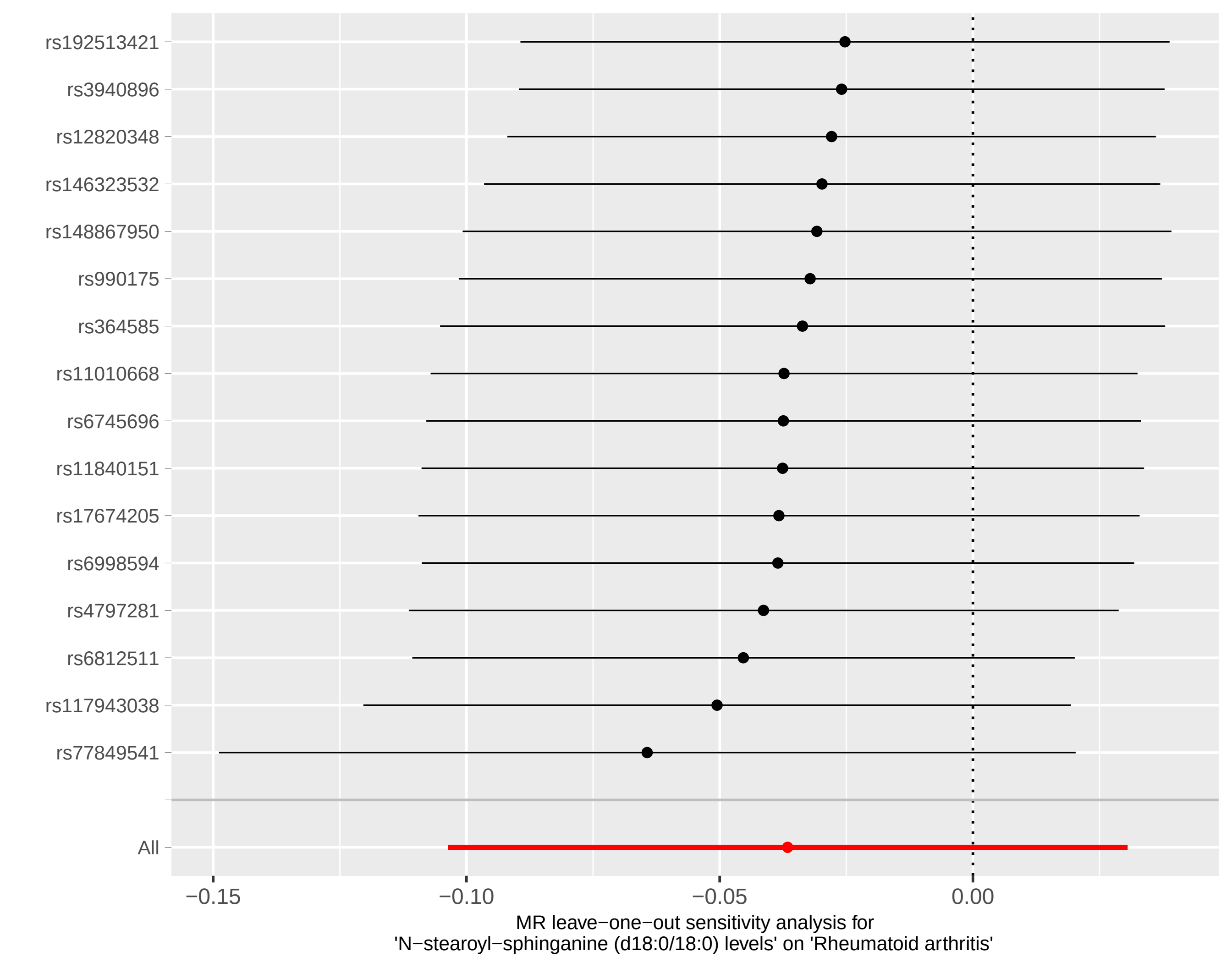
rs192513421
rs3940896
rs12820348
rs146323532
rs148867950
rs990175
rs364585
rs11010668
rs6745696
rs11840151
rs17674205
rs6998594
rs4797281
rs6812511
rs117943038
rs77849541
All
−0.15
−0.10
−0.05
0.00
MR leave−one−out sensitivity analysis for
'N−stearoyl−sphinganine (d18:0/18:0) levels' on 'Rheumatoid arthritis'

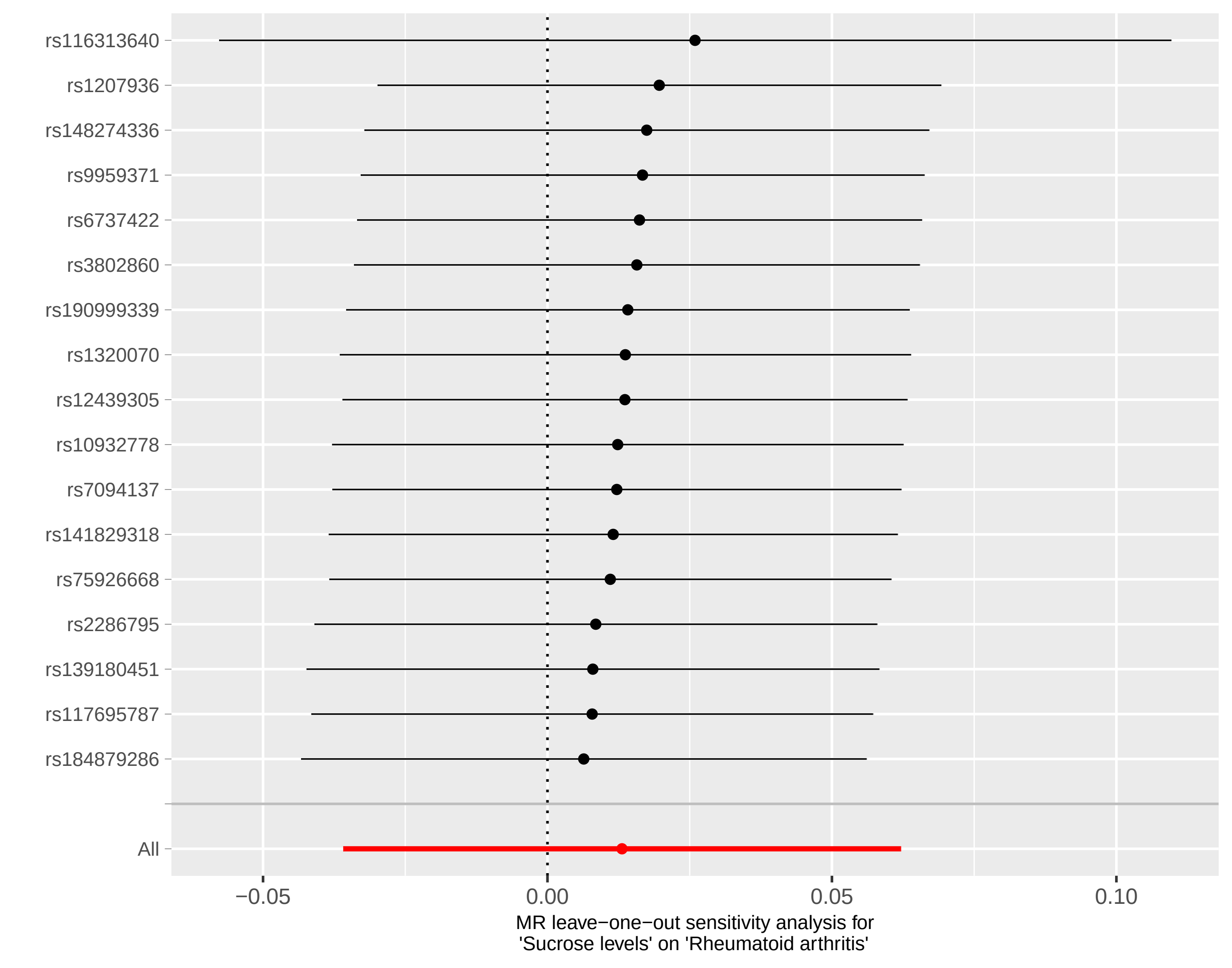
rs116313640
rs1207936
rs148274336
rs9959371
rs6737422
rs3802860
rs190999339
rs1320070
rs12439305
rs10932778
rs7094137
rs141829318
rs75926668
rs2286795
rs139180451
rs117695787
rs184879286
All
−0.05
0.00
0.05
0.10
MR leave−one−out sensitivity analysis for
'Sucrose levels' on 'Rheumatoid arthritis'

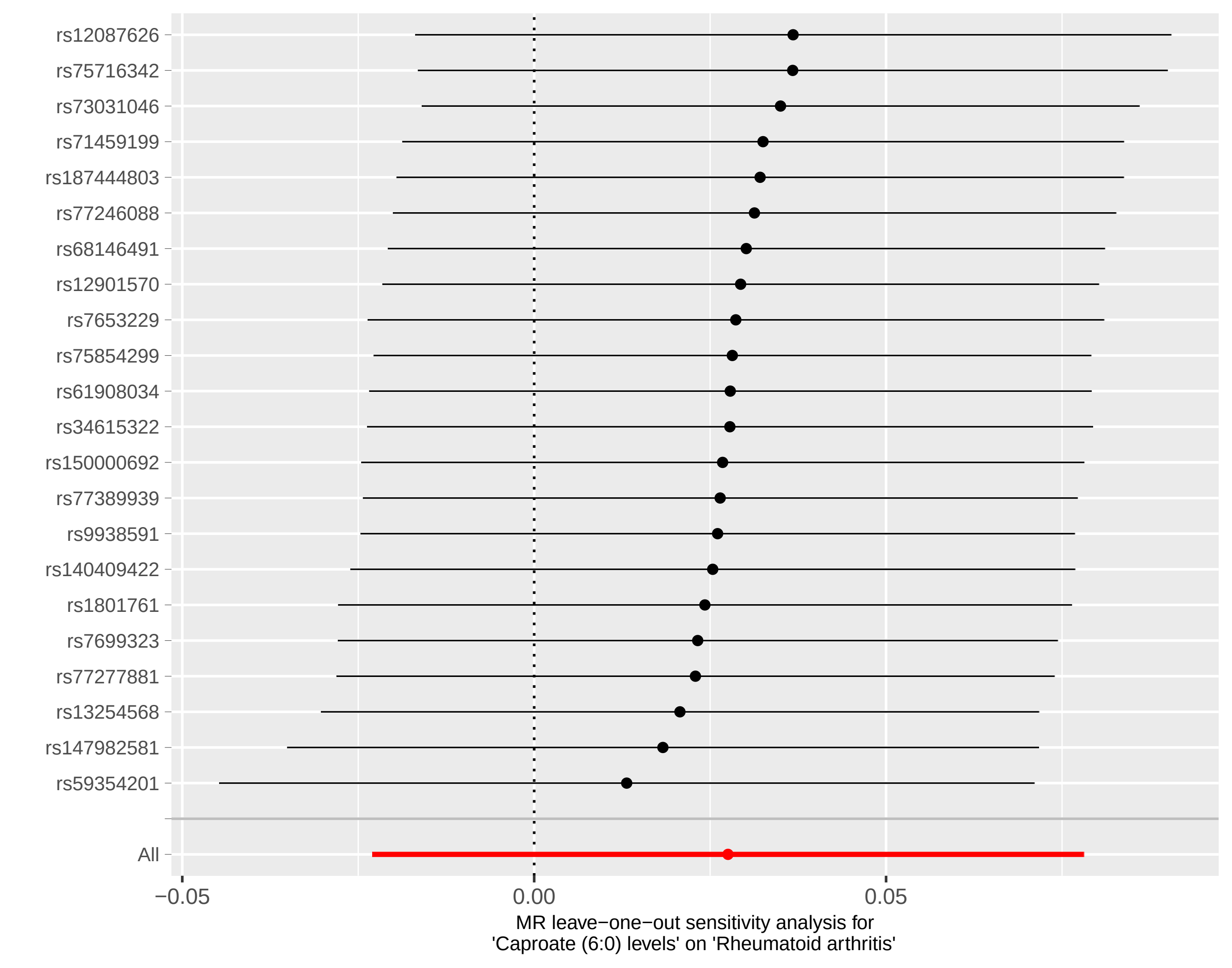
rs12087626
rs75716342
rs73031046
rs71459199
rs187444803
rs77246088
rs68146491
rs12901570
rs7653229
rs75854299
rs61908034
rs34615322
rs150000692
rs77389939
rs9938591
rs140409422
rs1801761
rs7699323
rs77277881
rs13254568
rs147982581
rs59354201
All
−0.05
0.00
0.05
MR leave−one−out sensitivity analysis for
'Caproate (6:0) levels' on 'Rheumatoid arthritis'

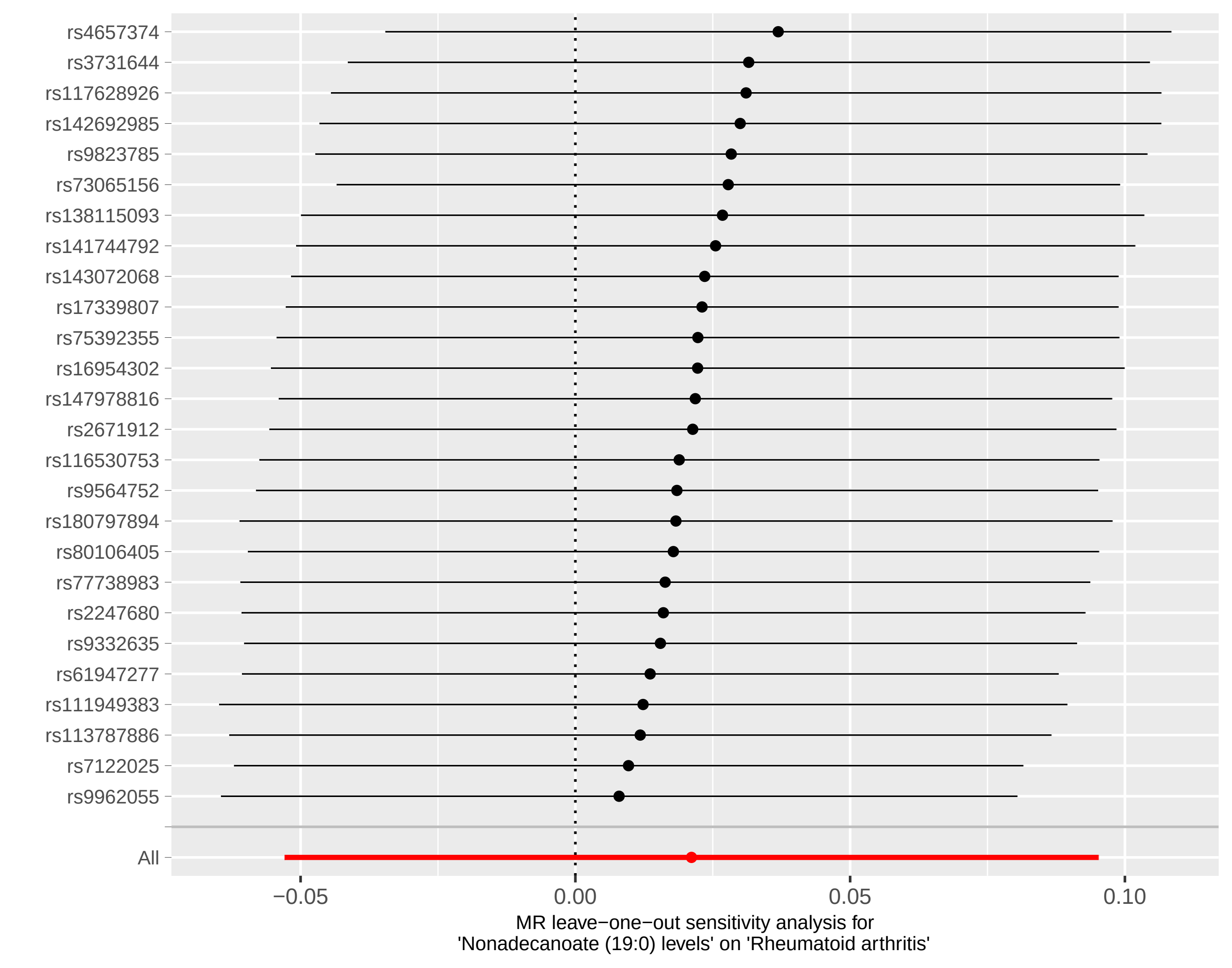
rs4657374
rs3731644
rs117628926
rs142692985
rs9823785
rs73065156
rs138115093
rs141744792
rs143072068
rs17339807
rs75392355
rs16954302
rs147978816
rs2671912
rs116530753
rs9564752
rs180797894
rs80106405
rs77738983
rs2247680
rs9332635
rs61947277
rs111949383
rs113787886
rs7122025
rs9962055
All
−0.05
0.00
0.05
0.10
MR leave−one−out sensitivity analysis for
'Nonadecanoate (19:0) levels' on 'Rheumatoid arthritis'

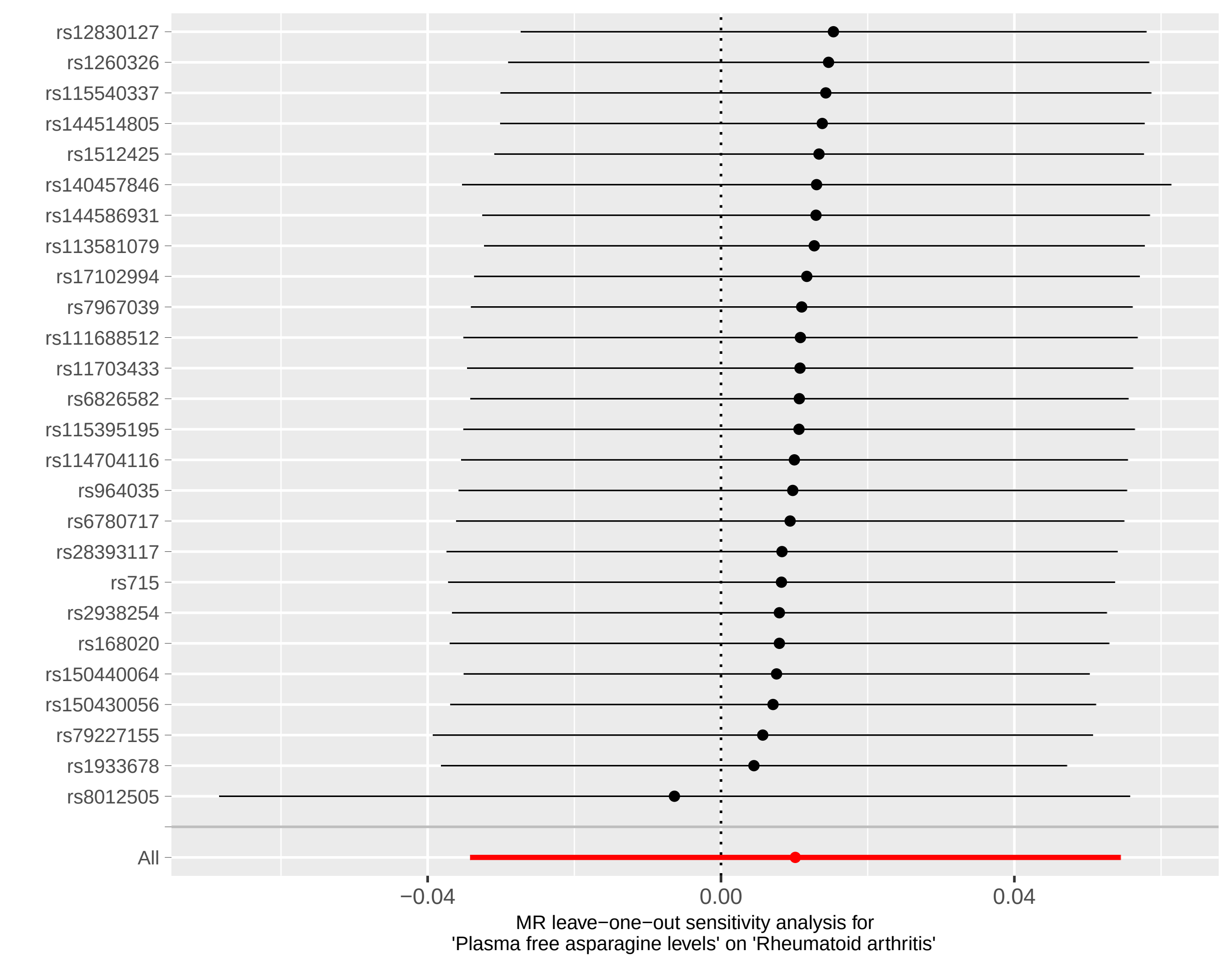
rs12830127
rs1260326
rs115540337
rs144514805
rs1512425
rs140457846
rs144586931
rs113581079
rs17102994
rs7967039
rs111688512
rs11703433
rs6826582
rs115395195
rs114704116
rs964035
rs6780717
rs28393117
rs715
rs2938254
rs168020
rs150440064
rs150430056
rs79227155
rs1933678
rs8012505
All
−0.04
0.00
0.04
MR leave−one−out sensitivity analysis for
'Plasma free asparagine levels' on 'Rheumatoid arthritis'

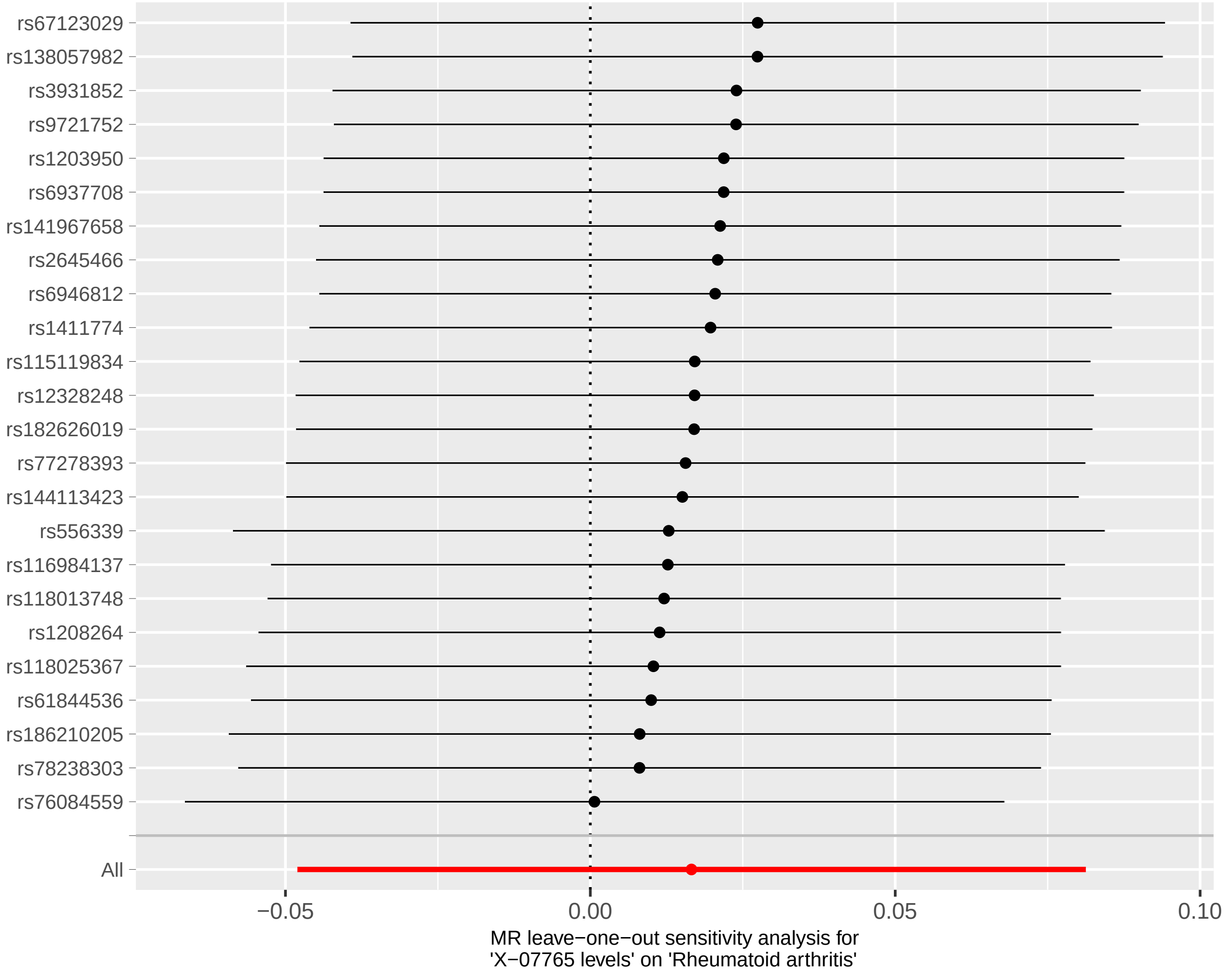

rs67123029
rs138057982
rs3931852
rs9721752
rs1203950
rs6937708
rs141967658
rs2645466
rs6946812
rs1411774
rs115119834
rs12328248
rs182626019
rs77278393
rs144113423
rs556339
rs116984137
rs118013748
rs1208264
rs118025367
rs61844536
rs186210205
rs78238303
rs76084559
All
−0.05
0.00
0.05
0.10
MR leave−one−out sensitivity analysis for
'X−07765 levels' on 'Rheumatoid arthritis'

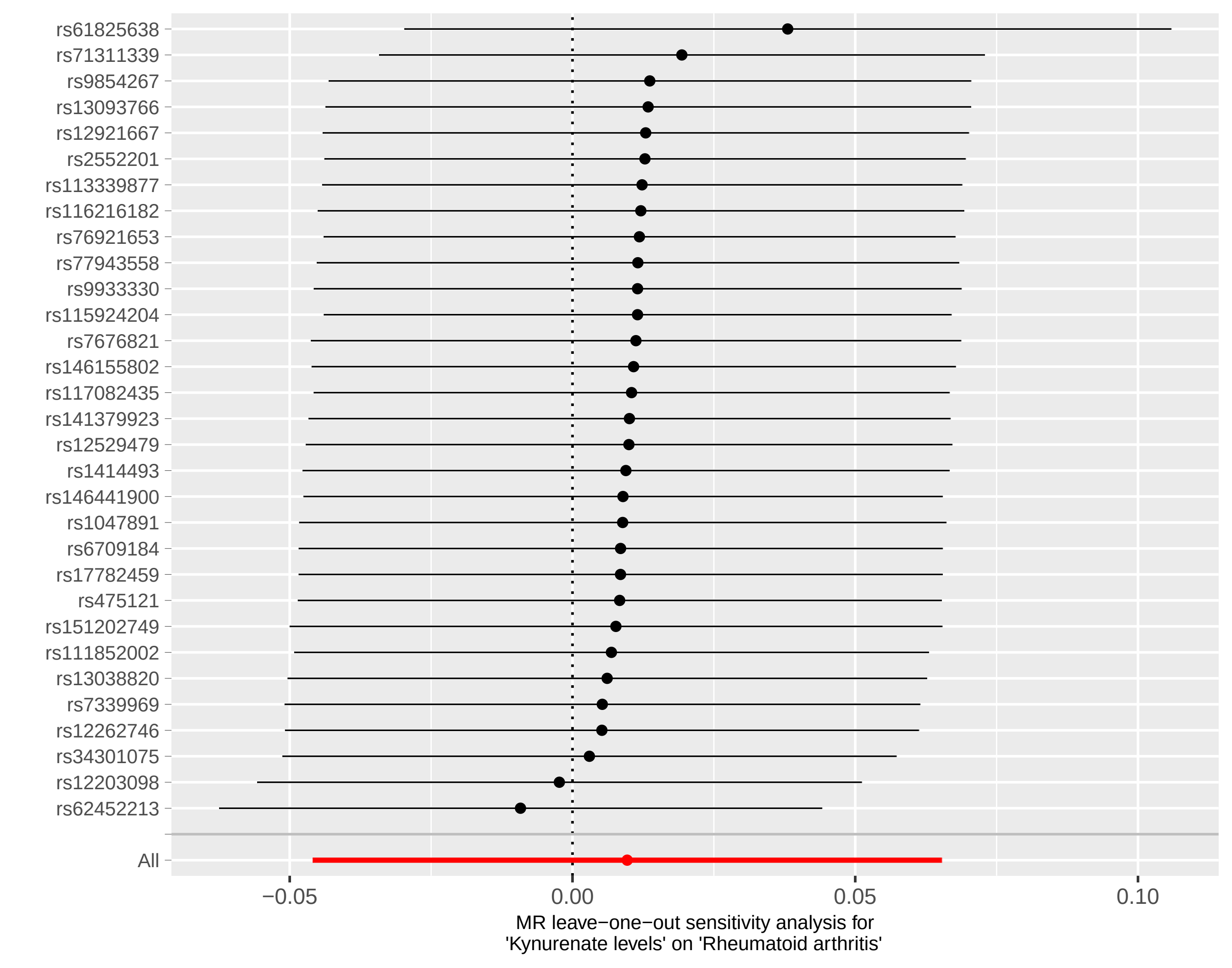

rs61825638
rs71311339
rs9854267
rs13093766
rs12921667
rs2552201
rs113339877
rs116216182
rs76921653
rs77943558
rs9933330
rs115924204
rs7676821
rs146155802
rs117082435
rs141379923
rs12529479
rs1414493
rs146441900
rs1047891
rs6709184
rs17782459
rs475121
rs151202749
rs111852002
rs13038820
rs7339969
rs12262746
rs34301075
rs12203098
rs62452213
All
−0.05
0.00
0.05
0.10
MR leave−one−out sensitivity analysis for
'Kynurenate levels' on 'Rheumatoid arthritis'

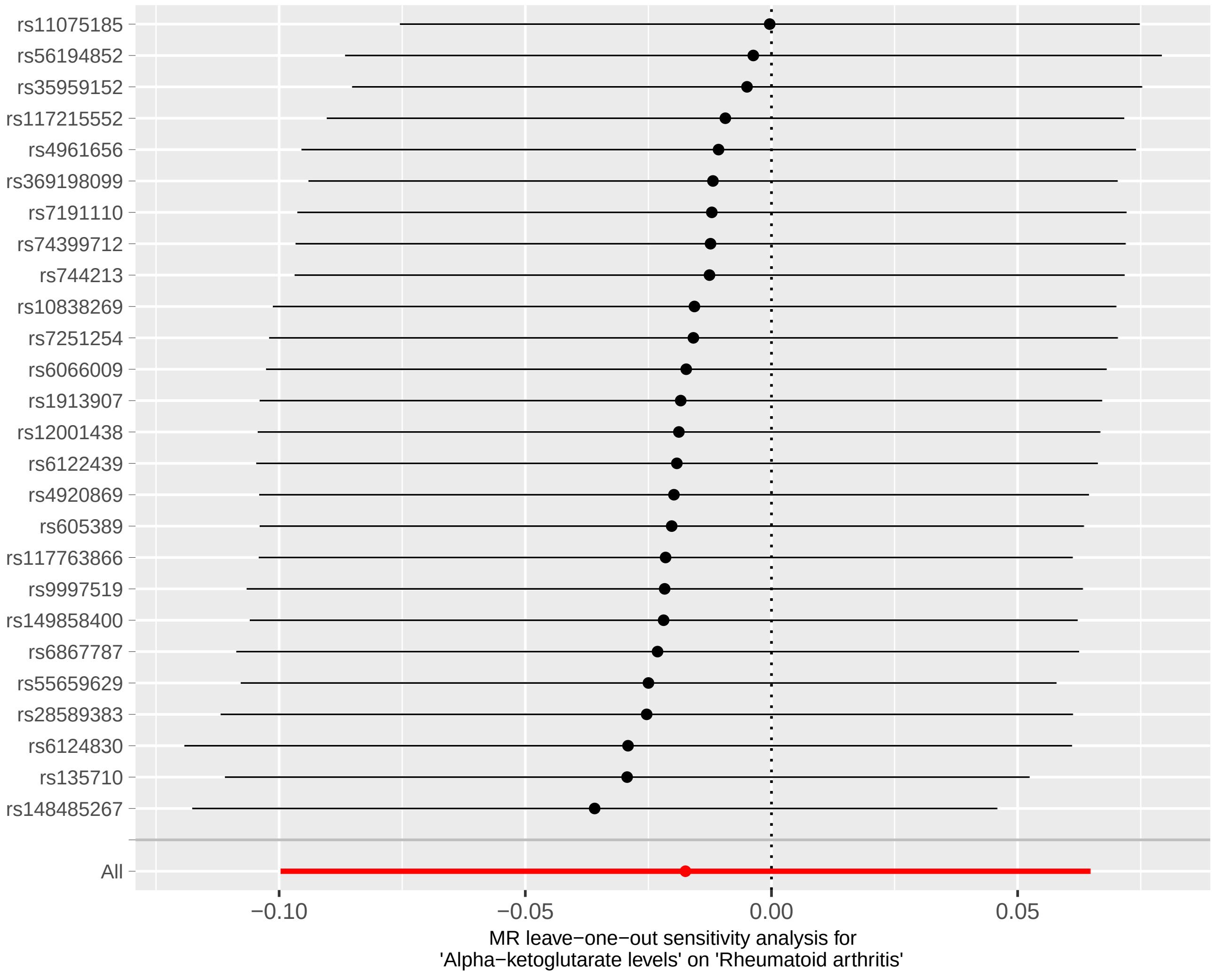
rs11075185
rs56194852
rs35959152
rs117215552
rs4961656
rs369198099
rs7191110
rs74399712
rs744213
rs10838269
rs7251254
rs6066009
rs1913907
rs12001438
rs6122439
rs4920869
rs605389
rs117763866
rs9997519
rs149858400
rs6867787
rs55659629
rs28589383
rs6124830
rs135710
rs148485267
All
−0.10
−0.05
0.00
0.05
MR leave−one−out sensitivity analysis for
'Alpha−ketoglutarate levels' on 'Rheumatoid arthritis'

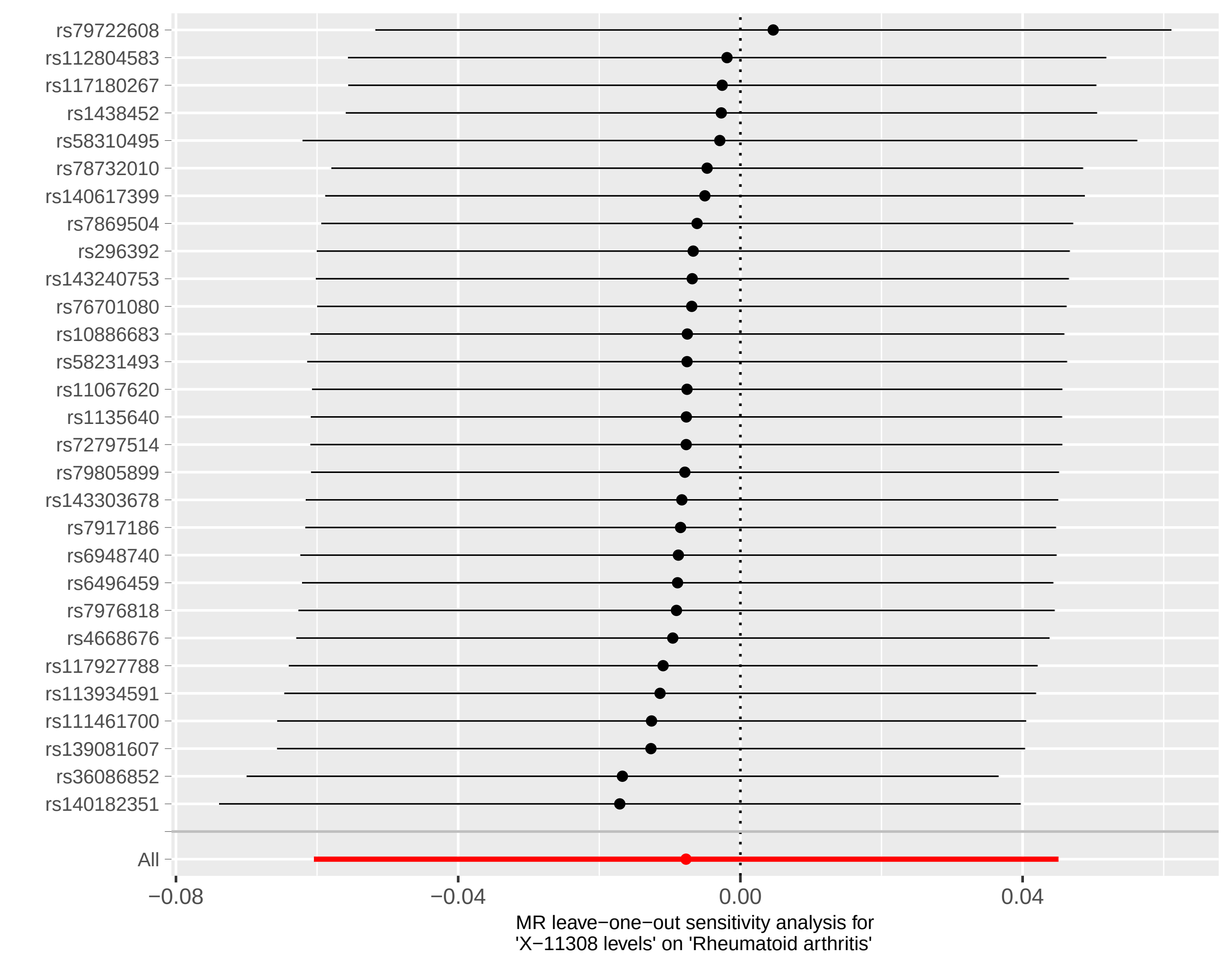
rs79722608
rs112804583
rs117180267
rs1438452
rs58310495
rs78732010
rs140617399
rs7869504
rs296392
rs143240753
rs76701080
rs10886683
rs58231493
rs11067620
rs1135640
rs72797514
rs79805899
rs143303678
rs7917186
rs6948740
rs6496459
rs7976818
rs4668676
rs117927788
rs113934591
rs111461700
rs139081607
rs36086852
rs140182351
All
−0.08
−0.04
0.00
0.04
MR leave−one−out sensitivity analysis for
'X−11308 levels' on 'Rheumatoid arthritis'

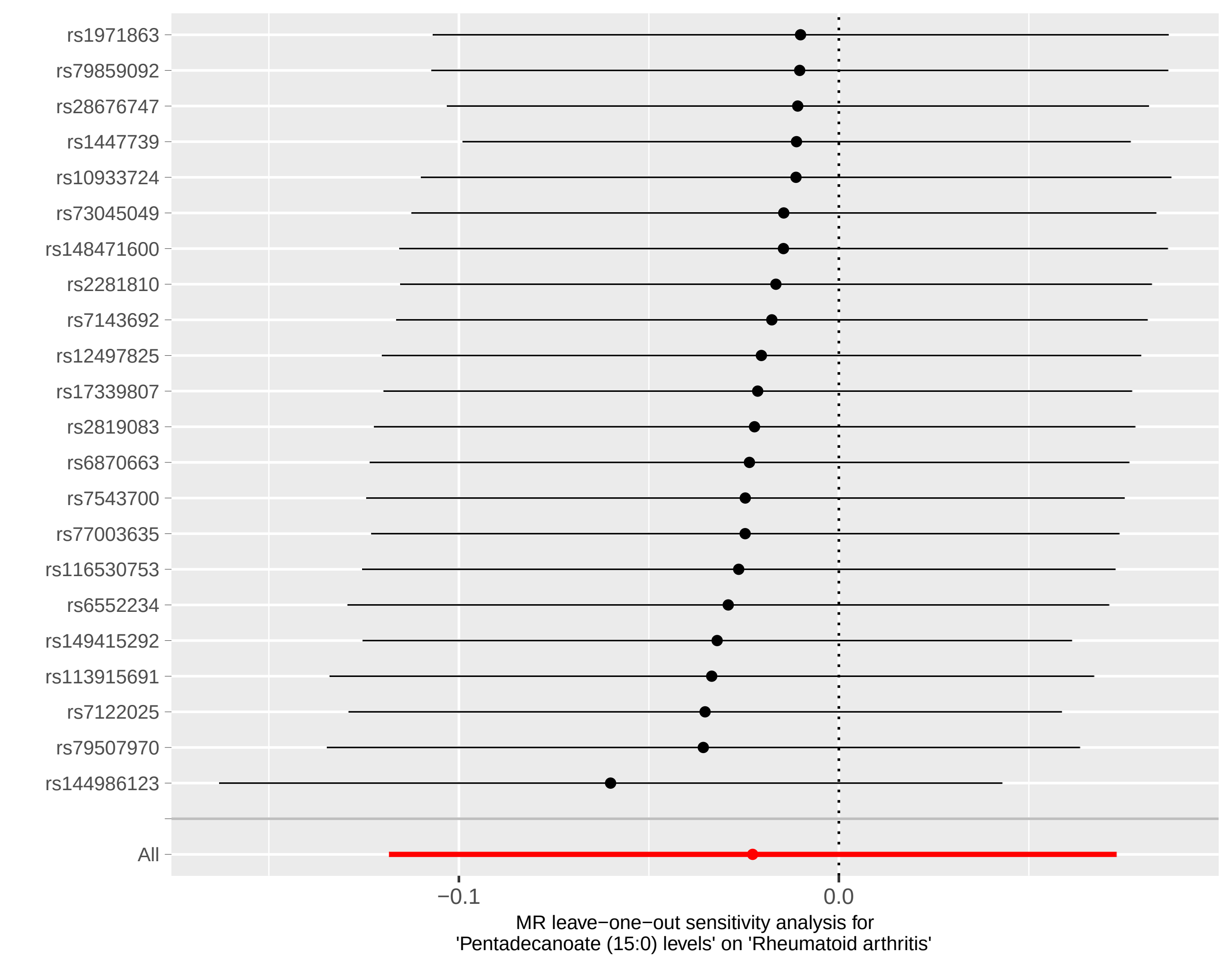
rs1971863
rs79859092
rs28676747
rs1447739
rs10933724
rs73045049
rs148471600
rs2281810
rs7143692
rs12497825
rs17339807
rs2819083
rs6870663
rs7543700
rs77003635
rs116530753
rs6552234
rs149415292
rs113915691
rs7122025
rs79507970
rs144986123
All
−0.1
0.0
MR leave−one−out sensitivity analysis for
'Pentadecanoate (15:0) levels' on 'Rheumatoid arthritis'

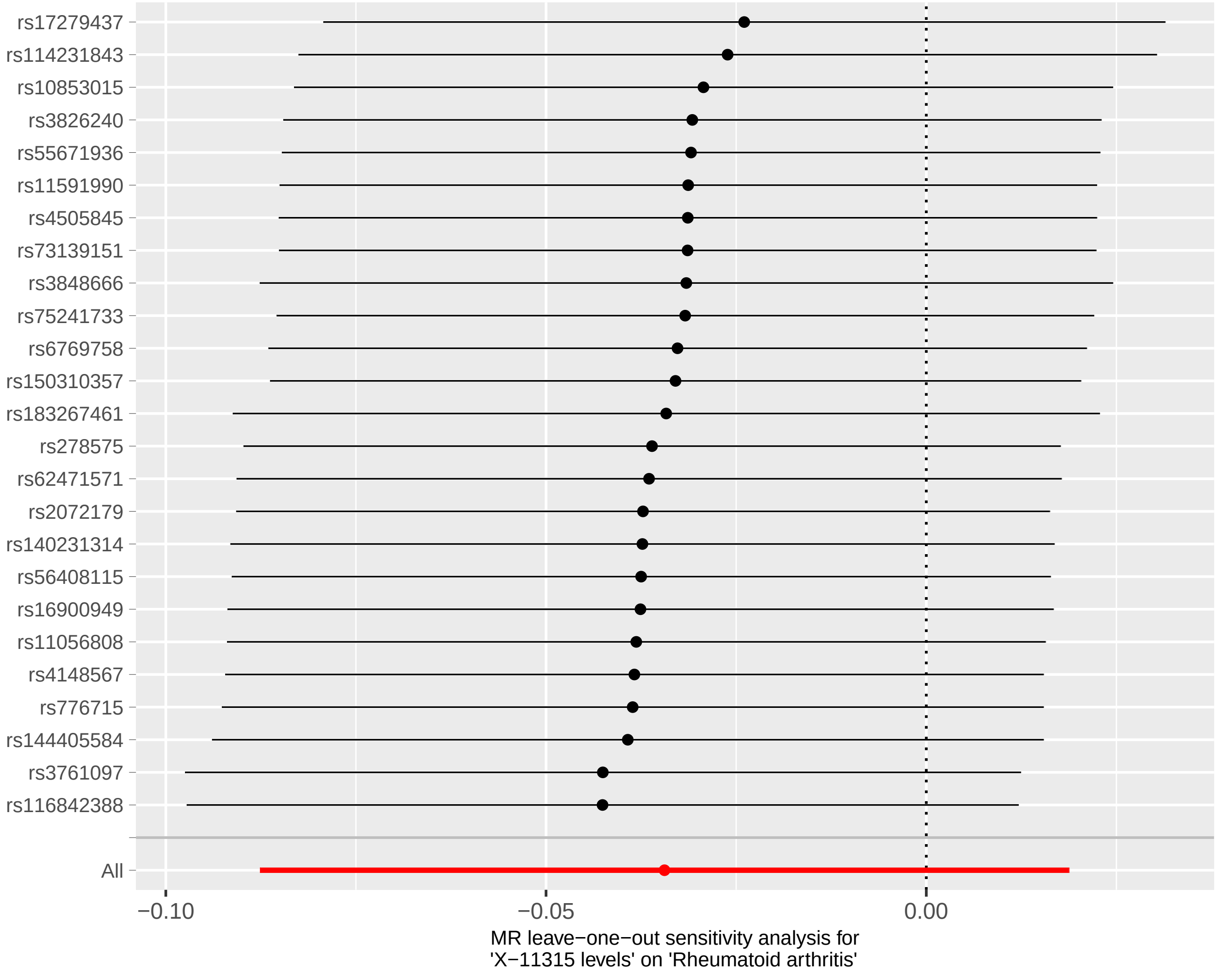
rs17279437
rs114231843
rs10853015
rs3826240
rs55671936
rs11591990
rs4505845
rs73139151
rs3848666
rs75241733
rs6769758
rs150310357
rs183267461
rs278575
rs62471571
rs2072179
rs140231314
rs56408115
rs16900949
rs11056808
rs4148567
rs776715
rs144405584
rs3761097
rs116842388
All
−0.10
−0.05
0.00
MR leave−one−out sensitivity analysis for
'X−11315 levels' on 'Rheumatoid arthritis'

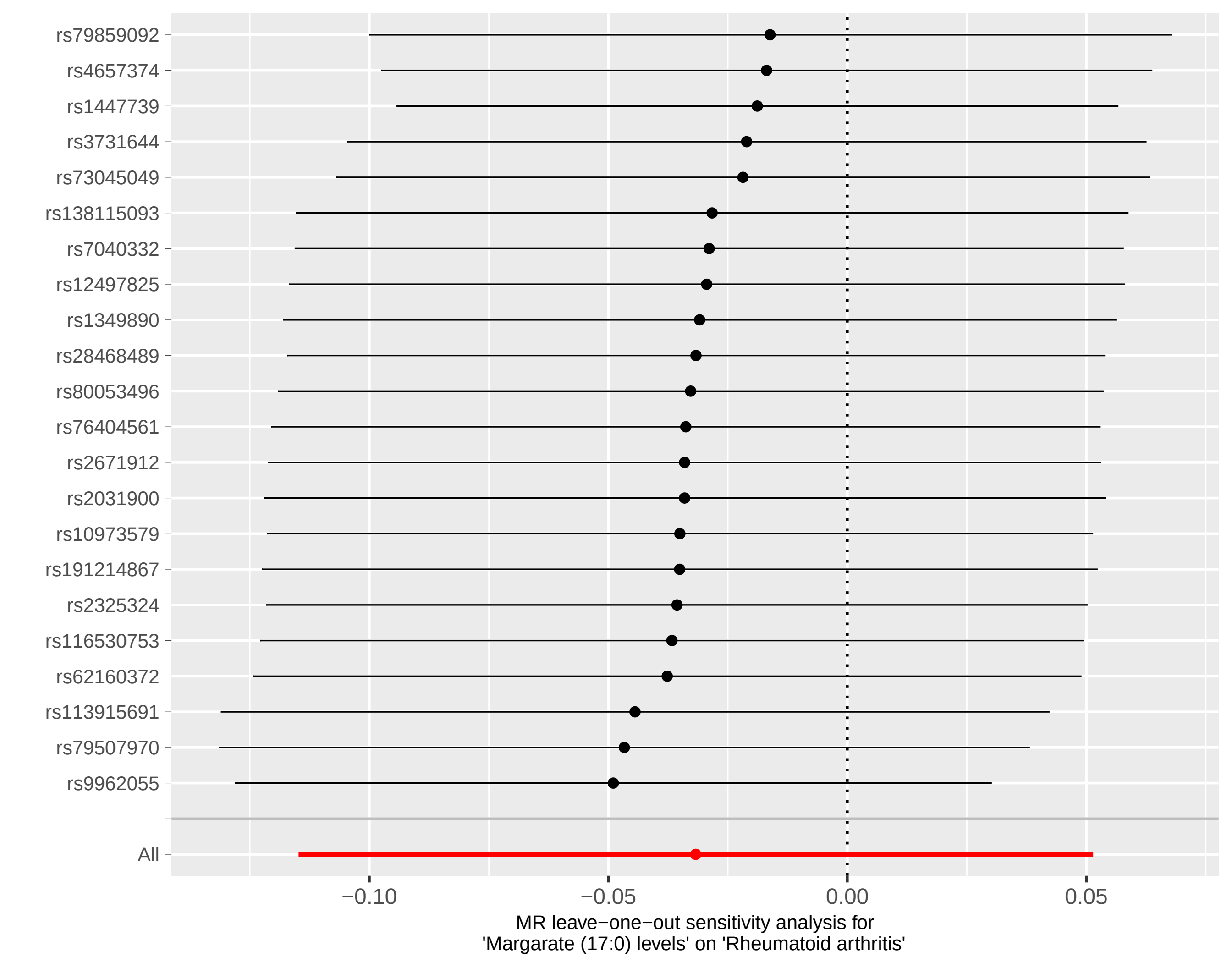
rs79859092
rs4657374
rs1447739
rs3731644
rs73045049
rs138115093
rs7040332
rs12497825
rs1349890
rs28468489
rs80053496
rs76404561
rs2671912
rs2031900
rs10973579
rs191214867
rs2325324
rs116530753
rs62160372
rs113915691
rs79507970
rs9962055
All
−0.10
−0.05
0.00
0.05
MR leave−one−out sensitivity analysis for
'Margarate (17:0) levels' on 'Rheumatoid arthritis'

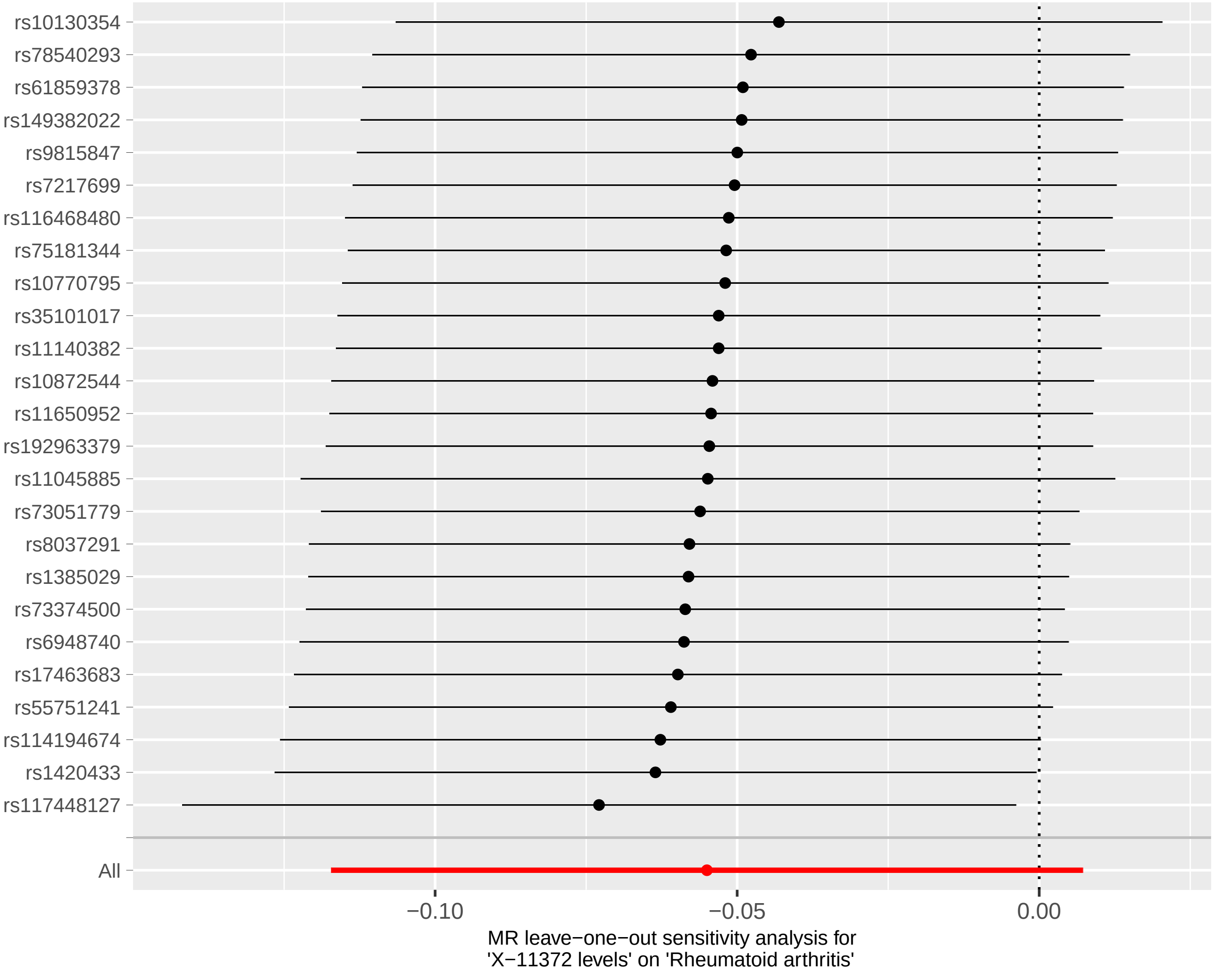

rs10130354
rs78540293
rs61859378
rs149382022
rs9815847
rs7217699
rs116468480
rs75181344
rs10770795
rs35101017
rs11140382
rs10872544
rs11650952
rs192963379
rs11045885
rs73051779
rs8037291
rs1385029
rs73374500
rs6948740
rs17463683
rs55751241
rs114194674
rs1420433
rs117448127
All
−0.10
−0.05
0.00
MR leave−one−out sensitivity analysis for
'X−11372 levels' on 'Rheumatoid arthritis'

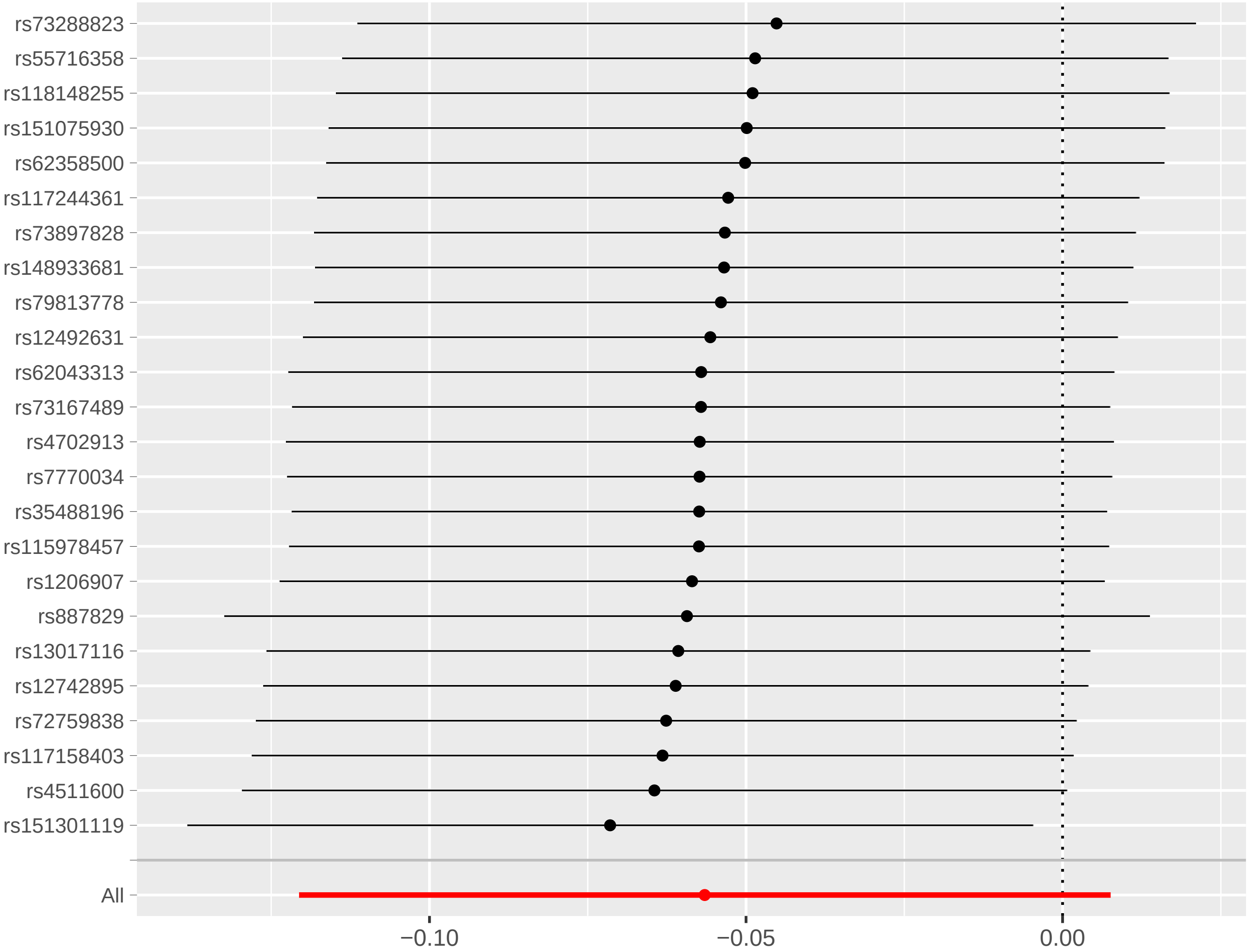

MR leave−one−out sensitivity analysis for
'X−10458 levels' on 'Rheumatoid arthritis'

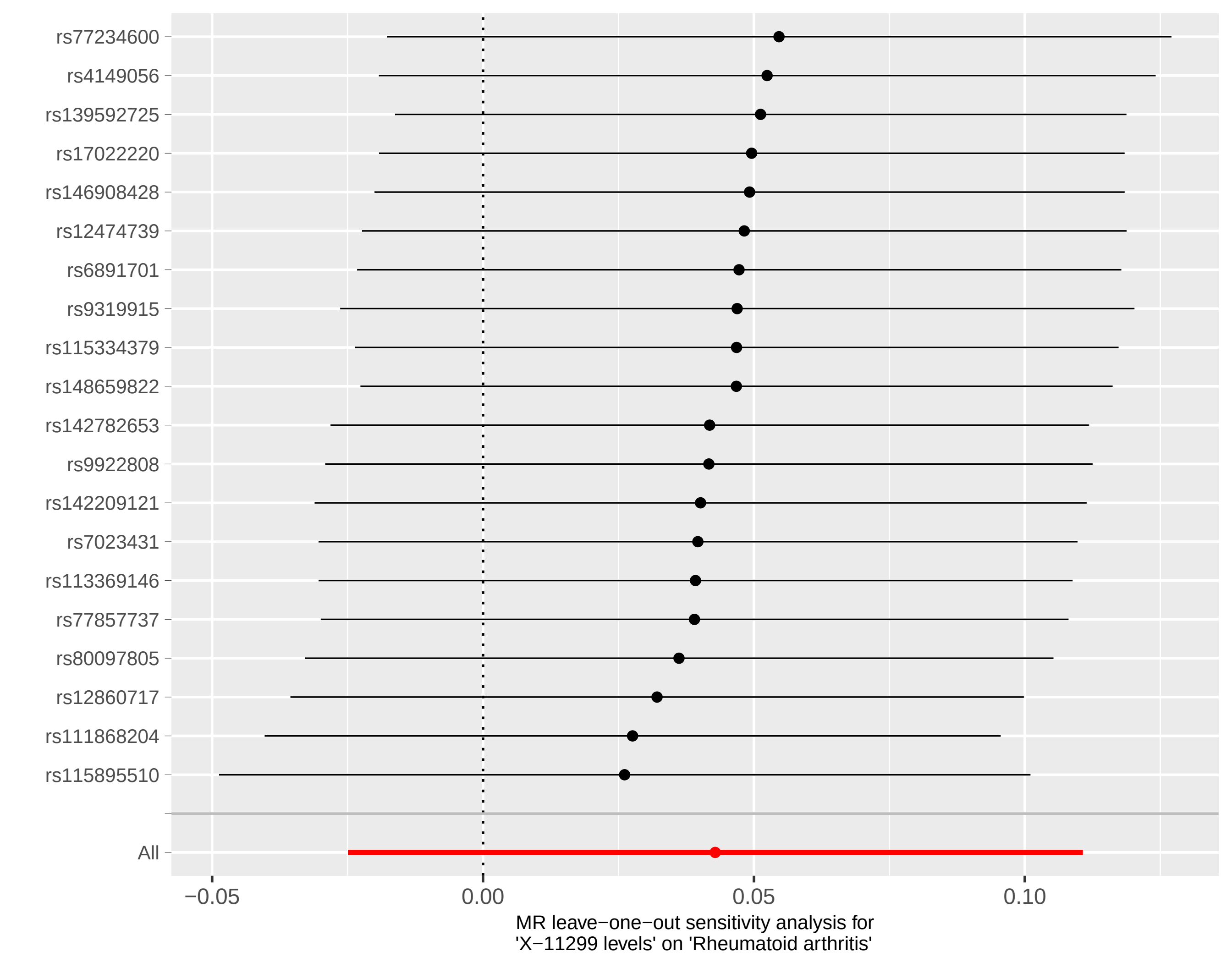

rs77234600
rs4149056
rs139592725
rs17022220
rs146908428
rs12474739
rs6891701
rs9319915
rs115334379
rs148659822
rs142782653
rs9922808
rs142209121
rs7023431
rs113369146
rs77857737
rs80097805
rs12860717
rs111868204
rs115895510
All
−0.05
0.00
0.05
0.10
MR leave−one−out sensitivity analysis for
'X−11299 levels' on 'Rheumatoid arthritis'

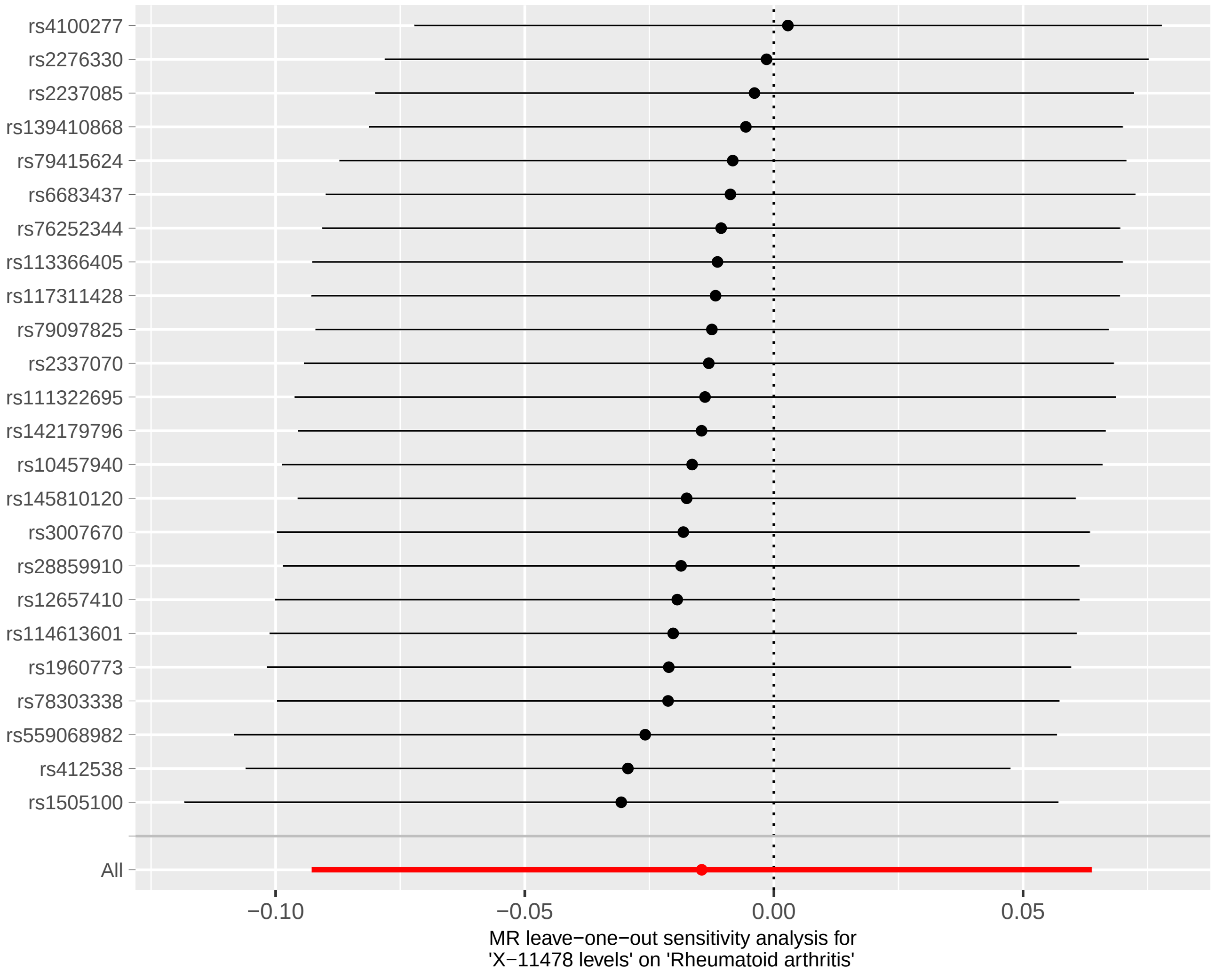

rs4100277
rs2276330
rs2237085
rs139410868
rs79415624
rs6683437
rs76252344
rs113366405
rs117311428
rs79097825
rs2337070
rs111322695
rs142179796
rs10457940
rs145810120
rs3007670
rs28859910
rs12657410
rs114613601
rs1960773
rs78303338
rs559068982
rs412538
rs1505100
All
−0.10
−0.05
0.00
0.05
MR leave−one−out sensitivity analysis for
'X−11478 levels' on 'Rheumatoid arthritis'

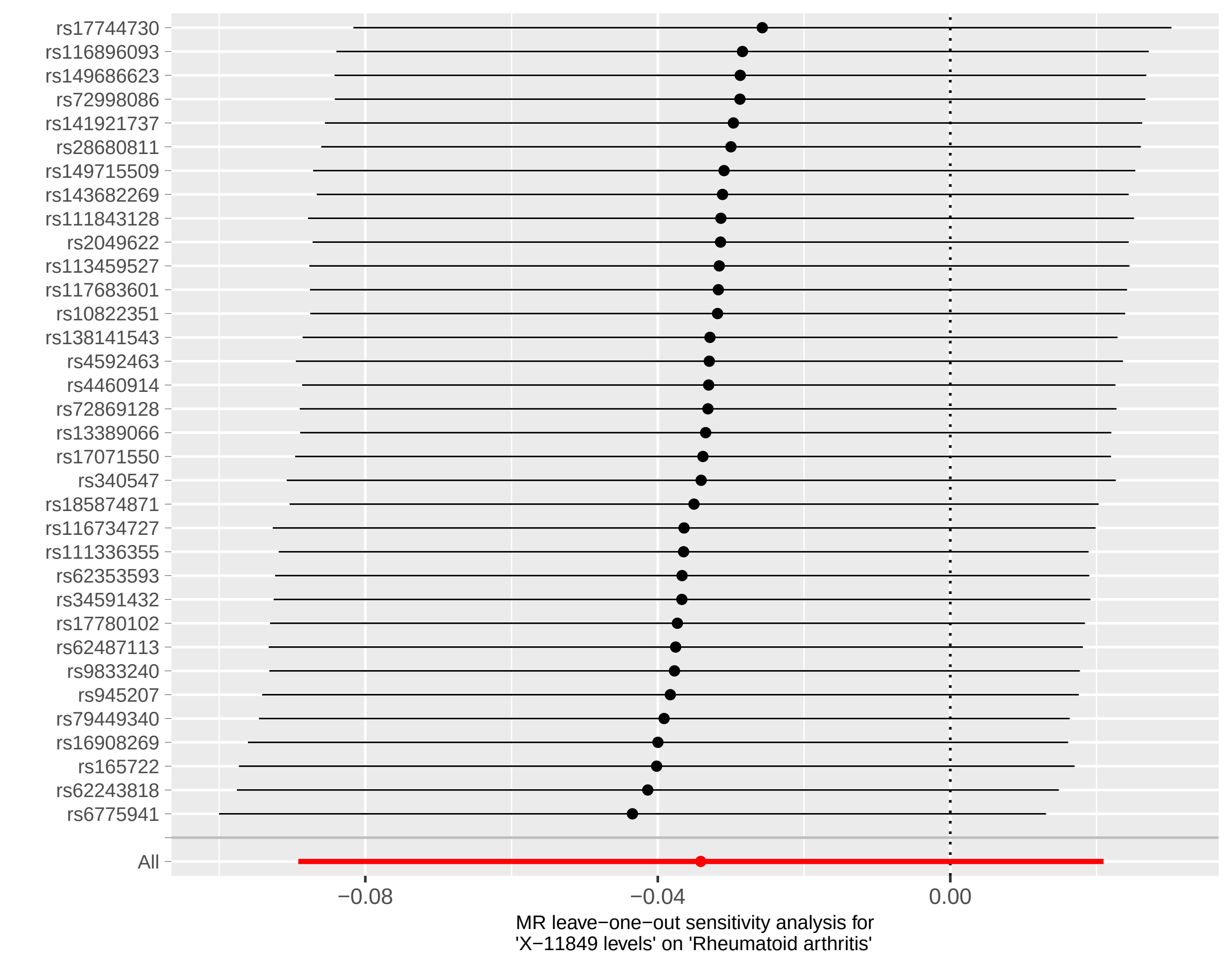
rs17744730
rs116896093
rs149686623
rs72998086
rs141921737
rs28680811
rs149715509
rs143682269
rs111843128
rs2049622
rs113459527
rs117683601
rs10822351
rs138141543
rs4592463
rs4460914
rs72869128
rs13389066
rs17071550
rs340547
rs185874871
rs116734727
rs111336355
rs62353593
rs34591432
rs17780102
rs62487113
rs9833240
rs945207
rs79449340
rs16908269
rs165722
rs62243818
rs6775941
All
−0.08
−0.04
0.00
MR leave−one−out sensitivity analysis for
'X−11849 levels' on 'Rheumatoid arthritis'

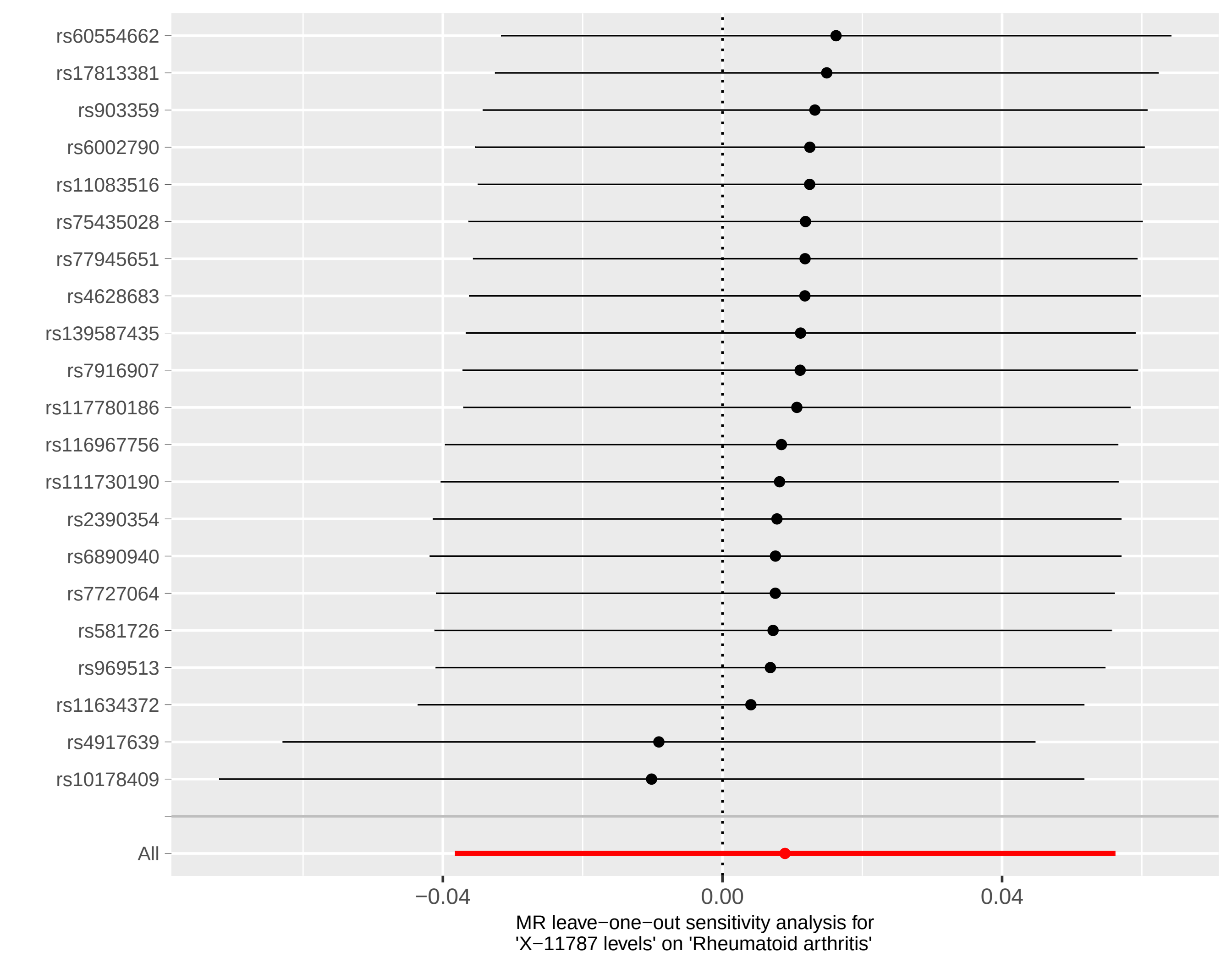
rs60554662
rs17813381
rs903359
rs6002790
rs11083516
rs75435028
rs77945651
rs4628683
rs139587435
rs7916907
rs117780186
rs116967756
rs111730190
rs2390354
rs6890940
rs7727064
rs581726
rs969513
rs11634372
rs4917639
rs10178409
All
−0.04
0.00
0.04
MR leave−one−out sensitivity analysis for
'X−11787 levels' on 'Rheumatoid arthritis'

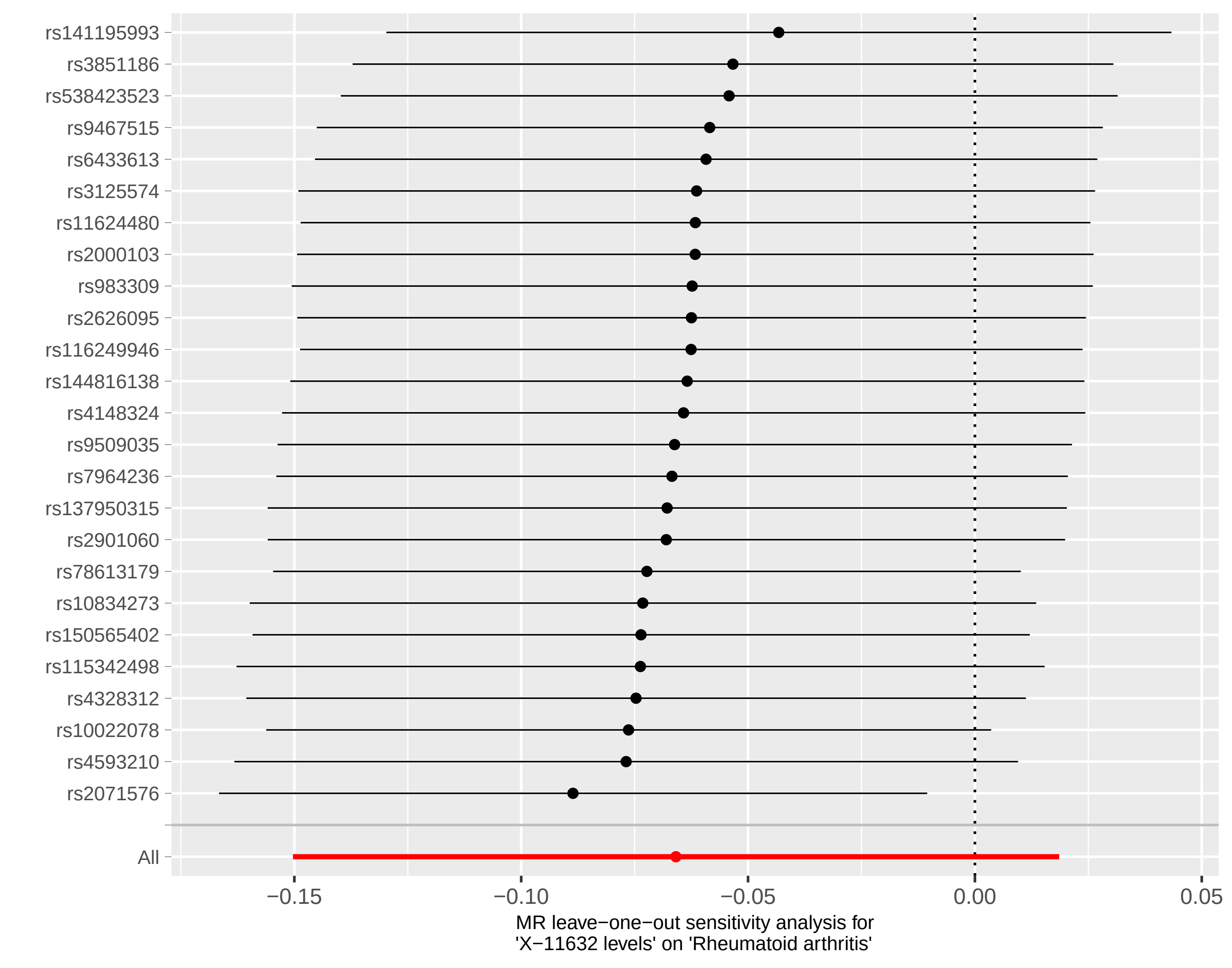

rs141195993
rs3851186
rs538423523
rs9467515
rs6433613
rs3125574
rs11624480
rs2000103
rs983309
rs2626095
rs116249946
rs144816138
rs4148324
rs9509035
rs7964236
rs137950315
rs2901060
rs78613179
rs10834273
rs150565402
rs115342498
rs4328312
rs10022078
rs4593210
rs2071576
All
−0.15
−0.10
−0.05
0.00
0.05
MR leave−one−out sensitivity analysis for
'X−11632 levels' on 'Rheumatoid arthritis'

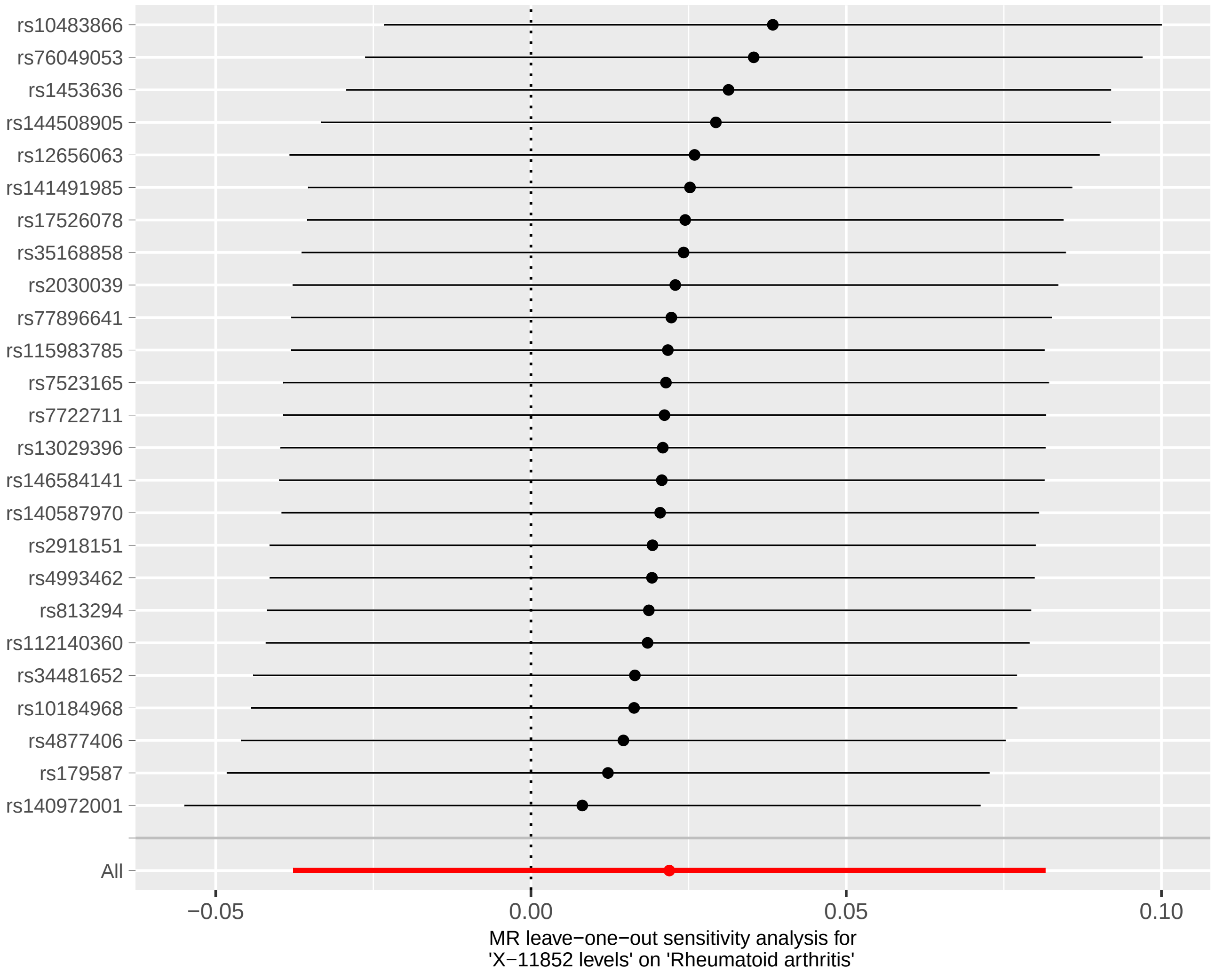
rs10483866
rs76049053
rs1453636
rs144508905
rs12656063
rs141491985
rs17526078
rs35168858
rs2030039
rs77896641
rs115983785
rs7523165
rs7722711
rs13029396
rs146584141
rs140587970
rs2918151
rs4993462
rs813294
rs112140360
rs34481652
rs10184968
rs4877406
rs179587
rs140972001
All
−0.05
0.00
0.05
0.10
MR leave−one−out sensitivity analysis for
'X−11852 levels' on 'Rheumatoid arthritis'

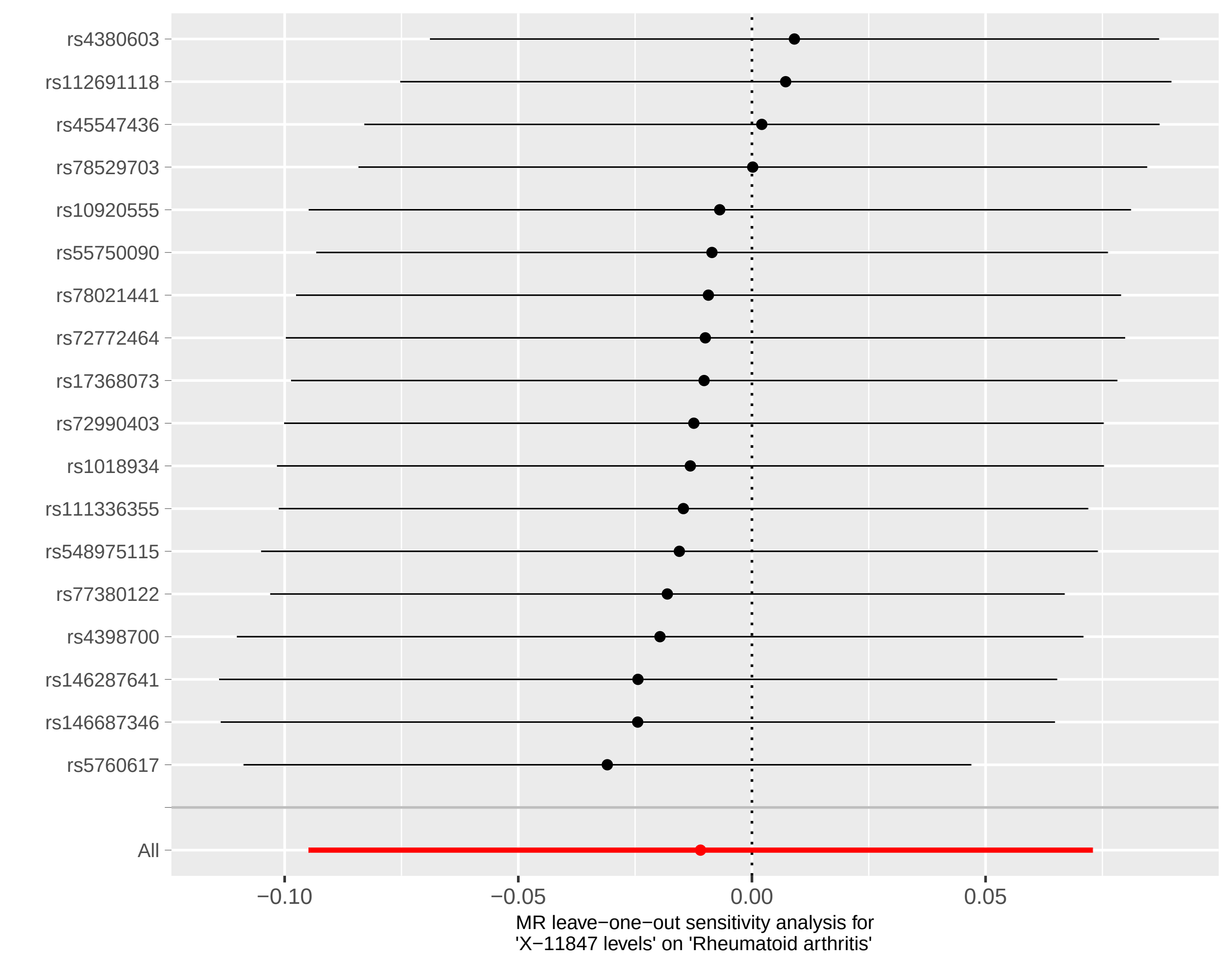
rs4380603
rs112691118
rs45547436
rs78529703
rs10920555
rs55750090
rs78021441
rs72772464
rs17368073
rs72990403
rs1018934
rs111336355
rs548975115
rs77380122
rs4398700
rs146287641
rs146687346
rs5760617
All
−0.10
−0.05
0.00
0.05
MR leave−one−out sensitivity analysis for
'X−11847 levels' on 'Rheumatoid arthritis'

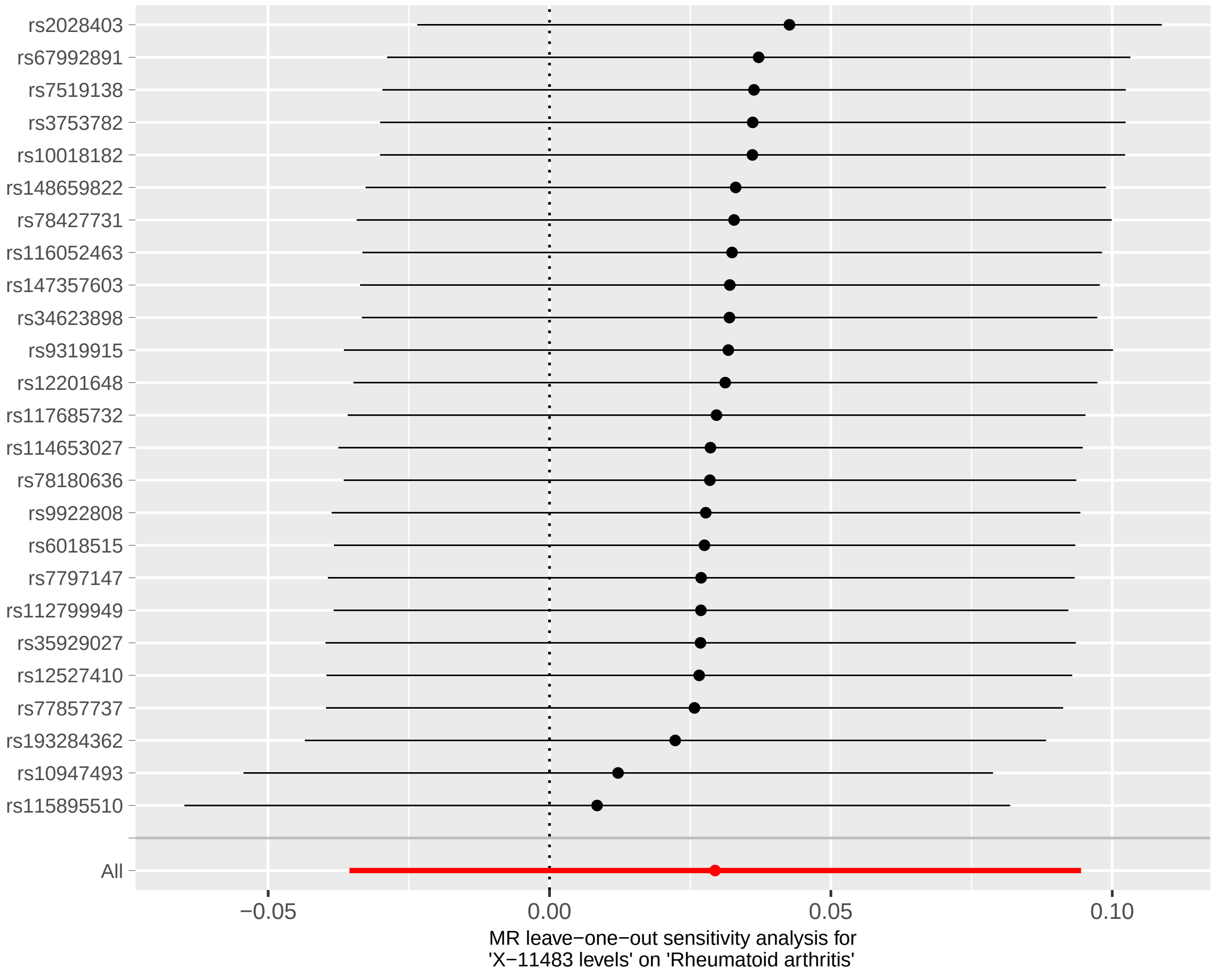

rs2028403
rs67992891
rs7519138
rs3753782
rs10018182
rs148659822
rs78427731
rs116052463
rs147357603
rs34623898
rs9319915
rs12201648
rs117685732
rs114653027
rs78180636
rs9922808
rs6018515
rs7797147
rs112799949
rs35929027
rs12527410
rs77857737
rs193284362
rs10947493
rs115895510
All
−0.05
0.00
0.05
0.10
MR leave−one−out sensitivity analysis for
'X−11483 levels' on 'Rheumatoid arthritis'

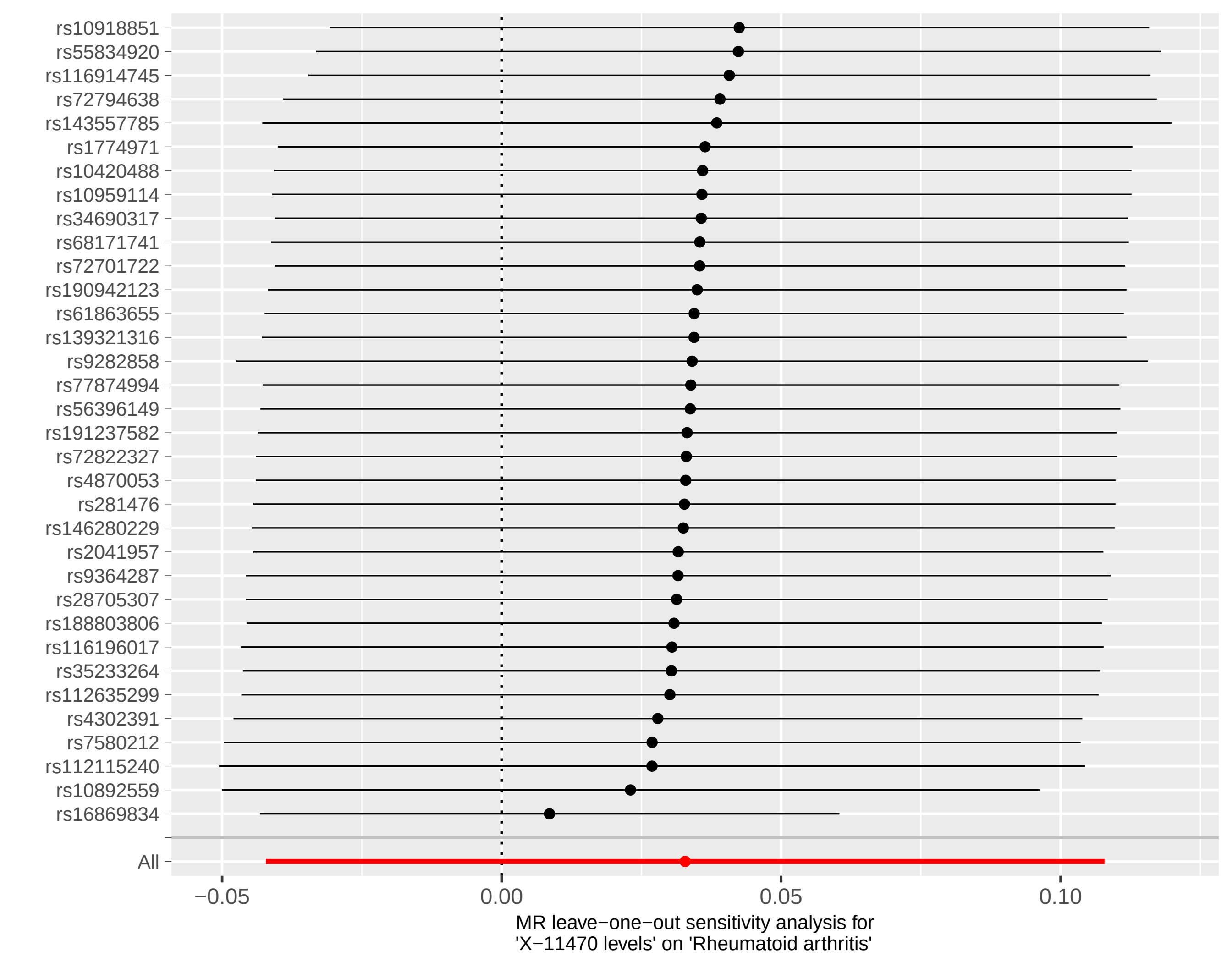
rs10918851
rs55834920
rs116914745
rs72794638
rs143557785
rs1774971
rs10420488
rs10959114
rs34690317
rs68171741
rs72701722
rs190942123
rs61863655
rs139321316
rs9282858
rs77874994
rs56396149
rs191237582
rs72822327
rs4870053
rs281476
rs146280229
rs2041957
rs9364287
rs28705307
rs188803806
rs116196017
rs35233264
rs112635299
rs4302391
rs7580212
rs112115240
rs10892559
rs16869834
All
−0.05
0.00
0.05
0.10
MR leave−one−out sensitivity analysis for
'X−11470 levels' on 'Rheumatoid arthritis'

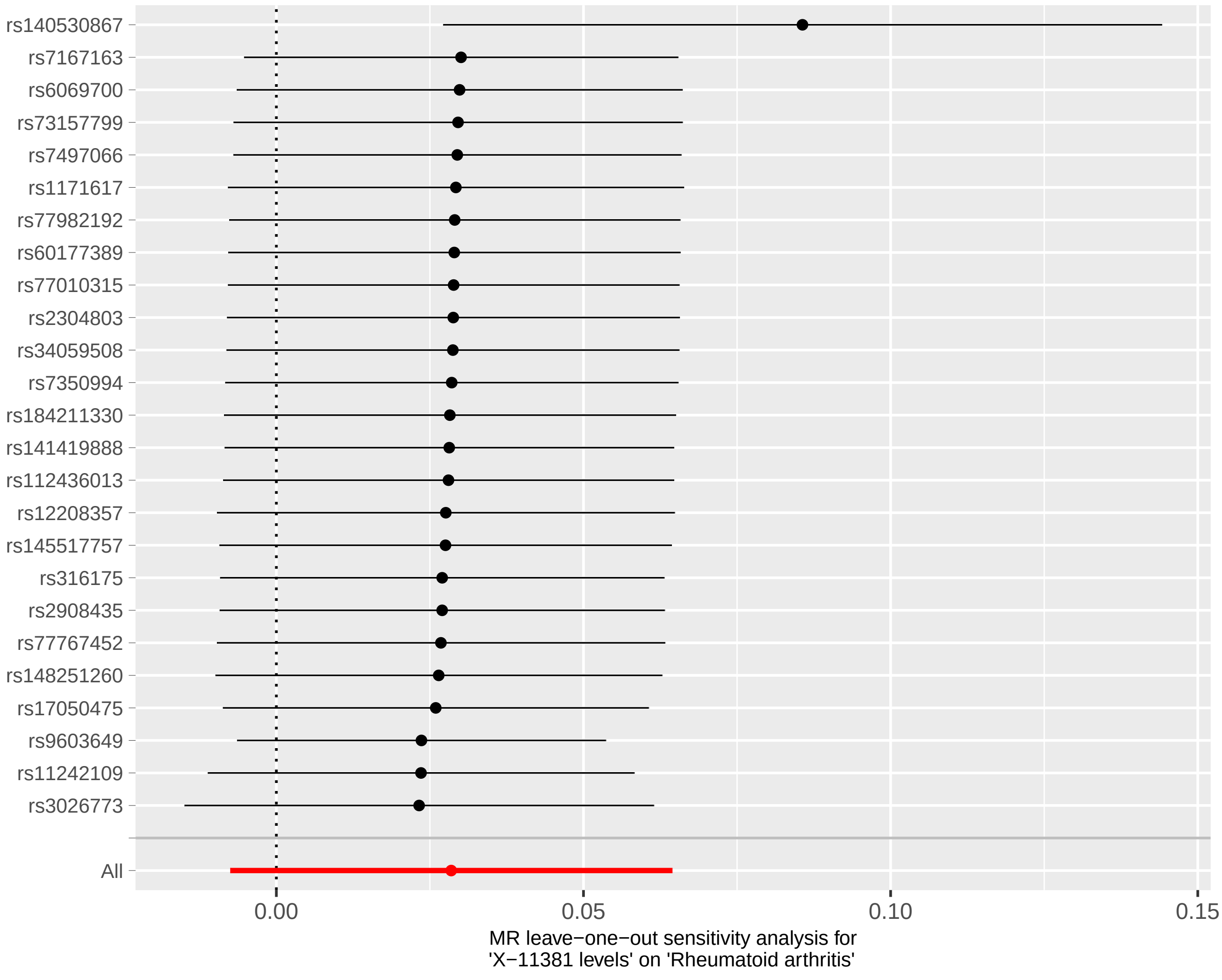

rs140530867
rs7167163
rs6069700
rs73157799
rs7497066
rs1171617
rs77982192
rs60177389
rs77010315
rs2304803
rs34059508
rs7350994
rs184211330
rs141419888
rs112436013
rs12208357
rs145517757
rs316175
rs2908435
rs77767452
rs148251260
rs17050475
rs9603649
rs11242109
rs3026773
All
0.00
0.05
0.10
0.15
MR leave−one−out sensitivity analysis for
'X−11381 levels' on 'Rheumatoid arthritis'

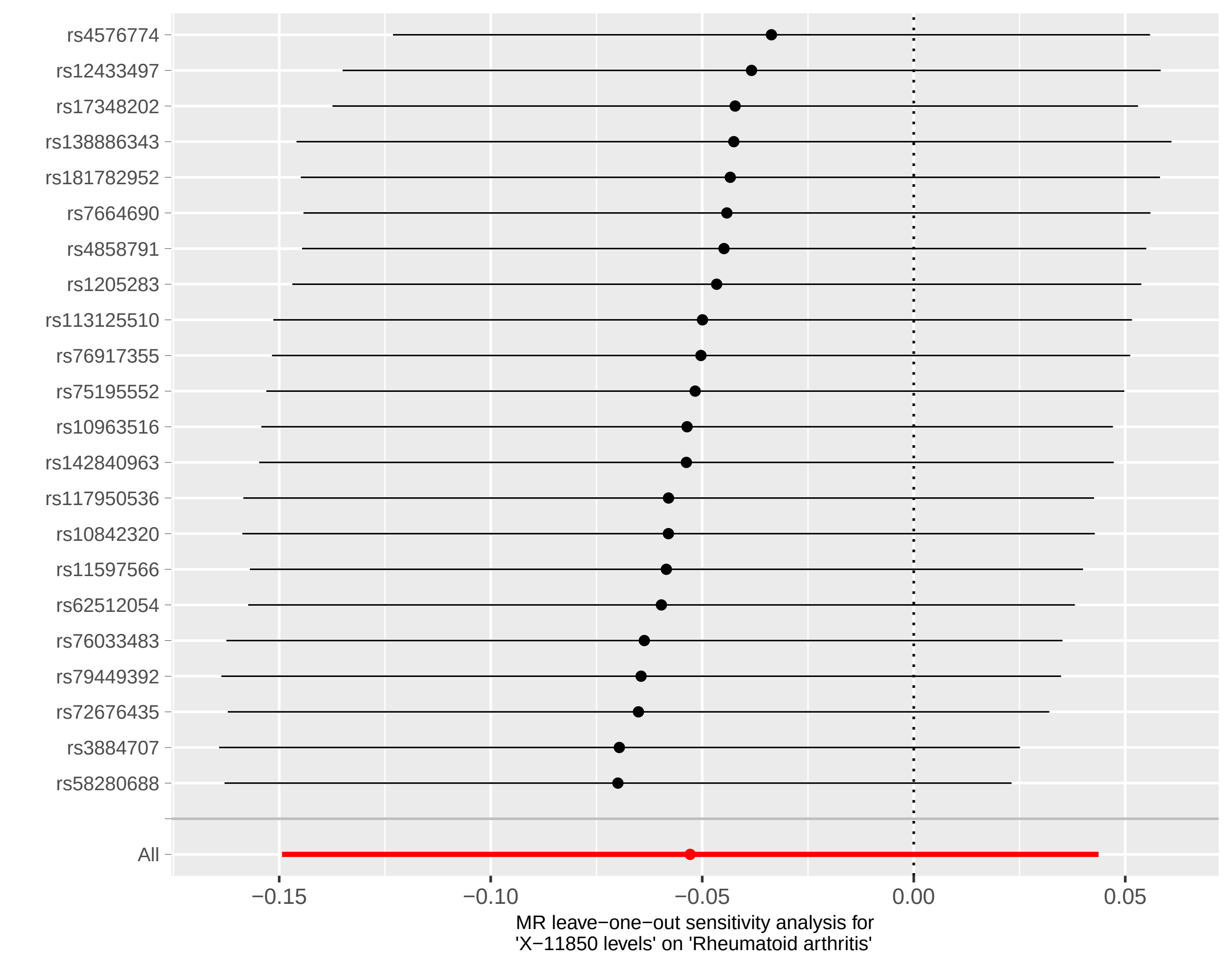
rs4576774
rs12433497
rs17348202
rs138886343
rs181782952
rs7664690
rs4858791
rs1205283
rs113125510
rs76917355
rs75195552
rs10963516
rs142840963
rs117950536
rs10842320
rs11597566
rs62512054
rs76033483
rs79449392
rs72676435
rs3884707
rs58280688
All
−0.15
−0.10
−0.05
0.00
0.05
MR leave−one−out sensitivity analysis for
'X−11850 levels' on 'Rheumatoid arthritis'

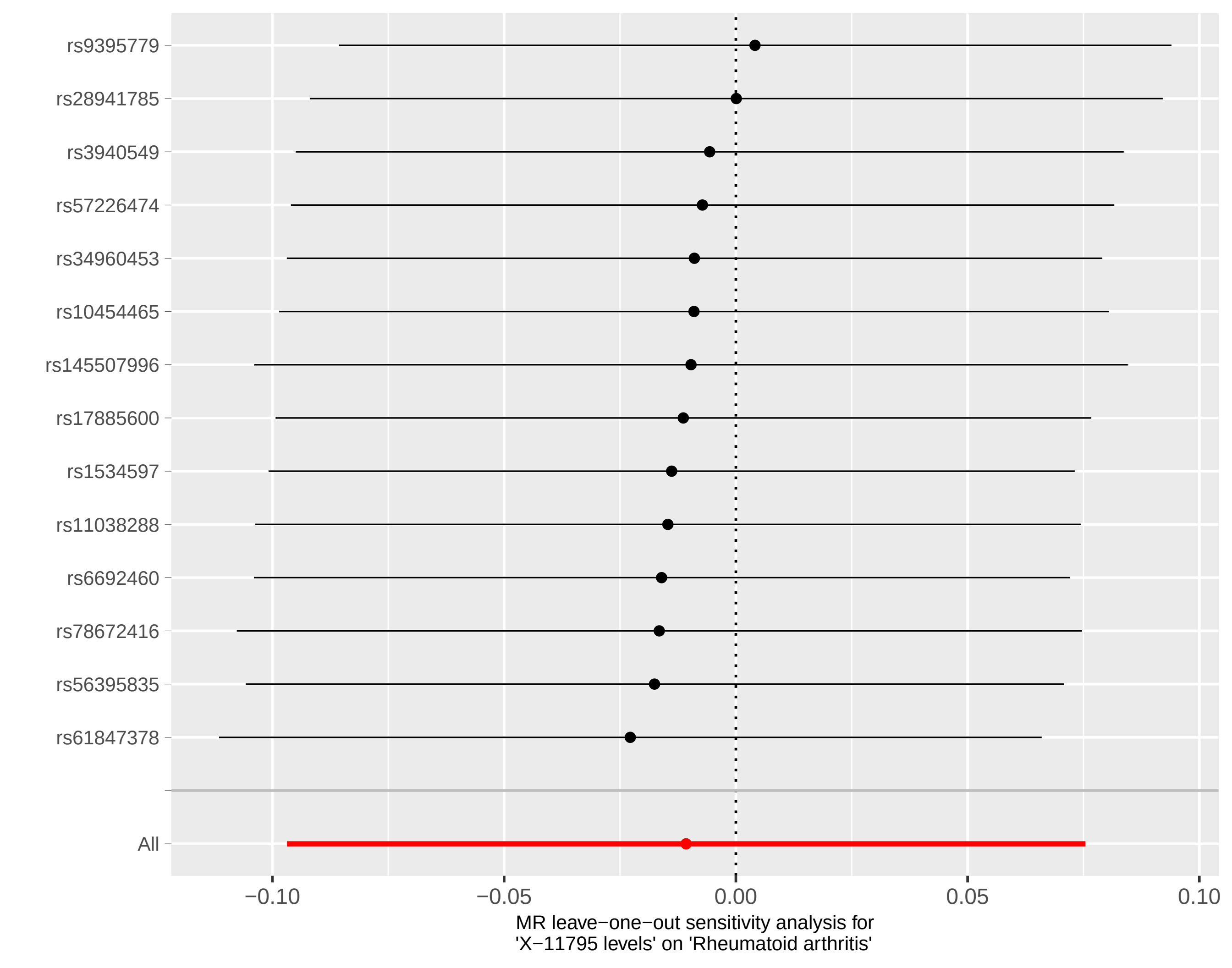

rs9395779
rs28941785
rs3940549
rs57226474
rs34960453
rs10454465
rs145507996
rs17885600
rs1534597
rs11038288
rs6692460
rs78672416
rs56395835
rs61847378
All
−0.10
−0.05
0.00
0.05
0.10
MR leave−one−out sensitivity analysis for
'X−11795 levels' on 'Rheumatoid arthritis'

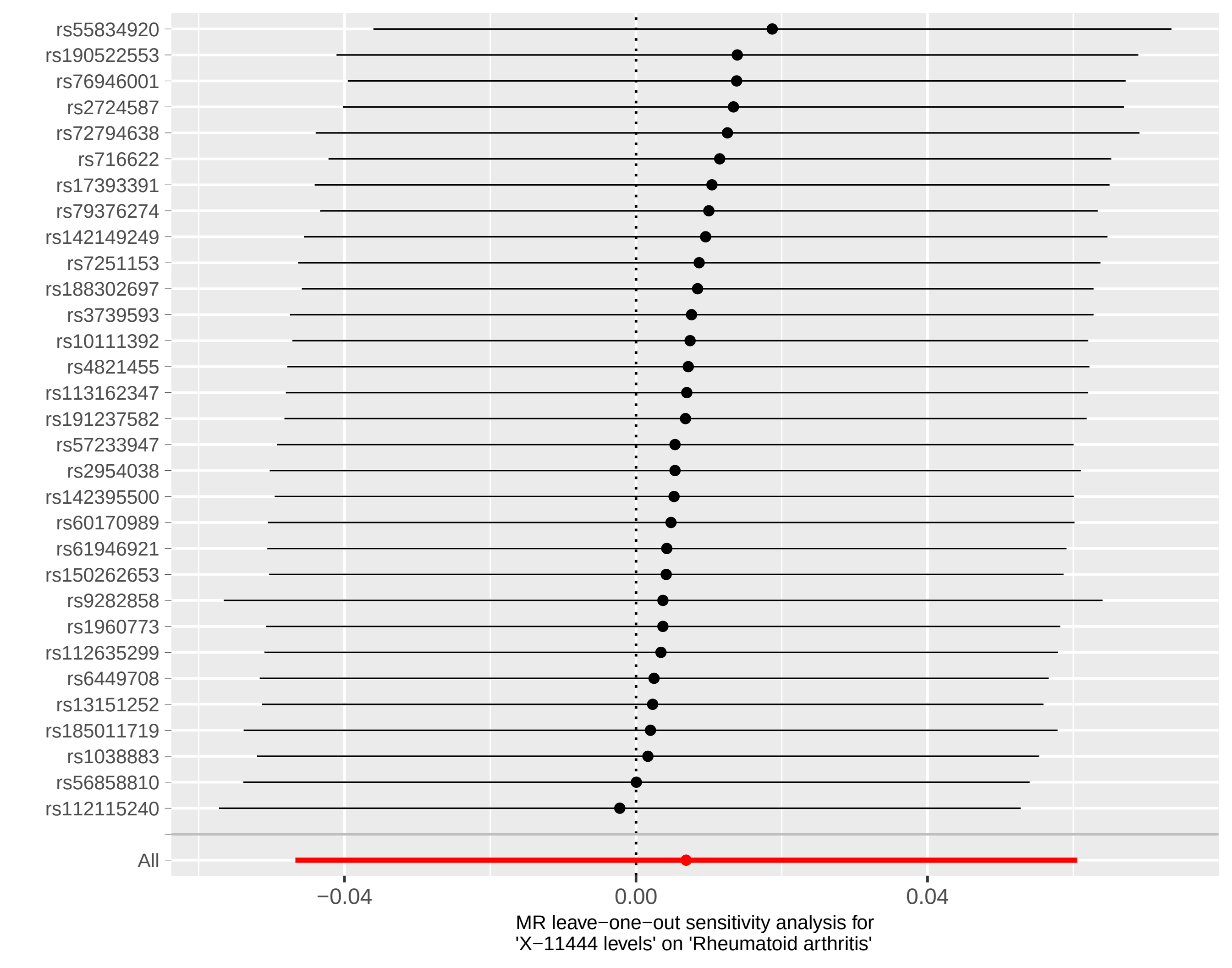
rs55834920
rs190522553
rs76946001
rs2724587
rs72794638
rs716622
rs17393391
rs79376274
rs142149249
rs7251153
rs188302697
rs3739593
rs10111392
rs4821455
rs113162347
rs191237582
rs57233947
rs2954038
rs142395500
rs60170989
rs61946921
rs150262653
rs9282858
rs1960773
rs112635299
rs6449708
rs13151252
rs185011719
rs1038883
rs56858810
rs112115240
All
−0.04
0.00
0.04
MR leave−one−out sensitivity analysis for
'X−11444 levels' on 'Rheumatoid arthritis'

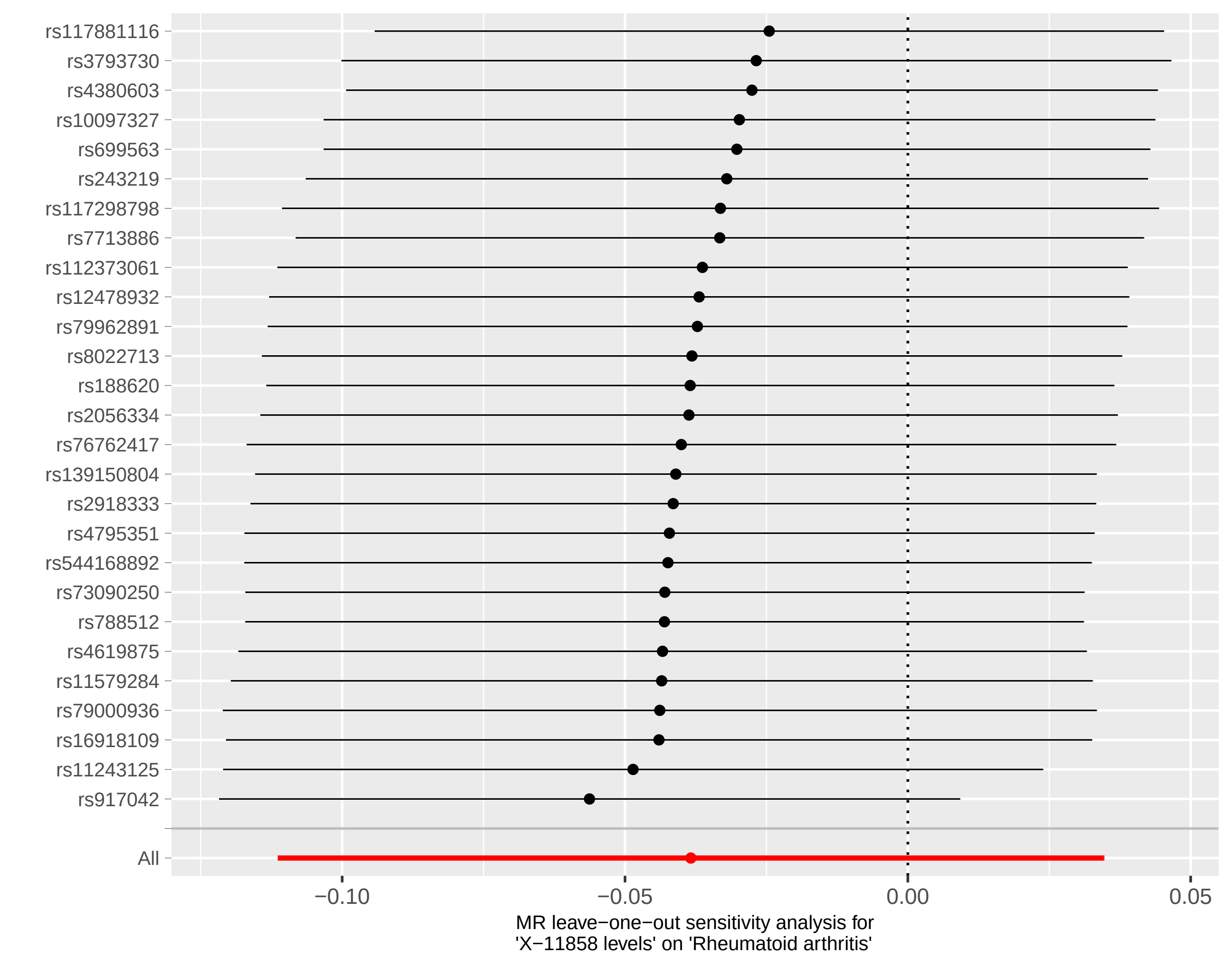
rs117881116
rs3793730
rs4380603
rs10097327
rs699563
rs243219
rs117298798
rs7713886
rs112373061
rs12478932
rs79962891
rs8022713
rs188620
rs2056334
rs76762417
rs139150804
rs2918333
rs4795351
rs544168892
rs73090250
rs788512
rs4619875
rs11579284
rs79000936
rs16918109
rs11243125
rs917042
All
−0.10
−0.05
0.00
0.05
MR leave−one−out sensitivity analysis for
'X−11858 levels' on 'Rheumatoid arthritis'

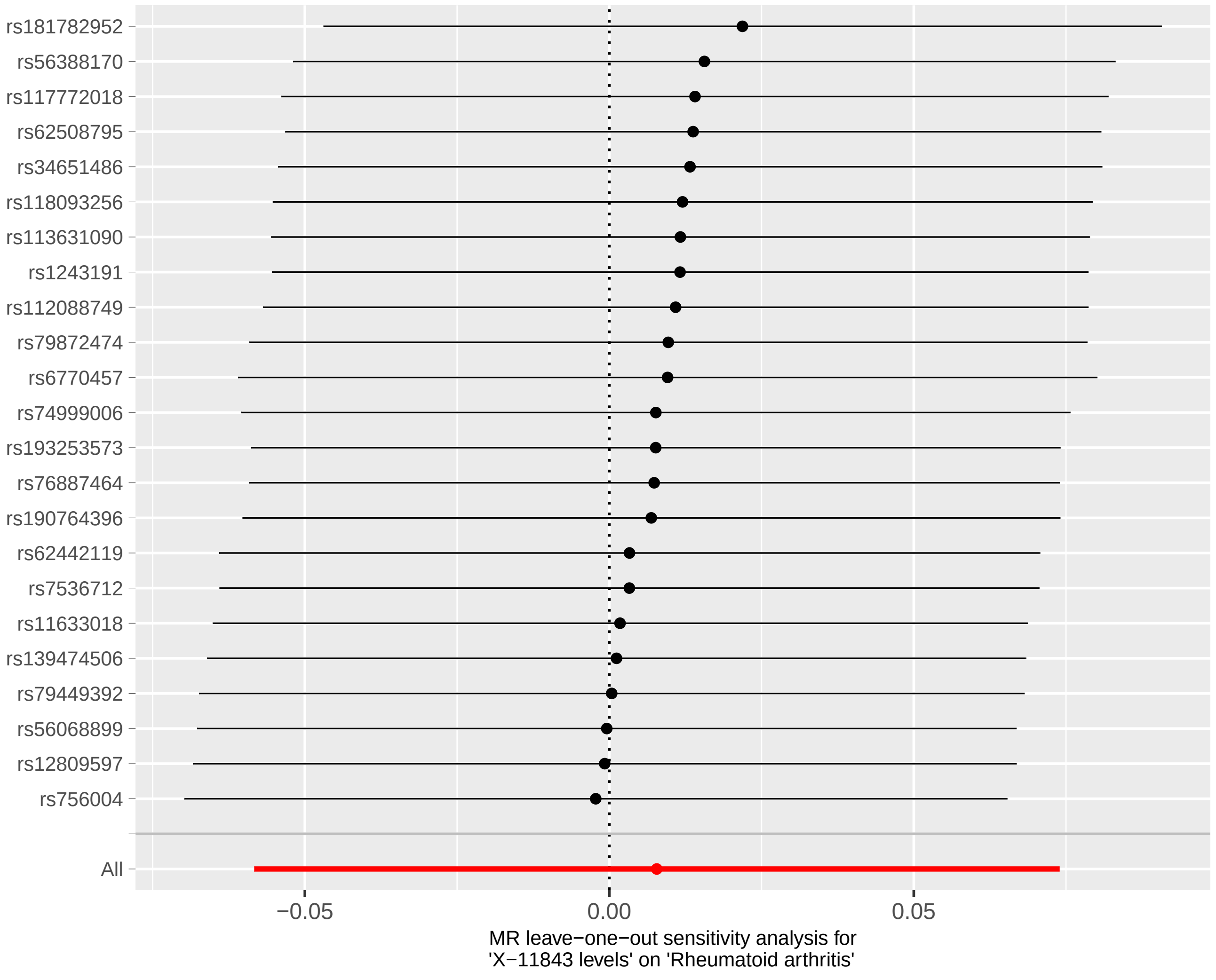
rs181782952
rs56388170
rs117772018
rs62508795
rs34651486
rs118093256
rs113631090
rs1243191
rs112088749
rs79872474
rs6770457
rs74999006
rs193253573
rs76887464
rs190764396
rs62442119
rs7536712
rs11633018
rs139474506
rs79449392
rs56068899
rs12809597
rs756004
All
−0.05
0.00
0.05
MR leave−one−out sensitivity analysis for
'X−11843 levels' on 'Rheumatoid arthritis'

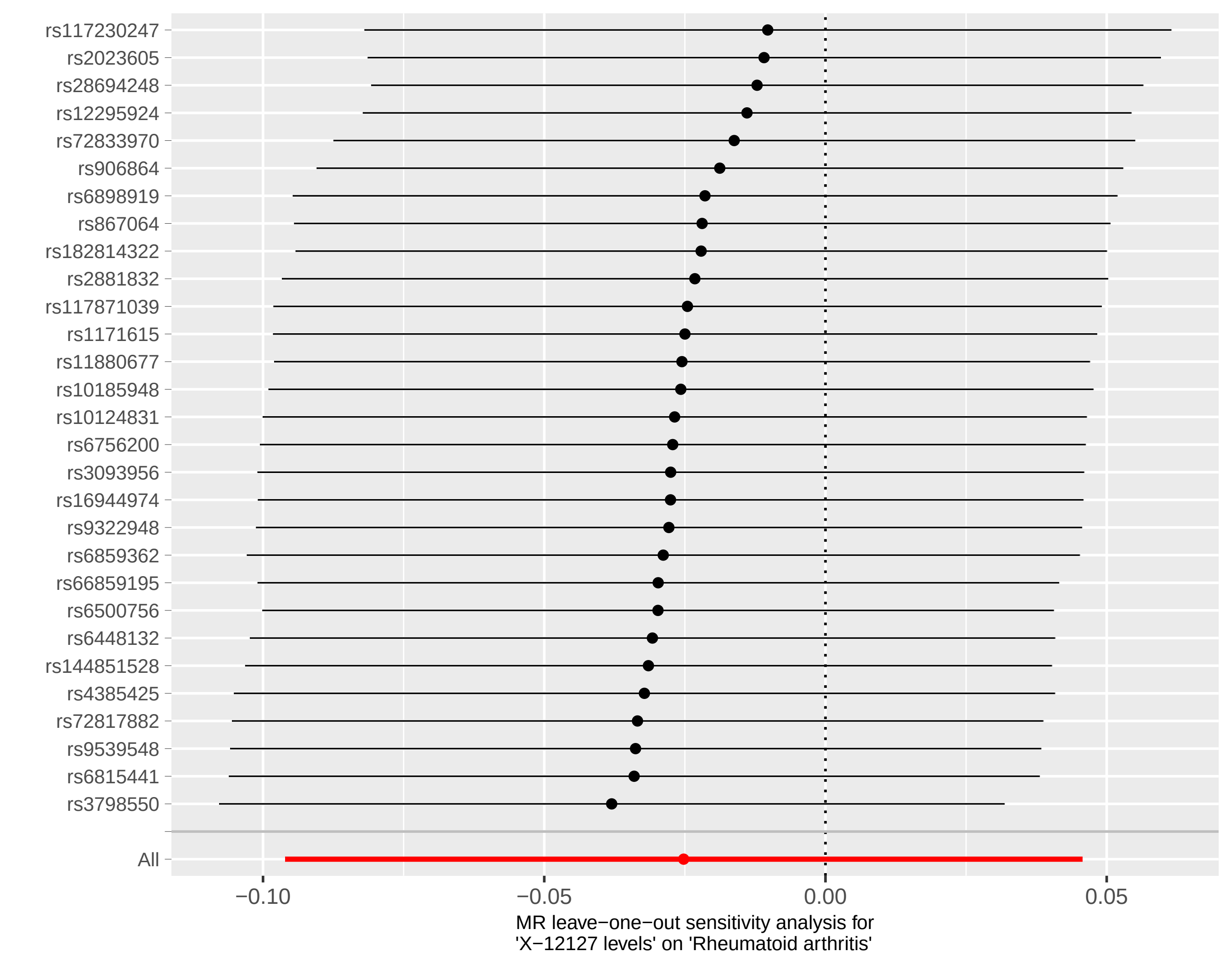
rs117230247
rs2023605
rs28694248
rs12295924
rs72833970
rs906864
rs6898919
rs867064
rs182814322
rs2881832
rs117871039
rs1171615
rs11880677
rs10185948
rs10124831
rs6756200
rs3093956
rs16944974
rs9322948
rs6859362
rs66859195
rs6500756
rs6448132
rs144851528
rs4385425
rs72817882
rs9539548
rs6815441
rs3798550
All
−0.10
−0.05
0.00
0.05
MR leave−one−out sensitivity analysis for
'X−12127 levels' on 'Rheumatoid arthritis'

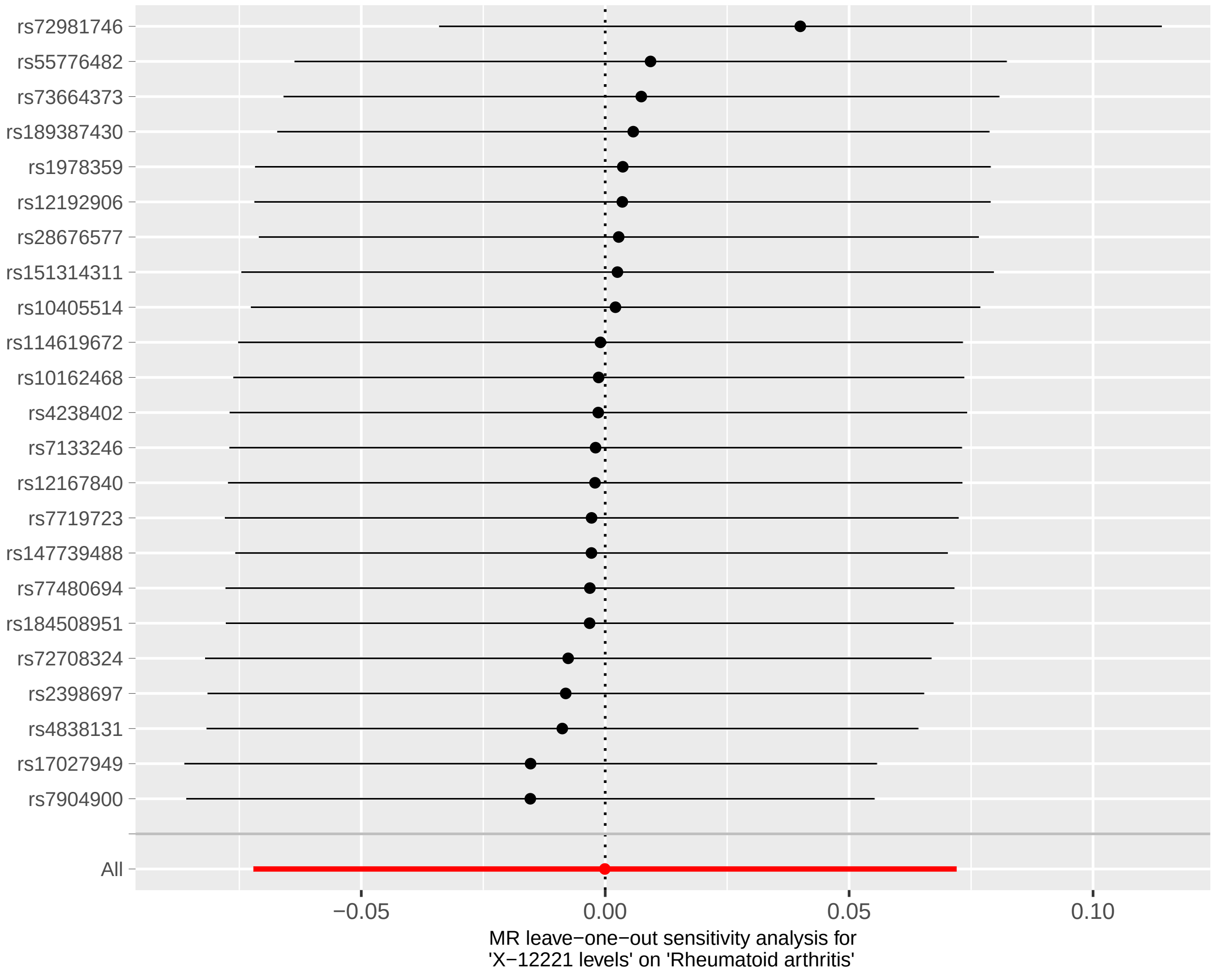
rs72981746
rs55776482
rs73664373
rs189387430
rs1978359
rs12192906
rs28676577
rs151314311
rs10405514
rs114619672
rs10162468
rs4238402
rs7133246
rs12167840
rs7719723
rs147739488
rs77480694
rs184508951
rs72708324
rs2398697
rs4838131
rs17027949
rs7904900
All
−0.05
0.00
0.05
0.10
MR leave−one−out sensitivity analysis for
'X−12221 levels' on 'Rheumatoid arthritis'

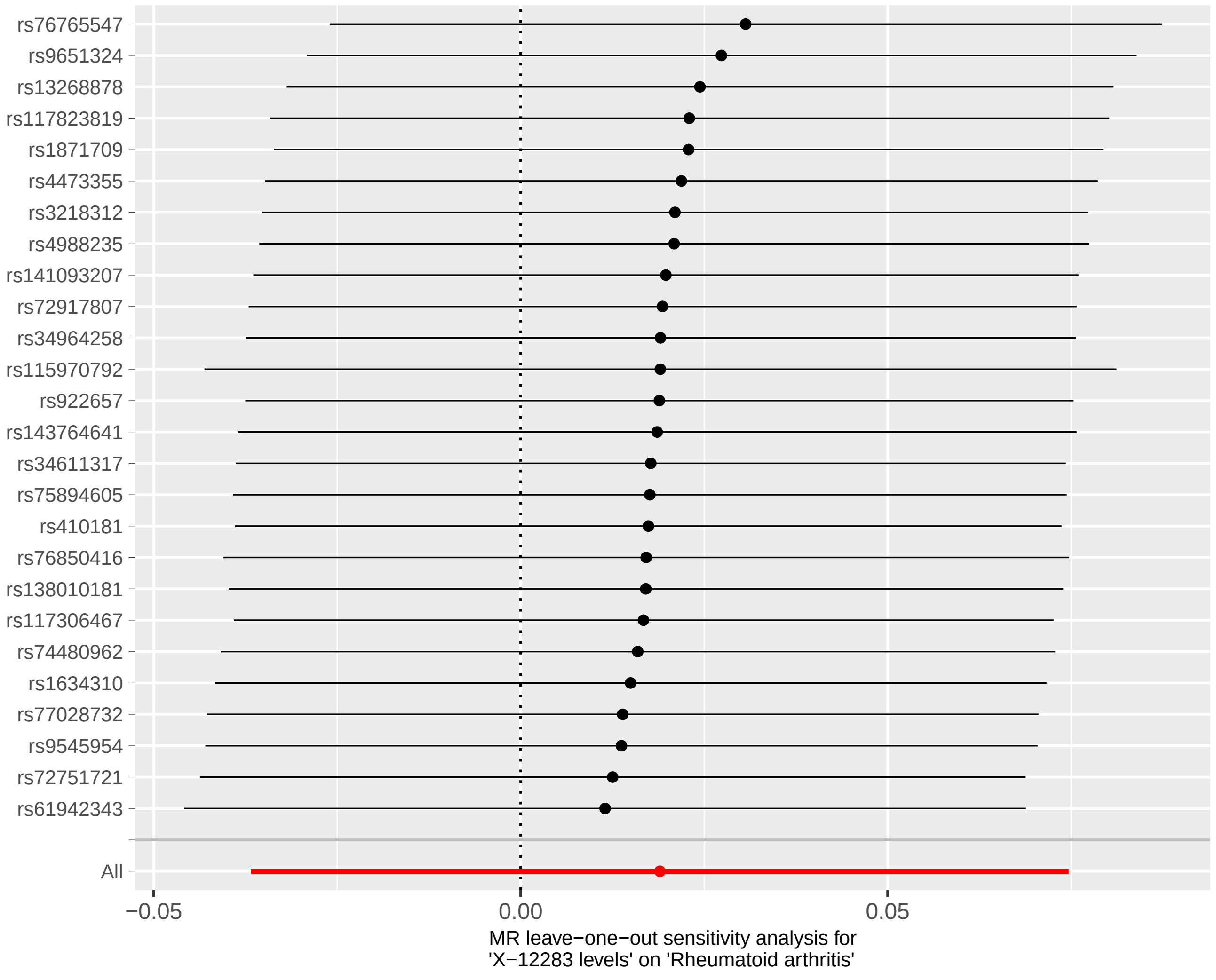
rs76765547
rs9651324
rs13268878
rs117823819
rs1871709
rs4473355
rs3218312
rs4988235
rs141093207
rs72917807
rs34964258
rs115970792
rs922657
rs143764641
rs34611317
rs75894605
rs410181
rs76850416
rs138010181
rs117306467
rs74480962
rs1634310
rs77028732
rs9545954
rs72751721
rs61942343
All
−0.05
0.00
0.05
MR leave−one−out sensitivity analysis for
'X−12283 levels' on 'Rheumatoid arthritis'

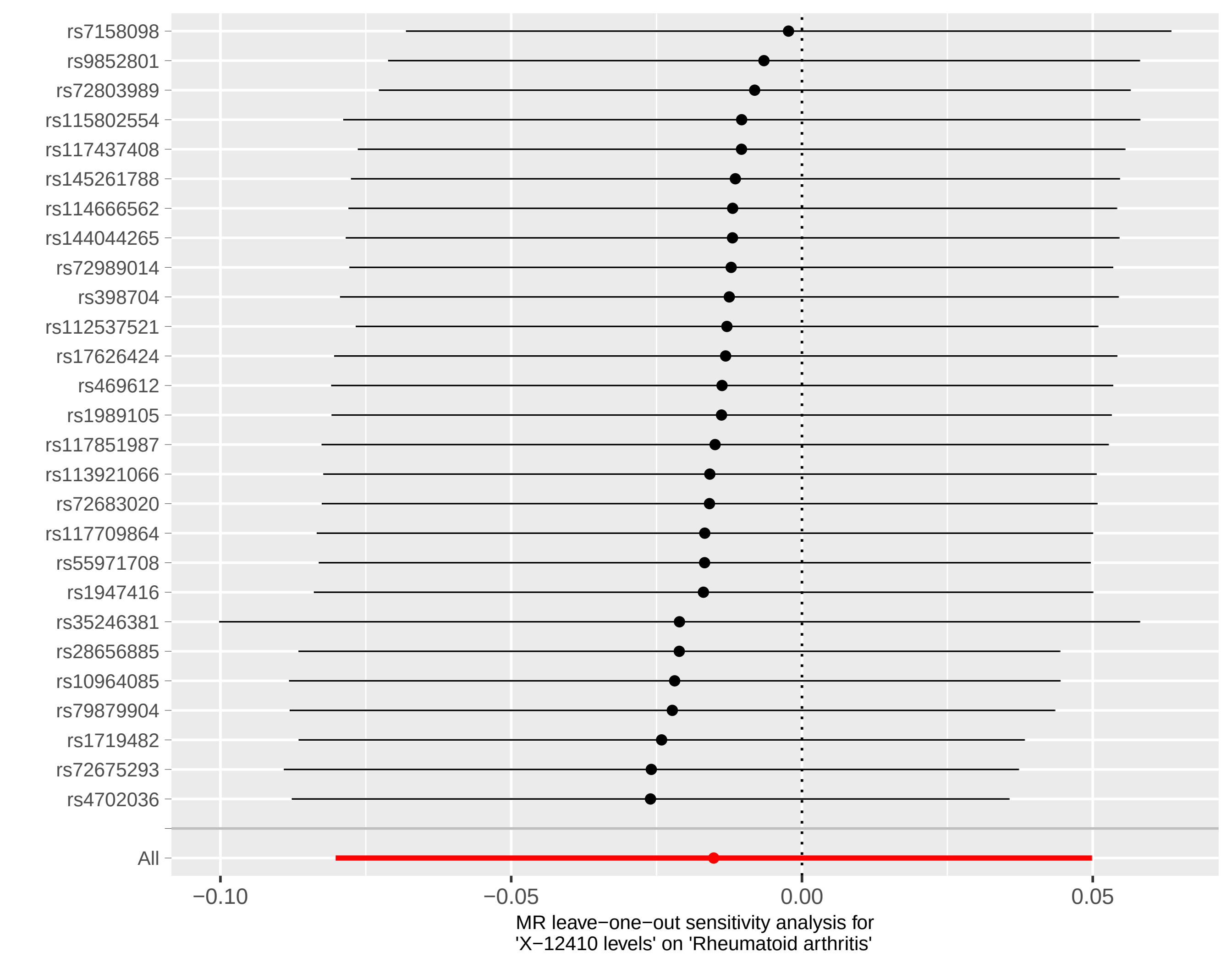
rs7158098
rs9852801
rs72803989
rs115802554
rs117437408
rs145261788
rs114666562
rs144044265
rs72989014
rs398704
rs112537521
rs17626424
rs469612
rs1989105
rs117851987
rs113921066
rs72683020
rs117709864
rs55971708
rs1947416
rs35246381
rs28656885
rs10964085
rs79879904
rs1719482
rs72675293
rs4702036
All
−0.10
−0.05
0.00
0.05
MR leave−one−out sensitivity analysis for
'X−12410 levels' on 'Rheumatoid arthritis'

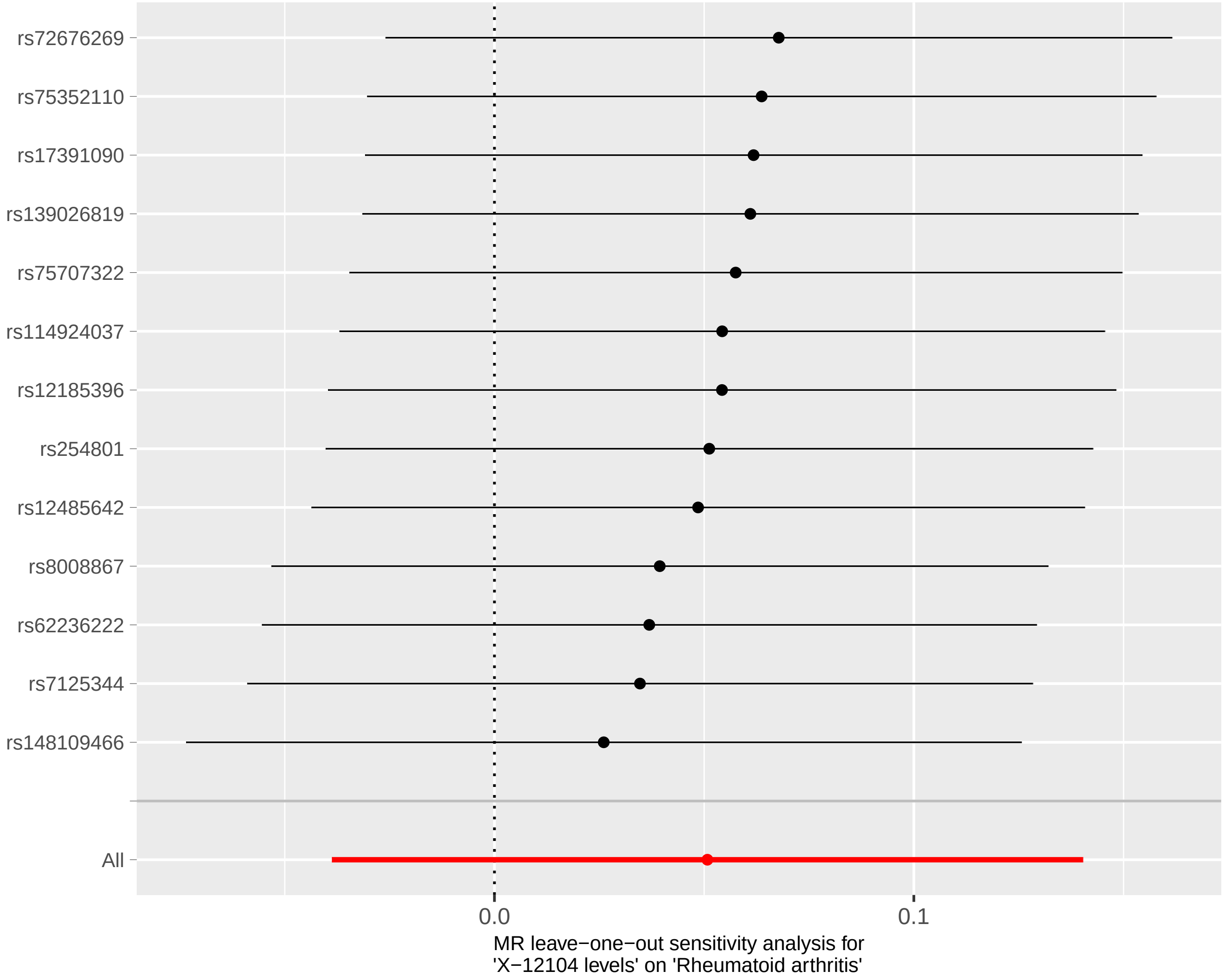
rs72676269
rs75352110
rs17391090
rs139026819
rs75707322
rs114924037
rs12185396
rs254801
rs12485642
rs8008867
rs62236222
rs7125344
rs148109466
All
0.0
0.1
MR leave−one−out sensitivity analysis for
'X−12104 levels' on 'Rheumatoid arthritis'

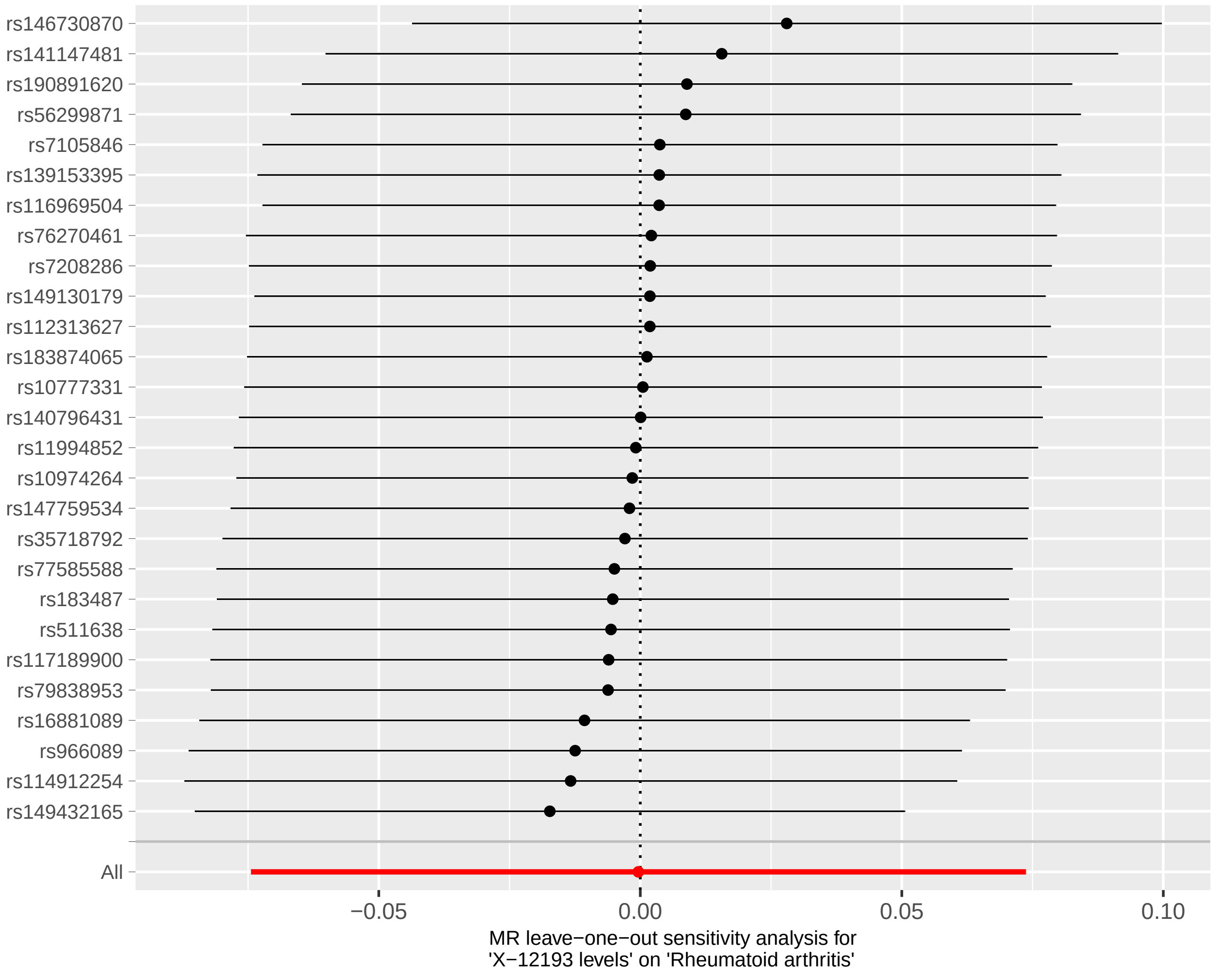
rs146730870
rs141147481
rs190891620
rs56299871
rs7105846
rs139153395
rs116969504
rs76270461
rs7208286
rs149130179
rs112313627
rs183874065
rs10777331
rs140796431
rs11994852
rs10974264
rs147759534
rs35718792
rs77585588
rs183487
rs511638
rs117189900
rs79838953
rs16881089
rs966089
rs114912254
rs149432165
All
−0.05
0.00
0.05
0.10
MR leave−one−out sensitivity analysis for
'X−12193 levels' on 'Rheumatoid arthritis'

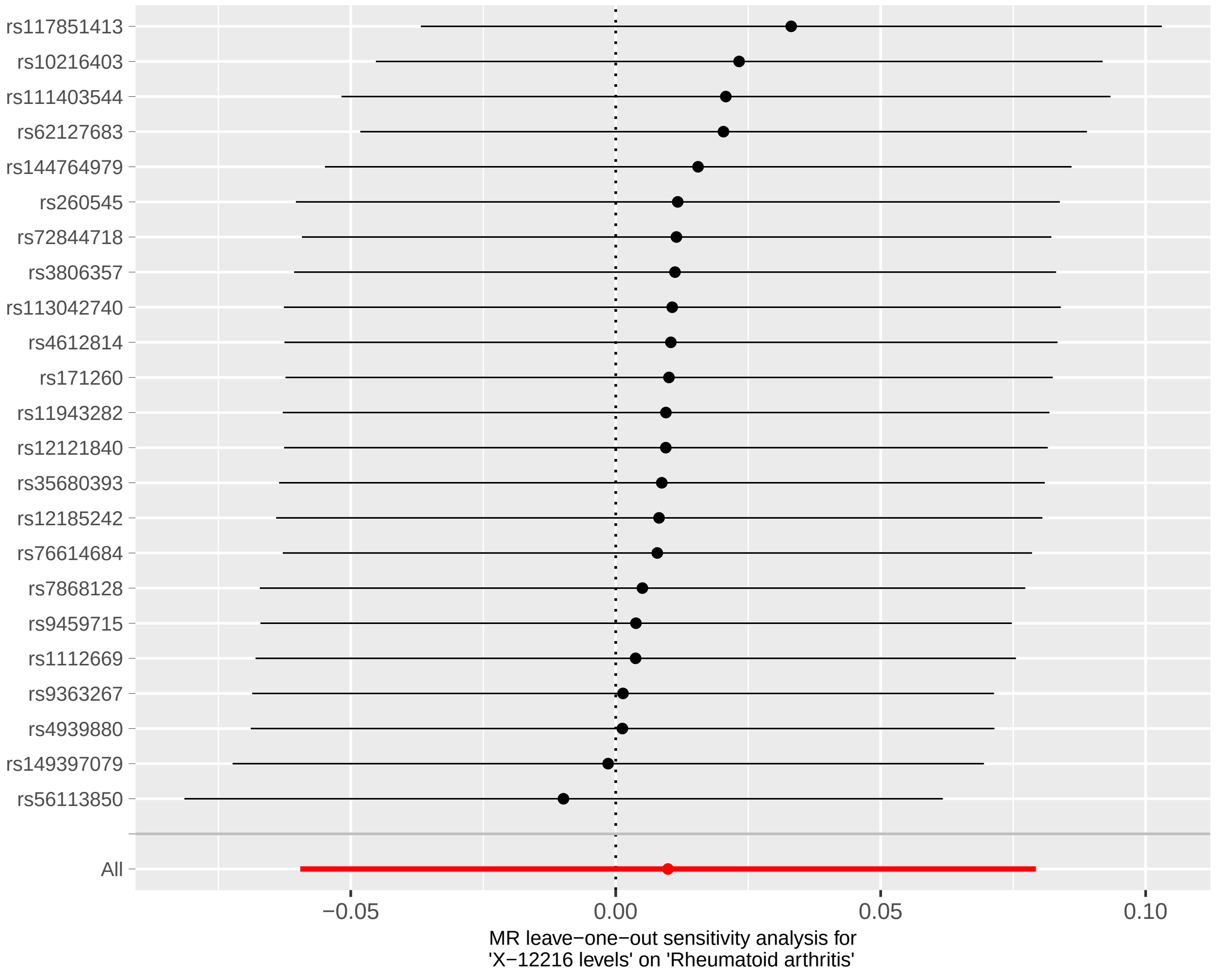

rs117851413
rs10216403
rs111403544
rs62127683
rs144764979
rs260545
rs72844718
rs3806357
rs113042740
rs4612814
rs171260
rs11943282
rs12121840
rs35680393
rs12185242
rs76614684
rs7868128
rs9459715
rs1112669
rs9363267
rs4939880
rs149397079
rs56113850
All
−0.05
0.00
0.05
0.10
MR leave−one−out sensitivity analysis for
'X−12216 levels' on 'Rheumatoid arthritis'

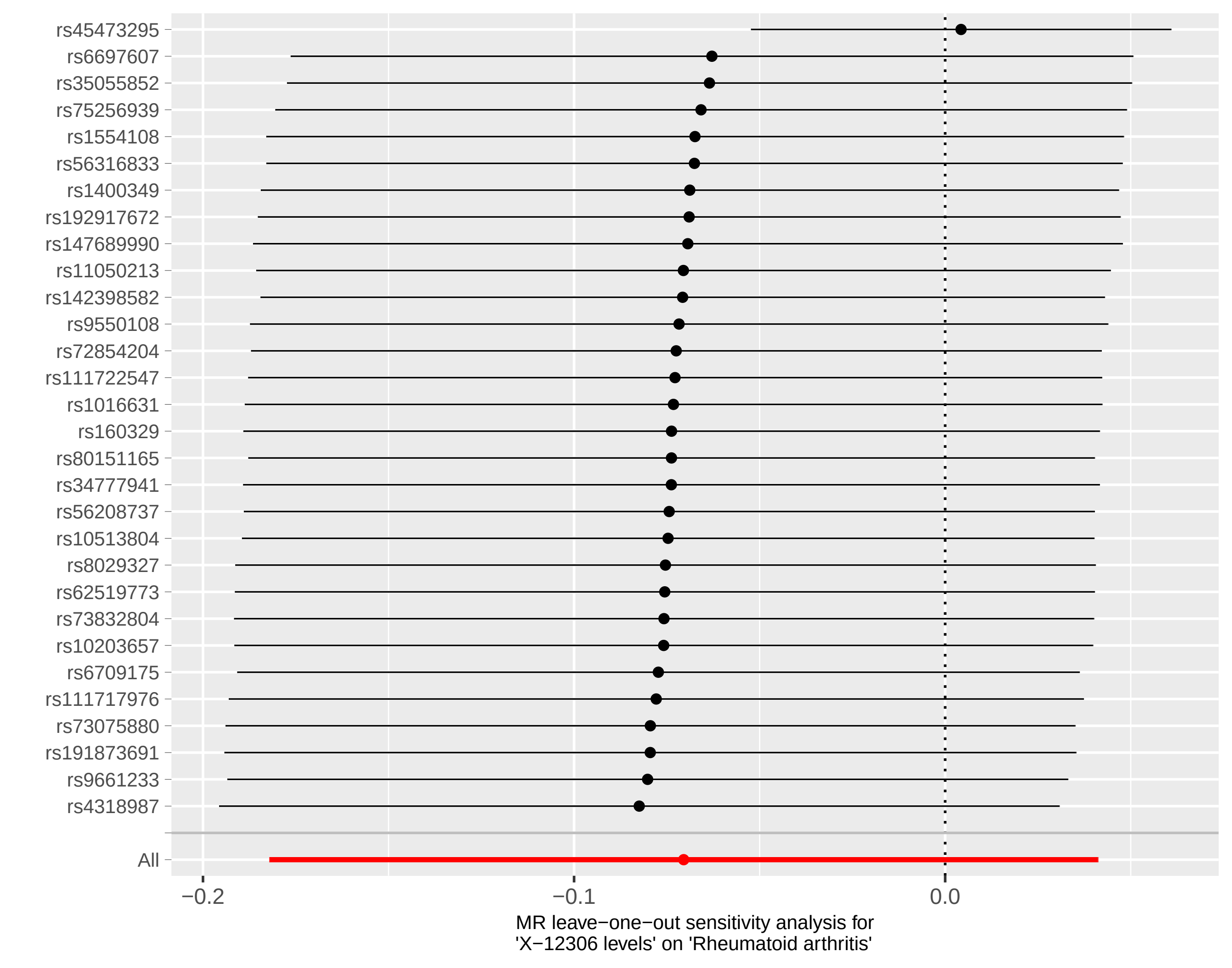
rs45473295
rs6697607
rs35055852
rs75256939
rs1554108
rs56316833
rs1400349
rs192917672
rs147689990
rs11050213
rs142398582
rs9550108
rs72854204
rs111722547
rs1016631
rs160329
rs80151165
rs34777941
rs56208737
rs10513804
rs8029327
rs62519773
rs73832804
rs10203657
rs6709175
rs111717976
rs73075880
rs191873691
rs9661233
rs4318987
All
−0.2
−0.1
0.0
MR leave−one−out sensitivity analysis for
'X−12306 levels' on 'Rheumatoid arthritis'

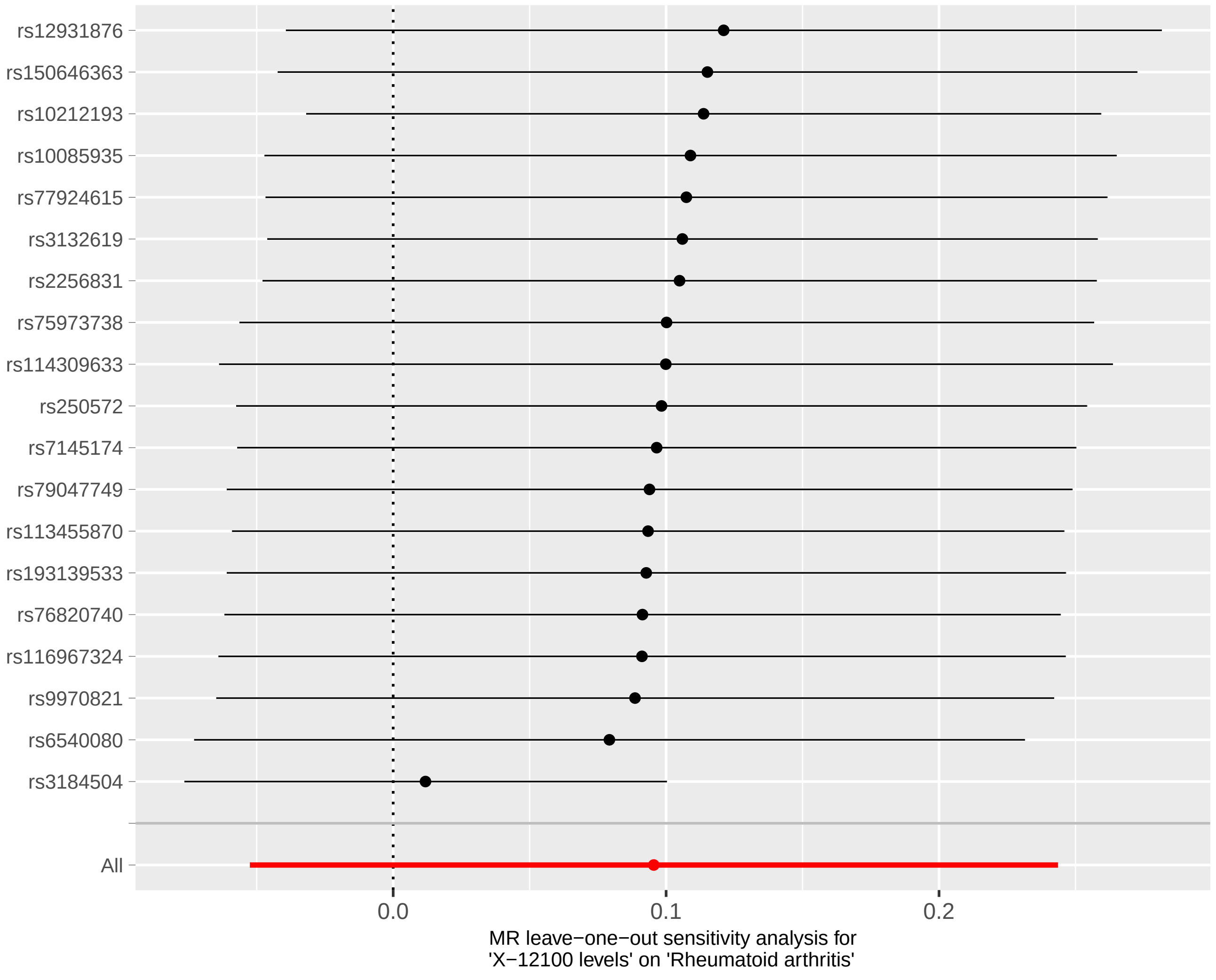

rs12931876
rs150646363
rs10212193
rs10085935
rs77924615
rs3132619
rs2256831
rs75973738
rs114309633
rs250572
rs7145174
rs79047749
rs113455870
rs193139533
rs76820740
rs116967324
rs9970821
rs6540080
rs3184504
All
0.0
0.1
0.2
MR leave−one−out sensitivity analysis for
'X−12100 levels' on 'Rheumatoid arthritis'

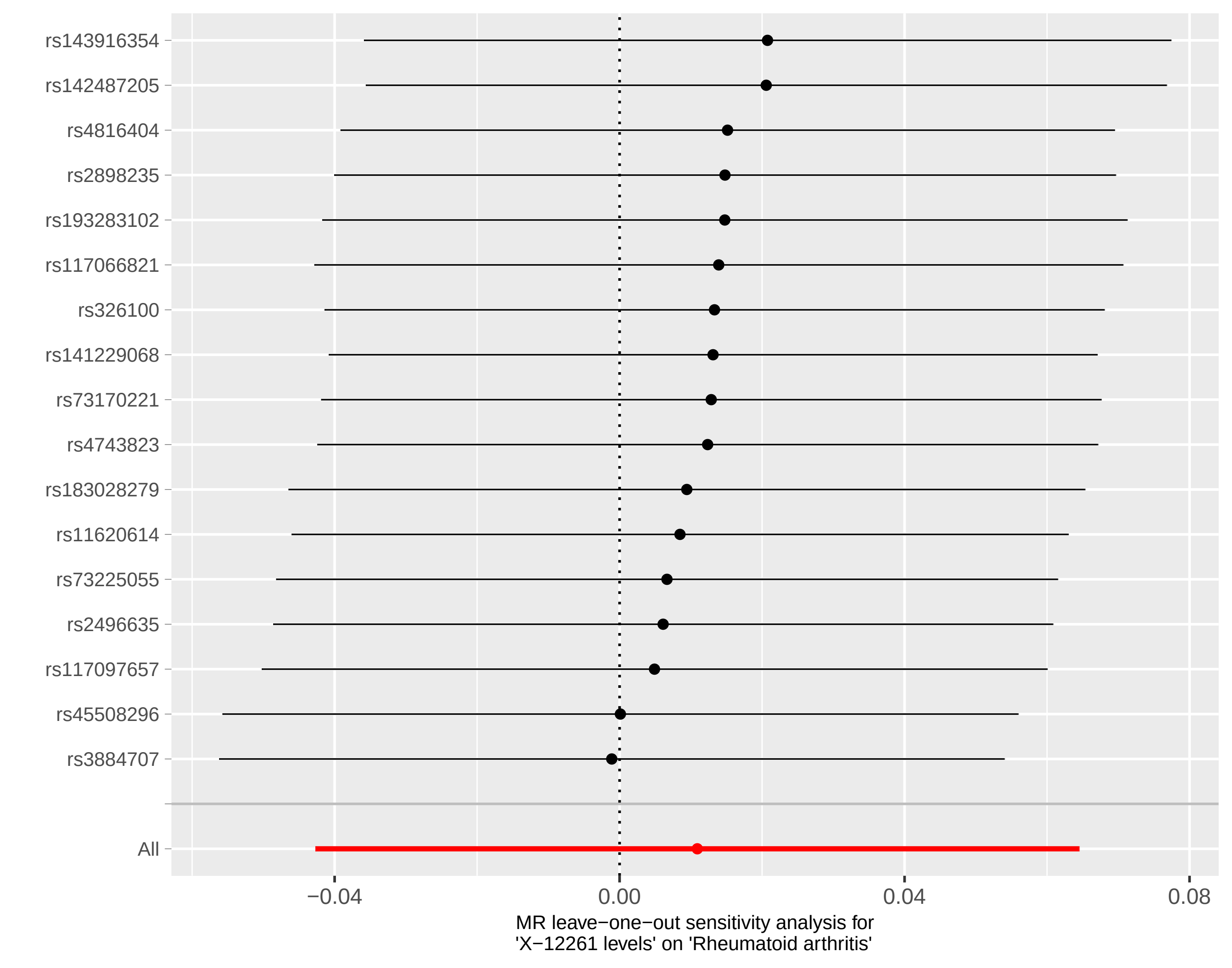

rs143916354
rs142487205
rs4816404
rs2898235
rs193283102
rs117066821
rs326100
rs141229068
rs73170221
rs4743823
rs183028279
rs11620614
rs73225055
rs2496635
rs117097657
rs45508296
rs3884707
All
−0.04
0.00
0.04
0.08
MR leave−one−out sensitivity analysis for
'X−12261 levels' on 'Rheumatoid arthritis'

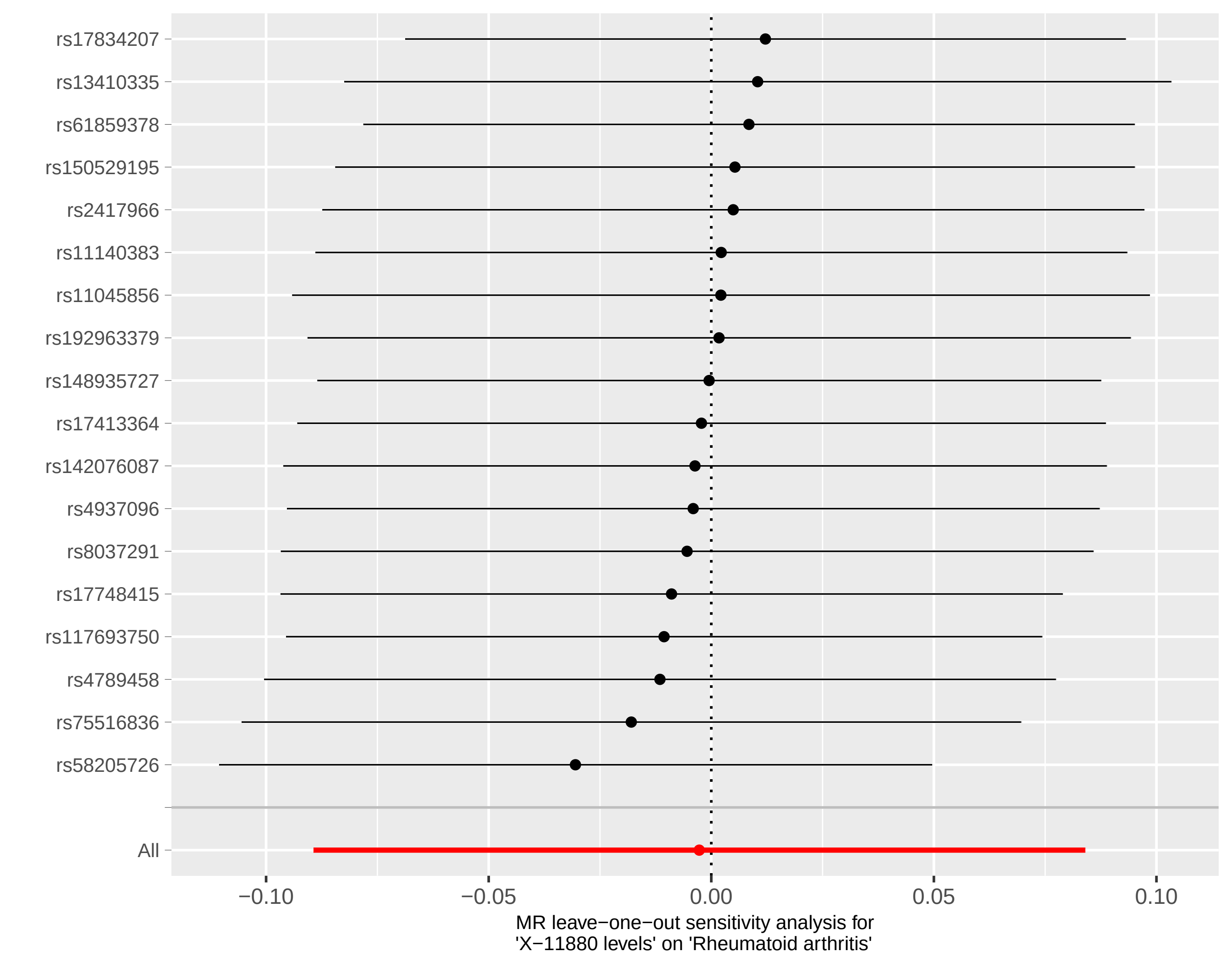
rs17834207
rs13410335
rs61859378
rs150529195
rs2417966
rs11140383
rs11045856
rs192963379
rs148935727
rs17413364
rs142076087
rs4937096
rs8037291
rs17748415
rs117693750
rs4789458
rs75516836
rs58205726
All
−0.10
−0.05
0.00
0.05
0.10
MR leave−one−out sensitivity analysis for
'X−11880 levels' on 'Rheumatoid arthritis'

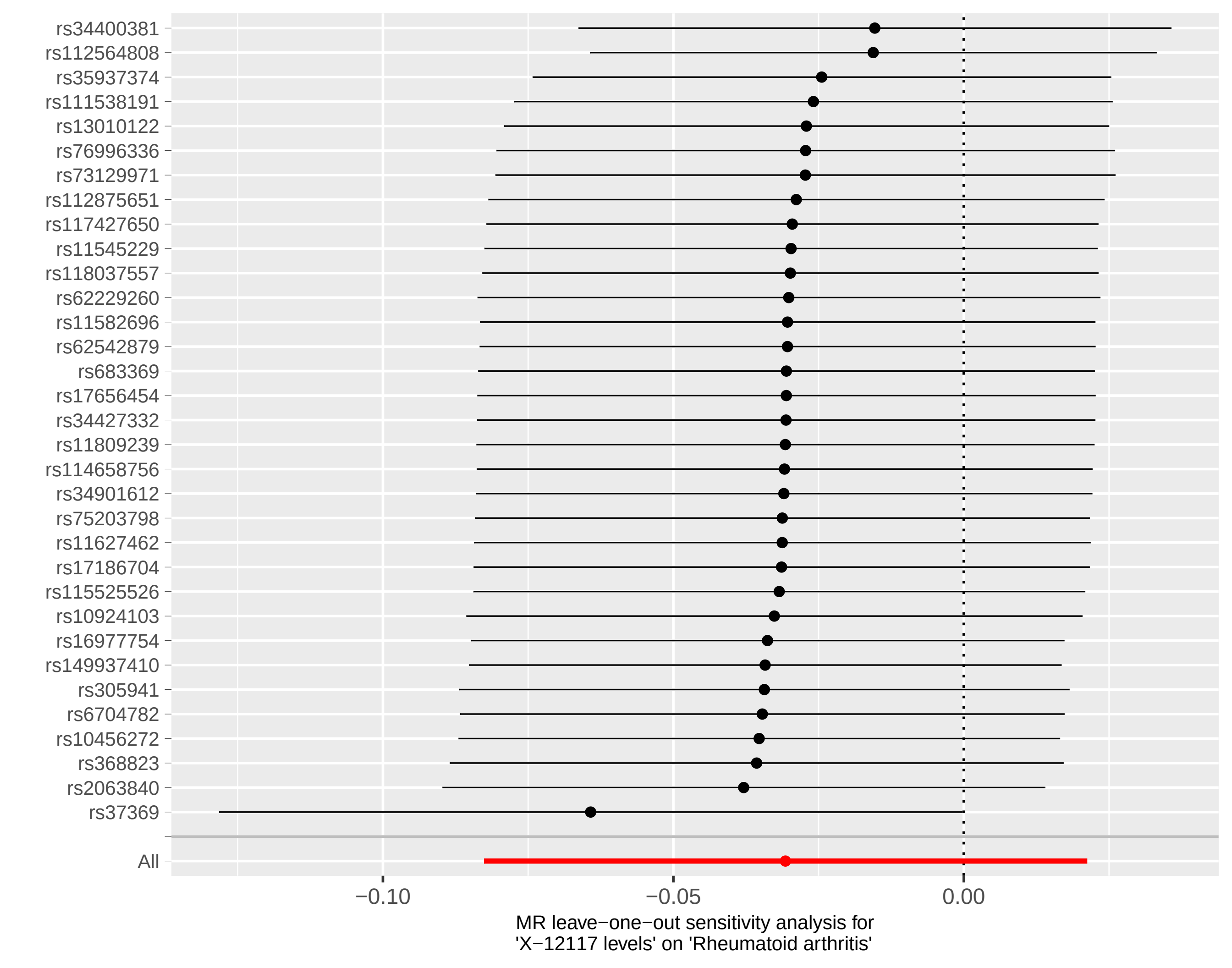
rs34400381
rs112564808
rs35937374
rs111538191
rs13010122
rs76996336
rs73129971
rs112875651
rs117427650
rs11545229
rs118037557
rs62229260
rs11582696
rs62542879
rs683369
rs17656454
rs34427332
rs11809239
rs114658756
rs34901612
rs75203798
rs11627462
rs17186704
rs115525526
rs10924103
rs16977754
rs149937410
rs305941
rs6704782
rs10456272
rs368823
rs2063840
rs37369
All
−0.10
−0.05
0.00
MR leave−one−out sensitivity analysis for
'X−12117 levels' on 'Rheumatoid arthritis'

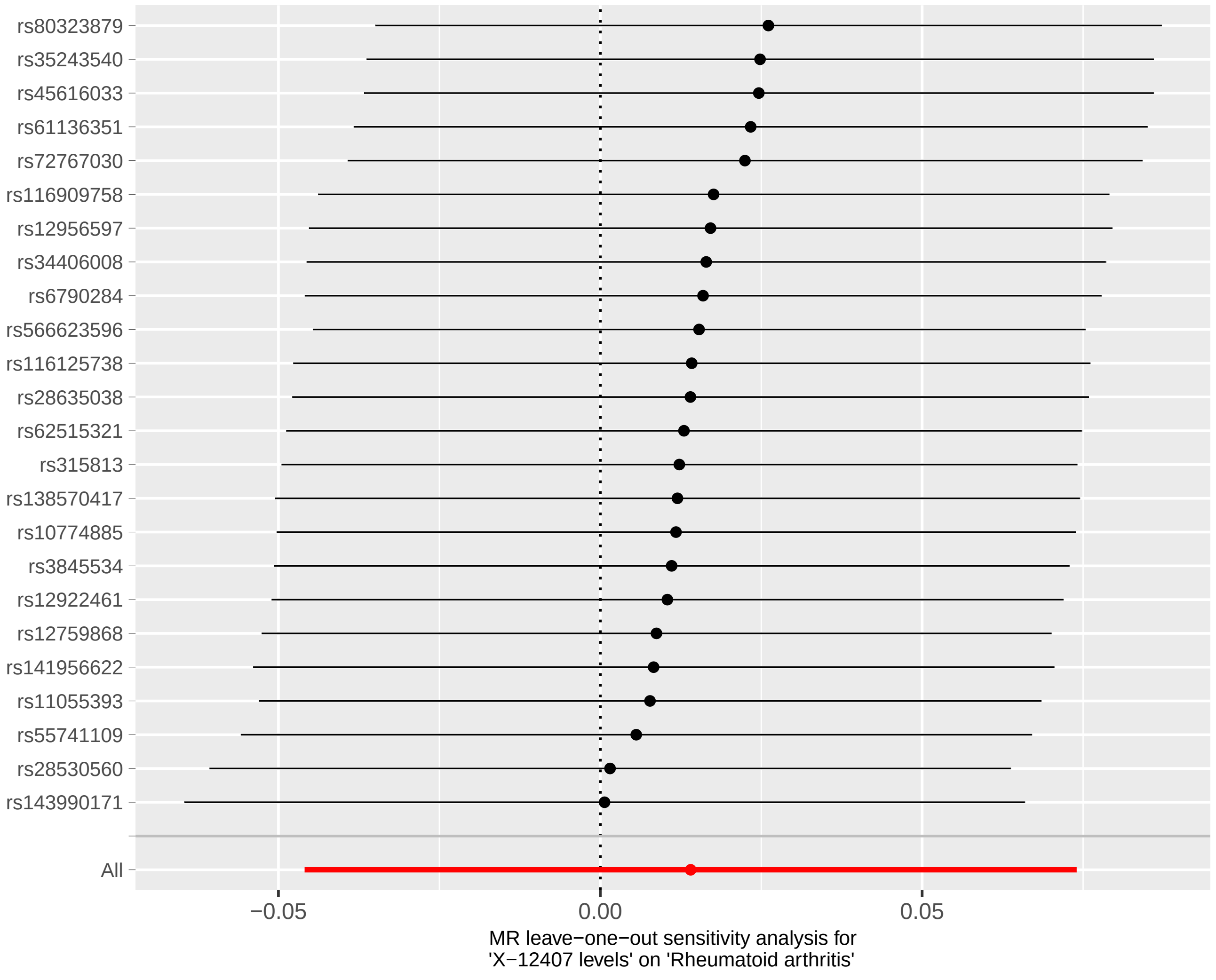

rs80323879
rs35243540
rs45616033
rs61136351
rs72767030
rs116909758
rs12956597
rs34406008
rs6790284
rs566623596
rs116125738
rs28635038
rs62515321
rs315813
rs138570417
rs10774885
rs3845534
rs12922461
rs12759868
rs141956622
rs11055393
rs55741109
rs28530560
rs143990171
All
−0.05
0.00
0.05
MR leave−one−out sensitivity analysis for
'X−12407 levels' on 'Rheumatoid arthritis'

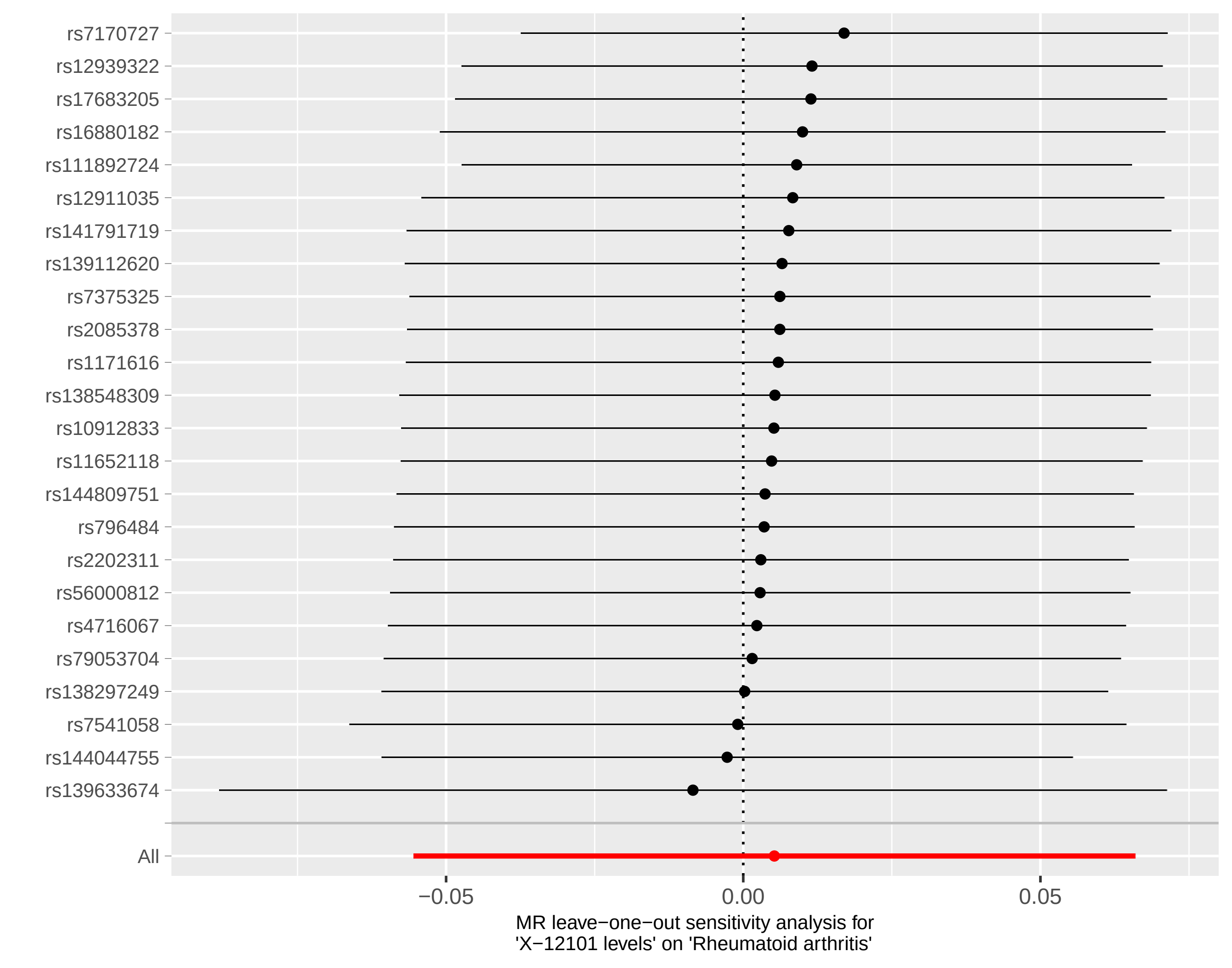

rs7170727
rs12939322
rs17683205
rs16880182
rs111892724
rs12911035
rs141791719
rs139112620
rs7375325
rs2085378
rs1171616
rs138548309
rs10912833
rs11652118
rs144809751
rs796484
rs2202311
rs56000812
rs4716067
rs79053704
rs138297249
rs7541058
rs144044755
rs139633674
All
−0.05
0.00
0.05
MR leave−one−out sensitivity analysis for
'X−12101 levels' on 'Rheumatoid arthritis'

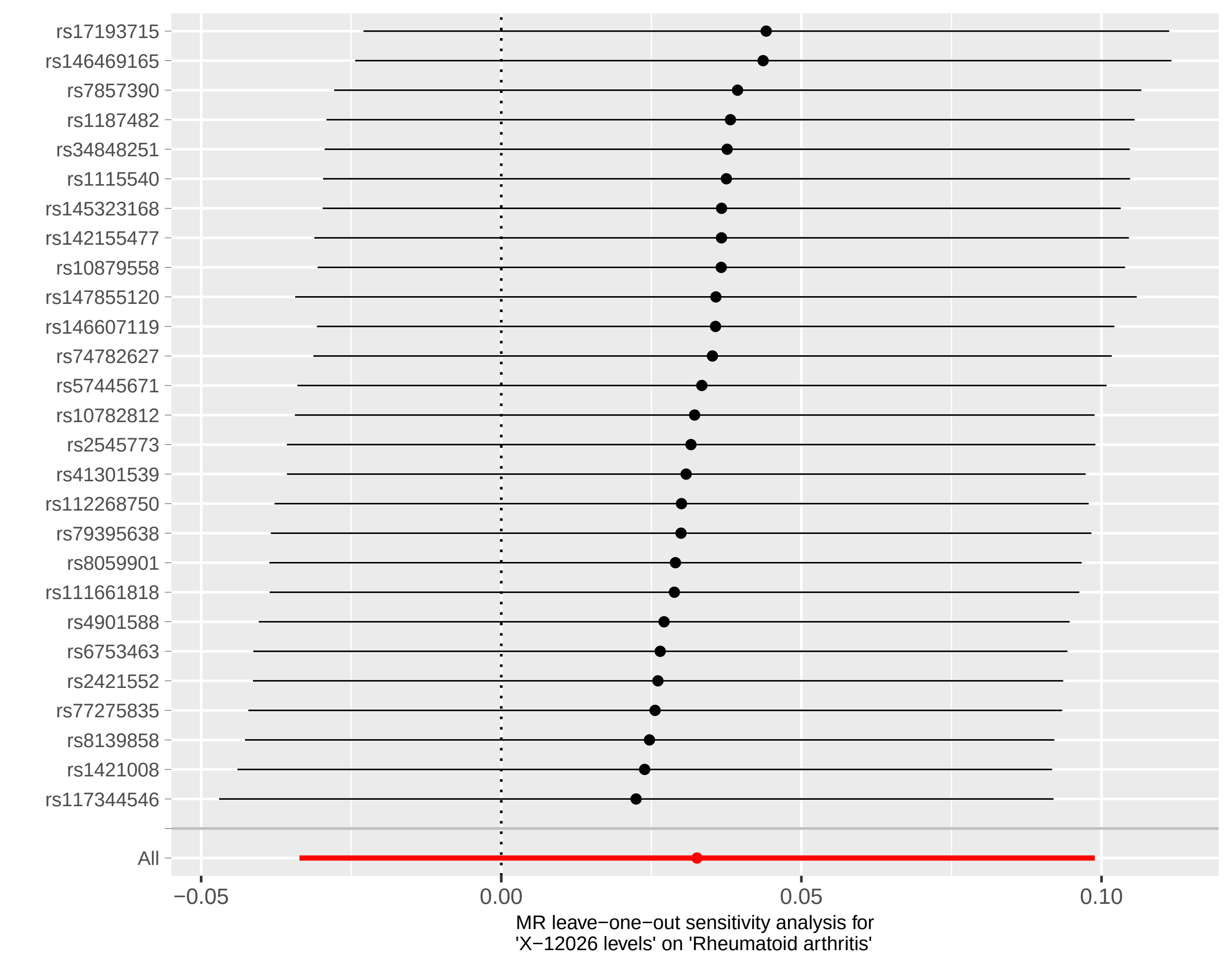
rs17193715
rs146469165
rs7857390
rs1187482
rs34848251
rs1115540
rs145323168
rs142155477
rs10879558
rs147855120
rs146607119
rs74782627
rs57445671
rs10782812
rs2545773
rs41301539
rs112268750
rs79395638
rs8059901
rs111661818
rs4901588
rs6753463
rs2421552
rs77275835
rs8139858
rs1421008
rs117344546
All
−0.05
0.00
0.05
0.10
MR leave−one−out sensitivity analysis for
'X−12026 levels' on 'Rheumatoid arthritis'

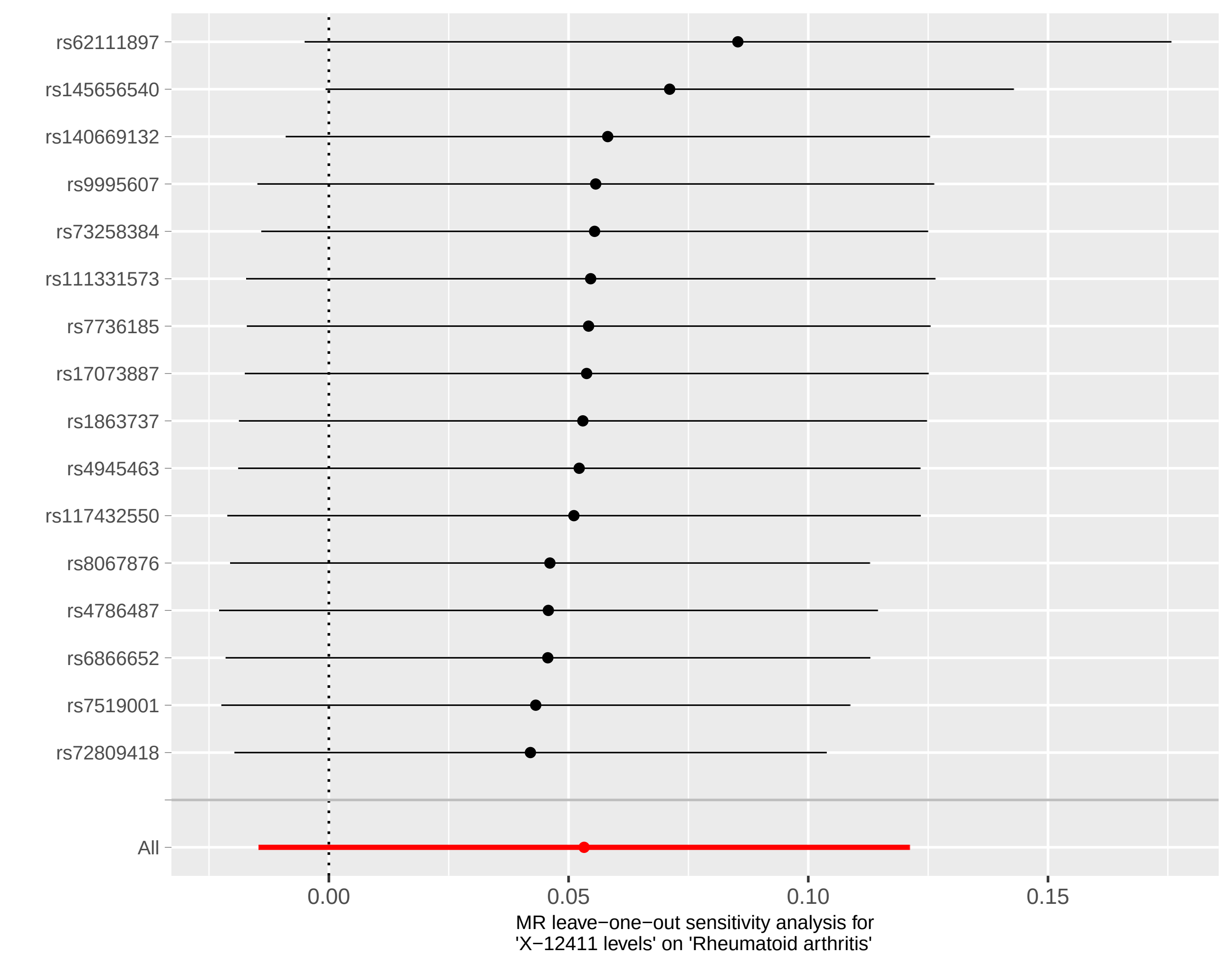
rs62111897
rs145656540
rs140669132
rs9995607
rs73258384
rs111331573
rs7736185
rs17073887
rs1863737
rs4945463
rs117432550
rs8067876
rs4786487
rs6866652
rs7519001
rs72809418
All
0.00
0.05
0.10
0.15
MR leave−one−out sensitivity analysis for
'X−12411 levels' on 'Rheumatoid arthritis'

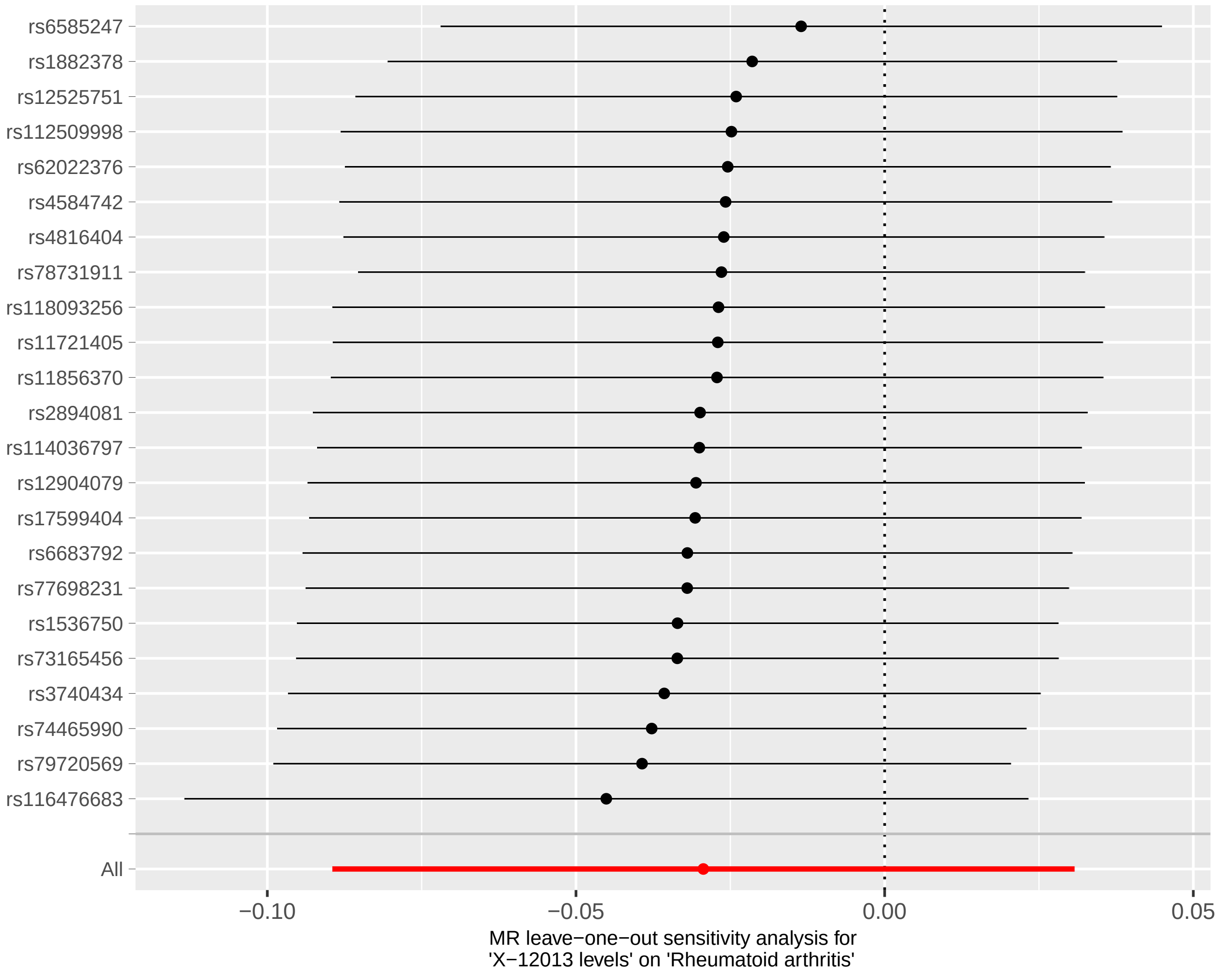
rs6585247
rs1882378
rs12525751
rs112509998
rs62022376
rs4584742
rs4816404
rs78731911
rs118093256
rs11721405
rs11856370
rs2894081
rs114036797
rs12904079
rs17599404
rs6683792
rs77698231
rs1536750
rs73165456
rs3740434
rs74465990
rs79720569
rs116476683
All
−0.10
−0.05
0.00
0.05
MR leave−one−out sensitivity analysis for
'X−12013 levels' on 'Rheumatoid arthritis'

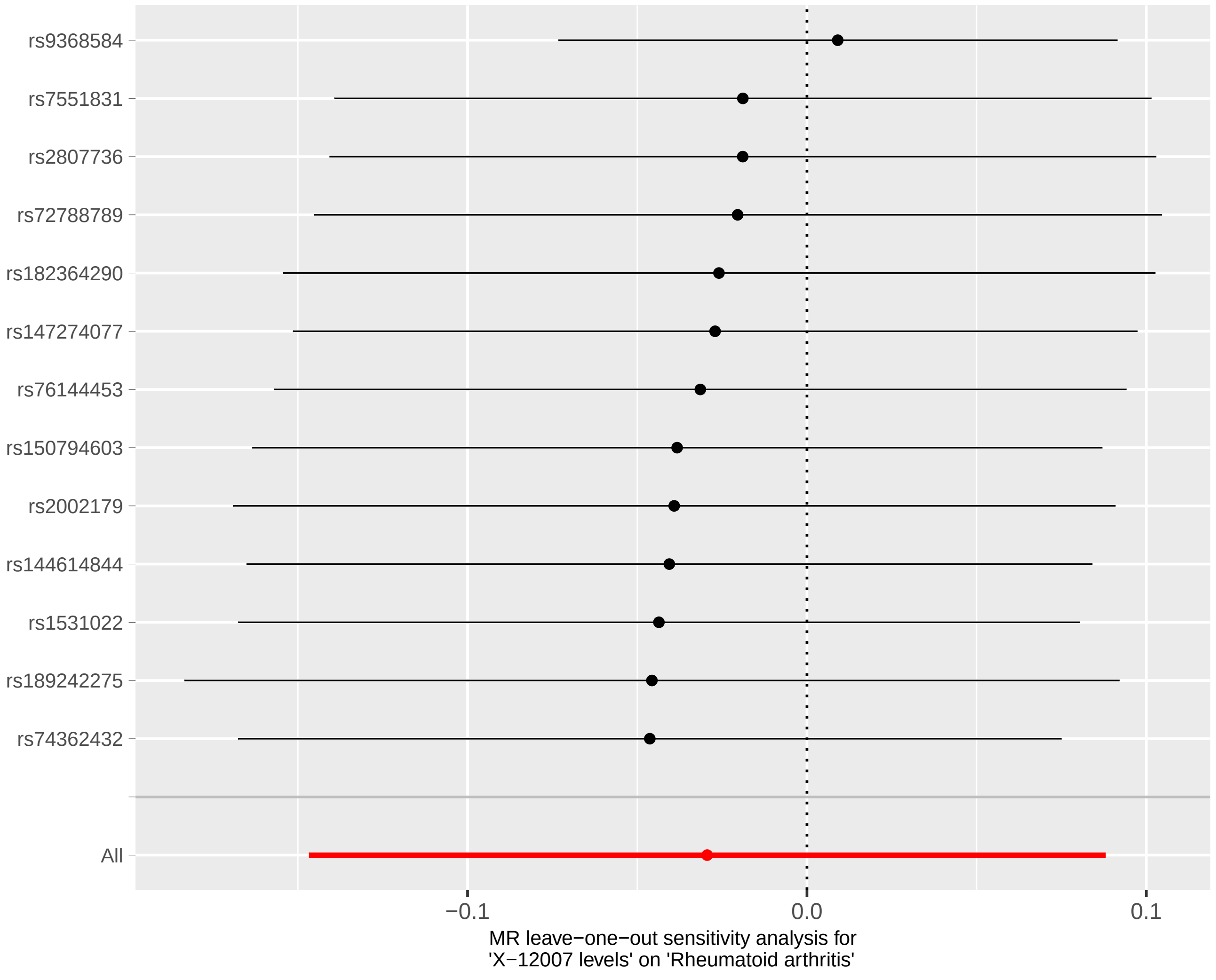

rs9368584
rs7551831
rs2807736
rs72788789
rs182364290
rs147274077
rs76144453
rs150794603
rs2002179
rs144614844
rs1531022
rs189242275
rs74362432
All
−0.1
0.0
0.1
MR leave−one−out sensitivity analysis for
'X−12007 levels' on 'Rheumatoid arthritis'

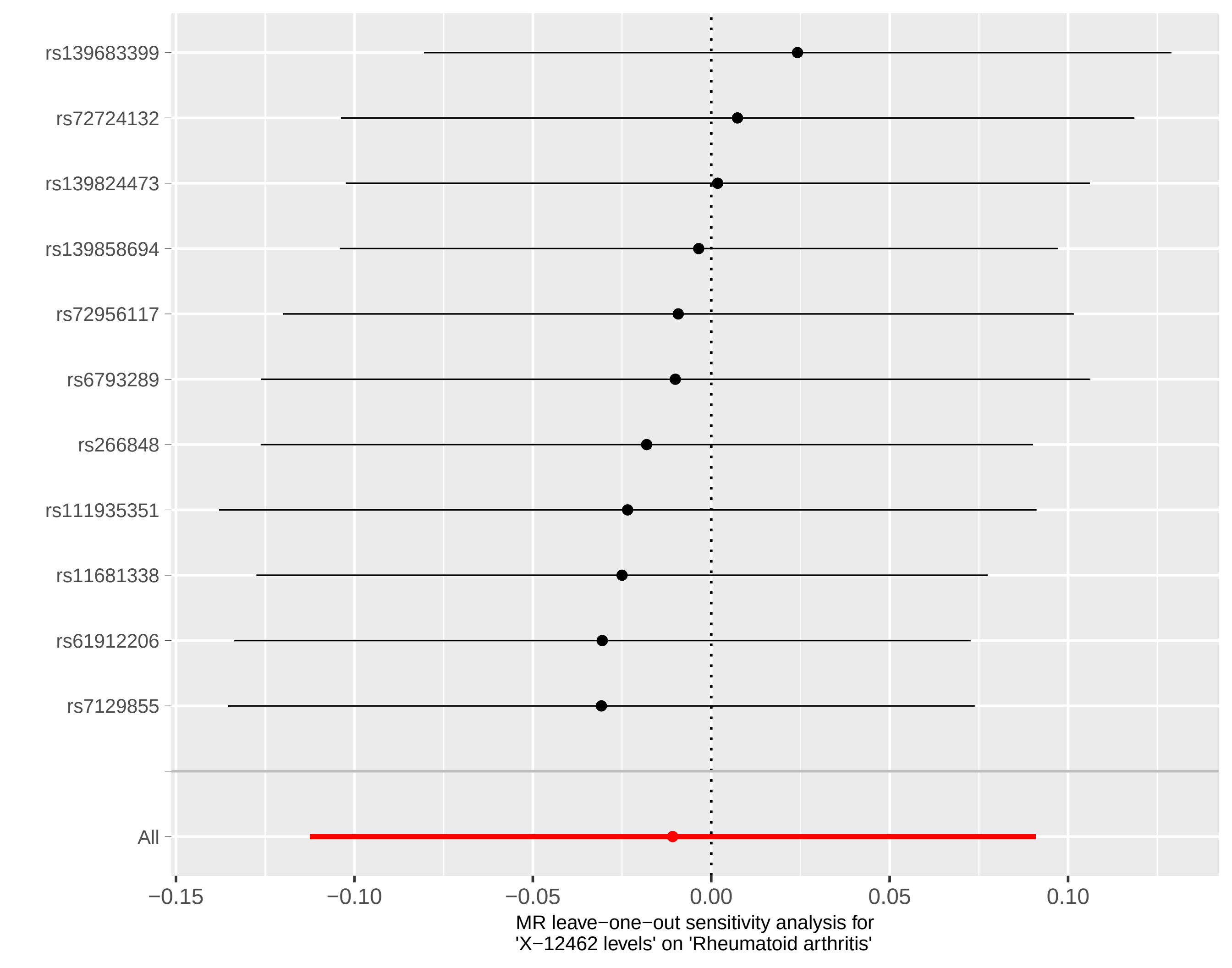
rs139683399
rs72724132
rs139824473
rs139858694
rs72956117
rs6793289
rs266848
rs111935351
rs11681338
rs61912206
rs7129855
All
−0.15
−0.10
−0.05
0.00
0.05
0.10
MR leave−one−out sensitivity analysis for
'X−12462 levels' on 'Rheumatoid arthritis'

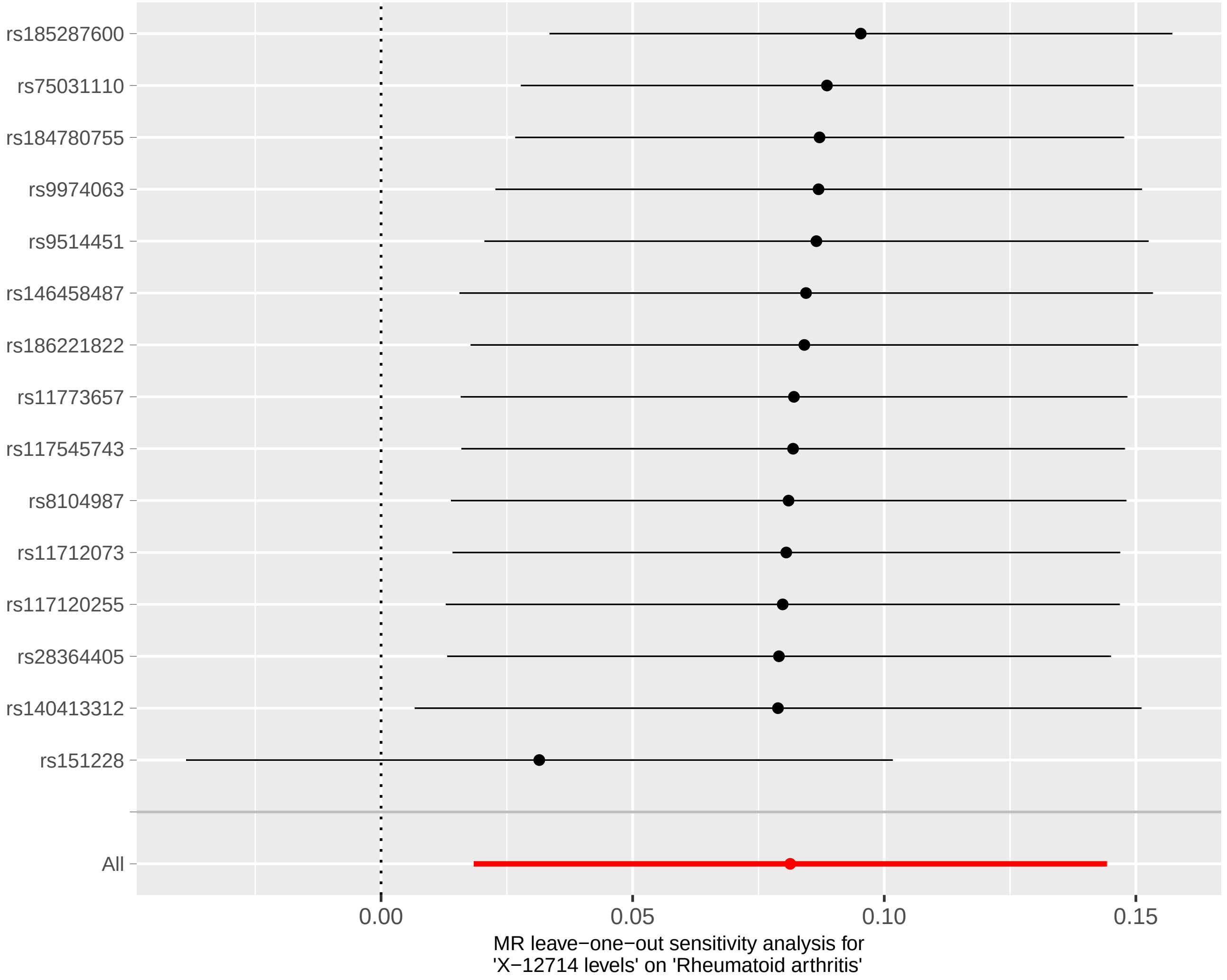

rs185287600
rs75031110
rs184780755
rs9974063
rs9514451
rs146458487
rs186221822
rs11773657
rs117545743
rs8104987
rs11712073
rs117120255
rs28364405
rs140413312
rs151228
All
0.00
0.05
0.10
0.15
MR leave−one−out sensitivity analysis for
'X−12714 levels' on 'Rheumatoid arthritis'

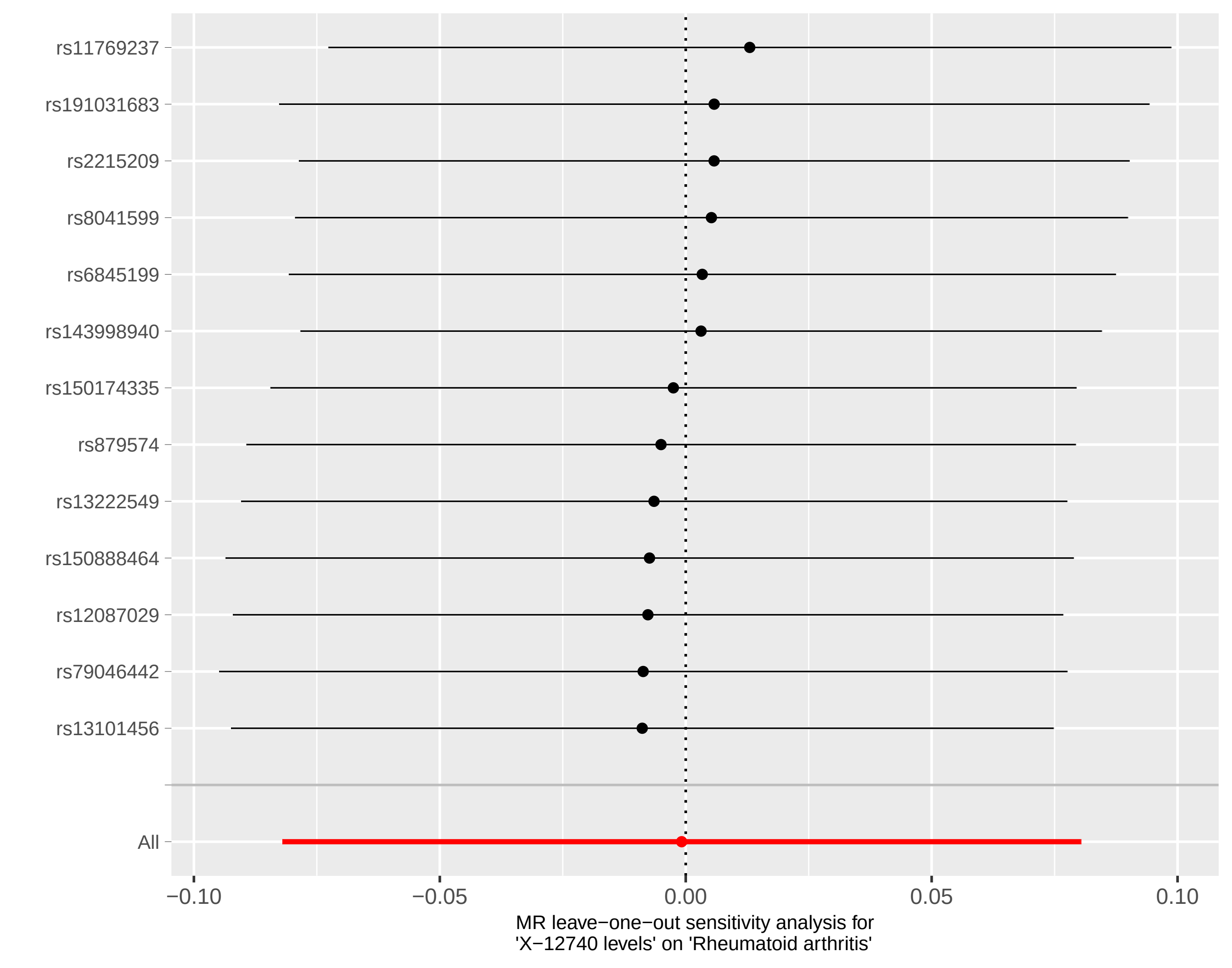

rs11769237
rs191031683
rs2215209
rs8041599
rs6845199
rs143998940
rs150174335
rs879574
rs13222549
rs150888464
rs12087029
rs79046442
rs13101456
All
−0.10
−0.05
0.00
0.05
0.10
MR leave−one−out sensitivity analysis for
'X−12740 levels' on 'Rheumatoid arthritis'

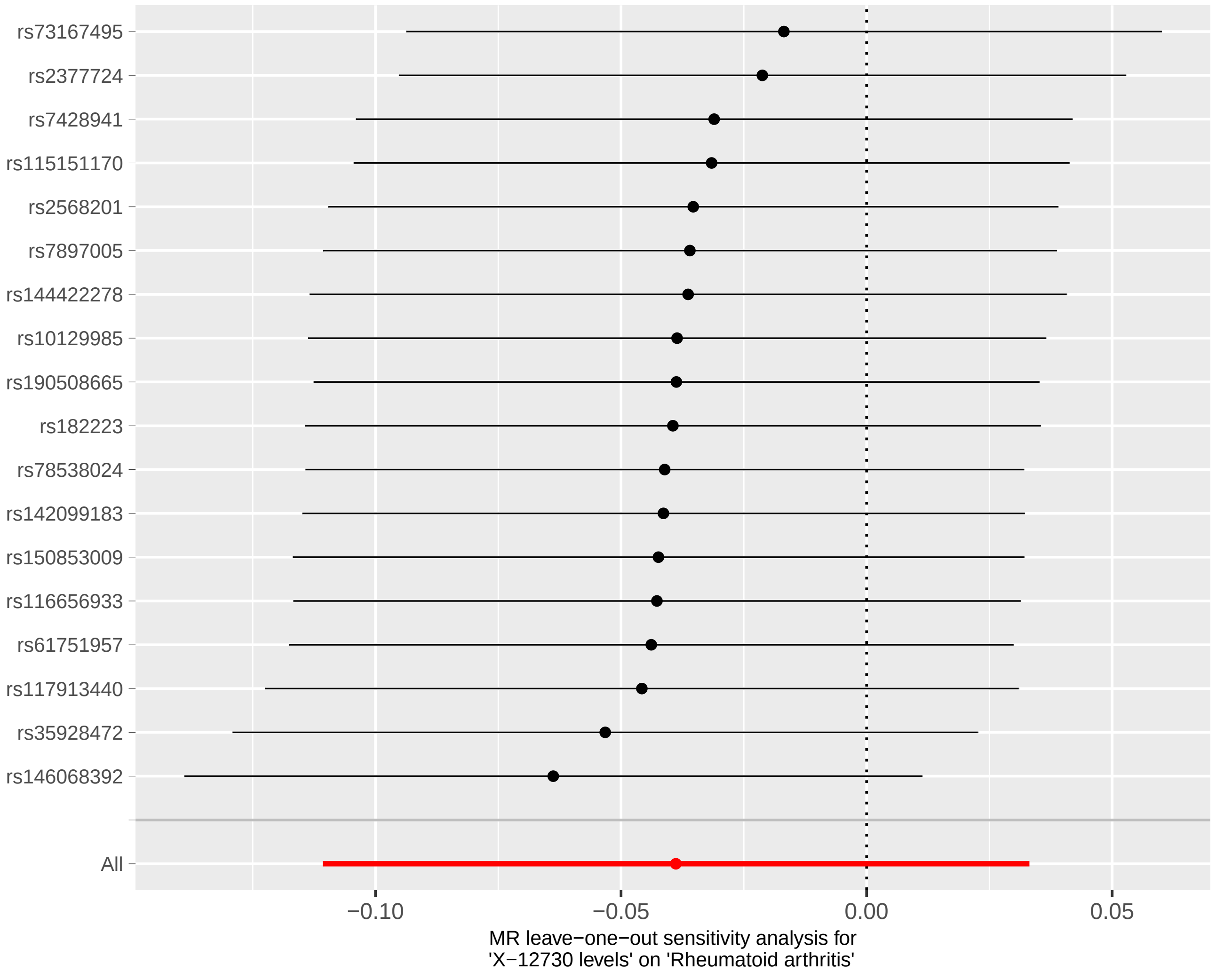
rs73167495
rs2377724
rs7428941
rs115151170
rs2568201
rs7897005
rs144422278
rs10129985
rs190508665
rs182223
rs78538024
rs142099183
rs150853009
rs116656933
rs61751957
rs117913440
rs35928472
rs146068392
All
−0.10
−0.05
0.00
0.05
MR leave−one−out sensitivity analysis for
'X−12730 levels' on 'Rheumatoid arthritis'

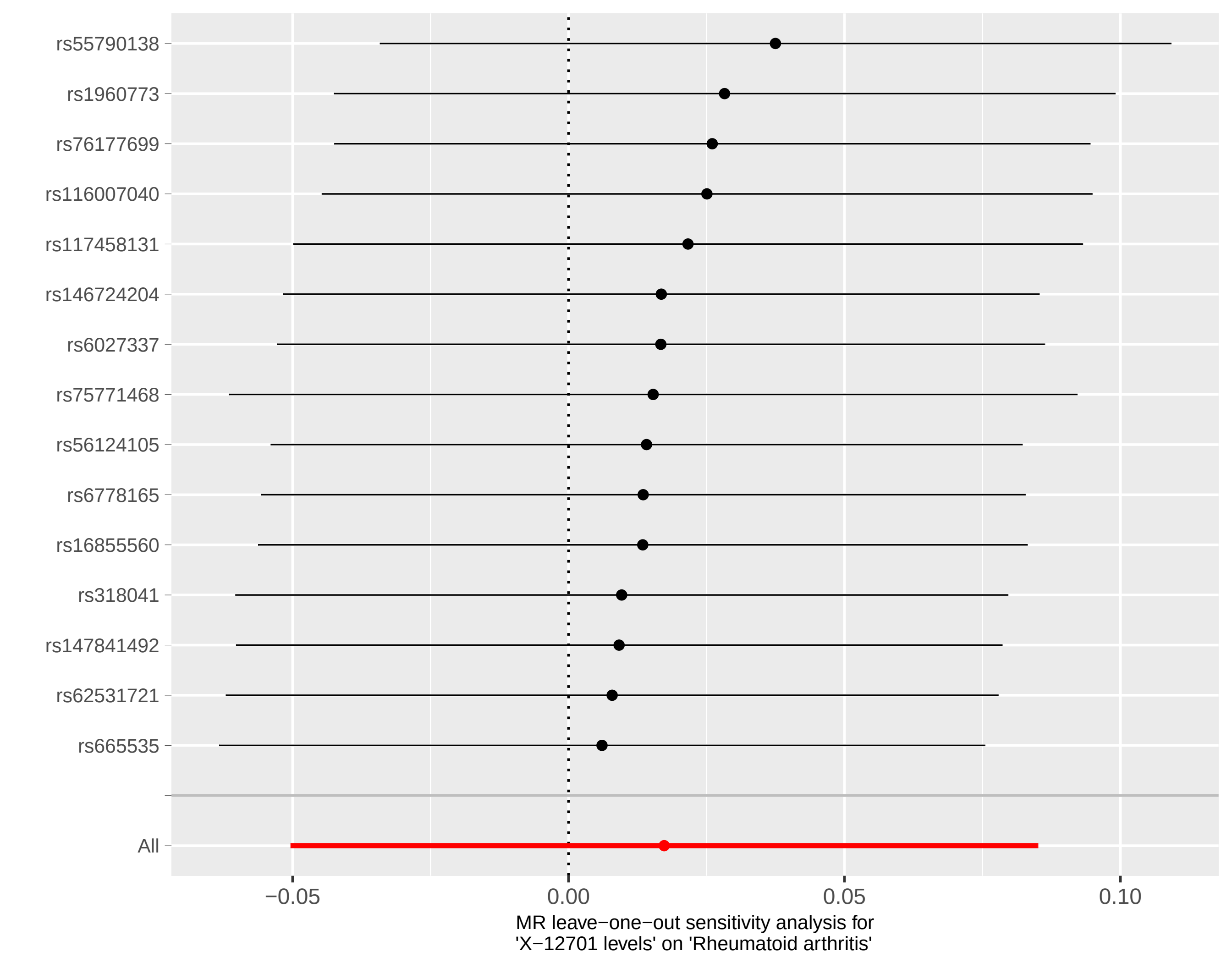

rs55790138
rs1960773
rs76177699
rs116007040
rs117458131
rs146724204
rs6027337
rs75771468
rs56124105
rs6778165
rs16855560
rs318041
rs147841492
rs62531721
rs665535
All
−0.05
0.00
0.05
0.10
MR leave−one−out sensitivity analysis for
'X−12701 levels' on 'Rheumatoid arthritis'

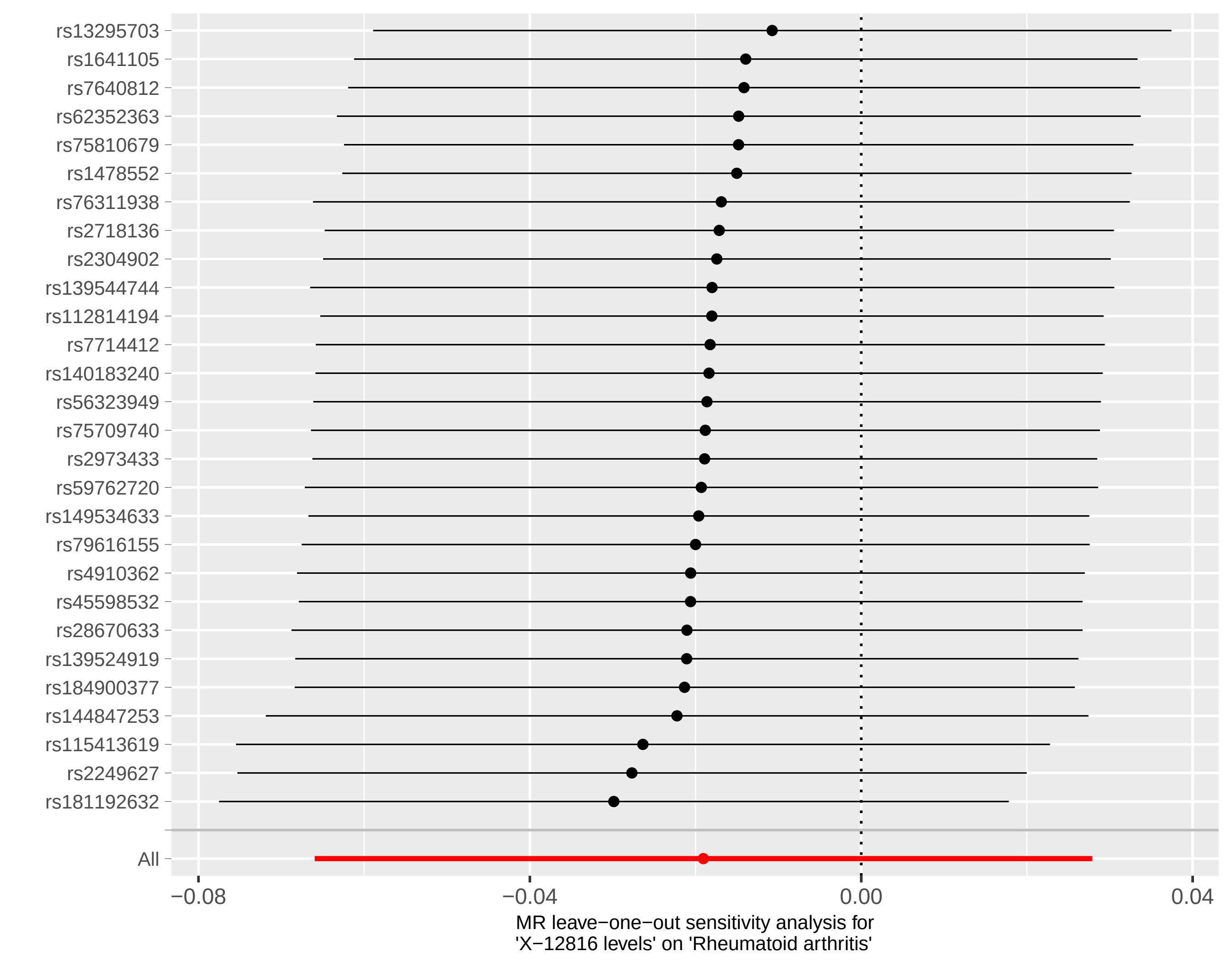

rs13295703
rs1641105
rs7640812
rs62352363
rs75810679
rs1478552
rs76311938
rs2718136
rs2304902
rs139544744
rs112814194
rs7714412
rs140183240
rs56323949
rs75709740
rs2973433
rs59762720
rs149534633
rs79616155
rs4910362
rs45598532
rs28670633
rs139524919
rs184900377
rs144847253
rs115413619
rs2249627
rs181192632
All
−0.08
−0.04
0.00
0.04
MR leave−one−out sensitivity analysis for
'X−12816 levels' on 'Rheumatoid arthritis'

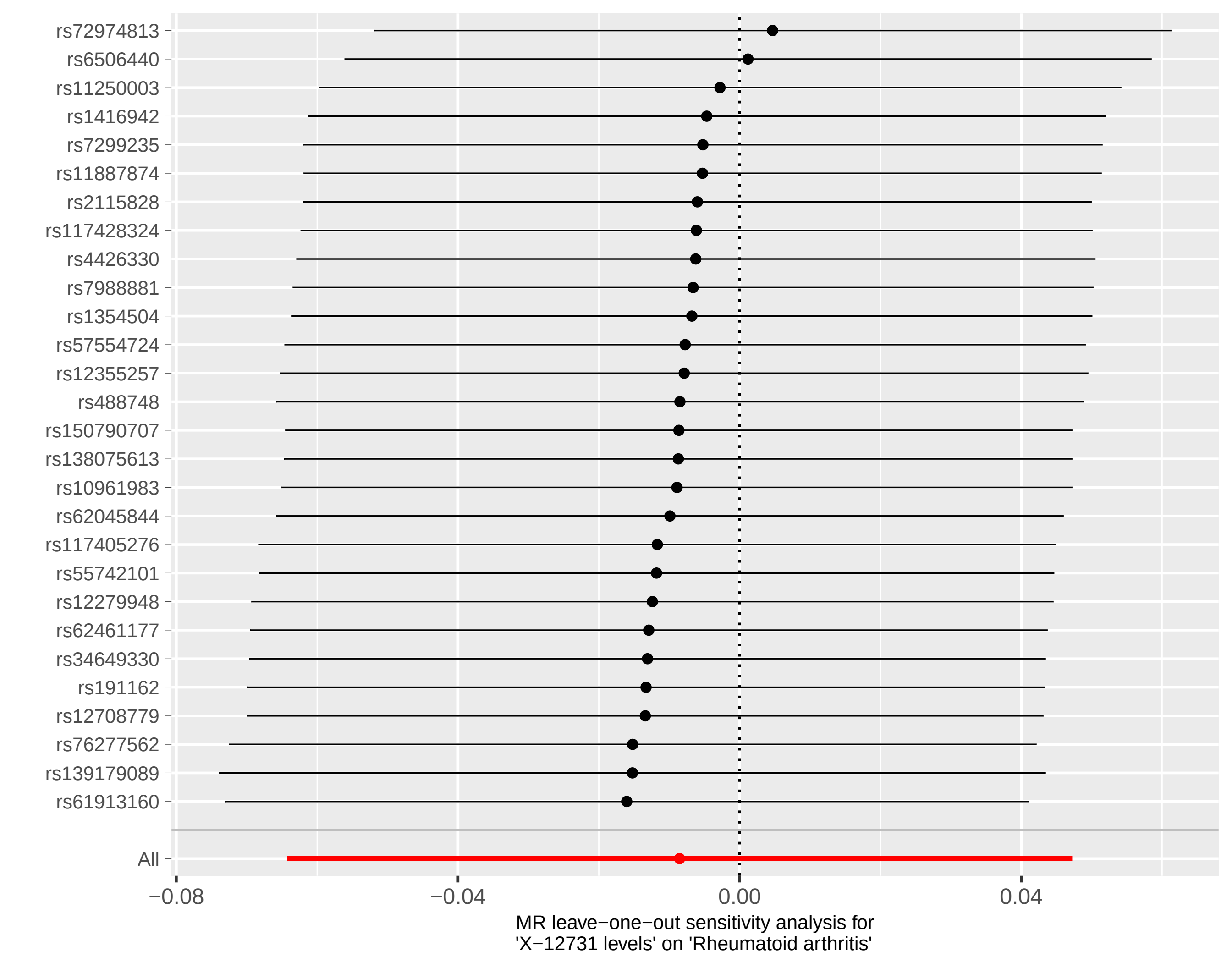
rs72974813
rs6506440
rs11250003
rs1416942
rs7299235
rs11887874
rs2115828
rs117428324
rs4426330
rs7988881
rs1354504
rs57554724
rs12355257
rs488748
rs150790707
rs138075613
rs10961983
rs62045844
rs117405276
rs55742101
rs12279948
rs62461177
rs34649330
rs191162
rs12708779
rs76277562
rs139179089
rs61913160
All
−0.08
−0.04
0.00
0.04
MR leave−one−out sensitivity analysis for
'X−12731 levels' on 'Rheumatoid arthritis'

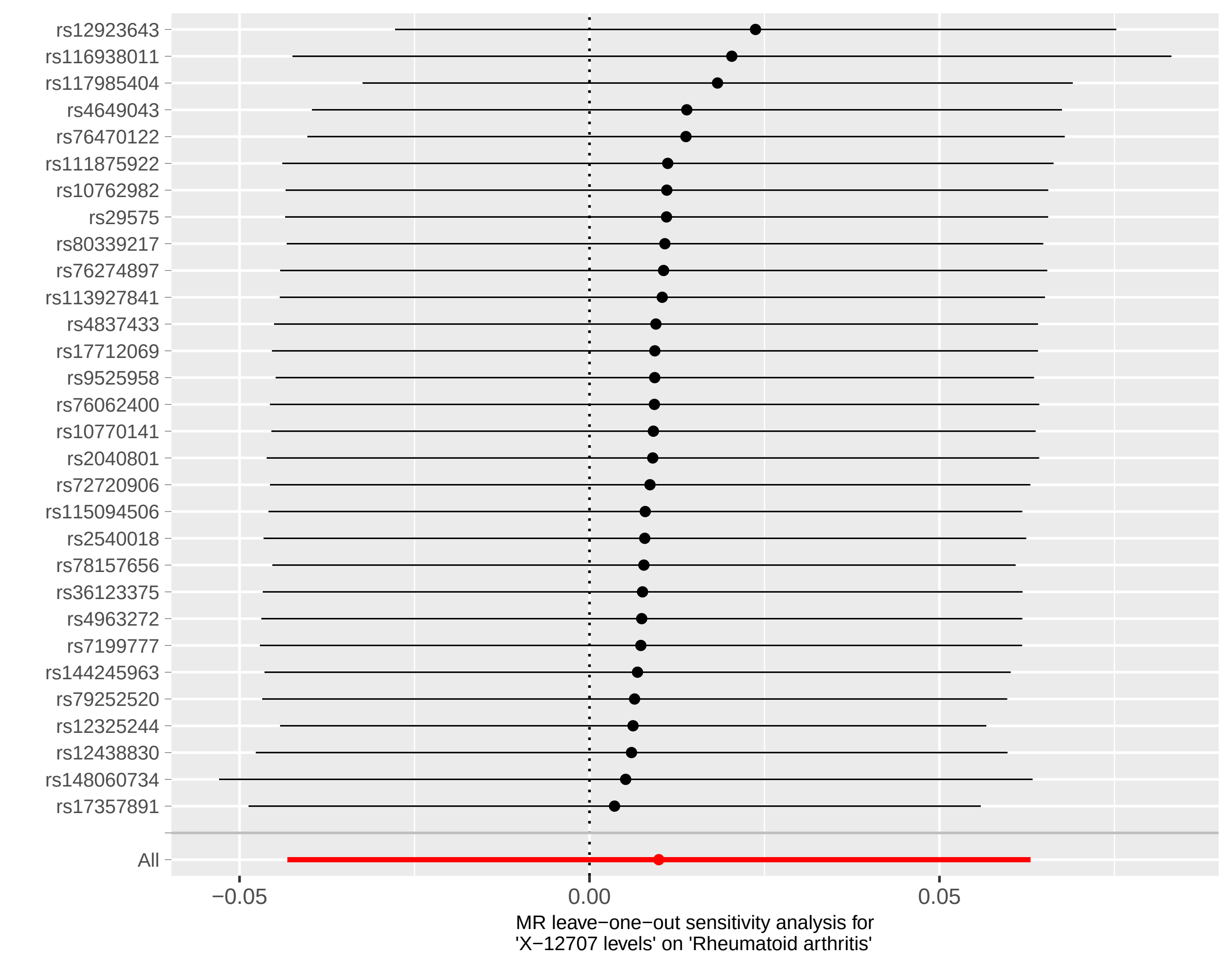
rs12923643
rs116938011
rs117985404
rs4649043
rs76470122
rs111875922
rs10762982
rs29575
rs80339217
rs76274897
rs113927841
rs4837433
rs17712069
rs9525958
rs76062400
rs10770141
rs2040801
rs72720906
rs115094506
rs2540018
rs78157656
rs36123375
rs4963272
rs7199777
rs144245963
rs79252520
rs12325244
rs12438830
rs148060734
rs17357891
All
−0.05
0.00
0.05
MR leave−one−out sensitivity analysis for
'X−12707 levels' on 'Rheumatoid arthritis'

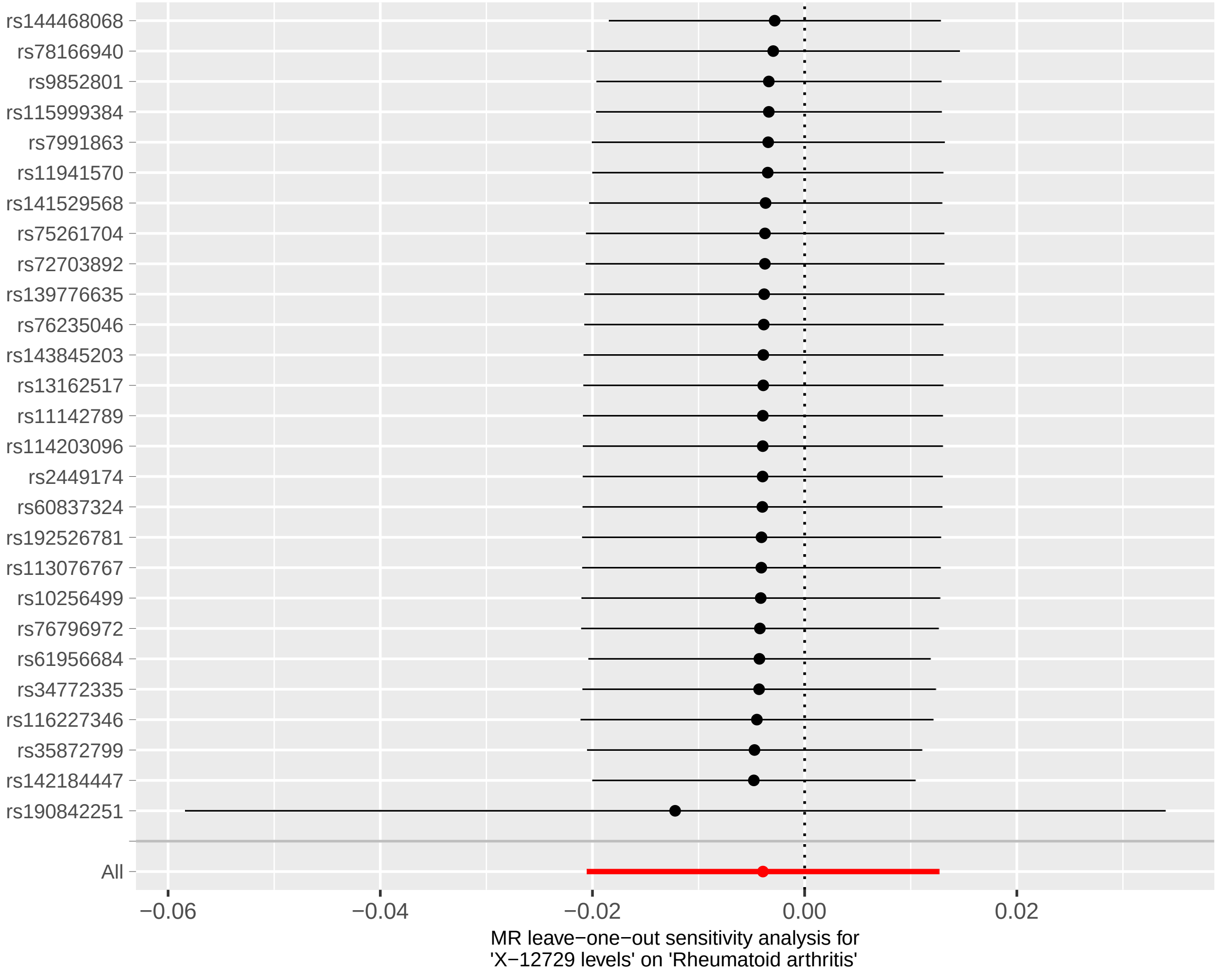
rs144468068
rs78166940
rs9852801
rs115999384
rs7991863
rs11941570
rs141529568
rs75261704
rs72703892
rs139776635
rs76235046
rs143845203
rs13162517
rs11142789
rs114203096
rs2449174
rs60837324
rs192526781
rs113076767
rs10256499
rs76796972
rs61956684
rs34772335
rs116227346
rs35872799
rs142184447
rs190842251
All
−0.06
−0.04
−0.02
0.00
0.02
MR leave−one−out sensitivity analysis for
'X−12729 levels' on 'Rheumatoid arthritis'

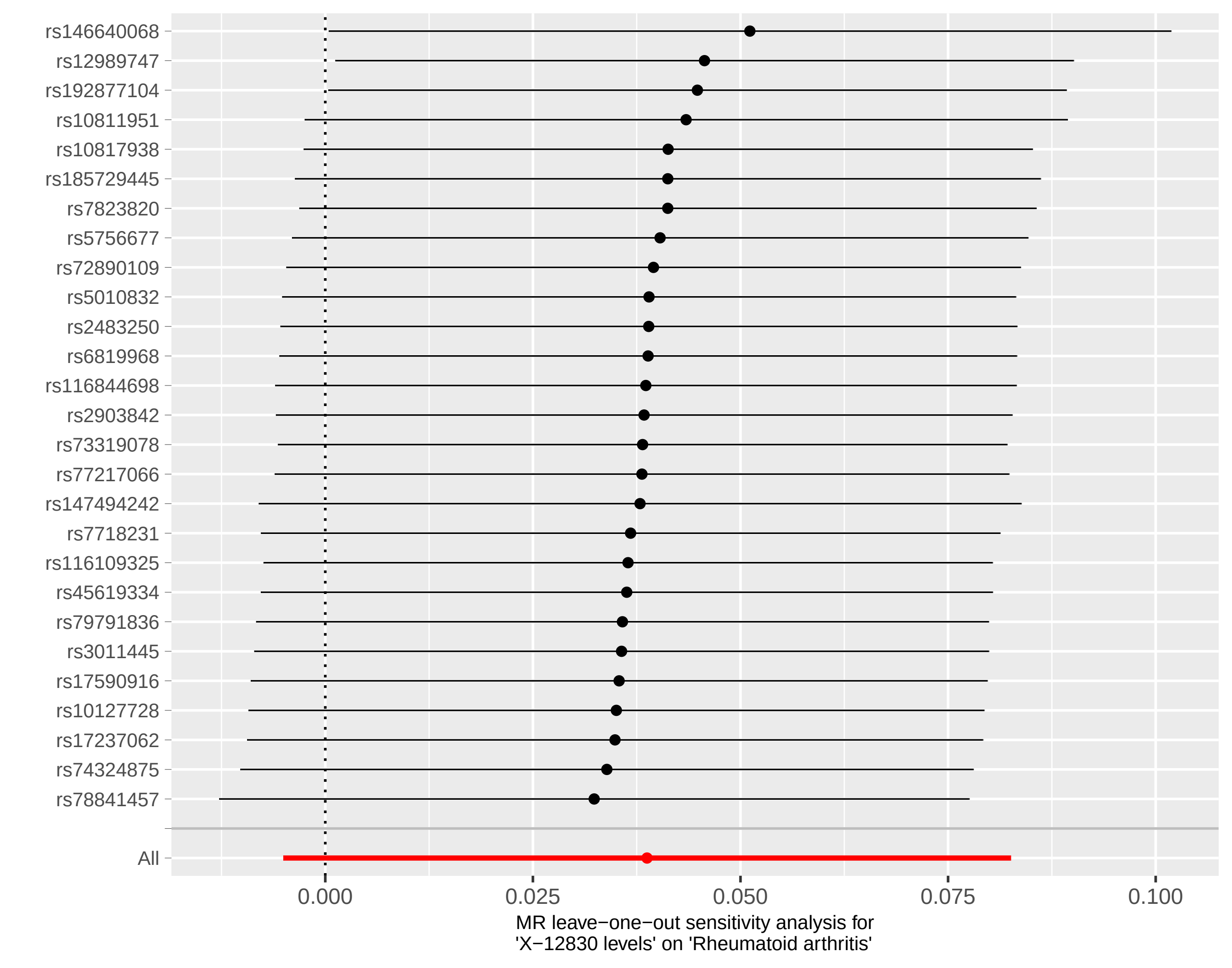
rs146640068
rs12989747
rs192877104
rs10811951
rs10817938
rs185729445
rs7823820
rs5756677
rs72890109
rs5010832
rs2483250
rs6819968
rs116844698
rs2903842
rs73319078
rs77217066
rs147494242
rs7718231
rs116109325
rs45619334
rs79791836
rs3011445
rs17590916
rs10127728
rs17237062
rs74324875
rs78841457
All
0.000
0.025
0.050
0.075
0.100
MR leave−one−out sensitivity analysis for
'X−12830 levels' on 'Rheumatoid arthritis'

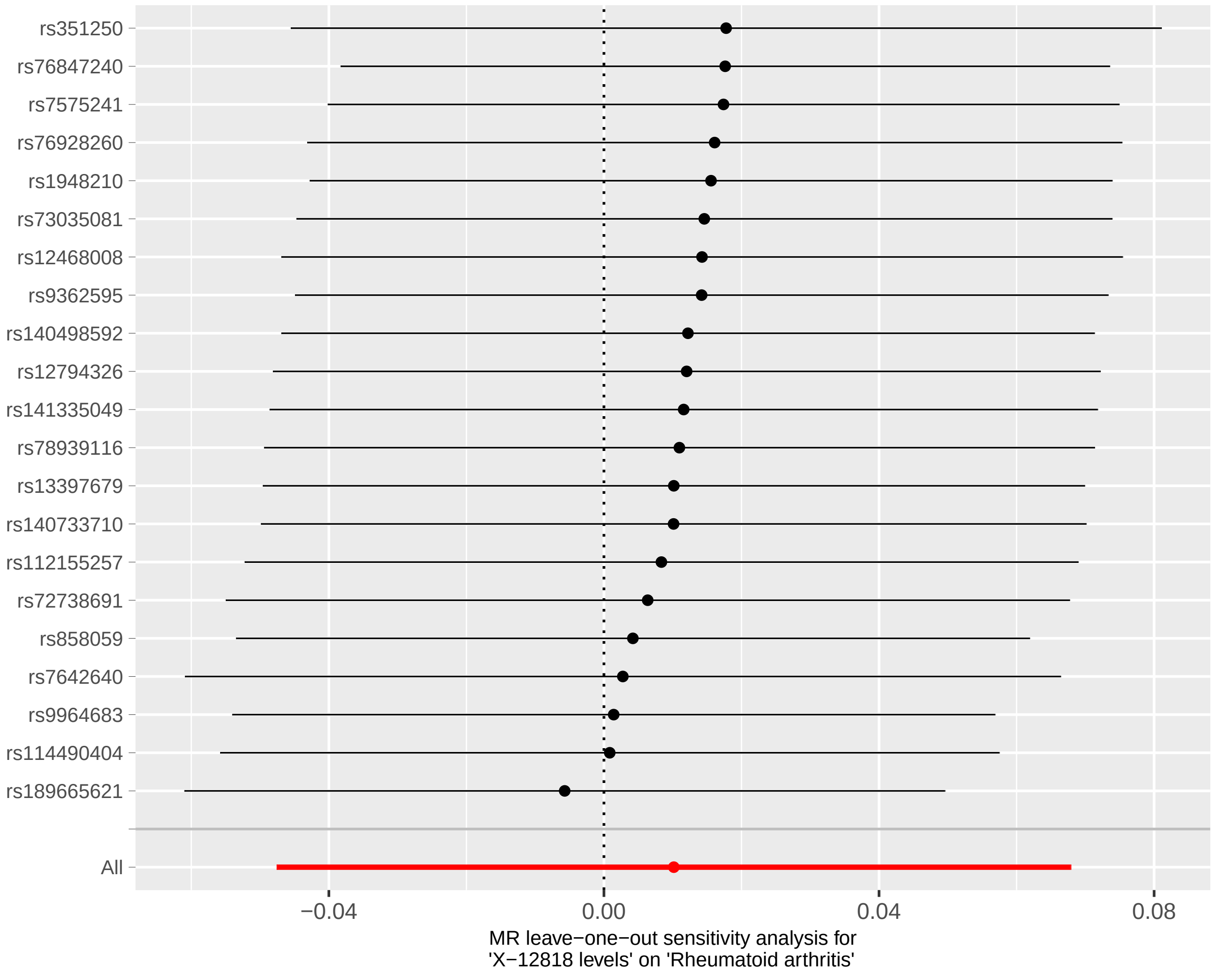
rs351250
rs76847240
rs7575241
rs76928260
rs1948210
rs73035081
rs12468008
rs9362595
rs140498592
rs12794326
rs141335049
rs78939116
rs13397679
rs140733710
rs112155257
rs72738691
rs858059
rs7642640
rs9964683
rs114490404
rs189665621
All
−0.04
0.00
0.04
0.08
MR leave−one−out sensitivity analysis for
'X−12818 levels' on 'Rheumatoid arthritis'

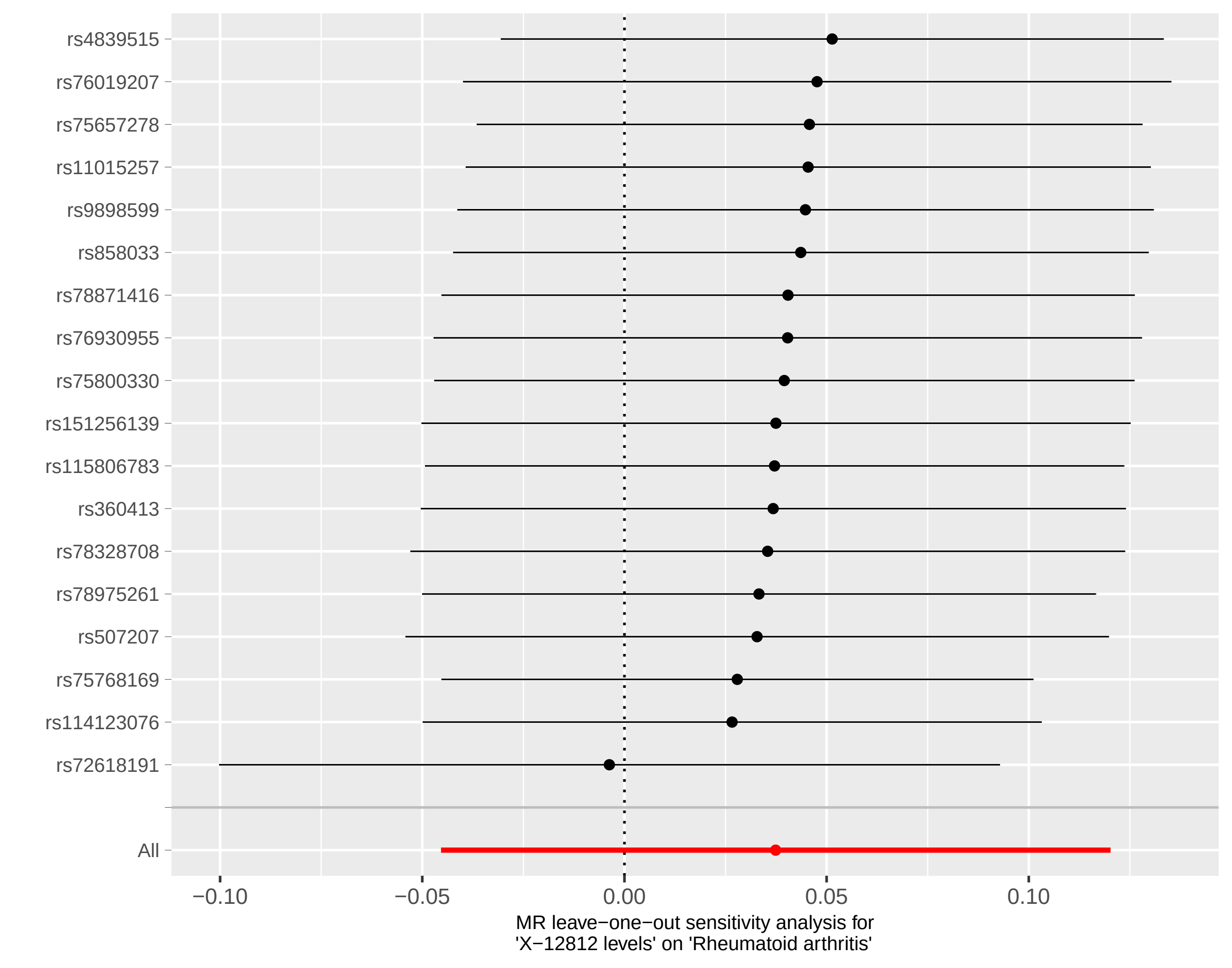
rs4839515
rs76019207
rs75657278
rs11015257
rs9898599
rs858033
rs78871416
rs76930955
rs75800330
rs151256139
rs115806783
rs360413
rs78328708
rs78975261
rs507207
rs75768169
rs114123076
rs72618191
All
−0.10
−0.05
0.00
0.05
0.10
MR leave−one−out sensitivity analysis for
'X−12812 levels' on 'Rheumatoid arthritis'

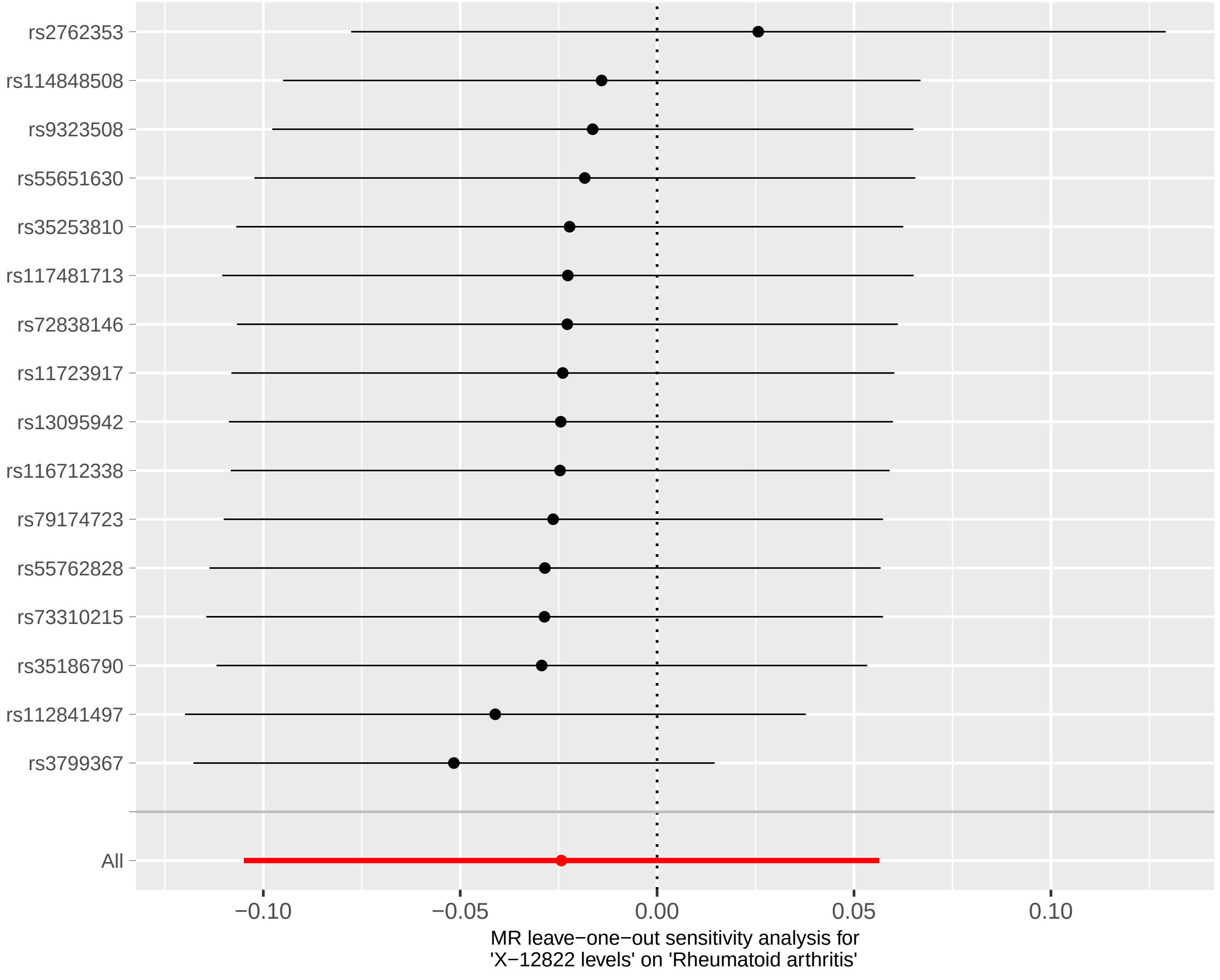
rs2762353
rs114848508
rs9323508
rs55651630
rs35253810
rs117481713
rs72838146
rs11723917
rs13095942
rs116712338
rs79174723
rs55762828
rs73310215
rs35186790
rs112841497
rs3799367
All
−0.10
−0.05
0.00
0.05
0.10
MR leave−one−out sensitivity analysis for
'X−12822 levels' on 'Rheumatoid arthritis'

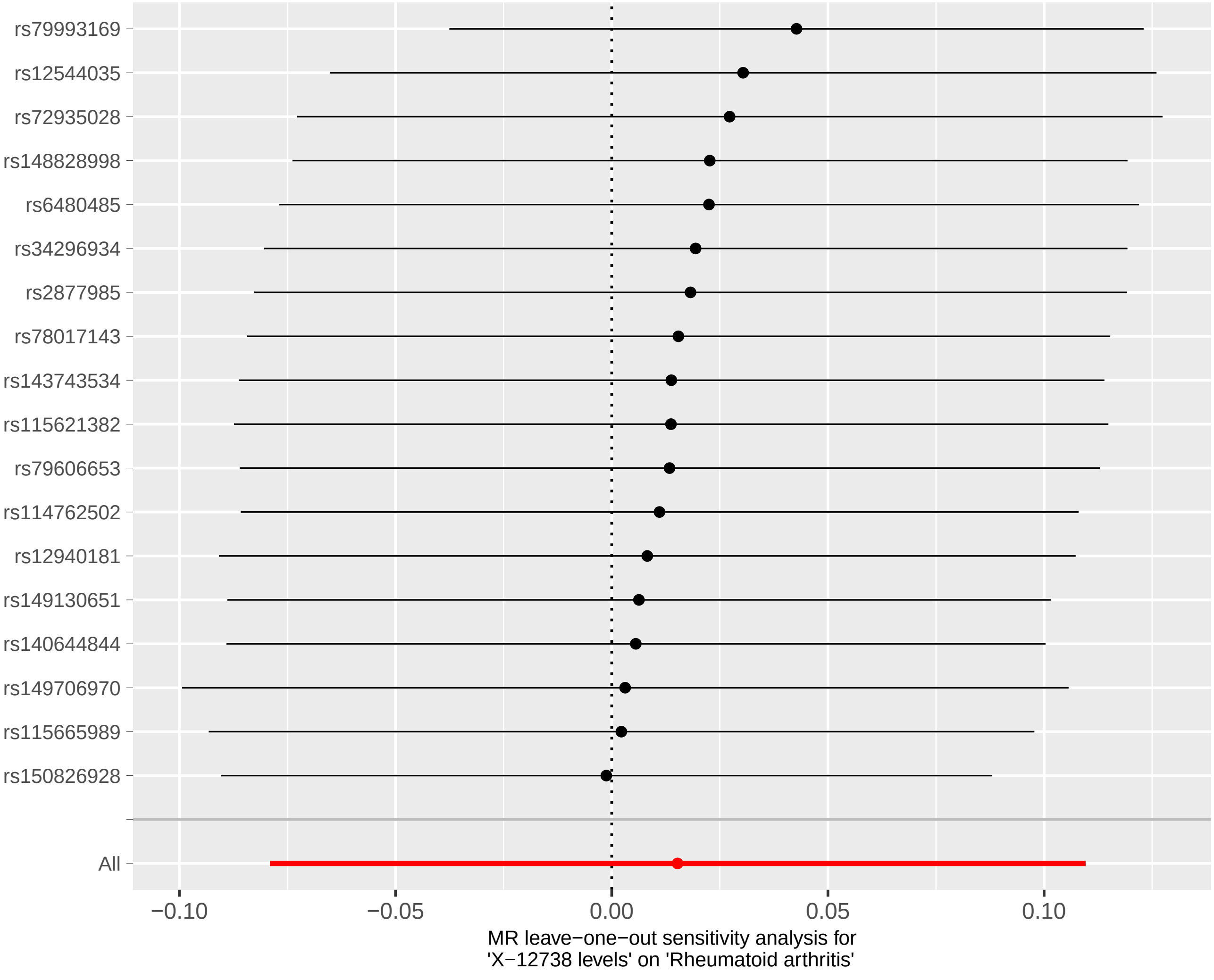
rs79993169
rs12544035
rs72935028
rs148828998
rs6480485
rs34296934
rs2877985
rs78017143
rs143743534
rs115621382
rs79606653
rs114762502
rs12940181
rs149130651
rs140644844
rs149706970
rs115665989
rs150826928
All
−0.10
−0.05
0.00
0.05
0.10
MR leave−one−out sensitivity analysis for
'X−12738 levels' on 'Rheumatoid arthritis'

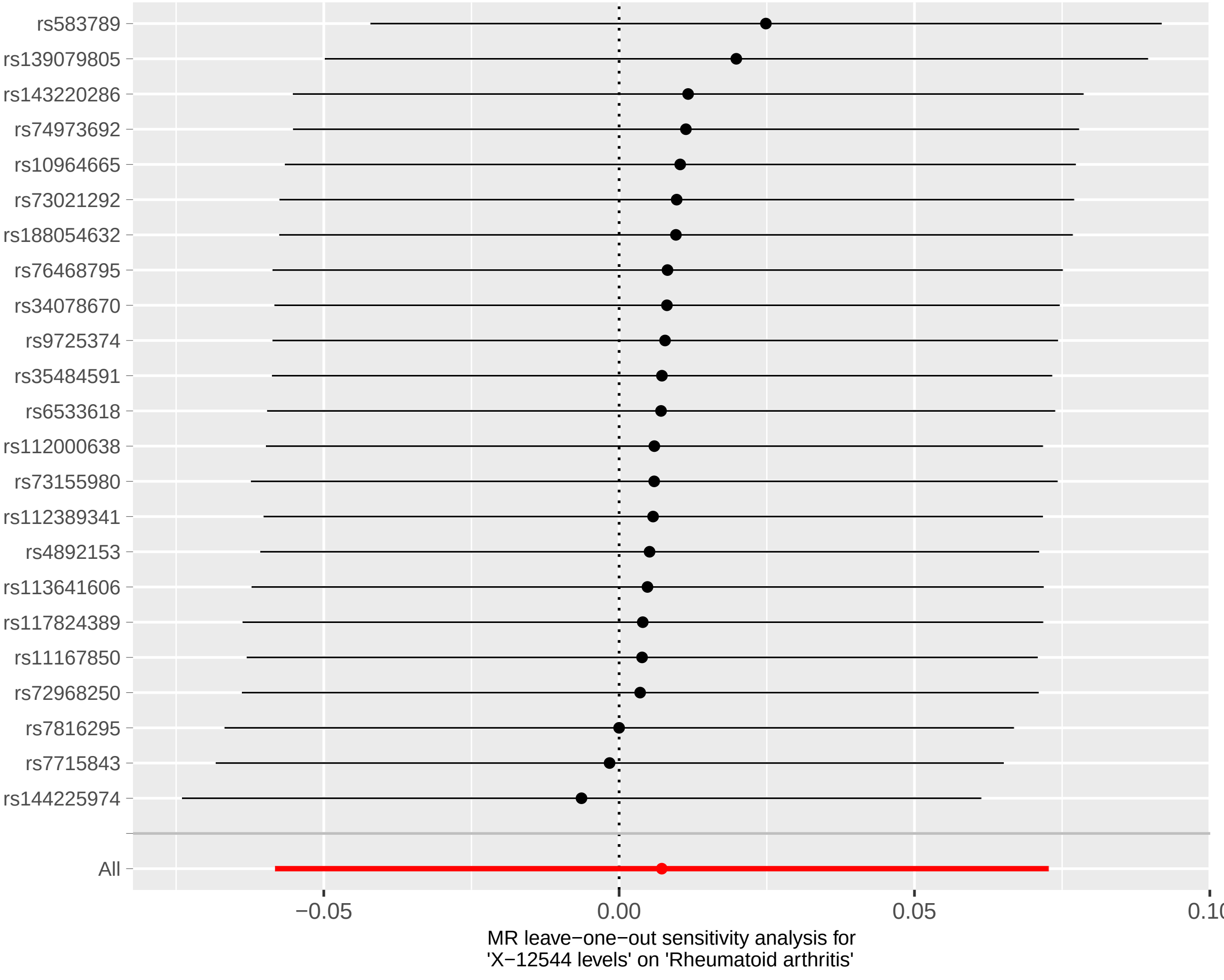
rs583789
rs139079805
rs143220286
rs74973692
rs10964665
rs73021292
rs188054632
rs76468795
rs34078670
rs9725374
rs35484591
rs6533618
rs112000638
rs73155980
rs112389341
rs4892153
rs113641606
rs117824389
rs11167850
rs72968250
rs7816295
rs7715843
rs144225974
All
−0.05
0.00
0.05
MR leave−one−out sensitivity analysis for
'X−12544 levels' on 'Rheumatoid arthritis'

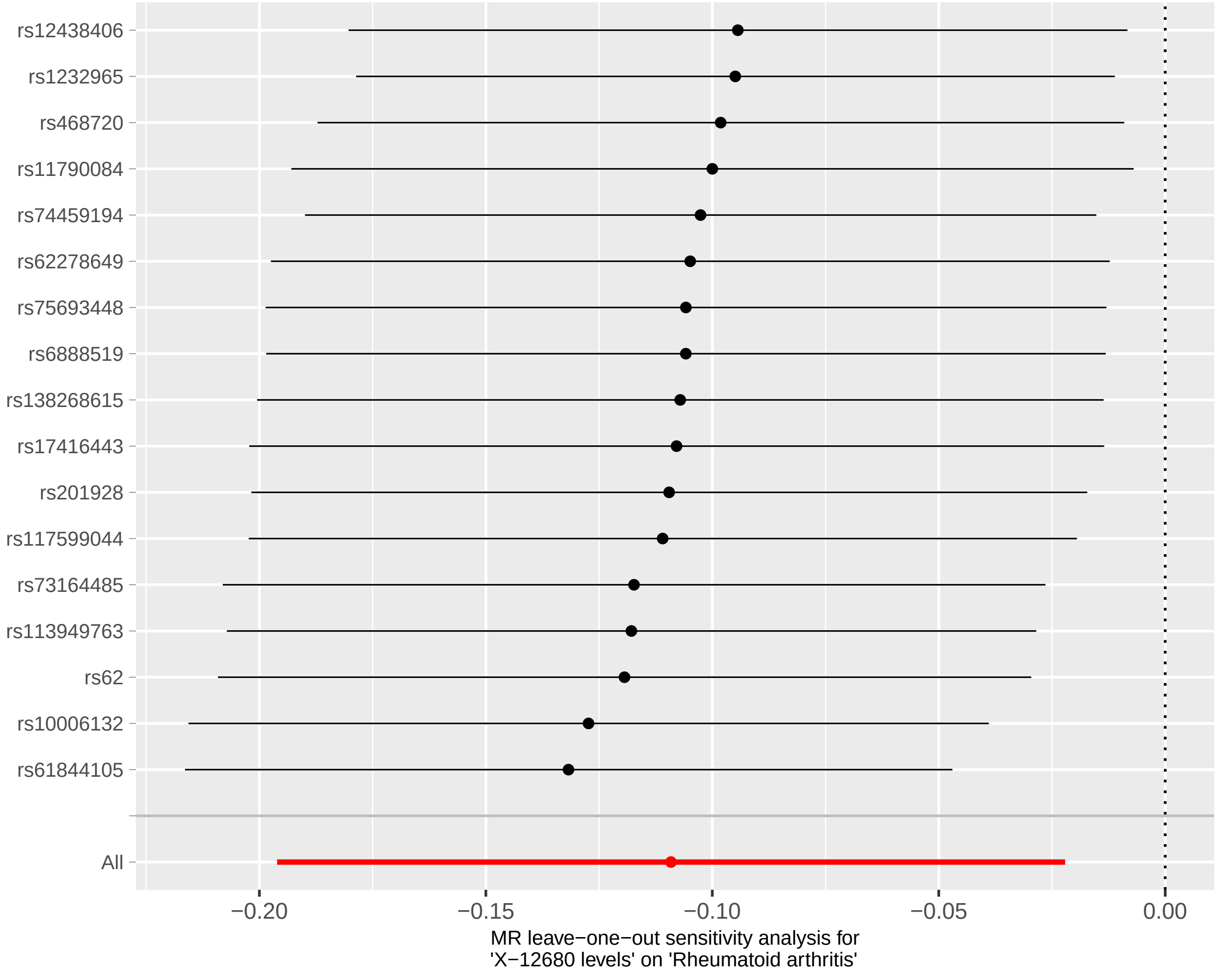
rs12438406
rs1232965
rs468720
rs11790084
rs74459194
rs62278649
rs75693448
rs6888519
rs138268615
rs17416443
rs201928
rs117599044
rs73164485
rs113949763
rs62
rs10006132
rs61844105
All
−0.20
−0.15
−0.10
−0.05
0.00
MR leave−one−out sensitivity analysis for
'X−12680 levels' on 'Rheumatoid arthritis'

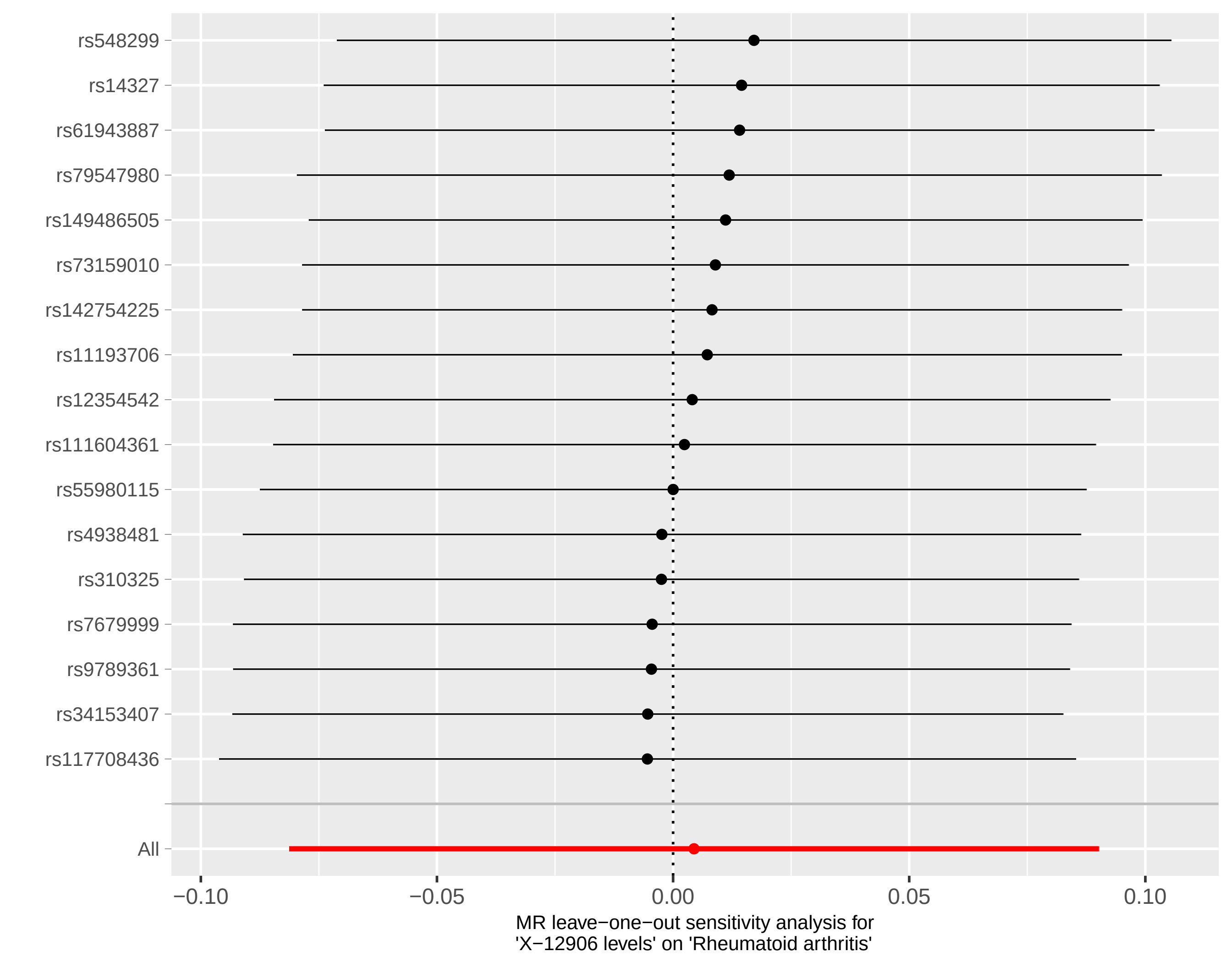

rs548299
rs14327
rs61943887
rs79547980
rs149486505
rs73159010
rs142754225
rs11193706
rs12354542
rs111604361
rs55980115
rs4938481
rs310325
rs7679999
rs9789361
rs34153407
rs117708436
All
−0.10
−0.05
0.00
0.05
0.10
MR leave−one−out sensitivity analysis for
'X−12906 levels' on 'Rheumatoid arthritis'

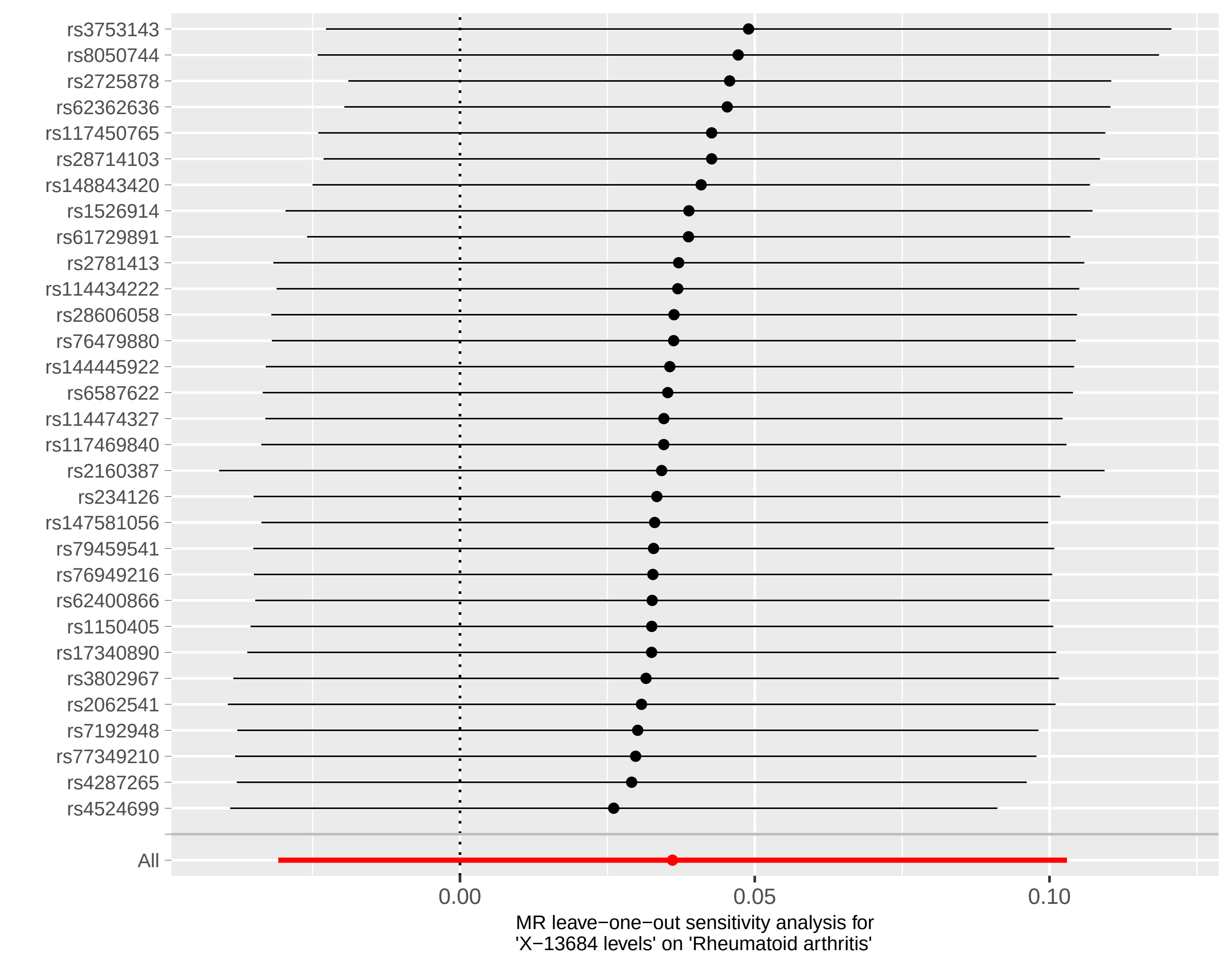
rs3753143
rs8050744
rs2725878
rs62362636
rs117450765
rs28714103
rs148843420
rs1526914
rs61729891
rs2781413
rs114434222
rs28606058
rs76479880
rs144445922
rs6587622
rs114474327
rs117469840
rs2160387
rs234126
rs147581056
rs79459541
rs76949216
rs62400866
rs1150405
rs17340890
rs3802967
rs2062541
rs7192948
rs77349210
rs4287265
rs4524699
All
0.00
0.05
0.10
MR leave−one−out sensitivity analysis for
'X−13684 levels' on 'Rheumatoid arthritis'

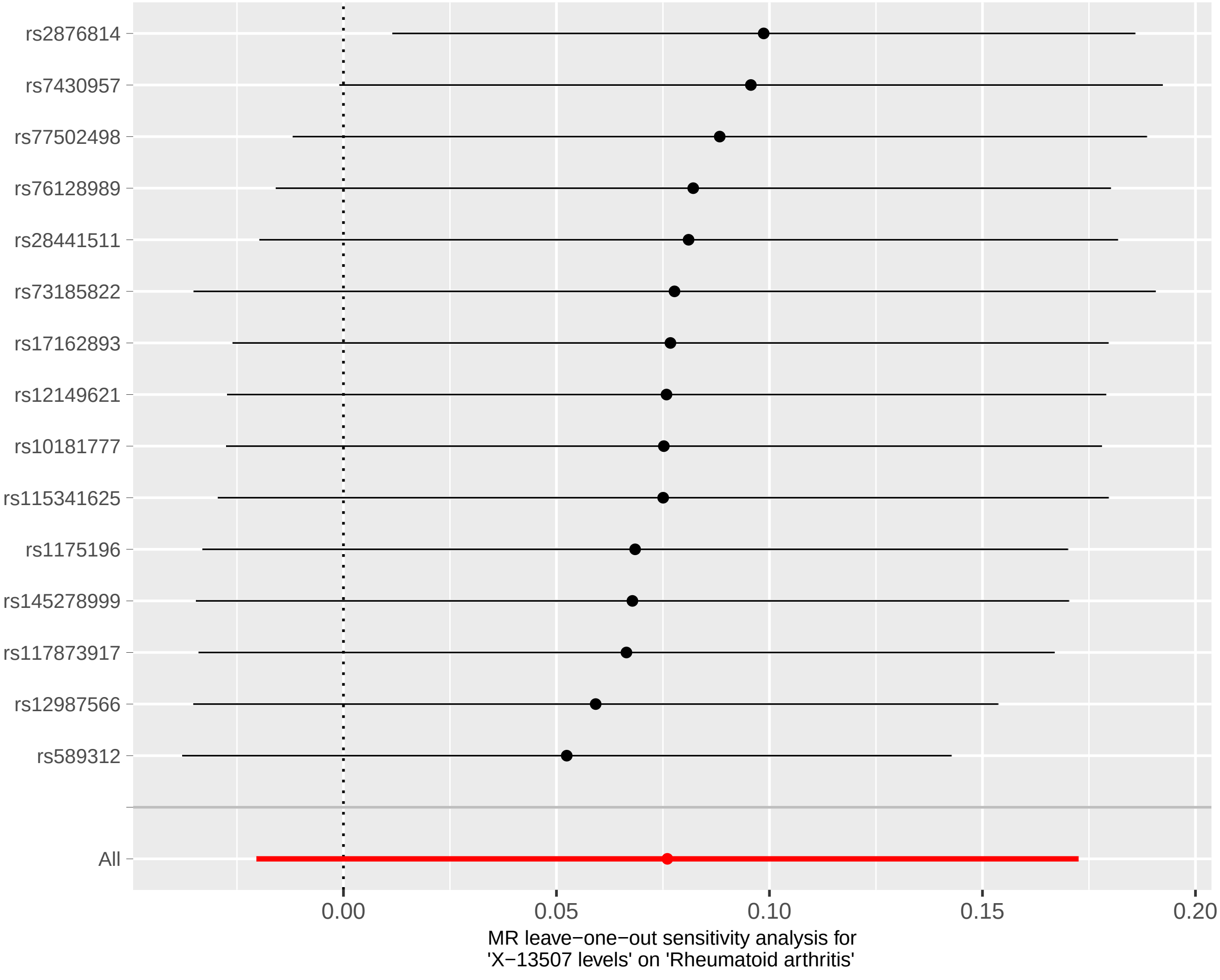

rs2876814
rs7430957
rs77502498
rs76128989
rs28441511
rs73185822
rs17162893
rs12149621
rs10181777
rs115341625
rs1175196
rs145278999
rs117873917
rs12987566
rs589312
All
0.00
0.05
0.10
0.15
0.20
MR leave−one−out sensitivity analysis for
'X−13507 levels' on 'Rheumatoid arthritis'

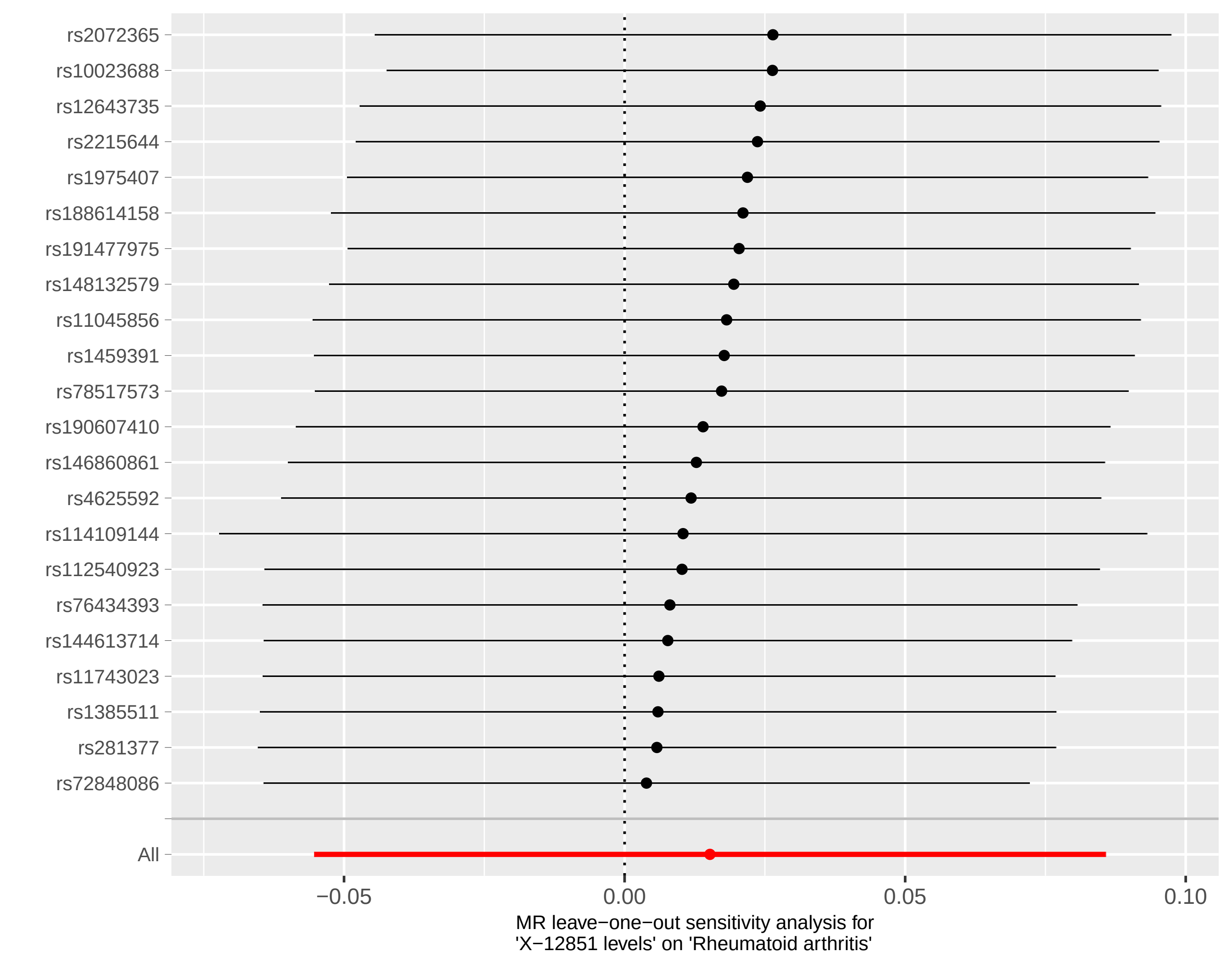
rs2072365
rs10023688
rs12643735
rs2215644
rs1975407
rs188614158
rs191477975
rs148132579
rs11045856
rs1459391
rs78517573
rs190607410
rs146860861
rs4625592
rs114109144
rs112540923
rs76434393
rs144613714
rs11743023
rs1385511
rs281377
rs72848086
All
−0.05
0.00
0.05
0.10
MR leave−one−out sensitivity analysis for
'X−12851 levels' on 'Rheumatoid arthritis'

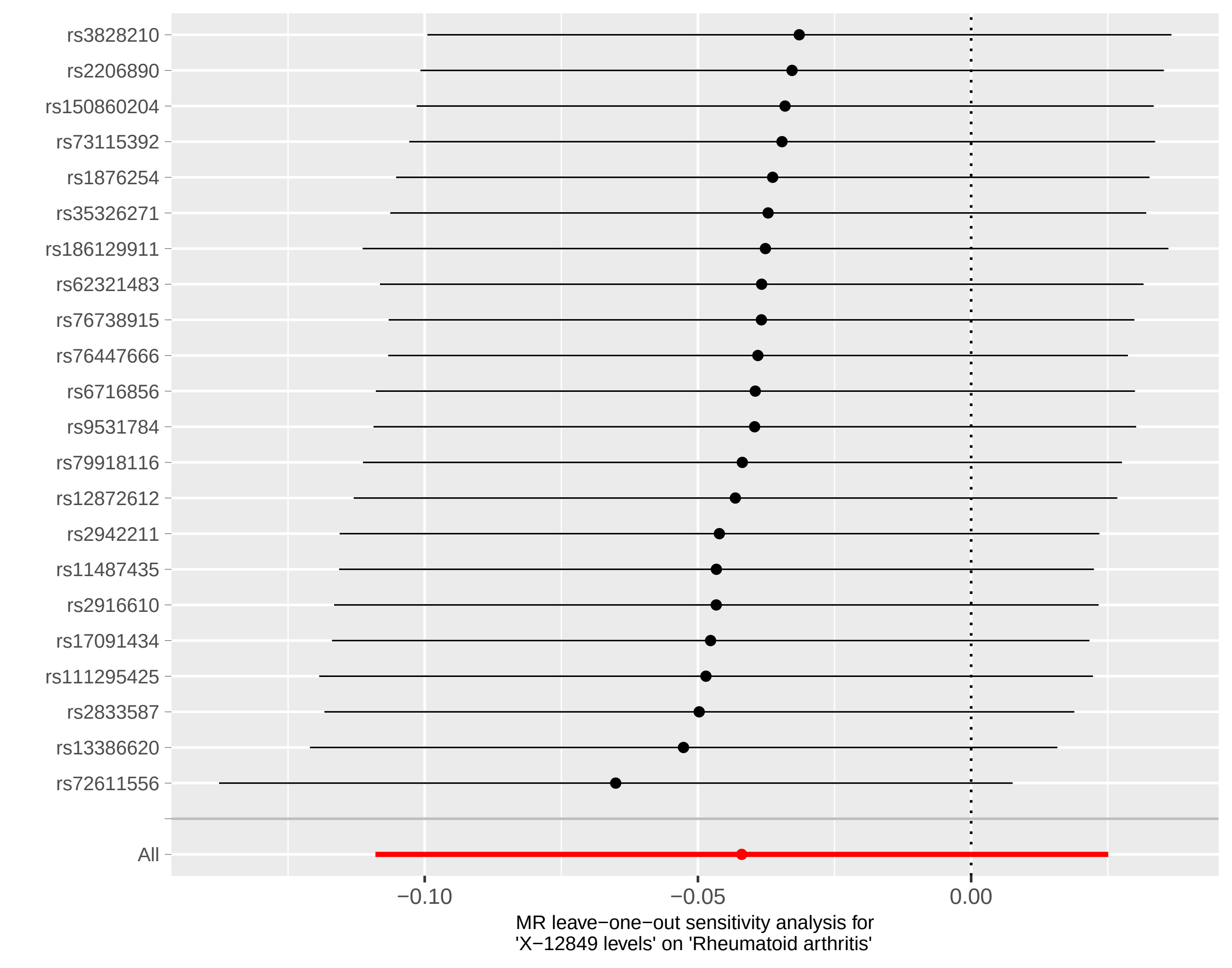
rs3828210
rs2206890
rs150860204
rs73115392
rs1876254
rs35326271
rs186129911
rs62321483
rs76738915
rs76447666
rs6716856
rs9531784
rs79918116
rs12872612
rs2942211
rs11487435
rs2916610
rs17091434
rs111295425
rs2833587
rs13386620
rs72611556
All
−0.10
−0.05
0.00
MR leave−one−out sensitivity analysis for
'X−12849 levels' on 'Rheumatoid arthritis'

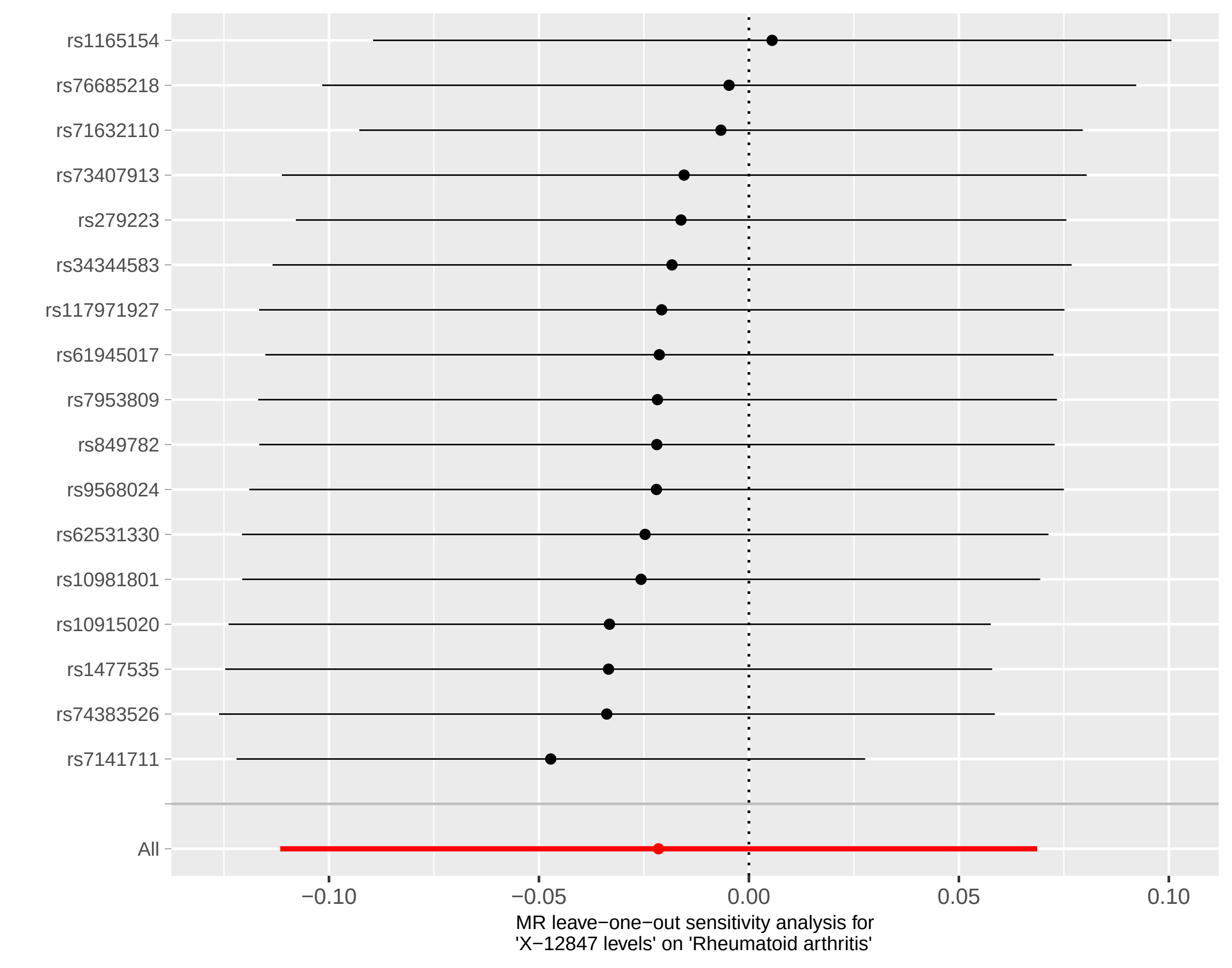
rs1165154
rs76685218
rs71632110
rs73407913
rs279223
rs34344583
rs117971927
rs61945017
rs7953809
rs849782
rs9568024
rs62531330
rs10981801
rs10915020
rs1477535
rs74383526
rs7141711
All
−0.10
−0.05
0.00
0.05
0.10
MR leave−one−out sensitivity analysis for
'X−12847 levels' on 'Rheumatoid arthritis'

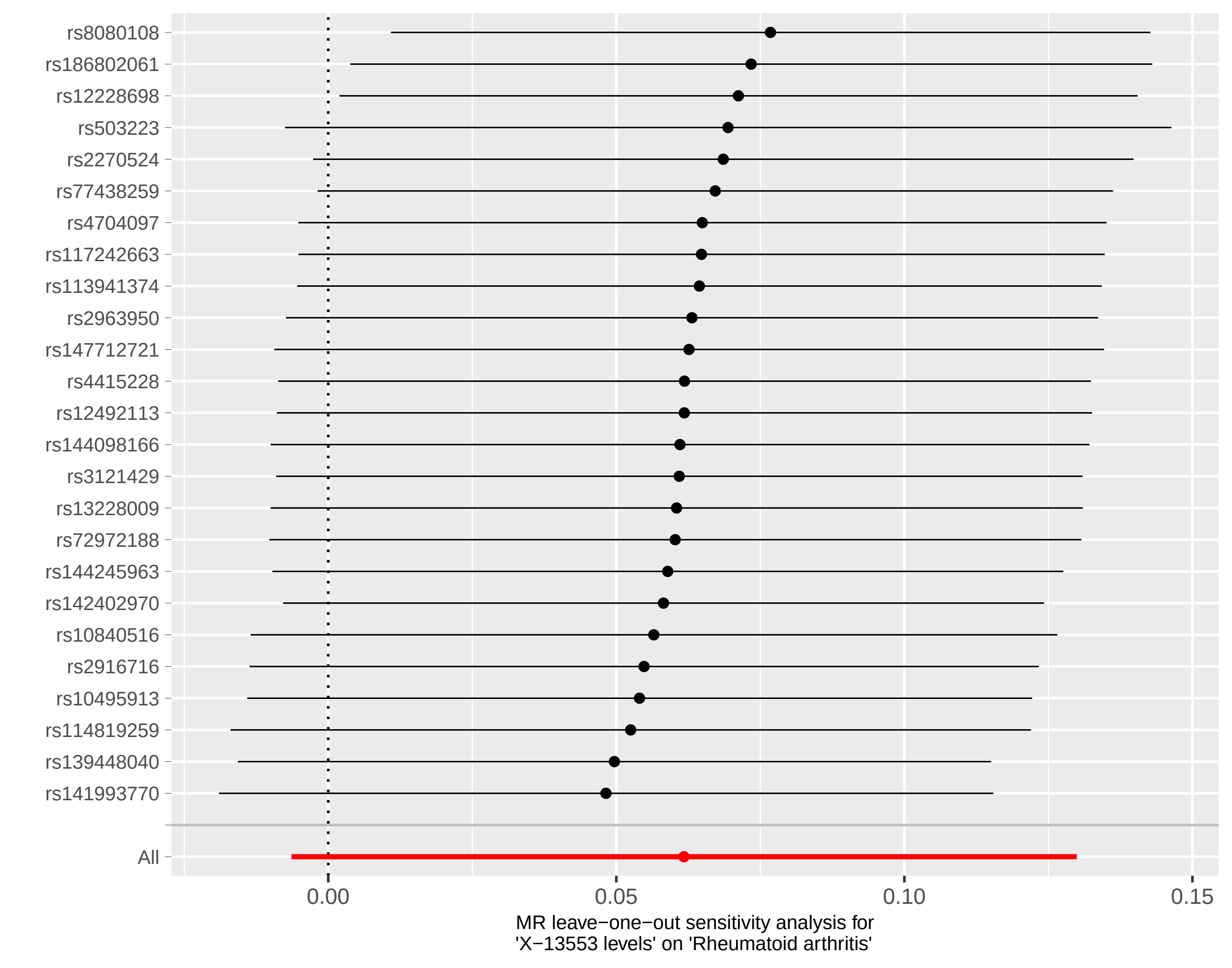
rs8080108
rs186802061
rs12228698
rs503223
rs2270524
rs77438259
rs4704097
rs117242663
rs113941374
rs2963950
rs147712721
rs4415228
rs12492113
rs144098166
rs3121429
rs13228009
rs72972188
rs144245963
rs142402970
rs10840516
rs2916716
rs10495913
rs114819259
rs139448040
rs141993770
All
0.00
0.05
0.10
0.15
MR leave−one−out sensitivity analysis for
'X−13553 levels' on 'Rheumatoid arthritis'

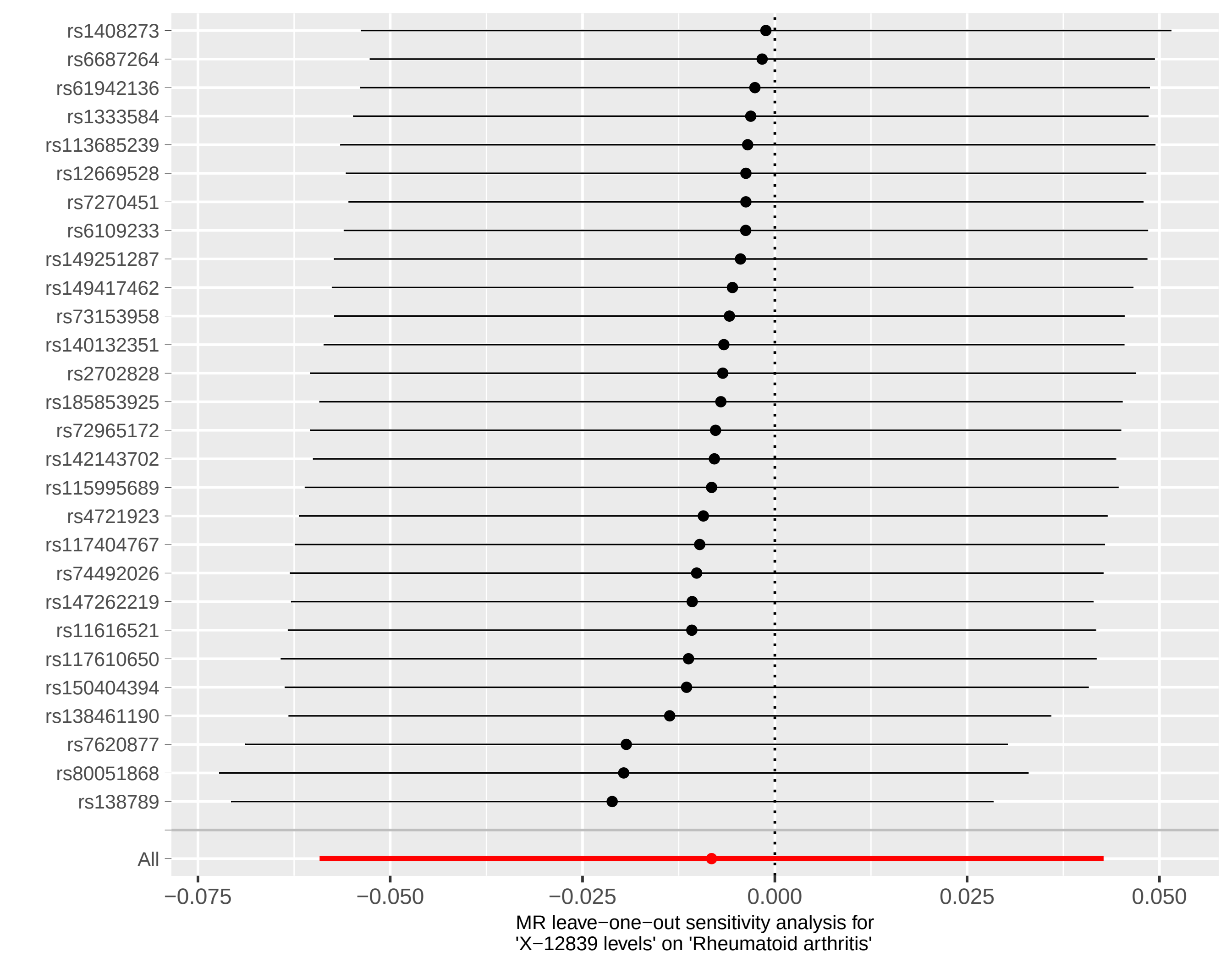

rs1408273
rs6687264
rs61942136
rs1333584
rs113685239
rs12669528
rs7270451
rs6109233
rs149251287
rs149417462
rs73153958
rs140132351
rs2702828
rs185853925
rs72965172
rs142143702
rs115995689
rs4721923
rs117404767
rs74492026
rs147262219
rs11616521
rs117610650
rs150404394
rs138461190
rs7620877
rs80051868
rs138789
All
−0.075
−0.050
−0.025
0.000
0.025
0.050
MR leave−one−out sensitivity analysis for
'X−12839 levels' on 'Rheumatoid arthritis'

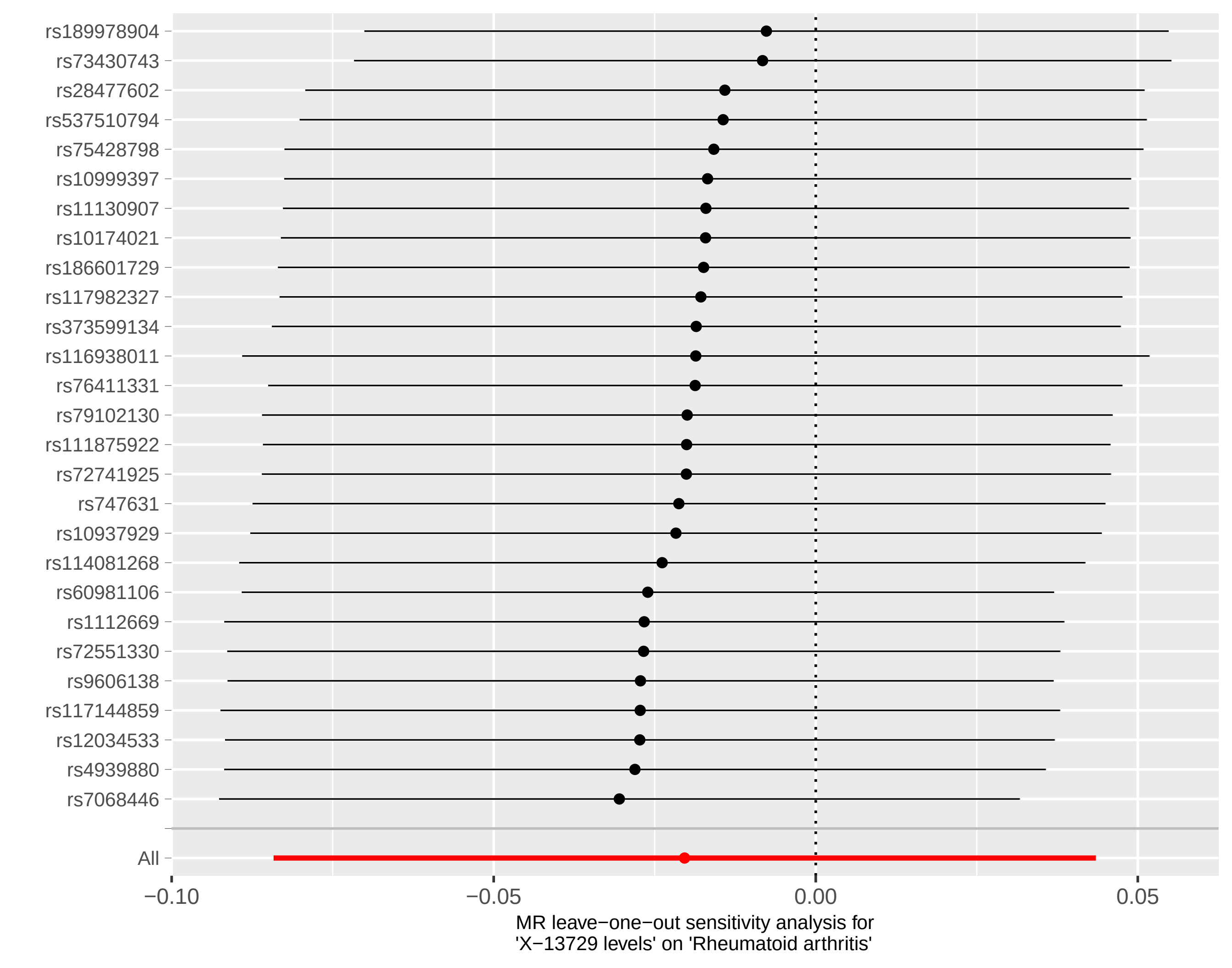
rs189978904
rs73430743
rs28477602
rs537510794
rs75428798
rs10999397
rs11130907
rs10174021
rs186601729
rs117982327
rs373599134
rs116938011
rs76411331
rs79102130
rs111875922
rs72741925
rs747631
rs10937929
rs114081268
rs60981106
rs1112669
rs72551330
rs9606138
rs117144859
rs12034533
rs4939880
rs7068446
All
−0.10
−0.05
0.00
0.05
MR leave−one−out sensitivity analysis for
'X−13729 levels' on 'Rheumatoid arthritis'

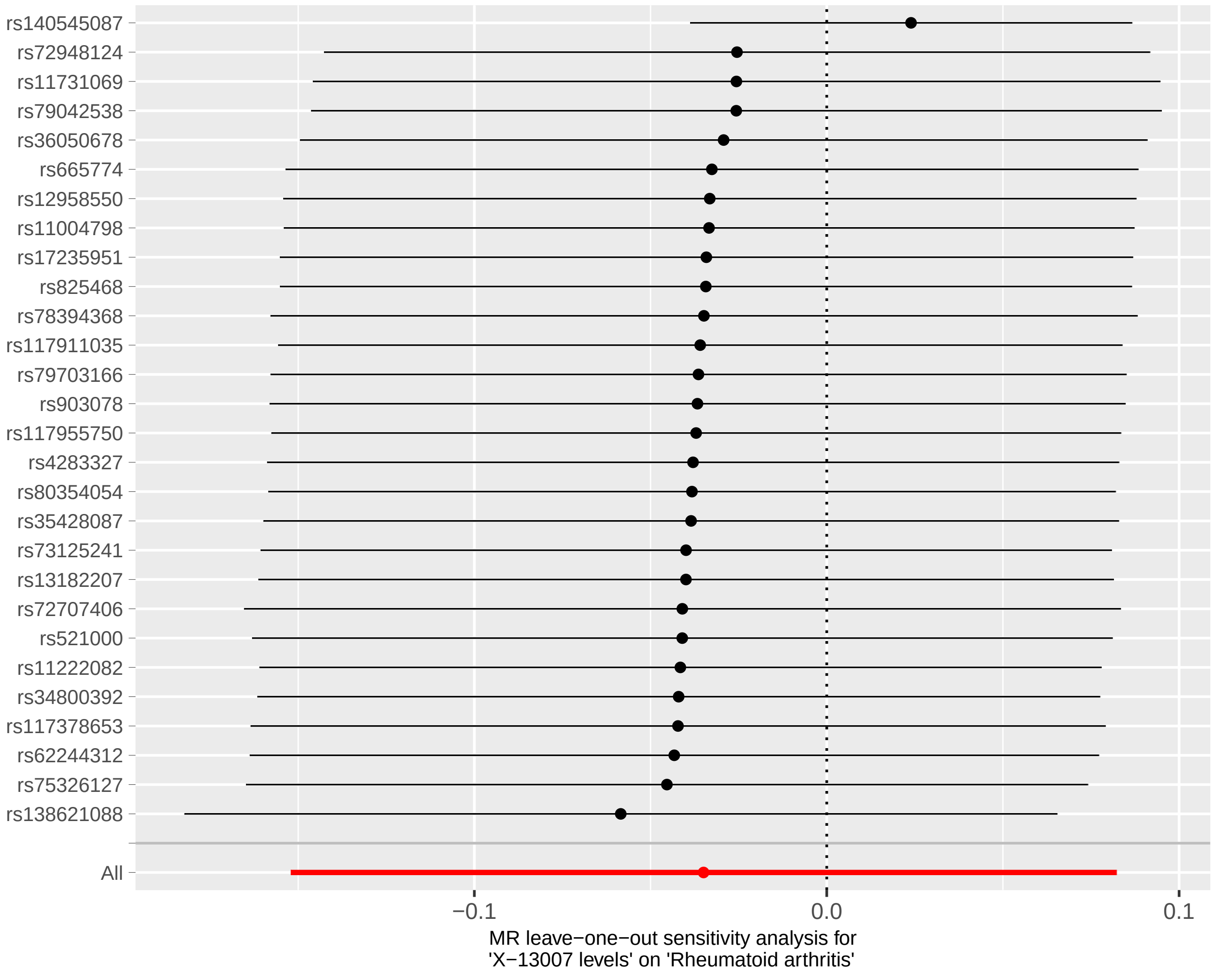
rs140545087
rs72948124
rs11731069
rs79042538
rs36050678
rs665774
rs12958550
rs11004798
rs17235951
rs825468
rs78394368
rs117911035
rs79703166
rs903078
rs117955750
rs4283327
rs80354054
rs35428087
rs73125241
rs13182207
rs72707406
rs521000
rs11222082
rs34800392
rs117378653
rs62244312
rs75326127
rs138621088
All
−0.1
0.0
0.1
MR leave−one−out sensitivity analysis for
'X−13007 levels' on 'Rheumatoid arthritis'

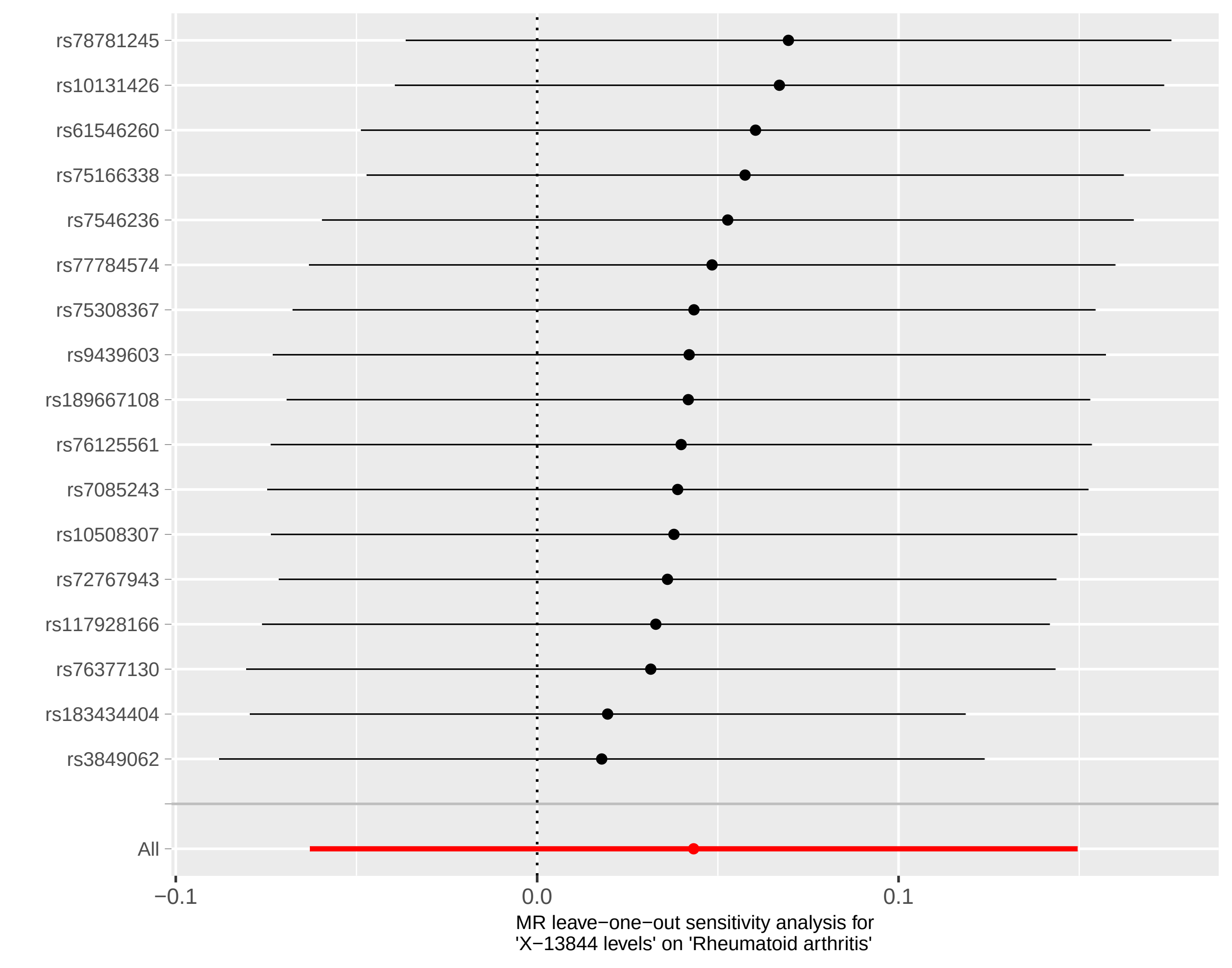
rs78781245
rs10131426
rs61546260
rs75166338
rs7546236
rs77784574
rs75308367
rs9439603
rs189667108
rs76125561
rs7085243
rs10508307
rs72767943
rs117928166
rs76377130
rs183434404
rs3849062
All
−0.1
0.0
0.1
MR leave−one−out sensitivity analysis for
'X−13844 levels' on 'Rheumatoid arthritis'

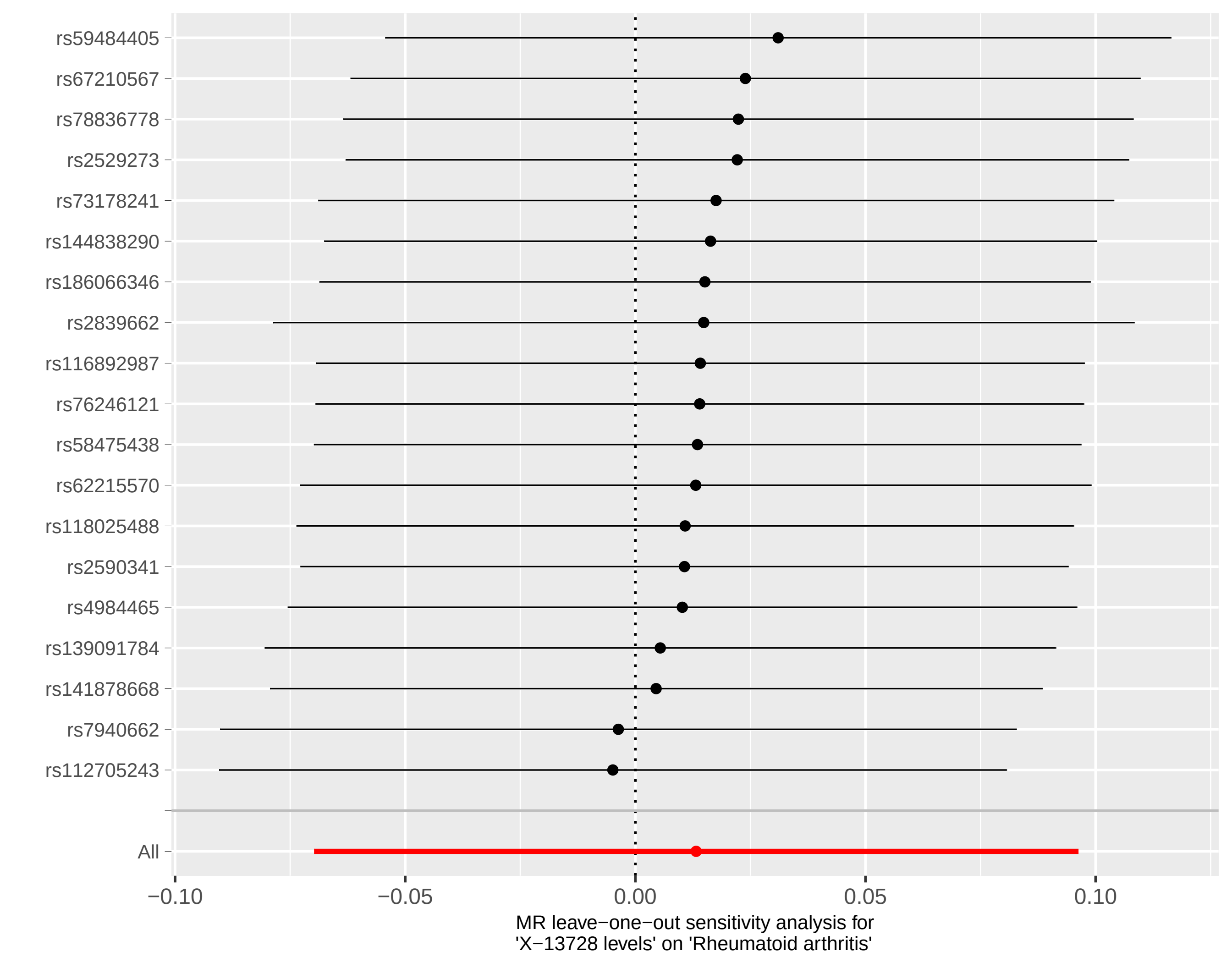
rs59484405
rs67210567
rs78836778
rs2529273
rs73178241
rs144838290
rs186066346
rs2839662
rs116892987
rs76246121
rs58475438
rs62215570
rs118025488
rs2590341
rs4984465
rs139091784
rs141878668
rs7940662
rs112705243
All
−0.10
−0.05
0.00
0.05
0.10
MR leave−one−out sensitivity analysis for
'X−13728 levels' on 'Rheumatoid arthritis'

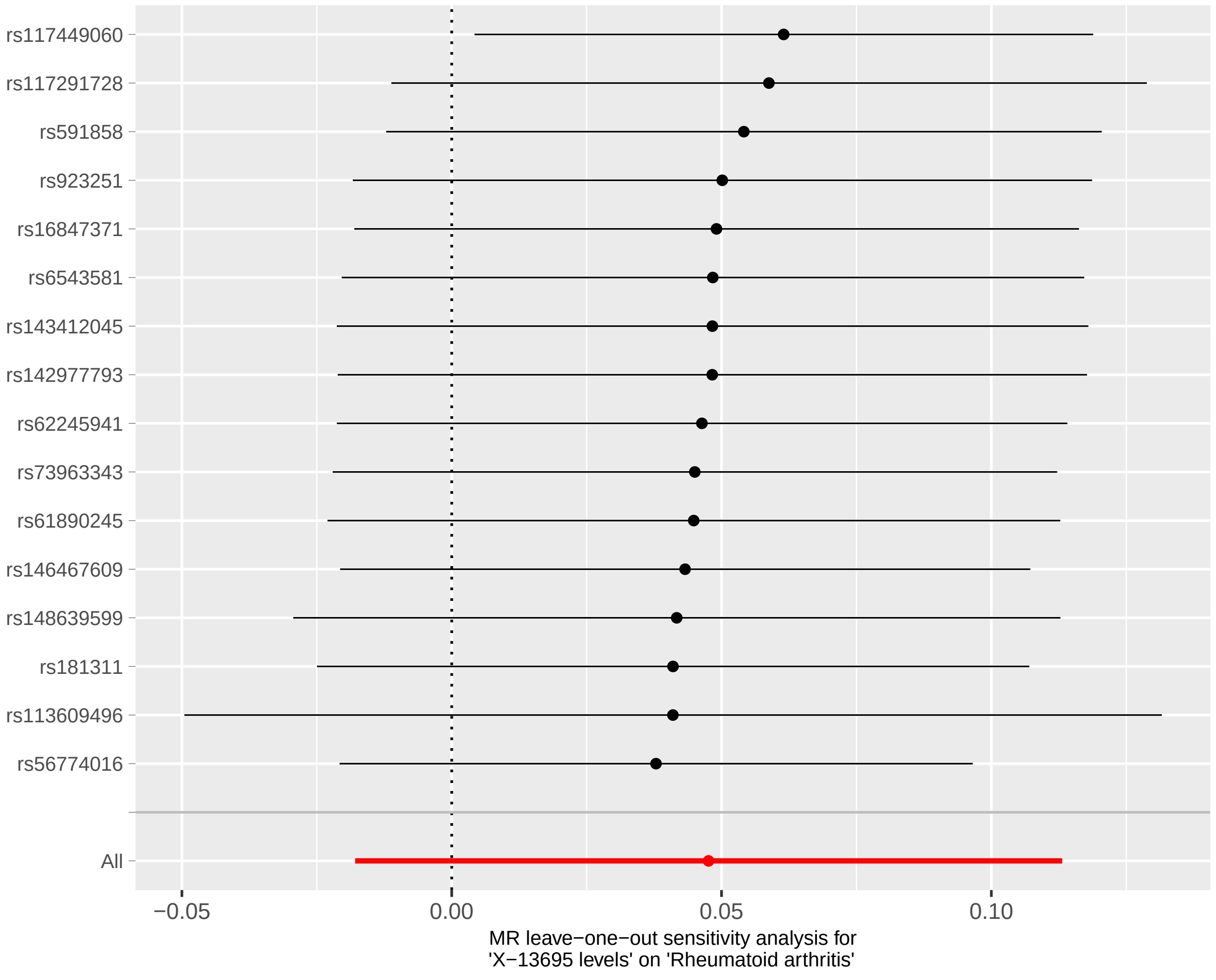
rs117449060
rs117291728
rs591858
rs923251
rs16847371
rs6543581
rs143412045
rs142977793
rs62245941
rs73963343
rs61890245
rs146467609
rs148639599
rs181311
rs113609496
rs56774016
All
−0.05
0.00
0.05
0.10
MR leave−one−out sensitivity analysis for
'X−13695 levels' on 'Rheumatoid arthritis'

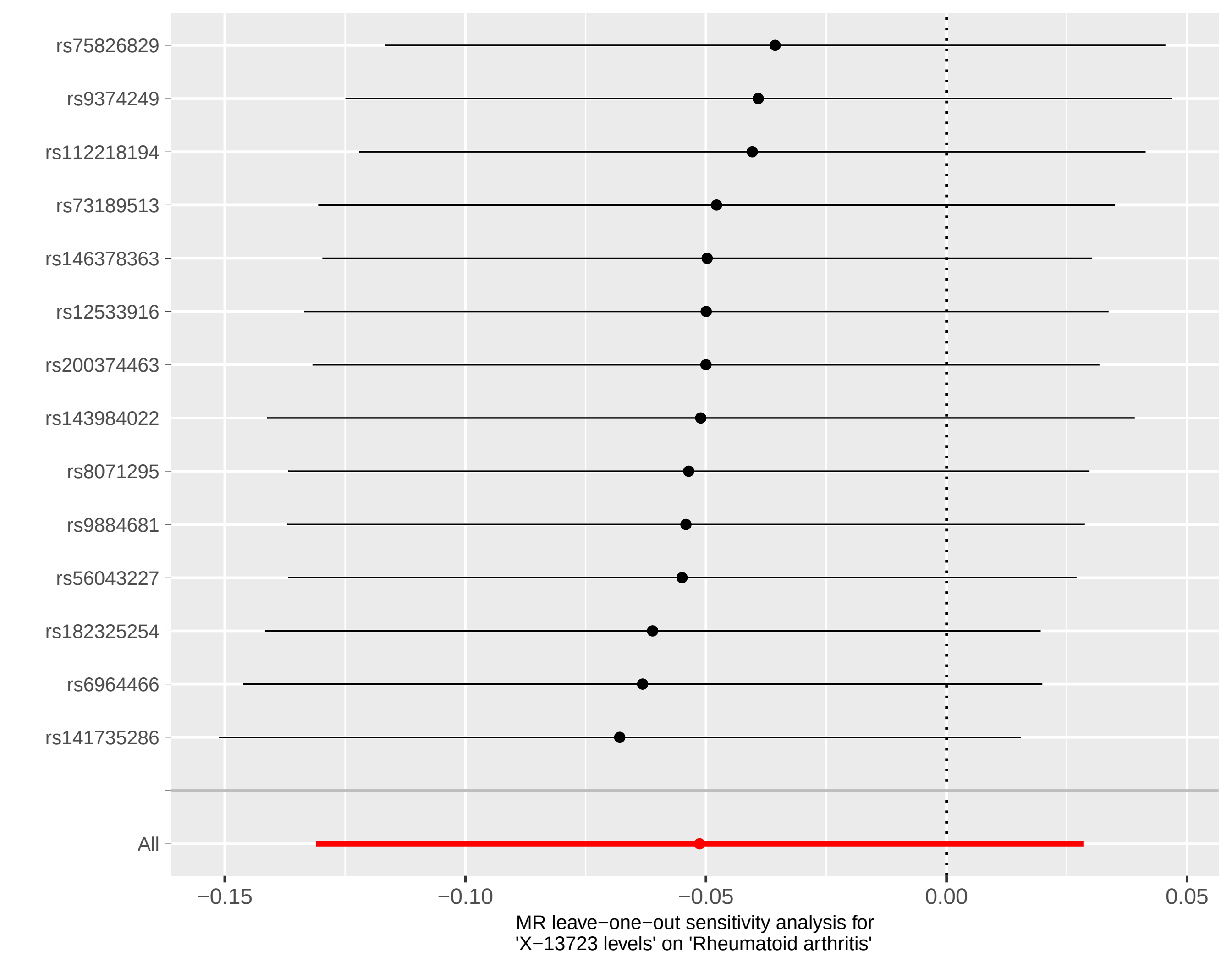

rs75826829
rs9374249
rs112218194
rs73189513
rs146378363
rs12533916
rs200374463
rs143984022
rs8071295
rs9884681
rs56043227
rs182325254
rs6964466
rs141735286
All
−0.15
−0.10
−0.05
0.00
0.05
MR leave−one−out sensitivity analysis for
'X−13723 levels' on 'Rheumatoid arthritis'

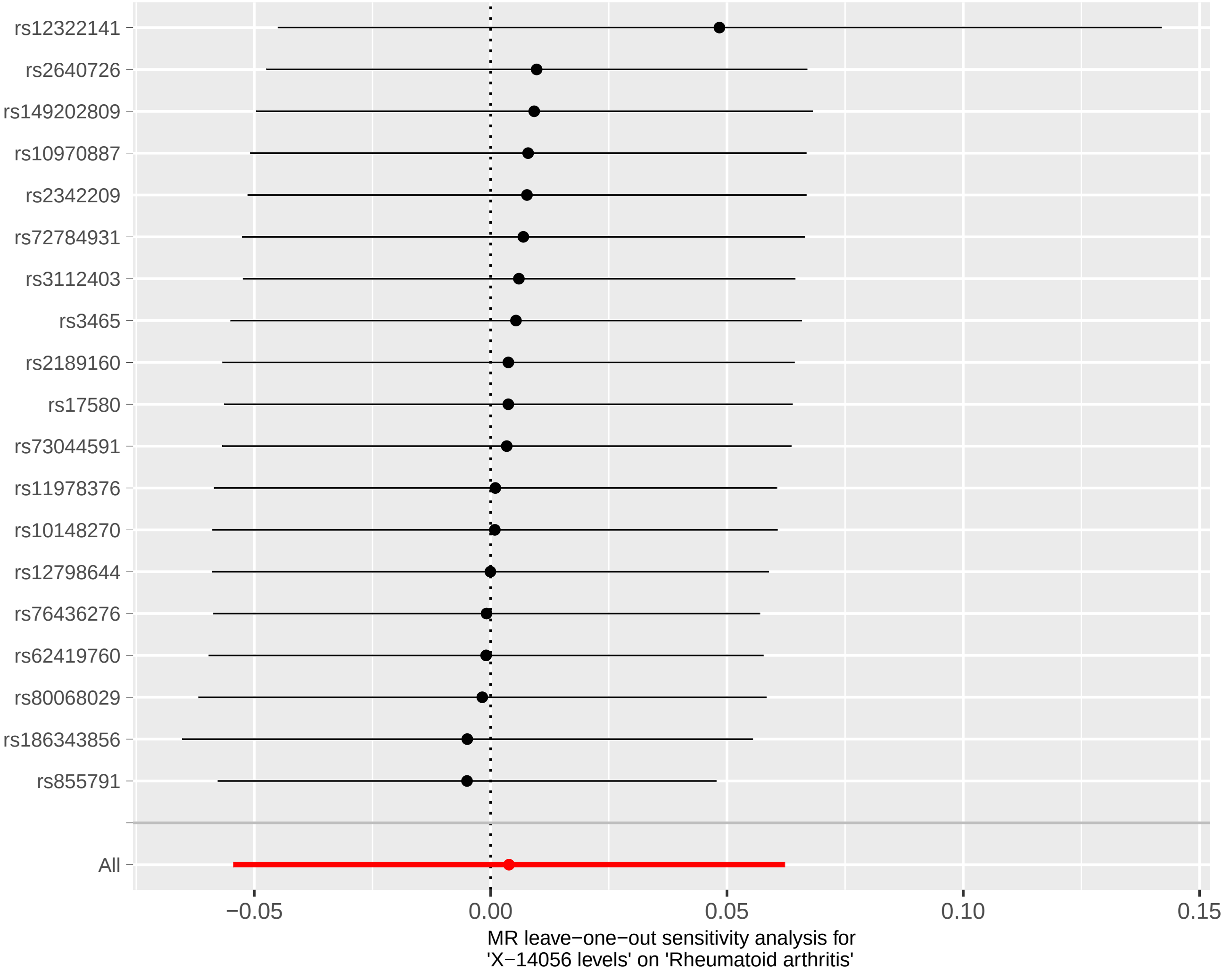

rs12322141
rs2640726
rs149202809
rs10970887
rs2342209
rs72784931
rs3112403
rs3465
rs2189160
rs17580
rs73044591
rs11978376
rs10148270
rs12798644
rs76436276
rs62419760
rs80068029
rs186343856
rs855791
All
−0.05
0.00
0.05
0.10
0.15
MR leave−one−out sensitivity analysis for
'X−14056 levels' on 'Rheumatoid arthritis'

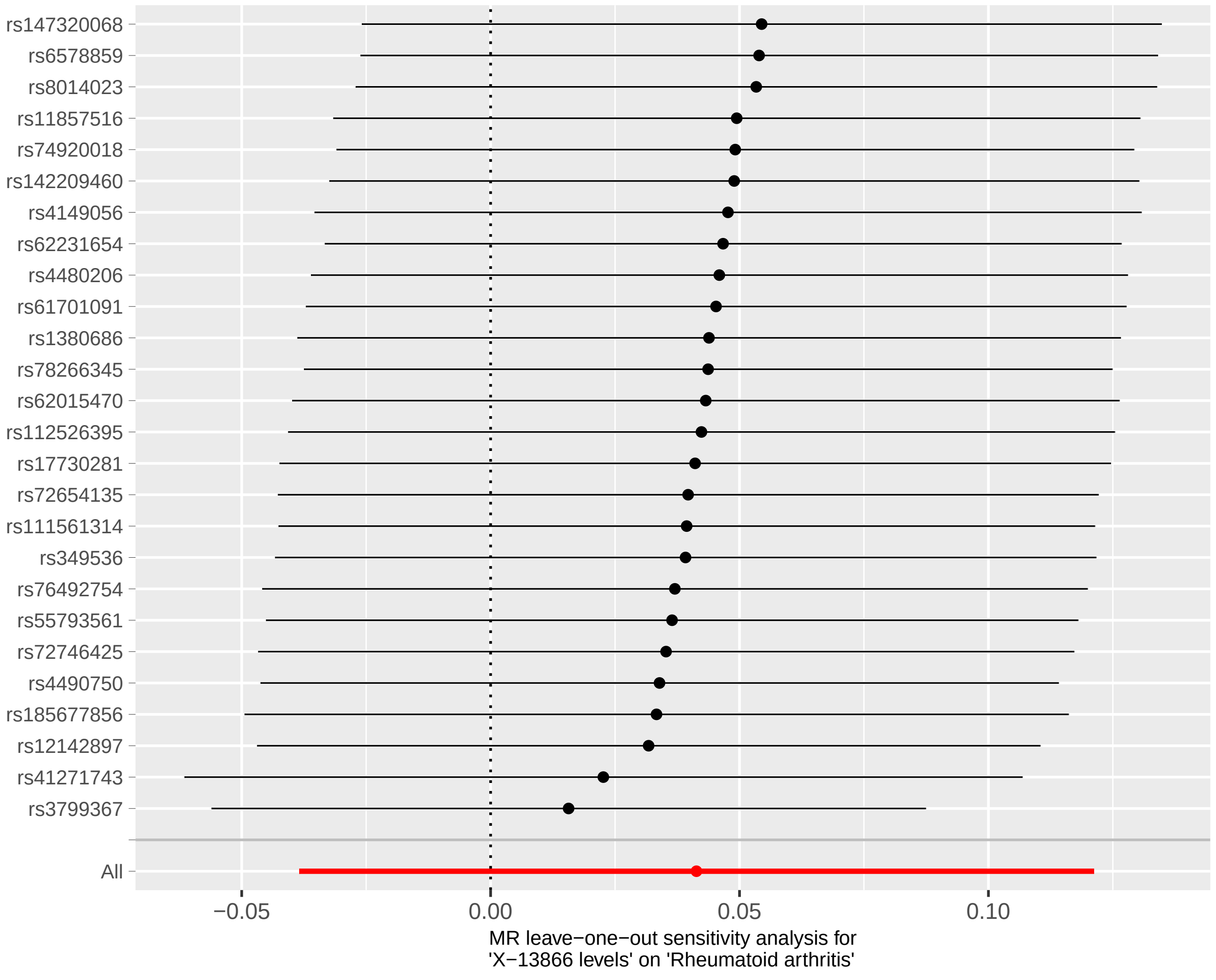

rs147320068
rs6578859
rs8014023
rs11857516
rs74920018
rs142209460
rs4149056
rs62231654
rs4480206
rs61701091
rs1380686
rs78266345
rs62015470
rs112526395
rs17730281
rs72654135
rs111561314
rs349536
rs76492754
rs55793561
rs72746425
rs4490750
rs185677856
rs12142897
rs41271743
rs3799367
All
−0.05
0.00
0.05
0.10
MR leave−one−out sensitivity analysis for
'X−13866 levels' on 'Rheumatoid arthritis'

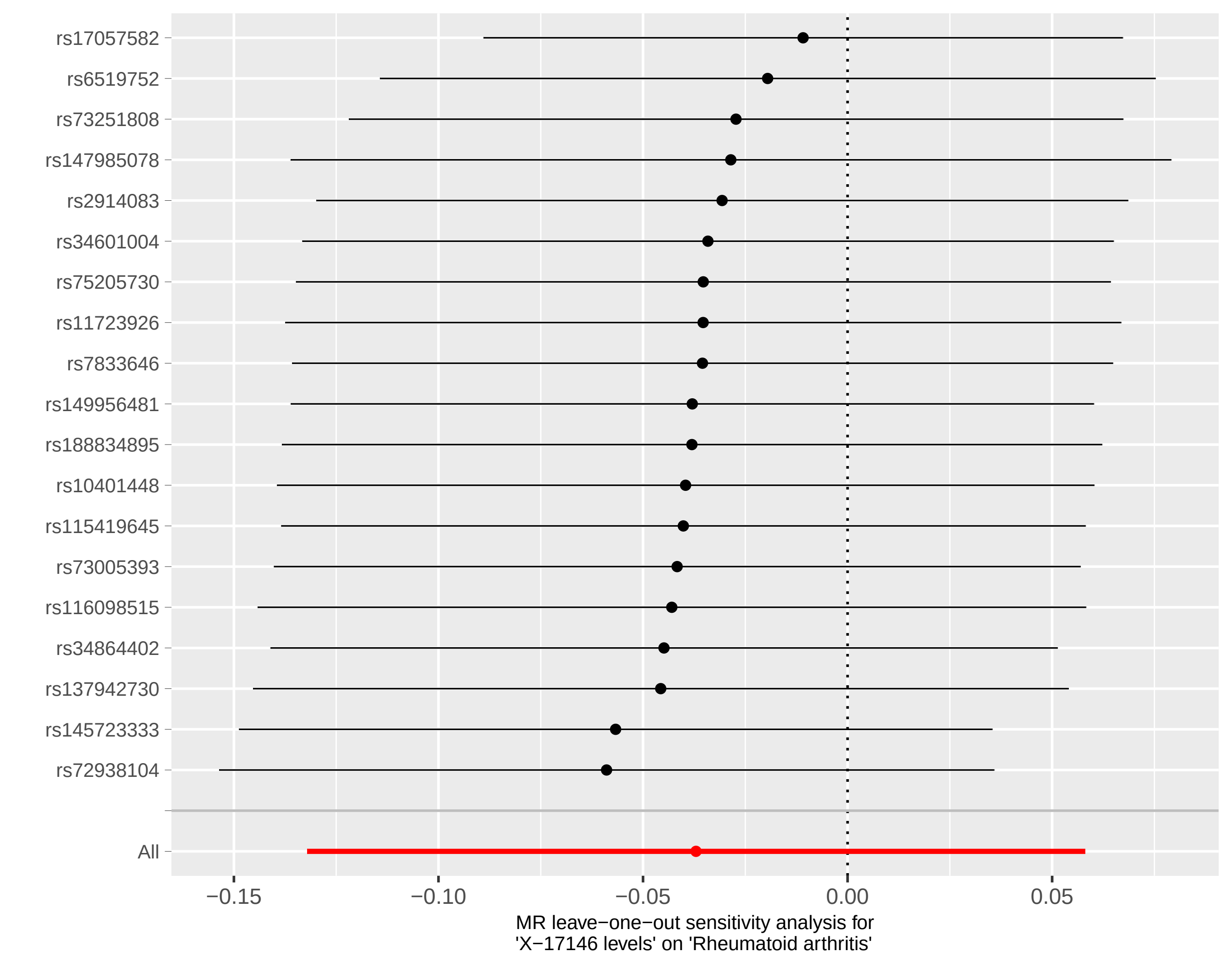
rs17057582
rs6519752
rs73251808
rs147985078
rs2914083
rs34601004
rs75205730
rs11723926
rs7833646
rs149956481
rs188834895
rs10401448
rs115419645
rs73005393
rs116098515
rs34864402
rs137942730
rs145723333
rs72938104
All
−0.15
−0.10
−0.05
0.00
0.05
MR leave−one−out sensitivity analysis for
'X−17146 levels' on 'Rheumatoid arthritis'

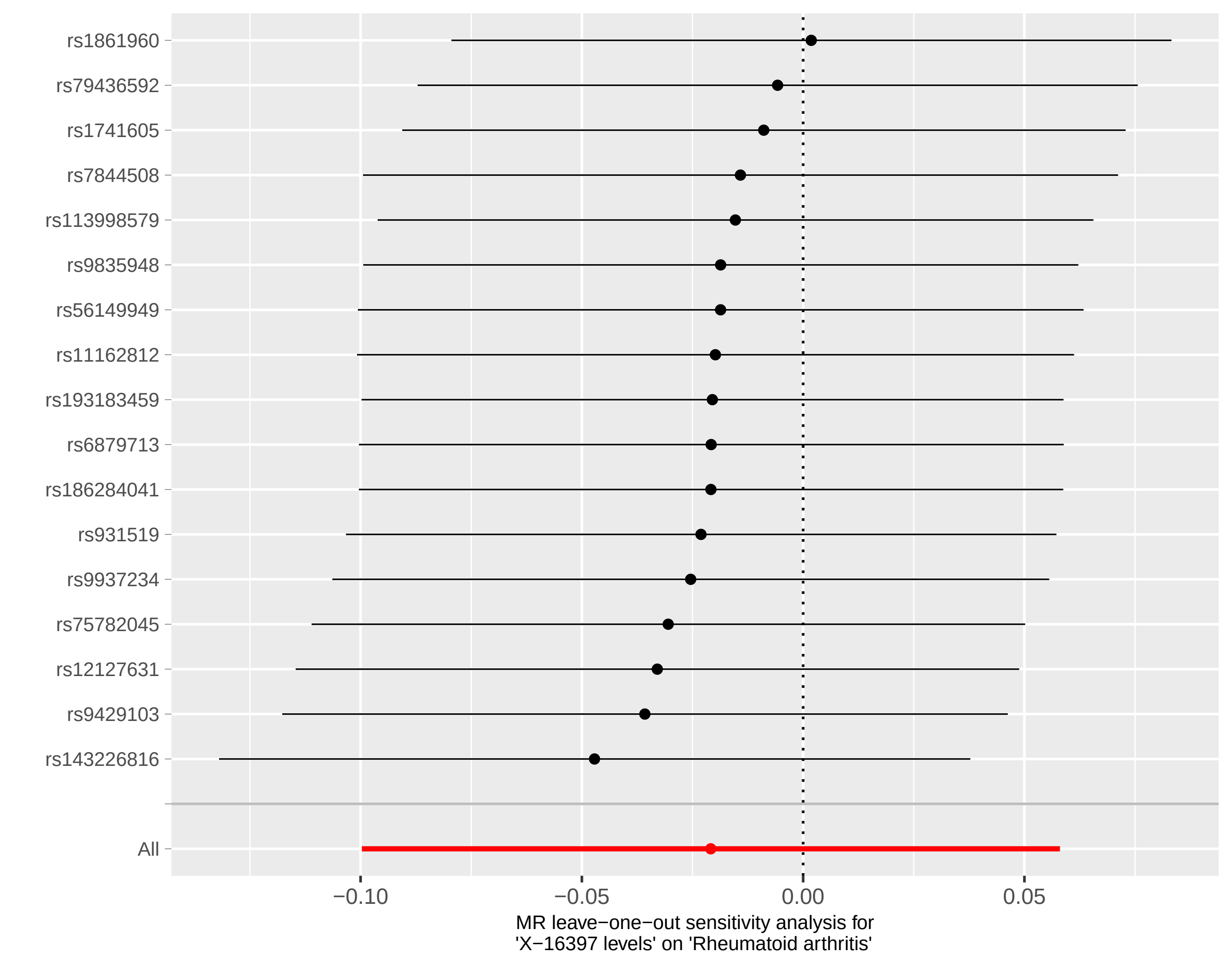

rs1861960
rs79436592
rs1741605
rs7844508
rs113998579
rs9835948
rs56149949
rs11162812
rs193183459
rs6879713
rs186284041
rs931519
rs9937234
rs75782045
rs12127631
rs9429103
rs143226816
All
−0.10
−0.05
0.00
0.05
MR leave−one−out sensitivity analysis for
'X−16397 levels' on 'Rheumatoid arthritis'

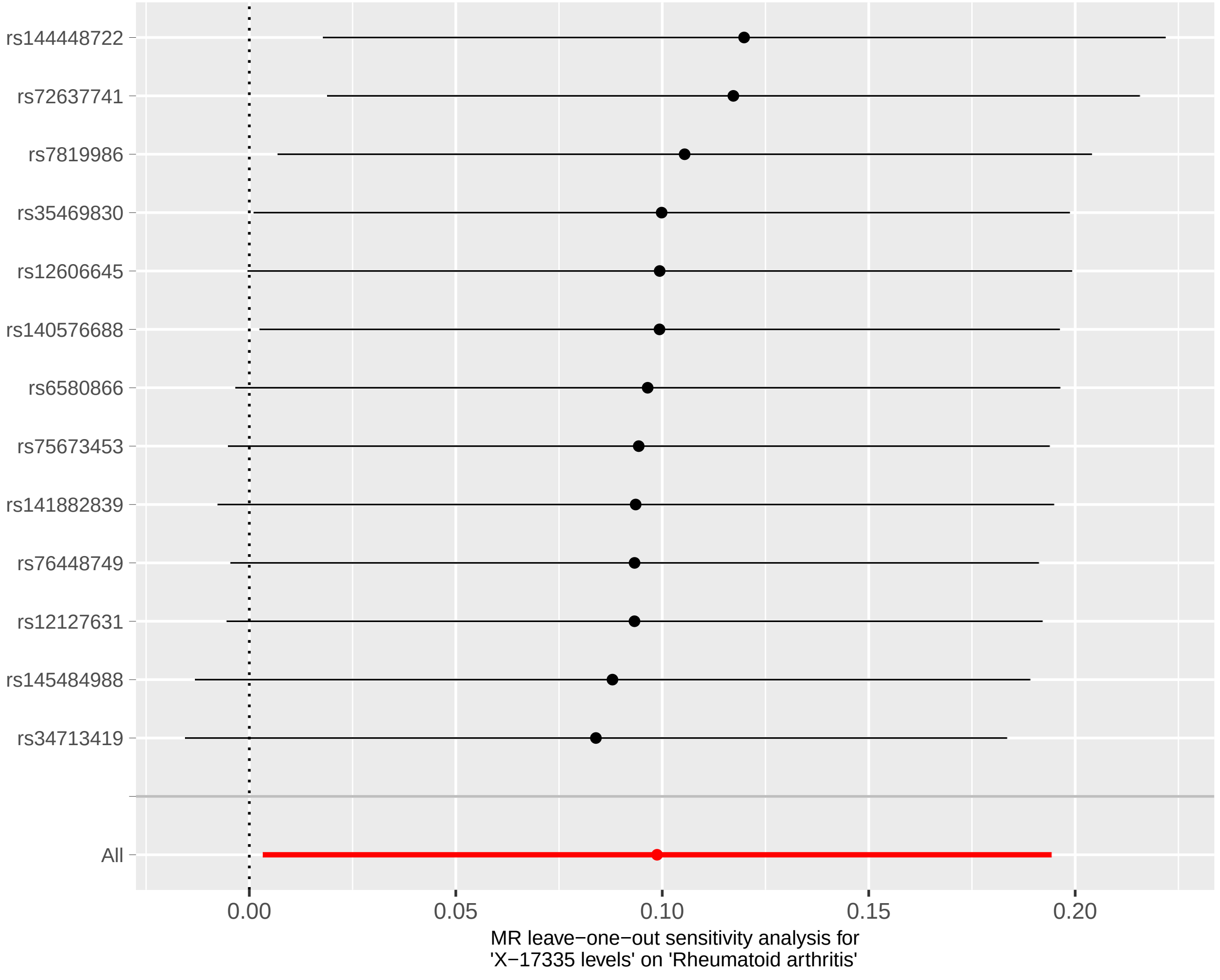

rs144448722
rs72637741
rs7819986
rs35469830
rs12606645
rs140576688
rs6580866
rs75673453
rs141882839
rs76448749
rs12127631
rs145484988
rs34713419
All
0.00
0.05
0.10
0.15
0.20
MR leave−one−out sensitivity analysis for
'X−17335 levels' on 'Rheumatoid arthritis'

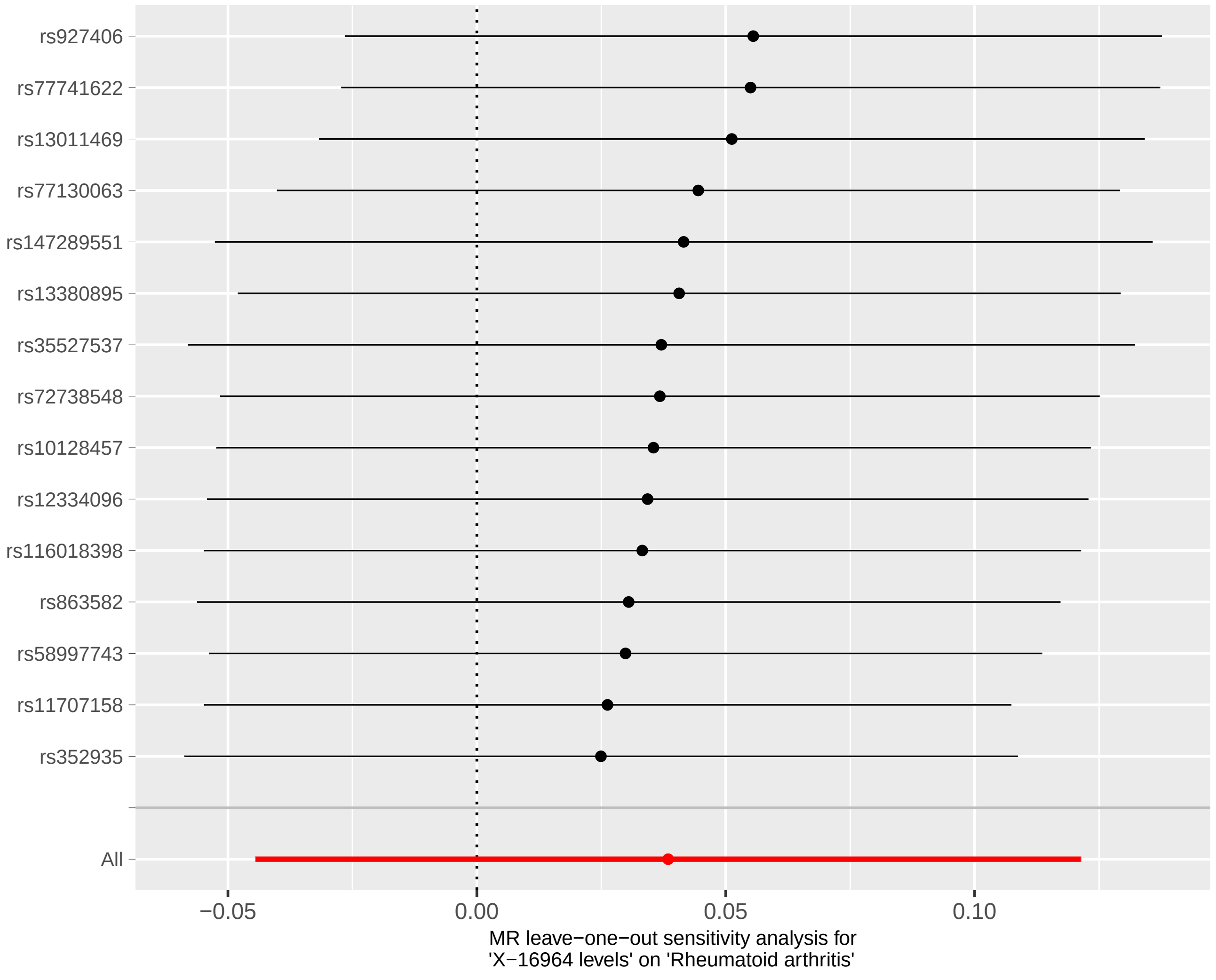

rs927406
rs77741622
rs13011469
rs77130063
rs147289551
rs13380895
rs35527537
rs72738548
rs10128457
rs12334096
rs116018398
rs863582
rs58997743
rs11707158
rs352935
All
−0.05
0.00
0.05
0.10
MR leave−one−out sensitivity analysis for
'X−16964 levels' on 'Rheumatoid arthritis'

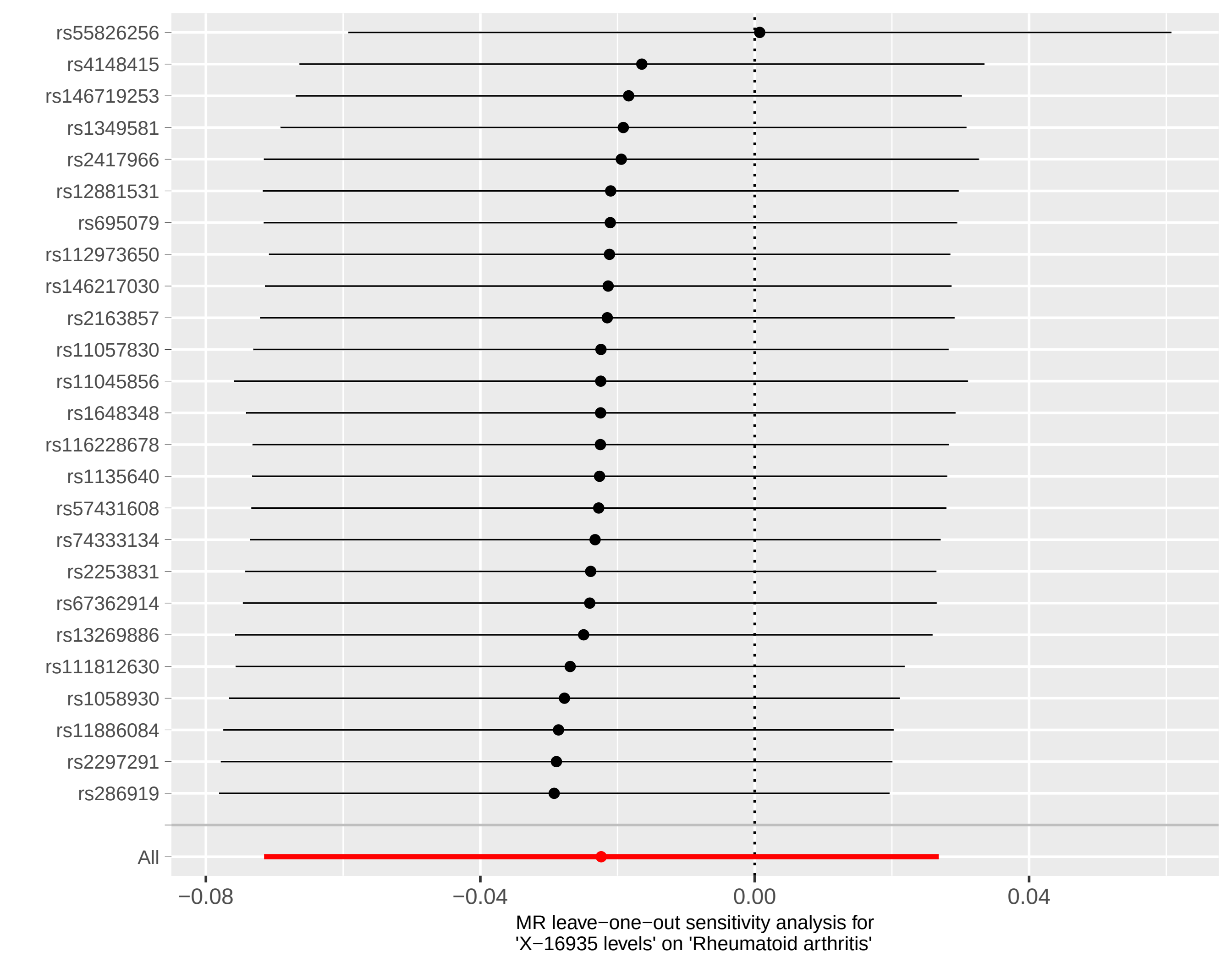

rs55826256
rs4148415
rs146719253
rs1349581
rs2417966
rs12881531
rs695079
rs112973650
rs146217030
rs2163857
rs11057830
rs11045856
rs1648348
rs116228678
rs1135640
rs57431608
rs74333134
rs2253831
rs67362914
rs13269886
rs111812630
rs1058930
rs11886084
rs2297291
rs286919
All
−0.08
−0.04
0.00
0.04
MR leave−one−out sensitivity analysis for
'X−16935 levels' on 'Rheumatoid arthritis'

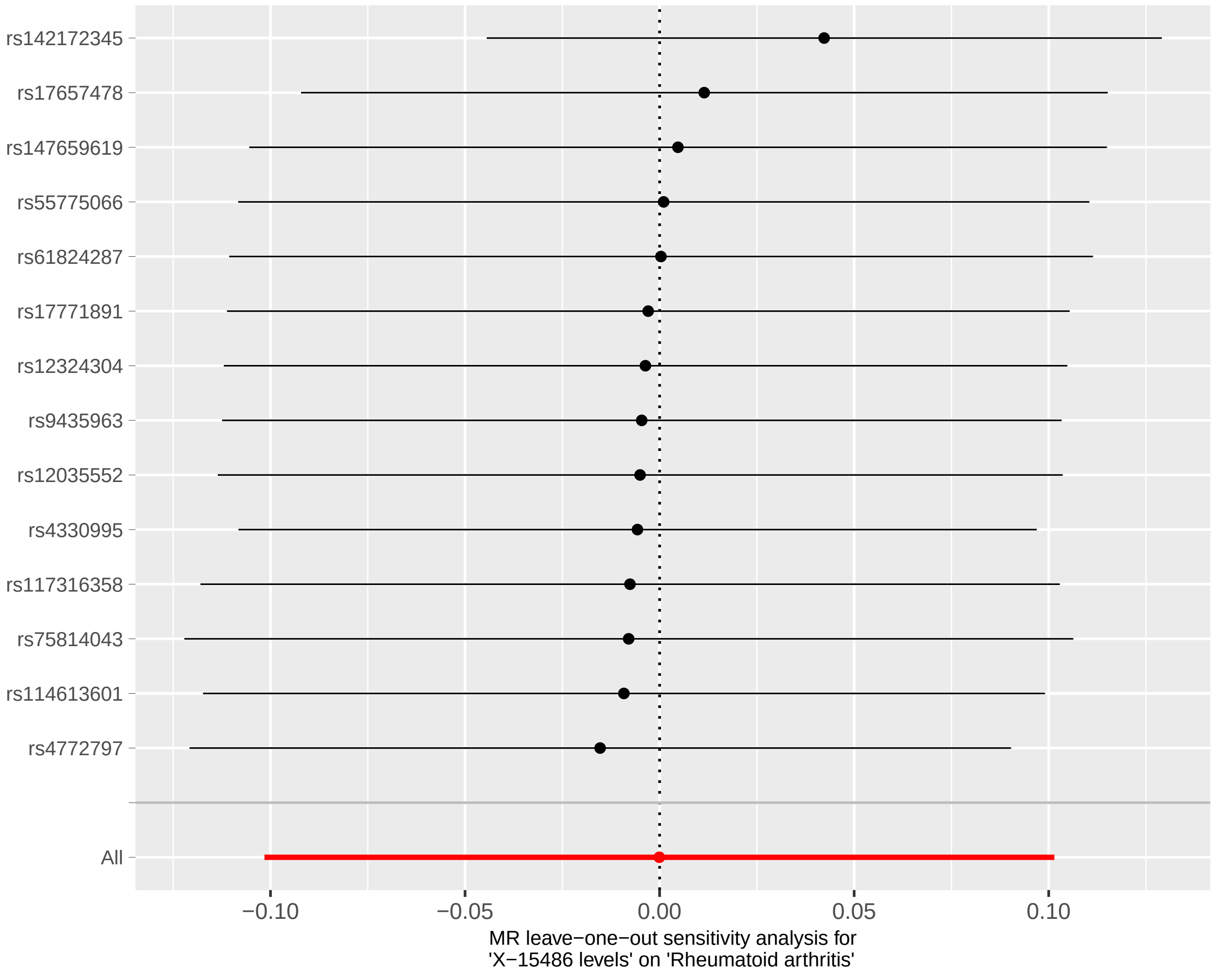
rs142172345
rs17657478
rs147659619
rs55775066
rs61824287
rs17771891
rs12324304
rs9435963
rs12035552
rs4330995
rs117316358
rs75814043
rs114613601
rs4772797
All
−0.10
−0.05
0.00
0.05
0.10
MR leave−one−out sensitivity analysis for
'X−15486 levels' on 'Rheumatoid arthritis'

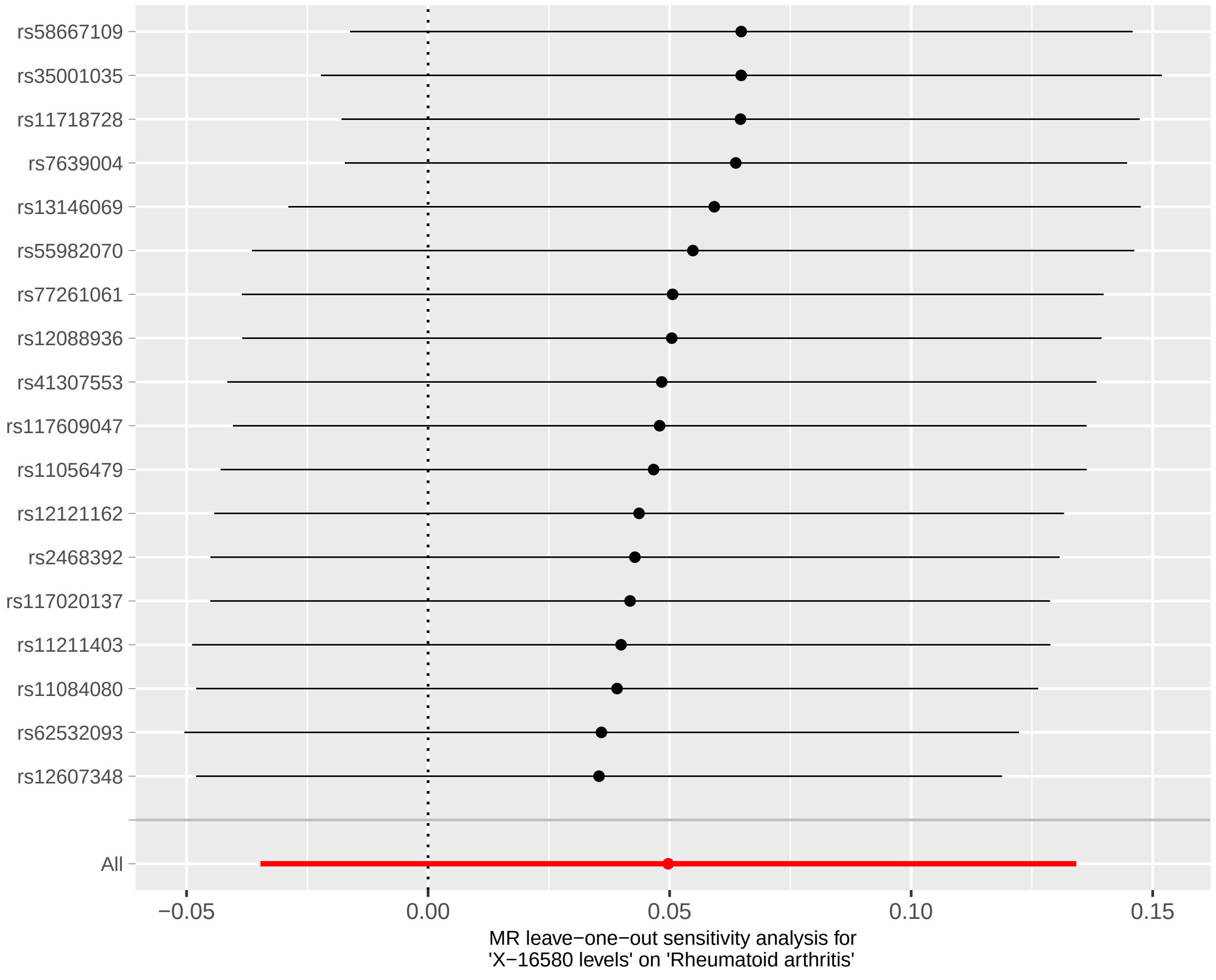
rs58667109
rs35001035
rs11718728
rs7639004
rs13146069
rs55982070
rs77261061
rs12088936
rs41307553
rs117609047
rs11056479
rs12121162
rs2468392
rs117020137
rs11211403
rs11084080
rs62532093
rs12607348
All
−0.05
0.00
0.05
0.10
0.15
MR leave−one−out sensitivity analysis for
'X−16580 levels' on 'Rheumatoid arthritis'

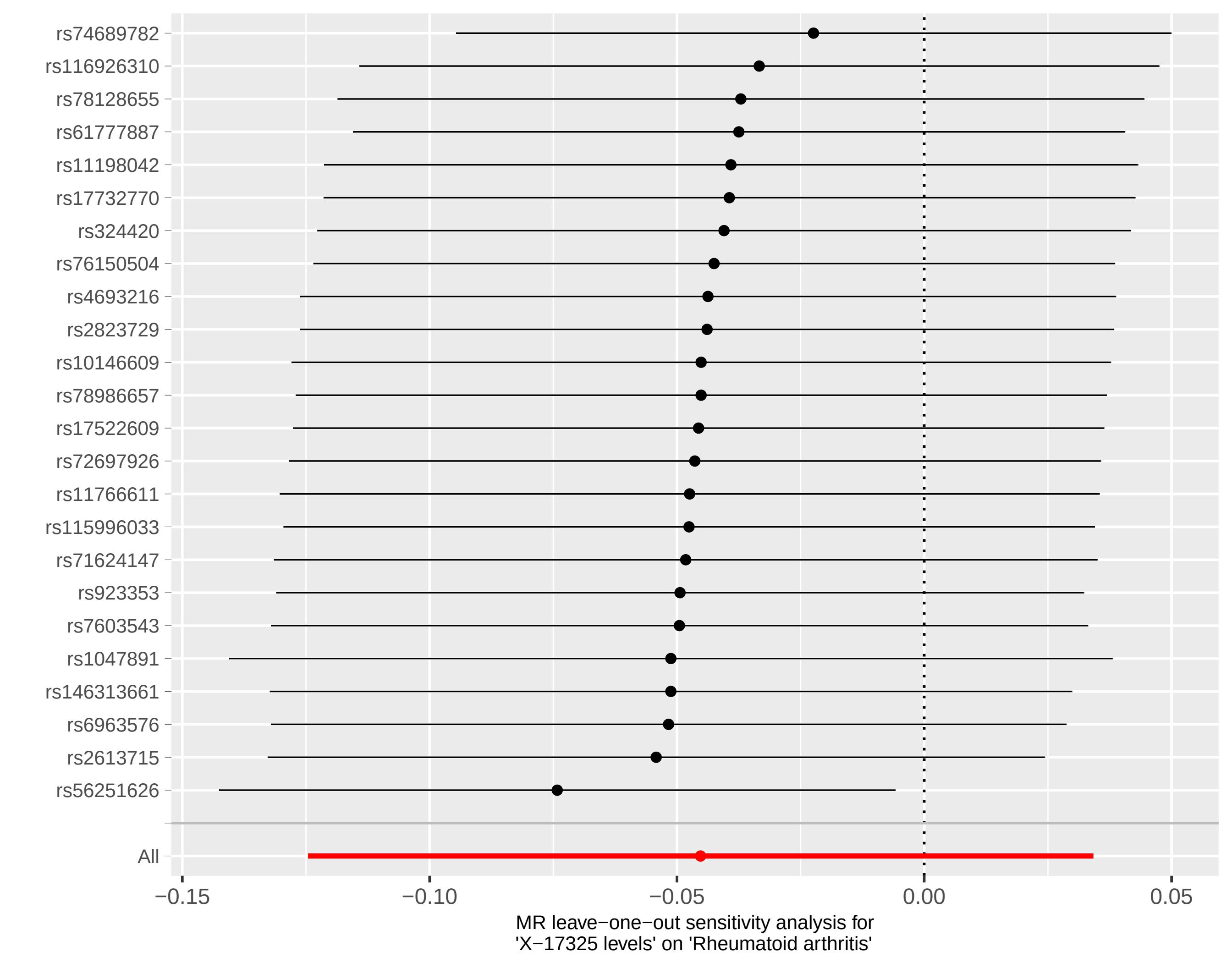
rs74689782
rs116926310
rs78128655
rs61777887
rs11198042
rs17732770
rs324420
rs76150504
rs4693216
rs2823729
rs10146609
rs78986657
rs17522609
rs72697926
rs11766611
rs115996033
rs71624147
rs923353
rs7603543
rs1047891
rs146313661
rs6963576
rs2613715
rs56251626
All
−0.15
−0.10
−0.05
0.00
0.05
MR leave−one−out sensitivity analysis for
'X−17325 levels' on 'Rheumatoid arthritis'

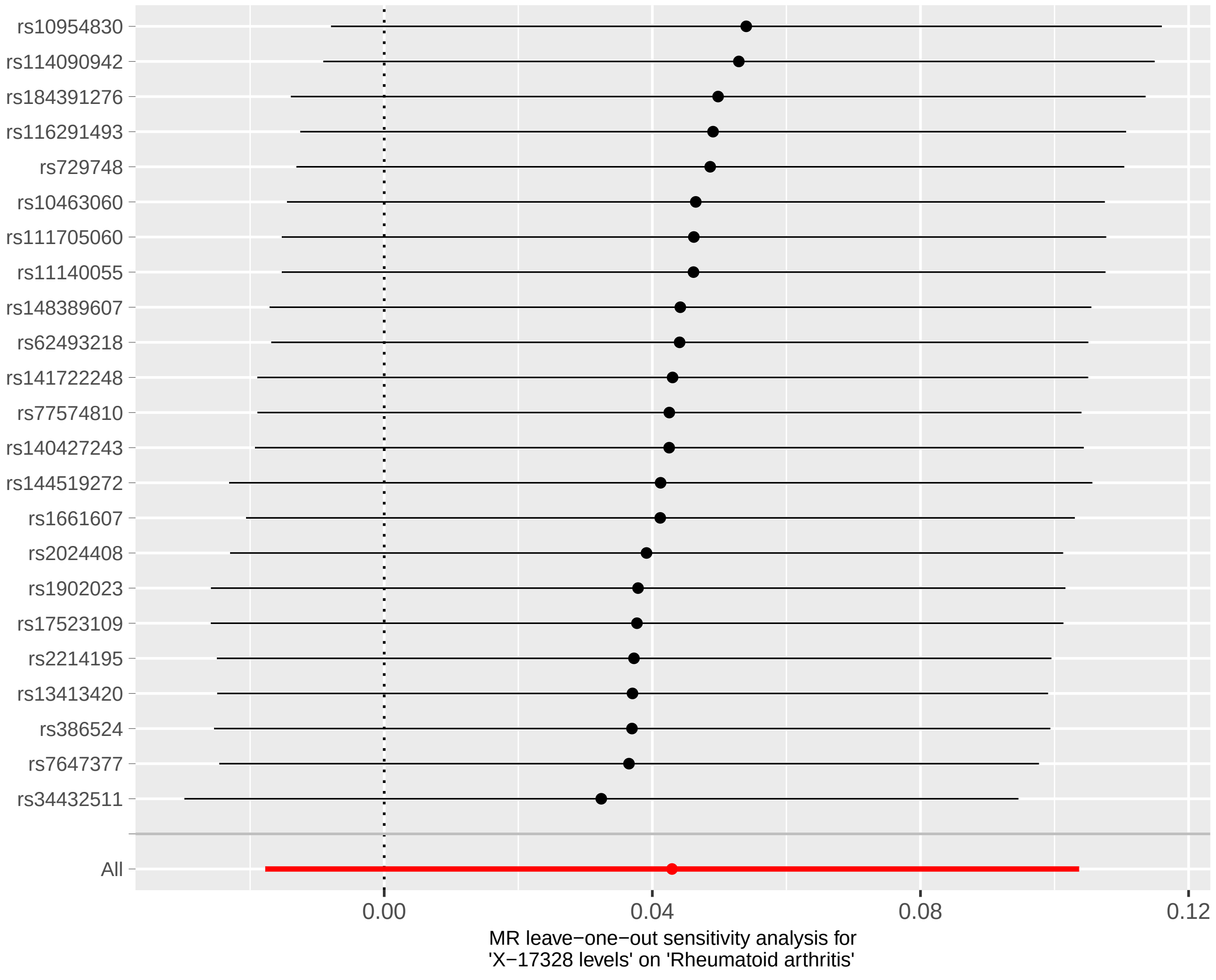
rs10954830
rs114090942
rs184391276
rs116291493
rs729748
rs10463060
rs111705060
rs11140055
rs148389607
rs62493218
rs141722248
rs77574810
rs140427243
rs144519272
rs1661607
rs2024408
rs1902023
rs17523109
rs2214195
rs13413420
rs386524
rs7647377
rs34432511
All
0.00
0.04
0.08
0.12
MR leave−one−out sensitivity analysis for
'X−17328 levels' on 'Rheumatoid arthritis'

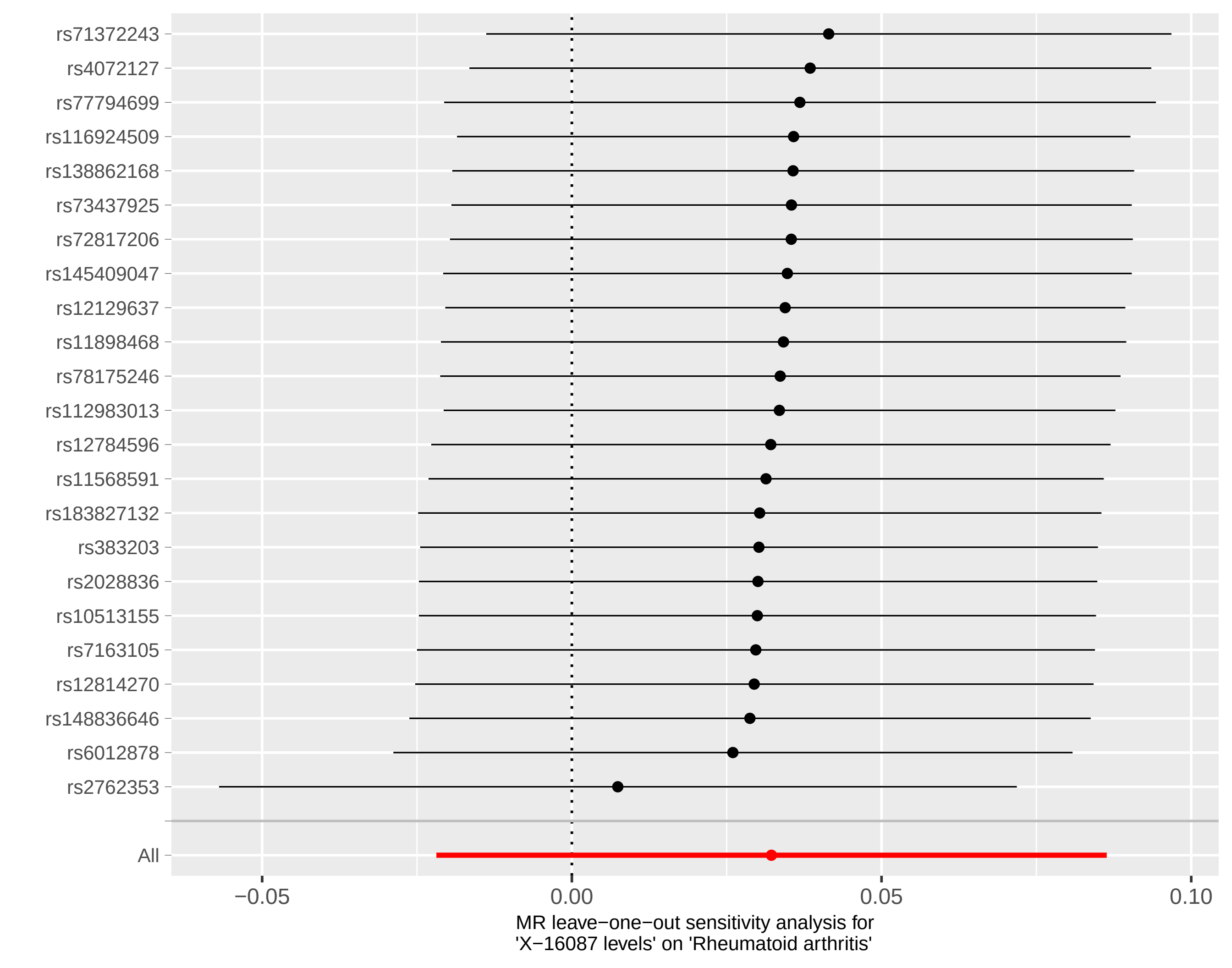
rs71372243
rs4072127
rs77794699
rs116924509
rs138862168
rs73437925
rs72817206
rs145409047
rs12129637
rs11898468
rs78175246
rs112983013
rs12784596
rs11568591
rs183827132
rs383203
rs2028836
rs10513155
rs7163105
rs12814270
rs148836646
rs6012878
rs2762353
All
−0.05
0.00
0.05
0.10
MR leave−one−out sensitivity analysis for
'X−16087 levels' on 'Rheumatoid arthritis'

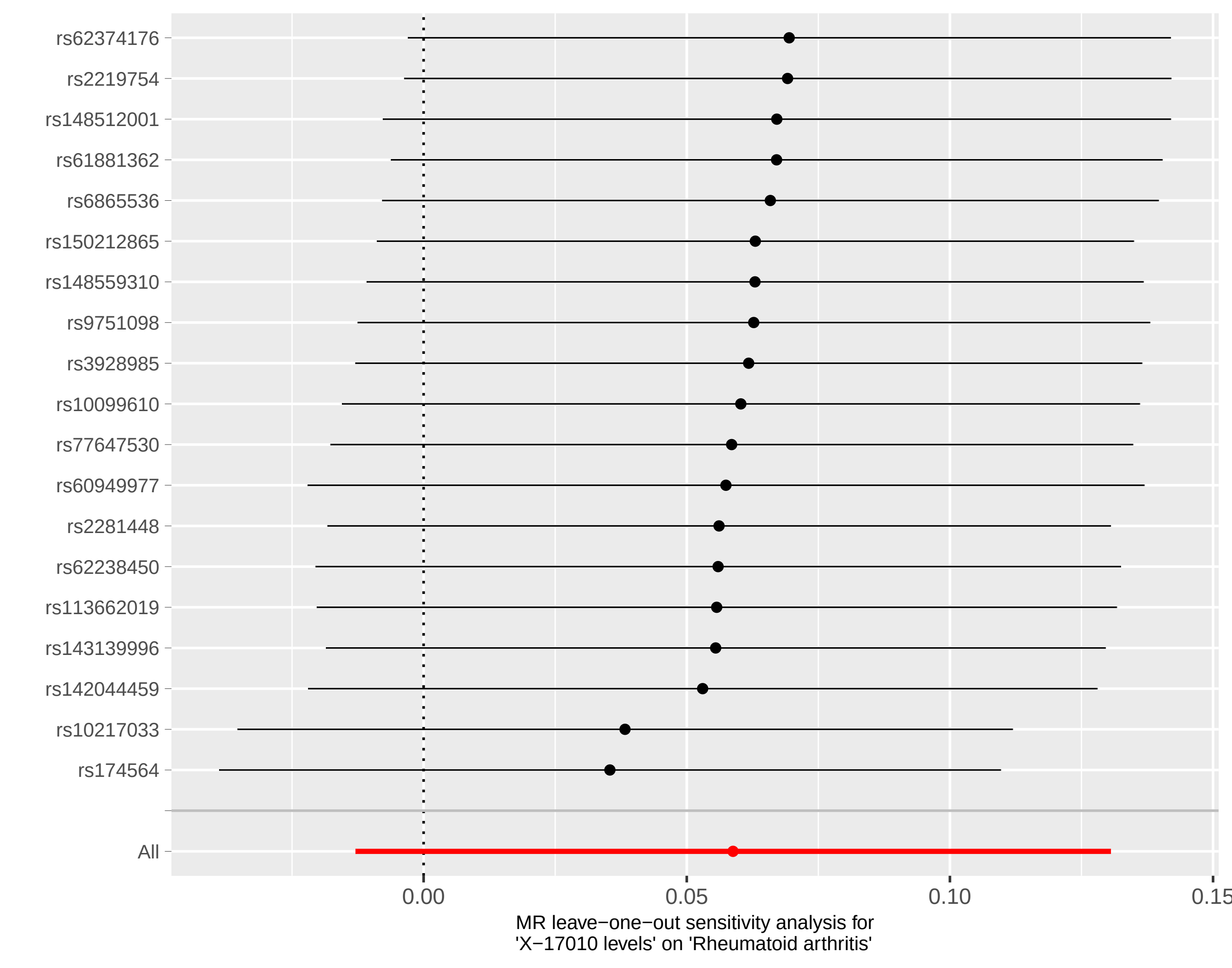
rs62374176
rs2219754
rs148512001
rs61881362
rs6865536
rs150212865
rs148559310
rs9751098
rs3928985
rs10099610
rs77647530
rs60949977
rs2281448
rs62238450
rs113662019
rs143139996
rs142044459
rs10217033
rs174564
All
0.00
0.05
0.10
0.15
MR leave−one−out sensitivity analysis for
'X−17010 levels' on 'Rheumatoid arthritis'

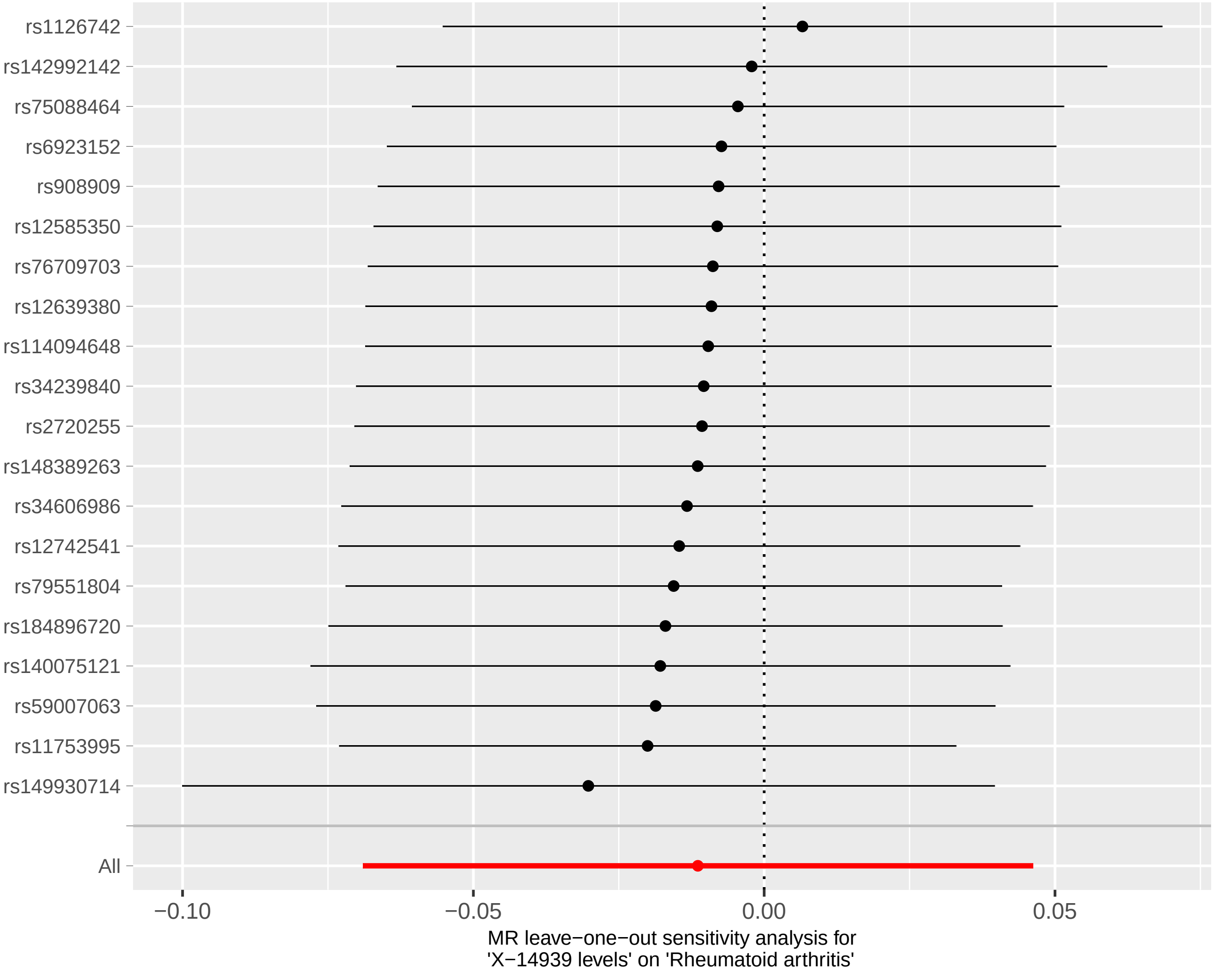
rs1126742
rs142992142
rs75088464
rs6923152
rs908909
rs12585350
rs76709703
rs12639380
rs114094648
rs34239840
rs2720255
rs148389263
rs34606986
rs12742541
rs79551804
rs184896720
rs140075121
rs59007063
rs11753995
rs149930714
All
−0.10
−0.05
0.00
0.05
MR leave−one−out sensitivity analysis for
'X−14939 levels' on 'Rheumatoid arthritis'

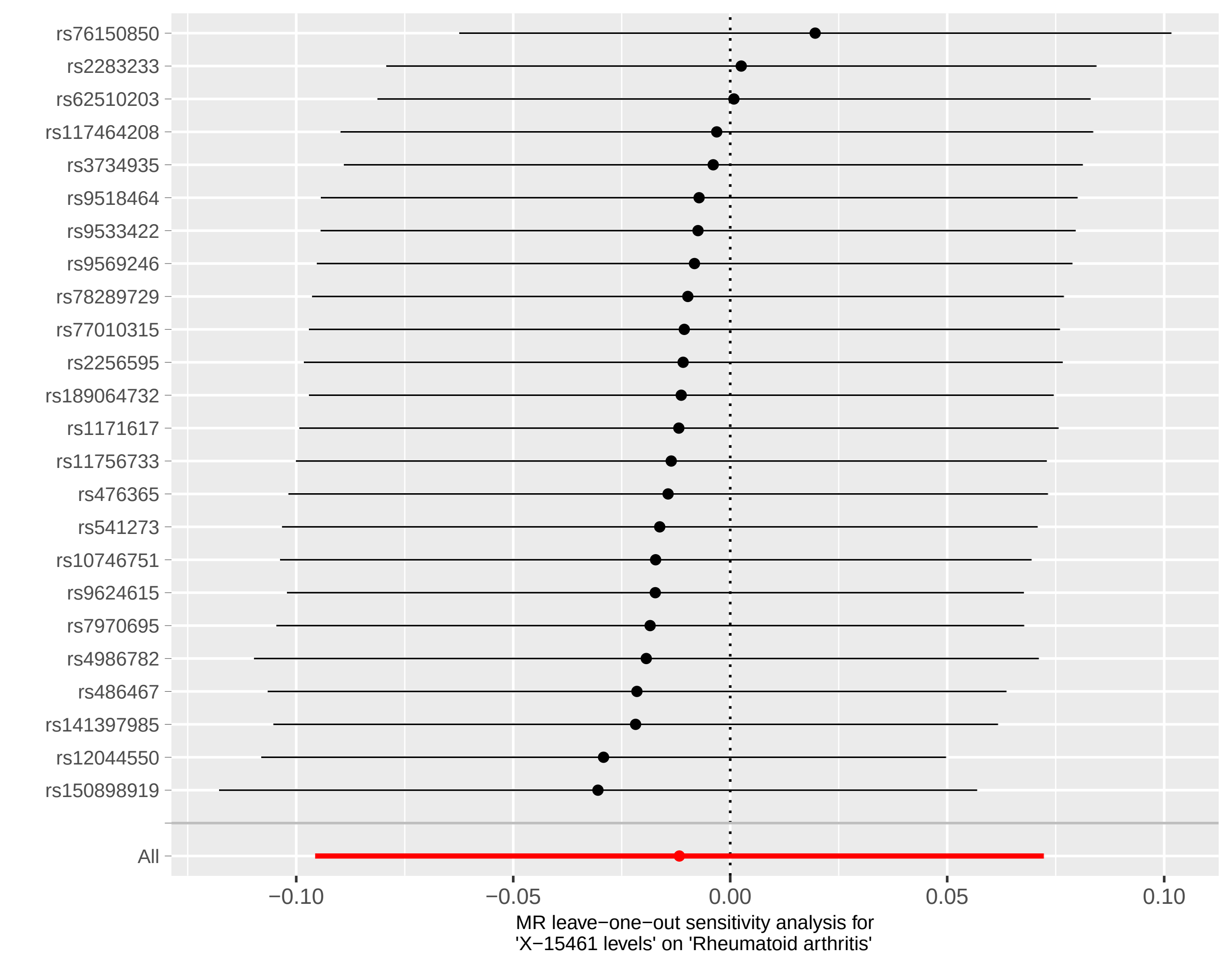

rs76150850
rs2283233
rs62510203
rs117464208
rs3734935
rs9518464
rs9533422
rs9569246
rs78289729
rs77010315
rs2256595
rs189064732
rs1171617
rs11756733
rs476365
rs541273
rs10746751
rs9624615
rs7970695
rs4986782
rs486467
rs141397985
rs12044550
rs150898919
All
−0.10
−0.05
0.00
0.05
0.10
MR leave−one−out sensitivity analysis for
'X−15461 levels' on 'Rheumatoid arthritis'

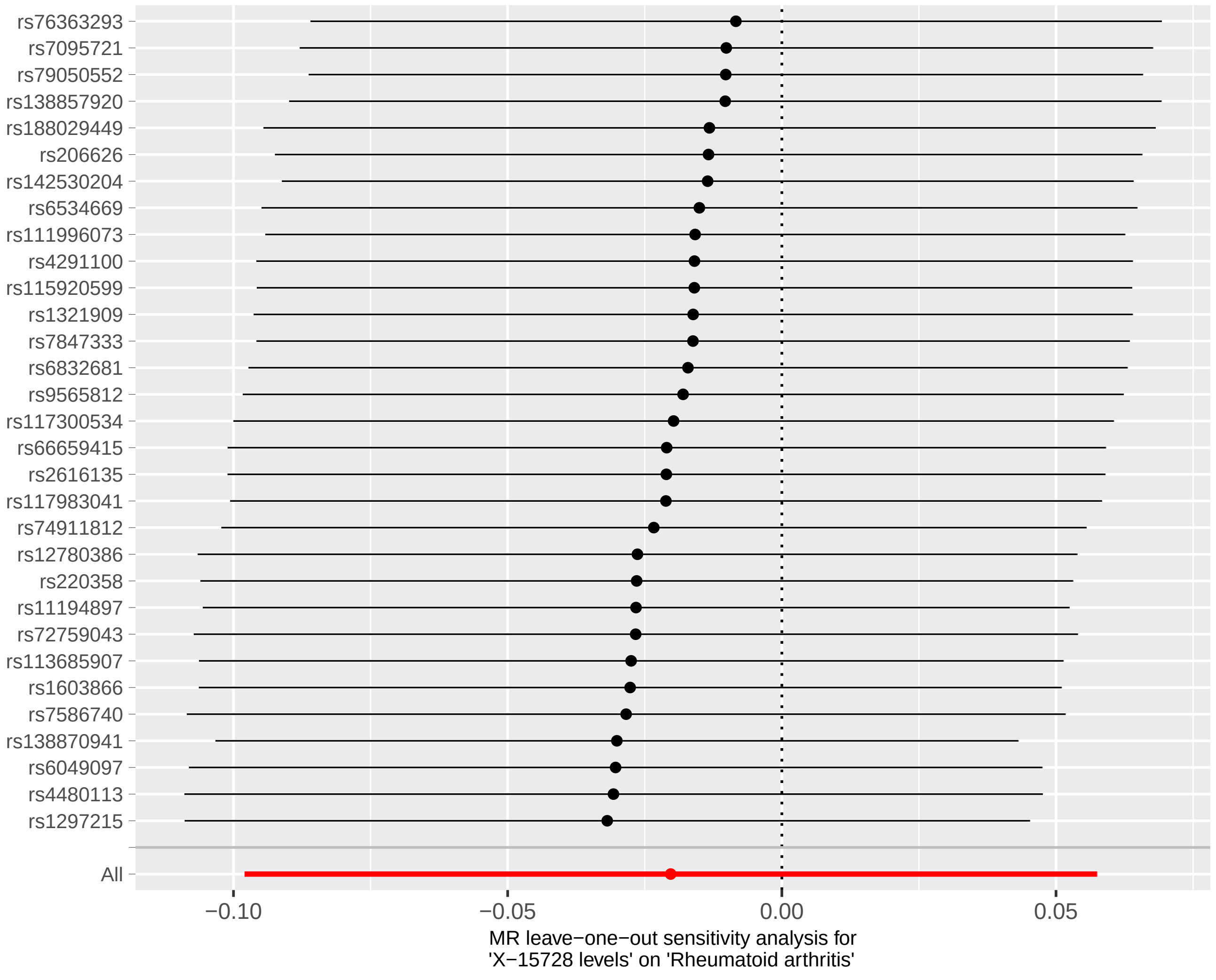

rs76363293
rs7095721
rs79050552
rs138857920
rs188029449
rs206626
rs142530204
rs6534669
rs111996073
rs4291100
rs115920599
rs1321909
rs7847333
rs6832681
rs9565812
rs117300534
rs66659415
rs2616135
rs117983041
rs74911812
rs12780386
rs220358
rs11194897
rs72759043
rs113685907
rs1603866
rs7586740
rs138870941
rs6049097
rs4480113
rs1297215
All
−0.10
−0.05
0.00
0.05
MR leave−one−out sensitivity analysis for
'X−15728 levels' on 'Rheumatoid arthritis'

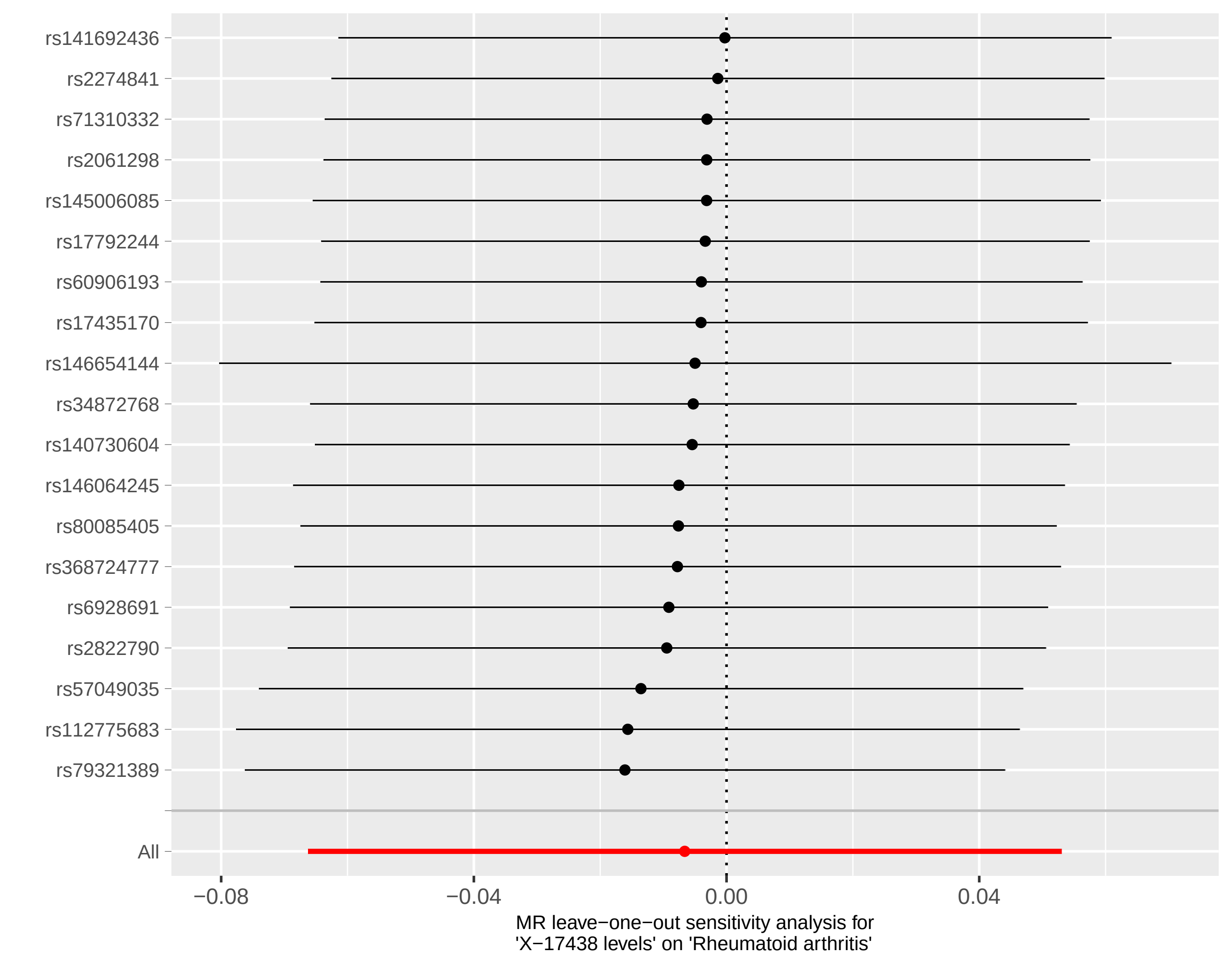
rs141692436
rs2274841
rs71310332
rs2061298
rs145006085
rs17792244
rs60906193
rs17435170
rs146654144
rs34872768
rs140730604
rs146064245
rs80085405
rs368724777
rs6928691
rs2822790
rs57049035
rs112775683
rs79321389
All
−0.08
−0.04
0.00
0.04
MR leave−one−out sensitivity analysis for
'X−17438 levels' on 'Rheumatoid arthritis'

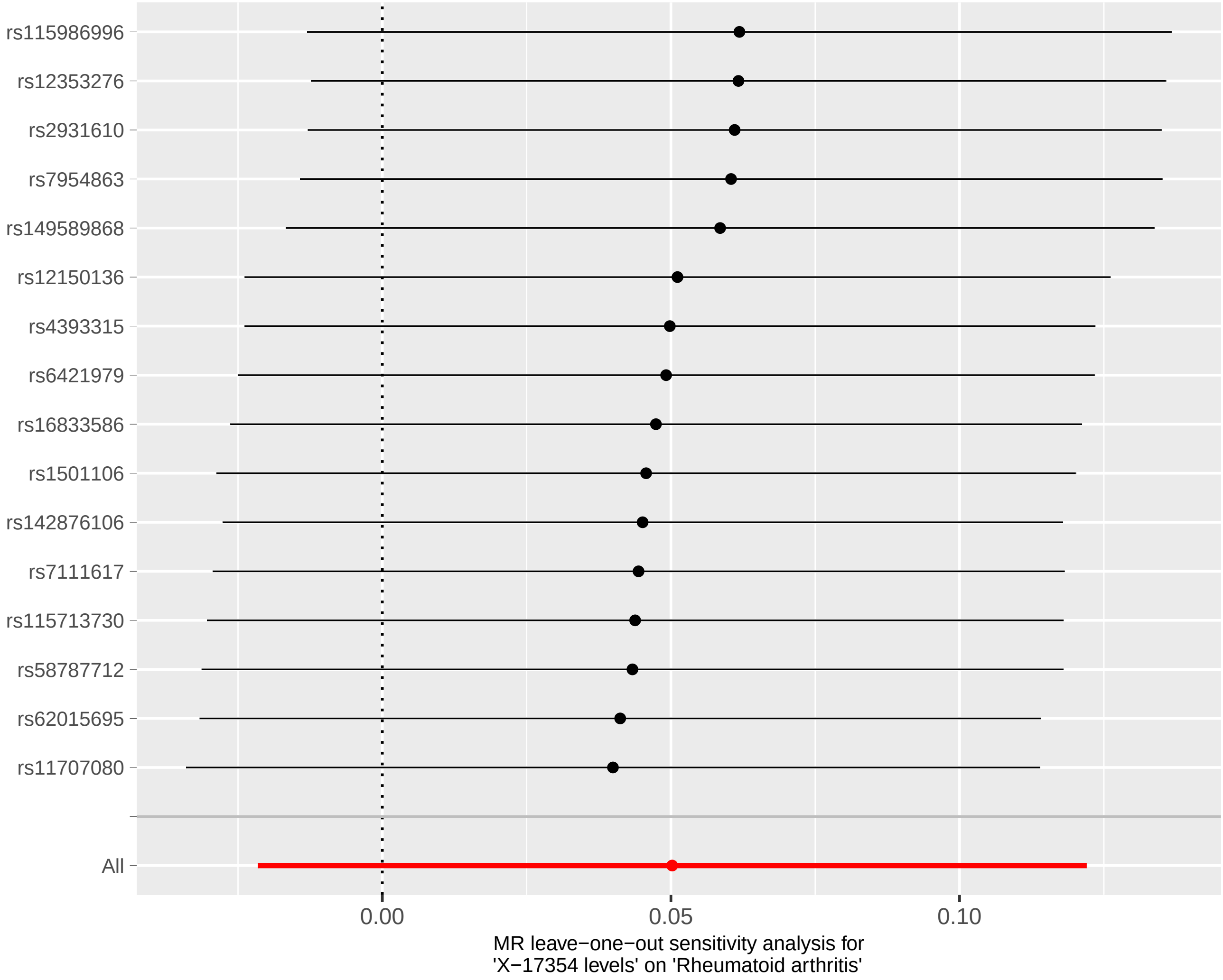
rs115986996
rs12353276
rs2931610
rs7954863
rs149589868
rs12150136
rs4393315
rs6421979
rs16833586
rs1501106
rs142876106
rs7111617
rs115713730
rs58787712
rs62015695
rs11707080
All
0.00
0.05
0.10
MR leave−one−out sensitivity analysis for
'X−17354 levels' on 'Rheumatoid arthritis'

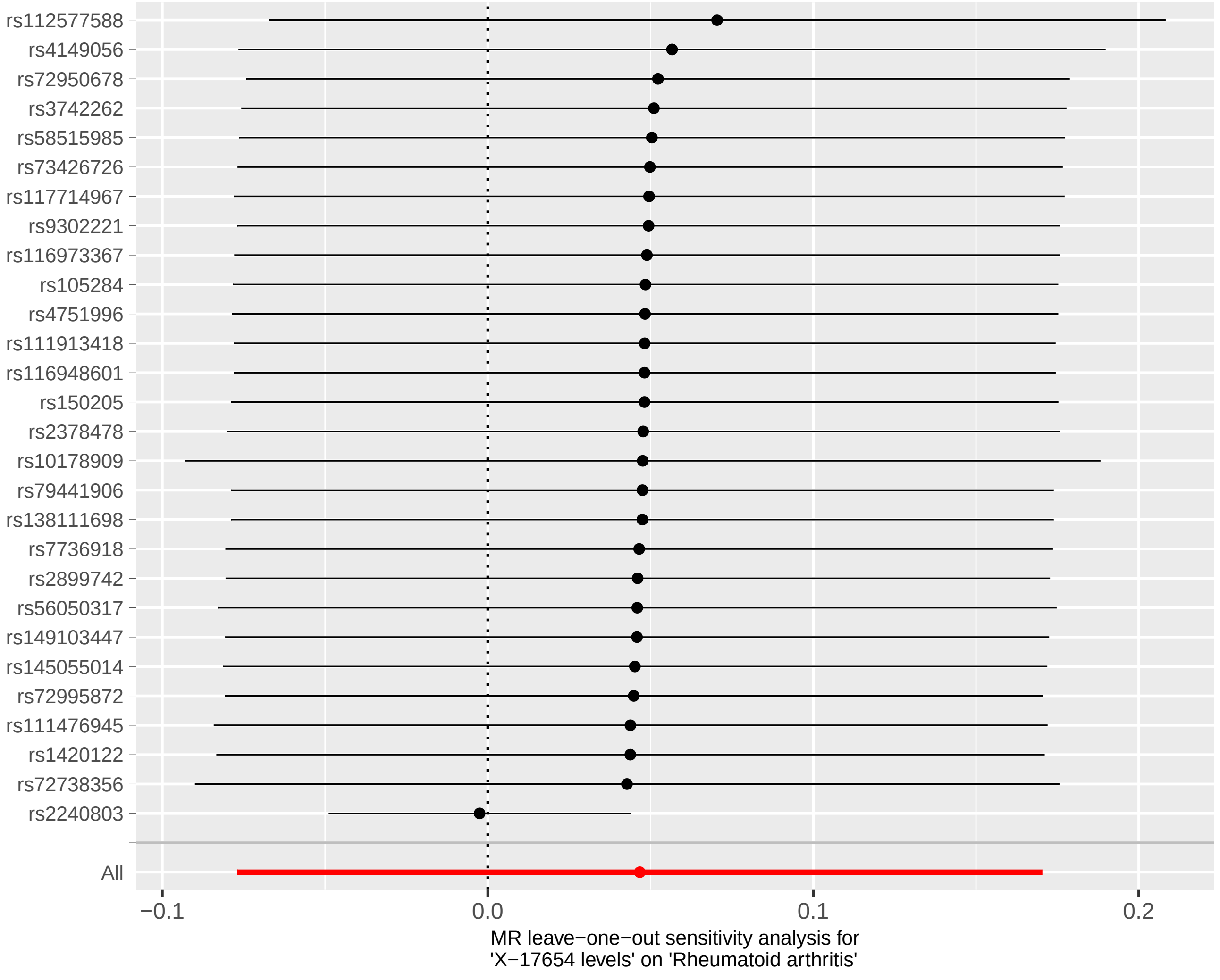
rs112577588
rs4149056
rs72950678
rs3742262
rs58515985
rs73426726
rs117714967
rs9302221
rs116973367
rs105284
rs4751996
rs111913418
rs116948601
rs150205
rs2378478
rs10178909
rs79441906
rs138111698
rs7736918
rs2899742
rs56050317
rs149103447
rs145055014
rs72995872
rs111476945
rs1420122
rs72738356
rs2240803
All
−0.1
0.0
0.1
0.2
MR leave−one−out sensitivity analysis for
'X−17654 levels' on 'Rheumatoid arthritis'

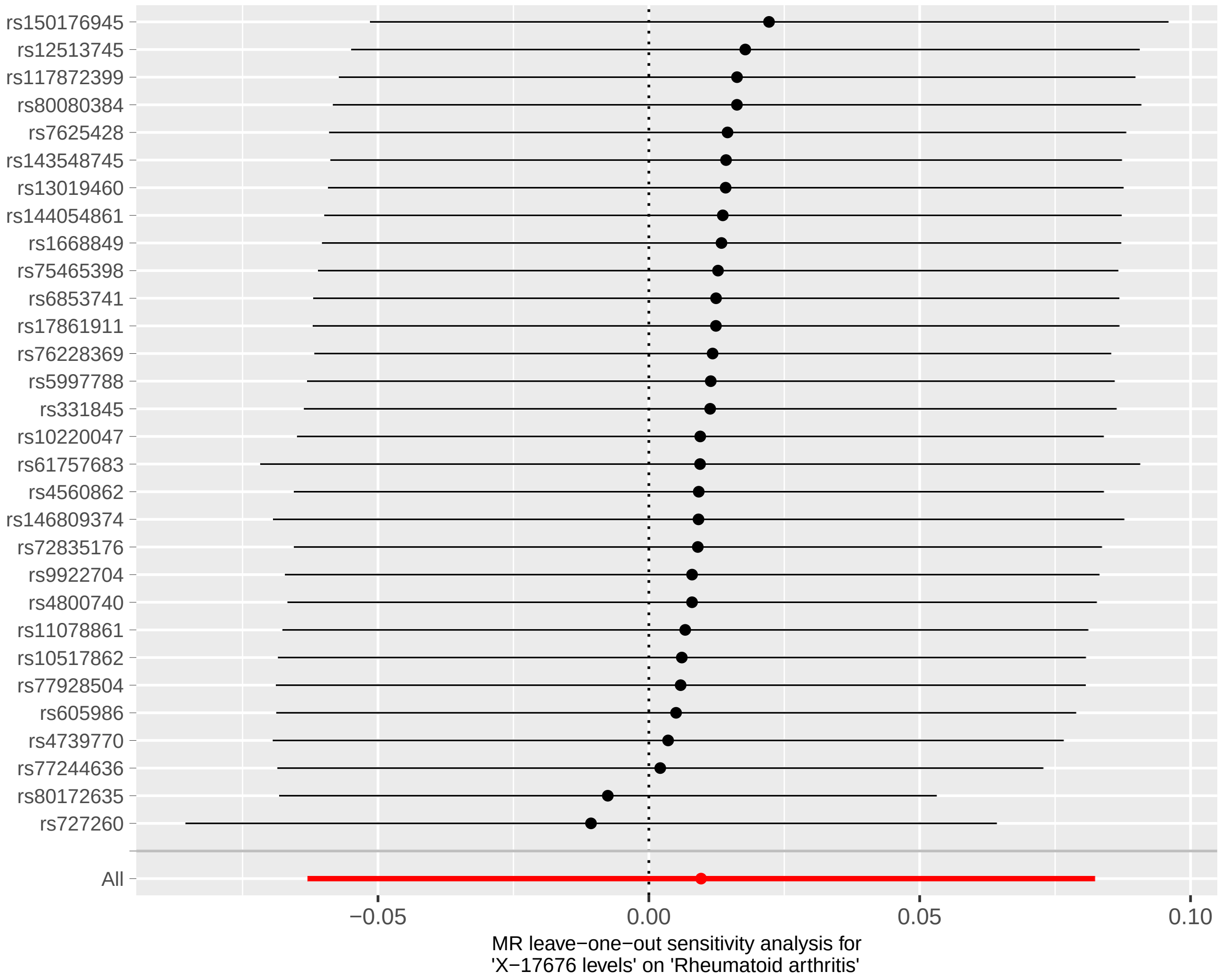
rs150176945
rs12513745
rs117872399
rs80080384
rs7625428
rs143548745
rs13019460
rs144054861
rs1668849
rs75465398
rs6853741
rs17861911
rs76228369
rs5997788
rs331845
rs10220047
rs61757683
rs4560862
rs146809374
rs72835176
rs9922704
rs4800740
rs11078861
rs10517862
rs77928504
rs605986
rs4739770
rs77244636
rs80172635
rs727260
All
−0.05
0.00
0.05
0.10
MR leave−one−out sensitivity analysis for
'X−17676 levels' on 'Rheumatoid arthritis'

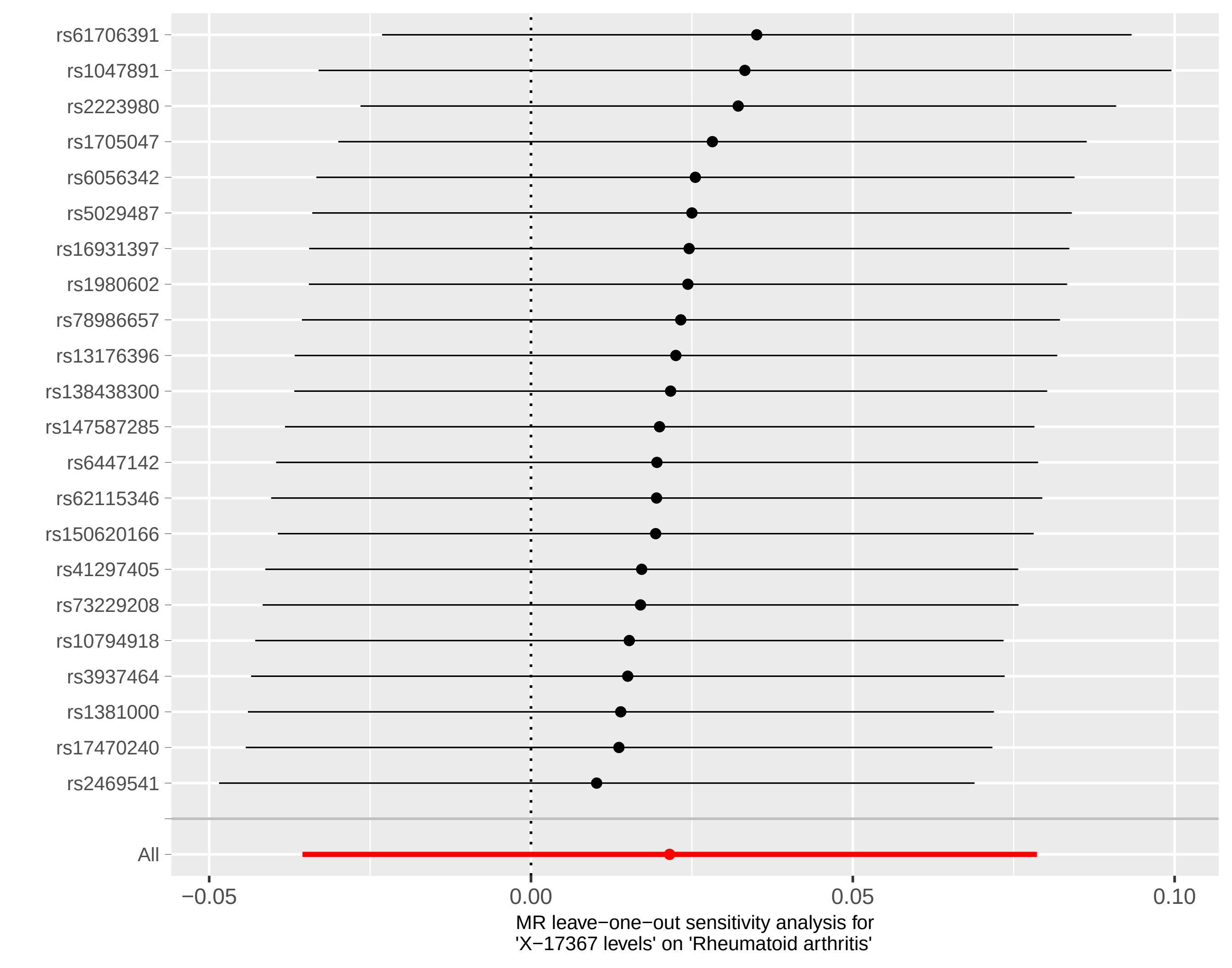

rs61706391
rs1047891
rs2223980
rs1705047
rs6056342
rs5029487
rs16931397
rs1980602
rs78986657
rs13176396
rs138438300
rs147587285
rs6447142
rs62115346
rs150620166
rs41297405
rs73229208
rs10794918
rs3937464
rs1381000
rs17470240
rs2469541
All
−0.05
0.00
0.05
0.10
MR leave−one−out sensitivity analysis for
'X−17367 levels' on 'Rheumatoid arthritis'

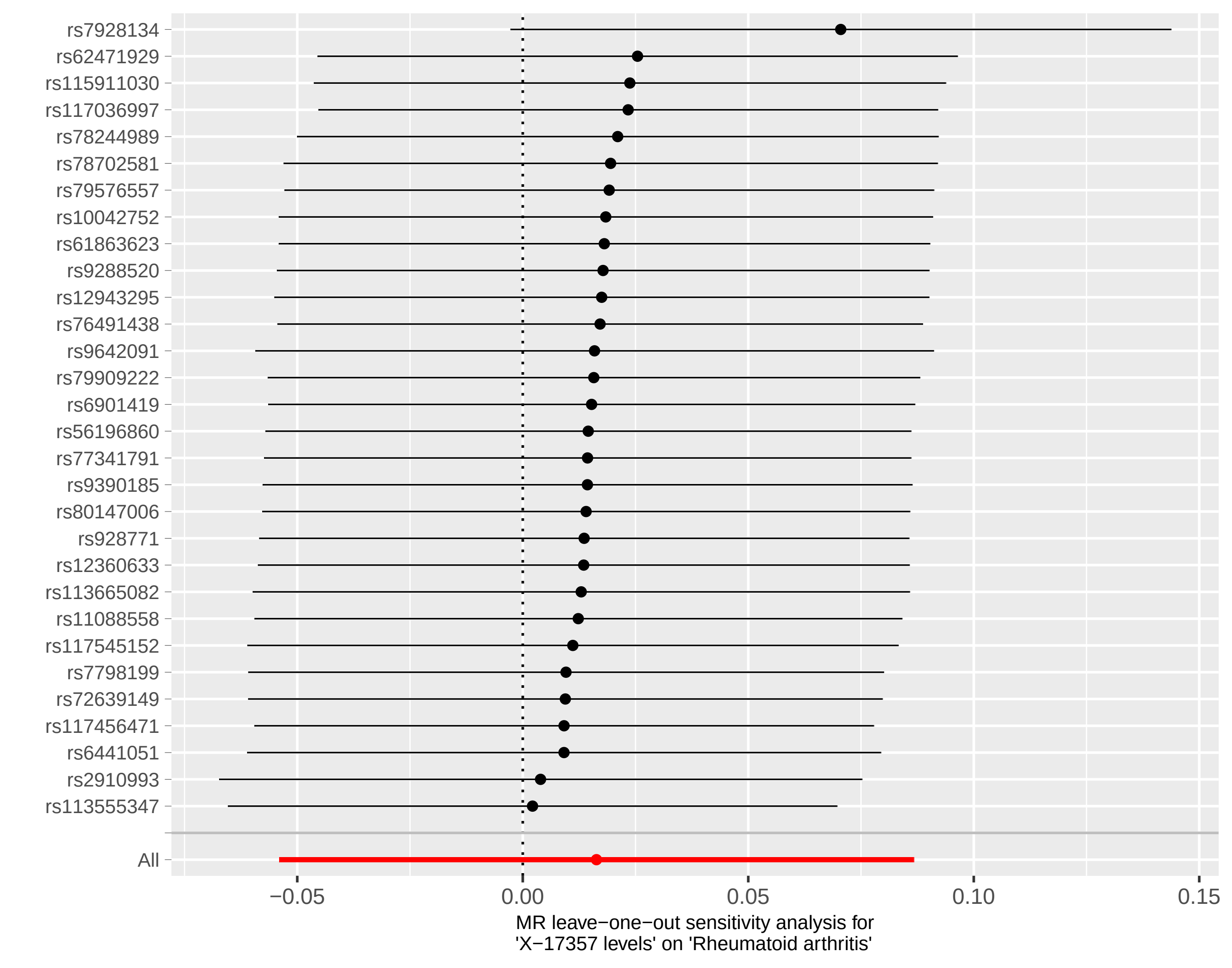
rs7928134
rs62471929
rs115911030
rs117036997
rs78244989
rs78702581
rs79576557
rs10042752
rs61863623
rs9288520
rs12943295
rs76491438
rs9642091
rs79909222
rs6901419
rs56196860
rs77341791
rs9390185
rs80147006
rs928771
rs12360633
rs113665082
rs11088558
rs117545152
rs7798199
rs72639149
rs117456471
rs6441051
rs2910993
rs113555347
All
−0.05
0.00
0.05
0.10
0.15
MR leave−one−out sensitivity analysis for
'X−17357 levels' on 'Rheumatoid arthritis'

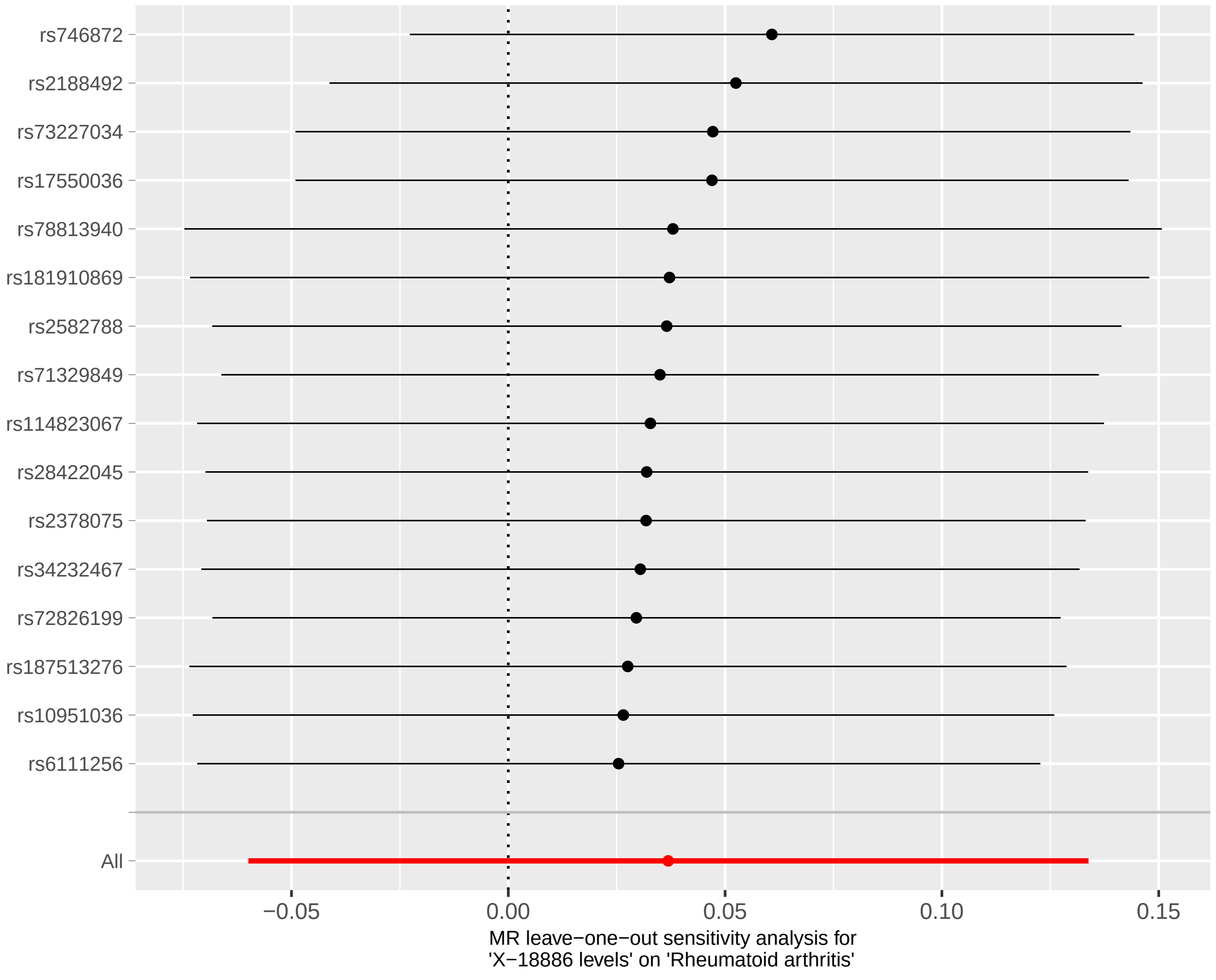
rs746872
rs2188492
rs73227034
rs17550036
rs78813940
rs181910869
rs2582788
rs71329849
rs114823067
rs28422045
rs2378075
rs34232467
rs72826199
rs187513276
rs10951036
rs6111256
All
−0.05
0.00
0.05
0.10
0.15
MR leave−one−out sensitivity analysis for
'X−18886 levels' on 'Rheumatoid arthritis'

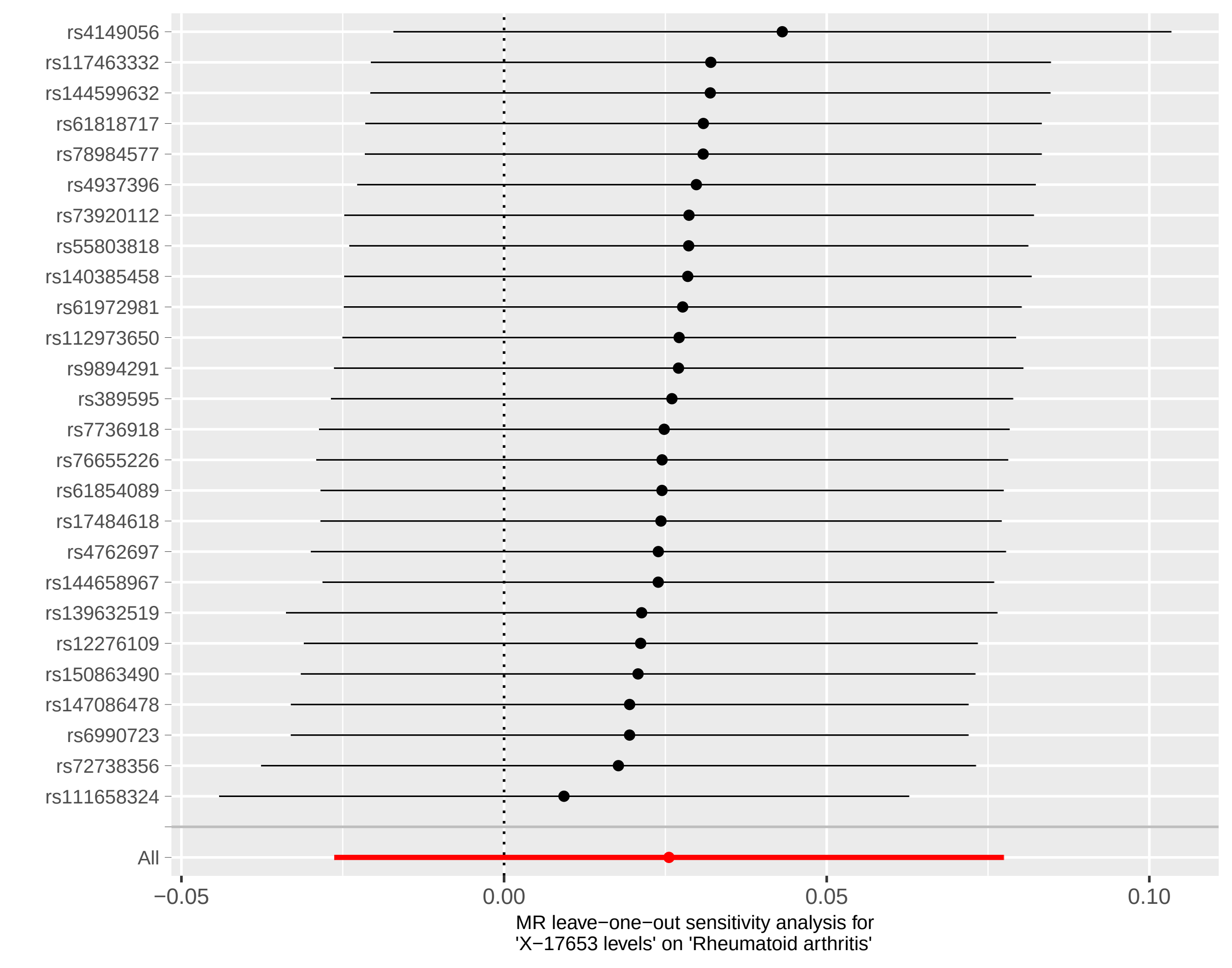

rs4149056
rs117463332
rs144599632
rs61818717
rs78984577
rs4937396
rs73920112
rs55803818
rs140385458
rs61972981
rs112973650
rs9894291
rs389595
rs7736918
rs76655226
rs61854089
rs17484618
rs4762697
rs144658967
rs139632519
rs12276109
rs150863490
rs147086478
rs6990723
rs72738356
rs111658324
All
−0.05
0.00
0.05
0.10
MR leave−one−out sensitivity analysis for
'X−17653 levels' on 'Rheumatoid arthritis'

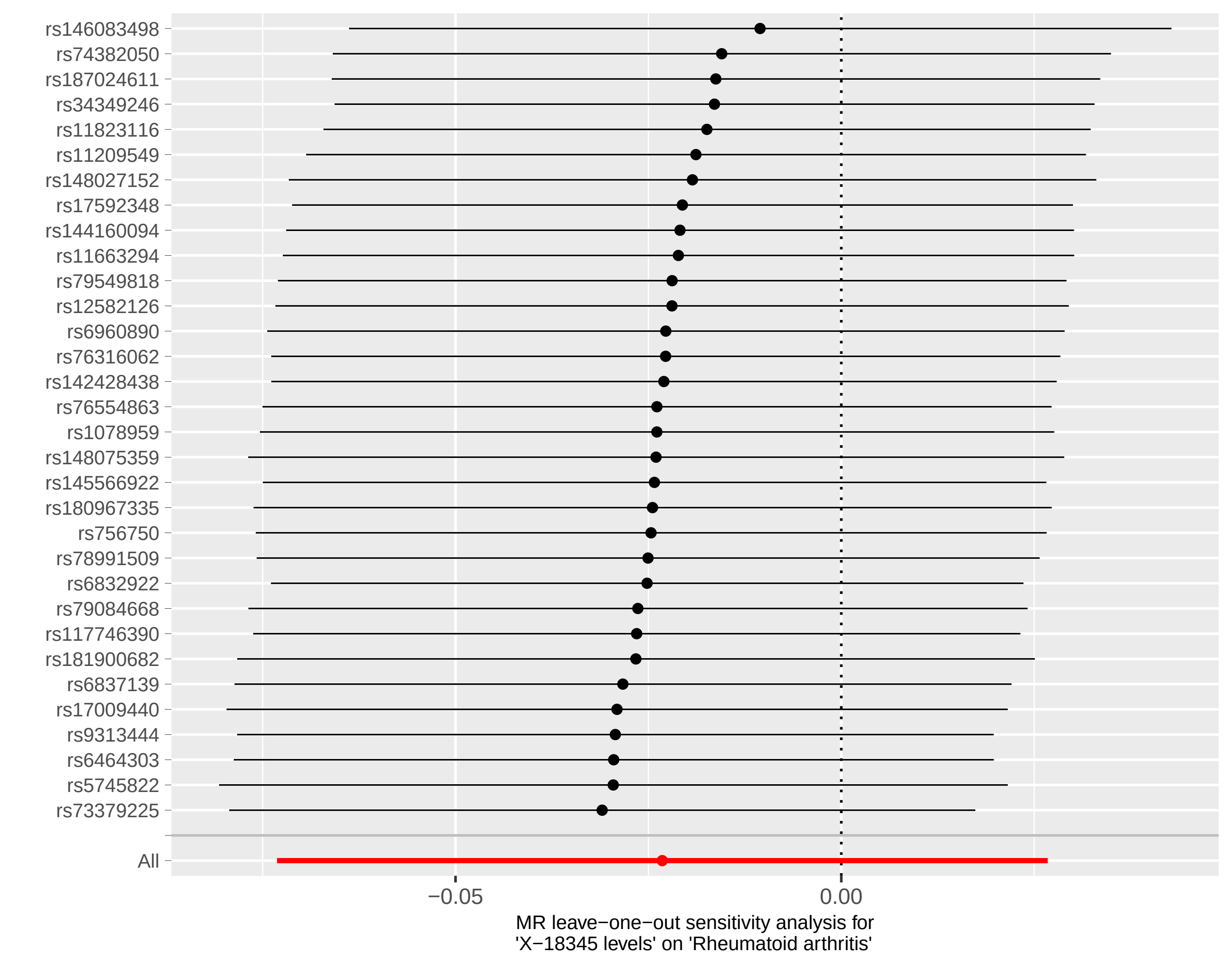

rs146083498
rs74382050
rs187024611
rs34349246
rs11823116
rs11209549
rs148027152
rs17592348
rs144160094
rs11663294
rs79549818
rs12582126
rs6960890
rs76316062
rs142428438
rs76554863
rs1078959
rs148075359
rs145566922
rs180967335
rs756750
rs78991509
rs6832922
rs79084668
rs117746390
rs181900682
rs6837139
rs17009440
rs9313444
rs6464303
rs5745822
rs73379225
All
−0.05
0.00
MR leave−one−out sensitivity analysis for
'X−18345 levels' on 'Rheumatoid arthritis'

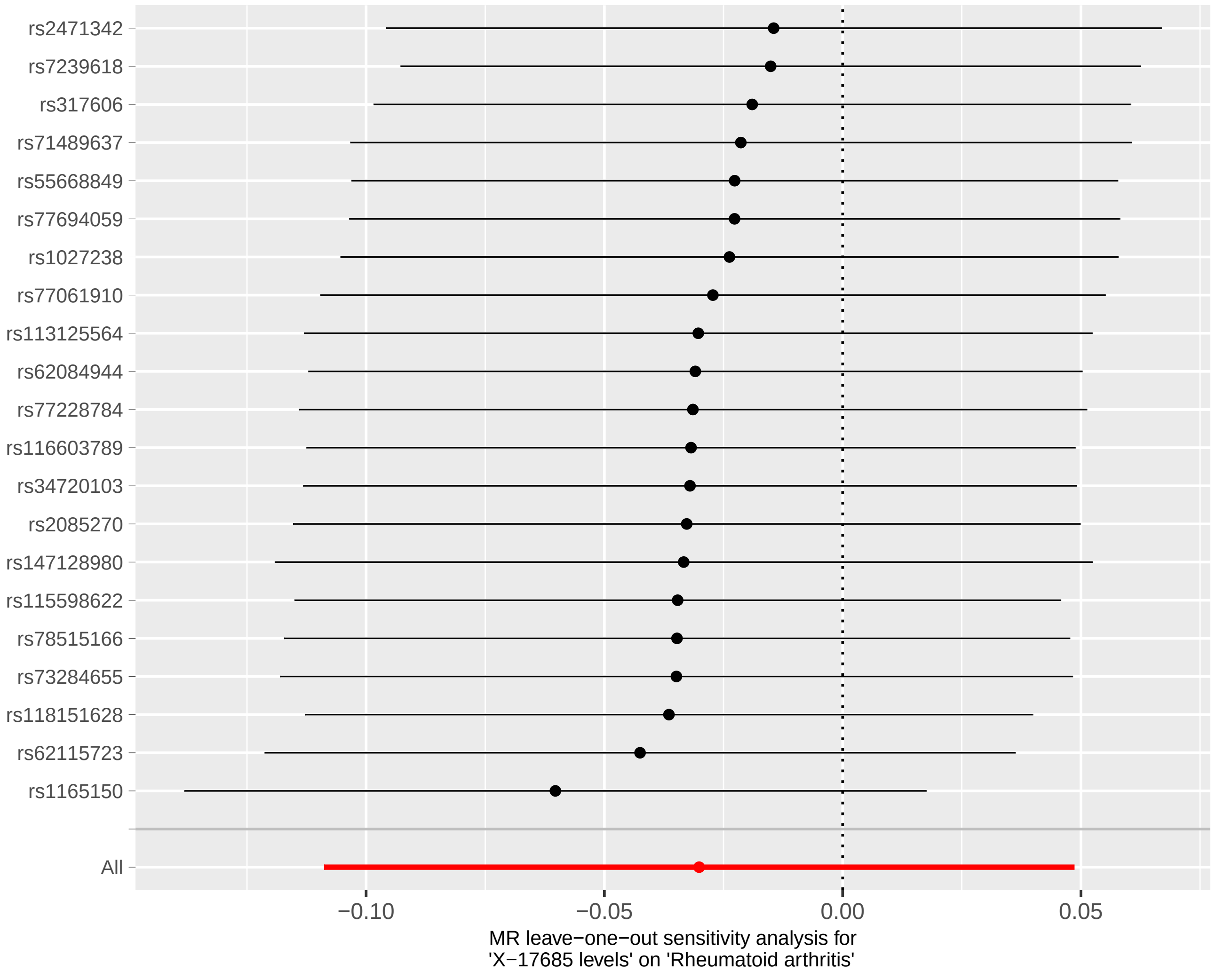
rs2471342
rs7239618
rs317606
rs71489637
rs55668849
rs77694059
rs1027238
rs77061910
rs113125564
rs62084944
rs77228784
rs116603789
rs34720103
rs2085270
rs147128980
rs115598622
rs78515166
rs73284655
rs118151628
rs62115723
rs1165150
All
−0.10
−0.05
0.00
0.05
MR leave−one−out sensitivity analysis for
'X−17685 levels' on 'Rheumatoid arthritis'

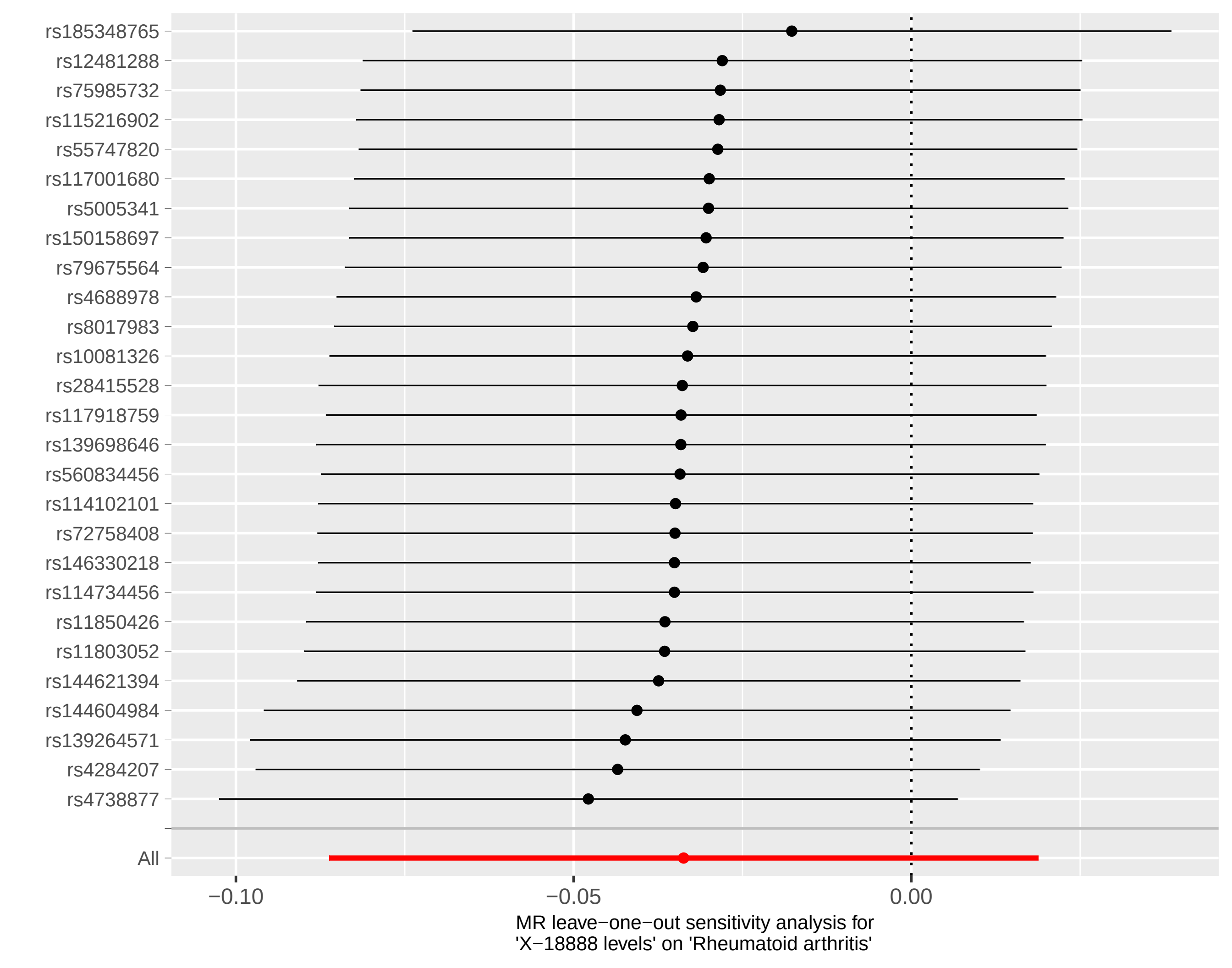
rs185348765
rs12481288
rs75985732
rs115216902
rs55747820
rs117001680
rs5005341
rs150158697
rs79675564
rs4688978
rs8017983
rs10081326
rs28415528
rs117918759
rs139698646
rs560834456
rs114102101
rs72758408
rs146330218
rs114734456
rs11850426
rs11803052
rs144621394
rs144604984
rs139264571
rs4284207
rs4738877
All
−0.10
−0.05
0.00
MR leave−one−out sensitivity analysis for
'X−18888 levels' on 'Rheumatoid arthritis'

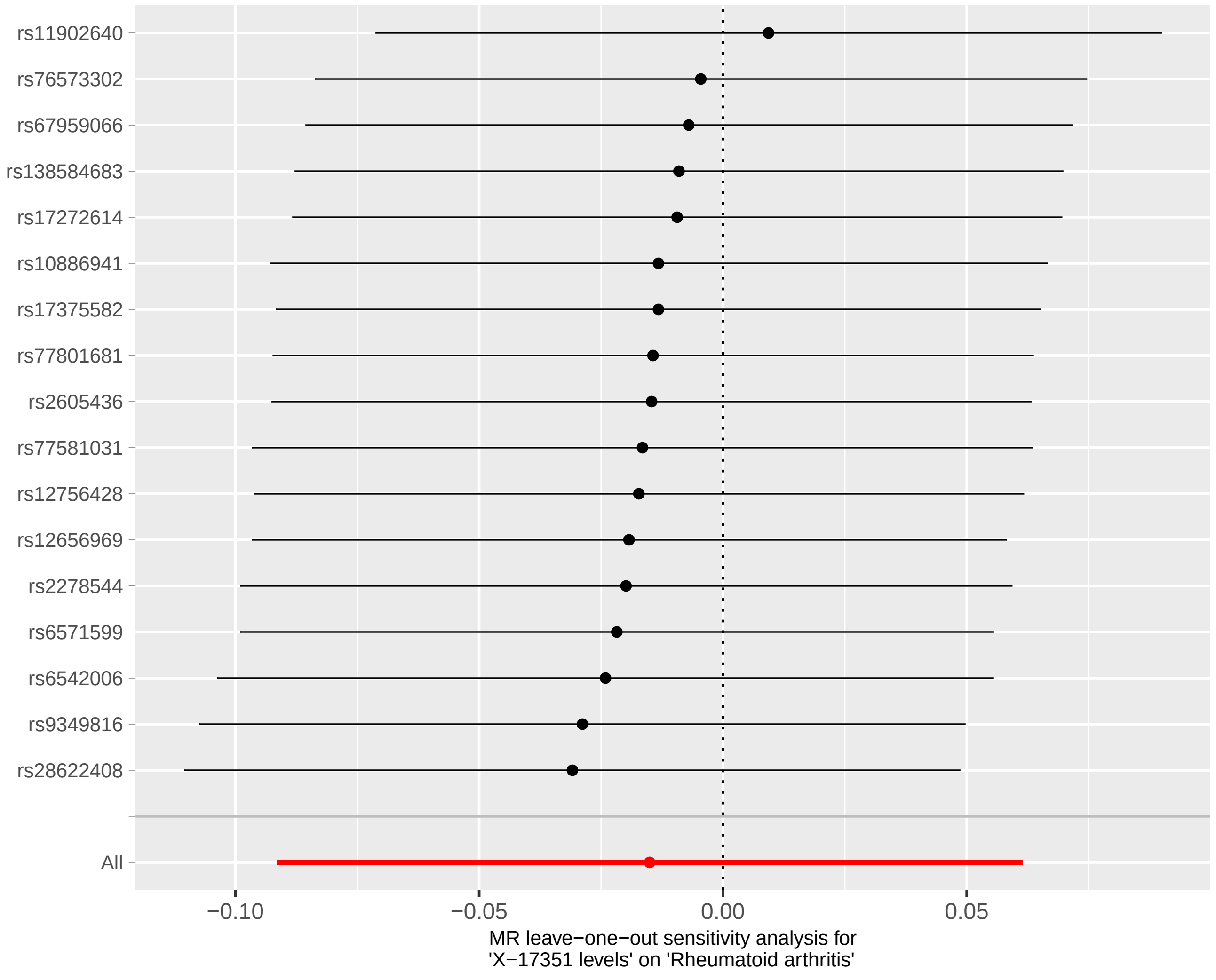
rs11902640
rs76573302
rs67959066
rs138584683
rs17272614
rs10886941
rs17375582
rs77801681
rs2605436
rs77581031
rs12756428
rs12656969
rs2278544
rs6571599
rs6542006
rs9349816
rs28622408
All
−0.10
−0.05
0.00
0.05
MR leave−one−out sensitivity analysis for
'X−17351 levels' on 'Rheumatoid arthritis'

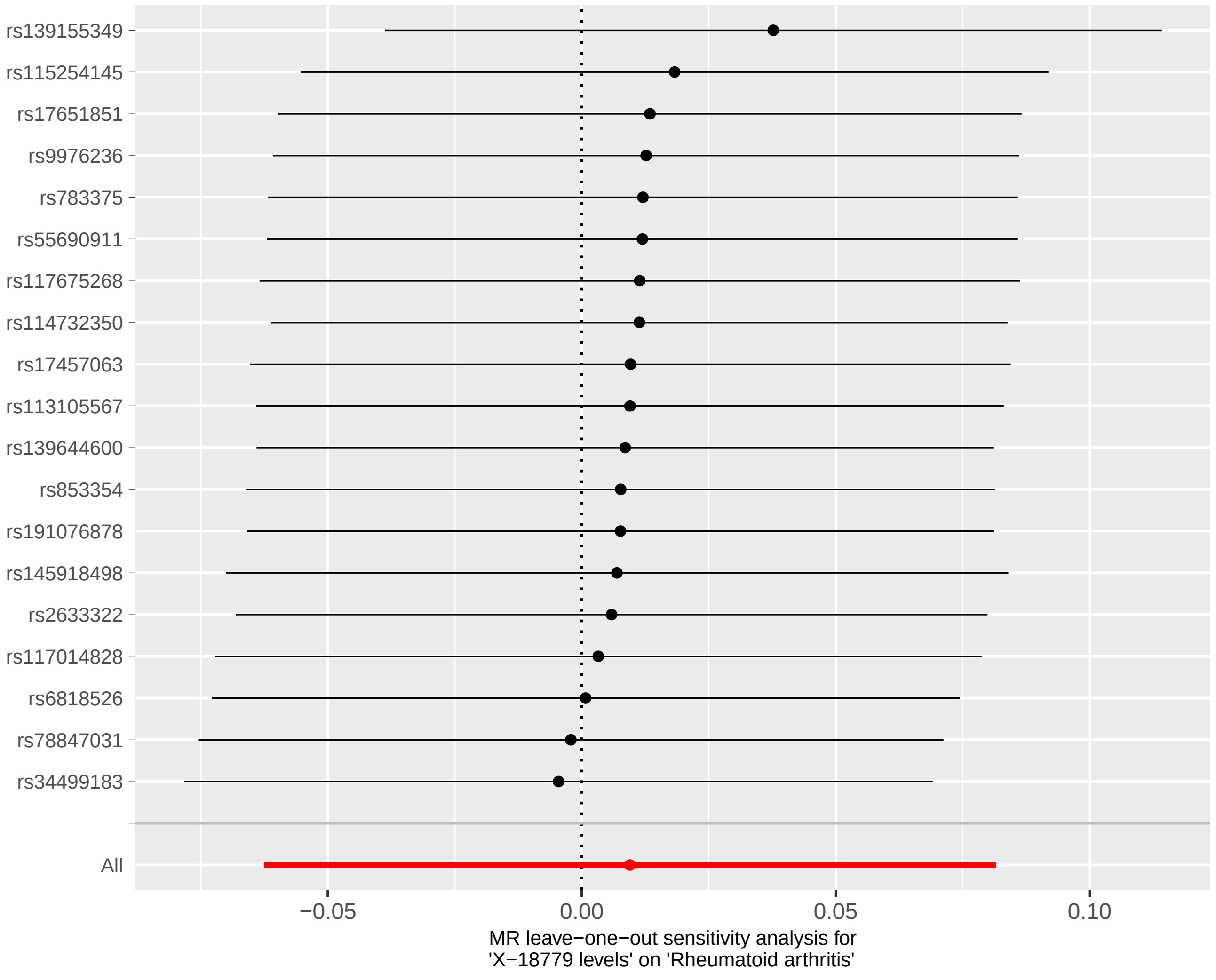
rs139155349
rs115254145
rs17651851
rs9976236
rs783375
rs55690911
rs117675268
rs114732350
rs17457063
rs113105567
rs139644600
rs853354
rs191076878
rs145918498
rs2633322
rs117014828
rs6818526
rs78847031
rs34499183
All
−0.05
0.00
0.05
0.10
MR leave−one−out sensitivity analysis for
'X−18779 levels' on 'Rheumatoid arthritis'

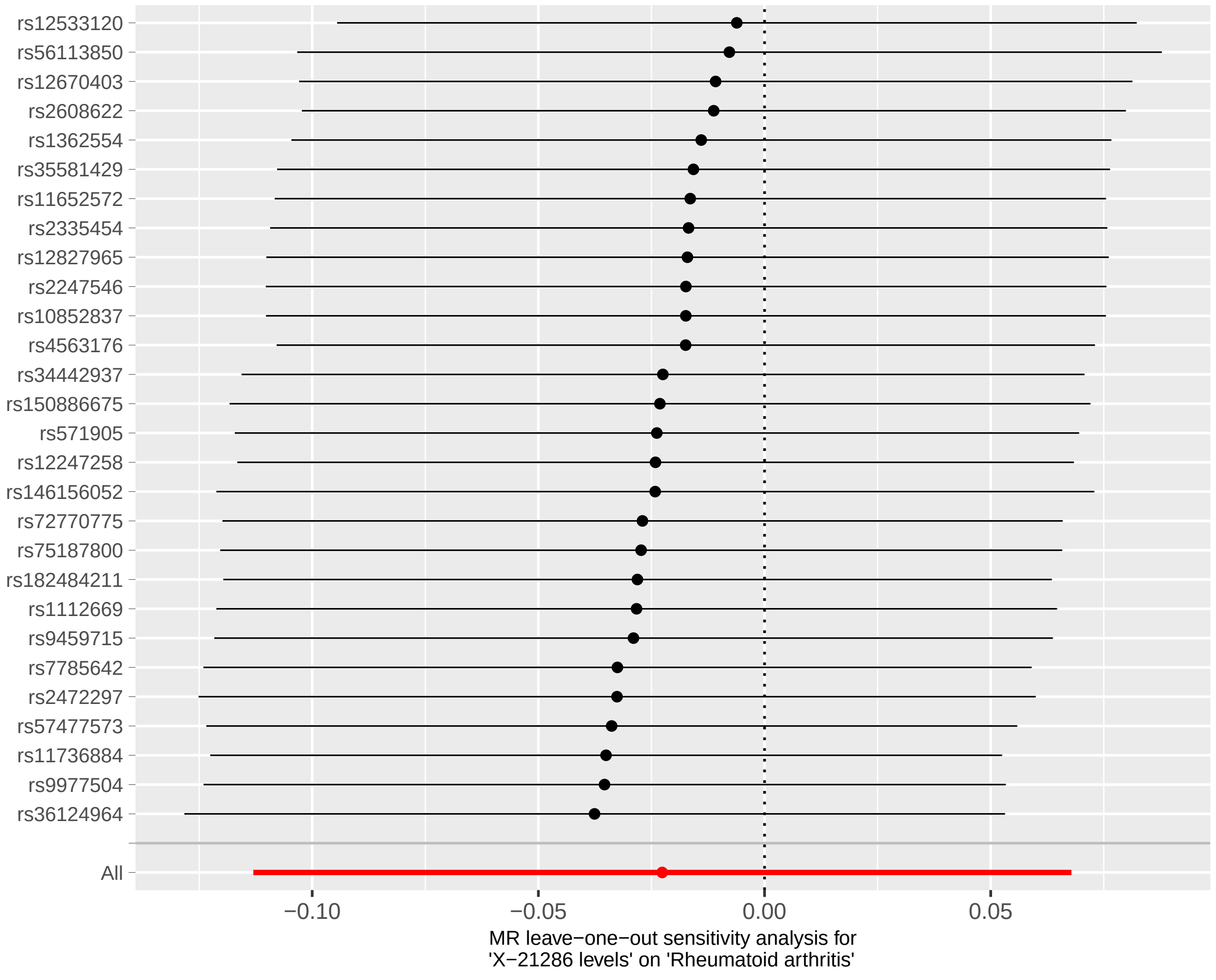
rs12533120
rs56113850
rs12670403
rs2608622
rs1362554
rs35581429
rs11652572
rs2335454
rs12827965
rs2247546
rs10852837
rs4563176
rs34442937
rs150886675
rs571905
rs12247258
rs146156052
rs72770775
rs75187800
rs182484211
rs1112669
rs9459715
rs7785642
rs2472297
rs57477573
rs11736884
rs9977504
rs36124964
All
−0.10
−0.05
0.00
0.05
MR leave−one−out sensitivity analysis for
'X−21286 levels' on 'Rheumatoid arthritis'

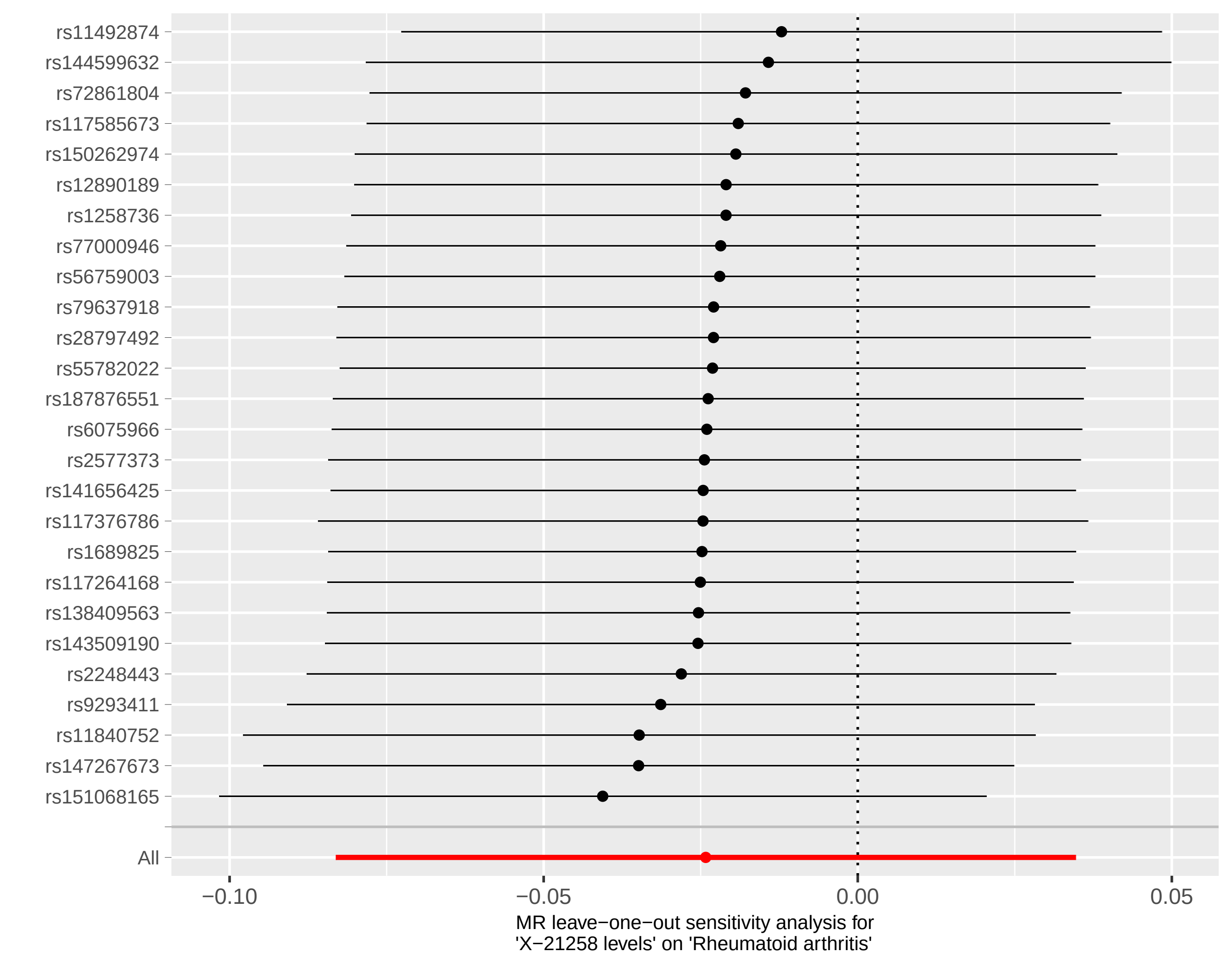
rs11492874
rs144599632
rs72861804
rs117585673
rs150262974
rs12890189
rs1258736
rs77000946
rs56759003
rs79637918
rs28797492
rs55782022
rs187876551
rs6075966
rs2577373
rs141656425
rs117376786
rs1689825
rs117264168
rs138409563
rs143509190
rs2248443
rs9293411
rs11840752
rs147267673
rs151068165
All
−0.10
−0.05
0.00
0.05
MR leave−one−out sensitivity analysis for
'X−21258 levels' on 'Rheumatoid arthritis'

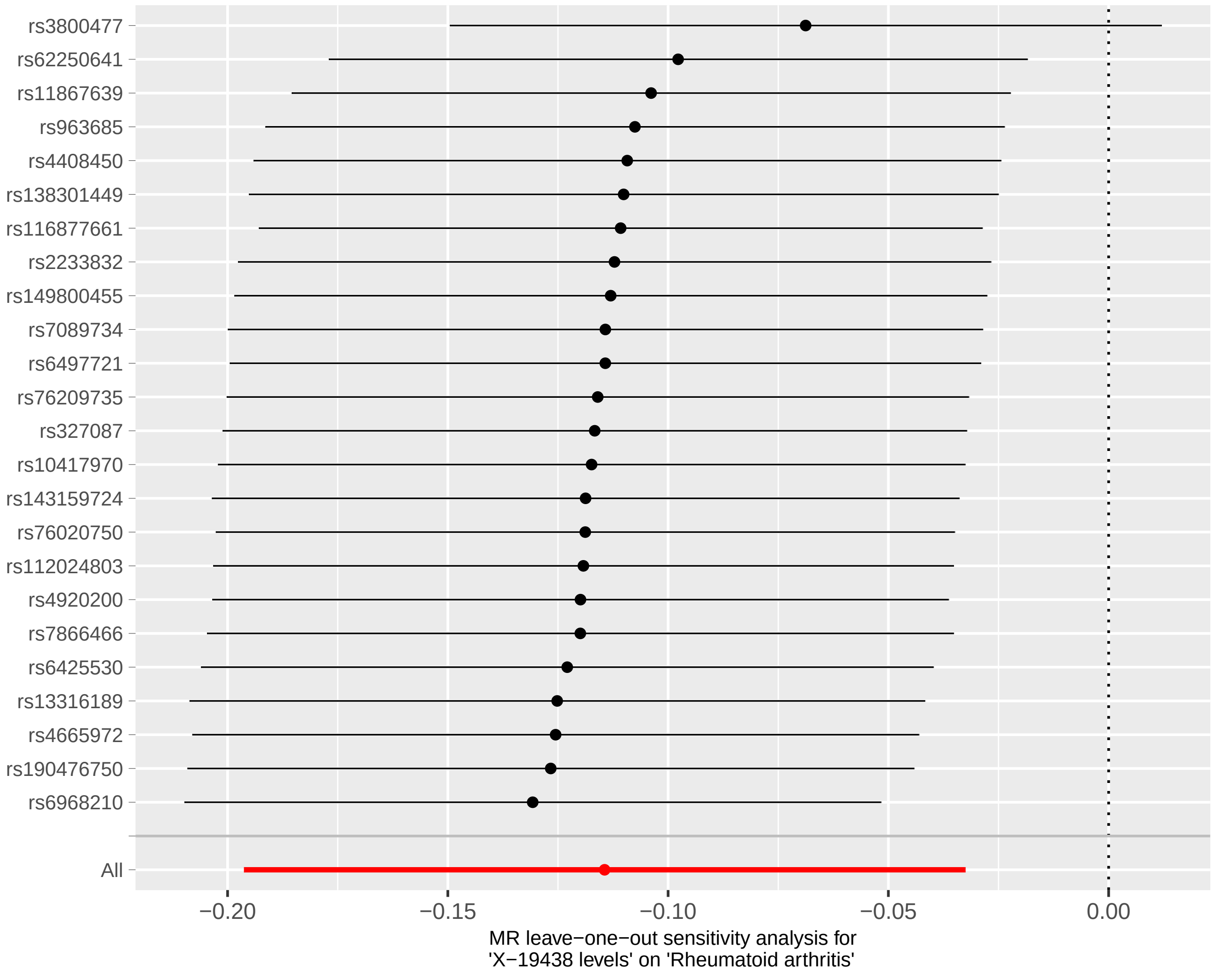
rs3800477
rs62250641
rs11867639
rs963685
rs4408450
rs138301449
rs116877661
rs2233832
rs149800455
rs7089734
rs6497721
rs76209735
rs327087
rs10417970
rs143159724
rs76020750
rs112024803
rs4920200
rs7866466
rs6425530
rs13316189
rs4665972
rs190476750
rs6968210
All
−0.20
−0.15
−0.10
−0.05
0.00
MR leave−one−out sensitivity analysis for
'X−19438 levels' on 'Rheumatoid arthritis'

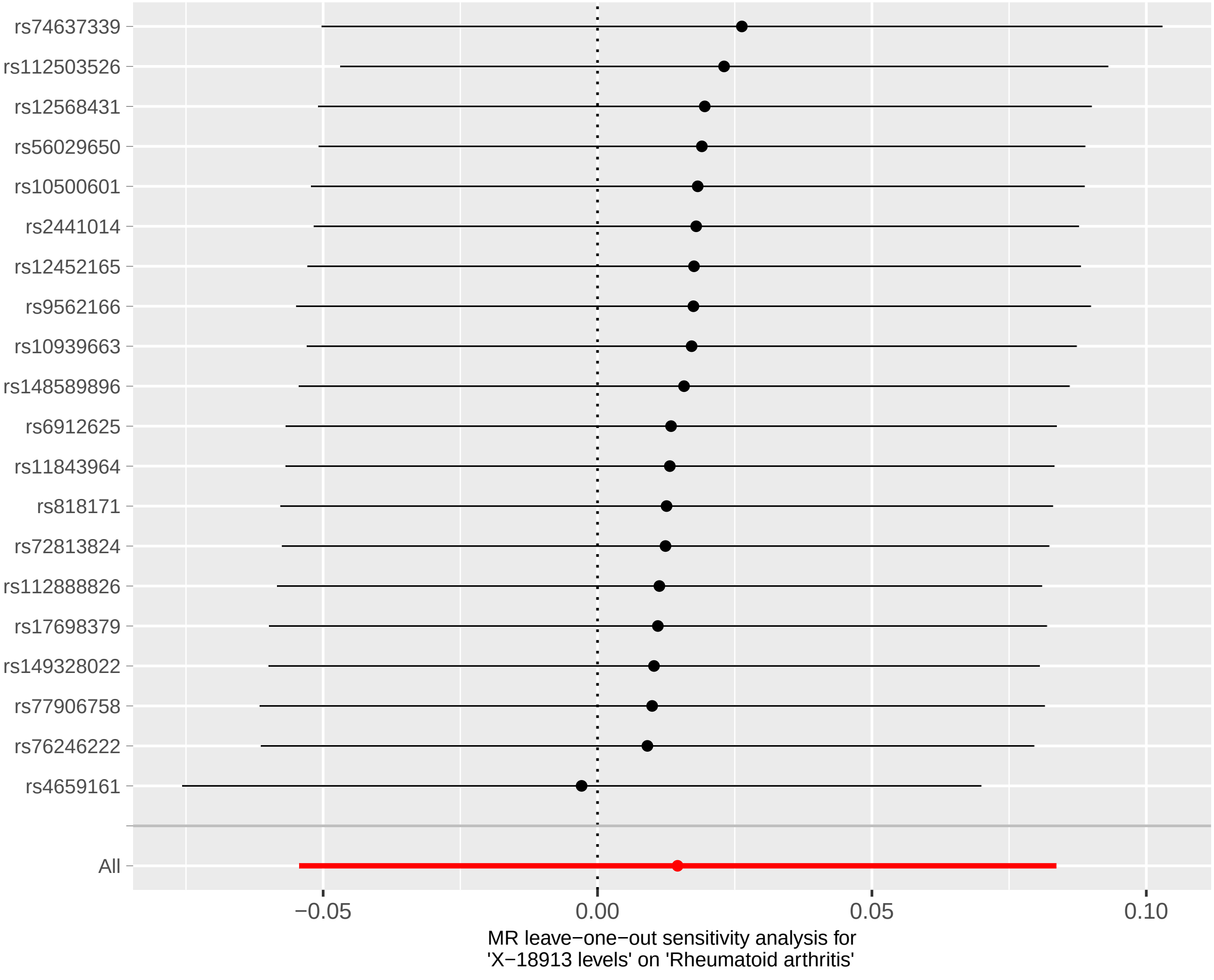
rs74637339
rs112503526
rs12568431
rs56029650
rs10500601
rs2441014
rs12452165
rs9562166
rs10939663
rs148589896
rs6912625
rs11843964
rs818171
rs72813824
rs112888826
rs17698379
rs149328022
rs77906758
rs76246222
rs4659161
All
−0.05
0.00
0.05
0.10
MR leave−one−out sensitivity analysis for
'X−18913 levels' on 'Rheumatoid arthritis'

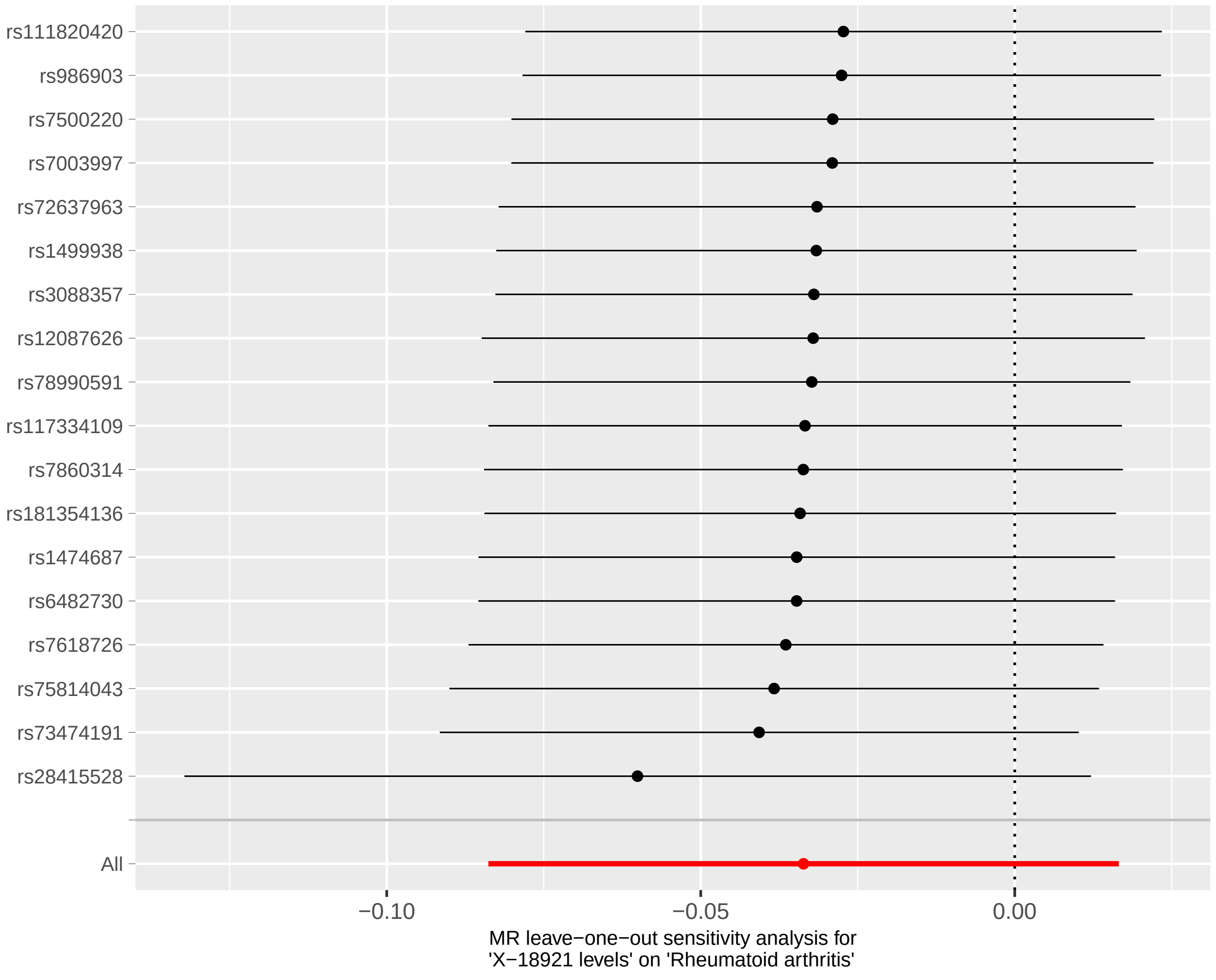
rs111820420
rs986903
rs7500220
rs7003997
rs72637963
rs1499938
rs3088357
rs12087626
rs78990591
rs117334109
rs7860314
rs181354136
rs1474687
rs6482730
rs7618726
rs75814043
rs73474191
rs28415528
All
−0.10
−0.05
0.00
MR leave−one−out sensitivity analysis for
'X−18921 levels' on 'Rheumatoid arthritis'

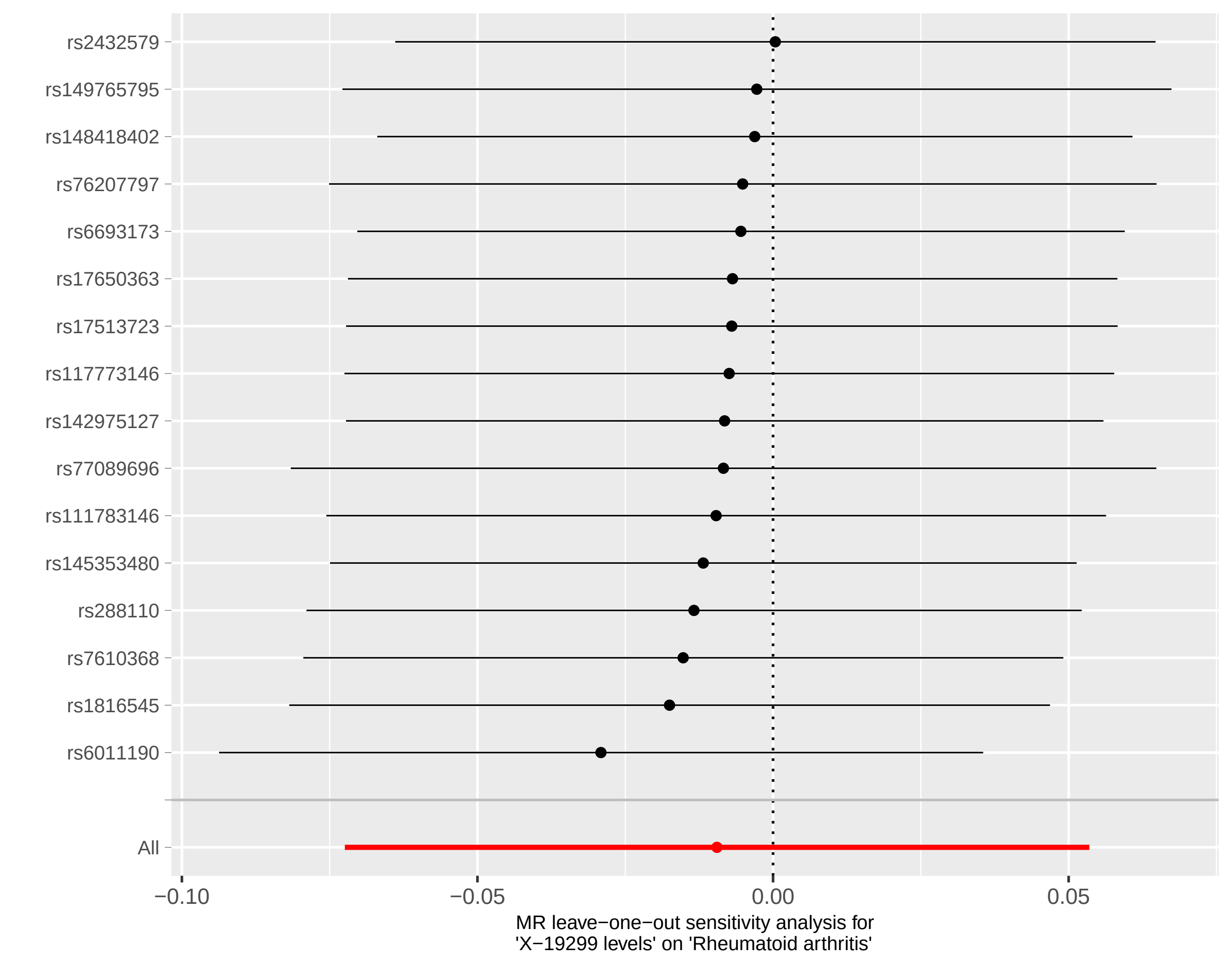
rs2432579
rs149765795
rs148418402
rs76207797
rs6693173
rs17650363
rs17513723
rs117773146
rs142975127
rs77089696
rs111783146
rs145353480
rs288110
rs7610368
rs1816545
rs6011190
All
−0.10
−0.05
0.00
0.05
MR leave−one−out sensitivity analysis for
'X−19299 levels' on 'Rheumatoid arthritis'

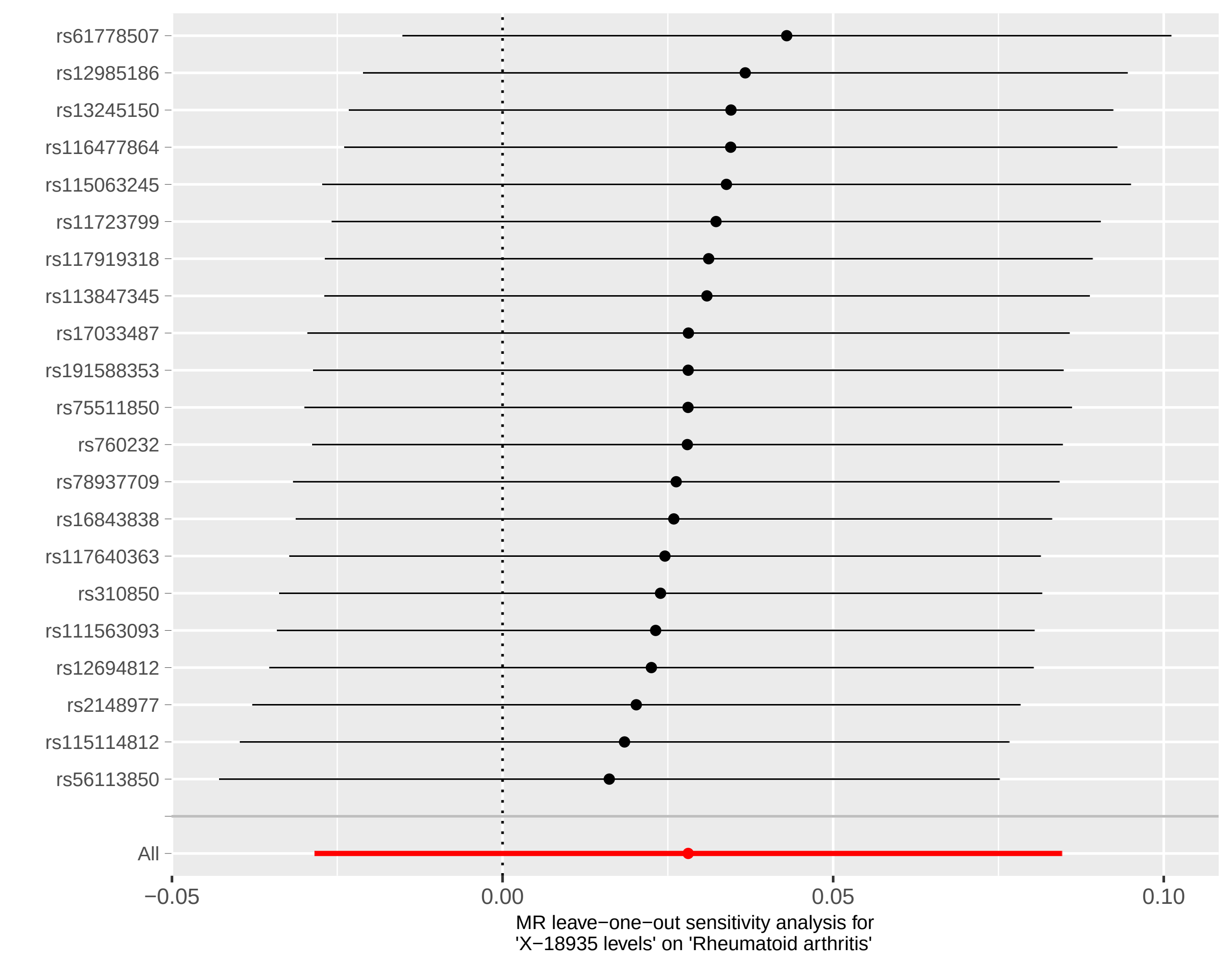

rs61778507
rs12985186
rs13245150
rs116477864
rs115063245
rs11723799
rs117919318
rs113847345
rs17033487
rs191588353
rs75511850
rs760232
rs78937709
rs16843838
rs117640363
rs310850
rs111563093
rs12694812
rs2148977
rs115114812
rs56113850
All
−0.05
0.00
0.05
0.10
MR leave−one−out sensitivity analysis for
'X−18935 levels' on 'Rheumatoid arthritis'

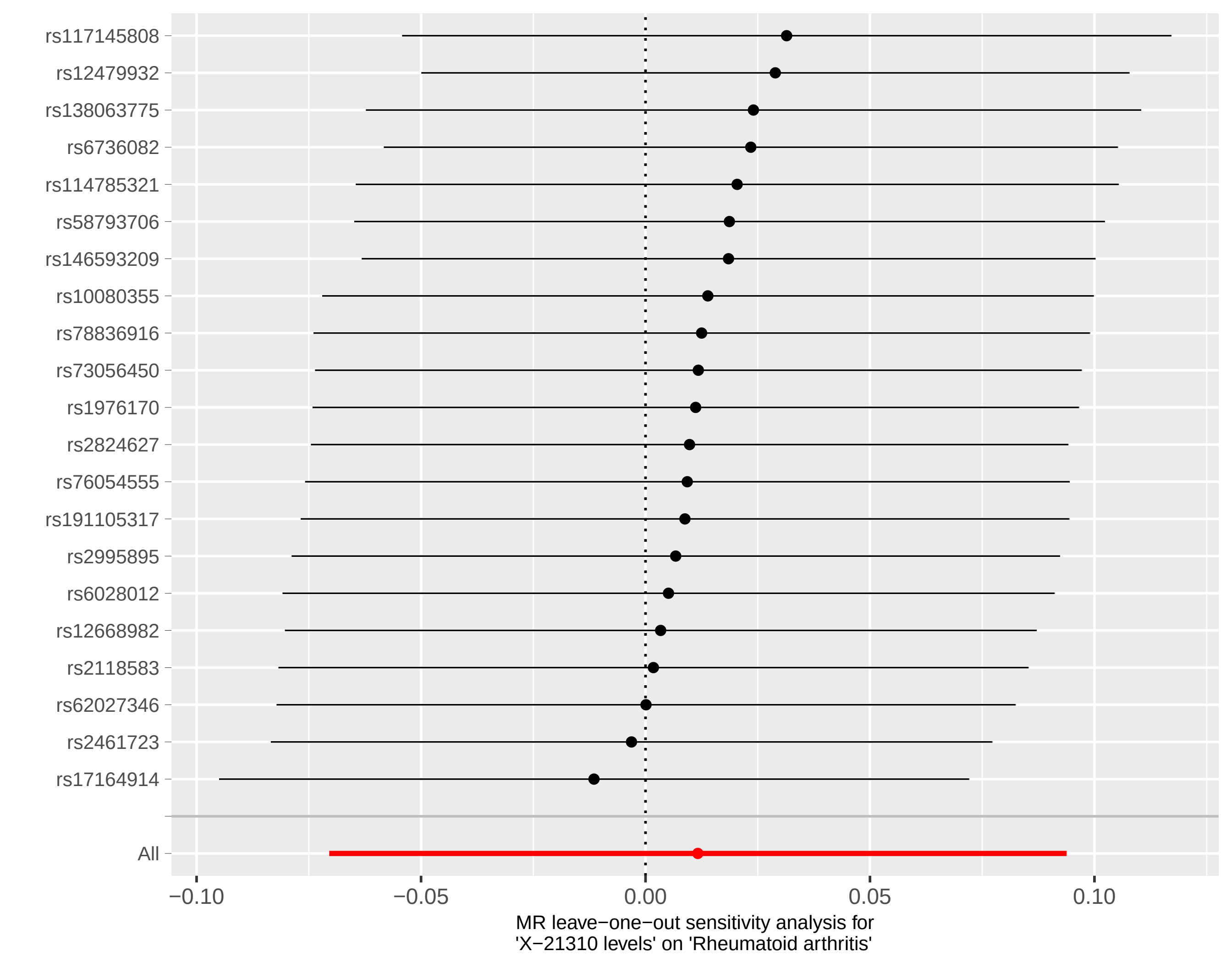
rs117145808
rs12479932
rs138063775
rs6736082
rs114785321
rs58793706
rs146593209
rs10080355
rs78836916
rs73056450
rs1976170
rs2824627
rs76054555
rs191105317
rs2995895
rs6028012
rs12668982
rs2118583
rs62027346
rs2461723
rs17164914
All
−0.10
−0.05
0.00
0.05
0.10
MR leave−one−out sensitivity analysis for
'X−21310 levels' on 'Rheumatoid arthritis'

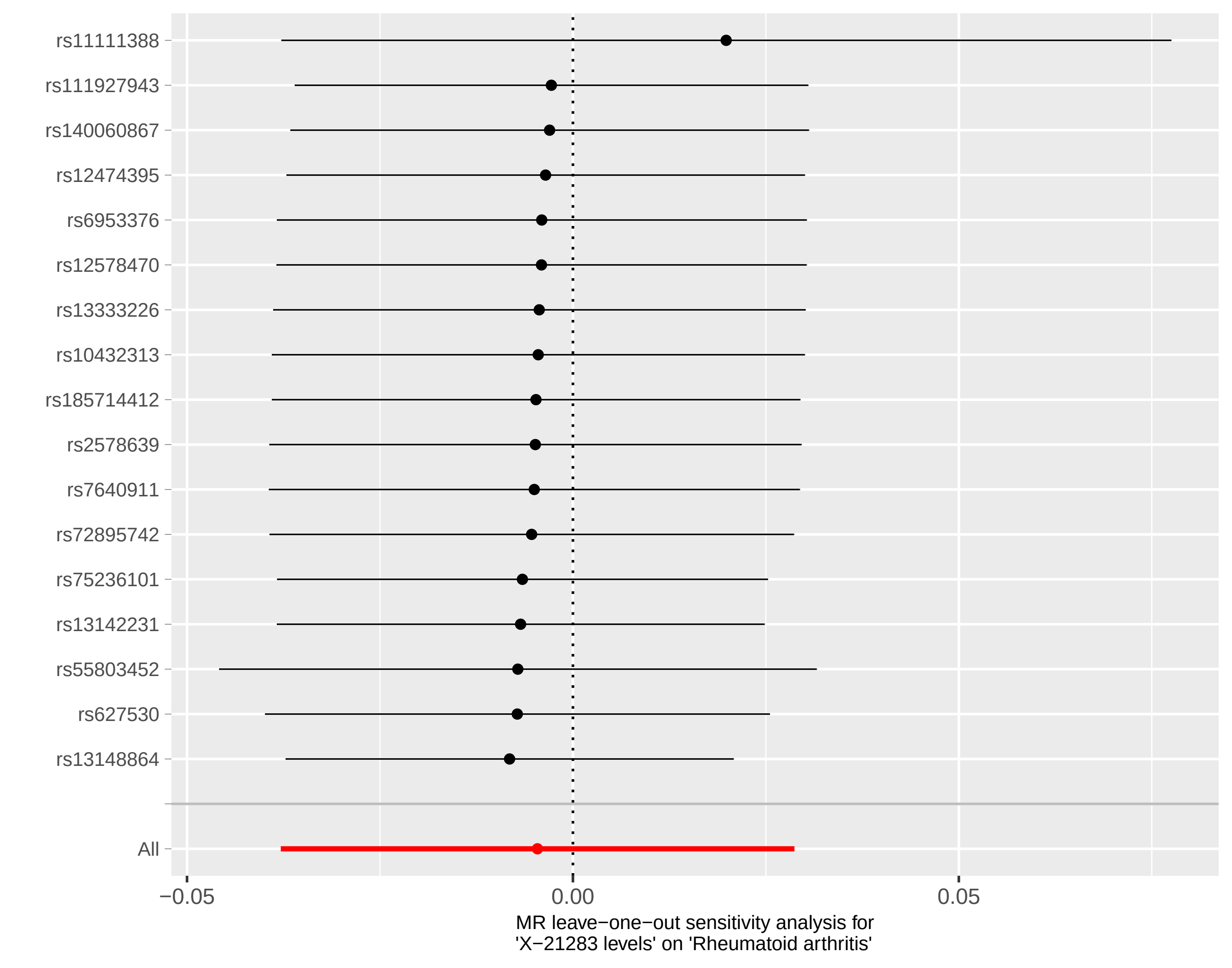
rs11111388
rs111927943
rs140060867
rs12474395
rs6953376
rs12578470
rs13333226
rs10432313
rs185714412
rs2578639
rs7640911
rs72895742
rs75236101
rs13142231
rs55803452
rs627530
rs13148864
All
−0.05
0.00
0.05
MR leave−one−out sensitivity analysis for
'X−21283 levels' on 'Rheumatoid arthritis'

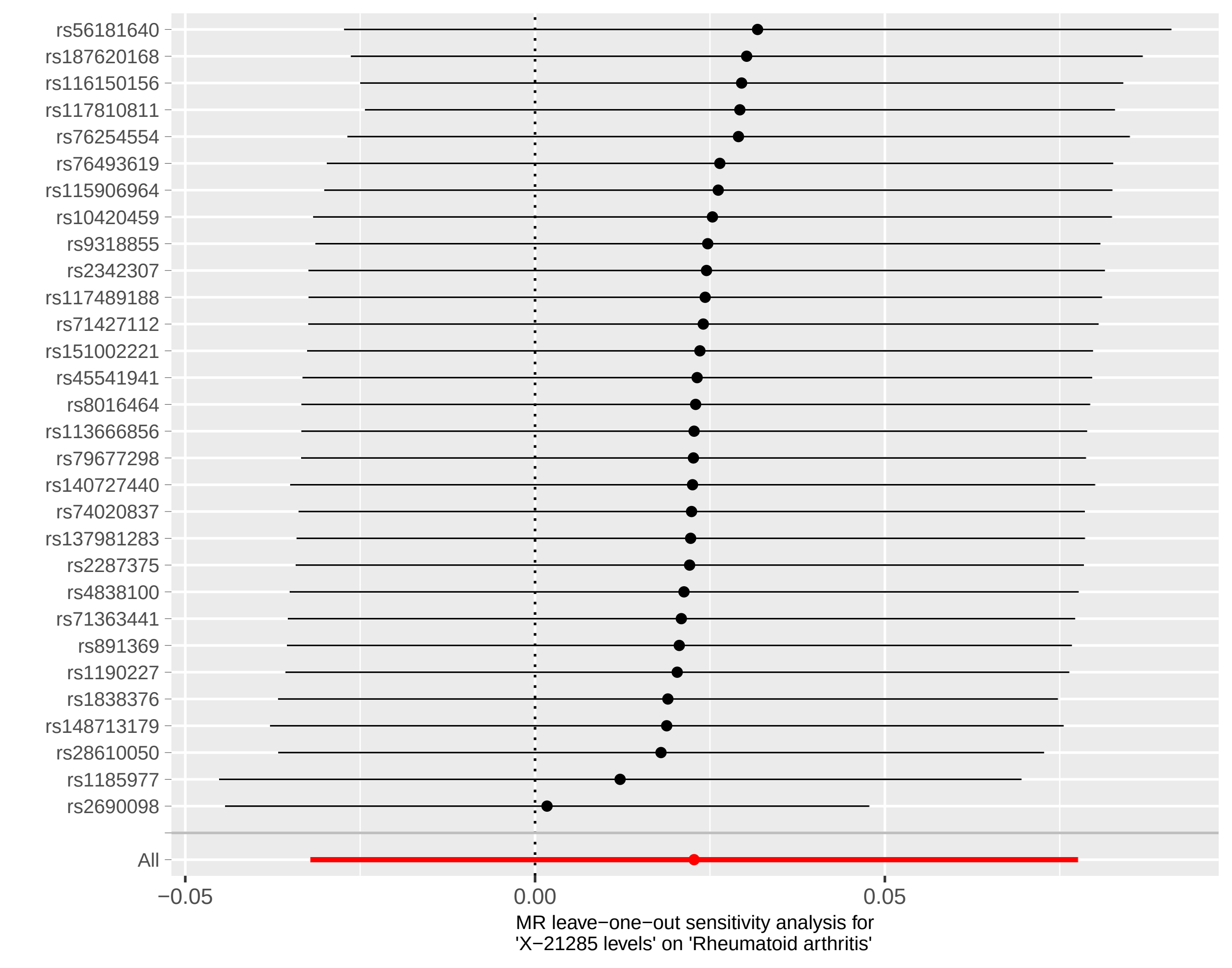
rs56181640
rs187620168
rs116150156
rs117810811
rs76254554
rs76493619
rs115906964
rs10420459
rs9318855
rs2342307
rs117489188
rs71427112
rs151002221
rs45541941
rs8016464
rs113666856
rs79677298
rs140727440
rs74020837
rs137981283
rs2287375
rs4838100
rs71363441
rs891369
rs1190227
rs1838376
rs148713179
rs28610050
rs1185977
rs2690098
All
−0.05
0.00
0.05
MR leave−one−out sensitivity analysis for
'X−21285 levels' on 'Rheumatoid arthritis'

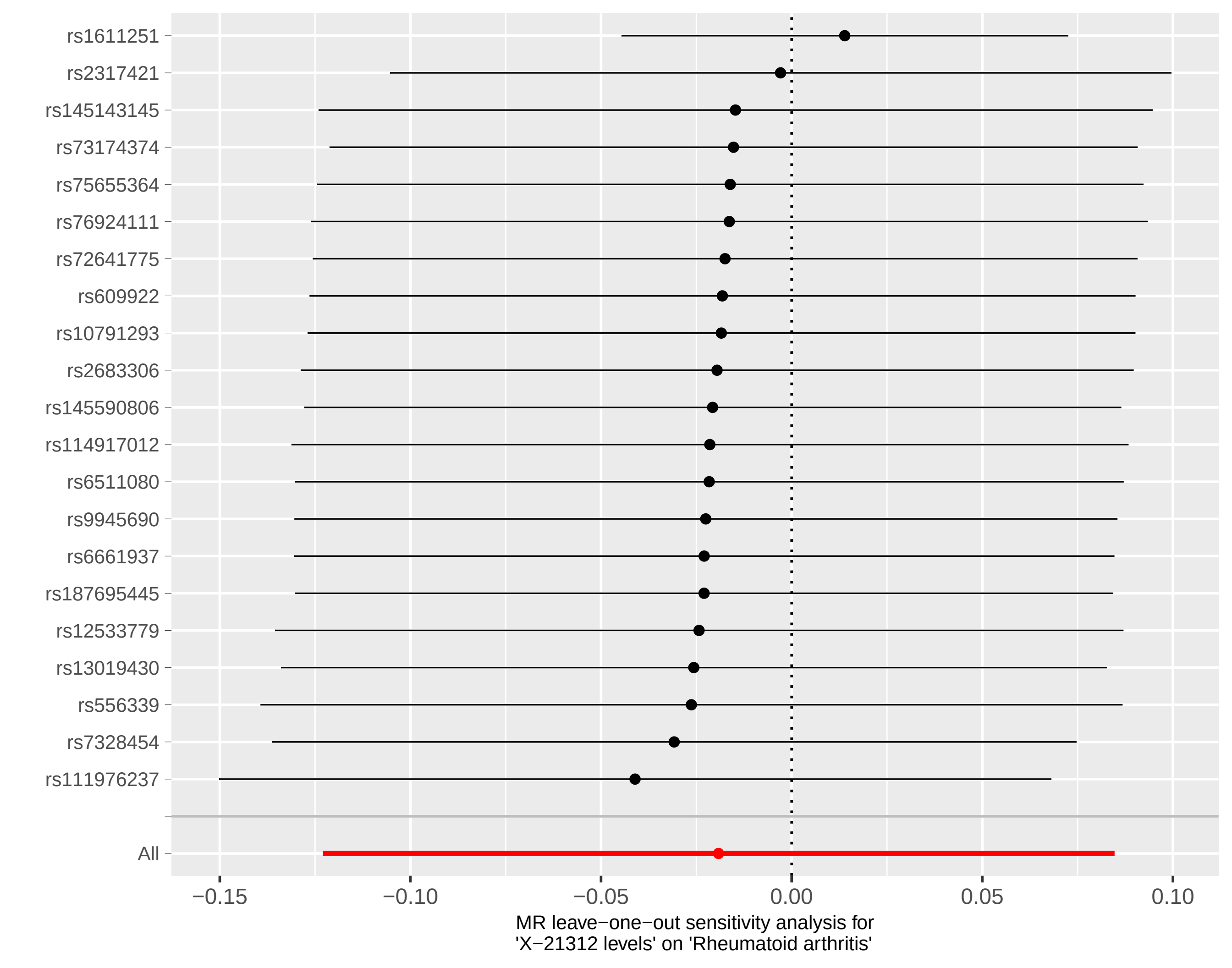
rs1611251
rs2317421
rs145143145
rs73174374
rs75655364
rs76924111
rs72641775
rs609922
rs10791293
rs2683306
rs145590806
rs114917012
rs6511080
rs9945690
rs6661937
rs187695445
rs12533779
rs13019430
rs556339
rs7328454
rs111976237
All
−0.15
−0.10
−0.05
0.00
0.05
0.10
MR leave−one−out sensitivity analysis for
'X−21312 levels' on 'Rheumatoid arthritis'

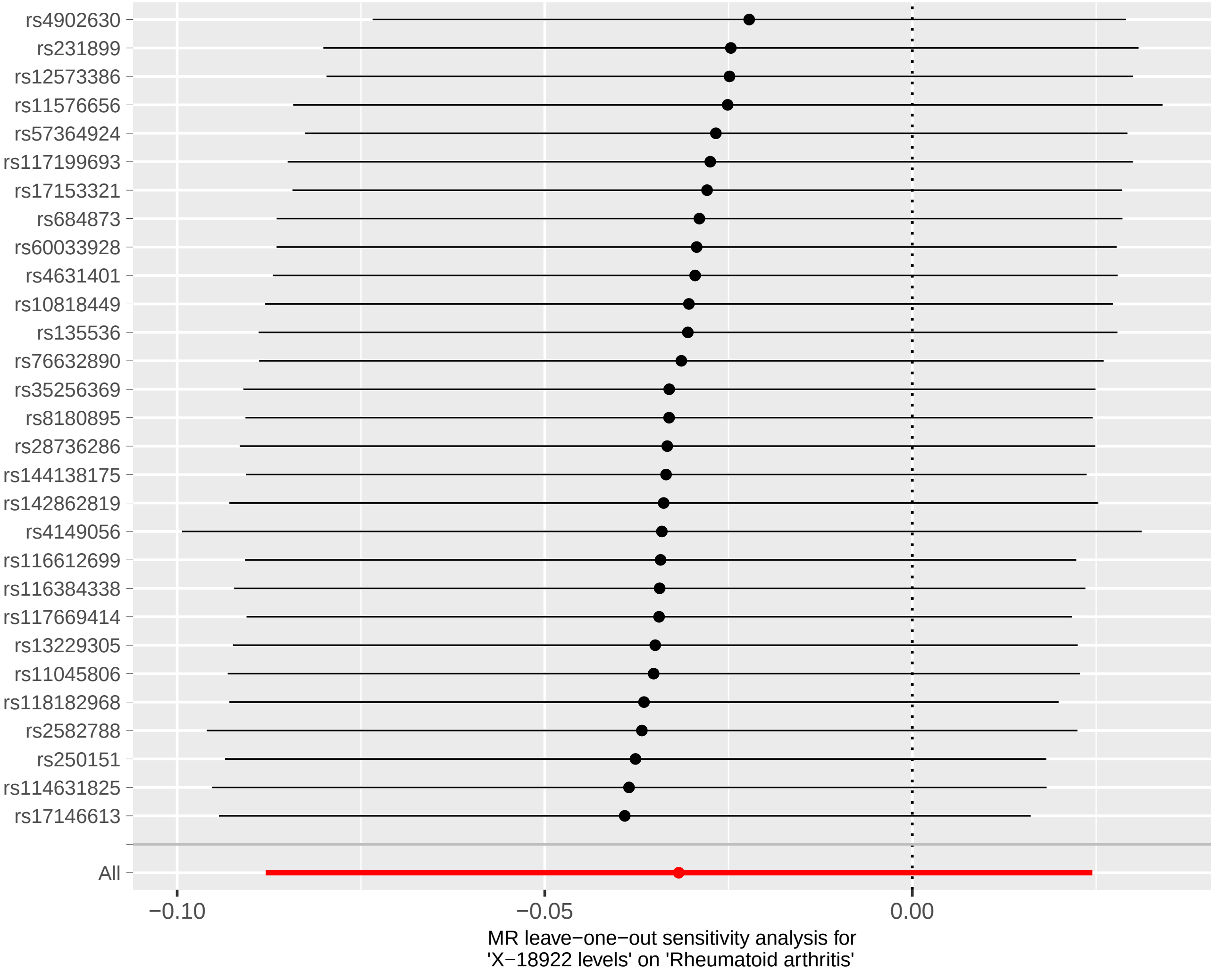
rs4902630
rs231899
rs12573386
rs11576656
rs57364924
rs117199693
rs17153321
rs684873
rs60033928
rs4631401
rs10818449
rs135536
rs76632890
rs35256369
rs8180895
rs28736286
rs144138175
rs142862819
rs4149056
rs116612699
rs116384338
rs117669414
rs13229305
rs11045806
rs118182968
rs2582788
rs250151
rs114631825
rs17146613
All
−0.10
−0.05
0.00
MR leave−one−out sensitivity analysis for
'X−18922 levels' on 'Rheumatoid arthritis'

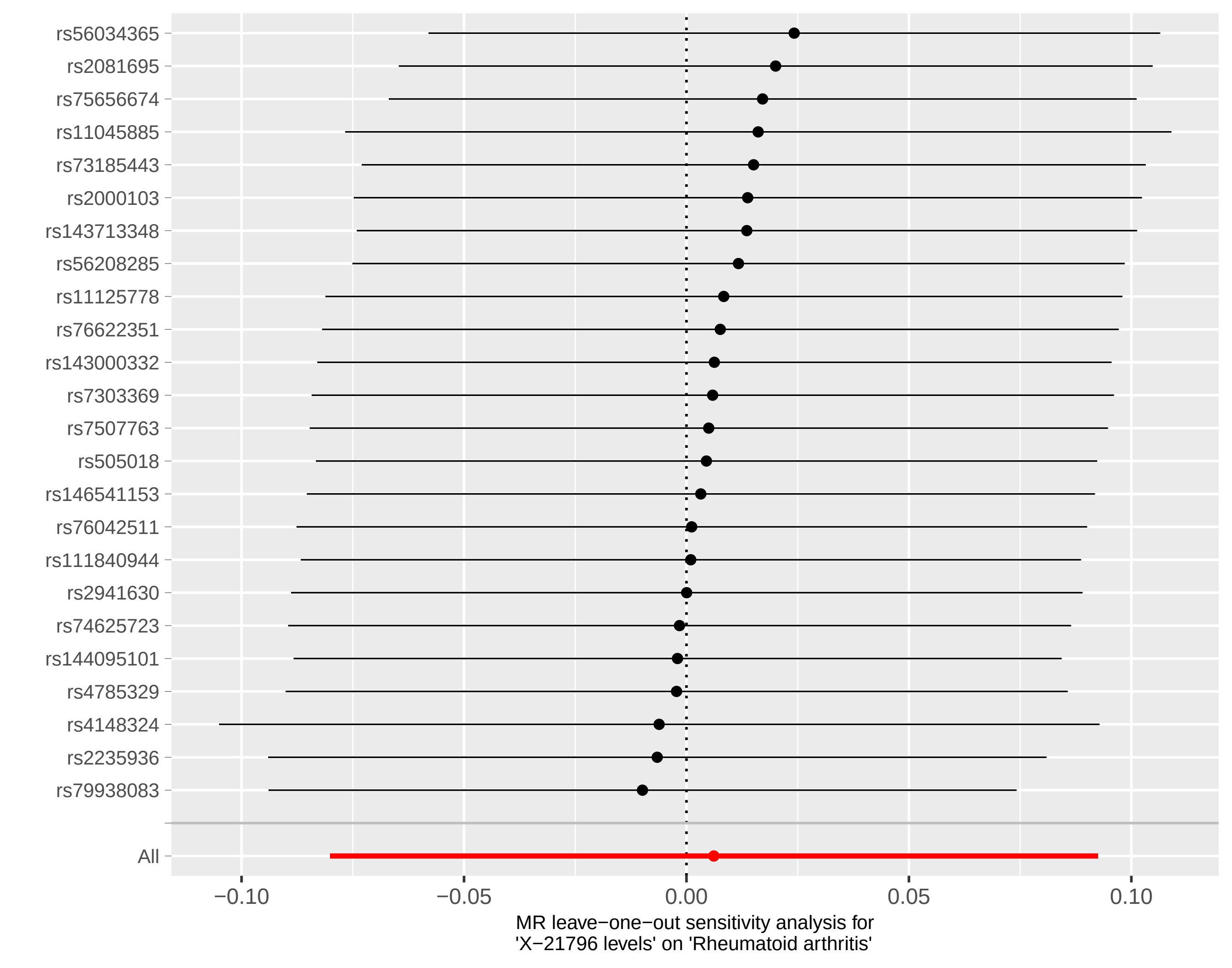
rs56034365
rs2081695
rs75656674
rs11045885
rs73185443
rs2000103
rs143713348
rs56208285
rs11125778
rs76622351
rs143000332
rs7303369
rs7507763
rs505018
rs146541153
rs76042511
rs111840944
rs2941630
rs74625723
rs144095101
rs4785329
rs4148324
rs2235936
rs79938083
All
−0.10
−0.05
0.00
0.05
0.10
MR leave−one−out sensitivity analysis for
'X−21796 levels' on 'Rheumatoid arthritis'

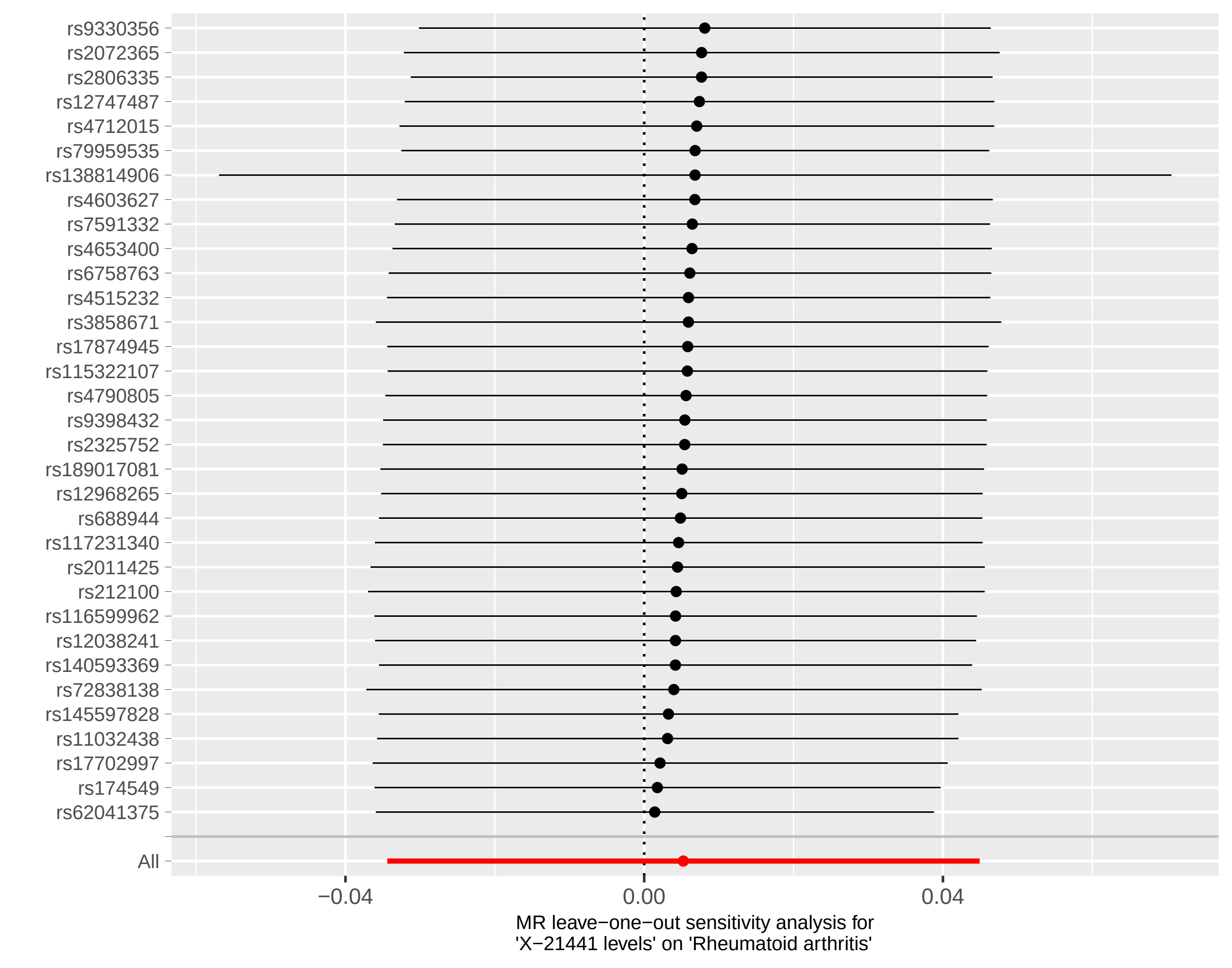

rs9330356
rs2072365
rs2806335
rs12747487
rs4712015
rs79959535
rs138814906
rs4603627
rs7591332
rs4653400
rs6758763
rs4515232
rs3858671
rs17874945
rs115322107
rs4790805
rs9398432
rs2325752
rs189017081
rs12968265
rs688944
rs117231340
rs2011425
rs212100
rs116599962
rs12038241
rs140593369
rs72838138
rs145597828
rs11032438
rs17702997
rs174549
rs62041375
All
−0.04
0.00
0.04
MR leave−one−out sensitivity analysis for
'X−21441 levels' on 'Rheumatoid arthritis'

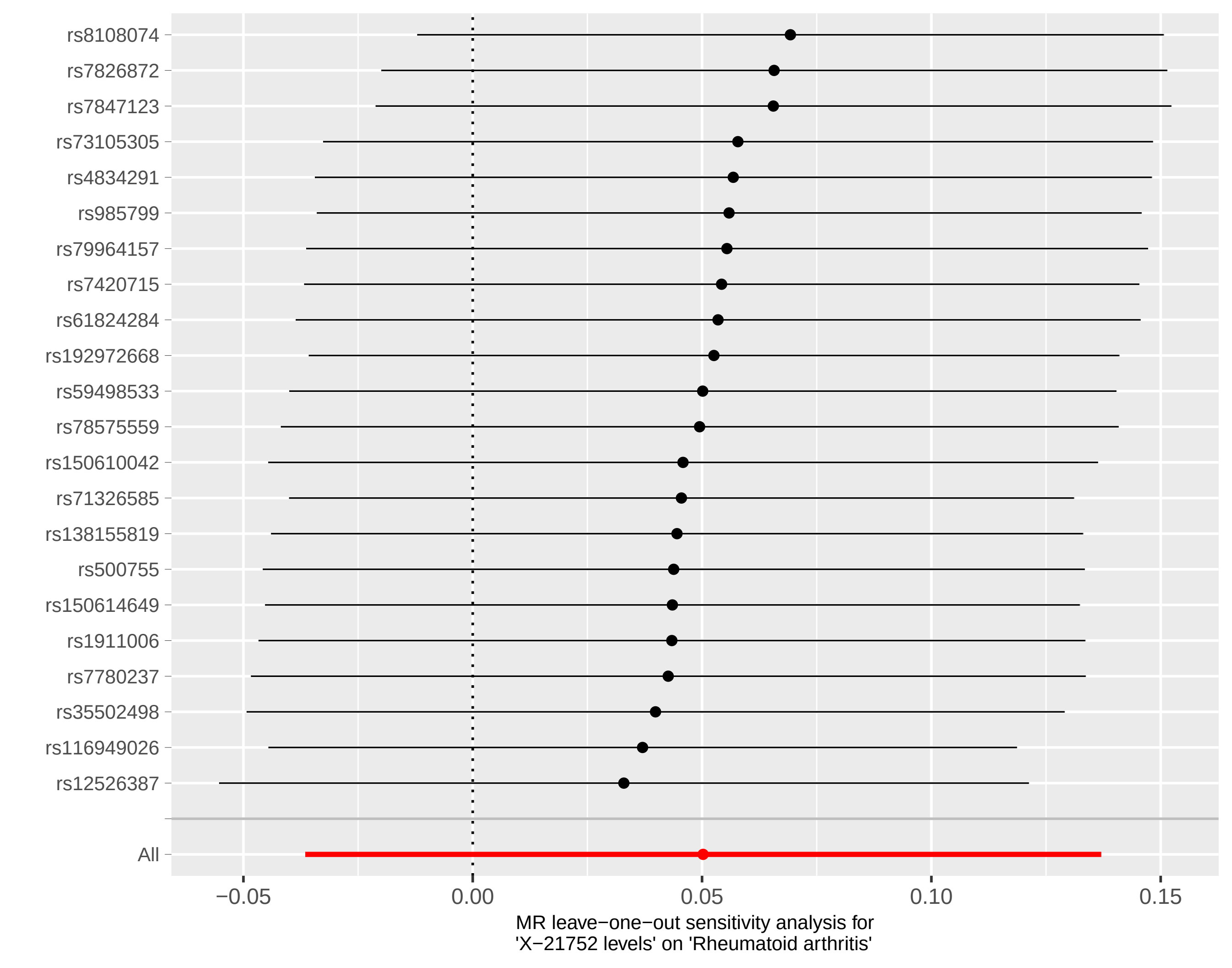
rs8108074
rs7826872
rs7847123
rs73105305
rs4834291
rs985799
rs79964157
rs7420715
rs61824284
rs192972668
rs59498533
rs78575559
rs150610042
rs71326585
rs138155819
rs500755
rs150614649
rs1911006
rs7780237
rs35502498
rs116949026
rs12526387
All
−0.05
0.00
0.05
0.10
0.15
MR leave−one−out sensitivity analysis for
'X−21752 levels' on 'Rheumatoid arthritis'

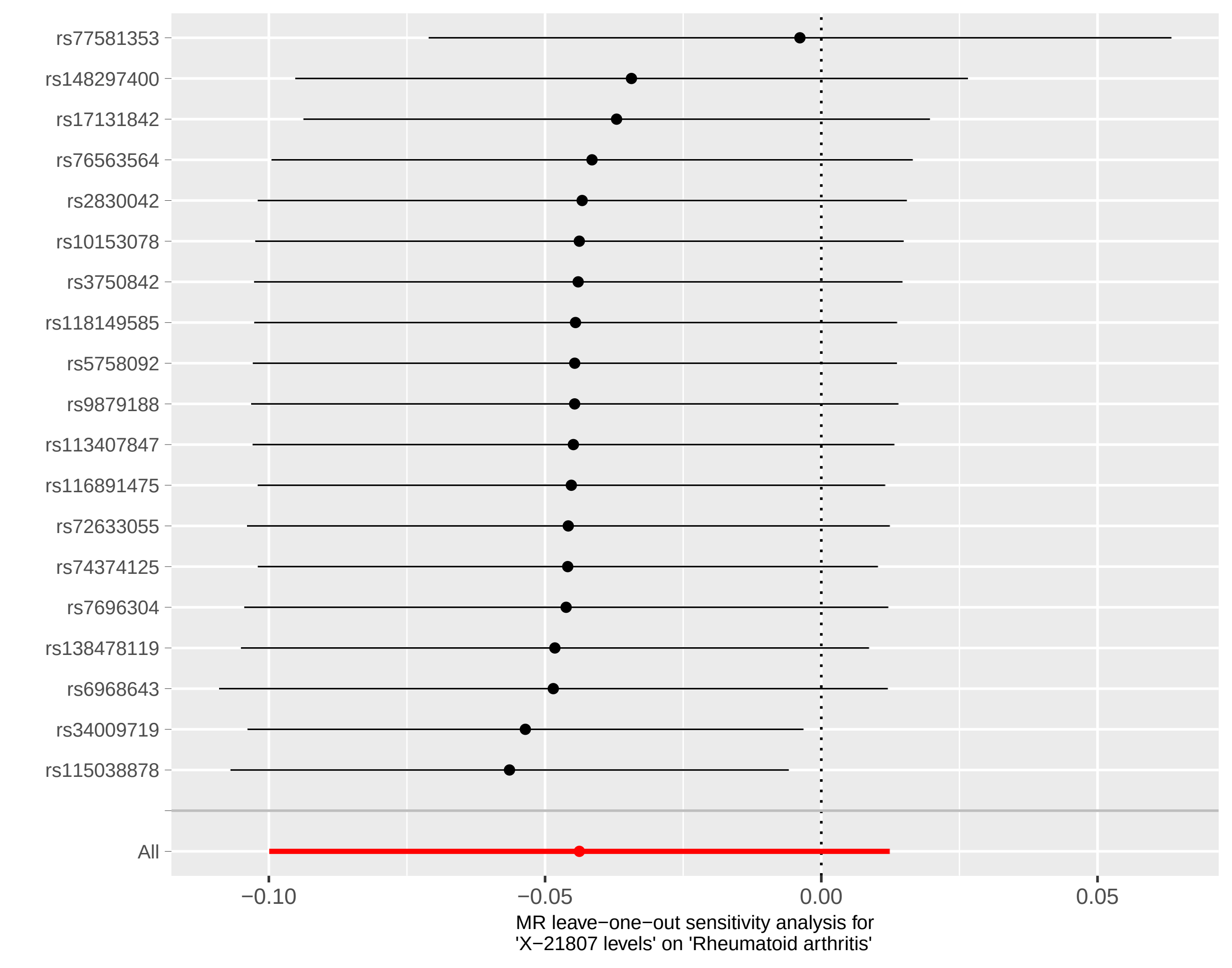
rs77581353
rs148297400
rs17131842
rs76563564
rs2830042
rs10153078
rs3750842
rs118149585
rs5758092
rs9879188
rs113407847
rs116891475
rs72633055
rs74374125
rs7696304
rs138478119
rs6968643
rs34009719
rs115038878
All
−0.10
−0.05
0.00
0.05
MR leave−one−out sensitivity analysis for
'X−21807 levels' on 'Rheumatoid arthritis'

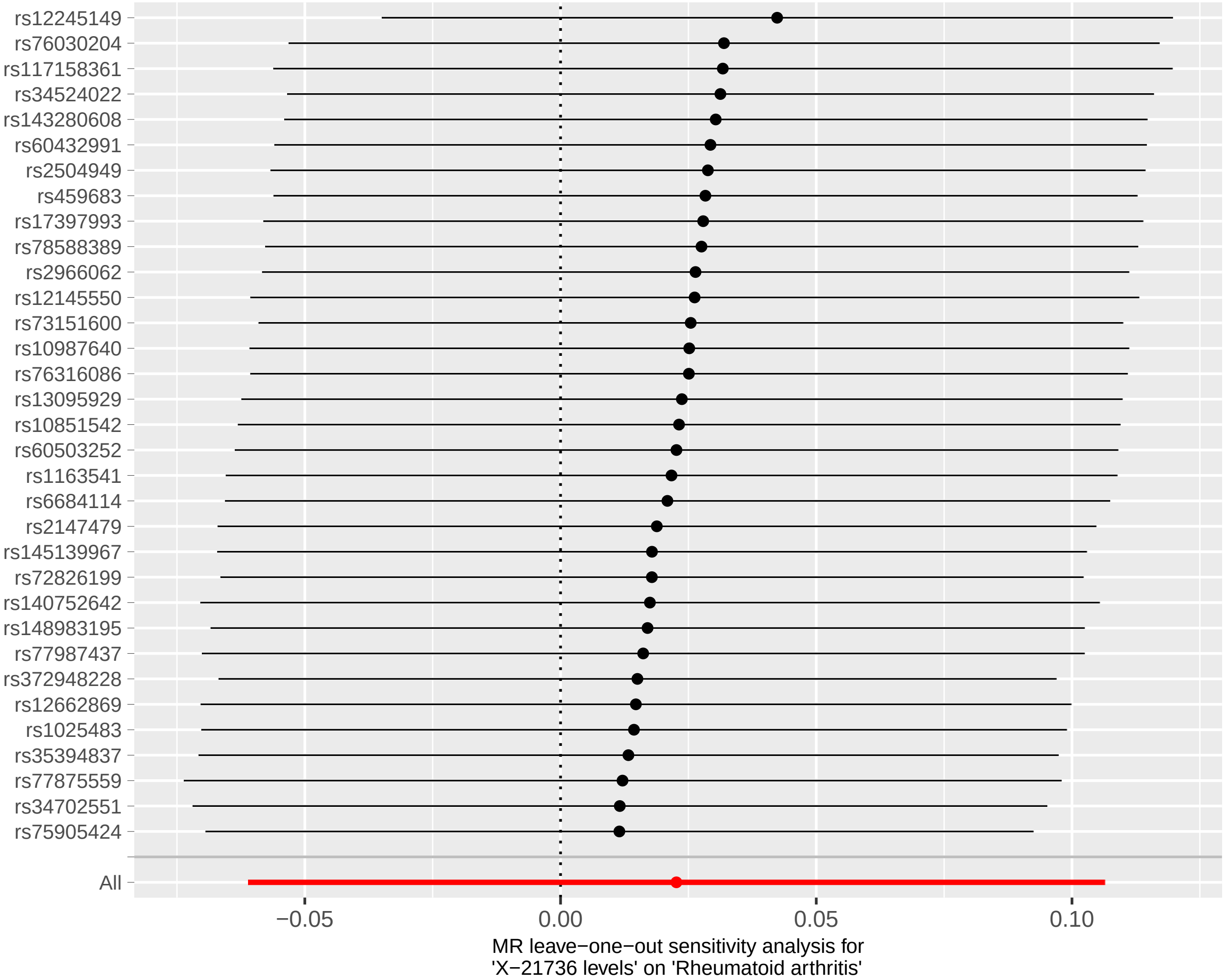
rs12245149
rs76030204
rs117158361
rs34524022
rs143280608
rs60432991
rs2504949
rs459683
rs17397993
rs78588389
rs2966062
rs12145550
rs73151600
rs10987640
rs76316086
rs13095929
rs10851542
rs60503252
rs1163541
rs6684114
rs2147479
rs145139967
rs72826199
rs140752642
rs148983195
rs77987437
rs372948228
rs12662869
rs1025483
rs35394837
rs77875559
rs34702551
rs75905424
All
−0.05
0.00
0.05
0.10
MR leave−one−out sensitivity analysis for
'X−21736 levels' on 'Rheumatoid arthritis'

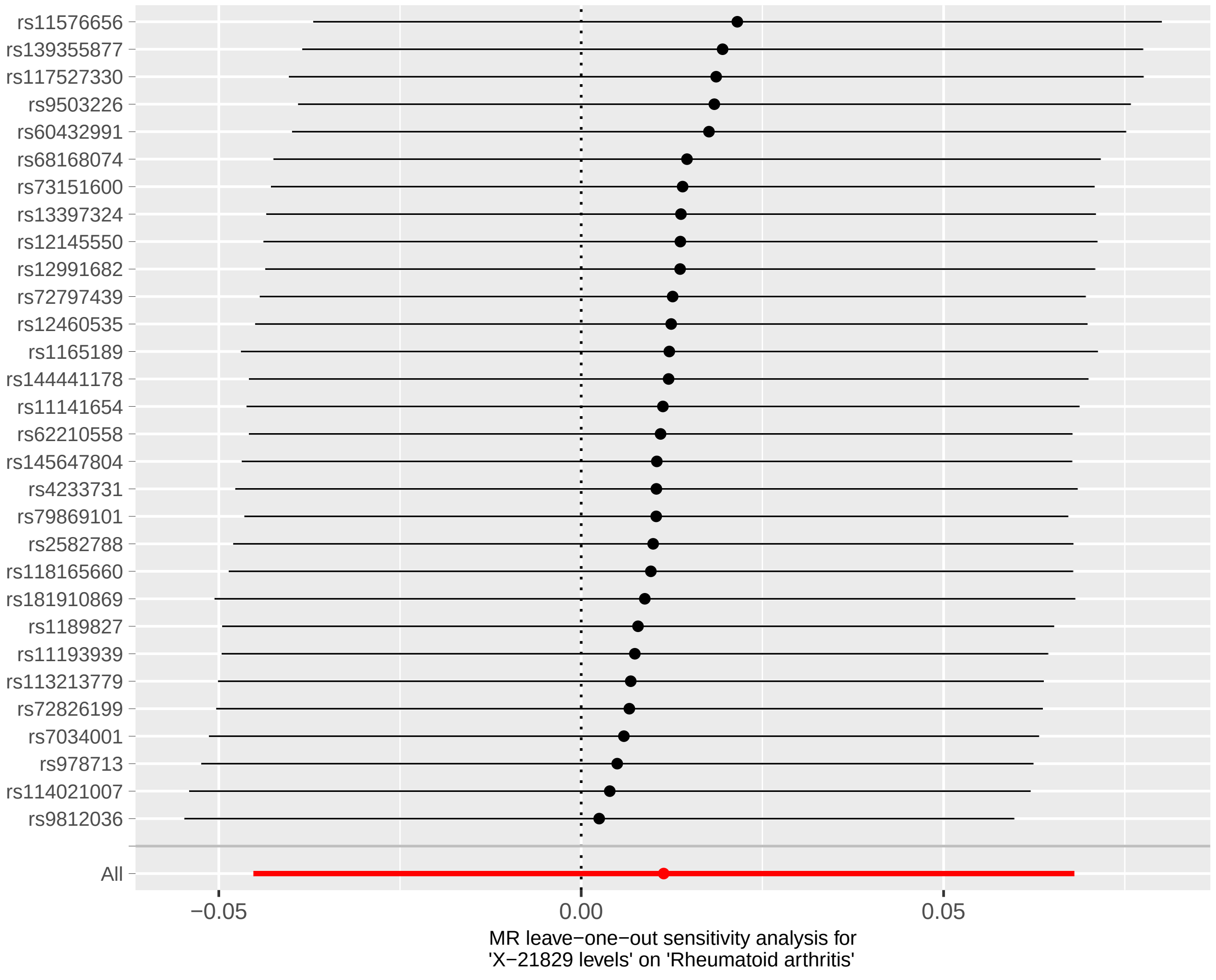
rs11576656
rs139355877
rs117527330
rs9503226
rs60432991
rs68168074
rs73151600
rs13397324
rs12145550
rs12991682
rs72797439
rs12460535
rs1165189
rs144441178
rs11141654
rs62210558
rs145647804
rs4233731
rs79869101
rs2582788
rs118165660
rs181910869
rs1189827
rs11193939
rs113213779
rs72826199
rs7034001
rs978713
rs114021007
rs9812036
All
−0.05
0.00
0.05
MR leave−one−out sensitivity analysis for
'X−21829 levels' on 'Rheumatoid arthritis'

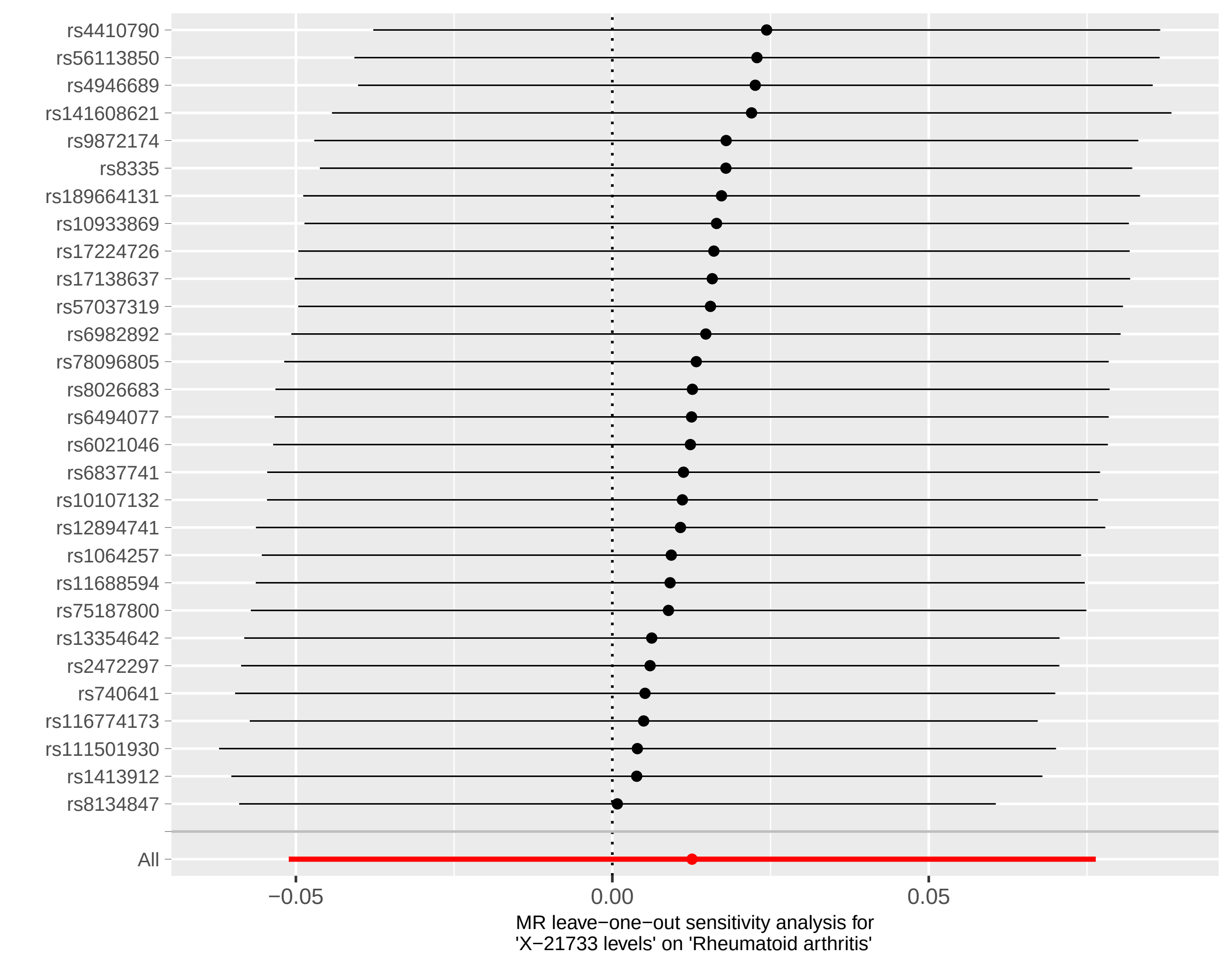
rs4410790
rs56113850
rs4946689
rs141608621
rs9872174
rs8335
rs189664131
rs10933869
rs17224726
rs17138637
rs57037319
rs6982892
rs78096805
rs8026683
rs6494077
rs6021046
rs6837741
rs10107132
rs12894741
rs1064257
rs11688594
rs75187800
rs13354642
rs2472297
rs740641
rs116774173
rs111501930
rs1413912
rs8134847
All
−0.05
0.00
0.05
MR leave−one−out sensitivity analysis for
'X−21733 levels' on 'Rheumatoid arthritis'

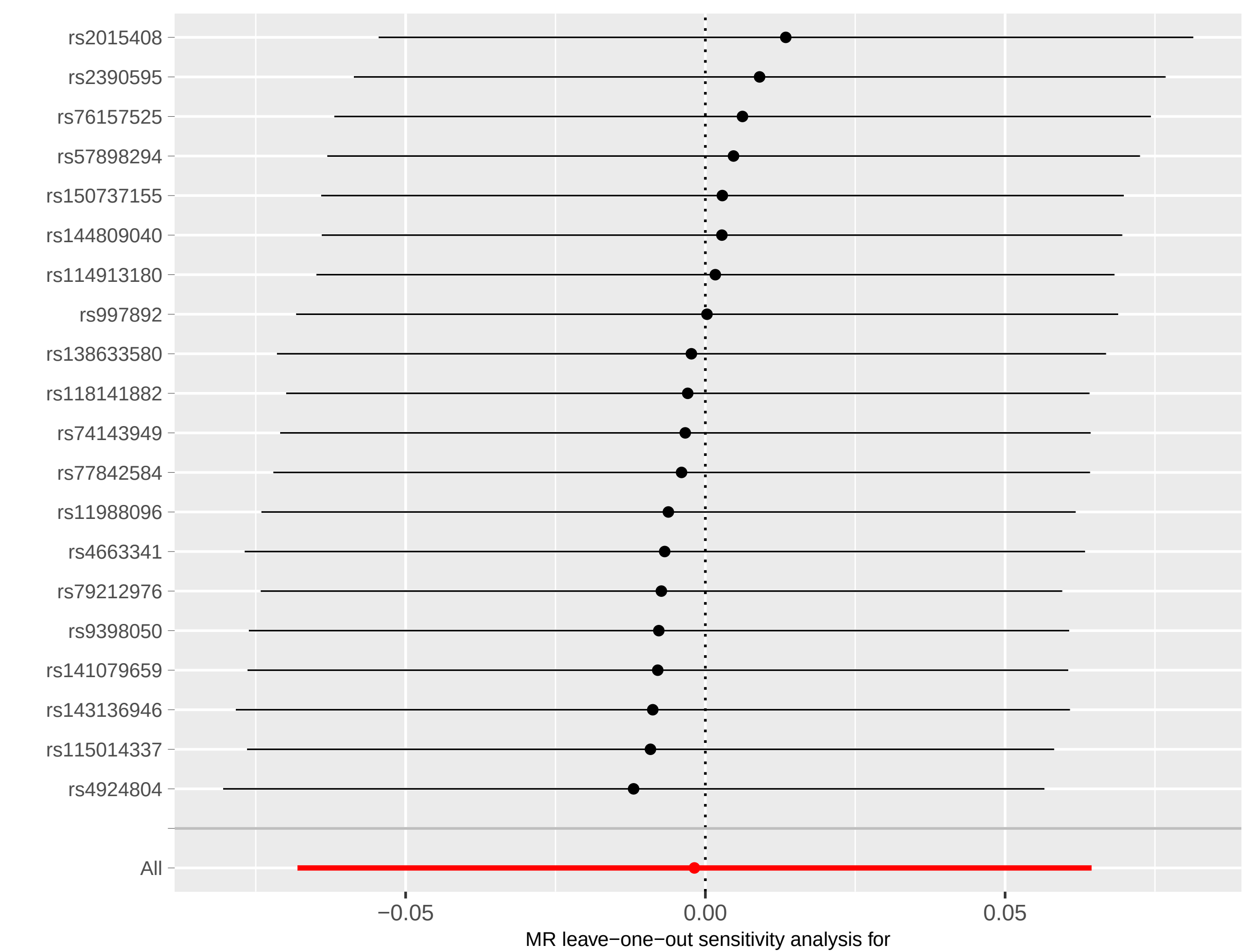

MR leave−one−out sensitivity analysis for
'X−21742 levels' on 'Rheumatoid arthritis'

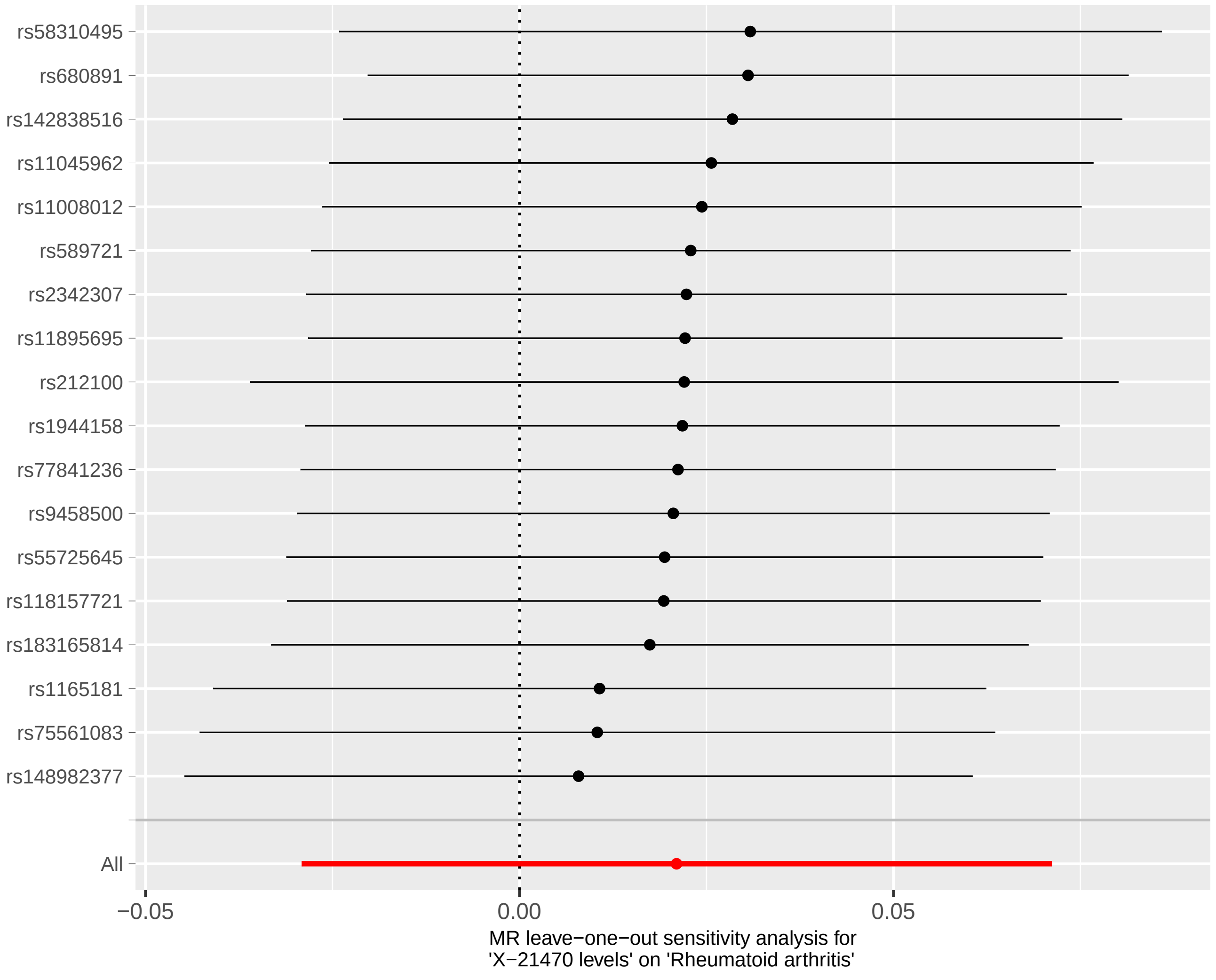

rs58310495
rs680891
rs142838516
rs11045962
rs11008012
rs589721
rs2342307
rs11895695
rs212100
rs1944158
rs77841236
rs9458500
rs55725645
rs118157721
rs183165814
rs1165181
rs75561083
rs148982377
All
−0.05
0.00
0.05
MR leave−one−out sensitivity analysis for
'X−21470 levels' on 'Rheumatoid arthritis'

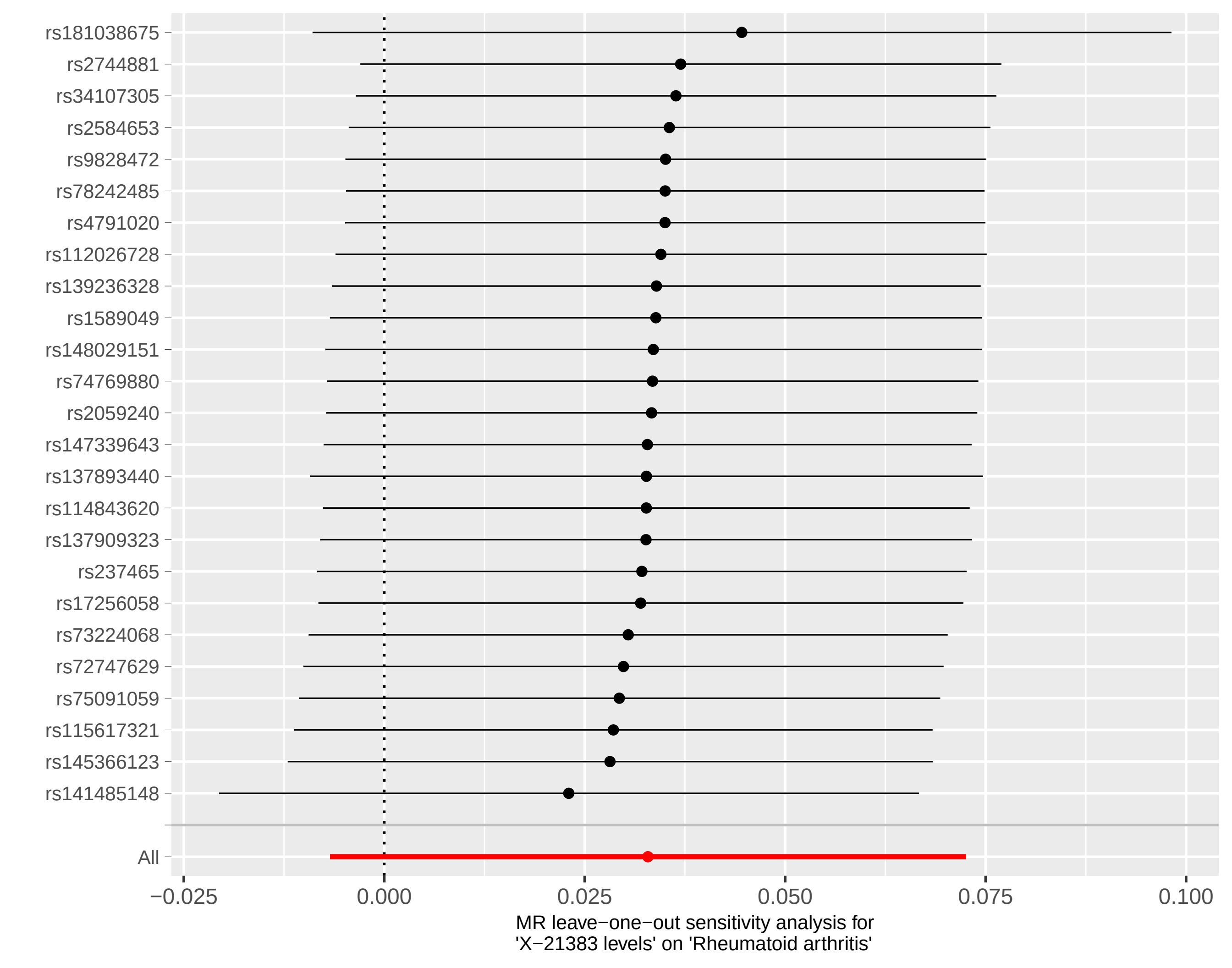

rs181038675
rs2744881
rs34107305
rs2584653
rs9828472
rs78242485
rs4791020
rs112026728
rs139236328
rs1589049
rs148029151
rs74769880
rs2059240
rs147339643
rs137893440
rs114843620
rs137909323
rs237465
rs17256058
rs73224068
rs72747629
rs75091059
rs115617321
rs145366123
rs141485148
All
−0.025
0.000
0.025
0.050
0.075
0.100
MR leave−one−out sensitivity analysis for
'X−21383 levels' on 'Rheumatoid arthritis'

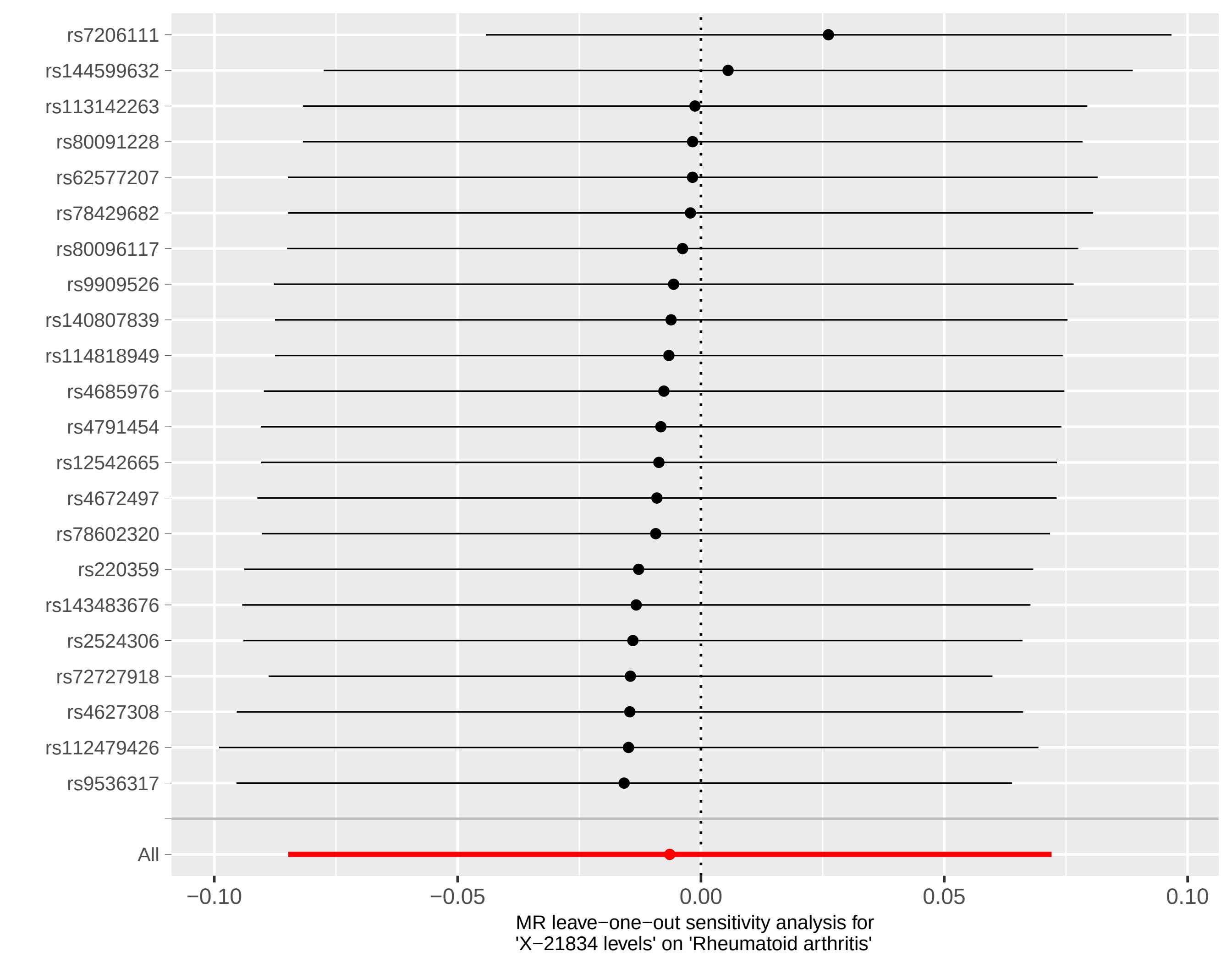
rs7206111
rs144599632
rs113142263
rs80091228
rs62577207
rs78429682
rs80096117
rs9909526
rs140807839
rs114818949
rs4685976
rs4791454
rs12542665
rs4672497
rs78602320
rs220359
rs143483676
rs2524306
rs72727918
rs4627308
rs112479426
rs9536317
All
−0.10
−0.05
0.00
0.05
0.10
MR leave−one−out sensitivity analysis for
'X−21834 levels' on 'Rheumatoid arthritis'

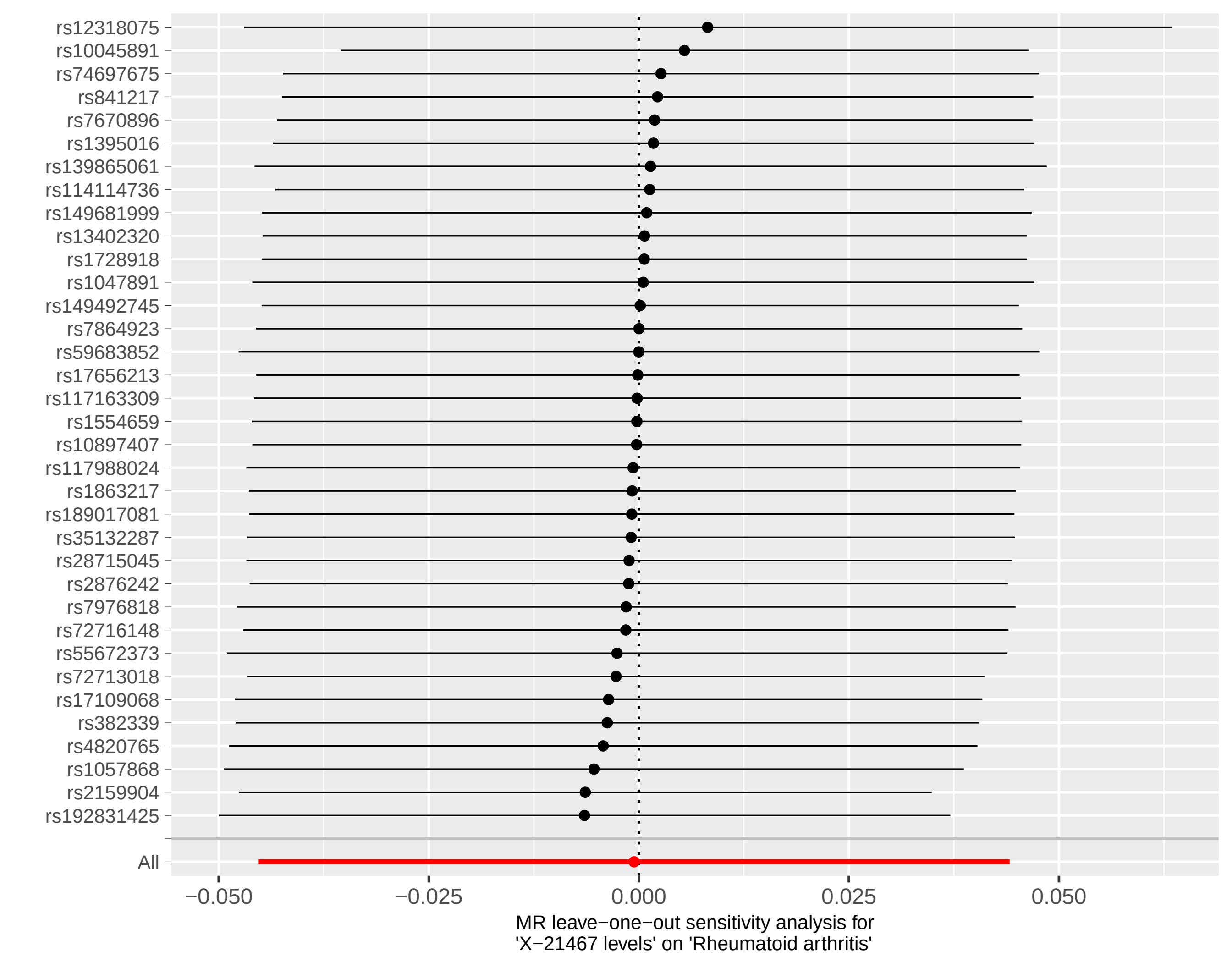
rs12318075
rs10045891
rs74697675
rs841217
rs7670896
rs1395016
rs139865061
rs114114736
rs149681999
rs13402320
rs1728918
rs1047891
rs149492745
rs7864923
rs59683852
rs17656213
rs117163309
rs1554659
rs10897407
rs117988024
rs1863217
rs189017081
rs35132287
rs28715045
rs2876242
rs7976818
rs72716148
rs55672373
rs72713018
rs17109068
rs382339
rs4820765
rs1057868
rs2159904
rs192831425
All
−0.050
−0.025
0.000
0.025
0.050
MR leave−one−out sensitivity analysis for
'X−21467 levels' on 'Rheumatoid arthritis'

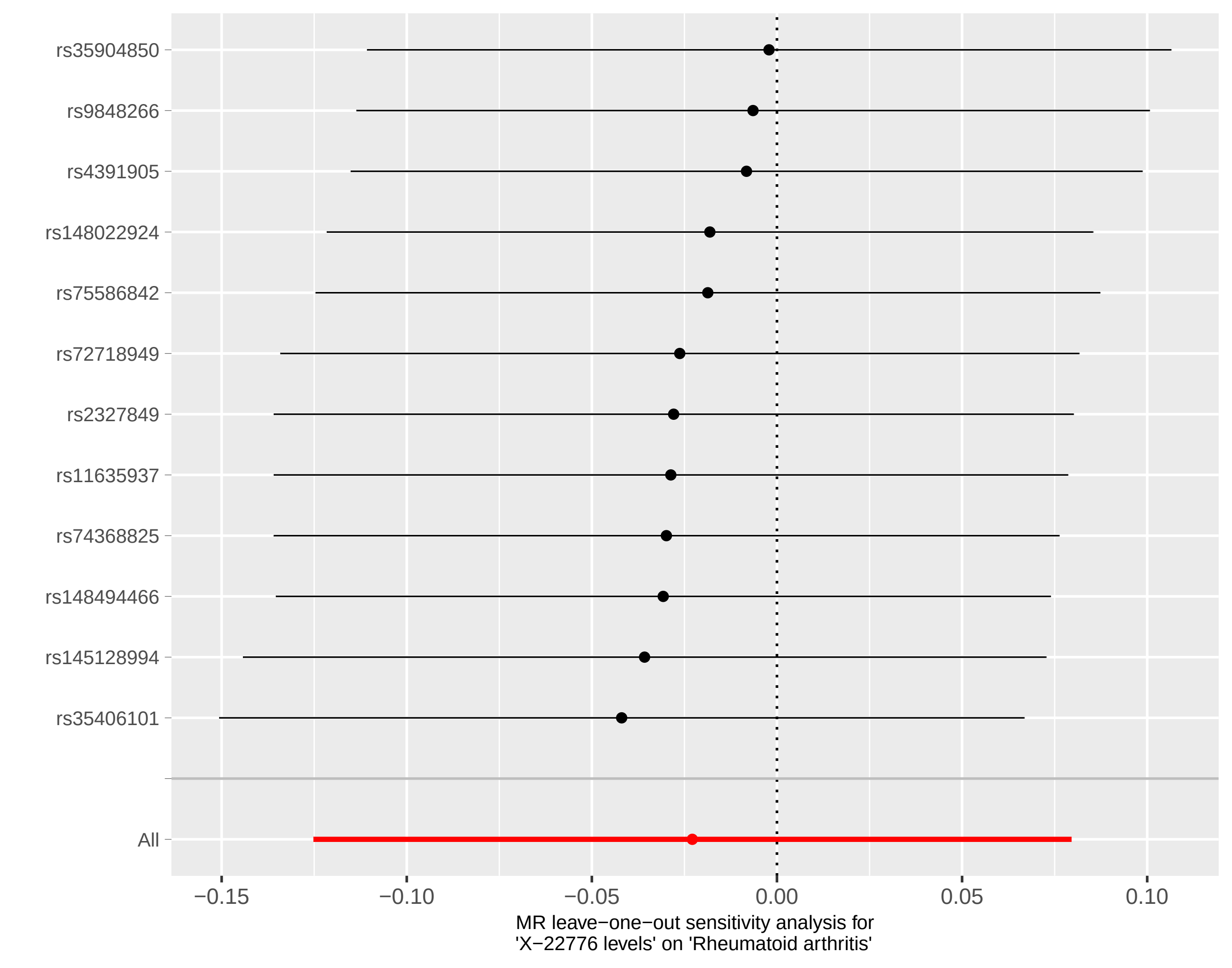
rs35904850
rs9848266
rs4391905
rs148022924
rs75586842
rs72718949
rs2327849
rs11635937
rs74368825
rs148494466
rs145128994
rs35406101
All
−0.15
−0.10
−0.05
0.00
0.05
0.10
MR leave−one−out sensitivity analysis for
'X−22776 levels' on 'Rheumatoid arthritis'

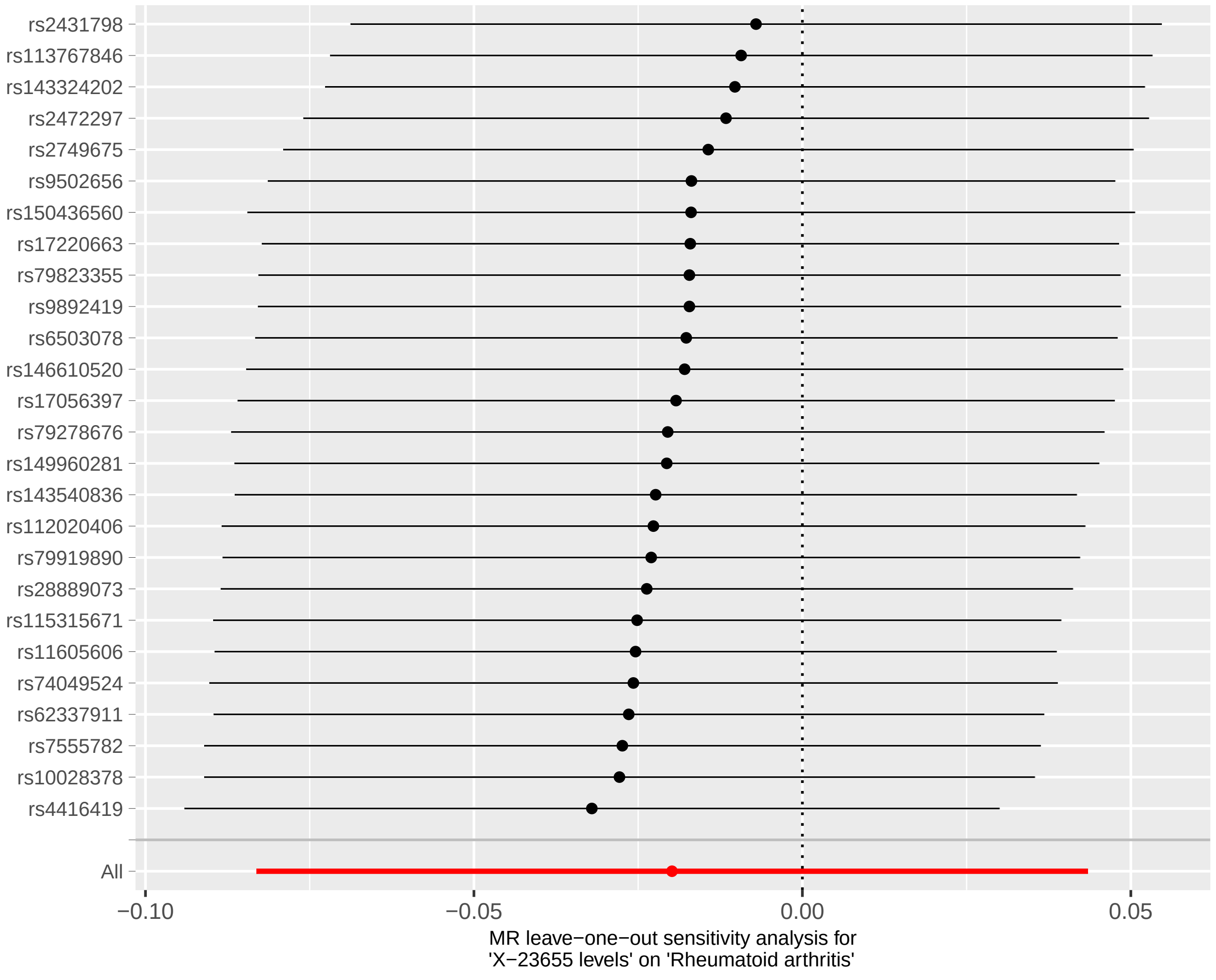

rs2431798
rs113767846
rs143324202
rs2472297
rs2749675
rs9502656
rs150436560
rs17220663
rs79823355
rs9892419
rs6503078
rs146610520
rs17056397
rs79278676
rs149960281
rs143540836
rs112020406
rs79919890
rs28889073
rs115315671
rs11605606
rs74049524
rs62337911
rs7555782
rs10028378
rs4416419
All
−0.10
−0.05
0.00
0.05
MR leave−one−out sensitivity analysis for
'X−23655 levels' on 'Rheumatoid arthritis'

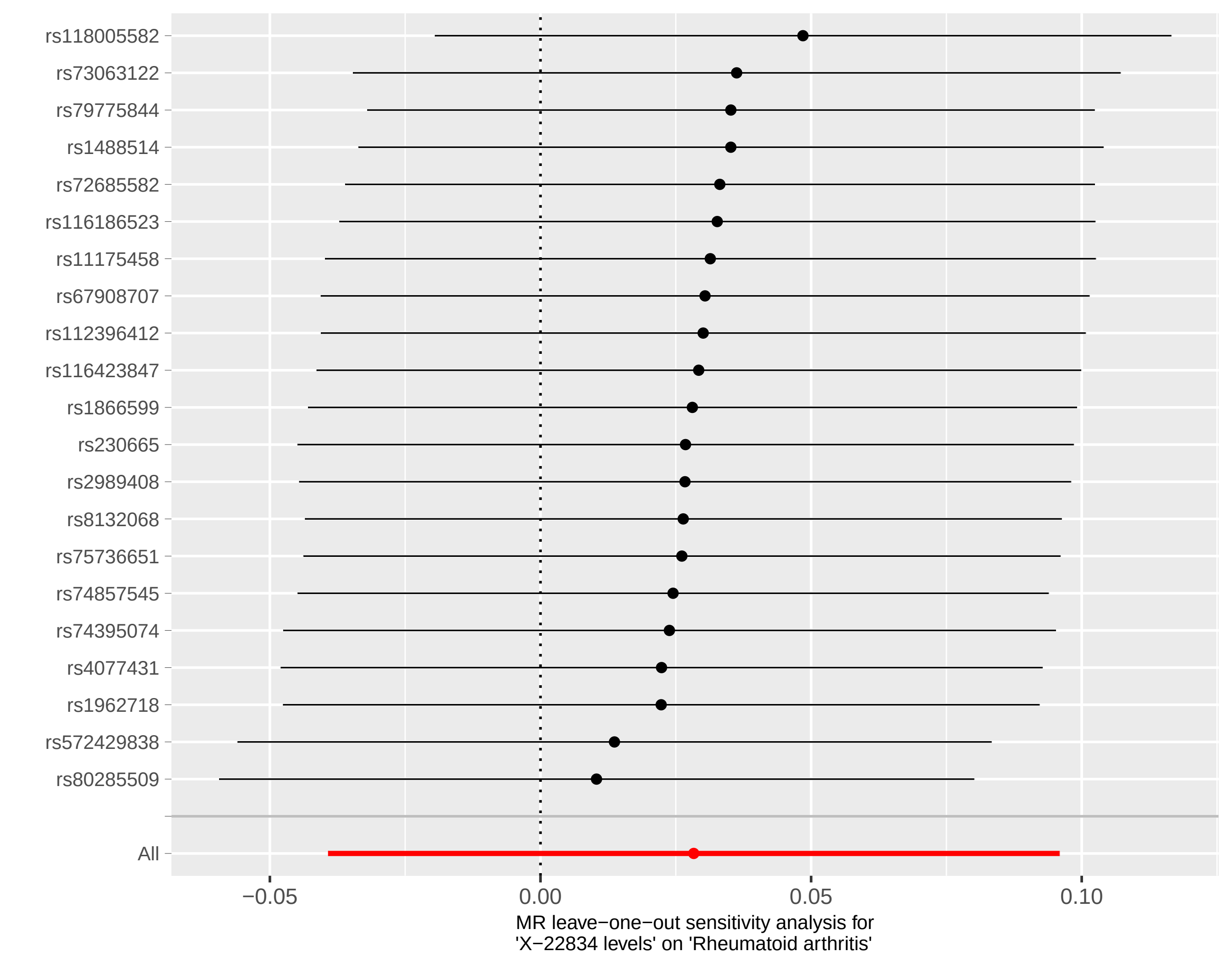
rs118005582
rs73063122
rs79775844
rs1488514
rs72685582
rs116186523
rs11175458
rs67908707
rs112396412
rs116423847
rs1866599
rs230665
rs2989408
rs8132068
rs75736651
rs74857545
rs74395074
rs4077431
rs1962718
rs572429838
rs80285509
All
−0.05
0.00
0.05
0.10
MR leave−one−out sensitivity analysis for
'X−22834 levels' on 'Rheumatoid arthritis'

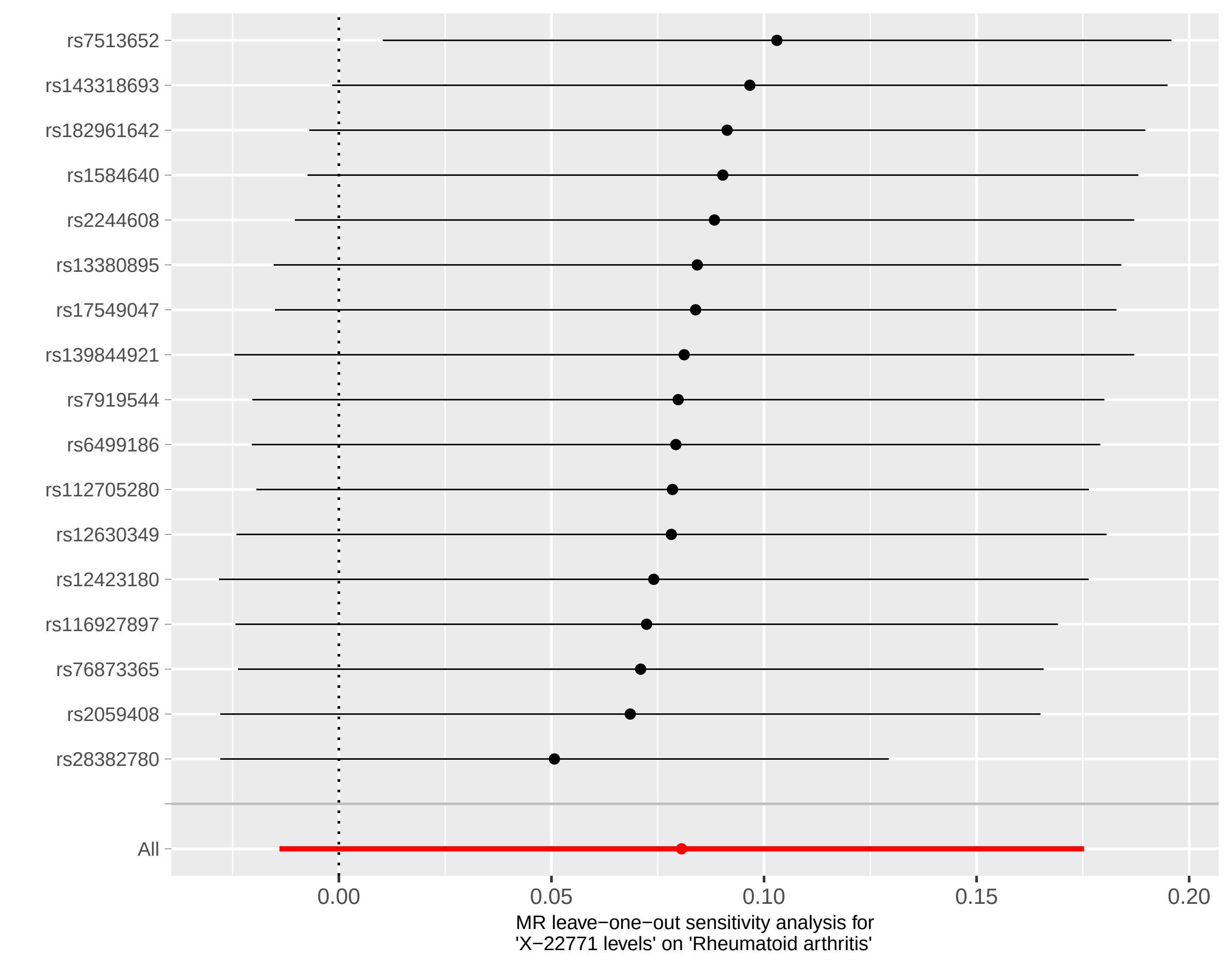
rs7513652
rs143318693
rs182961642
rs1584640
rs2244608
rs13380895
rs17549047
rs139844921
rs7919544
rs6499186
rs112705280
rs12630349
rs12423180
rs116927897
rs76873365
rs2059408
rs28382780
All
0.00
0.05
0.10
0.15
0.20
MR leave−one−out sensitivity analysis for
'X−22771 levels' on 'Rheumatoid arthritis'

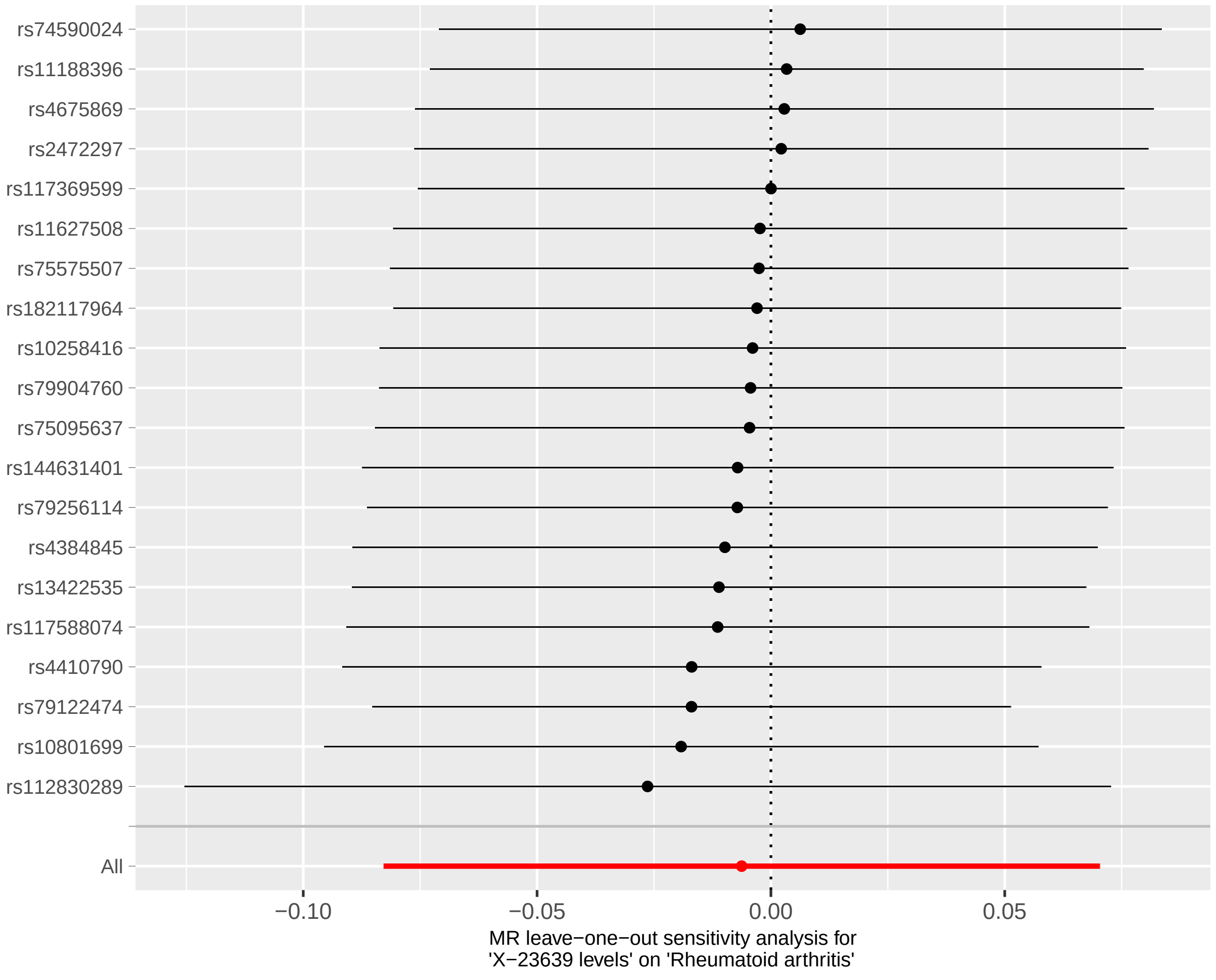

rs74590024
rs11188396
rs4675869
rs2472297
rs117369599
rs11627508
rs75575507
rs182117964
rs10258416
rs79904760
rs75095637
rs144631401
rs79256114
rs4384845
rs13422535
rs117588074
rs4410790
rs79122474
rs10801699
rs112830289
All
−0.10
−0.05
0.00
0.05
MR leave−one−out sensitivity analysis for
'X−23639 levels' on 'Rheumatoid arthritis'

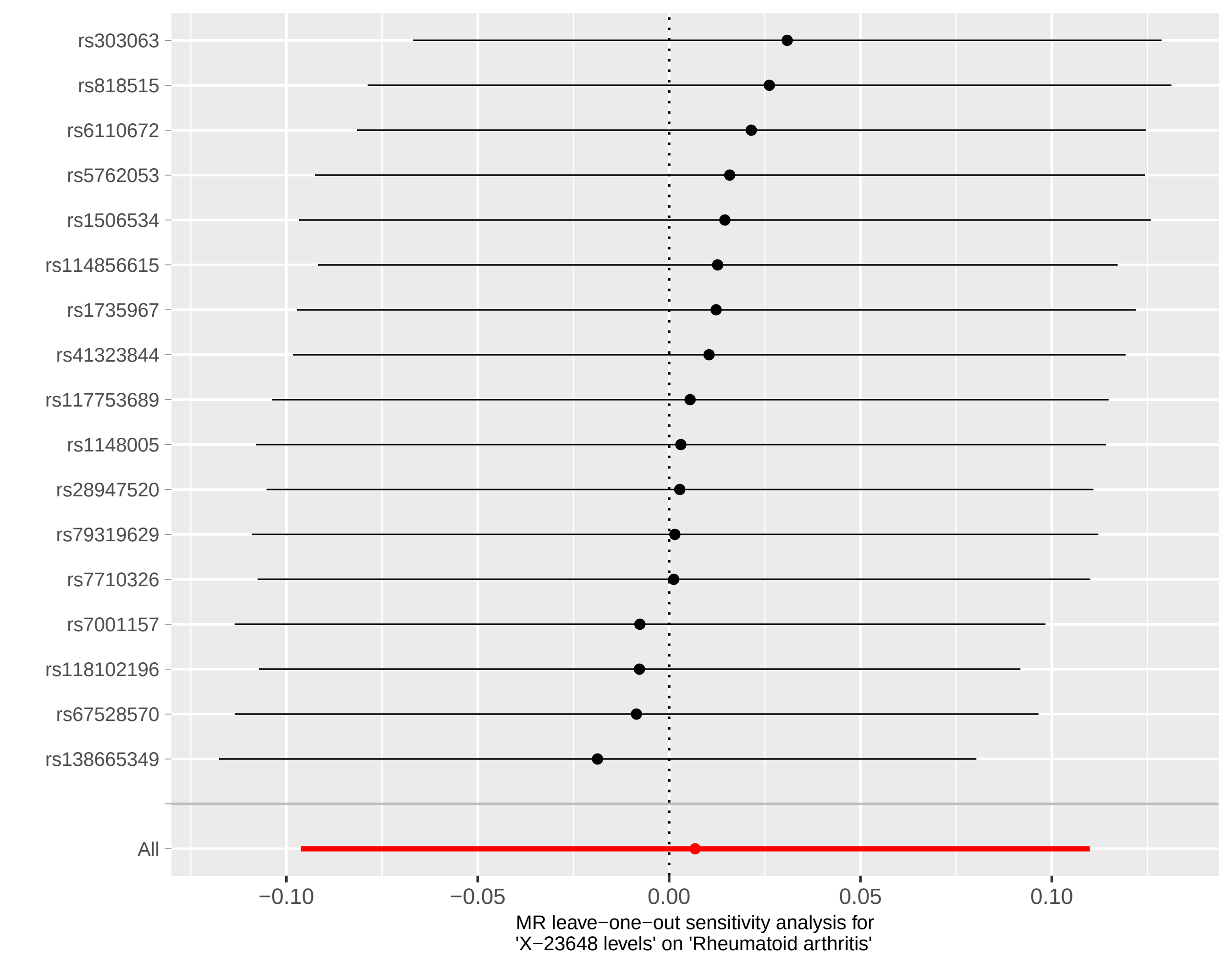

rs303063
rs818515
rs6110672
rs5762053
rs1506534
rs114856615
rs1735967
rs41323844
rs117753689
rs1148005
rs28947520
rs79319629
rs7710326
rs7001157
rs118102196
rs67528570
rs138665349
All
−0.10
−0.05
0.00
0.05
0.10
MR leave−one−out sensitivity analysis for
'X−23648 levels' on 'Rheumatoid arthritis'

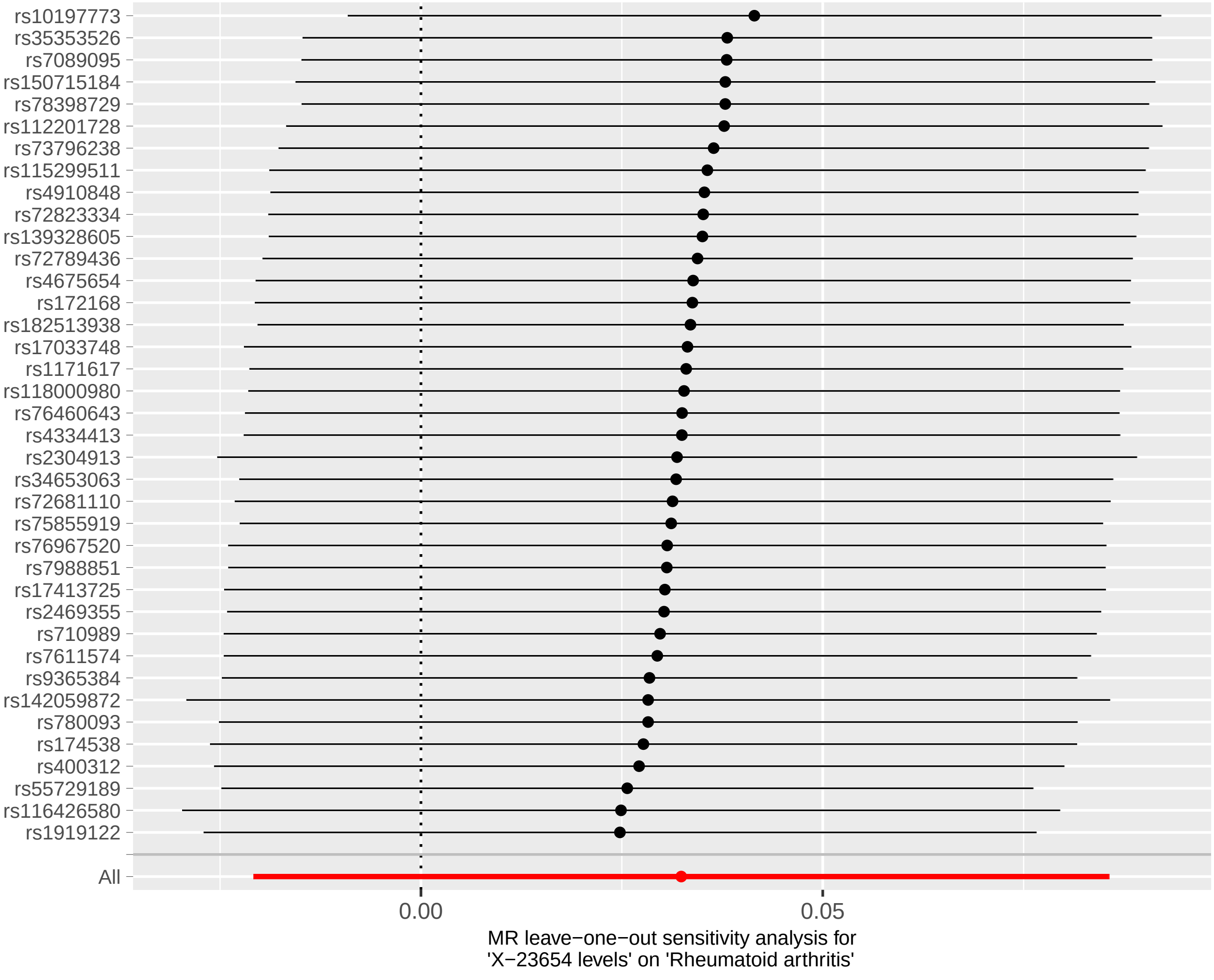
rs10197773
rs35353526
rs7089095
rs150715184
rs78398729
rs112201728
rs73796238
rs115299511
rs4910848
rs72823334
rs139328605
rs72789436
rs4675654
rs172168
rs182513938
rs17033748
rs1171617
rs118000980
rs76460643
rs4334413
rs2304913
rs34653063
rs72681110
rs75855919
rs76967520
rs7988851
rs17413725
rs2469355
rs710989
rs7611574
rs9365384
rs142059872
rs780093
rs174538
rs400312
rs55729189
rs116426580
rs1919122
All
0.00
0.05
MR leave−one−out sensitivity analysis for
'X−23654 levels' on 'Rheumatoid arthritis'

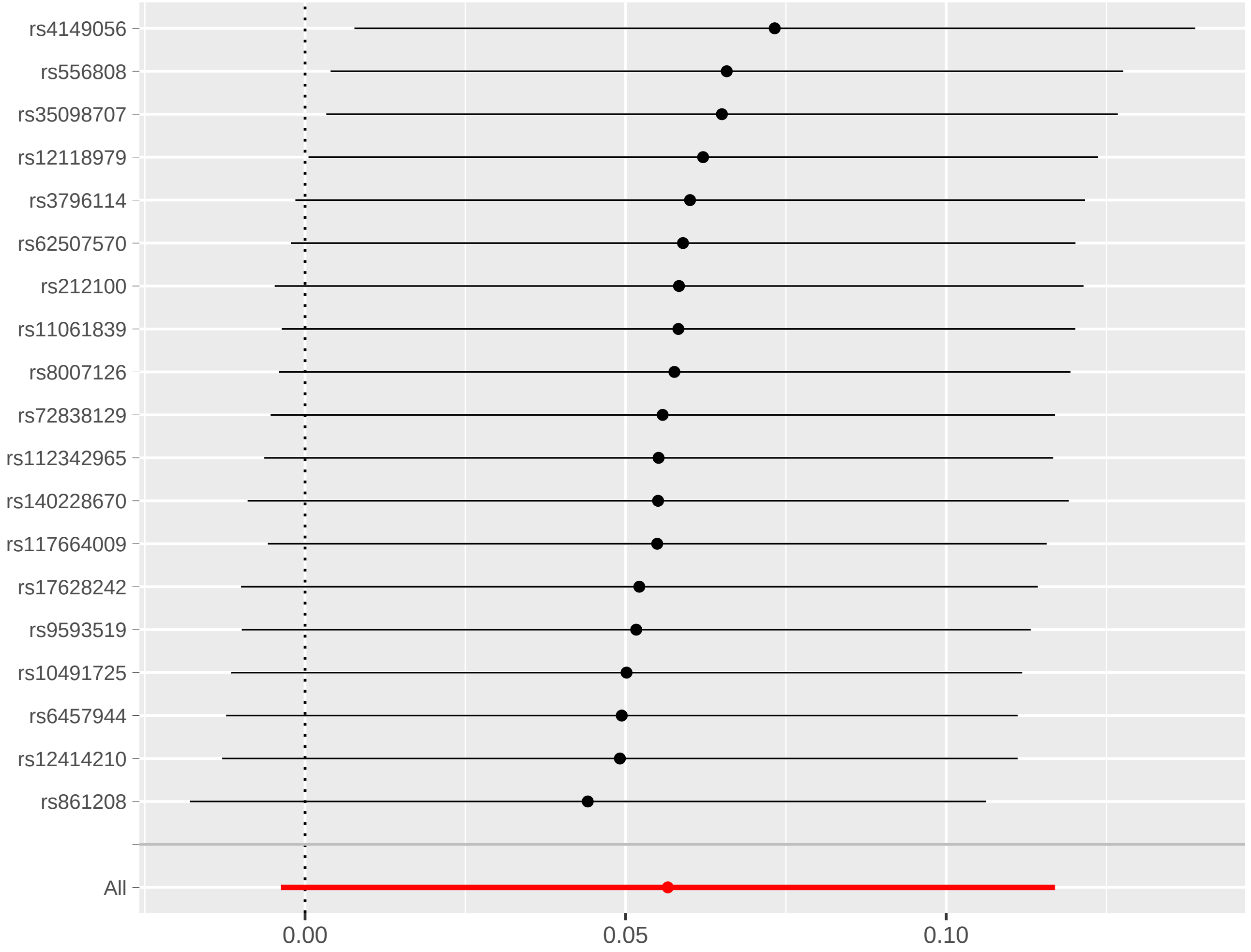

MR leave−one−out sensitivity analysis for
'X−22520 levels' on 'Rheumatoid arthritis'

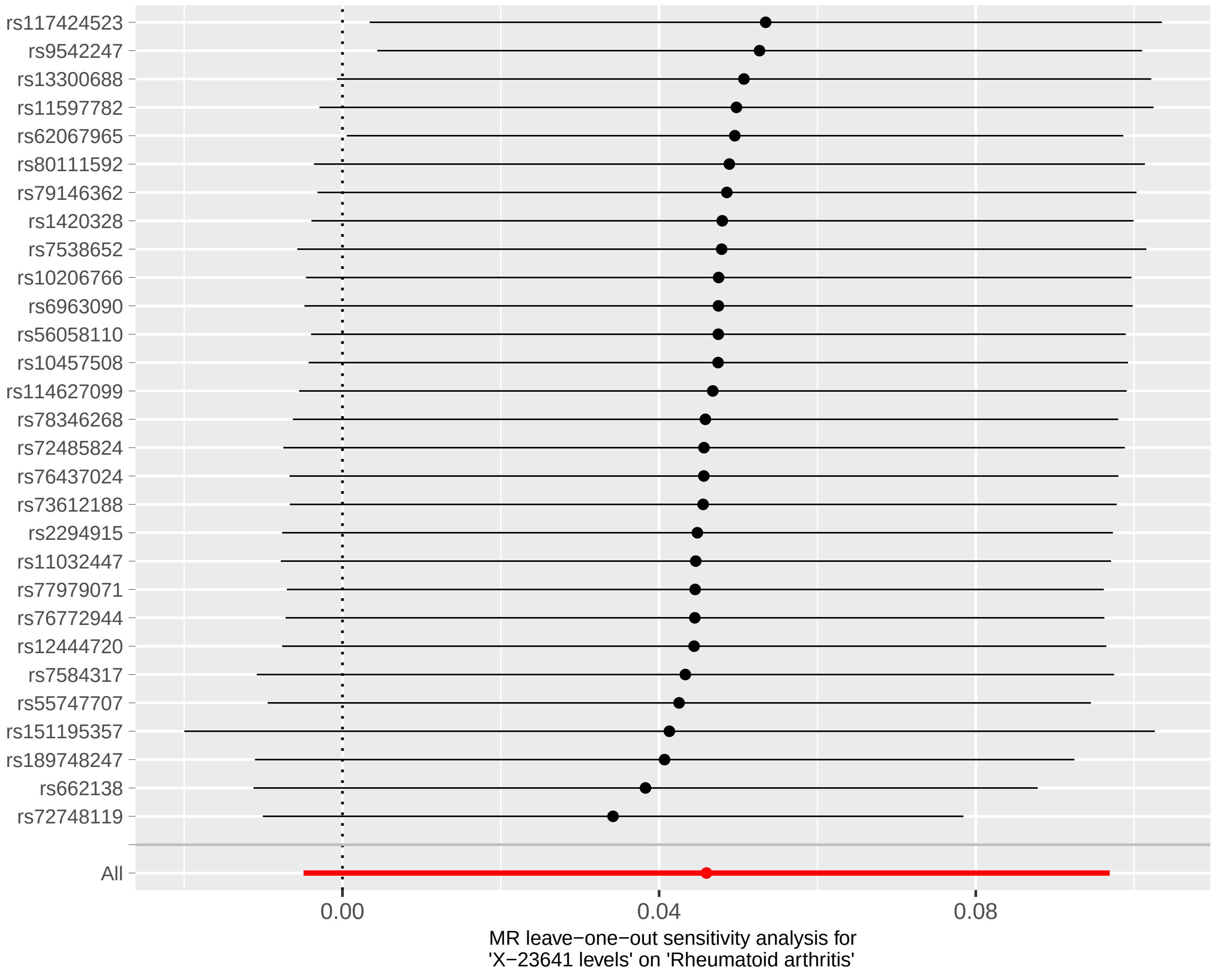

rs117424523
rs9542247
rs13300688
rs11597782
rs62067965
rs80111592
rs79146362
rs1420328
rs7538652
rs10206766
rs6963090
rs56058110
rs10457508
rs114627099
rs78346268
rs72485824
rs76437024
rs73612188
rs2294915
rs11032447
rs77979071
rs76772944
rs12444720
rs7584317
rs55747707
rs151195357
rs189748247
rs662138
rs72748119
All
0.00
0.04
0.08
MR leave−one−out sensitivity analysis for
'X−23641 levels' on 'Rheumatoid arthritis'

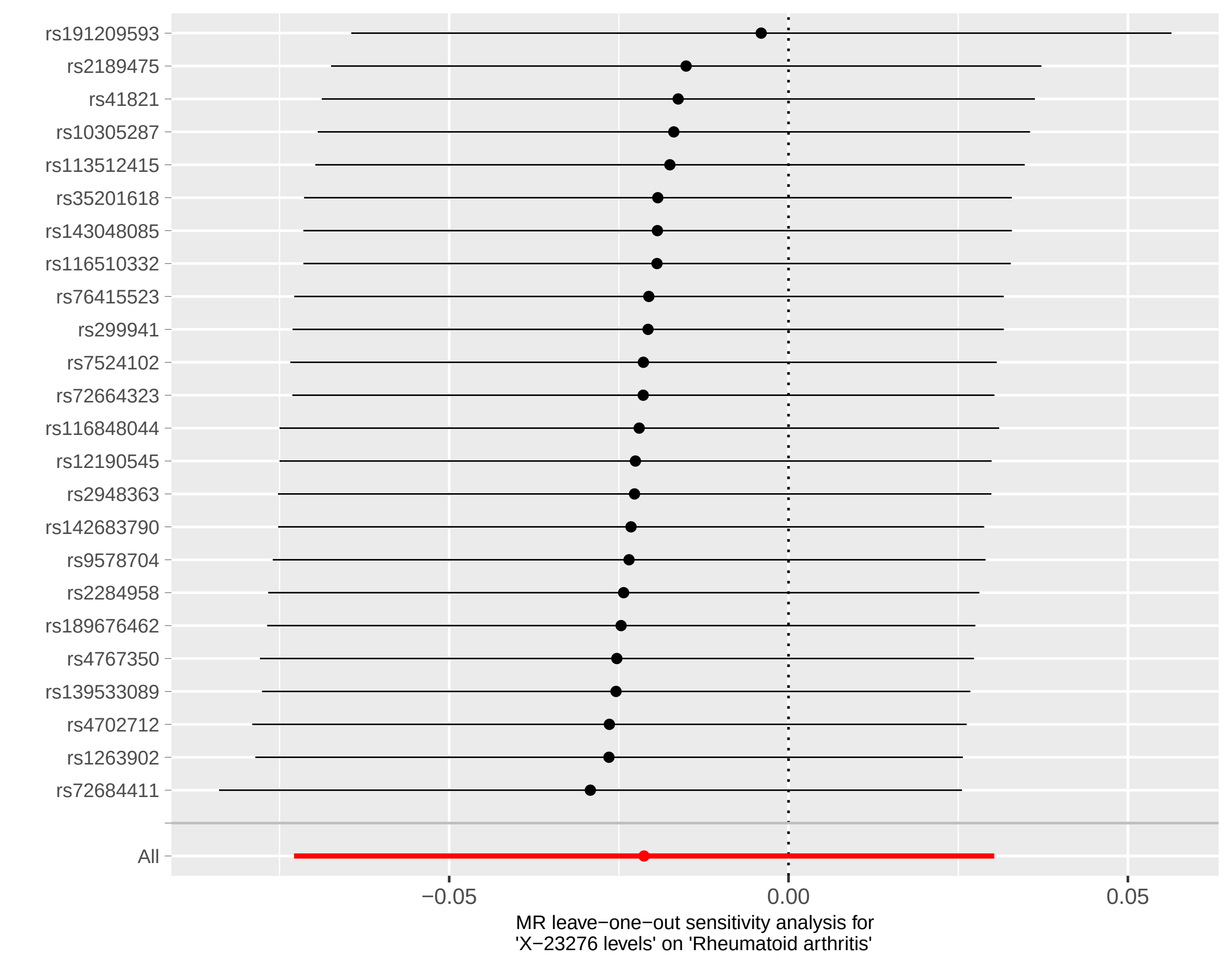
rs191209593
rs2189475
rs41821
rs10305287
rs113512415
rs35201618
rs143048085
rs116510332
rs76415523
rs299941
rs7524102
rs72664323
rs116848044
rs12190545
rs2948363
rs142683790
rs9578704
rs2284958
rs189676462
rs4767350
rs139533089
rs4702712
rs1263902
rs72684411
All
−0.05
0.00
0.05
MR leave−one−out sensitivity analysis for
'X−23276 levels' on 'Rheumatoid arthritis'

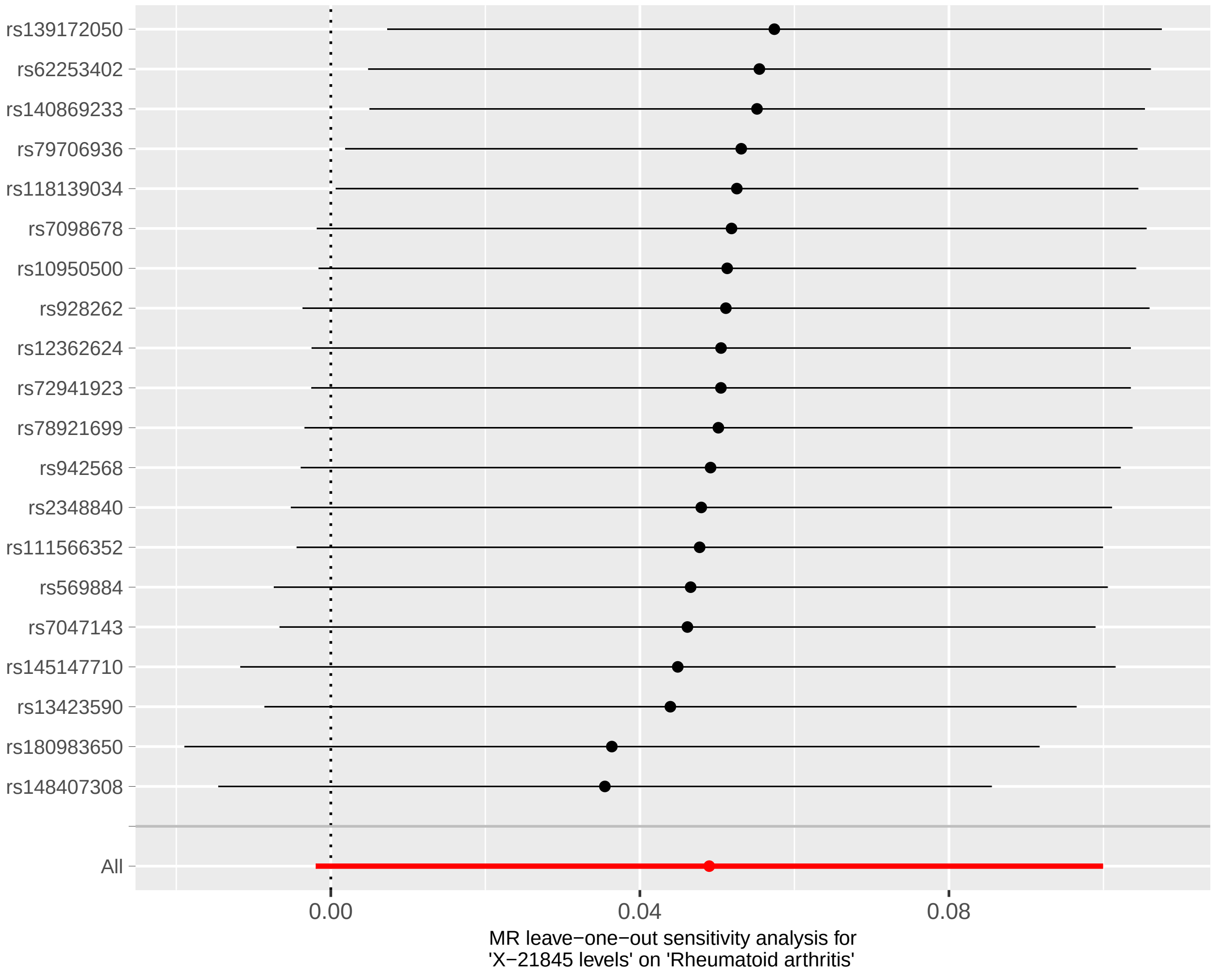
rs139172050
rs62253402
rs140869233
rs79706936
rs118139034
rs7098678
rs10950500
rs928262
rs12362624
rs72941923
rs78921699
rs942568
rs2348840
rs111566352
rs569884
rs7047143
rs145147710
rs13423590
rs180983650
rs148407308
All
0.00
0.04
0.08
MR leave−one−out sensitivity analysis for
'X−21845 levels' on 'Rheumatoid arthritis'

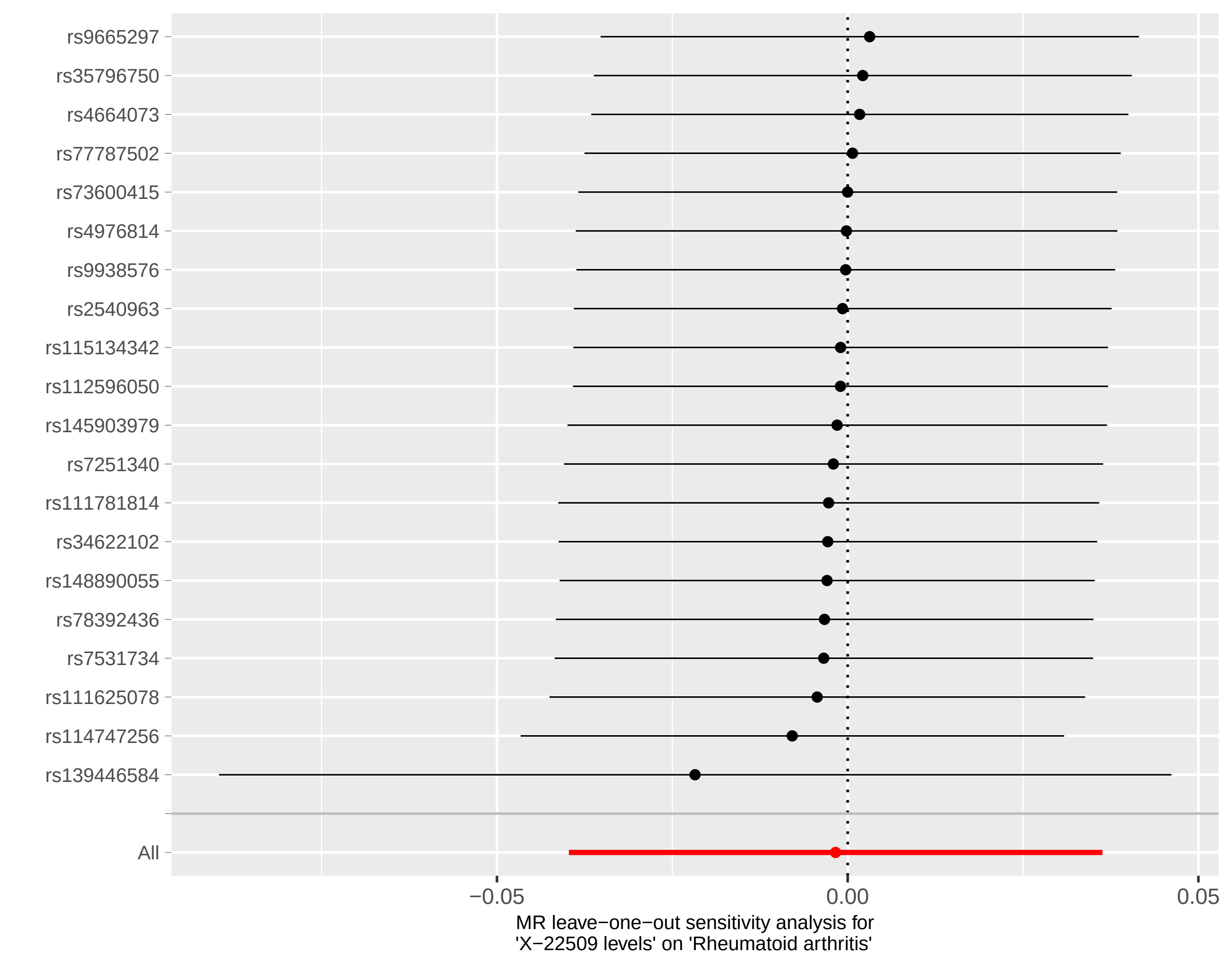
rs9665297
rs35796750
rs4664073
rs77787502
rs73600415
rs4976814
rs9938576
rs2540963
rs115134342
rs112596050
rs145903979
rs7251340
rs111781814
rs34622102
rs148890055
rs78392436
rs7531734
rs111625078
rs114747256
rs139446584
All
−0.05
0.00
0.05
MR leave−one−out sensitivity analysis for
'X−22509 levels' on 'Rheumatoid arthritis'

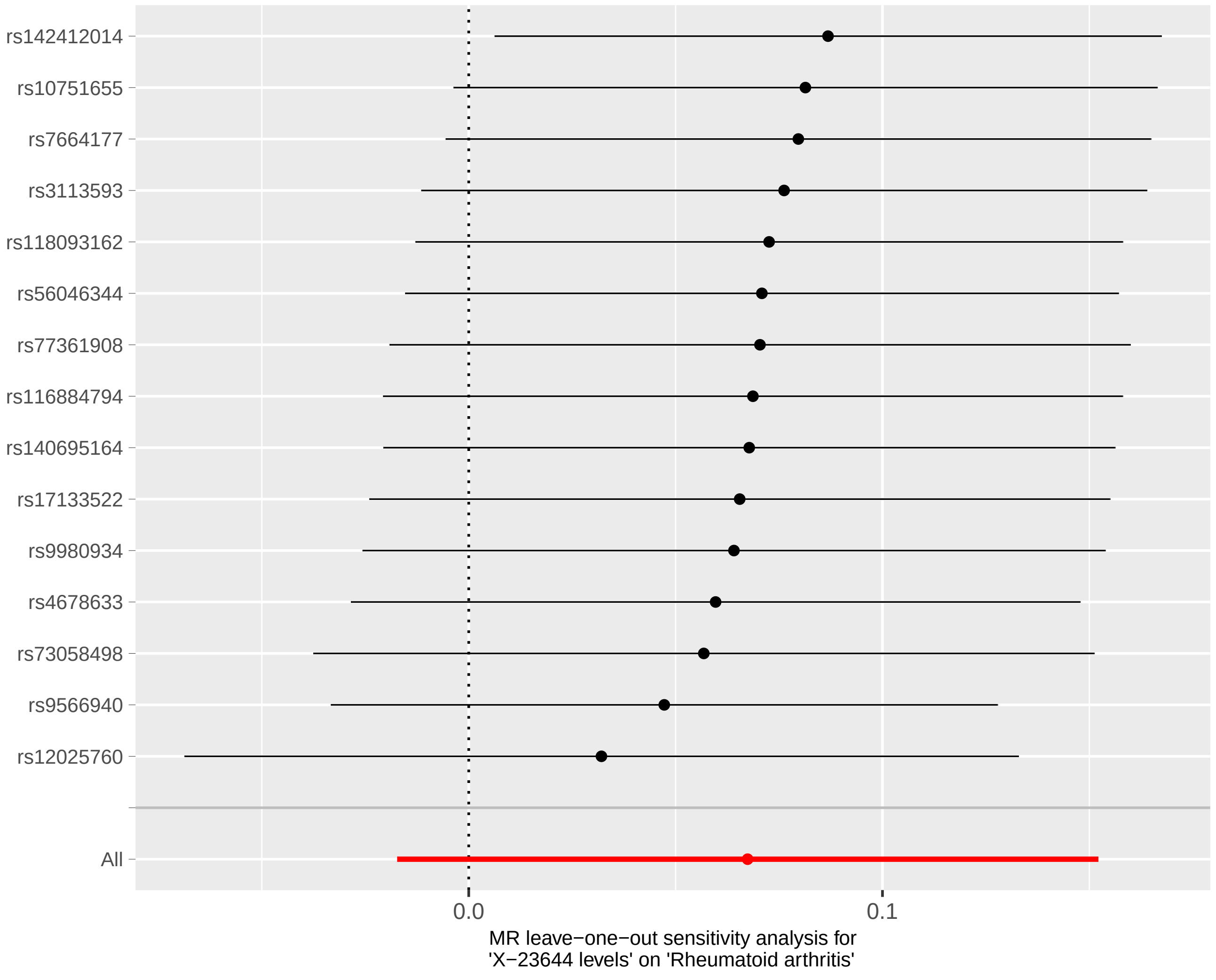
rs142412014
rs10751655
rs7664177
rs3113593
rs118093162
rs56046344
rs77361908
rs116884794
rs140695164
rs17133522
rs9980934
rs4678633
rs73058498
rs9566940
rs12025760
All
0.0
0.1
MR leave−one−out sensitivity analysis for
'X−23644 levels' on 'Rheumatoid arthritis'

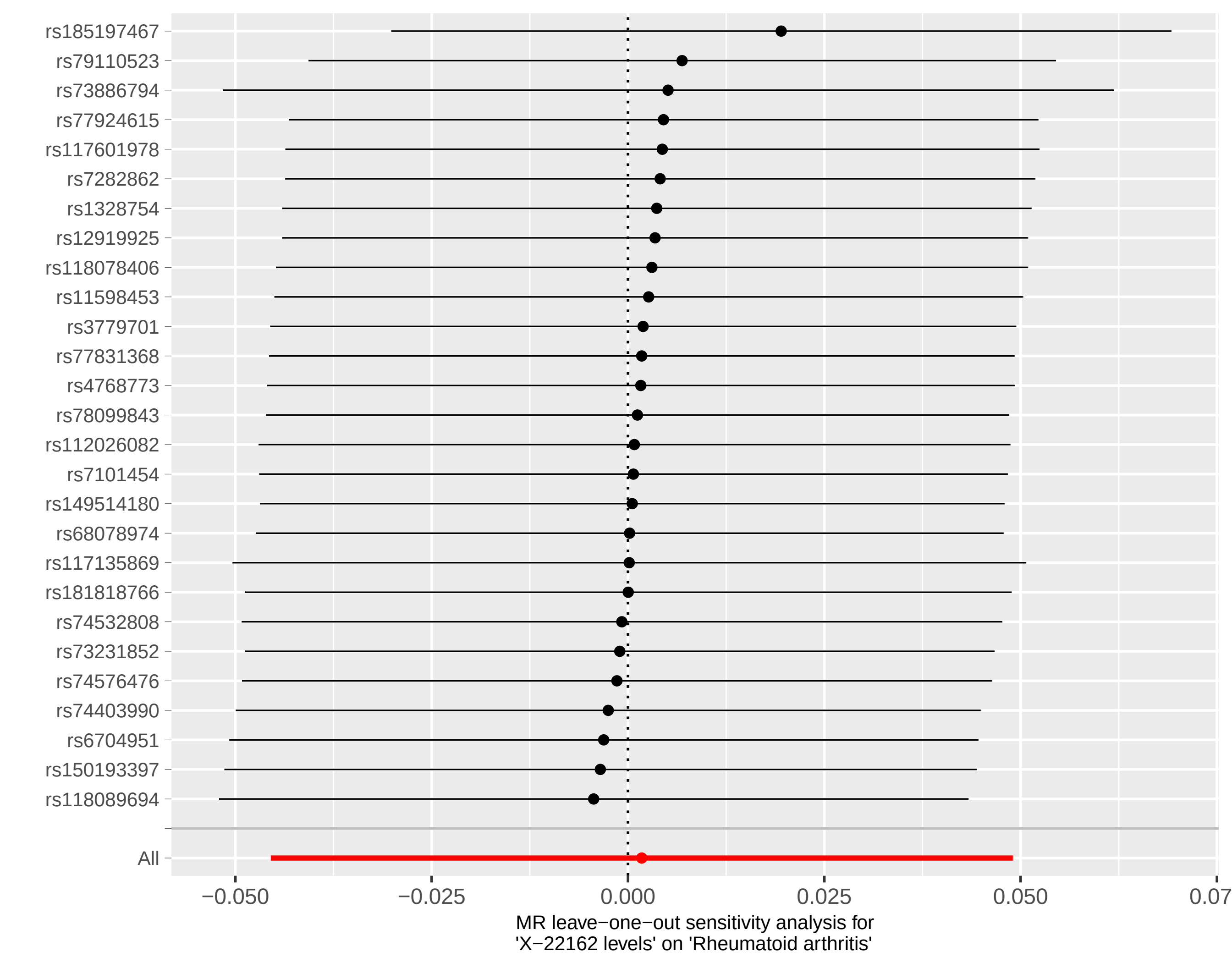
rs185197467
rs79110523
rs73886794
rs77924615
rs117601978
rs7282862
rs1328754
rs12919925
rs118078406
rs11598453
rs3779701
rs77831368
rs4768773
rs78099843
rs112026082
rs7101454
rs149514180
rs68078974
rs117135869
rs181818766
rs74532808
rs73231852
rs74576476
rs74403990
rs6704951
rs150193397
rs118089694
All
−0.050
−0.025
0.000
0.025
0.050
0.07
MR leave−one−out sensitivity analysis for
'X−22162 levels' on 'Rheumatoid arthritis'

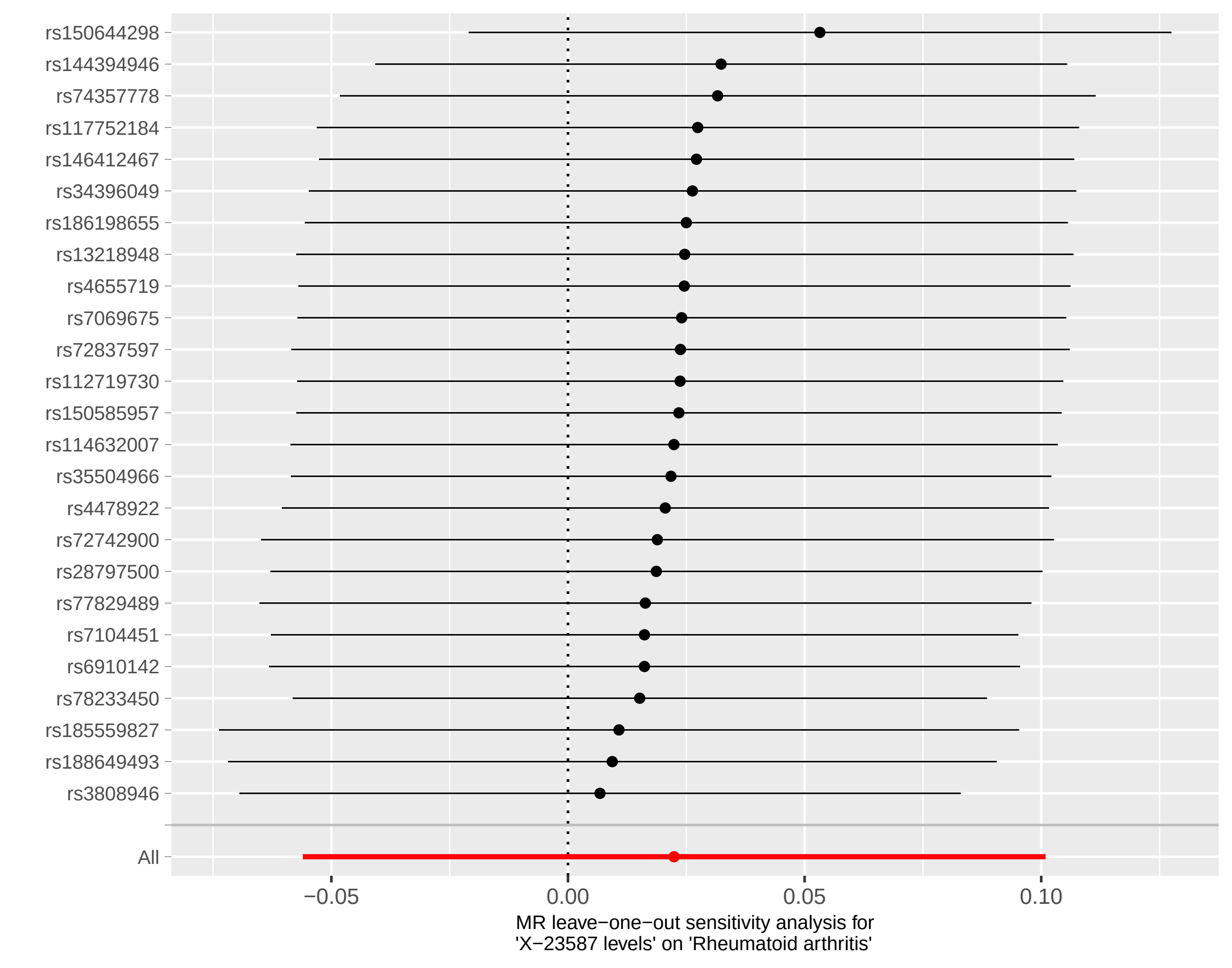
rs150644298
rs144394946
rs74357778
rs117752184
rs146412467
rs34396049
rs186198655
rs13218948
rs4655719
rs7069675
rs72837597
rs112719730
rs150585957
rs114632007
rs35504966
rs4478922
rs72742900
rs28797500
rs77829489
rs7104451
rs6910142
rs78233450
rs185559827
rs188649493
rs3808946
All
−0.05
0.00
0.05
0.10
MR leave−one−out sensitivity analysis for
'X−23587 levels' on 'Rheumatoid arthritis'

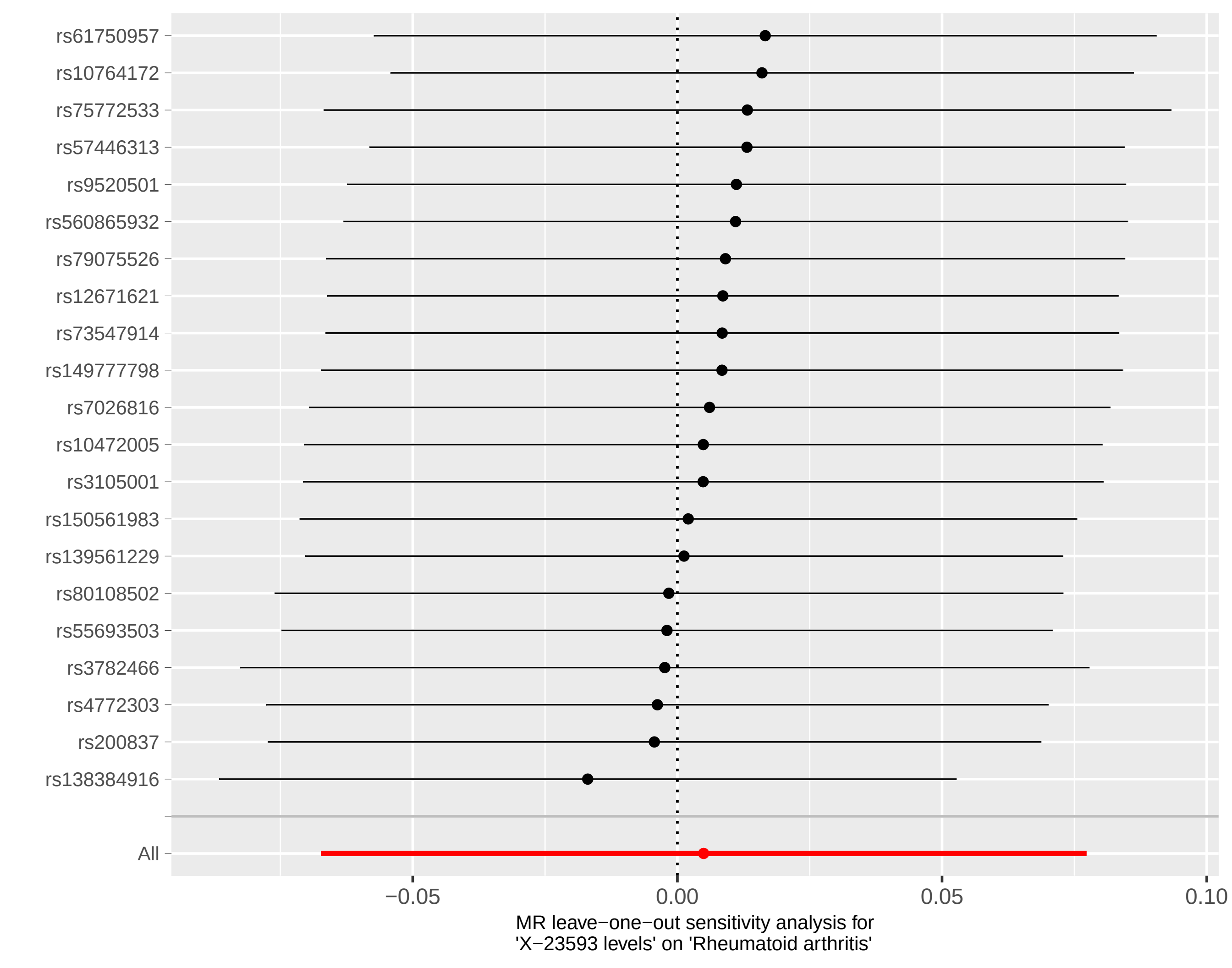
rs61750957
rs10764172
rs75772533
rs57446313
rs9520501
rs560865932
rs79075526
rs12671621
rs73547914
rs149777798
rs7026816
rs10472005
rs3105001
rs150561983
rs139561229
rs80108502
rs55693503
rs3782466
rs4772303
rs200837
rs138384916
All
−0.05
0.00
0.05
0.10
MR leave−one−out sensitivity analysis for
'X−23593 levels' on 'Rheumatoid arthritis'

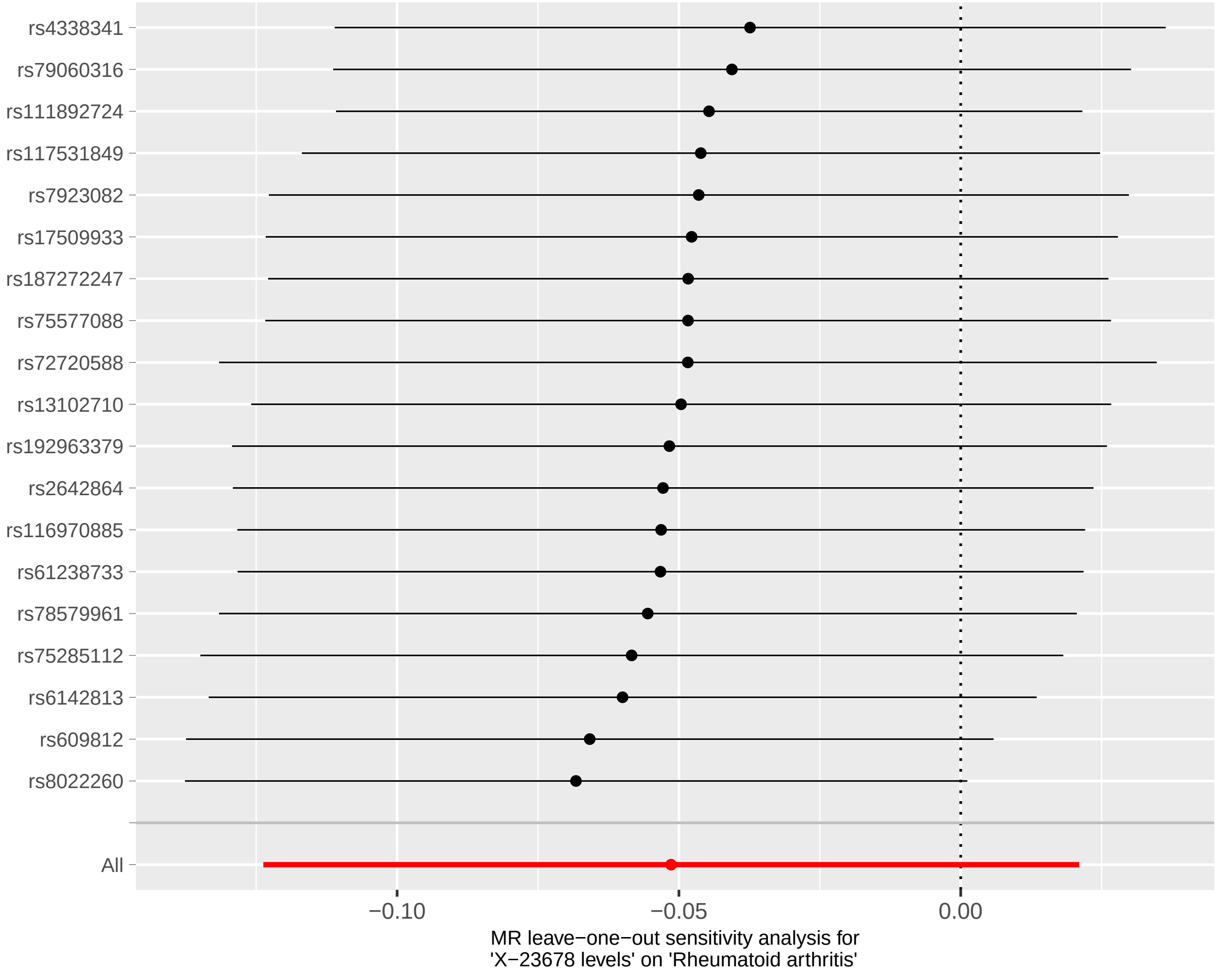
rs4338341
rs79060316
rs111892724
rs117531849
rs7923082
rs17509933
rs187272247
rs75577088
rs72720588
rs13102710
rs192963379
rs2642864
rs116970885
rs61238733
rs78579961
rs75285112
rs6142813
rs609812
rs8022260
All
−0.10
−0.05
0.00
MR leave−one−out sensitivity analysis for
'X−23678 levels' on 'Rheumatoid arthritis'

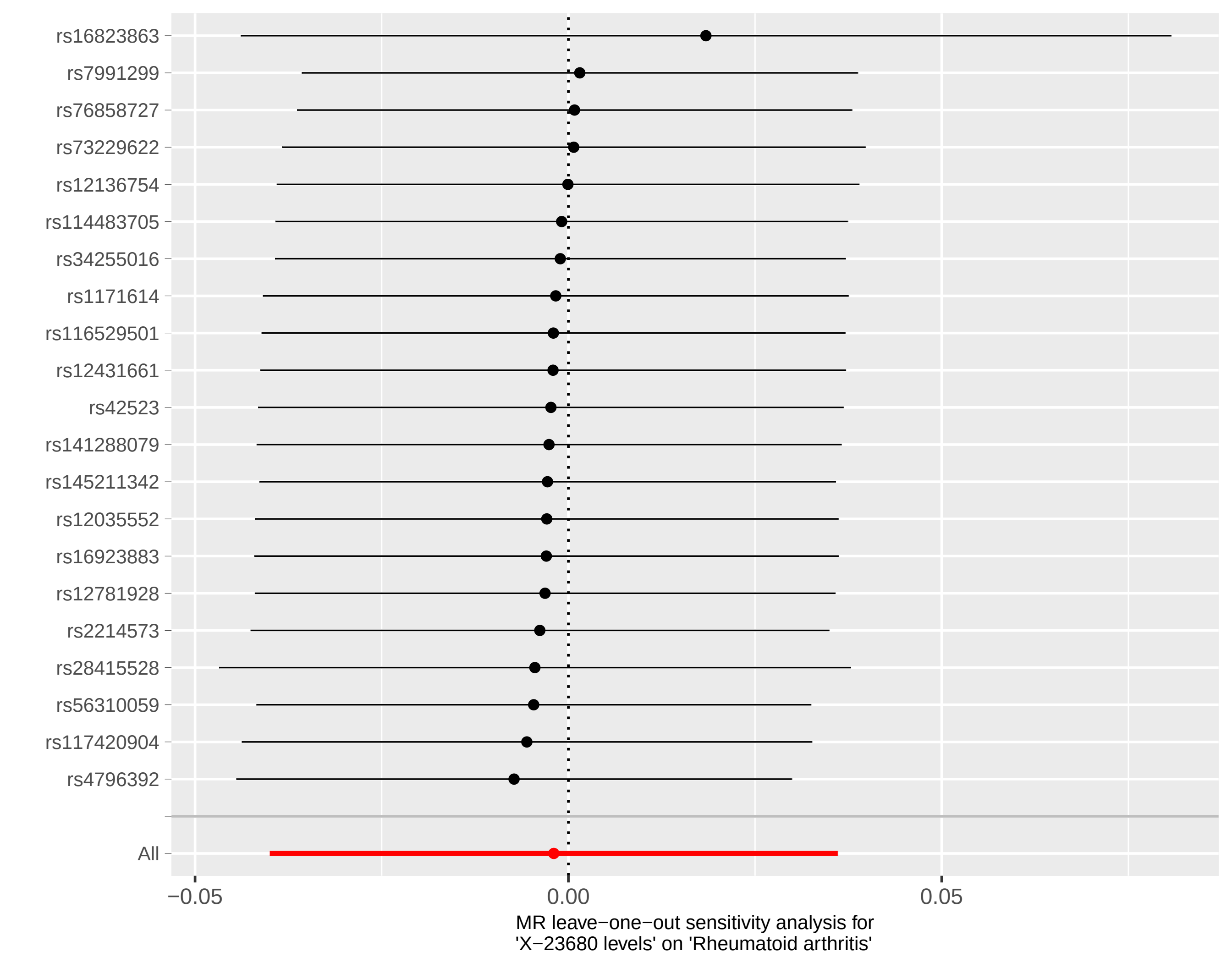
rs16823863
rs7991299
rs76858727
rs73229622
rs12136754
rs114483705
rs34255016
rs1171614
rs116529501
rs12431661
rs42523
rs141288079
rs145211342
rs12035552
rs16923883
rs12781928
rs2214573
rs28415528
rs56310059
rs117420904
rs4796392
All
−0.05
0.00
0.05
MR leave−one−out sensitivity analysis for
'X−23680 levels' on 'Rheumatoid arthritis'

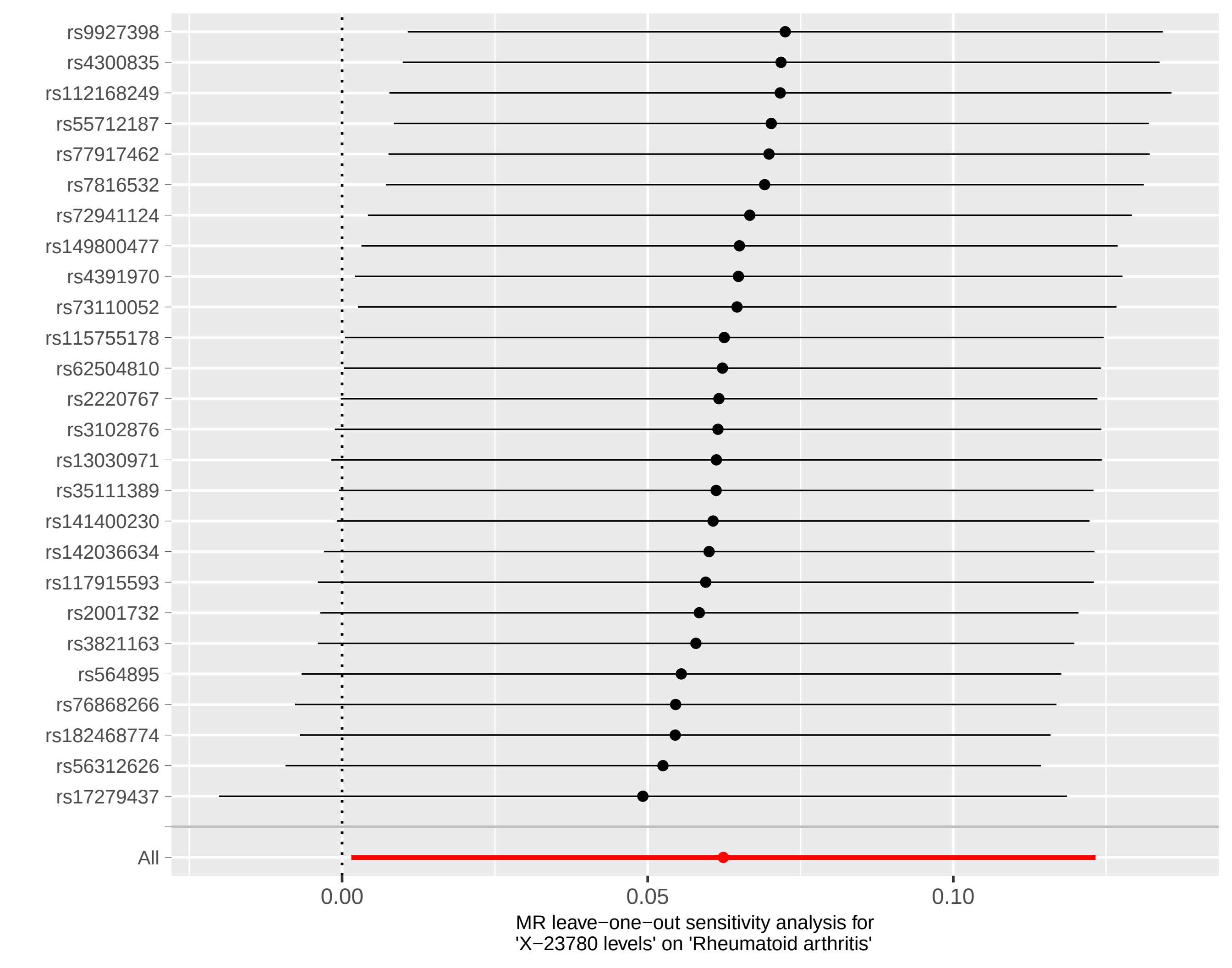
rs9927398
rs4300835
rs112168249
rs55712187
rs77917462
rs7816532
rs72941124
rs149800477
rs4391970
rs73110052
rs115755178
rs62504810
rs2220767
rs3102876
rs13030971
rs35111389
rs141400230
rs142036634
rs117915593
rs2001732
rs3821163
rs564895
rs76868266
rs182468774
rs56312626
rs17279437
All
0.00
0.05
0.10
MR leave−one−out sensitivity analysis for
'X−23780 levels' on 'Rheumatoid arthritis'

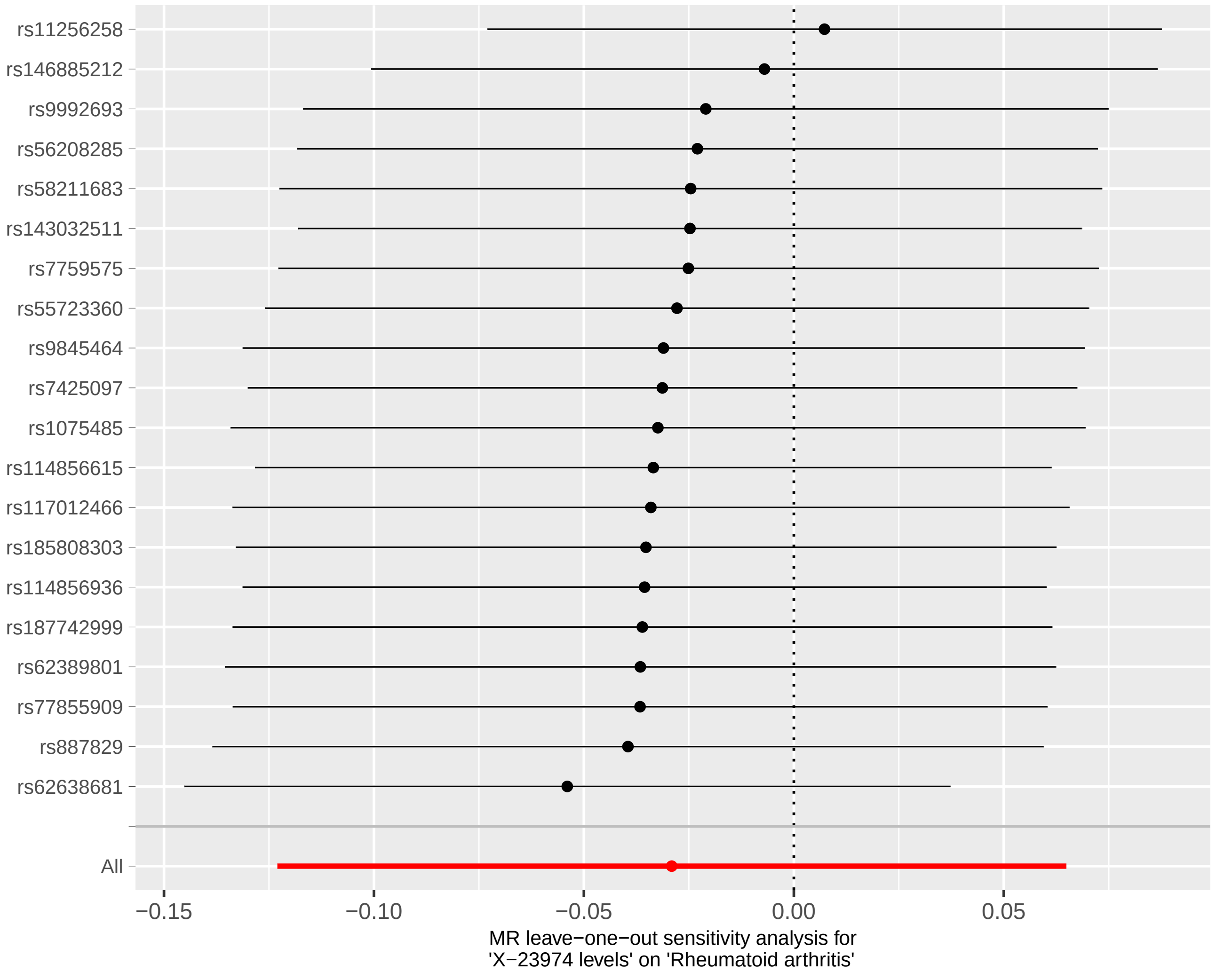
rs11256258
rs146885212
rs9992693
rs56208285
rs58211683
rs143032511
rs7759575
rs55723360
rs9845464
rs7425097
rs1075485
rs114856615
rs117012466
rs185808303
rs114856936
rs187742999
rs62389801
rs77855909
rs887829
rs62638681
All
−0.15
−0.10
−0.05
0.00
0.05
MR leave−one−out sensitivity analysis for
'X−23974 levels' on 'Rheumatoid arthritis'

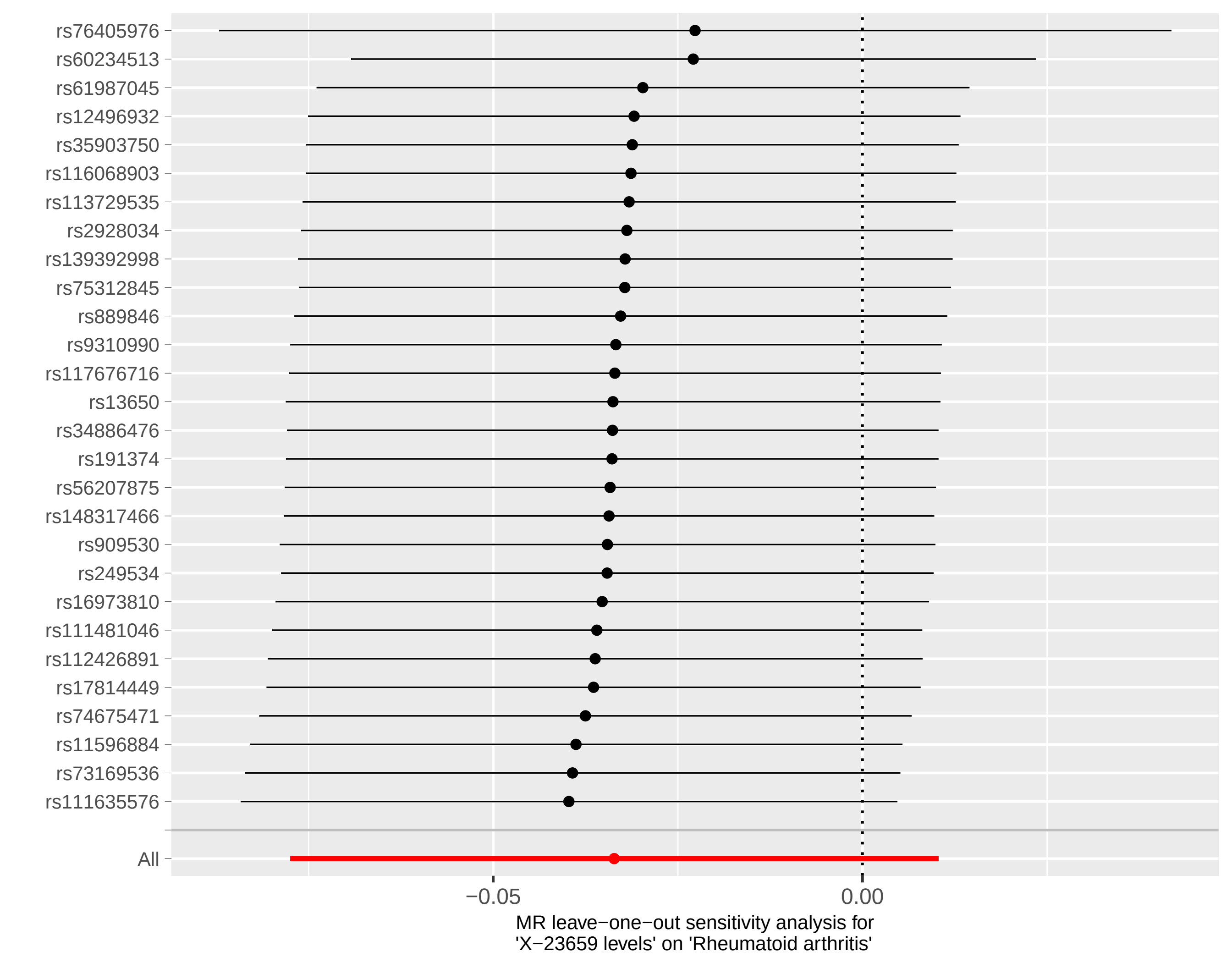
rs76405976
rs60234513
rs61987045
rs12496932
rs35903750
rs116068903
rs113729535
rs2928034
rs139392998
rs75312845
rs889846
rs9310990
rs117676716
rs13650
rs34886476
rs191374
rs56207875
rs148317466
rs909530
rs249534
rs16973810
rs111481046
rs112426891
rs17814449
rs74675471
rs11596884
rs73169536
rs111635576
All
−0.05
0.00
MR leave−one−out sensitivity analysis for
'X−23659 levels' on 'Rheumatoid arthritis'

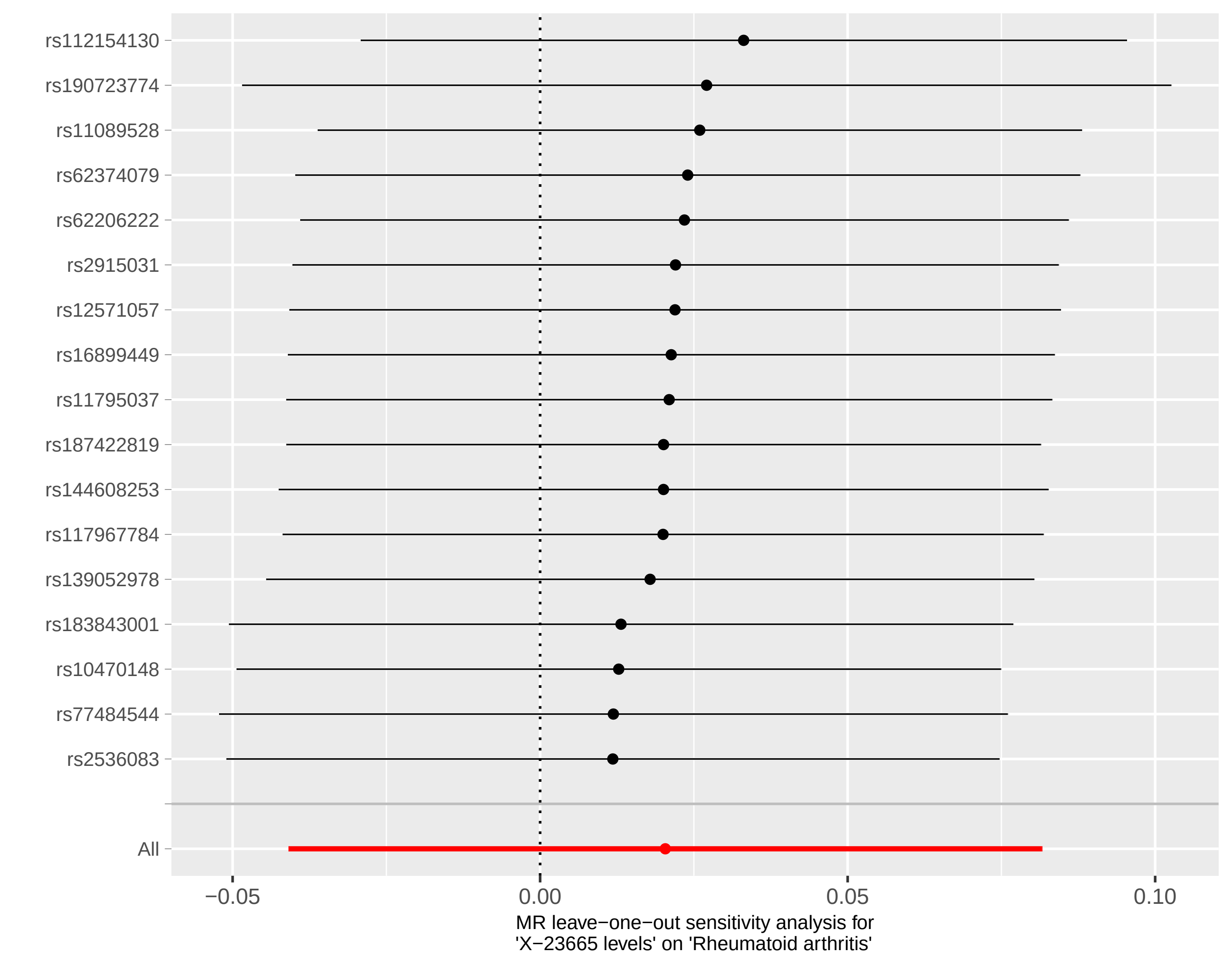
rs112154130
rs190723774
rs11089528
rs62374079
rs62206222
rs2915031
rs12571057
rs16899449
rs11795037
rs187422819
rs144608253
rs117967784
rs139052978
rs183843001
rs10470148
rs77484544
rs2536083
All
−0.05
0.00
0.05
0.10
MR leave−one−out sensitivity analysis for
'X−23665 levels' on 'Rheumatoid arthritis'

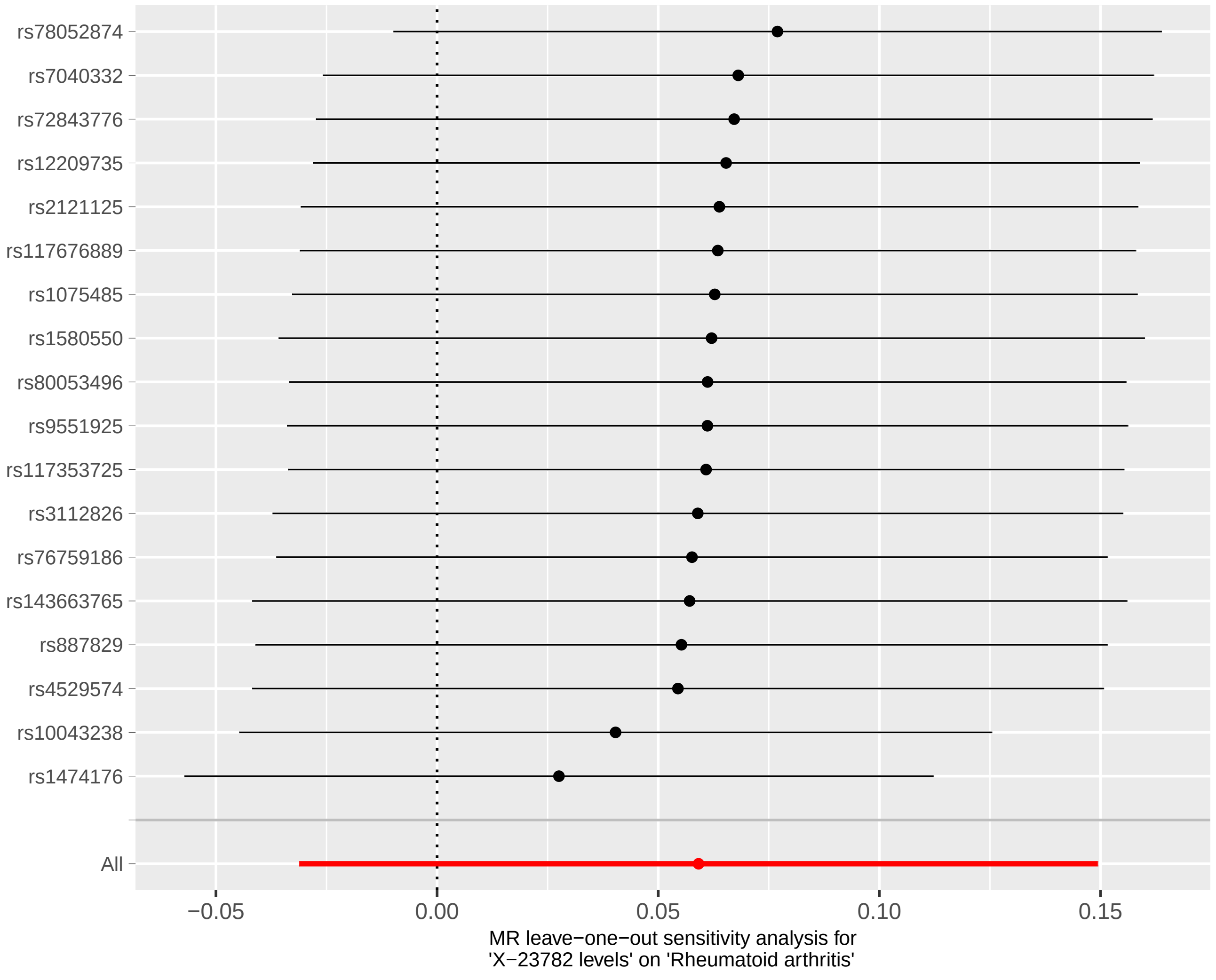
rs78052874
rs7040332
rs72843776
rs12209735
rs2121125
rs117676889
rs1075485
rs1580550
rs80053496
rs9551925
rs117353725
rs3112826
rs76759186
rs143663765
rs887829
rs4529574
rs10043238
rs1474176
All
−0.05
0.00
0.05
0.10
0.15
MR leave−one−out sensitivity analysis for
'X−23782 levels' on 'Rheumatoid arthritis'

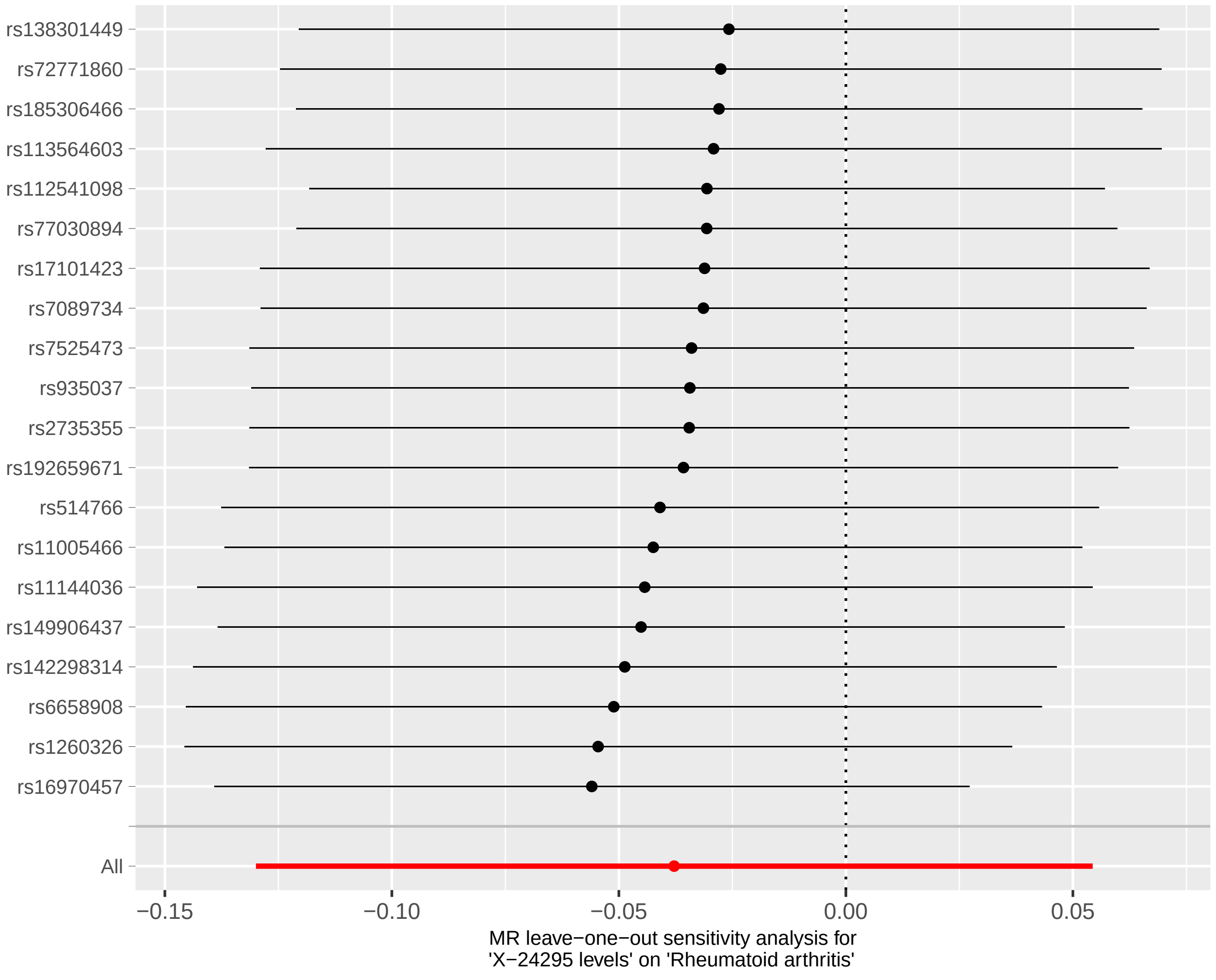

rs138301449
rs72771860
rs185306466
rs113564603
rs112541098
rs77030894
rs17101423
rs7089734
rs7525473
rs935037
rs2735355
rs192659671
rs514766
rs11005466
rs11144036
rs149906437
rs142298314
rs6658908
rs1260326
rs16970457
All
−0.15
−0.10
−0.05
0.00
0.05
MR leave−one−out sensitivity analysis for
'X−24295 levels' on 'Rheumatoid arthritis'

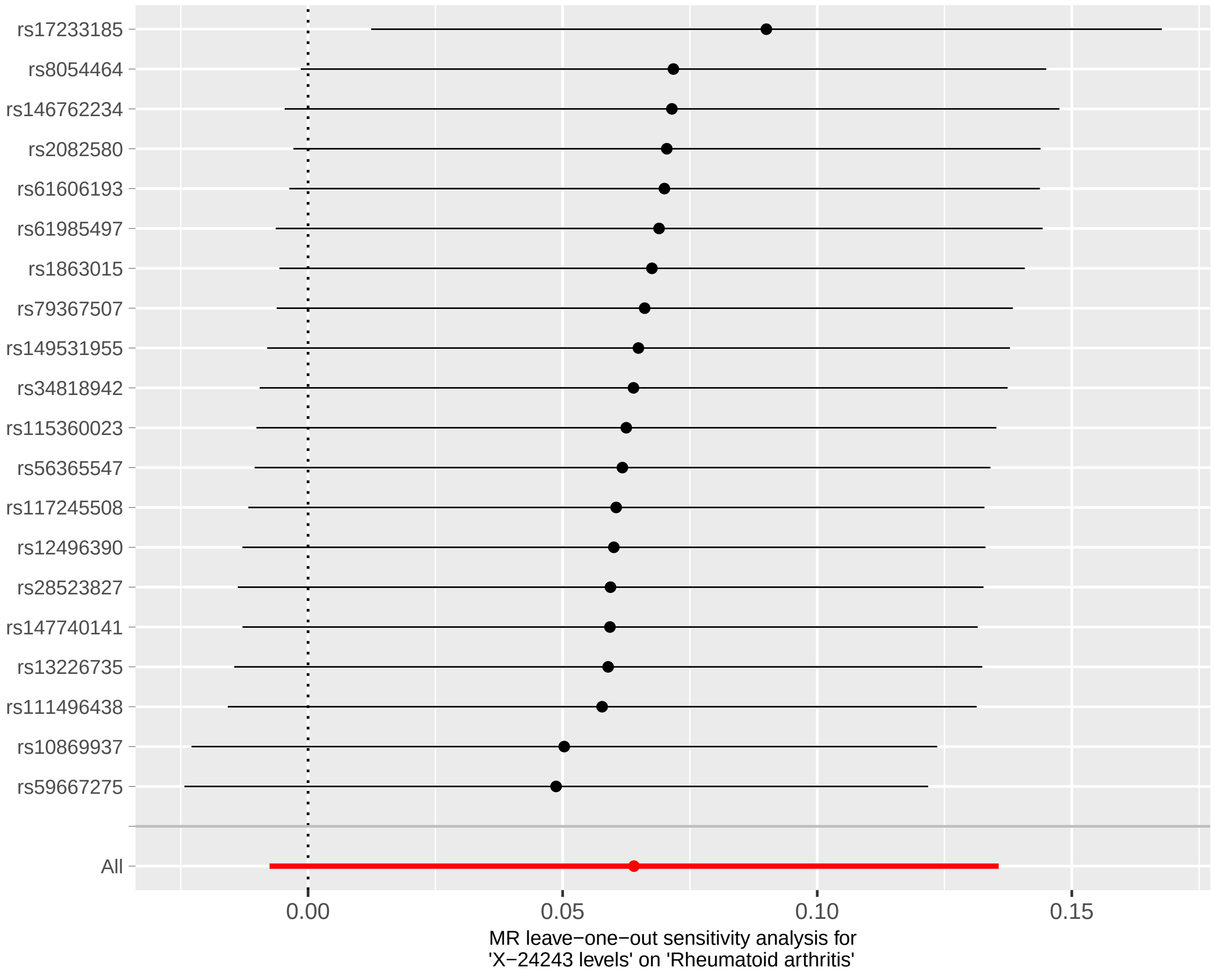
rs17233185
rs8054464
rs146762234
rs2082580
rs61606193
rs61985497
rs1863015
rs79367507
rs149531955
rs34818942
rs115360023
rs56365547
rs117245508
rs12496390
rs28523827
rs147740141
rs13226735
rs111496438
rs10869937
rs59667275
All
0.00
0.05
0.10
0.15
MR leave−one−out sensitivity analysis for
'X−24243 levels' on 'Rheumatoid arthritis'

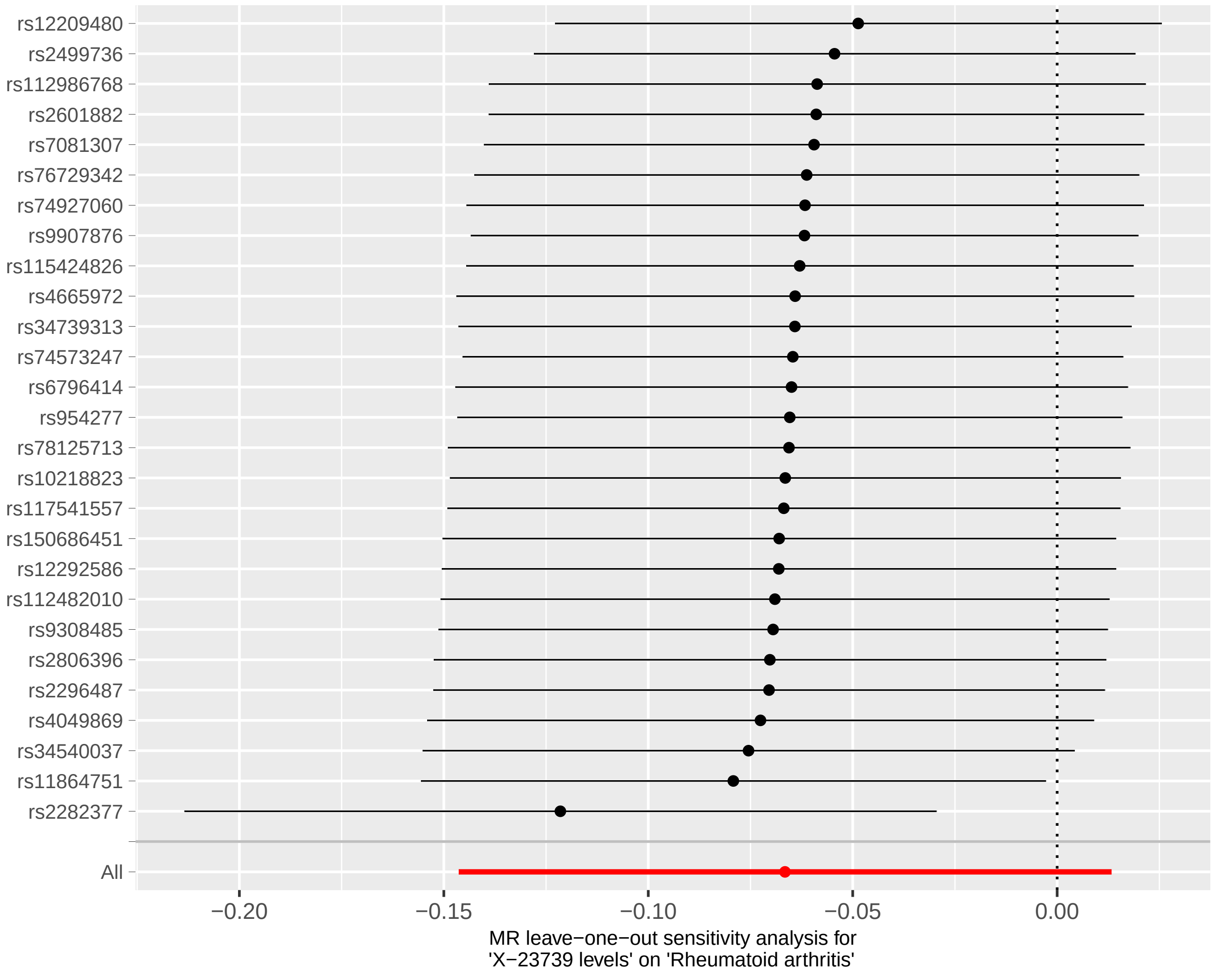
rs12209480
rs2499736
rs112986768
rs2601882
rs7081307
rs76729342
rs74927060
rs9907876
rs115424826
rs4665972
rs34739313
rs74573247
rs6796414
rs954277
rs78125713
rs10218823
rs117541557
rs150686451
rs12292586
rs112482010
rs9308485
rs2806396
rs2296487
rs4049869
rs34540037
rs11864751
rs2282377
All
−0.20
−0.15
−0.10
−0.05
0.00
MR leave−one−out sensitivity analysis for
'X−23739 levels' on 'Rheumatoid arthritis'

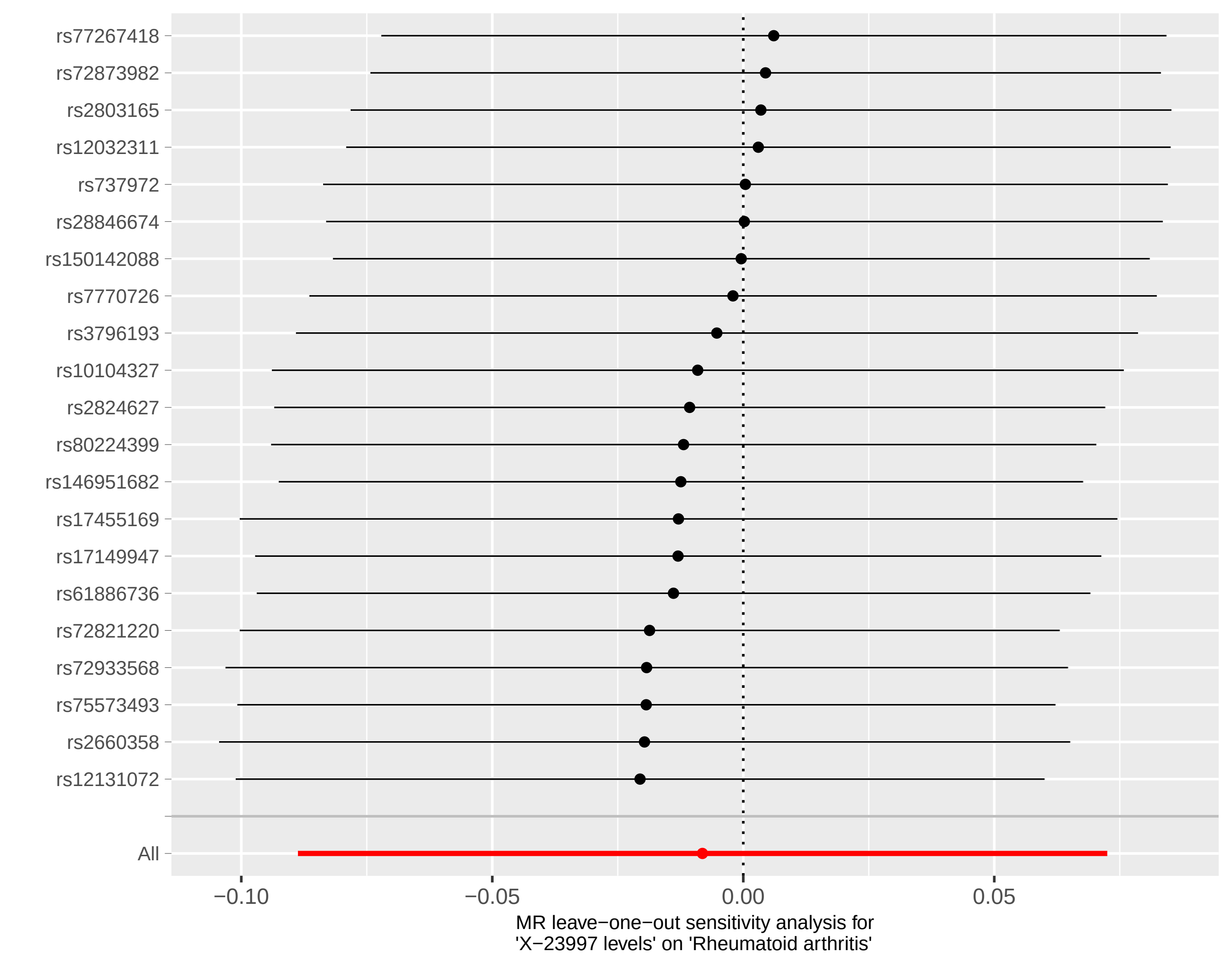
rs77267418
rs72873982
rs2803165
rs12032311
rs737972
rs28846674
rs150142088
rs7770726
rs3796193
rs10104327
rs2824627
rs80224399
rs146951682
rs17455169
rs17149947
rs61886736
rs72821220
rs72933568
rs75573493
rs2660358
rs12131072
All
−0.10
−0.05
0.00
0.05
MR leave−one−out sensitivity analysis for
'X−23997 levels' on 'Rheumatoid arthritis'

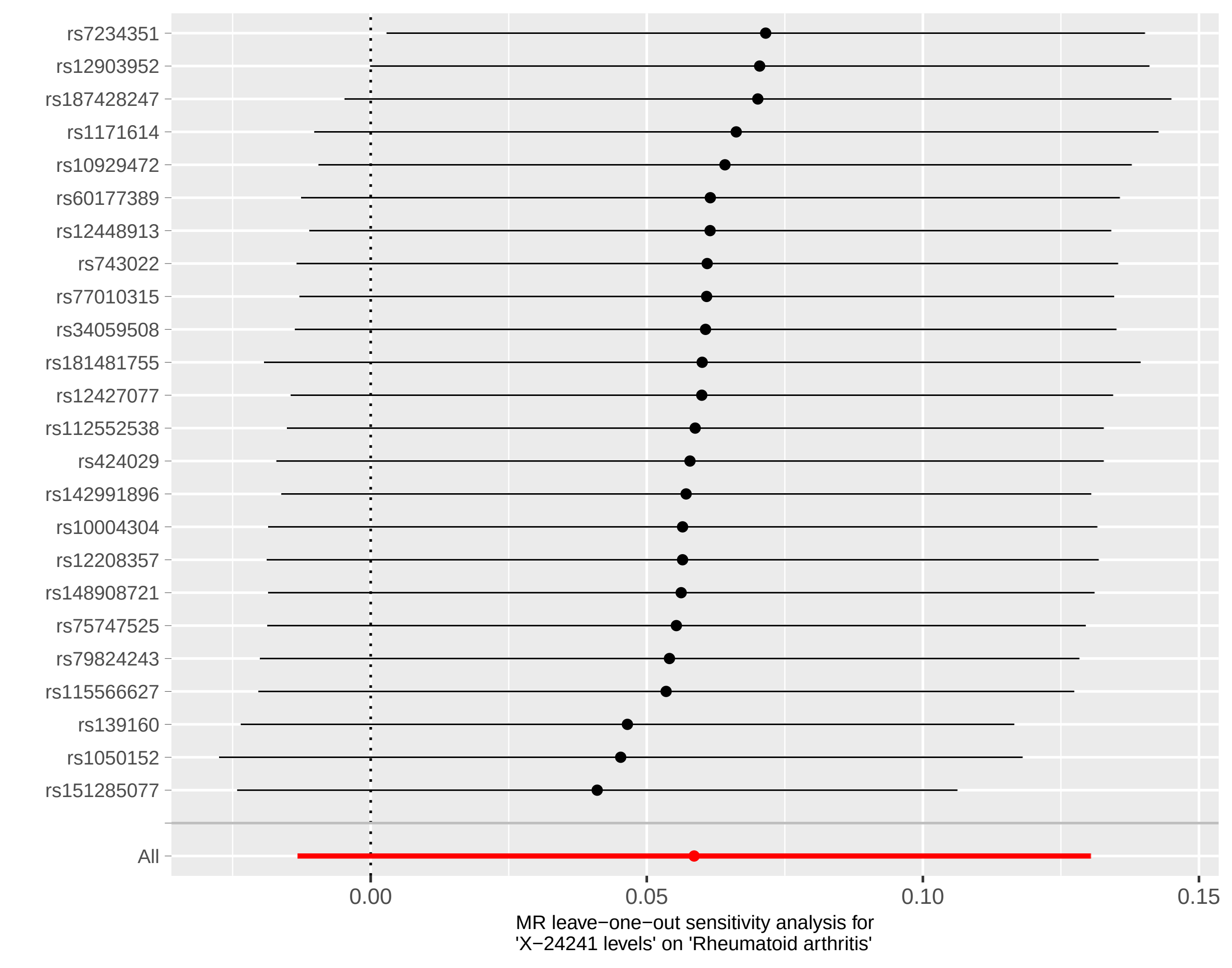
rs7234351
rs12903952
rs187428247
rs1171614
rs10929472
rs60177389
rs12448913
rs743022
rs77010315
rs34059508
rs181481755
rs12427077
rs112552538
rs424029
rs142991896
rs10004304
rs12208357
rs148908721
rs75747525
rs79824243
rs115566627
rs139160
rs1050152
rs151285077
All
0.00
0.05
0.10
0.15
MR leave−one−out sensitivity analysis for
'X−24241 levels' on 'Rheumatoid arthritis'

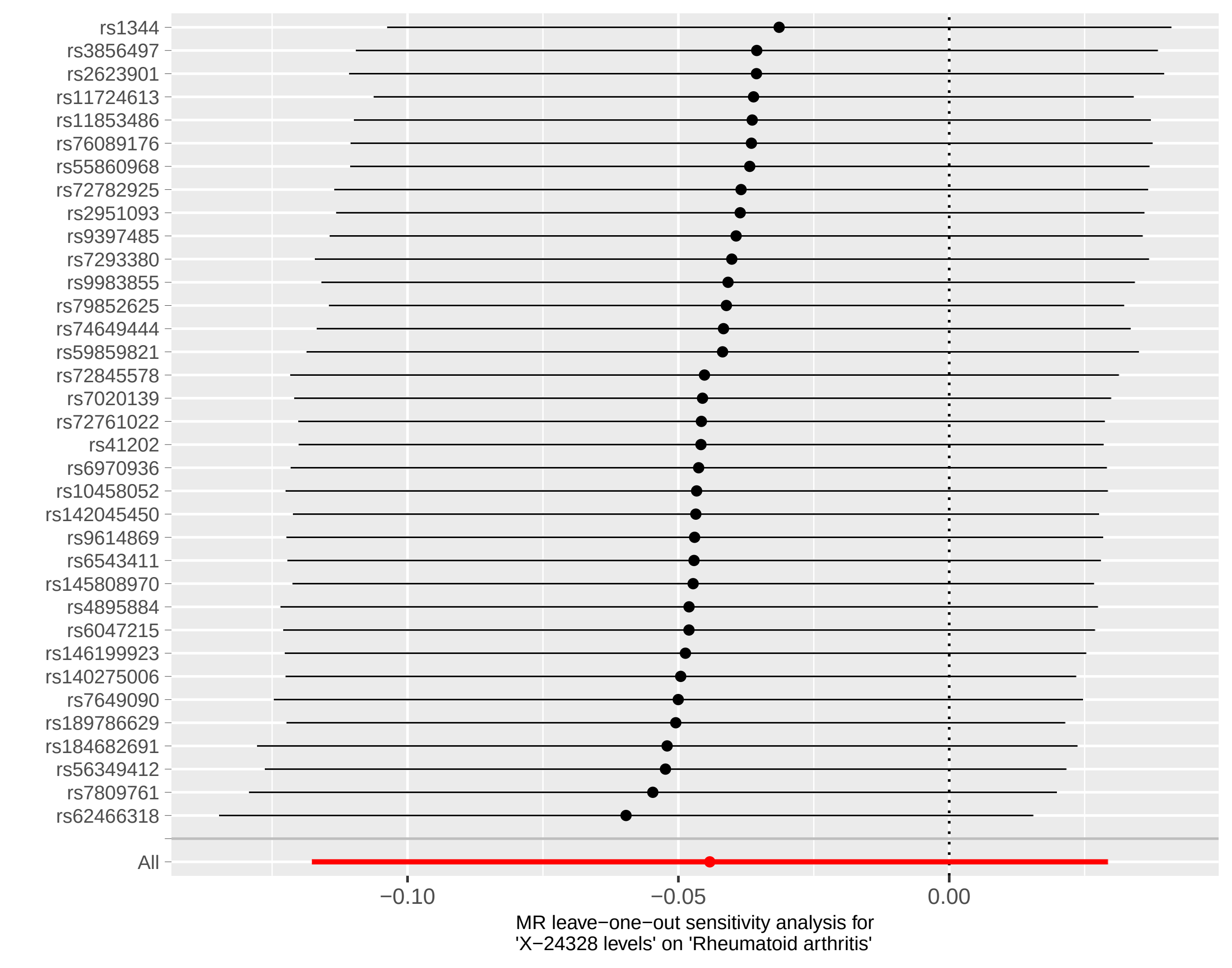
rs1344
rs3856497
rs2623901
rs11724613
rs11853486
rs76089176
rs55860968
rs72782925
rs2951093
rs9397485
rs7293380
rs9983855
rs79852625
rs74649444
rs59859821
rs72845578
rs7020139
rs72761022
rs41202
rs6970936
rs10458052
rs142045450
rs9614869
rs6543411
rs145808970
rs4895884
rs6047215
rs146199923
rs140275006
rs7649090
rs189786629
rs184682691
rs56349412
rs7809761
rs62466318
All
−0.10
−0.05
0.00
MR leave−one−out sensitivity analysis for
'X−24328 levels' on 'Rheumatoid arthritis'

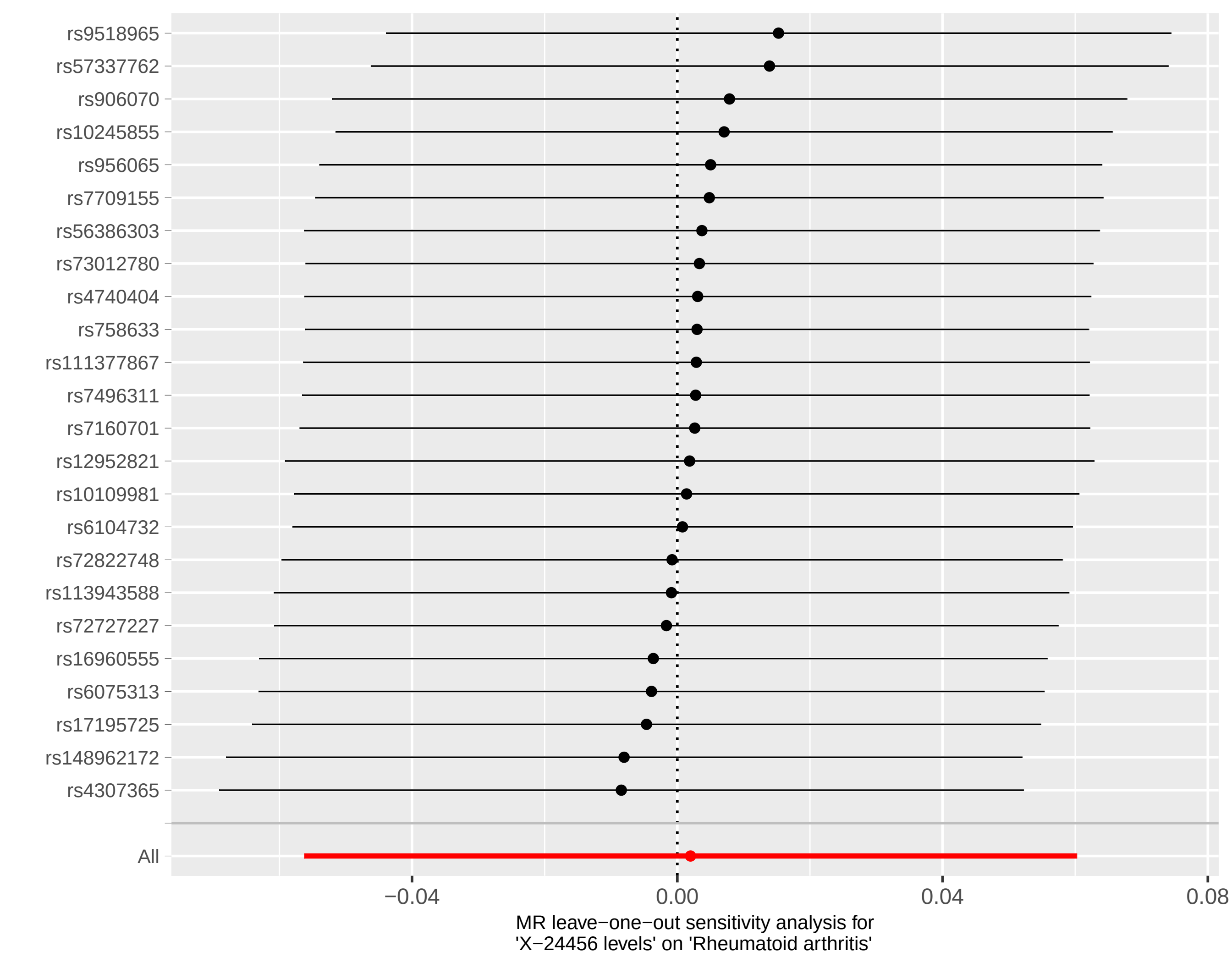

rs9518965
rs57337762
rs906070
rs10245855
rs956065
rs7709155
rs56386303
rs73012780
rs4740404
rs758633
rs111377867
rs7496311
rs7160701
rs12952821
rs10109981
rs6104732
rs72822748
rs113943588
rs72727227
rs16960555
rs6075313
rs17195725
rs148962172
rs4307365
All
−0.04
0.00
0.04
0.08
MR leave−one−out sensitivity analysis for
'X−24456 levels' on 'Rheumatoid arthritis'

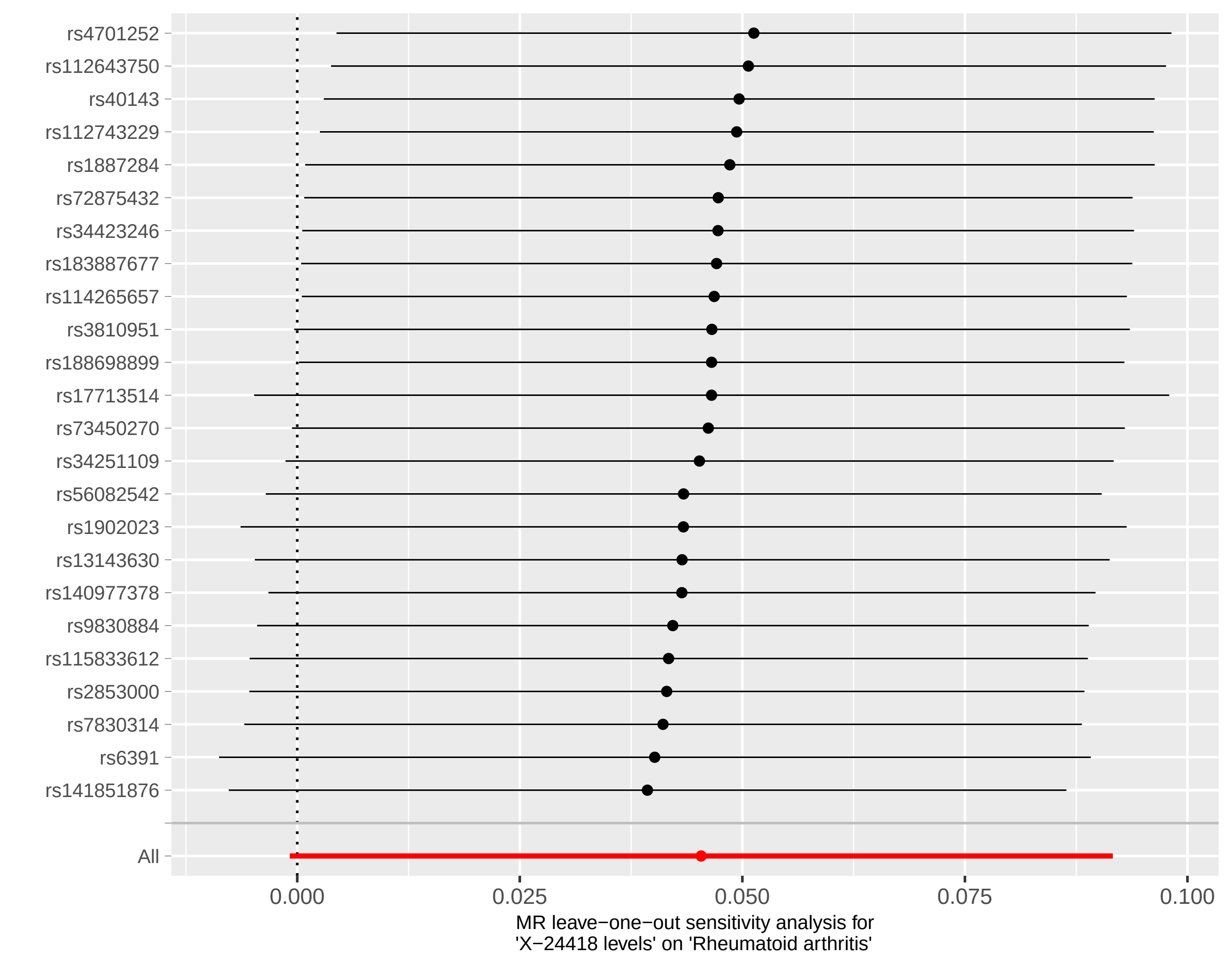

rs4701252
rs112643750
rs40143
rs112743229
rs1887284
rs72875432
rs34423246
rs183887677
rs114265657
rs3810951
rs188698899
rs17713514
rs73450270
rs34251109
rs56082542
rs1902023
rs13143630
rs140977378
rs9830884
rs115833612
rs2853000
rs7830314
rs6391
rs141851876
All
0.000
0.025
0.050
0.075
0.100
MR leave−one−out sensitivity analysis for
'X−24418 levels' on 'Rheumatoid arthritis'

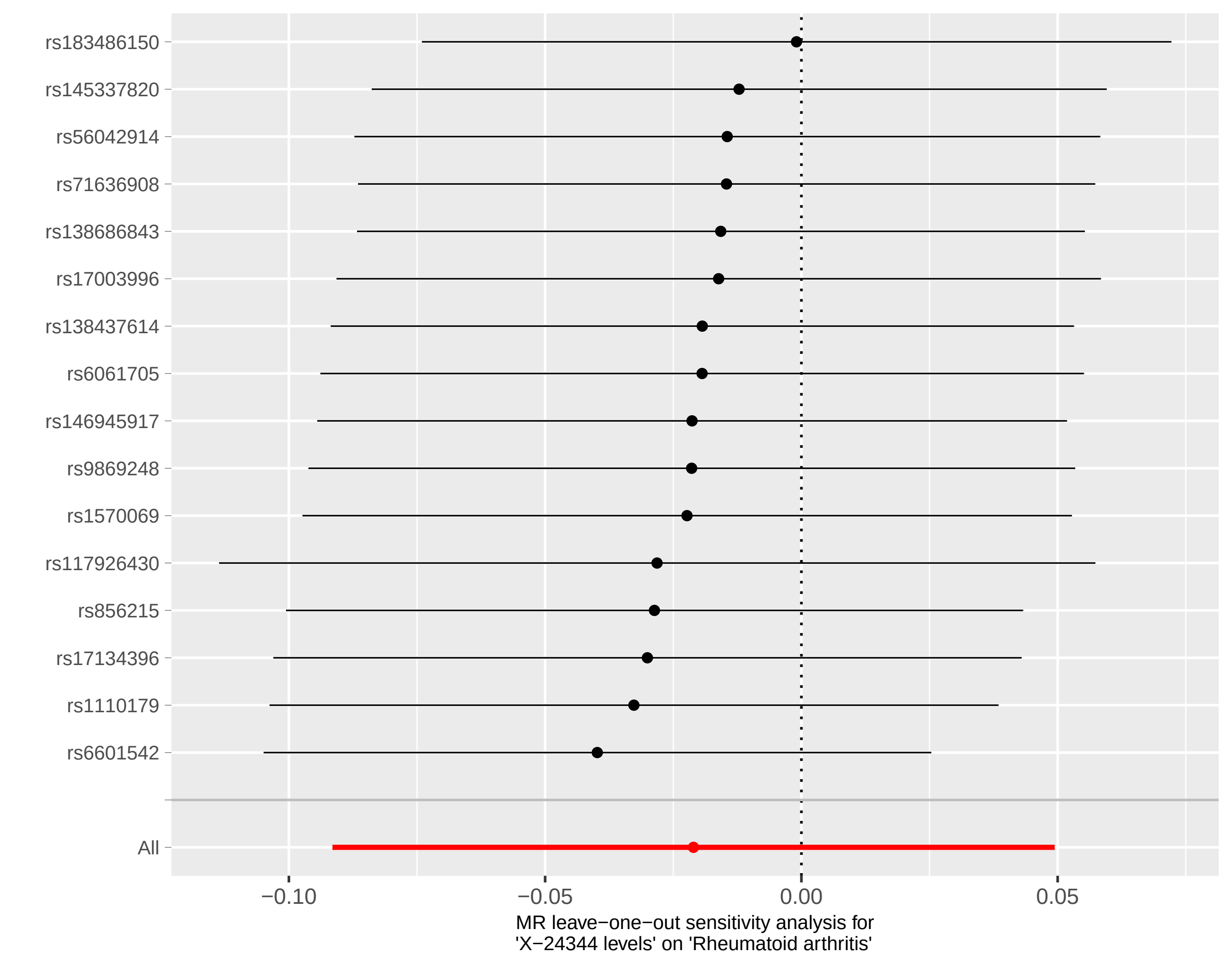
rs183486150
rs145337820
rs56042914
rs71636908
rs138686843
rs17003996
rs138437614
rs6061705
rs146945917
rs9869248
rs1570069
rs117926430
rs856215
rs17134396
rs1110179
rs6601542
All
−0.10
−0.05
0.00
0.05
MR leave−one−out sensitivity analysis for
'X−24344 levels' on 'Rheumatoid arthritis'

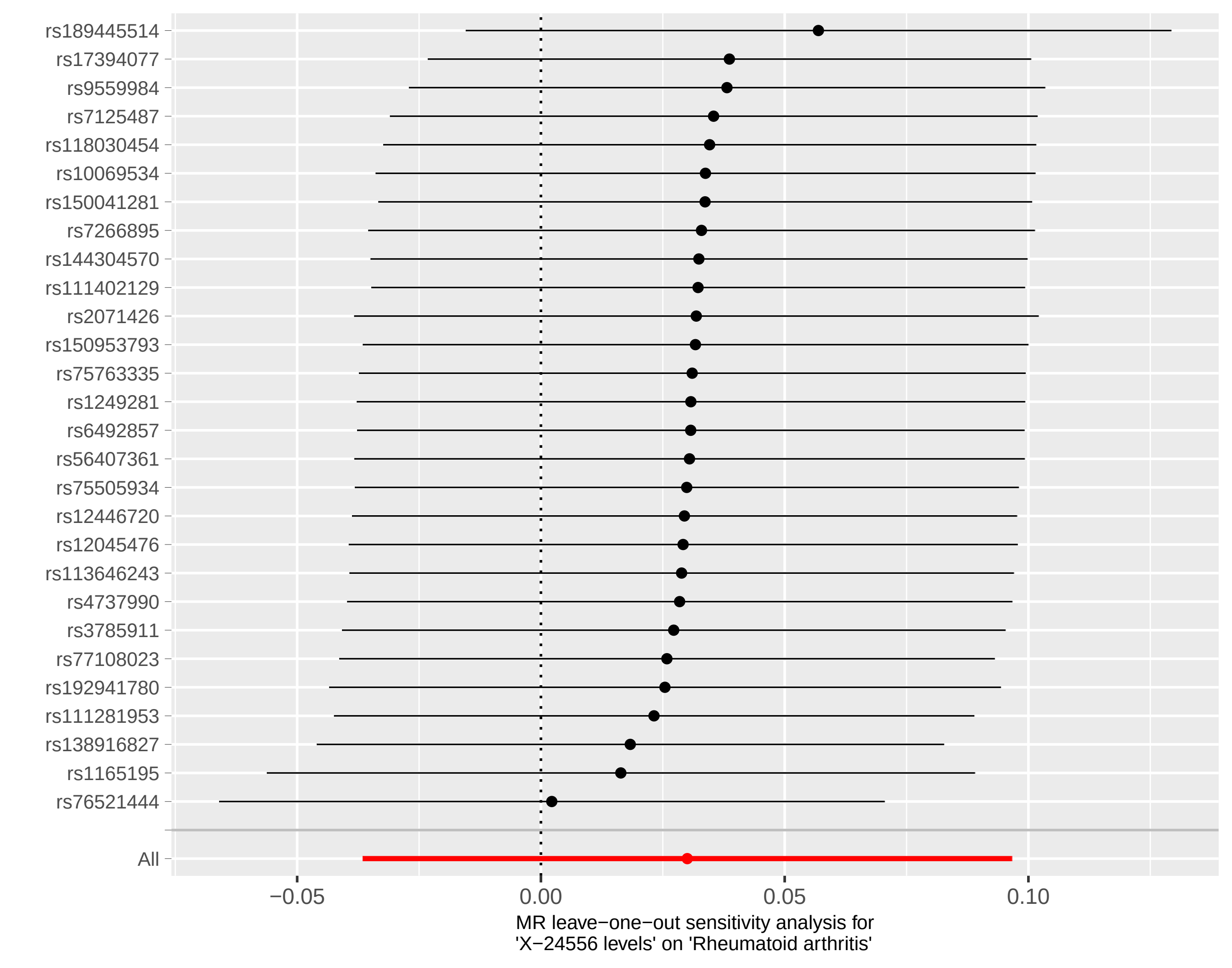
rs189445514
rs17394077
rs9559984
rs7125487
rs118030454
rs10069534
rs150041281
rs7266895
rs144304570
rs111402129
rs2071426
rs150953793
rs75763335
rs1249281
rs6492857
rs56407361
rs75505934
rs12446720
rs12045476
rs113646243
rs4737990
rs3785911
rs77108023
rs192941780
rs111281953
rs138916827
rs1165195
rs76521444
All
−0.05
0.00
0.05
0.10
MR leave−one−out sensitivity analysis for
'X−24556 levels' on 'Rheumatoid arthritis'

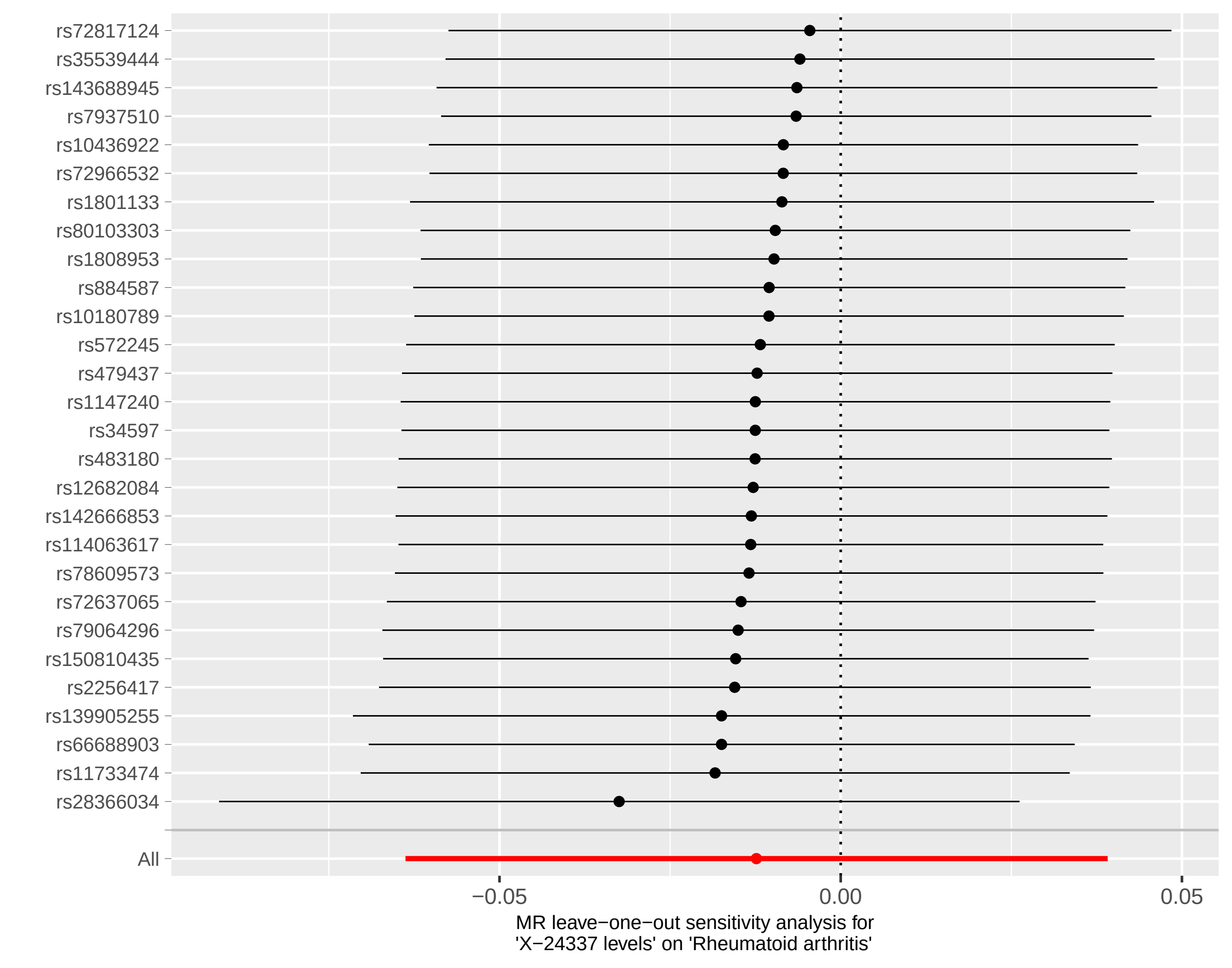

rs72817124
rs35539444
rs143688945
rs7937510
rs10436922
rs72966532
rs1801133
rs80103303
rs1808953
rs884587
rs10180789
rs572245
rs479437
rs1147240
rs34597
rs483180
rs12682084
rs142666853
rs114063617
rs78609573
rs72637065
rs79064296
rs150810435
rs2256417
rs139905255
rs66688903
rs11733474
rs28366034
All
−0.05
0.00
0.05
MR leave−one−out sensitivity analysis for
'X−24337 levels' on 'Rheumatoid arthritis'

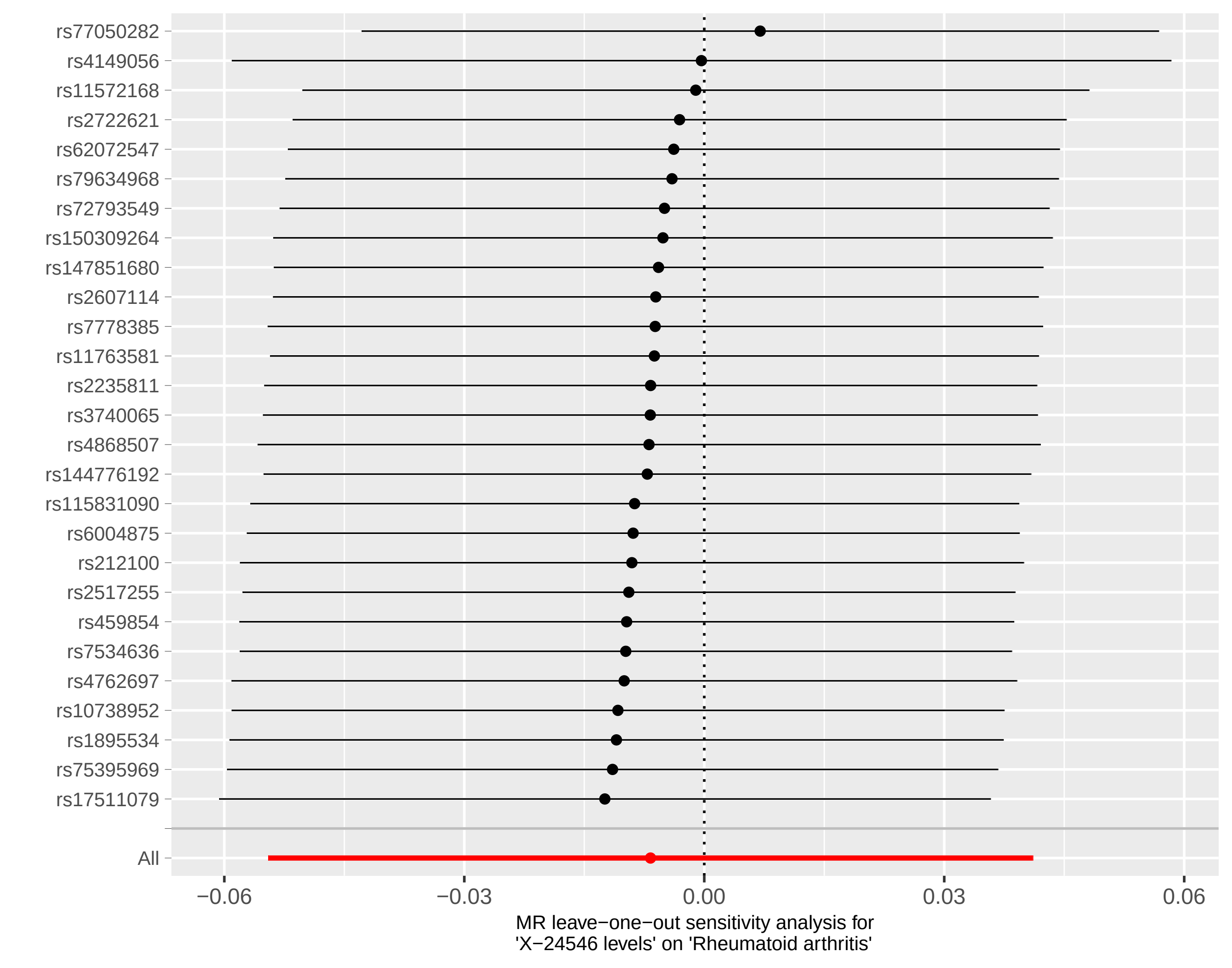
rs77050282
rs4149056
rs11572168
rs2722621
rs62072547
rs79634968
rs72793549
rs150309264
rs147851680
rs2607114
rs7778385
rs11763581
rs2235811
rs3740065
rs4868507
rs144776192
rs115831090
rs6004875
rs212100
rs2517255
rs459854
rs7534636
rs4762697
rs10738952
rs1895534
rs75395969
rs17511079
All
−0.06
−0.03
0.00
0.03
0.06
MR leave−one−out sensitivity analysis for
'X−24546 levels' on 'Rheumatoid arthritis'

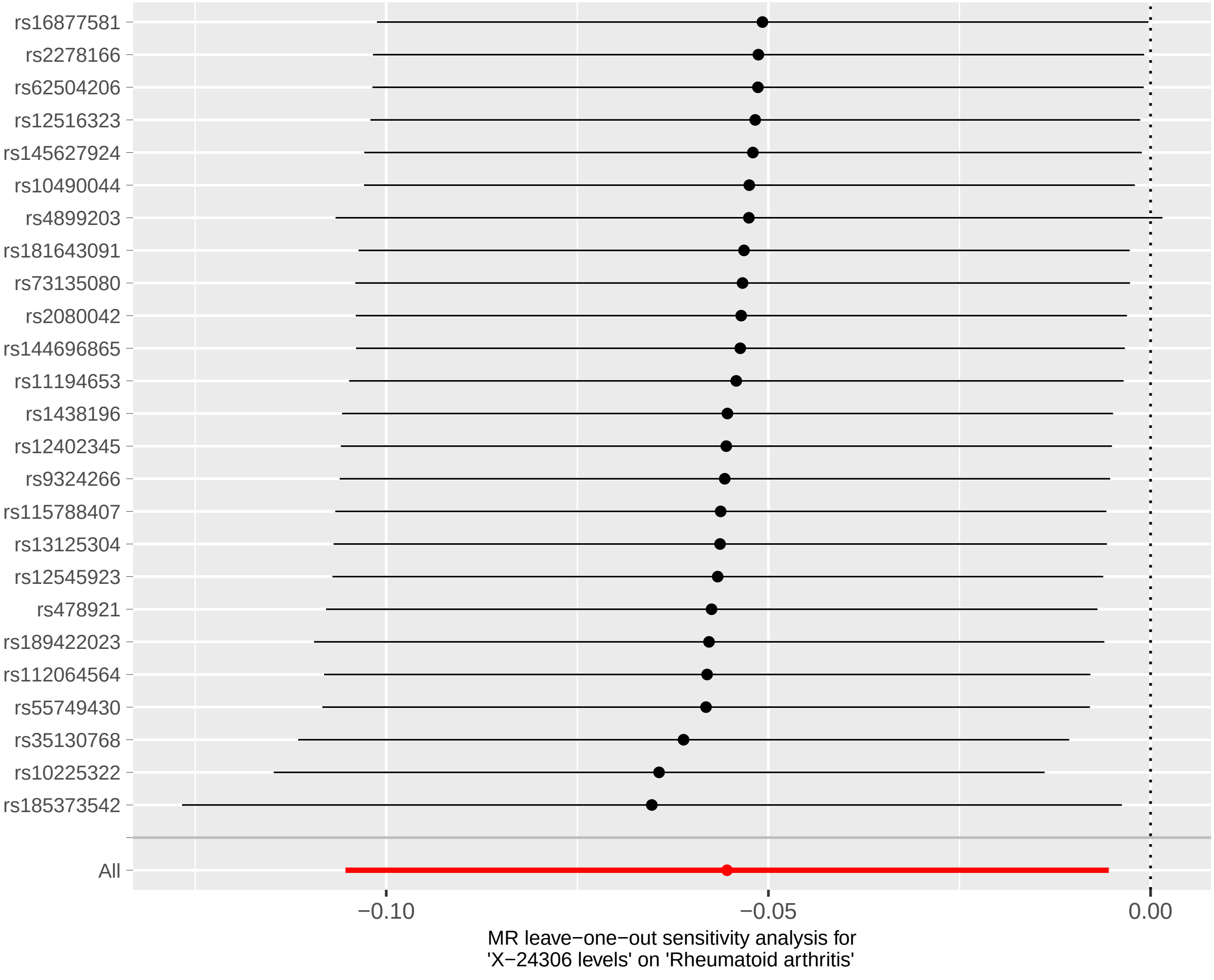

rs16877581
rs2278166
rs62504206
rs12516323
rs145627924
rs10490044
rs4899203
rs181643091
rs73135080
rs2080042
rs144696865
rs11194653
rs1438196
rs12402345
rs9324266
rs115788407
rs13125304
rs12545923
rs478921
rs189422023
rs112064564
rs55749430
rs35130768
rs10225322
rs185373542
All
−0.10
−0.05
0.00
MR leave−one−out sensitivity analysis for
'X−24306 levels' on 'Rheumatoid arthritis'

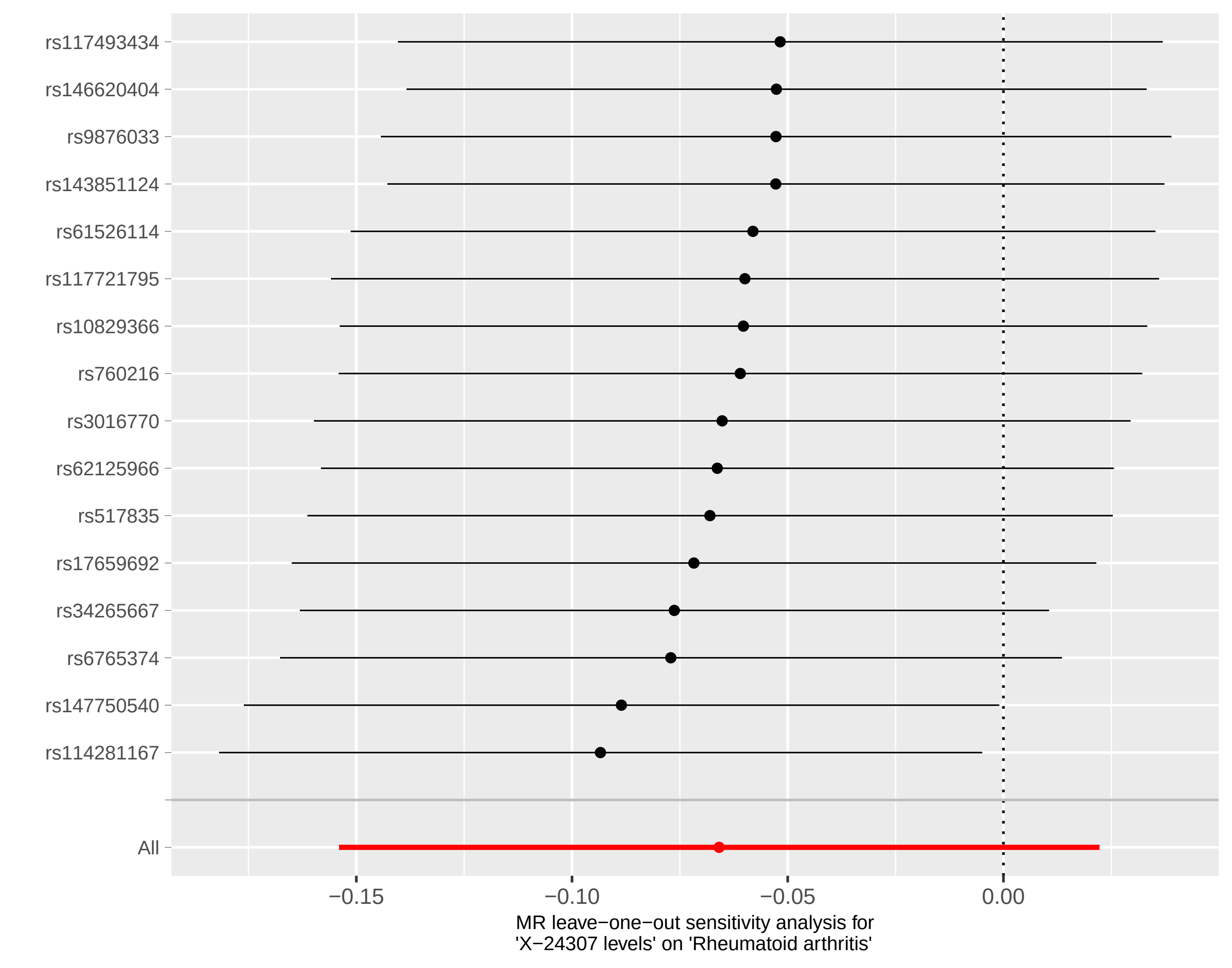
rs117493434
rs146620404
rs9876033
rs143851124
rs61526114
rs117721795
rs10829366
rs760216
rs3016770
rs62125966
rs517835
rs17659692
rs34265667
rs6765374
rs147750540
rs114281167
All
−0.15
−0.10
−0.05
0.00
MR leave−one−out sensitivity analysis for
'X−24307 levels' on 'Rheumatoid arthritis'

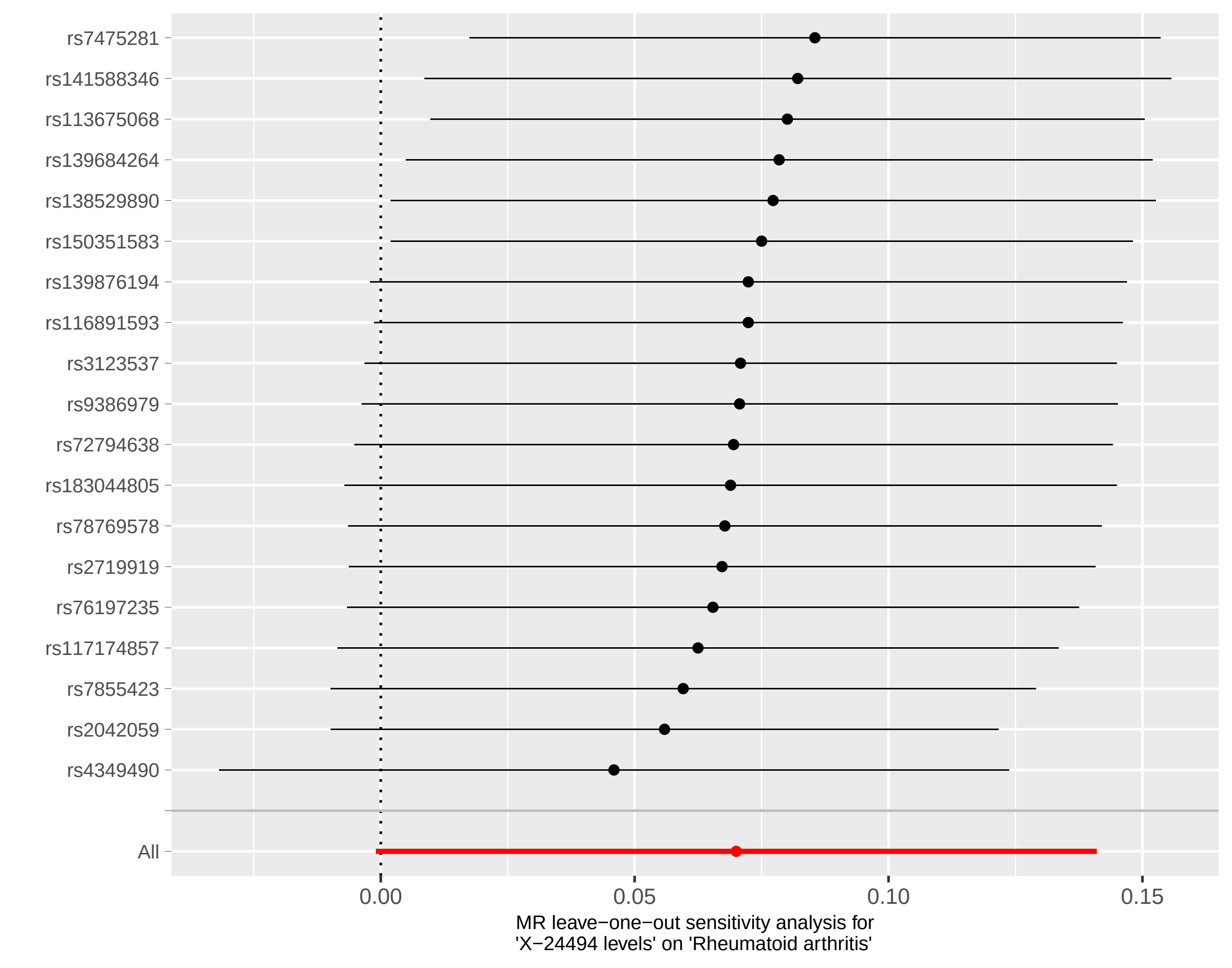

rs7475281
rs141588346
rs113675068
rs139684264
rs138529890
rs150351583
rs139876194
rs116891593
rs3123537
rs9386979
rs72794638
rs183044805
rs78769578
rs2719919
rs76197235
rs117174857
rs7855423
rs2042059
rs4349490
All
0.00
0.05
0.10
0.15
MR leave−one−out sensitivity analysis for
'X−24494 levels' on 'Rheumatoid arthritis'

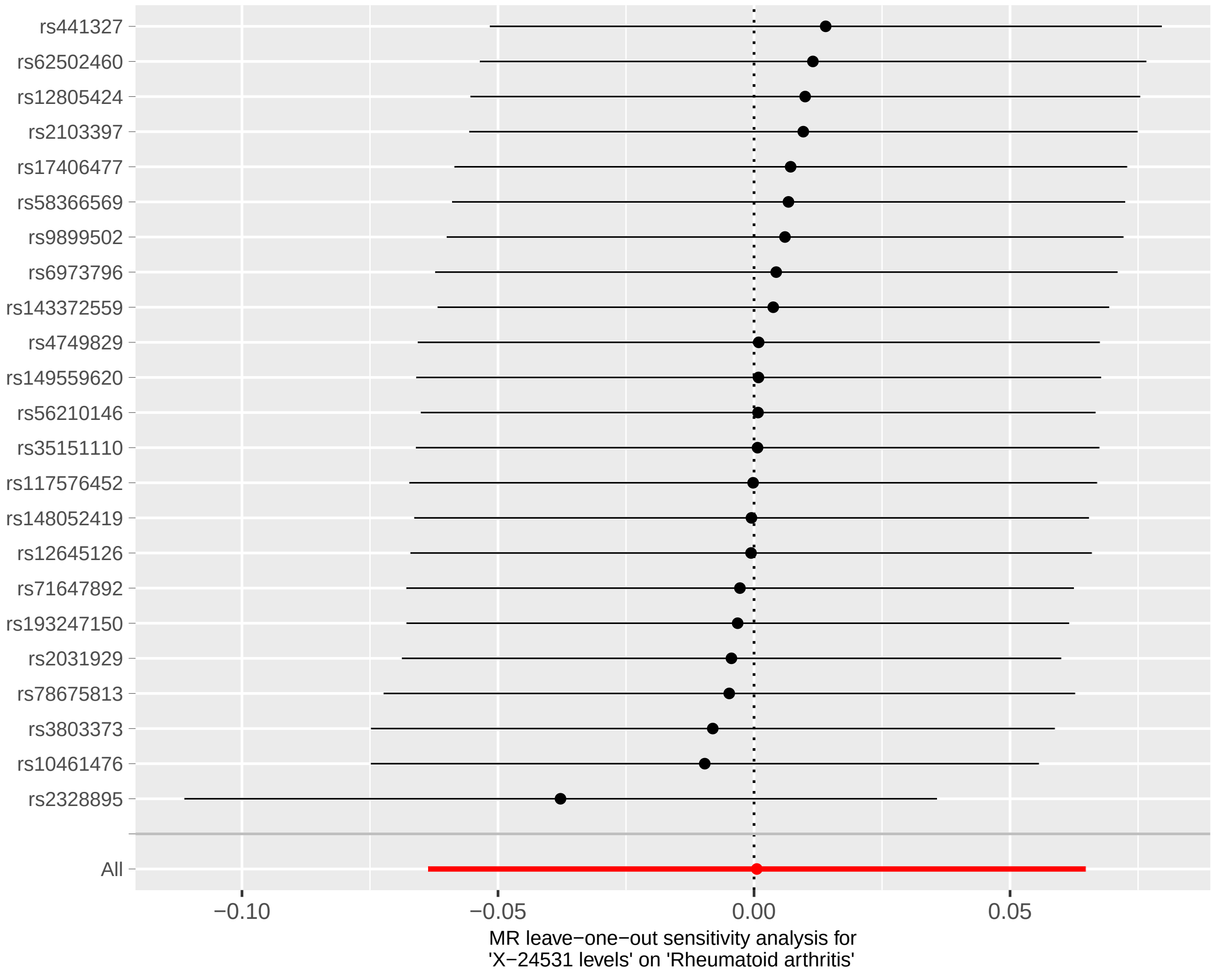

rs441327
rs62502460
rs12805424
rs2103397
rs17406477
rs58366569
rs9899502
rs6973796
rs143372559
rs4749829
rs149559620
rs56210146
rs35151110
rs117576452
rs148052419
rs12645126
rs71647892
rs193247150
rs2031929
rs78675813
rs3803373
rs10461476
rs2328895
All
−0.10
−0.05
0.00
0.05
MR leave−one−out sensitivity analysis for
'X−24531 levels' on 'Rheumatoid arthritis'

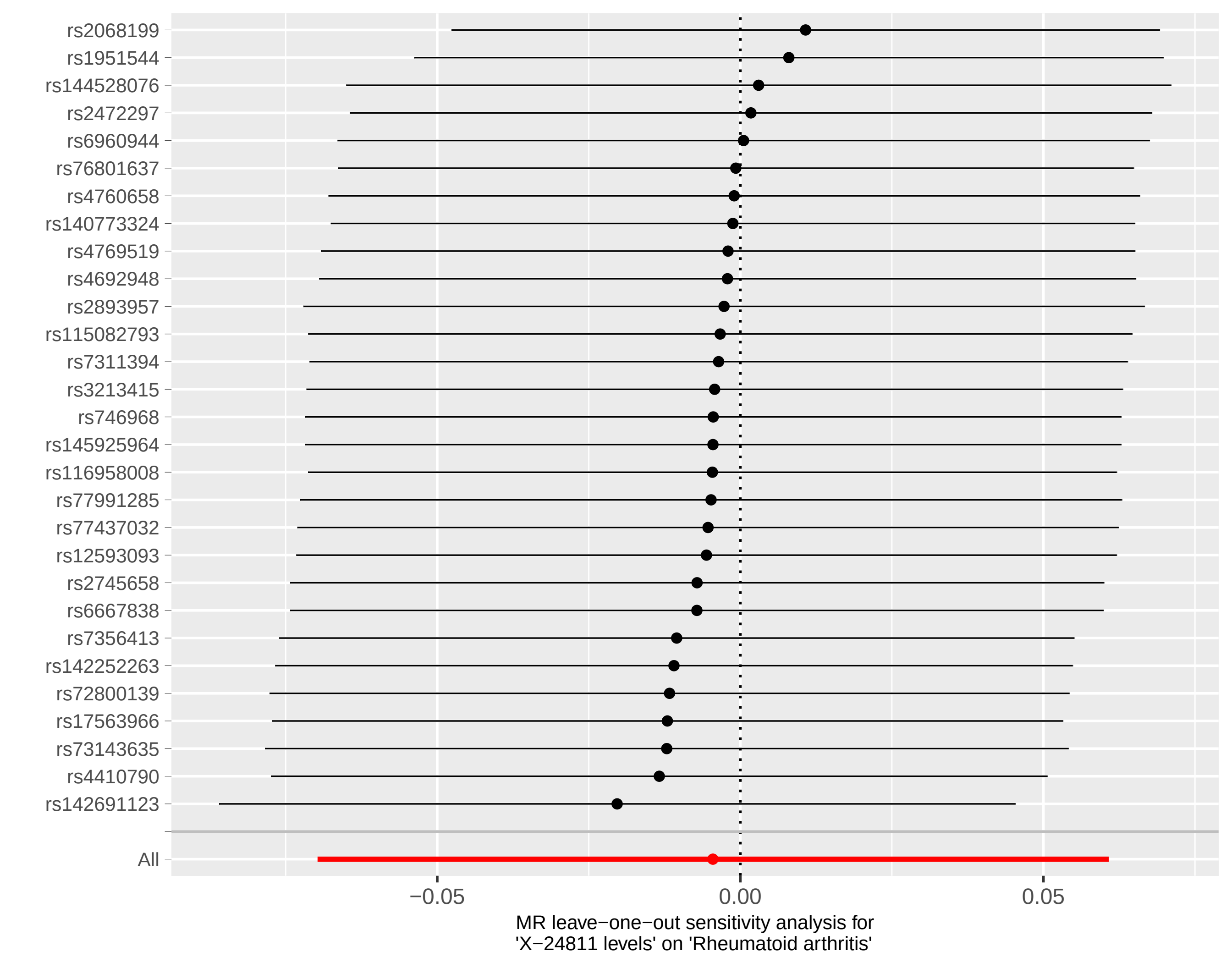
rs2068199
rs1951544
rs144528076
rs2472297
rs6960944
rs76801637
rs4760658
rs140773324
rs4769519
rs4692948
rs2893957
rs115082793
rs7311394
rs3213415
rs746968
rs145925964
rs116958008
rs77991285
rs77437032
rs12593093
rs2745658
rs6667838
rs7356413
rs142252263
rs72800139
rs17563966
rs73143635
rs4410790
rs142691123
All
−0.05
0.00
0.05
MR leave−one−out sensitivity analysis for
'X−24811 levels' on 'Rheumatoid arthritis'

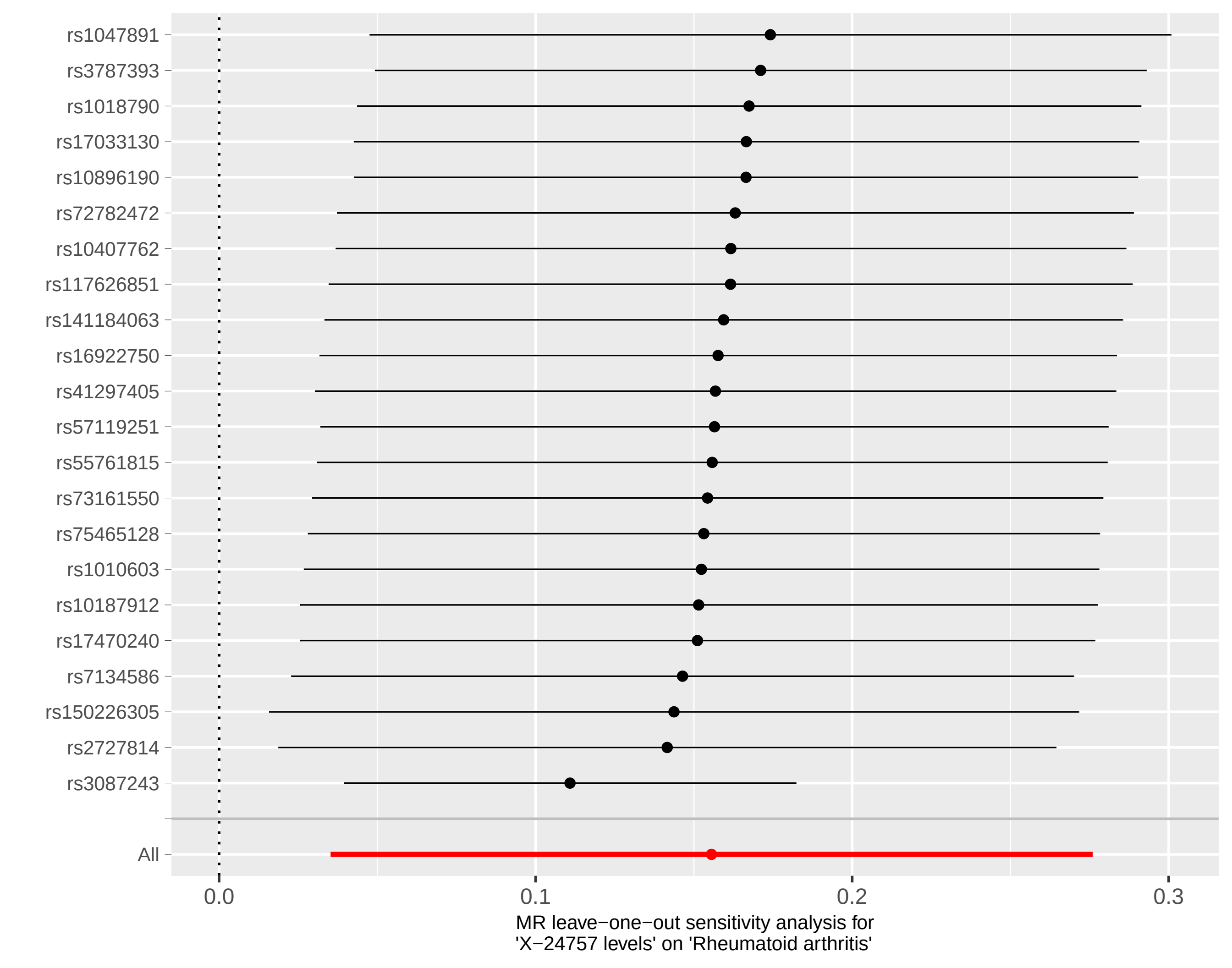
rs1047891
rs3787393
rs1018790
rs17033130
rs10896190
rs72782472
rs10407762
rs117626851
rs141184063
rs16922750
rs41297405
rs57119251
rs55761815
rs73161550
rs75465128
rs1010603
rs10187912
rs17470240
rs7134586
rs150226305
rs2727814
rs3087243
All
0.0
0.1
0.2
0.3
MR leave−one−out sensitivity analysis for
'X−24757 levels' on 'Rheumatoid arthritis'

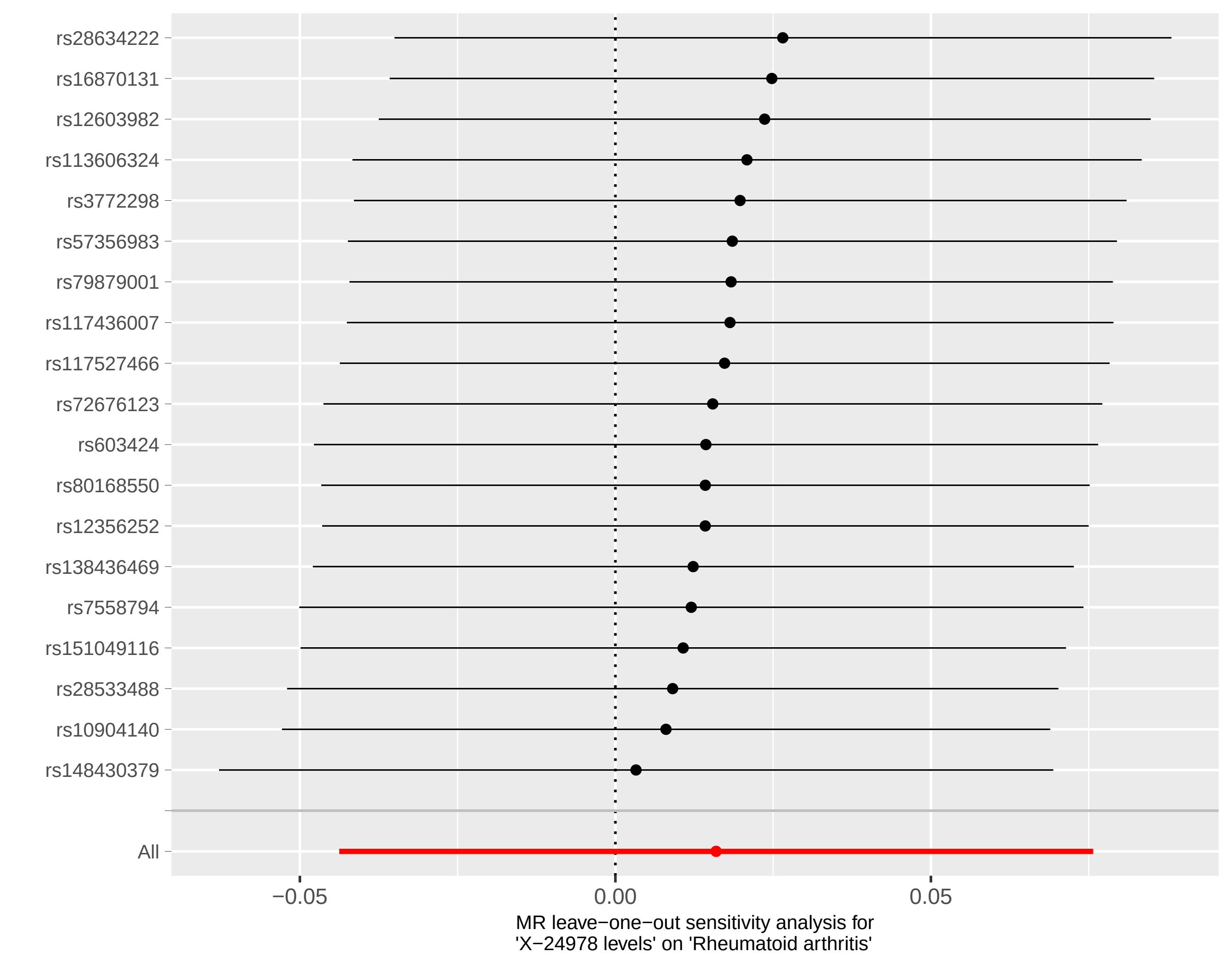
rs28634222
rs16870131
rs12603982
rs113606324
rs3772298
rs57356983
rs79879001
rs117436007
rs117527466
rs72676123
rs603424
rs80168550
rs12356252
rs138436469
rs7558794
rs151049116
rs28533488
rs10904140
rs148430379
All
−0.05
0.00
0.05
MR leave−one−out sensitivity analysis for
'X−24978 levels' on 'Rheumatoid arthritis'

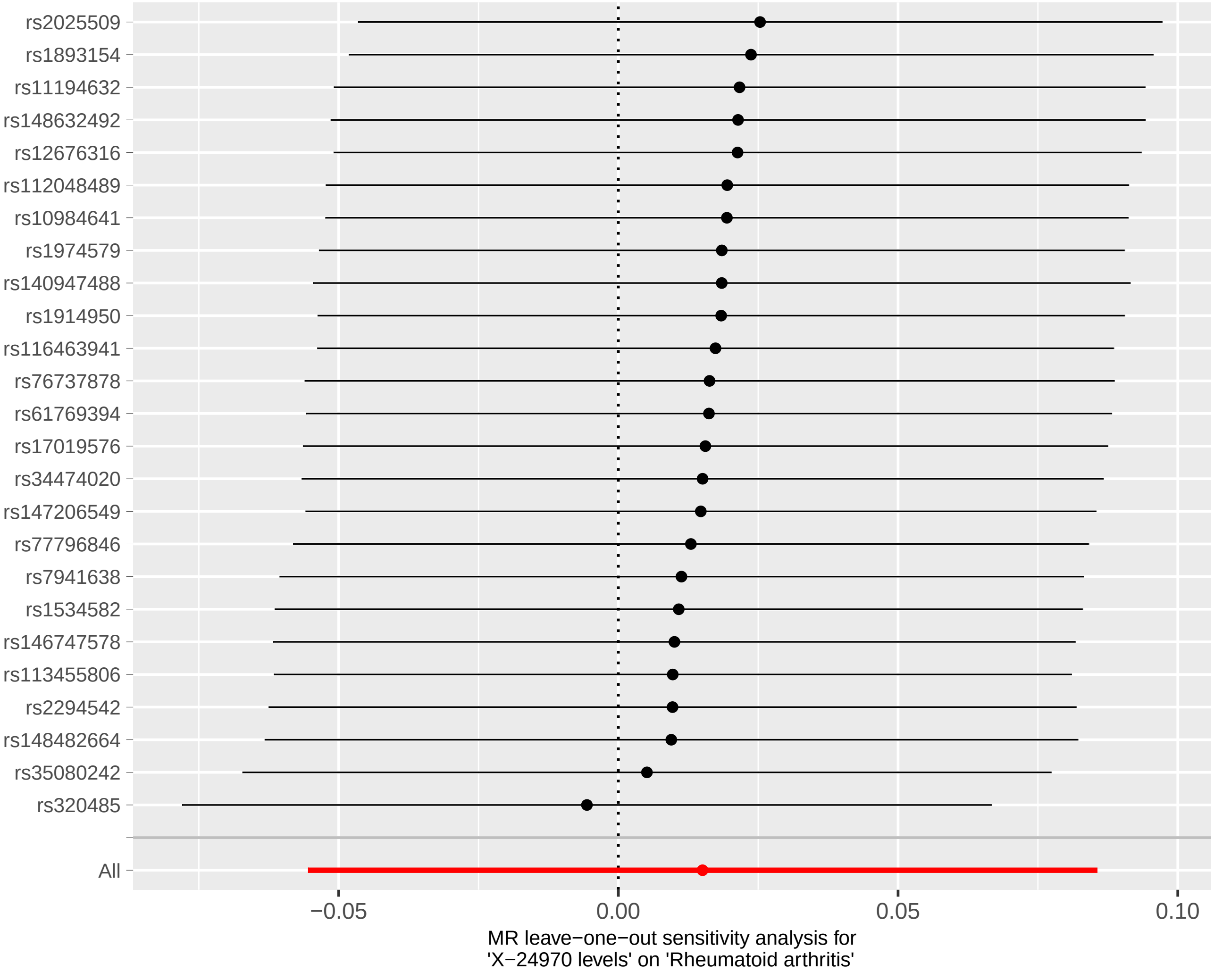
rs2025509
rs1893154
rs11194632
rs148632492
rs12676316
rs112048489
rs10984641
rs1974579
rs140947488
rs1914950
rs116463941
rs76737878
rs61769394
rs17019576
rs34474020
rs147206549
rs77796846
rs7941638
rs1534582
rs146747578
rs113455806
rs2294542
rs148482664
rs35080242
rs320485
All
−0.05
0.00
0.05
0.10
MR leave−one−out sensitivity analysis for
'X−24970 levels' on 'Rheumatoid arthritis'

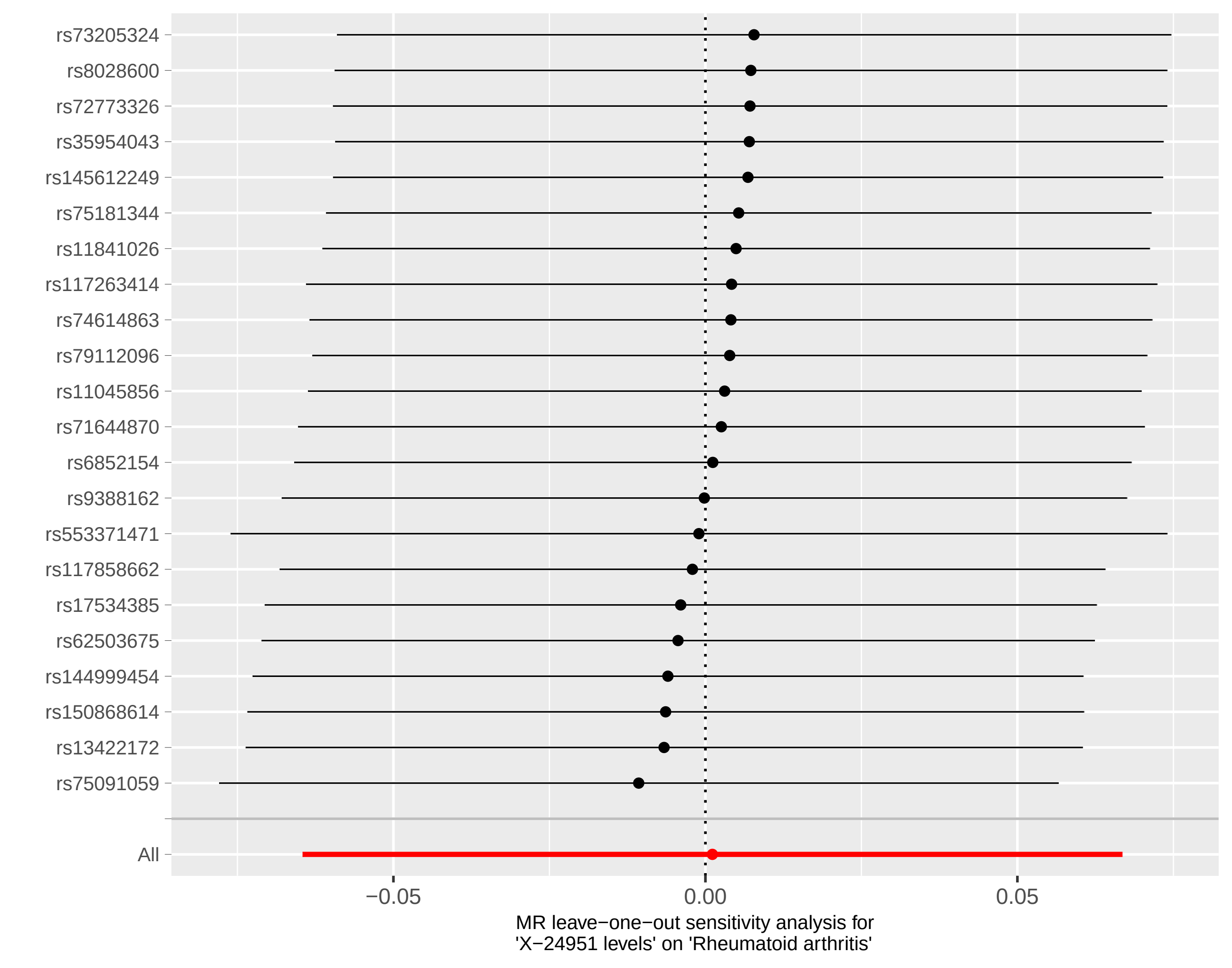
rs73205324
rs8028600
rs72773326
rs35954043
rs145612249
rs75181344
rs11841026
rs117263414
rs74614863
rs79112096
rs11045856
rs71644870
rs6852154
rs9388162
rs553371471
rs117858662
rs17534385
rs62503675
rs144999454
rs150868614
rs13422172
rs75091059
All
−0.05
0.00
0.05
MR leave−one−out sensitivity analysis for
'X−24951 levels' on 'Rheumatoid arthritis'

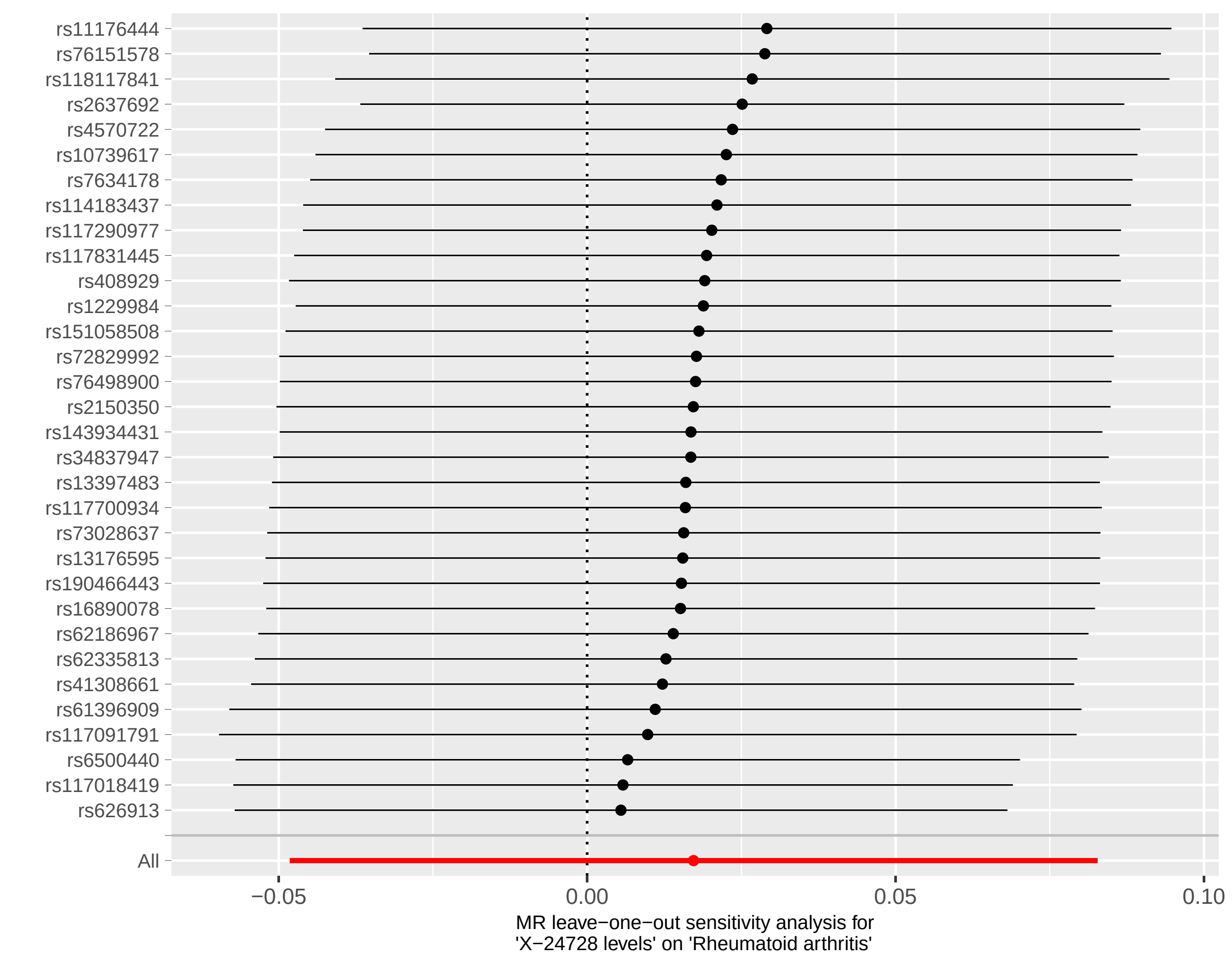
rs11176444
rs76151578
rs118117841
rs2637692
rs4570722
rs10739617
rs7634178
rs114183437
rs117290977
rs117831445
rs408929
rs1229984
rs151058508
rs72829992
rs76498900
rs2150350
rs143934431
rs34837947
rs13397483
rs117700934
rs73028637
rs13176595
rs190466443
rs16890078
rs62186967
rs62335813
rs41308661
rs61396909
rs117091791
rs6500440
rs117018419
rs626913
All
−0.05
0.00
0.05
0.10
MR leave−one−out sensitivity analysis for
'X−24728 levels' on 'Rheumatoid arthritis'

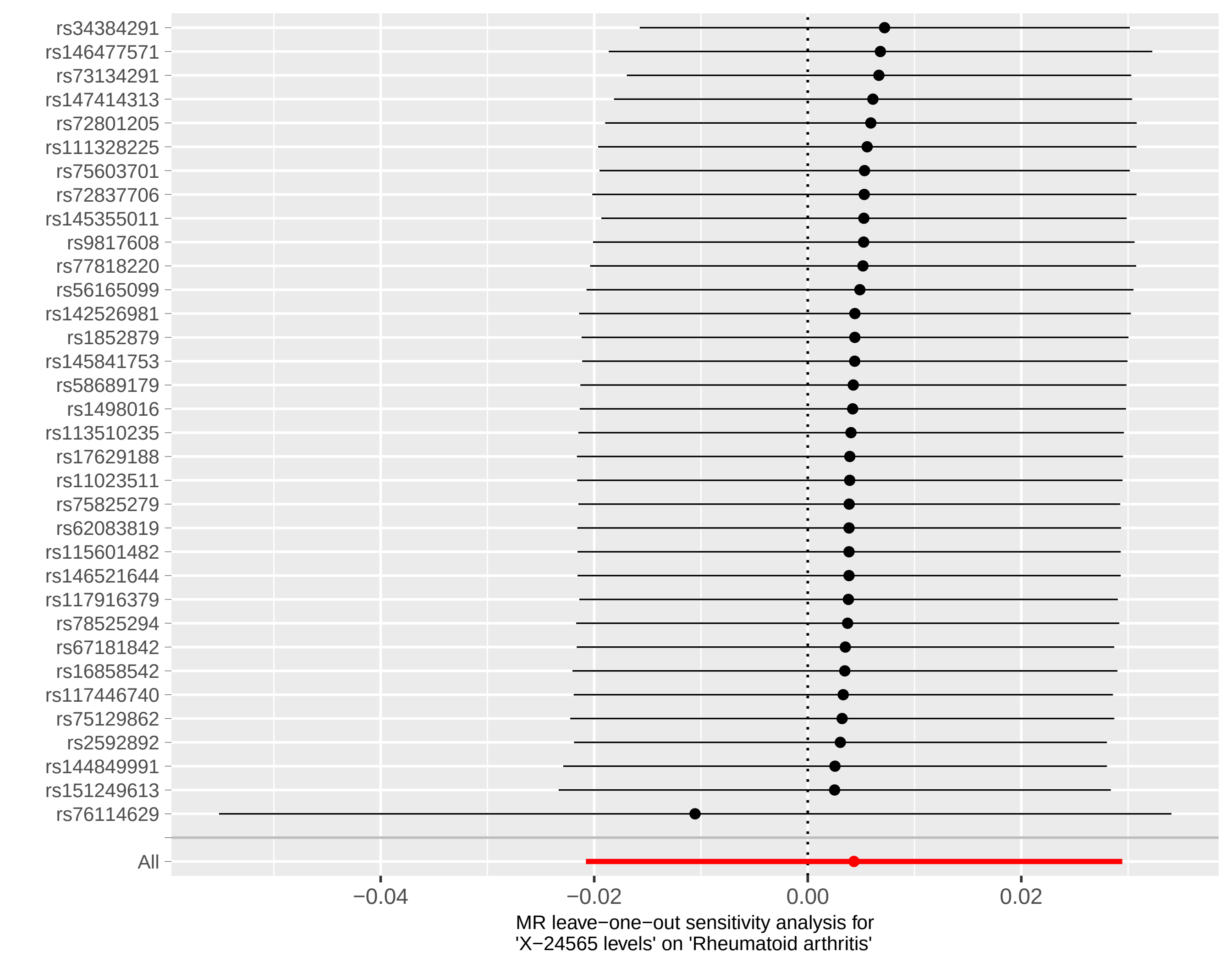
rs34384291
rs146477571
rs73134291
rs147414313
rs72801205
rs111328225
rs75603701
rs72837706
rs145355011
rs9817608
rs77818220
rs56165099
rs142526981
rs1852879
rs145841753
rs58689179
rs1498016
rs113510235
rs17629188
rs11023511
rs75825279
rs62083819
rs115601482
rs146521644
rs117916379
rs78525294
rs67181842
rs16858542
rs117446740
rs75129862
rs2592892
rs144849991
rs151249613
rs76114629
All
−0.04
−0.02
0.00
0.02
MR leave−one−out sensitivity analysis for
'X−24565 levels' on 'Rheumatoid arthritis'

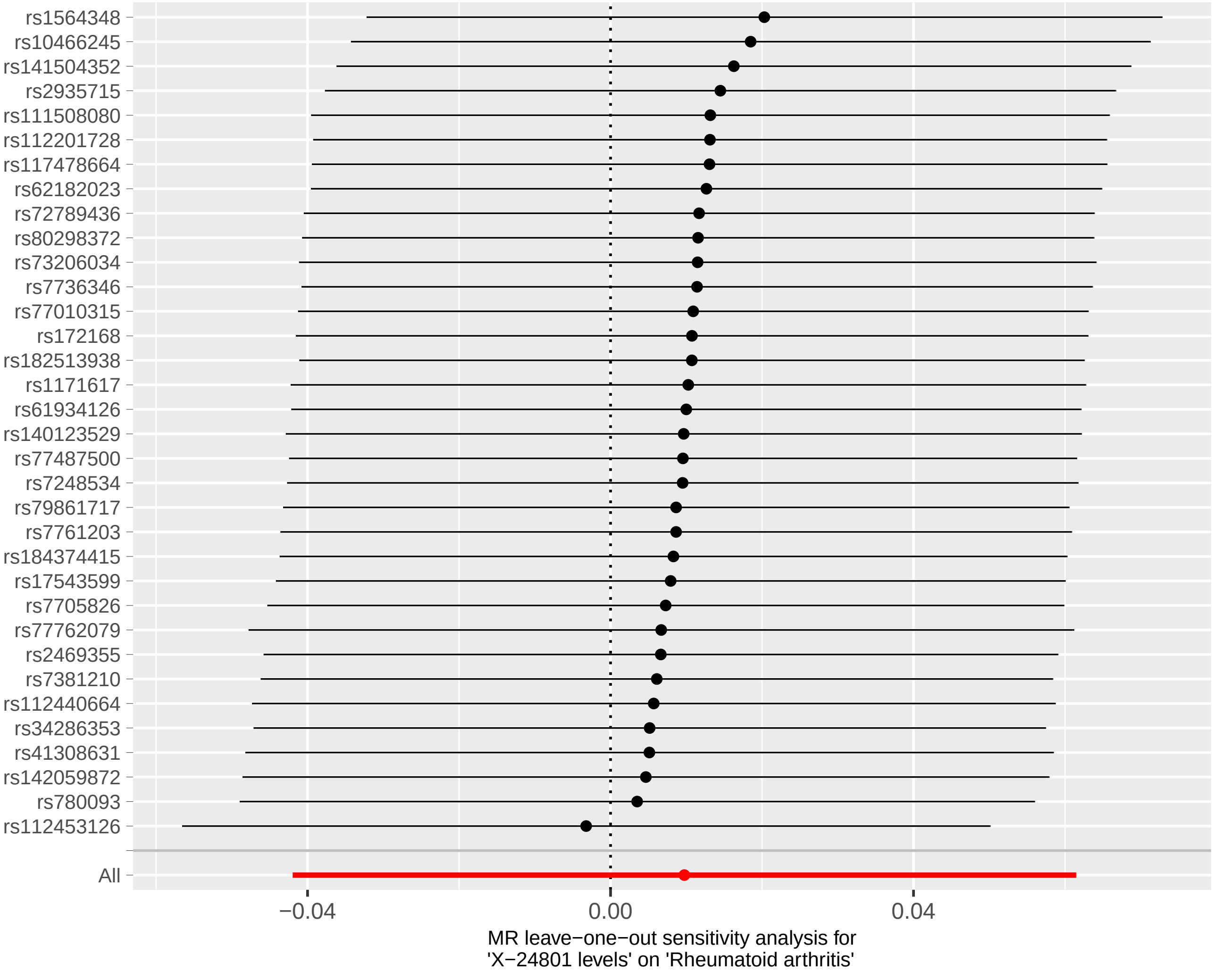
rs1564348
rs10466245
rs141504352
rs2935715
rs111508080
rs112201728
rs117478664
rs62182023
rs72789436
rs80298372
rs73206034
rs7736346
rs77010315
rs172168
rs182513938
rs1171617
rs61934126
rs140123529
rs77487500
rs7248534
rs79861717
rs7761203
rs184374415
rs17543599
rs7705826
rs77762079
rs2469355
rs7381210
rs112440664
rs34286353
rs41308631
rs142059872
rs780093
rs112453126
All
−0.04
0.00
0.04
MR leave−one−out sensitivity analysis for
'X−24801 levels' on 'Rheumatoid arthritis'

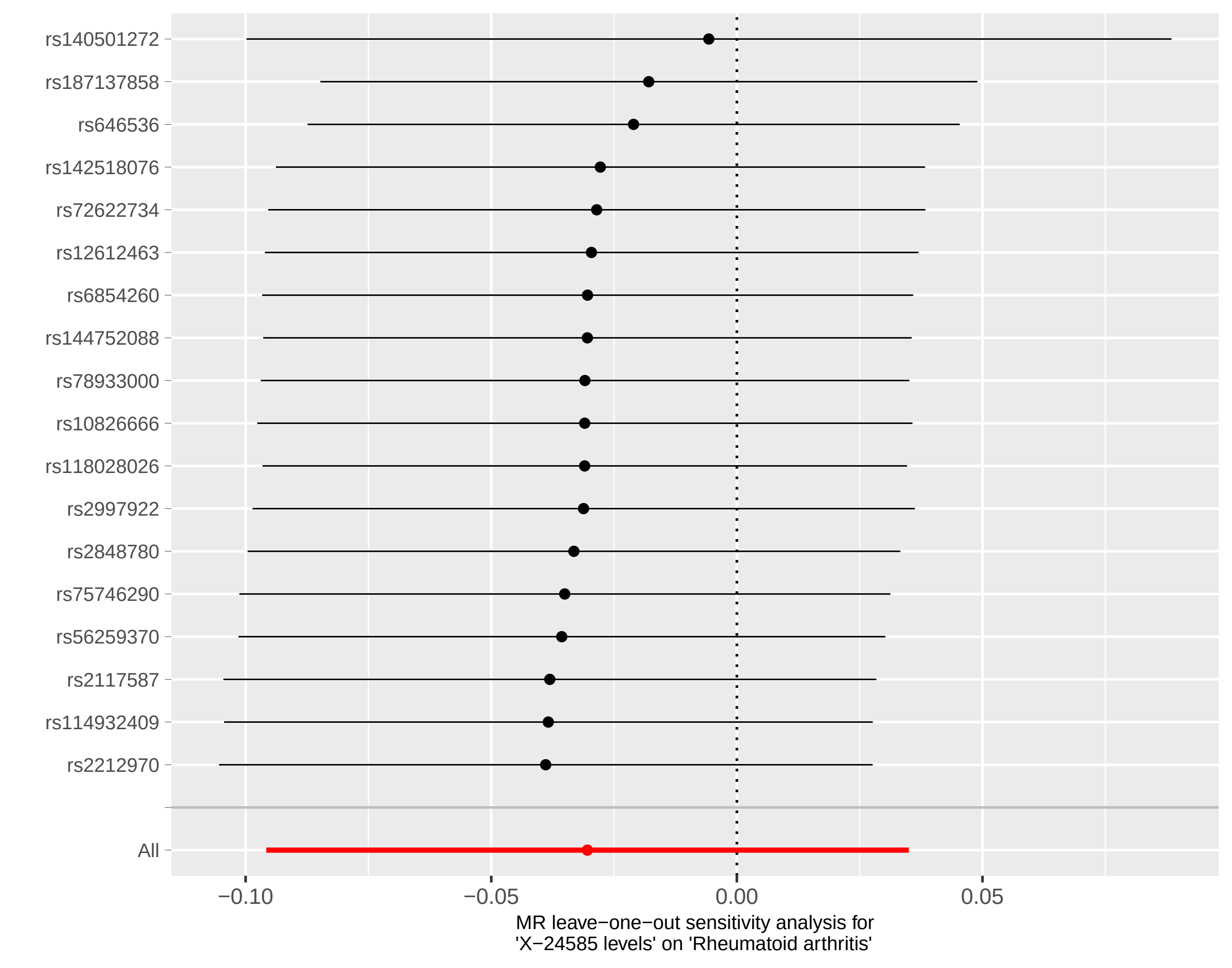

rs140501272
rs187137858
rs646536
rs142518076
rs72622734
rs12612463
rs6854260
rs144752088
rs78933000
rs10826666
rs118028026
rs2997922
rs2848780
rs75746290
rs56259370
rs2117587
rs114932409
rs2212970
All
−0.10
−0.05
0.00
0.05
MR leave−one−out sensitivity analysis for
'X−24585 levels' on 'Rheumatoid arthritis'

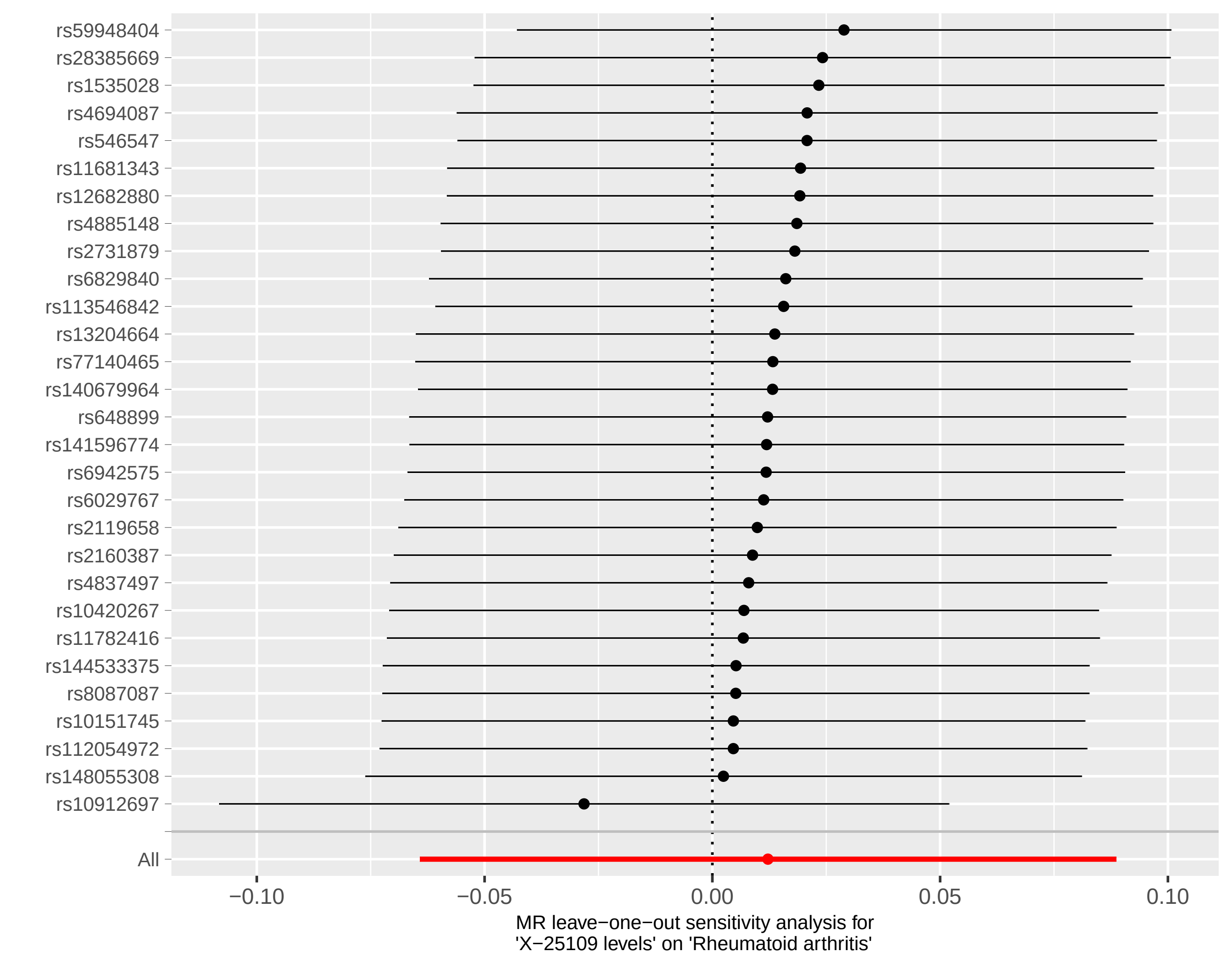
rs59948404
rs28385669
rs1535028
rs4694087
rs546547
rs11681343
rs12682880
rs4885148
rs2731879
rs6829840
rs113546842
rs13204664
rs77140465
rs140679964
rs648899
rs141596774
rs6942575
rs6029767
rs2119658
rs2160387
rs4837497
rs10420267
rs11782416
rs144533375
rs8087087
rs10151745
rs112054972
rs148055308
rs10912697
All
−0.10
−0.05
0.00
0.05
0.10
MR leave−one−out sensitivity analysis for
'X−25109 levels' on 'Rheumatoid arthritis'

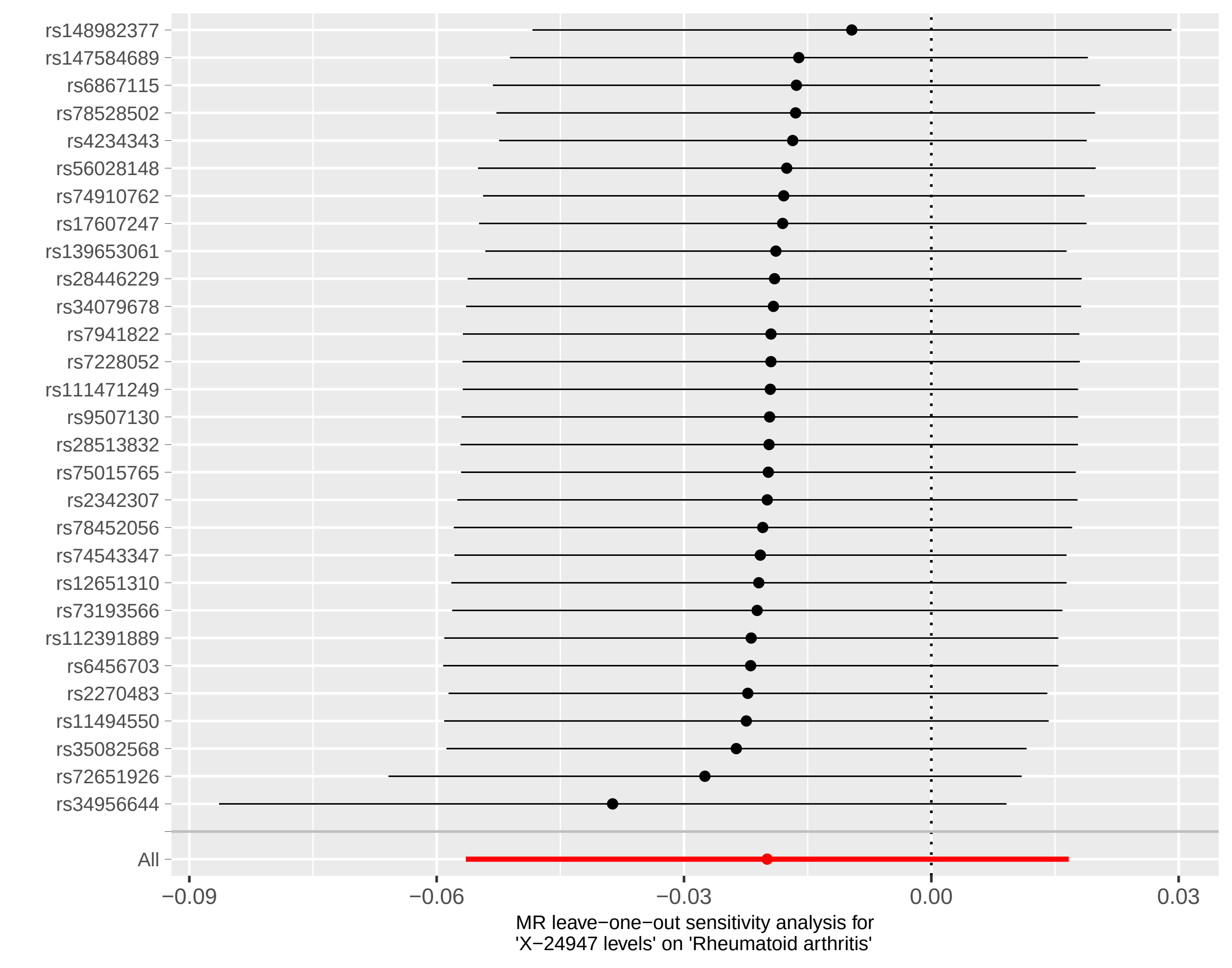

rs148982377
rs147584689
rs6867115
rs78528502
rs4234343
rs56028148
rs74910762
rs17607247
rs139653061
rs28446229
rs34079678
rs7941822
rs7228052
rs111471249
rs9507130
rs28513832
rs75015765
rs2342307
rs78452056
rs74543347
rs12651310
rs73193566
rs112391889
rs6456703
rs2270483
rs11494550
rs35082568
rs72651926
rs34956644
All
−0.09
−0.06
−0.03
0.00
0.03
MR leave−one−out sensitivity analysis for
'X−24947 levels' on 'Rheumatoid arthritis'

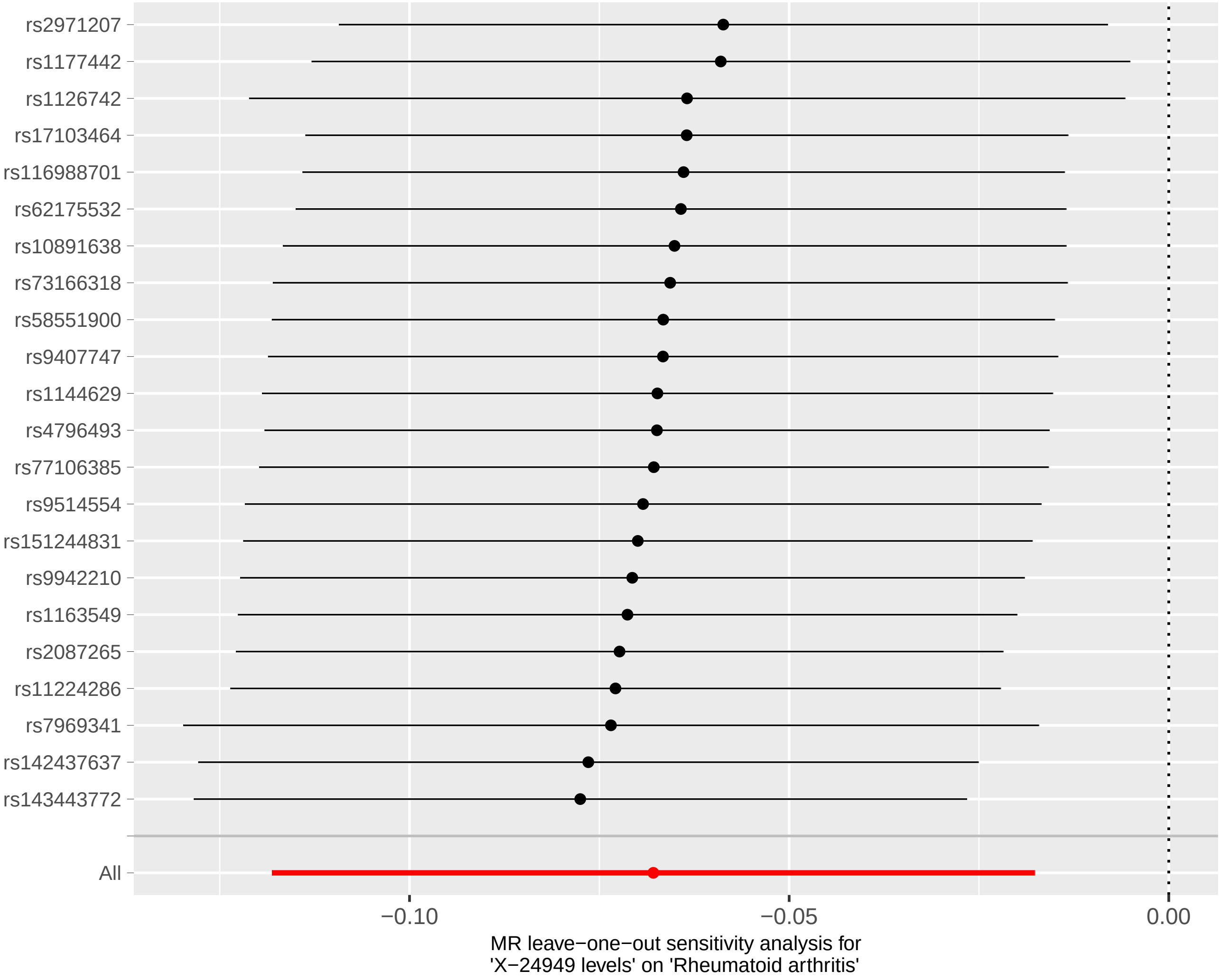

rs2971207
rs1177442
rs1126742
rs17103464
rs116988701
rs62175532
rs10891638
rs73166318
rs58551900
rs9407747
rs1144629
rs4796493
rs77106385
rs9514554
rs151244831
rs9942210
rs1163549
rs2087265
rs11224286
rs7969341
rs142437637
rs143443772
All
−0.10
−0.05
0.00
MR leave−one−out sensitivity analysis for
'X−24949 levels' on 'Rheumatoid arthritis'

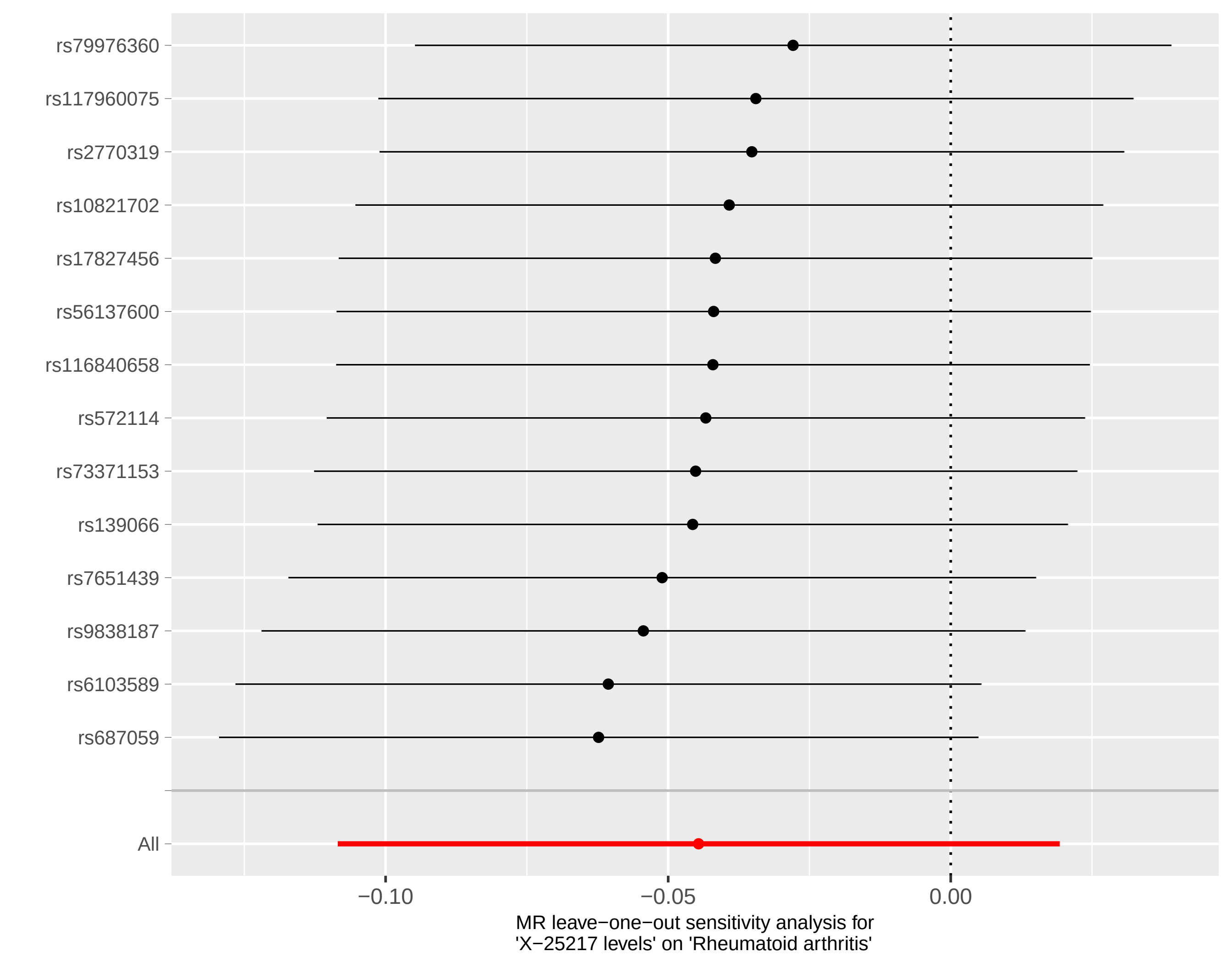

rs79976360
rs117960075
rs2770319
rs10821702
rs17827456
rs56137600
rs116840658
rs572114
rs73371153
rs139066
rs7651439
rs9838187
rs6103589
rs687059
All
−0.10
−0.05
0.00
MR leave−one−out sensitivity analysis for
'X−25217 levels' on 'Rheumatoid arthritis'

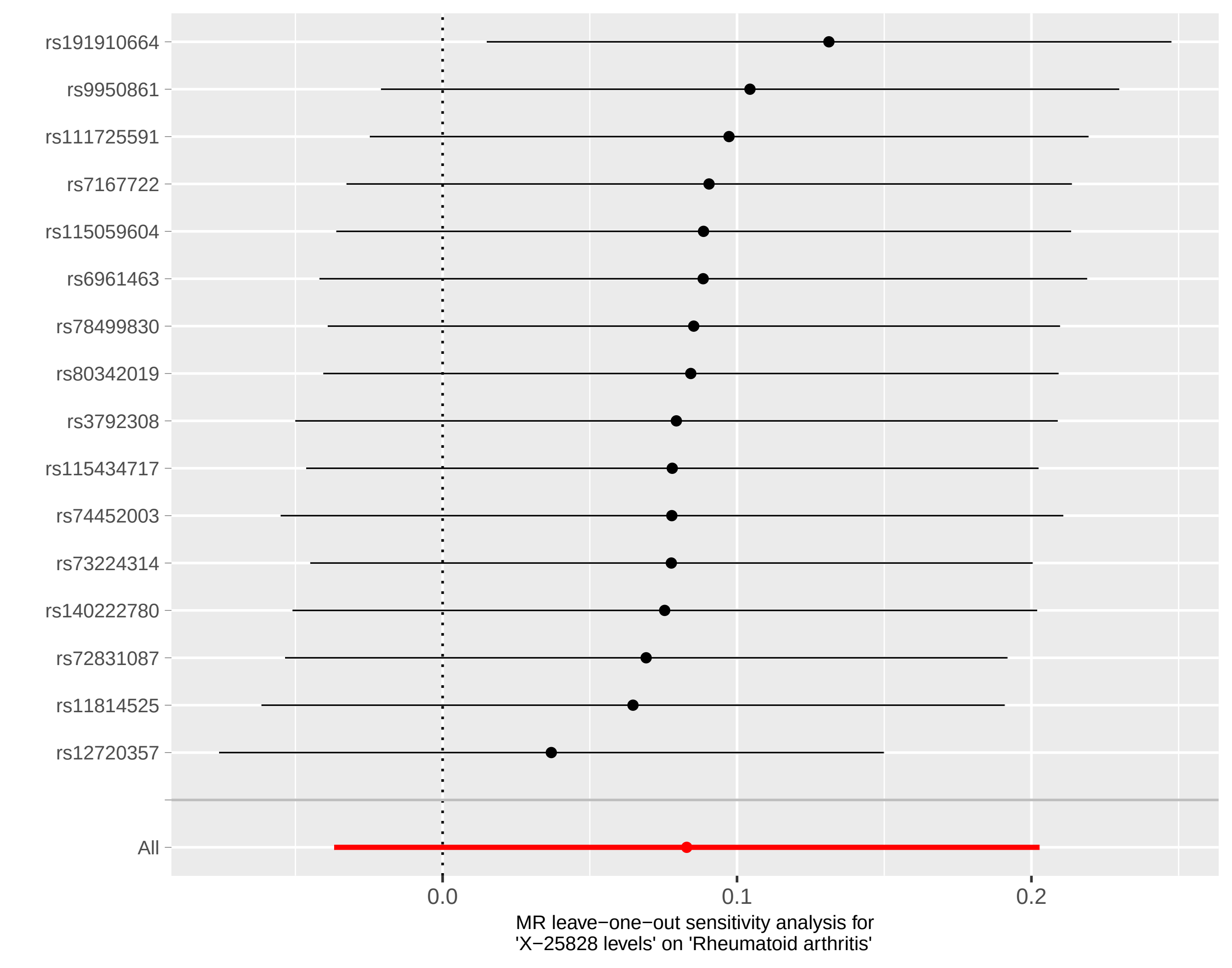
rs191910664
rs9950861
rs111725591
rs7167722
rs115059604
rs6961463
rs78499830
rs80342019
rs3792308
rs115434717
rs74452003
rs73224314
rs140222780
rs72831087
rs11814525
rs12720357
All
0.0
0.1
0.2
MR leave−one−out sensitivity analysis for
'X−25828 levels' on 'Rheumatoid arthritis'

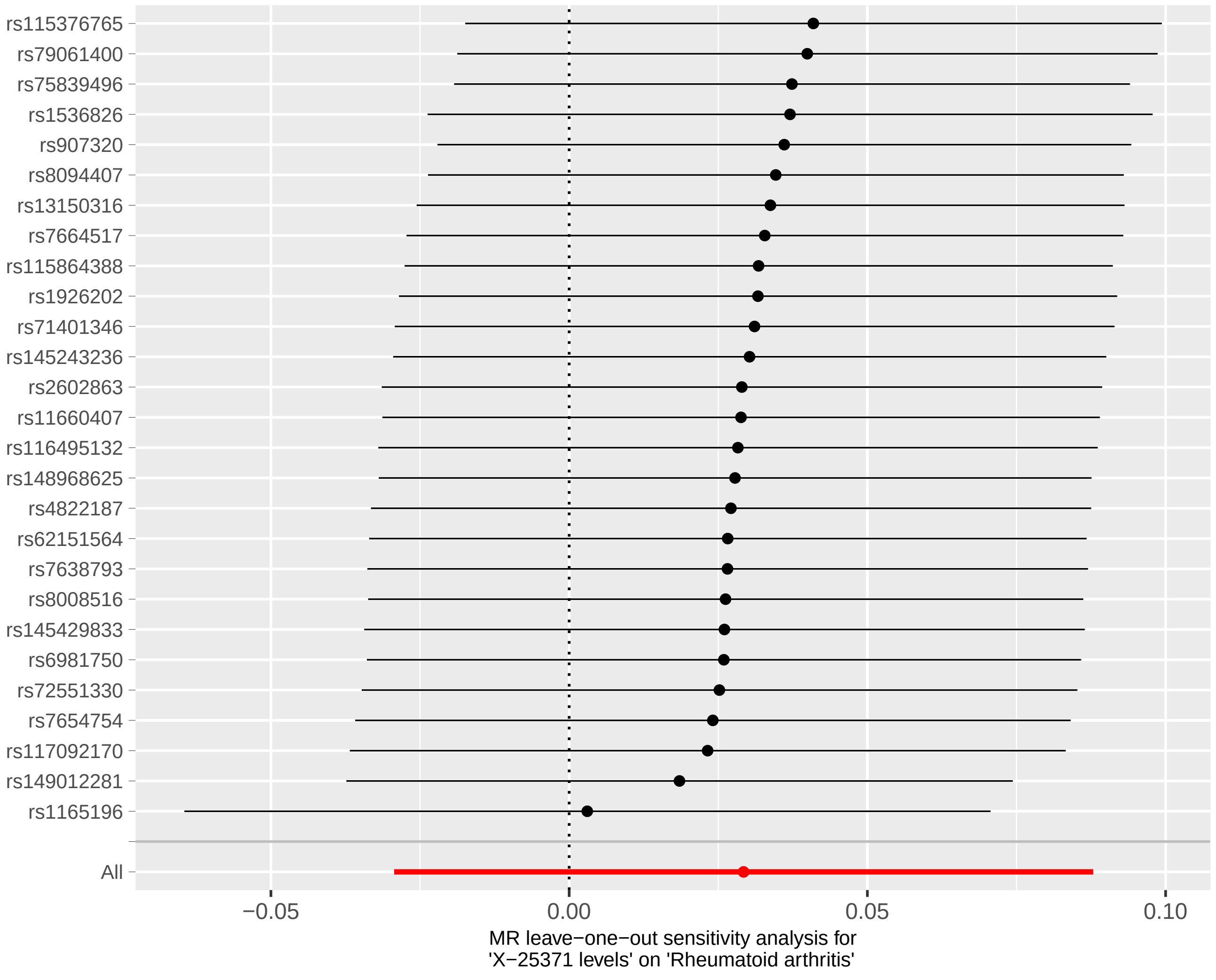
rs115376765
rs79061400
rs75839496
rs1536826
rs907320
rs8094407
rs13150316
rs7664517
rs115864388
rs1926202
rs71401346
rs145243236
rs2602863
rs11660407
rs116495132
rs148968625
rs4822187
rs62151564
rs7638793
rs8008516
rs145429833
rs6981750
rs72551330
rs7654754
rs117092170
rs149012281
rs1165196
All
−0.05
0.00
0.05
0.10
MR leave−one−out sensitivity analysis for
'X−25371 levels' on 'Rheumatoid arthritis'

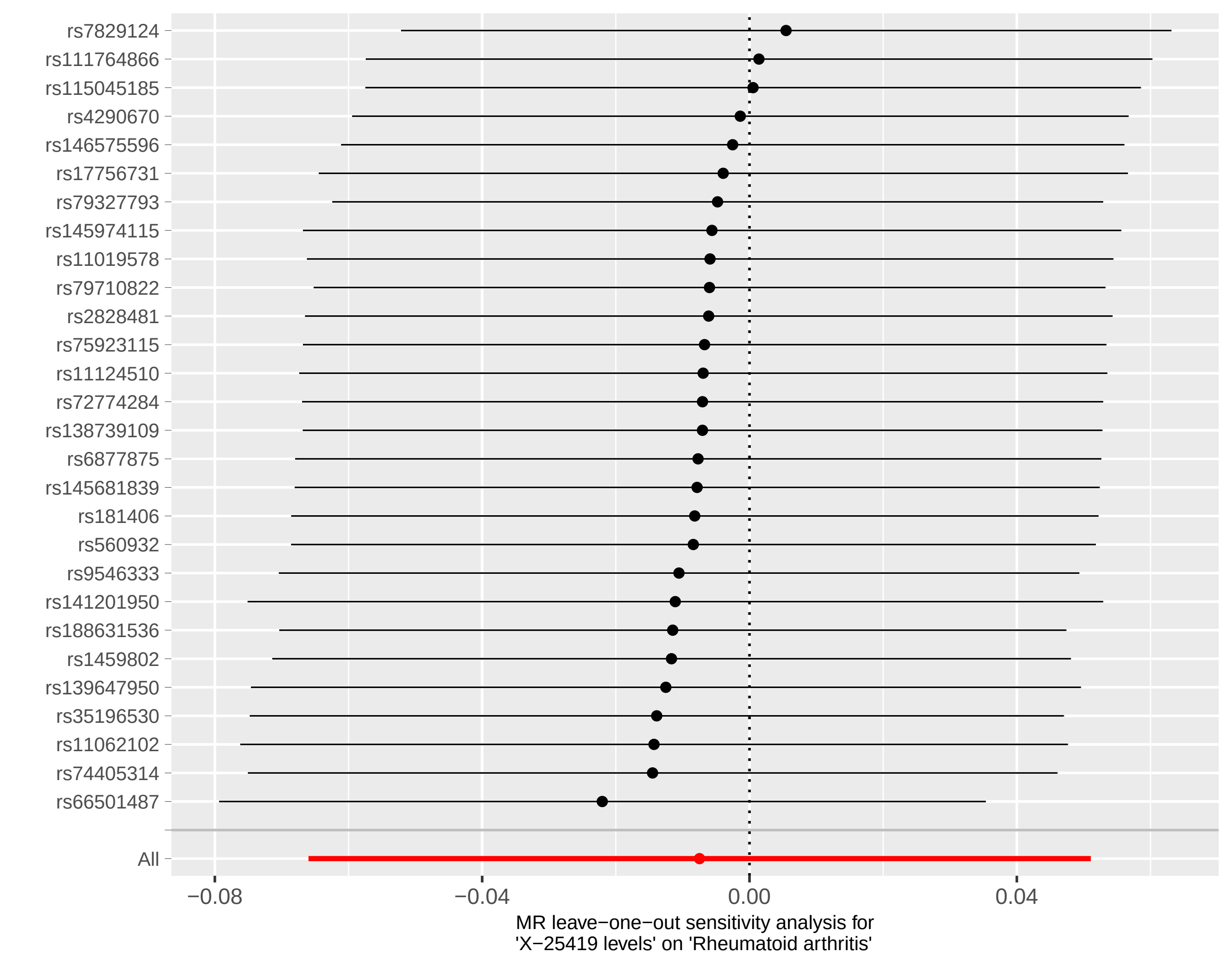
rs7829124
rs111764866
rs115045185
rs4290670
rs146575596
rs17756731
rs79327793
rs145974115
rs11019578
rs79710822
rs2828481
rs75923115
rs11124510
rs72774284
rs138739109
rs6877875
rs145681839
rs181406
rs560932
rs9546333
rs141201950
rs188631536
rs1459802
rs139647950
rs35196530
rs11062102
rs74405314
rs66501487
All
−0.08
−0.04
0.00
0.04
MR leave−one−out sensitivity analysis for
'X−25419 levels' on 'Rheumatoid arthritis'

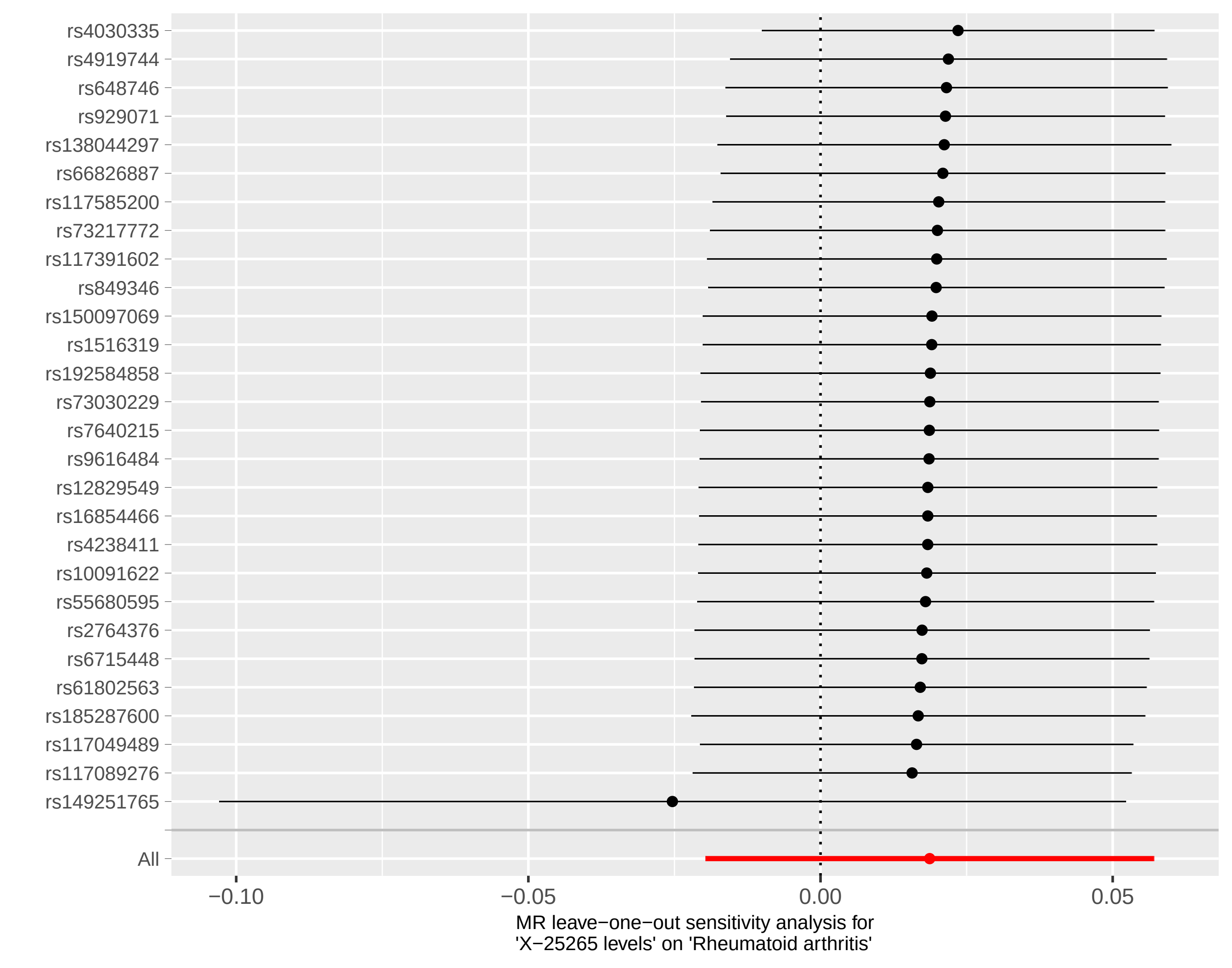
rs4030335
rs4919744
rs648746
rs929071
rs138044297
rs66826887
rs117585200
rs73217772
rs117391602
rs849346
rs150097069
rs1516319
rs192584858
rs73030229
rs7640215
rs9616484
rs12829549
rs16854466
rs4238411
rs10091622
rs55680595
rs2764376
rs6715448
rs61802563
rs185287600
rs117049489
rs117089276
rs149251765
All
−0.10
−0.05
0.00
0.05
MR leave−one−out sensitivity analysis for
'X−25265 levels' on 'Rheumatoid arthritis'

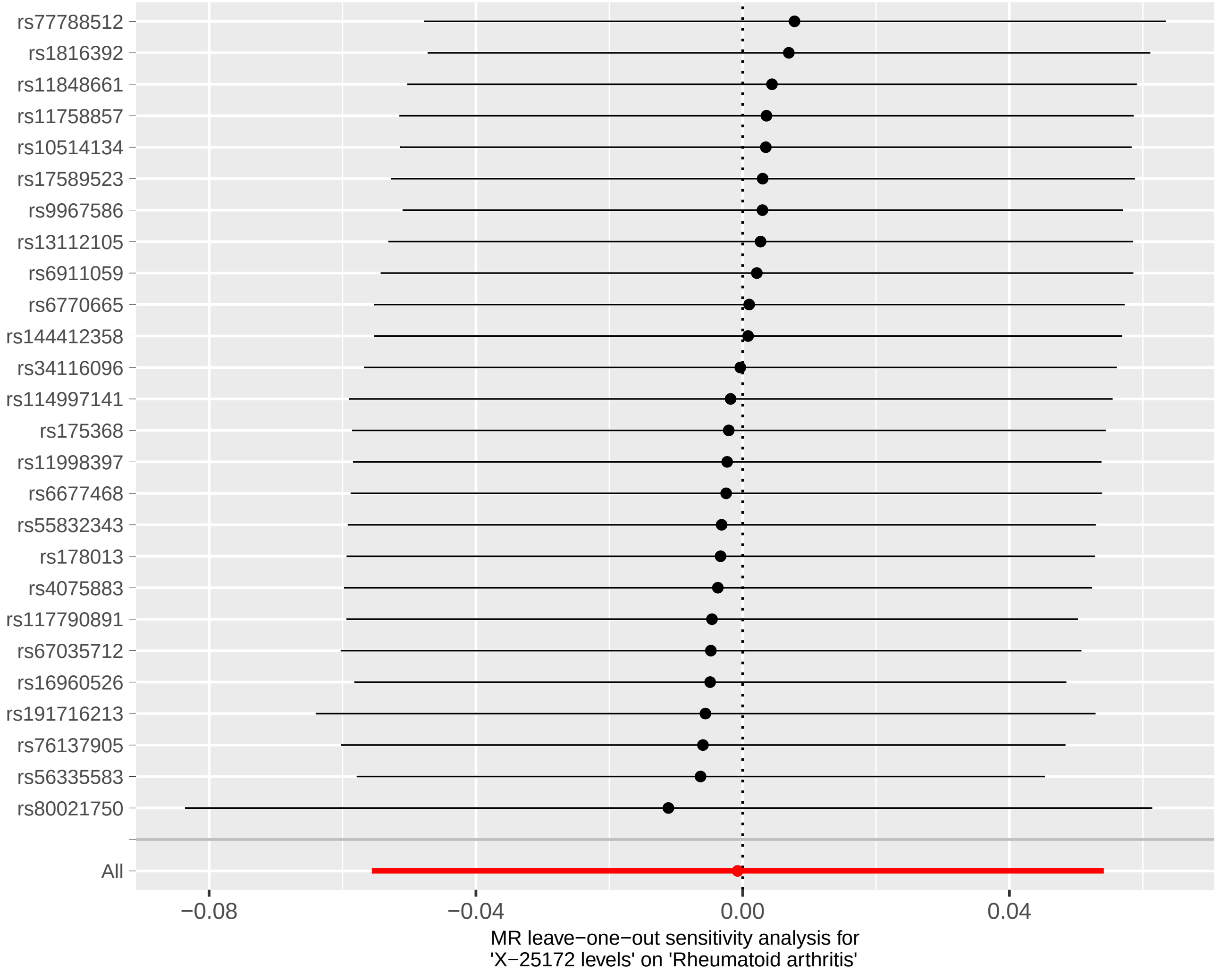
rs77788512
rs1816392
rs11848661
rs11758857
rs10514134
rs17589523
rs9967586
rs13112105
rs6911059
rs6770665
rs144412358
rs34116096
rs114997141
rs175368
rs11998397
rs6677468
rs55832343
rs178013
rs4075883
rs117790891
rs67035712
rs16960526
rs191716213
rs76137905
rs56335583
rs80021750
All
−0.08
−0.04
0.00
0.04
MR leave−one−out sensitivity analysis for
'X−25172 levels' on 'Rheumatoid arthritis'

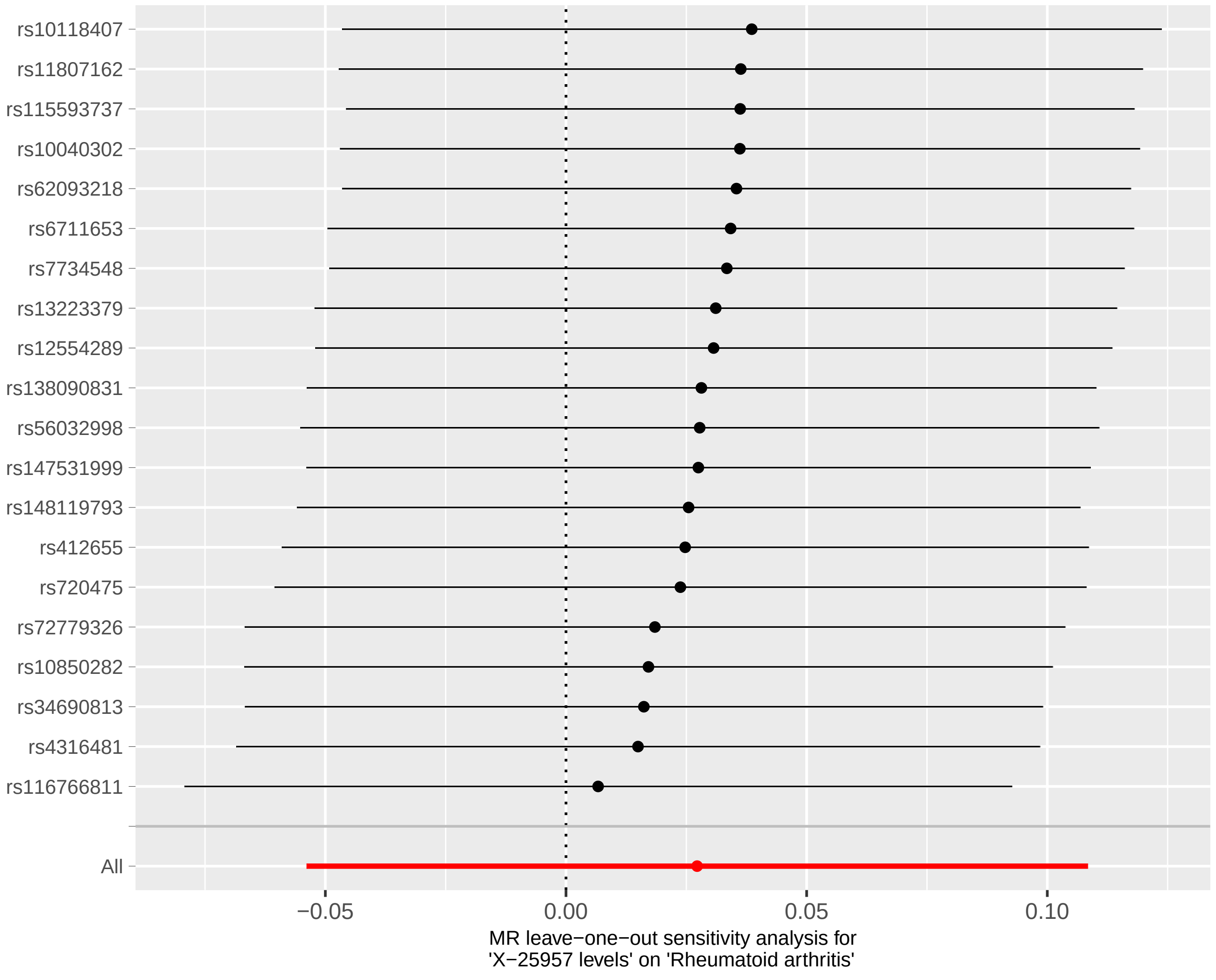
rs10118407
rs11807162
rs115593737
rs10040302
rs62093218
rs6711653
rs7734548
rs13223379
rs12554289
rs138090831
rs56032998
rs147531999
rs148119793
rs412655
rs720475
rs72779326
rs10850282
rs34690813
rs4316481
rs116766811
All
−0.05
0.00
0.05
0.10
MR leave−one−out sensitivity analysis for
'X−25957 levels' on 'Rheumatoid arthritis'

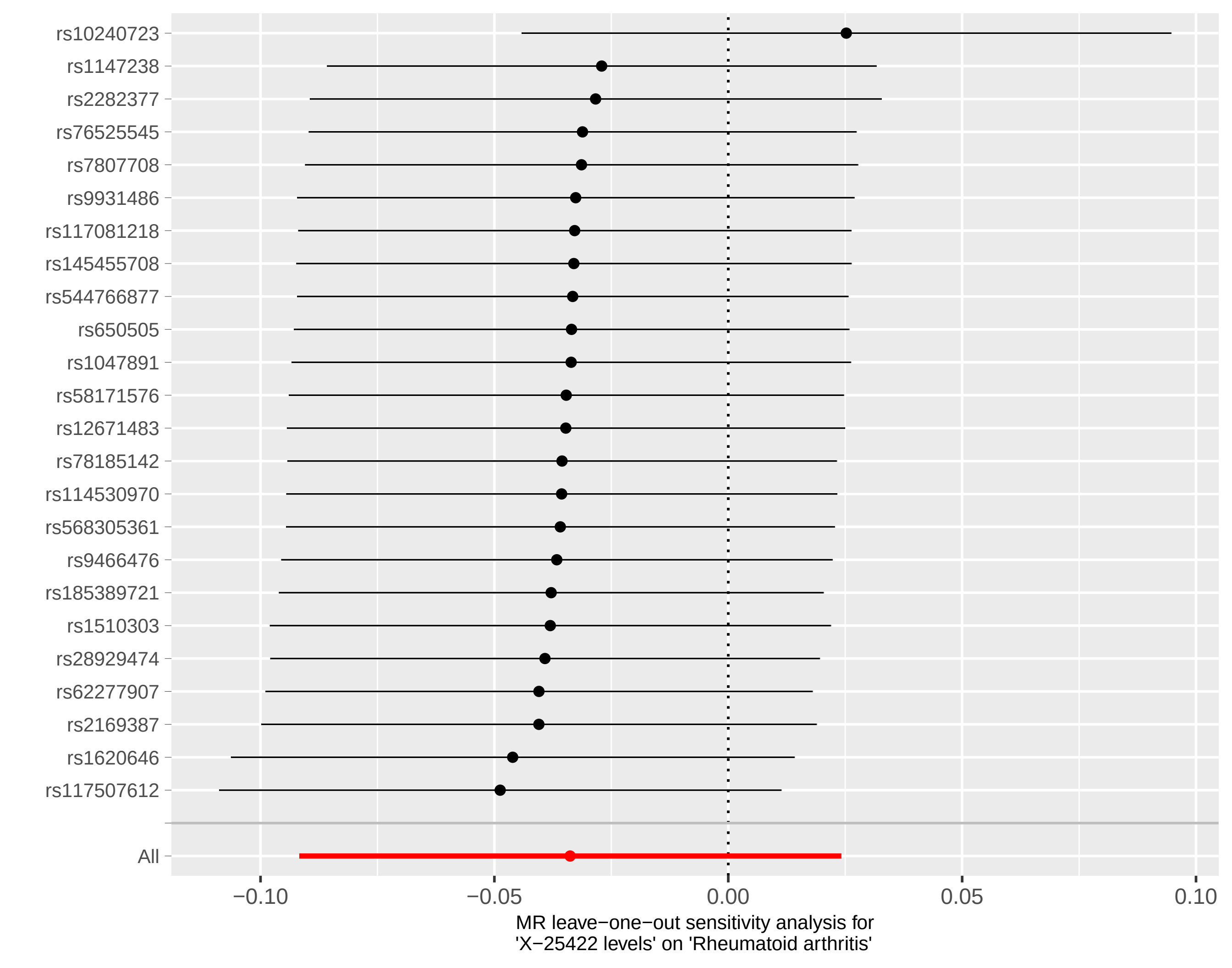
rs10240723
rs1147238
rs2282377
rs76525545
rs7807708
rs9931486
rs117081218
rs145455708
rs544766877
rs650505
rs1047891
rs58171576
rs12671483
rs78185142
rs114530970
rs568305361
rs9466476
rs185389721
rs1510303
rs28929474
rs62277907
rs2169387
rs1620646
rs117507612
All
−0.10
−0.05
0.00
0.05
0.10
MR leave−one−out sensitivity analysis for
'X−25422 levels' on 'Rheumatoid arthritis'

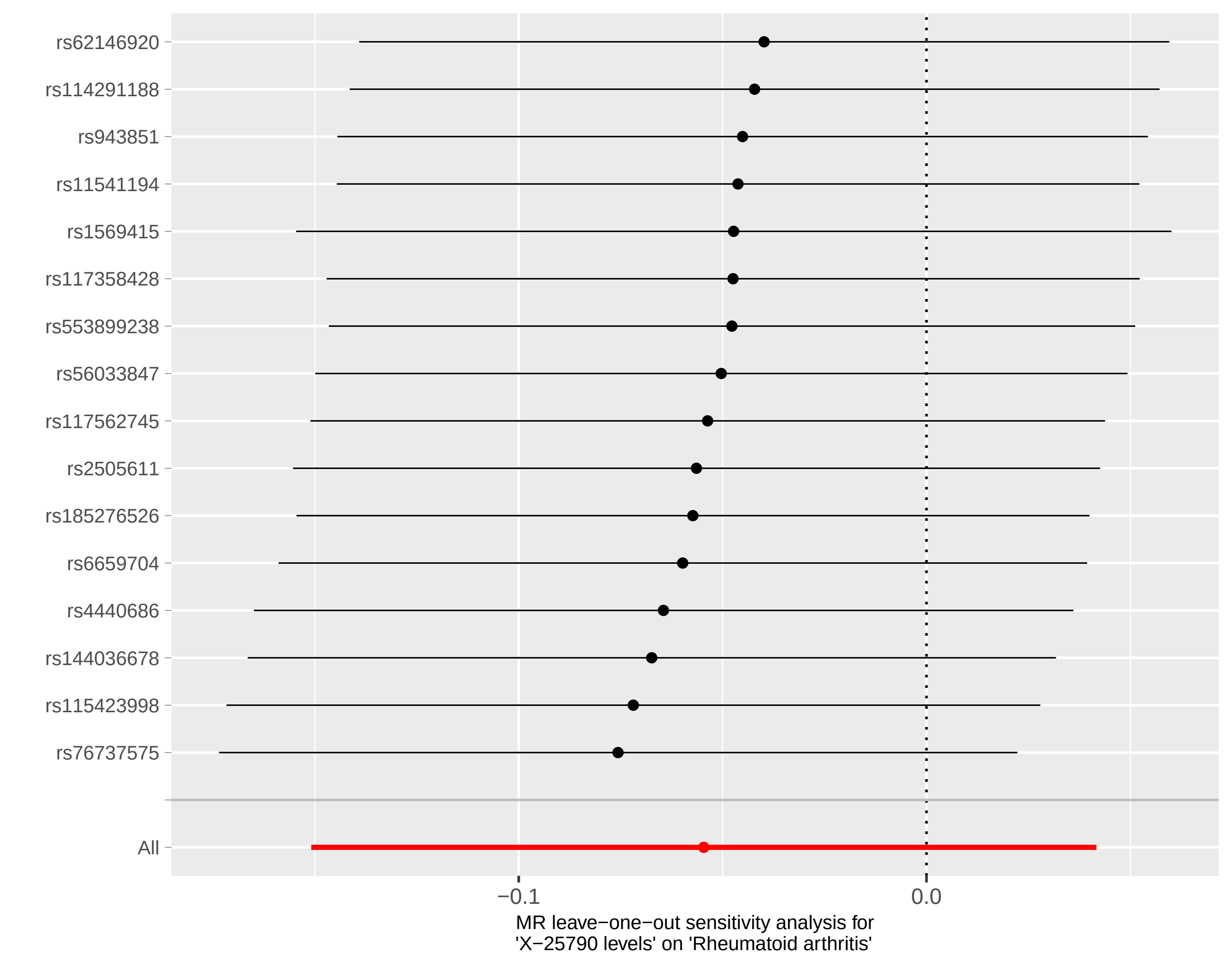

rs62146920
rs114291188
rs943851
rs11541194
rs1569415
rs117358428
rs553899238
rs56033847
rs117562745
rs2505611
rs185276526
rs6659704
rs4440686
rs144036678
rs115423998
rs76737575
All
−0.1
0.0
MR leave−one−out sensitivity analysis for
'X−25790 levels' on 'Rheumatoid arthritis'

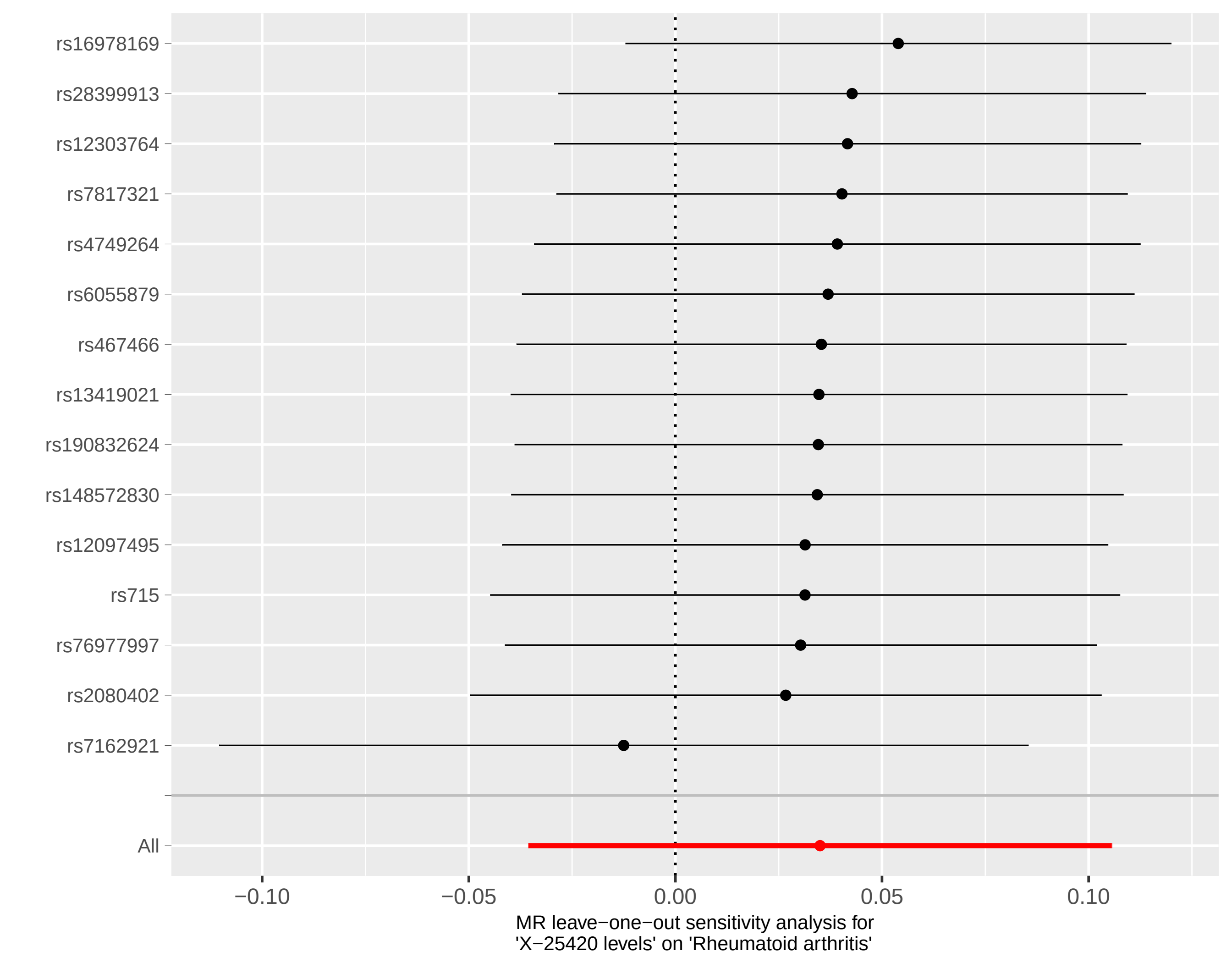

rs16978169
rs28399913
rs12303764
rs7817321
rs4749264
rs6055879
rs467466
rs13419021
rs190832624
rs148572830
rs12097495
rs715
rs76977997
rs2080402
rs7162921
All
−0.10
−0.05
0.00
0.05
0.10
MR leave−one−out sensitivity analysis for
'X−25420 levels' on 'Rheumatoid arthritis'

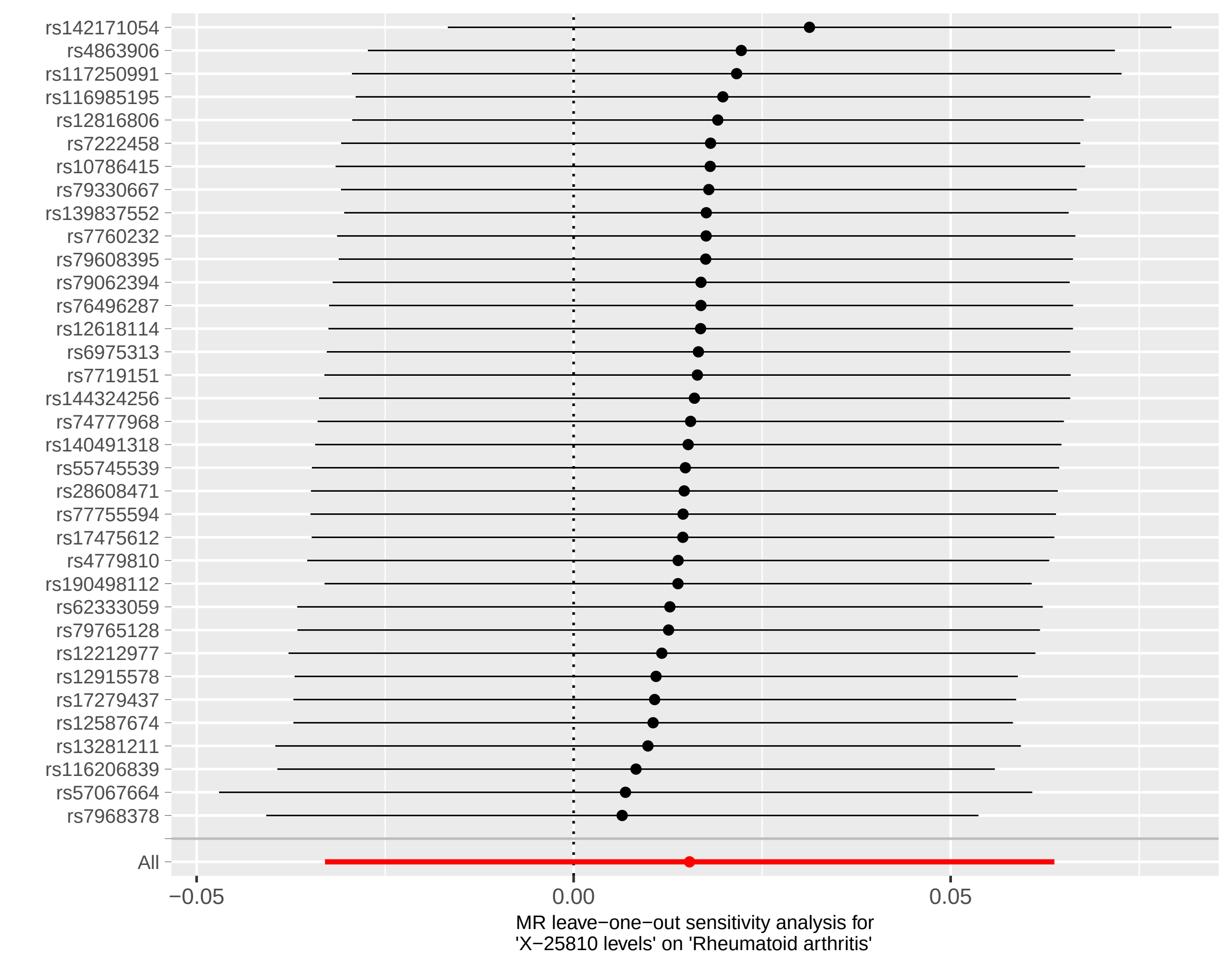
rs142171054
rs4863906
rs117250991
rs116985195
rs12816806
rs7222458
rs10786415
rs79330667
rs139837552
rs7760232
rs79608395
rs79062394
rs76496287
rs12618114
rs6975313
rs7719151
rs144324256
rs74777968
rs140491318
rs55745539
rs28608471
rs77755594
rs17475612
rs4779810
rs190498112
rs62333059
rs79765128
rs12212977
rs12915578
rs17279437
rs12587674
rs13281211
rs116206839
rs57067664
rs7968378
All
−0.05
0.00
0.05
MR leave−one−out sensitivity analysis for
'X−25810 levels' on 'Rheumatoid arthritis'

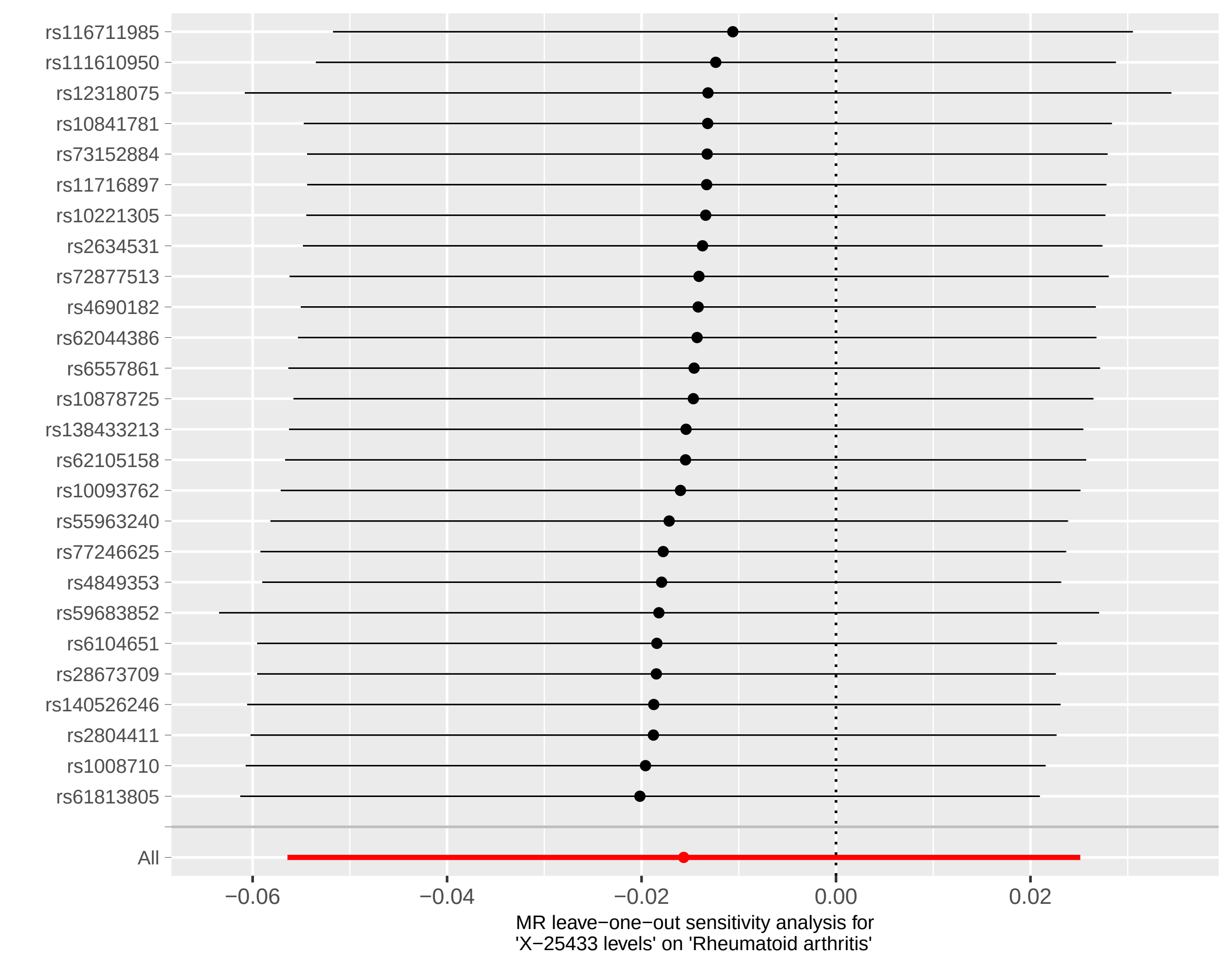

rs116711985
rs111610950
rs12318075
rs10841781
rs73152884
rs11716897
rs10221305
rs2634531
rs72877513
rs4690182
rs62044386
rs6557861
rs10878725
rs138433213
rs62105158
rs10093762
rs55963240
rs77246625
rs4849353
rs59683852
rs6104651
rs28673709
rs140526246
rs2804411
rs1008710
rs61813805
All
−0.06
−0.04
−0.02
0.00
0.02
MR leave−one−out sensitivity analysis for
'X−25433 levels' on 'Rheumatoid arthritis'

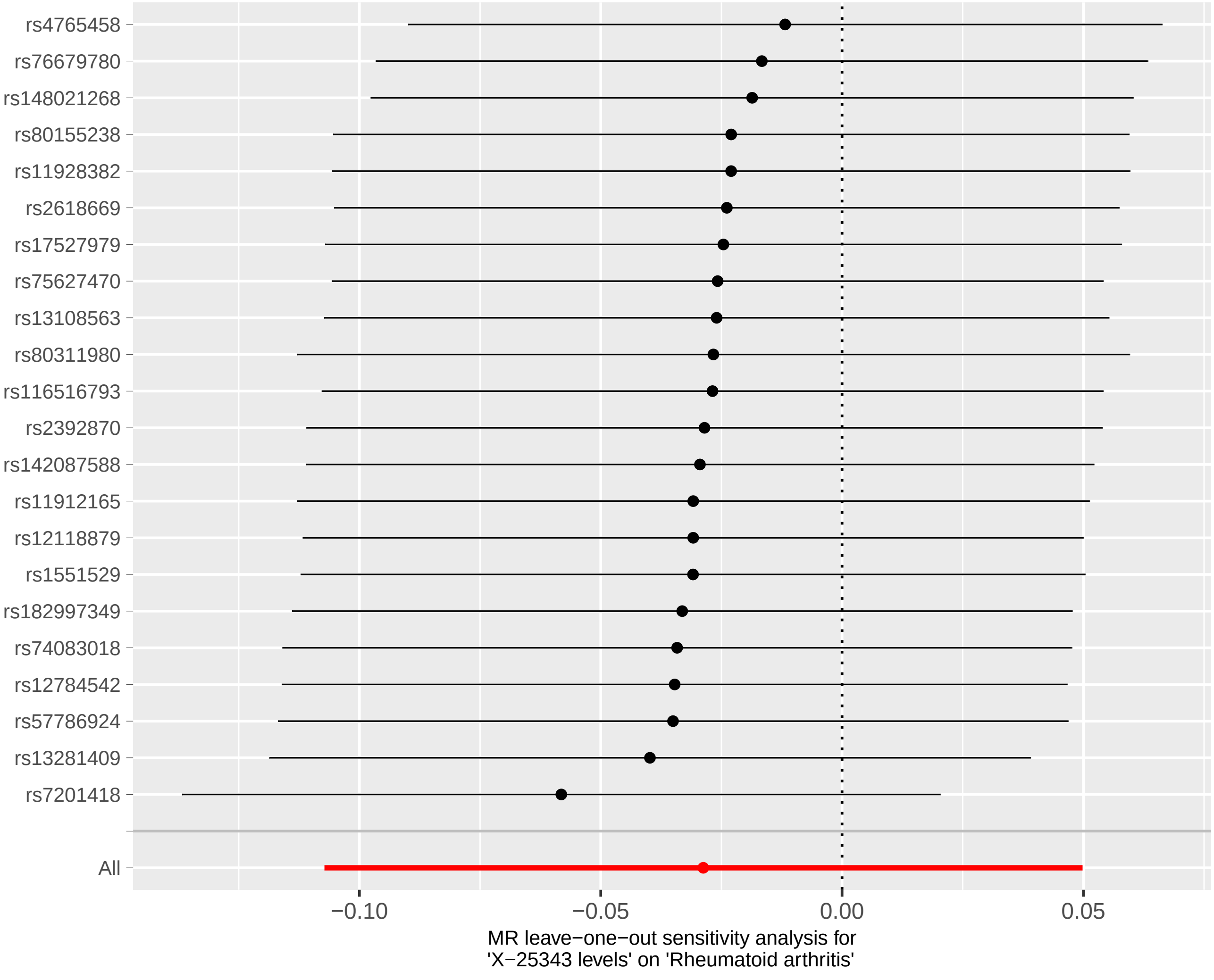
rs4765458
rs76679780
rs148021268
rs80155238
rs11928382
rs2618669
rs17527979
rs75627470
rs13108563
rs80311980
rs116516793
rs2392870
rs142087588
rs11912165
rs12118879
rs1551529
rs182997349
rs74083018
rs12784542
rs57786924
rs13281409
rs7201418
All
−0.10
−0.05
0.00
0.05
MR leave−one−out sensitivity analysis for
'X−25343 levels' on 'Rheumatoid arthritis'

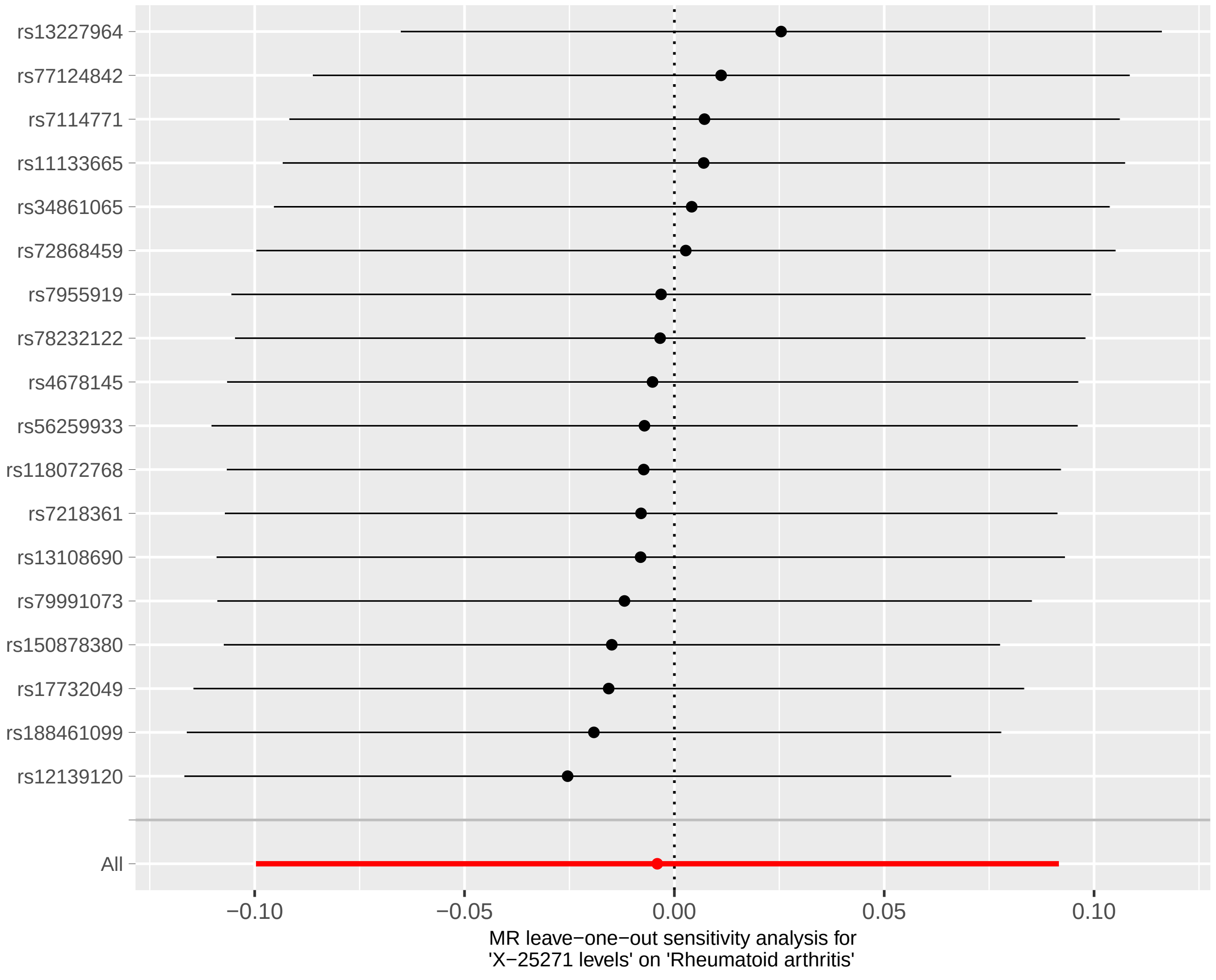

rs13227964
rs77124842
rs7114771
rs11133665
rs34861065
rs72868459
rs7955919
rs78232122
rs4678145
rs56259933
rs118072768
rs7218361
rs13108690
rs79991073
rs150878380
rs17732049
rs188461099
rs12139120
All
−0.10
−0.05
0.00
0.05
0.10
MR leave−one−out sensitivity analysis for
'X−25271 levels' on 'Rheumatoid arthritis'

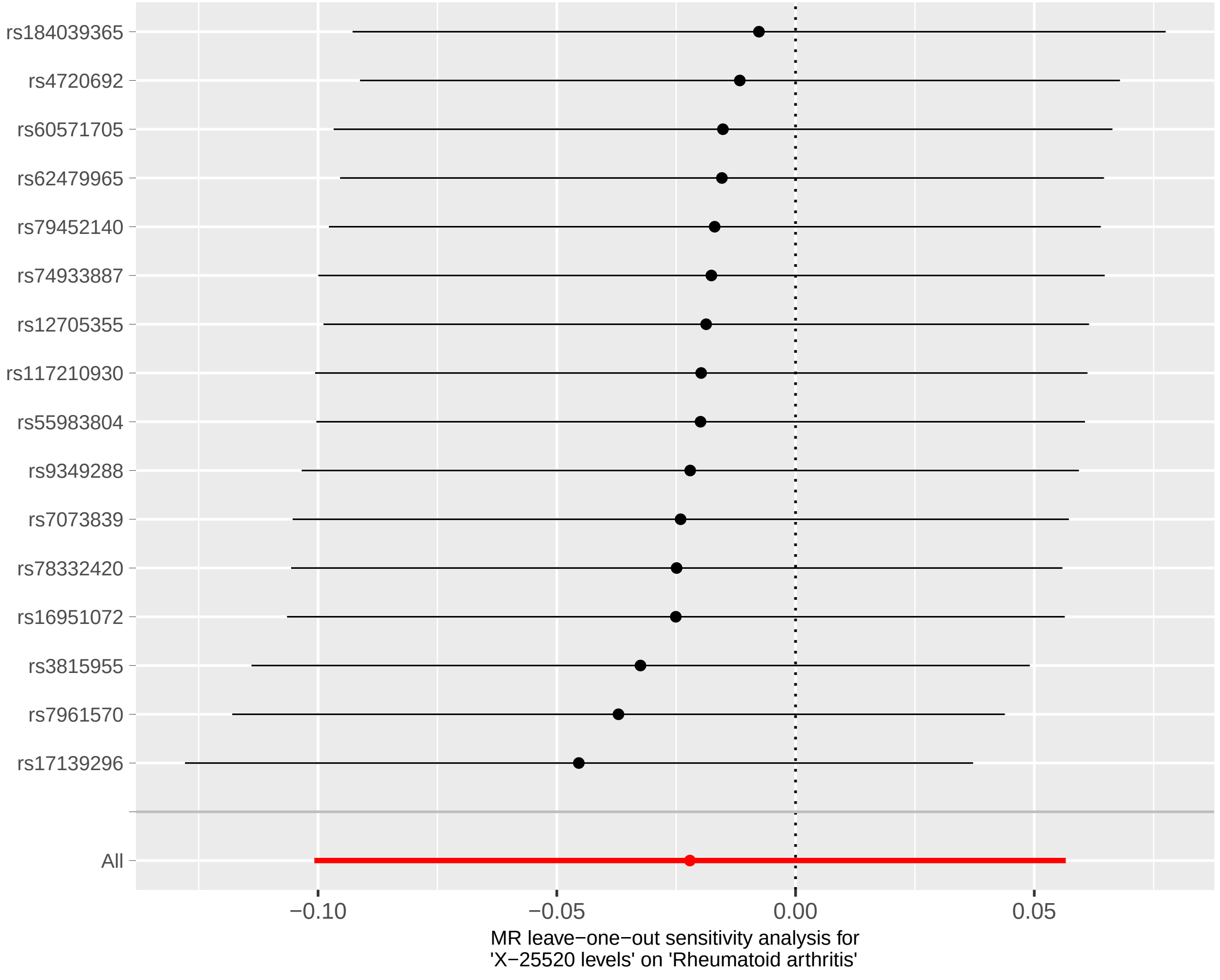

rs184039365
rs4720692
rs60571705
rs62479965
rs79452140
rs74933887
rs12705355
rs117210930
rs55983804
rs9349288
rs7073839
rs78332420
rs16951072
rs3815955
rs7961570
rs17139296
All
−0.10
−0.05
0.00
0.05
MR leave−one−out sensitivity analysis for
'X−25520 levels' on 'Rheumatoid arthritis'

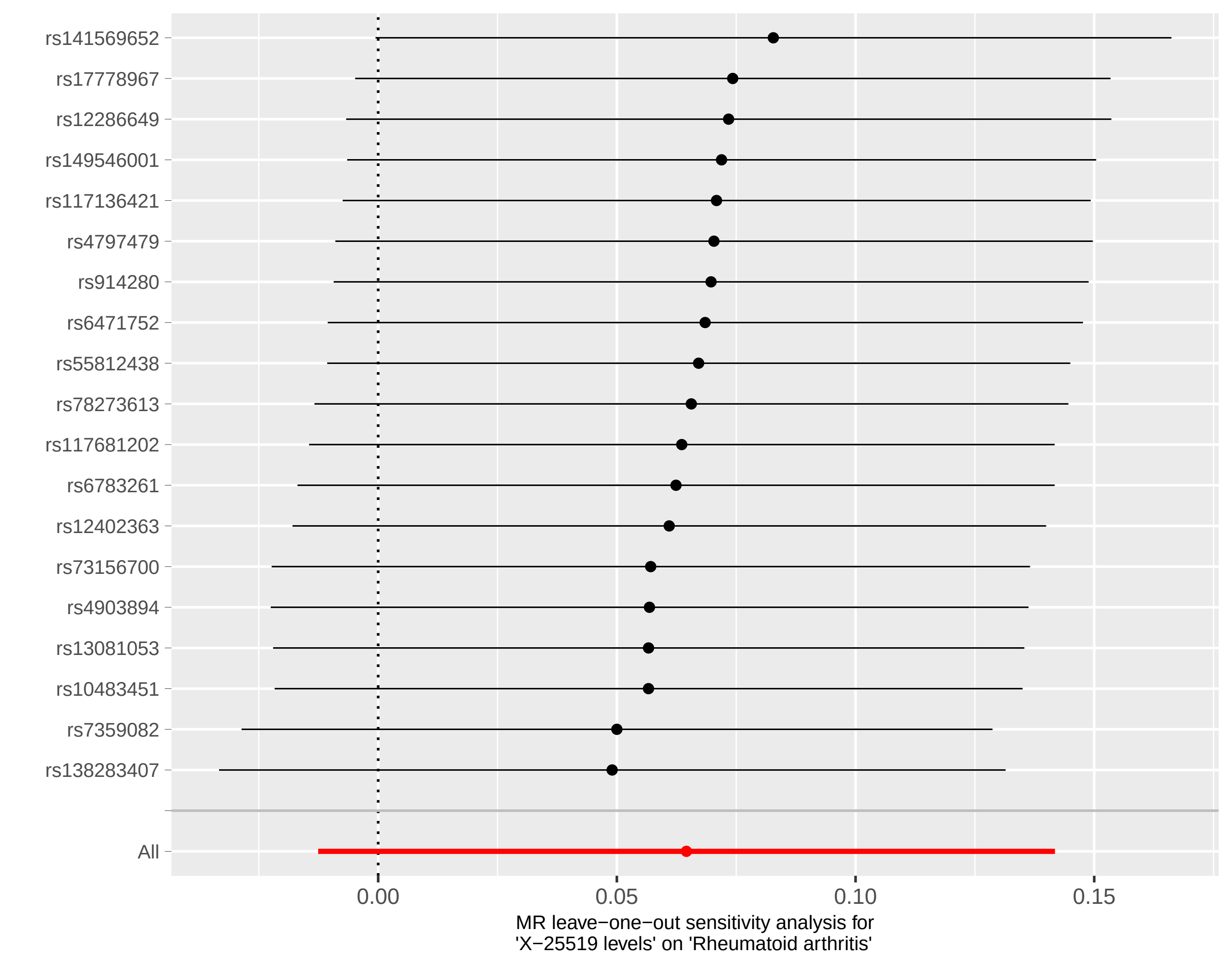
rs141569652
rs17778967
rs12286649
rs149546001
rs117136421
rs4797479
rs914280
rs6471752
rs55812438
rs78273613
rs117681202
rs6783261
rs12402363
rs73156700
rs4903894
rs13081053
rs10483451
rs7359082
rs138283407
All
0.00
0.05
0.10
0.15
MR leave−one−out sensitivity analysis for
'X−25519 levels' on 'Rheumatoid arthritis'

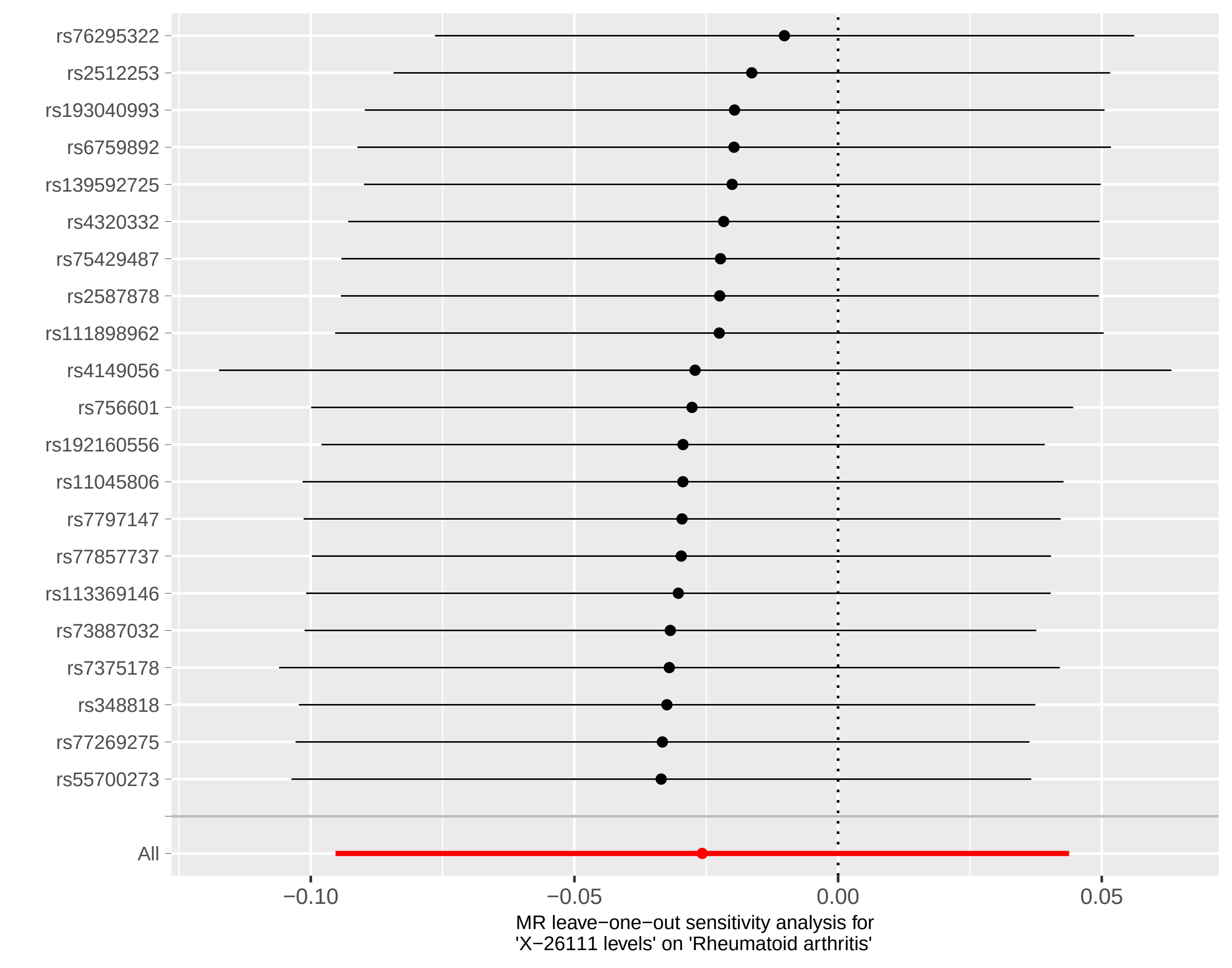
rs76295322
rs2512253
rs193040993
rs6759892
rs139592725
rs4320332
rs75429487
rs2587878
rs111898962
rs4149056
rs756601
rs192160556
rs11045806
rs7797147
rs77857737
rs113369146
rs73887032
rs7375178
rs348818
rs77269275
rs55700273
All
−0.10
−0.05
0.00
0.05
MR leave−one−out sensitivity analysis for
'X−26111 levels' on 'Rheumatoid arthritis'

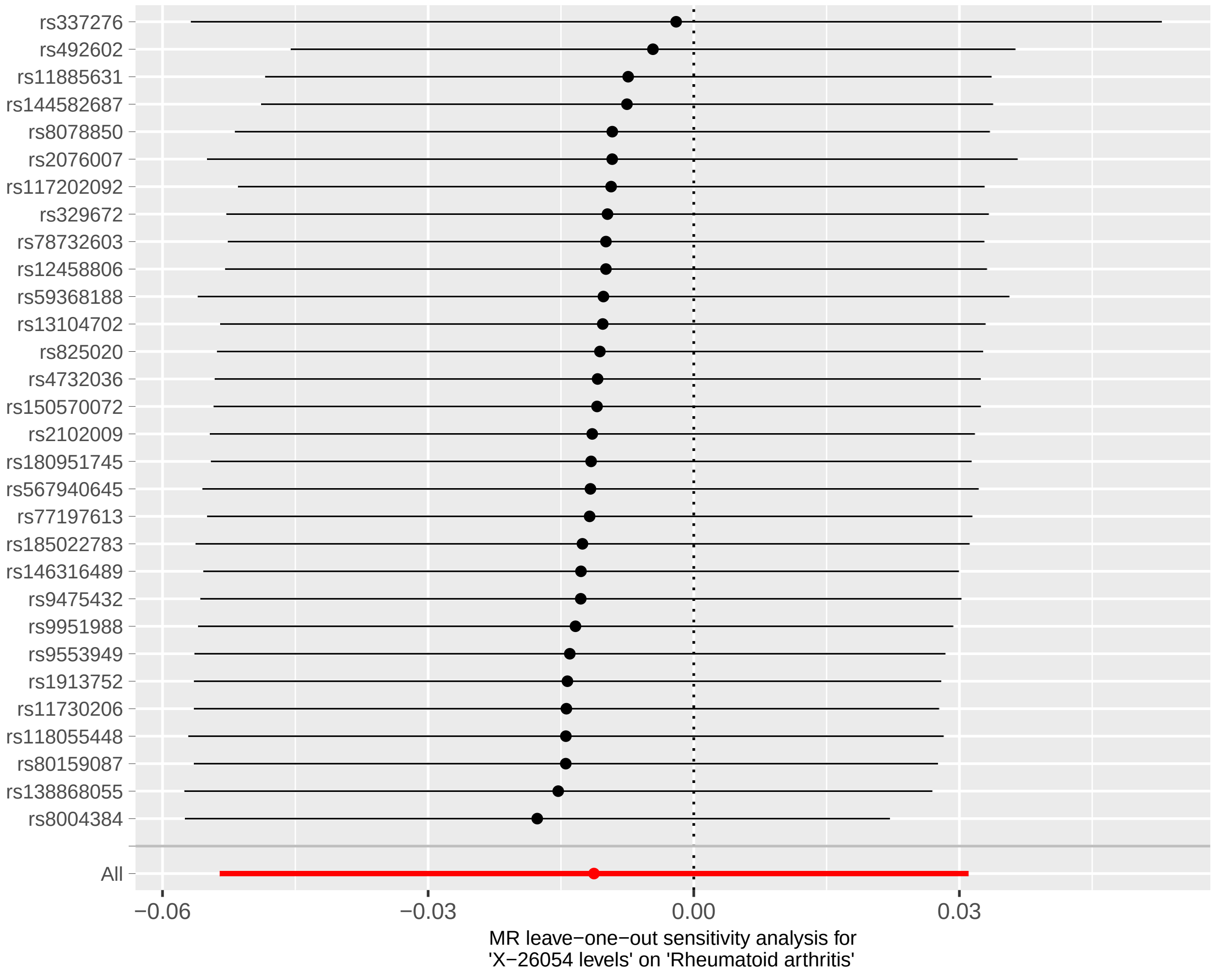

rs337276
rs492602
rs11885631
rs144582687
rs8078850
rs2076007
rs117202092
rs329672
rs78732603
rs12458806
rs59368188
rs13104702
rs825020
rs4732036
rs150570072
rs2102009
rs180951745
rs567940645
rs77197613
rs185022783
rs146316489
rs9475432
rs9951988
rs9553949
rs1913752
rs11730206
rs118055448
rs80159087
rs138868055
rs8004384
All
−0.06
−0.03
0.00
0.03
MR leave−one−out sensitivity analysis for
'X−26054 levels' on 'Rheumatoid arthritis'

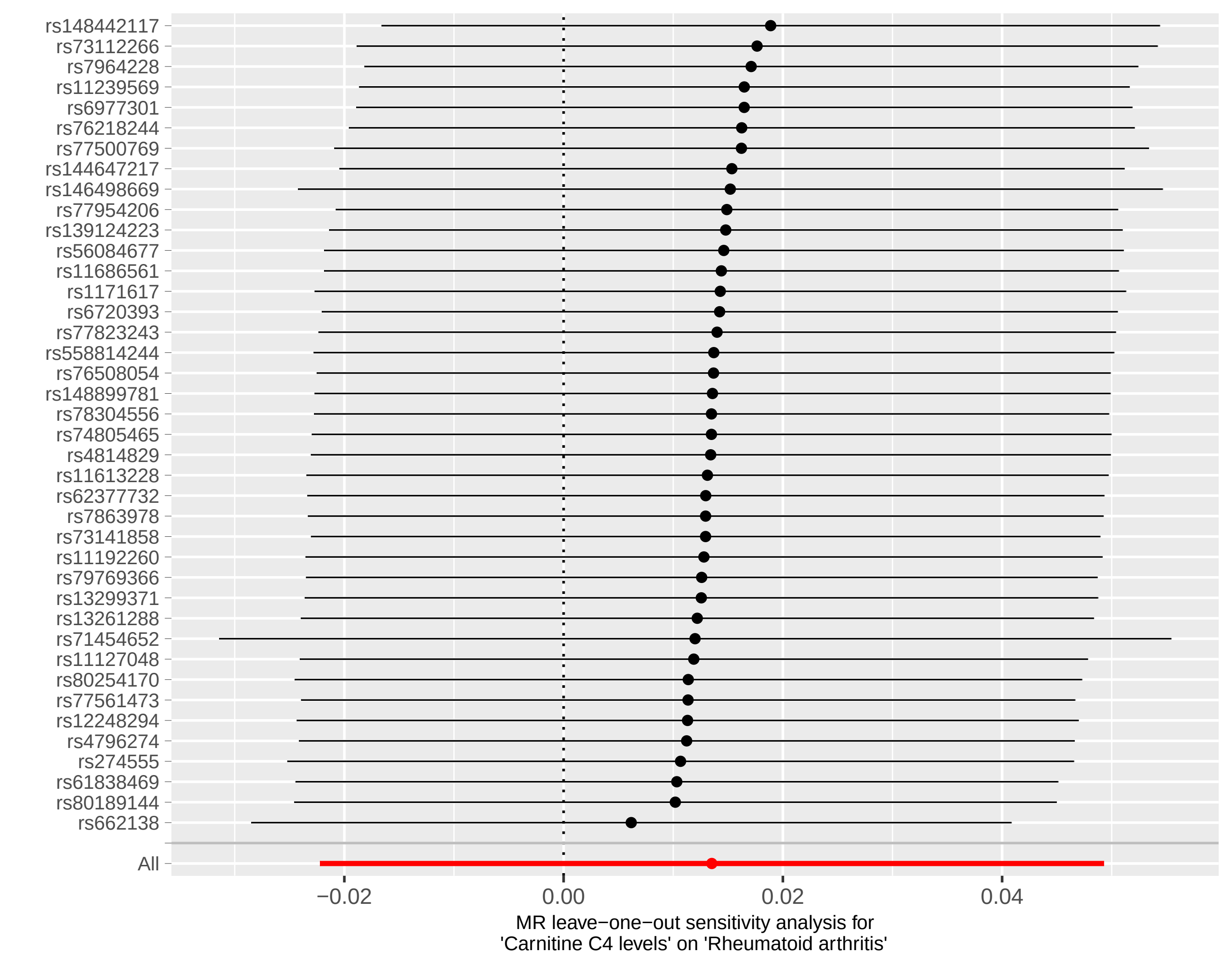
rs148442117
rs73112266
rs7964228
rs11239569
rs6977301
rs76218244
rs77500769
rs144647217
rs146498669
rs77954206
rs139124223
rs56084677
rs11686561
rs1171617
rs6720393
rs77823243
rs558814244
rs76508054
rs148899781
rs78304556
rs74805465
rs4814829
rs11613228
rs62377732
rs7863978
rs73141858
rs11192260
rs79769366
rs13299371
rs13261288
rs71454652
rs11127048
rs80254170
rs77561473
rs12248294
rs4796274
rs274555
rs61838469
rs80189144
rs662138
All
−0.02
0.00
0.02
0.04
MR leave−one−out sensitivity analysis for
'Carnitine C4 levels' on 'Rheumatoid arthritis'

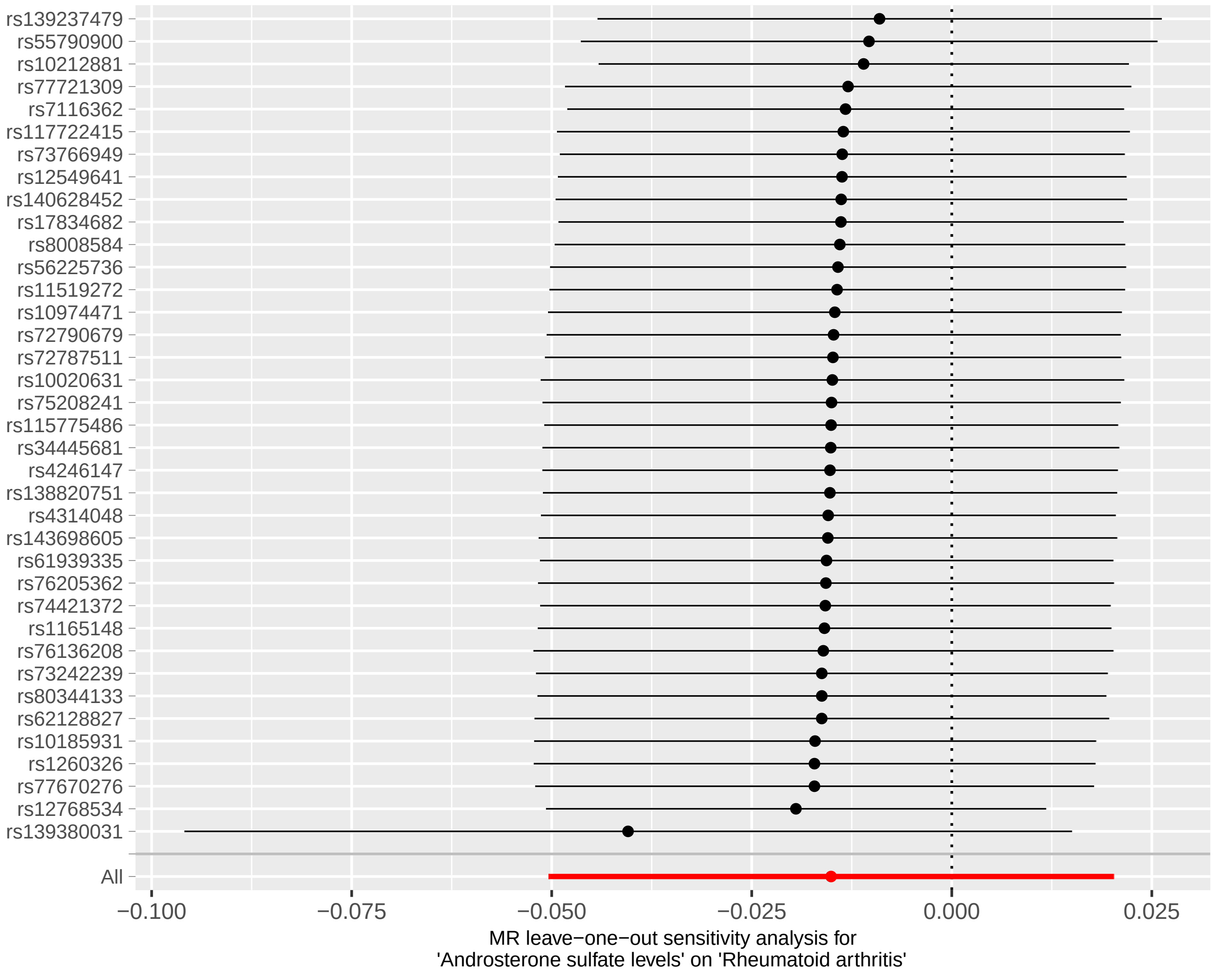

rs139237479
rs55790900
rs10212881
rs77721309
rs7116362
rs117722415
rs73766949
rs12549641
rs140628452
rs17834682
rs8008584
rs56225736
rs11519272
rs10974471
rs72790679
rs72787511
rs10020631
rs75208241
rs115775486
rs34445681
rs4246147
rs138820751
rs4314048
rs143698605
rs61939335
rs76205362
rs74421372
rs1165148
rs76136208
rs73242239
rs80344133
rs62128827
rs10185931
rs1260326
rs77670276
rs12768534
rs139380031
All
−0.100
−0.075
−0.050
−0.025
0.000
0.025
MR leave−one−out sensitivity analysis for
'Androsterone sulfate levels' on 'Rheumatoid arthritis'

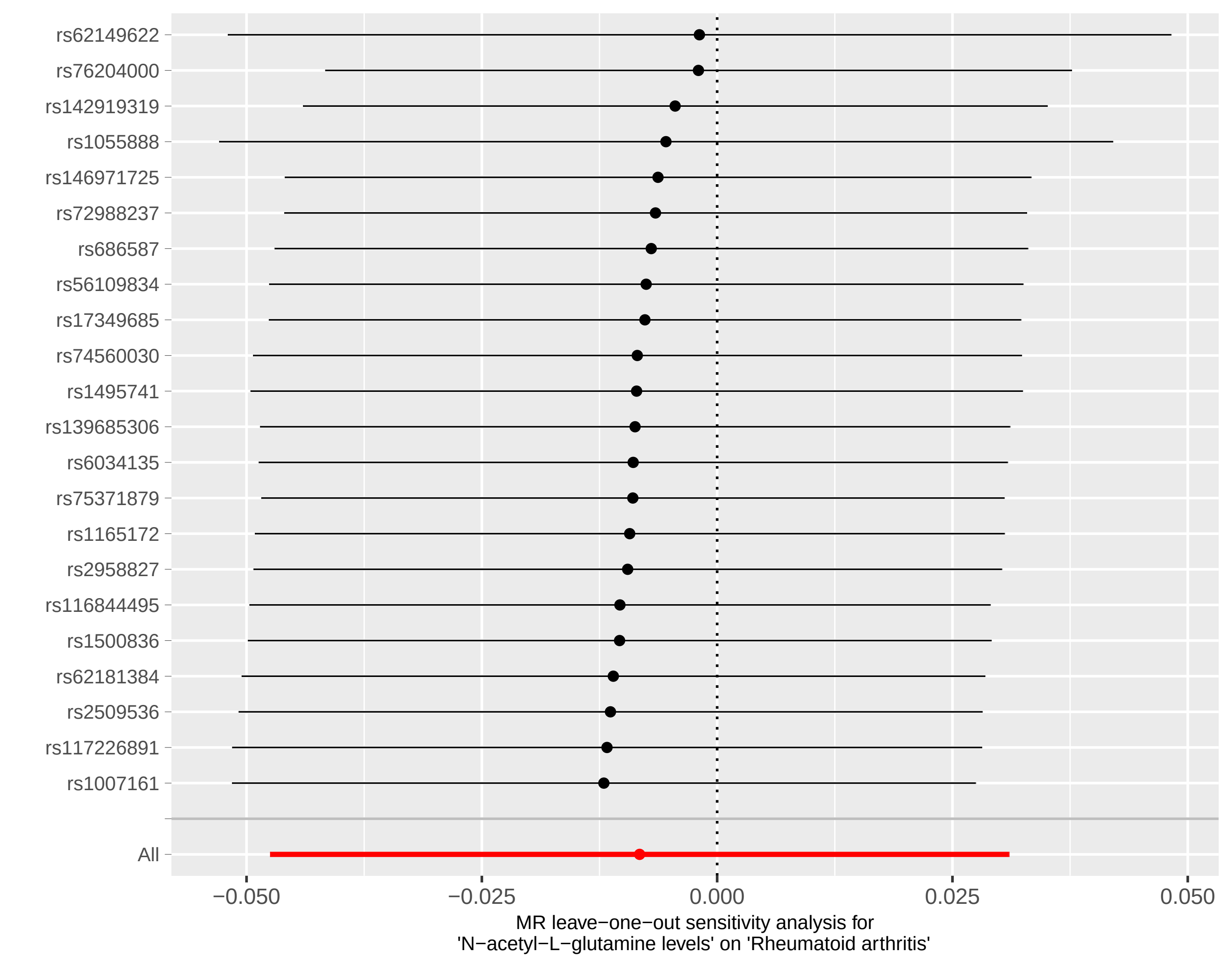
rs62149622
rs76204000
rs142919319
rs1055888
rs146971725
rs72988237
rs686587
rs56109834
rs17349685
rs74560030
rs1495741
rs139685306
rs6034135
rs75371879
rs1165172
rs2958827
rs116844495
rs1500836
rs62181384
rs2509536
rs117226891
rs1007161
All
−0.050
−0.025
0.000
0.025
0.050
MR leave−one−out sensitivity analysis for
'N−acetyl−L−glutamine levels' on 'Rheumatoid arthritis'

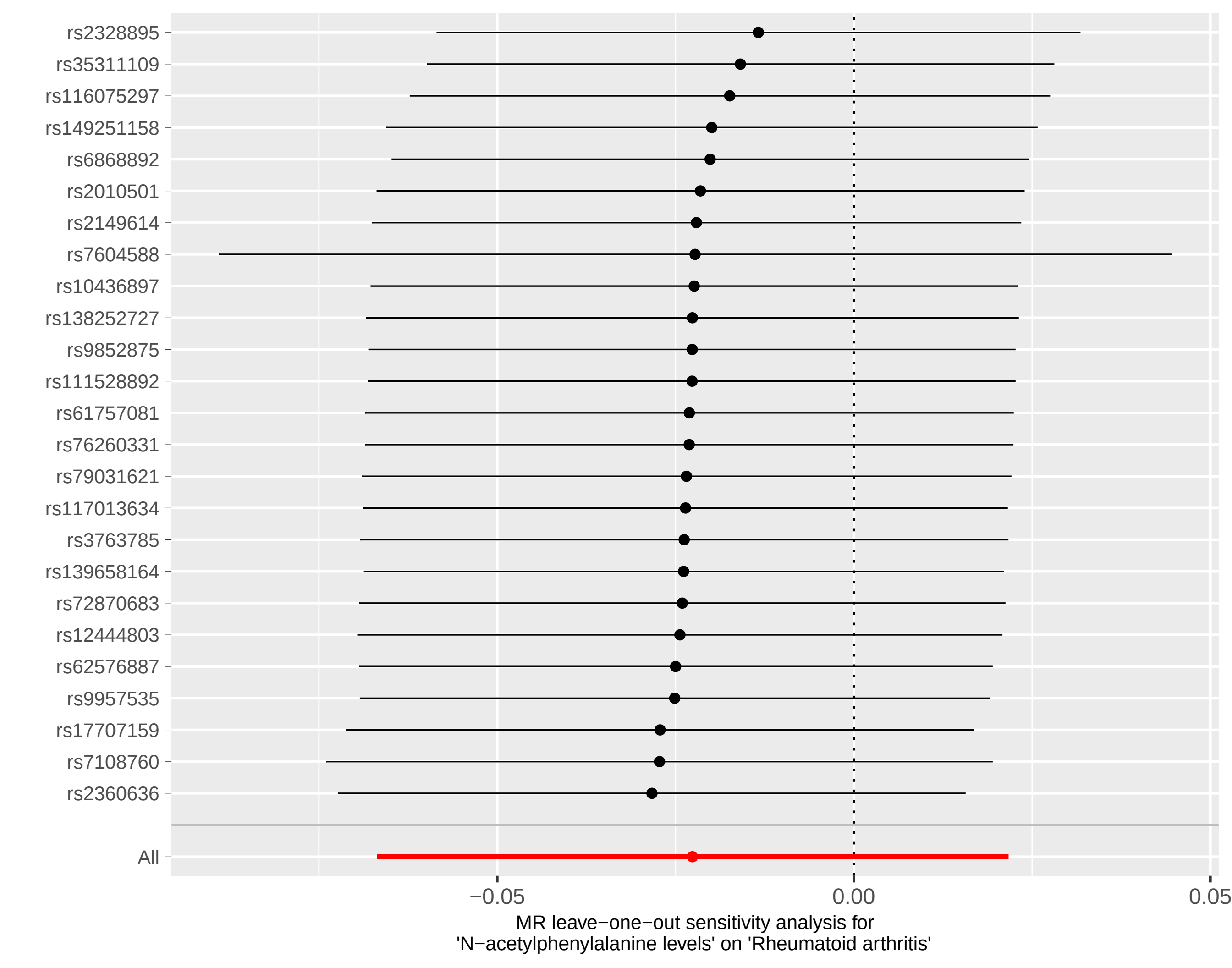

rs2328895
rs35311109
rs116075297
rs149251158
rs6868892
rs2010501
rs2149614
rs7604588
rs10436897
rs138252727
rs9852875
rs111528892
rs61757081
rs76260331
rs79031621
rs117013634
rs3763785
rs139658164
rs72870683
rs12444803
rs62576887
rs9957535
rs17707159
rs7108760
rs2360636
All
−0.05
0.00
0.05
MR leave−one−out sensitivity analysis for
'N−acetylphenylalanine levels' on 'Rheumatoid arthritis'

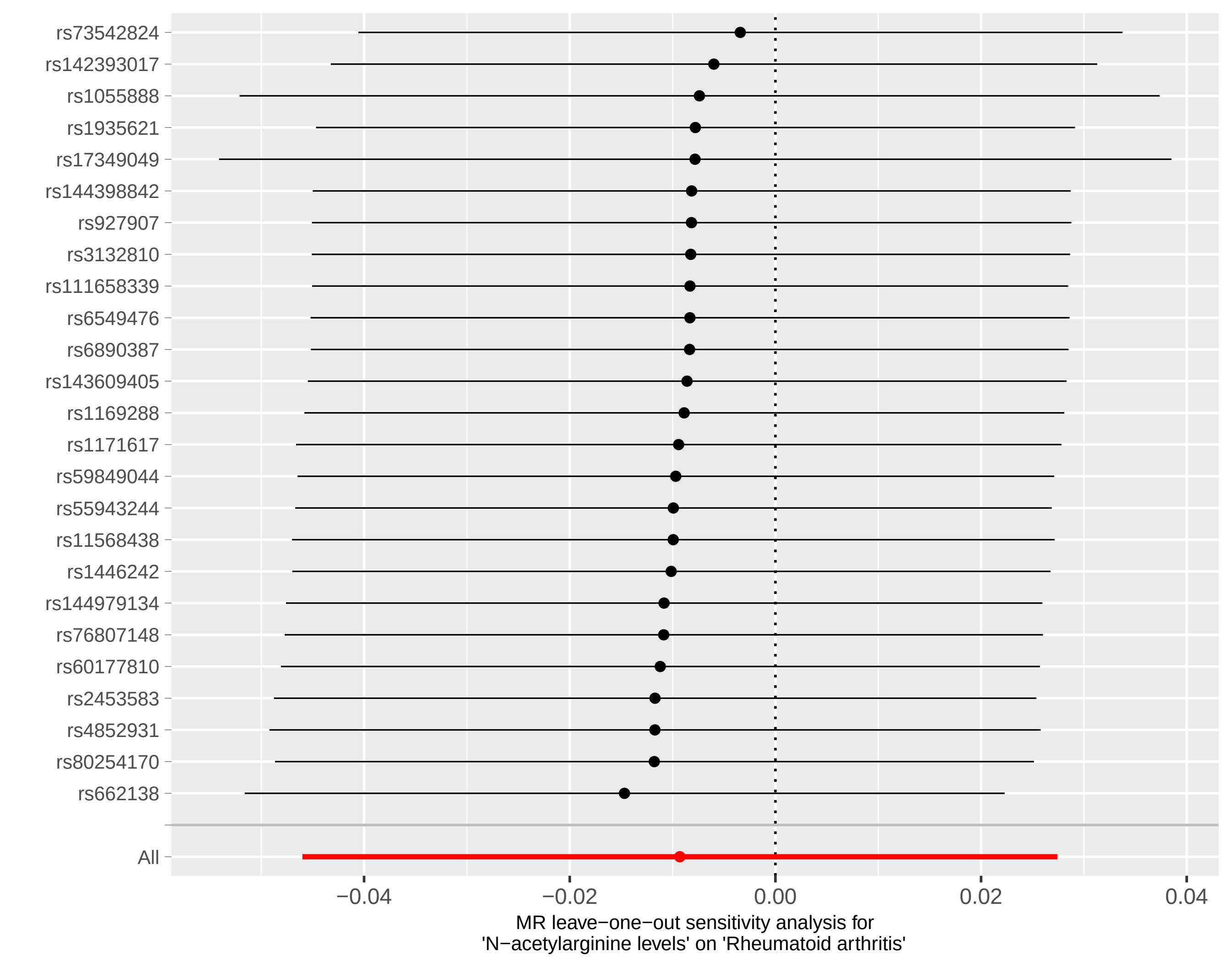

rs73542824
rs142393017
rs1055888
rs1935621
rs17349049
rs144398842
rs927907
rs3132810
rs111658339
rs6549476
rs6890387
rs143609405
rs1169288
rs1171617
rs59849044
rs55943244
rs11568438
rs1446242
rs144979134
rs76807148
rs60177810
rs2453583
rs4852931
rs80254170
rs662138
All
−0.04
−0.02
0.00
0.02
0.04
MR leave−one−out sensitivity analysis for
'N−acetylarginine levels' on 'Rheumatoid arthritis'

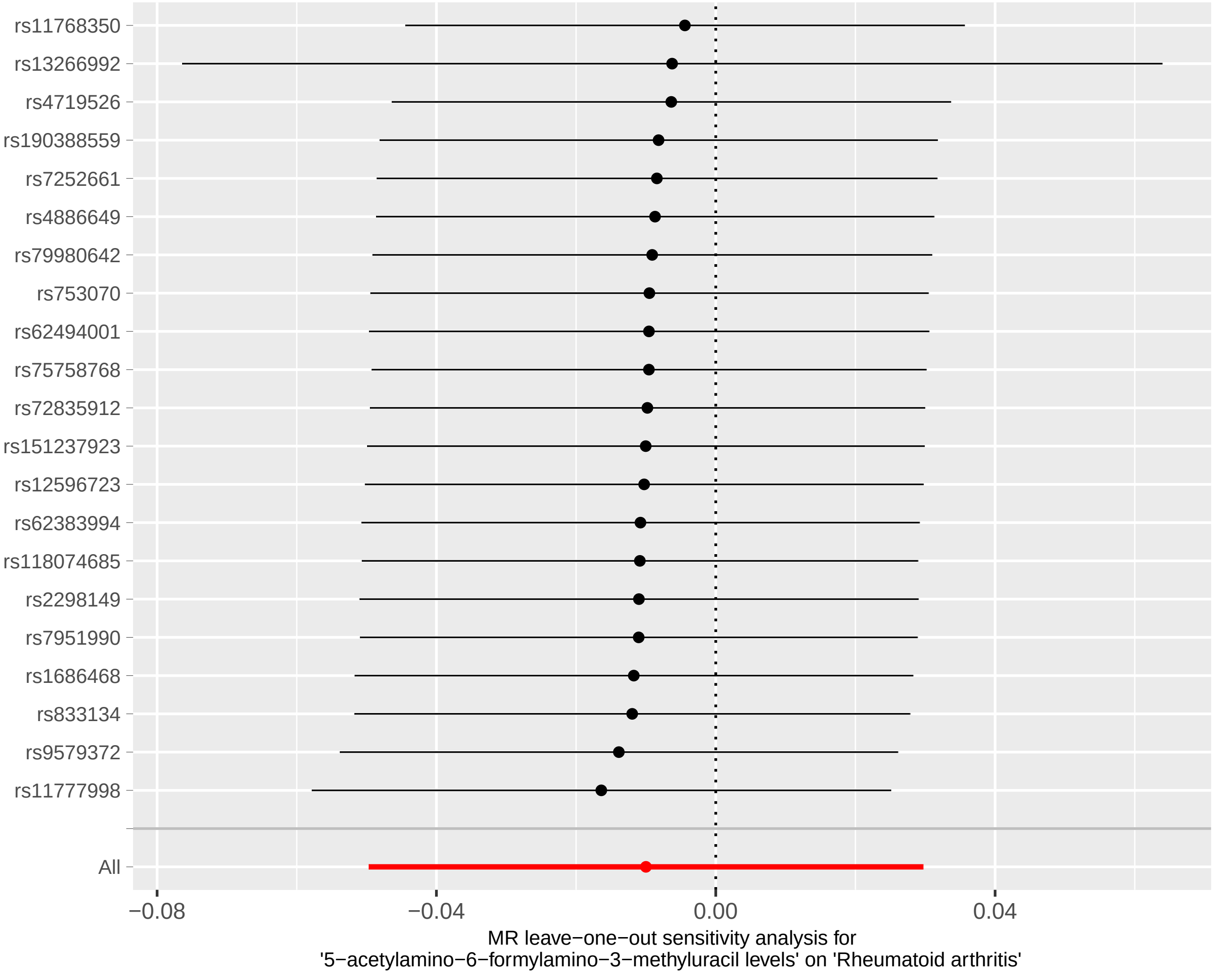
rs11768350
rs13266992
rs4719526
rs190388559
rs7252661
rs4886649
rs79980642
rs753070
rs62494001
rs75758768
rs72835912
rs151237923
rs12596723
rs62383994
rs118074685
rs2298149
rs7951990
rs1686468
rs833134
rs9579372
rs11777998
All
−0.08
−0.04
0.00
0.04
MR leave−one−out sensitivity analysis for
'5−acetylamino−6−formylamino−3−methyluracil levels' on 'Rheumatoid arthritis'

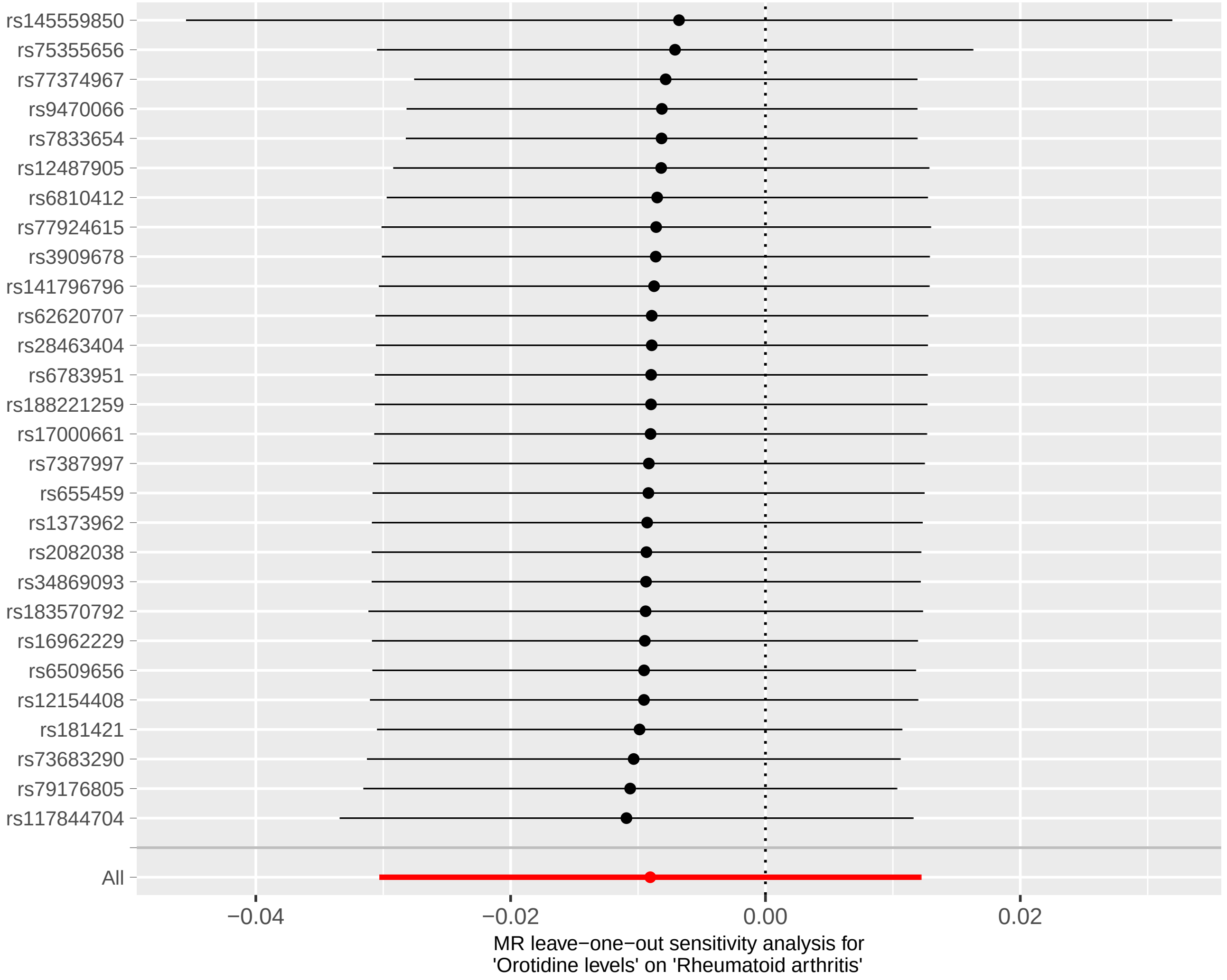
rs145559850
rs75355656
rs77374967
rs9470066
rs7833654
rs12487905
rs6810412
rs77924615
rs3909678
rs141796796
rs62620707
rs28463404
rs6783951
rs188221259
rs17000661
rs7387997
rs655459
rs1373962
rs2082038
rs34869093
rs183570792
rs16962229
rs6509656
rs12154408
rs181421
rs73683290
rs79176805
rs117844704
All
−0.04
−0.02
0.00
0.02
MR leave−one−out sensitivity analysis for
'Orotidine levels' on 'Rheumatoid arthritis'

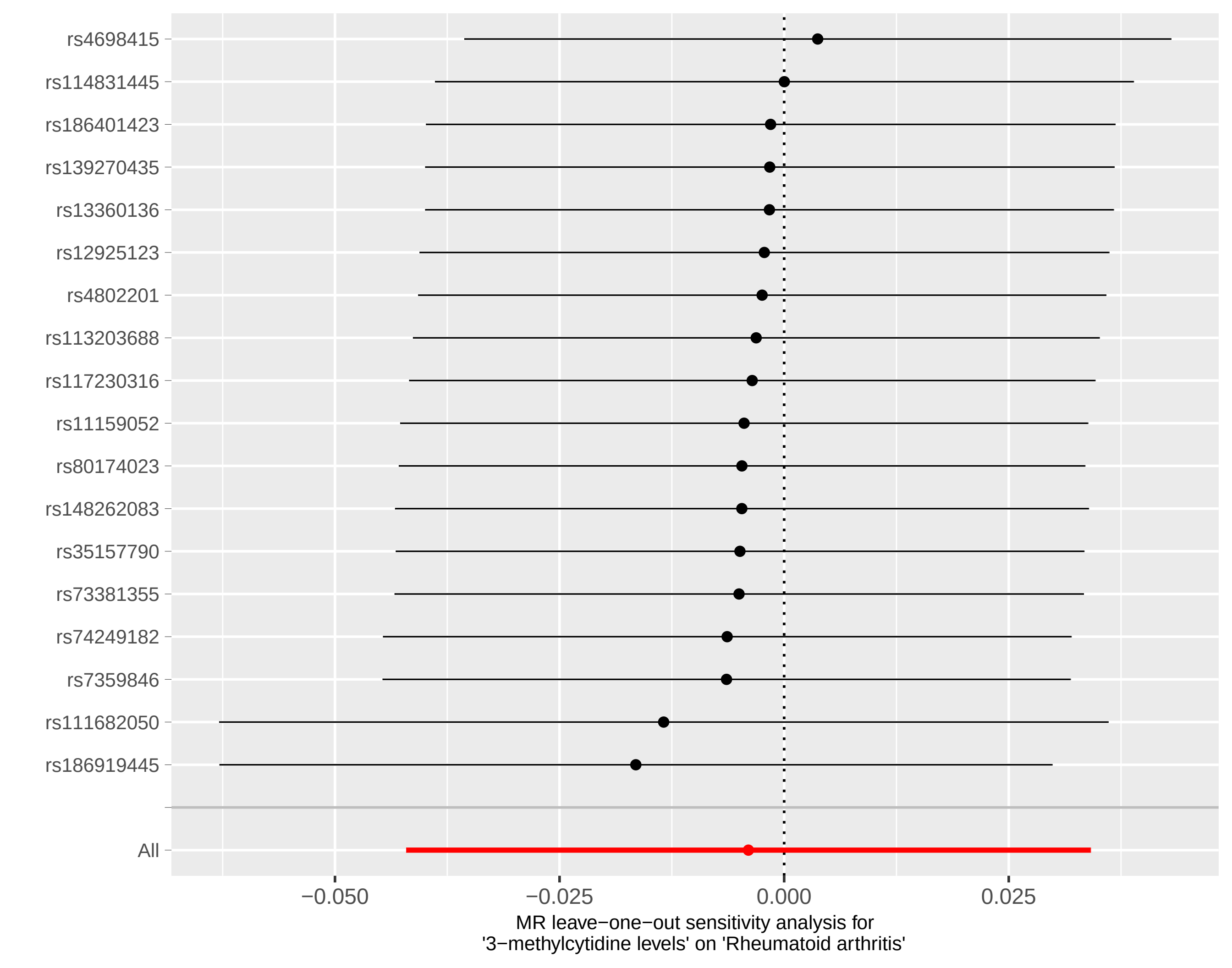

rs4698415
rs114831445
rs186401423
rs139270435
rs13360136
rs12925123
rs4802201
rs113203688
rs117230316
rs11159052
rs80174023
rs148262083
rs35157790
rs73381355
rs74249182
rs7359846
rs111682050
rs186919445
All
−0.050
−0.025
0.000
0.025
MR leave−one−out sensitivity analysis for
'3−methylcytidine levels' on 'Rheumatoid arthritis'

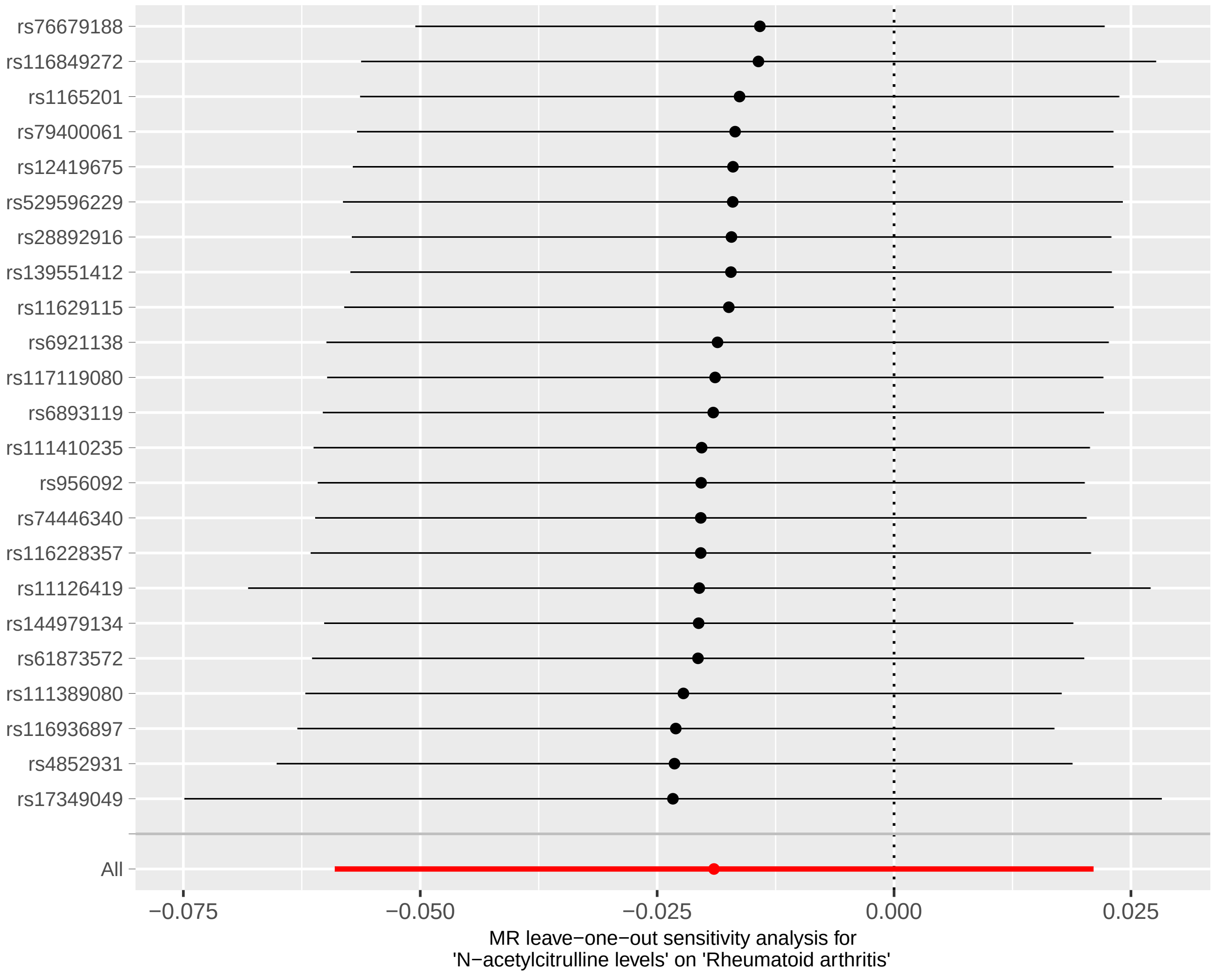
rs76679188
rs116849272
rs1165201
rs79400061
rs12419675
rs529596229
rs28892916
rs139551412
rs11629115
rs6921138
rs117119080
rs6893119
rs111410235
rs956092
rs74446340
rs116228357
rs11126419
rs144979134
rs61873572
rs111389080
rs116936897
rs4852931
rs17349049
All
−0.075
−0.050
−0.025
0.000
0.025
MR leave−one−out sensitivity analysis for
'N−acetylcitrulline levels' on 'Rheumatoid arthritis'

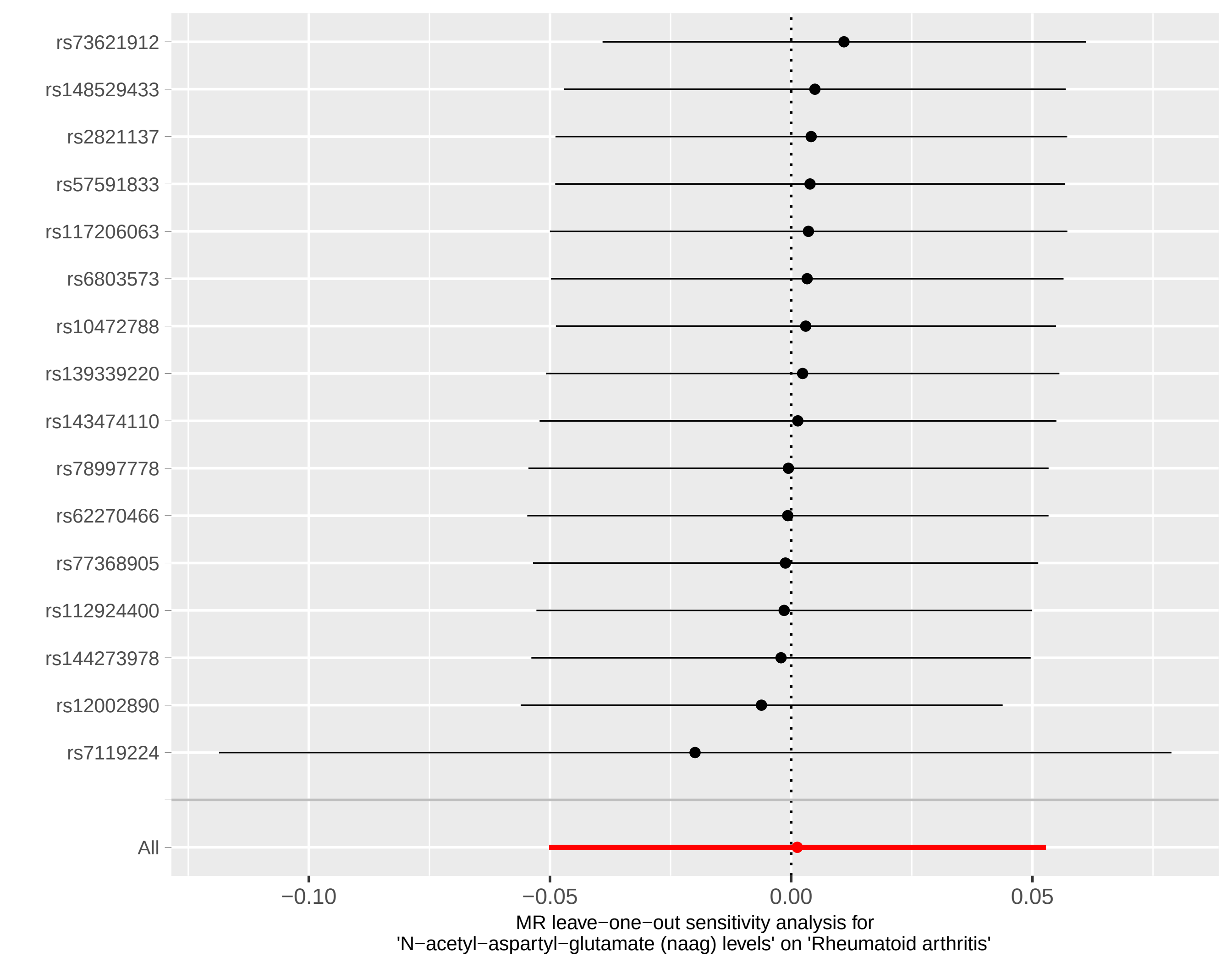
rs73621912
rs148529433
rs2821137
rs57591833
rs117206063
rs6803573
rs10472788
rs139339220
rs143474110
rs78997778
rs62270466
rs77368905
rs112924400
rs144273978
rs12002890
rs7119224
All
−0.10
−0.05
0.00
0.05
MR leave−one−out sensitivity analysis for
'N−acetyl−aspartyl−glutamate (naag) levels' on 'Rheumatoid arthritis'

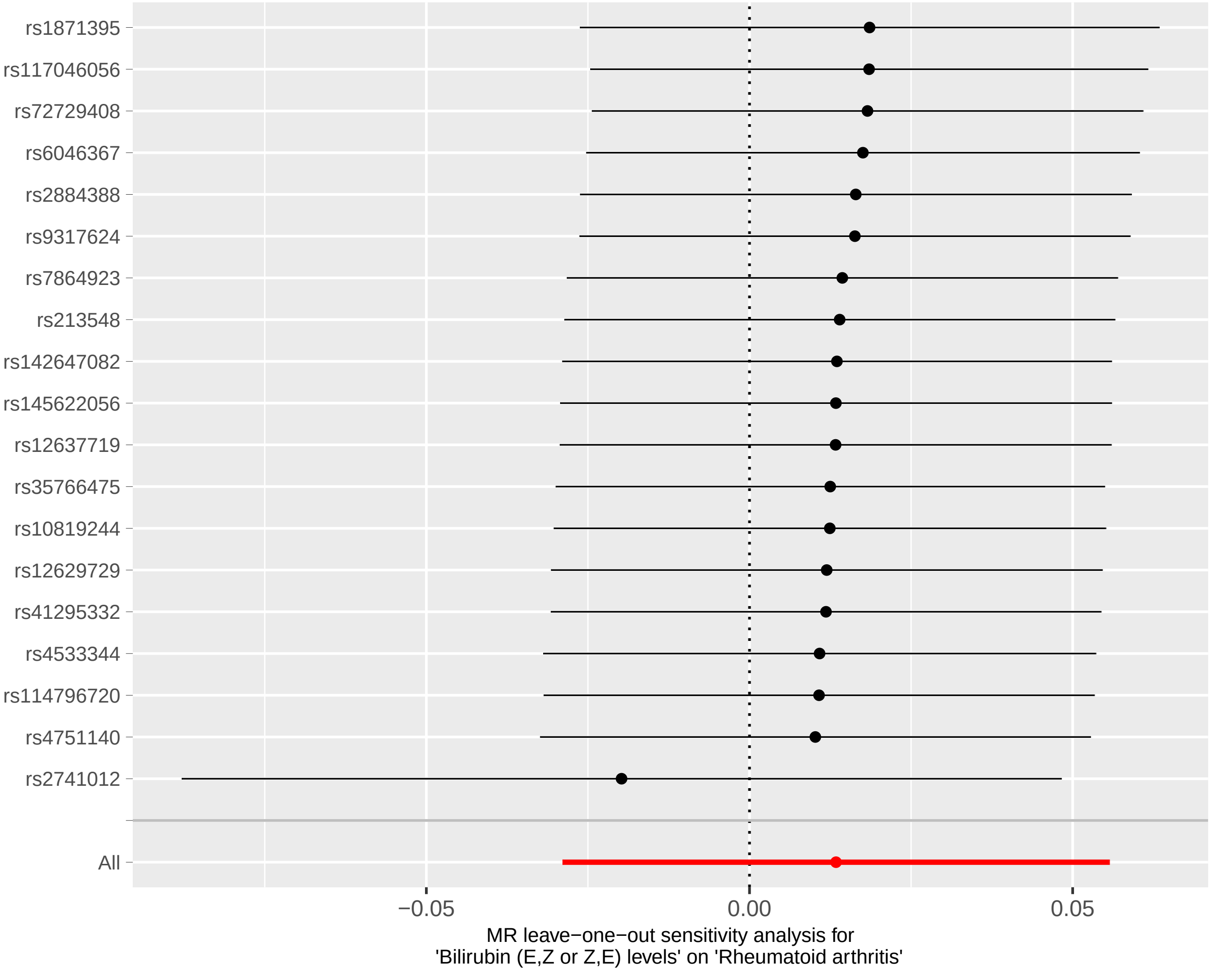

rs1871395
rs117046056
rs72729408
rs6046367
rs2884388
rs9317624
rs7864923
rs213548
rs142647082
rs145622056
rs12637719
rs35766475
rs10819244
rs12629729
rs41295332
rs4533344
rs114796720
rs4751140
rs2741012
All
−0.05
0.00
0.05
MR leave−one−out sensitivity analysis for
'Bilirubin (E,Z or Z,E) levels' on 'Rheumatoid arthritis'

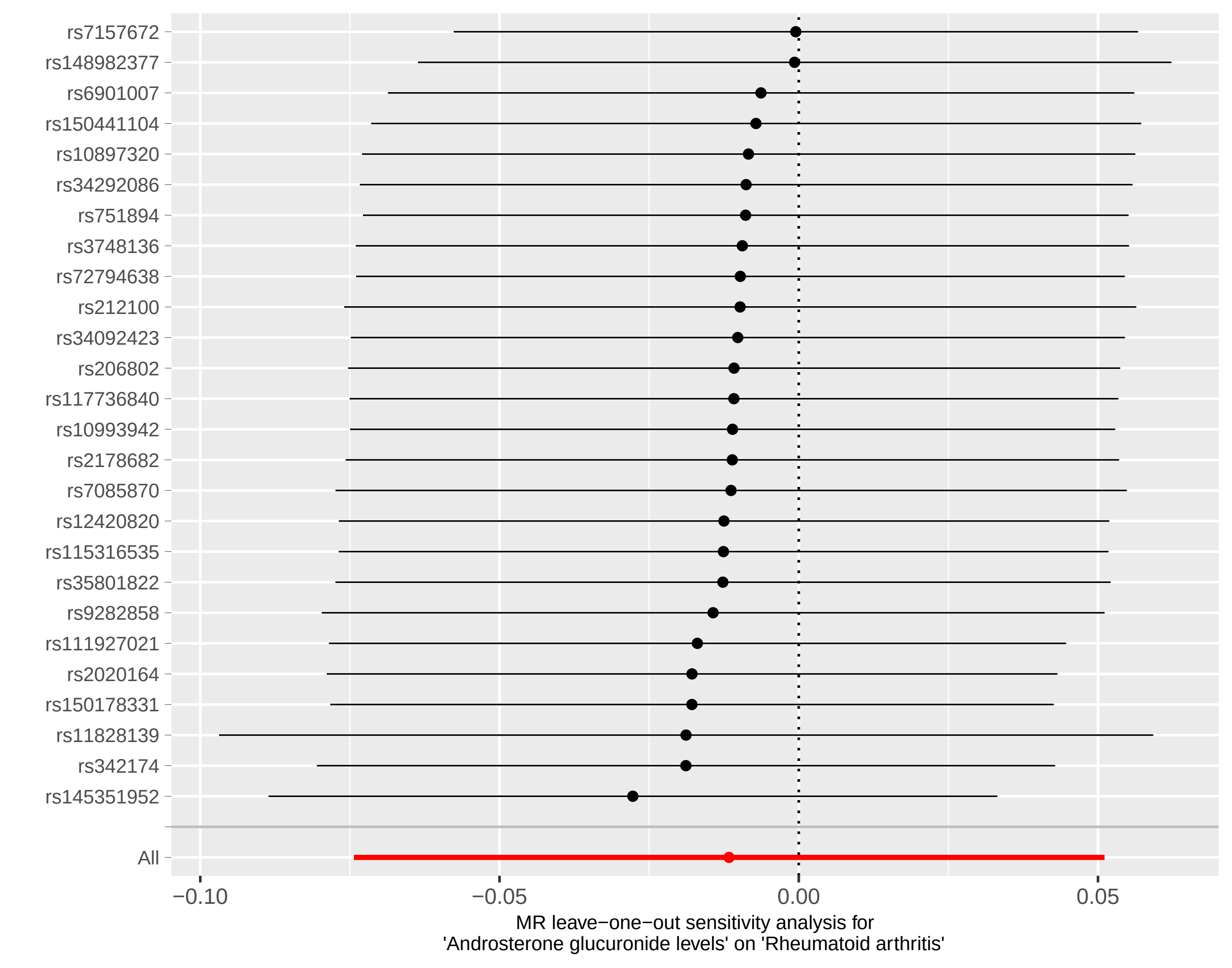

rs7157672
rs148982377
rs6901007
rs150441104
rs10897320
rs34292086
rs751894
rs3748136
rs72794638
rs212100
rs34092423
rs206802
rs117736840
rs10993942
rs2178682
rs7085870
rs12420820
rs115316535
rs35801822
rs9282858
rs111927021
rs2020164
rs150178331
rs11828139
rs342174
rs145351952
All
−0.10
−0.05
0.00
0.05
MR leave−one−out sensitivity analysis for
'Androsterone glucuronide levels' on 'Rheumatoid arthritis'

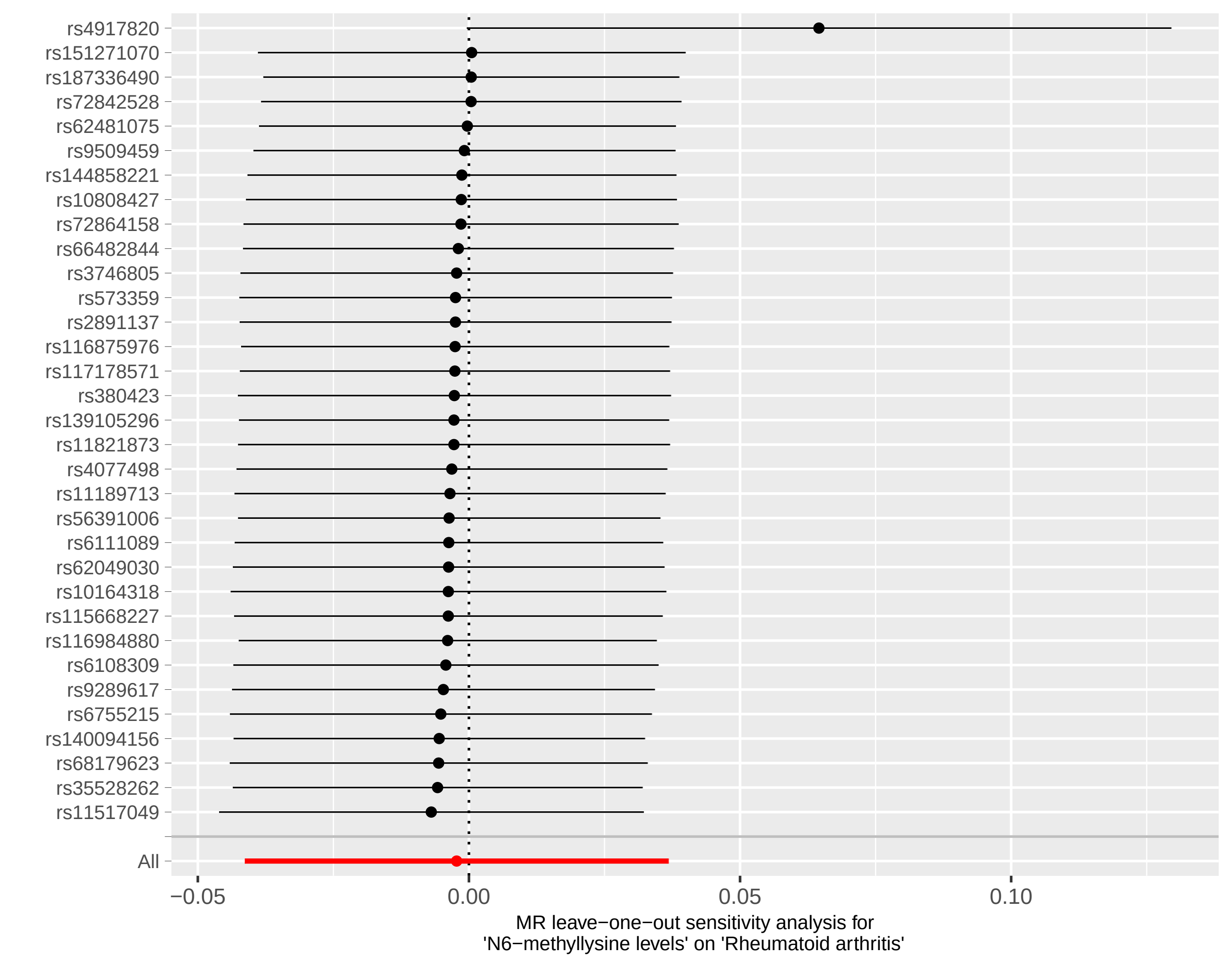
rs4917820
rs151271070
rs187336490
rs72842528
rs62481075
rs9509459
rs144858221
rs10808427
rs72864158
rs66482844
rs3746805
rs573359
rs2891137
rs116875976
rs117178571
rs380423
rs139105296
rs11821873
rs4077498
rs11189713
rs56391006
rs6111089
rs62049030
rs10164318
rs115668227
rs116984880
rs6108309
rs9289617
rs6755215
rs140094156
rs68179623
rs35528262
rs11517049
All
−0.05
0.00
0.05
0.10
MR leave−one−out sensitivity analysis for
'N6−methyllysine levels' on 'Rheumatoid arthritis'

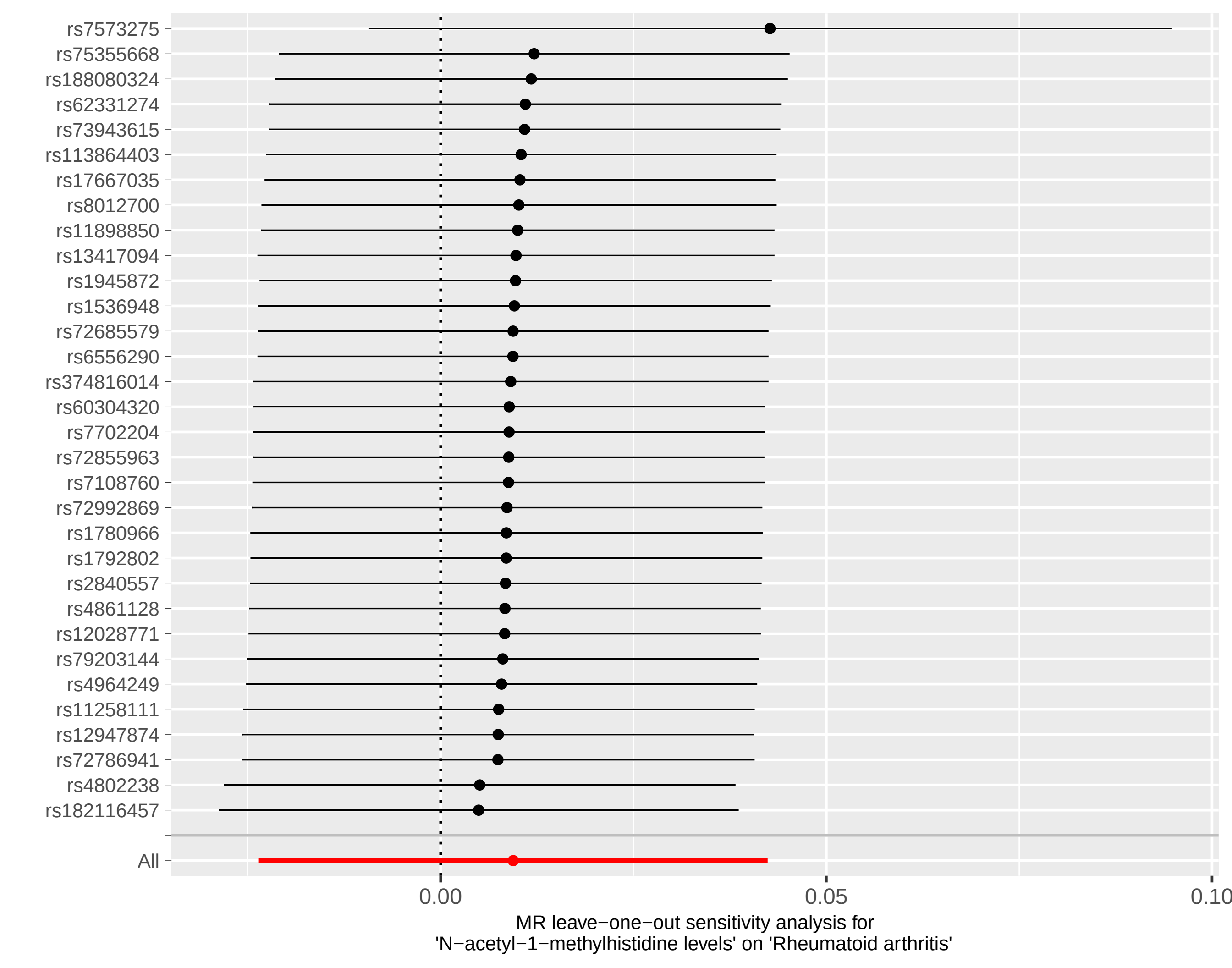
rs7573275
rs75355668
rs188080324
rs62331274
rs73943615
rs113864403
rs17667035
rs8012700
rs11898850
rs13417094
rs1945872
rs1536948
rs72685579
rs6556290
rs374816014
rs60304320
rs7702204
rs72855963
rs7108760
rs72992869
rs1780966
rs1792802
rs2840557
rs4861128
rs12028771
rs79203144
rs4964249
rs11258111
rs12947874
rs72786941
rs4802238
rs182116457
All
0.00
0.05
0.10
MR leave−one−out sensitivity analysis for
'N−acetyl−1−methylhistidine levels' on 'Rheumatoid arthritis'

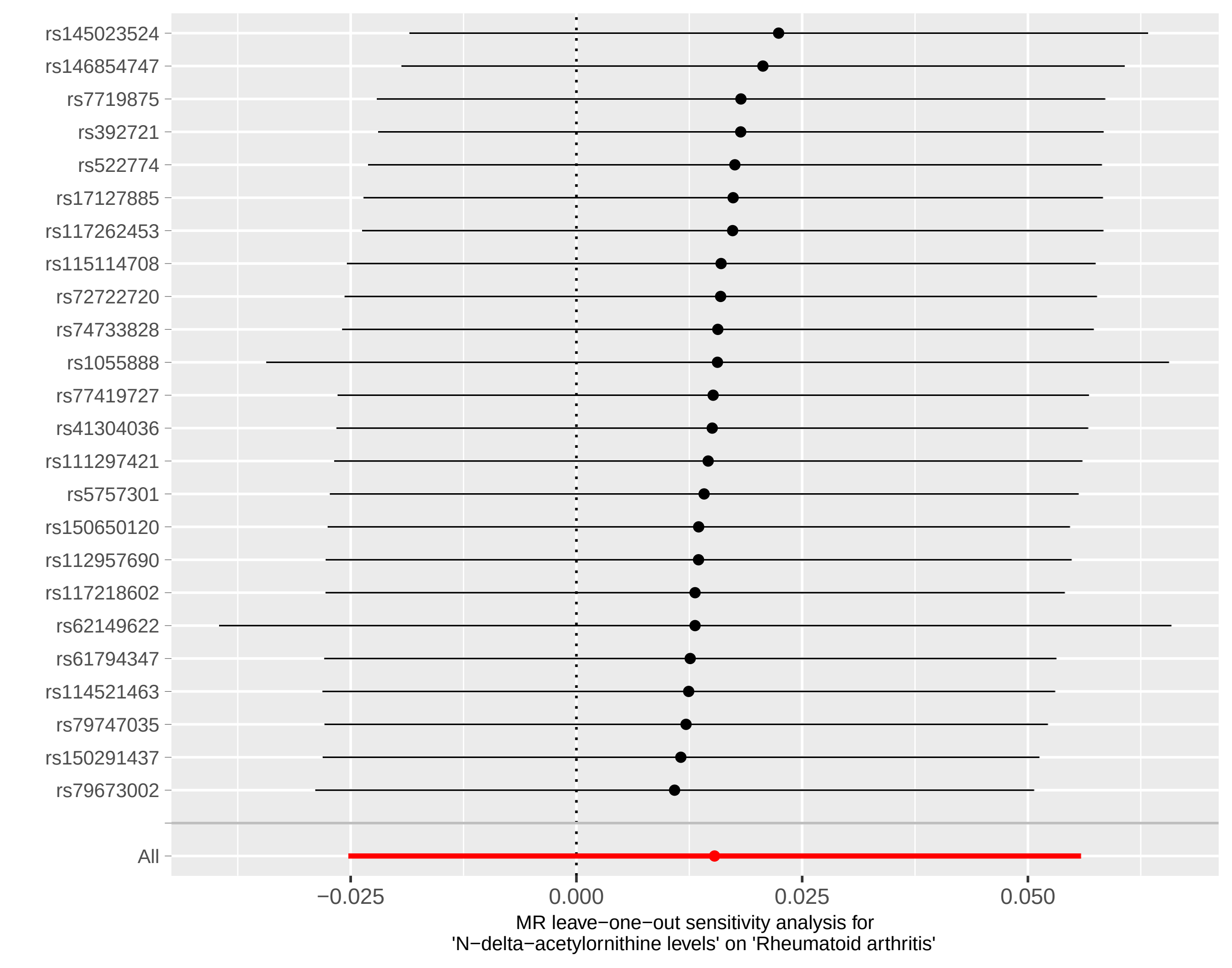
rs145023524
rs146854747
rs7719875
rs392721
rs522774
rs17127885
rs117262453
rs115114708
rs72722720
rs74733828
rs1055888
rs77419727
rs41304036
rs111297421
rs5757301
rs150650120
rs112957690
rs117218602
rs62149622
rs61794347
rs114521463
rs79747035
rs150291437
rs79673002
All
−0.025
0.000
0.025
0.050
MR leave−one−out sensitivity analysis for
'N−delta−acetylornithine levels' on 'Rheumatoid arthritis'

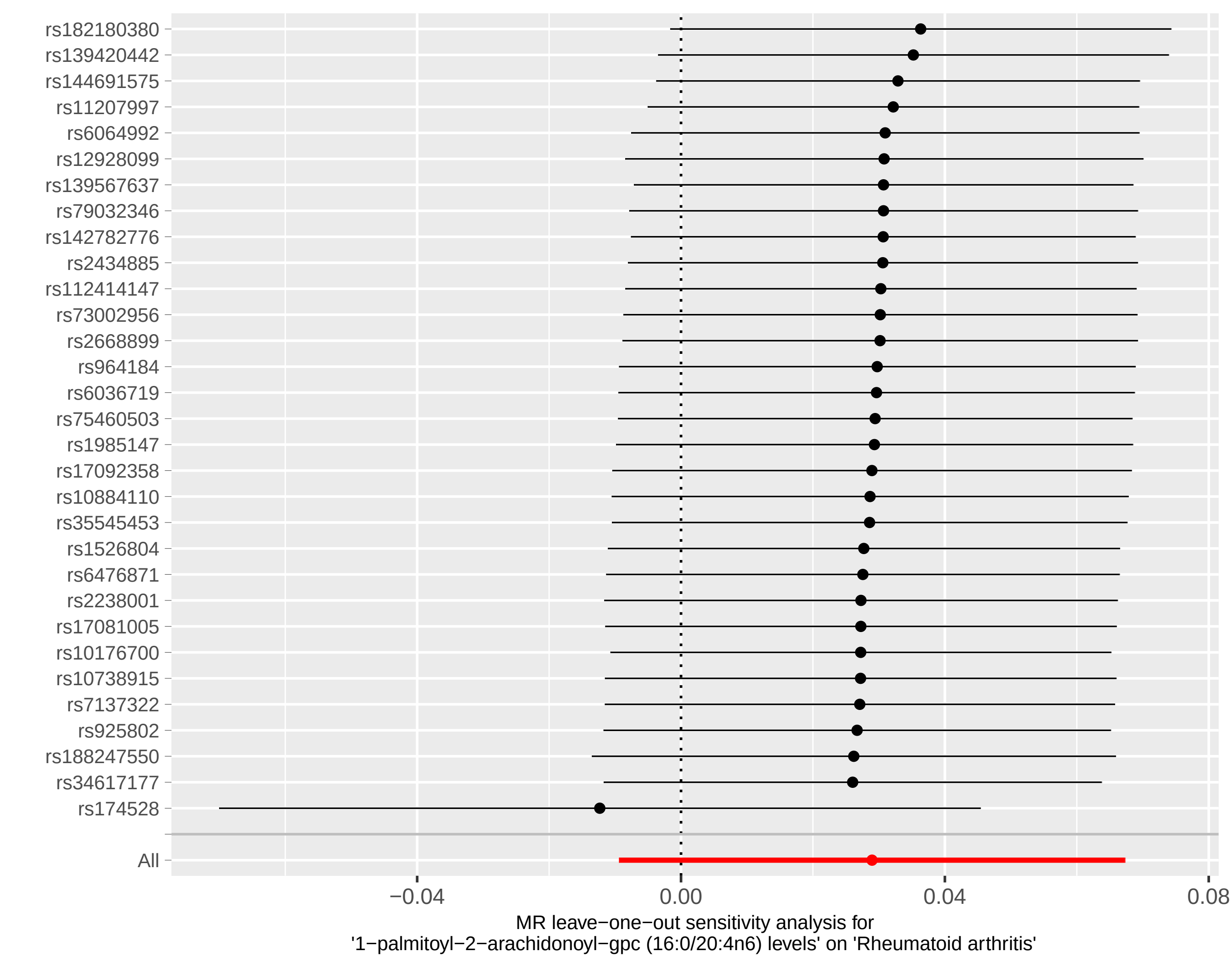
rs182180380
rs139420442
rs144691575
rs11207997
rs6064992
rs12928099
rs139567637
rs79032346
rs142782776
rs2434885
rs112414147
rs73002956
rs2668899
rs964184
rs6036719
rs75460503
rs1985147
rs17092358
rs10884110
rs35545453
rs1526804
rs6476871
rs2238001
rs17081005
rs10176700
rs10738915
rs7137322
rs925802
rs188247550
rs34617177
rs174528
All
−0.04
0.00
0.04
0.08
MR leave−one−out sensitivity analysis for
'1−palmitoyl−2−arachidonoyl−gpc (16:0/20:4n6) levels' on 'Rheumatoid arthritis'

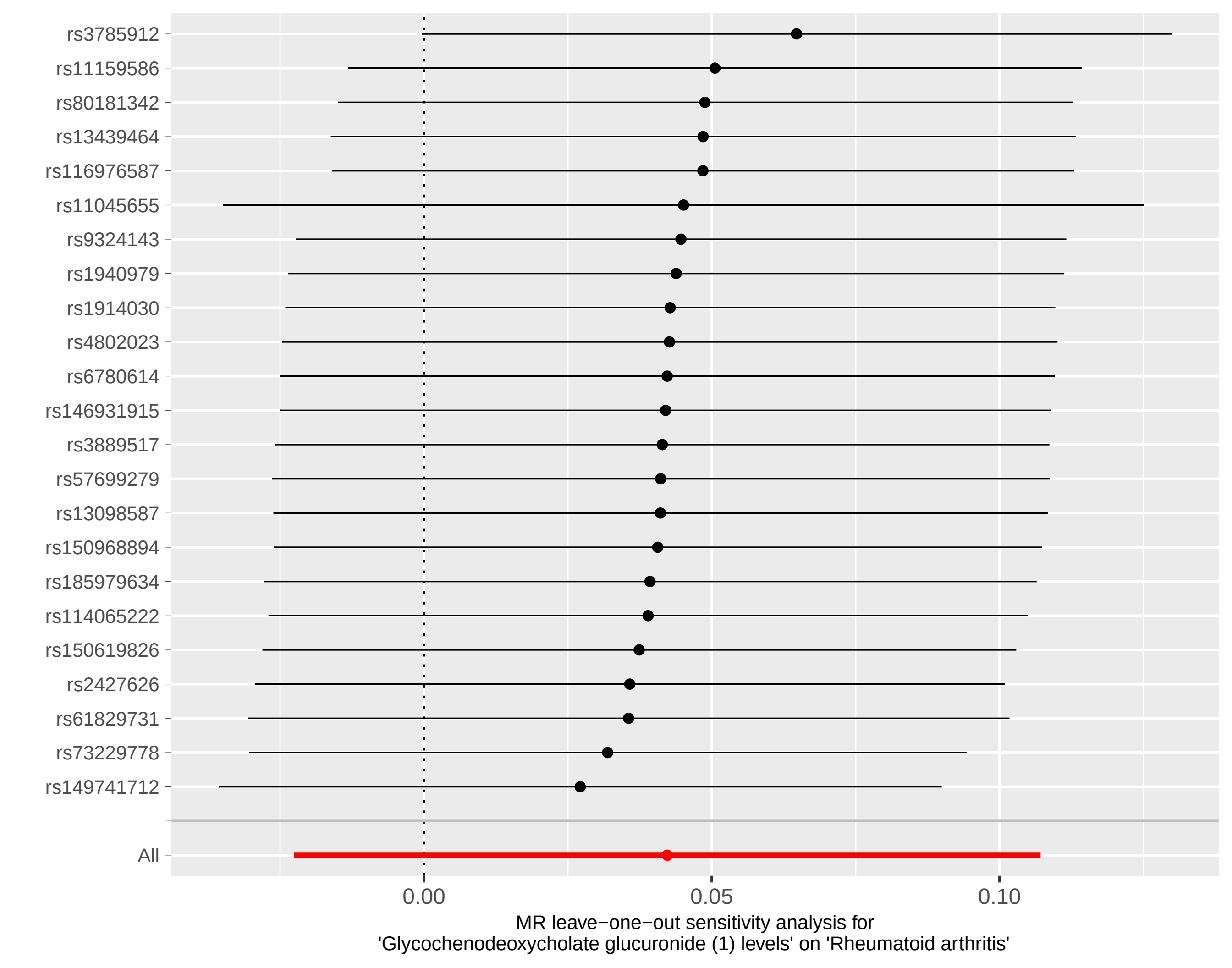
rs3785912
rs11159586
rs80181342
rs13439464
rs116976587
rs11045655
rs9324143
rs1940979
rs1914030
rs4802023
rs6780614
rs146931915
rs3889517
rs57699279
rs13098587
rs150968894
rs185979634
rs114065222
rs150619826
rs2427626
rs61829731
rs73229778
rs149741712
All
0.00
0.05
0.10
MR leave−one−out sensitivity analysis for
'Glycochenodeoxycholate glucuronide (1) levels' on 'Rheumatoid arthritis'

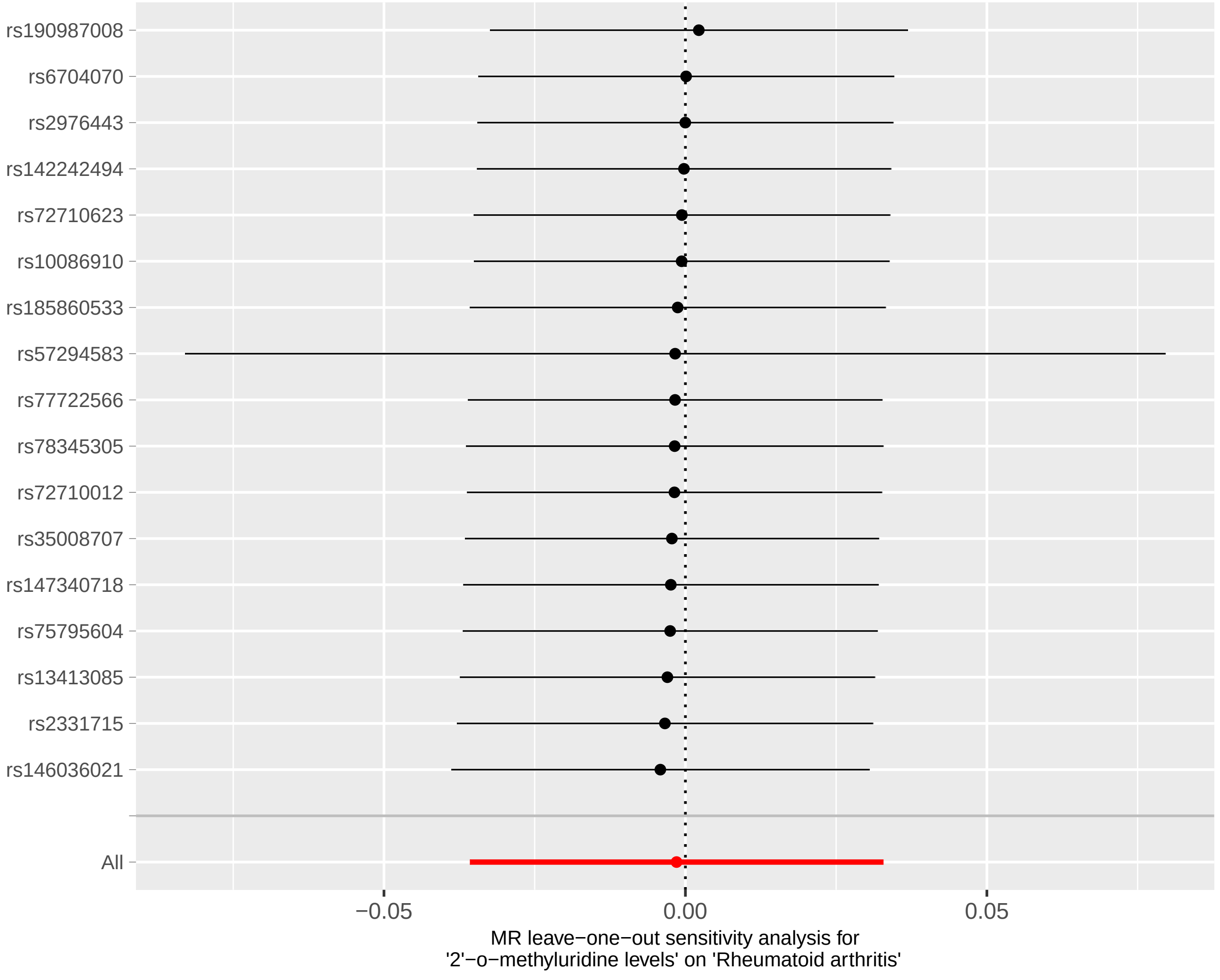
rs190987008
rs6704070
rs2976443
rs142242494
rs72710623
rs10086910
rs185860533
rs57294583
rs77722566
rs78345305
rs72710012
rs35008707
rs147340718
rs75795604
rs13413085
rs2331715
rs146036021
All
−0.05
0.00
0.05
MR leave−one−out sensitivity analysis for
'2'−o−methyluridine levels' on 'Rheumatoid arthritis'

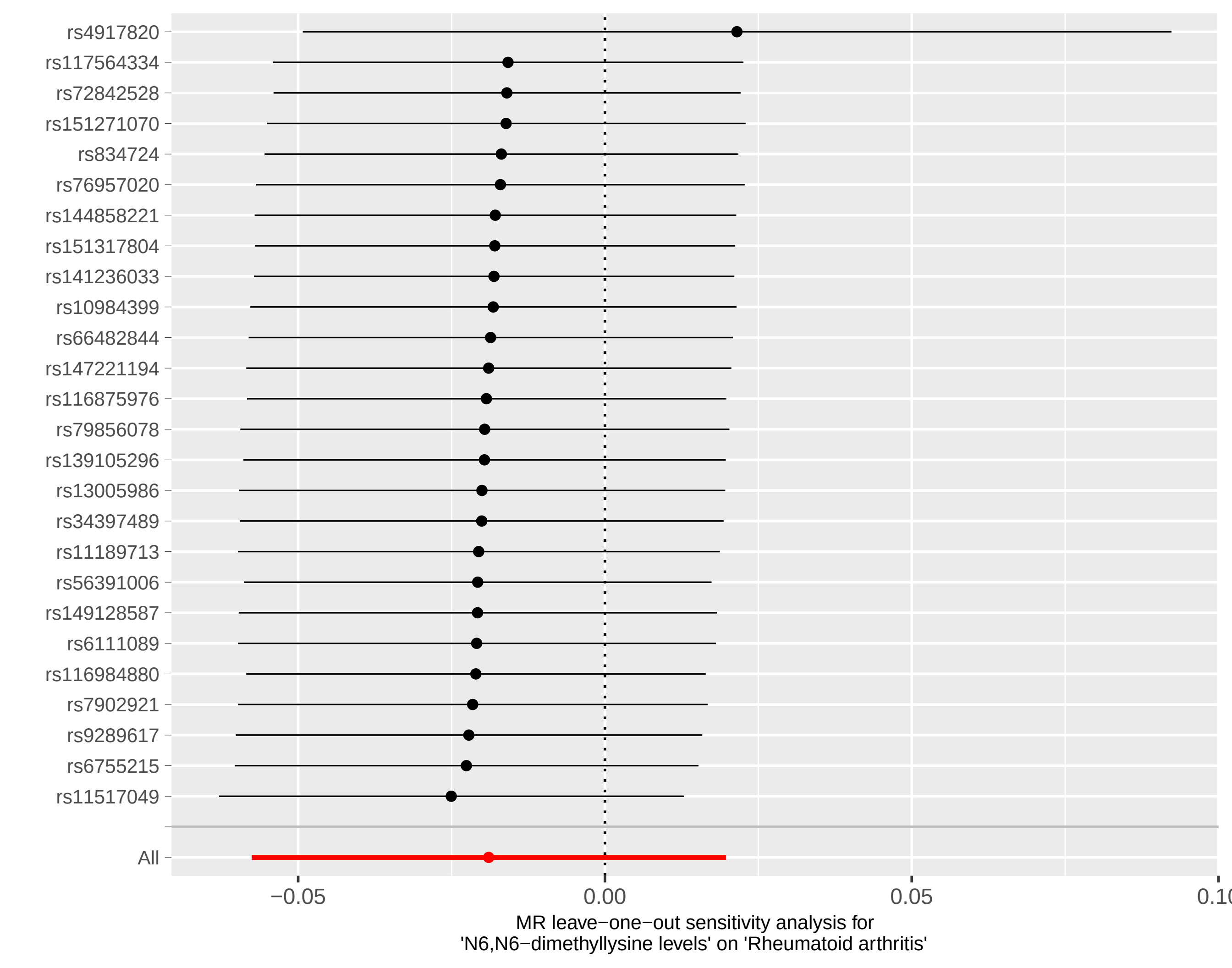

rs4917820
rs117564334
rs72842528
rs151271070
rs834724
rs76957020
rs144858221
rs151317804
rs141236033
rs10984399
rs66482844
rs147221194
rs116875976
rs79856078
rs139105296
rs13005986
rs34397489
rs11189713
rs56391006
rs149128587
rs6111089
rs116984880
rs7902921
rs9289617
rs6755215
rs11517049
All
−0.05
0.00
0.05
MR leave−one−out sensitivity analysis for
'N6,N6−dimethyllysine levels' on 'Rheumatoid arthritis'

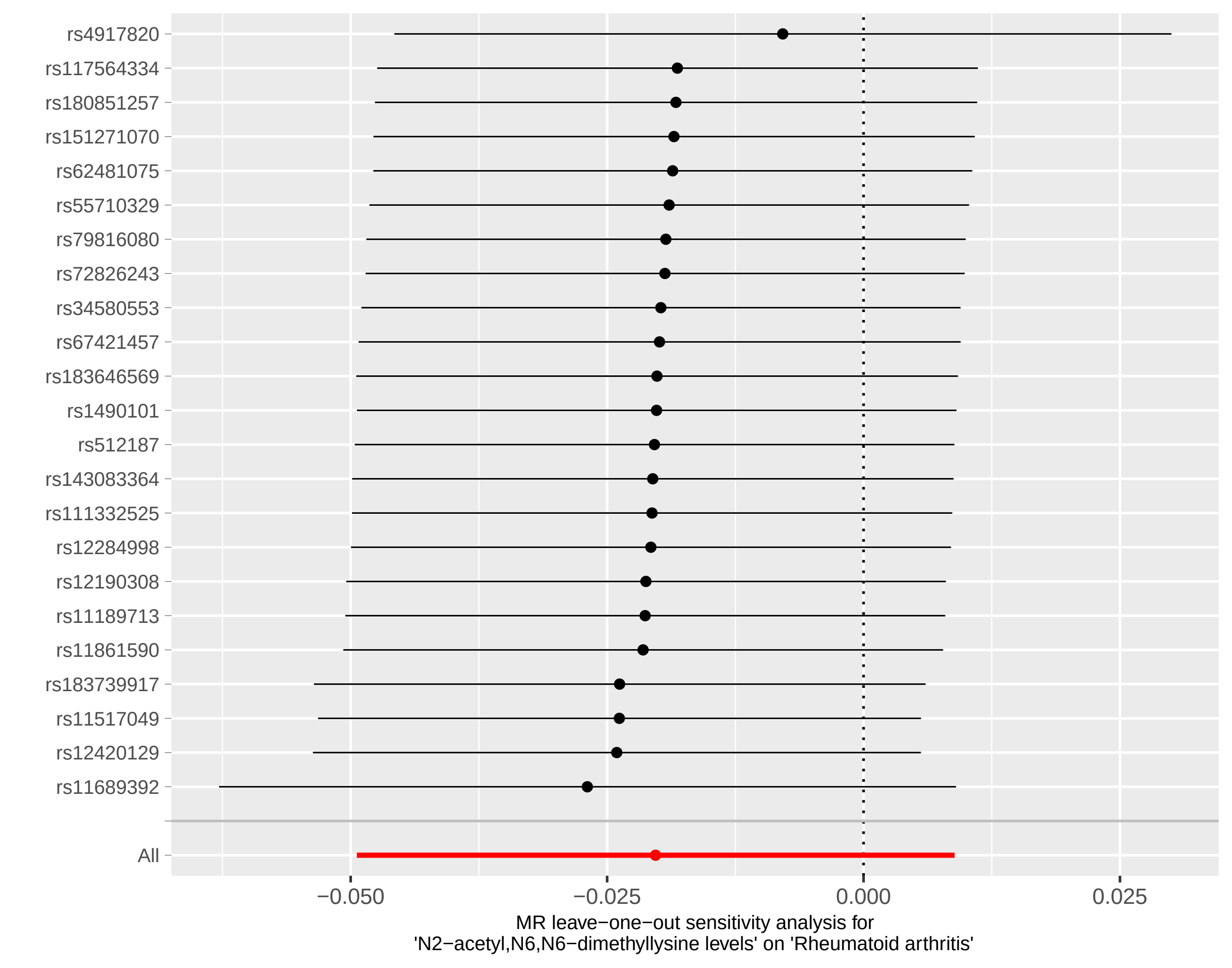
rs4917820
rs117564334
rs180851257
rs151271070
rs62481075
rs55710329
rs79816080
rs72826243
rs34580553
rs67421457
rs183646569
rs1490101
rs512187
rs143083364
rs111332525
rs12284998
rs12190308
rs11189713
rs11861590
rs183739917
rs11517049
rs12420129
rs11689392
All
−0.050
−0.025
0.000
0.025
MR leave−one−out sensitivity analysis for
'N2−acetyl,N6,N6−dimethyllysine levels' on 'Rheumatoid arthritis'

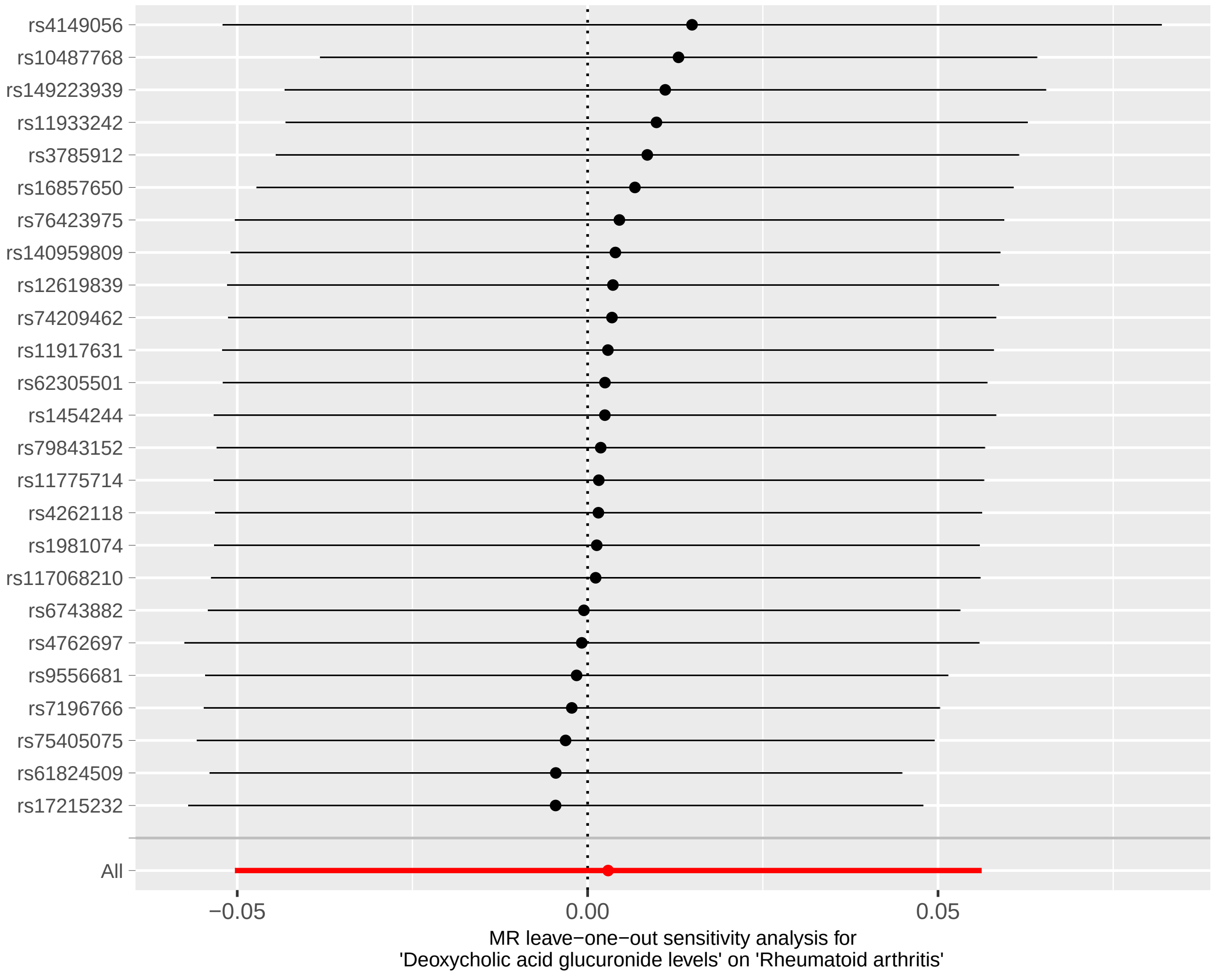
rs4149056
rs10487768
rs149223939
rs11933242
rs3785912
rs16857650
rs76423975
rs140959809
rs12619839
rs74209462
rs11917631
rs62305501
rs1454244
rs79843152
rs11775714
rs4262118
rs1981074
rs117068210
rs6743882
rs4762697
rs9556681
rs7196766
rs75405075
rs61824509
rs17215232
All
−0.05
0.00
0.05
MR leave−one−out sensitivity analysis for
'Deoxycholic acid glucuronide levels' on 'Rheumatoid arthritis'

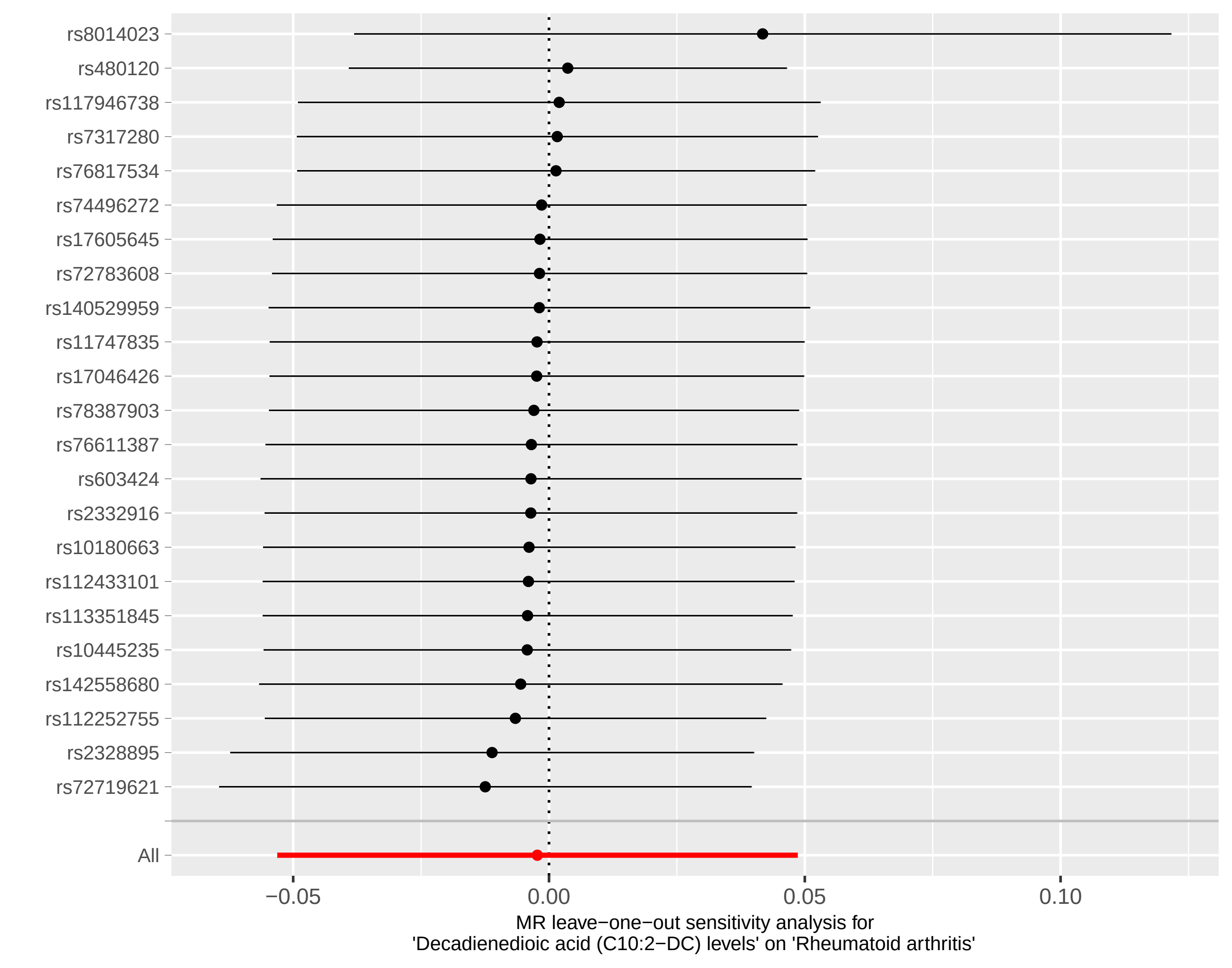
rs8014023
rs480120
rs117946738
rs7317280
rs76817534
rs74496272
rs17605645
rs72783608
rs140529959
rs11747835
rs17046426
rs78387903
rs76611387
rs603424
rs2332916
rs10180663
rs112433101
rs113351845
rs10445235
rs142558680
rs112252755
rs2328895
rs72719621
All
−0.05
0.00
0.05
0.10
MR leave−one−out sensitivity analysis for
'Decadienedioic acid (C10:2−DC) levels' on 'Rheumatoid arthritis'

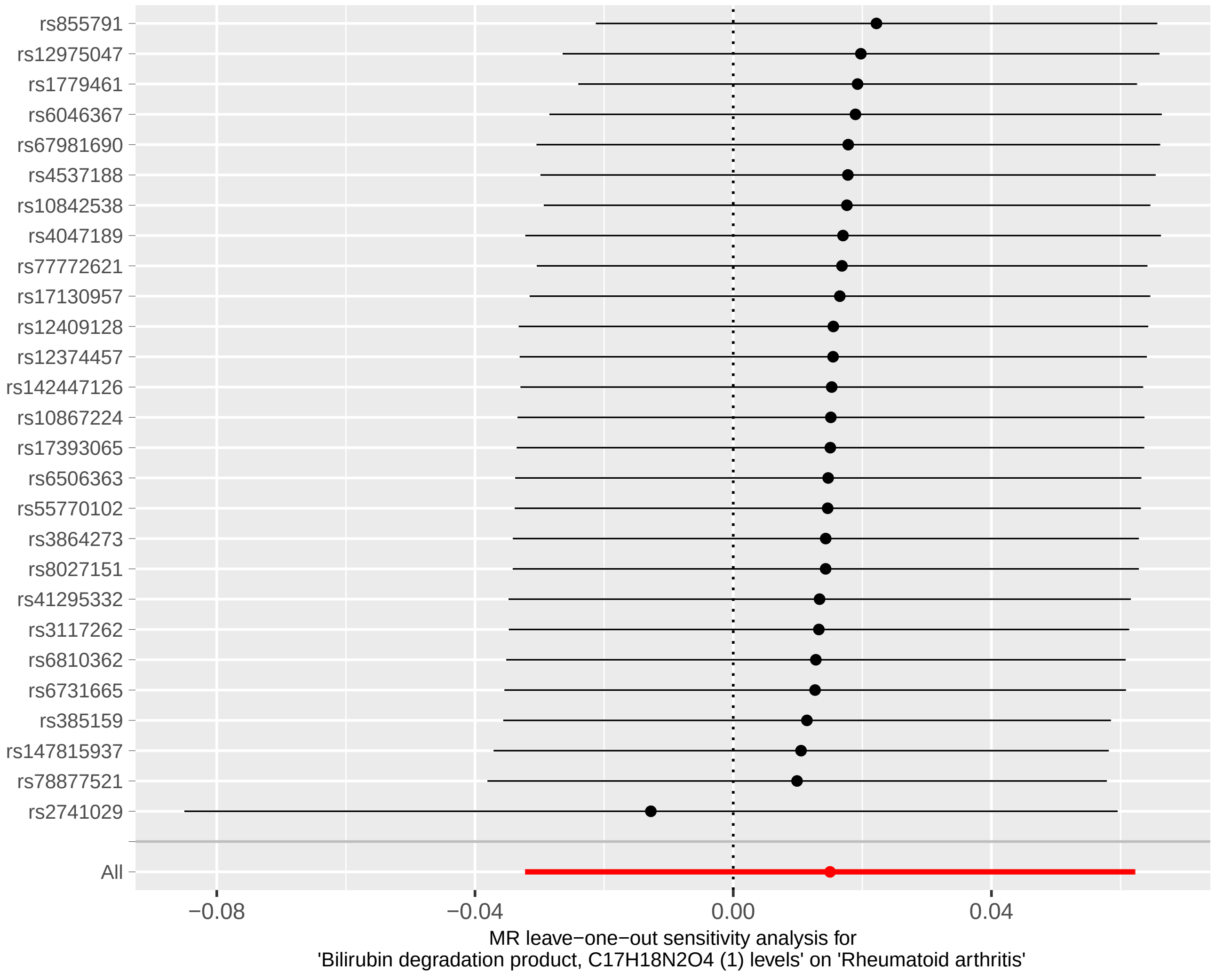
rs855791
rs12975047
rs1779461
rs6046367
rs67981690
rs4537188
rs10842538
rs4047189
rs77772621
rs17130957
rs12409128
rs12374457
rs142447126
rs10867224
rs17393065
rs6506363
rs55770102
rs3864273
rs8027151
rs41295332
rs3117262
rs6810362
rs6731665
rs385159
rs147815937
rs78877521
rs2741029
All
−0.08
−0.04
0.00
0.04
MR leave−one−out sensitivity analysis for
'Bilirubin degradation product, C17H18N2O4 (1) levels' on 'Rheumatoid arthritis'

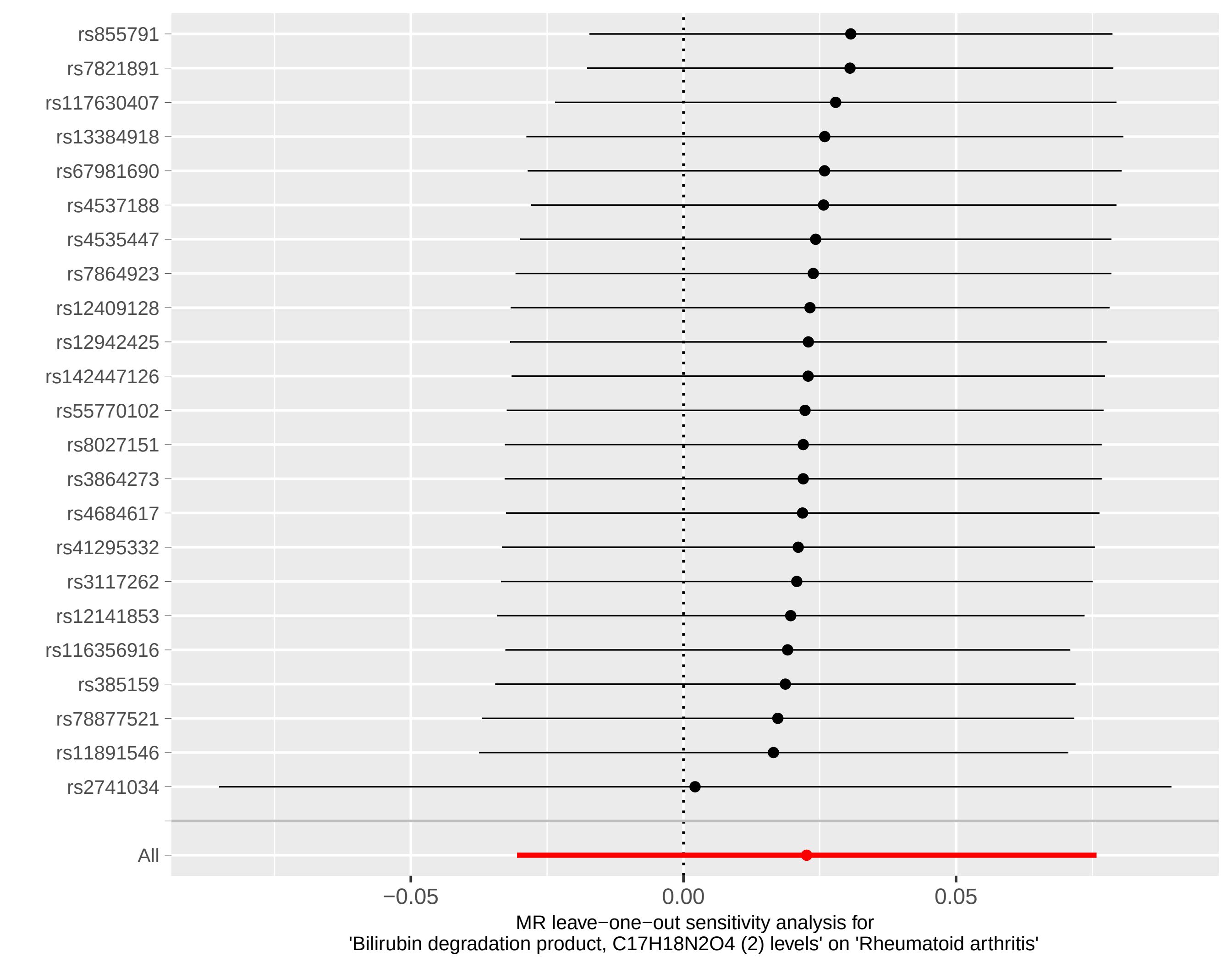
rs855791
rs7821891
rs117630407
rs13384918
rs67981690
rs4537188
rs4535447
rs7864923
rs12409128
rs12942425
rs142447126
rs55770102
rs8027151
rs3864273
rs4684617
rs41295332
rs3117262
rs12141853
rs116356916
rs385159
rs78877521
rs11891546
rs2741034
All
−0.05
0.00
0.05
MR leave−one−out sensitivity analysis for
'Bilirubin degradation product, C17H18N2O4 (2) levels' on 'Rheumatoid arthritis'

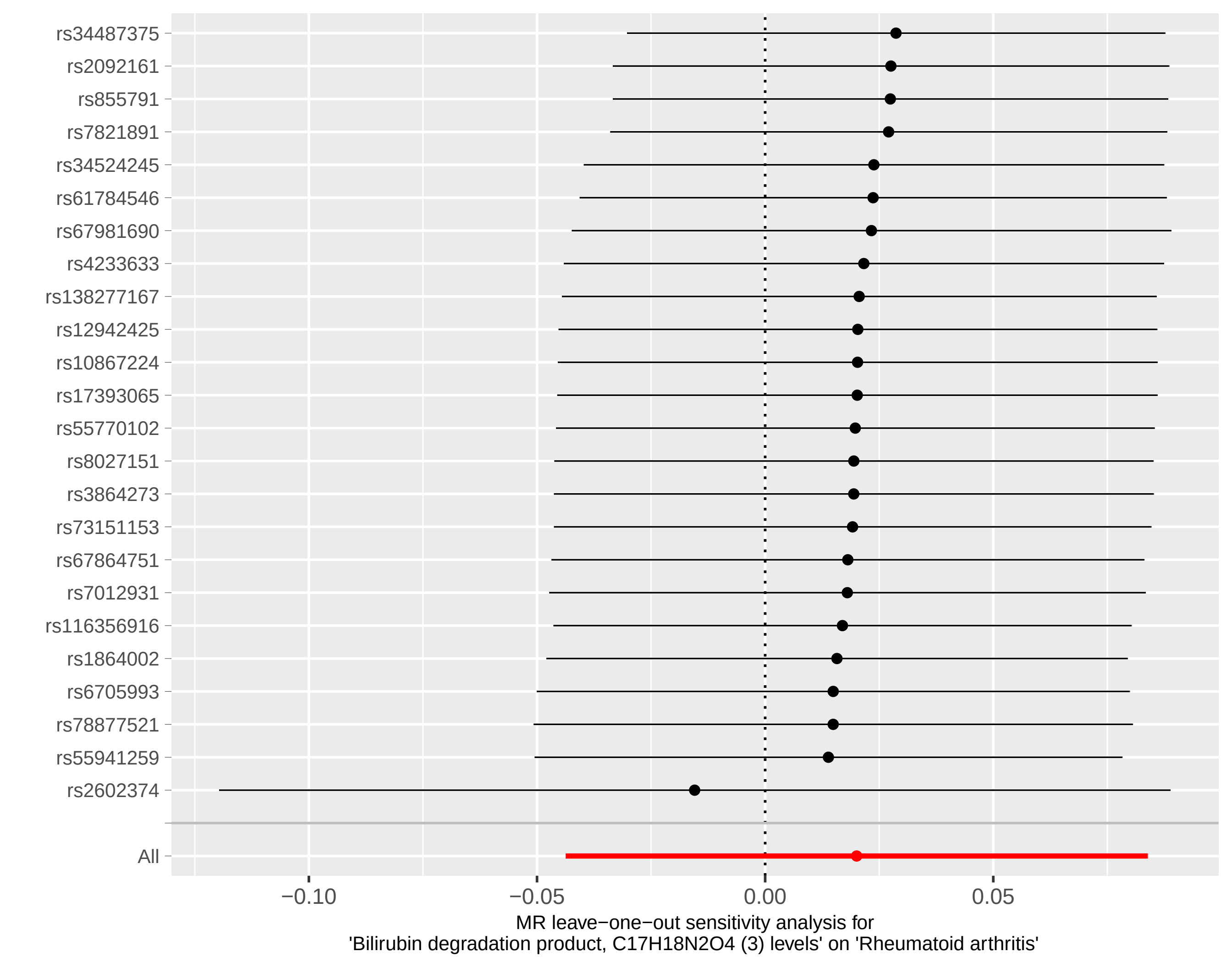
rs34487375
rs2092161
rs855791
rs7821891
rs34524245
rs61784546
rs67981690
rs4233633
rs138277167
rs12942425
rs10867224
rs17393065
rs55770102
rs8027151
rs3864273
rs73151153
rs67864751
rs7012931
rs116356916
rs1864002
rs6705993
rs78877521
rs55941259
rs2602374
All
−0.10
−0.05
0.00
0.05
MR leave−one−out sensitivity analysis for
'Bilirubin degradation product, C17H18N2O4 (3) levels' on 'Rheumatoid arthritis'

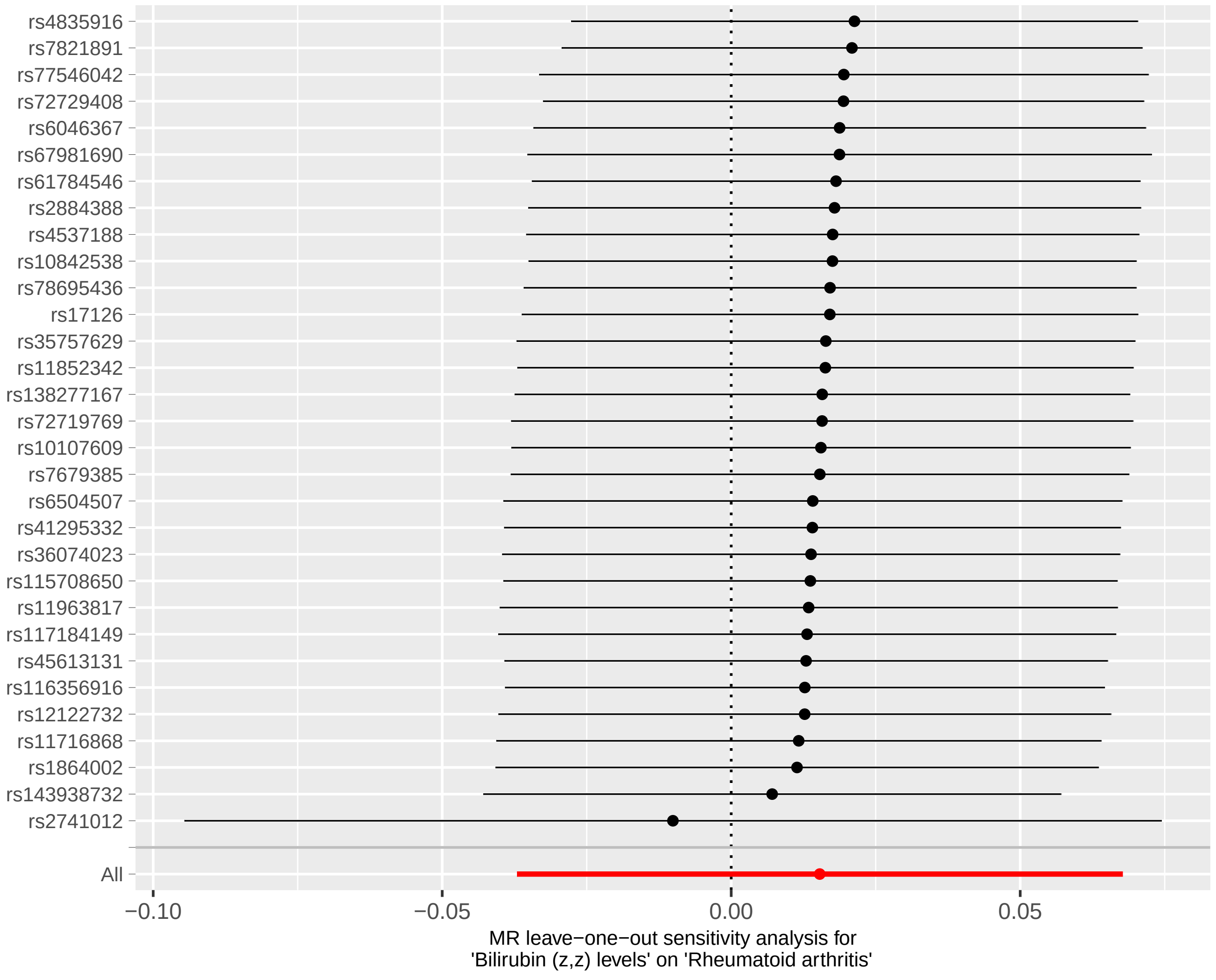
rs4835916
rs7821891
rs77546042
rs72729408
rs6046367
rs67981690
rs61784546
rs2884388
rs4537188
rs10842538
rs78695436
rs17126
rs35757629
rs11852342
rs138277167
rs72719769
rs10107609
rs7679385
rs6504507
rs41295332
rs36074023
rs115708650
rs11963817
rs117184149
rs45613131
rs116356916
rs12122732
rs11716868
rs1864002
rs143938732
rs2741012
All
−0.10
−0.05
0.00
0.05
MR leave−one−out sensitivity analysis for
'Bilirubin (z,z) levels' on 'Rheumatoid arthritis'

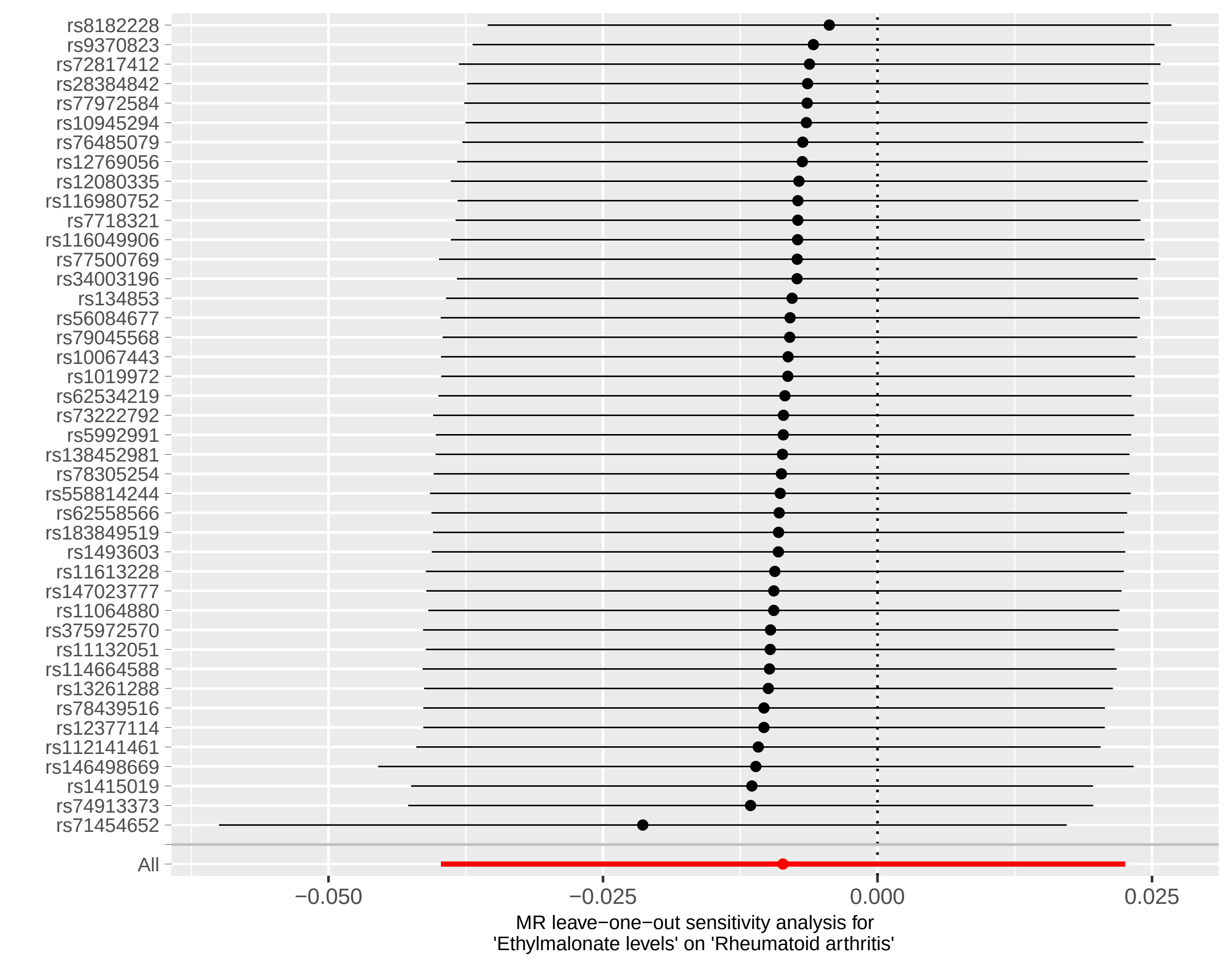

rs8182228
rs9370823
rs72817412
rs28384842
rs77972584
rs10945294
rs76485079
rs12769056
rs12080335
rs116980752
rs7718321
rs116049906
rs77500769
rs34003196
rs134853
rs56084677
rs79045568
rs10067443
rs1019972
rs62534219
rs73222792
rs5992991
rs138452981
rs78305254
rs558814244
rs62558566
rs183849519
rs1493603
rs11613228
rs147023777
rs11064880
rs375972570
rs11132051
rs114664588
rs13261288
rs78439516
rs12377114
rs112141461
rs146498669
rs1415019
rs74913373
rs71454652
All
−0.050
−0.025
0.000
0.025
MR leave−one−out sensitivity analysis for
'Ethylmalonate levels' on 'Rheumatoid arthritis'

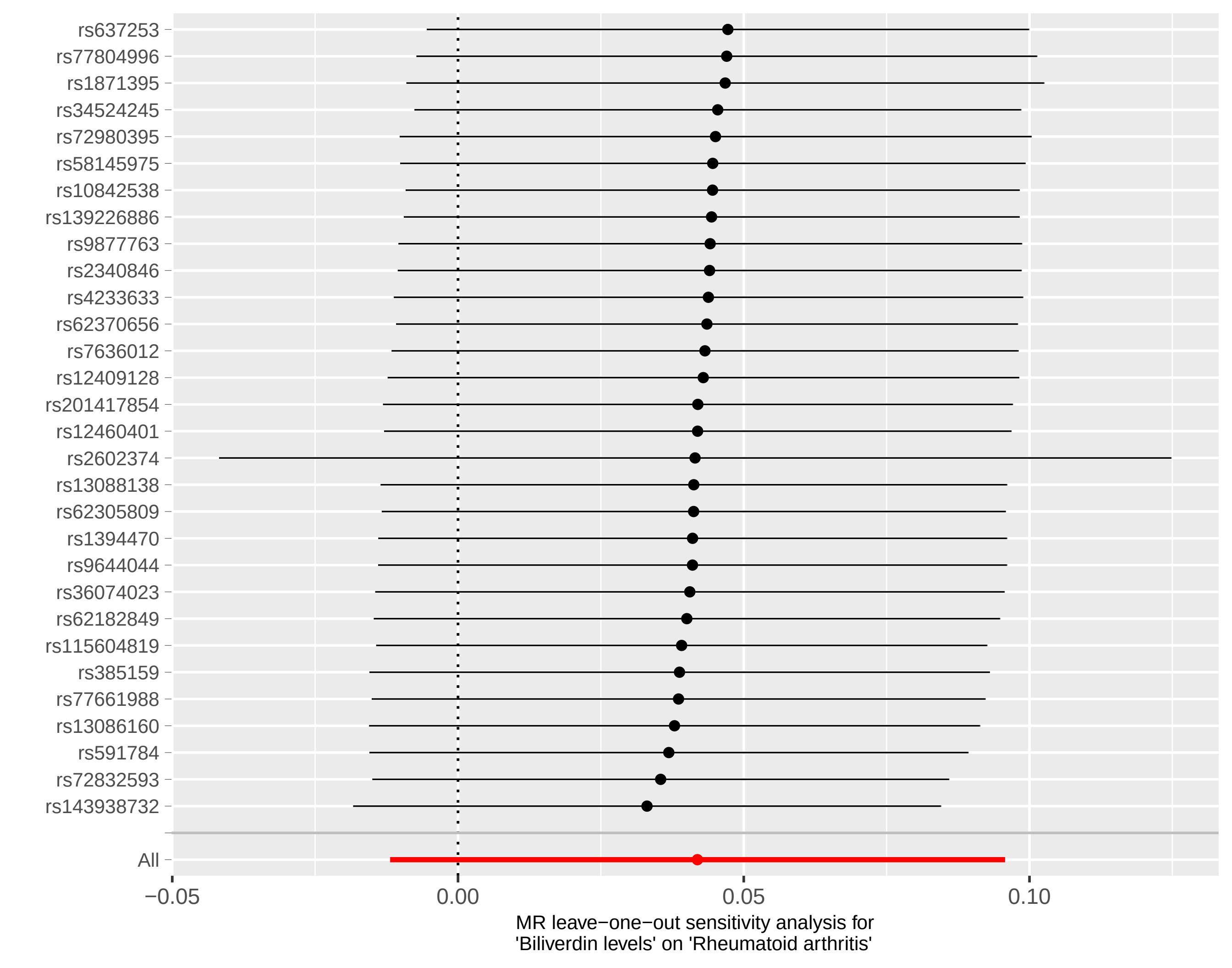
rs637253
rs77804996
rs1871395
rs34524245
rs72980395
rs58145975
rs10842538
rs139226886
rs9877763
rs2340846
rs4233633
rs62370656
rs7636012
rs12409128
rs201417854
rs12460401
rs2602374
rs13088138
rs62305809
rs1394470
rs9644044
rs36074023
rs62182849
rs115604819
rs385159
rs77661988
rs13086160
rs591784
rs72832593
rs143938732
All
−0.05
0.00
0.05
0.10
MR leave−one−out sensitivity analysis for
'Biliverdin levels' on 'Rheumatoid arthritis'

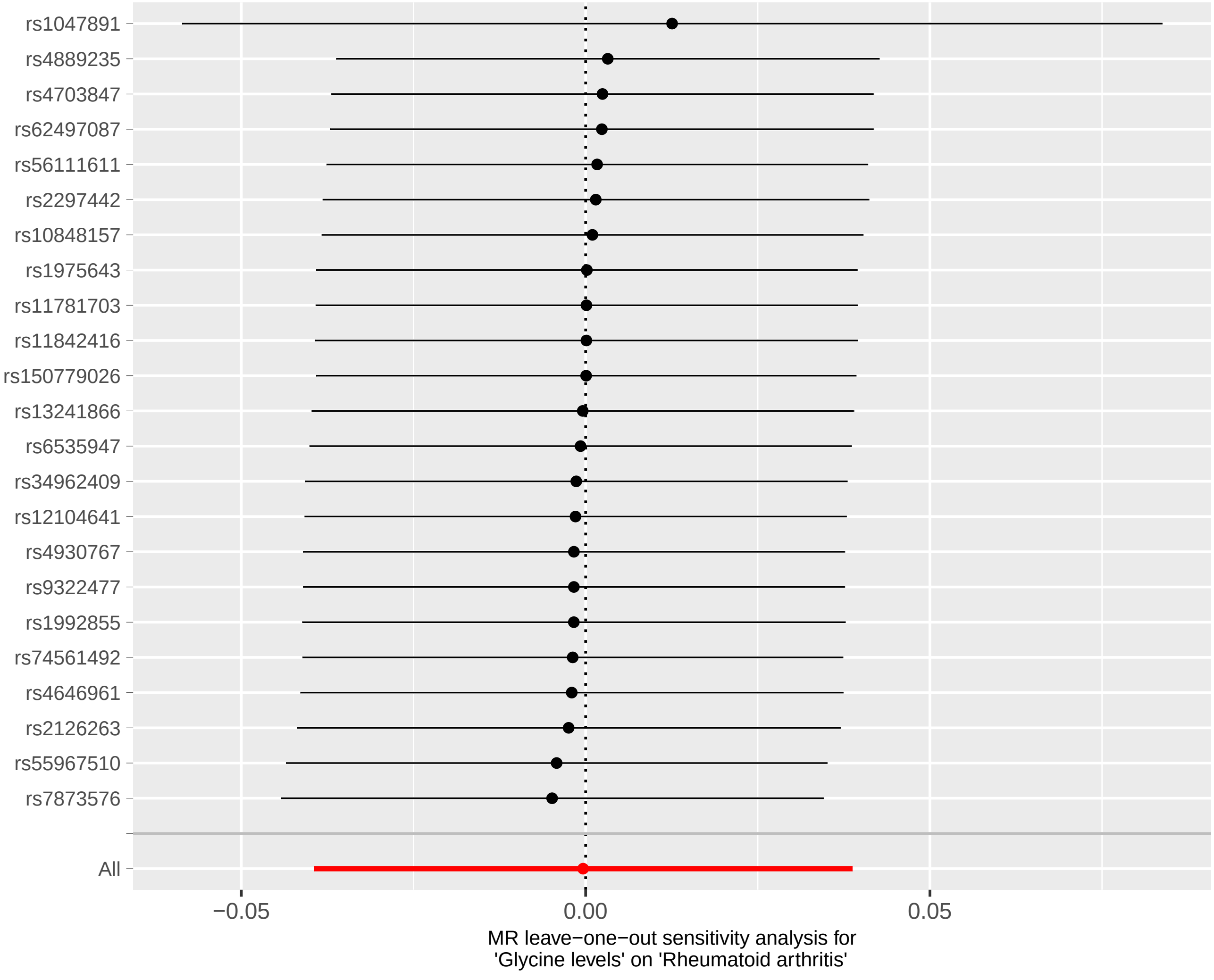
rs1047891
rs4889235
rs4703847
rs62497087
rs56111611
rs2297442
rs10848157
rs1975643
rs11781703
rs11842416
rs150779026
rs13241866
rs6535947
rs34962409
rs12104641
rs4930767
rs9322477
rs1992855
rs74561492
rs4646961
rs2126263
rs55967510
rs7873576
All
−0.05
0.00
0.05
MR leave−one−out sensitivity analysis for
'Glycine levels' on 'Rheumatoid arthritis'

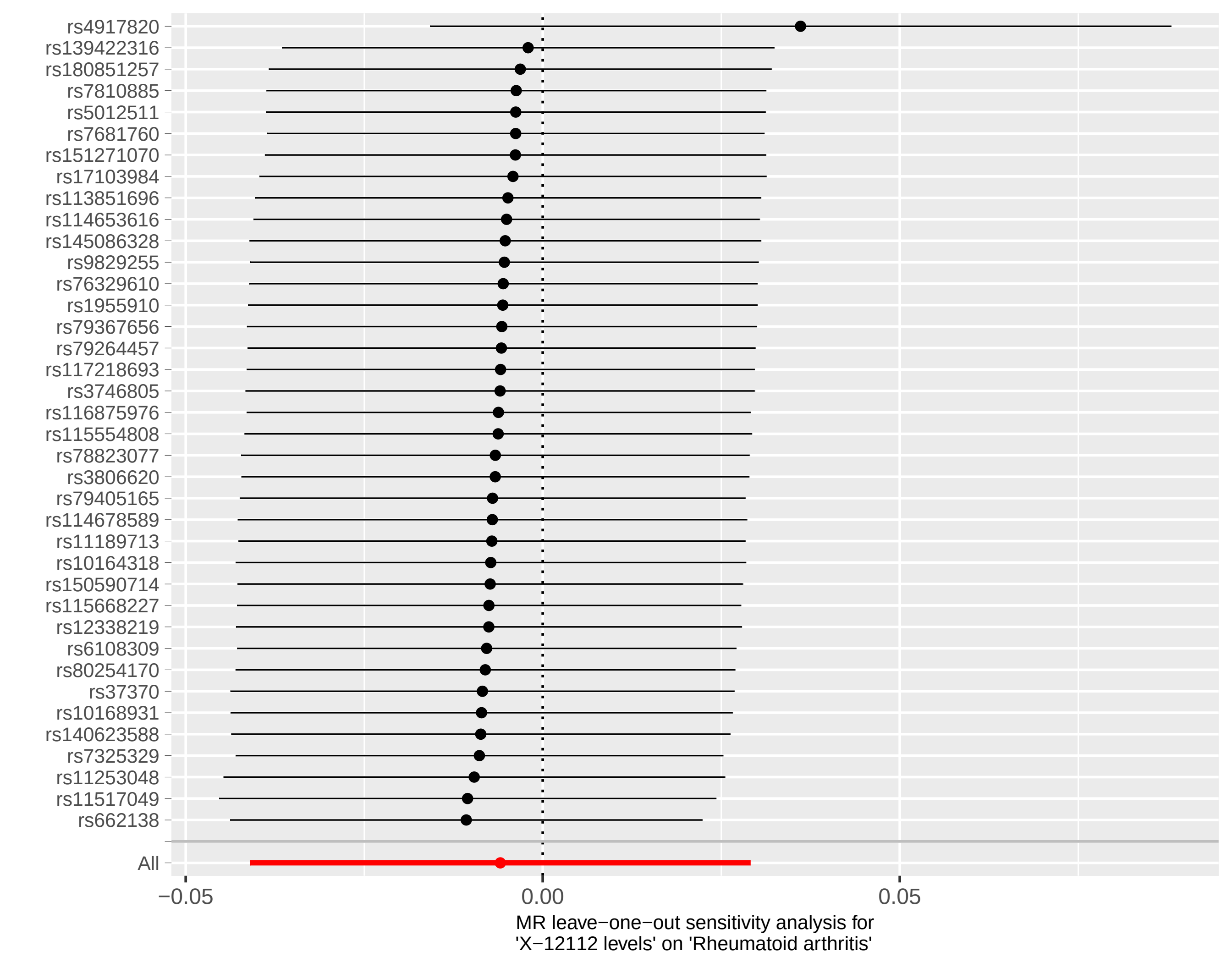
rs4917820
rs139422316
rs180851257
rs7810885
rs5012511
rs7681760
rs151271070
rs17103984
rs113851696
rs114653616
rs145086328
rs9829255
rs76329610
rs1955910
rs79367656
rs79264457
rs117218693
rs3746805
rs116875976
rs115554808
rs78823077
rs3806620
rs79405165
rs114678589
rs11189713
rs10164318
rs150590714
rs115668227
rs12338219
rs6108309
rs80254170
rs37370
rs10168931
rs140623588
rs7325329
rs11253048
rs11517049
rs662138
All
−0.05
0.00
0.05
MR leave−one−out sensitivity analysis for
'X−12112 levels' on 'Rheumatoid arthritis'

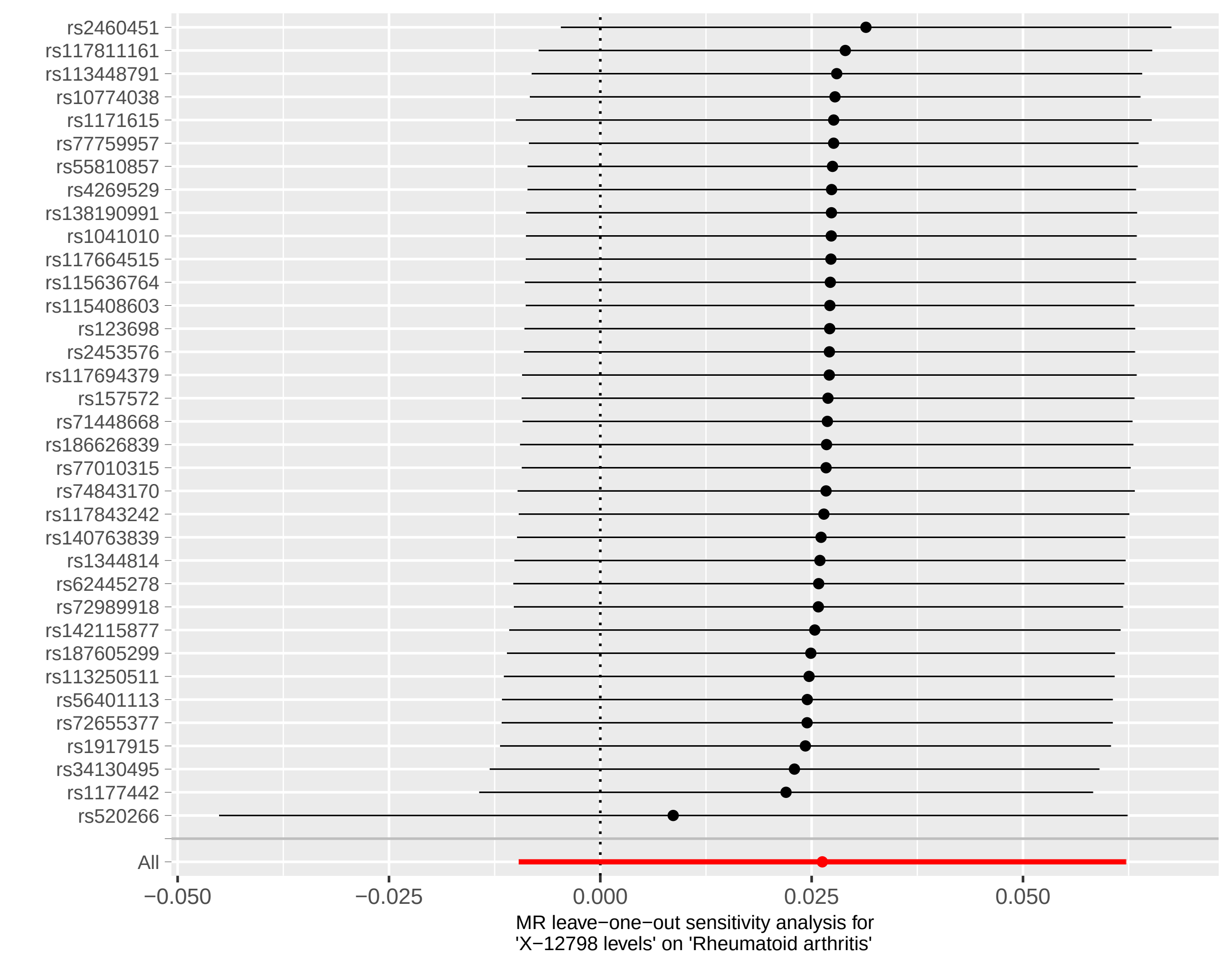
rs2460451
rs117811161
rs113448791
rs10774038
rs1171615
rs77759957
rs55810857
rs4269529
rs138190991
rs1041010
rs117664515
rs115636764
rs115408603
rs123698
rs2453576
rs117694379
rs157572
rs71448668
rs186626839
rs77010315
rs74843170
rs117843242
rs140763839
rs1344814
rs62445278
rs72989918
rs142115877
rs187605299
rs113250511
rs56401113
rs72655377
rs1917915
rs34130495
rs1177442
rs520266
All
−0.050
−0.025
0.000
0.025
0.050
MR leave−one−out sensitivity analysis for
'X−12798 levels' on 'Rheumatoid arthritis'

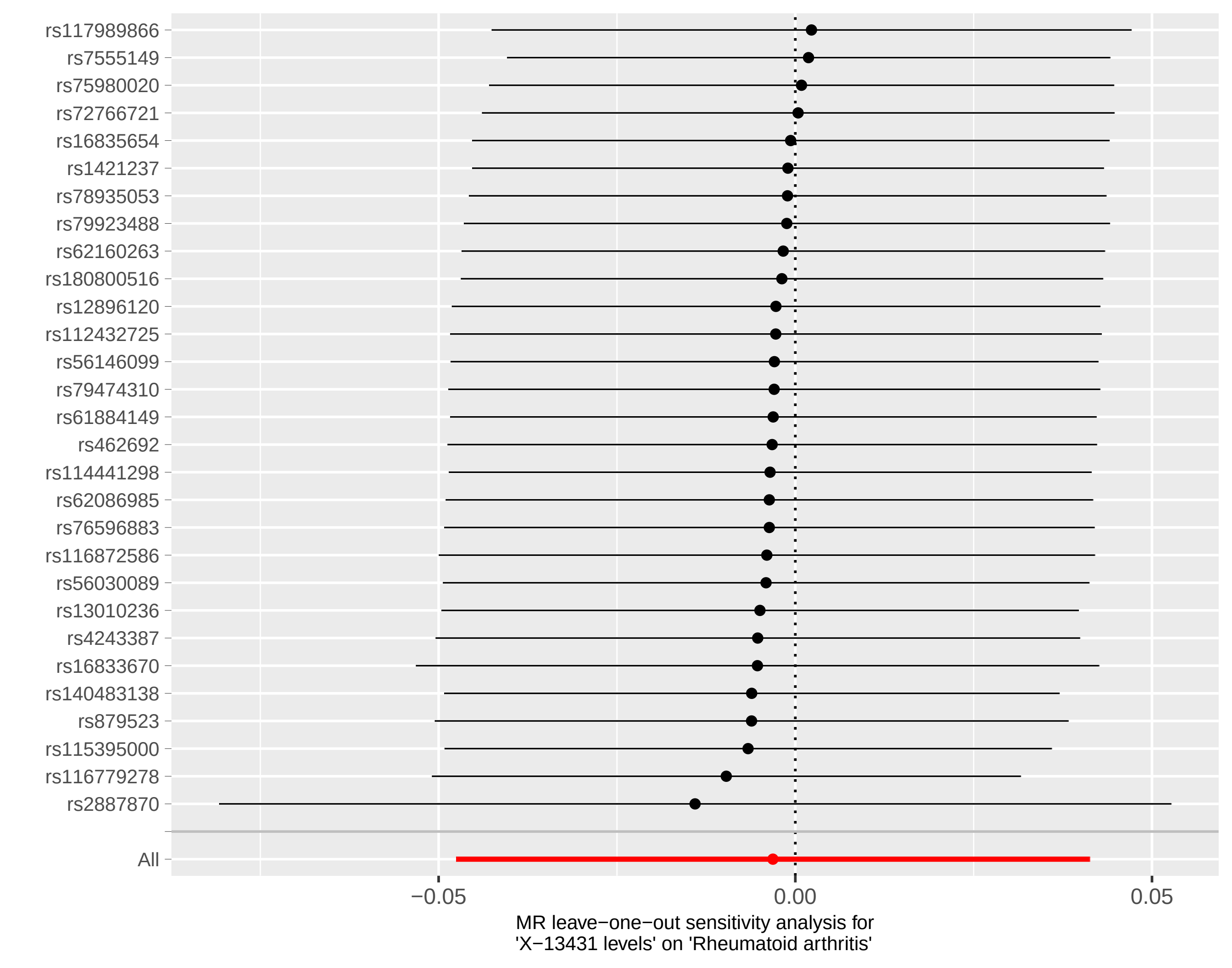

rs117989866
rs7555149
rs75980020
rs72766721
rs16835654
rs1421237
rs78935053
rs79923488
rs62160263
rs180800516
rs12896120
rs112432725
rs56146099
rs79474310
rs61884149
rs462692
rs114441298
rs62086985
rs76596883
rs116872586
rs56030089
rs13010236
rs4243387
rs16833670
rs140483138
rs879523
rs115395000
rs116779278
rs2887870
All
−0.05
0.00
0.05
MR leave−one−out sensitivity analysis for
'X−13431 levels' on 'Rheumatoid arthritis'

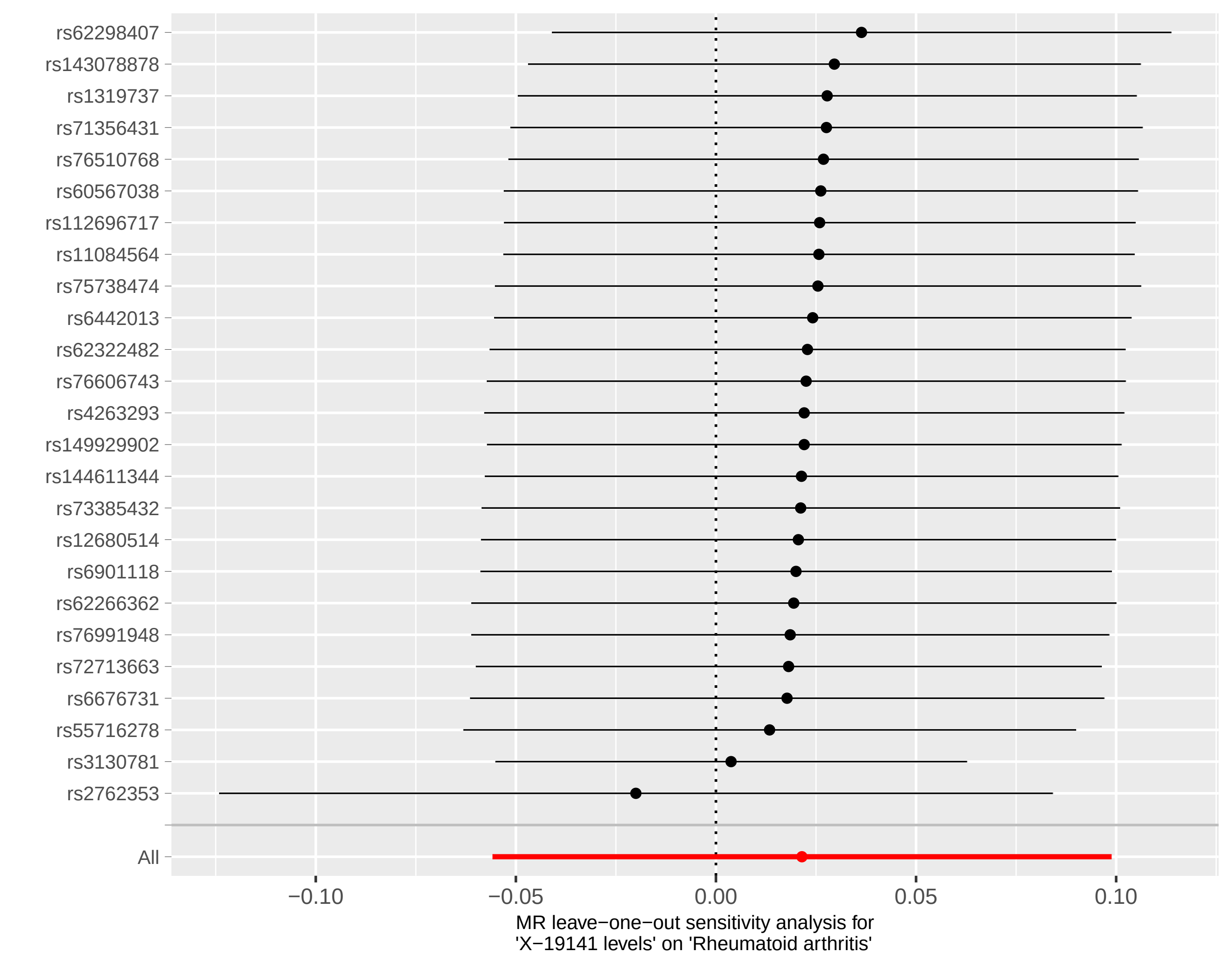
rs62298407
rs143078878
rs1319737
rs71356431
rs76510768
rs60567038
rs112696717
rs11084564
rs75738474
rs6442013
rs62322482
rs76606743
rs4263293
rs149929902
rs144611344
rs73385432
rs12680514
rs6901118
rs62266362
rs76991948
rs72713663
rs6676731
rs55716278
rs3130781
rs2762353
All
−0.10
−0.05
0.00
0.05
0.10
MR leave−one−out sensitivity analysis for
'X−19141 levels' on 'Rheumatoid arthritis'

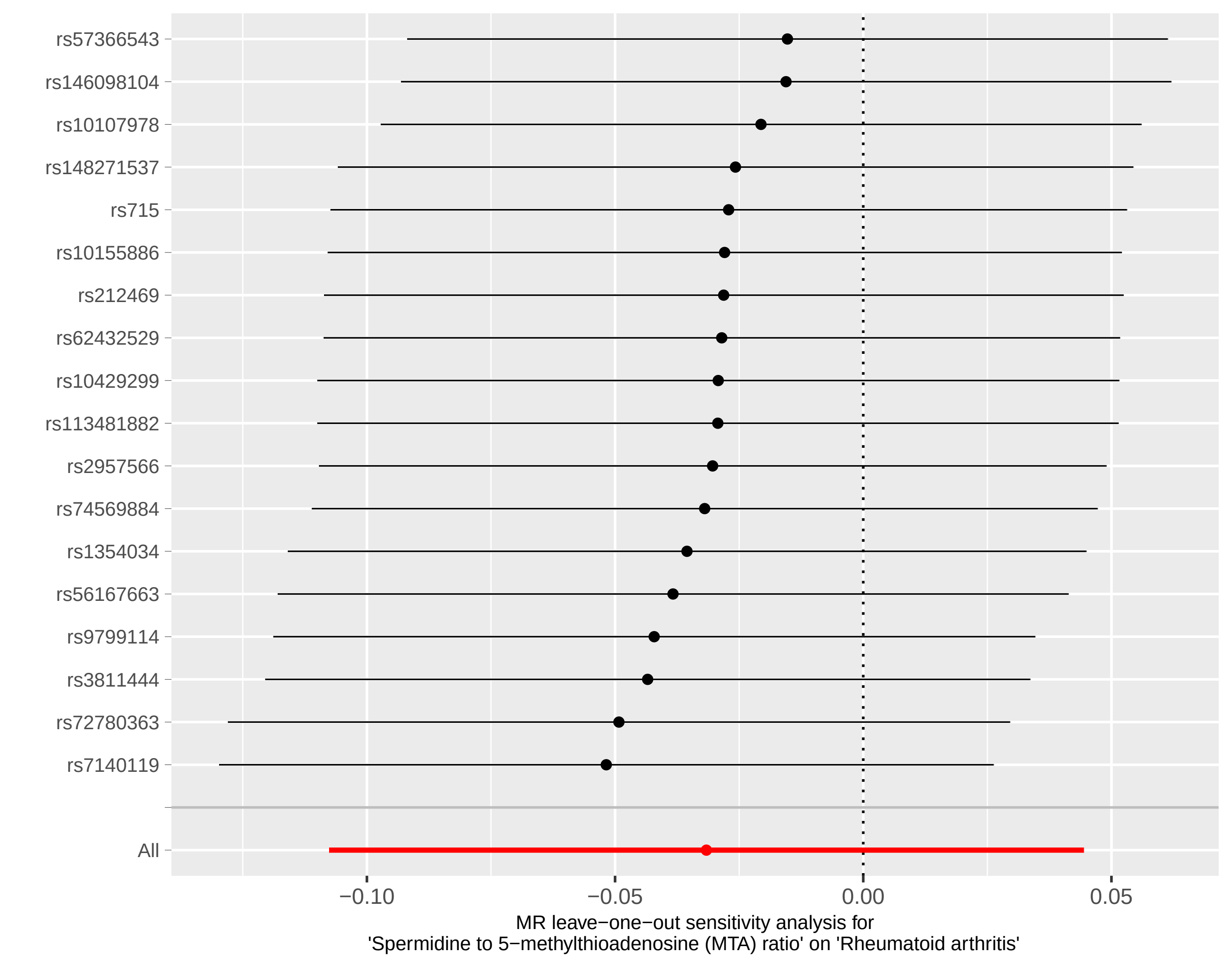

rs57366543
rs146098104
rs10107978
rs148271537
rs715
rs10155886
rs212469
rs62432529
rs10429299
rs113481882
rs2957566
rs74569884
rs1354034
rs56167663
rs9799114
rs3811444
rs72780363
rs7140119
All
−0.10
−0.05
0.00
0.05
MR leave−one−out sensitivity analysis for
'Spermidine to 5−methylthioadenosine (MTA) ratio' on 'Rheumatoid arthritis'

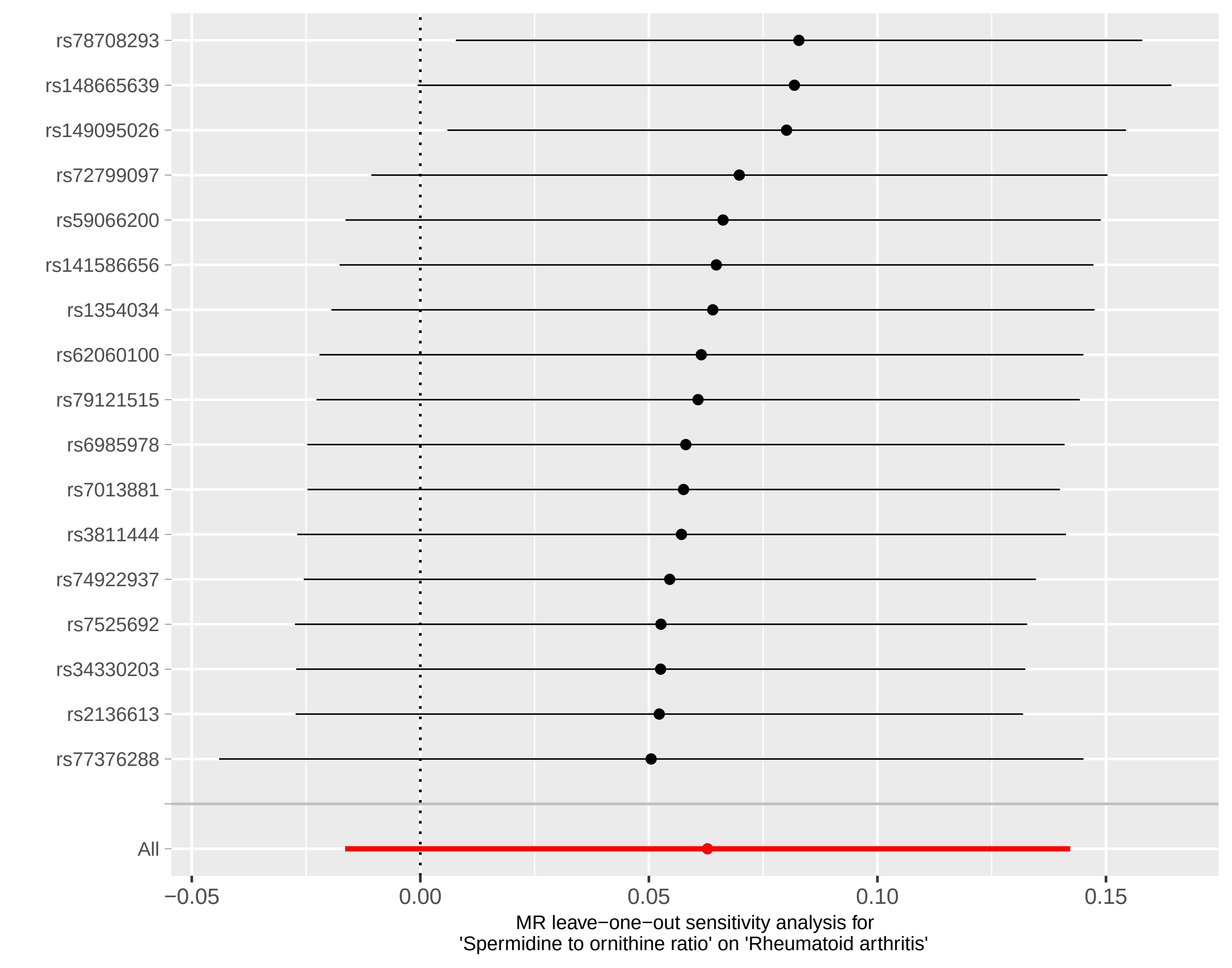
rs78708293
rs148665639
rs149095026
rs72799097
rs59066200
rs141586656
rs1354034
rs62060100
rs79121515
rs6985978
rs7013881
rs3811444
rs74922937
rs7525692
rs34330203
rs2136613
rs77376288
All
−0.05
0.00
0.05
0.10
0.15
MR leave−one−out sensitivity analysis for
'Spermidine to ornithine ratio' on 'Rheumatoid arthritis'

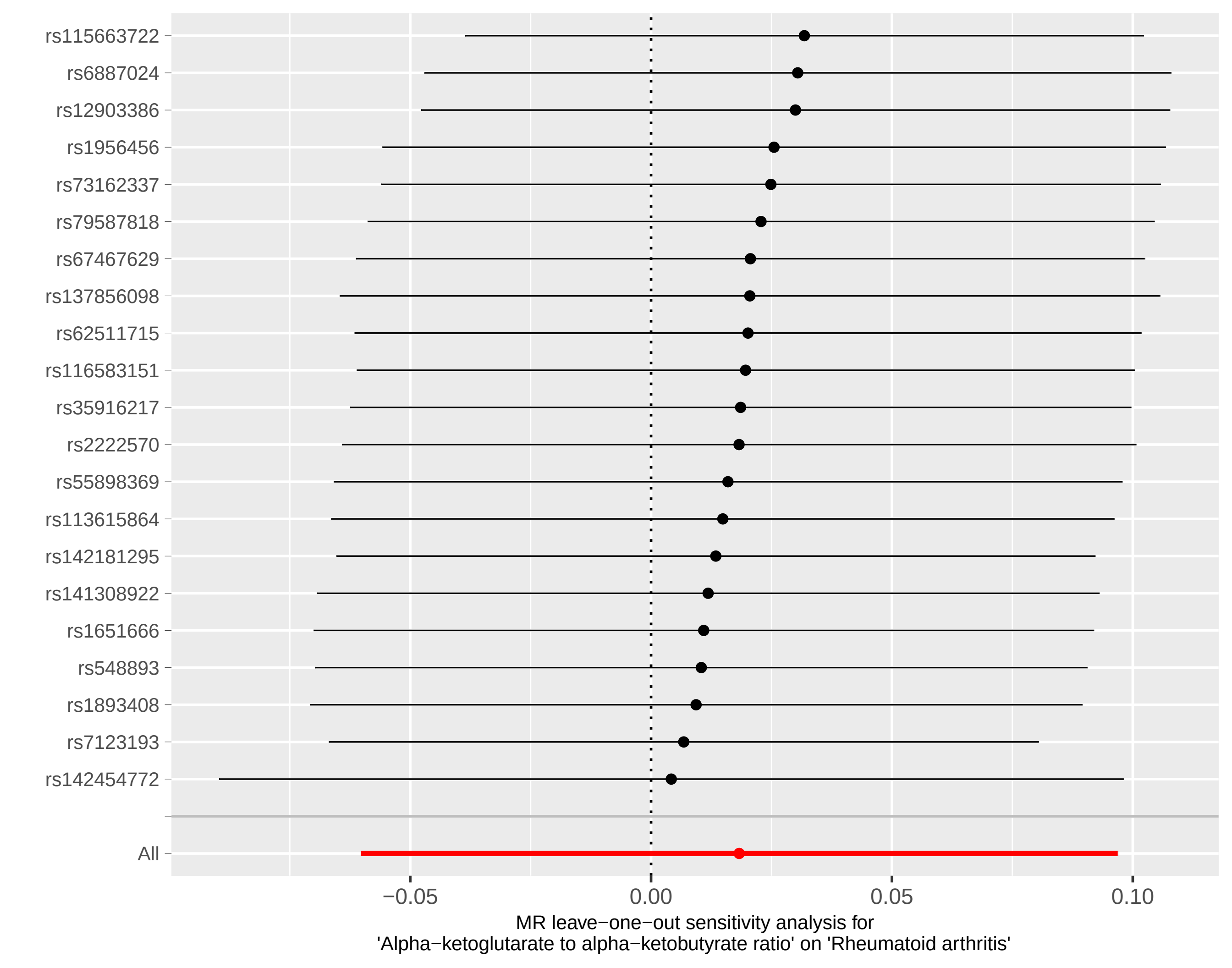

rs115663722
rs6887024
rs12903386
rs1956456
rs73162337
rs79587818
rs67467629
rs137856098
rs62511715
rs116583151
rs35916217
rs2222570
rs55898369
rs113615864
rs142181295
rs141308922
rs1651666
rs548893
rs1893408
rs7123193
rs142454772
All
−0.05
0.00
0.05
0.10
MR leave−one−out sensitivity analysis for
'Alpha−ketoglutarate to alpha−ketobutyrate ratio' on 'Rheumatoid arthritis'

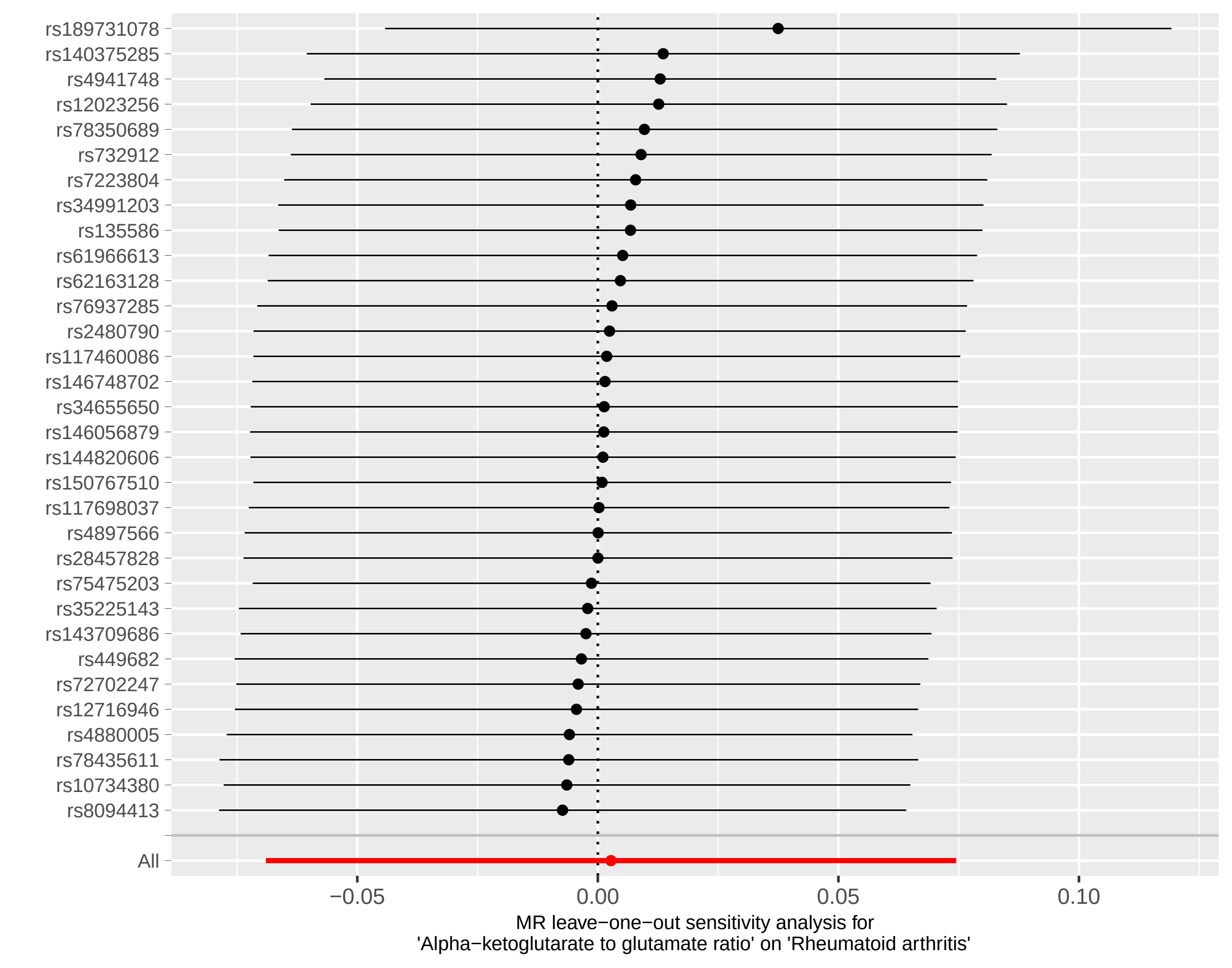
rs189731078
rs140375285
rs4941748
rs12023256
rs78350689
rs732912
rs7223804
rs34991203
rs135586
rs61966613
rs62163128
rs76937285
rs2480790
rs117460086
rs146748702
rs34655650
rs146056879
rs144820606
rs150767510
rs117698037
rs4897566
rs28457828
rs75475203
rs35225143
rs143709686
rs449682
rs72702247
rs12716946
rs4880005
rs78435611
rs10734380
rs8094413
All
−0.05
0.00
0.05
0.10
MR leave−one−out sensitivity analysis for
'Alpha−ketoglutarate to glutamate ratio' on 'Rheumatoid arthritis'

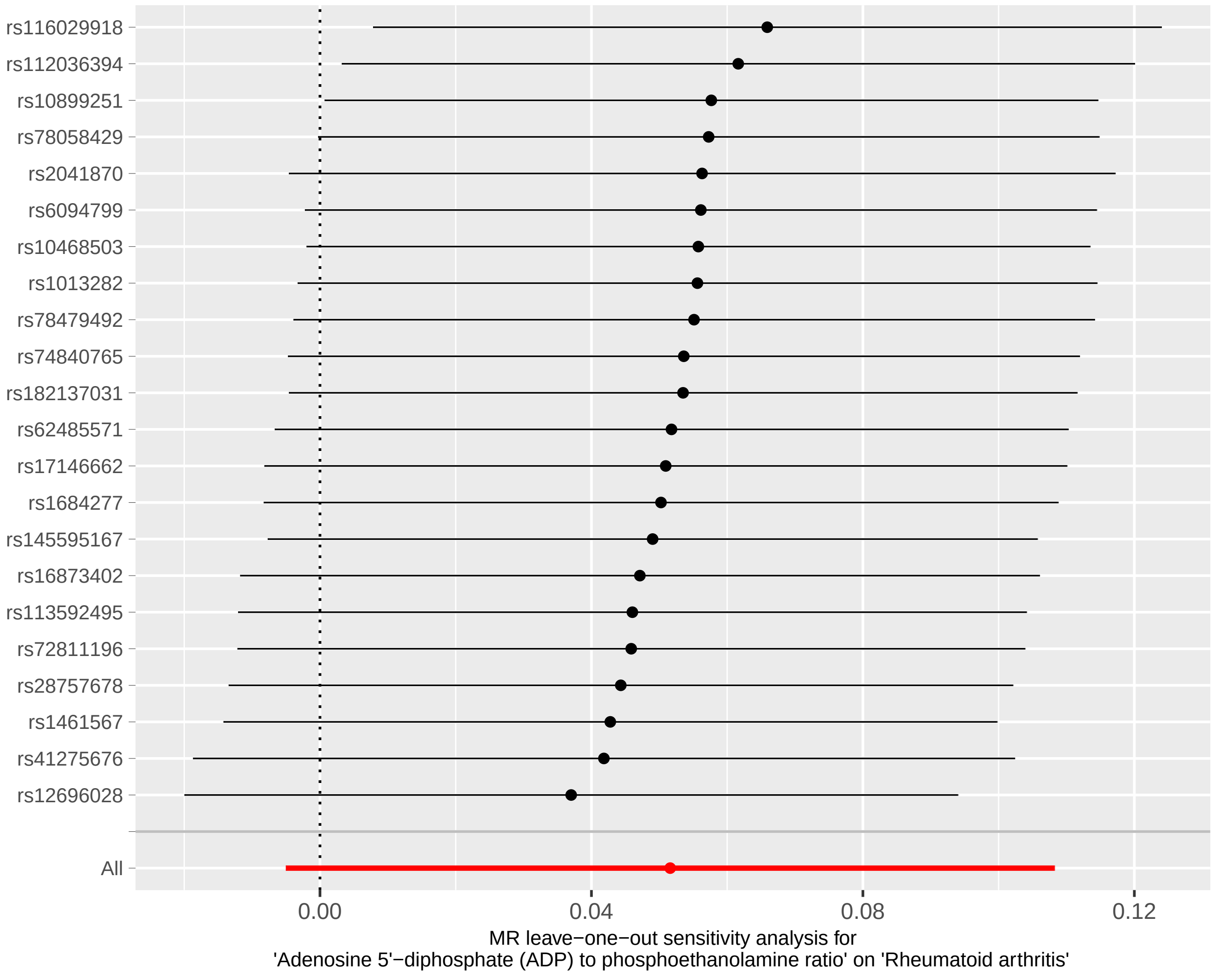

rs116029918
rs112036394
rs10899251
rs78058429
rs2041870
rs6094799
rs10468503
rs1013282
rs78479492
rs74840765
rs182137031
rs62485571
rs17146662
rs1684277
rs145595167
rs16873402
rs113592495
rs72811196
rs28757678
rs1461567
rs41275676
rs12696028
All
0.00
0.04
0.08
0.12
MR leave−one−out sensitivity analysis for
'Adenosine 5'−diphosphate (ADP) to phosphoethanolamine ratio' on 'Rheumatoid arthritis'

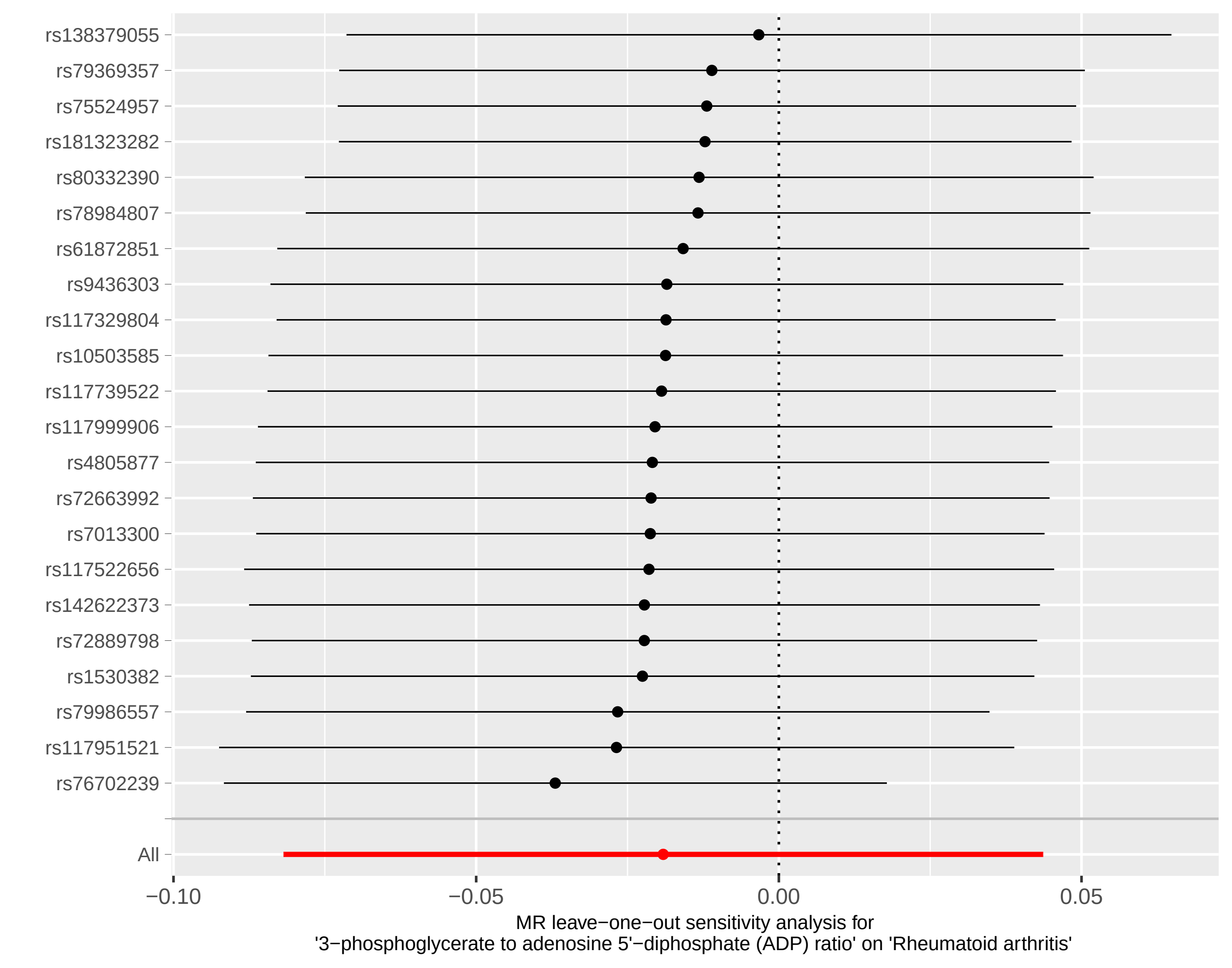

rs138379055
rs79369357
rs75524957
rs181323282
rs80332390
rs78984807
rs61872851
rs9436303
rs117329804
rs10503585
rs117739522
rs117999906
rs4805877
rs72663992
rs7013300
rs117522656
rs142622373
rs72889798
rs1530382
rs79986557
rs117951521
rs76702239
All
−0.10
−0.05
0.00
0.05
MR leave−one−out sensitivity analysis for
'3−phosphoglycerate to adenosine 5'−diphosphate (ADP) ratio' on 'Rheumatoid arthritis'

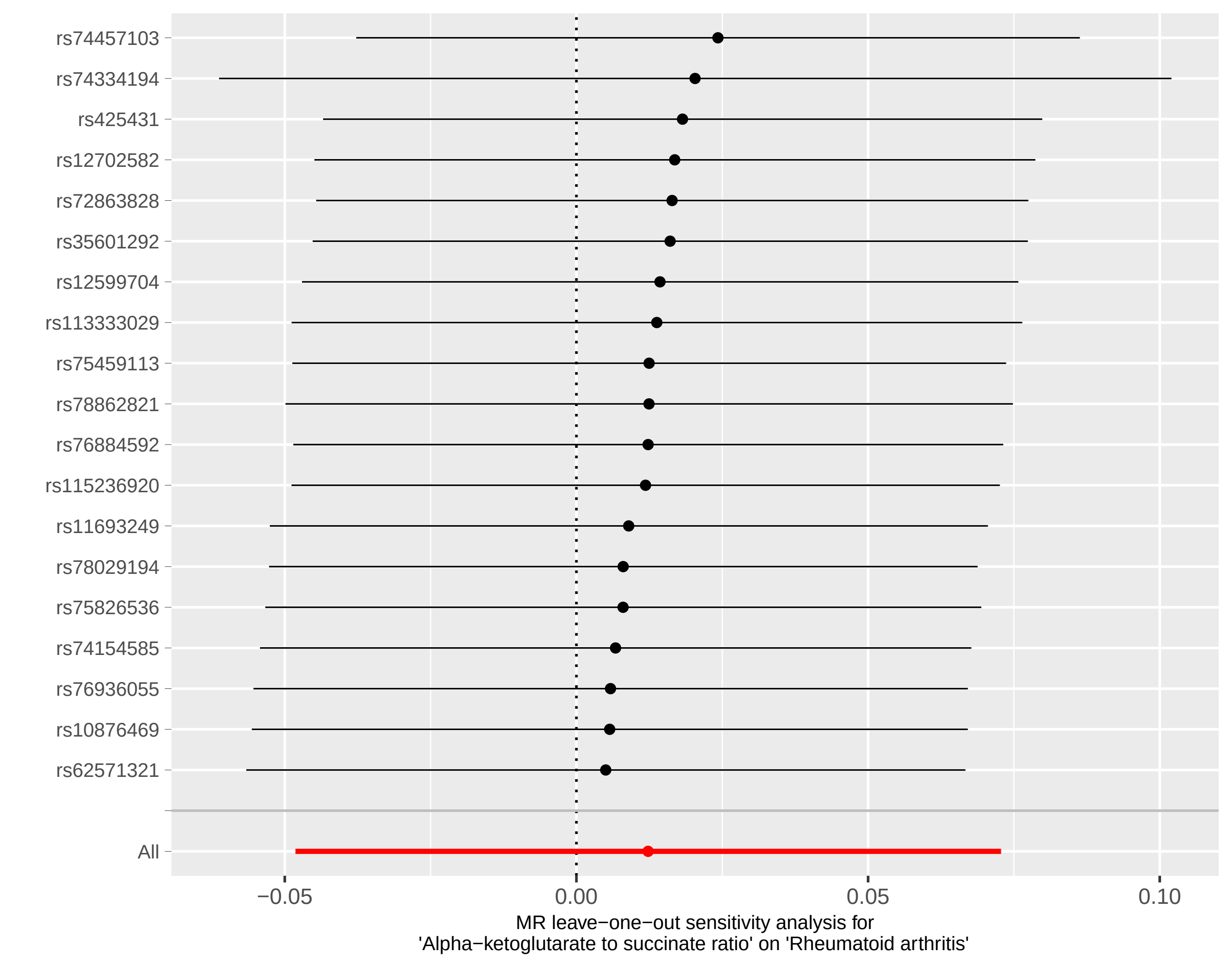
rs74457103
rs74334194
rs425431
rs12702582
rs72863828
rs35601292
rs12599704
rs113333029
rs75459113
rs78862821
rs76884592
rs115236920
rs11693249
rs78029194
rs75826536
rs74154585
rs76936055
rs10876469
rs62571321
All
−0.05
0.00
0.05
0.10
MR leave−one−out sensitivity analysis for
'Alpha−ketoglutarate to succinate ratio' on 'Rheumatoid arthritis'

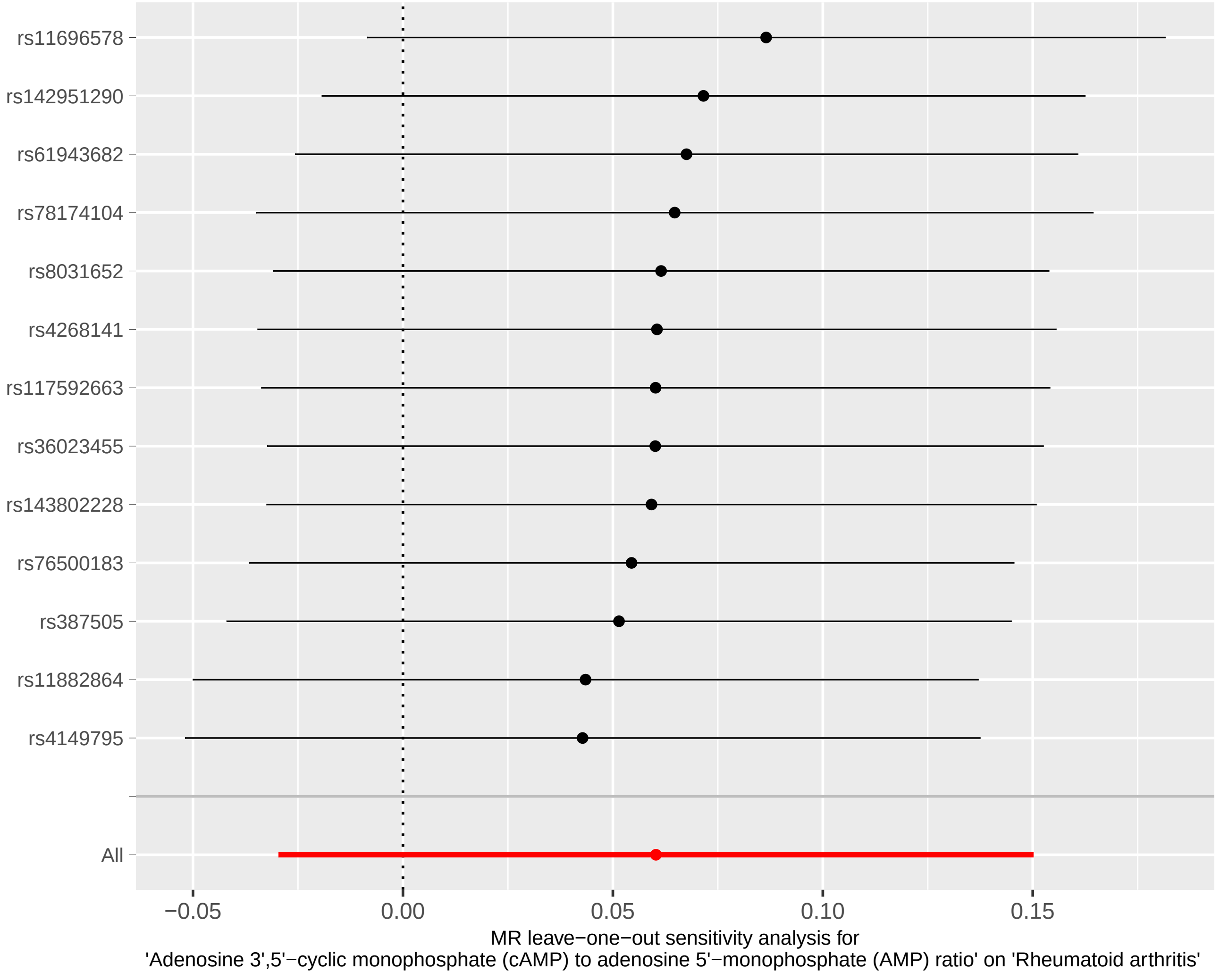
rs11696578
rs142951290
rs61943682
rs78174104
rs8031652
rs4268141
rs117592663
rs36023455
rs143802228
rs76500183
rs387505
rs11882864
rs4149795
All
−0.05
0.00
0.05
0.10
0.15
MR leave−one−out sensitivity analysis for
'Adenosine 3',5'−cyclic monophosphate (cAMP) to adenosine 5'−monophosphate (AMP) ratio' on 'Rheumatoid arthritis'

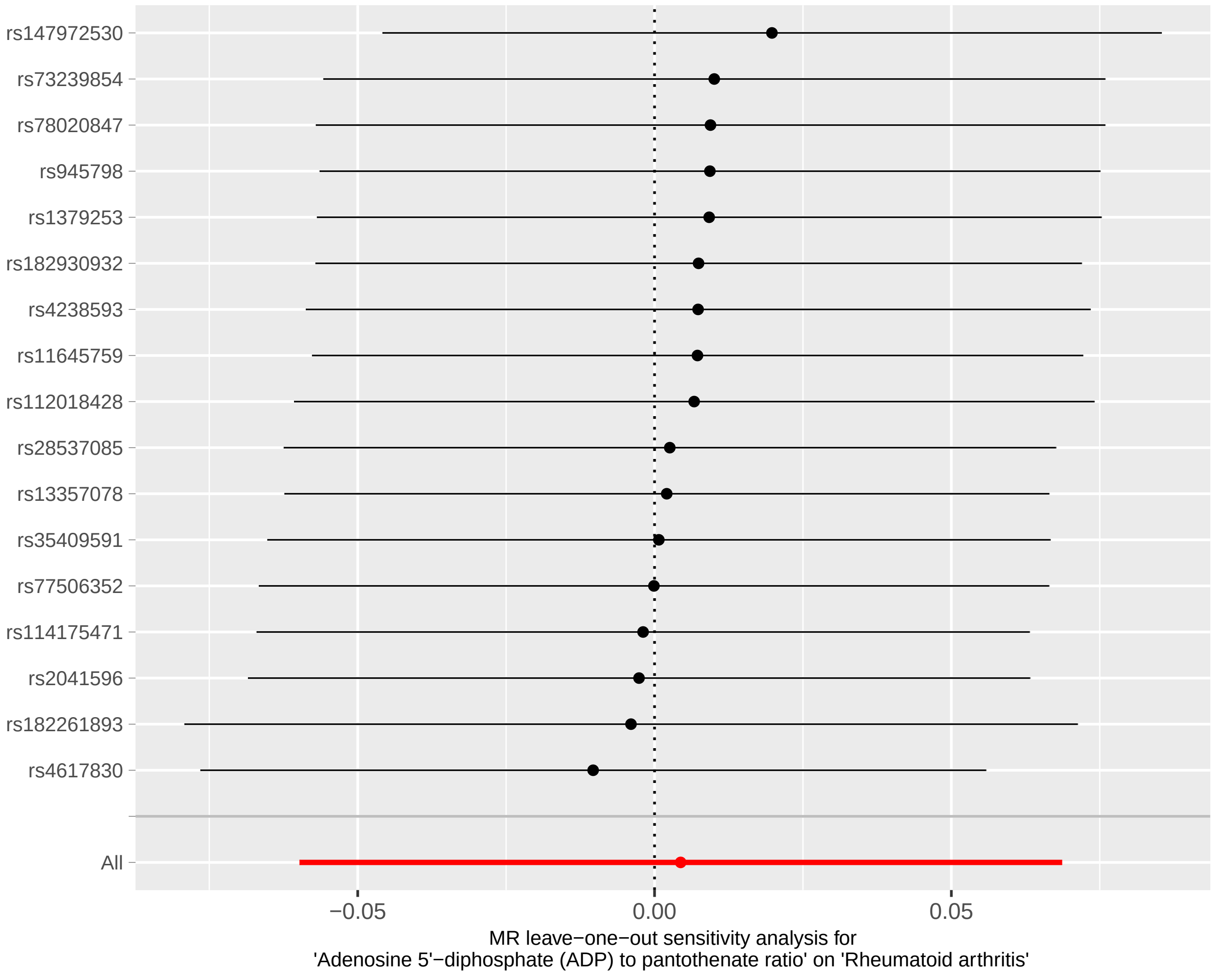
rs147972530
rs73239854
rs78020847
rs945798
rs1379253
rs182930932
rs4238593
rs11645759
rs112018428
rs28537085
rs13357078
rs35409591
rs77506352
rs114175471
rs2041596
rs182261893
rs4617830
All
−0.05
0.00
0.05
MR leave−one−out sensitivity analysis for
'Adenosine 5'−diphosphate (ADP) to pantothenate ratio' on 'Rheumatoid arthritis'

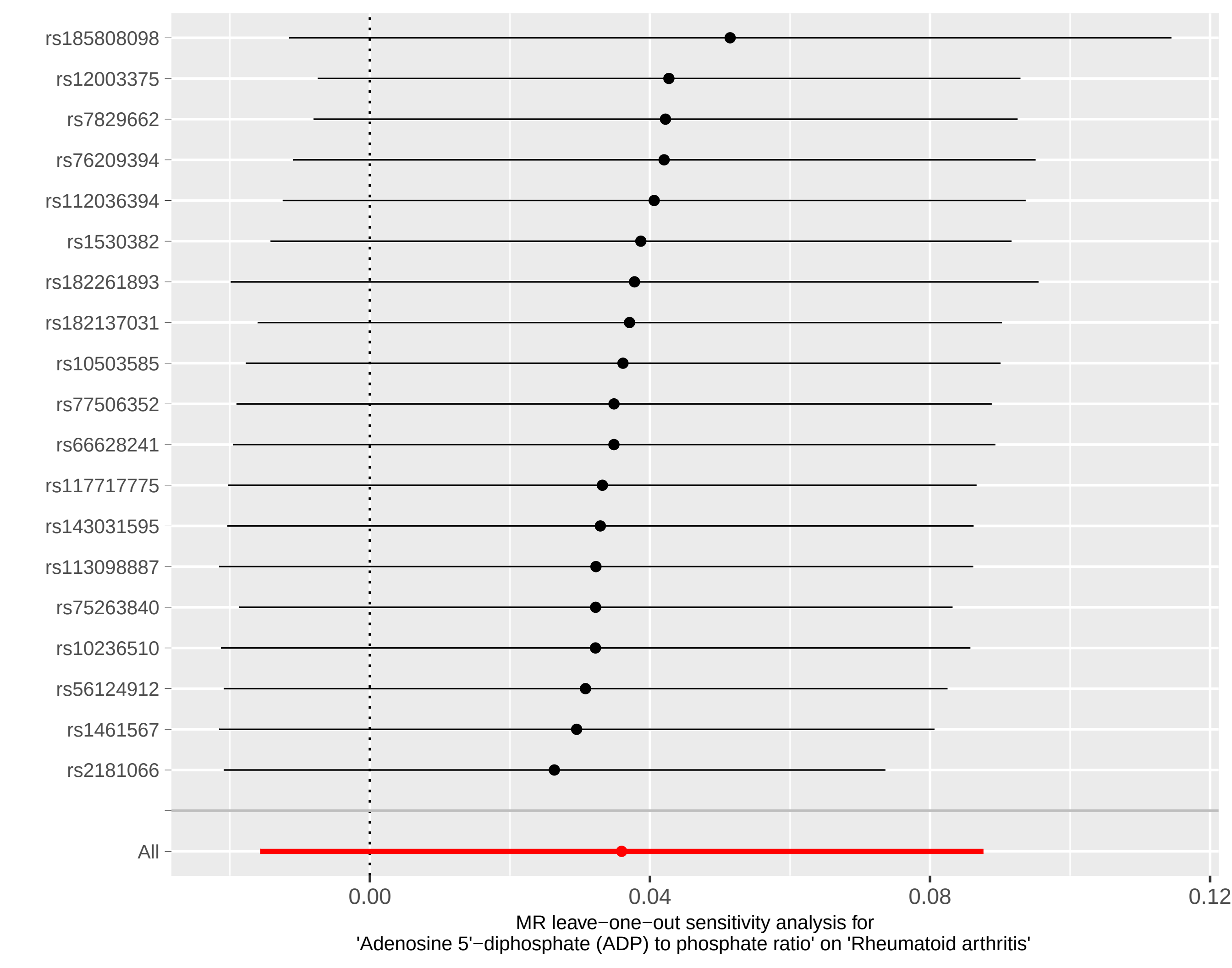
rs185808098
rs12003375
rs7829662
rs76209394
rs112036394
rs1530382
rs182261893
rs182137031
rs10503585
rs77506352
rs66628241
rs117717775
rs143031595
rs113098887
rs75263840
rs10236510
rs56124912
rs1461567
rs2181066
All
0.00
0.04
0.08
0.12
MR leave−one−out sensitivity analysis for
'Adenosine 5'−diphosphate (ADP) to phosphate ratio' on 'Rheumatoid arthritis'

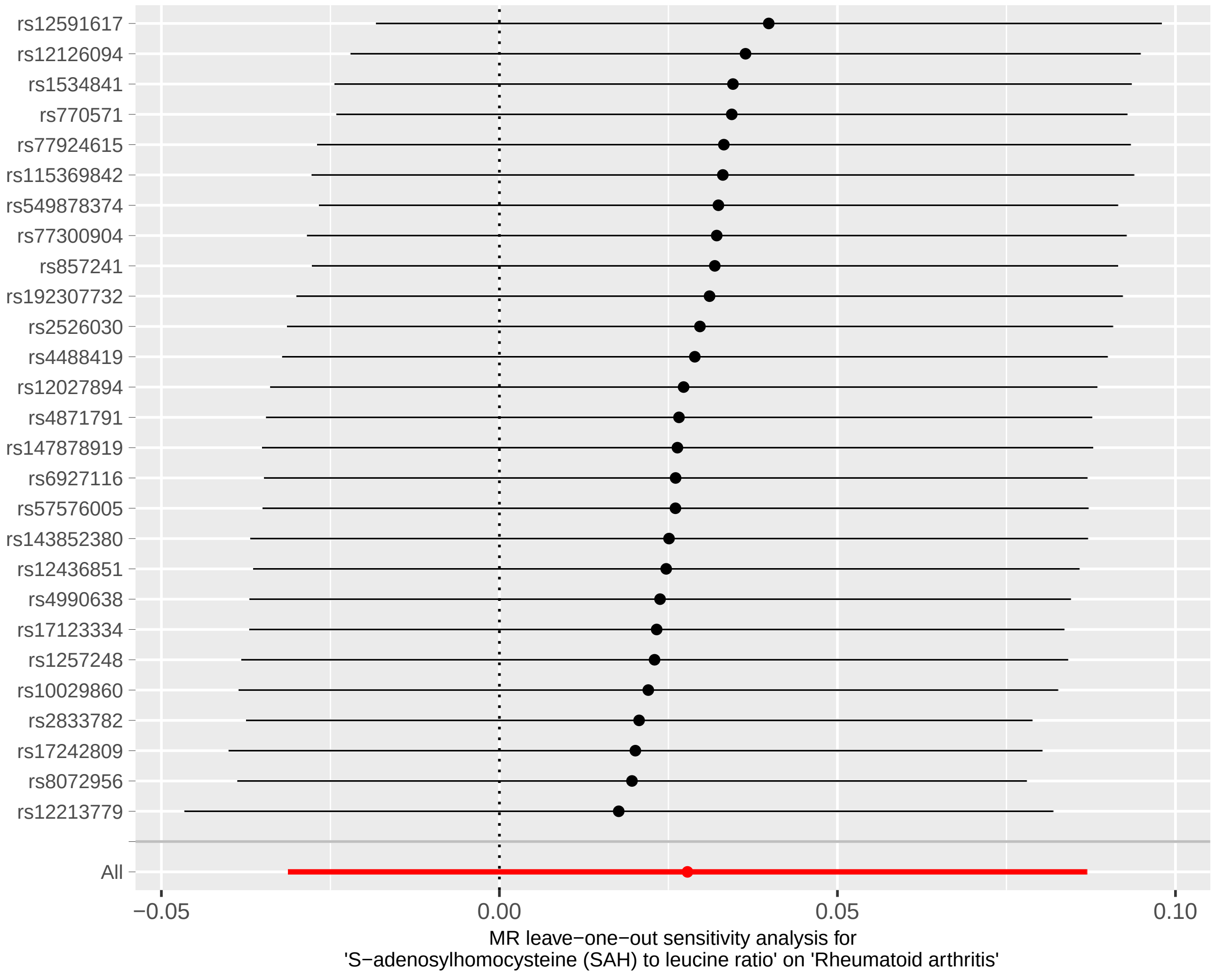
rs12591617
rs12126094
rs1534841
rs770571
rs77924615
rs115369842
rs549878374
rs77300904
rs857241
rs192307732
rs2526030
rs4488419
rs12027894
rs4871791
rs147878919
rs6927116
rs57576005
rs143852380
rs12436851
rs4990638
rs17123334
rs1257248
rs10029860
rs2833782
rs17242809
rs8072956
rs12213779
All
−0.05
0.00
0.05
0.10
MR leave−one−out sensitivity analysis for
'S−adenosylhomocysteine (SAH) to leucine ratio' on 'Rheumatoid arthritis'

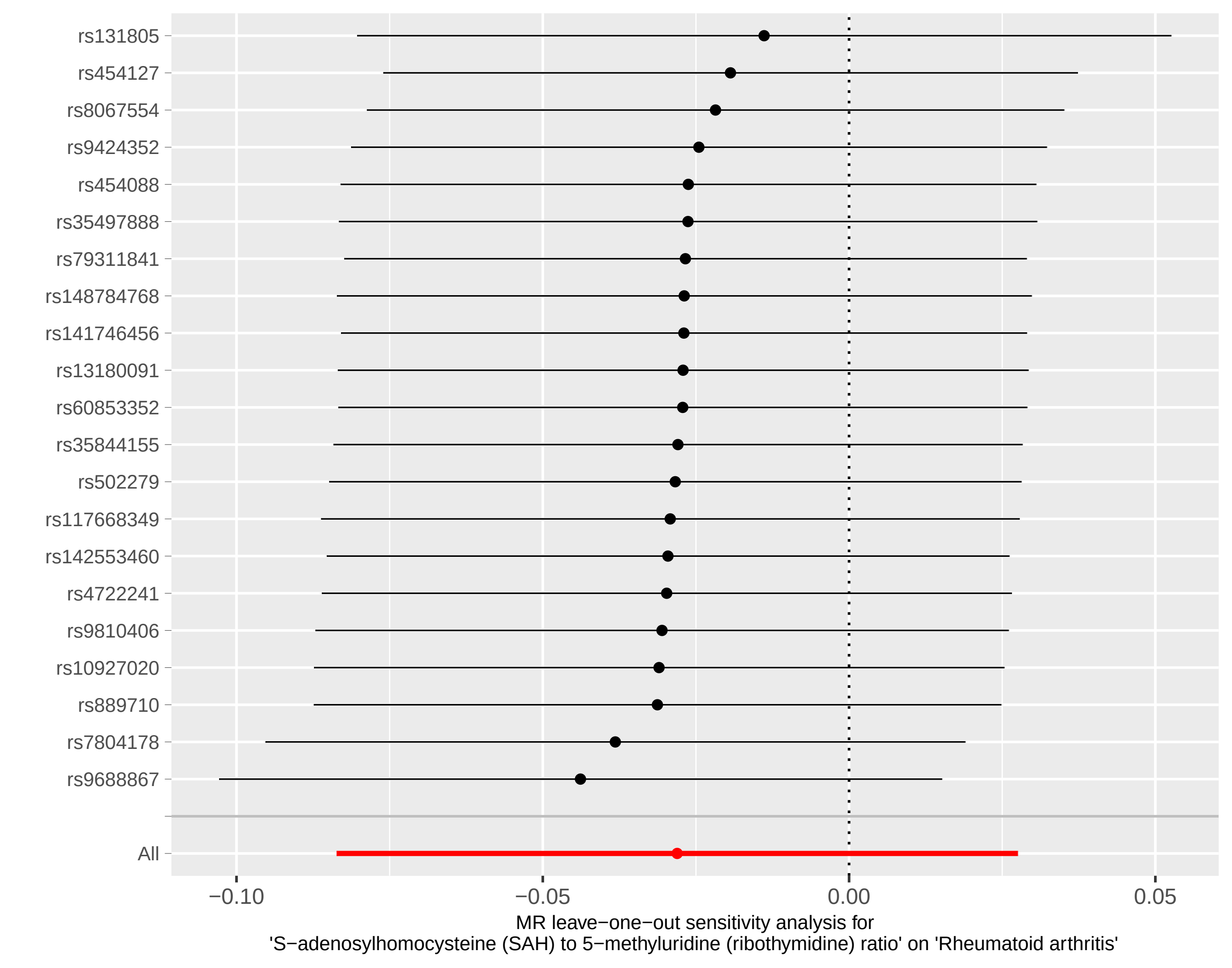
rs131805
rs454127
rs8067554
rs9424352
rs454088
rs35497888
rs79311841
rs148784768
rs141746456
rs13180091
rs60853352
rs35844155
rs502279
rs117668349
rs142553460
rs4722241
rs9810406
rs10927020
rs889710
rs7804178
rs9688867
All
−0.10
−0.05
0.00
0.05
MR leave−one−out sensitivity analysis for
'S−adenosylhomocysteine (SAH) to 5−methyluridine (ribothymidine) ratio' on 'Rheumatoid arthritis'

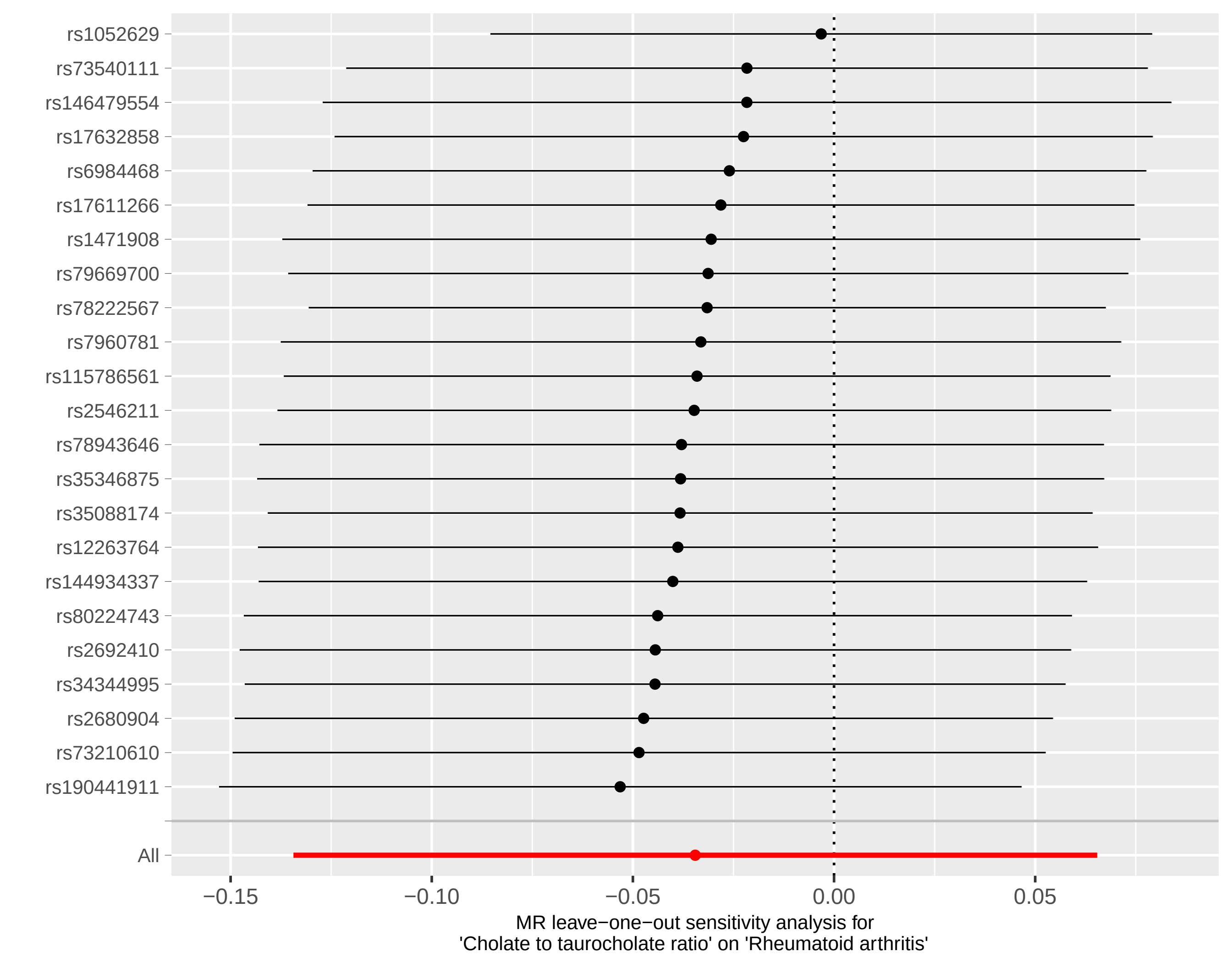
rs1052629
rs73540111
rs146479554
rs17632858
rs6984468
rs17611266
rs1471908
rs79669700
rs78222567
rs7960781
rs115786561
rs2546211
rs78943646
rs35346875
rs35088174
rs12263764
rs144934337
rs80224743
rs2692410
rs34344995
rs2680904
rs73210610
rs190441911
All
−0.15
−0.10
−0.05
0.00
0.05
MR leave−one−out sensitivity analysis for
'Cholate to taurocholate ratio' on 'Rheumatoid arthritis'

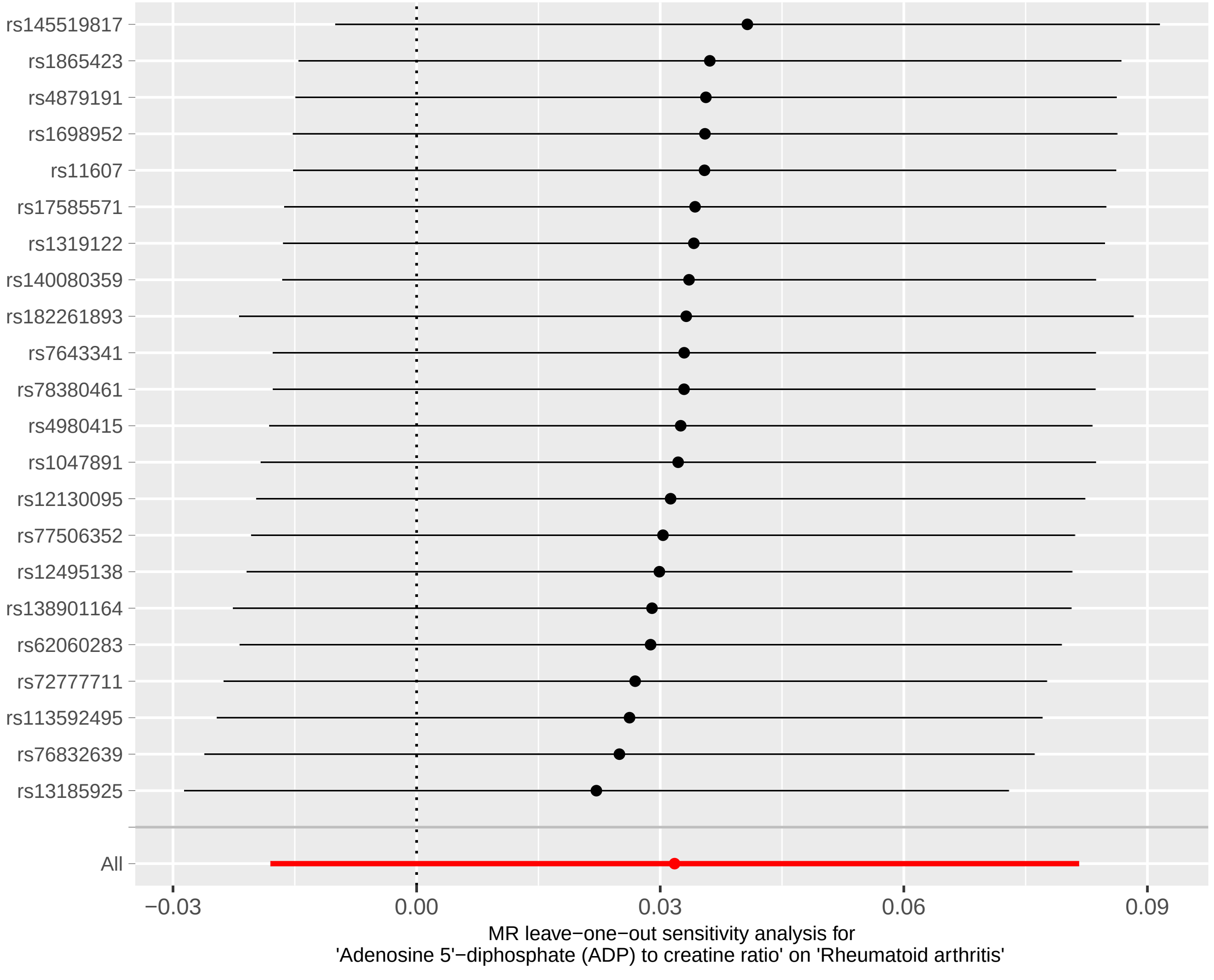
rs145519817
rs1865423
rs4879191
rs1698952
rs11607
rs17585571
rs1319122
rs140080359
rs182261893
rs7643341
rs78380461
rs4980415
rs1047891
rs12130095
rs77506352
rs12495138
rs138901164
rs62060283
rs72777711
rs113592495
rs76832639
rs13185925
All
−0.03
0.00
0.03
0.06
0.09
MR leave−one−out sensitivity analysis for
'Adenosine 5'−diphosphate (ADP) to creatine ratio' on 'Rheumatoid arthritis'

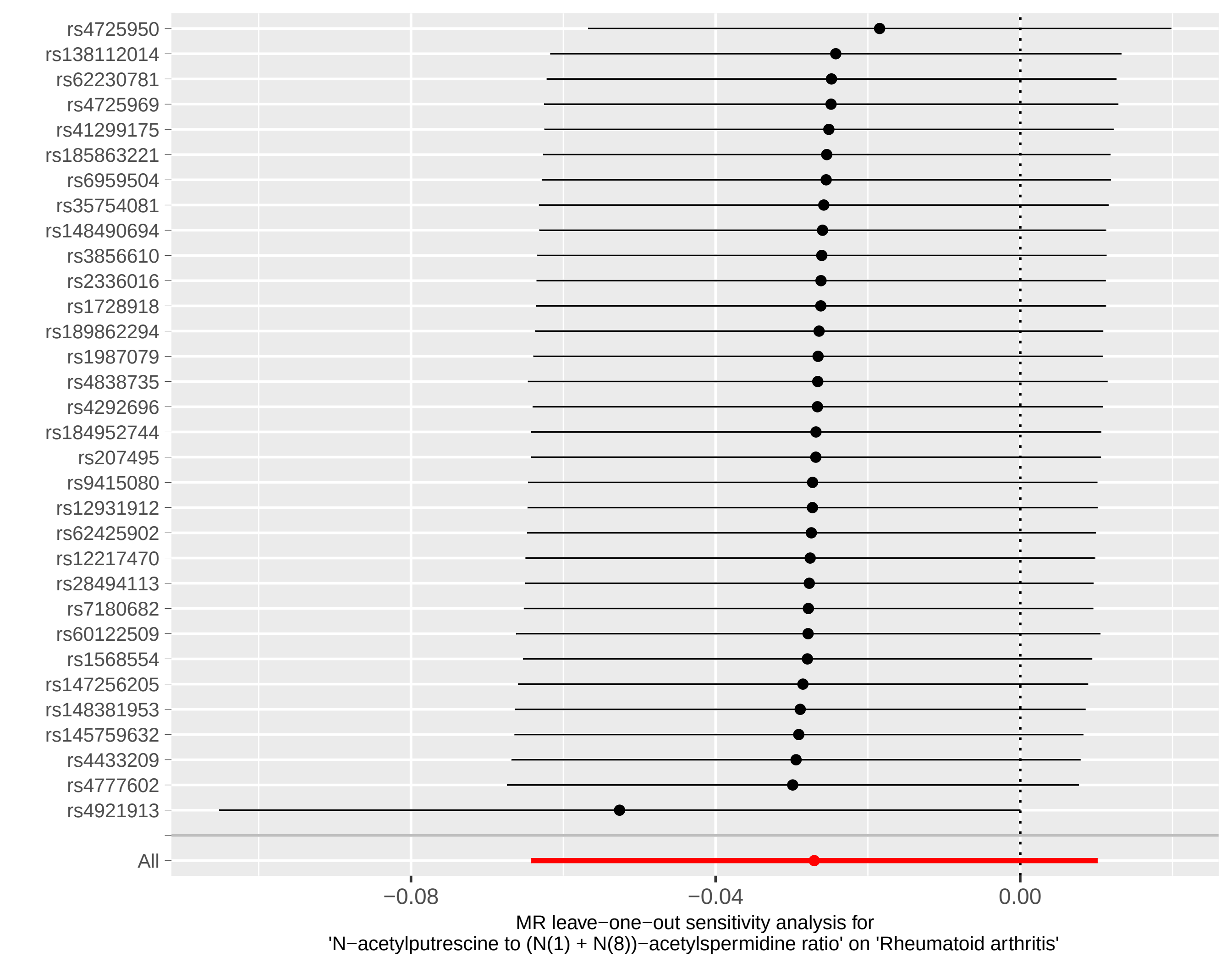

rs4725950
rs138112014
rs62230781
rs4725969
rs41299175
rs185863221
rs6959504
rs35754081
rs148490694
rs3856610
rs2336016
rs1728918
rs189862294
rs1987079
rs4838735
rs4292696
rs184952744
rs207495
rs9415080
rs12931912
rs62425902
rs12217470
rs28494113
rs7180682
rs60122509
rs1568554
rs147256205
rs148381953
rs145759632
rs4433209
rs4777602
rs4921913
All
−0.08
−0.04
0.00
MR leave−one−out sensitivity analysis for
'N−acetylputrescine to (N(1) + N(8))−acetylspermidine ratio' on 'Rheumatoid arthritis'

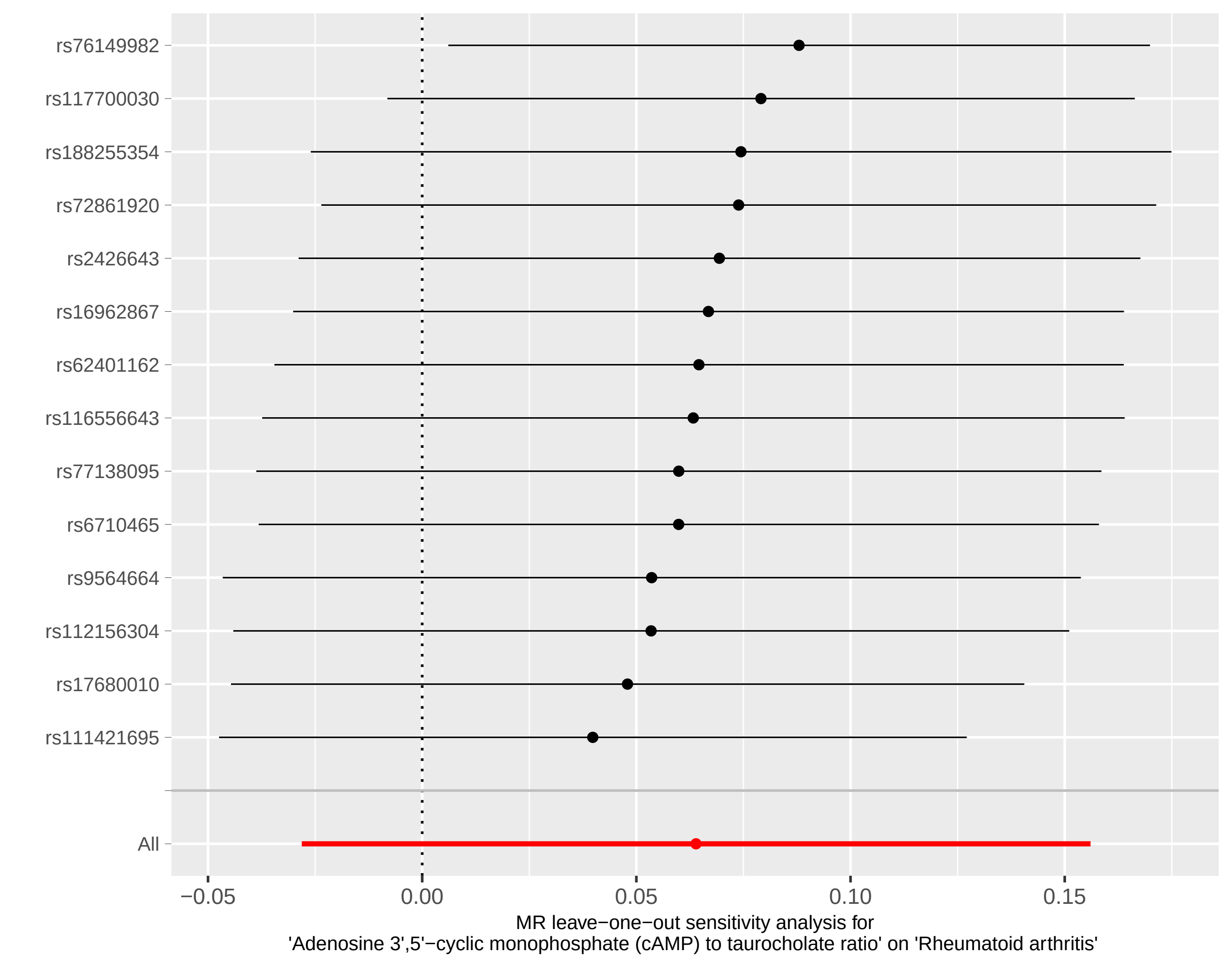
rs76149982
rs117700030
rs188255354
rs72861920
rs2426643
rs16962867
rs62401162
rs116556643
rs77138095
rs6710465
rs9564664
rs112156304
rs17680010
rs111421695
All
−0.05
0.00
0.05
0.10
0.15
MR leave−one−out sensitivity analysis for
'Adenosine 3',5'−cyclic monophosphate (cAMP) to taurocholate ratio' on 'Rheumatoid arthritis'

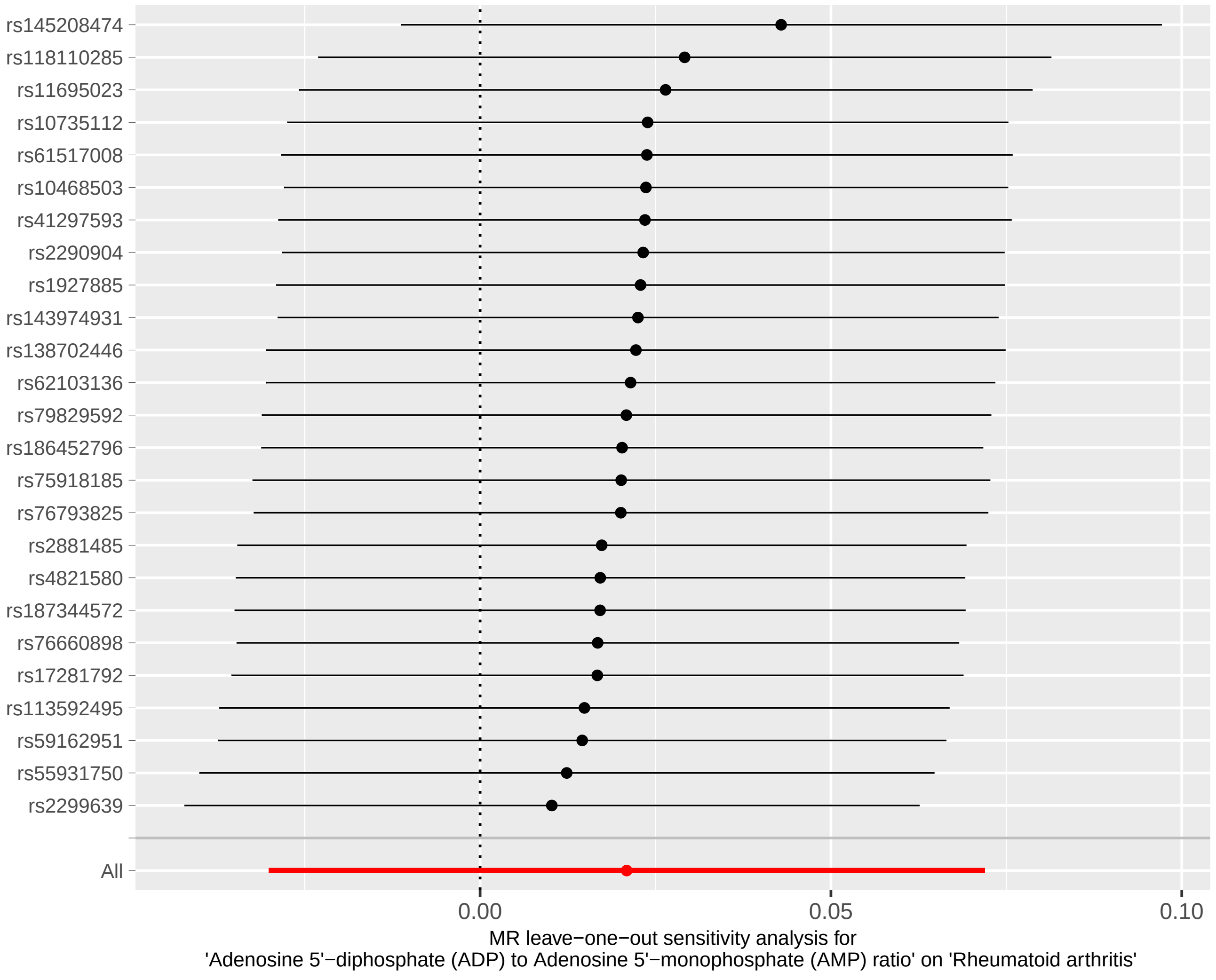

rs145208474
rs118110285
rs11695023
rs10735112
rs61517008
rs10468503
rs41297593
rs2290904
rs1927885
rs143974931
rs138702446
rs62103136
rs79829592
rs186452796
rs75918185
rs76793825
rs2881485
rs4821580
rs187344572
rs76660898
rs17281792
rs113592495
rs59162951
rs55931750
rs2299639
All
0.00
0.05
0.10
MR leave−one−out sensitivity analysis for
'Adenosine 5'−diphosphate (ADP) to Adenosine 5'−monophosphate (AMP) ratio' on 'Rheumatoid arthritis'

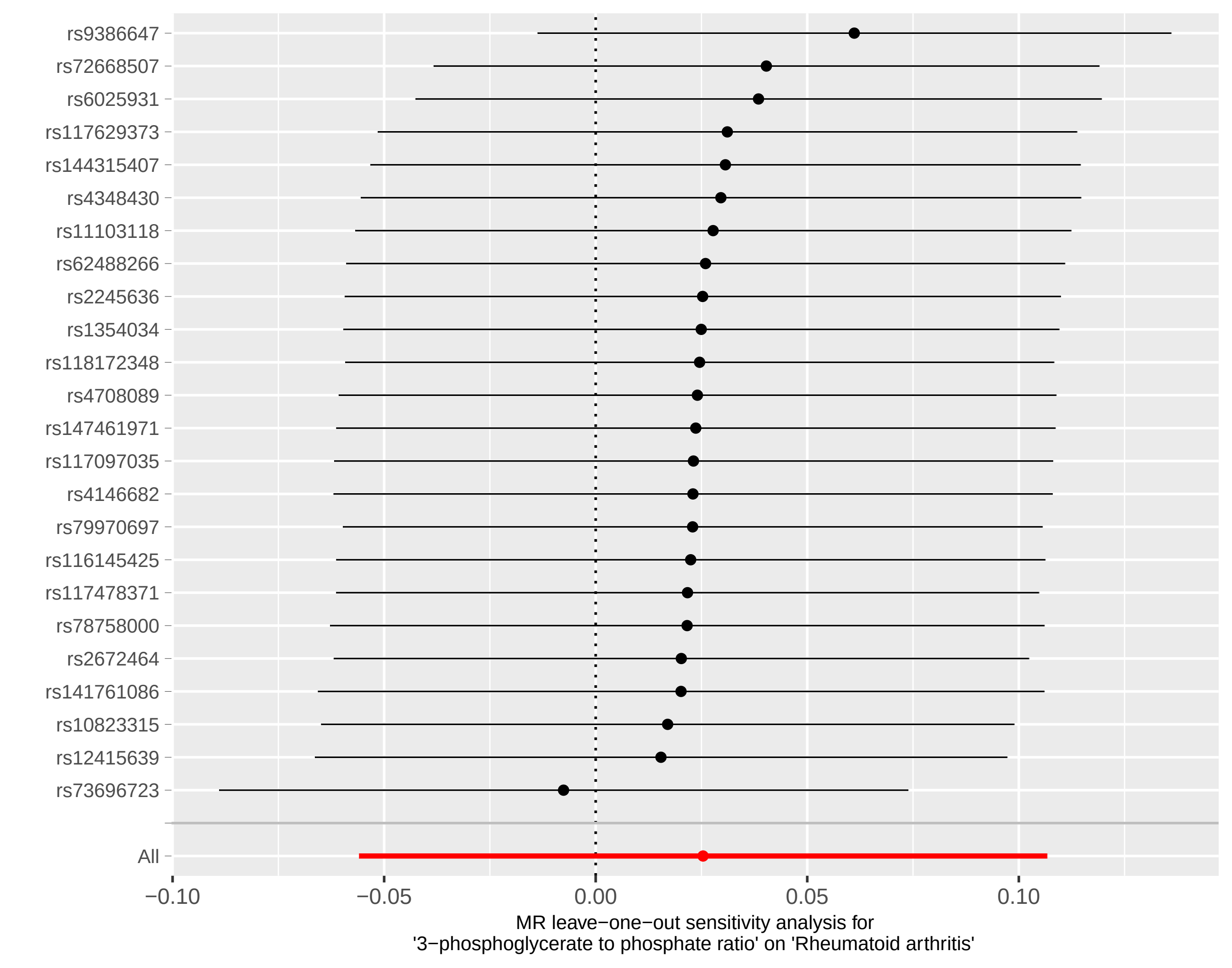

rs9386647
rs72668507
rs6025931
rs117629373
rs144315407
rs4348430
rs11103118
rs62488266
rs2245636
rs1354034
rs118172348
rs4708089
rs147461971
rs117097035
rs4146682
rs79970697
rs116145425
rs117478371
rs78758000
rs2672464
rs141761086
rs10823315
rs12415639
rs73696723
All
−0.10
−0.05
0.00
0.05
0.10
MR leave−one−out sensitivity analysis for
'3−phosphoglycerate to phosphate ratio' on 'Rheumatoid arthritis'

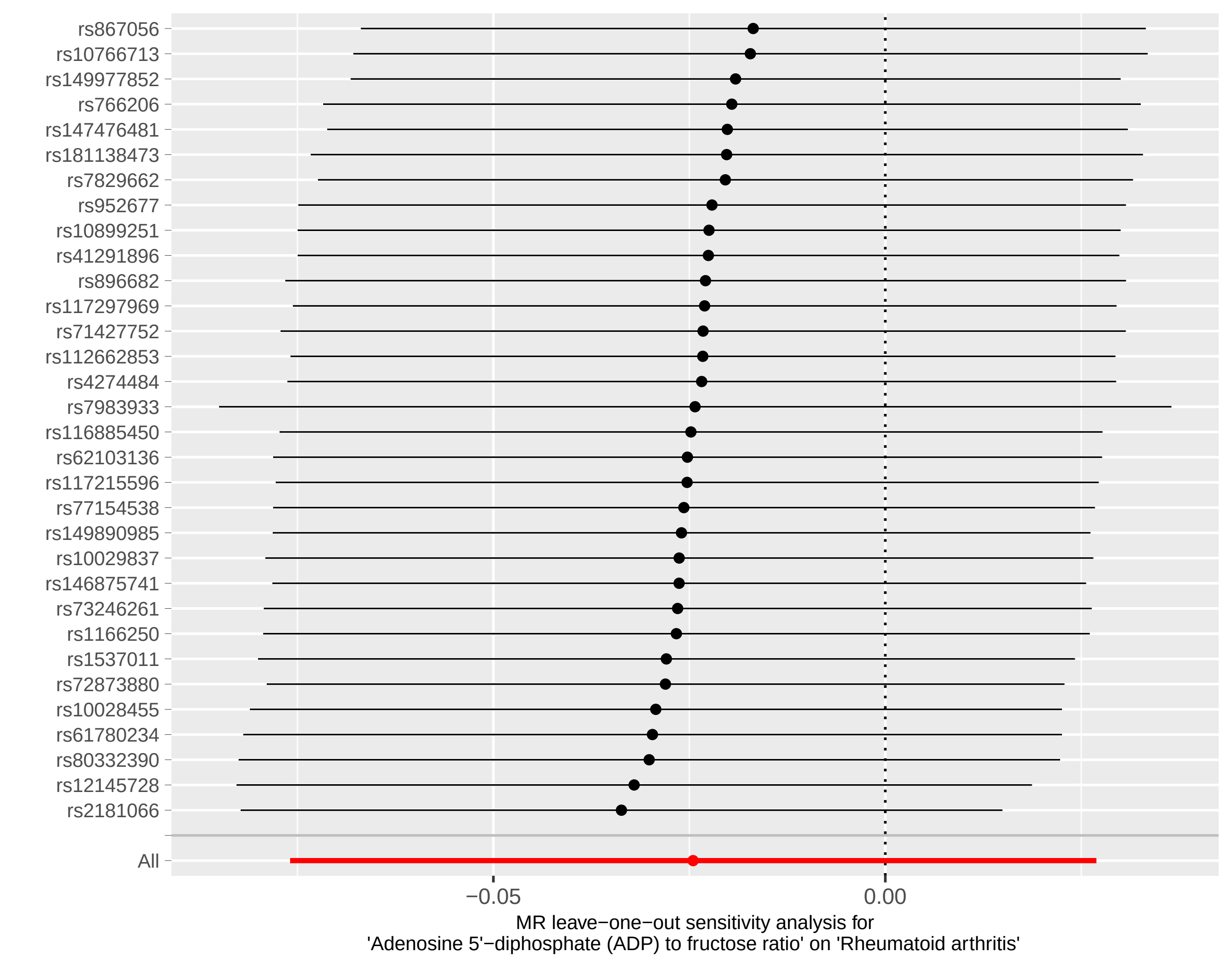
rs867056
rs10766713
rs149977852
rs766206
rs147476481
rs181138473
rs7829662
rs952677
rs10899251
rs41291896
rs896682
rs117297969
rs71427752
rs112662853
rs4274484
rs7983933
rs116885450
rs62103136
rs117215596
rs77154538
rs149890985
rs10029837
rs146875741
rs73246261
rs1166250
rs1537011
rs72873880
rs10028455
rs61780234
rs80332390
rs12145728
rs2181066
All
−0.05
0.00
MR leave−one−out sensitivity analysis for
'Adenosine 5'−diphosphate (ADP) to fructose ratio' on 'Rheumatoid arthritis'

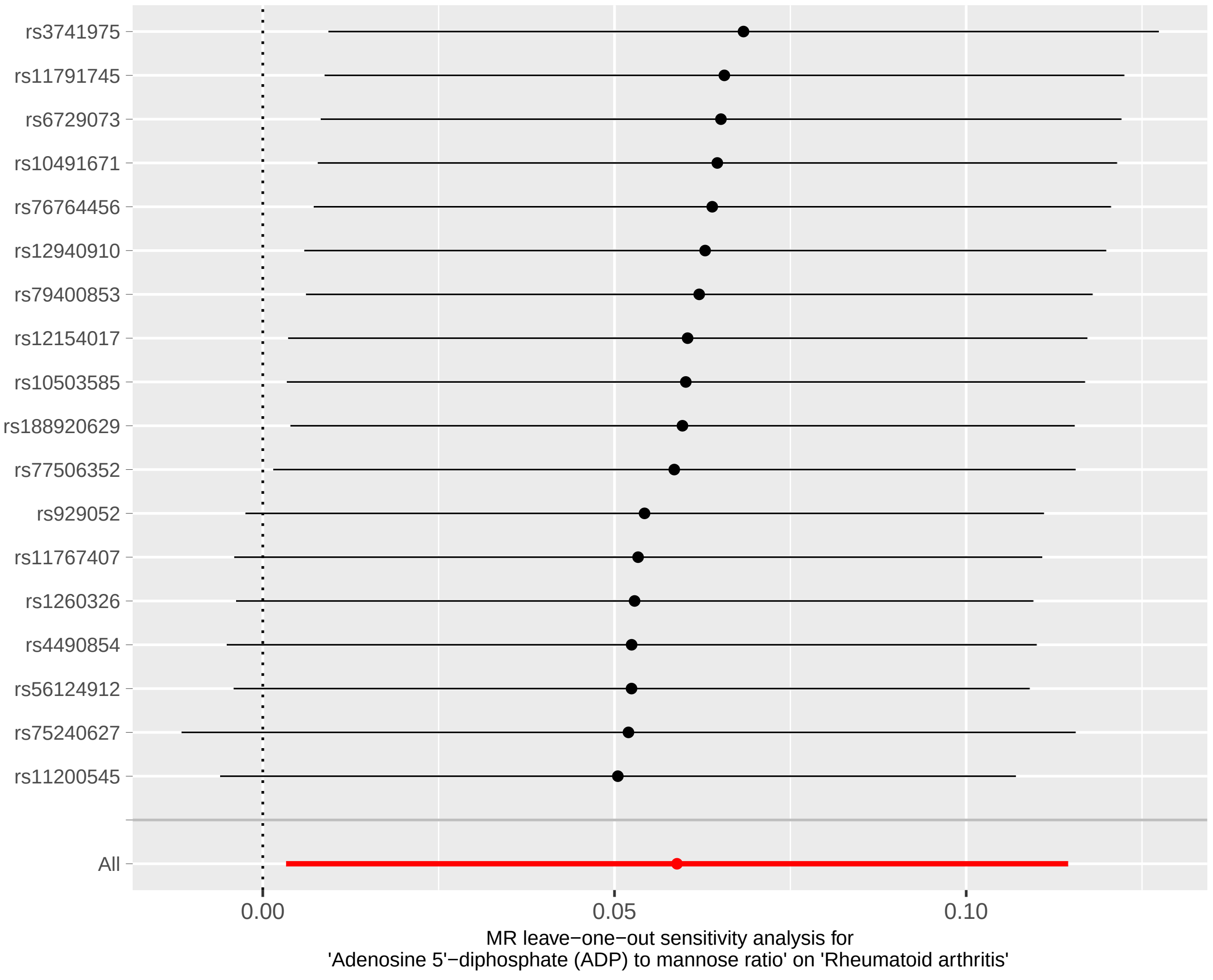
rs3741975
rs11791745
rs6729073
rs10491671
rs76764456
rs12940910
rs79400853
rs12154017
rs10503585
rs188920629
rs77506352
rs929052
rs11767407
rs1260326
rs4490854
rs56124912
rs75240627
rs11200545
All
0.00
0.05
0.10
MR leave−one−out sensitivity analysis for
'Adenosine 5'−diphosphate (ADP) to mannose ratio' on 'Rheumatoid arthritis'

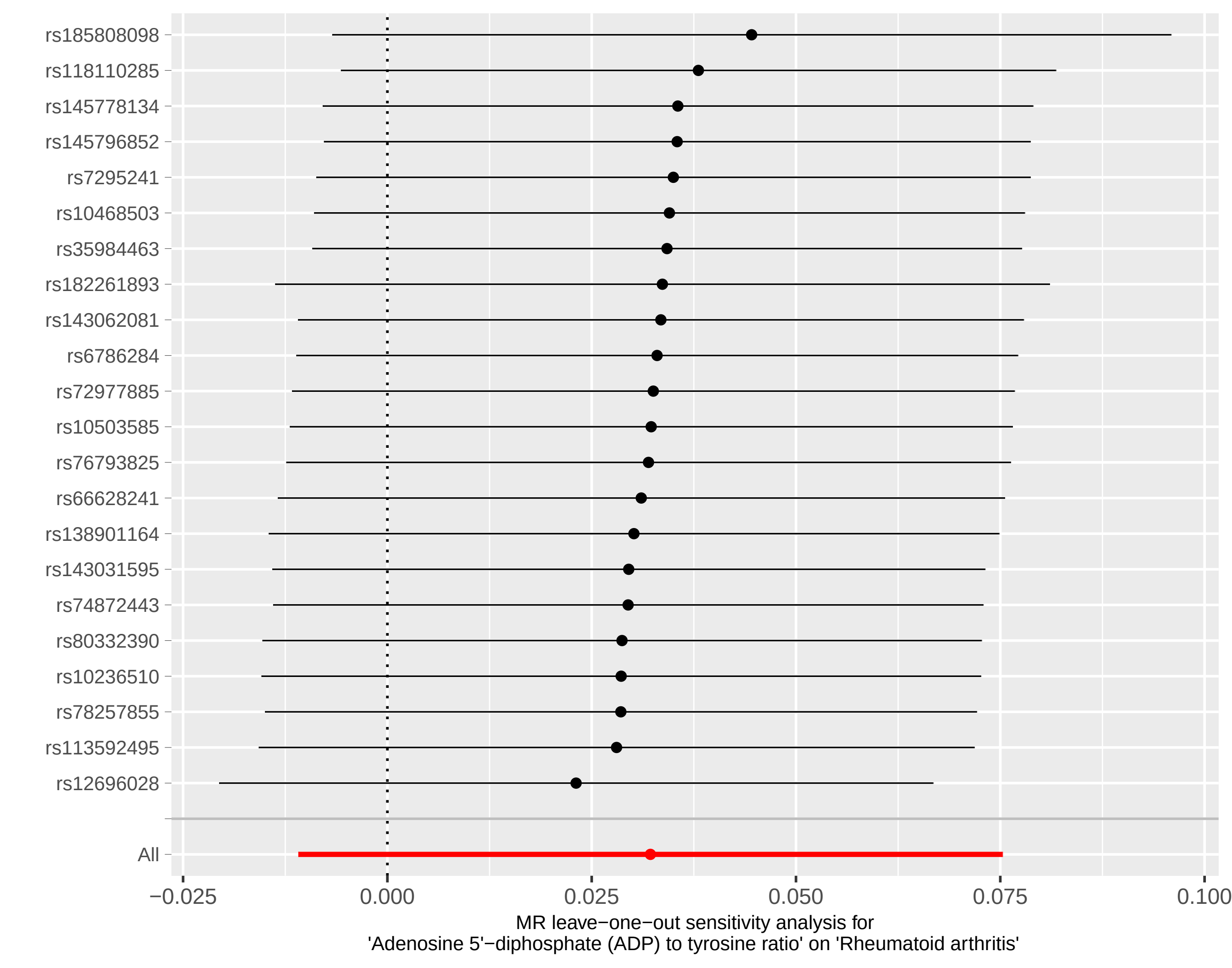
rs185808098
rs118110285
rs145778134
rs145796852
rs7295241
rs10468503
rs35984463
rs182261893
rs143062081
rs6786284
rs72977885
rs10503585
rs76793825
rs66628241
rs138901164
rs143031595
rs74872443
rs80332390
rs10236510
rs78257855
rs113592495
rs12696028
All
−0.025
0.000
0.025
0.050
0.075
0.100
MR leave−one−out sensitivity analysis for
'Adenosine 5'−diphosphate (ADP) to tyrosine ratio' on 'Rheumatoid arthritis'

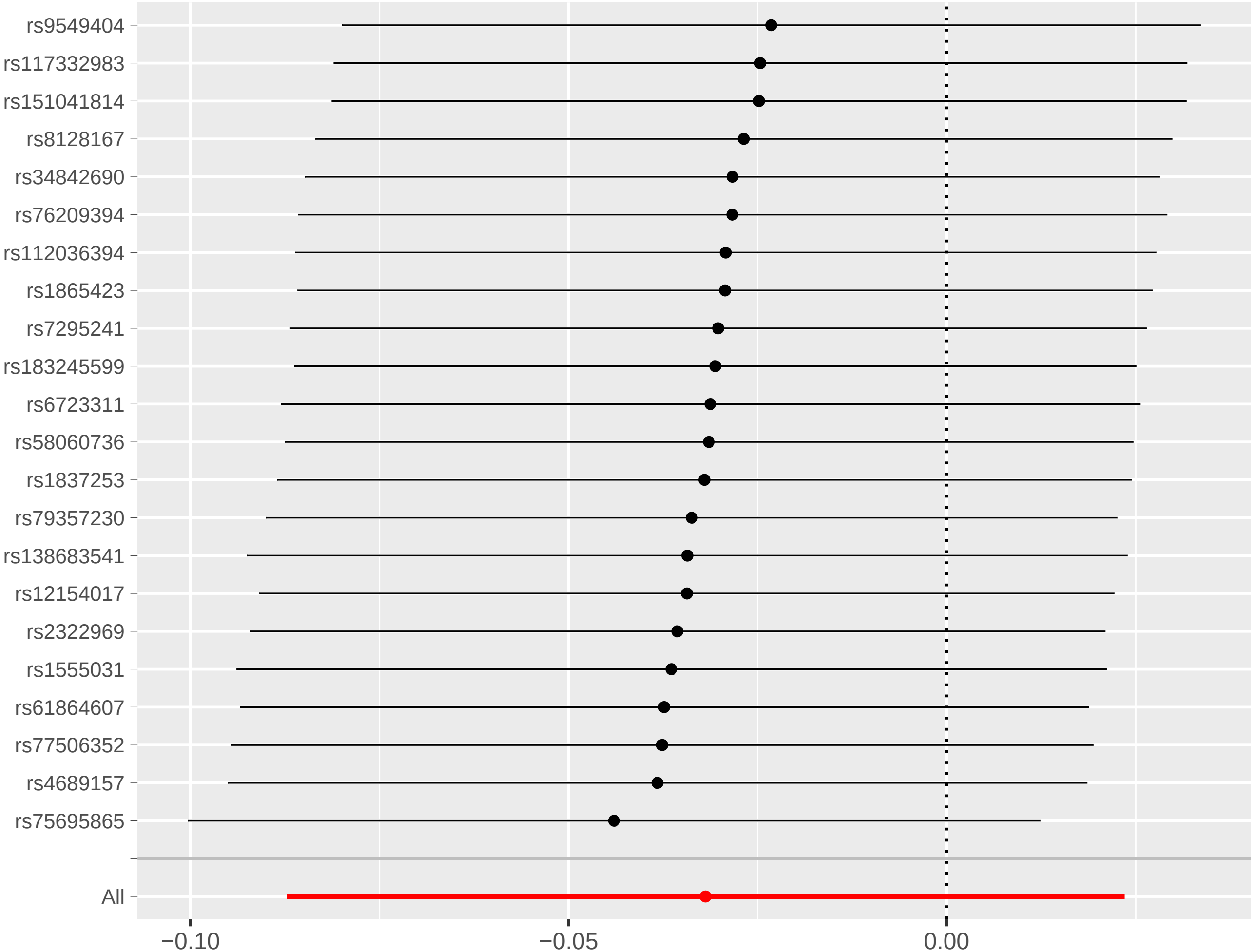

MR leave−one−out sensitivity analysis for
'Adenosine 5'−diphosphate (ADP) to N−acetylglucosamine to N−acetylgalactosamine ratio' on 'Rheumatoid arthritis'

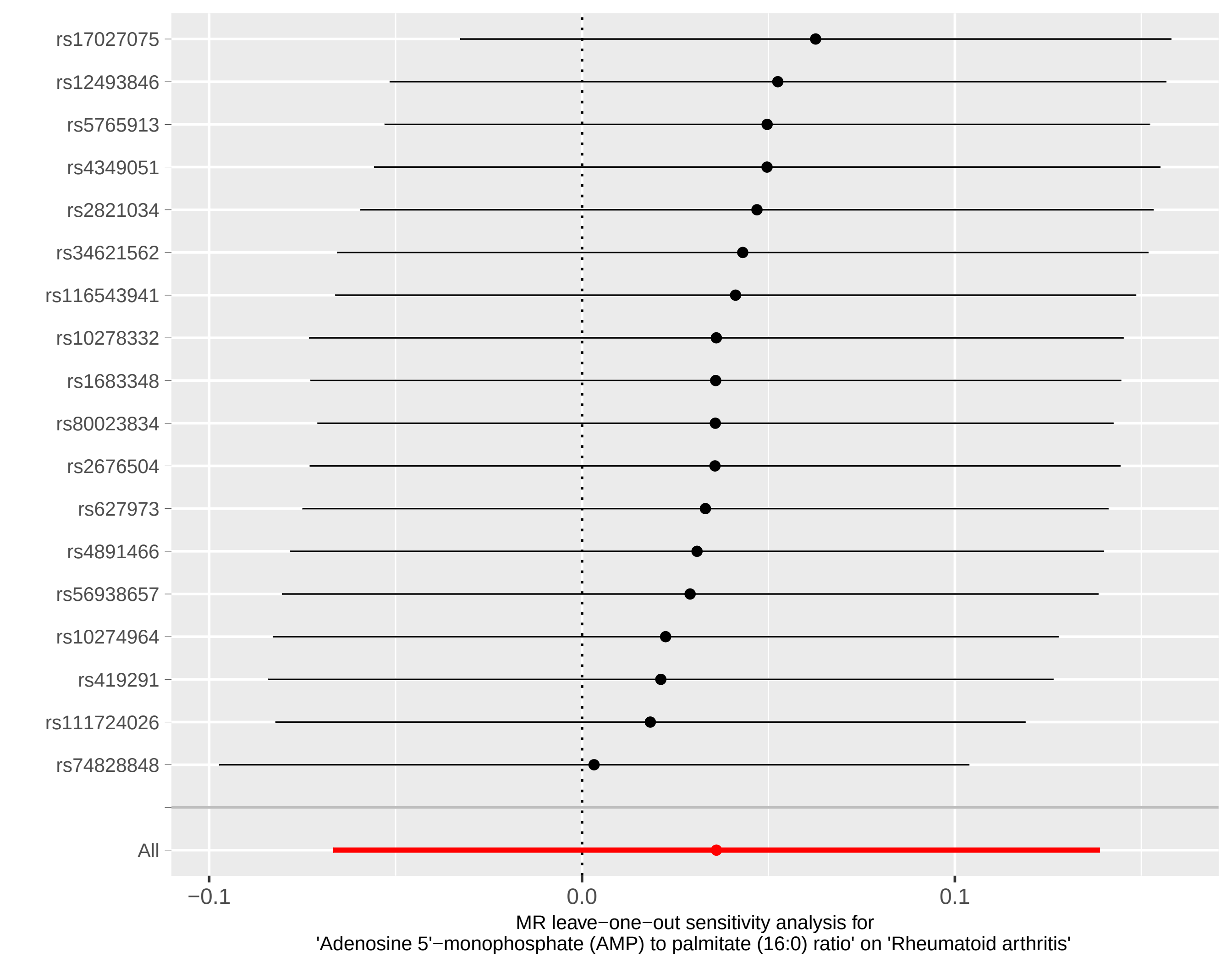
rs17027075
rs12493846
rs5765913
rs4349051
rs2821034
rs34621562
rs116543941
rs10278332
rs1683348
rs80023834
rs2676504
rs627973
rs4891466
rs56938657
rs10274964
rs419291
rs111724026
rs74828848
All
−0.1
0.0
0.1
MR leave−one−out sensitivity analysis for
'Adenosine 5'−monophosphate (AMP) to palmitate (16:0) ratio' on 'Rheumatoid arthritis'

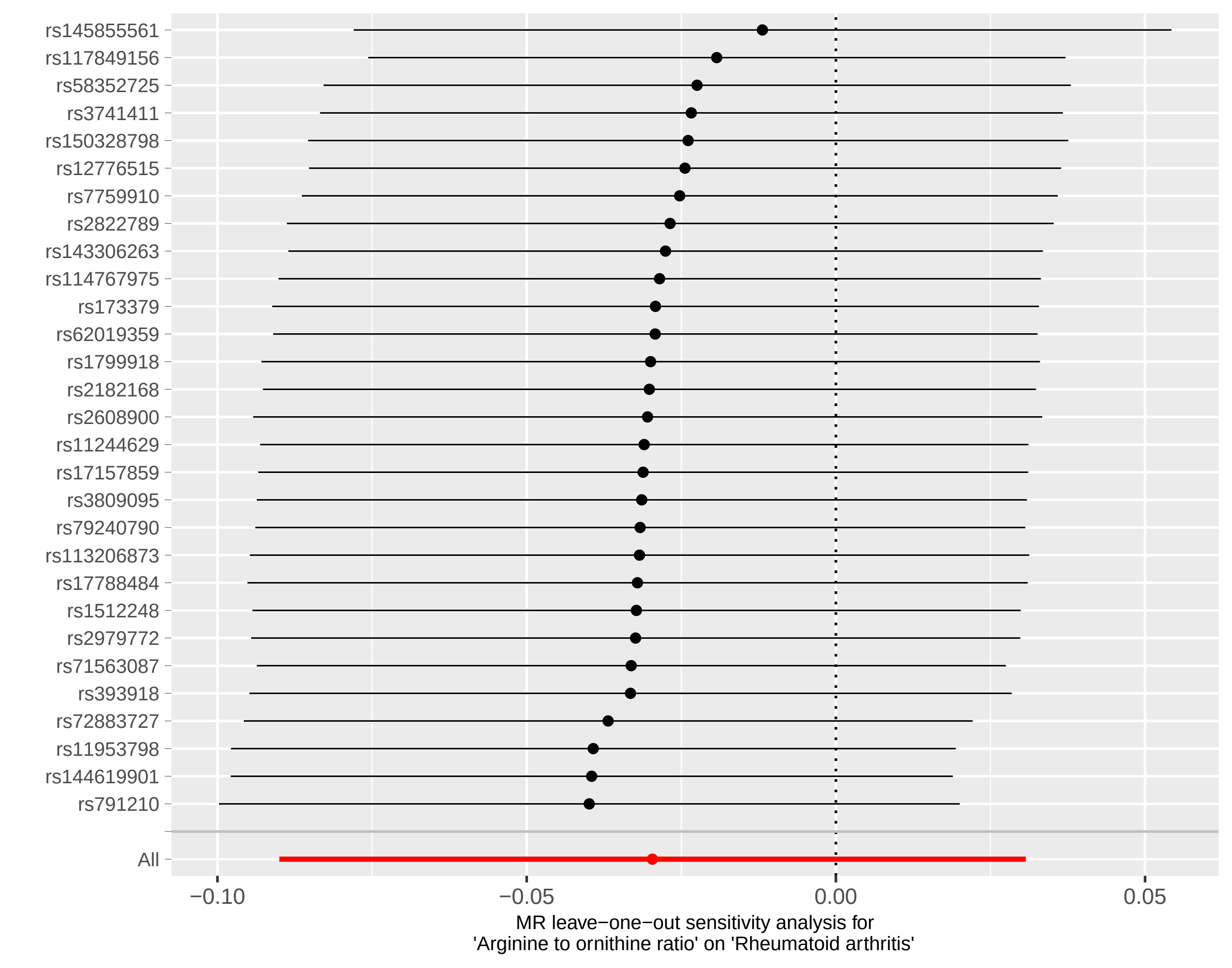
rs145855561
rs117849156
rs58352725
rs3741411
rs150328798
rs12776515
rs7759910
rs2822789
rs143306263
rs114767975
rs173379
rs62019359
rs1799918
rs2182168
rs2608900
rs11244629
rs17157859
rs3809095
rs79240790
rs113206873
rs17788484
rs1512248
rs2979772
rs71563087
rs393918
rs72883727
rs11953798
rs144619901
rs791210
All
−0.10
−0.05
0.00
0.05
MR leave−one−out sensitivity analysis for
'Arginine to ornithine ratio' on 'Rheumatoid arthritis'

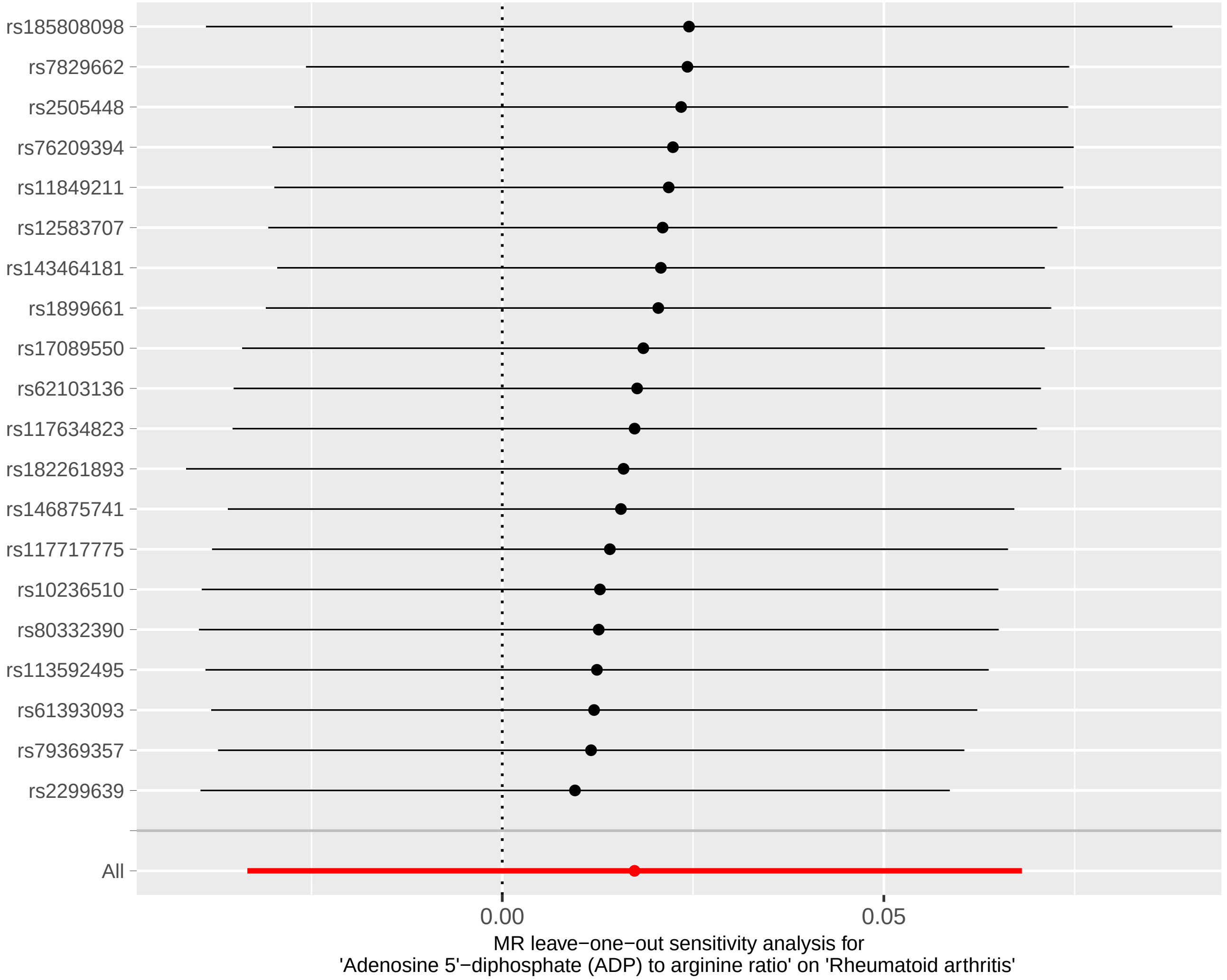

rs185808098
rs7829662
rs2505448
rs76209394
rs11849211
rs12583707
rs143464181
rs1899661
rs17089550
rs62103136
rs117634823
rs182261893
rs146875741
rs117717775
rs10236510
rs80332390
rs113592495
rs61393093
rs79369357
rs2299639
All
0.00
0.05
MR leave−one−out sensitivity analysis for
'Adenosine 5'−diphosphate (ADP) to arginine ratio' on 'Rheumatoid arthritis'

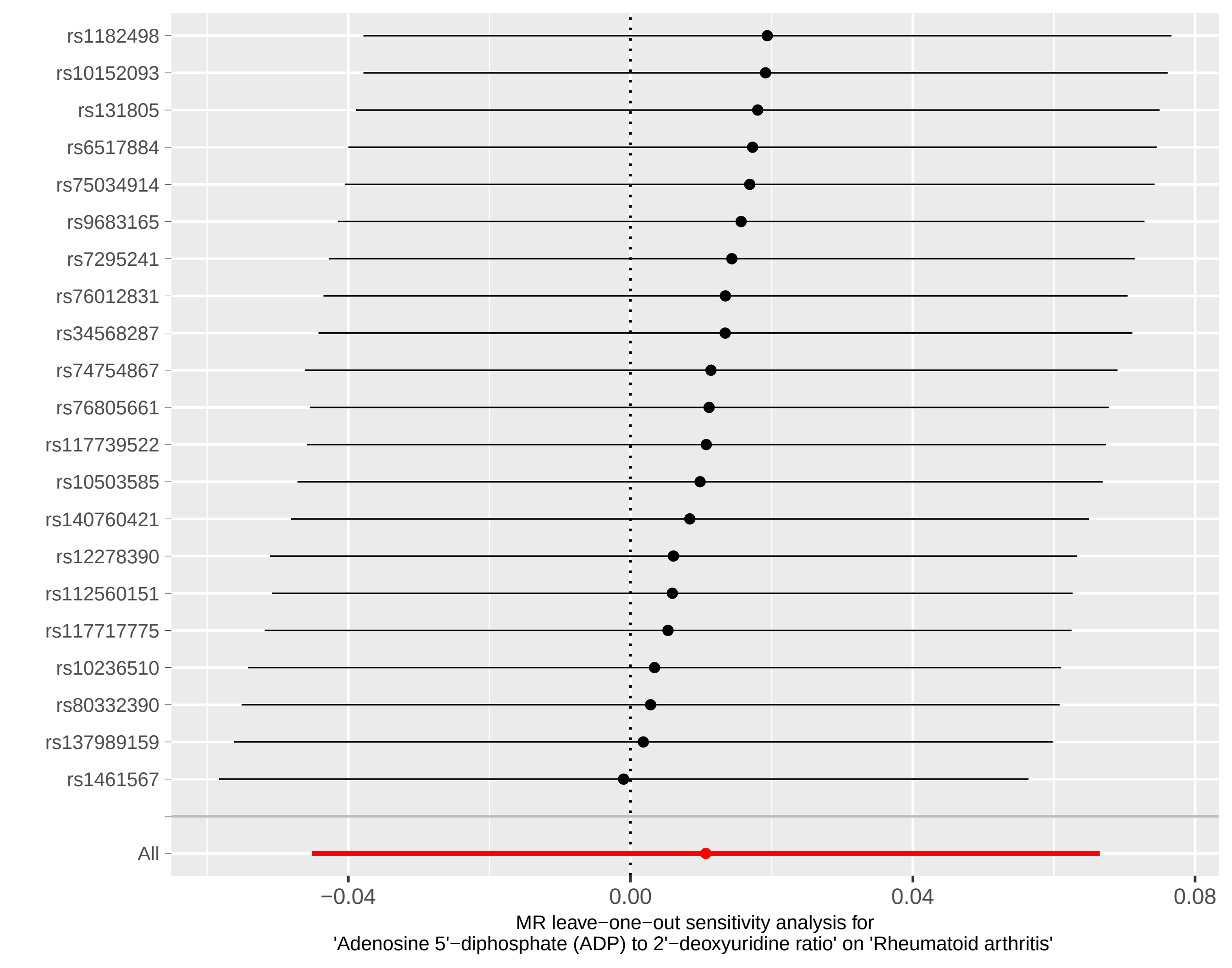
rs1182498
rs10152093
rs131805
rs6517884
rs75034914
rs9683165
rs7295241
rs76012831
rs34568287
rs74754867
rs76805661
rs117739522
rs10503585
rs140760421
rs12278390
rs112560151
rs117717775
rs10236510
rs80332390
rs137989159
rs1461567
All
−0.04
0.00
0.04
0.08
MR leave−one−out sensitivity analysis for
'Adenosine 5'−diphosphate (ADP) to 2'−deoxyuridine ratio' on 'Rheumatoid arthritis'

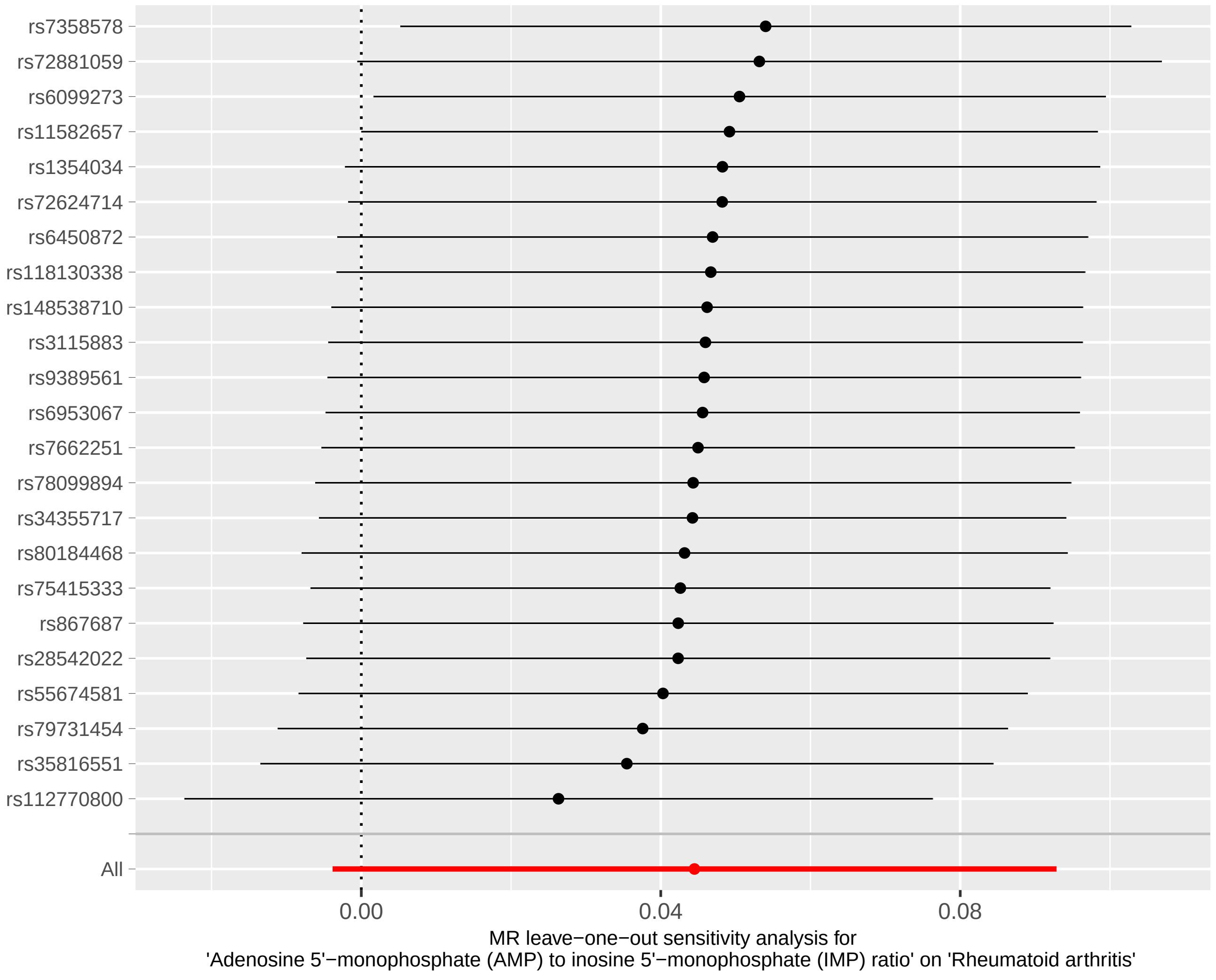
rs7358578
rs72881059
rs6099273
rs11582657
rs1354034
rs72624714
rs6450872
rs118130338
rs148538710
rs3115883
rs9389561
rs6953067
rs7662251
rs78099894
rs34355717
rs80184468
rs75415333
rs867687
rs28542022
rs55674581
rs79731454
rs35816551
rs112770800
All
0.00
0.04
0.08
MR leave−one−out sensitivity analysis for
'Adenosine 5'−monophosphate (AMP) to inosine 5'−monophosphate (IMP) ratio' on 'Rheumatoid arthritis'

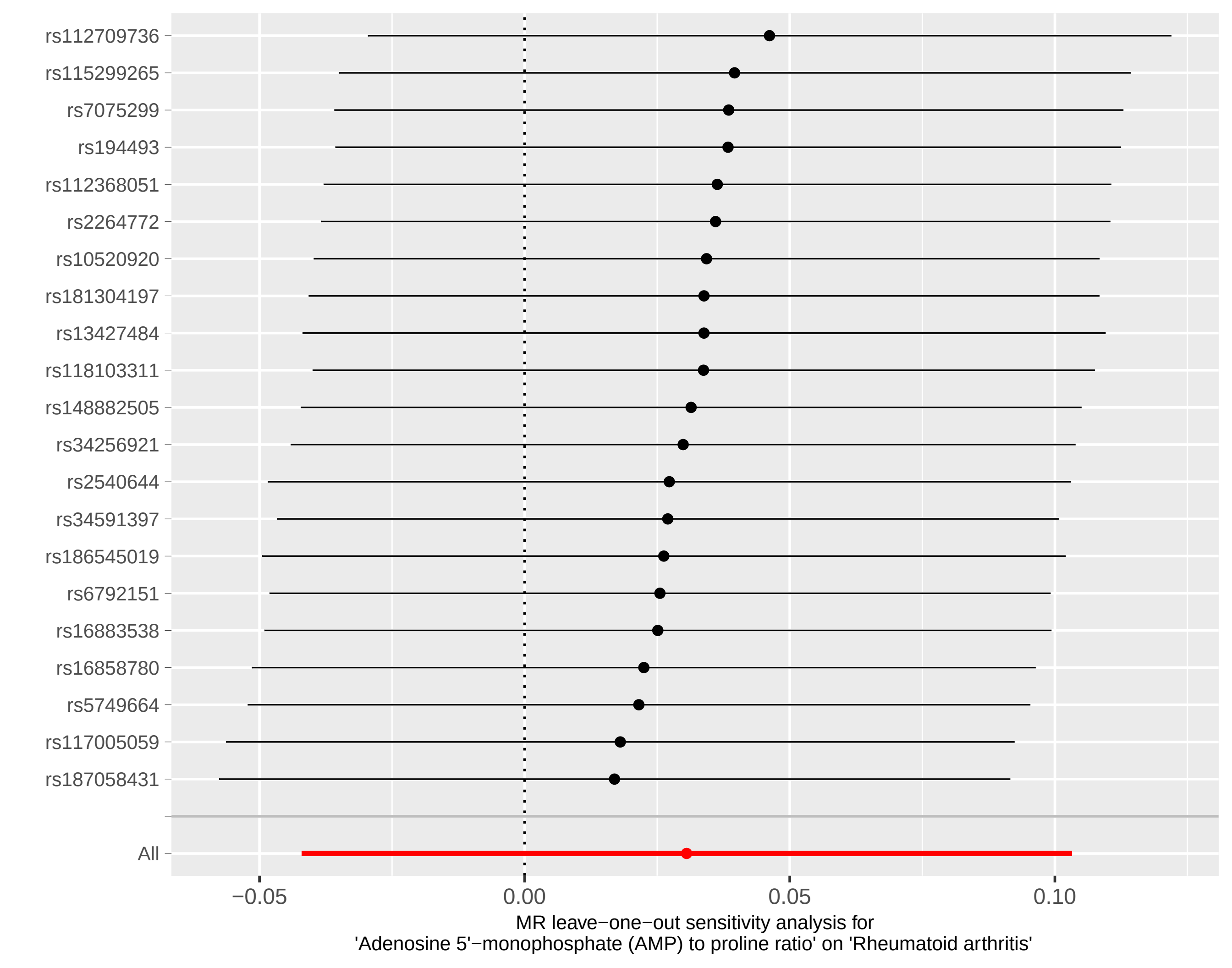
rs112709736
rs115299265
rs7075299
rs194493
rs112368051
rs2264772
rs10520920
rs181304197
rs13427484
rs118103311
rs148882505
rs34256921
rs2540644
rs34591397
rs186545019
rs6792151
rs16883538
rs16858780
rs5749664
rs117005059
rs187058431
All
−0.05
0.00
0.05
0.10
MR leave−one−out sensitivity analysis for
'Adenosine 5'−monophosphate (AMP) to proline ratio' on 'Rheumatoid arthritis'

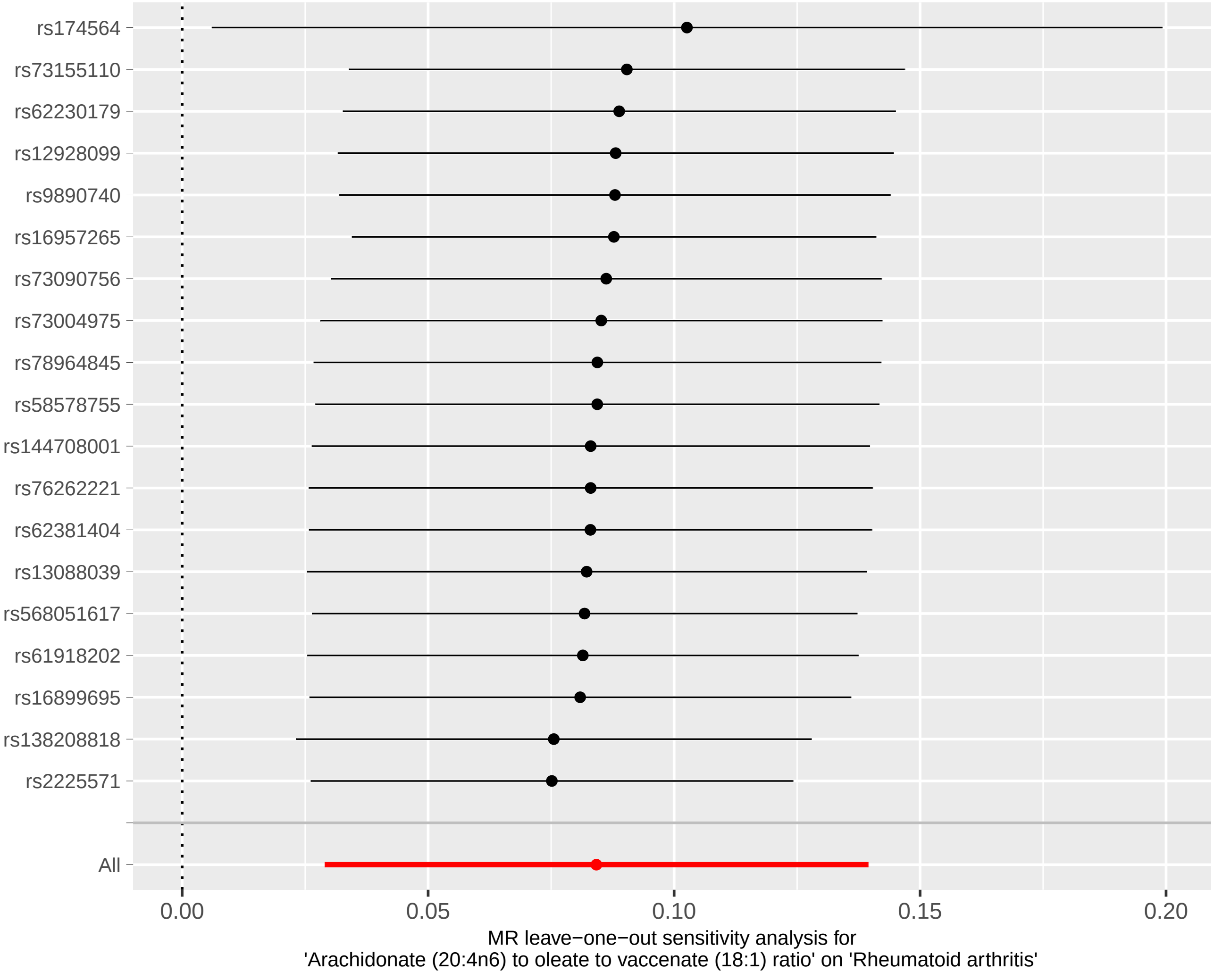

rs174564
rs73155110
rs62230179
rs12928099
rs9890740
rs16957265
rs73090756
rs73004975
rs78964845
rs58578755
rs144708001
rs76262221
rs62381404
rs13088039
rs568051617
rs61918202
rs16899695
rs138208818
rs2225571
All
0.00
0.05
0.10
0.15
0.20
MR leave−one−out sensitivity analysis for
'Arachidonate (20:4n6) to oleate to vaccenate (18:1) ratio' on 'Rheumatoid arthritis'

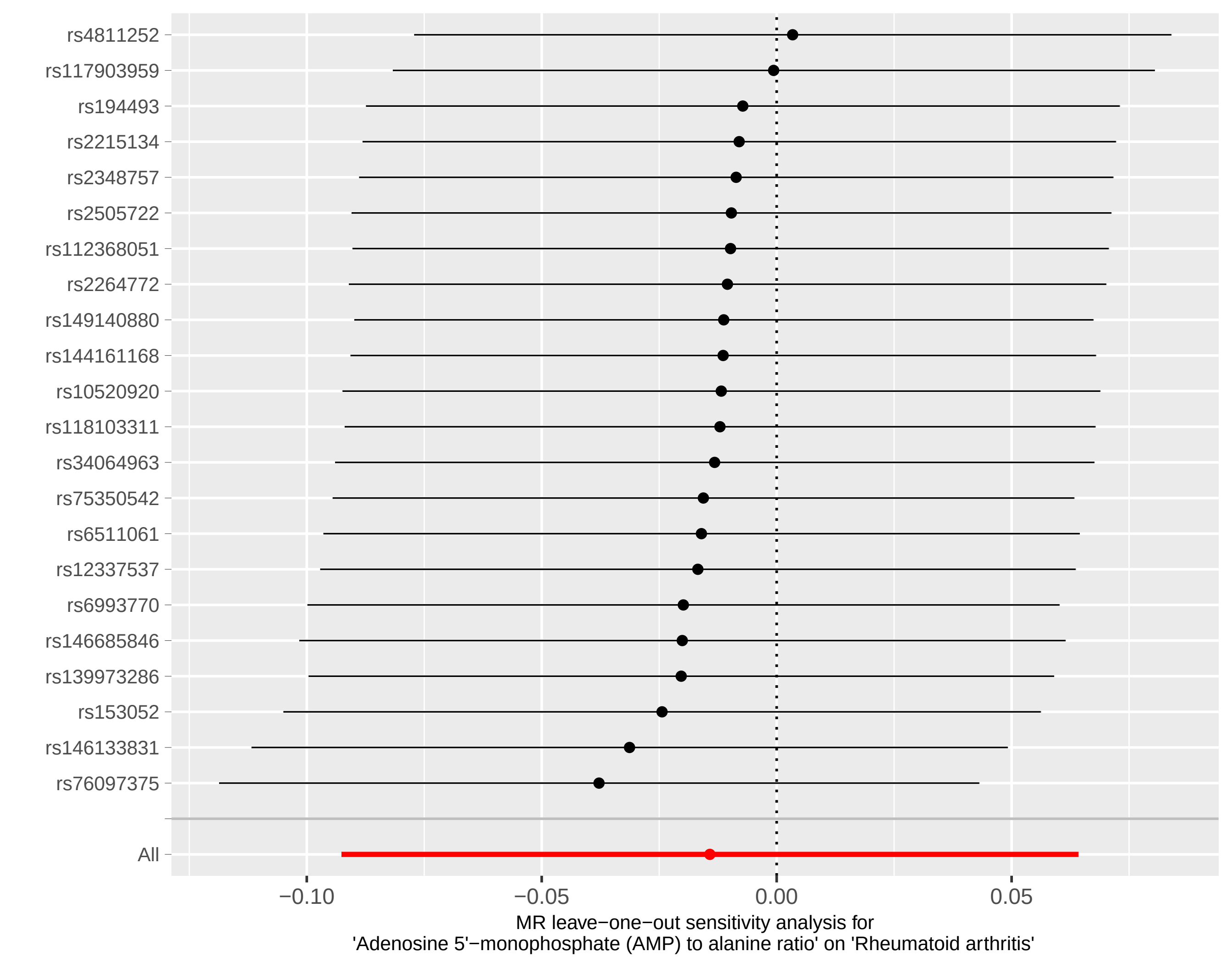
rs4811252
rs117903959
rs194493
rs2215134
rs2348757
rs2505722
rs112368051
rs2264772
rs149140880
rs144161168
rs10520920
rs118103311
rs34064963
rs75350542
rs6511061
rs12337537
rs6993770
rs146685846
rs139973286
rs153052
rs146133831
rs76097375
All
−0.10
−0.05
0.00
0.05
MR leave−one−out sensitivity analysis for
'Adenosine 5'−monophosphate (AMP) to alanine ratio' on 'Rheumatoid arthritis'

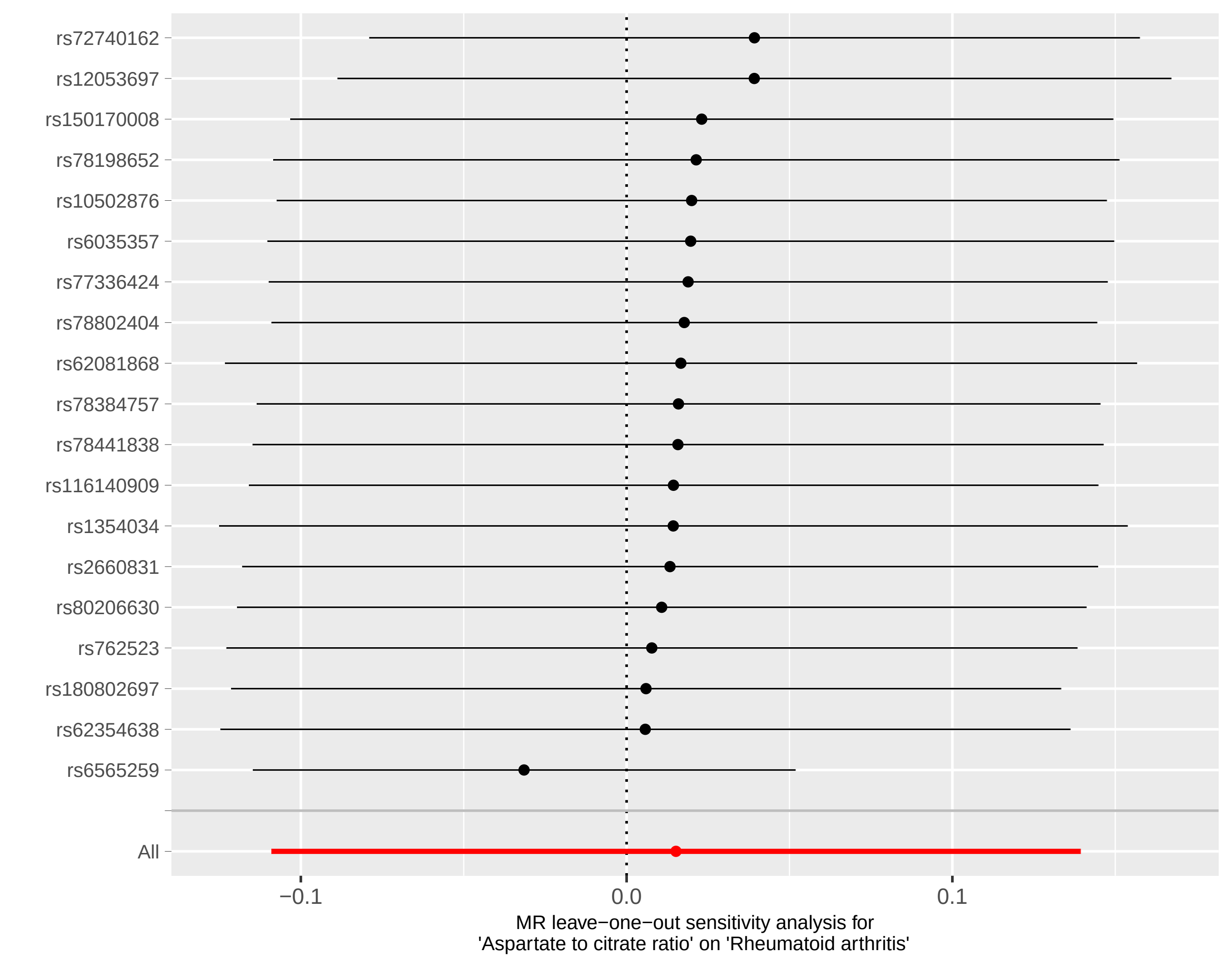

rs72740162
rs12053697
rs150170008
rs78198652
rs10502876
rs6035357
rs77336424
rs78802404
rs62081868
rs78384757
rs78441838
rs116140909
rs1354034
rs2660831
rs80206630
rs762523
rs180802697
rs62354638
rs6565259
All
−0.1
0.0
0.1
MR leave−one−out sensitivity analysis for
'Aspartate to citrate ratio' on 'Rheumatoid arthritis'

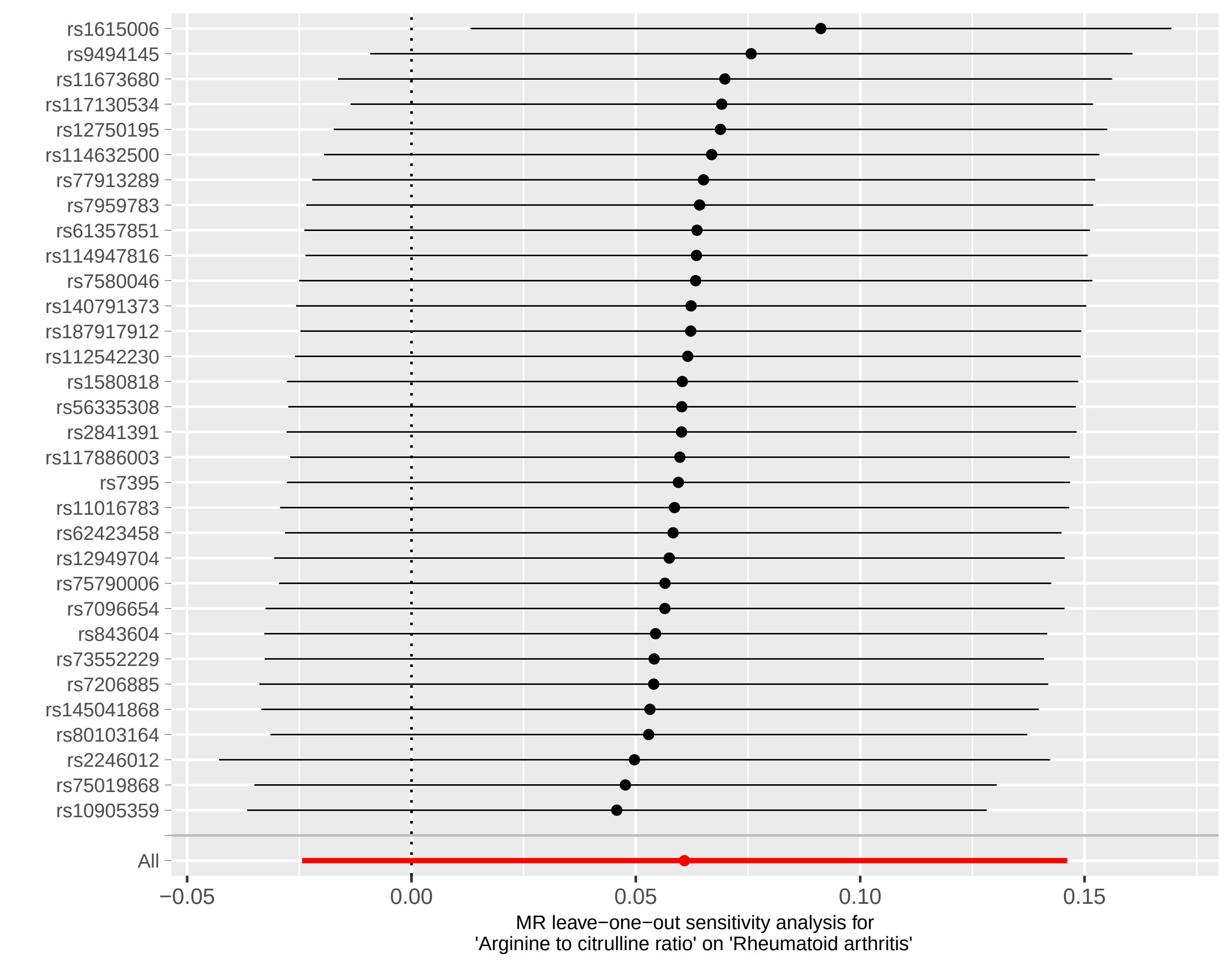

rs1615006
rs9494145
rs11673680
rs117130534
rs12750195
rs114632500
rs77913289
rs7959783
rs61357851
rs114947816
rs7580046
rs140791373
rs187917912
rs112542230
rs1580818
rs56335308
rs2841391
rs117886003
rs7395
rs11016783
rs62423458
rs12949704
rs75790006
rs7096654
rs843604
rs73552229
rs7206885
rs145041868
rs80103164
rs2246012
rs75019868
rs10905359
All
−0.05
0.00
0.05
0.10
0.15
MR leave−one−out sensitivity analysis for
'Arginine to citrulline ratio' on 'Rheumatoid arthritis'

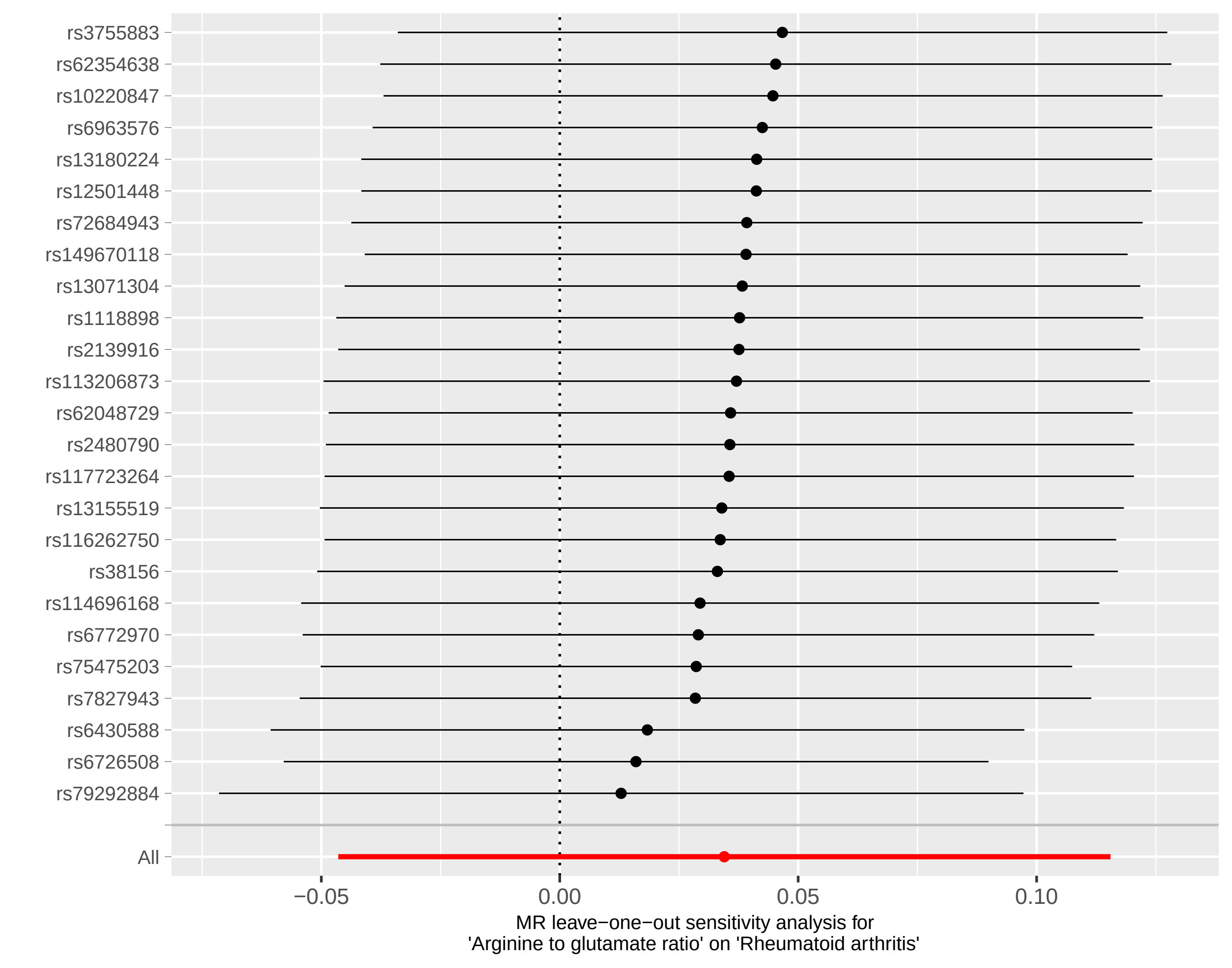
rs3755883
rs62354638
rs10220847
rs6963576
rs13180224
rs12501448
rs72684943
rs149670118
rs13071304
rs1118898
rs2139916
rs113206873
rs62048729
rs2480790
rs117723264
rs13155519
rs116262750
rs38156
rs114696168
rs6772970
rs75475203
rs7827943
rs6430588
rs6726508
rs79292884
All
−0.05
0.00
0.05
0.10
MR leave−one−out sensitivity analysis for
'Arginine to glutamate ratio' on 'Rheumatoid arthritis'

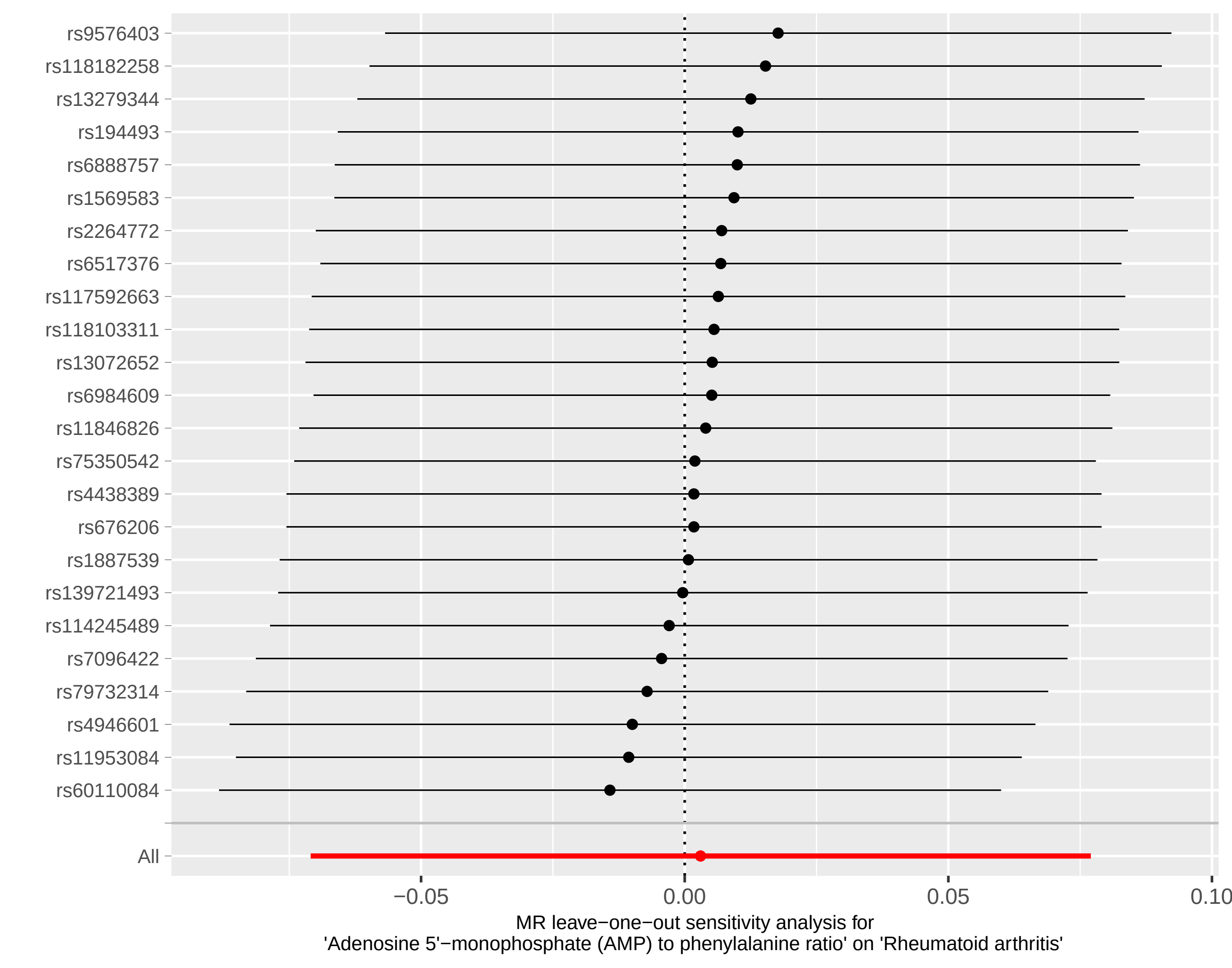
rs9576403
rs118182258
rs13279344
rs194493
rs6888757
rs1569583
rs2264772
rs6517376
rs117592663
rs118103311
rs13072652
rs6984609
rs11846826
rs75350542
rs4438389
rs676206
rs1887539
rs139721493
rs114245489
rs7096422
rs79732314
rs4946601
rs11953084
rs60110084
All
−0.05
0.00
0.05
0.10
MR leave−one−out sensitivity analysis for
'Adenosine 5'−monophosphate (AMP) to phenylalanine ratio' on 'Rheumatoid arthritis'

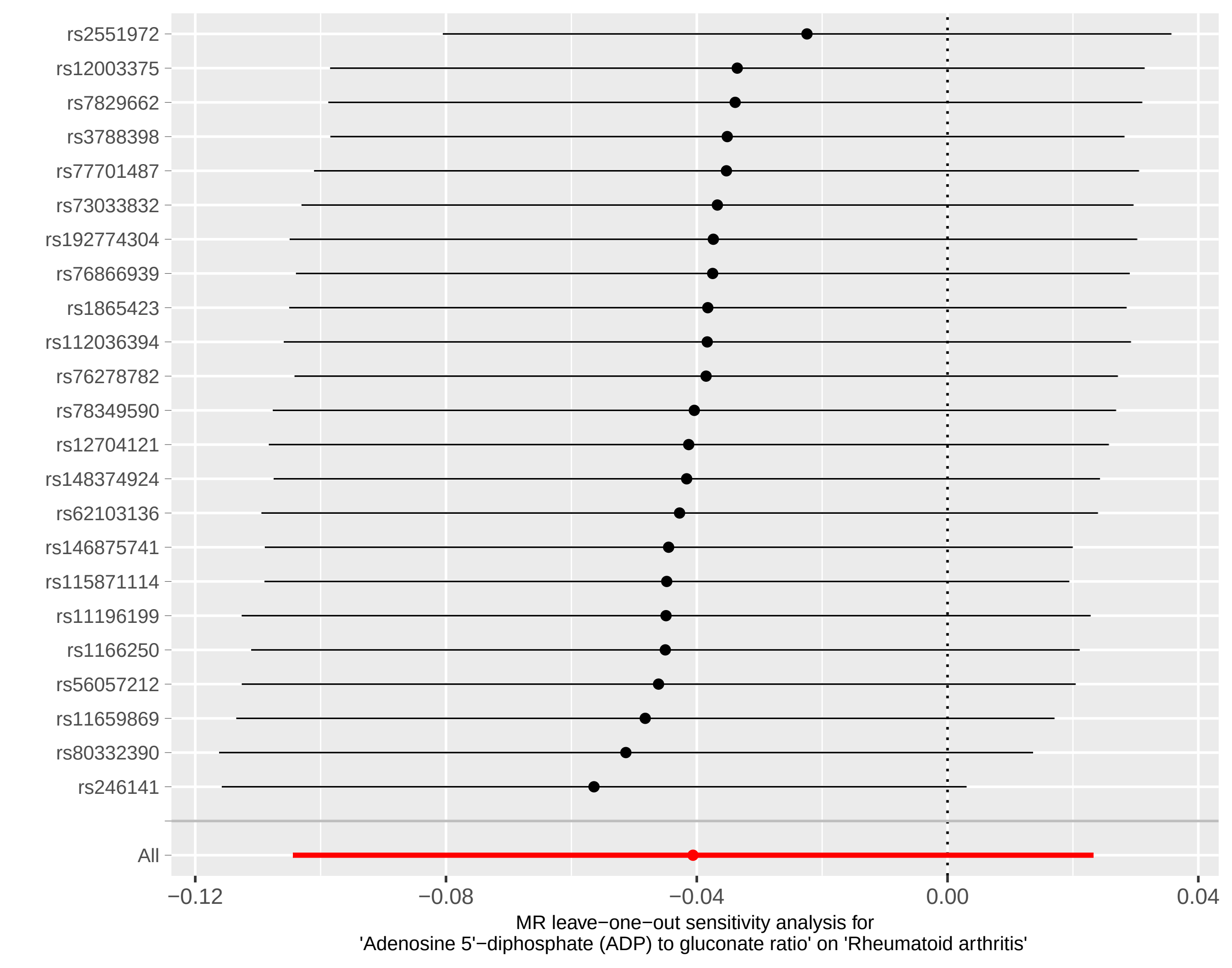
rs2551972
rs12003375
rs7829662
rs3788398
rs77701487
rs73033832
rs192774304
rs76866939
rs1865423
rs112036394
rs76278782
rs78349590
rs12704121
rs148374924
rs62103136
rs146875741
rs115871114
rs11196199
rs1166250
rs56057212
rs11659869
rs80332390
rs246141
All
−0.12
−0.08
−0.04
0.00
0.04
MR leave−one−out sensitivity analysis for
'Adenosine 5'−diphosphate (ADP) to gluconate ratio' on 'Rheumatoid arthritis'

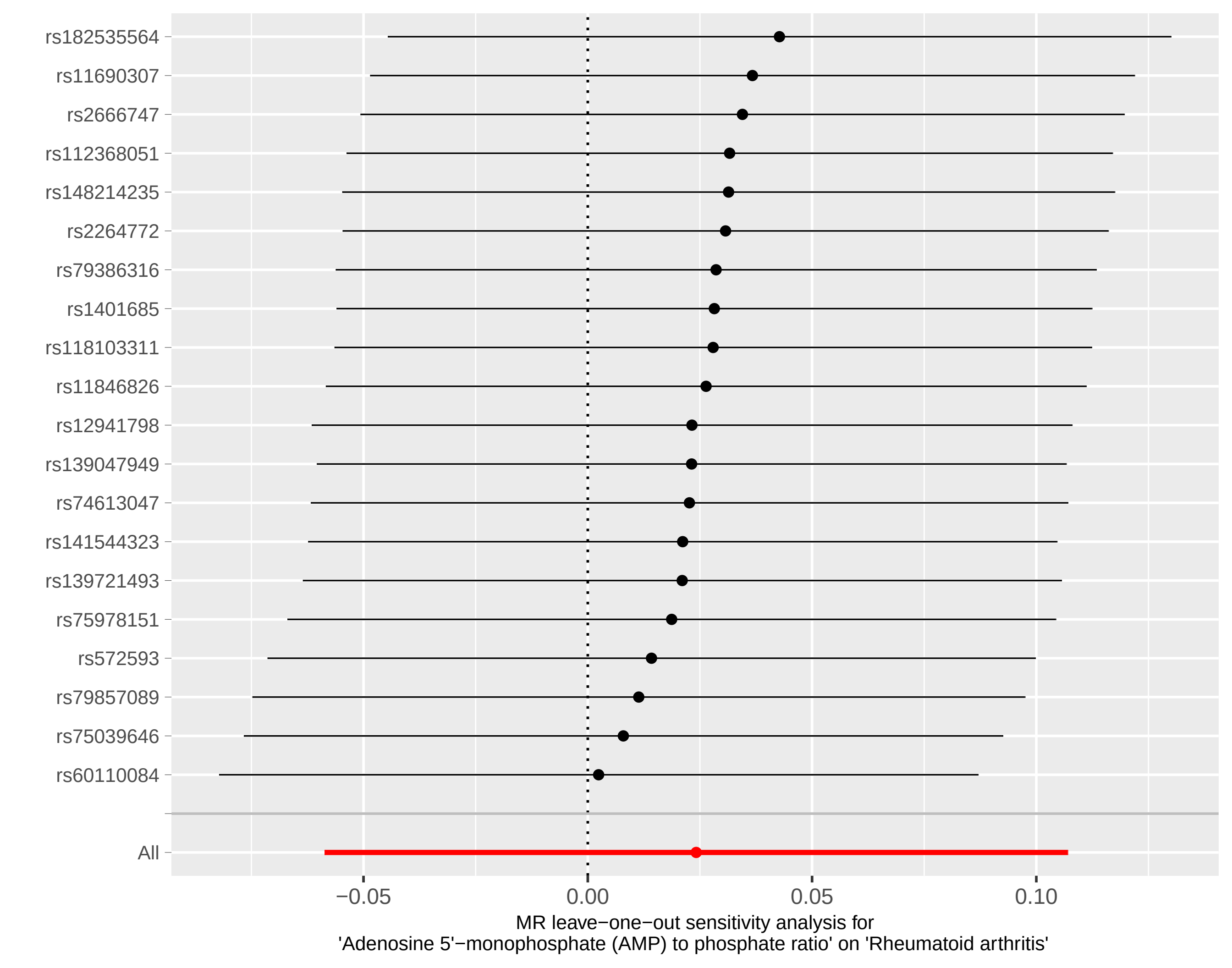
rs182535564
rs11690307
rs2666747
rs112368051
rs148214235
rs2264772
rs79386316
rs1401685
rs118103311
rs11846826
rs12941798
rs139047949
rs74613047
rs141544323
rs139721493
rs75978151
rs572593
rs79857089
rs75039646
rs60110084
All
−0.05
0.00
0.05
0.10
MR leave−one−out sensitivity analysis for
'Adenosine 5'−monophosphate (AMP) to phosphate ratio' on 'Rheumatoid arthritis'

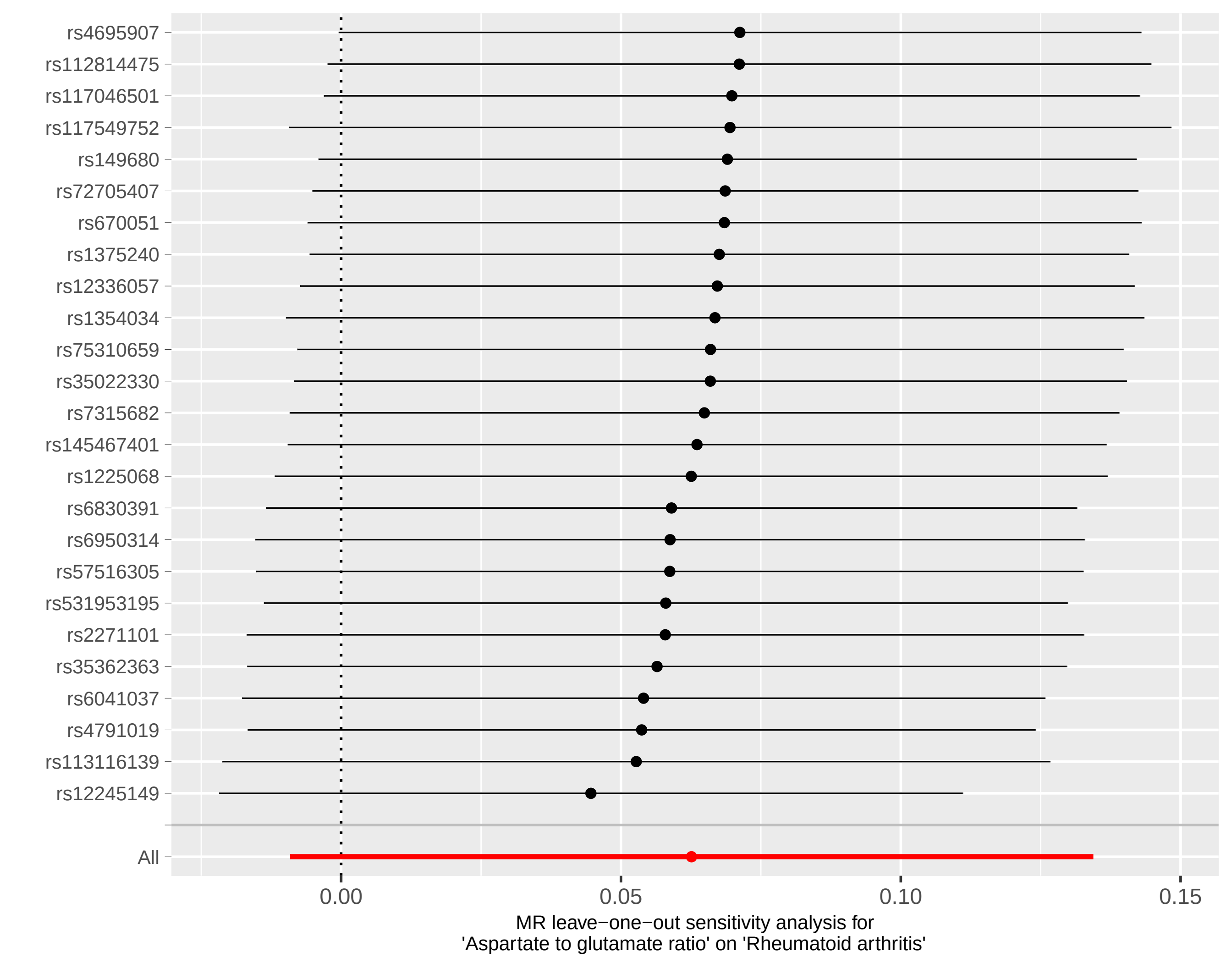
rs4695907
rs112814475
rs117046501
rs117549752
rs149680
rs72705407
rs670051
rs1375240
rs12336057
rs1354034
rs75310659
rs35022330
rs7315682
rs145467401
rs1225068
rs6830391
rs6950314
rs57516305
rs531953195
rs2271101
rs35362363
rs6041037
rs4791019
rs113116139
rs12245149
All
0.00
0.05
0.10
0.15
MR leave−one−out sensitivity analysis for
'Aspartate to glutamate ratio' on 'Rheumatoid arthritis'

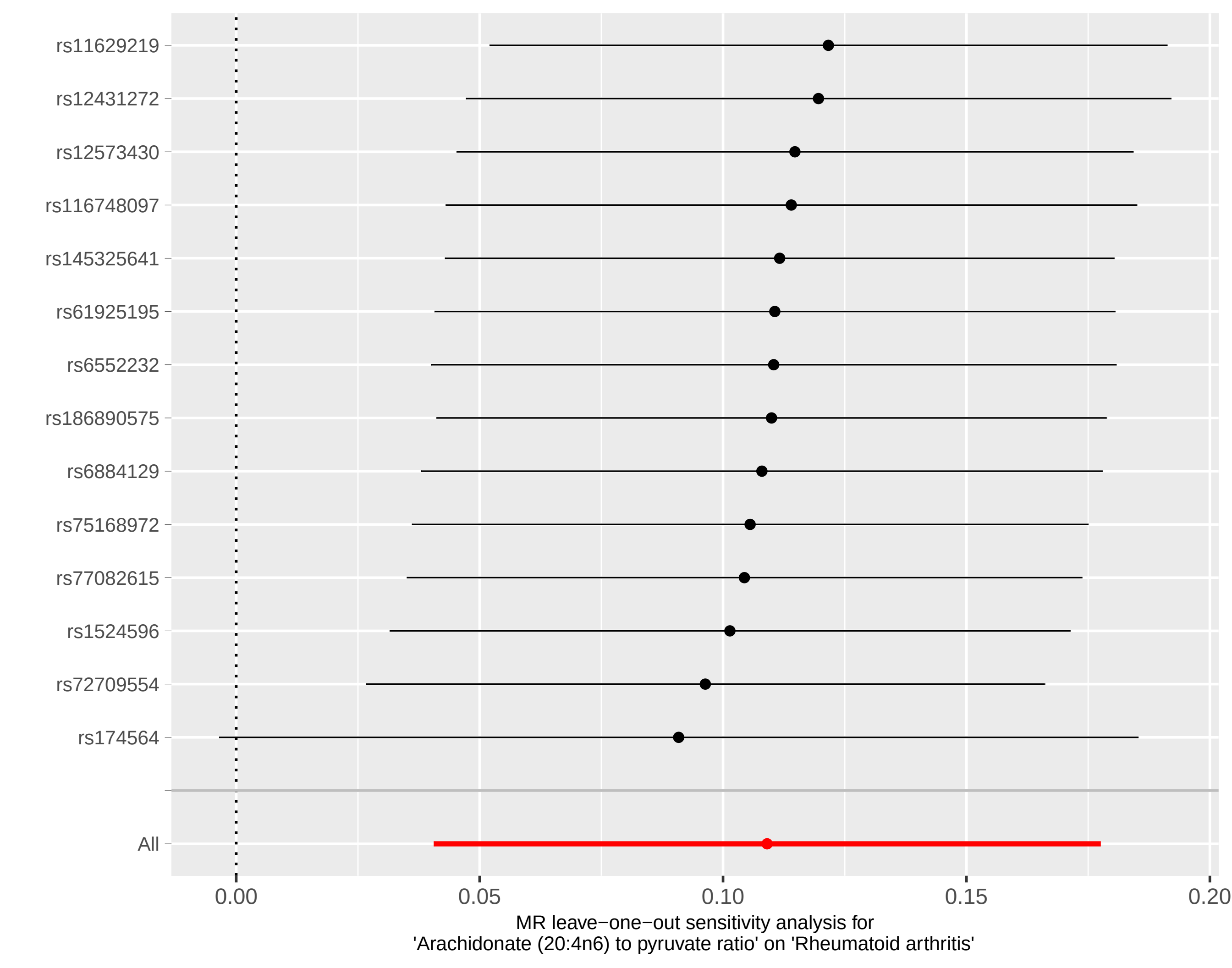
rs11629219
rs12431272
rs12573430
rs116748097
rs145325641
rs61925195
rs6552232
rs186890575
rs6884129
rs75168972
rs77082615
rs1524596
rs72709554
rs174564
All
0.00
0.05
0.10
0.15
0.20
MR leave−one−out sensitivity analysis for
'Arachidonate (20:4n6) to pyruvate ratio' on 'Rheumatoid arthritis'

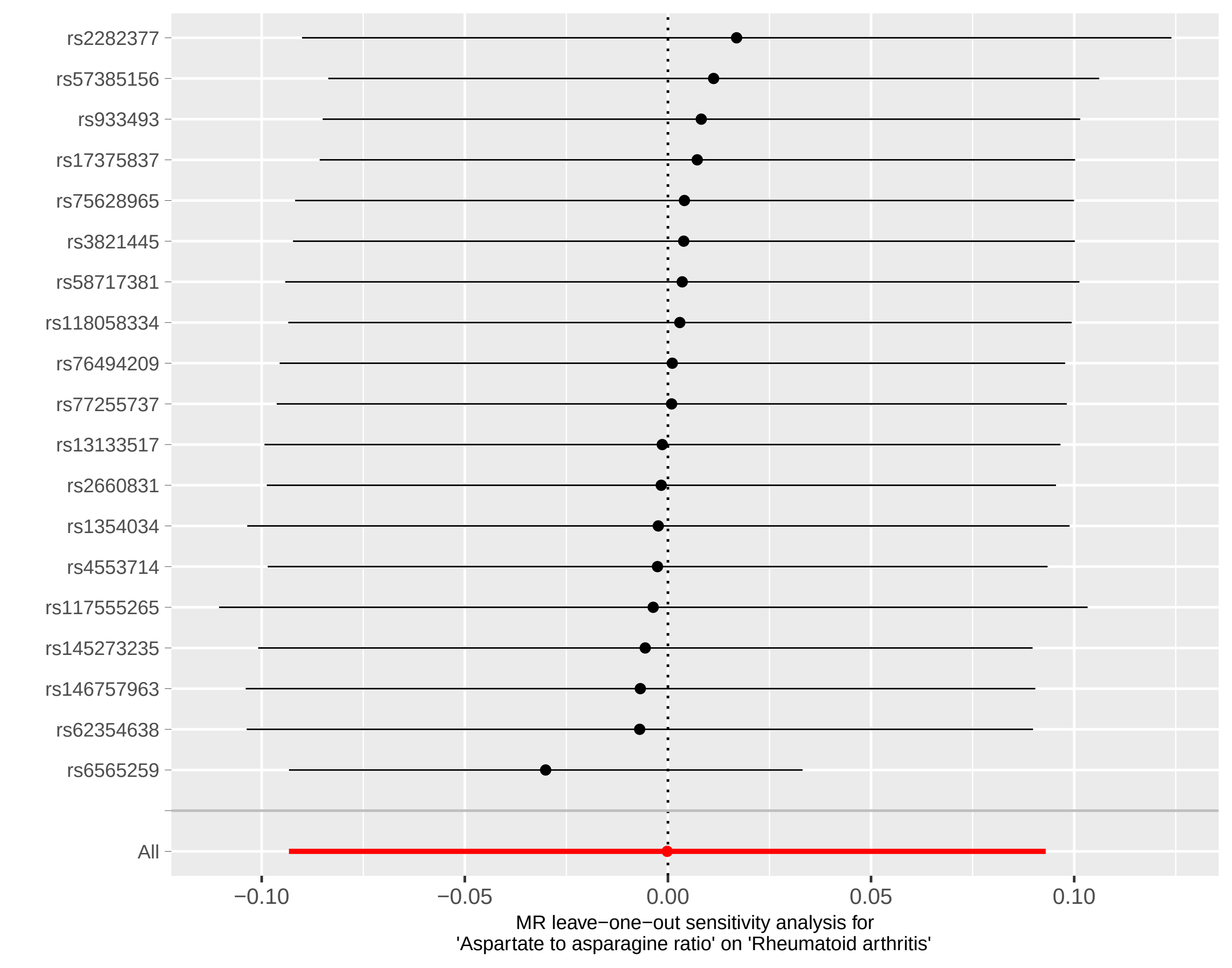
rs2282377
rs57385156
rs933493
rs17375837
rs75628965
rs3821445
rs58717381
rs118058334
rs76494209
rs77255737
rs13133517
rs2660831
rs1354034
rs4553714
rs117555265
rs145273235
rs146757963
rs62354638
rs6565259
All
−0.10
−0.05
0.00
0.05
0.10
MR leave−one−out sensitivity analysis for
'Aspartate to asparagine ratio' on 'Rheumatoid arthritis'

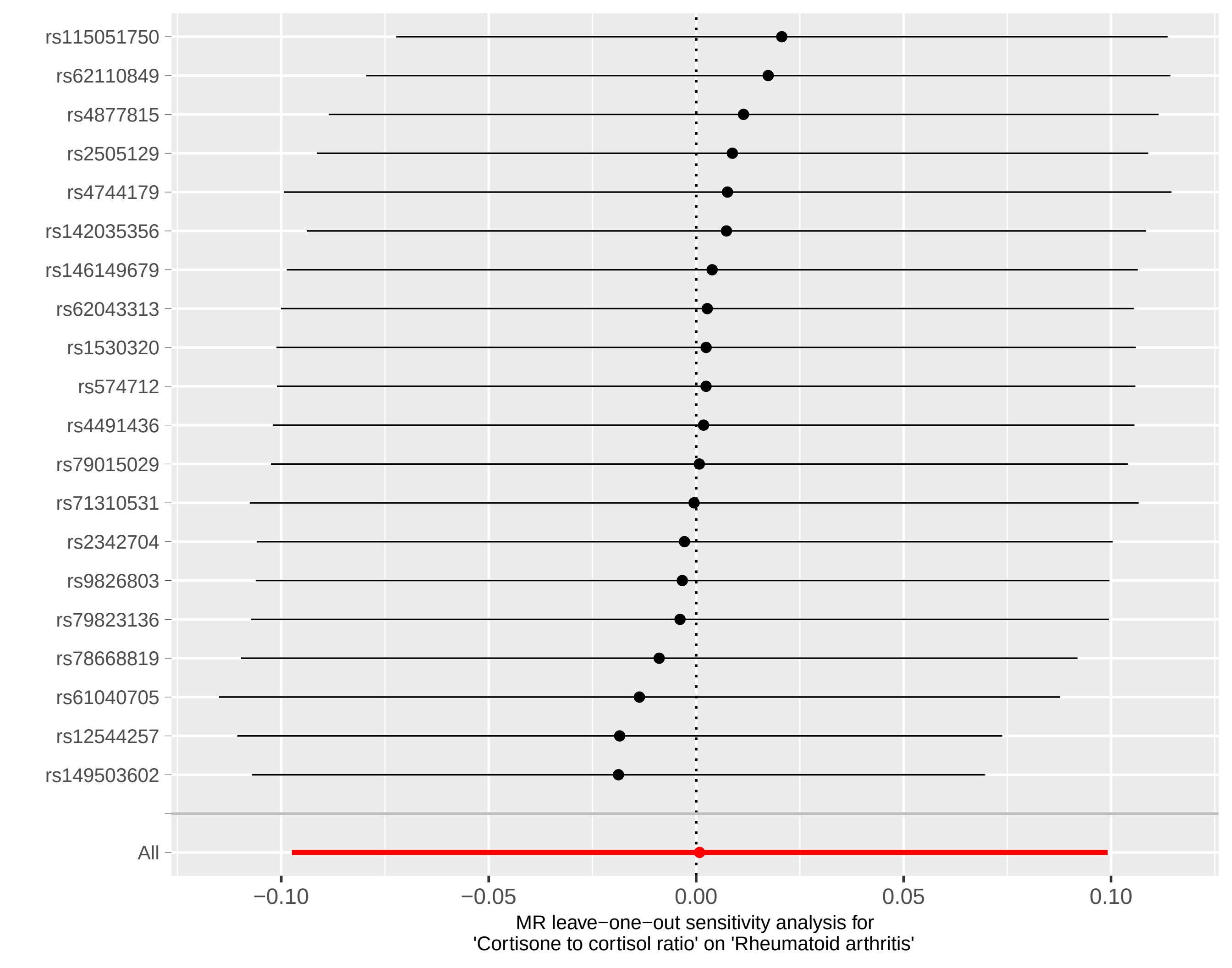
rs115051750
rs62110849
rs4877815
rs2505129
rs4744179
rs142035356
rs146149679
rs62043313
rs1530320
rs574712
rs4491436
rs79015029
rs71310531
rs2342704
rs9826803
rs79823136
rs78668819
rs61040705
rs12544257
rs149503602
All
−0.10
−0.05
0.00
0.05
0.10
MR leave−one−out sensitivity analysis for
'Cortisone to cortisol ratio' on 'Rheumatoid arthritis'

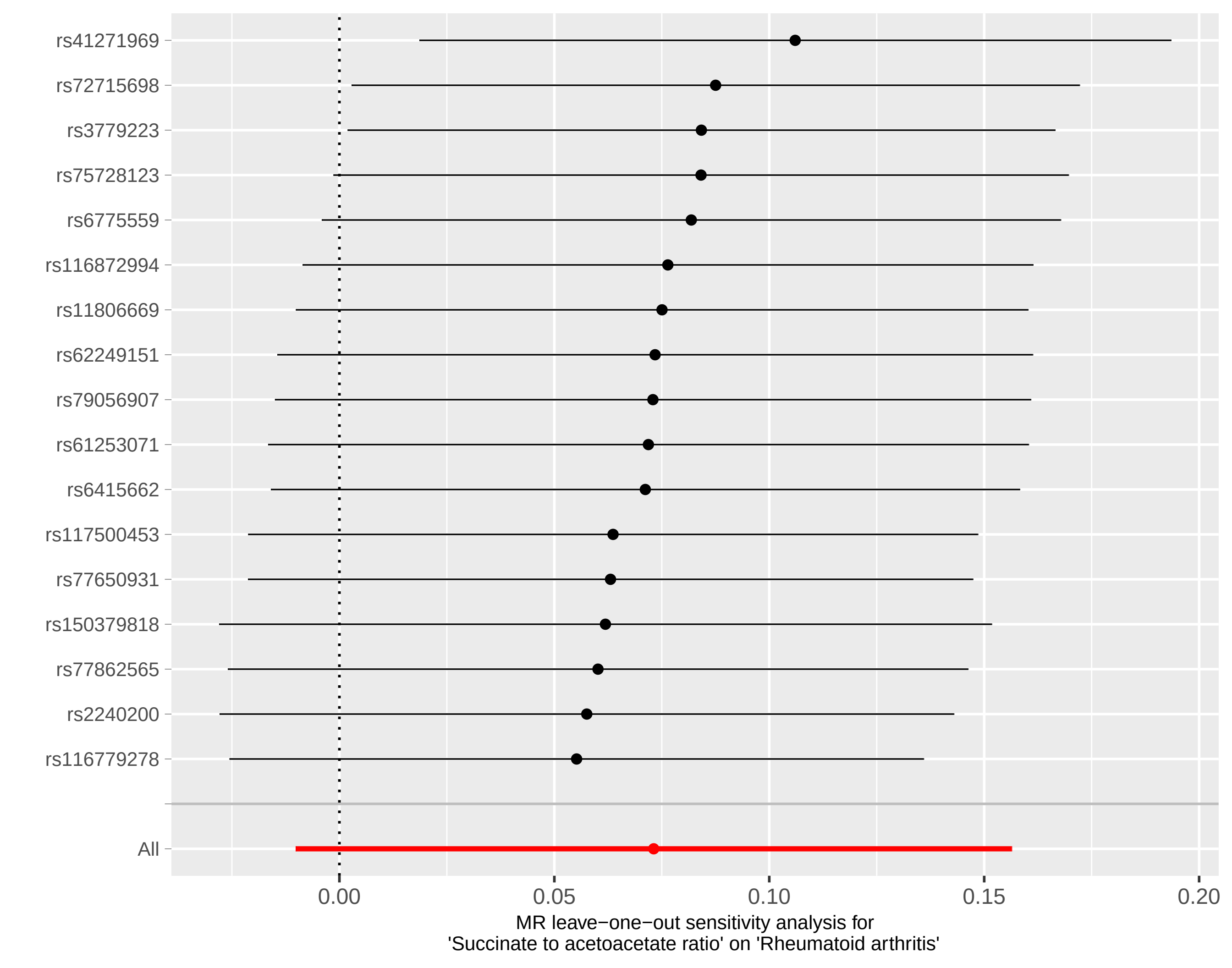
rs41271969
rs72715698
rs3779223
rs75728123
rs6775559
rs116872994
rs11806669
rs62249151
rs79056907
rs61253071
rs6415662
rs117500453
rs77650931
rs150379818
rs77862565
rs2240200
rs116779278
All
0.00
0.05
0.10
0.15
0.20
MR leave−one−out sensitivity analysis for
'Succinate to acetoacetate ratio' on 'Rheumatoid arthritis'

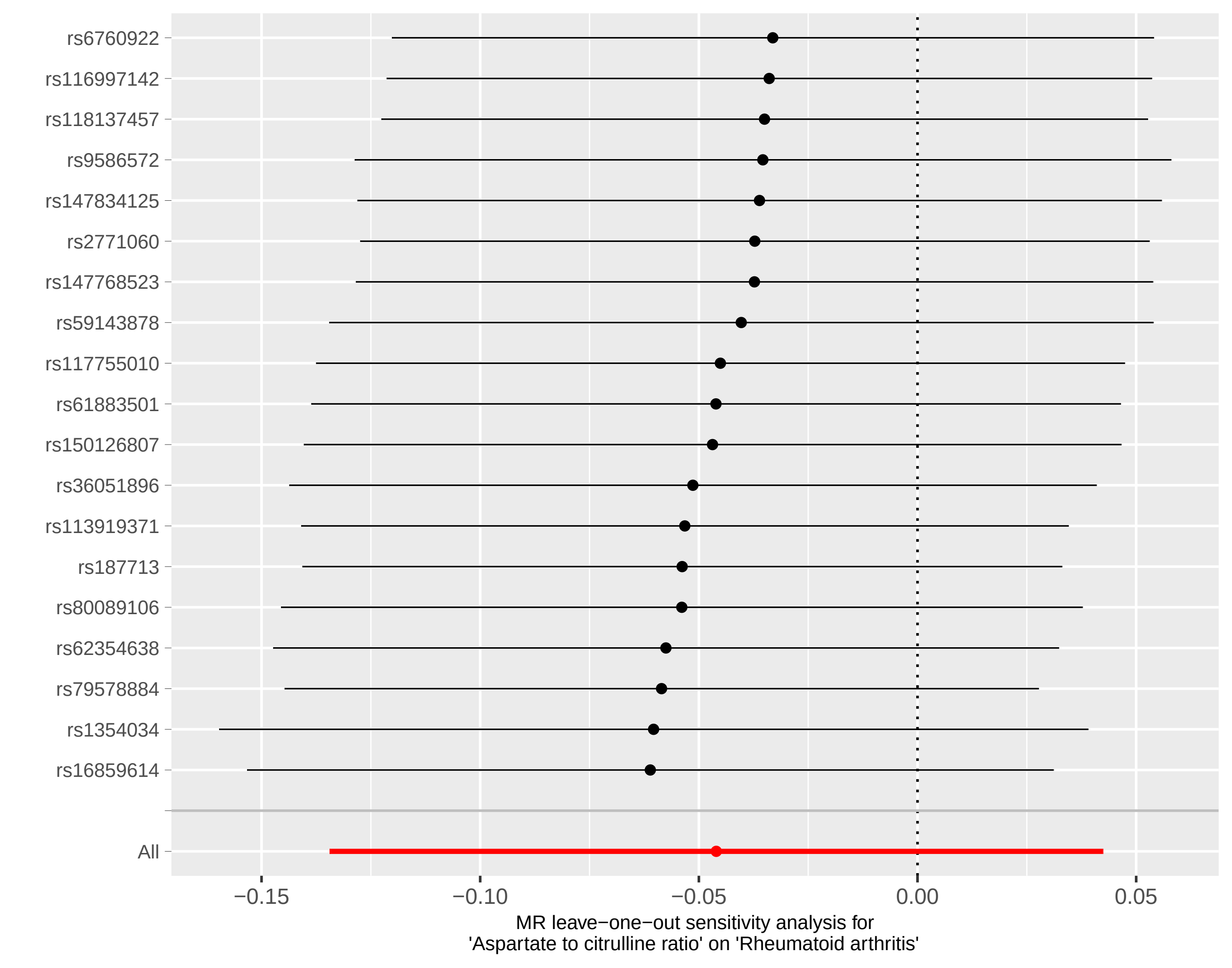
rs6760922
rs116997142
rs118137457
rs9586572
rs147834125
rs2771060
rs147768523
rs59143878
rs117755010
rs61883501
rs150126807
rs36051896
rs113919371
rs187713
rs80089106
rs62354638
rs79578884
rs1354034
rs16859614
All
−0.15
−0.10
−0.05
0.00
0.05
MR leave−one−out sensitivity analysis for
'Aspartate to citrulline ratio' on 'Rheumatoid arthritis'

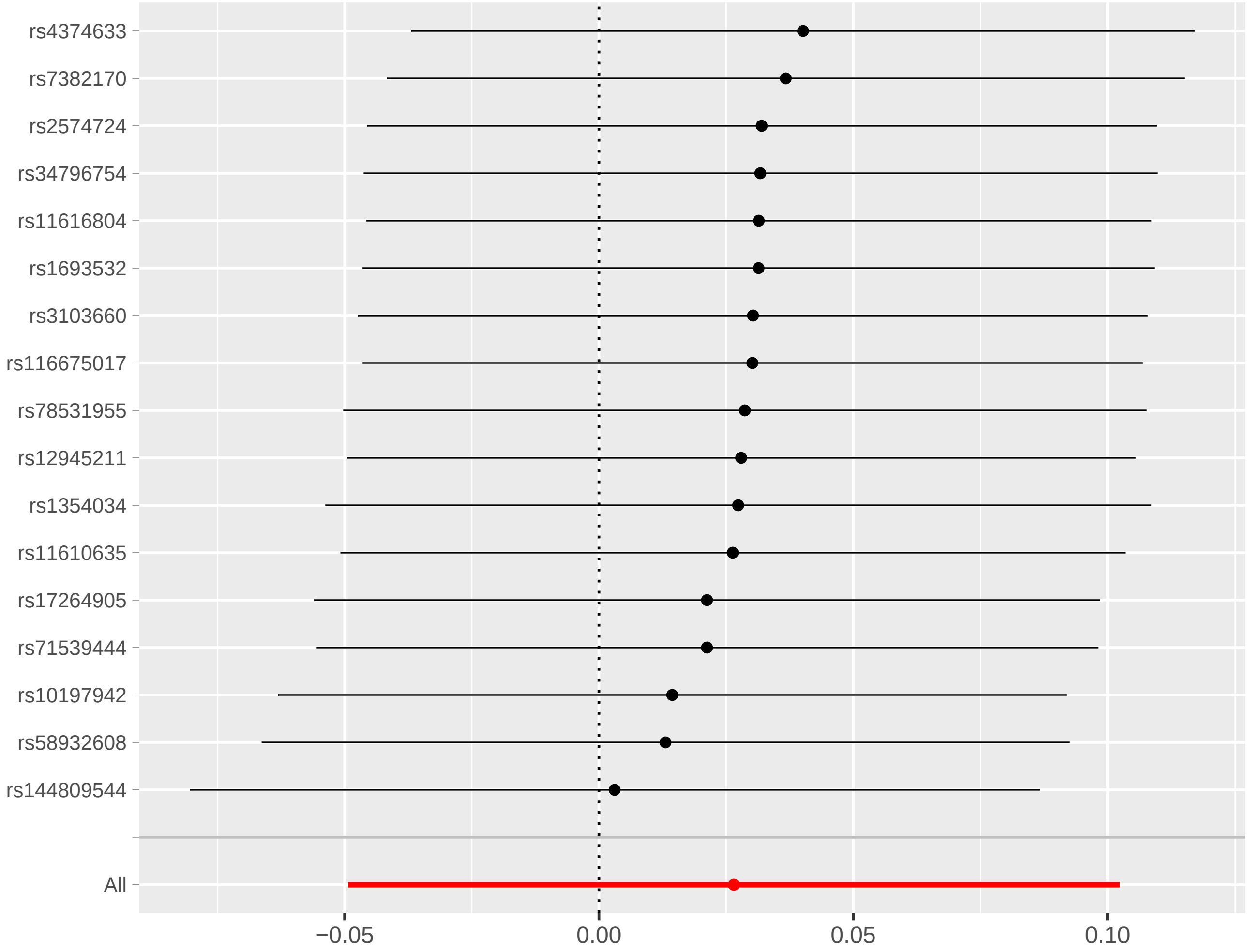

MR leave−one−out sensitivity analysis for
'Sphingosine to phosphate ratio' on 'Rheumatoid arthritis'

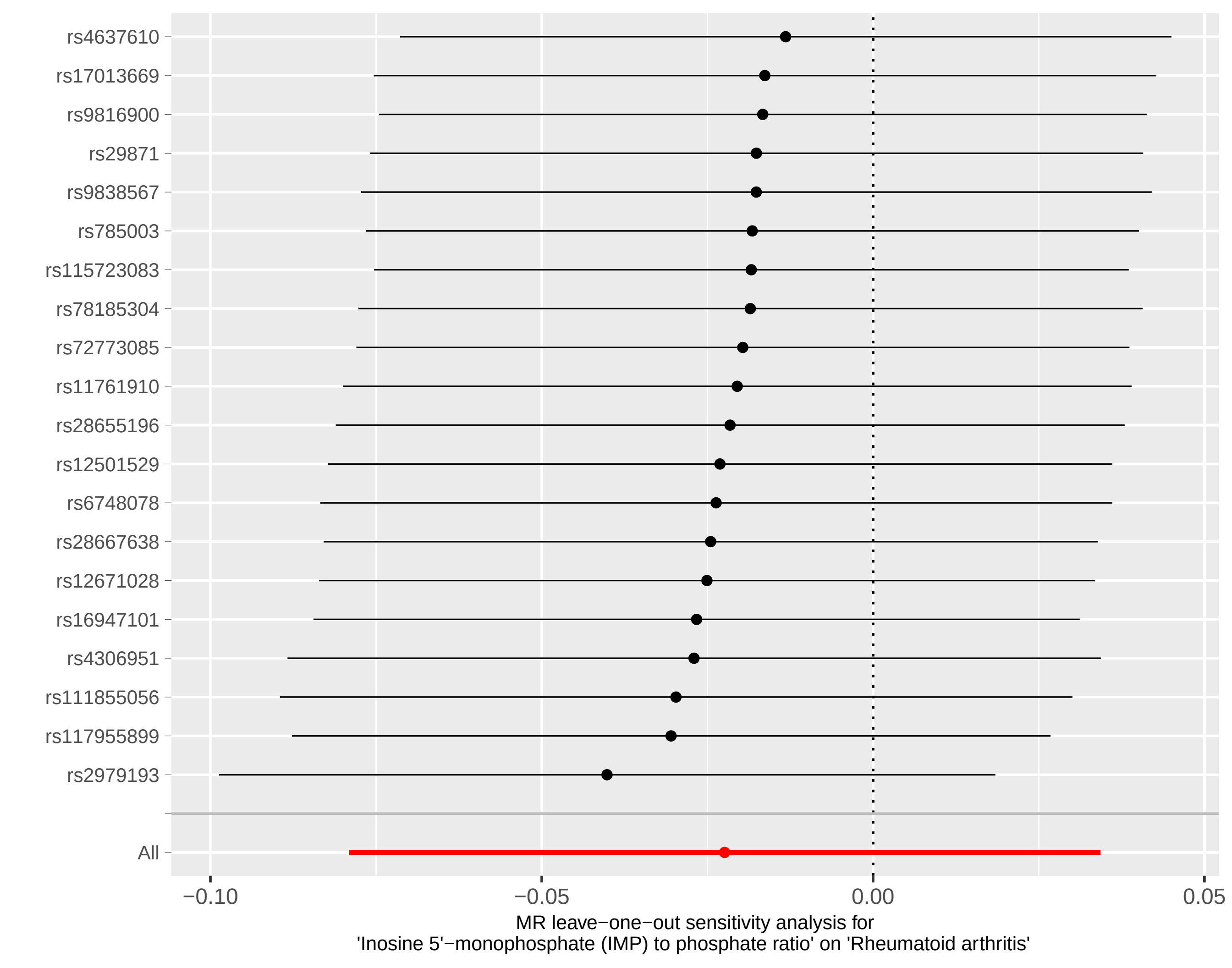

rs4637610
rs17013669
rs9816900
rs29871
rs9838567
rs785003
rs115723083
rs78185304
rs72773085
rs11761910
rs28655196
rs12501529
rs6748078
rs28667638
rs12671028
rs16947101
rs4306951
rs111855056
rs117955899
rs2979193
All
−0.10
−0.05
0.00
0.05
MR leave−one−out sensitivity analysis for
'Inosine 5'−monophosphate (IMP) to phosphate ratio' on 'Rheumatoid arthritis'

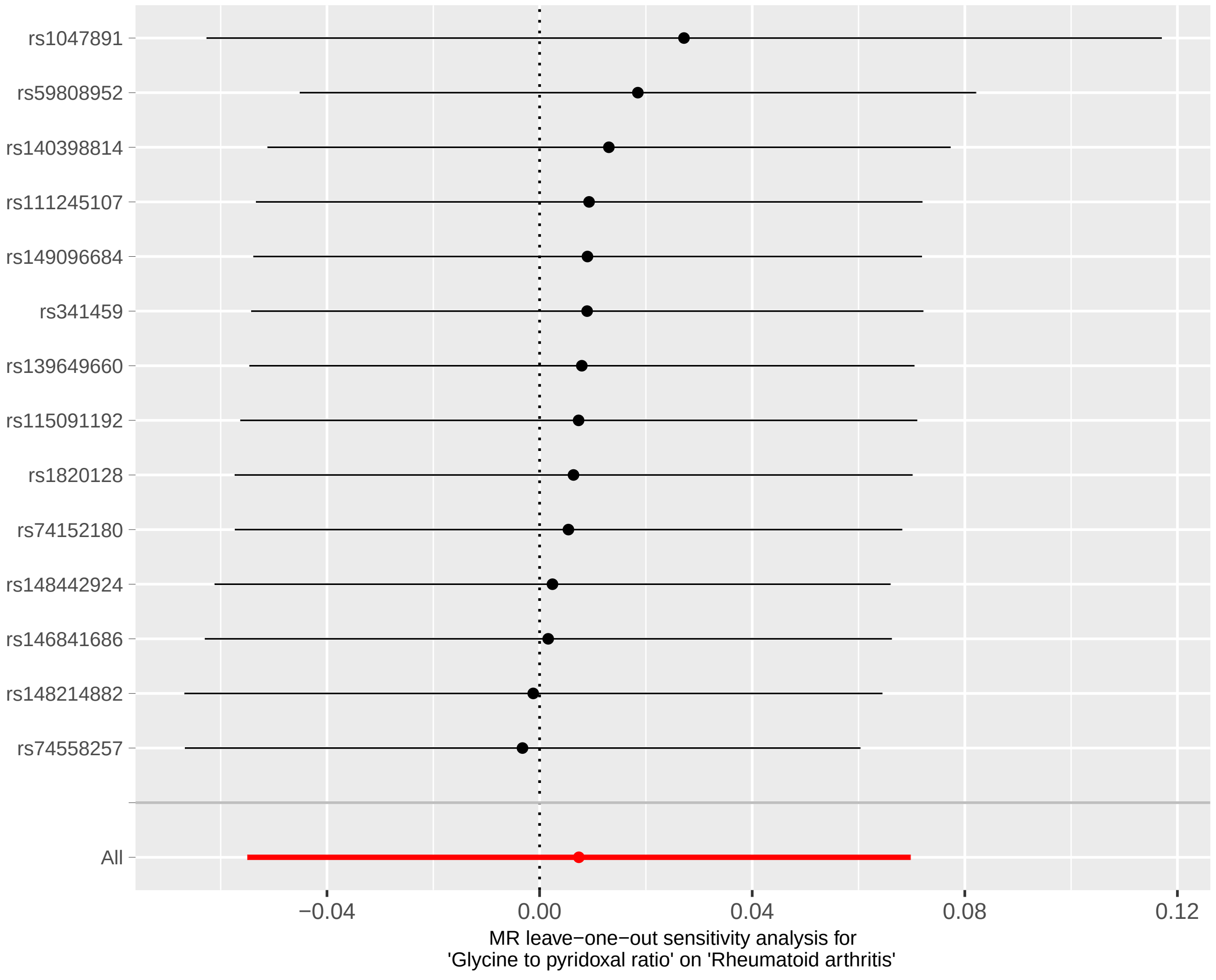
rs1047891
rs59808952
rs140398814
rs111245107
rs149096684
rs341459
rs139649660
rs115091192
rs1820128
rs74152180
rs148442924
rs146841686
rs148214882
rs74558257
All
−0.04
0.00
0.04
0.08
0.12
MR leave−one−out sensitivity analysis for
'Glycine to pyridoxal ratio' on 'Rheumatoid arthritis'

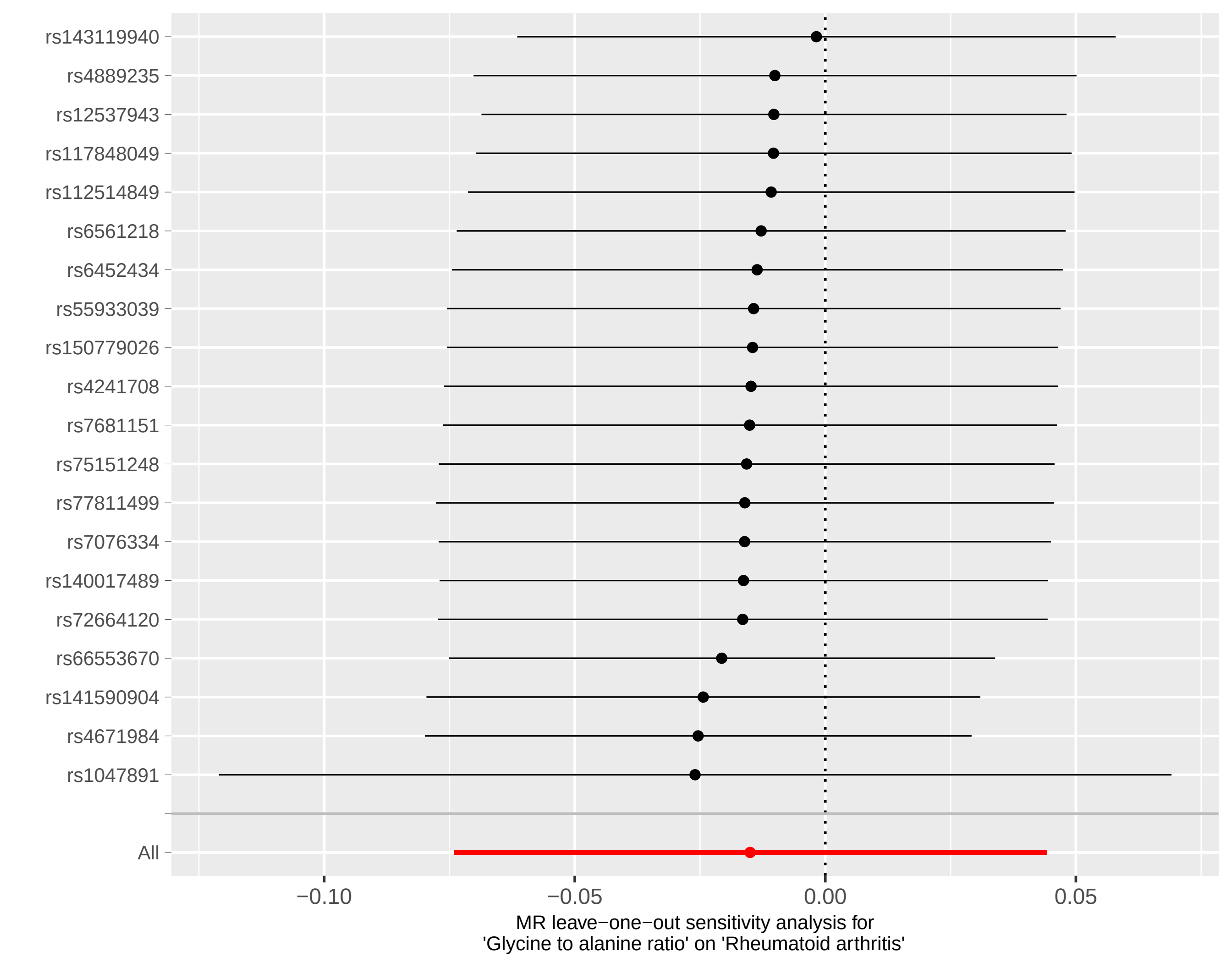
rs143119940
rs4889235
rs12537943
rs117848049
rs112514849
rs6561218
rs6452434
rs55933039
rs150779026
rs4241708
rs7681151
rs75151248
rs77811499
rs7076334
rs140017489
rs72664120
rs66553670
rs141590904
rs4671984
rs1047891
All
−0.10
−0.05
0.00
0.05
MR leave−one−out sensitivity analysis for
'Glycine to alanine ratio' on 'Rheumatoid arthritis'

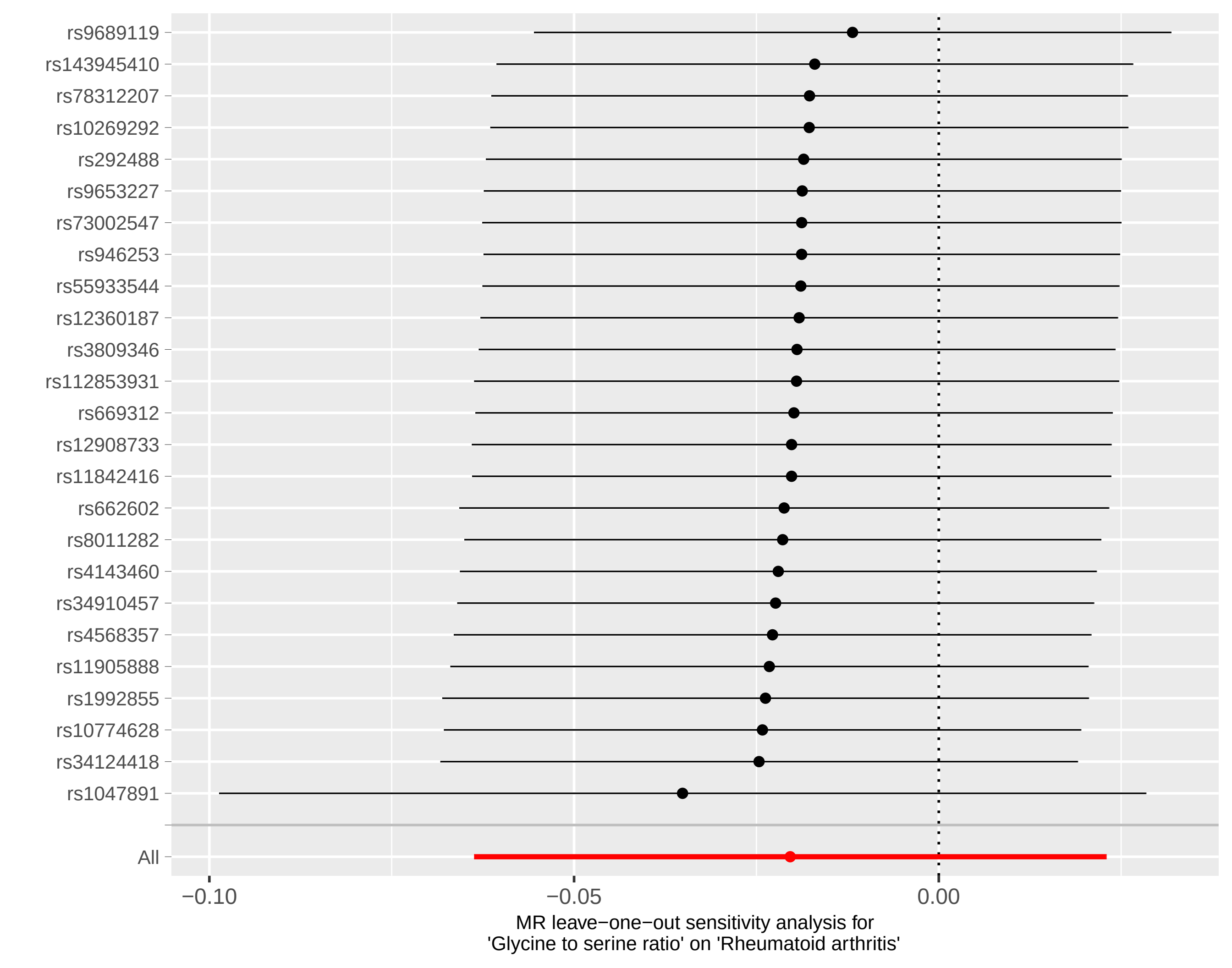

rs9689119
rs143945410
rs78312207
rs10269292
rs292488
rs9653227
rs73002547
rs946253
rs55933544
rs12360187
rs3809346
rs112853931
rs669312
rs12908733
rs11842416
rs662602
rs8011282
rs4143460
rs34910457
rs4568357
rs11905888
rs1992855
rs10774628
rs34124418
rs1047891
All
−0.10
−0.05
0.00
MR leave−one−out sensitivity analysis for
'Glycine to serine ratio' on 'Rheumatoid arthritis'

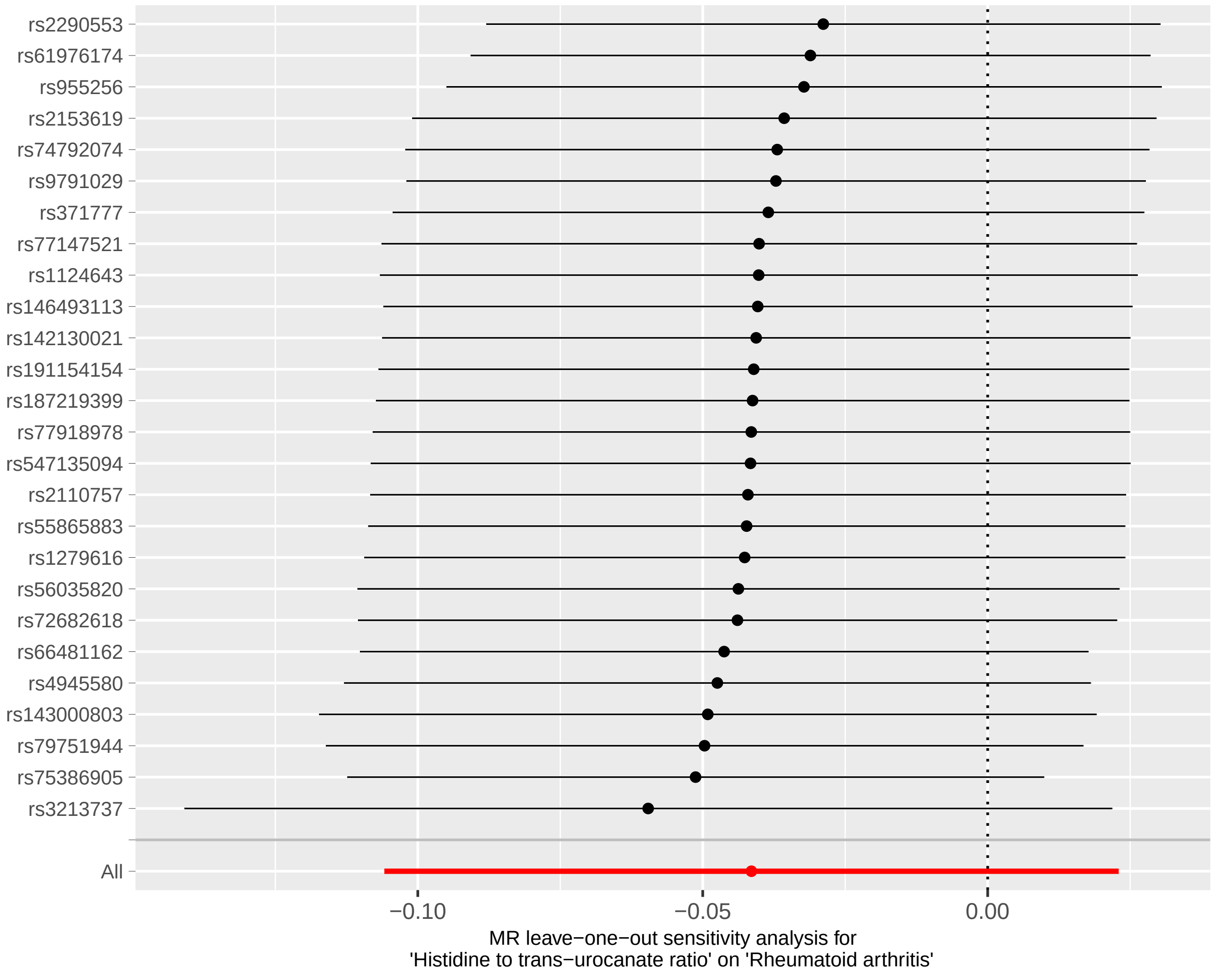

rs2290553
rs61976174
rs955256
rs2153619
rs74792074
rs9791029
rs371777
rs77147521
rs1124643
rs146493113
rs142130021
rs191154154
rs187219399
rs77918978
rs547135094
rs2110757
rs55865883
rs1279616
rs56035820
rs72682618
rs66481162
rs4945580
rs143000803
rs79751944
rs75386905
rs3213737
All
−0.10
−0.05
0.00
MR leave−one−out sensitivity analysis for
'Histidine to trans−urocanate ratio' on 'Rheumatoid arthritis'

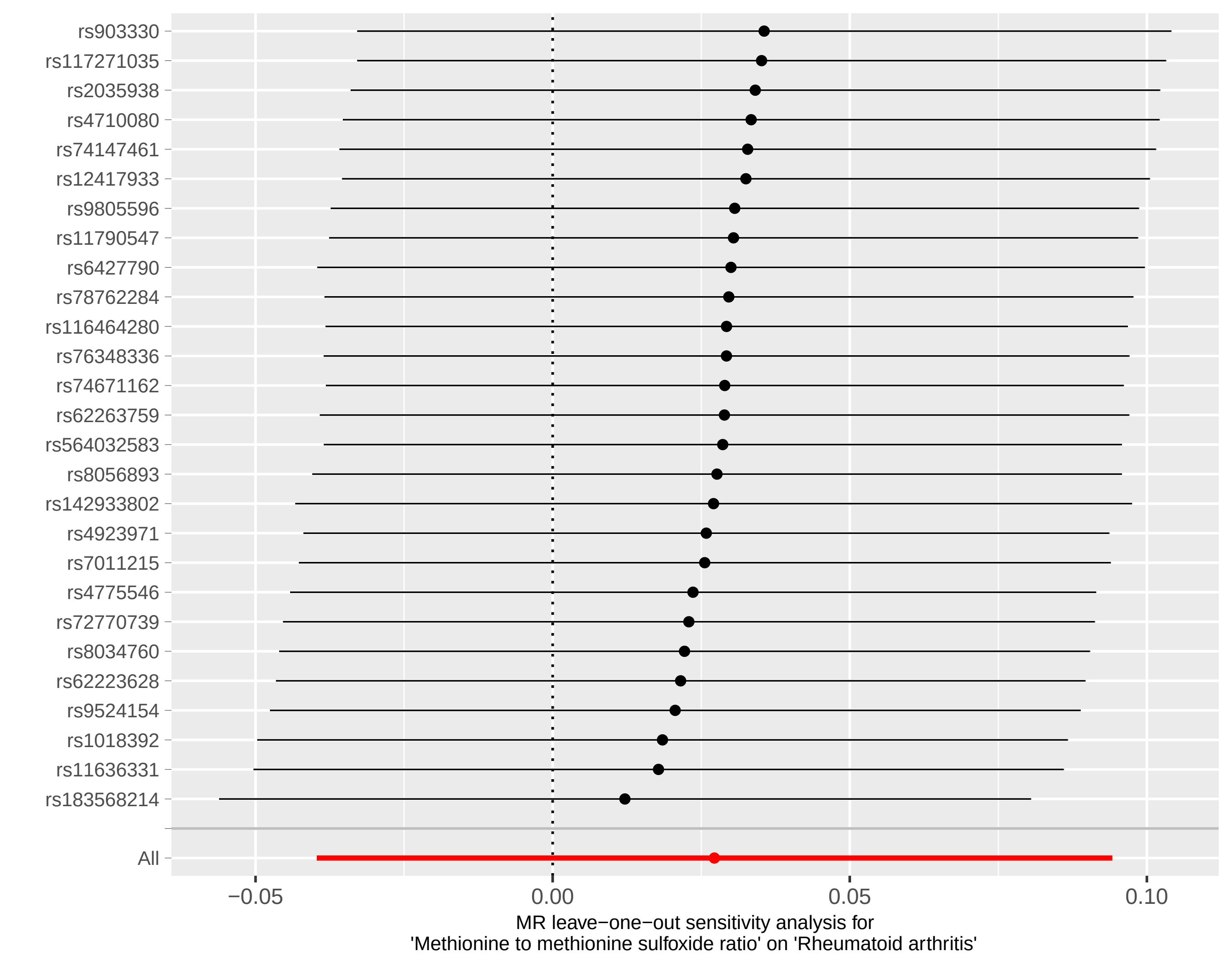
rs903330
rs117271035
rs2035938
rs4710080
rs74147461
rs12417933
rs9805596
rs11790547
rs6427790
rs78762284
rs116464280
rs76348336
rs74671162
rs62263759
rs564032583
rs8056893
rs142933802
rs4923971
rs7011215
rs4775546
rs72770739
rs8034760
rs62223628
rs9524154
rs1018392
rs11636331
rs183568214
All
−0.05
0.00
0.05
0.10
MR leave−one−out sensitivity analysis for
'Methionine to methionine sulfoxide ratio' on 'Rheumatoid arthritis'

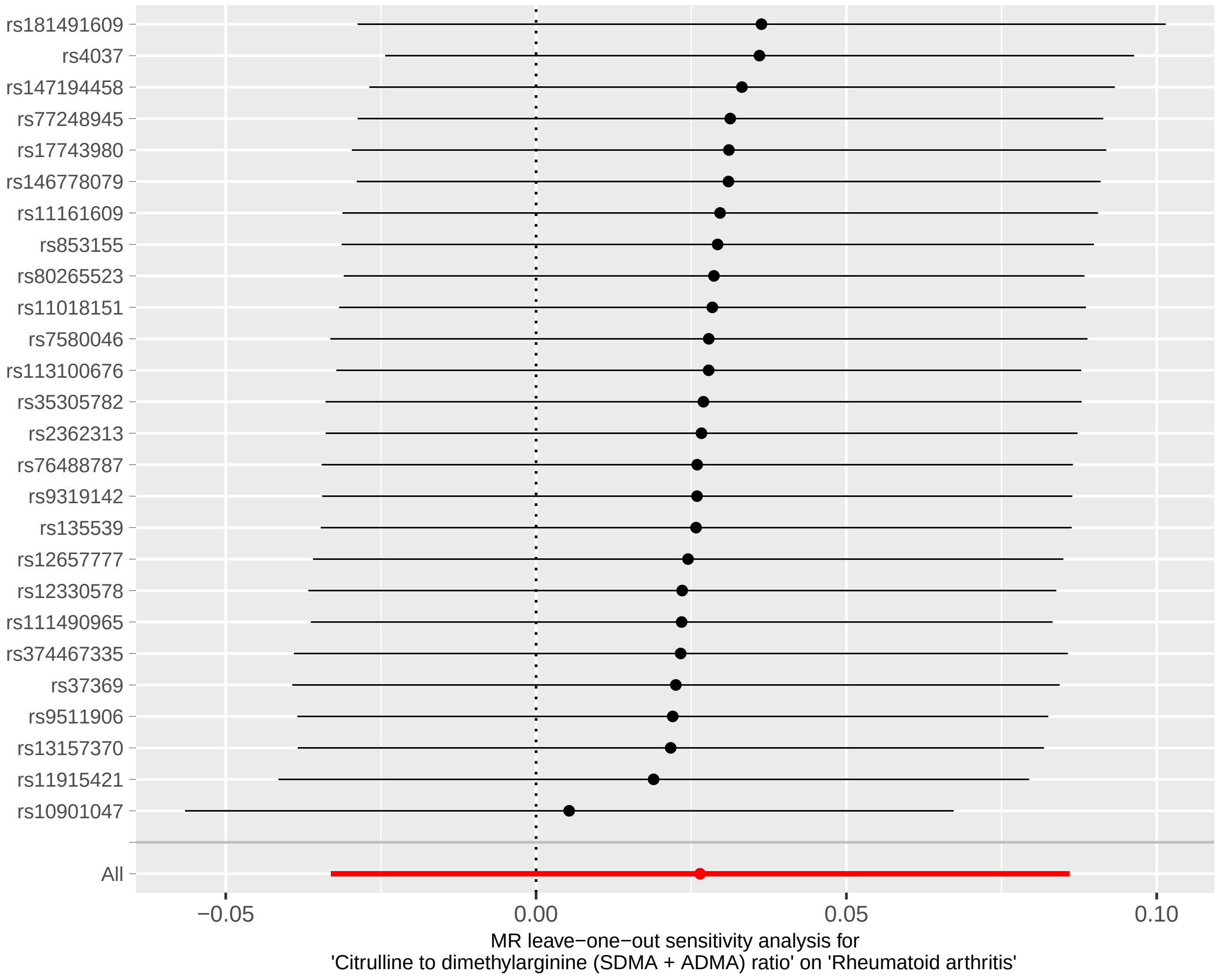
rs181491609
rs4037
rs147194458
rs77248945
rs17743980
rs146778079
rs11161609
rs853155
rs80265523
rs11018151
rs7580046
rs113100676
rs35305782
rs2362313
rs76488787
rs9319142
rs135539
rs12657777
rs12330578
rs111490965
rs374467335
rs37369
rs9511906
rs13157370
rs11915421
rs10901047
All
−0.05
0.00
0.05
0.10
MR leave−one−out sensitivity analysis for
'Citrulline to dimethylarginine (SDMA + ADMA) ratio' on 'Rheumatoid arthritis'

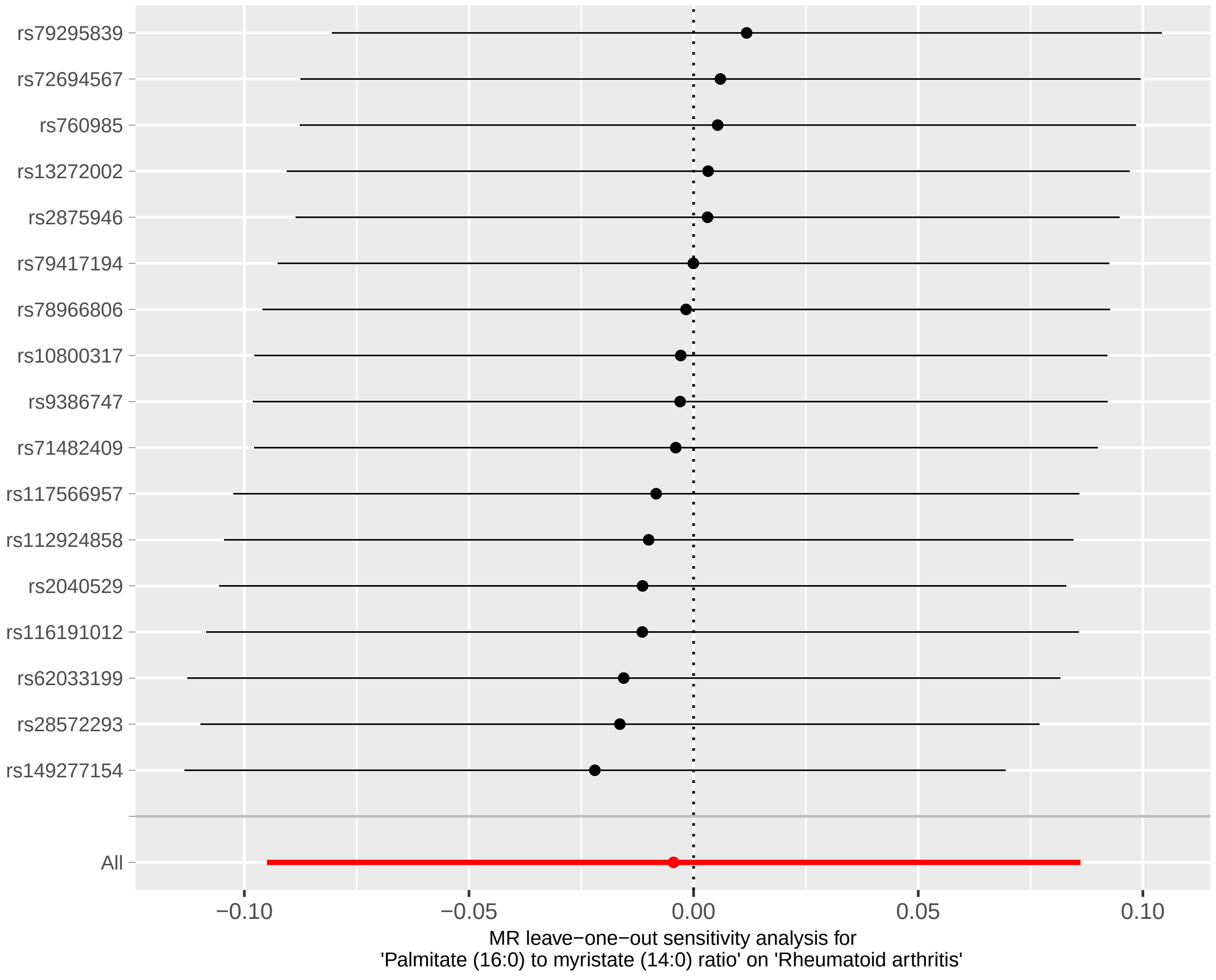
rs79295839
rs72694567
rs760985
rs13272002
rs2875946
rs79417194
rs78966806
rs10800317
rs9386747
rs71482409
rs117566957
rs112924858
rs2040529
rs116191012
rs62033199
rs28572293
rs149277154
All
−0.10
−0.05
0.00
0.05
0.10
MR leave−one−out sensitivity analysis for
'Palmitate (16:0) to myristate (14:0) ratio' on 'Rheumatoid arthritis'

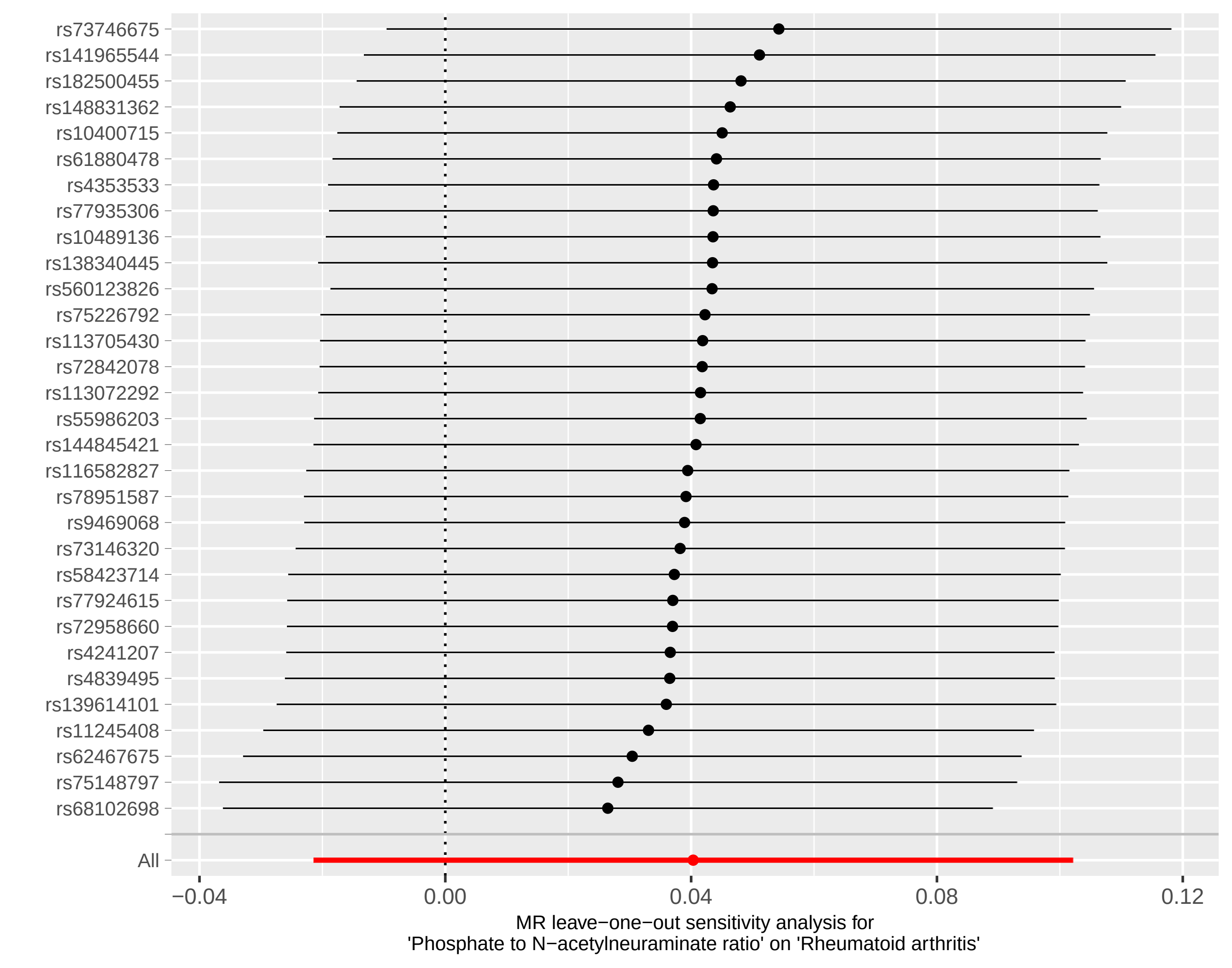

rs73746675
rs141965544
rs182500455
rs148831362
rs10400715
rs61880478
rs4353533
rs77935306
rs10489136
rs138340445
rs560123826
rs75226792
rs113705430
rs72842078
rs113072292
rs55986203
rs144845421
rs116582827
rs78951587
rs9469068
rs73146320
rs58423714
rs77924615
rs72958660
rs4241207
rs4839495
rs139614101
rs11245408
rs62467675
rs75148797
rs68102698
All
−0.04
0.00
0.04
0.08
0.12
MR leave−one−out sensitivity analysis for
'Phosphate to N−acetylneuraminate ratio' on 'Rheumatoid arthritis'

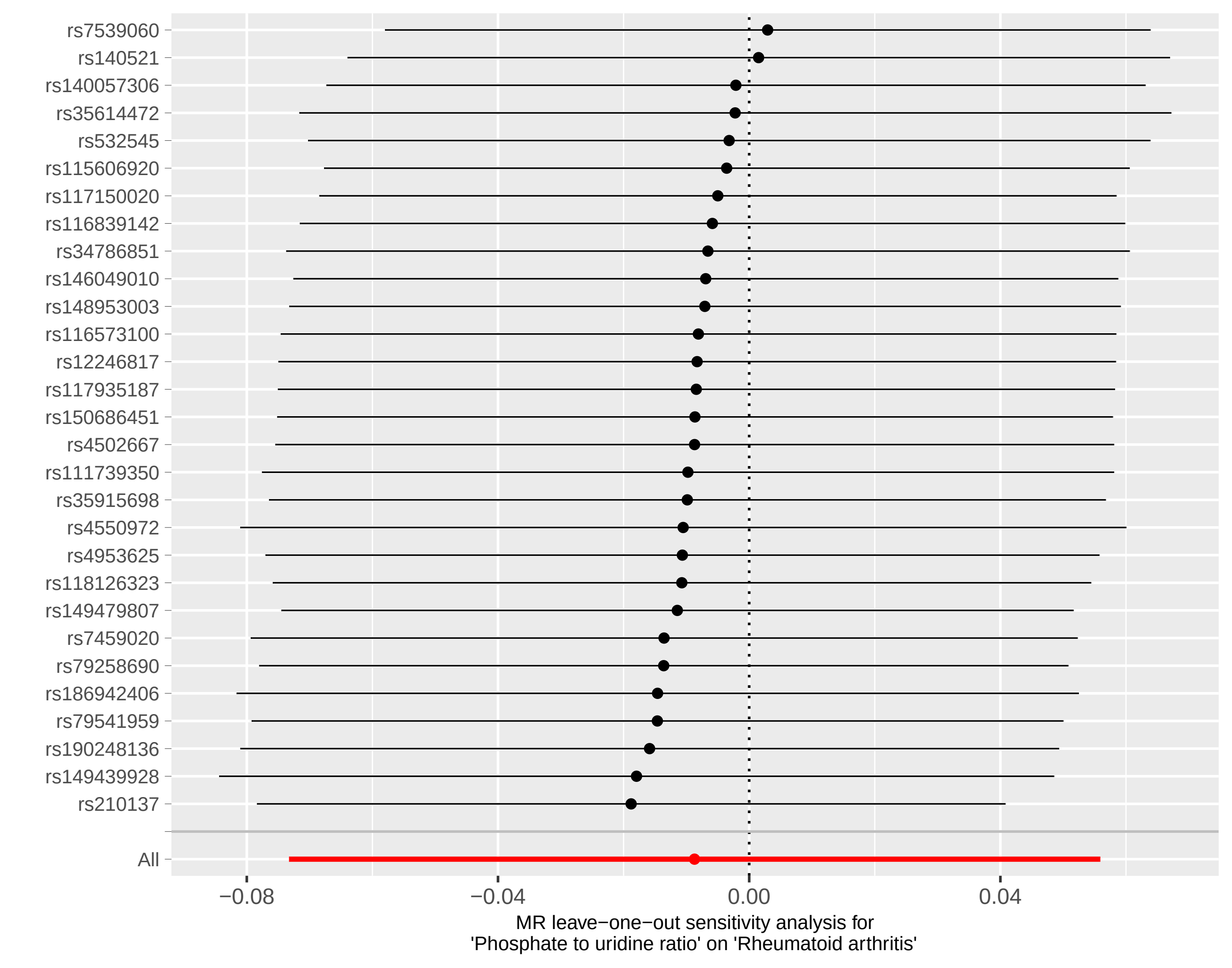
rs7539060
rs140521
rs140057306
rs35614472
rs532545
rs115606920
rs117150020
rs116839142
rs34786851
rs146049010
rs148953003
rs116573100
rs12246817
rs117935187
rs150686451
rs4502667
rs111739350
rs35915698
rs4550972
rs4953625
rs118126323
rs149479807
rs7459020
rs79258690
rs186942406
rs79541959
rs190248136
rs149439928
rs210137
All
−0.08
−0.04
0.00
0.04
MR leave−one−out sensitivity analysis for
'Phosphate to uridine ratio' on 'Rheumatoid arthritis'

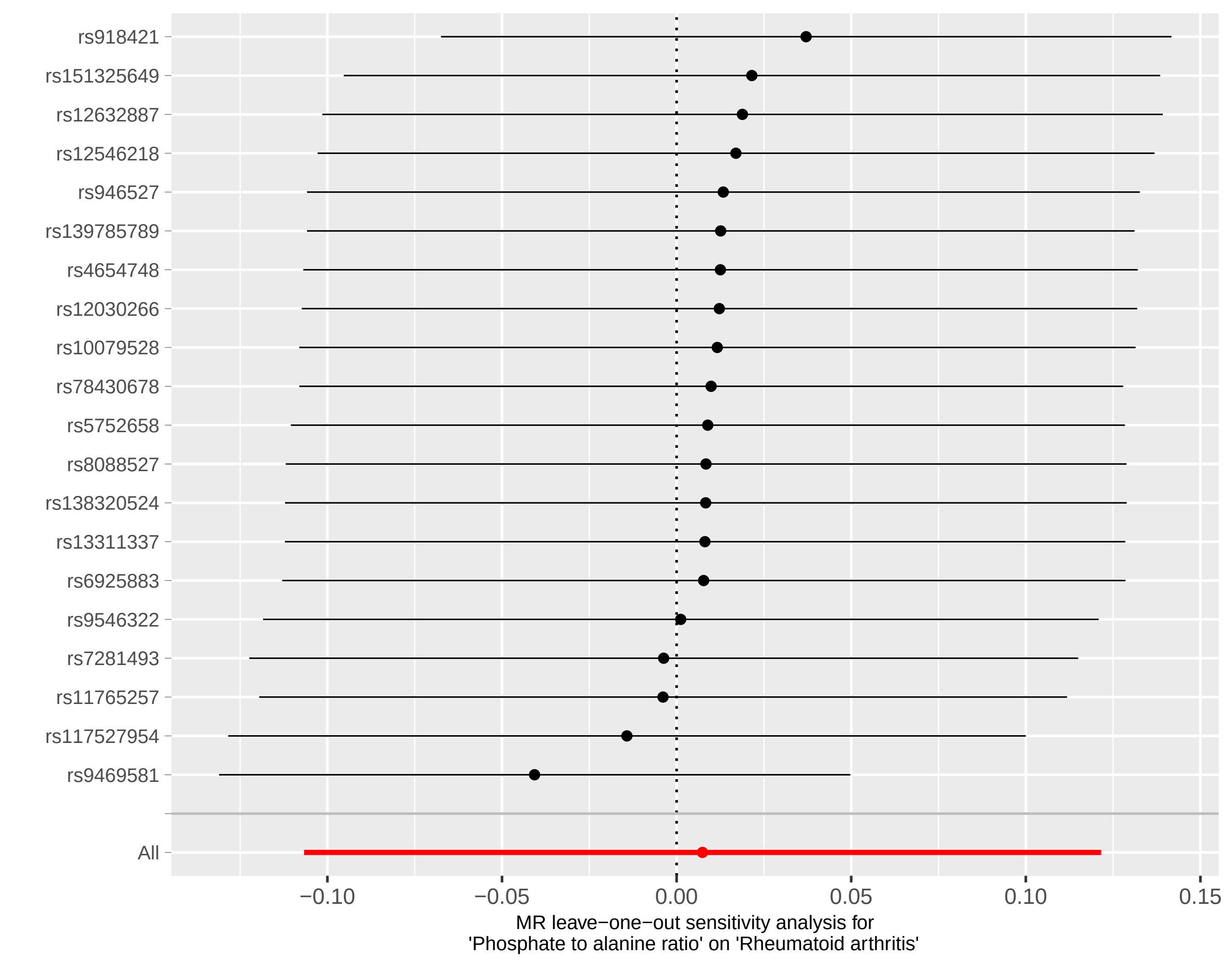

rs918421
rs151325649
rs12632887
rs12546218
rs946527
rs139785789
rs4654748
rs12030266
rs10079528
rs78430678
rs5752658
rs8088527
rs138320524
rs13311337
rs6925883
rs9546322
rs7281493
rs11765257
rs117527954
rs9469581
All
−0.10
−0.05
0.00
0.05
0.10
0.15
MR leave−one−out sensitivity analysis for
'Phosphate to alanine ratio' on 'Rheumatoid arthritis'

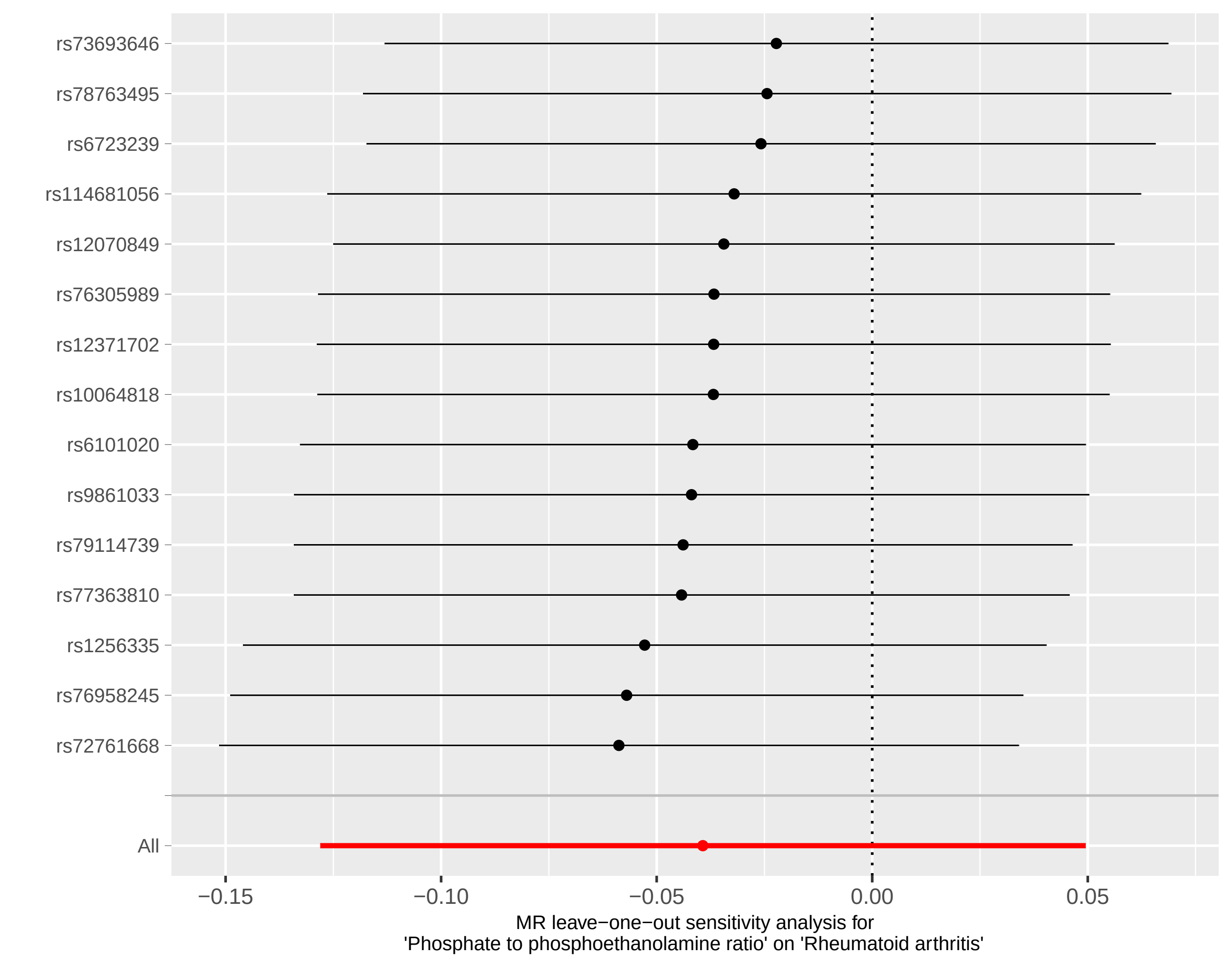
rs73693646
rs78763495
rs6723239
rs114681056
rs12070849
rs76305989
rs12371702
rs10064818
rs6101020
rs9861033
rs79114739
rs77363810
rs1256335
rs76958245
rs72761668
All
−0.15
−0.10
−0.05
0.00
0.05
MR leave−one−out sensitivity analysis for
'Phosphate to phosphoethanolamine ratio' on 'Rheumatoid arthritis'

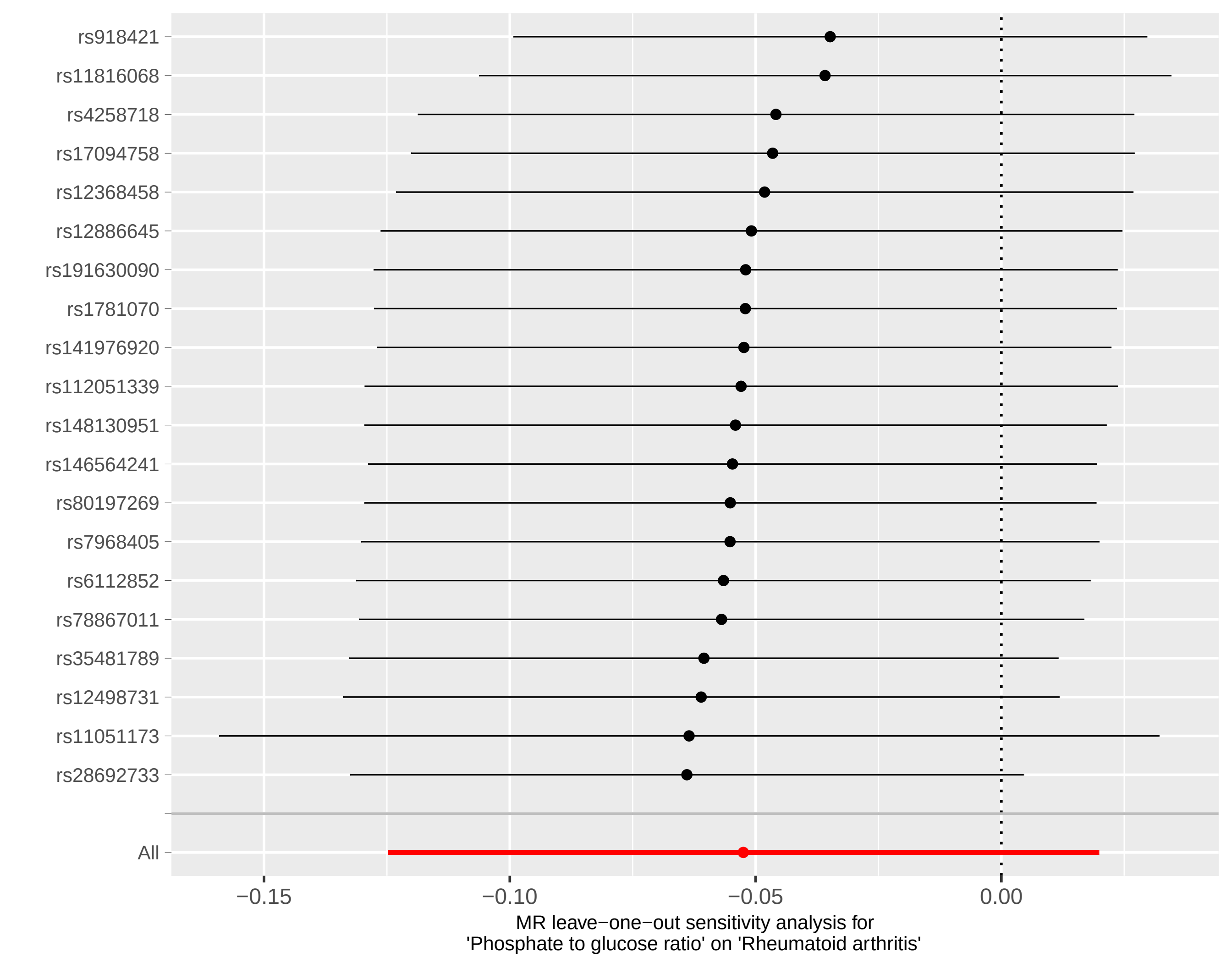
rs918421
rs11816068
rs4258718
rs17094758
rs12368458
rs12886645
rs191630090
rs1781070
rs141976920
rs112051339
rs148130951
rs146564241
rs80197269
rs7968405
rs6112852
rs78867011
rs35481789
rs12498731
rs11051173
rs28692733
All
−0.15
−0.10
−0.05
0.00
MR leave−one−out sensitivity analysis for
'Phosphate to glucose ratio' on 'Rheumatoid arthritis'

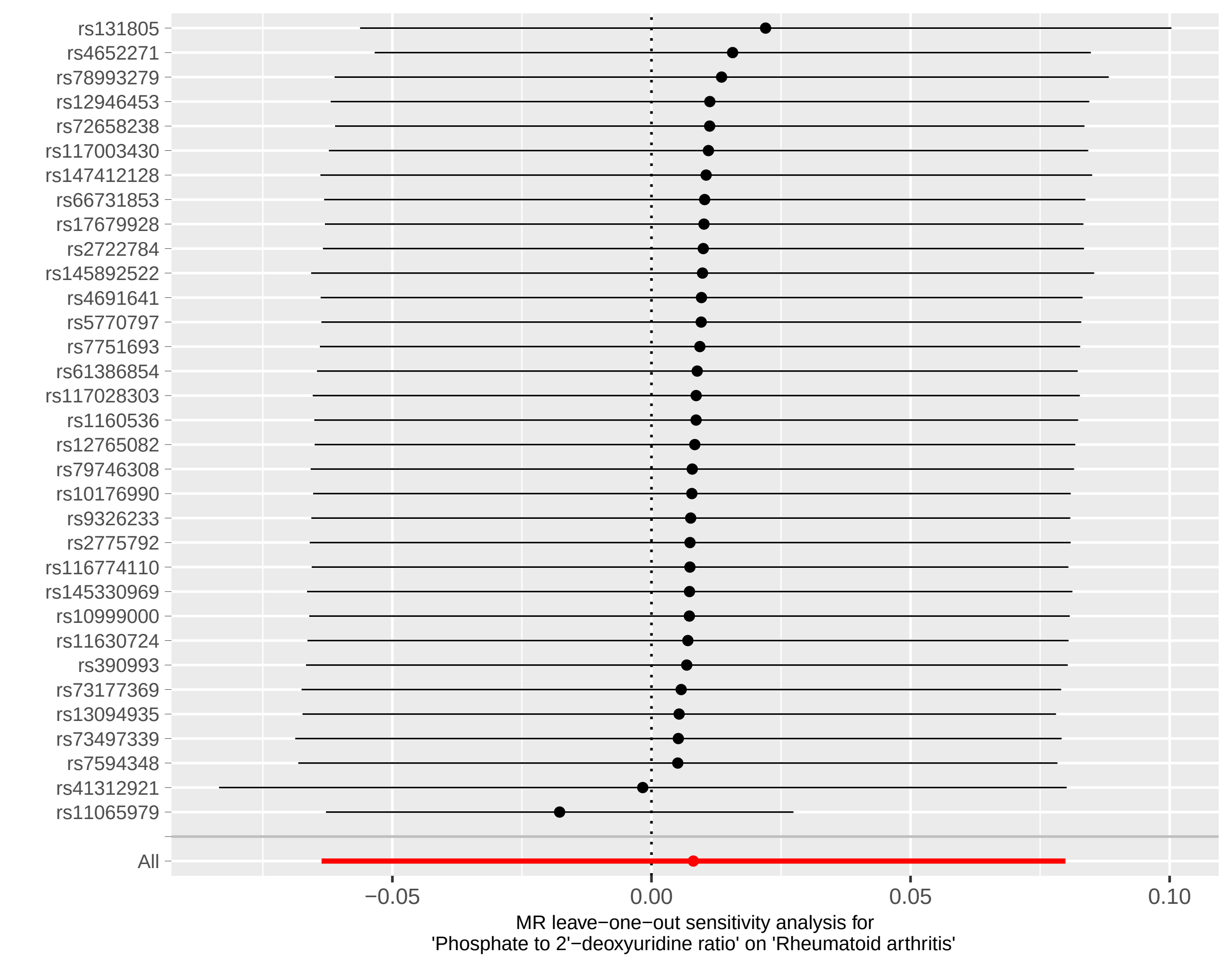
rs131805
rs4652271
rs78993279
rs12946453
rs72658238
rs117003430
rs147412128
rs66731853
rs17679928
rs2722784
rs145892522
rs4691641
rs5770797
rs7751693
rs61386854
rs117028303
rs1160536
rs12765082
rs79746308
rs10176990
rs9326233
rs2775792
rs116774110
rs145330969
rs10999000
rs11630724
rs390993
rs73177369
rs13094935
rs73497339
rs7594348
rs41312921
rs11065979
All
−0.05
0.00
0.05
0.10
MR leave−one−out sensitivity analysis for
'Phosphate to 2'−deoxyuridine ratio' on 'Rheumatoid arthritis'

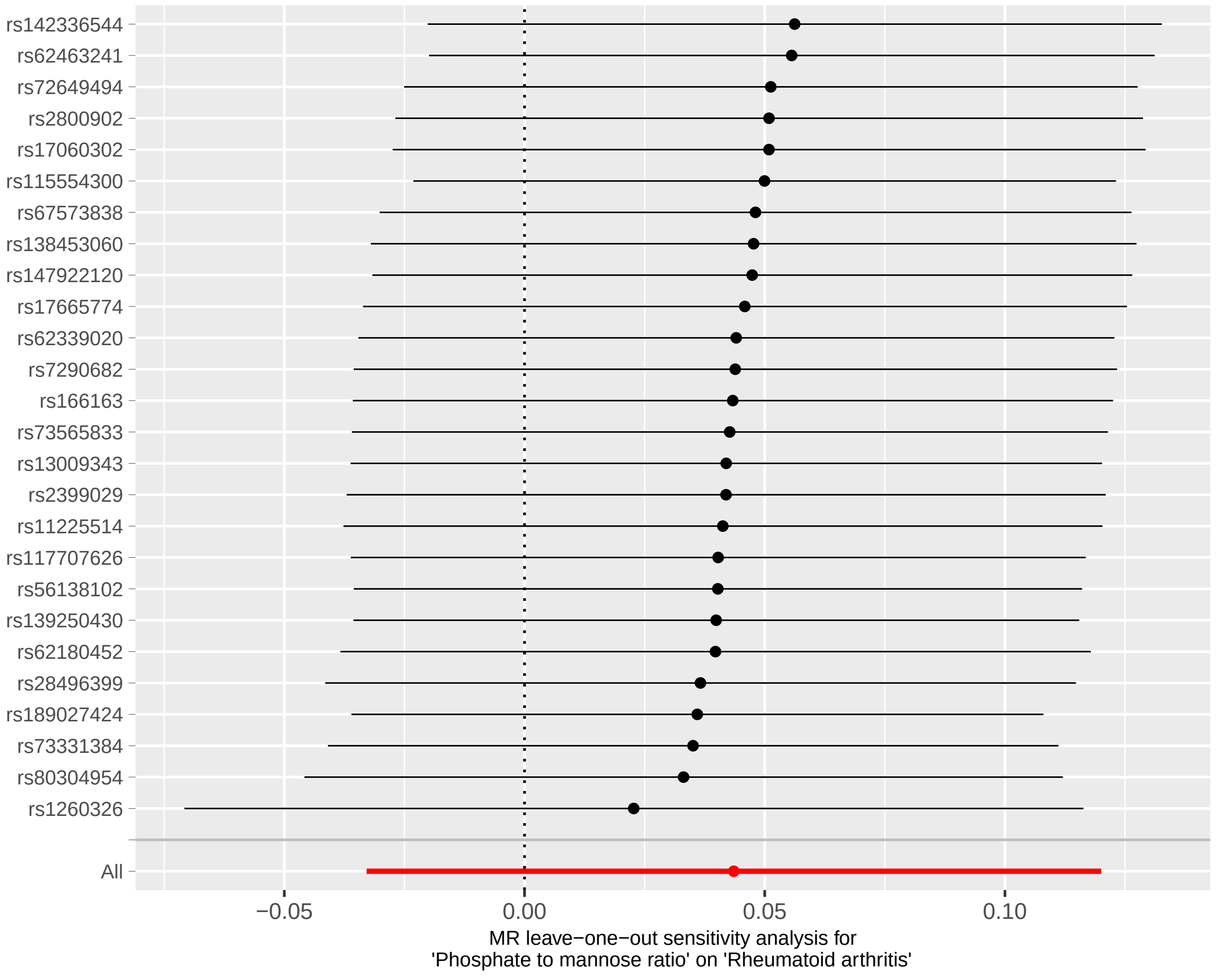
rs142336544
rs62463241
rs72649494
rs2800902
rs17060302
rs115554300
rs67573838
rs138453060
rs147922120
rs17665774
rs62339020
rs7290682
rs166163
rs73565833
rs13009343
rs2399029
rs11225514
rs117707626
rs56138102
rs139250430
rs62180452
rs28496399
rs189027424
rs73331384
rs80304954
rs1260326
All
−0.05
0.00
0.05
0.10
MR leave−one−out sensitivity analysis for
'Phosphate to mannose ratio' on 'Rheumatoid arthritis'

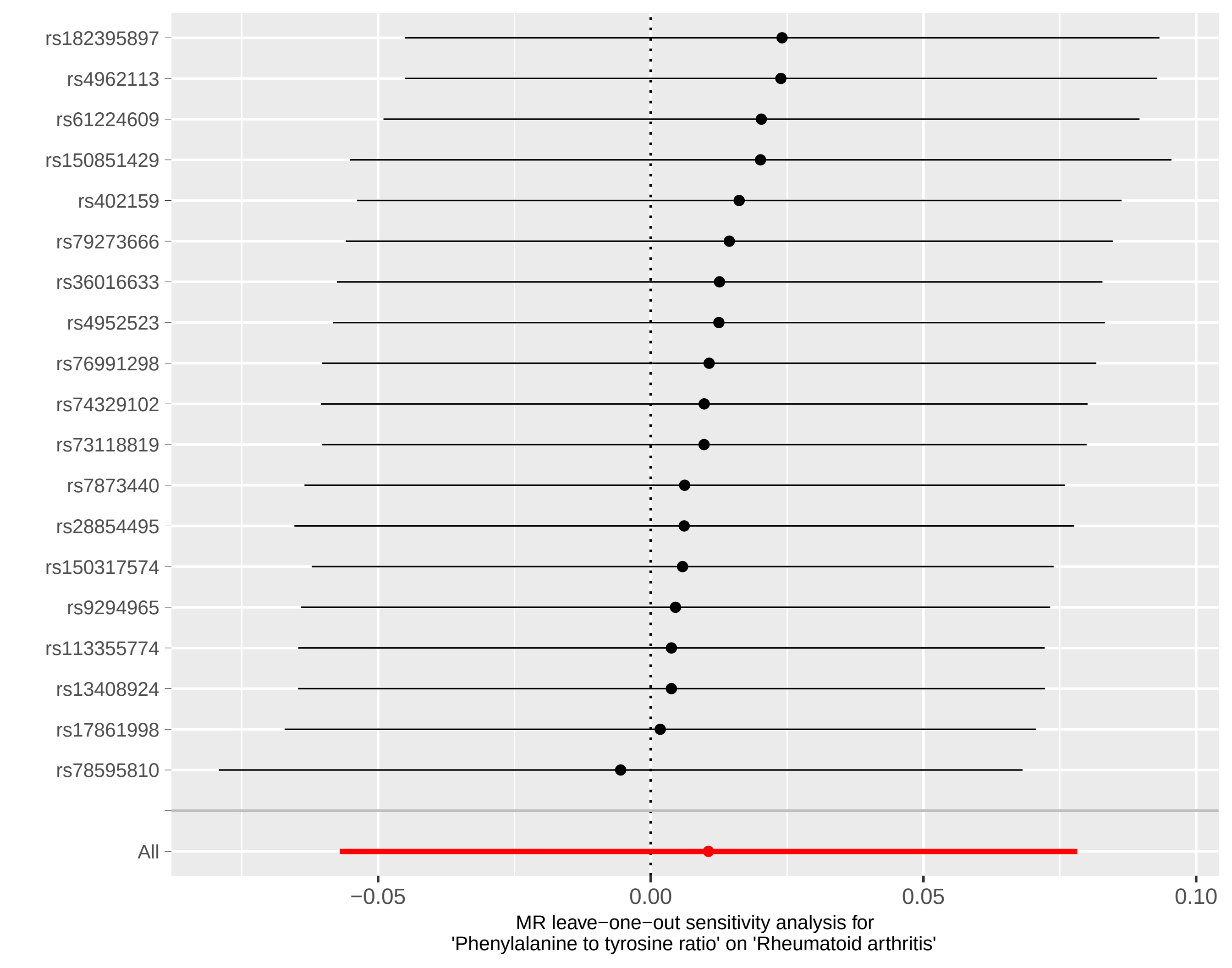
rs182395897
rs4962113
rs61224609
rs150851429
rs402159
rs79273666
rs36016633
rs4952523
rs76991298
rs74329102
rs73118819
rs7873440
rs28854495
rs150317574
rs9294965
rs113355774
rs13408924
rs17861998
rs78595810
All
−0.05
0.00
0.05
0.10
MR leave−one−out sensitivity analysis for
'Phenylalanine to tyrosine ratio' on 'Rheumatoid arthritis'

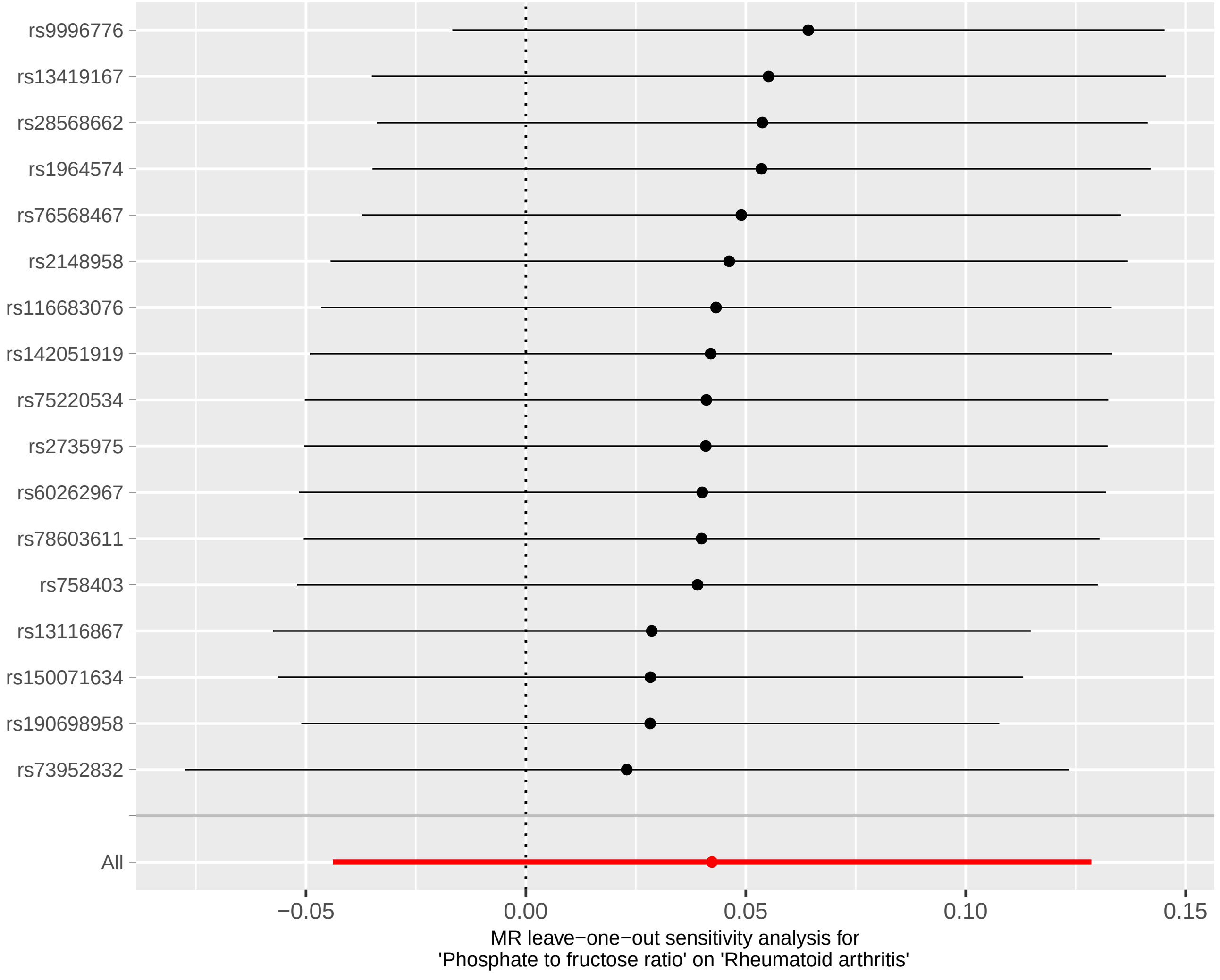

rs9996776
rs13419167
rs28568662
rs1964574
rs76568467
rs2148958
rs116683076
rs142051919
rs75220534
rs2735975
rs60262967
rs78603611
rs758403
rs13116867
rs150071634
rs190698958
rs73952832
All
−0.05
0.00
0.05
0.10
0.15
MR leave−one−out sensitivity analysis for
'Phosphate to fructose ratio' on 'Rheumatoid arthritis'

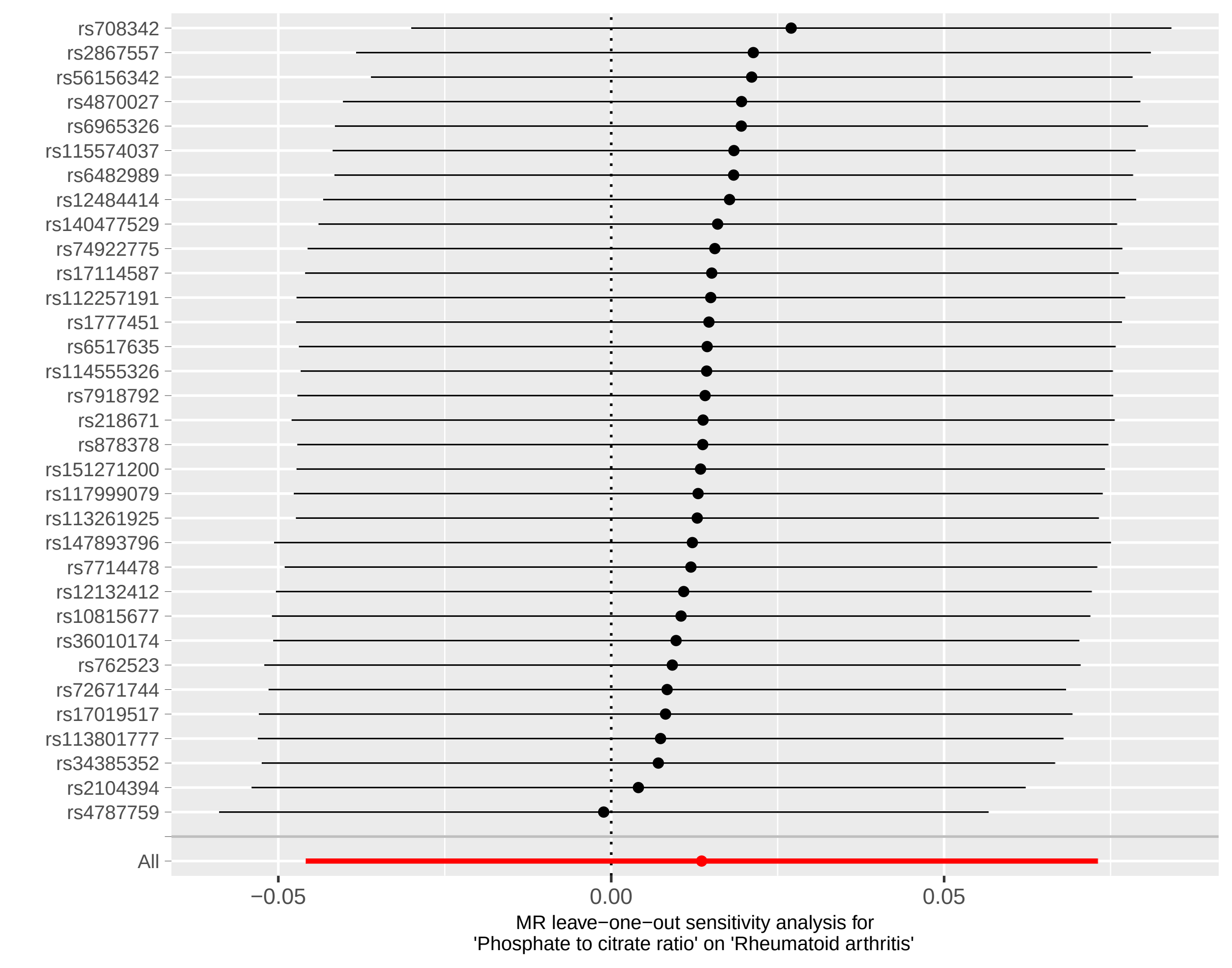
rs708342
rs2867557
rs56156342
rs4870027
rs6965326
rs115574037
rs6482989
rs12484414
rs140477529
rs74922775
rs17114587
rs112257191
rs1777451
rs6517635
rs114555326
rs7918792
rs218671
rs878378
rs151271200
rs117999079
rs113261925
rs147893796
rs7714478
rs12132412
rs10815677
rs36010174
rs762523
rs72671744
rs17019517
rs113801777
rs34385352
rs2104394
rs4787759
All
−0.05
0.00
0.05
MR leave−one−out sensitivity analysis for
'Phosphate to citrate ratio' on 'Rheumatoid arthritis'

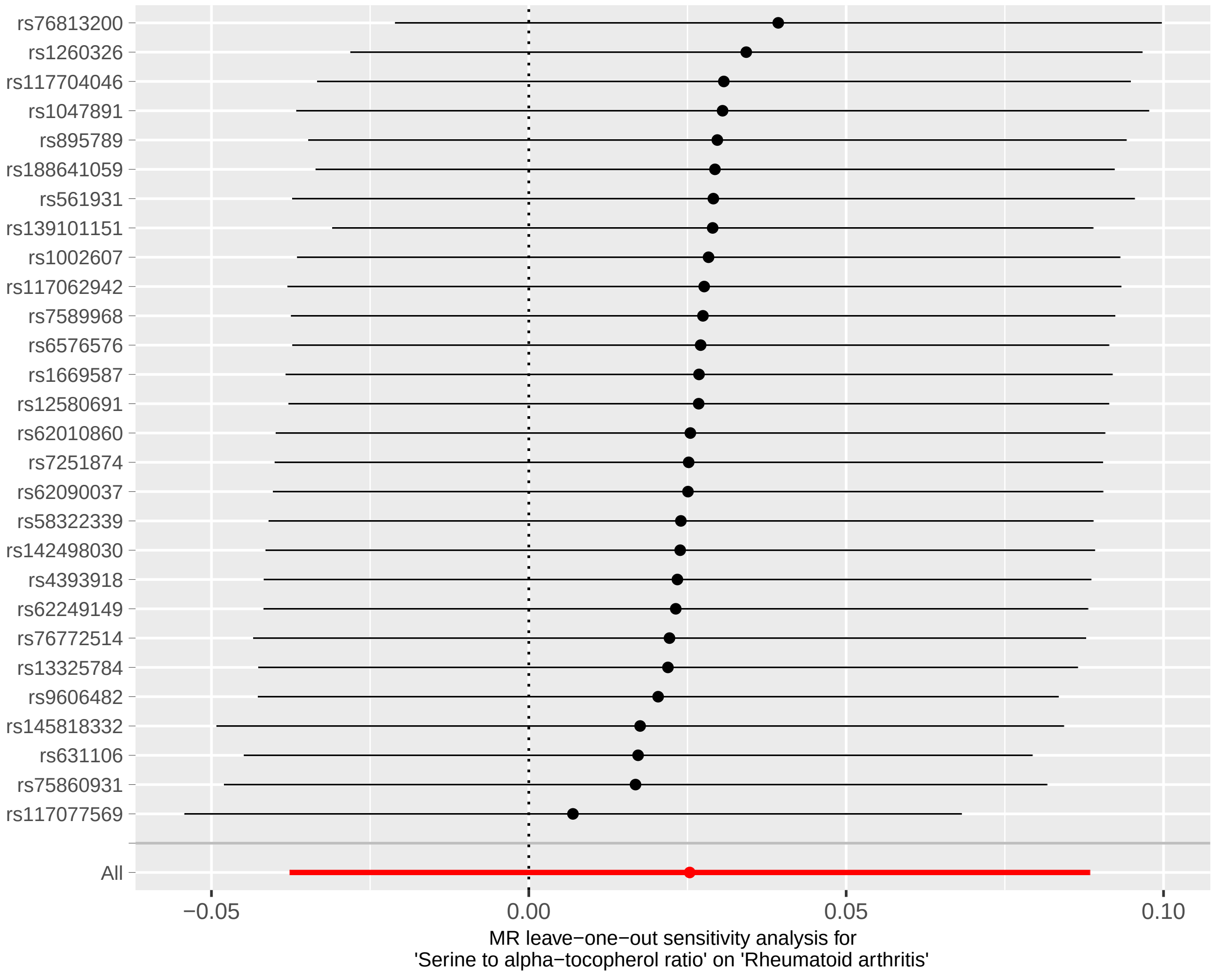
rs76813200
rs1260326
rs117704046
rs1047891
rs895789
rs188641059
rs561931
rs139101151
rs1002607
rs117062942
rs7589968
rs6576576
rs1669587
rs12580691
rs62010860
rs7251874
rs62090037
rs58322339
rs142498030
rs4393918
rs62249149
rs76772514
rs13325784
rs9606482
rs145818332
rs631106
rs75860931
rs117077569
All
−0.05
0.00
0.05
0.10
MR leave−one−out sensitivity analysis for
'Serine to alpha−tocopherol ratio' on 'Rheumatoid arthritis'

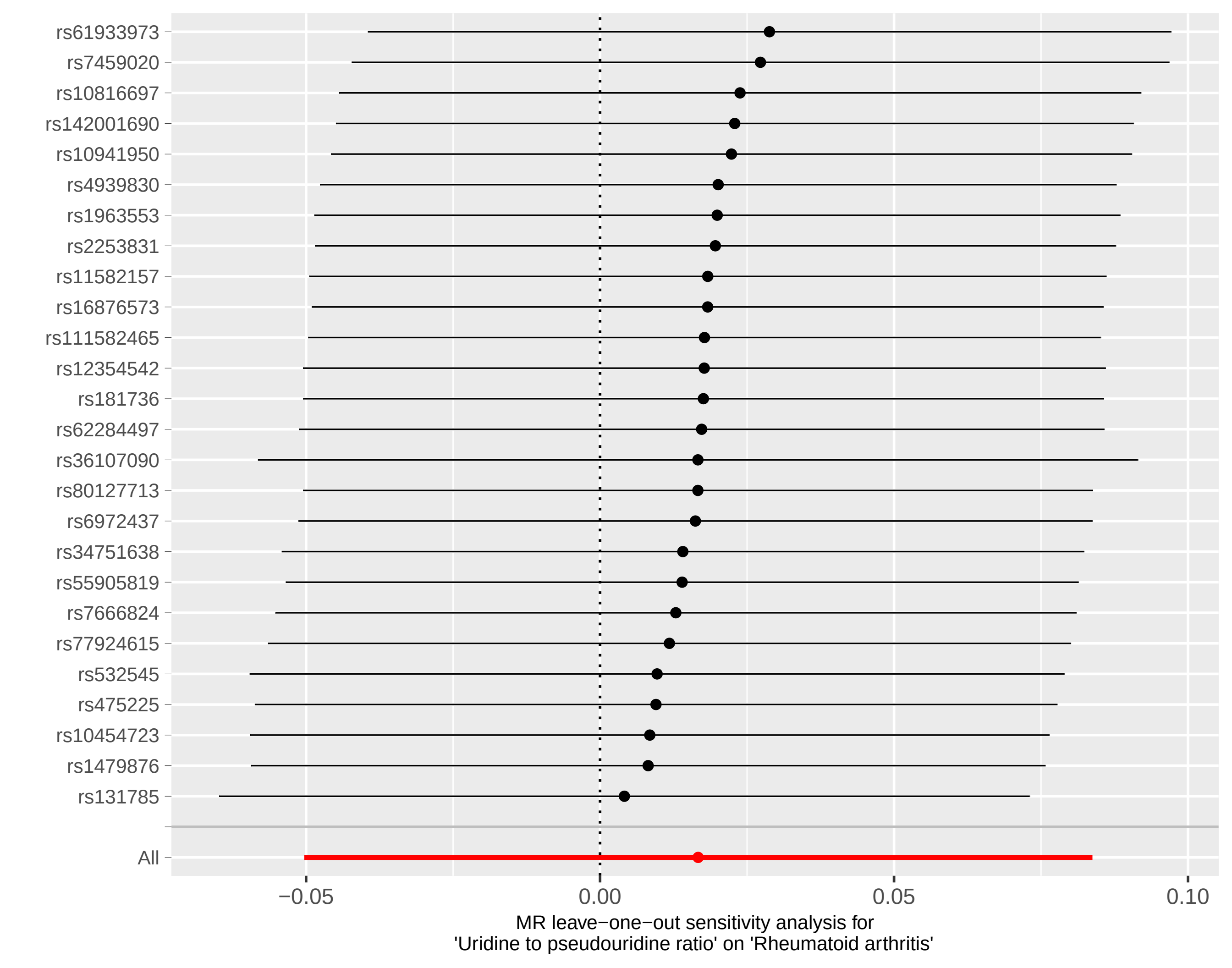
rs61933973
rs7459020
rs10816697
rs142001690
rs10941950
rs4939830
rs1963553
rs2253831
rs11582157
rs16876573
rs111582465
rs12354542
rs181736
rs62284497
rs36107090
rs80127713
rs6972437
rs34751638
rs55905819
rs7666824
rs77924615
rs532545
rs475225
rs10454723
rs1479876
rs131785
All
−0.05
0.00
0.05
0.10
MR leave−one−out sensitivity analysis for
'Uridine to pseudouridine ratio' on 'Rheumatoid arthritis'

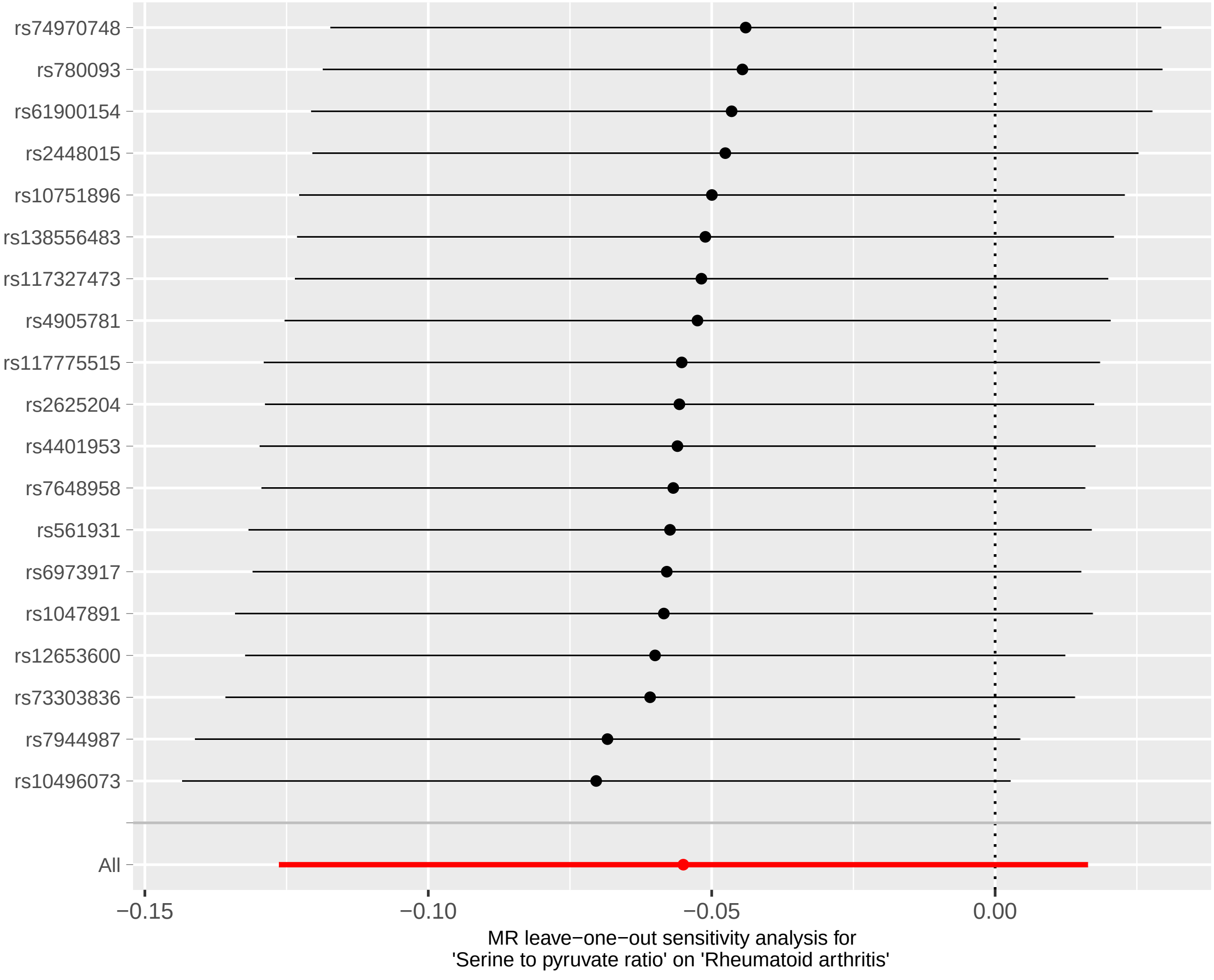
rs74970748
rs780093
rs61900154
rs2448015
rs10751896
rs138556483
rs117327473
rs4905781
rs117775515
rs2625204
rs4401953
rs7648958
rs561931
rs6973917
rs1047891
rs12653600
rs73303836
rs7944987
rs10496073
All
−0.15
−0.10
−0.05
0.00
MR leave−one−out sensitivity analysis for
'Serine to pyruvate ratio' on 'Rheumatoid arthritis'

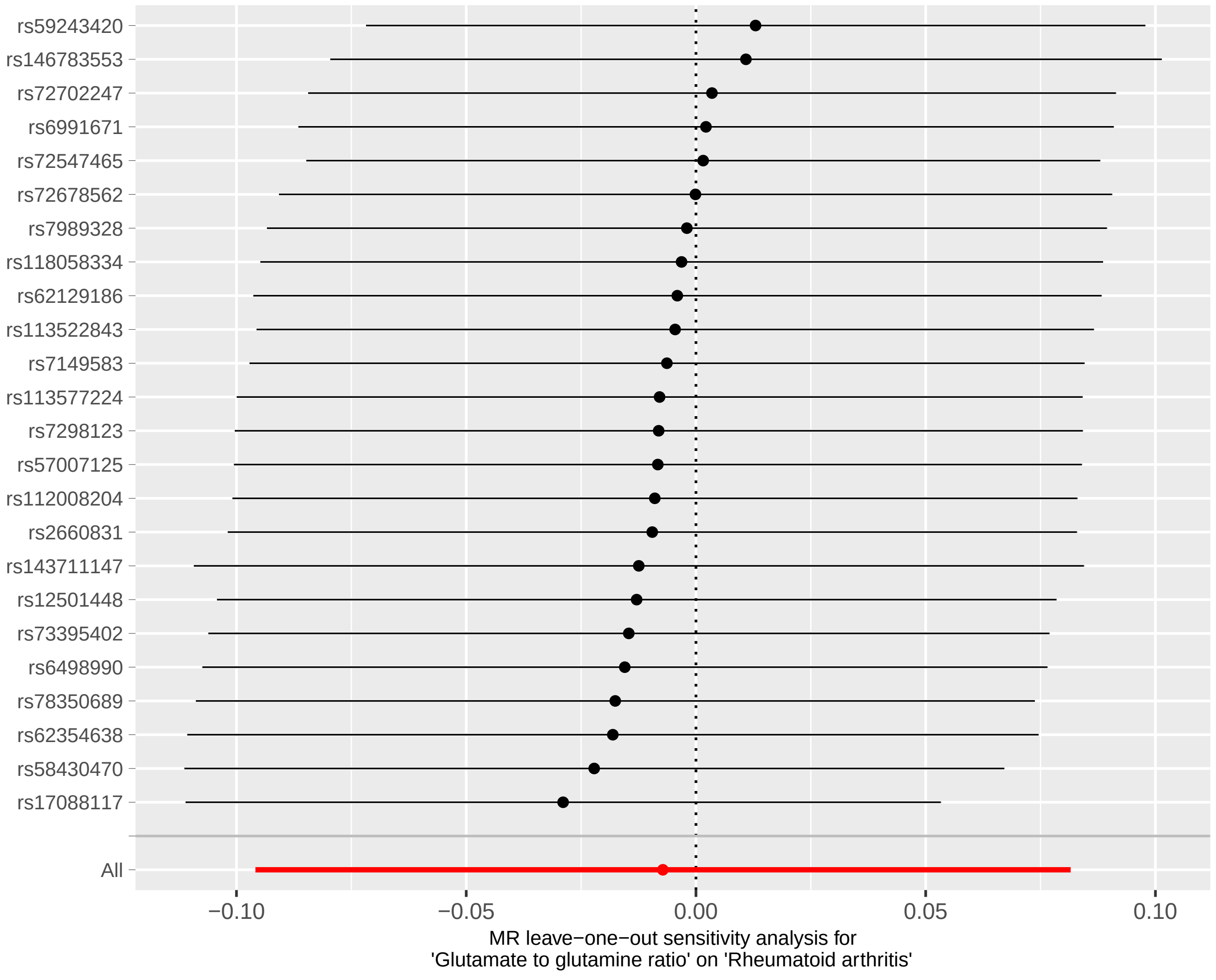
rs59243420
rs146783553
rs72702247
rs6991671
rs72547465
rs72678562
rs7989328
rs118058334
rs62129186
rs113522843
rs7149583
rs113577224
rs7298123
rs57007125
rs112008204
rs2660831
rs143711147
rs12501448
rs73395402
rs6498990
rs78350689
rs62354638
rs58430470
rs17088117
All
−0.10
−0.05
0.00
0.05
0.10
MR leave−one−out sensitivity analysis for
'Glutamate to glutamine ratio' on 'Rheumatoid arthritis'

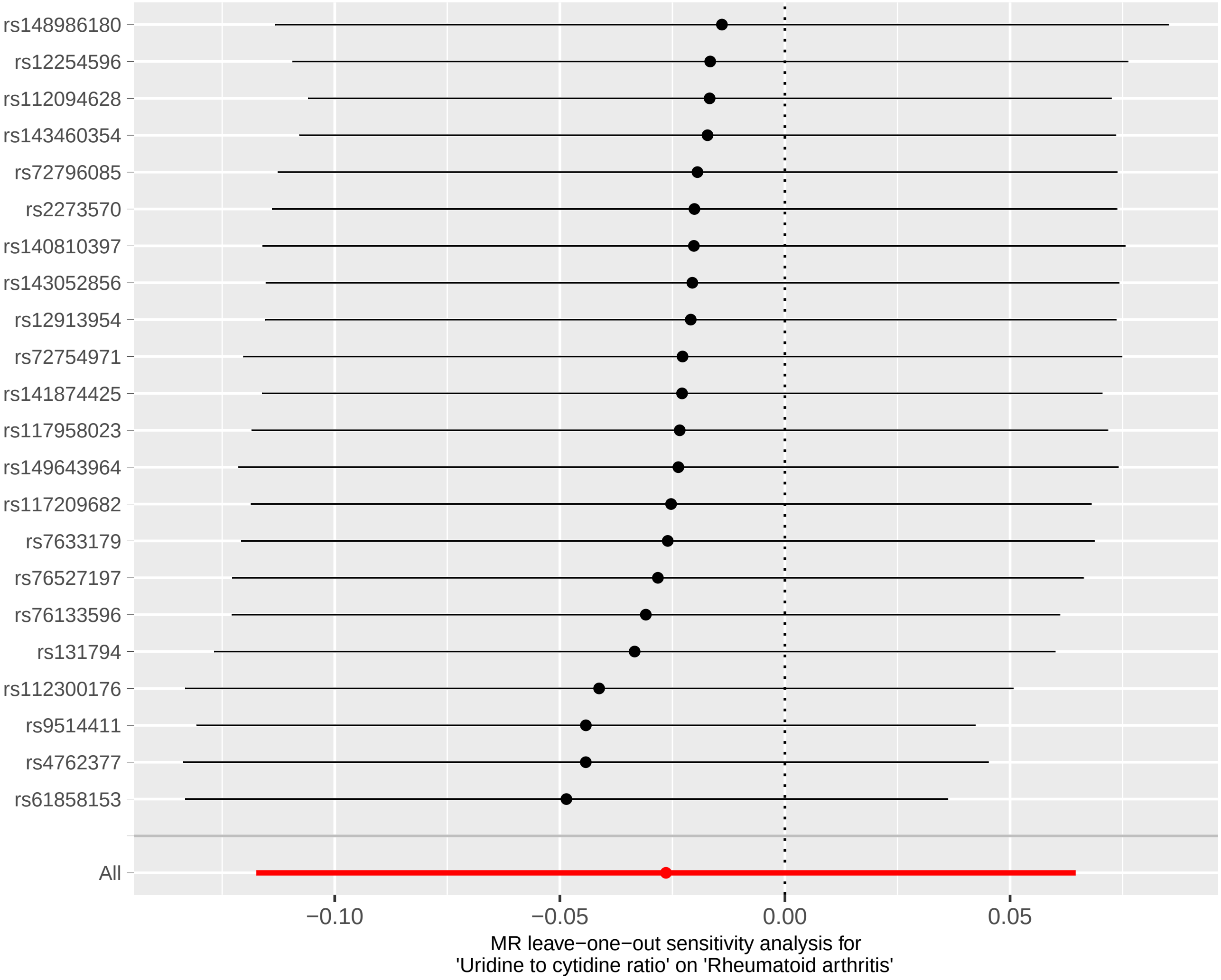
rs148986180
rs12254596
rs112094628
rs143460354
rs72796085
rs2273570
rs140810397
rs143052856
rs12913954
rs72754971
rs141874425
rs117958023
rs149643964
rs117209682
rs7633179
rs76527197
rs76133596
rs131794
rs112300176
rs9514411
rs4762377
rs61858153
All
−0.10
−0.05
0.00
0.05
MR leave−one−out sensitivity analysis for
'Uridine to cytidine ratio' on 'Rheumatoid arthritis'

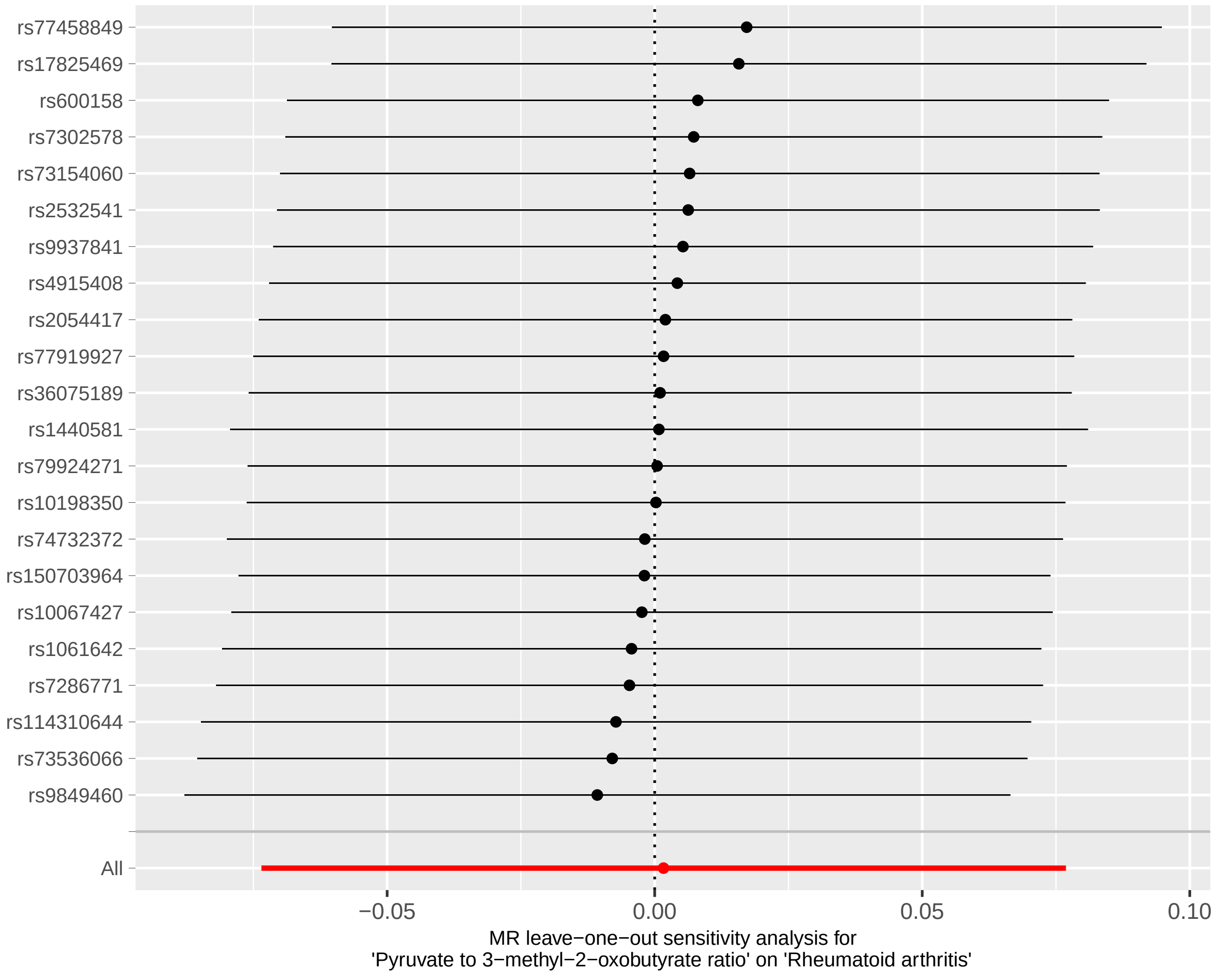

rs77458849
rs17825469
rs600158
rs7302578
rs73154060
rs2532541
rs9937841
rs4915408
rs2054417
rs77919927
rs36075189
rs1440581
rs79924271
rs10198350
rs74732372
rs150703964
rs10067427
rs1061642
rs7286771
rs114310644
rs73536066
rs9849460
All
−0.05
0.00
0.05
0.10
MR leave−one−out sensitivity analysis for
'Pyruvate to 3−methyl−2−oxobutyrate ratio' on 'Rheumatoid arthritis'

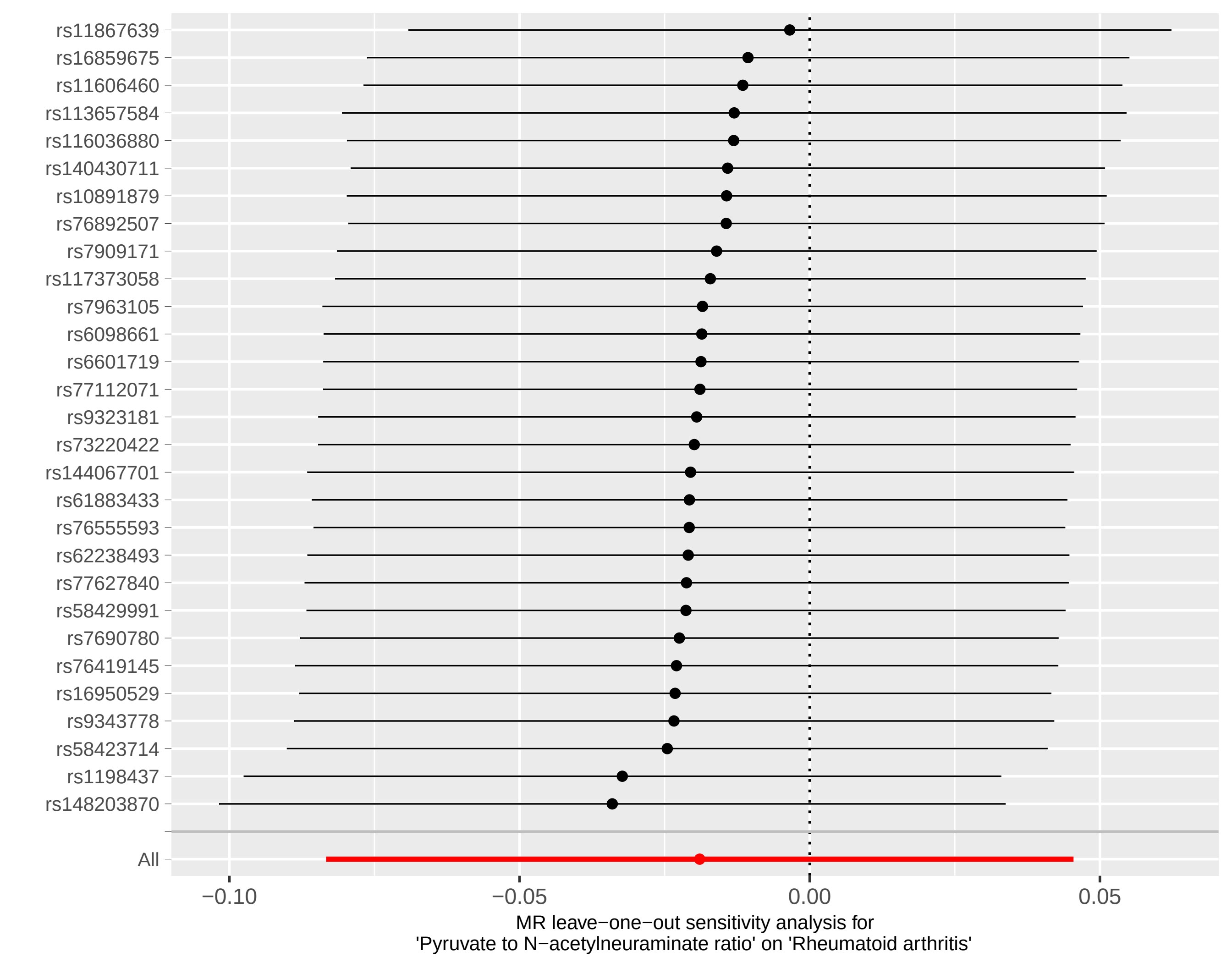
rs11867639
rs16859675
rs11606460
rs113657584
rs116036880
rs140430711
rs10891879
rs76892507
rs7909171
rs117373058
rs7963105
rs6098661
rs6601719
rs77112071
rs9323181
rs73220422
rs144067701
rs61883433
rs76555593
rs62238493
rs77627840
rs58429991
rs7690780
rs76419145
rs16950529
rs9343778
rs58423714
rs1198437
rs148203870
All
−0.10
−0.05
0.00
0.05
MR leave−one−out sensitivity analysis for
'Pyruvate to N−acetylneuraminate ratio' on 'Rheumatoid arthritis'

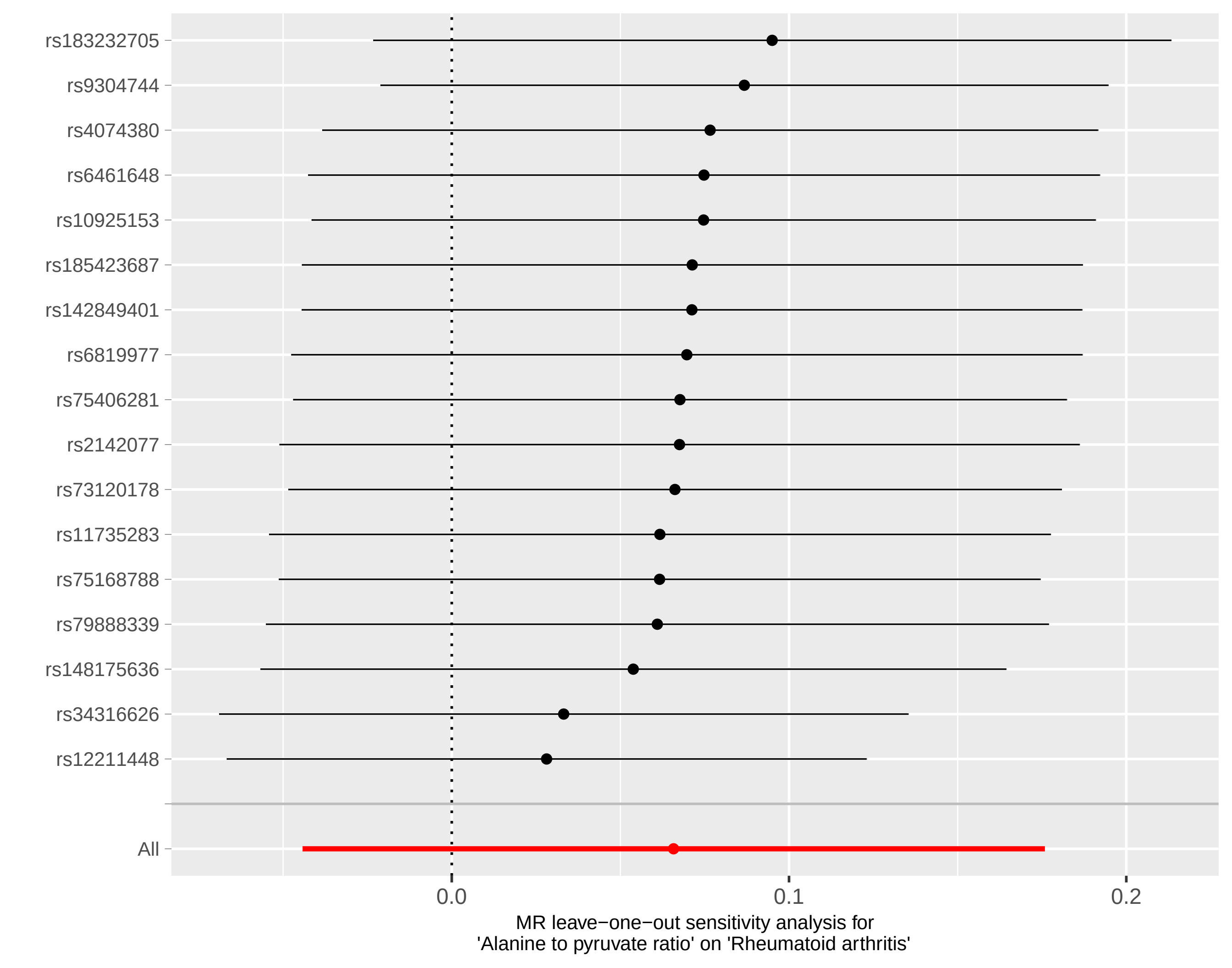
rs183232705
rs9304744
rs4074380
rs6461648
rs10925153
rs185423687
rs142849401
rs6819977
rs75406281
rs2142077
rs73120178
rs11735283
rs75168788
rs79888339
rs148175636
rs34316626
rs12211448
All
0.0
0.1
0.2
MR leave−one−out sensitivity analysis for
'Alanine to pyruvate ratio' on 'Rheumatoid arthritis'

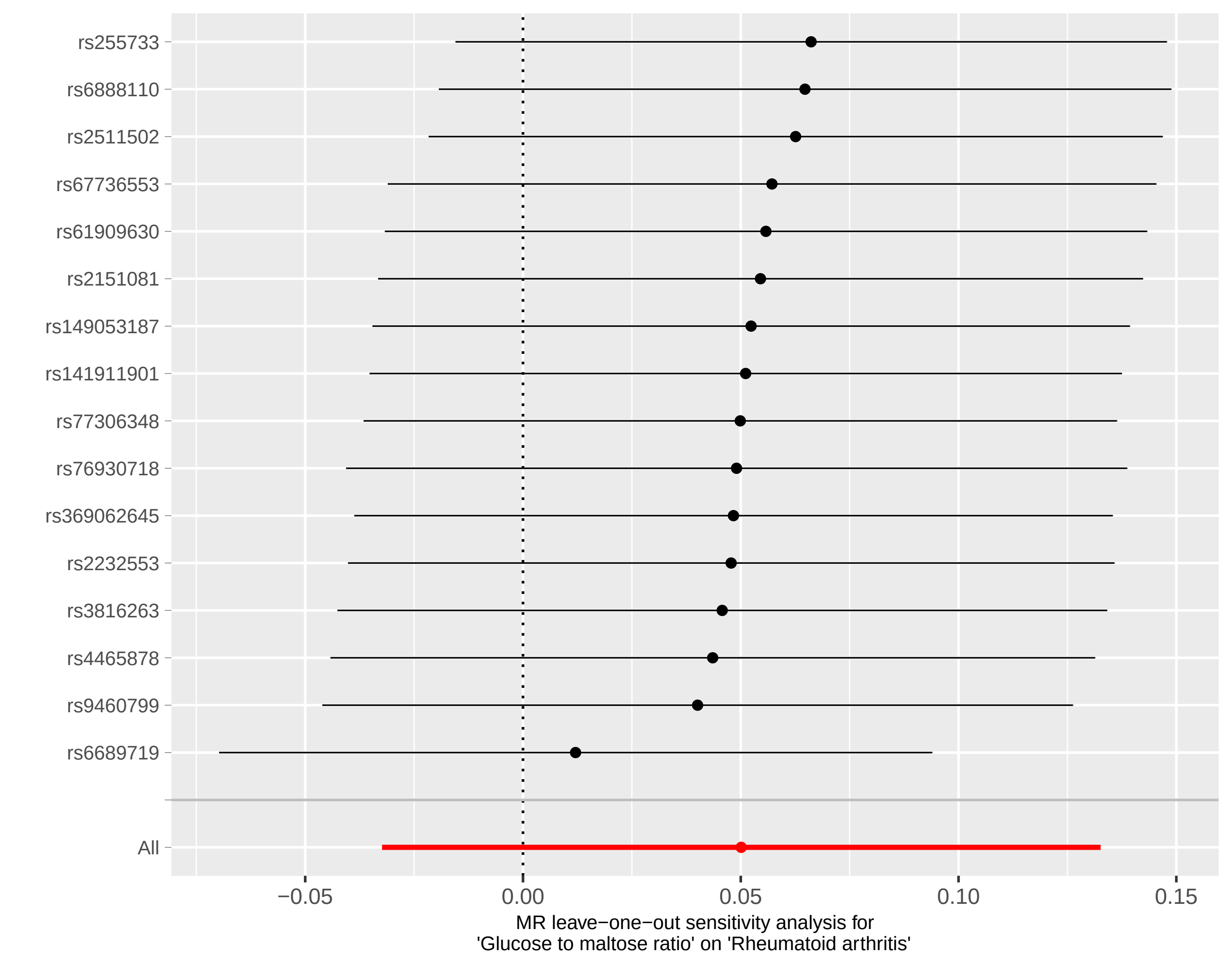

rs255733
rs6888110
rs2511502
rs67736553
rs61909630
rs2151081
rs149053187
rs141911901
rs77306348
rs76930718
rs369062645
rs2232553
rs3816263
rs4465878
rs9460799
rs6689719
All
−0.05
0.00
0.05
0.10
0.15
MR leave−one−out sensitivity analysis for
'Glucose to maltose ratio' on 'Rheumatoid arthritis'

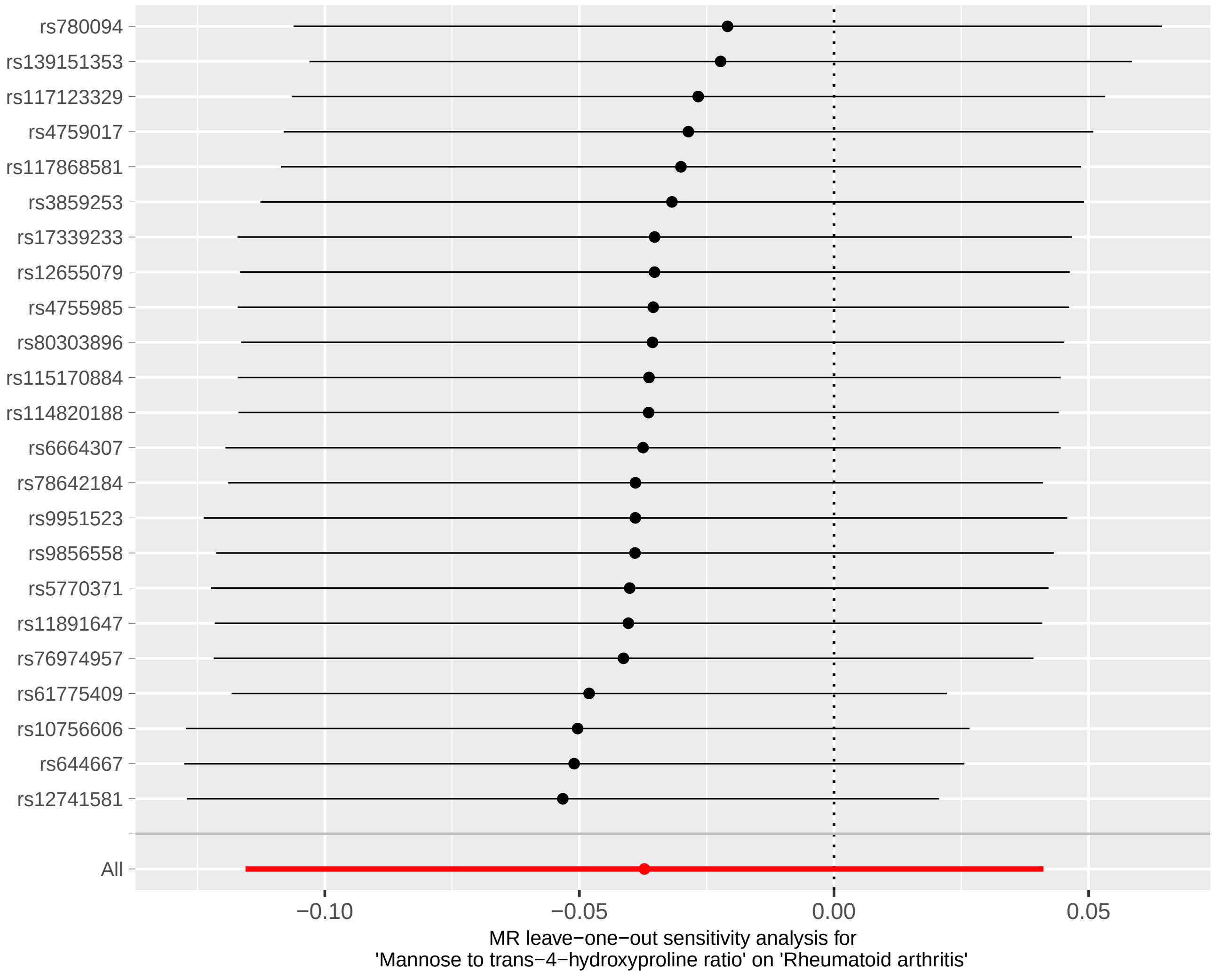

rs780094
rs139151353
rs117123329
rs4759017
rs117868581
rs3859253
rs17339233
rs12655079
rs4755985
rs80303896
rs115170884
rs114820188
rs6664307
rs78642184
rs9951523
rs9856558
rs5770371
rs11891647
rs76974957
rs61775409
rs10756606
rs644667
rs12741581
All
−0.10
−0.05
0.00
0.05
MR leave−one−out sensitivity analysis for
'Mannose to trans−4−hydroxyproline ratio' on 'Rheumatoid arthritis'

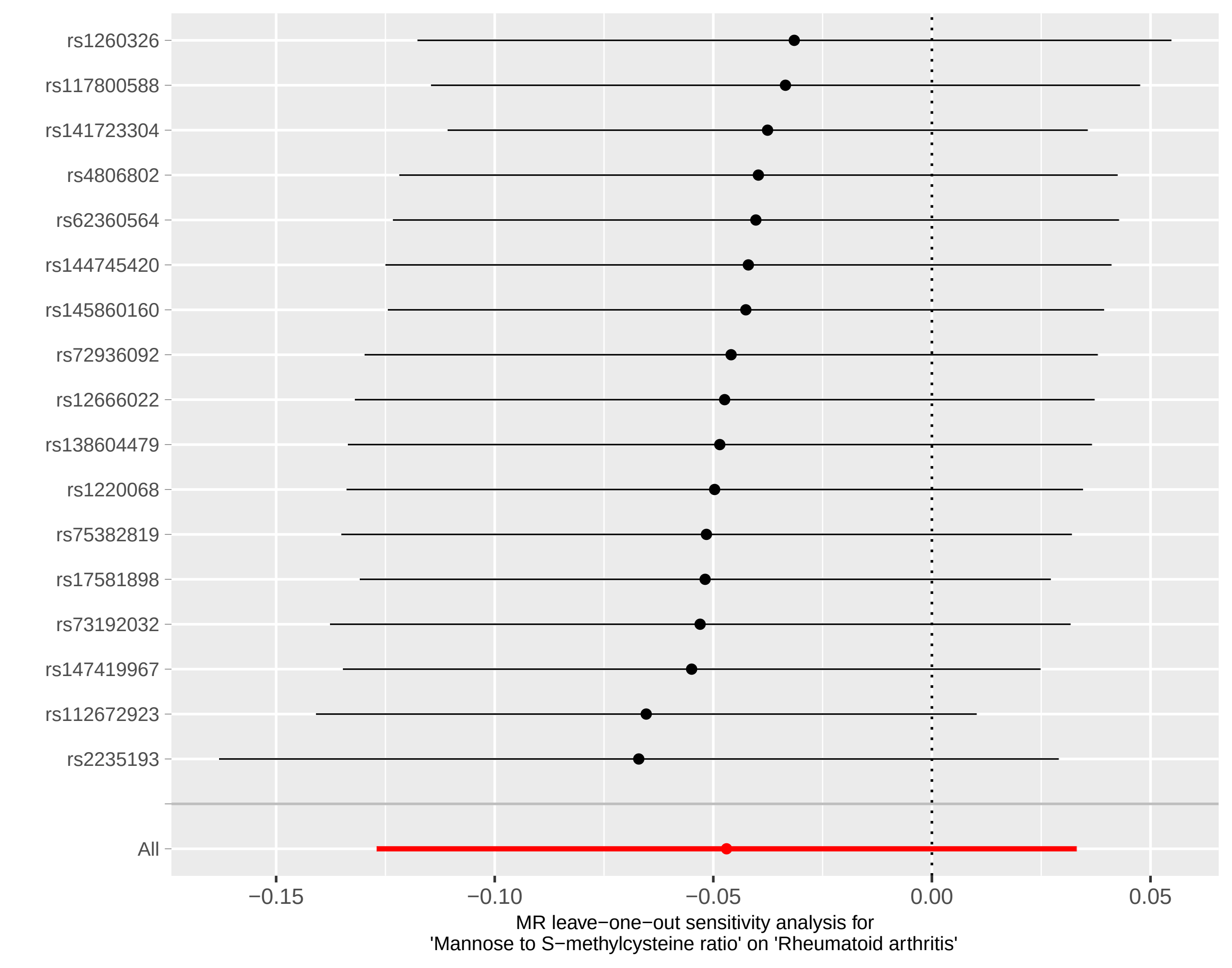

rs1260326
rs117800588
rs141723304
rs4806802
rs62360564
rs144745420
rs145860160
rs72936092
rs12666022
rs138604479
rs1220068
rs75382819
rs17581898
rs73192032
rs147419967
rs112672923
rs2235193
All
−0.15
−0.10
−0.05
0.00
0.05
MR leave−one−out sensitivity analysis for
'Mannose to S−methylcysteine ratio' on 'Rheumatoid arthritis'

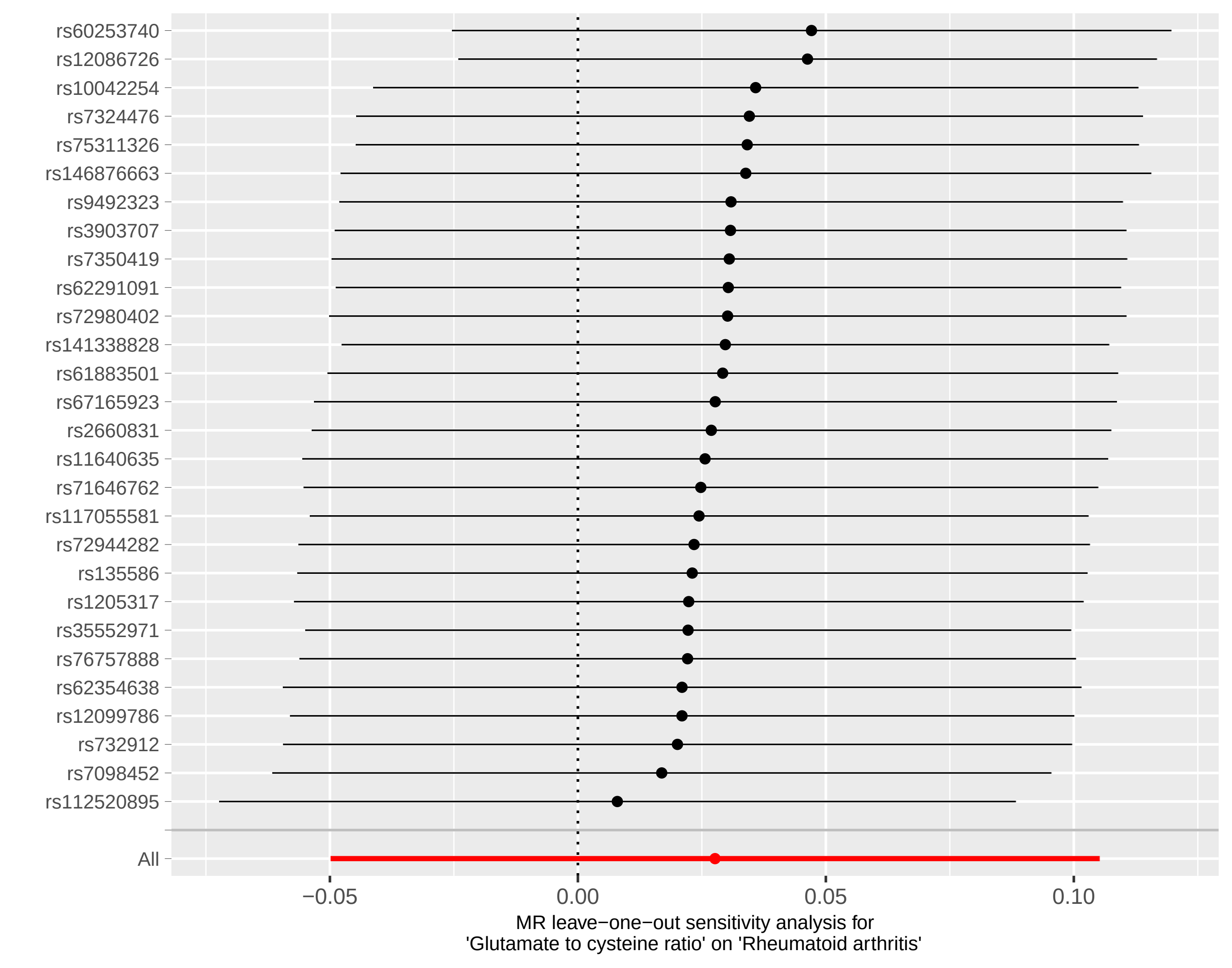
rs60253740
rs12086726
rs10042254
rs7324476
rs75311326
rs146876663
rs9492323
rs3903707
rs7350419
rs62291091
rs72980402
rs141338828
rs61883501
rs67165923
rs2660831
rs11640635
rs71646762
rs117055581
rs72944282
rs135586
rs1205317
rs35552971
rs76757888
rs62354638
rs12099786
rs732912
rs7098452
rs112520895
All
−0.05
0.00
0.05
0.10
MR leave−one−out sensitivity analysis for
'Glutamate to cysteine ratio' on 'Rheumatoid arthritis'

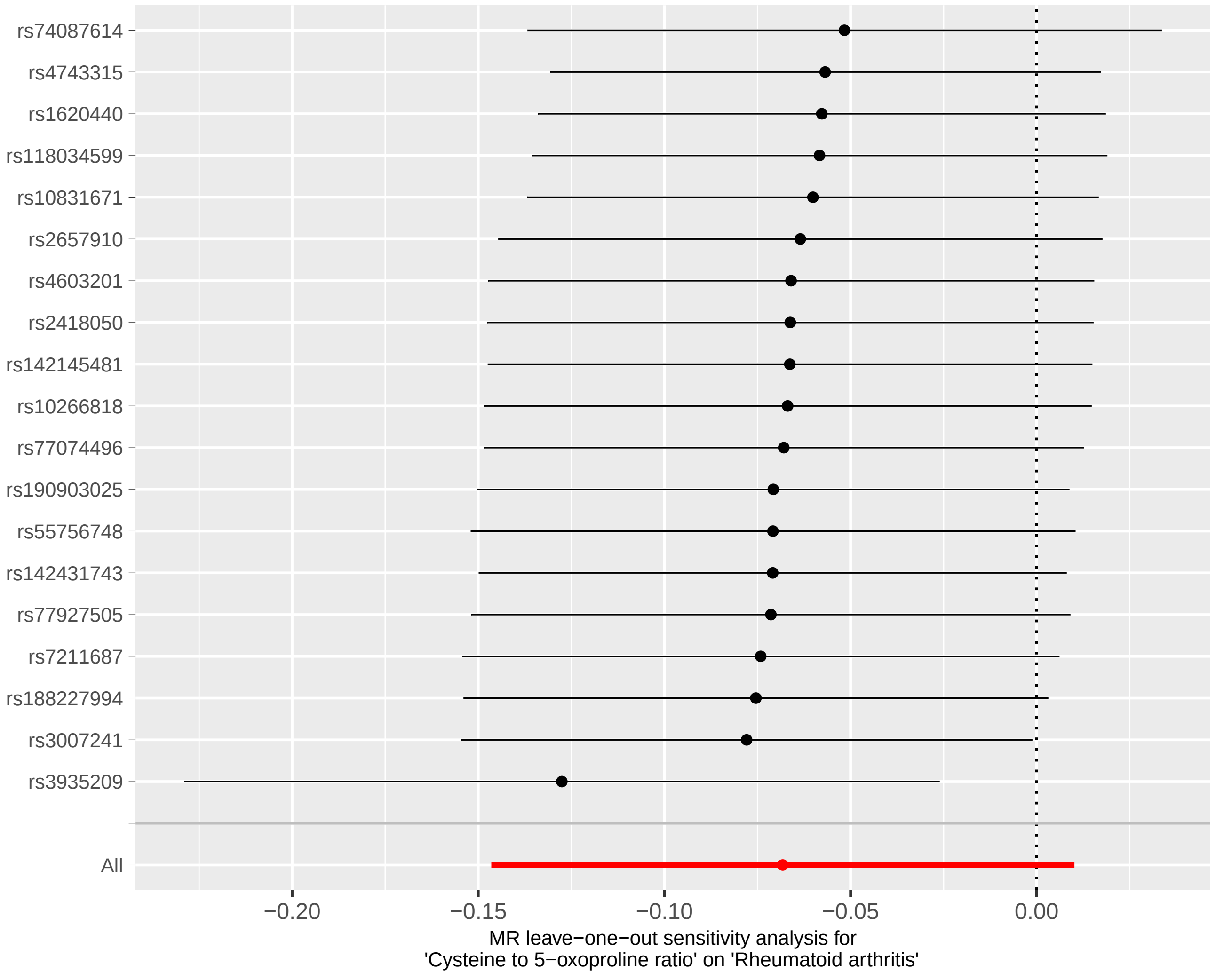
rs74087614
rs4743315
rs1620440
rs118034599
rs10831671
rs2657910
rs4603201
rs2418050
rs142145481
rs10266818
rs77074496
rs190903025
rs55756748
rs142431743
rs77927505
rs7211687
rs188227994
rs3007241
rs3935209
All
−0.20
−0.15
−0.10
−0.05
0.00
MR leave−one−out sensitivity analysis for
'Cysteine to 5−oxoproline ratio' on 'Rheumatoid arthritis'

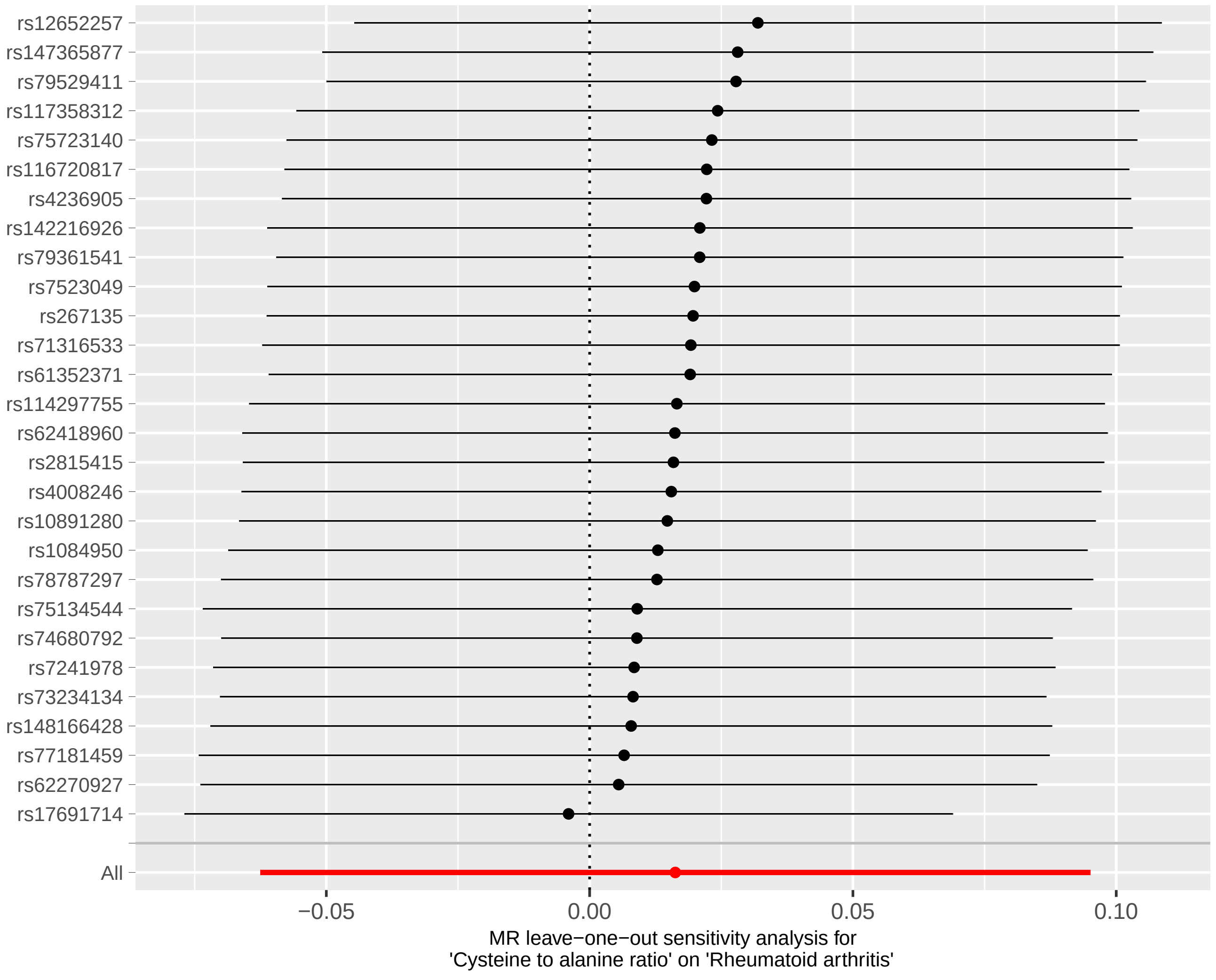
rs12652257
rs147365877
rs79529411
rs117358312
rs75723140
rs116720817
rs4236905
rs142216926
rs79361541
rs7523049
rs267135
rs71316533
rs61352371
rs114297755
rs62418960
rs2815415
rs4008246
rs10891280
rs1084950
rs78787297
rs75134544
rs74680792
rs7241978
rs73234134
rs148166428
rs77181459
rs62270927
rs17691714
All
−0.05
0.00
0.05
0.10
MR leave−one−out sensitivity analysis for
'Cysteine to alanine ratio' on 'Rheumatoid arthritis'

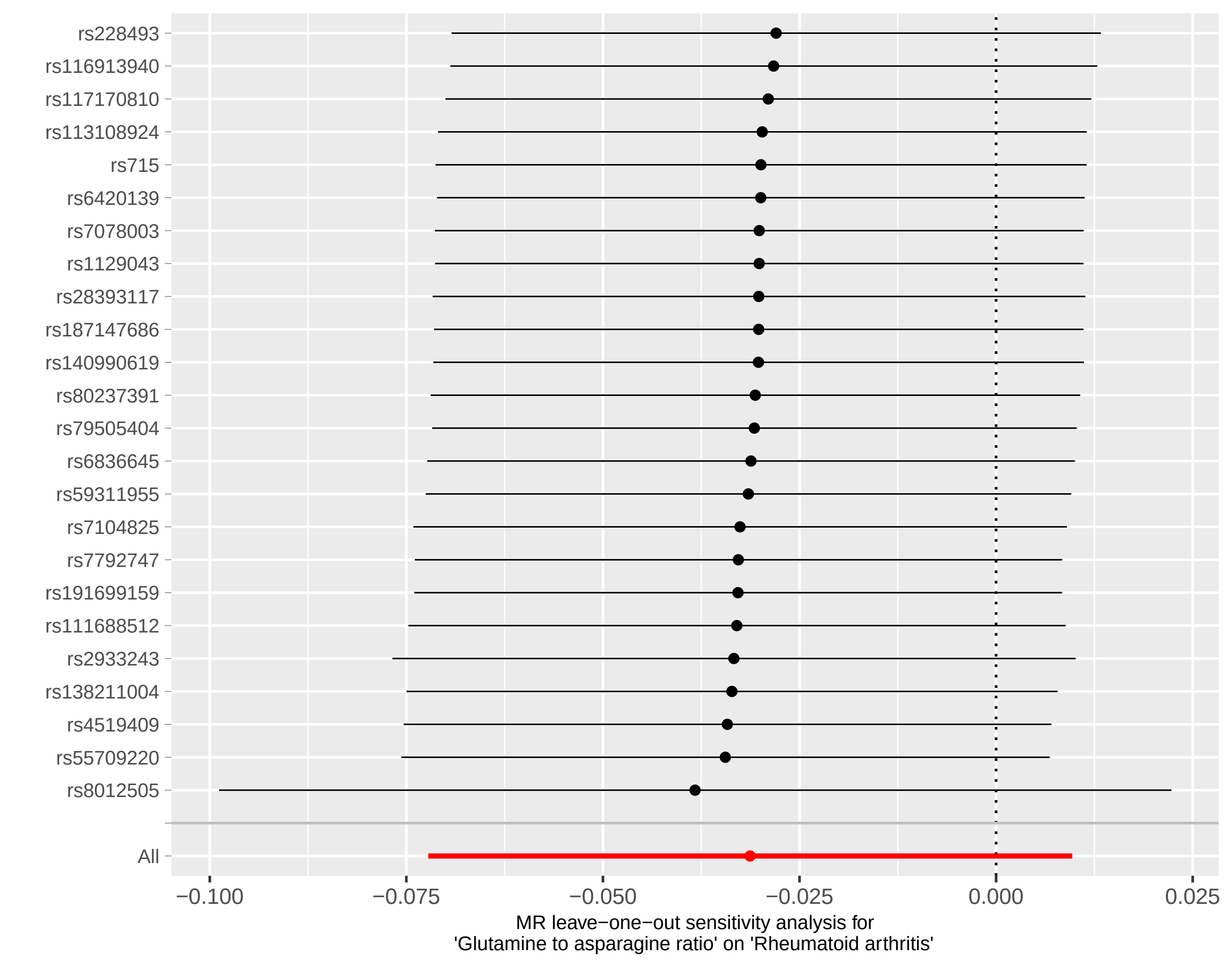

rs228493
rs116913940
rs117170810
rs113108924
rs715
rs6420139
rs7078003
rs1129043
rs28393117
rs187147686
rs140990619
rs80237391
rs79505404
rs6836645
rs59311955
rs7104825
rs7792747
rs191699159
rs111688512
rs2933243
rs138211004
rs4519409
rs55709220
rs8012505
All
−0.100
−0.075
−0.050
−0.025
0.000
0.025
MR leave−one−out sensitivity analysis for
'Glutamine to asparagine ratio' on 'Rheumatoid arthritis'

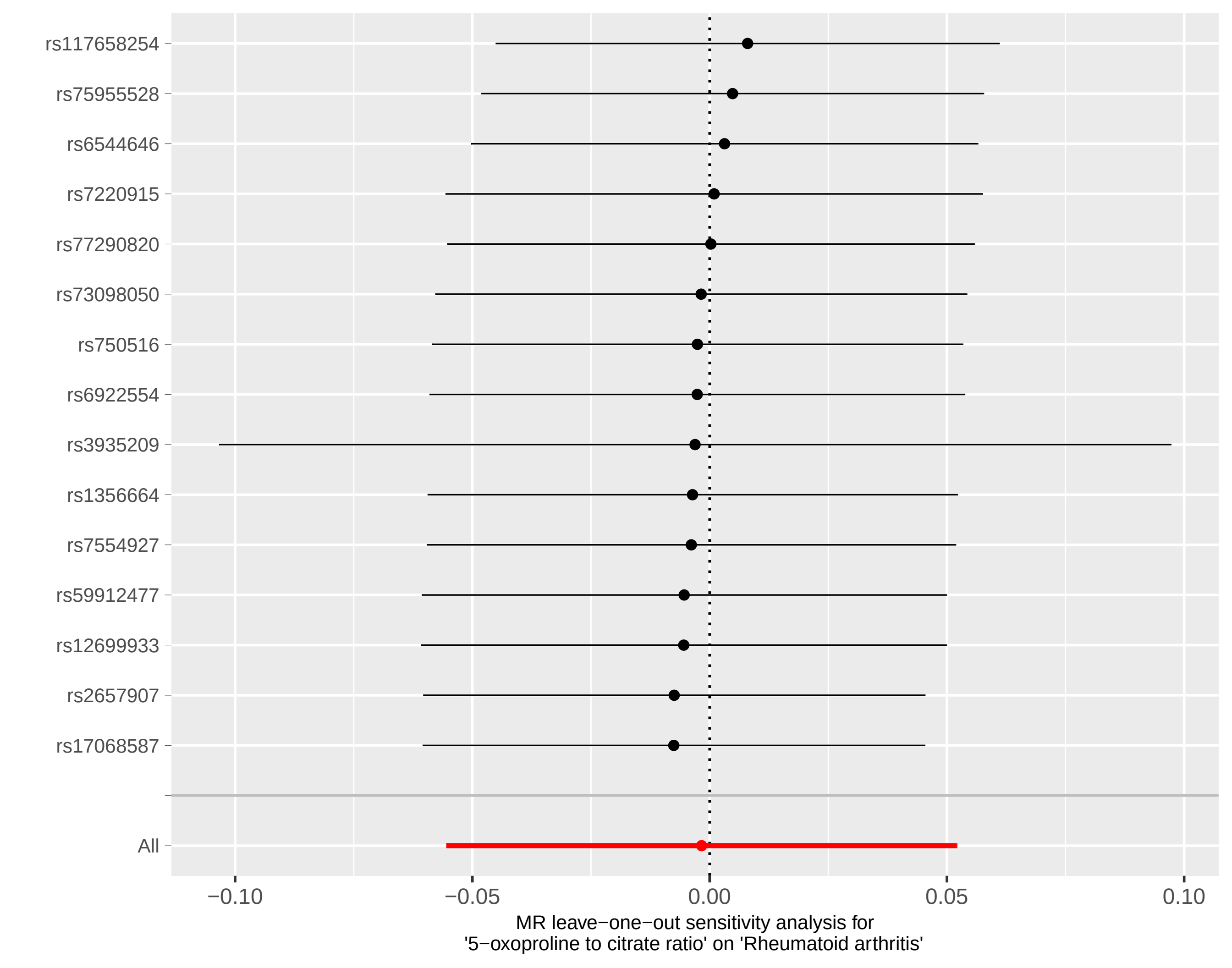
rs117658254
rs75955528
rs6544646
rs7220915
rs77290820
rs73098050
rs750516
rs6922554
rs3935209
rs1356664
rs7554927
rs59912477
rs12699933
rs2657907
rs17068587
All
−0.10
−0.05
0.00
0.05
0.10
MR leave−one−out sensitivity analysis for
'5−oxoproline to citrate ratio' on 'Rheumatoid arthritis'

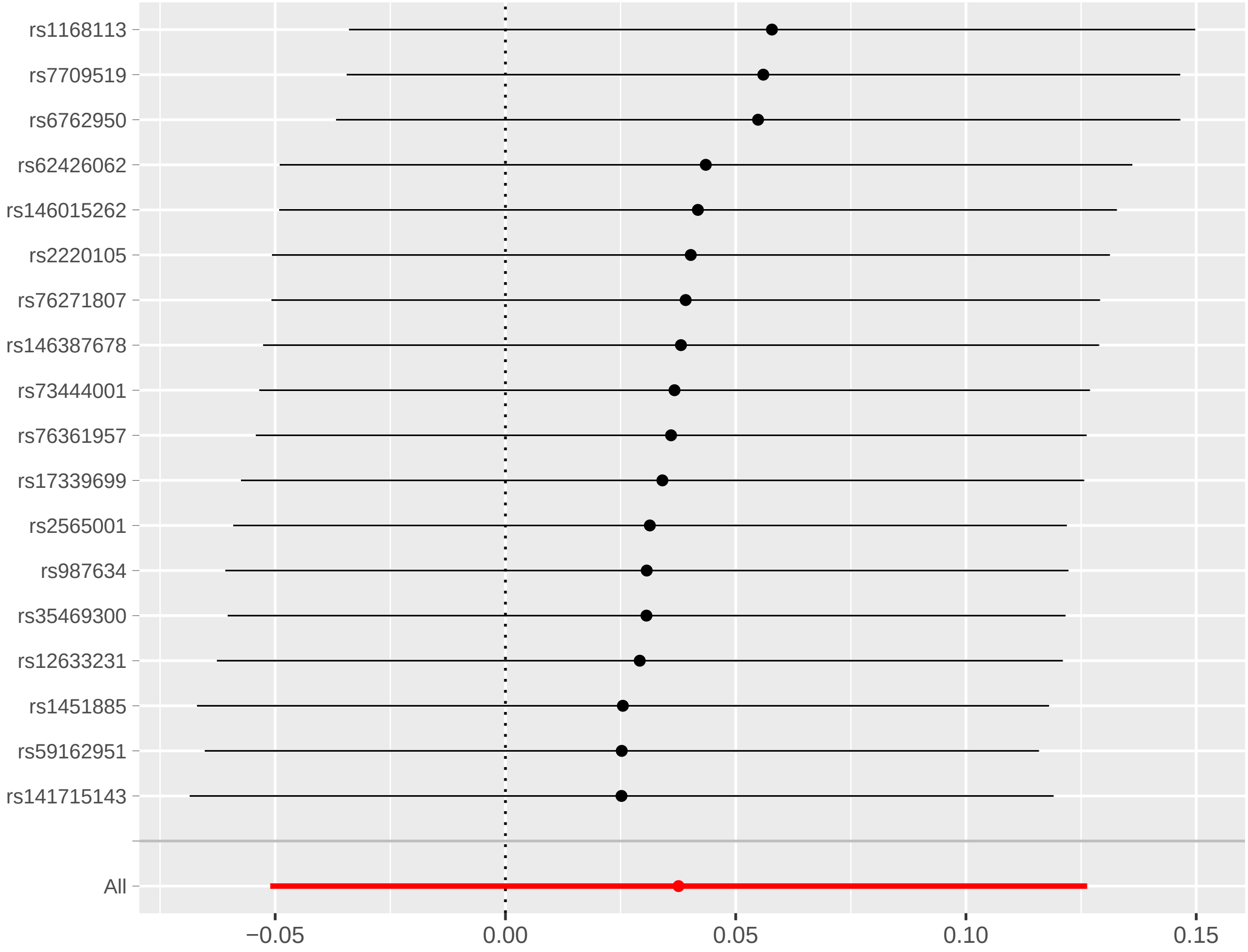

MR leave−one−out sensitivity analysis for
'Alpha−tocopherol to sulfate ratio' on 'Rheumatoid arthritis'

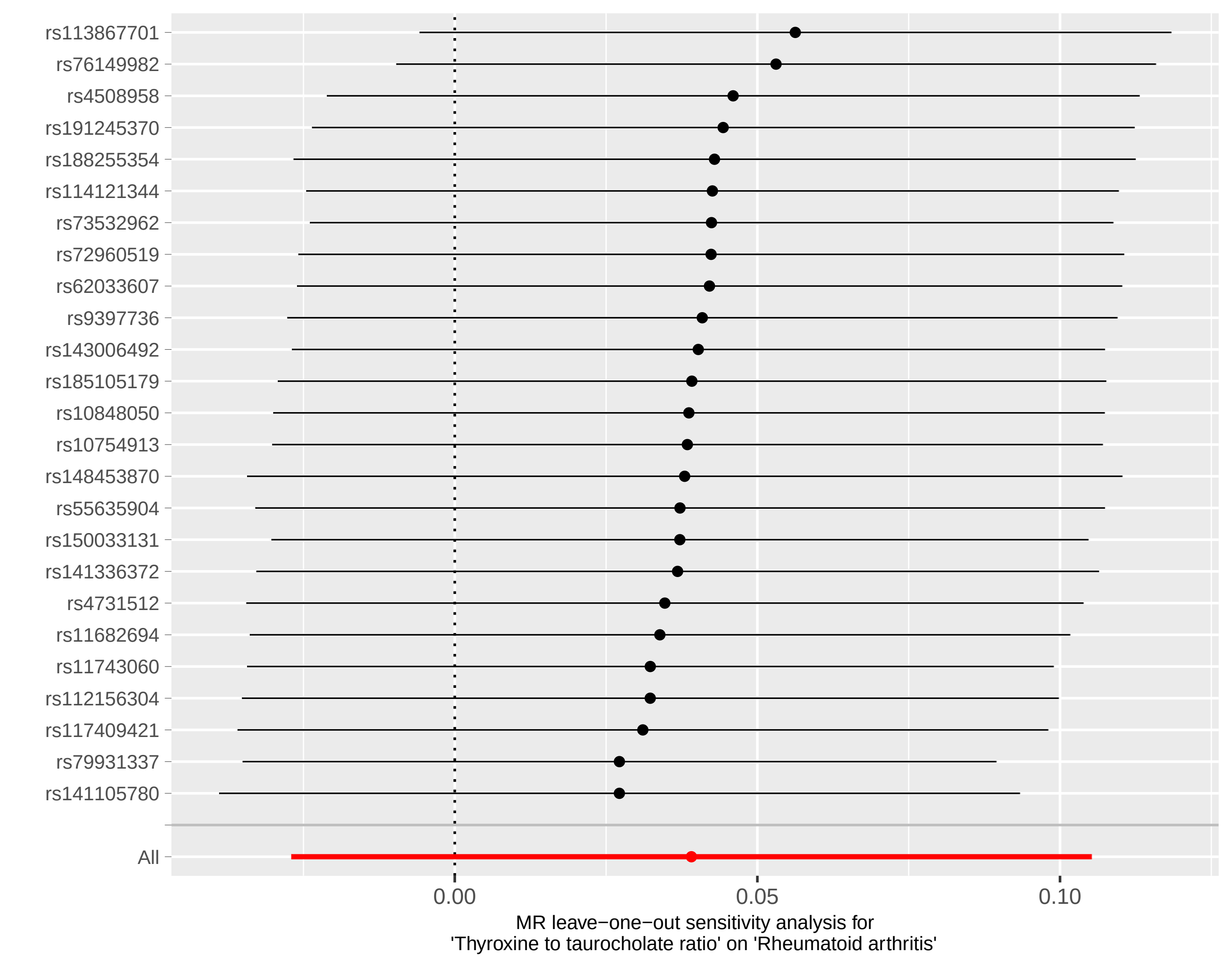
rs113867701
rs76149982
rs4508958
rs191245370
rs188255354
rs114121344
rs73532962
rs72960519
rs62033607
rs9397736
rs143006492
rs185105179
rs10848050
rs10754913
rs148453870
rs55635904
rs150033131
rs141336372
rs4731512
rs11682694
rs11743060
rs112156304
rs117409421
rs79931337
rs141105780
All
0.00
0.05
0.10
MR leave−one−out sensitivity analysis for
'Thyroxine to taurocholate ratio' on 'Rheumatoid arthritis'

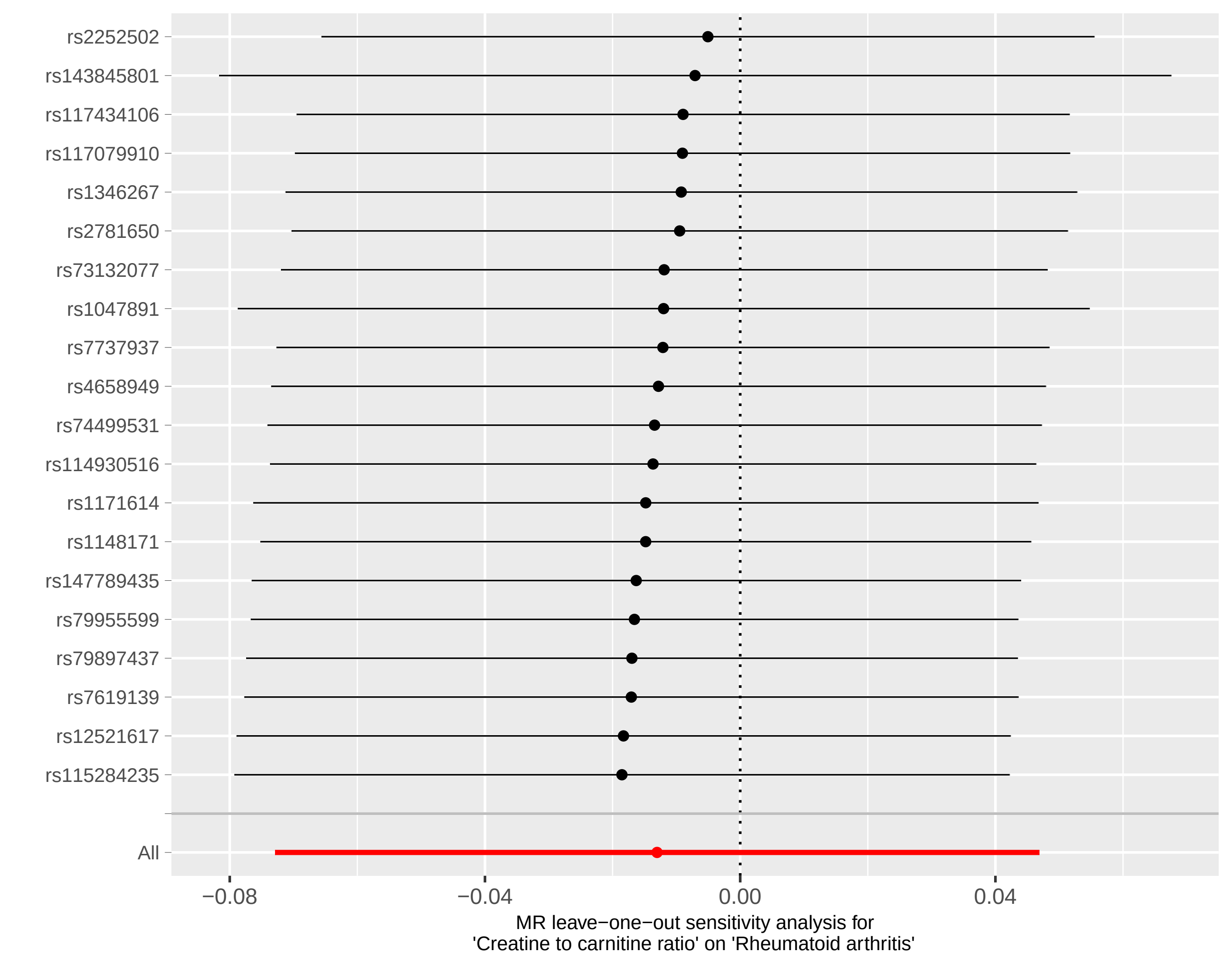

rs2252502
rs143845801
rs117434106
rs117079910
rs1346267
rs2781650
rs73132077
rs1047891
rs7737937
rs4658949
rs74499531
rs114930516
rs1171614
rs1148171
rs147789435
rs79955599
rs79897437
rs7619139
rs12521617
rs115284235
All
−0.08
−0.04
0.00
0.04
MR leave−one−out sensitivity analysis for
'Creatine to carnitine ratio' on 'Rheumatoid arthritis'

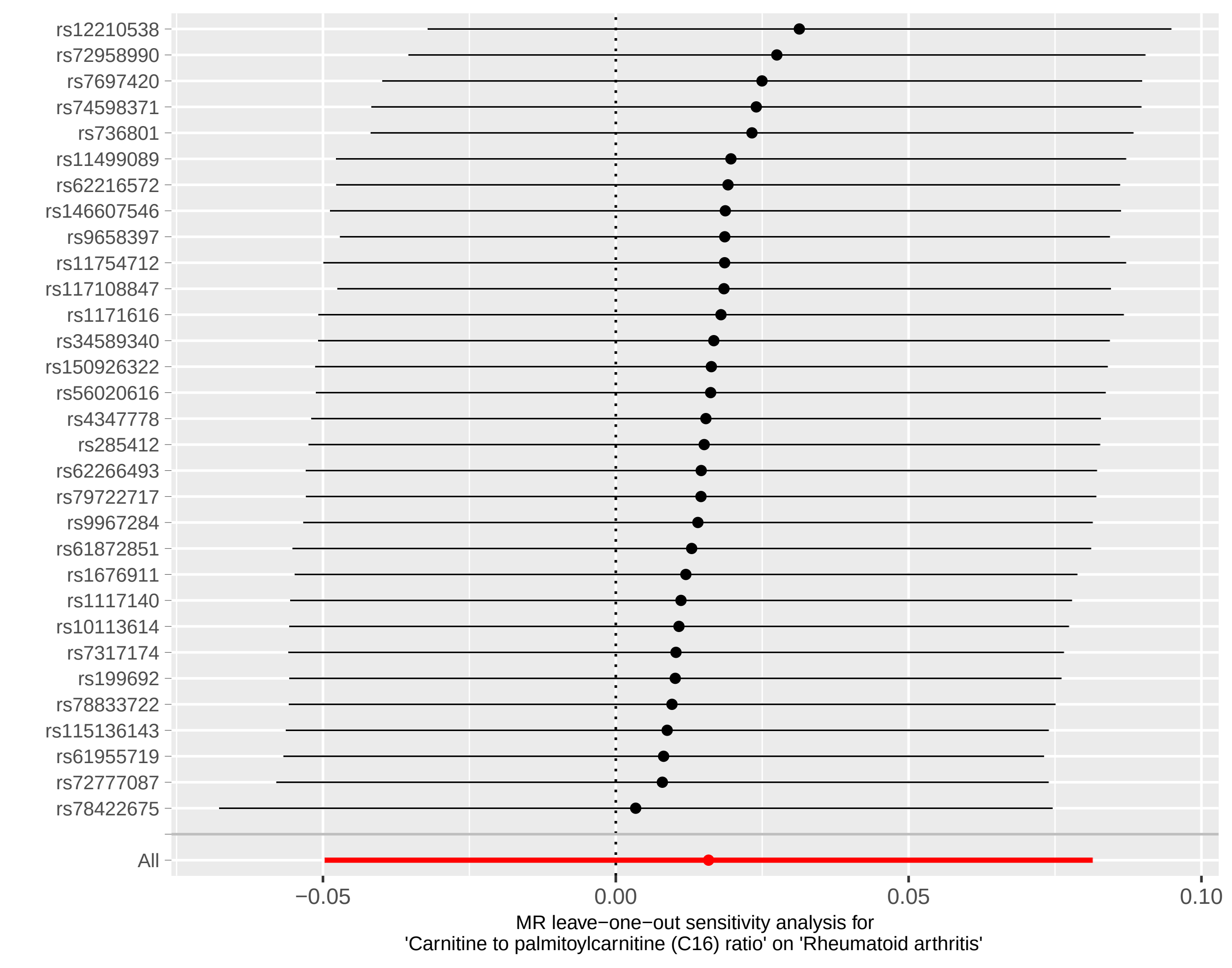
rs12210538
rs72958990
rs7697420
rs74598371
rs736801
rs11499089
rs62216572
rs146607546
rs9658397
rs11754712
rs117108847
rs1171616
rs34589340
rs150926322
rs56020616
rs4347778
rs285412
rs62266493
rs79722717
rs9967284
rs61872851
rs1676911
rs1117140
rs10113614
rs7317174
rs199692
rs78833722
rs115136143
rs61955719
rs72777087
rs78422675
All
−0.05
0.00
0.05
0.10
MR leave−one−out sensitivity analysis for
'Carnitine to palmitoylcarnitine (C16) ratio' on 'Rheumatoid arthritis'

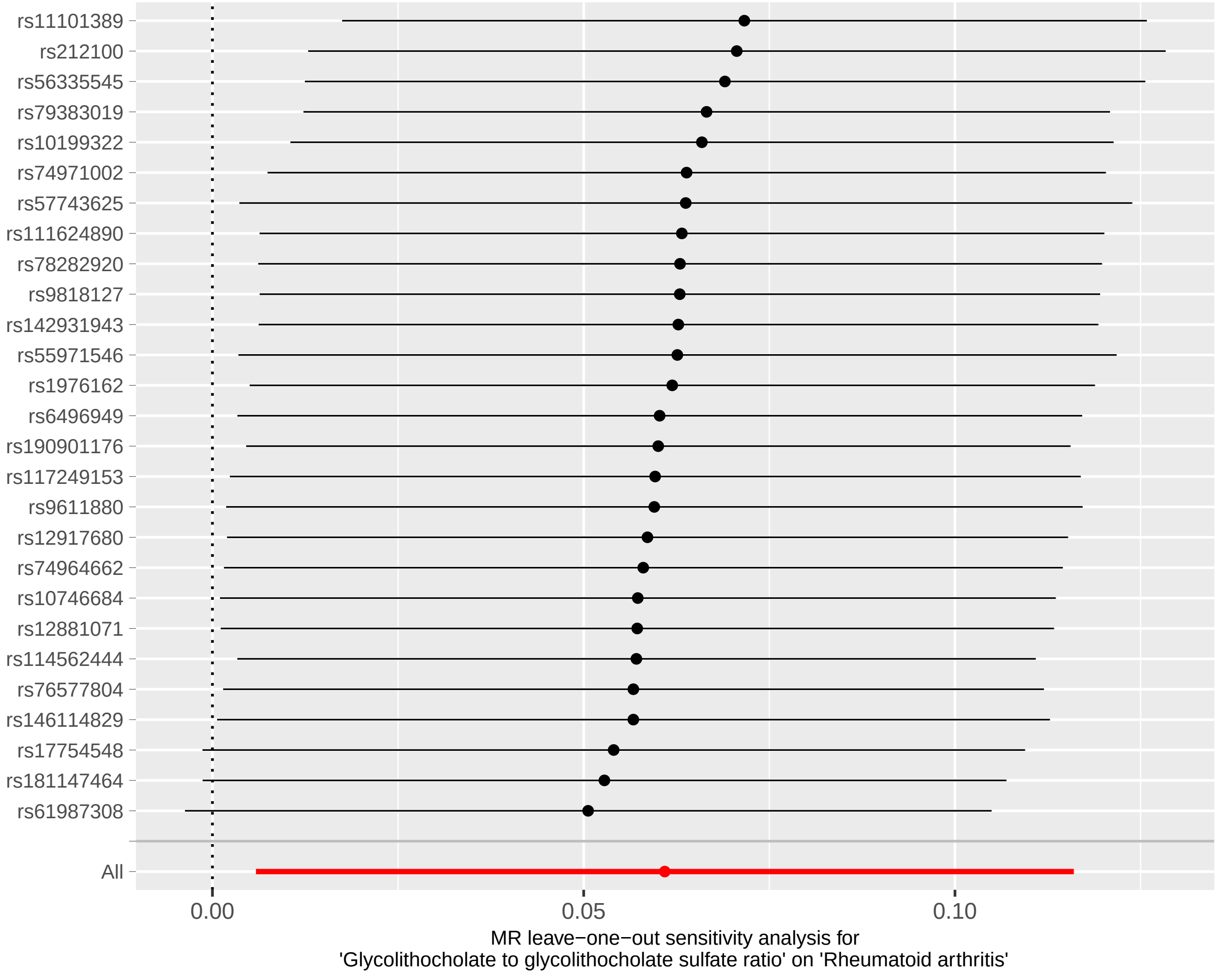
rs11101389
rs212100
rs56335545
rs79383019
rs10199322
rs74971002
rs57743625
rs111624890
rs78282920
rs9818127
rs142931943
rs55971546
rs1976162
rs6496949
rs190901176
rs117249153
rs9611880
rs12917680
rs74964662
rs10746684
rs12881071
rs114562444
rs76577804
rs146114829
rs17754548
rs181147464
rs61987308
All
0.00
0.05
0.10
MR leave−one−out sensitivity analysis for
'Glycolithocholate to glycolithocholate sulfate ratio' on 'Rheumatoid arthritis'

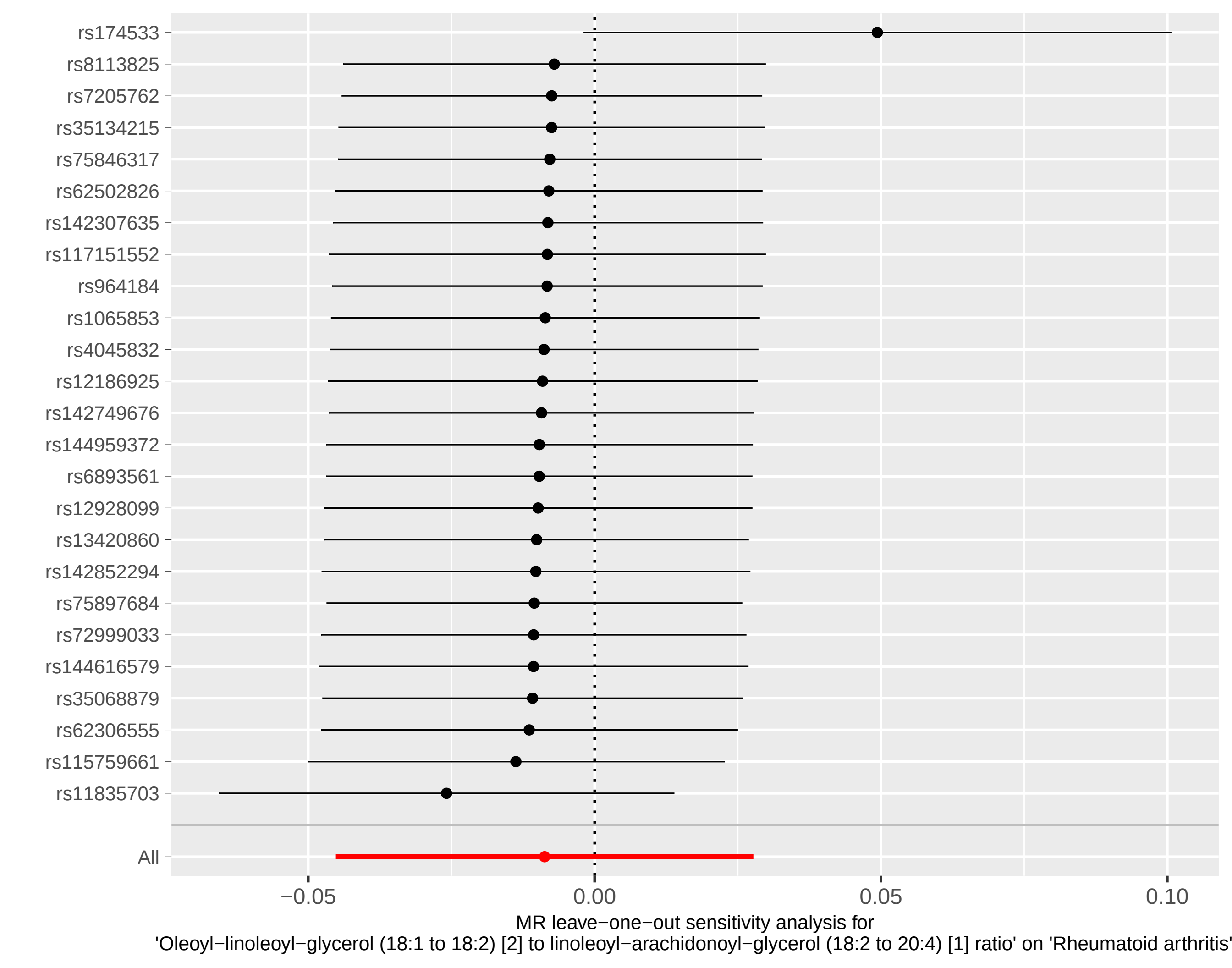
rs174533
rs8113825
rs7205762
rs35134215
rs75846317
rs62502826
rs142307635
rs117151552
rs964184
rs1065853
rs4045832
rs12186925
rs142749676
rs144959372
rs6893561
rs12928099
rs13420860
rs142852294
rs75897684
rs72999033
rs144616579
rs35068879
rs62306555
rs115759661
rs11835703
All
−0.05
0.00
0.05
0.10
MR leave−one−out sensitivity analysis for
'Oleoyl−linoleoyl−glycerol (18:1 to 18:2) [2] to linoleoyl−arachidonoyl−glycerol (18:2 to 20:4) [1] ratio' on 'Rheumatoid arthritis'

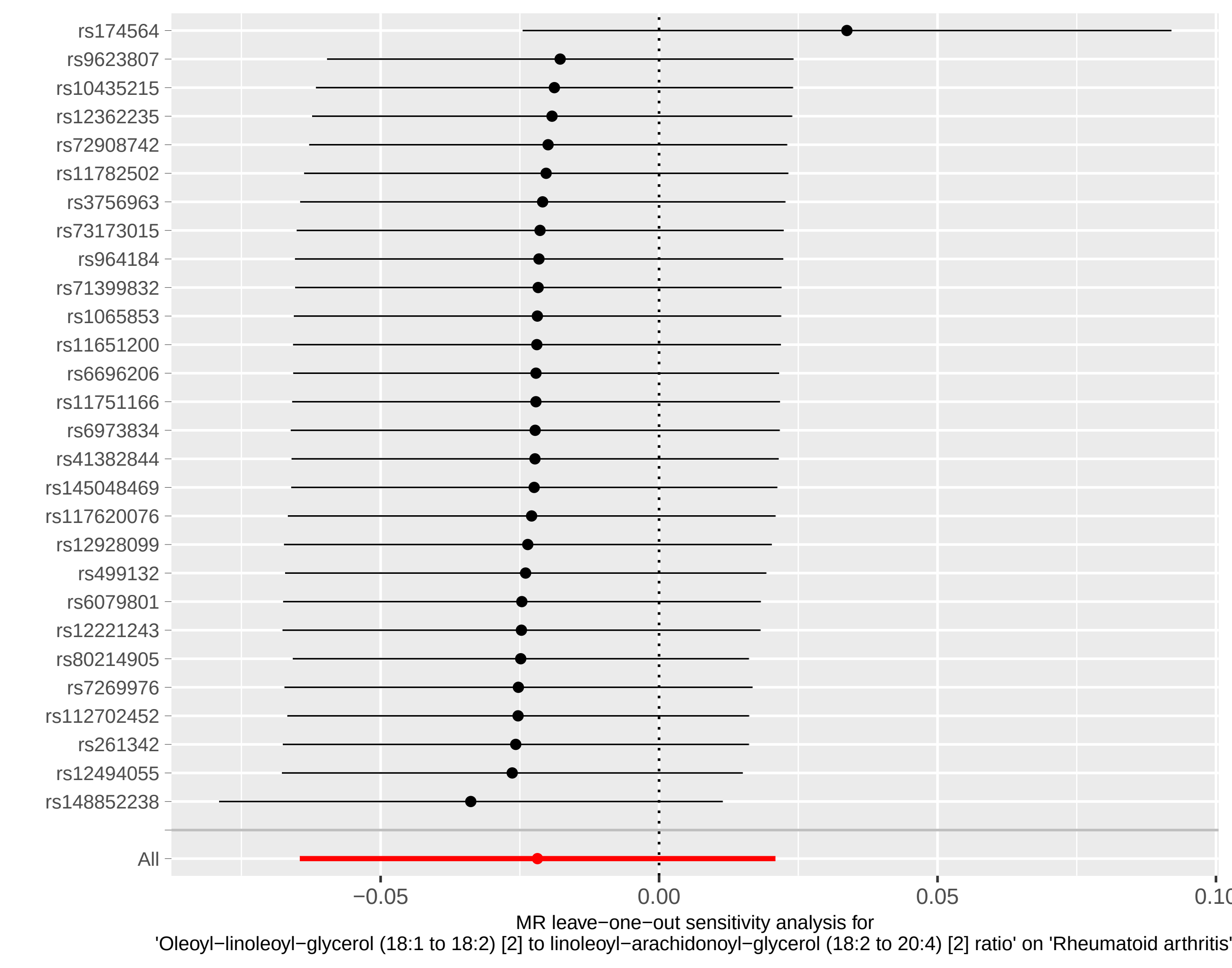
rs174564
rs9623807
rs10435215
rs12362235
rs72908742
rs11782502
rs3756963
rs73173015
rs964184
rs71399832
rs1065853
rs11651200
rs6696206
rs11751166
rs6973834
rs41382844
rs145048469
rs117620076
rs12928099
rs499132
rs6079801
rs12221243
rs80214905
rs7269976
rs112702452
rs261342
rs12494055
rs148852238
All
−0.05
0.00
0.05
0.10
MR leave−one−out sensitivity analysis for
'Oleoyl−linoleoyl−glycerol (18:1 to 18:2) [2] to linoleoyl−arachidonoyl−glycerol (18:2 to 20:4) [2] ratio' on 'Rheumatoid arthritis'

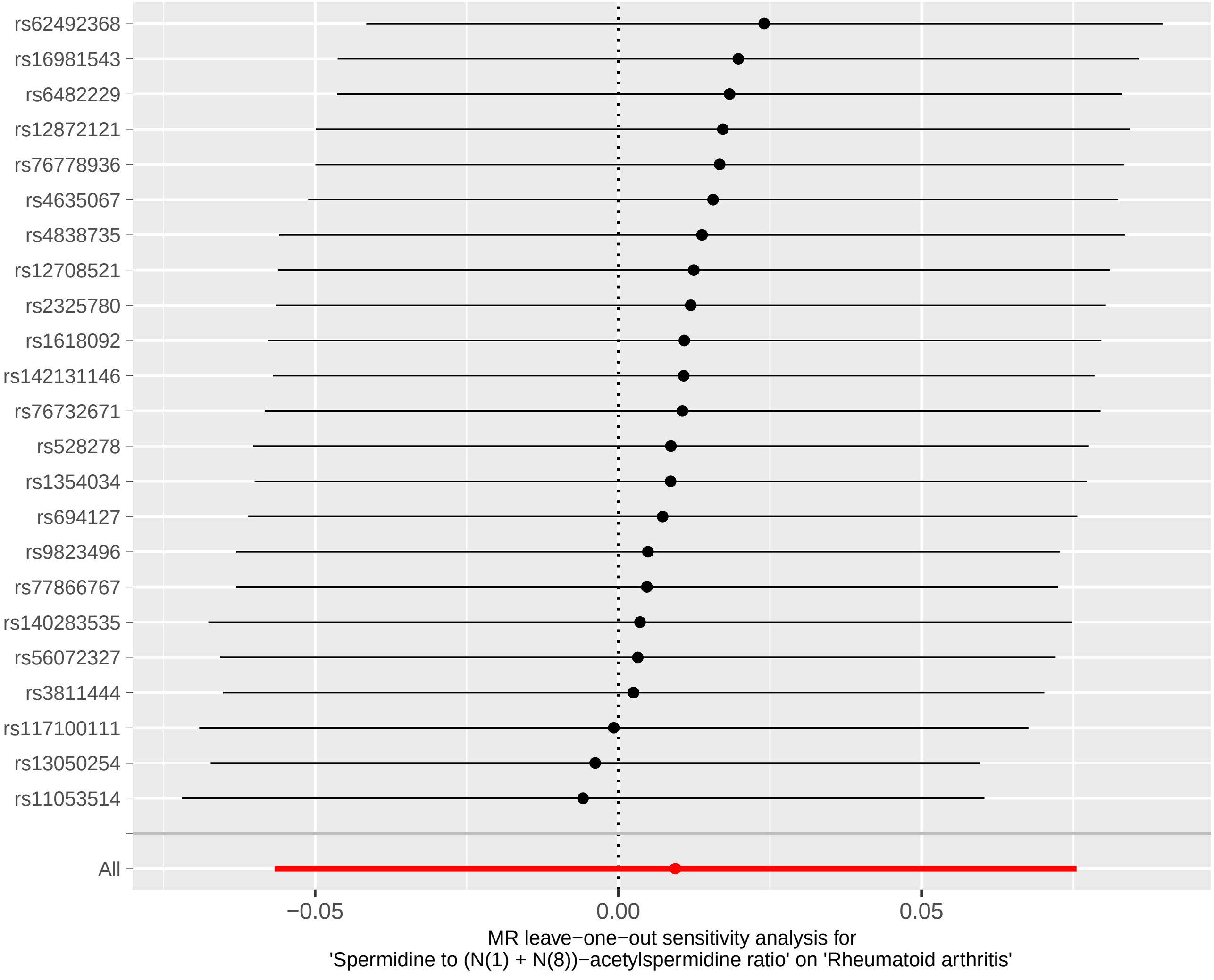
rs62492368
rs16981543
rs6482229
rs12872121
rs76778936
rs4635067
rs4838735
rs12708521
rs2325780
rs1618092
rs142131146
rs76732671
rs528278
rs1354034
rs694127
rs9823496
rs77866767
rs140283535
rs56072327
rs3811444
rs117100111
rs13050254
rs11053514
All
−0.05
0.00
0.05
MR leave−one−out sensitivity analysis for
'Spermidine to (N(1) + N(8))−acetylspermidine ratio' on 'Rheumatoid arthritis'

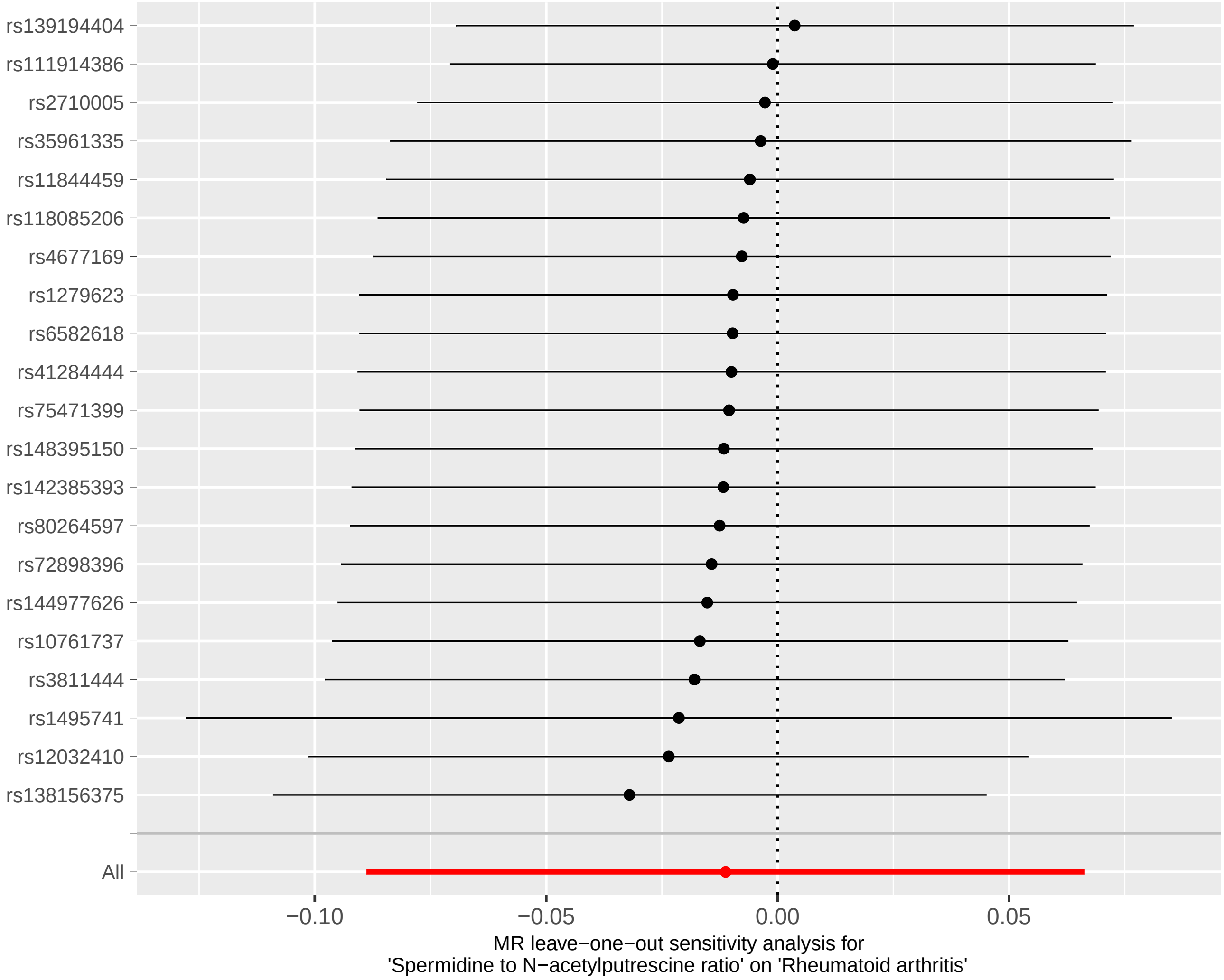
rs139194404
rs111914386
rs2710005
rs35961335
rs11844459
rs118085206
rs4677169
rs1279623
rs6582618
rs41284444
rs75471399
rs148395150
rs142385393
rs80264597
rs72898396
rs144977626
rs10761737
rs3811444
rs1495741
rs12032410
rs138156375
All
−0.10
−0.05
0.00
0.05
MR leave−one−out sensitivity analysis for
'Spermidine to N−acetylputrescine ratio' on 'Rheumatoid arthritis'

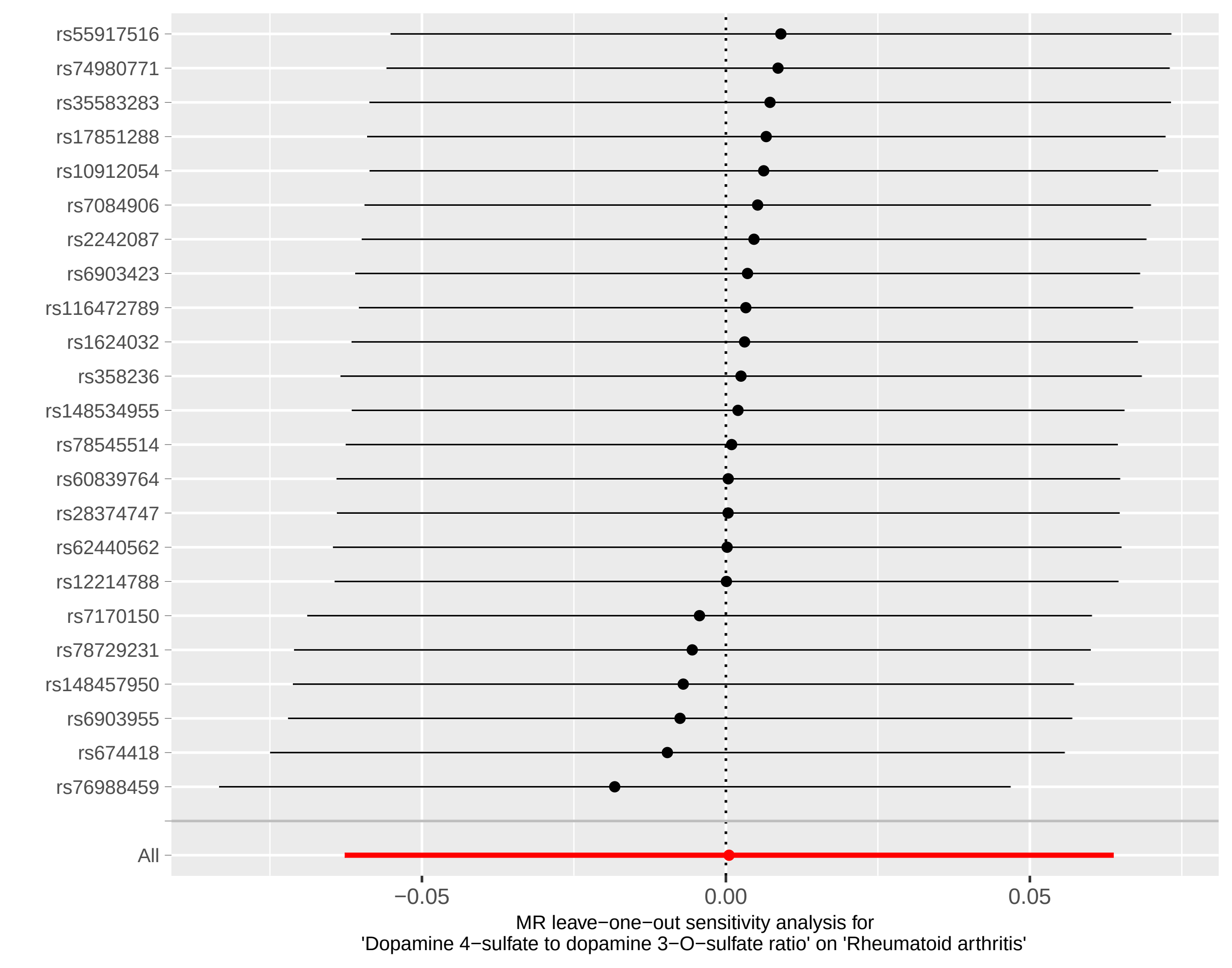
rs55917516
rs74980771
rs35583283
rs17851288
rs10912054
rs7084906
rs2242087
rs6903423
rs116472789
rs1624032
rs358236
rs148534955
rs78545514
rs60839764
rs28374747
rs62440562
rs12214788
rs7170150
rs78729231
rs148457950
rs6903955
rs674418
rs76988459
All
−0.05
0.00
0.05
MR leave−one−out sensitivity analysis for
'Dopamine 4−sulfate to dopamine 3−O−sulfate ratio' on 'Rheumatoid arthritis'

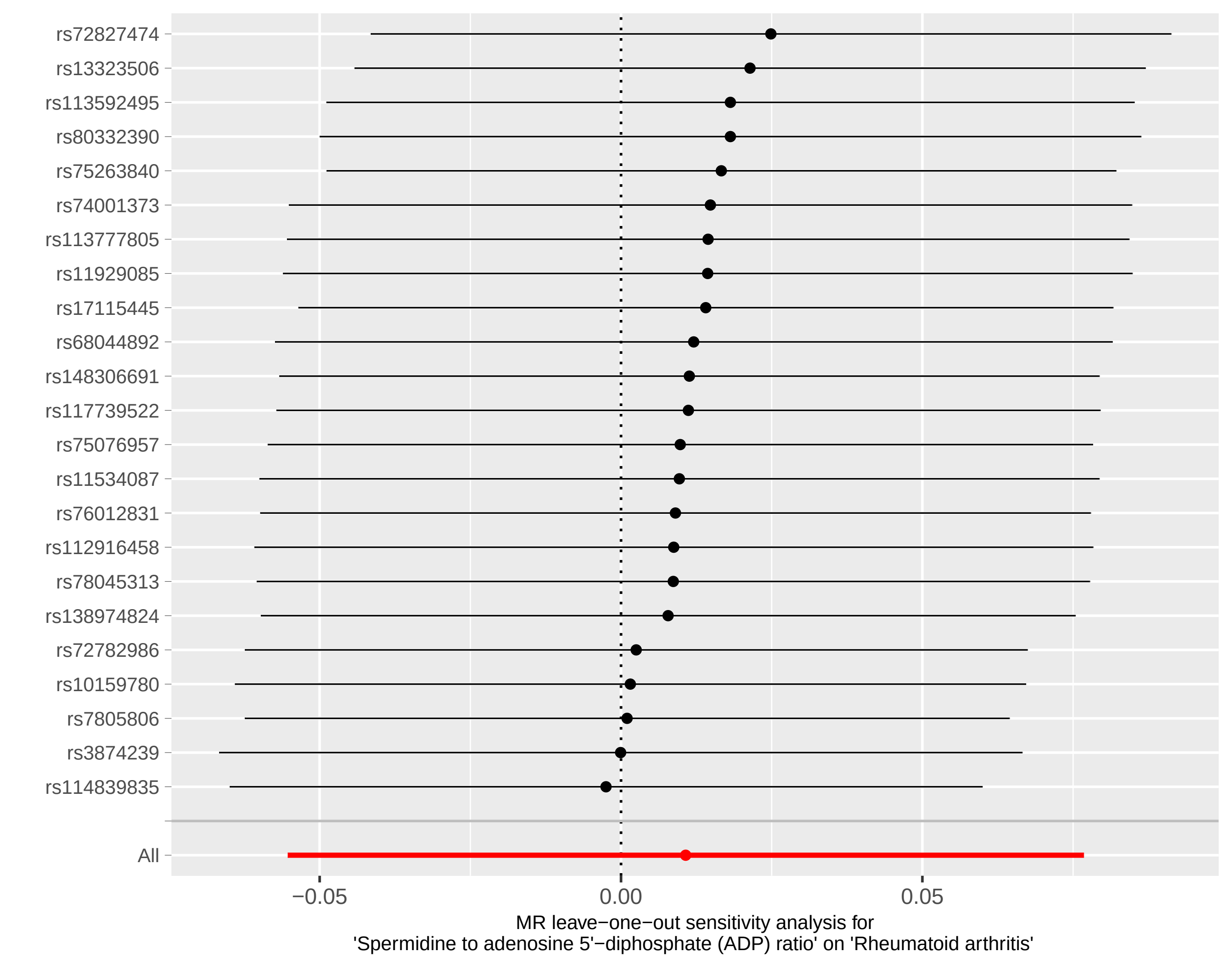
rs72827474
rs13323506
rs113592495
rs80332390
rs75263840
rs74001373
rs113777805
rs11929085
rs17115445
rs68044892
rs148306691
rs117739522
rs75076957
rs11534087
rs76012831
rs112916458
rs78045313
rs138974824
rs72782986
rs10159780
rs7805806
rs3874239
rs114839835
All
−0.05
0.00
0.05
MR leave−one−out sensitivity analysis for
'Spermidine to adenosine 5'−diphosphate (ADP) ratio' on 'Rheumatoid arthritis'

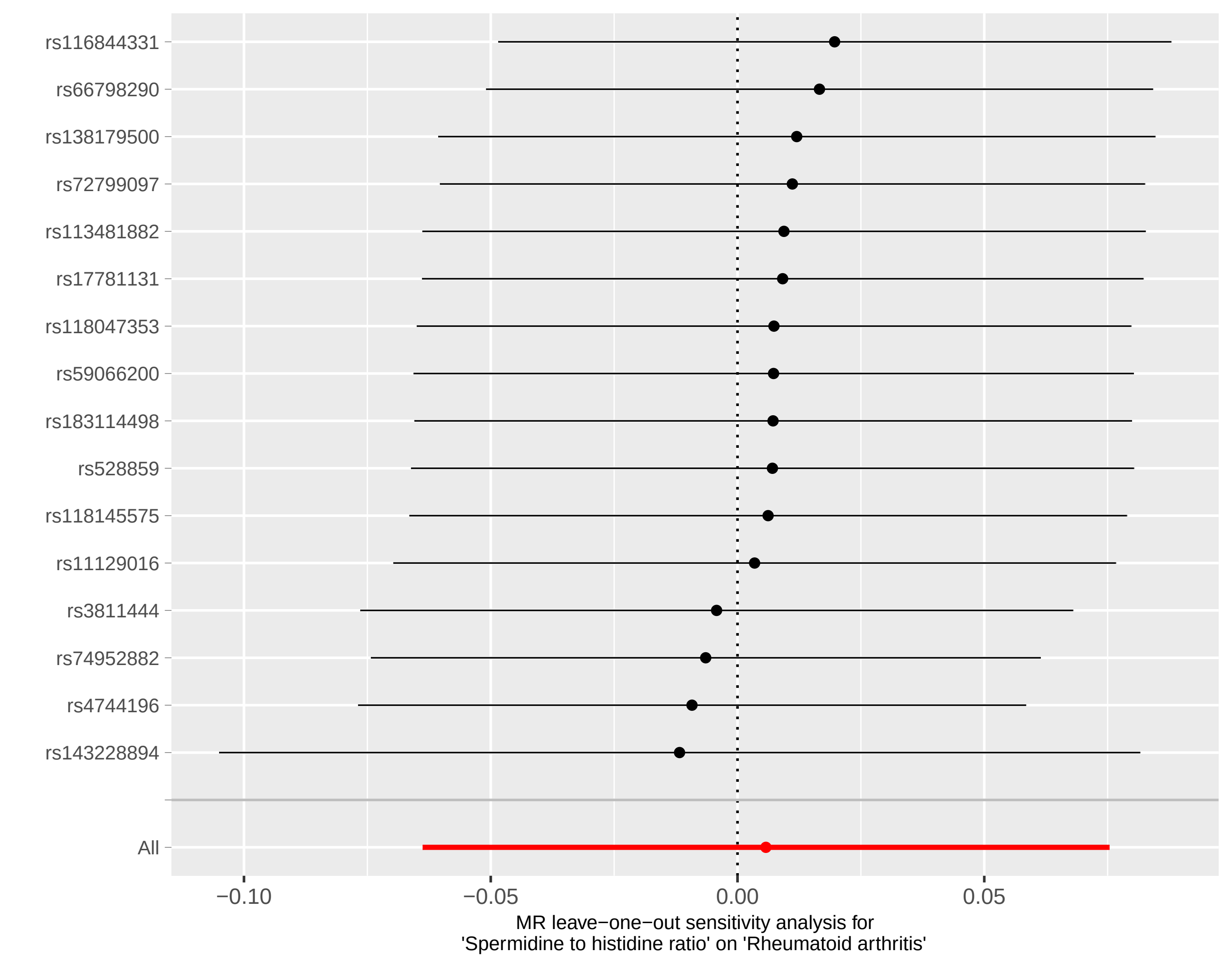
rs116844331
rs66798290
rs138179500
rs72799097
rs113481882
rs17781131
rs118047353
rs59066200
rs183114498
rs528859
rs118145575
rs11129016
rs3811444
rs74952882
rs4744196
rs143228894
All
−0.10
−0.05
0.00
0.05
MR leave−one−out sensitivity analysis for
'Spermidine to histidine ratio' on 'Rheumatoid arthritis'

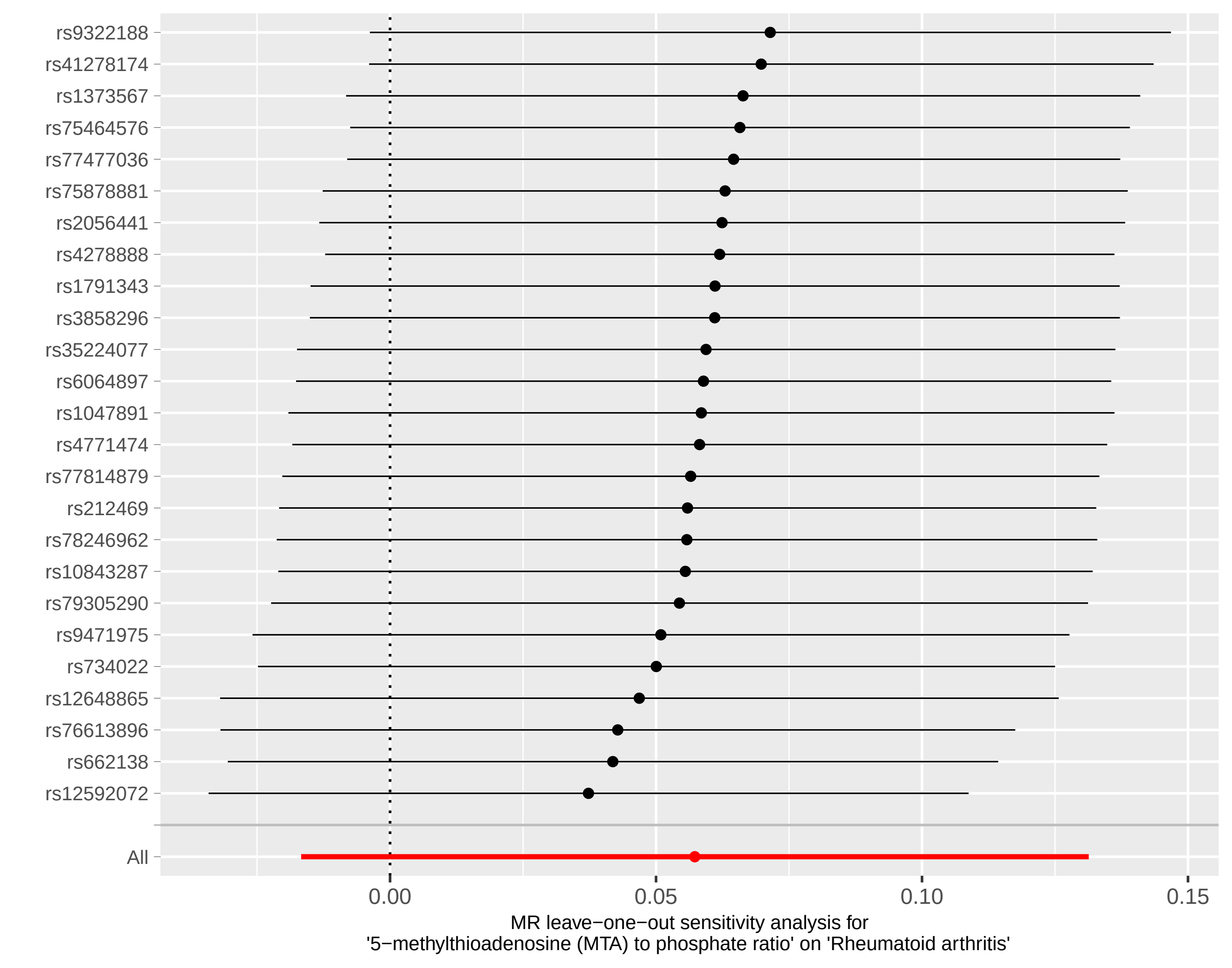
rs9322188
rs41278174
rs1373567
rs75464576
rs77477036
rs75878881
rs2056441
rs4278888
rs1791343
rs3858296
rs35224077
rs6064897
rs1047891
rs4771474
rs77814879
rs212469
rs78246962
rs10843287
rs79305290
rs9471975
rs734022
rs12648865
rs76613896
rs662138
rs12592072
All
0.00
0.05
0.10
0.15
MR leave−one−out sensitivity analysis for
'5−methylthioadenosine (MTA) to phosphate ratio' on 'Rheumatoid arthritis'

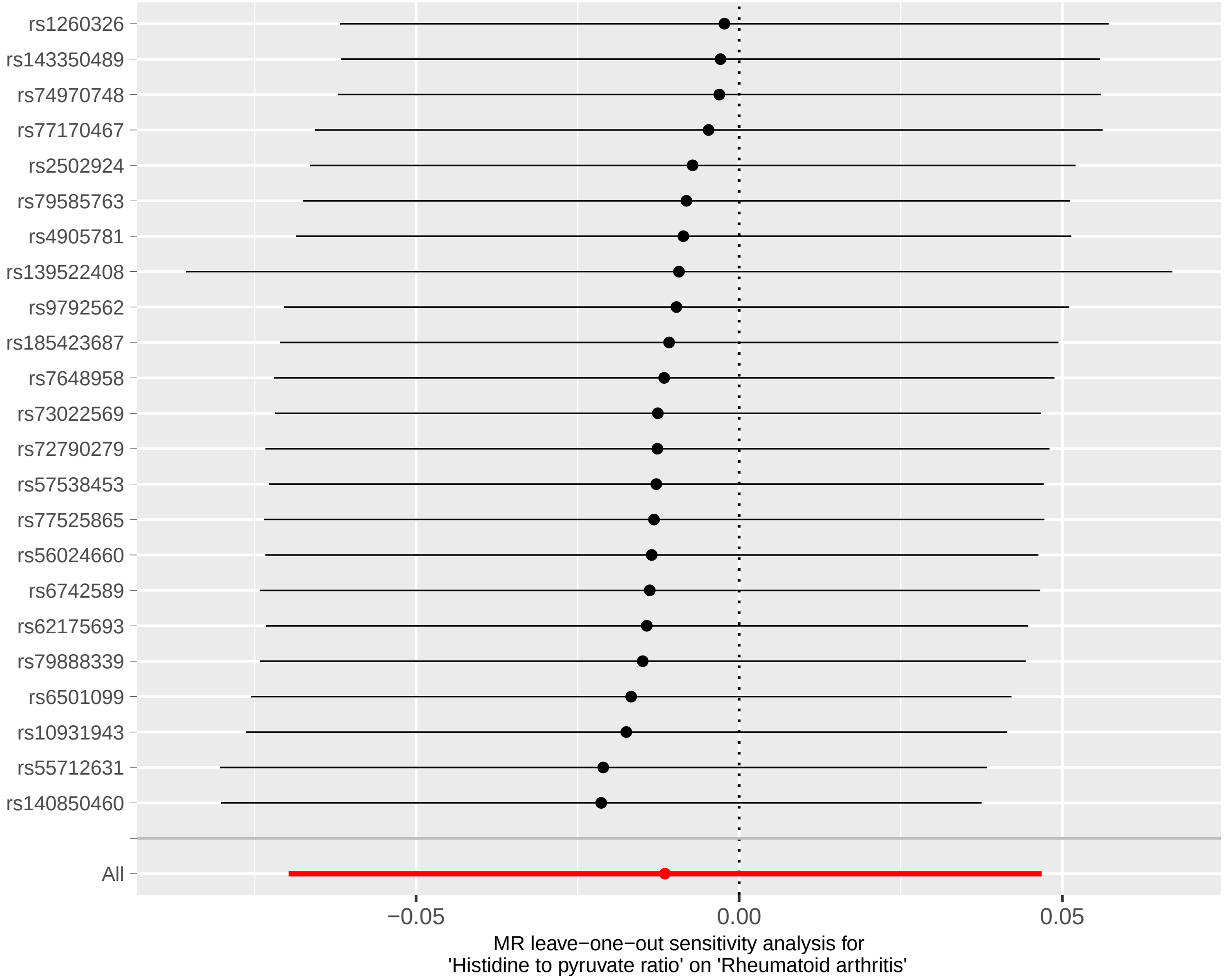

rs1260326
rs143350489
rs74970748
rs77170467
rs2502924
rs79585763
rs4905781
rs139522408
rs9792562
rs185423687
rs7648958
rs73022569
rs72790279
rs57538453
rs77525865
rs56024660
rs6742589
rs62175693
rs79888339
rs6501099
rs10931943
rs55712631
rs140850460
All
−0.05
0.00
0.05
MR leave−one−out sensitivity analysis for
'Histidine to pyruvate ratio' on 'Rheumatoid arthritis'

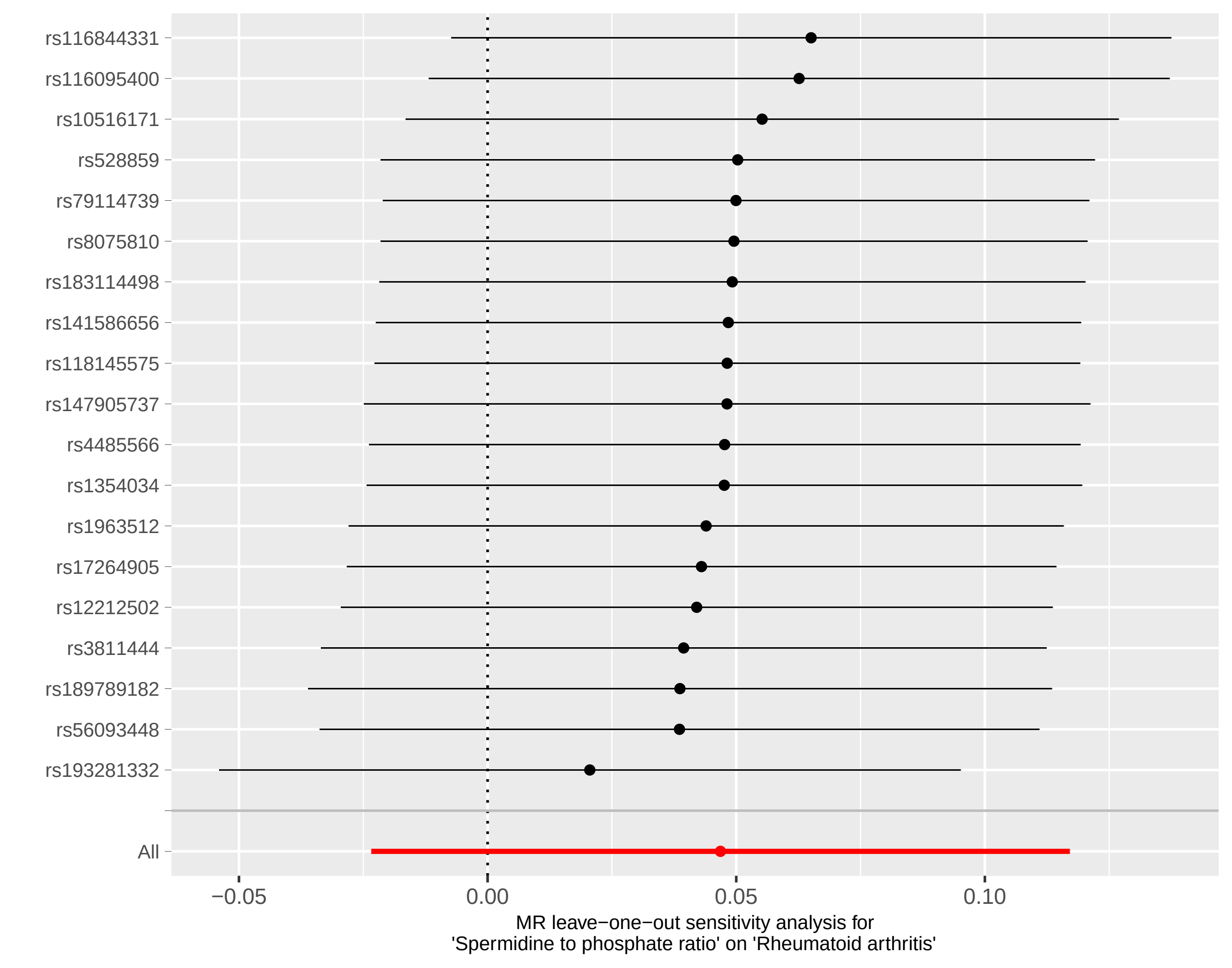

rs116844331
rs116095400
rs10516171
rs528859
rs79114739
rs8075810
rs183114498
rs141586656
rs118145575
rs147905737
rs4485566
rs1354034
rs1963512
rs17264905
rs12212502
rs3811444
rs189789182
rs56093448
rs193281332
All
−0.05
0.00
0.05
0.10
MR leave−one−out sensitivity analysis for
'Spermidine to phosphate ratio' on 'Rheumatoid arthritis'

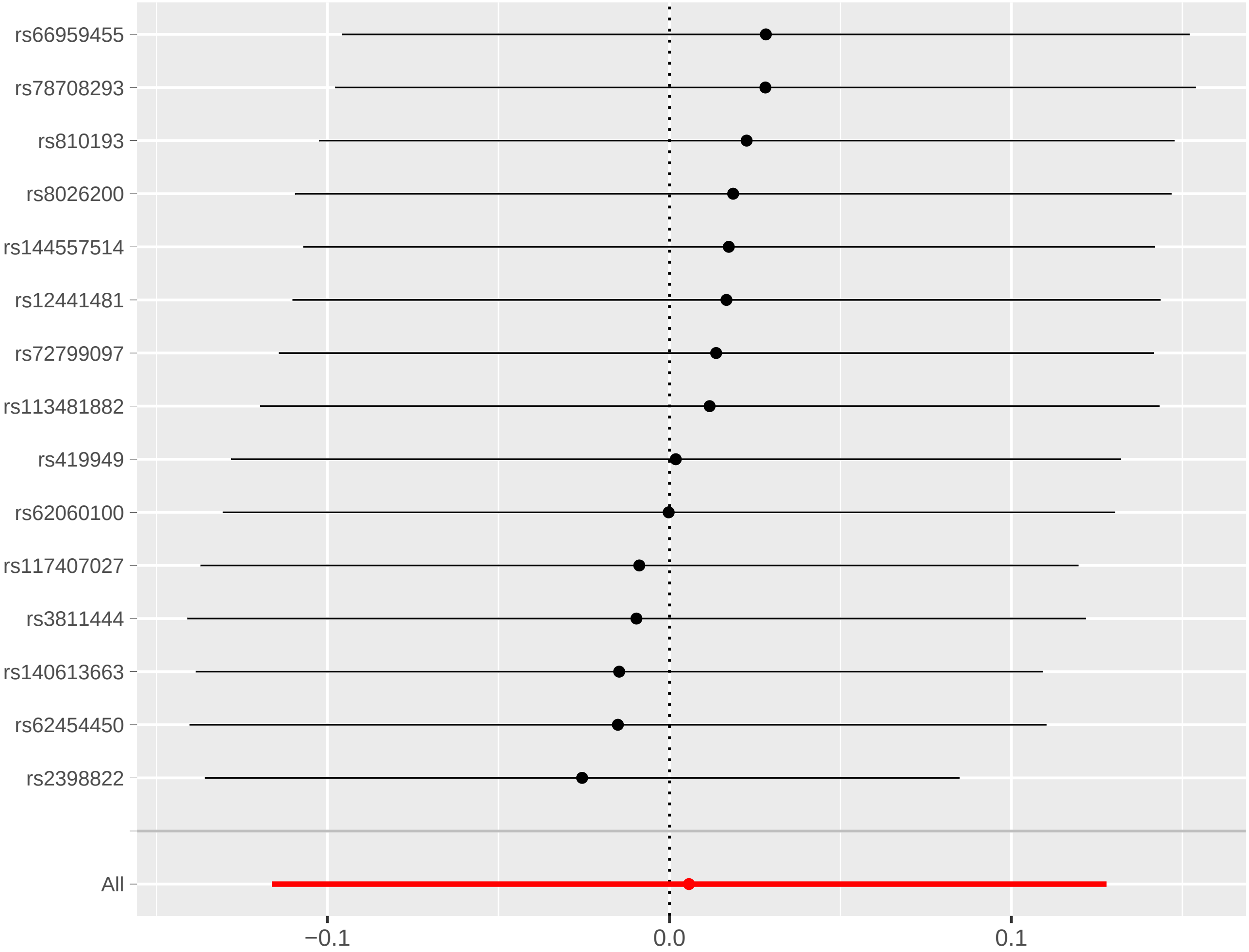

MR leave−one−out sensitivity analysis for
'Spermidine to pyruvate ratio' on 'Rheumatoid arthritis'

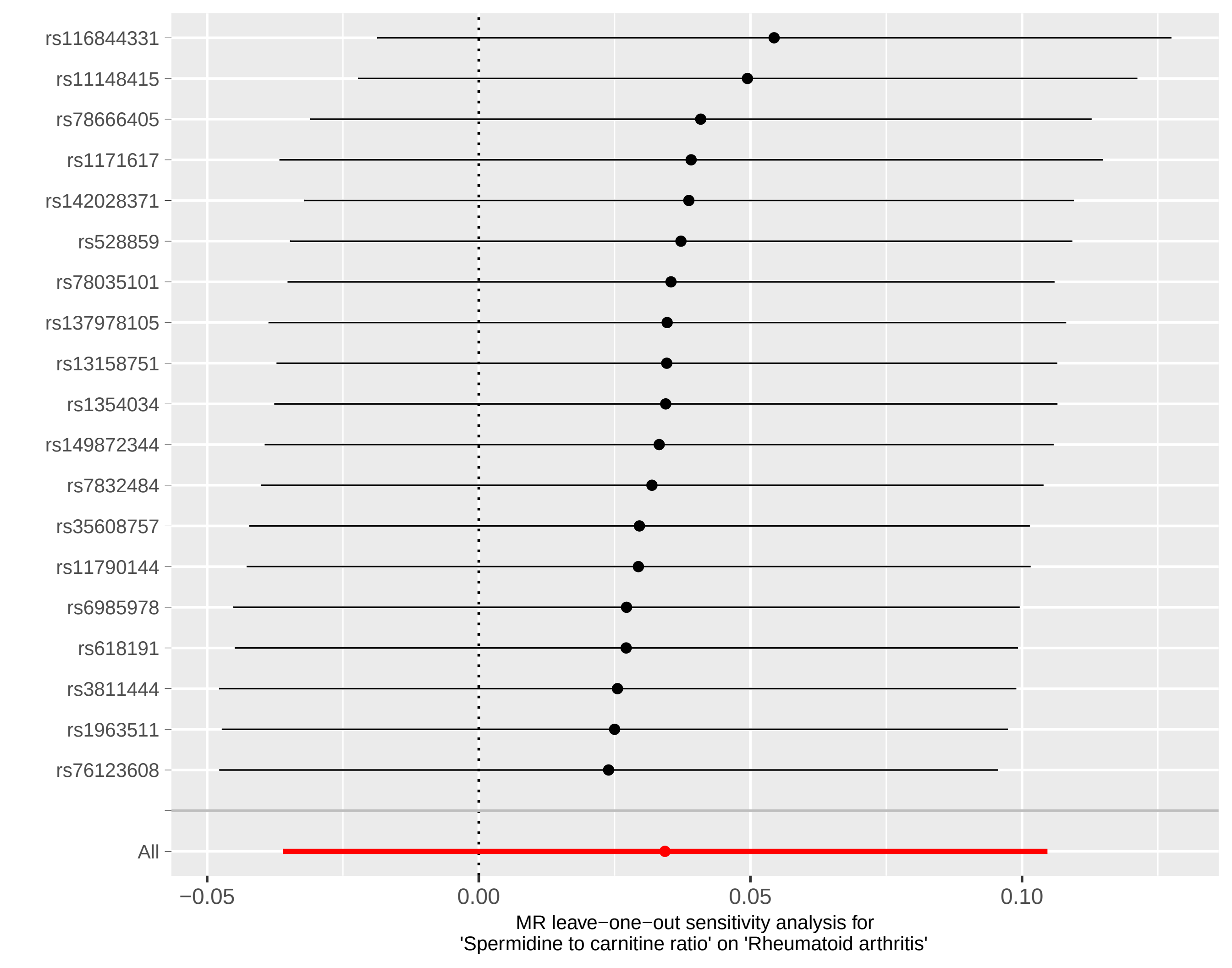
rs116844331
rs11148415
rs78666405
rs1171617
rs142028371
rs528859
rs78035101
rs137978105
rs13158751
rs1354034
rs149872344
rs7832484
rs35608757
rs11790144
rs6985978
rs618191
rs3811444
rs1963511
rs76123608
All
−0.05
0.00
0.05
0.10
MR leave−one−out sensitivity analysis for
'Spermidine to carnitine ratio' on 'Rheumatoid arthritis'

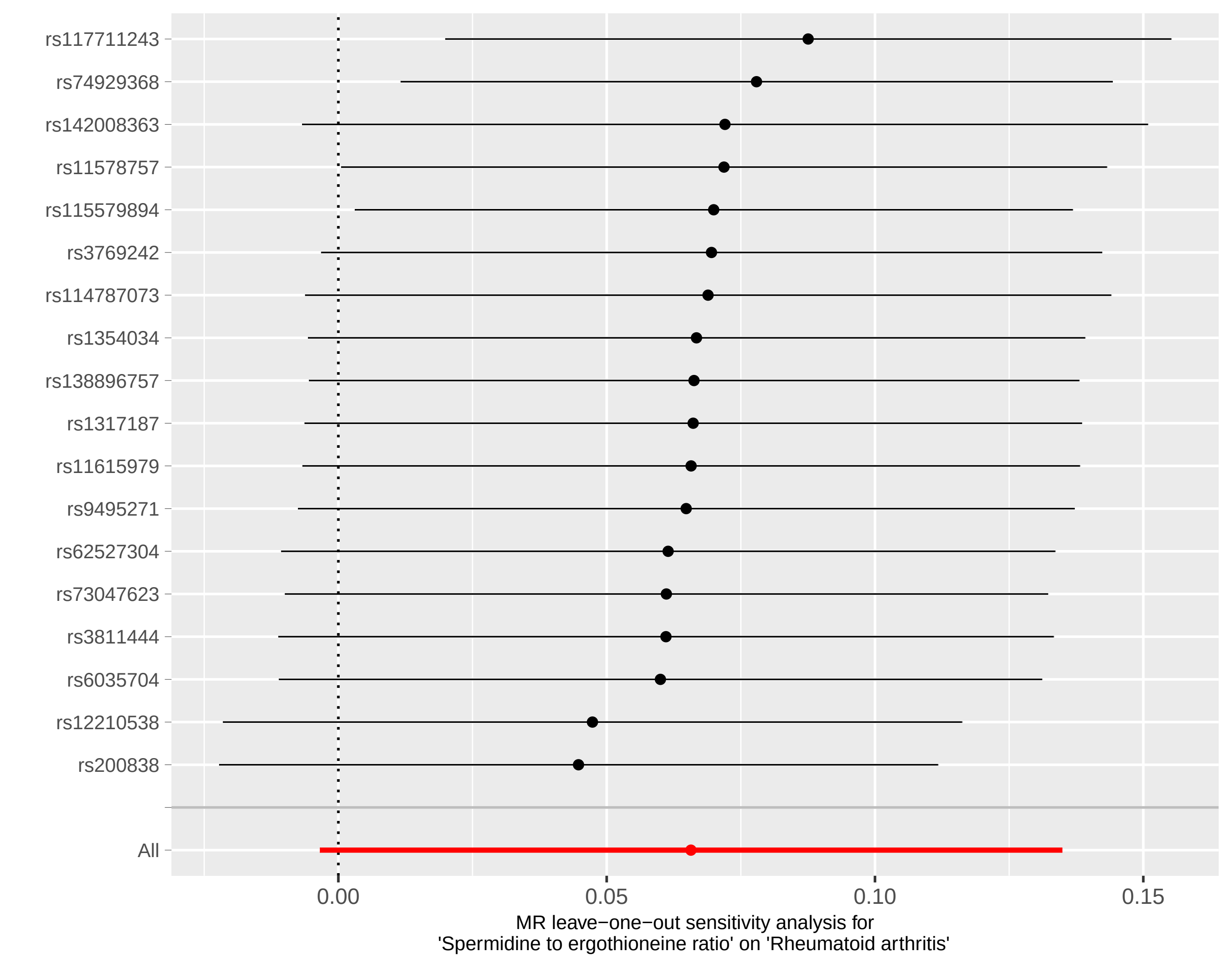
rs117711243
rs74929368
rs142008363
rs11578757
rs115579894
rs3769242
rs114787073
rs1354034
rs138896757
rs1317187
rs11615979
rs9495271
rs62527304
rs73047623
rs3811444
rs6035704
rs12210538
rs200838
All
0.00
0.05
0.10
0.15
MR leave−one−out sensitivity analysis for
'Spermidine to ergothioneine ratio' on 'Rheumatoid arthritis'

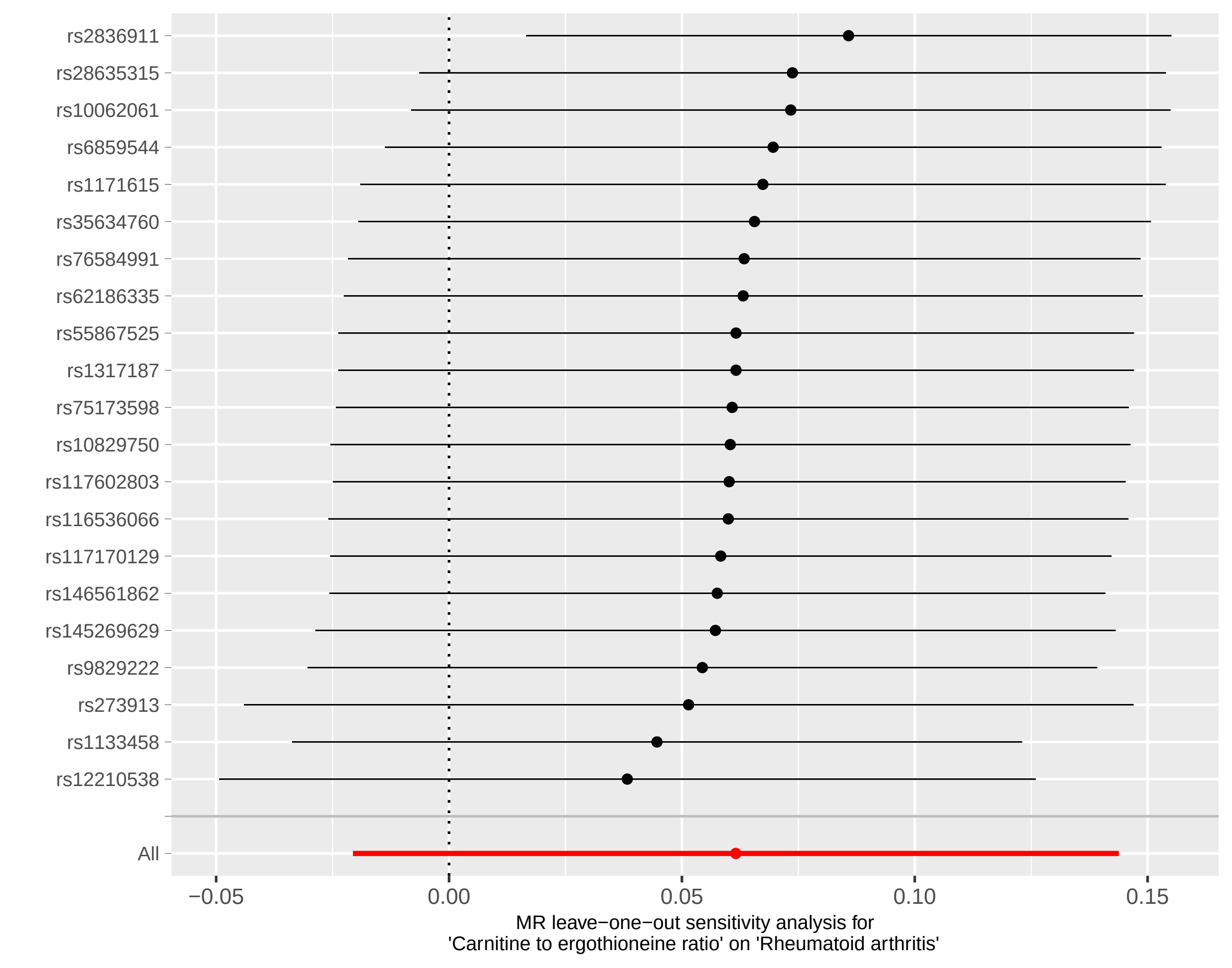

rs2836911
rs28635315
rs10062061
rs6859544
rs1171615
rs35634760
rs76584991
rs62186335
rs55867525
rs1317187
rs75173598
rs10829750
rs117602803
rs116536066
rs117170129
rs146561862
rs145269629
rs9829222
rs273913
rs1133458
rs12210538
All
−0.05
0.00
0.05
0.10
0.15
MR leave−one−out sensitivity analysis for
'Carnitine to ergothioneine ratio' on 'Rheumatoid arthritis'

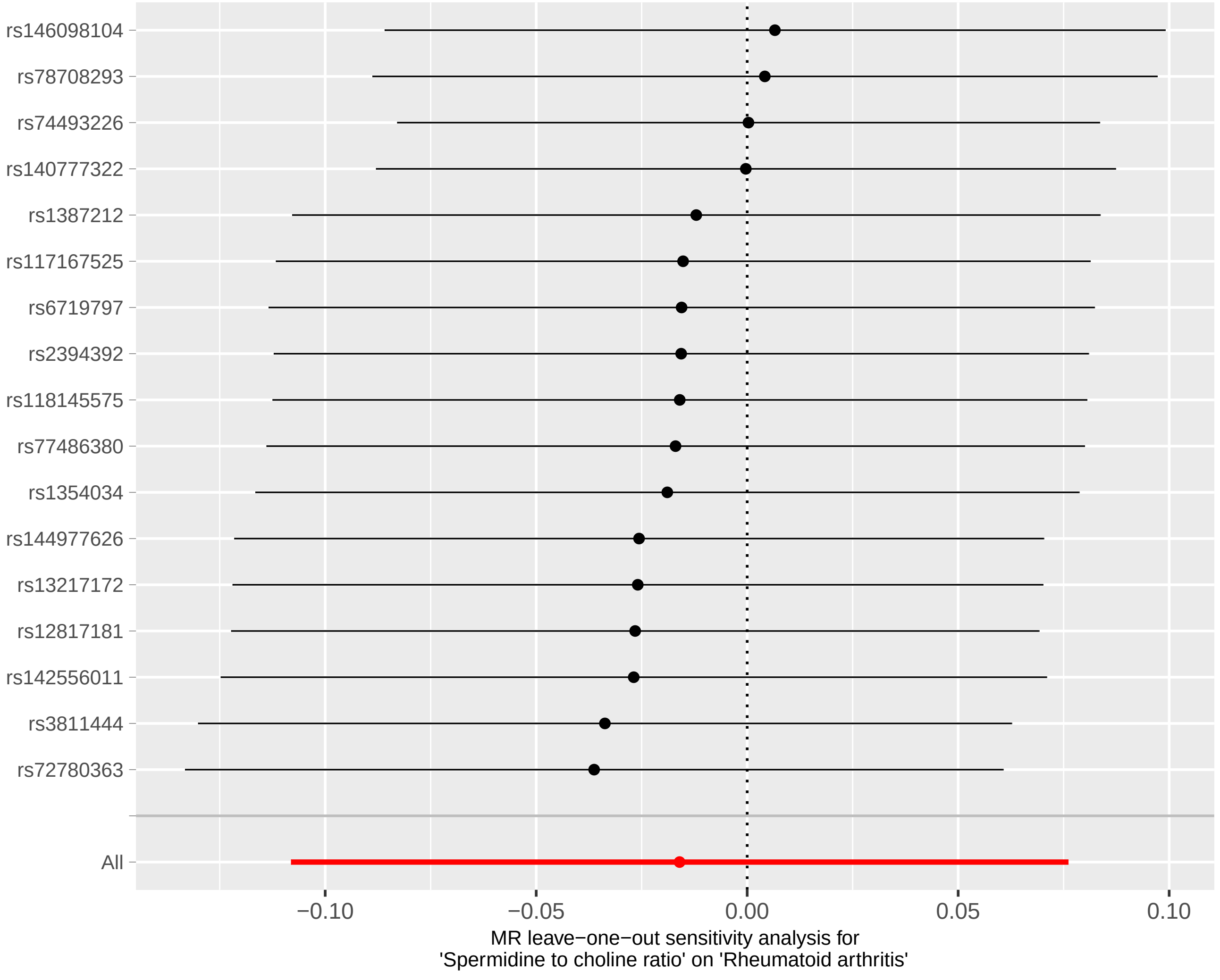
rs146098104
rs78708293
rs74493226
rs140777322
rs1387212
rs117167525
rs6719797
rs2394392
rs118145575
rs77486380
rs1354034
rs144977626
rs13217172
rs12817181
rs142556011
rs3811444
rs72780363
All
−0.10
−0.05
0.00
0.05
0.10
MR leave−one−out sensitivity analysis for
'Spermidine to choline ratio' on 'Rheumatoid arthritis'

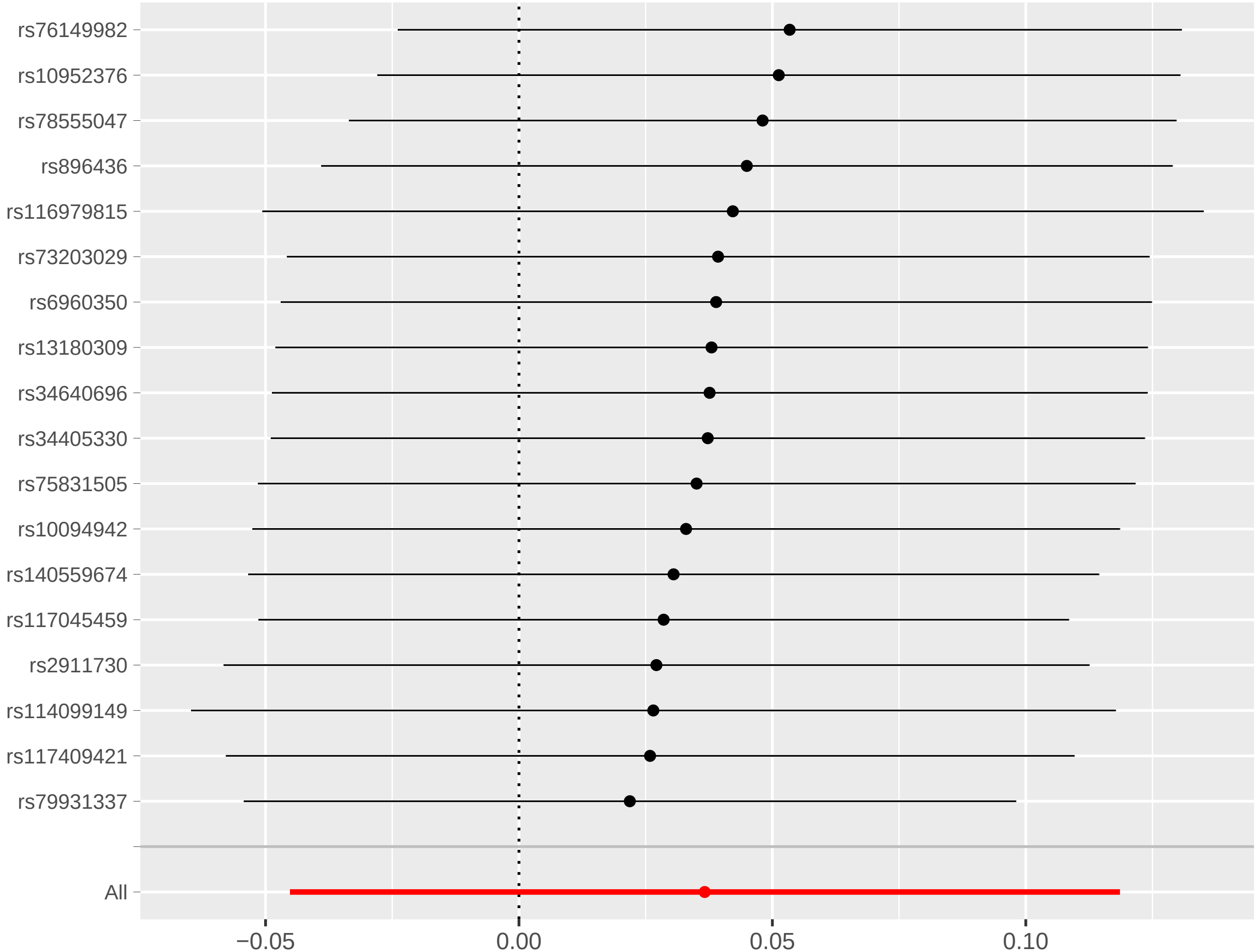

MR leave−one−out sensitivity analysis for
'Spermidine to taurocholate ratio' on 'Rheumatoid arthritis'

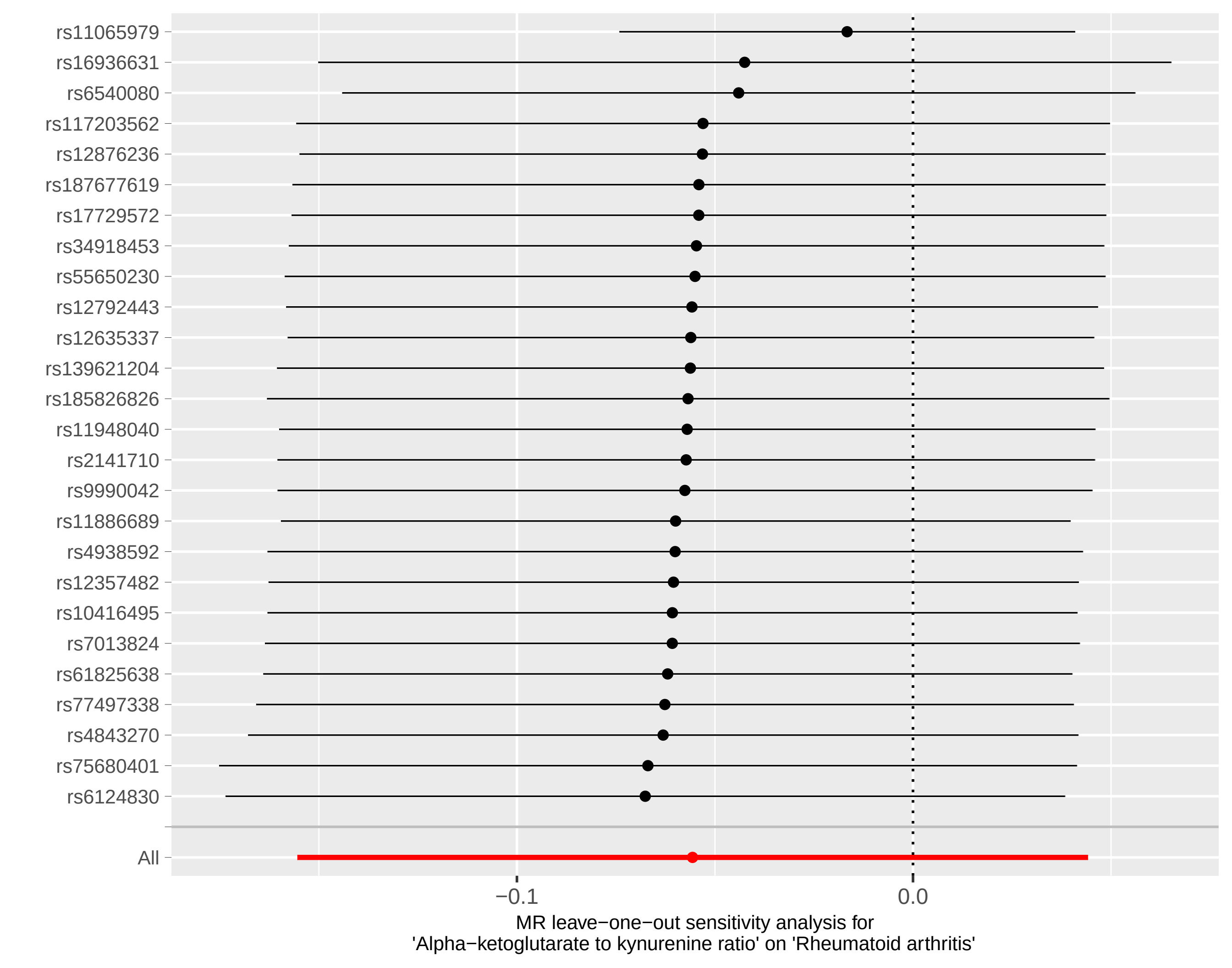
rs11065979
rs16936631
rs6540080
rs117203562
rs12876236
rs187677619
rs17729572
rs34918453
rs55650230
rs12792443
rs12635337
rs139621204
rs185826826
rs11948040
rs2141710
rs9990042
rs11886689
rs4938592
rs12357482
rs10416495
rs7013824
rs61825638
rs77497338
rs4843270
rs75680401
rs6124830
All
−0.1
0.0
MR leave−one−out sensitivity analysis for
'Alpha−ketoglutarate to kynurenine ratio' on 'Rheumatoid arthritis'

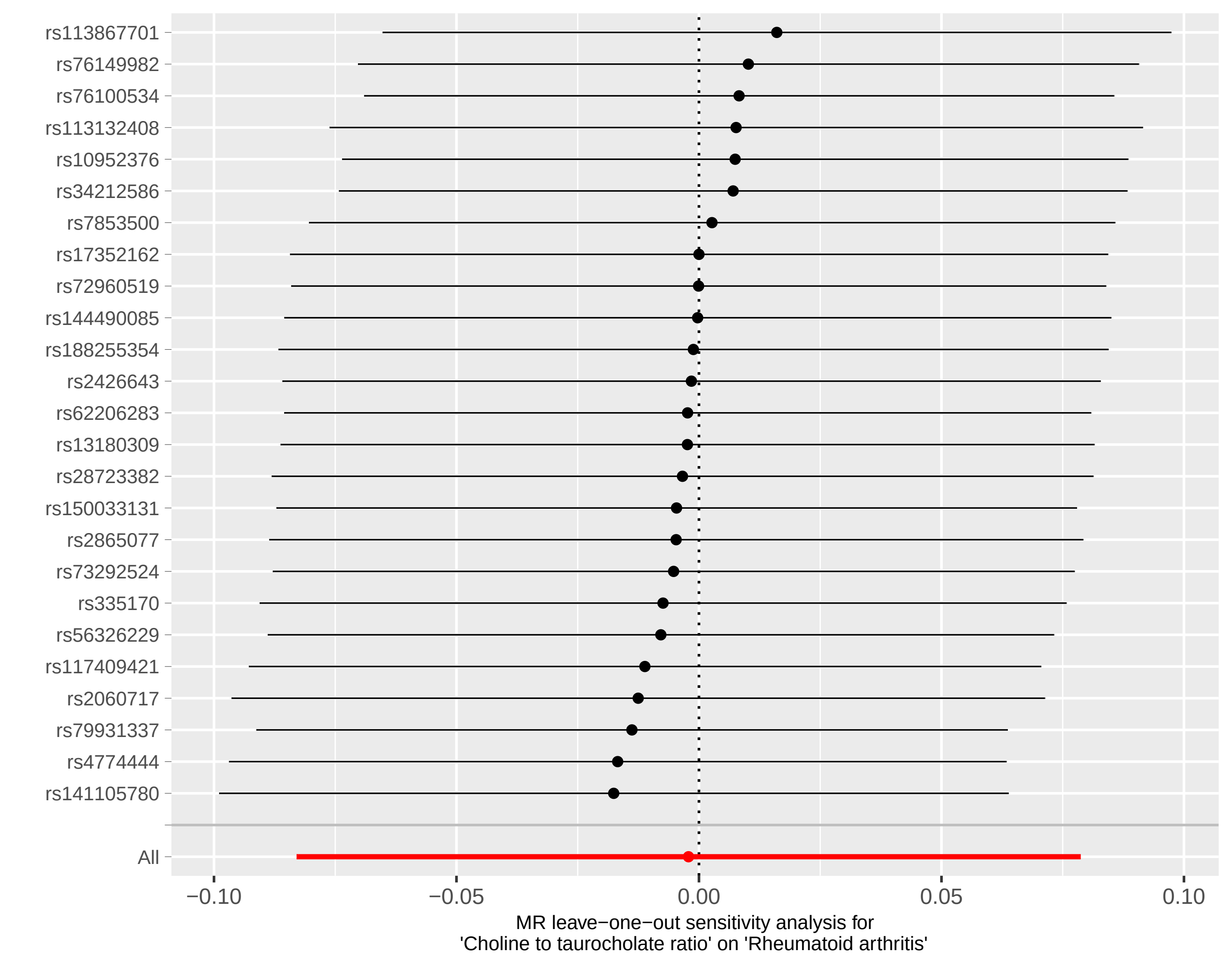
rs113867701
rs76149982
rs76100534
rs113132408
rs10952376
rs34212586
rs7853500
rs17352162
rs72960519
rs144490085
rs188255354
rs2426643
rs62206283
rs13180309
rs28723382
rs150033131
rs2865077
rs73292524
rs335170
rs56326229
rs117409421
rs2060717
rs79931337
rs4774444
rs141105780
All
−0.10
−0.05
0.00
0.05
0.10
MR leave−one−out sensitivity analysis for
'Choline to taurocholate ratio' on 'Rheumatoid arthritis'

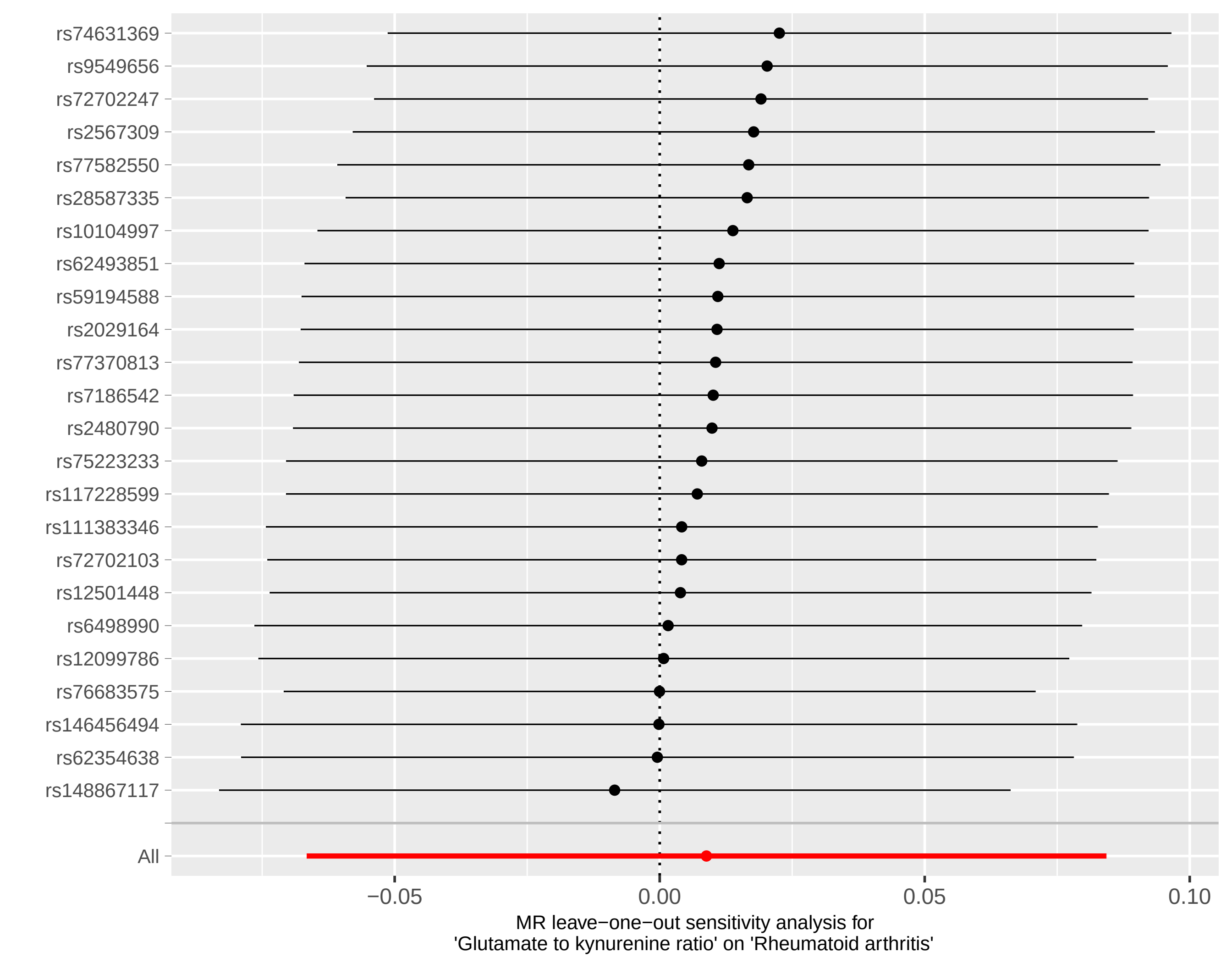
rs74631369
rs9549656
rs72702247
rs2567309
rs77582550
rs28587335
rs10104997
rs62493851
rs59194588
rs2029164
rs77370813
rs7186542
rs2480790
rs75223233
rs117228599
rs111383346
rs72702103
rs12501448
rs6498990
rs12099786
rs76683575
rs146456494
rs62354638
rs148867117
All
−0.05
0.00
0.05
0.10
MR leave−one−out sensitivity analysis for
'Glutamate to kynurenine ratio' on 'Rheumatoid arthritis'

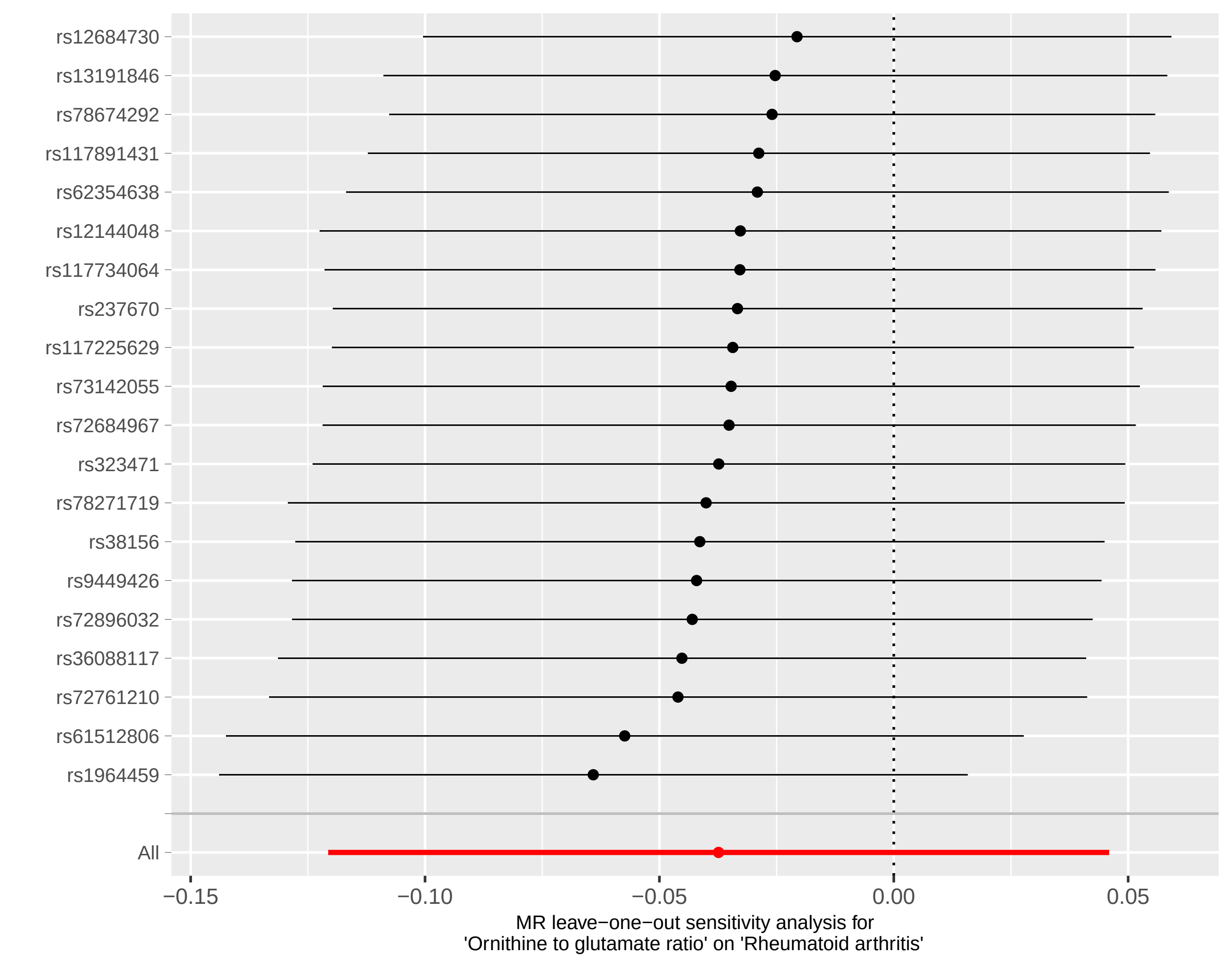
rs12684730
rs13191846
rs78674292
rs117891431
rs62354638
rs12144048
rs117734064
rs237670
rs117225629
rs73142055
rs72684967
rs323471
rs78271719
rs38156
rs9449426
rs72896032
rs36088117
rs72761210
rs61512806
rs1964459
All
−0.15
−0.10
−0.05
0.00
0.05
MR leave−one−out sensitivity analysis for
'Ornithine to glutamate ratio' on 'Rheumatoid arthritis'

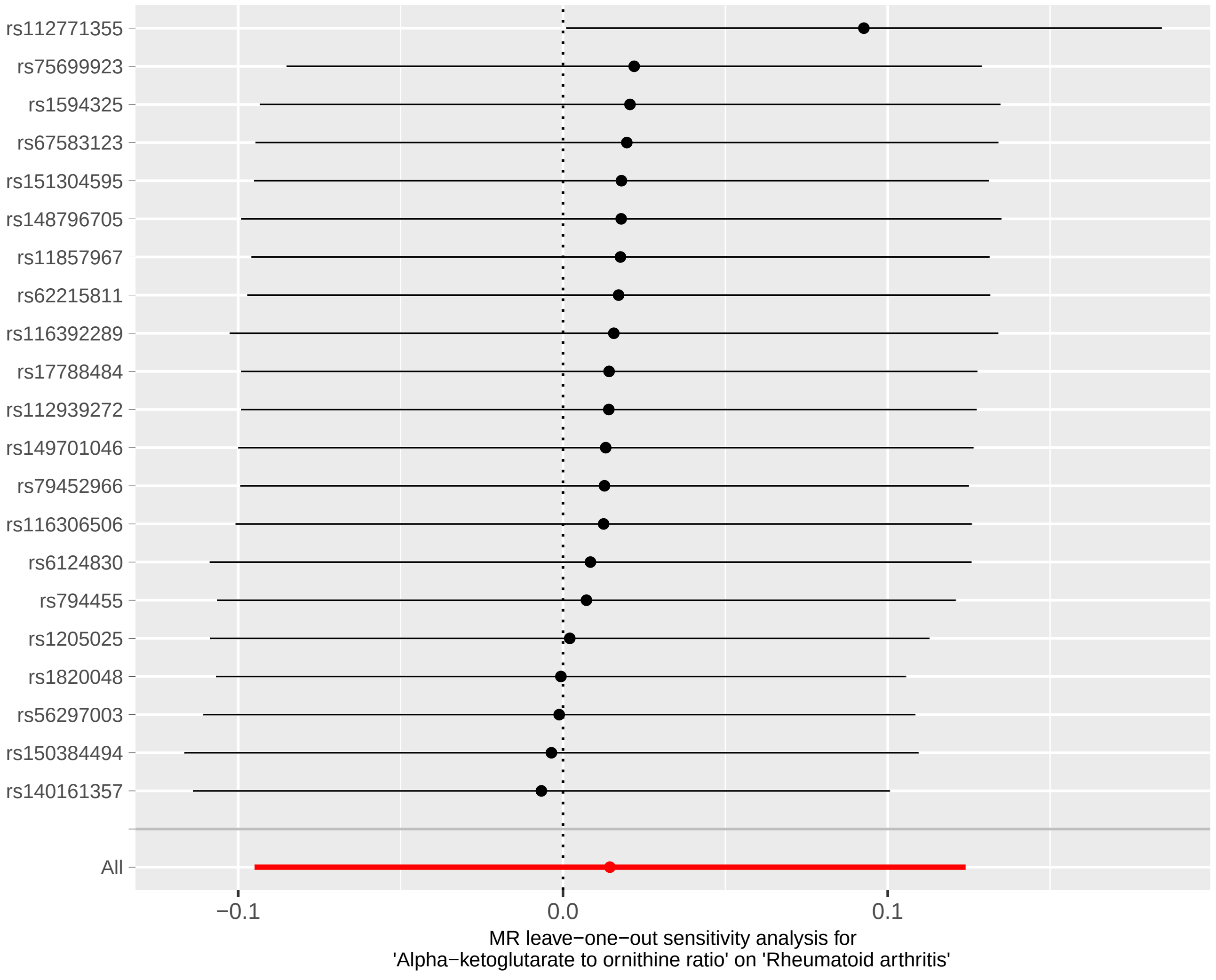
rs112771355
rs75699923
rs1594325
rs67583123
rs151304595
rs148796705
rs11857967
rs62215811
rs116392289
rs17788484
rs112939272
rs149701046
rs79452966
rs116306506
rs6124830
rs794455
rs1205025
rs1820048
rs56297003
rs150384494
rs140161357
All
−0.1
0.0
0.1
MR leave−one−out sensitivity analysis for
'Alpha−ketoglutarate to ornithine ratio' on 'Rheumatoid arthritis'

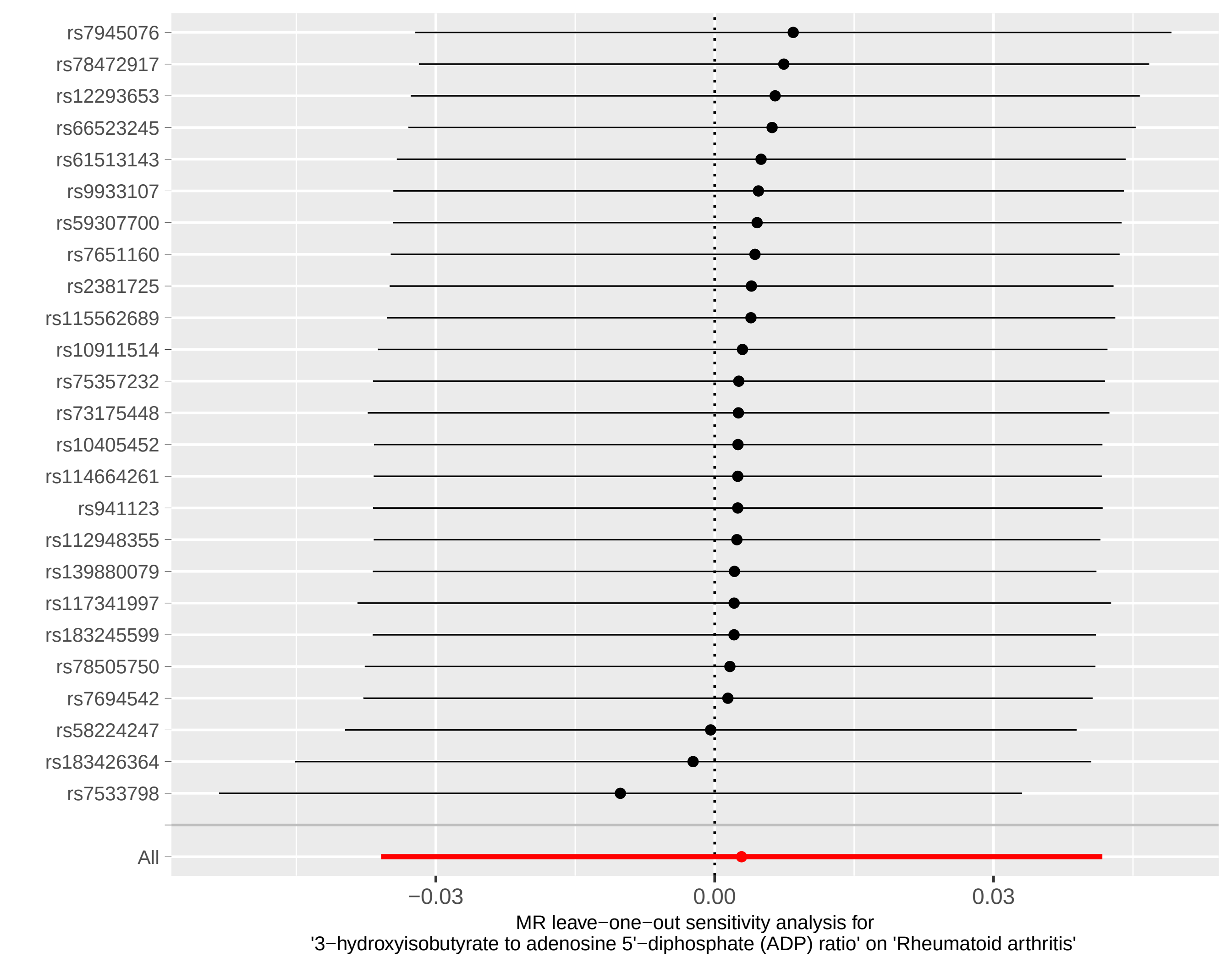
rs7945076
rs78472917
rs12293653
rs66523245
rs61513143
rs9933107
rs59307700
rs7651160
rs2381725
rs115562689
rs10911514
rs75357232
rs73175448
rs10405452
rs114664261
rs941123
rs112948355
rs139880079
rs117341997
rs183245599
rs78505750
rs7694542
rs58224247
rs183426364
rs7533798
All
−0.03
0.00
0.03
MR leave−one−out sensitivity analysis for
'3−hydroxyisobutyrate to adenosine 5'−diphosphate (ADP) ratio' on 'Rheumatoid arthritis'

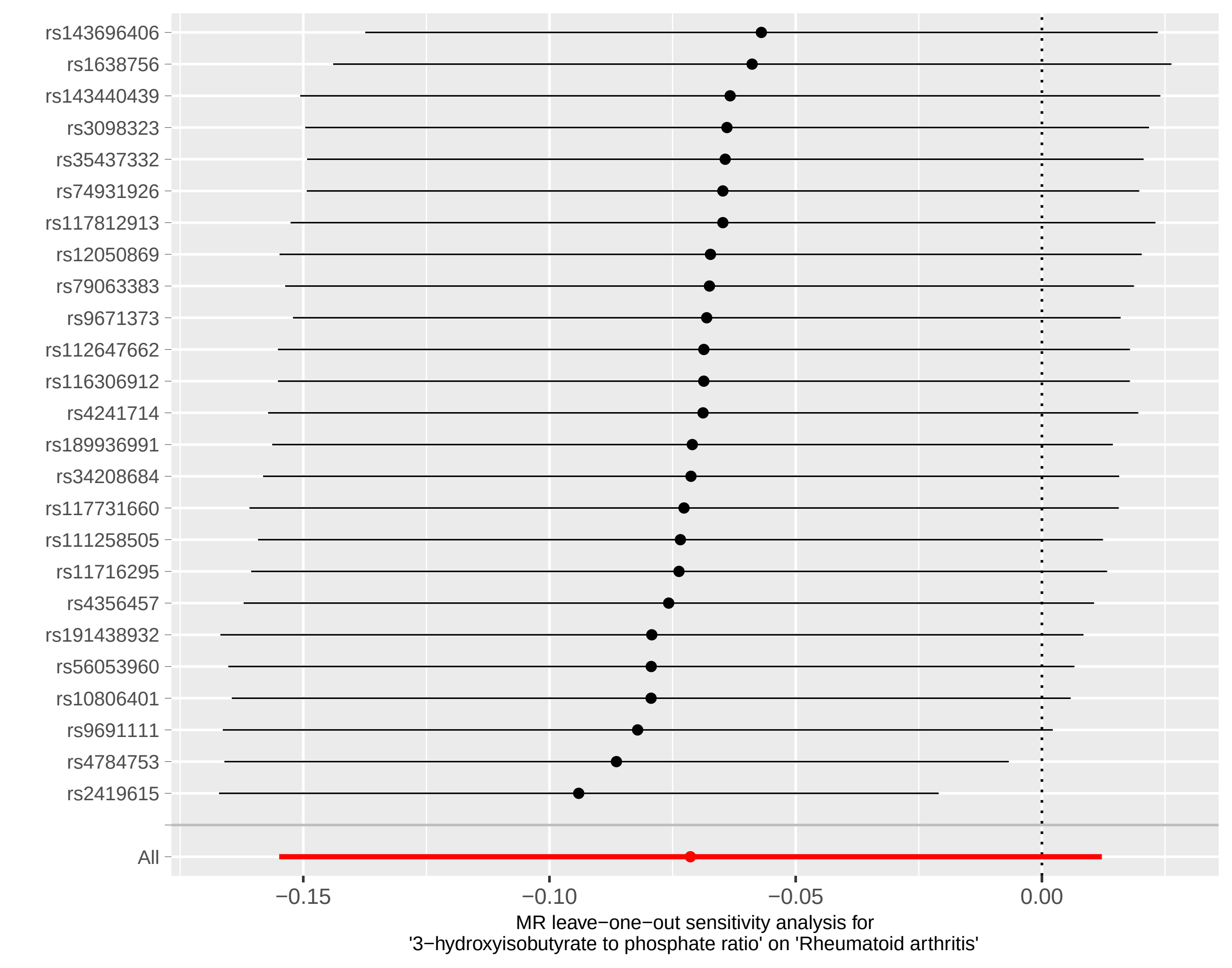

rs143696406
rs1638756
rs143440439
rs3098323
rs35437332
rs74931926
rs117812913
rs12050869
rs79063383
rs9671373
rs112647662
rs116306912
rs4241714
rs189936991
rs34208684
rs117731660
rs111258505
rs11716295
rs4356457
rs191438932
rs56053960
rs10806401
rs9691111
rs4784753
rs2419615
All
−0.15
−0.10
−0.05
0.00
MR leave−one−out sensitivity analysis for
'3−hydroxyisobutyrate to phosphate ratio' on 'Rheumatoid arthritis'

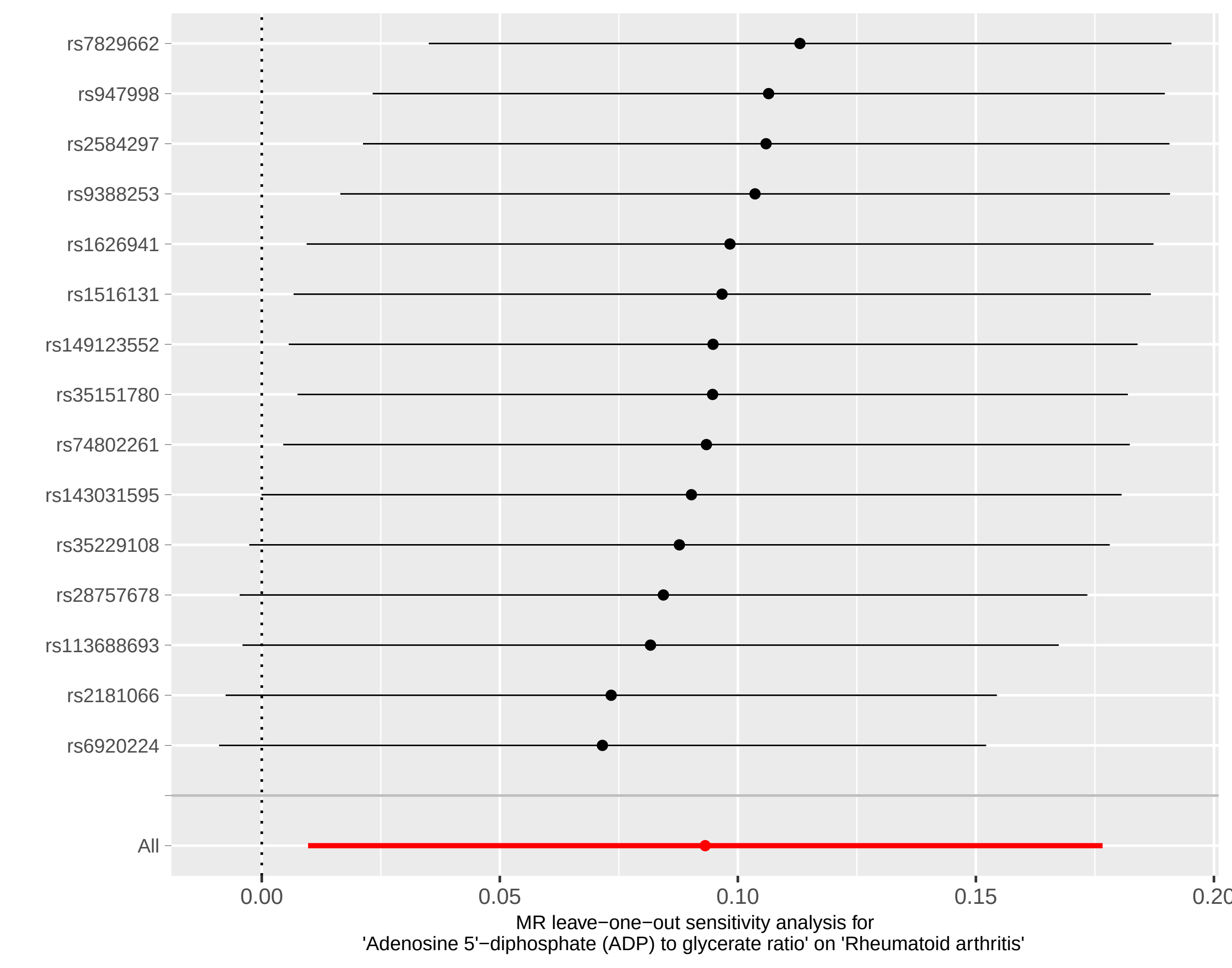
rs7829662
rs947998
rs2584297
rs9388253
rs1626941
rs1516131
rs149123552
rs35151780
rs74802261
rs143031595
rs35229108
rs28757678
rs113688693
rs2181066
rs6920224
All
0.00
0.05
0.10
0.15
0.20
MR leave−one−out sensitivity analysis for
'Adenosine 5'−diphosphate (ADP) to glycerate ratio' on 'Rheumatoid arthritis'

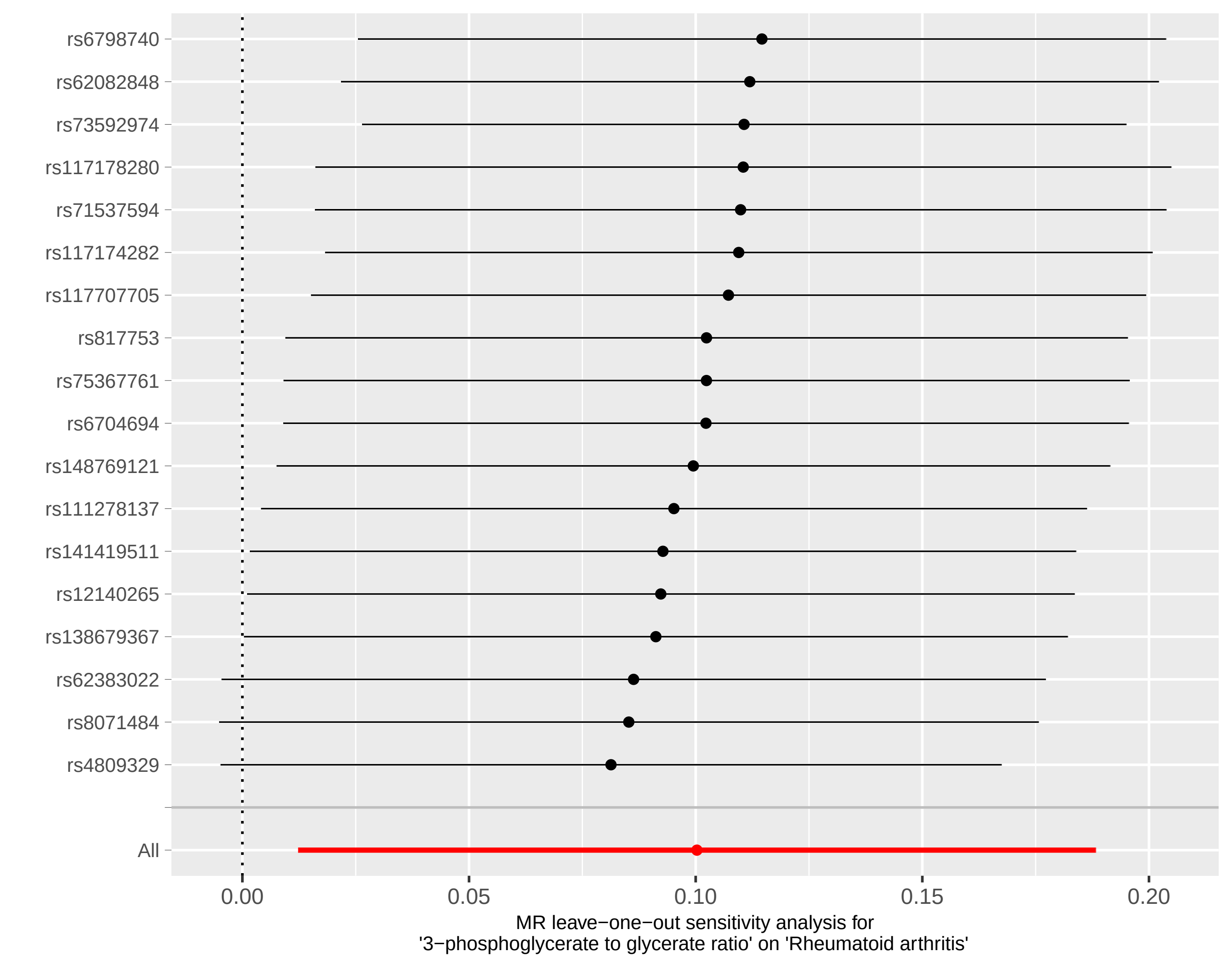
rs6798740
rs62082848
rs73592974
rs117178280
rs71537594
rs117174282
rs117707705
rs817753
rs75367761
rs6704694
rs148769121
rs111278137
rs141419511
rs12140265
rs138679367
rs62383022
rs8071484
rs4809329
All
0.00
0.05
0.10
0.15
0.20
MR leave−one−out sensitivity analysis for
'3−phosphoglycerate to glycerate ratio' on 'Rheumatoid arthritis'

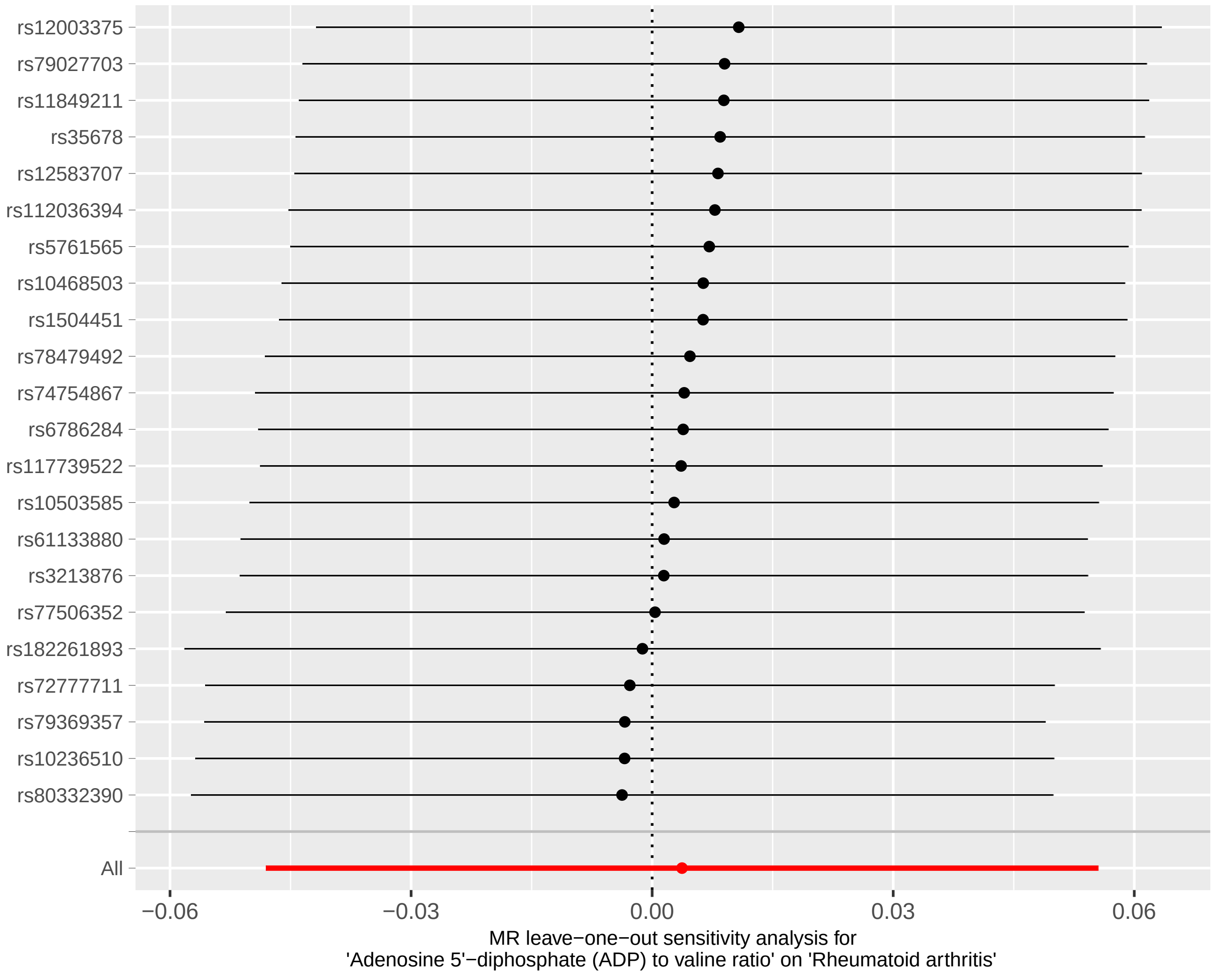
rs12003375
rs79027703
rs11849211
rs35678
rs12583707
rs112036394
rs5761565
rs10468503
rs1504451
rs78479492
rs74754867
rs6786284
rs117739522
rs10503585
rs61133880
rs3213876
rs77506352
rs182261893
rs72777711
rs79369357
rs10236510
rs80332390
All
−0.06
−0.03
0.00
0.03
0.06
MR leave−one−out sensitivity analysis for
'Adenosine 5'−diphosphate (ADP) to valine ratio' on 'Rheumatoid arthritis'

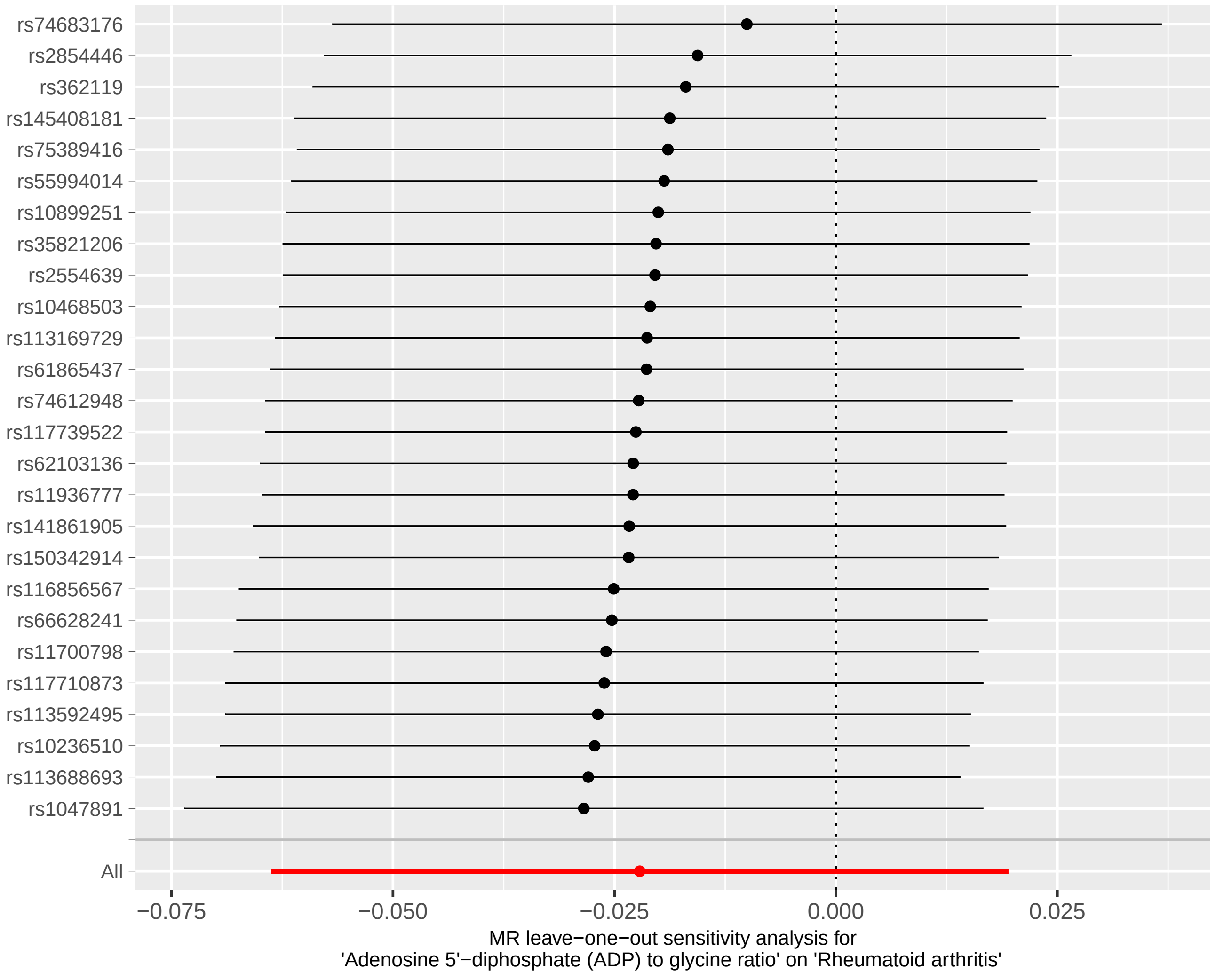
rs74683176
rs2854446
rs362119
rs145408181
rs75389416
rs55994014
rs10899251
rs35821206
rs2554639
rs10468503
rs113169729
rs61865437
rs74612948
rs117739522
rs62103136
rs11936777
rs141861905
rs150342914
rs116856567
rs66628241
rs11700798
rs117710873
rs113592495
rs10236510
rs113688693
rs1047891
All
−0.075
−0.050
−0.025
0.000
0.025
MR leave−one−out sensitivity analysis for
'Adenosine 5'−diphosphate (ADP) to glycine ratio' on 'Rheumatoid arthritis'

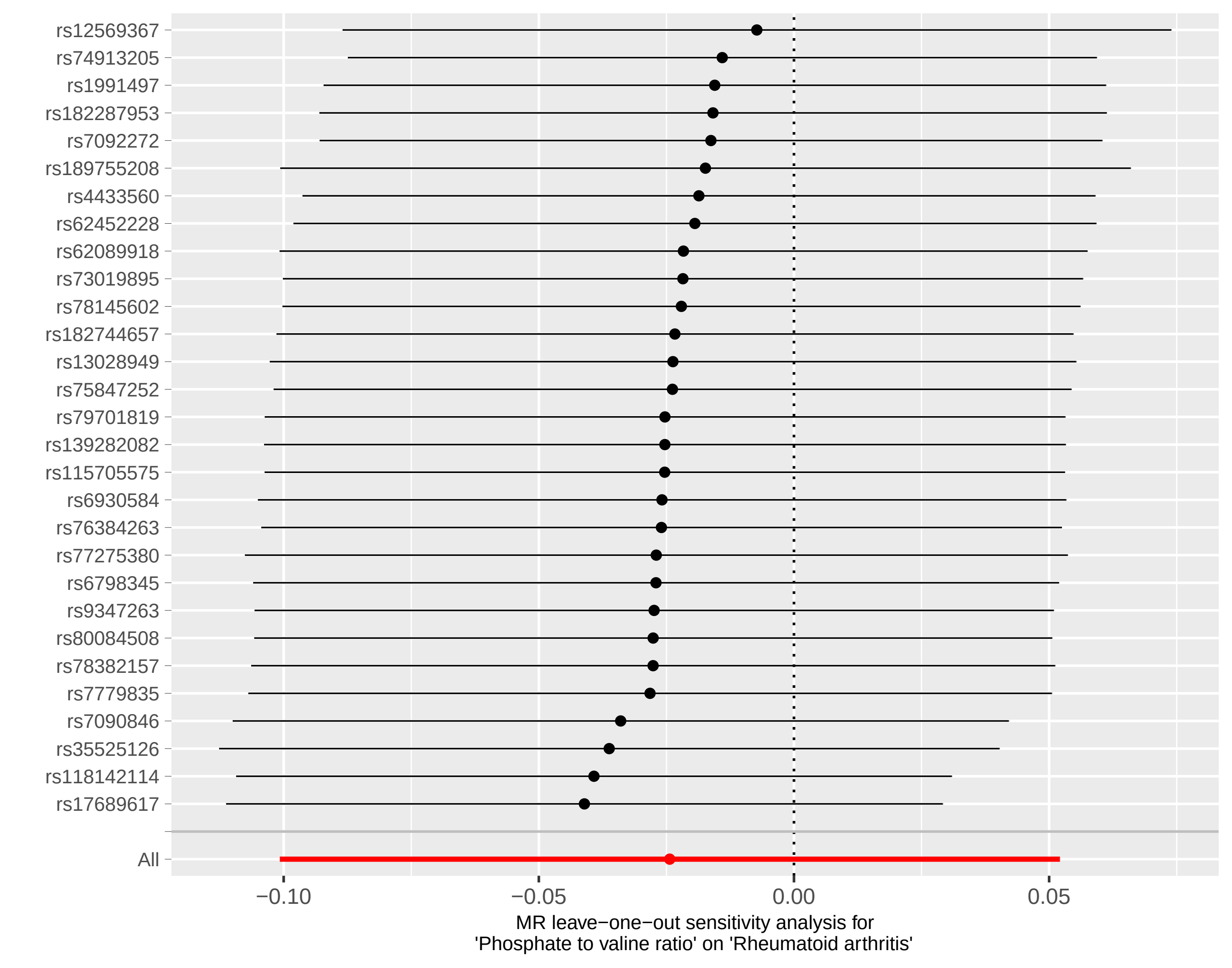

rs12569367
rs74913205
rs1991497
rs182287953
rs7092272
rs189755208
rs4433560
rs62452228
rs62089918
rs73019895
rs78145602
rs182744657
rs13028949
rs75847252
rs79701819
rs139282082
rs115705575
rs6930584
rs76384263
rs77275380
rs6798345
rs9347263
rs80084508
rs78382157
rs7779835
rs7090846
rs35525126
rs118142114
rs17689617
All
−0.10
−0.05
0.00
0.05
MR leave−one−out sensitivity analysis for
'Phosphate to valine ratio' on 'Rheumatoid arthritis'

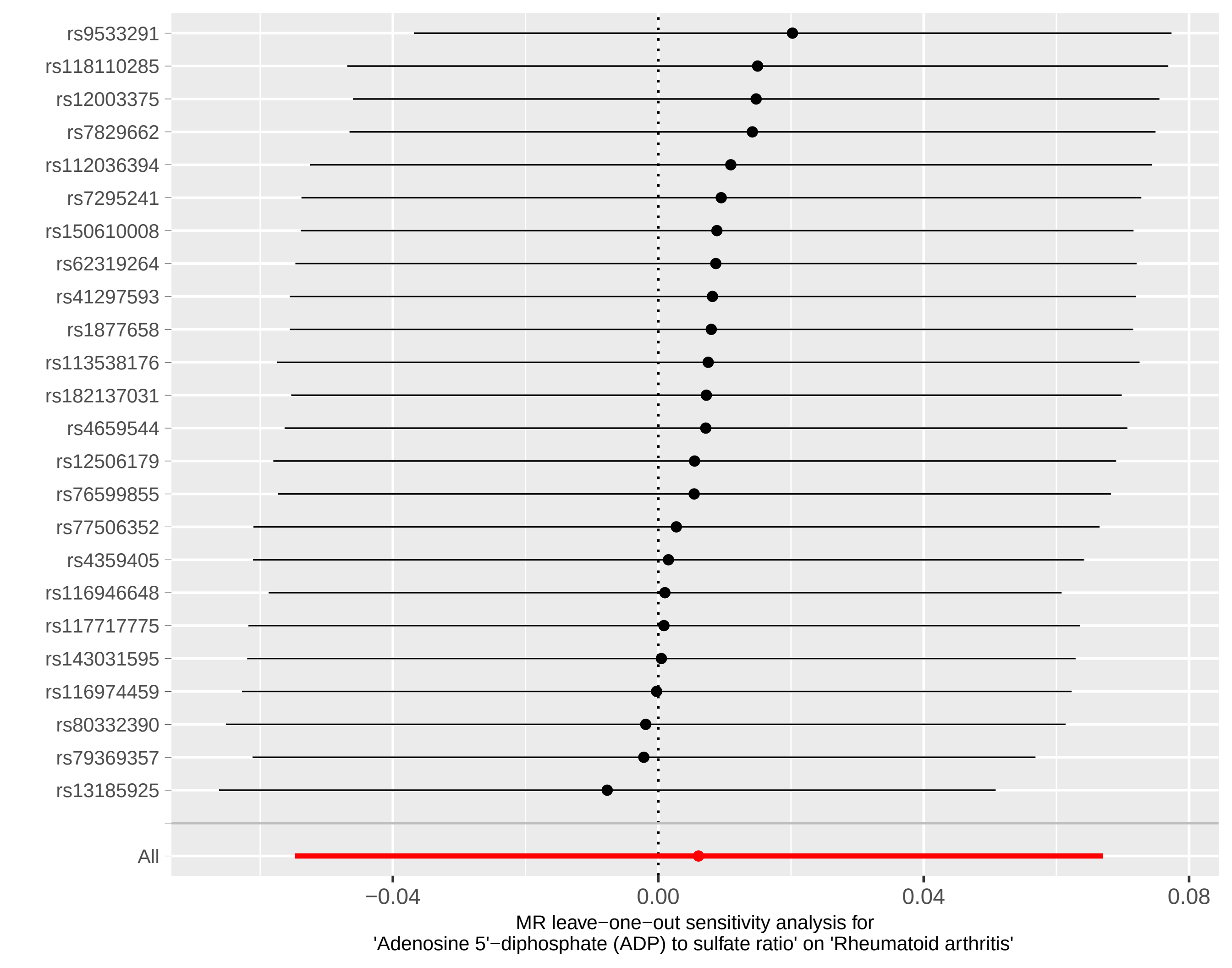
rs9533291
rs118110285
rs12003375
rs7829662
rs112036394
rs7295241
rs150610008
rs62319264
rs41297593
rs1877658
rs113538176
rs182137031
rs4659544
rs12506179
rs76599855
rs77506352
rs4359405
rs116946648
rs117717775
rs143031595
rs116974459
rs80332390
rs79369357
rs13185925
All
−0.04
0.00
0.04
0.08
MR leave−one−out sensitivity analysis for
'Adenosine 5'−diphosphate (ADP) to sulfate ratio' on 'Rheumatoid arthritis'

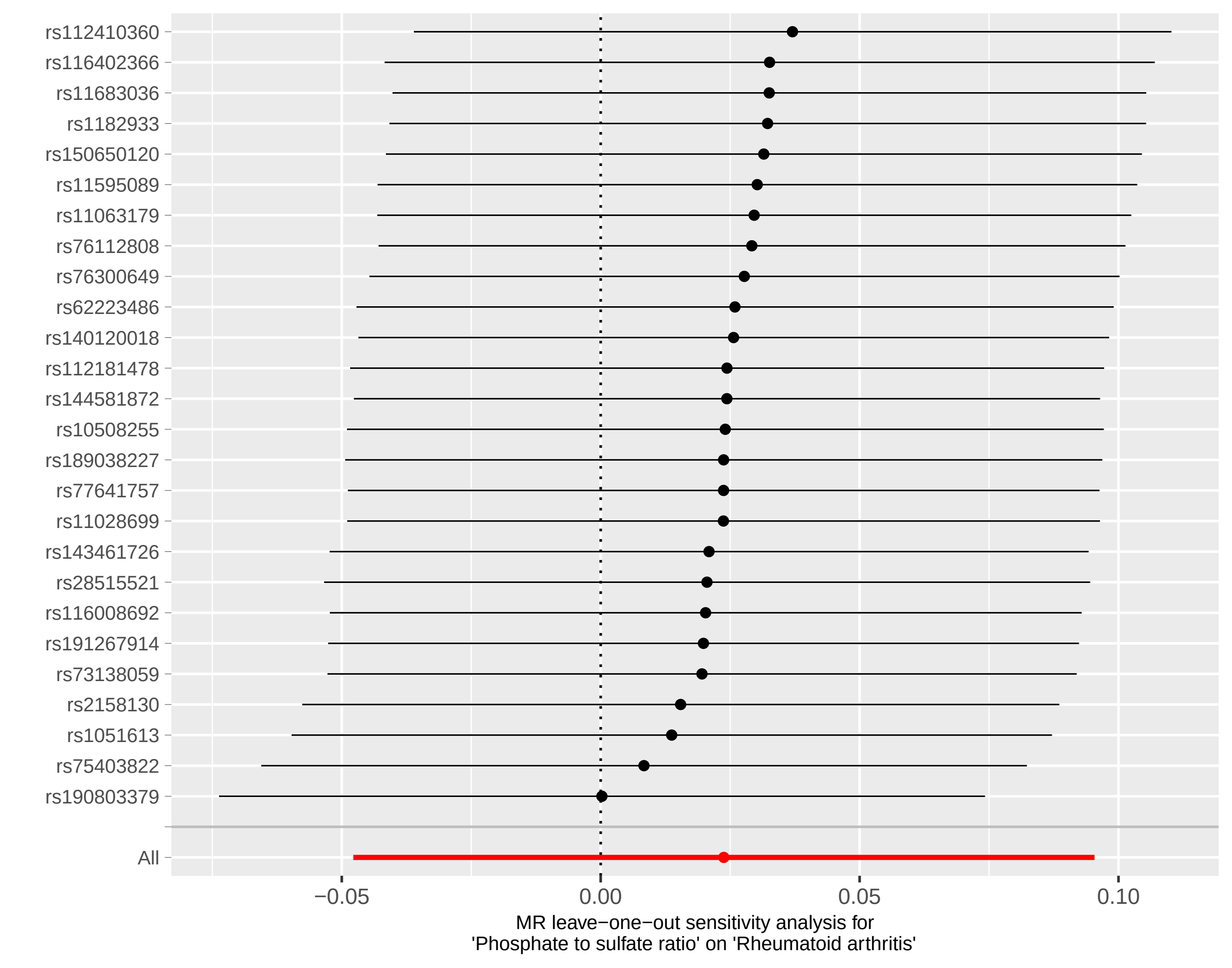

rs112410360
rs116402366
rs11683036
rs1182933
rs150650120
rs11595089
rs11063179
rs76112808
rs76300649
rs62223486
rs140120018
rs112181478
rs144581872
rs10508255
rs189038227
rs77641757
rs11028699
rs143461726
rs28515521
rs116008692
rs191267914
rs73138059
rs2158130
rs1051613
rs75403822
rs190803379
All
−0.05
0.00
0.05
0.10
MR leave−one−out sensitivity analysis for
'Phosphate to sulfate ratio' on 'Rheumatoid arthritis'

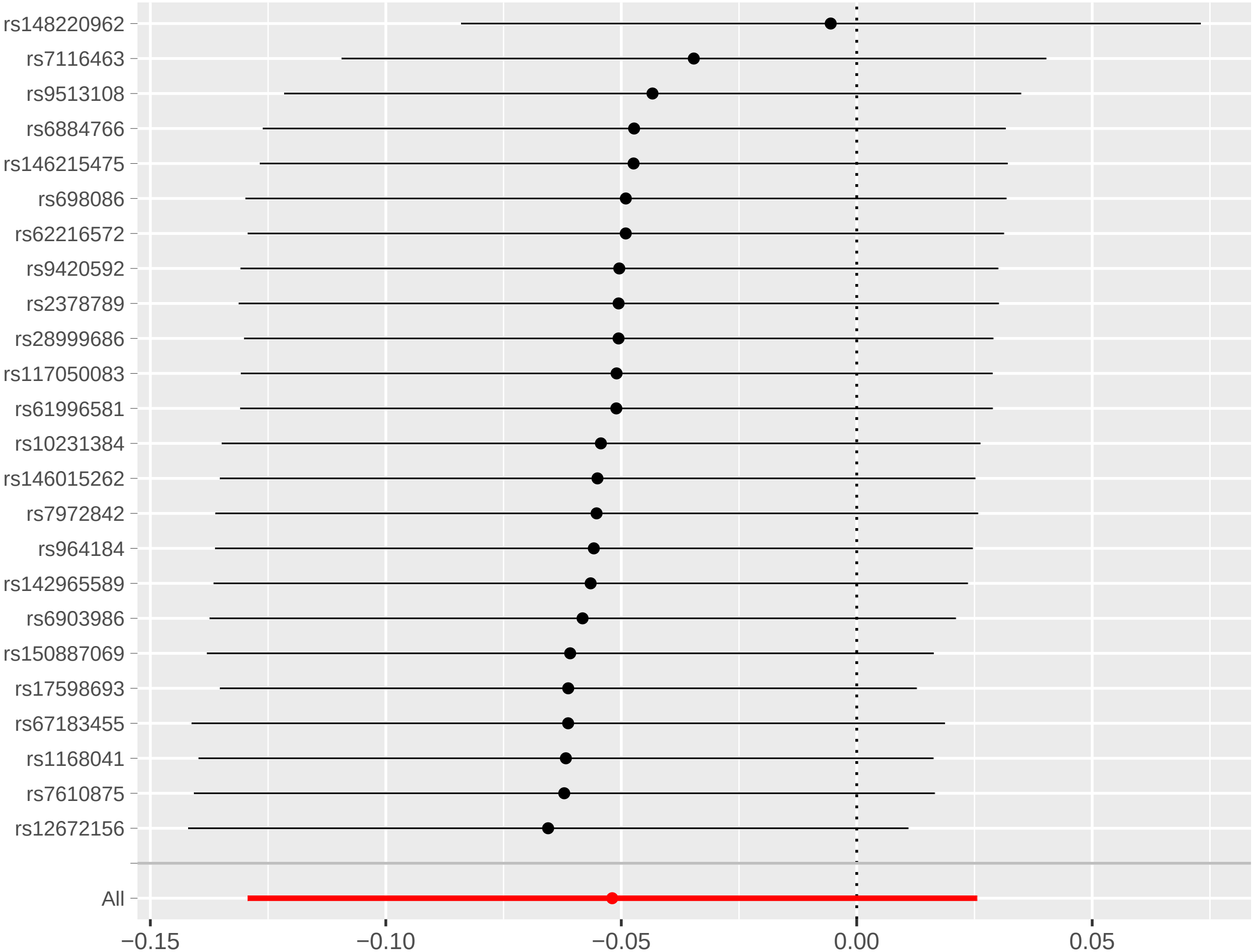

MR leave−one−out sensitivity analysis for
'Phosphate to N−palmitoyl−sphingosine (d18:1 to 16:0) ratio' on 'Rheumatoid arthritis'

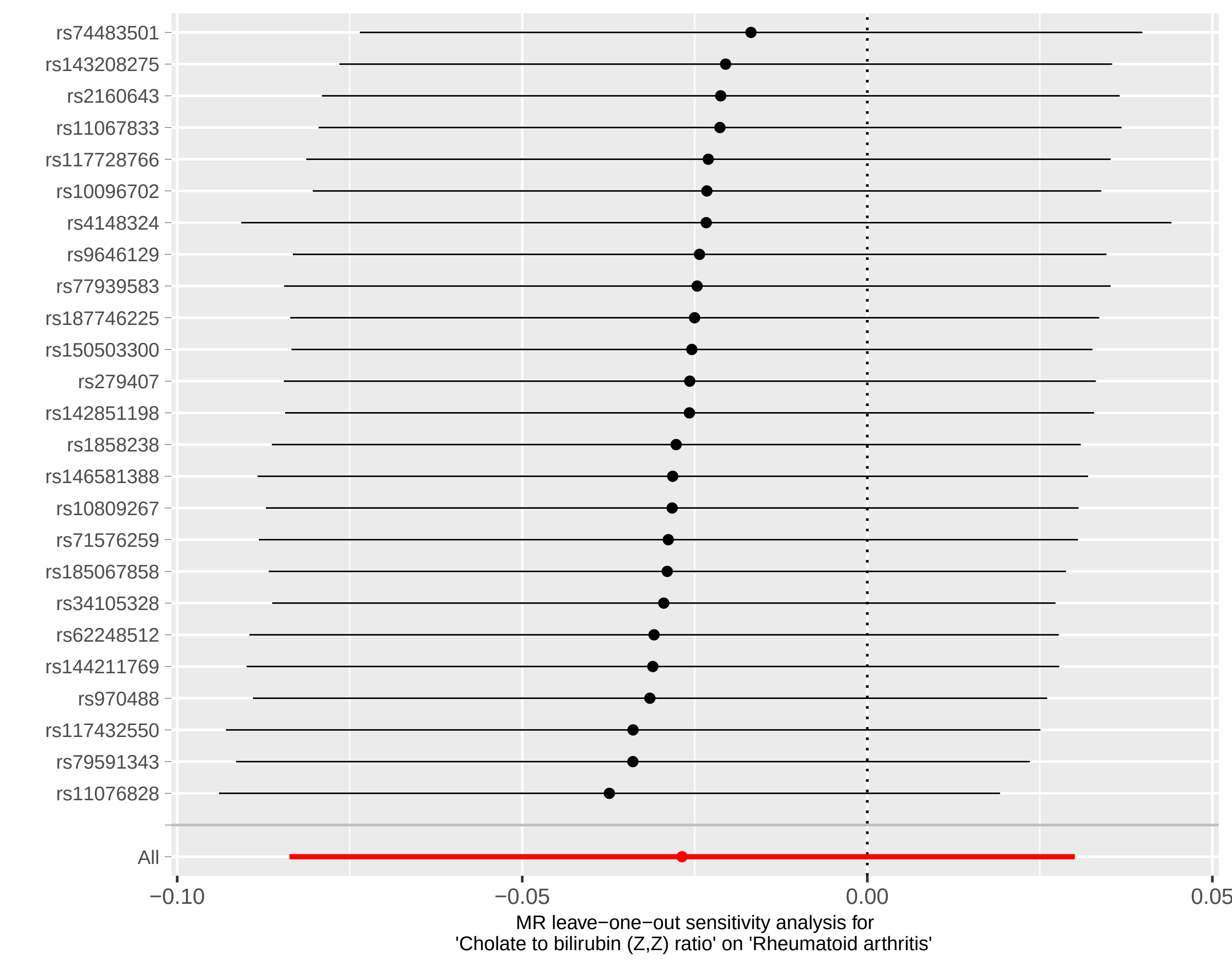
rs74483501
rs143208275
rs2160643
rs11067833
rs117728766
rs10096702
rs4148324
rs9646129
rs77939583
rs187746225
rs150503300
rs279407
rs142851198
rs1858238
rs146581388
rs10809267
rs71576259
rs185067858
rs34105328
rs62248512
rs144211769
rs970488
rs117432550
rs79591343
rs11076828
All
−0.10
−0.05
0.00
0.05
MR leave−one−out sensitivity analysis for
'Cholate to bilirubin (Z,Z) ratio' on 'Rheumatoid arthritis'

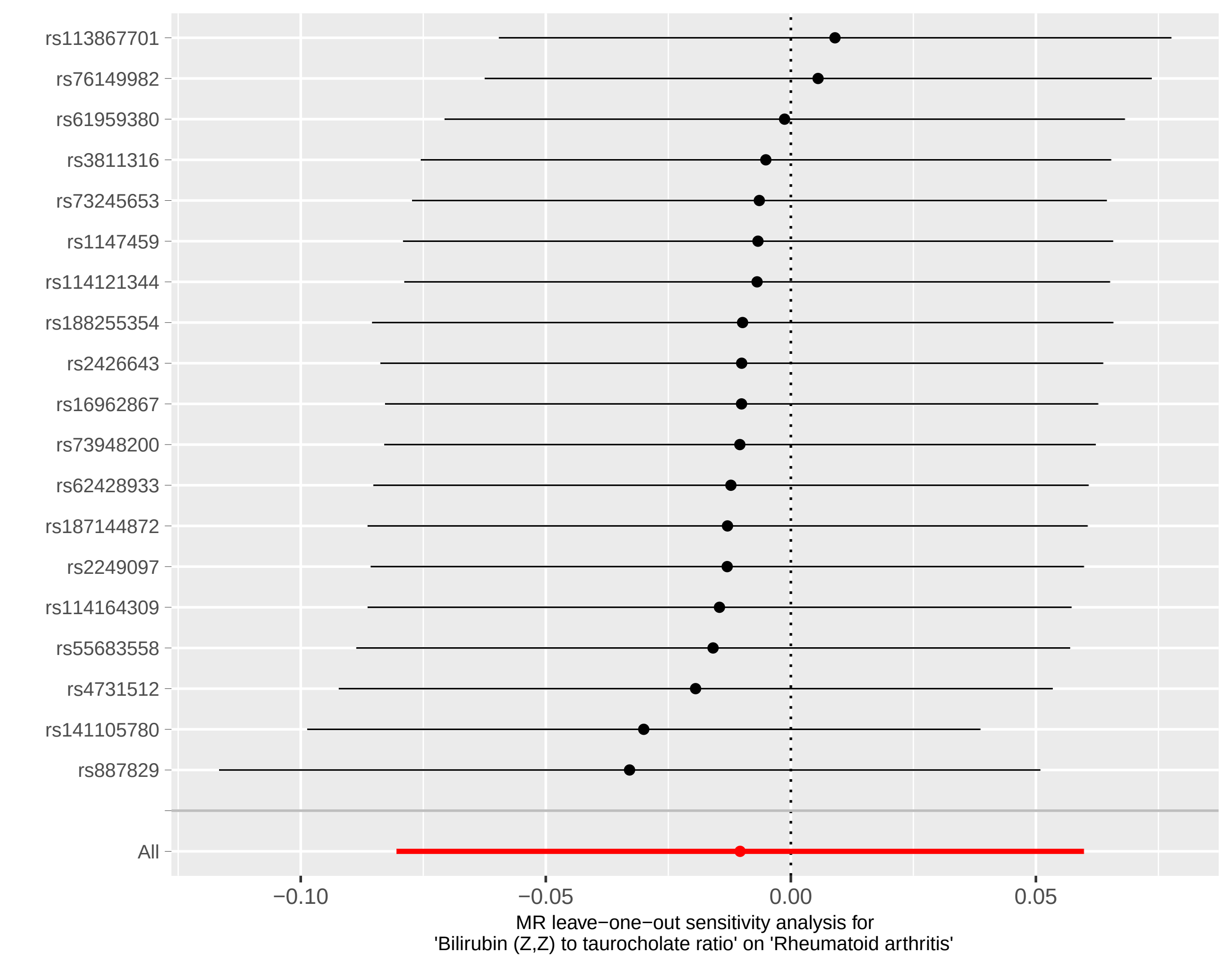

rs113867701
rs76149982
rs61959380
rs3811316
rs73245653
rs1147459
rs114121344
rs188255354
rs2426643
rs16962867
rs73948200
rs62428933
rs187144872
rs2249097
rs114164309
rs55683558
rs4731512
rs141105780
rs887829
All
−0.10
−0.05
0.00
0.05
MR leave−one−out sensitivity analysis for
'Bilirubin (Z,Z) to taurocholate ratio' on 'Rheumatoid arthritis'

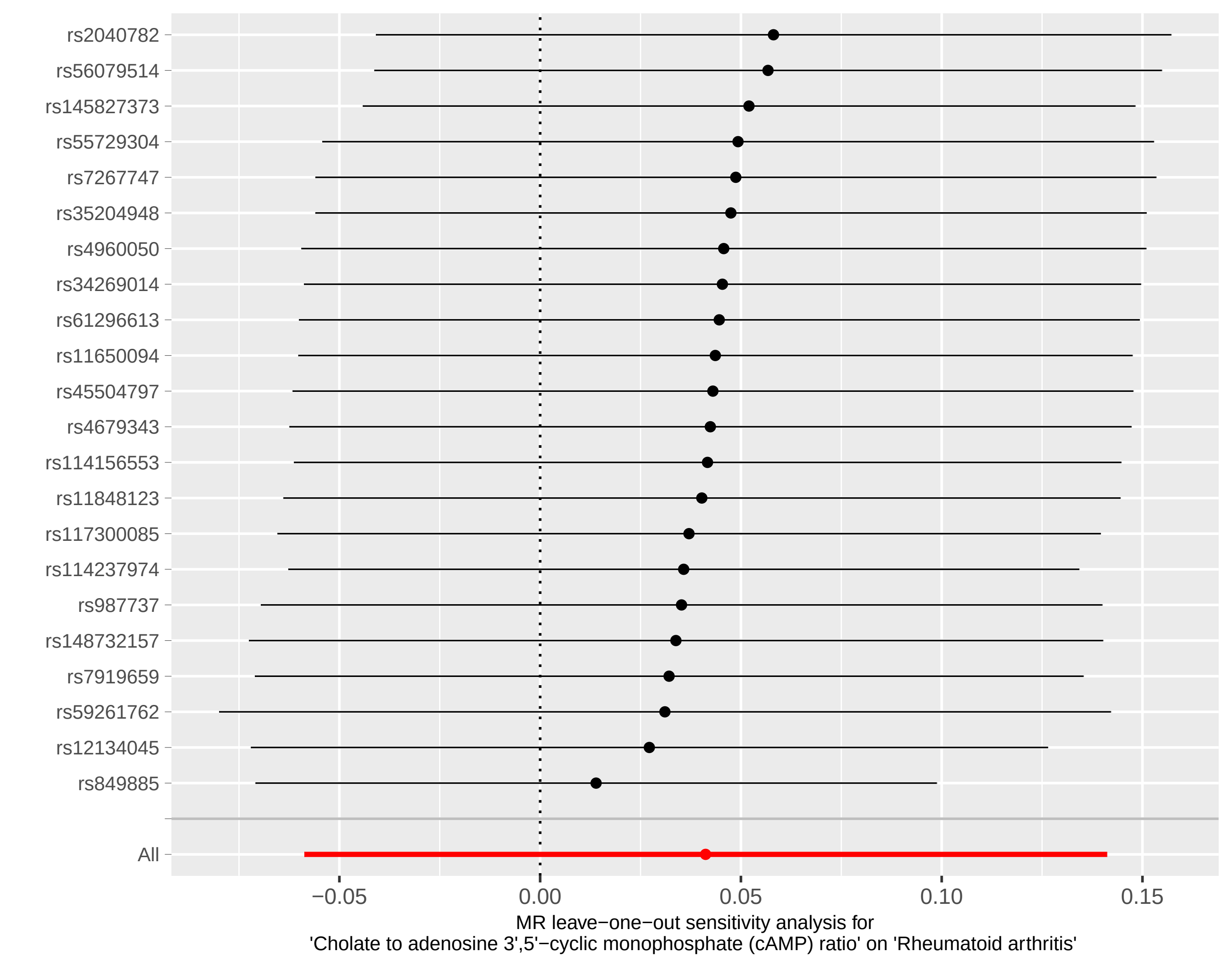

rs2040782
rs56079514
rs145827373
rs55729304
rs7267747
rs35204948
rs4960050
rs34269014
rs61296613
rs11650094
rs45504797
rs4679343
rs114156553
rs11848123
rs117300085
rs114237974
rs987737
rs148732157
rs7919659
rs59261762
rs12134045
rs849885
All
−0.05
0.00
0.05
0.10
0.15
MR leave−one−out sensitivity analysis for
'Cholate to adenosine 3',5'−cyclic monophosphate (cAMP) ratio' on 'Rheumatoid arthritis'

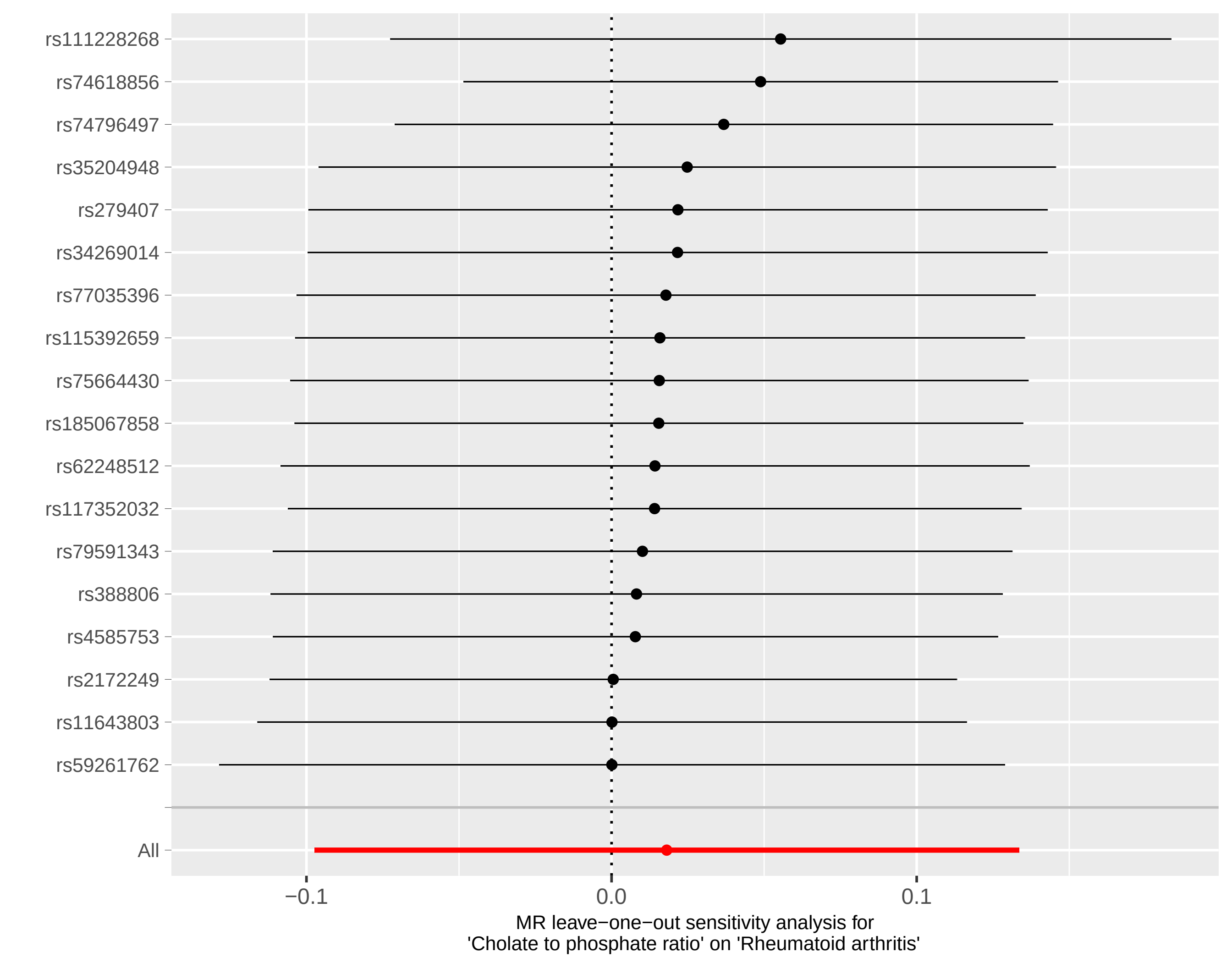
rs111228268
rs74618856
rs74796497
rs35204948
rs279407
rs34269014
rs77035396
rs115392659
rs75664430
rs185067858
rs62248512
rs117352032
rs79591343
rs388806
rs4585753
rs2172249
rs11643803
rs59261762
All
−0.1
0.0
0.1
MR leave−one−out sensitivity analysis for
'Cholate to phosphate ratio' on 'Rheumatoid arthritis'

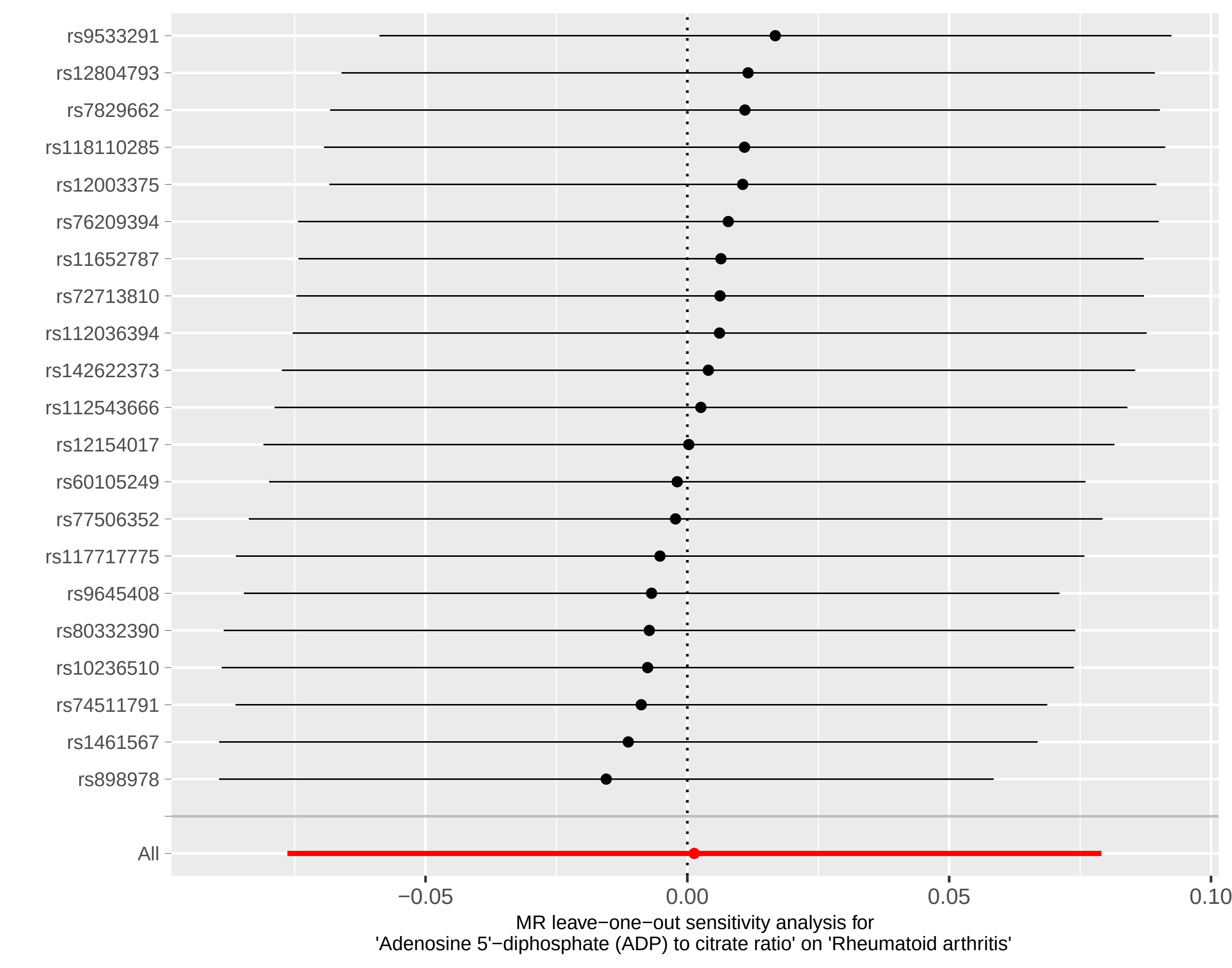
rs9533291
rs12804793
rs7829662
rs118110285
rs12003375
rs76209394
rs11652787
rs72713810
rs112036394
rs142622373
rs112543666
rs12154017
rs60105249
rs77506352
rs117717775
rs9645408
rs80332390
rs10236510
rs74511791
rs1461567
rs898978
All
−0.05
0.00
0.05
0.10
MR leave−one−out sensitivity analysis for
'Adenosine 5'−diphosphate (ADP) to citrate ratio' on 'Rheumatoid arthritis'

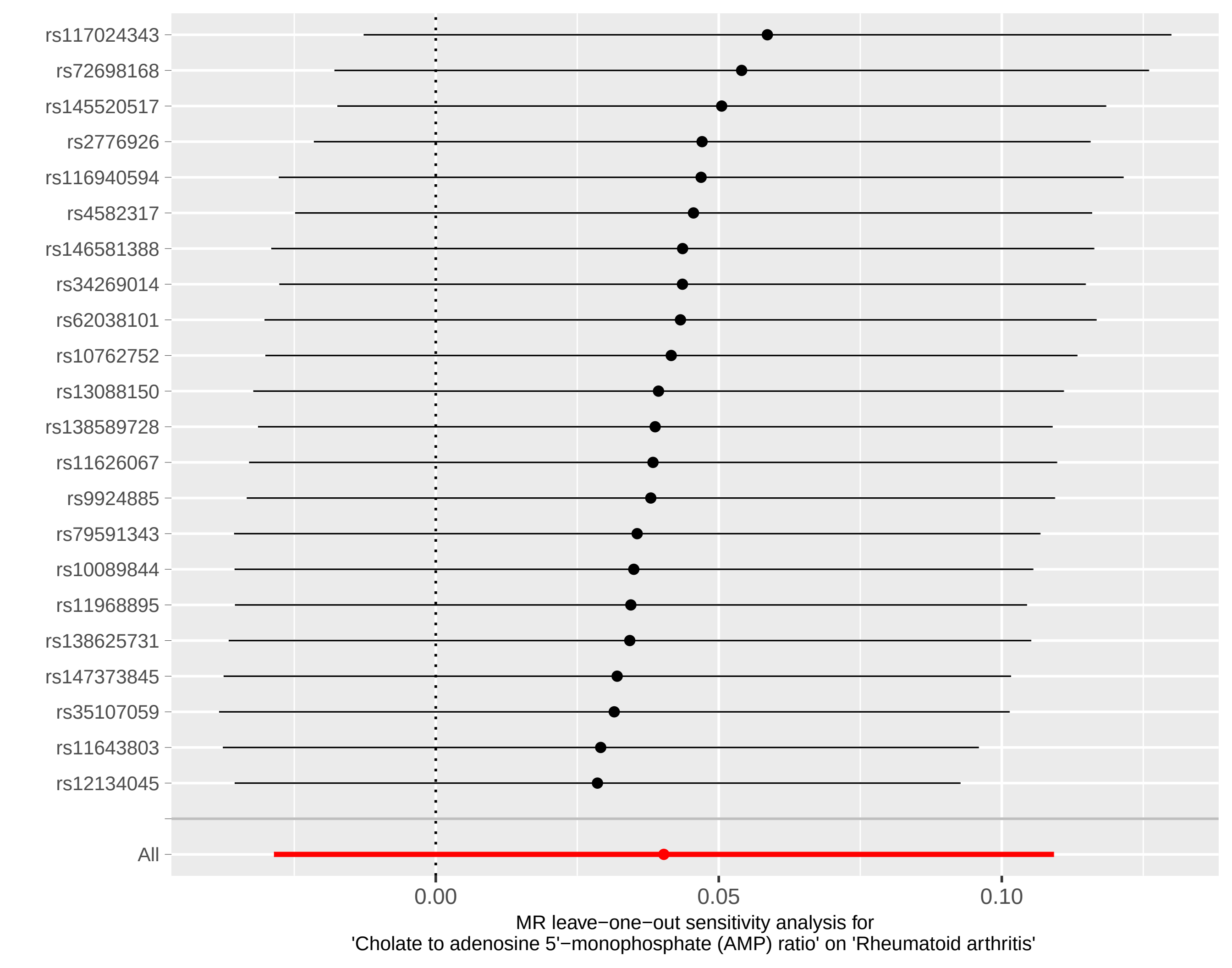

rs117024343
rs72698168
rs145520517
rs2776926
rs116940594
rs4582317
rs146581388
rs34269014
rs62038101
rs10762752
rs13088150
rs138589728
rs11626067
rs9924885
rs79591343
rs10089844
rs11968895
rs138625731
rs147373845
rs35107059
rs11643803
rs12134045
All
0.00
0.05
0.10
MR leave−one−out sensitivity analysis for
'Cholate to adenosine 5'−monophosphate (AMP) ratio' on 'Rheumatoid arthritis'

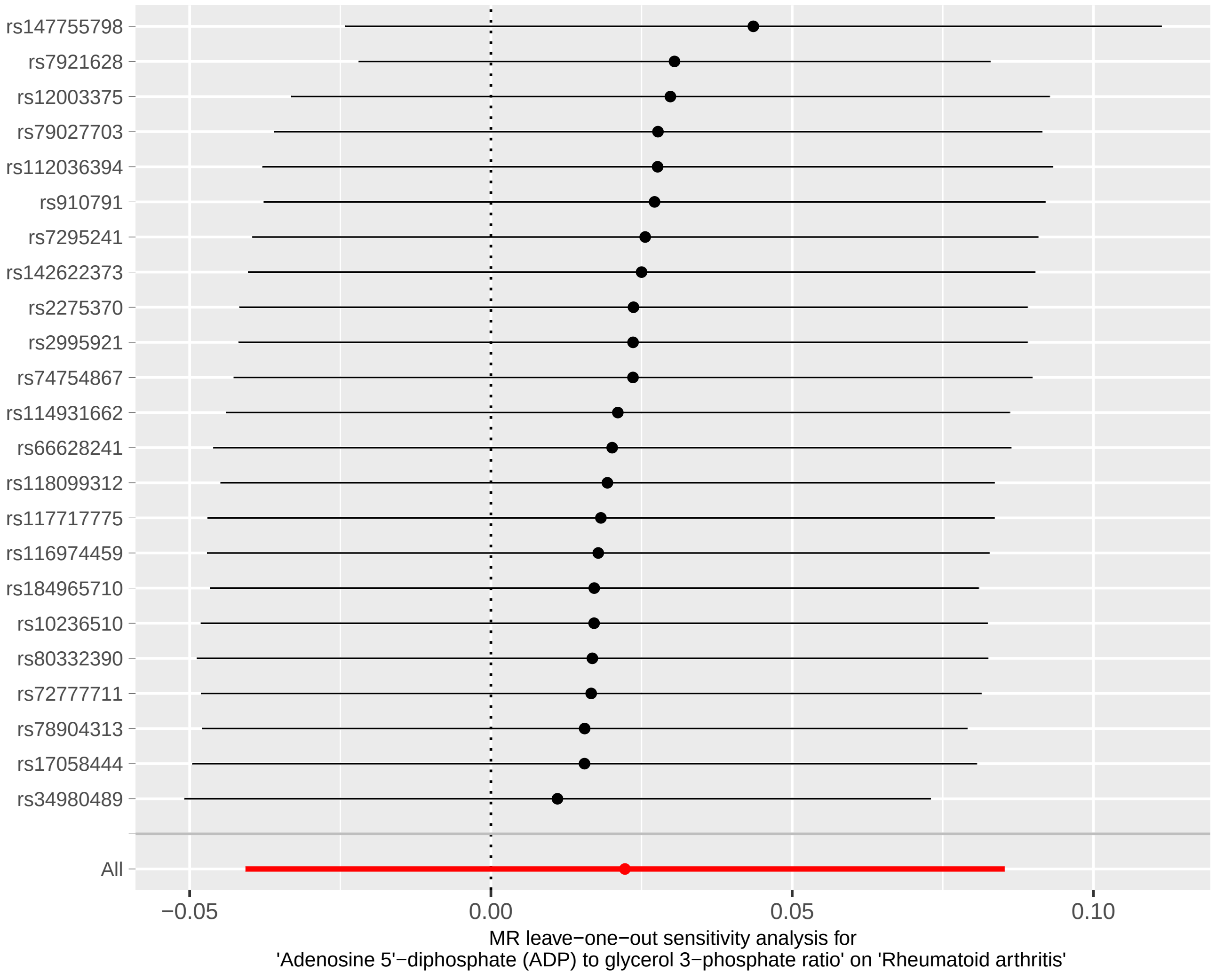

rs147755798
rs7921628
rs12003375
rs79027703
rs112036394
rs910791
rs7295241
rs142622373
rs2275370
rs2995921
rs74754867
rs114931662
rs66628241
rs118099312
rs117717775
rs116974459
rs184965710
rs10236510
rs80332390
rs72777711
rs78904313
rs17058444
rs34980489
All
−0.05
0.00
0.05
0.10
MR leave−one−out sensitivity analysis for
'Adenosine 5'−diphosphate (ADP) to glycerol 3−phosphate ratio' on 'Rheumatoid arthritis'

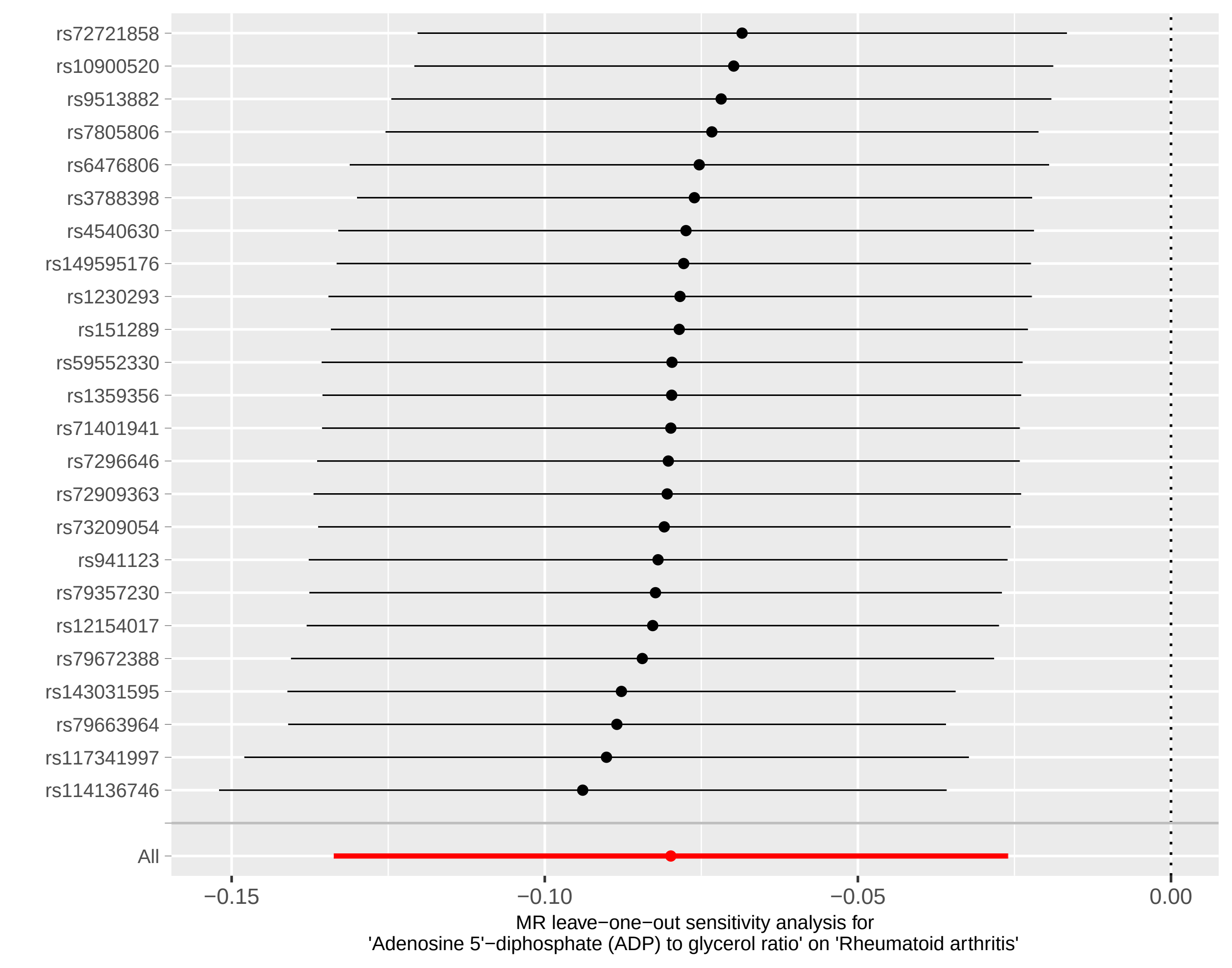

rs72721858
rs10900520
rs9513882
rs7805806
rs6476806
rs3788398
rs4540630
rs149595176
rs1230293
rs151289
rs59552330
rs1359356
rs71401941
rs7296646
rs72909363
rs73209054
rs941123
rs79357230
rs12154017
rs79672388
rs143031595
rs79663964
rs117341997
rs114136746
All
−0.15
−0.10
−0.05
0.00
MR leave−one−out sensitivity analysis for
'Adenosine 5'−diphosphate (ADP) to glycerol ratio' on 'Rheumatoid arthritis'

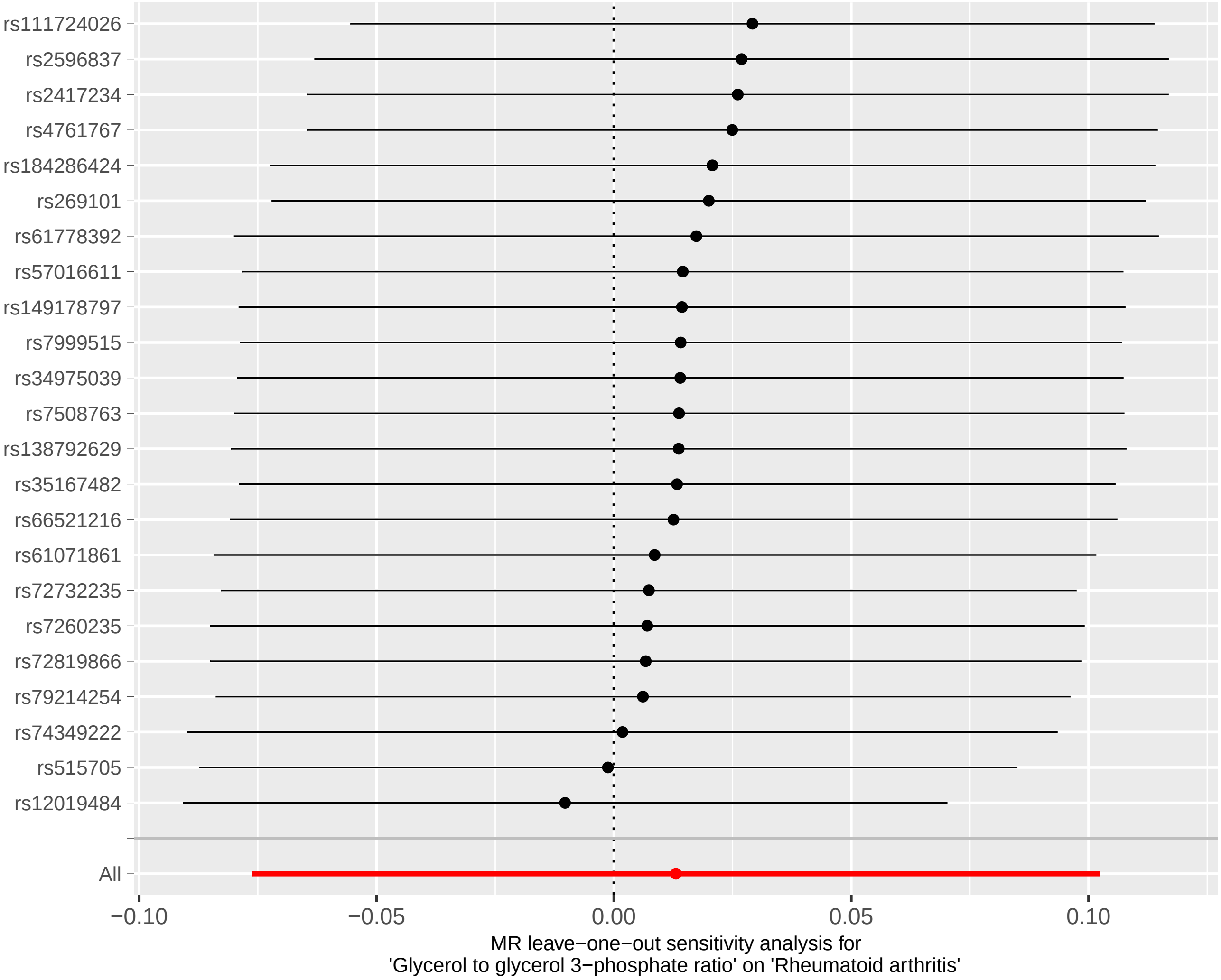
rs111724026
rs2596837
rs2417234
rs4761767
rs184286424
rs269101
rs61778392
rs57016611
rs149178797
rs7999515
rs34975039
rs7508763
rs138792629
rs35167482
rs66521216
rs61071861
rs72732235
rs7260235
rs72819866
rs79214254
rs74349222
rs515705
rs12019484
All
−0.10
−0.05
0.00
0.05
0.10
MR leave−one−out sensitivity analysis for
'Glycerol to glycerol 3−phosphate ratio' on 'Rheumatoid arthritis'

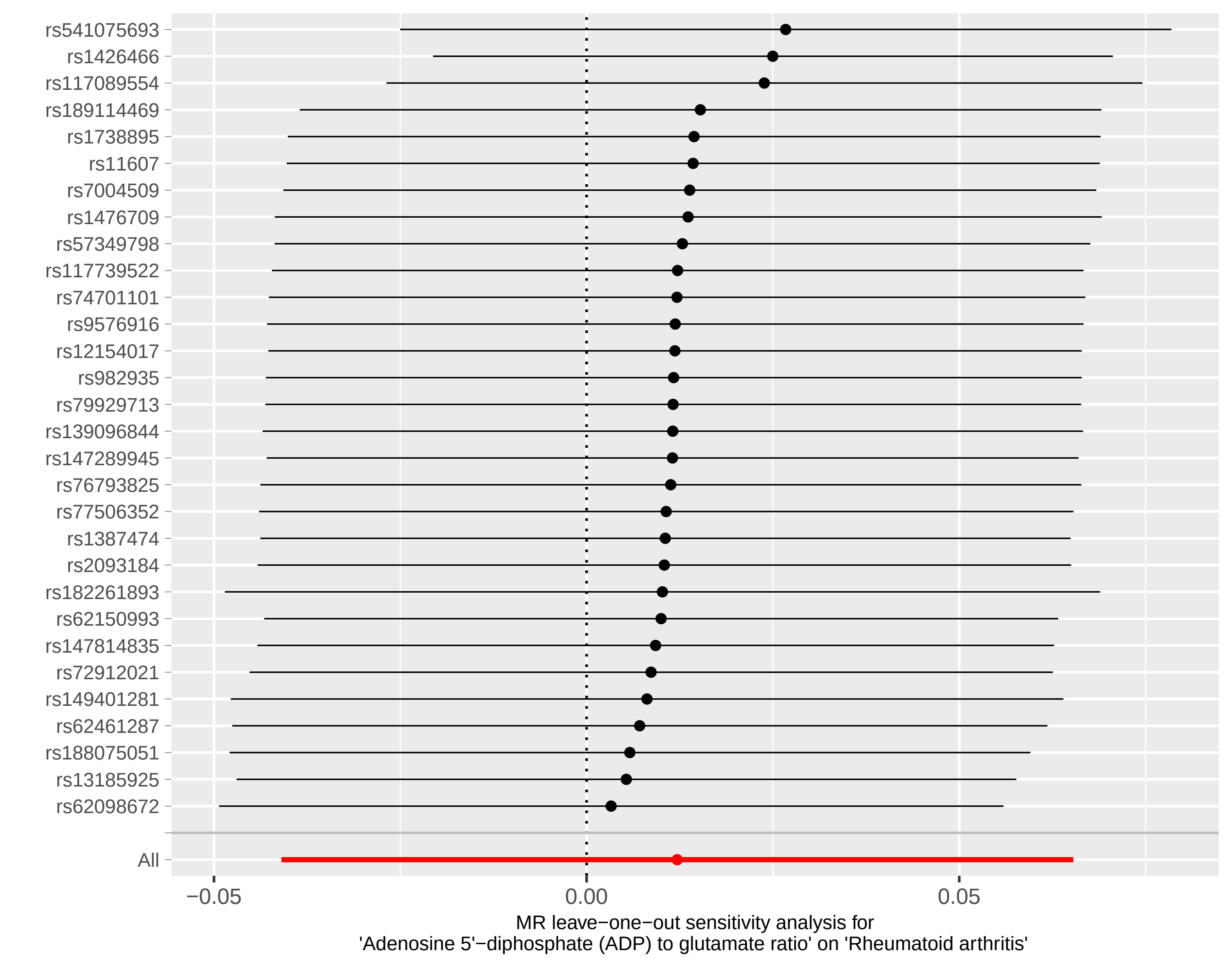
rs541075693
rs1426466
rs117089554
rs189114469
rs1738895
rs11607
rs7004509
rs1476709
rs57349798
rs117739522
rs74701101
rs9576916
rs12154017
rs982935
rs79929713
rs139096844
rs147289945
rs76793825
rs77506352
rs1387474
rs2093184
rs182261893
rs62150993
rs147814835
rs72912021
rs149401281
rs62461287
rs188075051
rs13185925
rs62098672
All
−0.05
0.00
0.05
MR leave−one−out sensitivity analysis for
'Adenosine 5'−diphosphate (ADP) to glutamate ratio' on 'Rheumatoid arthritis'

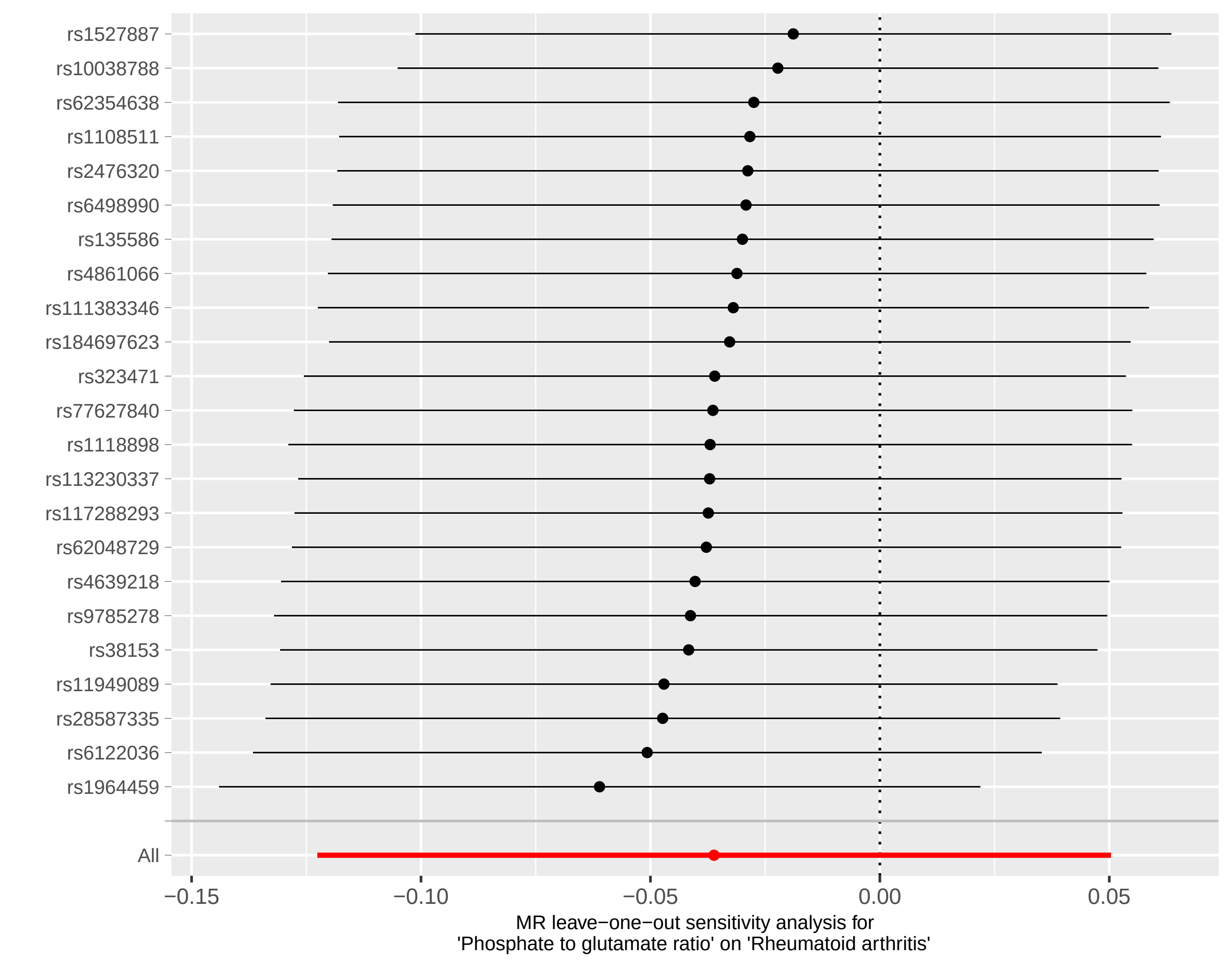

rs1527887
rs10038788
rs62354638
rs1108511
rs2476320
rs6498990
rs135586
rs4861066
rs111383346
rs184697623
rs323471
rs77627840
rs1118898
rs113230337
rs117288293
rs62048729
rs4639218
rs9785278
rs38153
rs11949089
rs28587335
rs6122036
rs1964459
All
−0.15
−0.10
−0.05
0.00
0.05
MR leave−one−out sensitivity analysis for
'Phosphate to glutamate ratio' on 'Rheumatoid arthritis'

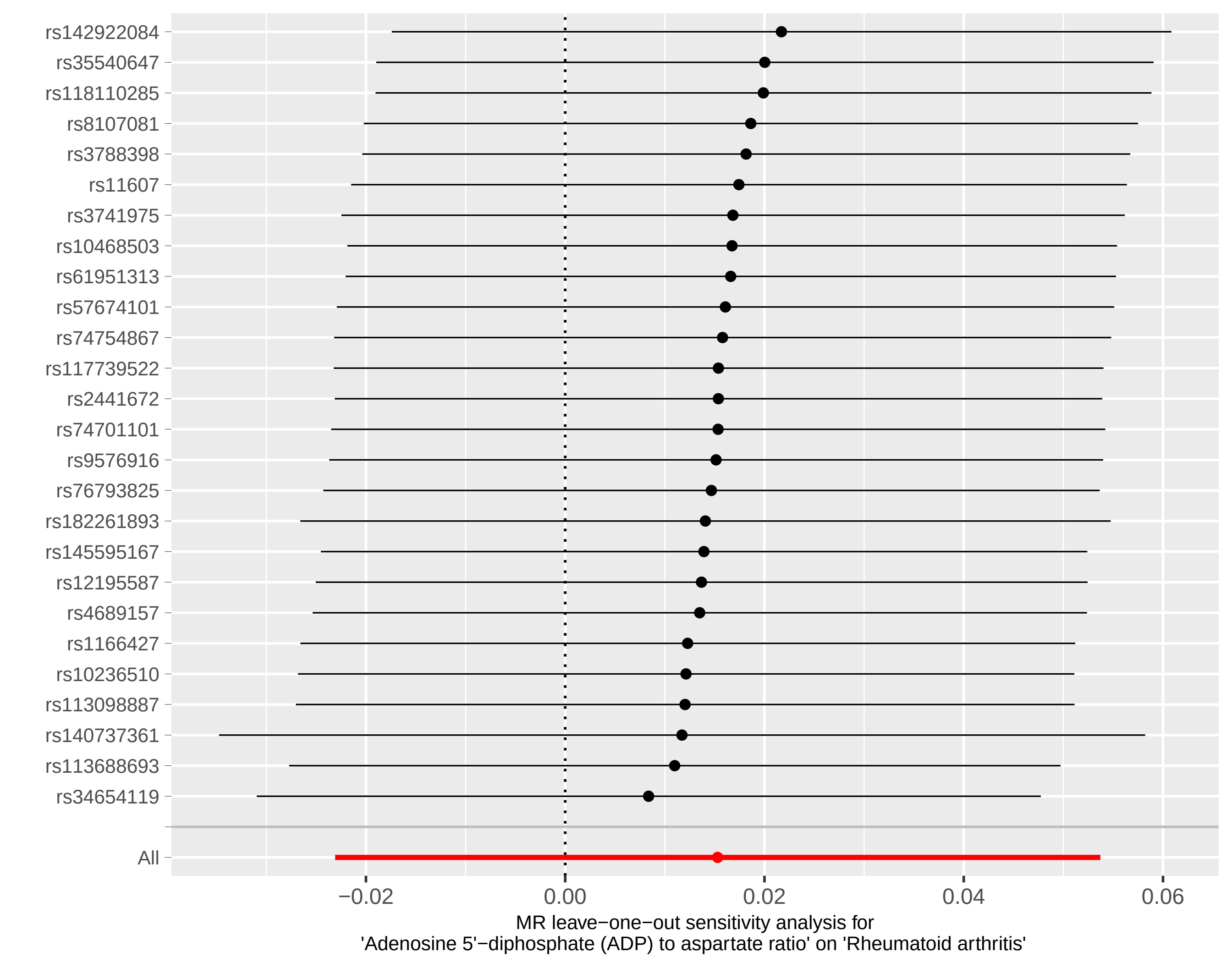

rs142922084
rs35540647
rs118110285
rs8107081
rs3788398
rs11607
rs3741975
rs10468503
rs61951313
rs57674101
rs74754867
rs117739522
rs2441672
rs74701101
rs9576916
rs76793825
rs182261893
rs145595167
rs12195587
rs4689157
rs1166427
rs10236510
rs113098887
rs140737361
rs113688693
rs34654119
All
−0.02
0.00
0.02
0.04
0.06
MR leave−one−out sensitivity analysis for
'Adenosine 5'−diphosphate (ADP) to aspartate ratio' on 'Rheumatoid arthritis'

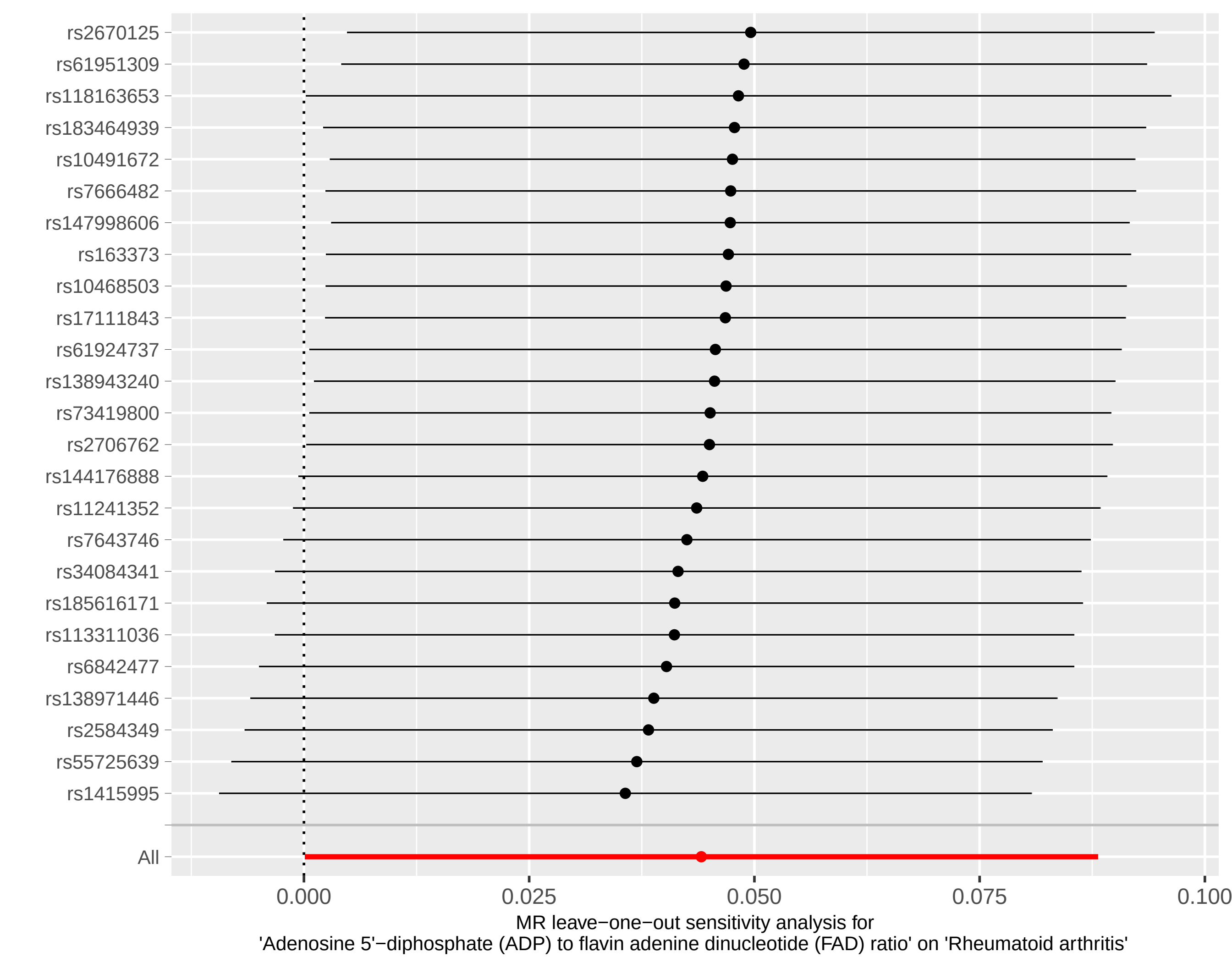
rs2670125
rs61951309
rs118163653
rs183464939
rs10491672
rs7666482
rs147998606
rs163373
rs10468503
rs17111843
rs61924737
rs138943240
rs73419800
rs2706762
rs144176888
rs11241352
rs7643746
rs34084341
rs185616171
rs113311036
rs6842477
rs138971446
rs2584349
rs55725639
rs1415995
All
0.000
0.025
0.050
0.075
0.100
MR leave−one−out sensitivity analysis for
'Adenosine 5'−diphosphate (ADP) to flavin adenine dinucleotide (FAD) ratio' on 'Rheumatoid arthritis'

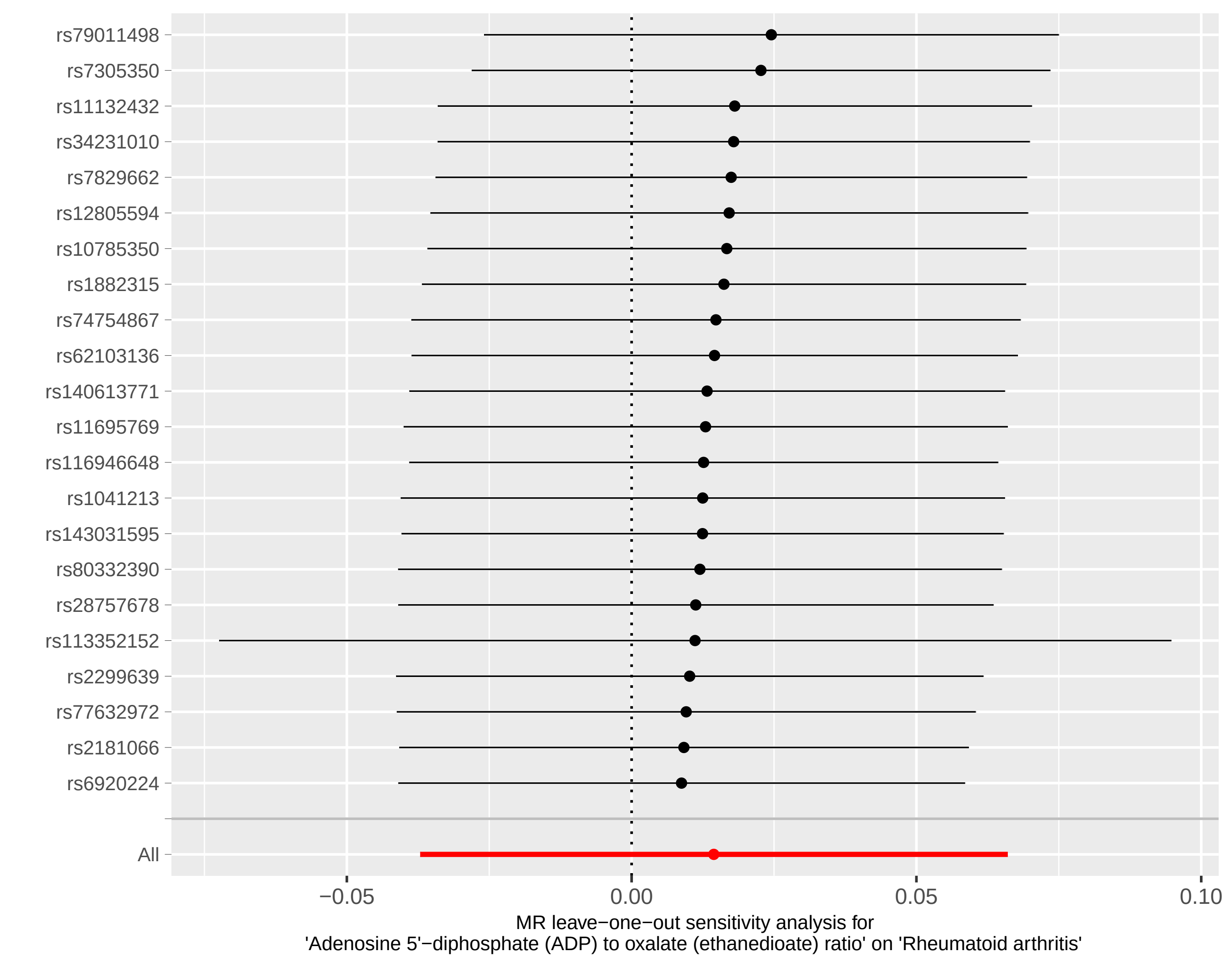
rs79011498
rs7305350
rs11132432
rs34231010
rs7829662
rs12805594
rs10785350
rs1882315
rs74754867
rs62103136
rs140613771
rs11695769
rs116946648
rs1041213
rs143031595
rs80332390
rs28757678
rs113352152
rs2299639
rs77632972
rs2181066
rs6920224
All
−0.05
0.00
0.05
0.10
MR leave−one−out sensitivity analysis for
'Adenosine 5'−diphosphate (ADP) to oxalate (ethanedioate) ratio' on 'Rheumatoid arthritis'

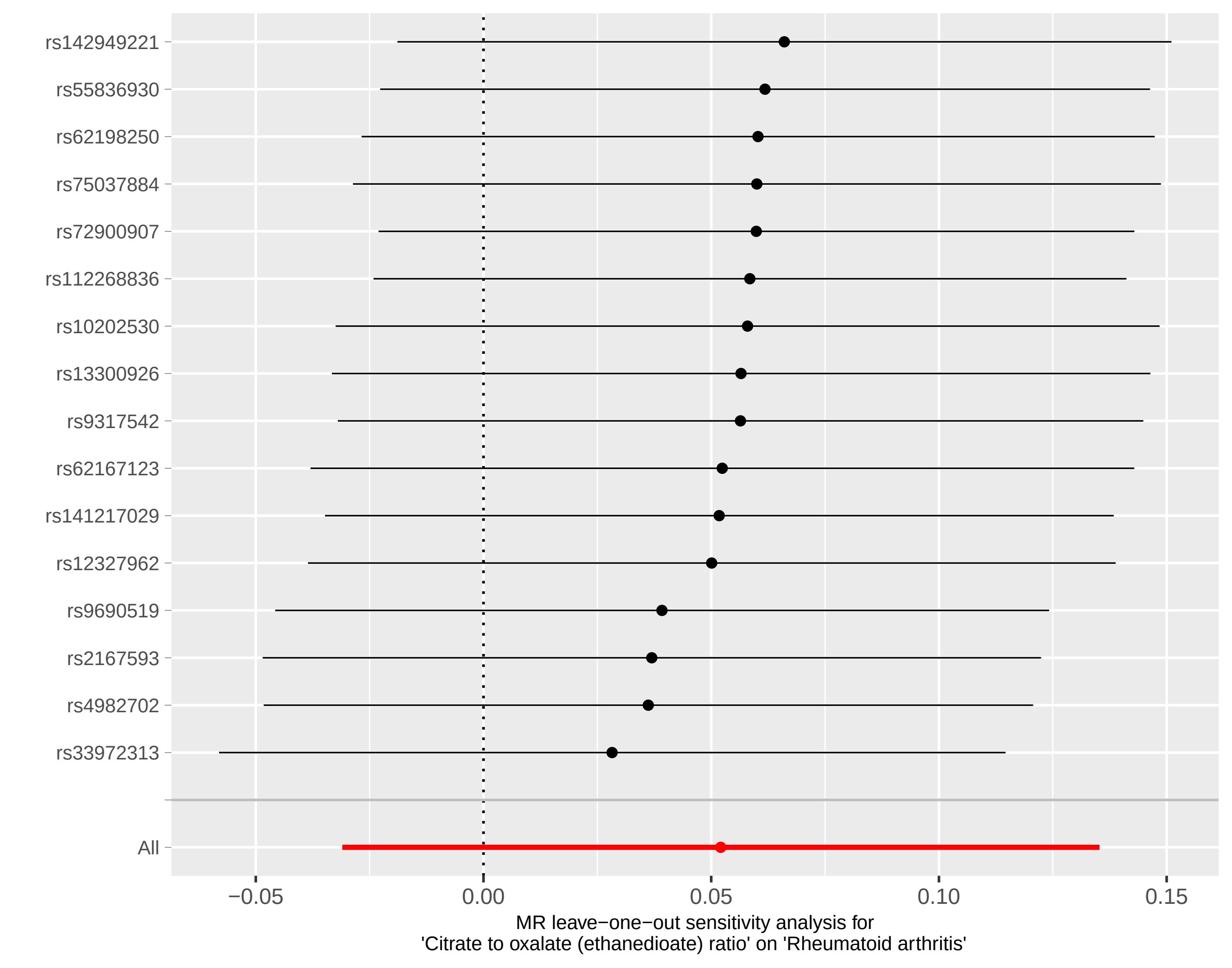

rs142949221
rs55836930
rs62198250
rs75037884
rs72900907
rs112268836
rs10202530
rs13300926
rs9317542
rs62167123
rs141217029
rs12327962
rs9690519
rs2167593
rs4982702
rs33972313
All
−0.05
0.00
0.05
0.10
0.15
MR leave−one−out sensitivity analysis for
'Citrate to oxalate (ethanedioate) ratio' on 'Rheumatoid arthritis'

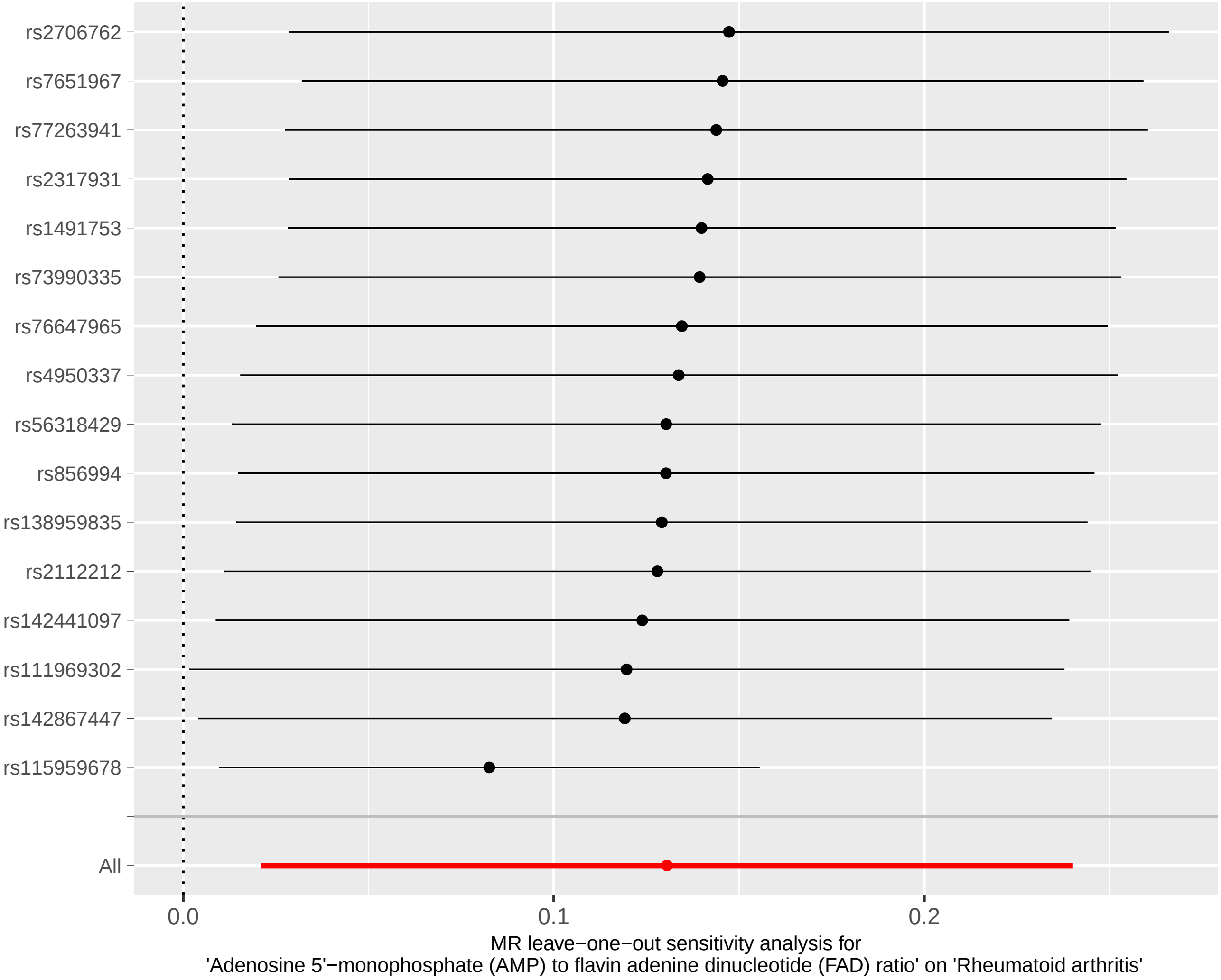

rs2706762
rs7651967
rs77263941
rs2317931
rs1491753
rs73990335
rs76647965
rs4950337
rs56318429
rs856994
rs138959835
rs2112212
rs142441097
rs111969302
rs142867447
rs115959678
All
0.0
0.1
0.2
MR leave−one−out sensitivity analysis for
'Adenosine 5'−monophosphate (AMP) to flavin adenine dinucleotide (FAD) ratio' on 'Rheumatoid arthritis'

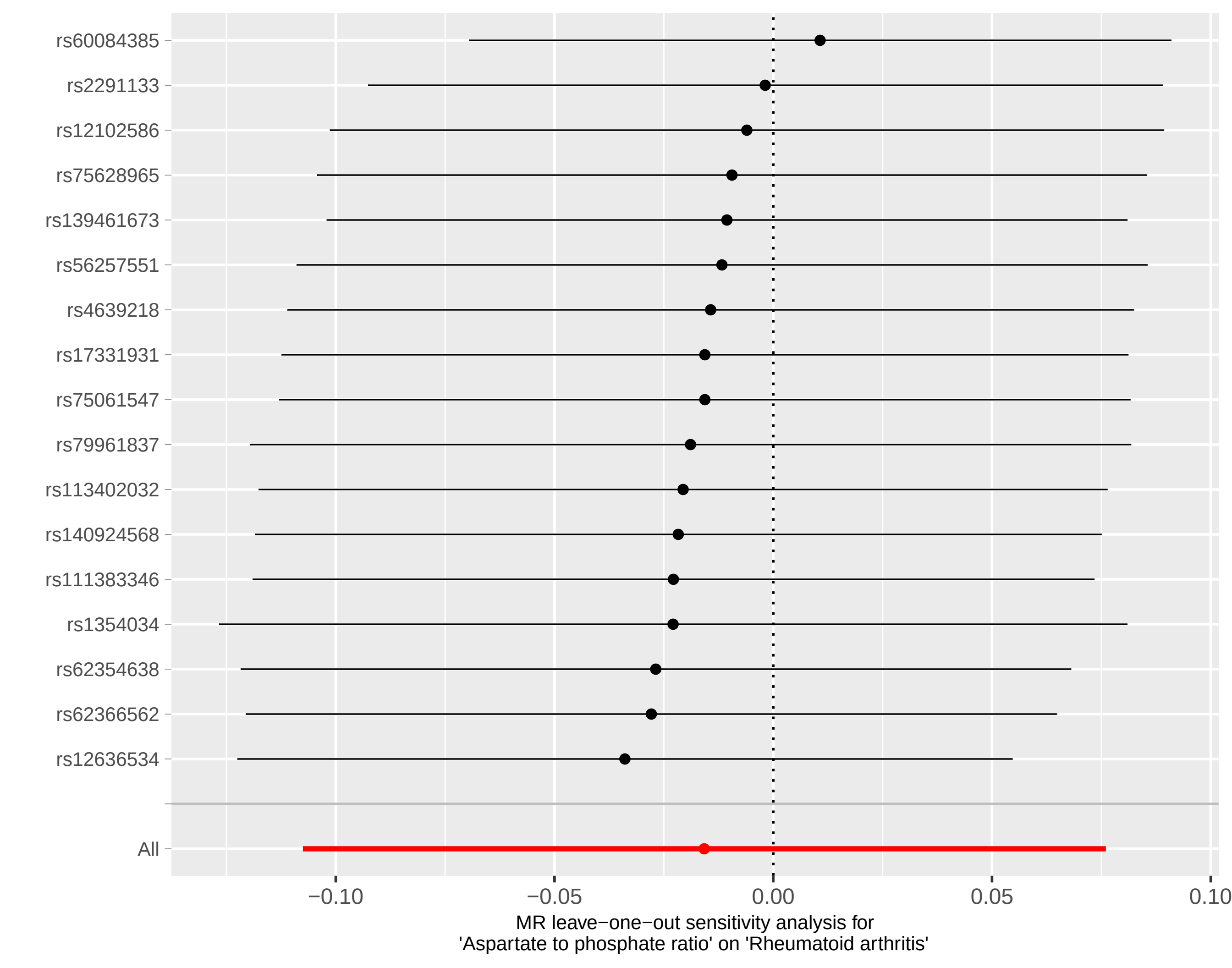
rs60084385
rs2291133
rs12102586
rs75628965
rs139461673
rs56257551
rs4639218
rs17331931
rs75061547
rs79961837
rs113402032
rs140924568
rs111383346
rs1354034
rs62354638
rs62366562
rs12636534
All
−0.10
−0.05
0.00
0.05
0.10
MR leave−one−out sensitivity analysis for
'Aspartate to phosphate ratio' on 'Rheumatoid arthritis'

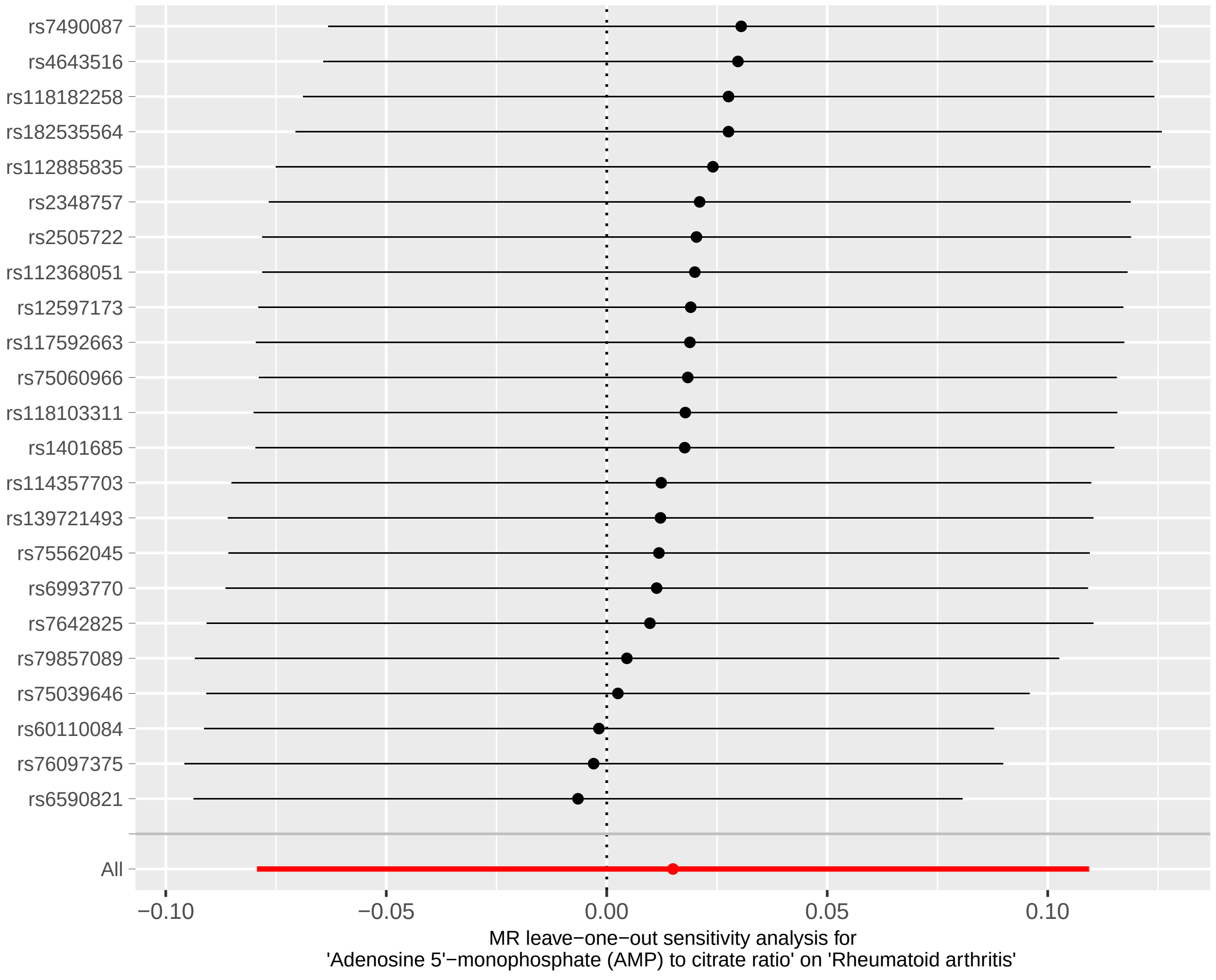
rs7490087
rs4643516
rs118182258
rs182535564
rs112885835
rs2348757
rs2505722
rs112368051
rs12597173
rs117592663
rs75060966
rs118103311
rs1401685
rs114357703
rs139721493
rs75562045
rs6993770
rs7642825
rs79857089
rs75039646
rs60110084
rs76097375
rs6590821
All
−0.10
−0.05
0.00
0.05
0.10
MR leave−one−out sensitivity analysis for
'Adenosine 5'−monophosphate (AMP) to citrate ratio' on 'Rheumatoid arthritis'

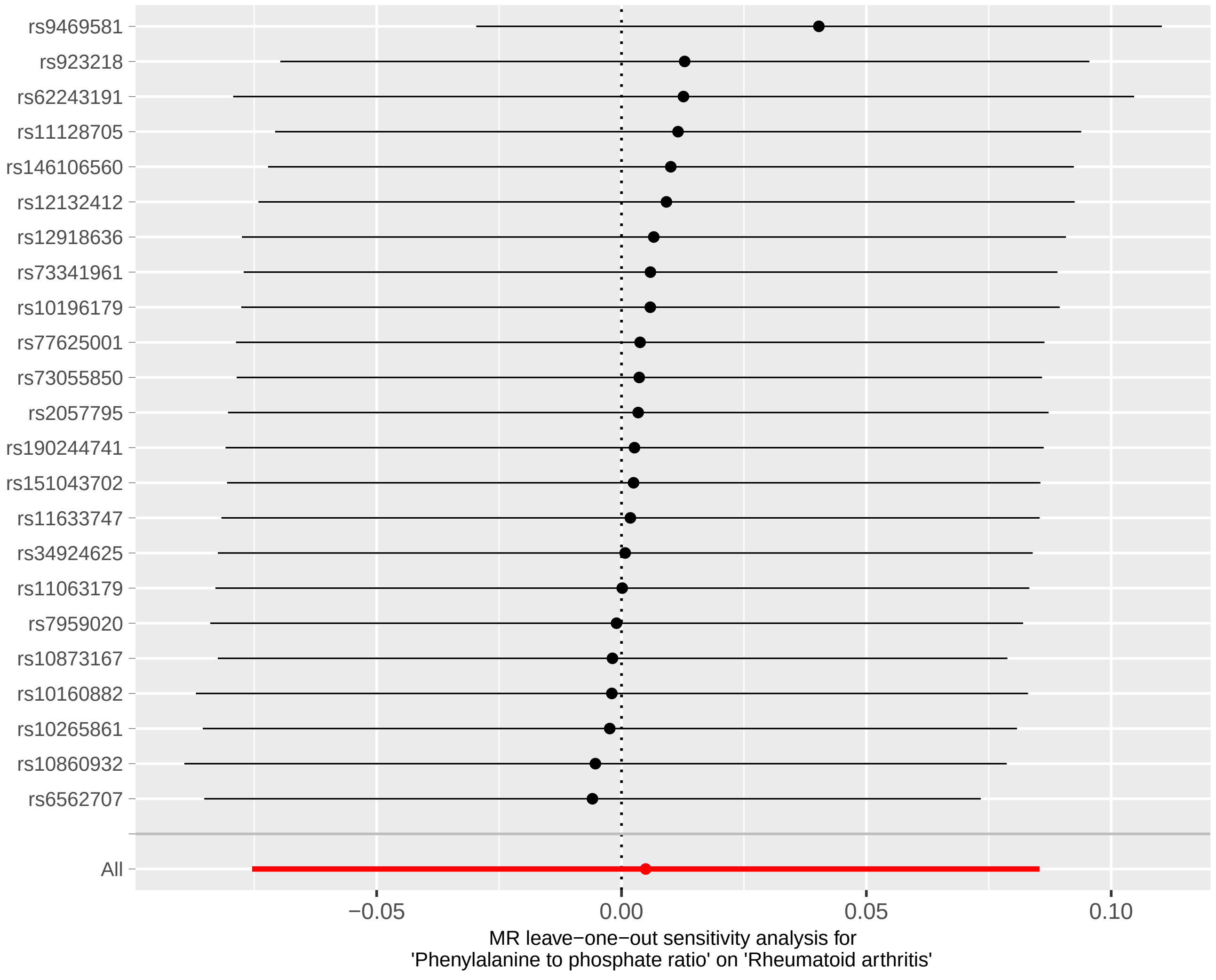
rs9469581
rs923218
rs62243191
rs11128705
rs146106560
rs12132412
rs12918636
rs73341961
rs10196179
rs77625001
rs73055850
rs2057795
rs190244741
rs151043702
rs11633747
rs34924625
rs11063179
rs7959020
rs10873167
rs10160882
rs10265861
rs10860932
rs6562707
All
−0.05
0.00
0.05
0.10
MR leave−one−out sensitivity analysis for
'Phenylalanine to phosphate ratio' on 'Rheumatoid arthritis'

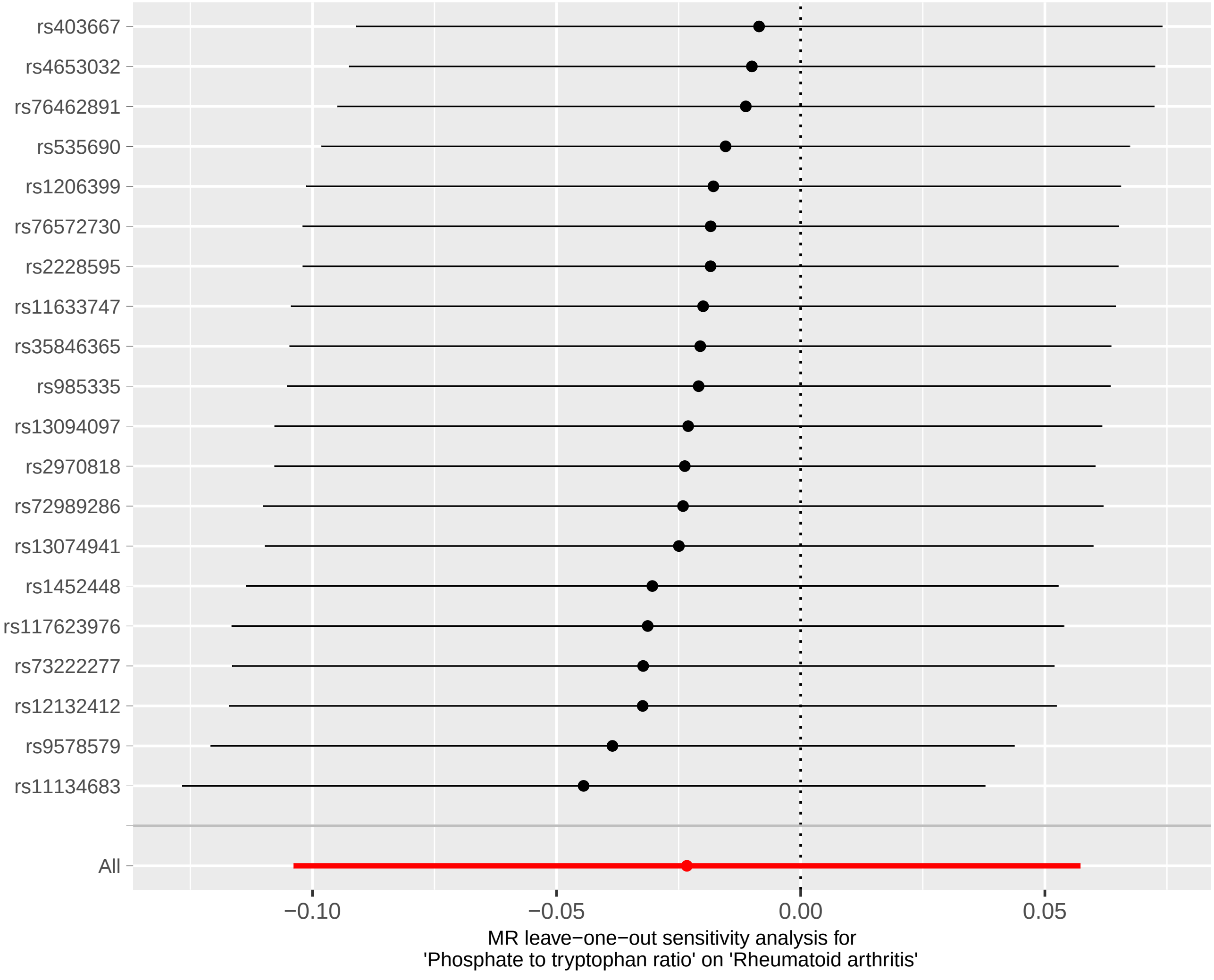
rs403667
rs4653032
rs76462891
rs535690
rs1206399
rs76572730
rs2228595
rs11633747
rs35846365
rs985335
rs13094097
rs2970818
rs72989286
rs13074941
rs1452448
rs117623976
rs73222277
rs12132412
rs9578579
rs11134683
All
−0.10
−0.05
0.00
0.05
MR leave−one−out sensitivity analysis for
'Phosphate to tryptophan ratio' on 'Rheumatoid arthritis'

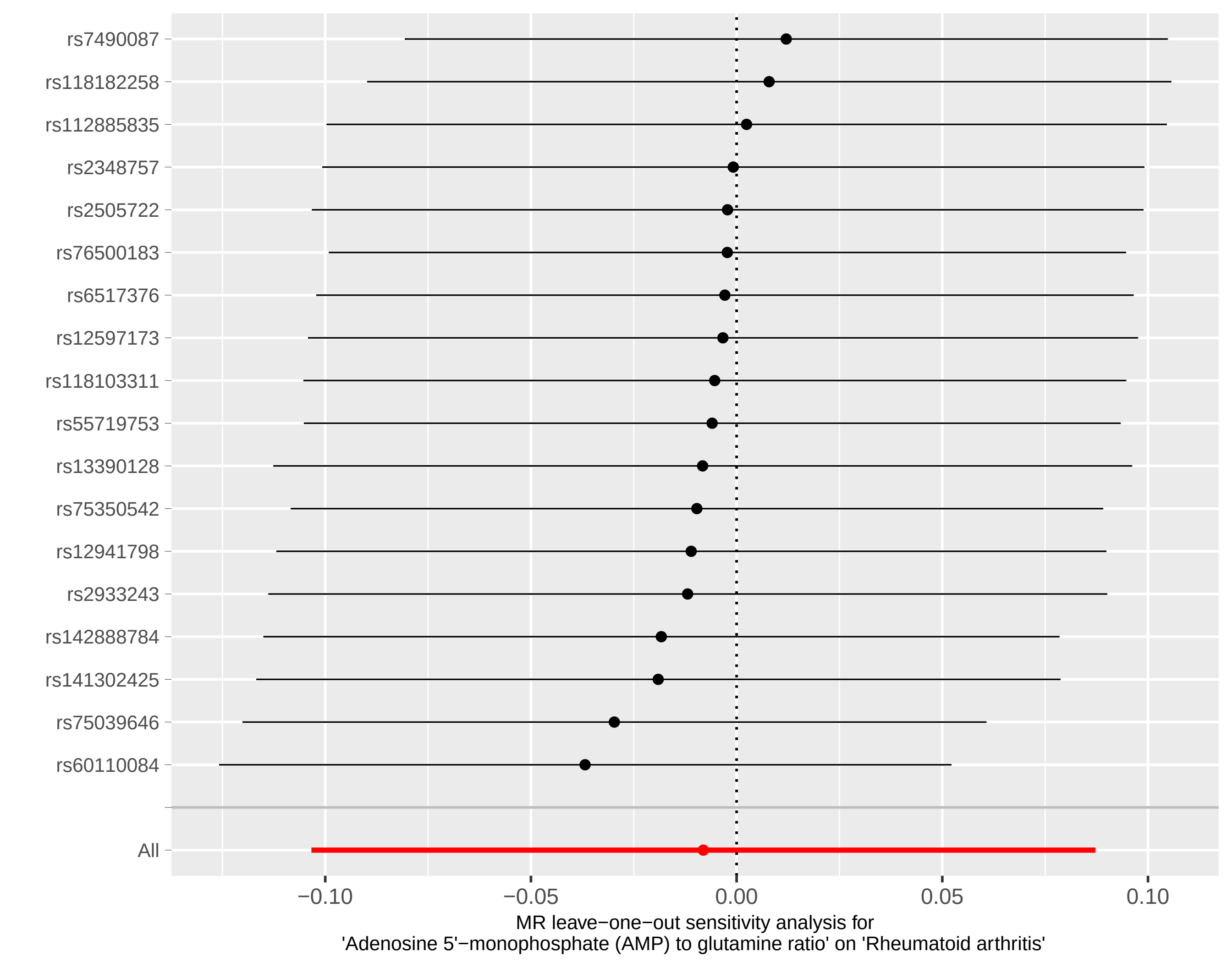

rs7490087
rs118182258
rs112885835
rs2348757
rs2505722
rs76500183
rs6517376
rs12597173
rs118103311
rs55719753
rs13390128
rs75350542
rs12941798
rs2933243
rs142888784
rs141302425
rs75039646
rs60110084
All
−0.10
−0.05
0.00
0.05
0.10
MR leave−one−out sensitivity analysis for
'Adenosine 5'−monophosphate (AMP) to glutamine ratio' on 'Rheumatoid arthritis'

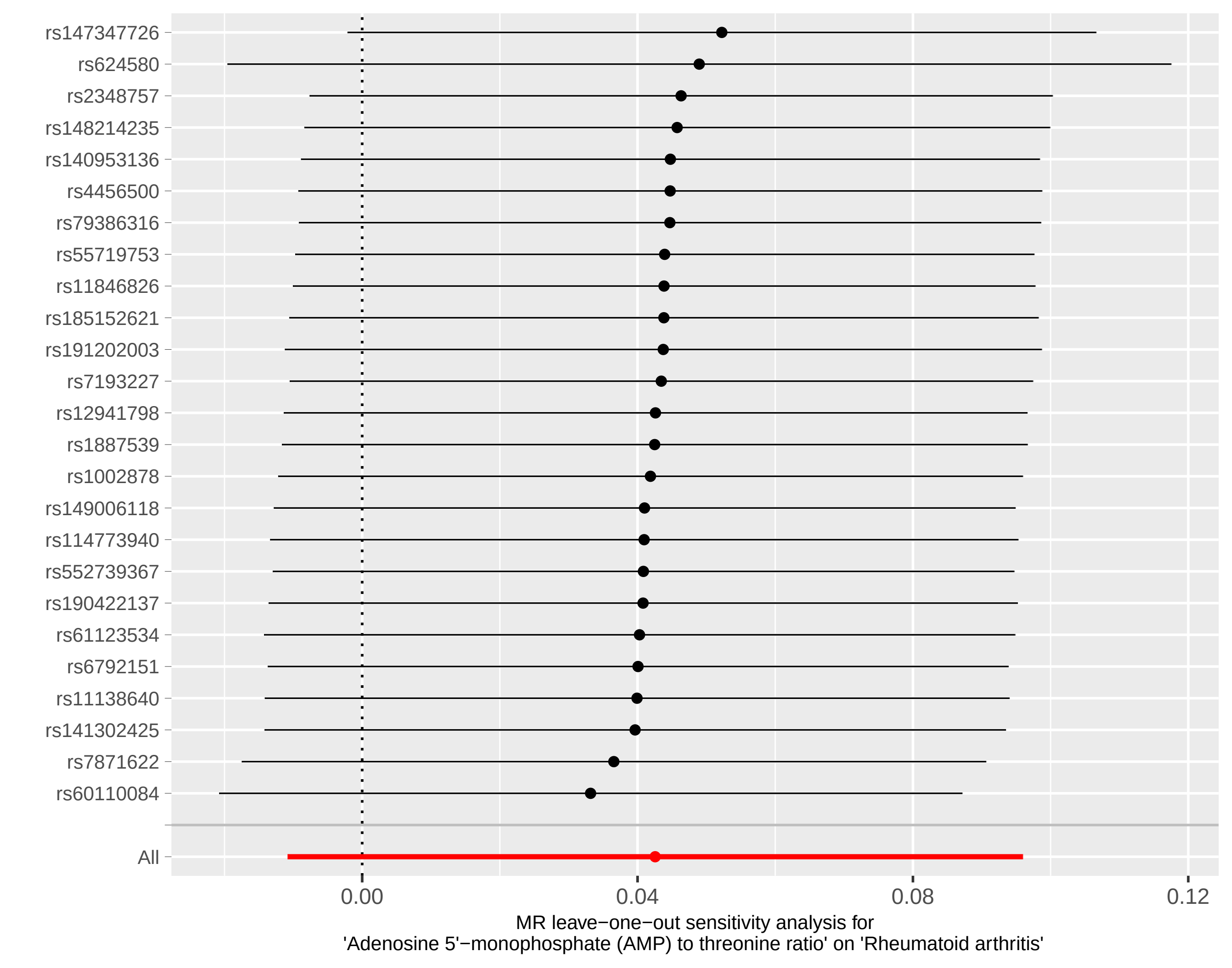

rs147347726
rs624580
rs2348757
rs148214235
rs140953136
rs4456500
rs79386316
rs55719753
rs11846826
rs185152621
rs191202003
rs7193227
rs12941798
rs1887539
rs1002878
rs149006118
rs114773940
rs552739367
rs190422137
rs61123534
rs6792151
rs11138640
rs141302425
rs7871622
rs60110084
All
0.00
0.04
0.08
0.12
MR leave−one−out sensitivity analysis for
'Adenosine 5'−monophosphate (AMP) to threonine ratio' on 'Rheumatoid arthritis'

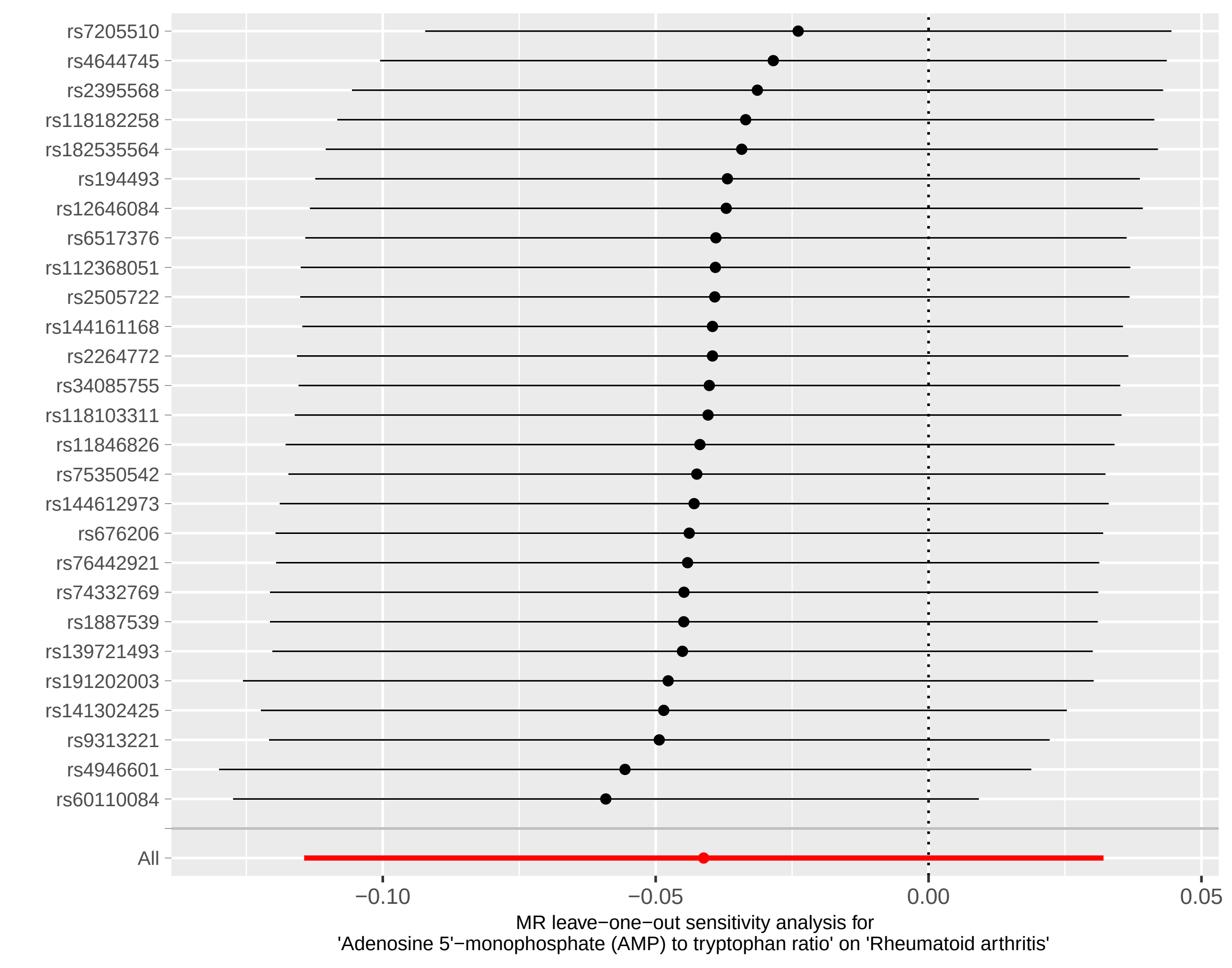

rs7205510
rs4644745
rs2395568
rs118182258
rs182535564
rs194493
rs12646084
rs6517376
rs112368051
rs2505722
rs144161168
rs2264772
rs34085755
rs118103311
rs11846826
rs75350542
rs144612973
rs676206
rs76442921
rs74332769
rs1887539
rs139721493
rs191202003
rs141302425
rs9313221
rs4946601
rs60110084
All
−0.10
−0.05
0.00
0.05
MR leave−one−out sensitivity analysis for
'Adenosine 5'−monophosphate (AMP) to tryptophan ratio' on 'Rheumatoid arthritis'

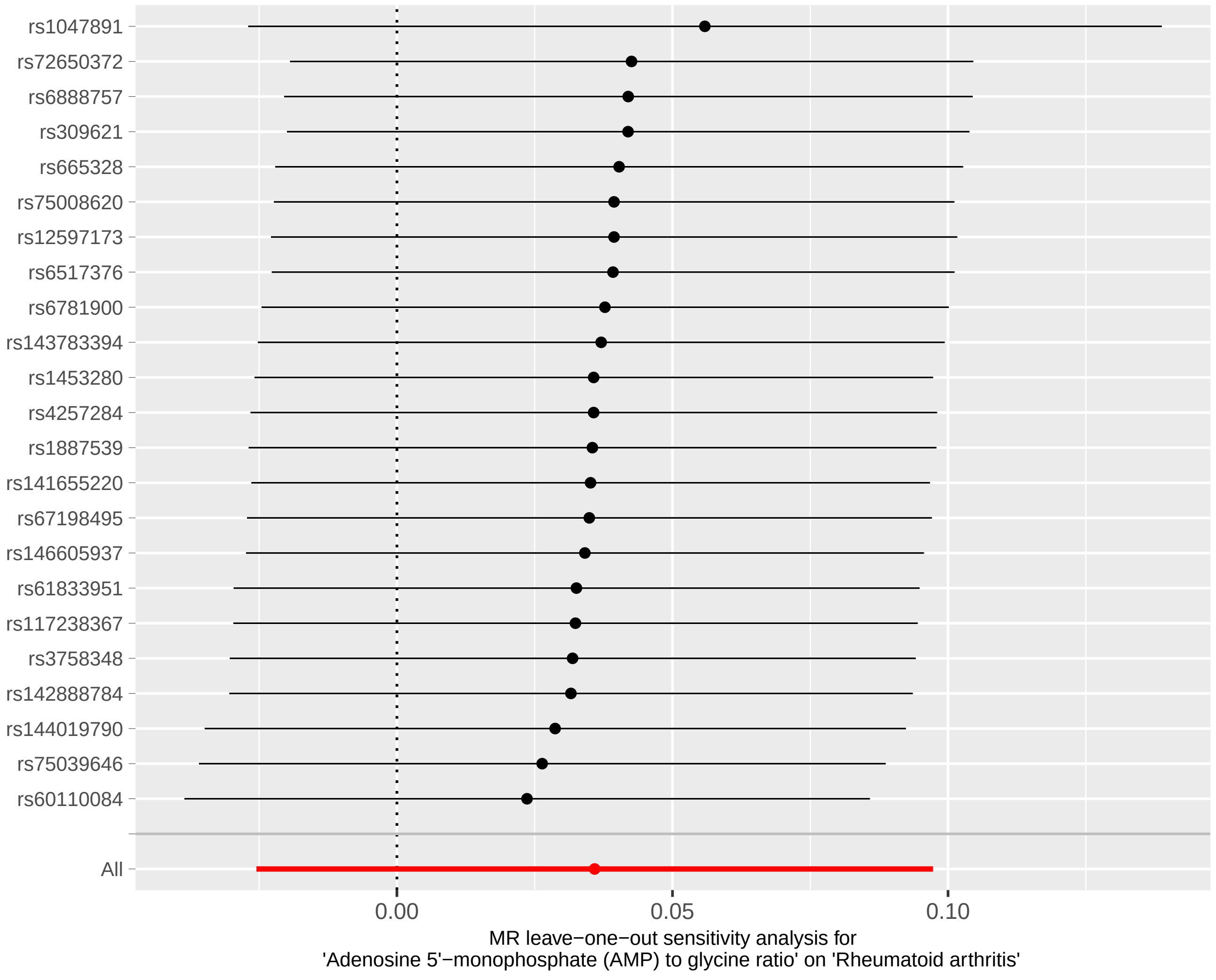
rs1047891
rs72650372
rs6888757
rs309621
rs665328
rs75008620
rs12597173
rs6517376
rs6781900
rs143783394
rs1453280
rs4257284
rs1887539
rs141655220
rs67198495
rs146605937
rs61833951
rs117238367
rs3758348
rs142888784
rs144019790
rs75039646
rs60110084
All
0.00
0.05
0.10
MR leave−one−out sensitivity analysis for
'Adenosine 5'−monophosphate (AMP) to glycine ratio' on 'Rheumatoid arthritis'

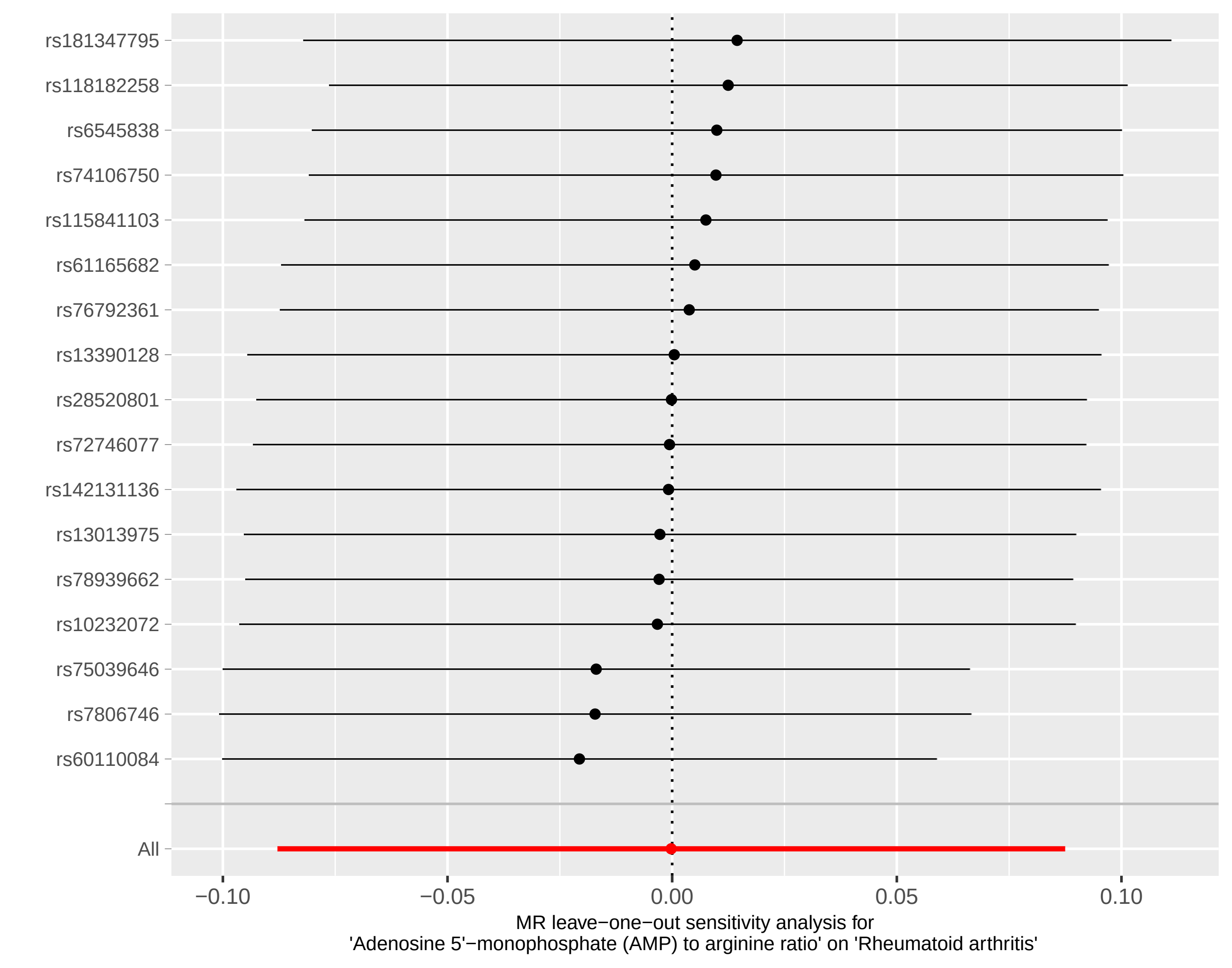
rs181347795
rs118182258
rs6545838
rs74106750
rs115841103
rs61165682
rs76792361
rs13390128
rs28520801
rs72746077
rs142131136
rs13013975
rs78939662
rs10232072
rs75039646
rs7806746
rs60110084
All
−0.10
−0.05
0.00
0.05
0.10
MR leave−one−out sensitivity analysis for
'Adenosine 5'−monophosphate (AMP) to arginine ratio' on 'Rheumatoid arthritis'

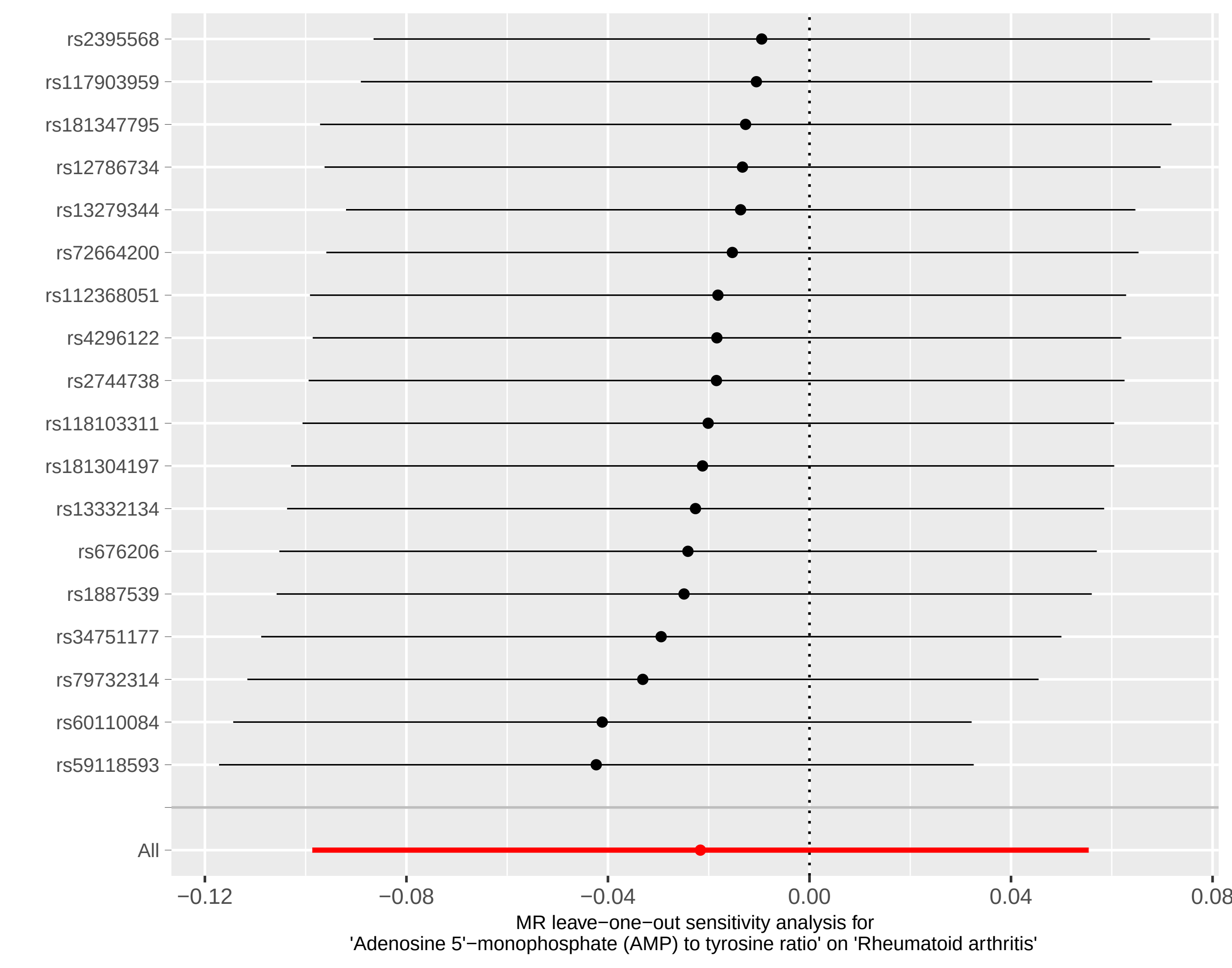
rs2395568
rs117903959
rs181347795
rs12786734
rs13279344
rs72664200
rs112368051
rs4296122
rs2744738
rs118103311
rs181304197
rs13332134
rs676206
rs1887539
rs34751177
rs79732314
rs60110084
rs59118593
All
−0.12
−0.08
−0.04
0.00
0.04
0.08
MR leave−one−out sensitivity analysis for
'Adenosine 5'−monophosphate (AMP) to tyrosine ratio' on 'Rheumatoid arthritis'

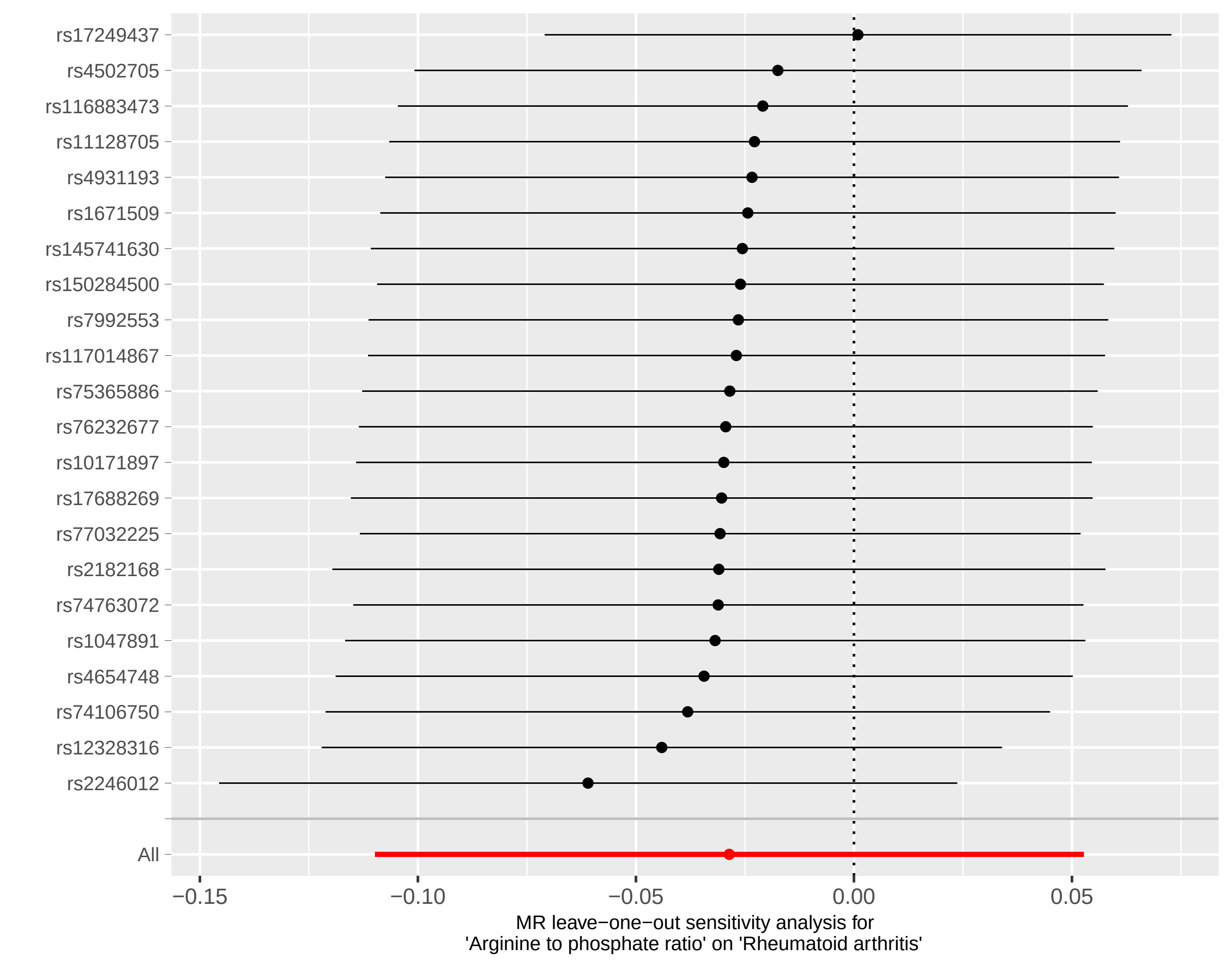
rs17249437
rs4502705
rs116883473
rs11128705
rs4931193
rs1671509
rs145741630
rs150284500
rs7992553
rs117014867
rs75365886
rs76232677
rs10171897
rs17688269
rs77032225
rs2182168
rs74763072
rs1047891
rs4654748
rs74106750
rs12328316
rs2246012
All
−0.15
−0.10
−0.05
0.00
0.05
MR leave−one−out sensitivity analysis for
'Arginine to phosphate ratio' on 'Rheumatoid arthritis'

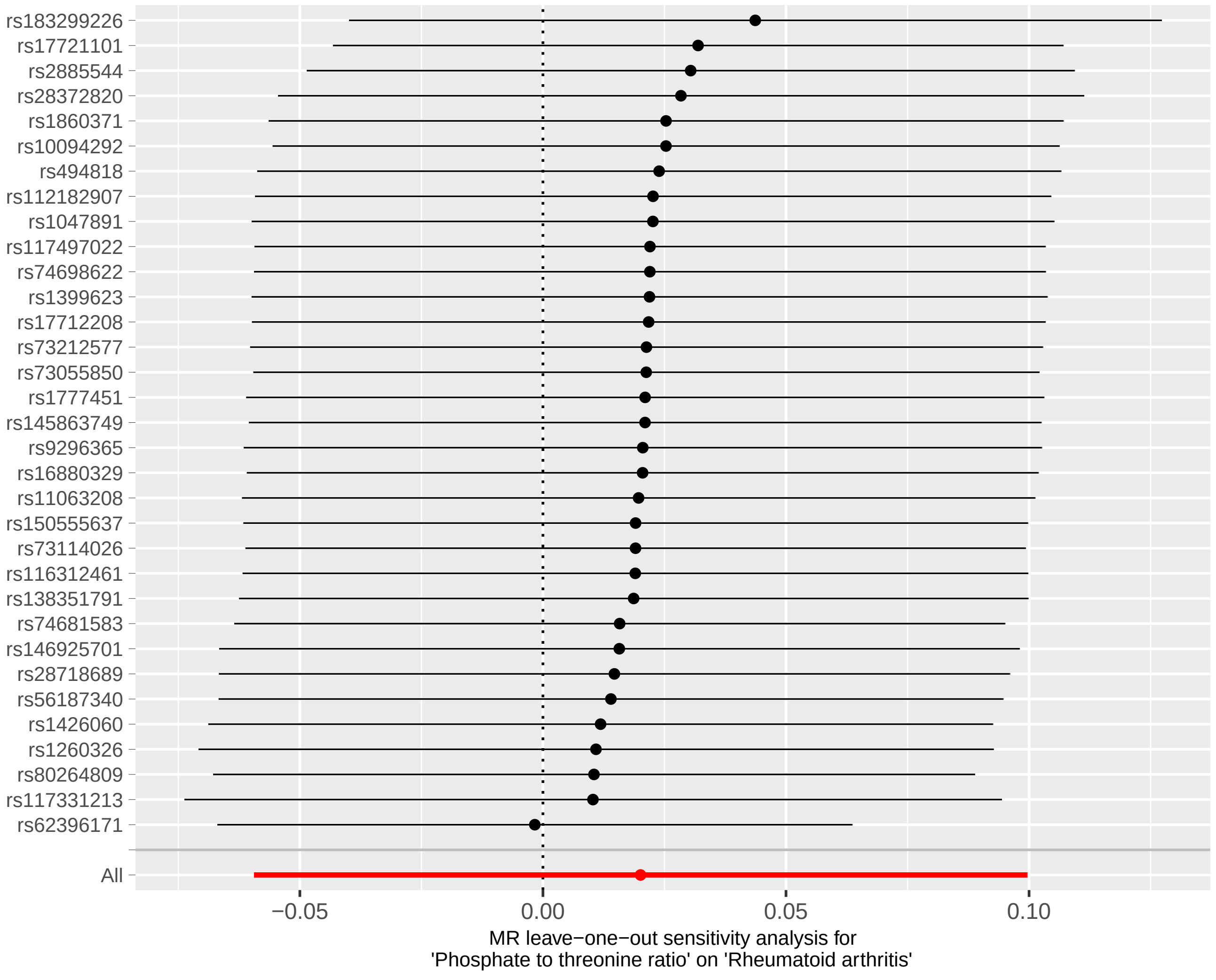
rs183299226
rs17721101
rs2885544
rs28372820
rs1860371
rs10094292
rs494818
rs112182907
rs1047891
rs117497022
rs74698622
rs1399623
rs17712208
rs73212577
rs73055850
rs1777451
rs145863749
rs9296365
rs16880329
rs11063208
rs150555637
rs73114026
rs116312461
rs138351791
rs74681583
rs146925701
rs28718689
rs56187340
rs1426060
rs1260326
rs80264809
rs117331213
rs62396171
All
−0.05
0.00
0.05
0.10
MR leave−one−out sensitivity analysis for
'Phosphate to threonine ratio' on 'Rheumatoid arthritis'

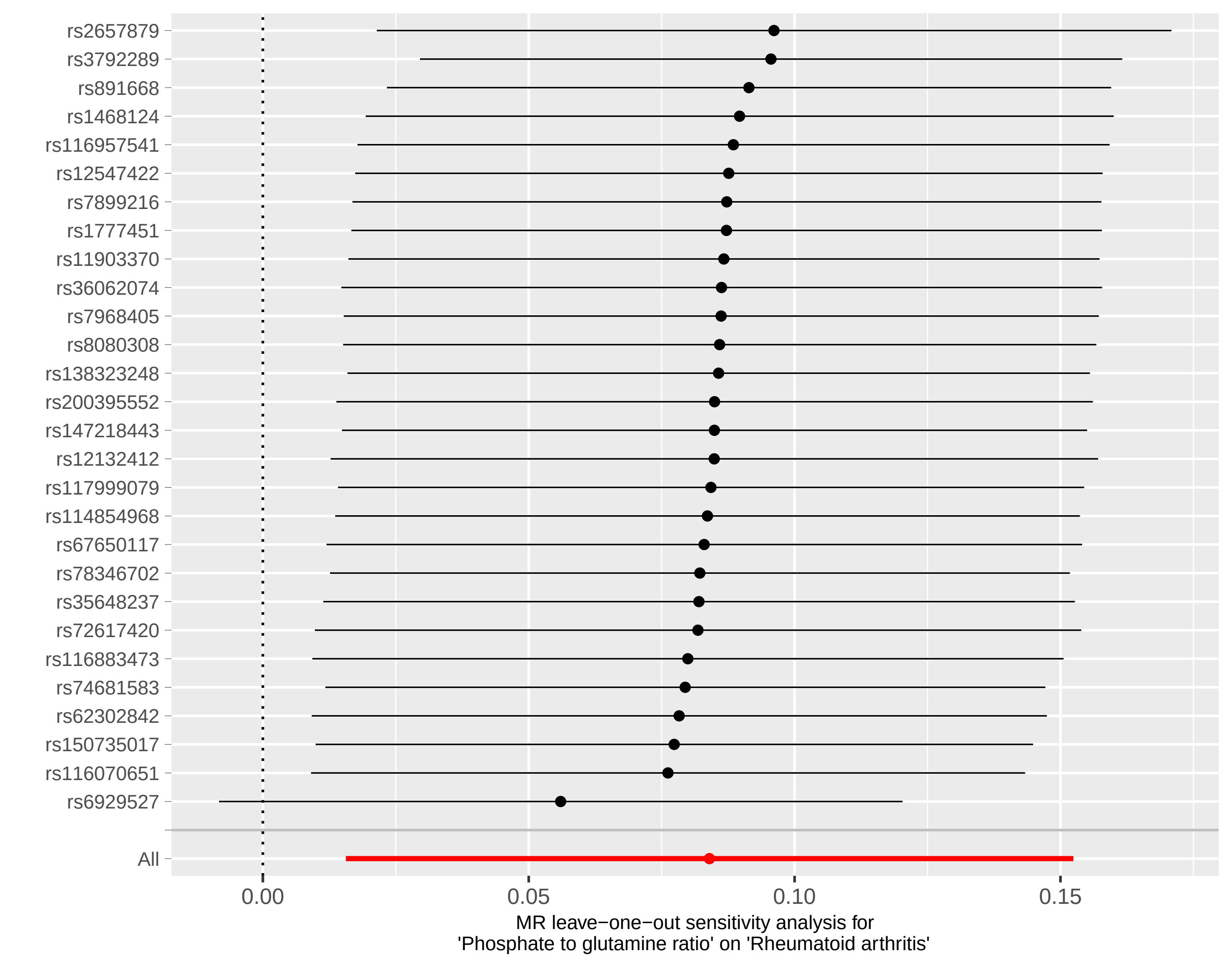
rs2657879
rs3792289
rs891668
rs1468124
rs116957541
rs12547422
rs7899216
rs1777451
rs11903370
rs36062074
rs7968405
rs8080308
rs138323248
rs200395552
rs147218443
rs12132412
rs117999079
rs114854968
rs67650117
rs78346702
rs35648237
rs72617420
rs116883473
rs74681583
rs62302842
rs150735017
rs116070651
rs6929527
All
0.00
0.05
0.10
0.15
MR leave−one−out sensitivity analysis for
'Phosphate to glutamine ratio' on 'Rheumatoid arthritis'

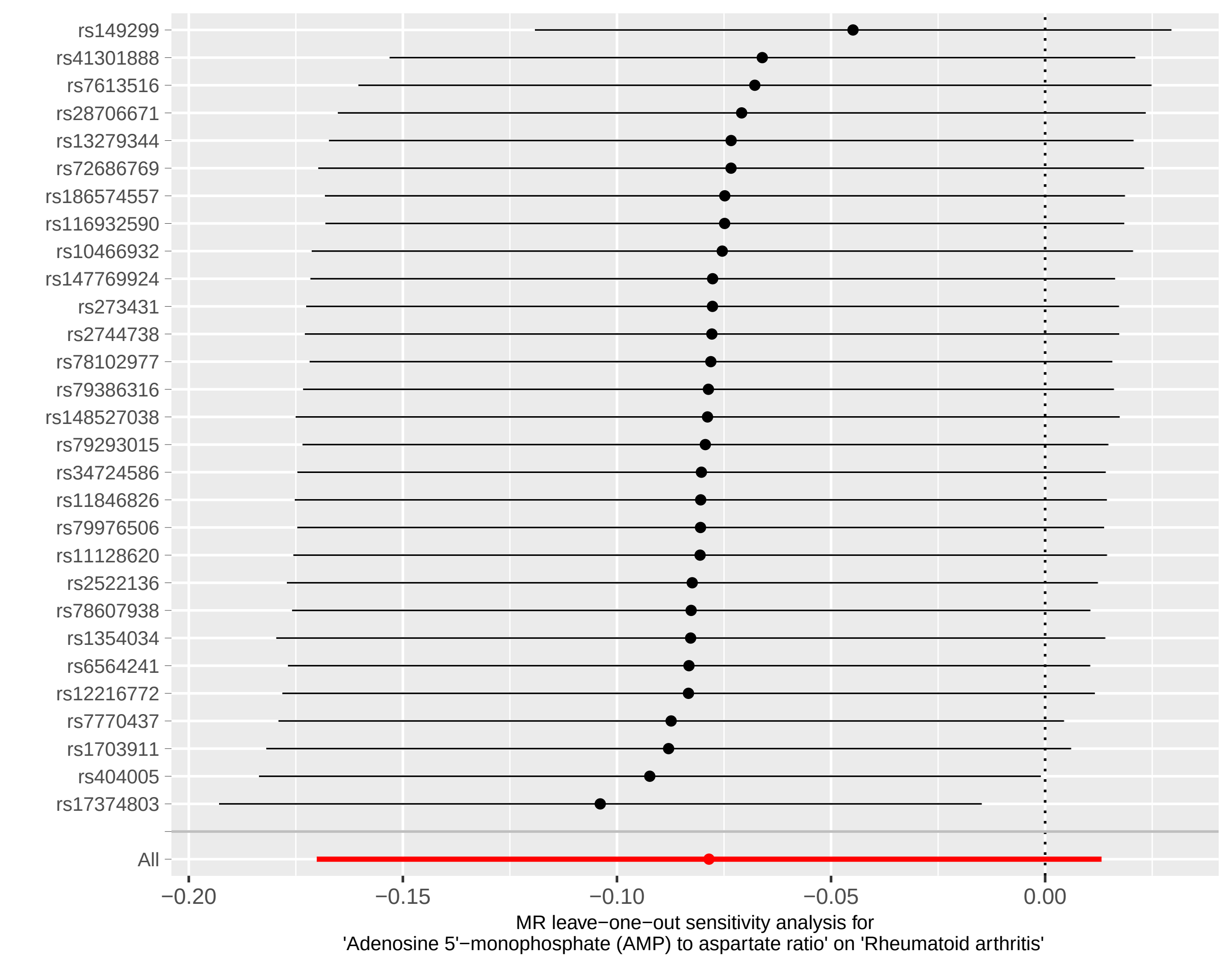
rs149299
rs41301888
rs7613516
rs28706671
rs13279344
rs72686769
rs186574557
rs116932590
rs10466932
rs147769924
rs273431
rs2744738
rs78102977
rs79386316
rs148527038
rs79293015
rs34724586
rs11846826
rs79976506
rs11128620
rs2522136
rs78607938
rs1354034
rs6564241
rs12216772
rs7770437
rs1703911
rs404005
rs17374803
All
−0.20
−0.15
−0.10
−0.05
0.00
MR leave−one−out sensitivity analysis for
'Adenosine 5'−monophosphate (AMP) to aspartate ratio' on 'Rheumatoid arthritis'

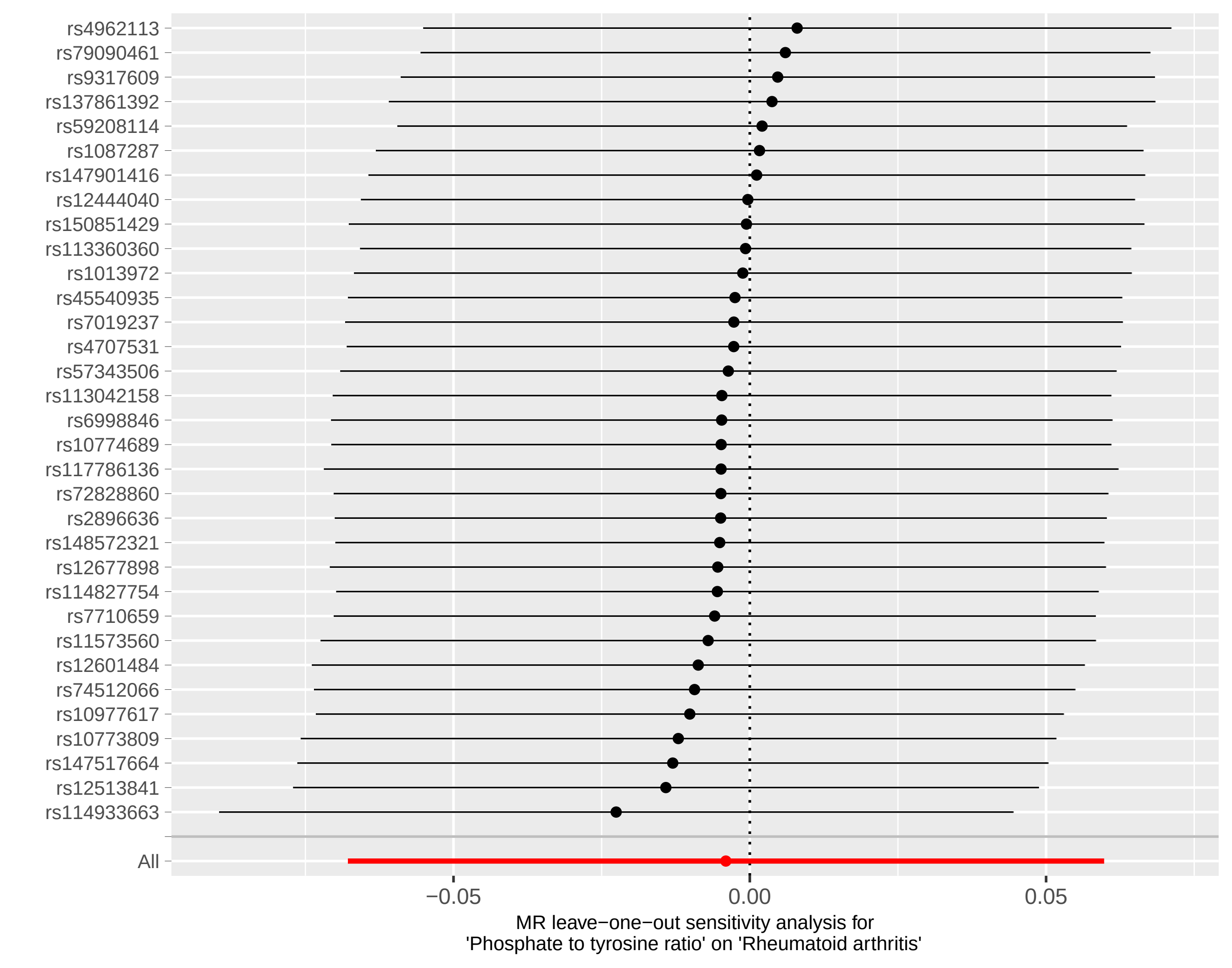
rs4962113
rs79090461
rs9317609
rs137861392
rs59208114
rs1087287
rs147901416
rs12444040
rs150851429
rs113360360
rs1013972
rs45540935
rs7019237
rs4707531
rs57343506
rs113042158
rs6998846
rs10774689
rs117786136
rs72828860
rs2896636
rs148572321
rs12677898
rs114827754
rs7710659
rs11573560
rs12601484
rs74512066
rs10977617
rs10773809
rs147517664
rs12513841
rs114933663
All
−0.05
0.00
0.05
MR leave−one−out sensitivity analysis for
'Phosphate to tyrosine ratio' on 'Rheumatoid arthritis'

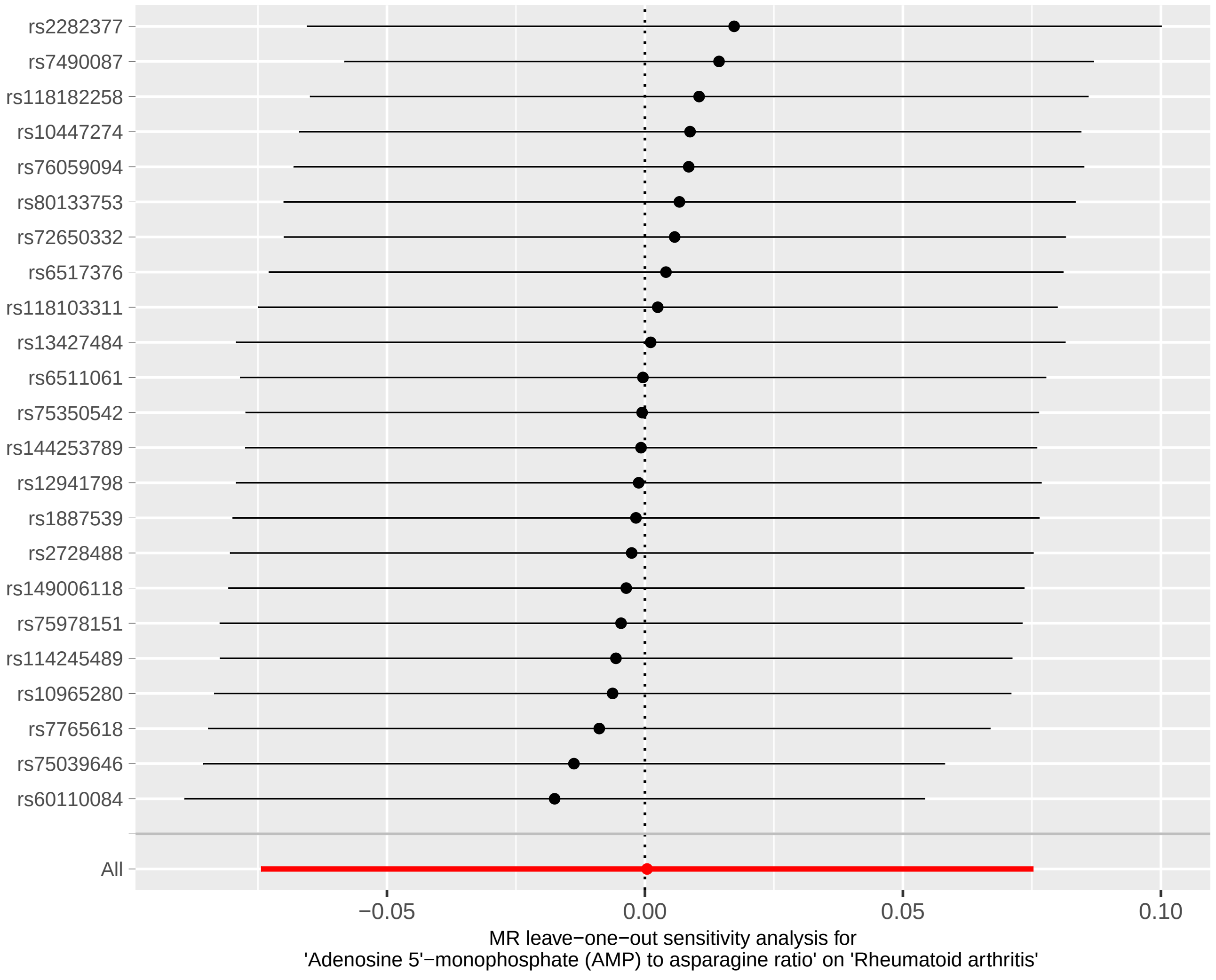

rs2282377
rs7490087
rs118182258
rs10447274
rs76059094
rs80133753
rs72650332
rs6517376
rs118103311
rs13427484
rs6511061
rs75350542
rs144253789
rs12941798
rs1887539
rs2728488
rs149006118
rs75978151
rs114245489
rs10965280
rs7765618
rs75039646
rs60110084
All
−0.05
0.00
0.05
0.10
MR leave−one−out sensitivity analysis for
'Adenosine 5'−monophosphate (AMP) to asparagine ratio' on 'Rheumatoid arthritis'

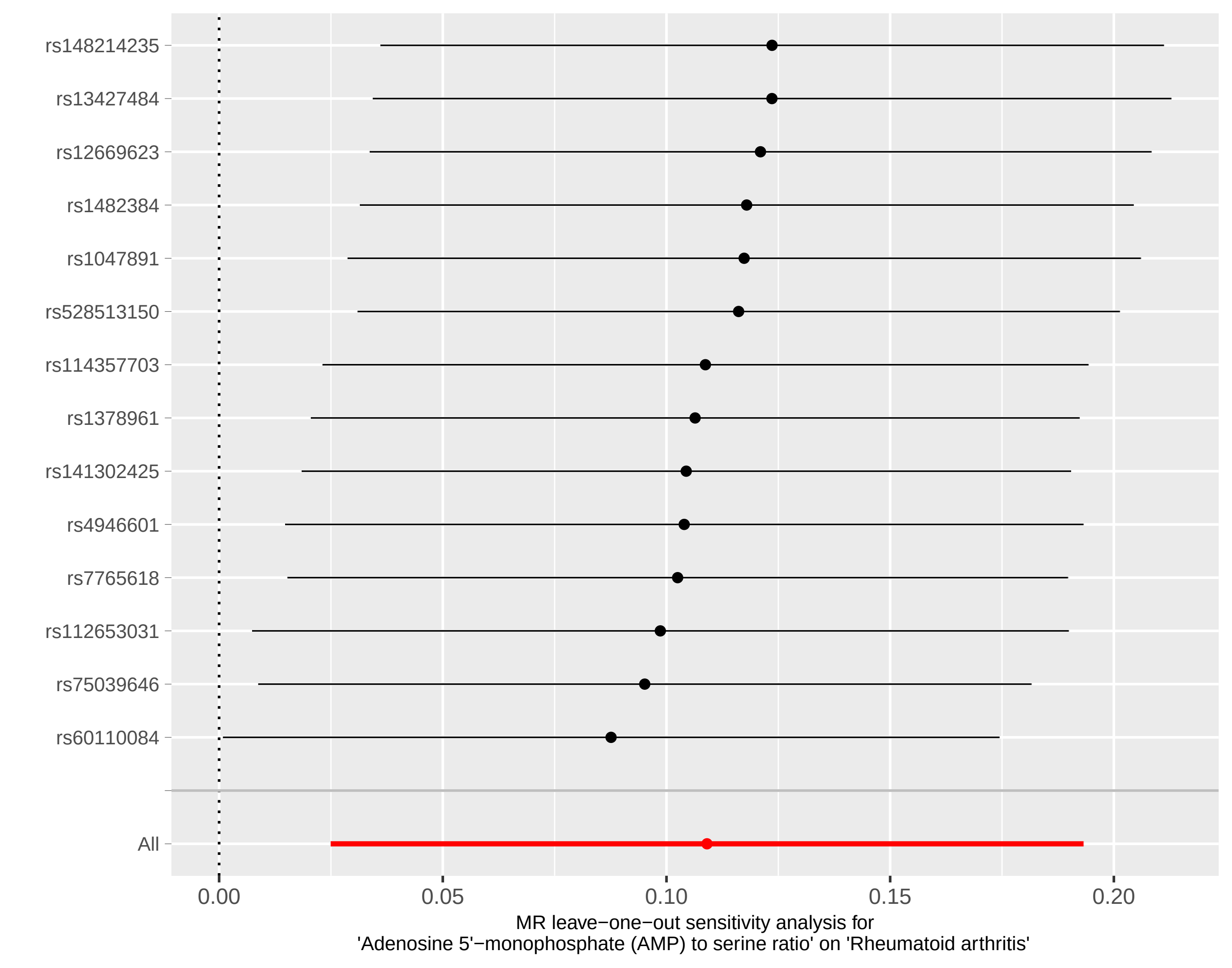
rs148214235
rs13427484
rs12669623
rs1482384
rs1047891
rs528513150
rs114357703
rs1378961
rs141302425
rs4946601
rs7765618
rs112653031
rs75039646
rs60110084
All
0.00
0.05
0.10
0.15
0.20
MR leave−one−out sensitivity analysis for
'Adenosine 5'−monophosphate (AMP) to serine ratio' on 'Rheumatoid arthritis'

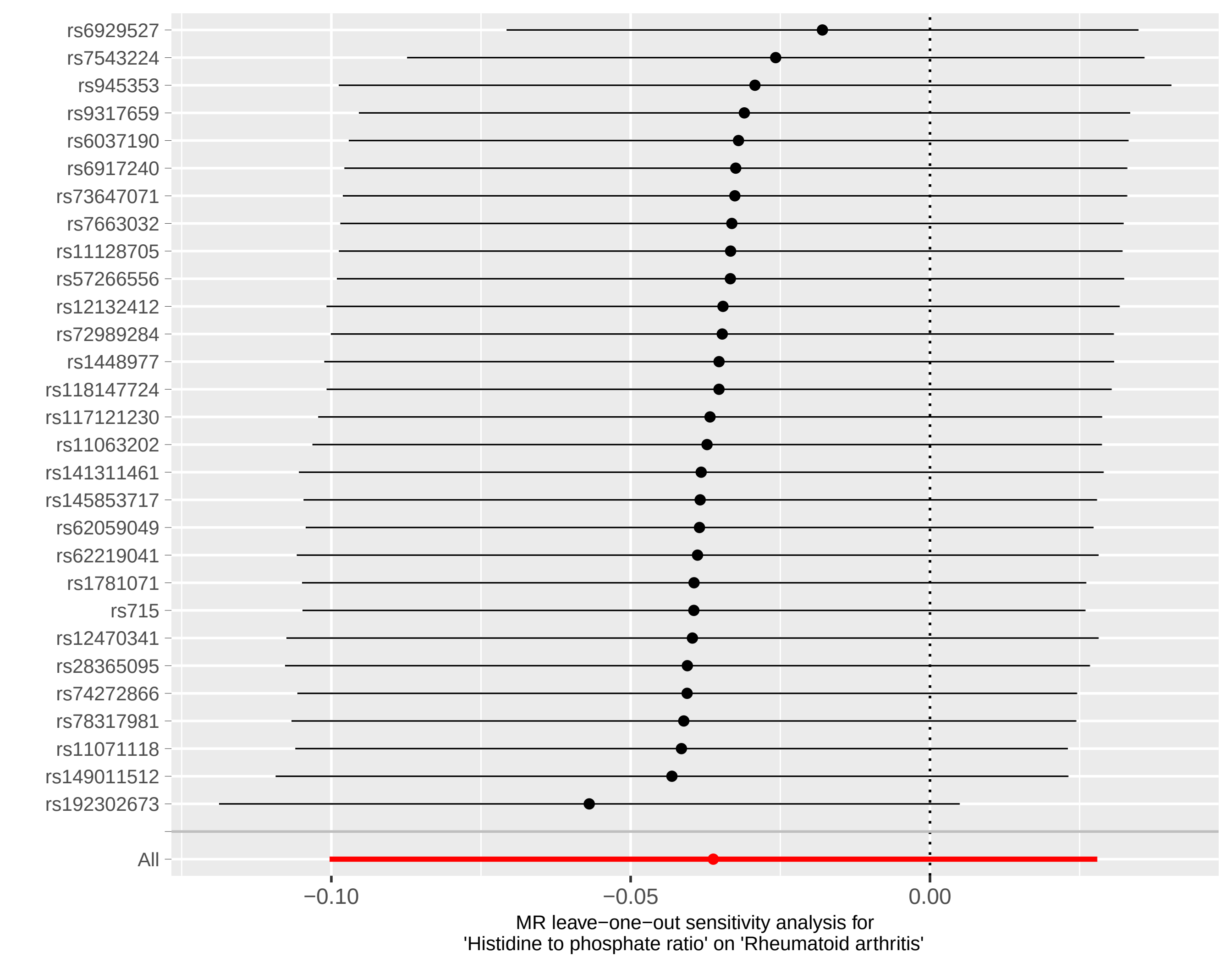

rs6929527
rs7543224
rs945353
rs9317659
rs6037190
rs6917240
rs73647071
rs7663032
rs11128705
rs57266556
rs12132412
rs72989284
rs1448977
rs118147724
rs117121230
rs11063202
rs141311461
rs145853717
rs62059049
rs62219041
rs1781071
rs715
rs12470341
rs28365095
rs74272866
rs78317981
rs11071118
rs149011512
rs192302673
All
−0.10
−0.05
0.00
MR leave−one−out sensitivity analysis for
'Histidine to phosphate ratio' on 'Rheumatoid arthritis'

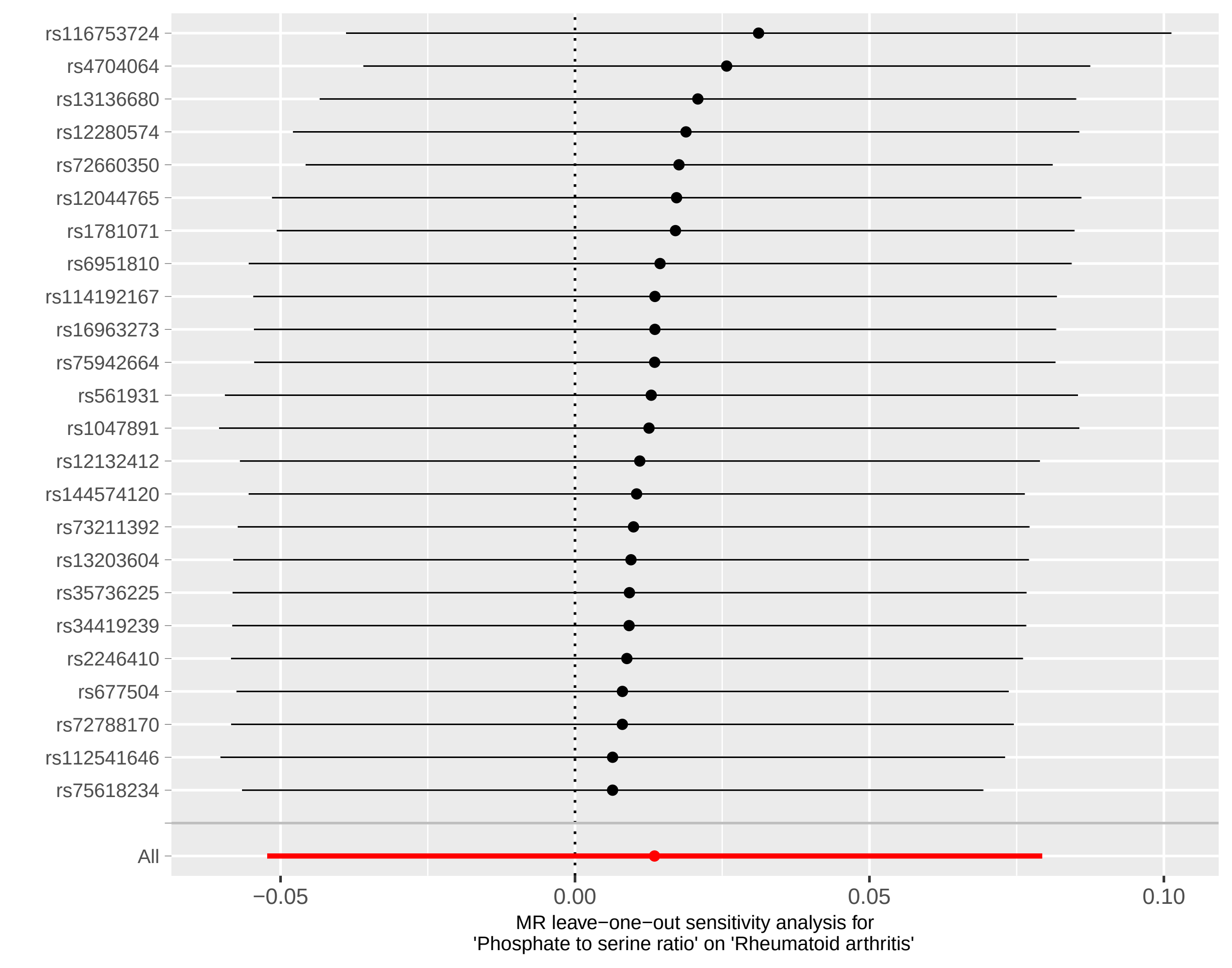

rs116753724
rs4704064
rs13136680
rs12280574
rs72660350
rs12044765
rs1781071
rs6951810
rs114192167
rs16963273
rs75942664
rs561931
rs1047891
rs12132412
rs144574120
rs73211392
rs13203604
rs35736225
rs34419239
rs2246410
rs677504
rs72788170
rs112541646
rs75618234
All
−0.05
0.00
0.05
0.10
MR leave−one−out sensitivity analysis for
'Phosphate to serine ratio' on 'Rheumatoid arthritis'

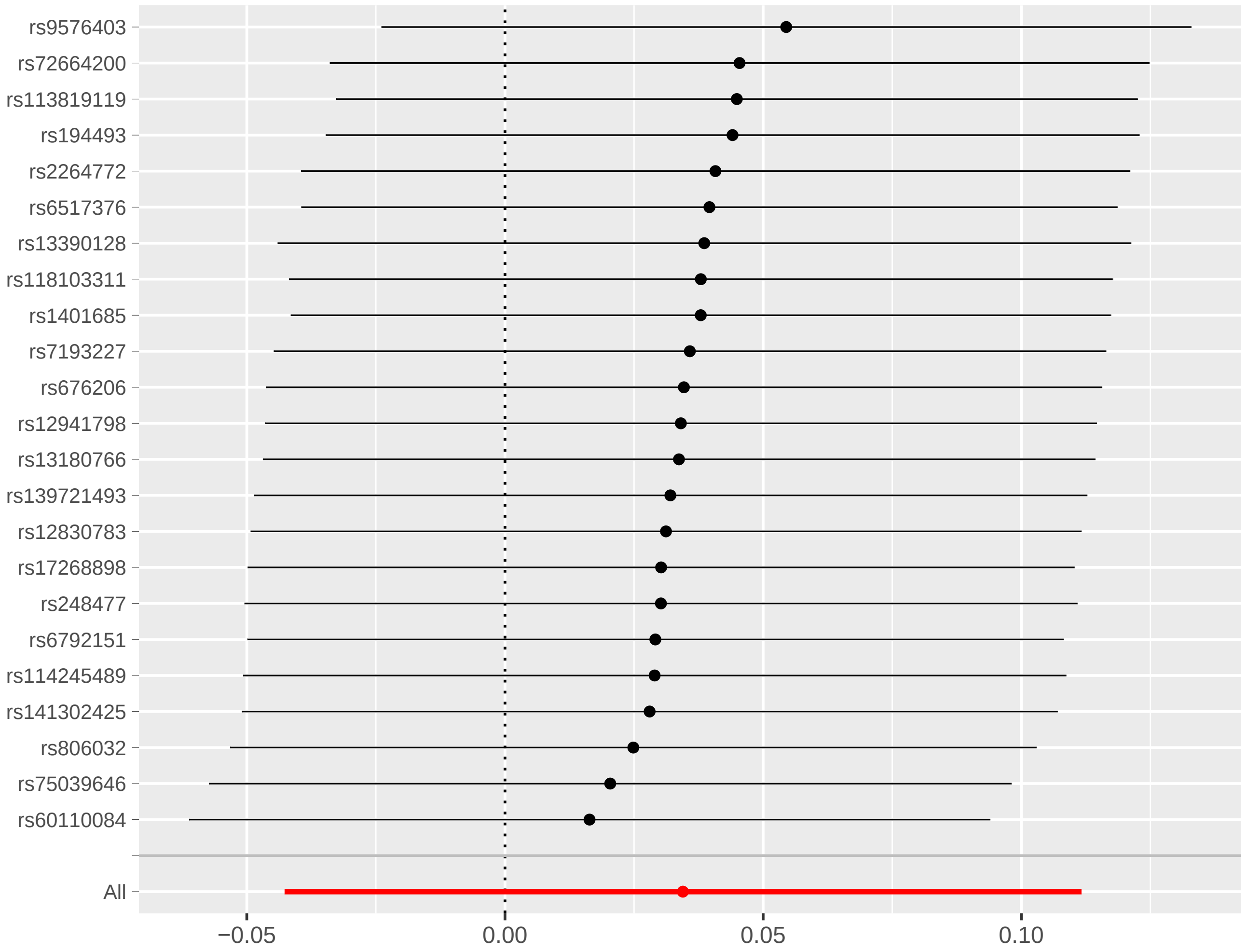

MR leave−one−out sensitivity analysis for
'Adenosine 5'−monophosphate (AMP) to histidine ratio' on 'Rheumatoid arthritis'

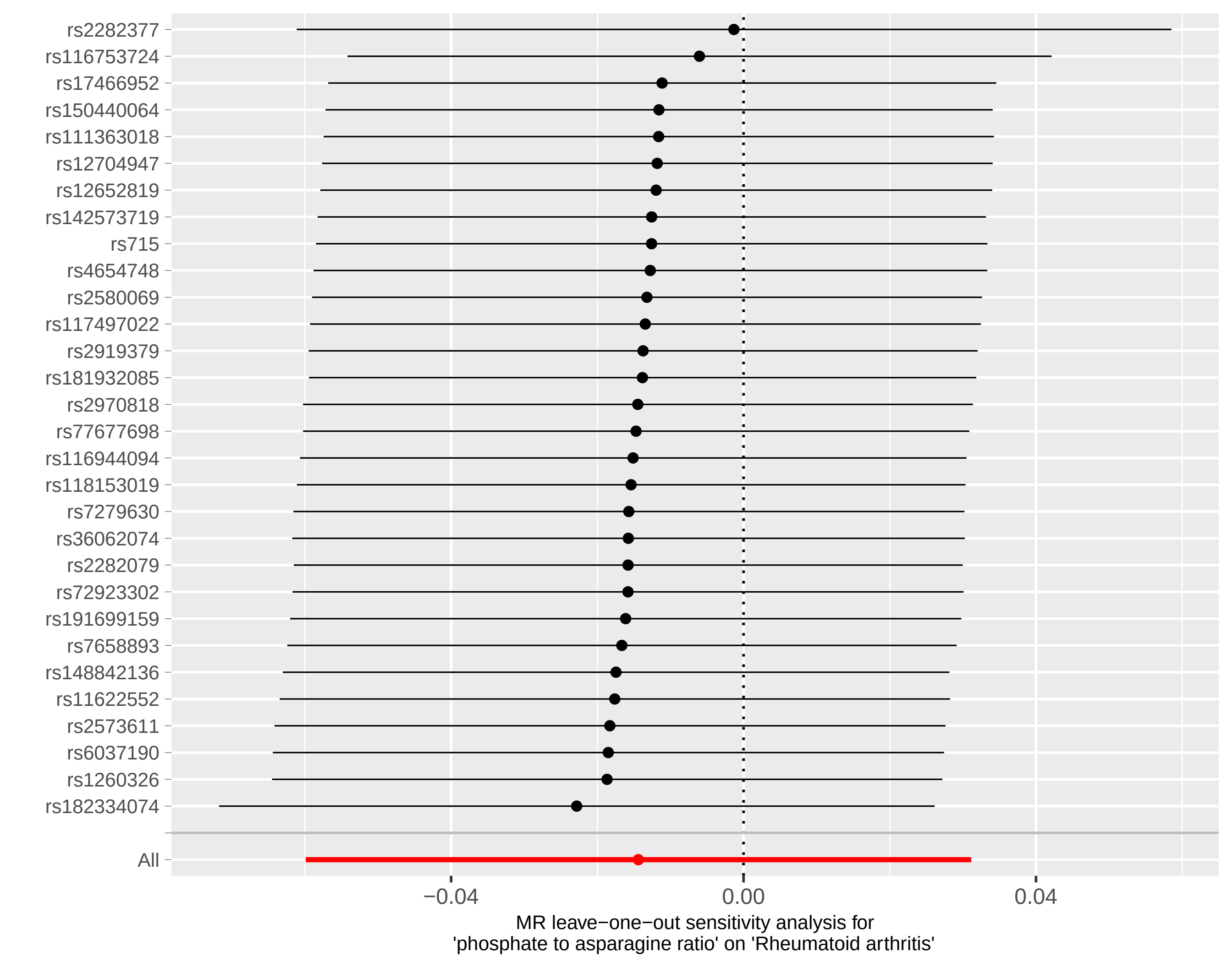
rs2282377
rs116753724
rs17466952
rs150440064
rs111363018
rs12704947
rs12652819
rs142573719
rs715
rs4654748
rs2580069
rs117497022
rs2919379
rs181932085
rs2970818
rs77677698
rs116944094
rs118153019
rs7279630
rs36062074
rs2282079
rs72923302
rs191699159
rs7658893
rs148842136
rs11622552
rs2573611
rs6037190
rs1260326
rs182334074
All
−0.04
0.00
0.04
MR leave−one−out sensitivity analysis for
'phosphate to asparagine ratio' on 'Rheumatoid arthritis'

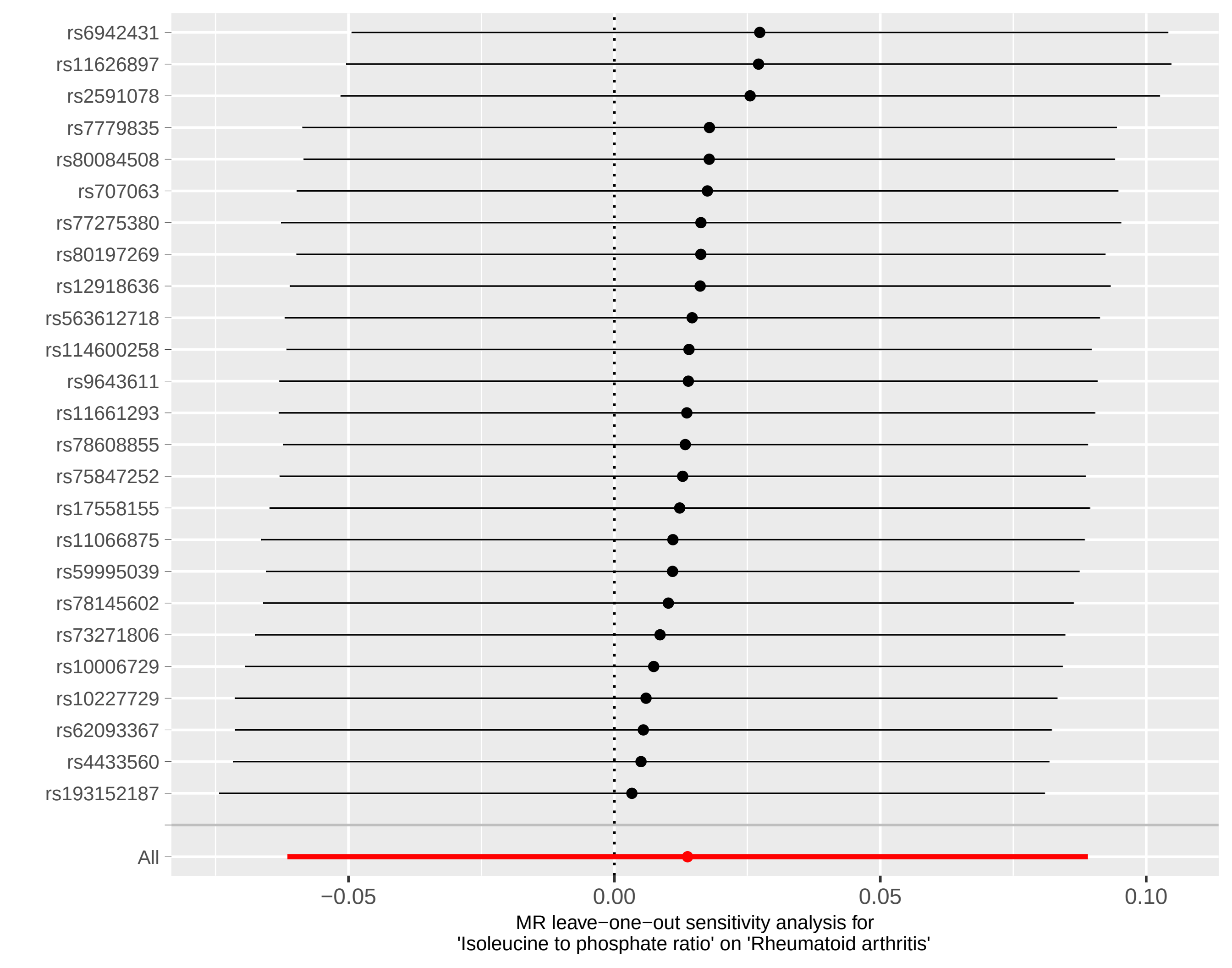
rs6942431
rs11626897
rs2591078
rs7779835
rs80084508
rs707063
rs77275380
rs80197269
rs12918636
rs563612718
rs114600258
rs9643611
rs11661293
rs78608855
rs75847252
rs17558155
rs11066875
rs59995039
rs78145602
rs73271806
rs10006729
rs10227729
rs62093367
rs4433560
rs193152187
All
−0.05
0.00
0.05
0.10
MR leave−one−out sensitivity analysis for
'Isoleucine to phosphate ratio' on 'Rheumatoid arthritis'

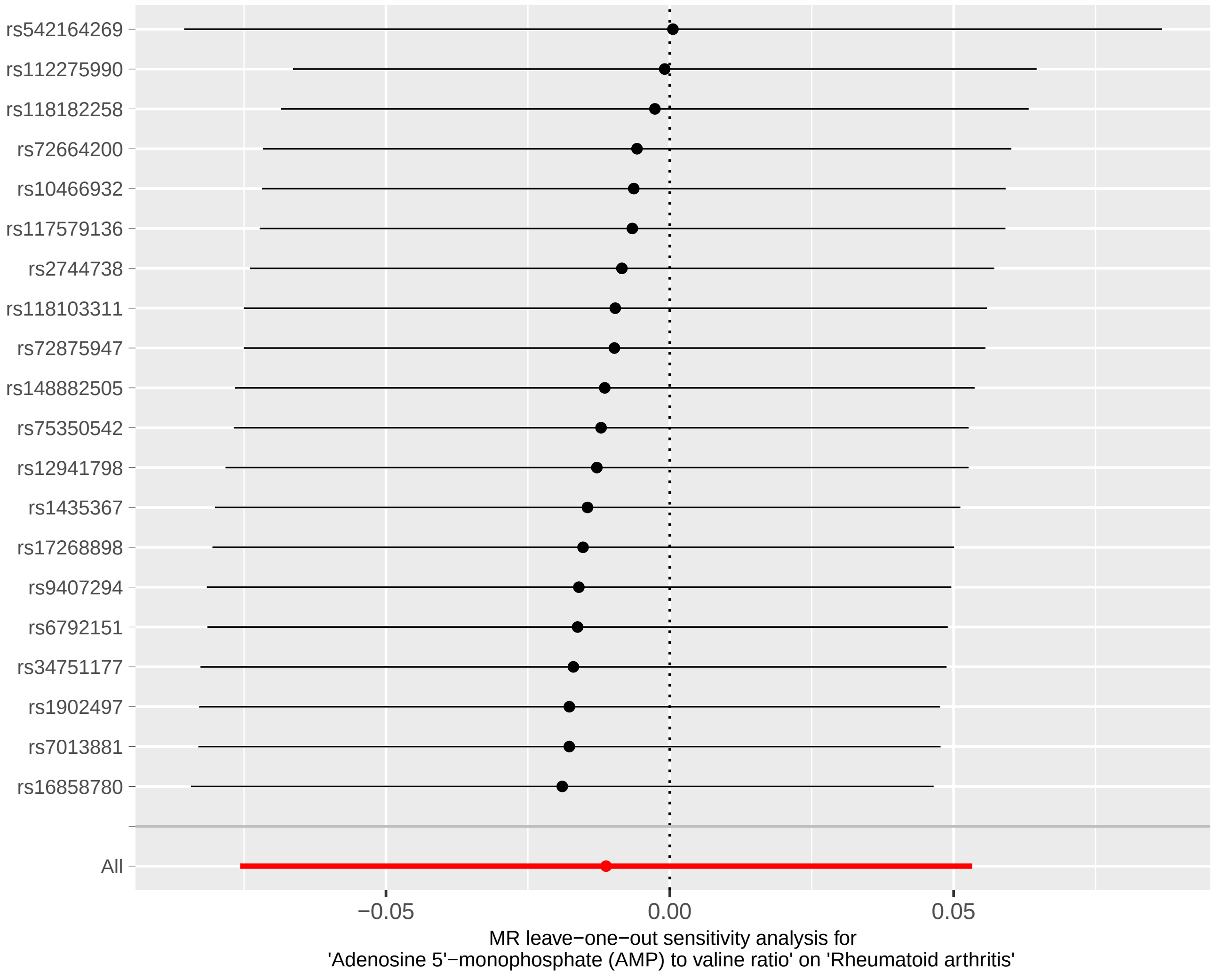

rs542164269
rs112275990
rs118182258
rs72664200
rs10466932
rs117579136
rs2744738
rs118103311
rs72875947
rs148882505
rs75350542
rs12941798
rs1435367
rs17268898
rs9407294
rs6792151
rs34751177
rs1902497
rs7013881
rs16858780
All
−0.05
0.00
0.05
MR leave−one−out sensitivity analysis for
'Adenosine 5'−monophosphate (AMP) to valine ratio' on 'Rheumatoid arthritis'

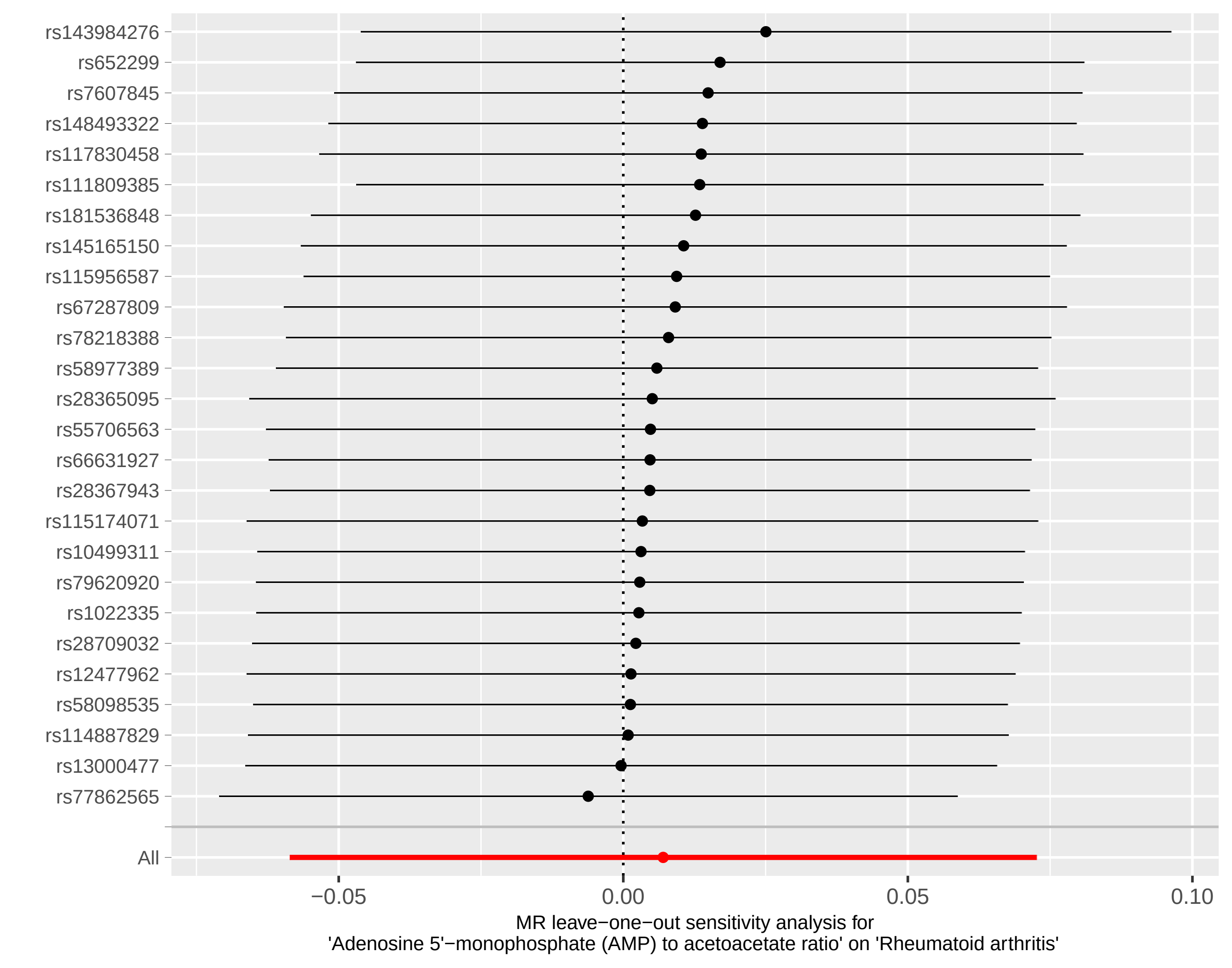
rs143984276
rs652299
rs7607845
rs148493322
rs117830458
rs111809385
rs181536848
rs145165150
rs115956587
rs67287809
rs78218388
rs58977389
rs28365095
rs55706563
rs66631927
rs28367943
rs115174071
rs10499311
rs79620920
rs1022335
rs28709032
rs12477962
rs58098535
rs114887829
rs13000477
rs77862565
All
−0.05
0.00
0.05
0.10
MR leave−one−out sensitivity analysis for
'Adenosine 5'−monophosphate (AMP) to acetoacetate ratio' on 'Rheumatoid arthritis'

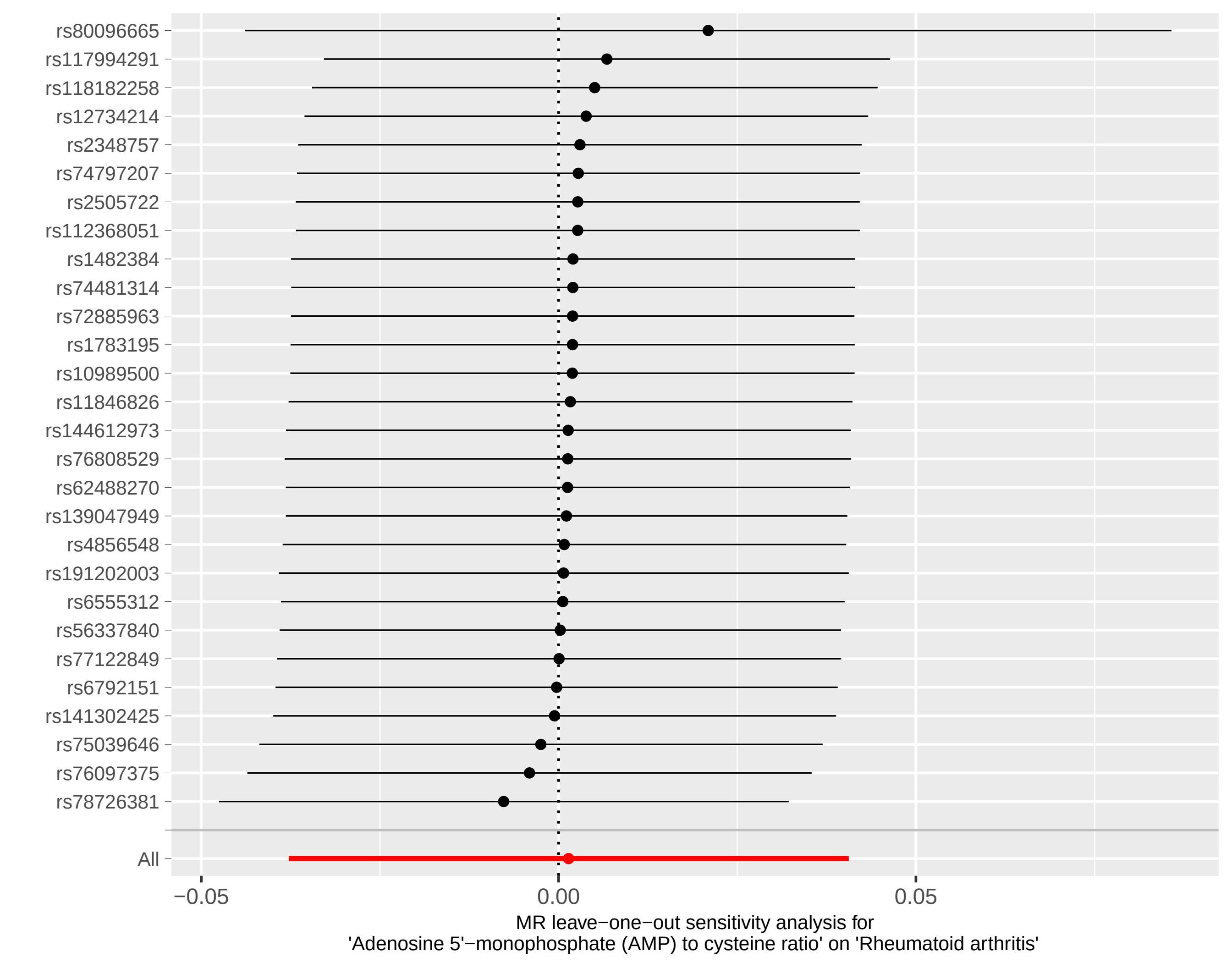
rs80096665
rs117994291
rs118182258
rs12734214
rs2348757
rs74797207
rs2505722
rs112368051
rs1482384
rs74481314
rs72885963
rs1783195
rs10989500
rs11846826
rs144612973
rs76808529
rs62488270
rs139047949
rs4856548
rs191202003
rs6555312
rs56337840
rs77122849
rs6792151
rs141302425
rs75039646
rs76097375
rs78726381
All
−0.05
0.00
0.05
MR leave−one−out sensitivity analysis for
'Adenosine 5'−monophosphate (AMP) to cysteine ratio' on 'Rheumatoid arthritis'

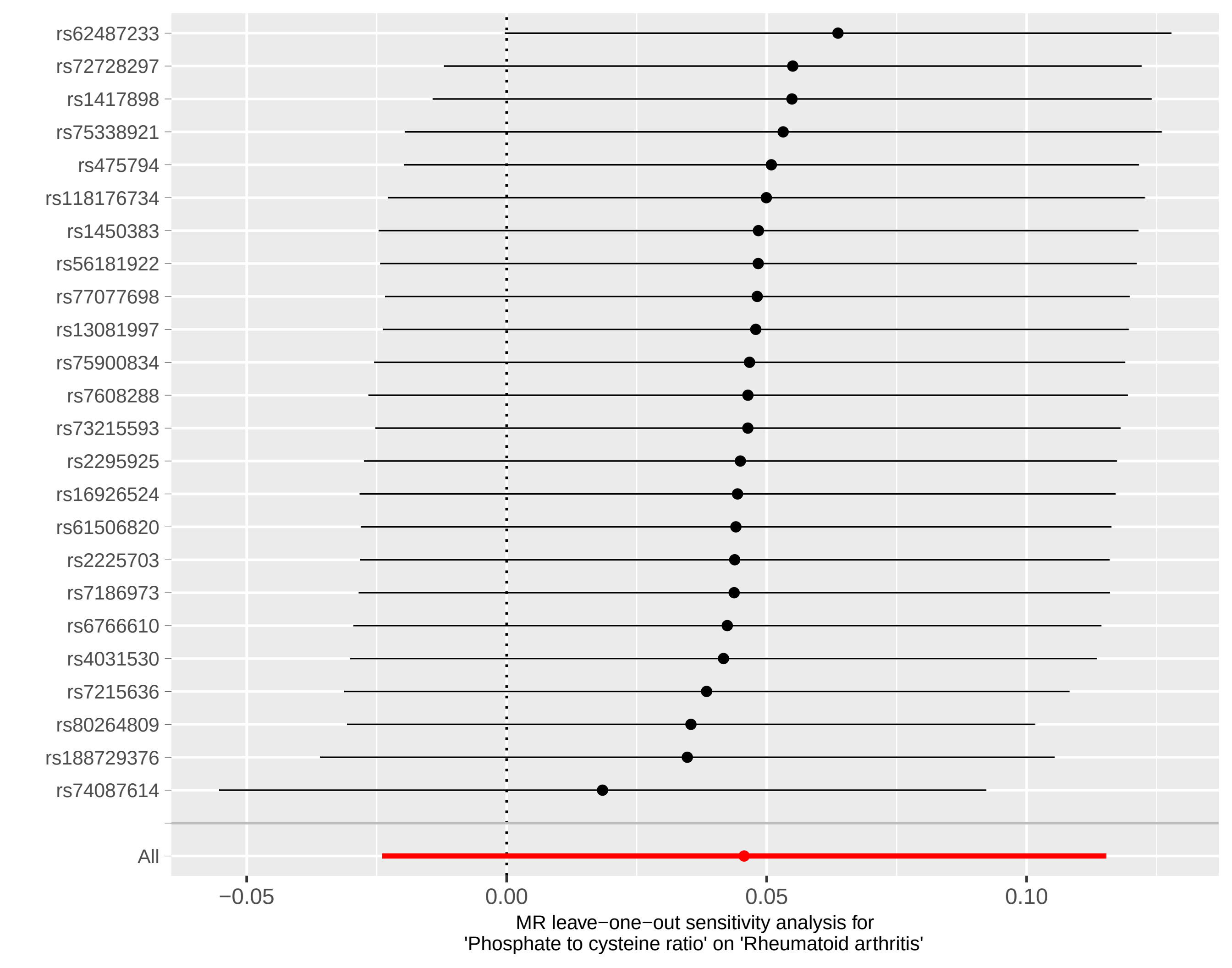
rs62487233
rs72728297
rs1417898
rs75338921
rs475794
rs118176734
rs1450383
rs56181922
rs77077698
rs13081997
rs75900834
rs7608288
rs73215593
rs2295925
rs16926524
rs61506820
rs2225703
rs7186973
rs6766610
rs4031530
rs7215636
rs80264809
rs188729376
rs74087614
All
−0.05
0.00
0.05
0.10
MR leave−one−out sensitivity analysis for
'Phosphate to cysteine ratio' on 'Rheumatoid arthritis'

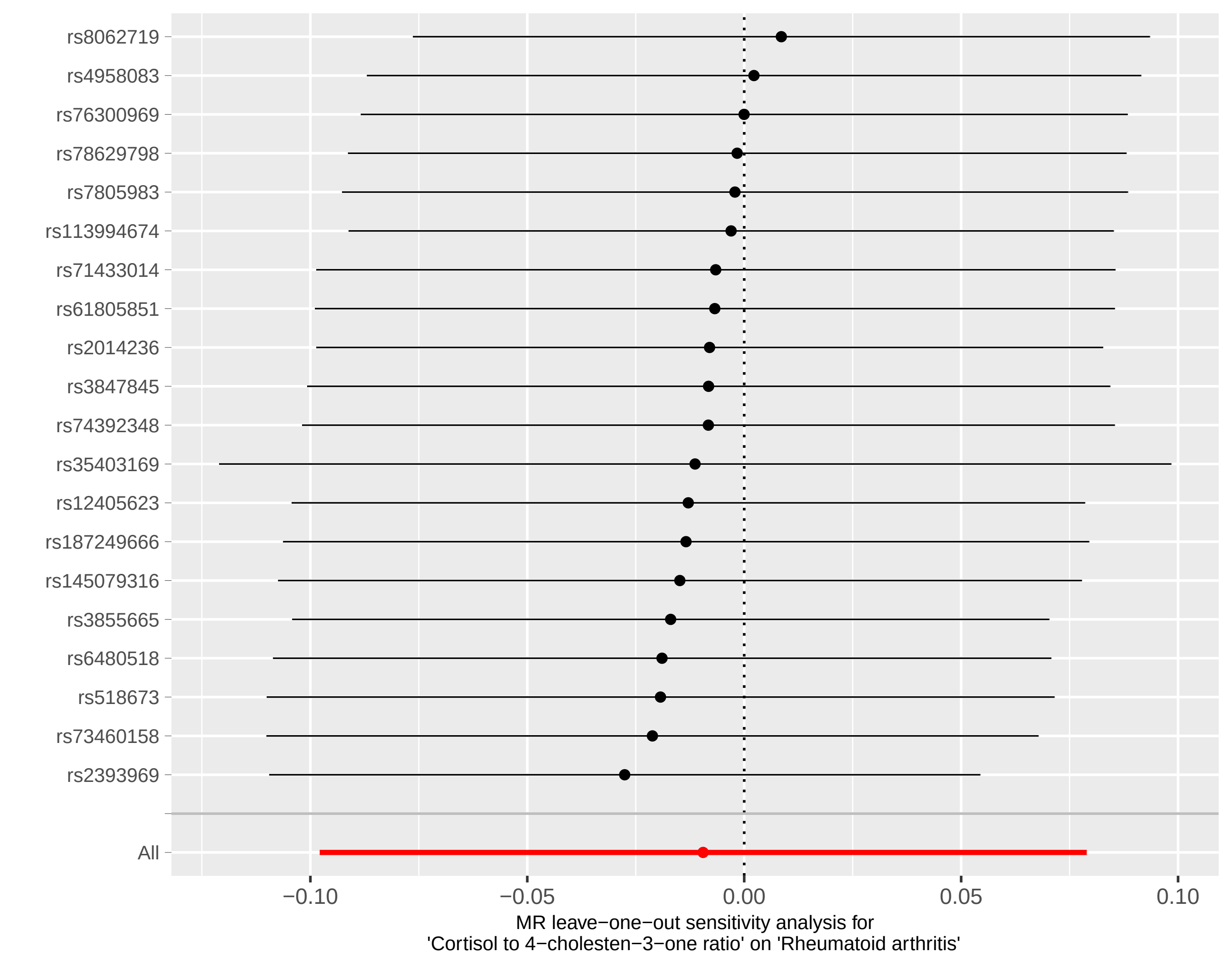
rs8062719
rs4958083
rs76300969
rs78629798
rs7805983
rs113994674
rs71433014
rs61805851
rs2014236
rs3847845
rs74392348
rs35403169
rs12405623
rs187249666
rs145079316
rs3855665
rs6480518
rs518673
rs73460158
rs2393969
All
−0.10
−0.05
0.00
0.05
0.10
MR leave−one−out sensitivity analysis for
'Cortisol to 4−cholesten−3−one ratio' on 'Rheumatoid arthritis'

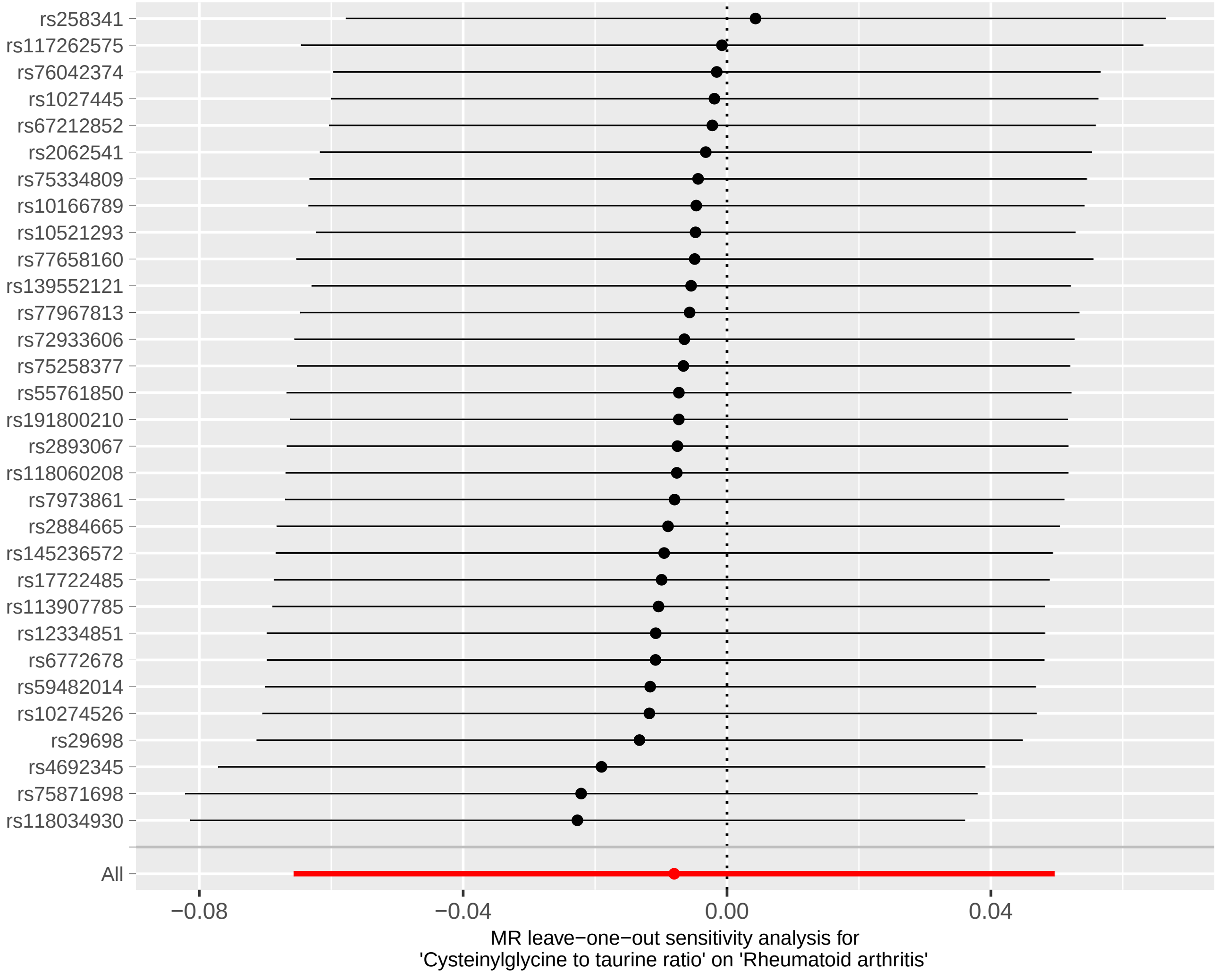

rs258341
rs117262575
rs76042374
rs1027445
rs67212852
rs2062541
rs75334809
rs10166789
rs10521293
rs77658160
rs139552121
rs77967813
rs72933606
rs75258377
rs55761850
rs191800210
rs2893067
rs118060208
rs7973861
rs2884665
rs145236572
rs17722485
rs113907785
rs12334851
rs6772678
rs59482014
rs10274526
rs29698
rs4692345
rs75871698
rs118034930
All
−0.08
−0.04
0.00
0.04
MR leave−one−out sensitivity analysis for
'Cysteinylglycine to taurine ratio' on 'Rheumatoid arthritis'

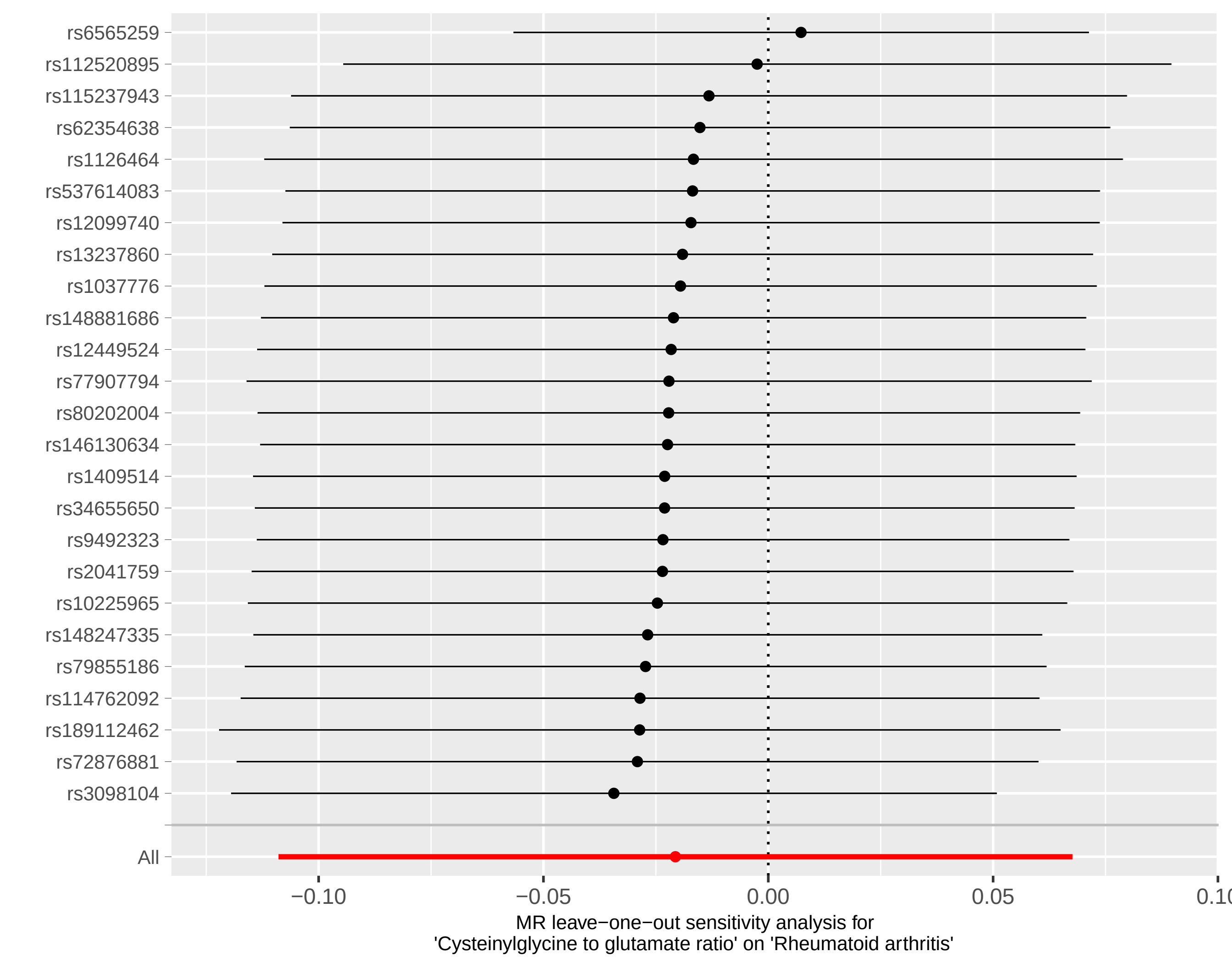

rs6565259
rs112520895
rs115237943
rs62354638
rs1126464
rs537614083
rs12099740
rs13237860
rs1037776
rs148881686
rs12449524
rs77907794
rs80202004
rs146130634
rs1409514
rs34655650
rs9492323
rs2041759
rs10225965
rs148247335
rs79855186
rs114762092
rs189112462
rs72876881
rs3098104
All
−0.10
−0.05
0.00
0.05
0.10
MR leave−one−out sensitivity analysis for
'Cysteinylglycine to glutamate ratio' on 'Rheumatoid arthritis'

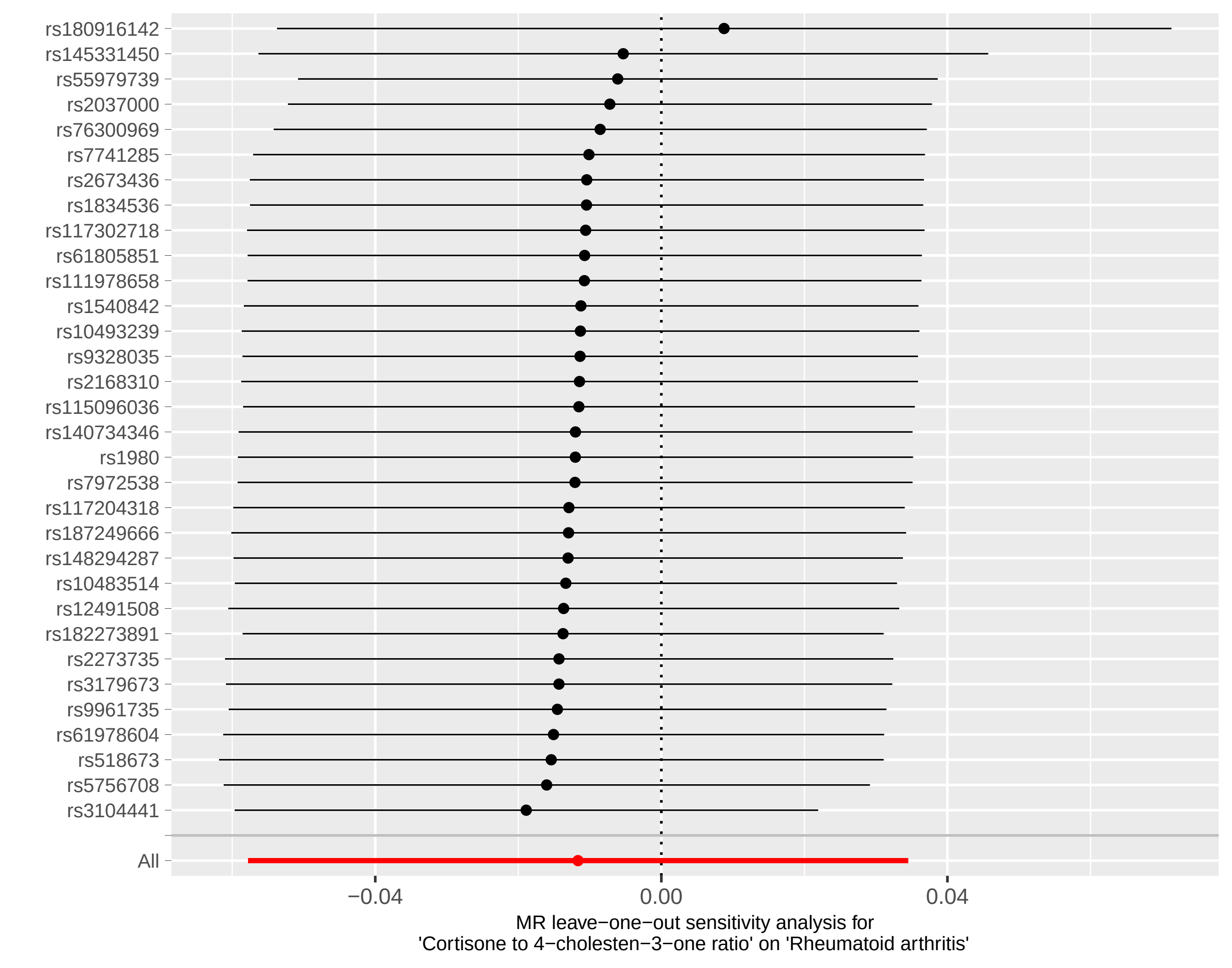
rs180916142
rs145331450
rs55979739
rs2037000
rs76300969
rs7741285
rs2673436
rs1834536
rs117302718
rs61805851
rs111978658
rs1540842
rs10493239
rs9328035
rs2168310
rs115096036
rs140734346
rs1980
rs7972538
rs117204318
rs187249666
rs148294287
rs10483514
rs12491508
rs182273891
rs2273735
rs3179673
rs9961735
rs61978604
rs518673
rs5756708
rs3104441
All
−0.04
0.00
0.04
MR leave−one−out sensitivity analysis for
'Cortisone to 4−cholesten−3−one ratio' on 'Rheumatoid arthritis'

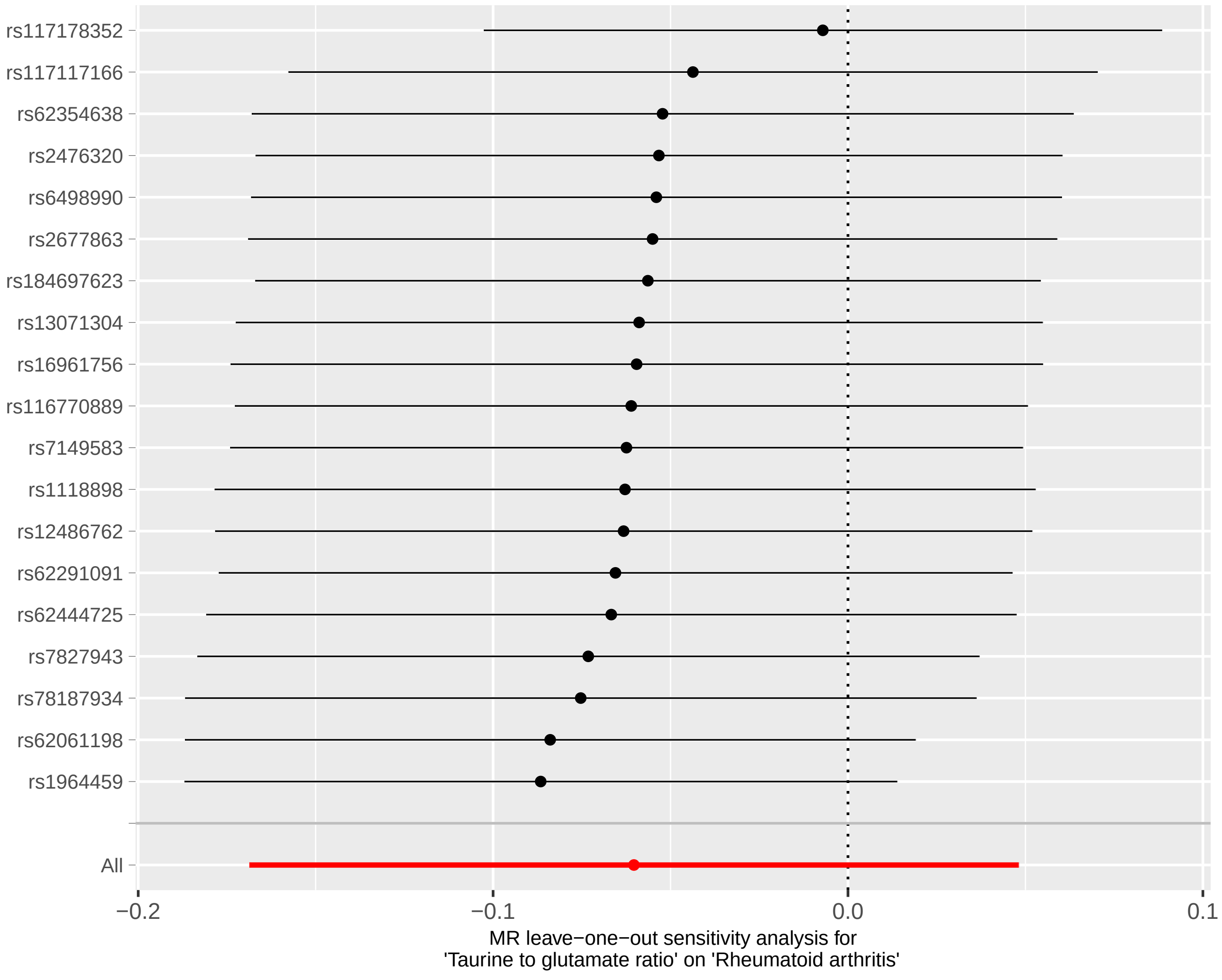
rs117178352
rs117117166
rs62354638
rs2476320
rs6498990
rs2677863
rs184697623
rs13071304
rs16961756
rs116770889
rs7149583
rs1118898
rs12486762
rs62291091
rs62444725
rs7827943
rs78187934
rs62061198
rs1964459
All
−0.2
−0.1
0.0
0.1
MR leave−one−out sensitivity analysis for
'Taurine to glutamate ratio' on 'Rheumatoid arthritis'

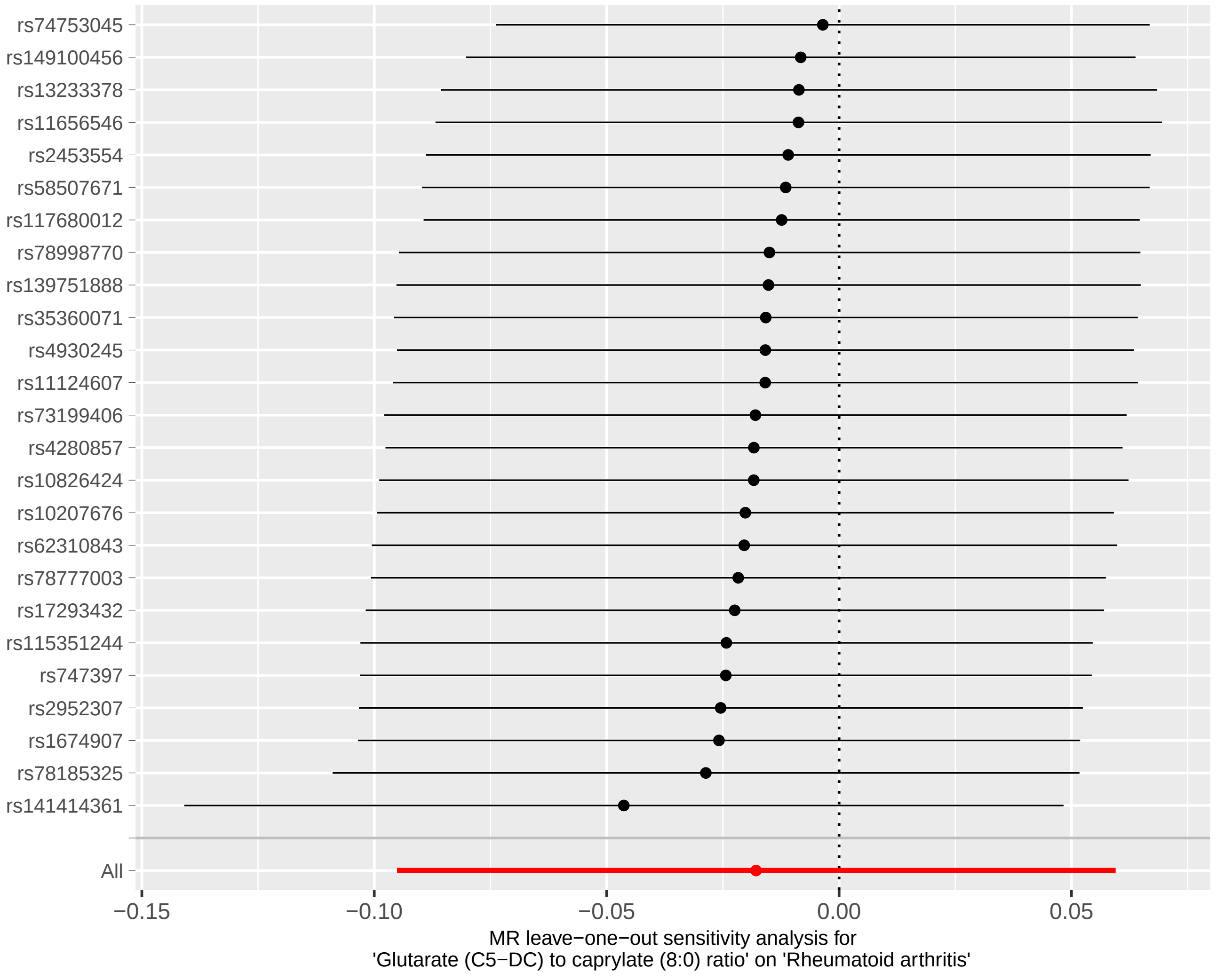
rs74753045
rs149100456
rs13233378
rs11656546
rs2453554
rs58507671
rs117680012
rs78998770
rs139751888
rs35360071
rs4930245
rs11124607
rs73199406
rs4280857
rs10826424
rs10207676
rs62310843
rs78777003
rs17293432
rs115351244
rs747397
rs2952307
rs1674907
rs78185325
rs141414361
All
−0.15
−0.10
−0.05
0.00
0.05
MR leave−one−out sensitivity analysis for
'Glutarate (C5−DC) to caprylate (8:0) ratio' on 'Rheumatoid arthritis'

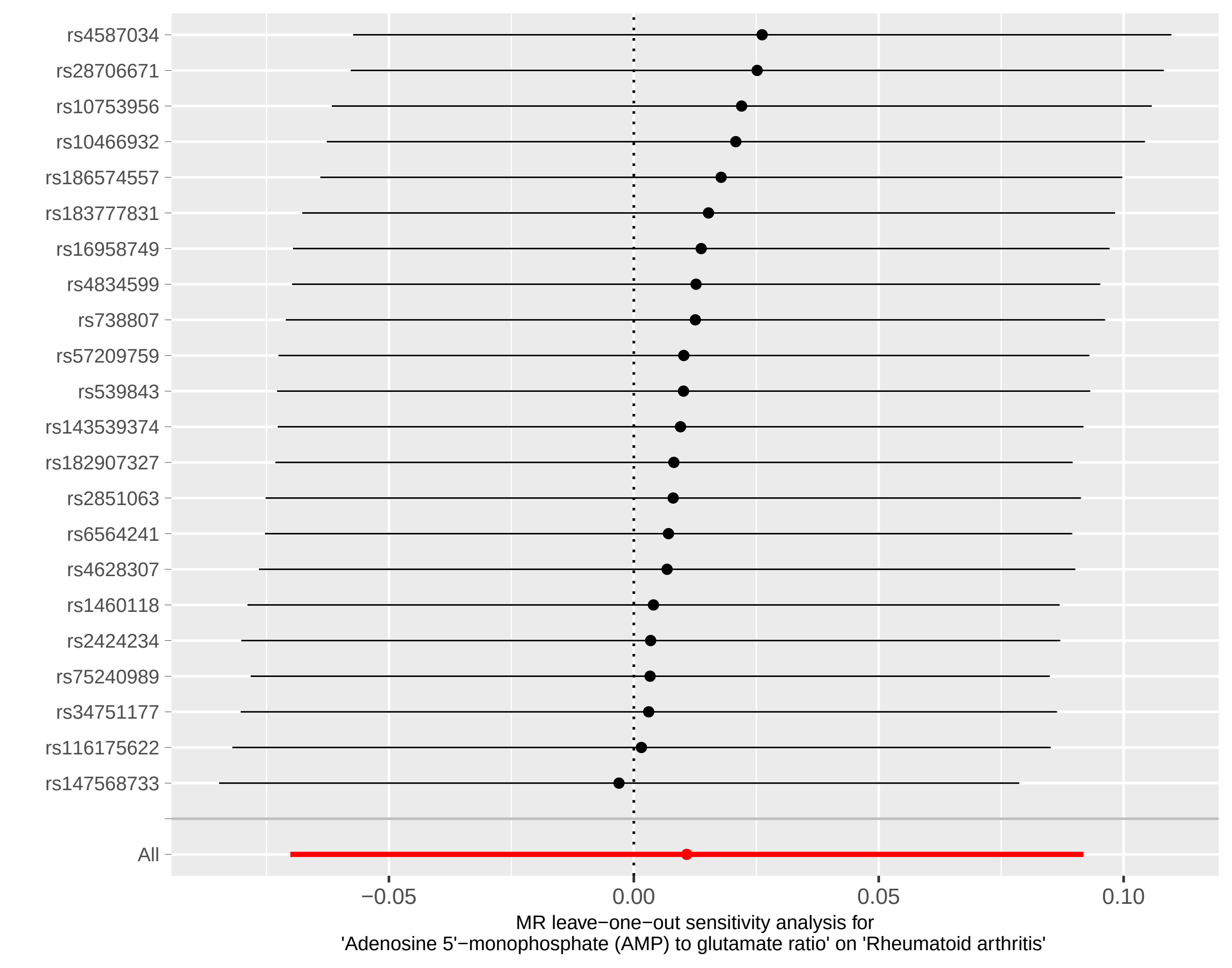

rs4587034
rs28706671
rs10753956
rs10466932
rs186574557
rs183777831
rs16958749
rs4834599
rs738807
rs57209759
rs539843
rs143539374
rs182907327
rs2851063
rs6564241
rs4628307
rs1460118
rs2424234
rs75240989
rs34751177
rs116175622
rs147568733
All
−0.05
0.00
0.05
0.10
MR leave−one−out sensitivity analysis for
'Adenosine 5'−monophosphate (AMP) to glutamate ratio' on 'Rheumatoid arthritis'

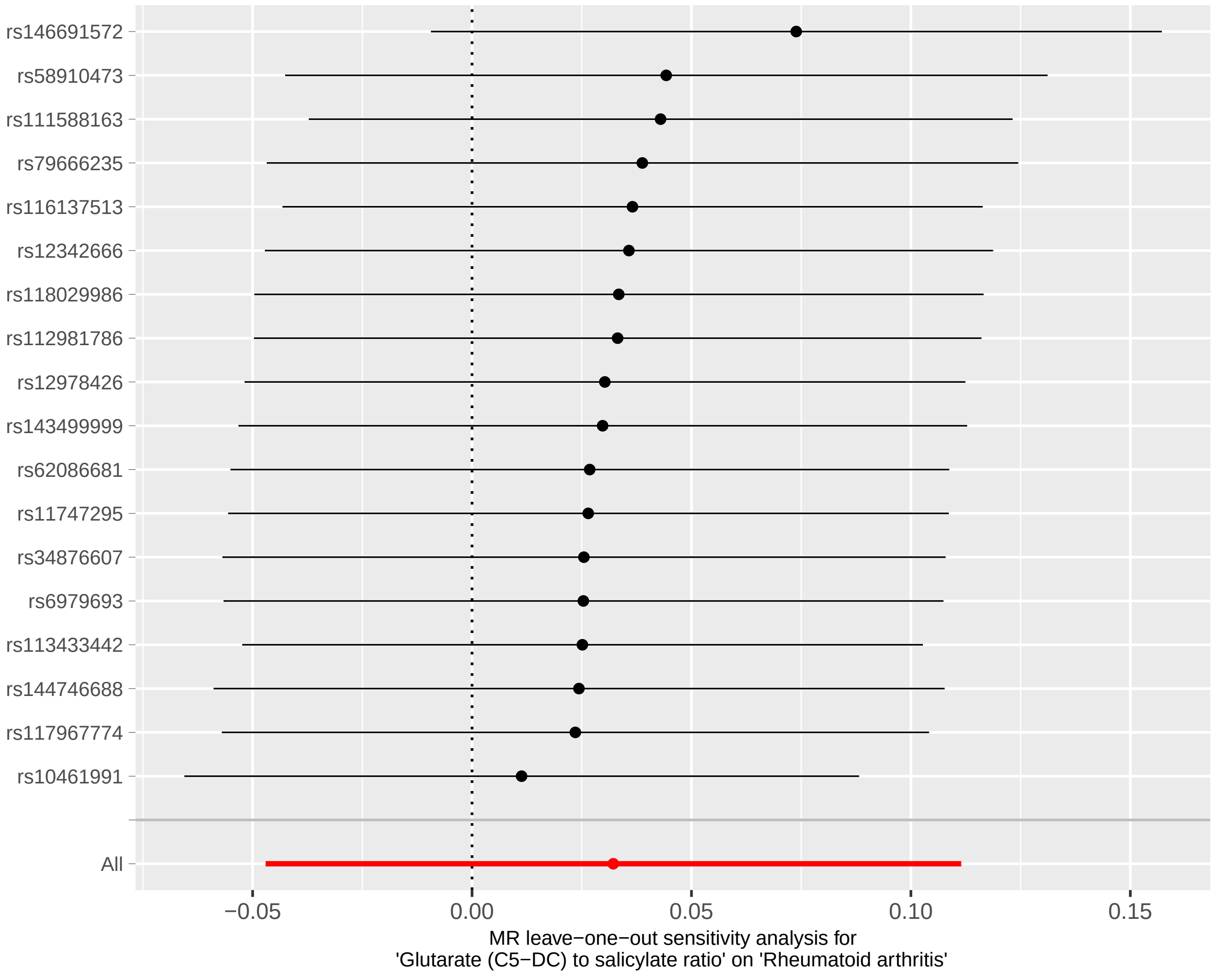

rs146691572
rs58910473
rs111588163
rs79666235
rs116137513
rs12342666
rs118029986
rs112981786
rs12978426
rs143499999
rs62086681
rs11747295
rs34876607
rs6979693
rs113433442
rs144746688
rs117967774
rs10461991
All
−0.05
0.00
0.05
0.10
0.15
MR leave−one−out sensitivity analysis for
'Glutarate (C5−DC) to salicylate ratio' on 'Rheumatoid arthritis'

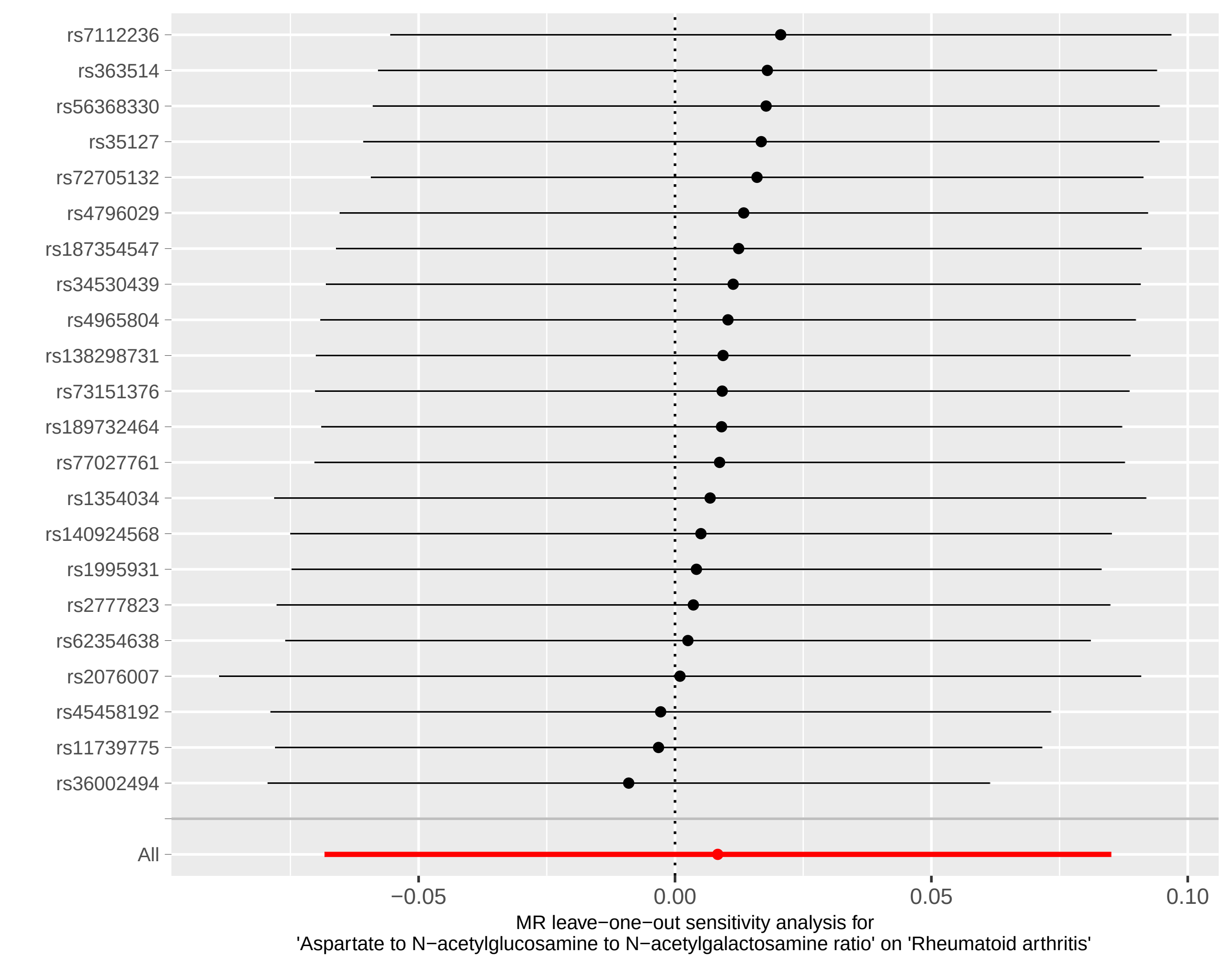
rs7112236
rs363514
rs56368330
rs35127
rs72705132
rs4796029
rs187354547
rs34530439
rs4965804
rs138298731
rs73151376
rs189732464
rs77027761
rs1354034
rs140924568
rs1995931
rs2777823
rs62354638
rs2076007
rs45458192
rs11739775
rs36002494
All
−0.05
0.00
0.05
0.10
MR leave−one−out sensitivity analysis for
'Aspartate to N−acetylglucosamine to N−acetylgalactosamine ratio' on 'Rheumatoid arthritis'

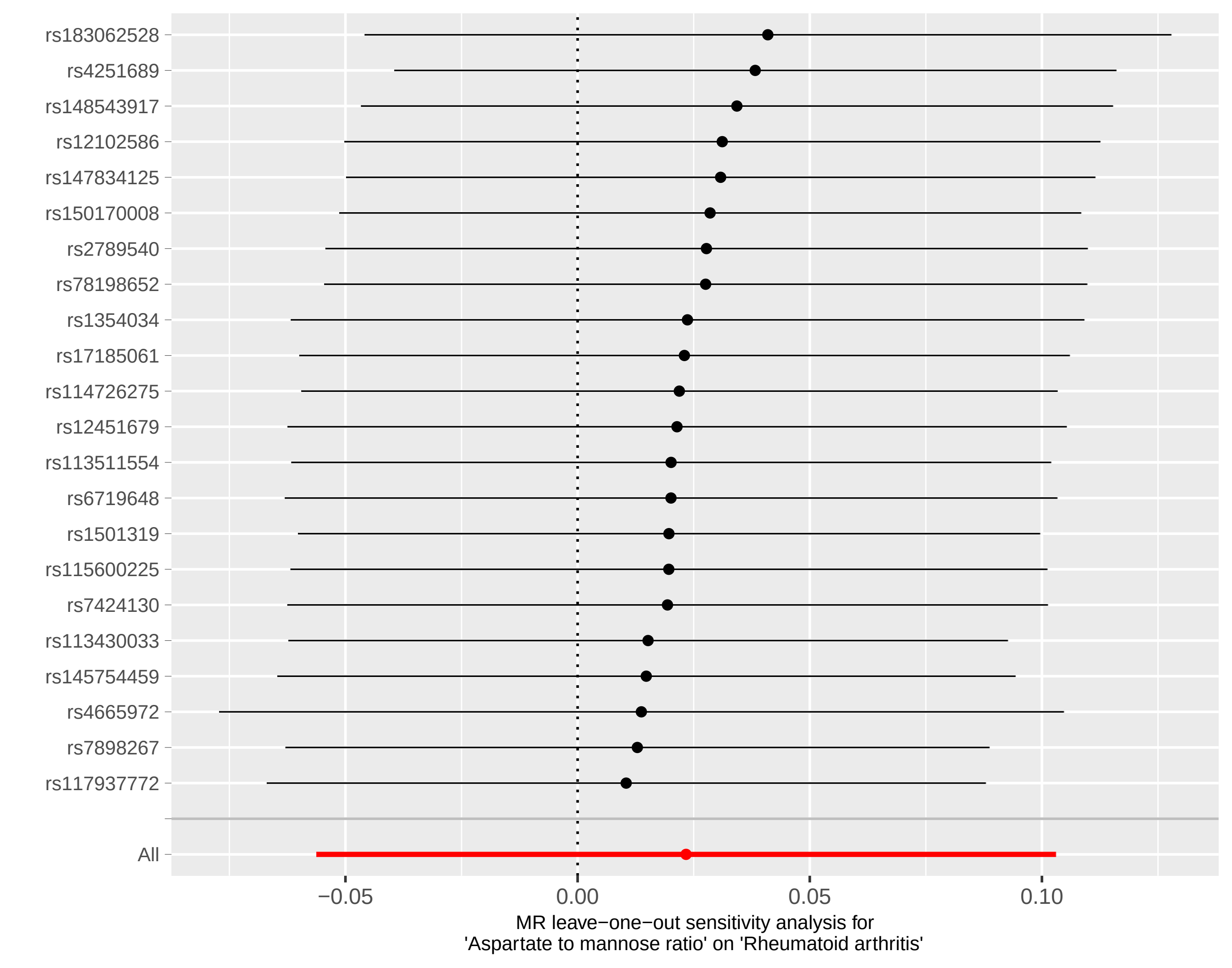

rs183062528
rs4251689
rs148543917
rs12102586
rs147834125
rs150170008
rs2789540
rs78198652
rs1354034
rs17185061
rs114726275
rs12451679
rs113511554
rs6719648
rs1501319
rs115600225
rs7424130
rs113430033
rs145754459
rs4665972
rs7898267
rs117937772
All
−0.05
0.00
0.05
0.10
MR leave−one−out sensitivity analysis for
'Aspartate to mannose ratio' on 'Rheumatoid arthritis'

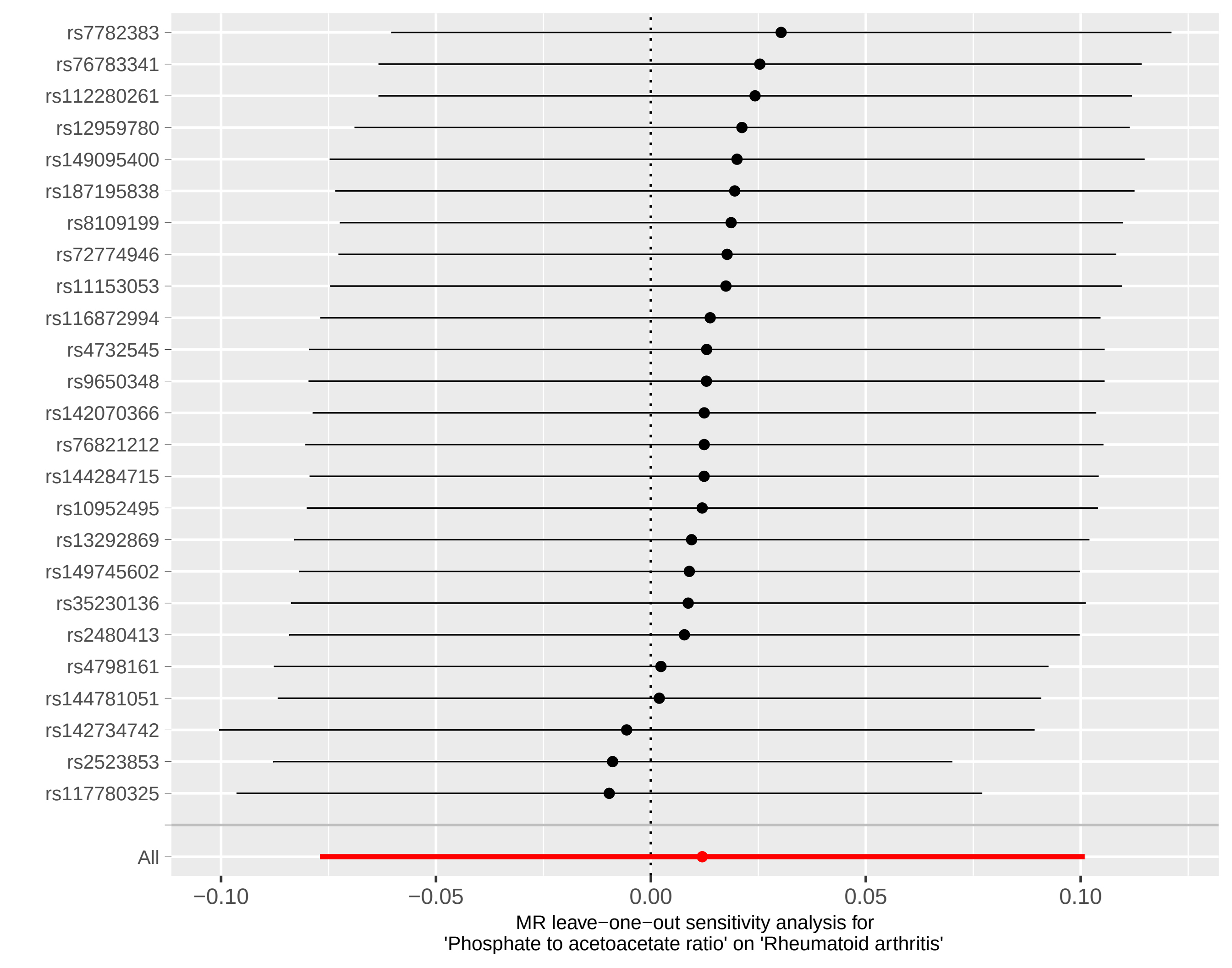
rs7782383
rs76783341
rs112280261
rs12959780
rs149095400
rs187195838
rs8109199
rs72774946
rs11153053
rs116872994
rs4732545
rs9650348
rs142070366
rs76821212
rs144284715
rs10952495
rs13292869
rs149745602
rs35230136
rs2480413
rs4798161
rs144781051
rs142734742
rs2523853
rs117780325
All
−0.10
−0.05
0.00
0.05
0.10
MR leave−one−out sensitivity analysis for
'Phosphate to acetoacetate ratio' on 'Rheumatoid arthritis'

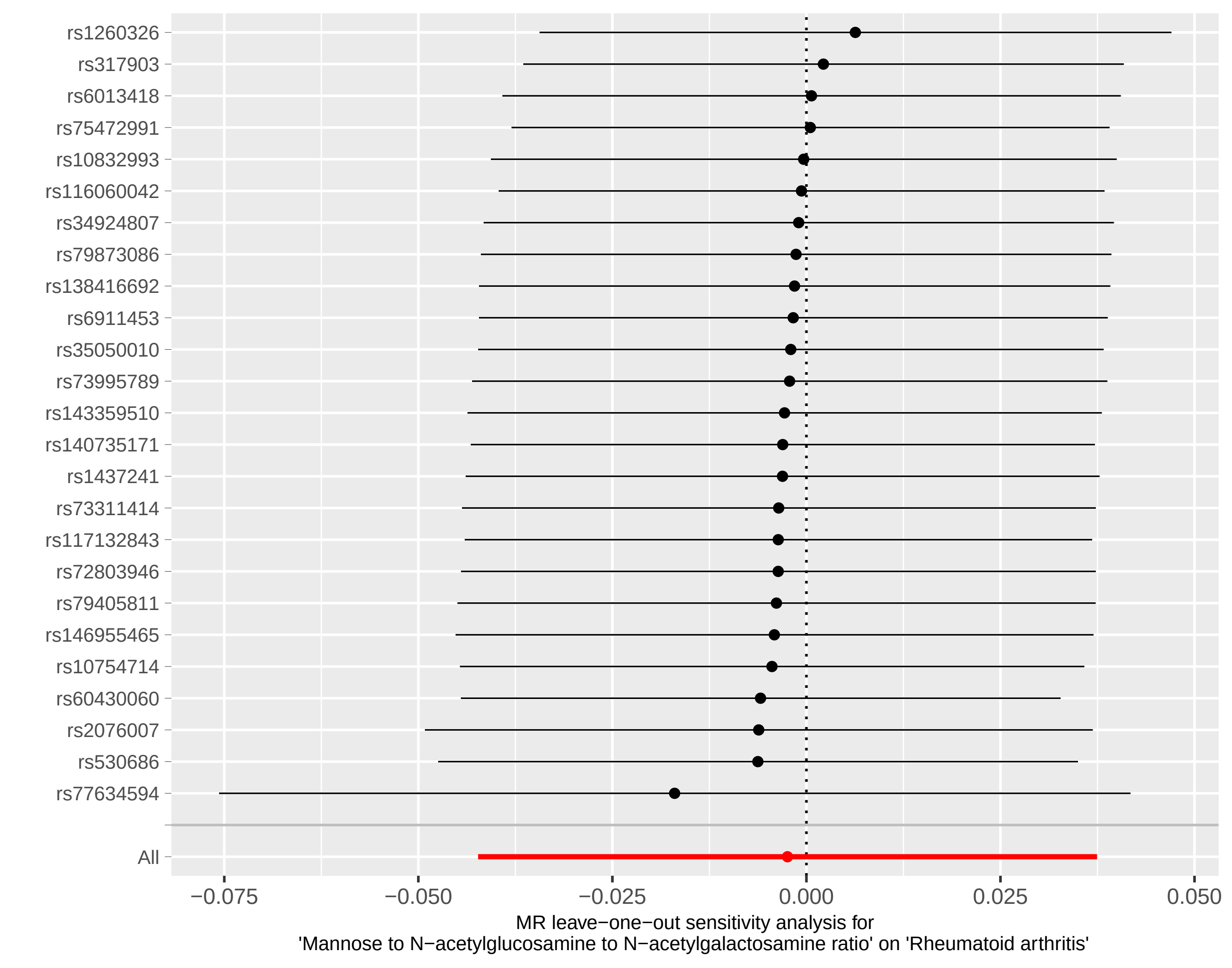
rs1260326
rs317903
rs6013418
rs75472991
rs10832993
rs116060042
rs34924807
rs79873086
rs138416692
rs6911453
rs35050010
rs73995789
rs143359510
rs140735171
rs1437241
rs73311414
rs117132843
rs72803946
rs79405811
rs146955465
rs10754714
rs60430060
rs2076007
rs530686
rs77634594
All
−0.075
−0.050
−0.025
0.000
0.025
0.050
MR leave−one−out sensitivity analysis for
'Mannose to N−acetylglucosamine to N−acetylgalactosamine ratio' on 'Rheumatoid arthritis'

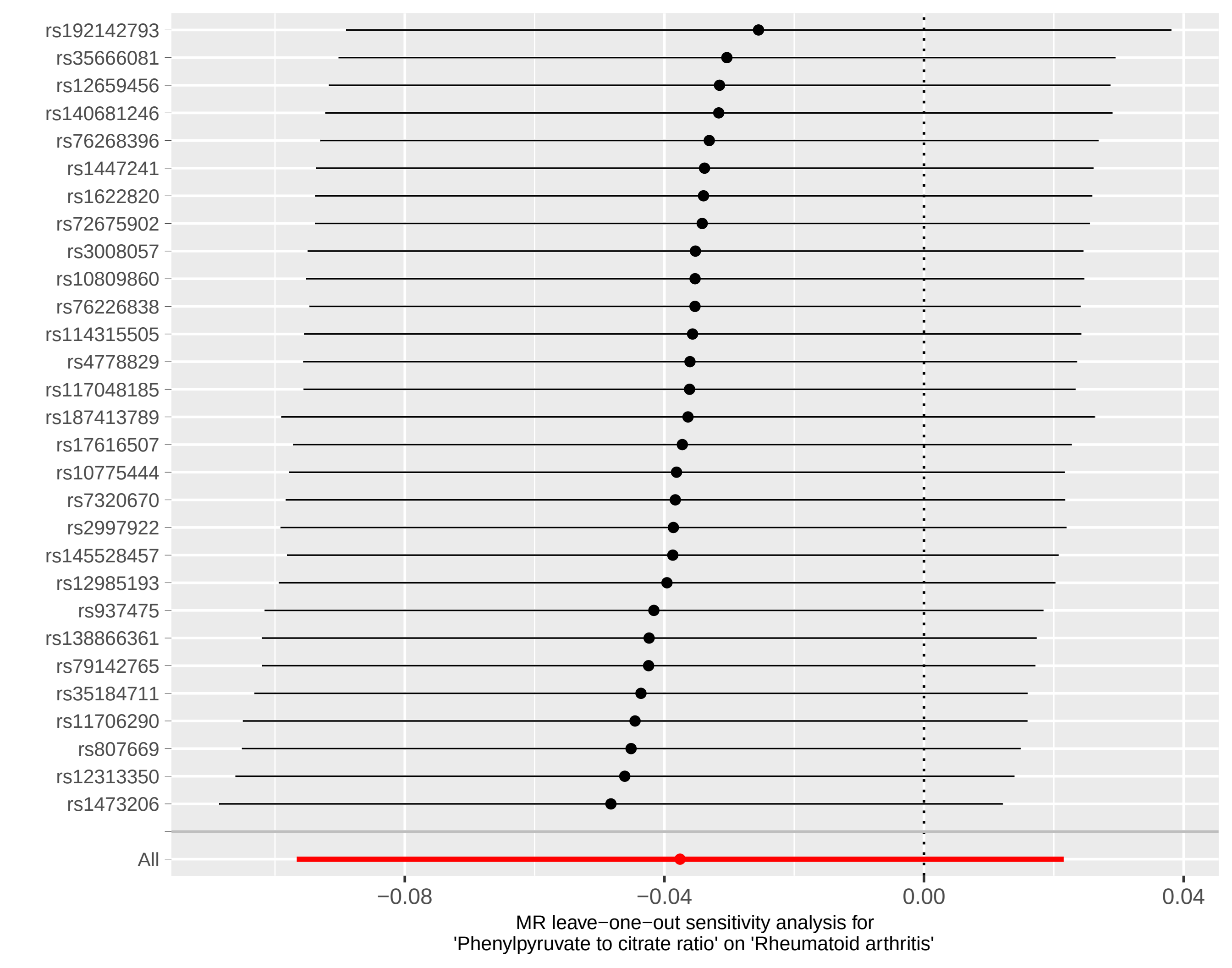
rs192142793
rs35666081
rs12659456
rs140681246
rs76268396
rs1447241
rs1622820
rs72675902
rs3008057
rs10809860
rs76226838
rs114315505
rs4778829
rs117048185
rs187413789
rs17616507
rs10775444
rs7320670
rs2997922
rs145528457
rs12985193
rs937475
rs138866361
rs79142765
rs35184711
rs11706290
rs807669
rs12313350
rs1473206
All
−0.08
−0.04
0.00
0.04
MR leave−one−out sensitivity analysis for
'Phenylpyruvate to citrate ratio' on 'Rheumatoid arthritis'

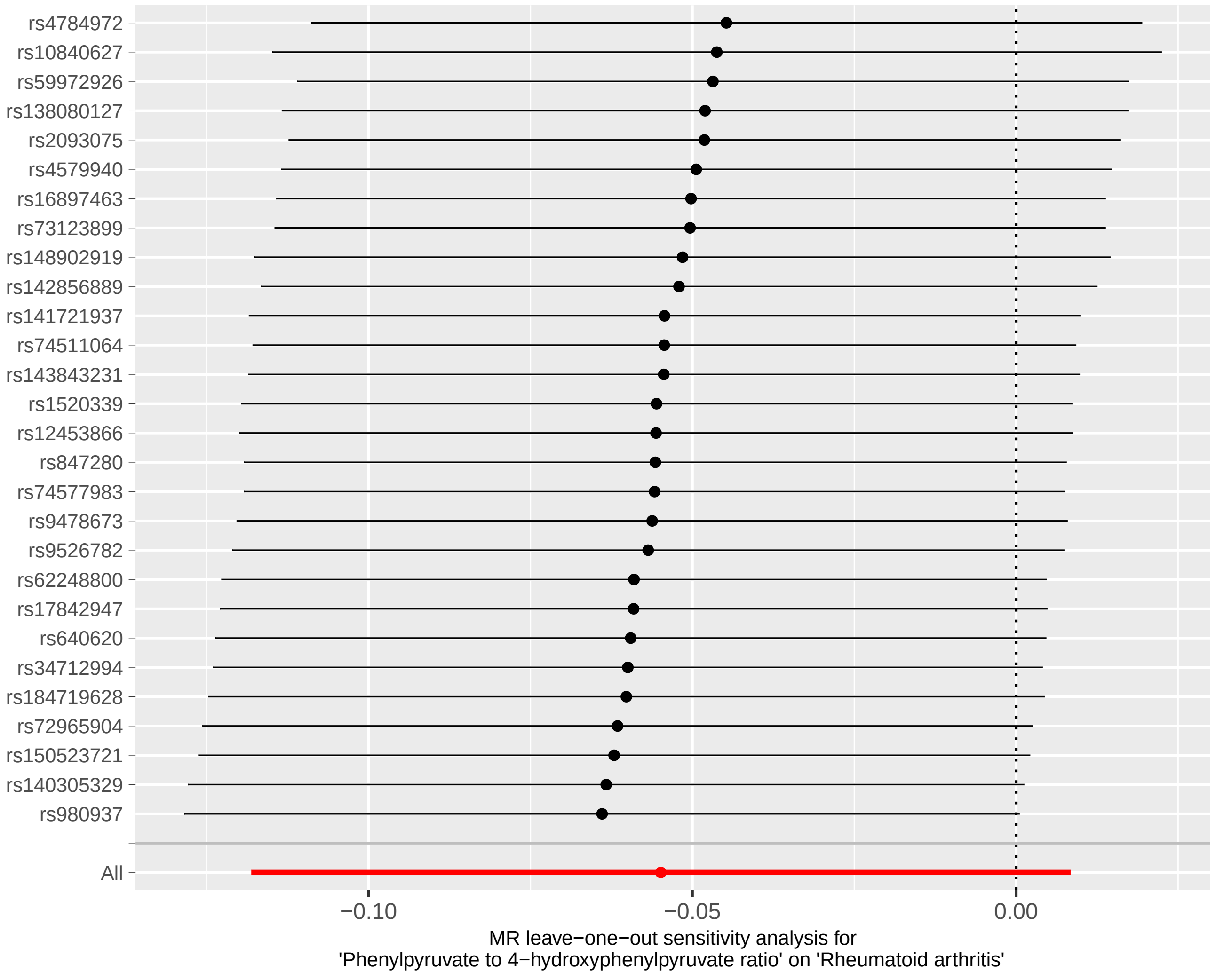
rs4784972
rs10840627
rs59972926
rs138080127
rs2093075
rs4579940
rs16897463
rs73123899
rs148902919
rs142856889
rs141721937
rs74511064
rs143843231
rs1520339
rs12453866
rs847280
rs74577983
rs9478673
rs9526782
rs62248800
rs17842947
rs640620
rs34712994
rs184719628
rs72965904
rs150523721
rs140305329
rs980937
All
−0.10
−0.05
0.00
MR leave−one−out sensitivity analysis for
'Phenylpyruvate to 4−hydroxyphenylpyruvate ratio' on 'Rheumatoid arthritis'

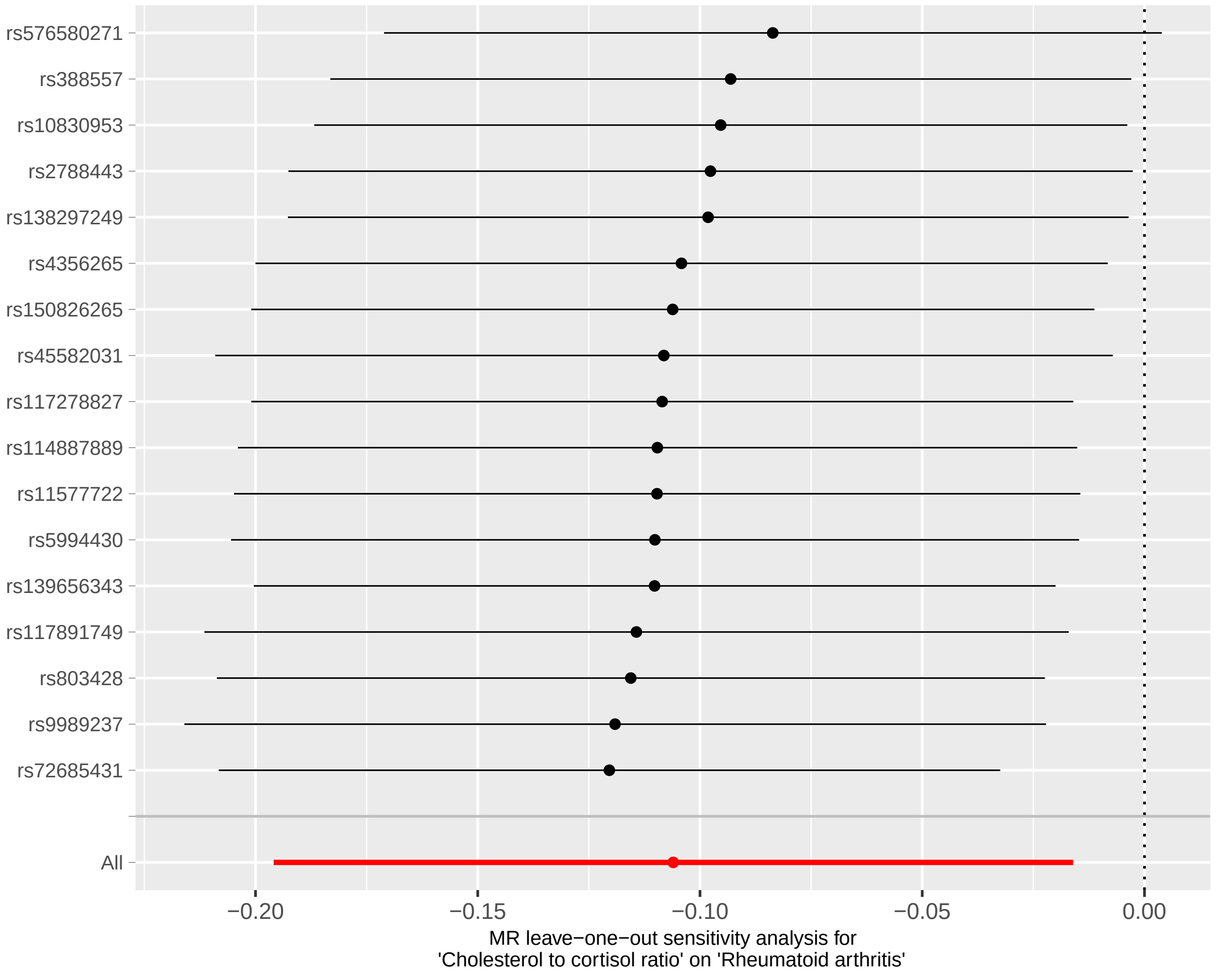
rs576580271
rs388557
rs10830953
rs2788443
rs138297249
rs4356265
rs150826265
rs45582031
rs117278827
rs114887889
rs11577722
rs5994430
rs139656343
rs117891749
rs803428
rs9989237
rs72685431
All
−0.20
−0.15
−0.10
−0.05
0.00
MR leave−one−out sensitivity analysis for
'Cholesterol to cortisol ratio' on 'Rheumatoid arthritis'

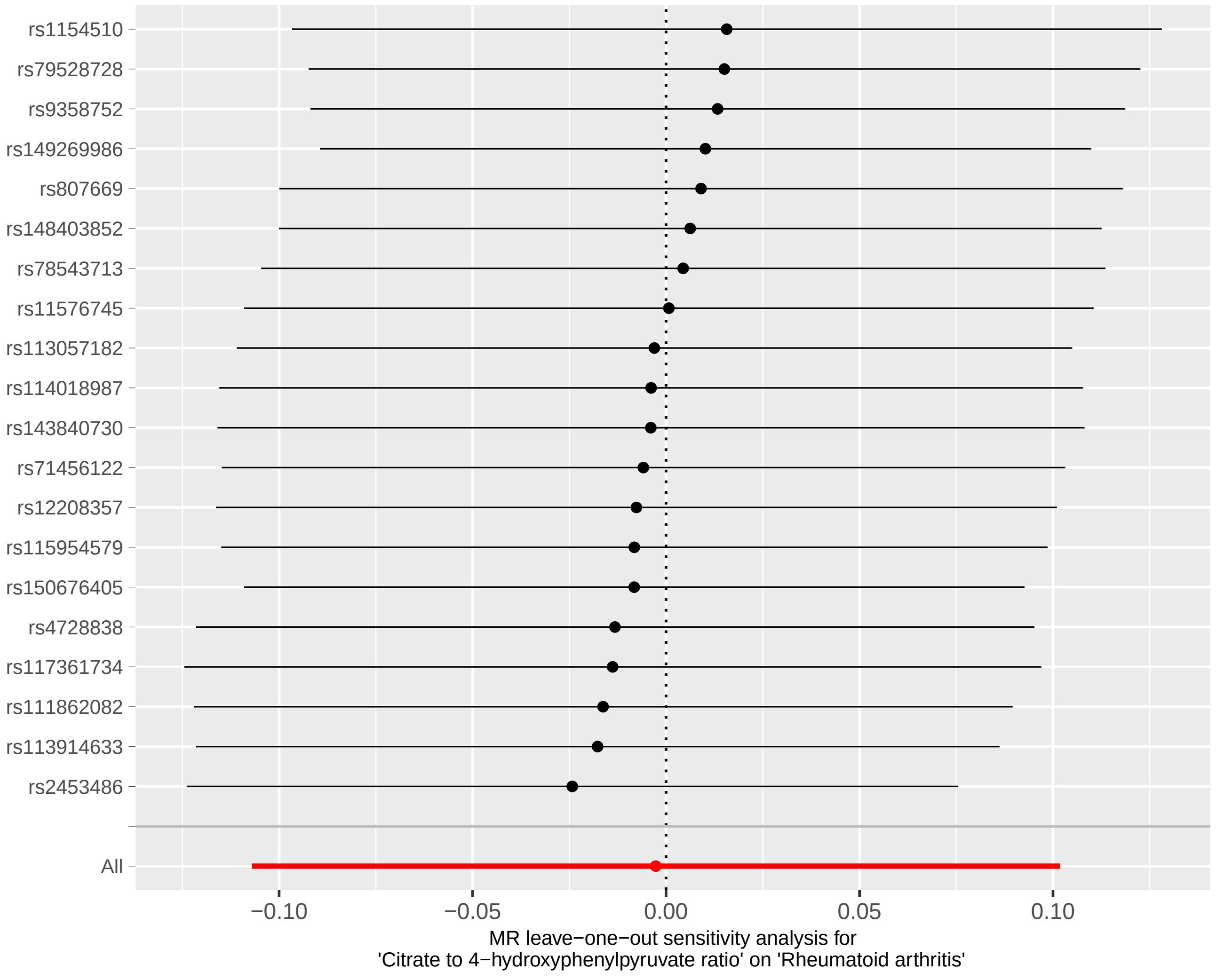
rs1154510
rs79528728
rs9358752
rs149269986
rs807669
rs148403852
rs78543713
rs11576745
rs113057182
rs114018987
rs143840730
rs71456122
rs12208357
rs115954579
rs150676405
rs4728838
rs117361734
rs111862082
rs113914633
rs2453486
All
−0.10
−0.05
0.00
0.05
0.10
MR leave−one−out sensitivity analysis for
'Citrate to 4−hydroxyphenylpyruvate ratio' on 'Rheumatoid arthritis'

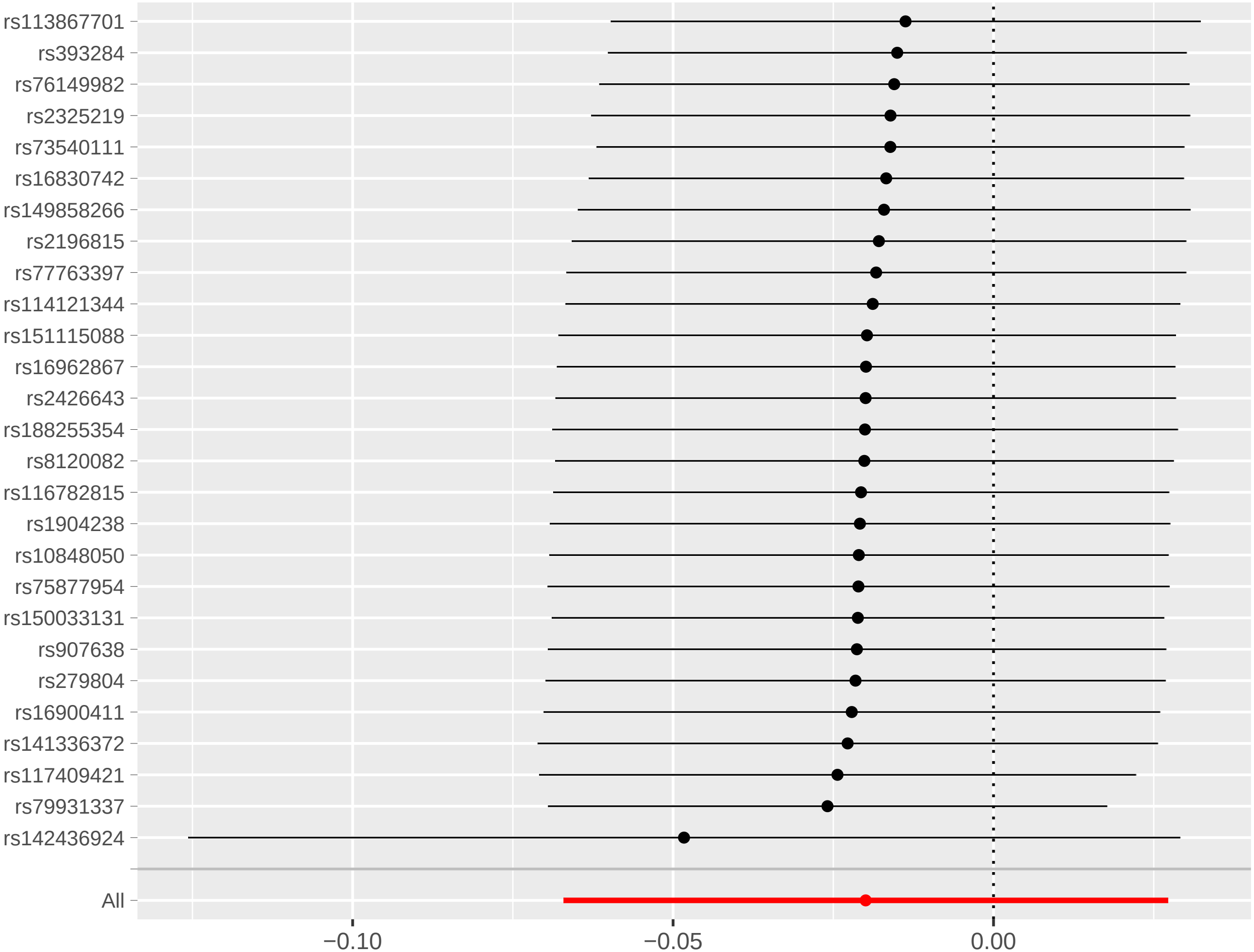

MR leave−one−out sensitivity analysis for
'Cholesterol to taurocholate ratio' on 'Rheumatoid arthritis'

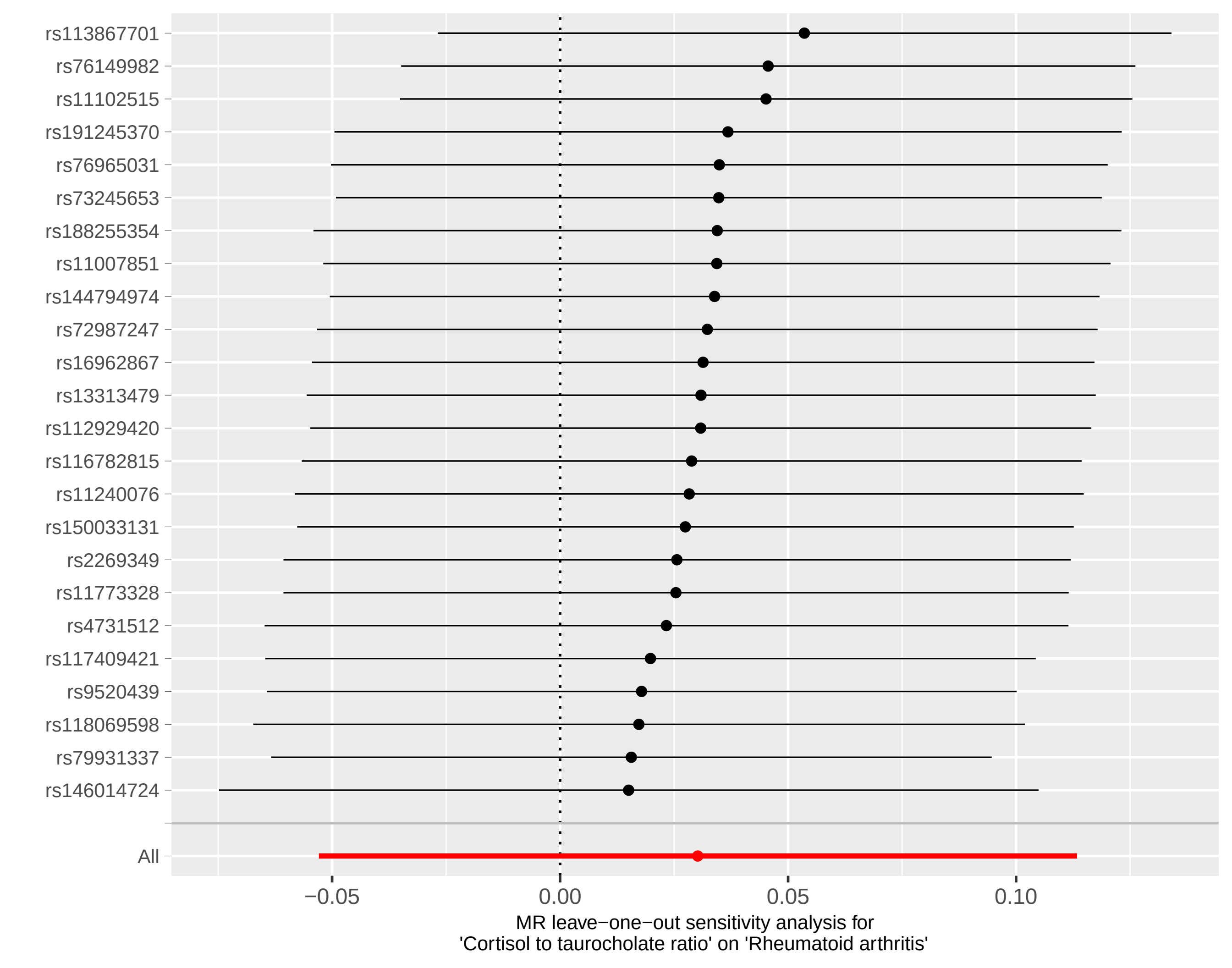
rs113867701
rs76149982
rs11102515
rs191245370
rs76965031
rs73245653
rs188255354
rs11007851
rs144794974
rs72987247
rs16962867
rs13313479
rs112929420
rs116782815
rs11240076
rs150033131
rs2269349
rs11773328
rs4731512
rs117409421
rs9520439
rs118069598
rs79931337
rs146014724
All
−0.05
0.00
0.05
0.10
MR leave−one−out sensitivity analysis for
'Cortisol to taurocholate ratio' on 'Rheumatoid arthritis'

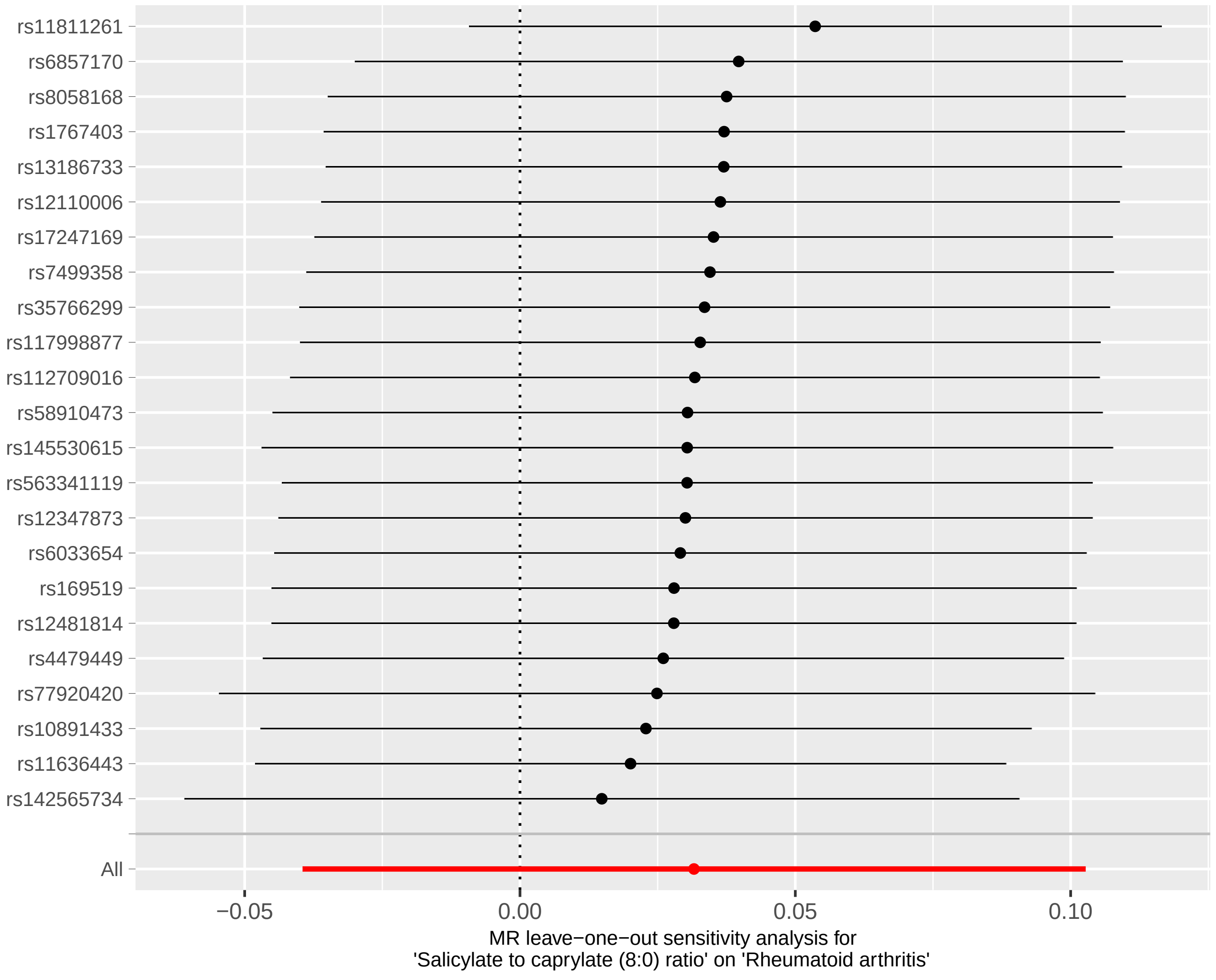
rs11811261
rs6857170
rs8058168
rs1767403
rs13186733
rs12110006
rs17247169
rs7499358
rs35766299
rs117998877
rs112709016
rs58910473
rs145530615
rs563341119
rs12347873
rs6033654
rs169519
rs12481814
rs4479449
rs77920420
rs10891433
rs11636443
rs142565734
All
−0.05
0.00
0.05
0.10
MR leave−one−out sensitivity analysis for
'Salicylate to caprylate (8:0) ratio' on 'Rheumatoid arthritis'

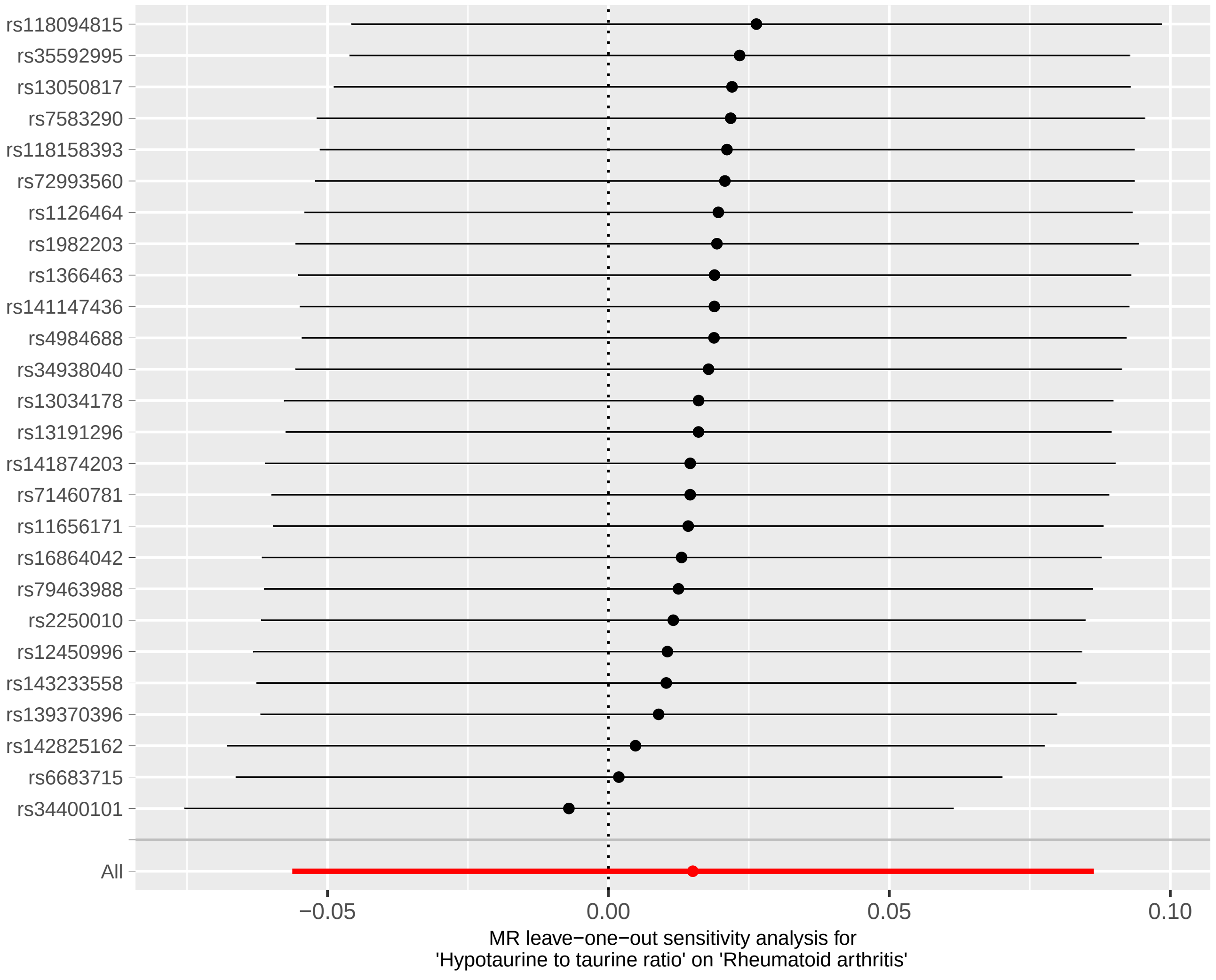
rs118094815
rs35592995
rs13050817
rs7583290
rs118158393
rs72993560
rs1126464
rs1982203
rs1366463
rs141147436
rs4984688
rs34938040
rs13034178
rs13191296
rs141874203
rs71460781
rs11656171
rs16864042
rs79463988
rs2250010
rs12450996
rs143233558
rs139370396
rs142825162
rs6683715
rs34400101
All
−0.05
0.00
0.05
0.10
MR leave−one−out sensitivity analysis for
'Hypotaurine to taurine ratio' on 'Rheumatoid arthritis'

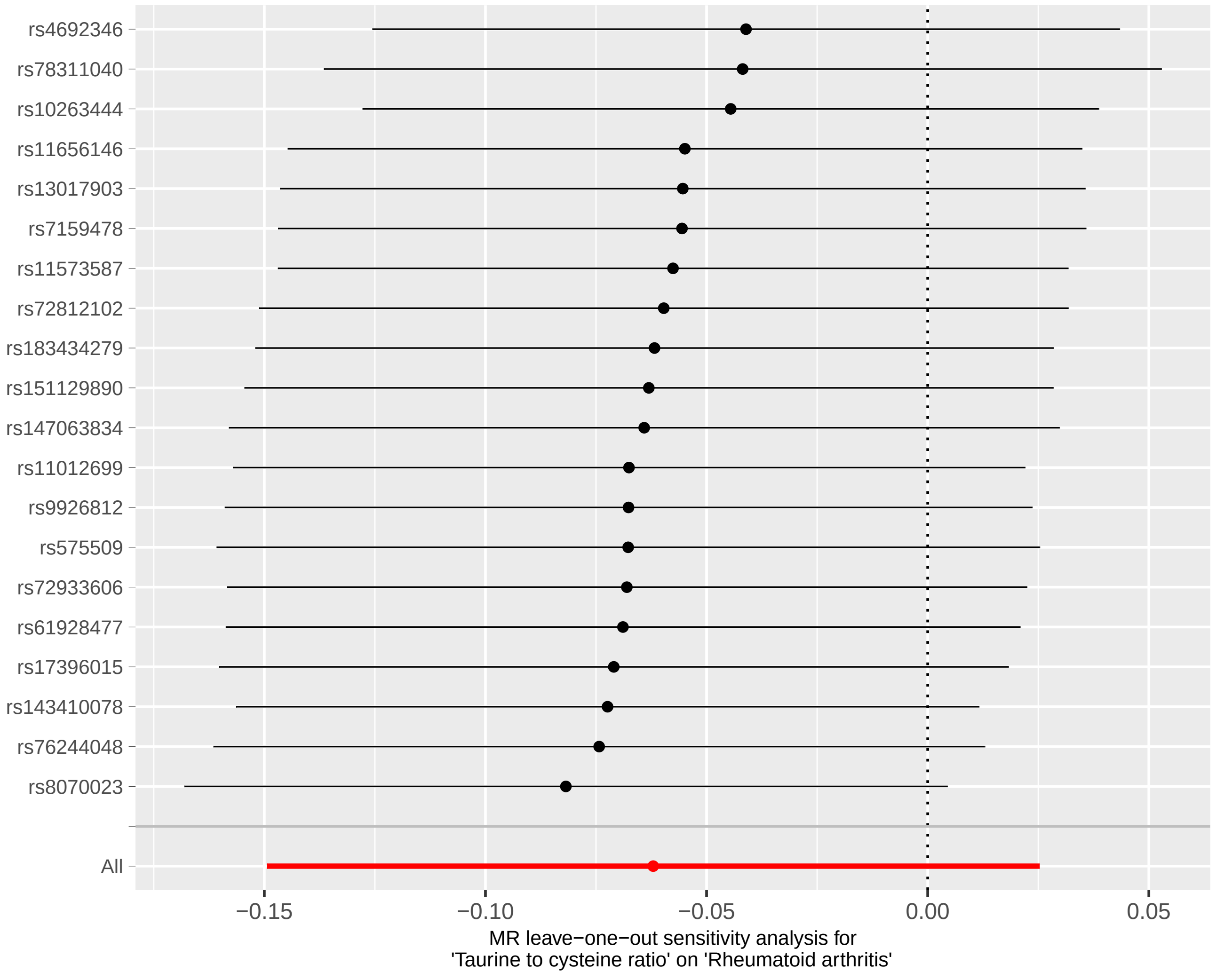
rs4692346
rs78311040
rs10263444
rs11656146
rs13017903
rs7159478
rs11573587
rs72812102
rs183434279
rs151129890
rs147063834
rs11012699
rs9926812
rs575509
rs72933606
rs61928477
rs17396015
rs143410078
rs76244048
rs8070023
All
−0.15
−0.10
−0.05
0.00
0.05
MR leave−one−out sensitivity analysis for
'Taurine to cysteine ratio' on 'Rheumatoid arthritis'

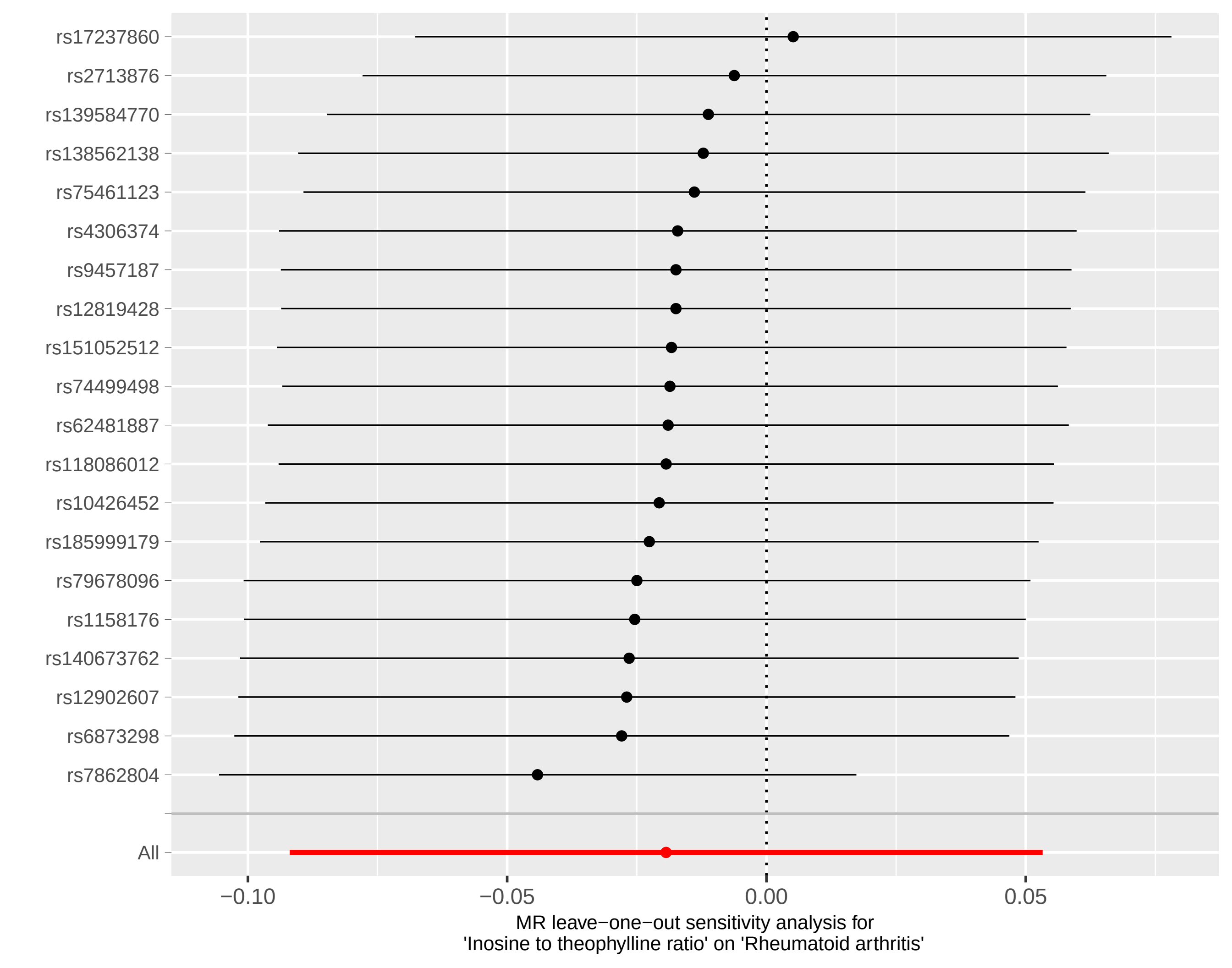

rs17237860
rs2713876
rs139584770
rs138562138
rs75461123
rs4306374
rs9457187
rs12819428
rs151052512
rs74499498
rs62481887
rs118086012
rs10426452
rs185999179
rs79678096
rs1158176
rs140673762
rs12902607
rs6873298
rs7862804
All
−0.10
−0.05
0.00
0.05
MR leave−one−out sensitivity analysis for
'Inosine to theophylline ratio' on 'Rheumatoid arthritis'

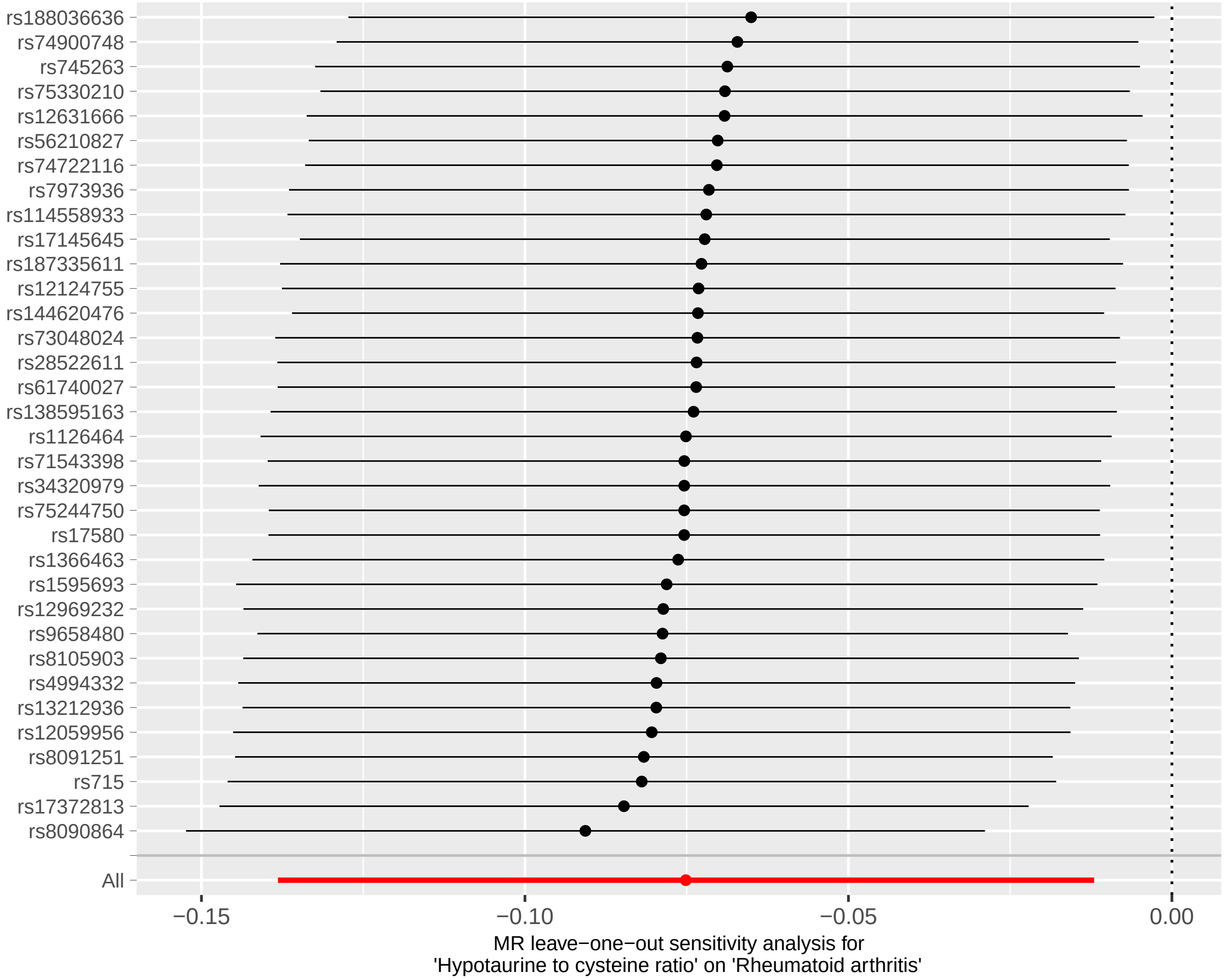

rs188036636
rs74900748
rs745263
rs75330210
rs12631666
rs56210827
rs74722116
rs7973936
rs114558933
rs17145645
rs187335611
rs12124755
rs144620476
rs73048024
rs28522611
rs61740027
rs138595163
rs1126464
rs71543398
rs34320979
rs75244750
rs17580
rs1366463
rs1595693
rs12969232
rs9658480
rs8105903
rs4994332
rs13212936
rs12059956
rs8091251
rs715
rs17372813
rs8090864
All
−0.15
−0.10
−0.05
0.00
MR leave−one−out sensitivity analysis for
'Hypotaurine to cysteine ratio' on 'Rheumatoid arthritis'

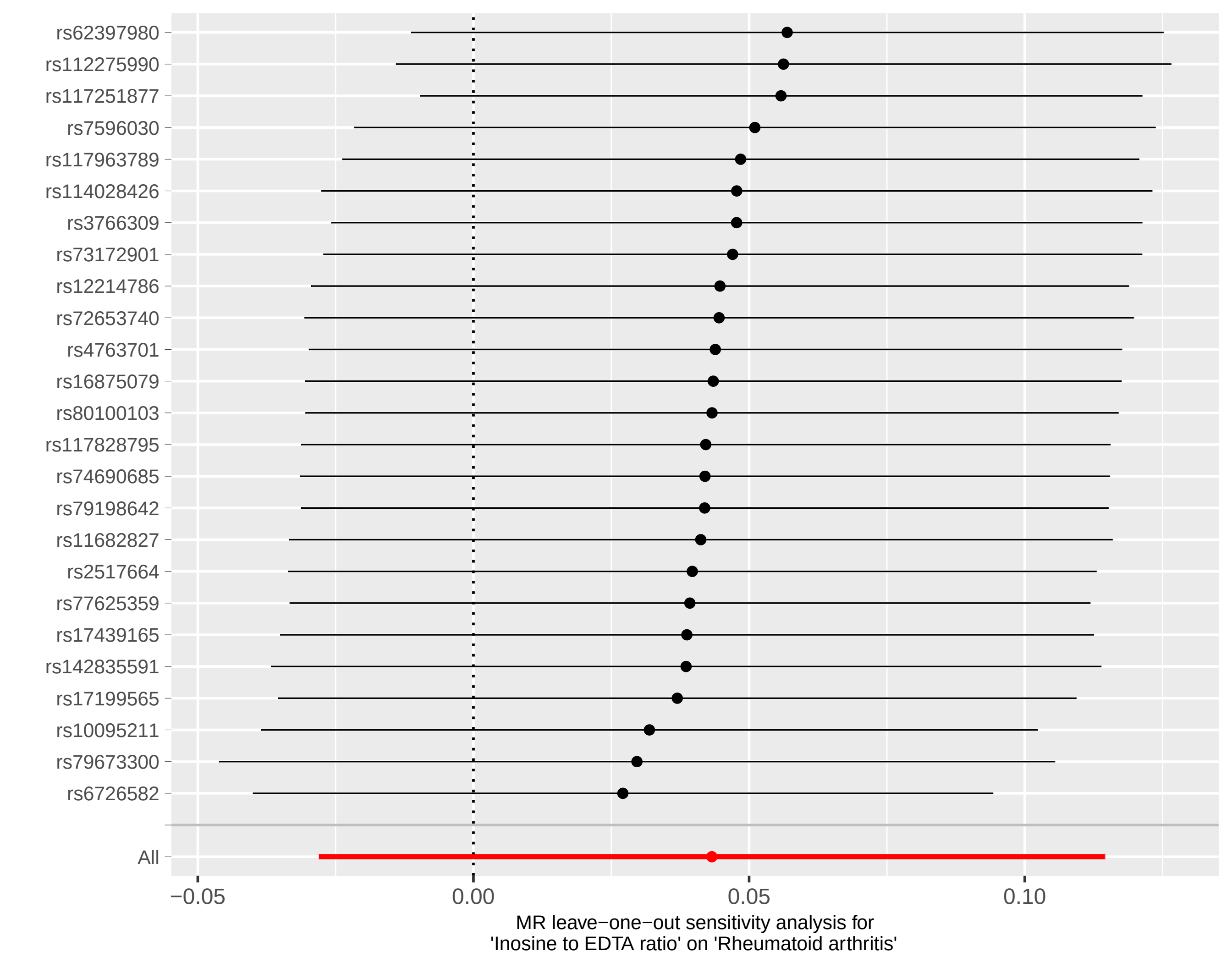
rs62397980
rs112275990
rs117251877
rs7596030
rs117963789
rs114028426
rs3766309
rs73172901
rs12214786
rs72653740
rs4763701
rs16875079
rs80100103
rs117828795
rs74690685
rs79198642
rs11682827
rs2517664
rs77625359
rs17439165
rs142835591
rs17199565
rs10095211
rs79673300
rs6726582
All
−0.05
0.00
0.05
0.10
MR leave−one−out sensitivity analysis for
'Inosine to EDTA ratio' on 'Rheumatoid arthritis'

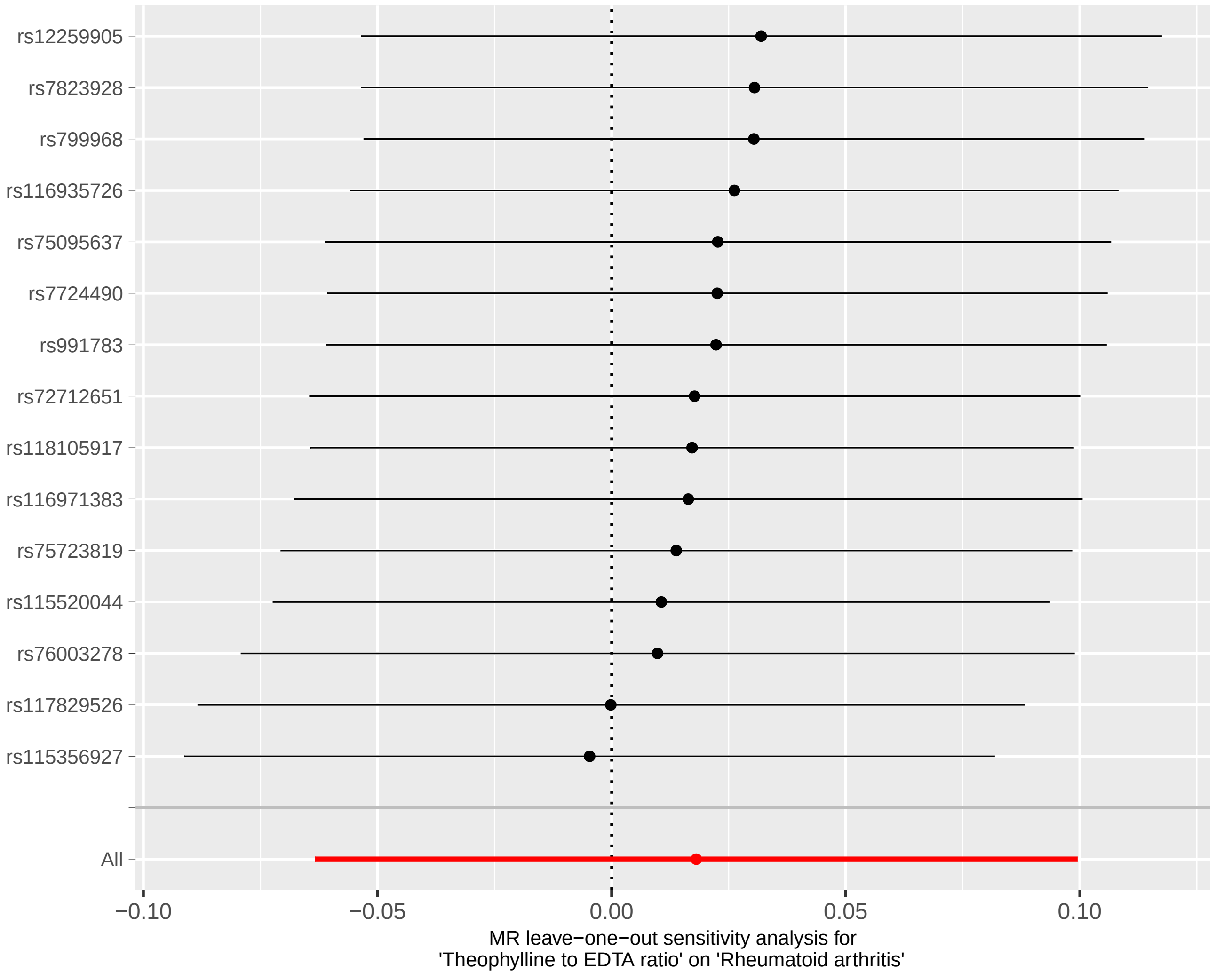
rs12259905
rs7823928
rs799968
rs116935726
rs75095637
rs7724490
rs991783
rs72712651
rs118105917
rs116971383
rs75723819
rs115520044
rs76003278
rs117829526
rs115356927
All
−0.10
−0.05
0.00
0.05
0.10
MR leave−one−out sensitivity analysis for
'Theophylline to EDTA ratio' on 'Rheumatoid arthritis'

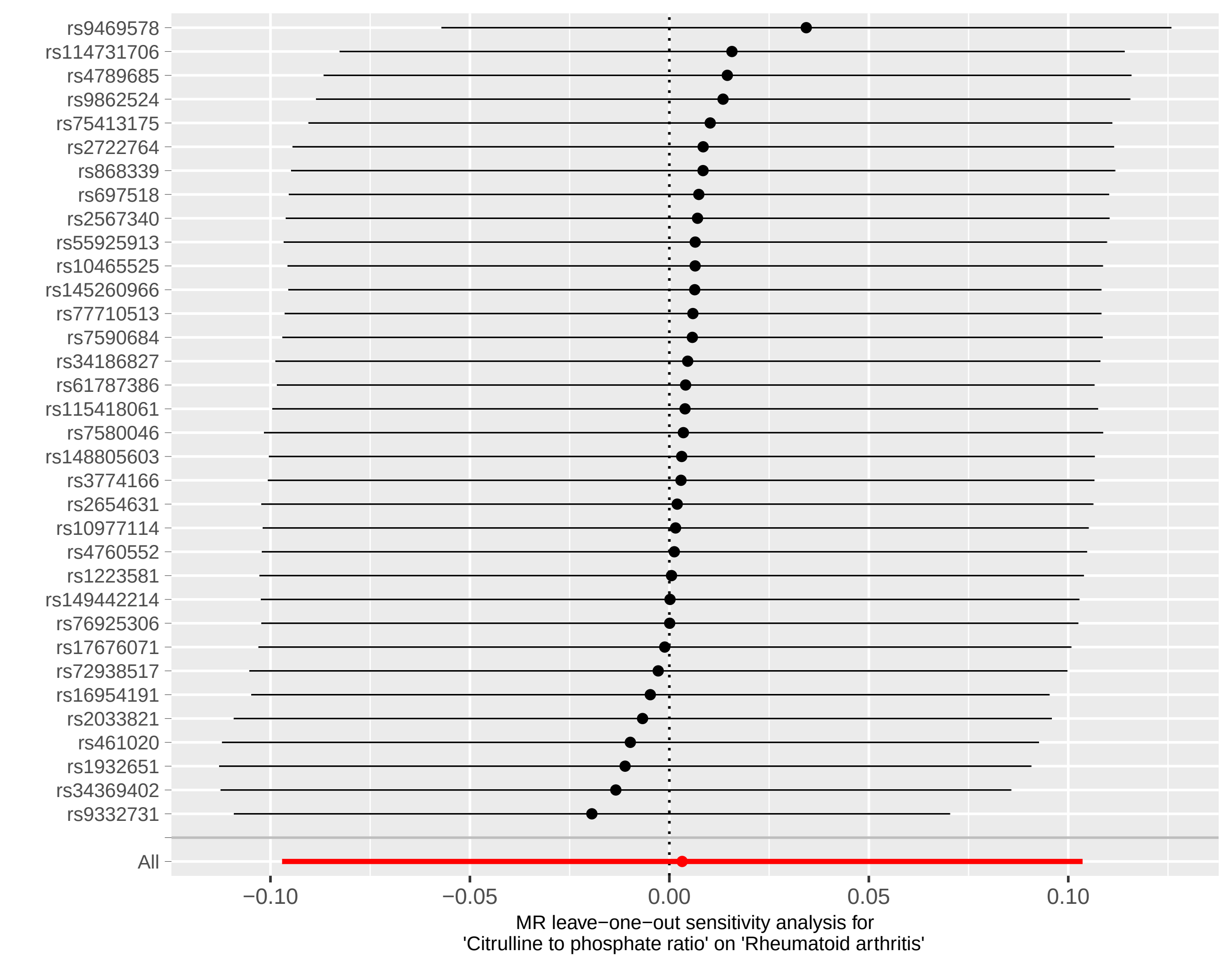

rs9469578
rs114731706
rs4789685
rs9862524
rs75413175
rs2722764
rs868339
rs697518
rs2567340
rs55925913
rs10465525
rs145260966
rs77710513
rs7590684
rs34186827
rs61787386
rs115418061
rs7580046
rs148805603
rs3774166
rs2654631
rs10977114
rs4760552
rs1223581
rs149442214
rs76925306
rs17676071
rs72938517
rs16954191
rs2033821
rs461020
rs1932651
rs34369402
rs9332731
All
−0.10
−0.05
0.00
0.05
0.10
MR leave−one−out sensitivity analysis for
'Citrulline to phosphate ratio' on 'Rheumatoid arthritis'

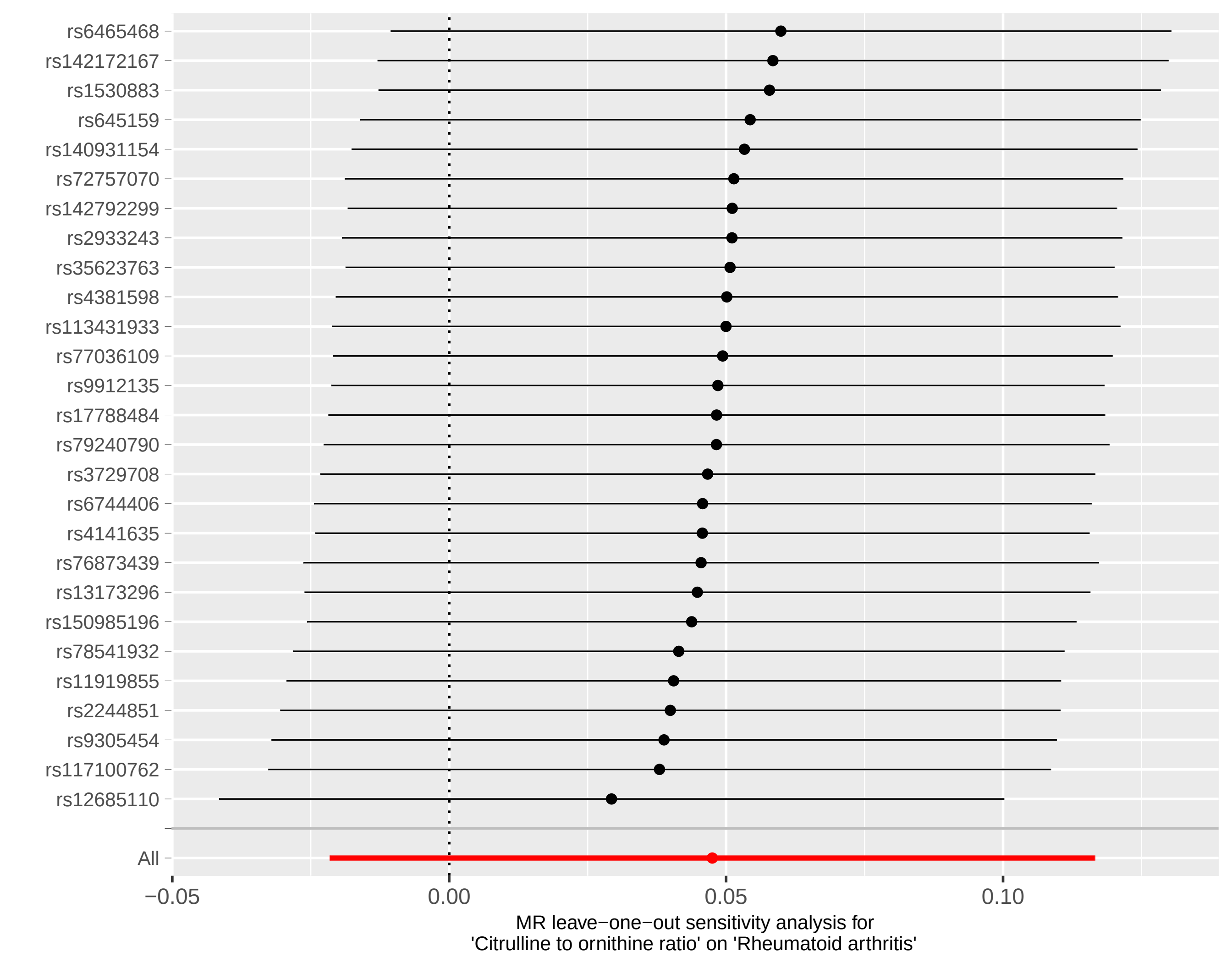
rs6465468
rs142172167
rs1530883
rs645159
rs140931154
rs72757070
rs142792299
rs2933243
rs35623763
rs4381598
rs113431933
rs77036109
rs9912135
rs17788484
rs79240790
rs3729708
rs6744406
rs4141635
rs76873439
rs13173296
rs150985196
rs78541932
rs11919855
rs2244851
rs9305454
rs117100762
rs12685110
All
−0.05
0.00
0.05
0.10
MR leave−one−out sensitivity analysis for
'Citrulline to ornithine ratio' on 'Rheumatoid arthritis'

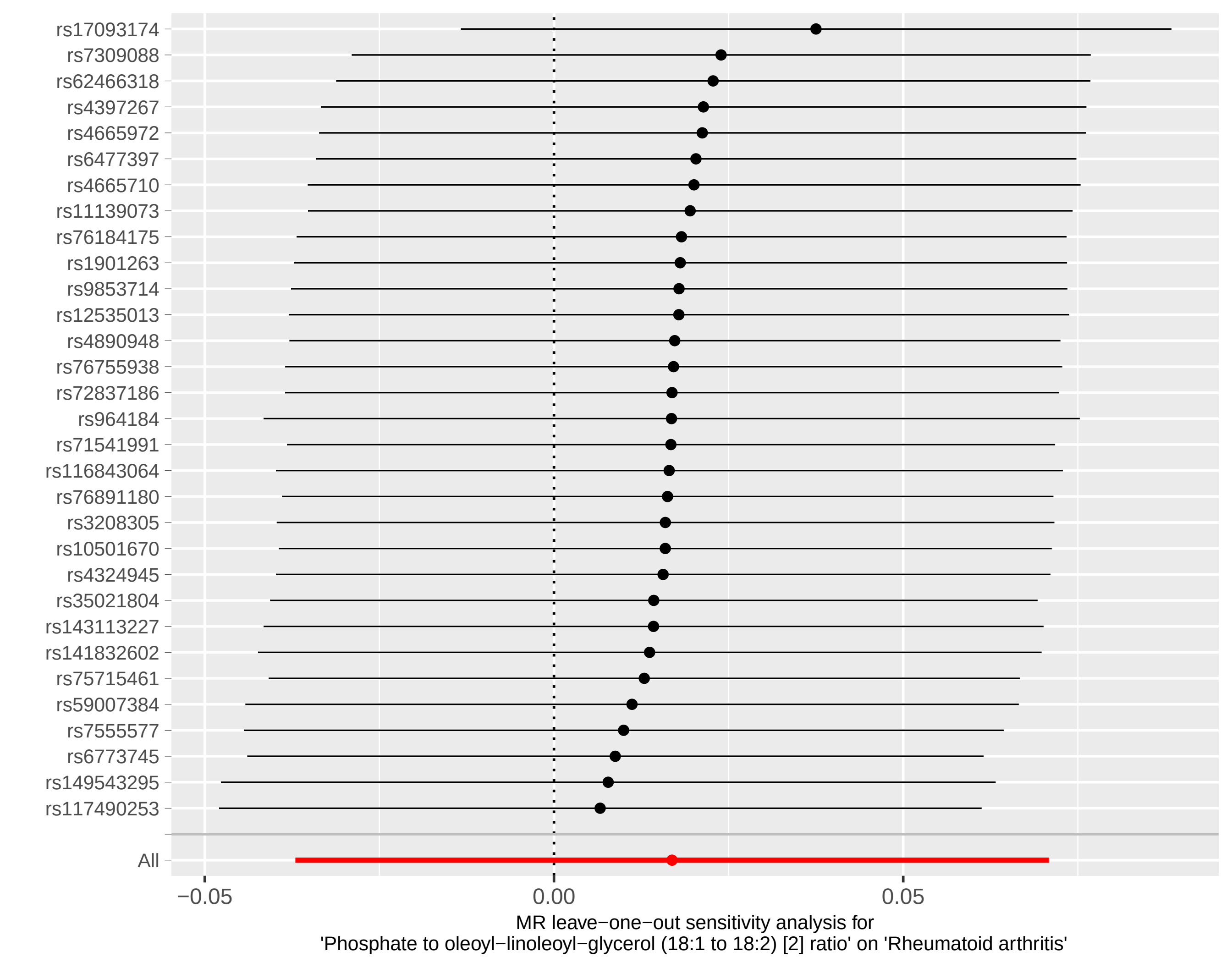
rs17093174
rs7309088
rs62466318
rs4397267
rs4665972
rs6477397
rs4665710
rs11139073
rs76184175
rs1901263
rs9853714
rs12535013
rs4890948
rs76755938
rs72837186
rs964184
rs71541991
rs116843064
rs76891180
rs3208305
rs10501670
rs4324945
rs35021804
rs143113227
rs141832602
rs75715461
rs59007384
rs7555577
rs6773745
rs149543295
rs117490253
All
−0.05
0.00
0.05
MR leave−one−out sensitivity analysis for
'Phosphate to oleoyl−linoleoyl−glycerol (18:1 to 18:2) [2] ratio' on 'Rheumatoid arthritis'

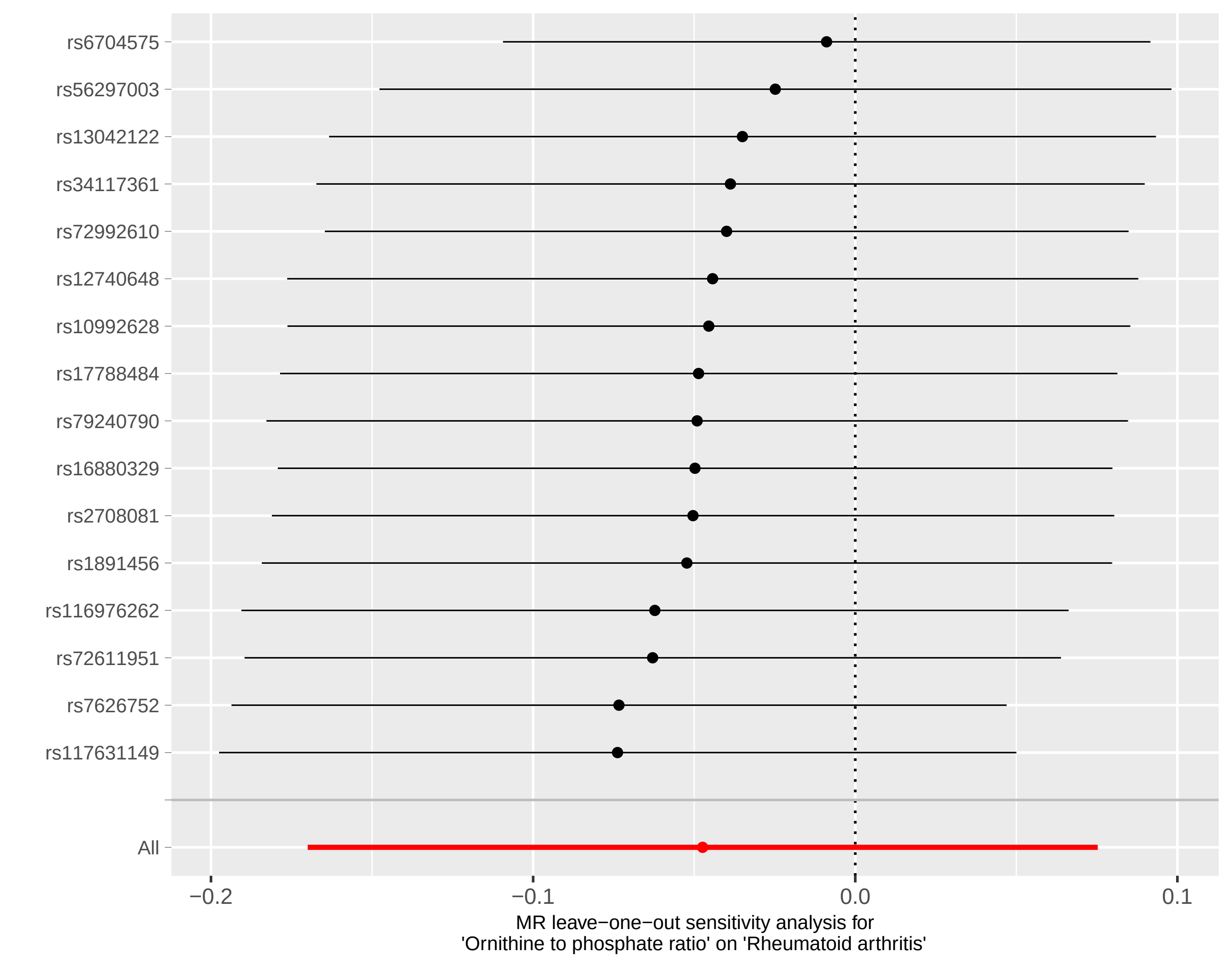
rs6704575
rs56297003
rs13042122
rs34117361
rs72992610
rs12740648
rs10992628
rs17788484
rs79240790
rs16880329
rs2708081
rs1891456
rs116976262
rs72611951
rs7626752
rs117631149
All
−0.2
−0.1
0.0
0.1
MR leave−one−out sensitivity analysis for
'Ornithine to phosphate ratio' on 'Rheumatoid arthritis'

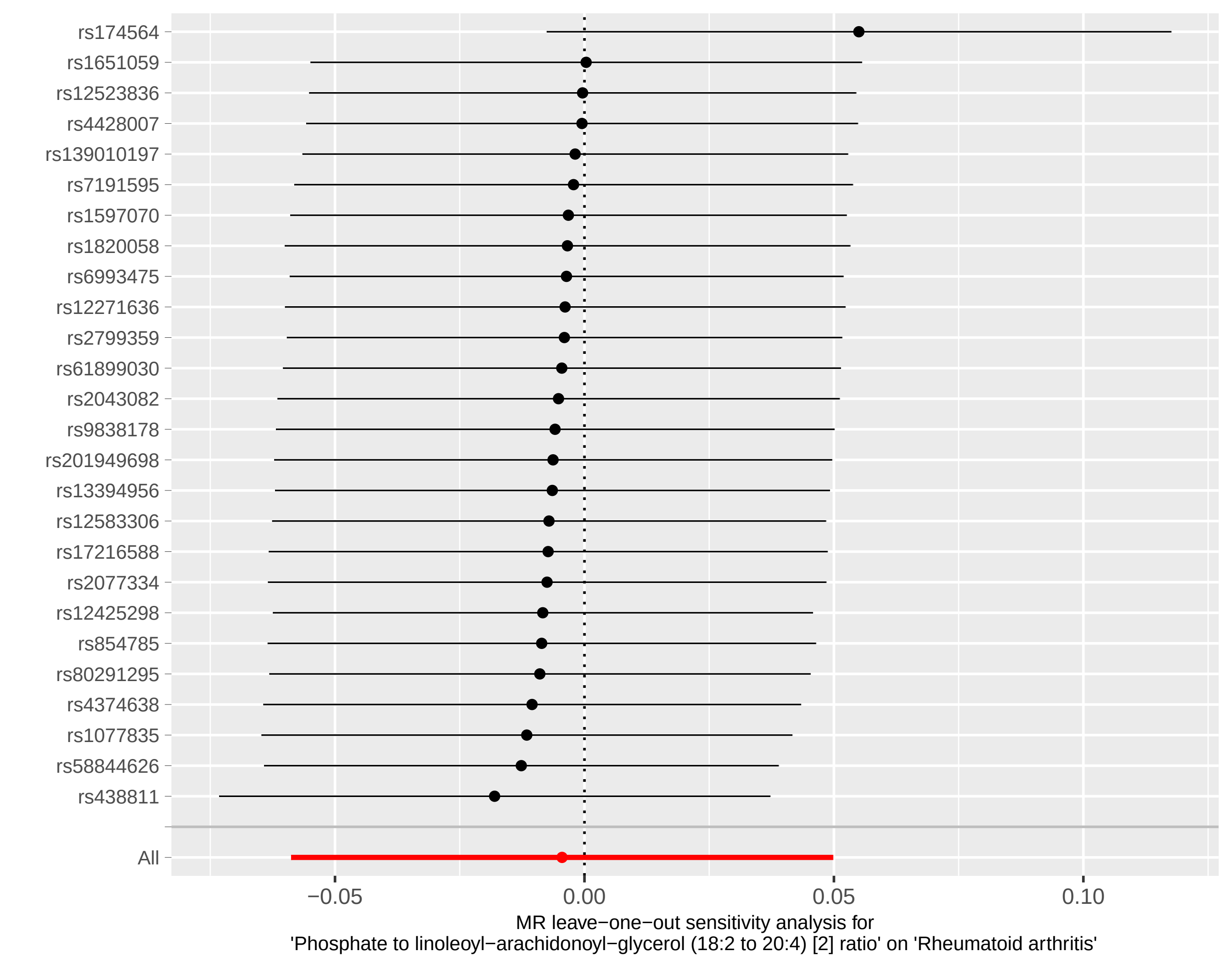
rs174564
rs1651059
rs12523836
rs4428007
rs139010197
rs7191595
rs1597070
rs1820058
rs6993475
rs12271636
rs2799359
rs61899030
rs2043082
rs9838178
rs201949698
rs13394956
rs12583306
rs17216588
rs2077334
rs12425298
rs854785
rs80291295
rs4374638
rs1077835
rs58844626
rs438811
All
−0.05
0.00
0.05
0.10
MR leave−one−out sensitivity analysis for
'Phosphate to linoleoyl−arachidonoyl−glycerol (18:2 to 20:4) [2] ratio' on 'Rheumatoid arthritis'

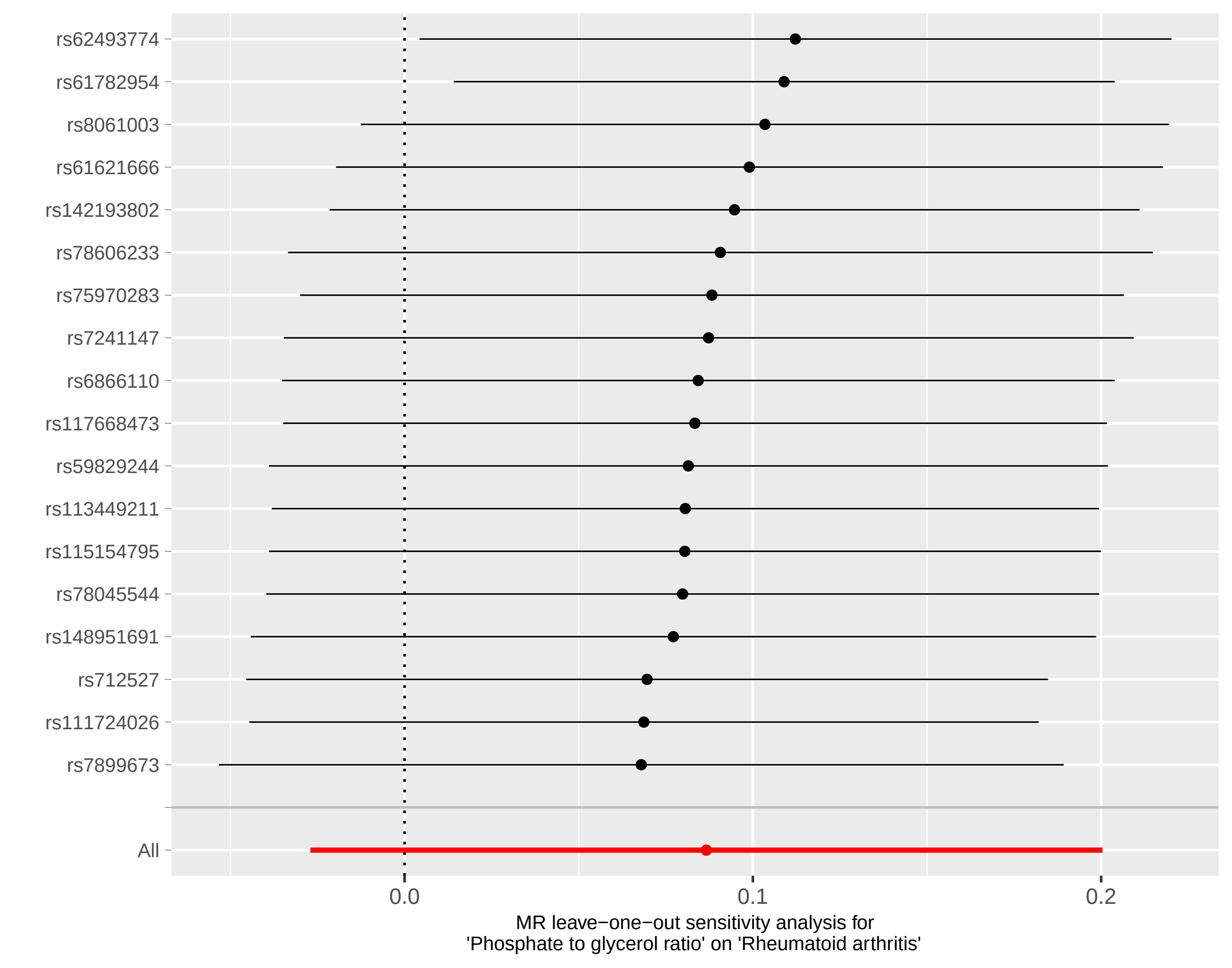
rs62493774
rs61782954
rs8061003
rs61621666
rs142193802
rs78606233
rs75970283
rs7241147
rs6866110
rs117668473
rs59829244
rs113449211
rs115154795
rs78045544
rs148951691
rs712527
rs111724026
rs7899673
All
0.0
0.1
0.2
MR leave−one−out sensitivity analysis for
'Phosphate to glycerol ratio' on 'Rheumatoid arthritis'

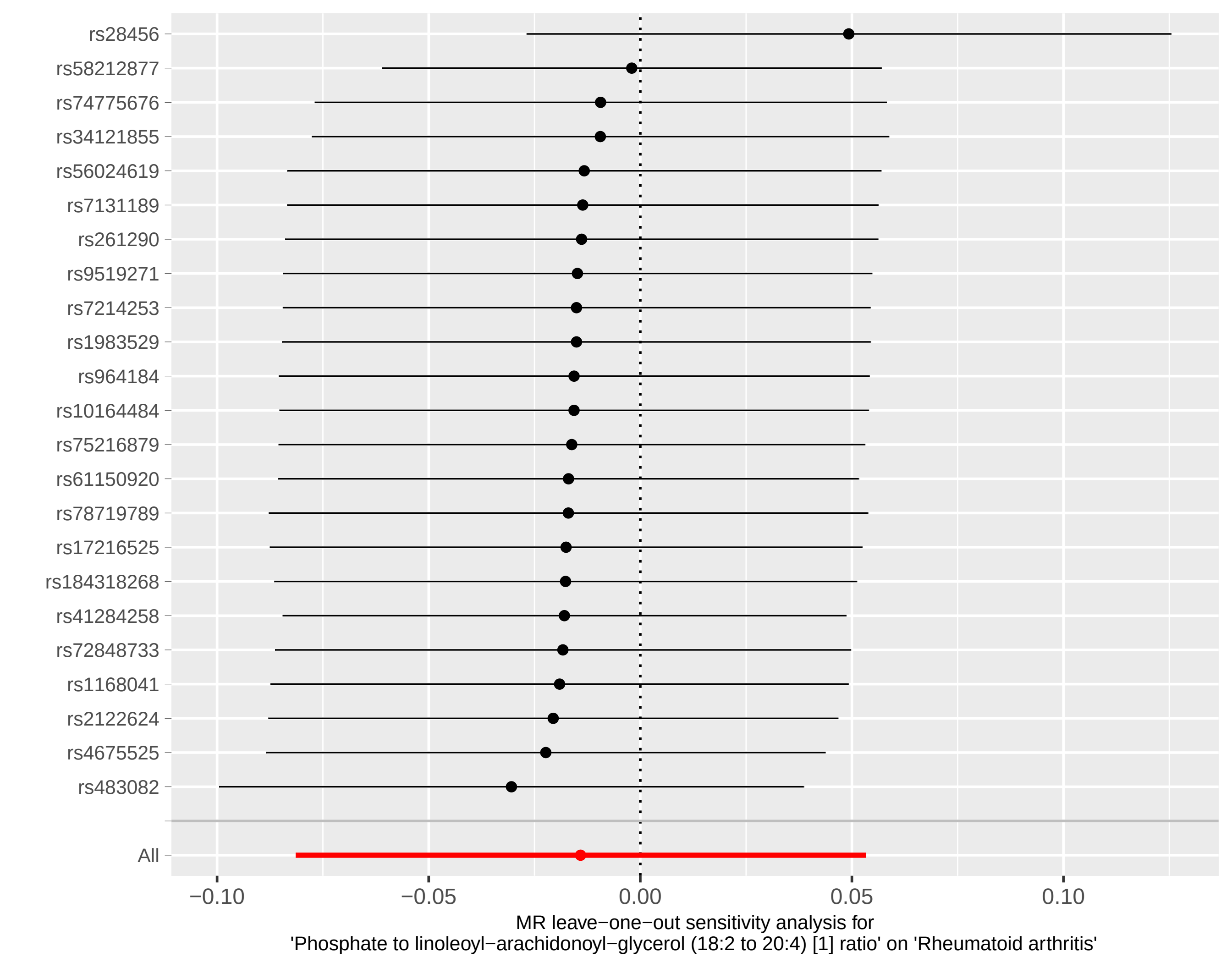

rs28456
rs58212877
rs74775676
rs34121855
rs56024619
rs7131189
rs261290
rs9519271
rs7214253
rs1983529
rs964184
rs10164484
rs75216879
rs61150920
rs78719789
rs17216525
rs184318268
rs41284258
rs72848733
rs1168041
rs2122624
rs4675525
rs483082
All
−0.10
−0.05
0.00
0.05
0.10
MR leave−one−out sensitivity analysis for
'Phosphate to linoleoyl−arachidonoyl−glycerol (18:2 to 20:4) [1] ratio' on 'Rheumatoid arthritis'

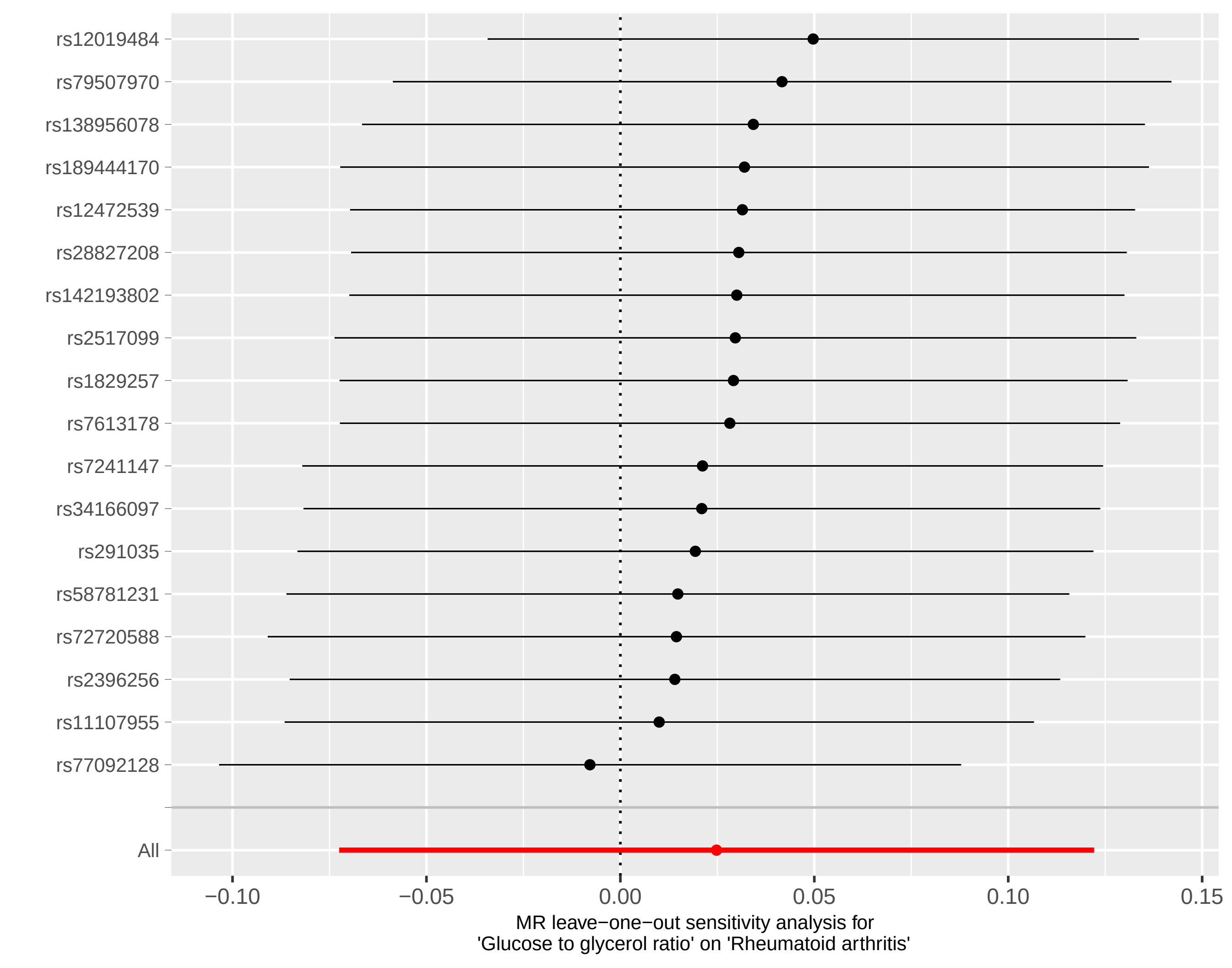
rs12019484
rs79507970
rs138956078
rs189444170
rs12472539
rs28827208
rs142193802
rs2517099
rs1829257
rs7613178
rs7241147
rs34166097
rs291035
rs58781231
rs72720588
rs2396256
rs11107955
rs77092128
All
−0.10
−0.05
0.00
0.05
0.10
0.15
MR leave−one−out sensitivity analysis for
'Glucose to glycerol ratio' on 'Rheumatoid arthritis'

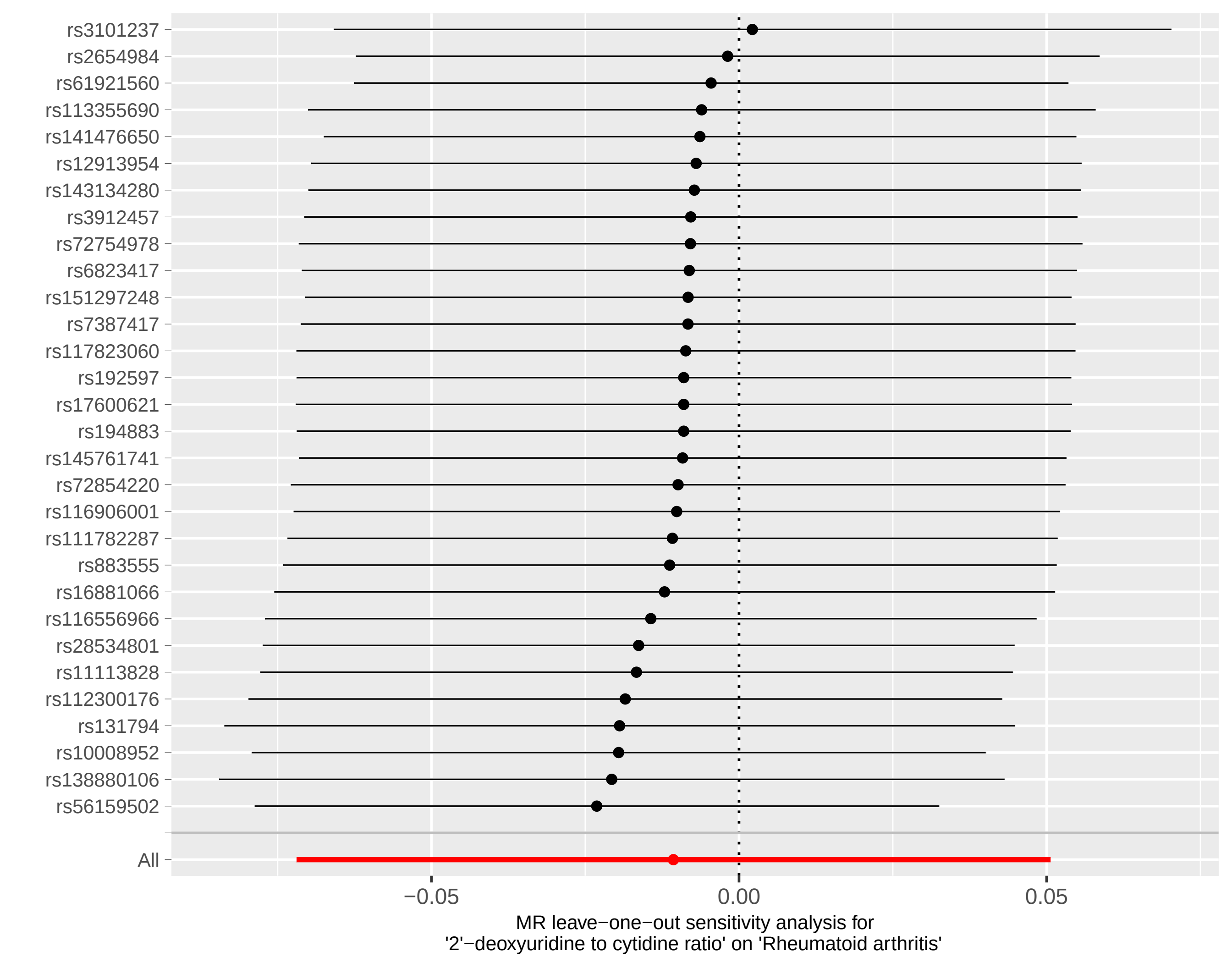
rs3101237
rs2654984
rs61921560
rs113355690
rs141476650
rs12913954
rs143134280
rs3912457
rs72754978
rs6823417
rs151297248
rs7387417
rs117823060
rs192597
rs17600621
rs194883
rs145761741
rs72854220
rs116906001
rs111782287
rs883555
rs16881066
rs116556966
rs28534801
rs11113828
rs112300176
rs131794
rs10008952
rs138880106
rs56159502
All
−0.05
0.00
0.05
MR leave−one−out sensitivity analysis for
'2'−deoxyuridine to cytidine ratio' on 'Rheumatoid arthritis'

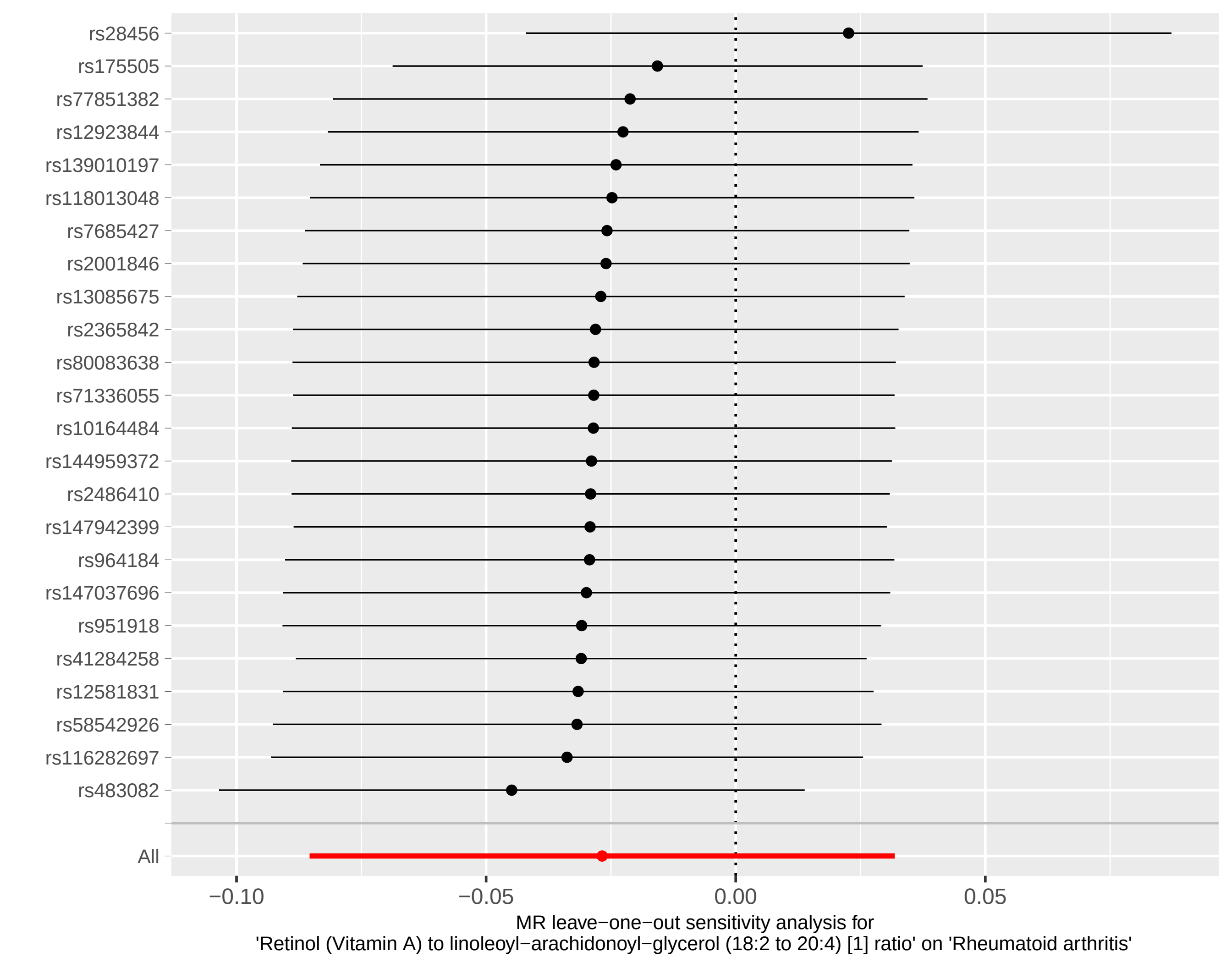
rs28456
rs175505
rs77851382
rs12923844
rs139010197
rs118013048
rs7685427
rs2001846
rs13085675
rs2365842
rs80083638
rs71336055
rs10164484
rs144959372
rs2486410
rs147942399
rs964184
rs147037696
rs951918
rs41284258
rs12581831
rs58542926
rs116282697
rs483082
All
−0.10
−0.05
0.00
0.05
MR leave−one−out sensitivity analysis for
'Retinol (Vitamin A) to linoleoyl−arachidonoyl−glycerol (18:2 to 20:4) [1] ratio' on 'Rheumatoid arthritis'

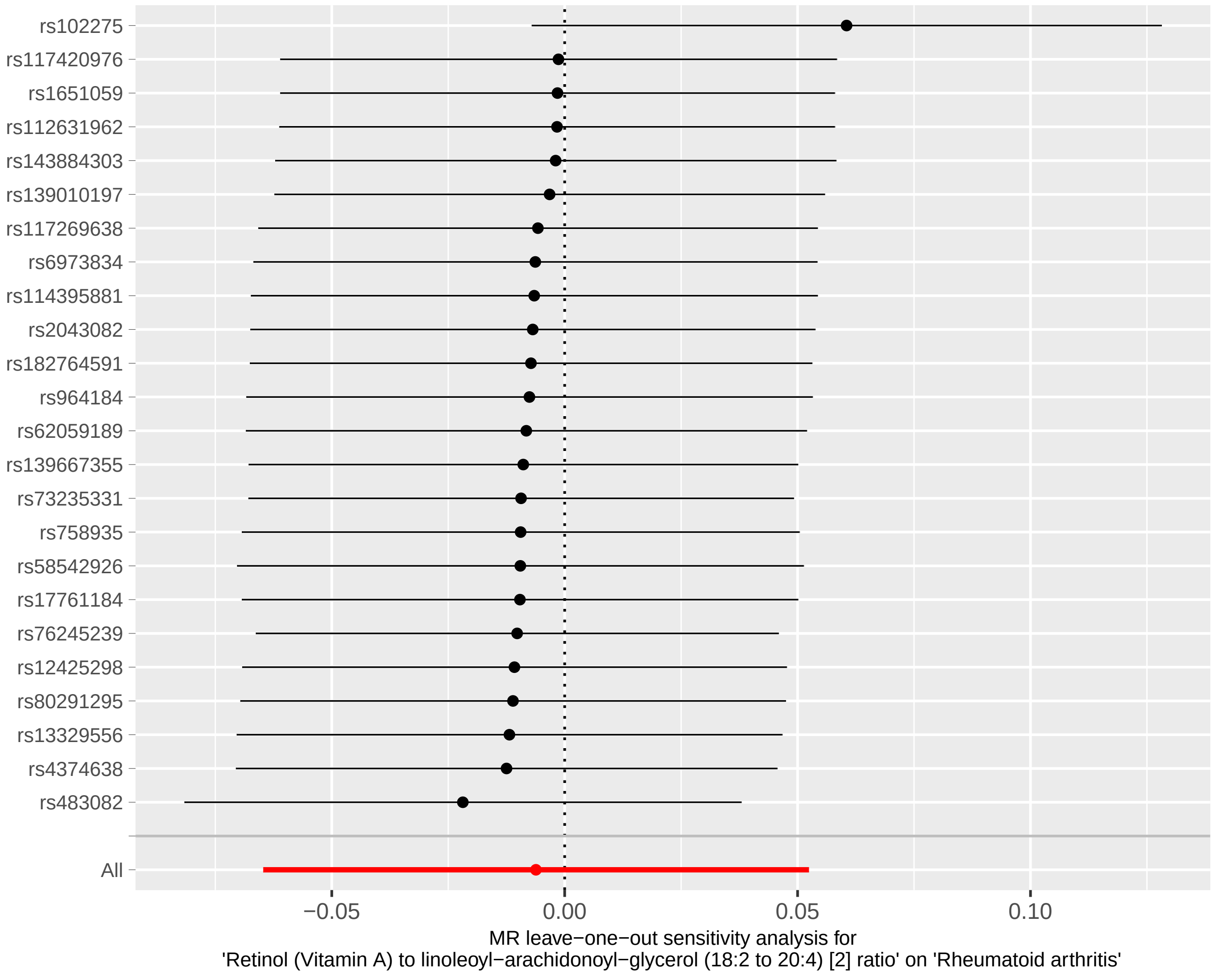

rs102275
rs117420976
rs1651059
rs112631962
rs143884303
rs139010197
rs117269638
rs6973834
rs114395881
rs2043082
rs182764591
rs964184
rs62059189
rs139667355
rs73235331
rs758935
rs58542926
rs17761184
rs76245239
rs12425298
rs80291295
rs13329556
rs4374638
rs483082
All
−0.05
0.00
0.05
0.10
MR leave−one−out sensitivity analysis for
'Retinol (Vitamin A) to linoleoyl−arachidonoyl−glycerol (18:2 to 20:4) [2] ratio' on 'Rheumatoid arthritis'

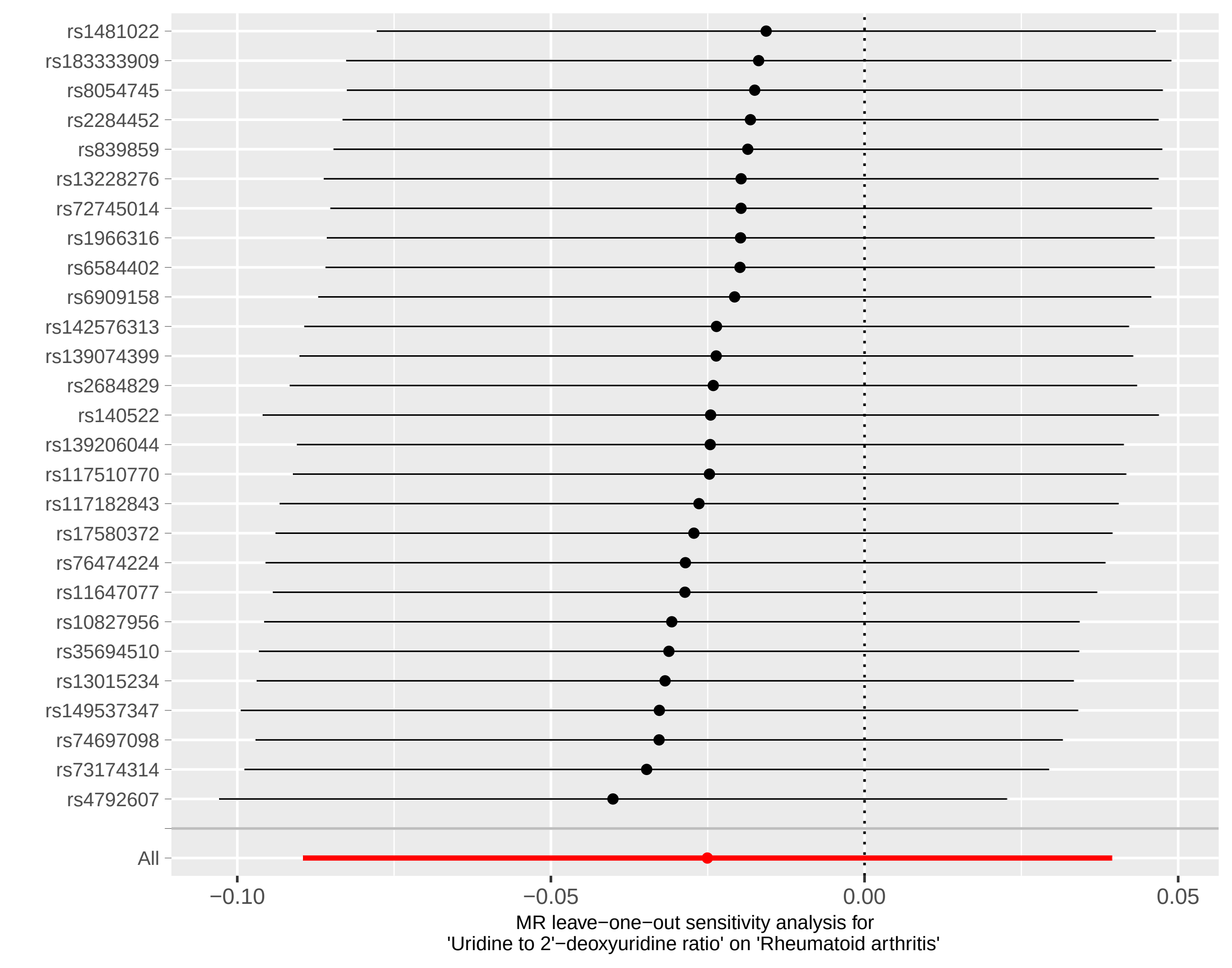
rs1481022
rs183333909
rs8054745
rs2284452
rs839859
rs13228276
rs72745014
rs1966316
rs6584402
rs6909158
rs142576313
rs139074399
rs2684829
rs140522
rs139206044
rs117510770
rs117182843
rs17580372
rs76474224
rs11647077
rs10827956
rs35694510
rs13015234
rs149537347
rs74697098
rs73174314
rs4792607
All
−0.10
−0.05
0.00
0.05
MR leave−one−out sensitivity analysis for
'Uridine to 2'−deoxyuridine ratio' on 'Rheumatoid arthritis'

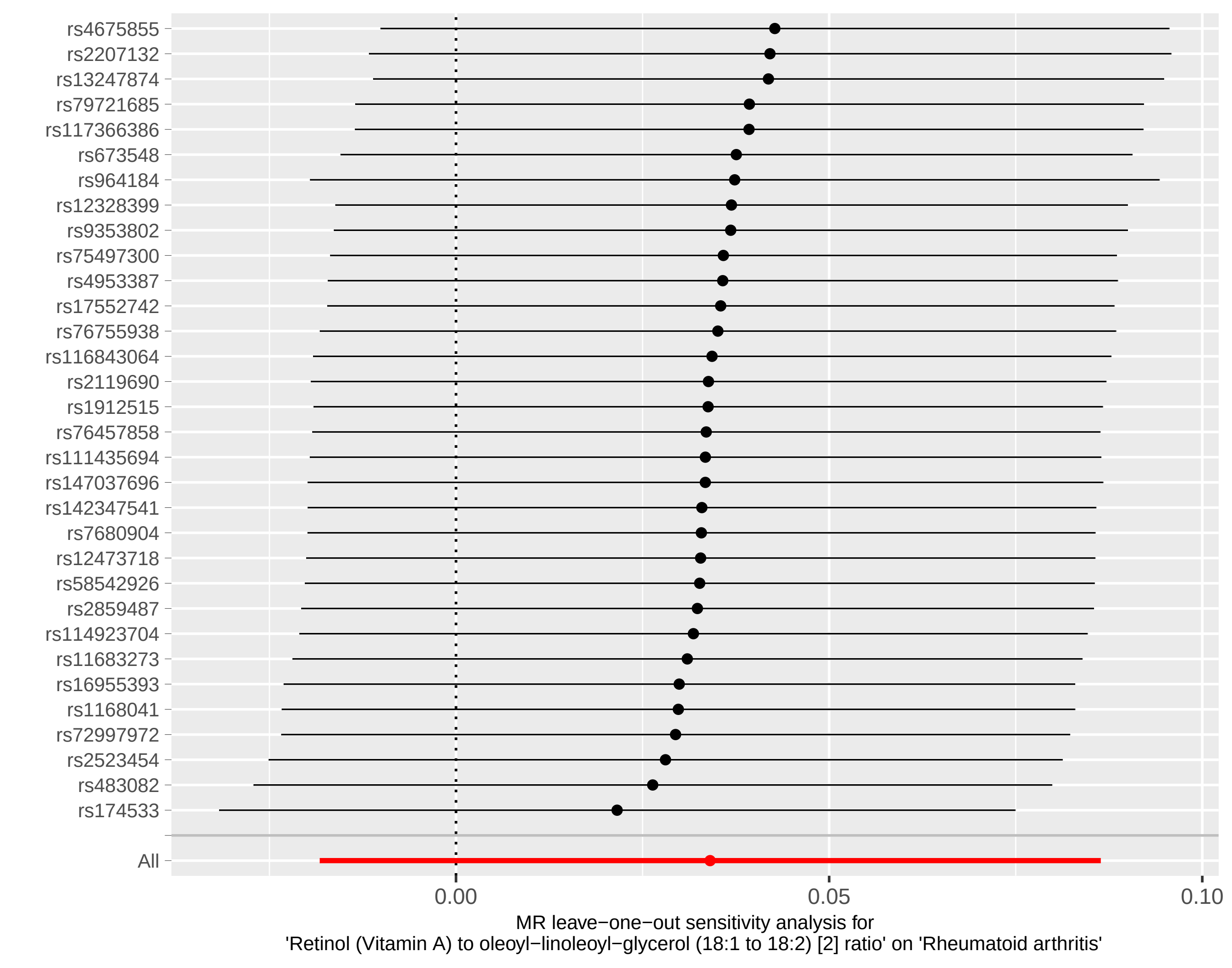
rs4675855
rs2207132
rs13247874
rs79721685
rs117366386
rs673548
rs964184
rs12328399
rs9353802
rs75497300
rs4953387
rs17552742
rs76755938
rs116843064
rs2119690
rs1912515
rs76457858
rs111435694
rs147037696
rs142347541
rs7680904
rs12473718
rs58542926
rs2859487
rs114923704
rs11683273
rs16955393
rs1168041
rs72997972
rs2523454
rs483082
rs174533
All
0.00
0.05
0.10
MR leave−one−out sensitivity analysis for
'Retinol (Vitamin A) to oleoyl−linoleoyl−glycerol (18:1 to 18:2) [2] ratio' on 'Rheumatoid arthritis'

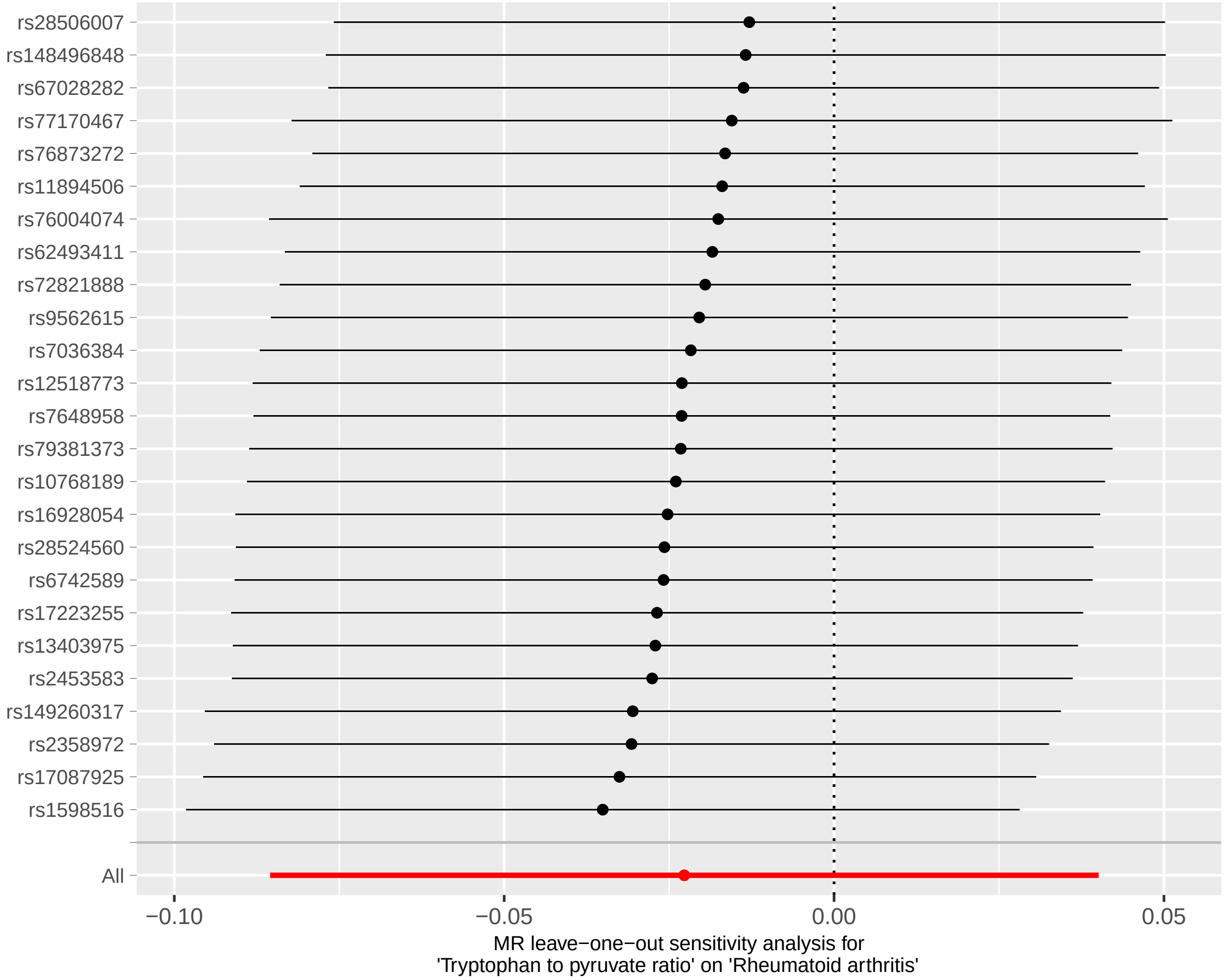
rs28506007
rs148496848
rs67028282
rs77170467
rs76873272
rs11894506
rs76004074
rs62493411
rs72821888
rs9562615
rs7036384
rs12518773
rs7648958
rs79381373
rs10768189
rs16928054
rs28524560
rs6742589
rs17223255
rs13403975
rs2453583
rs149260317
rs2358972
rs17087925
rs1598516
All
−0.10
−0.05
0.00
0.05
MR leave−one−out sensitivity analysis for
'Tryptophan to pyruvate ratio' on 'Rheumatoid arthritis'

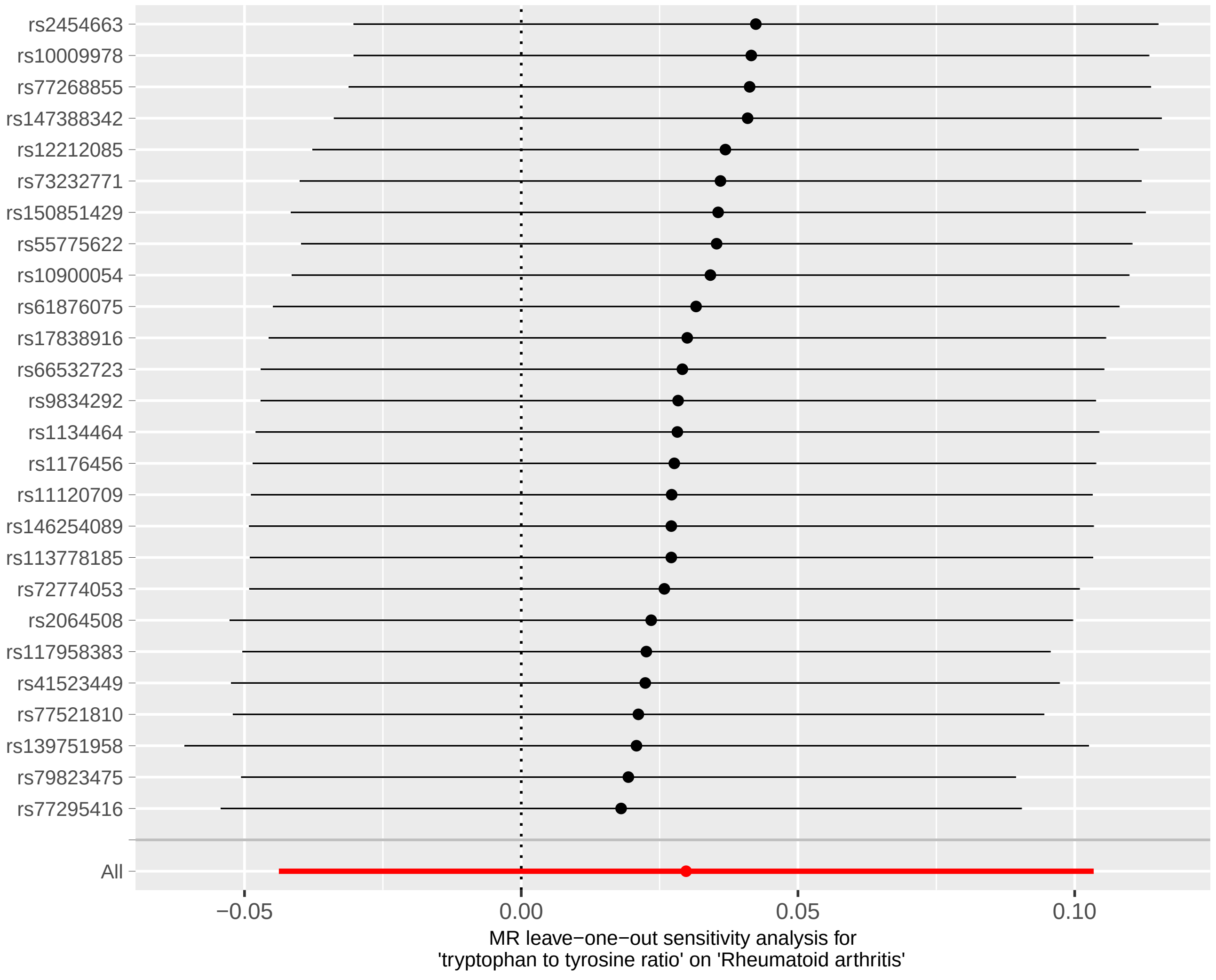
rs2454663
rs10009978
rs77268855
rs147388342
rs12212085
rs73232771
rs150851429
rs55775622
rs10900054
rs61876075
rs17838916
rs66532723
rs9834292
rs1134464
rs1176456
rs11120709
rs146254089
rs113778185
rs72774053
rs2064508
rs117958383
rs41523449
rs77521810
rs139751958
rs79823475
rs77295416
All
−0.05
0.00
0.05
0.10
MR leave−one−out sensitivity analysis for
'tryptophan to tyrosine ratio' on 'Rheumatoid arthritis'

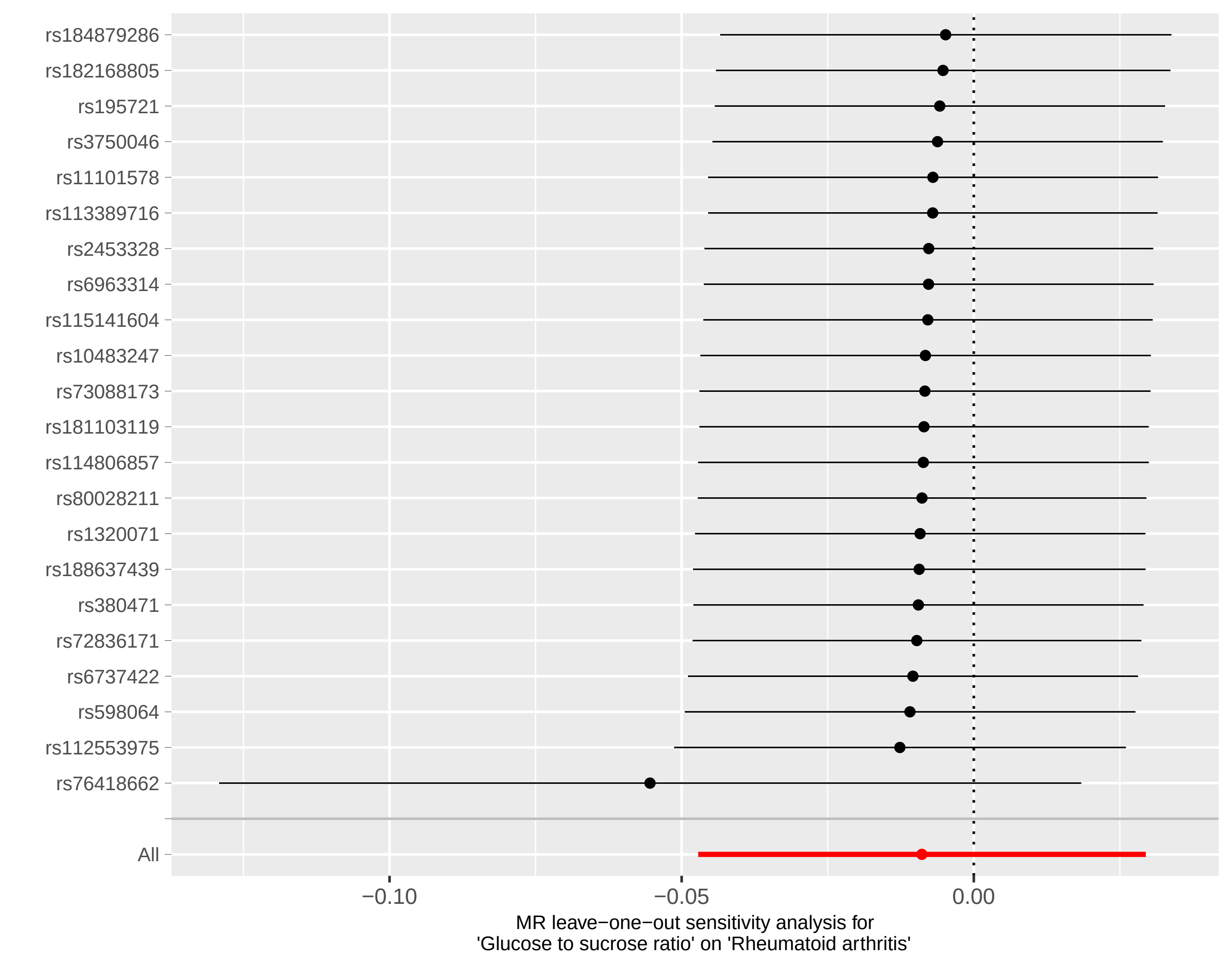

rs184879286
rs182168805
rs195721
rs3750046
rs11101578
rs113389716
rs2453328
rs6963314
rs115141604
rs10483247
rs73088173
rs181103119
rs114806857
rs80028211
rs1320071
rs188637439
rs380471
rs72836171
rs6737422
rs598064
rs112553975
rs76418662
All
−0.10
−0.05
0.00
MR leave−one−out sensitivity analysis for
'Glucose to sucrose ratio' on 'Rheumatoid arthritis'

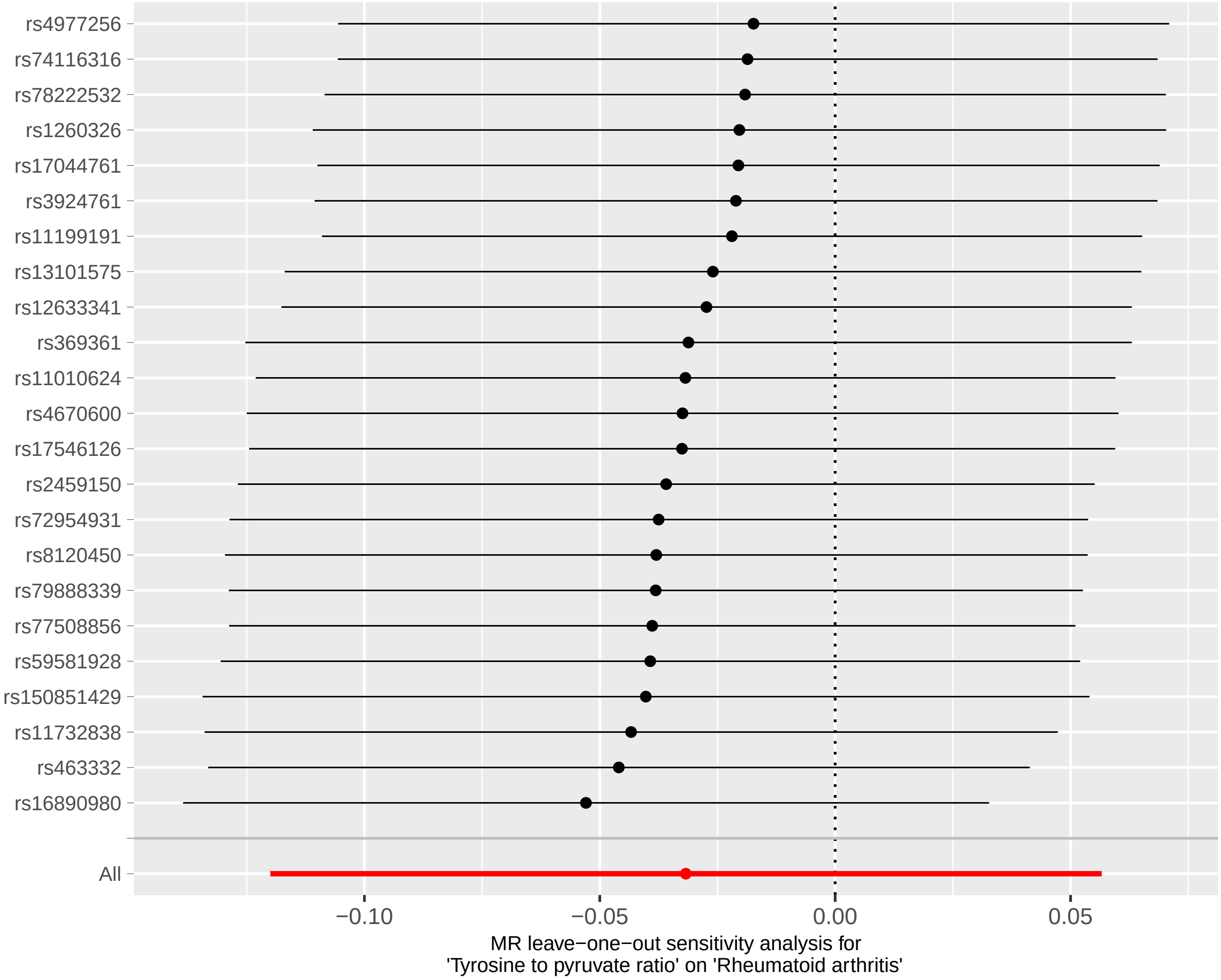

rs4977256
rs74116316
rs78222532
rs1260326
rs17044761
rs3924761
rs11199191
rs13101575
rs12633341
rs369361
rs11010624
rs4670600
rs17546126
rs2459150
rs72954931
rs8120450
rs79888339
rs77508856
rs59581928
rs150851429
rs11732838
rs463332
rs16890980
All
−0.10
−0.05
0.00
0.05
MR leave−one−out sensitivity analysis for
'Tyrosine to pyruvate ratio' on 'Rheumatoid arthritis'

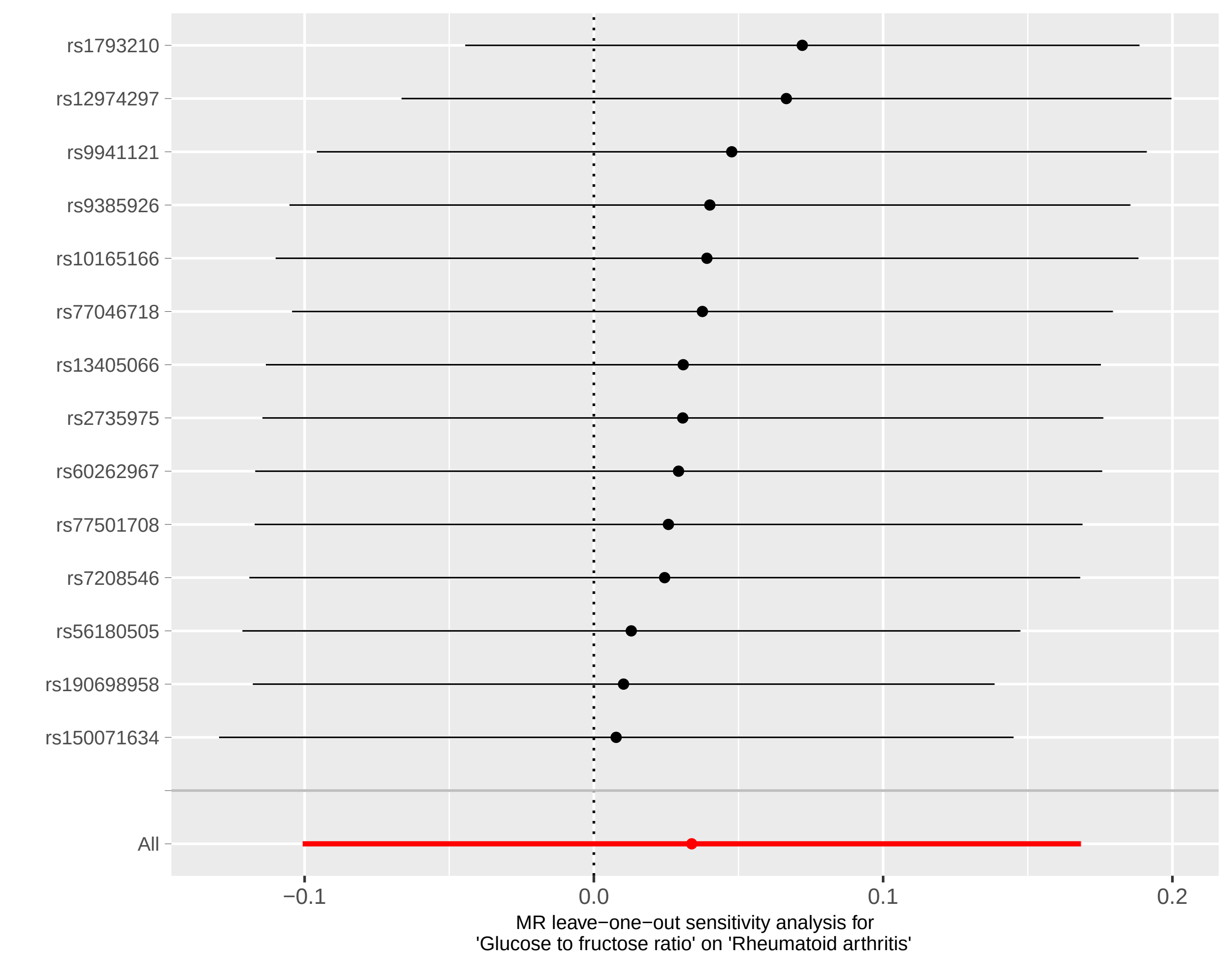

rs1793210
rs12974297
rs9941121
rs9385926
rs10165166
rs77046718
rs13405066
rs2735975
rs60262967
rs77501708
rs7208546
rs56180505
rs190698958
rs150071634
All
−0.1
0.0
0.1
0.2
MR leave−one−out sensitivity analysis for
'Glucose to fructose ratio' on 'Rheumatoid arthritis'

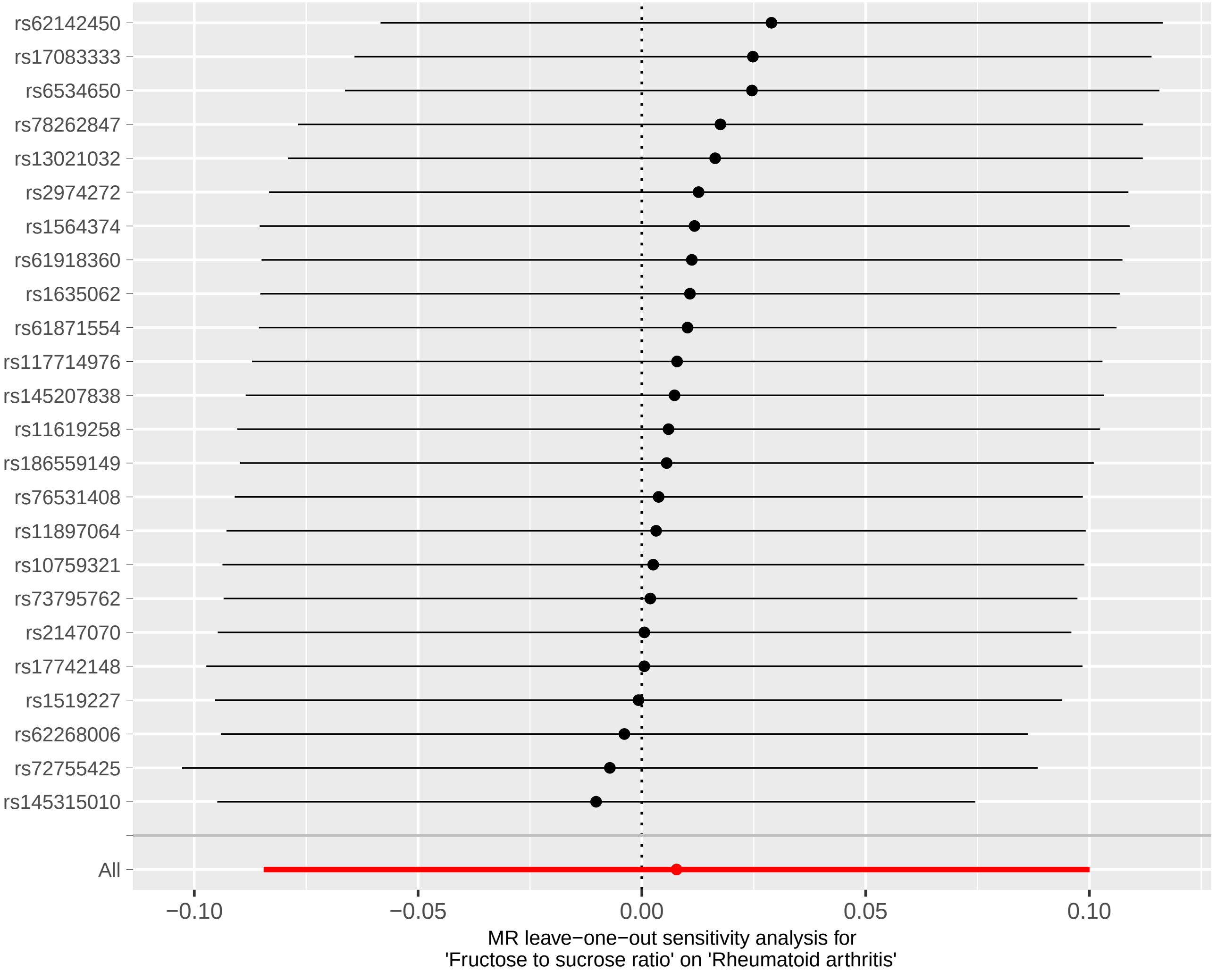

rs62142450
rs17083333
rs6534650
rs78262847
rs13021032
rs2974272
rs1564374
rs61918360
rs1635062
rs61871554
rs117714976
rs145207838
rs11619258
rs186559149
rs76531408
rs11897064
rs10759321
rs73795762
rs2147070
rs17742148
rs1519227
rs62268006
rs72755425
rs145315010
All
−0.10
−0.05
0.00
0.05
0.10
MR leave−one−out sensitivity analysis for
'Fructose to sucrose ratio' on 'Rheumatoid arthritis'

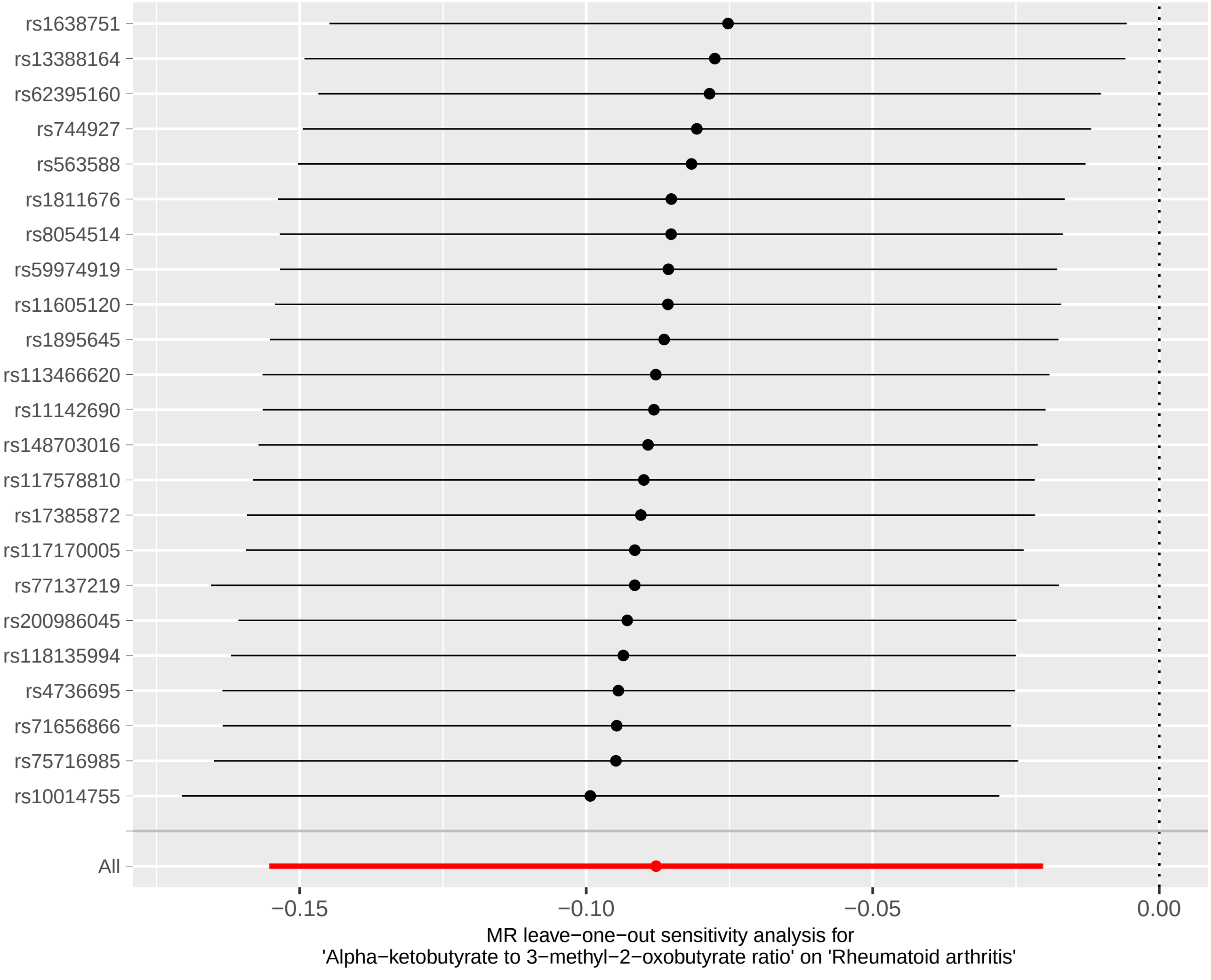
rs1638751
rs13388164
rs62395160
rs744927
rs563588
rs1811676
rs8054514
rs59974919
rs11605120
rs1895645
rs113466620
rs11142690
rs148703016
rs117578810
rs17385872
rs117170005
rs77137219
rs200986045
rs118135994
rs4736695
rs71656866
rs75716985
rs10014755
All
−0.15
−0.10
−0.05
0.00
MR leave−one−out sensitivity analysis for
'Alpha−ketobutyrate to 3−methyl−2−oxobutyrate ratio' on 'Rheumatoid arthritis'

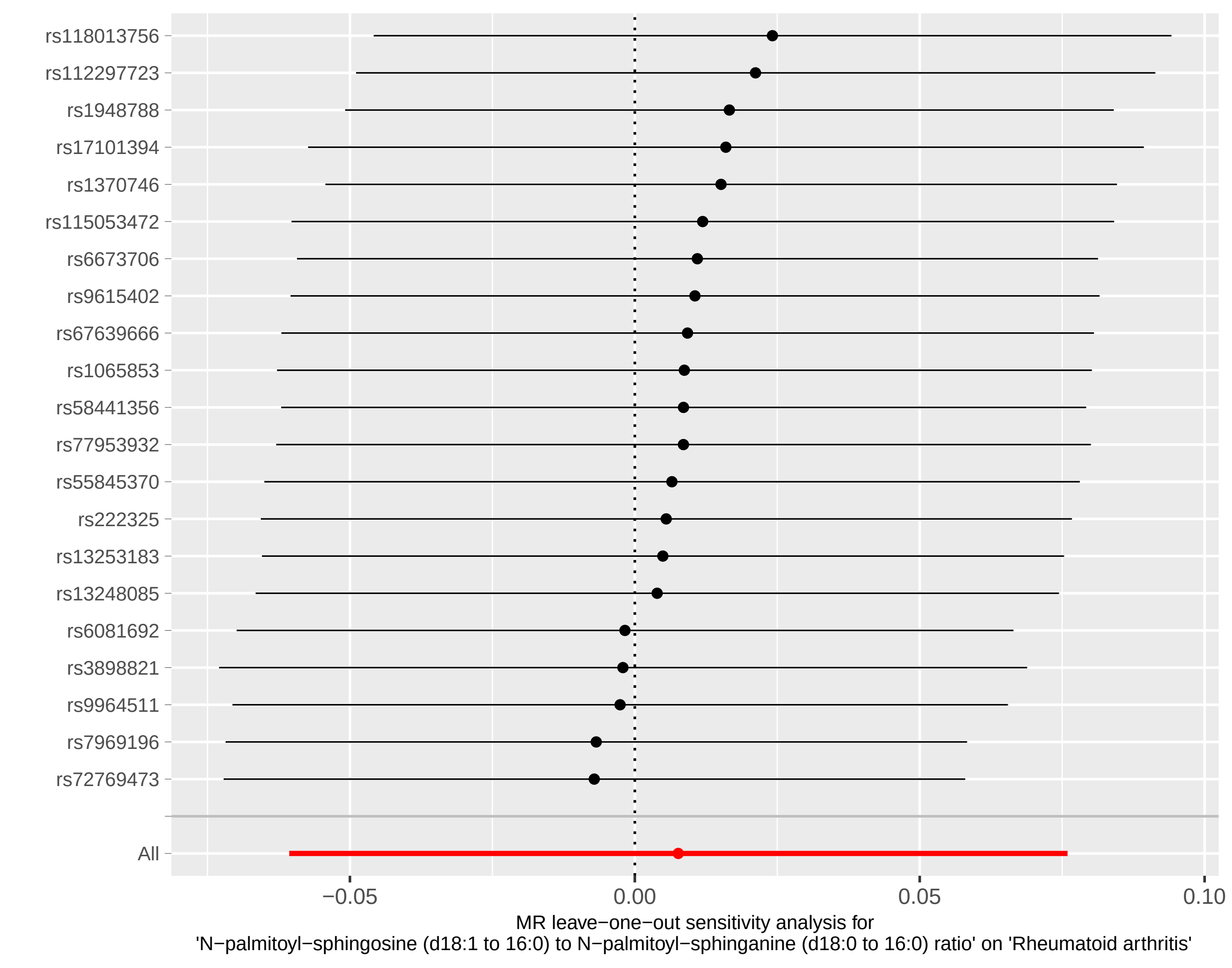
rs118013756
rs112297723
rs1948788
rs17101394
rs1370746
rs115053472
rs6673706
rs9615402
rs67639666
rs1065853
rs58441356
rs77953932
rs55845370
rs222325
rs13253183
rs13248085
rs6081692
rs3898821
rs9964511
rs7969196
rs72769473
All
−0.05
0.00
0.05
0.10
MR leave−one−out sensitivity analysis for
'N−palmitoyl−sphingosine (d18:1 to 16:0) to N−palmitoyl−sphinganine (d18:0 to 16:0) ratio' on 'Rheumatoid arthritis'

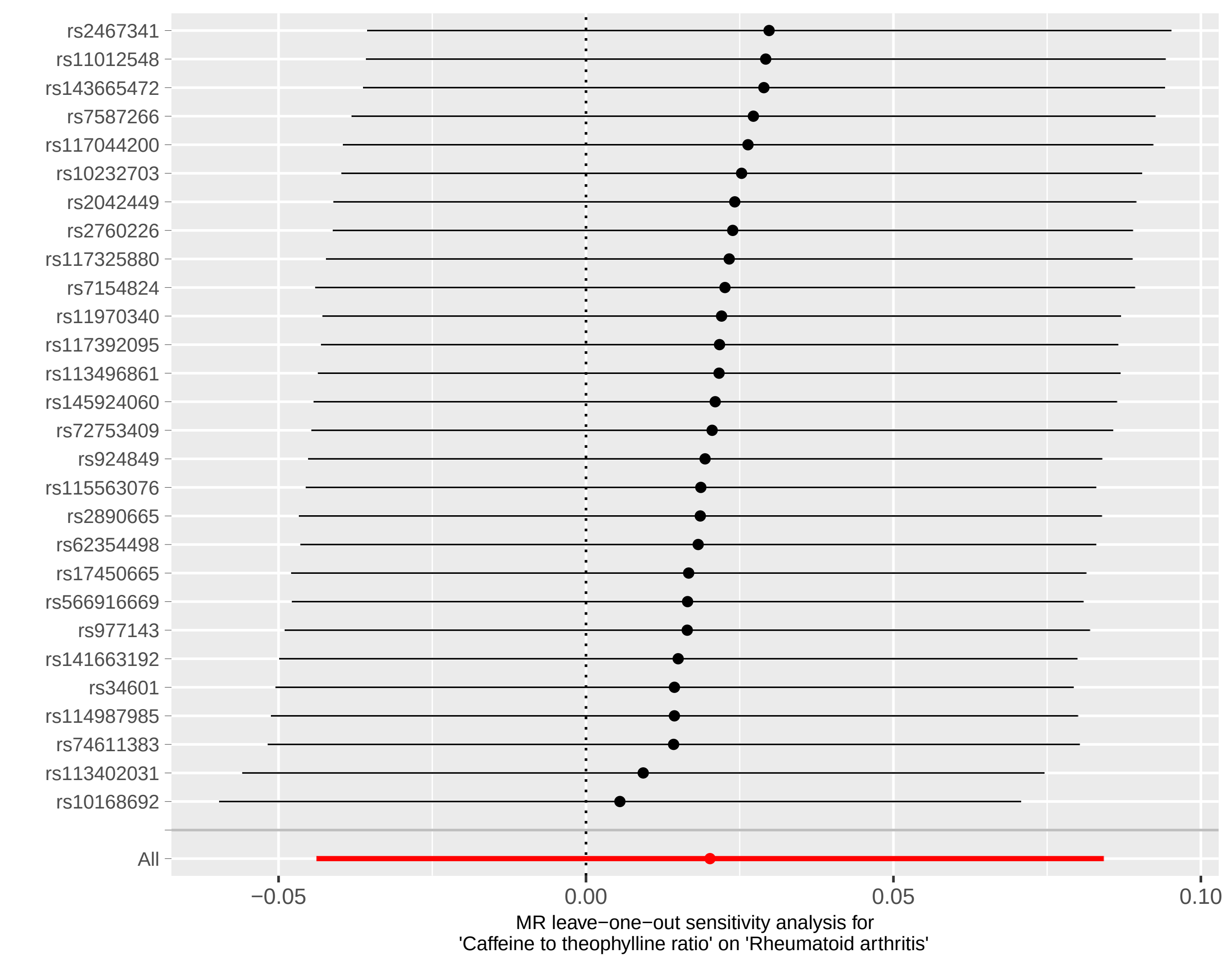
rs2467341
rs11012548
rs143665472
rs7587266
rs117044200
rs10232703
rs2042449
rs2760226
rs117325880
rs7154824
rs11970340
rs117392095
rs113496861
rs145924060
rs72753409
rs924849
rs115563076
rs2890665
rs62354498
rs17450665
rs566916669
rs977143
rs141663192
rs34601
rs114987985
rs74611383
rs113402031
rs10168692
All
−0.05
0.00
0.05
0.10
MR leave−one−out sensitivity analysis for
'Caffeine to theophylline ratio' on 'Rheumatoid arthritis'

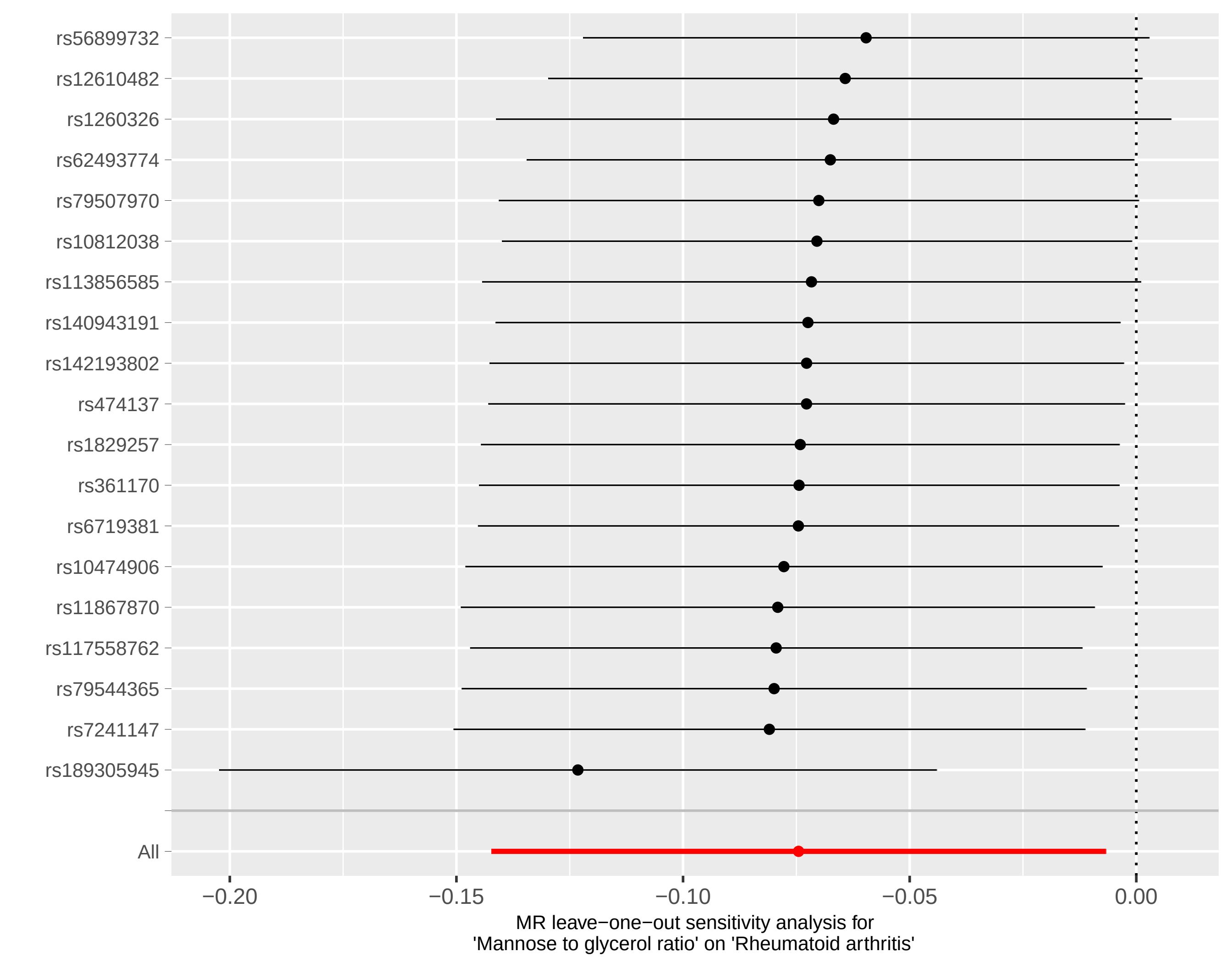
rs56899732
rs12610482
rs1260326
rs62493774
rs79507970
rs10812038
rs113856585
rs140943191
rs142193802
rs474137
rs1829257
rs361170
rs6719381
rs10474906
rs11867870
rs117558762
rs79544365
rs7241147
rs189305945
All
−0.20
−0.15
−0.10
−0.05
0.00
MR leave−one−out sensitivity analysis for
'Mannose to glycerol ratio' on 'Rheumatoid arthritis'

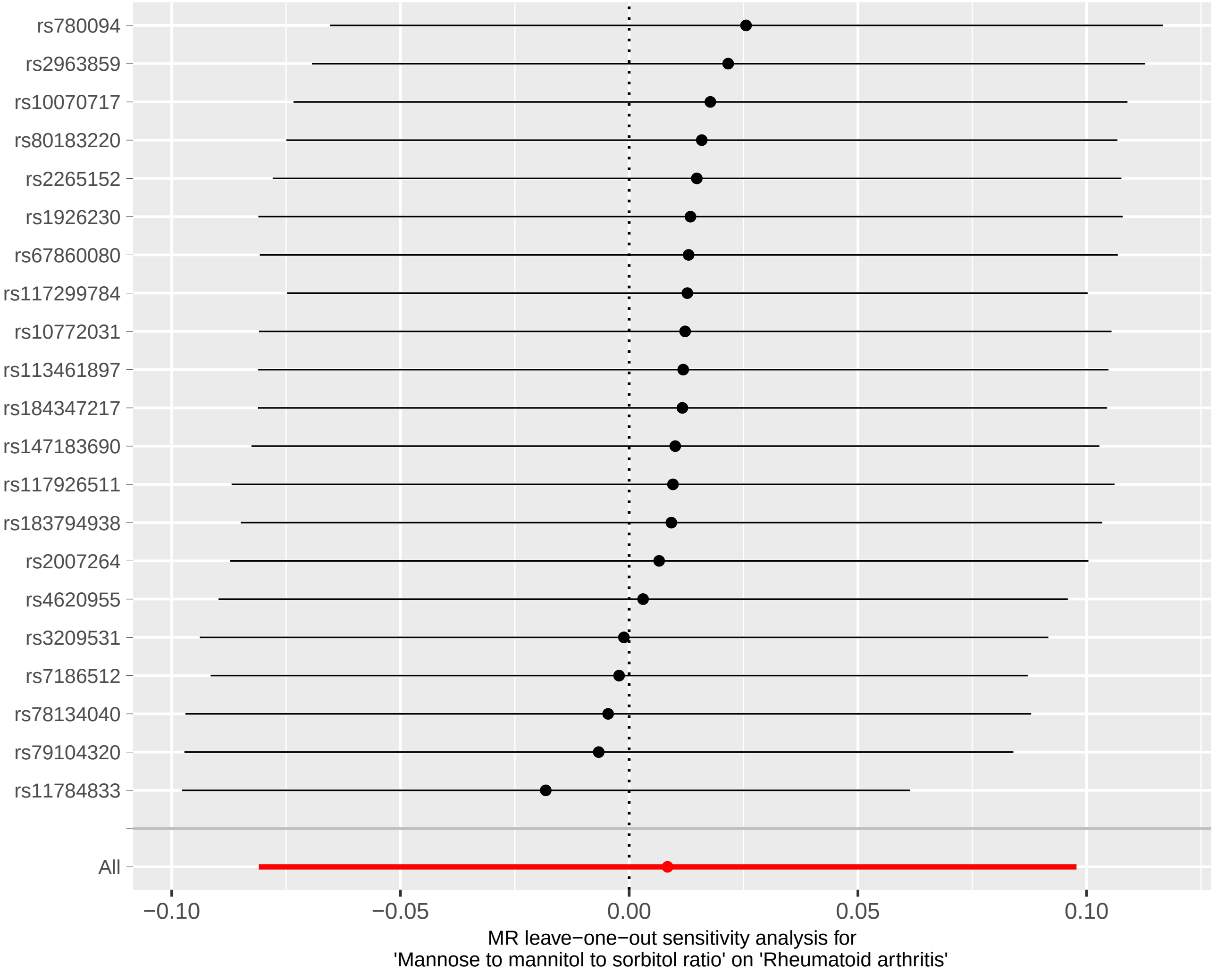
rs780094
rs2963859
rs10070717
rs80183220
rs2265152
rs1926230
rs67860080
rs117299784
rs10772031
rs113461897
rs184347217
rs147183690
rs117926511
rs183794938
rs2007264
rs4620955
rs3209531
rs7186512
rs78134040
rs79104320
rs11784833
All
−0.10
−0.05
0.00
0.05
0.10
MR leave−one−out sensitivity analysis for
'Mannose to mannitol to sorbitol ratio' on 'Rheumatoid arthritis'

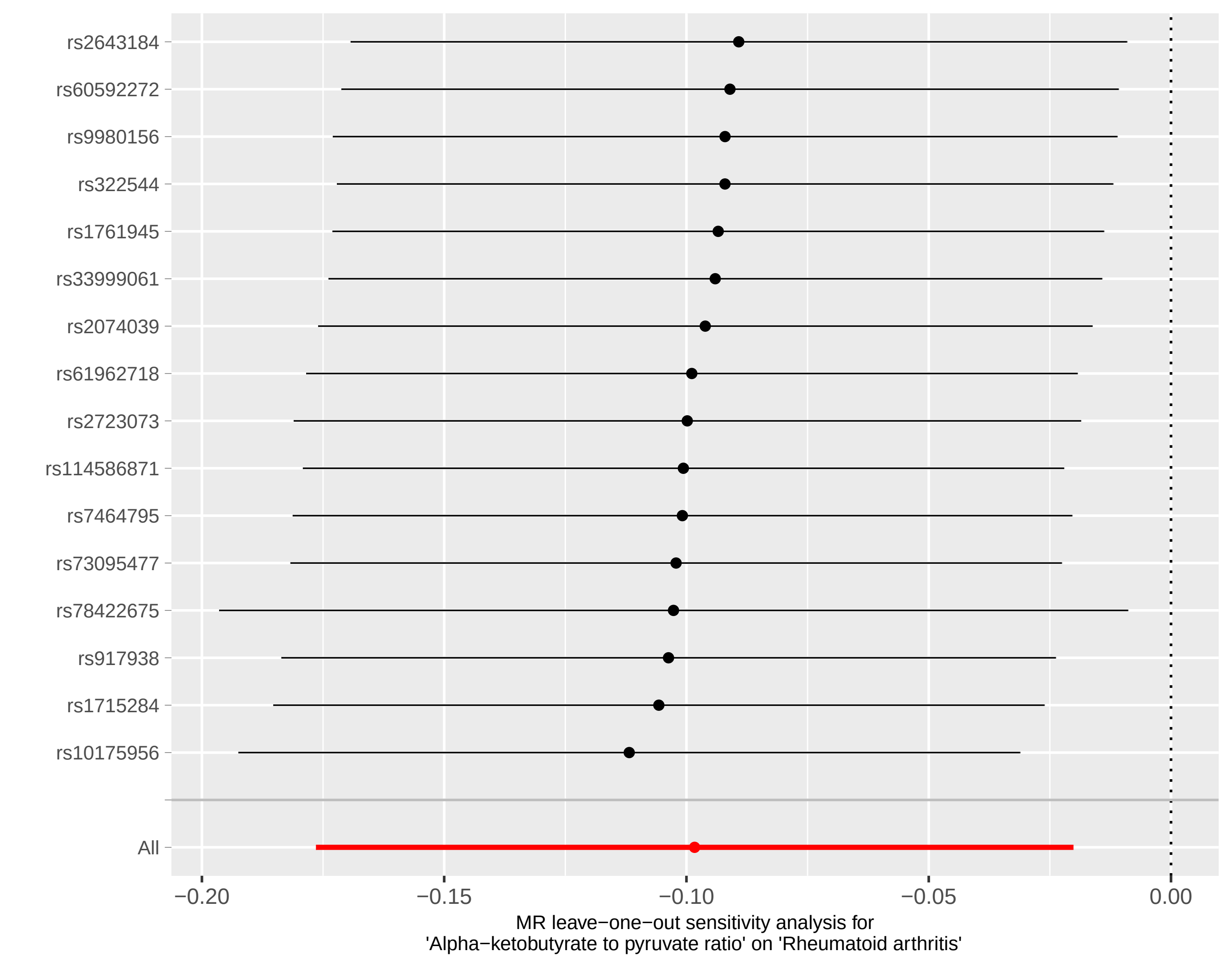
rs2643184
rs60592272
rs9980156
rs322544
rs1761945
rs33999061
rs2074039
rs61962718
rs2723073
rs114586871
rs7464795
rs73095477
rs78422675
rs917938
rs1715284
rs10175956
All
−0.20
−0.15
−0.10
−0.05
0.00
MR leave−one−out sensitivity analysis for
'Alpha−ketobutyrate to pyruvate ratio' on 'Rheumatoid arthritis'

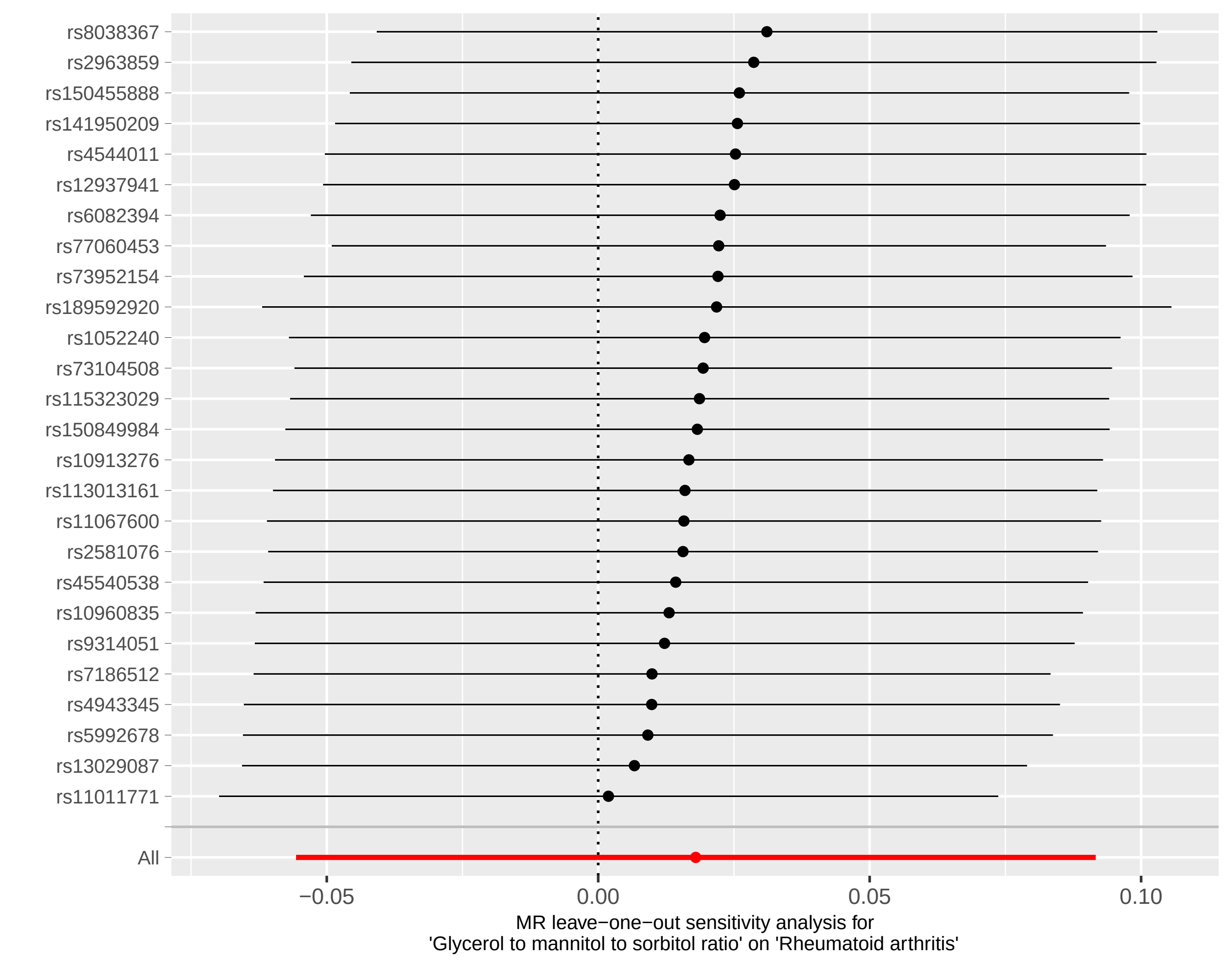

rs8038367
rs2963859
rs150455888
rs141950209
rs4544011
rs12937941
rs6082394
rs77060453
rs73952154
rs189592920
rs1052240
rs73104508
rs115323029
rs150849984
rs10913276
rs113013161
rs11067600
rs2581076
rs45540538
rs10960835
rs9314051
rs7186512
rs4943345
rs5992678
rs13029087
rs11011771
All
−0.05
0.00
0.05
0.10
MR leave−one−out sensitivity analysis for
'Glycerol to mannitol to sorbitol ratio' on 'Rheumatoid arthritis'

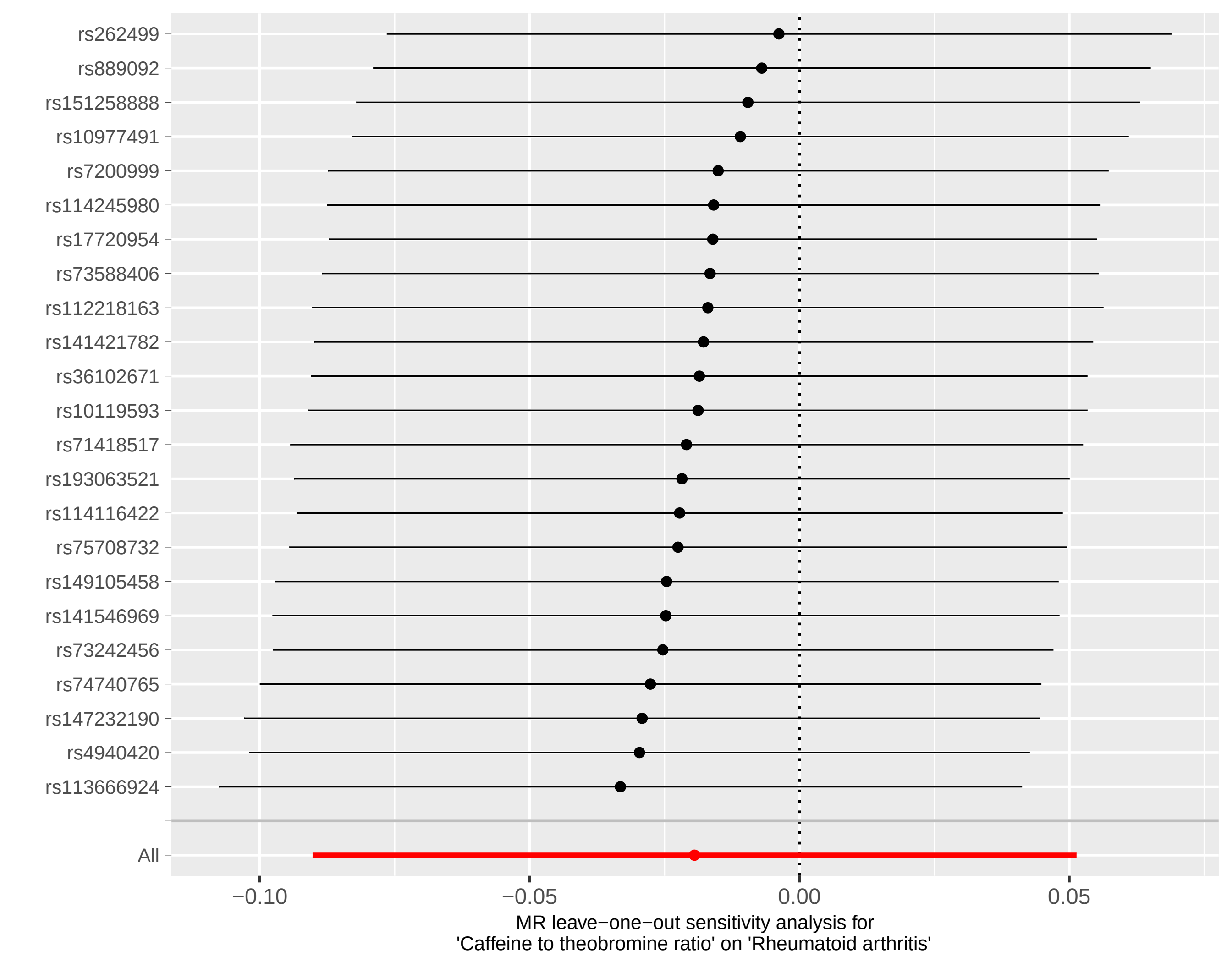
rs262499
rs889092
rs151258888
rs10977491
rs7200999
rs114245980
rs17720954
rs73588406
rs112218163
rs141421782
rs36102671
rs10119593
rs71418517
rs193063521
rs114116422
rs75708732
rs149105458
rs141546969
rs73242456
rs74740765
rs147232190
rs4940420
rs113666924
All
−0.10
−0.05
0.00
0.05
MR leave−one−out sensitivity analysis for
'Caffeine to theobromine ratio' on 'Rheumatoid arthritis'

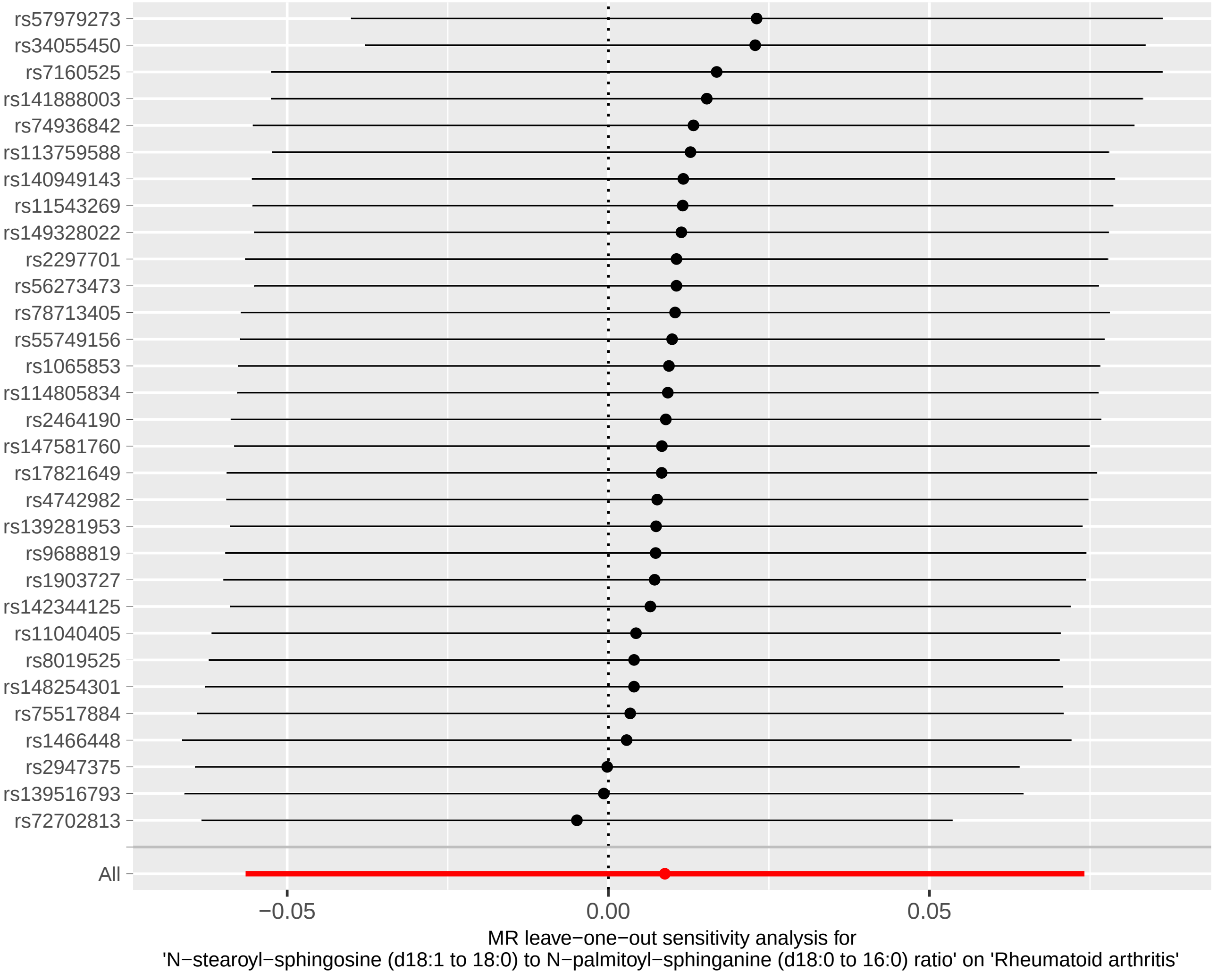
rs57979273
rs34055450
rs7160525
rs141888003
rs74936842
rs113759588
rs140949143
rs11543269
rs149328022
rs2297701
rs56273473
rs78713405
rs55749156
rs1065853
rs114805834
rs2464190
rs147581760
rs17821649
rs4742982
rs139281953
rs9688819
rs1903727
rs142344125
rs11040405
rs8019525
rs148254301
rs75517884
rs1466448
rs2947375
rs139516793
rs72702813
All
−0.05
0.00
0.05
MR leave−one−out sensitivity analysis for
'N−stearoyl−sphingosine (d18:1 to 18:0) to N−palmitoyl−sphinganine (d18:0 to 16:0) ratio' on 'Rheumatoid arthritis'

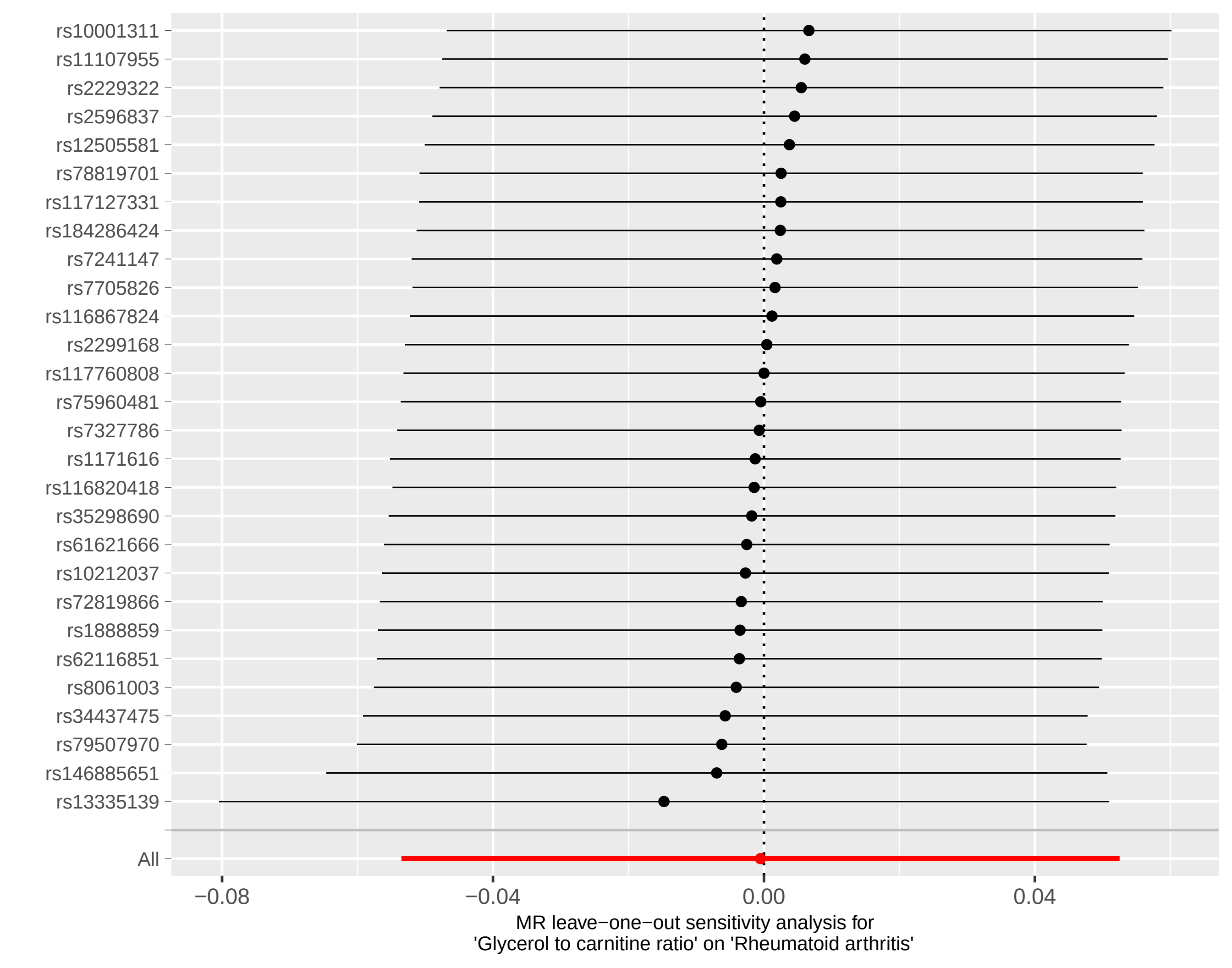
rs10001311
rs11107955
rs2229322
rs2596837
rs12505581
rs78819701
rs117127331
rs184286424
rs7241147
rs7705826
rs116867824
rs2299168
rs117760808
rs75960481
rs7327786
rs1171616
rs116820418
rs35298690
rs61621666
rs10212037
rs72819866
rs1888859
rs62116851
rs8061003
rs34437475
rs79507970
rs146885651
rs13335139
All
−0.08
−0.04
0.00
0.04
MR leave−one−out sensitivity analysis for
'Glycerol to carnitine ratio' on 'Rheumatoid arthritis'

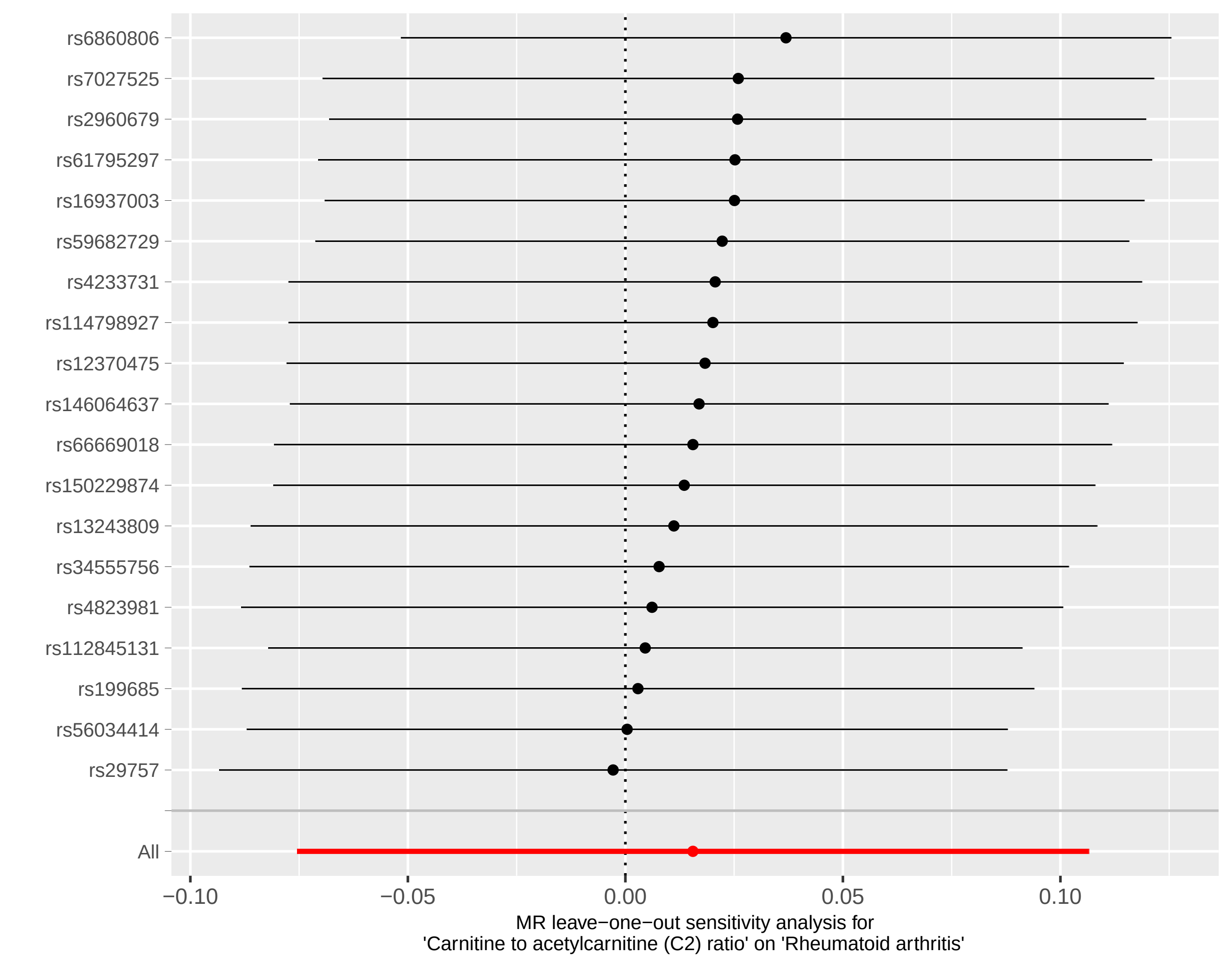
rs6860806
rs7027525
rs2960679
rs61795297
rs16937003
rs59682729
rs4233731
rs114798927
rs12370475
rs146064637
rs66669018
rs150229874
rs13243809
rs34555756
rs4823981
rs112845131
rs199685
rs56034414
rs29757
All
−0.10
−0.05
0.00
0.05
0.10
MR leave−one−out sensitivity analysis for
'Carnitine to acetylcarnitine (C2) ratio' on 'Rheumatoid arthritis'

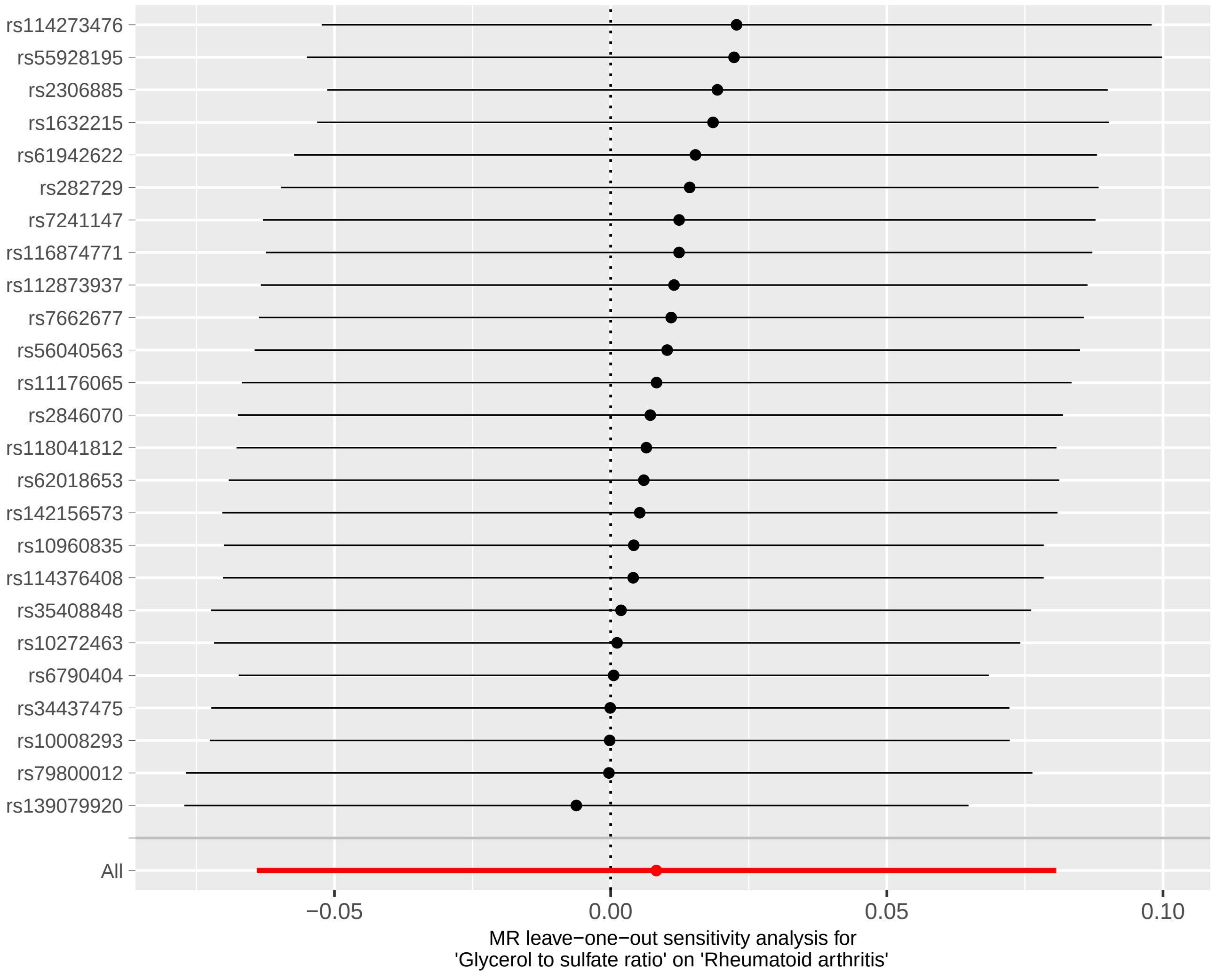
rs114273476
rs55928195
rs2306885
rs1632215
rs61942622
rs282729
rs7241147
rs116874771
rs112873937
rs7662677
rs56040563
rs11176065
rs2846070
rs118041812
rs62018653
rs142156573
rs10960835
rs114376408
rs35408848
rs10272463
rs6790404
rs34437475
rs10008293
rs79800012
rs139079920
All
−0.05
0.00
0.05
0.10
MR leave−one−out sensitivity analysis for
'Glycerol to sulfate ratio' on 'Rheumatoid arthritis'

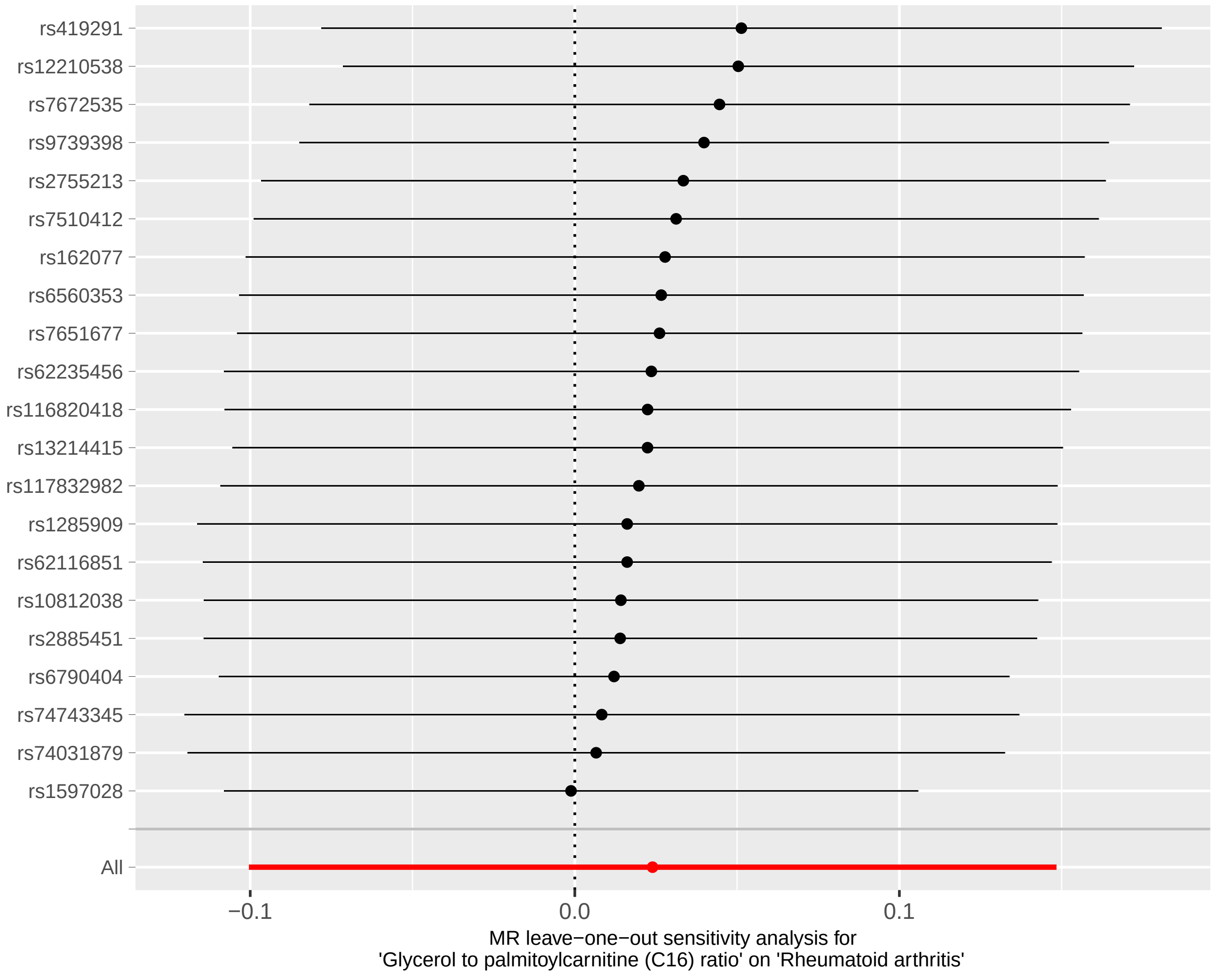
rs419291
rs12210538
rs7672535
rs9739398
rs2755213
rs7510412
rs162077
rs6560353
rs7651677
rs62235456
rs116820418
rs13214415
rs117832982
rs1285909
rs62116851
rs10812038
rs2885451
rs6790404
rs74743345
rs74031879
rs1597028
All
−0.1
0.0
0.1
MR leave−one−out sensitivity analysis for
'Glycerol to palmitoylcarnitine (C16) ratio' on 'Rheumatoid arthritis'

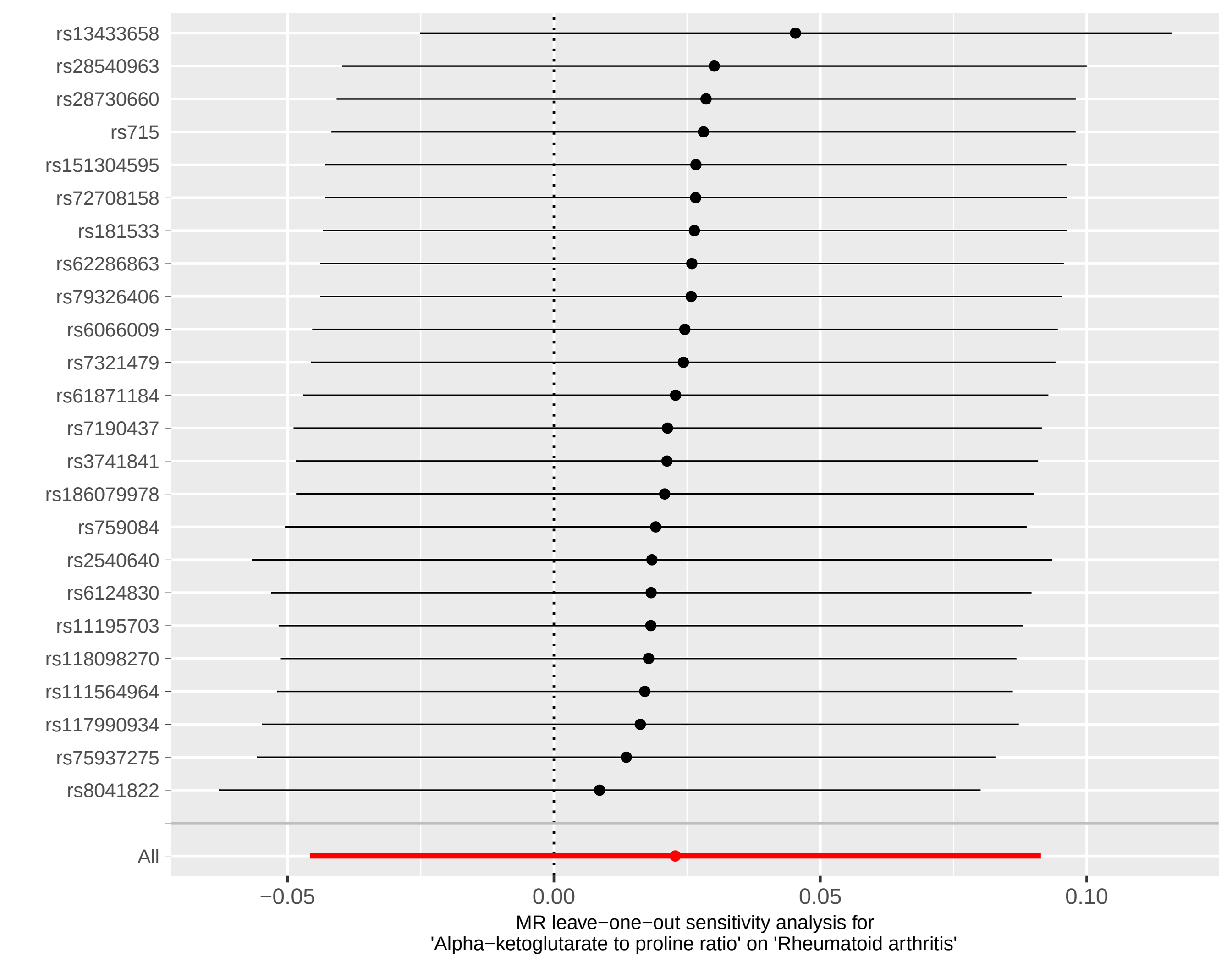

rs13433658
rs28540963
rs28730660
rs715
rs151304595
rs72708158
rs181533
rs62286863
rs79326406
rs6066009
rs7321479
rs61871184
rs7190437
rs3741841
rs186079978
rs759084
rs2540640
rs6124830
rs11195703
rs118098270
rs111564964
rs117990934
rs75937275
rs8041822
All
−0.05
0.00
0.05
0.10
MR leave−one−out sensitivity analysis for
'Alpha−ketoglutarate to proline ratio' on 'Rheumatoid arthritis'

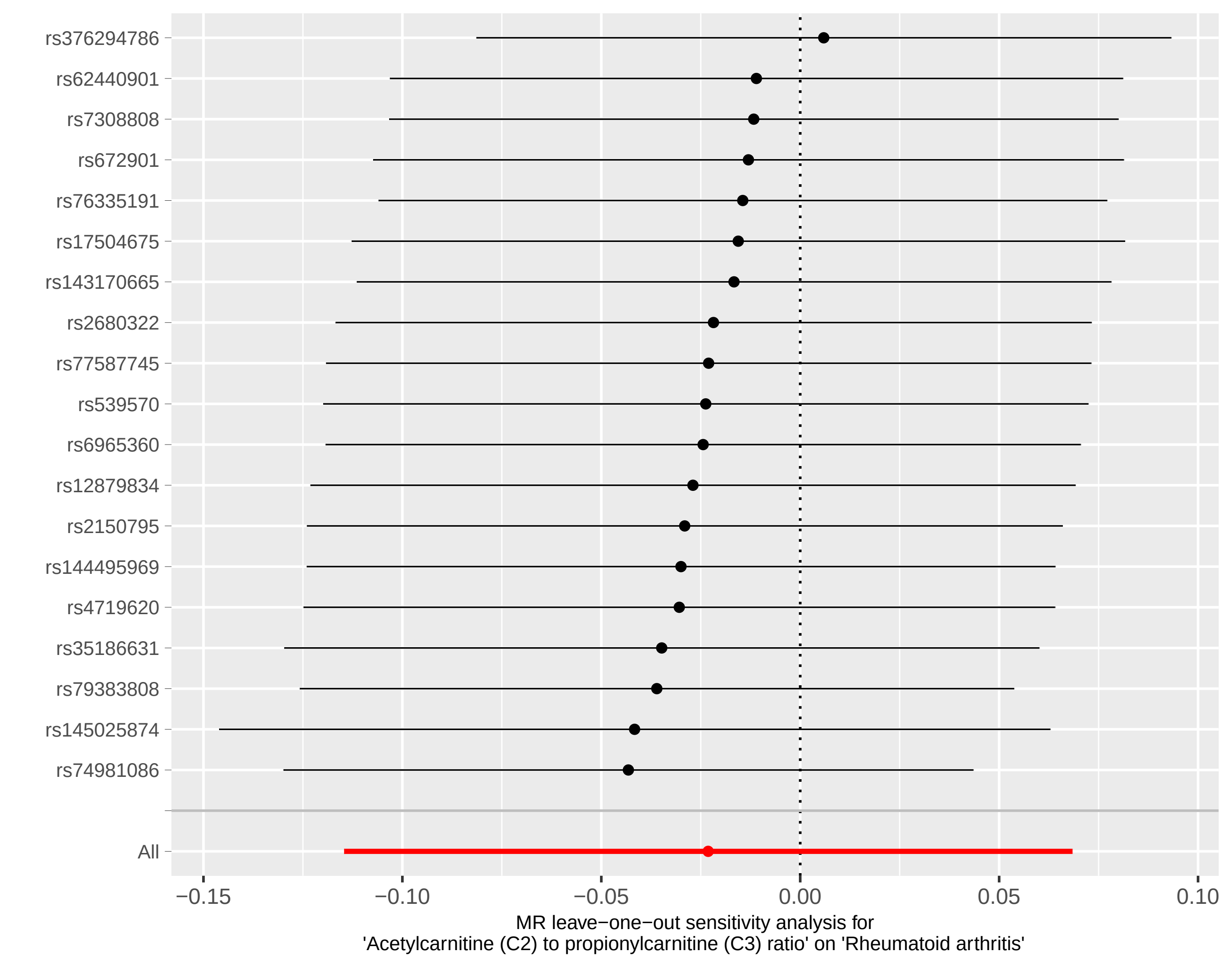
rs376294786
rs62440901
rs7308808
rs672901
rs76335191
rs17504675
rs143170665
rs2680322
rs77587745
rs539570
rs6965360
rs12879834
rs2150795
rs144495969
rs4719620
rs35186631
rs79383808
rs145025874
rs74981086
All
−0.15
−0.10
−0.05
0.00
0.05
0.10
MR leave−one−out sensitivity analysis for
'Acetylcarnitine (C2) to propionylcarnitine (C3) ratio' on 'Rheumatoid arthritis'

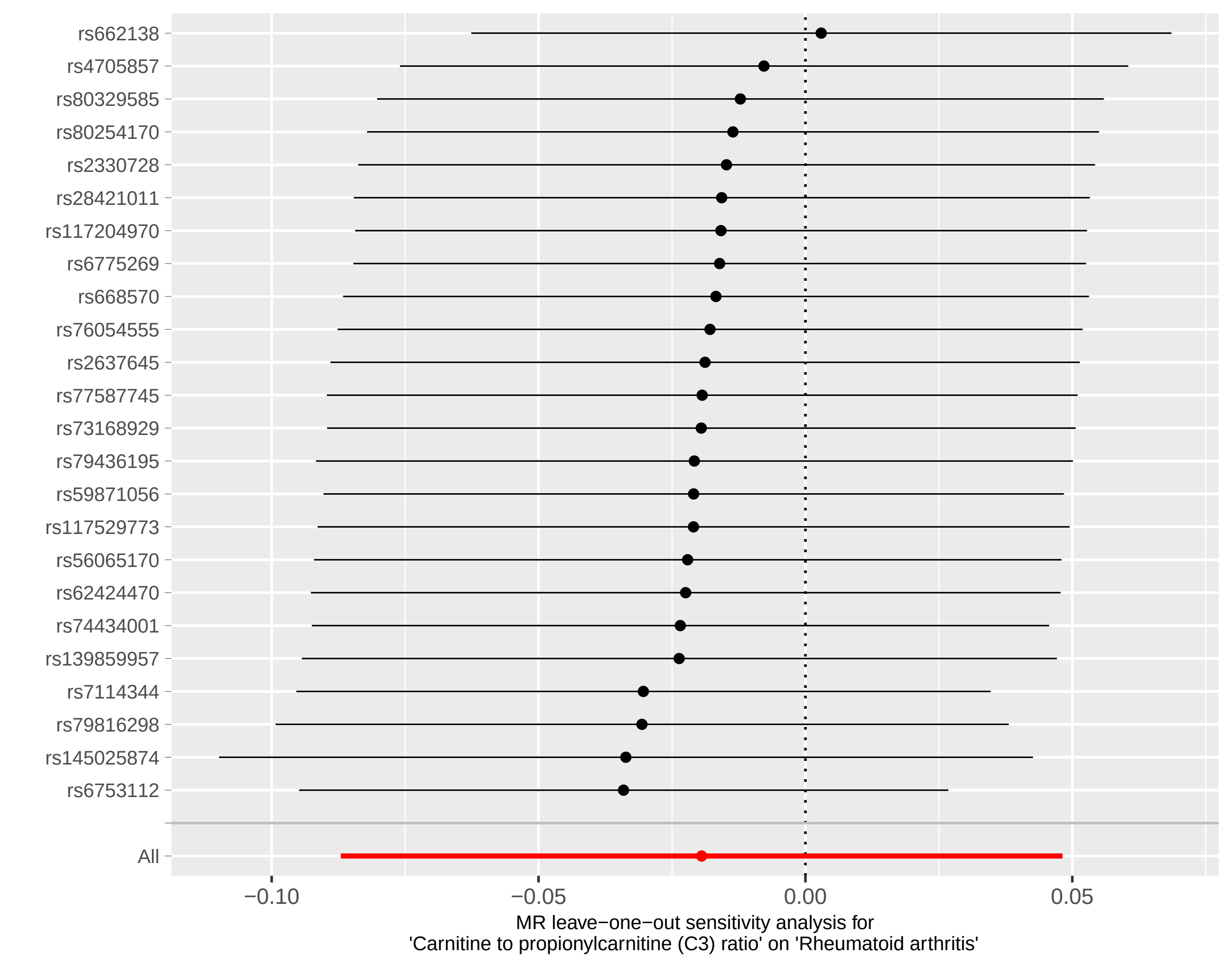
rs662138
rs4705857
rs80329585
rs80254170
rs2330728
rs28421011
rs117204970
rs6775269
rs668570
rs76054555
rs2637645
rs77587745
rs73168929
rs79436195
rs59871056
rs117529773
rs56065170
rs62424470
rs74434001
rs139859957
rs7114344
rs79816298
rs145025874
rs6753112
All
−0.10
−0.05
0.00
0.05
MR leave−one−out sensitivity analysis for
'Carnitine to propionylcarnitine (C3) ratio' on 'Rheumatoid arthritis'

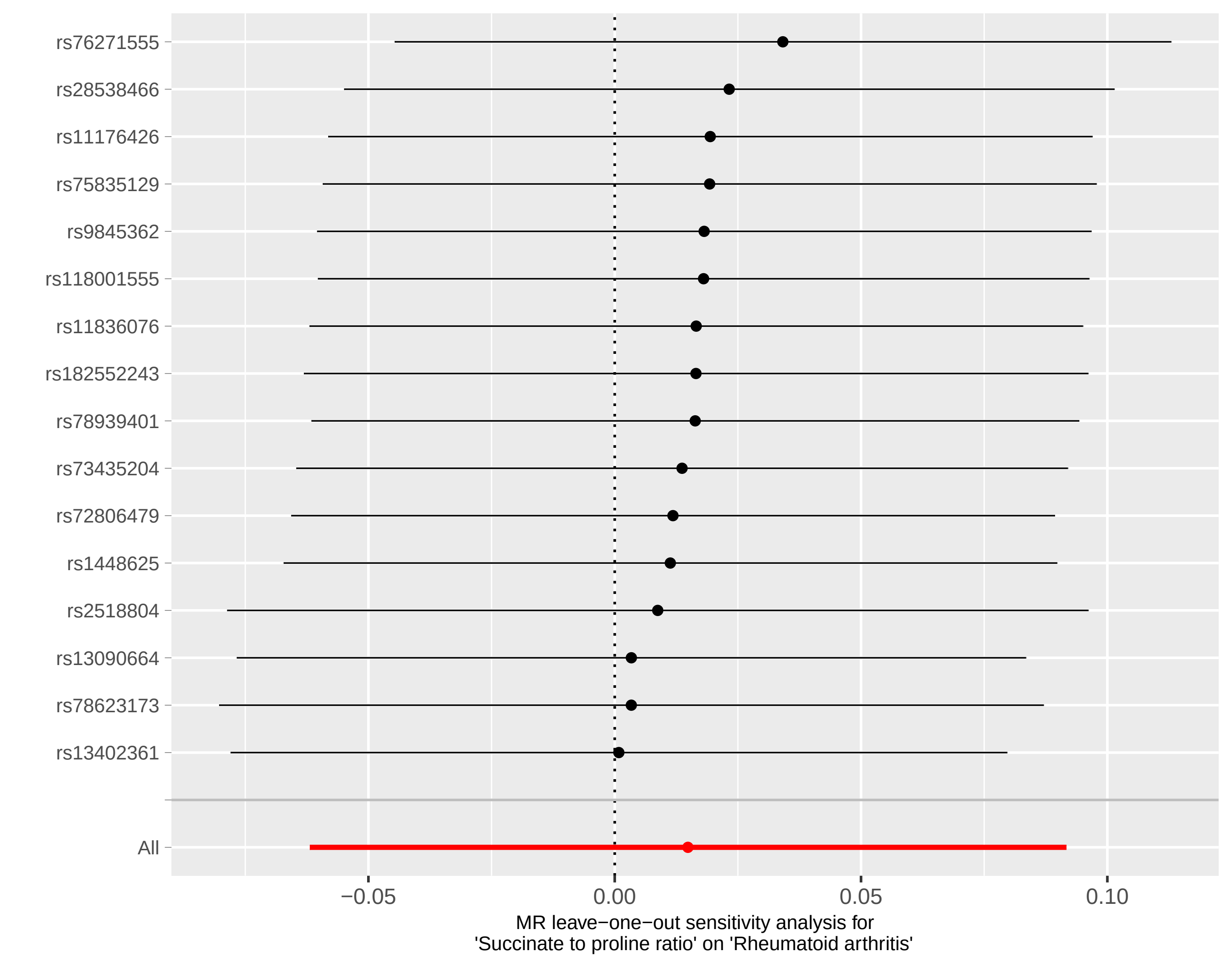
rs76271555
rs28538466
rs11176426
rs75835129
rs9845362
rs118001555
rs11836076
rs182552243
rs78939401
rs73435204
rs72806479
rs1448625
rs2518804
rs13090664
rs78623173
rs13402361
All
−0.05
0.00
0.05
0.10
MR leave−one−out sensitivity analysis for
'Succinate to proline ratio' on 'Rheumatoid arthritis'

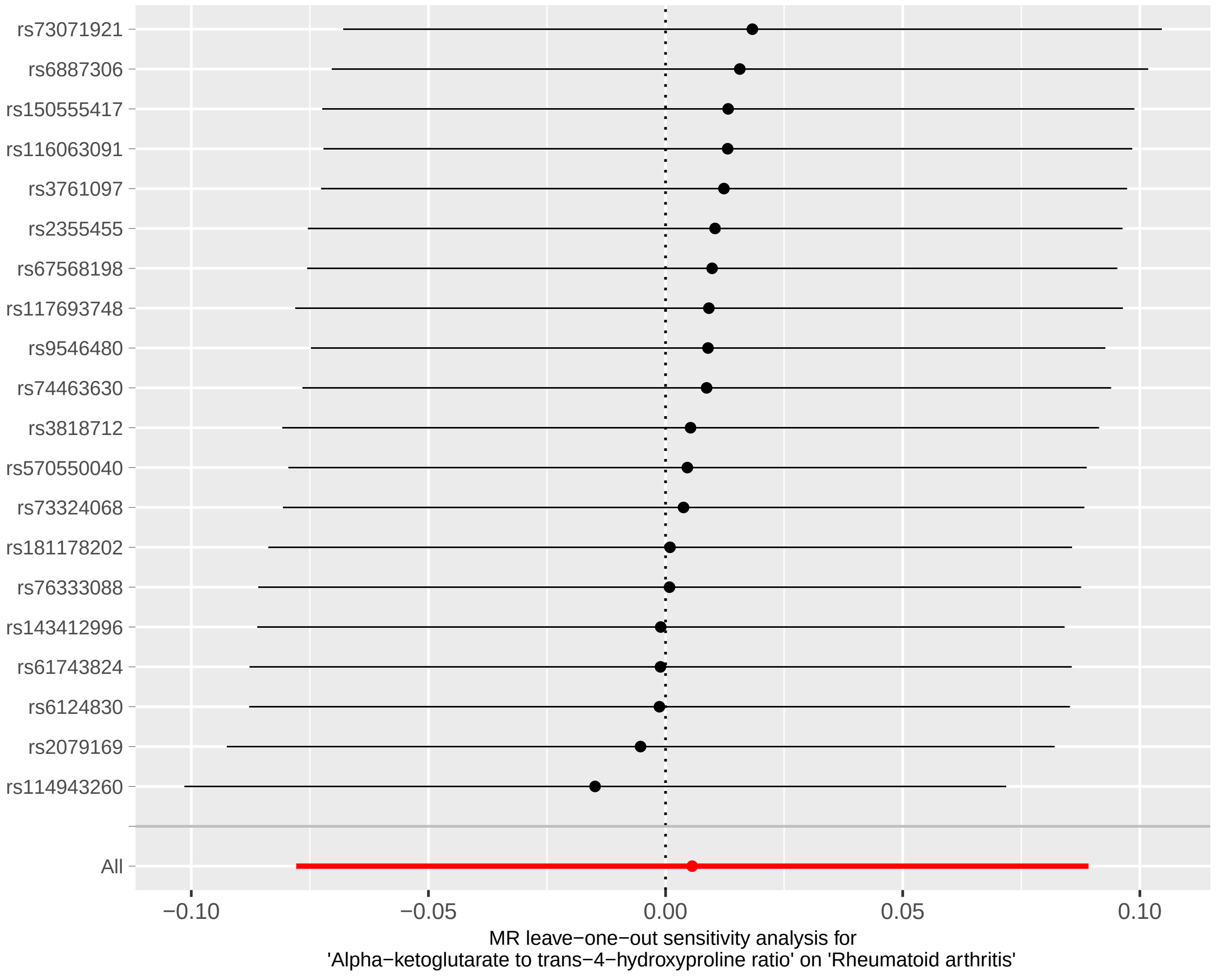
rs73071921
rs6887306
rs150555417
rs116063091
rs3761097
rs2355455
rs67568198
rs117693748
rs9546480
rs74463630
rs3818712
rs570550040
rs73324068
rs181178202
rs76333088
rs143412996
rs61743824
rs6124830
rs2079169
rs114943260
All
−0.10
−0.05
0.00
0.05
0.10
MR leave−one−out sensitivity analysis for
'Alpha−ketoglutarate to trans−4−hydroxyproline ratio' on 'Rheumatoid arthritis'

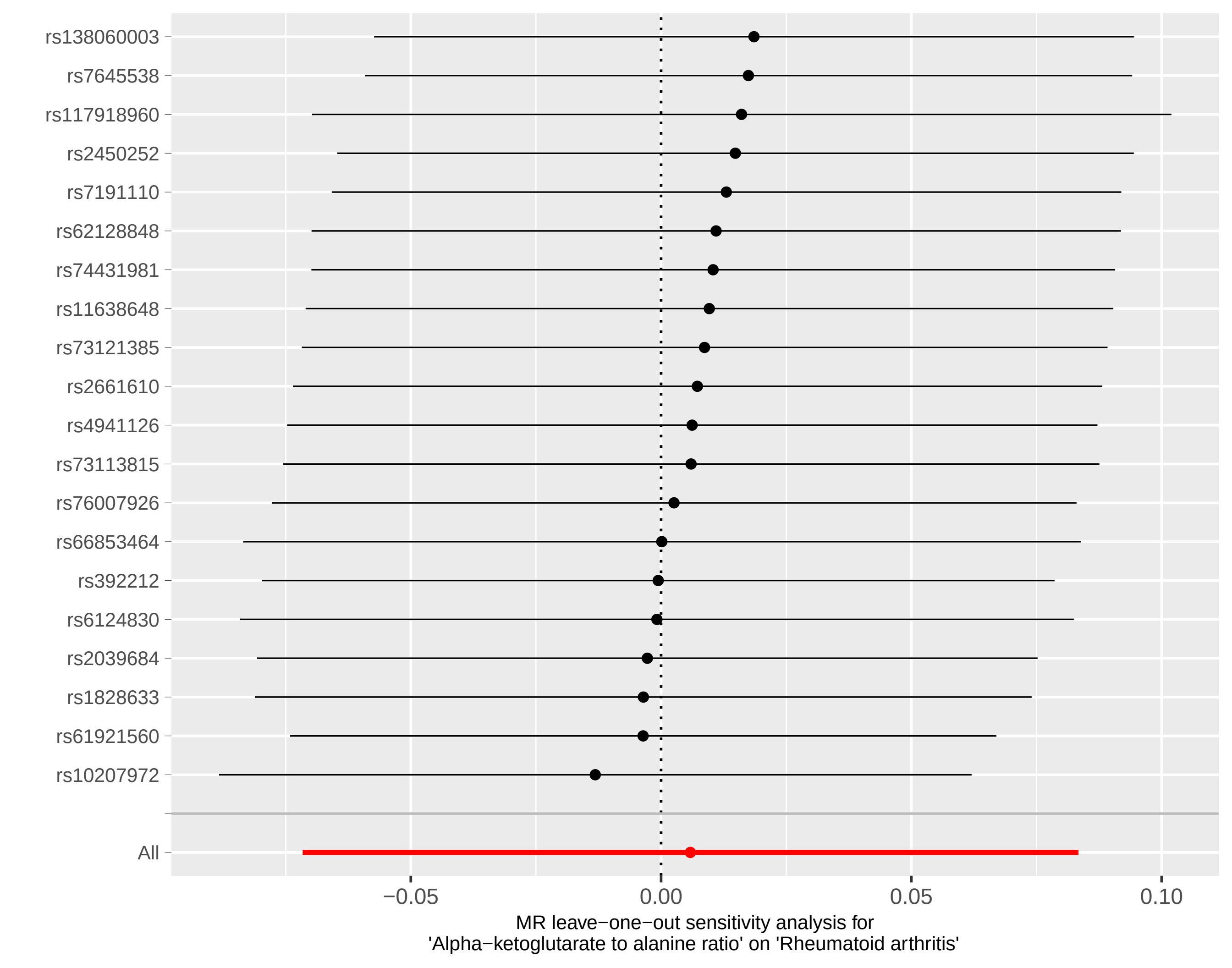
rs138060003
rs7645538
rs117918960
rs2450252
rs7191110
rs62128848
rs74431981
rs11638648
rs73121385
rs2661610
rs4941126
rs73113815
rs76007926
rs66853464
rs392212
rs6124830
rs2039684
rs1828633
rs61921560
rs10207972
All
−0.05
0.00
0.05
0.10
MR leave−one−out sensitivity analysis for
'Alpha−ketoglutarate to alanine ratio' on 'Rheumatoid arthritis'

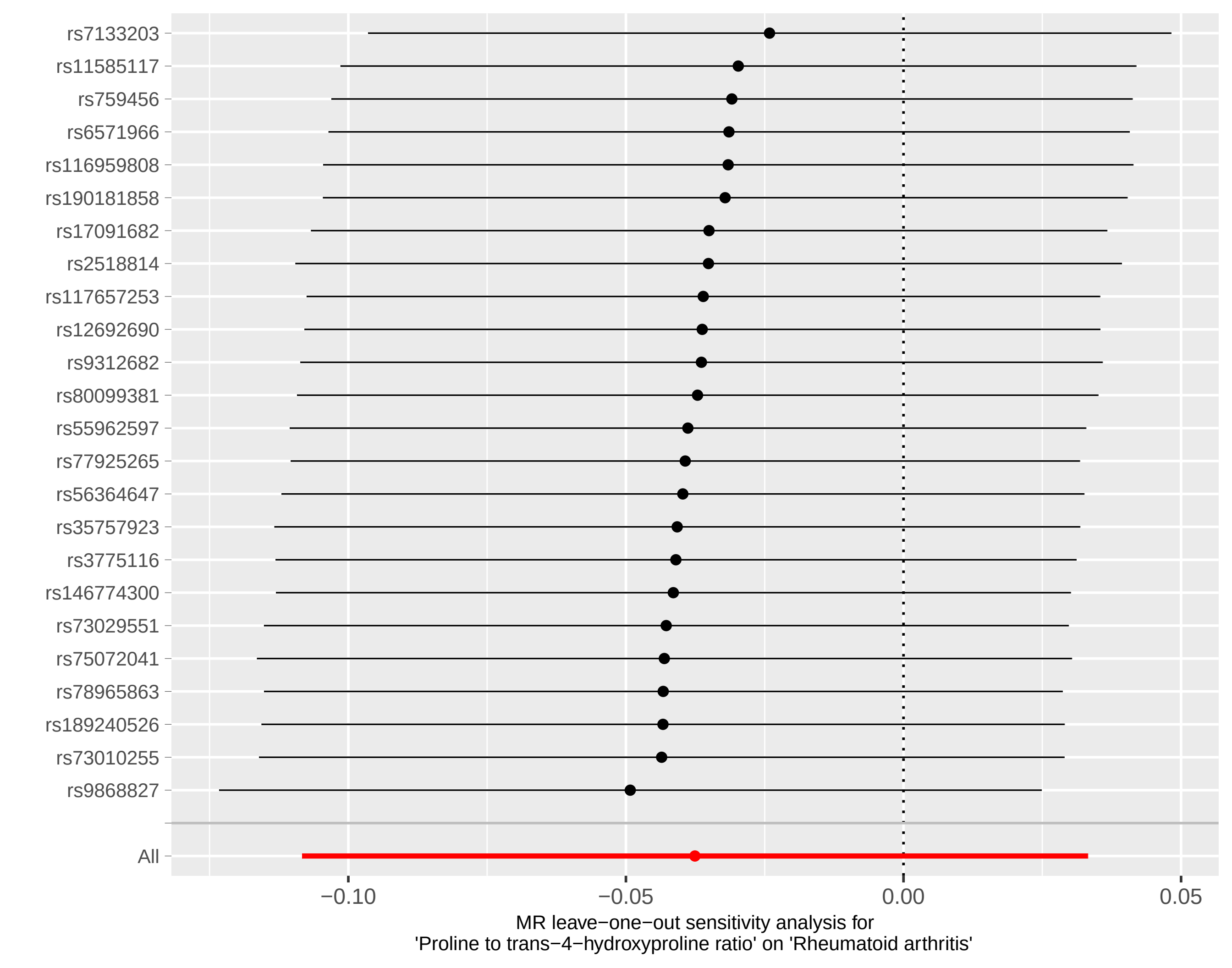
rs7133203
rs11585117
rs759456
rs6571966
rs116959808
rs190181858
rs17091682
rs2518814
rs117657253
rs12692690
rs9312682
rs80099381
rs55962597
rs77925265
rs56364647
rs35757923
rs3775116
rs146774300
rs73029551
rs75072041
rs78965863
rs189240526
rs73010255
rs9868827
All
−0.10
−0.05
0.00
0.05
MR leave−one−out sensitivity analysis for
'Proline to trans−4−hydroxyproline ratio' on 'Rheumatoid arthritis'

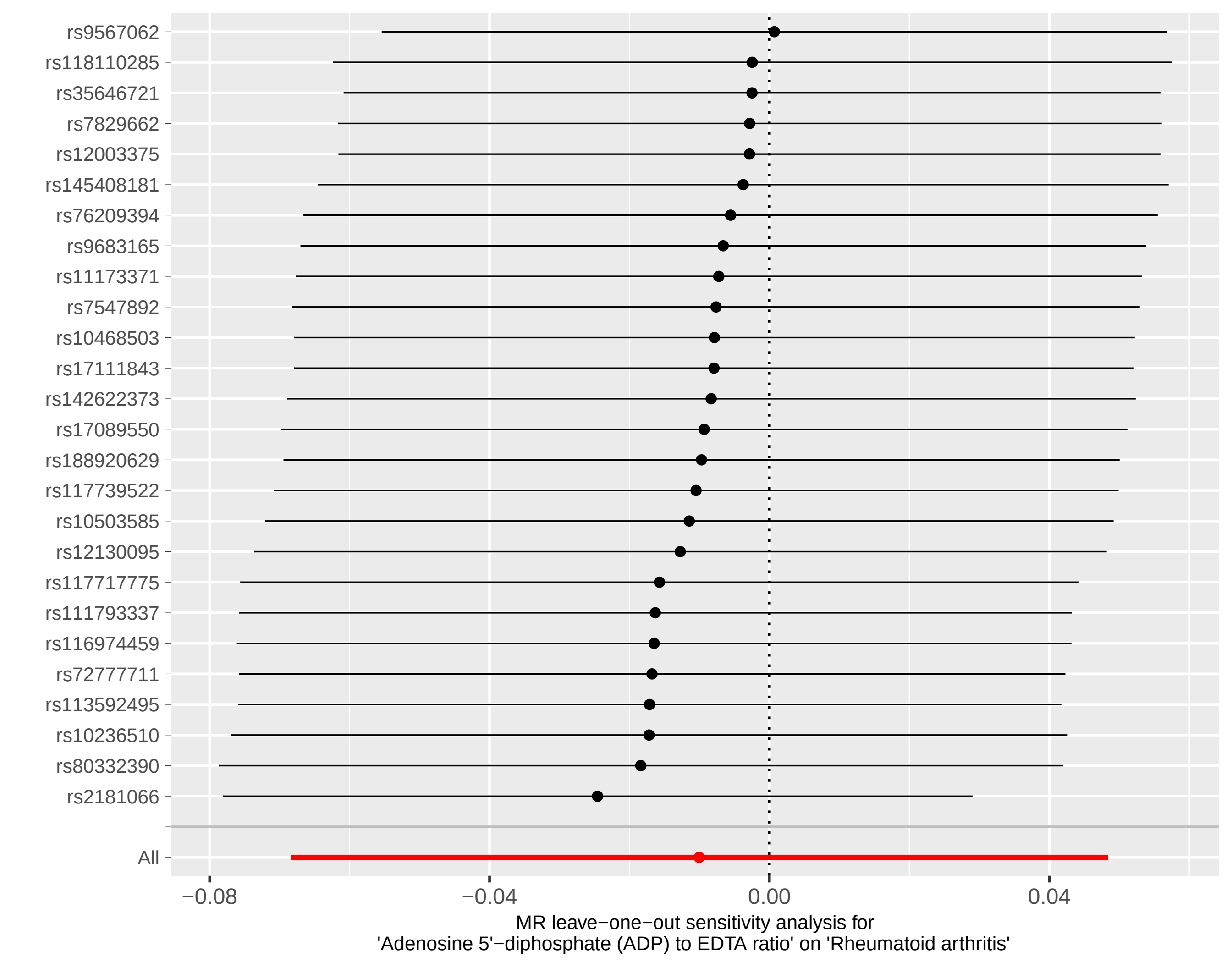
rs9567062
rs118110285
rs35646721
rs7829662
rs12003375
rs145408181
rs76209394
rs9683165
rs11173371
rs7547892
rs10468503
rs17111843
rs142622373
rs17089550
rs188920629
rs117739522
rs10503585
rs12130095
rs117717775
rs111793337
rs116974459
rs72777711
rs113592495
rs10236510
rs80332390
rs2181066
All
−0.08
−0.04
0.00
0.04
MR leave−one−out sensitivity analysis for
'Adenosine 5'−diphosphate (ADP) to EDTA ratio' on 'Rheumatoid arthritis'

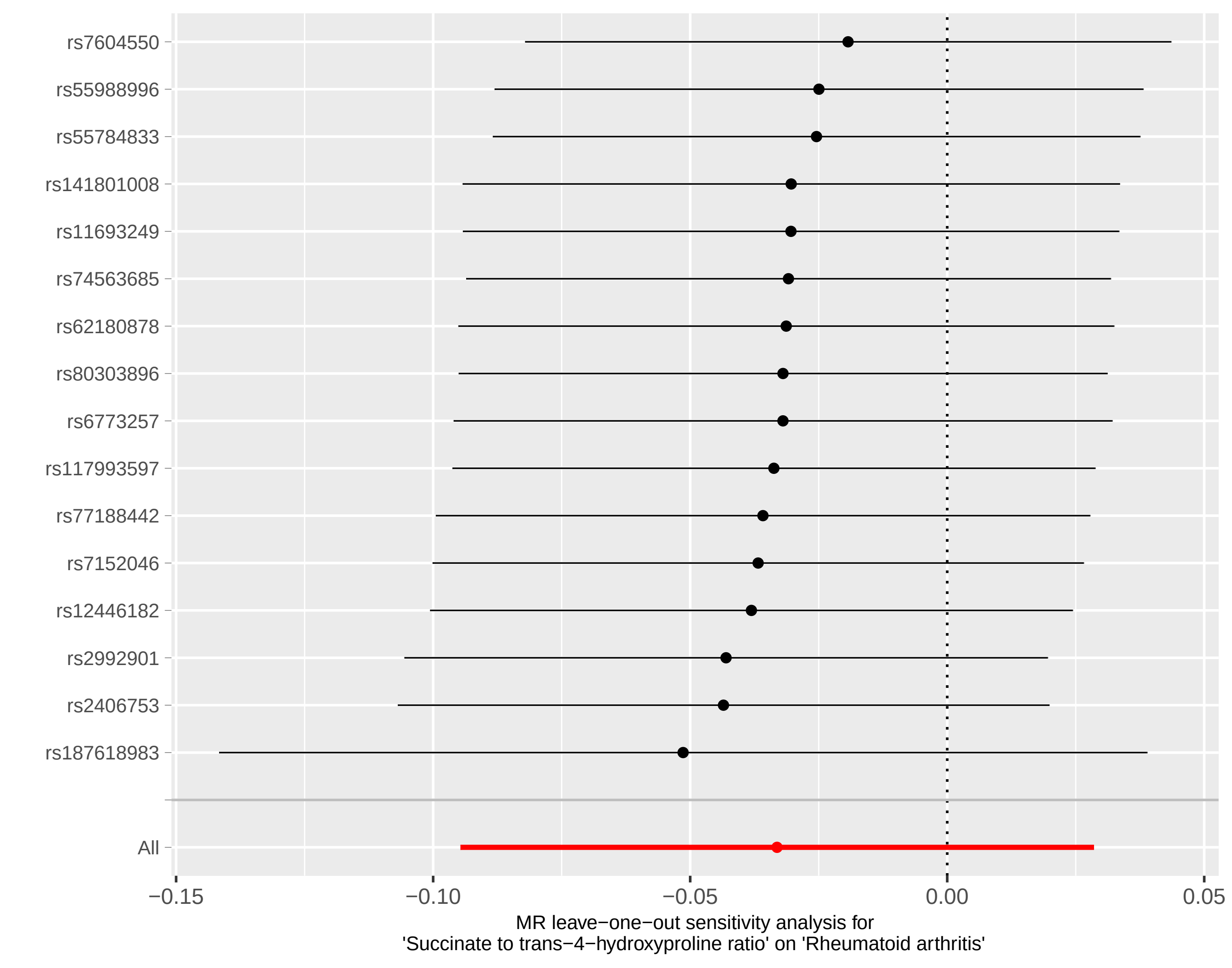
rs7604550
rs55988996
rs55784833
rs141801008
rs11693249
rs74563685
rs62180878
rs80303896
rs6773257
rs117993597
rs77188442
rs7152046
rs12446182
rs2992901
rs2406753
rs187618983
All
−0.15
−0.10
−0.05
0.00
0.05
MR leave−one−out sensitivity analysis for
'Succinate to trans−4−hydroxyproline ratio' on 'Rheumatoid arthritis'

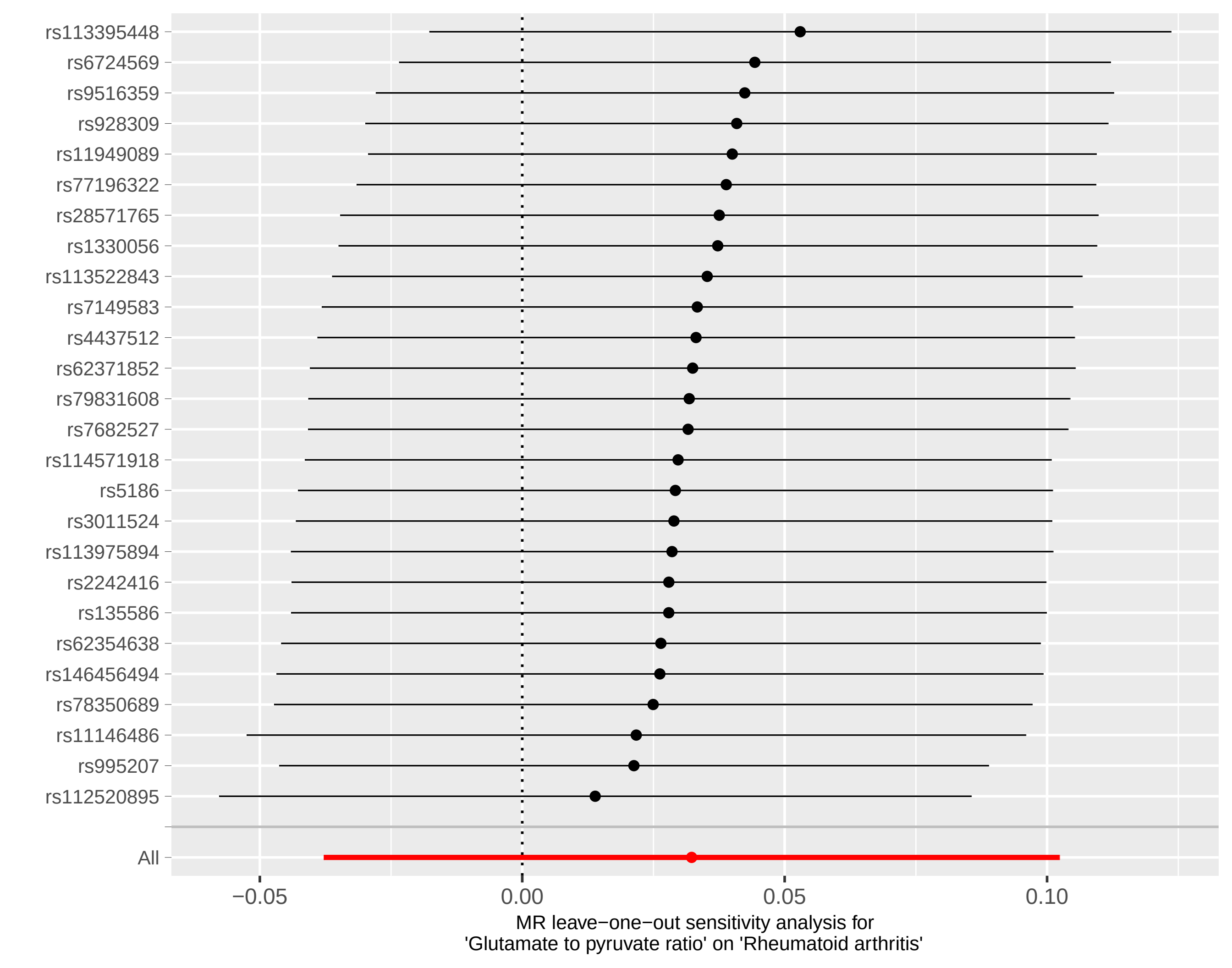
rs113395448
rs6724569
rs9516359
rs928309
rs11949089
rs77196322
rs28571765
rs1330056
rs113522843
rs7149583
rs4437512
rs62371852
rs79831608
rs7682527
rs114571918
rs5186
rs3011524
rs113975894
rs2242416
rs135586
rs62354638
rs146456494
rs78350689
rs11146486
rs995207
rs112520895
All
−0.05
0.00
0.05
0.10
MR leave−one−out sensitivity analysis for
'Glutamate to pyruvate ratio' on 'Rheumatoid arthritis'

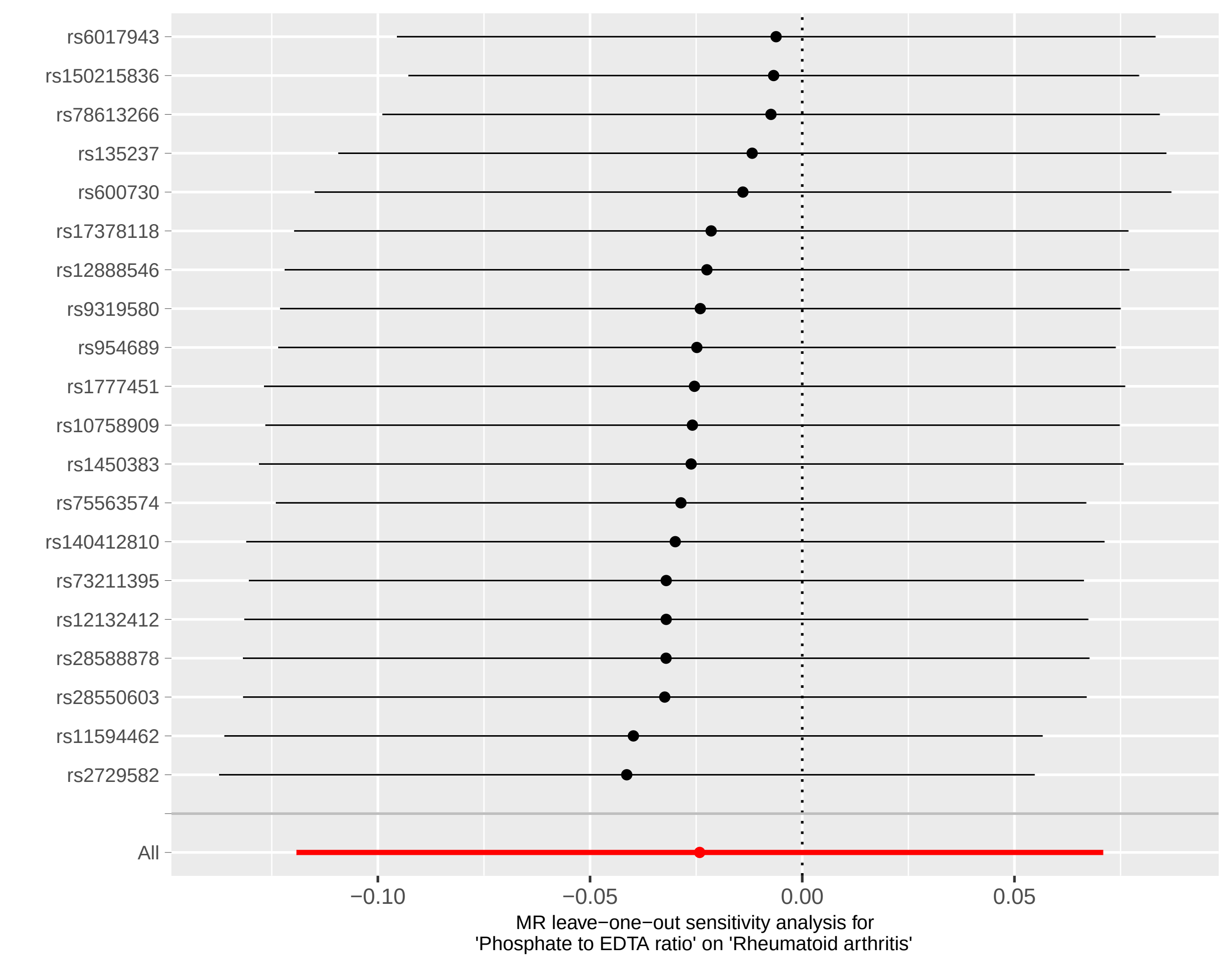
rs6017943
rs150215836
rs78613266
rs135237
rs600730
rs17378118
rs12888546
rs9319580
rs954689
rs1777451
rs10758909
rs1450383
rs75563574
rs140412810
rs73211395
rs12132412
rs28588878
rs28550603
rs11594462
rs2729582
All
−0.10
−0.05
0.00
0.05
MR leave−one−out sensitivity analysis for
'Phosphate to EDTA ratio' on 'Rheumatoid arthritis'

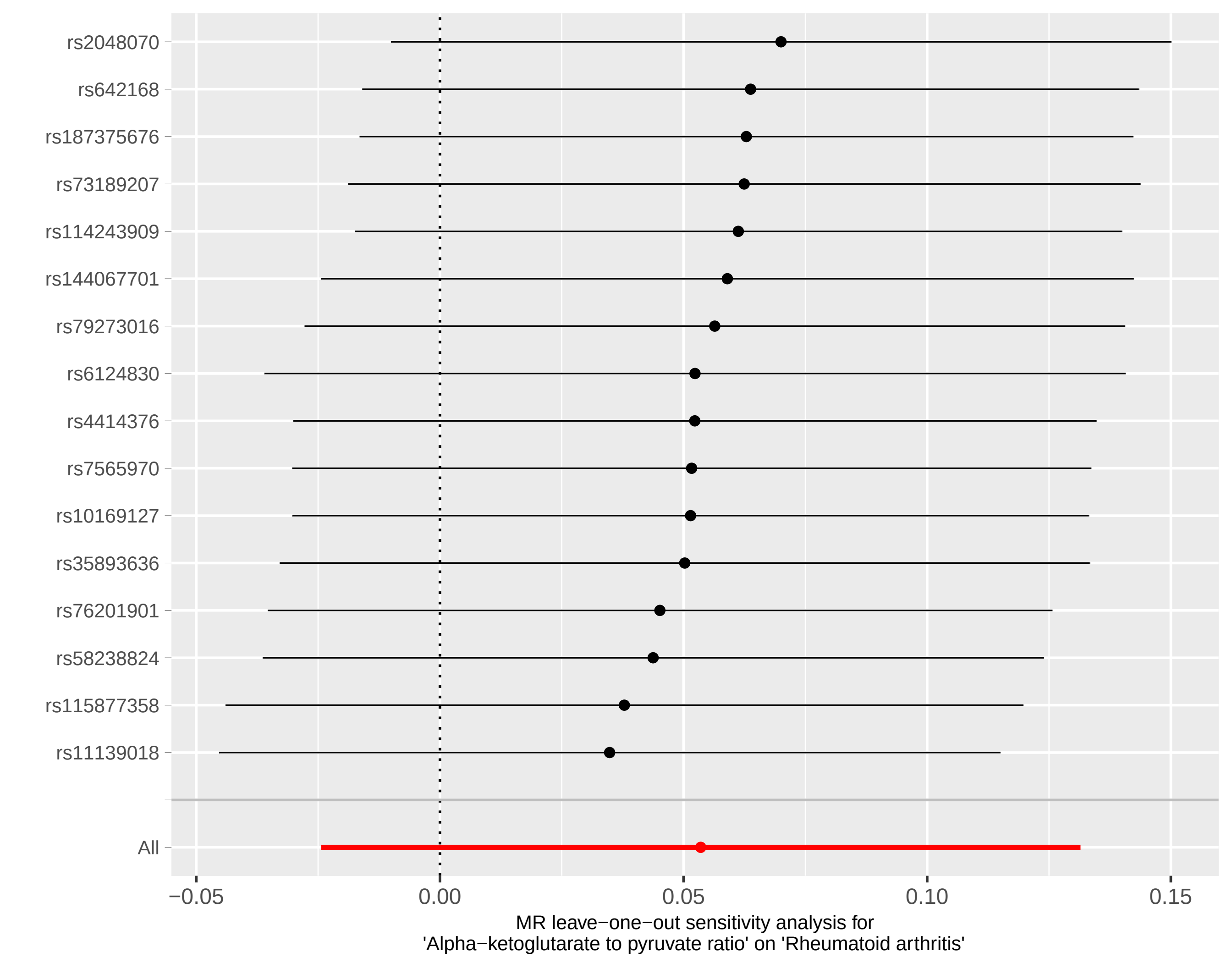

rs2048070
rs642168
rs187375676
rs73189207
rs114243909
rs144067701
rs79273016
rs6124830
rs4414376
rs7565970
rs10169127
rs35893636
rs76201901
rs58238824
rs115877358
rs11139018
All
−0.05
0.00
0.05
0.10
0.15
MR leave−one−out sensitivity analysis for
'Alpha−ketoglutarate to pyruvate ratio' on 'Rheumatoid arthritis'

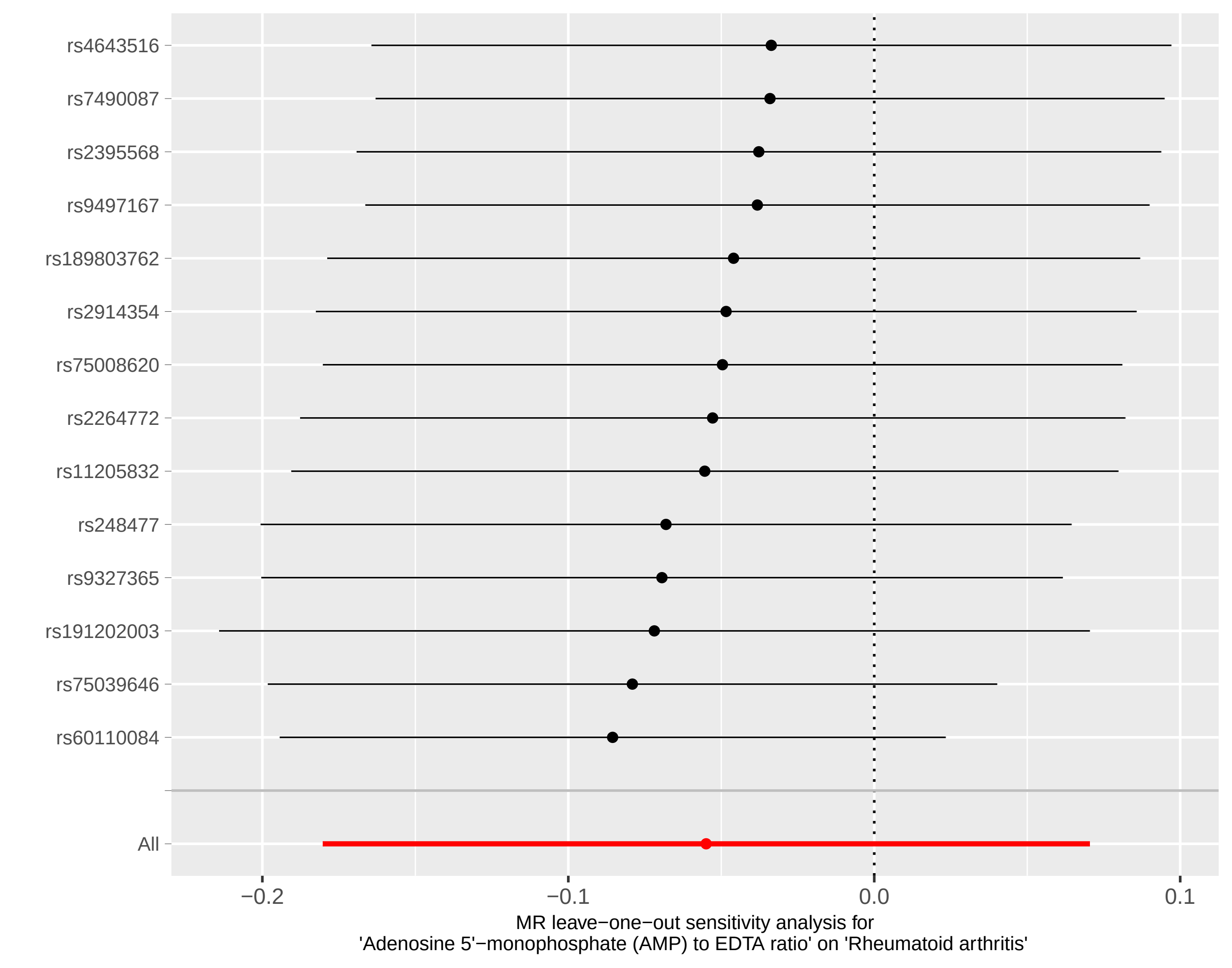
rs4643516
rs7490087
rs2395568
rs9497167
rs189803762
rs2914354
rs75008620
rs2264772
rs11205832
rs248477
rs9327365
rs191202003
rs75039646
rs60110084
All
−0.2
−0.1
0.0
0.1
MR leave−one−out sensitivity analysis for
'Adenosine 5'−monophosphate (AMP) to EDTA ratio' on 'Rheumatoid arthritis'

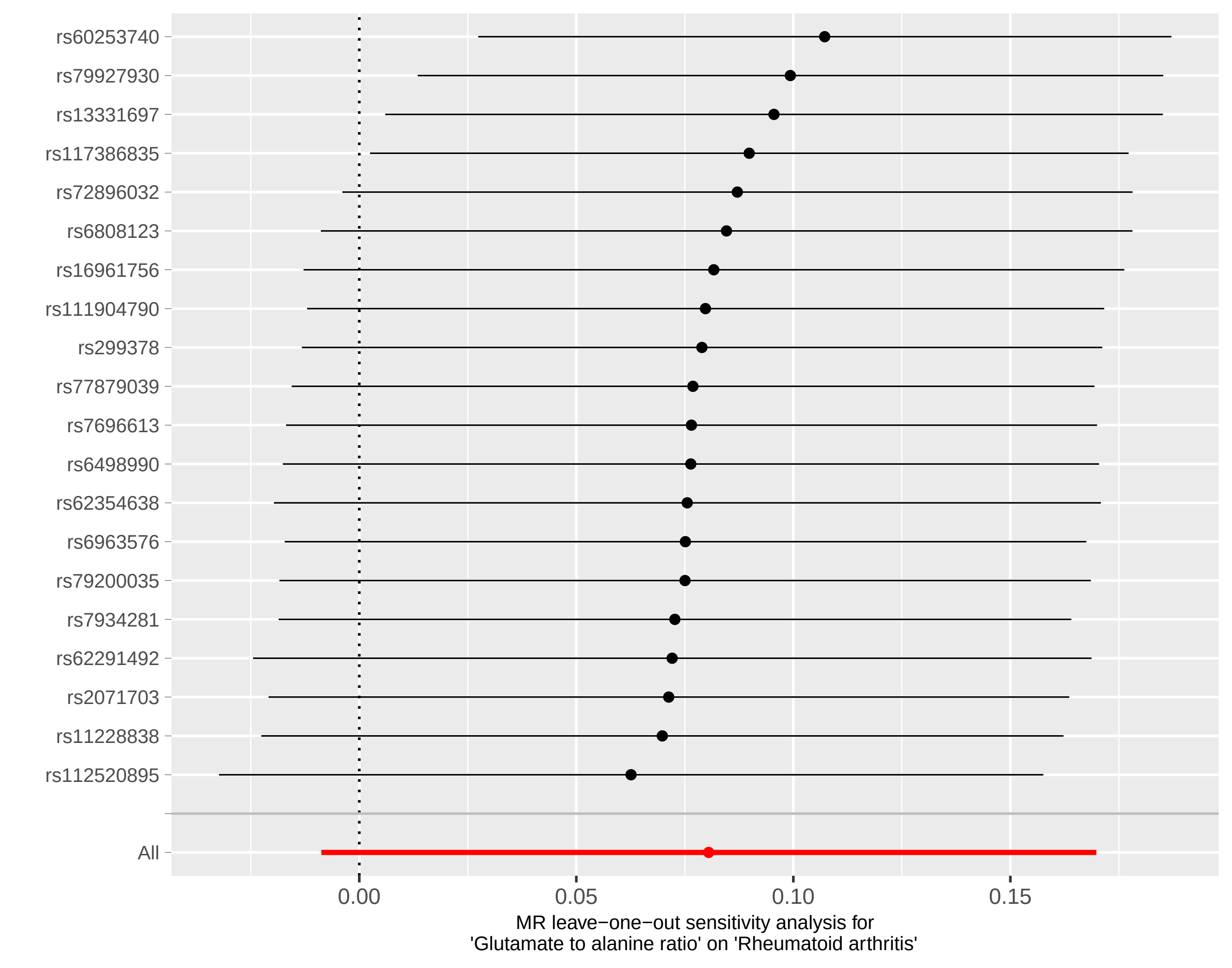

rs60253740
rs79927930
rs13331697
rs117386835
rs72896032
rs6808123
rs16961756
rs111904790
rs299378
rs77879039
rs7696613
rs6498990
rs62354638
rs6963576
rs79200035
rs7934281
rs62291492
rs2071703
rs11228838
rs112520895
All
0.00
0.05
0.10
0.15
MR leave−one−out sensitivity analysis for
'Glutamate to alanine ratio' on 'Rheumatoid arthritis'

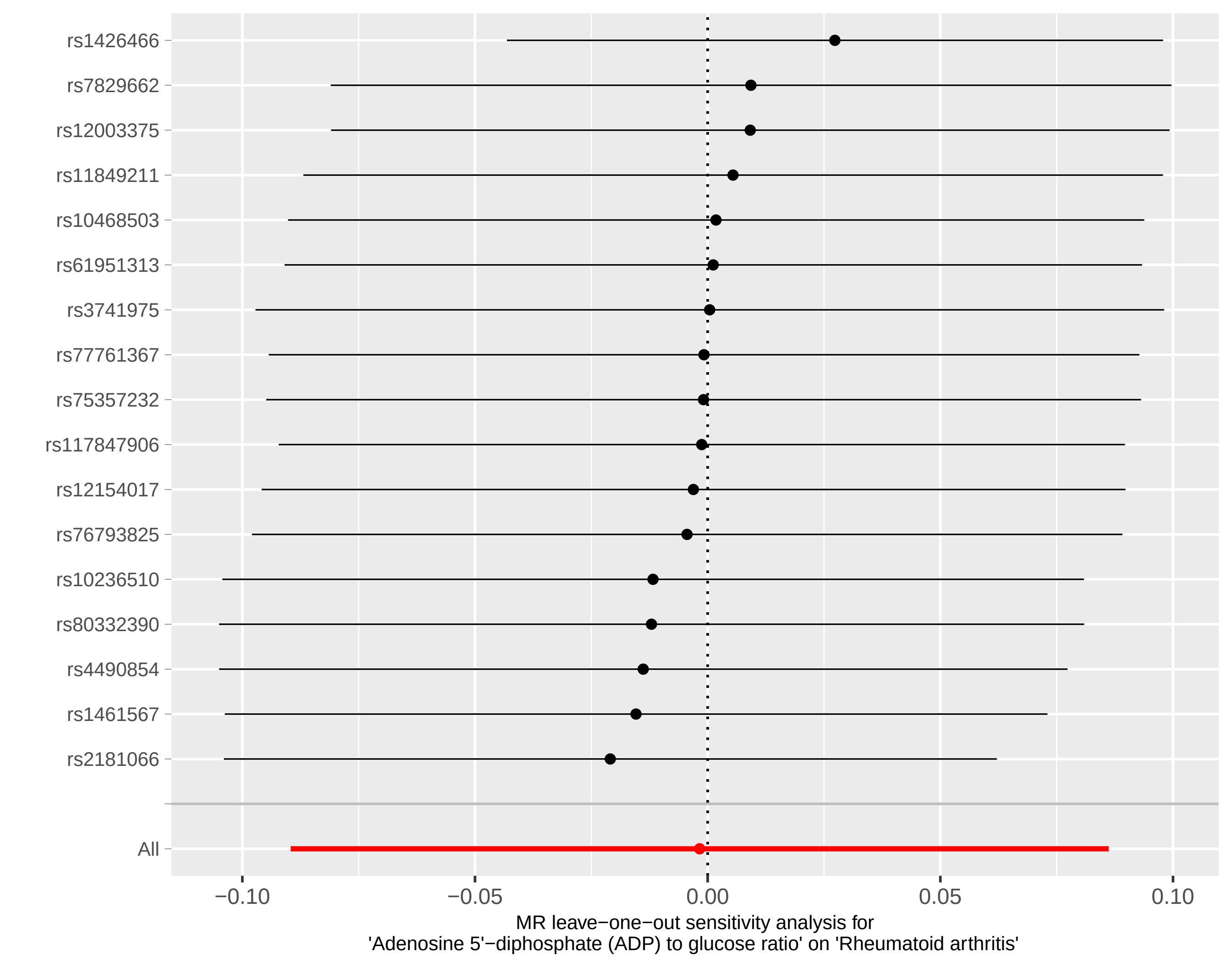
rs1426466
rs7829662
rs12003375
rs11849211
rs10468503
rs61951313
rs3741975
rs77761367
rs75357232
rs117847906
rs12154017
rs76793825
rs10236510
rs80332390
rs4490854
rs1461567
rs2181066
All
−0.10
−0.05
0.00
0.05
0.10
MR leave−one−out sensitivity analysis for
'Adenosine 5'−diphosphate (ADP) to glucose ratio' on 'Rheumatoid arthritis'

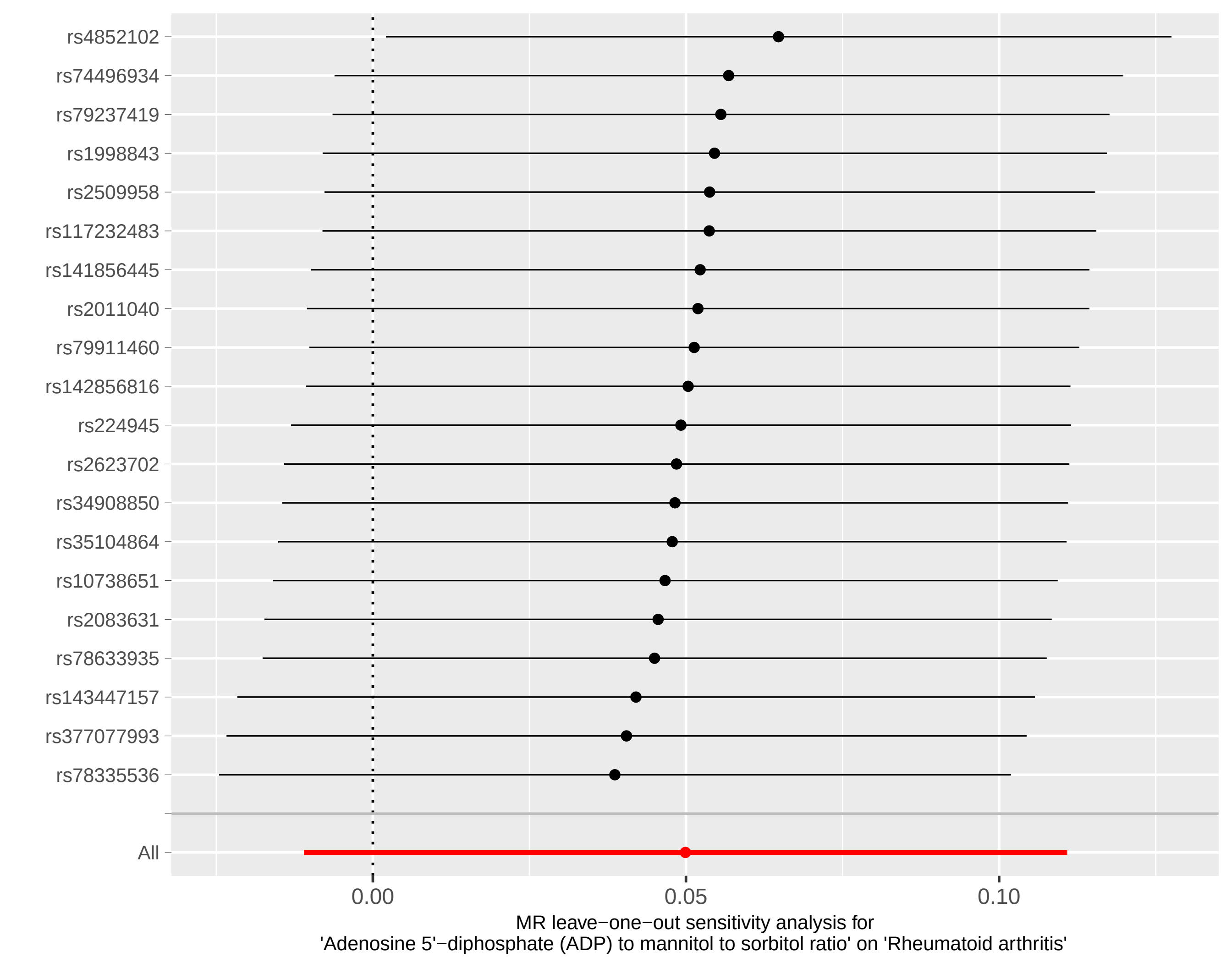

rs4852102
rs74496934
rs79237419
rs1998843
rs2509958
rs117232483
rs141856445
rs2011040
rs79911460
rs142856816
rs224945
rs2623702
rs34908850
rs35104864
rs10738651
rs2083631
rs78633935
rs143447157
rs377077993
rs78335536
All
0.00
0.05
0.10
MR leave−one−out sensitivity analysis for
'Adenosine 5'−diphosphate (ADP) to mannitol to sorbitol ratio' on 'Rheumatoid arthritis'

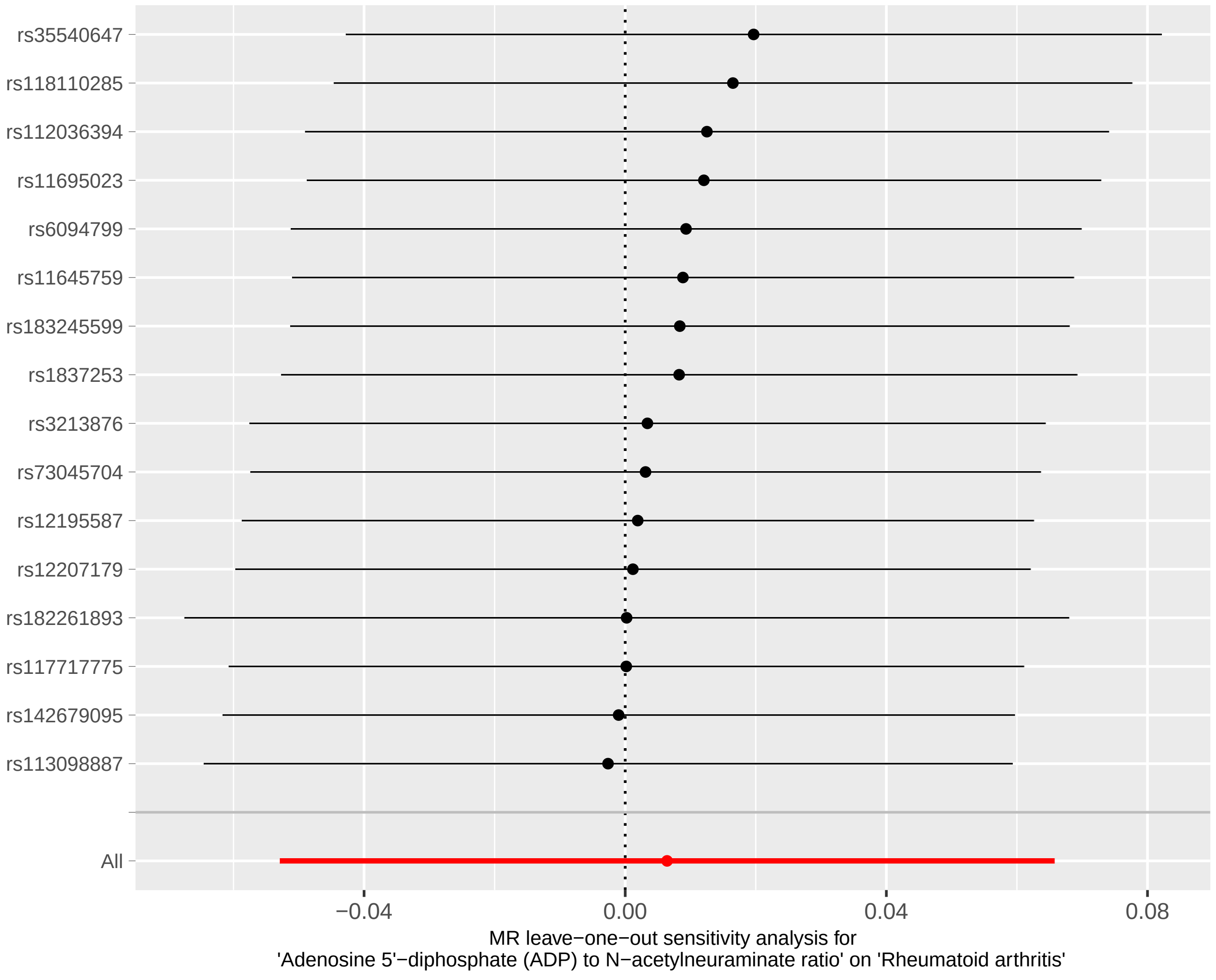
rs35540647
rs118110285
rs112036394
rs11695023
rs6094799
rs11645759
rs183245599
rs1837253
rs3213876
rs73045704
rs12195587
rs12207179
rs182261893
rs117717775
rs142679095
rs113098887
All
−0.04
0.00
0.04
0.08
MR leave−one−out sensitivity analysis for
'Adenosine 5'−diphosphate (ADP) to N−acetylneuraminate ratio' on 'Rheumatoid arthritis'

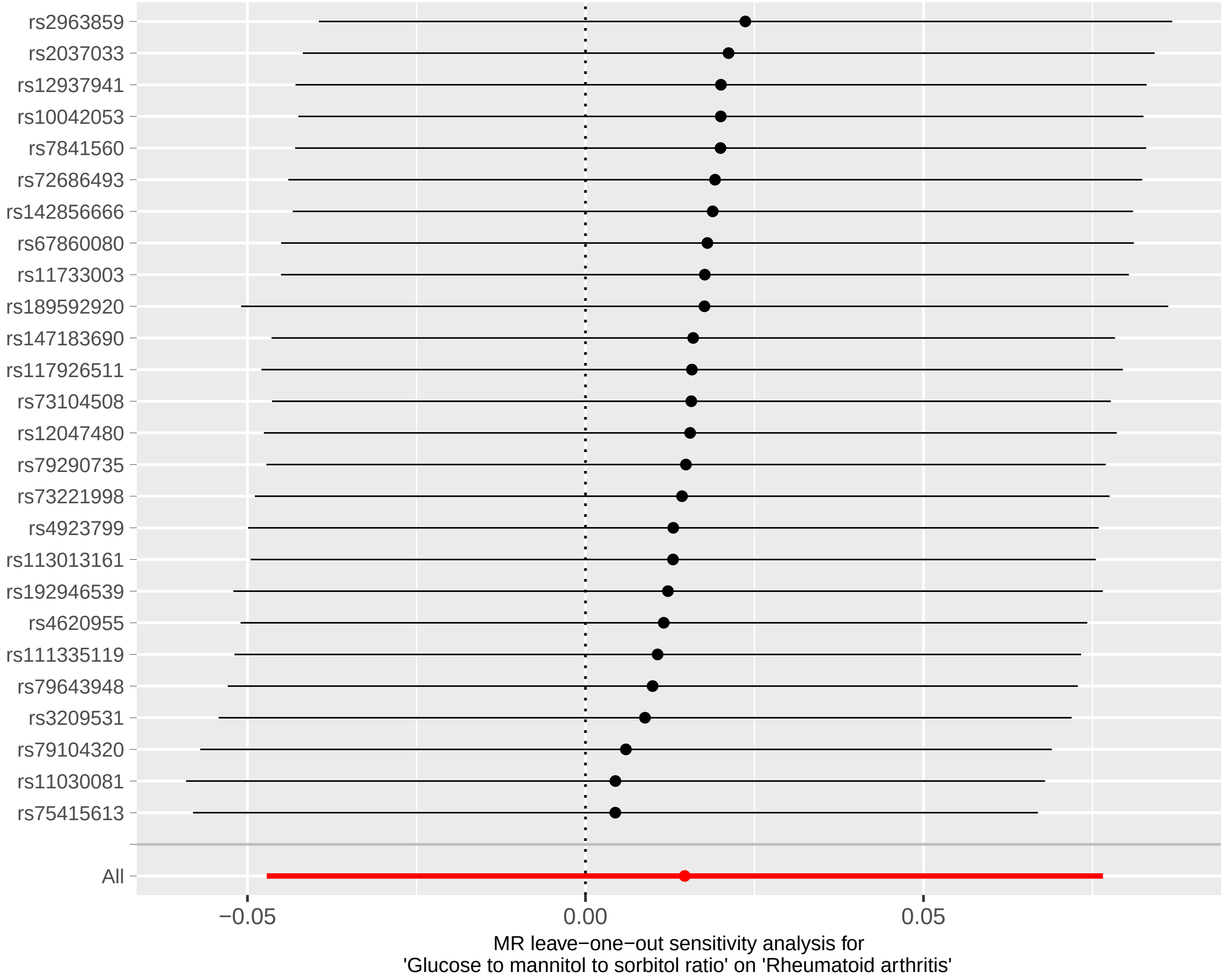

rs2963859
rs2037033
rs12937941
rs10042053
rs7841560
rs72686493
rs142856666
rs67860080
rs11733003
rs189592920
rs147183690
rs117926511
rs73104508
rs12047480
rs79290735
rs73221998
rs4923799
rs113013161
rs192946539
rs4620955
rs111335119
rs79643948
rs3209531
rs79104320
rs11030081
rs75415613
All
−0.05
0.00
0.05
MR leave−one−out sensitivity analysis for
'Glucose to mannitol to sorbitol ratio' on 'Rheumatoid arthritis'

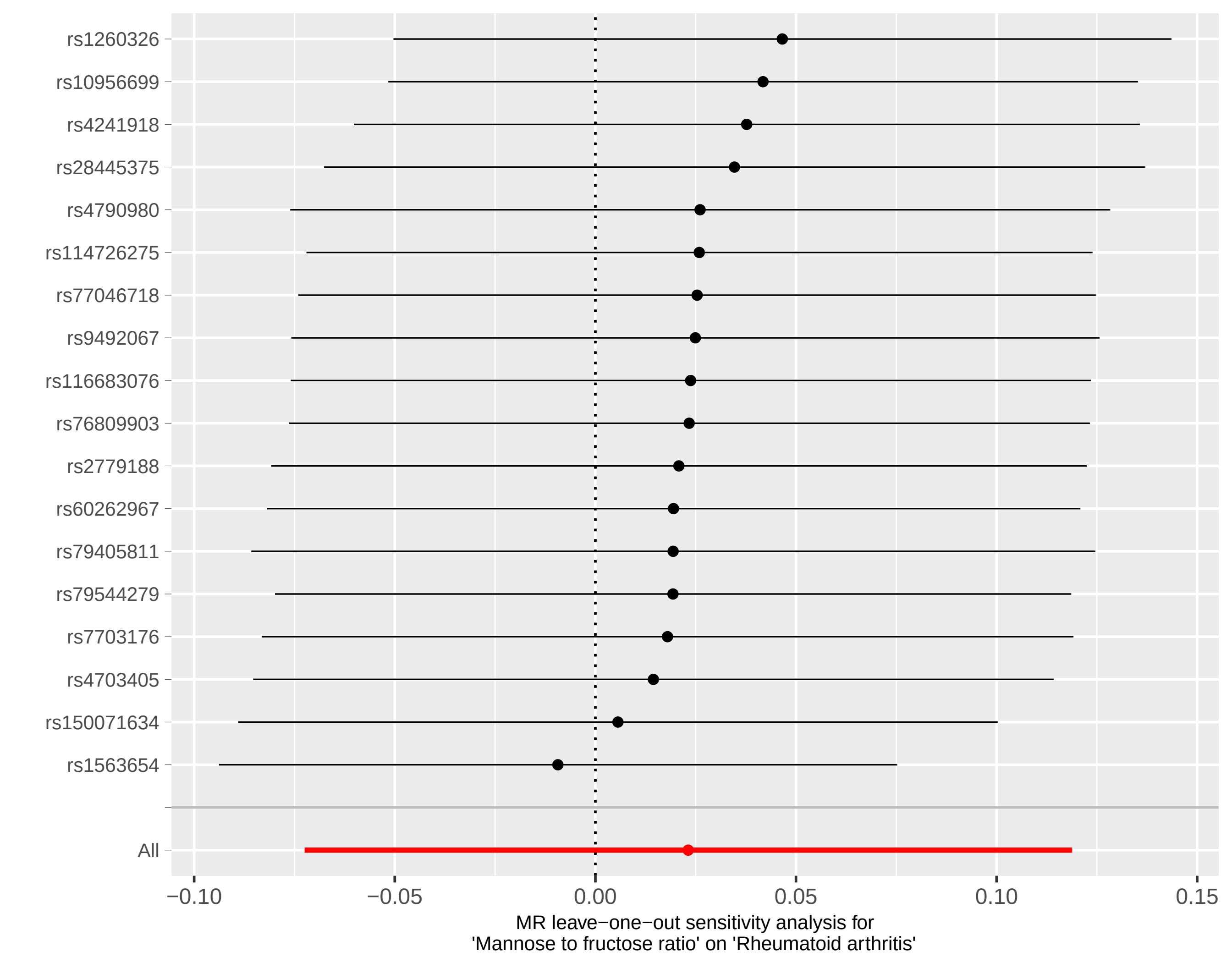
rs1260326
rs10956699
rs4241918
rs28445375
rs4790980
rs114726275
rs77046718
rs9492067
rs116683076
rs76809903
rs2779188
rs60262967
rs79405811
rs79544279
rs7703176
rs4703405
rs150071634
rs1563654
All
−0.10
−0.05
0.00
0.05
0.10
0.15
MR leave−one−out sensitivity analysis for
'Mannose to fructose ratio' on 'Rheumatoid arthritis'

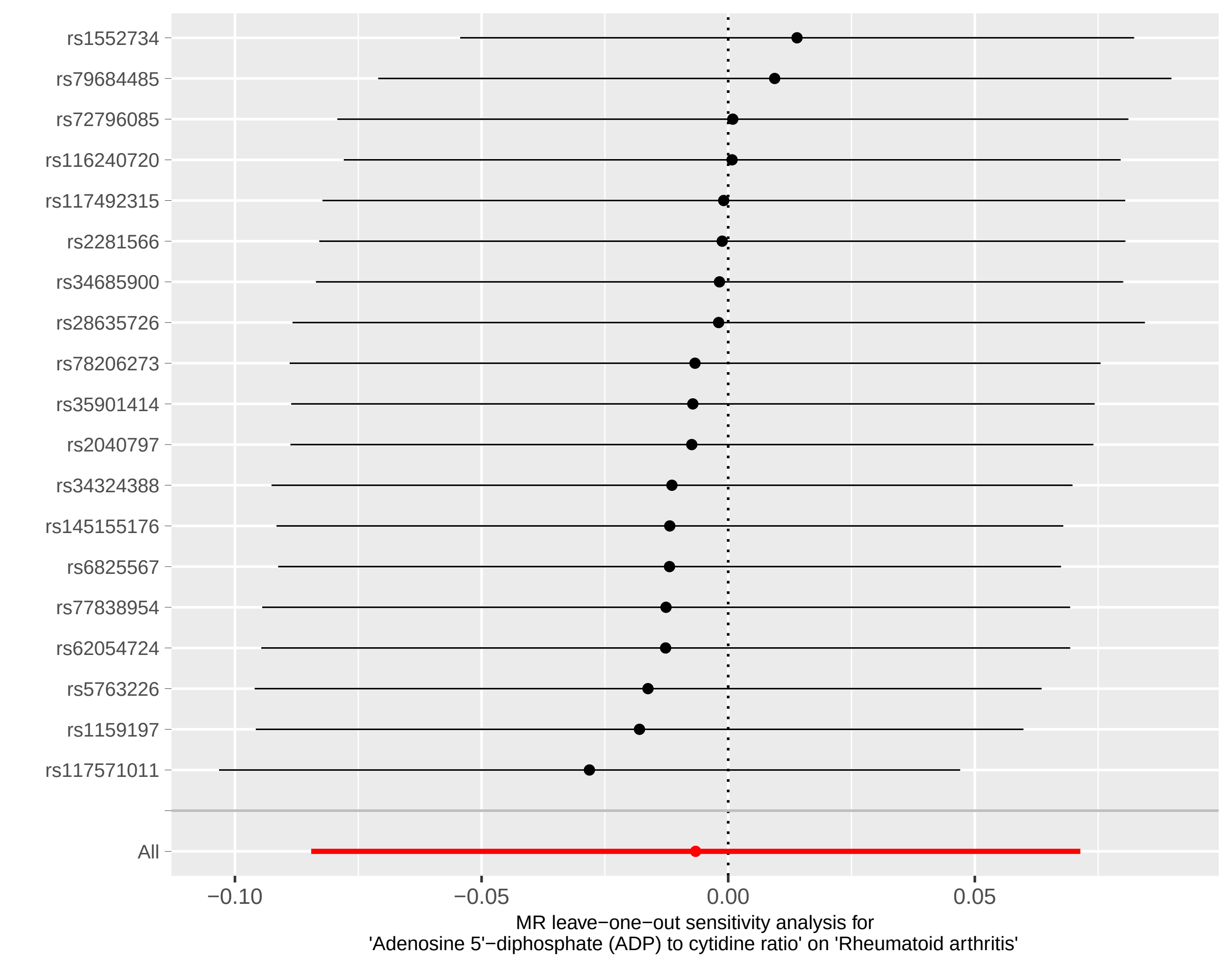

rs1552734
rs79684485
rs72796085
rs116240720
rs117492315
rs2281566
rs34685900
rs28635726
rs78206273
rs35901414
rs2040797
rs34324388
rs145155176
rs6825567
rs77838954
rs62054724
rs5763226
rs1159197
rs117571011
All
−0.10
−0.05
0.00
0.05
MR leave−one−out sensitivity analysis for
'Adenosine 5'−diphosphate (ADP) to cytidine ratio' on 'Rheumatoid arthritis'

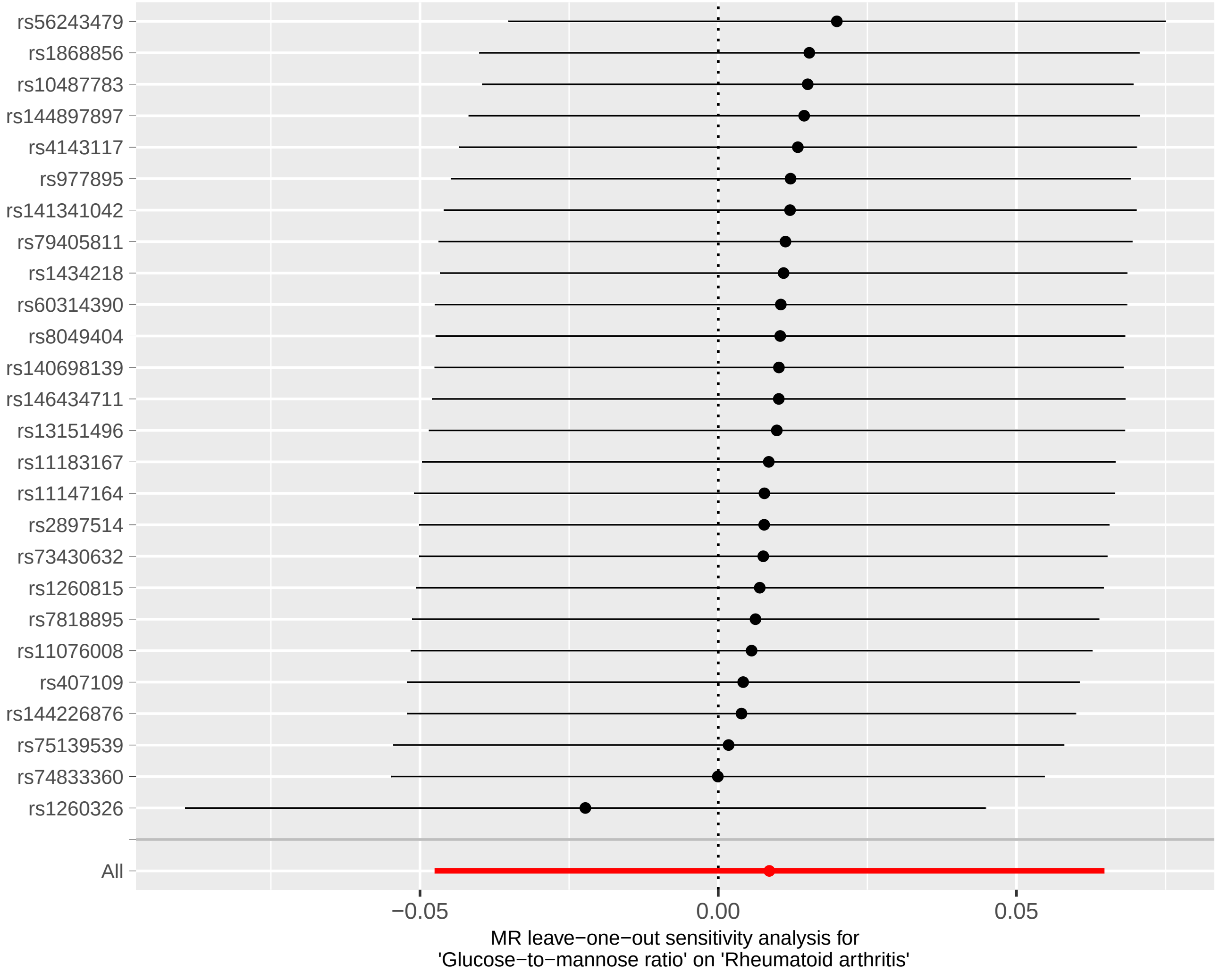

rs56243479
rs1868856
rs10487783
rs144897897
rs4143117
rs977895
rs141341042
rs79405811
rs1434218
rs60314390
rs8049404
rs140698139
rs146434711
rs13151496
rs11183167
rs11147164
rs2897514
rs73430632
rs1260815
rs7818895
rs11076008
rs407109
rs144226876
rs75139539
rs74833360
rs1260326
All
−0.05
0.00
0.05
MR leave−one−out sensitivity analysis for
'Glucose−to−mannose ratio' on 'Rheumatoid arthritis'

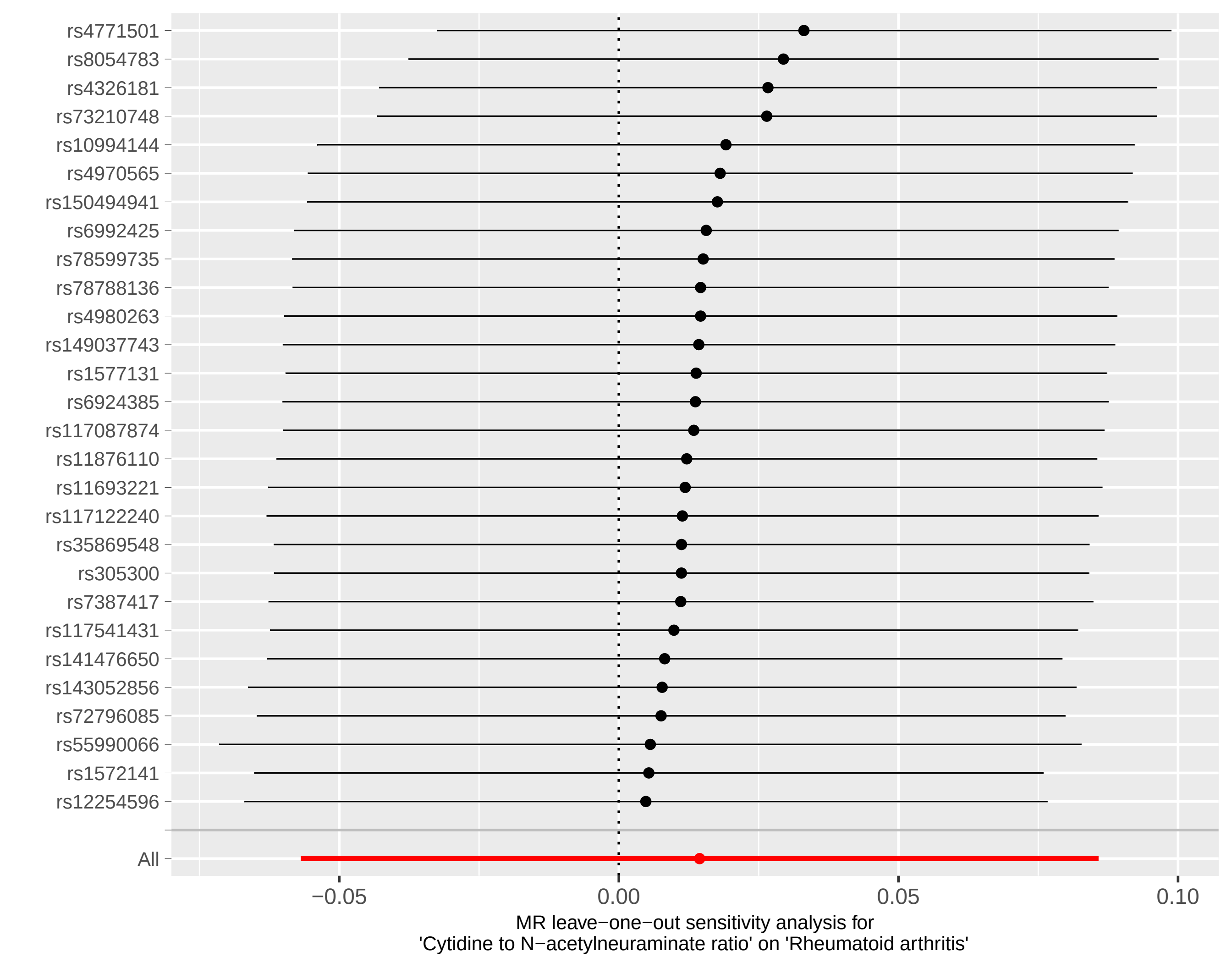

rs4771501
rs8054783
rs4326181
rs73210748
rs10994144
rs4970565
rs150494941
rs6992425
rs78599735
rs78788136
rs4980263
rs149037743
rs1577131
rs6924385
rs117087874
rs11876110
rs11693221
rs117122240
rs35869548
rs305300
rs7387417
rs117541431
rs141476650
rs143052856
rs72796085
rs55990066
rs1572141
rs12254596
All
−0.05
0.00
0.05
0.10
MR leave−one−out sensitivity analysis for
'Cytidine to N−acetylneuraminate ratio' on 'Rheumatoid arthritis'

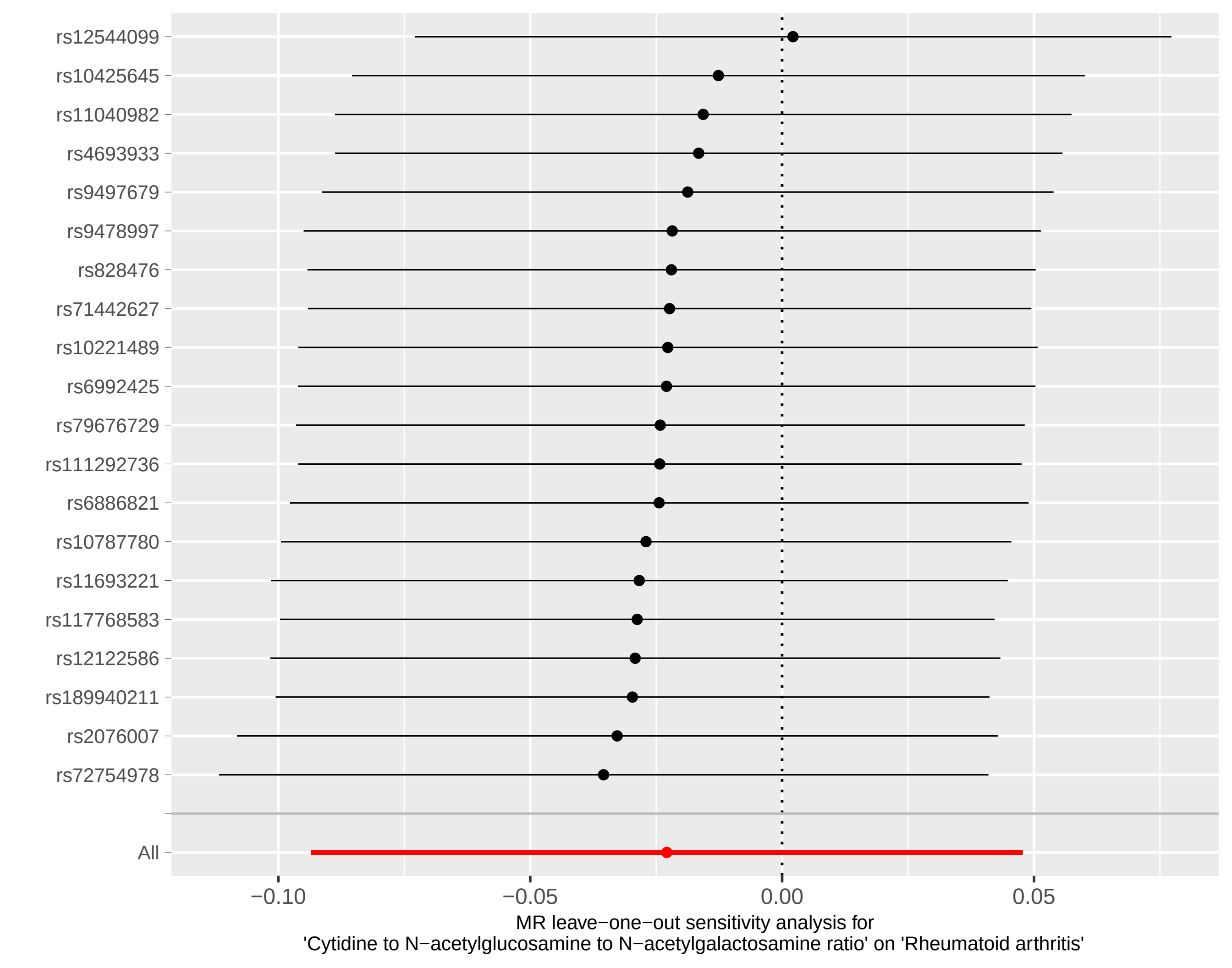

rs12544099
rs10425645
rs11040982
rs4693933
rs9497679
rs9478997
rs828476
rs71442627
rs10221489
rs6992425
rs79676729
rs111292736
rs6886821
rs10787780
rs11693221
rs117768583
rs12122586
rs189940211
rs2076007
rs72754978
All
−0.10
−0.05
0.00
0.05
MR leave−one−out sensitivity analysis for
'Cytidine to N−acetylglucosamine to N−acetylgalactosamine ratio' on 'Rheumatoid arthritis'

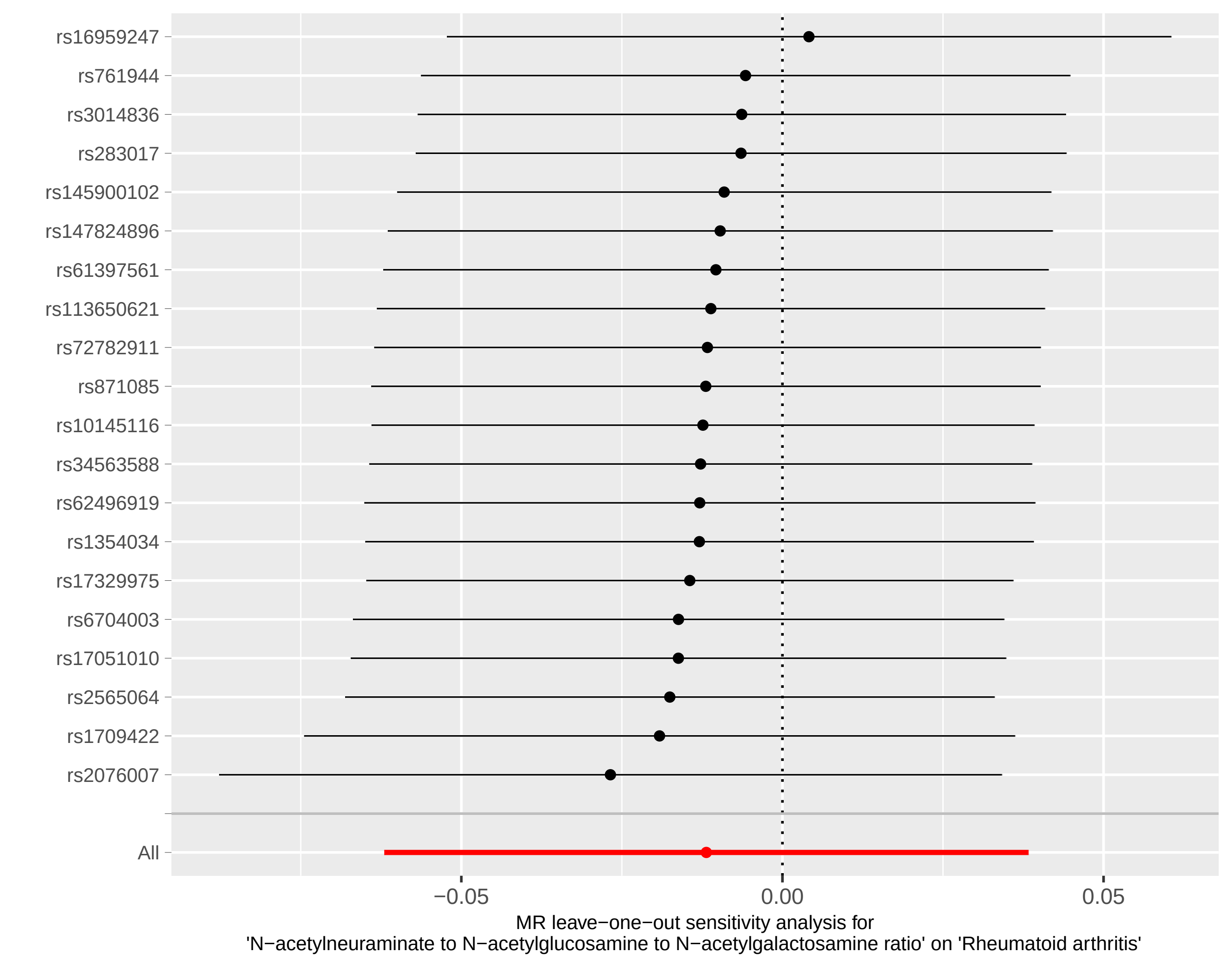

rs16959247
rs761944
rs3014836
rs283017
rs145900102
rs147824896
rs61397561
rs113650621
rs72782911
rs871085
rs10145116
rs34563588
rs62496919
rs1354034
rs17329975
rs6704003
rs17051010
rs2565064
rs1709422
rs2076007
All
−0.05
0.00
0.05
MR leave−one−out sensitivity analysis for
'N−acetylneuraminate to N−acetylglucosamine to N−acetylgalactosamine ratio' on 'Rheumatoid arthritis'

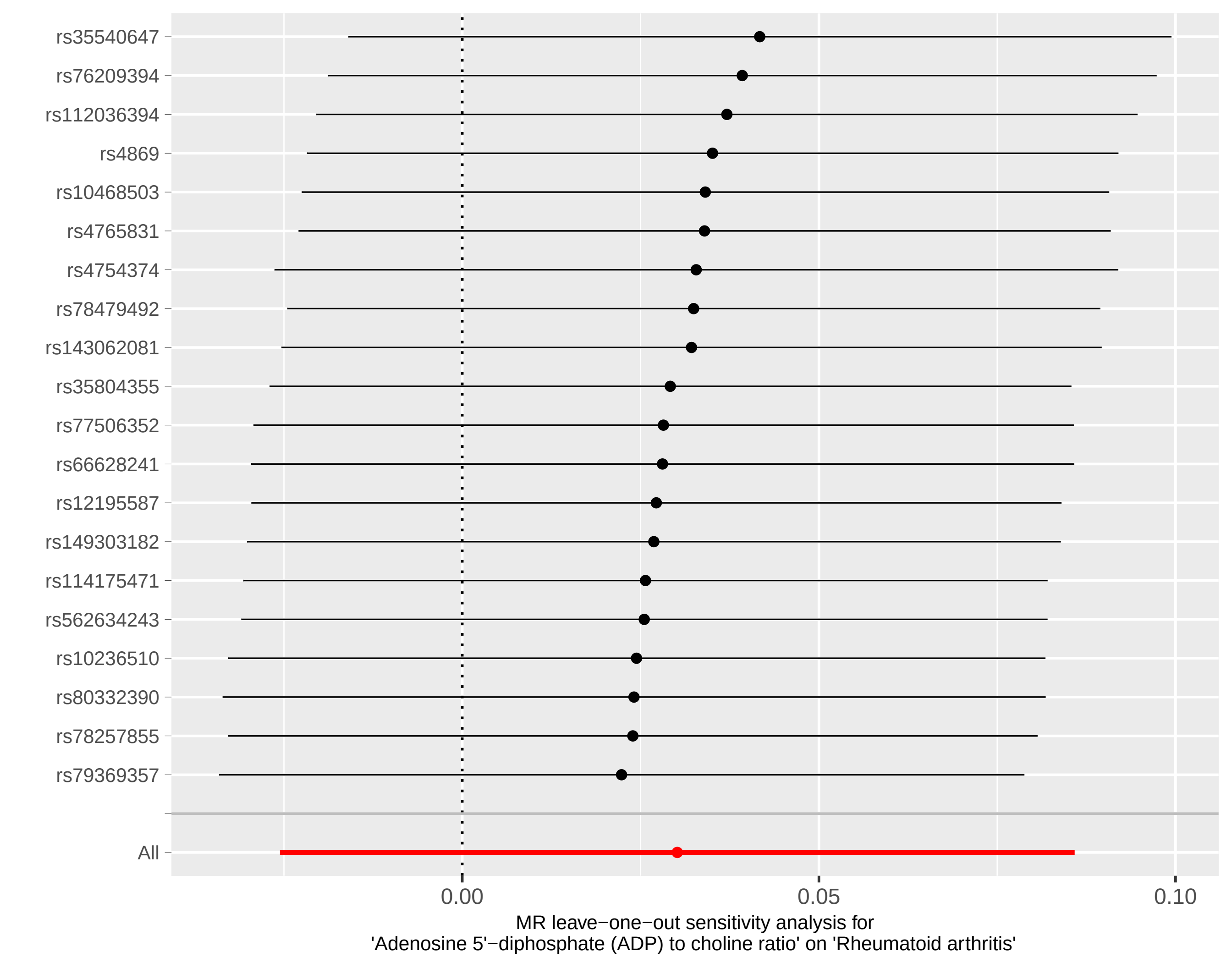
rs35540647
rs76209394
rs112036394
rs4869
rs10468503
rs4765831
rs4754374
rs78479492
rs143062081
rs35804355
rs77506352
rs66628241
rs12195587
rs149303182
rs114175471
rs562634243
rs10236510
rs80332390
rs78257855
rs79369357
All
0.00
0.05
0.10
MR leave−one−out sensitivity analysis for
'Adenosine 5'−diphosphate (ADP) to choline ratio' on 'Rheumatoid arthritis'

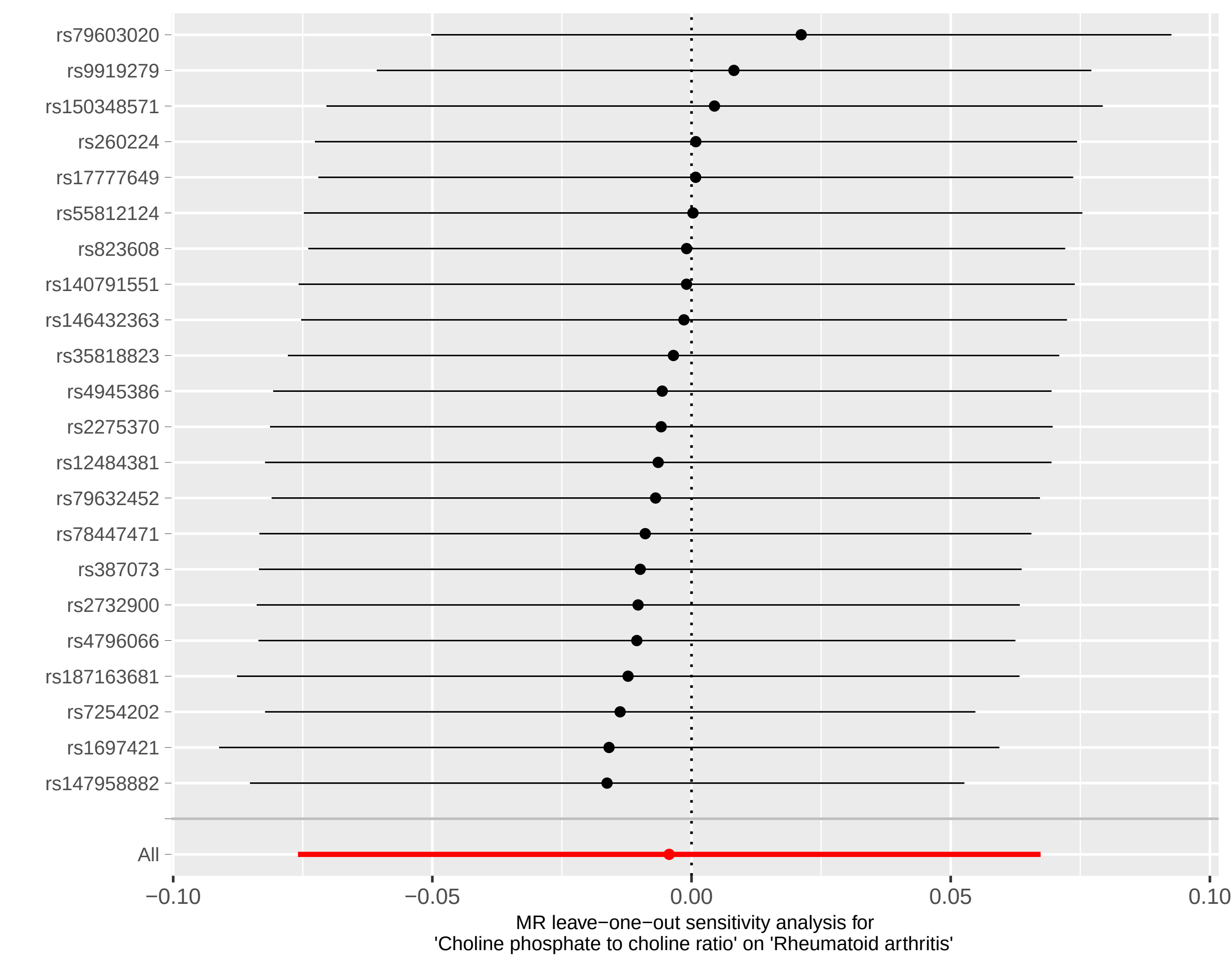
rs79603020
rs9919279
rs150348571
rs260224
rs17777649
rs55812124
rs823608
rs140791551
rs146432363
rs35818823
rs4945386
rs2275370
rs12484381
rs79632452
rs78447471
rs387073
rs2732900
rs4796066
rs187163681
rs7254202
rs1697421
rs147958882
All
−0.10
−0.05
0.00
0.05
0.10
MR leave−one−out sensitivity analysis for
'Choline phosphate to choline ratio' on 'Rheumatoid arthritis'

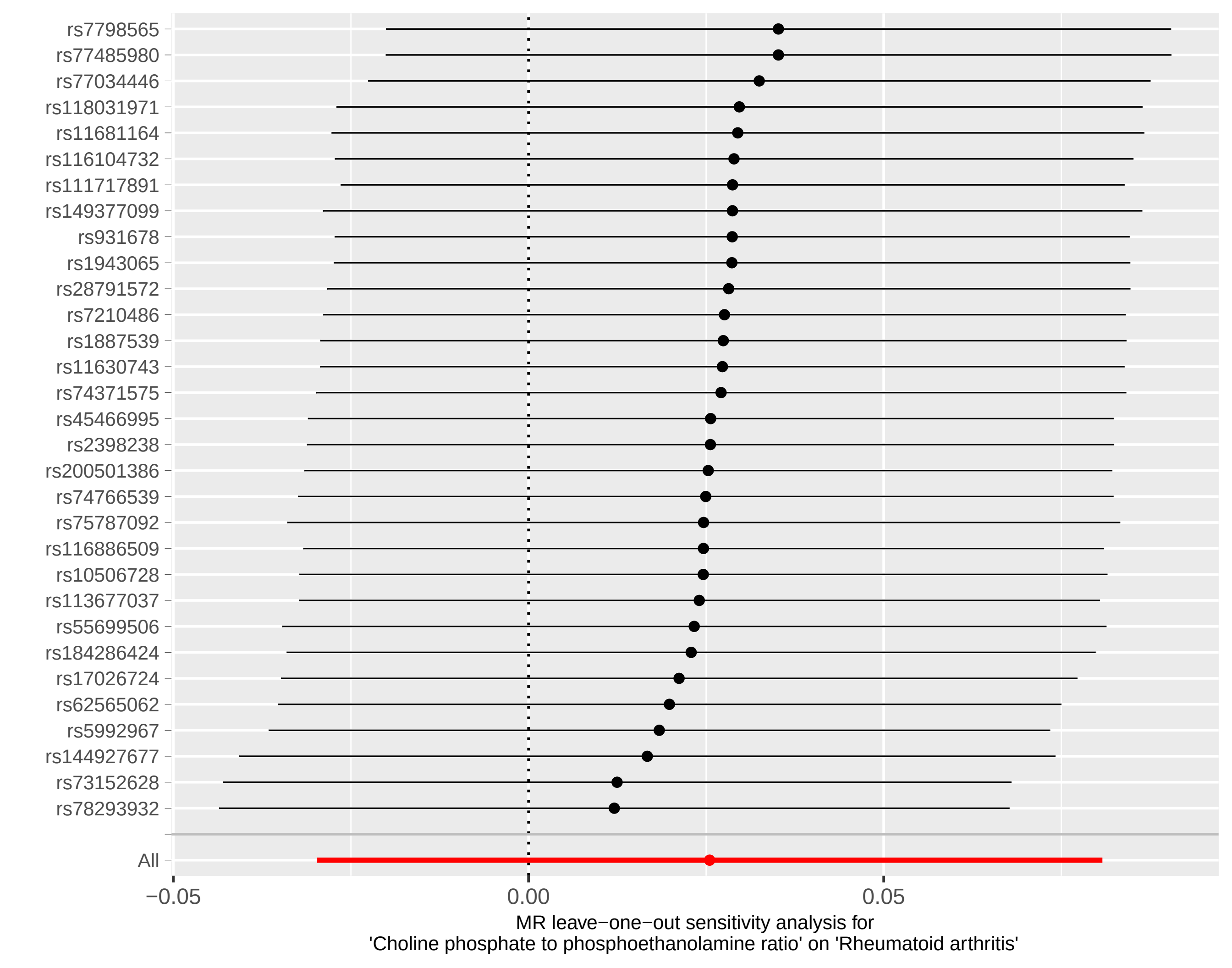
rs7798565
rs77485980
rs77034446
rs118031971
rs11681164
rs116104732
rs111717891
rs149377099
rs931678
rs1943065
rs28791572
rs7210486
rs1887539
rs11630743
rs74371575
rs45466995
rs2398238
rs200501386
rs74766539
rs75787092
rs116886509
rs10506728
rs113677037
rs55699506
rs184286424
rs17026724
rs62565062
rs5992967
rs144927677
rs73152628
rs78293932
All
−0.05
0.00
0.05
MR leave−one−out sensitivity analysis for
'Choline phosphate to phosphoethanolamine ratio' on 'Rheumatoid arthritis'

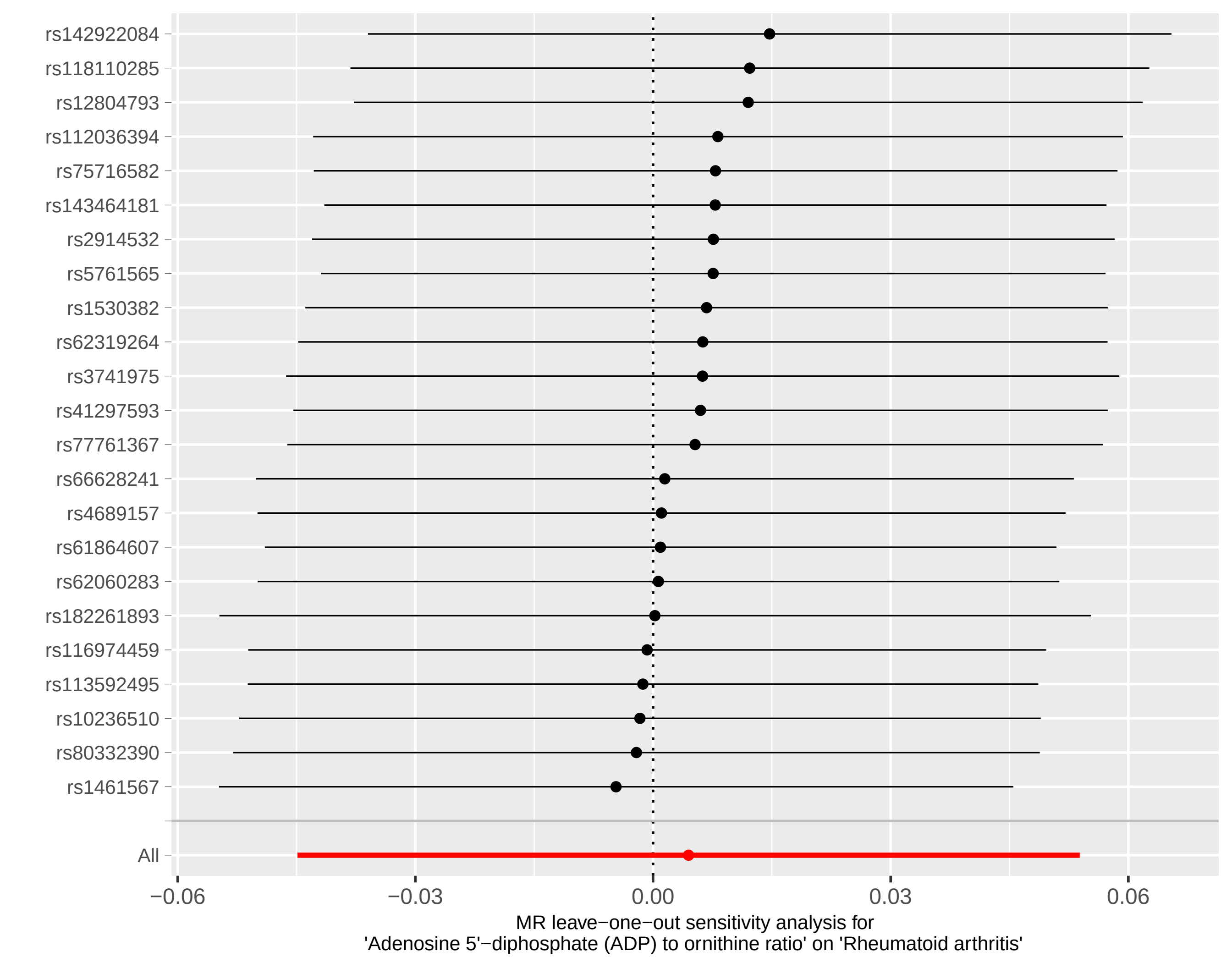
rs142922084
rs118110285
rs12804793
rs112036394
rs75716582
rs143464181
rs2914532
rs5761565
rs1530382
rs62319264
rs3741975
rs41297593
rs77761367
rs66628241
rs4689157
rs61864607
rs62060283
rs182261893
rs116974459
rs113592495
rs10236510
rs80332390
rs1461567
All
−0.06
−0.03
0.00
0.03
0.06
MR leave−one−out sensitivity analysis for
'Adenosine 5'−diphosphate (ADP) to ornithine ratio' on 'Rheumatoid arthritis'

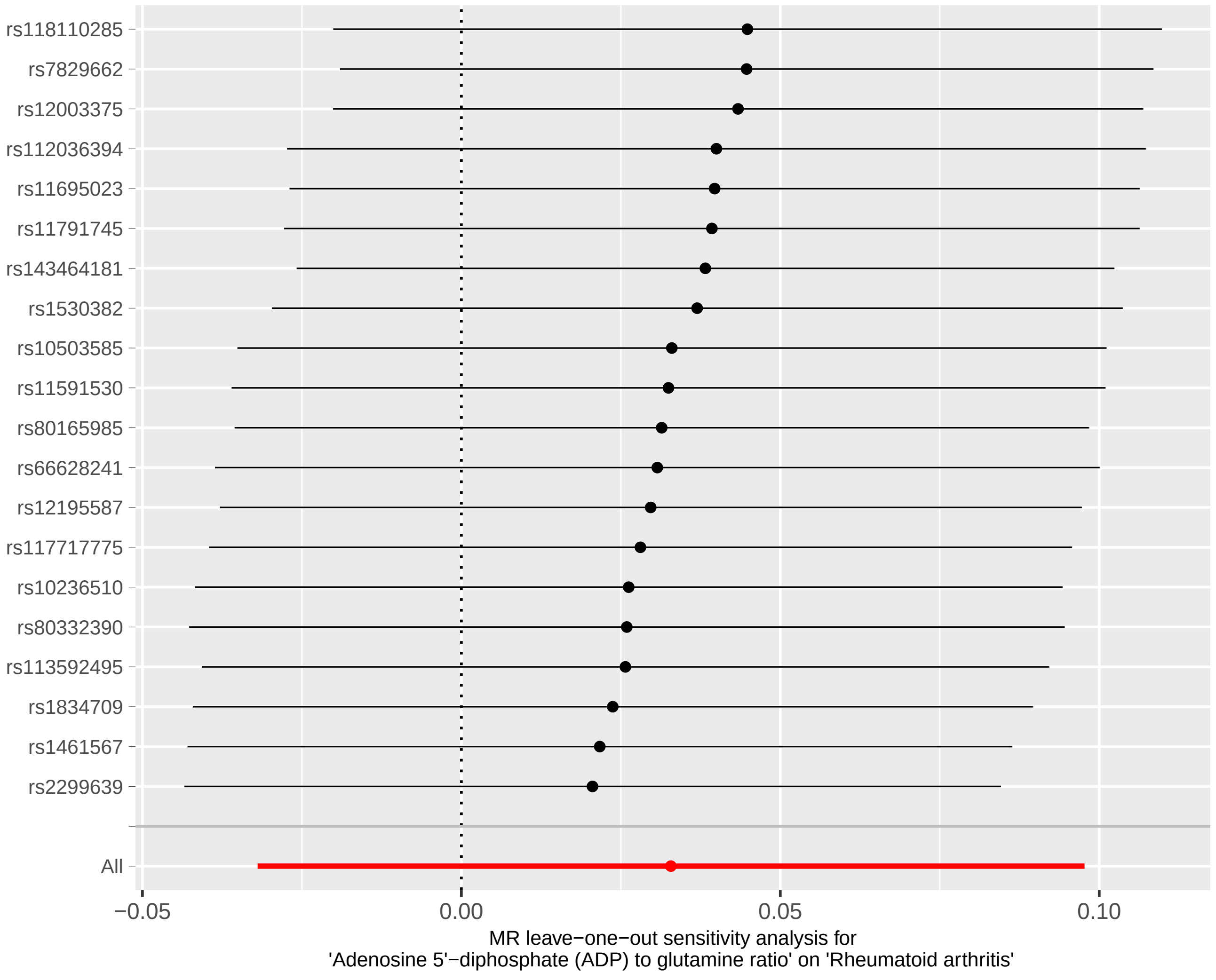

rs118110285
rs7829662
rs12003375
rs112036394
rs11695023
rs11791745
rs143464181
rs1530382
rs10503585
rs11591530
rs80165985
rs66628241
rs12195587
rs117717775
rs10236510
rs80332390
rs113592495
rs1834709
rs1461567
rs2299639
All
−0.05
0.00
0.05
0.10
MR leave−one−out sensitivity analysis for
'Adenosine 5'−diphosphate (ADP) to glutamine ratio' on 'Rheumatoid arthritis'

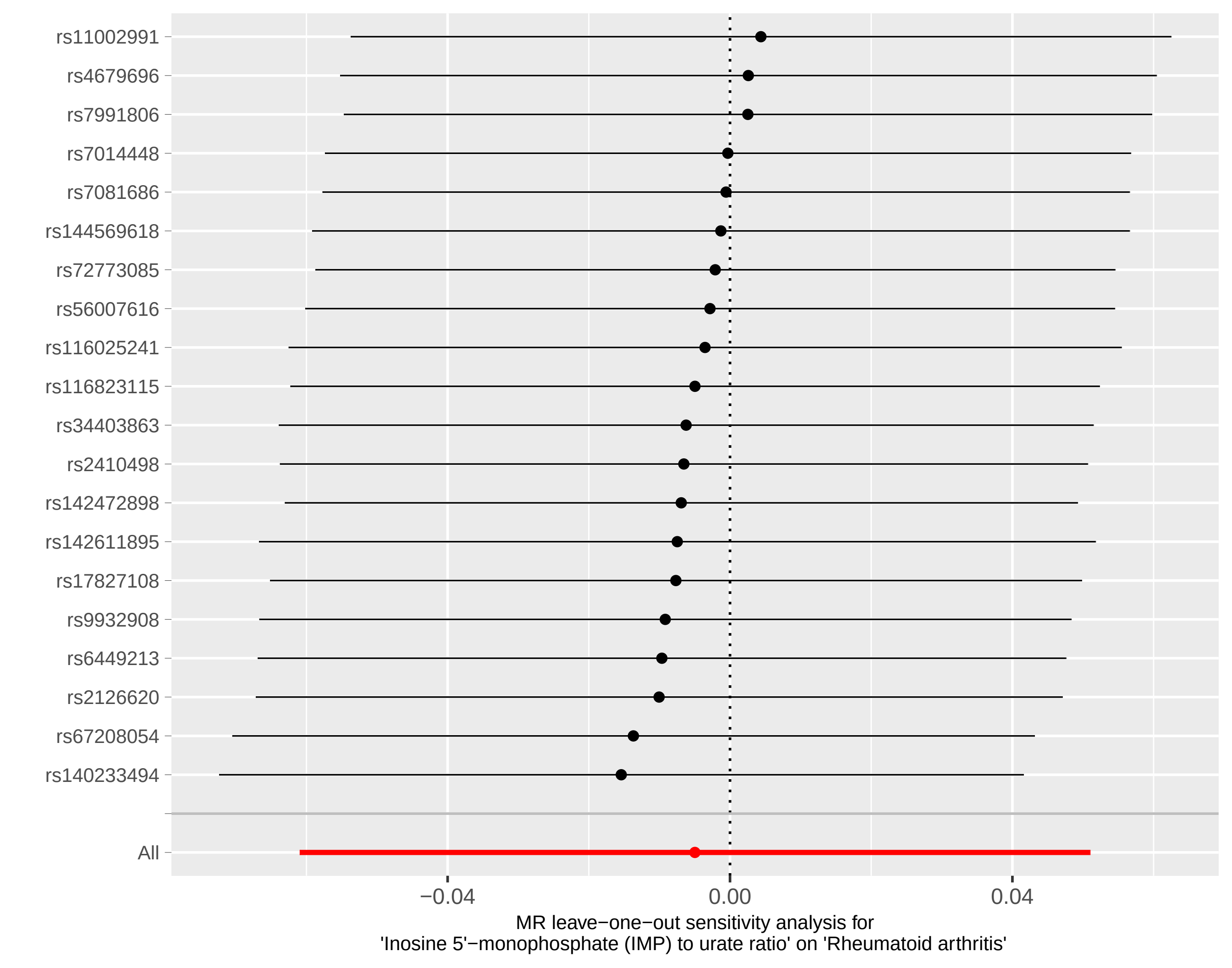

rs11002991
rs4679696
rs7991806
rs7014448
rs7081686
rs144569618
rs72773085
rs56007616
rs116025241
rs116823115
rs34403863
rs2410498
rs142472898
rs142611895
rs17827108
rs9932908
rs6449213
rs2126620
rs67208054
rs140233494
All
−0.04
0.00
0.04
MR leave−one−out sensitivity analysis for
'Inosine 5'−monophosphate (IMP) to urate ratio' on 'Rheumatoid arthritis'

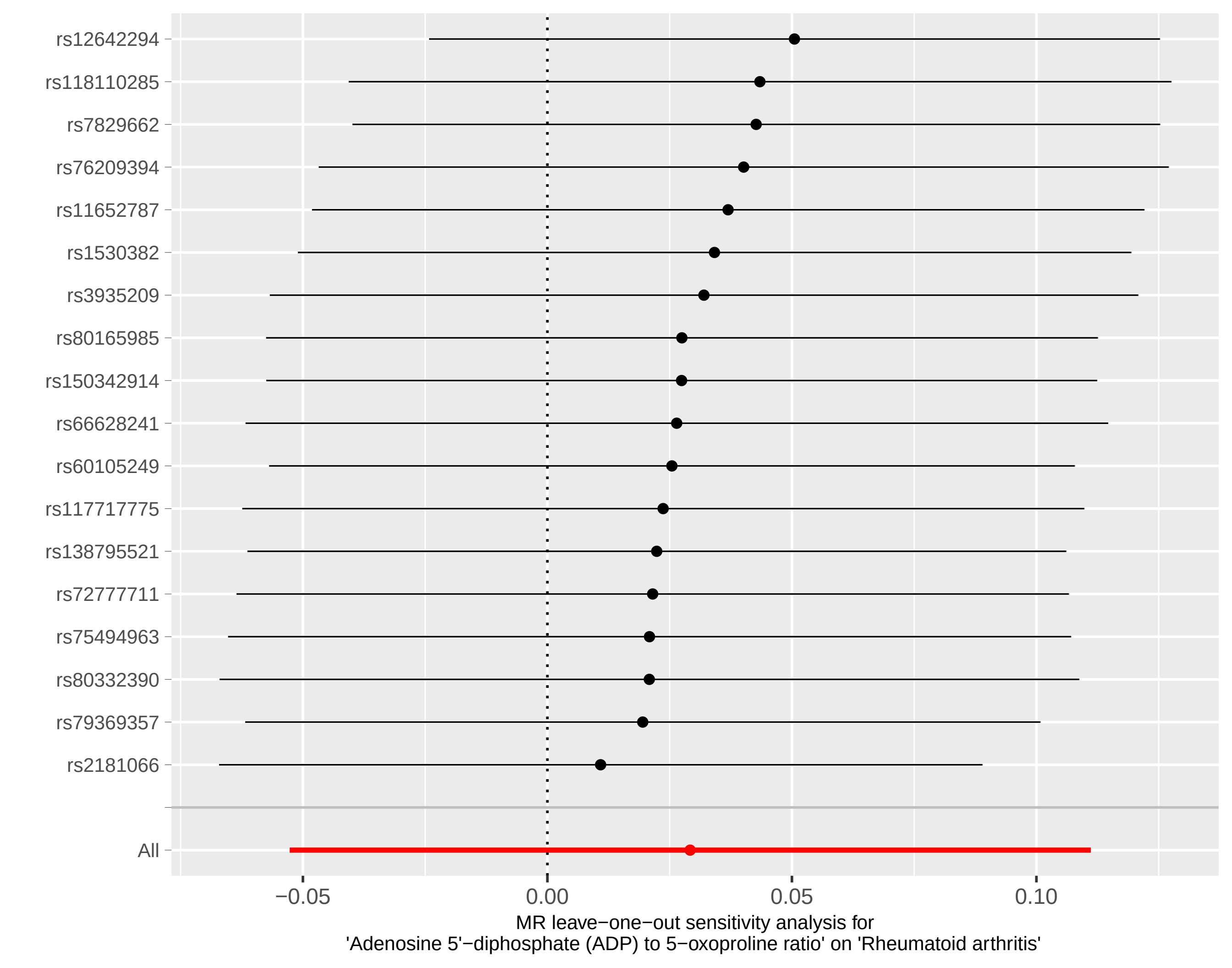
rs12642294
rs118110285
rs7829662
rs76209394
rs11652787
rs1530382
rs3935209
rs80165985
rs150342914
rs66628241
rs60105249
rs117717775
rs138795521
rs72777711
rs75494963
rs80332390
rs79369357
rs2181066
All
−0.05
0.00
0.05
0.10
MR leave−one−out sensitivity analysis for
'Adenosine 5'−diphosphate (ADP) to 5−oxoproline ratio' on 'Rheumatoid arthritis'

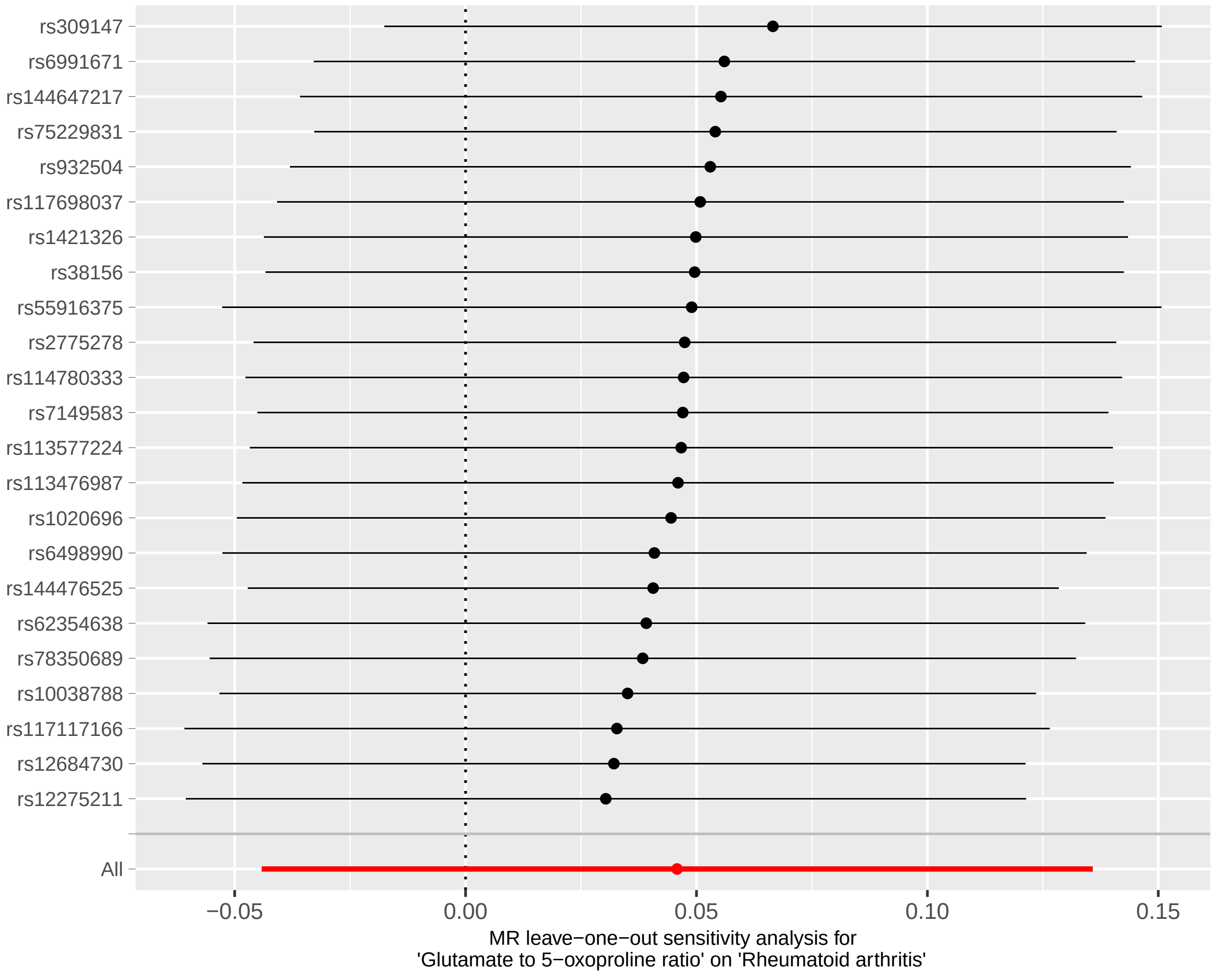
rs309147
rs6991671
rs144647217
rs75229831
rs932504
rs117698037
rs1421326
rs38156
rs55916375
rs2775278
rs114780333
rs7149583
rs113577224
rs113476987
rs1020696
rs6498990
rs144476525
rs62354638
rs78350689
rs10038788
rs117117166
rs12684730
rs12275211
All
−0.05
0.00
0.05
0.10
0.15
MR leave−one−out sensitivity analysis for
'Glutamate to 5−oxoproline ratio' on 'Rheumatoid arthritis'

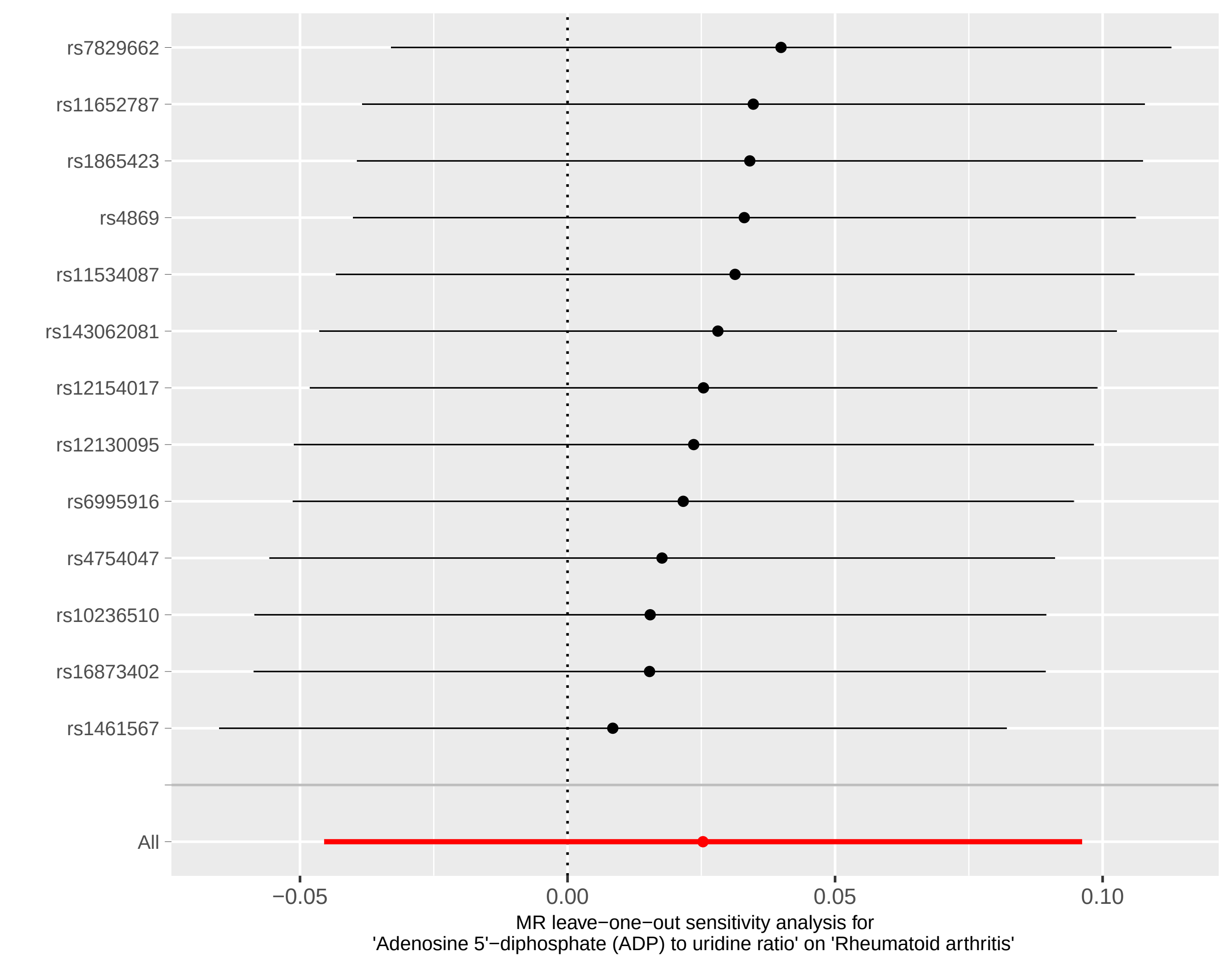

rs7829662
rs11652787
rs1865423
rs4869
rs11534087
rs143062081
rs12154017
rs12130095
rs6995916
rs4754047
rs10236510
rs16873402
rs1461567
All
−0.05
0.00
0.05
0.10
MR leave−one−out sensitivity analysis for
'Adenosine 5'−diphosphate (ADP) to uridine ratio' on 'Rheumatoid arthritis'

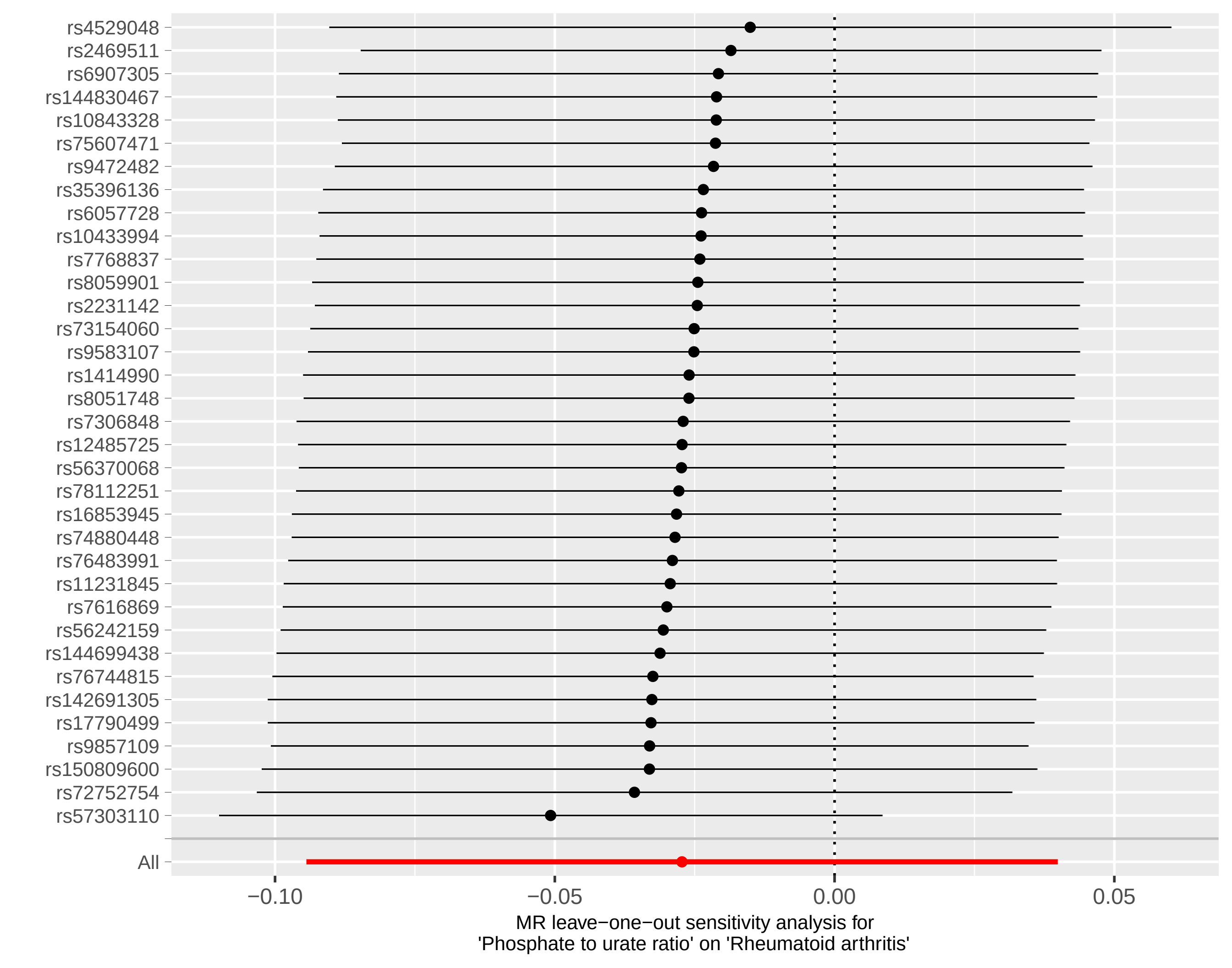

rs4529048
rs2469511
rs6907305
rs144830467
rs10843328
rs75607471
rs9472482
rs35396136
rs6057728
rs10433994
rs7768837
rs8059901
rs2231142
rs73154060
rs9583107
rs1414990
rs8051748
rs7306848
rs12485725
rs56370068
rs78112251
rs16853945
rs74880448
rs76483991
rs11231845
rs7616869
rs56242159
rs144699438
rs76744815
rs142691305
rs17790499
rs9857109
rs150809600
rs72752754
rs57303110
All
−0.10
−0.05
0.00
0.05
MR leave−one−out sensitivity analysis for
'Phosphate to urate ratio' on 'Rheumatoid arthritis'

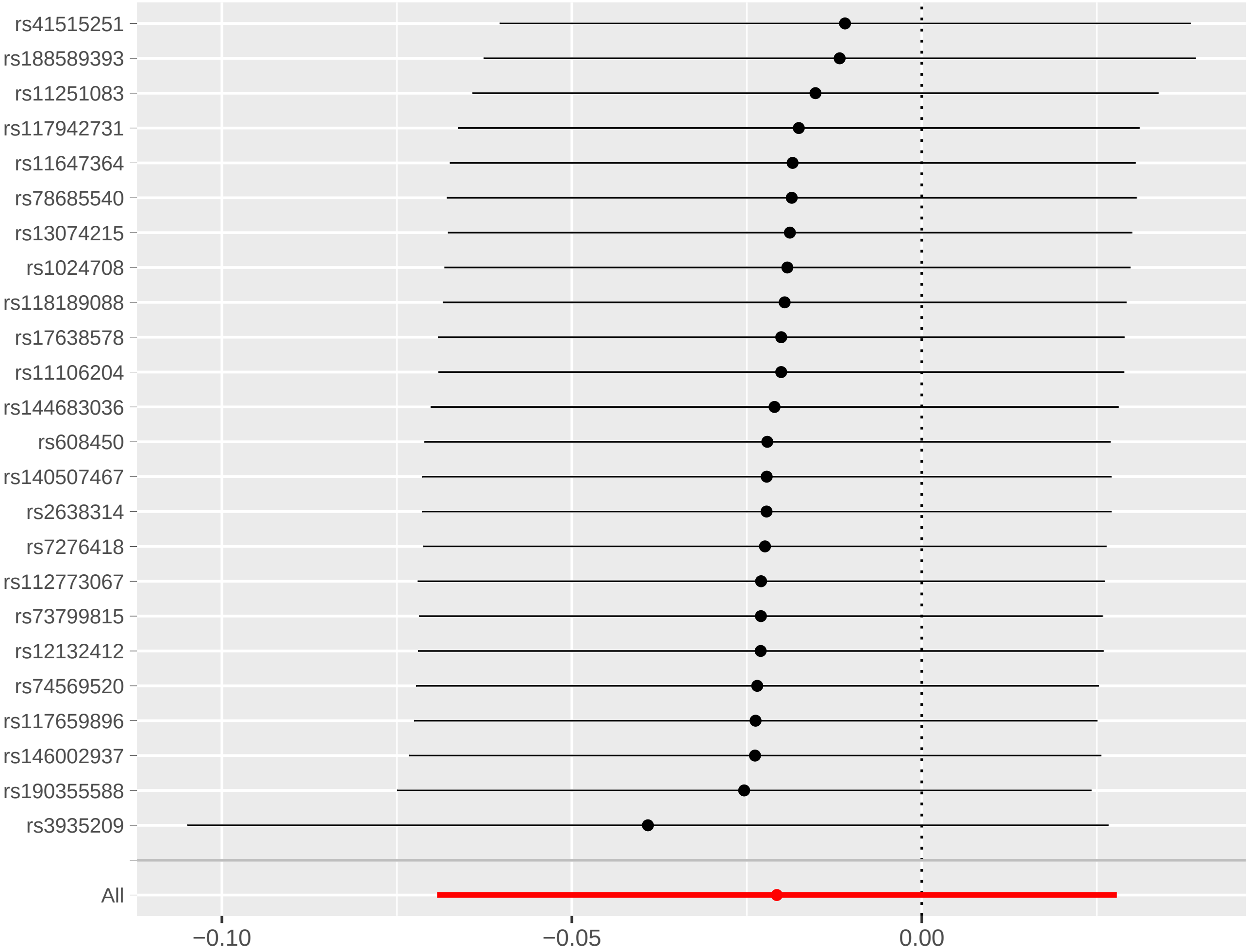

MR leave−one−out sensitivity analysis for
'Phosphate to 5−oxoproline ratio' on 'Rheumatoid arthritis'

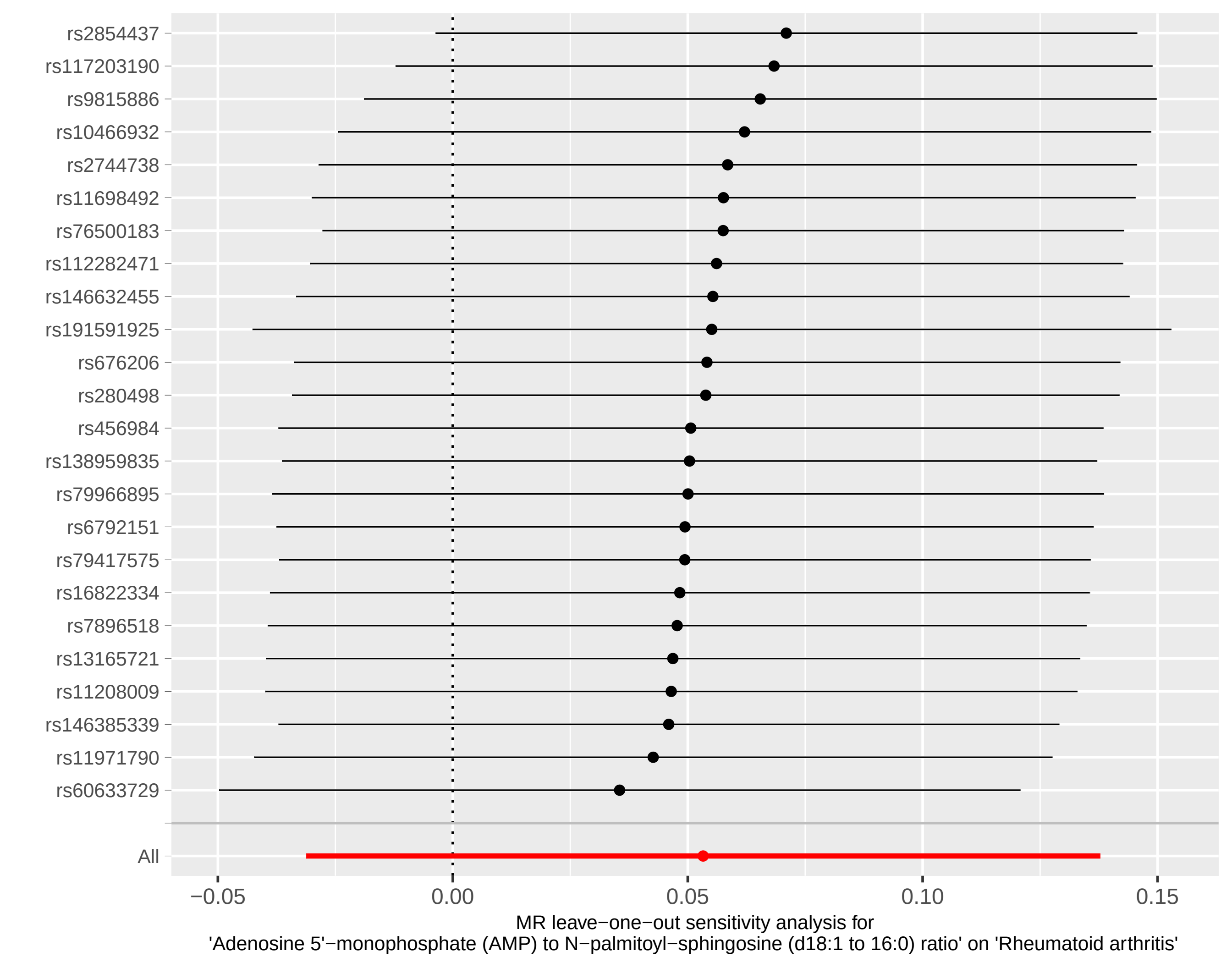

rs2854437
rs117203190
rs9815886
rs10466932
rs2744738
rs11698492
rs76500183
rs112282471
rs146632455
rs191591925
rs676206
rs280498
rs456984
rs138959835
rs79966895
rs6792151
rs79417575
rs16822334
rs7896518
rs13165721
rs11208009
rs146385339
rs11971790
rs60633729
All
−0.05
0.00
0.05
0.10
0.15
MR leave−one−out sensitivity analysis for
'Adenosine 5'−monophosphate (AMP) to N−palmitoyl−sphingosine (d18:1 to 16:0) ratio' on 'Rheumatoid arthritis'

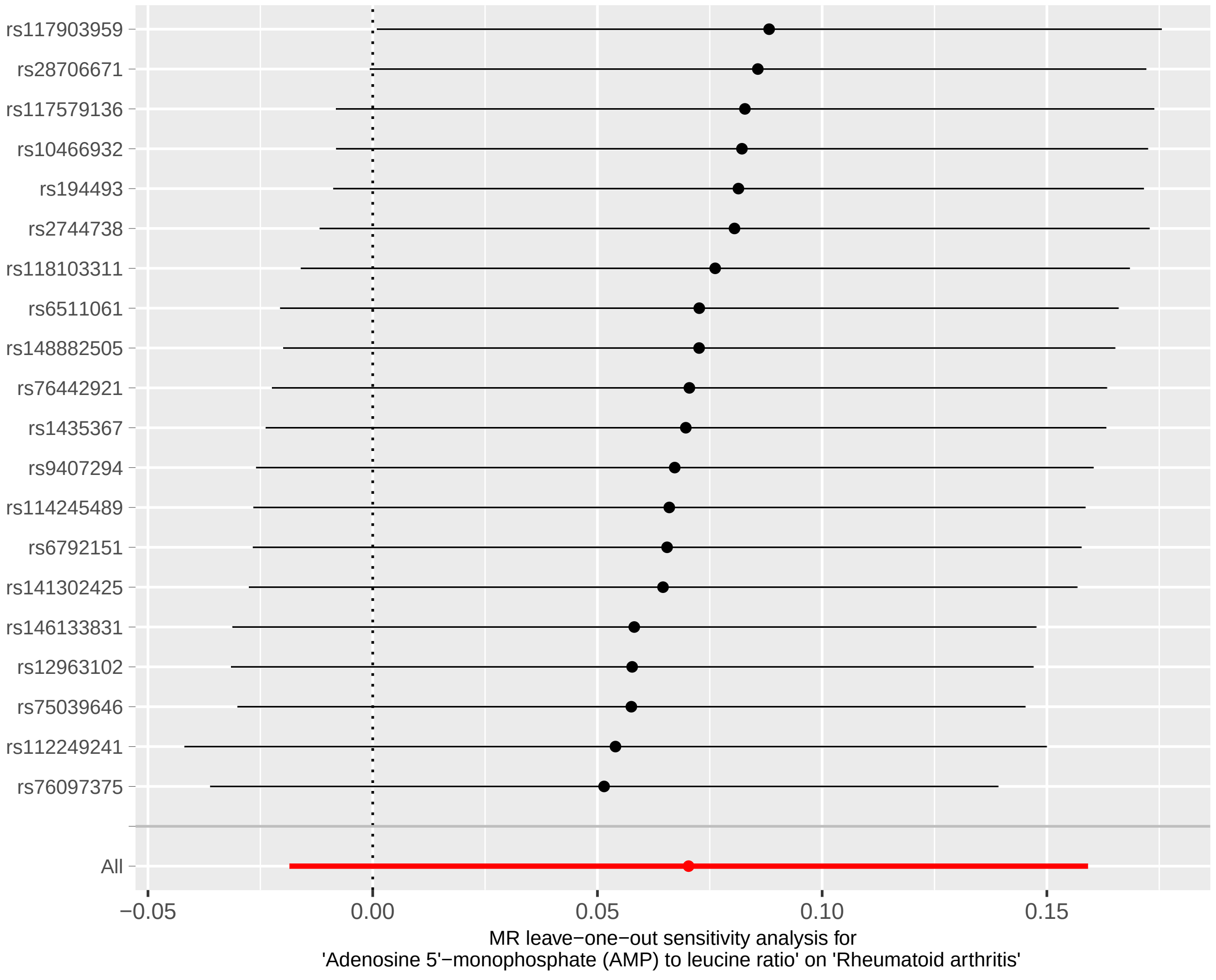

rs117903959
rs28706671
rs117579136
rs10466932
rs194493
rs2744738
rs118103311
rs6511061
rs148882505
rs76442921
rs1435367
rs9407294
rs114245489
rs6792151
rs141302425
rs146133831
rs12963102
rs75039646
rs112249241
rs76097375
All
−0.05
0.00
0.05
0.10
0.15
MR leave−one−out sensitivity analysis for
'Adenosine 5'−monophosphate (AMP) to leucine ratio' on 'Rheumatoid arthritis'

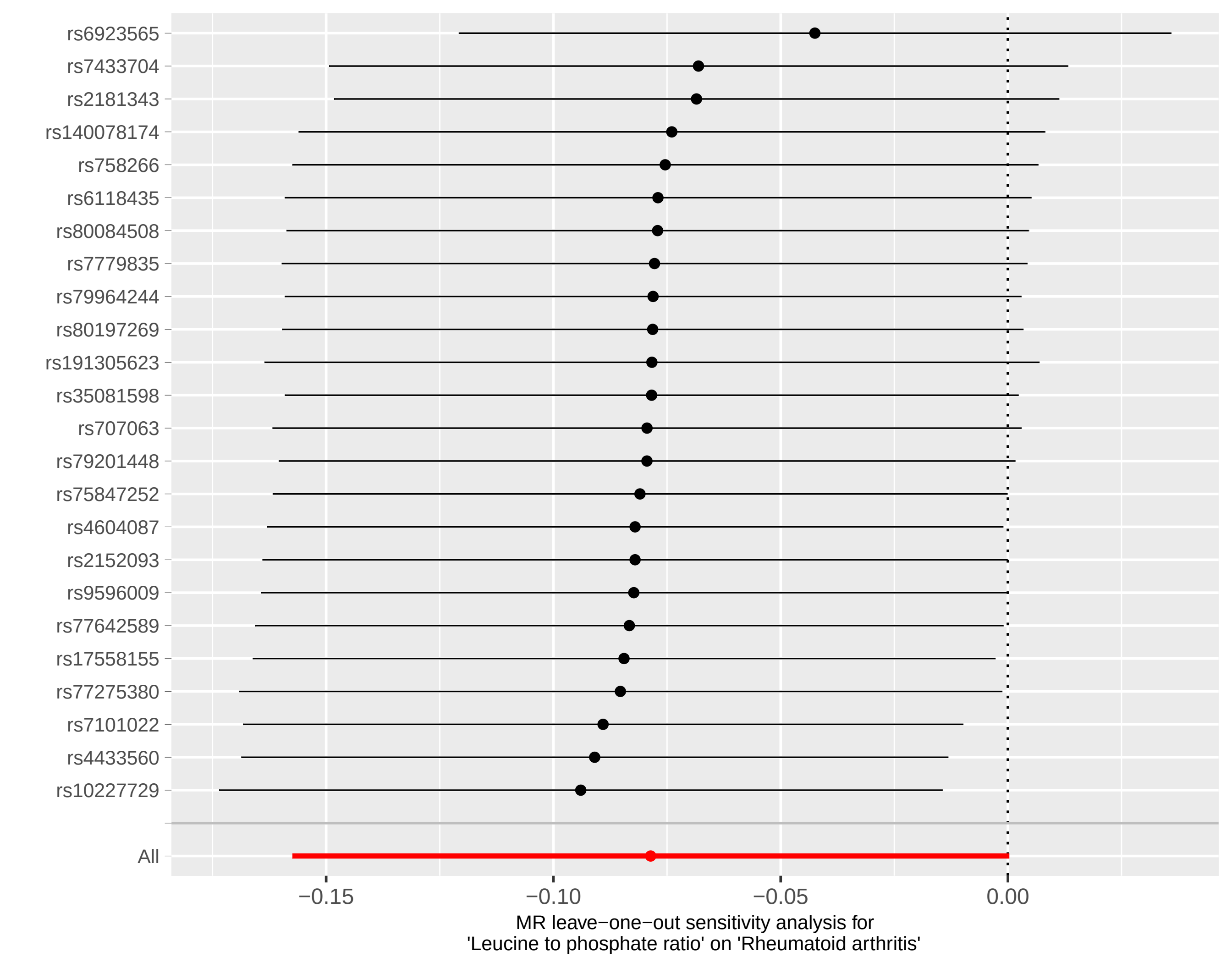
rs6923565
rs7433704
rs2181343
rs140078174
rs758266
rs6118435
rs80084508
rs7779835
rs79964244
rs80197269
rs191305623
rs35081598
rs707063
rs79201448
rs75847252
rs4604087
rs2152093
rs9596009
rs77642589
rs17558155
rs77275380
rs7101022
rs4433560
rs10227729
All
−0.15
−0.10
−0.05
0.00
MR leave−one−out sensitivity analysis for
'Leucine to phosphate ratio' on 'Rheumatoid arthritis'

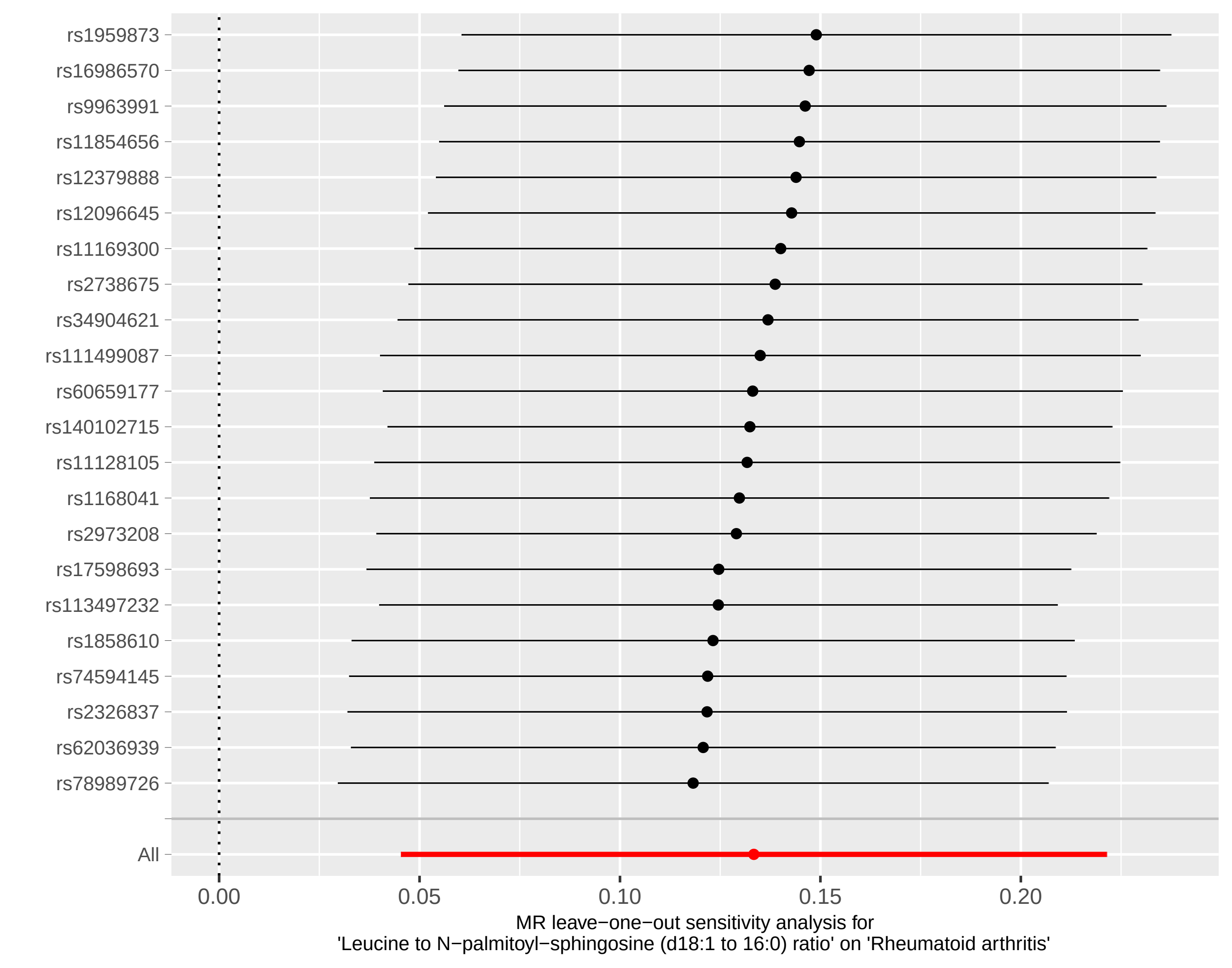
rs1959873
rs16986570
rs9963991
rs11854656
rs12379888
rs12096645
rs11169300
rs2738675
rs34904621
rs111499087
rs60659177
rs140102715
rs11128105
rs1168041
rs2973208
rs17598693
rs113497232
rs1858610
rs74594145
rs2326837
rs62036939
rs78989726
All
0.00
0.05
0.10
0.15
0.20
MR leave−one−out sensitivity analysis for
'Leucine to N−palmitoyl−sphingosine (d18:1 to 16:0) ratio' on 'Rheumatoid arthritis'

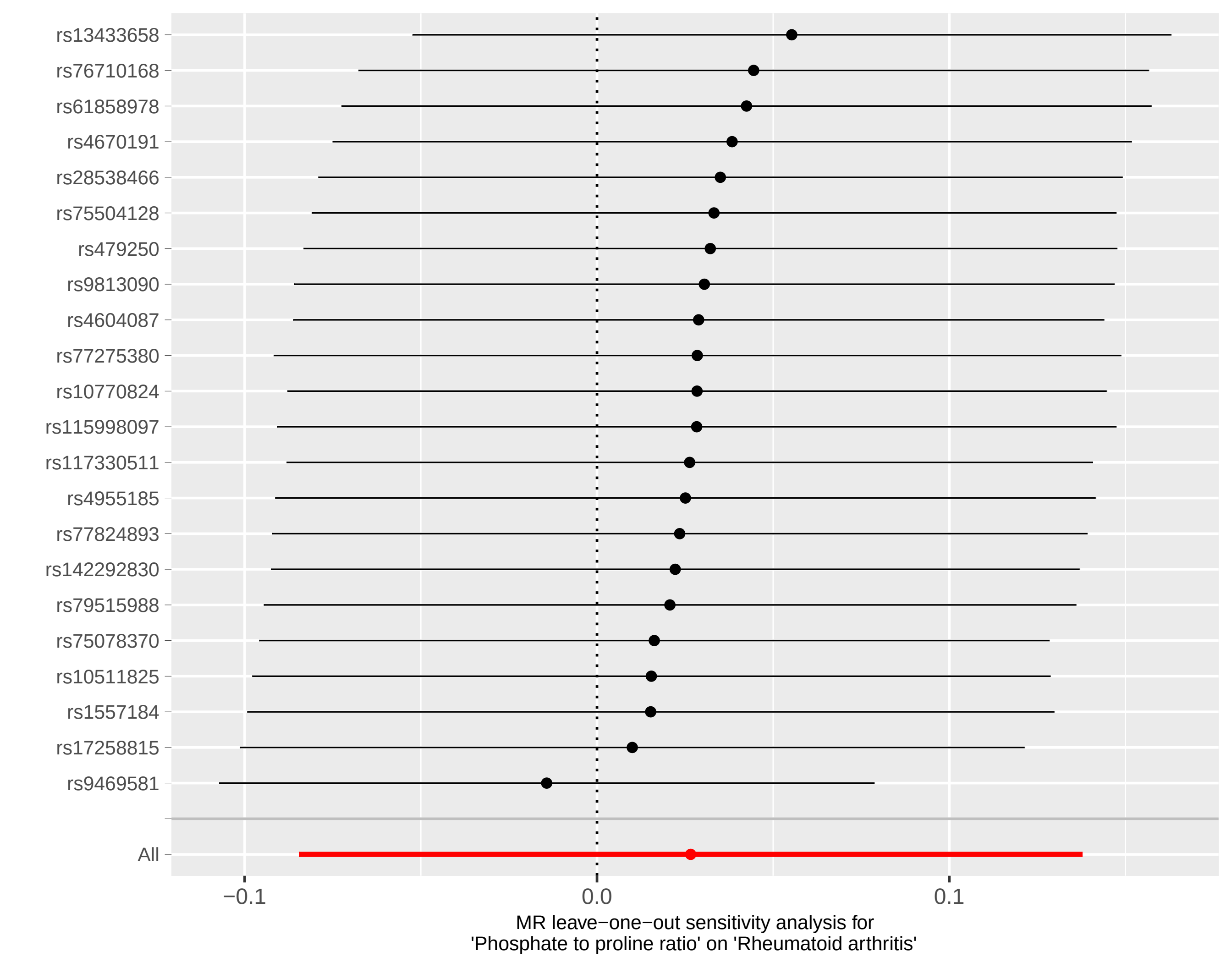
rs13433658
rs76710168
rs61858978
rs4670191
rs28538466
rs75504128
rs479250
rs9813090
rs4604087
rs77275380
rs10770824
rs115998097
rs117330511
rs4955185
rs77824893
rs142292830
rs79515988
rs75078370
rs10511825
rs1557184
rs17258815
rs9469581
All
−0.1
0.0
0.1
MR leave−one−out sensitivity analysis for
'Phosphate to proline ratio' on 'Rheumatoid arthritis'

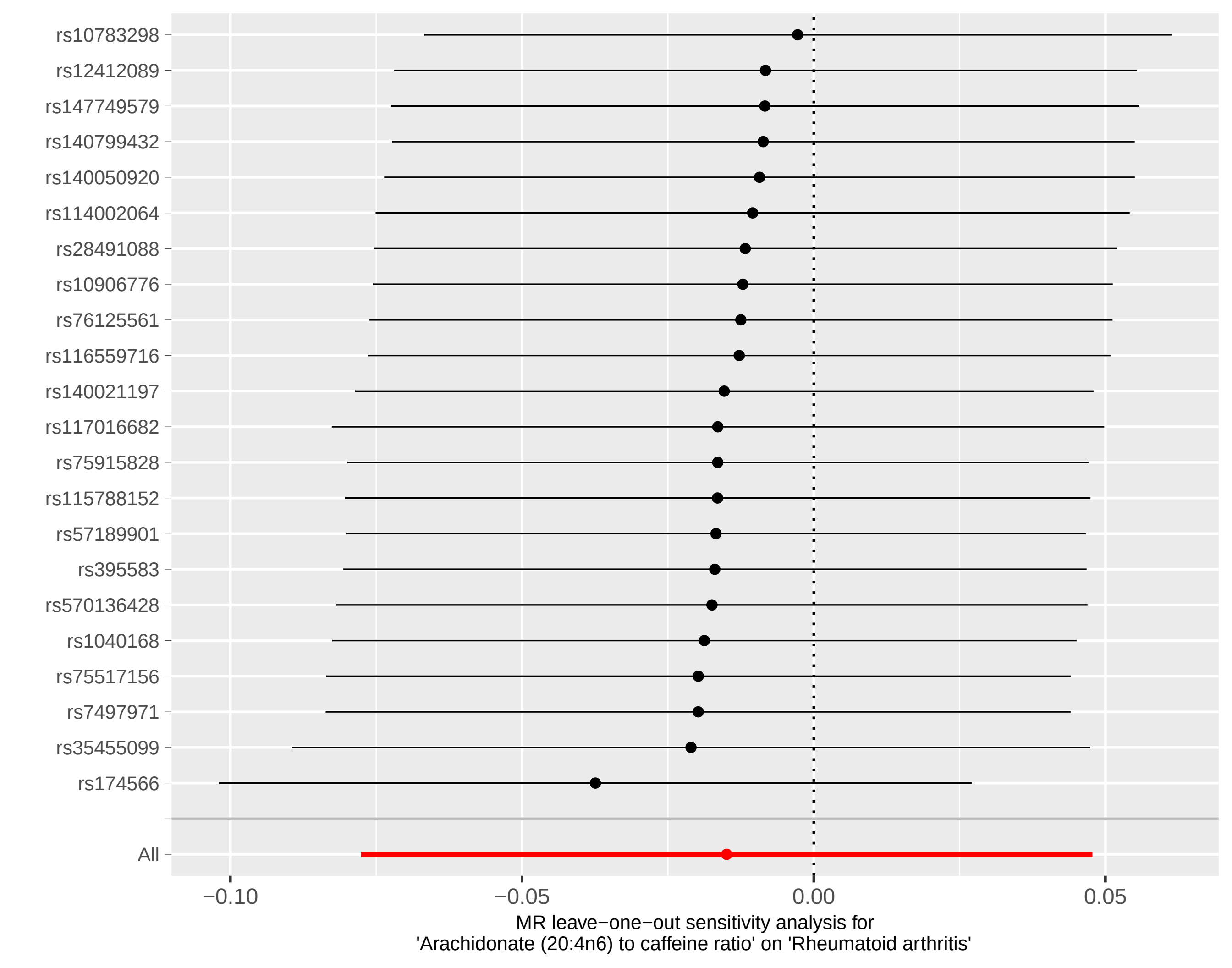

rs10783298
rs12412089
rs147749579
rs140799432
rs140050920
rs114002064
rs28491088
rs10906776
rs76125561
rs116559716
rs140021197
rs117016682
rs75915828
rs115788152
rs57189901
rs395583
rs570136428
rs1040168
rs75517156
rs7497971
rs35455099
rs174566
All
−0.10
−0.05
0.00
0.05
MR leave−one−out sensitivity analysis for
'Arachidonate (20:4n6) to caffeine ratio' on 'Rheumatoid arthritis'

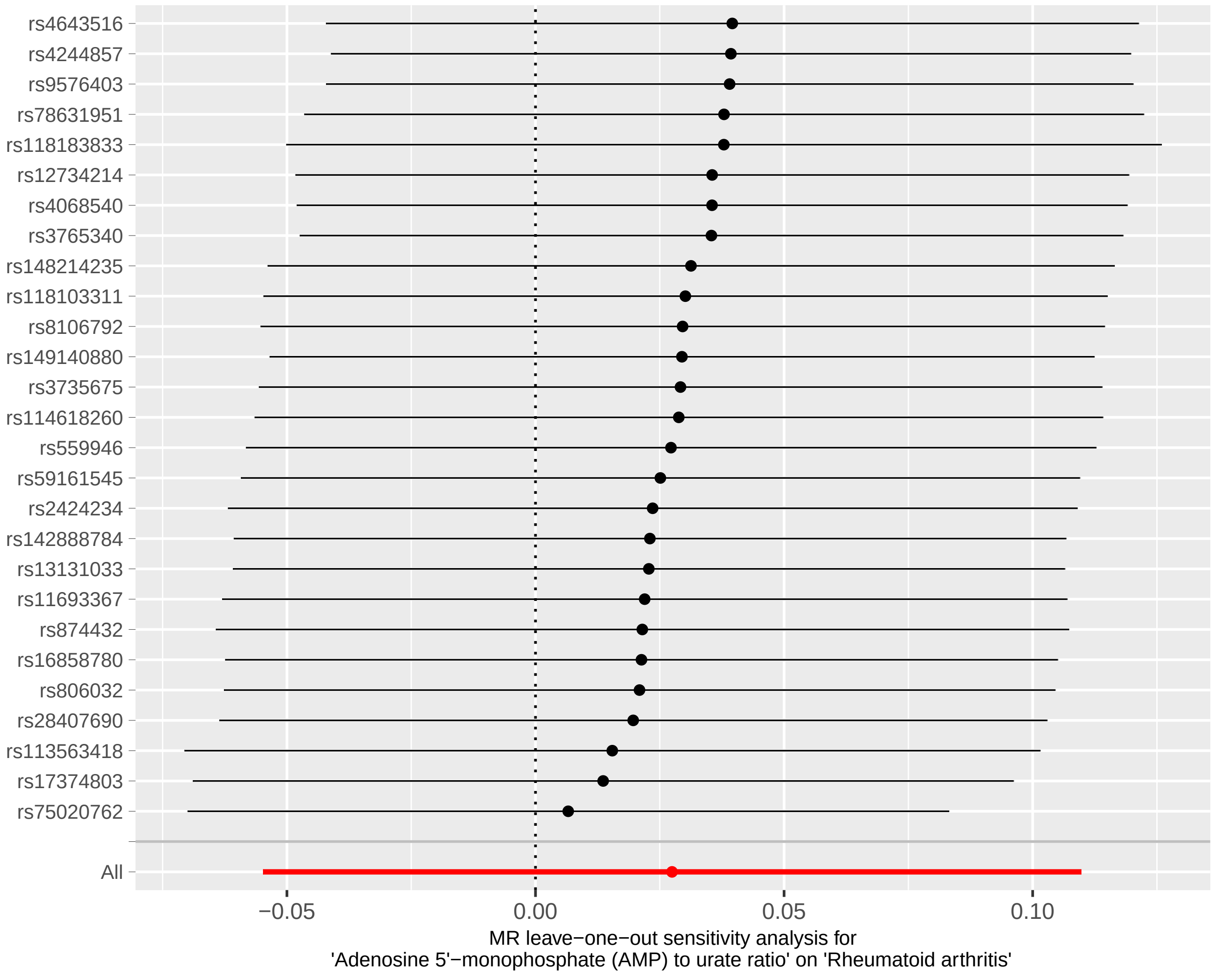

rs4643516
rs4244857
rs9576403
rs78631951
rs118183833
rs12734214
rs4068540
rs3765340
rs148214235
rs118103311
rs8106792
rs149140880
rs3735675
rs114618260
rs559946
rs59161545
rs2424234
rs142888784
rs13131033
rs11693367
rs874432
rs16858780
rs806032
rs28407690
rs113563418
rs17374803
rs75020762
All
−0.05
0.00
0.05
0.10
MR leave−one−out sensitivity analysis for
'Adenosine 5'−monophosphate (AMP) to urate ratio' on 'Rheumatoid arthritis'

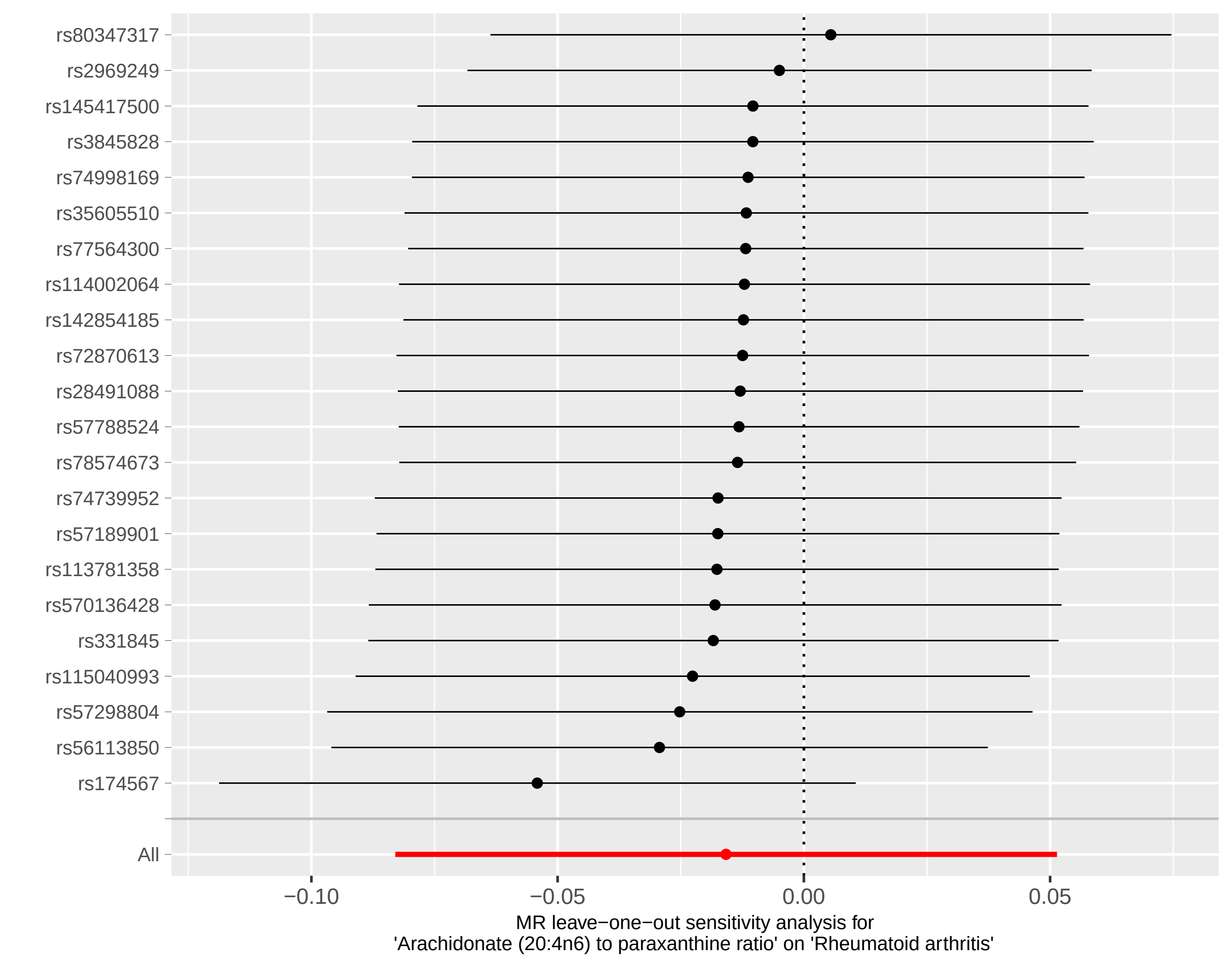
rs80347317
rs2969249
rs145417500
rs3845828
rs74998169
rs35605510
rs77564300
rs114002064
rs142854185
rs72870613
rs28491088
rs57788524
rs78574673
rs74739952
rs57189901
rs113781358
rs570136428
rs331845
rs115040993
rs57298804
rs56113850
rs174567
All
−0.10
−0.05
0.00
0.05
MR leave−one−out sensitivity analysis for
'Arachidonate (20:4n6) to paraxanthine ratio' on 'Rheumatoid arthritis'

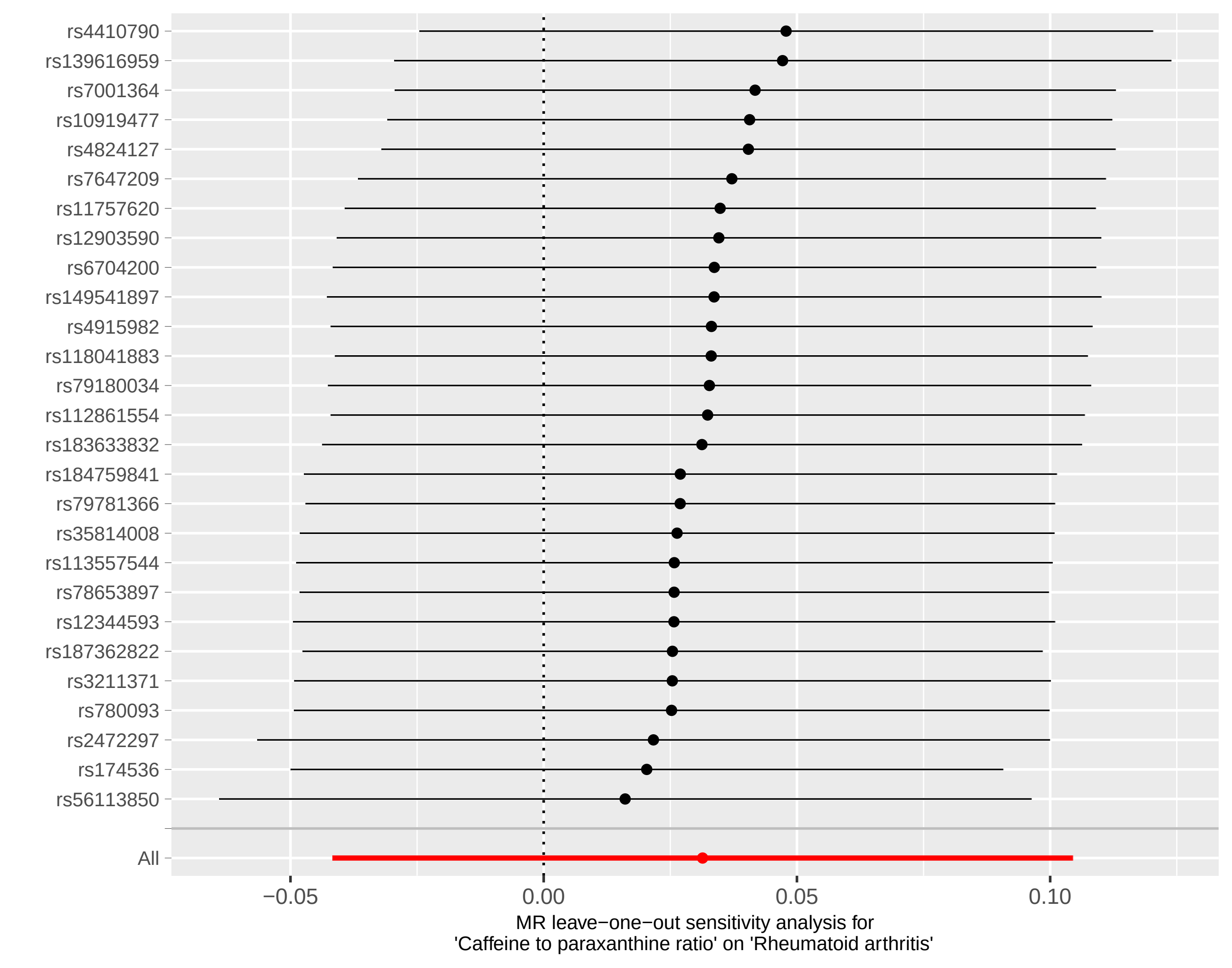
rs4410790
rs139616959
rs7001364
rs10919477
rs4824127
rs7647209
rs11757620
rs12903590
rs6704200
rs149541897
rs4915982
rs118041883
rs79180034
rs112861554
rs183633832
rs184759841
rs79781366
rs35814008
rs113557544
rs78653897
rs12344593
rs187362822
rs3211371
rs780093
rs2472297
rs174536
rs56113850
All
−0.05
0.00
0.05
0.10
MR leave−one−out sensitivity analysis for
'Caffeine to paraxanthine ratio' on 'Rheumatoid arthritis'

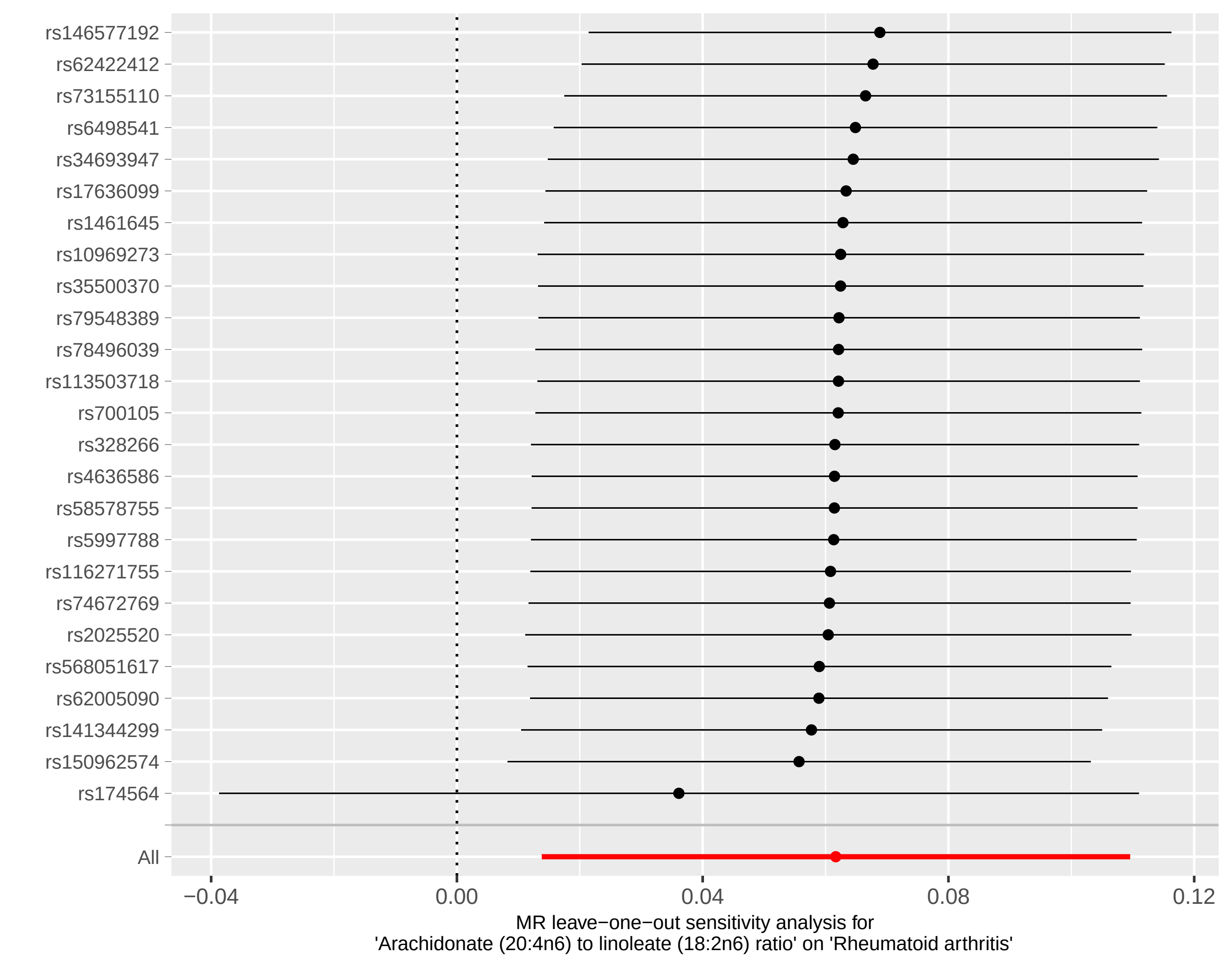
rs146577192
rs62422412
rs73155110
rs6498541
rs34693947
rs17636099
rs1461645
rs10969273
rs35500370
rs79548389
rs78496039
rs113503718
rs700105
rs328266
rs4636586
rs58578755
rs5997788
rs116271755
rs74672769
rs2025520
rs568051617
rs62005090
rs141344299
rs150962574
rs174564
All
−0.04
0.00
0.04
0.08
0.12
MR leave−one−out sensitivity analysis for
'Arachidonate (20:4n6) to linoleate (18:2n6) ratio' on 'Rheumatoid arthritis'

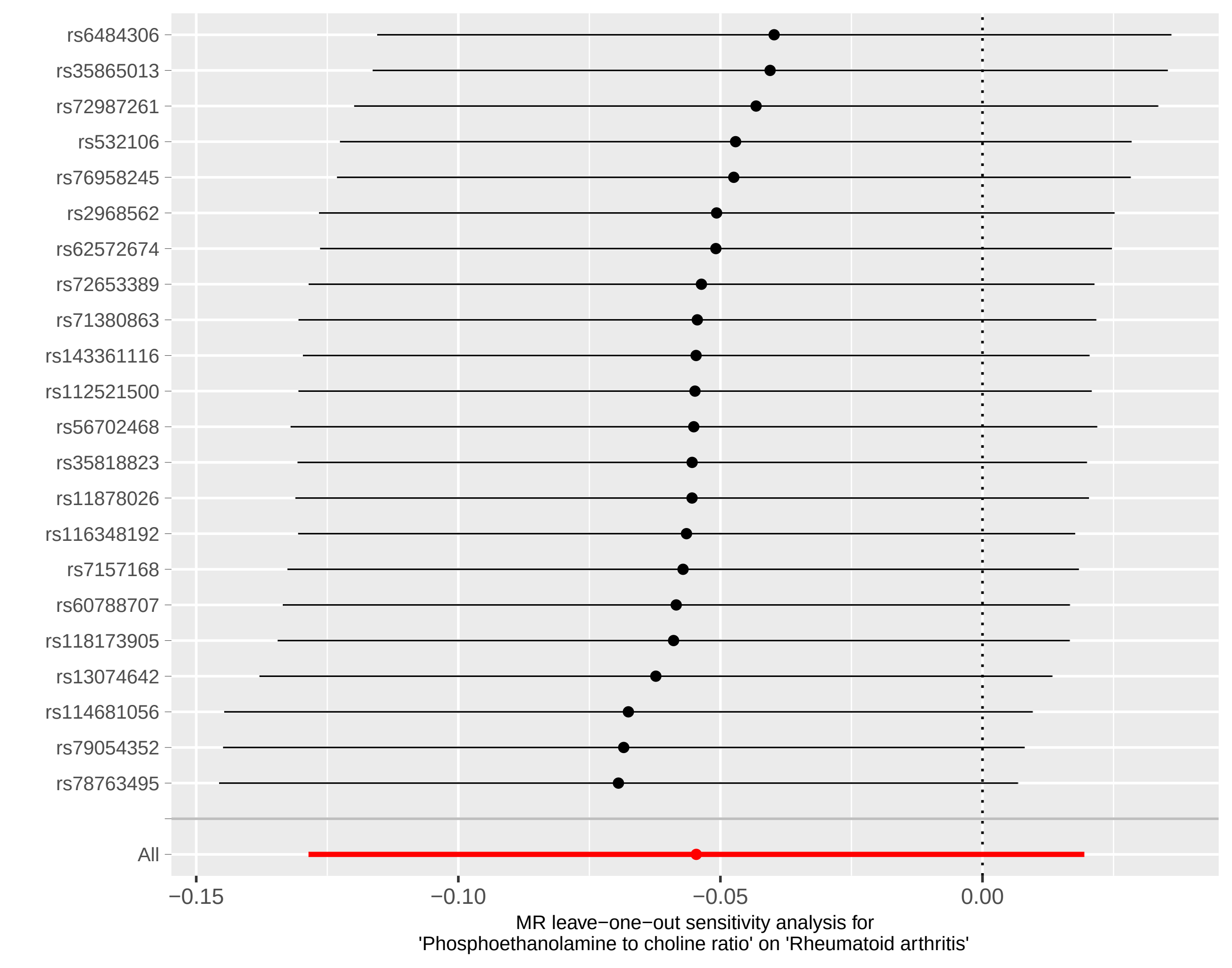
rs6484306
rs35865013
rs72987261
rs532106
rs76958245
rs2968562
rs62572674
rs72653389
rs71380863
rs143361116
rs112521500
rs56702468
rs35818823
rs11878026
rs116348192
rs7157168
rs60788707
rs118173905
rs13074642
rs114681056
rs79054352
rs78763495
All
−0.15
−0.10
−0.05
0.00
MR leave−one−out sensitivity analysis for
'Phosphoethanolamine to choline ratio' on 'Rheumatoid arthritis'

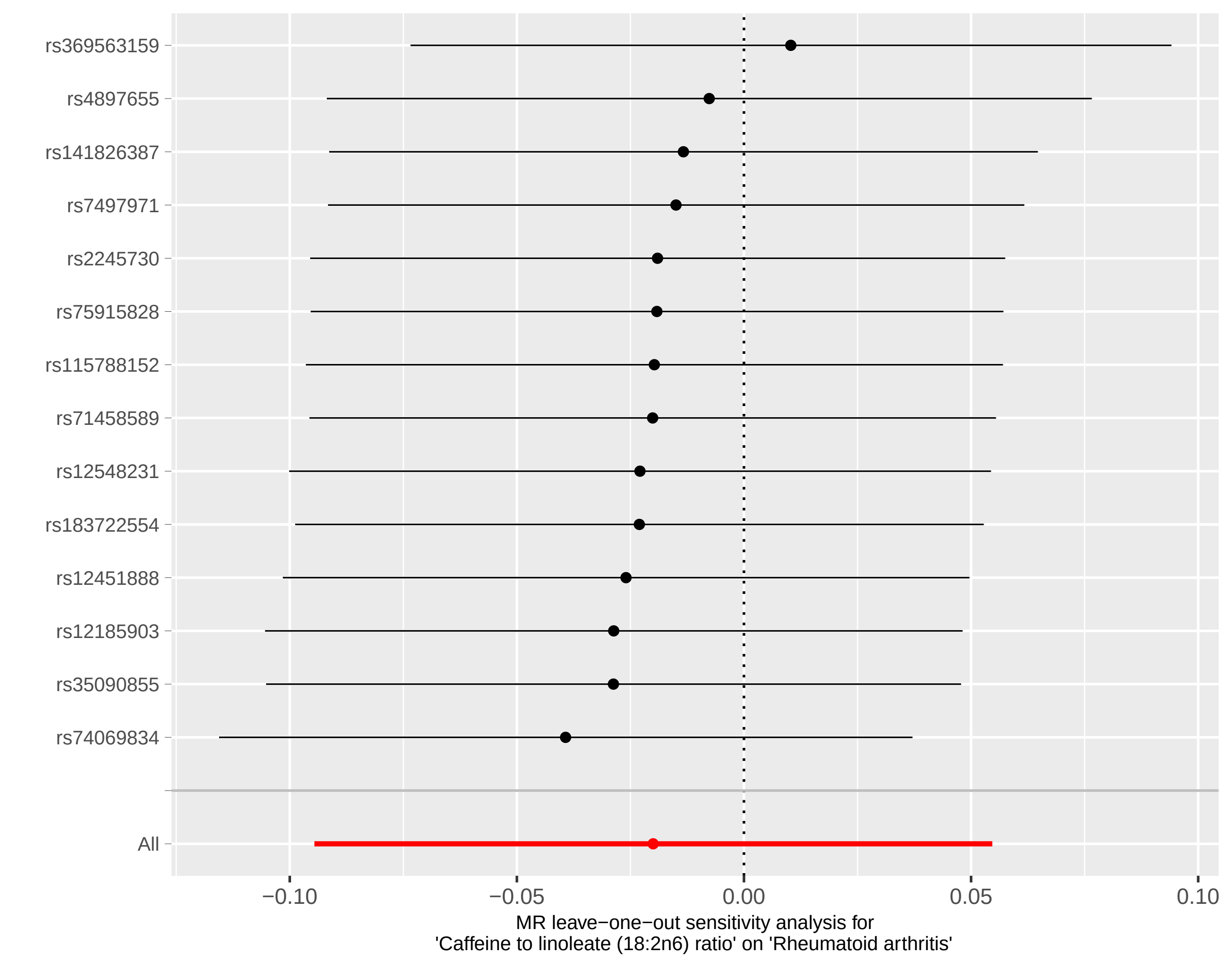
rs369563159
rs4897655
rs141826387
rs7497971
rs2245730
rs75915828
rs115788152
rs71458589
rs12548231
rs183722554
rs12451888
rs12185903
rs35090855
rs74069834
All
−0.10
−0.05
0.00
0.05
0.10
MR leave−one−out sensitivity analysis for
'Caffeine to linoleate (18:2n6) ratio' on 'Rheumatoid arthritis'

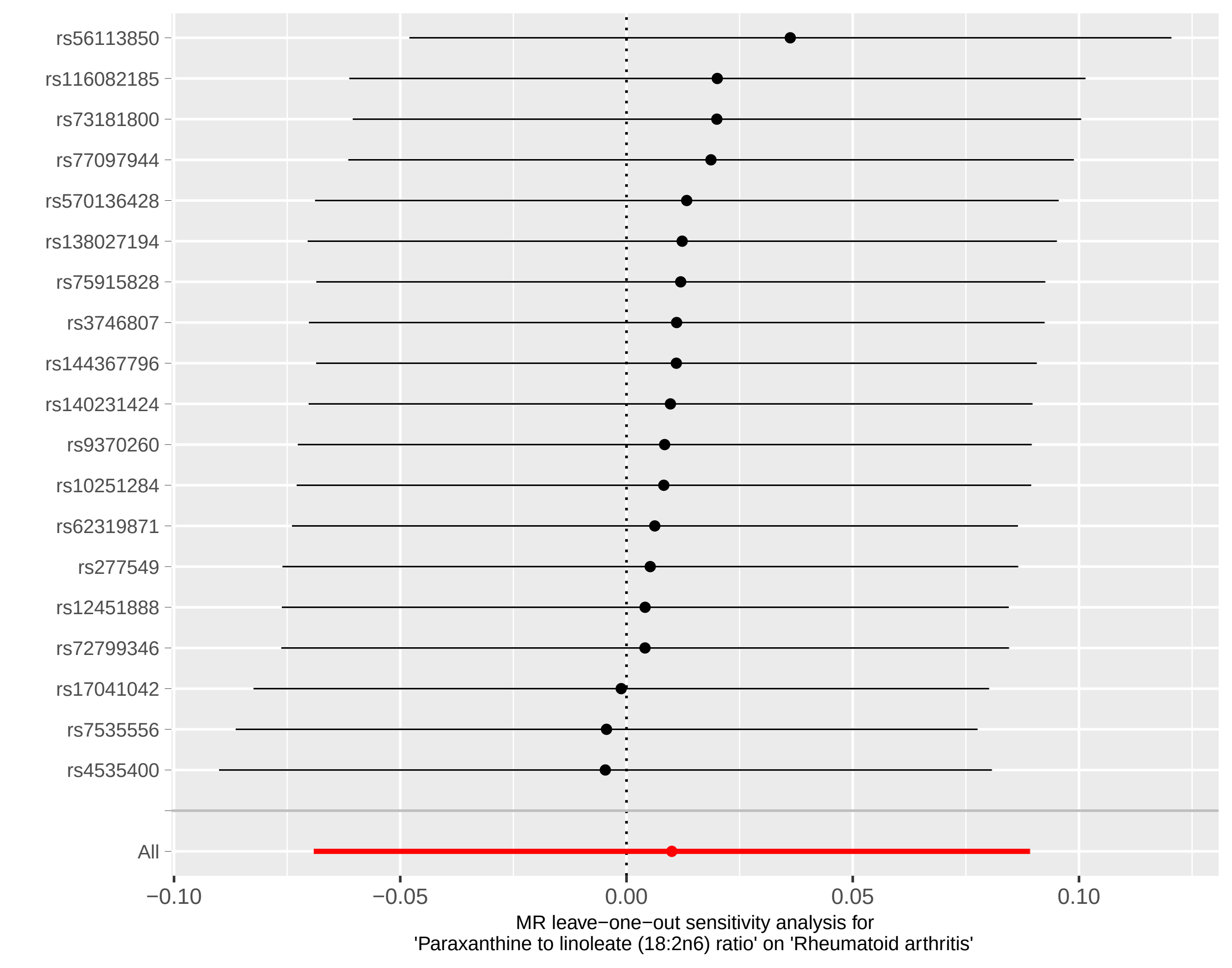
rs56113850
rs116082185
rs73181800
rs77097944
rs570136428
rs138027194
rs75915828
rs3746807
rs144367796
rs140231424
rs9370260
rs10251284
rs62319871
rs277549
rs12451888
rs72799346
rs17041042
rs7535556
rs4535400
All
−0.10
−0.05
0.00
0.05
0.10
MR leave−one−out sensitivity analysis for
'Paraxanthine to linoleate (18:2n6) ratio' on 'Rheumatoid arthritis'

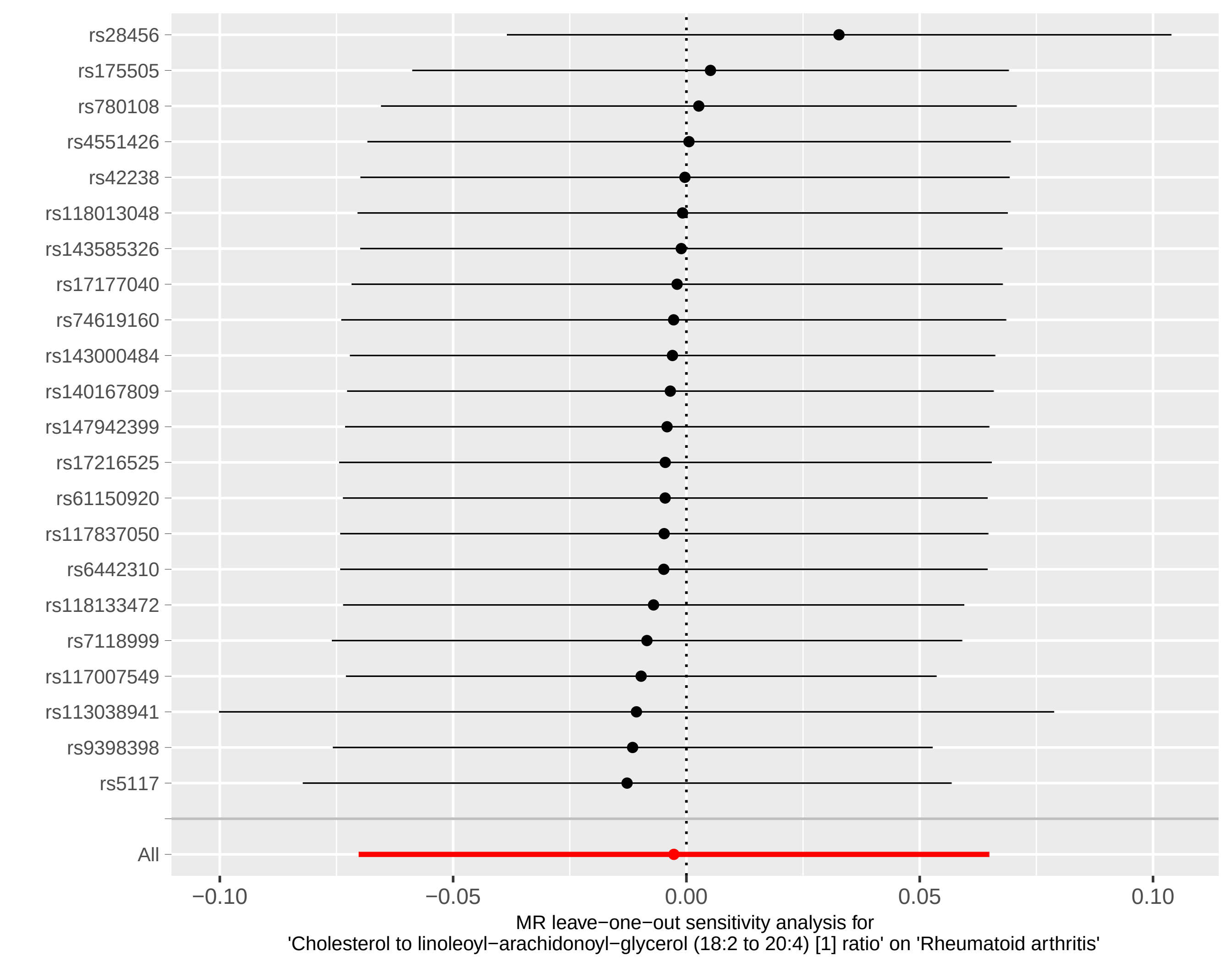
rs28456
rs175505
rs780108
rs4551426
rs42238
rs118013048
rs143585326
rs17177040
rs74619160
rs143000484
rs140167809
rs147942399
rs17216525
rs61150920
rs117837050
rs6442310
rs118133472
rs7118999
rs117007549
rs113038941
rs9398398
rs5117
All
−0.10
−0.05
0.00
0.05
0.10
MR leave−one−out sensitivity analysis for
'Cholesterol to linoleoyl−arachidonoyl−glycerol (18:2 to 20:4) [1] ratio' on 'Rheumatoid arthritis'

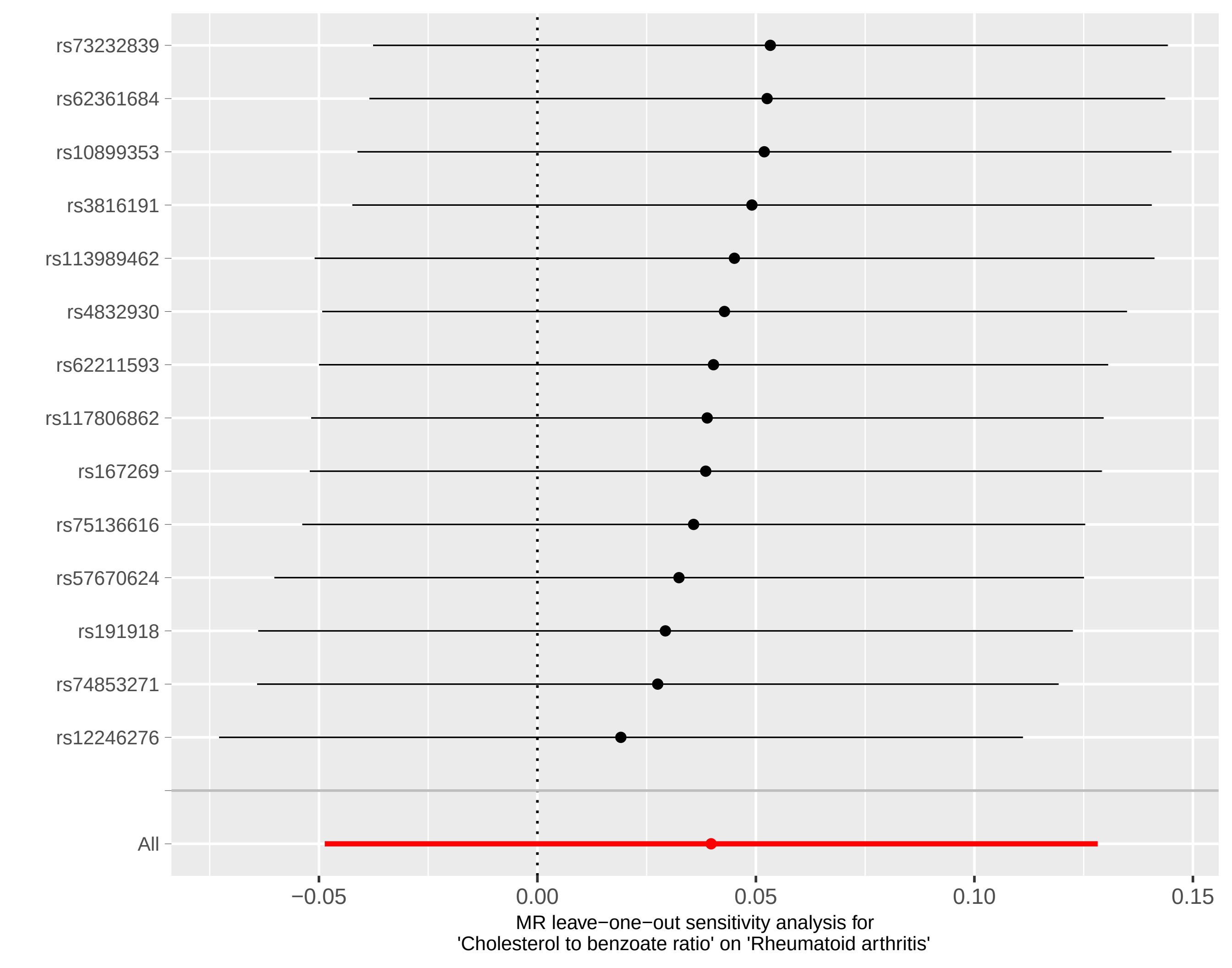
rs73232839
rs62361684
rs10899353
rs3816191
rs113989462
rs4832930
rs62211593
rs117806862
rs167269
rs75136616
rs57670624
rs191918
rs74853271
rs12246276
All
−0.05
0.00
0.05
0.10
0.15
MR leave−one−out sensitivity analysis for
'Cholesterol to benzoate ratio' on 'Rheumatoid arthritis'

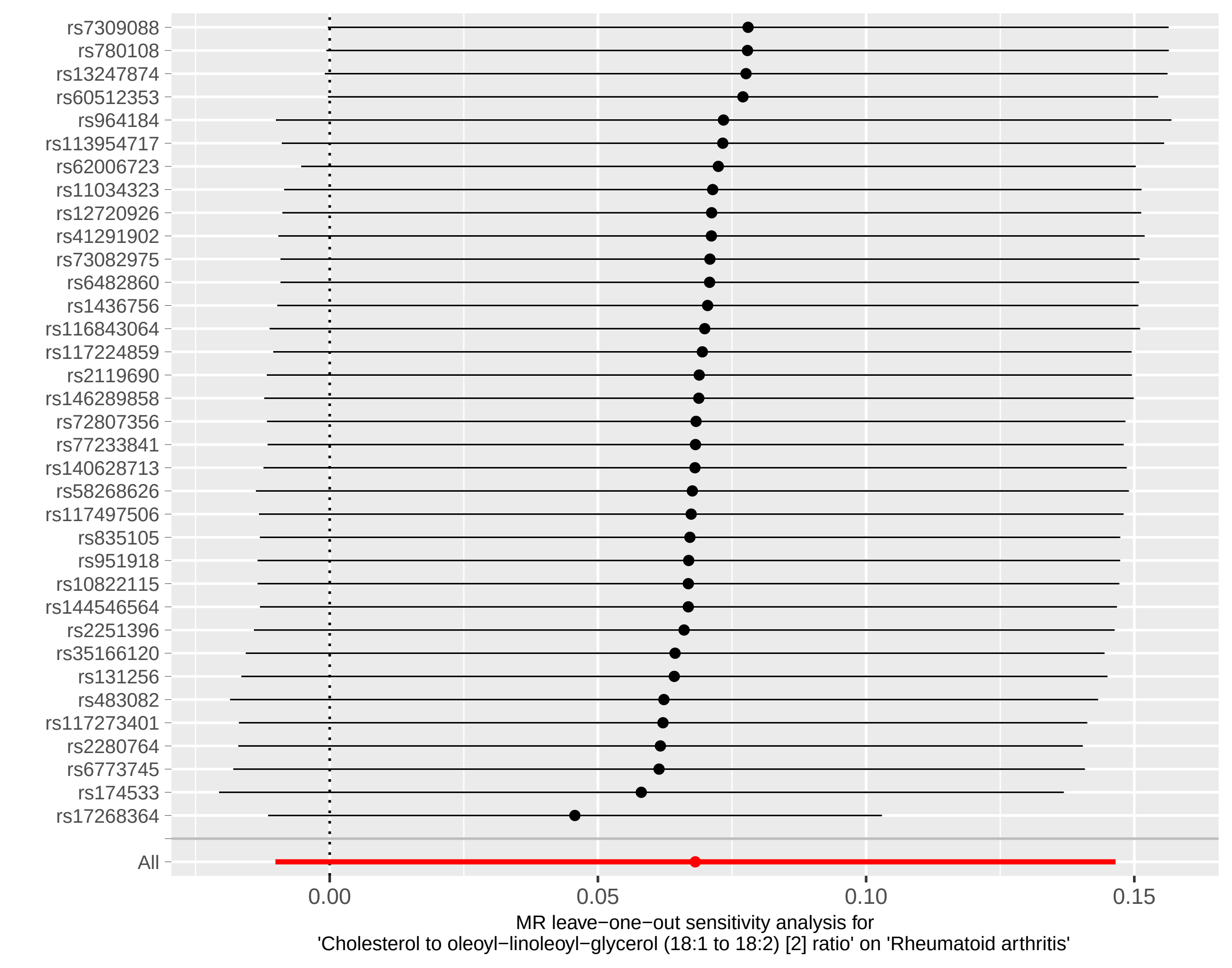
rs7309088
rs780108
rs13247874
rs60512353
rs964184
rs113954717
rs62006723
rs11034323
rs12720926
rs41291902
rs73082975
rs6482860
rs1436756
rs116843064
rs117224859
rs2119690
rs146289858
rs72807356
rs77233841
rs140628713
rs58268626
rs117497506
rs835105
rs951918
rs10822115
rs144546564
rs2251396
rs35166120
rs131256
rs483082
rs117273401
rs2280764
rs6773745
rs174533
rs17268364
All
0.00
0.05
0.10
0.15
MR leave−one−out sensitivity analysis for
'Cholesterol to oleoyl−linoleoyl−glycerol (18:1 to 18:2) [2] ratio' on 'Rheumatoid arthritis'

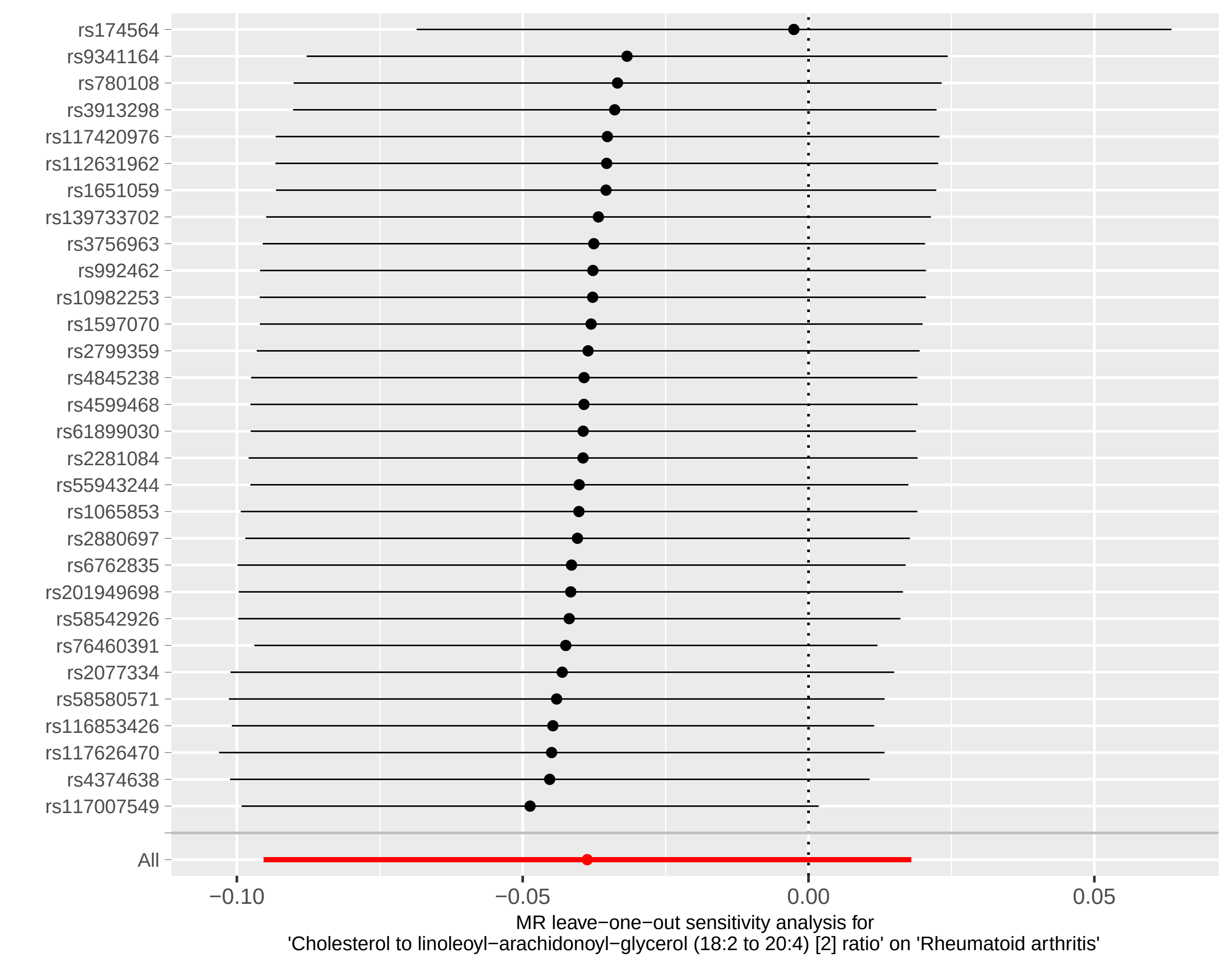

rs174564
rs9341164
rs780108
rs3913298
rs117420976
rs112631962
rs1651059
rs139733702
rs3756963
rs992462
rs10982253
rs1597070
rs2799359
rs4845238
rs4599468
rs61899030
rs2281084
rs55943244
rs1065853
rs2880697
rs6762835
rs201949698
rs58542926
rs76460391
rs2077334
rs58580571
rs116853426
rs117626470
rs4374638
rs117007549
All
−0.10
−0.05
0.00
0.05
MR leave−one−out sensitivity analysis for
'Cholesterol to linoleoyl−arachidonoyl−glycerol (18:2 to 20:4) [2] ratio' on 'Rheumatoid arthritis'

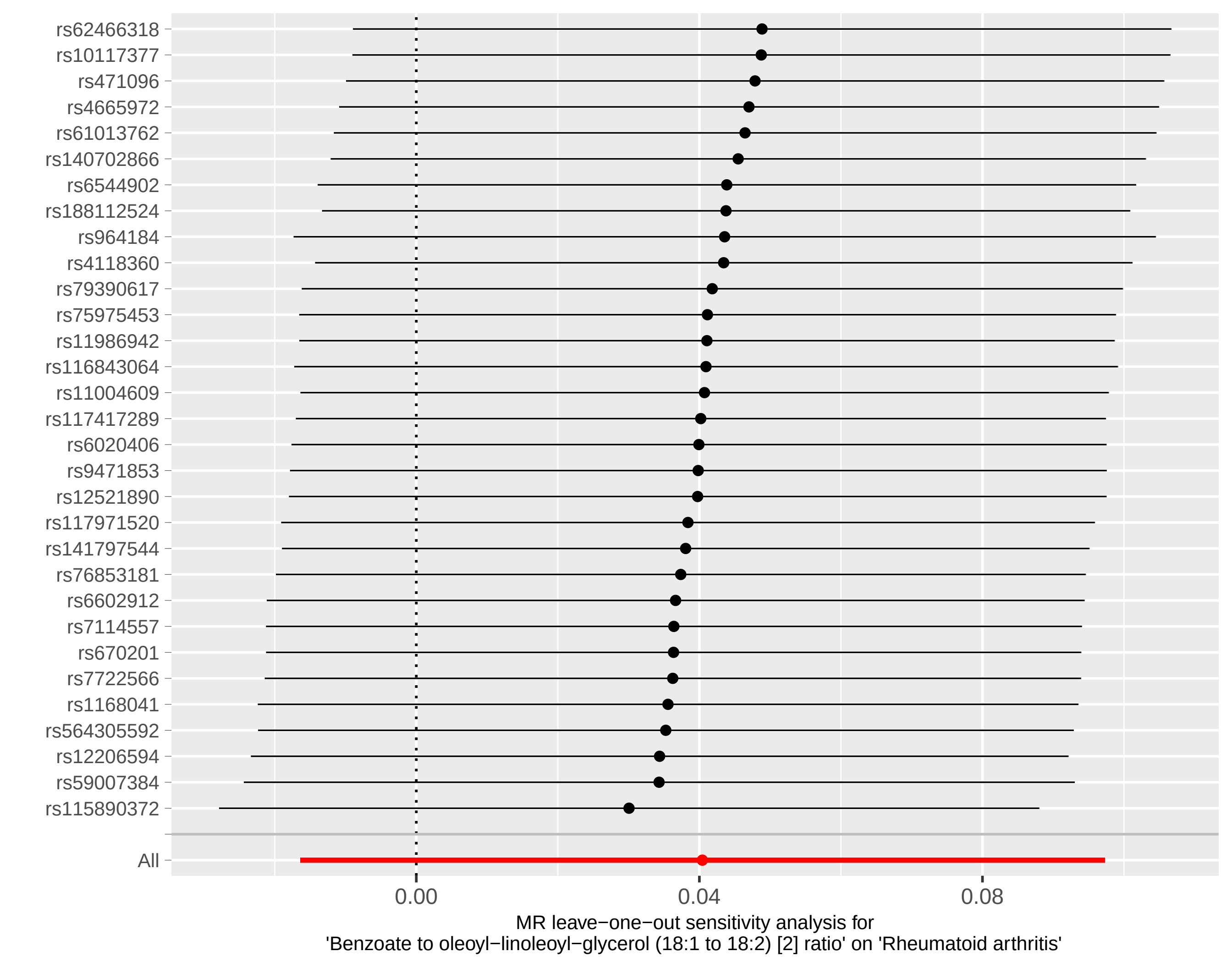
rs62466318
rs10117377
rs471096
rs4665972
rs61013762
rs140702866
rs6544902
rs188112524
rs964184
rs4118360
rs79390617
rs75975453
rs11986942
rs116843064
rs11004609
rs117417289
rs6020406
rs9471853
rs12521890
rs117971520
rs141797544
rs76853181
rs6602912
rs7114557
rs670201
rs7722566
rs1168041
rs564305592
rs12206594
rs59007384
rs115890372
All
0.00
0.04
0.08
MR leave−one−out sensitivity analysis for
'Benzoate to oleoyl−linoleoyl−glycerol (18:1 to 18:2) [2] ratio' on 'Rheumatoid arthritis'

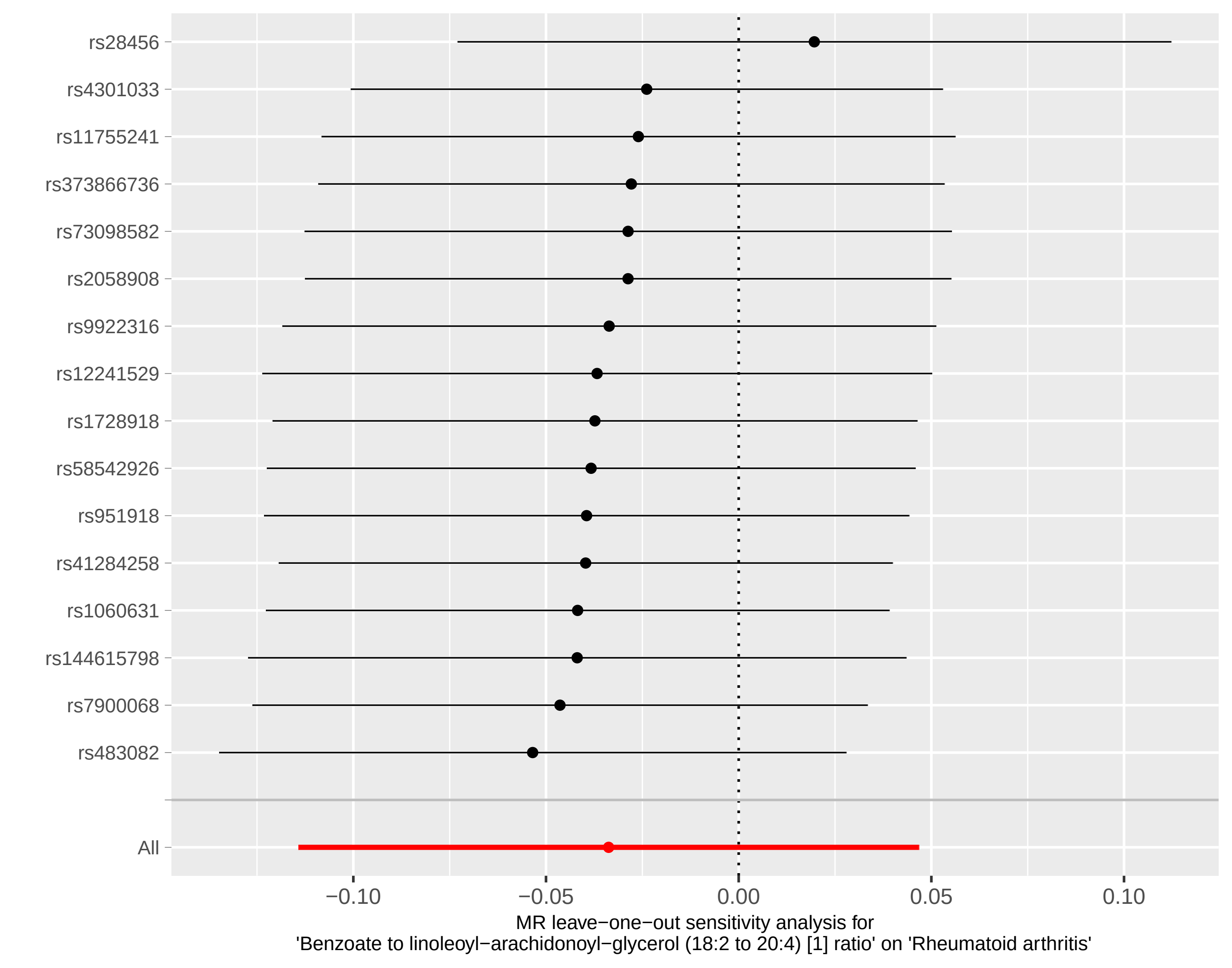

rs28456
rs4301033
rs11755241
rs373866736
rs73098582
rs2058908
rs9922316
rs12241529
rs1728918
rs58542926
rs951918
rs41284258
rs1060631
rs144615798
rs7900068
rs483082
All
−0.10
−0.05
0.00
0.05
0.10
MR leave−one−out sensitivity analysis for
'Benzoate to linoleoyl−arachidonoyl−glycerol (18:2 to 20:4) [1] ratio' on 'Rheumatoid arthritis'

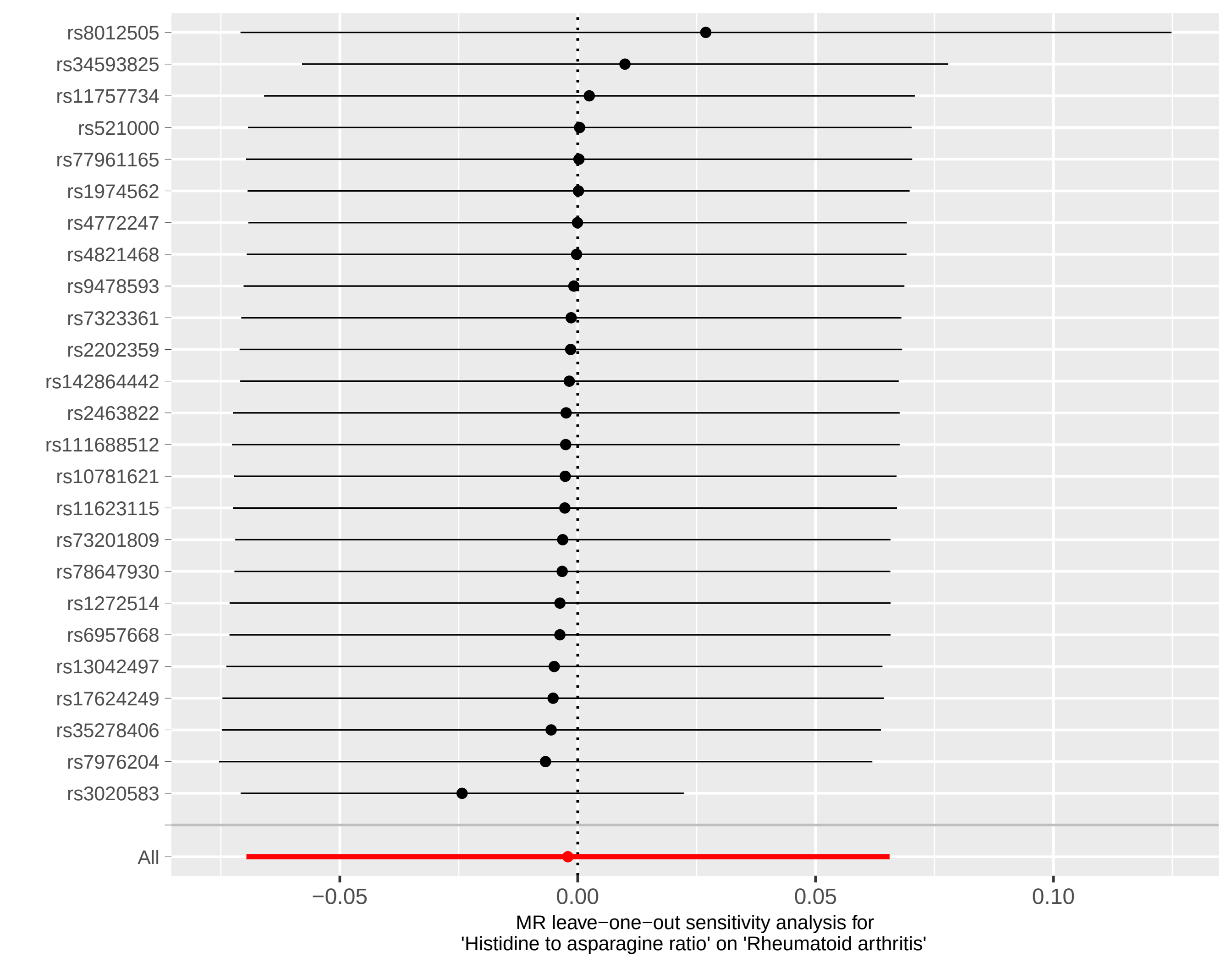
rs8012505
rs34593825
rs11757734
rs521000
rs77961165
rs1974562
rs4772247
rs4821468
rs9478593
rs7323361
rs2202359
rs142864442
rs2463822
rs111688512
rs10781621
rs11623115
rs73201809
rs78647930
rs1272514
rs6957668
rs13042497
rs17624249
rs35278406
rs7976204
rs3020583
All
−0.05
0.00
0.05
0.10
MR leave−one−out sensitivity analysis for
'Histidine to asparagine ratio' on 'Rheumatoid arthritis'

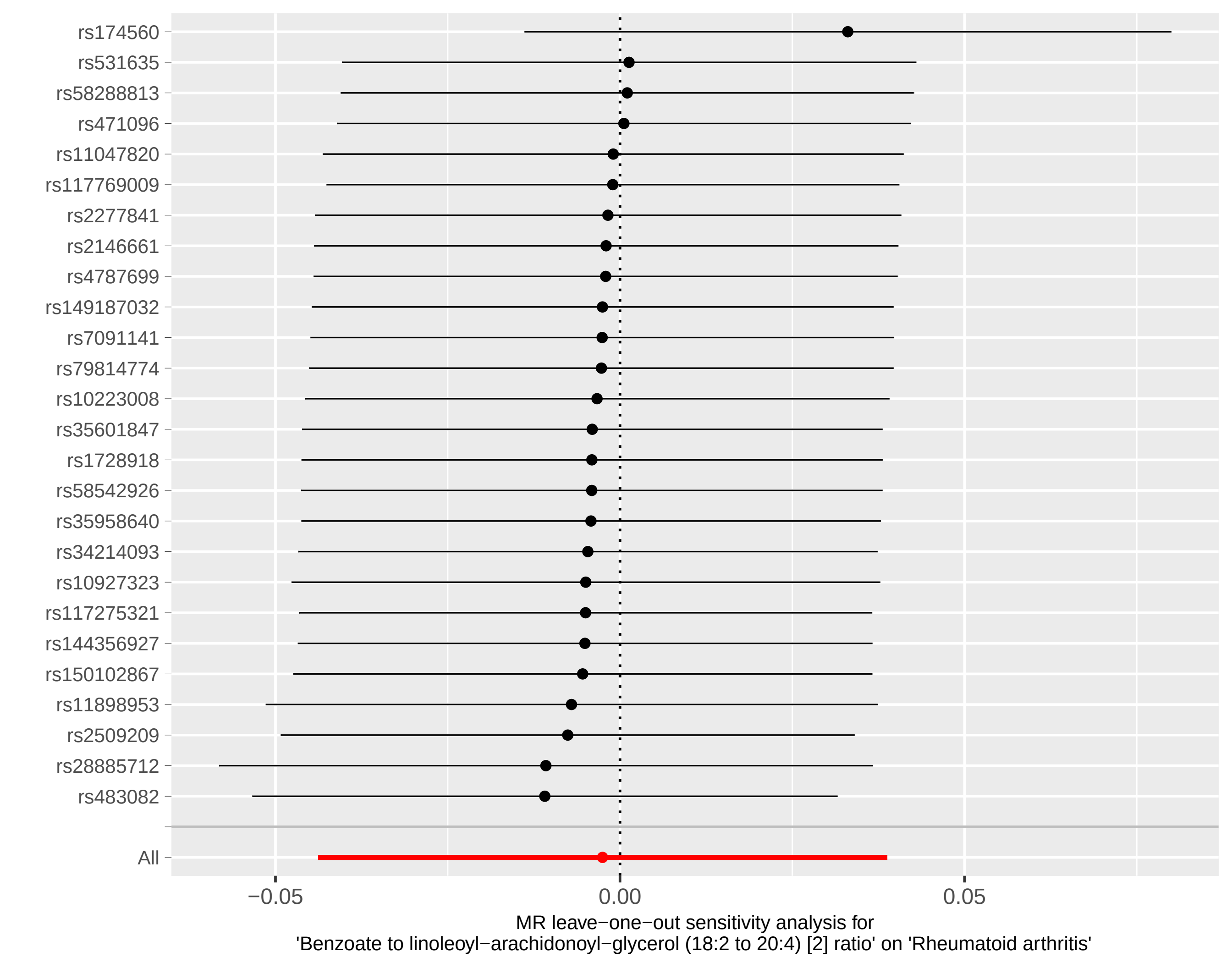

rs174560
rs531635
rs58288813
rs471096
rs11047820
rs117769009
rs2277841
rs2146661
rs4787699
rs149187032
rs7091141
rs79814774
rs10223008
rs35601847
rs1728918
rs58542926
rs35958640
rs34214093
rs10927323
rs117275321
rs144356927
rs150102867
rs11898953
rs2509209
rs28885712
rs483082
All
−0.05
0.00
0.05
MR leave−one−out sensitivity analysis for
'Benzoate to linoleoyl−arachidonoyl−glycerol (18:2 to 20:4) [2] ratio' on 'Rheumatoid arthritis'

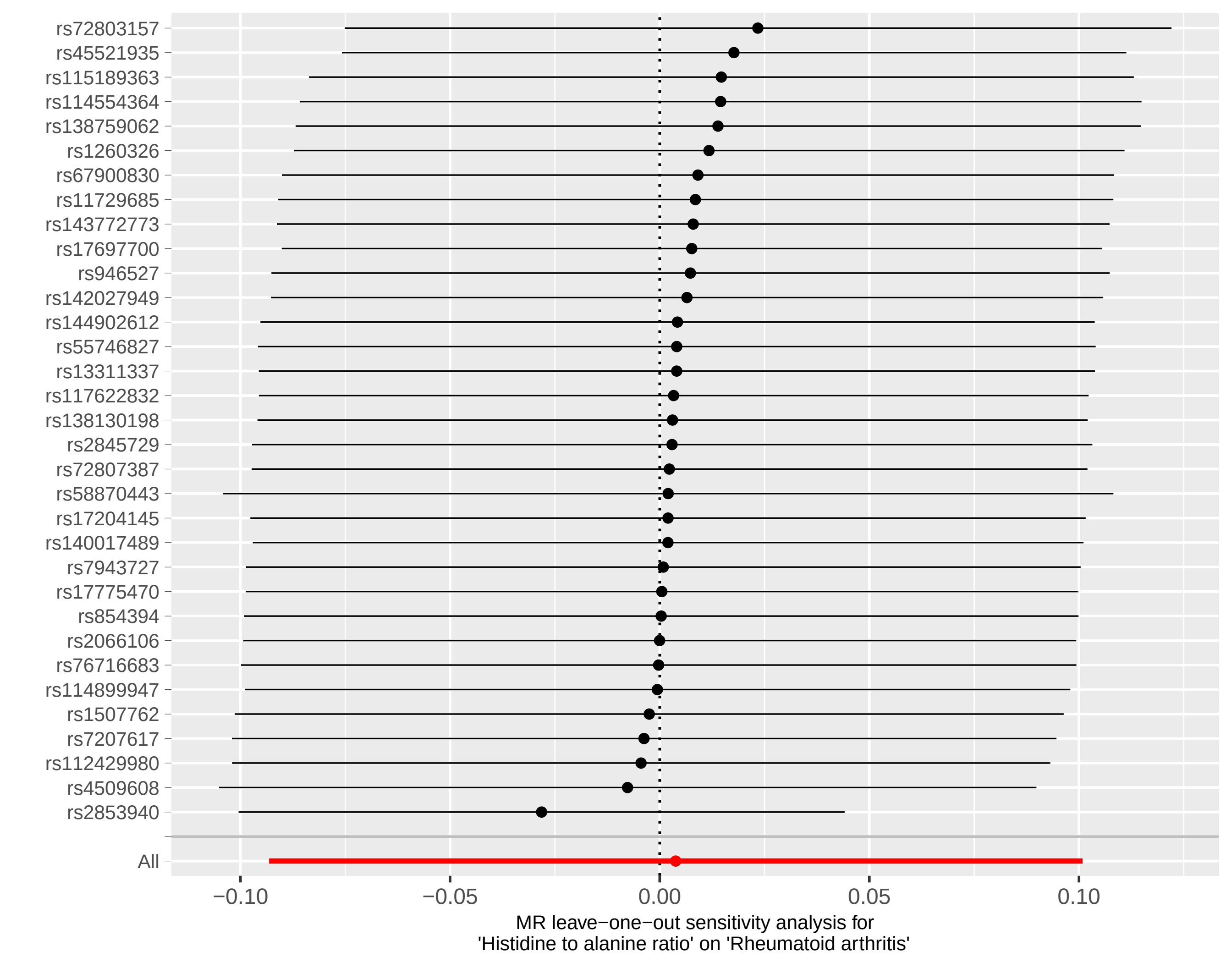
rs72803157
rs45521935
rs115189363
rs114554364
rs138759062
rs1260326
rs67900830
rs11729685
rs143772773
rs17697700
rs946527
rs142027949
rs144902612
rs55746827
rs13311337
rs117622832
rs138130198
rs2845729
rs72807387
rs58870443
rs17204145
rs140017489
rs7943727
rs17775470
rs854394
rs2066106
rs76716683
rs114899947
rs1507762
rs7207617
rs112429980
rs4509608
rs2853940
All
−0.10
−0.05
0.00
0.05
0.10
MR leave−one−out sensitivity analysis for
'Histidine to alanine ratio' on 'Rheumatoid arthritis'

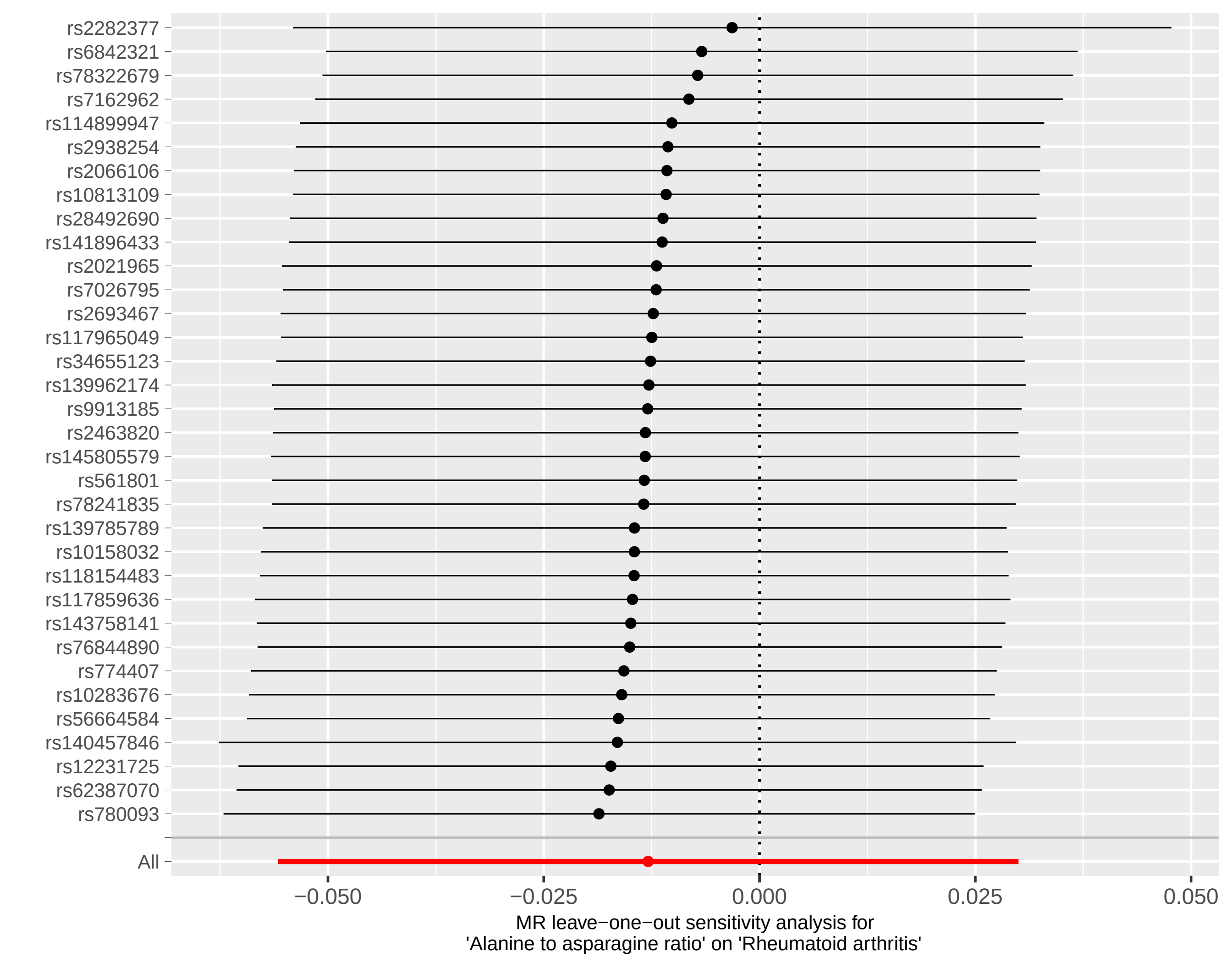
rs2282377
rs6842321
rs78322679
rs7162962
rs114899947
rs2938254
rs2066106
rs10813109
rs28492690
rs141896433
rs2021965
rs7026795
rs2693467
rs117965049
rs34655123
rs139962174
rs9913185
rs2463820
rs145805579
rs561801
rs78241835
rs139785789
rs10158032
rs118154483
rs117859636
rs143758141
rs76844890
rs774407
rs10283676
rs56664584
rs140457846
rs12231725
rs62387070
rs780093
All
−0.050
−0.025
0.000
0.025
0.050
MR leave−one−out sensitivity analysis for
'Alanine to asparagine ratio' on 'Rheumatoid arthritis'

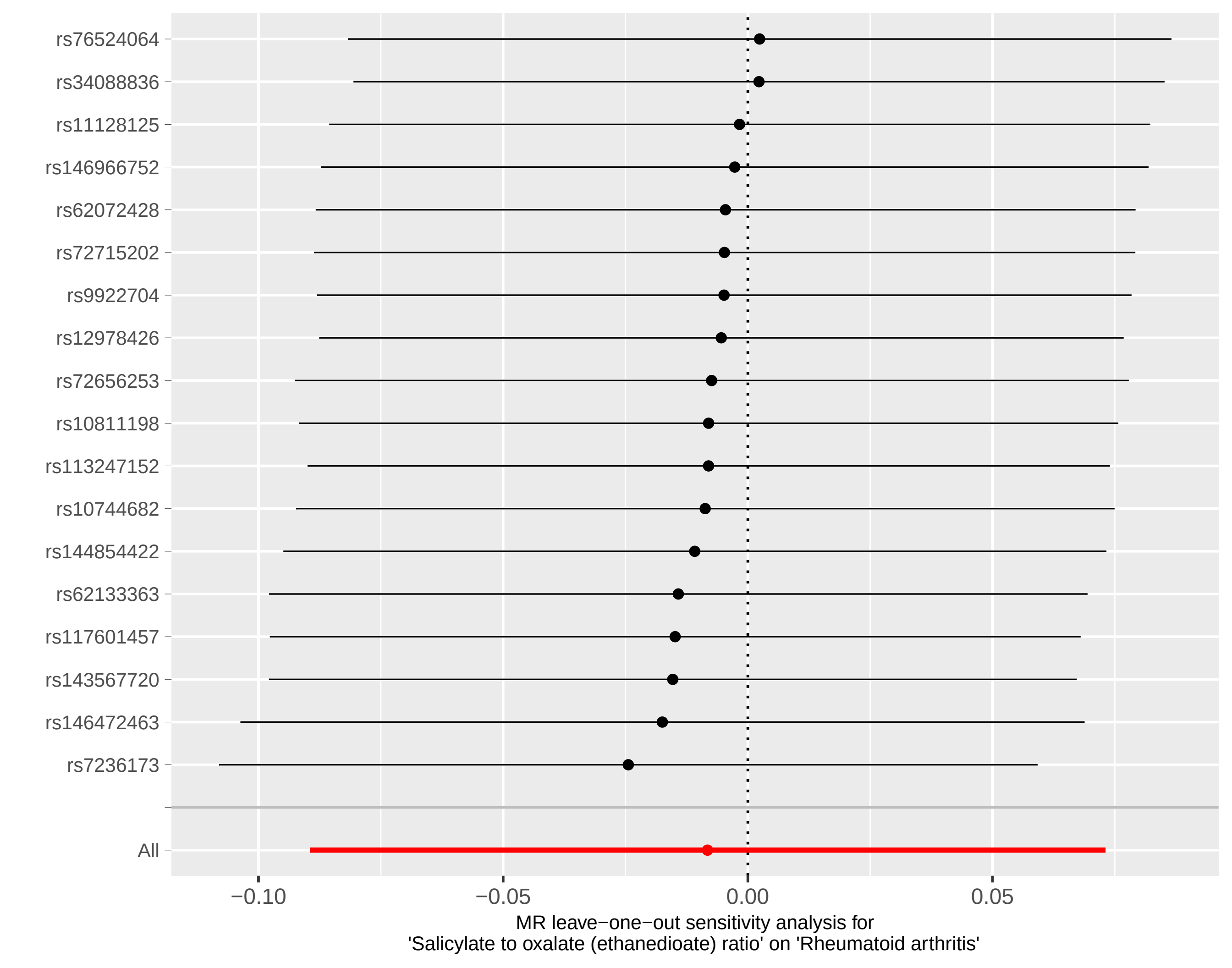
rs76524064
rs34088836
rs11128125
rs146966752
rs62072428
rs72715202
rs9922704
rs12978426
rs72656253
rs10811198
rs113247152
rs10744682
rs144854422
rs62133363
rs117601457
rs143567720
rs146472463
rs7236173
All
−0.10
−0.05
0.00
0.05
MR leave−one−out sensitivity analysis for
'Salicylate to oxalate (ethanedioate) ratio' on 'Rheumatoid arthritis'

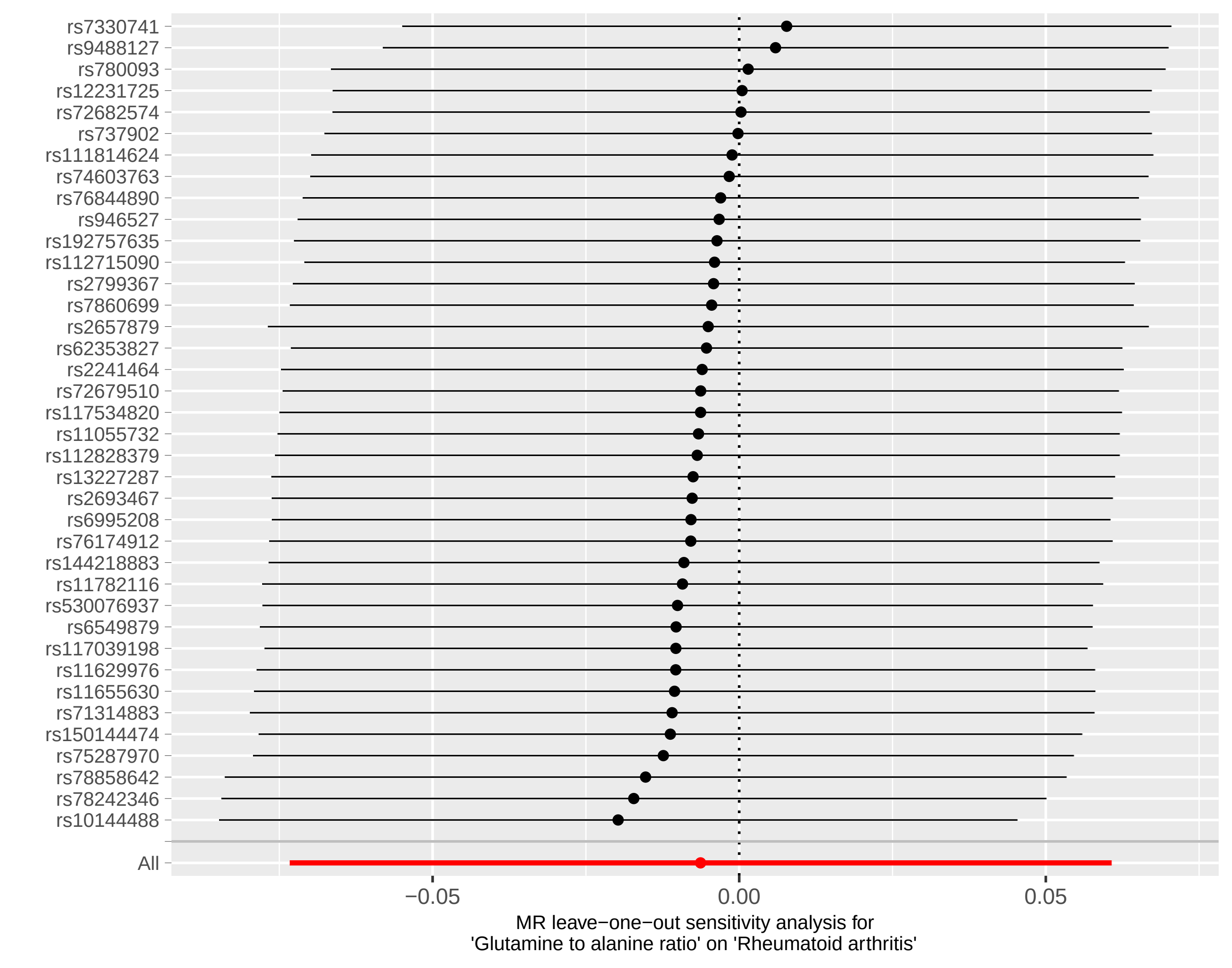
rs7330741
rs9488127
rs780093
rs12231725
rs72682574
rs737902
rs111814624
rs74603763
rs76844890
rs946527
rs192757635
rs112715090
rs2799367
rs7860699
rs2657879
rs62353827
rs2241464
rs72679510
rs117534820
rs11055732
rs112828379
rs13227287
rs2693467
rs6995208
rs76174912
rs144218883
rs11782116
rs530076937
rs6549879
rs117039198
rs11629976
rs11655630
rs71314883
rs150144474
rs75287970
rs78858642
rs78242346
rs10144488
All
−0.05
0.00
0.05
MR leave−one−out sensitivity analysis for
'Glutamine to alanine ratio' on 'Rheumatoid arthritis'

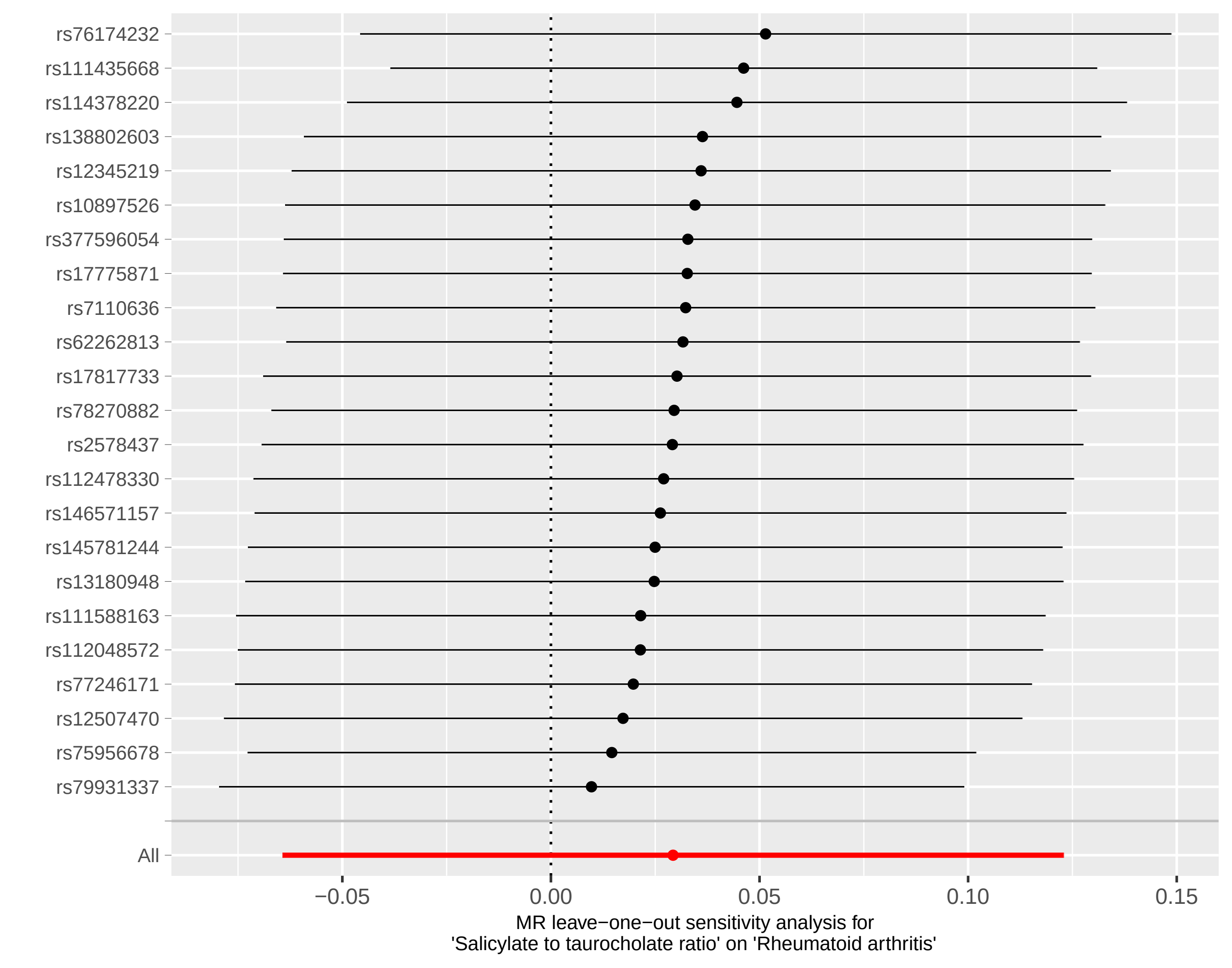

rs76174232
rs111435668
rs114378220
rs138802603
rs12345219
rs10897526
rs377596054
rs17775871
rs7110636
rs62262813
rs17817733
rs78270882
rs2578437
rs112478330
rs146571157
rs145781244
rs13180948
rs111588163
rs112048572
rs77246171
rs12507470
rs75956678
rs79931337
All
−0.05
0.00
0.05
0.10
0.15
MR leave−one−out sensitivity analysis for
'Salicylate to taurocholate ratio' on 'Rheumatoid arthritis'

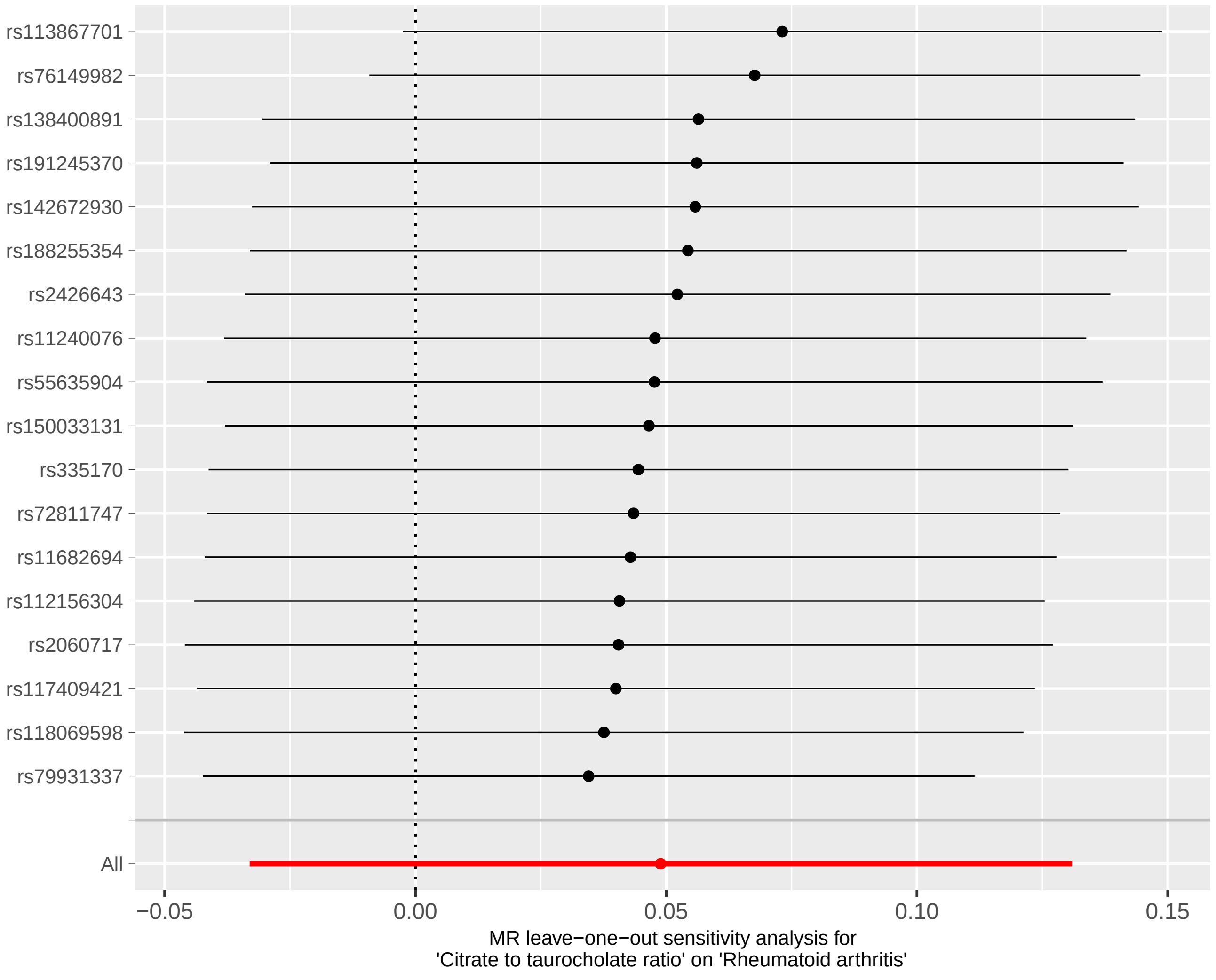
rs113867701
rs76149982
rs138400891
rs191245370
rs142672930
rs188255354
rs2426643
rs11240076
rs55635904
rs150033131
rs335170
rs72811747
rs11682694
rs112156304
rs2060717
rs117409421
rs118069598
rs79931337
All
−0.05
0.00
0.05
0.10
0.15
MR leave−one−out sensitivity analysis for
'Citrate to taurocholate ratio' on 'Rheumatoid arthritis'

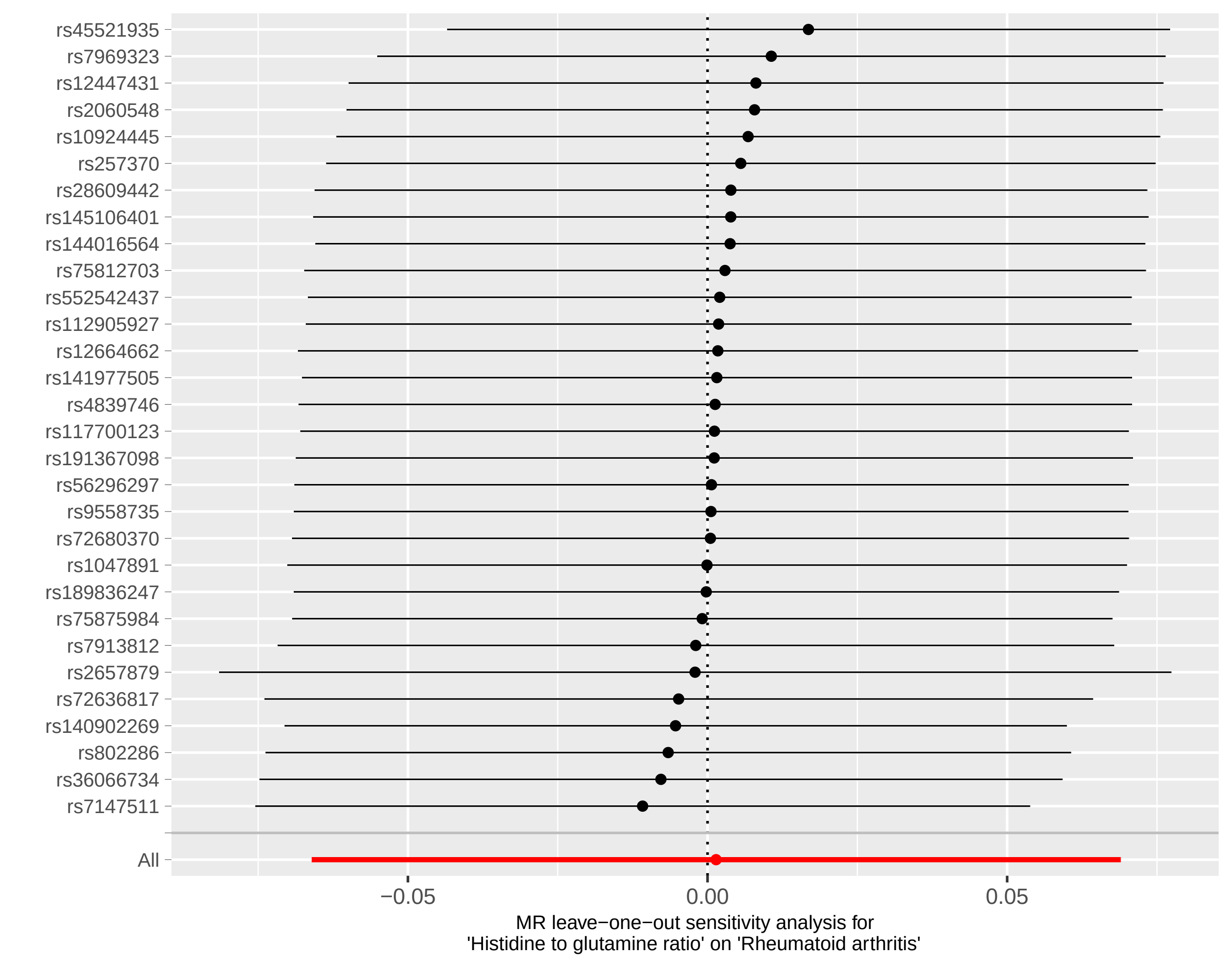
rs45521935
rs7969323
rs12447431
rs2060548
rs10924445
rs257370
rs28609442
rs145106401
rs144016564
rs75812703
rs552542437
rs112905927
rs12664662
rs141977505
rs4839746
rs117700123
rs191367098
rs56296297
rs9558735
rs72680370
rs1047891
rs189836247
rs75875984
rs7913812
rs2657879
rs72636817
rs140902269
rs802286
rs36066734
rs7147511
All
−0.05
0.00
0.05
MR leave−one−out sensitivity analysis for
'Histidine to glutamine ratio' on 'Rheumatoid arthritis'

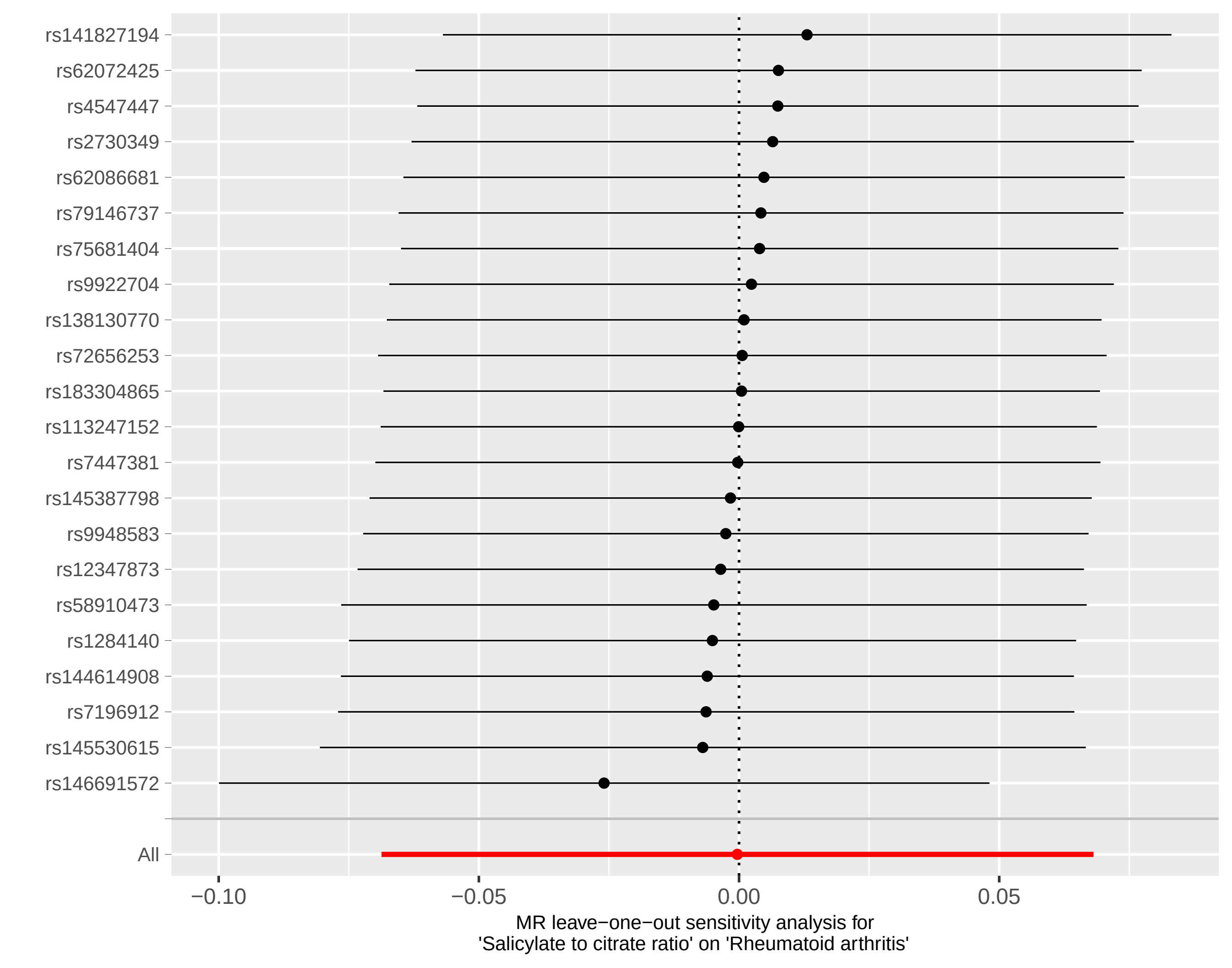
rs141827194
rs62072425
rs4547447
rs2730349
rs62086681
rs79146737
rs75681404
rs9922704
rs138130770
rs72656253
rs183304865
rs113247152
rs7447381
rs145387798
rs9948583
rs12347873
rs58910473
rs1284140
rs144614908
rs7196912
rs145530615
rs146691572
All
−0.10
−0.05
0.00
0.05
MR leave−one−out sensitivity analysis for
'Salicylate to citrate ratio' on 'Rheumatoid arthritis'

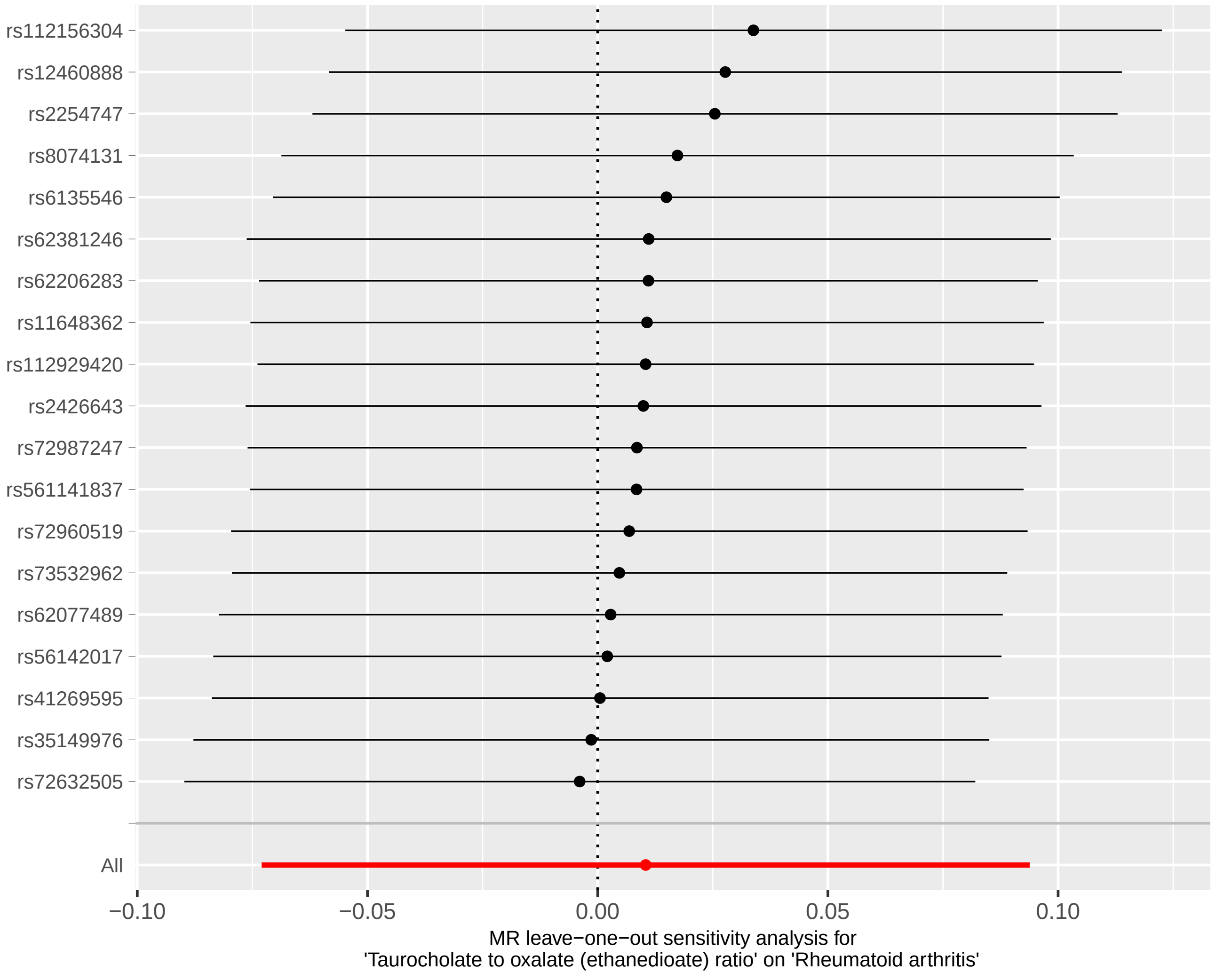
rs112156304
rs12460888
rs2254747
rs8074131
rs6135546
rs62381246
rs62206283
rs11648362
rs112929420
rs2426643
rs72987247
rs561141837
rs72960519
rs73532962
rs62077489
rs56142017
rs41269595
rs35149976
rs72632505
All
−0.10
−0.05
0.00
0.05
0.10
MR leave−one−out sensitivity analysis for
'Taurocholate to oxalate (ethanedioate) ratio' on 'Rheumatoid arthritis'

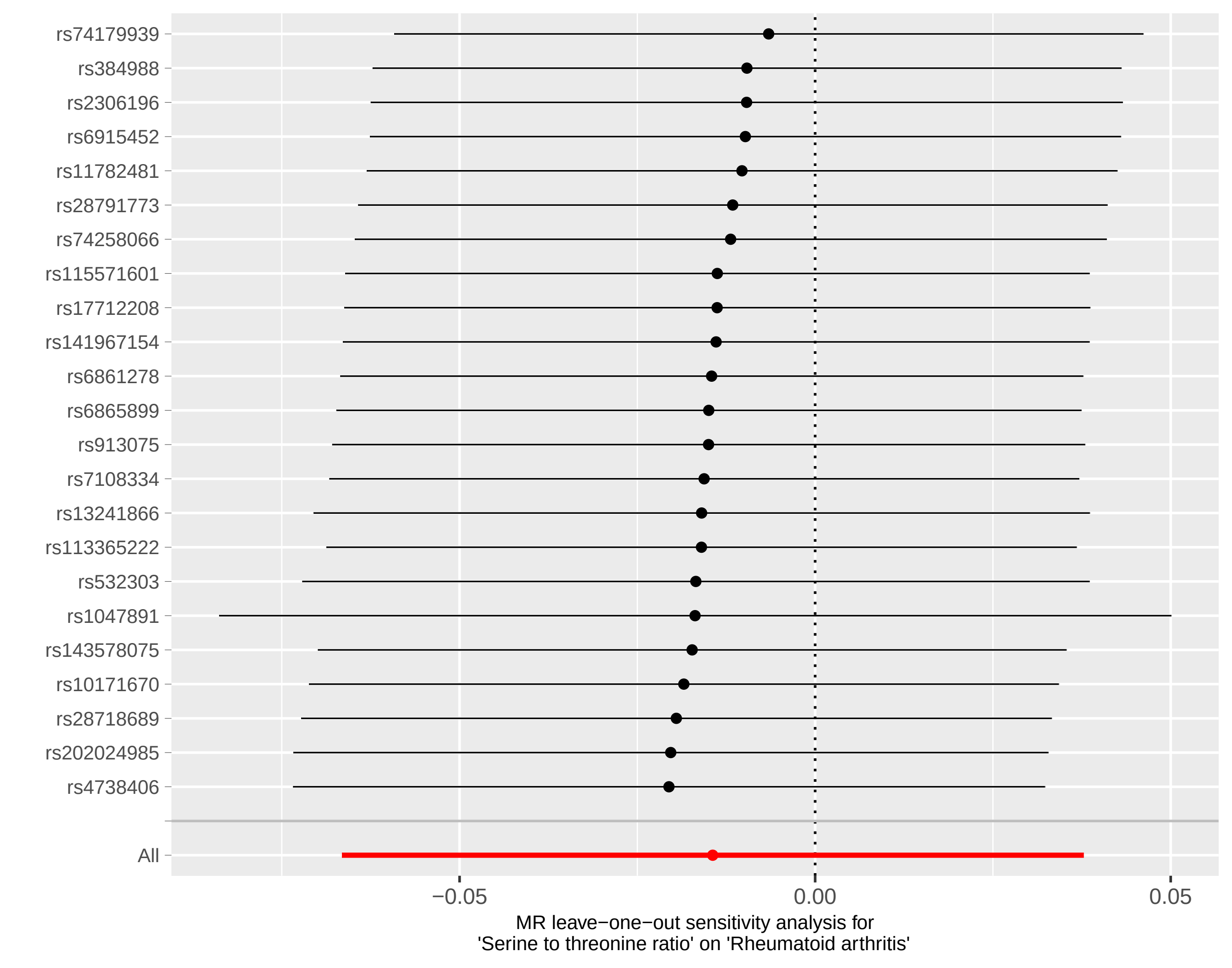
rs74179939
rs384988
rs2306196
rs6915452
rs11782481
rs28791773
rs74258066
rs115571601
rs17712208
rs141967154
rs6861278
rs6865899
rs913075
rs7108334
rs13241866
rs113365222
rs532303
rs1047891
rs143578075
rs10171670
rs28718689
rs202024985
rs4738406
All
−0.05
0.00
0.05
MR leave−one−out sensitivity analysis for
'Serine to threonine ratio' on 'Rheumatoid arthritis'

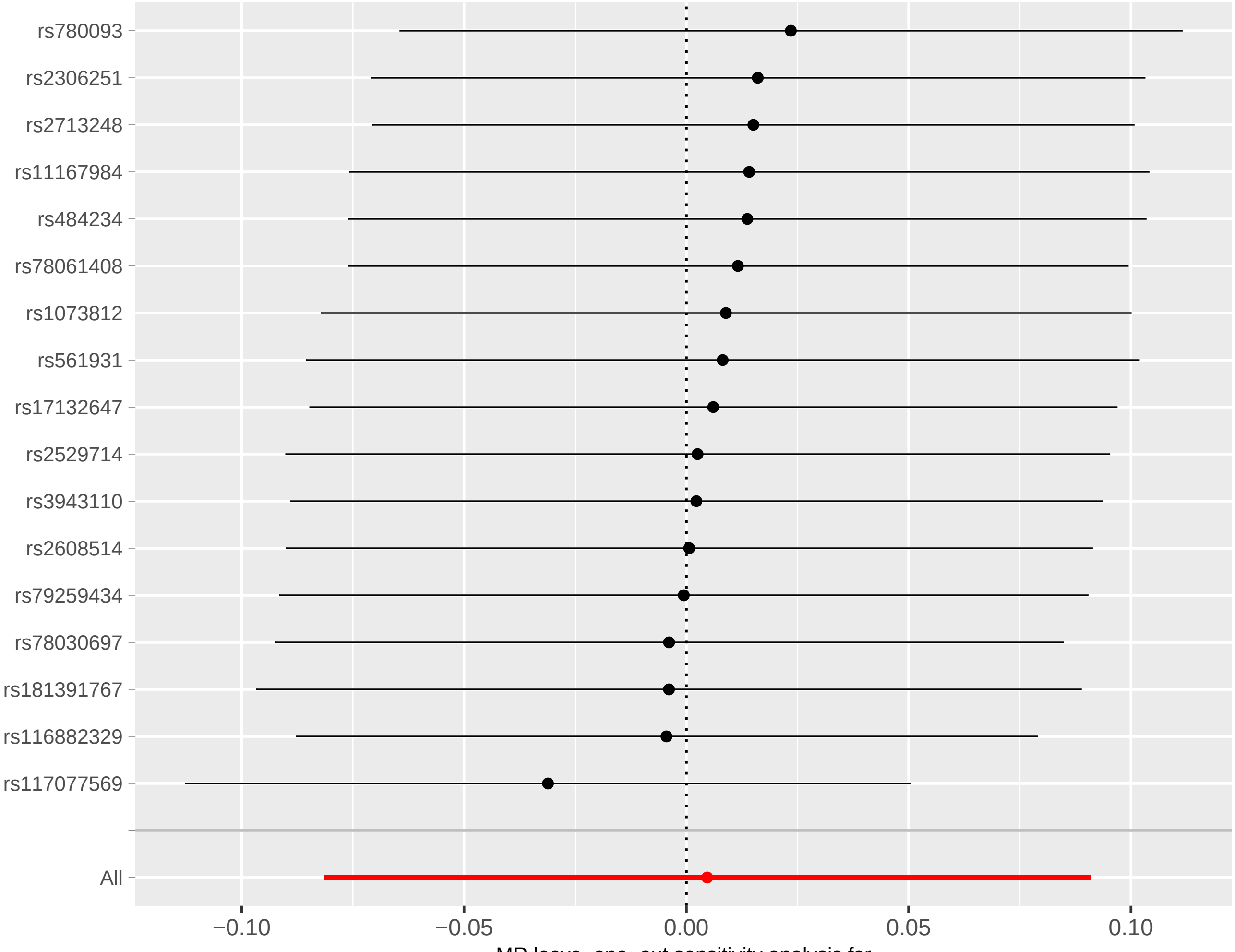

MR leave−one−out sensitivity analysis for
'Serine to alpha−ketobutyrate ratio' on 'Rheumatoid arthritis'

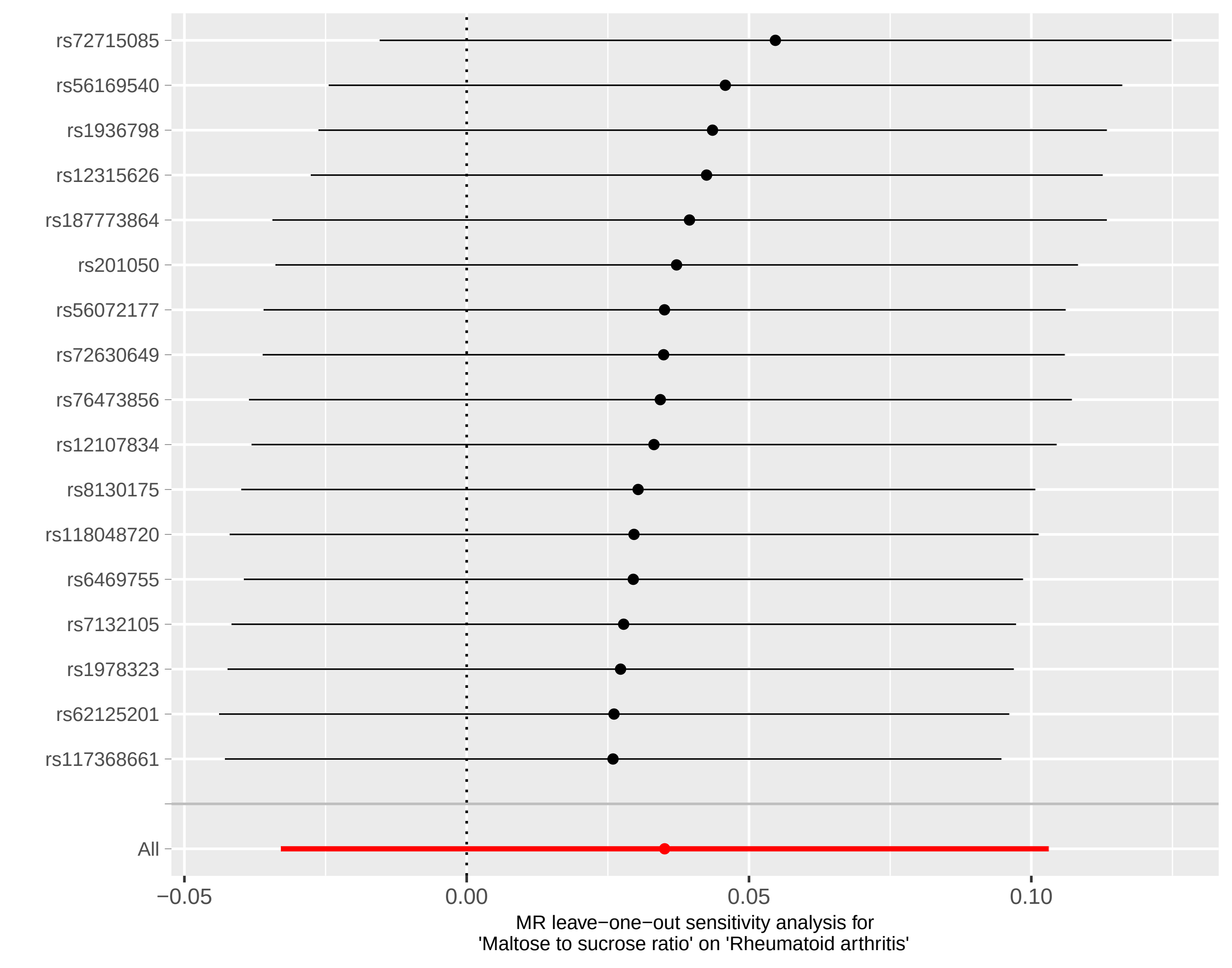
rs72715085
rs56169540
rs1936798
rs12315626
rs187773864
rs201050
rs56072177
rs72630649
rs76473856
rs12107834
rs8130175
rs118048720
rs6469755
rs7132105
rs1978323
rs62125201
rs117368661
All
−0.05
0.00
0.05
0.10
MR leave−one−out sensitivity analysis for
'Maltose to sucrose ratio' on 'Rheumatoid arthritis'

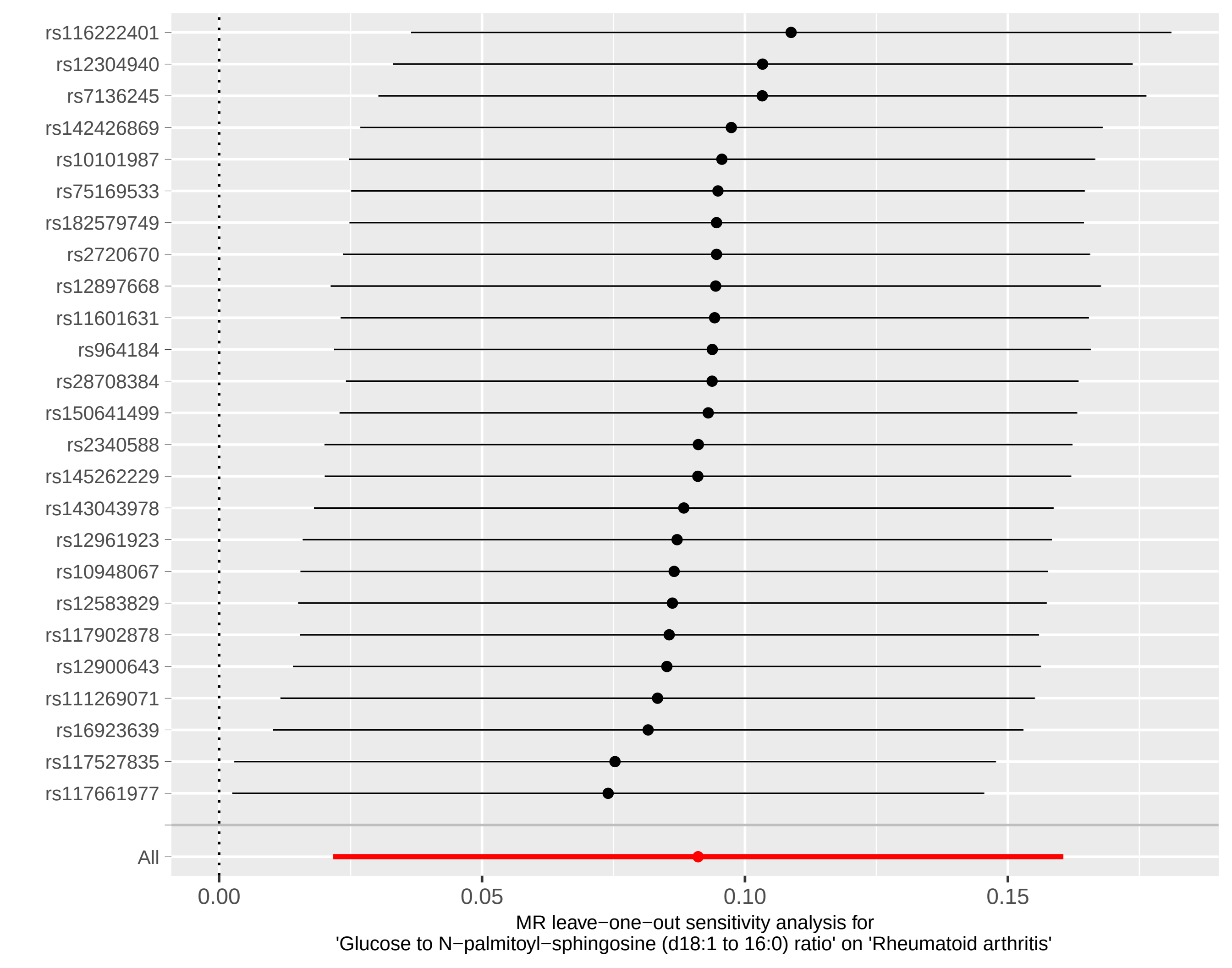

rs116222401
rs12304940
rs7136245
rs142426869
rs10101987
rs75169533
rs182579749
rs2720670
rs12897668
rs11601631
rs964184
rs28708384
rs150641499
rs2340588
rs145262229
rs143043978
rs12961923
rs10948067
rs12583829
rs117902878
rs12900643
rs111269071
rs16923639
rs117527835
rs117661977
All
0.00
0.05
0.10
0.15
MR leave−one−out sensitivity analysis for
'Glucose to N−palmitoyl−sphingosine (d18:1 to 16:0) ratio' on 'Rheumatoid arthritis'

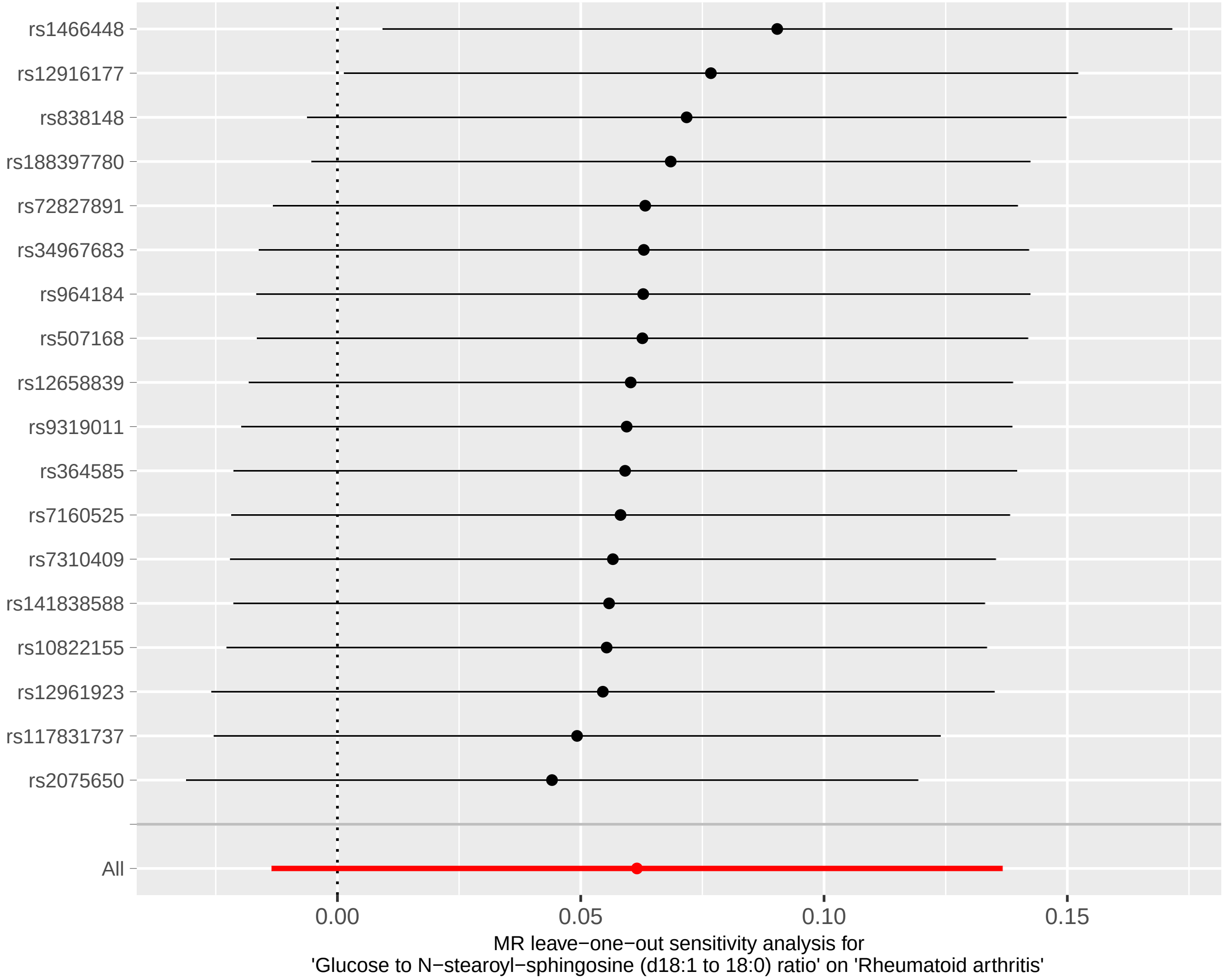
rs1466448
rs12916177
rs838148
rs188397780
rs72827891
rs34967683
rs964184
rs507168
rs12658839
rs9319011
rs364585
rs7160525
rs7310409
rs141838588
rs10822155
rs12961923
rs117831737
rs2075650
All
0.00
0.05
0.10
0.15
MR leave−one−out sensitivity analysis for
'Glucose to N−stearoyl−sphingosine (d18:1 to 18:0) ratio' on 'Rheumatoid arthritis'

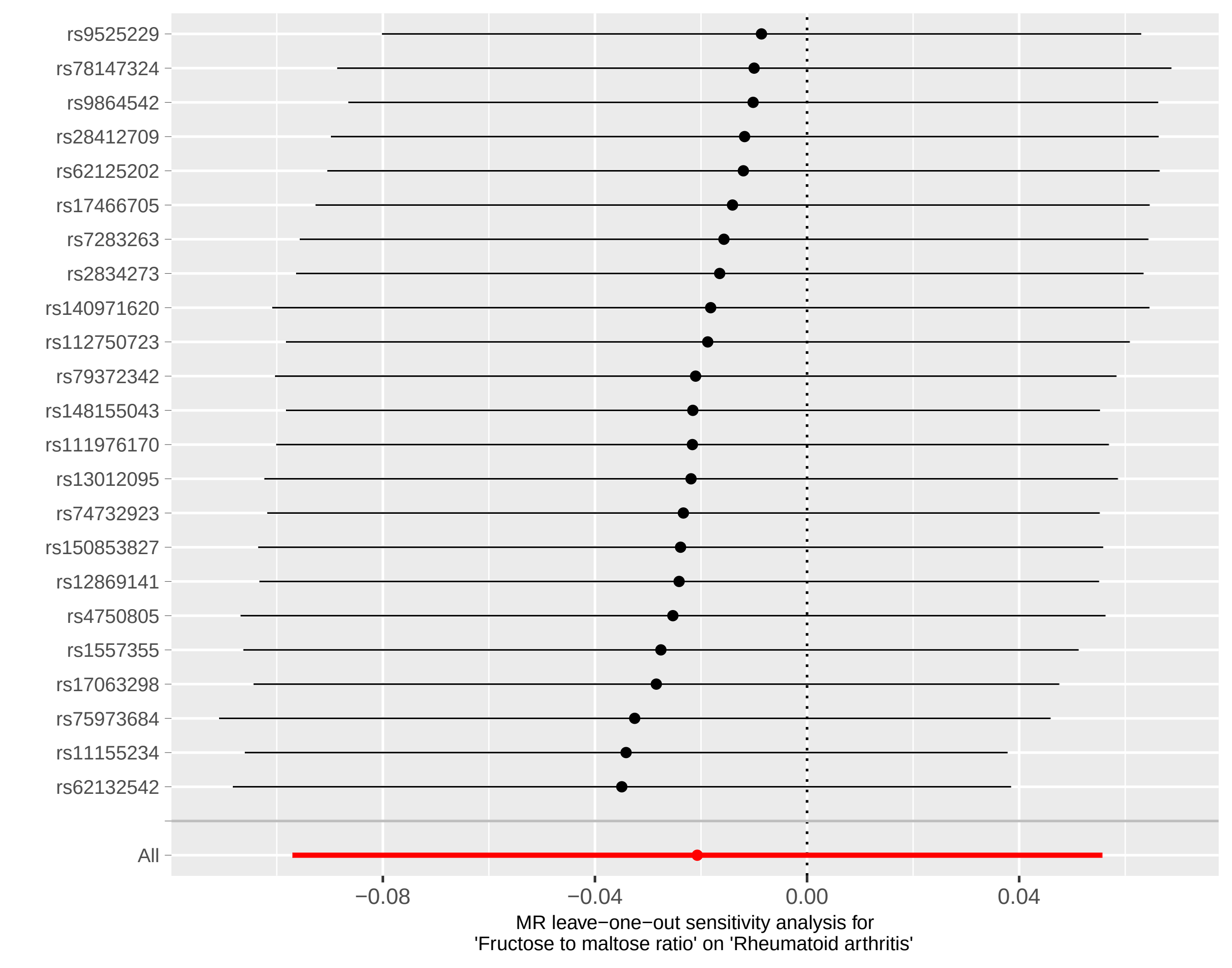
rs9525229
rs78147324
rs9864542
rs28412709
rs62125202
rs17466705
rs7283263
rs2834273
rs140971620
rs112750723
rs79372342
rs148155043
rs111976170
rs13012095
rs74732923
rs150853827
rs12869141
rs4750805
rs1557355
rs17063298
rs75973684
rs11155234
rs62132542
All
−0.08
−0.04
0.00
0.04
MR leave−one−out sensitivity analysis for
'Fructose to maltose ratio' on 'Rheumatoid arthritis'

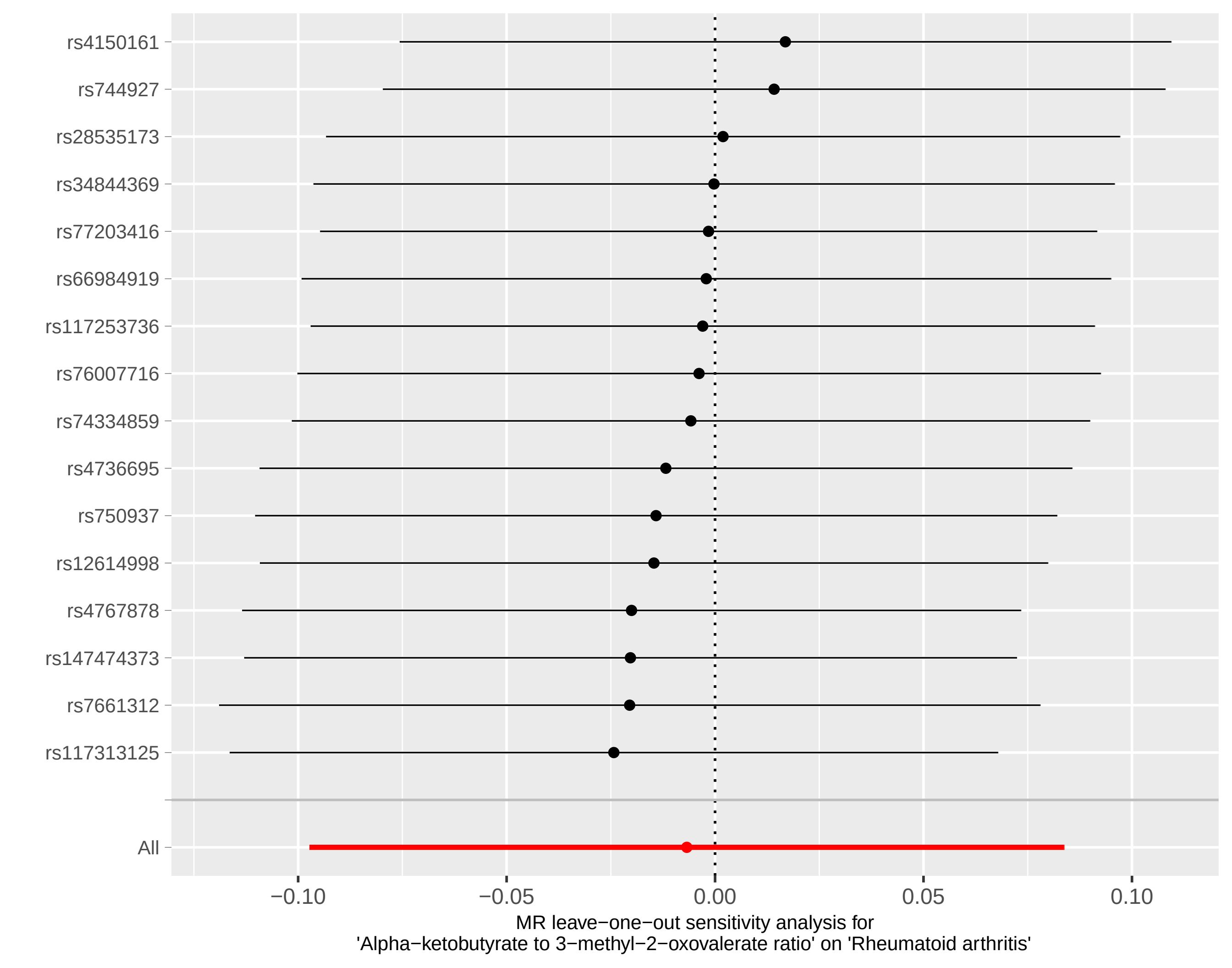
rs4150161
rs744927
rs28535173
rs34844369
rs77203416
rs66984919
rs117253736
rs76007716
rs74334859
rs4736695
rs750937
rs12614998
rs4767878
rs147474373
rs7661312
rs117313125
All
−0.10
−0.05
0.00
0.05
0.10
MR leave−one−out sensitivity analysis for
'Alpha−ketobutyrate to 3−methyl−2−oxovalerate ratio' on 'Rheumatoid arthritis'

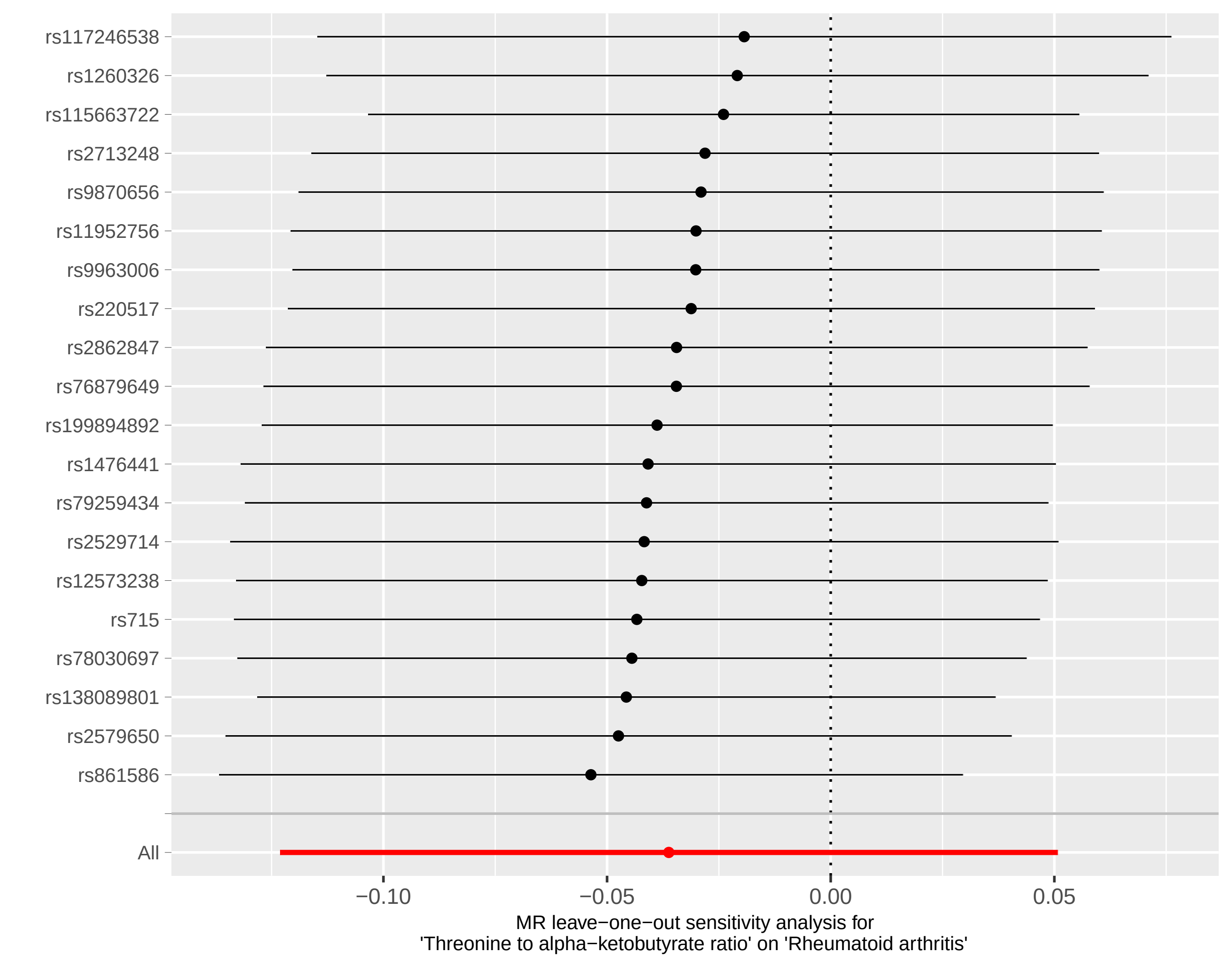

rs117246538
rs1260326
rs115663722
rs2713248
rs9870656
rs11952756
rs9963006
rs220517
rs2862847
rs76879649
rs199894892
rs1476441
rs79259434
rs2529714
rs12573238
rs715
rs78030697
rs138089801
rs2579650
rs861586
All
−0.10
−0.05
0.00
0.05
MR leave−one−out sensitivity analysis for
'Threonine to alpha−ketobutyrate ratio' on 'Rheumatoid arthritis'

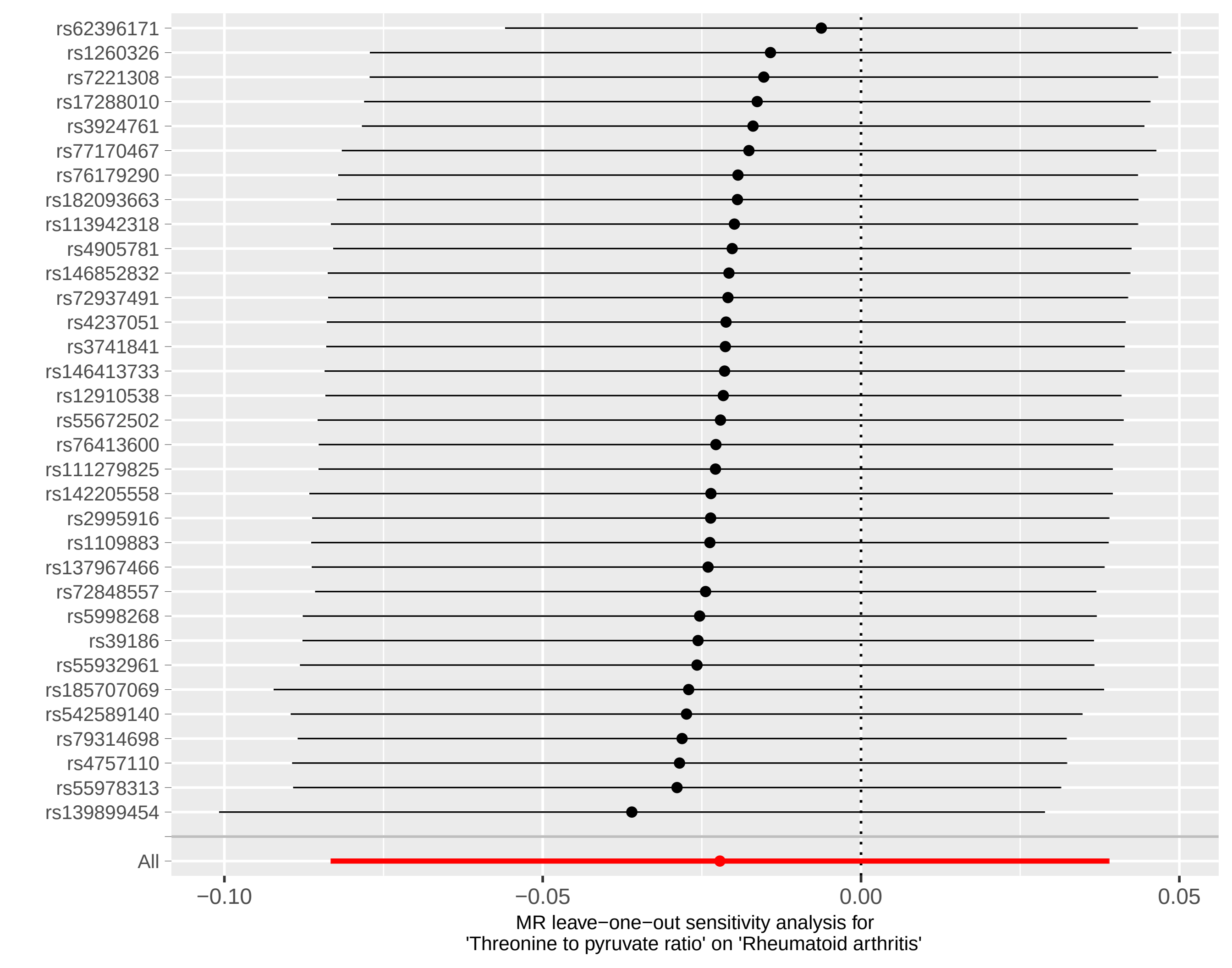
rs62396171
rs1260326
rs7221308
rs17288010
rs3924761
rs77170467
rs76179290
rs182093663
rs113942318
rs4905781
rs146852832
rs72937491
rs4237051
rs3741841
rs146413733
rs12910538
rs55672502
rs76413600
rs111279825
rs142205558
rs2995916
rs1109883
rs137967466
rs72848557
rs5998268
rs39186
rs55932961
rs185707069
rs542589140
rs79314698
rs4757110
rs55978313
rs139899454
All
−0.10
−0.05
0.00
0.05
MR leave−one−out sensitivity analysis for
'Threonine to pyruvate ratio' on 'Rheumatoid arthritis'

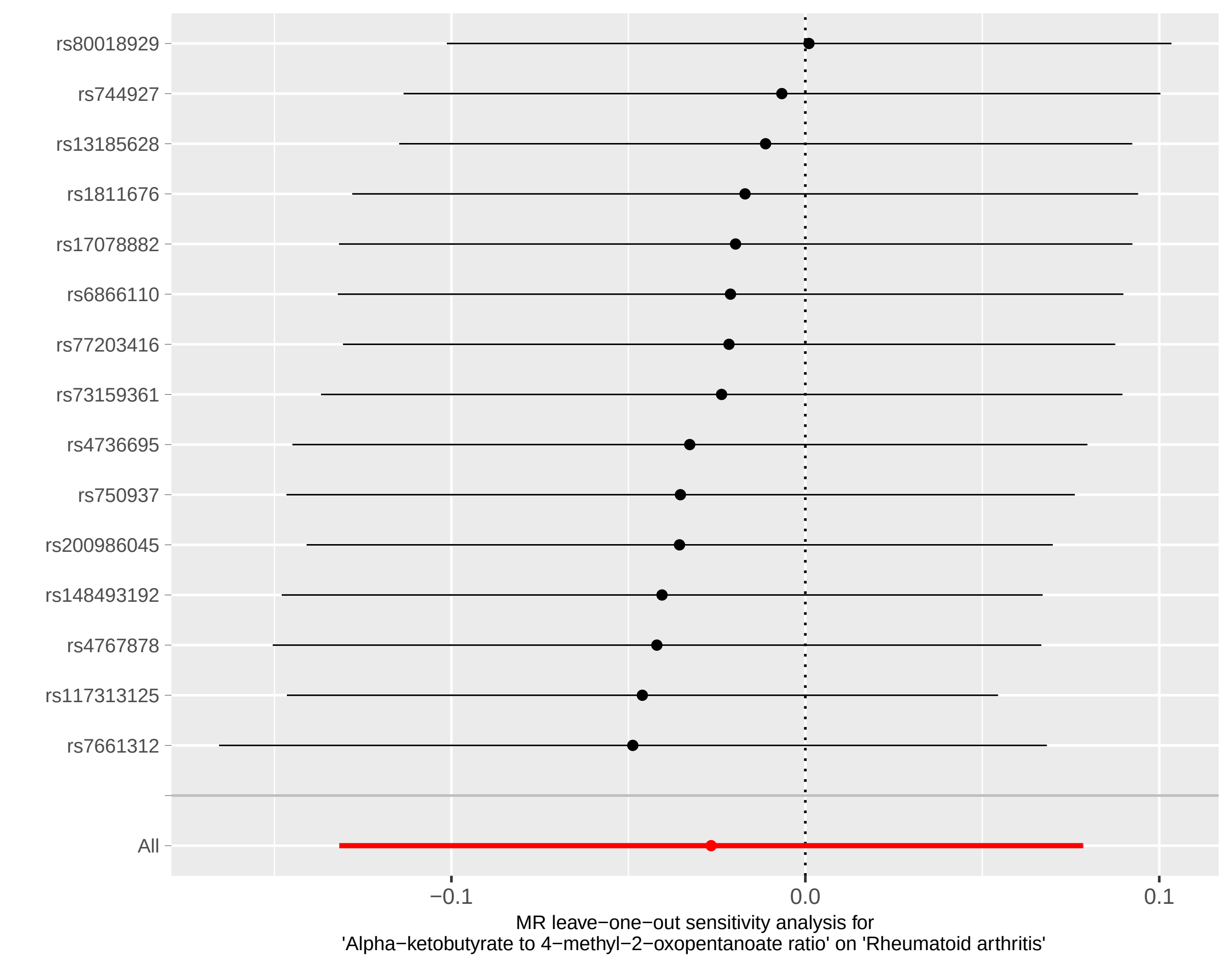
rs80018929
rs744927
rs13185628
rs1811676
rs17078882
rs6866110
rs77203416
rs73159361
rs4736695
rs750937
rs200986045
rs148493192
rs4767878
rs117313125
rs7661312
All
−0.1
0.0
0.1
MR leave−one−out sensitivity analysis for
'Alpha−ketobutyrate to 4−methyl−2−oxopentanoate ratio' on 'Rheumatoid arthritis'

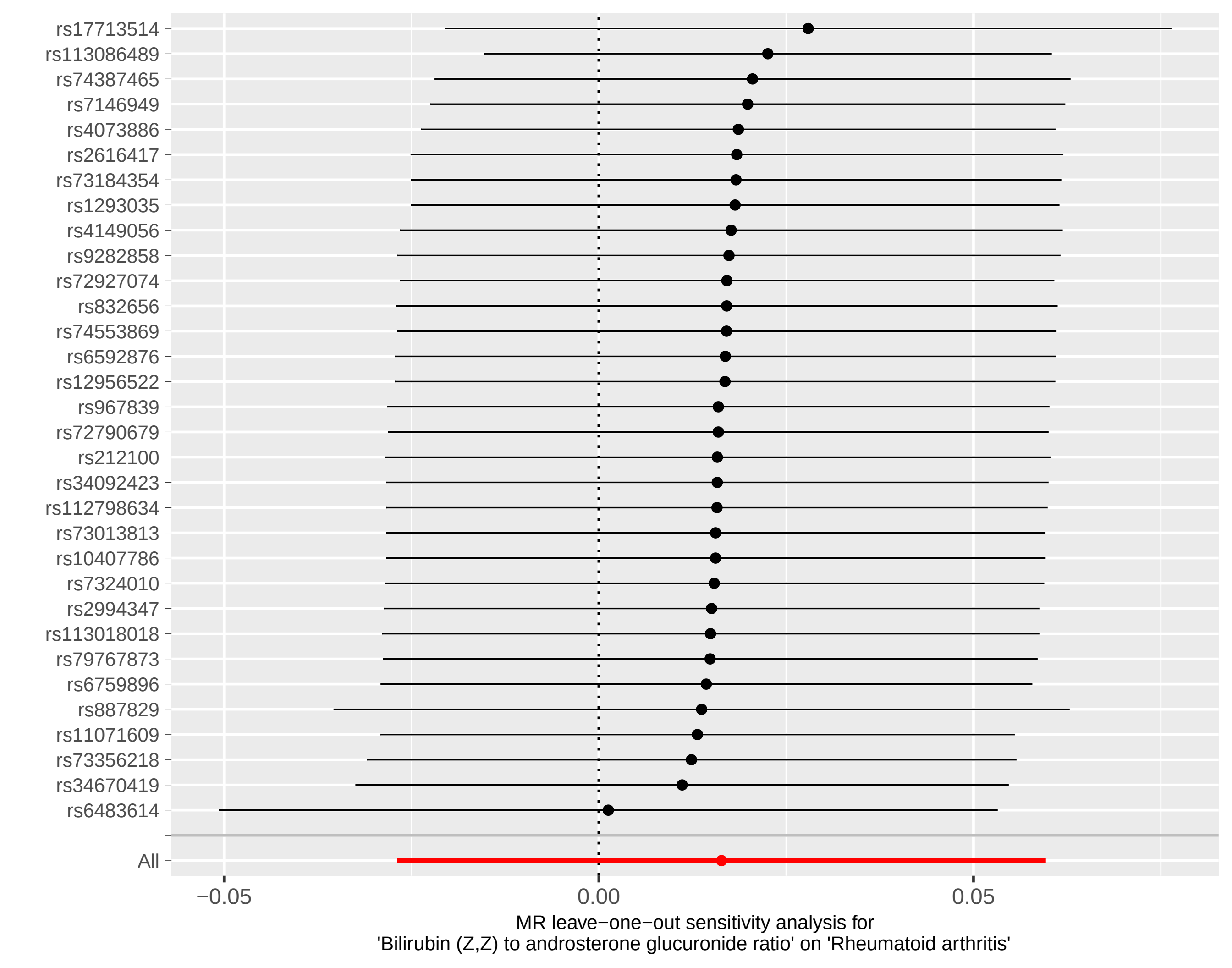
rs17713514
rs113086489
rs74387465
rs7146949
rs4073886
rs2616417
rs73184354
rs1293035
rs4149056
rs9282858
rs72927074
rs832656
rs74553869
rs6592876
rs12956522
rs967839
rs72790679
rs212100
rs34092423
rs112798634
rs73013813
rs10407786
rs7324010
rs2994347
rs113018018
rs79767873
rs6759896
rs887829
rs11071609
rs73356218
rs34670419
rs6483614
All
−0.05
0.00
0.05
MR leave−one−out sensitivity analysis for
'Bilirubin (Z,Z) to androsterone glucuronide ratio' on 'Rheumatoid arthritis'

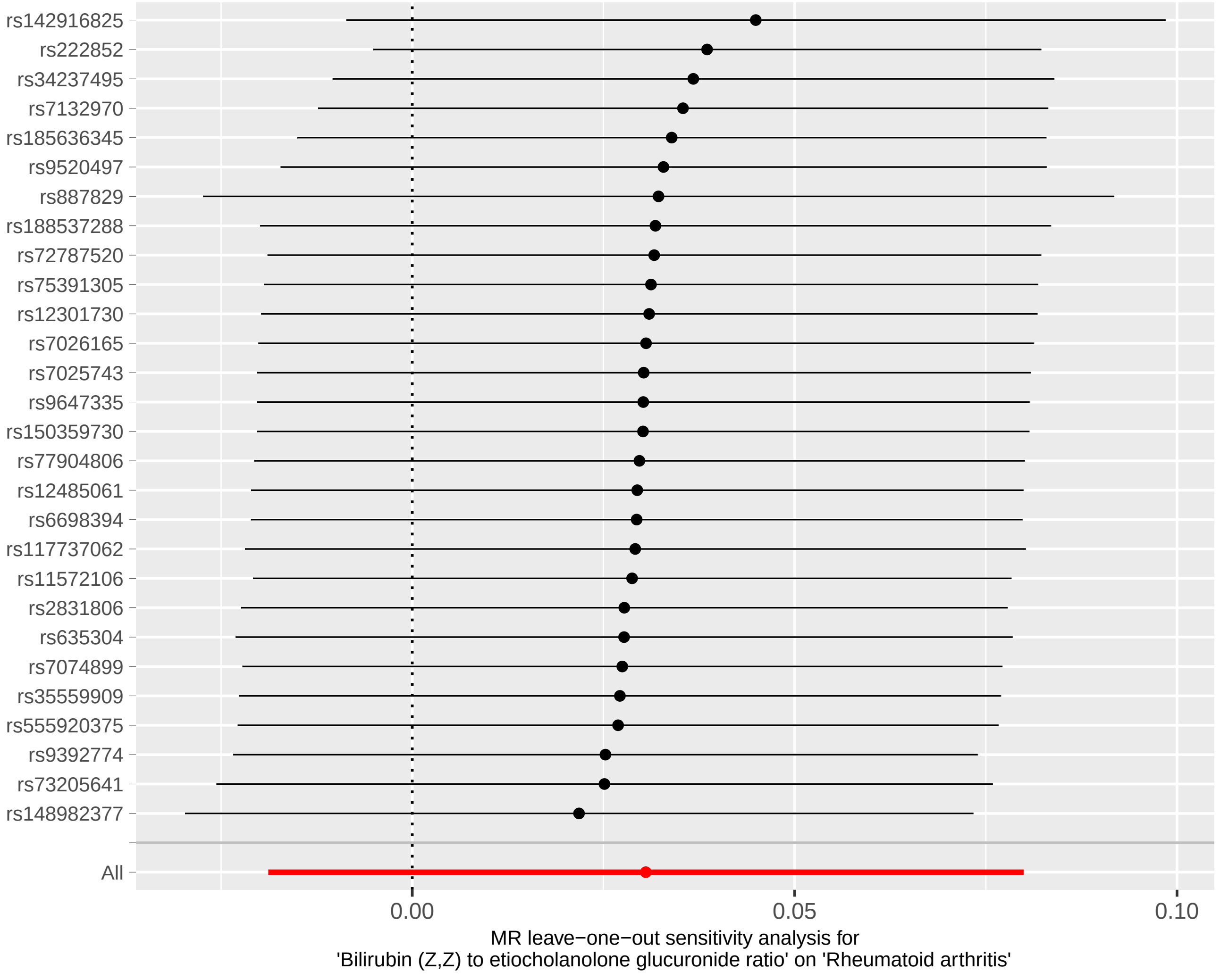

rs142916825
rs222852
rs34237495
rs7132970
rs185636345
rs9520497
rs887829
rs188537288
rs72787520
rs75391305
rs12301730
rs7026165
rs7025743
rs9647335
rs150359730
rs77904806
rs12485061
rs6698394
rs117737062
rs11572106
rs2831806
rs635304
rs7074899
rs35559909
rs555920375
rs9392774
rs73205641
rs148982377
All
0.00
0.05
0.10
MR leave−one−out sensitivity analysis for
'Bilirubin (Z,Z) to etiocholanolone glucuronide ratio' on 'Rheumatoid arthritis'

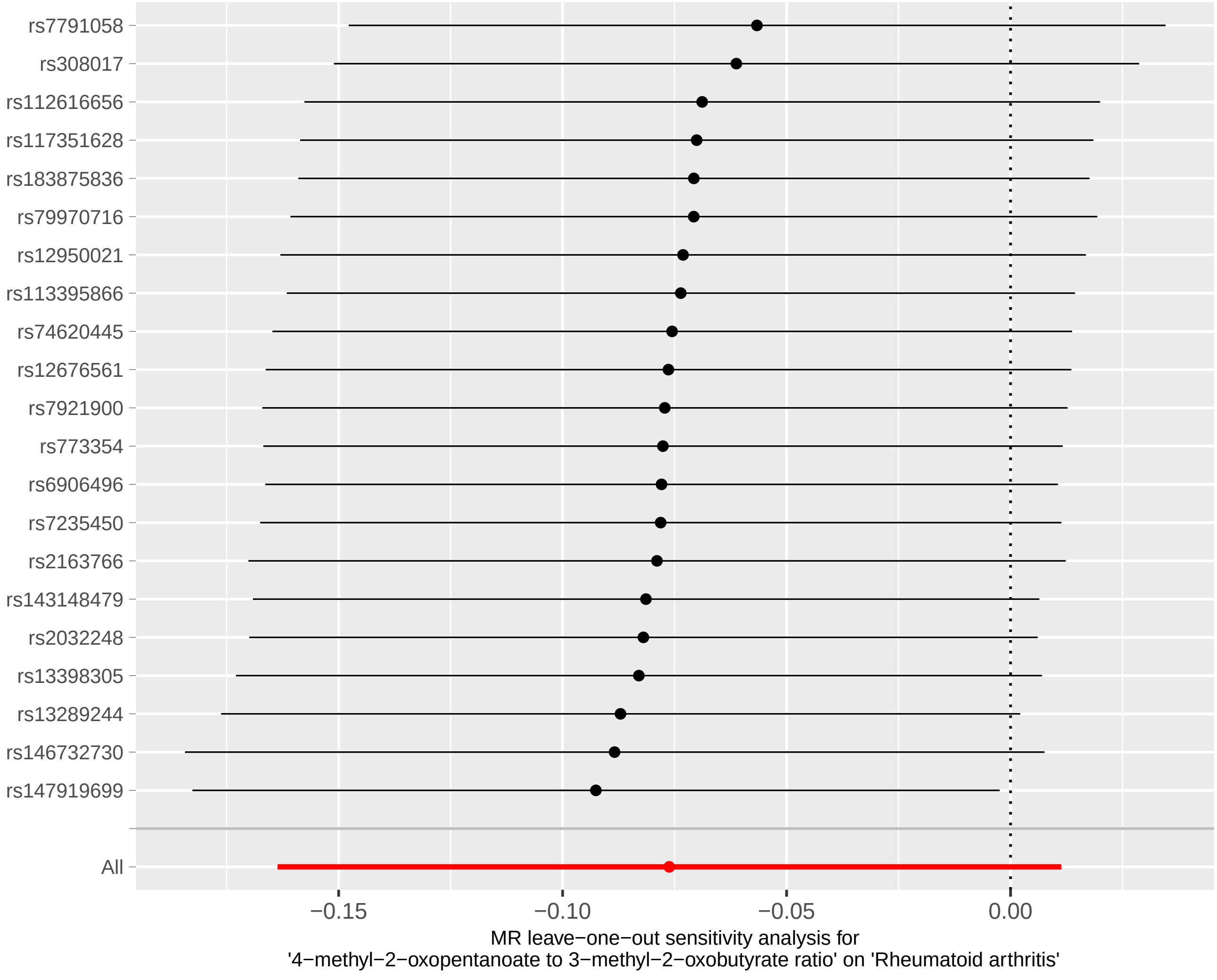

rs7791058
rs308017
rs112616656
rs117351628
rs183875836
rs79970716
rs12950021
rs113395866
rs74620445
rs12676561
rs7921900
rs773354
rs6906496
rs7235450
rs2163766
rs143148479
rs2032248
rs13398305
rs13289244
rs146732730
rs147919699
All
−0.15
−0.10
−0.05
0.00
MR leave−one−out sensitivity analysis for
'4−methyl−2−oxopentanoate to 3−methyl−2−oxobutyrate ratio' on 'Rheumatoid arthritis'

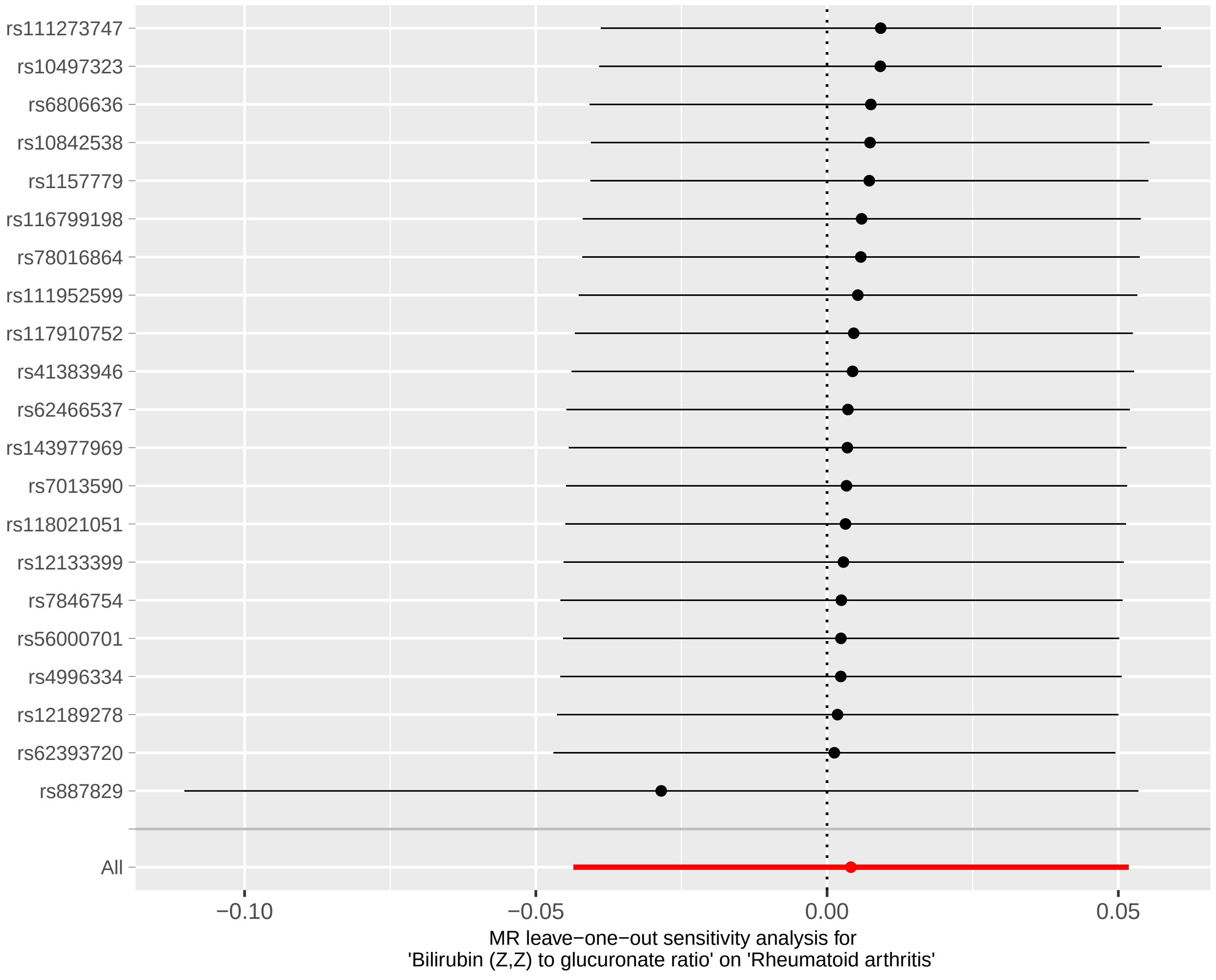
rs111273747
rs10497323
rs6806636
rs10842538
rs1157779
rs116799198
rs78016864
rs111952599
rs117910752
rs41383946
rs62466537
rs143977969
rs7013590
rs118021051
rs12133399
rs7846754
rs56000701
rs4996334
rs12189278
rs62393720
rs887829
All
−0.10
−0.05
0.00
0.05
MR leave−one−out sensitivity analysis for
'Bilirubin (Z,Z) to glucuronate ratio' on 'Rheumatoid arthritis'

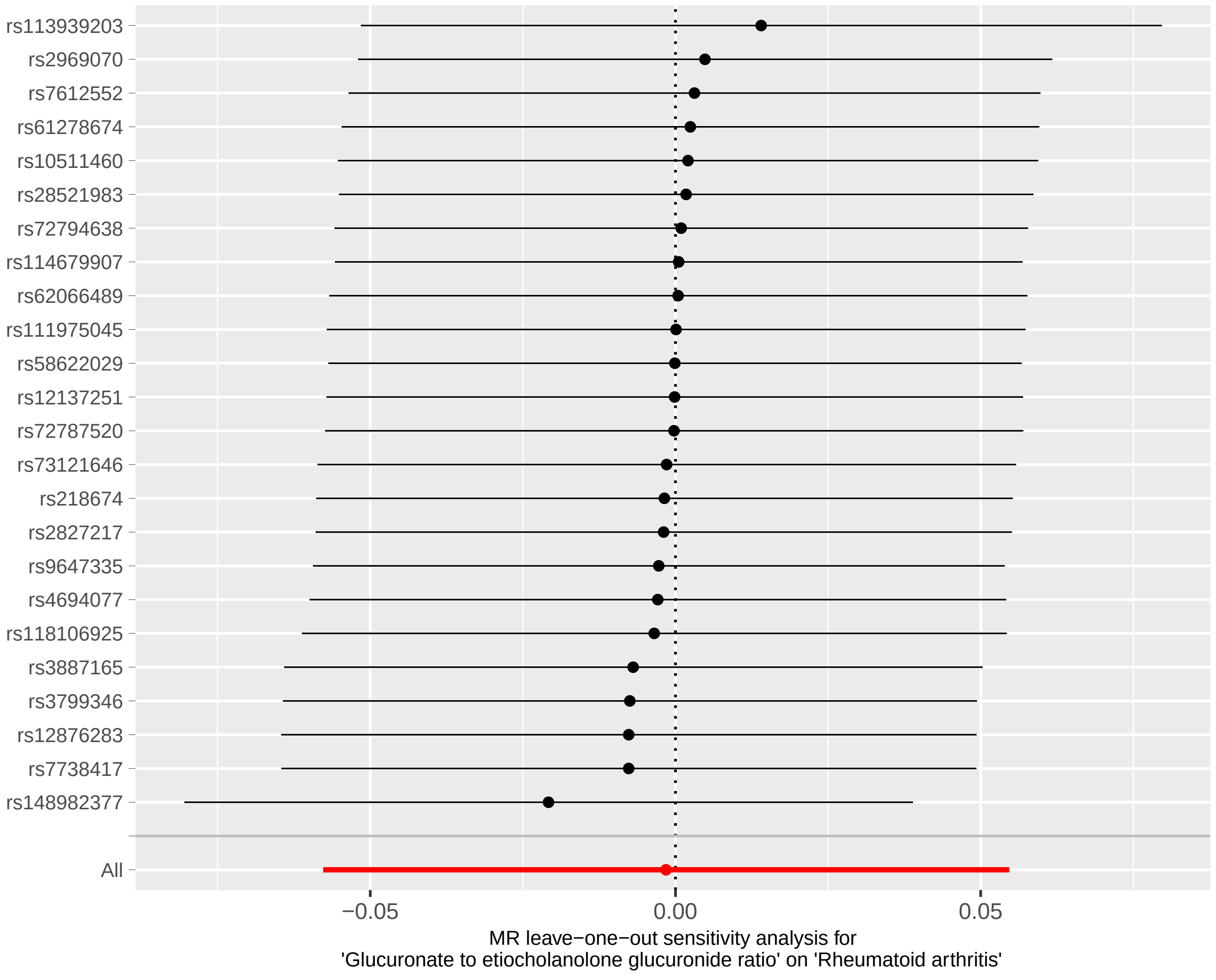
rs113939203
rs2969070
rs7612552
rs61278674
rs10511460
rs28521983
rs72794638
rs114679907
rs62066489
rs111975045
rs58622029
rs12137251
rs72787520
rs73121646
rs218674
rs2827217
rs9647335
rs4694077
rs118106925
rs3887165
rs3799346
rs12876283
rs7738417
rs148982377
All
−0.05
0.00
0.05
MR leave−one−out sensitivity analysis for
'Glucuronate to etiocholanolone glucuronide ratio' on 'Rheumatoid arthritis'

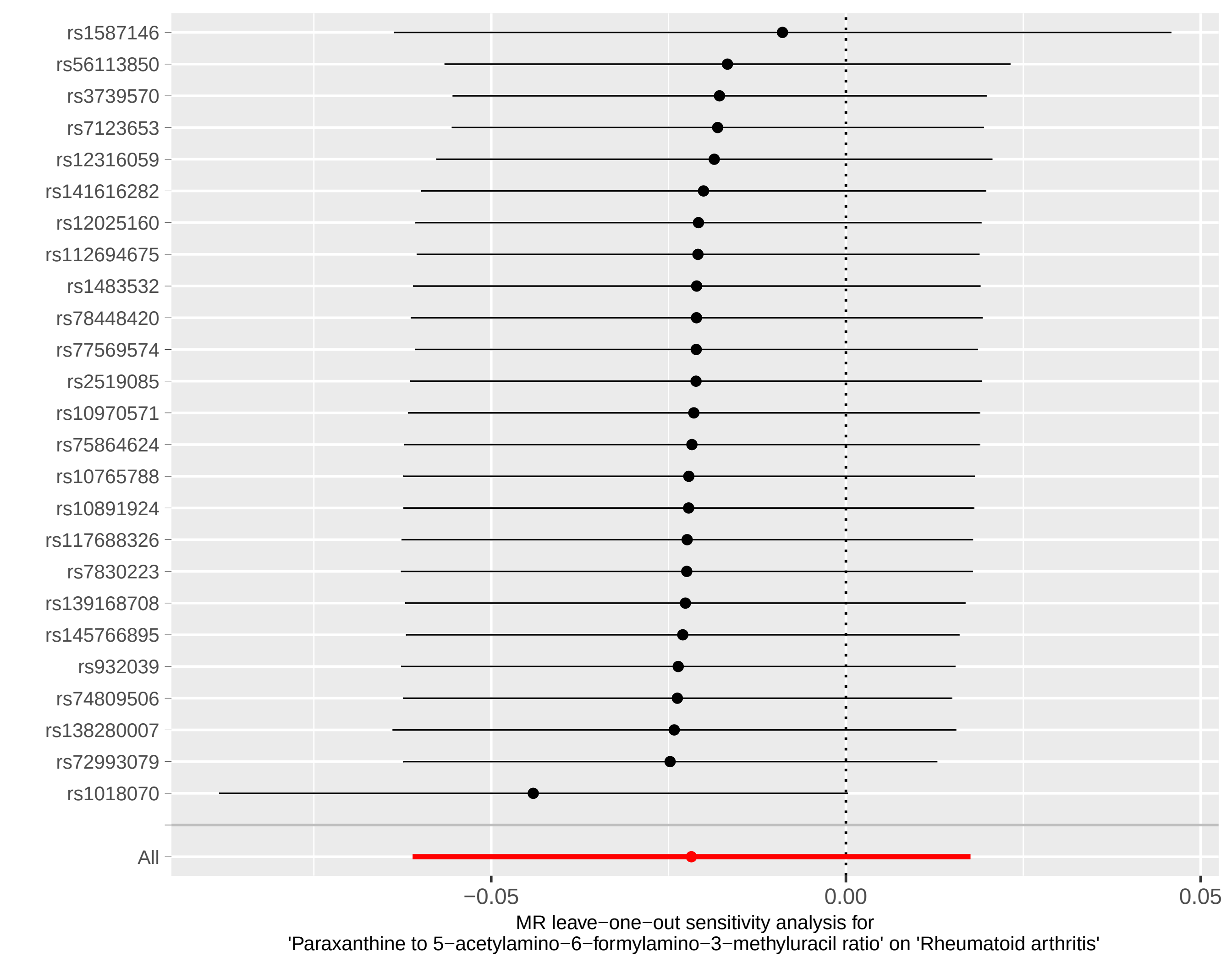
rs1587146
rs56113850
rs3739570
rs7123653
rs12316059
rs141616282
rs12025160
rs112694675
rs1483532
rs78448420
rs77569574
rs2519085
rs10970571
rs75864624
rs10765788
rs10891924
rs117688326
rs7830223
rs139168708
rs145766895
rs932039
rs74809506
rs138280007
rs72993079
rs1018070
All
−0.05
0.00
0.05
MR leave−one−out sensitivity analysis for
'Paraxanthine to 5−acetylamino−6−formylamino−3−methyluracil ratio' on 'Rheumatoid arthritis'

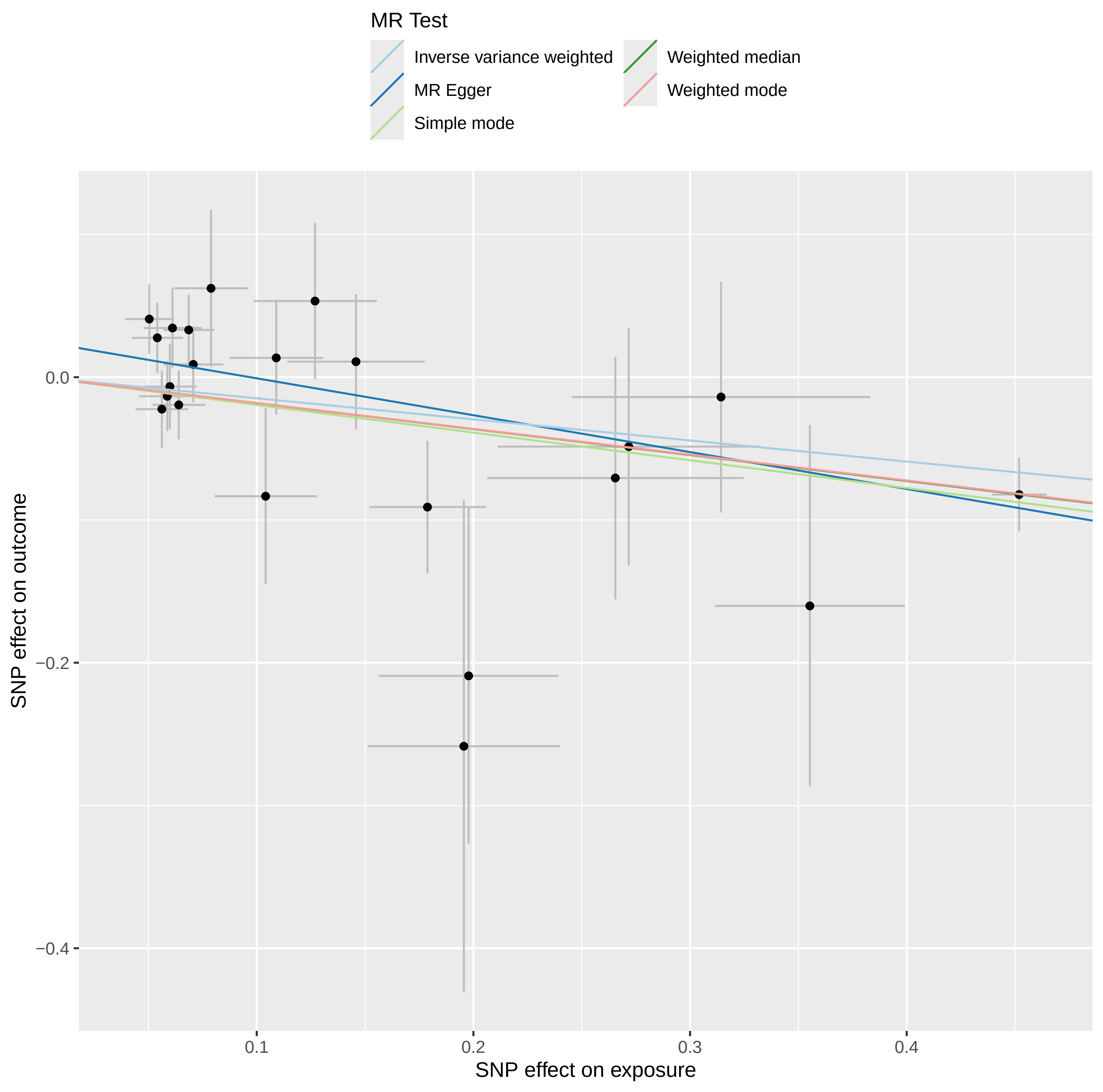

MR Test
Inverse variance weighted
MR Egger
Simple mode
Weighted median
Weighted mode
SNP effect on outcome
0.0
−0.2
−0.4
0.1
0.2
0.3
0.4
SNP effect on exposure

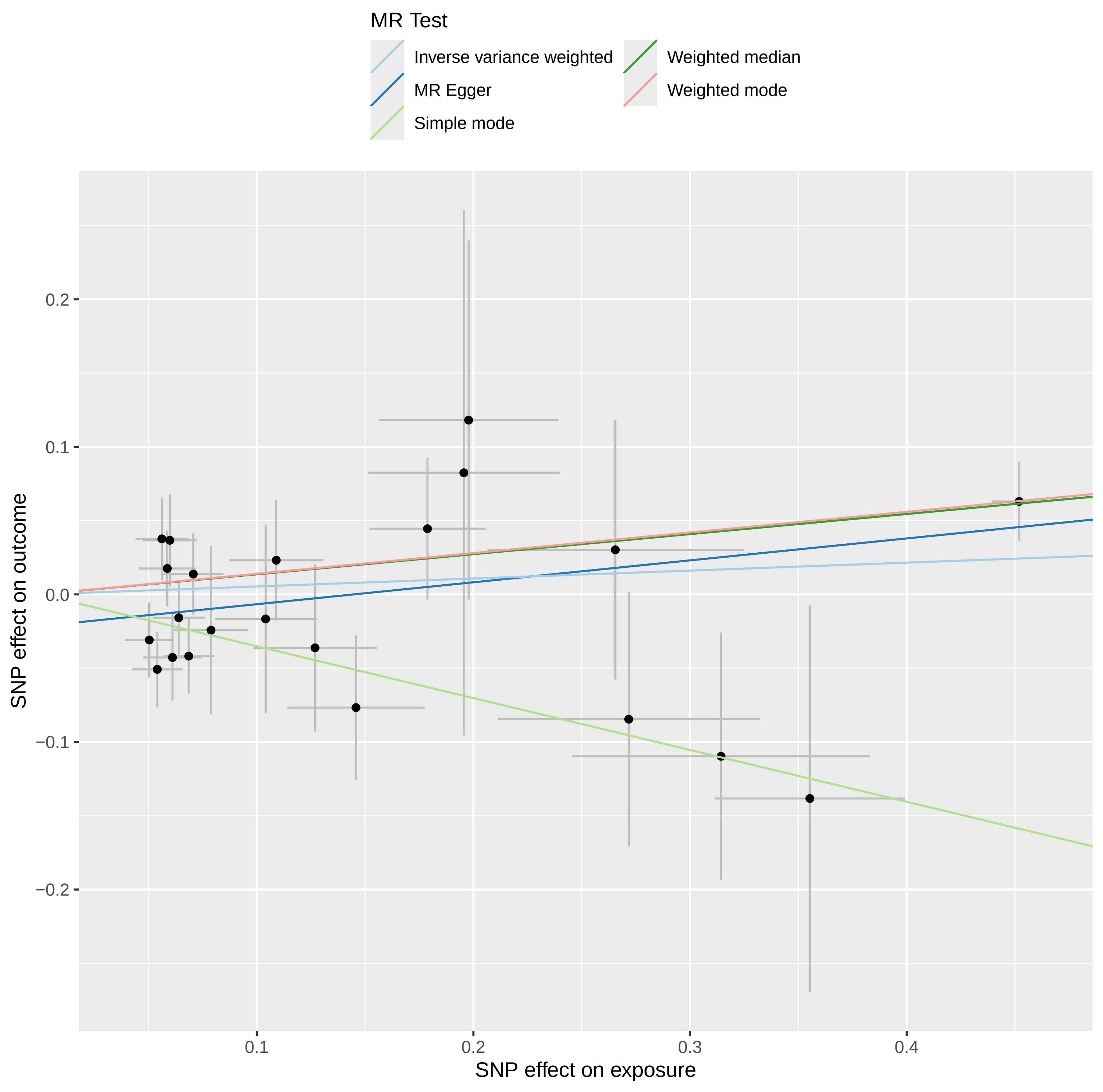
MR Test
Inverse variance weighted
MR Egger
Simple mode
Weighted median
Weighted mode
SNP effect on outcome
0.2
0.1
0.0
−0.1
−0.2
0.1
0.2
0.3
0.4
SNP effect on exposure

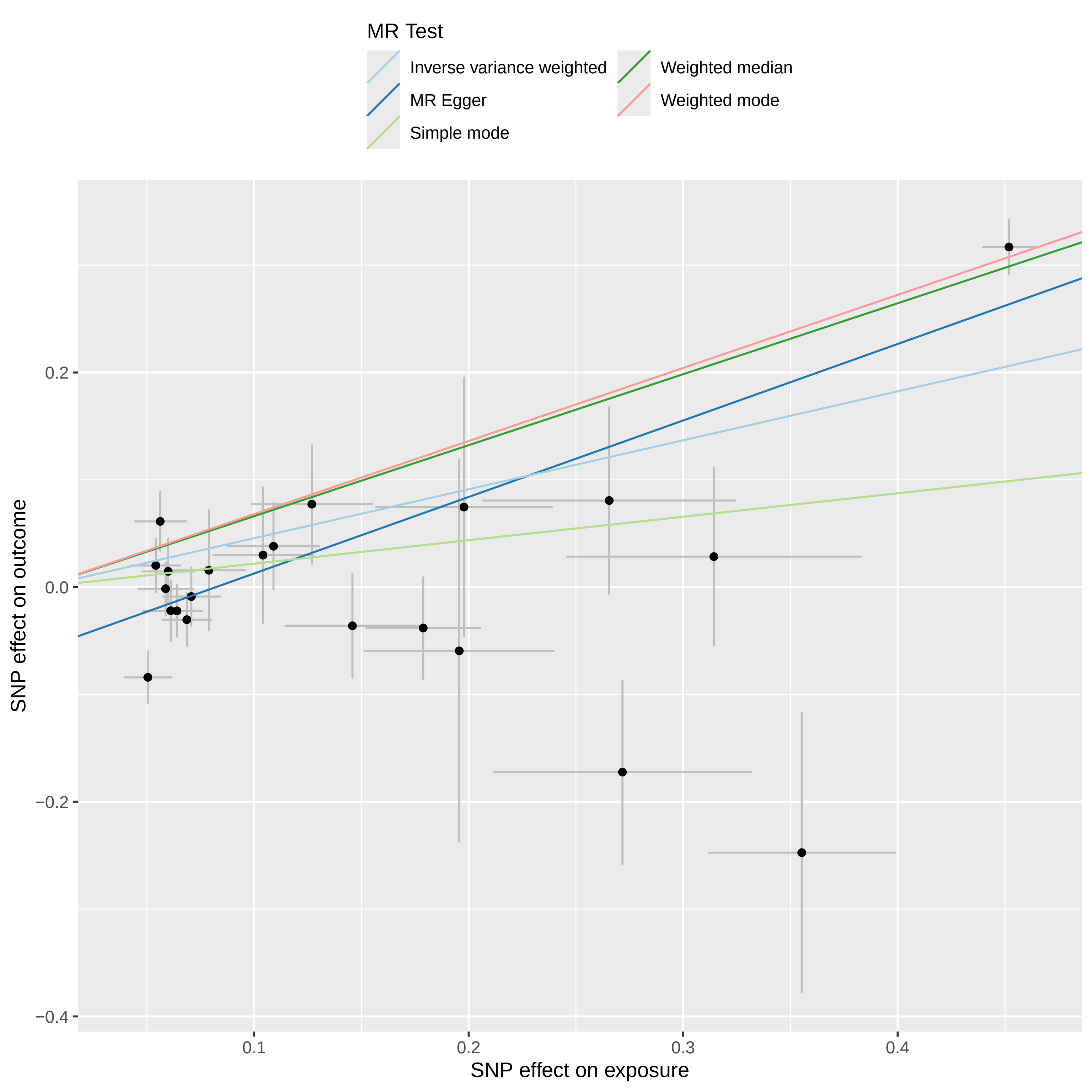
MR Test
Inverse variance weighted
MR Egger
Simple mode
Weighted median
Weighted mode
SNP effect on outcome
0.2
0.0
−0.2
−0.4
0.1
0.2
0.3
0.4
SNP effect on exposure

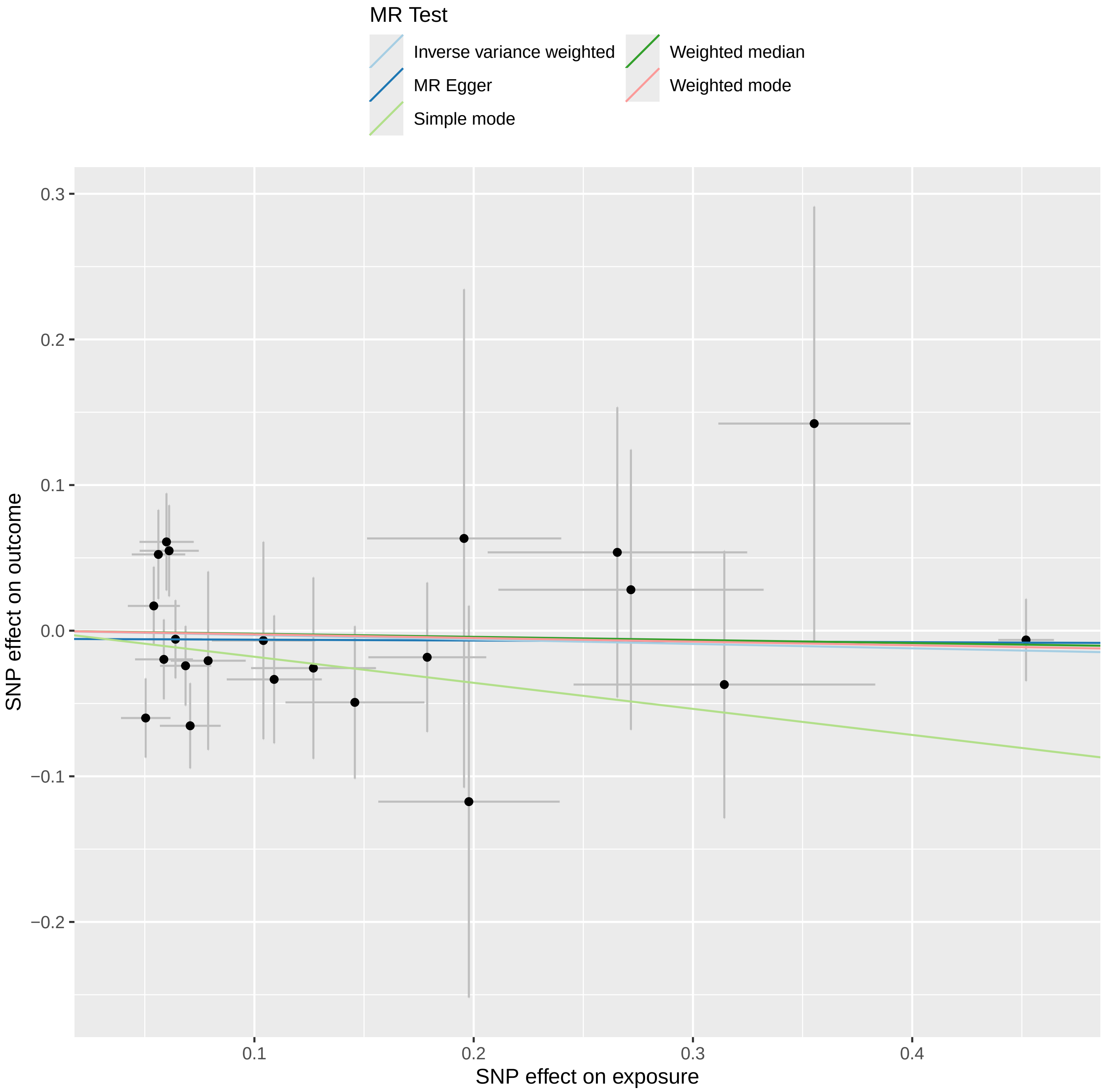
MR Test
Inverse variance weighted
MR Egger
Simple mode
Weighted median
Weighted mode
0.3
0.2
0.1
0.0
−0.1
−0.2
SNP effect on outcome
0.1
0.2
0.3
0.4
SNP effect on exposure

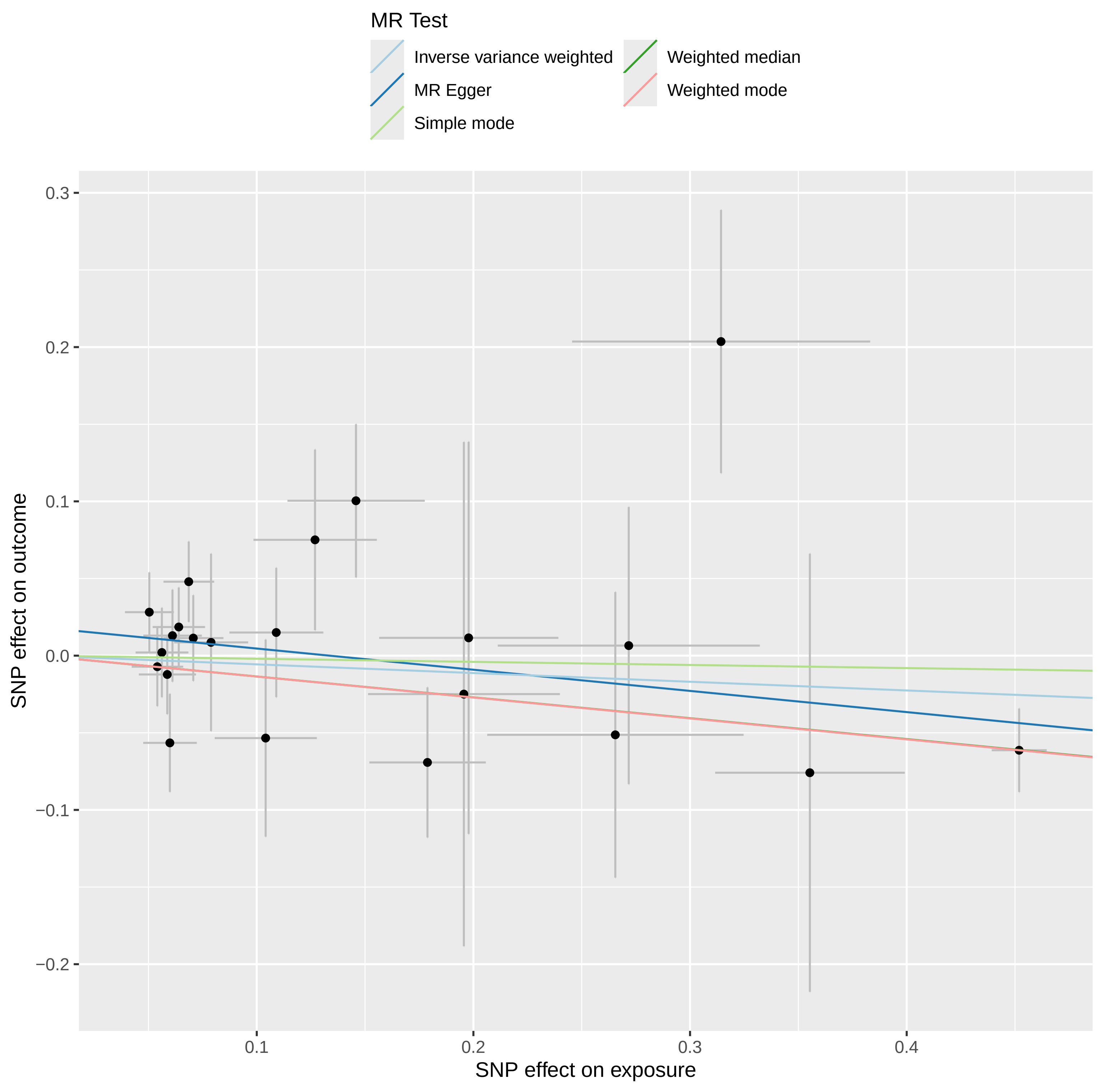

MR Test
Inverse variance weighted
MR Egger
Simple mode
Weighted median
Weighted mode
0.3
0.2
0.1
0.0
−0.1
−0.2
SNP effect on outcome
0.1
0.2
0.3
0.4
SNP effect on exposure

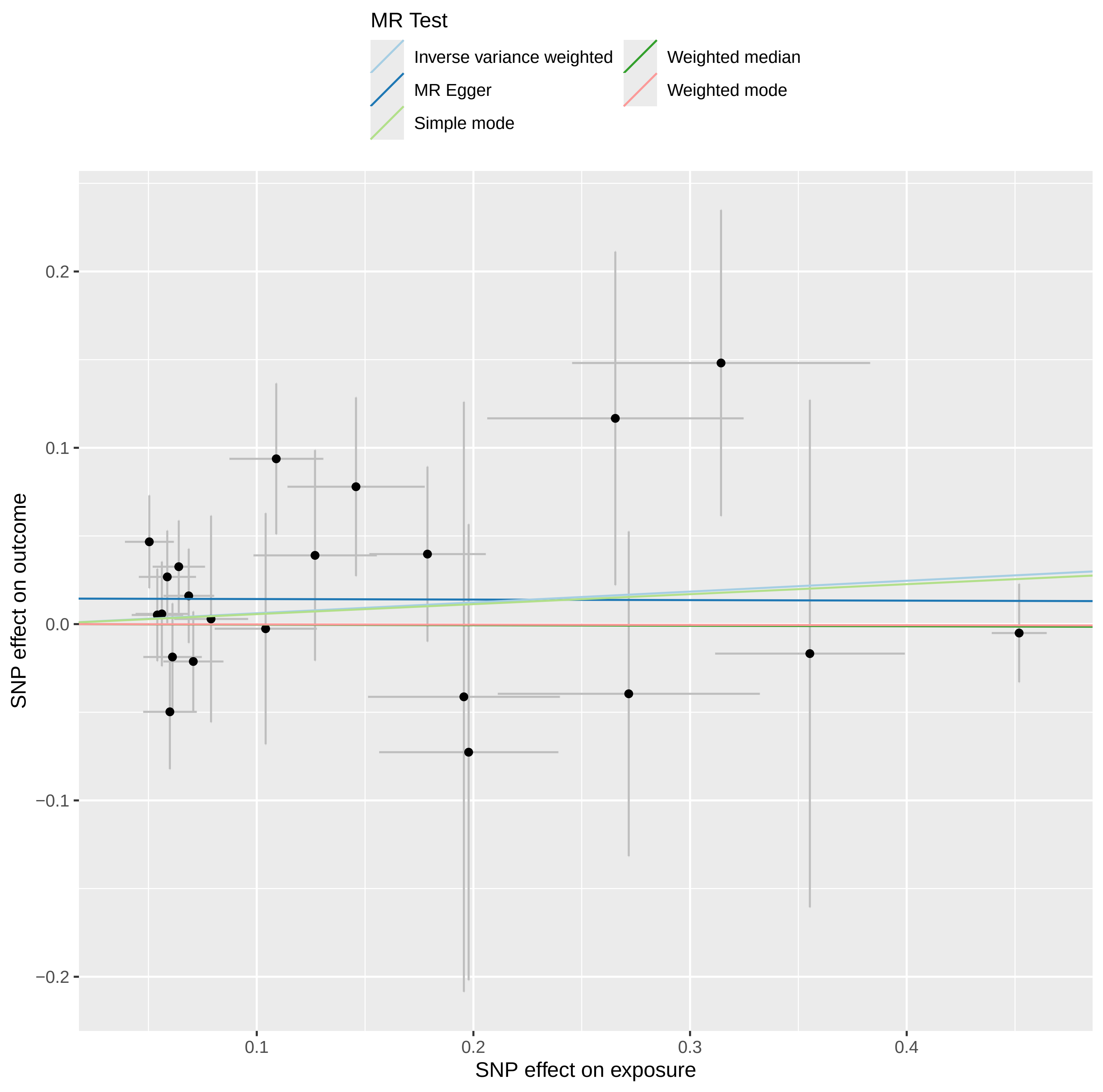
MR Test
Inverse variance weighted
MR Egger
Simple mode
Weighted median
Weighted mode
SNP effect on outcome
0.2
0.1
0.0
−0.1
−0.2
0.1
0.2
0.3
0.4
SNP effect on exposure

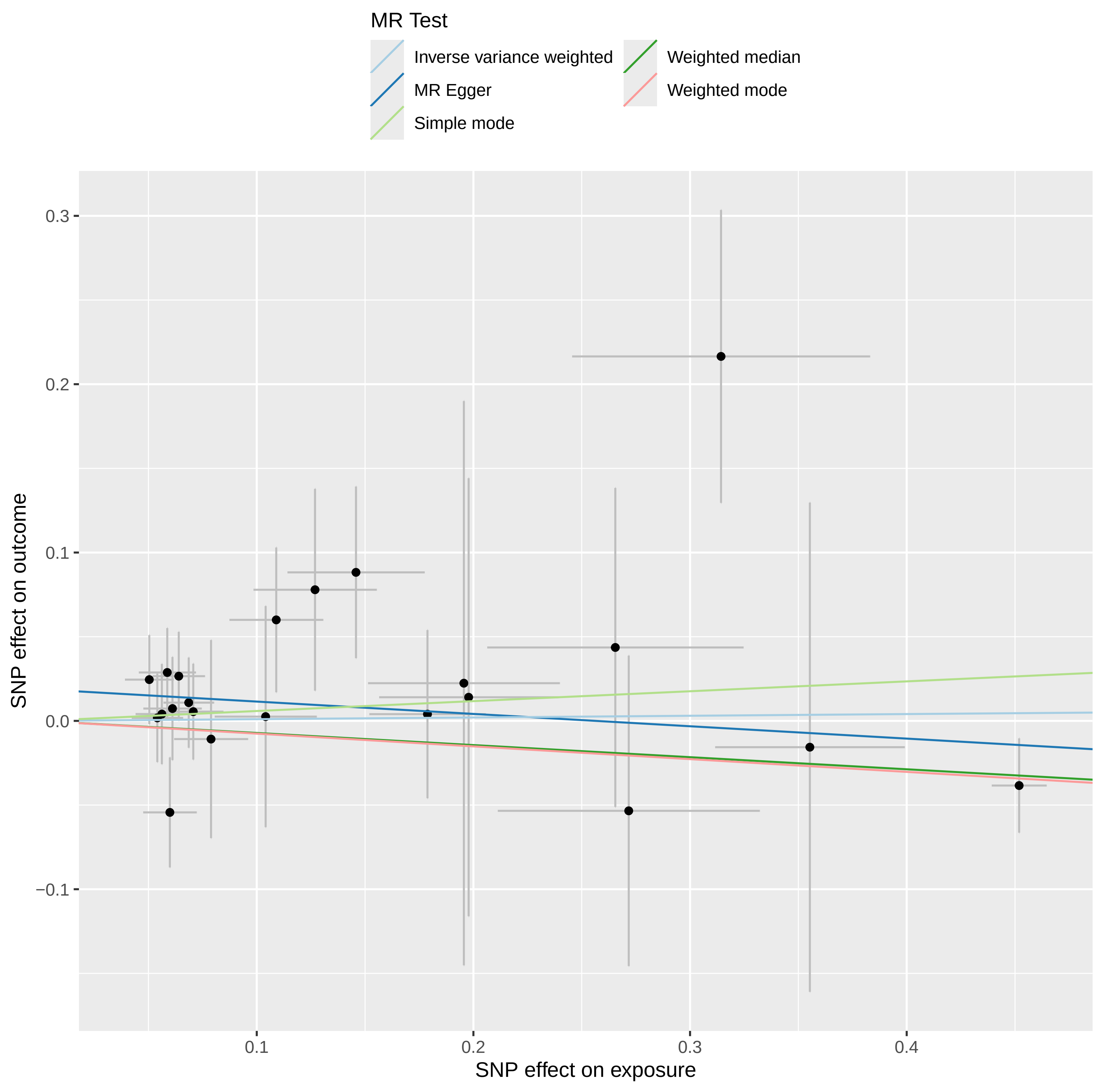
MR Test
Inverse variance weighted
MR Egger
Simple mode
Weighted median
Weighted mode
SNP effect on outcome
SNP effect on exposure
0.3
0.2
0.1
0.0
−0.1
0.1
0.2
0.3
0.4

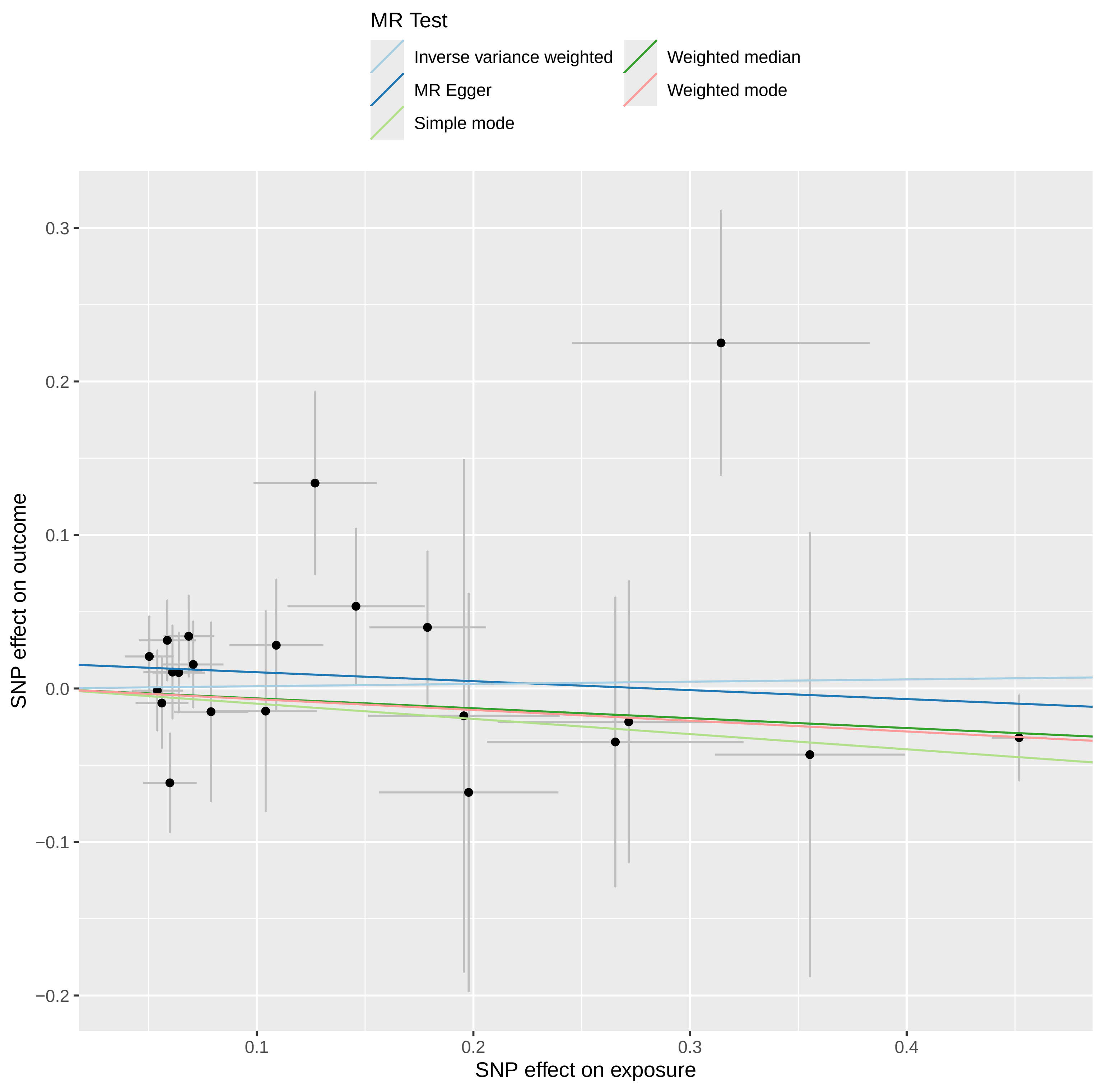
MR Test
Inverse variance weighted
MR Egger
Simple mode
Weighted median
Weighted mode
SNP effect on outcome
0.3
0.2
0.1
0.0
−0.1
−0.2
0.1
0.2
0.3
0.4
SNP effect on exposure

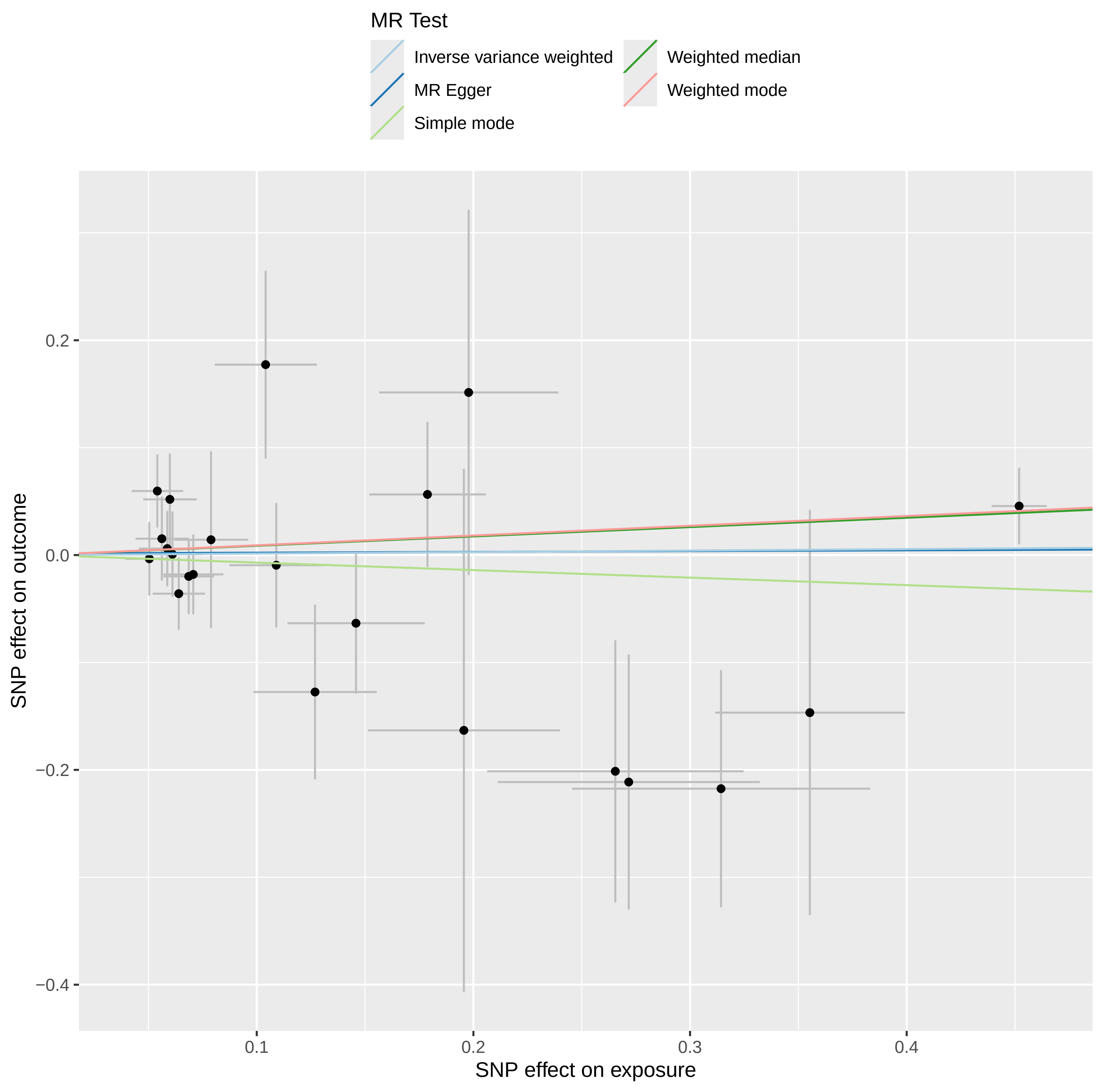
MR Test
Inverse variance weighted
MR Egger
Simple mode
Weighted median
Weighted mode
0.2
0.0
−0.2
−0.4
0.1
0.2
0.3
0.4
SNP effect on exposure
SNP effect on outcome

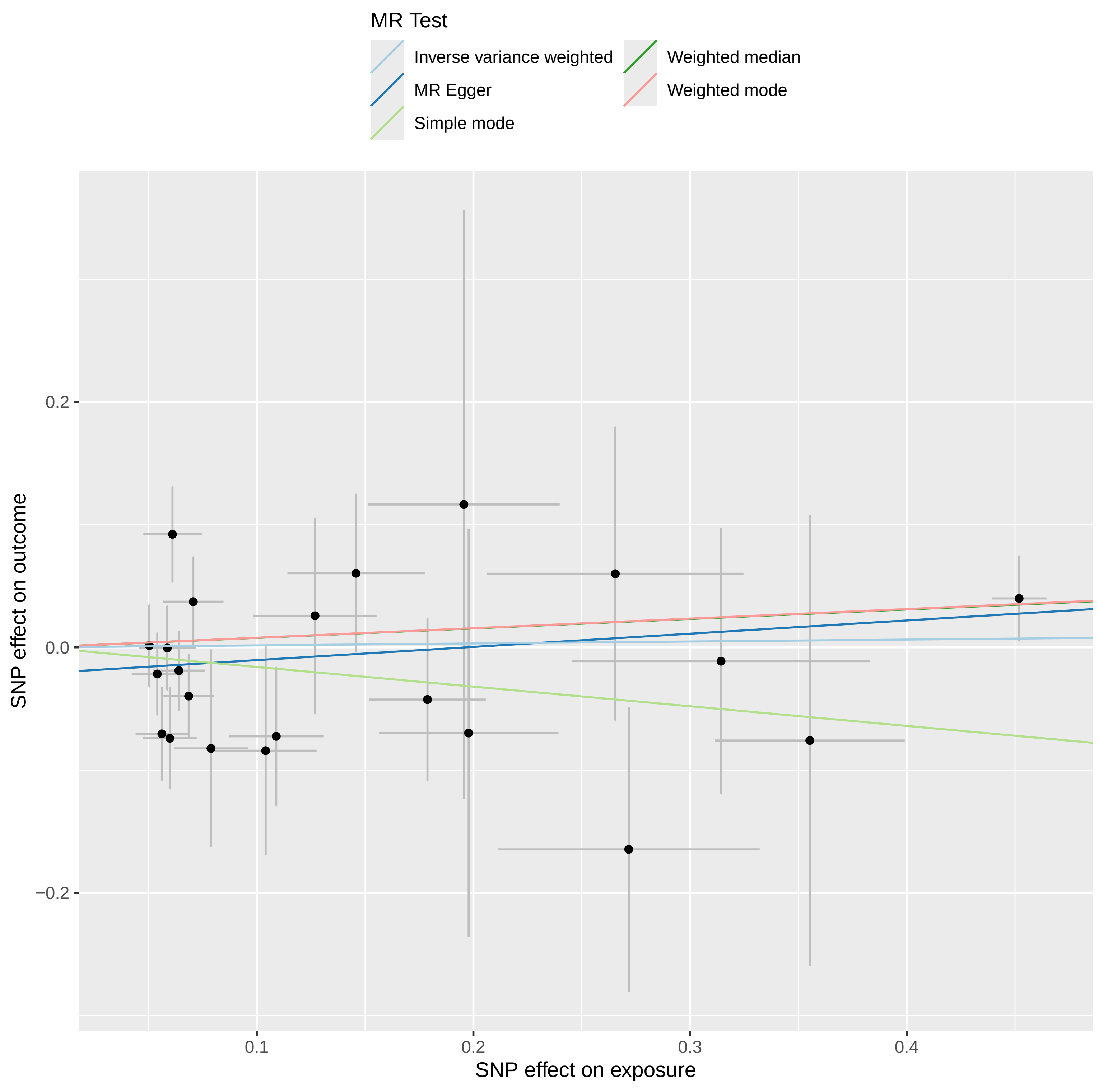
MR Test
Inverse variance weighted
MR Egger
Simple mode
Weighted median
Weighted mode
0.2
0.0
−0.2
0.1
0.2
0.3
0.4
SNP effect on outcome
SNP effect on exposure

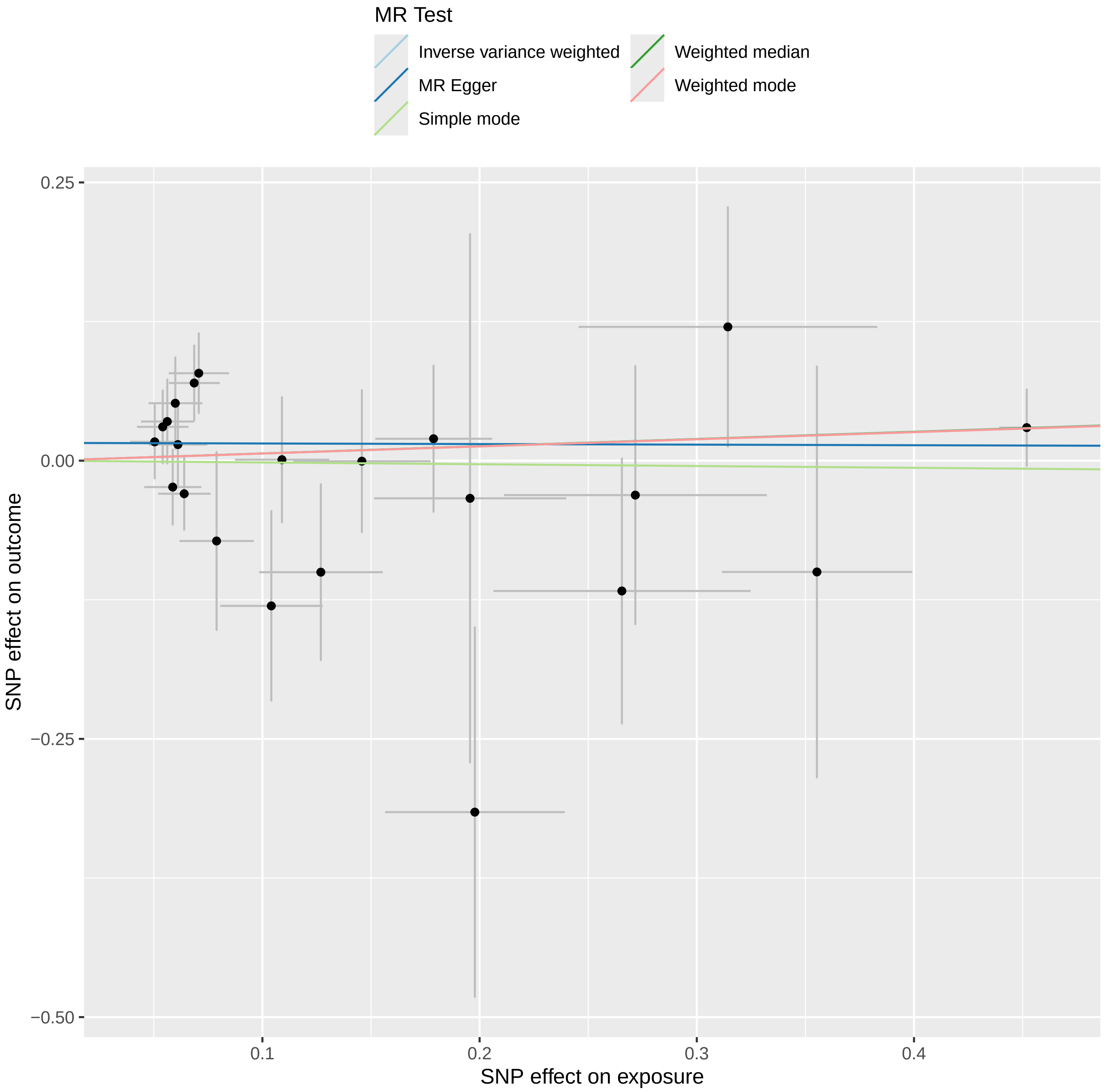
MR Test
Inverse variance weighted
MR Egger
Simple mode
Weighted median
Weighted mode
0.25
0.00
−0.25
−0.50
SNP effect on outcome
0.1
0.2
0.3
0.4
SNP effect on exposure

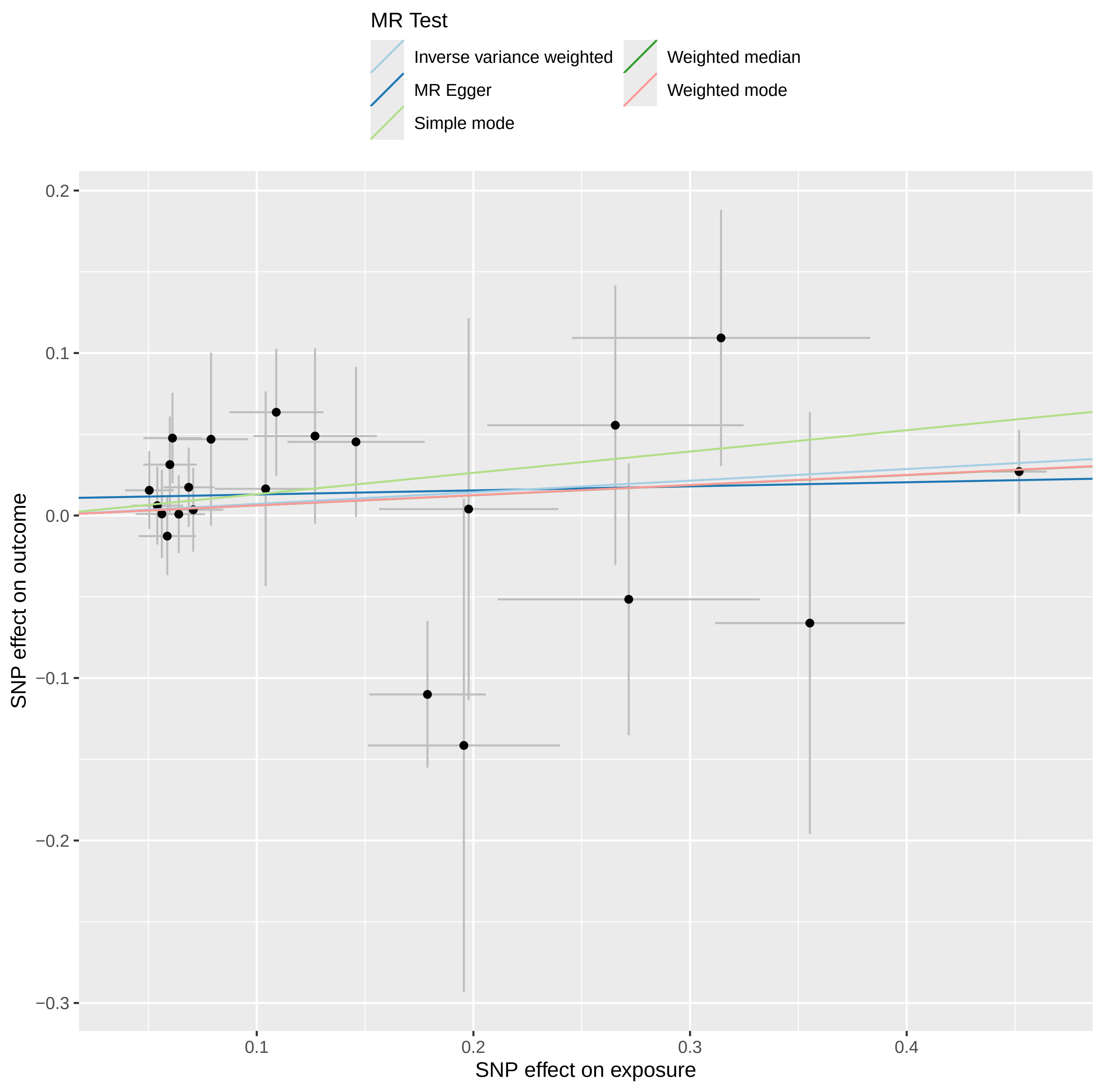
MR Test
Inverse variance weighted
MR Egger
Simple mode
Weighted median
Weighted mode
0.2
0.1
0.0
−0.1
−0.2
−0.3
SNP effect on outcome
0.1
0.2
0.3
0.4
SNP effect on exposure

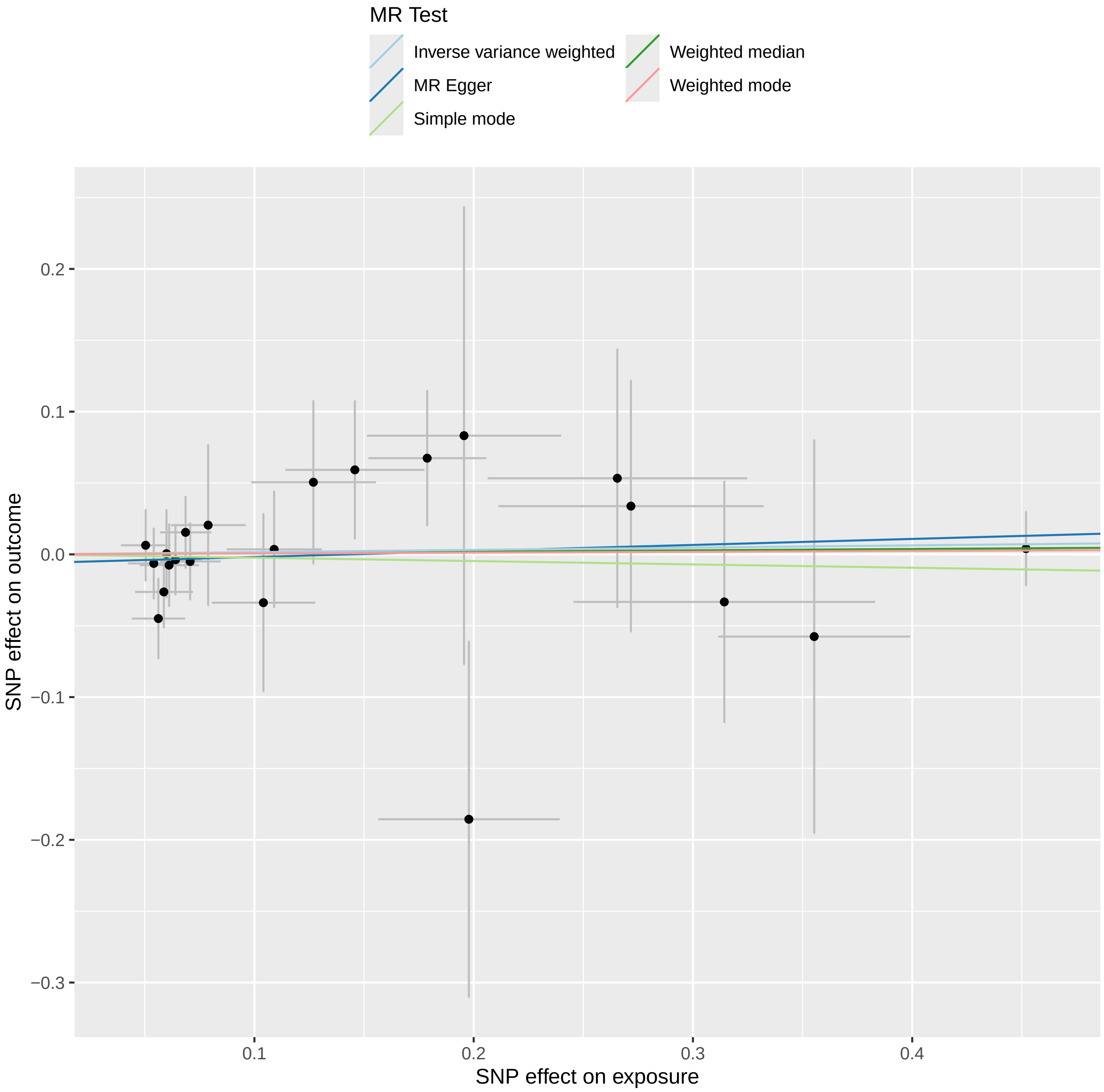
MR Test
Inverse variance weighted
MR Egger
Simple mode
Weighted median
Weighted mode
0.2
0.1
0.0
−0.1
−0.2
−0.3
SNP effect on outcome
0.1
0.2
0.3
0.4
SNP effect on exposure

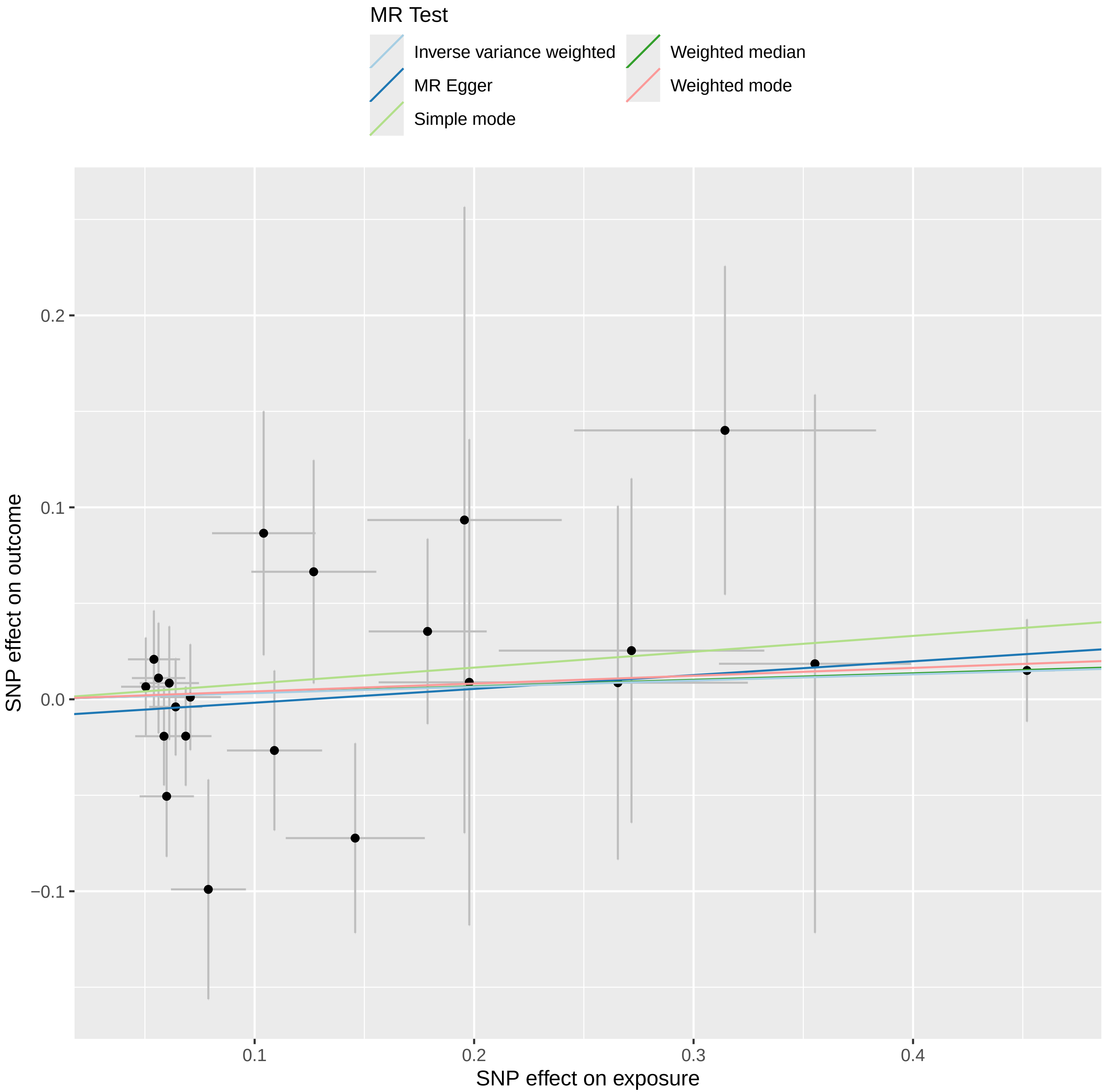
MR Test
Inverse variance weighted
MR Egger
Simple mode
Weighted median
Weighted mode
0.2
0.1
0.0
−0.1
0.1
0.2
0.3
0.4
SNP effect on outcome
SNP effect on exposure

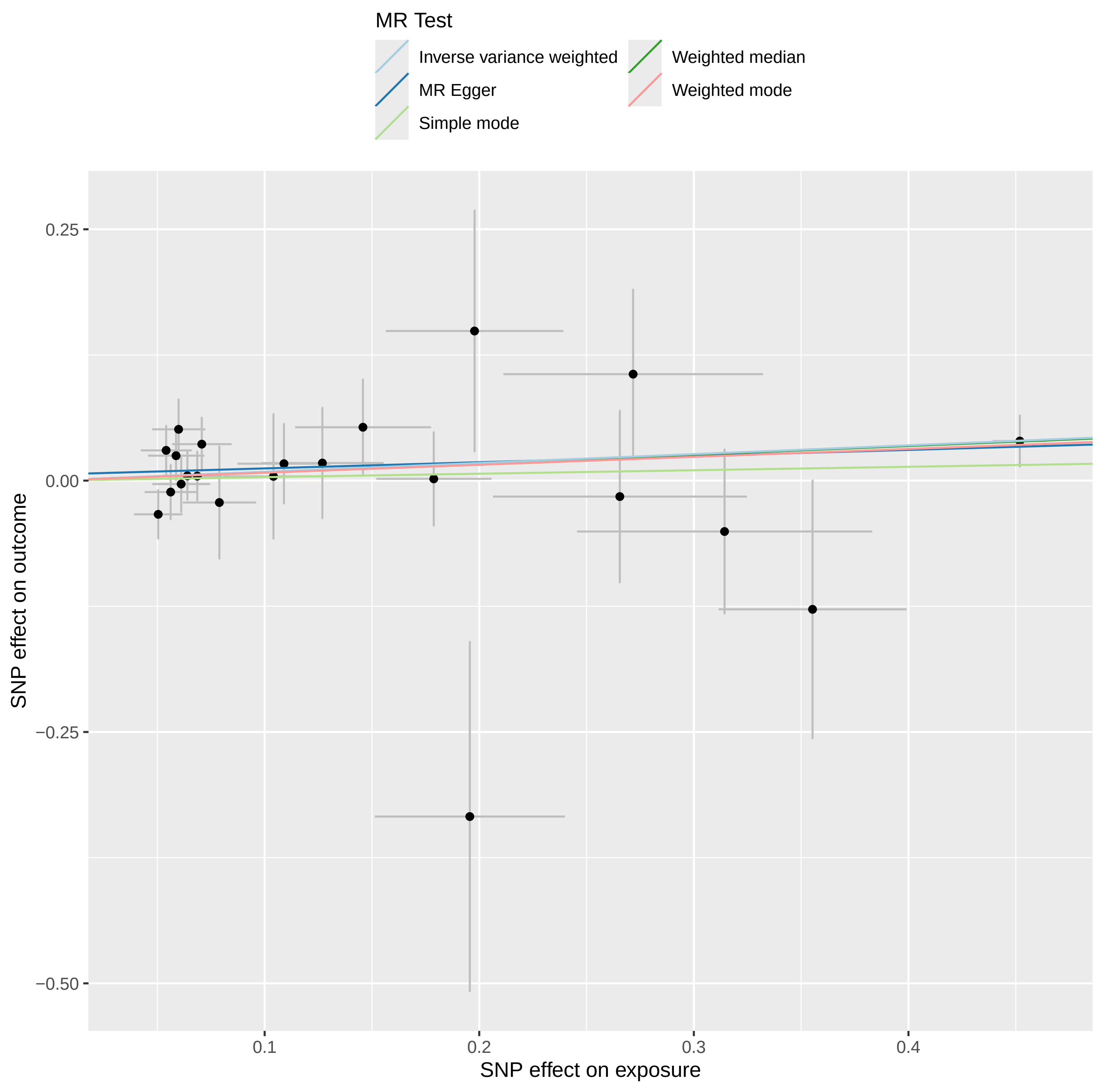
MR Test
Inverse variance weighted
MR Egger
Simple mode
Weighted median
Weighted mode
0.25
0.00
−0.25
−0.50
0.1
0.2
0.3
0.4
SNP effect on exposure
SNP effect on outcome

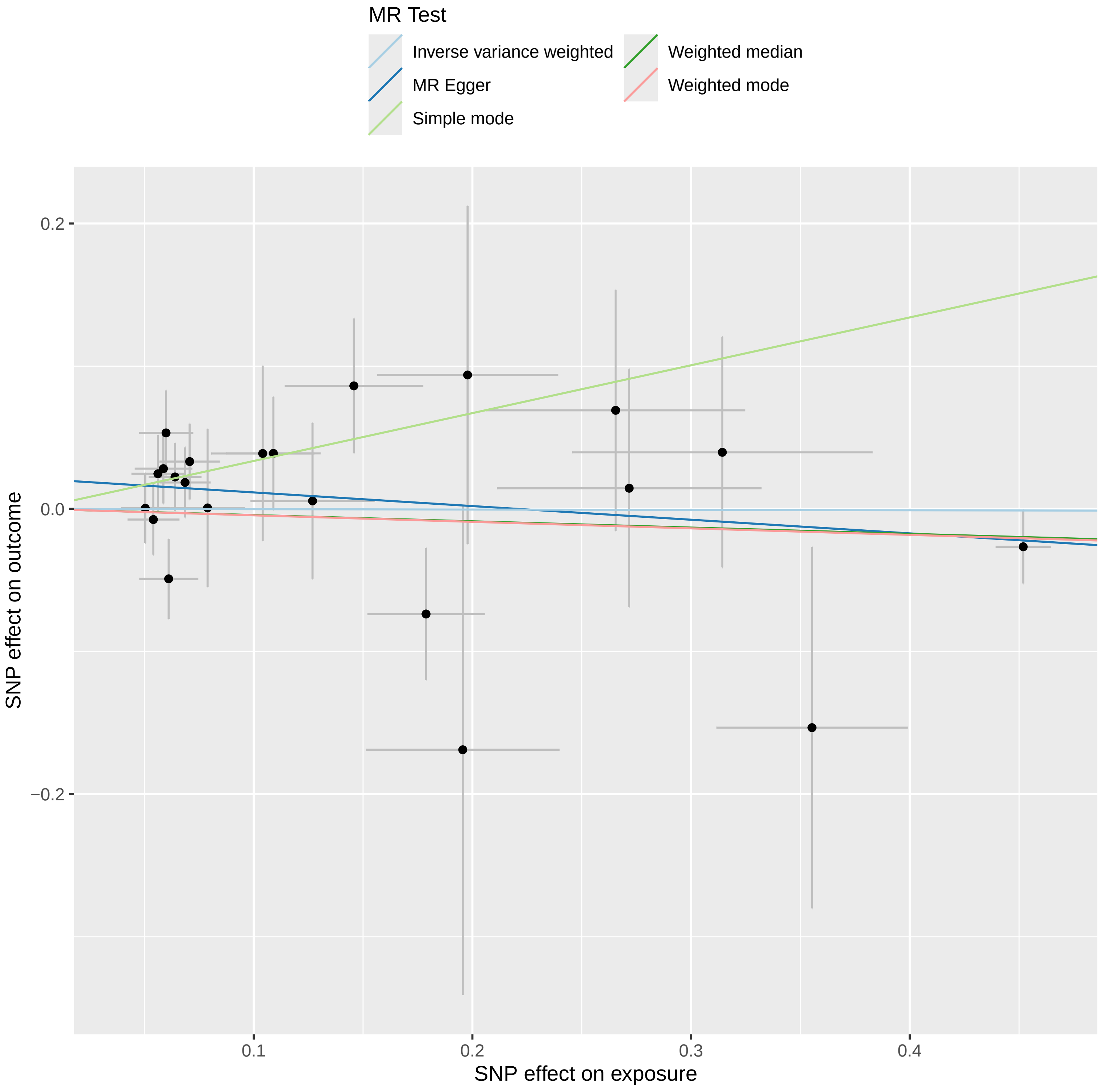

MR Test
Inverse variance weighted
MR Egger
Simple mode
Weighted median
Weighted mode
0.2
0.0
−0.2
0.1
0.2
0.3
0.4
SNP effect on outcome
SNP effect on exposure

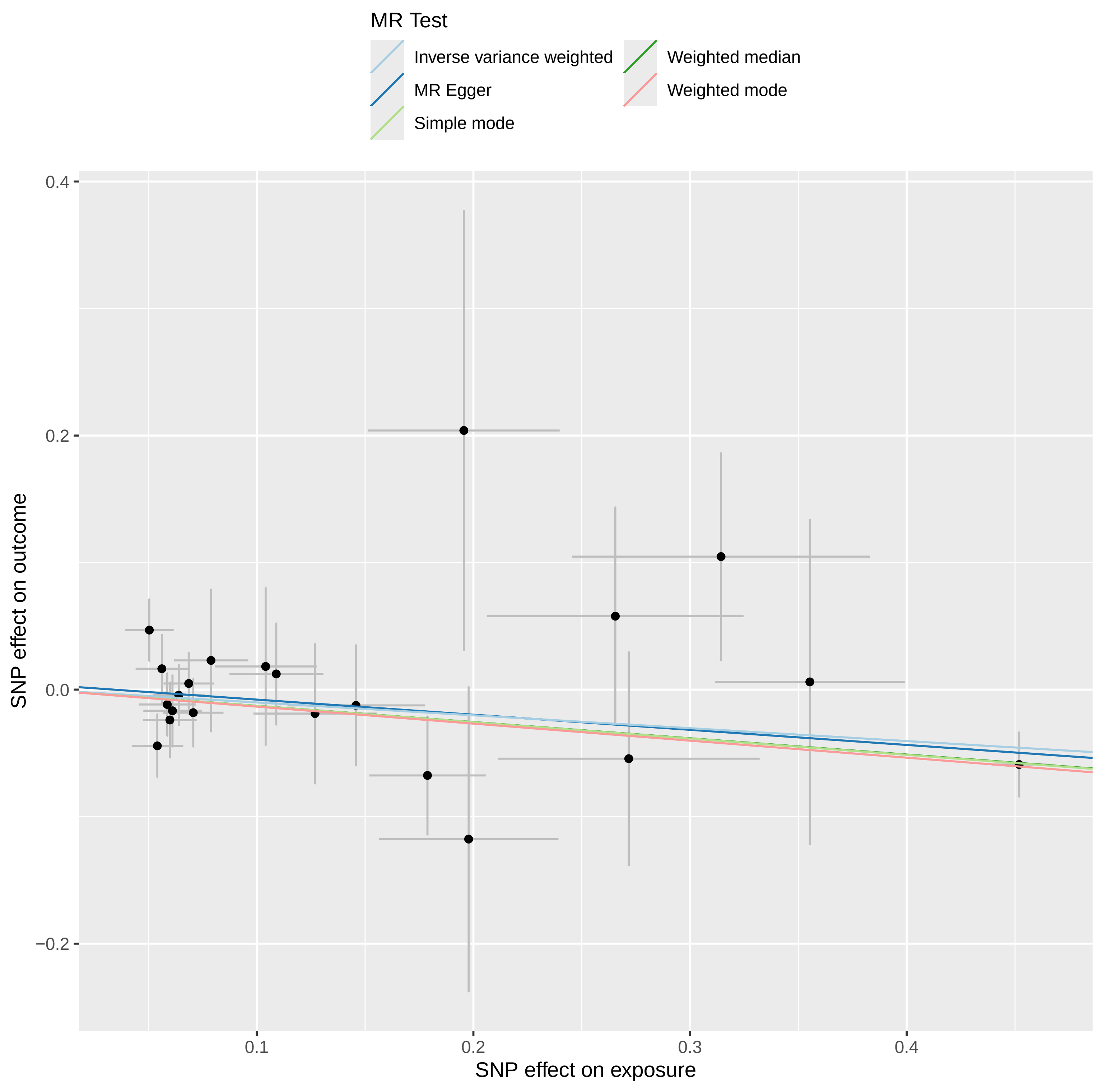

MR Test
Inverse variance weighted
MR Egger
Simple mode
Weighted median
Weighted mode
0.4
0.2
0.0
−0.2
0.1
0.2
0.3
0.4
SNP effect on outcome
SNP effect on exposure

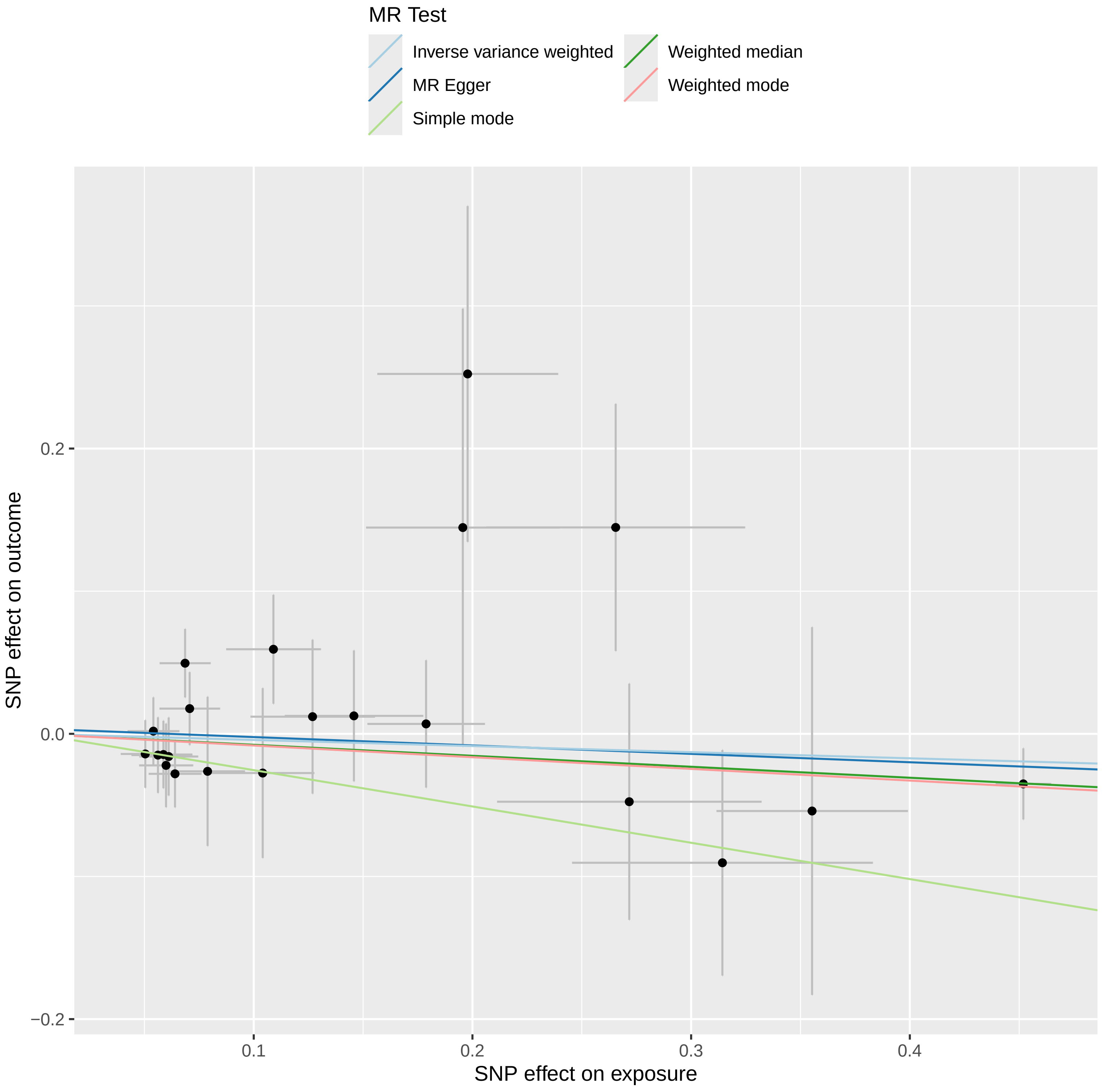

MR Test
Inverse variance weighted
MR Egger
Simple mode
Weighted median
Weighted mode
0.2
0.0
−0.2
0.1
0.2
0.3
0.4
SNP effect on outcome
SNP effect on exposure

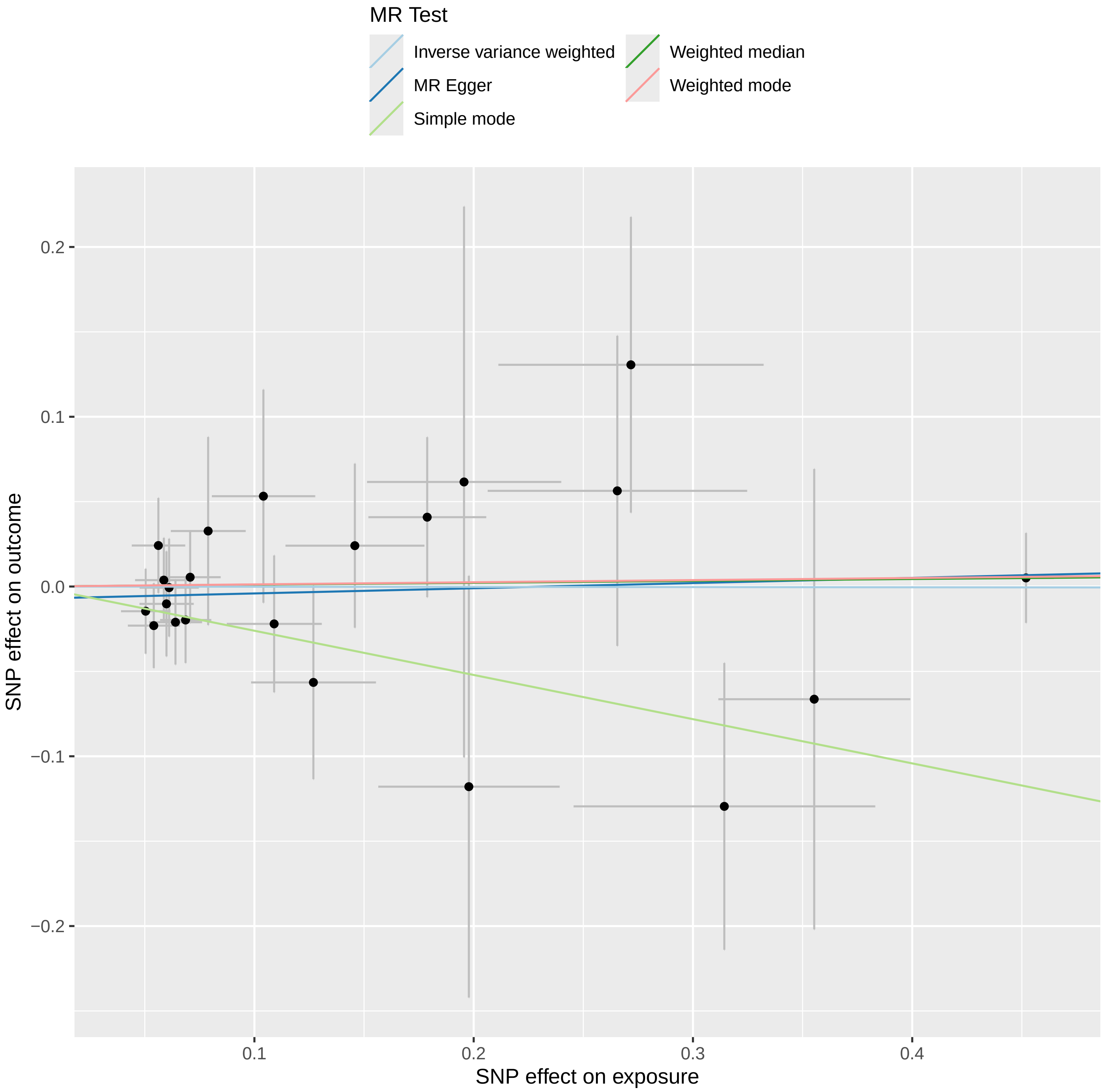

MR Test
Inverse variance weighted
MR Egger
Simple mode
Weighted median
Weighted mode
0.2
0.1
0.0
−0.1
−0.2
SNP effect on outcome
0.1
0.2
0.3
0.4
SNP effect on exposure

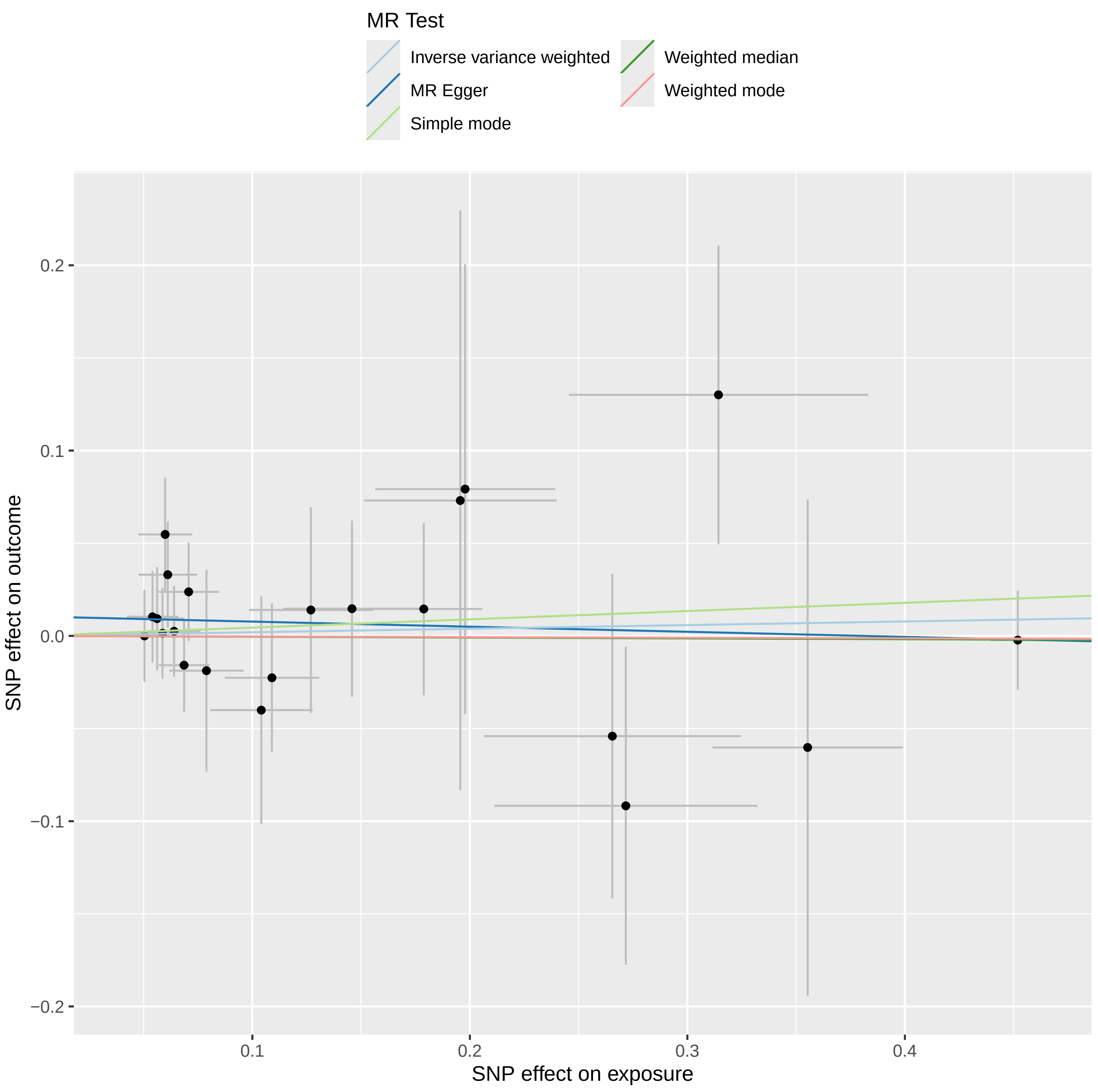

MR Test
Inverse variance weighted
MR Egger
Simple mode
Weighted median
Weighted mode
0.2
0.1
0.0
−0.1
−0.2
SNP effect on outcome
0.1
0.2
0.3
0.4
SNP effect on exposure

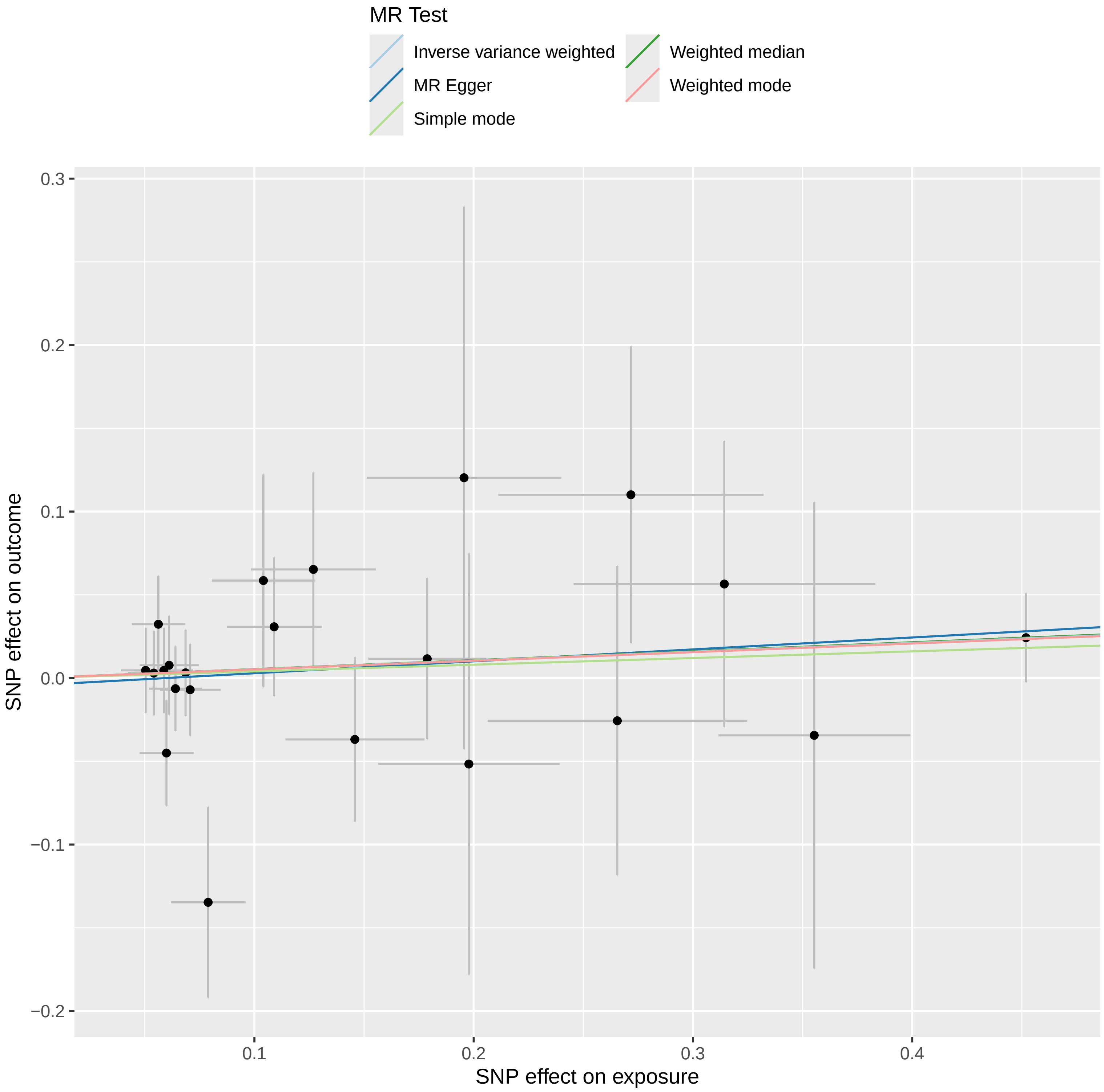

MR Test
Inverse variance weighted
MR Egger
Simple mode
Weighted median
Weighted mode
0.3
0.2
0.1
0.0
−0.1
−0.2
SNP effect on outcome
0.1
0.2
0.3
0.4
SNP effect on exposure

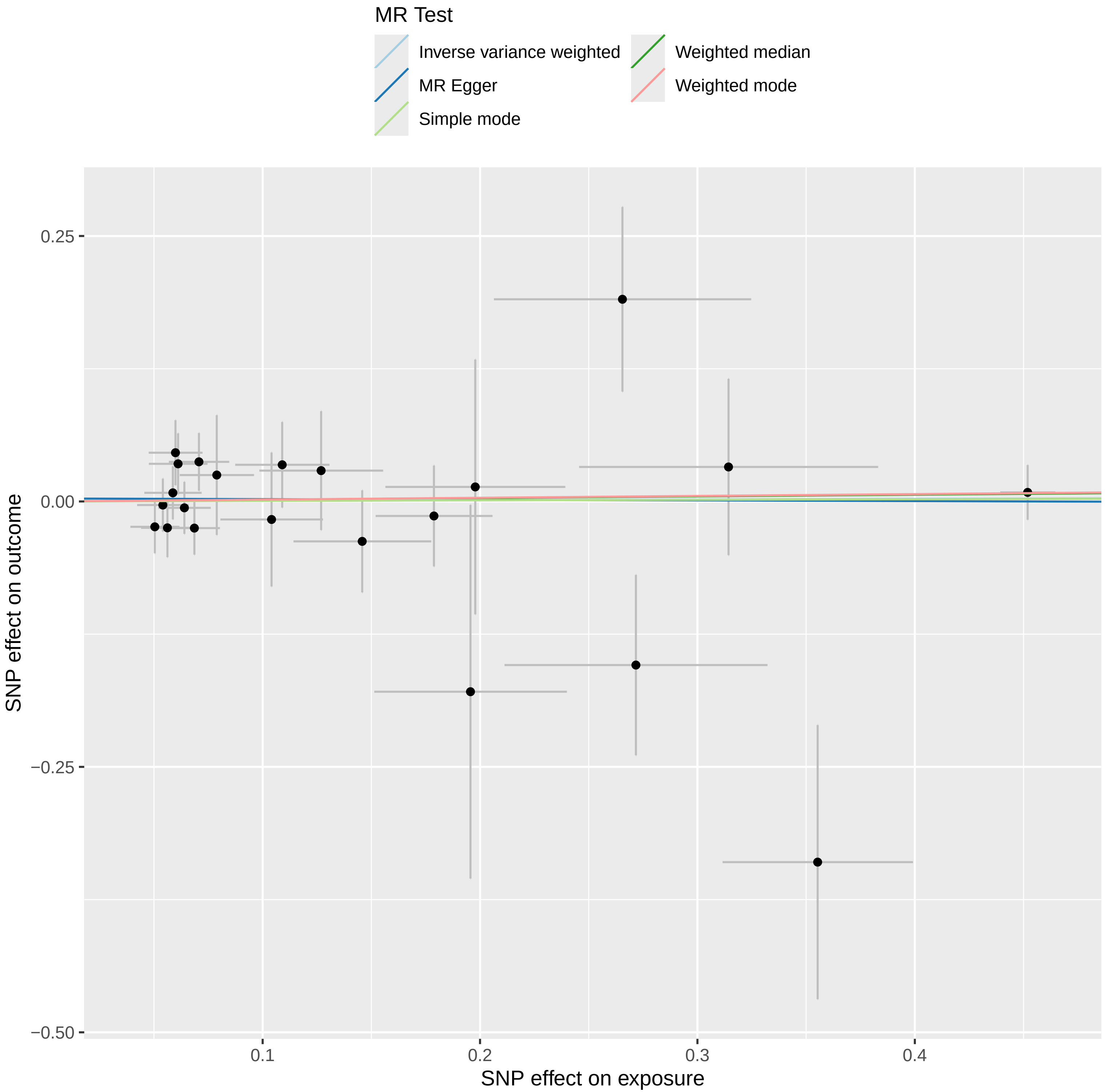
MR Test
Inverse variance weighted
MR Egger
Simple mode
Weighted median
Weighted mode
0.25
0.00
−0.25
−0.50
0.1
0.2
0.3
0.4
SNP effect on exposure
SNP effect on outcome

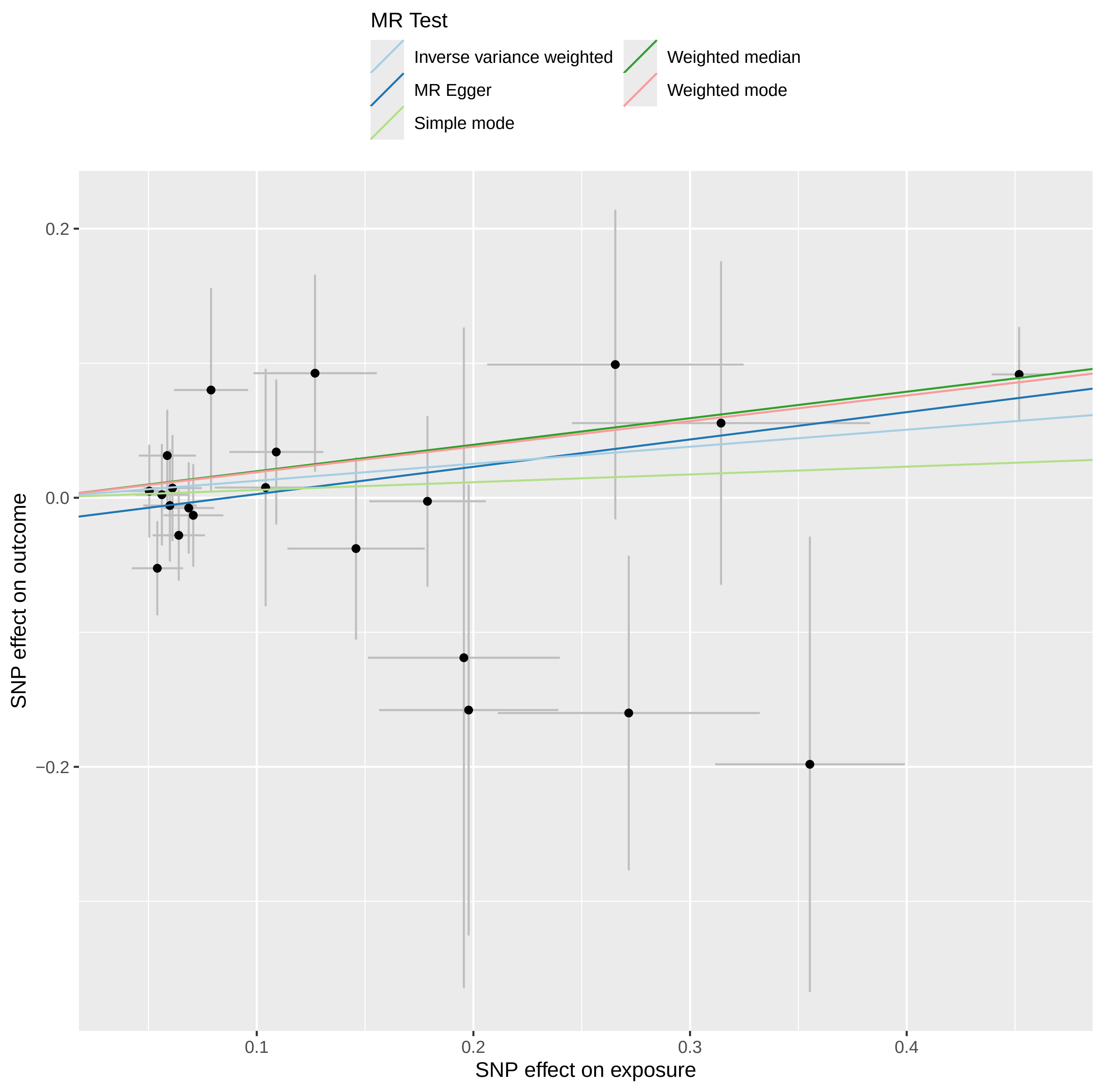
MR Test
Inverse variance weighted
MR Egger
Simple mode
Weighted median
Weighted mode
0.2
0.0
−0.2
0.1
0.2
0.3
0.4
SNP effect on outcome
SNP effect on exposure

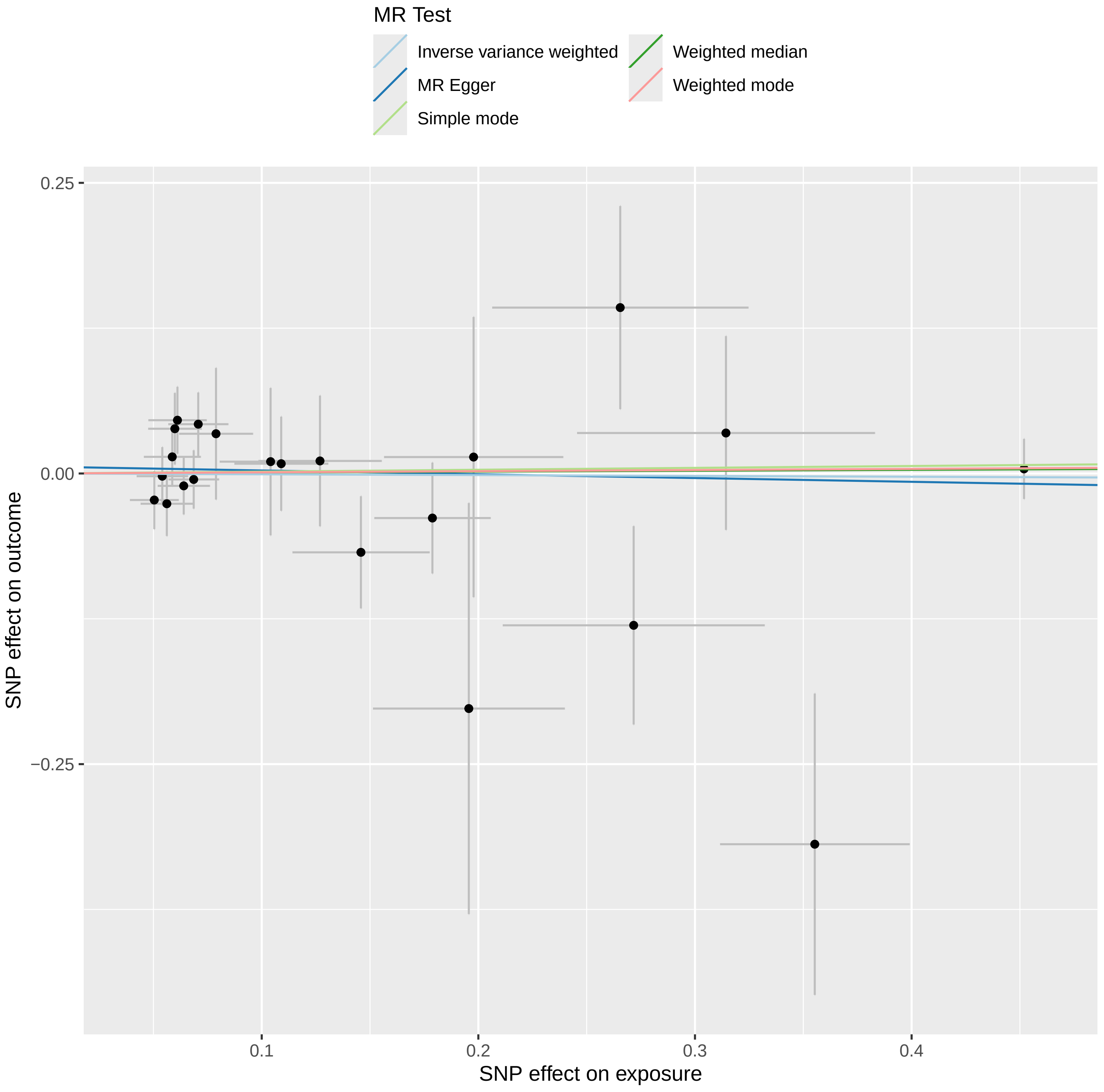

MR Test
Inverse variance weighted
MR Egger
Simple mode
Weighted median
Weighted mode
0.25
0.00
−0.25
0.1
0.2
0.3
0.4
SNP effect on exposure
SNP effect on outcome

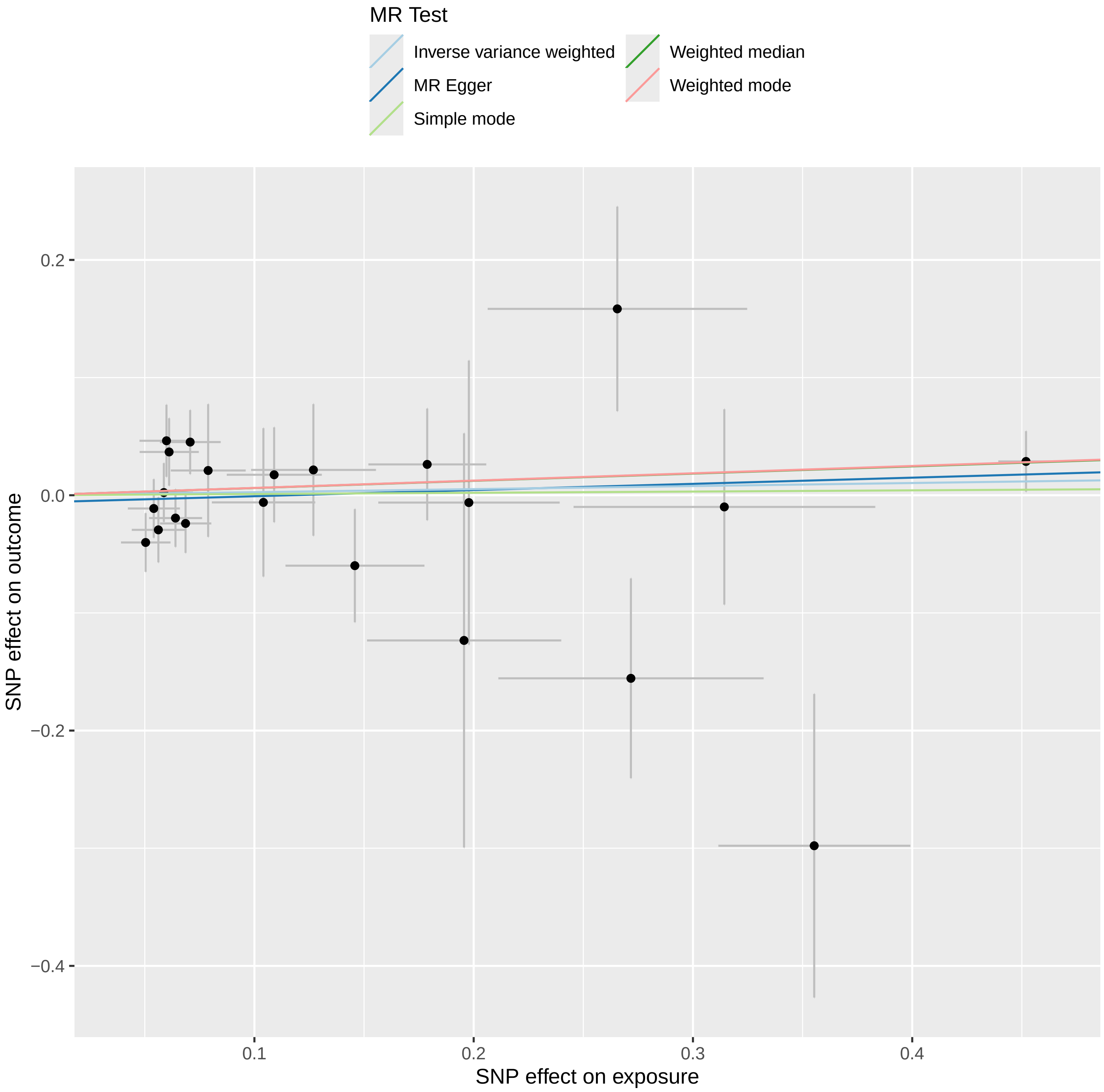
MR Test
Inverse variance weighted
MR Egger
Simple mode
Weighted median
Weighted mode
0.2
0.0
−0.2
−0.4
SNP effect on outcome
0.1
0.2
0.3
0.4
SNP effect on exposure

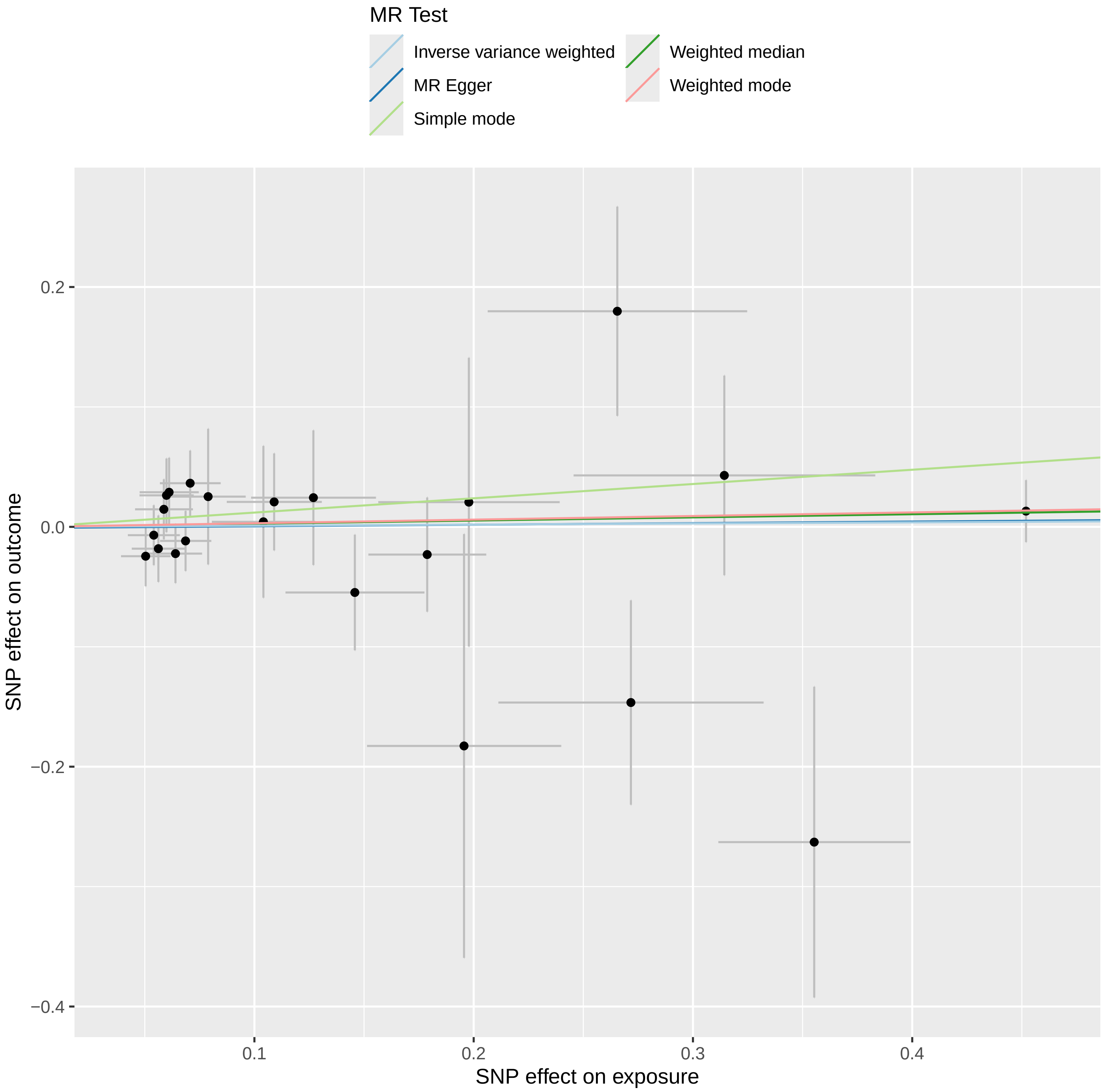
MR Test
Inverse variance weighted
MR Egger
Simple mode
Weighted median
Weighted mode
0.2
0.0
−0.2
−0.4
SNP effect on outcome
0.1
0.2
0.3
0.4
SNP effect on exposure

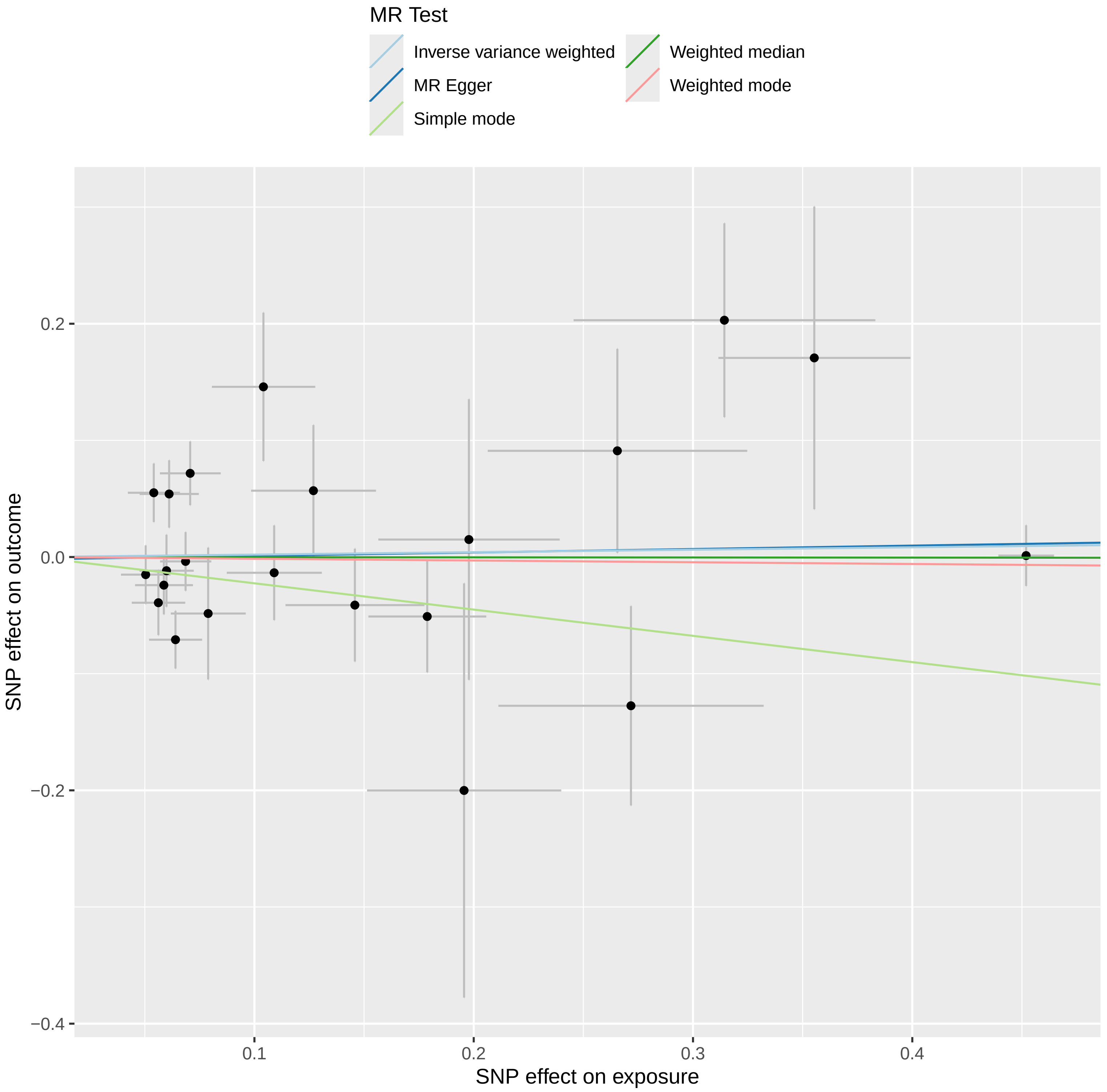
MR Test
Inverse variance weighted
MR Egger
Simple mode
Weighted median
Weighted mode
SNP effect on outcome
0.2
0.0
−0.2
−0.4
0.1
0.2
0.3
0.4
SNP effect on exposure

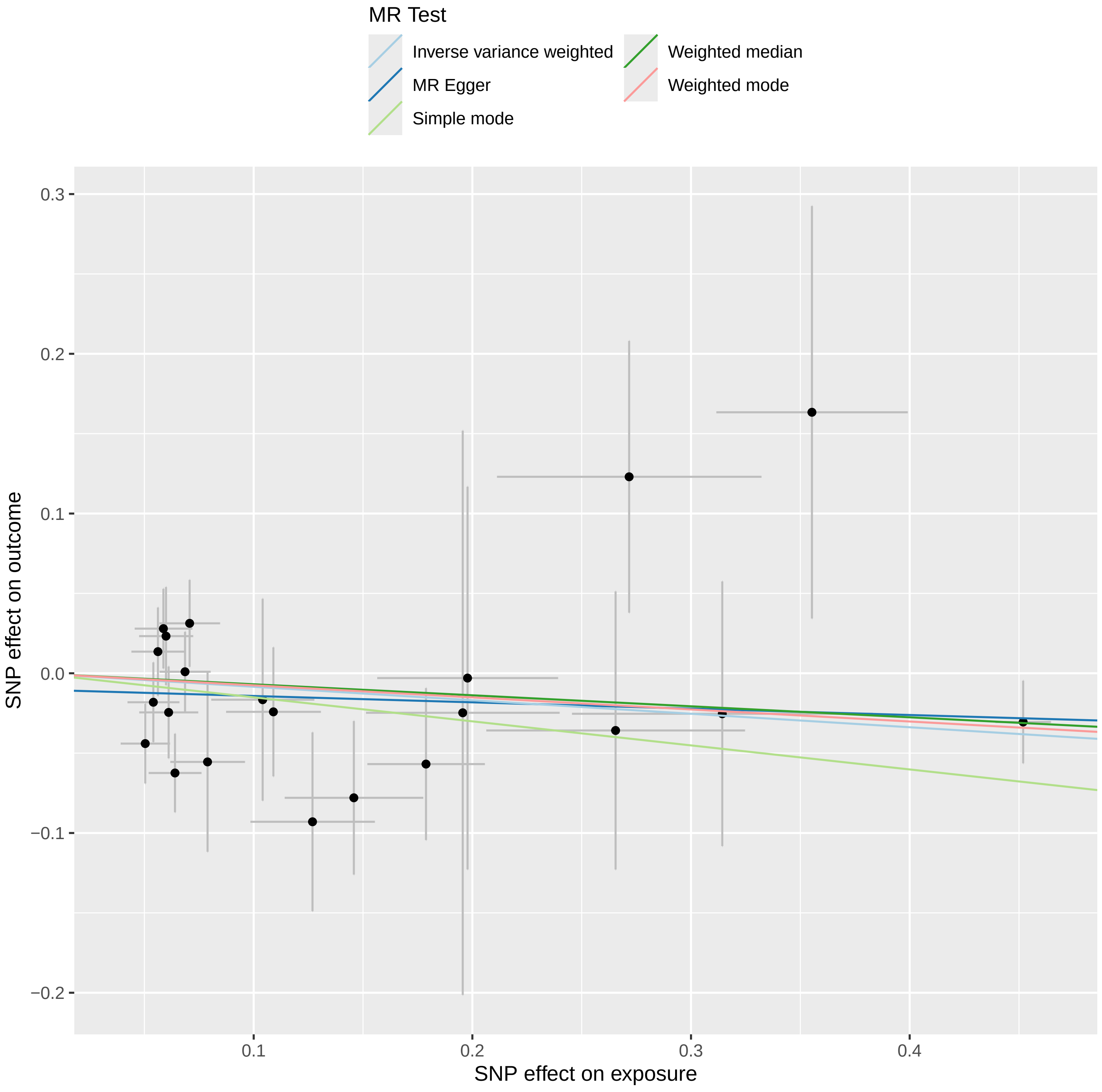
MR Test
Inverse variance weighted
MR Egger
Simple mode
Weighted median
Weighted mode
0.3
0.2
0.1
0.0
−0.1
−0.2
0.1
0.2
0.3
0.4
SNP effect on outcome
SNP effect on exposure

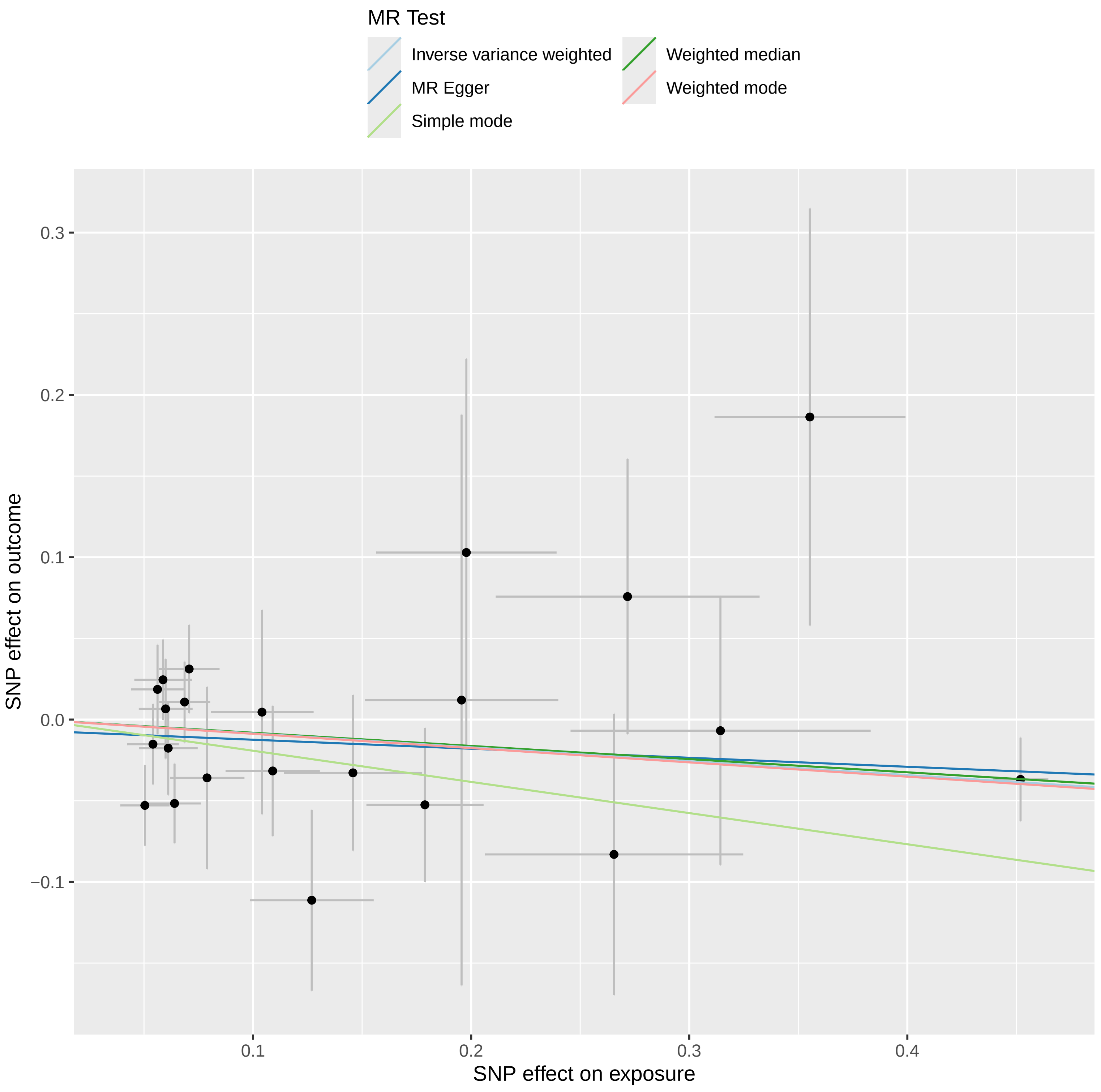
MR Test
Inverse variance weighted
MR Egger
Simple mode
Weighted median
Weighted mode
SNP effect on outcome
0.3
0.2
0.1
0.0
−0.1
0.1
0.2
0.3
0.4
SNP effect on exposure

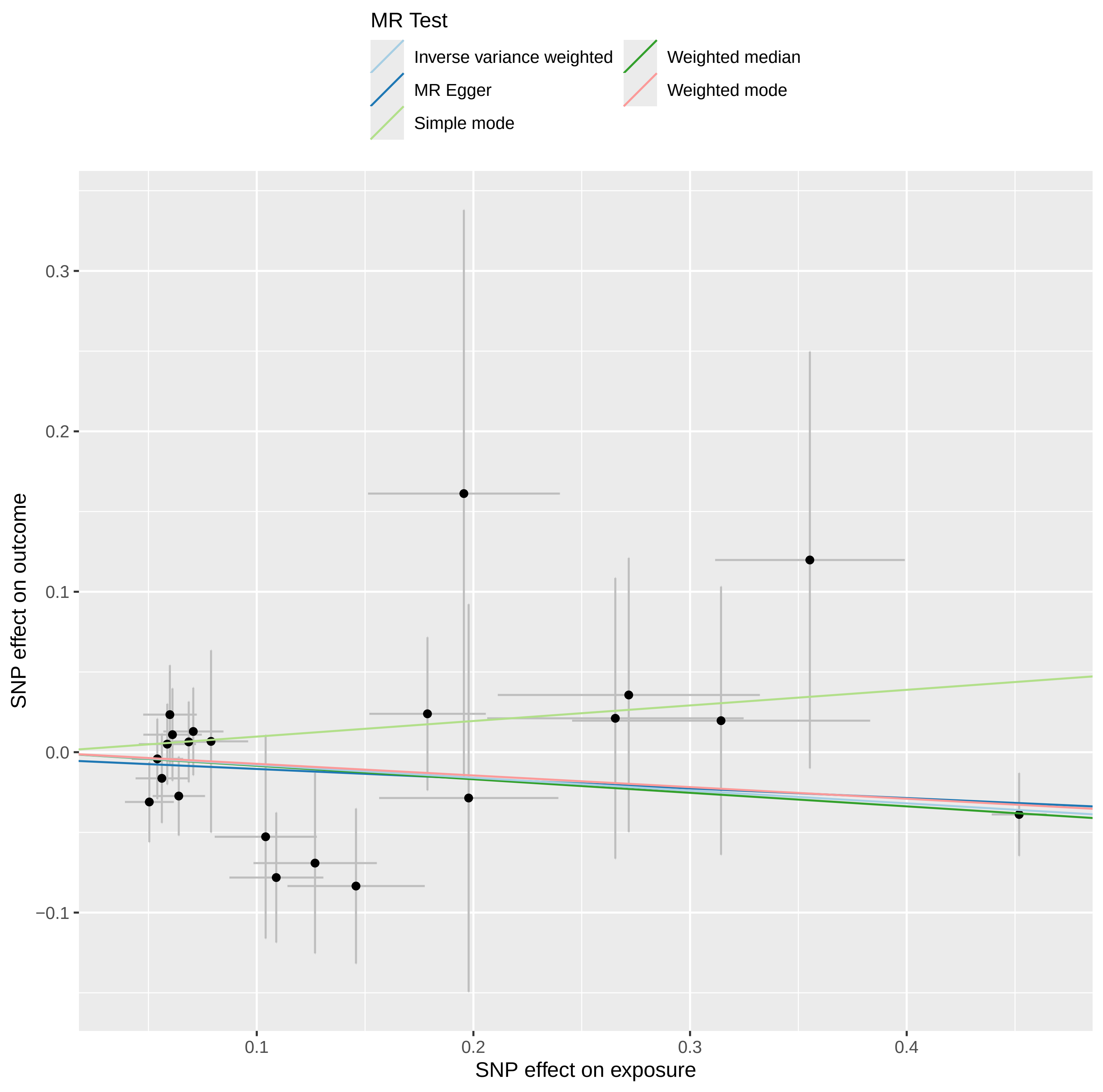
MR Test
Inverse variance weighted
MR Egger
Simple mode
Weighted median
Weighted mode
0.3
0.2
0.1
0.0
−0.1
SNP effect on outcome
0.1
0.2
0.3
0.4
SNP effect on exposure

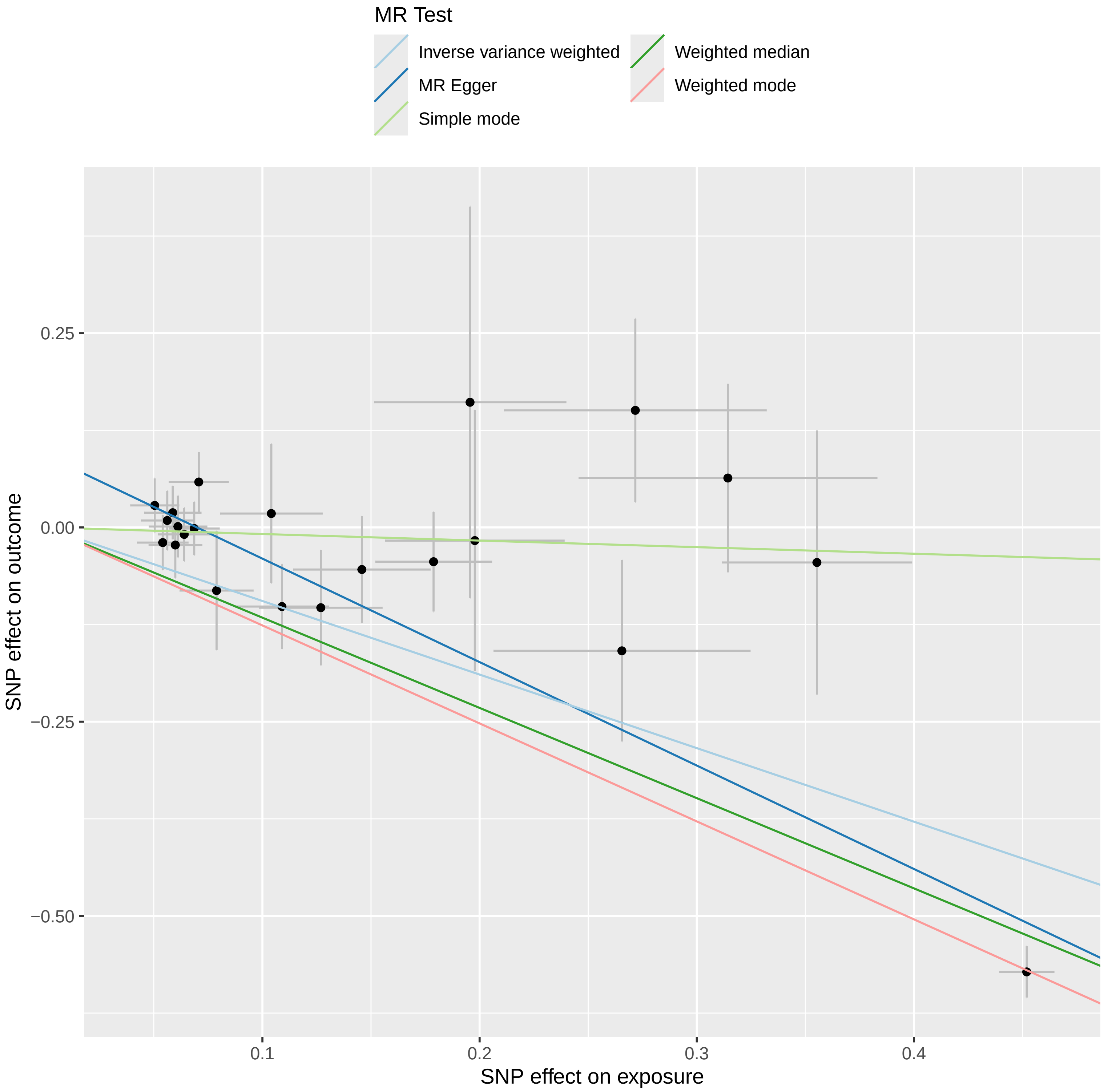
MR Test
Inverse variance weighted
MR Egger
Simple mode
Weighted median
Weighted mode
0.25
0.00
−0.25
−0.50
SNP effect on outcome
0.1
0.2
0.3
0.4
SNP effect on exposure

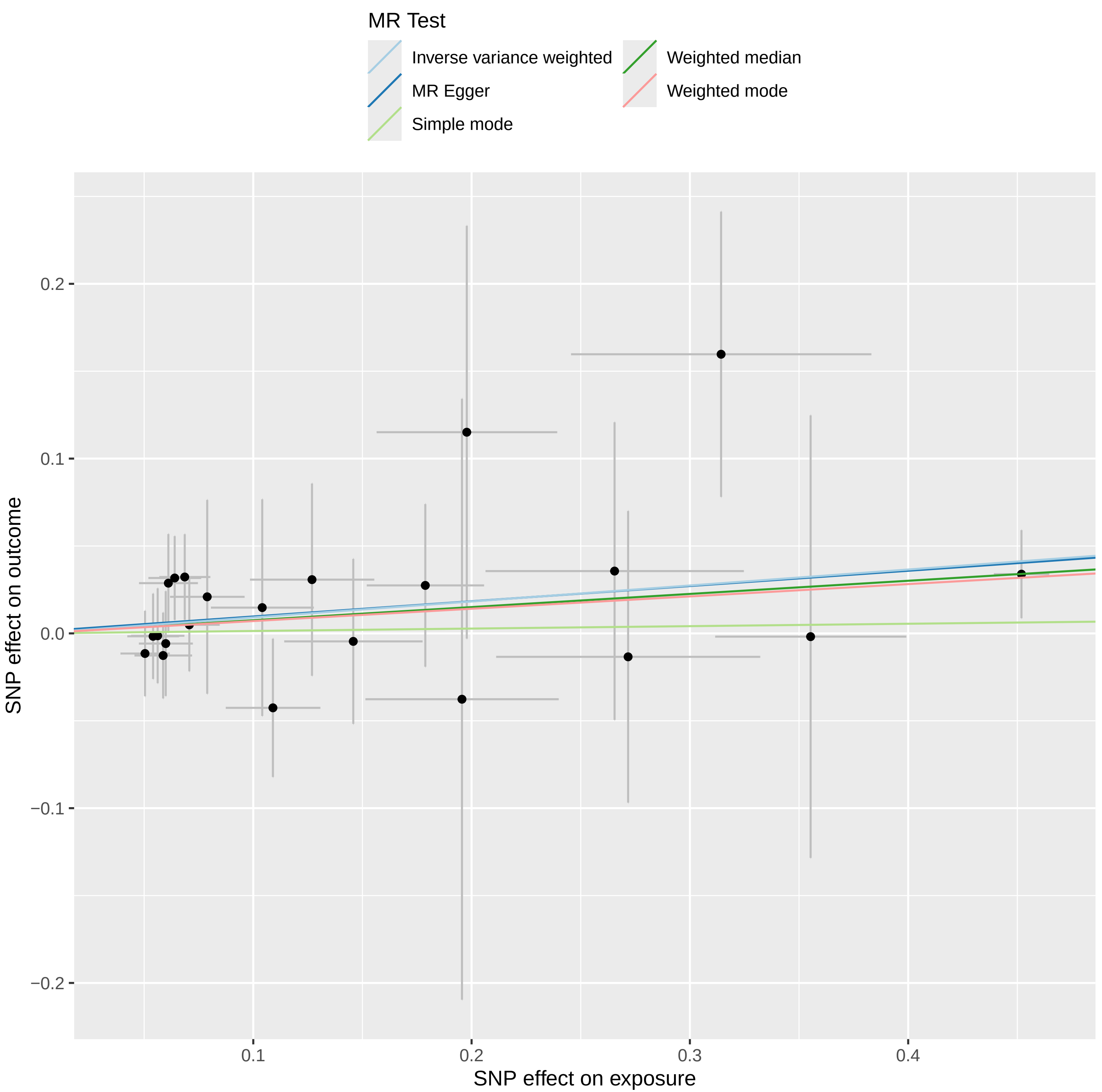

MR Test
Inverse variance weighted
MR Egger
Simple mode
Weighted median
Weighted mode
SNP effect on outcome
0.2
0.1
0.0
−0.1
−0.2
0.1
0.2
0.3
0.4
SNP effect on exposure

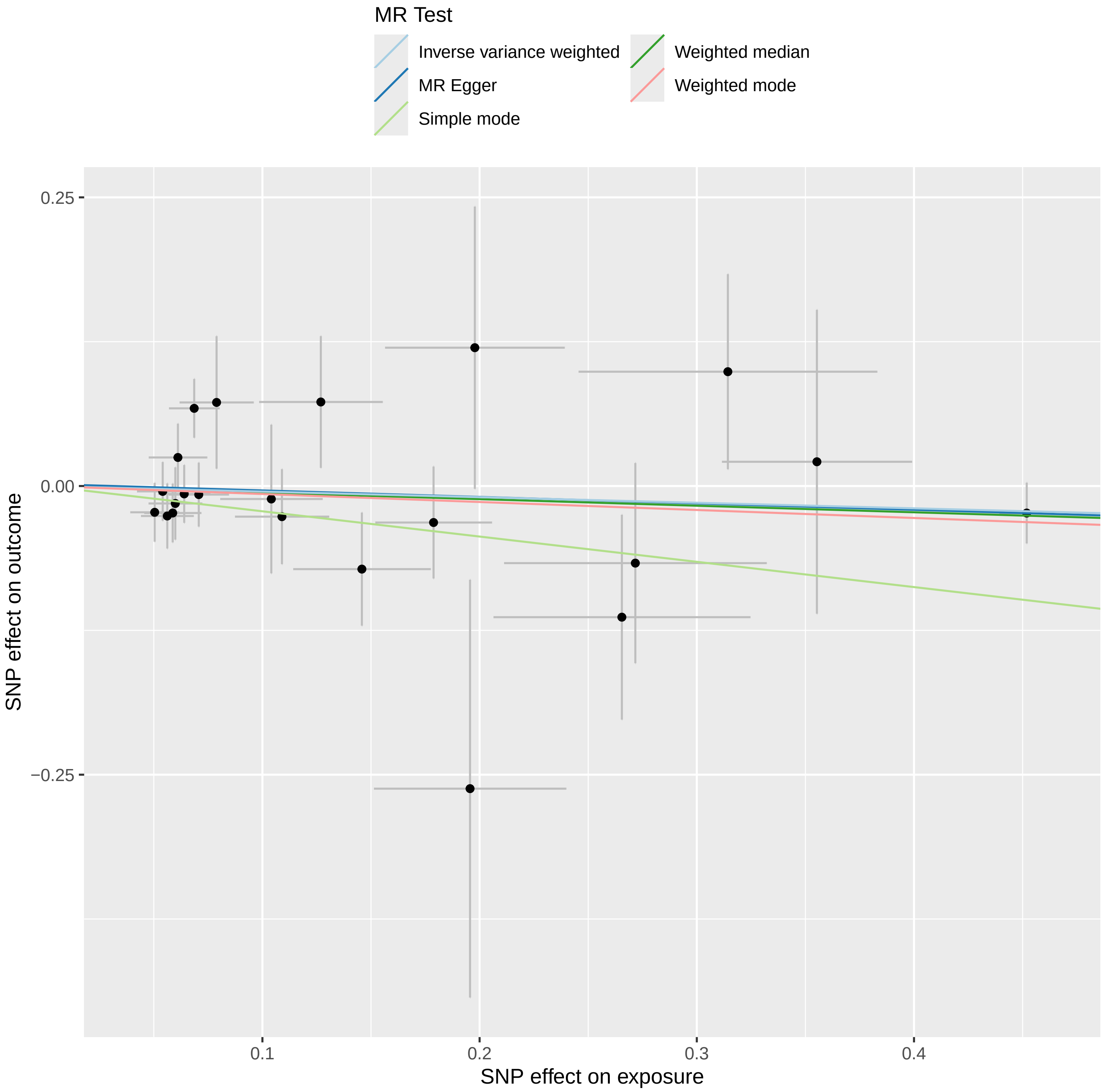
MR Test
Inverse variance weighted
MR Egger
Simple mode
Weighted median
Weighted mode
SNP effect on outcome
0.25
0.00
−0.25
SNP effect on exposure
0.1
0.2
0.3
0.4

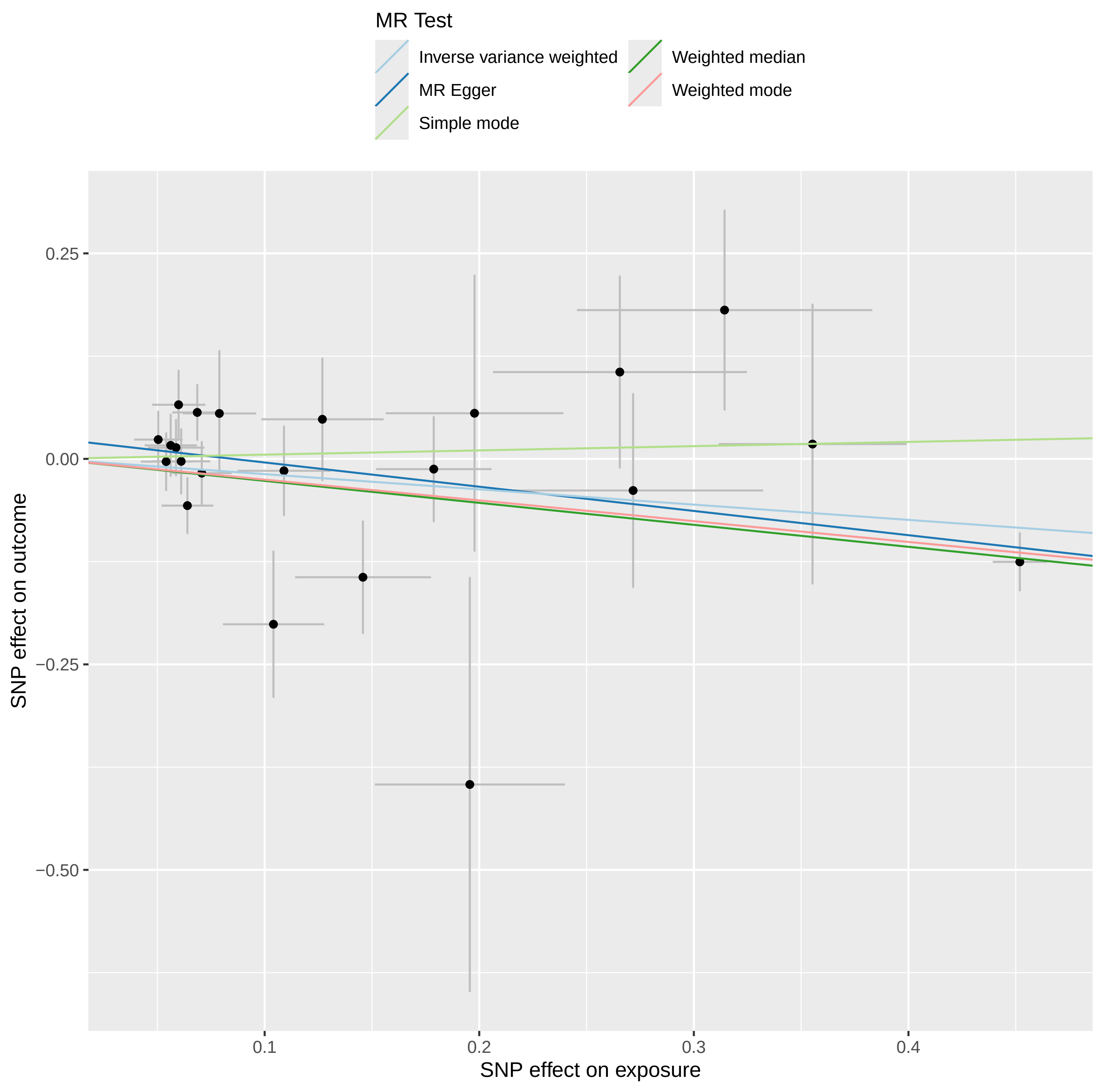

MR Test
Inverse variance weighted
MR Egger
Simple mode
Weighted median
Weighted mode
0.25
0.00
−0.25
−0.50
SNP effect on outcome
0.1
0.2
0.3
0.4
SNP effect on exposure

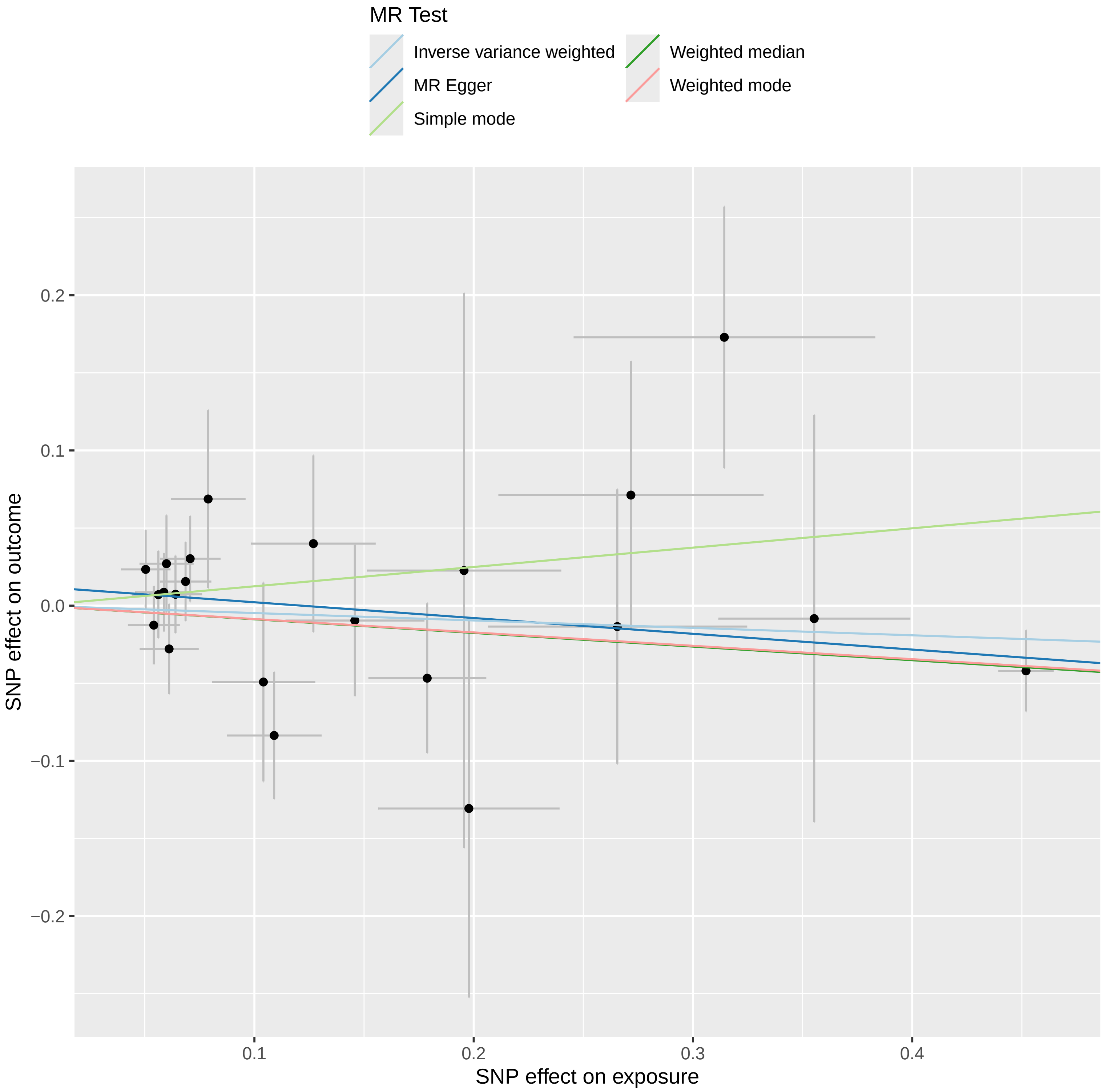

MR Test
Inverse variance weighted
MR Egger
Simple mode
Weighted median
Weighted mode
0.2
0.1
0.0
−0.1
−0.2
SNP effect on outcome
0.1
0.2
0.3
0.4
SNP effect on exposure

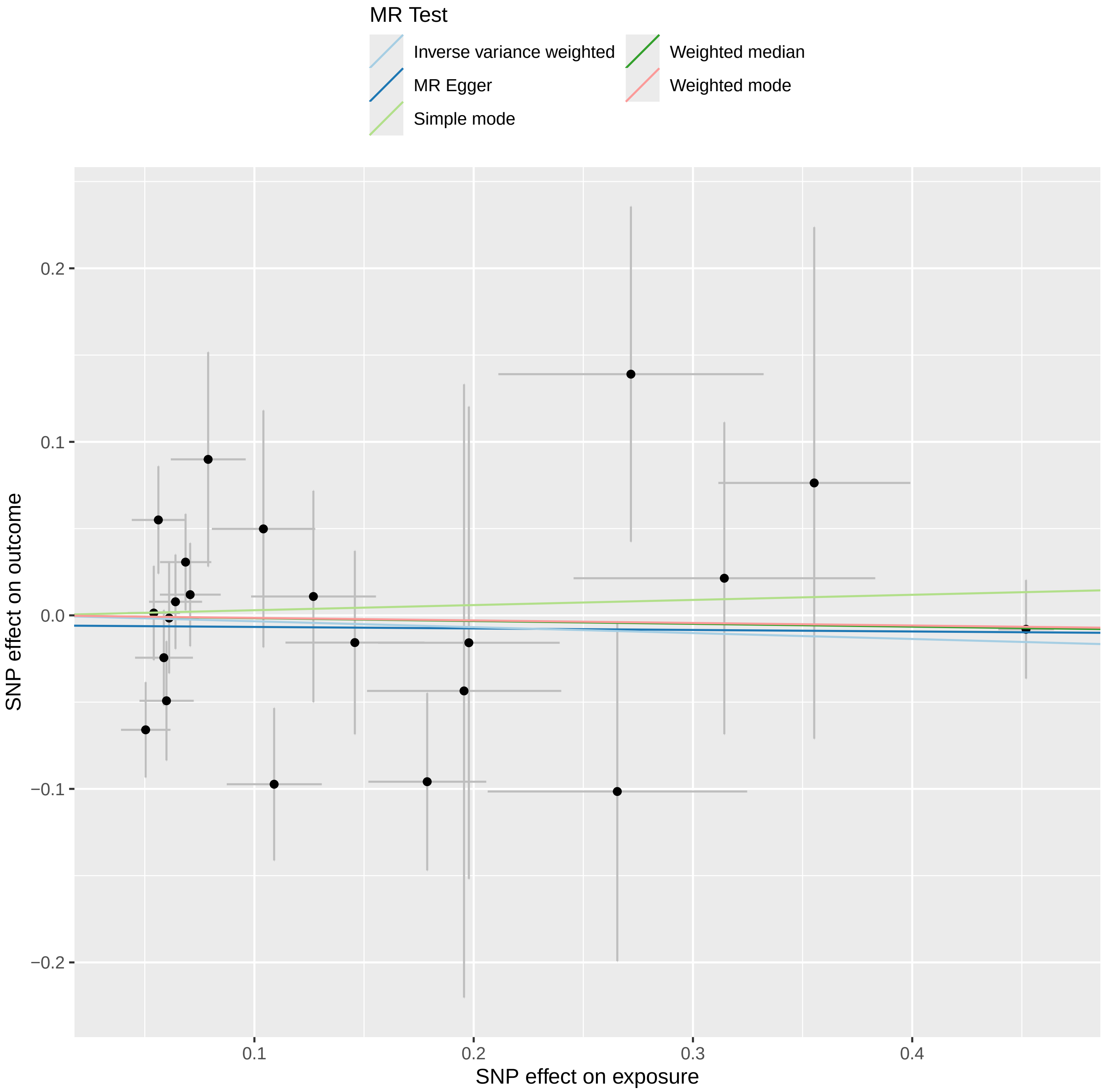

MR Test
Inverse variance weighted
MR Egger
Simple mode
Weighted median
Weighted mode
0.2
0.1
0.0
−0.1
−0.2
SNP effect on outcome
0.1
0.2
0.3
0.4
SNP effect on exposure

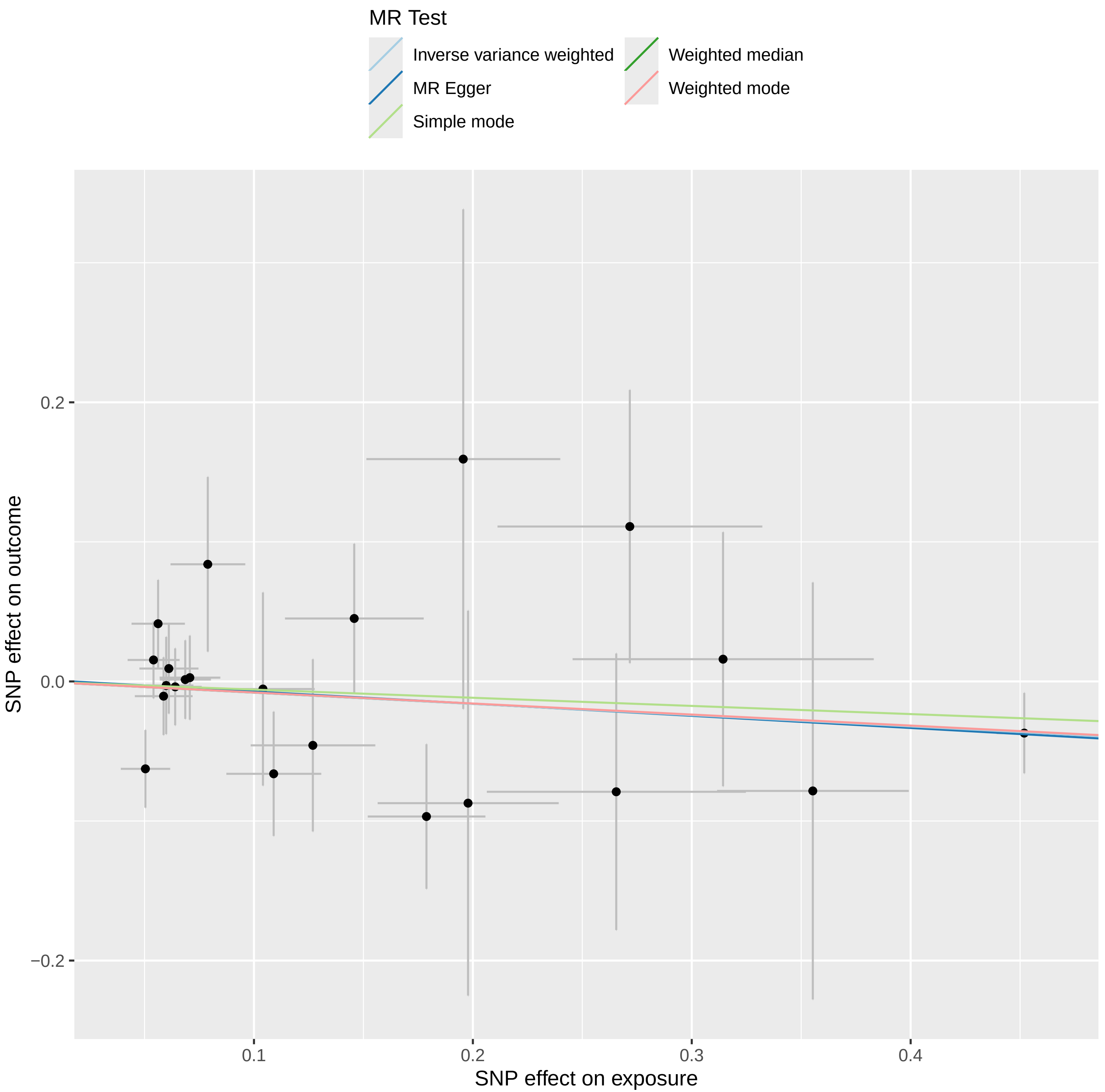

MR Test
Inverse variance weighted
MR Egger
Simple mode
Weighted median
Weighted mode
0.2
0.0
−0.2
0.1
0.2
0.3
0.4
SNP effect on outcome
SNP effect on exposure

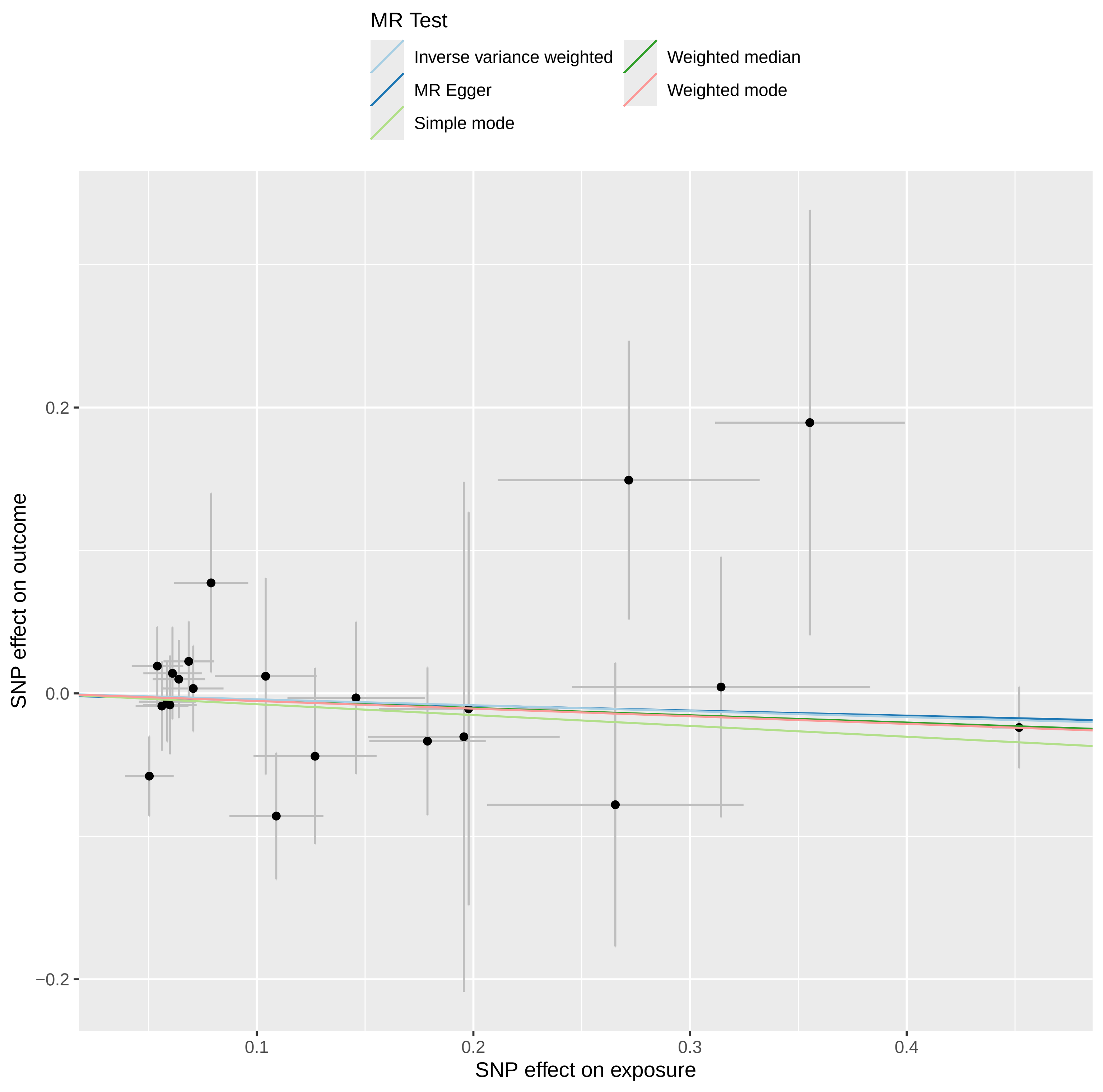

MR Test
Inverse variance weighted
MR Egger
Simple mode
Weighted median
Weighted mode
0.2
0.0
−0.2
0.1
0.2
0.3
0.4
SNP effect on outcome
SNP effect on exposure

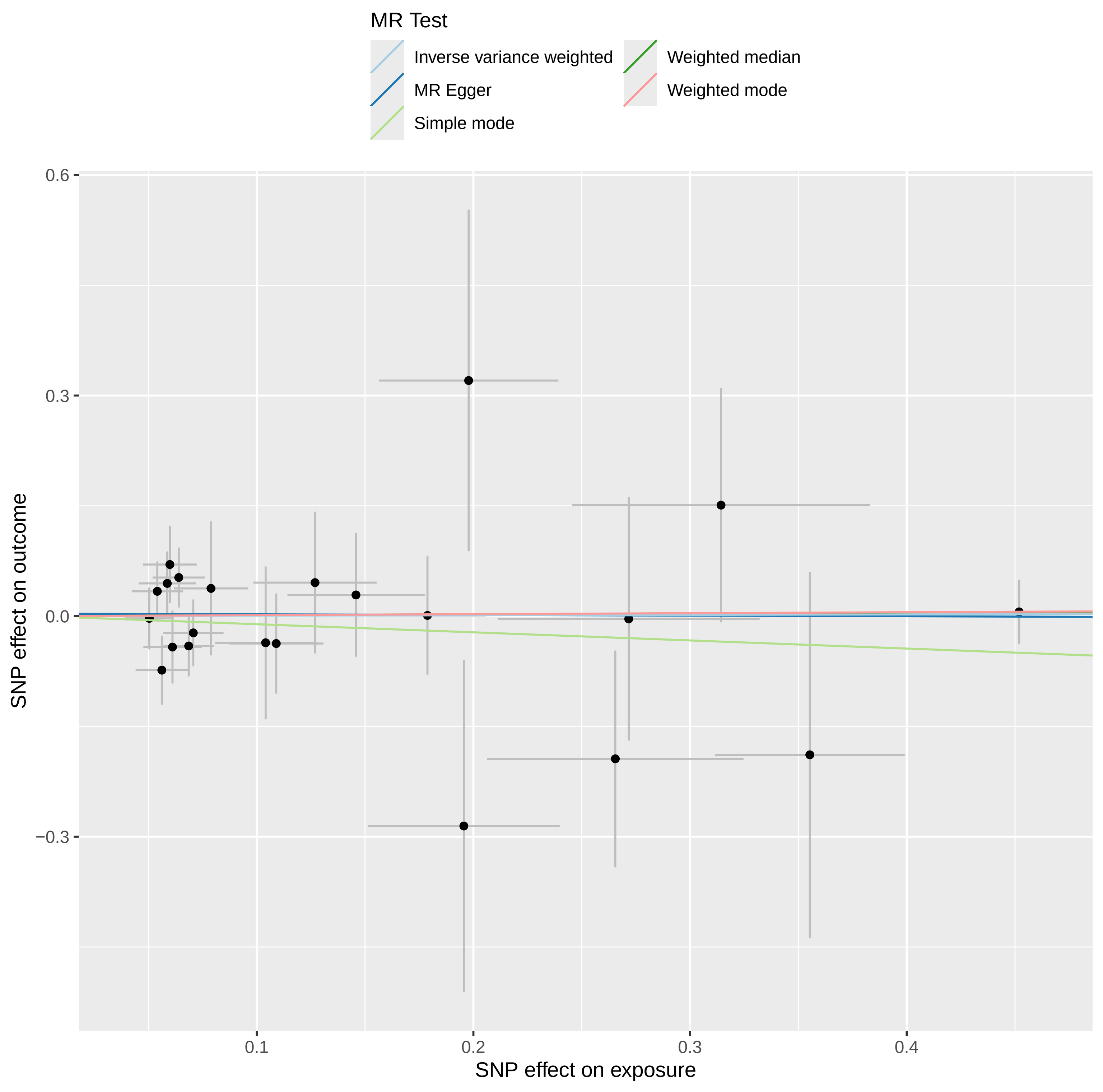

MR Test
Inverse variance weighted
MR Egger
Simple mode
Weighted median
Weighted mode
0.6
0.3
0.0
−0.3
0.1
0.2
0.3
0.4
SNP effect on outcome
SNP effect on exposure

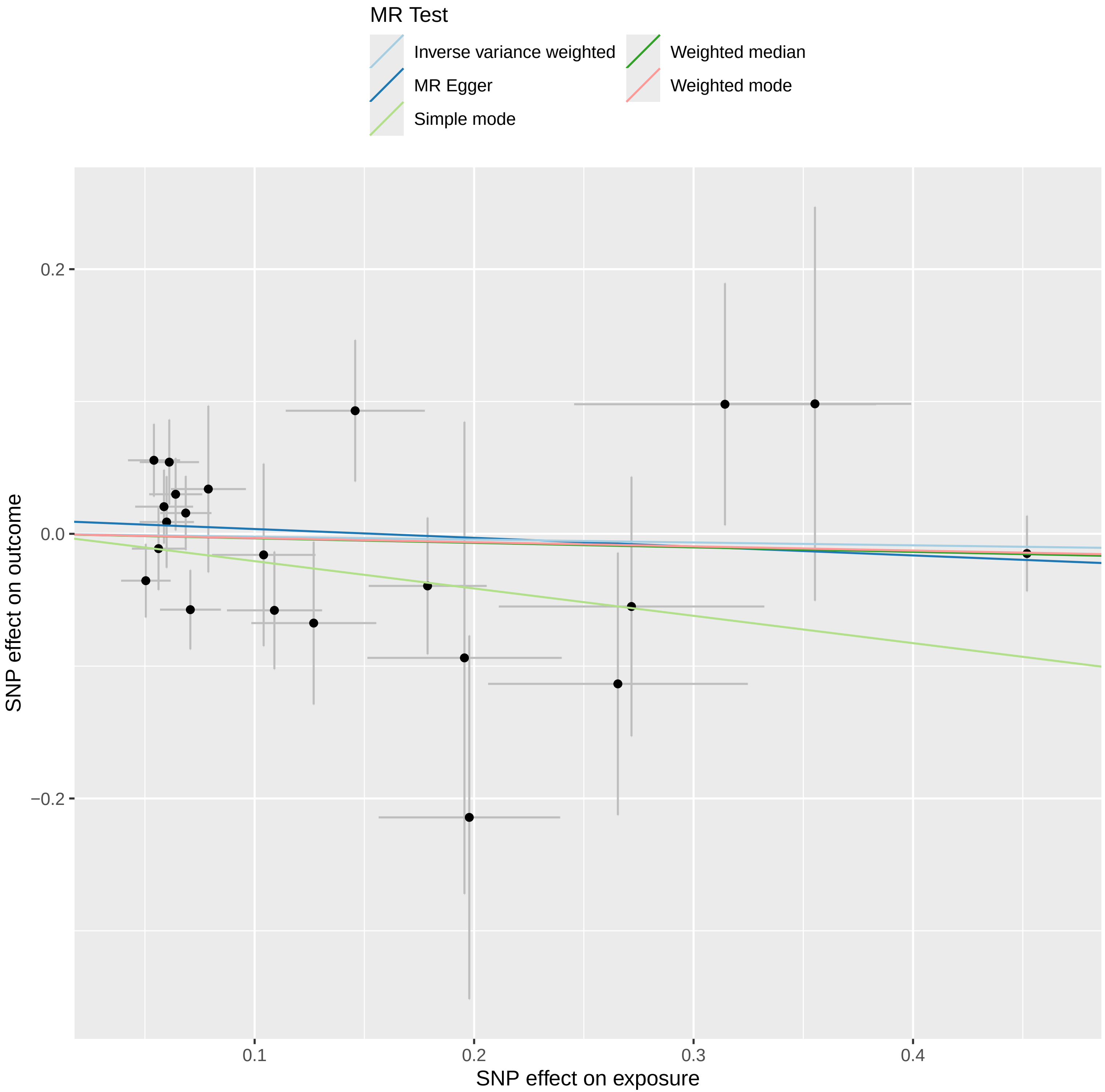
MR Test
Inverse variance weighted
MR Egger
Simple mode
Weighted median
Weighted mode
0.2
0.0
−0.2
0.1
0.2
0.3
0.4
SNP effect on outcome
SNP effect on exposure

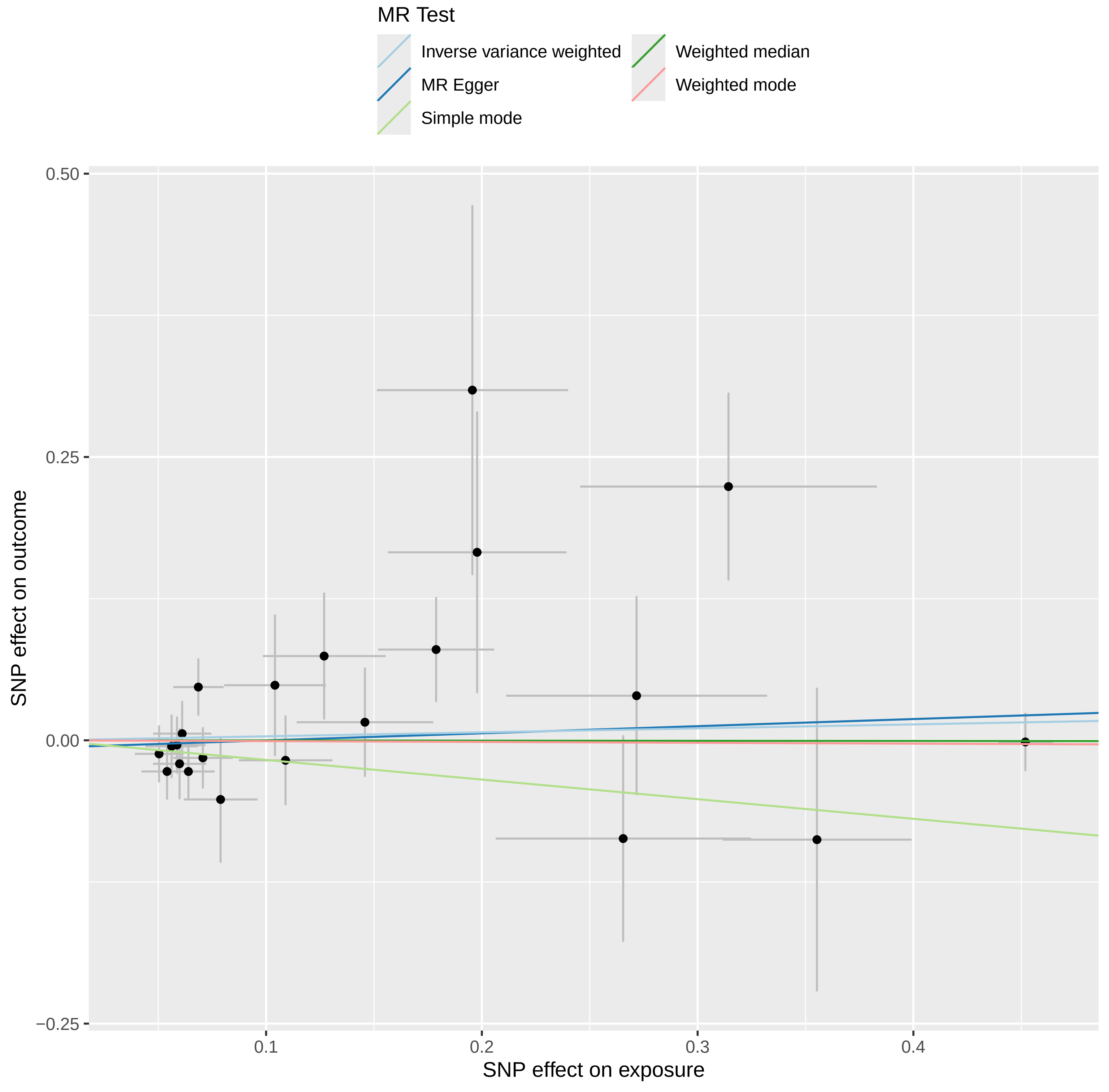
MR Test
Inverse variance weighted
MR Egger
Simple mode
Weighted median
Weighted mode
0.50
0.25
0.00
−0.25
0.1
0.2
0.3
0.4
SNP effect on exposure
SNP effect on outcome

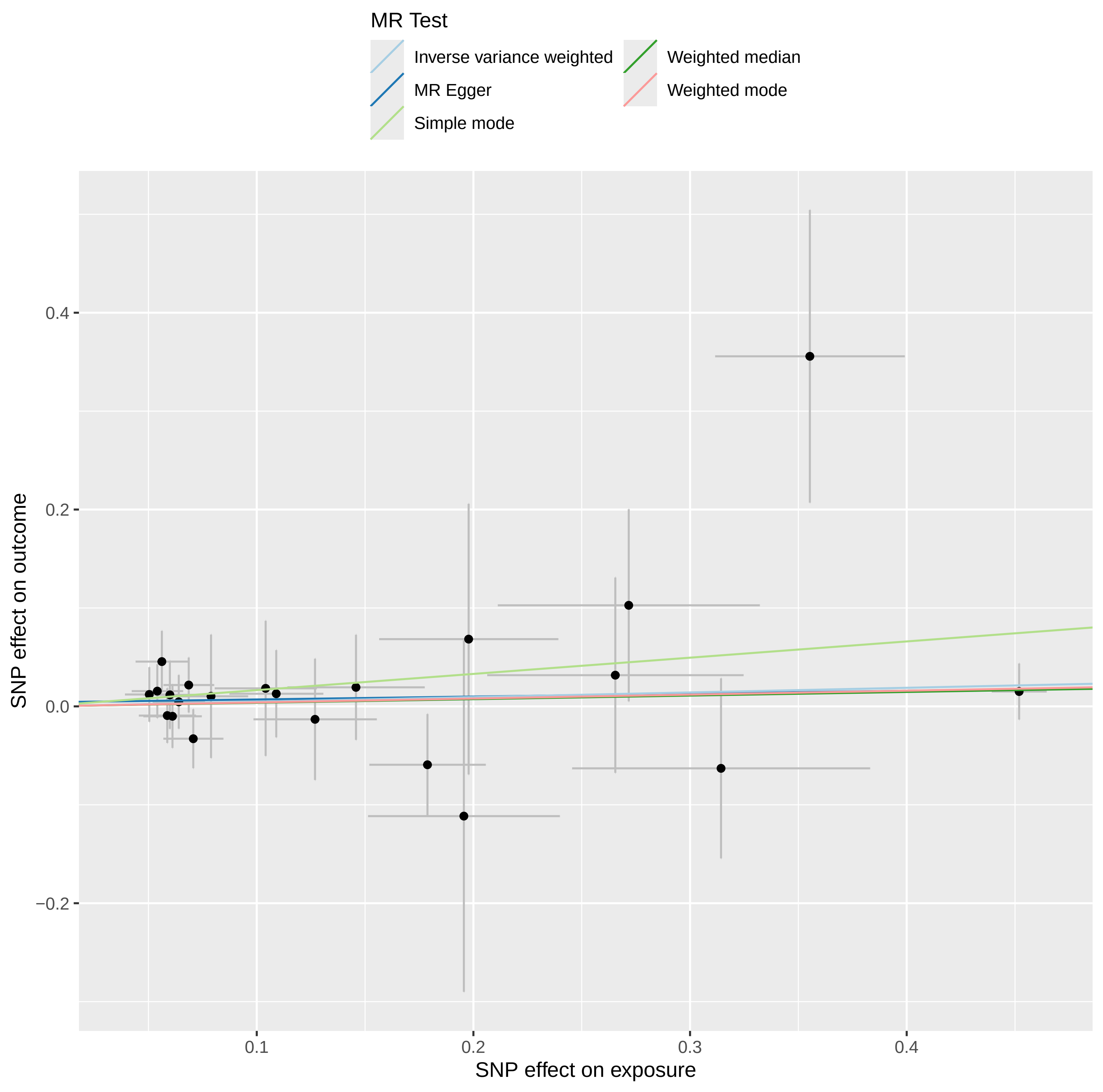
MR Test
Inverse variance weighted
MR Egger
Simple mode
Weighted median
Weighted mode
0.4
0.2
0.0
−0.2
SNP effect on outcome
0.1
0.2
0.3
0.4
SNP effect on exposure

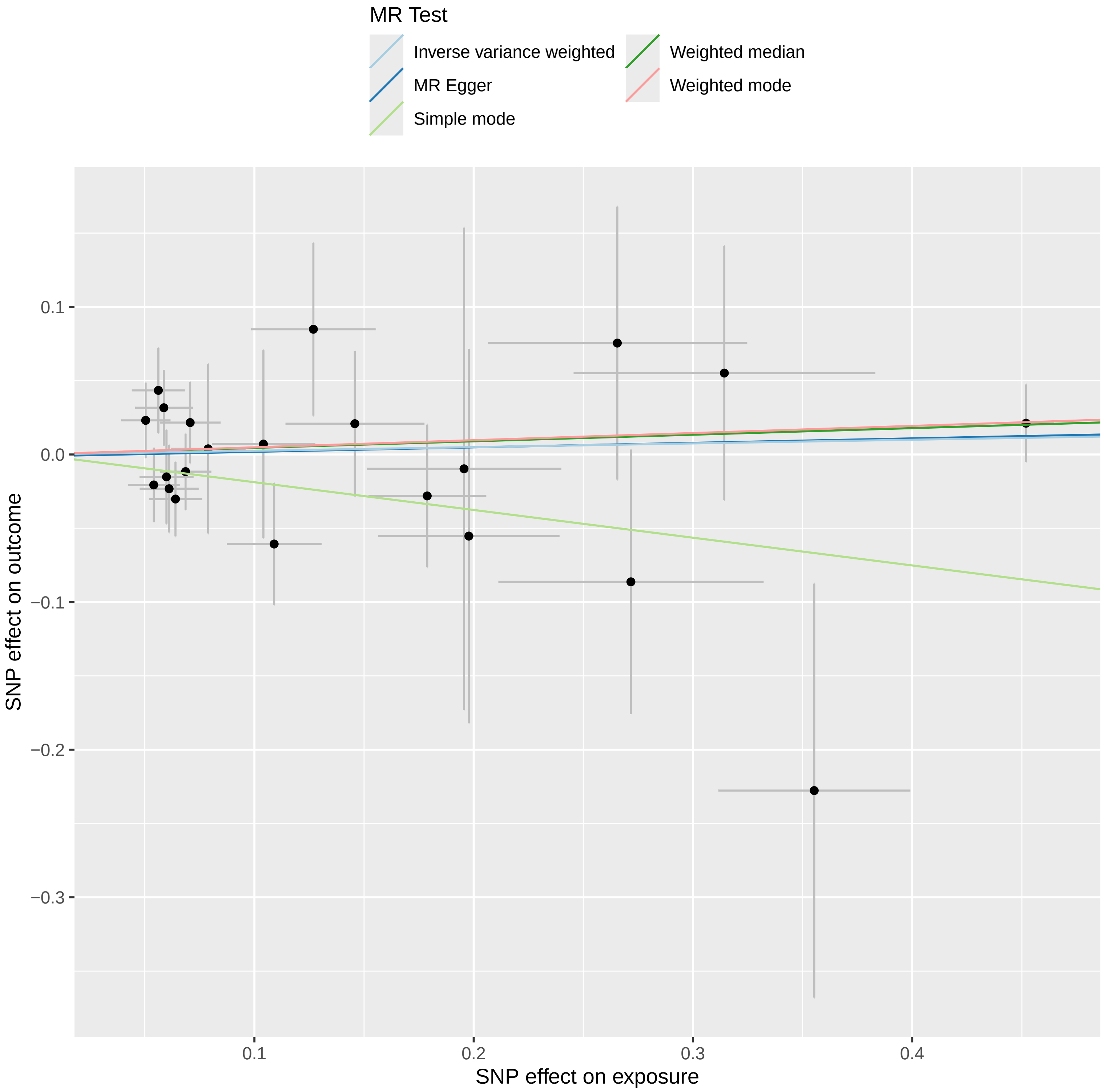

MR Test
Inverse variance weighted
MR Egger
Simple mode
Weighted median
Weighted mode
0.1
0.0
−0.1
−0.2
−0.3
0.1
0.2
0.3
0.4
SNP effect on exposure
SNP effect on outcome

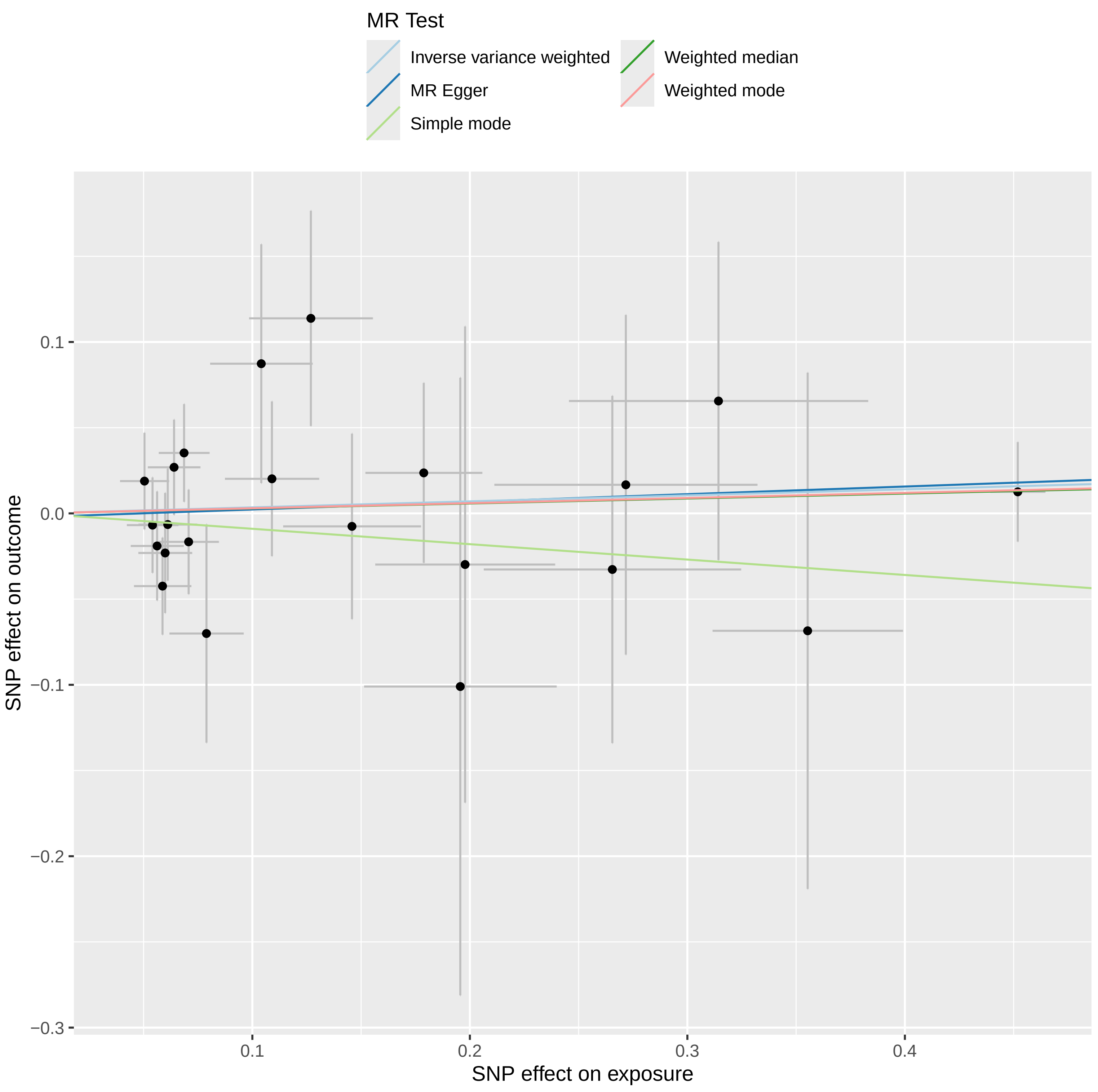
MR Test
Inverse variance weighted
MR Egger
Simple mode
Weighted median
Weighted mode
0.1
0.0
−0.1
−0.2
−0.3
0.1
0.2
0.3
0.4
SNP effect on outcome
SNP effect on exposure

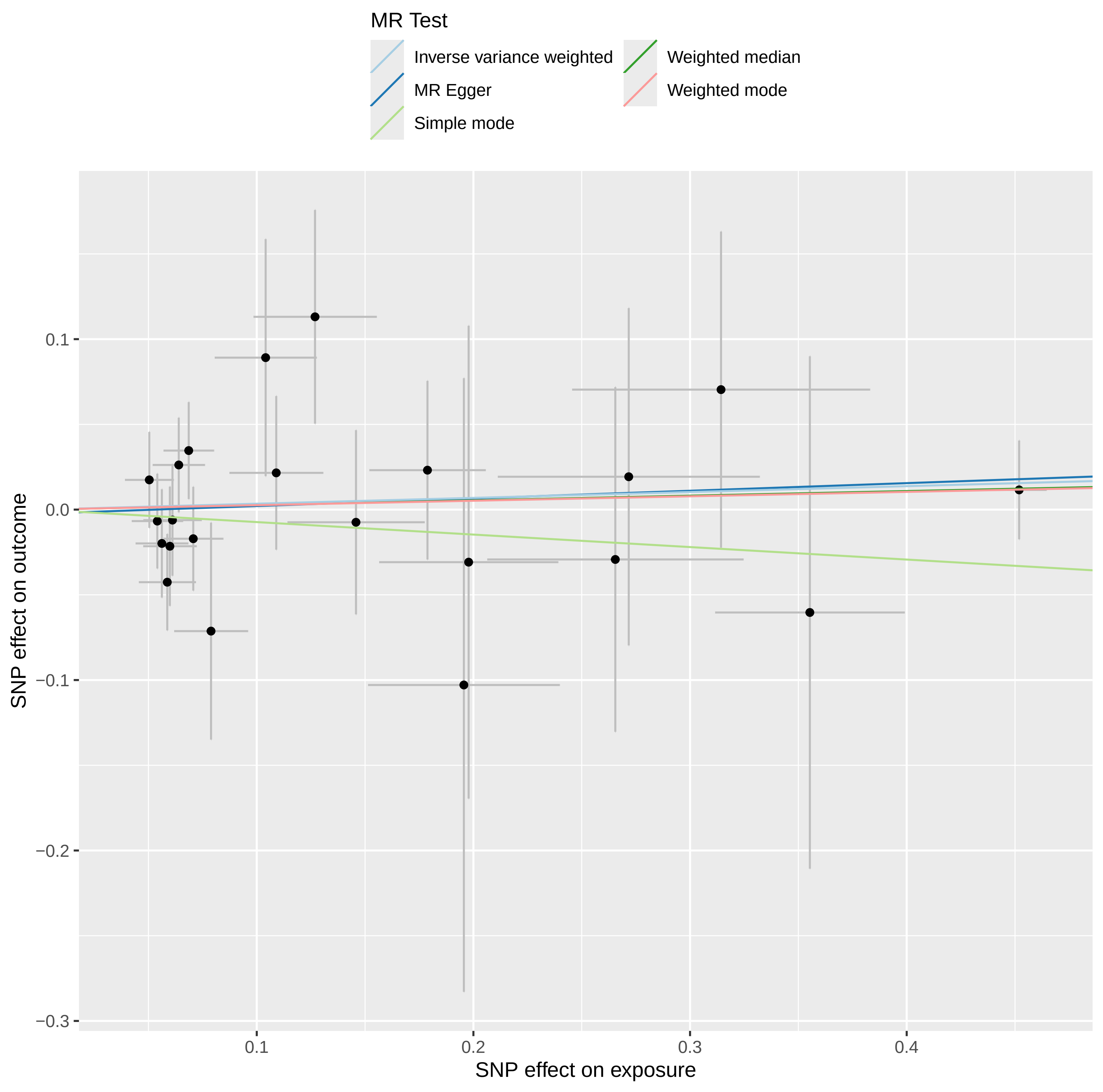
MR Test
Inverse variance weighted
MR Egger
Simple mode
Weighted median
Weighted mode
0.1
0.0
−0.1
−0.2
−0.3
SNP effect on outcome
0.1
0.2
0.3
0.4
SNP effect on exposure

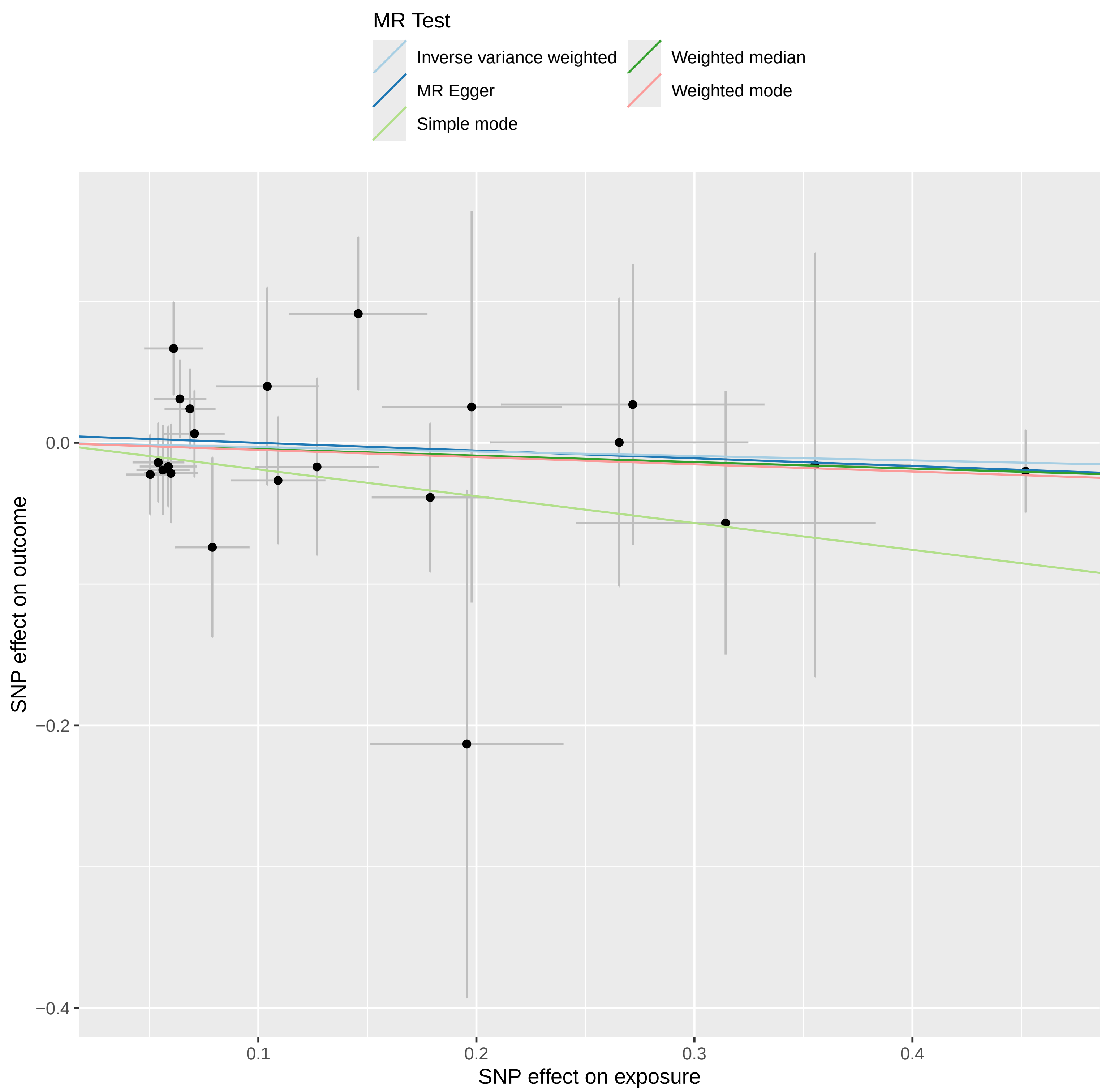

MR Test
Inverse variance weighted
MR Egger
Simple mode
Weighted median
Weighted mode
0.0
−0.2
−0.4
0.1
0.2
0.3
0.4
SNP effect on exposure
SNP effect on outcome

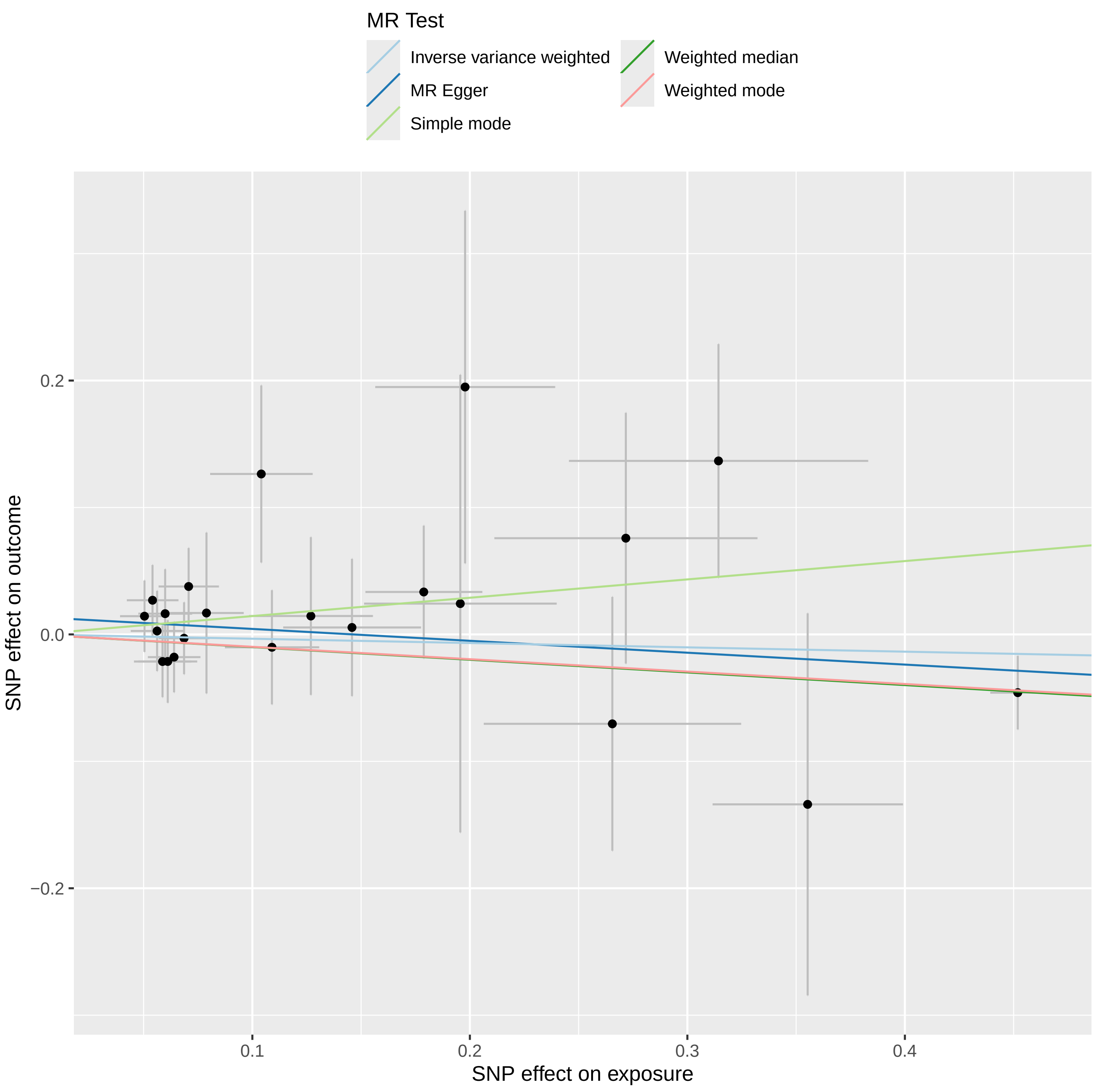
MR Test
Inverse variance weighted
MR Egger
Simple mode
Weighted median
Weighted mode
0.2
0.0
−0.2
0.1
0.2
0.3
0.4
SNP effect on exposure
SNP effect on outcome

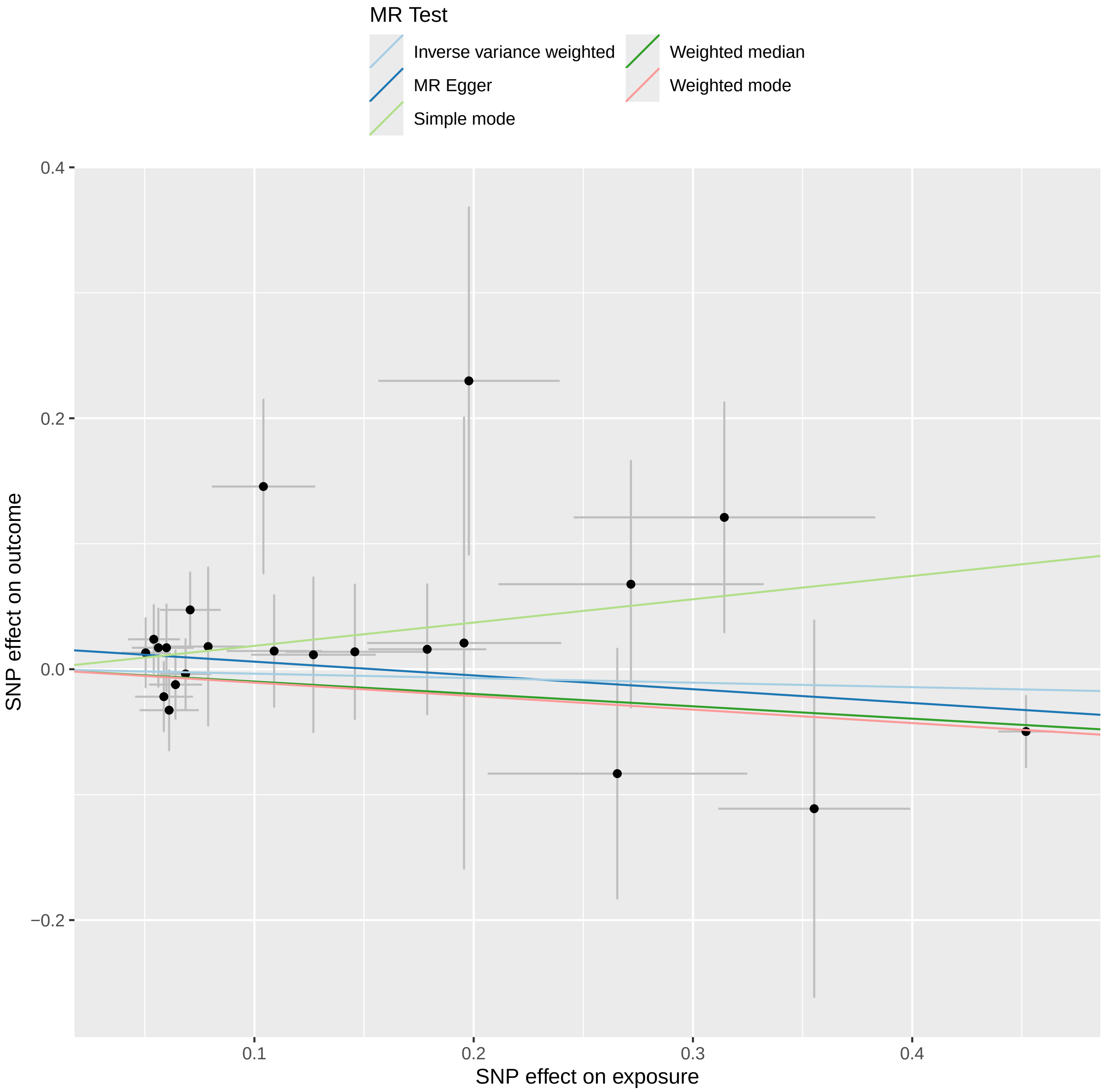
MR Test
Inverse variance weighted
MR Egger
Simple mode
Weighted median
Weighted mode
0.4
0.2
0.0
−0.2
0.1
0.2
0.3
0.4
SNP effect on outcome
SNP effect on exposure

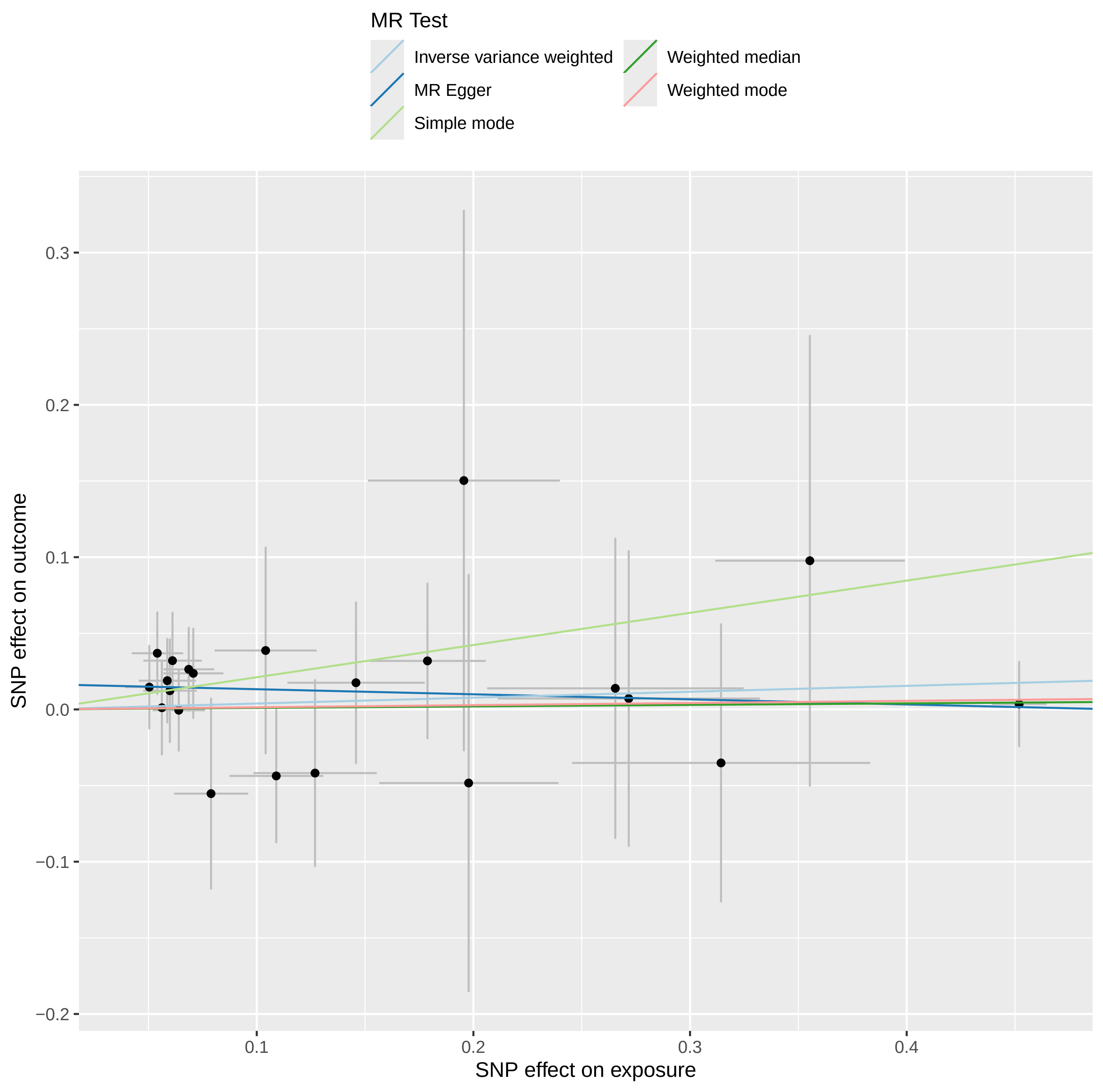
MR Test
Inverse variance weighted
MR Egger
Simple mode
Weighted median
Weighted mode
SNP effect on outcome
SNP effect on exposure
0.3
0.2
0.1
0.0
−0.1
−0.2
0.1
0.2
0.3
0.4

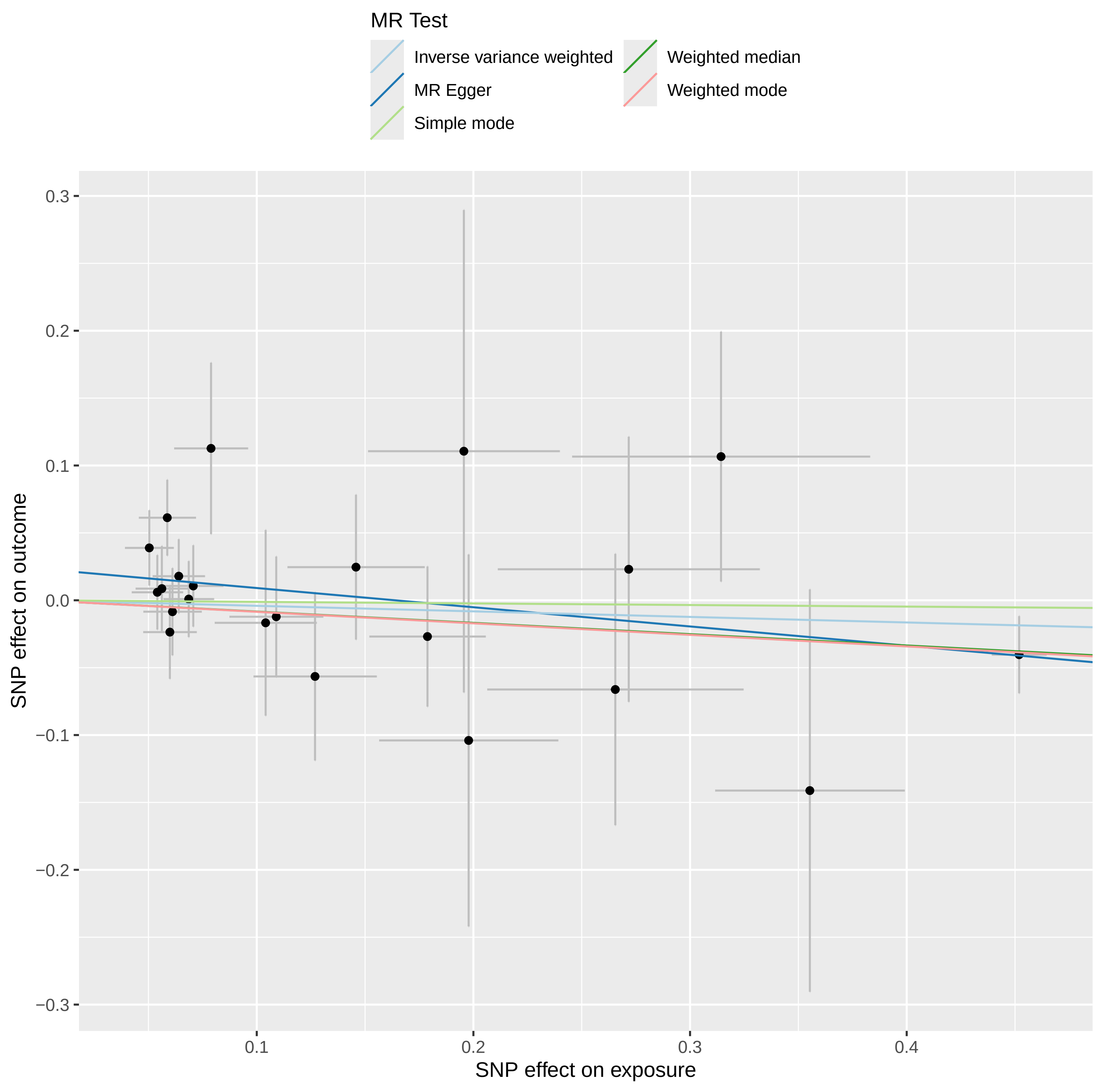
MR Test
Inverse variance weighted
MR Egger
Simple mode
Weighted median
Weighted mode
SNP effect on outcome
0.3
0.2
0.1
0.0
−0.1
−0.2
−0.3
0.1
0.2
0.3
0.4
SNP effect on exposure

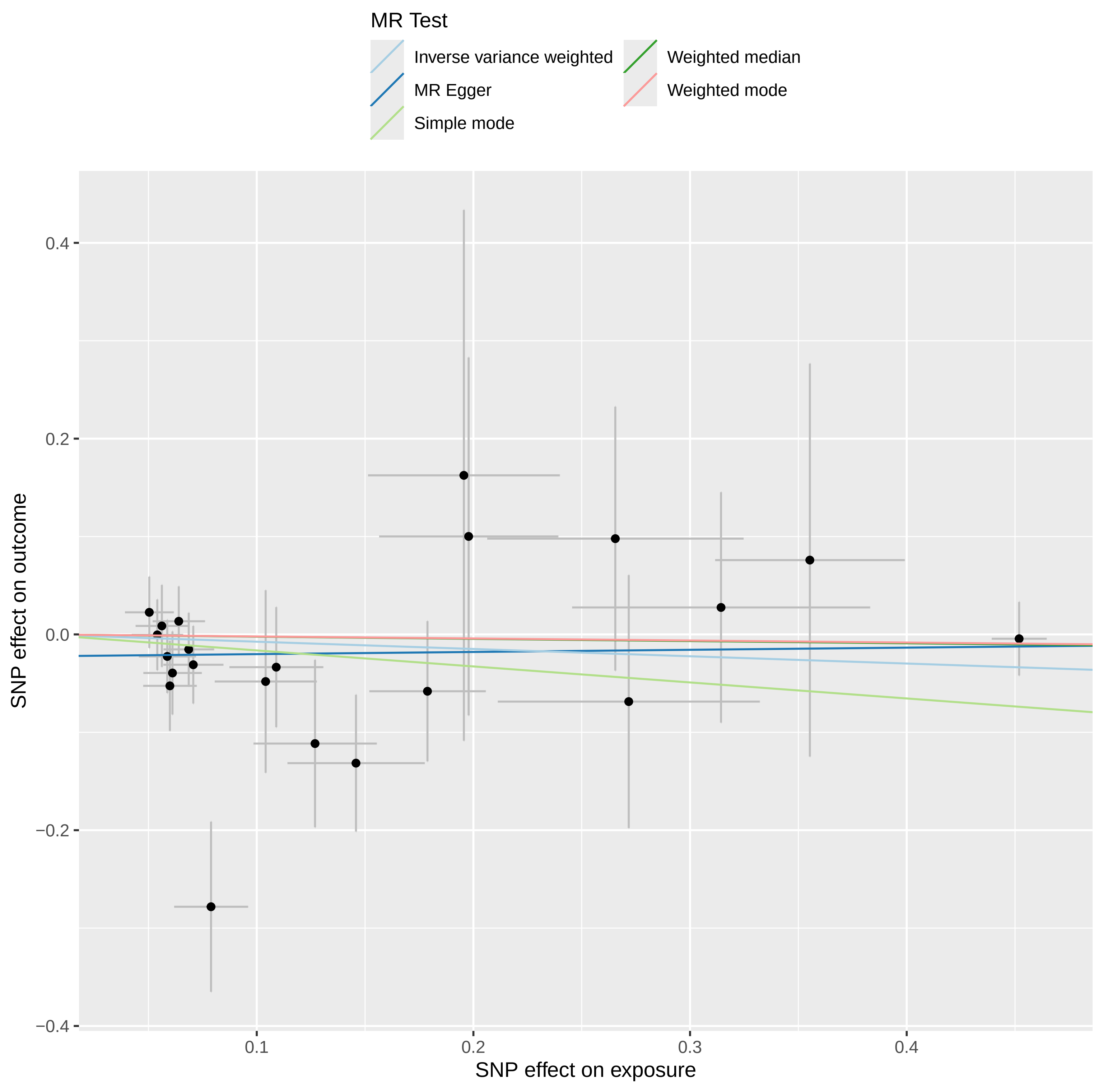
MR Test
Inverse variance weighted
MR Egger
Simple mode
Weighted median
Weighted mode
0.4
0.2
0.0
−0.2
−0.4
SNP effect on outcome
0.1
0.2
0.3
0.4
SNP effect on exposure

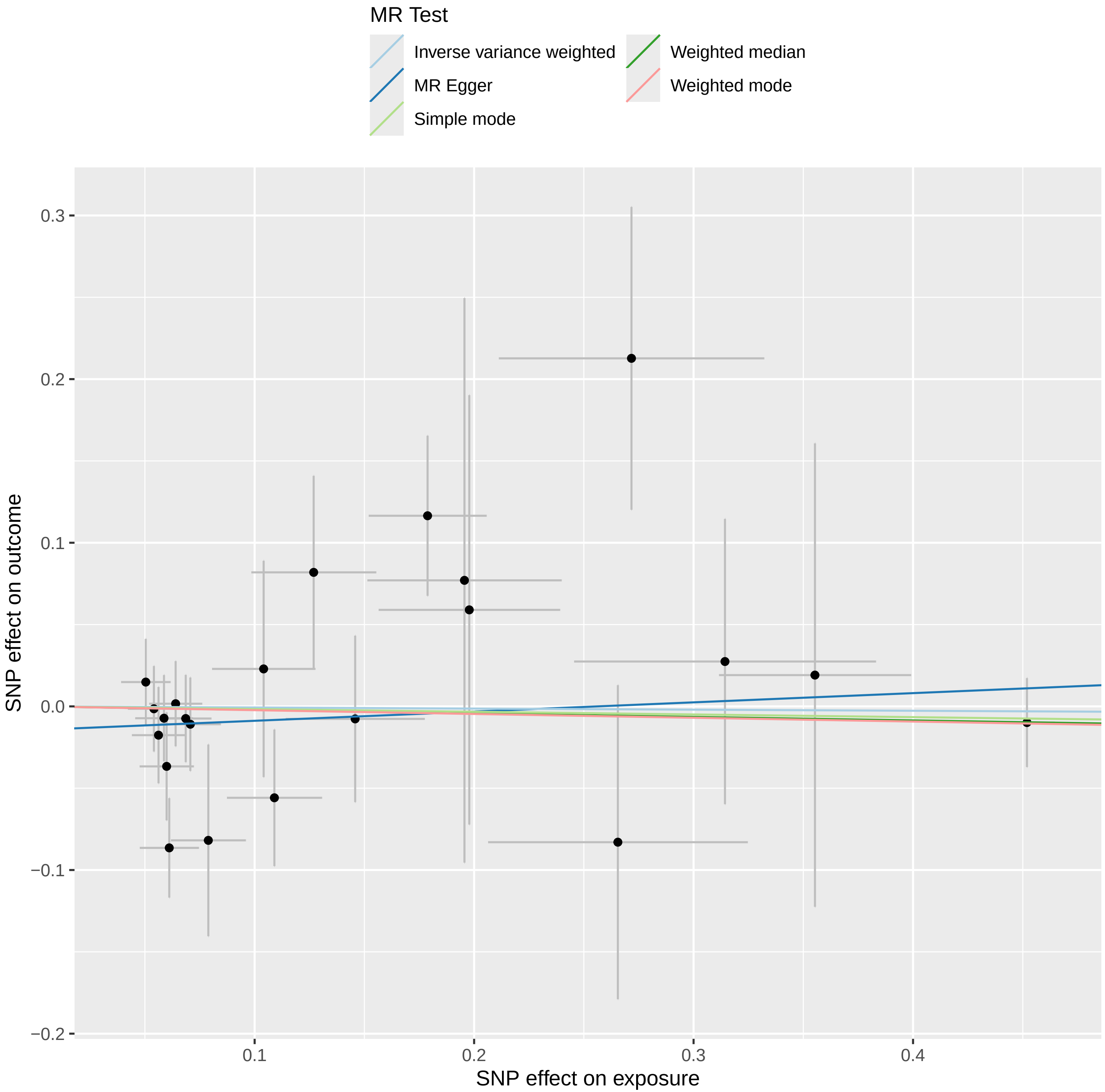
MR Test
Inverse variance weighted
MR Egger
Simple mode
Weighted median
Weighted mode
SNP effect on outcome
0.3
0.2
0.1
0.0
−0.1
−0.2
0.1
0.2
0.3
0.4
SNP effect on exposure

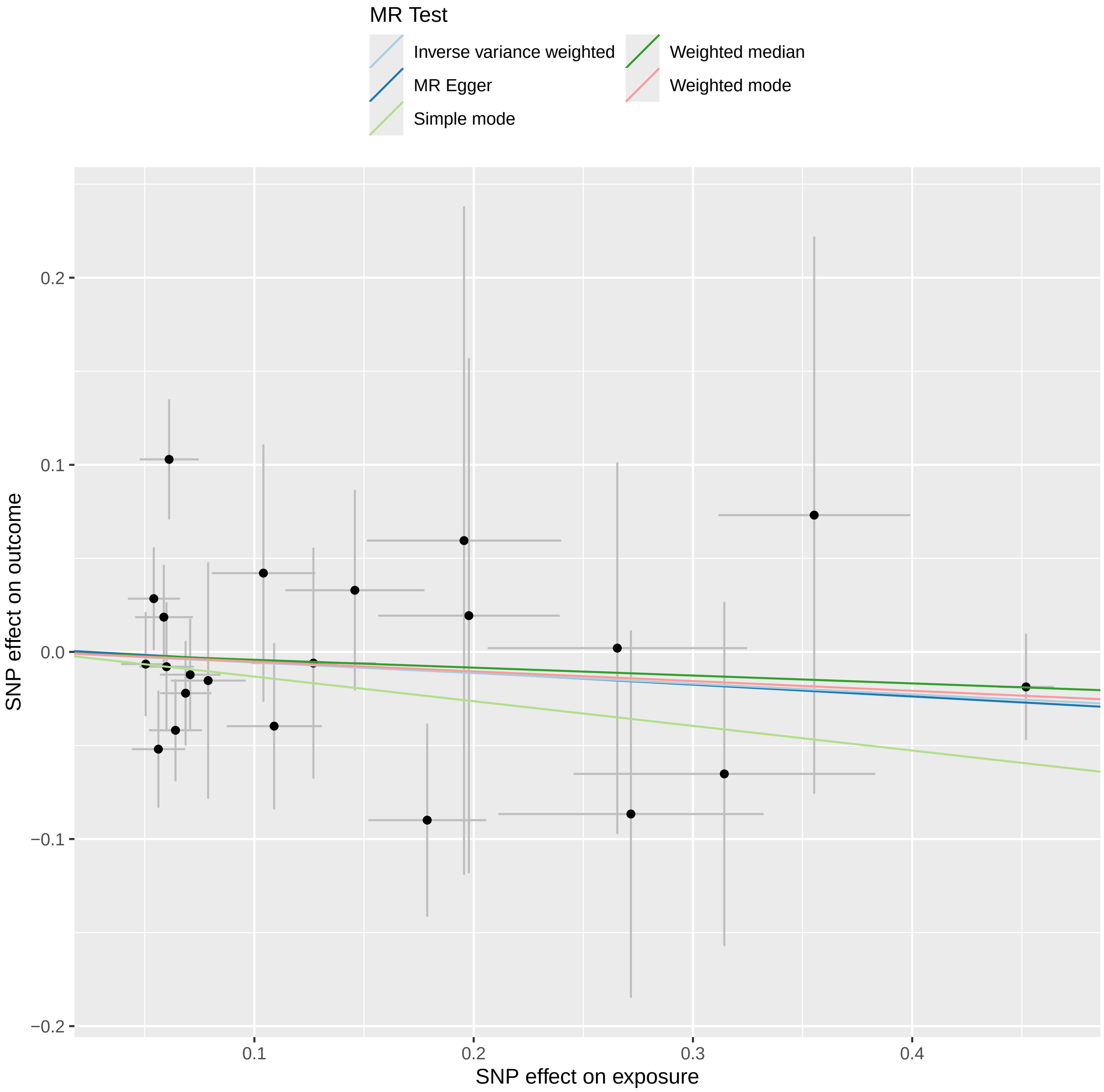
MR Test
Inverse variance weighted
MR Egger
Simple mode
Weighted median
Weighted mode
0.2
0.1
0.0
−0.1
−0.2
SNP effect on outcome
0.1
0.2
0.3
0.4
SNP effect on exposure

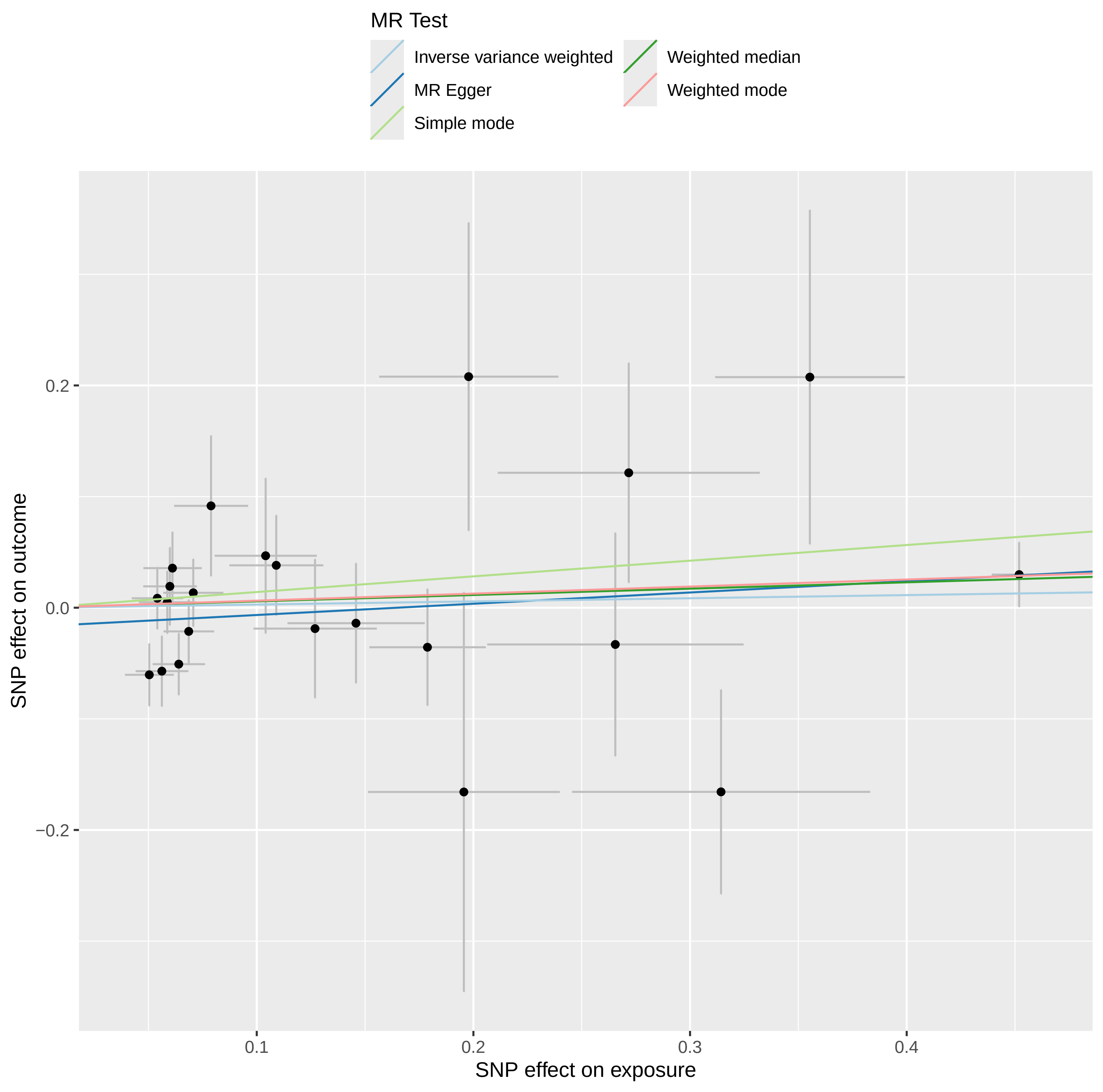

MR Test
Inverse variance weighted
MR Egger
Simple mode
Weighted median
Weighted mode
0.2
0.0
−0.2
0.1
0.2
0.3
0.4
SNP effect on outcome
SNP effect on exposure

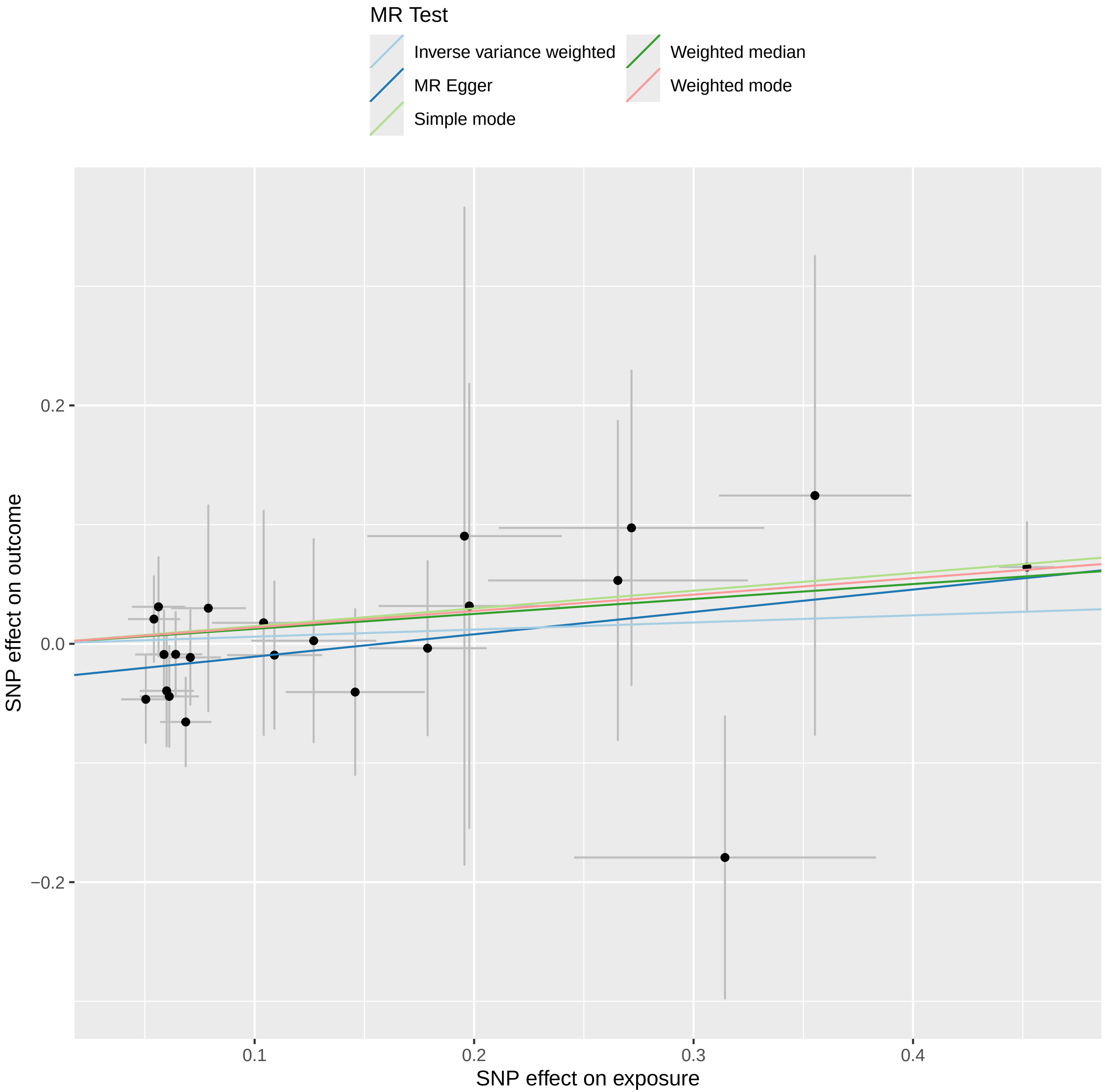

MR Test
Inverse variance weighted
MR Egger
Simple mode
Weighted median
Weighted mode
SNP effect on outcome
0.2
0.0
−0.2
SNP effect on exposure
0.1
0.2
0.3
0.4

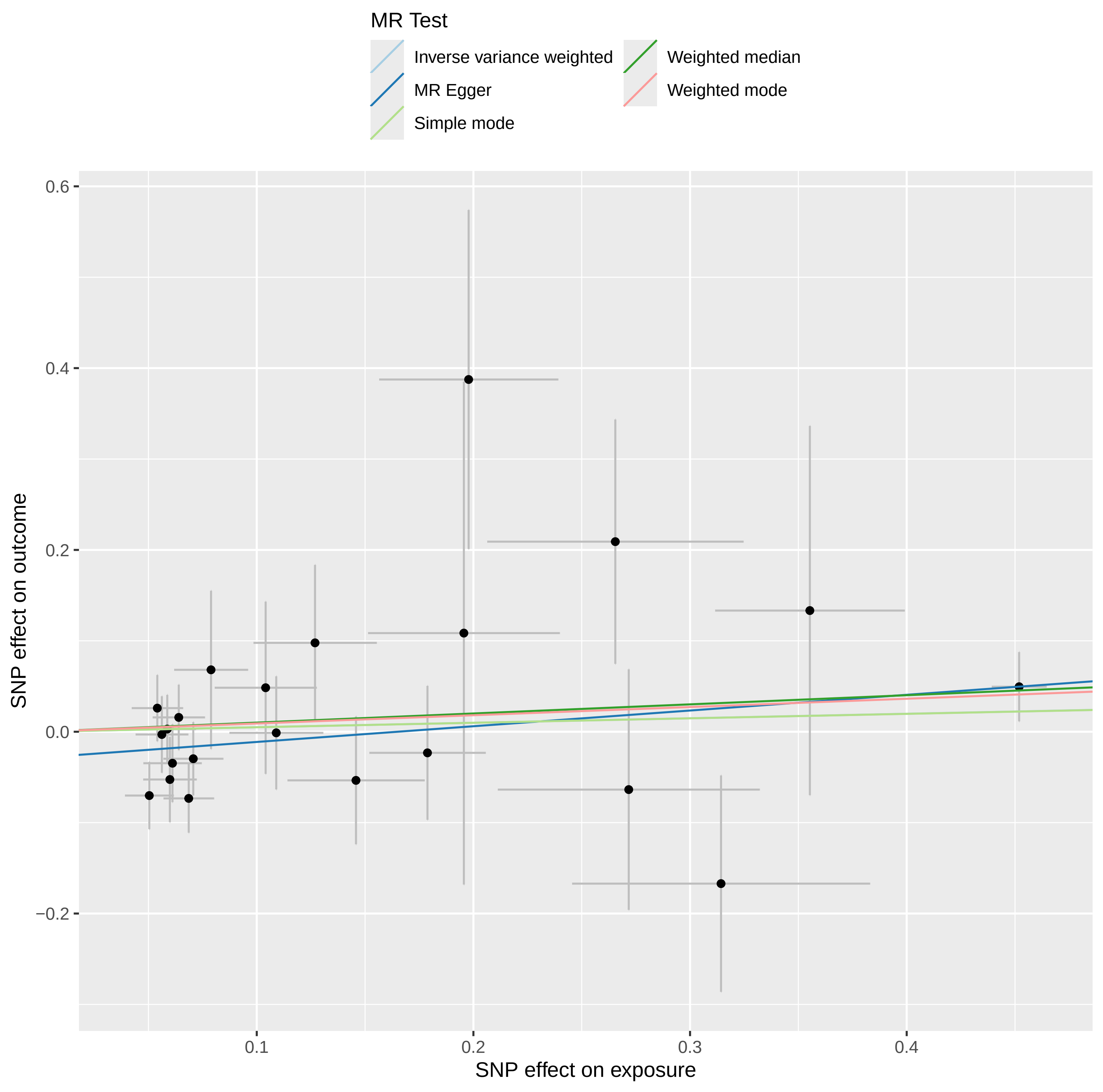
MR Test
Inverse variance weighted
MR Egger
Simple mode
Weighted median
Weighted mode
SNP effect on outcome
0.6
0.4
0.2
0.0
−0.2
0.1
0.2
0.3
0.4
SNP effect on exposure

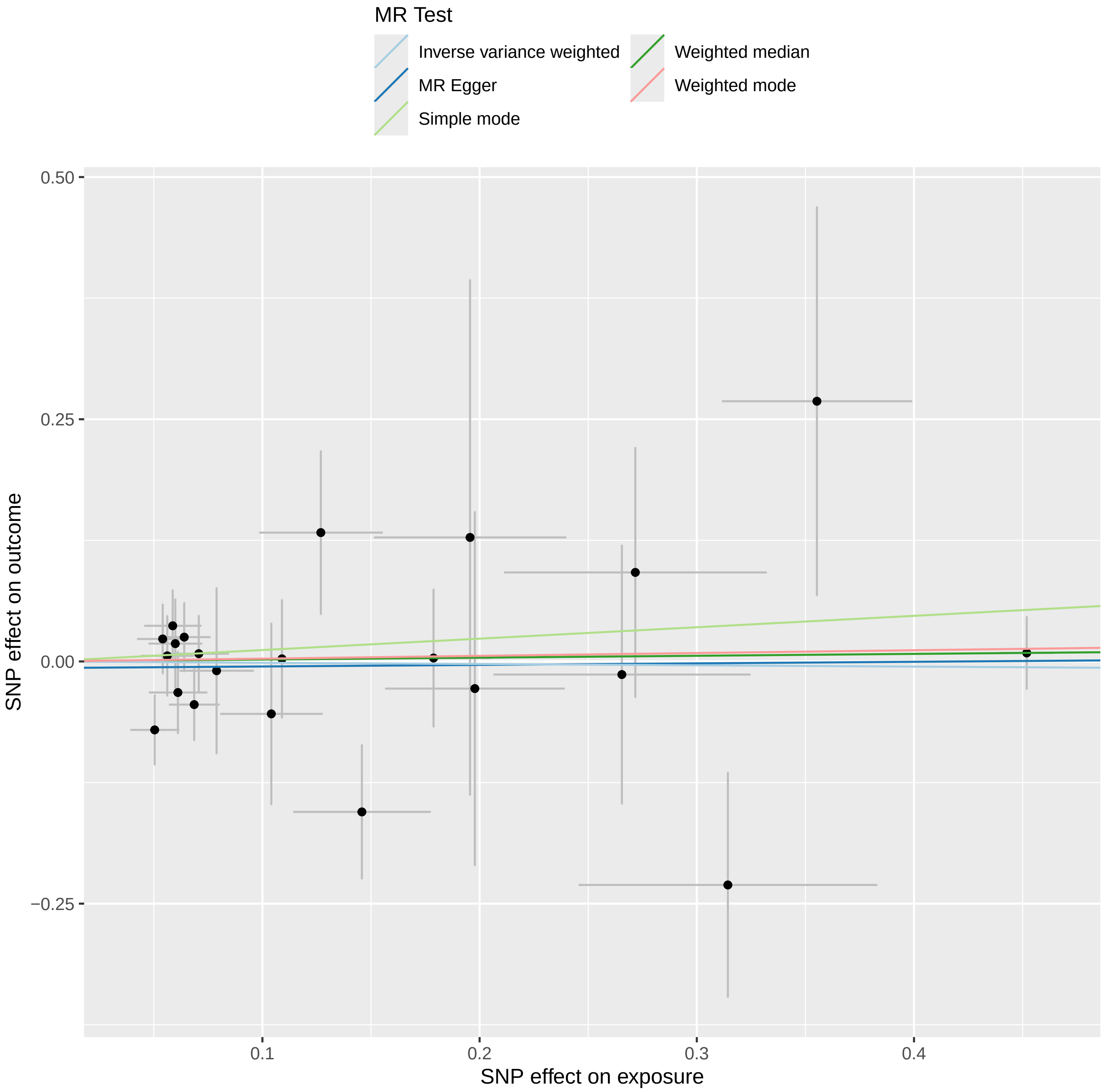
MR Test
Inverse variance weighted
MR Egger
Simple mode
Weighted median
Weighted mode
0.50
0.25
0.00
−0.25
SNP effect on outcome
0.1
0.2
0.3
0.4
SNP effect on exposure

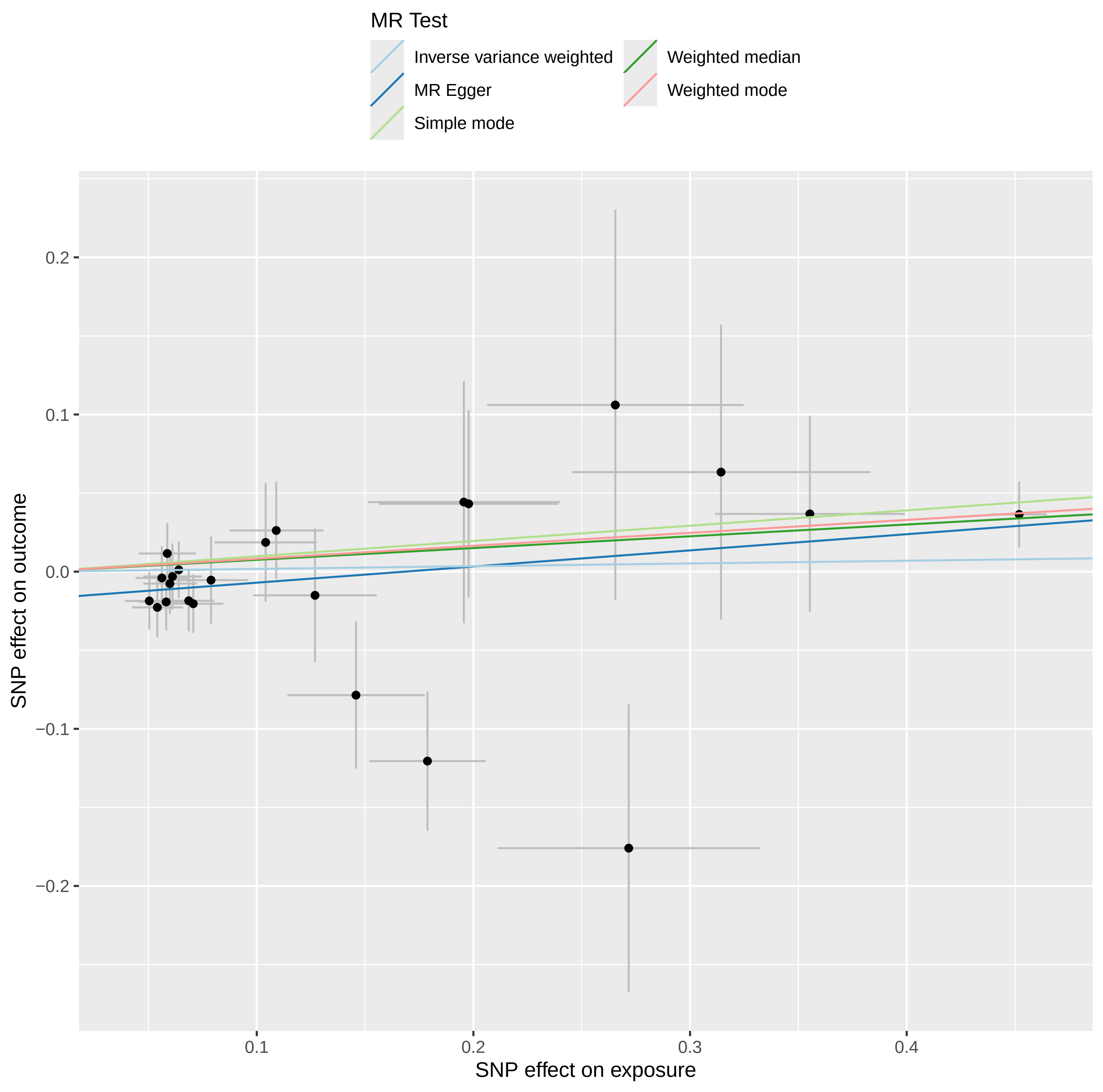

MR Test
Inverse variance weighted
MR Egger
Simple mode
Weighted median
Weighted mode
0.2
0.1
0.0
−0.1
−0.2
0.1
0.2
0.3
0.4
SNP effect on exposure
SNP effect on outcome

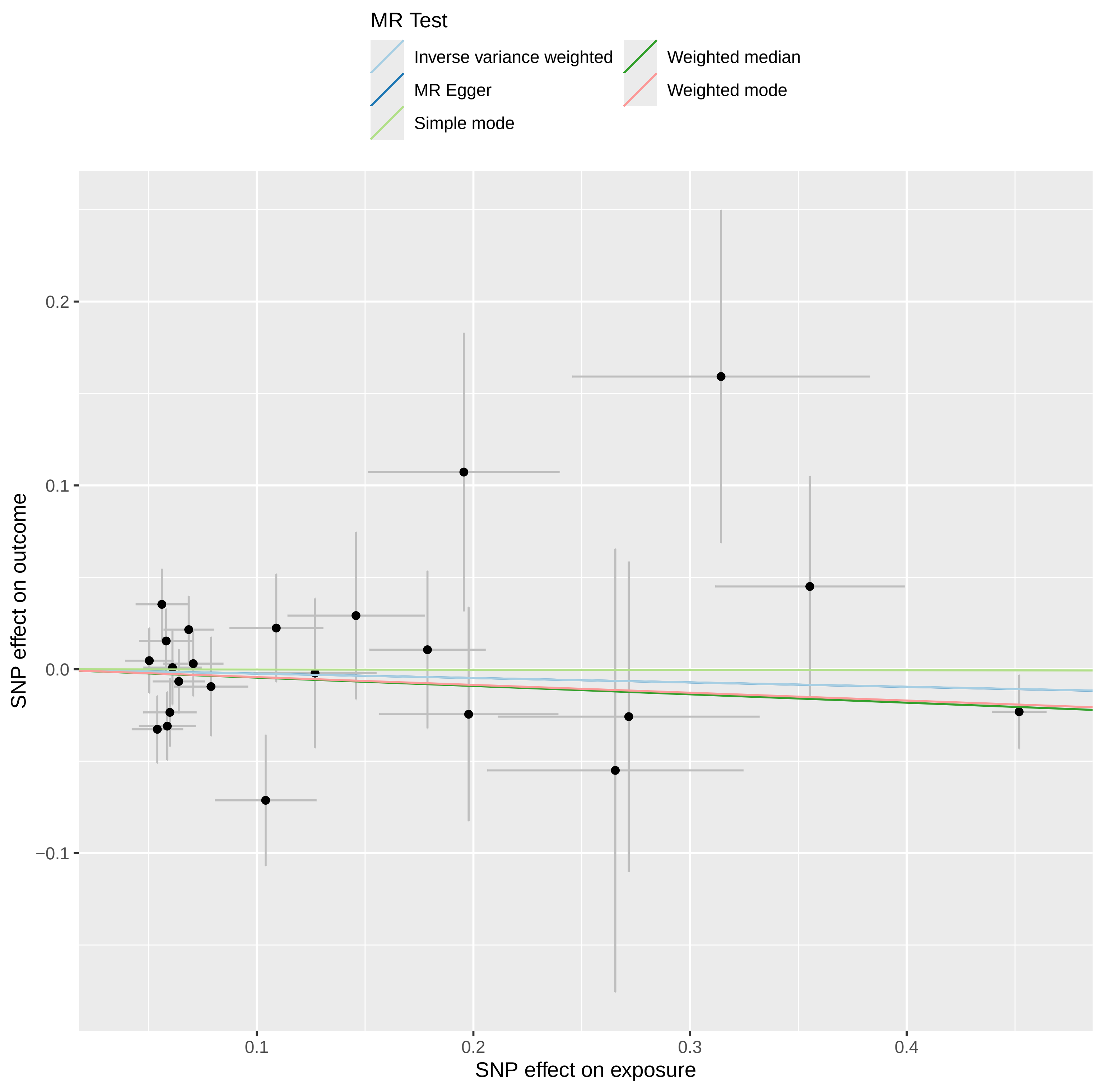

MR Test
Inverse variance weighted
MR Egger
Simple mode
Weighted median
Weighted mode
0.2
0.1
0.0
−0.1
0.1
0.2
0.3
0.4
SNP effect on outcome
SNP effect on exposure

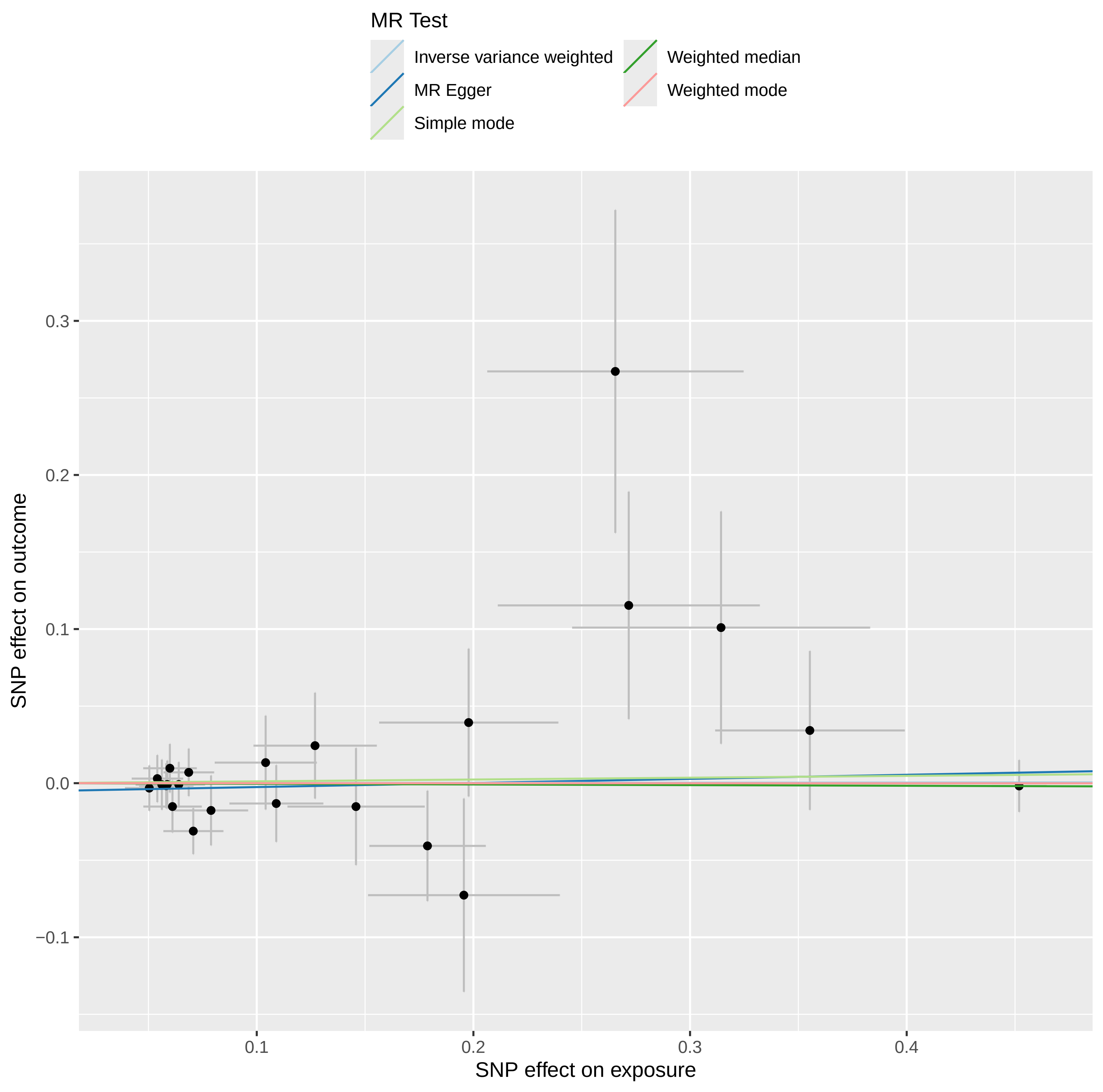
MR Test
Inverse variance weighted
MR Egger
Simple mode
Weighted median
Weighted mode
SNP effect on outcome
SNP effect on exposure
0.3
0.2
0.1
0.0
−0.1
0.1
0.2
0.3
0.4

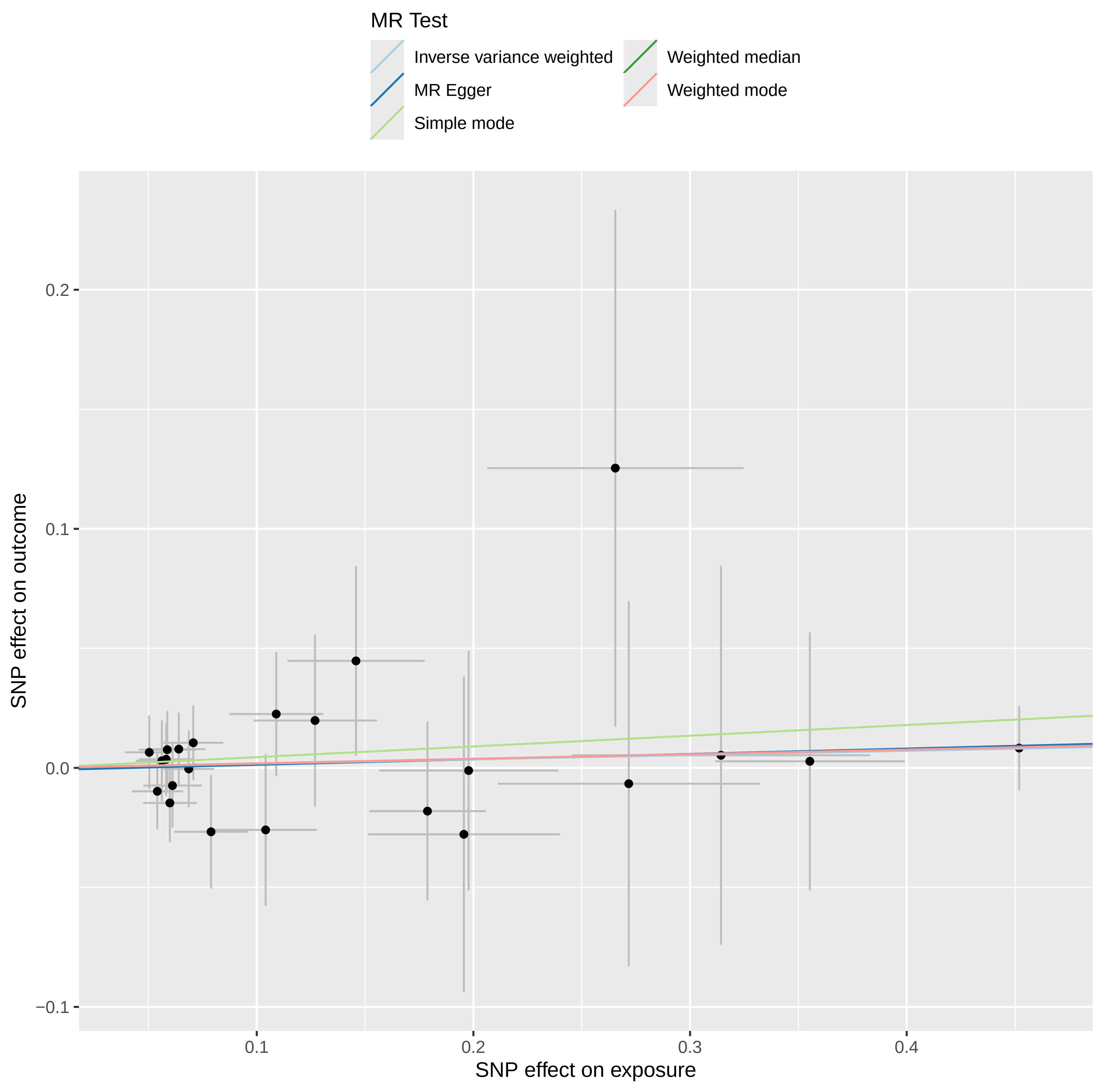

MR Test
Inverse variance weighted
MR Egger
Simple mode
Weighted median
Weighted mode
0.2
0.1
0.0
−0.1
0.1
0.2
0.3
0.4
SNP effect on outcome
SNP effect on exposure

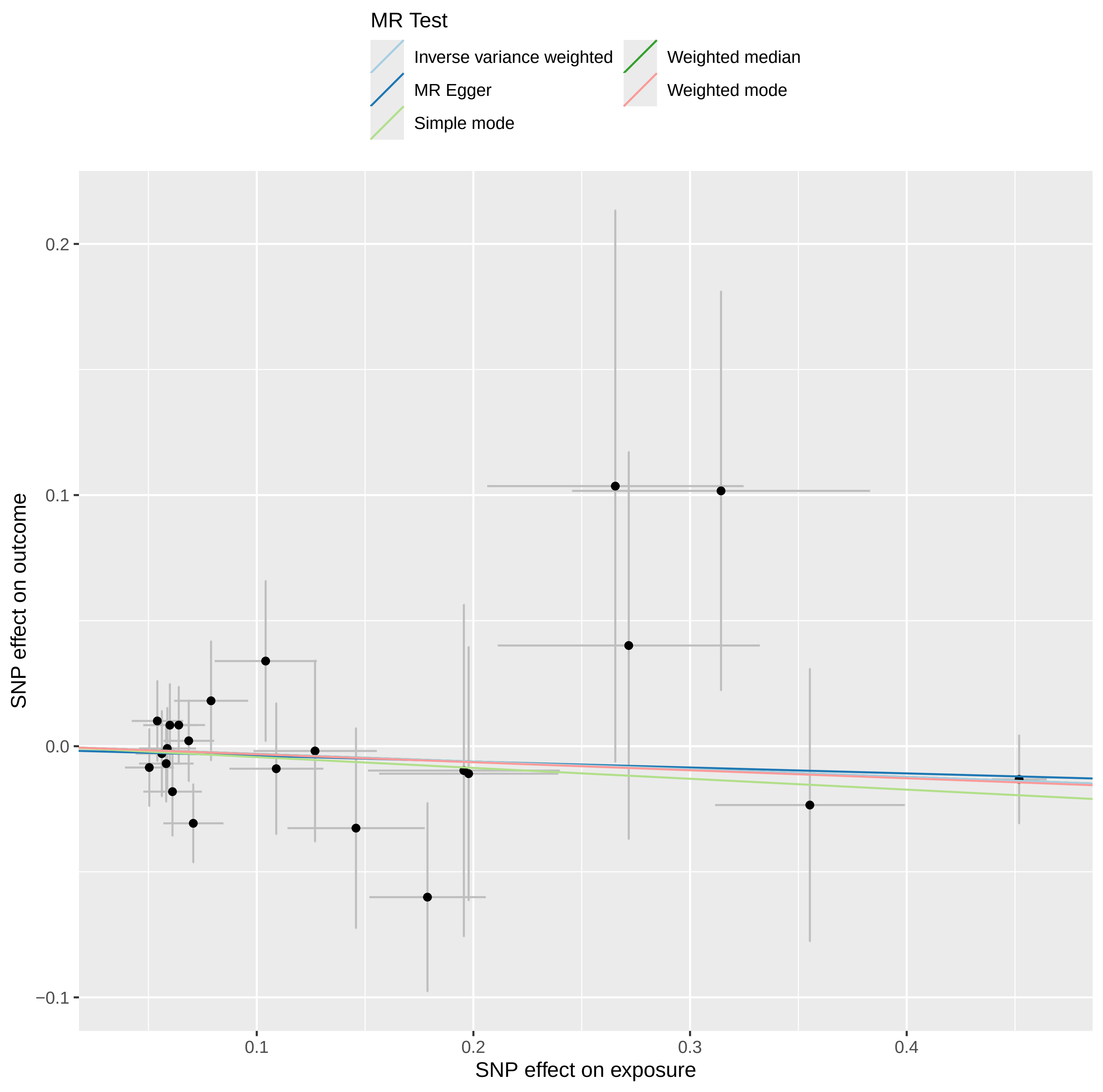
MR Test
Inverse variance weighted
MR Egger
Simple mode
Weighted median
Weighted mode
SNP effect on outcome
0.2
0.1
0.0
−0.1
0.1
0.2
0.3
0.4
SNP effect on exposure

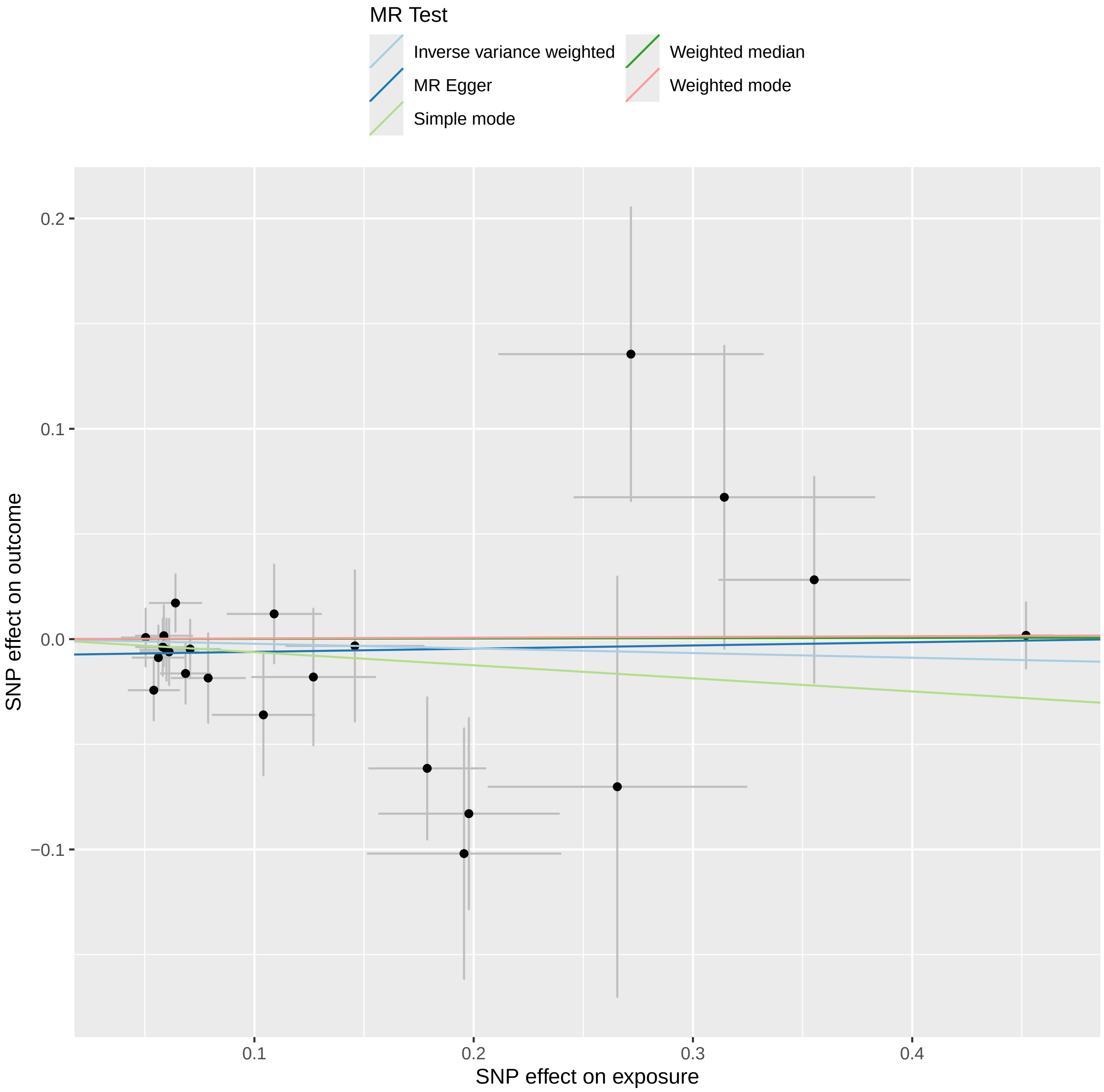
MR Test
Inverse variance weighted
MR Egger
Simple mode
Weighted median
Weighted mode
SNP effect on outcome
0.2
0.1
0.0
−0.1
SNP effect on exposure
0.1
0.2
0.3
0.4

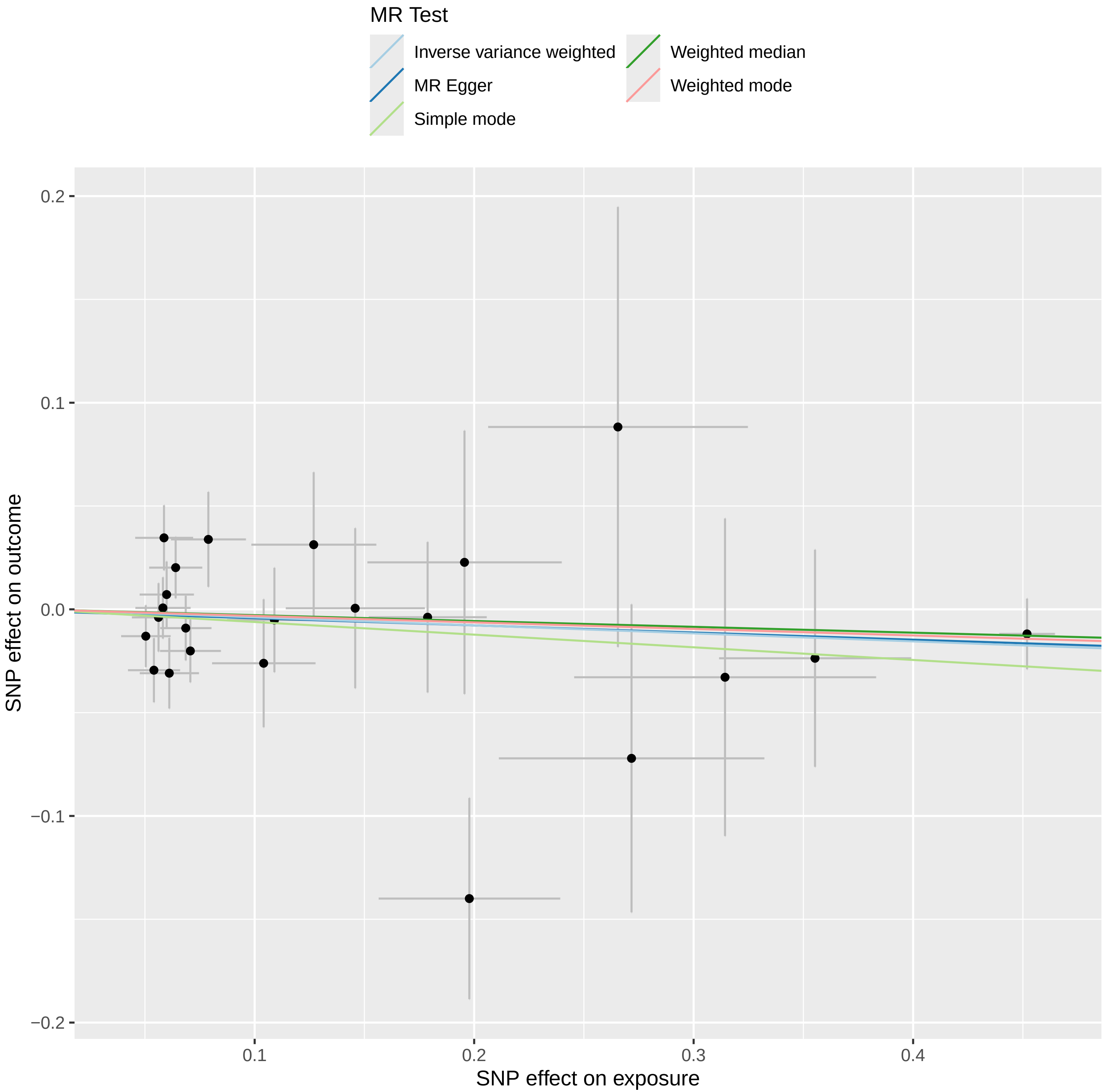

MR Test
Inverse variance weighted
MR Egger
Simple mode
Weighted median
Weighted mode
0.2
0.1
0.0
−0.1
−0.2
SNP effect on outcome
0.1
0.2
0.3
0.4
SNP effect on exposure

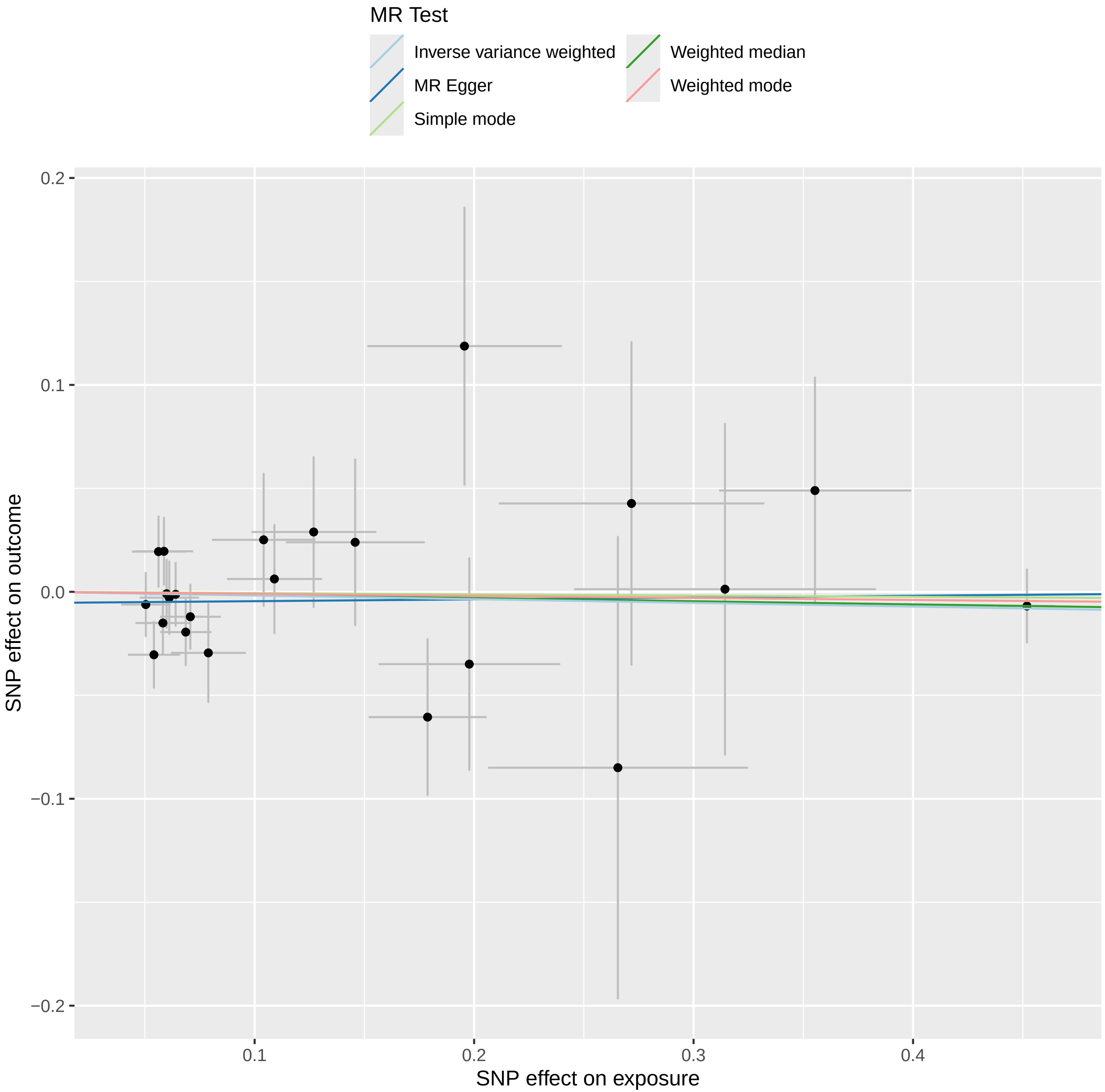
MR Test
Inverse variance weighted
MR Egger
Simple mode
Weighted median
Weighted mode
SNP effect on outcome
SNP effect on exposure
0.2
0.1
0.0
−0.1
−0.2
0.1
0.2
0.3
0.4

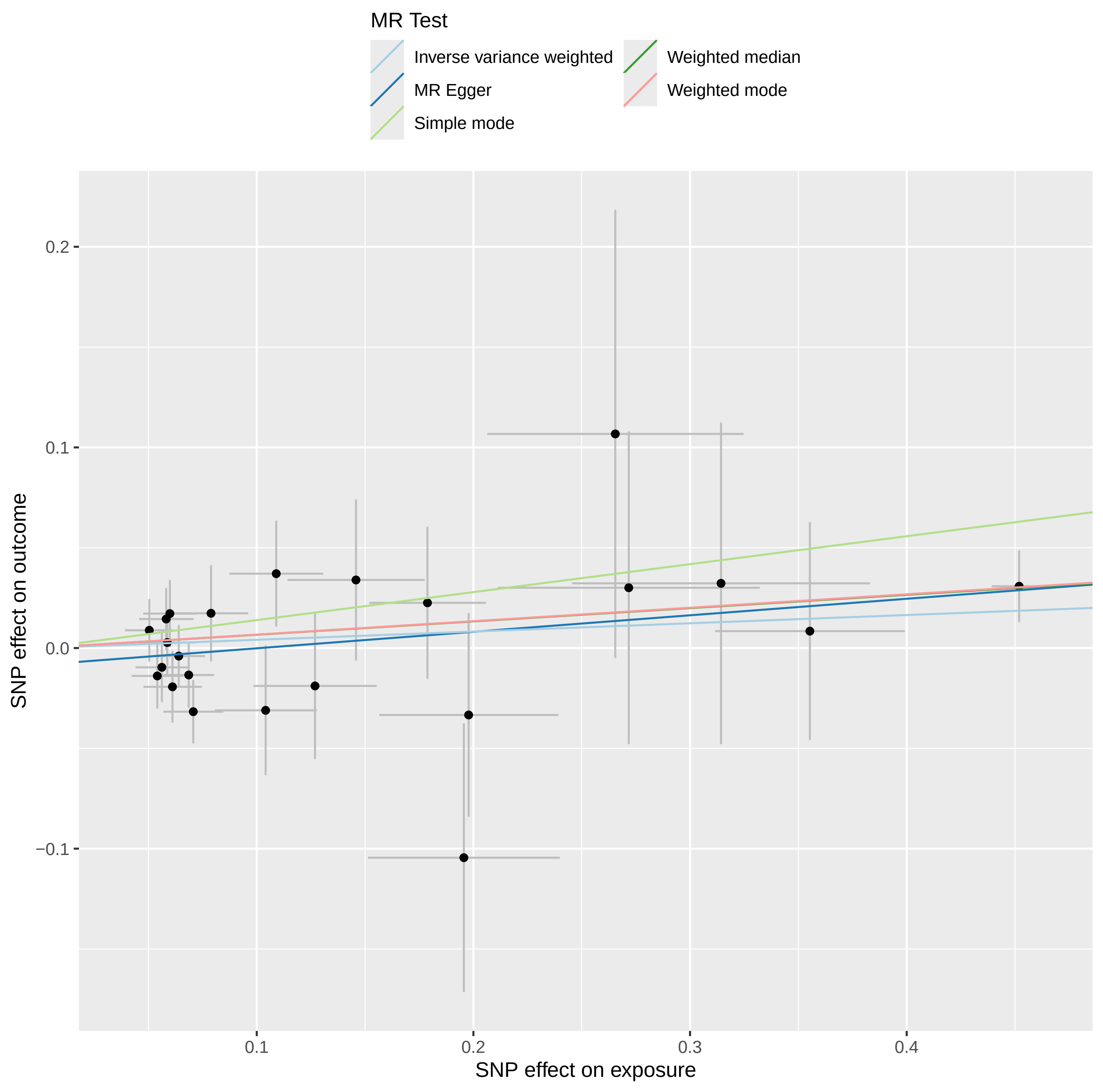
MR Test
Inverse variance weighted
MR Egger
Simple mode
Weighted median
Weighted mode
0.2
0.1
0.0
−0.1
SNP effect on outcome
0.1
0.2
0.3
0.4
SNP effect on exposure

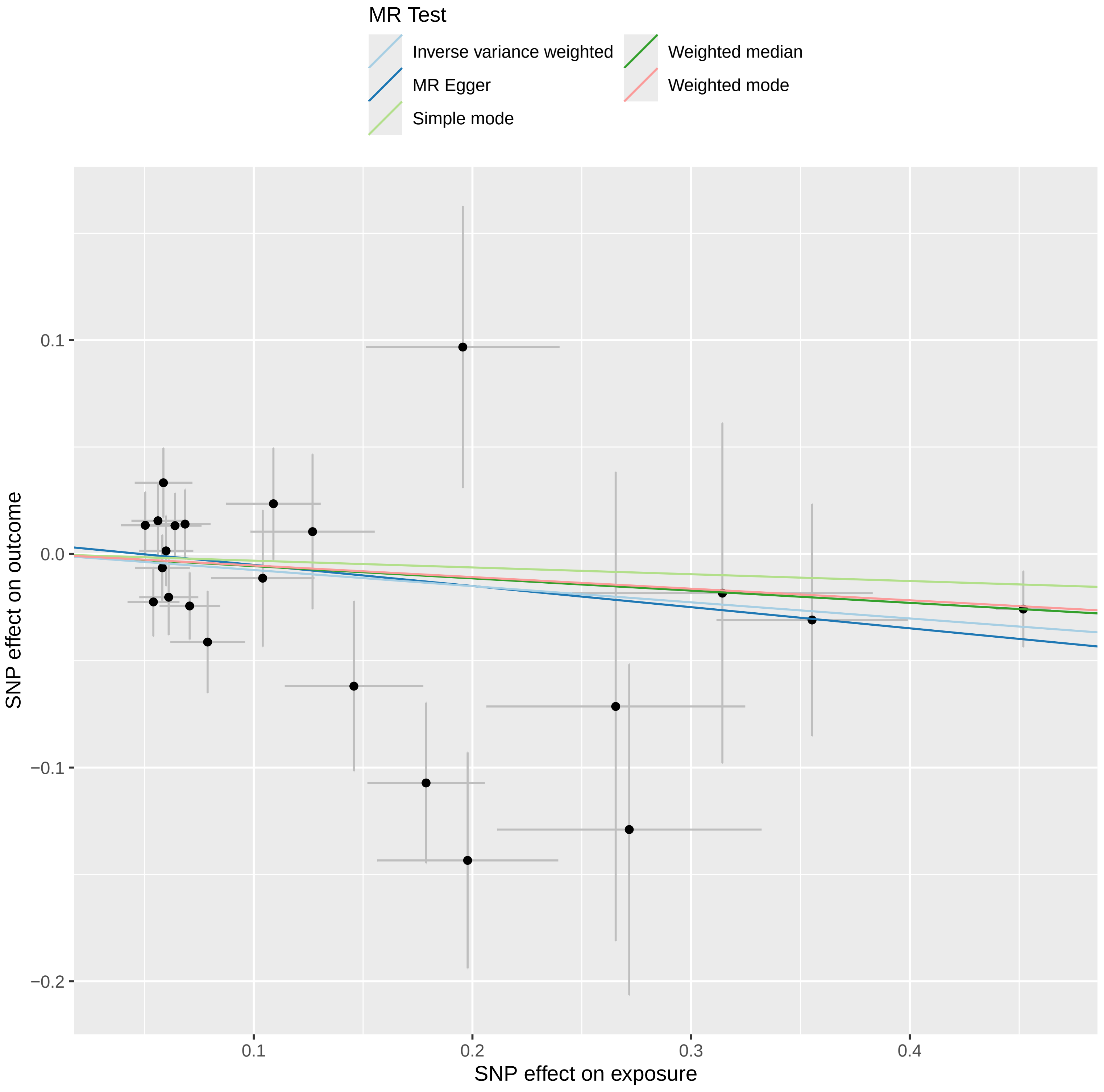

MR Test
Inverse variance weighted
MR Egger
Simple mode
Weighted median
Weighted mode
0.1
0.0
−0.1
−0.2
SNP effect on outcome
0.1
0.2
0.3
0.4
SNP effect on exposure

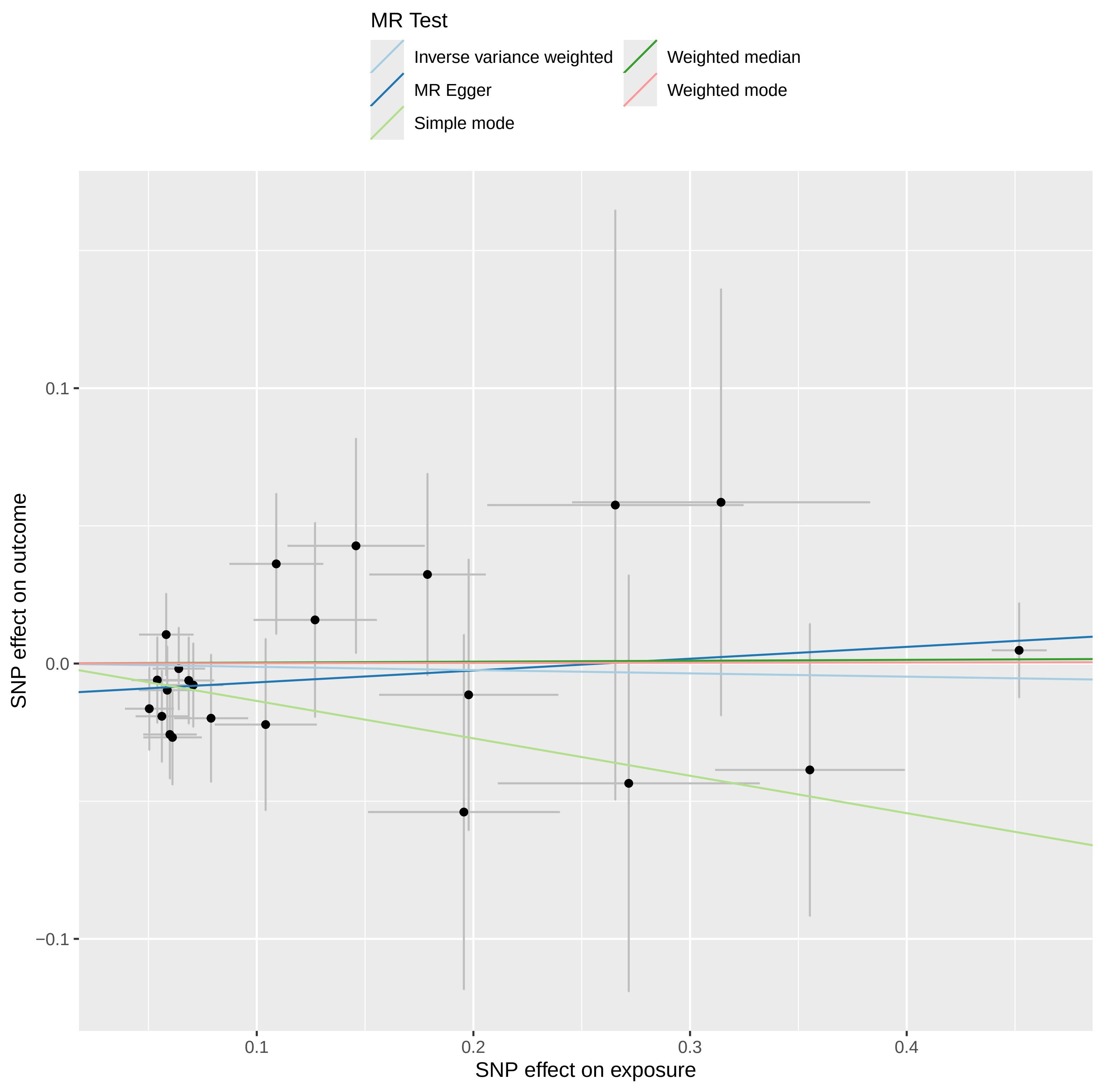
MR Test
Inverse variance weighted
MR Egger
Simple mode
Weighted median
Weighted mode
SNP effect on outcome
0.1
0.0
−0.1
0.1
0.2
0.3
0.4
SNP effect on exposure

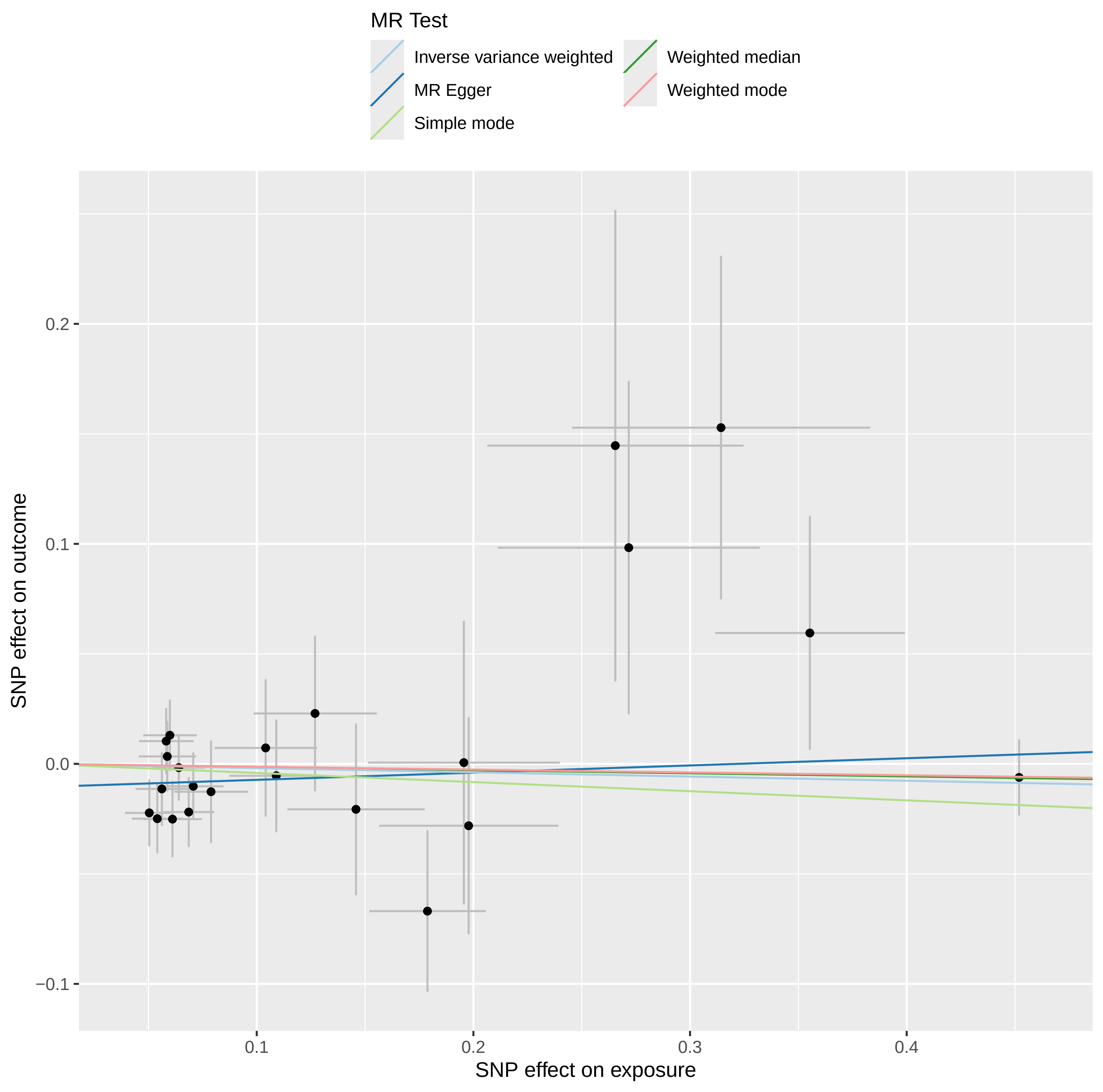
MR Test
Inverse variance weighted
MR Egger
Simple mode
Weighted median
Weighted mode
SNP effect on outcome
0.2
0.1
0.0
−0.1
0.1
0.2
0.3
0.4
SNP effect on exposure

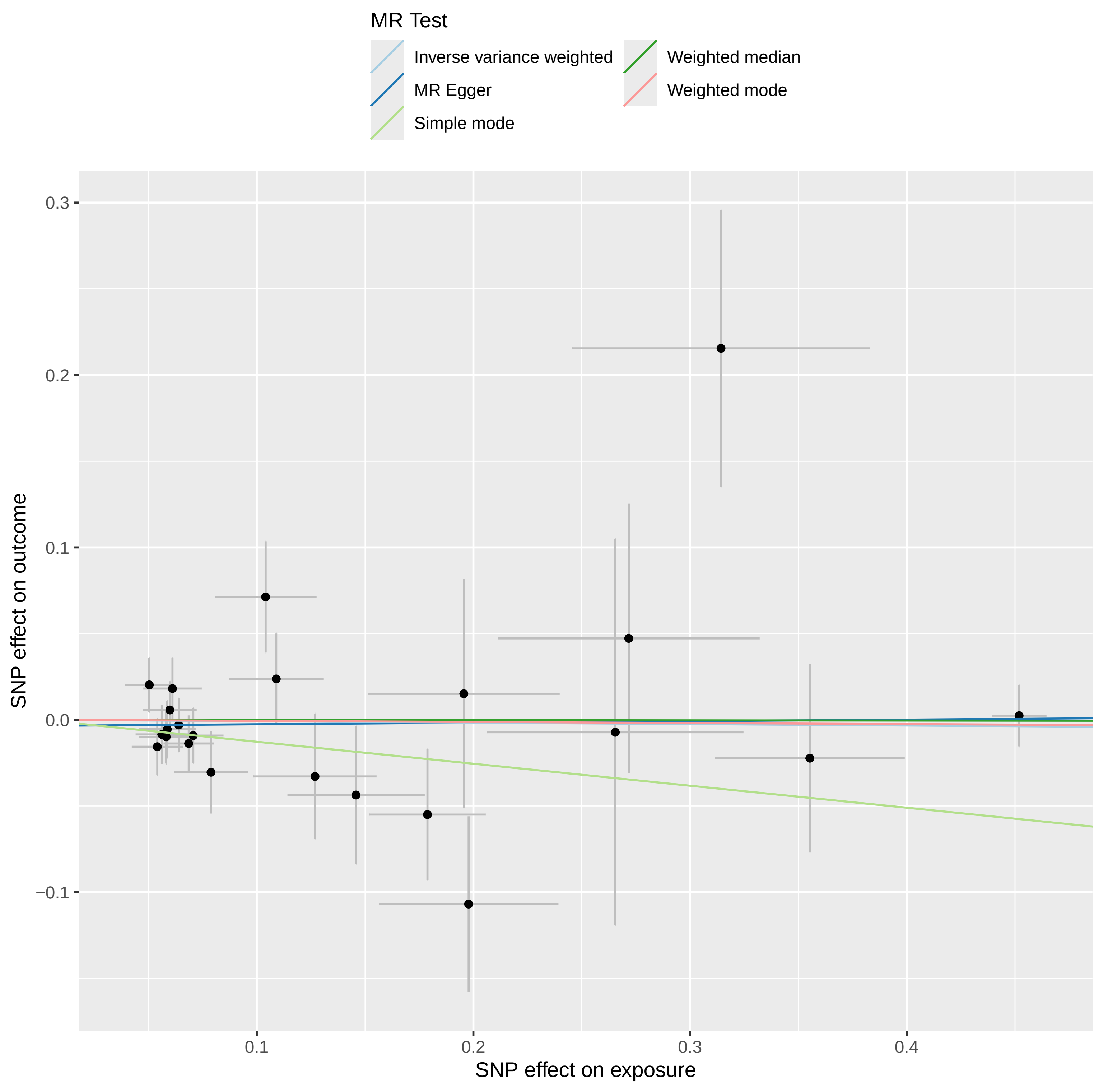

MR Test
Inverse variance weighted
MR Egger
Simple mode
Weighted median
Weighted mode
0.3
0.2
0.1
0.0
−0.1
SNP effect on outcome
0.1
0.2
0.3
0.4
SNP effect on exposure

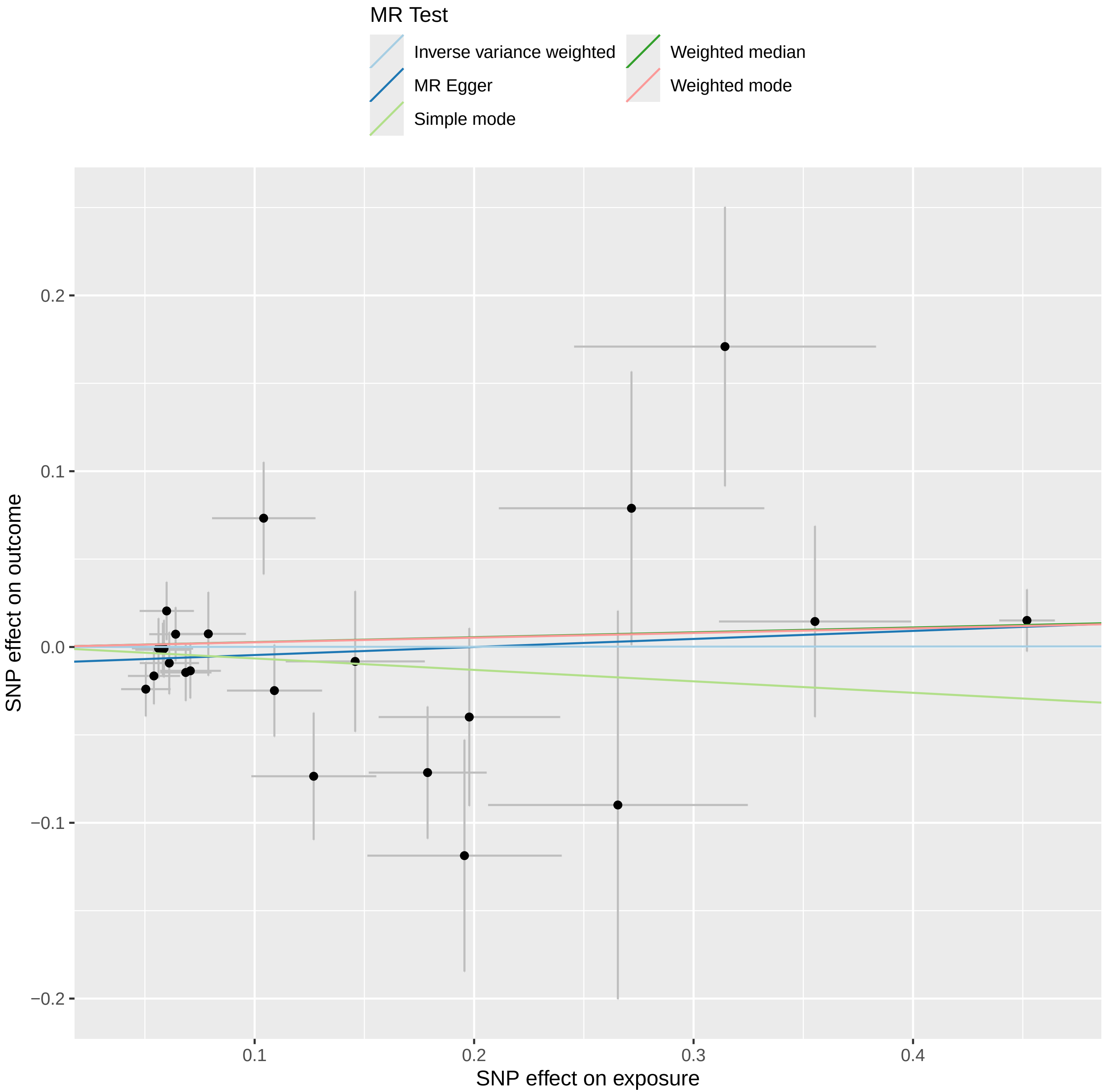

MR Test
Inverse variance weighted
MR Egger
Simple mode
Weighted median
Weighted mode
0.2
0.1
0.0
−0.1
−0.2
SNP effect on outcome
0.1
0.2
0.3
0.4
SNP effect on exposure

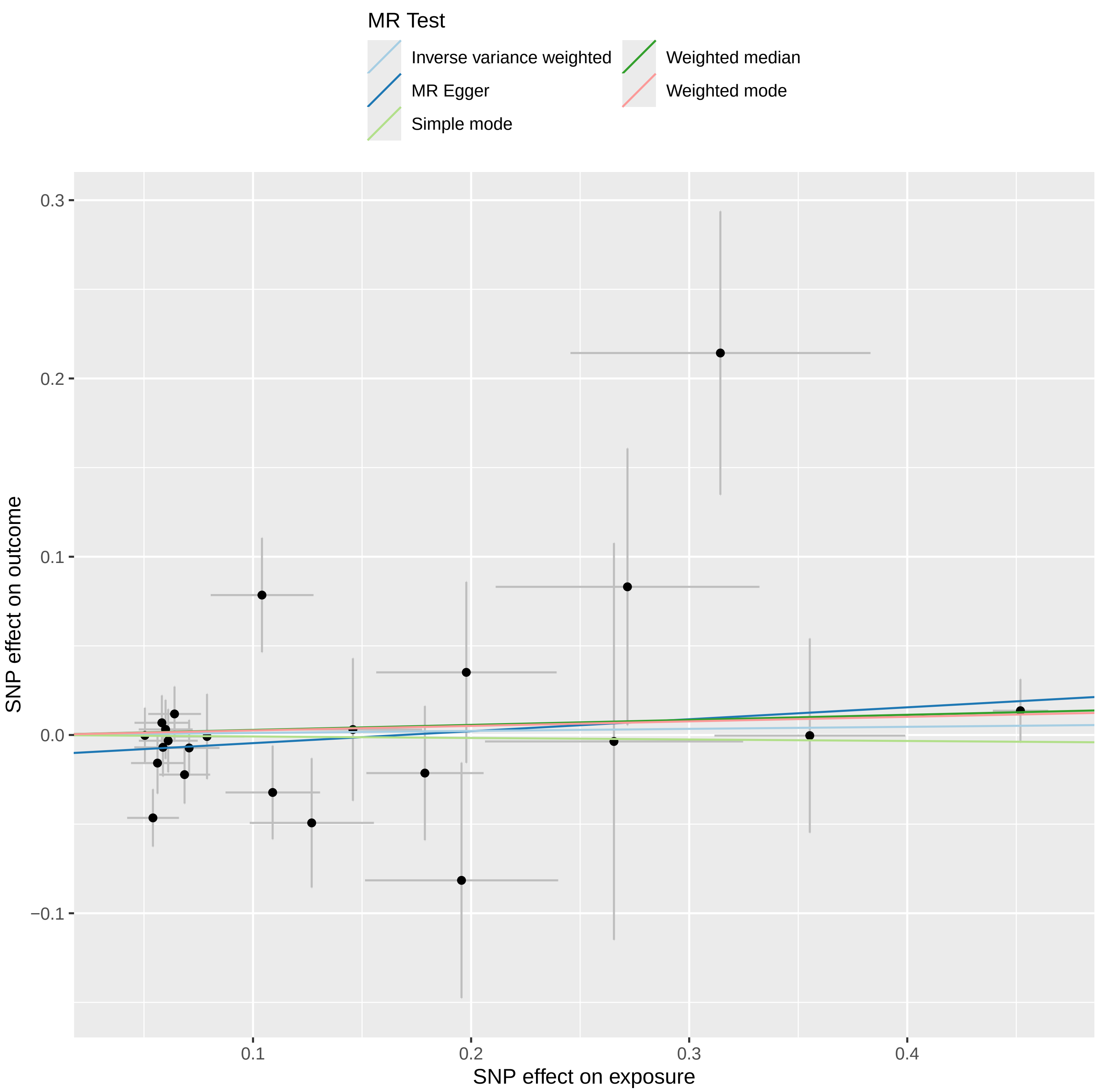

MR Test
Inverse variance weighted
MR Egger
Simple mode
Weighted median
Weighted mode
0.3
0.2
0.1
0.0
−0.1
0.1
0.2
0.3
0.4
SNP effect on outcome
SNP effect on exposure

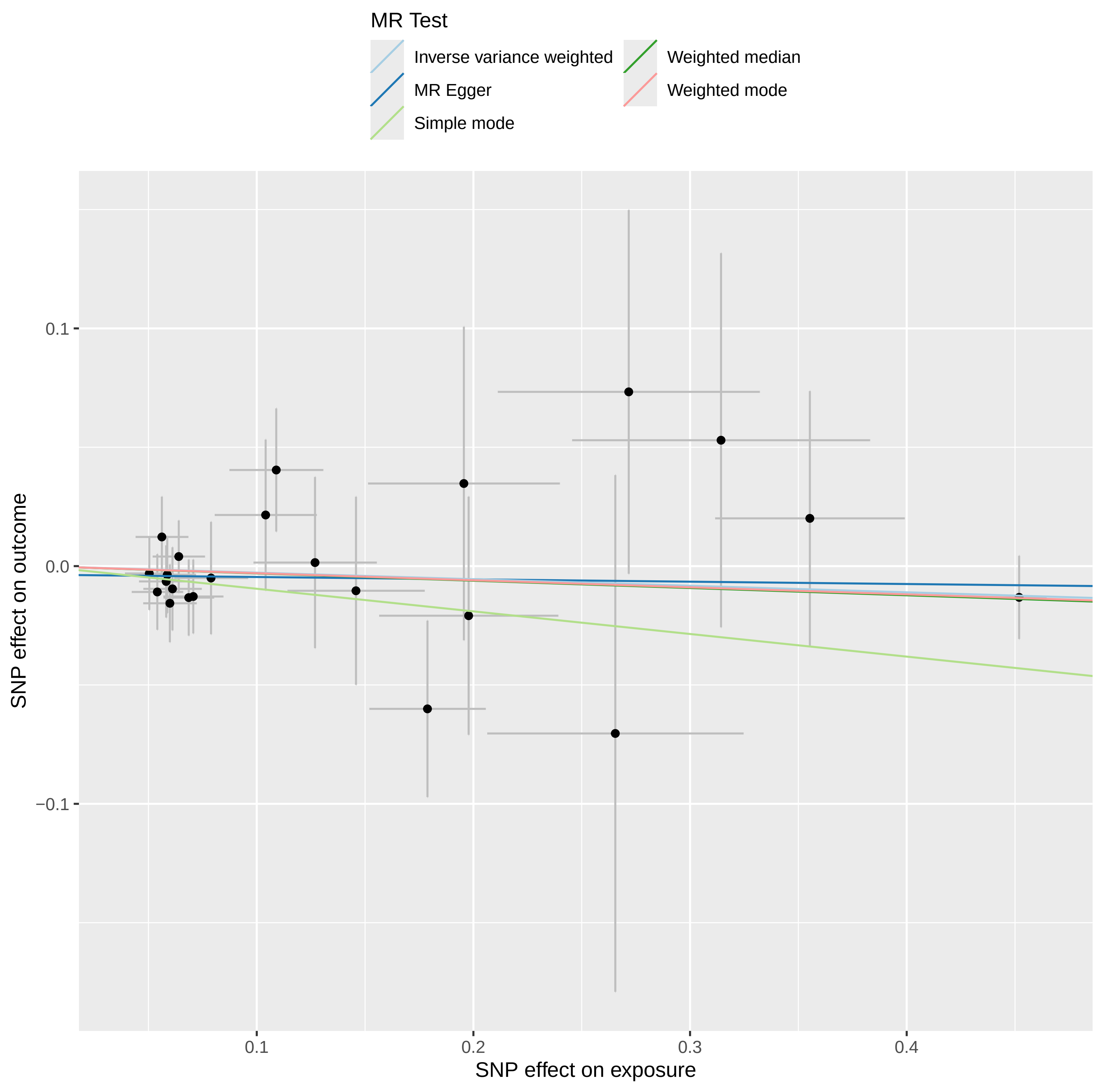

MR Test
Inverse variance weighted
MR Egger
Simple mode
Weighted median
Weighted mode
0.1
0.0
−0.1
SNP effect on outcome
0.1
0.2
0.3
0.4
SNP effect on exposure

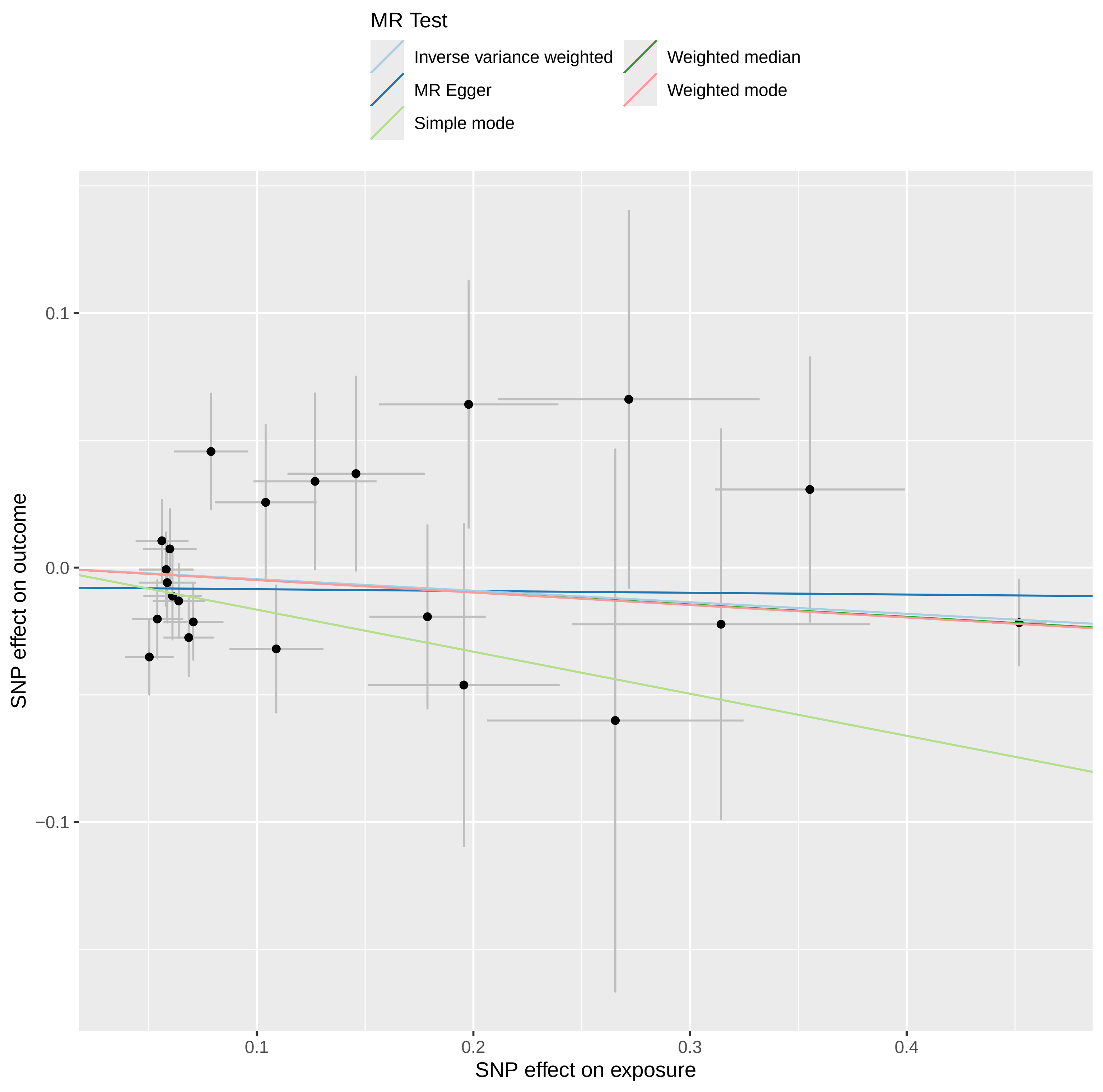

MR Test
Inverse variance weighted
MR Egger
Simple mode
Weighted median
Weighted mode
0.1
0.0
−0.1
SNP effect on outcome
0.1
0.2
0.3
0.4
SNP effect on exposure

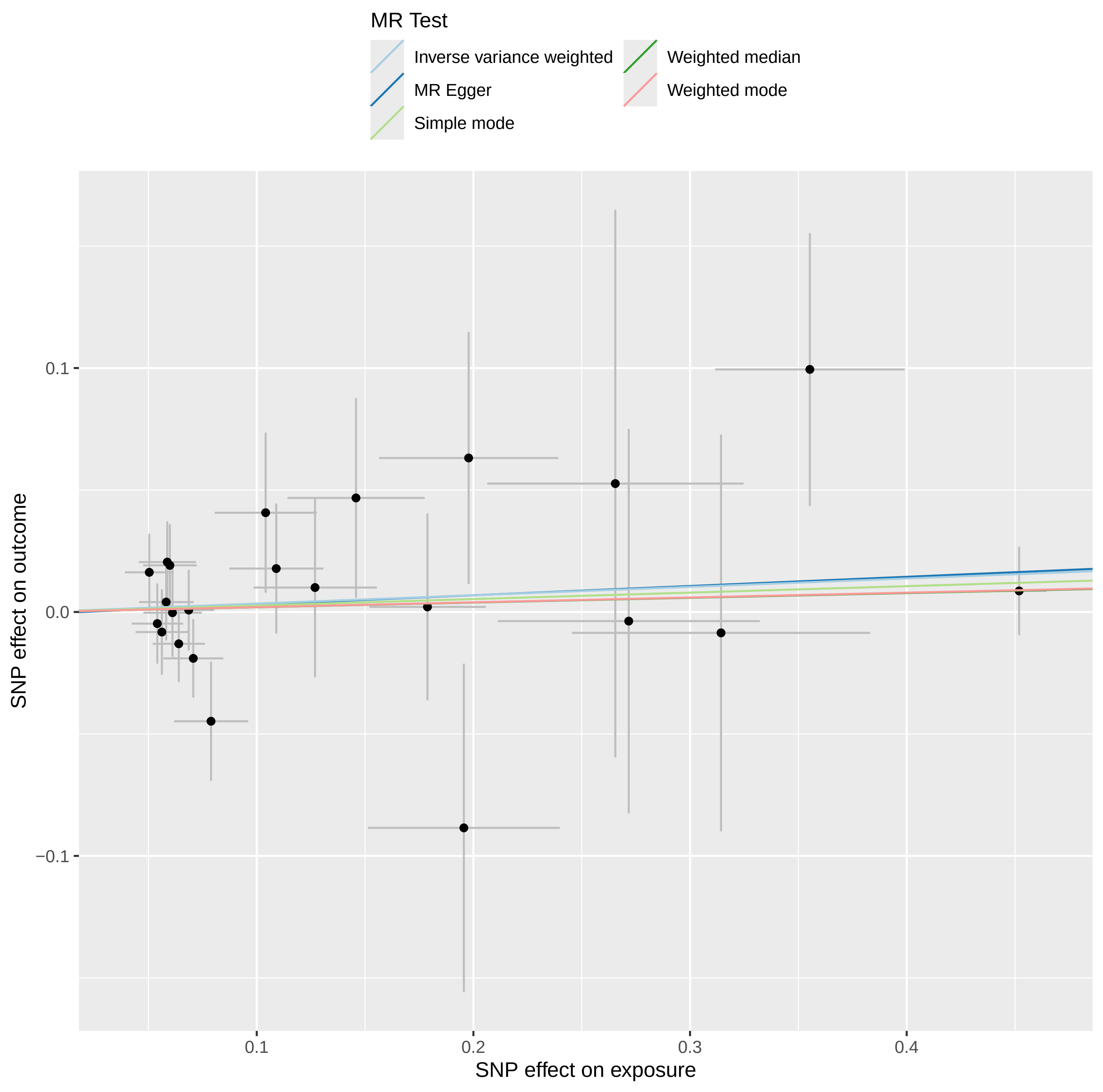

MR Test
Inverse variance weighted
MR Egger
Simple mode
Weighted median
Weighted mode
0.1
0.0
−0.1
SNP effect on outcome
0.1
0.2
0.3
0.4
SNP effect on exposure

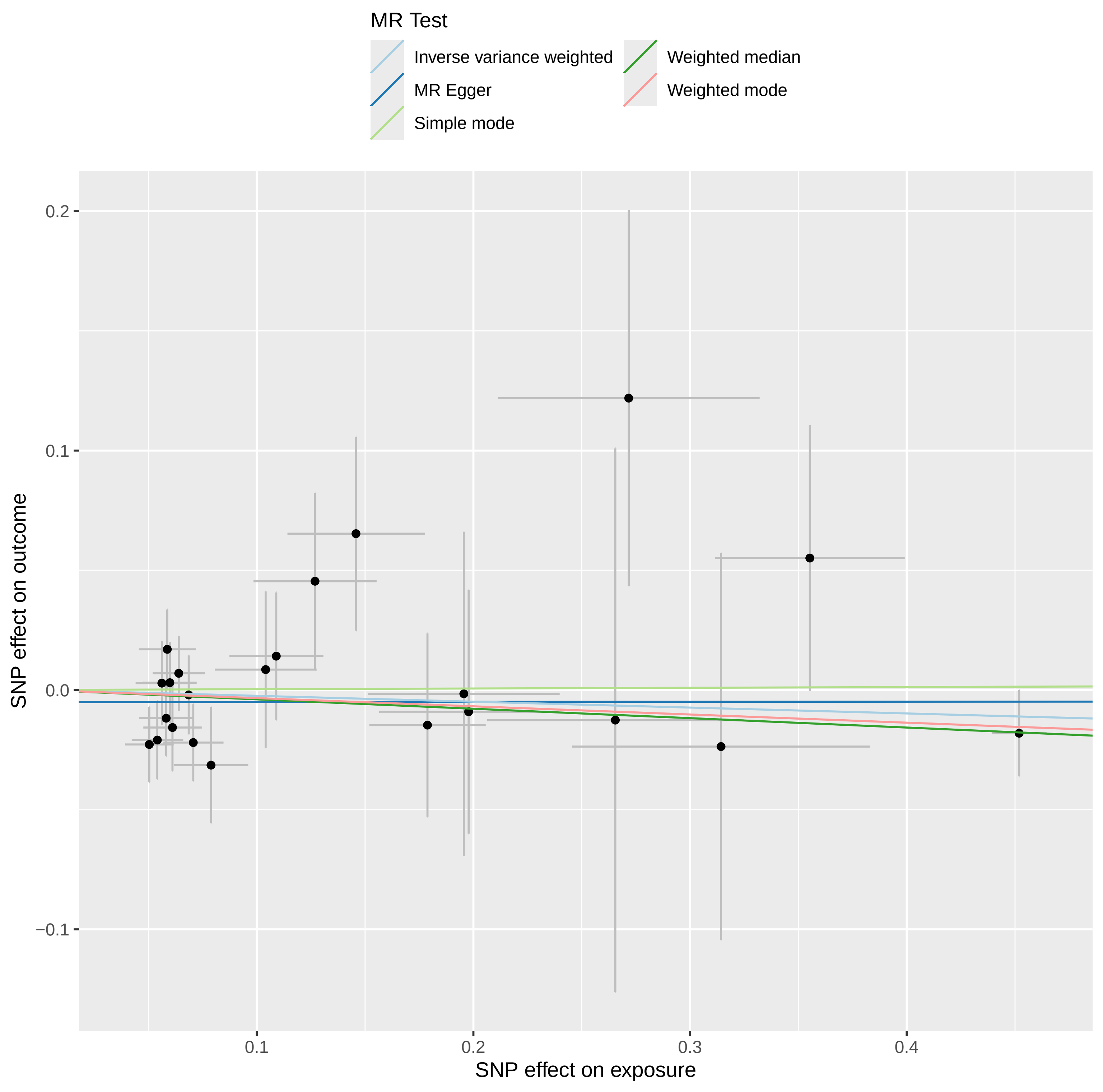
MR Test
Inverse variance weighted
MR Egger
Simple mode
Weighted median
Weighted mode
0.2
0.1
0.0
−0.1
SNP effect on outcome
0.1
0.2
0.3
0.4
SNP effect on exposure

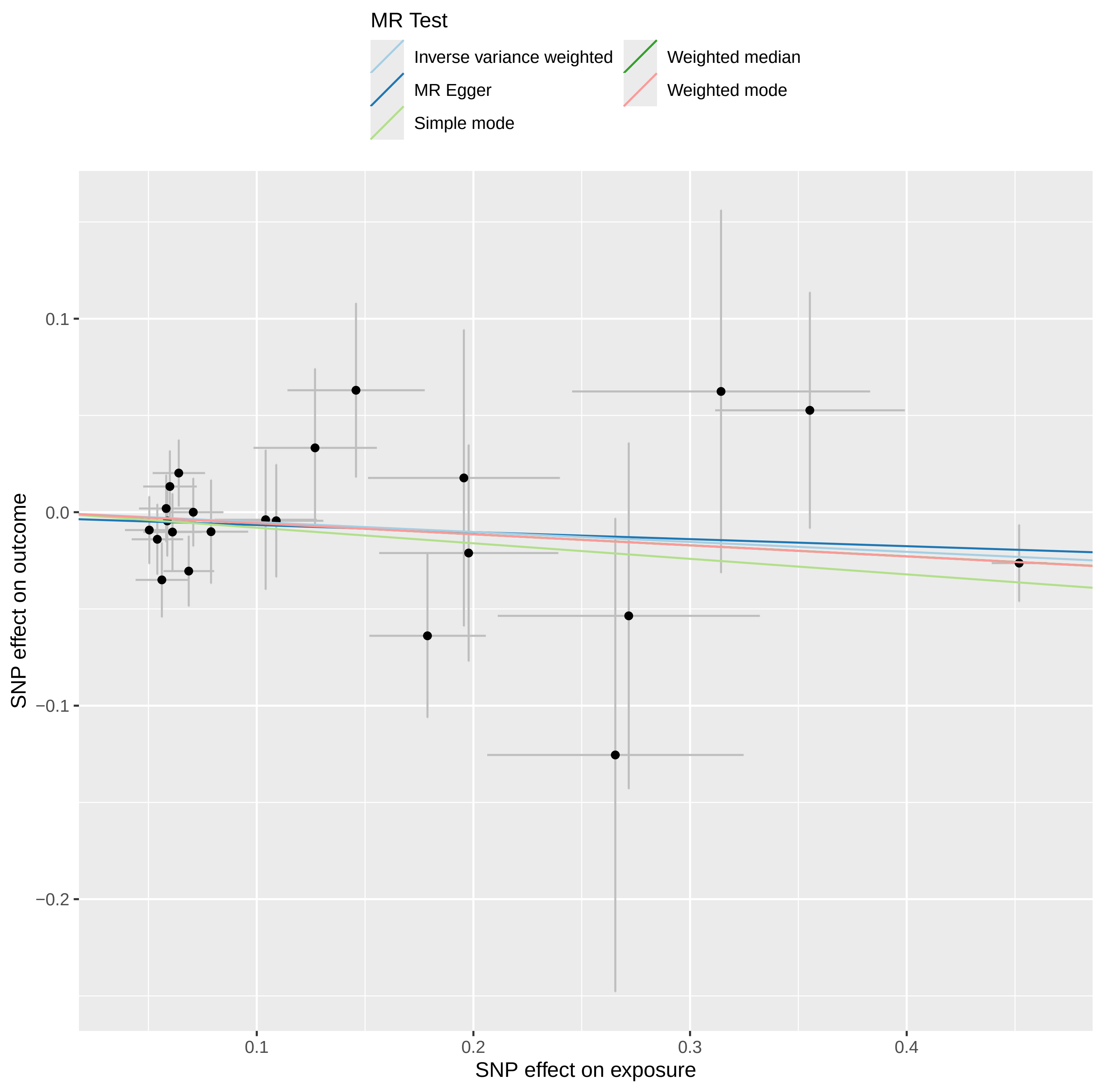
MR Test
Inverse variance weighted
MR Egger
Simple mode
Weighted median
Weighted mode
SNP effect on outcome
SNP effect on exposure
0.1
0.0
−0.1
−0.2
0.1
0.2
0.3
0.4

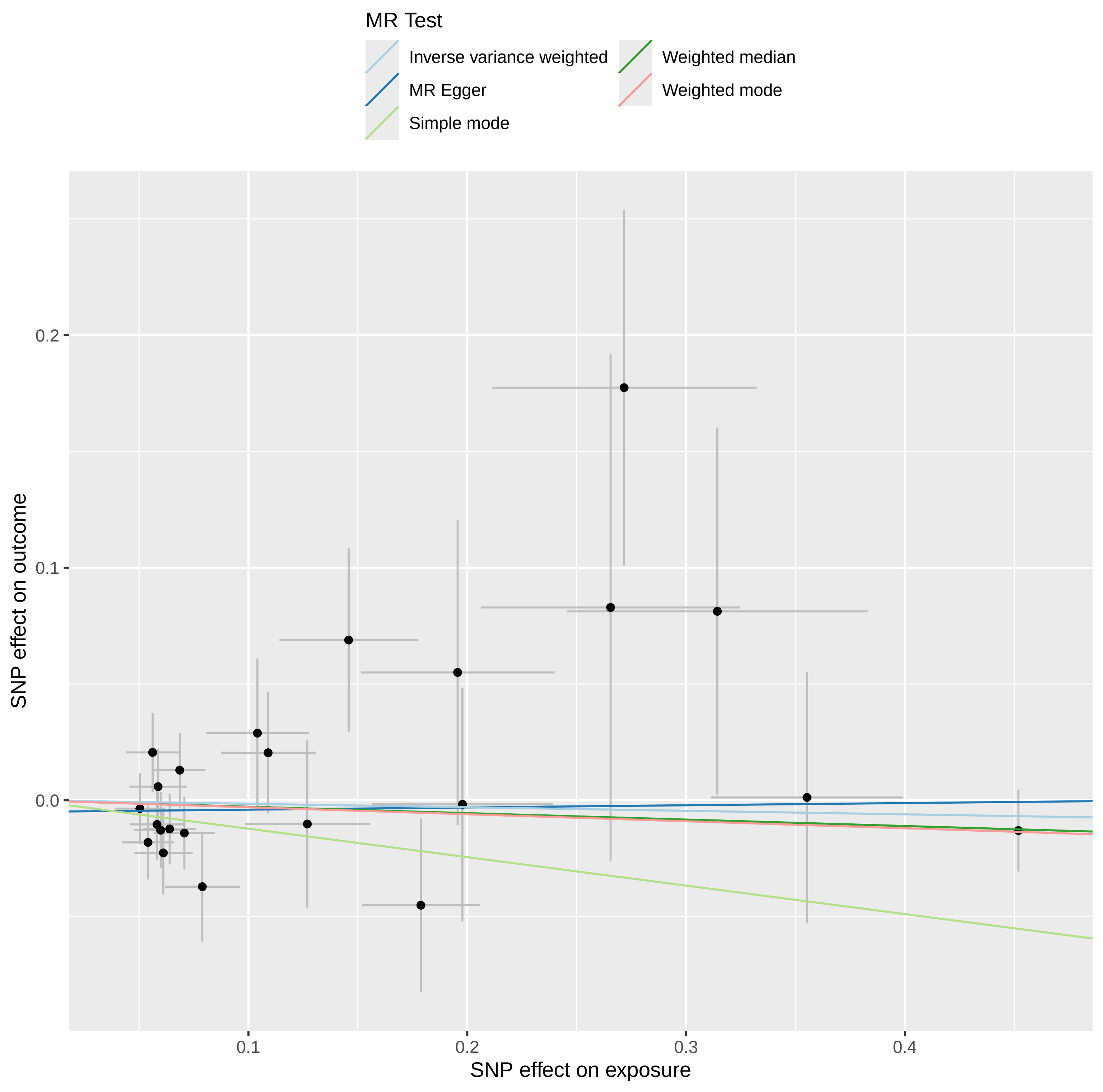

MR Test
Inverse variance weighted
MR Egger
Simple mode
Weighted median
Weighted mode
SNP effect on outcome
0.2
0.1
0.0
0.1
0.2
0.3
0.4
SNP effect on exposure

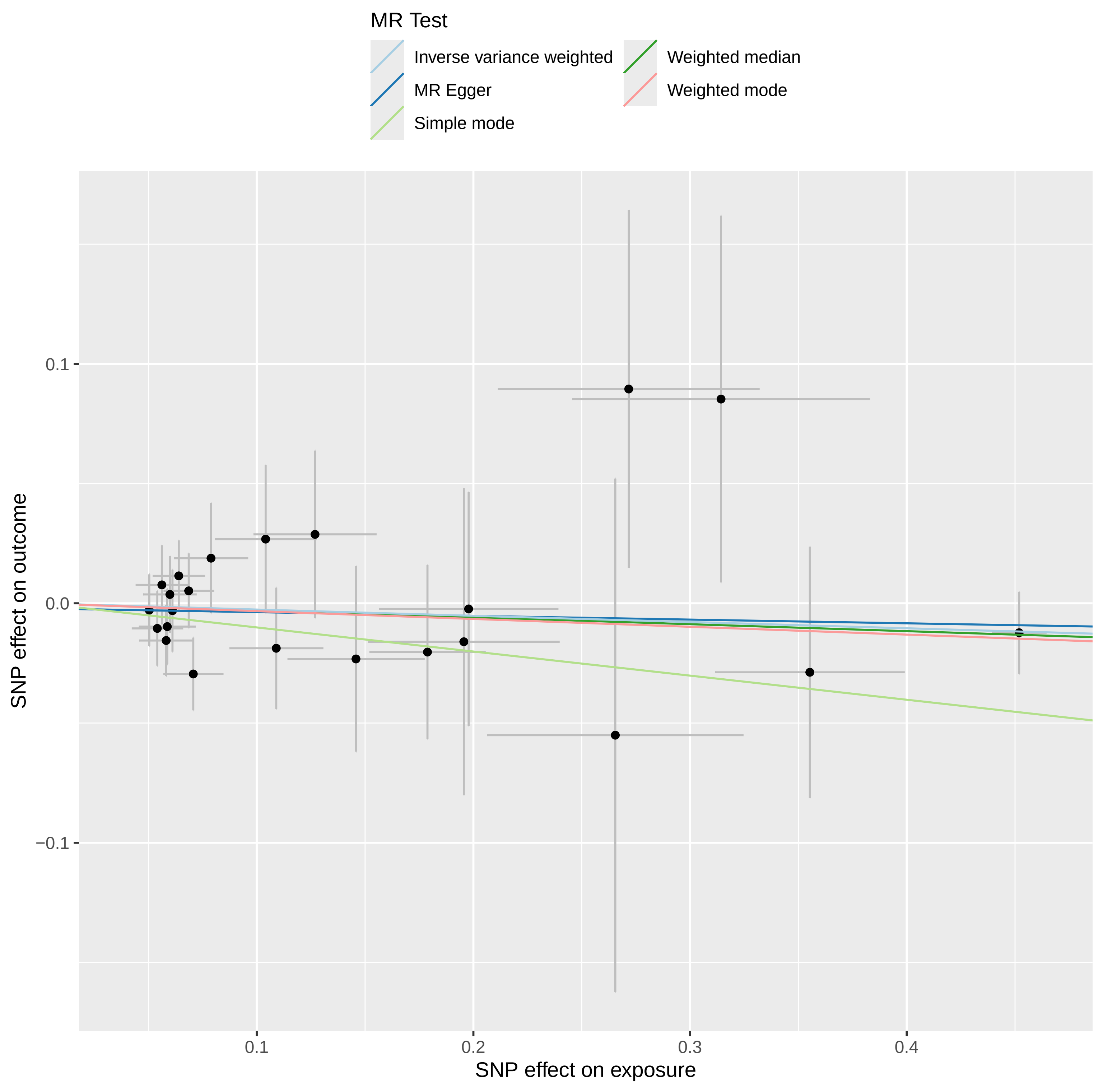

MR Test
Inverse variance weighted
MR Egger
Simple mode
Weighted median
Weighted mode
0.1
0.0
−0.1
SNP effect on outcome
0.1
0.2
0.3
0.4
SNP effect on exposure

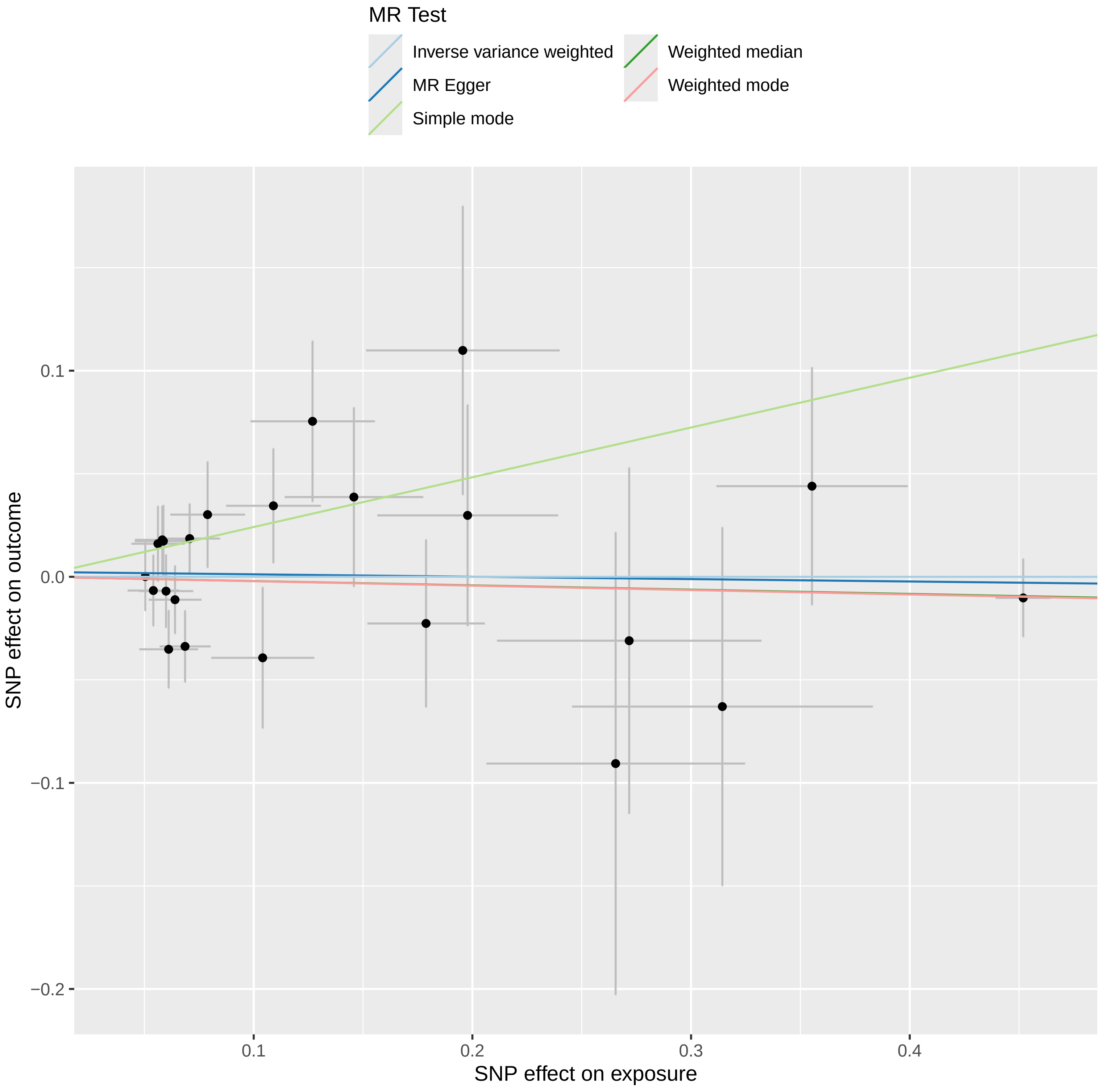

MR Test
Inverse variance weighted
MR Egger
Simple mode
Weighted median
Weighted mode
0.1
0.0
−0.1
−0.2
SNP effect on outcome
0.1
0.2
0.3
0.4
SNP effect on exposure

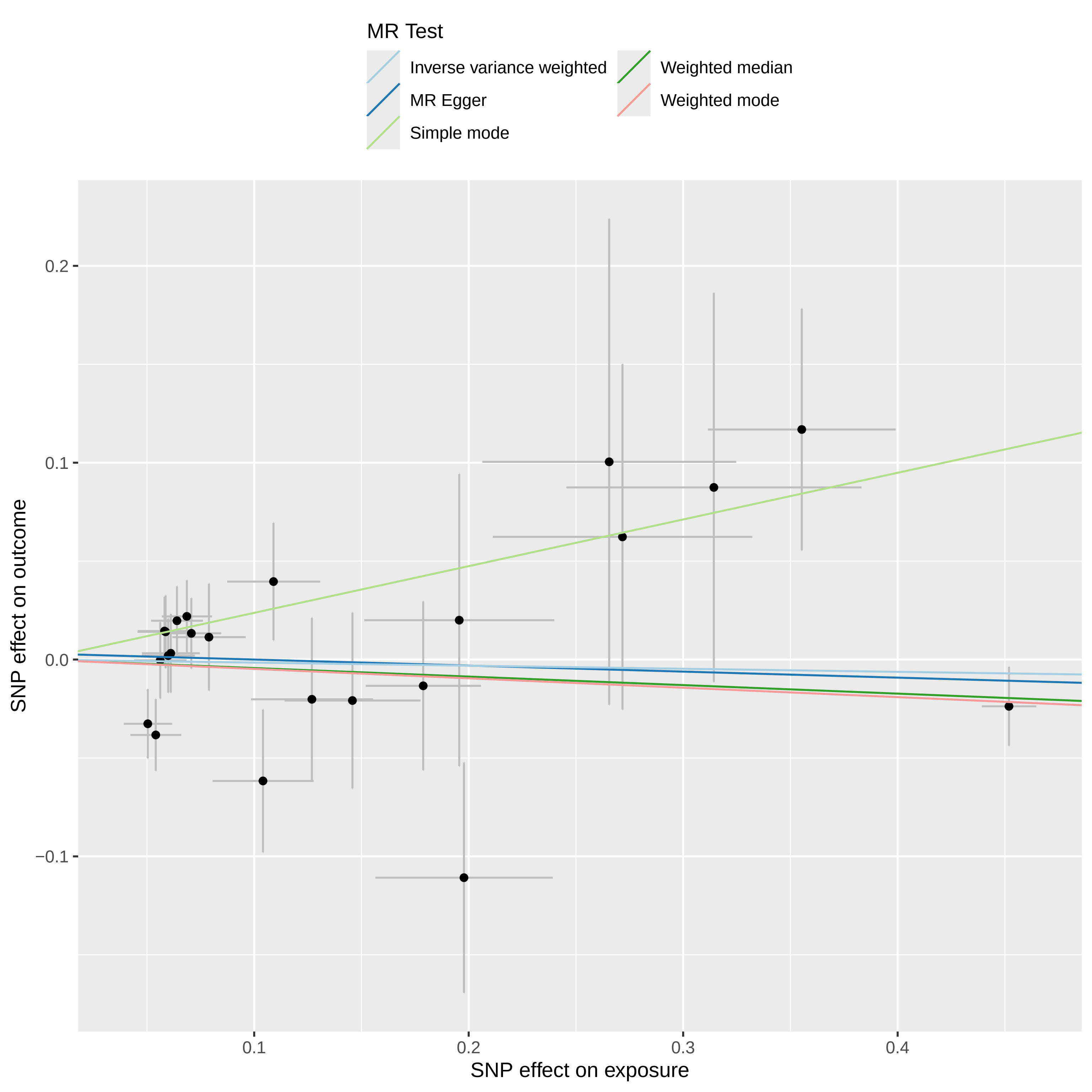
MR Test
Inverse variance weighted
MR Egger
Simple mode
Weighted median
Weighted mode
SNP effect on outcome
0.2
0.1
0.0
−0.1
0.1
0.2
0.3
0.4
SNP effect on exposure

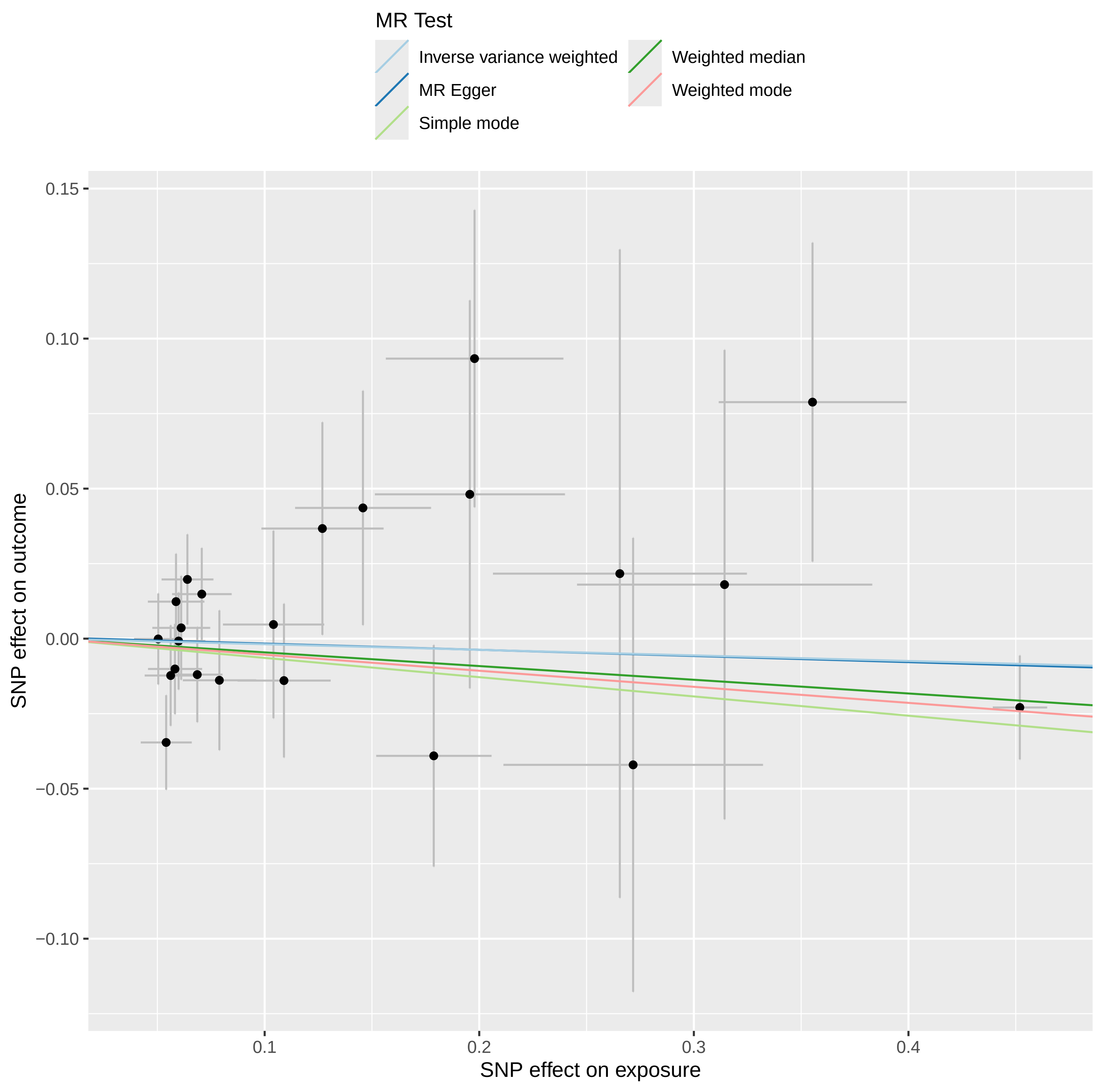
MR Test
Inverse variance weighted
MR Egger
Simple mode
Weighted median
Weighted mode
0.15
0.10
0.05
0.00
−0.05
−0.10
SNP effect on outcome
0.1
0.2
0.3
0.4
SNP effect on exposure

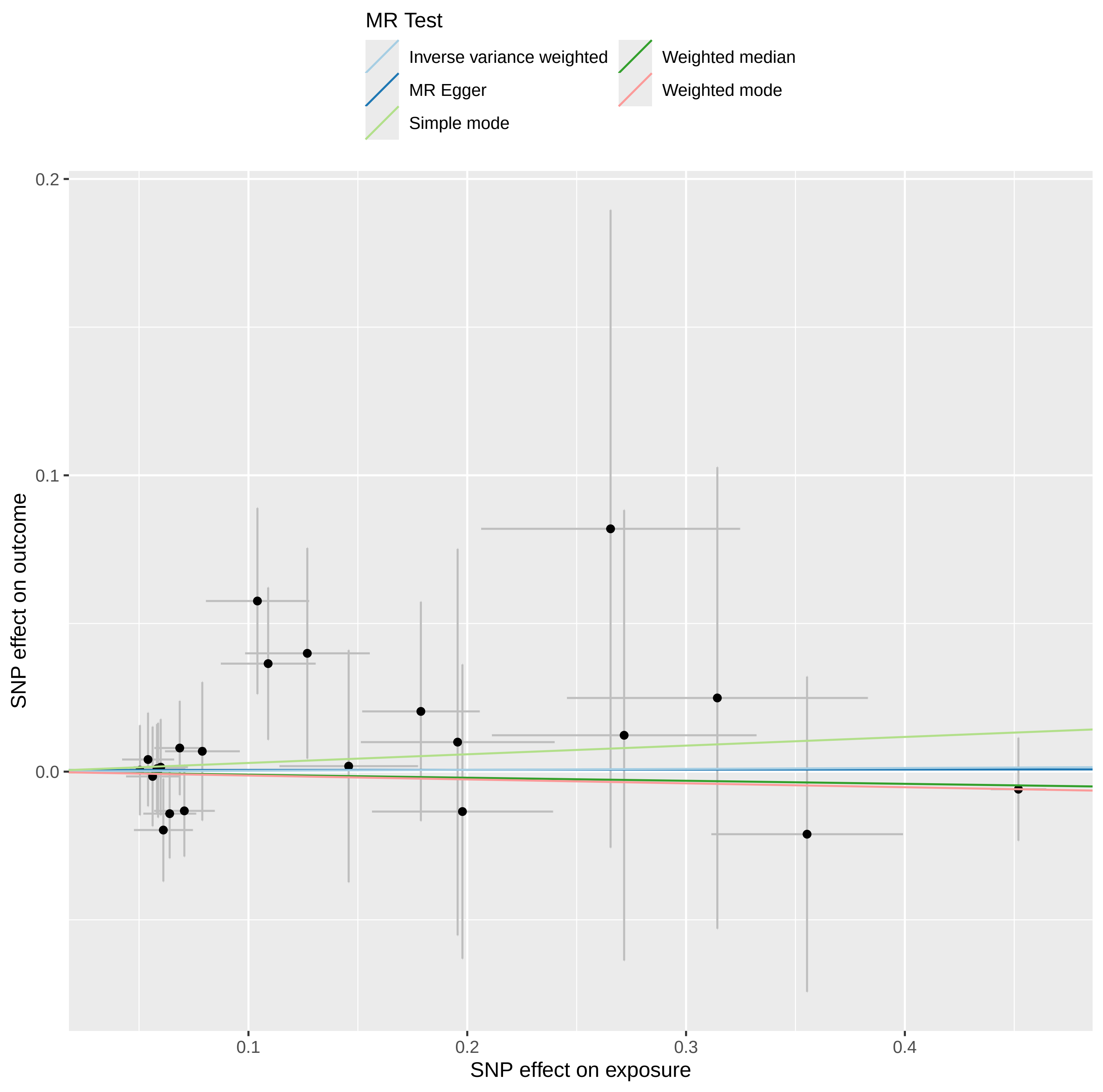

MR Test
Inverse variance weighted
MR Egger
Simple mode
Weighted median
Weighted mode
0.2
0.1
0.0
0.1
0.2
0.3
0.4
SNP effect on outcome
SNP effect on exposure

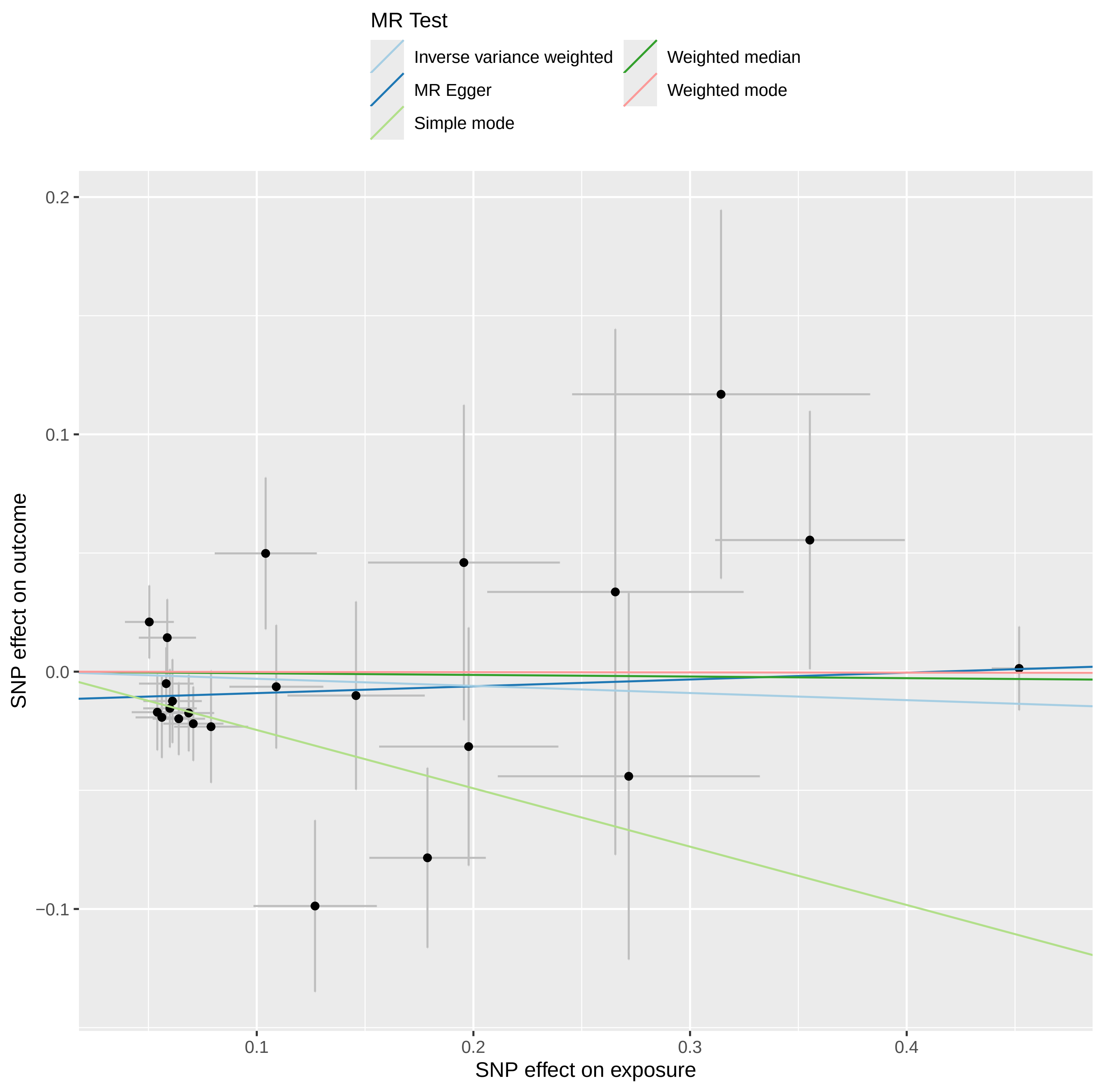
MR Test
Inverse variance weighted
MR Egger
Simple mode
Weighted median
Weighted mode
0.2
0.1
0.0
−0.1
0.1
0.2
0.3
0.4
SNP effect on outcome
SNP effect on exposure

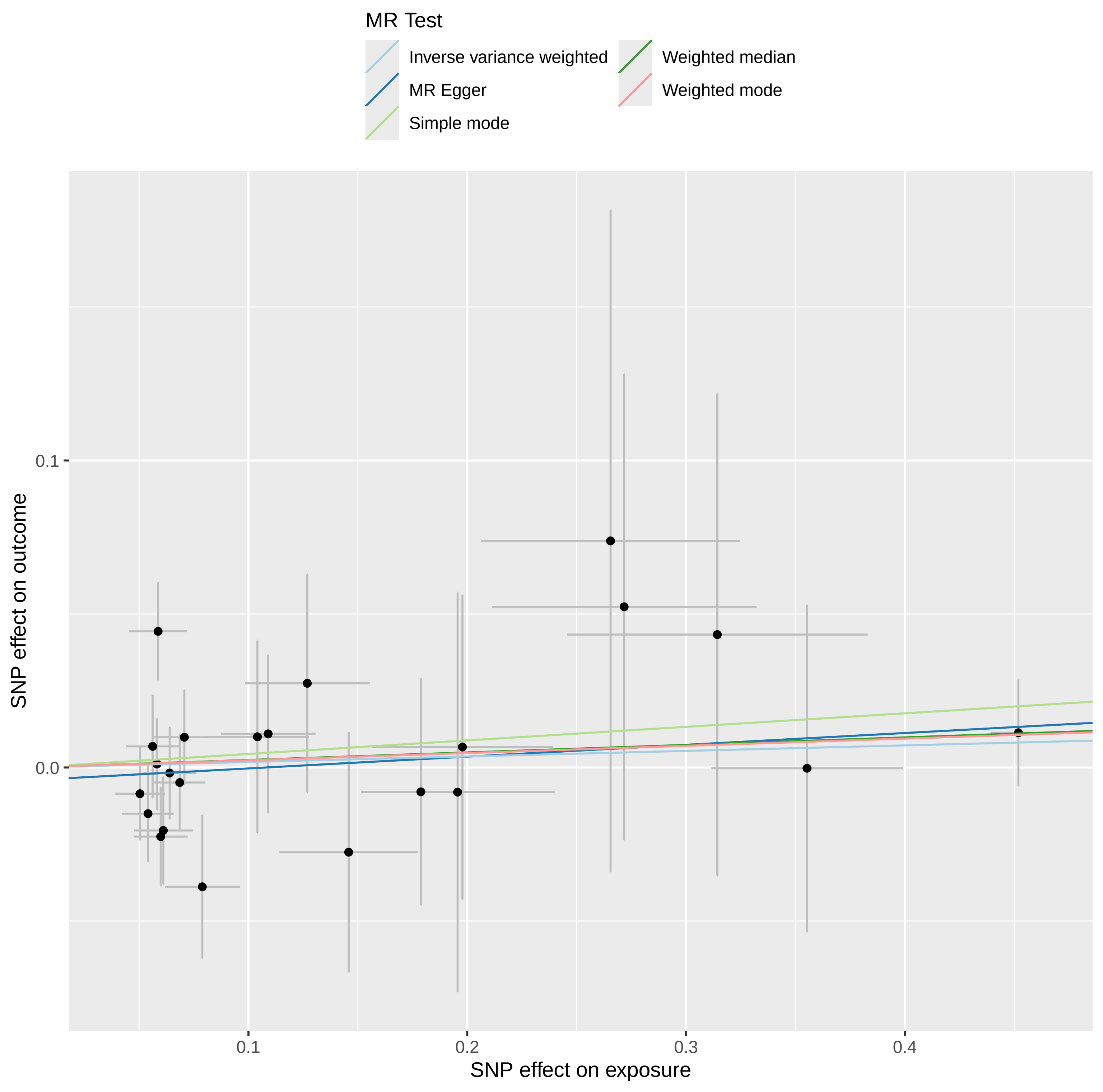

MR Test
Inverse variance weighted
MR Egger
Simple mode
Weighted median
Weighted mode
SNP effect on outcome
0.1
0.0
SNP effect on exposure
0.1
0.2
0.3
0.4

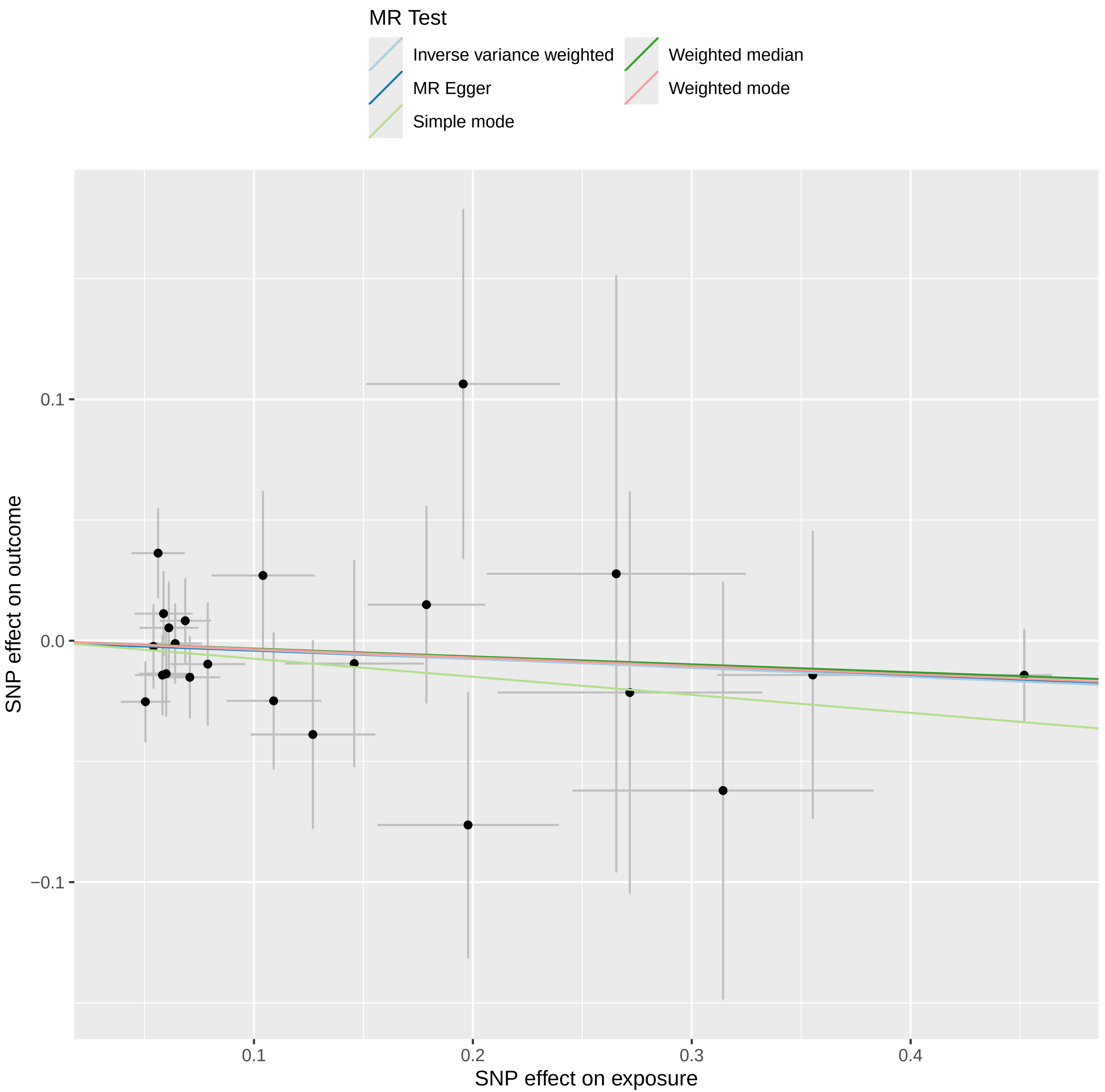
MR Test
Inverse variance weighted
MR Egger
Simple mode
Weighted median
Weighted mode
SNP effect on outcome
0.1
0.0
−0.1
0.1
0.2
0.3
0.4
SNP effect on exposure

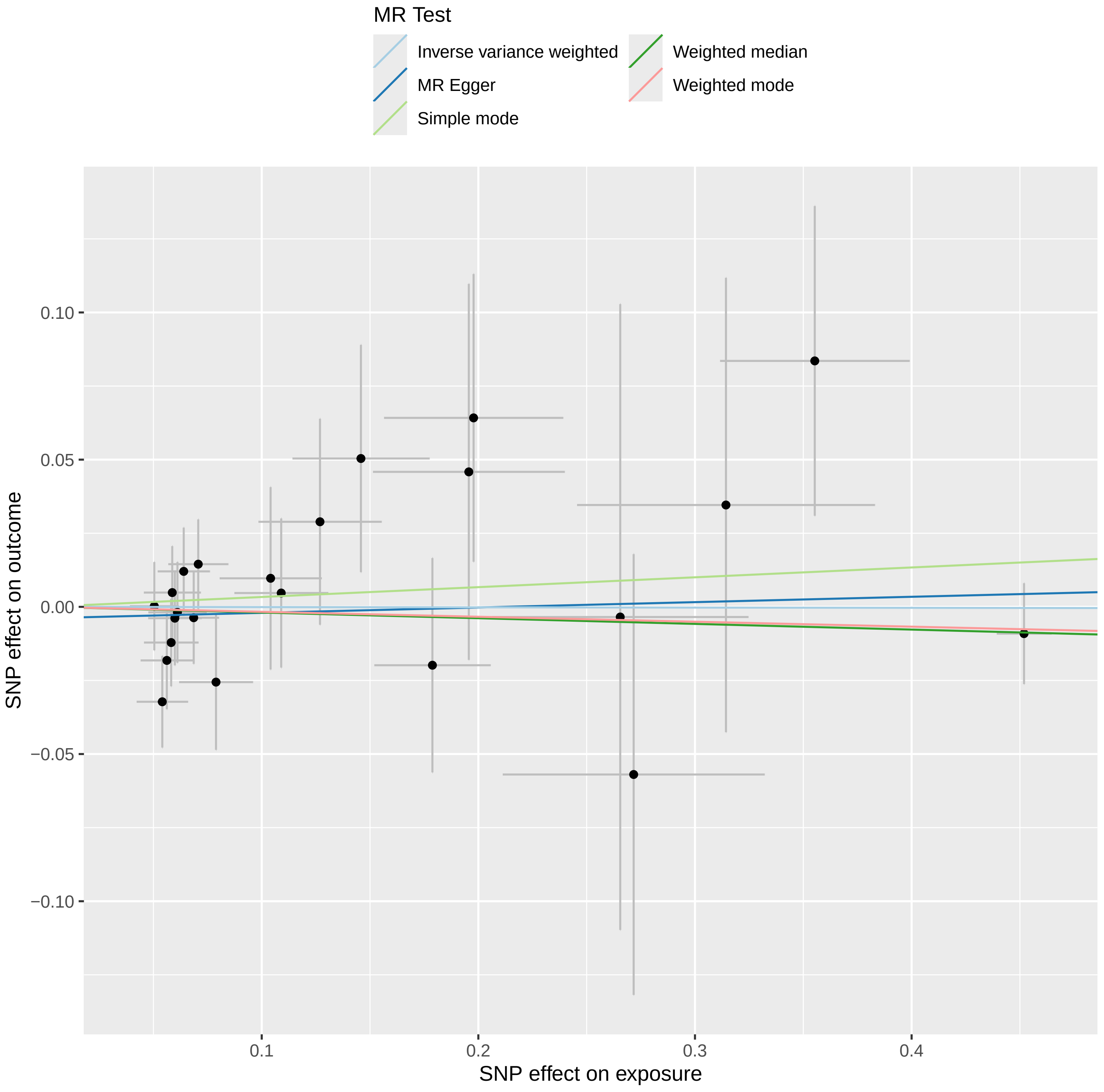

MR Test
Inverse variance weighted
MR Egger
Simple mode
Weighted median
Weighted mode
0.10
0.05
0.00
−0.05
−0.10
SNP effect on outcome
0.1
0.2
0.3
0.4
SNP effect on exposure

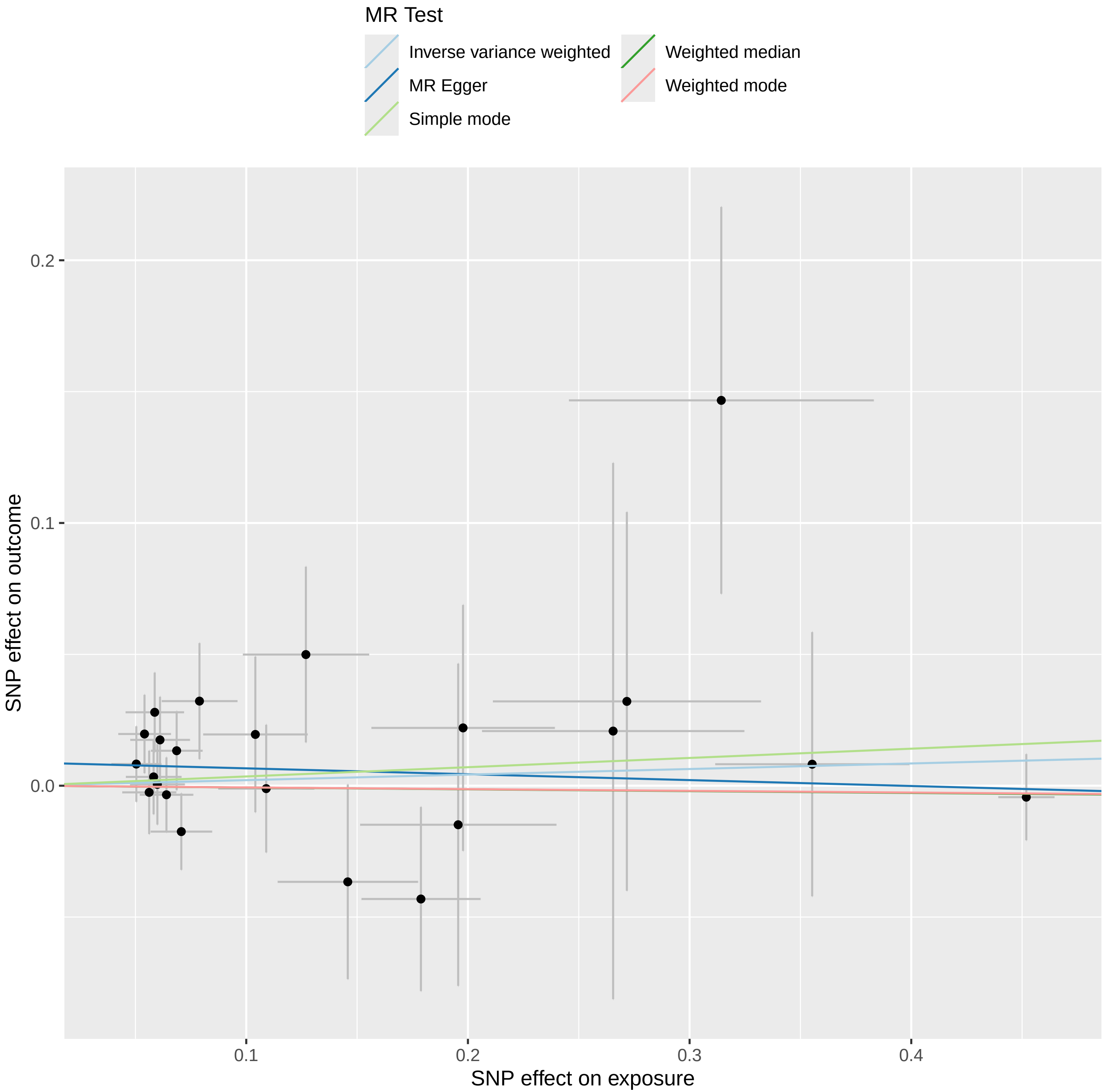
MR Test
Inverse variance weighted
MR Egger
Simple mode
Weighted median
Weighted mode
SNP effect on outcome
SNP effect on exposure
0.0
0.1
0.2
0.1
0.2
0.3
0.4

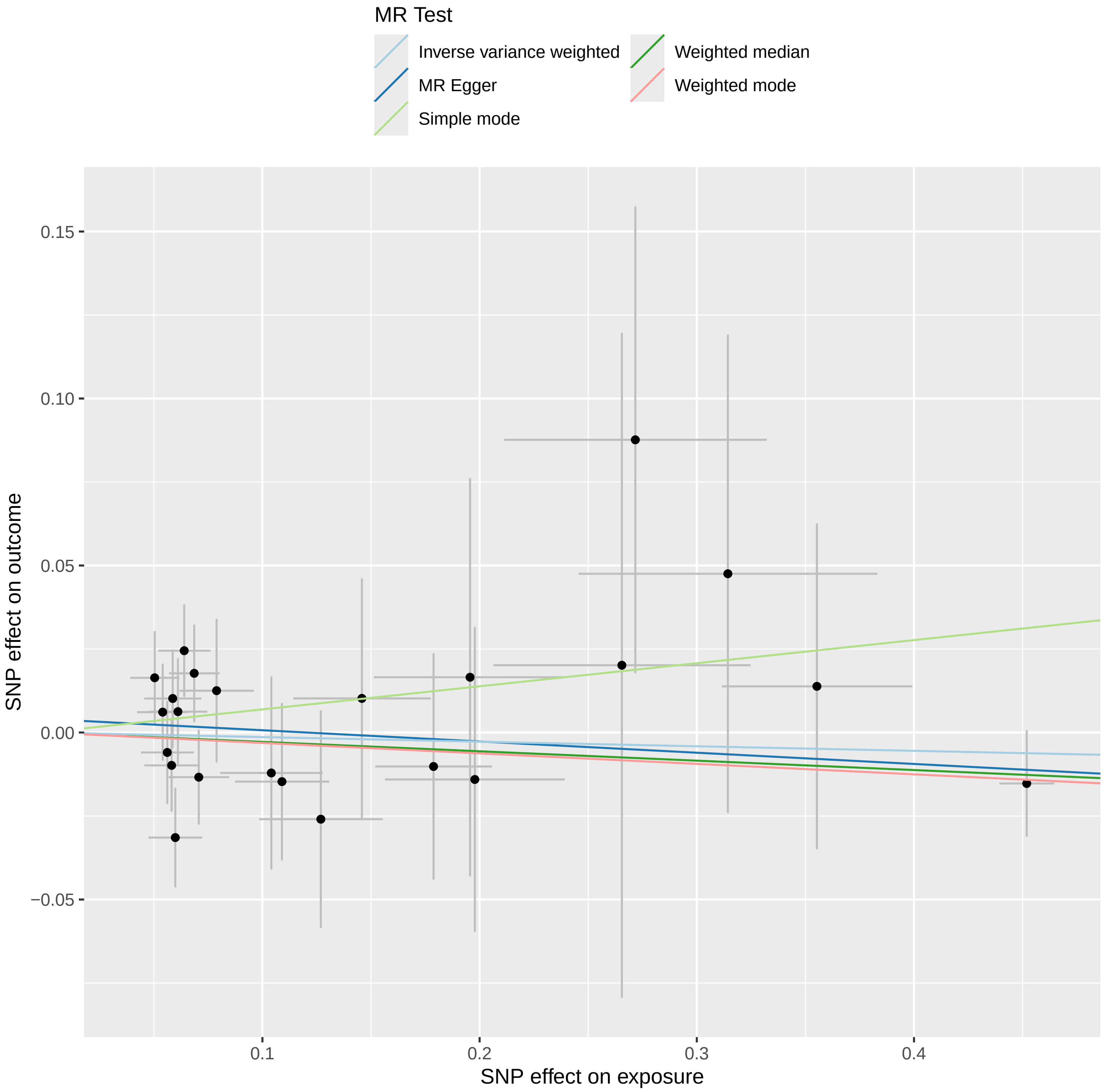
MR Test
Inverse variance weighted
MR Egger
Simple mode
Weighted median
Weighted mode
0.15
0.10
0.05
0.00
−0.05
SNP effect on outcome
0.1
0.2
0.3
0.4
SNP effect on exposure

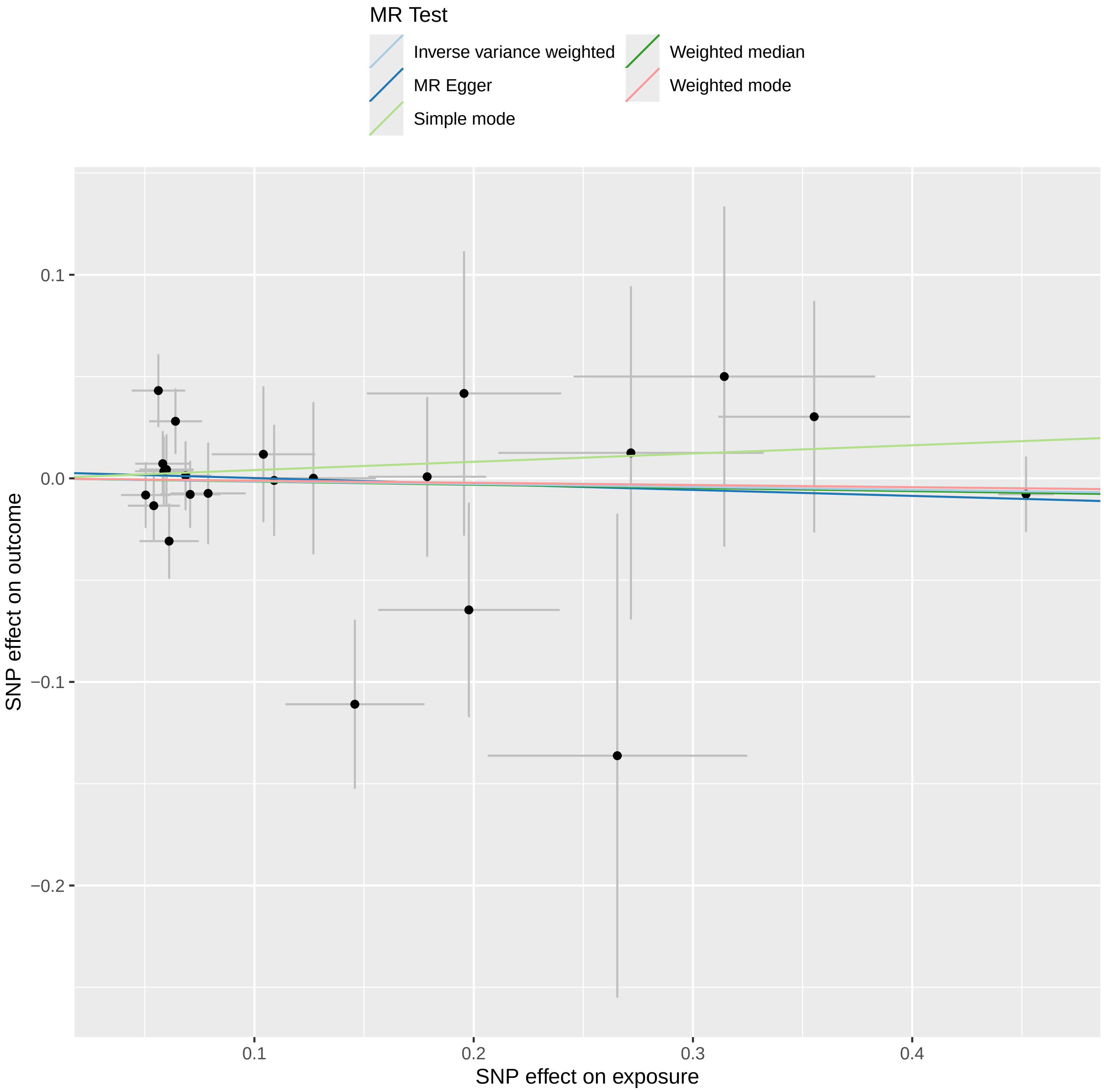
MR Test
Inverse variance weighted
MR Egger
Simple mode
Weighted median
Weighted mode
0.1
0.0
−0.1
−0.2
SNP effect on outcome
0.1
0.2
0.3
0.4
SNP effect on exposure

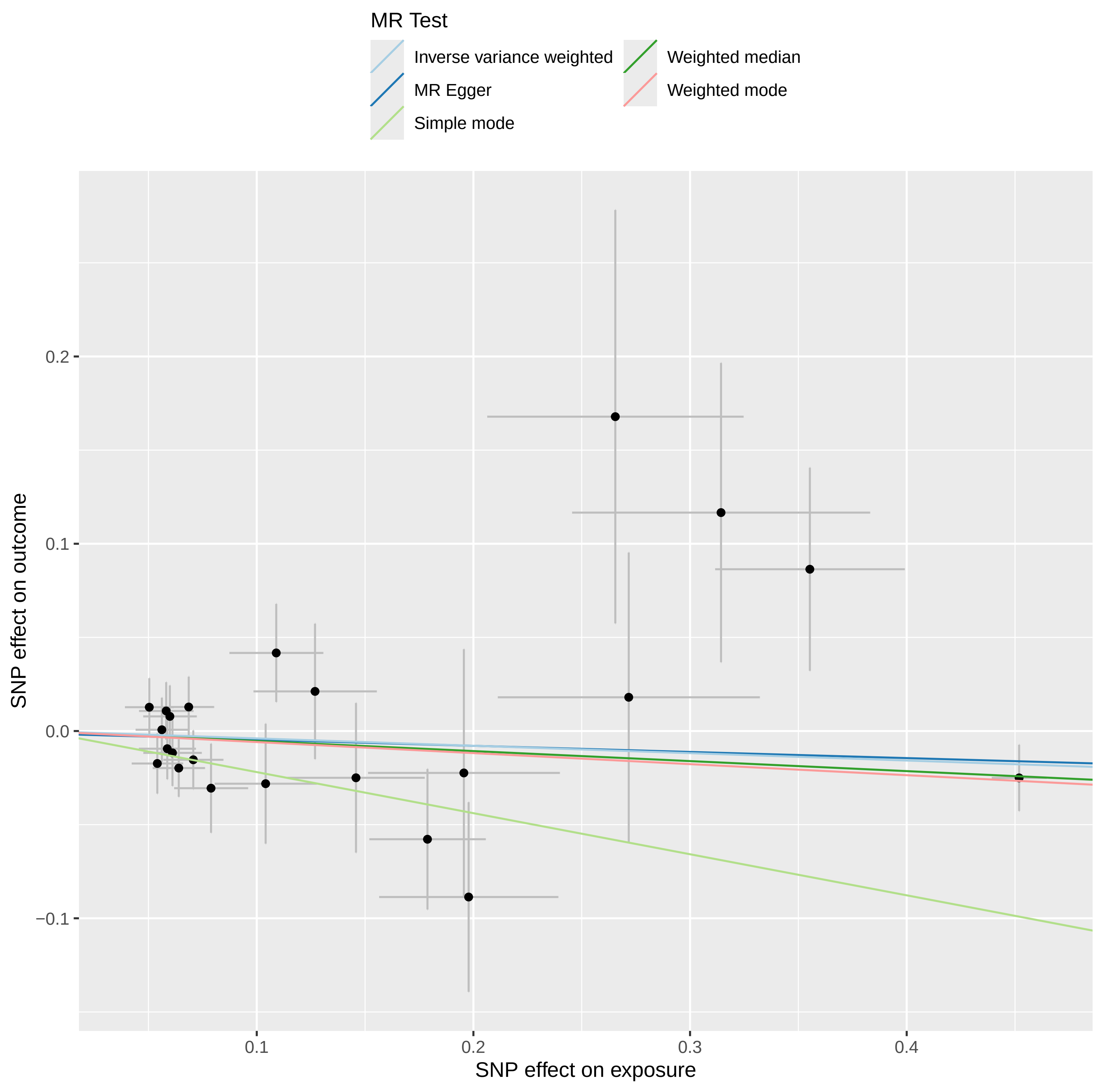
MR Test
Inverse variance weighted
MR Egger
Simple mode
Weighted median
Weighted mode
0.2
0.1
0.0
−0.1
0.1
0.2
0.3
0.4
SNP effect on outcome
SNP effect on exposure

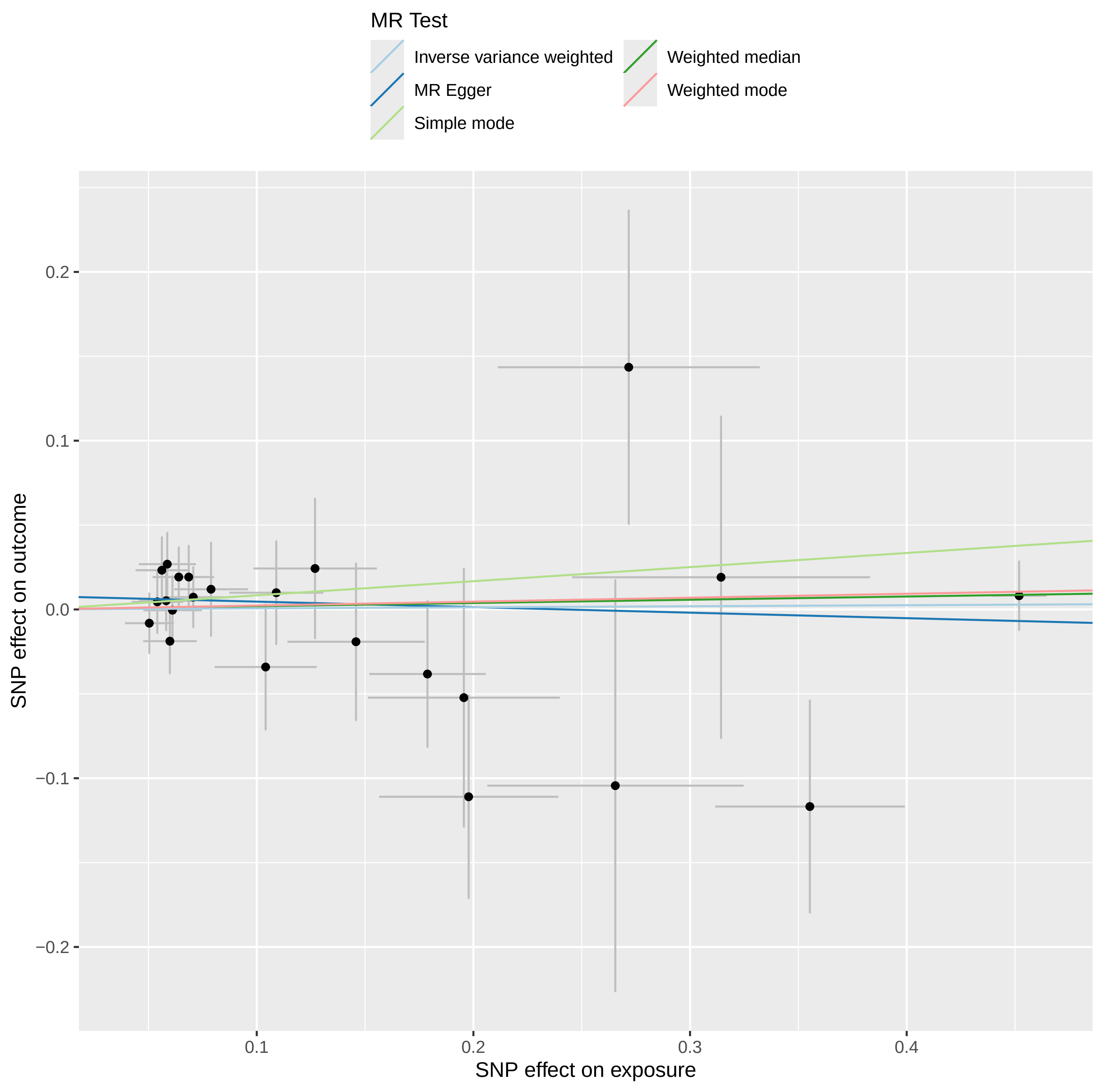
MR Test
Inverse variance weighted
MR Egger
Simple mode
Weighted median
Weighted mode
SNP effect on outcome
SNP effect on exposure
0.2
0.1
0.0
−0.1
−0.2
0.1
0.2
0.3
0.4

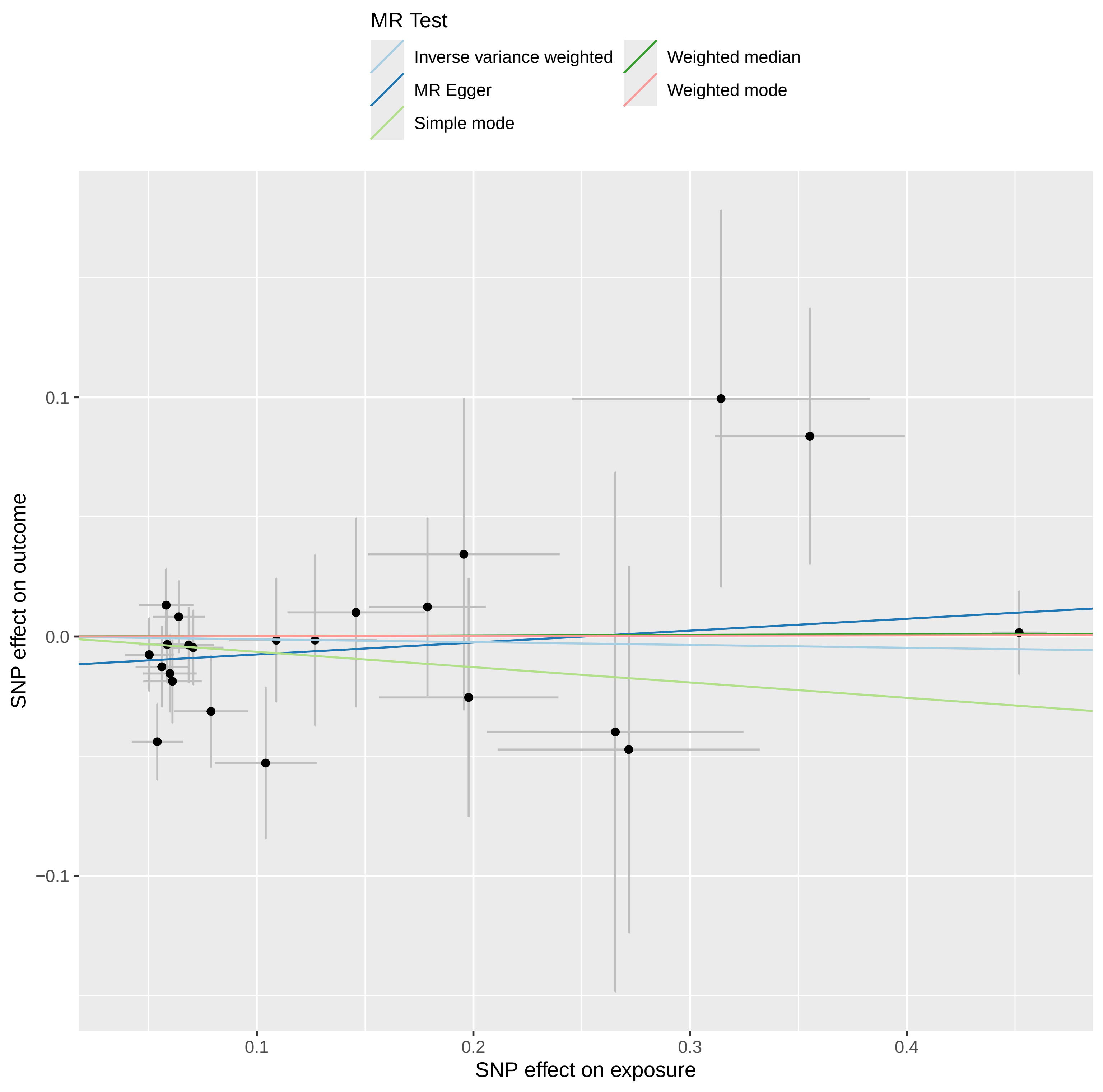

MR Test
Inverse variance weighted
MR Egger
Simple mode
Weighted median
Weighted mode
0.1
0.0
−0.1
SNP effect on outcome
0.1
0.2
0.3
0.4
SNP effect on exposure

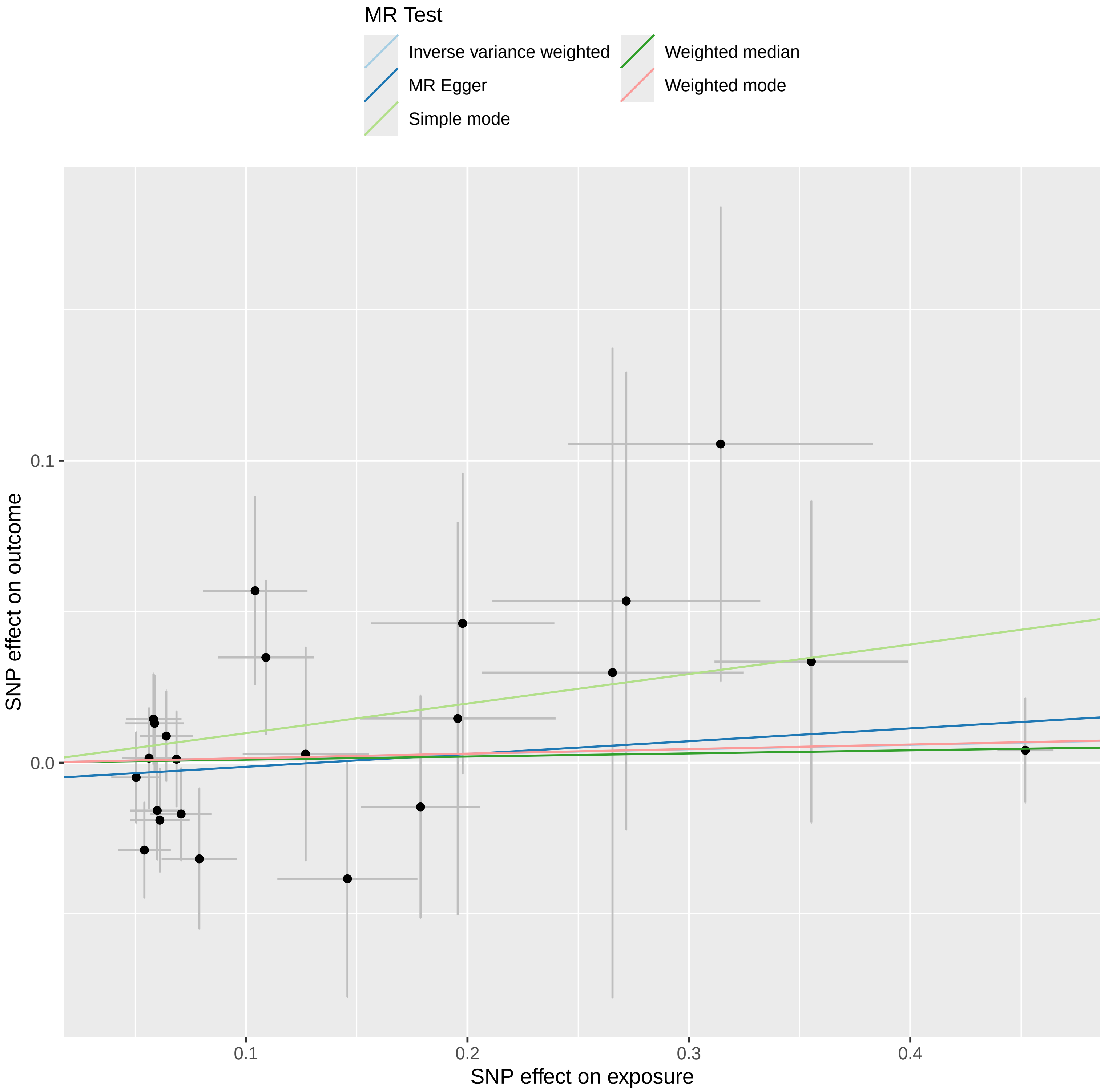
MR Test
Inverse variance weighted
MR Egger
Simple mode
Weighted median
Weighted mode
0.1
0.0
SNP effect on outcome
0.1
0.2
0.3
0.4
SNP effect on exposure

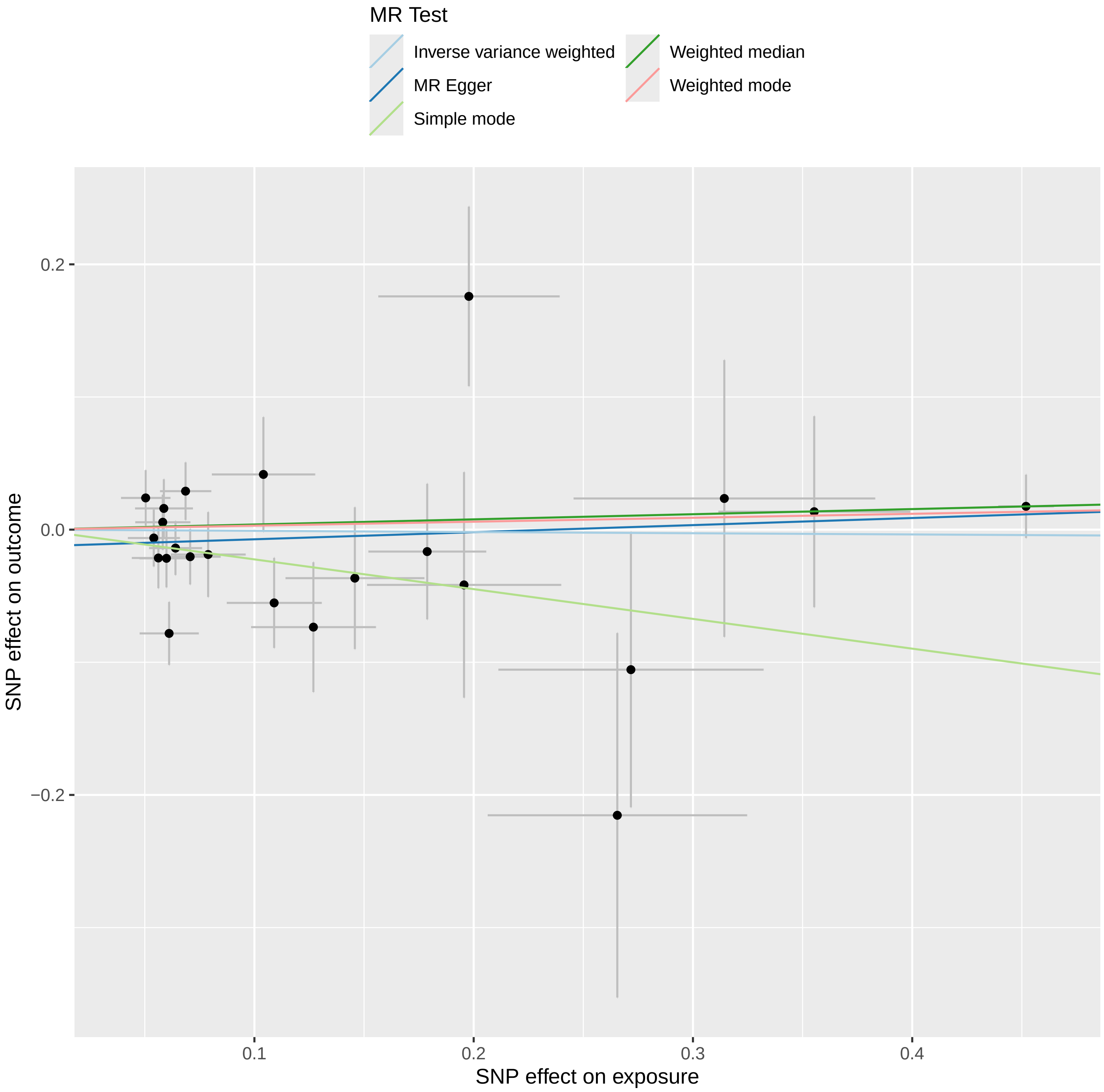

MR Test
Inverse variance weighted
MR Egger
Simple mode
Weighted median
Weighted mode
SNP effect on outcome
0.2
0.0
−0.2
0.1
0.2
0.3
0.4
SNP effect on exposure

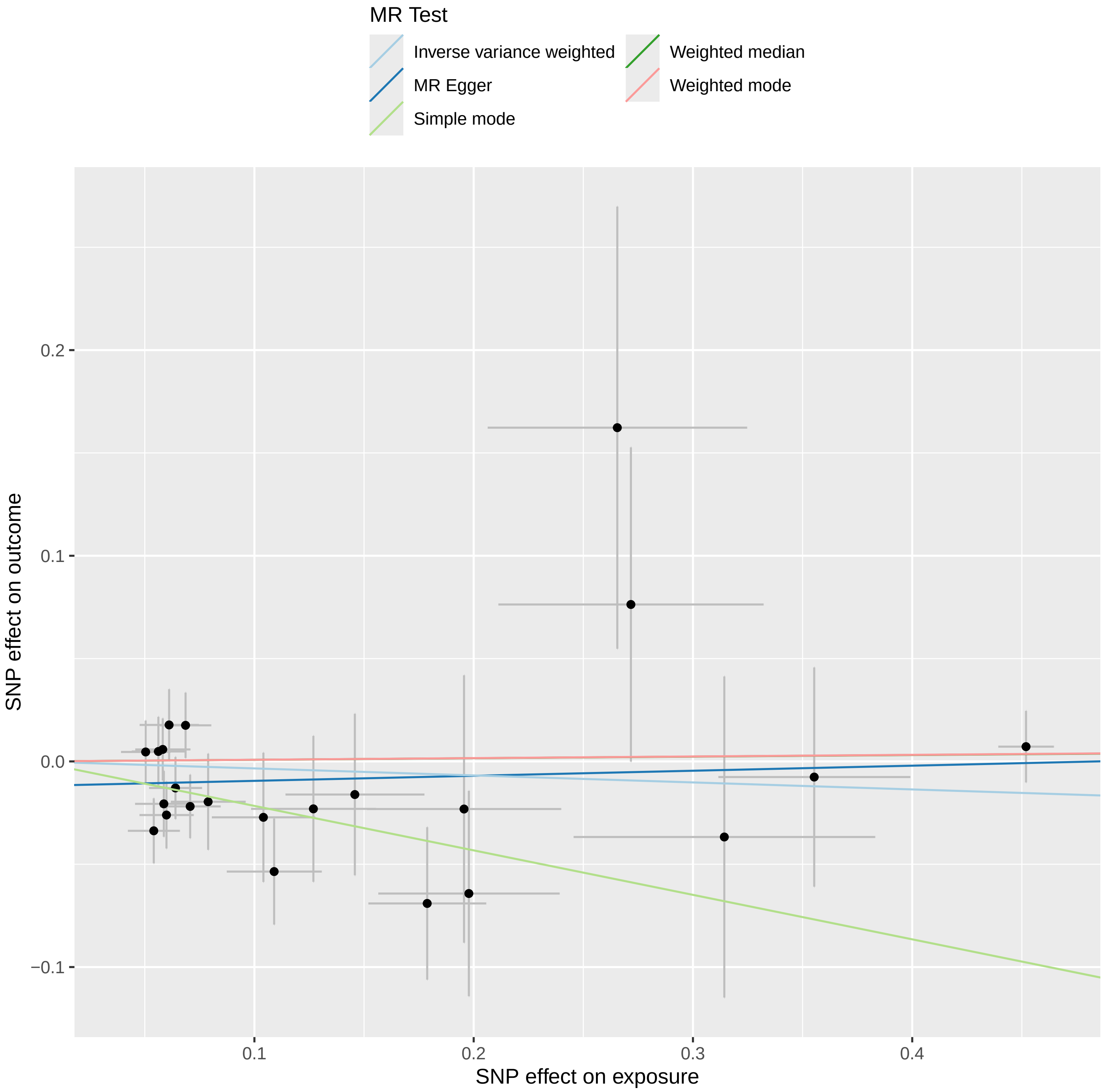

MR Test
Inverse variance weighted
MR Egger
Simple mode
Weighted median
Weighted mode
0.2
0.1
0.0
−0.1
SNP effect on outcome
0.1
0.2
0.3
0.4
SNP effect on exposure

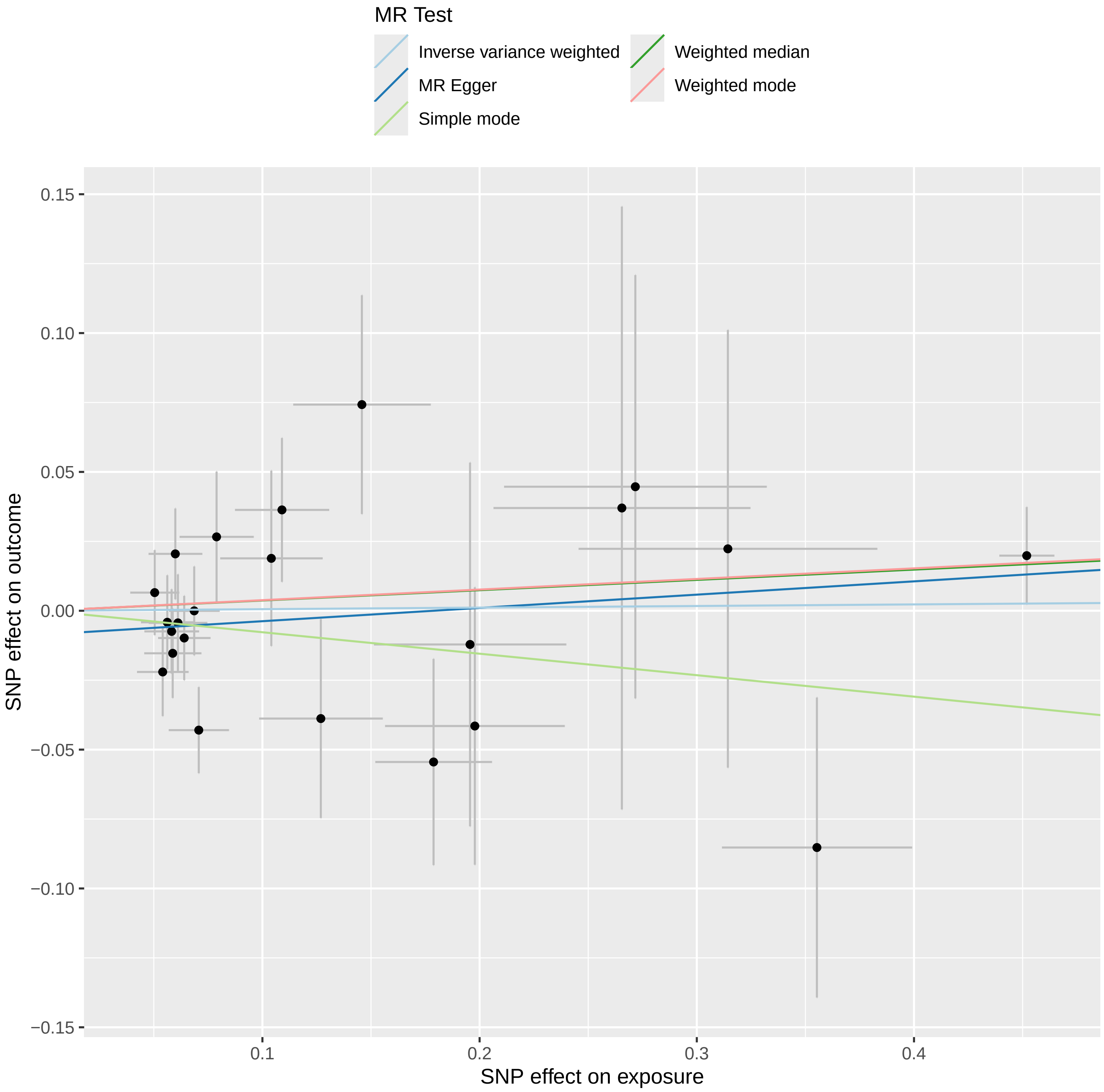

MR Test
Inverse variance weighted
MR Egger
Simple mode
Weighted median
Weighted mode
SNP effect on outcome
SNP effect on exposure
0.15
0.10
0.05
0.00
−0.05
−0.10
−0.15
0.1
0.2
0.3
0.4

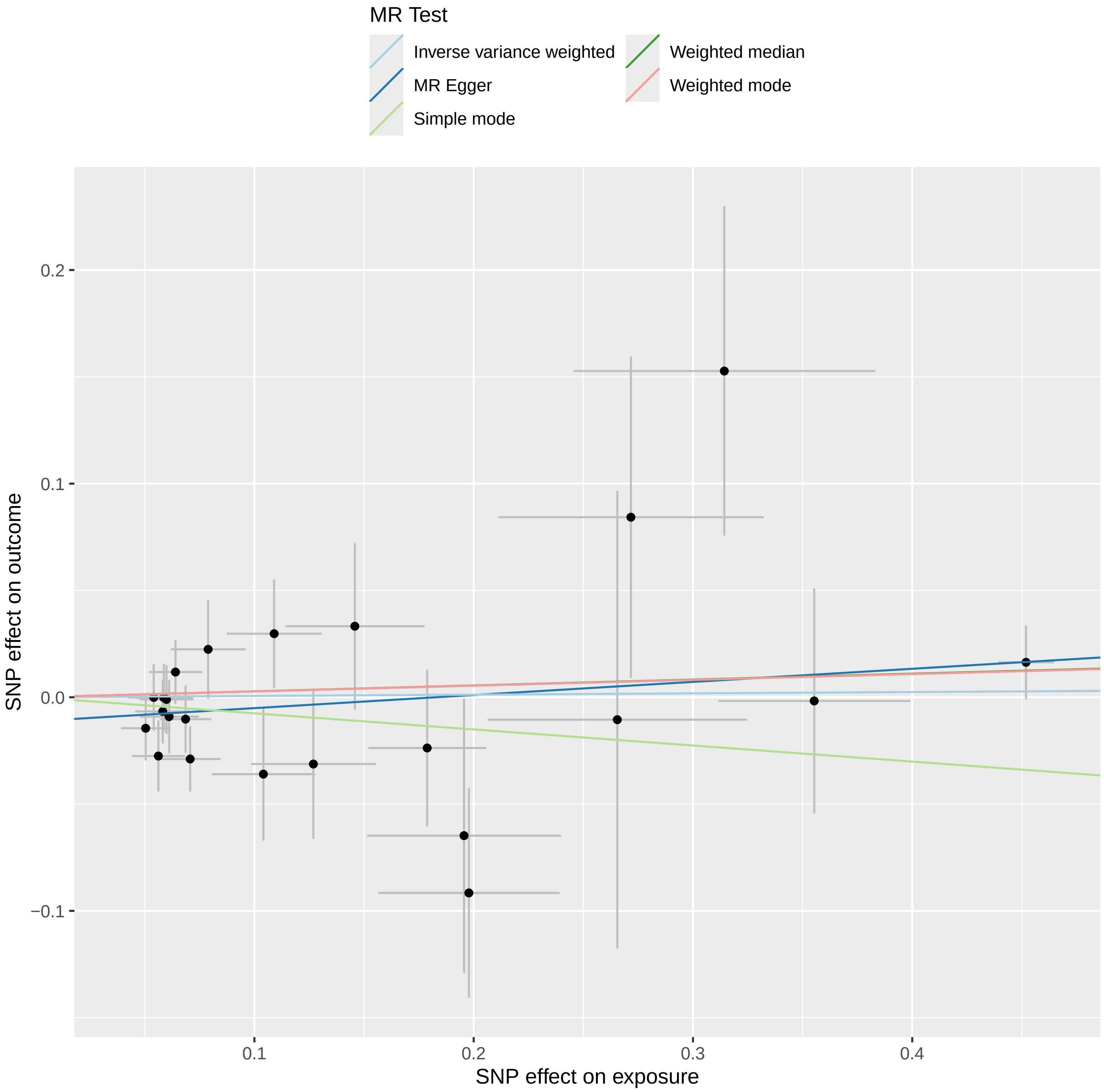

MR Test
Inverse variance weighted
MR Egger
Simple mode
Weighted median
Weighted mode
SNP effect on outcome
SNP effect on exposure
0.2
0.1
0.0
−0.1
0.1
0.2
0.3
0.4

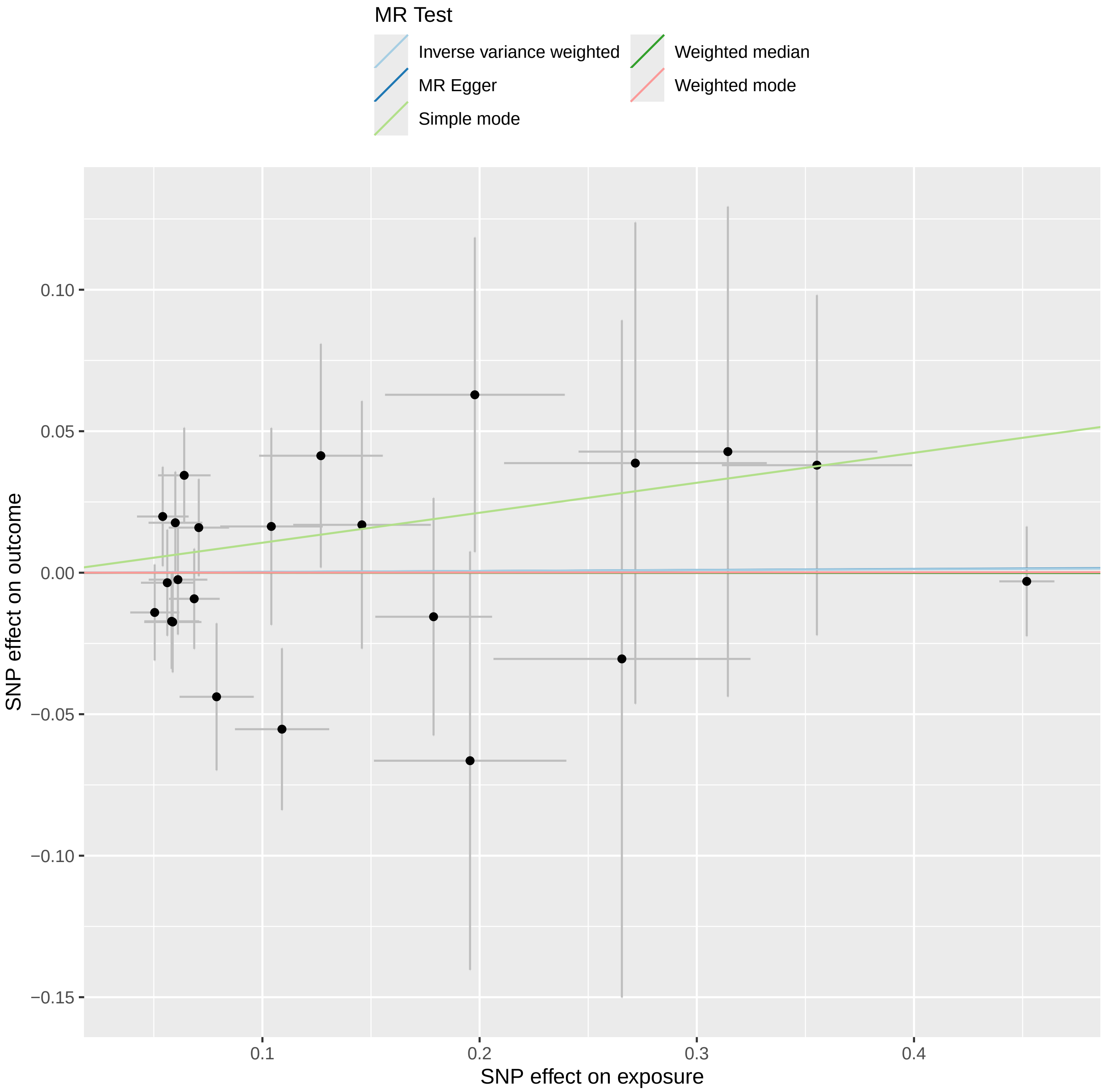
MR Test
Inverse variance weighted
MR Egger
Simple mode
Weighted median
Weighted mode
0.10
0.05
0.00
−0.05
−0.10
−0.15
SNP effect on outcome
0.1
0.2
0.3
0.4
SNP effect on exposure

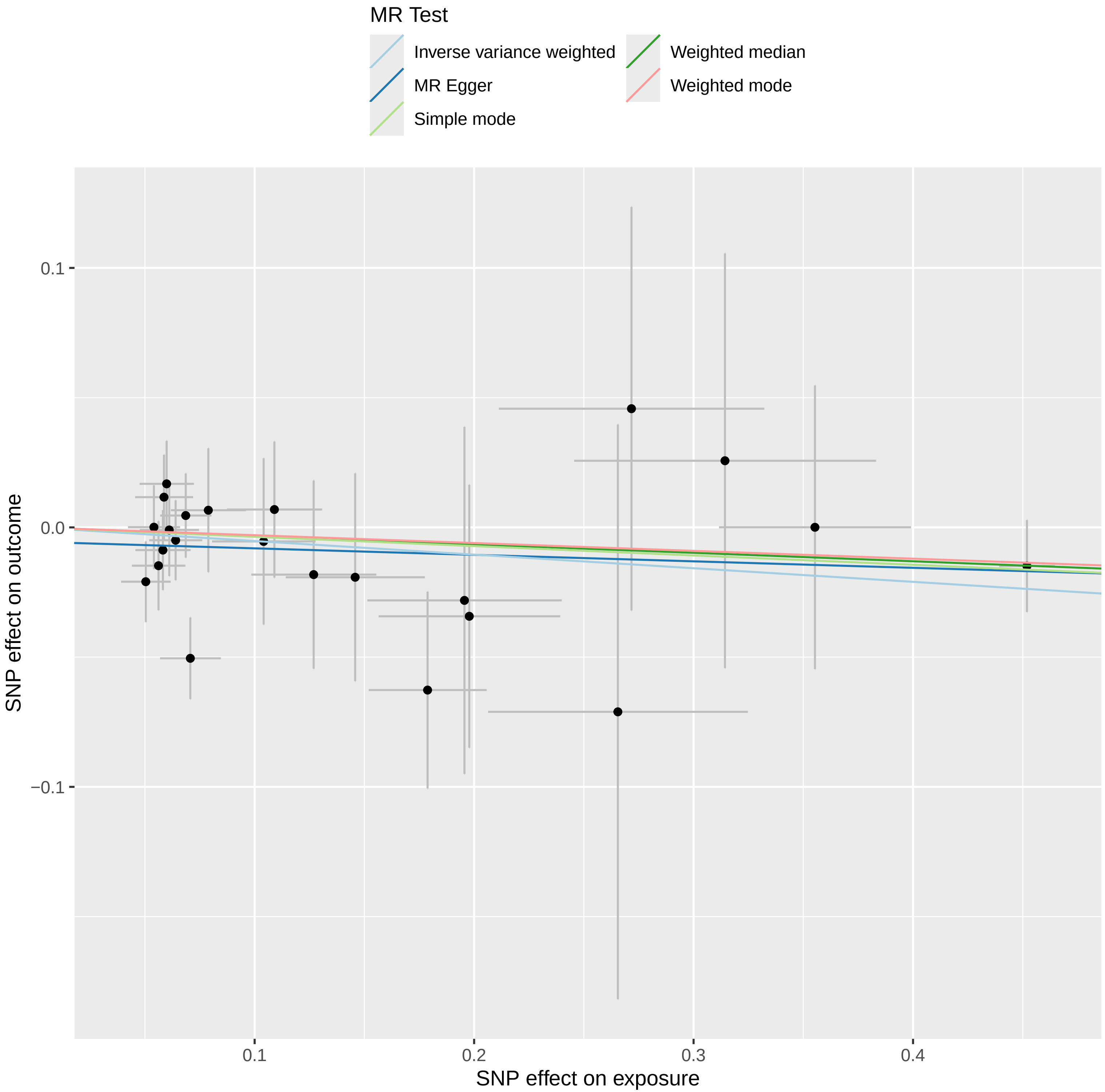

MR Test
Inverse variance weighted
MR Egger
Simple mode
Weighted median
Weighted mode
0.1
0.0
−0.1
SNP effect on outcome
0.1
0.2
0.3
0.4
SNP effect on exposure

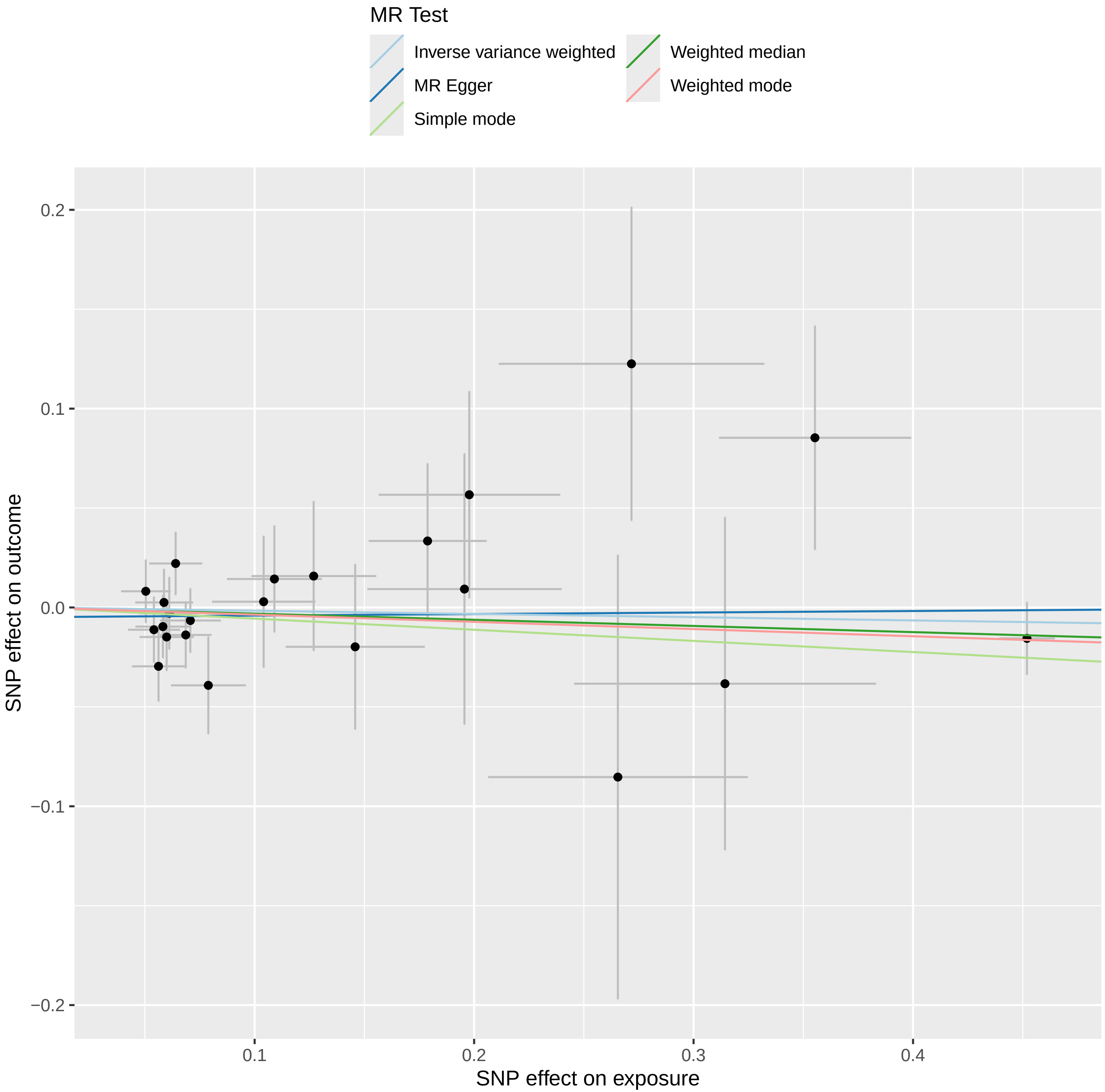
MR Test
Inverse variance weighted
MR Egger
Simple mode
Weighted median
Weighted mode
0.2
0.1
0.0
−0.1
−0.2
SNP effect on outcome
0.1
0.2
0.3
0.4
SNP effect on exposure

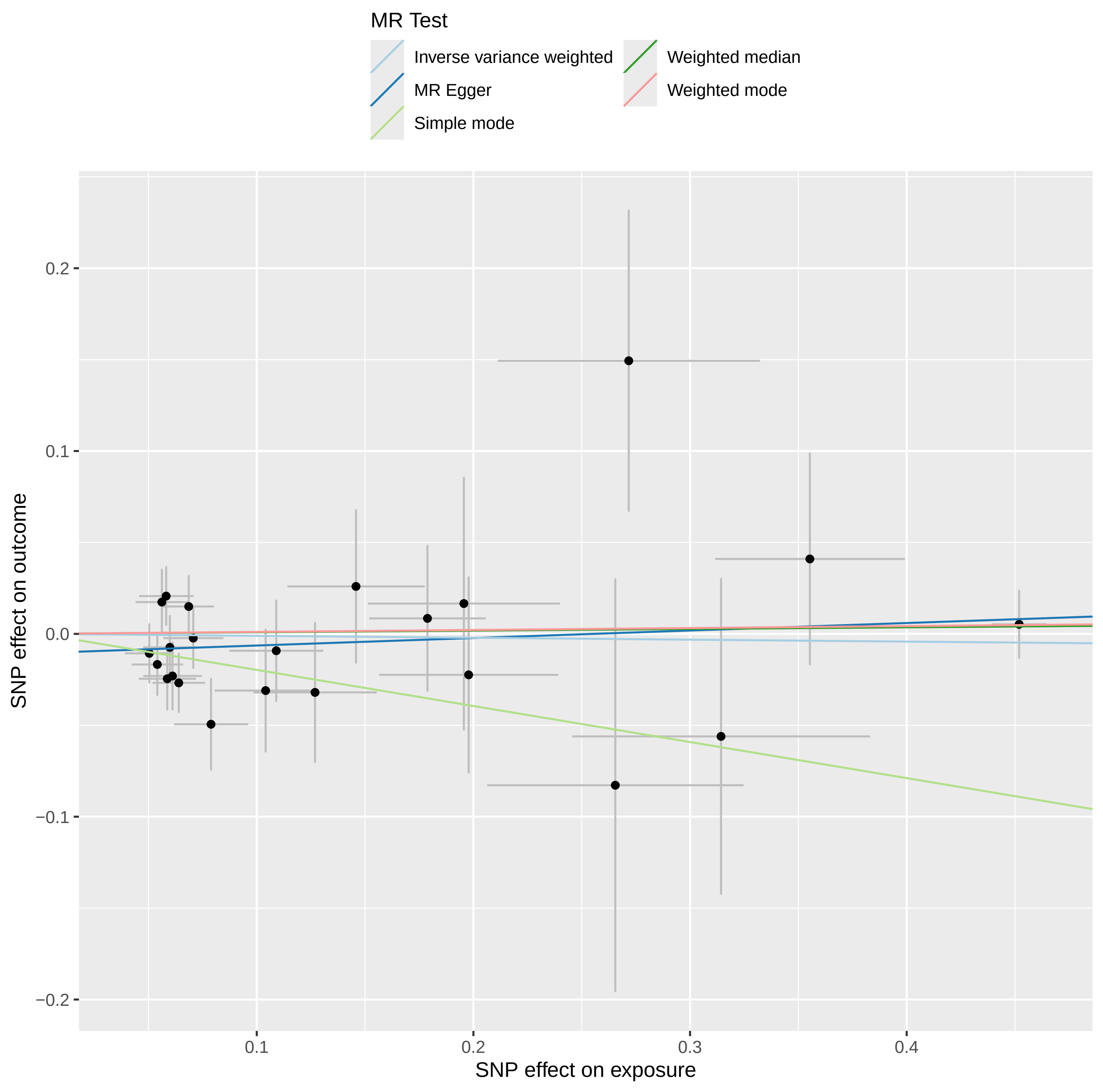
MR Test
Inverse variance weighted
MR Egger
Simple mode
Weighted median
Weighted mode
0.2
0.1
0.0
−0.1
−0.2
SNP effect on outcome
0.1
0.2
0.3
0.4
SNP effect on exposure

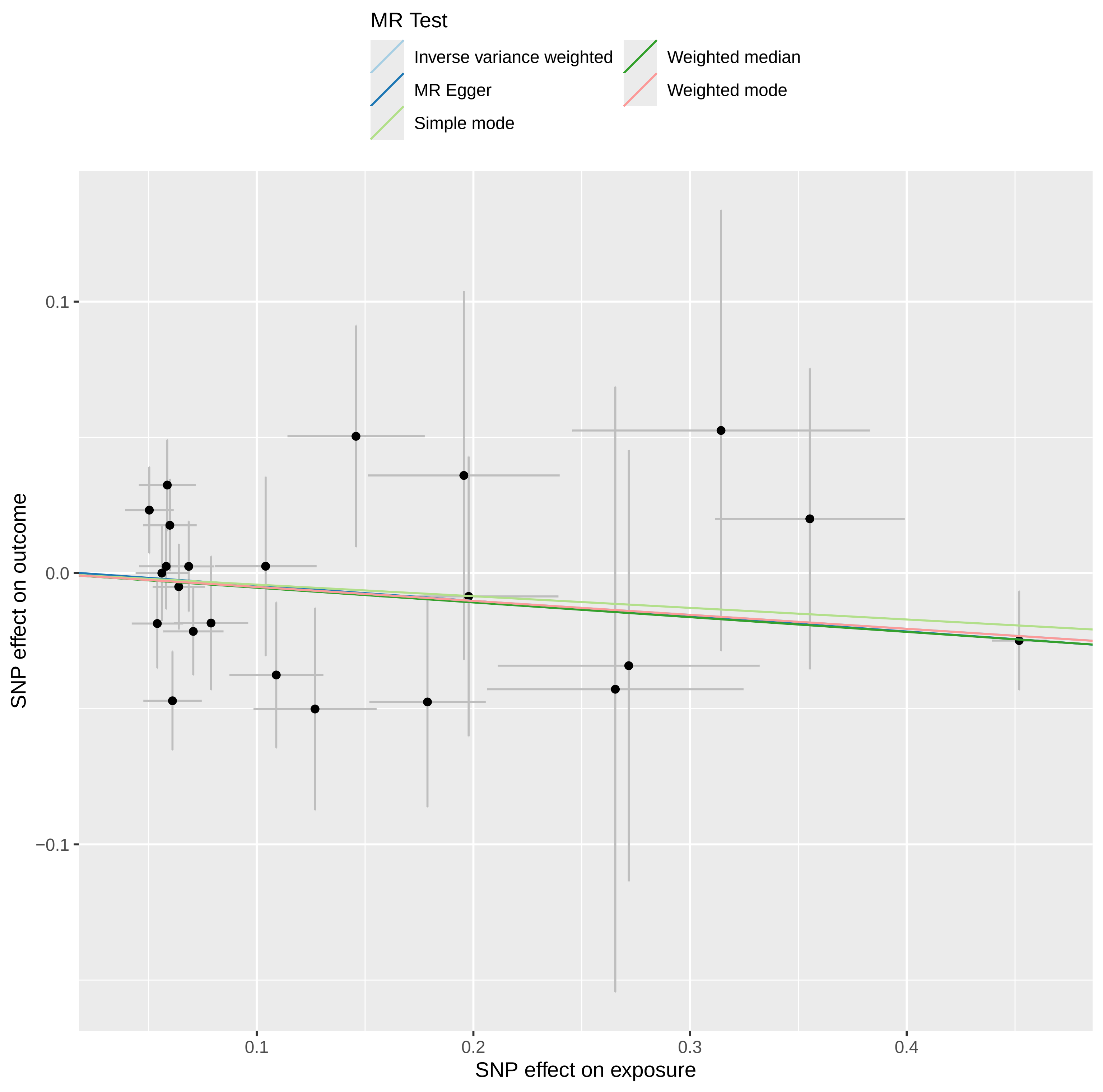

MR Test
Inverse variance weighted
MR Egger
Simple mode
Weighted median
Weighted mode
SNP effect on outcome
0.1
0.0
−0.1
0.1
0.2
0.3
0.4
SNP effect on exposure

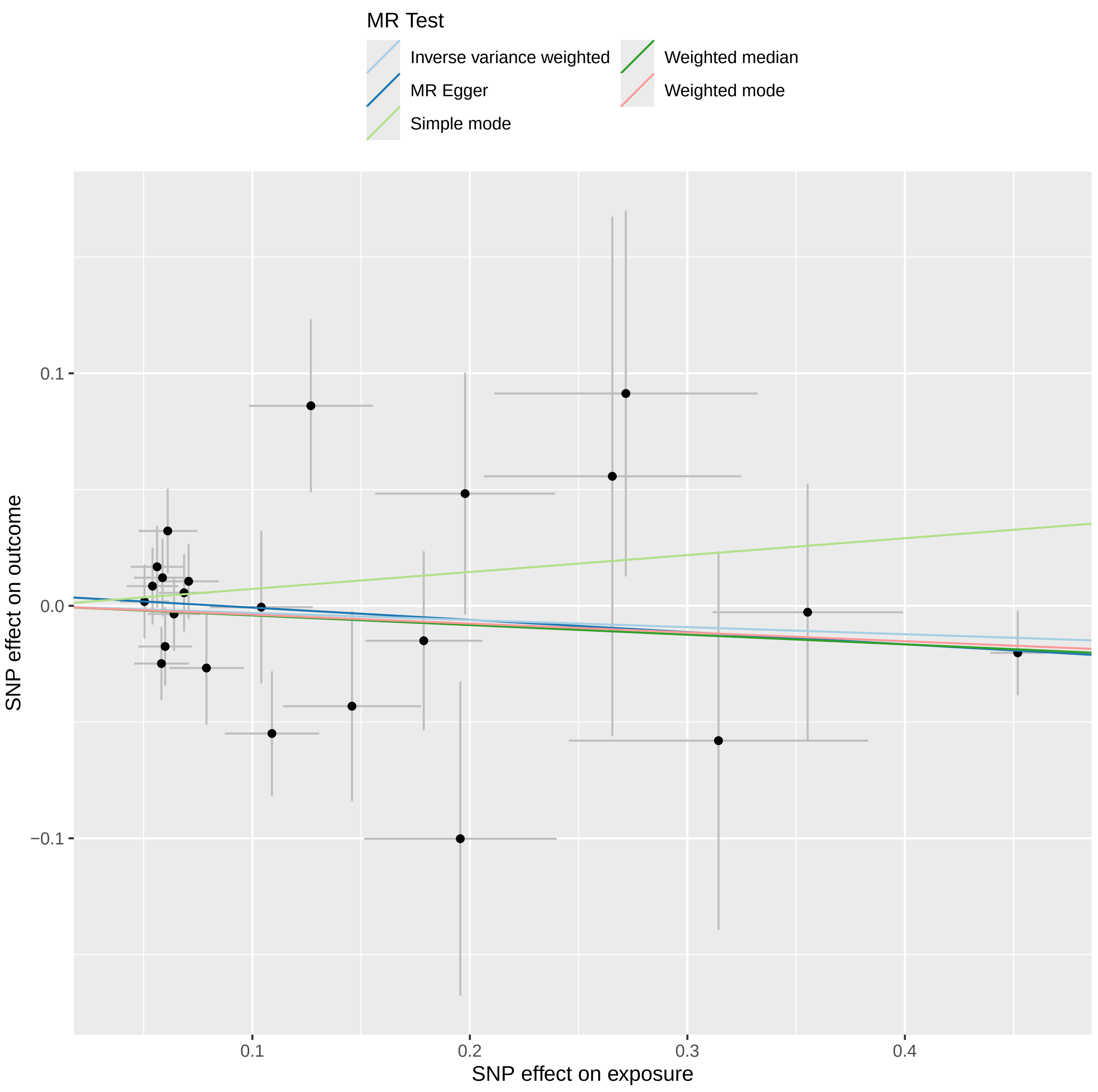
MR Test
Inverse variance weighted
MR Egger
Simple mode
Weighted median
Weighted mode
SNP effect on outcome
0.1
0.0
−0.1
0.1
0.2
0.3
0.4
SNP effect on exposure

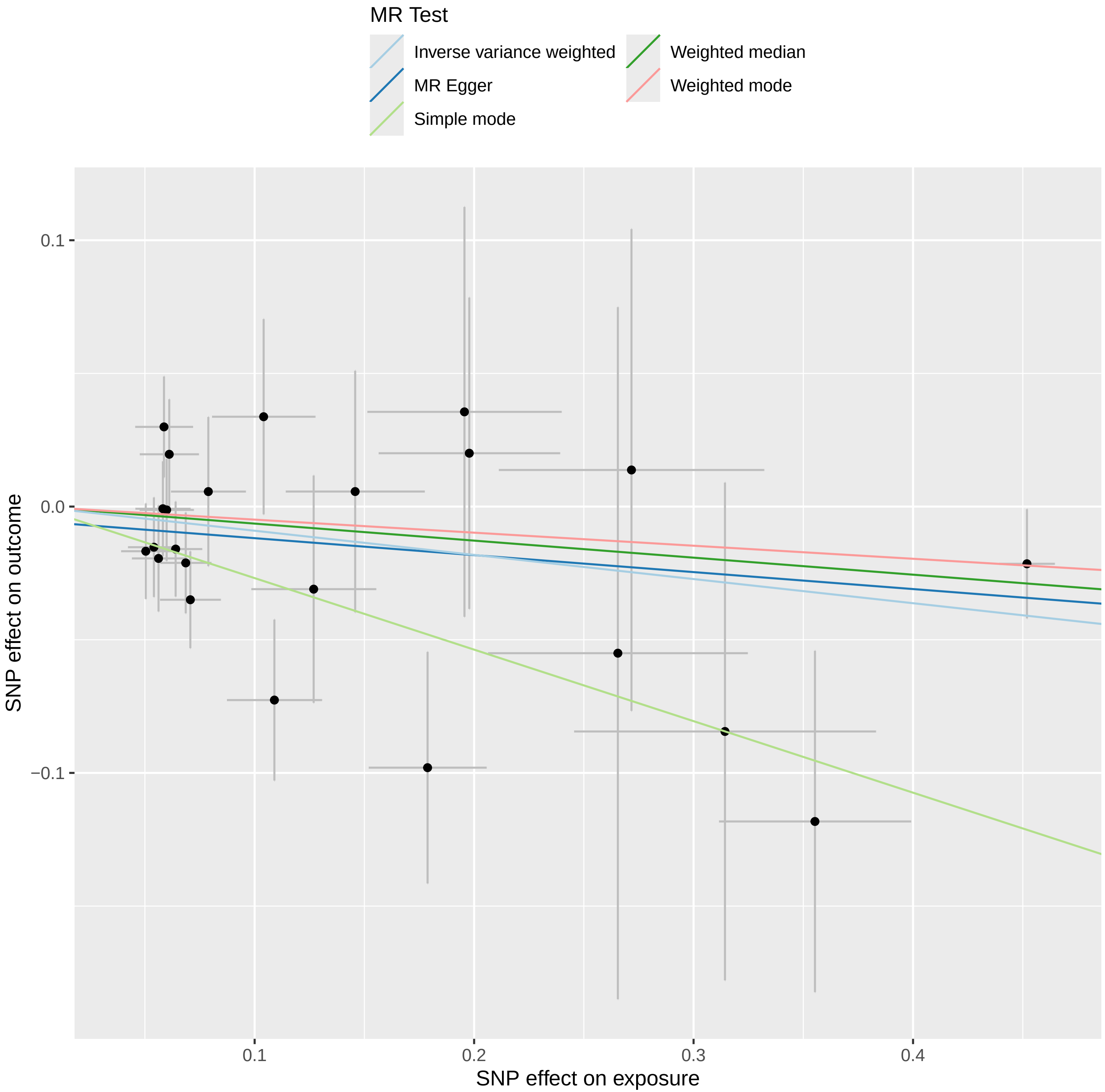

MR Test
Inverse variance weighted
MR Egger
Simple mode
Weighted median
Weighted mode
0.1
0.0
−0.1
SNP effect on outcome
0.1
0.2
0.3
0.4
SNP effect on exposure

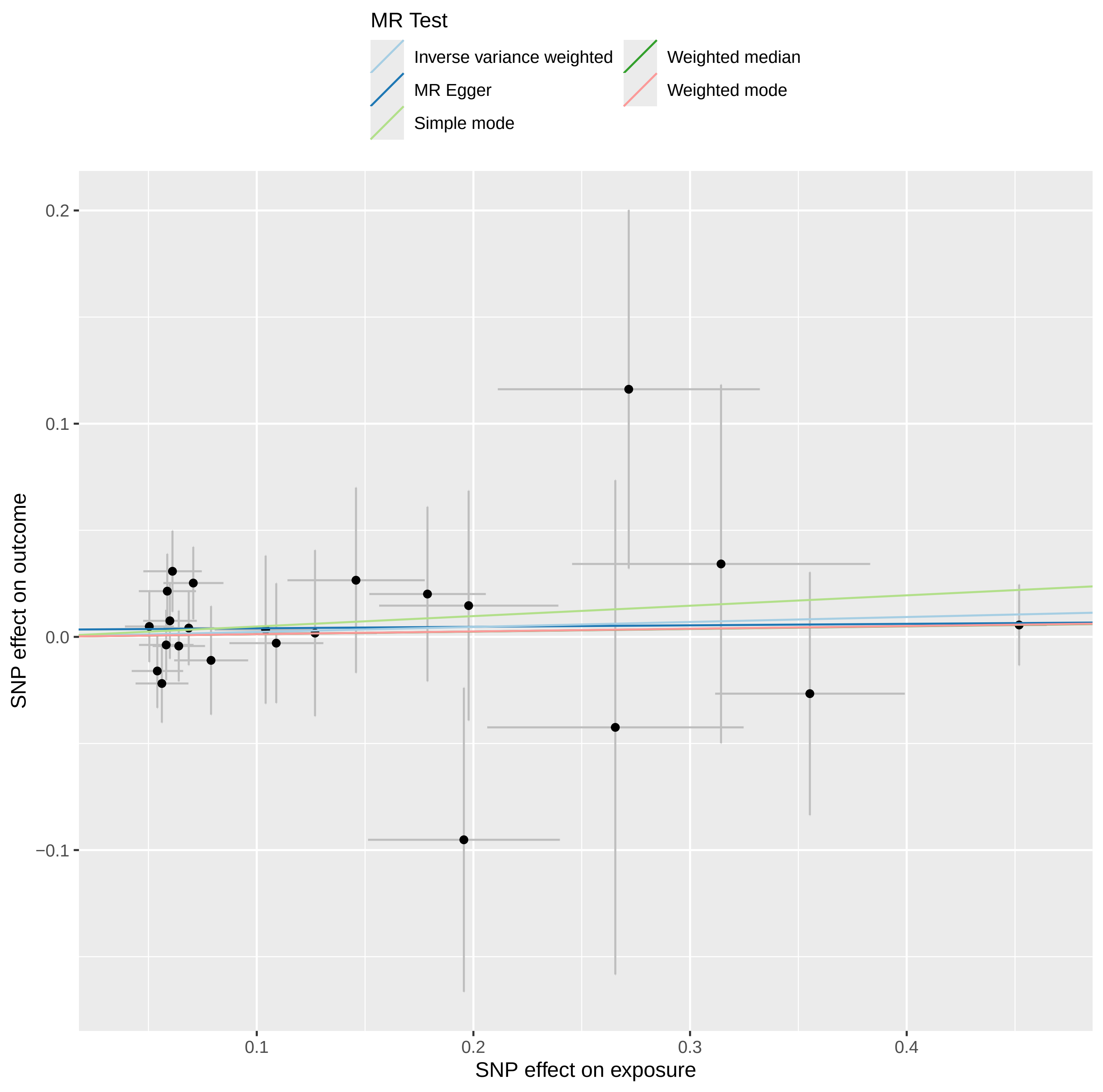

MR Test
Inverse variance weighted
MR Egger
Simple mode
Weighted median
Weighted mode
0.2
0.1
0.0
−0.1
0.1
0.2
0.3
0.4
SNP effect on outcome
SNP effect on exposure

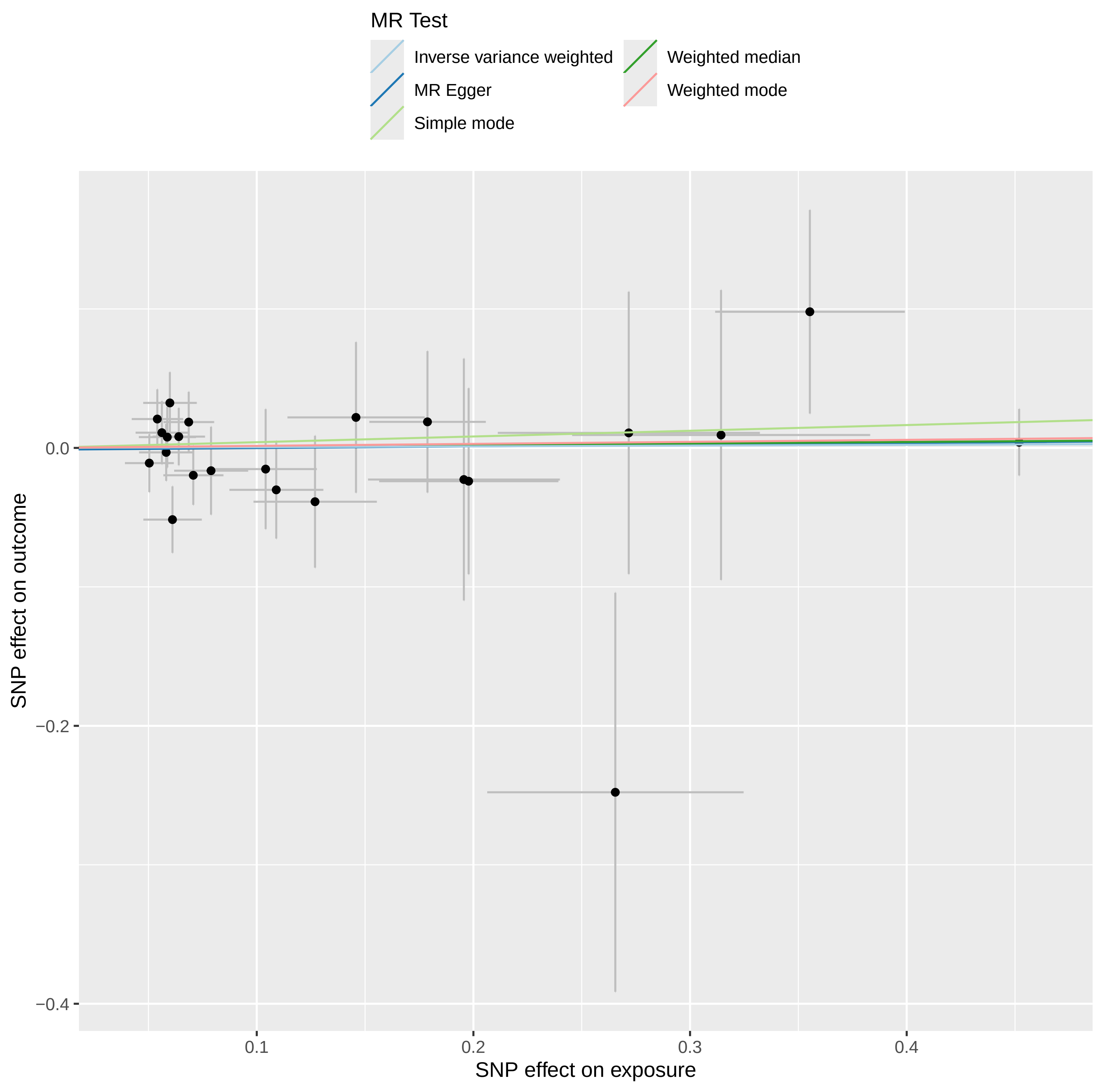

MR Test
Inverse variance weighted
MR Egger
Simple mode
Weighted median
Weighted mode
0.0
−0.2
−0.4
0.1
0.2
0.3
0.4
SNP effect on outcome
SNP effect on exposure

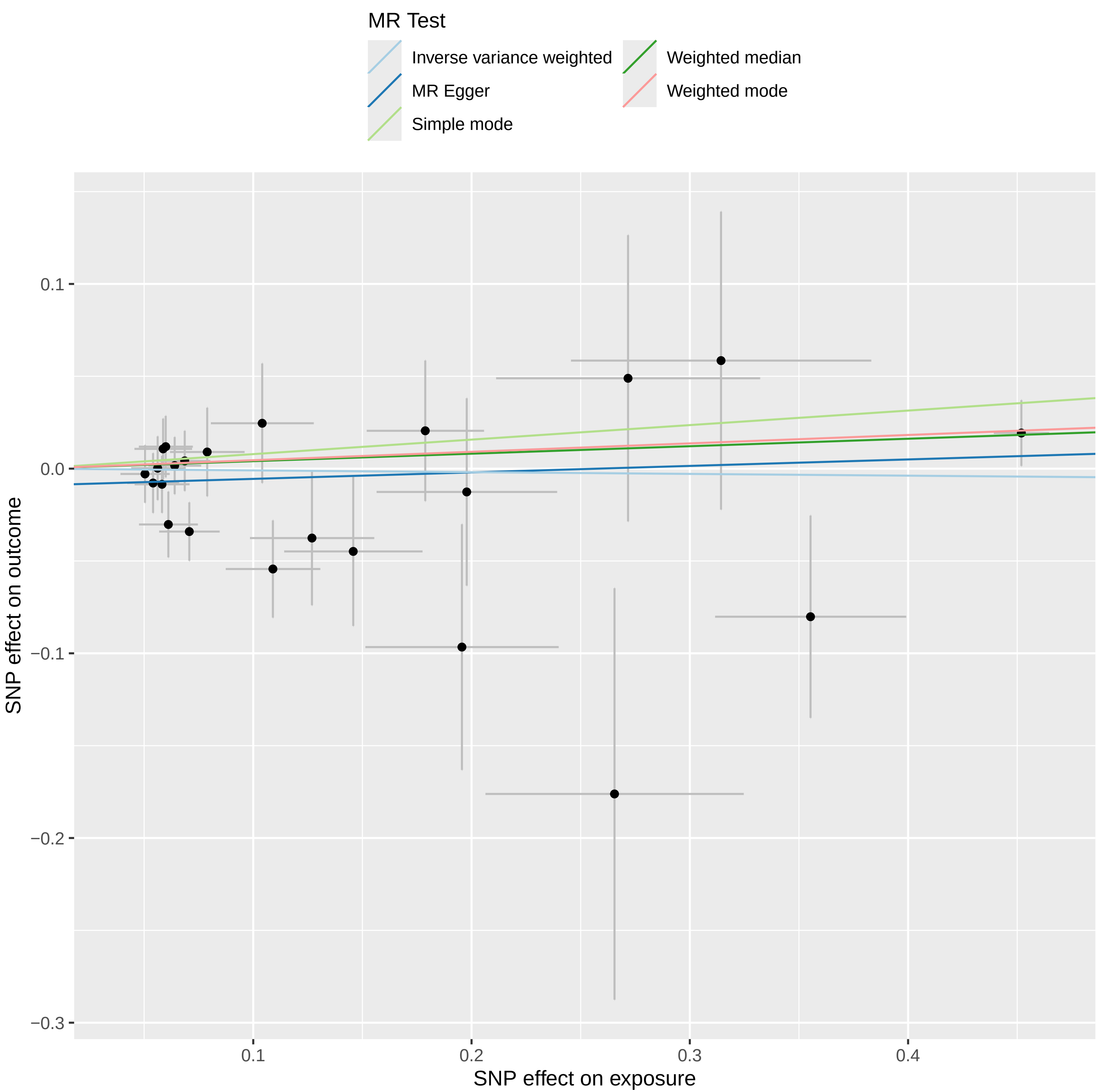
MR Test
Inverse variance weighted
MR Egger
Simple mode
Weighted median
Weighted mode
0.1
0.0
−0.1
−0.2
−0.3
0.1
0.2
0.3
0.4
SNP effect on outcome
SNP effect on exposure

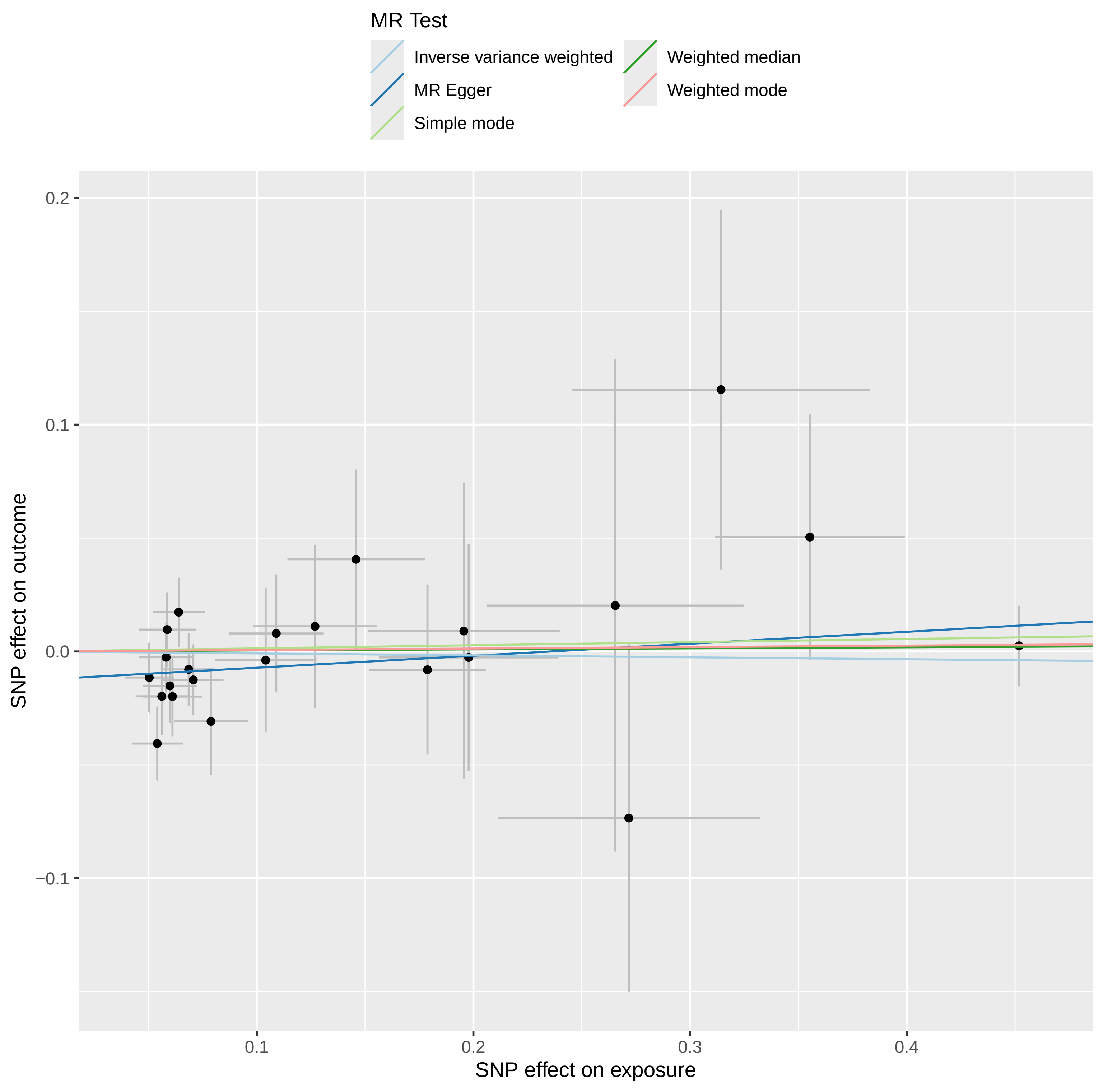

MR Test
Inverse variance weighted
MR Egger
Simple mode
Weighted median
Weighted mode
0.2
0.1
0.0
−0.1
0.1
0.2
0.3
0.4
SNP effect on outcome
SNP effect on exposure

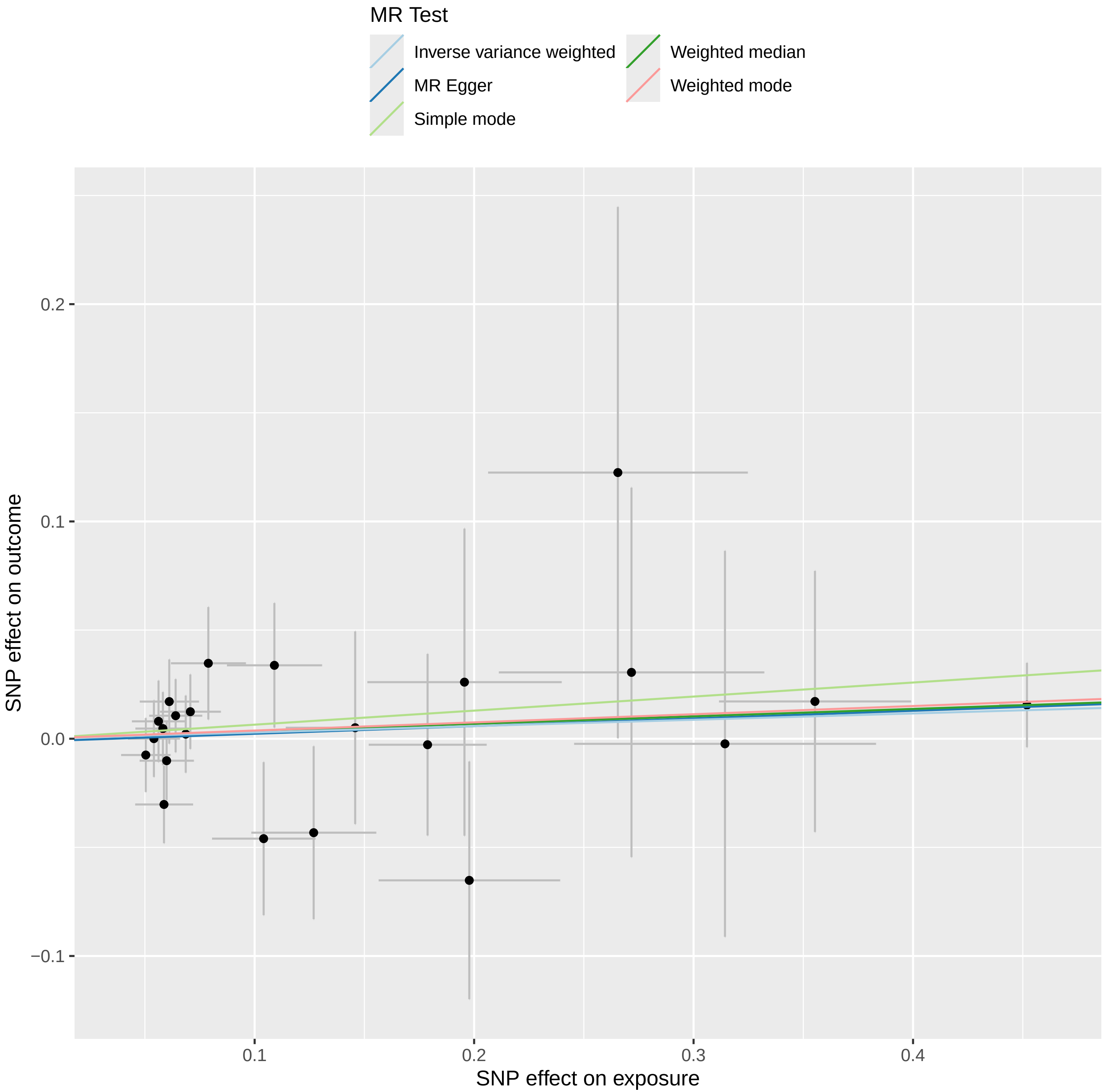
MR Test
Inverse variance weighted
MR Egger
Simple mode
Weighted median
Weighted mode
0.2
0.1
0.0
−0.1
SNP effect on outcome
0.1
0.2
0.3
0.4
SNP effect on exposure

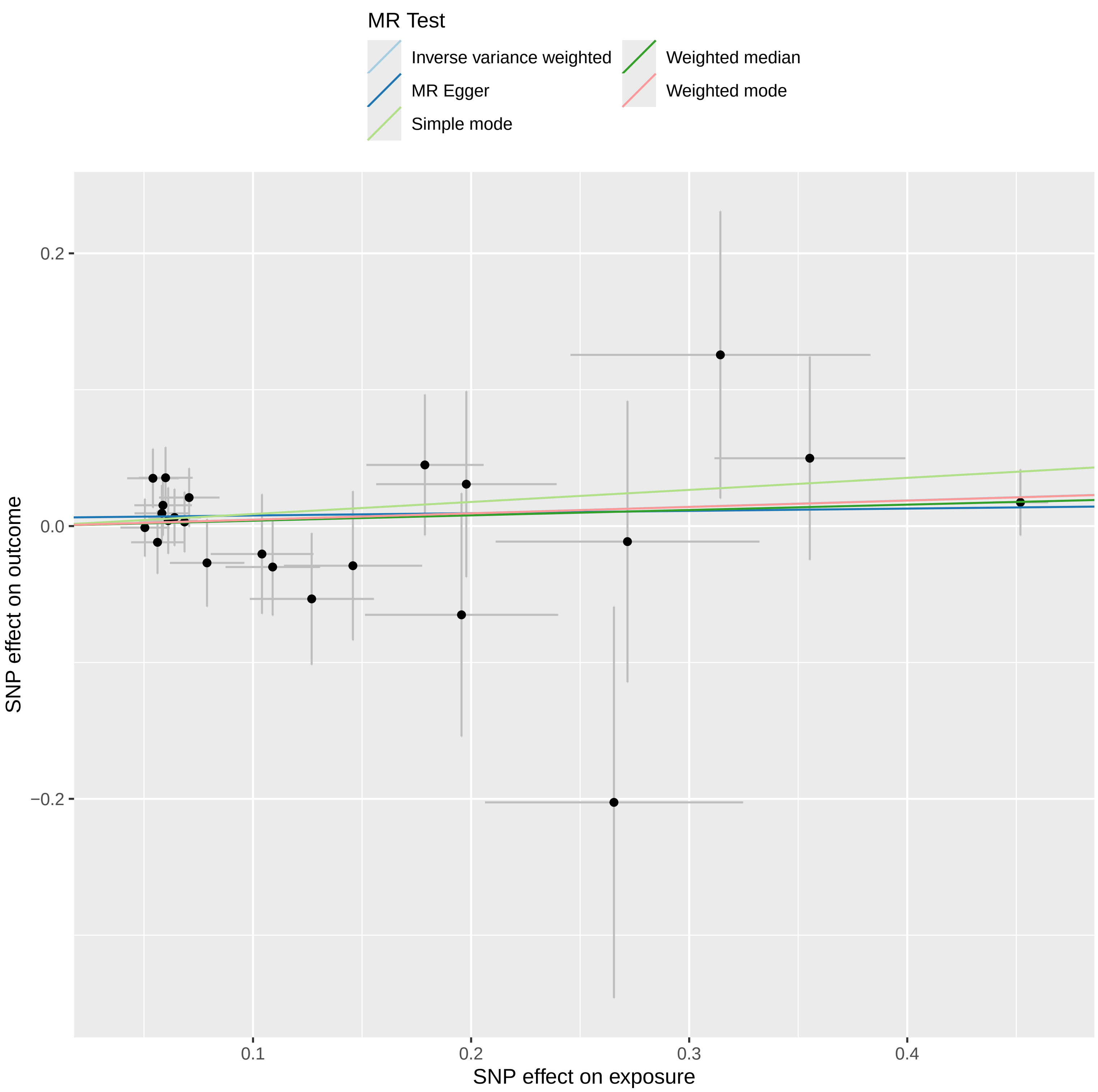

MR Test
Inverse variance weighted
MR Egger
Simple mode
Weighted median
Weighted mode
0.2
0.0
−0.2
0.1
0.2
0.3
0.4
SNP effect on outcome
SNP effect on exposure

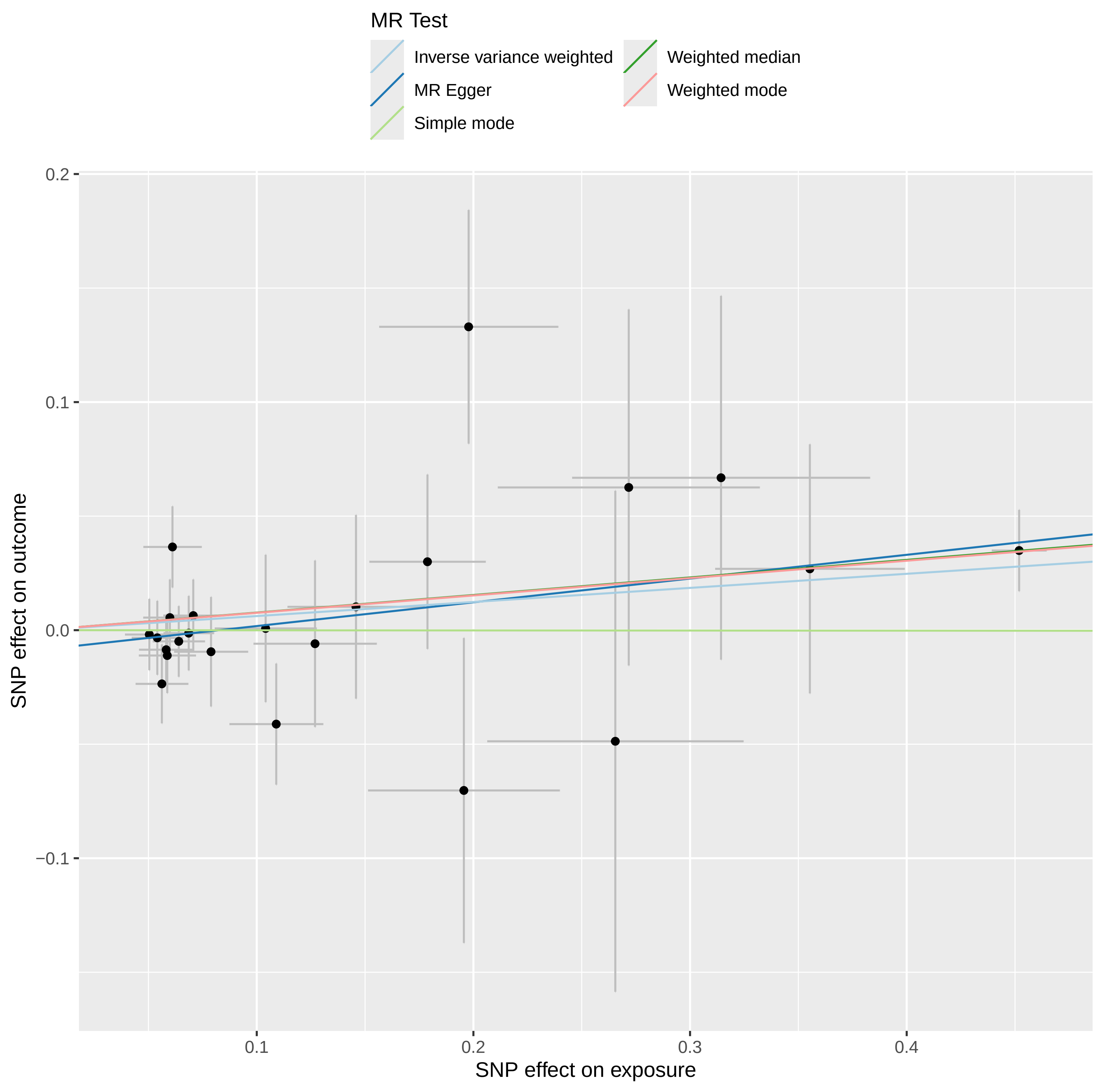

MR Test
Inverse variance weighted
MR Egger
Simple mode
Weighted median
Weighted mode
0.2
0.1
0.0
−0.1
SNP effect on outcome
0.1
0.2
0.3
0.4
SNP effect on exposure

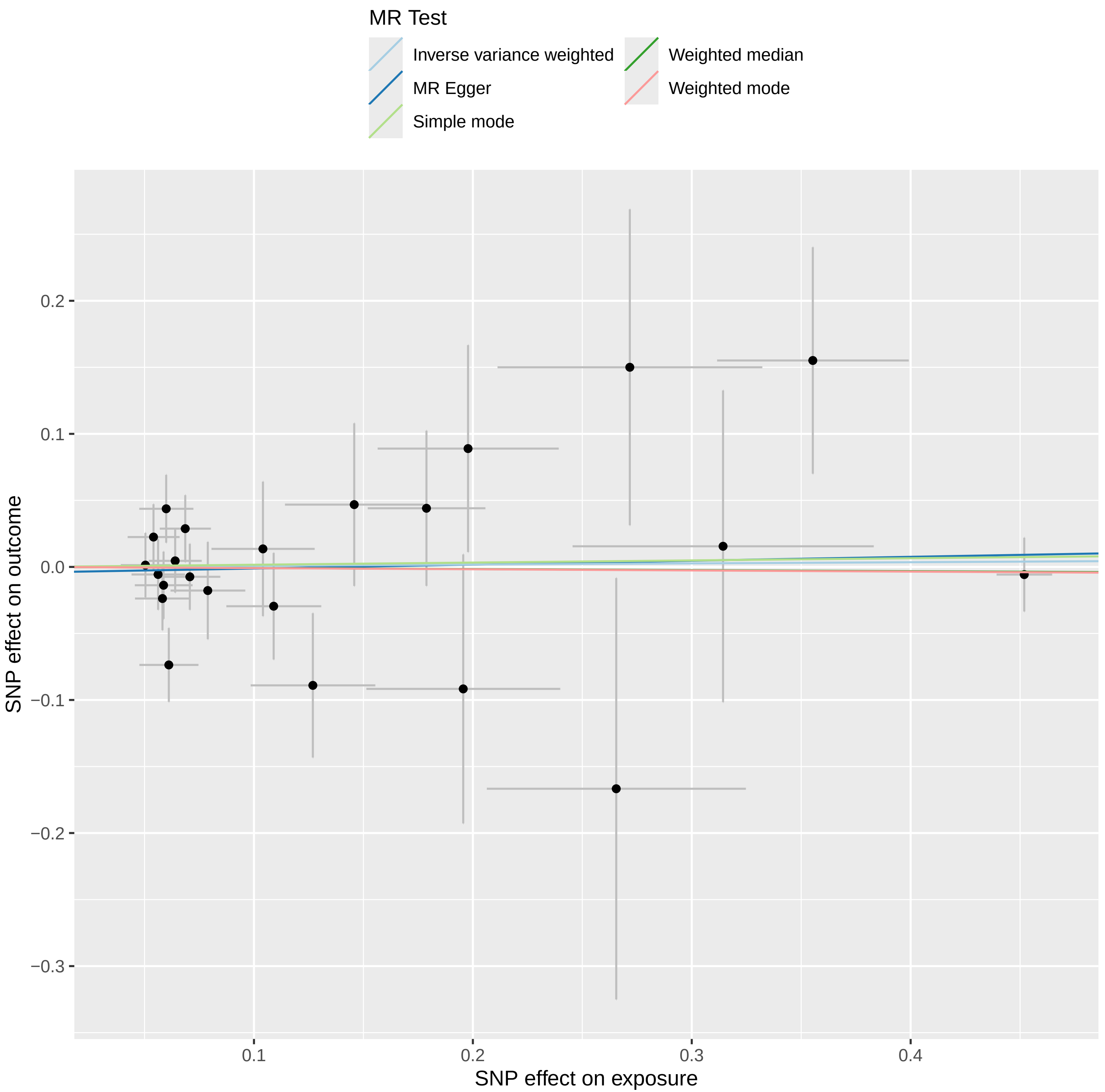
MR Test
Inverse variance weighted
MR Egger
Simple mode
Weighted median
Weighted mode
0.2
0.1
0.0
−0.1
−0.2
−0.3
SNP effect on outcome
0.1
0.2
0.3
0.4
SNP effect on exposure

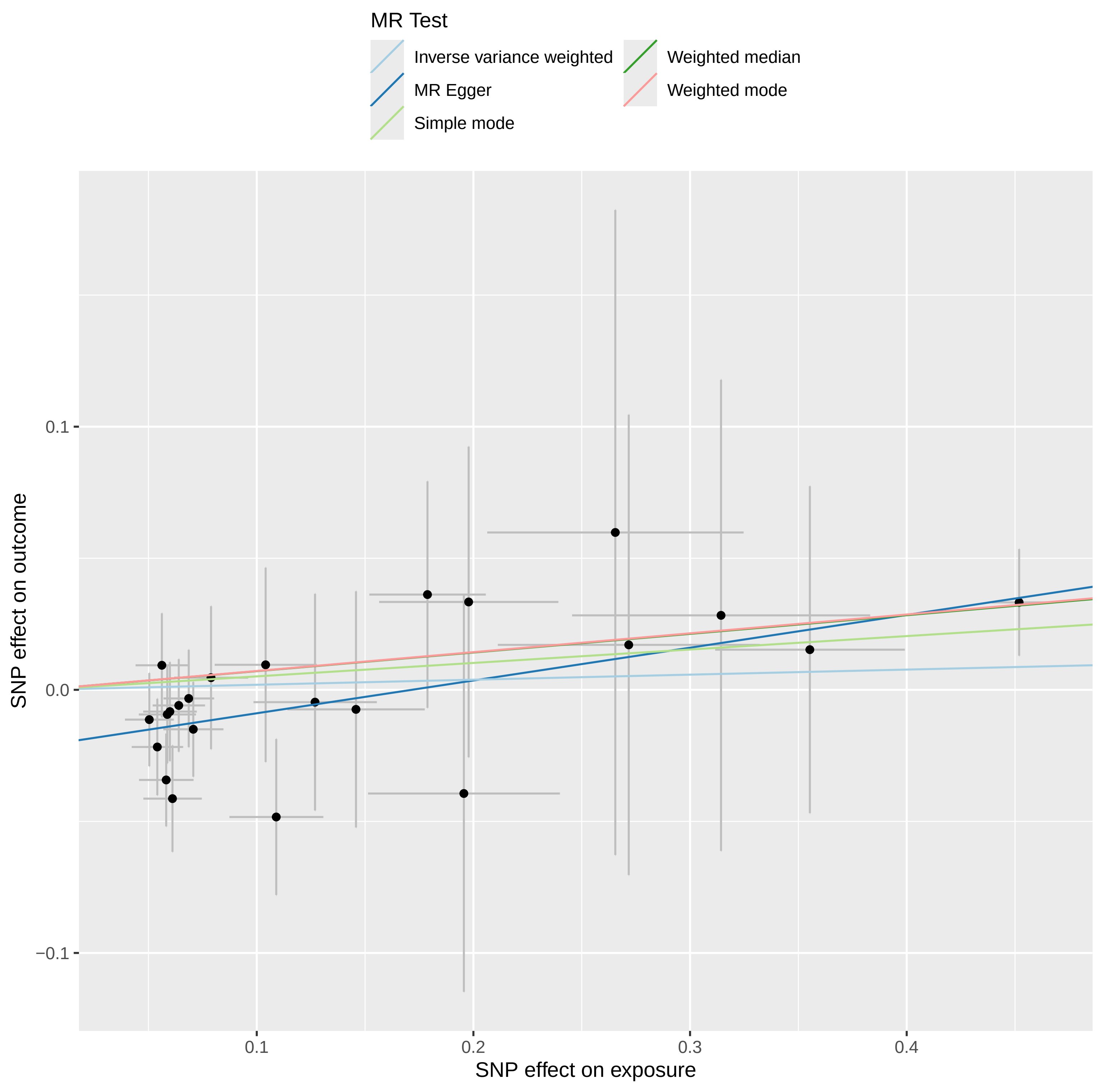

MR Test
Inverse variance weighted
MR Egger
Simple mode
Weighted median
Weighted mode
SNP effect on outcome
0.1
0.0
−0.1
0.1
0.2
0.3
0.4
SNP effect on exposure

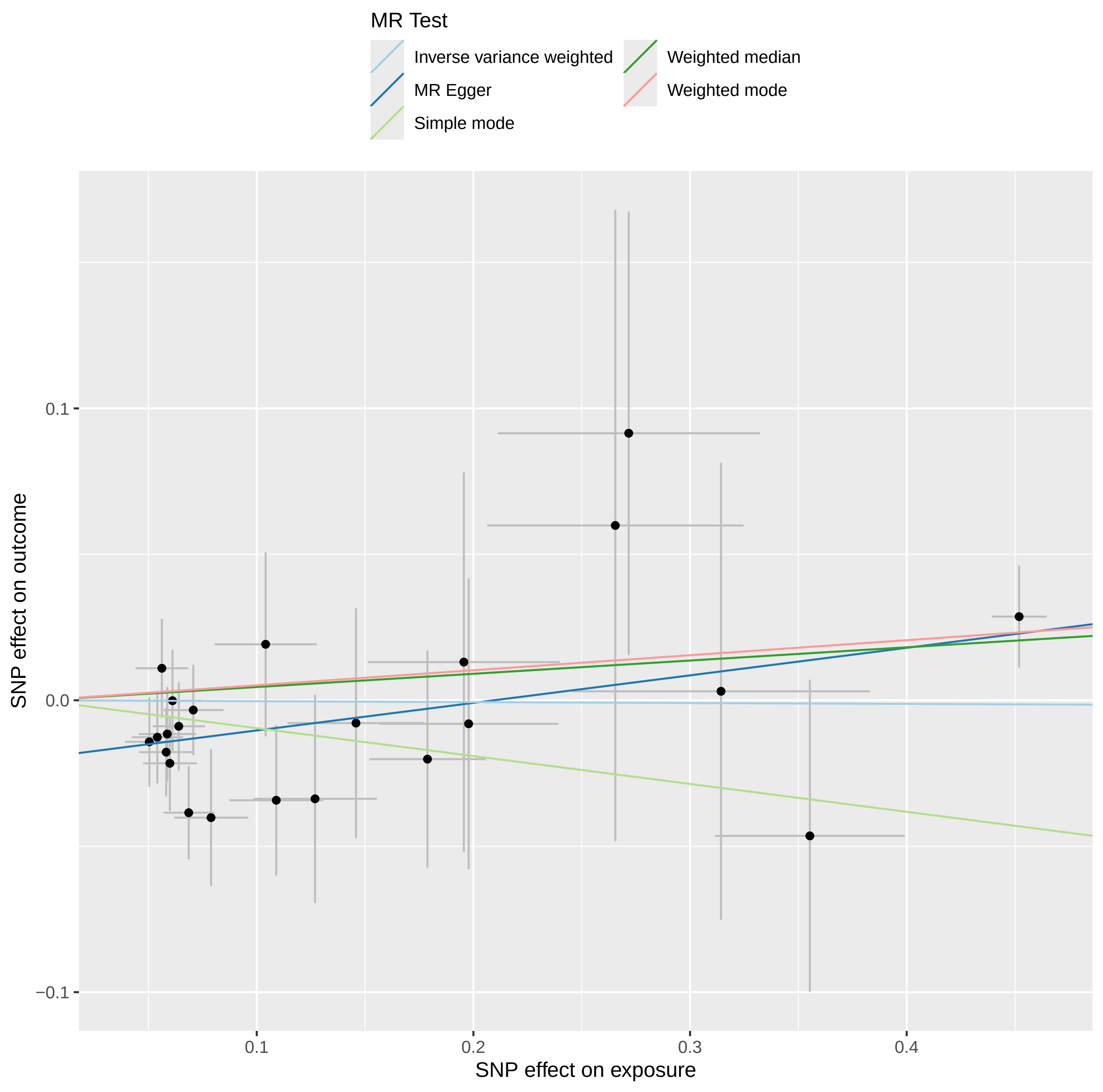

MR Test
Inverse variance weighted
MR Egger
Simple mode
Weighted median
Weighted mode
0.1
0.0
−0.1
0.1
0.2
0.3
0.4
SNP effect on outcome
SNP effect on exposure

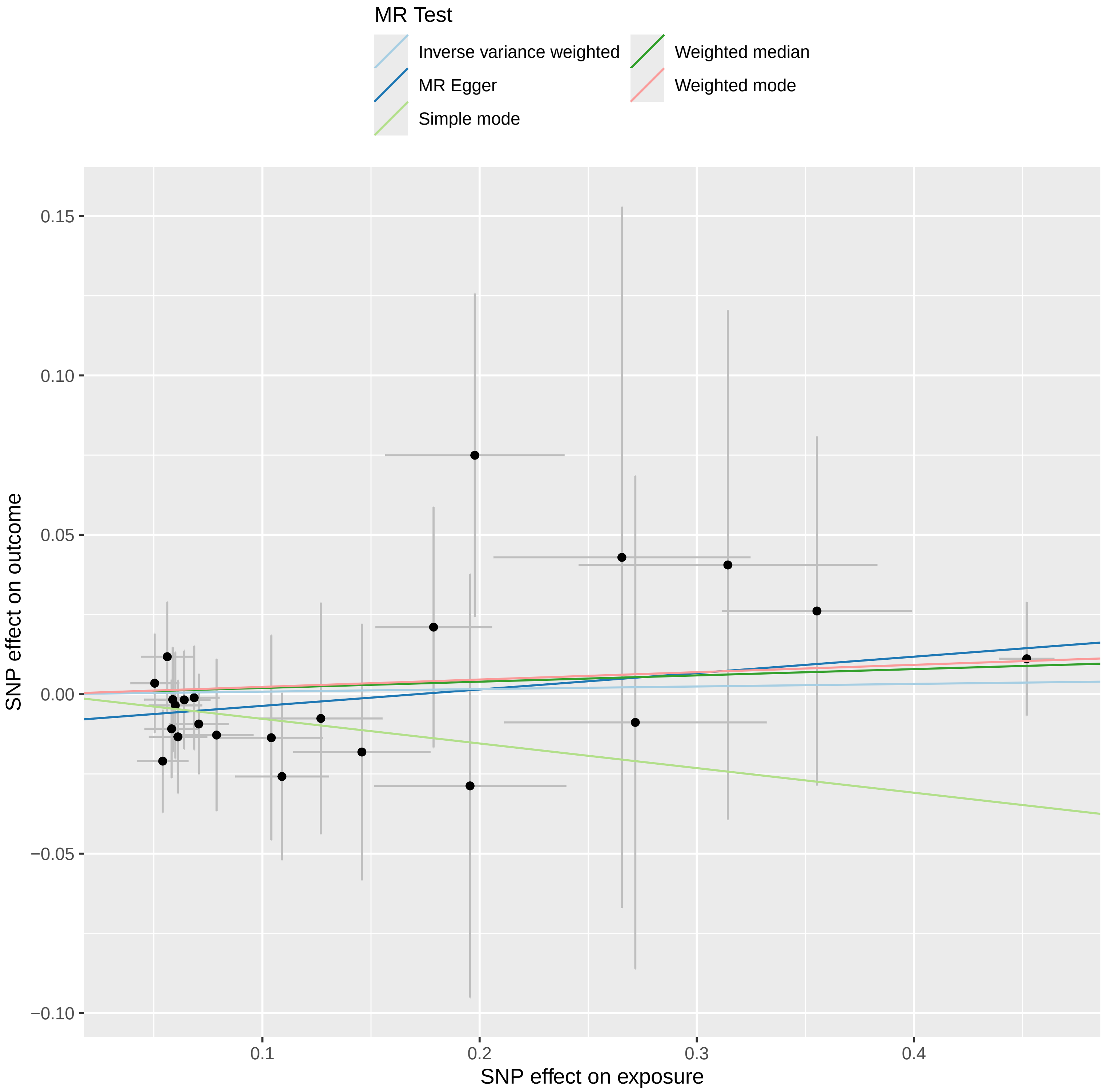
MR Test
Inverse variance weighted
MR Egger
Simple mode
Weighted median
Weighted mode
SNP effect on outcome
0.15
0.10
0.05
0.00
−0.05
−0.10
0.1
0.2
0.3
0.4
SNP effect on exposure

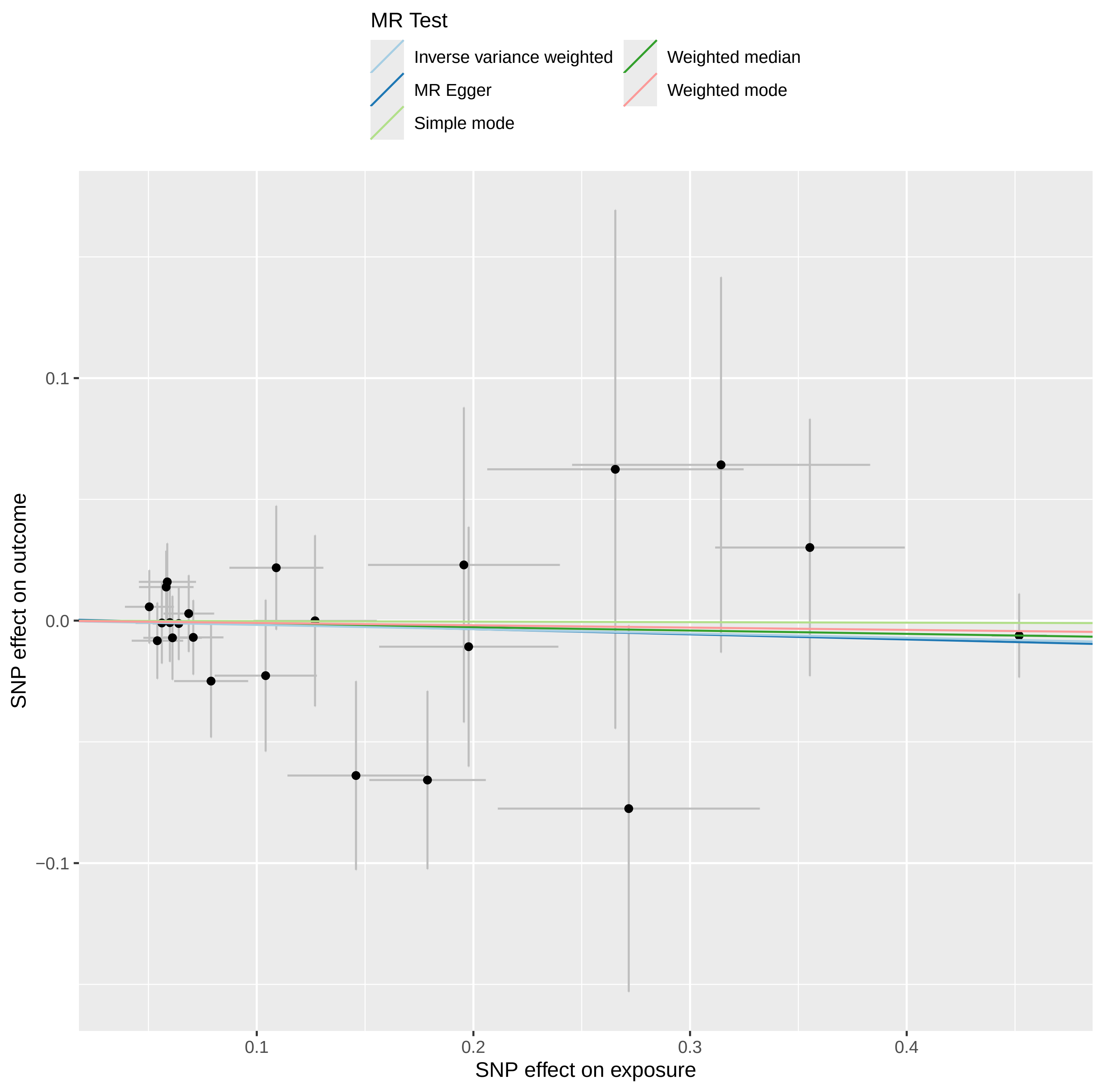

MR Test
Inverse variance weighted
MR Egger
Simple mode
Weighted median
Weighted mode
SNP effect on outcome
0.1
0.0
−0.1
SNP effect on exposure
0.1
0.2
0.3
0.4

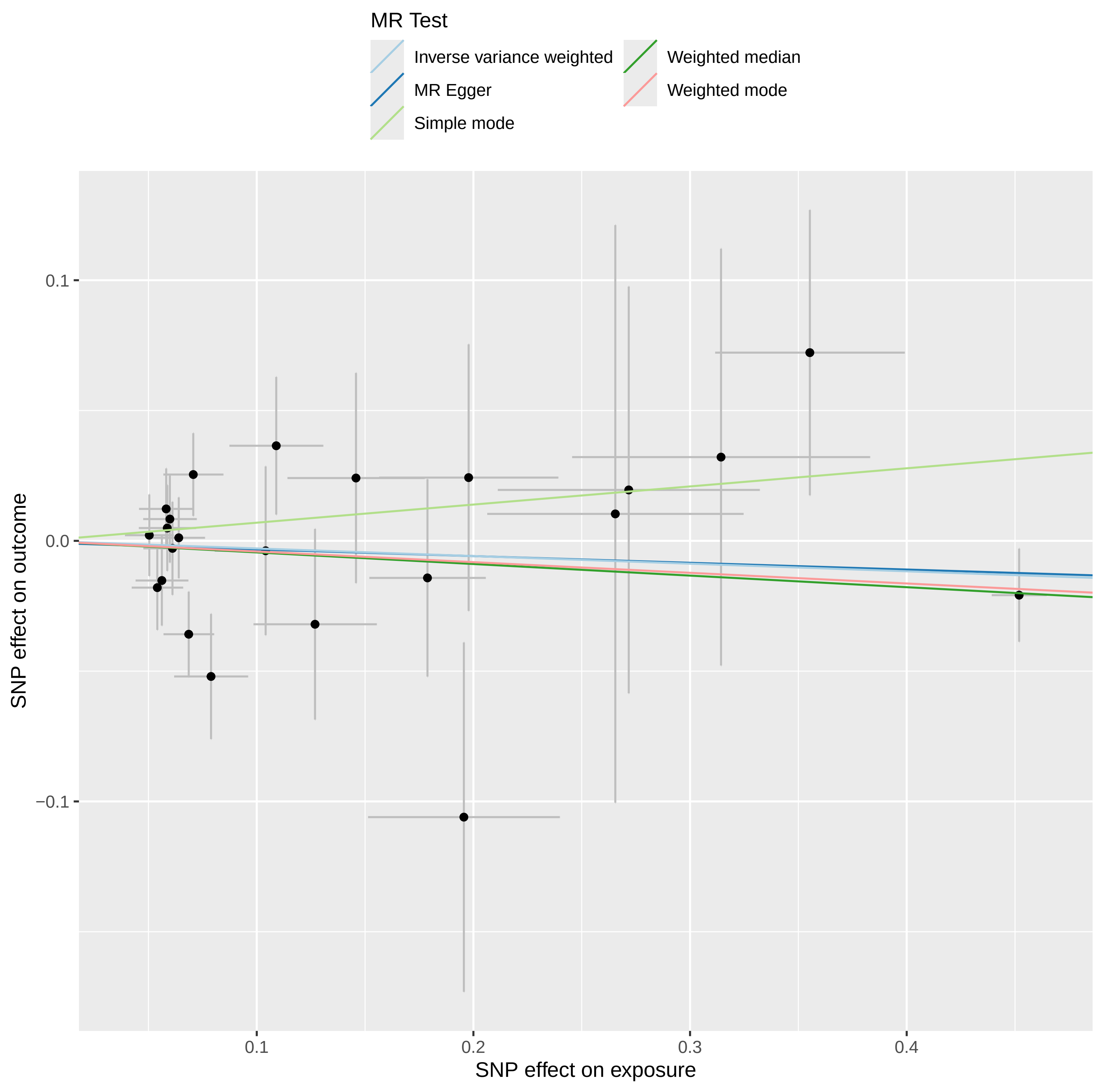

MR Test
Inverse variance weighted
MR Egger
Simple mode
Weighted median
Weighted mode
0.1
0.0
−0.1
SNP effect on outcome
0.1
0.2
0.3
0.4
SNP effect on exposure

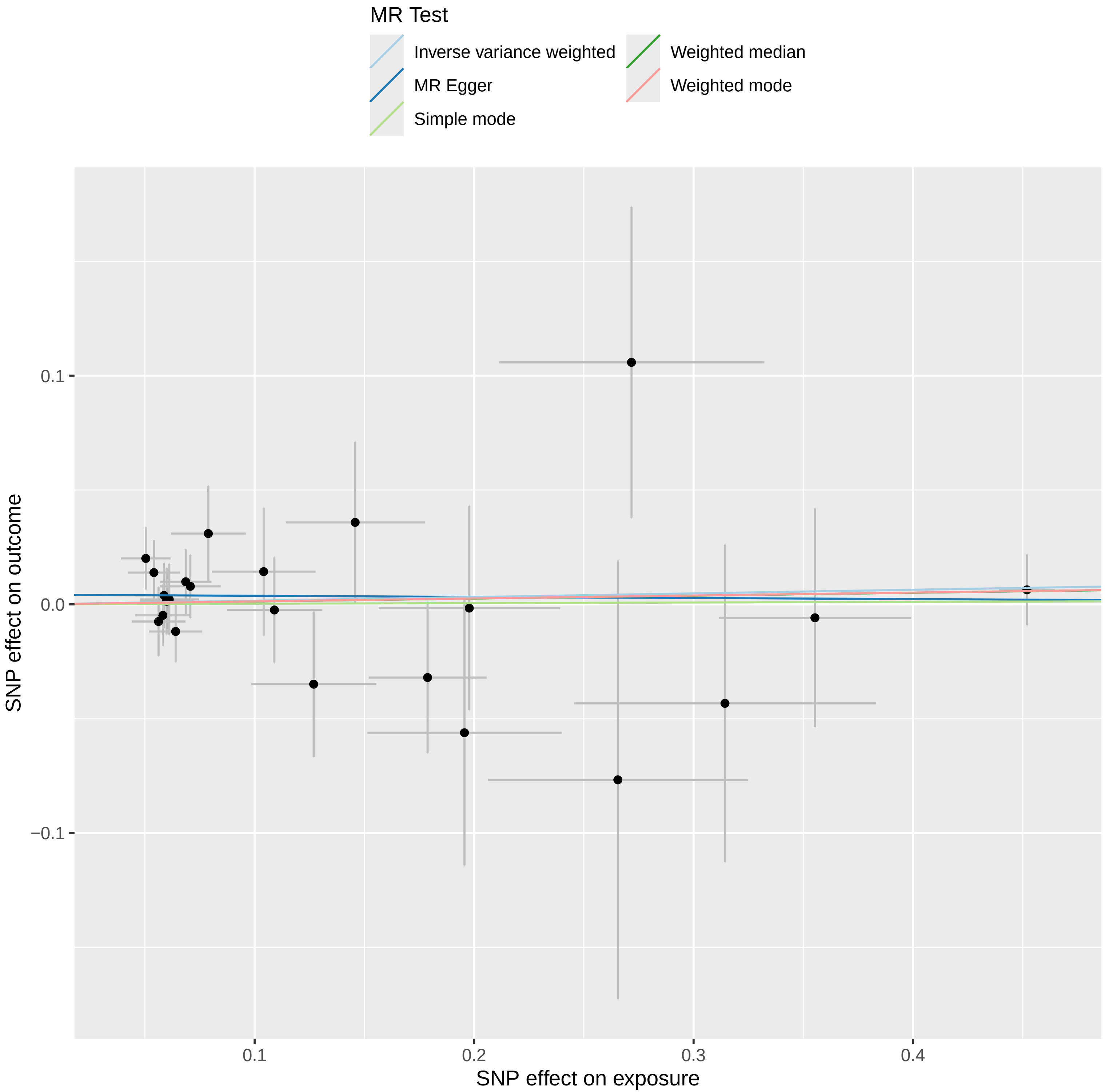
MR Test
Inverse variance weighted
MR Egger
Simple mode
Weighted median
Weighted mode
SNP effect on outcome
0.1
0.0
−0.1
0.1
0.2
0.3
0.4
SNP effect on exposure

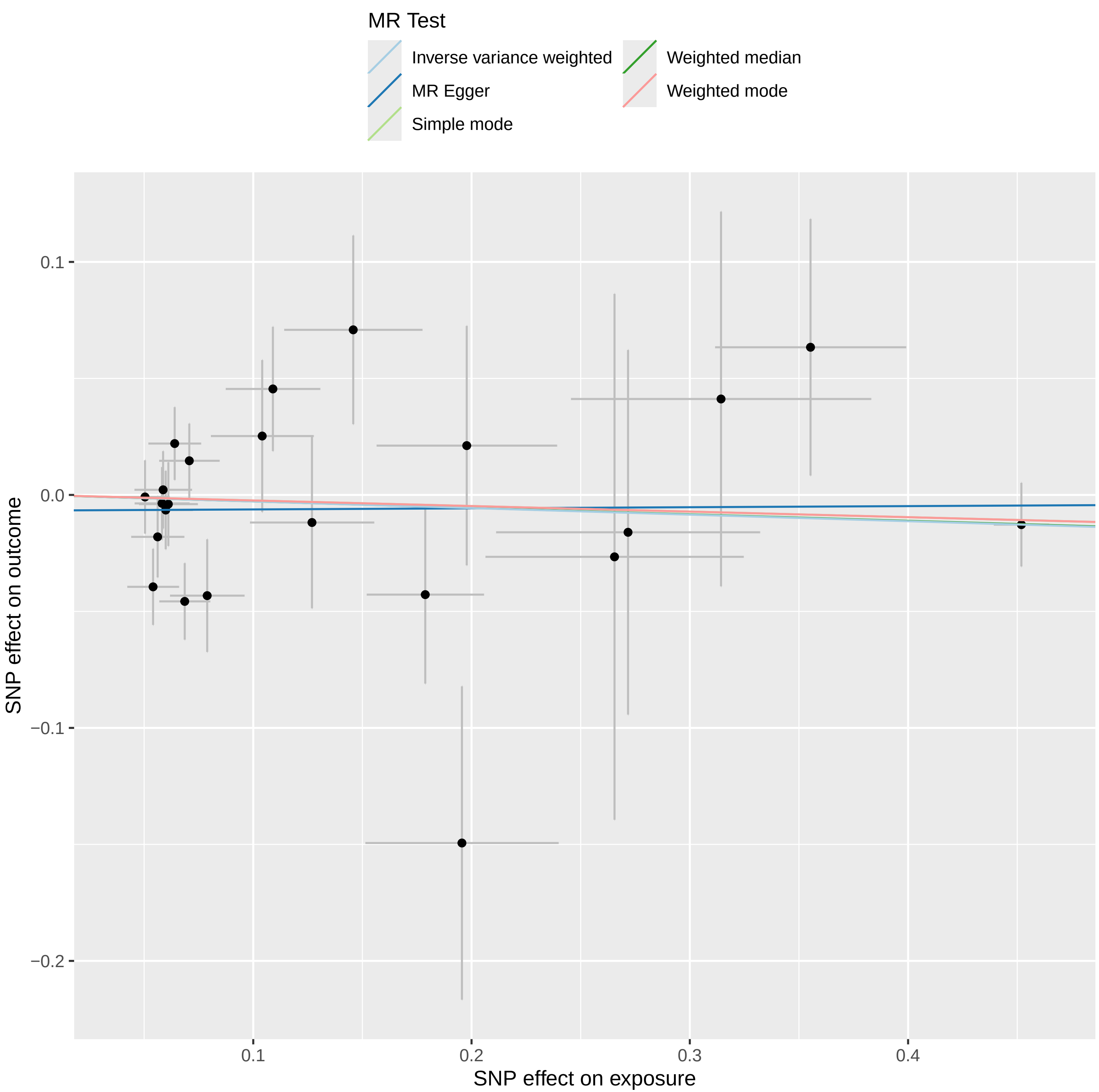
MR Test
Inverse variance weighted
MR Egger
Simple mode
Weighted median
Weighted mode
SNP effect on outcome
0.1
0.0
−0.1
−0.2
SNP effect on exposure
0.1
0.2
0.3
0.4

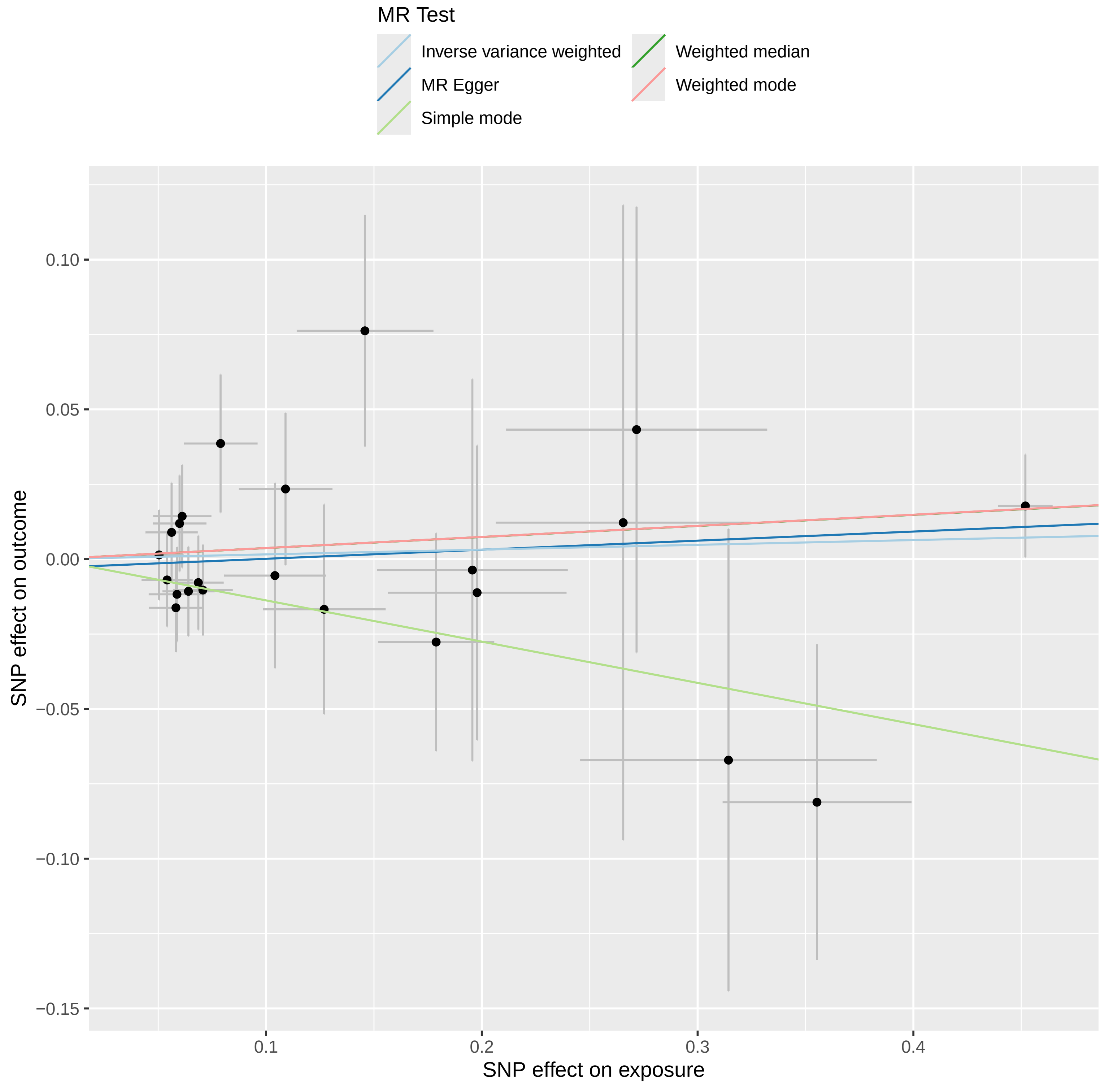

MR Test
Inverse variance weighted
MR Egger
Simple mode
Weighted median
Weighted mode
SNP effect on outcome
SNP effect on exposure
0.10
0.05
0.00
−0.05
−0.10
−0.15
0.1
0.2
0.3
0.4

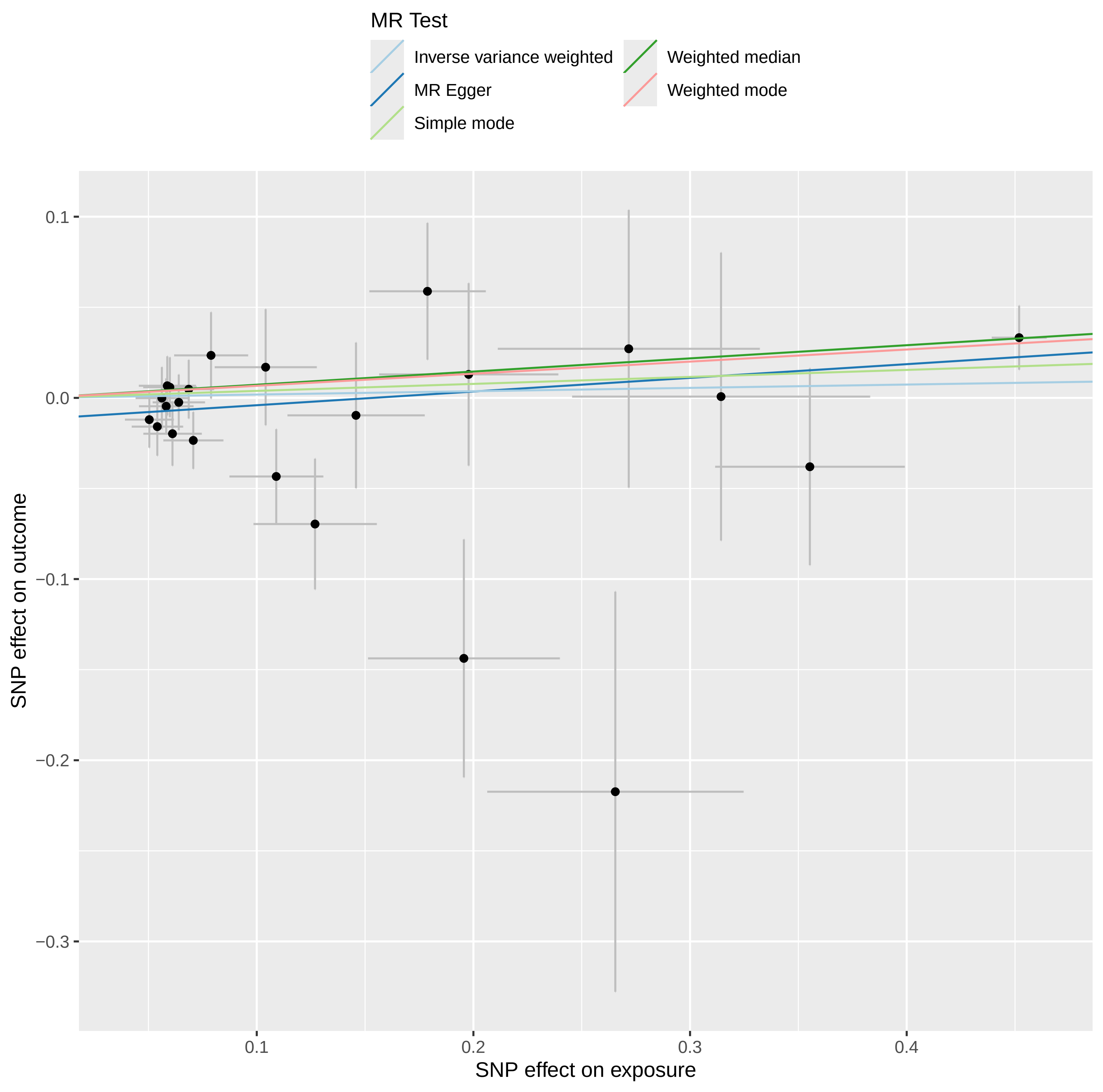

MR Test
Inverse variance weighted
MR Egger
Simple mode
Weighted median
Weighted mode
0.1
0.0
−0.1
−0.2
−0.3
SNP effect on outcome
0.1
0.2
0.3
0.4
SNP effect on exposure

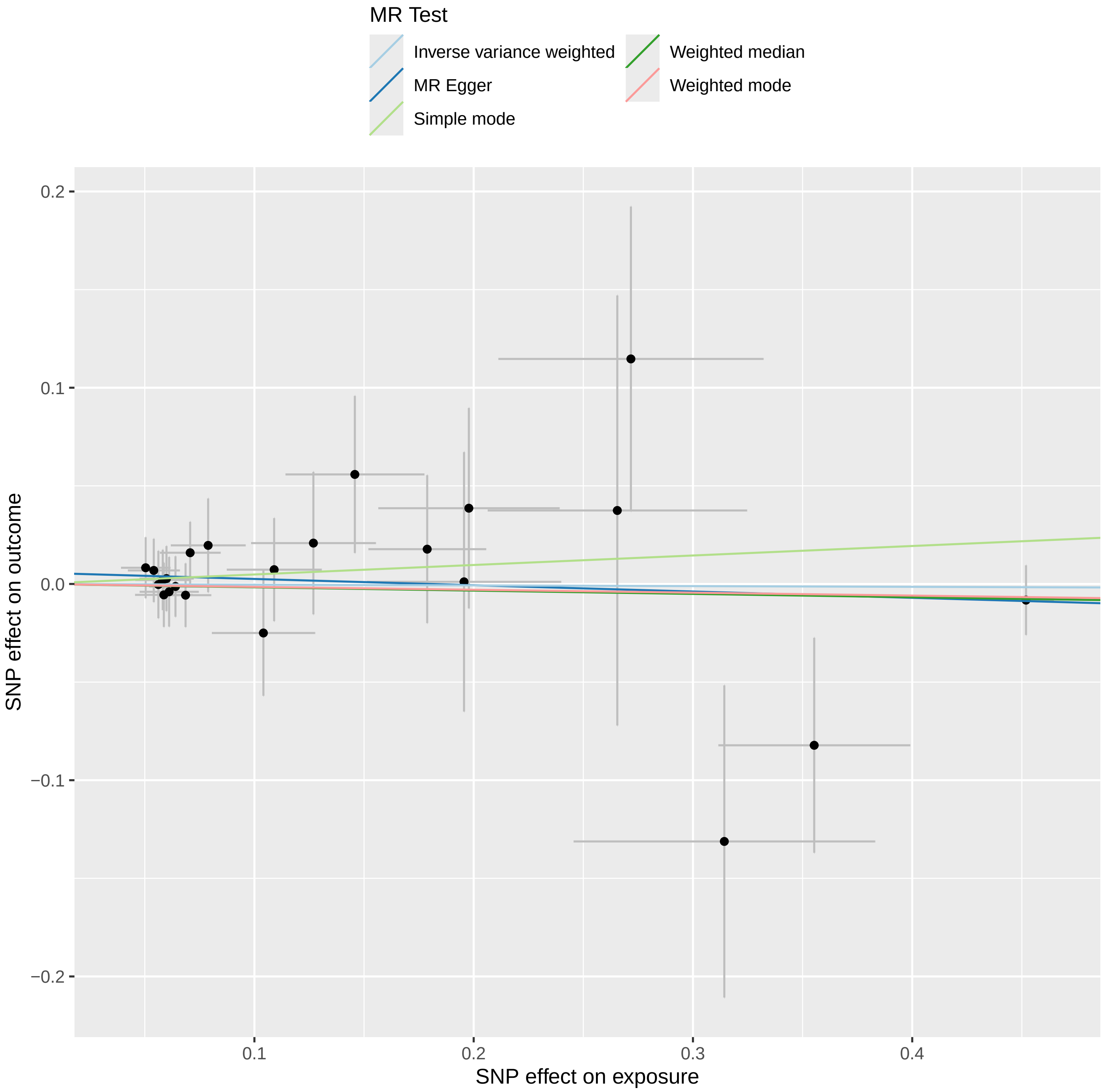
MR Test
Inverse variance weighted
MR Egger
Simple mode
Weighted median
Weighted mode
0.2
0.1
0.0
−0.1
−0.2
SNP effect on outcome
0.1
0.2
0.3
0.4
SNP effect on exposure

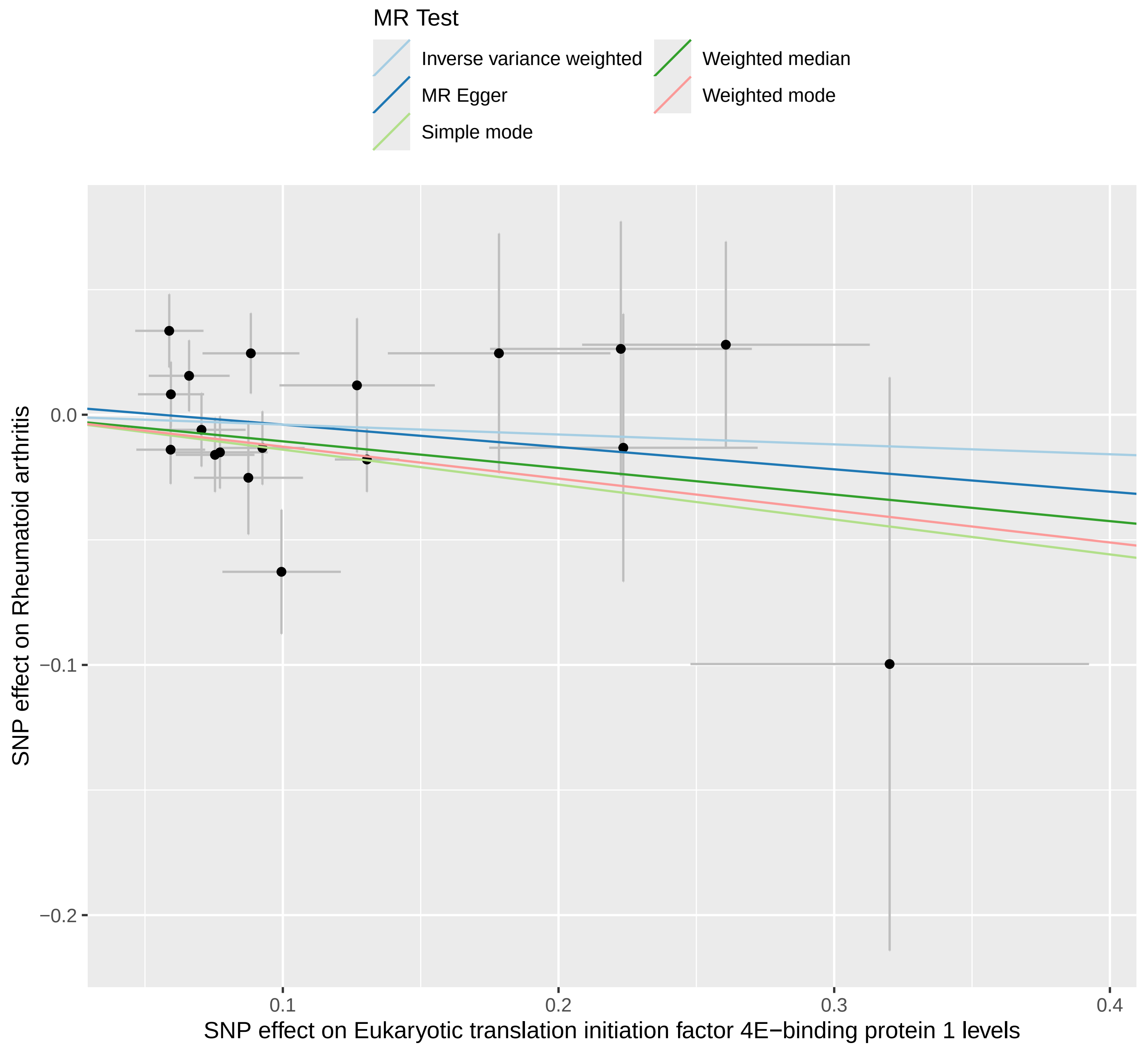
MR Test
Inverse variance weighted
MR Egger
Simple mode
Weighted median
Weighted mode
0.0
−0.1
−0.2
0.1
0.2
0.3
0.4
SNP effect on Rheumatoid arthritis
SNP effect on Eukaryotic translation initiation factor 4E−binding protein 1 levels

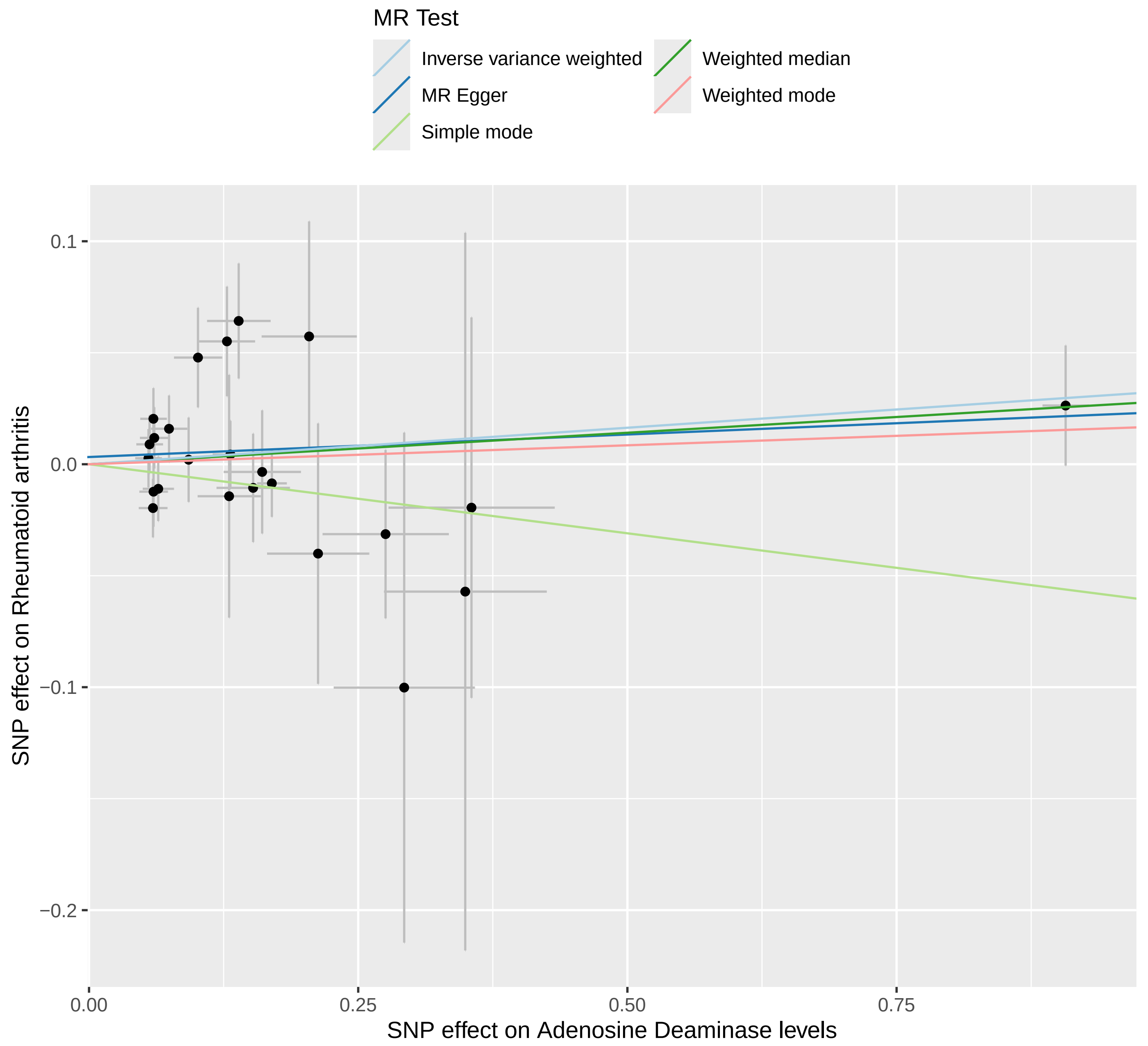

MR Test
Inverse variance weighted
MR Egger
Simple mode
Weighted median
Weighted mode
0.1
0.0
−0.1
−0.2
0.00
0.25
0.50
0.75
SNP effect on Adenosine Deaminase levels
SNP effect on Rheumatoid arthritis

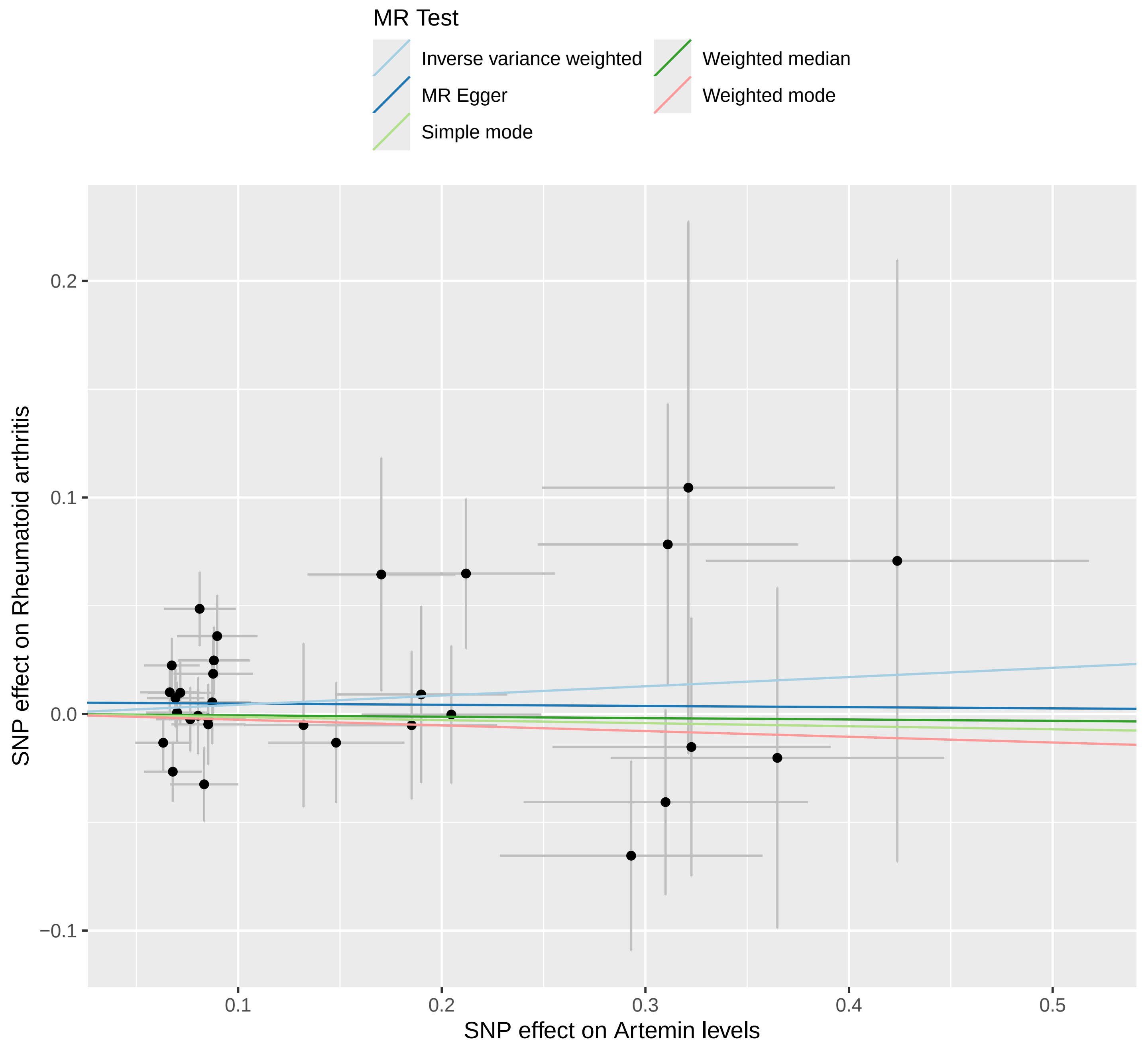

MR Test
Inverse variance weighted
MR Egger
Simple mode
Weighted median
Weighted mode
0.2
0.1
0.0
−0.1
SNP effect on Rheumatoid arthritis
0.1
0.2
0.3
0.4
0.5
SNP effect on Artemin levels

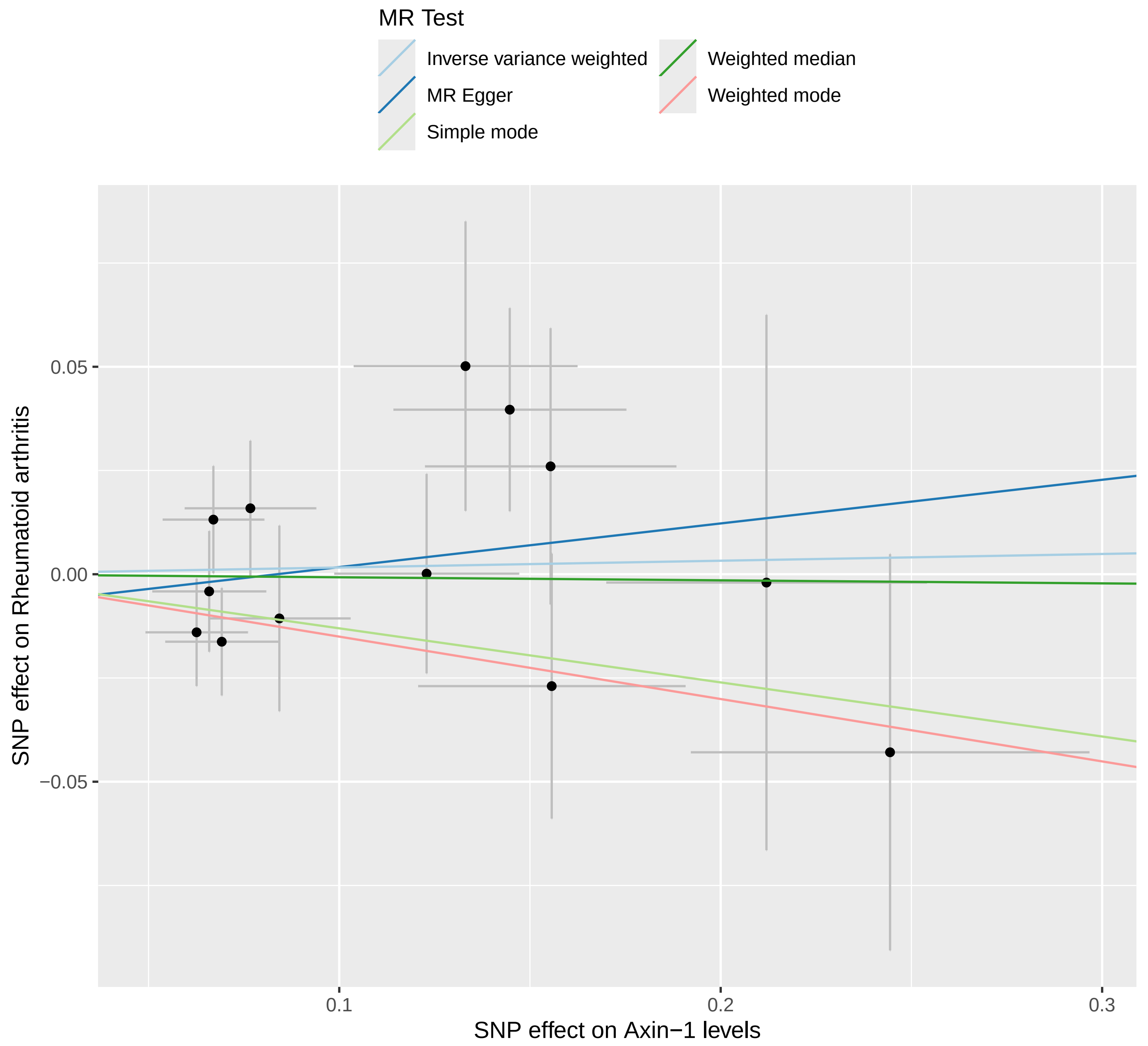

MR Test
Inverse variance weighted
MR Egger
Simple mode
Weighted median
Weighted mode
0.05
0.00
−0.05
SNP effect on Rheumatoid arthritis
0.1
0.2
0.3
SNP effect on Axin−1 levels

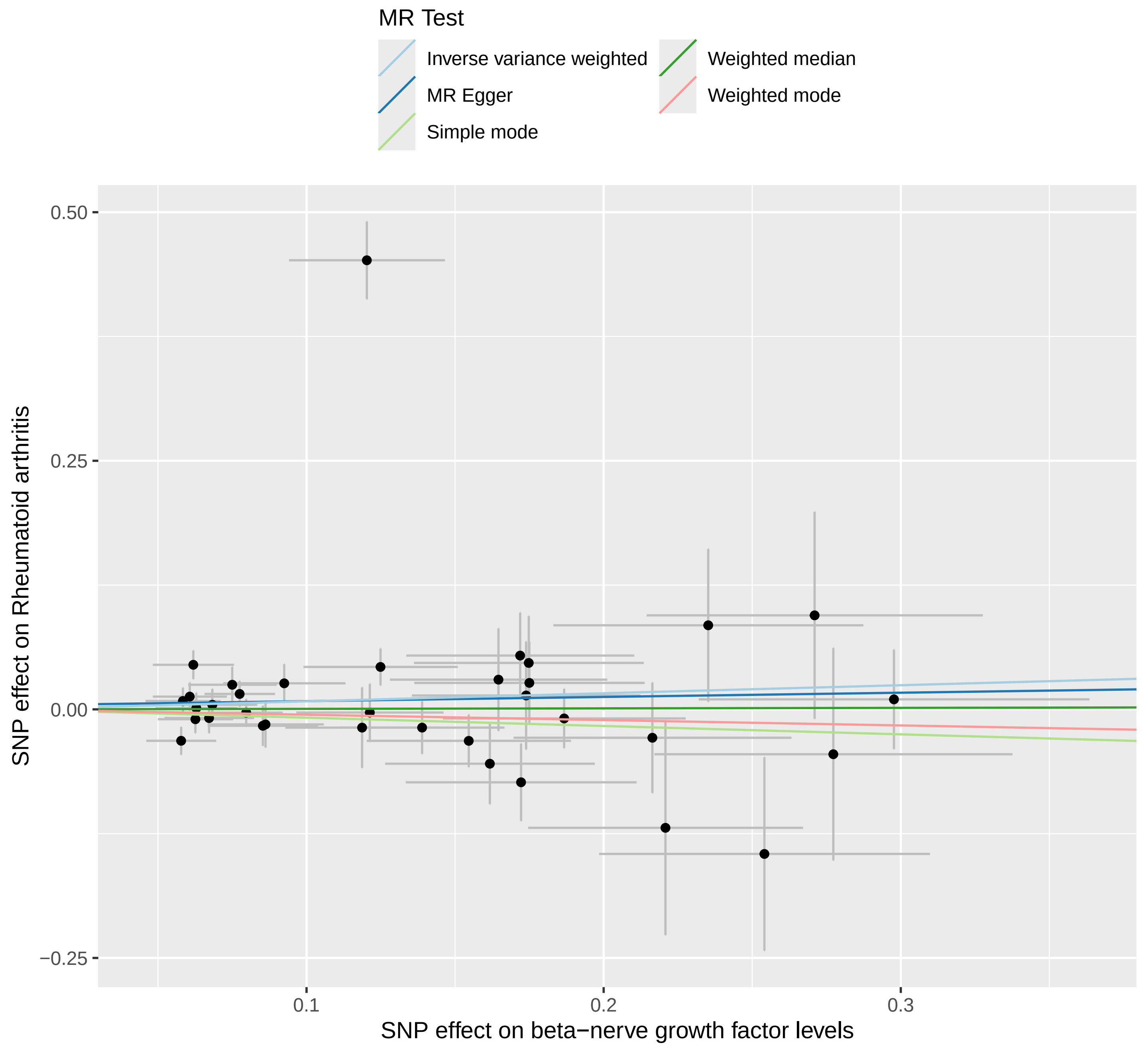
MR Test
Inverse variance weighted
MR Egger
Simple mode
Weighted median
Weighted mode
0.50
0.25
0.00
−0.25
0.1
0.2
0.3
SNP effect on Rheumatoid arthritis
SNP effect on beta−nerve growth factor levels

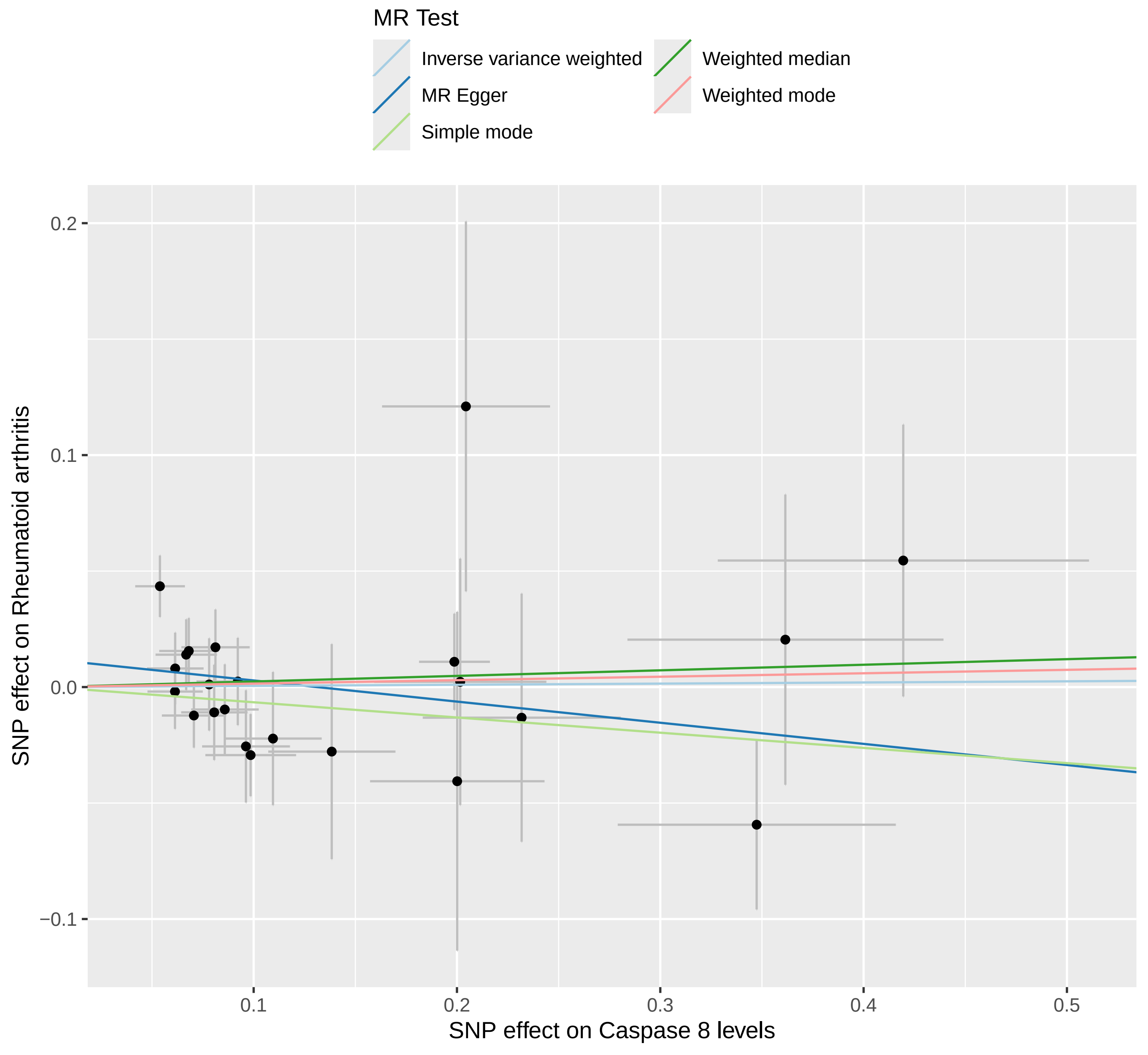
MR Test
Inverse variance weighted
MR Egger
Simple mode
Weighted median
Weighted mode
0.2
0.1
0.0
−0.1
0.1
0.2
0.3
0.4
0.5
SNP effect on Caspase 8 levels
SNP effect on Rheumatoid arthritis

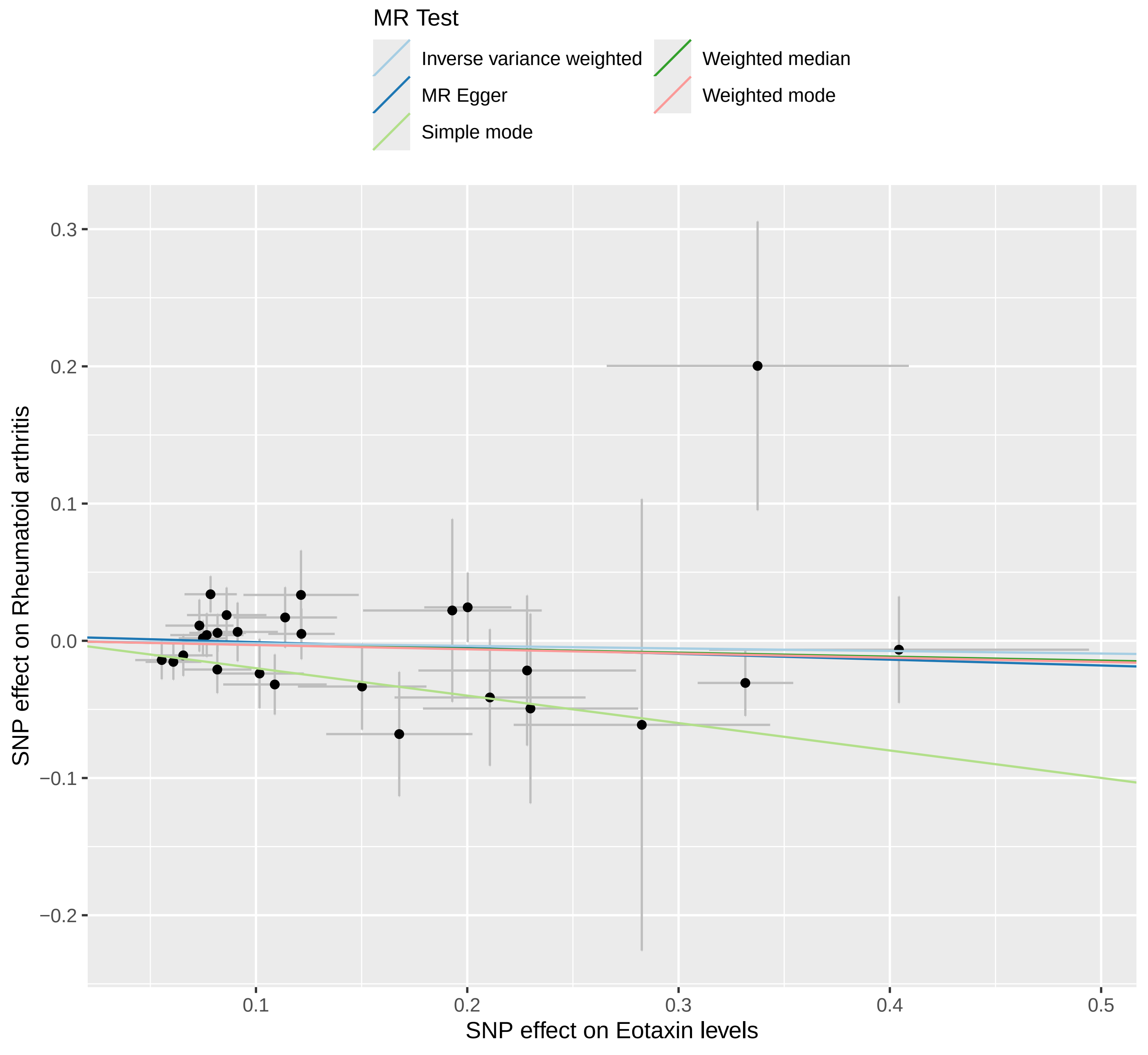
MR Test
Inverse variance weighted
MR Egger
Simple mode
Weighted median
Weighted mode
SNP effect on Rheumatoid arthritis
0.3
0.2
0.1
0.0
−0.1
−0.2
0.1
0.2
0.3
0.4
0.5
SNP effect on Eotaxin levels

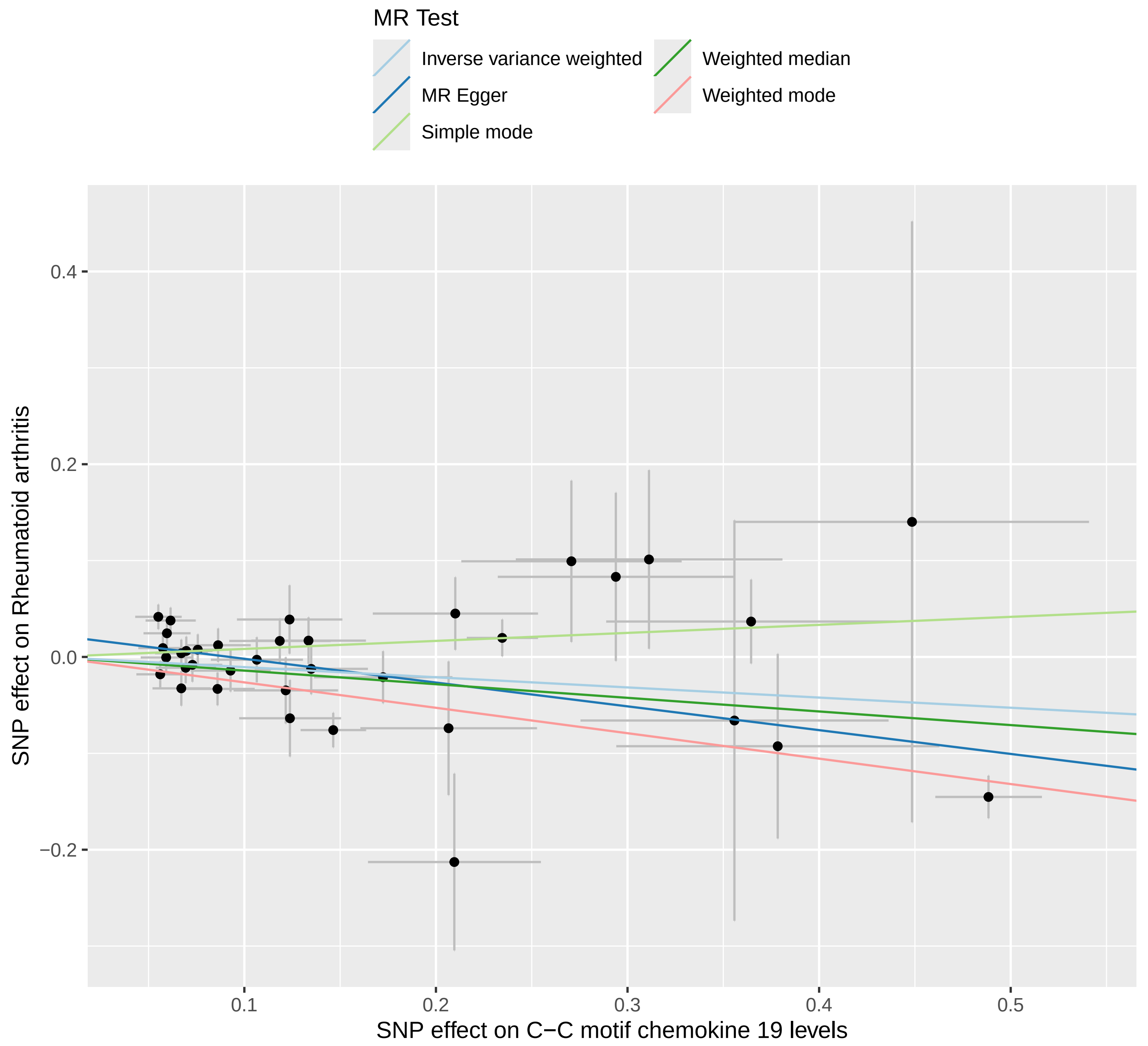
MR Test
Inverse variance weighted
MR Egger
Simple mode
Weighted median
Weighted mode
0.4
0.2
0.0
−0.2
SNP effect on Rheumatoid arthritis
0.1
0.2
0.3
0.4
0.5
SNP effect on C−C motif chemokine 19 levels

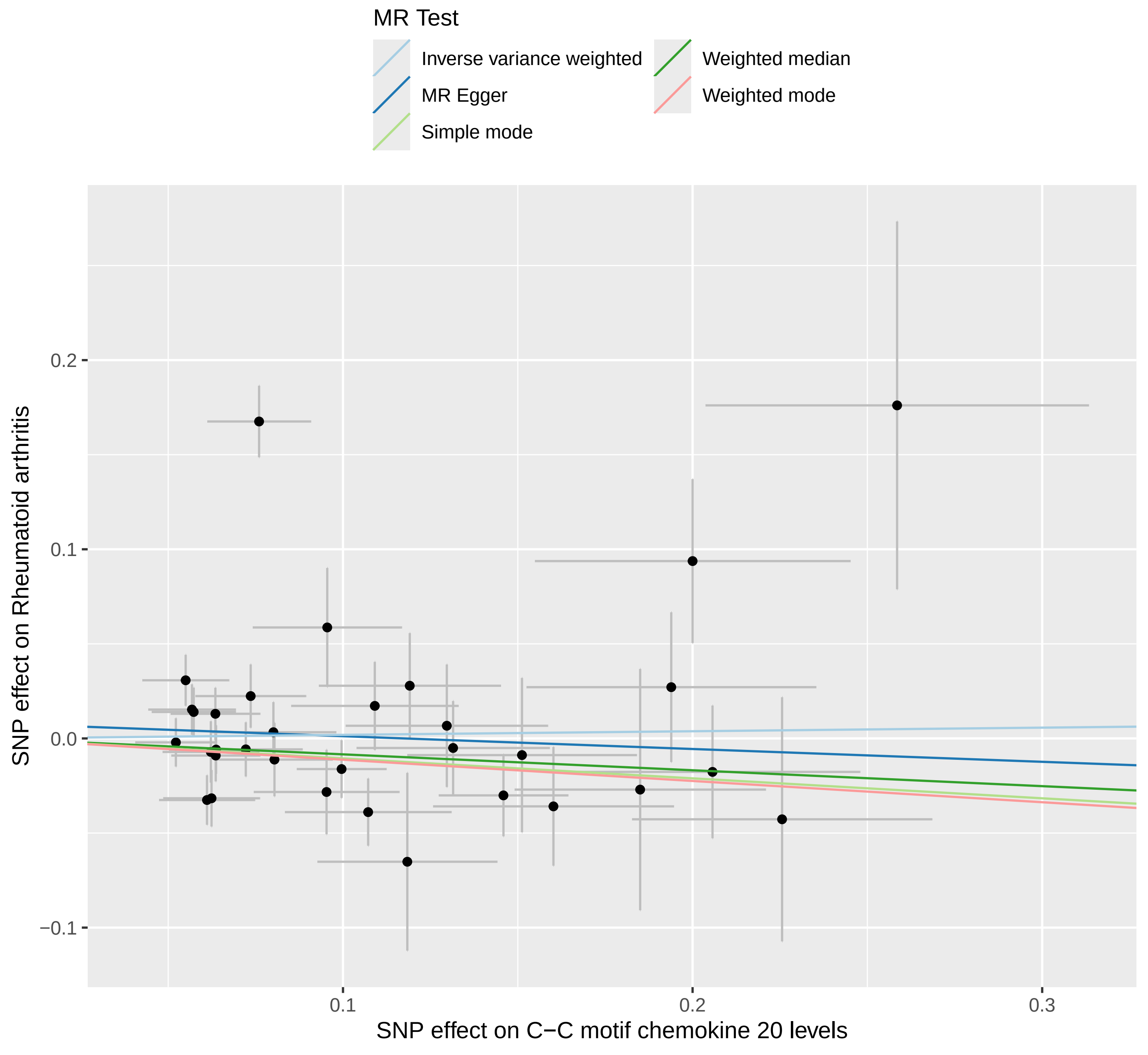
MR Test
Inverse variance weighted
MR Egger
Simple mode
Weighted median
Weighted mode
SNP effect on Rheumatoid arthritis
0.2
0.1
0.0
−0.1
0.1
0.2
0.3
SNP effect on C−C motif chemokine 20 levels

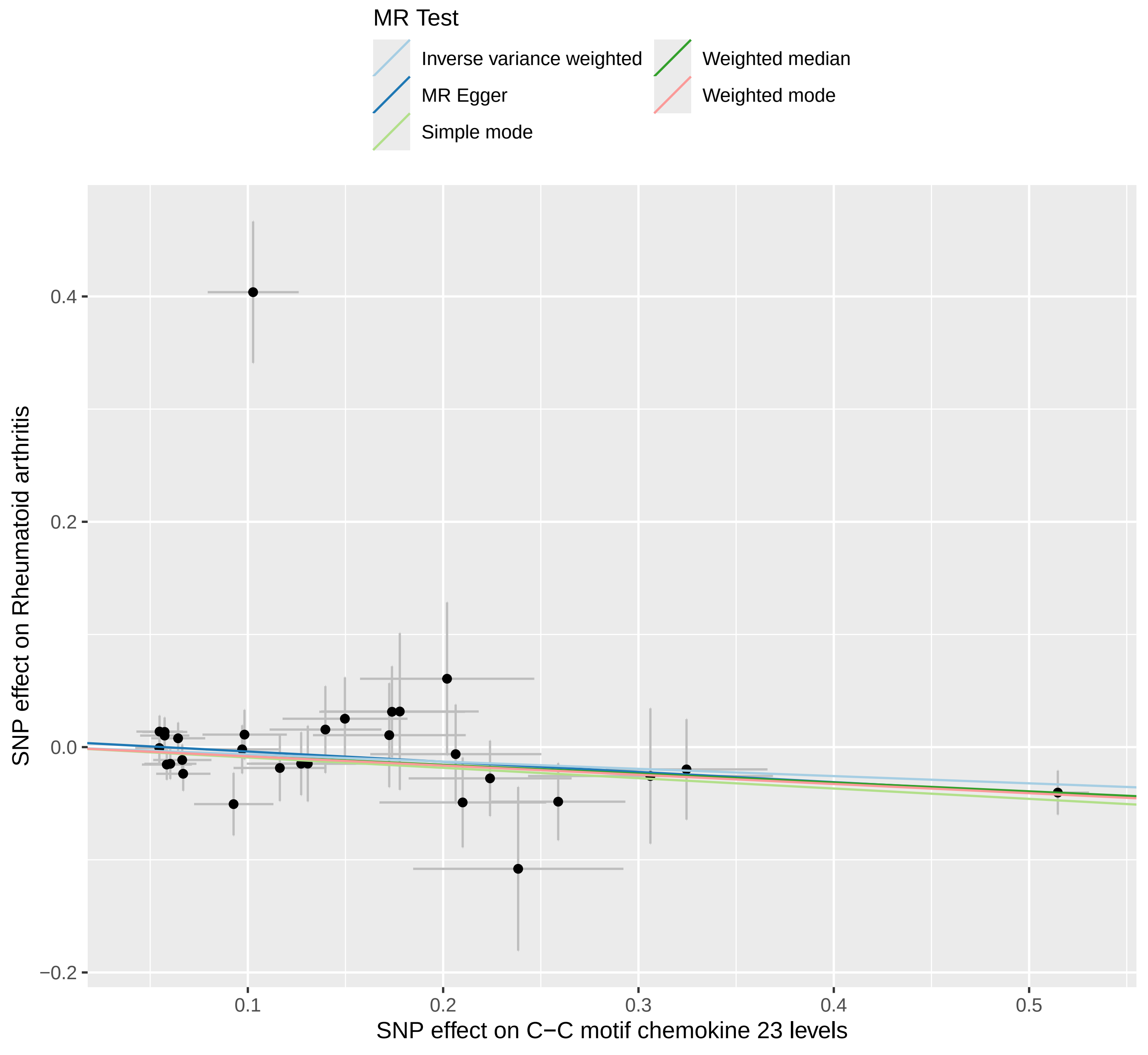
MR Test
Inverse variance weighted
MR Egger
Simple mode
Weighted median
Weighted mode
SNP effect on Rheumatoid arthritis
0.4
0.2
0.0
−0.2
0.1
0.2
0.3
0.4
0.5
SNP effect on C−C motif chemokine 23 levels

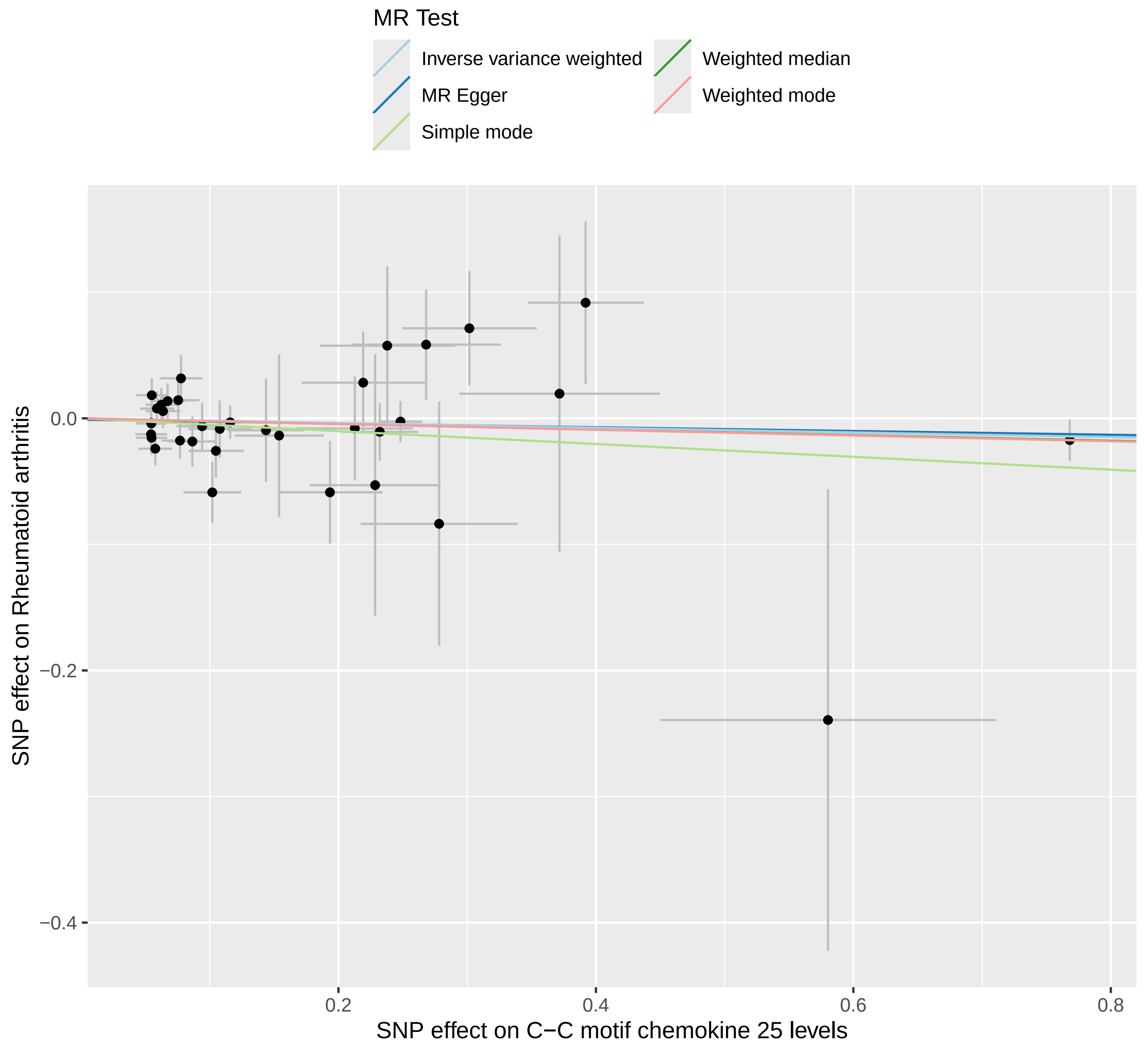
MR Test
Inverse variance weighted
MR Egger
Simple mode
Weighted median
Weighted mode
SNP effect on Rheumatoid arthritis
0.0
−0.2
−0.4
0.2
0.4
0.6
0.8
SNP effect on C−C motif chemokine 25 levels

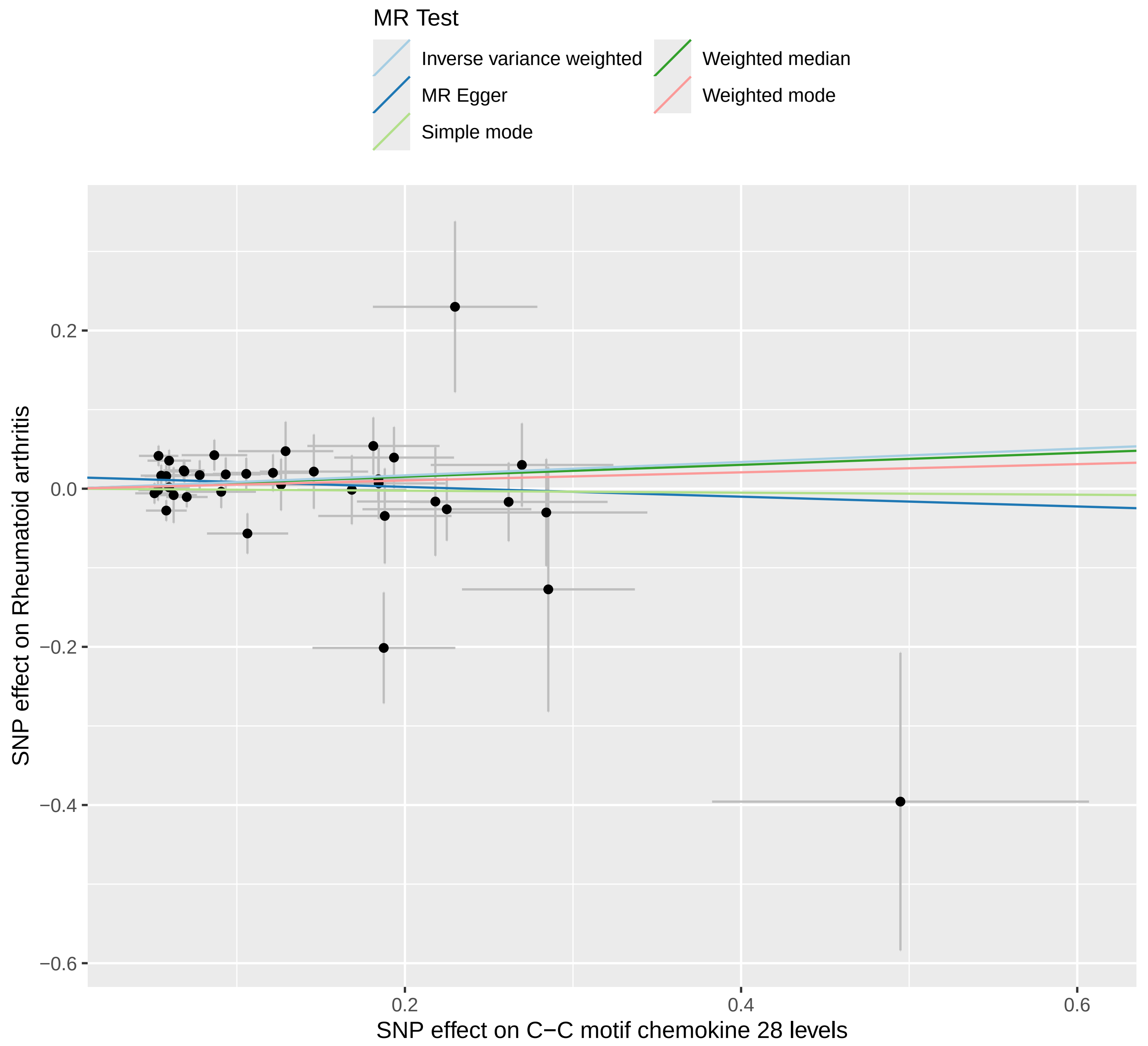
MR Test
Inverse variance weighted
MR Egger
Simple mode
Weighted median
Weighted mode
SNP effect on Rheumatoid arthritis
0.2
0.0
−0.2
−0.4
−0.6
0.2
0.4
0.6
SNP effect on C−C motif chemokine 28 levels

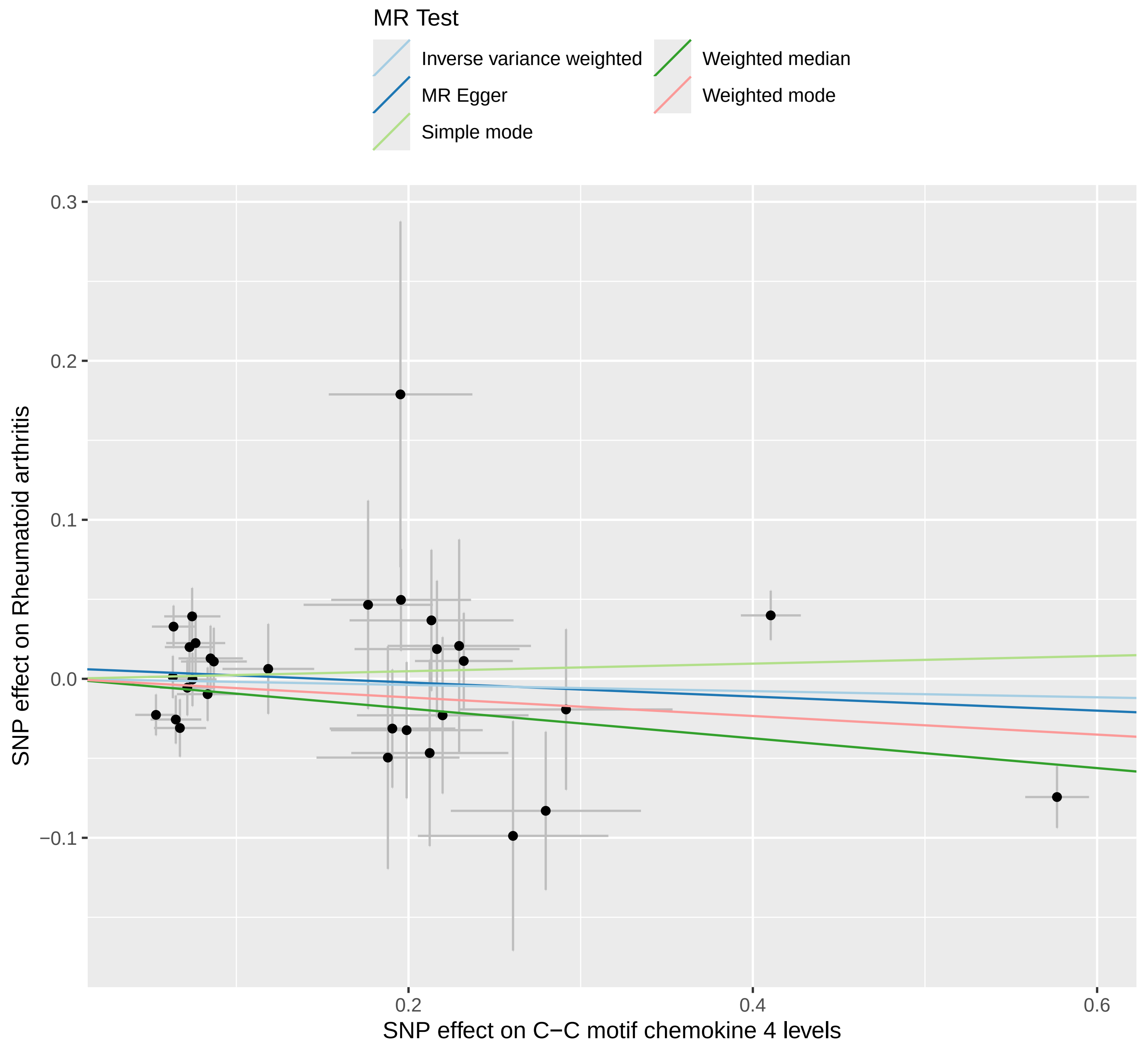
MR Test
Inverse variance weighted
MR Egger
Simple mode
Weighted median
Weighted mode
0.3
0.2
0.1
0.0
−0.1
0.2
0.4
0.6
SNP effect on Rheumatoid arthritis
SNP effect on C−C motif chemokine 4 levels

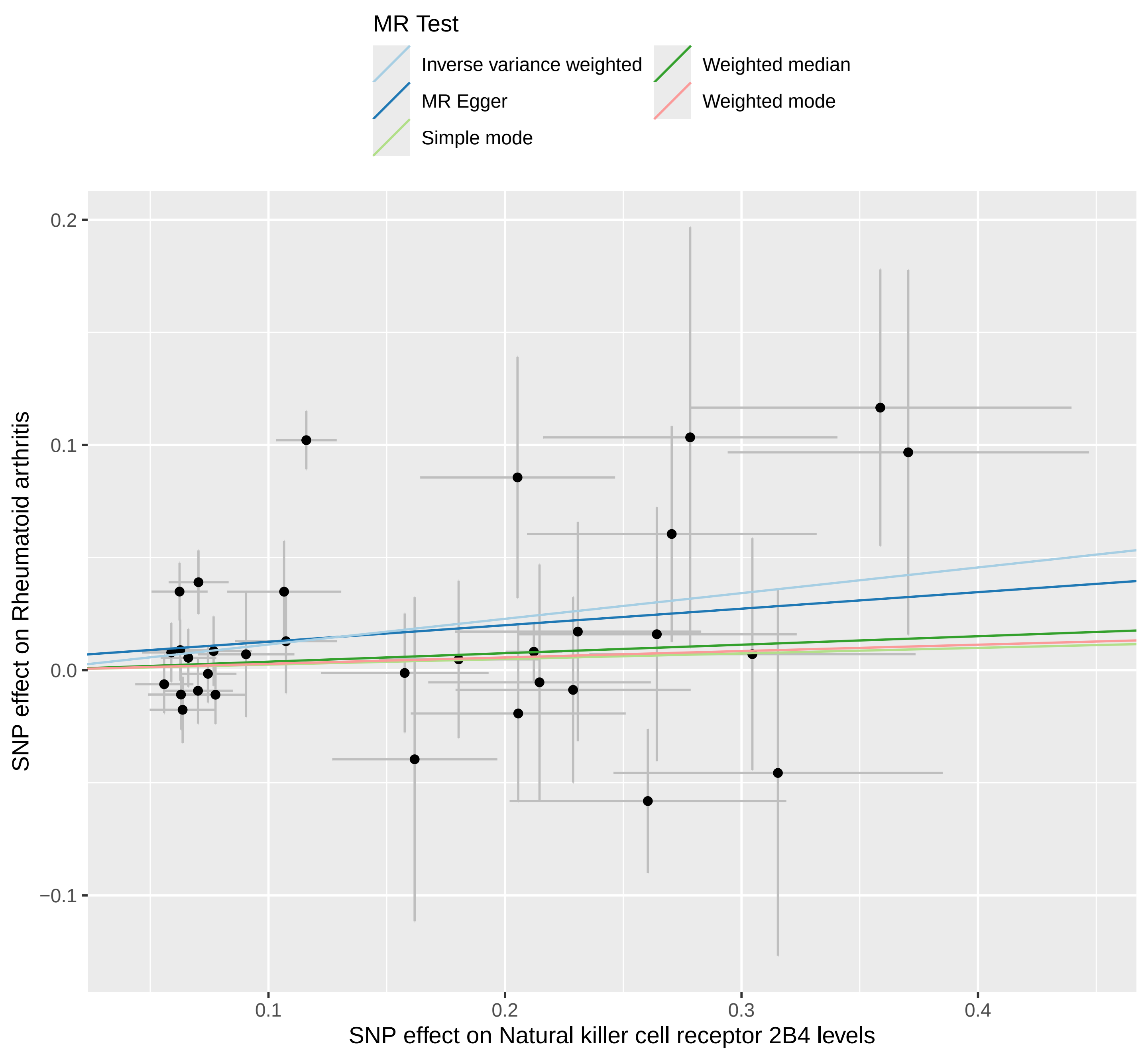
MR Test
Inverse variance weighted
MR Egger
Simple mode
Weighted median
Weighted mode
0.2
0.1
0.0
−0.1
0.1
0.2
0.3
0.4
SNP effect on Rheumatoid arthritis
SNP effect on Natural killer cell receptor 2B4 levels

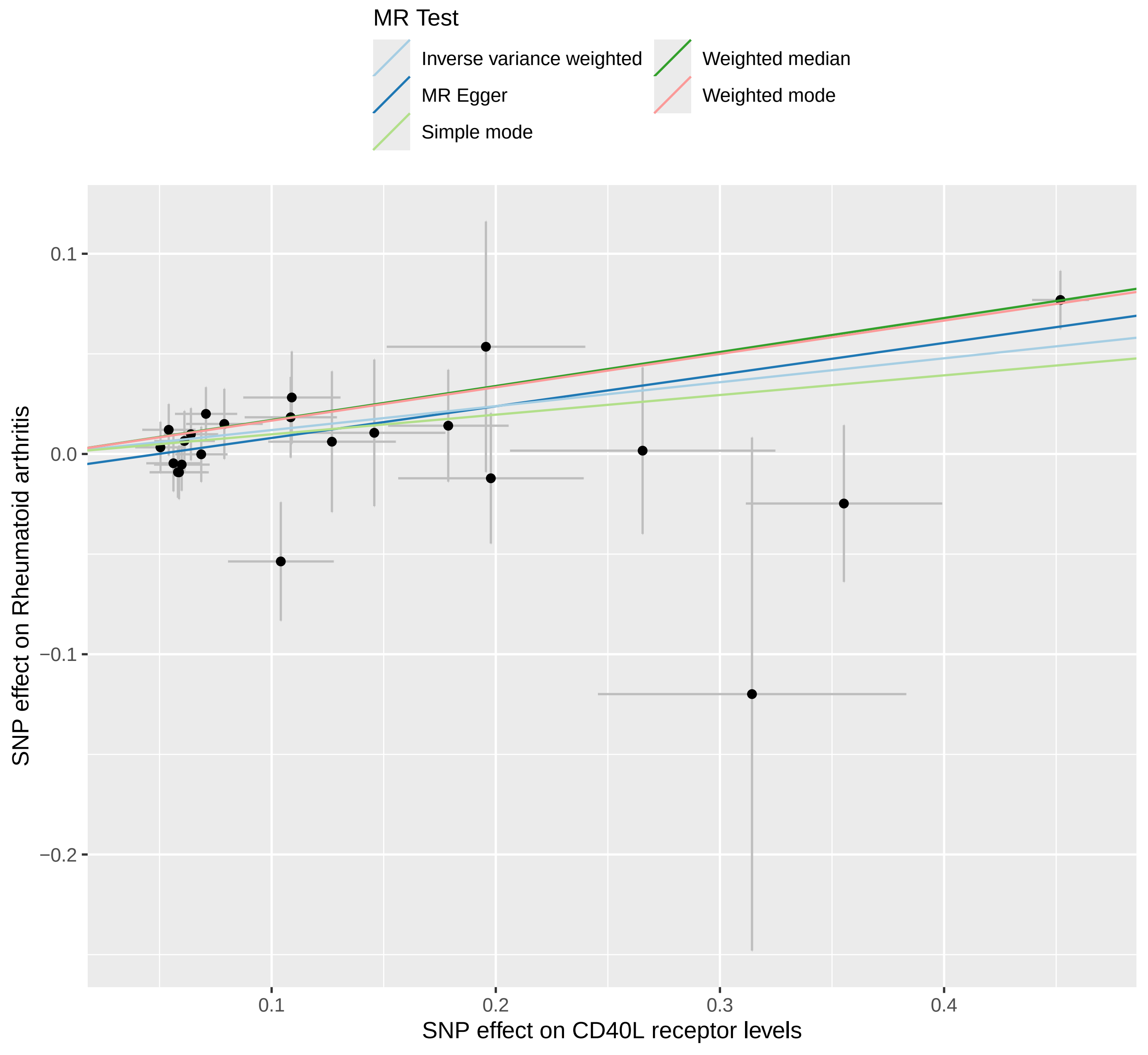

MR Test
Inverse variance weighted
MR Egger
Simple mode
Weighted median
Weighted mode
0.1
0.0
−0.1
−0.2
0.1
0.2
0.3
0.4
SNP effect on Rheumatoid arthritis
SNP effect on CD40L receptor levels

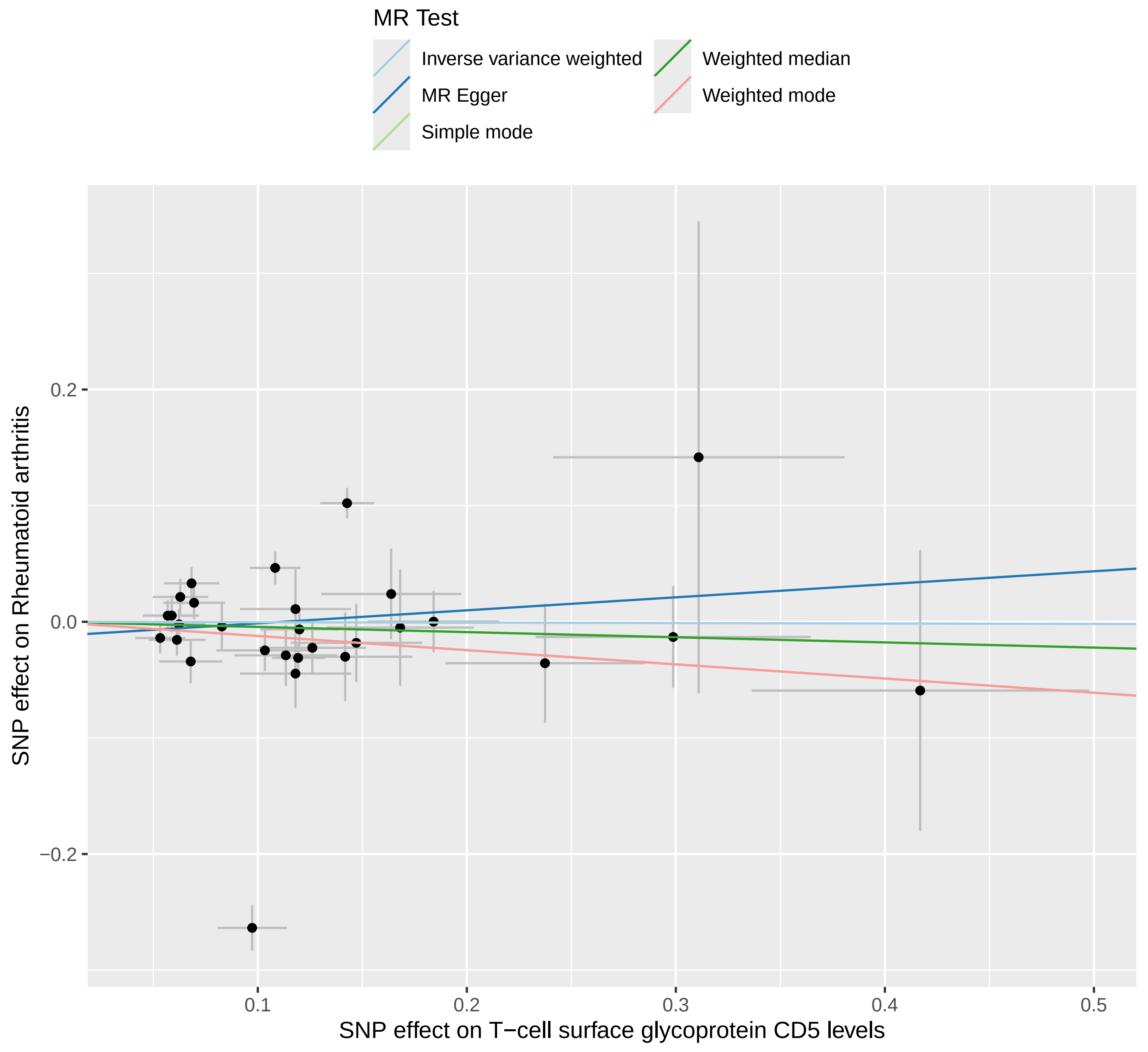

MR Test
Inverse variance weighted
MR Egger
Simple mode
Weighted median
Weighted mode
0.2
0.0
−0.2
SNP effect on Rheumatoid arthritis
0.1
0.2
0.3
0.4
0.5
SNP effect on T−cell surface glycoprotein CD5 levels

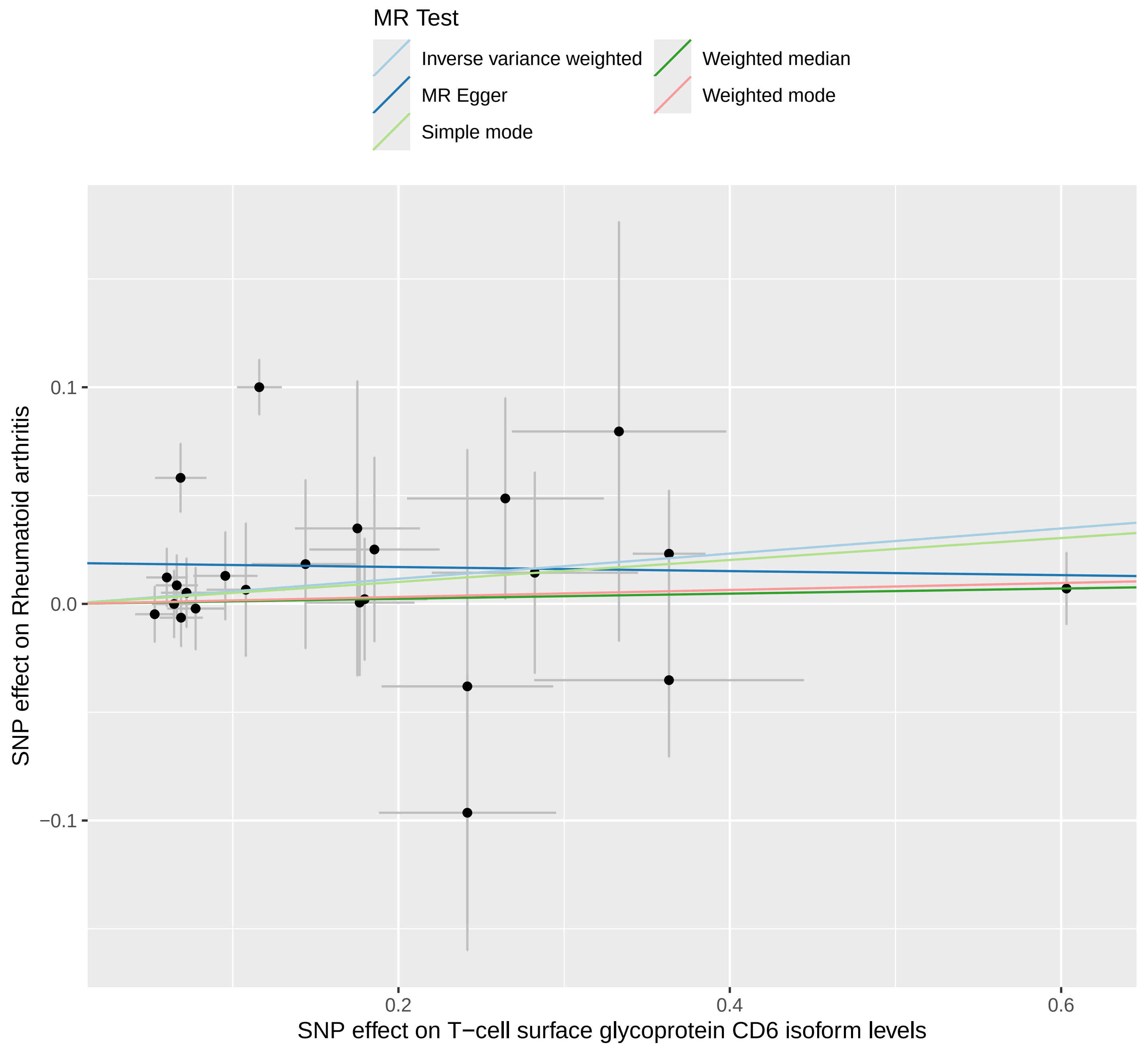
MR Test
Inverse variance weighted
MR Egger
Simple mode
Weighted median
Weighted mode
SNP effect on Rheumatoid arthritis
0.1
0.0
−0.1
0.2
0.4
0.6
SNP effect on T−cell surface glycoprotein CD6 isoform levels

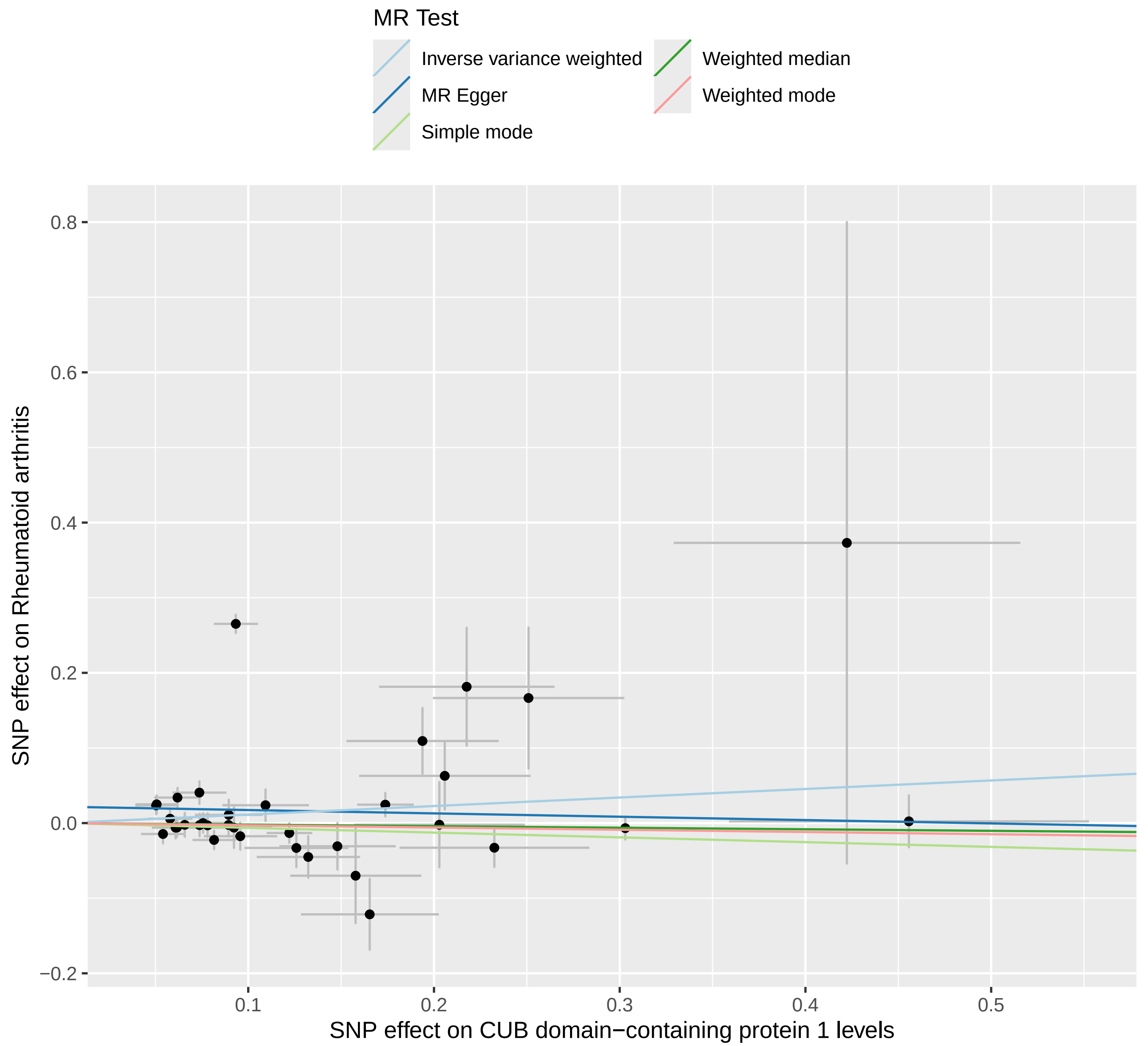
MR Test
Inverse variance weighted
MR Egger
Simple mode
Weighted median
Weighted mode
0.8
0.6
0.4
0.2
0.0
−0.2
0.1
0.2
0.3
0.4
0.5
SNP effect on Rheumatoid arthritis
SNP effect on CUB domain−containing protein 1 levels

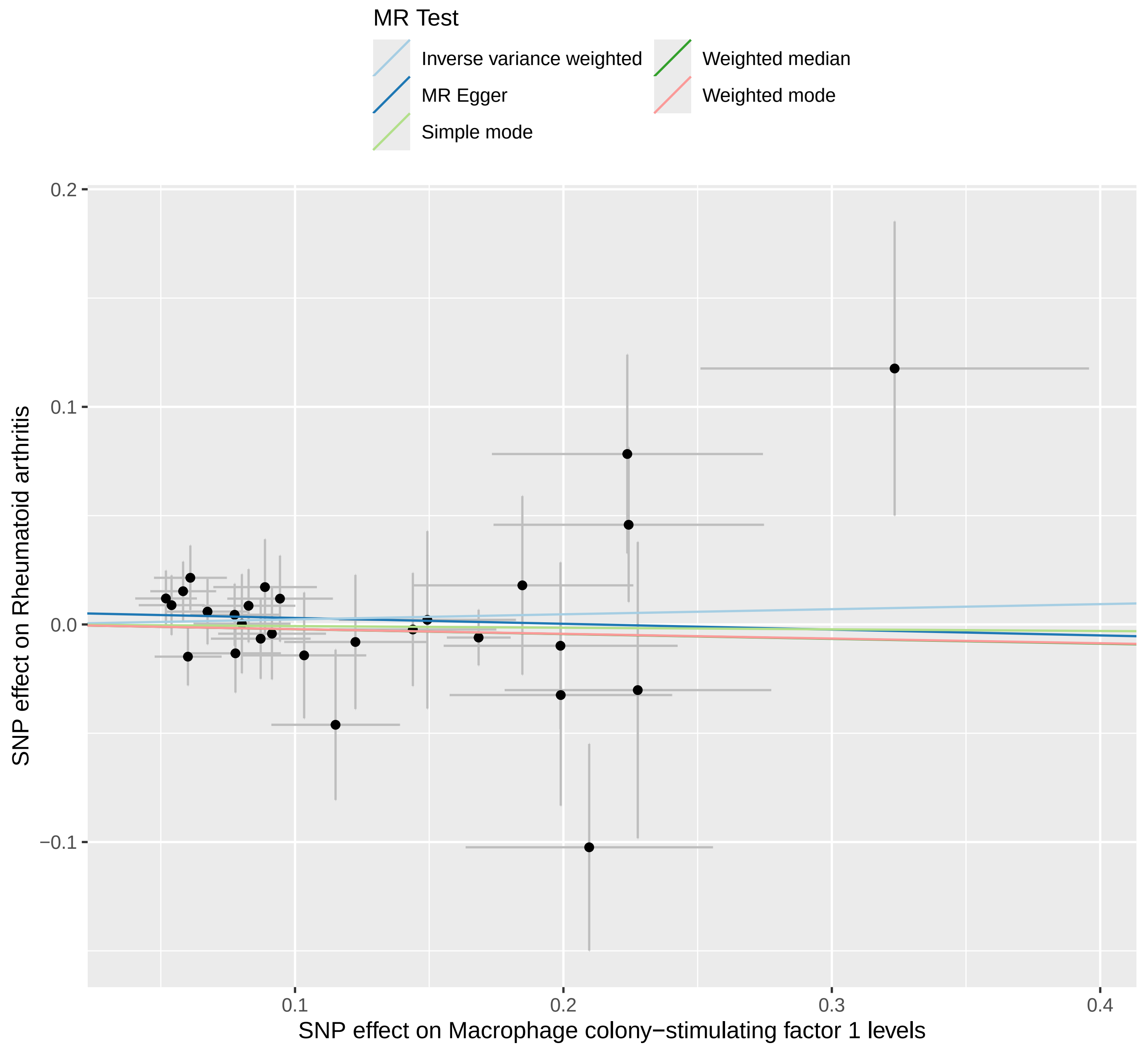

MR Test
Inverse variance weighted
MR Egger
Simple mode
Weighted median
Weighted mode
0.2
0.1
0.0
−0.1
0.1
0.2
0.3
0.4
SNP effect on Rheumatoid arthritis
SNP effect on Macrophage colony−stimulating factor 1 levels

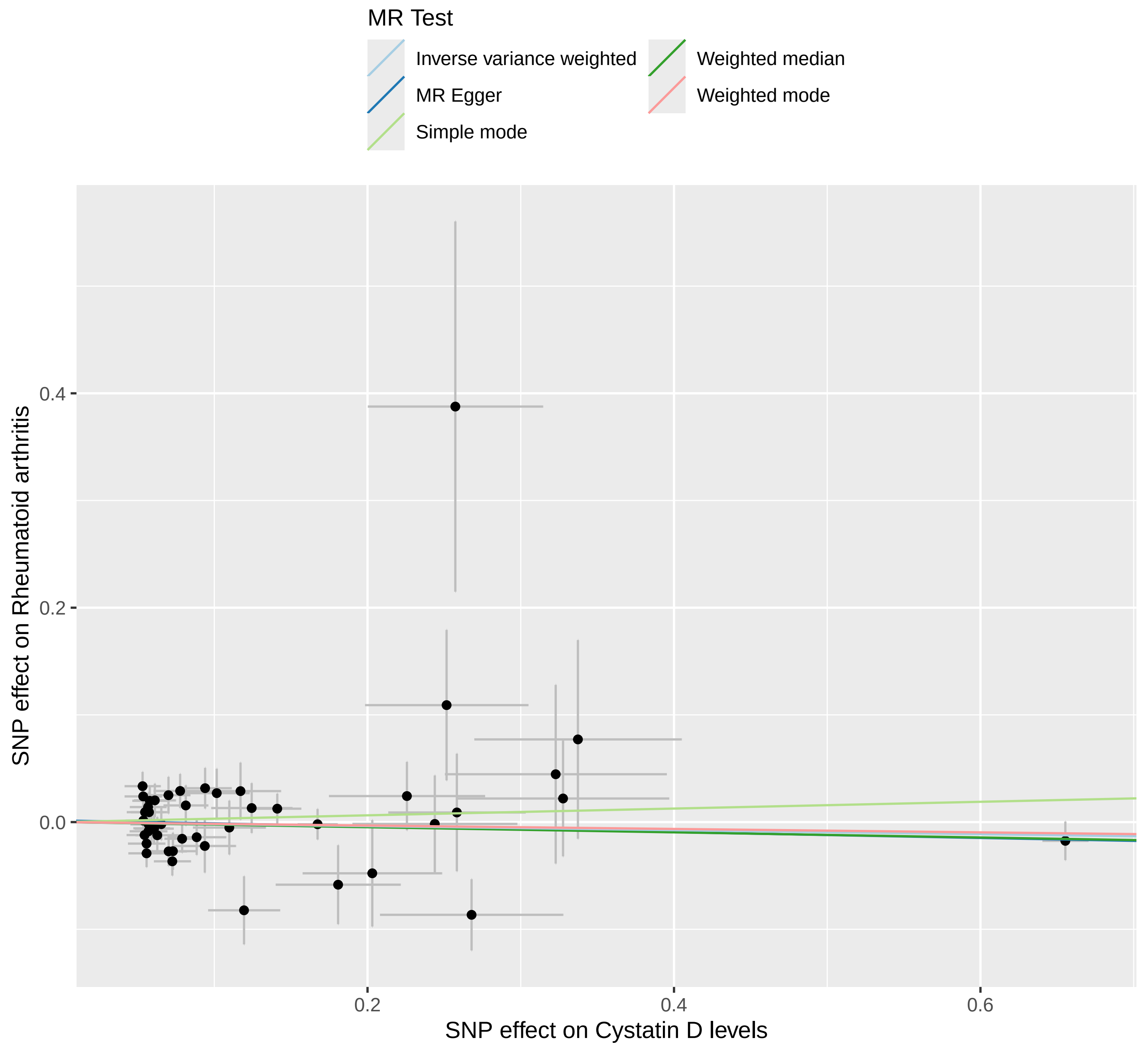
MR Test
Inverse variance weighted
MR Egger
Simple mode
Weighted median
Weighted mode
0.4
0.2
0.0
0.2
0.4
0.6
SNP effect on Rheumatoid arthritis
SNP effect on Cystatin D levels

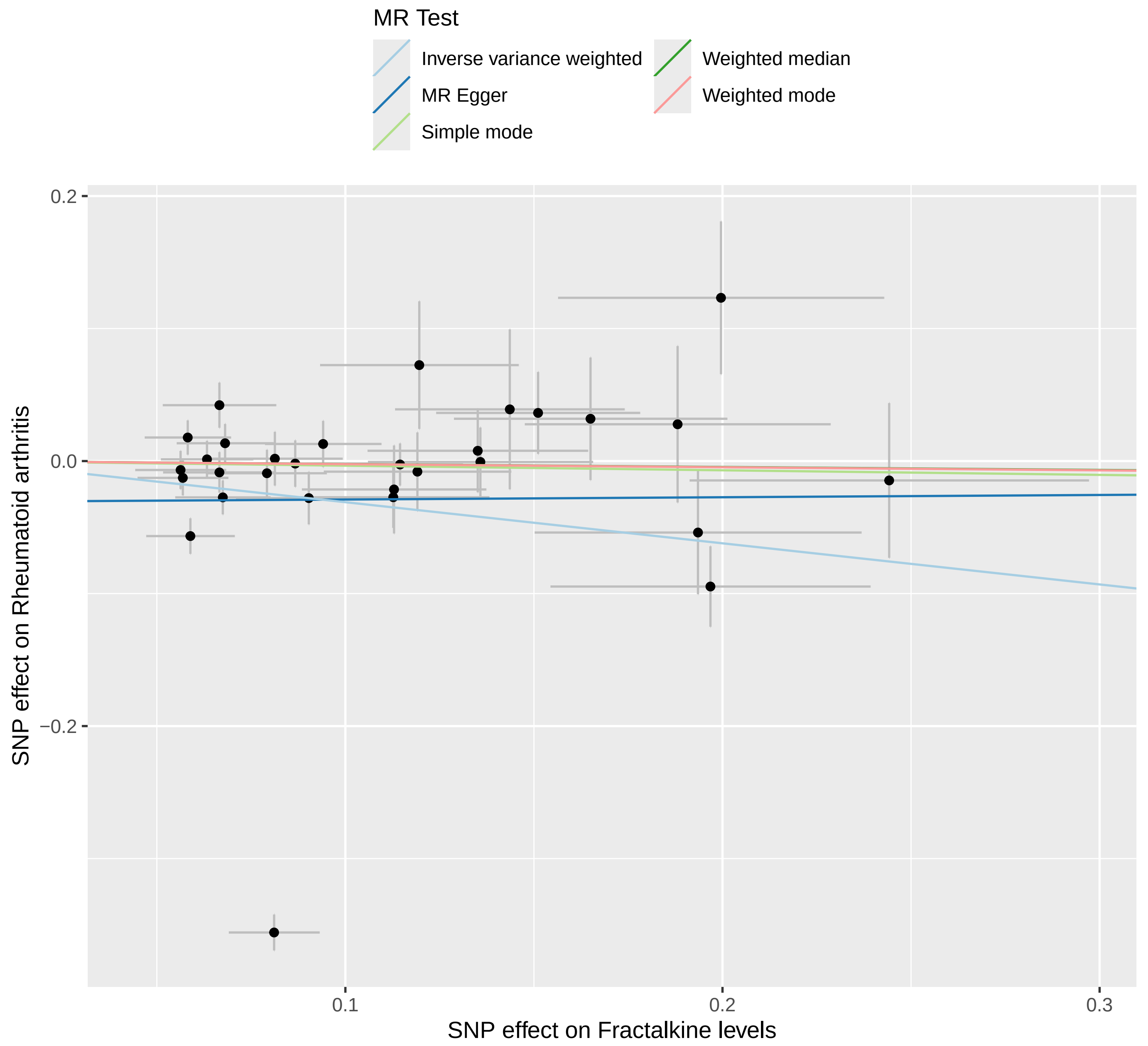
MR Test
Inverse variance weighted
MR Egger
Simple mode
Weighted median
Weighted mode
0.2
0.0
−0.2
0.1
0.2
0.3
SNP effect on Rheumatoid arthritis
SNP effect on Fractalkine levels

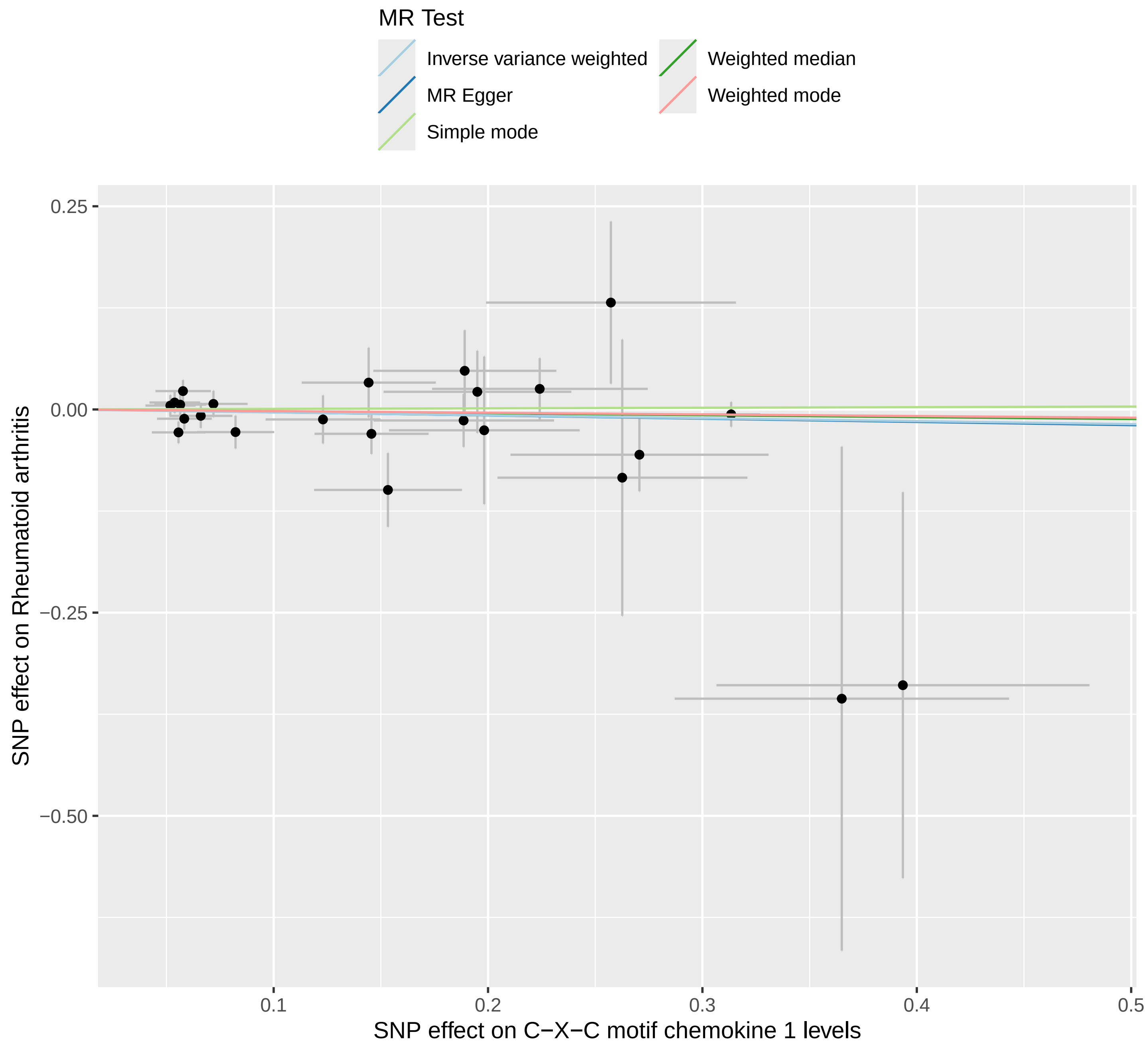
MR Test
Inverse variance weighted
MR Egger
Simple mode
Weighted median
Weighted mode
0.25
0.00
−0.25
−0.50
0.1
0.2
0.3
0.4
0.5
SNP effect on Rheumatoid arthritis
SNP effect on C−X−C motif chemokine 1 levels

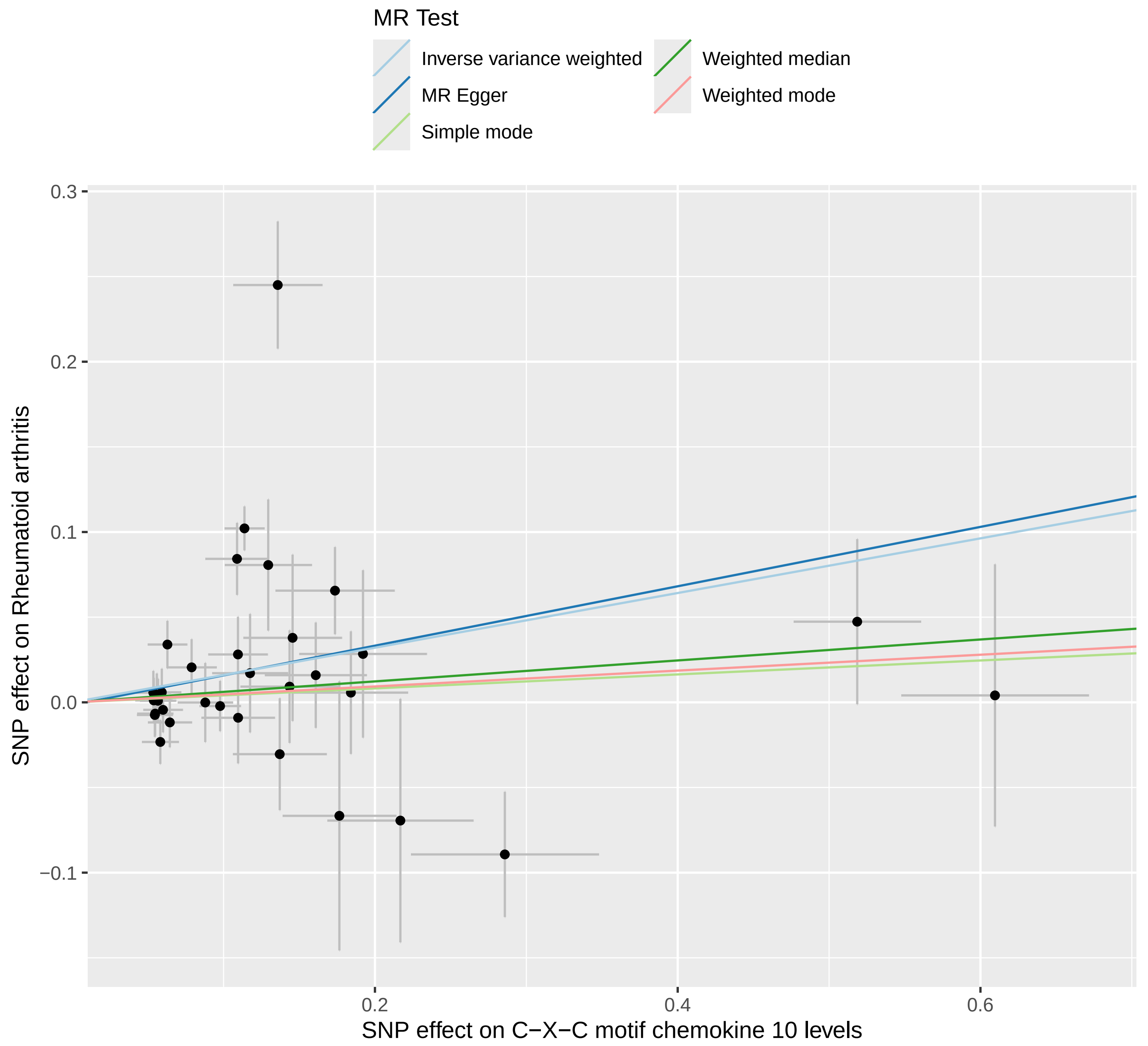

MR Test
Inverse variance weighted
MR Egger
Simple mode
Weighted median
Weighted mode
0.3
0.2
0.1
0.0
−0.1
0.2
0.4
0.6
SNP effect on Rheumatoid arthritis
SNP effect on C−X−C motif chemokine 10 levels

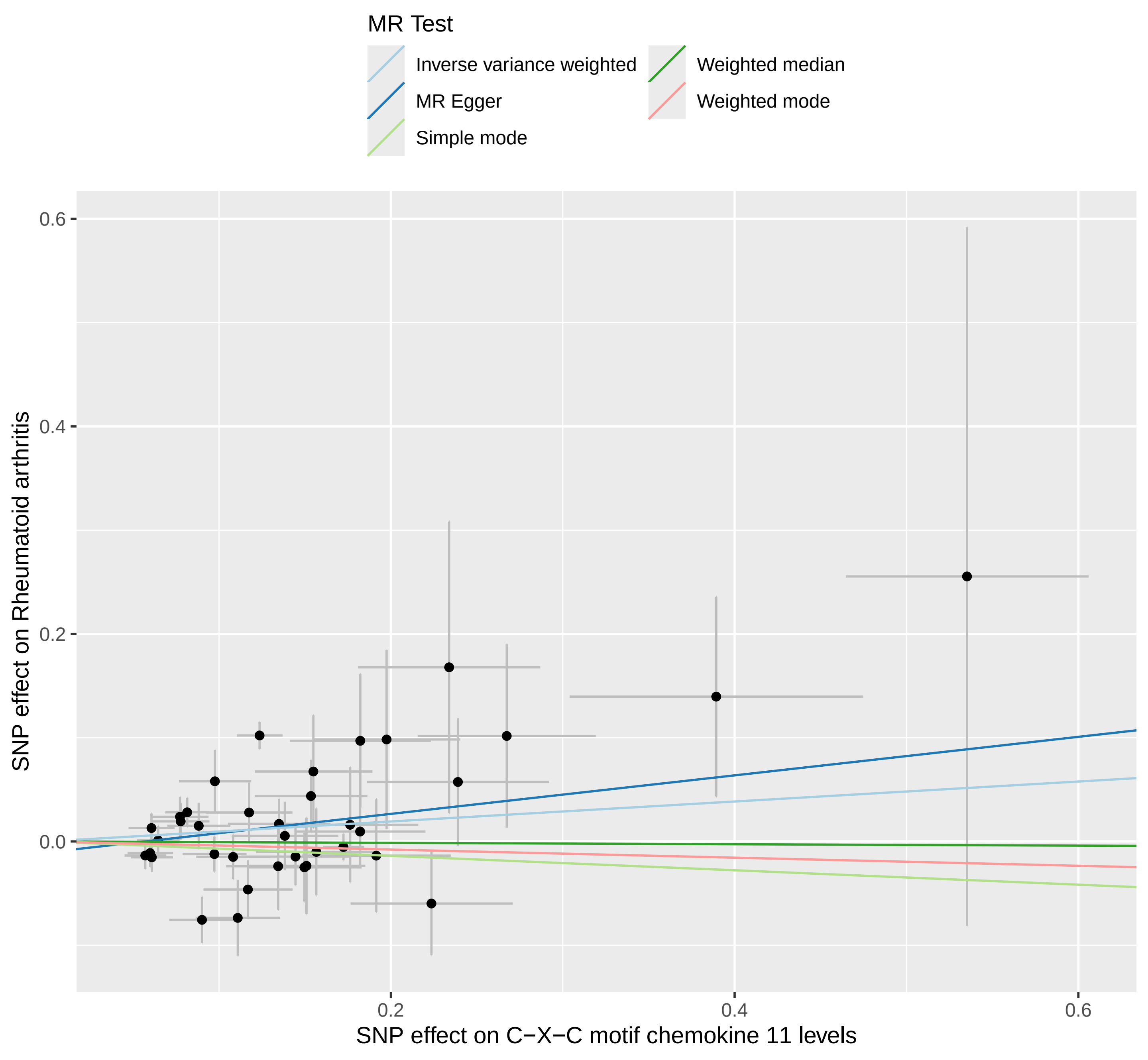

MR Test
Inverse variance weighted
MR Egger
Simple mode
Weighted median
Weighted mode
0.6
0.4
0.2
0.0
0.2
0.4
0.6
SNP effect on Rheumatoid arthritis
SNP effect on C−X−C motif chemokine 11 levels

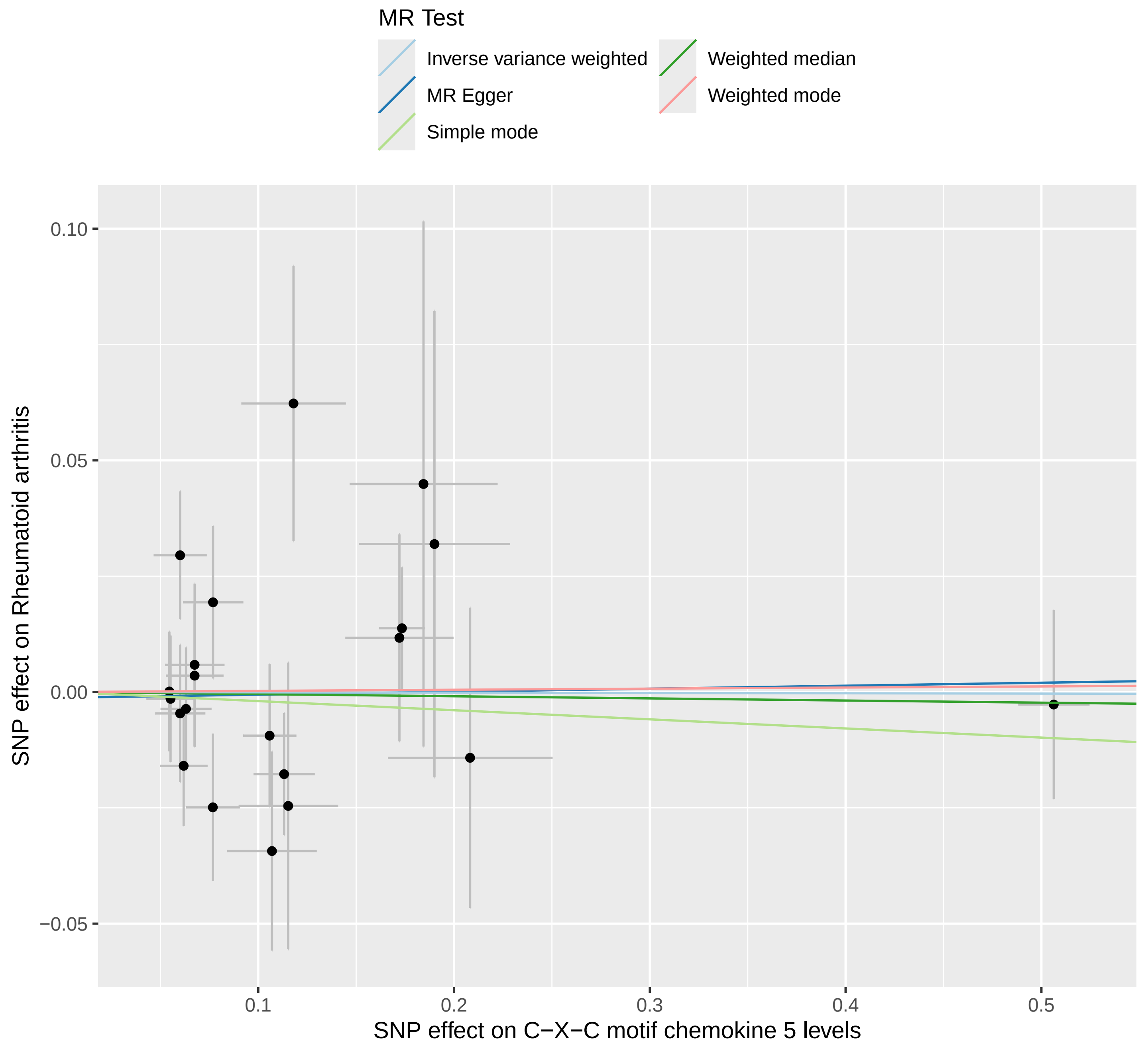
MR Test
Inverse variance weighted
MR Egger
Simple mode
Weighted median
Weighted mode
0.10
0.05
0.00
−0.05
0.1
0.2
0.3
0.4
0.5
SNP effect on Rheumatoid arthritis
SNP effect on C−X−C motif chemokine 5 levels

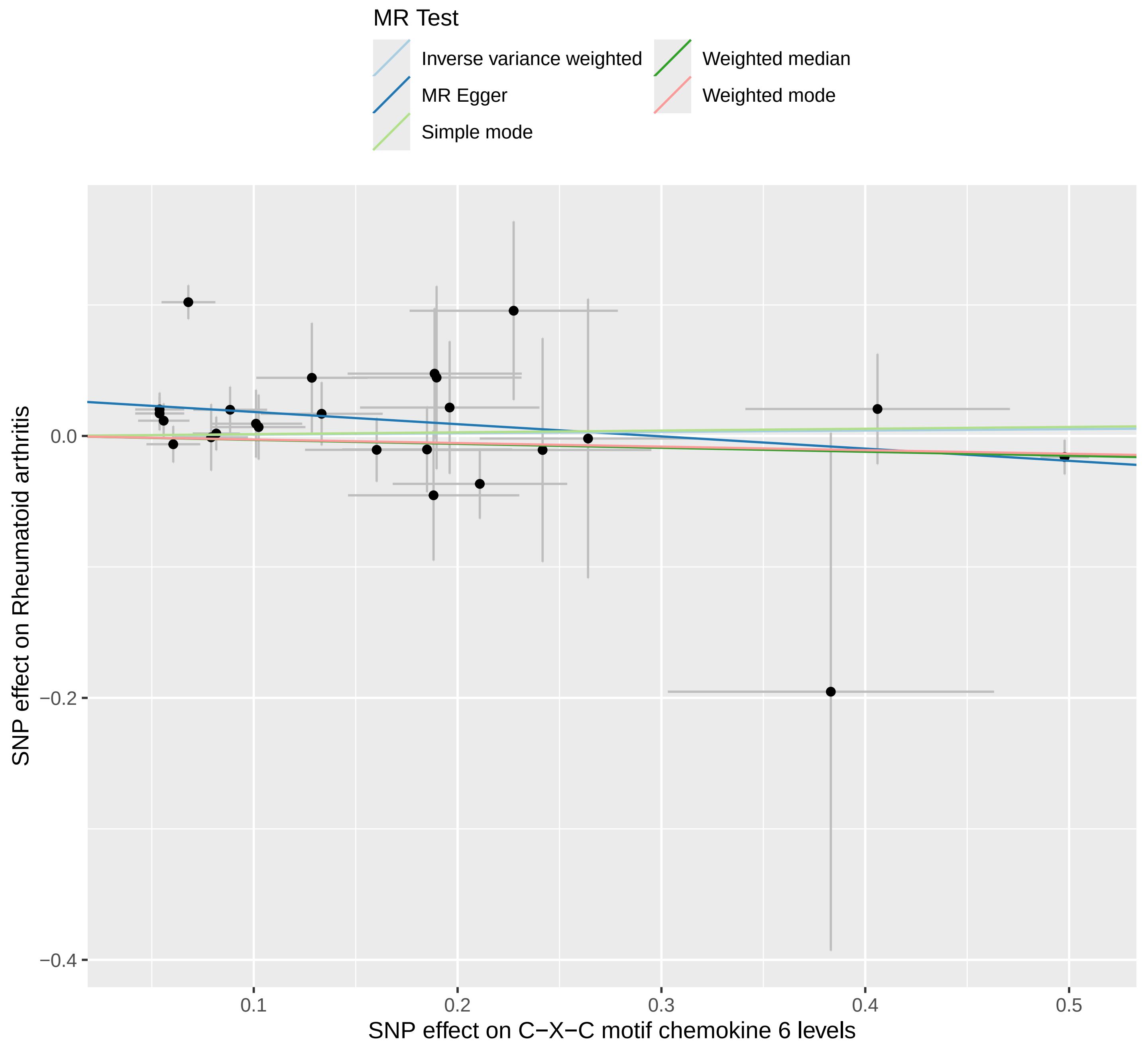

MR Test
Inverse variance weighted
MR Egger
Simple mode
Weighted median
Weighted mode
0.0
−0.2
−0.4
0.1
0.2
0.3
0.4
0.5
SNP effect on C−X−C motif chemokine 6 levels
SNP effect on Rheumatoid arthritis

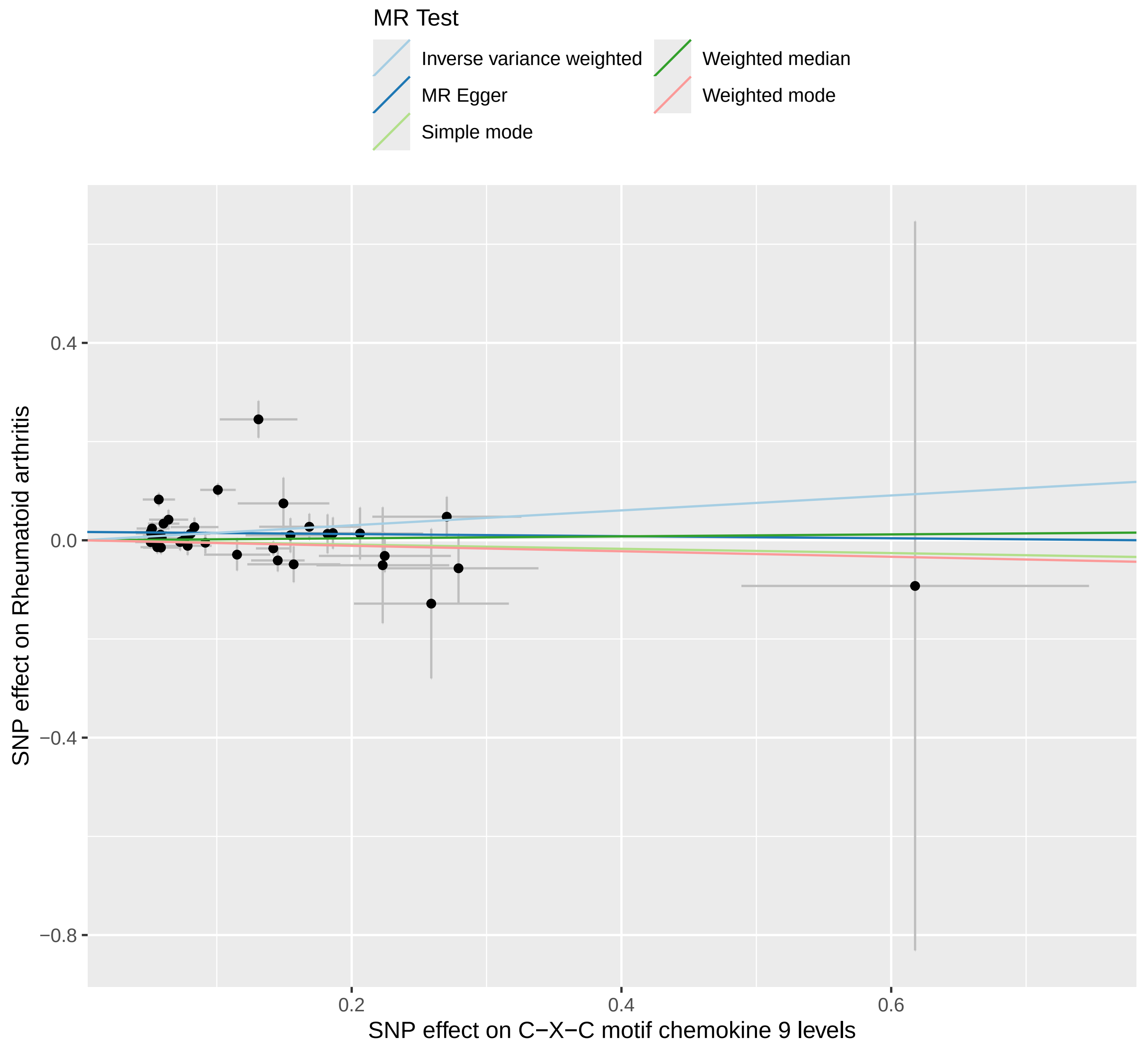
MR Test
Inverse variance weighted
MR Egger
Simple mode
Weighted median
Weighted mode
0.4
0.0
−0.4
−0.8
0.2
0.4
0.6
SNP effect on Rheumatoid arthritis
SNP effect on C−X−C motif chemokine 9 levels

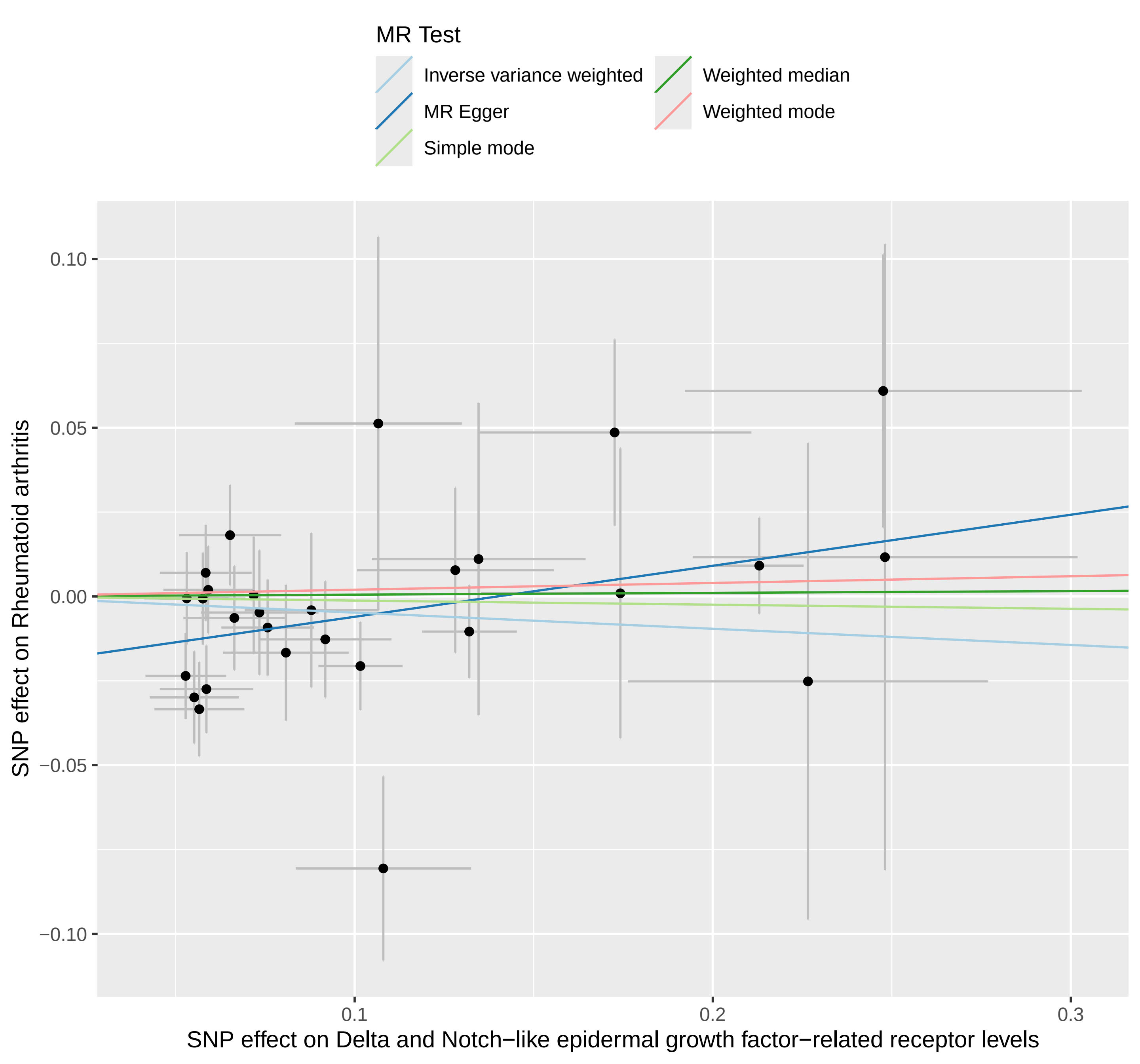
MR Test
Inverse variance weighted
MR Egger
Simple mode
Weighted median
Weighted mode
SNP effect on Rheumatoid arthritis
0.10
0.05
0.00
−0.05
−0.10
0.1
0.2
0.3
SNP effect on Delta and Notch−like epidermal growth factor−related receptor levels

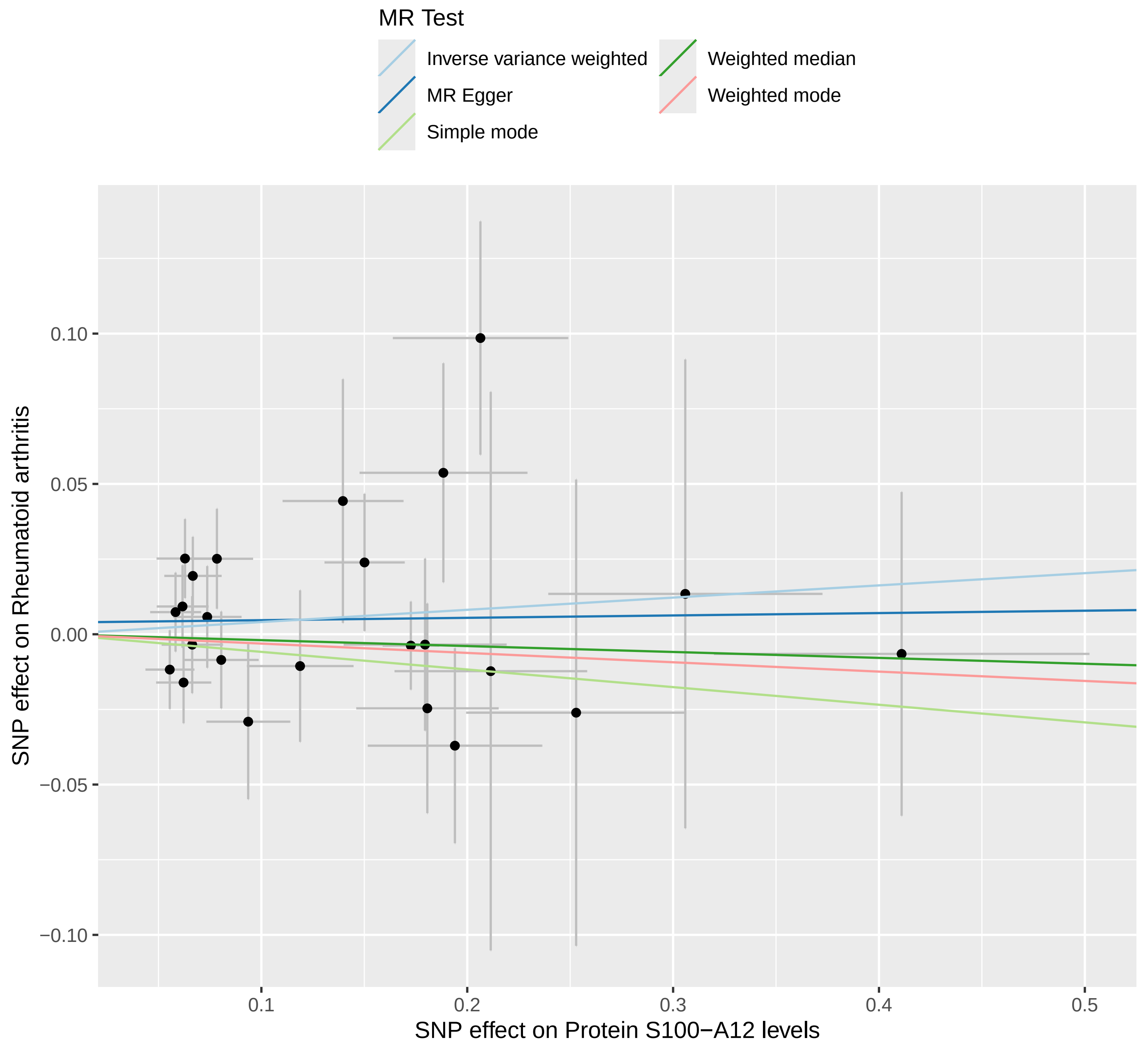
MR Test
Inverse variance weighted
MR Egger
Simple mode
Weighted median
Weighted mode
0.10
0.05
0.00
−0.05
−0.10
SNP effect on Rheumatoid arthritis
0.1
0.2
0.3
0.4
0.5
SNP effect on Protein S100−A12 levels

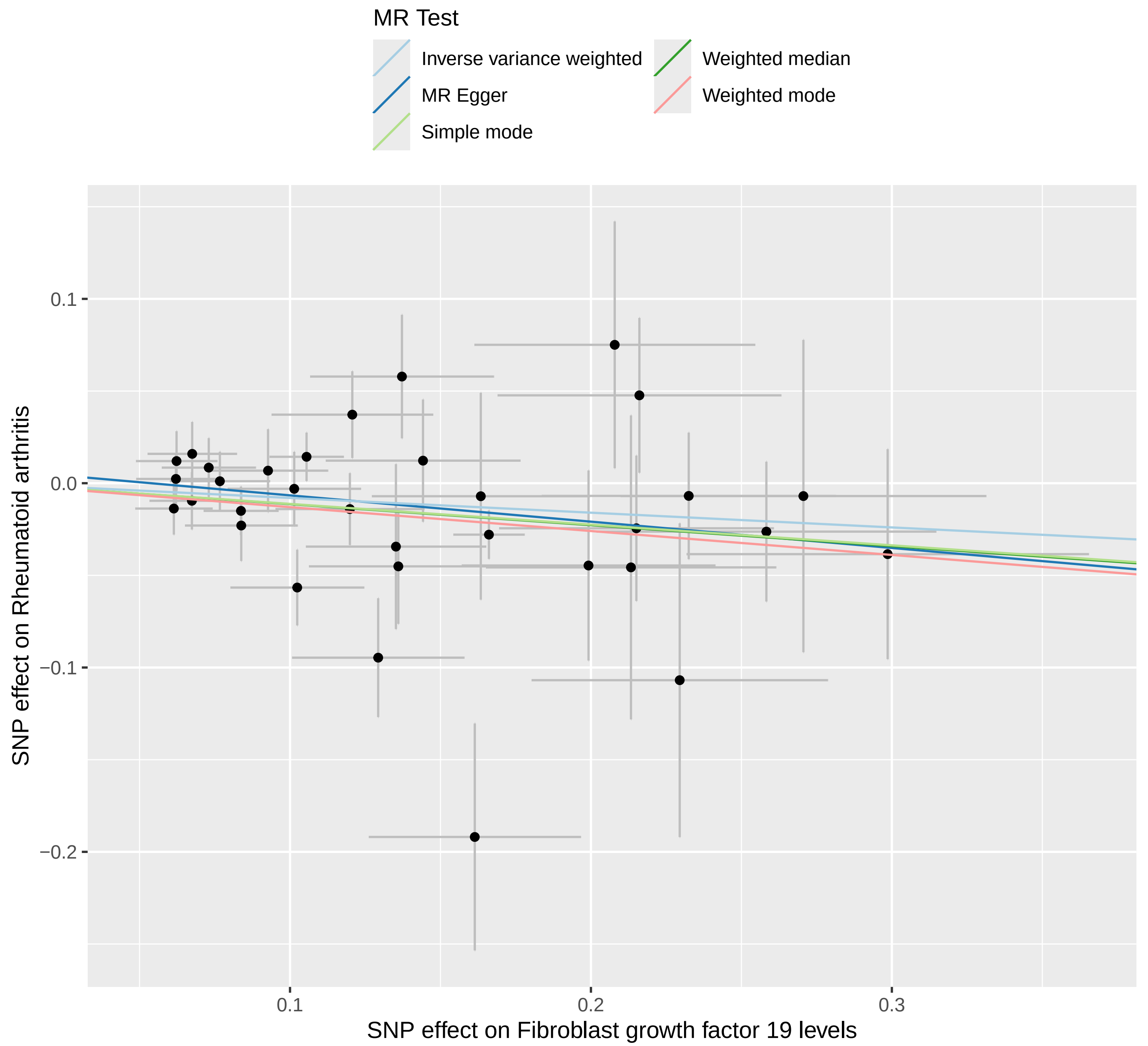
MR Test
Inverse variance weighted
Weighted median
MR Egger
Weighted mode
Simple mode
0.1
0.0
−0.1
−0.2
0.1
0.2
0.3
SNP effect on Rheumatoid arthritis
SNP effect on Fibroblast growth factor 19 levels

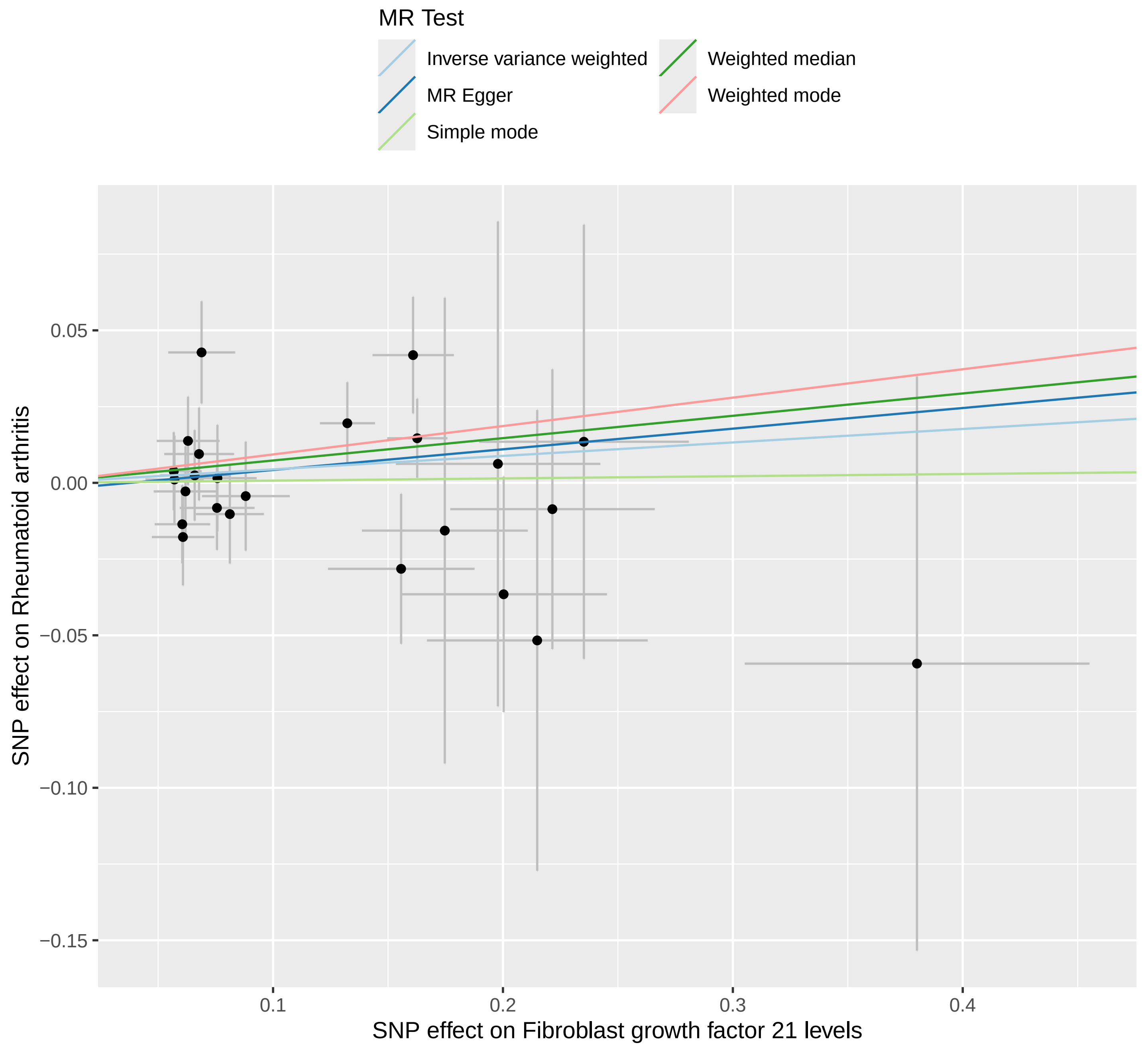

MR Test
Inverse variance weighted
MR Egger
Simple mode
Weighted median
Weighted mode
0.05
0.00
−0.05
−0.10
−0.15
0.1
0.2
0.3
0.4
SNP effect on Rheumatoid arthritis
SNP effect on Fibroblast growth factor 21 levels

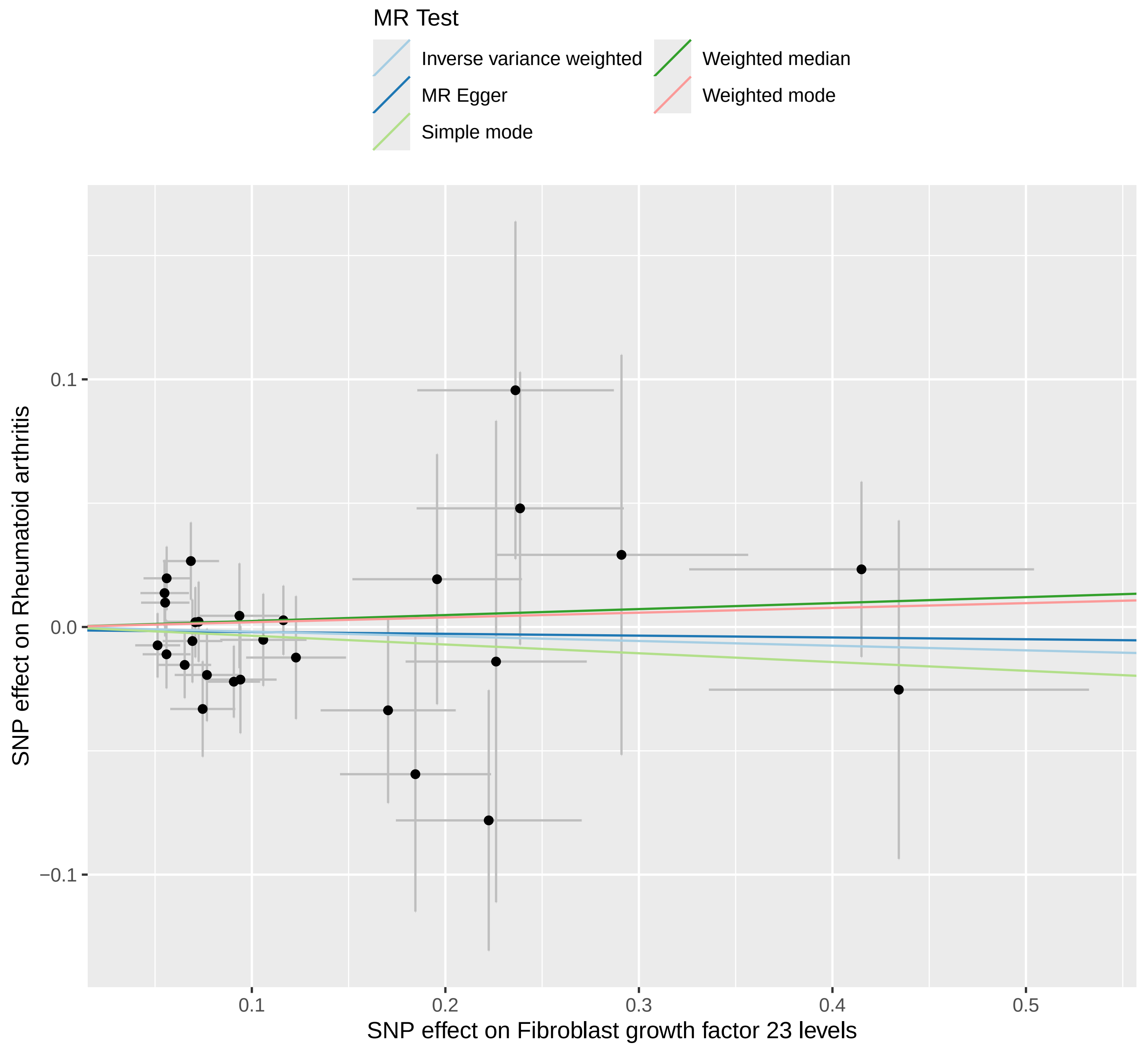
MR Test
Inverse variance weighted
MR Egger
Simple mode
Weighted median
Weighted mode
SNP effect on Rheumatoid arthritis
0.1
0.0
−0.1
0.1
0.2
0.3
0.4
0.5
SNP effect on Fibroblast growth factor 23 levels

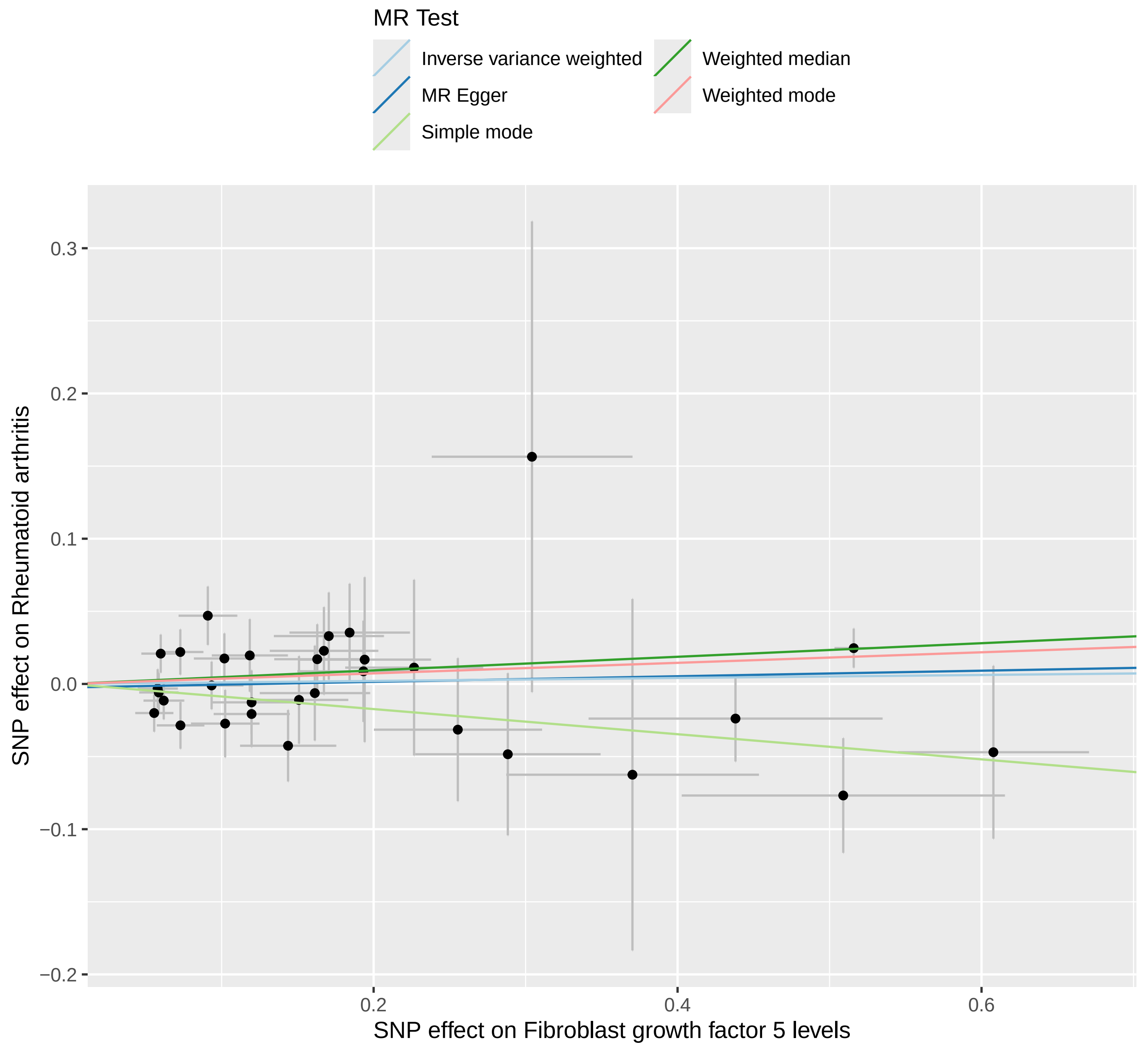
MR Test
Inverse variance weighted
MR Egger
Simple mode
Weighted median
Weighted mode
0.3
0.2
0.1
0.0
−0.1
−0.2
SNP effect on Rheumatoid arthritis
0.2
0.4
0.6
SNP effect on Fibroblast growth factor 5 levels

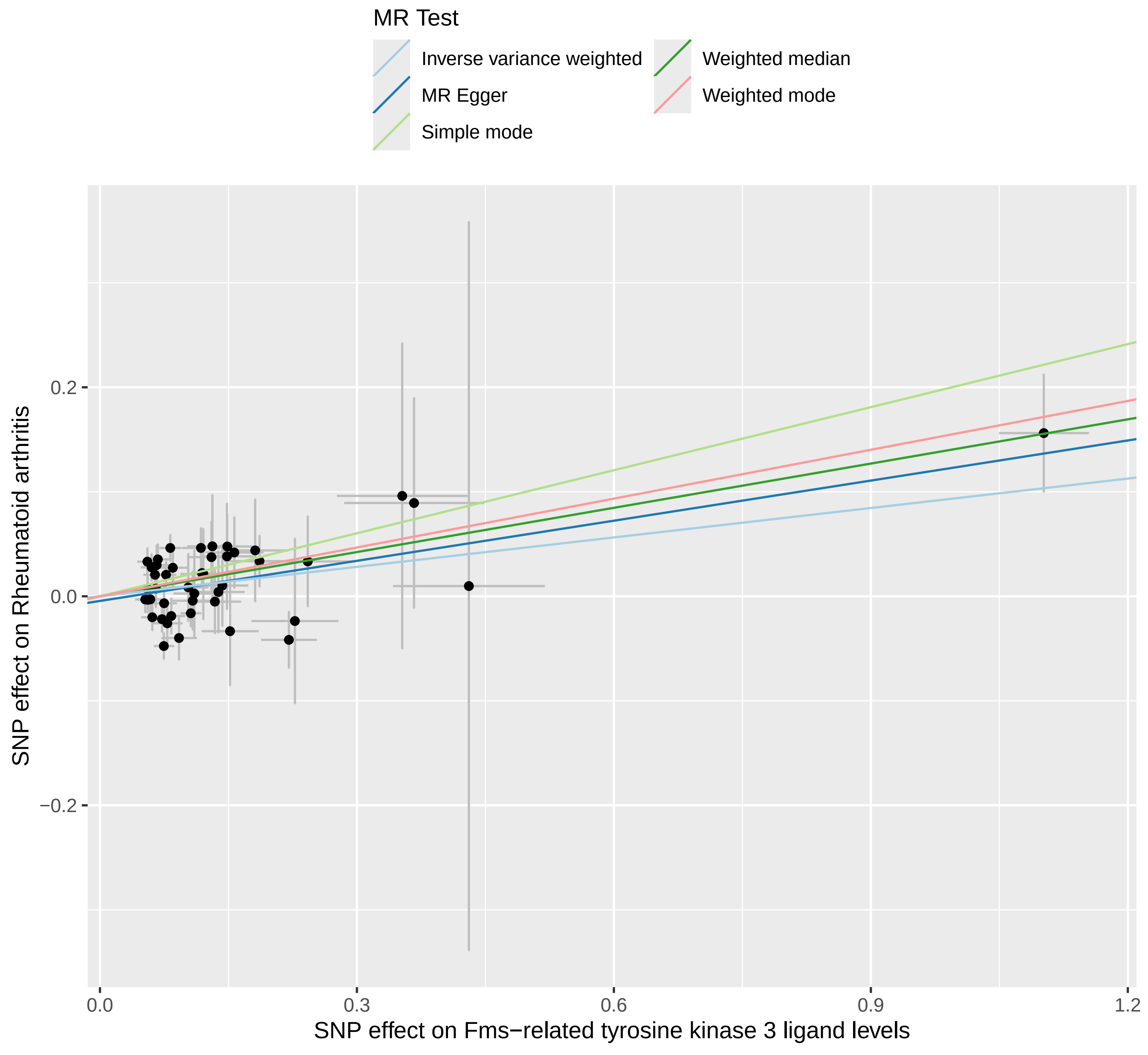

MR Test
Inverse variance weighted
MR Egger
Simple mode
Weighted median
Weighted mode
0.2
0.0
−0.2
0.0
0.3
0.6
0.9
1.2
SNP effect on Rheumatoid arthritis
SNP effect on Fms−related tyrosine kinase 3 ligand levels

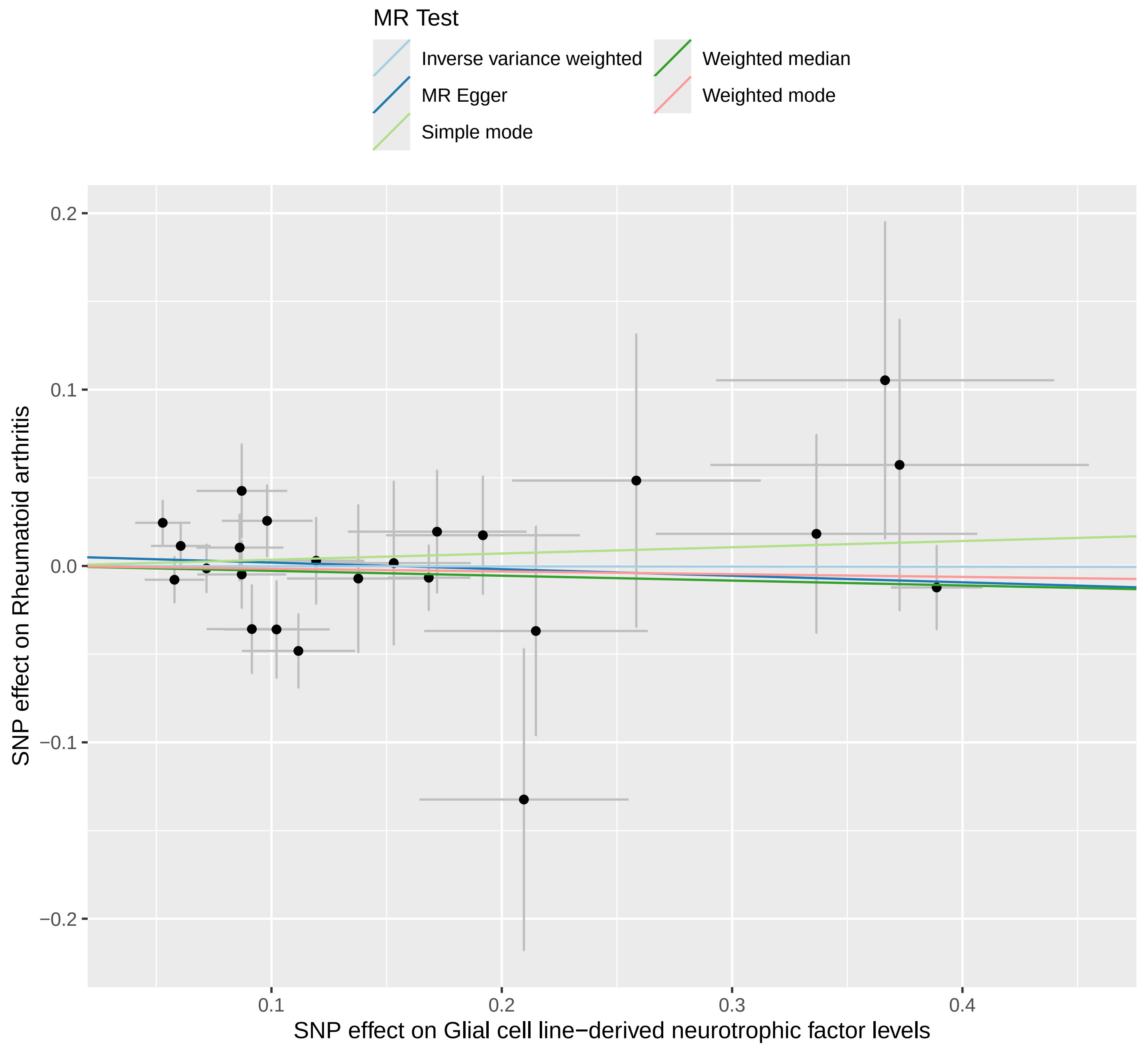
MR Test
Inverse variance weighted
MR Egger
Simple mode
Weighted median
Weighted mode
SNP effect on Rheumatoid arthritis
0.2
0.1
0.0
−0.1
−0.2
0.1
0.2
0.3
0.4
SNP effect on Glial cell line−derived neurotrophic factor levels

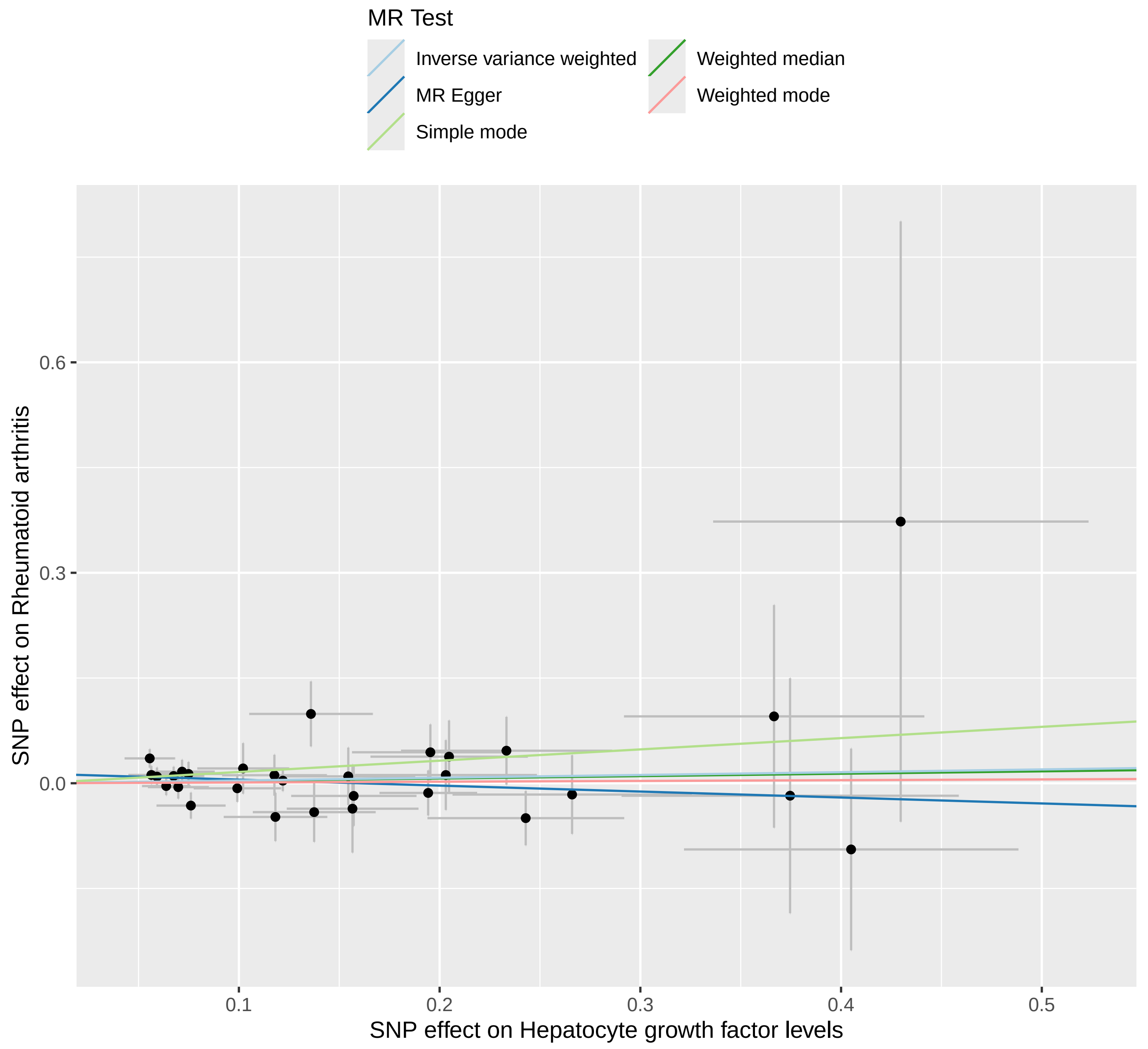

MR Test
Inverse variance weighted
MR Egger
Simple mode
Weighted median
Weighted mode
SNP effect on Rheumatoid arthritis
0.6
0.3
0.0
0.1
0.2
0.3
0.4
0.5
SNP effect on Hepatocyte growth factor levels

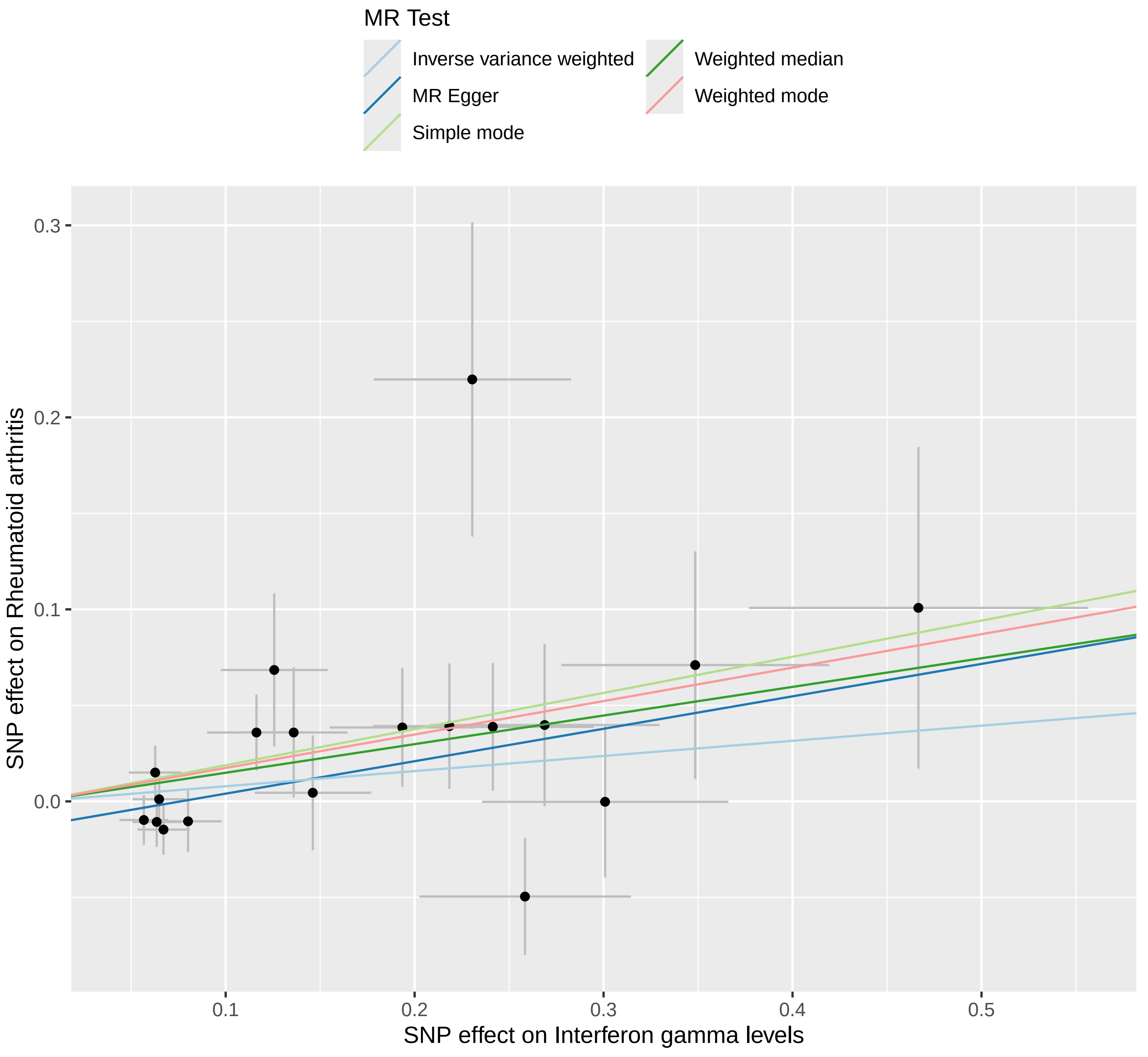
MR Test
Inverse variance weighted
MR Egger
Simple mode
Weighted median
Weighted mode
SNP effect on Rheumatoid arthritis
0.3
0.2
0.1
0.0
0.1
0.2
0.3
0.4
0.5
SNP effect on Interferon gamma levels

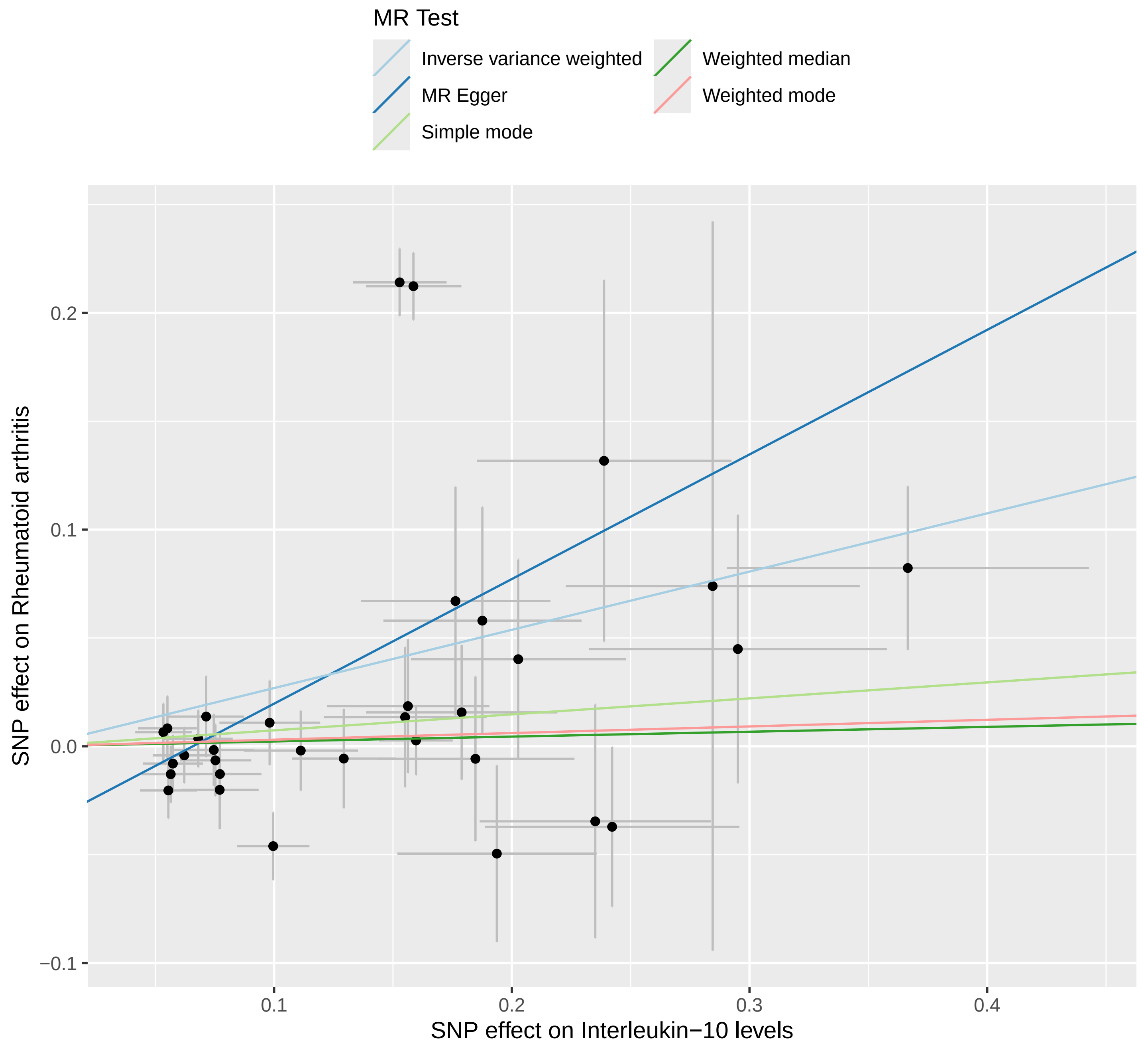
MR Test
Inverse variance weighted
MR Egger
Simple mode
Weighted median
Weighted mode
0.2
0.1
0.0
−0.1
0.1
0.2
0.3
0.4
SNP effect on Rheumatoid arthritis
SNP effect on Interleukin−10 levels

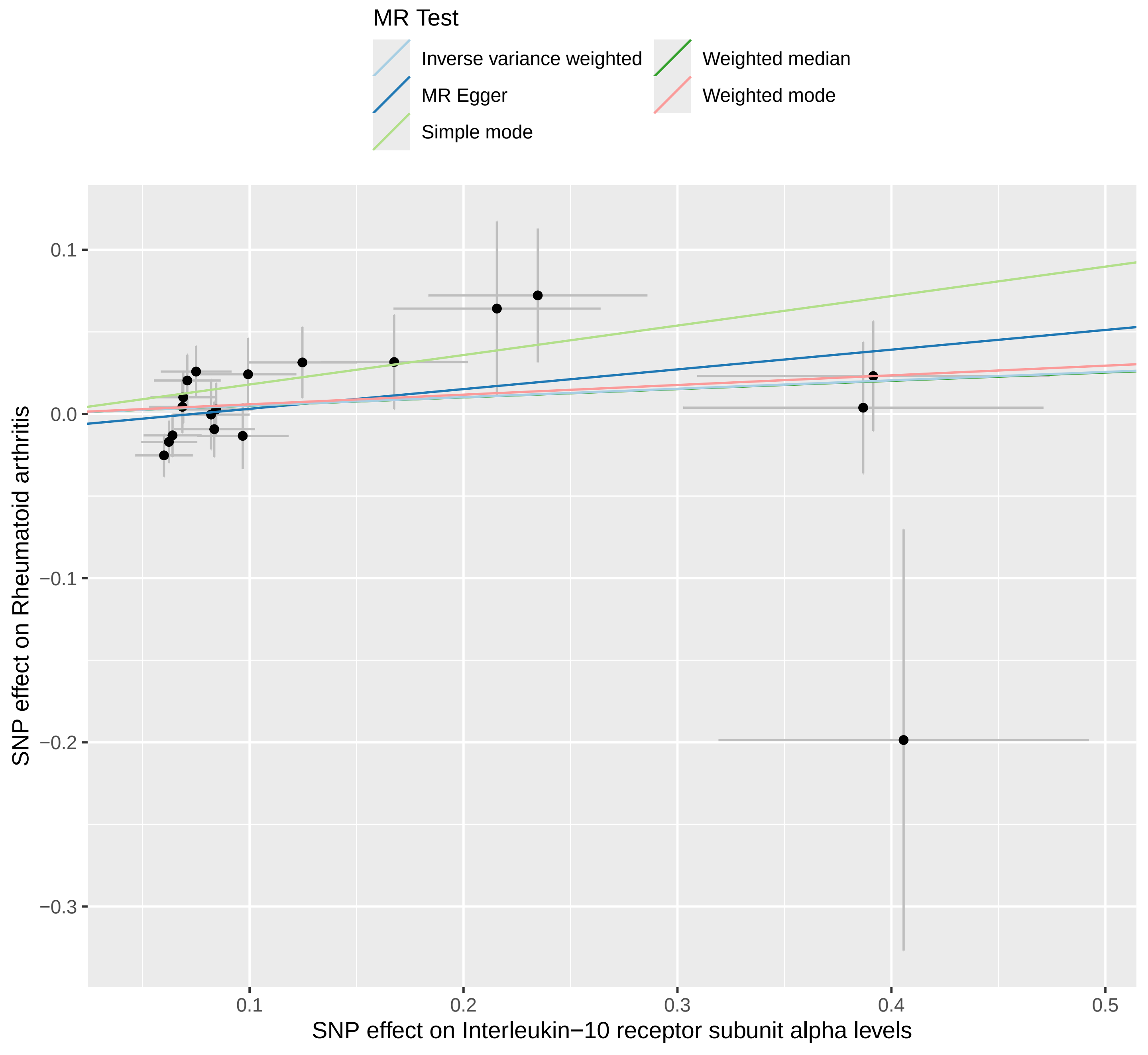
MR Test
Inverse variance weighted
MR Egger
Simple mode
Weighted median
Weighted mode
0.1
0.0
−0.1
−0.2
−0.3
SNP effect on Rheumatoid arthritis
0.1
0.2
0.3
0.4
0.5
SNP effect on Interleukin−10 receptor subunit alpha levels

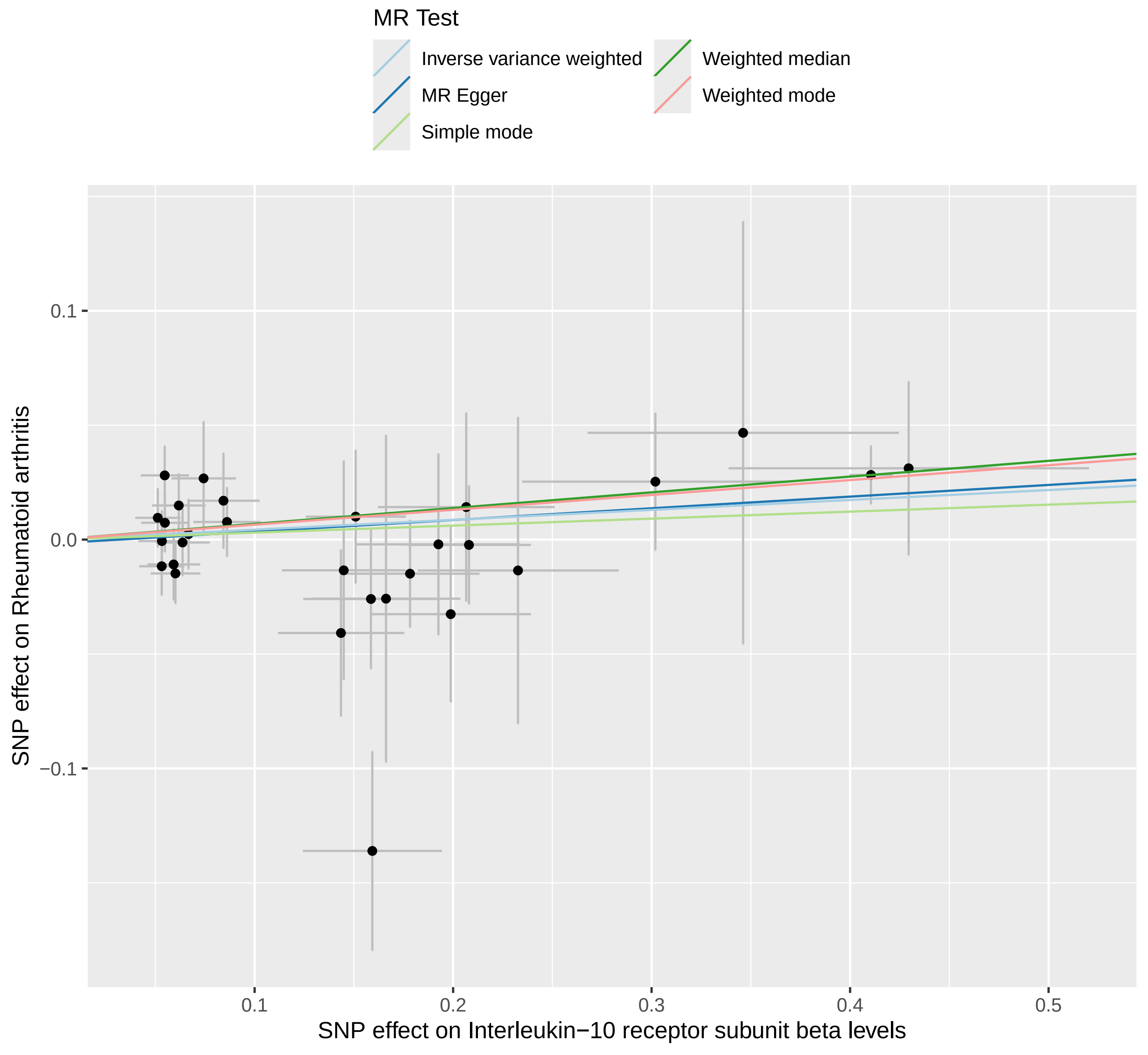
MR Test
Inverse variance weighted
MR Egger
Simple mode
Weighted median
Weighted mode
0.1
0.0
−0.1
SNP effect on Rheumatoid arthritis
0.1
0.2
0.3
0.4
0.5
SNP effect on Interleukin−10 receptor subunit beta levels

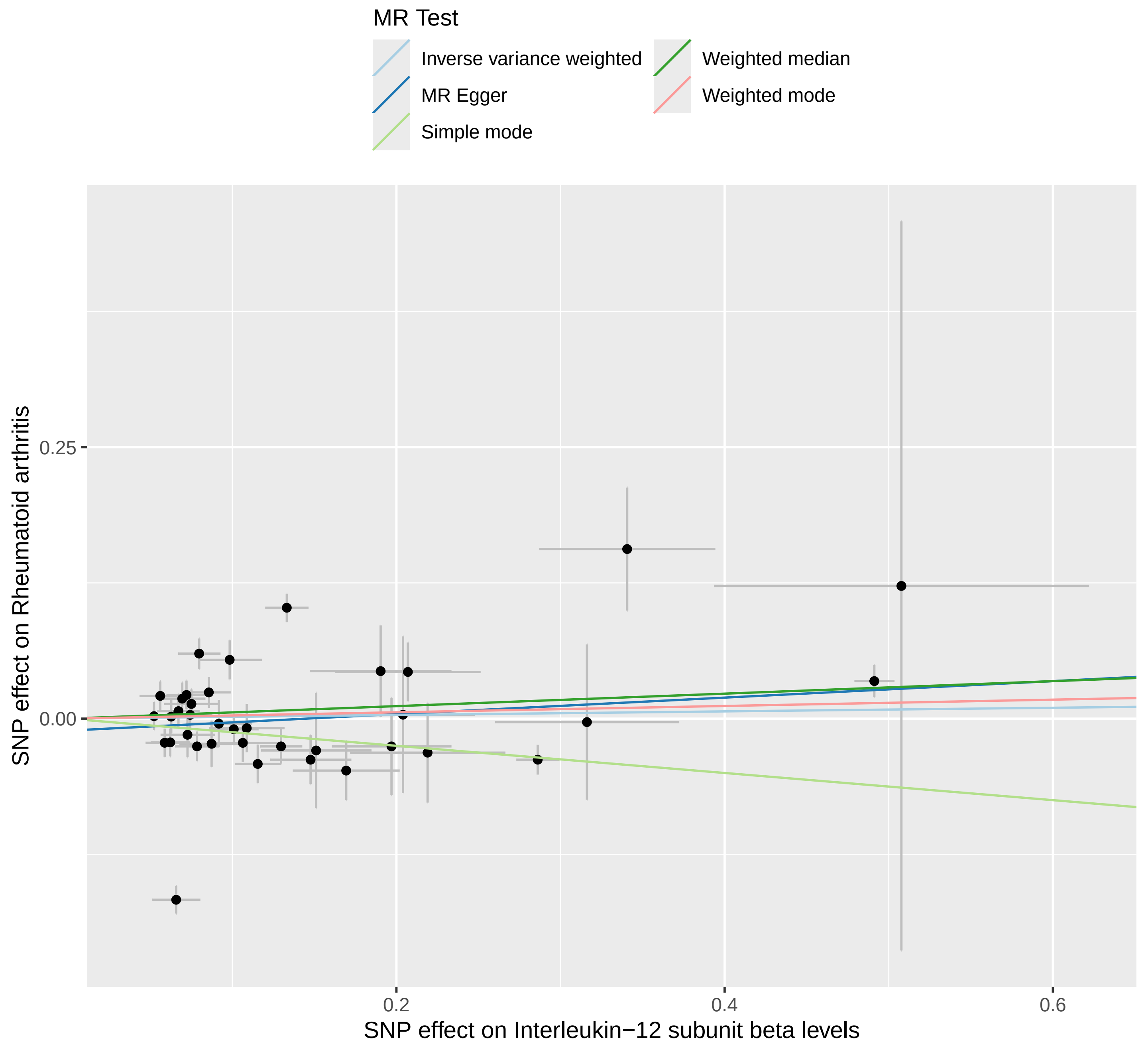
MR Test
Inverse variance weighted
MR Egger
Simple mode
Weighted median
Weighted mode
SNP effect on Rheumatoid arthritis
0.25
0.00
0.2
0.4
0.6
SNP effect on Interleukin−12 subunit beta levels

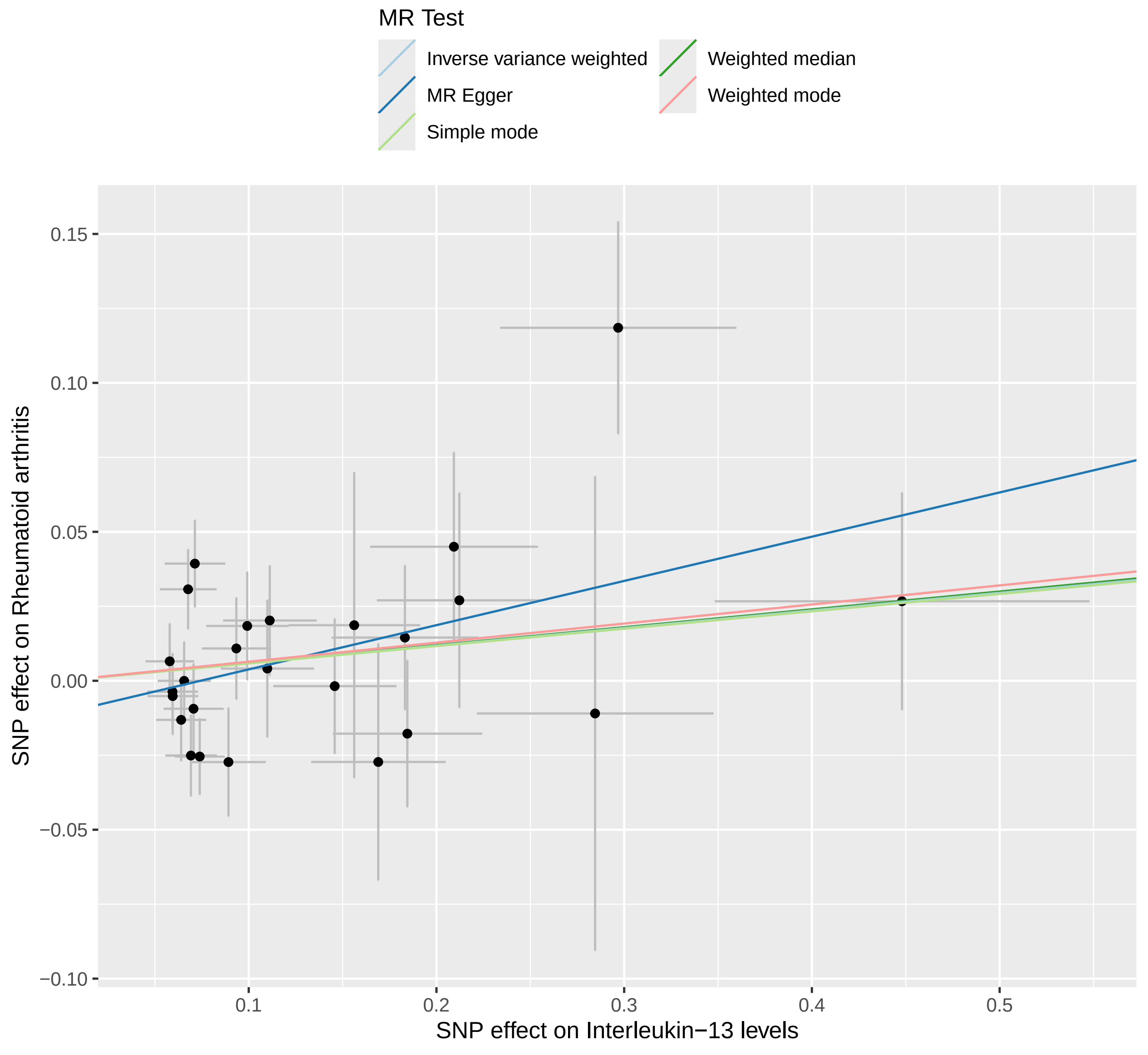

MR Test
Inverse variance weighted
MR Egger
Simple mode
Weighted median
Weighted mode
0.15
0.10
0.05
0.00
−0.05
−0.10
0.1
0.2
0.3
0.4
0.5
SNP effect on Rheumatoid arthritis
SNP effect on Interleukin−13 levels

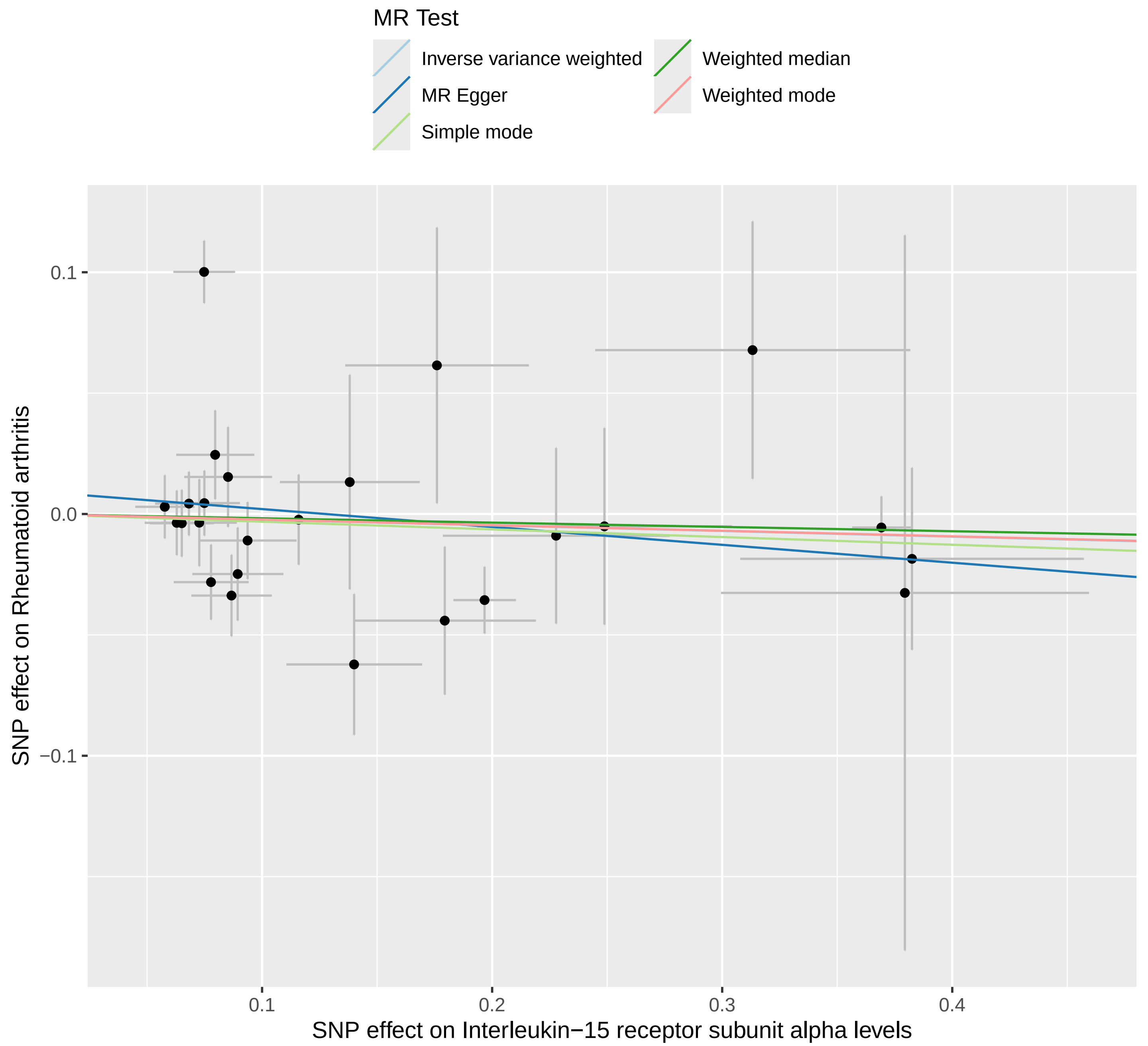
MR Test
Inverse variance weighted
MR Egger
Simple mode
Weighted median
Weighted mode
0.1
0.0
−0.1
0.1
0.2
0.3
0.4
SNP effect on Rheumatoid arthritis
SNP effect on Interleukin−15 receptor subunit alpha levels

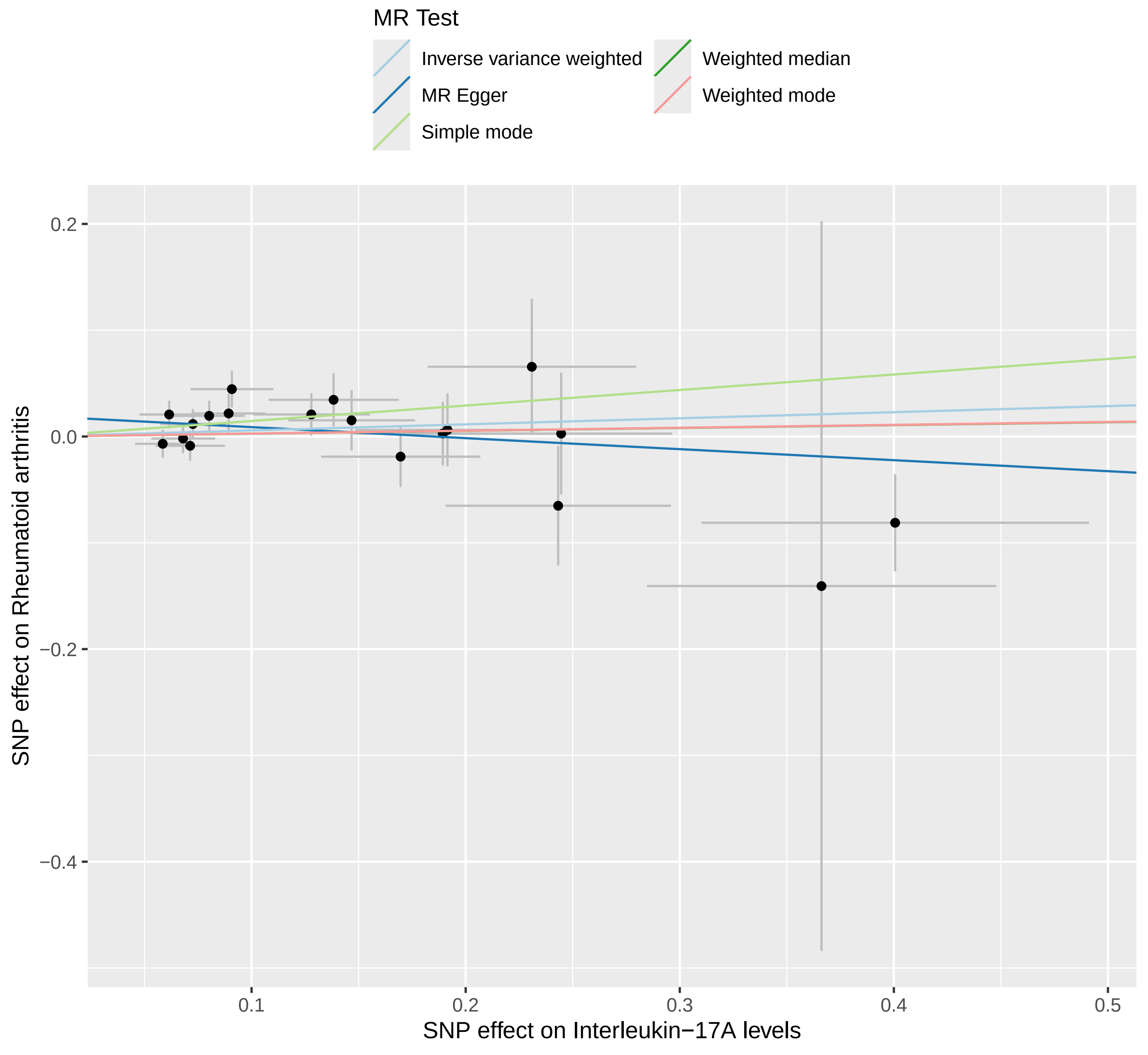
MR Test
Inverse variance weighted
MR Egger
Simple mode
Weighted median
Weighted mode
0.2
0.0
−0.2
−0.4
0.1
0.2
0.3
0.4
0.5
SNP effect on Rheumatoid arthritis
SNP effect on Interleukin−17A levels

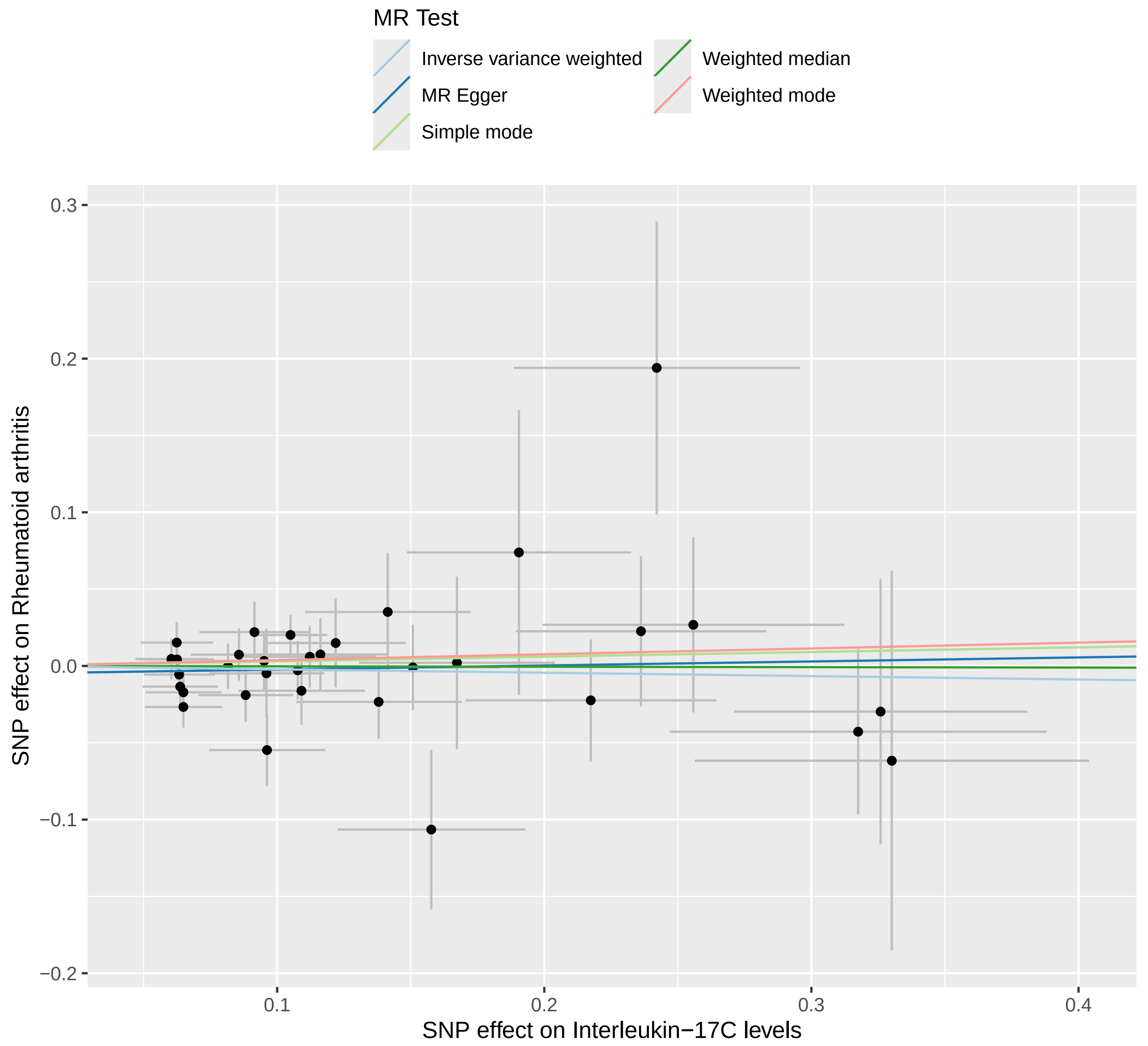

MR Test
Inverse variance weighted
MR Egger
Simple mode
Weighted median
Weighted mode
SNP effect on Rheumatoid arthritis
0.3
0.2
0.1
0.0
−0.1
−0.2
0.1
0.2
0.3
0.4
SNP effect on Interleukin−17C levels

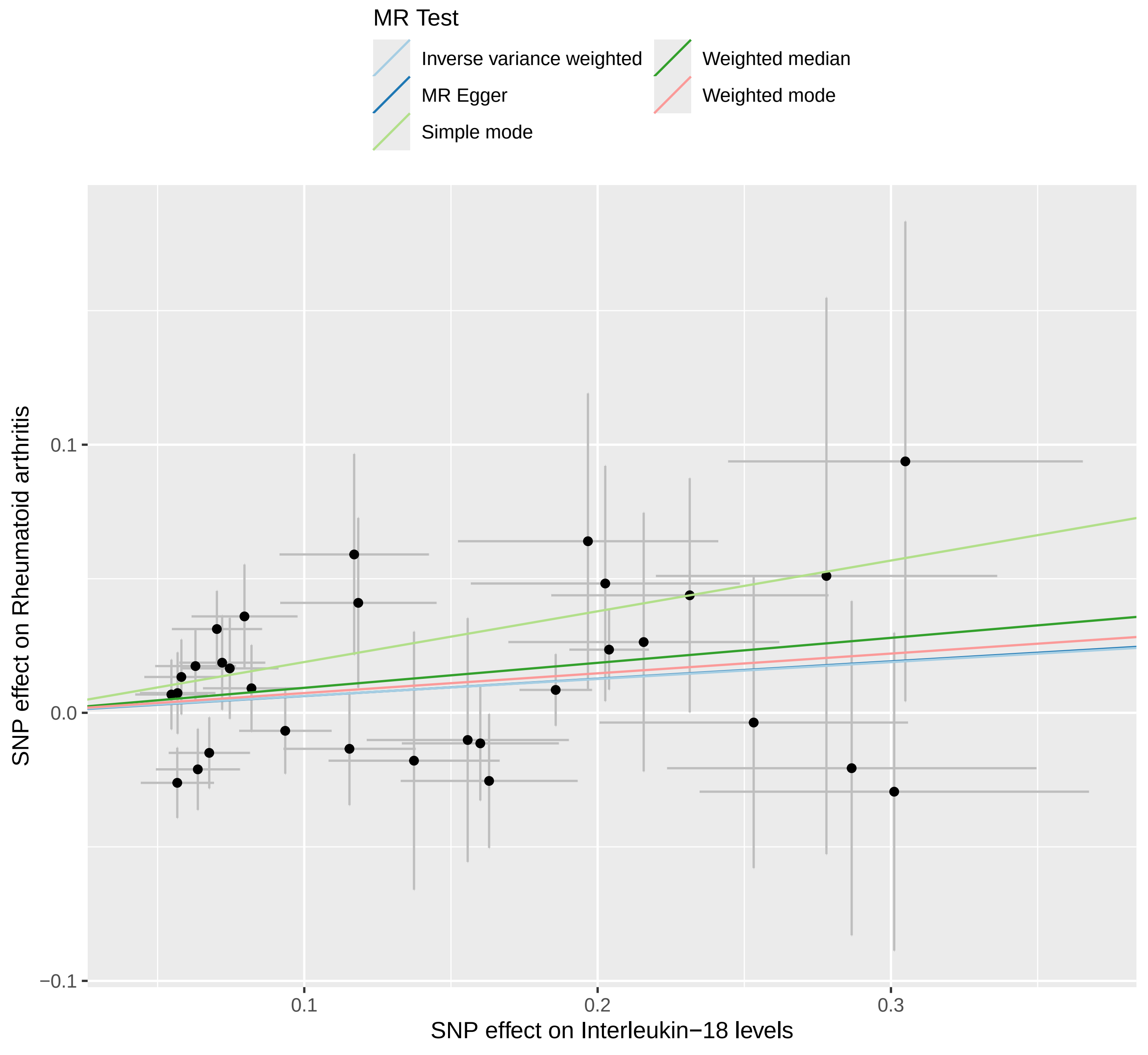
MR Test
Inverse variance weighted
MR Egger
Simple mode
Weighted median
Weighted mode
0.1
0.0
−0.1
0.1
0.2
0.3
SNP effect on Rheumatoid arthritis
SNP effect on Interleukin−18 levels

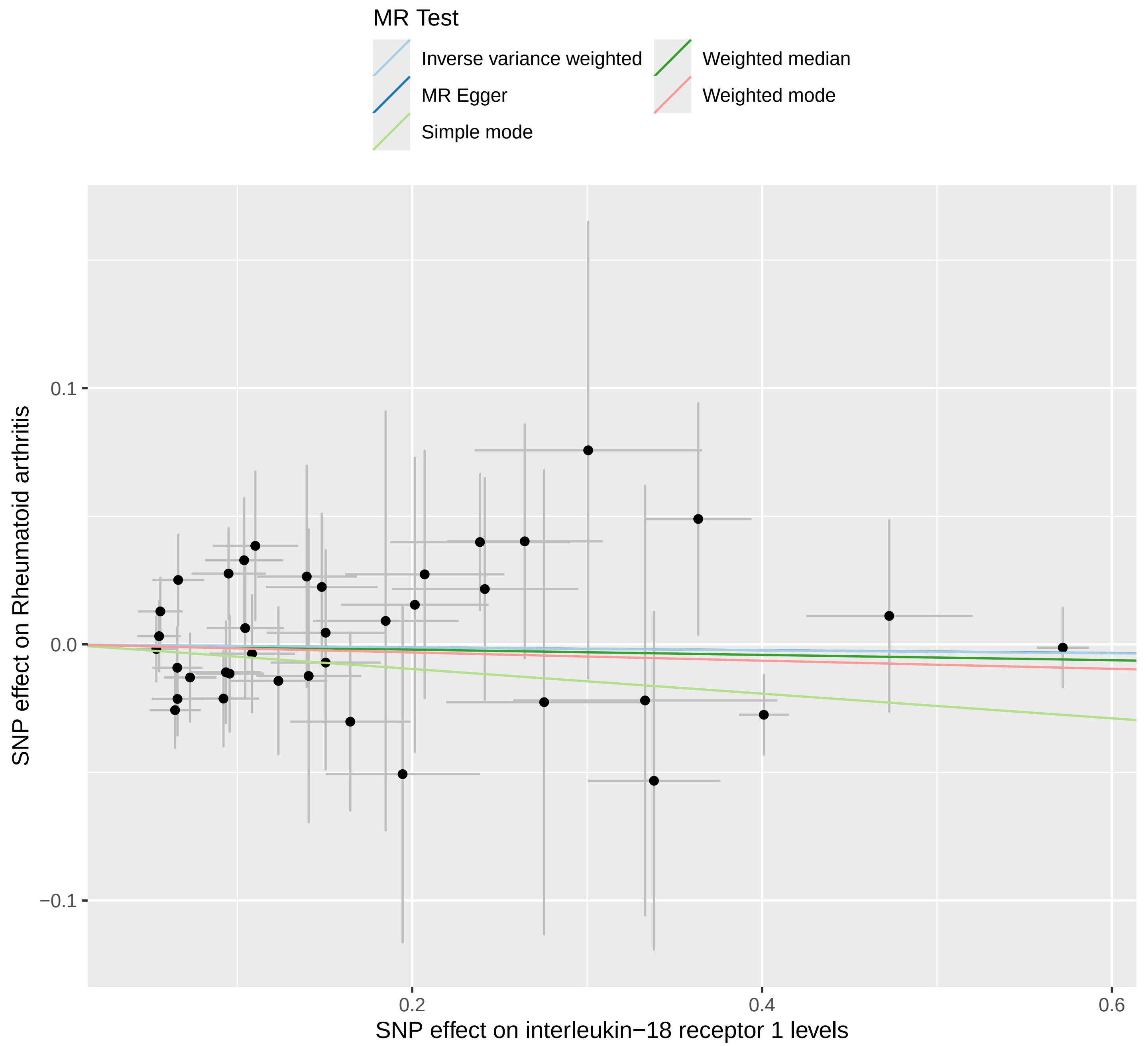
MR Test
Inverse variance weighted
MR Egger
Simple mode
Weighted median
Weighted mode
0.1
0.0
−0.1
0.2
0.4
0.6
SNP effect on Rheumatoid arthritis
SNP effect on interleukin−18 receptor 1 levels

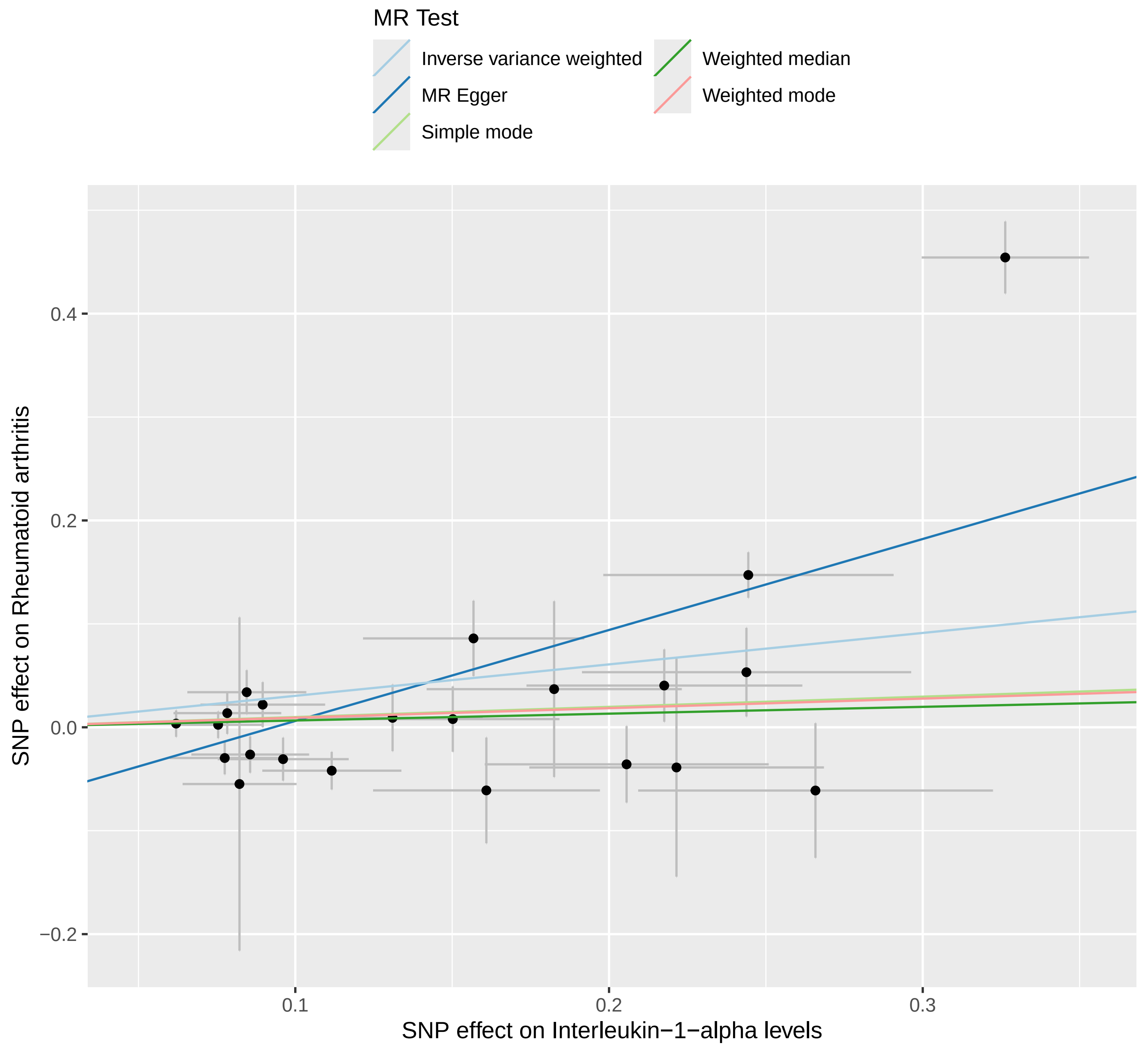

MR Test
Inverse variance weighted
MR Egger
Simple mode
Weighted median
Weighted mode
0.4
0.2
0.0
−0.2
0.1
0.2
0.3
SNP effect on Rheumatoid arthritis
SNP effect on Interleukin−1−alpha levels

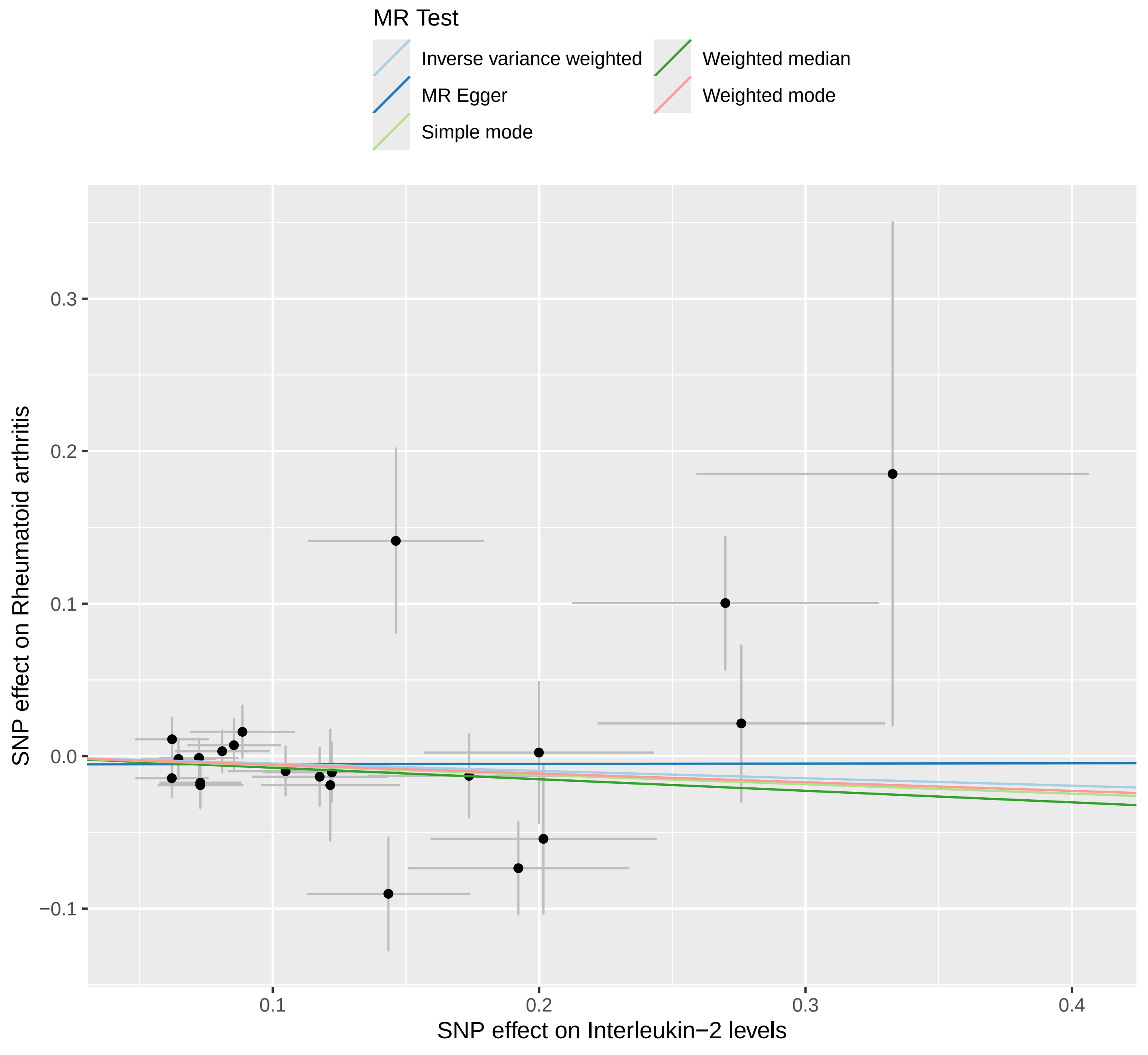

MR Test
Inverse variance weighted
MR Egger
Simple mode
Weighted median
Weighted mode
SNP effect on Rheumatoid arthritis
0.3
0.2
0.1
0.0
−0.1
0.1
0.2
0.3
0.4
SNP effect on Interleukin−2 levels

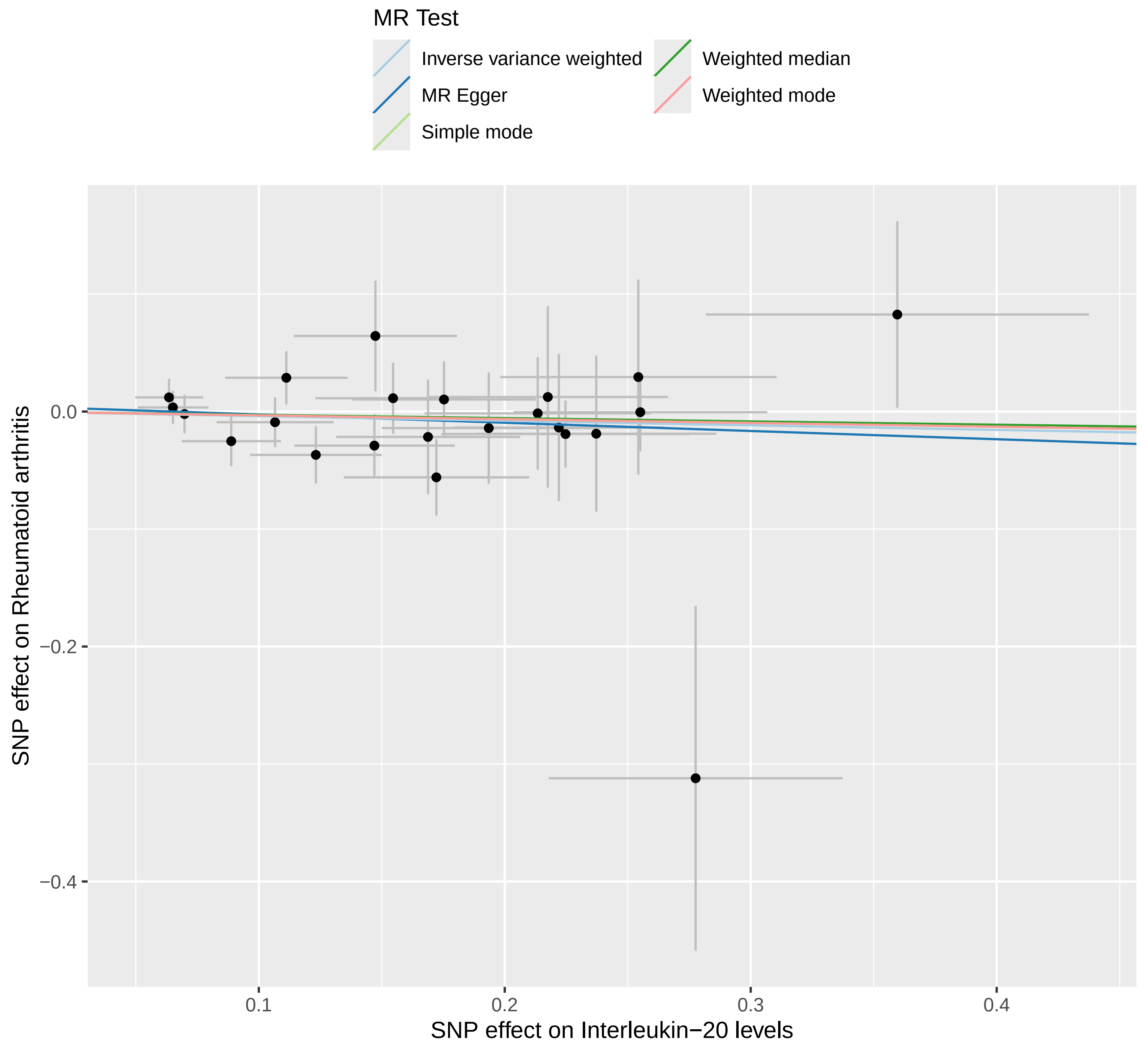
MR Test
Inverse variance weighted
MR Egger
Simple mode
Weighted median
Weighted mode
SNP effect on Rheumatoid arthritis
0.0
−0.2
−0.4
0.1
0.2
0.3
0.4
SNP effect on Interleukin−20 levels

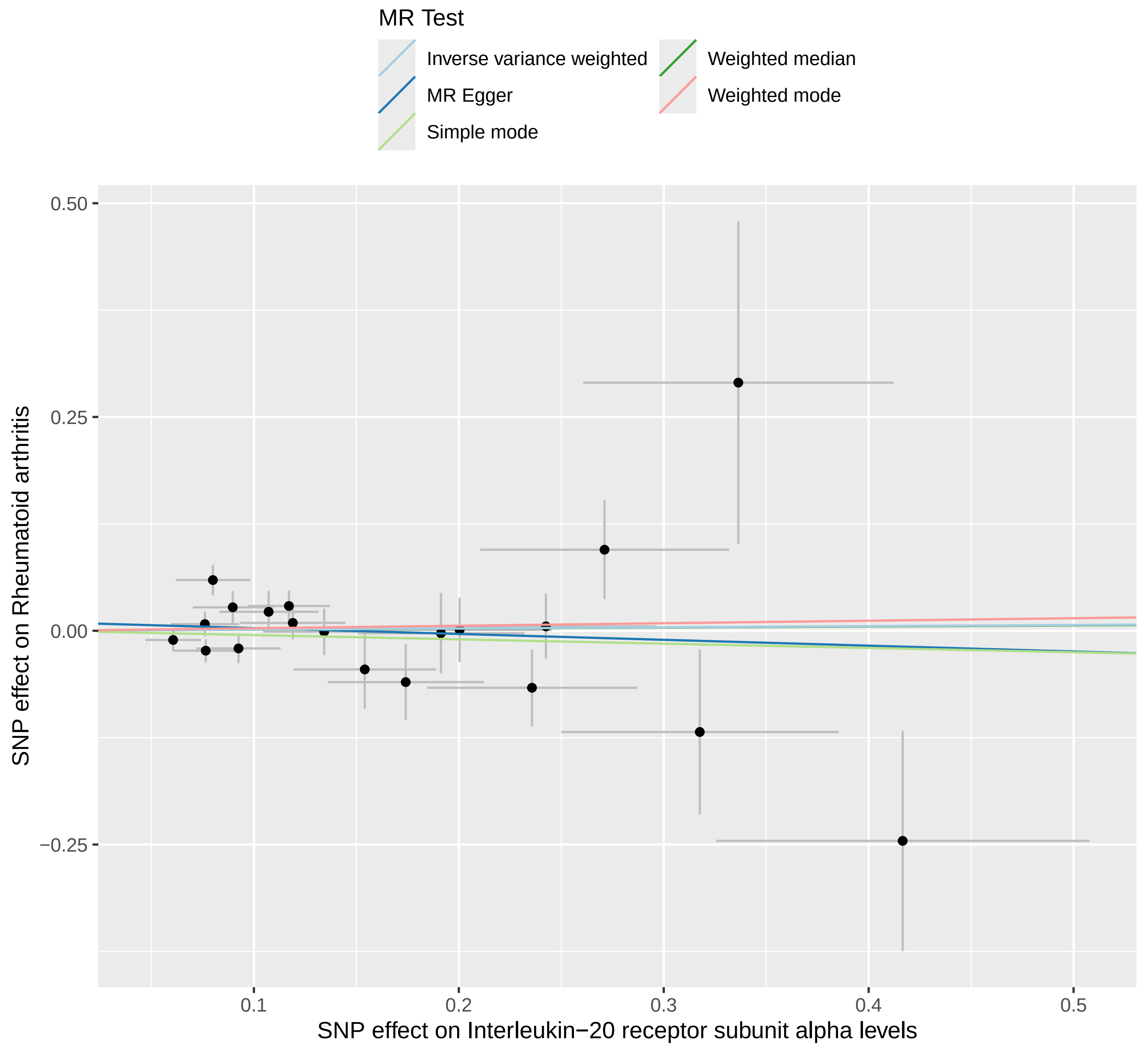
MR Test
Inverse variance weighted
MR Egger
Simple mode
Weighted median
Weighted mode
0.50
0.25
0.00
−0.25
0.1
0.2
0.3
0.4
0.5
SNP effect on Rheumatoid arthritis
SNP effect on Interleukin−20 receptor subunit alpha levels

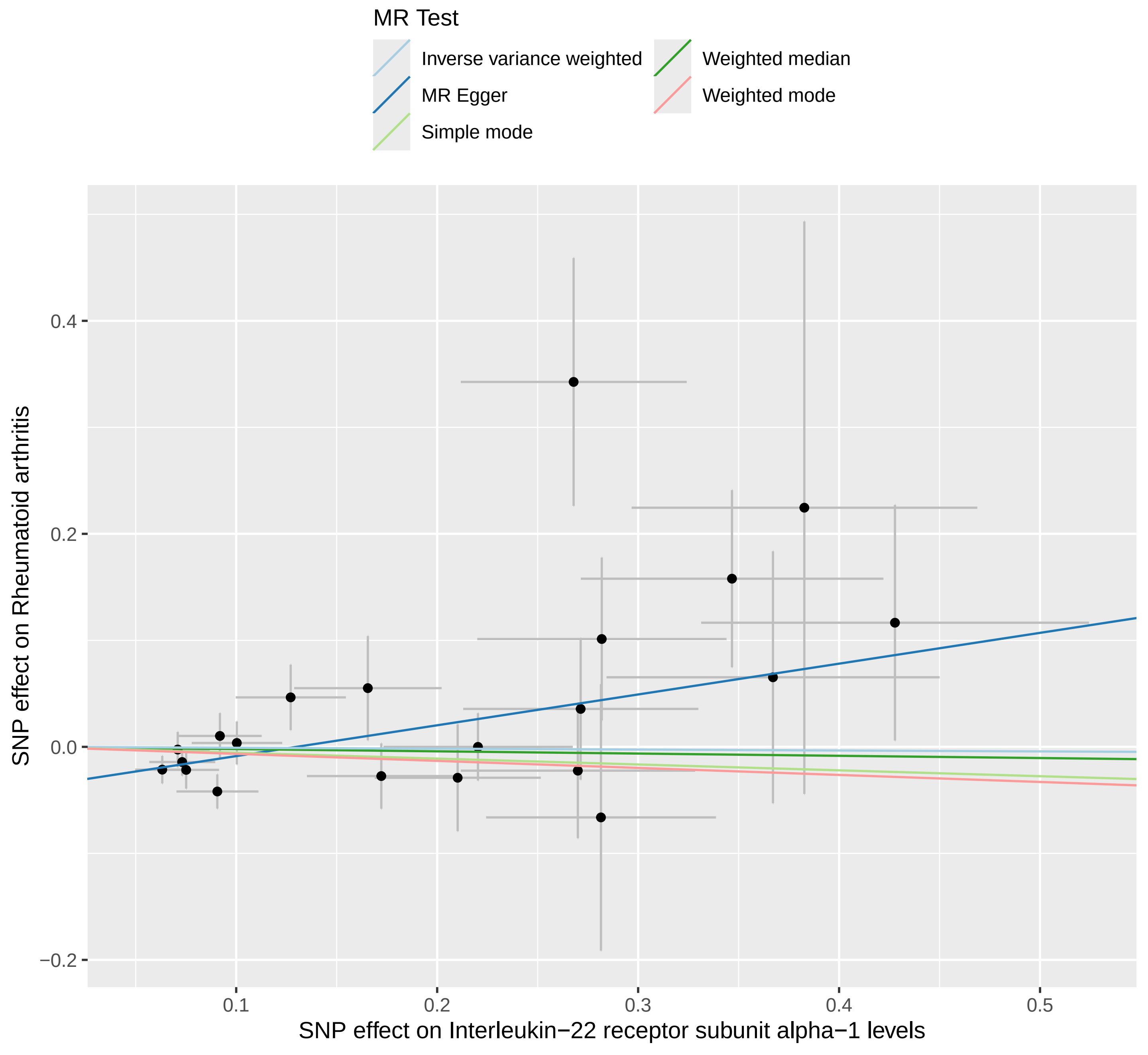
MR Test
Inverse variance weighted
MR Egger
Simple mode
Weighted median
Weighted mode
0.4
0.2
0.0
−0.2
0.1
0.2
0.3
0.4
0.5
SNP effect on Rheumatoid arthritis
SNP effect on Interleukin−22 receptor subunit alpha−1 levels

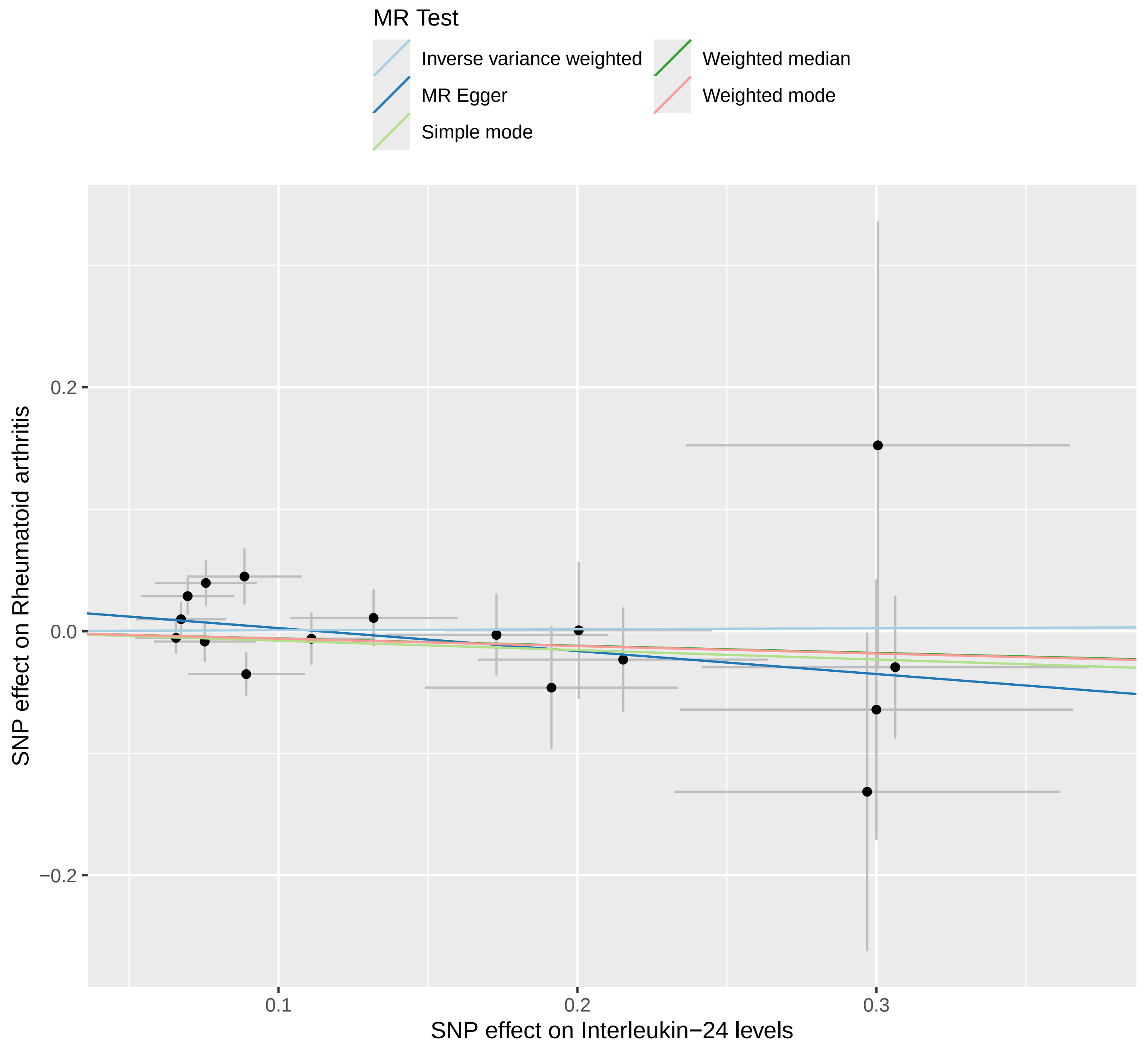

MR Test
Inverse variance weighted
MR Egger
Simple mode
Weighted median
Weighted mode
0.2
0.0
−0.2
0.1
0.2
0.3
SNP effect on Rheumatoid arthritis
SNP effect on Interleukin−24 levels

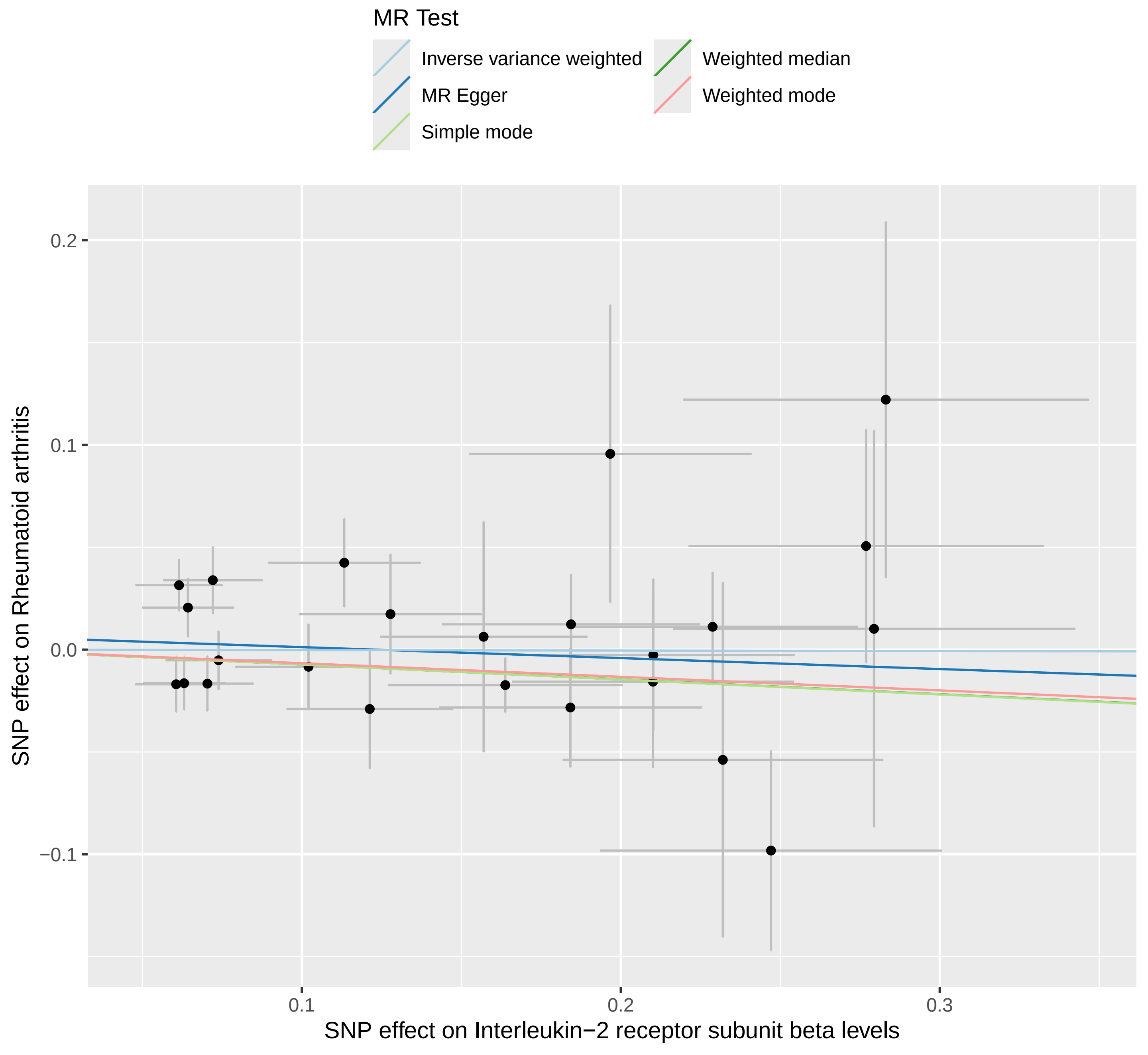
MR Test
Inverse variance weighted
MR Egger
Simple mode
Weighted median
Weighted mode
SNP effect on Rheumatoid arthritis
0.2
0.1
0.0
−0.1
0.1
0.2
0.3
SNP effect on Interleukin−2 receptor subunit beta levels

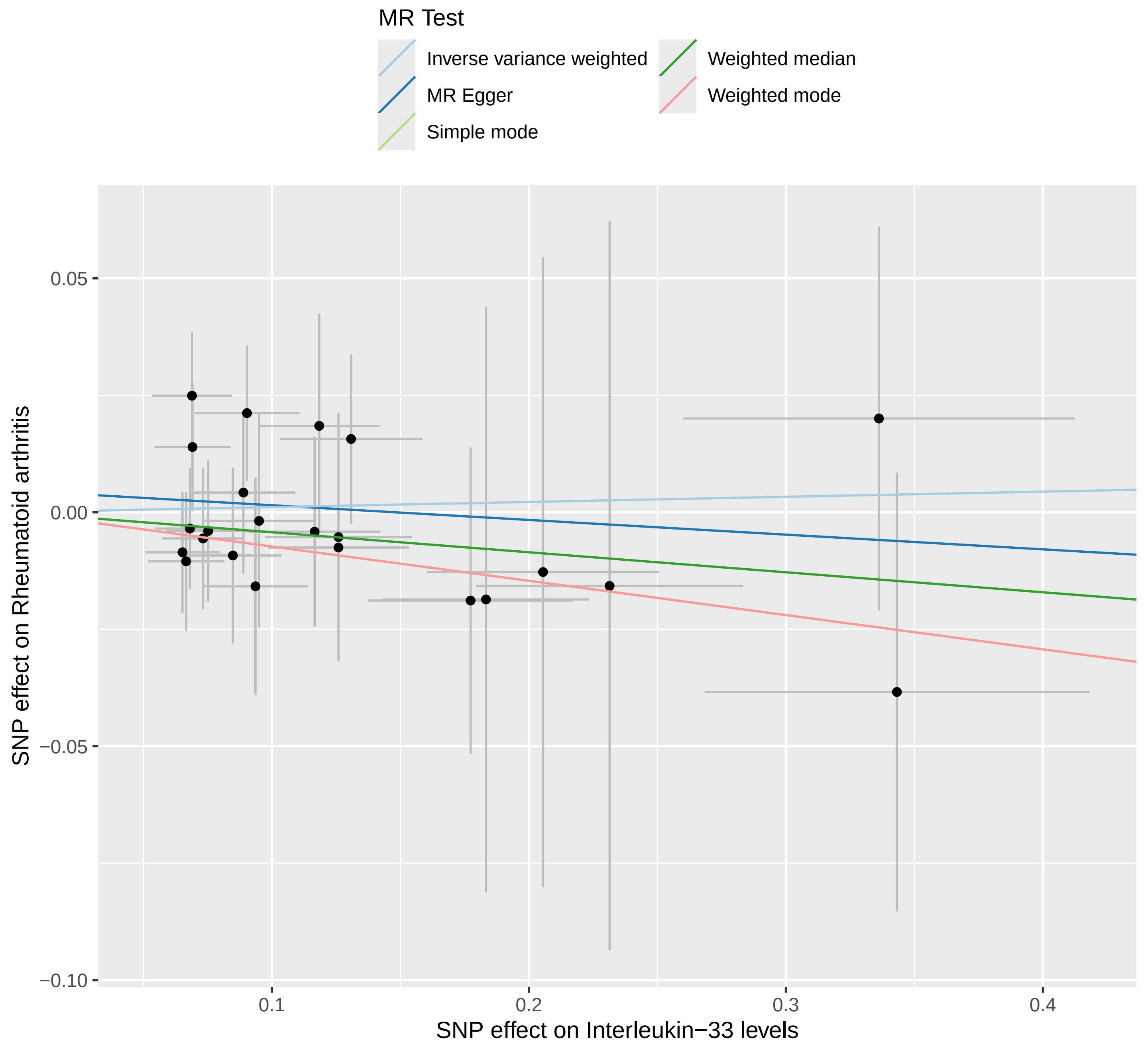

MR Test
Inverse variance weighted
MR Egger
Simple mode
Weighted median
Weighted mode
0.05
0.00
−0.05
−0.10
0.1
0.2
0.3
0.4
SNP effect on Rheumatoid arthritis
SNP effect on Interleukin−33 levels

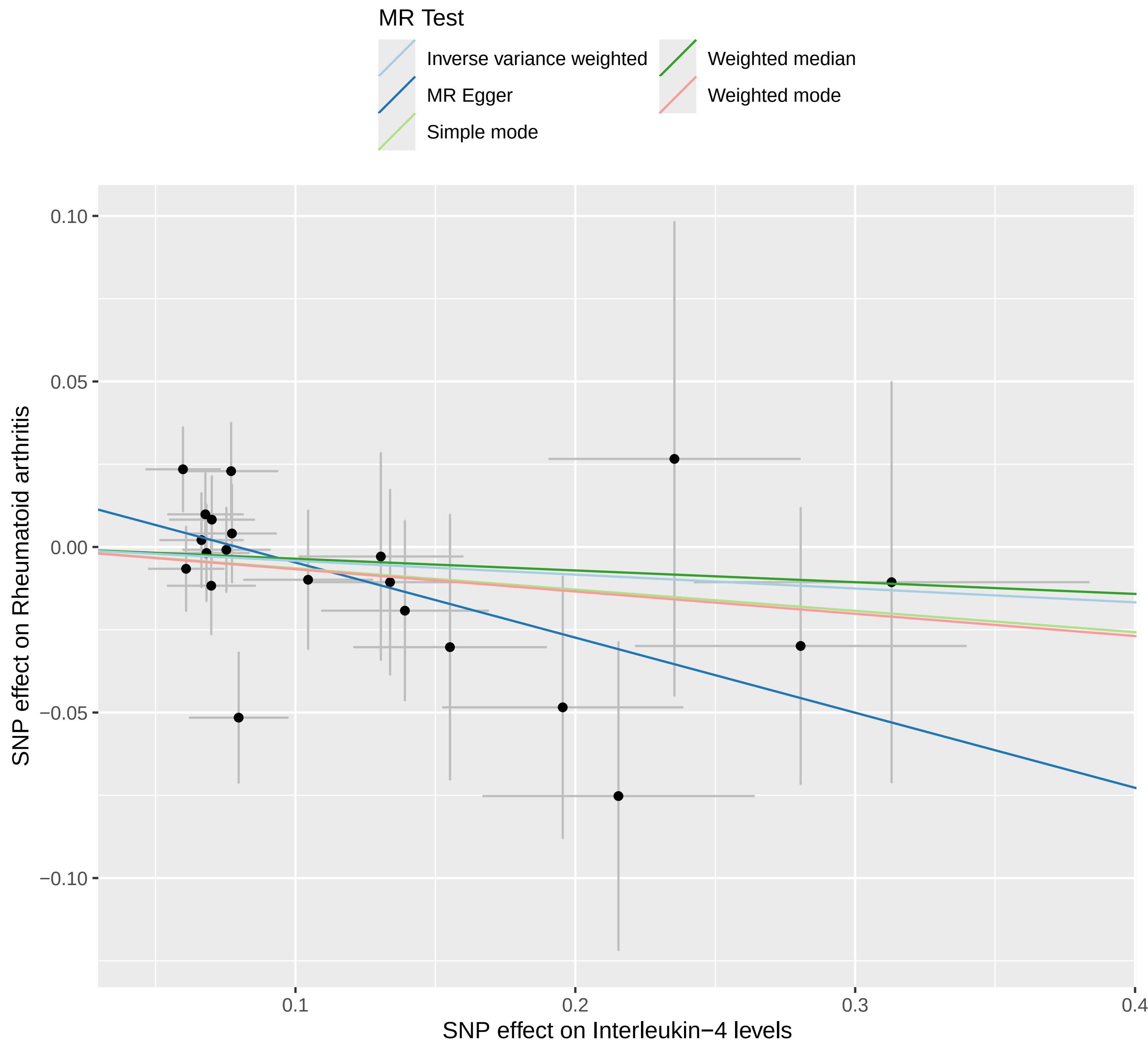

MR Test
Inverse variance weighted
MR Egger
Simple mode
Weighted median
Weighted mode
0.10
0.05
0.00
−0.05
−0.10
SNP effect on Rheumatoid arthritis
0.1
0.2
0.3
0.4
SNP effect on Interleukin−4 levels

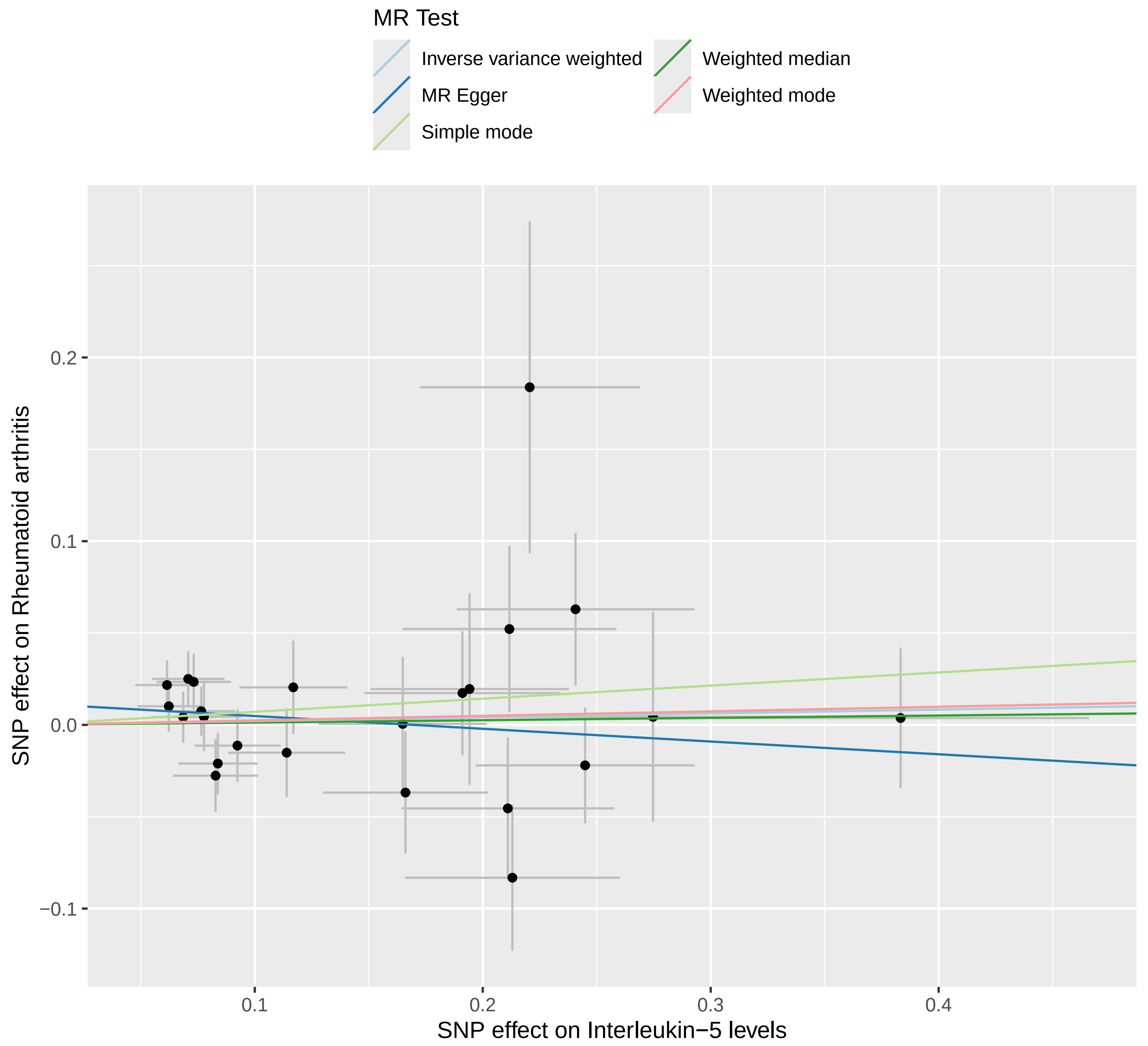
MR Test
Inverse variance weighted
MR Egger
Simple mode
Weighted median
Weighted mode
SNP effect on Rheumatoid arthritis
0.2
0.1
0.0
−0.1
0.1
0.2
0.3
0.4
SNP effect on Interleukin−5 levels

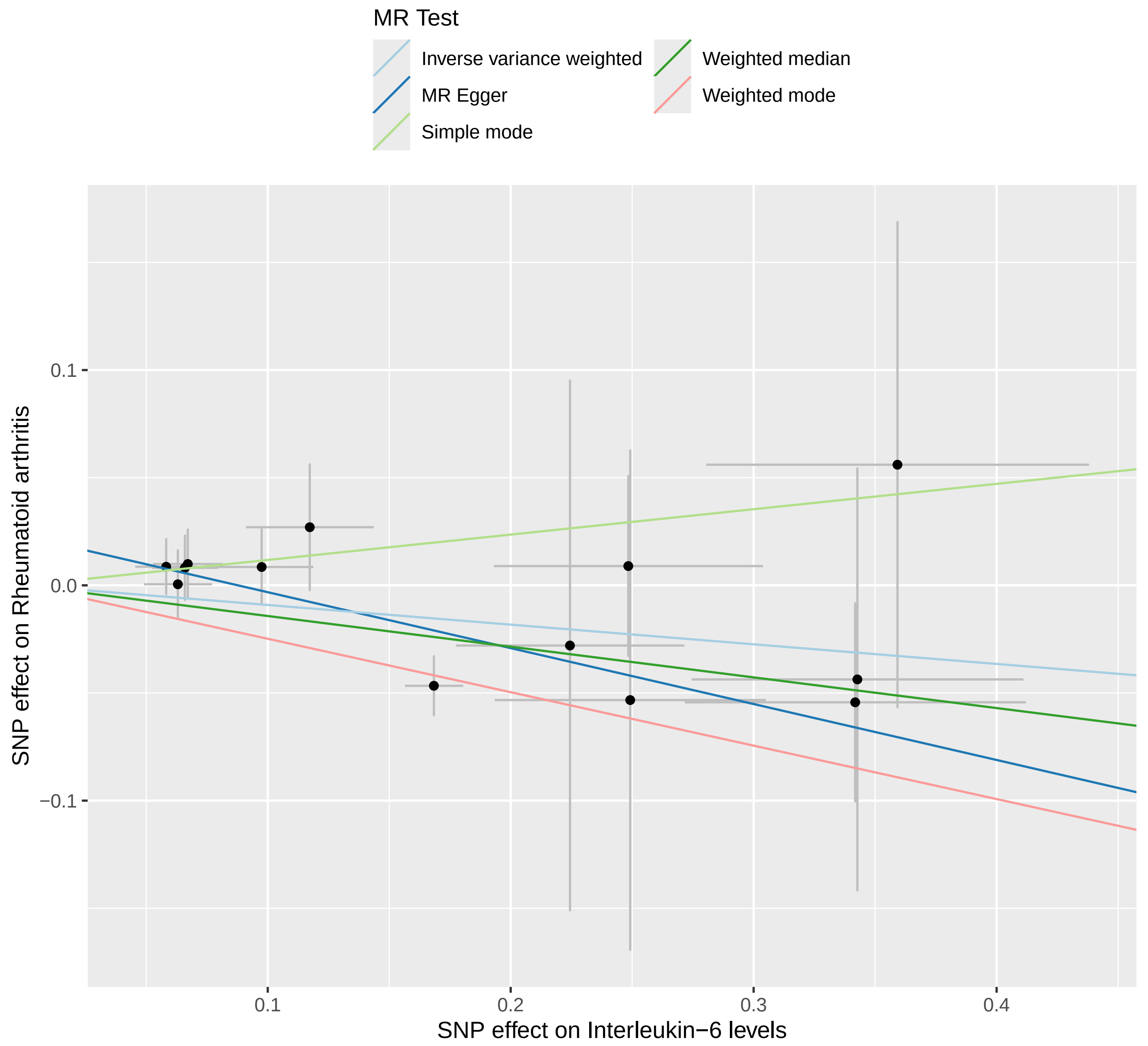

MR Test
Inverse variance weighted
MR Egger
Simple mode
Weighted median
Weighted mode
0.1
0.0
−0.1
SNP effect on Rheumatoid arthritis
0.1
0.2
0.3
0.4
SNP effect on Interleukin−6 levels

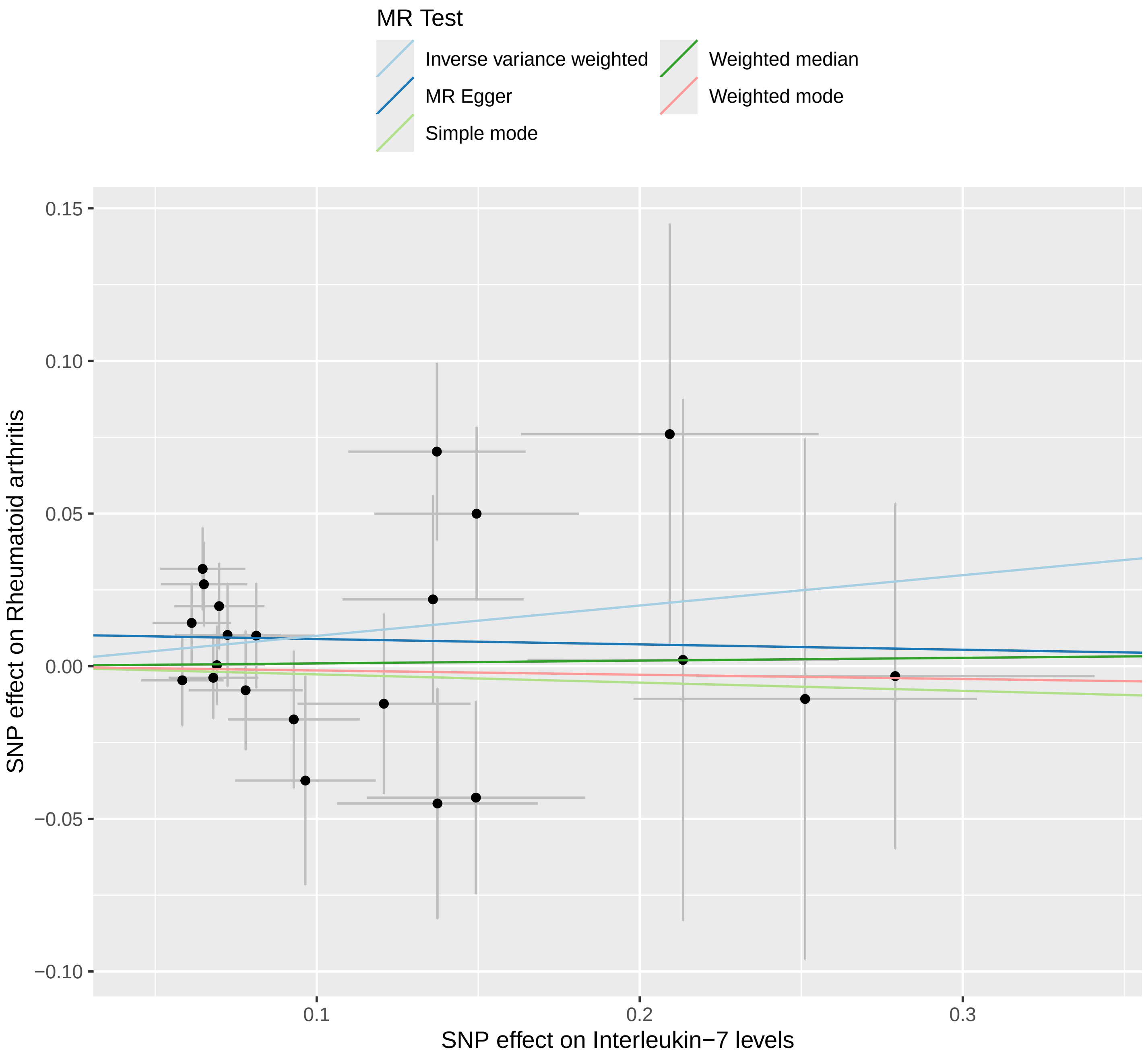
MR Test
Inverse variance weighted
MR Egger
Simple mode
Weighted median
Weighted mode
0.15
0.10
0.05
0.00
−0.05
−0.10
0.1
0.2
0.3
SNP effect on Rheumatoid arthritis
SNP effect on Interleukin−7 levels

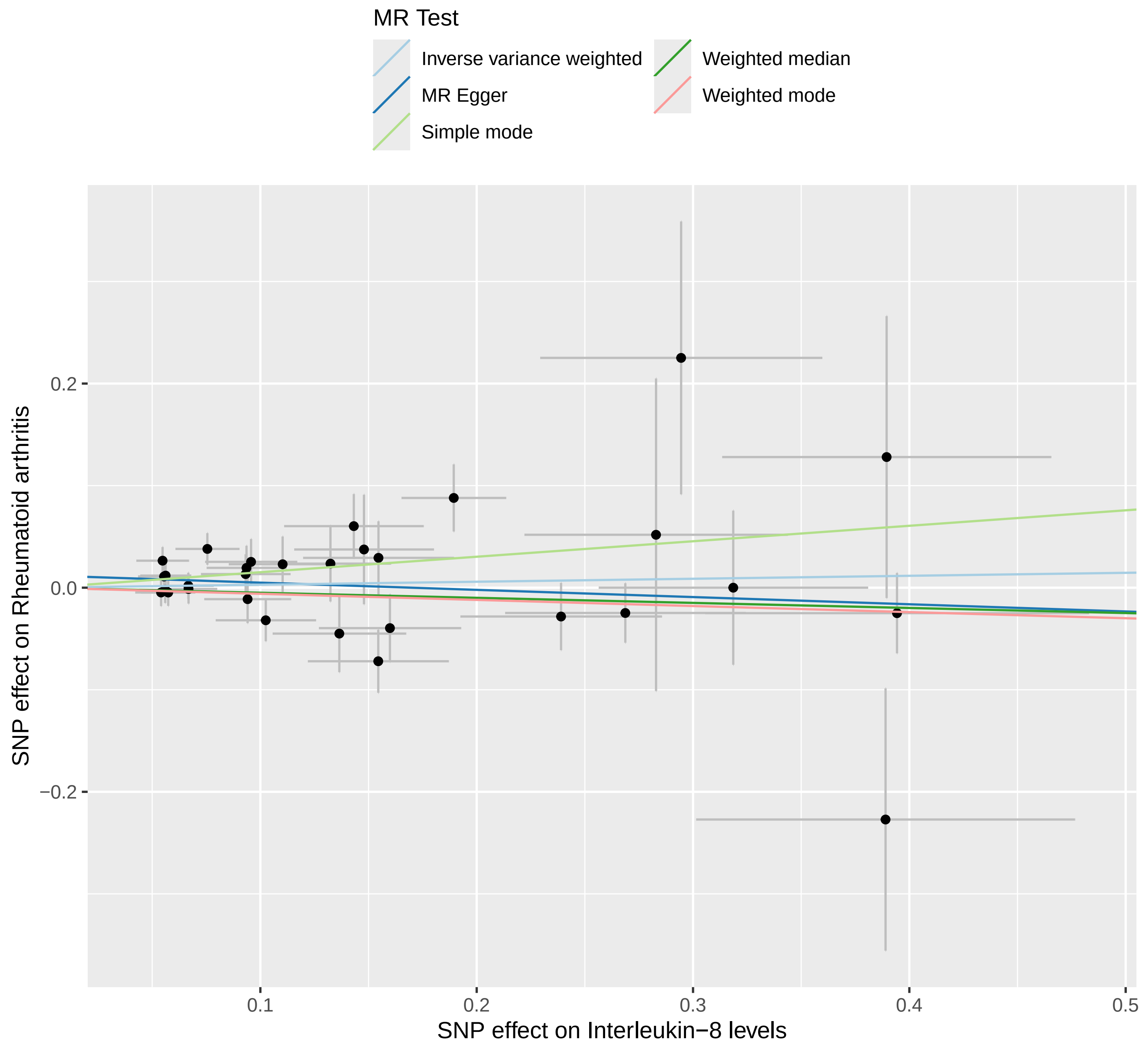
MR Test
Inverse variance weighted
MR Egger
Simple mode
Weighted median
Weighted mode
0.2
0.0
−0.2
0.1
0.2
0.3
0.4
0.5
SNP effect on Rheumatoid arthritis
SNP effect on Interleukin−8 levels

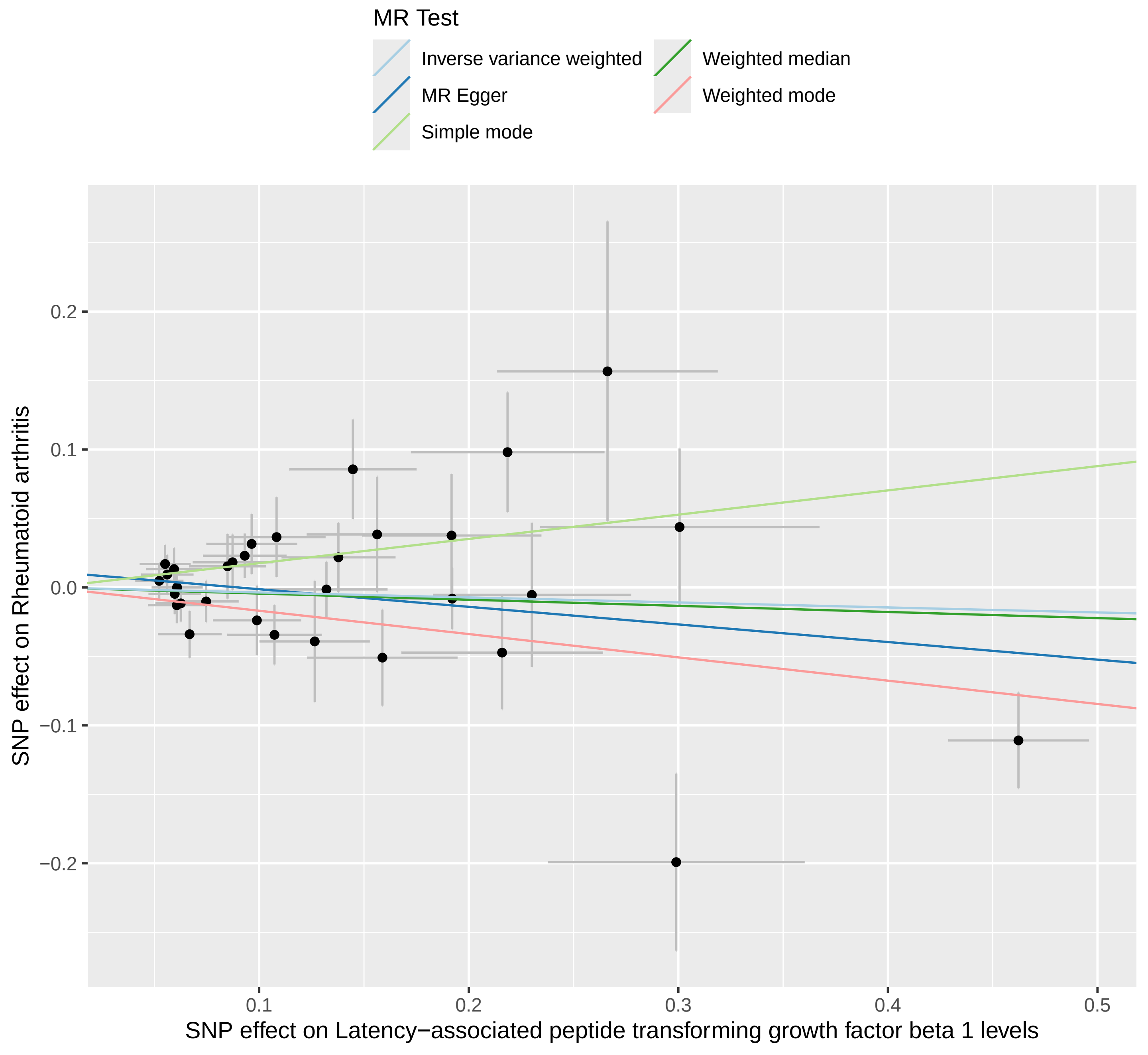
MR Test
Inverse variance weighted
MR Egger
Simple mode
Weighted median
Weighted mode
SNP effect on Rheumatoid arthritis
0.2
0.1
0.0
−0.1
−0.2
0.1
0.2
0.3
0.4
0.5
SNP effect on Latency−associated peptide transforming growth factor beta 1 levels

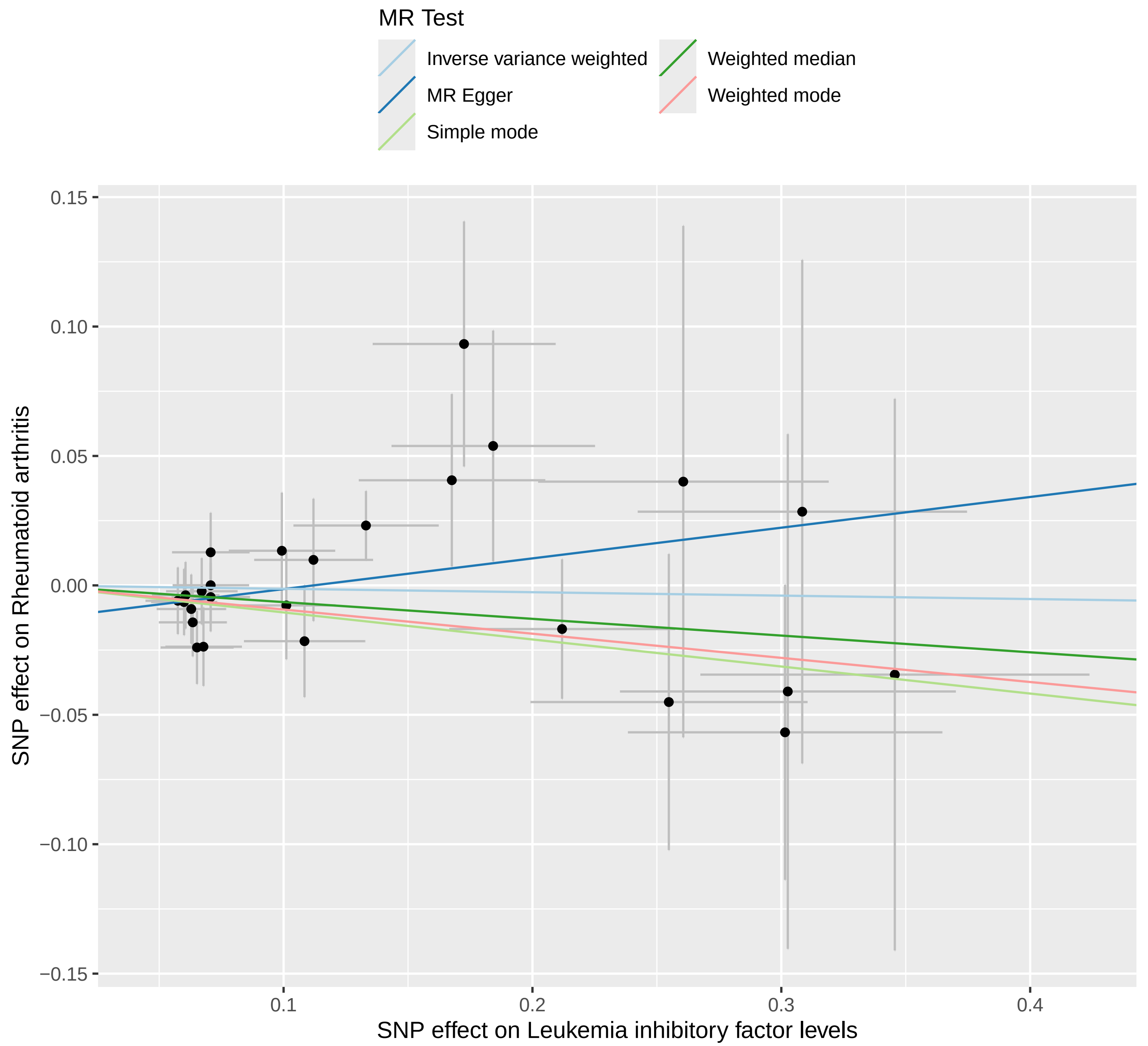
MR Test
Inverse variance weighted
MR Egger
Simple mode
Weighted median
Weighted mode
0.15
0.10
0.05
0.00
−0.05
−0.10
−0.15
0.1
0.2
0.3
0.4
SNP effect on Rheumatoid arthritis
SNP effect on Leukemia inhibitory factor levels

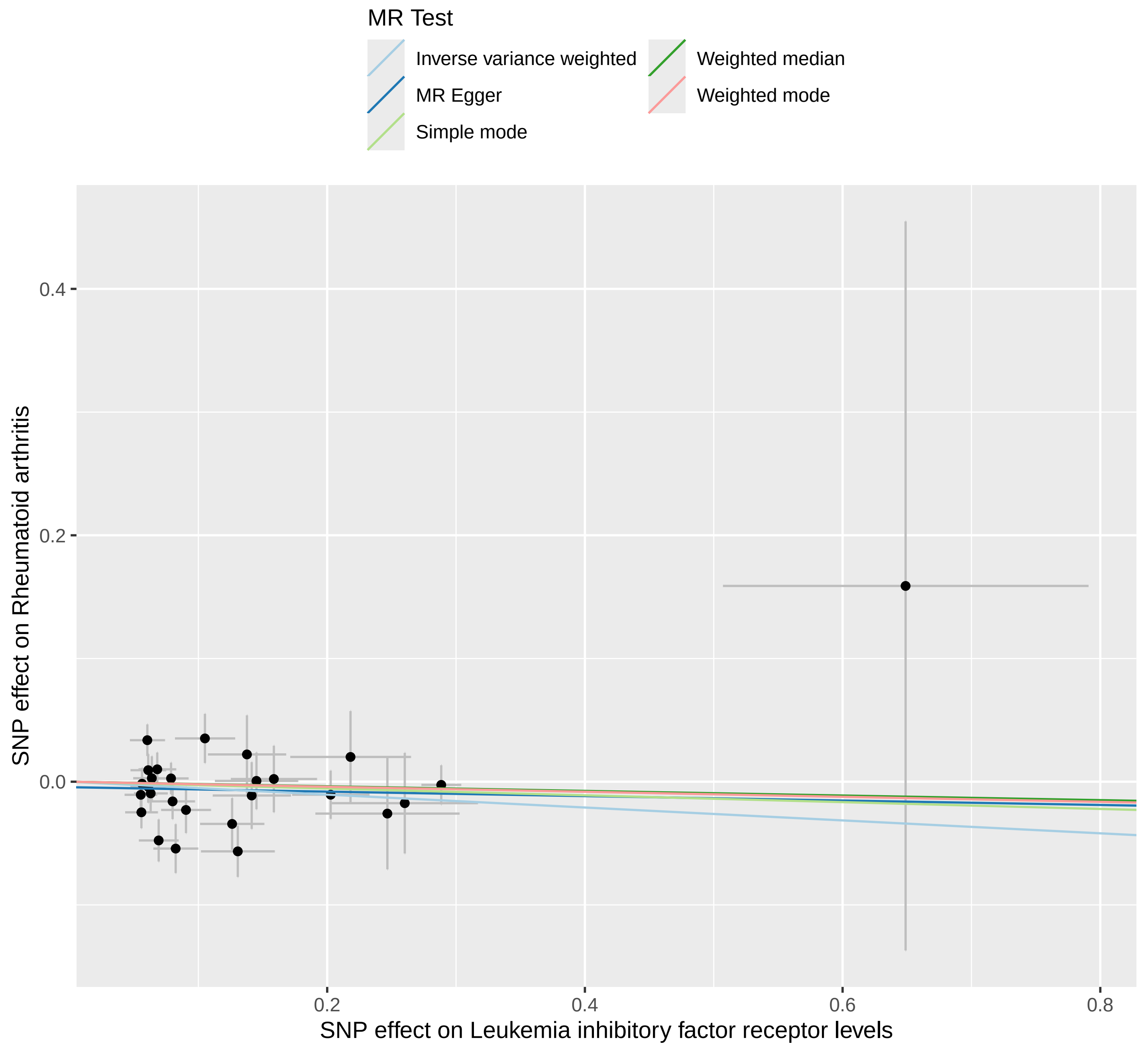
MR Test
Inverse variance weighted
MR Egger
Simple mode
Weighted median
Weighted mode
SNP effect on Rheumatoid arthritis
0.4
0.2
0.0
0.2
0.4
0.6
0.8
SNP effect on Leukemia inhibitory factor receptor levels

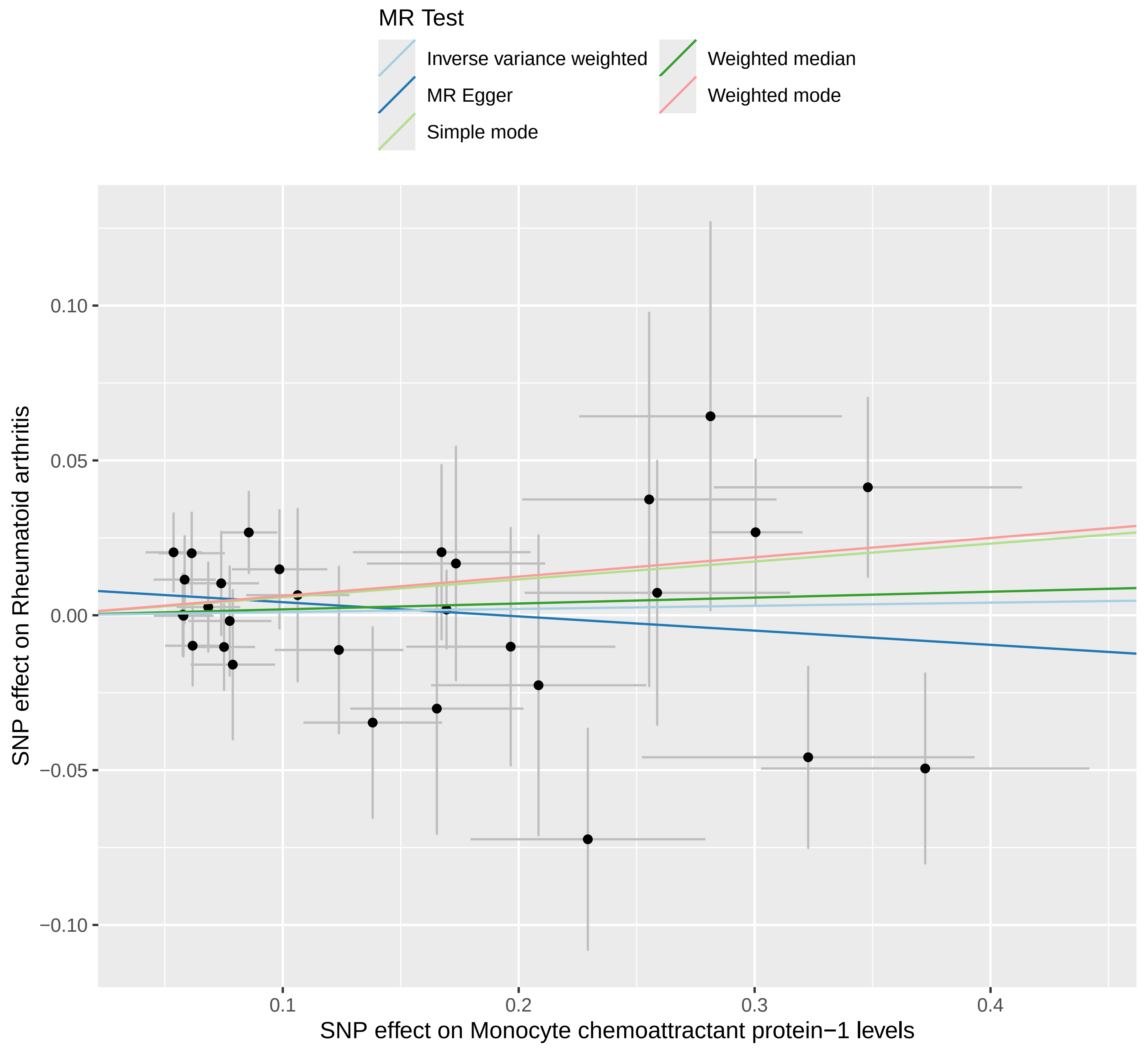

MR Test
Inverse variance weighted
MR Egger
Simple mode
Weighted median
Weighted mode
SNP effect on Rheumatoid arthritis
0.10
0.05
0.00
−0.05
−0.10
0.1
0.2
0.3
0.4
SNP effect on Monocyte chemoattractant protein−1 levels

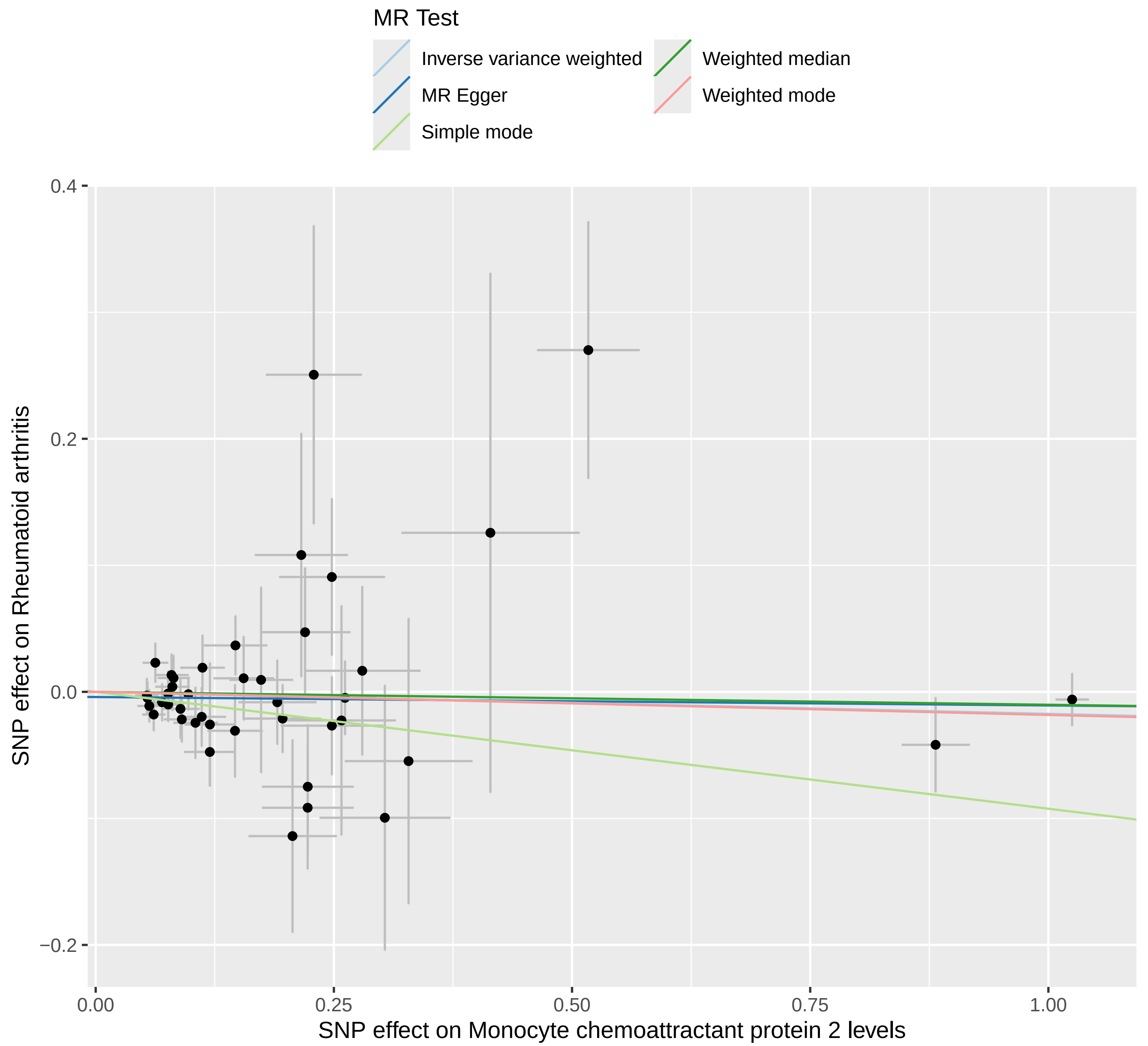
MR Test
Inverse variance weighted
MR Egger
Simple mode
Weighted median
Weighted mode
0.4
0.2
0.0
−0.2
0.00
0.25
0.50
0.75
1.00
SNP effect on Rheumatoid arthritis
SNP effect on Monocyte chemoattractant protein 2 levels

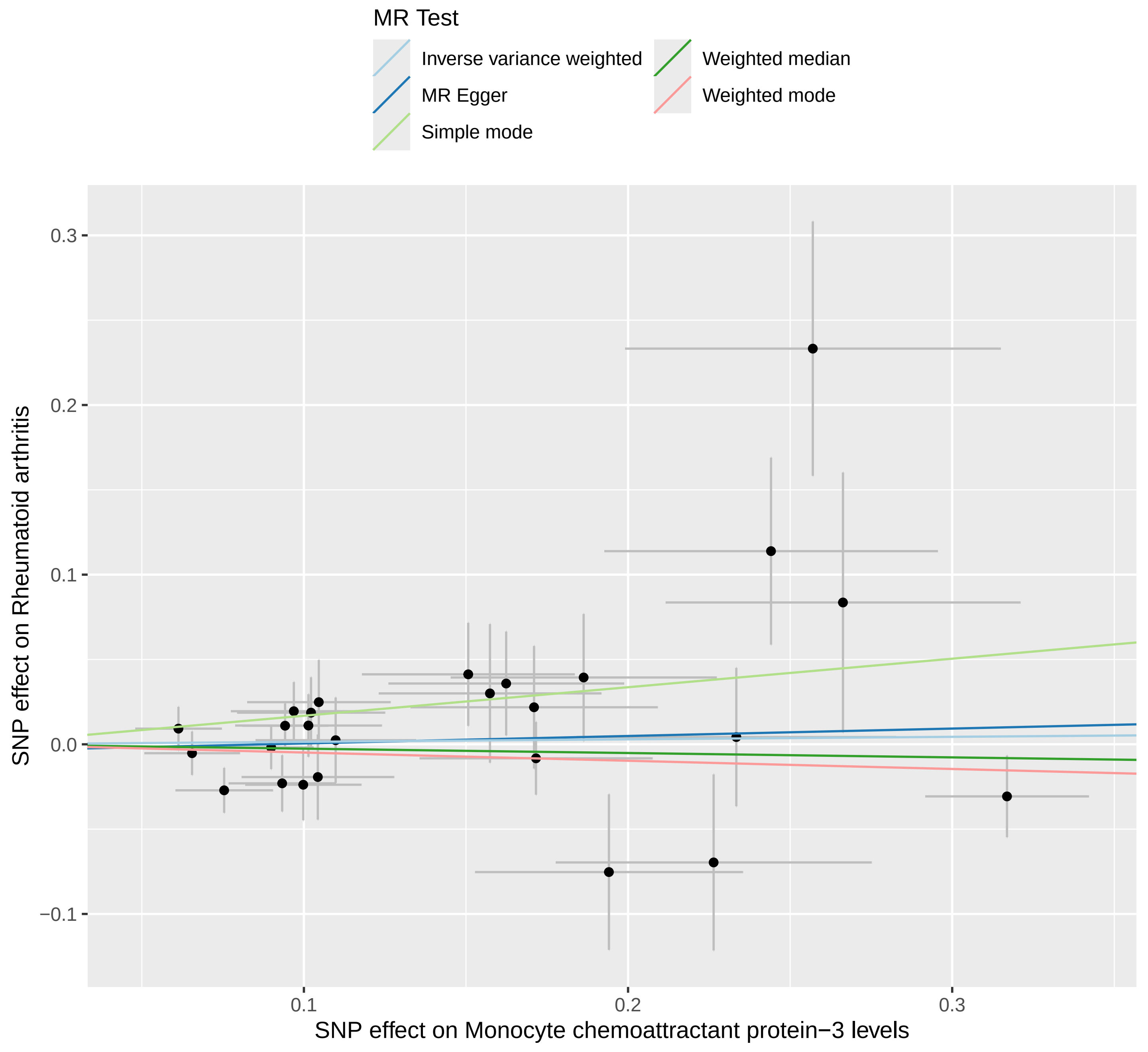
MR Test
Inverse variance weighted
MR Egger
Simple mode
Weighted median
Weighted mode
0.3
0.2
0.1
0.0
−0.1
0.1
0.2
0.3
SNP effect on Rheumatoid arthritis
SNP effect on Monocyte chemoattractant protein−3 levels

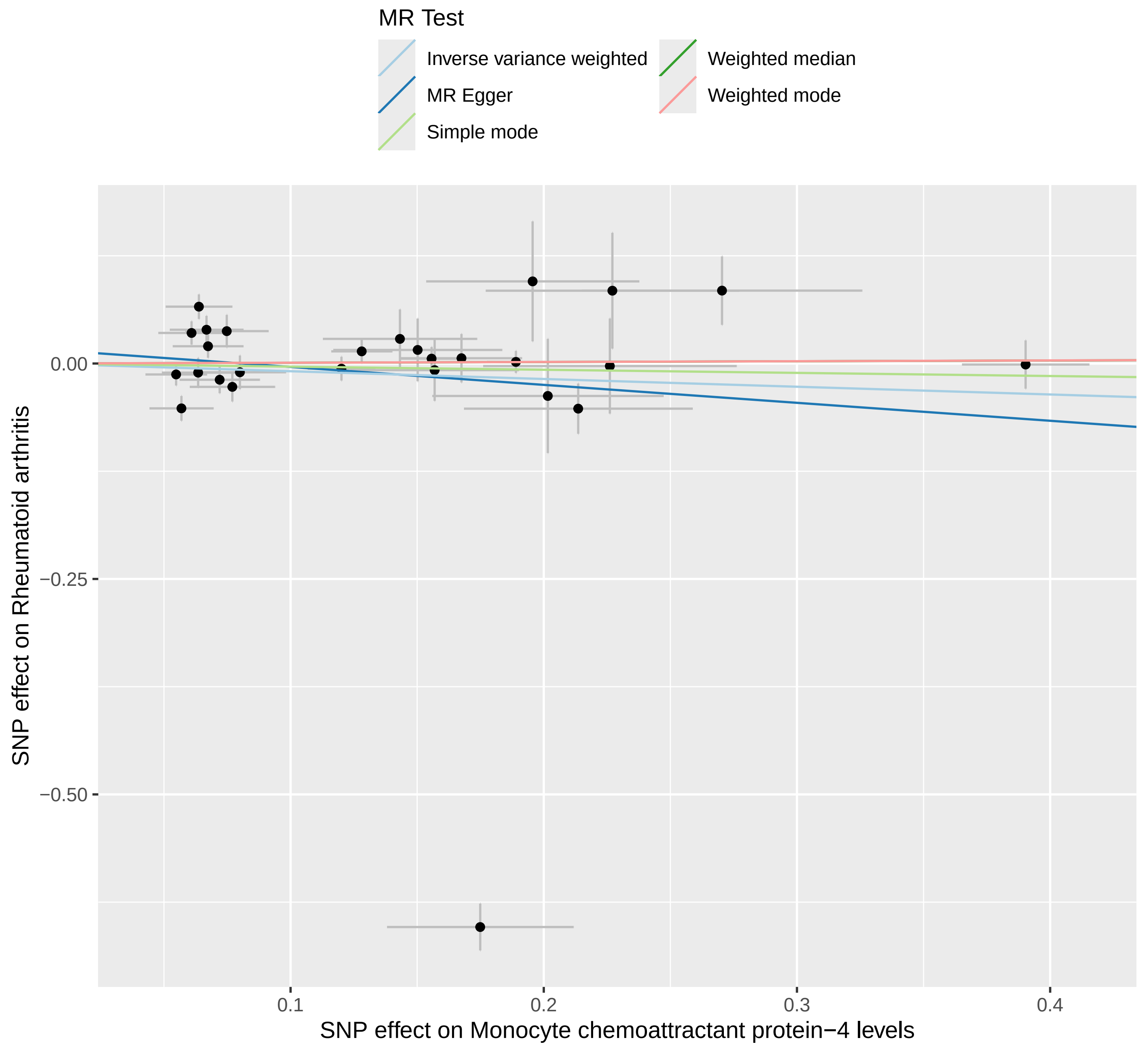

MR Test
Inverse variance weighted
MR Egger
Simple mode
Weighted median
Weighted mode
SNP effect on Rheumatoid arthritis
0.00
−0.25
−0.50
0.1
0.2
0.3
0.4
SNP effect on Monocyte chemoattractant protein−4 levels

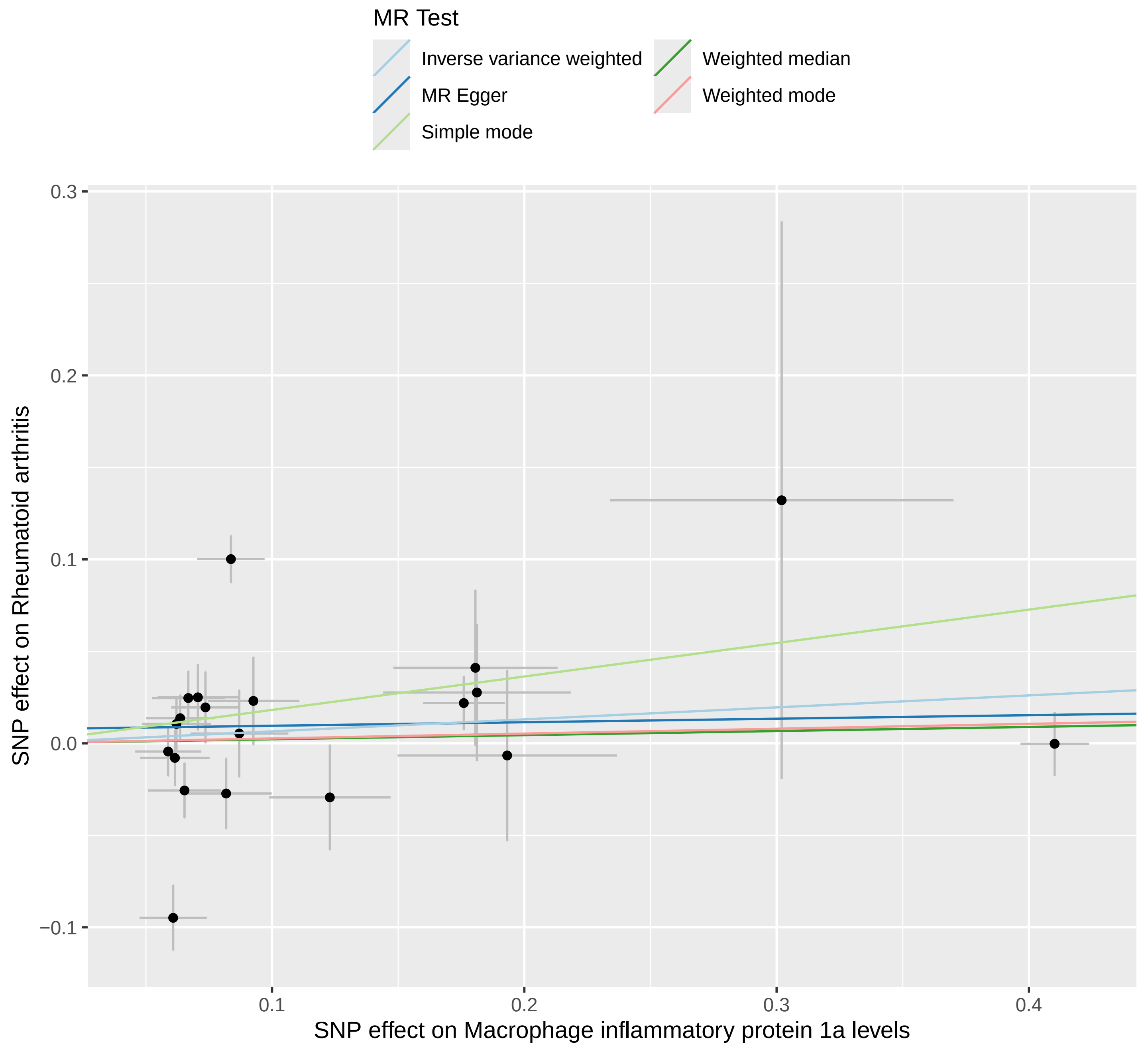
MR Test
Inverse variance weighted
MR Egger
Simple mode
Weighted median
Weighted mode
0.3
0.2
0.1
0.0
−0.1
0.1
0.2
0.3
0.4
SNP effect on Rheumatoid arthritis
SNP effect on Macrophage inflammatory protein 1a levels

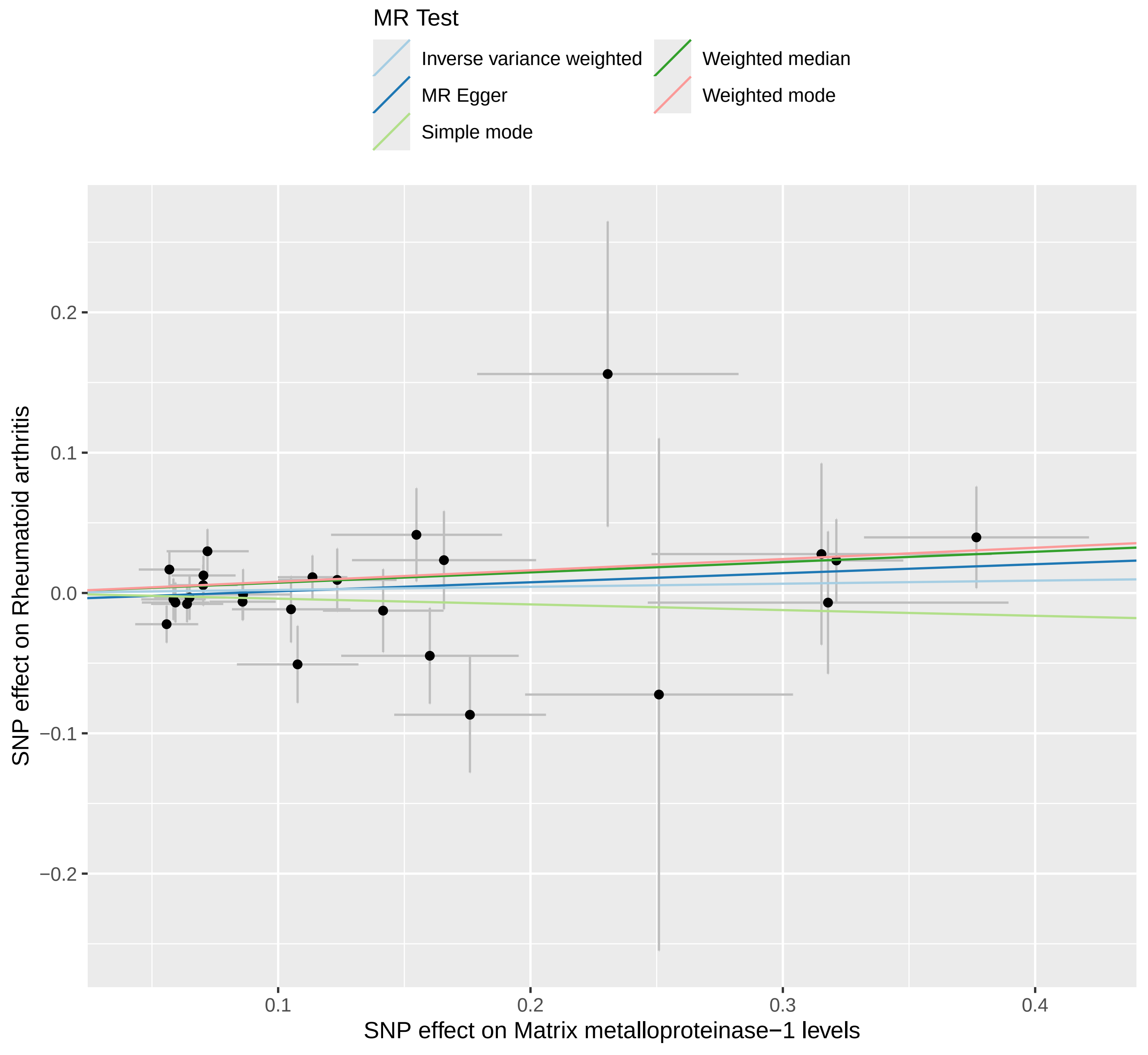
MR Test
Inverse variance weighted
MR Egger
Simple mode
Weighted median
Weighted mode
SNP effect on Rheumatoid arthritis
0.2
0.1
0.0
−0.1
−0.2
0.1
0.2
0.3
0.4
SNP effect on Matrix metalloproteinase−1 levels

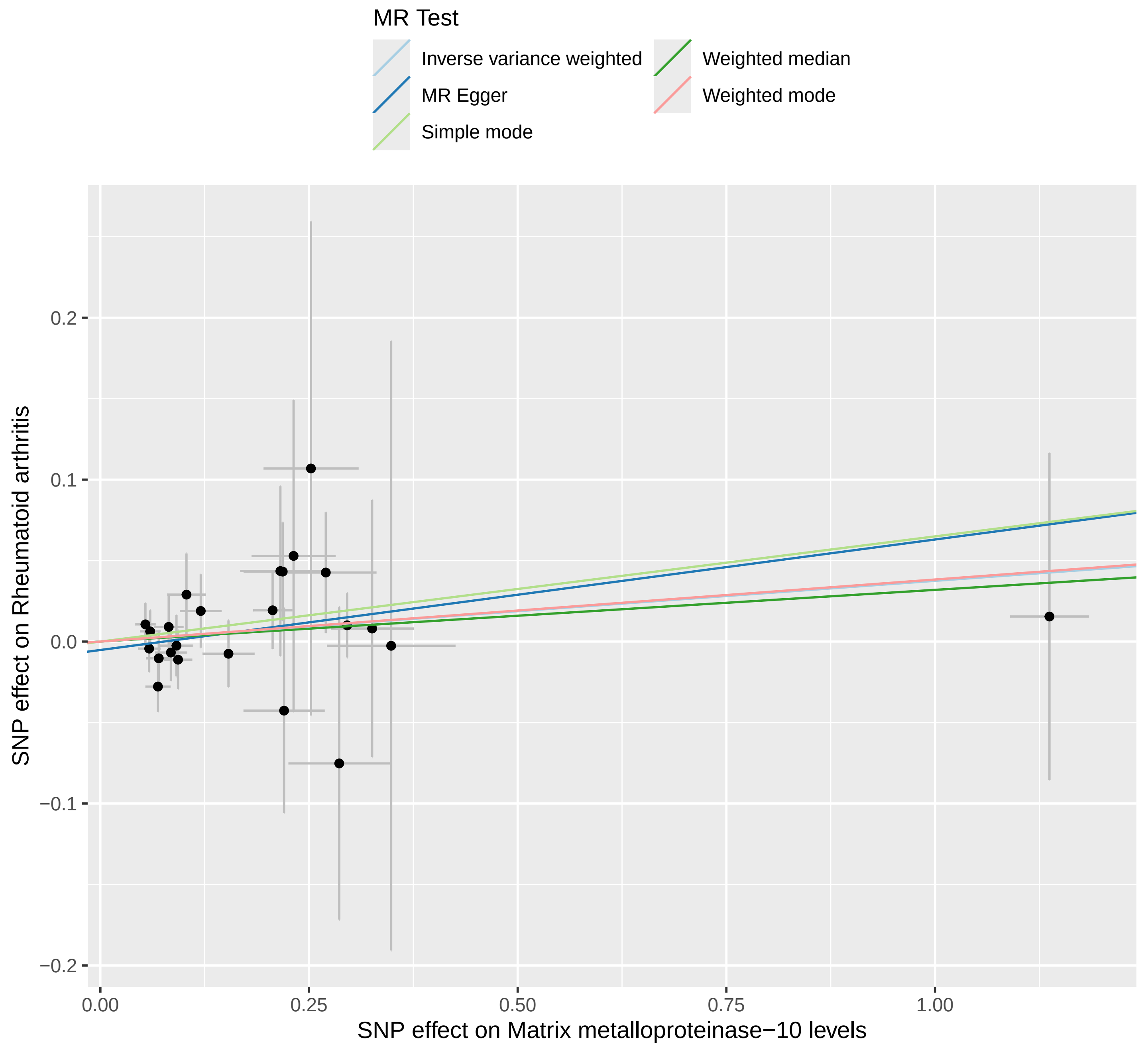

MR Test
Inverse variance weighted
MR Egger
Simple mode
Weighted median
Weighted mode
0.2
0.1
0.0
−0.1
−0.2
SNP effect on Rheumatoid arthritis
0.00
0.25
0.50
0.75
1.00
SNP effect on Matrix metalloproteinase−10 levels

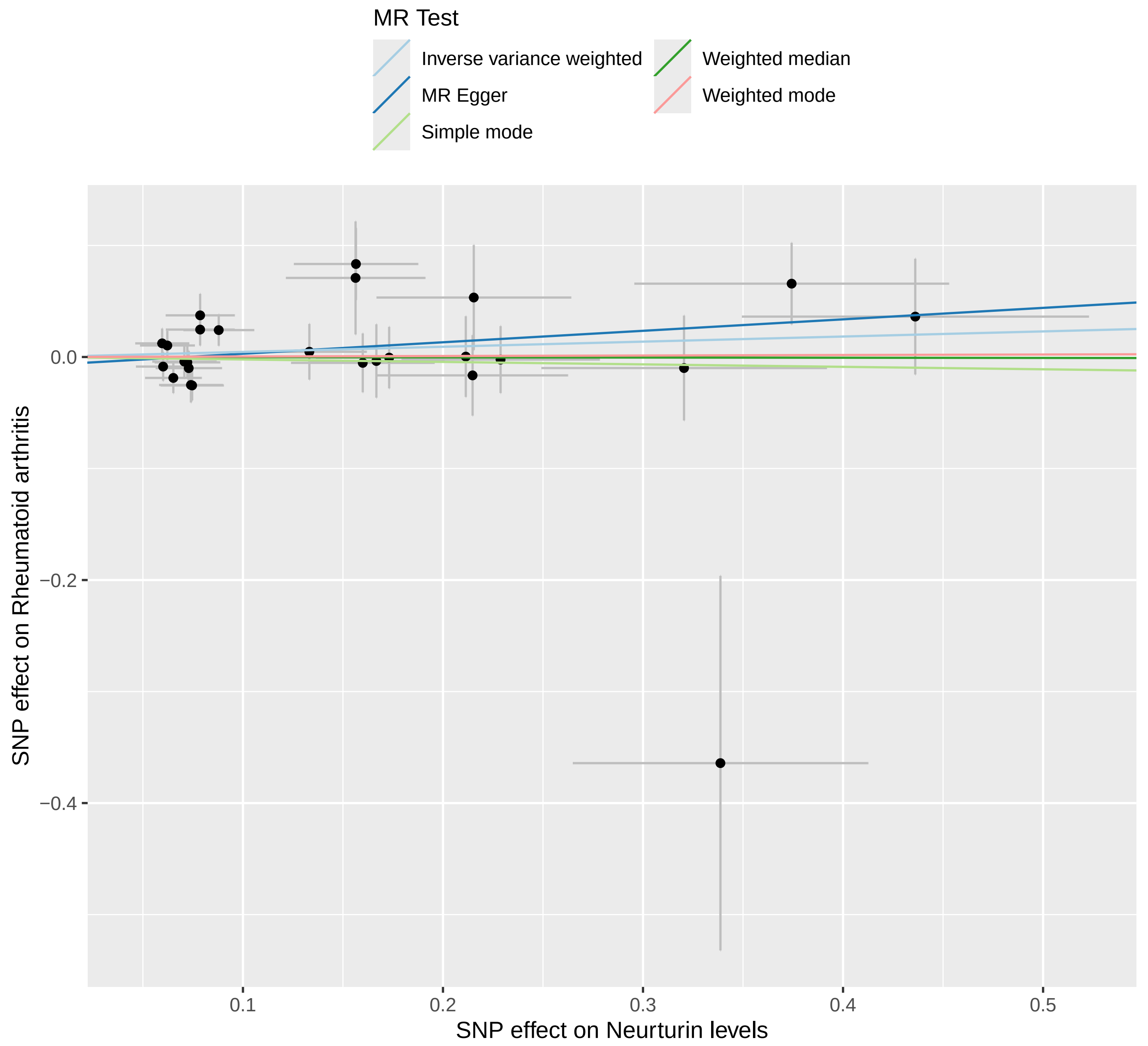
MR Test
Inverse variance weighted
MR Egger
Simple mode
Weighted median
Weighted mode
SNP effect on Rheumatoid arthritis
0.0
−0.2
−0.4
0.1
0.2
0.3
0.4
0.5
SNP effect on Neurturin levels

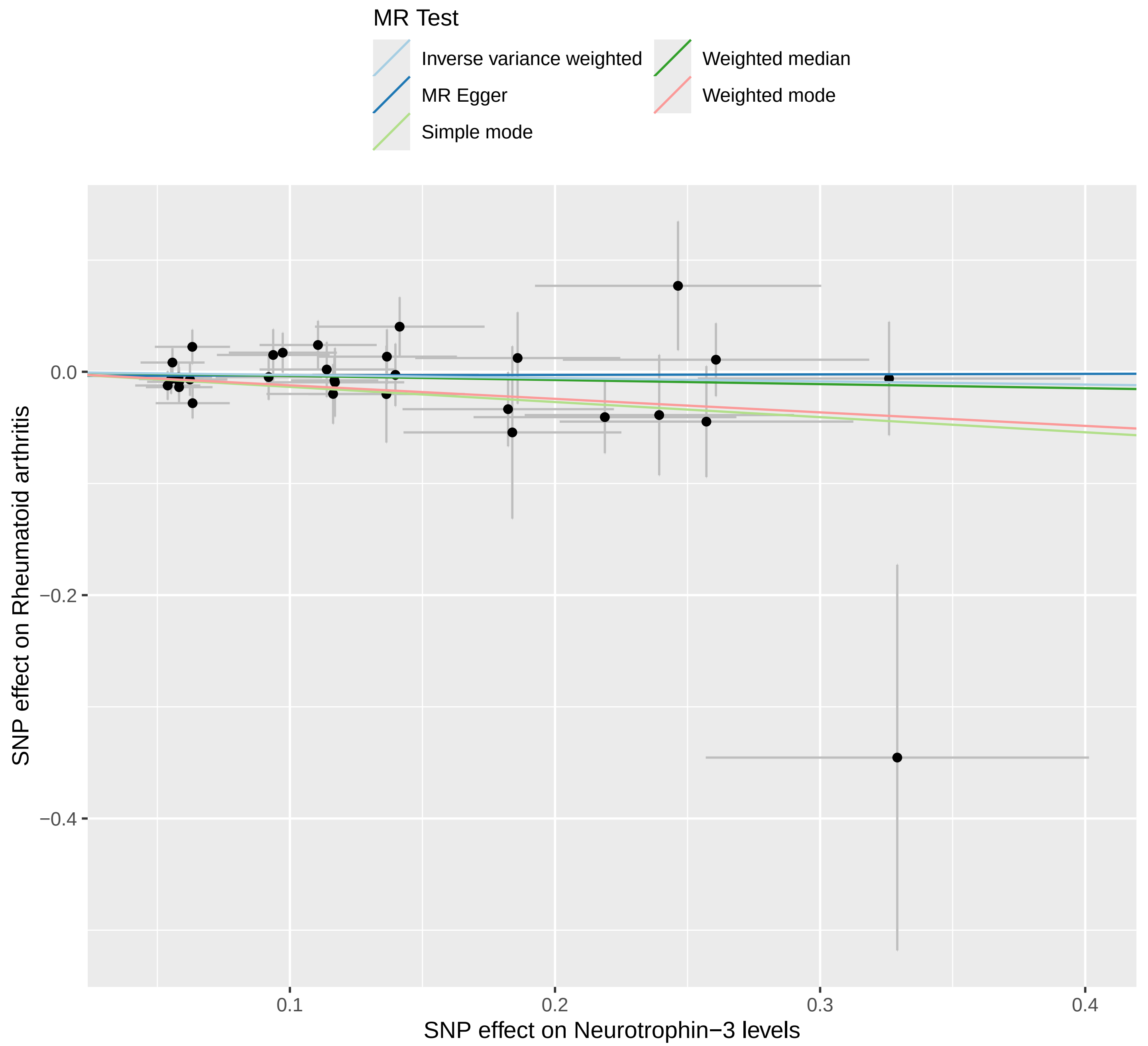
MR Test
Inverse variance weighted
MR Egger
Simple mode
Weighted median
Weighted mode
0.0
−0.2
−0.4
0.1
0.2
0.3
0.4
SNP effect on Rheumatoid arthritis
SNP effect on Neurotrophin−3 levels

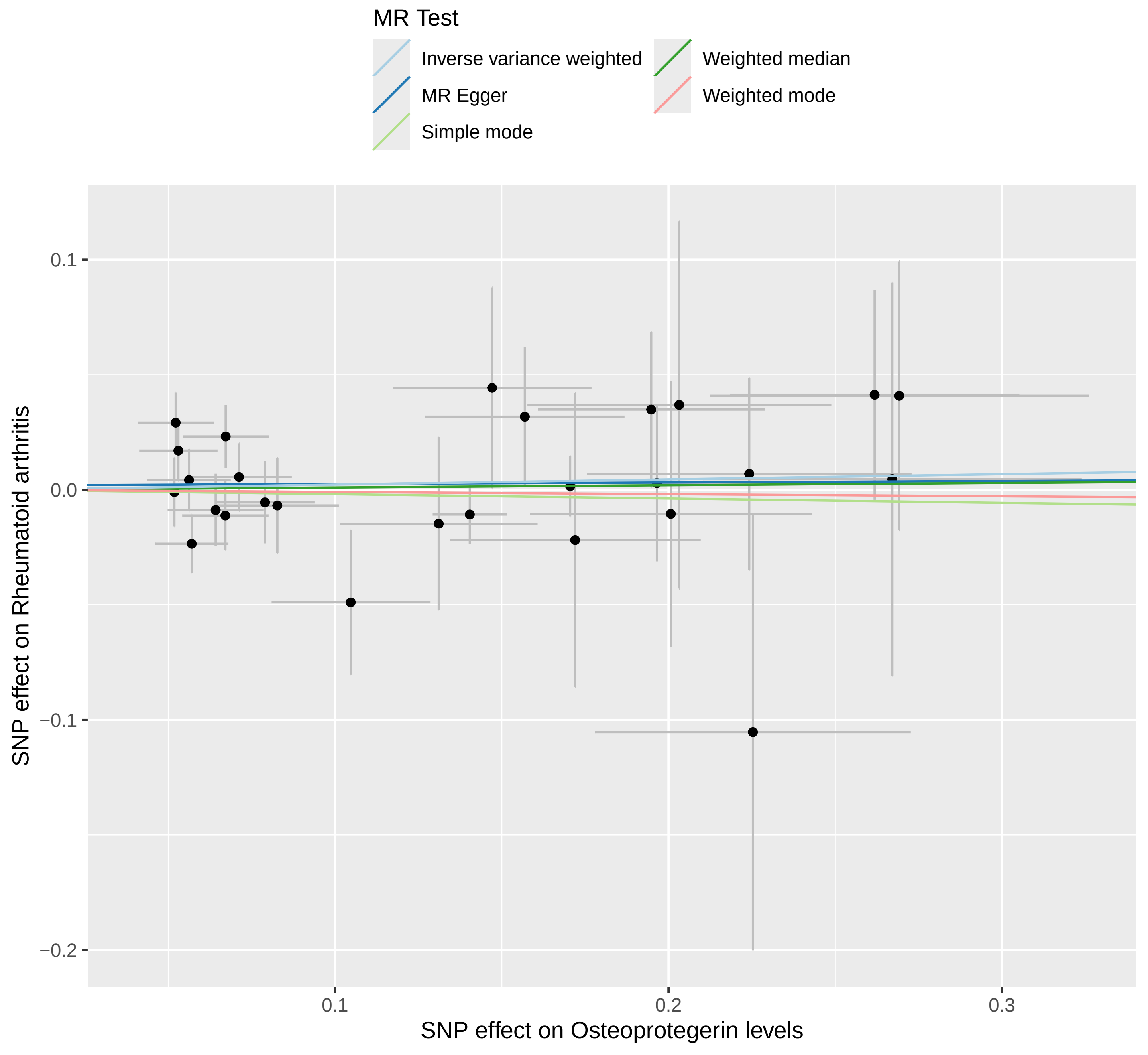
MR Test
Inverse variance weighted
MR Egger
Simple mode
Weighted median
Weighted mode
0.1
0.0
−0.1
−0.2
0.1
0.2
0.3
SNP effect on Rheumatoid arthritis
SNP effect on Osteoprotegerin levels

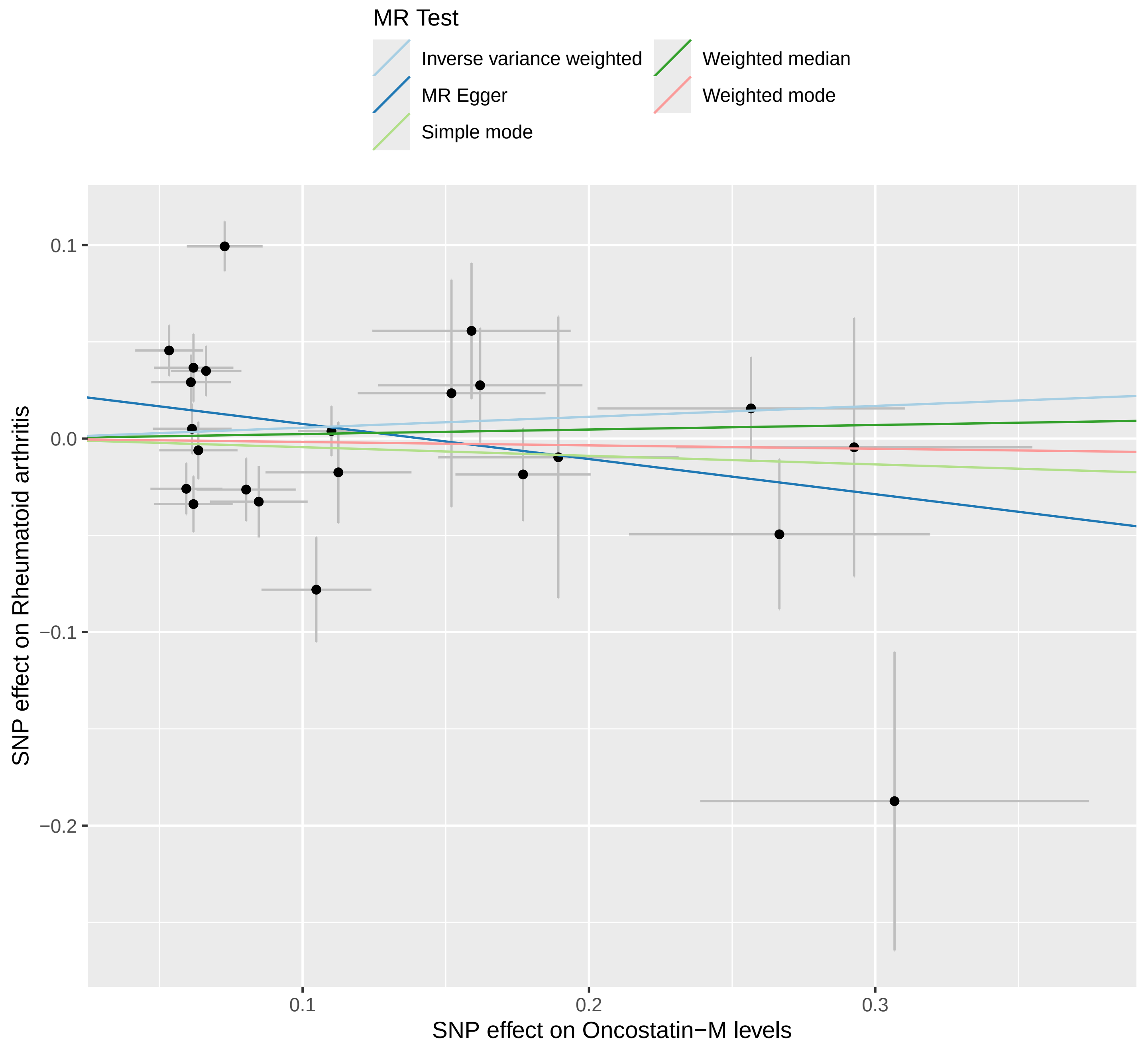
MR Test
Inverse variance weighted
MR Egger
Simple mode
Weighted median
Weighted mode
0.1
0.0
−0.1
−0.2
SNP effect on Rheumatoid arthritis
0.1
0.2
0.3
SNP effect on Oncostatin−M levels

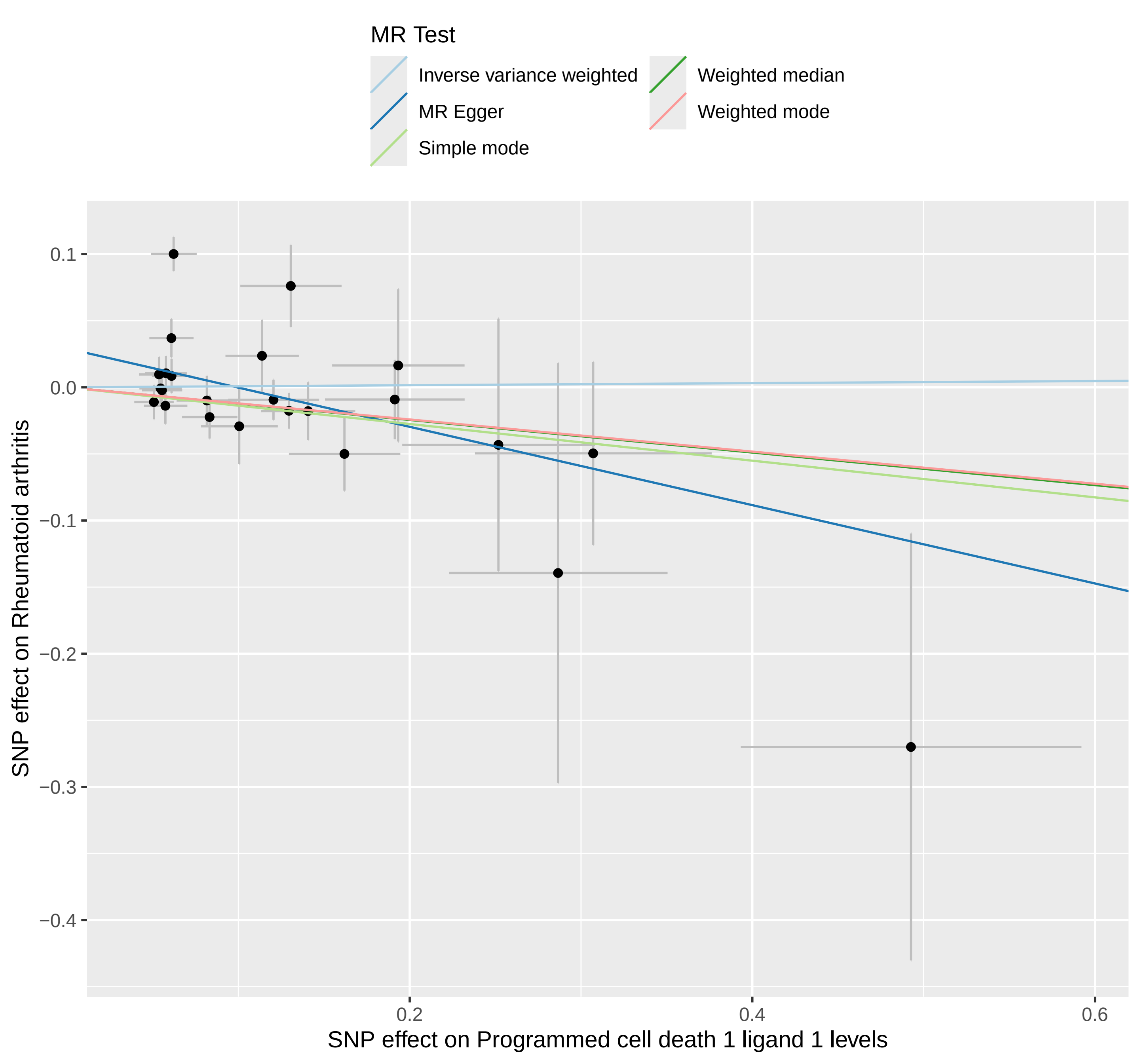
MR Test
Inverse variance weighted
MR Egger
Simple mode
Weighted median
Weighted mode
0.1
0.0
−0.1
−0.2
−0.3
−0.4
0.2
0.4
0.6
SNP effect on Rheumatoid arthritis
SNP effect on Programmed cell death 1 ligand 1 levels

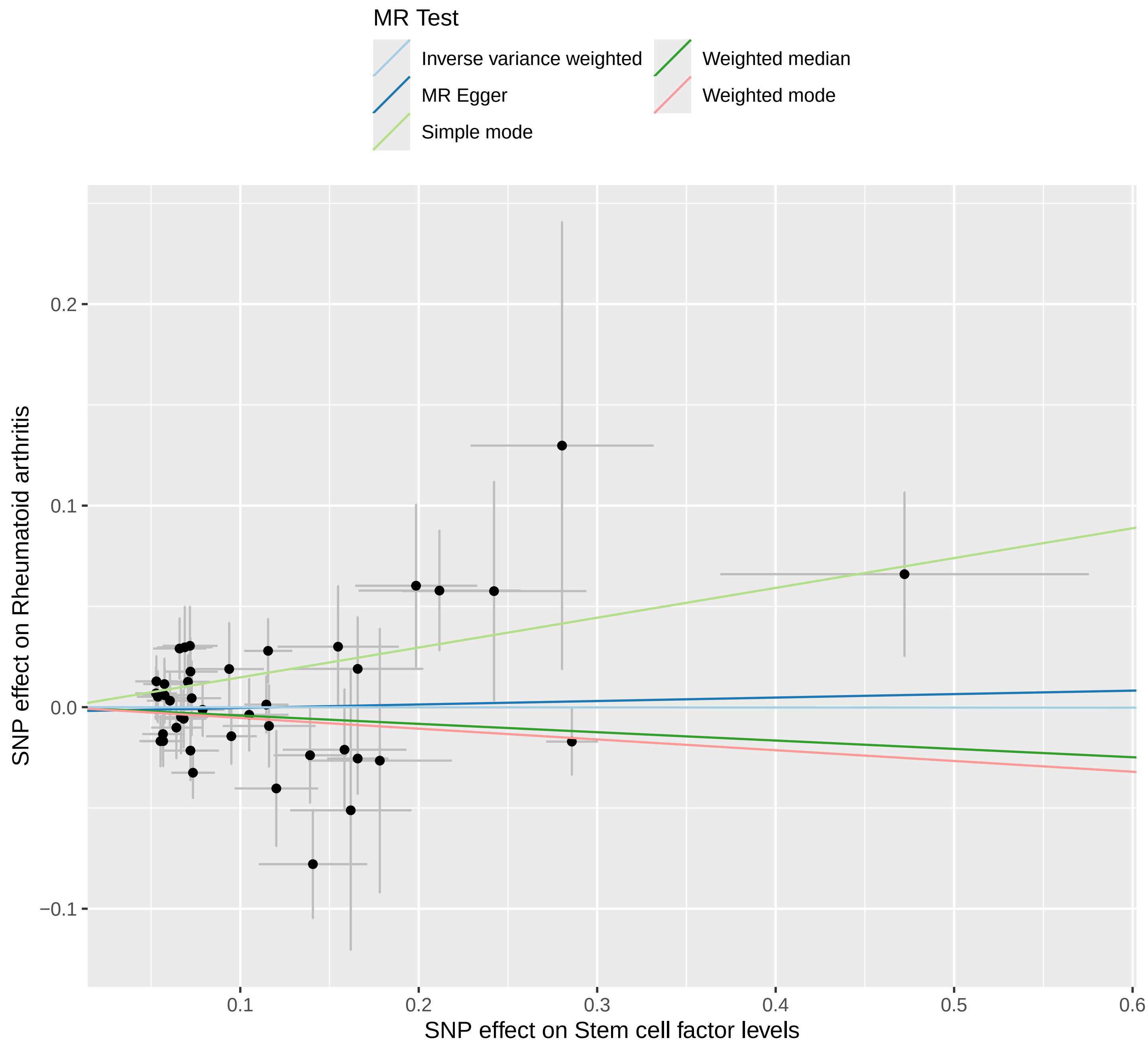

MR Test
Inverse variance weighted
MR Egger
Simple mode
Weighted median
Weighted mode
SNP effect on Rheumatoid arthritis
0.2
0.1
0.0
−0.1
0.1
0.2
0.3
0.4
0.5
0.6
SNP effect on Stem cell factor levels

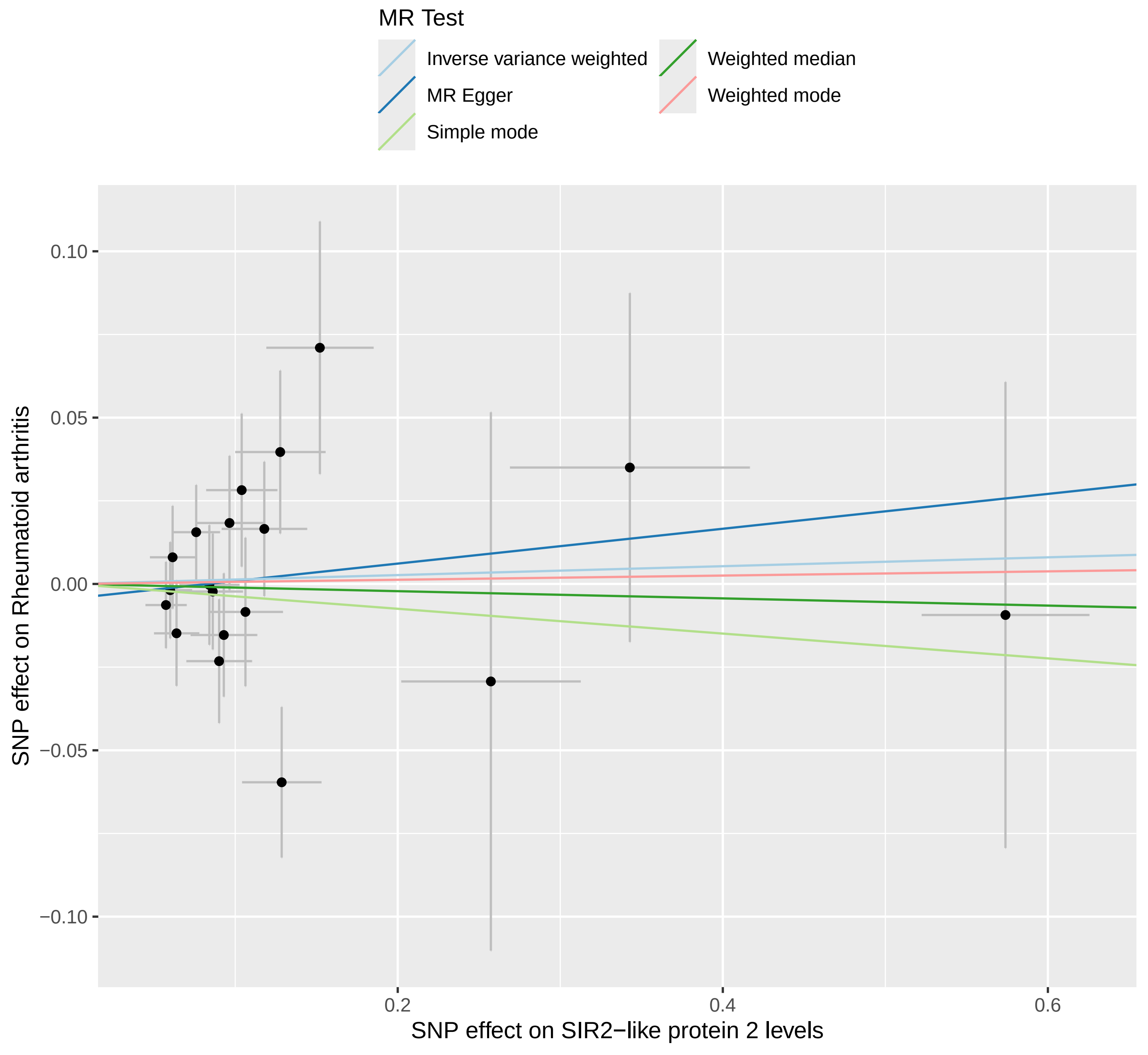
MR Test
Inverse variance weighted
MR Egger
Simple mode
Weighted median
Weighted mode
0.10
0.05
0.00
−0.05
−0.10
0.2
0.4
0.6
SNP effect on Rheumatoid arthritis
SNP effect on SIR2−like protein 2 levels

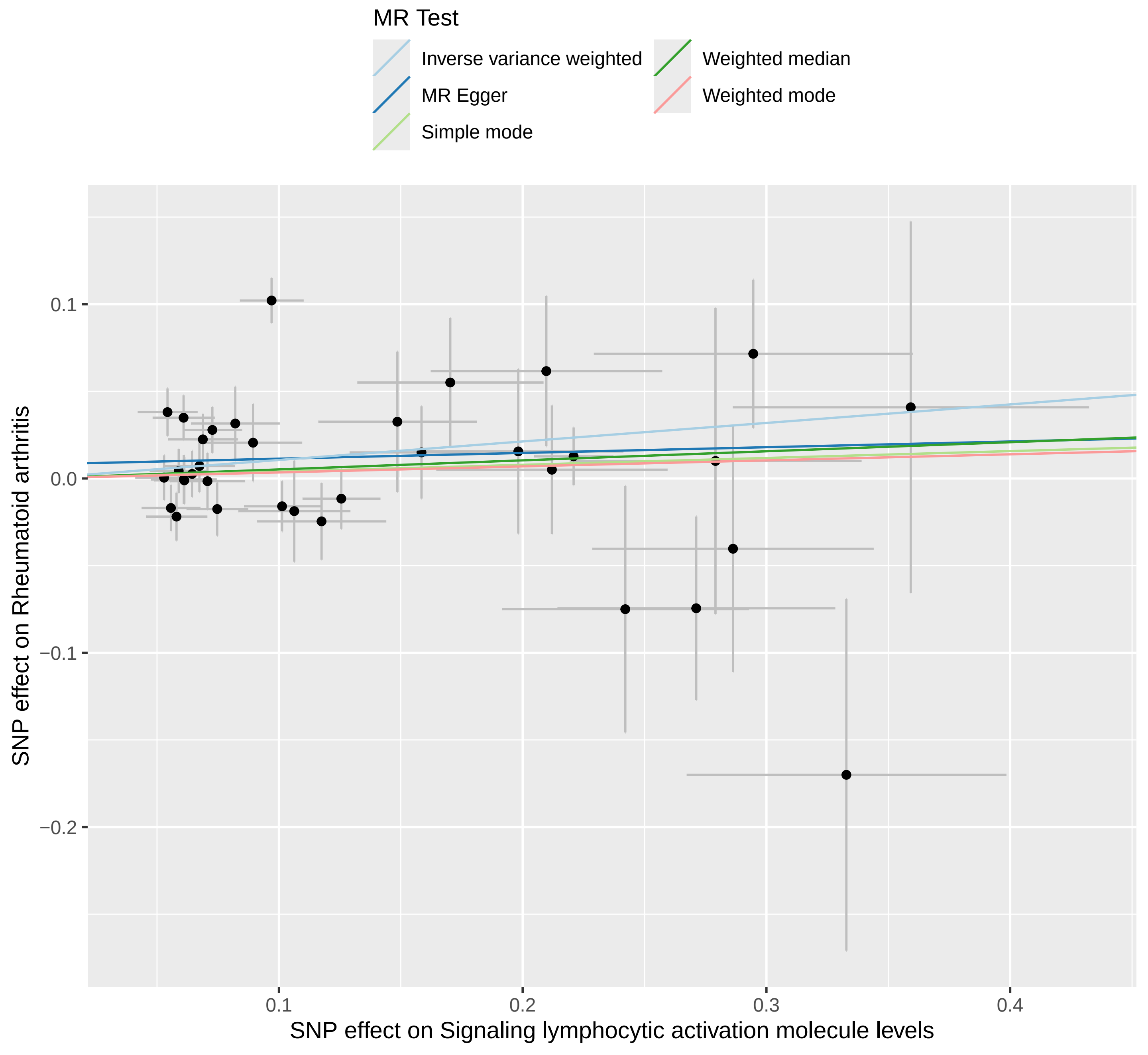

MR Test
Inverse variance weighted
MR Egger
Simple mode
Weighted median
Weighted mode
SNP effect on Rheumatoid arthritis
0.1
0.0
−0.1
−0.2
0.1
0.2
0.3
0.4
SNP effect on Signaling lymphocytic activation molecule levels

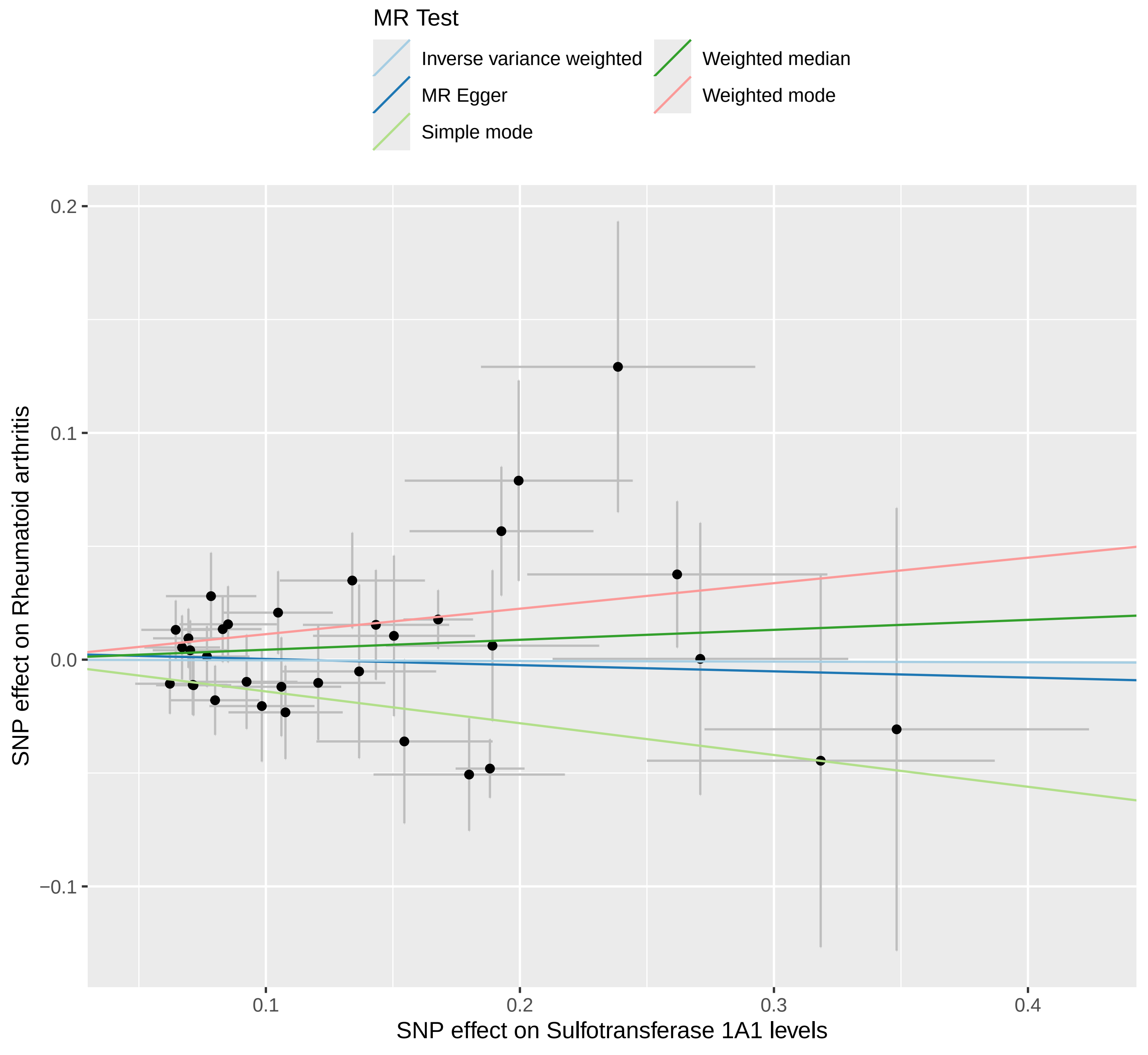
MR Test
Inverse variance weighted
MR Egger
Simple mode
Weighted median
Weighted mode
SNP effect on Rheumatoid arthritis
0.2
0.1
0.0
−0.1
0.1
0.2
0.3
0.4
SNP effect on Sulfotransferase 1A1 levels

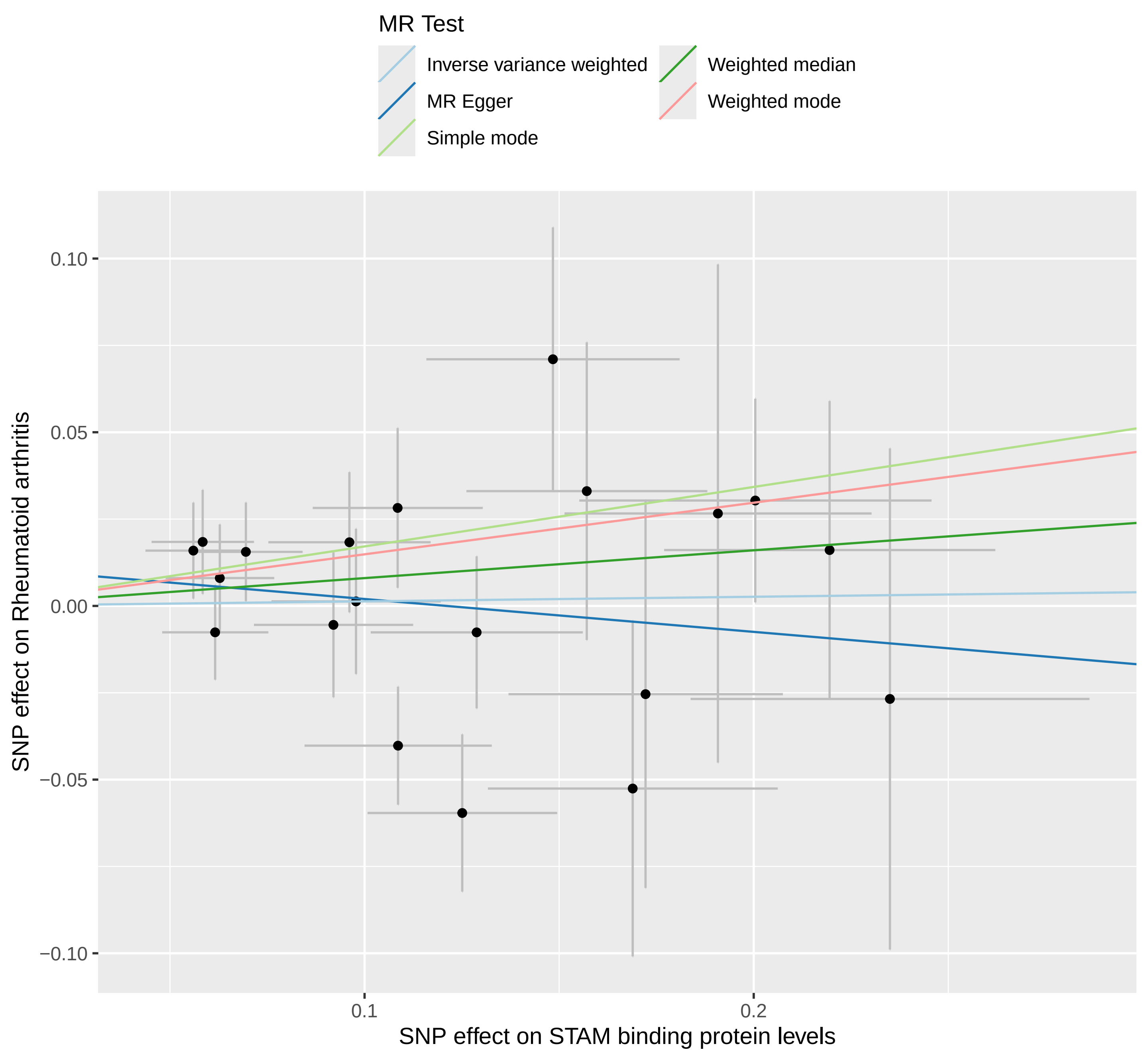
MR Test
Inverse variance weighted
MR Egger
Simple mode
Weighted median
Weighted mode
0.10
0.05
0.00
−0.05
−0.10
0.1
0.2
SNP effect on Rheumatoid arthritis
SNP effect on STAM binding protein levels

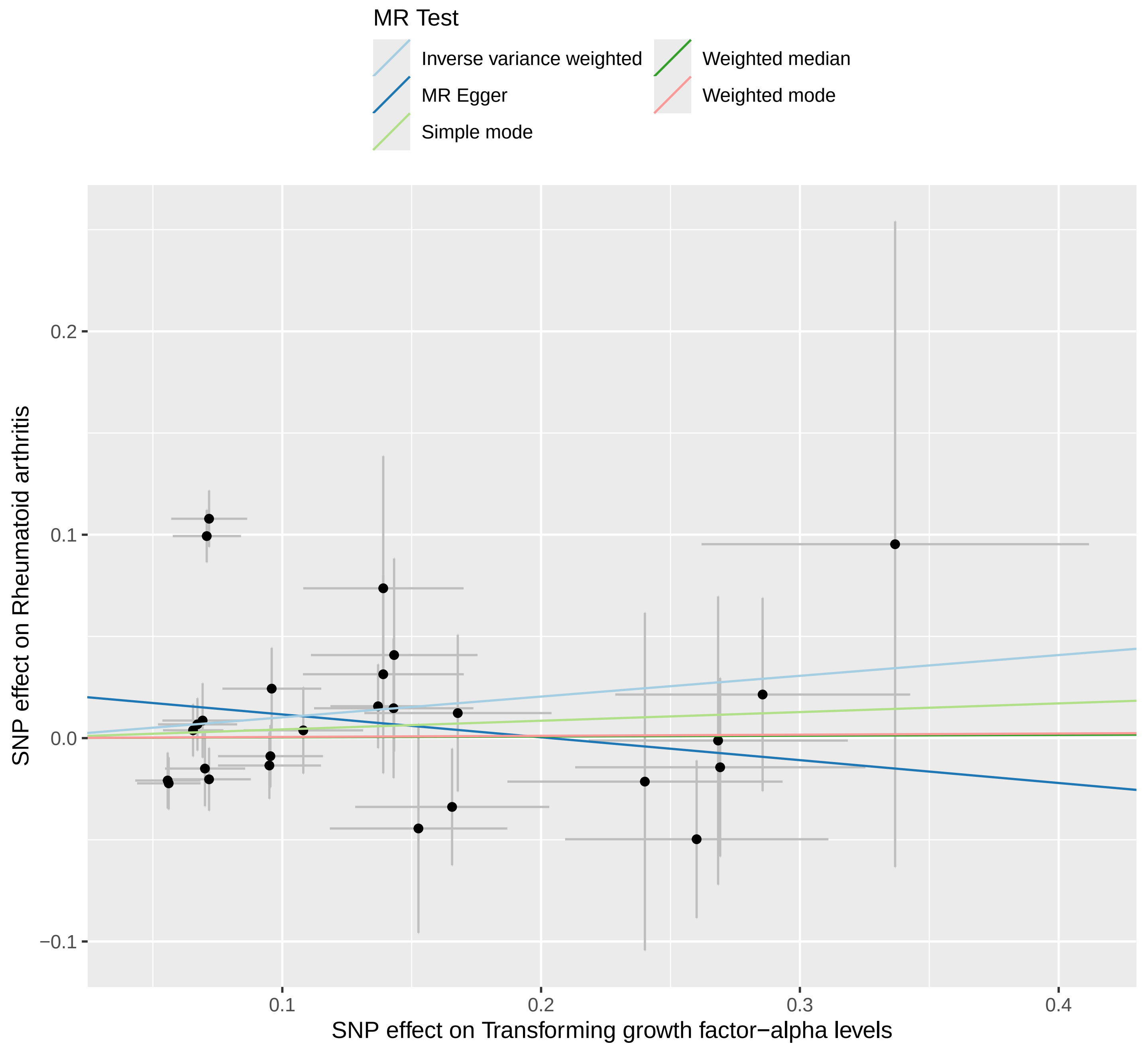

MR Test
Inverse variance weighted
MR Egger
Simple mode
Weighted median
Weighted mode
0.2
0.1
0.0
−0.1
0.1
0.2
0.3
0.4
SNP effect on Rheumatoid arthritis
SNP effect on Transforming growth factor−alpha levels

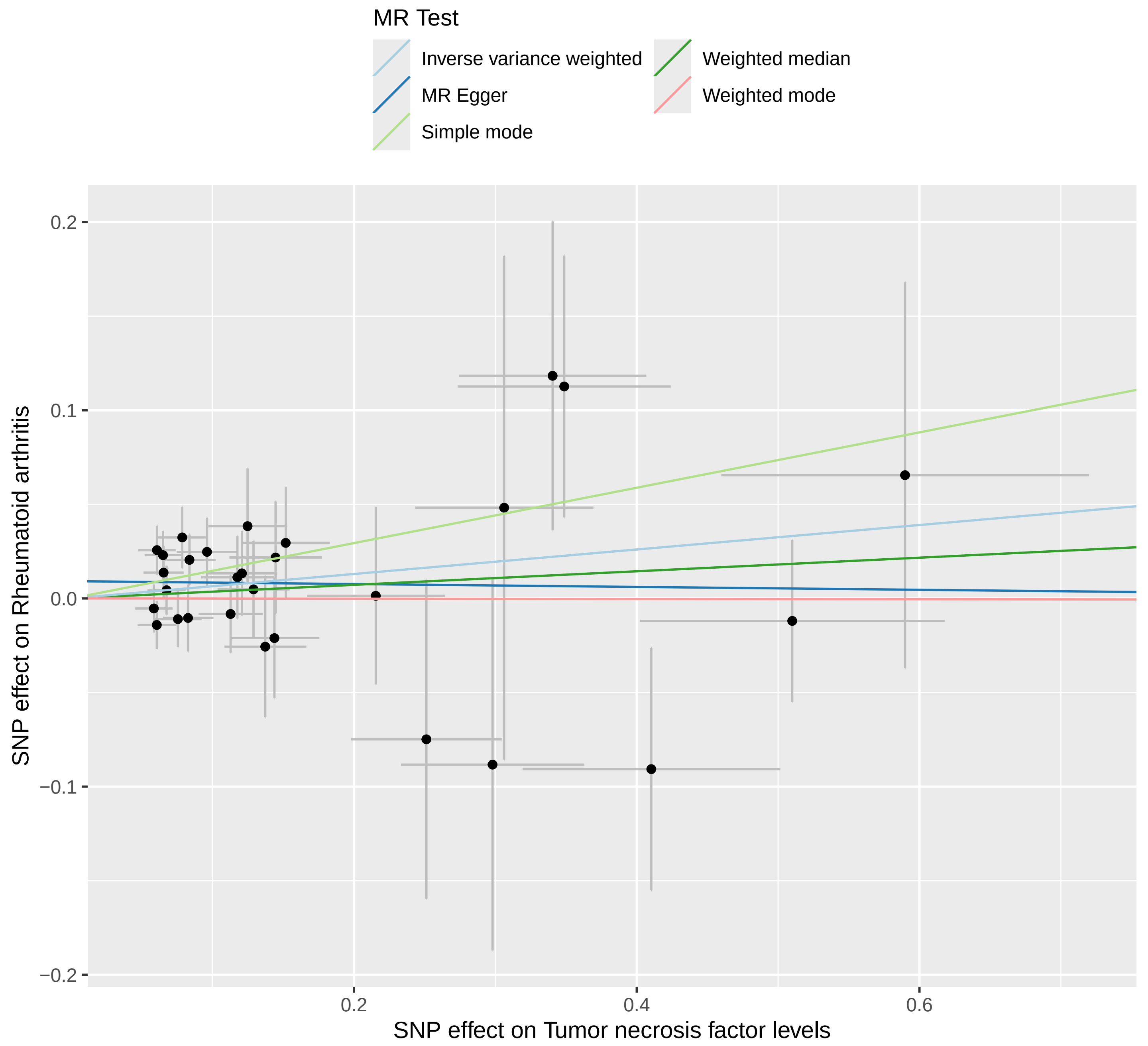

MR Test
Inverse variance weighted
MR Egger
Simple mode
Weighted median
Weighted mode
0.2
0.1
0.0
−0.1
−0.2
SNP effect on Rheumatoid arthritis
0.2
0.4
0.6
SNP effect on Tumor necrosis factor levels

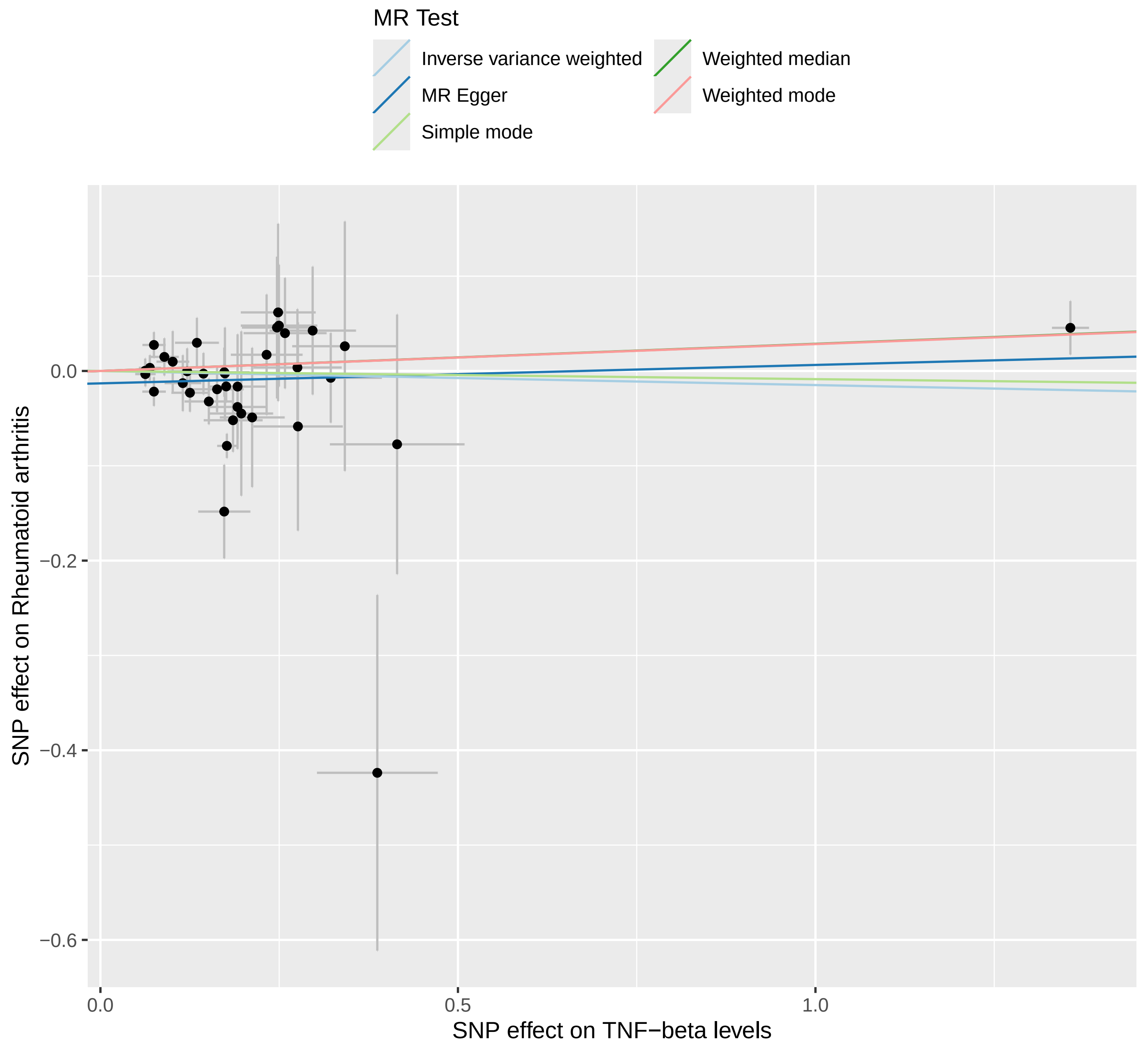

MR Test
Inverse variance weighted
MR Egger
Simple mode
Weighted median
Weighted mode
SNP effect on Rheumatoid arthritis
0.0
−0.2
−0.4
−0.6
0.0
0.5
1.0
SNP effect on TNF−beta levels

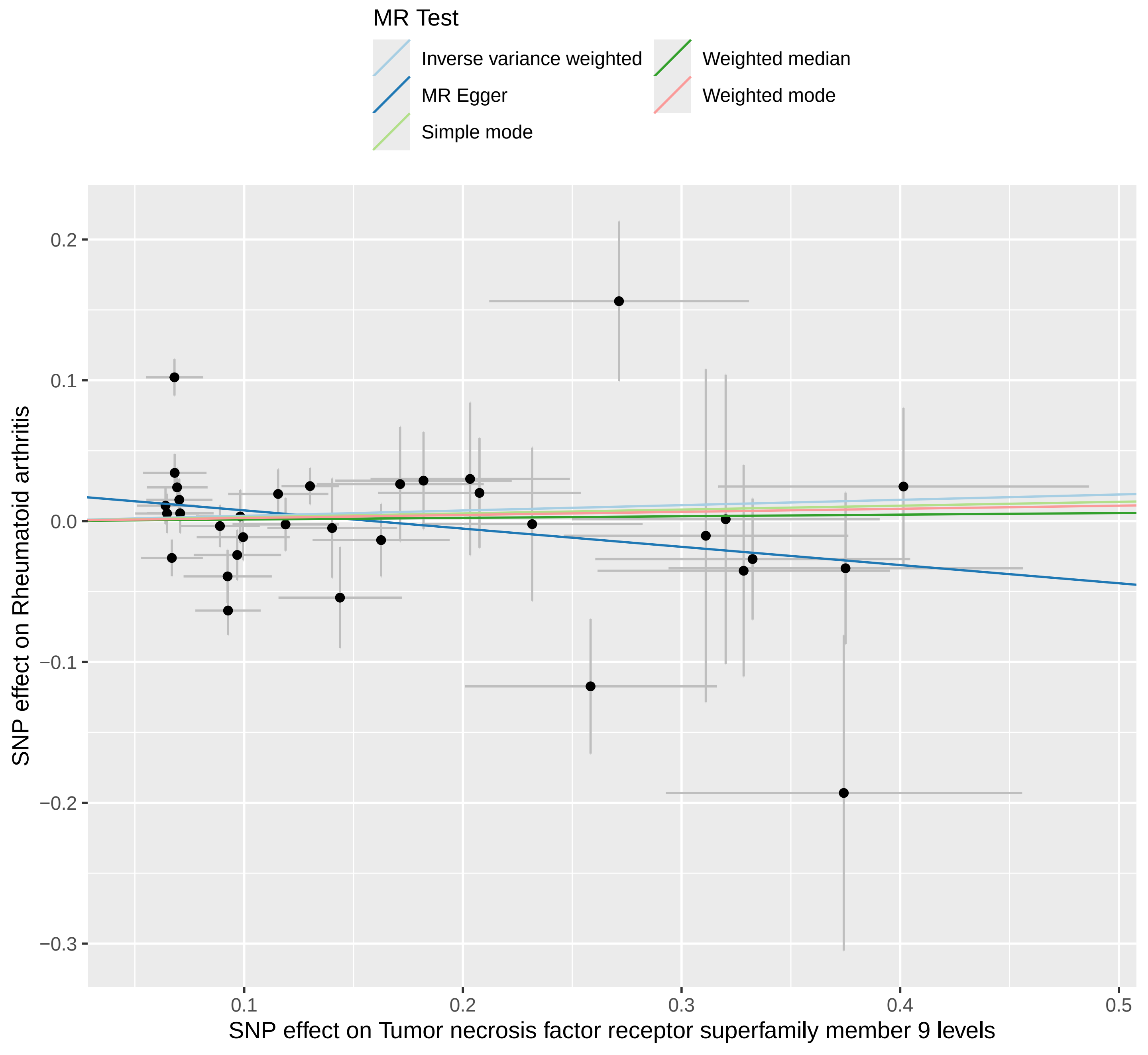

MR Test
Inverse variance weighted
MR Egger
Simple mode
Weighted median
Weighted mode
0.2
0.1
0.0
−0.1
−0.2
−0.3
SNP effect on Rheumatoid arthritis
0.1
0.2
0.3
0.4
0.5
SNP effect on Tumor necrosis factor receptor superfamily member 9 levels

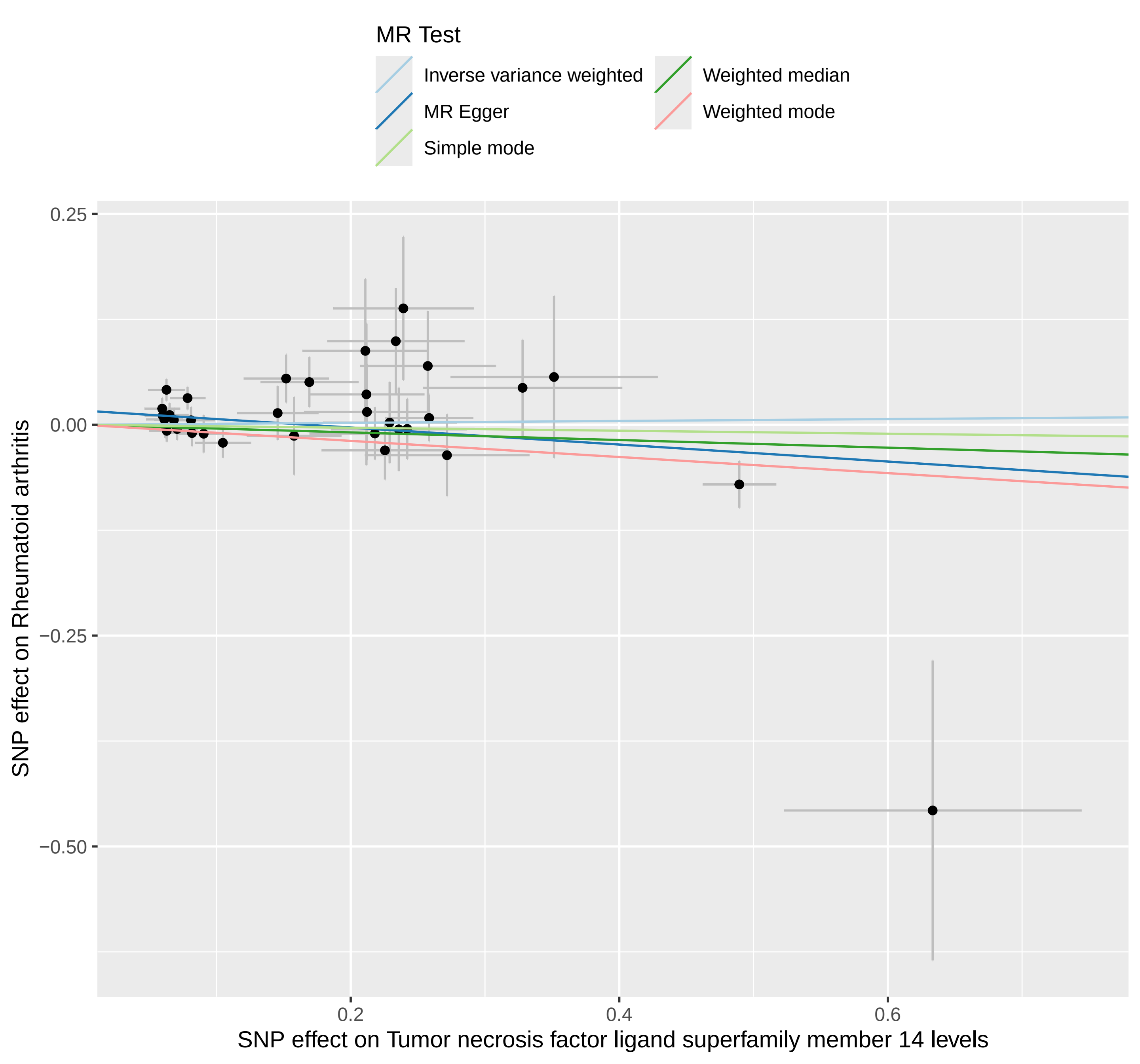
MR Test
Inverse variance weighted
MR Egger
Simple mode
Weighted median
Weighted mode
0.25
0.00
−0.25
−0.50
SNP effect on Rheumatoid arthritis
0.2
0.4
0.6
SNP effect on Tumor necrosis factor ligand superfamily member 14 levels

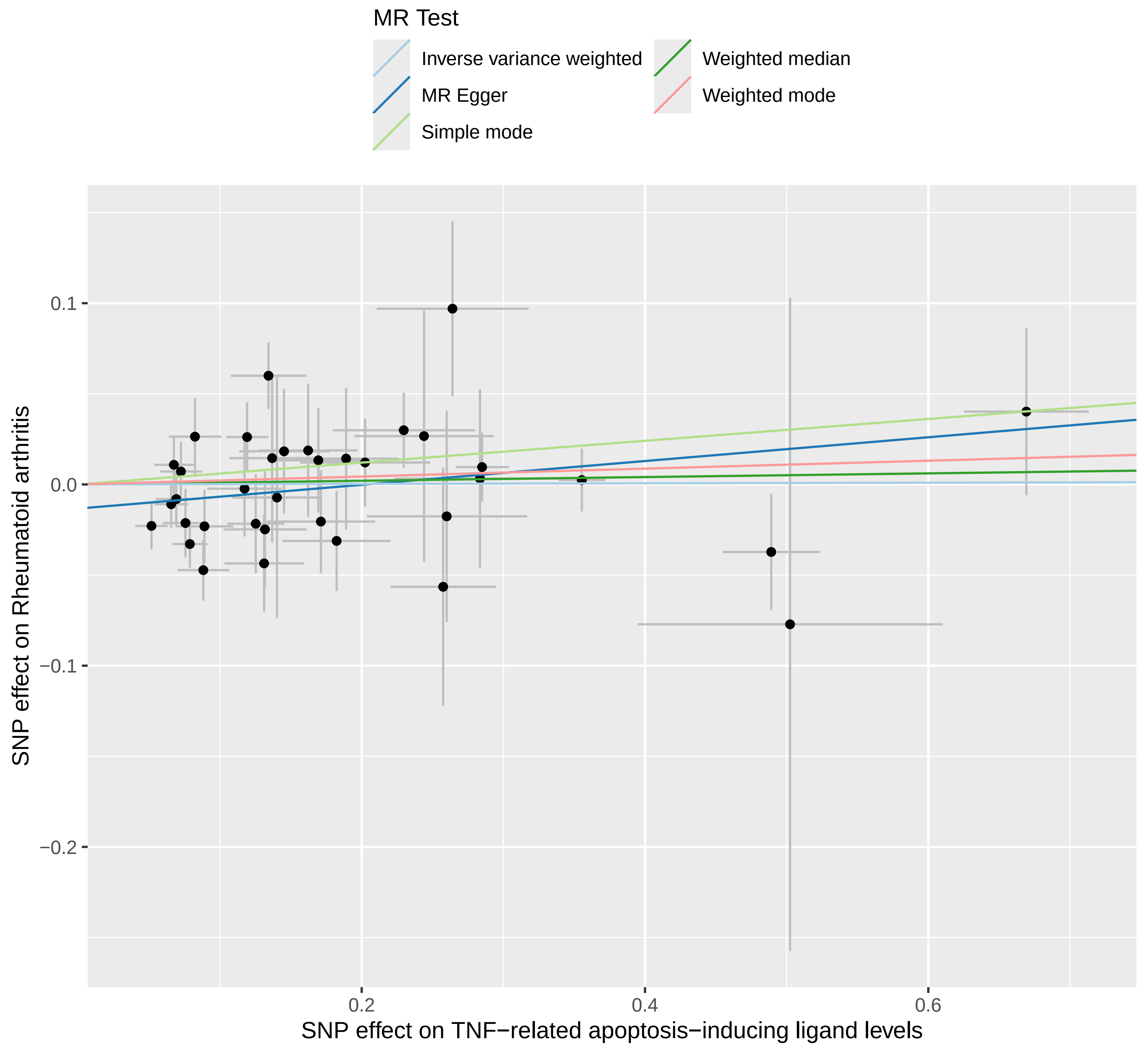
MR Test
Inverse variance weighted
MR Egger
Simple mode
Weighted median
Weighted mode
0.1
0.0
−0.1
−0.2
0.2
0.4
0.6
SNP effect on Rheumatoid arthritis
SNP effect on TNF−related apoptosis−inducing ligand levels

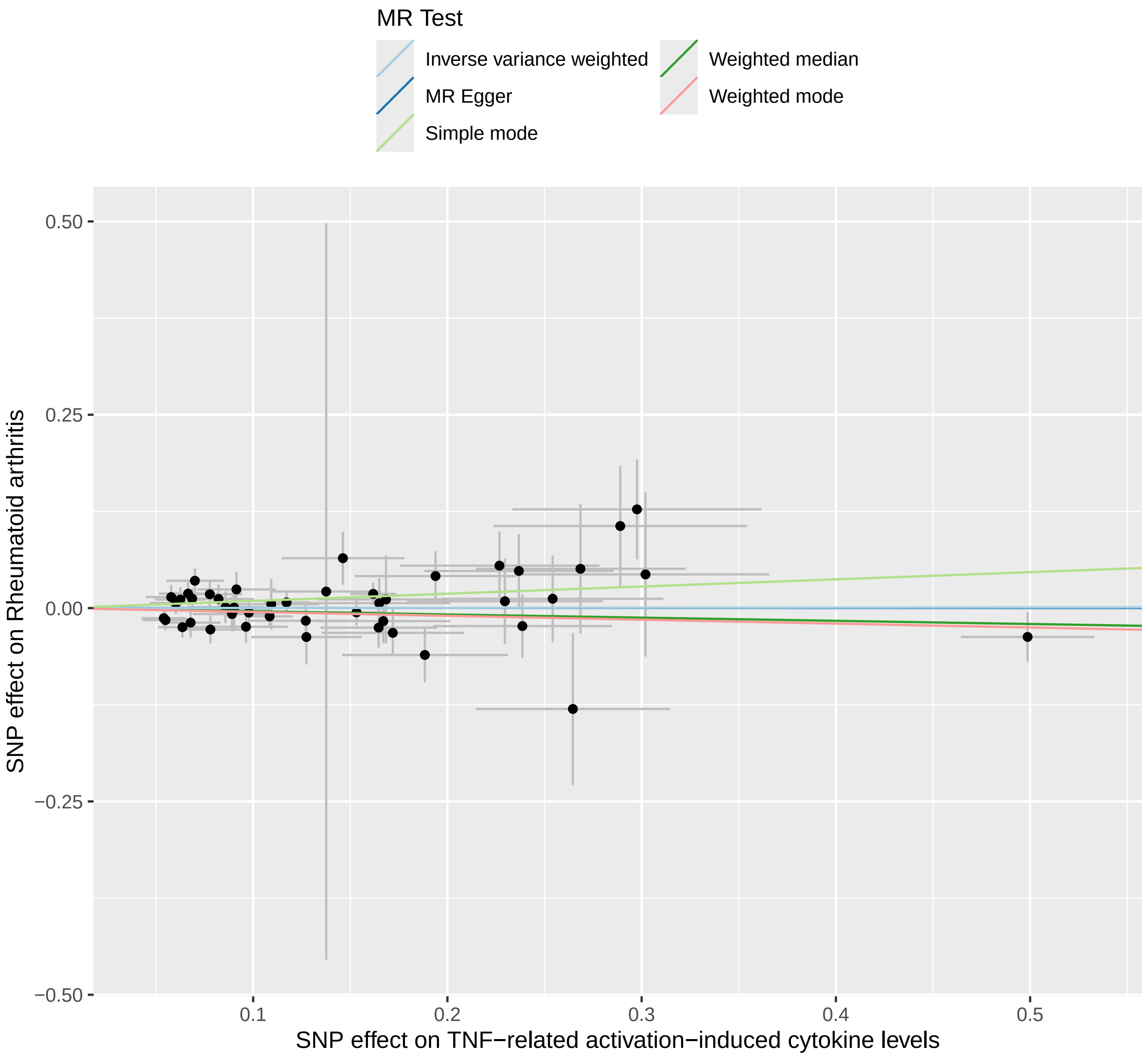
MR Test
Inverse variance weighted
MR Egger
Simple mode
Weighted median
Weighted mode
0.50
0.25
0.00
−0.25
−0.50
0.1
0.2
0.3
0.4
0.5
SNP effect on Rheumatoid arthritis
SNP effect on TNF−related activation−induced cytokine levels

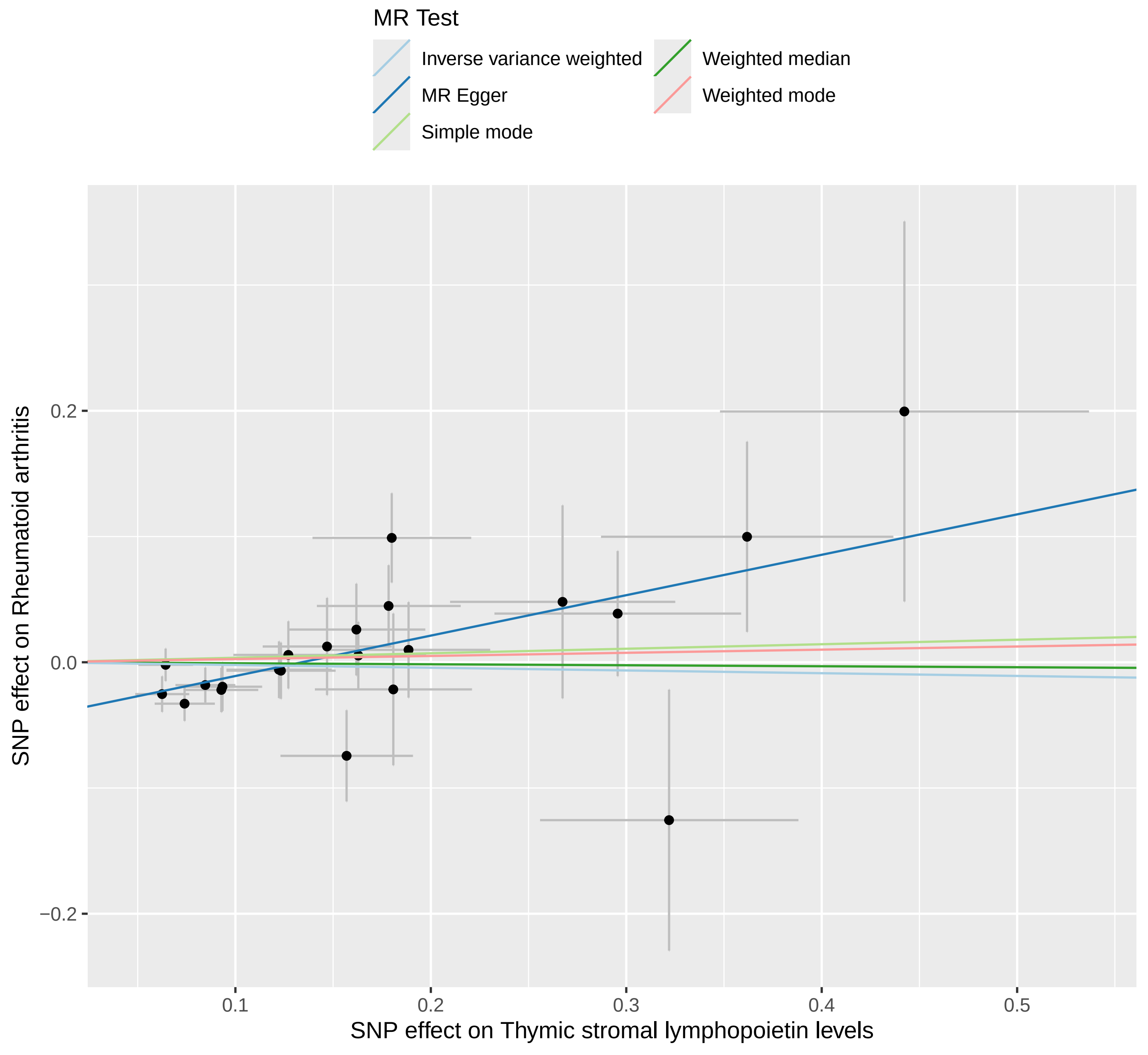

MR Test
Inverse variance weighted
MR Egger
Simple mode
Weighted median
Weighted mode
0.2
0.0
−0.2
0.1
0.2
0.3
0.4
0.5
SNP effect on Rheumatoid arthritis
SNP effect on Thymic stromal lymphopoietin levels

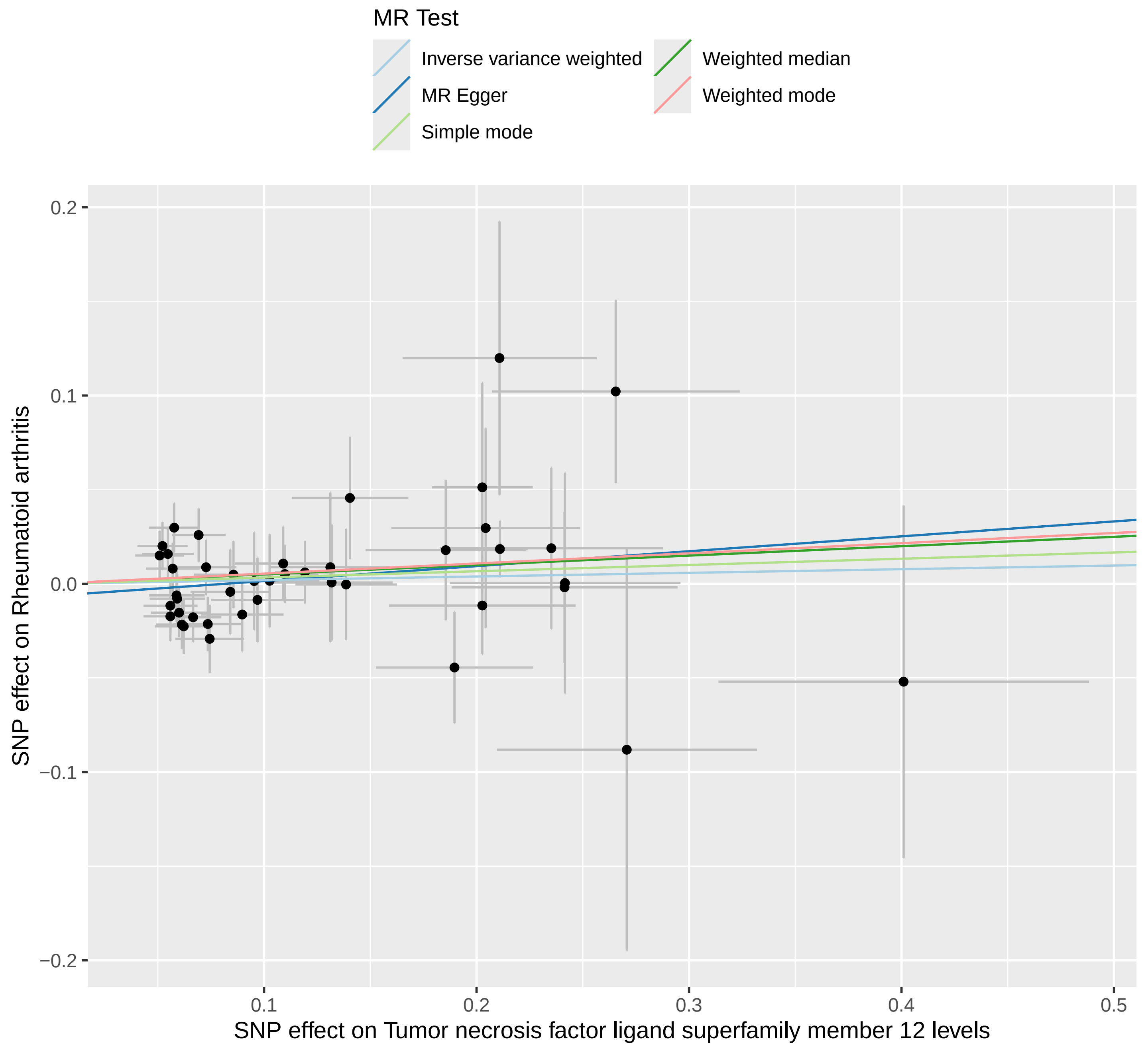

MR Test
Inverse variance weighted
MR Egger
Simple mode
Weighted median
Weighted mode
0.2
0.1
0.0
−0.1
−0.2
SNP effect on Rheumatoid arthritis
0.1
0.2
0.3
0.4
0.5
SNP effect on Tumor necrosis factor ligand superfamily member 12 levels

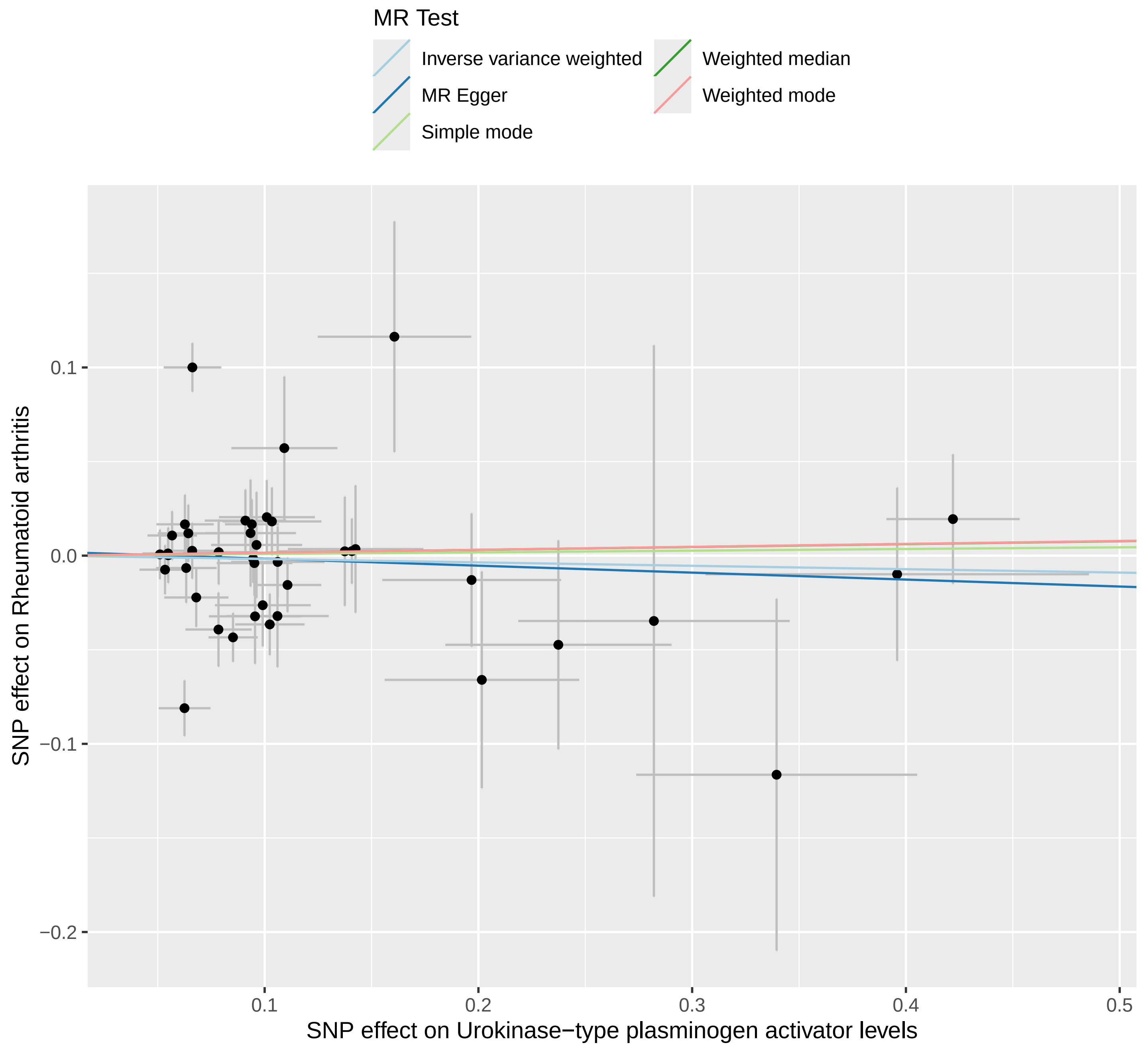
MR Test
Inverse variance weighted
MR Egger
Simple mode
Weighted median
Weighted mode
0.1
0.0
−0.1
−0.2
0.1
0.2
0.3
0.4
0.5
SNP effect on Rheumatoid arthritis
SNP effect on Urokinase−type plasminogen activator levels

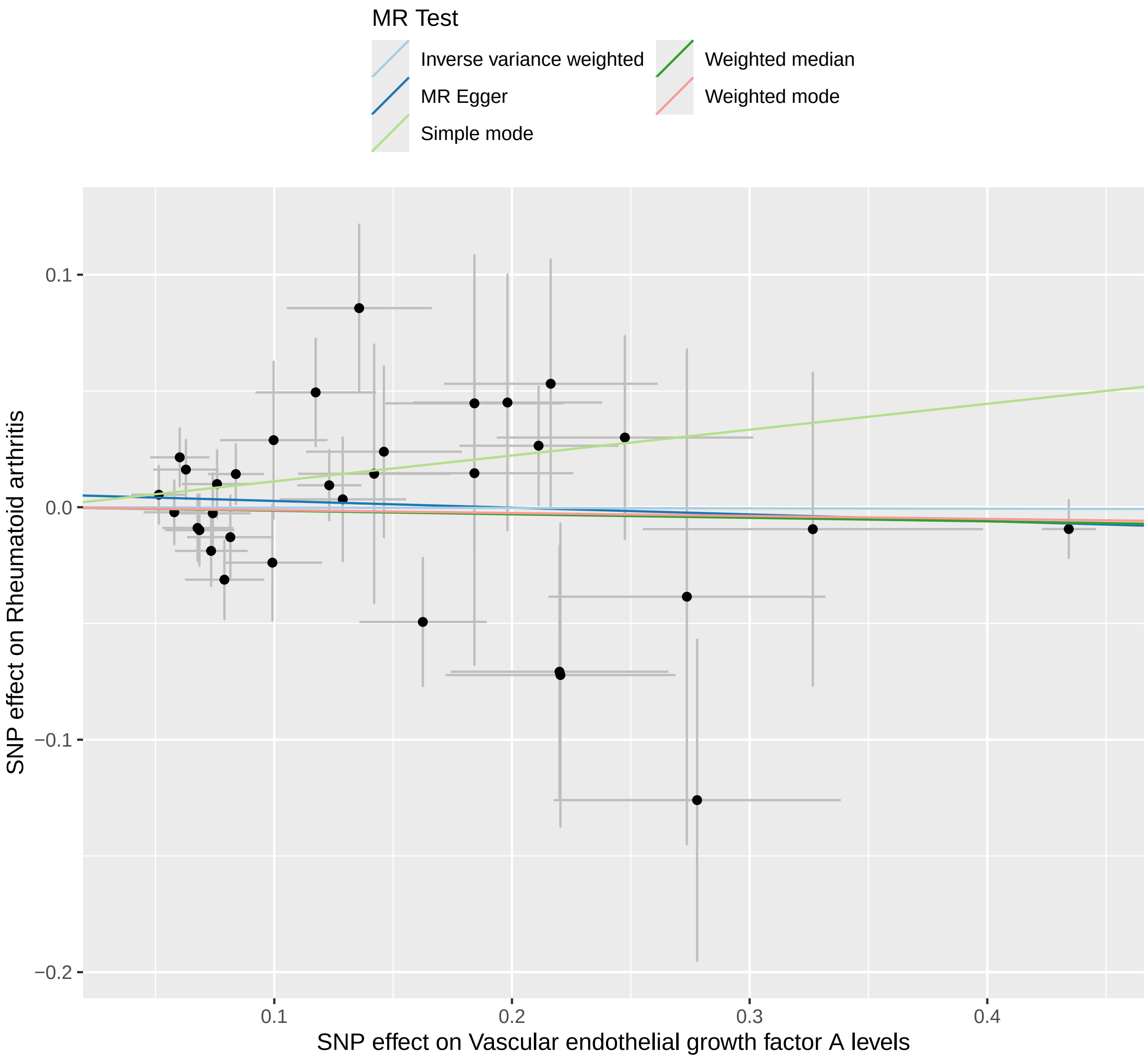

MR Test
Inverse variance weighted
MR Egger
Simple mode
Weighted median
Weighted mode
0.1
0.0
−0.1
−0.2
SNP effect on Rheumatoid arthritis
0.1
0.2
0.3
0.4
SNP effect on Vascular endothelial growth factor A levels

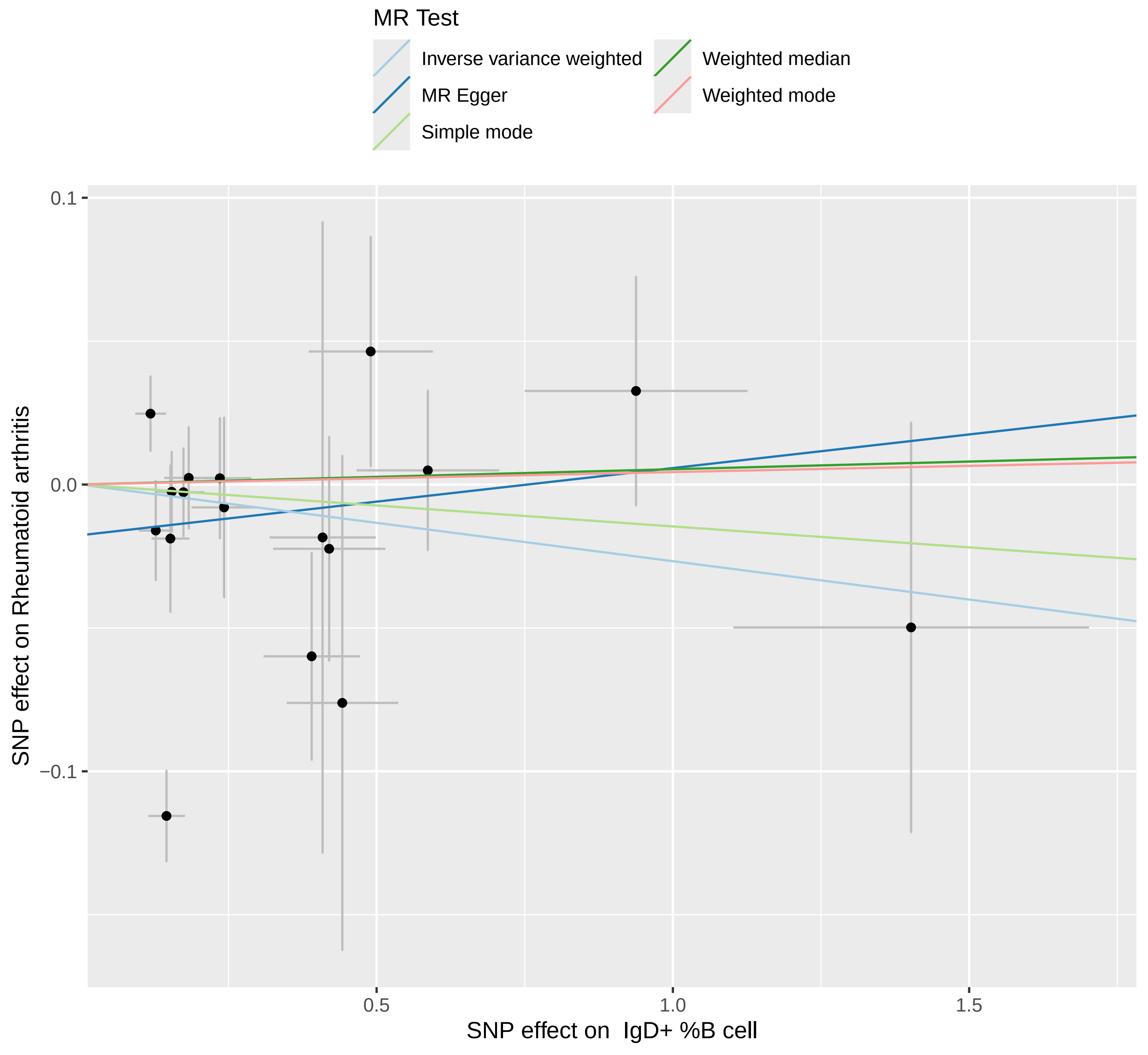
MR Test
Inverse variance weighted
MR Egger
Simple mode
Weighted median
Weighted mode
0.1
0.0
−0.1
0.5
1.0
1.5
SNP effect on Rheumatoid arthritis
SNP effect on IgD+ %B cell

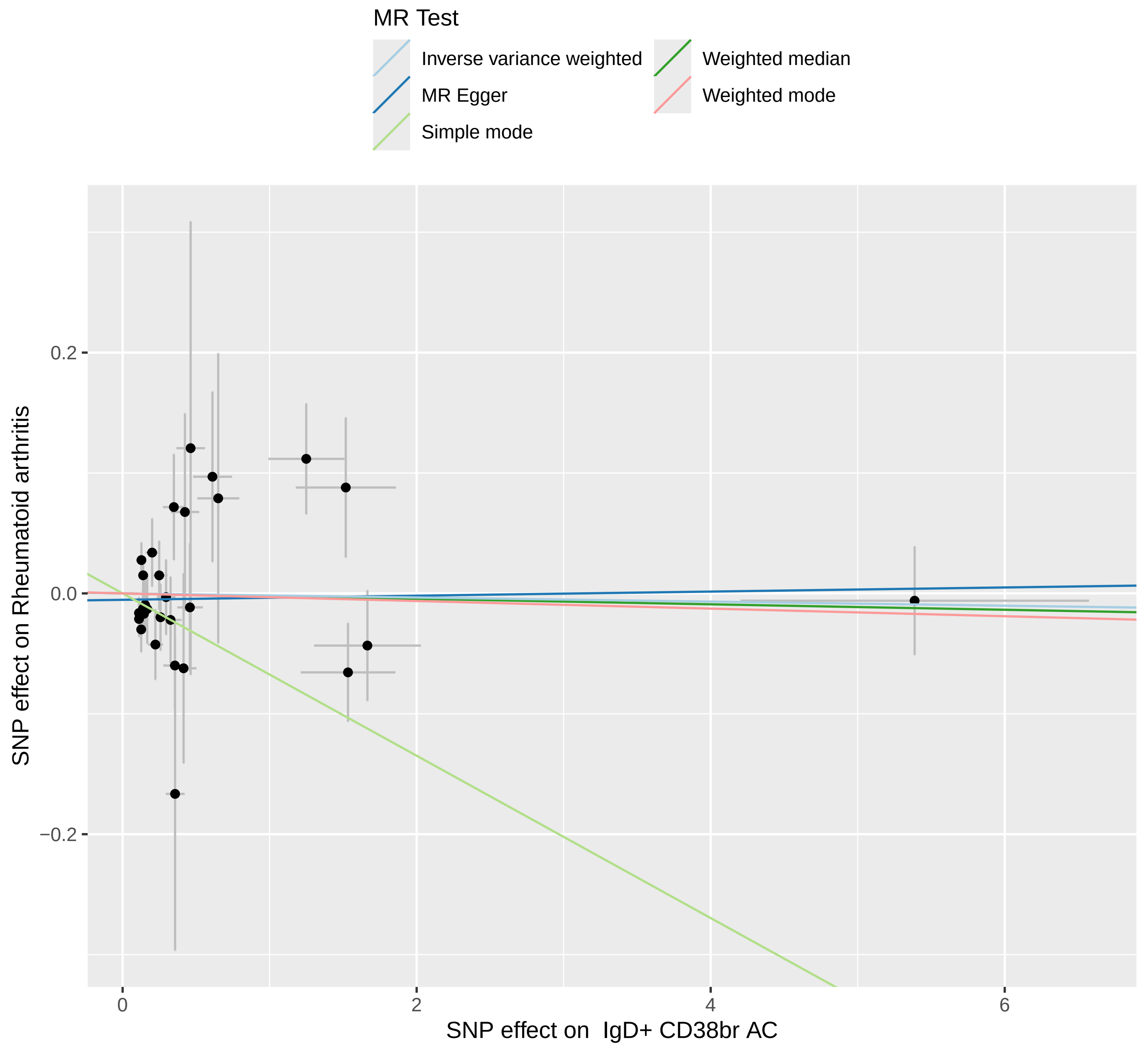

MR Test
Inverse variance weighted
MR Egger
Simple mode
Weighted median
Weighted mode
0.2
0.0
−0.2
0
2
4
6
SNP effect on Rheumatoid arthritis
SNP effect on IgD+ CD38br AC

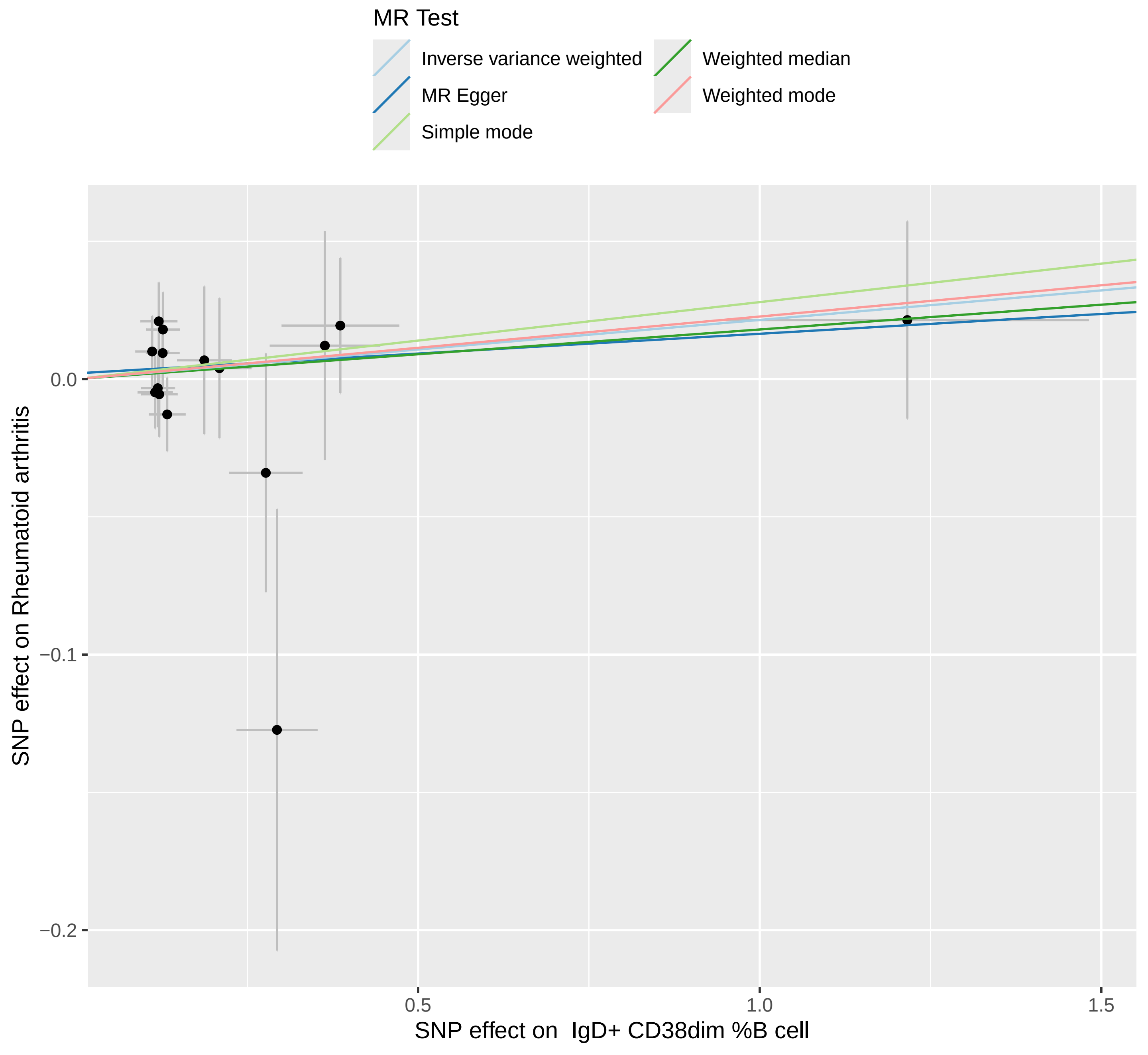

MR Test
Inverse variance weighted
MR Egger
Simple mode
Weighted median
Weighted mode
0.0
−0.1
−0.2
0.5
1.0
1.5
SNP effect on Rheumatoid arthritis
SNP effect on IgD+ CD38dim %B cell

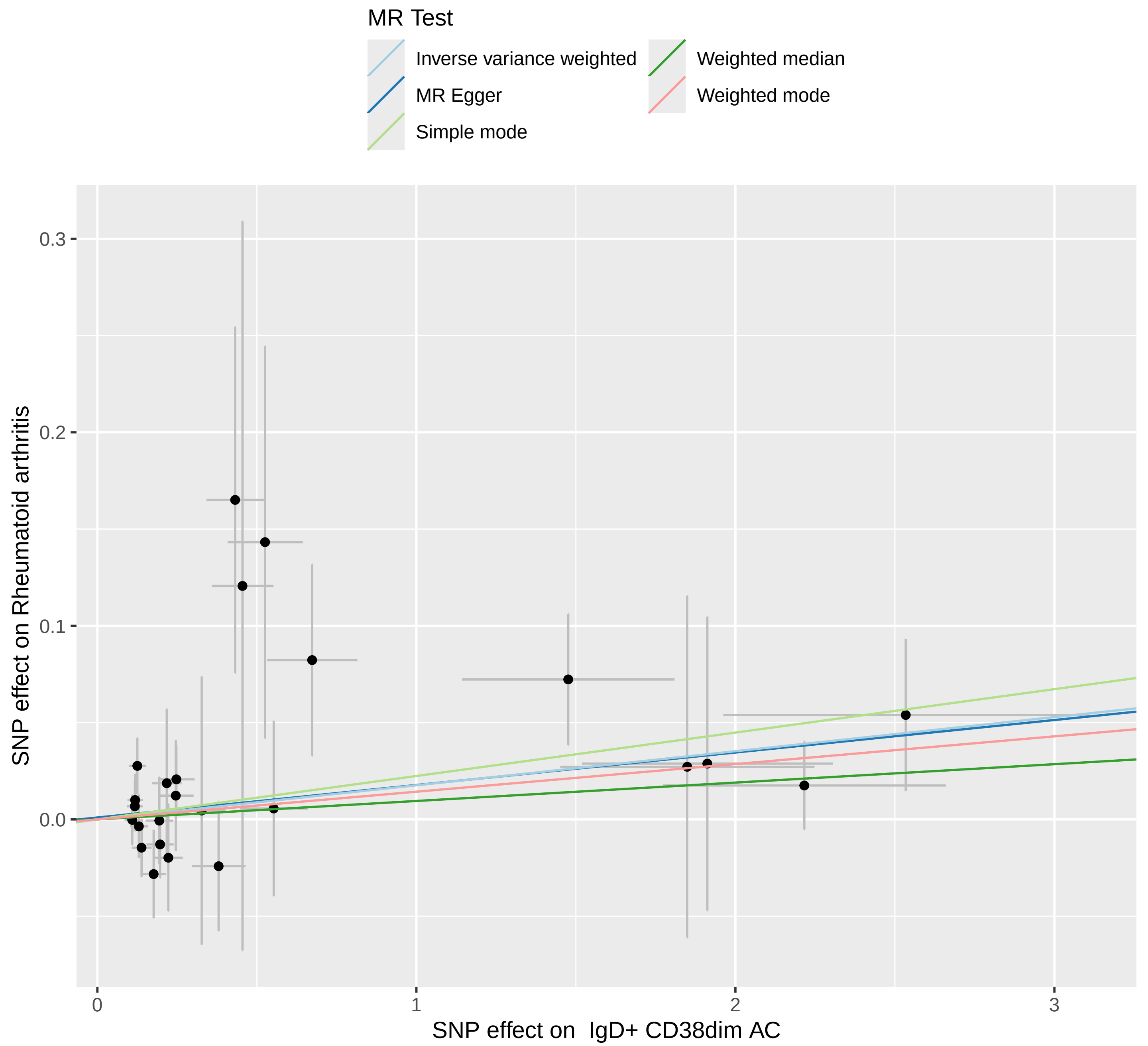

MR Test
Inverse variance weighted
MR Egger
Simple mode
Weighted median
Weighted mode
0.3
0.2
0.1
0.0
0
1
2
3
SNP effect on Rheumatoid arthritis
SNP effect on IgD+ CD38dim AC

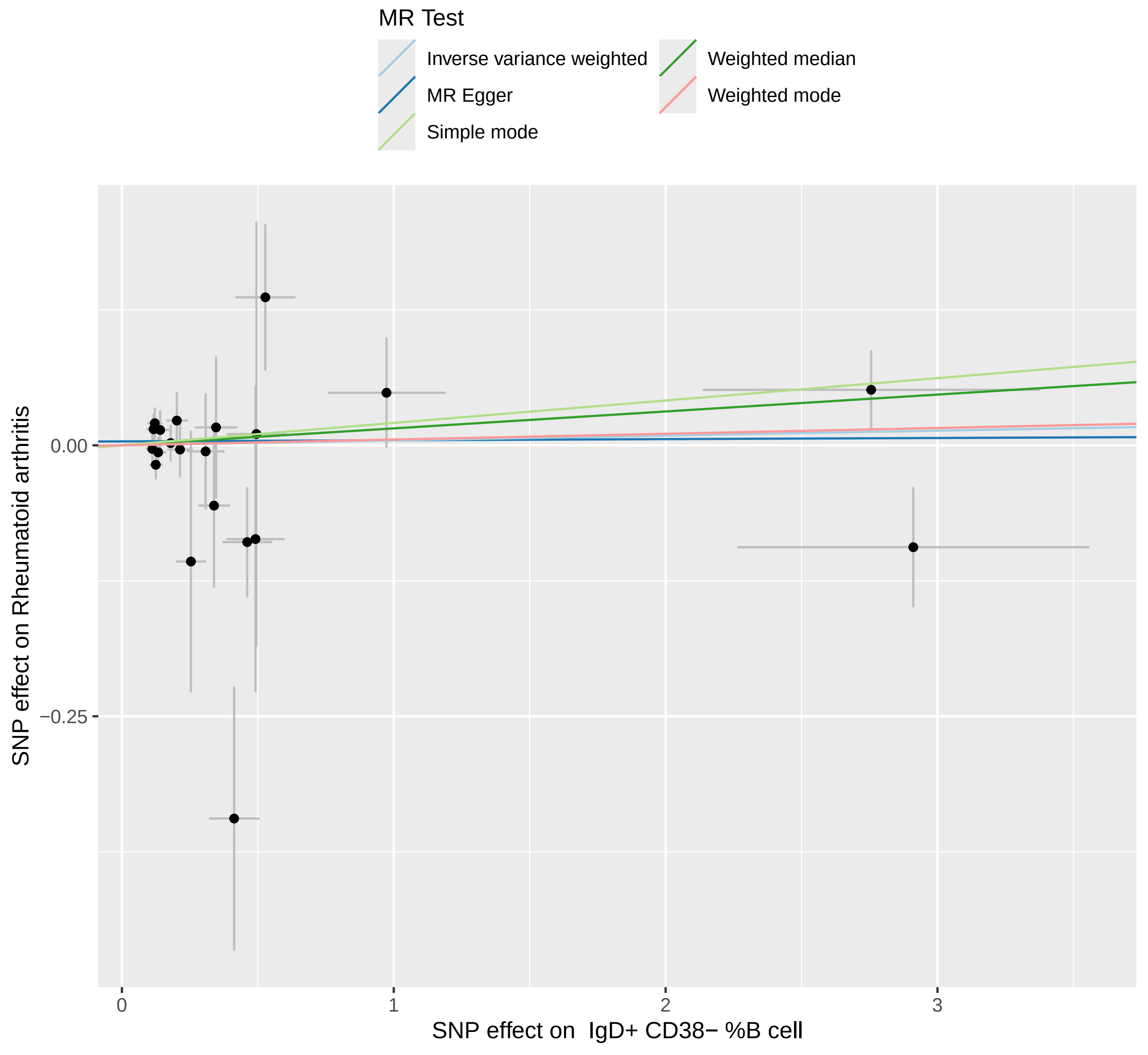
MR Test
Inverse variance weighted
MR Egger
Simple mode
Weighted median
Weighted mode
SNP effect on Rheumatoid arthritis
0.00
−0.25
0
1
2
3
SNP effect on IgD+ CD38− %B cell

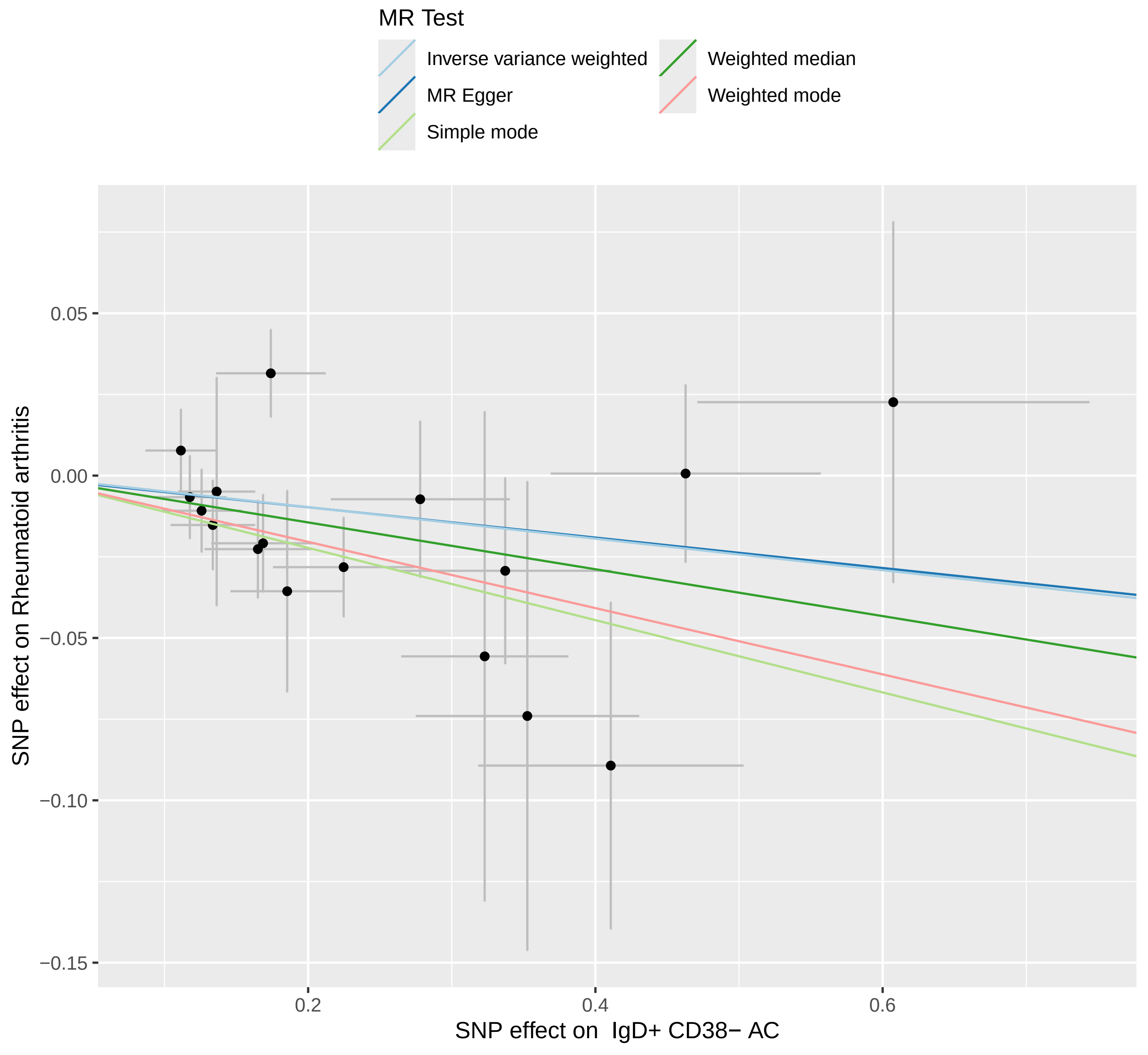

MR Test
Inverse variance weighted
MR Egger
Simple mode
Weighted median
Weighted mode
0.05
0.00
−0.05
−0.10
−0.15
SNP effect on Rheumatoid arthritis
0.2
0.4
0.6
SNP effect on IgD+ CD38− AC

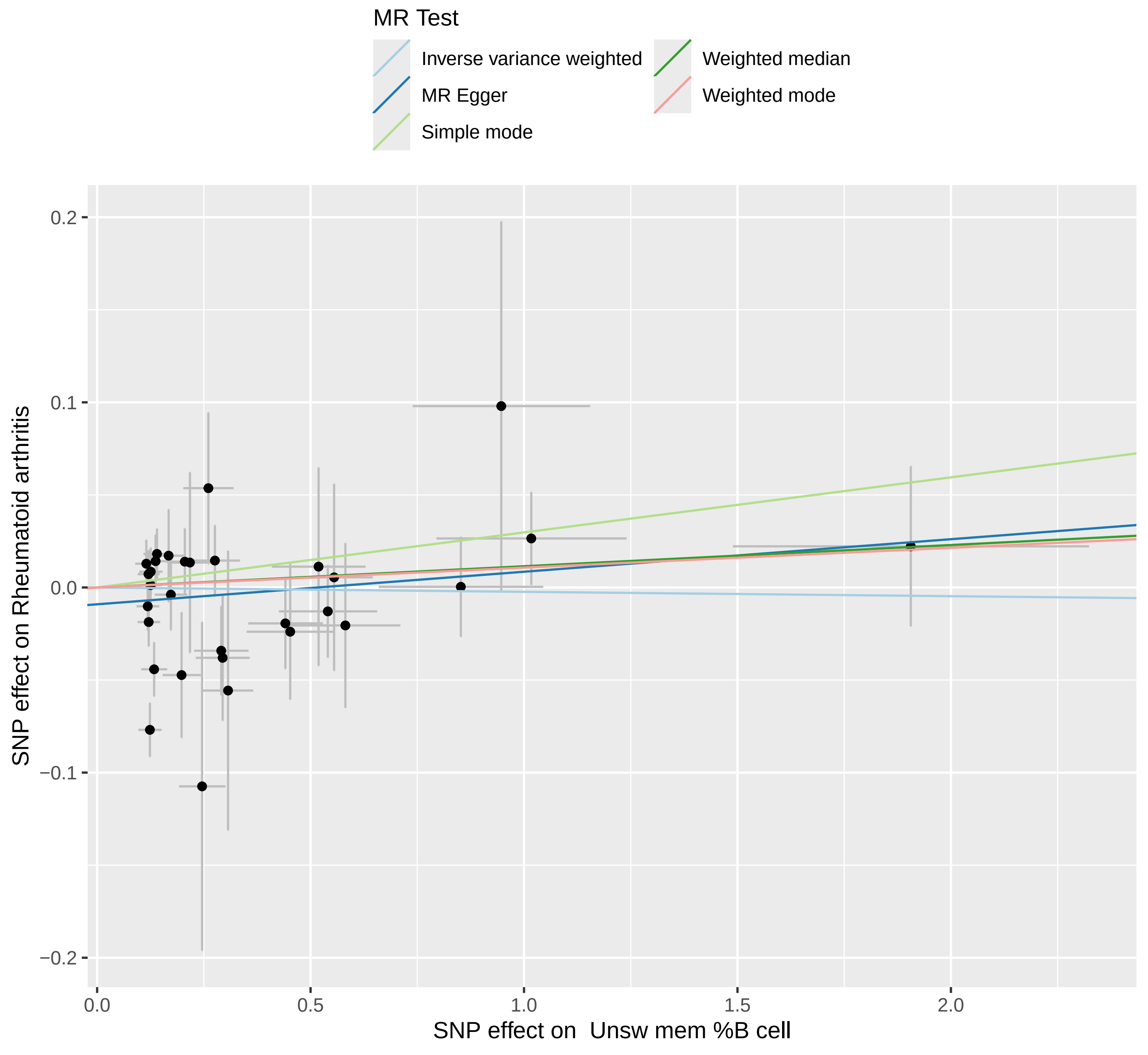

MR Test
Inverse variance weighted
MR Egger
Simple mode
Weighted median
Weighted mode
0.2
0.1
0.0
−0.1
−0.2
SNP effect on Rheumatoid arthritis
0.0
0.5
1.0
1.5
2.0
SNP effect on Unsw mem %B cell

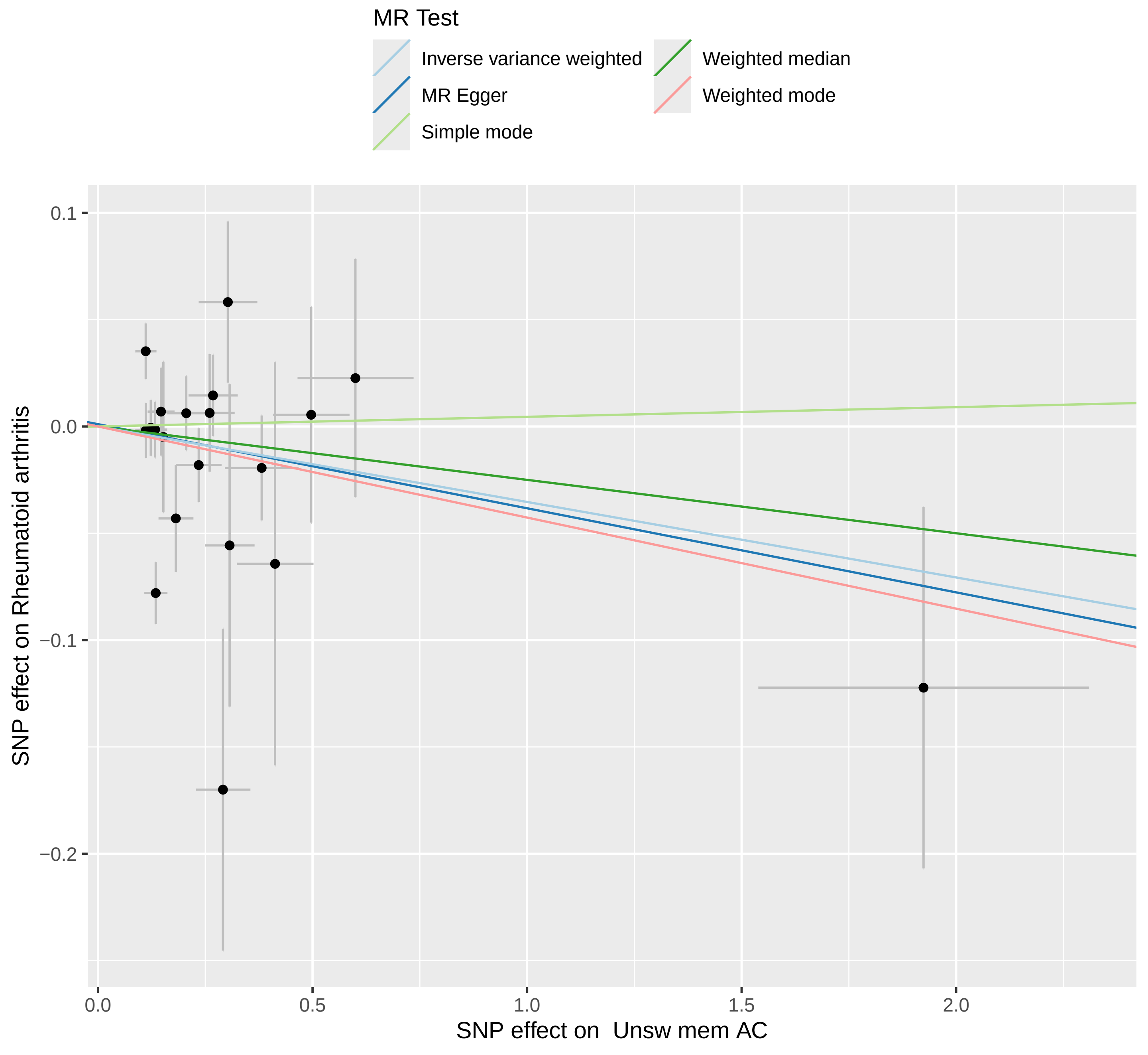

MR Test
Inverse variance weighted
MR Egger
Simple mode
Weighted median
Weighted mode
0.1
0.0
−0.1
−0.2
0.0
0.5
1.0
1.5
2.0
SNP effect on Rheumatoid arthritis
SNP effect on Unsw mem AC

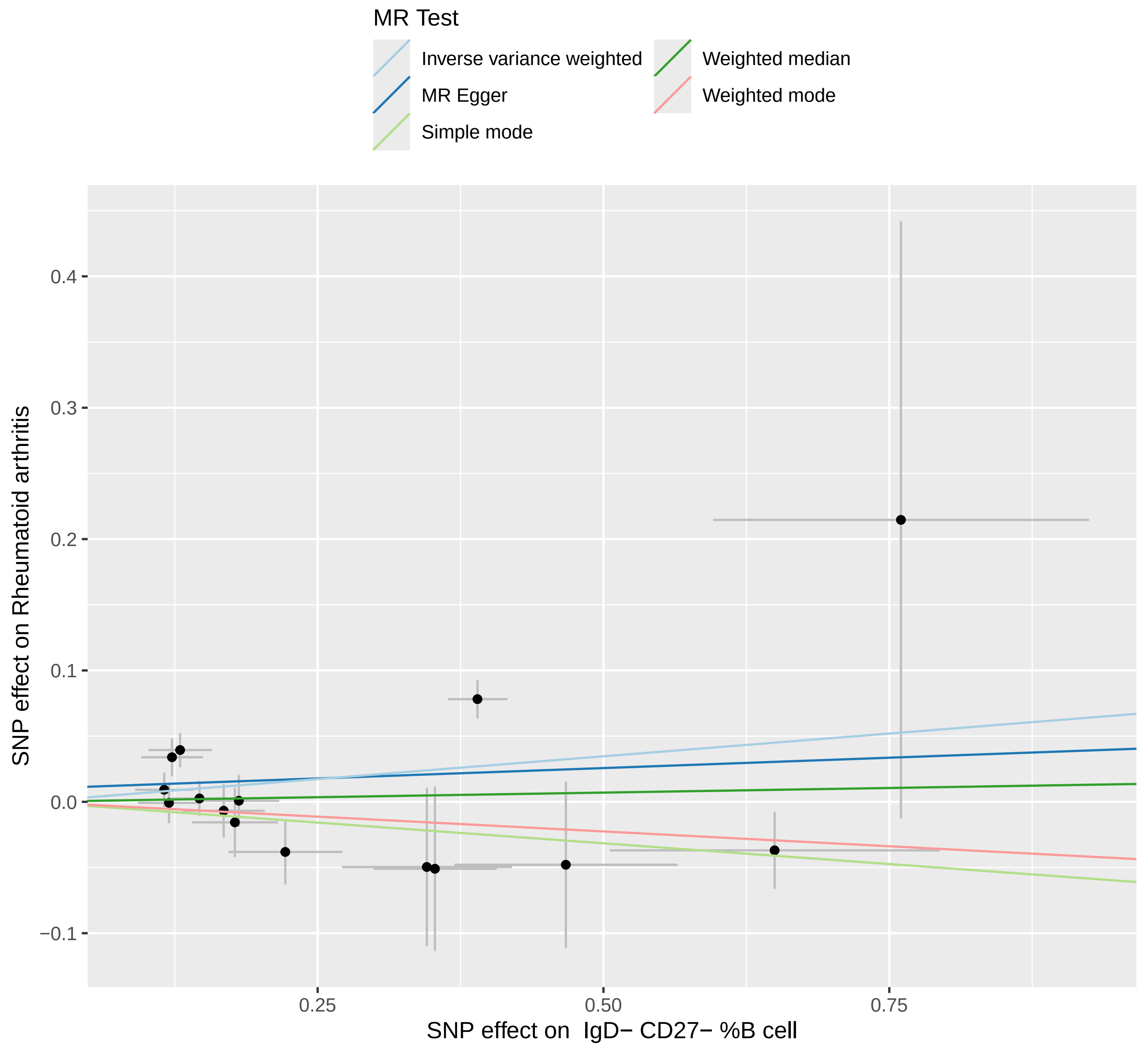
MR Test
Inverse variance weighted
MR Egger
Simple mode
Weighted median
Weighted mode
SNP effect on Rheumatoid arthritis
0.4
0.3
0.2
0.1
0.0
−0.1
0.25
0.50
0.75
SNP effect on IgD− CD27− %B cell

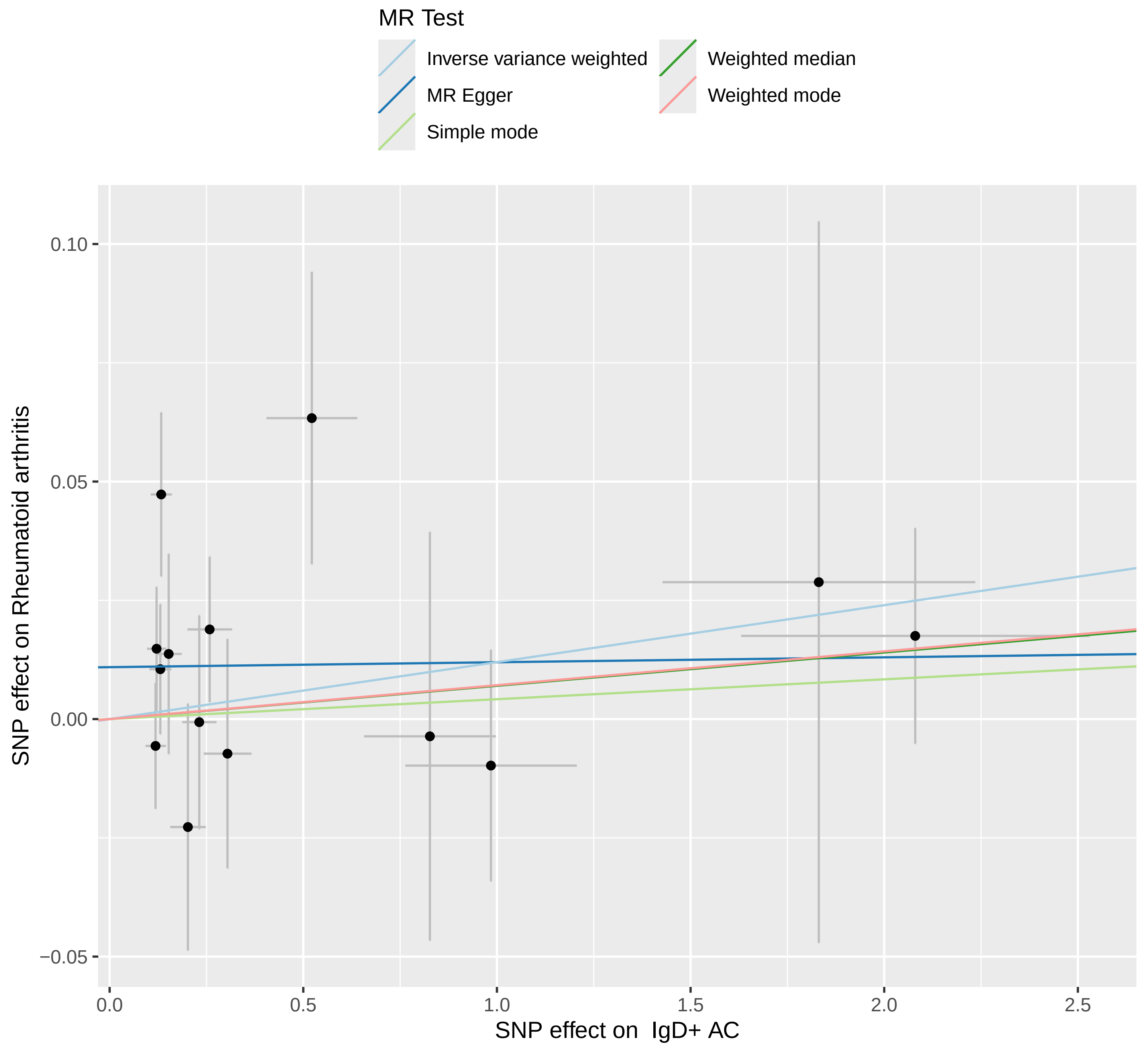

MR Test
Inverse variance weighted
MR Egger
Simple mode
Weighted median
Weighted mode
0.10
0.05
0.00
−0.05
0.0
0.5
1.0
1.5
2.0
2.5
SNP effect on Rheumatoid arthritis
SNP effect on IgD+ AC

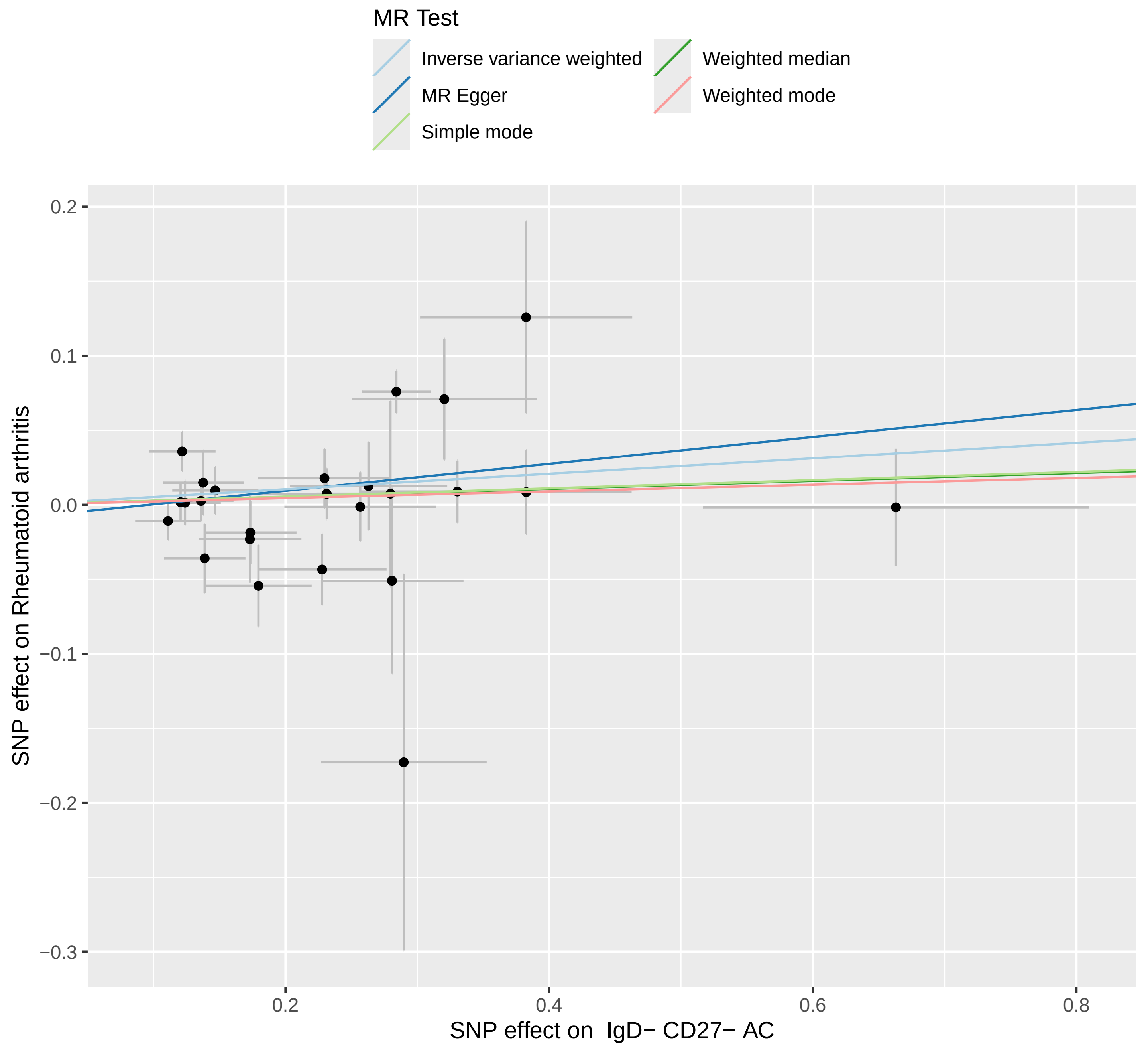
MR Test
Inverse variance weighted
MR Egger
Simple mode
Weighted median
Weighted mode
0.2
0.1
0.0
−0.1
−0.2
−0.3
SNP effect on Rheumatoid arthritis
0.2
0.4
0.6
0.8
SNP effect on IgD− CD27− AC

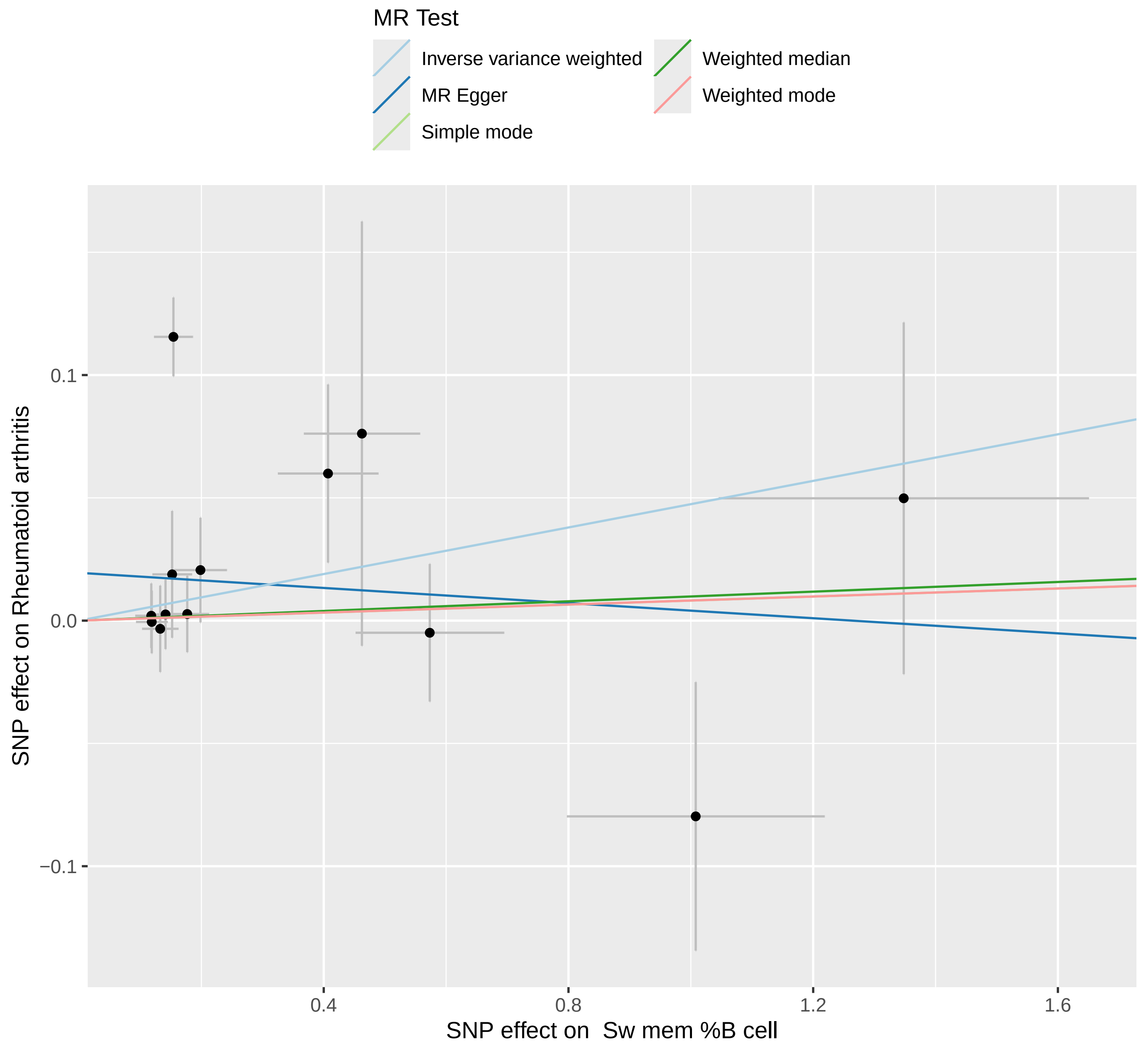
MR Test
Inverse variance weighted
MR Egger
Simple mode
Weighted median
Weighted mode
0.1
0.0
−0.1
SNP effect on Rheumatoid arthritis
0.4
0.8
1.2
1.6
SNP effect on Sw mem %B cell

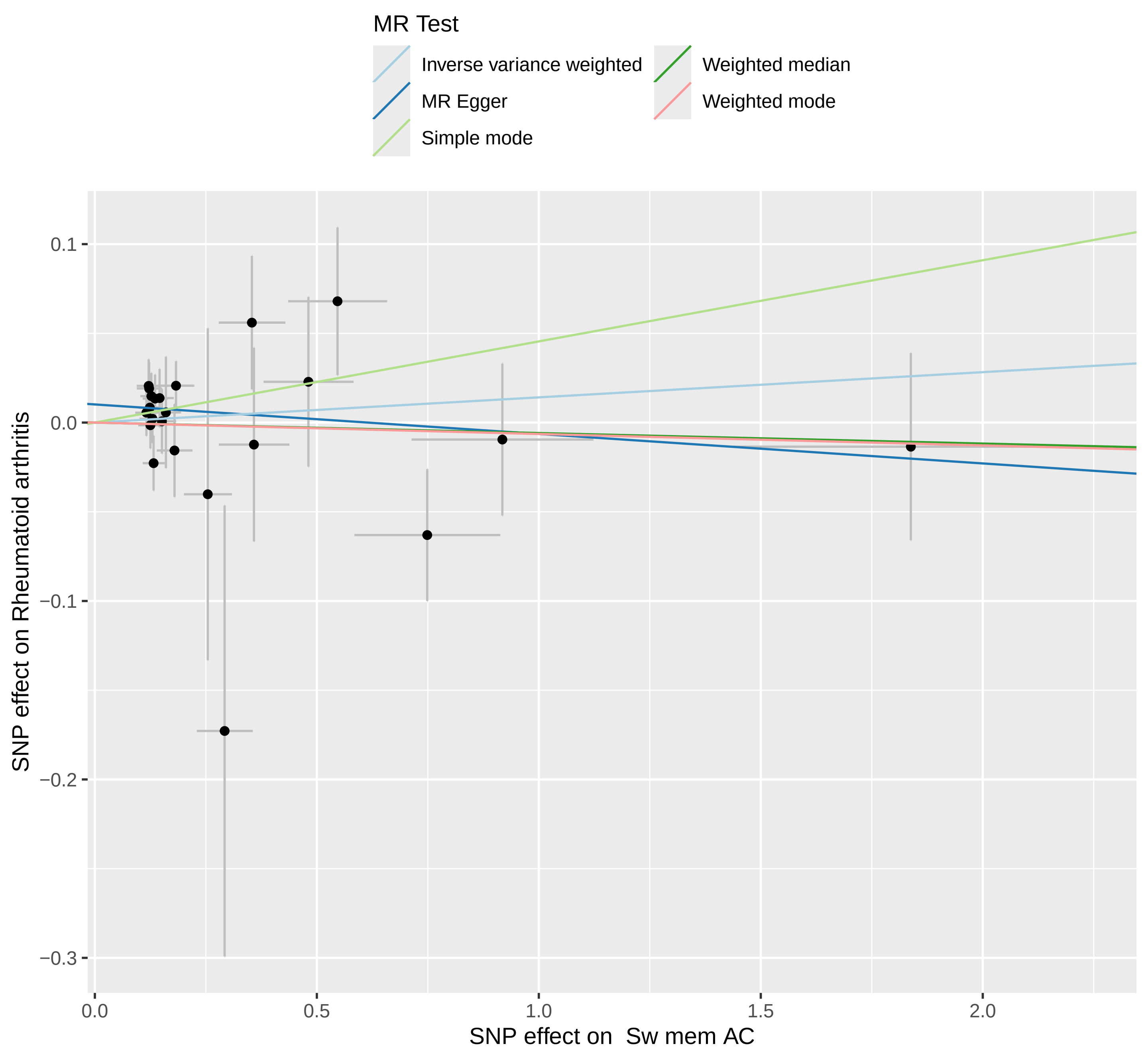
MR Test
Inverse variance weighted
MR Egger
Simple mode
Weighted median
Weighted mode
SNP effect on Rheumatoid arthritis
0.1
0.0
−0.1
−0.2
−0.3
0.0
0.5
1.0
1.5
2.0
SNP effect on Sw mem AC

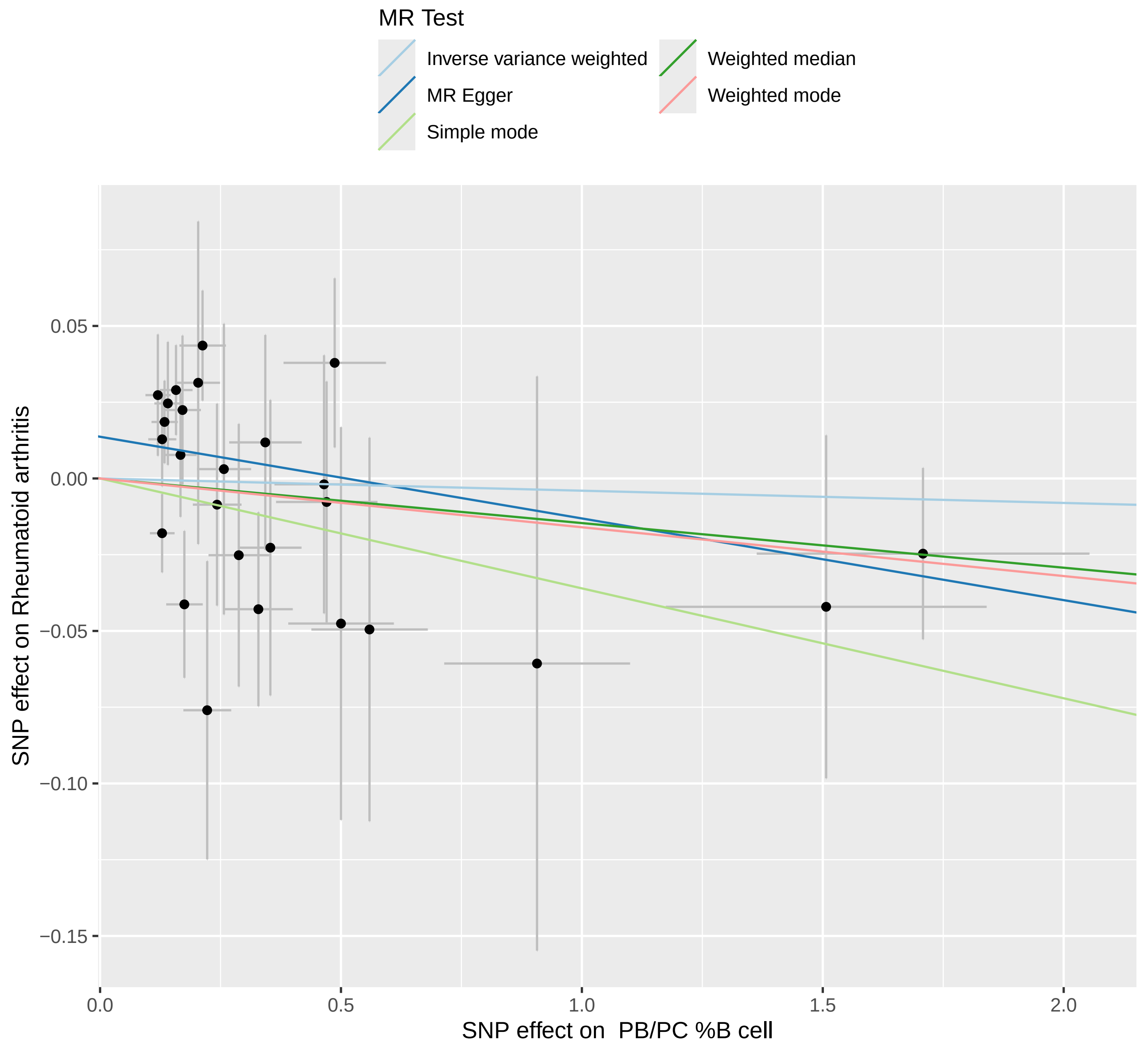

MR Test
Inverse variance weighted
MR Egger
Simple mode
Weighted median
Weighted mode
SNP effect on Rheumatoid arthritis
0.05
0.00
−0.05
−0.10
−0.15
0.0
0.5
1.0
1.5
2.0
SNP effect on PB/PC %B cell

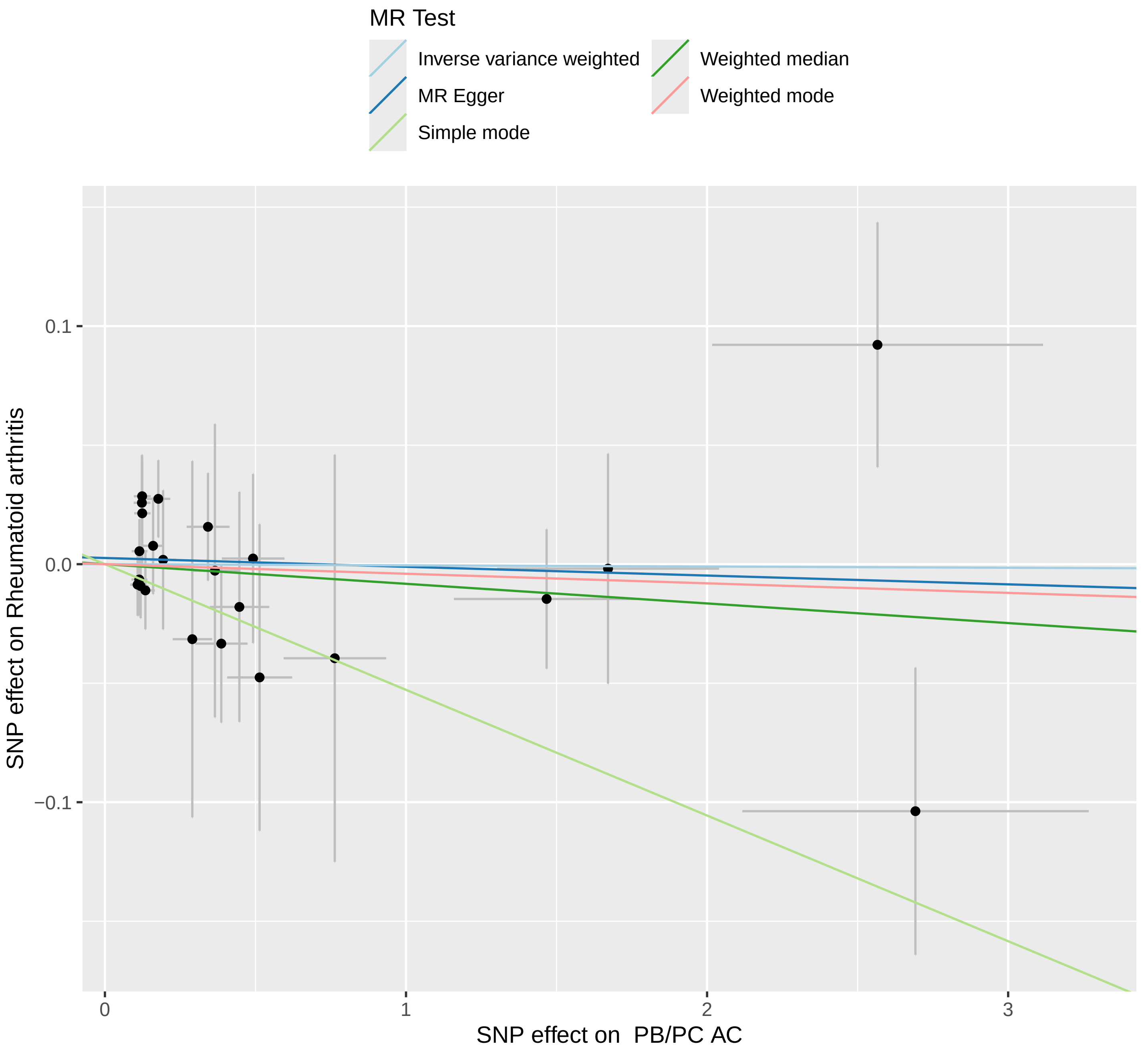
MR Test
Inverse variance weighted
Weighted median
MR Egger
Weighted mode
Simple mode
SNP effect on Rheumatoid arthritis
0.1
0.0
−0.1
0
1
2
3
SNP effect on PB/PC AC

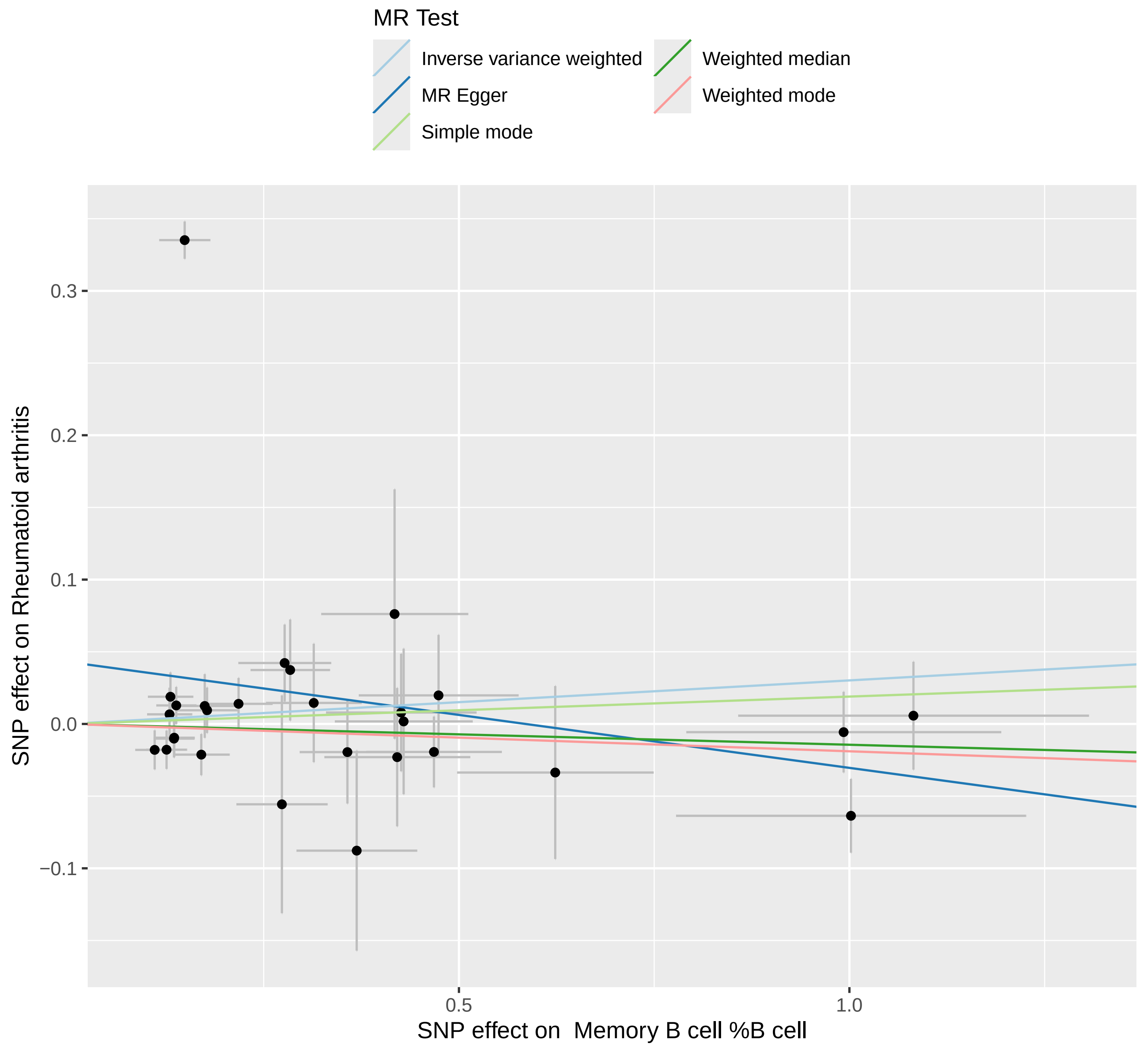
MR Test
Inverse variance weighted
MR Egger
Simple mode
Weighted median
Weighted mode
SNP effect on Rheumatoid arthritis
0.3
0.2
0.1
0.0
−0.1
0.5
1.0
SNP effect on Memory B cell %B cell

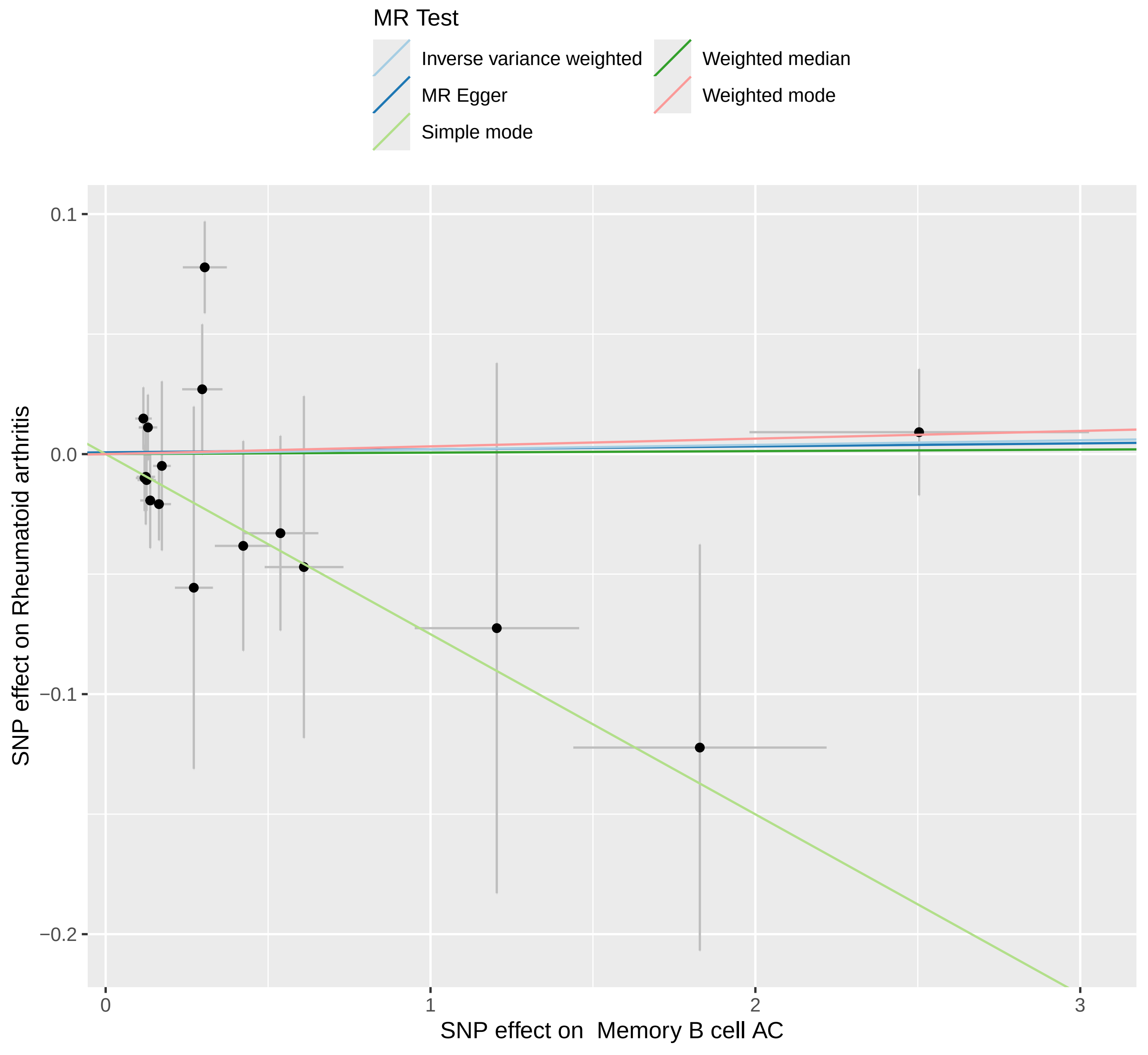
MR Test
Inverse variance weighted
MR Egger
Simple mode
Weighted median
Weighted mode
0.1
0.0
−0.1
−0.2
0
1
2
3
SNP effect on Rheumatoid arthritis
SNP effect on Memory B cell AC

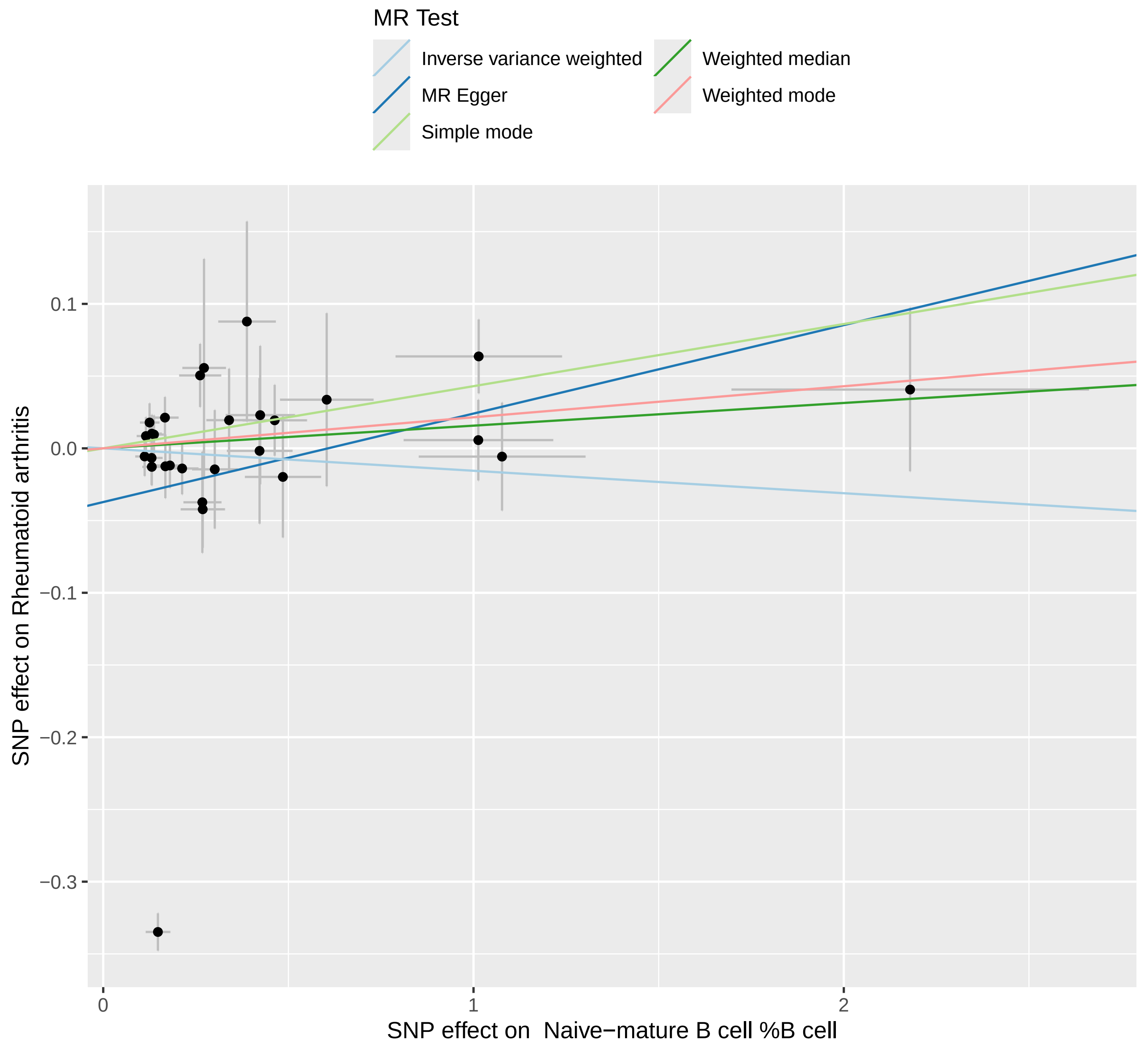
MR Test
Inverse variance weighted
MR Egger
Simple mode
Weighted median
Weighted mode
SNP effect on Rheumatoid arthritis
0.1
0.0
−0.1
−0.2
−0.3
0
1
2
SNP effect on Naive−mature B cell %B cell

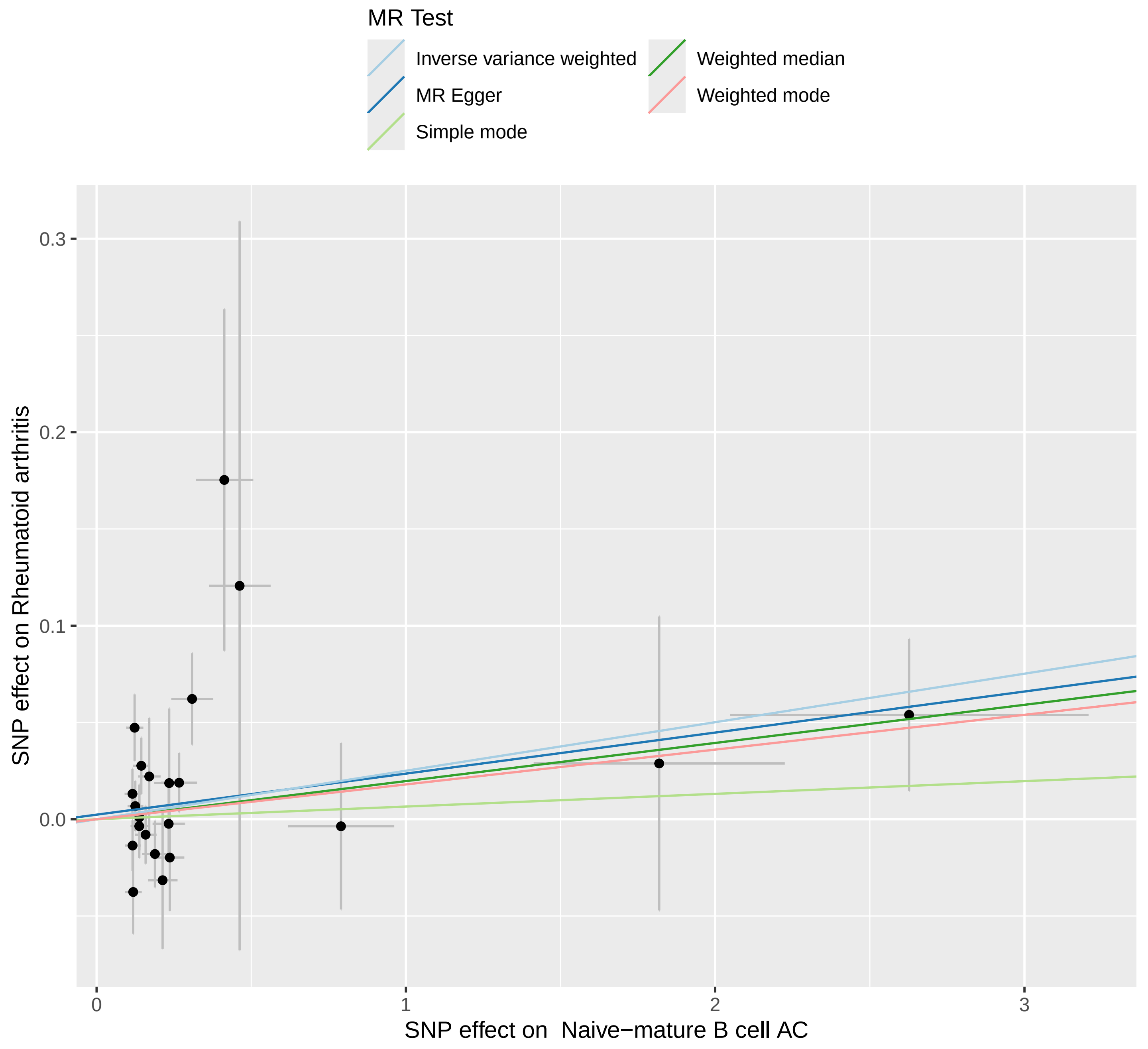

MR Test
Inverse variance weighted
MR Egger
Simple mode
Weighted median
Weighted mode
0.3
0.2
0.1
0.0
0
1
2
3
SNP effect on Rheumatoid arthritis
SNP effect on Naive−mature B cell AC

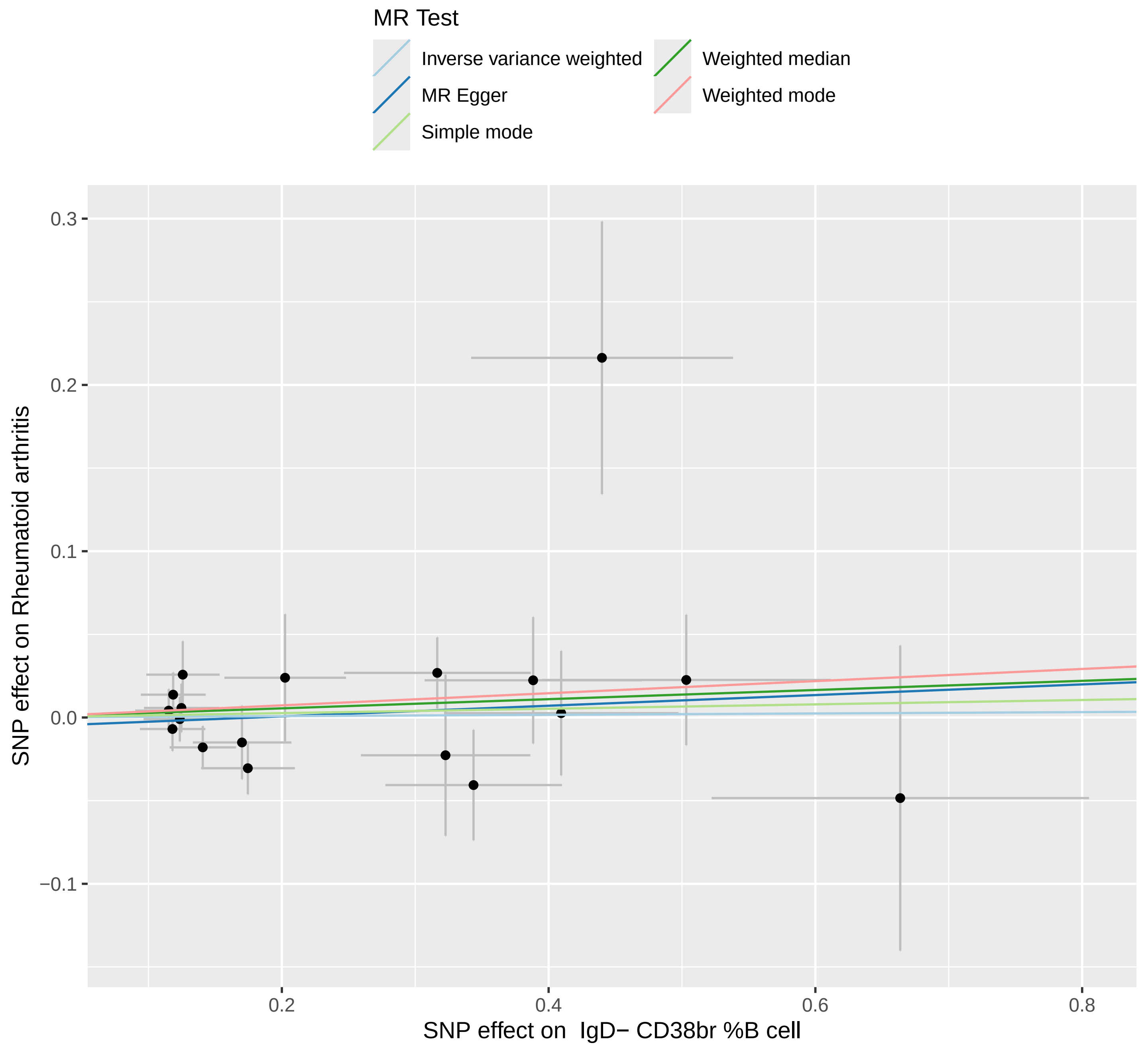

MR Test
Inverse variance weighted
MR Egger
Simple mode
Weighted median
Weighted mode
0.3
0.2
0.1
0.0
−0.1
0.2
0.4
0.6
0.8
SNP effect on Rheumatoid arthritis
SNP effect on IgD− CD38br %B cell

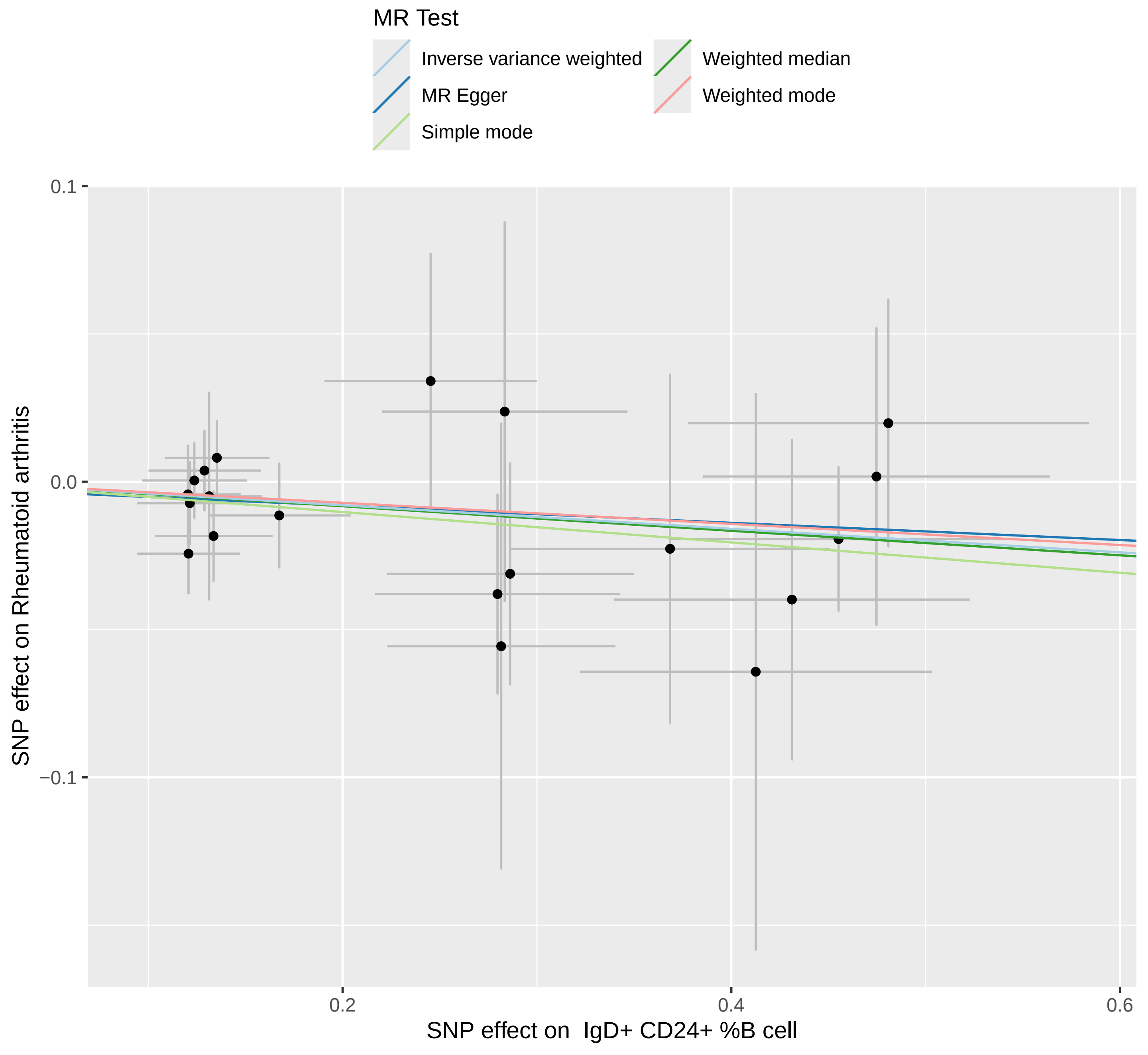
MR Test
Inverse variance weighted
MR Egger
Simple mode
Weighted median
Weighted mode
0.1
0.0
−0.1
0.2
0.4
0.6
SNP effect on Rheumatoid arthritis
SNP effect on IgD+ CD24+ %B cell

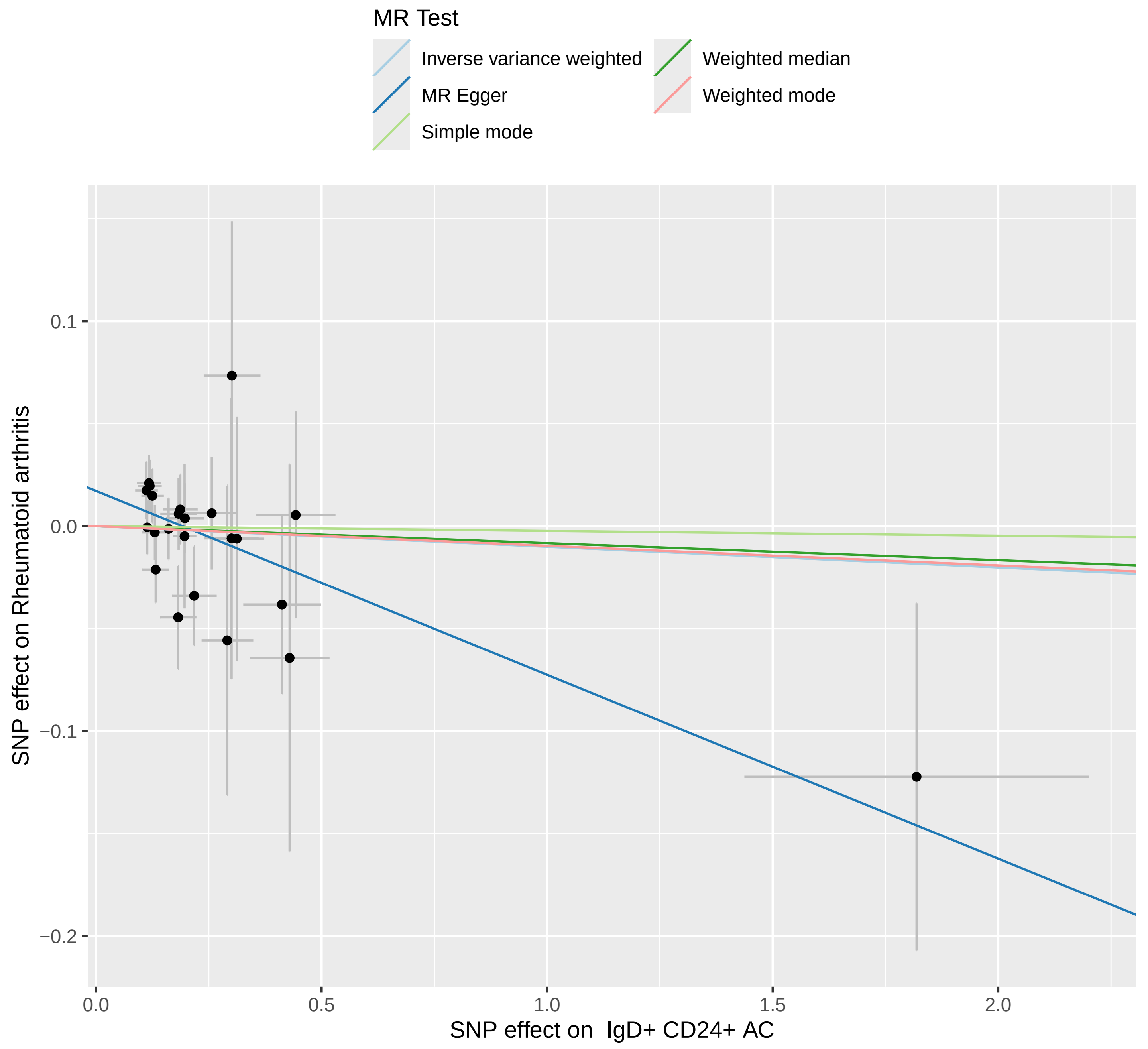
MR Test
Inverse variance weighted
MR Egger
Simple mode
Weighted median
Weighted mode
SNP effect on Rheumatoid arthritis
0.1
0.0
−0.1
−0.2
0.0
0.5
1.0
1.5
2.0
SNP effect on IgD+ CD24+ AC

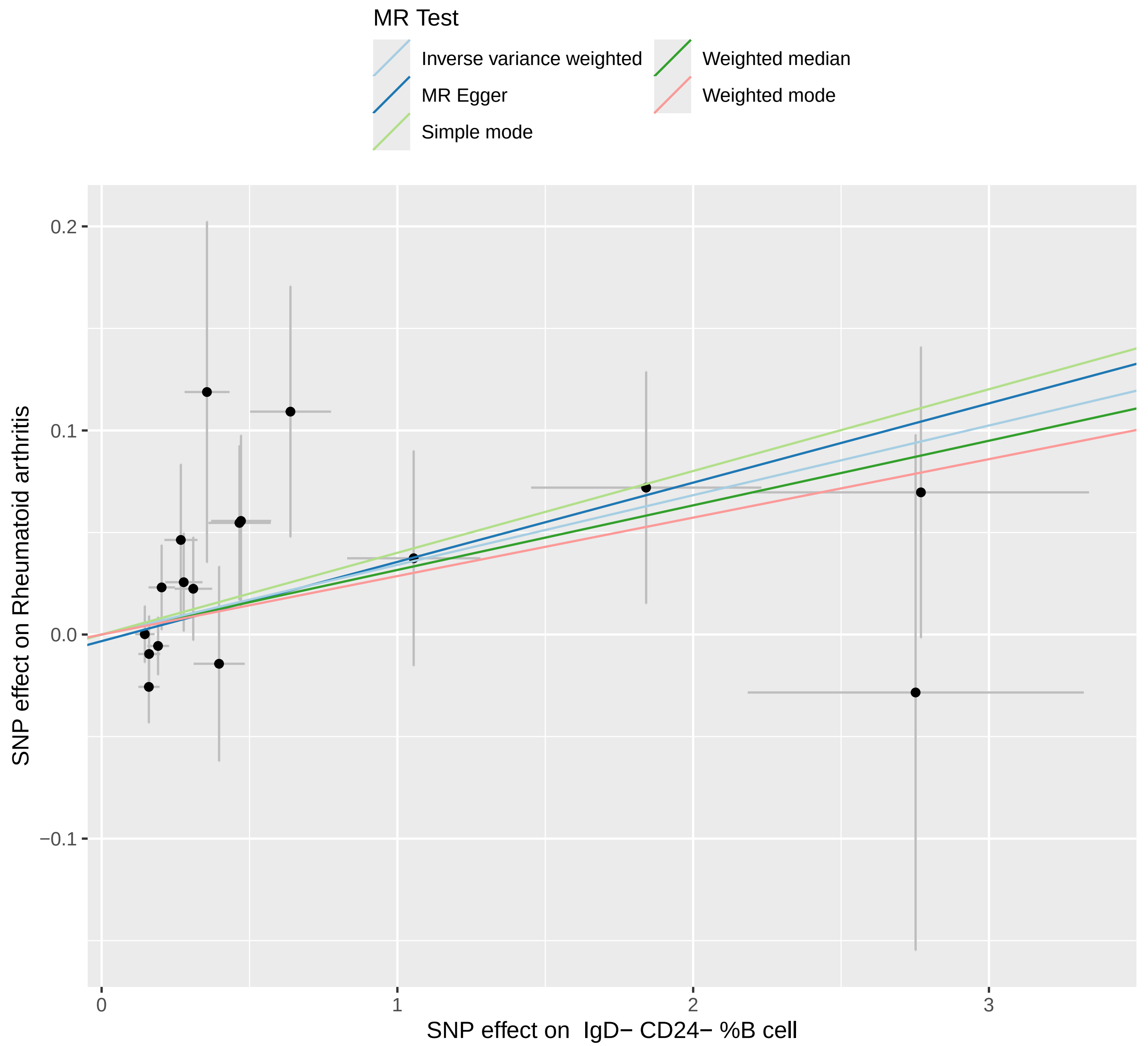

MR Test
Inverse variance weighted
MR Egger
Simple mode
Weighted median
Weighted mode
0.2
0.1
0.0
−0.1
0
1
2
3
SNP effect on Rheumatoid arthritis
SNP effect on IgD− CD24− %B cell

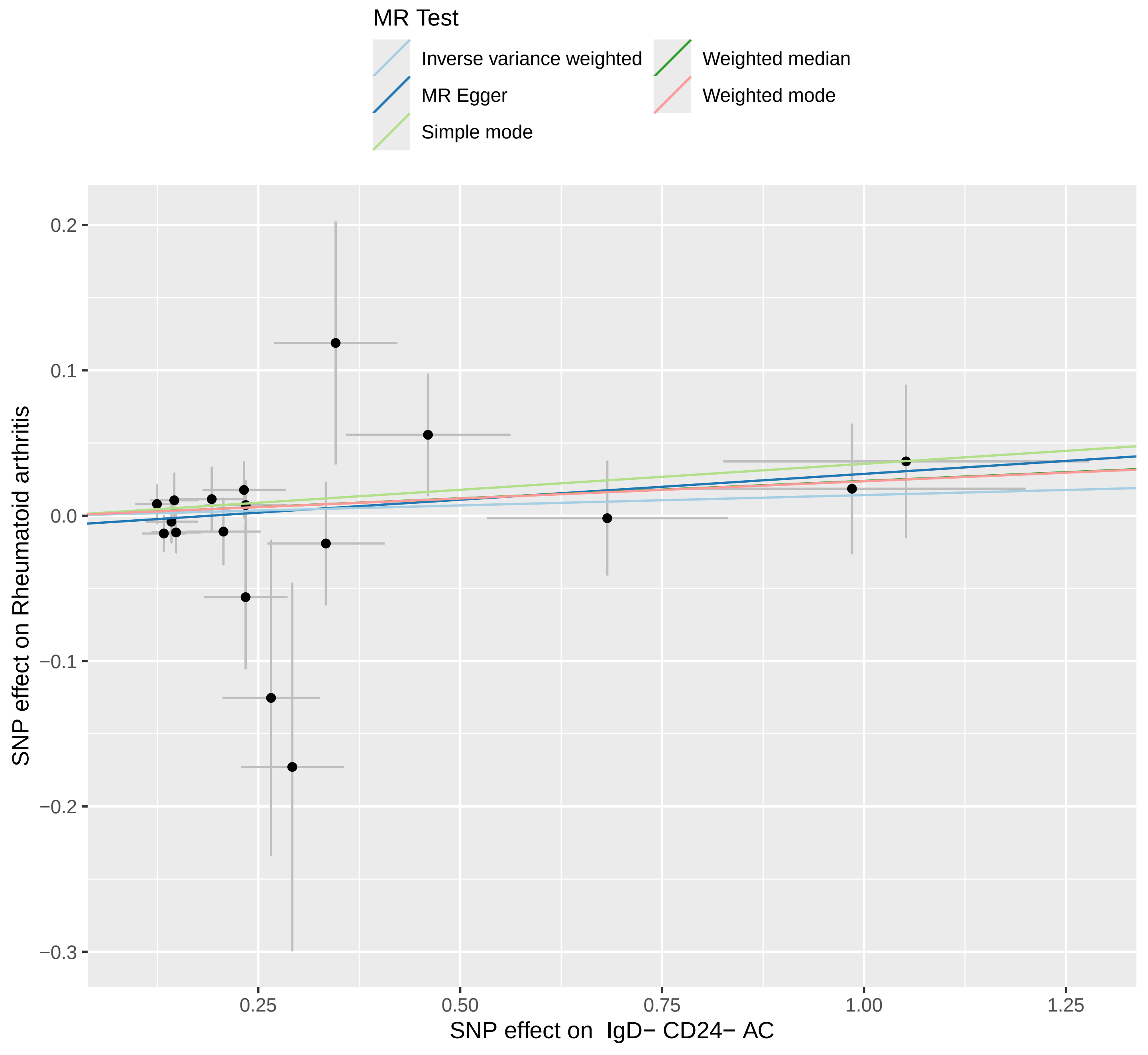

MR Test
Inverse variance weighted
MR Egger
Simple mode
Weighted median
Weighted mode
0.2
0.1
0.0
−0.1
−0.2
−0.3
SNP effect on Rheumatoid arthritis
0.25
0.50
0.75
1.00
1.25
SNP effect on IgD− CD24− AC

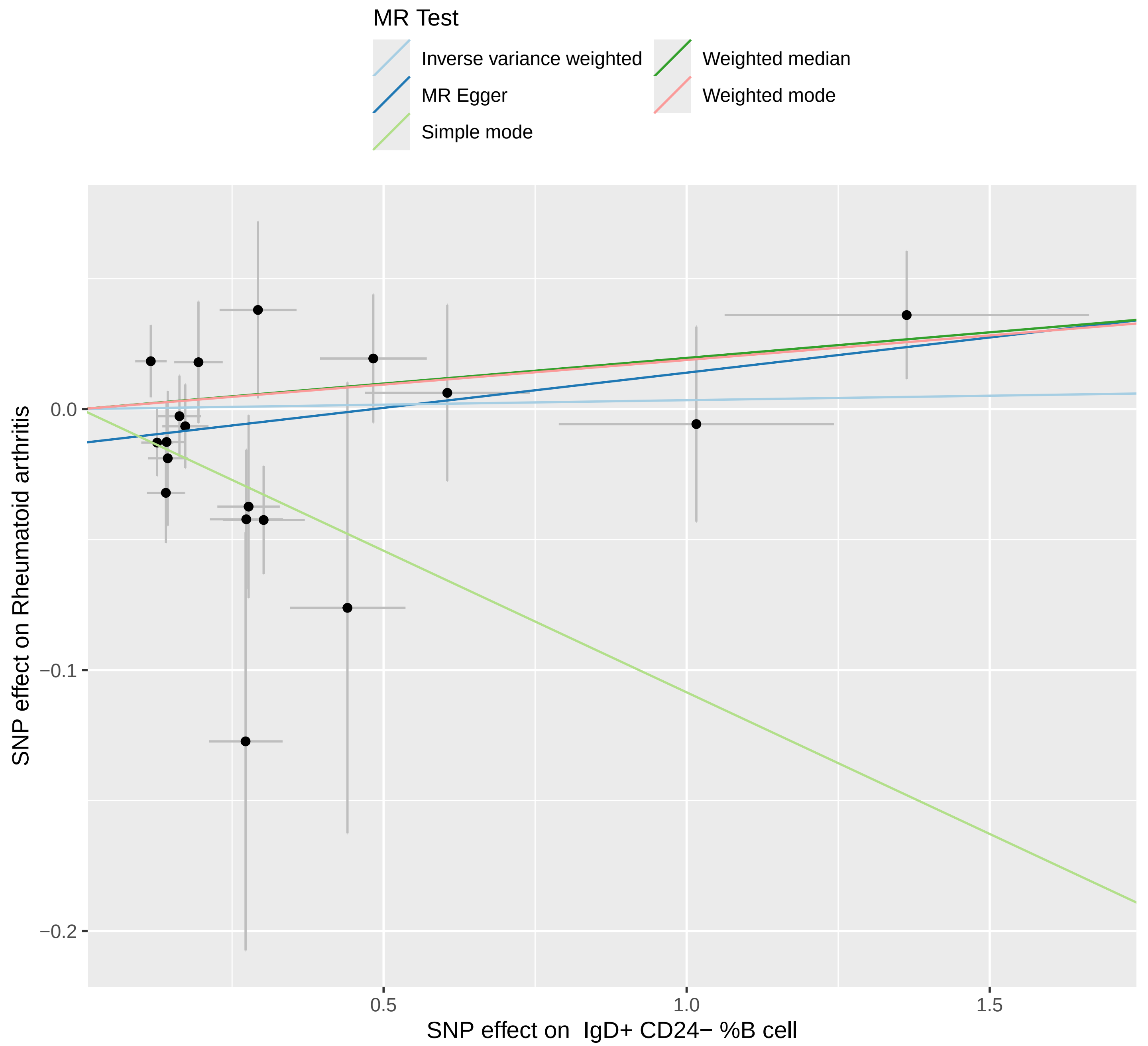

MR Test
Inverse variance weighted
MR Egger
Simple mode
Weighted median
Weighted mode
SNP effect on Rheumatoid arthritis
0.0
−0.1
−0.2
0.5
1.0
1.5
SNP effect on IgD+ CD24− %B cell

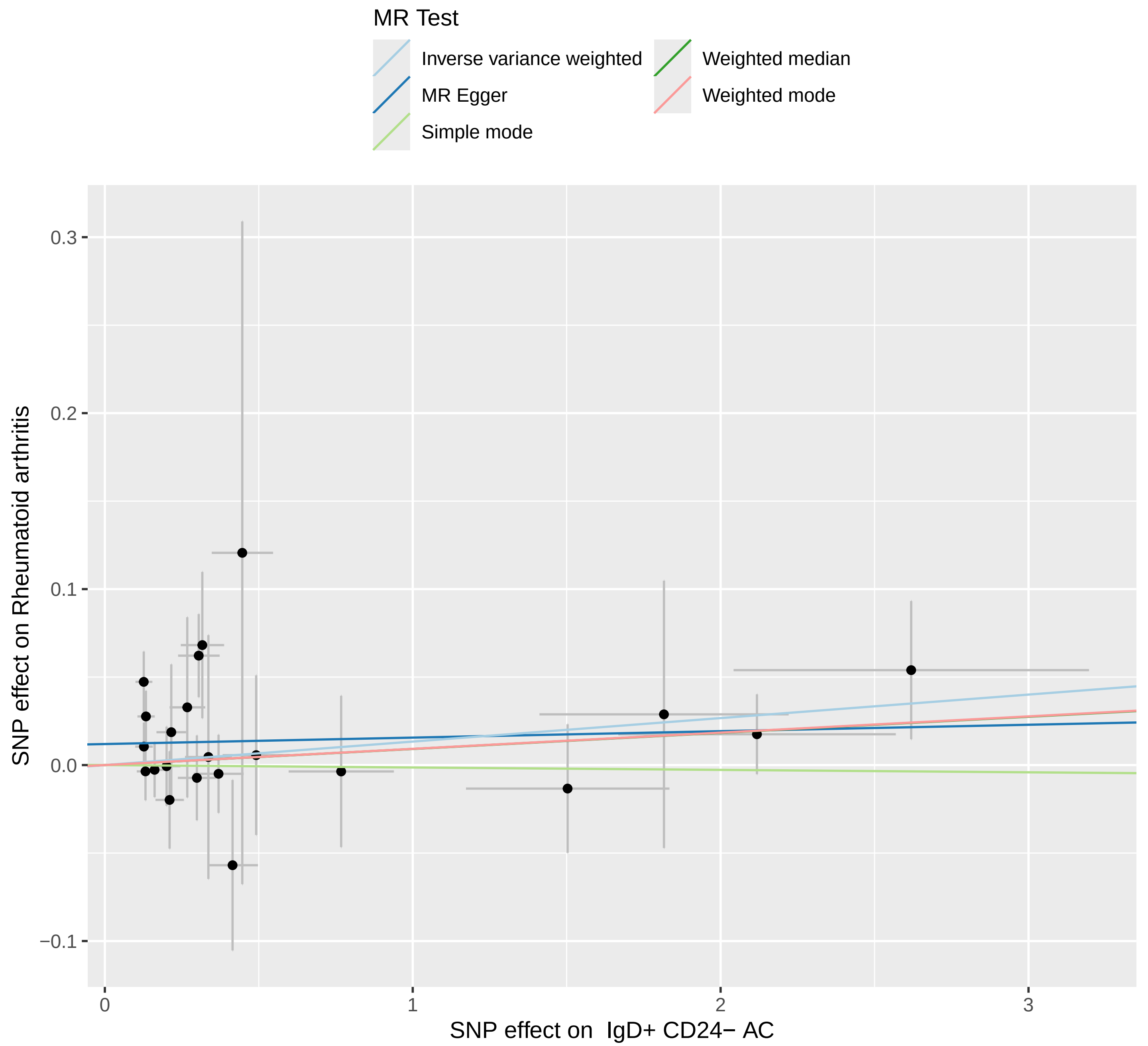

MR Test
Inverse variance weighted
MR Egger
Simple mode
Weighted median
Weighted mode
SNP effect on Rheumatoid arthritis
0.3
0.2
0.1
0.0
−0.1
0
1
2
3
SNP effect on IgD+ CD24− AC

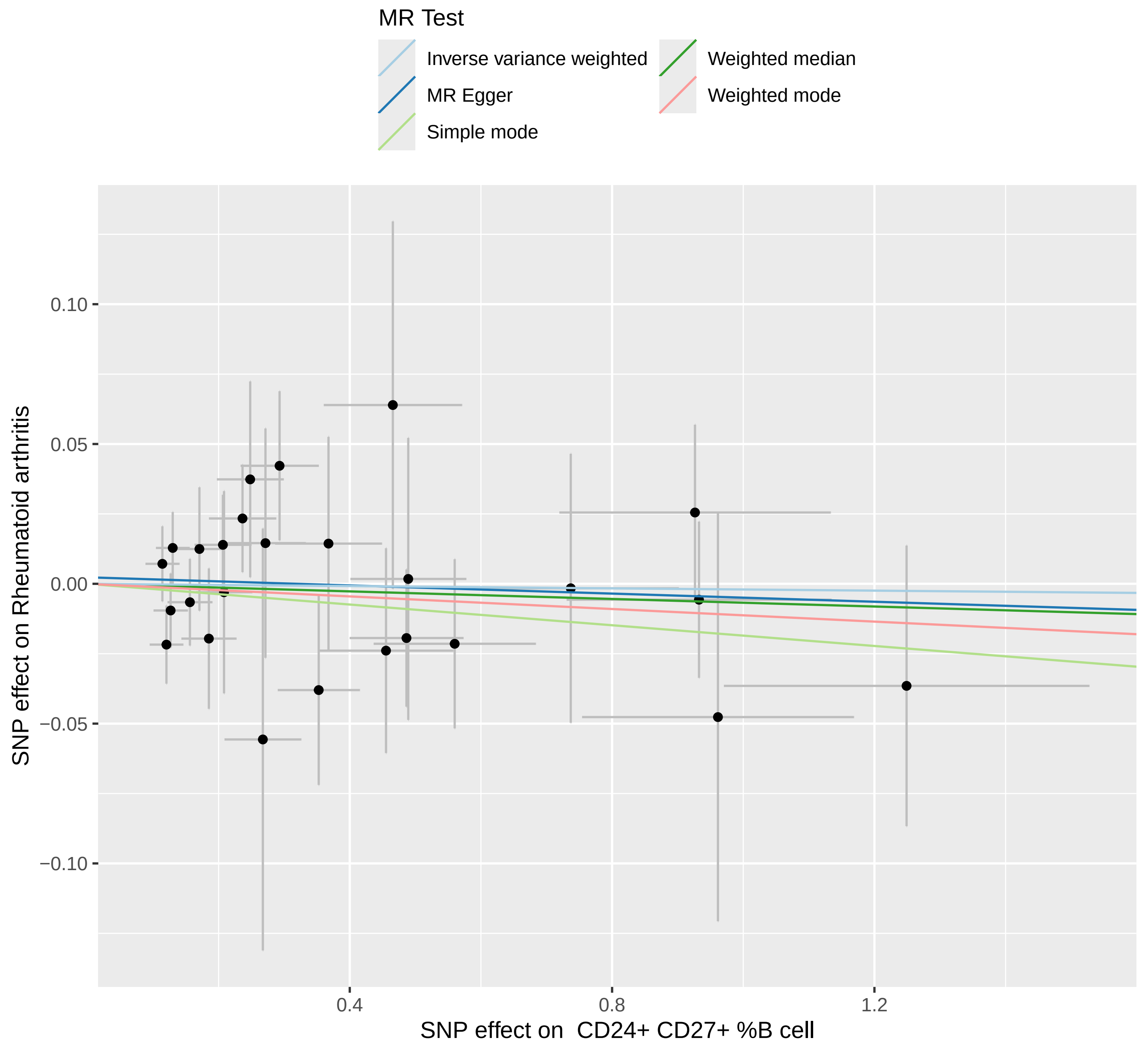
MR Test
Inverse variance weighted
MR Egger
Simple mode
Weighted median
Weighted mode
0.10
0.05
0.00
−0.05
−0.10
0.4
0.8
1.2
SNP effect on Rheumatoid arthritis
SNP effect on CD24+ CD27+ %B cell

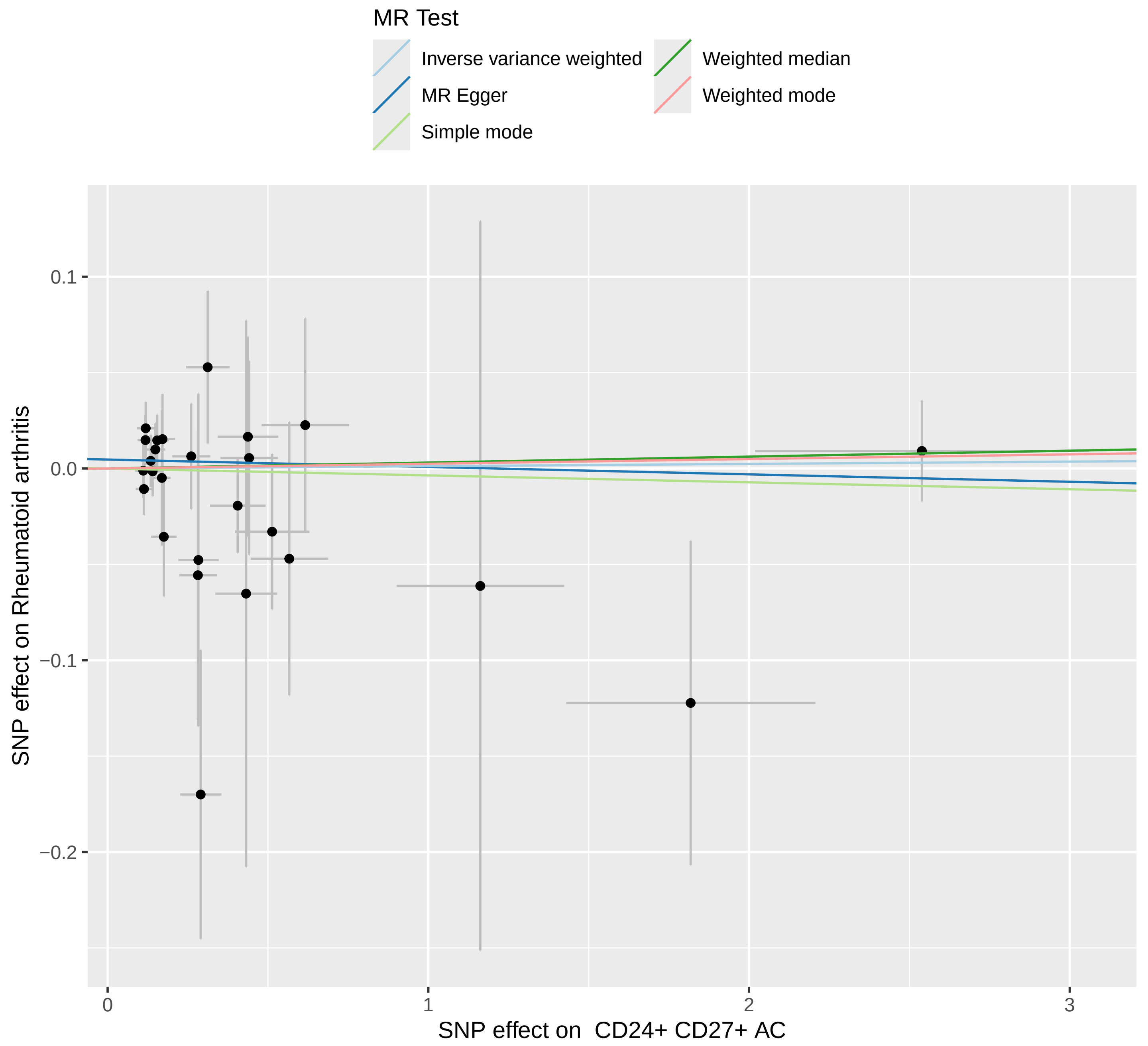
MR Test
Inverse variance weighted
MR Egger
Simple mode
Weighted median
Weighted mode
0.1
0.0
−0.1
−0.2
SNP effect on Rheumatoid arthritis
0
1
2
3
SNP effect on CD24+ CD27+ AC

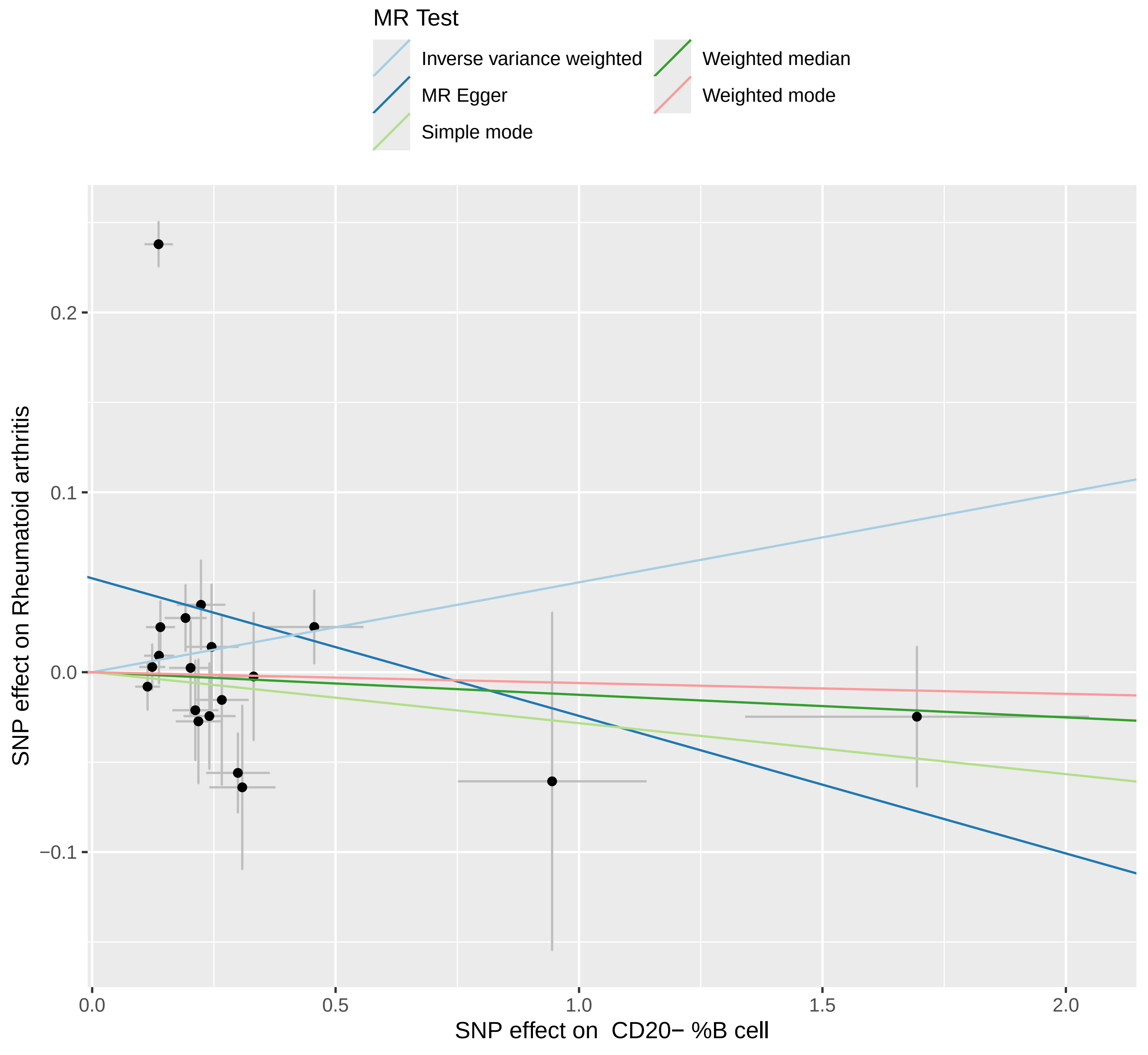
MR Test
Inverse variance weighted
MR Egger
Simple mode
Weighted median
Weighted mode
SNP effect on Rheumatoid arthritis
0.2
0.1
0.0
−0.1
0.0
0.5
1.0
1.5
2.0
SNP effect on CD20− %B cell

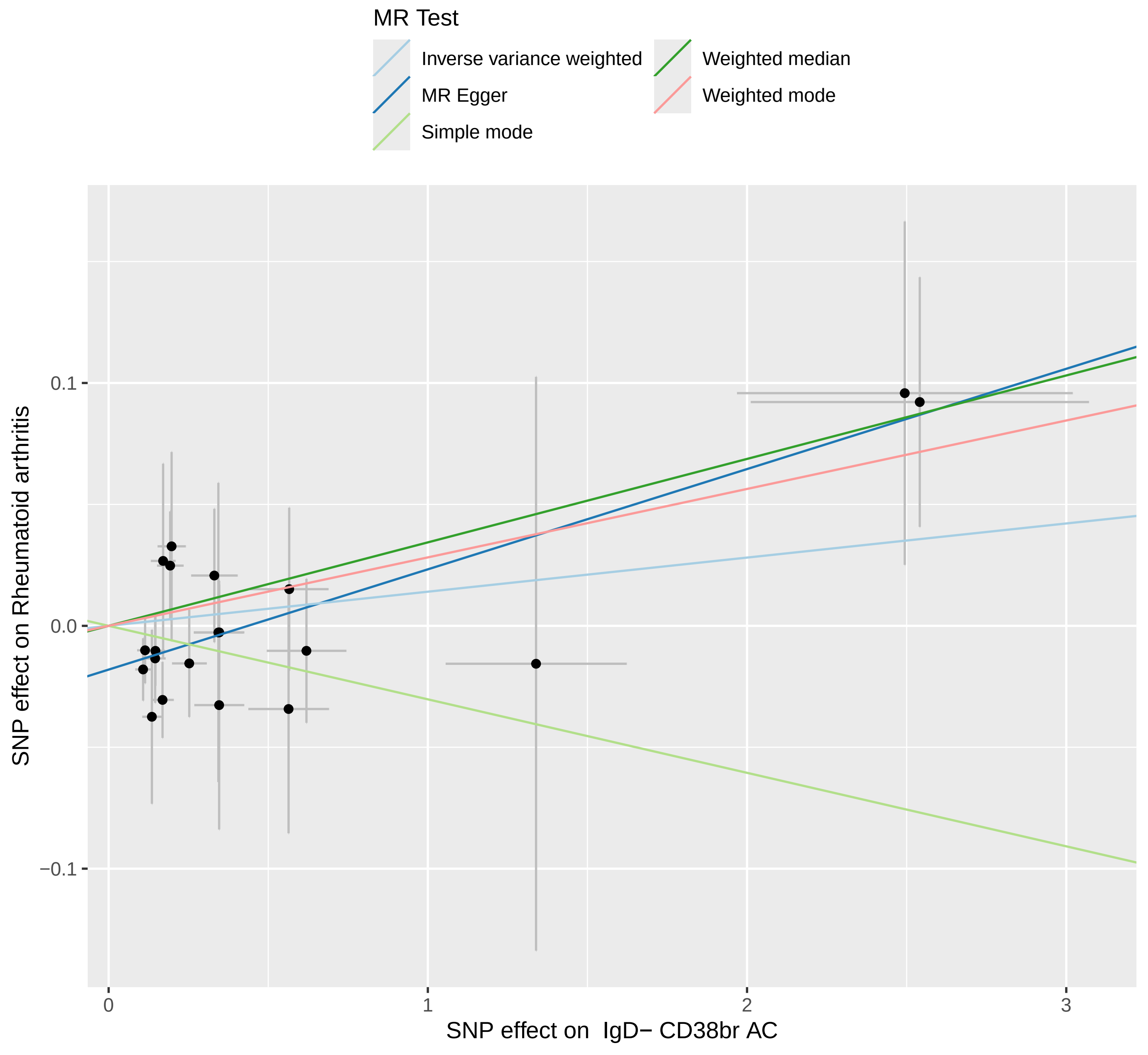

MR Test
Inverse variance weighted
MR Egger
Simple mode
Weighted median
Weighted mode
0.1
0.0
−0.1
0
1
2
3
SNP effect on Rheumatoid arthritis
SNP effect on IgD− CD38br AC

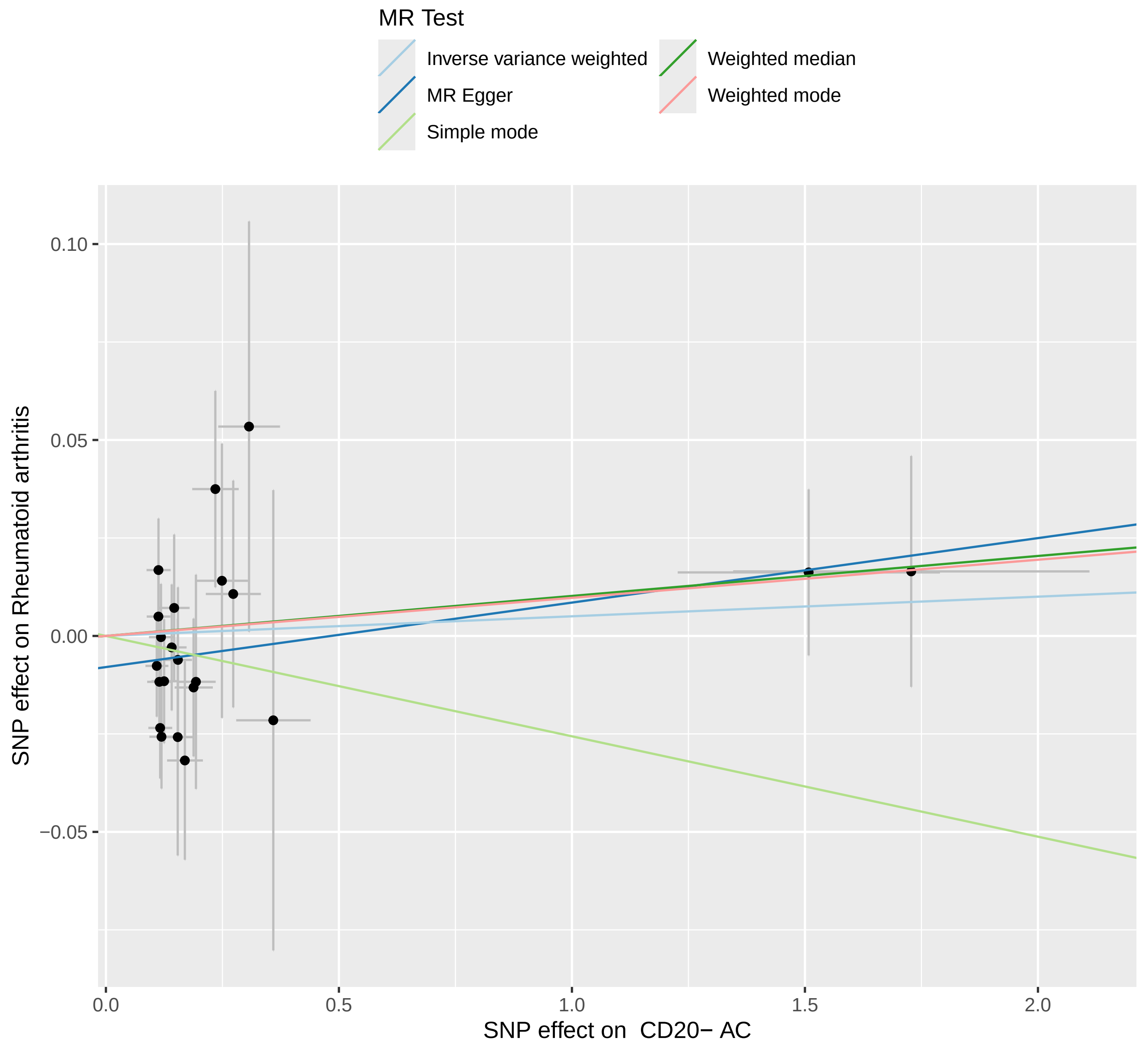

MR Test
Inverse variance weighted
MR Egger
Simple mode
Weighted median
Weighted mode
SNP effect on Rheumatoid arthritis
0.10
0.05
0.00
−0.05
0.0
0.5
1.0
1.5
2.0
SNP effect on CD20− AC

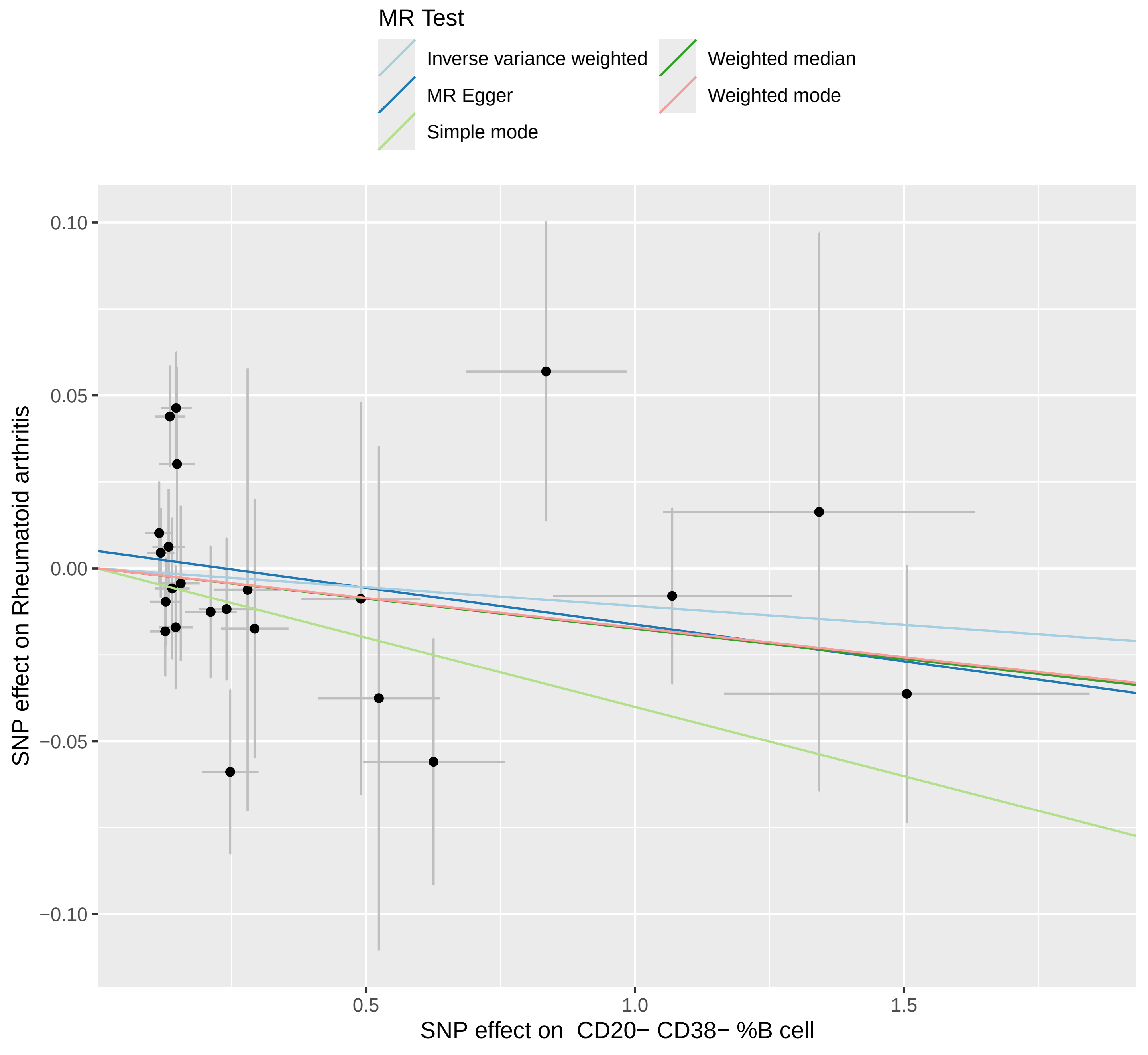

MR Test
Inverse variance weighted
MR Egger
Simple mode
Weighted median
Weighted mode
0.10
0.05
0.00
−0.05
−0.10
SNP effect on Rheumatoid arthritis
0.5
1.0
1.5
SNP effect on CD20− CD38− %B cell

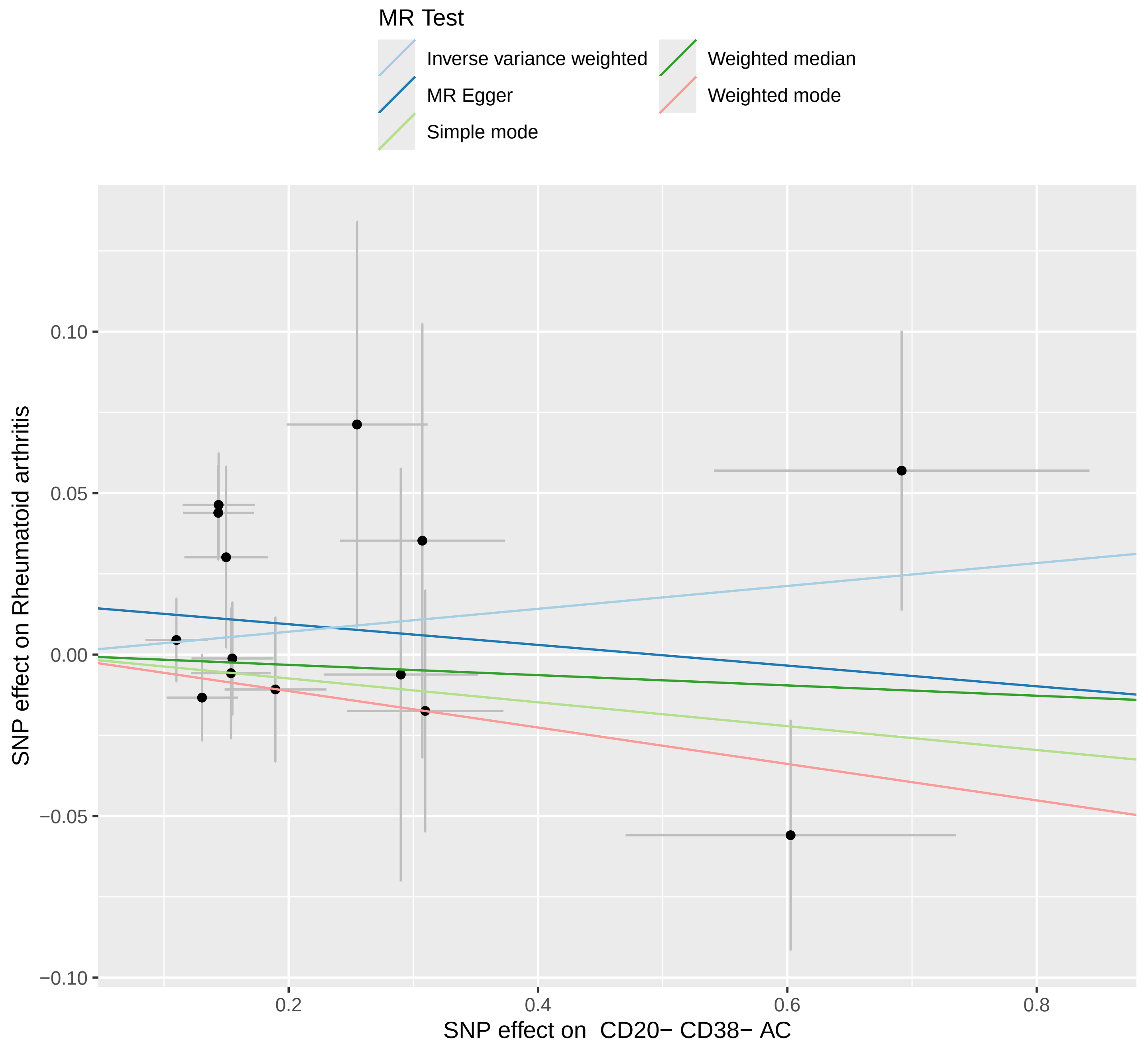
MR Test
Inverse variance weighted
MR Egger
Simple mode
Weighted median
Weighted mode
SNP effect on Rheumatoid arthritis
0.10
0.05
0.00
−0.05
−0.10
0.2
0.4
0.6
0.8
SNP effect on CD20− CD38− AC

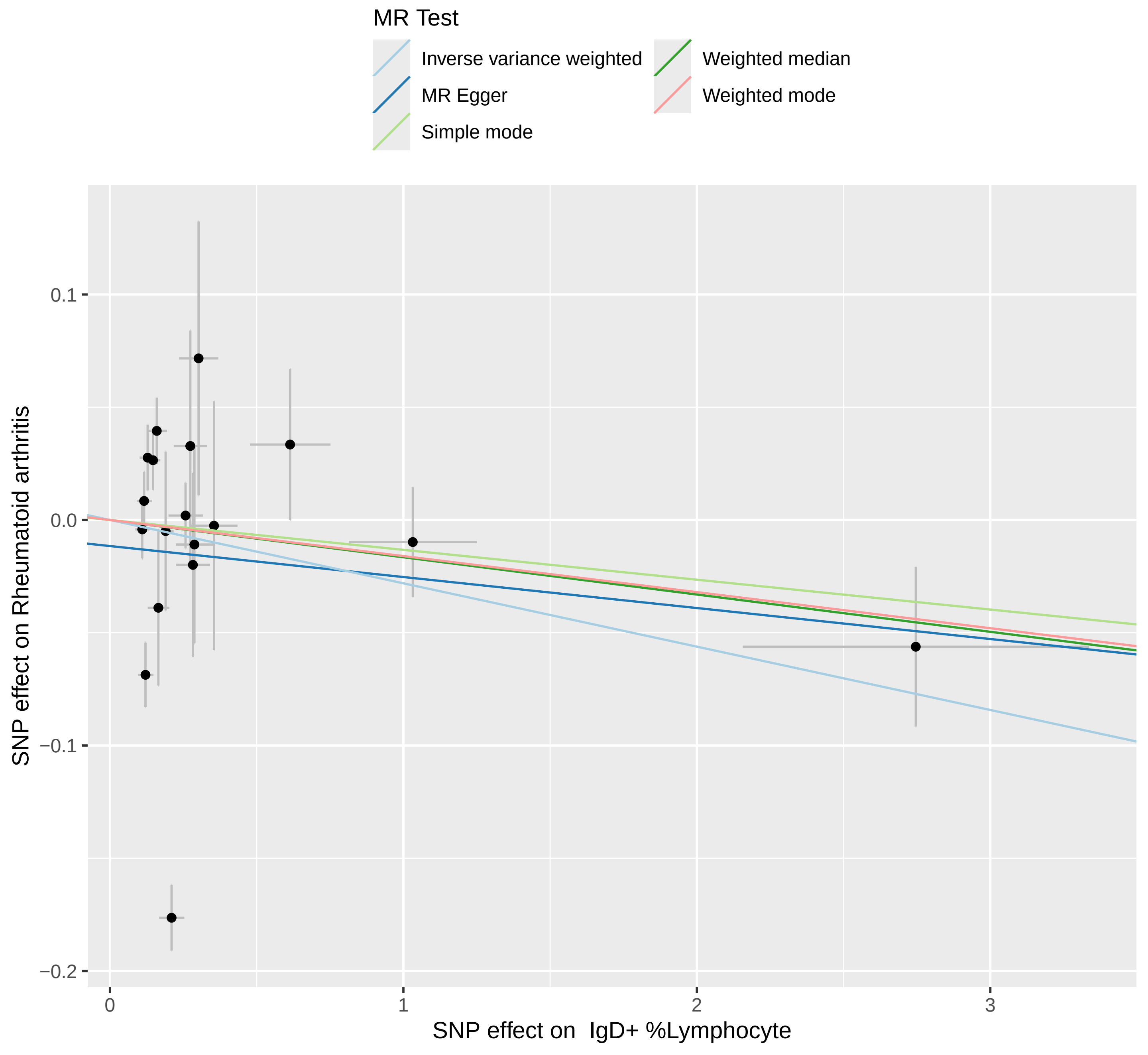

MR Test
Inverse variance weighted
MR Egger
Simple mode
Weighted median
Weighted mode
0.1
0.0
−0.1
−0.2
0
1
2
3
SNP effect on Rheumatoid arthritis
SNP effect on IgD+ %Lymphocyte

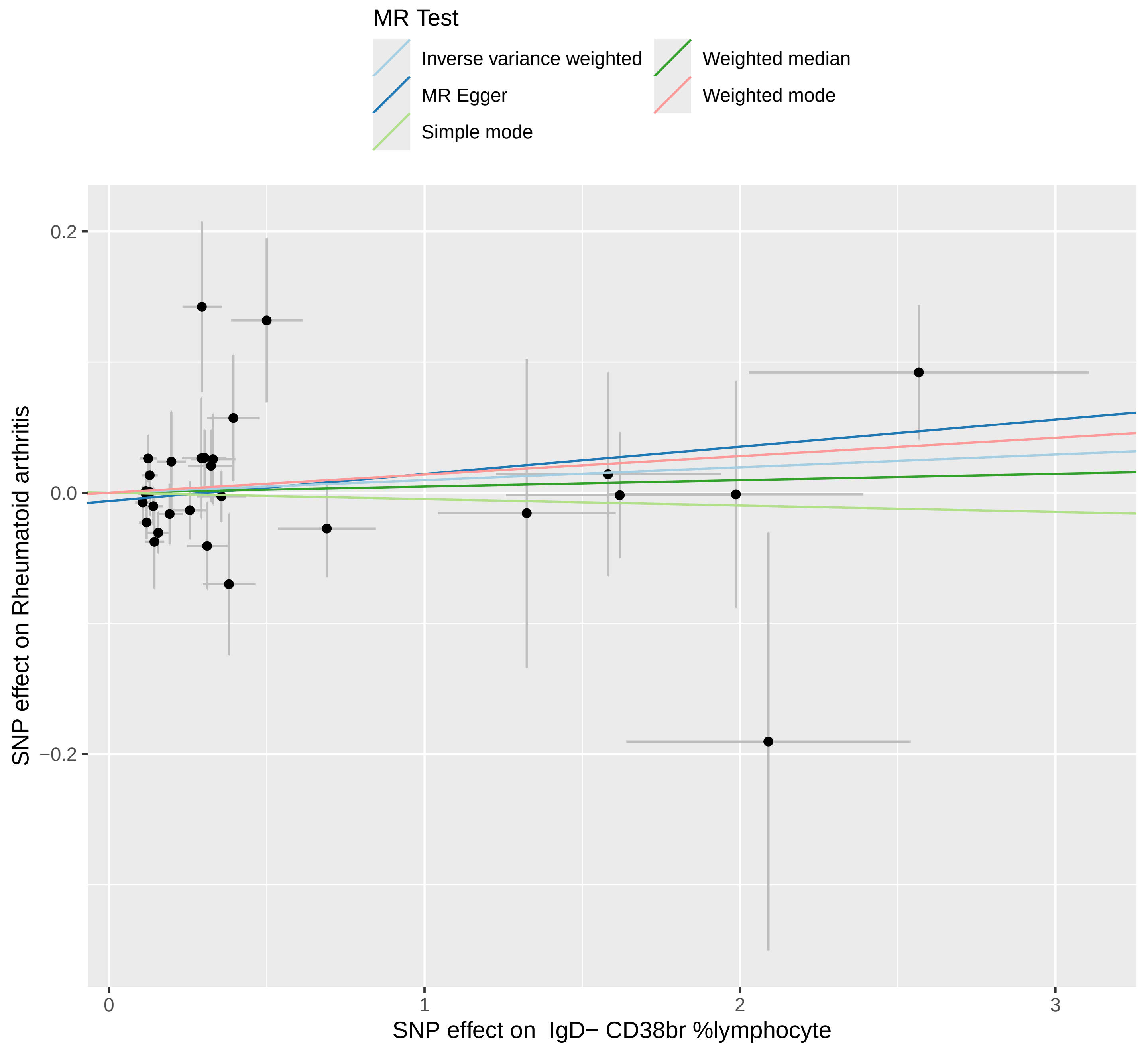
MR Test
Inverse variance weighted
MR Egger
Simple mode
Weighted median
Weighted mode
0.2
0.0
−0.2
0
1
2
3
SNP effect on Rheumatoid arthritis
SNP effect on IgD− CD38br %lymphocyte

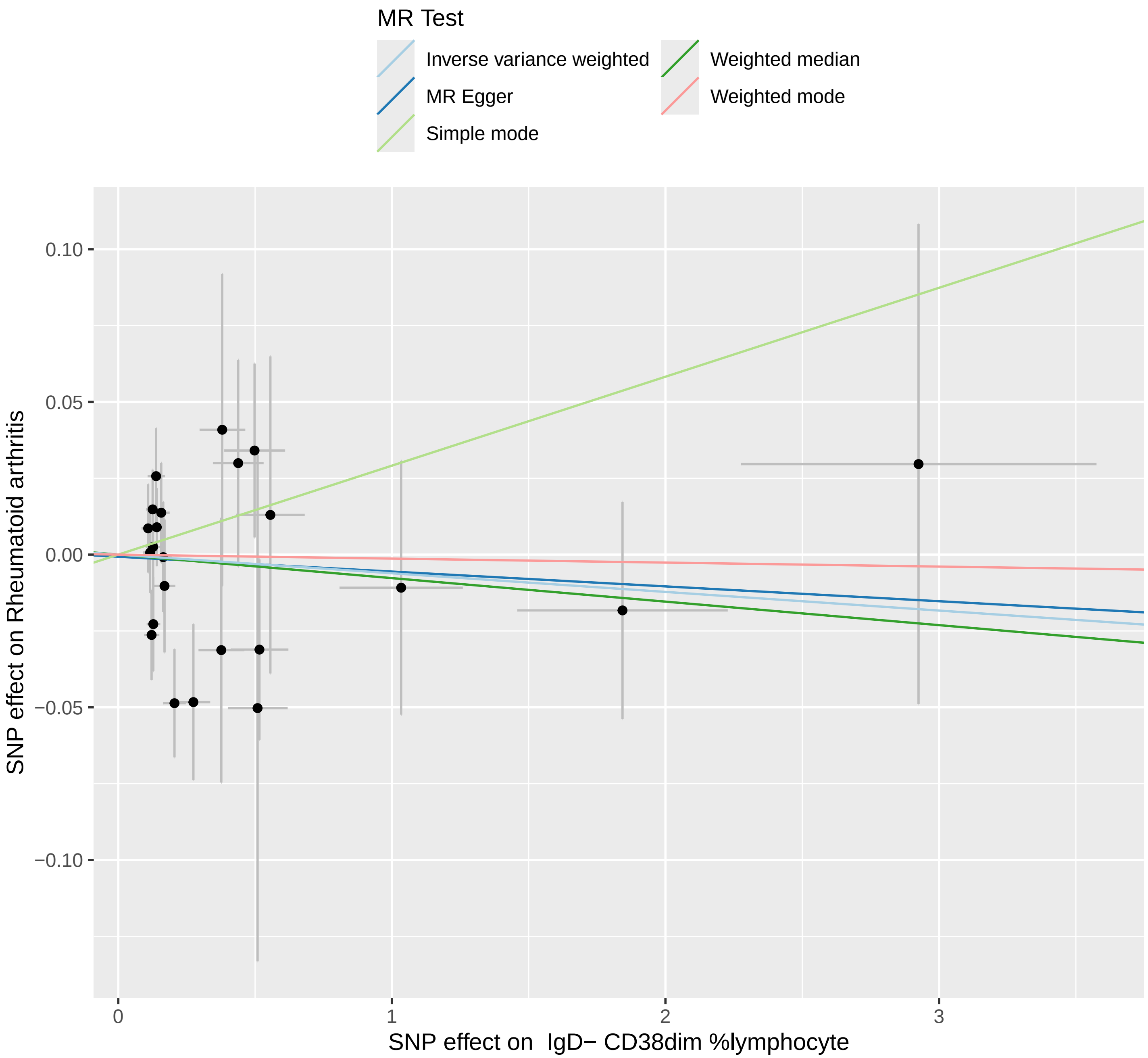

MR Test
Inverse variance weighted
MR Egger
Simple mode
Weighted median
Weighted mode
0.10
0.05
0.00
−0.05
−0.10
SNP effect on Rheumatoid arthritis
0
1
2
3
SNP effect on IgD− CD38dim %lymphocyte

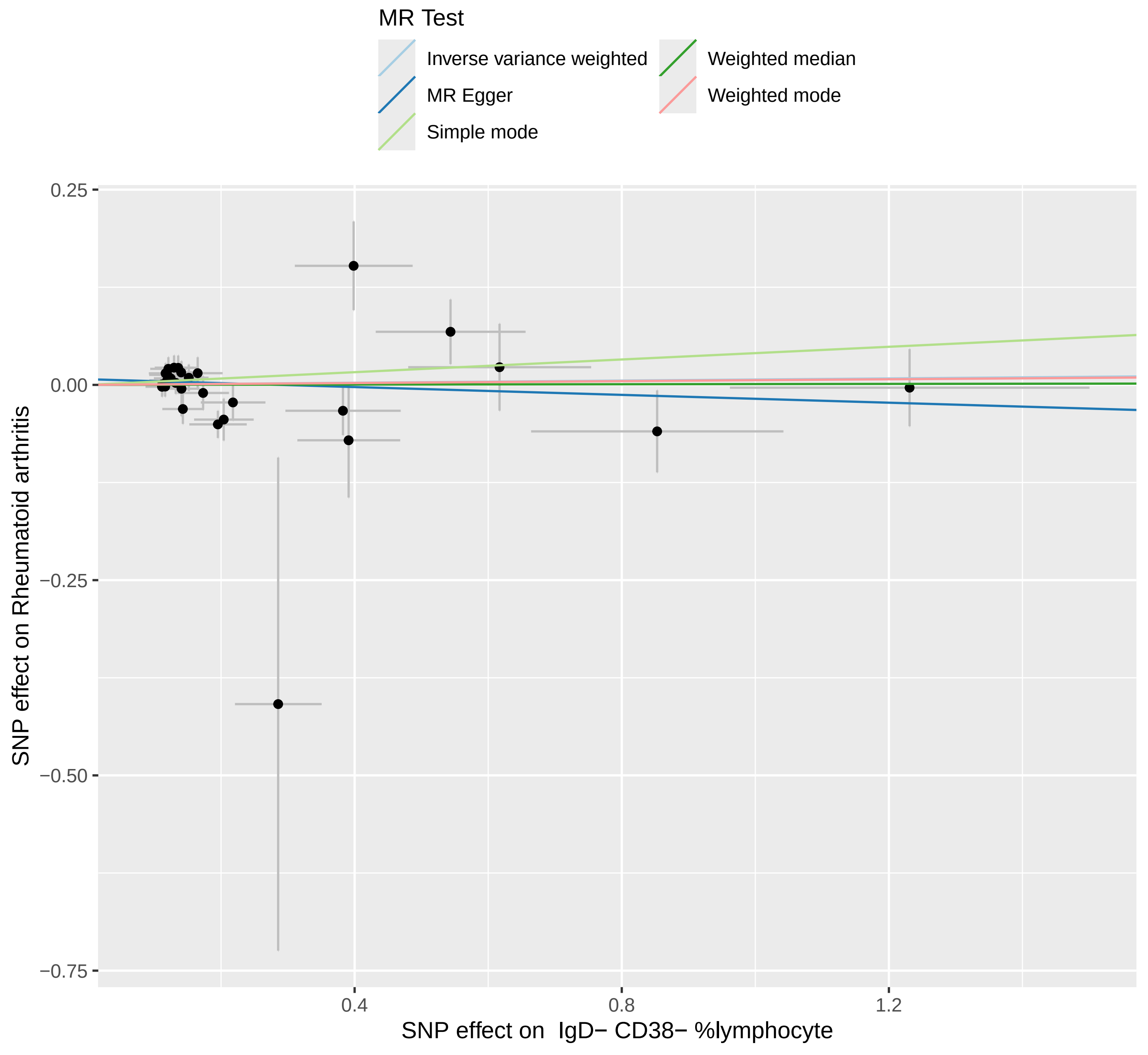
MR Test
Inverse variance weighted
MR Egger
Simple mode
Weighted median
Weighted mode
0.25
0.00
−0.25
−0.50
−0.75
0.4
0.8
1.2
SNP effect on Rheumatoid arthritis
SNP effect on IgD− CD38− %lymphocyte

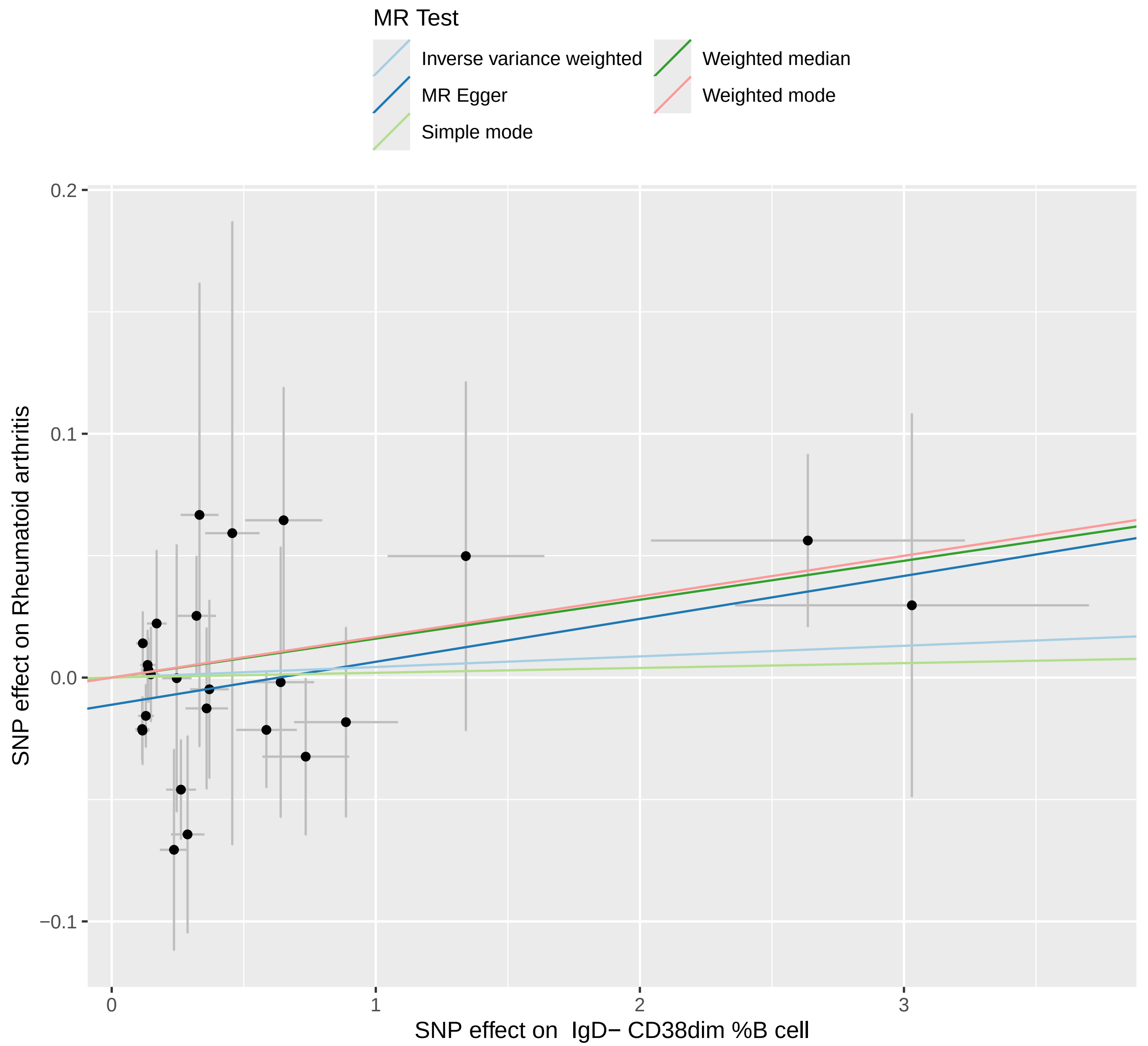
MR Test
Inverse variance weighted
MR Egger
Simple mode
Weighted median
Weighted mode
0.2
0.1
0.0
−0.1
0
1
2
3
SNP effect on Rheumatoid arthritis
SNP effect on IgD− CD38dim %B cell

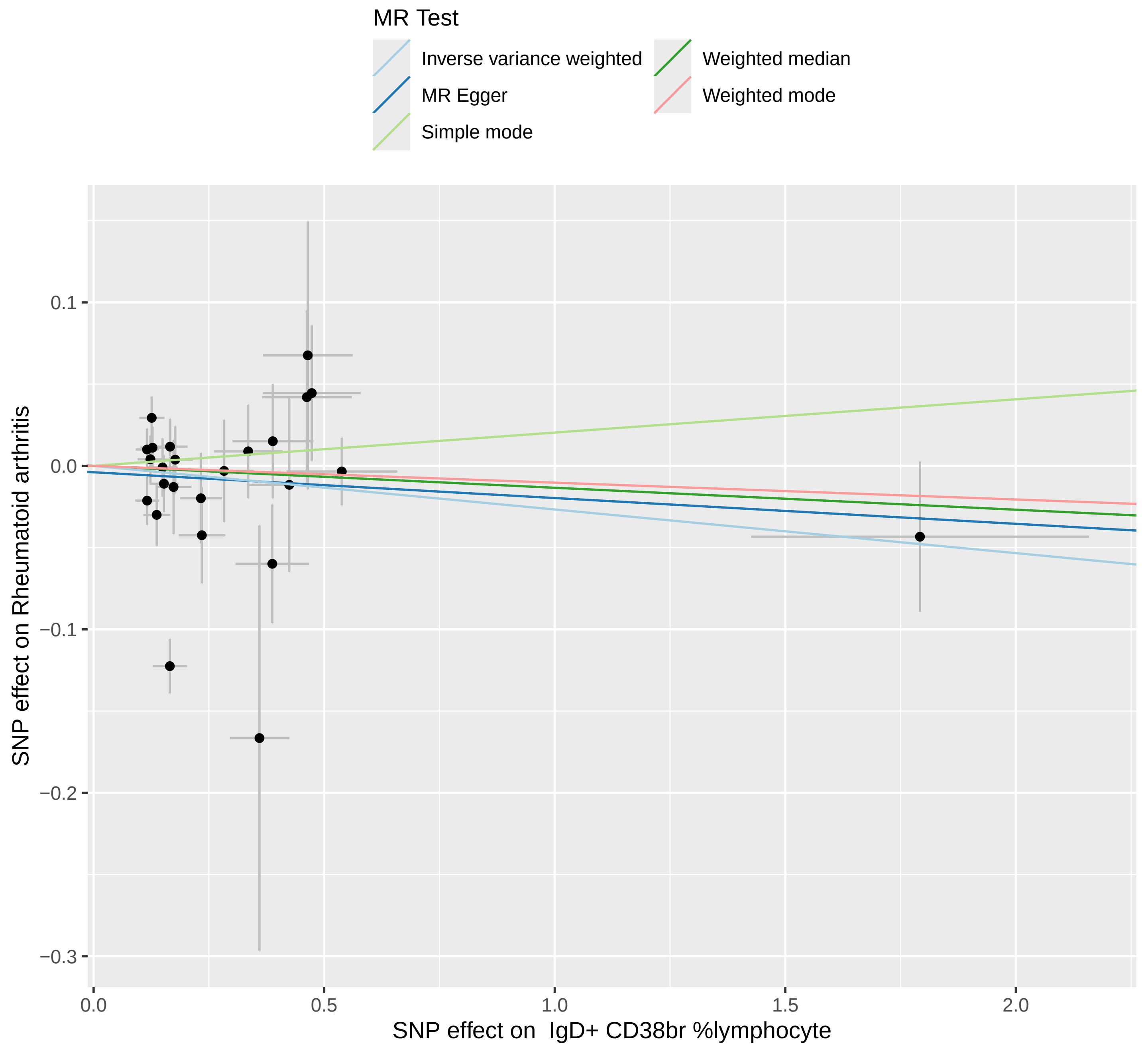

MR Test
Inverse variance weighted
MR Egger
Simple mode
Weighted median
Weighted mode
0.1
0.0
−0.1
−0.2
−0.3
0.0
0.5
1.0
1.5
2.0
SNP effect on Rheumatoid arthritis
SNP effect on IgD+ CD38br %lymphocyte

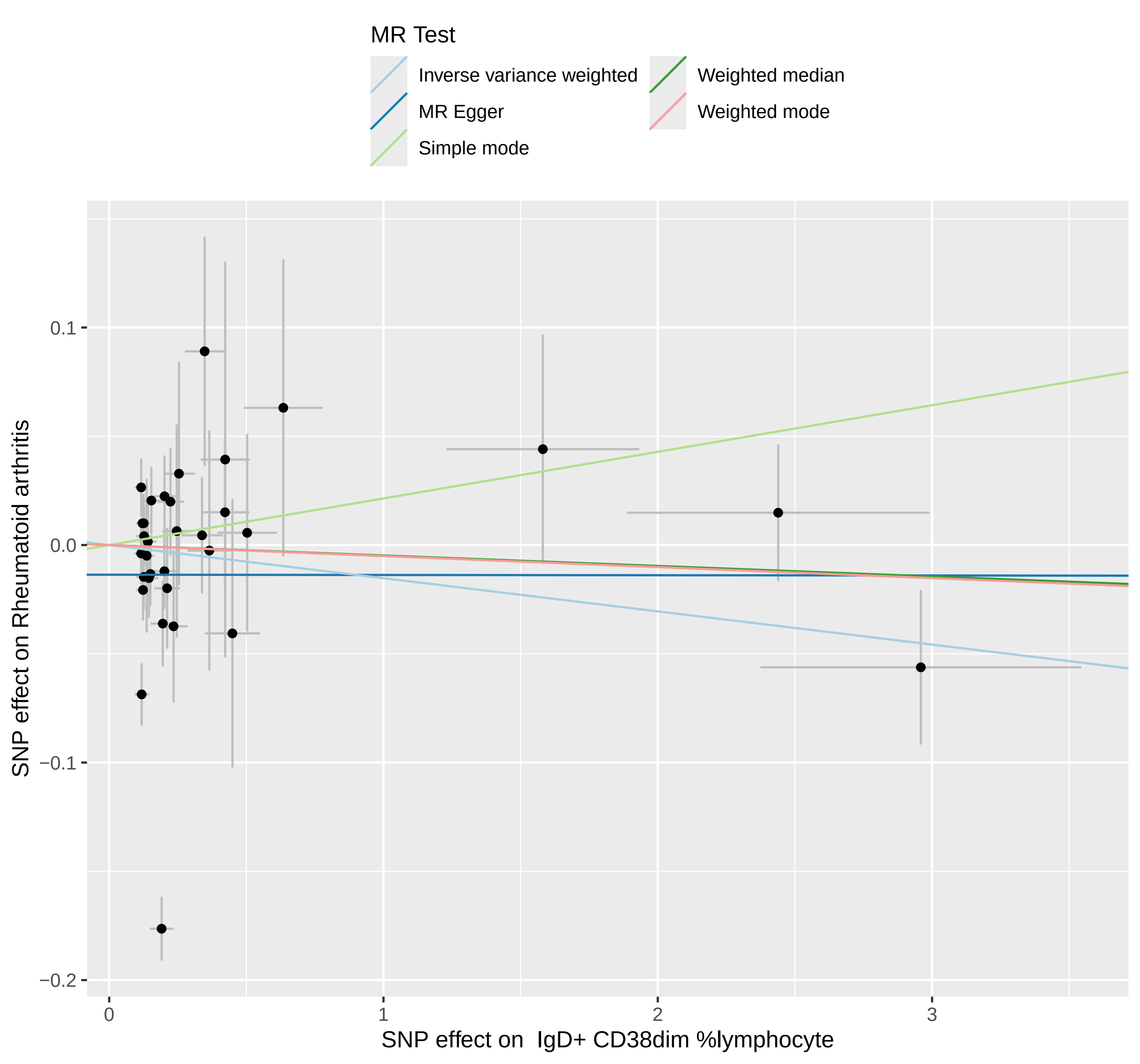

MR Test
Inverse variance weighted
MR Egger
Simple mode
Weighted median
Weighted mode
0.1
0.0
−0.1
−0.2
0
1
2
3
SNP effect on Rheumatoid arthritis
SNP effect on IgD+ CD38dim %lymphocyte

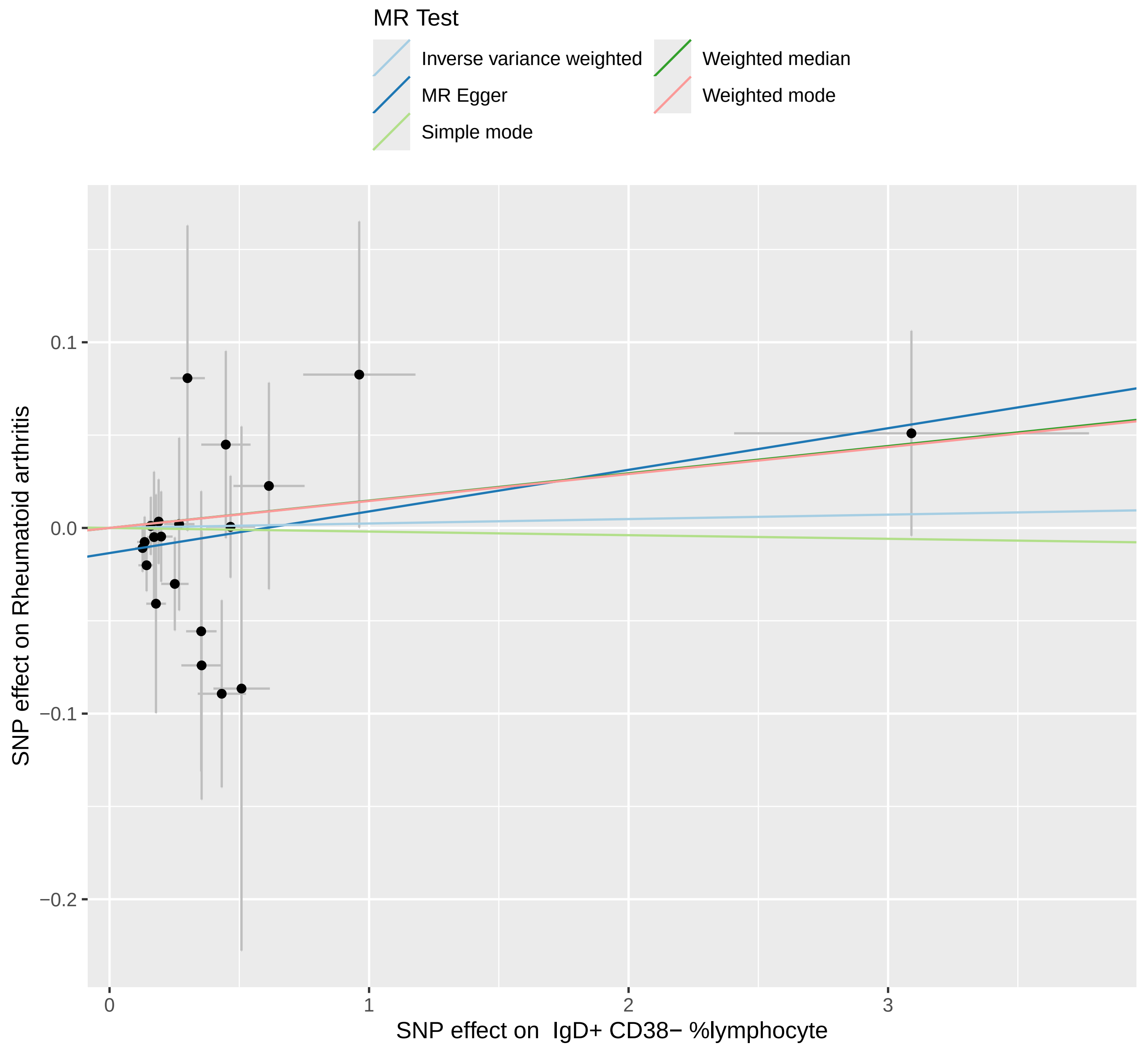
MR Test
Inverse variance weighted
MR Egger
Simple mode
Weighted median
Weighted mode
0.1
0.0
−0.1
−0.2
0
1
2
3
SNP effect on IgD+ CD38− %lymphocyte
SNP effect on Rheumatoid arthritis

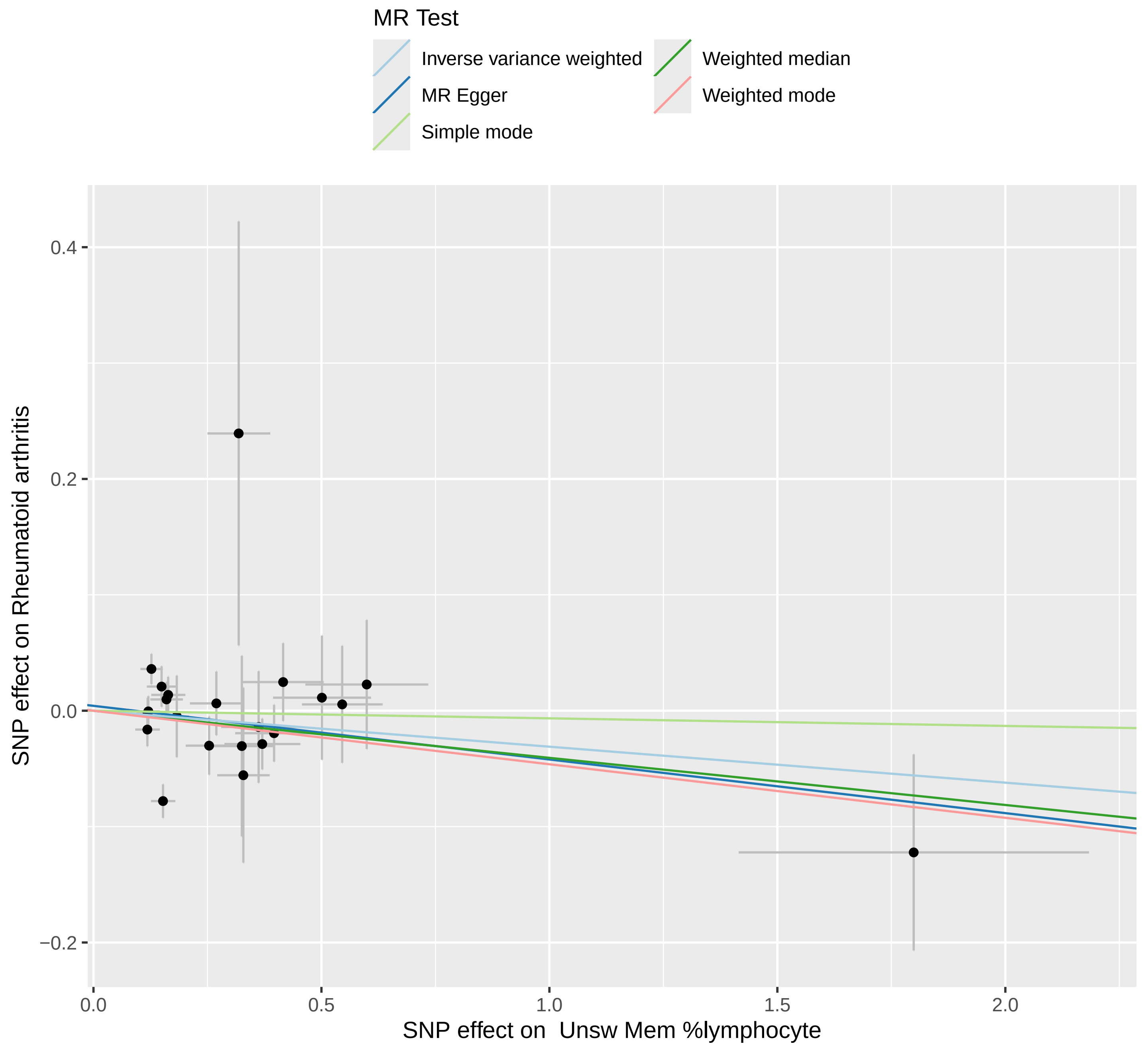

MR Test
Inverse variance weighted
MR Egger
Simple mode
Weighted median
Weighted mode
0.4
0.2
0.0
−0.2
0.0
0.5
1.0
1.5
2.0
SNP effect on Rheumatoid arthritis
SNP effect on Unsw Mem %lymphocyte

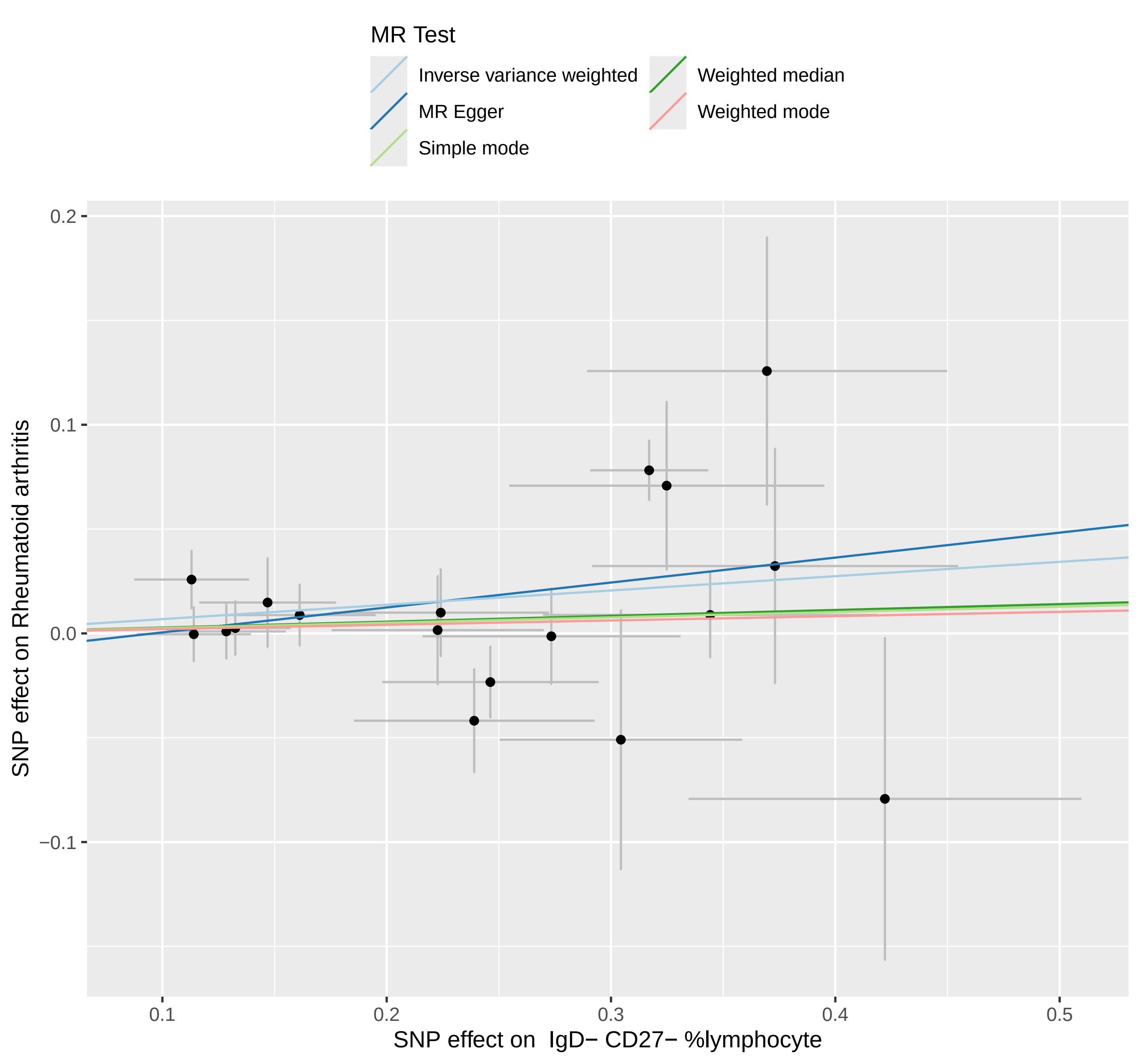

MR Test
Inverse variance weighted
MR Egger
Simple mode
Weighted median
Weighted mode
0.2
0.1
0.0
−0.1
0.1
0.2
0.3
0.4
0.5
SNP effect on Rheumatoid arthritis
SNP effect on IgD− CD27− %lymphocyte

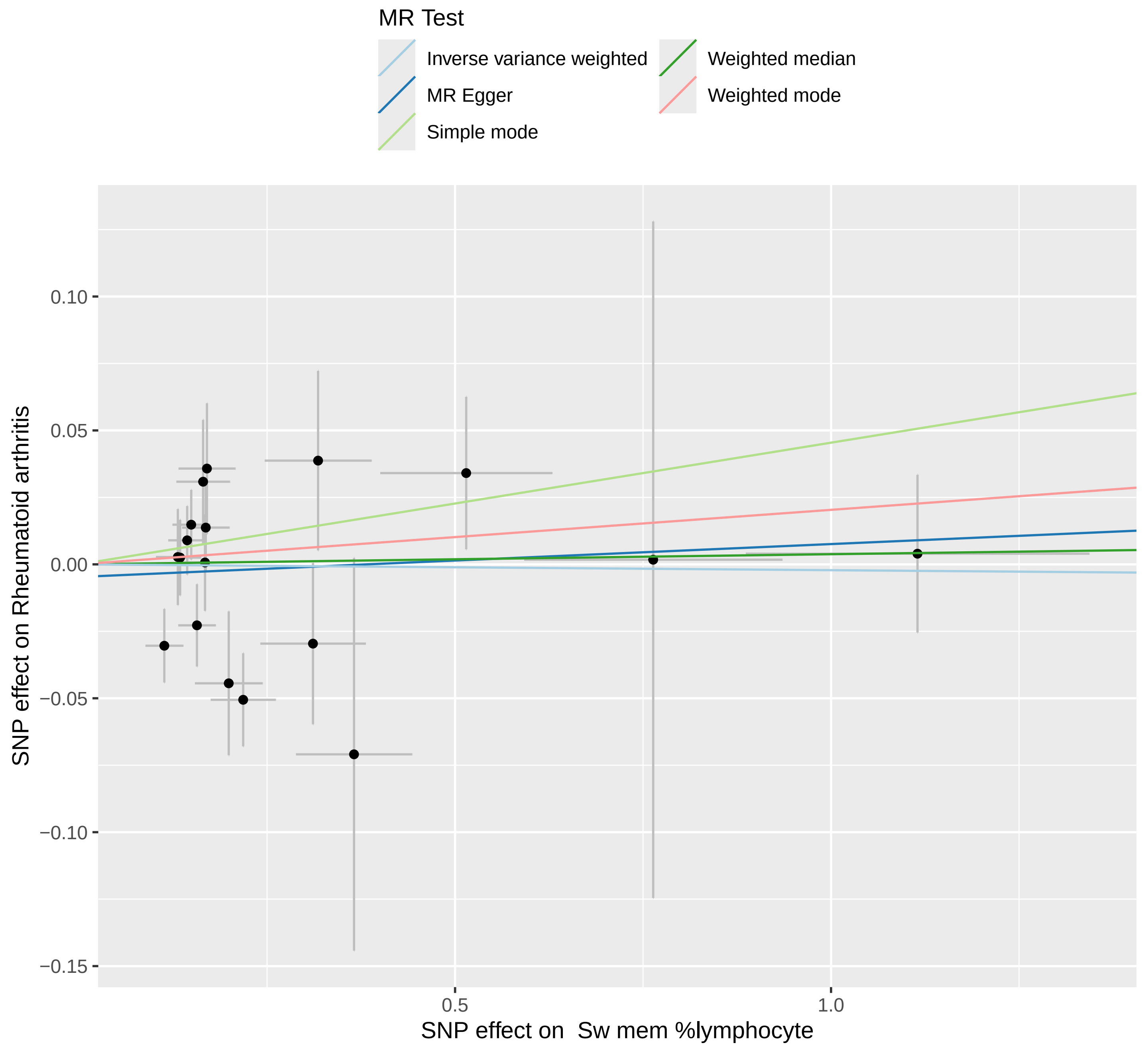
MR Test
Inverse variance weighted
MR Egger
Simple mode
Weighted median
Weighted mode
0.10
0.05
0.00
−0.05
−0.10
−0.15
SNP effect on Rheumatoid arthritis
0.5
1.0
SNP effect on Sw mem %lymphocyte

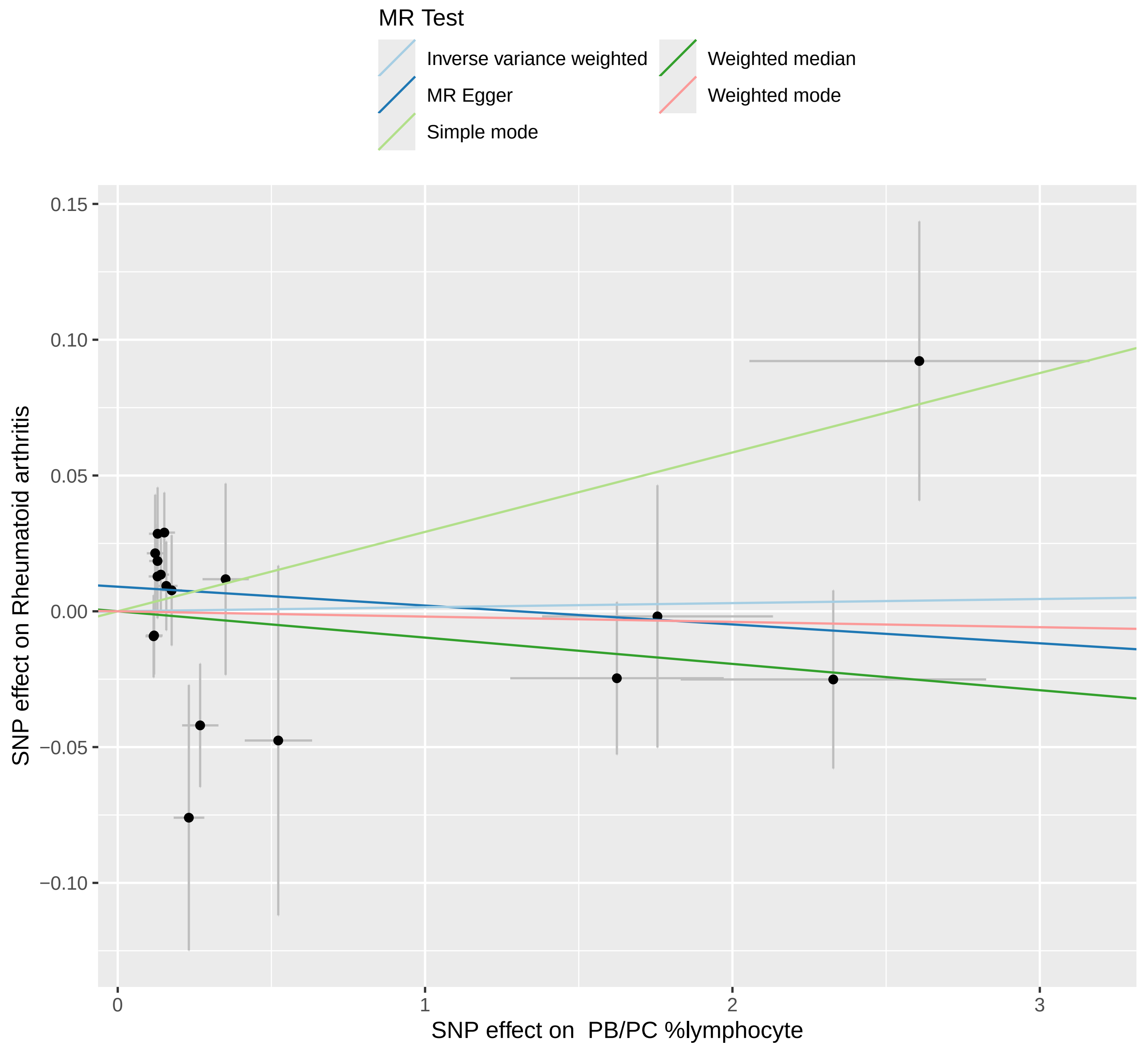

MR Test
Inverse variance weighted
MR Egger
Simple mode
Weighted median
Weighted mode
0.15
0.10
0.05
0.00
−0.05
−0.10
SNP effect on Rheumatoid arthritis
0
1
2
3
SNP effect on PB/PC %lymphocyte

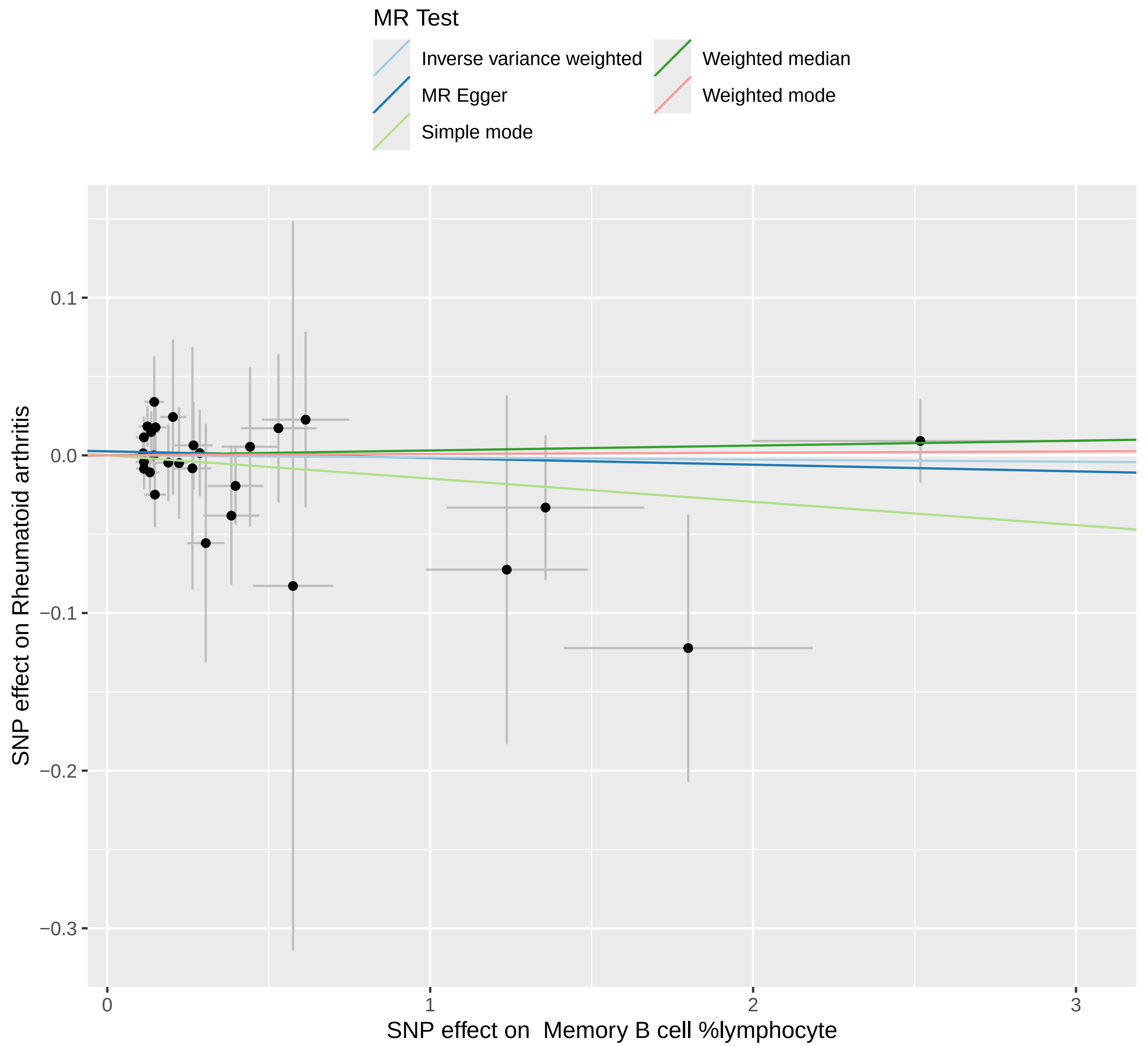
MR Test
Inverse variance weighted
MR Egger
Simple mode
Weighted median
Weighted mode
0.1
0.0
−0.1
−0.2
−0.3
0
1
2
3
SNP effect on Rheumatoid arthritis
SNP effect on Memory B cell %lymphocyte

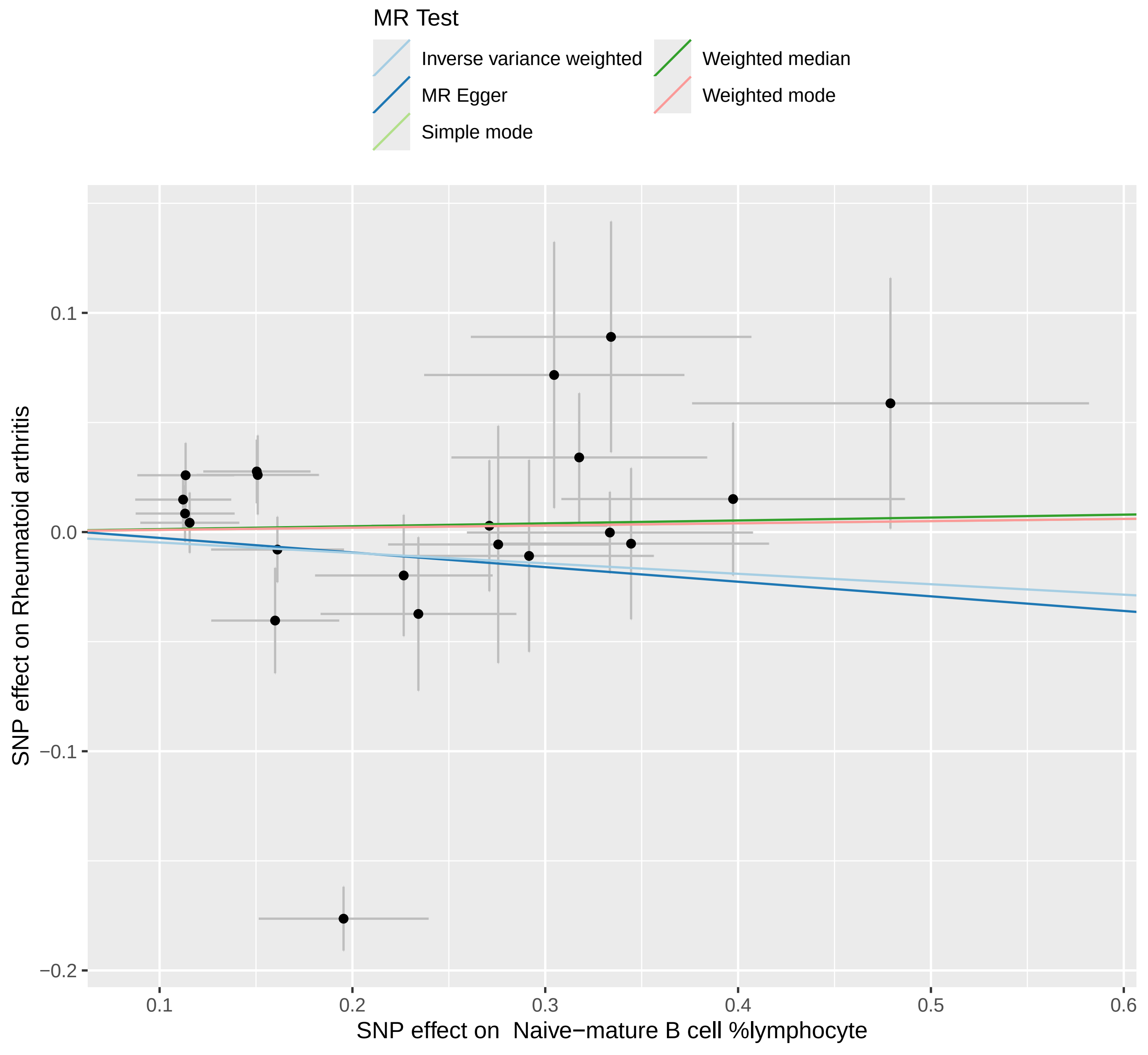
MR Test
Inverse variance weighted
MR Egger
Simple mode
Weighted median
Weighted mode
SNP effect on Rheumatoid arthritis
0.1
0.0
−0.1
−0.2
0.1
0.2
0.3
0.4
0.5
0.6
SNP effect on Naive−mature B cell %lymphocyte

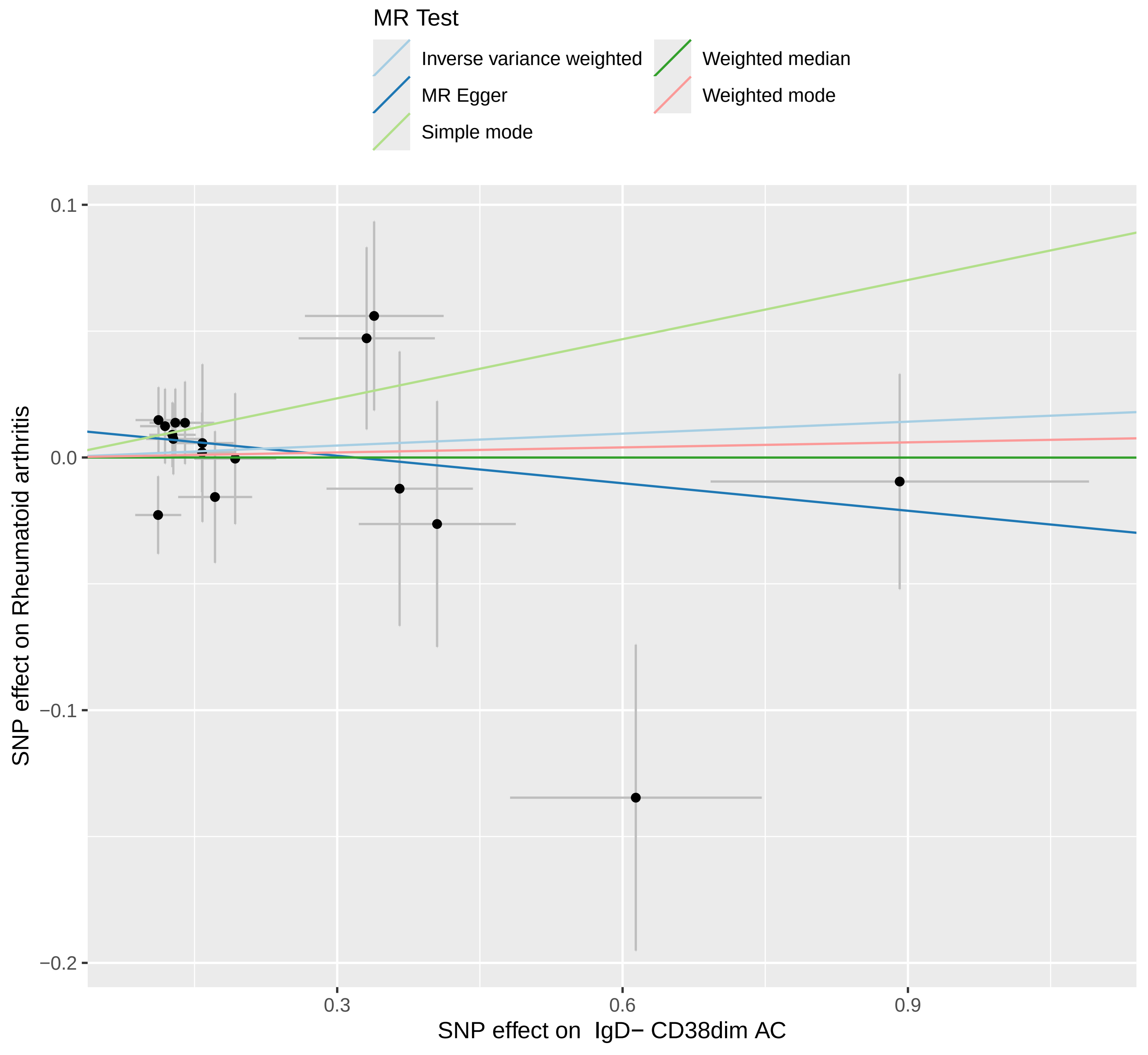
MR Test
Inverse variance weighted
MR Egger
Simple mode
Weighted median
Weighted mode
0.1
0.0
−0.1
−0.2
SNP effect on Rheumatoid arthritis
0.3
0.6
0.9
SNP effect on IgD− CD38dim AC

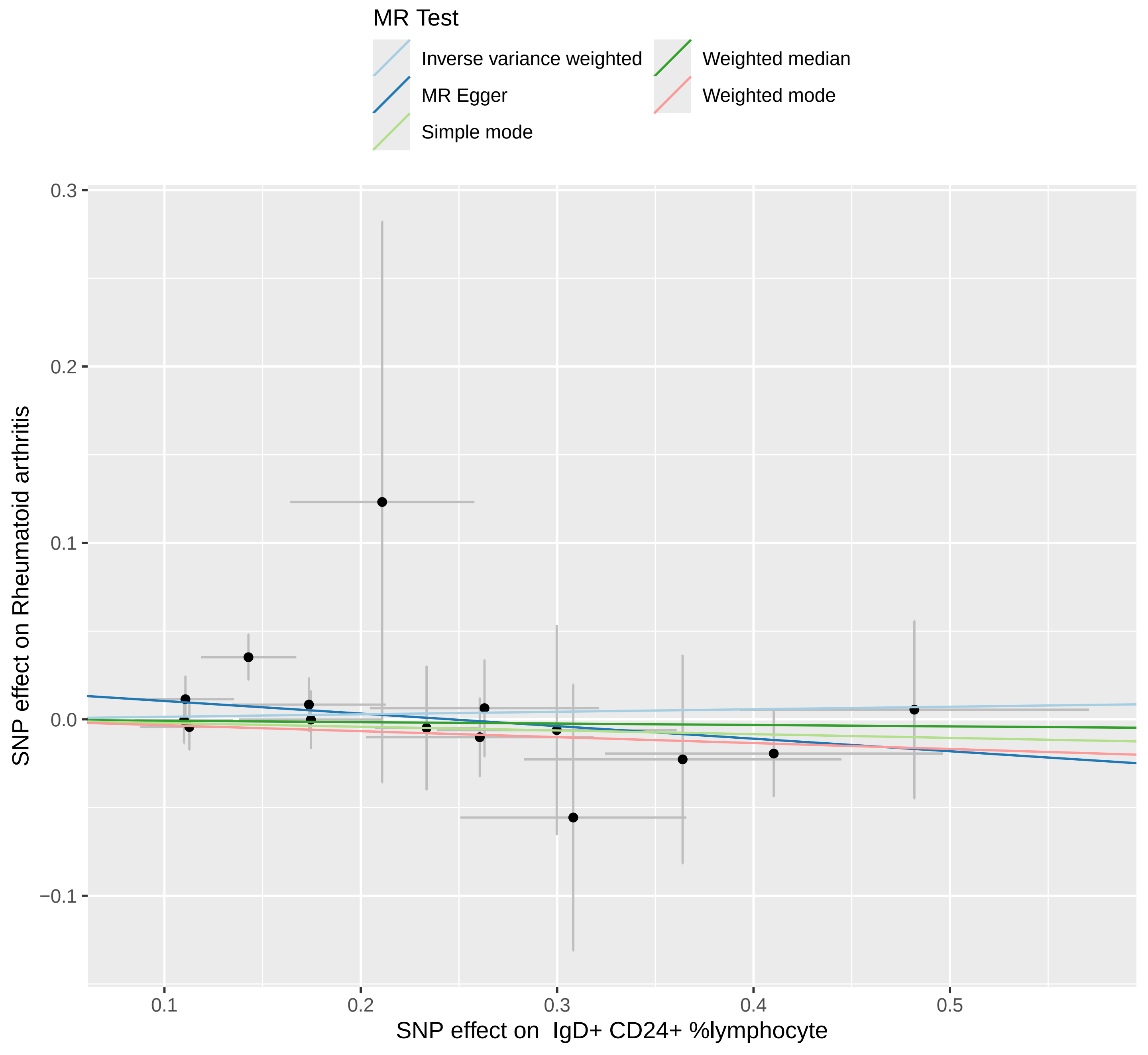
MR Test
Inverse variance weighted
MR Egger
Simple mode
Weighted median
Weighted mode
0.3
0.2
0.1
0.0
−0.1
SNP effect on Rheumatoid arthritis
0.1
0.2
0.3
0.4
0.5
SNP effect on IgD+ CD24+ %lymphocyte

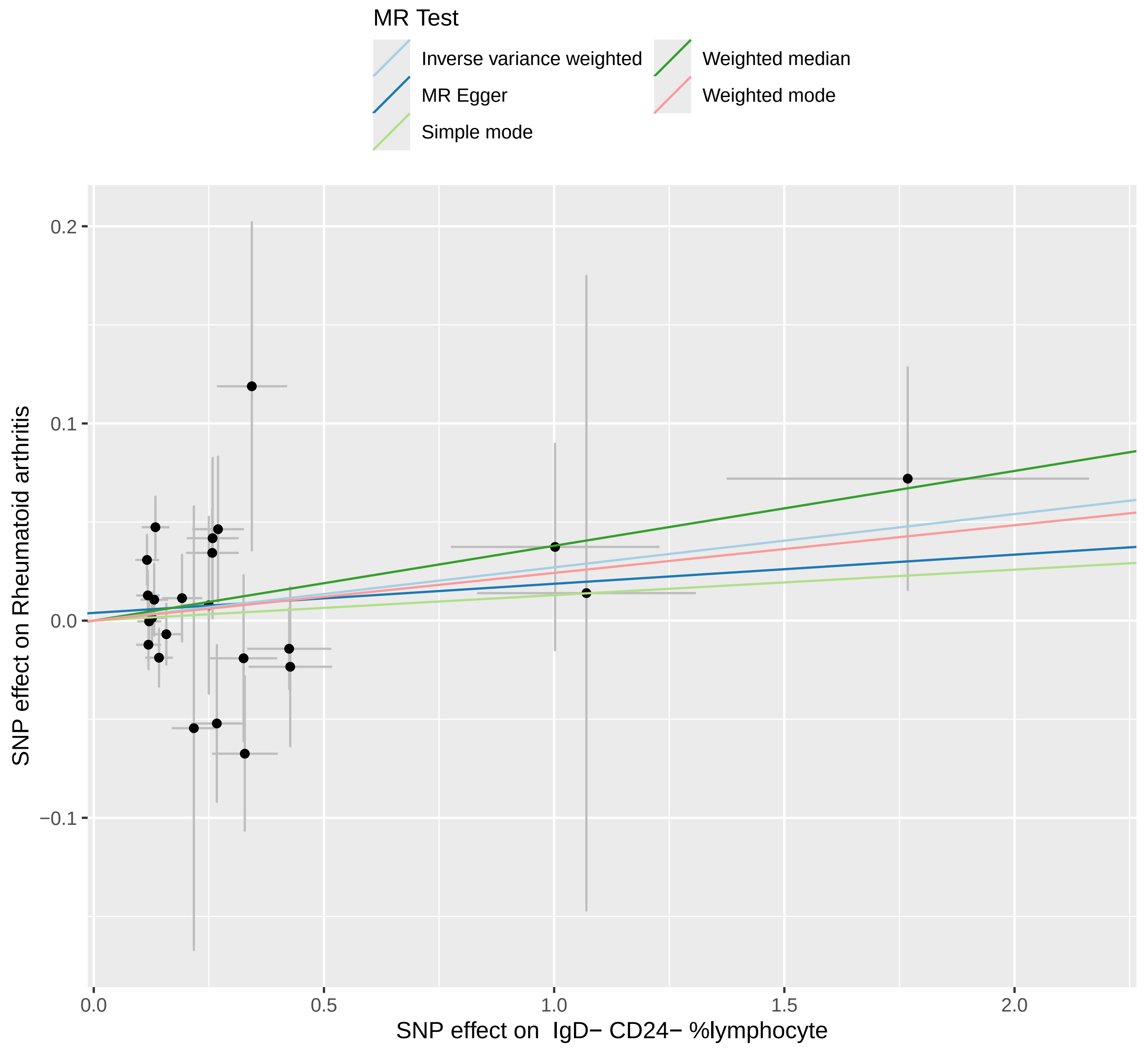
MR Test
Inverse variance weighted
MR Egger
Simple mode
Weighted median
Weighted mode
0.2
0.1
0.0
−0.1
0.0
0.5
1.0
1.5
2.0
SNP effect on Rheumatoid arthritis
SNP effect on IgD− CD24− %lymphocyte

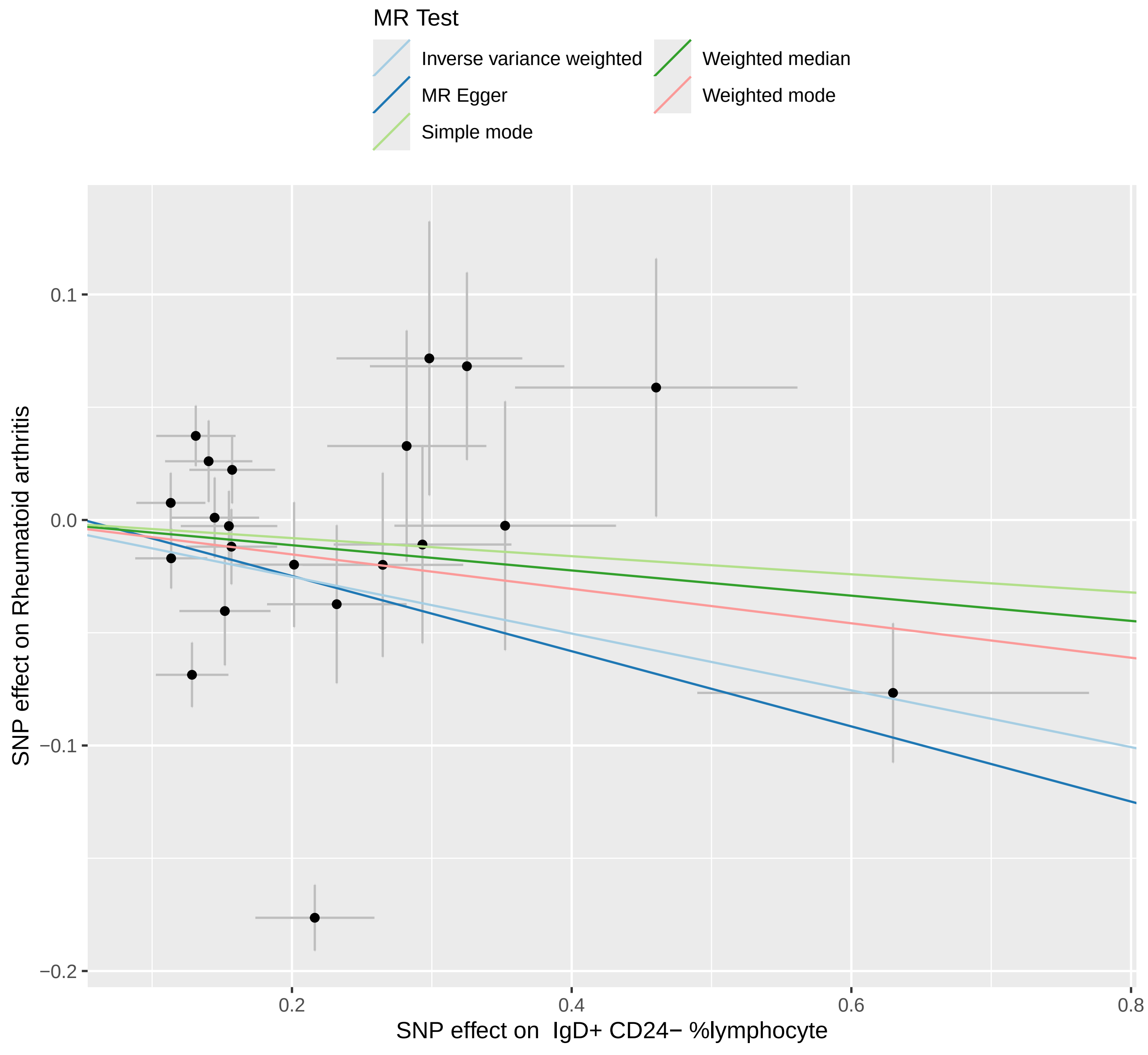

MR Test
Inverse variance weighted
MR Egger
Simple mode
Weighted median
Weighted mode
0.1
0.0
−0.1
−0.2
0.2
0.4
0.6
0.8
SNP effect on Rheumatoid arthritis
SNP effect on IgD+ CD24− %lymphocyte

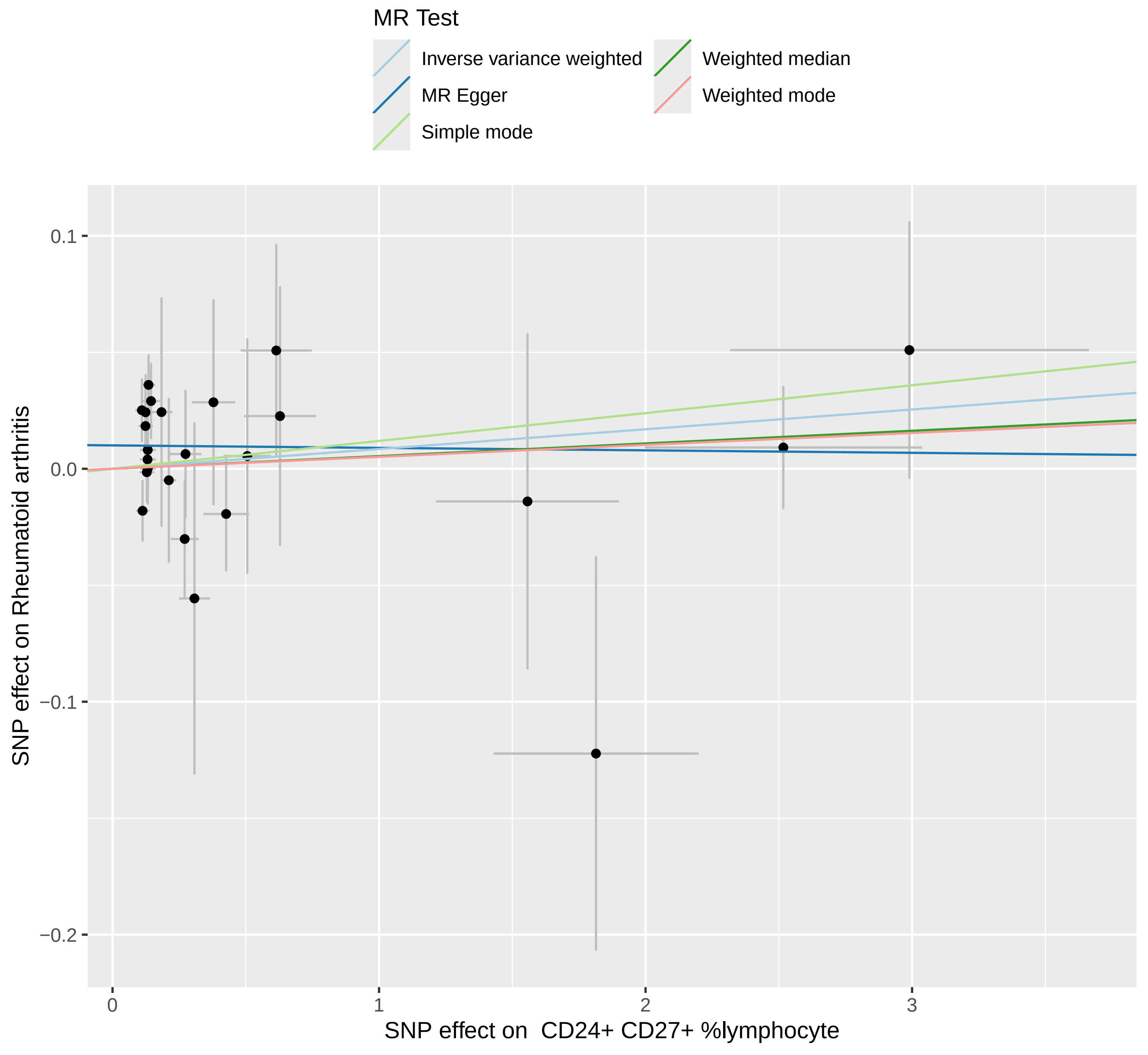

MR Test
Inverse variance weighted
MR Egger
Simple mode
Weighted median
Weighted mode
0.1
0.0
−0.1
−0.2
0
1
2
3
SNP effect on Rheumatoid arthritis
SNP effect on CD24+ CD27+ %lymphocyte

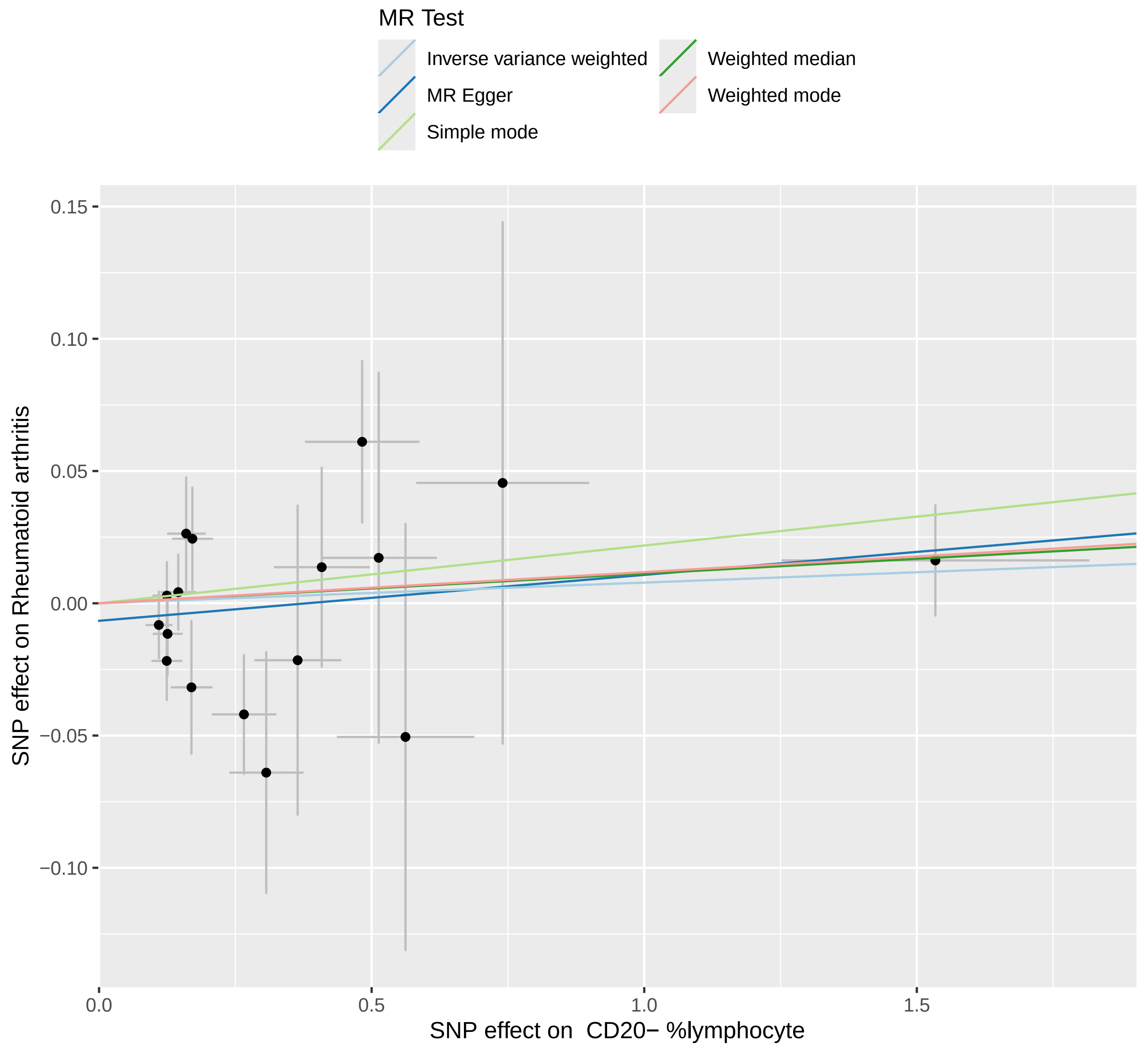
MR Test
Inverse variance weighted
MR Egger
Simple mode
Weighted median
Weighted mode
0.15
0.10
0.05
0.00
−0.05
−0.10
0.0
0.5
1.0
1.5
SNP effect on Rheumatoid arthritis
SNP effect on CD20− %lymphocyte

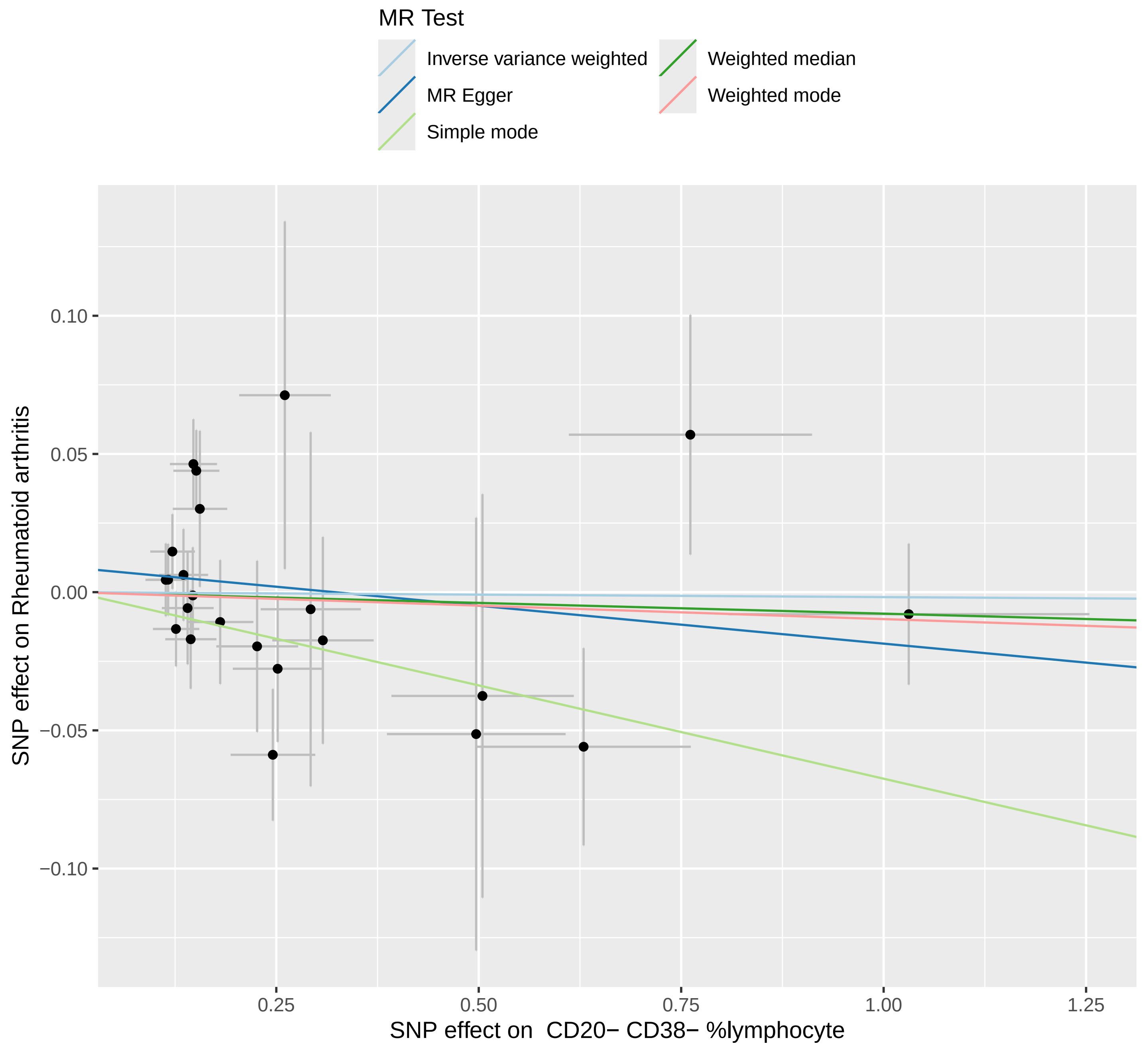

MR Test
Inverse variance weighted
MR Egger
Simple mode
Weighted median
Weighted mode
0.10
0.05
0.00
−0.05
−0.10
SNP effect on Rheumatoid arthritis
0.25
0.50
0.75
1.00
1.25
SNP effect on CD20− CD38− %lymphocyte

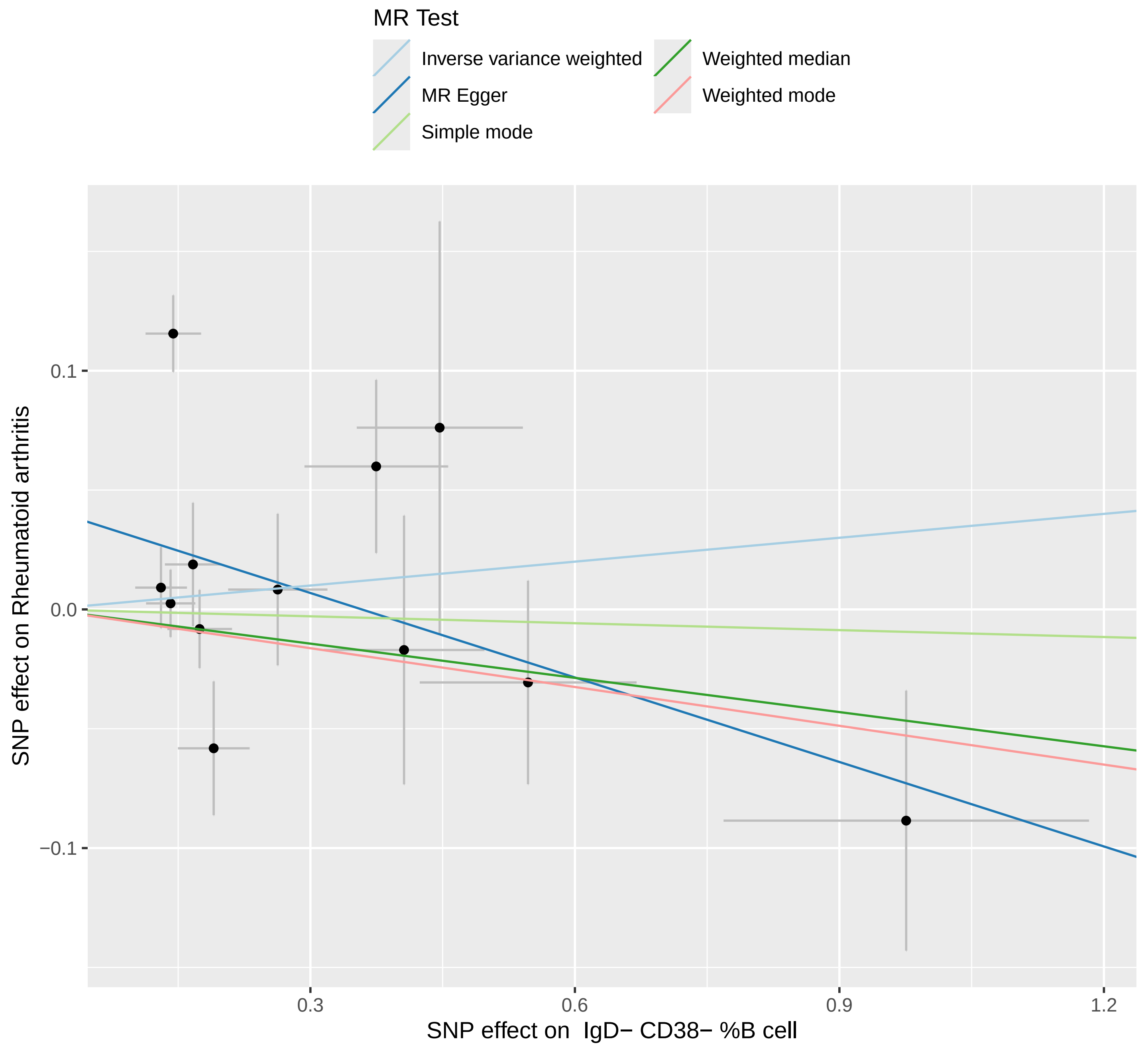
MR Test
Inverse variance weighted
MR Egger
Simple mode
Weighted median
Weighted mode
SNP effect on Rheumatoid arthritis
0.1
0.0
−0.1
0.3
0.6
0.9
1.2
SNP effect on IgD− CD38− %B cell

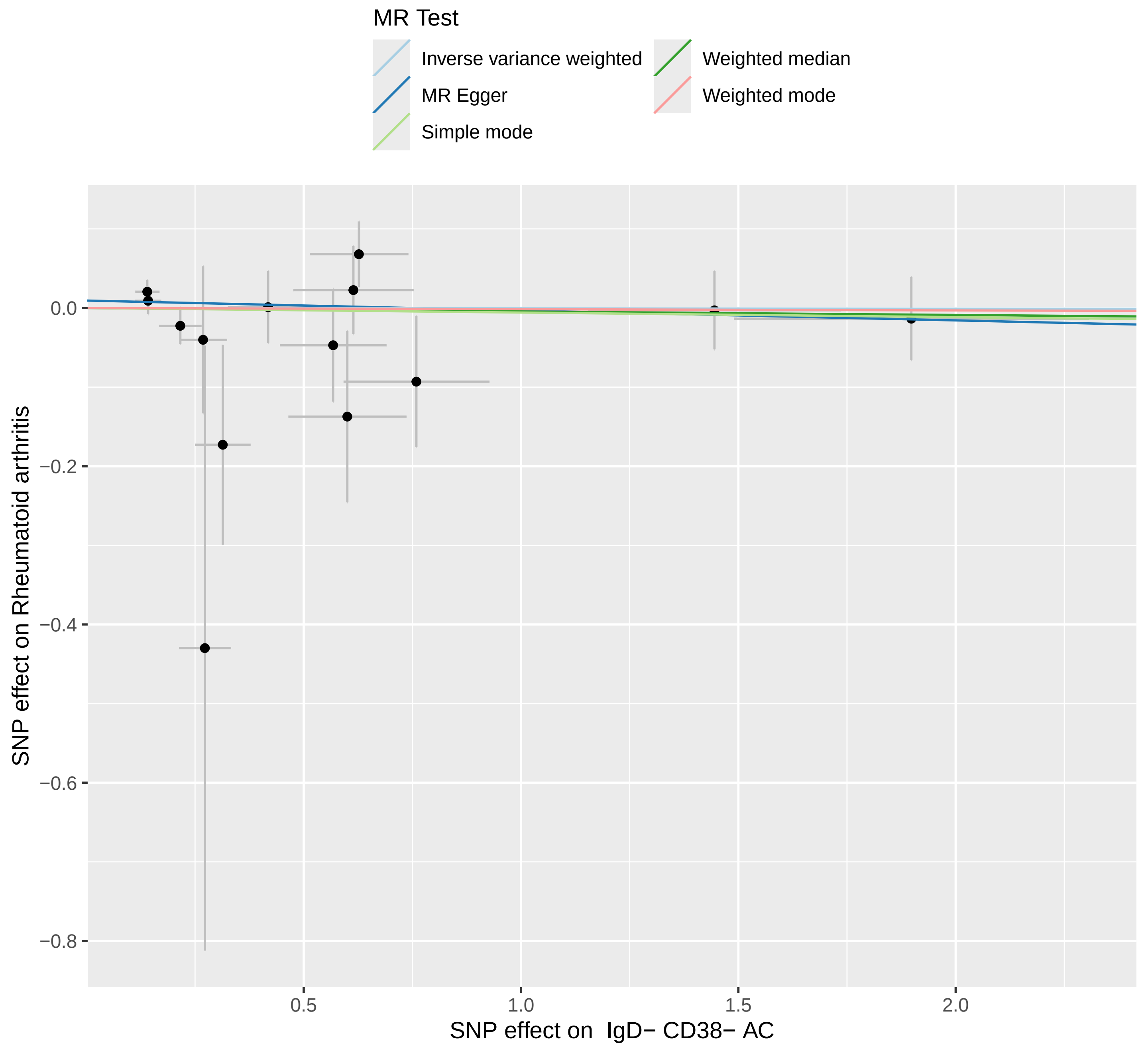
MR Test
Inverse variance weighted
MR Egger
Simple mode
Weighted median
Weighted mode
0.0
−0.2
−0.4
−0.6
−0.8
0.5
1.0
1.5
2.0
SNP effect on Rheumatoid arthritis
SNP effect on IgD− CD38− AC

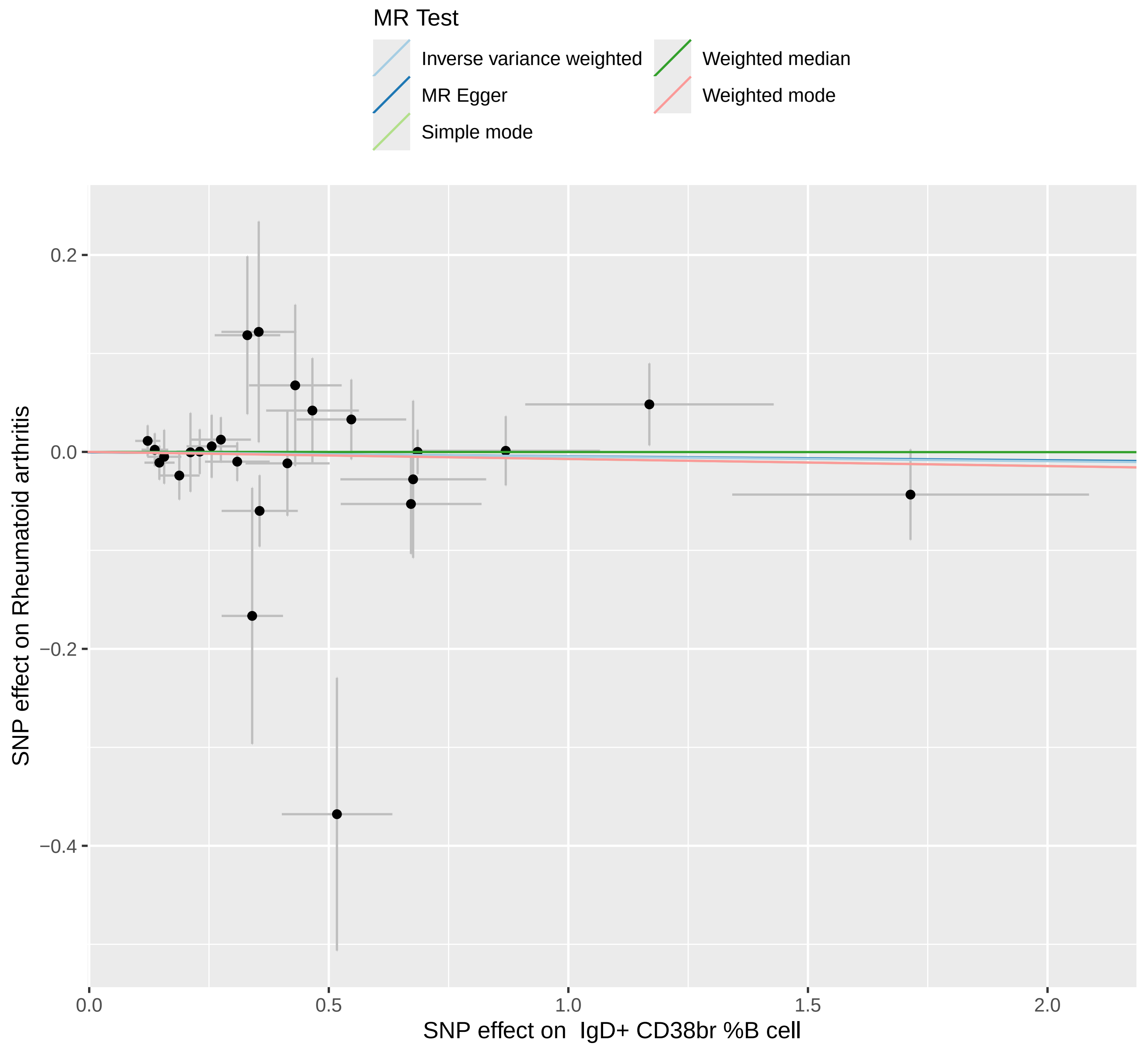

MR Test
Inverse variance weighted
MR Egger
Simple mode
Weighted median
Weighted mode
SNP effect on Rheumatoid arthritis
0.2
0.0
−0.2
−0.4
0.0
0.5
1.0
1.5
2.0
SNP effect on IgD+ CD38br %B cell

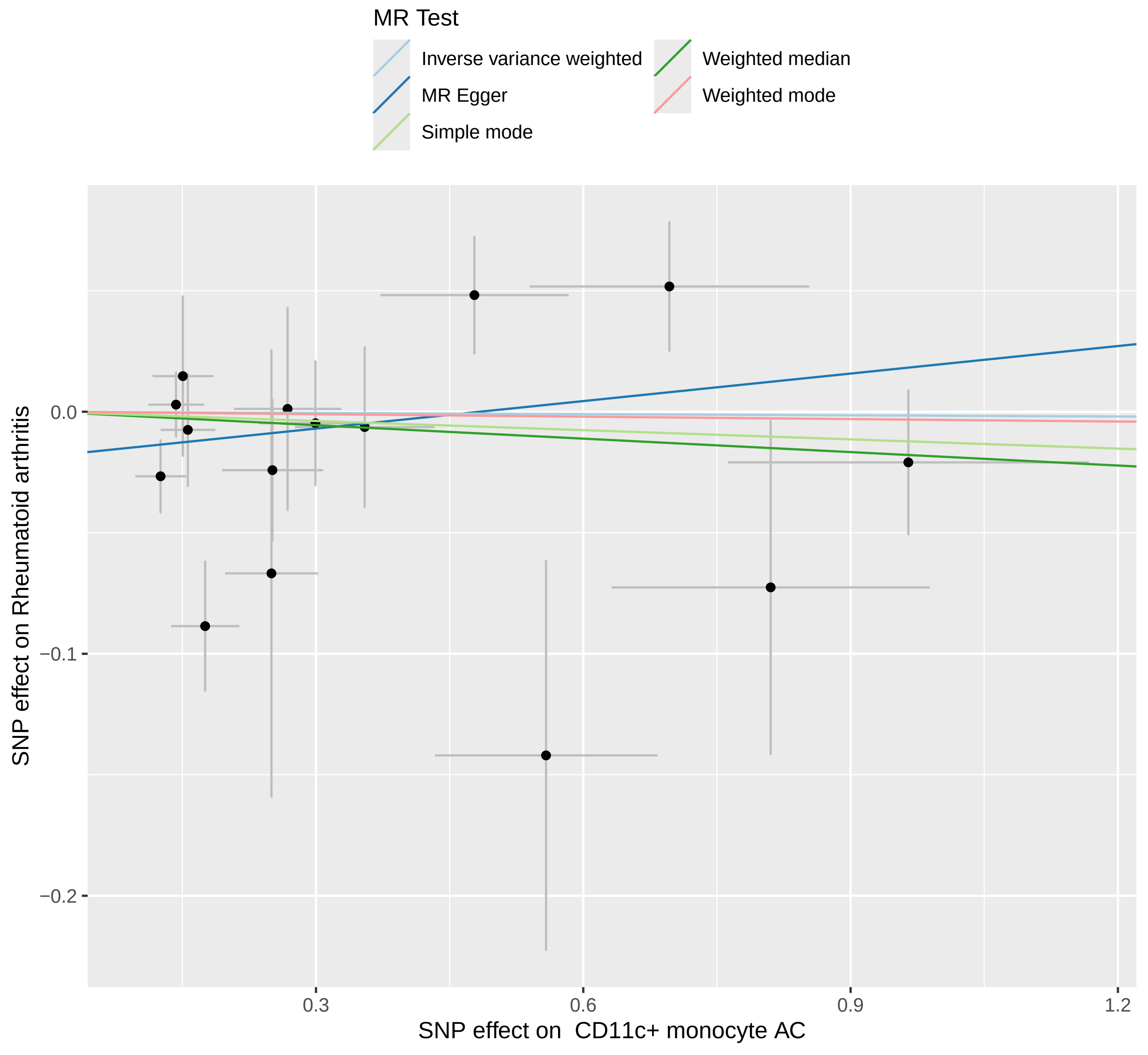

MR Test
Inverse variance weighted
MR Egger
Simple mode
Weighted median
Weighted mode
SNP effect on Rheumatoid arthritis
0.0
−0.1
−0.2
0.3
0.6
0.9
1.2
SNP effect on CD11c+ monocyte AC

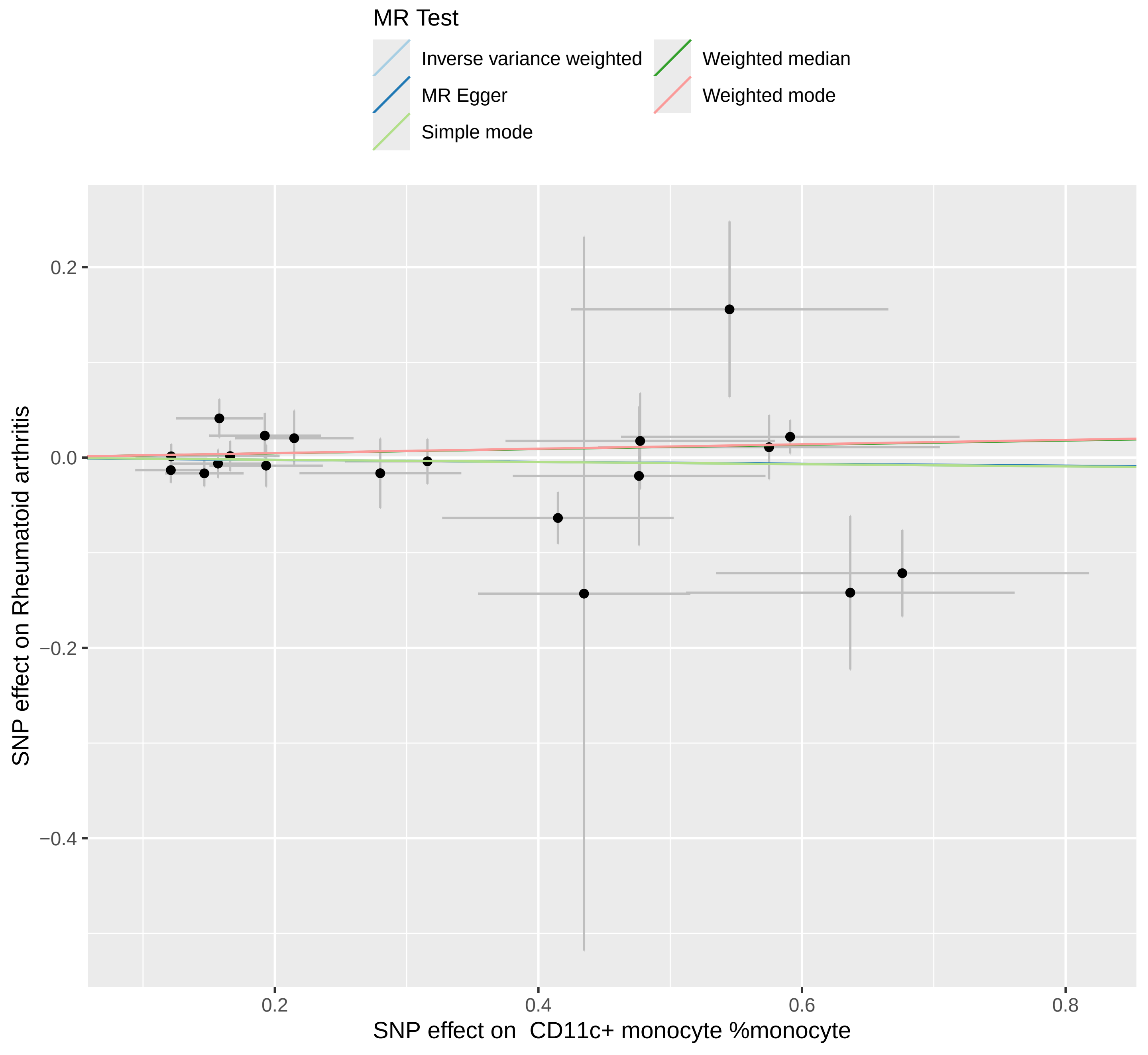
MR Test
Inverse variance weighted
MR Egger
Simple mode
Weighted median
Weighted mode
SNP effect on Rheumatoid arthritis
0.2
0.0
−0.2
−0.4
0.2
0.4
0.6
0.8
SNP effect on CD11c+ monocyte %monocyte

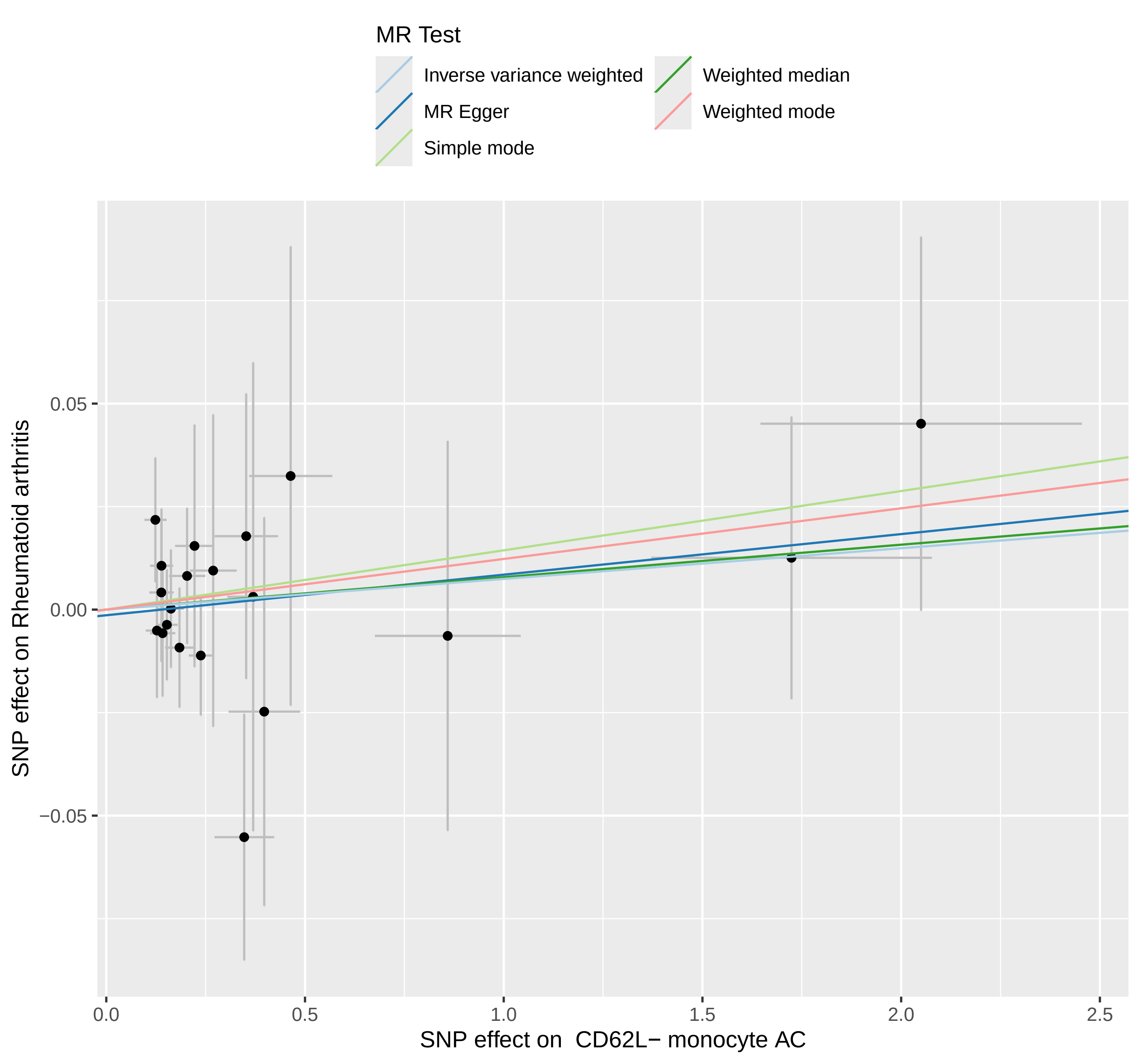
MR Test
Inverse variance weighted
MR Egger
Simple mode
Weighted median
Weighted mode
0.05
0.00
−0.05
0.0
0.5
1.0
1.5
2.0
2.5
SNP effect on Rheumatoid arthritis
SNP effect on CD62L− monocyte AC

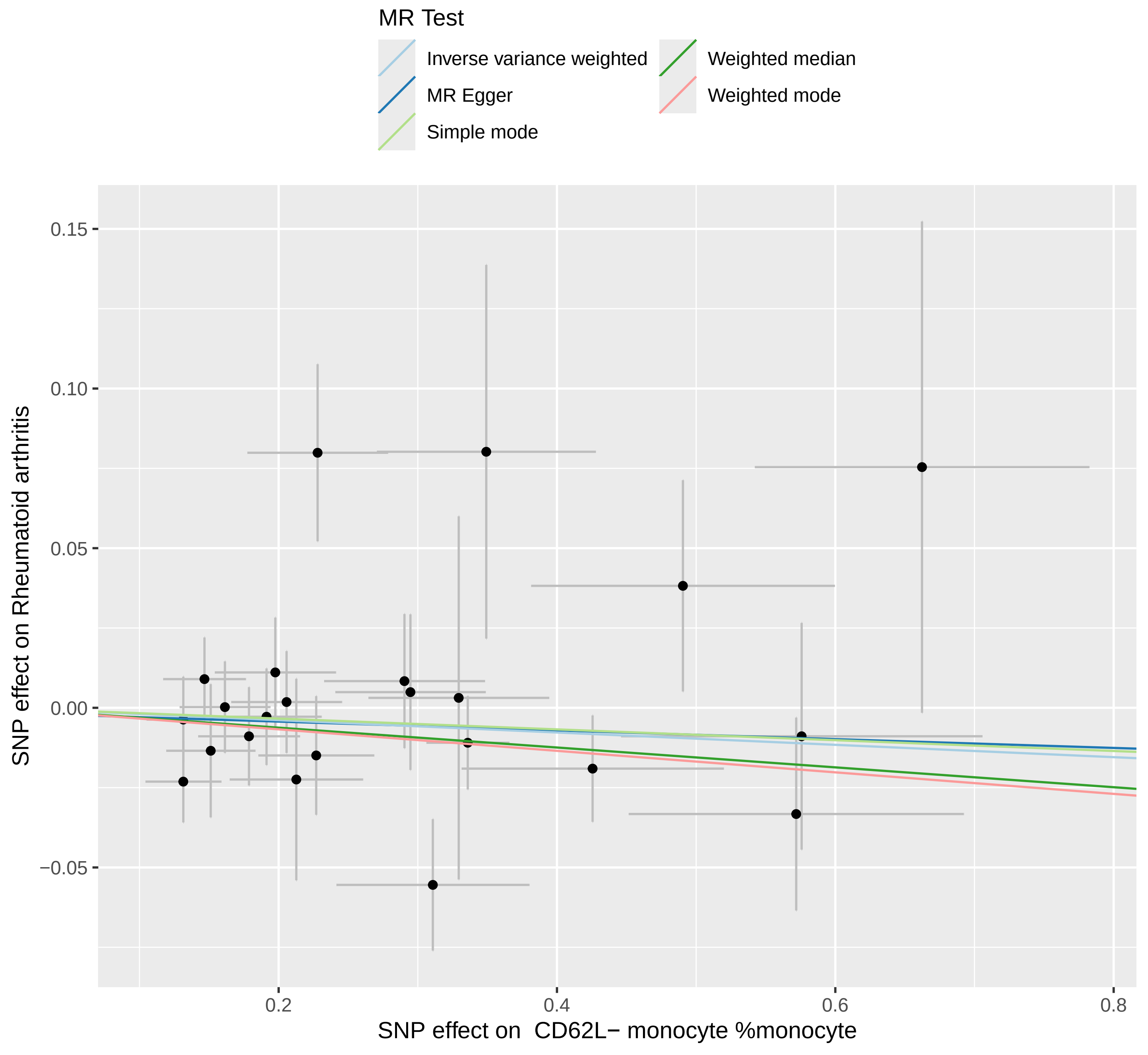
MR Test
Inverse variance weighted
MR Egger
Simple mode
Weighted median
Weighted mode
SNP effect on Rheumatoid arthritis
0.15
0.10
0.05
0.00
−0.05
0.2
0.4
0.6
0.8
SNP effect on CD62L− monocyte %monocyte

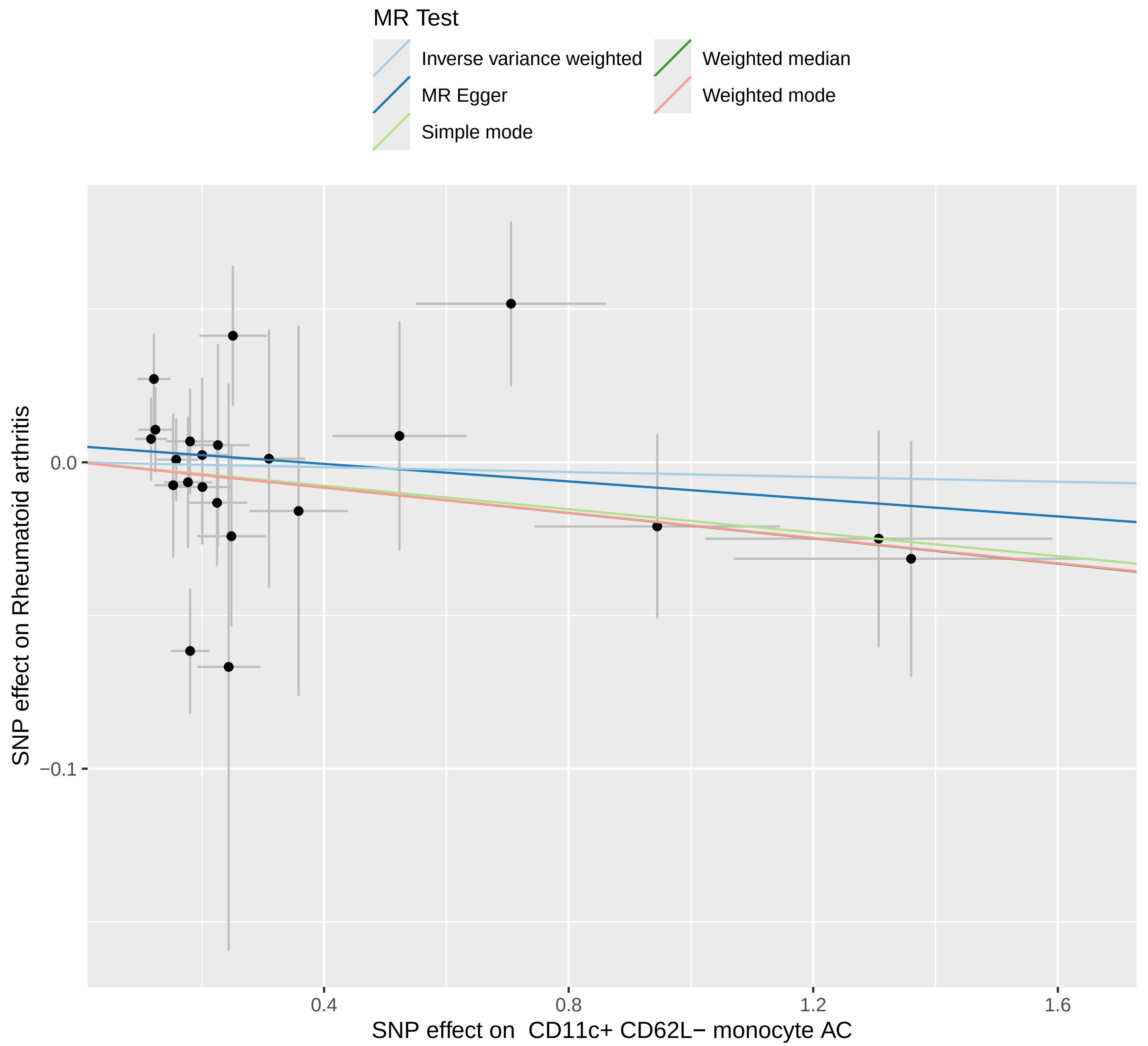
MR Test
Inverse variance weighted
MR Egger
Simple mode
Weighted median
Weighted mode
SNP effect on Rheumatoid arthritis
0.0
−0.1
0.4
0.8
1.2
1.6
SNP effect on CD11c+ CD62L− monocyte AC

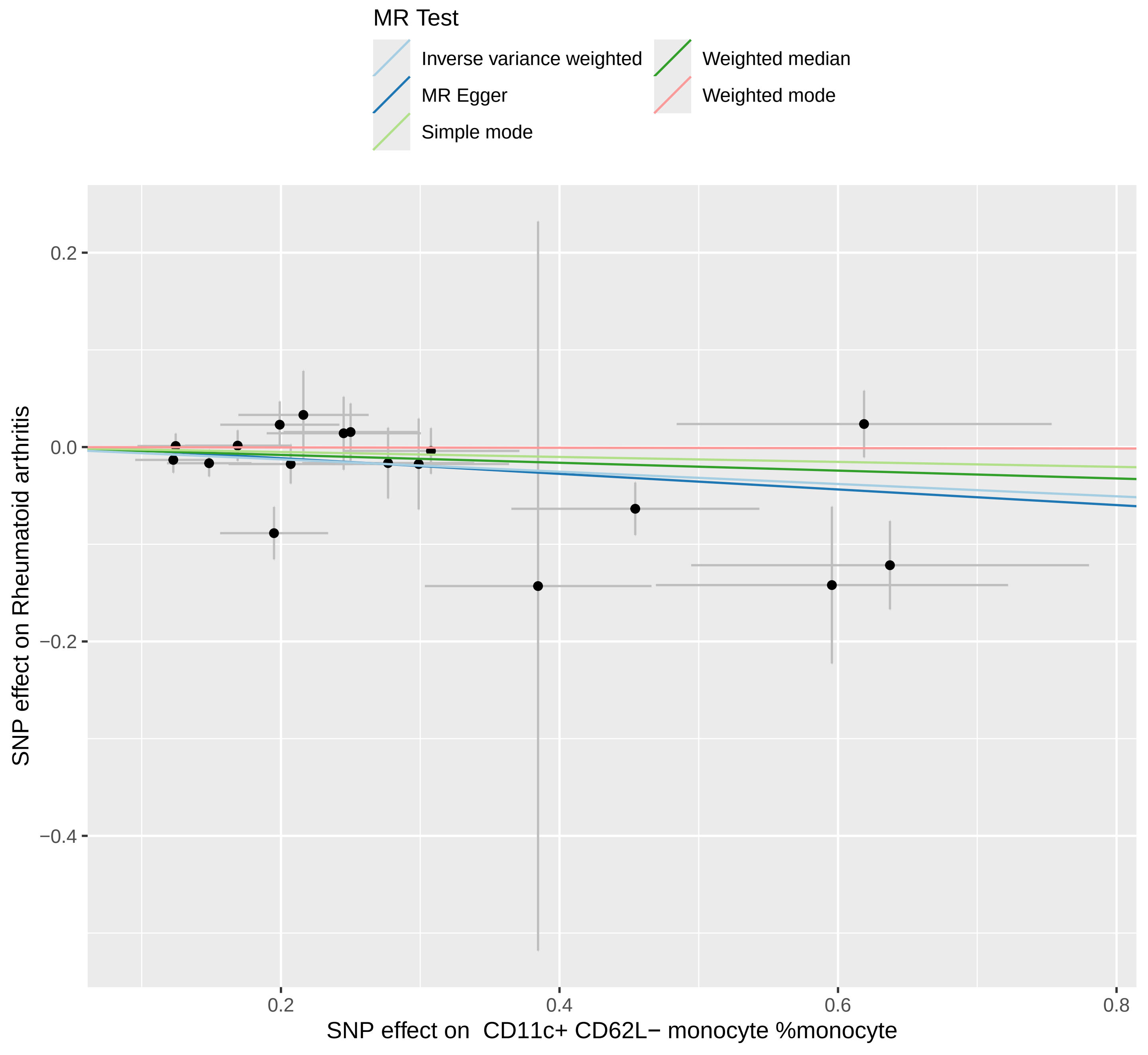

MR Test
Inverse variance weighted
MR Egger
Simple mode
Weighted median
Weighted mode
0.2
0.0
−0.2
−0.4
0.2
0.4
0.6
0.8
SNP effect on Rheumatoid arthritis
SNP effect on CD11c+ CD62L− monocyte %monocyte

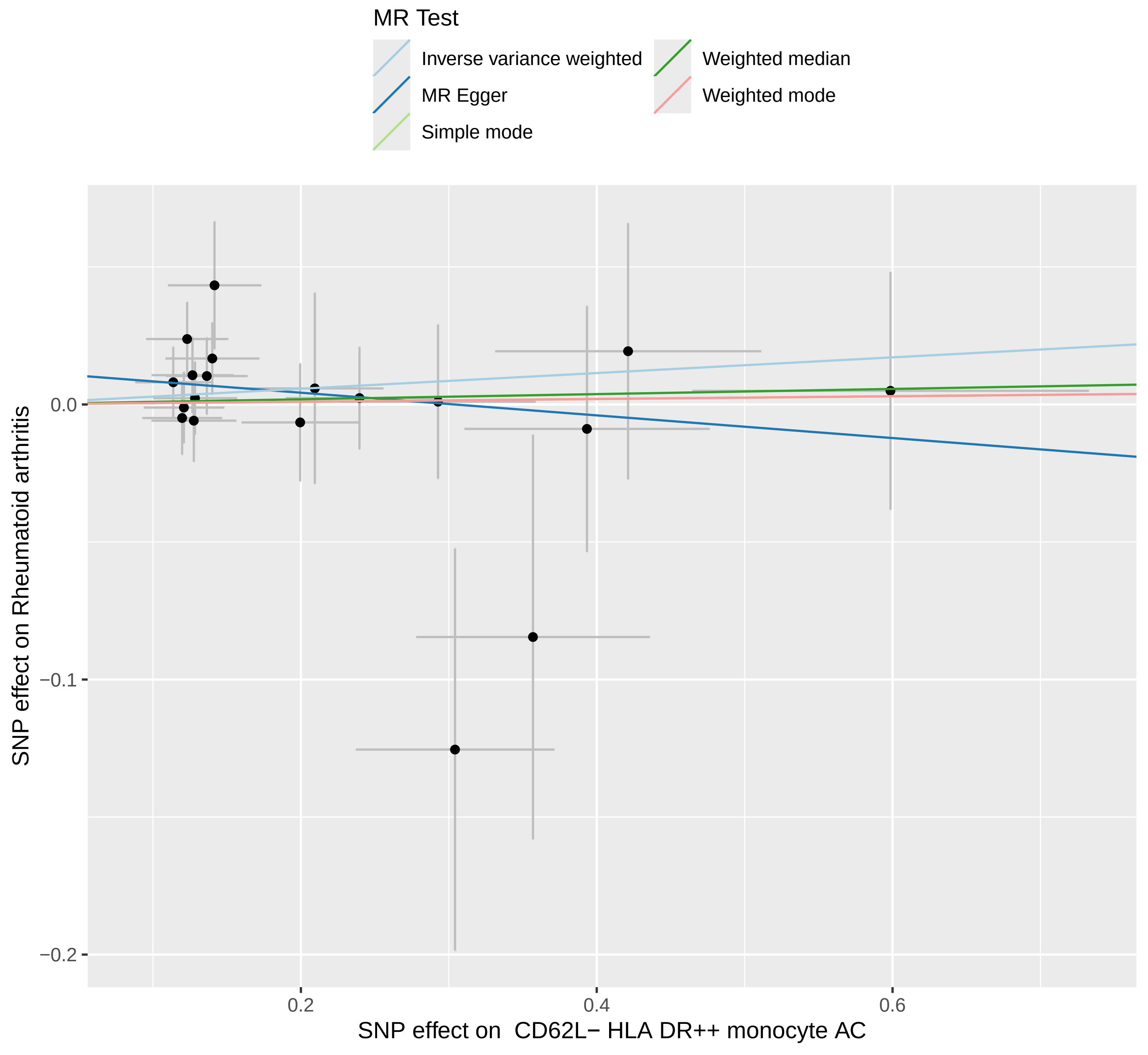

MR Test
Inverse variance weighted
MR Egger
Simple mode
Weighted median
Weighted mode
SNP effect on Rheumatoid arthritis
0.0
−0.1
−0.2
0.2
0.4
0.6
SNP effect on CD62L− HLA DR++ monocyte AC

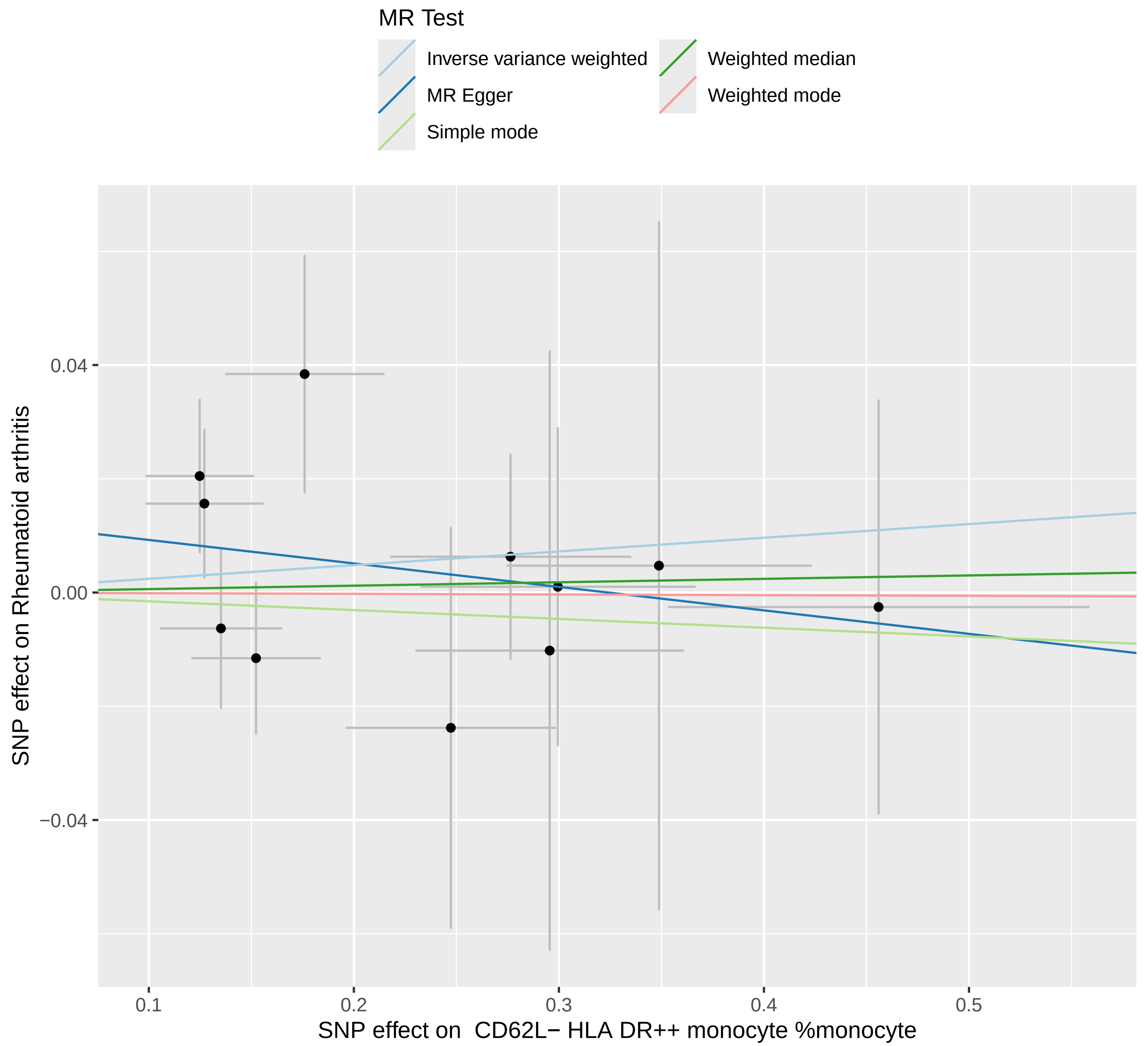

MR Test
Inverse variance weighted
MR Egger
Simple mode
Weighted median
Weighted mode
0.04
0.00
−0.04
0.1
0.2
0.3
0.4
0.5
SNP effect on Rheumatoid arthritis
SNP effect on CD62L− HLA DR++ monocyte %monocyte

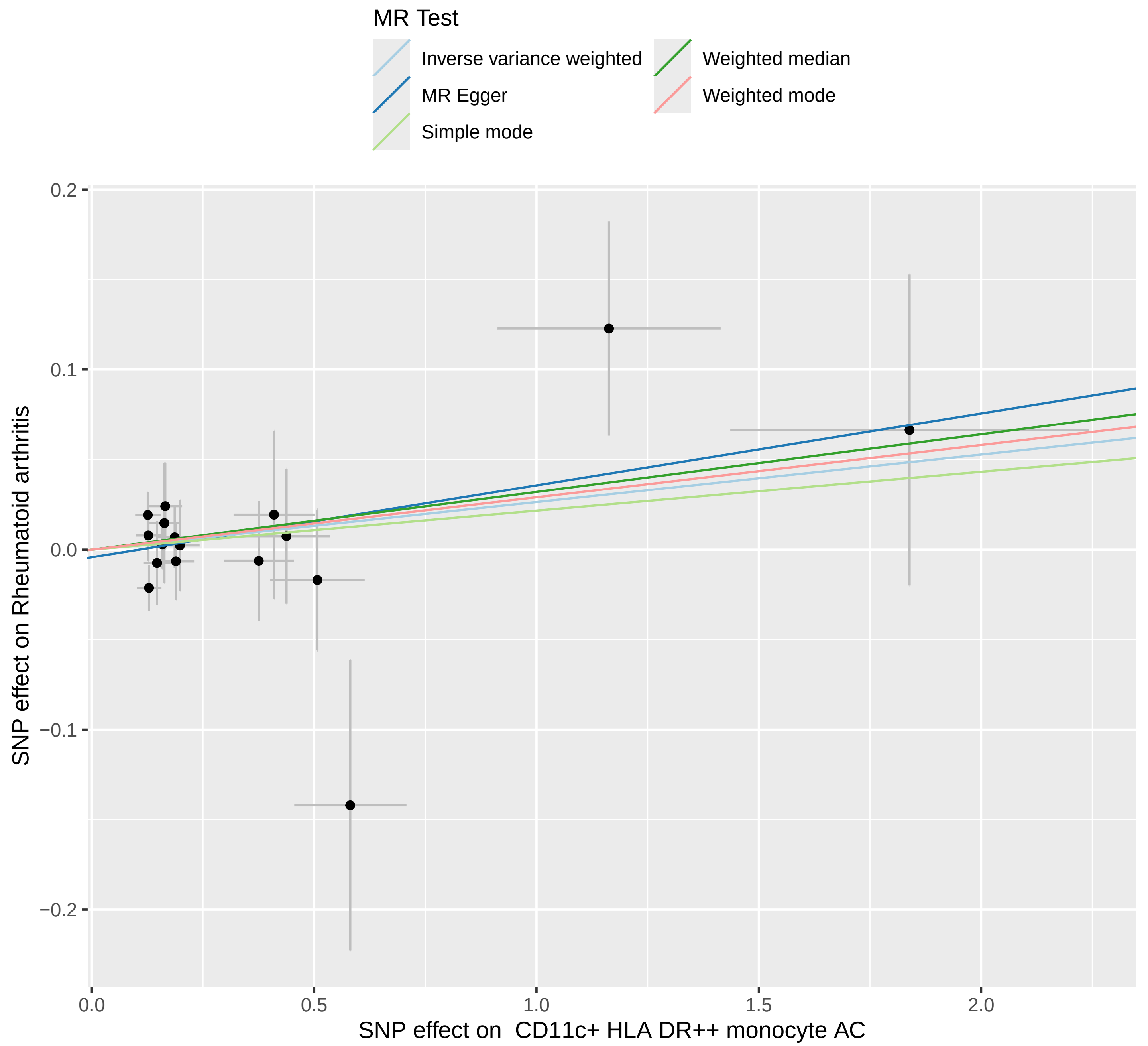

MR Test
Inverse variance weighted
MR Egger
Simple mode
Weighted median
Weighted mode
0.2
0.1
0.0
−0.1
−0.2
SNP effect on Rheumatoid arthritis
0.0
0.5
1.0
1.5
2.0
SNP effect on CD11c+ HLA DR++ monocyte AC

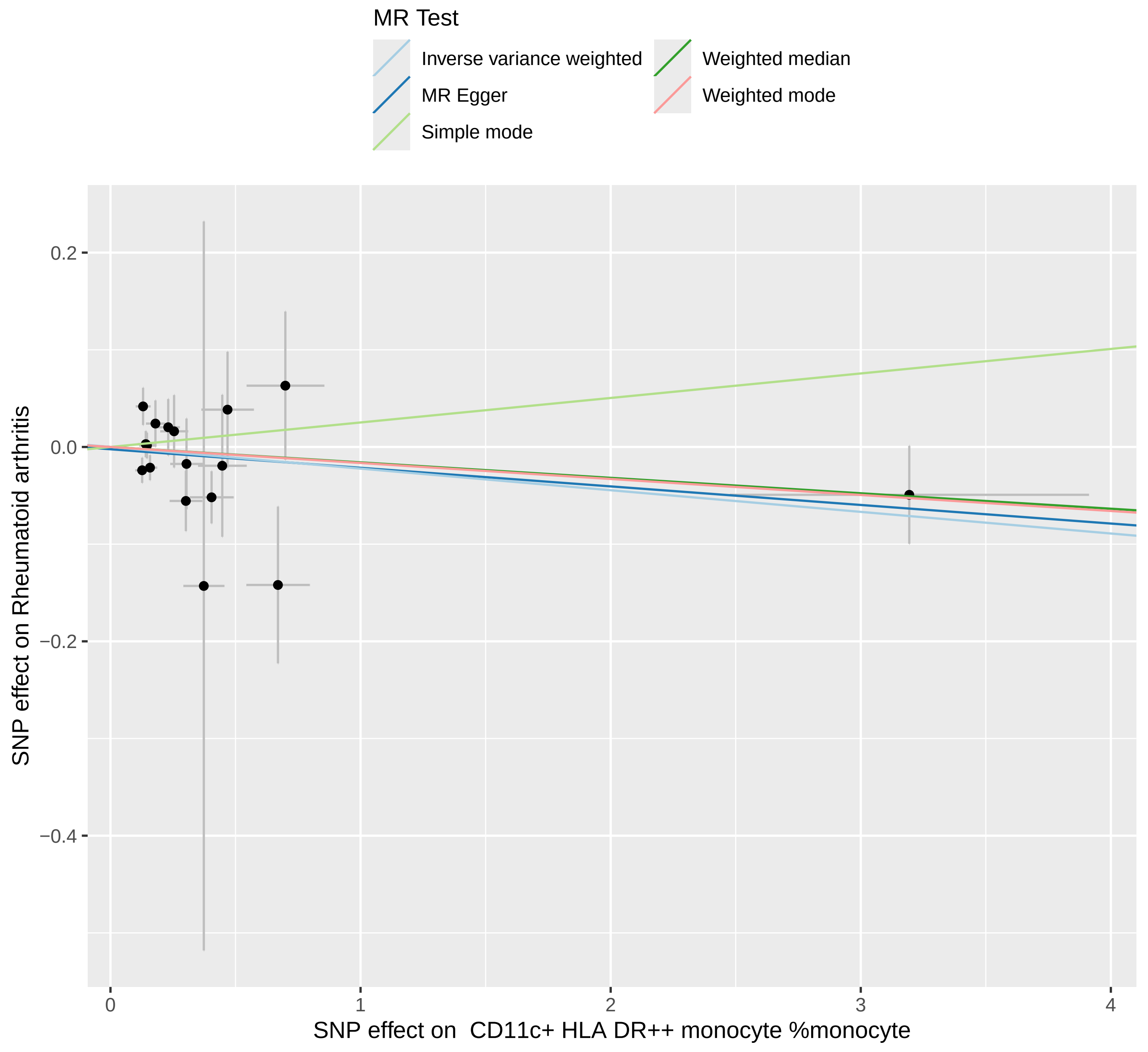
MR Test
Inverse variance weighted
MR Egger
Simple mode
Weighted median
Weighted mode
0.2
0.0
−0.2
−0.4
0
1
2
3
4
SNP effect on Rheumatoid arthritis
SNP effect on CD11c+ HLA DR++ monocyte %monocyte

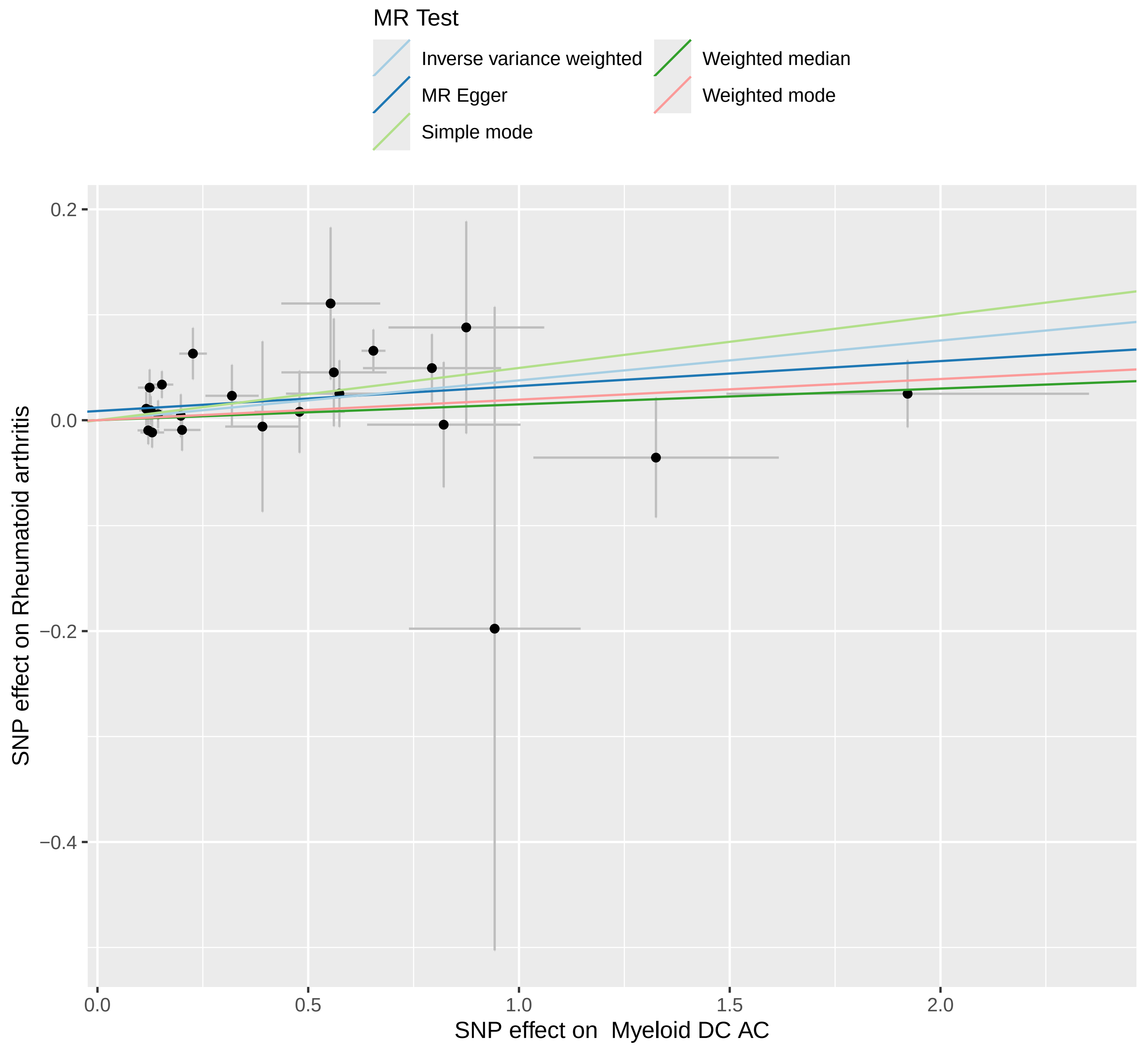

MR Test
Inverse variance weighted
MR Egger
Simple mode
Weighted median
Weighted mode
0.2
0.0
−0.2
−0.4
0.0
0.5
1.0
1.5
2.0
SNP effect on Rheumatoid arthritis
SNP effect on Myeloid DC AC

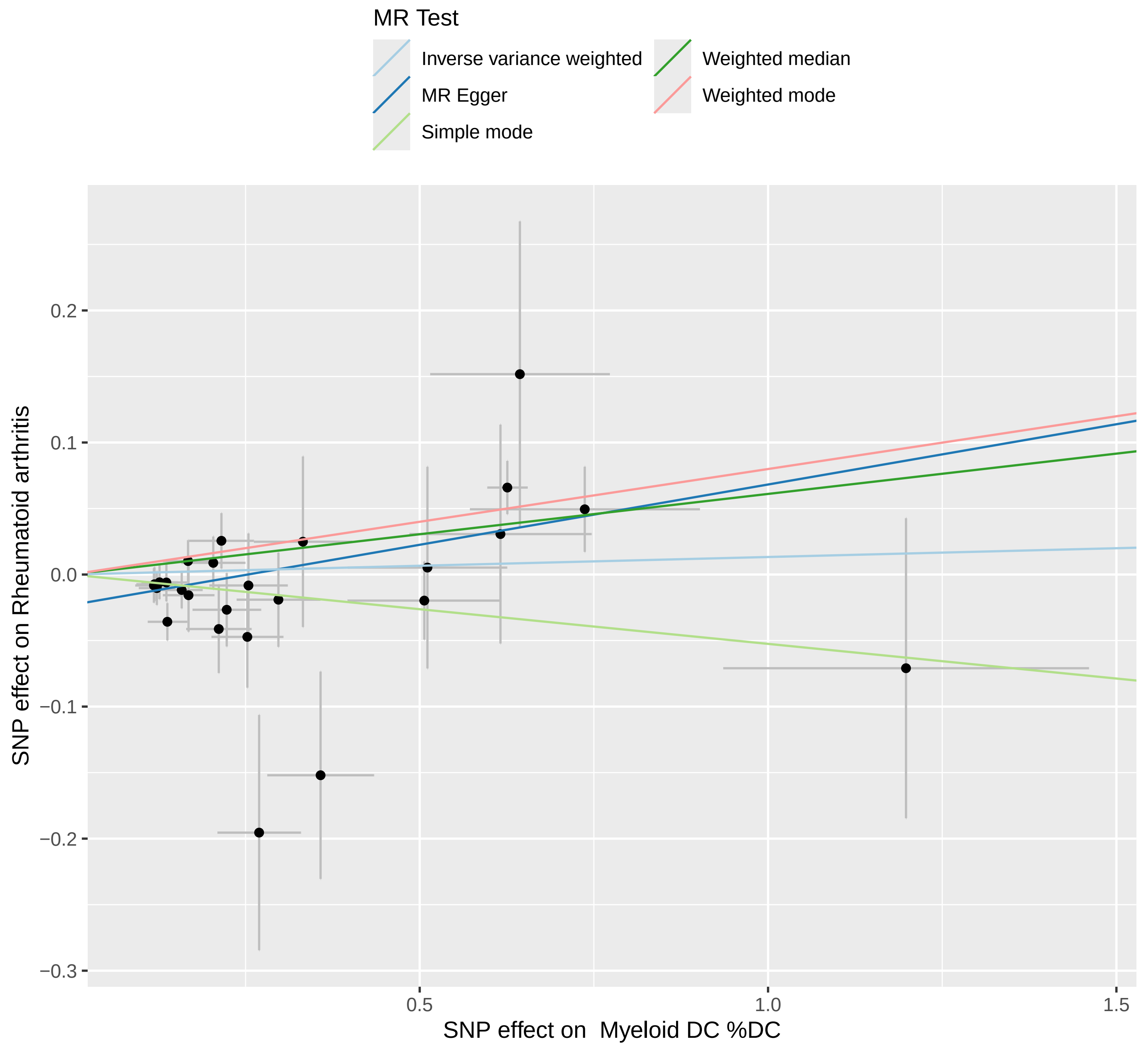
MR Test
Inverse variance weighted
MR Egger
Simple mode
Weighted median
Weighted mode
SNP effect on Rheumatoid arthritis
0.2
0.1
0.0
−0.1
−0.2
−0.3
0.5
1.0
1.5
SNP effect on Myeloid DC %DC

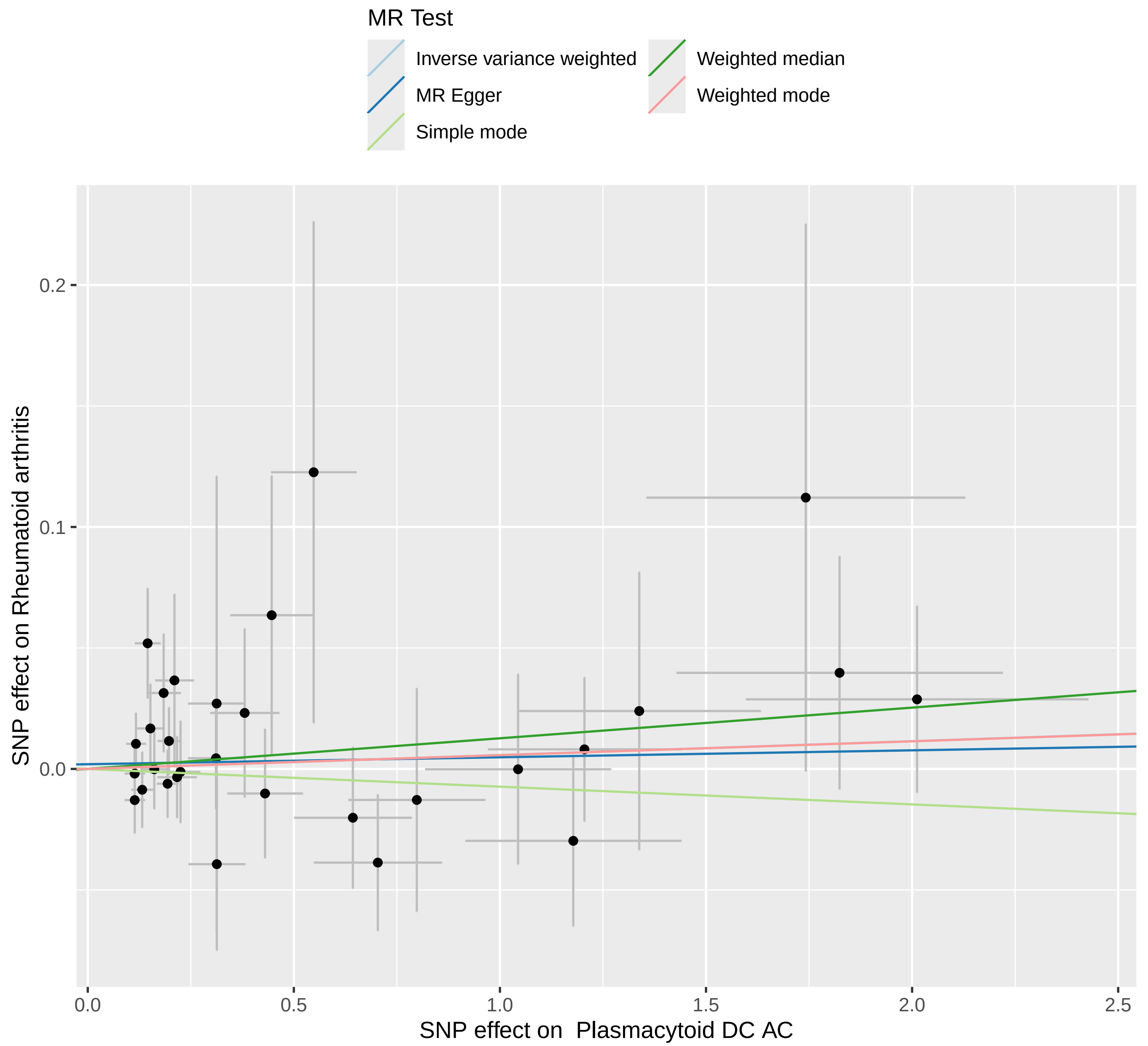
MR Test
Inverse variance weighted
MR Egger
Simple mode
Weighted median
Weighted mode
0.2
0.1
0.0
0.0
0.5
1.0
1.5
2.0
2.5
SNP effect on Rheumatoid arthritis
SNP effect on Plasmacytoid DC AC

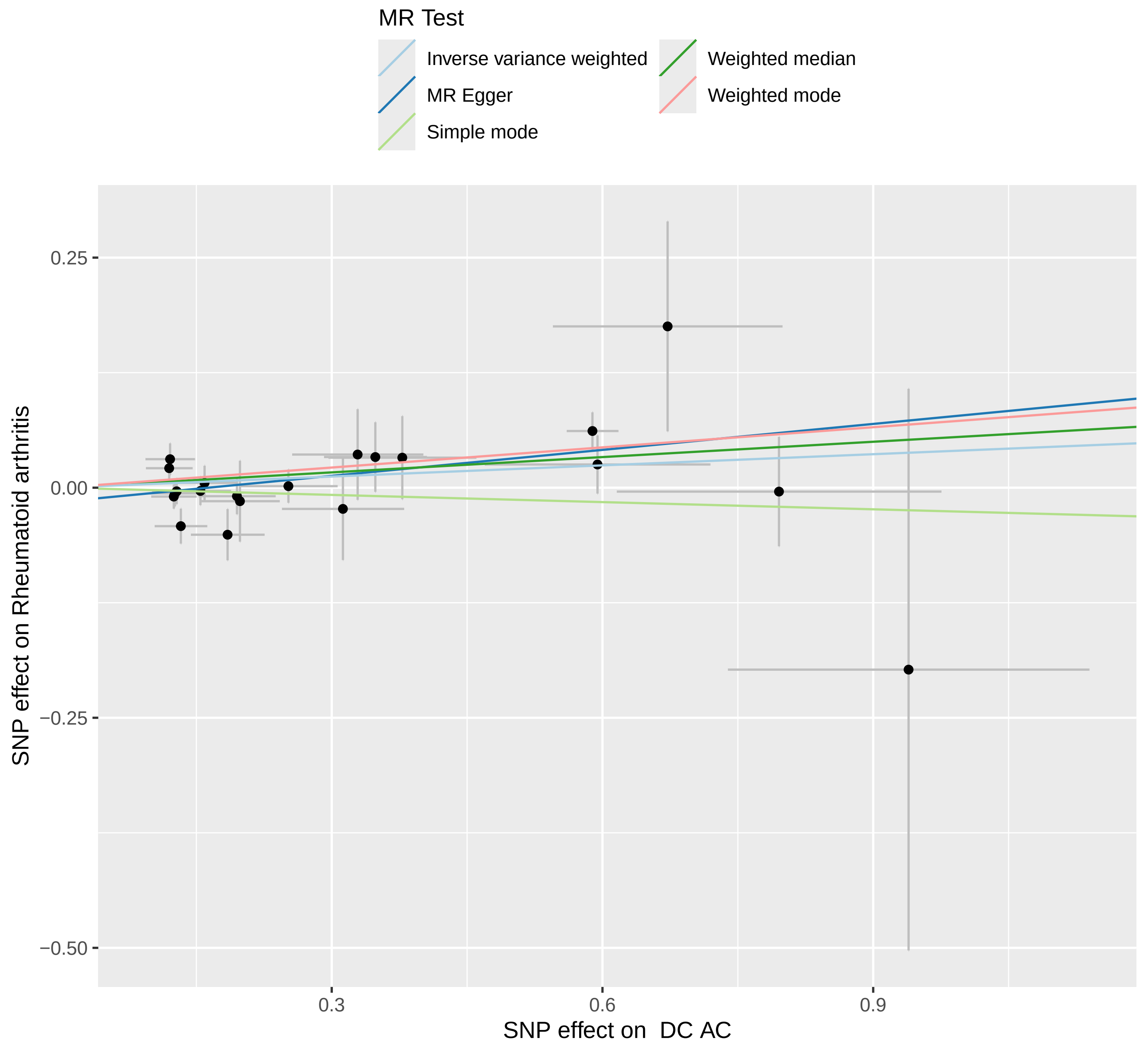

MR Test
Inverse variance weighted
MR Egger
Simple mode
Weighted median
Weighted mode
0.25
0.00
−0.25
−0.50
0.3
0.6
0.9
SNP effect on Rheumatoid arthritis
SNP effect on DC AC

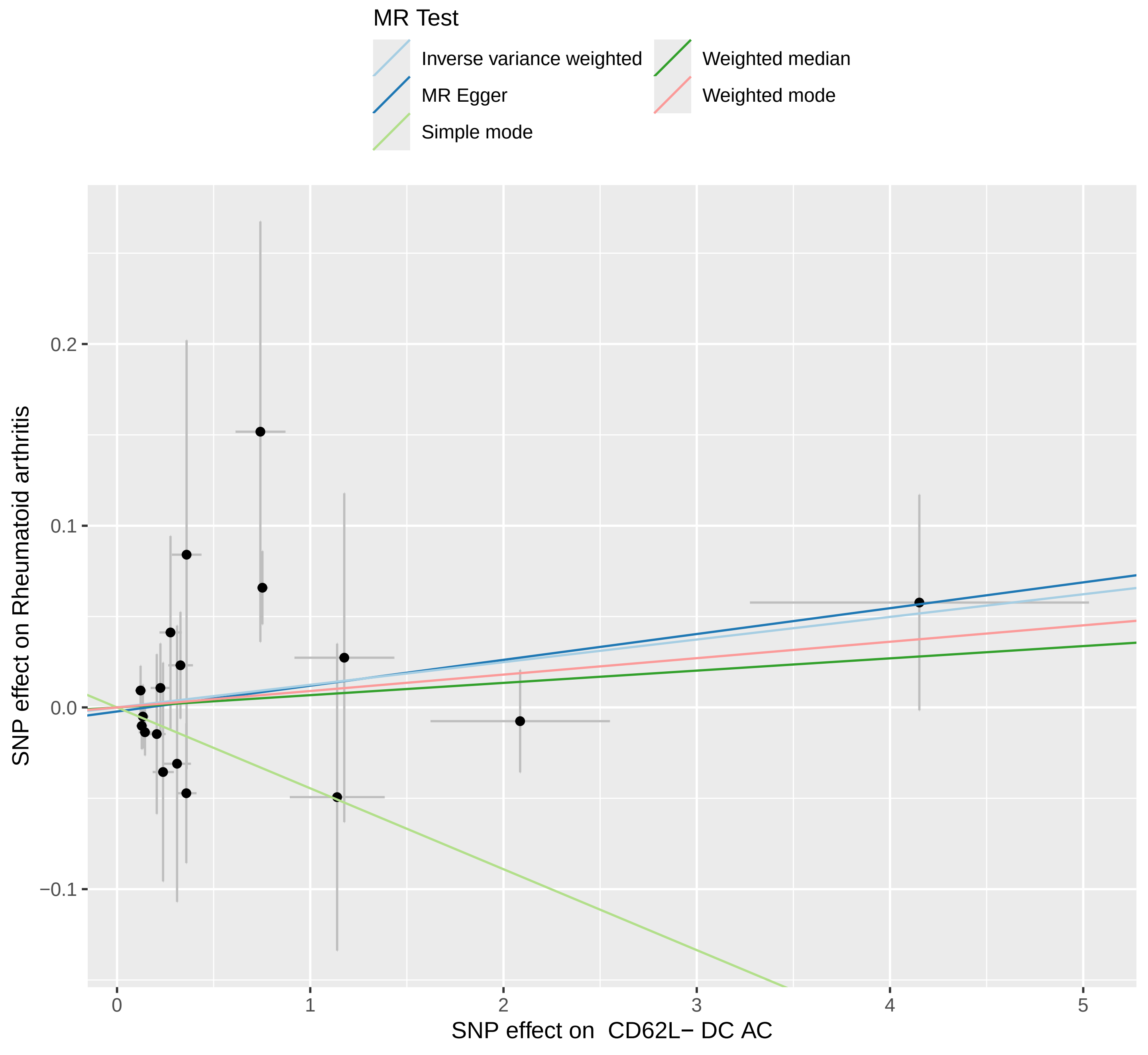

MR Test
Inverse variance weighted
MR Egger
Simple mode
Weighted median
Weighted mode
SNP effect on Rheumatoid arthritis
0.2
0.1
0.0
−0.1
0
1
2
3
4
5
SNP effect on CD62L− DC AC

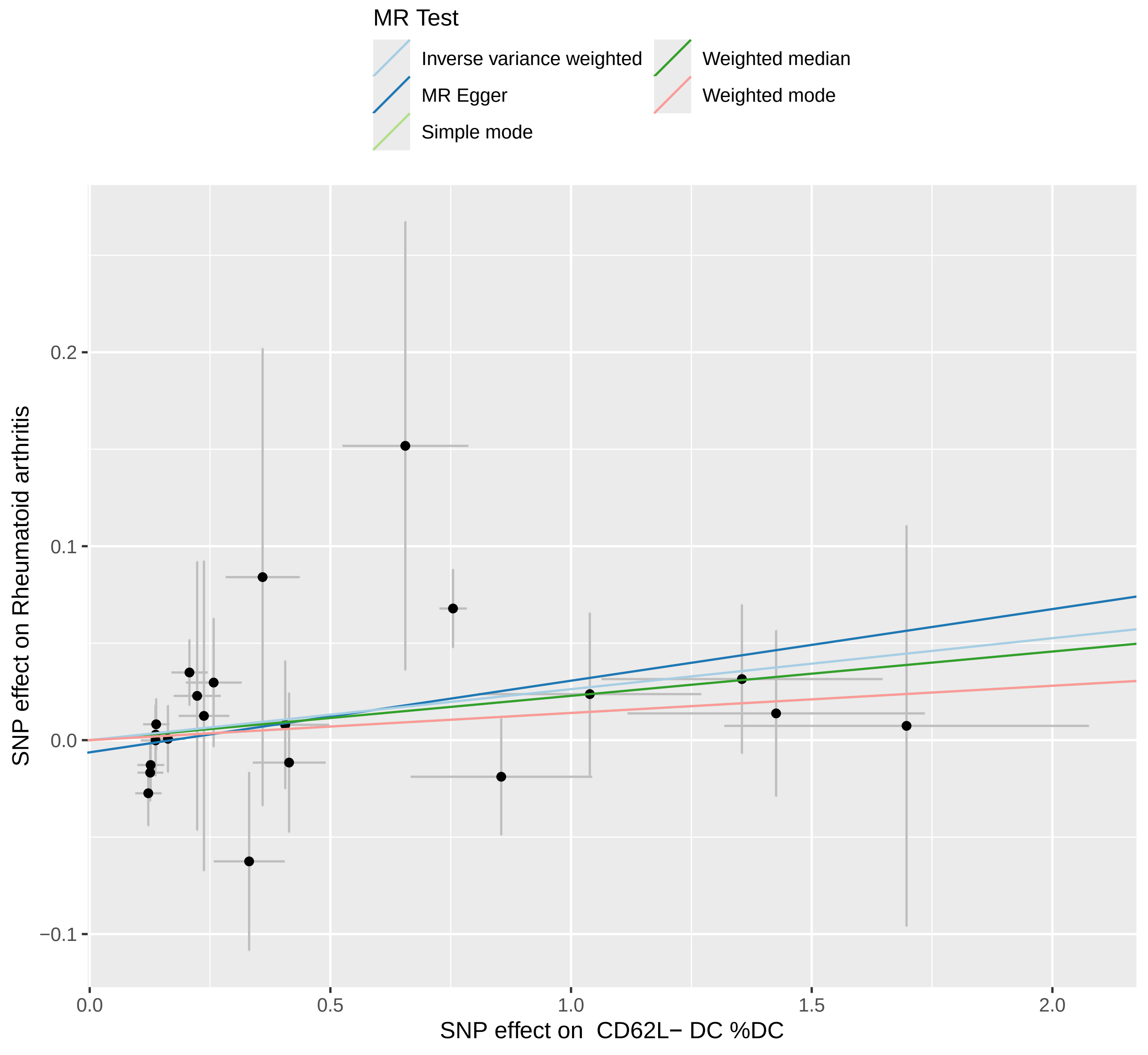
MR Test
Inverse variance weighted
MR Egger
Simple mode
Weighted median
Weighted mode
SNP effect on Rheumatoid arthritis
0.2
0.1
0.0
−0.1
0.0
0.5
1.0
1.5
2.0
SNP effect on CD62L− DC %DC

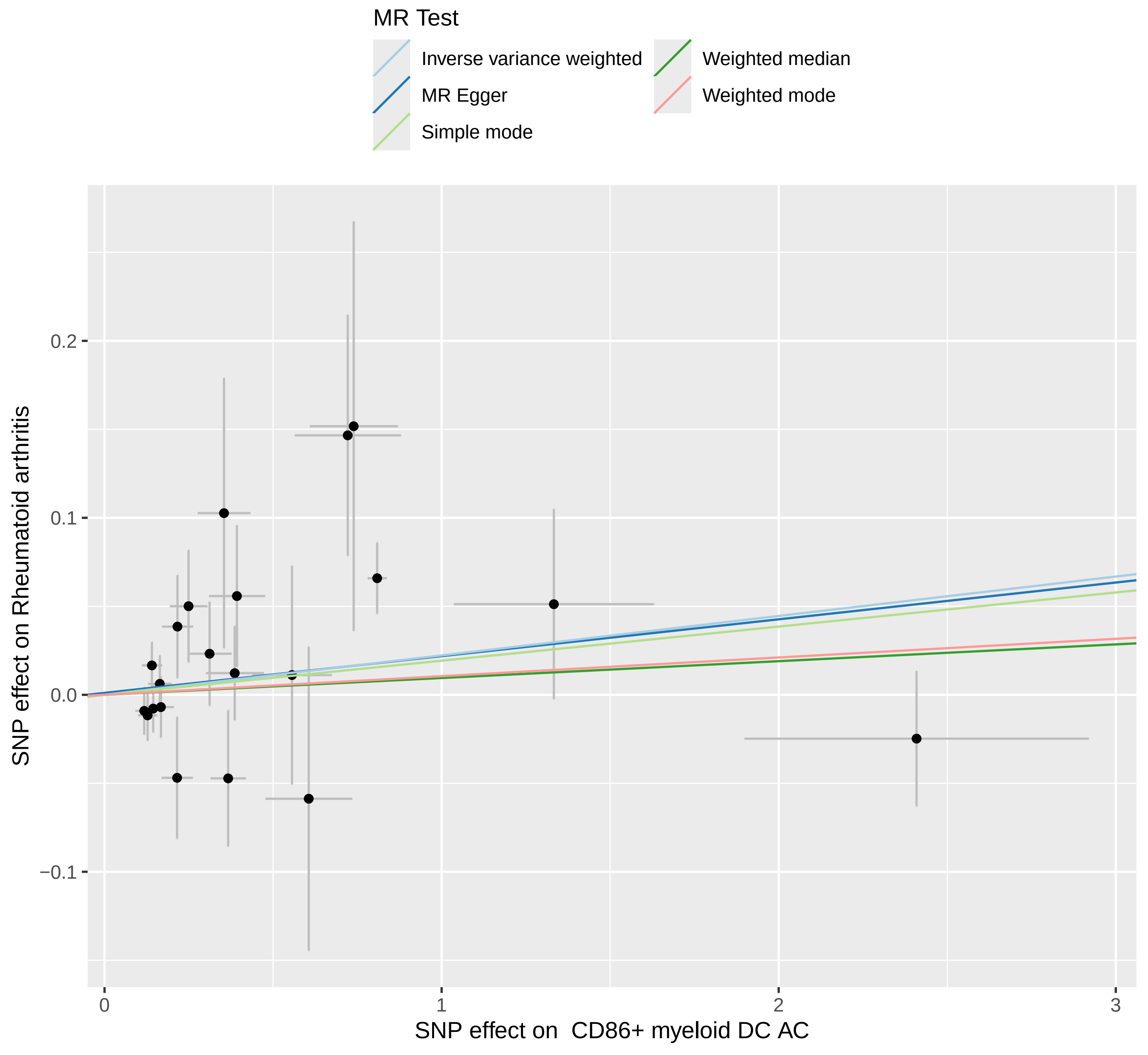
MR Test
Inverse variance weighted
MR Egger
Simple mode
Weighted median
Weighted mode
SNP effect on Rheumatoid arthritis
0.2
0.1
0.0
−0.1
0
1
2
3
SNP effect on CD86+ myeloid DC AC

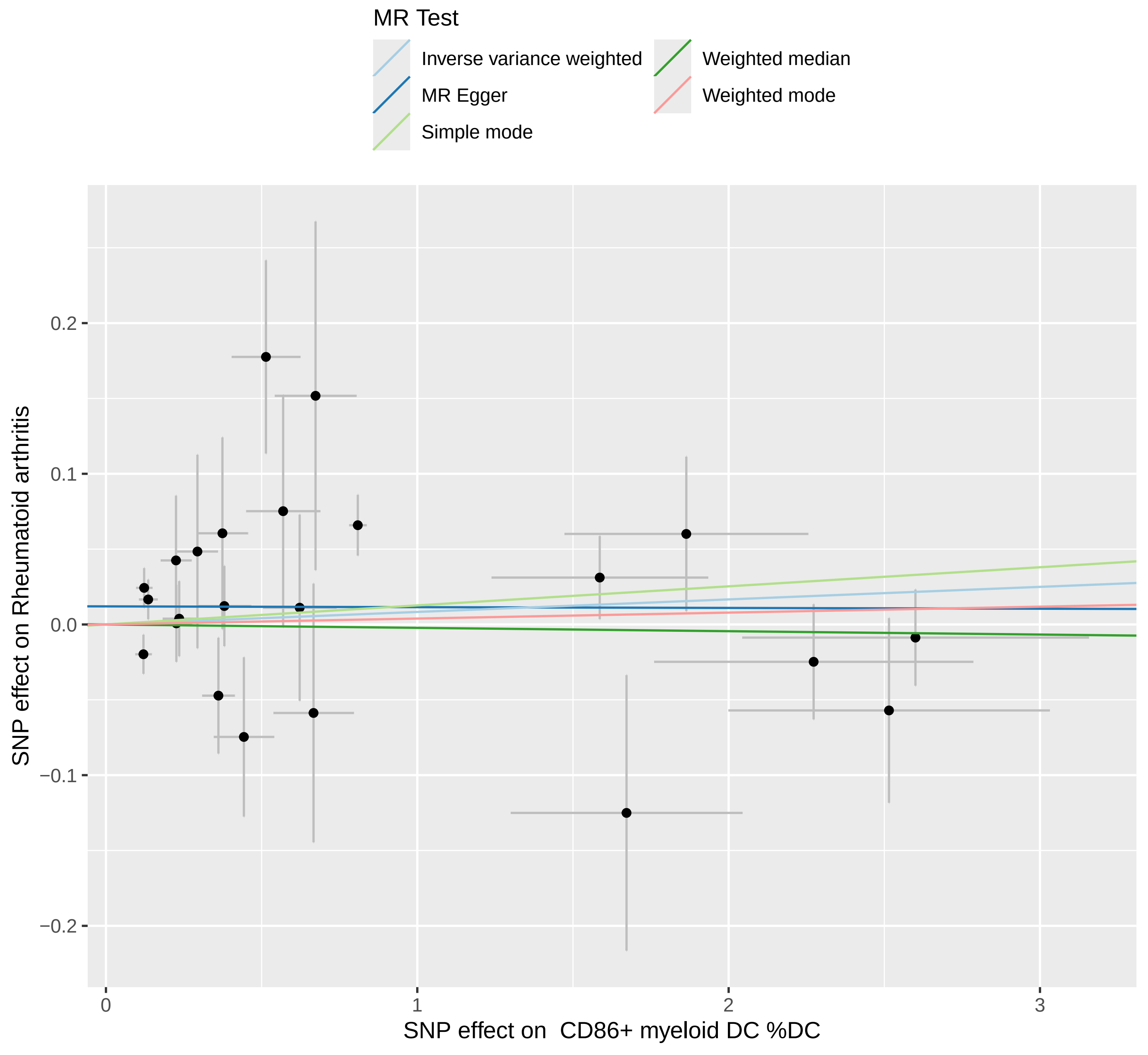

MR Test
Inverse variance weighted
MR Egger
Simple mode
Weighted median
Weighted mode
SNP effect on Rheumatoid arthritis
0.2
0.1
0.0
−0.1
−0.2
0
1
2
3
SNP effect on CD86+ myeloid DC %DC

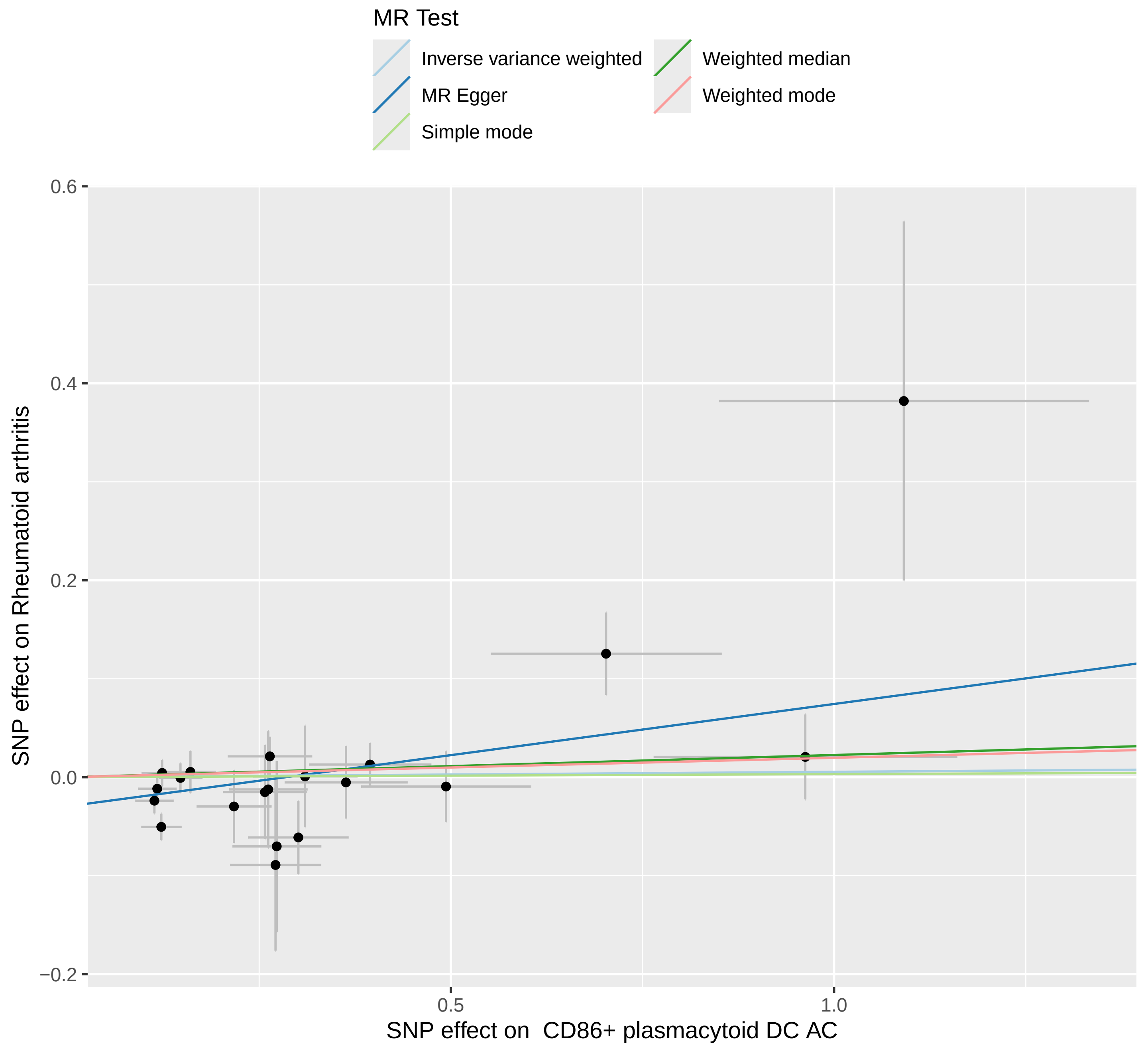
MR Test
Inverse variance weighted
MR Egger
Simple mode
Weighted median
Weighted mode
0.6
0.4
0.2
0.0
−0.2
0.5
1.0
SNP effect on Rheumatoid arthritis
SNP effect on CD86+ plasmacytoid DC AC

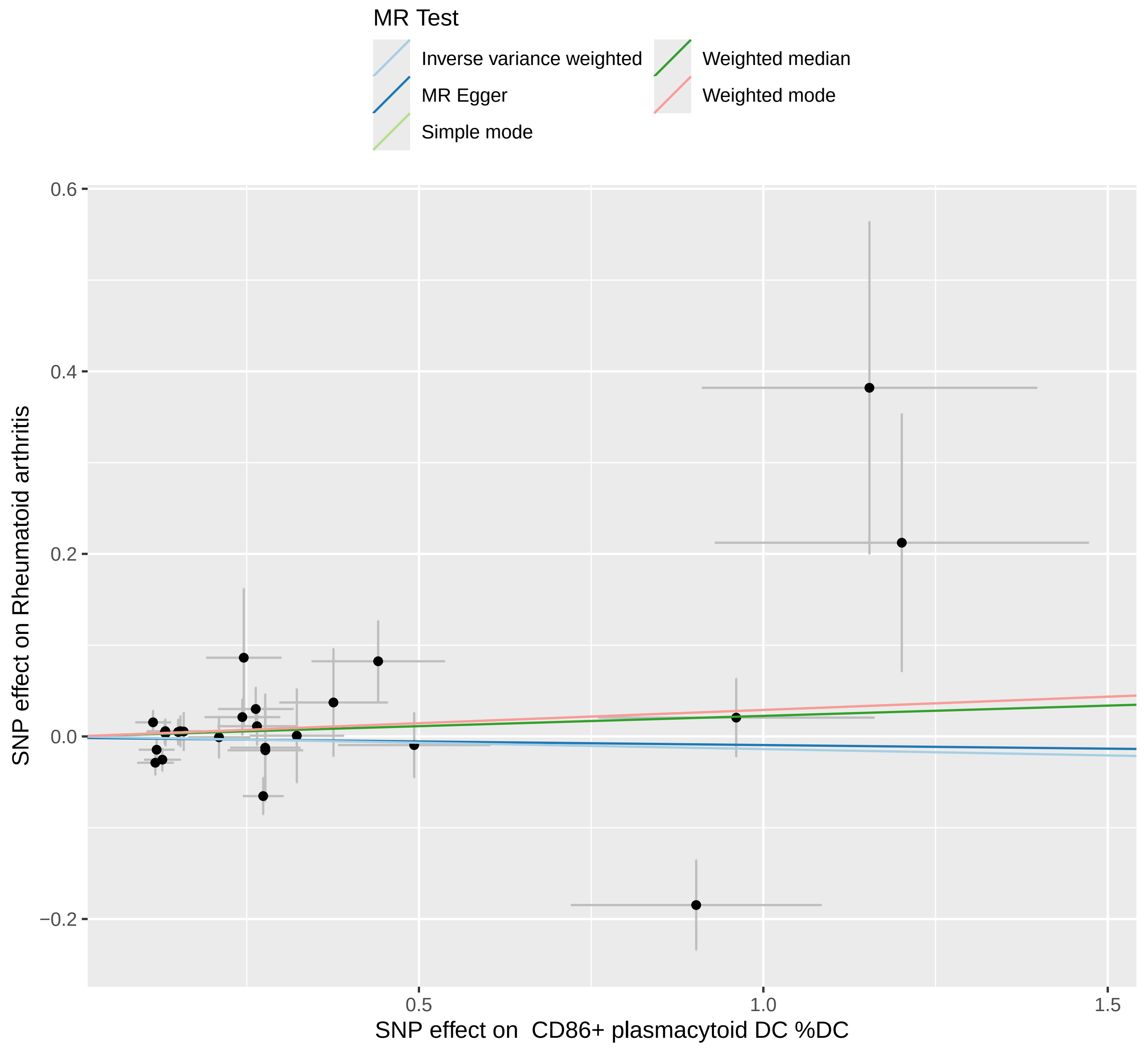

MR Test
Inverse variance weighted
Weighted median
MR Egger
Weighted mode
Simple mode
0.6
0.4
0.2
0.0
−0.2
0.5
1.0
1.5
SNP effect on Rheumatoid arthritis
SNP effect on CD86+ plasmacytoid DC %DC

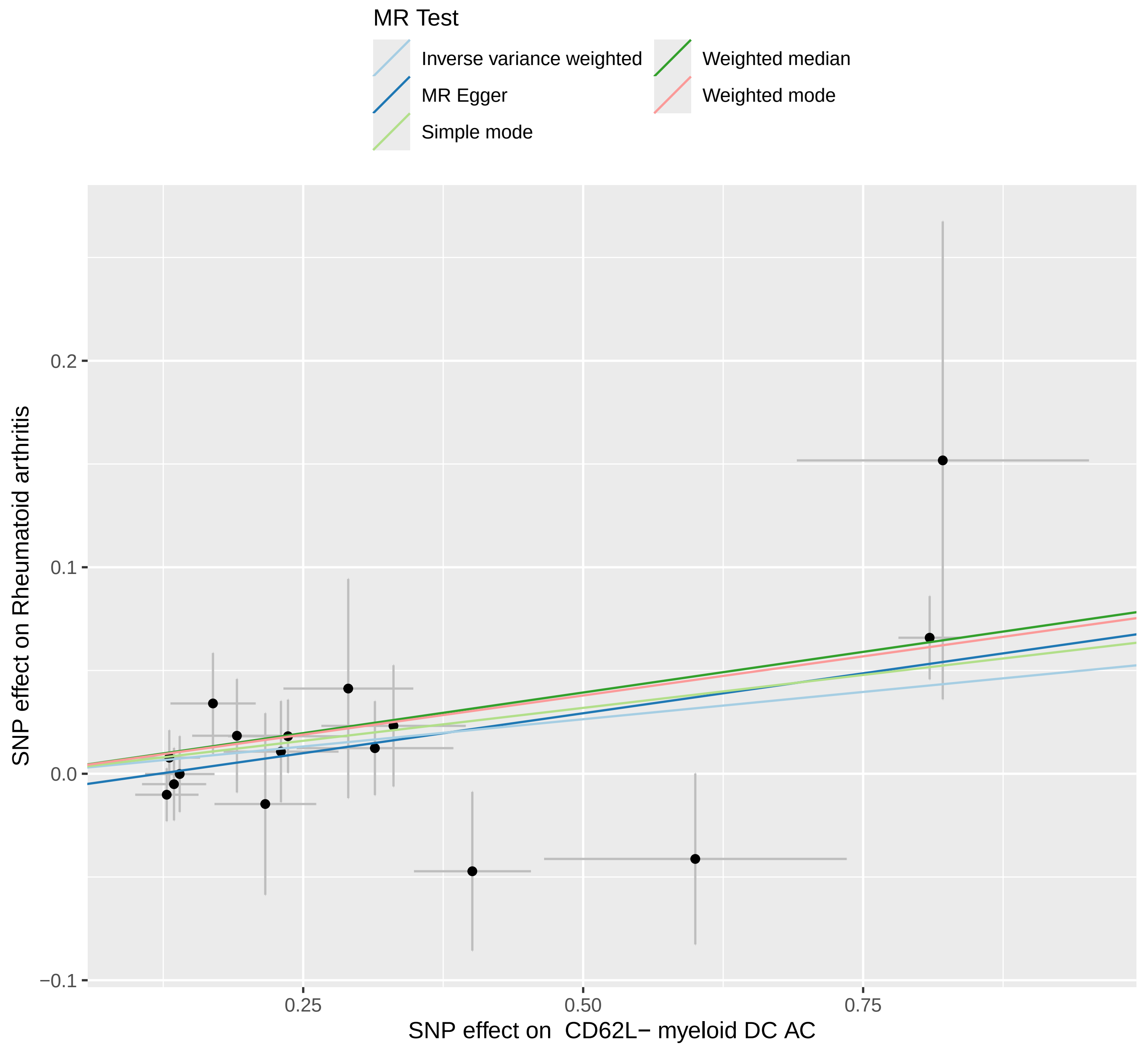
MR Test
Inverse variance weighted
MR Egger
Simple mode
Weighted median
Weighted mode
SNP effect on Rheumatoid arthritis
0.2
0.1
0.0
−0.1
0.25
0.50
0.75
SNP effect on CD62L− myeloid DC AC

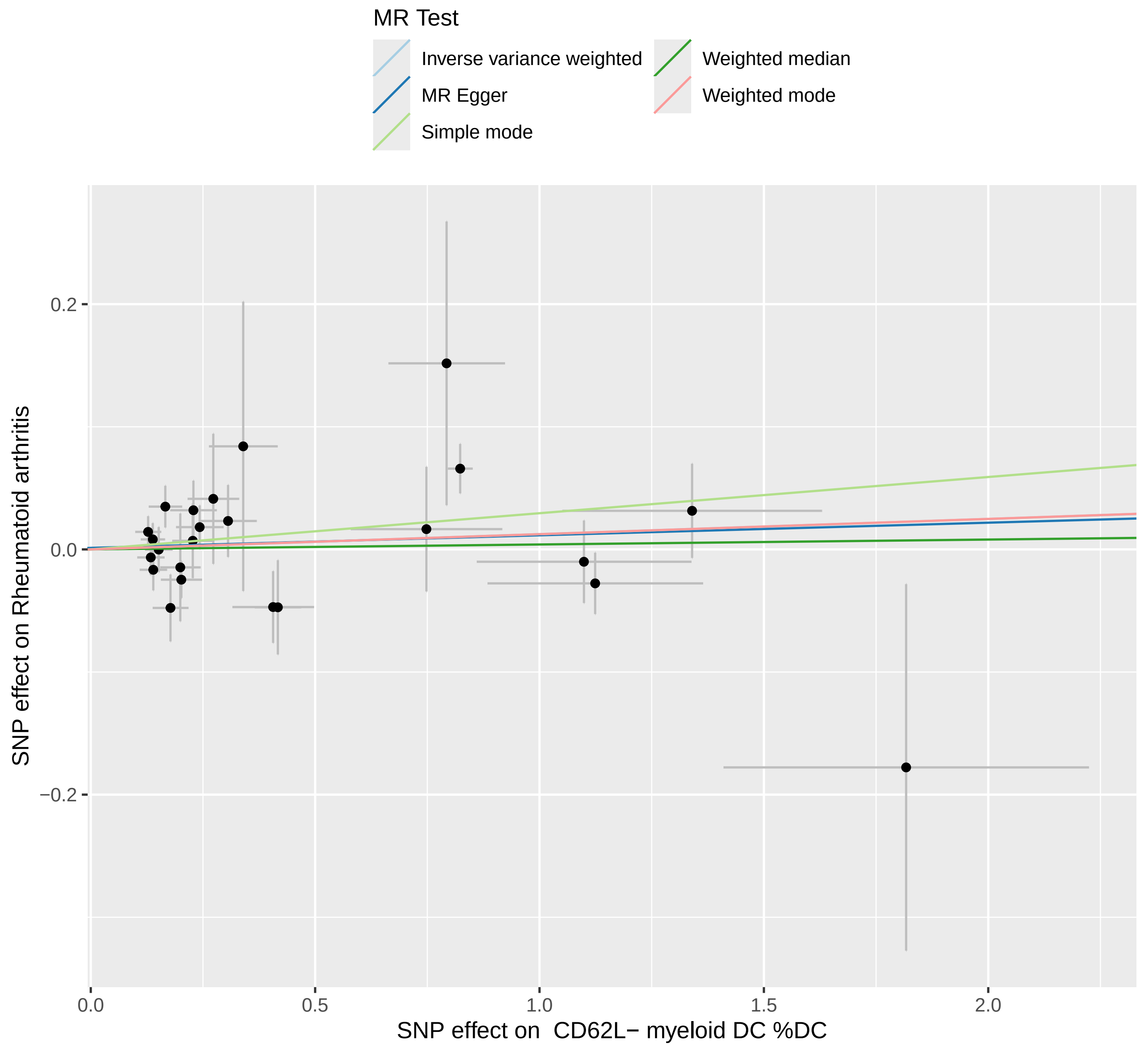
MR Test
Inverse variance weighted
MR Egger
Simple mode
Weighted median
Weighted mode
SNP effect on Rheumatoid arthritis
0.2
0.0
−0.2
0.0
0.5
1.0
1.5
2.0
SNP effect on CD62L− myeloid DC %DC

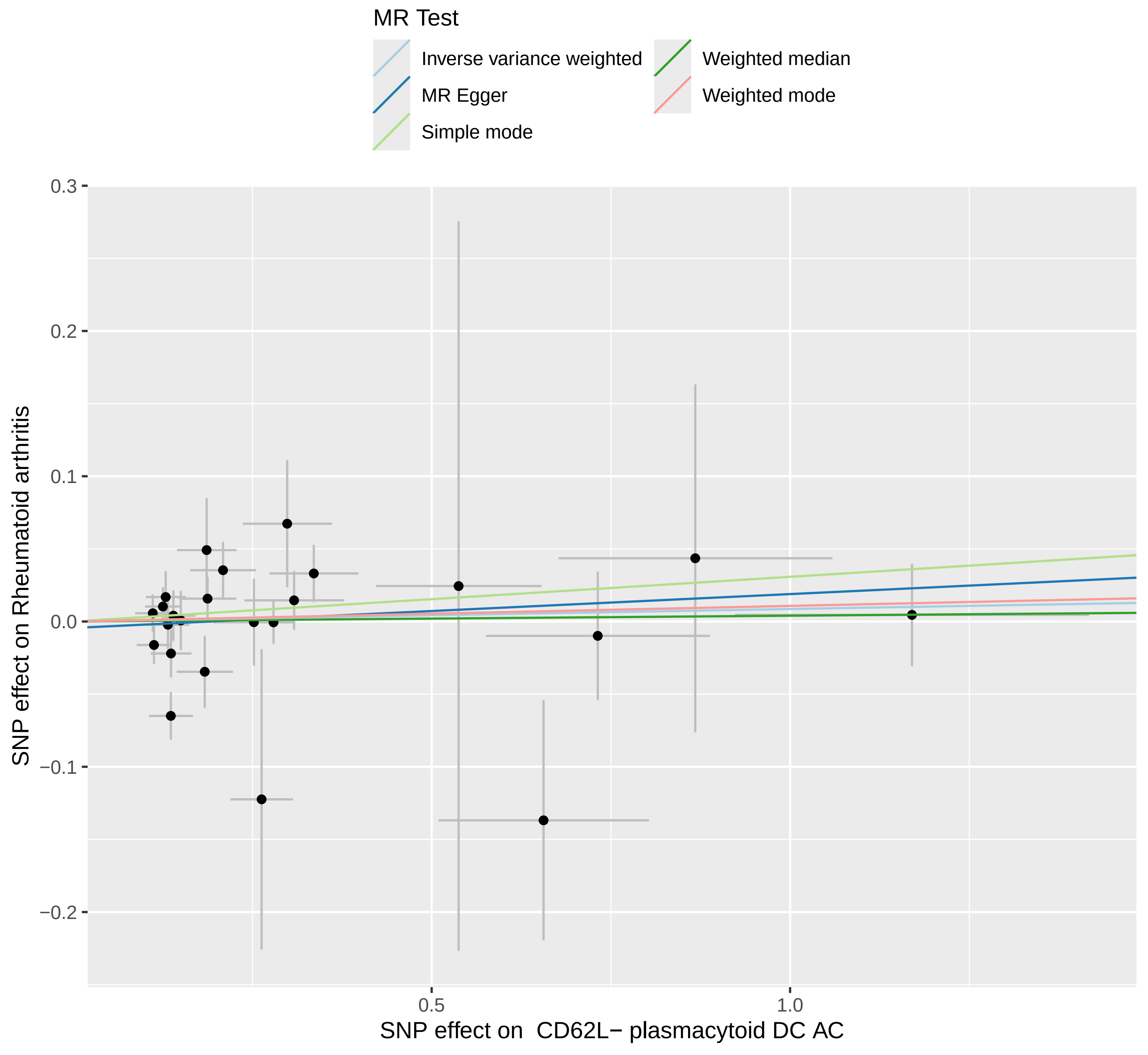

MR Test
Inverse variance weighted
MR Egger
Simple mode
Weighted median
Weighted mode
0.3
0.2
0.1
0.0
−0.1
−0.2
SNP effect on Rheumatoid arthritis
0.5
1.0
SNP effect on CD62L− plasmacytoid DC AC

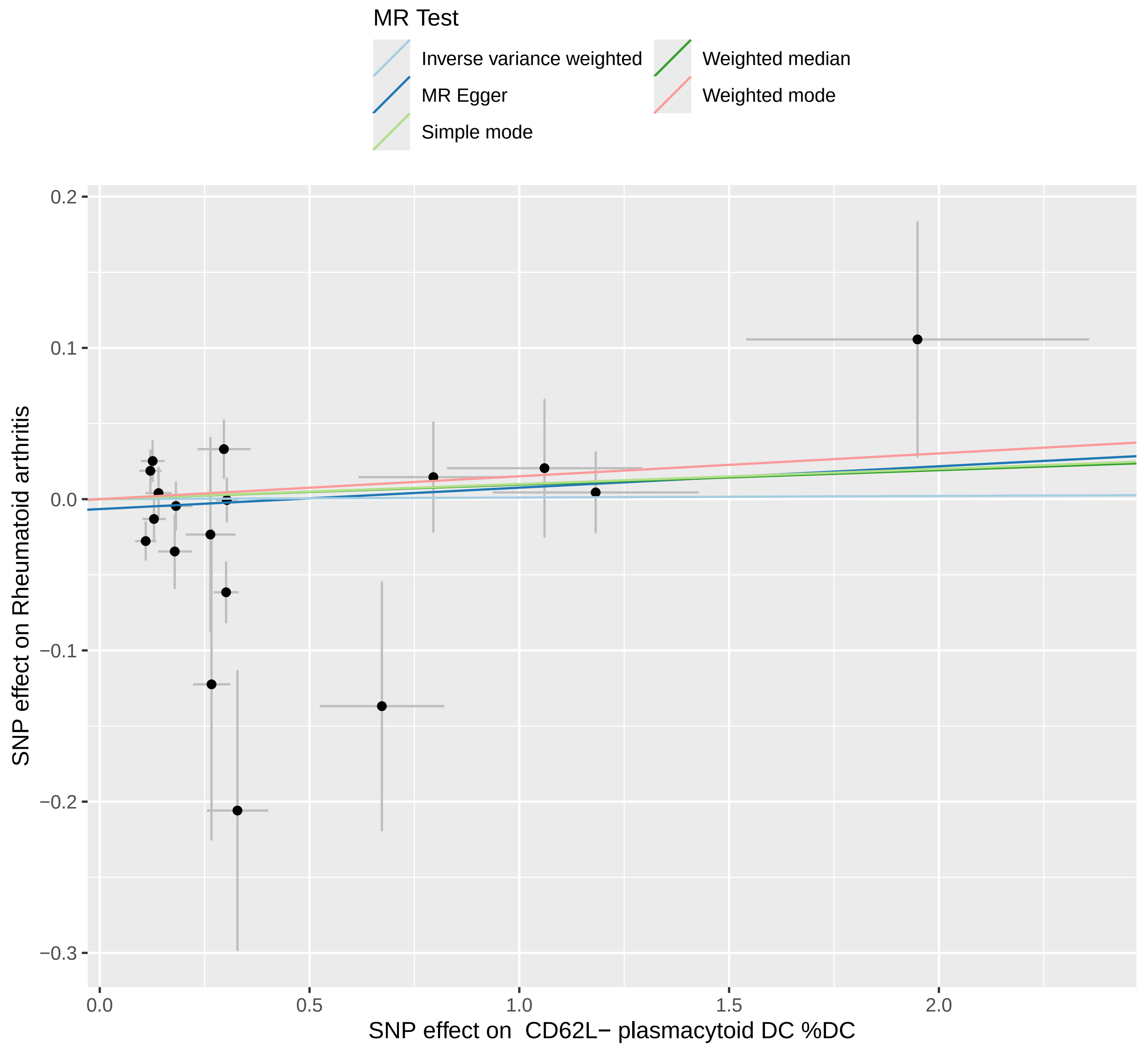

MR Test
Inverse variance weighted
MR Egger
Simple mode
Weighted median
Weighted mode
0.2
0.1
0.0
−0.1
−0.2
−0.3
SNP effect on Rheumatoid arthritis
0.0
0.5
1.0
1.5
2.0
SNP effect on CD62L− plasmacytoid DC %DC

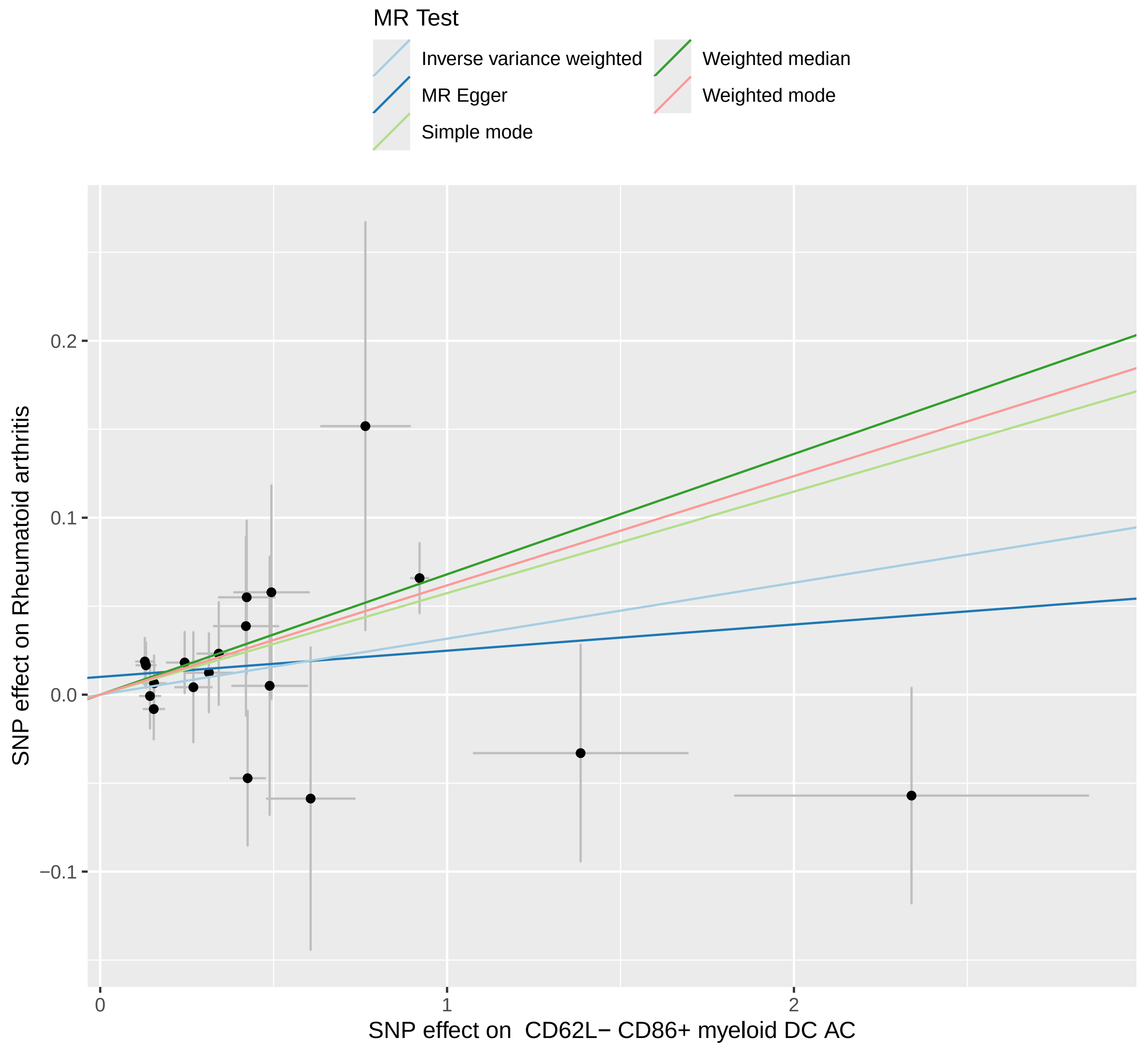
MR Test
Inverse variance weighted
MR Egger
Simple mode
Weighted median
Weighted mode
0.2
0.1
0.0
−0.1
SNP effect on Rheumatoid arthritis
0
1
2
SNP effect on CD62L− CD86+ myeloid DC AC

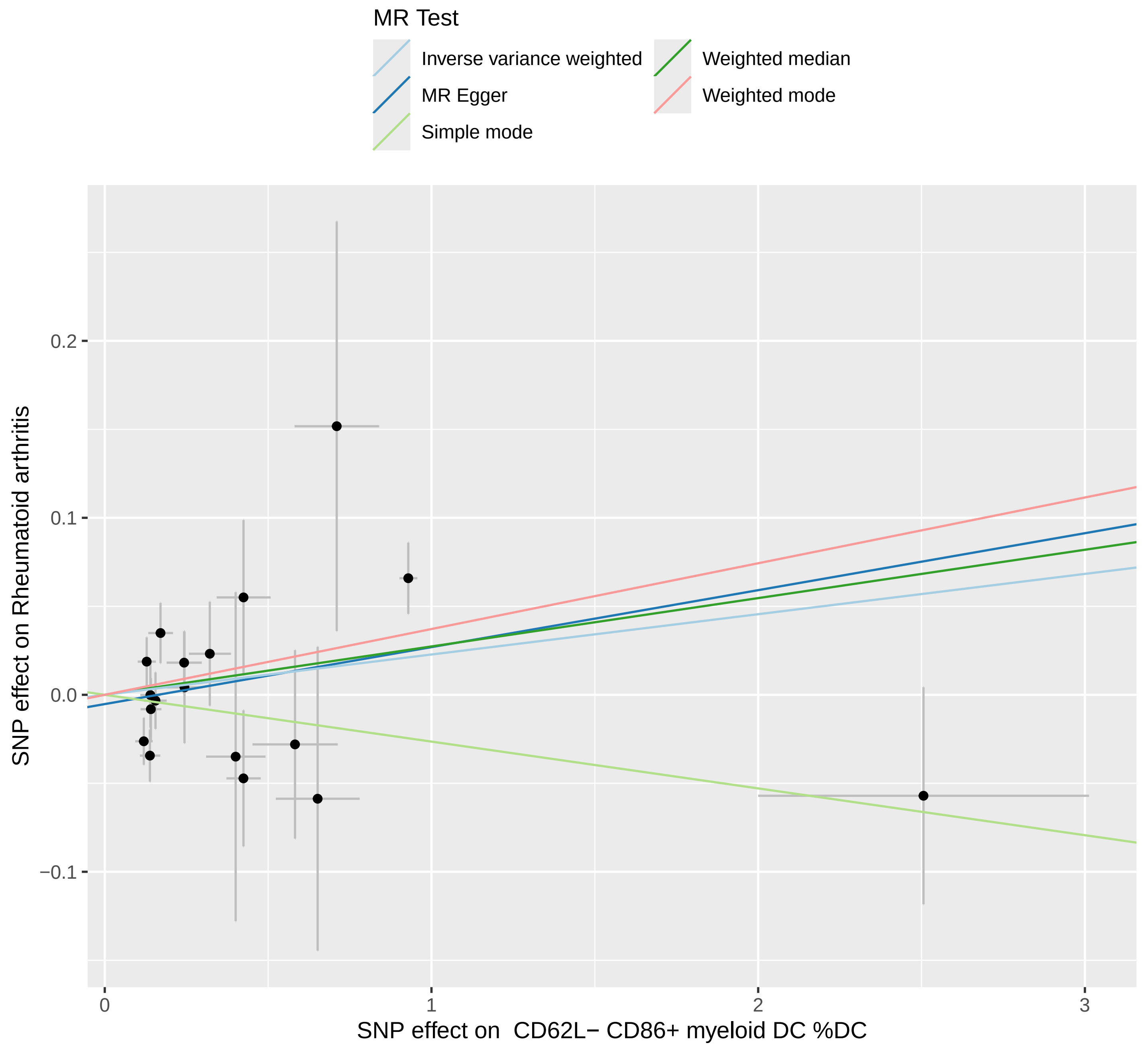

MR Test
Inverse variance weighted
MR Egger
Simple mode
Weighted median
Weighted mode
SNP effect on Rheumatoid arthritis
0.2
0.1
0.0
−0.1
0
1
2
3
SNP effect on CD62L− CD86+ myeloid DC %DC

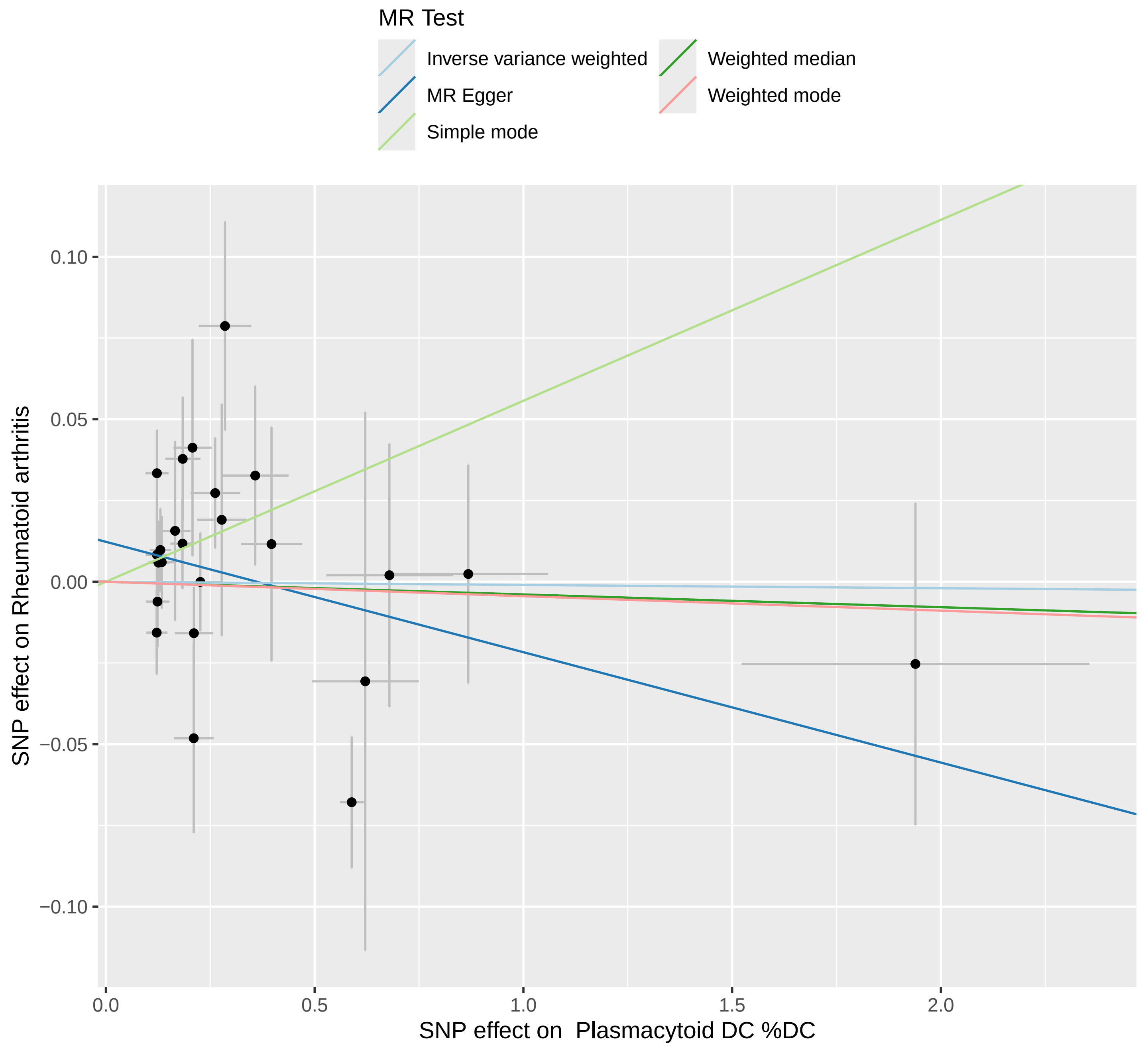

MR Test
Inverse variance weighted
MR Egger
Simple mode
Weighted median
Weighted mode
SNP effect on Rheumatoid arthritis
0.10
0.05
0.00
−0.05
−0.10
0.0
0.5
1.0
1.5
2.0
SNP effect on Plasmacytoid DC %DC

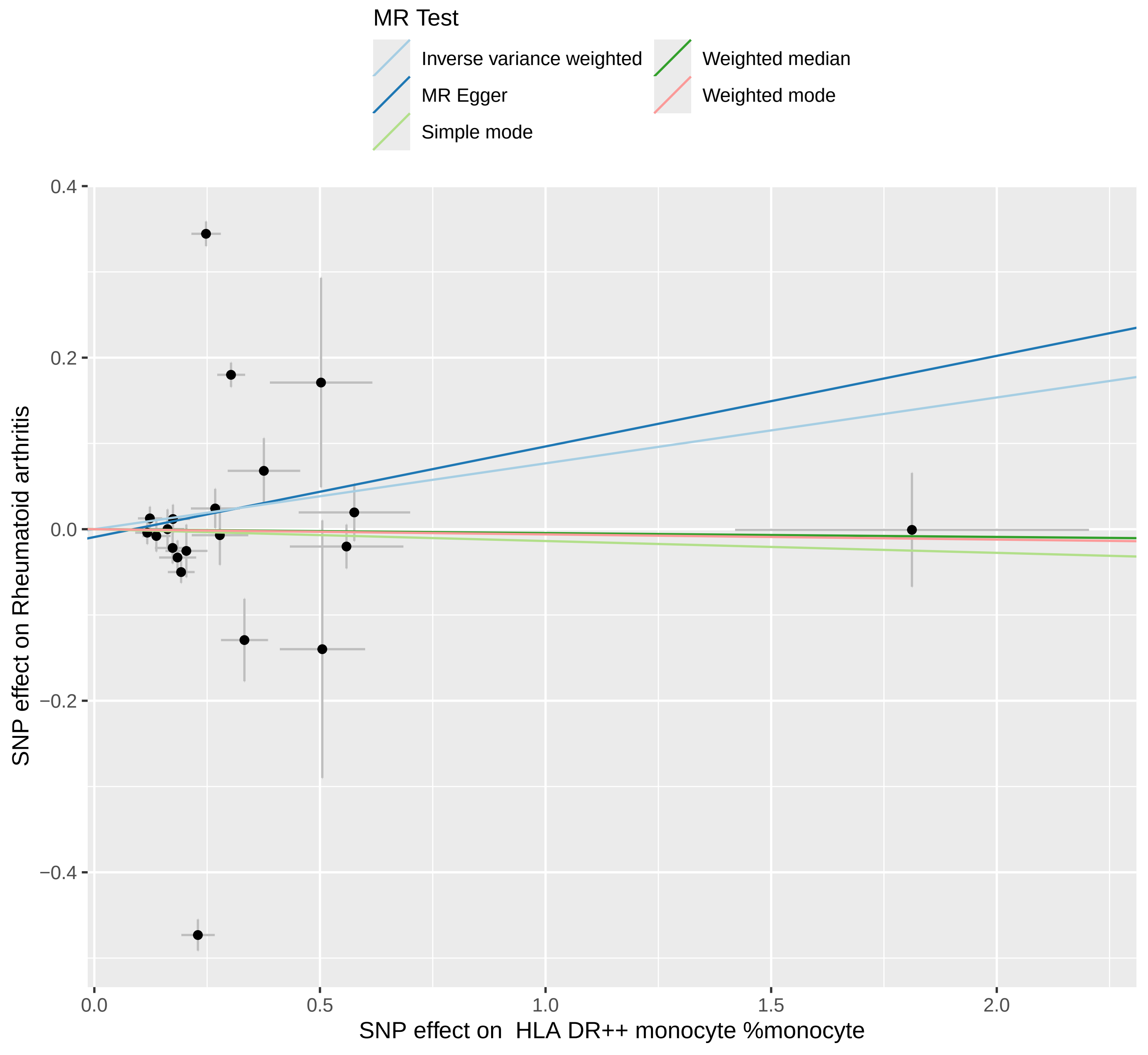

MR Test
Inverse variance weighted
MR Egger
Simple mode
Weighted median
Weighted mode
0.4
0.2
0.0
−0.2
−0.4
SNP effect on Rheumatoid arthritis
0.0
0.5
1.0
1.5
2.0
SNP effect on HLA DR++ monocyte %monocyte

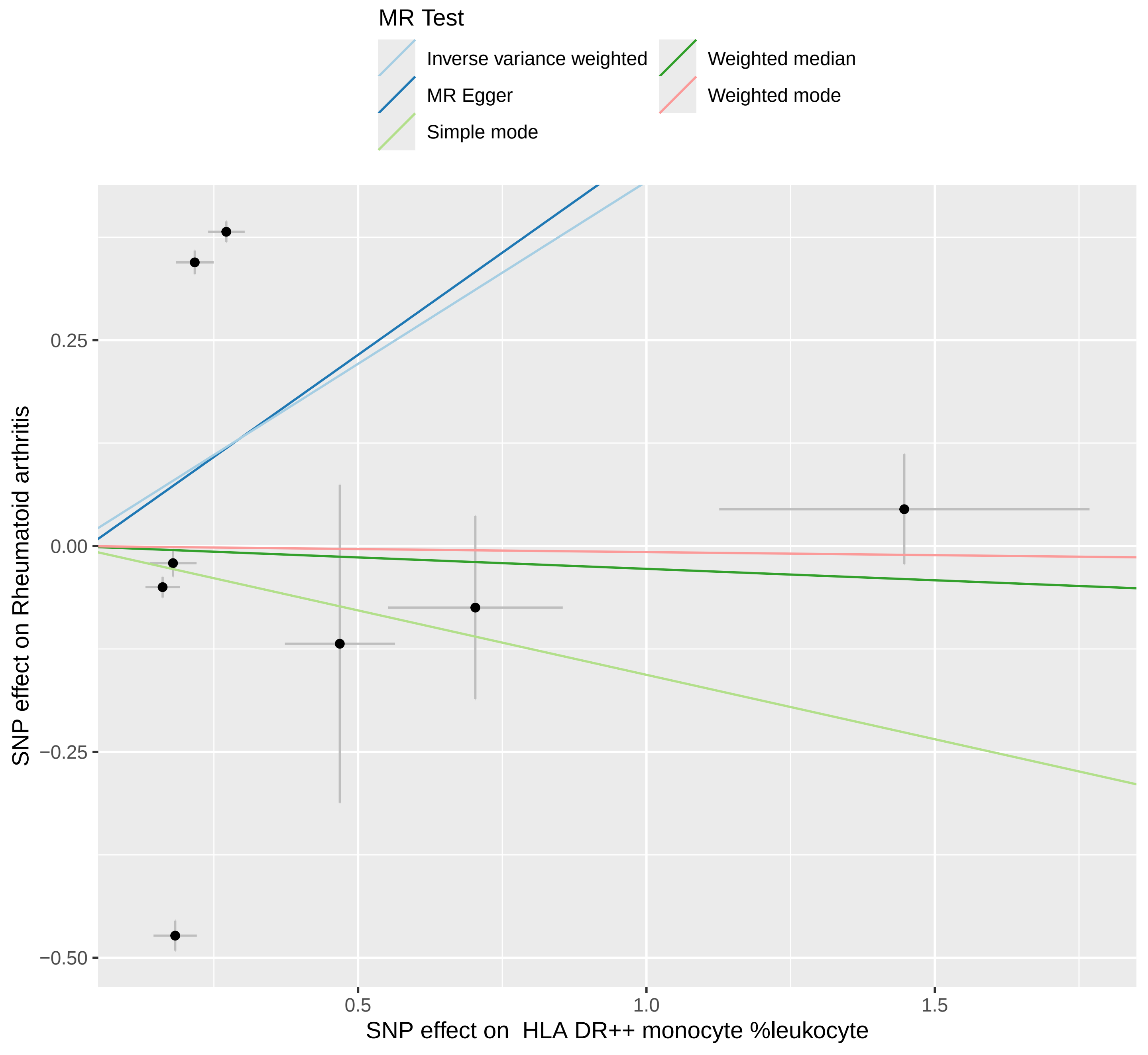
MR Test
Inverse variance weighted
MR Egger
Simple mode
Weighted median
Weighted mode
0.25
0.00
−0.25
−0.50
0.5
1.0
1.5
SNP effect on Rheumatoid arthritis
SNP effect on HLA DR++ monocyte %leukocyte

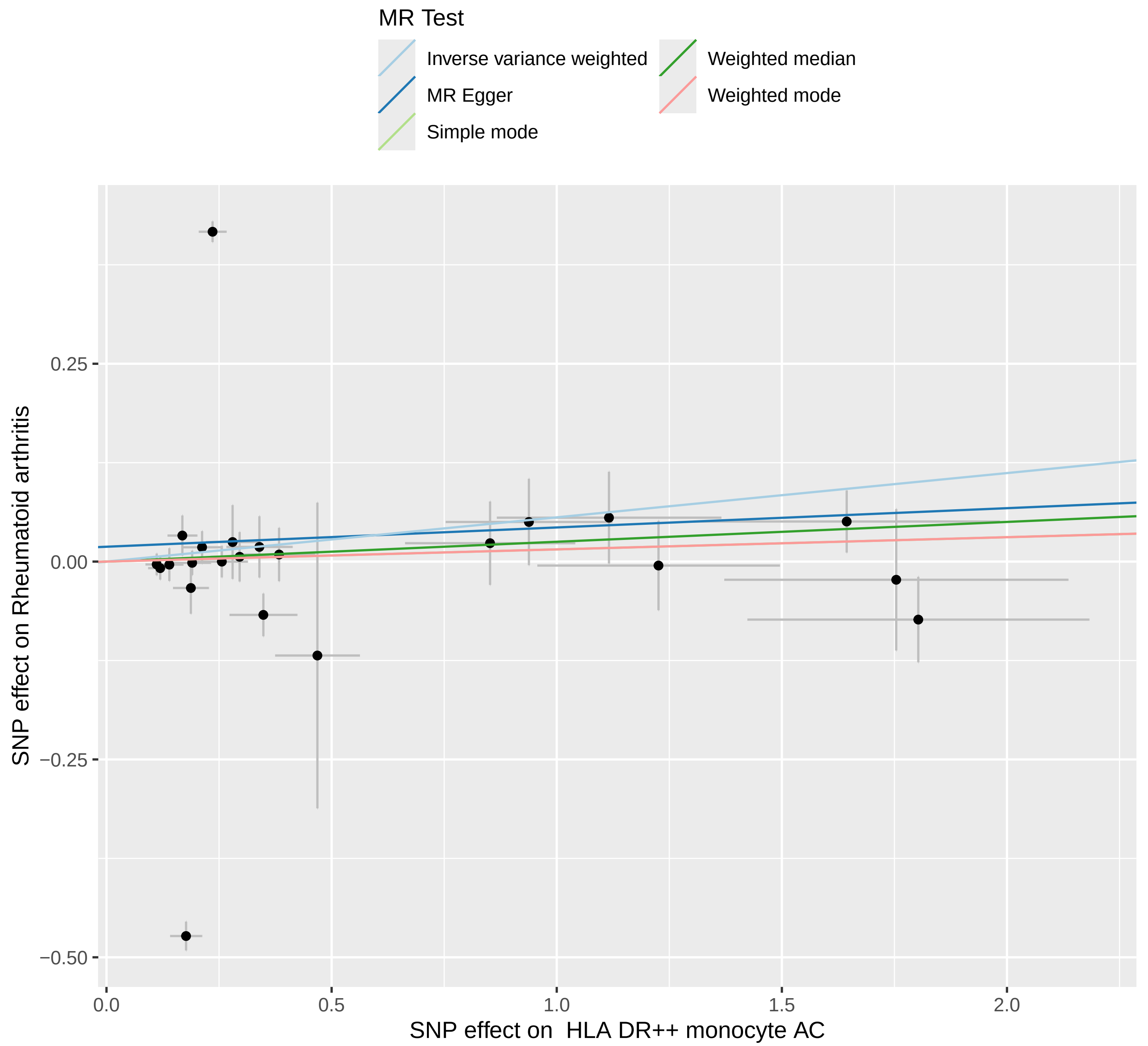

MR Test
Inverse variance weighted
MR Egger
Simple mode
Weighted median
Weighted mode
SNP effect on Rheumatoid arthritis
0.25
0.00
−0.25
−0.50
0.0
0.5
1.0
1.5
2.0
SNP effect on HLA DR++ monocyte AC

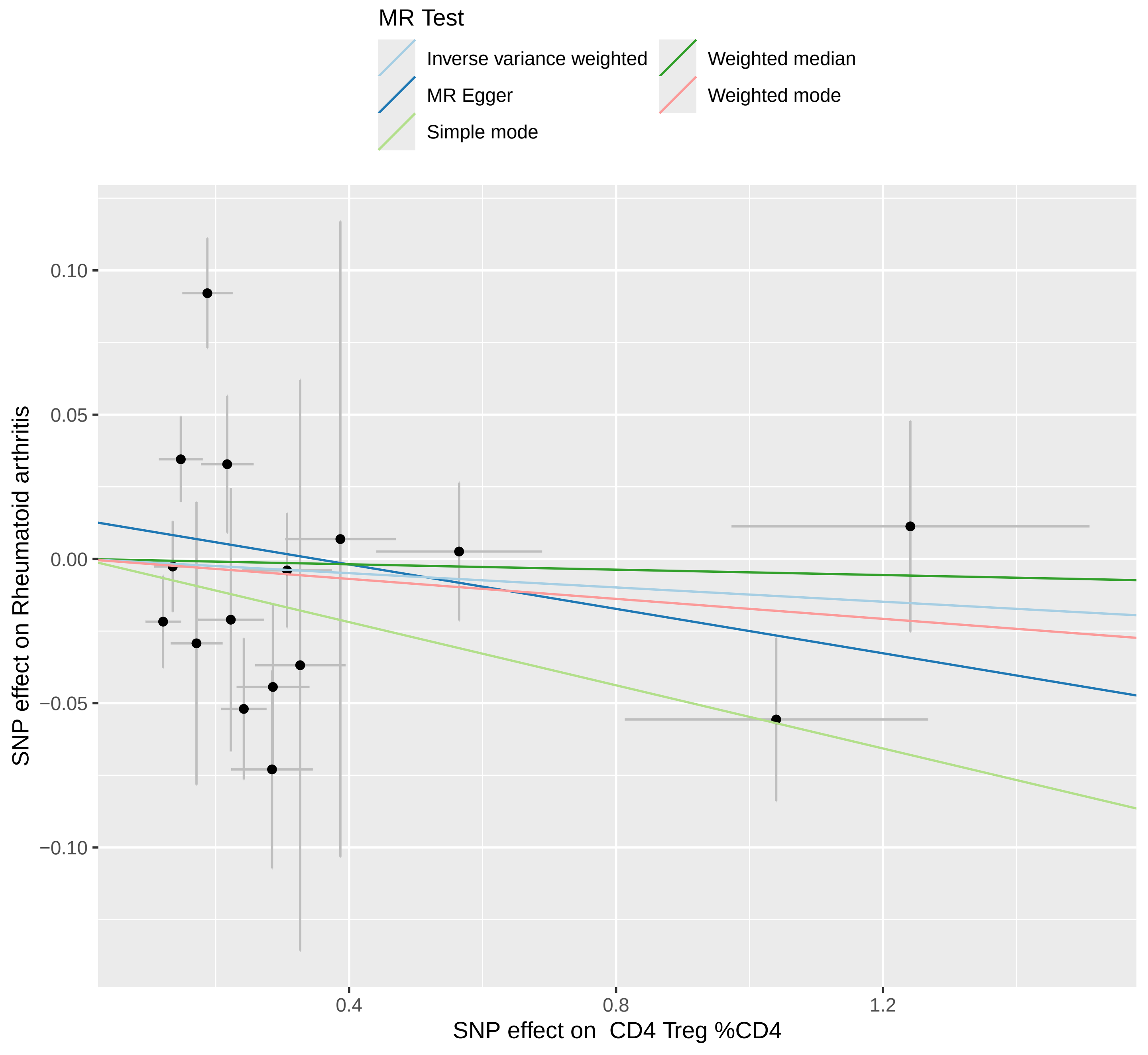
MR Test
Inverse variance weighted
MR Egger
Simple mode
Weighted median
Weighted mode
0.10
0.05
0.00
−0.05
−0.10
SNP effect on Rheumatoid arthritis
0.4
0.8
1.2
SNP effect on CD4 Treg %CD4

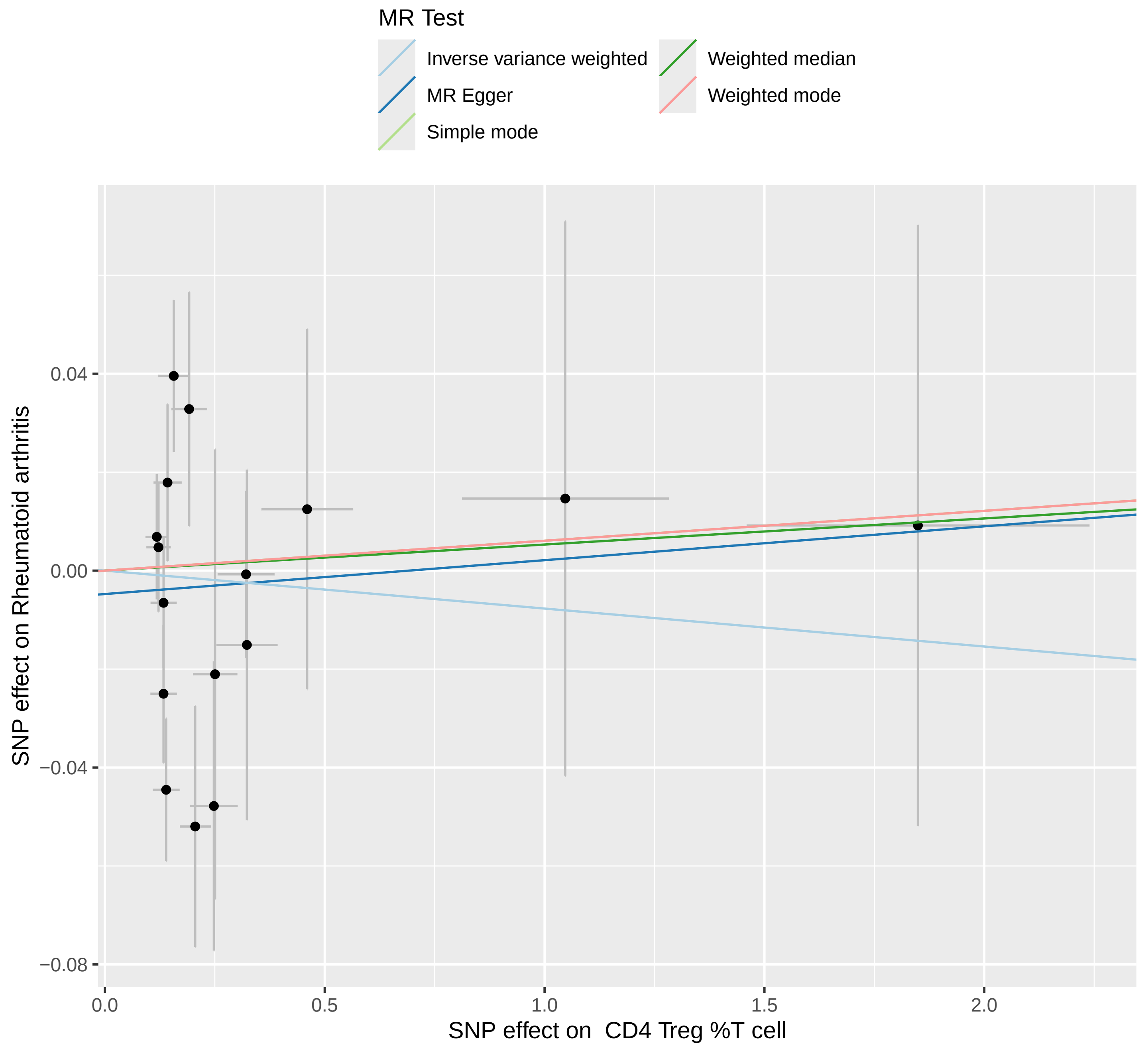

MR Test
Inverse variance weighted
MR Egger
Simple mode
Weighted median
Weighted mode
SNP effect on Rheumatoid arthritis
0.04
0.00
−0.04
−0.08
0.0
0.5
1.0
1.5
2.0
SNP effect on CD4 Treg %T cell

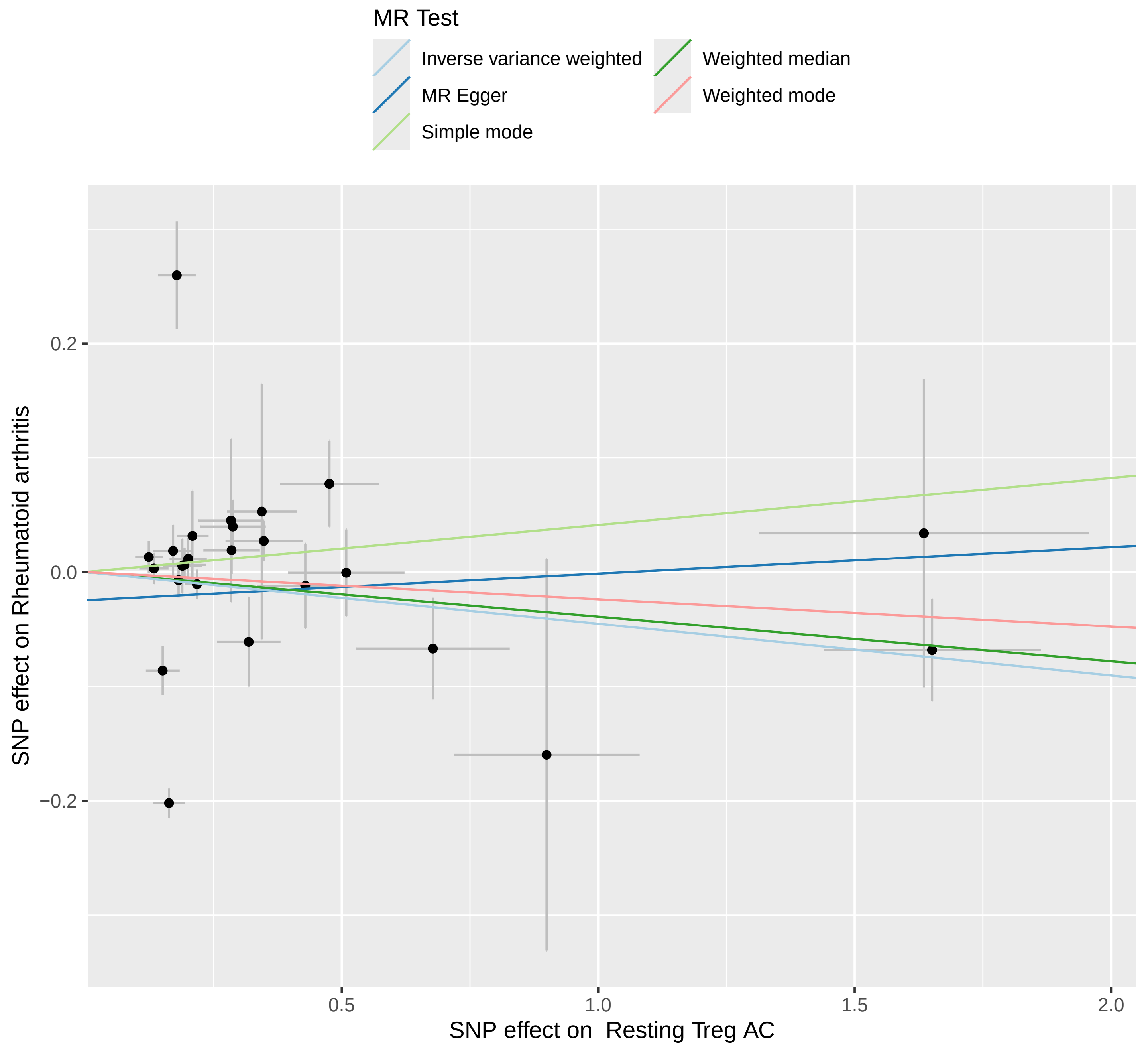
MR Test
Inverse variance weighted
MR Egger
Simple mode
Weighted median
Weighted mode
0.2
0.0
−0.2
0.5
1.0
1.5
2.0
SNP effect on Rheumatoid arthritis
SNP effect on Resting Treg AC

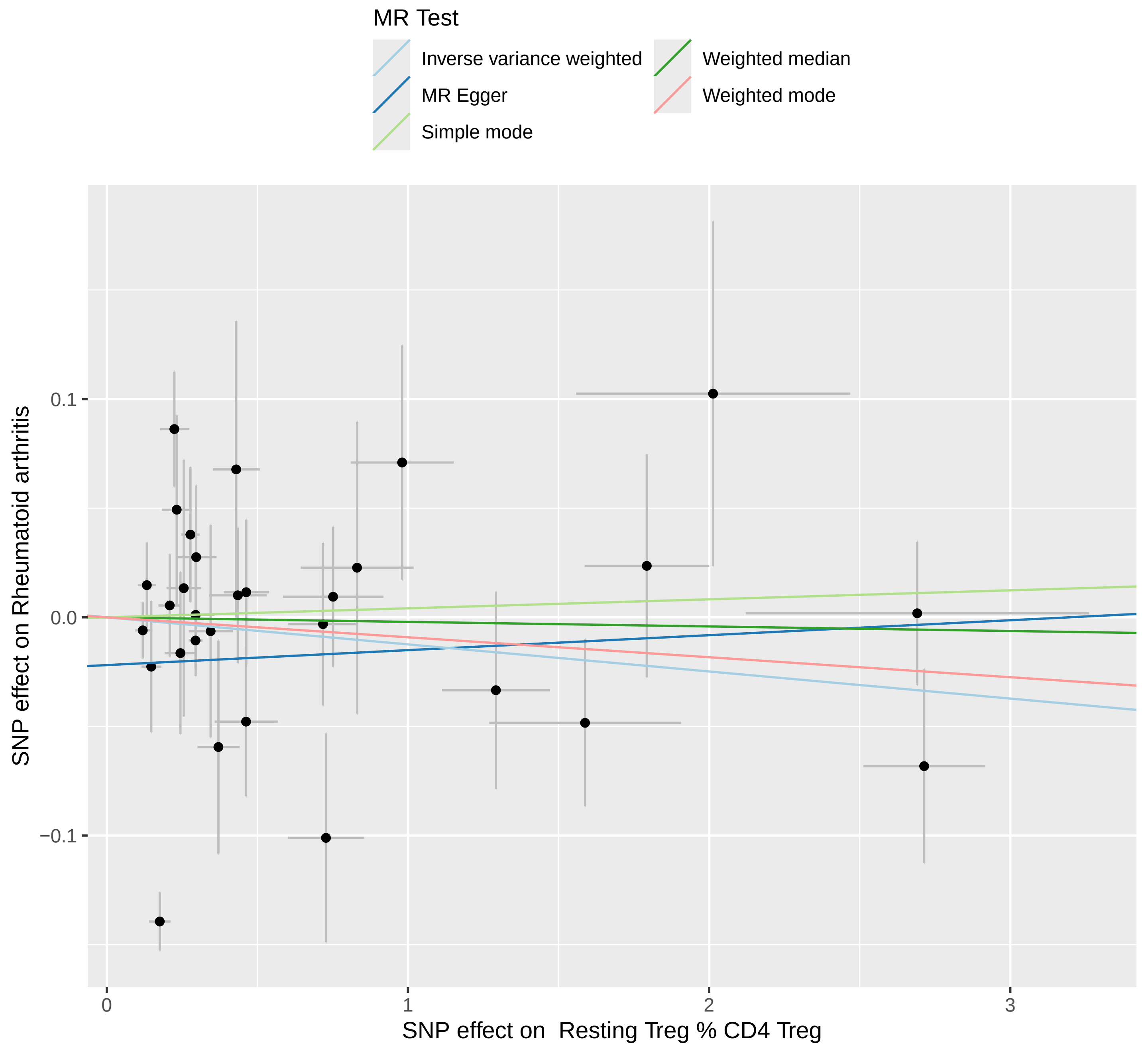
MR Test
Inverse variance weighted
MR Egger
Simple mode
Weighted median
Weighted mode
0.1
0.0
−0.1
0
1
2
3
SNP effect on Rheumatoid arthritis
SNP effect on Resting Treg % CD4 Treg

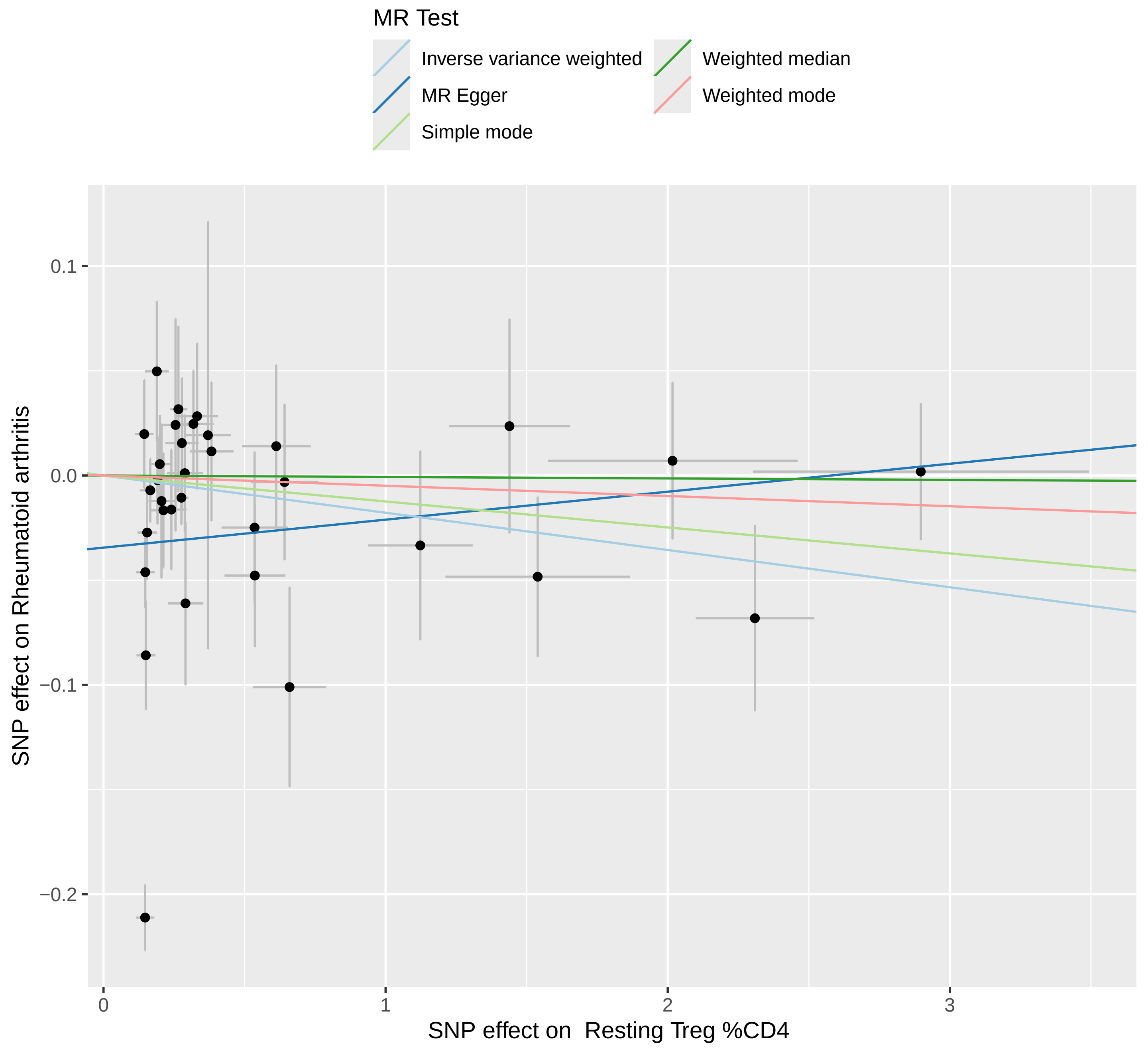
MR Test
Inverse variance weighted
MR Egger
Simple mode
Weighted median
Weighted mode
SNP effect on Rheumatoid arthritis
0.1
0.0
−0.1
−0.2
0
1
2
3
SNP effect on Resting Treg %CD4

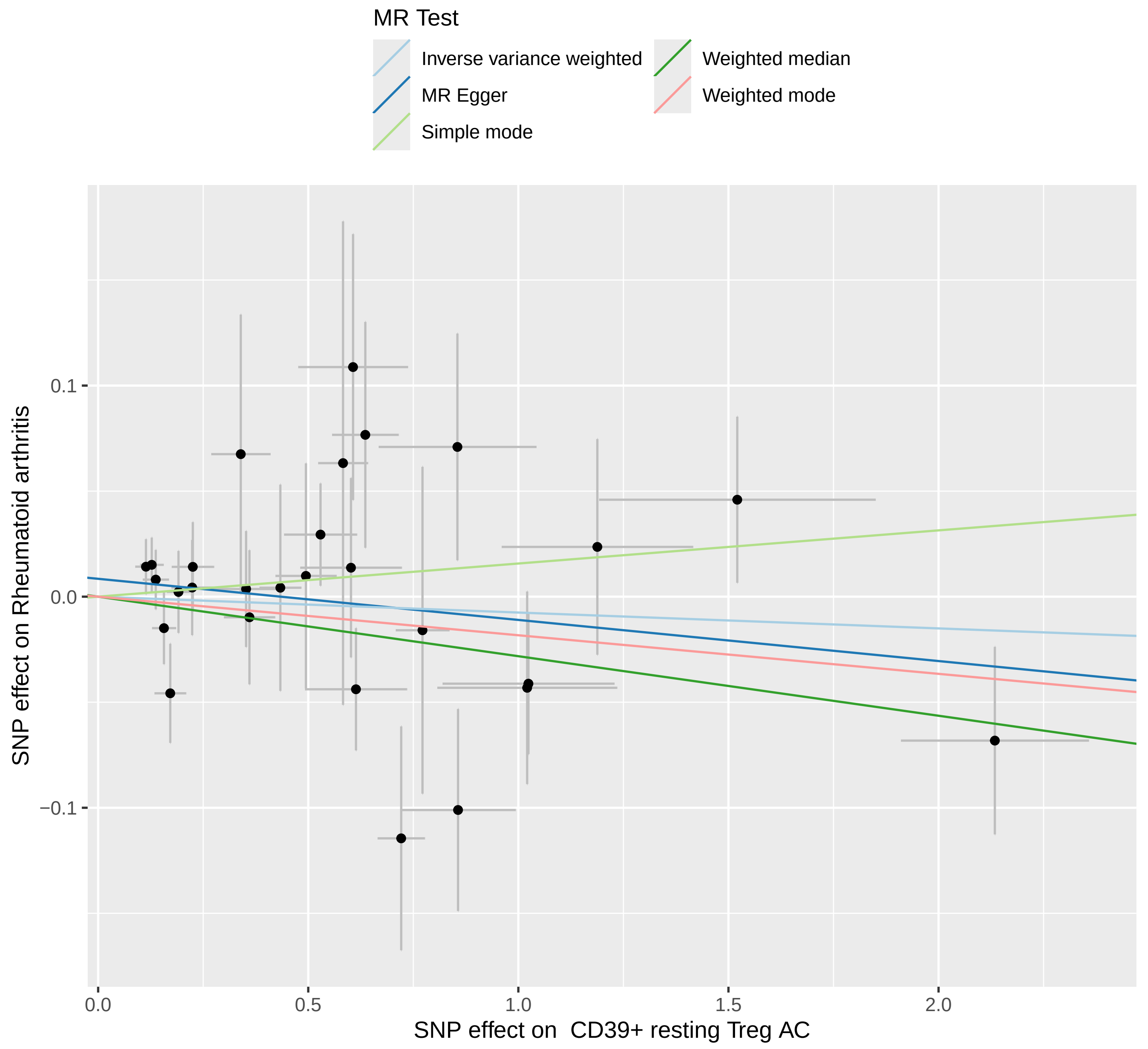
MR Test
Inverse variance weighted
MR Egger
Simple mode
Weighted median
Weighted mode
SNP effect on Rheumatoid arthritis
0.1
0.0
−0.1
0.0
0.5
1.0
1.5
2.0
SNP effect on CD39+ resting Treg AC

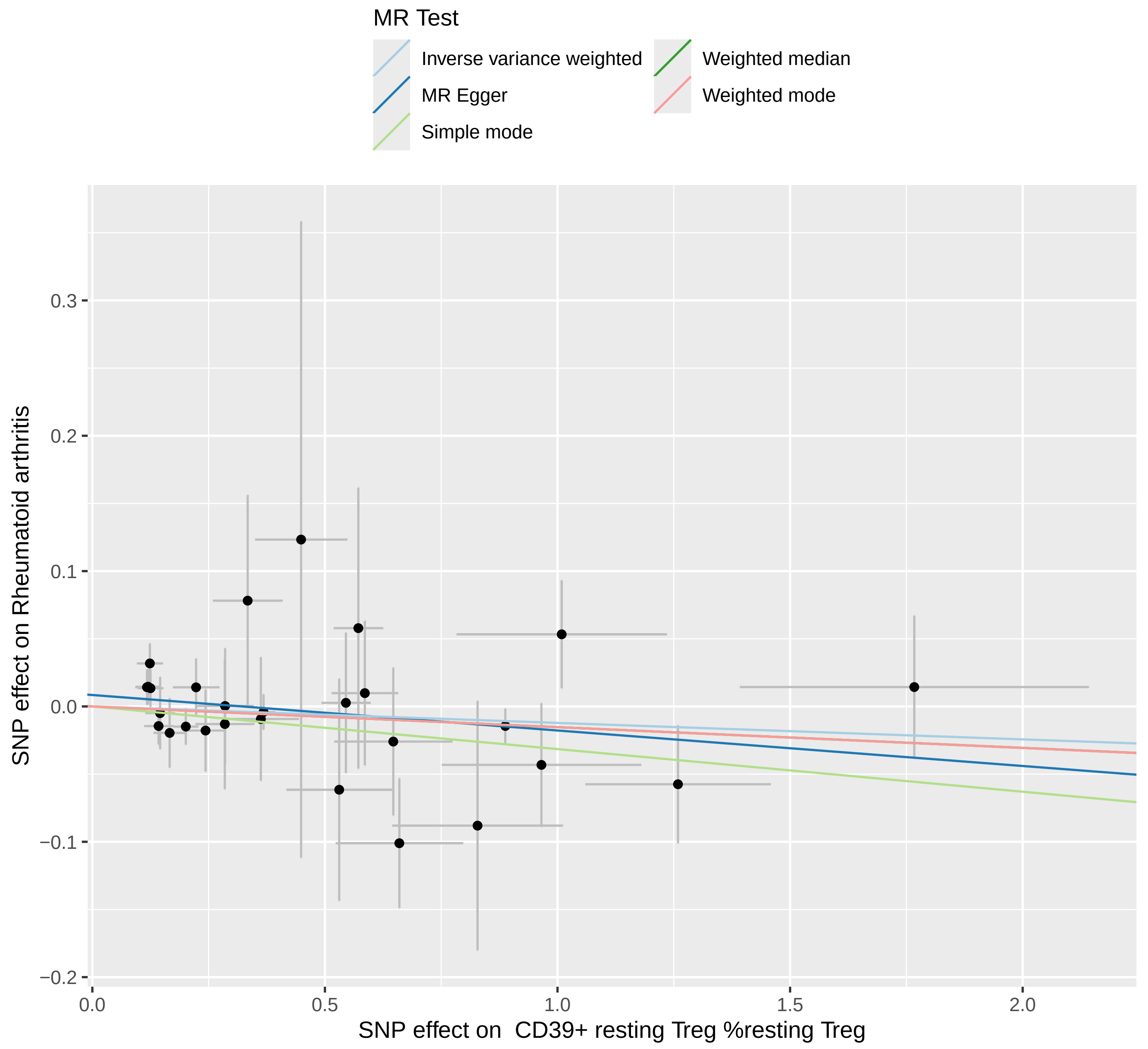

MR Test
Inverse variance weighted
MR Egger
Simple mode
Weighted median
Weighted mode
SNP effect on Rheumatoid arthritis
0.3
0.2
0.1
0.0
−0.1
−0.2
0.0
0.5
1.0
1.5
2.0
SNP effect on CD39+ resting Treg %resting Treg

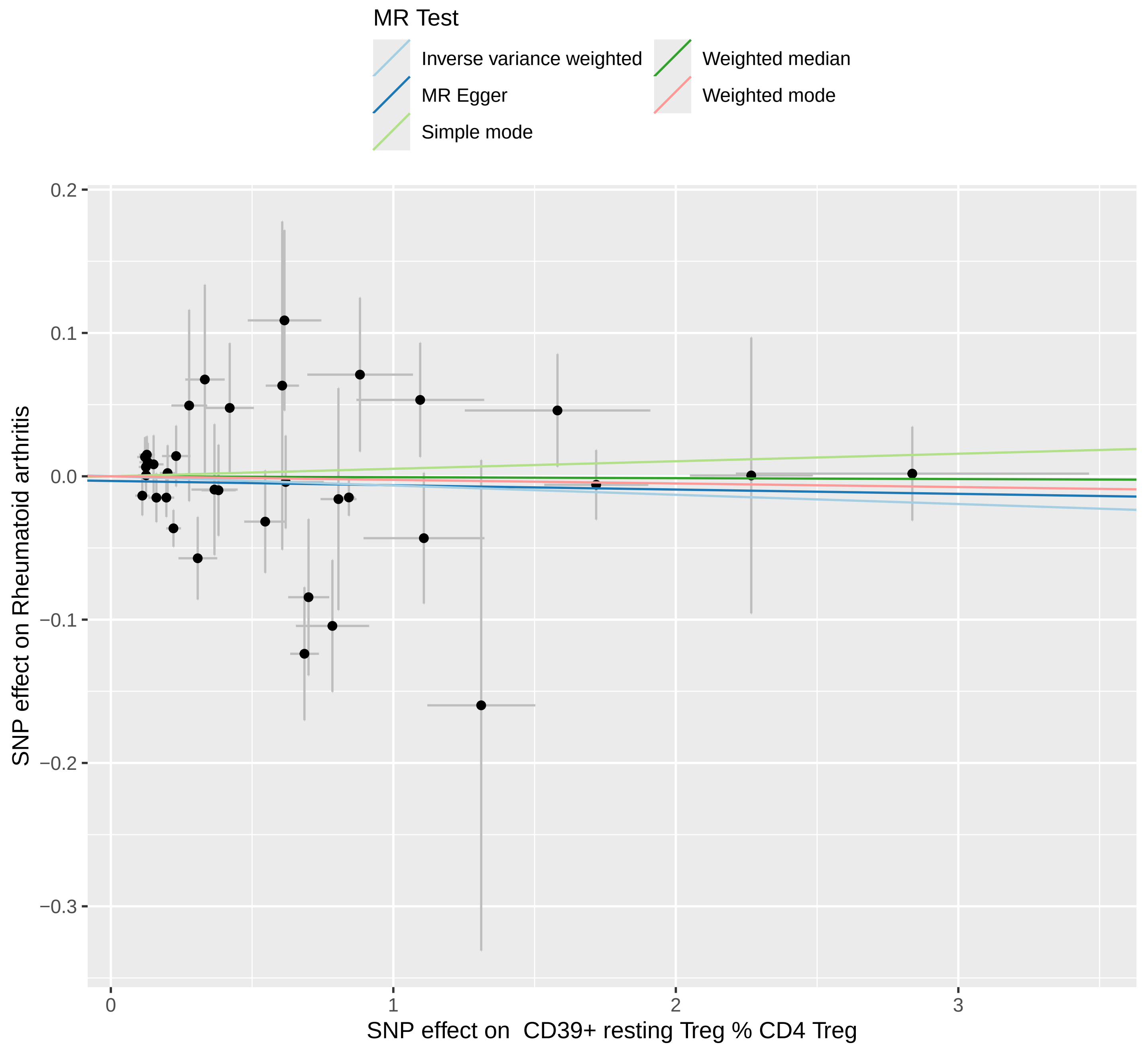

MR Test
Inverse variance weighted
MR Egger
Simple mode
Weighted median
Weighted mode
0.2
0.1
0.0
−0.1
−0.2
−0.3
0
1
2
3
SNP effect on Rheumatoid arthritis
SNP effect on CD39+ resting Treg % CD4 Treg

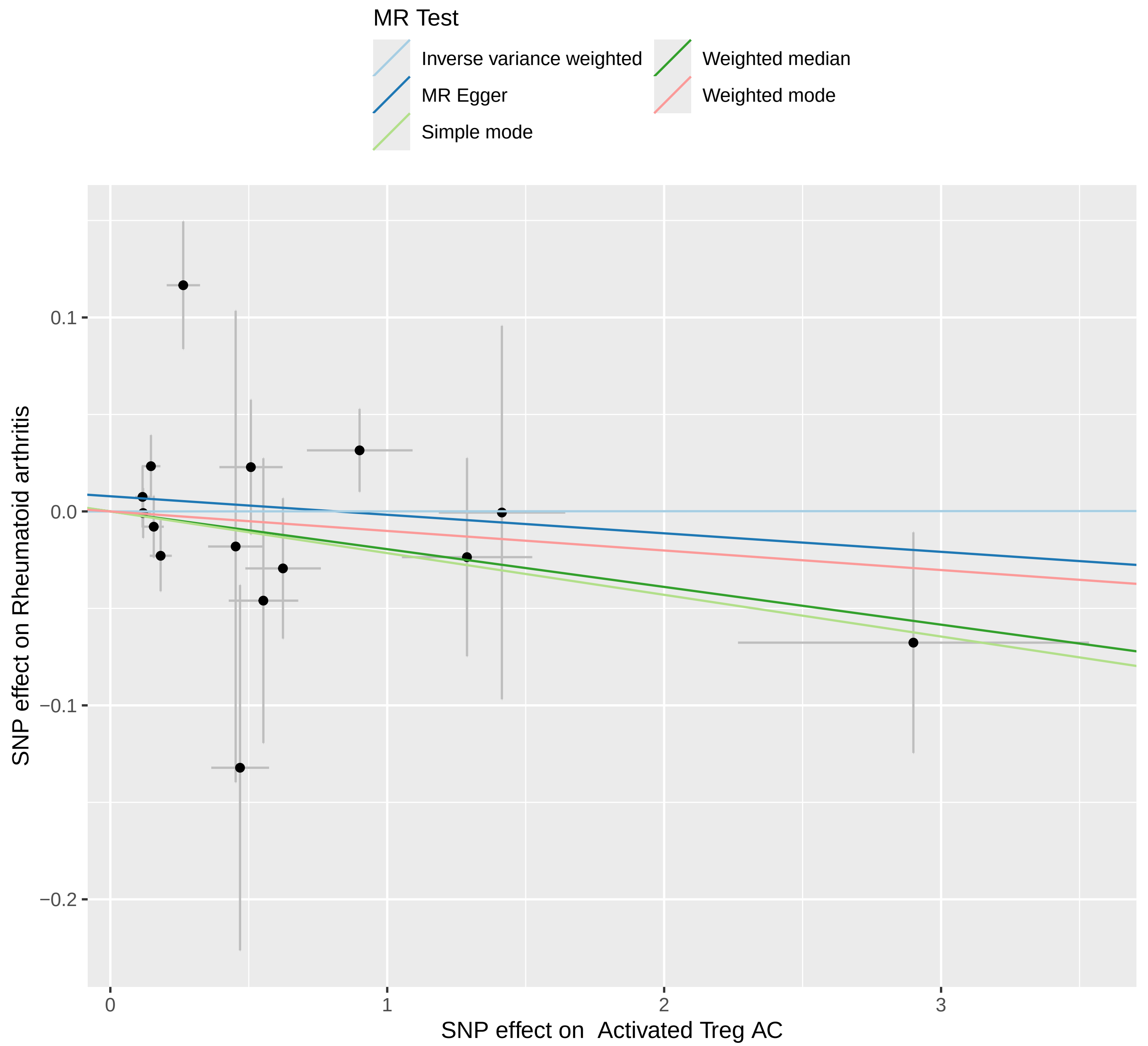

MR Test
Inverse variance weighted
MR Egger
Simple mode
Weighted median
Weighted mode
0.1
0.0
−0.1
−0.2
0
1
2
3
SNP effect on Rheumatoid arthritis
SNP effect on Activated Treg AC

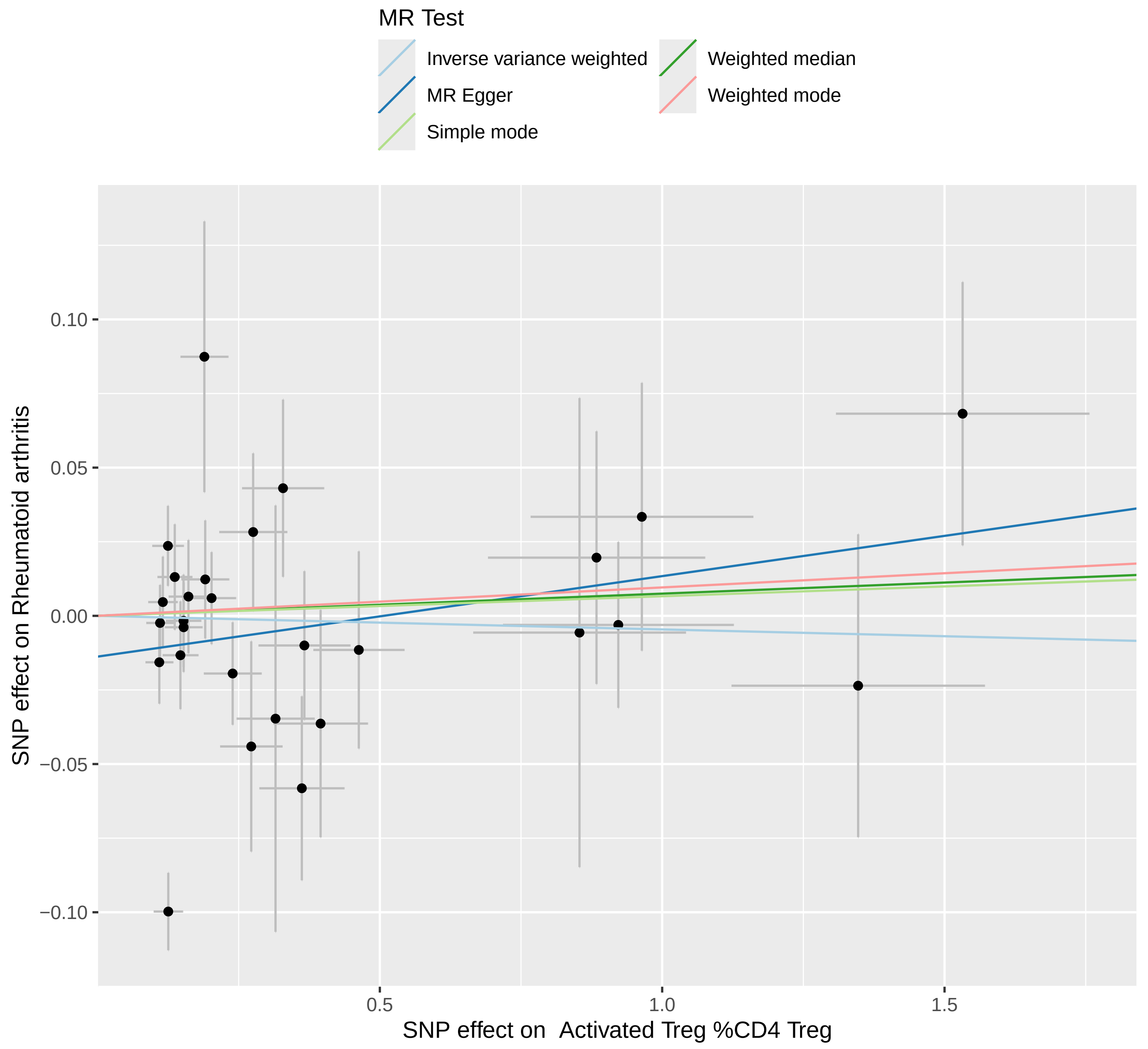
MR Test
Inverse variance weighted
MR Egger
Simple mode
Weighted median
Weighted mode
SNP effect on Rheumatoid arthritis
0.10
0.05
0.00
−0.05
−0.10
0.5
1.0
1.5
SNP effect on Activated Treg %CD4 Treg

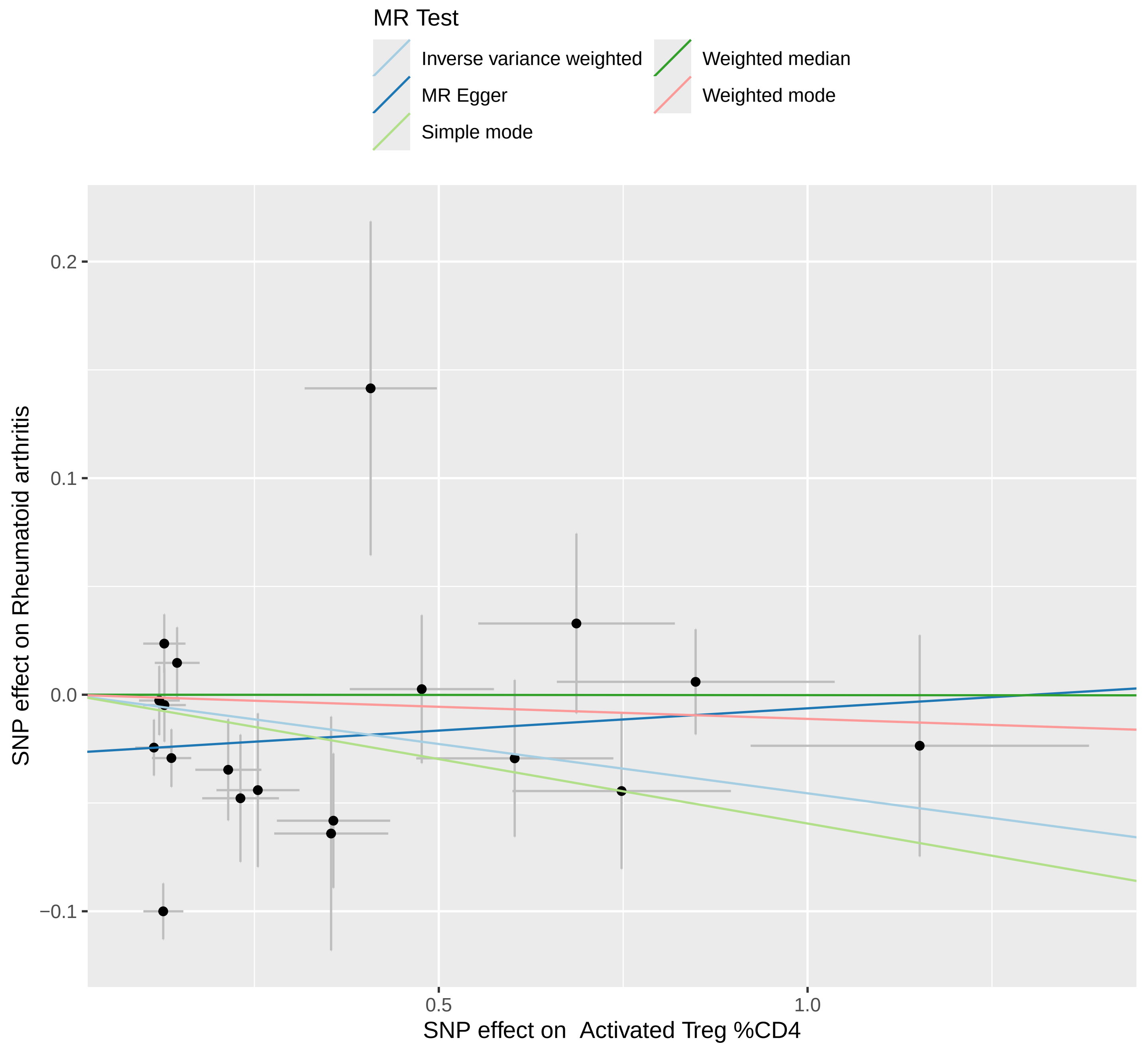
MR Test
Inverse variance weighted
MR Egger
Simple mode
Weighted median
Weighted mode
0.2
0.1
0.0
−0.1
SNP effect on Rheumatoid arthritis
0.5
1.0
SNP effect on Activated Treg %CD4

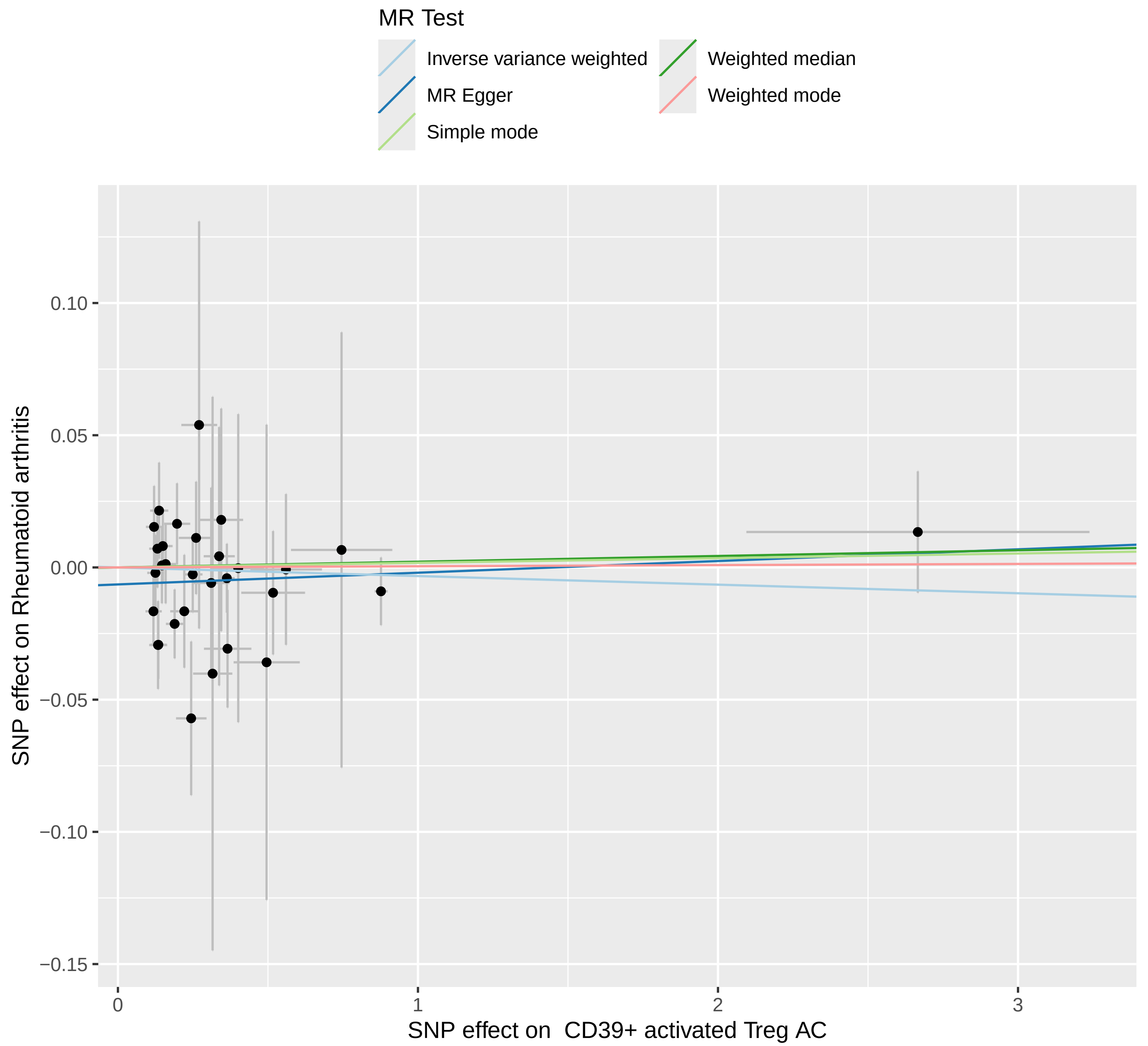
MR Test
Inverse variance weighted
MR Egger
Simple mode
Weighted median
Weighted mode
0.10
0.05
0.00
−0.05
−0.10
−0.15
SNP effect on Rheumatoid arthritis
0
1
2
3
SNP effect on CD39+ activated Treg AC

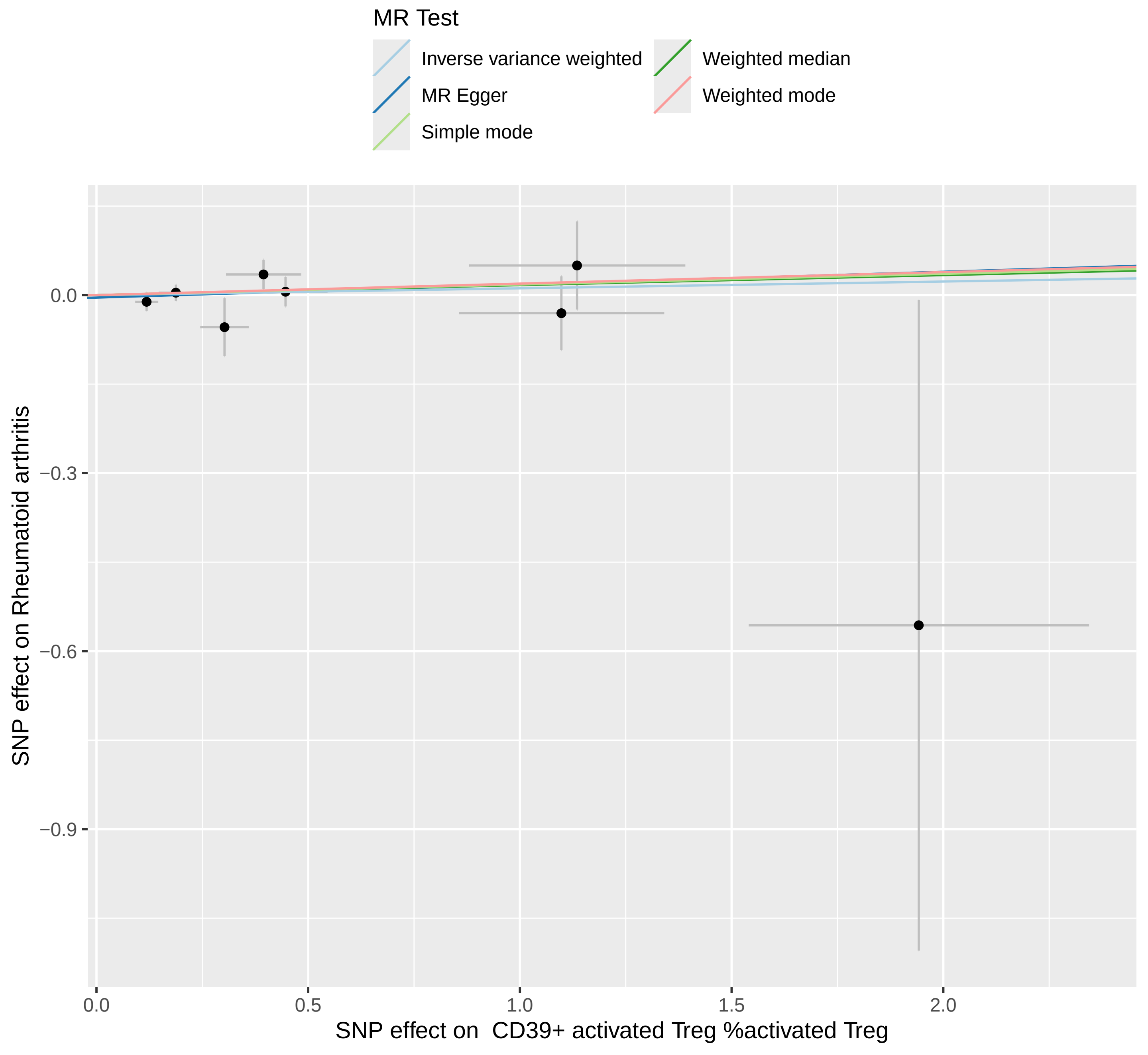

MR Test
Inverse variance weighted
MR Egger
Simple mode
Weighted median
Weighted mode
SNP effect on Rheumatoid arthritis
0.0
−0.3
−0.6
−0.9
0.0
0.5
1.0
1.5
2.0
SNP effect on CD39+ activated Treg %activated Treg

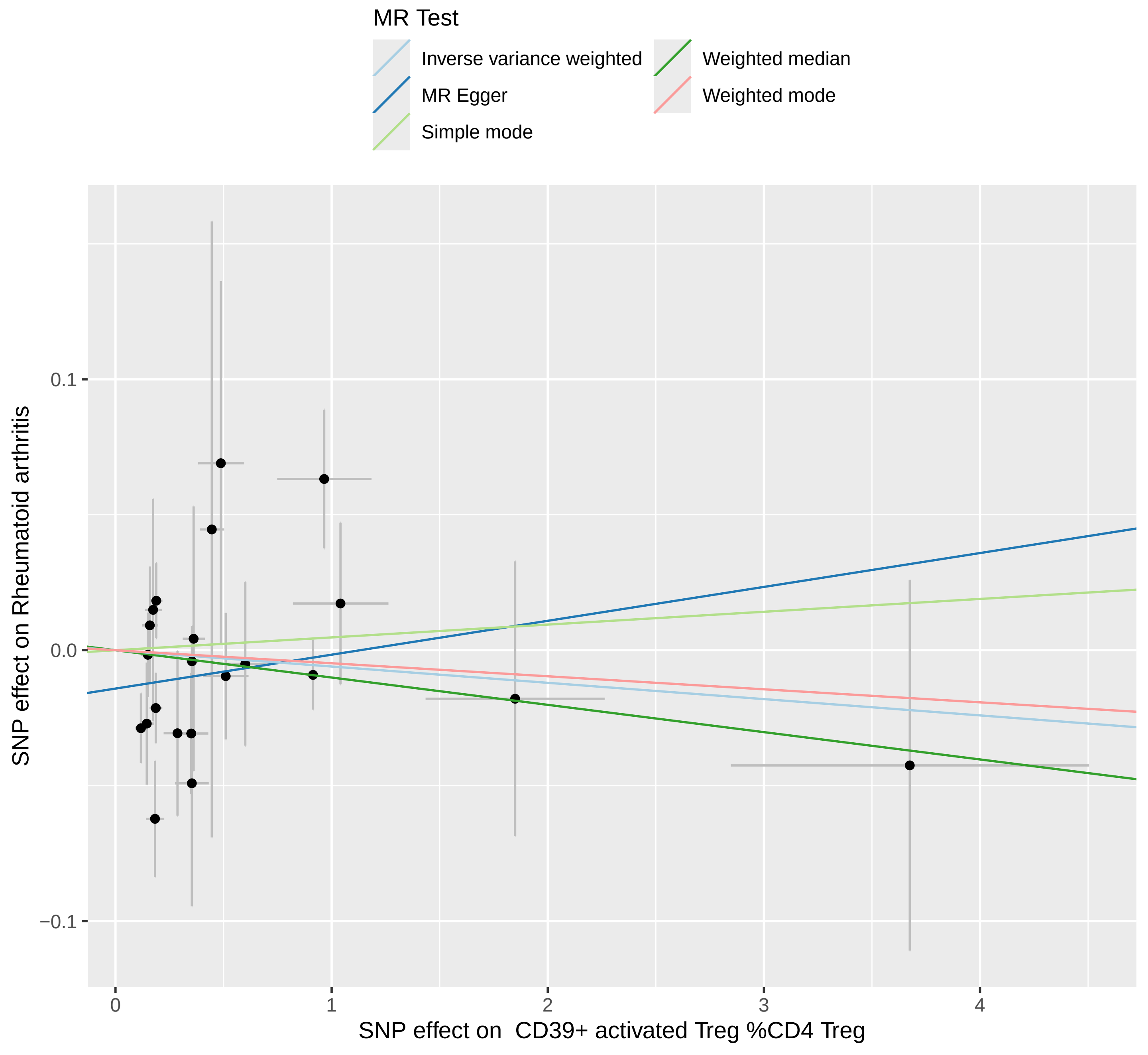

MR Test
Inverse variance weighted
MR Egger
Simple mode
Weighted median
Weighted mode
SNP effect on Rheumatoid arthritis
0.1
0.0
−0.1
0
1
2
3
4
SNP effect on CD39+ activated Treg %CD4 Treg

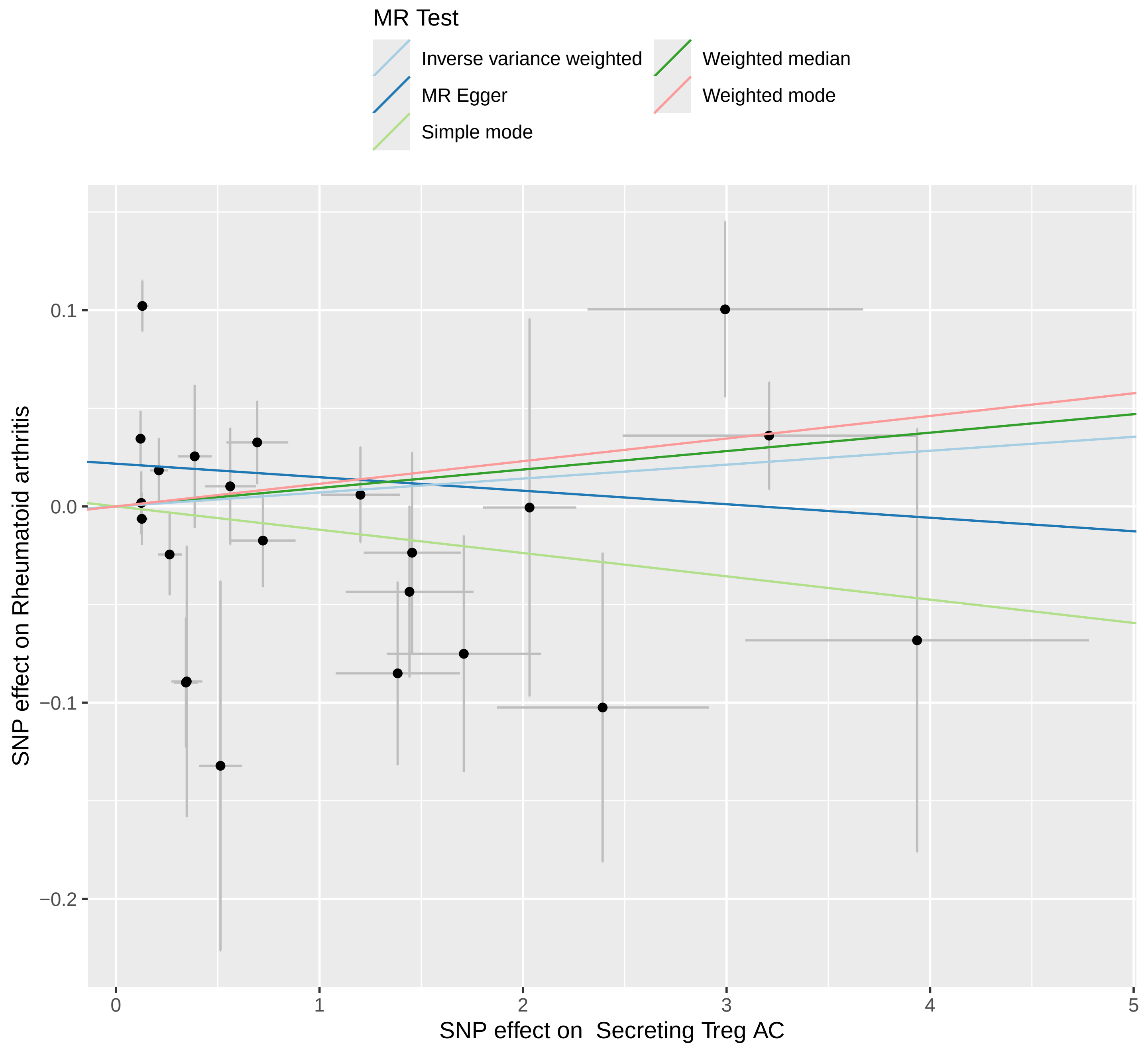
MR Test
Inverse variance weighted
MR Egger
Simple mode
Weighted median
Weighted mode
SNP effect on Rheumatoid arthritis
0.1
0.0
−0.1
−0.2
0
1
2
3
4
5
SNP effect on Secreting Treg AC

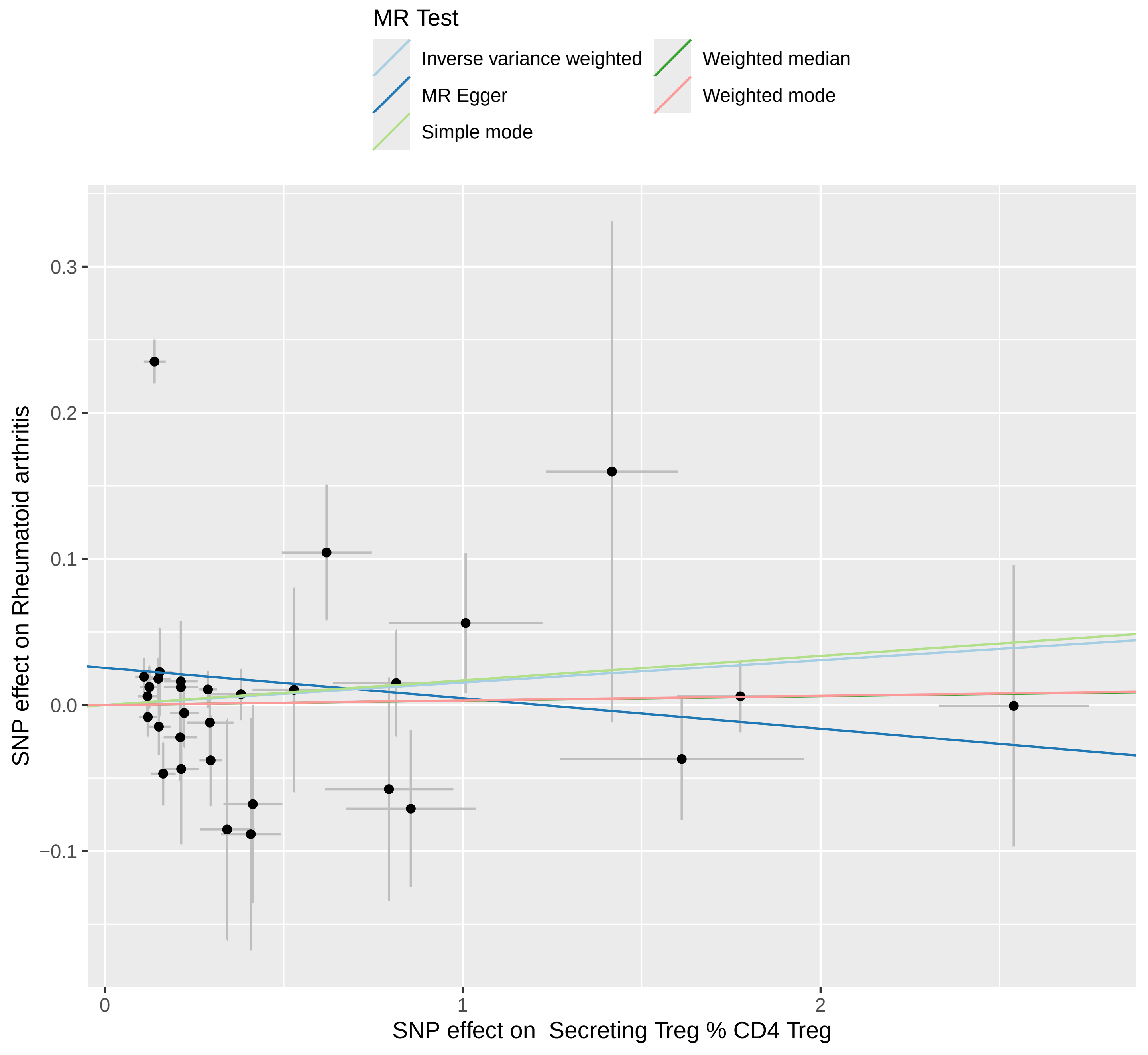
MR Test
Inverse variance weighted
MR Egger
Simple mode
Weighted median
Weighted mode
0.3
0.2
0.1
0.0
−0.1
0
1
2
SNP effect on Rheumatoid arthritis
SNP effect on Secreting Treg % CD4 Treg

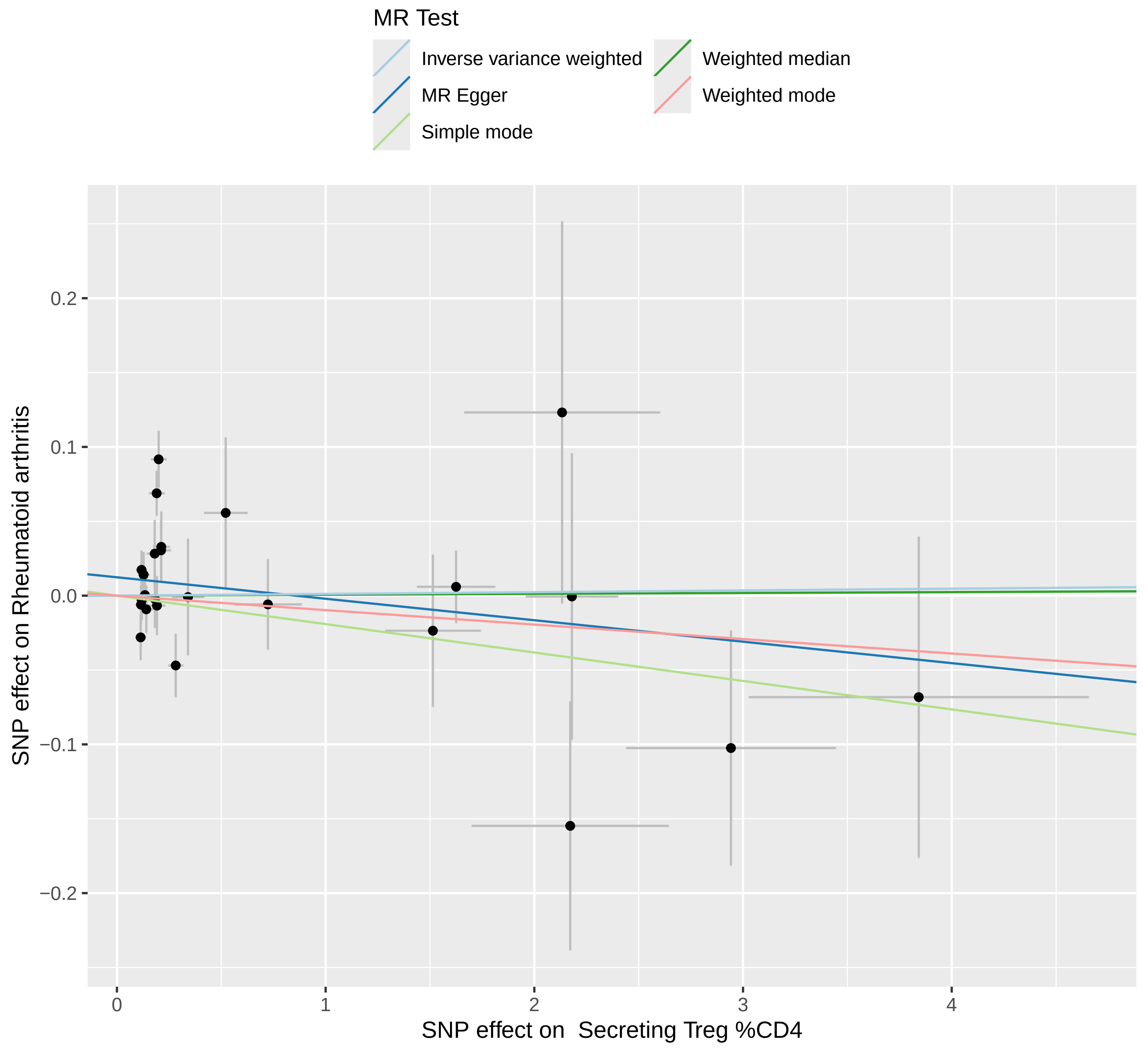
MR Test
Inverse variance weighted
MR Egger
Simple mode
Weighted median
Weighted mode
0.2
0.1
0.0
−0.1
−0.2
0
1
2
3
4
SNP effect on Rheumatoid arthritis
SNP effect on Secreting Treg %CD4

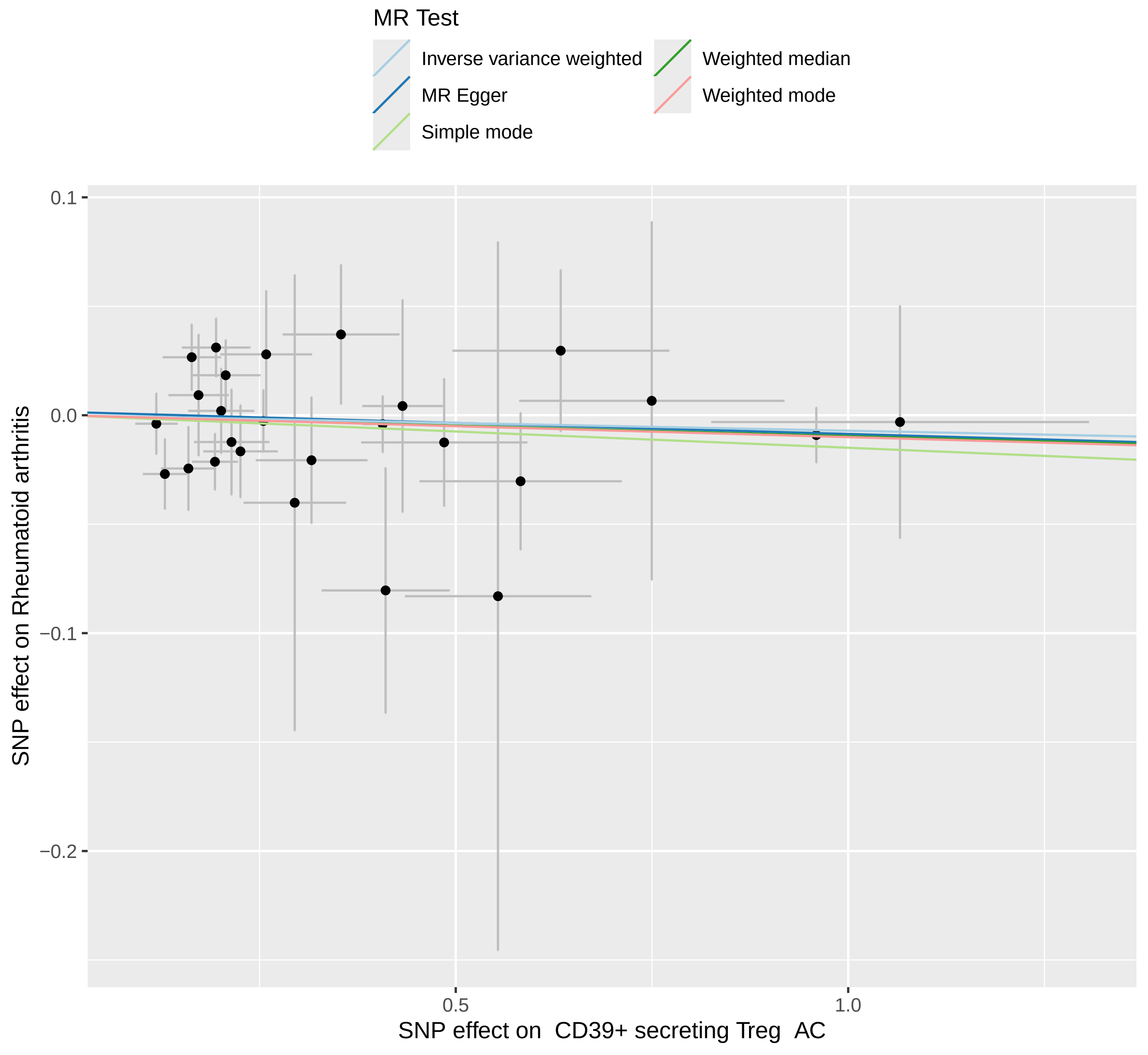

MR Test
Inverse variance weighted
MR Egger
Simple mode
Weighted median
Weighted mode
0.1
0.0
−0.1
−0.2
0.5
1.0
SNP effect on Rheumatoid arthritis
SNP effect on CD39+ secreting Treg AC

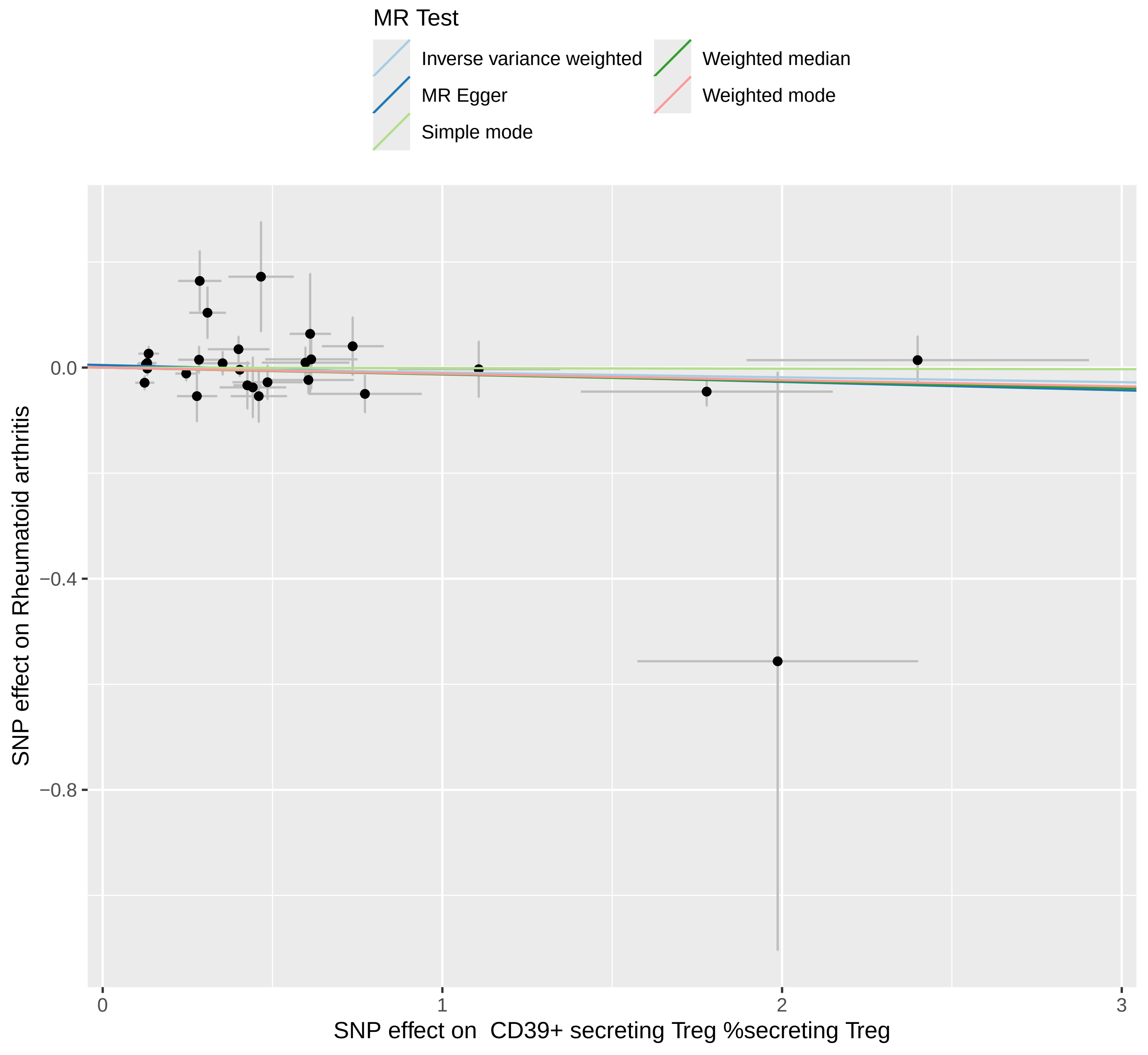

MR Test
Inverse variance weighted
MR Egger
Simple mode
Weighted median
Weighted mode
SNP effect on Rheumatoid arthritis
0.0
−0.4
−0.8
0
1
2
3
SNP effect on CD39+ secreting Treg %secreting Treg

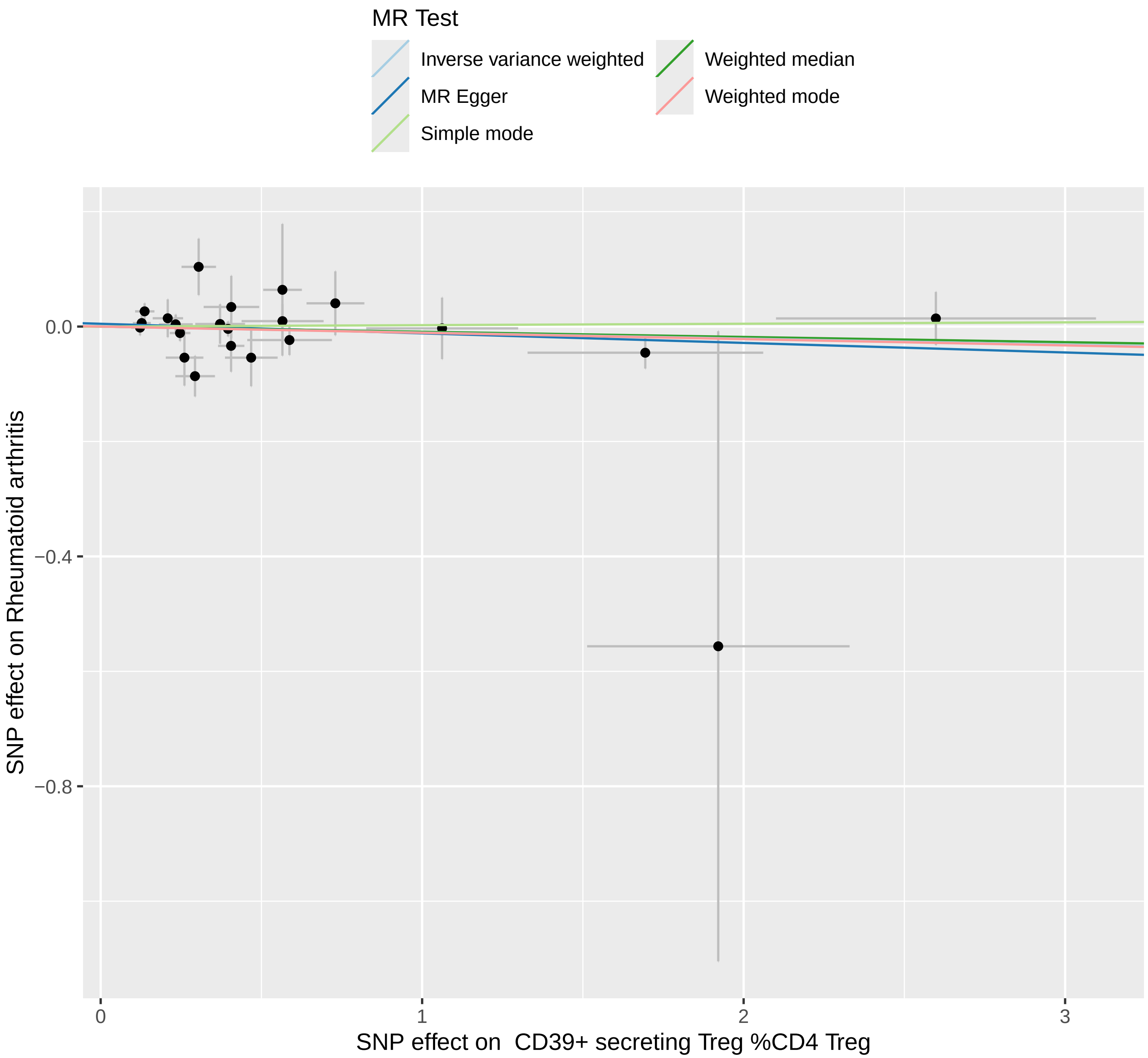
MR Test
Inverse variance weighted
MR Egger
Simple mode
Weighted median
Weighted mode
SNP effect on Rheumatoid arthritis
0.0
−0.4
−0.8
0
1
2
3
SNP effect on CD39+ secreting Treg %CD4 Treg

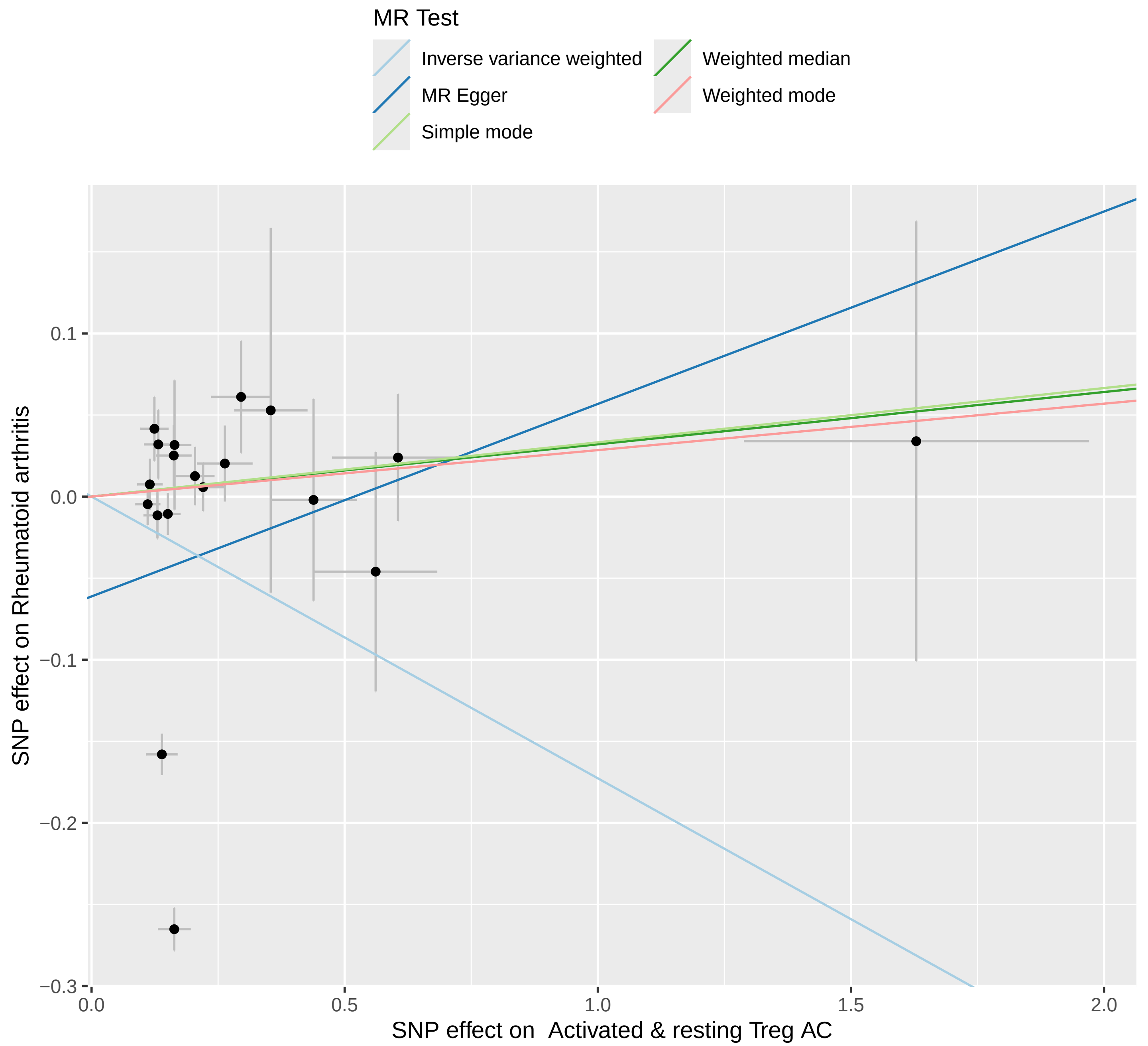

MR Test
Inverse variance weighted
MR Egger
Simple mode
Weighted median
Weighted mode
SNP effect on Rheumatoid arthritis
0.1
0.0
−0.1
−0.2
−0.3
0.0
0.5
1.0
1.5
2.0
SNP effect on Activated & resting Treg AC

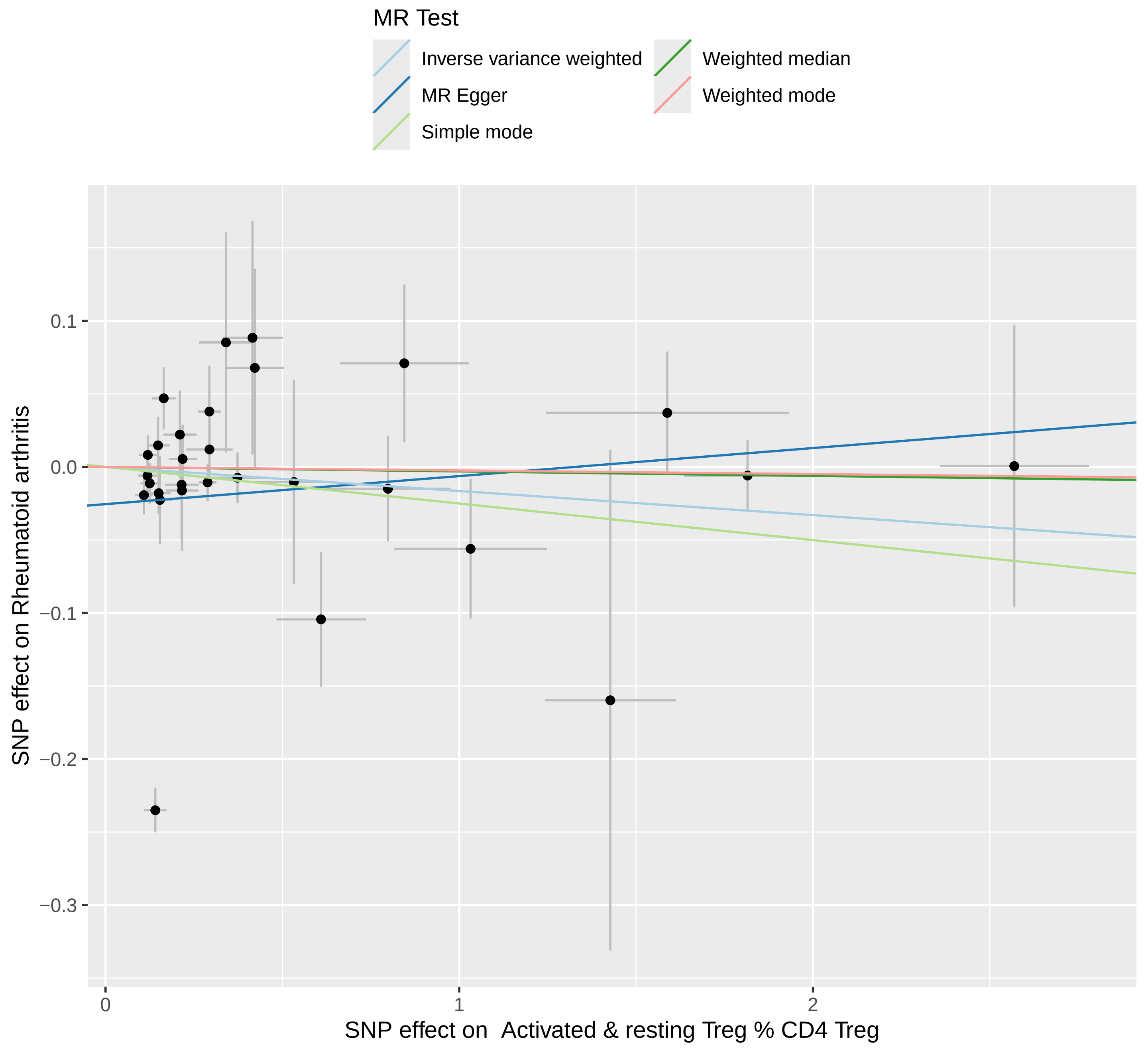

MR Test
Inverse variance weighted
MR Egger
Simple mode
Weighted median
Weighted mode
0.1
0.0
−0.1
−0.2
−0.3
0
1
2
SNP effect on Rheumatoid arthritis
SNP effect on Activated & resting Treg % CD4 Treg

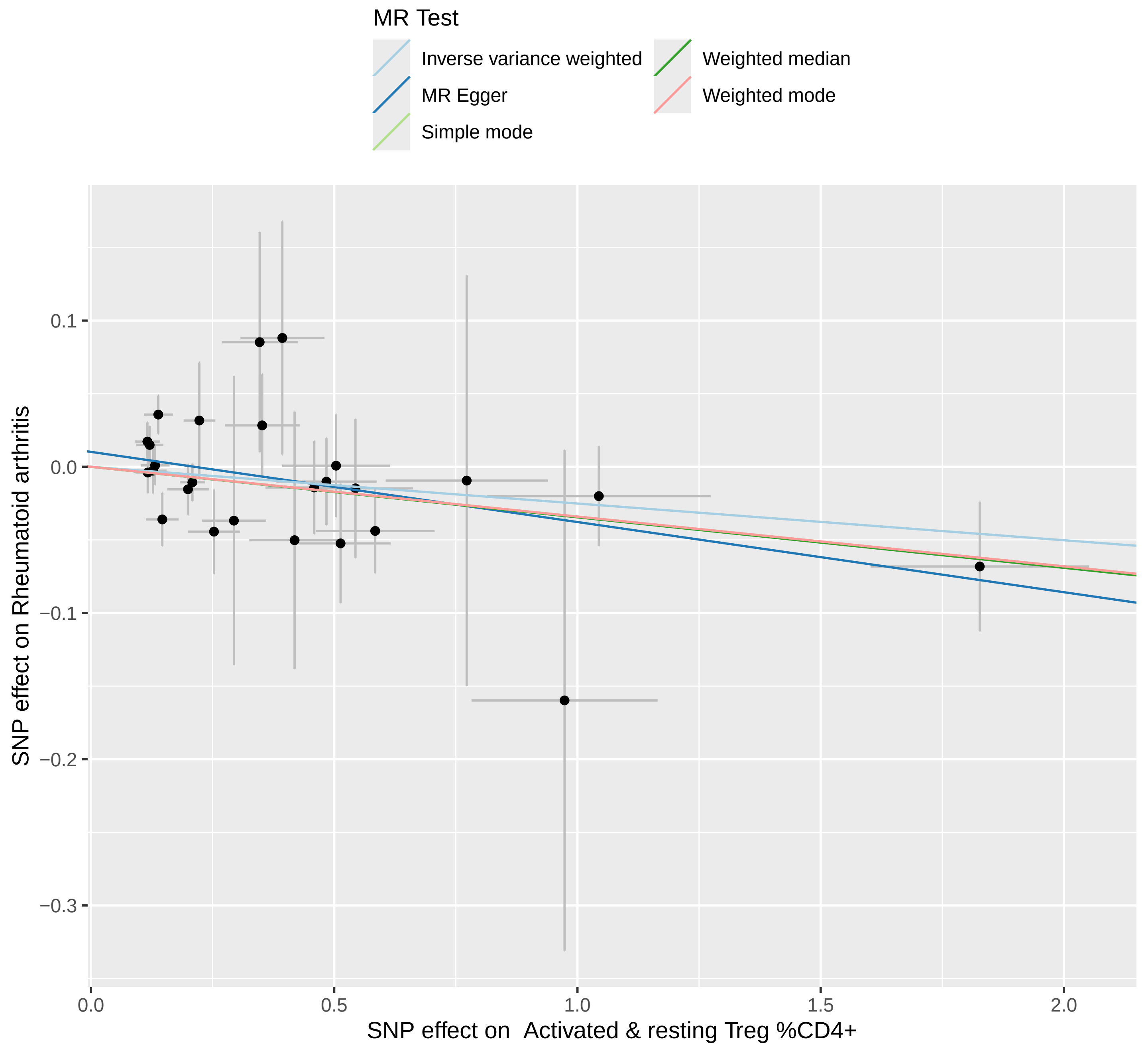

MR Test
Inverse variance weighted
MR Egger
Simple mode
Weighted median
Weighted mode
0.1
0.0
−0.1
−0.2
−0.3
SNP effect on Rheumatoid arthritis
0.0
0.5
1.0
1.5
2.0
SNP effect on Activated & resting Treg %CD4+

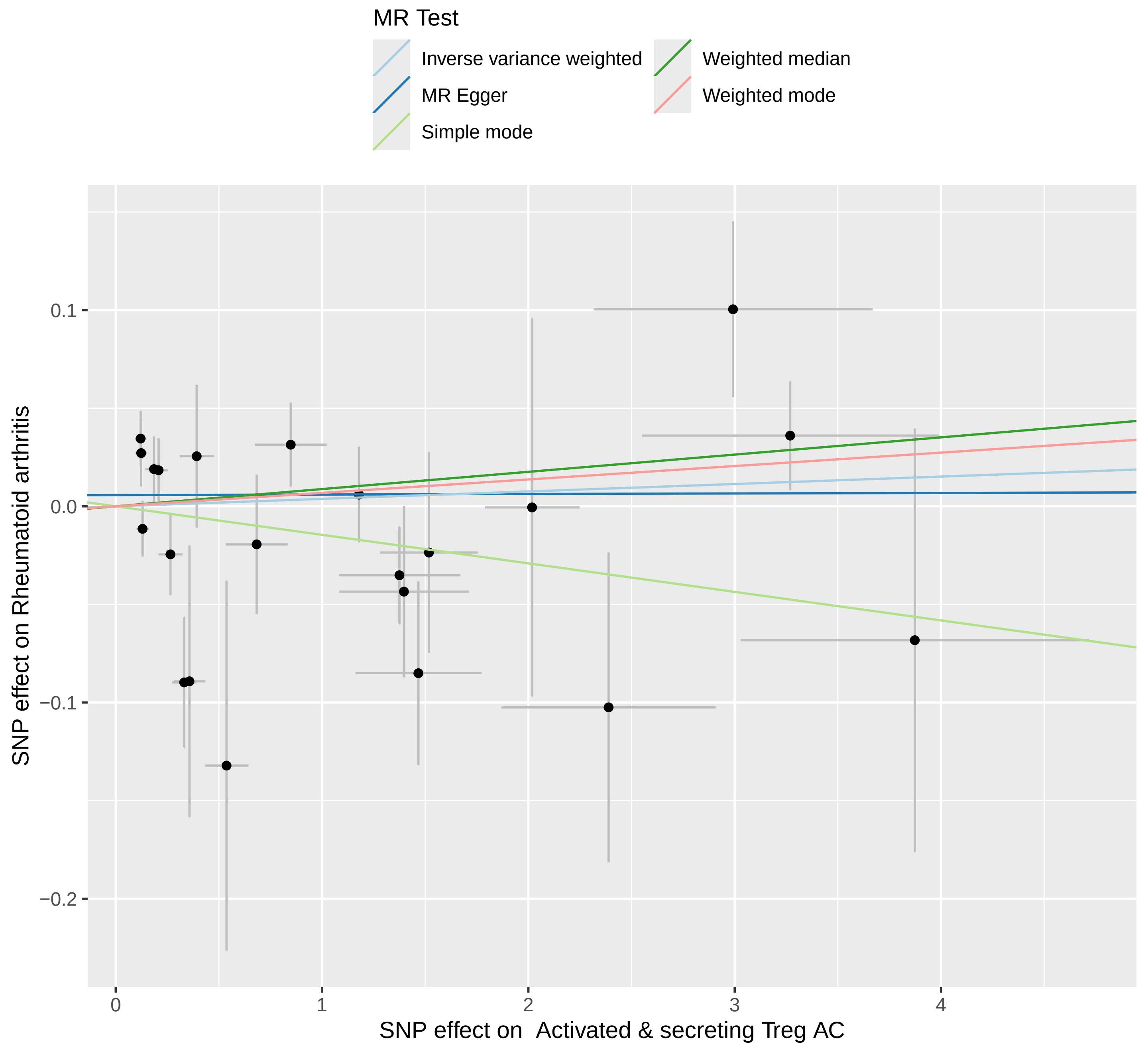
MR Test
Inverse variance weighted
MR Egger
Simple mode
Weighted median
Weighted mode
0.1
0.0
−0.1
−0.2
0
1
2
3
4
SNP effect on Rheumatoid arthritis
SNP effect on Activated & secreting Treg AC

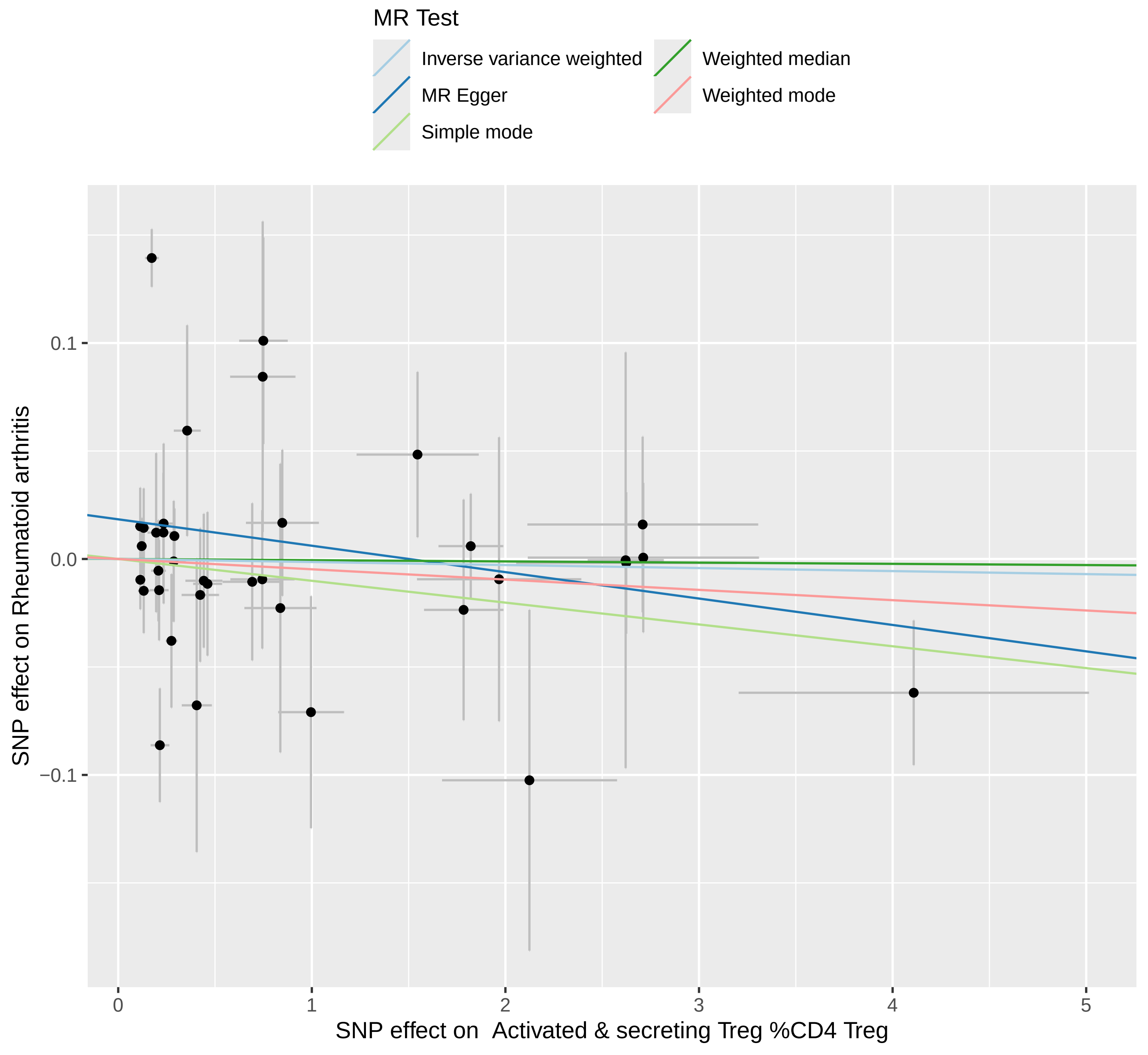
MR Test
Inverse variance weighted
Weighted median
MR Egger
Weighted mode
Simple mode
0.1
0.0
−0.1
SNP effect on Rheumatoid arthritis
0
1
2
3
4
5
SNP effect on Activated & secreting Treg %CD4 Treg

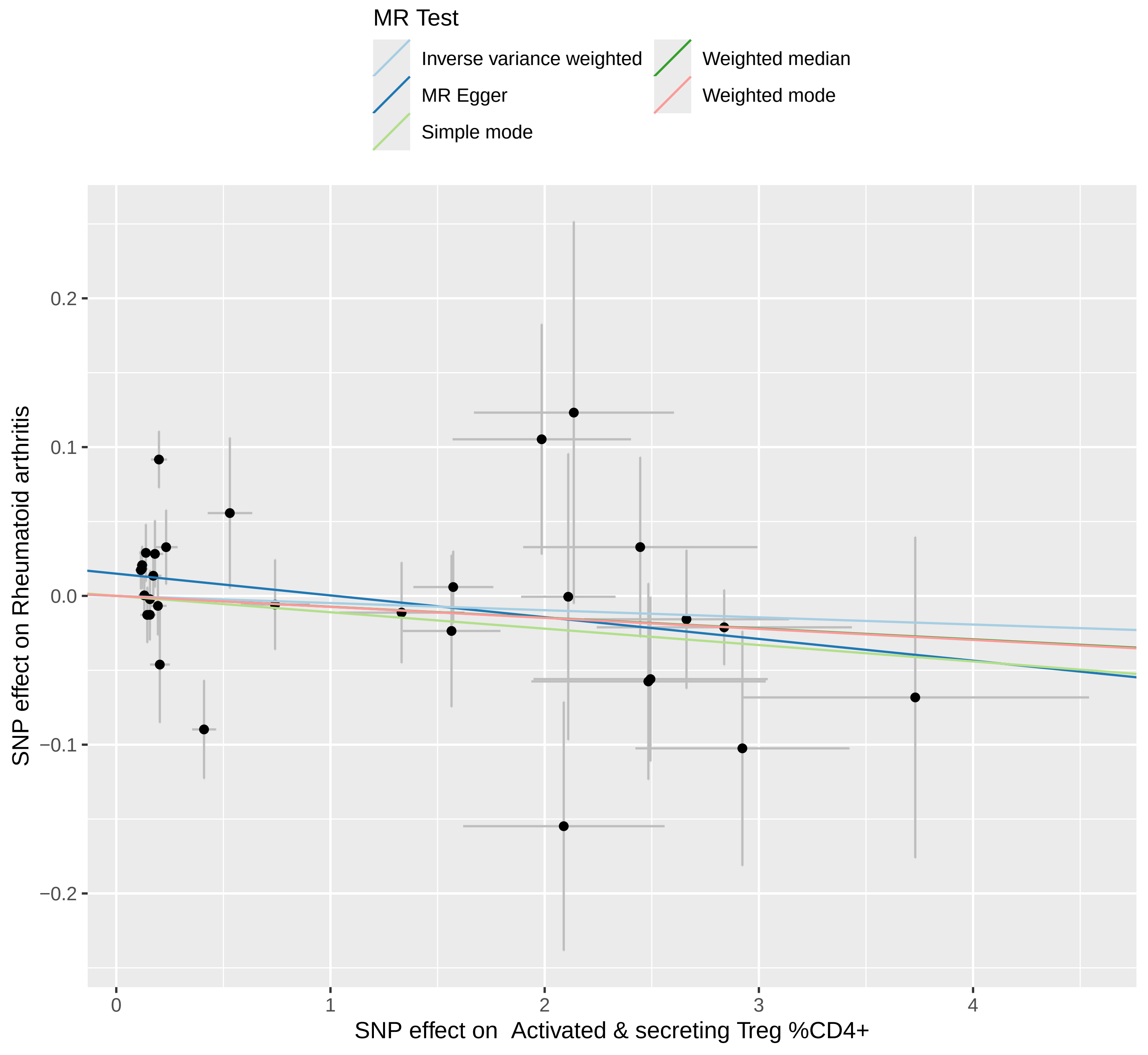
MR Test
Inverse variance weighted
MR Egger
Simple mode
Weighted median
Weighted mode
0.2
0.1
0.0
−0.1
−0.2
SNP effect on Rheumatoid arthritis
0
1
2
3
4
SNP effect on Activated & secreting Treg %CD4+

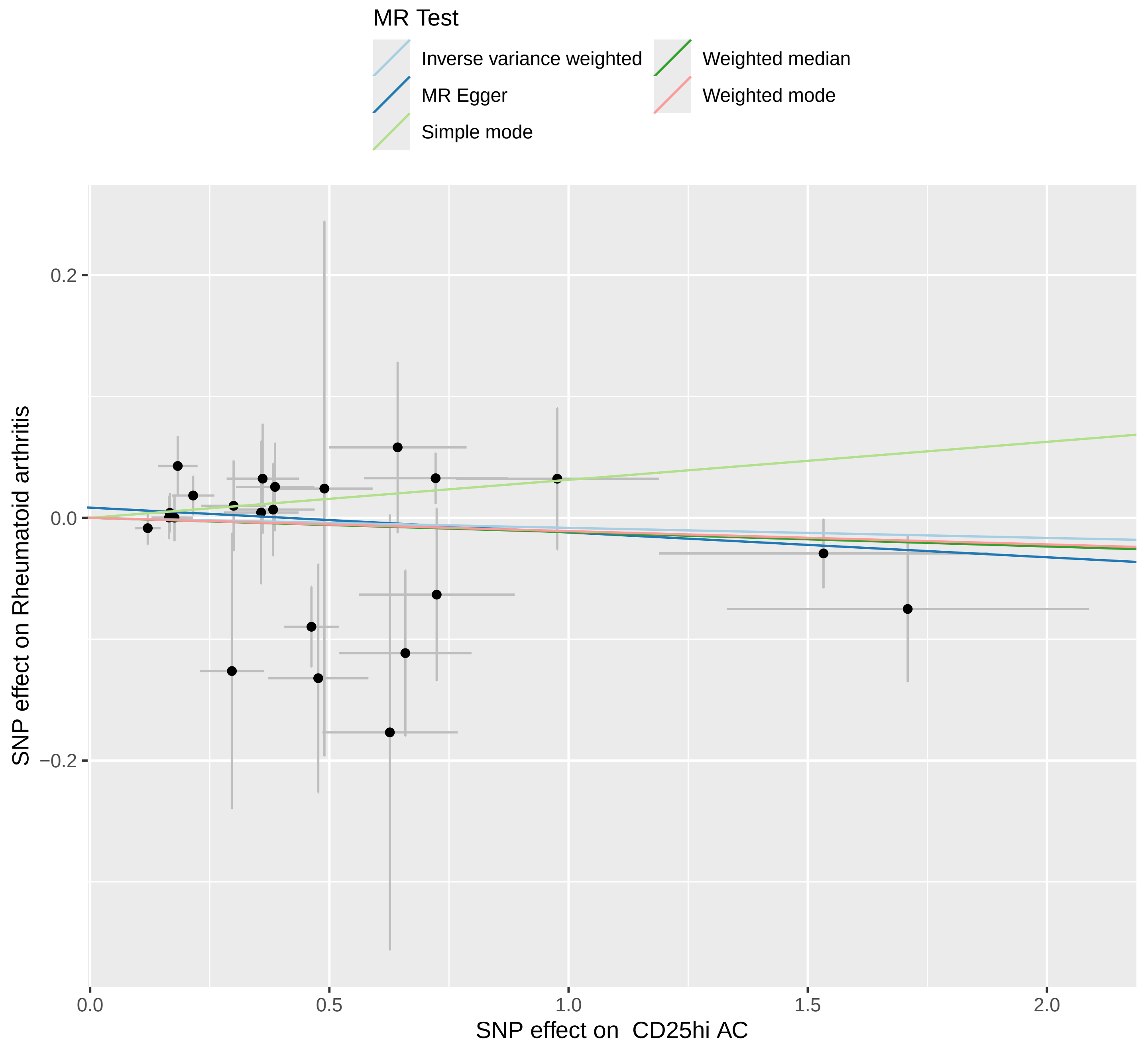

MR Test
Inverse variance weighted
MR Egger
Simple mode
Weighted median
Weighted mode
0.2
0.0
−0.2
SNP effect on Rheumatoid arthritis
0.0
0.5
1.0
1.5
2.0
SNP effect on CD25hi AC

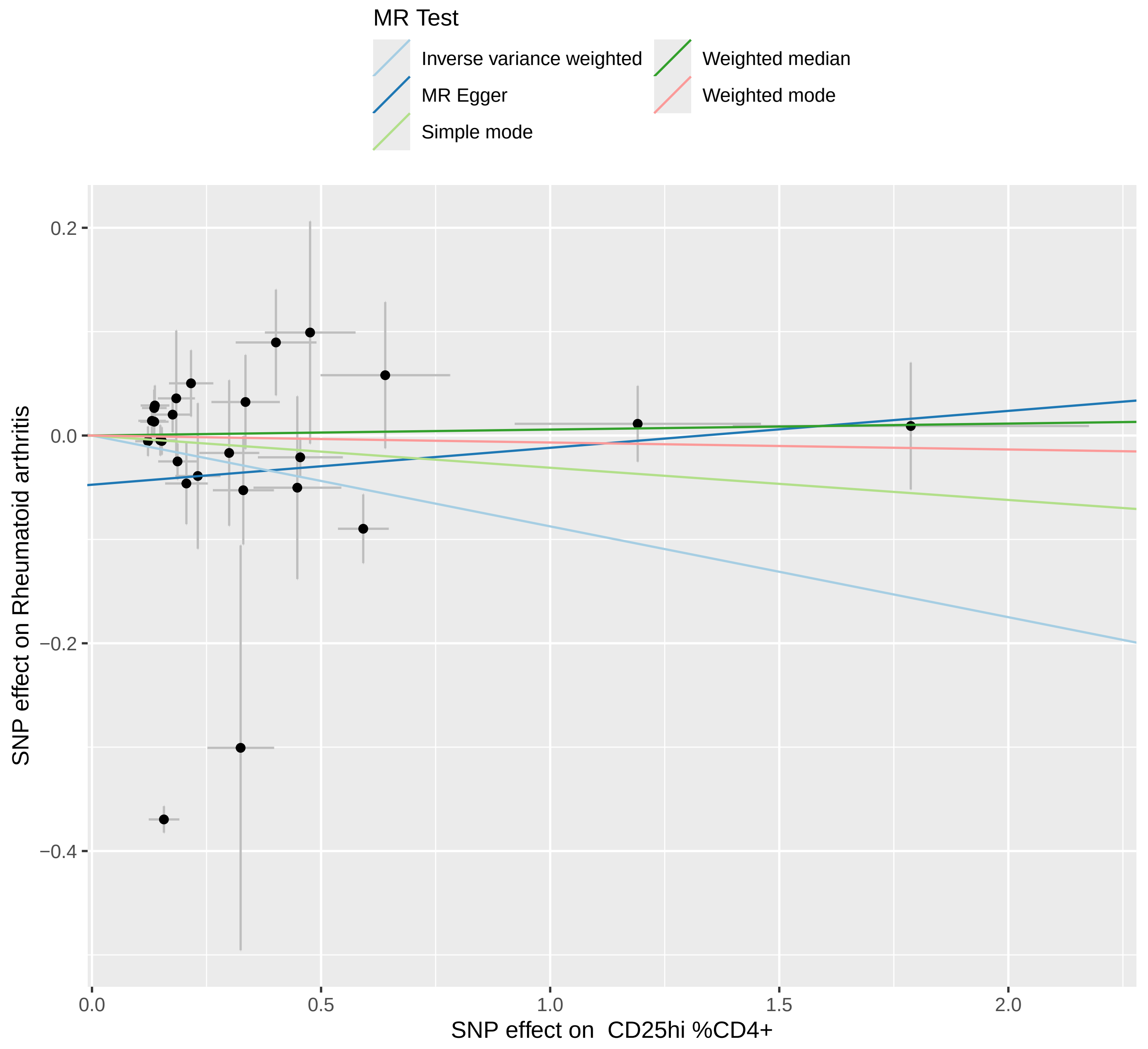
MR Test
Inverse variance weighted
MR Egger
Simple mode
Weighted median
Weighted mode
0.2
0.0
−0.2
−0.4
0.0
0.5
1.0
1.5
2.0
SNP effect on Rheumatoid arthritis
SNP effect on CD25hi %CD4+

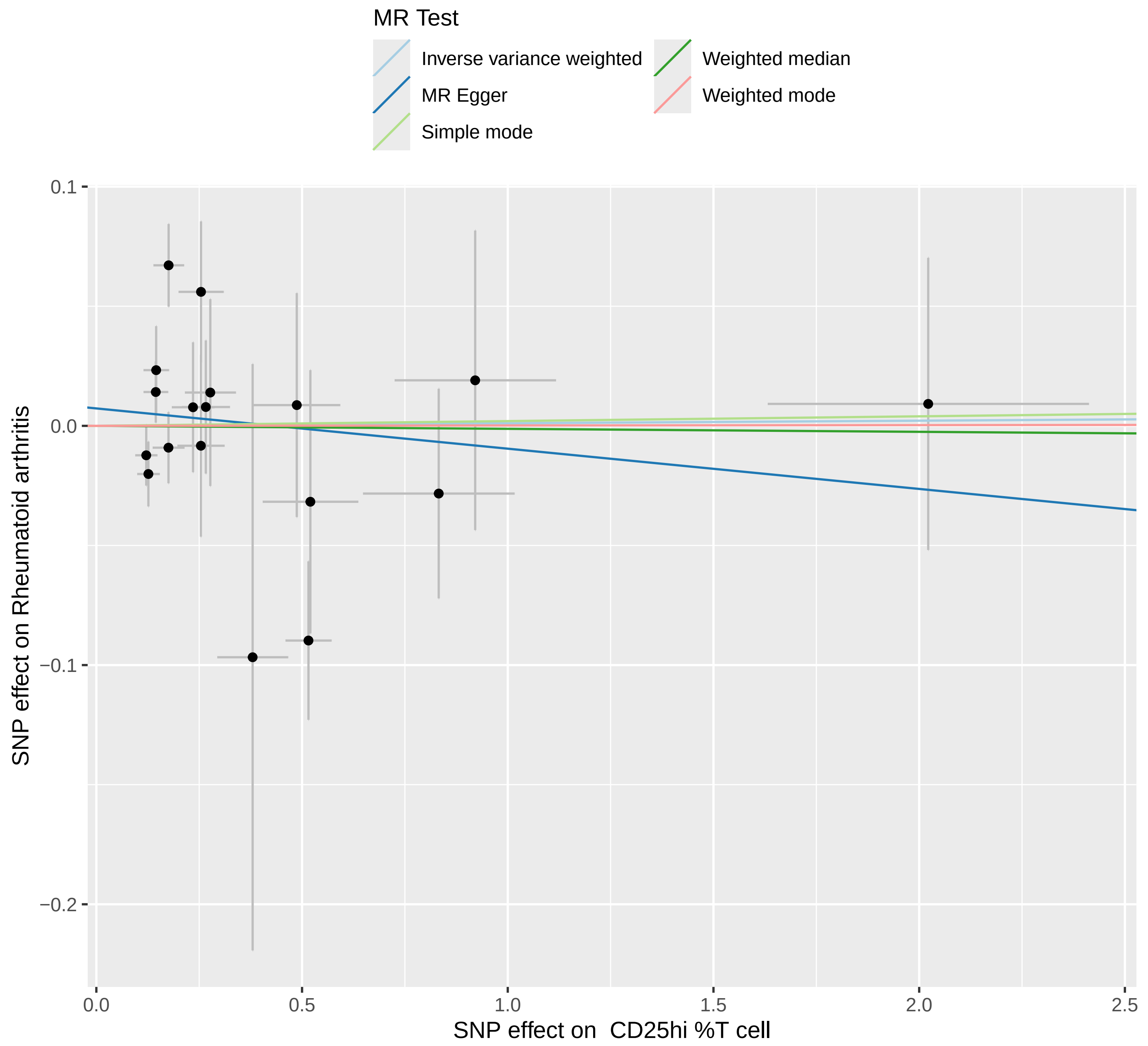
MR Test
Inverse variance weighted
MR Egger
Simple mode
Weighted median
Weighted mode
0.1
0.0
−0.1
−0.2
0.0
0.5
1.0
1.5
2.0
2.5
SNP effect on Rheumatoid arthritis
SNP effect on CD25hi %T cell

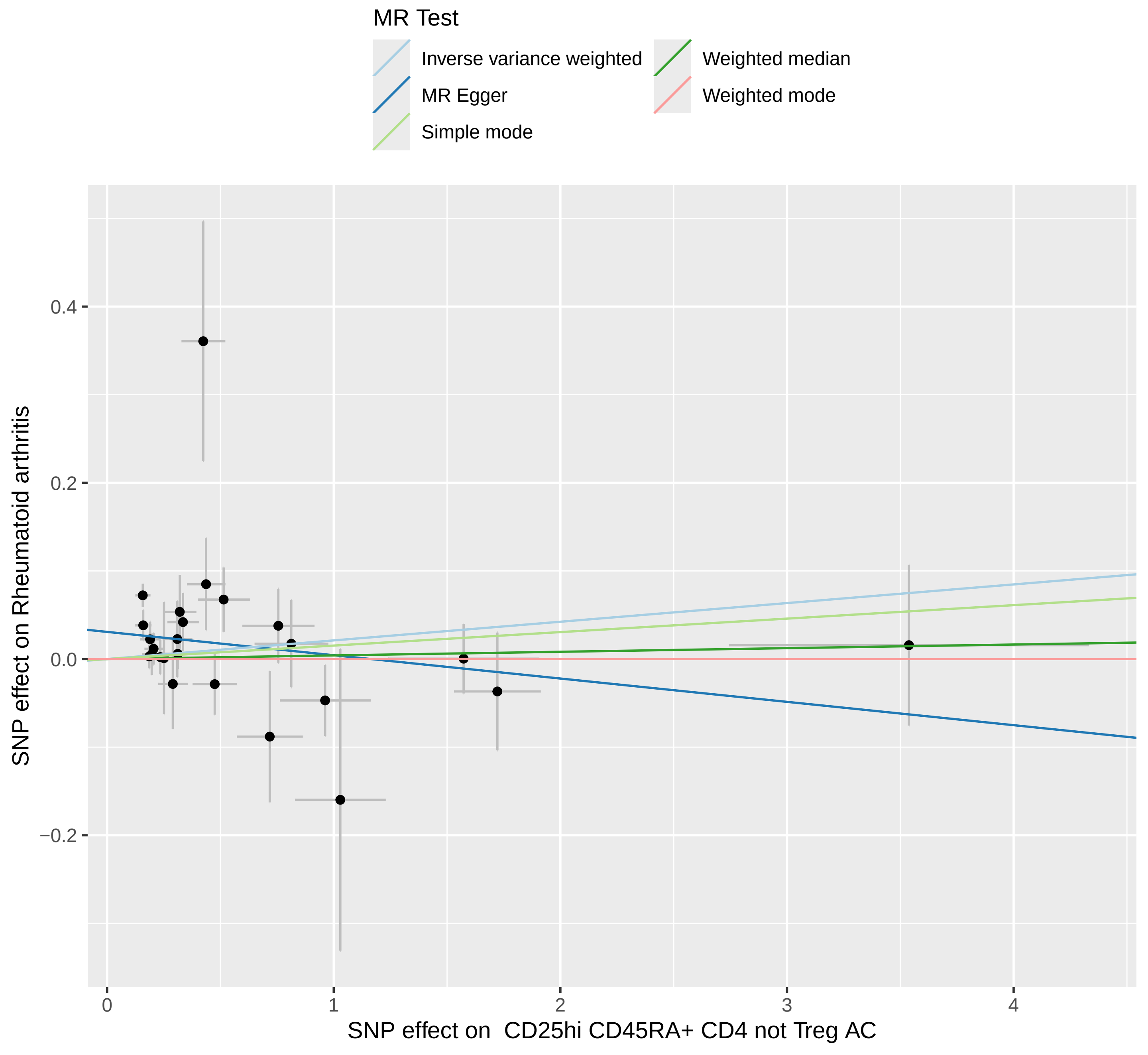
MR Test
Inverse variance weighted
Weighted median
MR Egger
Weighted mode
Simple mode
0.4
0.2
0.0
−0.2
0
1
2
3
4
SNP effect on Rheumatoid arthritis
SNP effect on CD25hi CD45RA+ CD4 not Treg AC

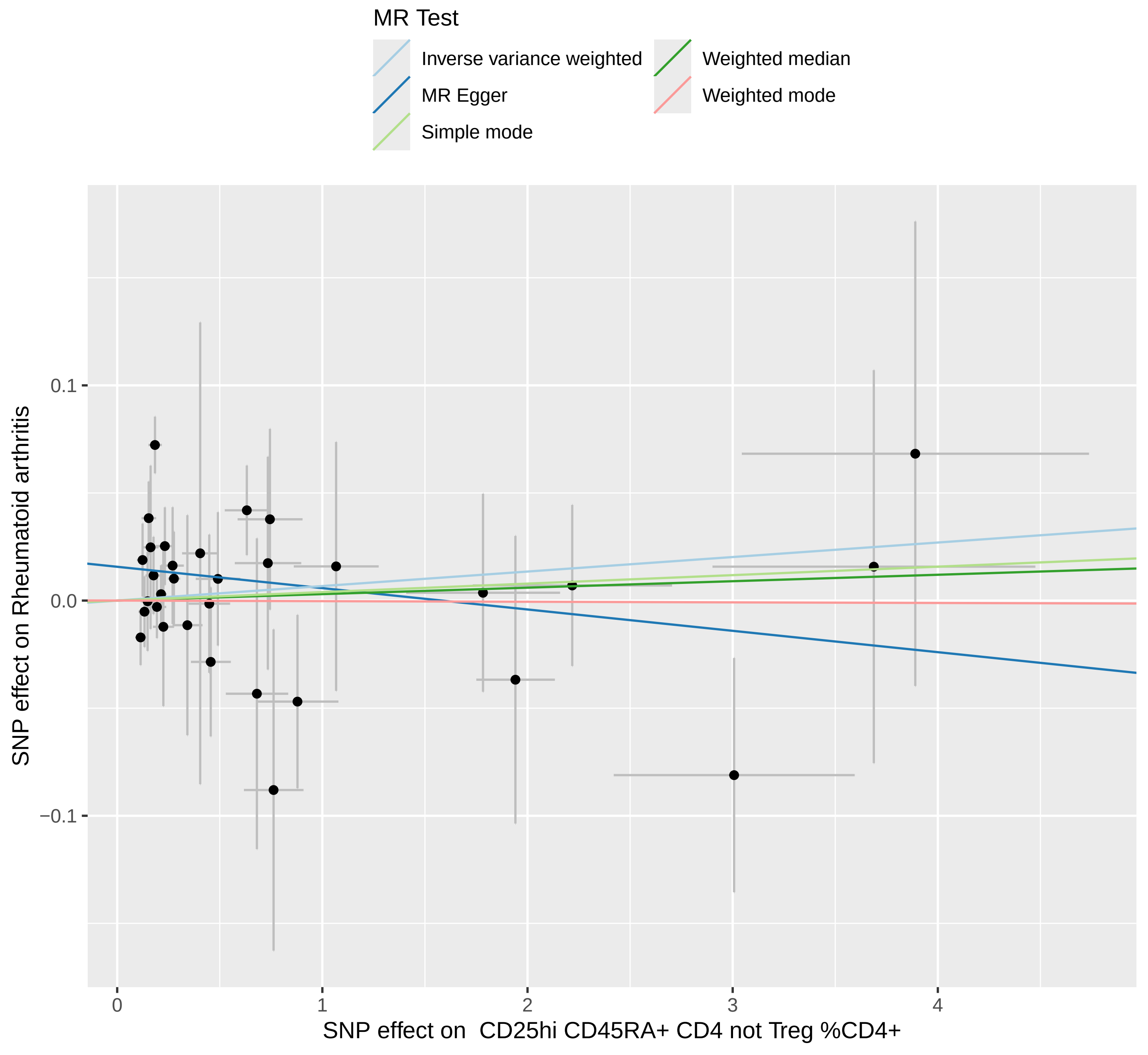

MR Test
Inverse variance weighted
MR Egger
Simple mode
Weighted median
Weighted mode
0.1
0.0
−0.1
0
1
2
3
4
SNP effect on Rheumatoid arthritis
SNP effect on CD25hi CD45RA+ CD4 not Treg %CD4+

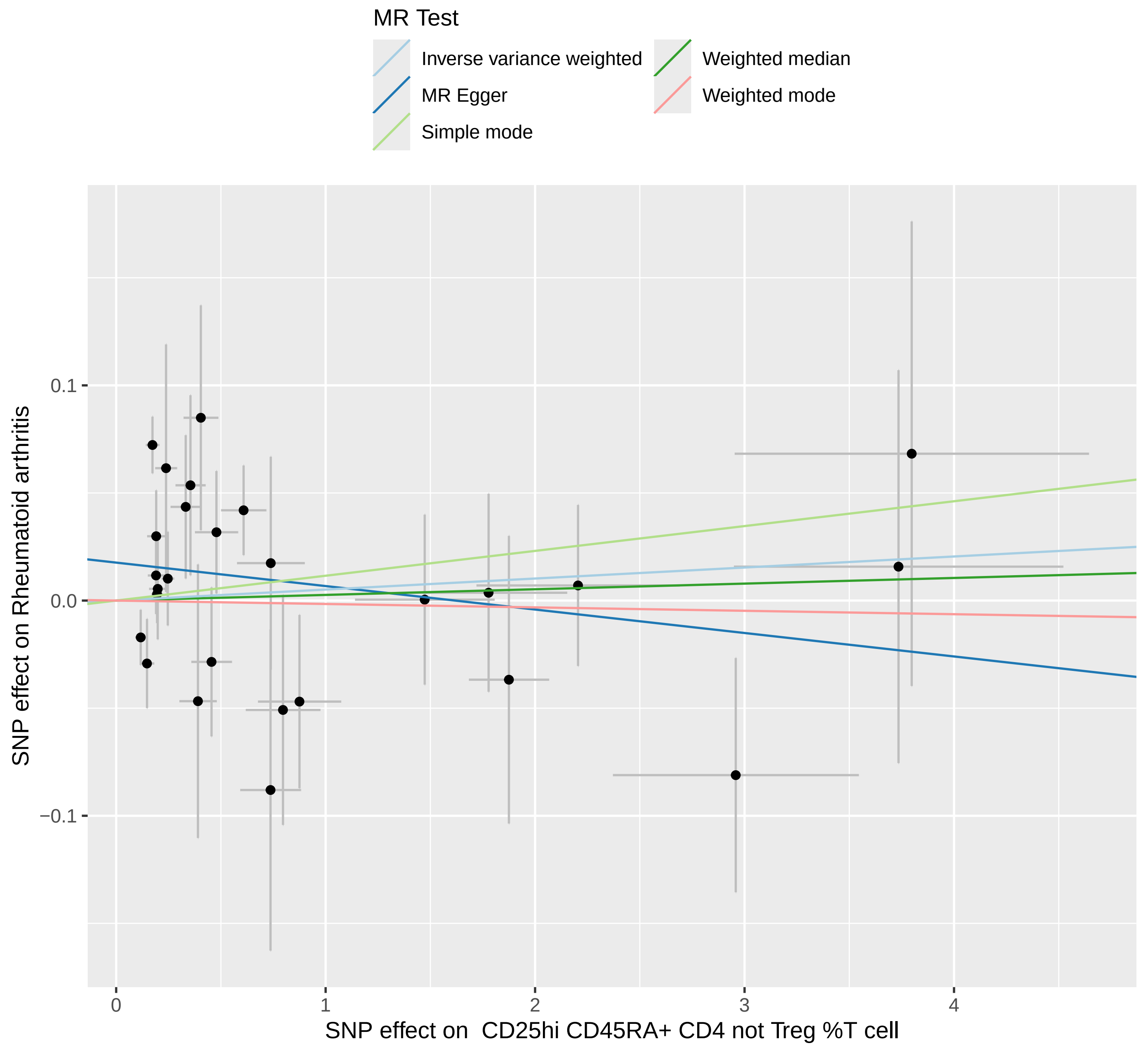

MR Test
Inverse variance weighted
MR Egger
Simple mode
Weighted median
Weighted mode
0.1
0.0
−0.1
0
1
2
3
4
SNP effect on Rheumatoid arthritis
SNP effect on CD25hi CD45RA+ CD4 not Treg %T cell

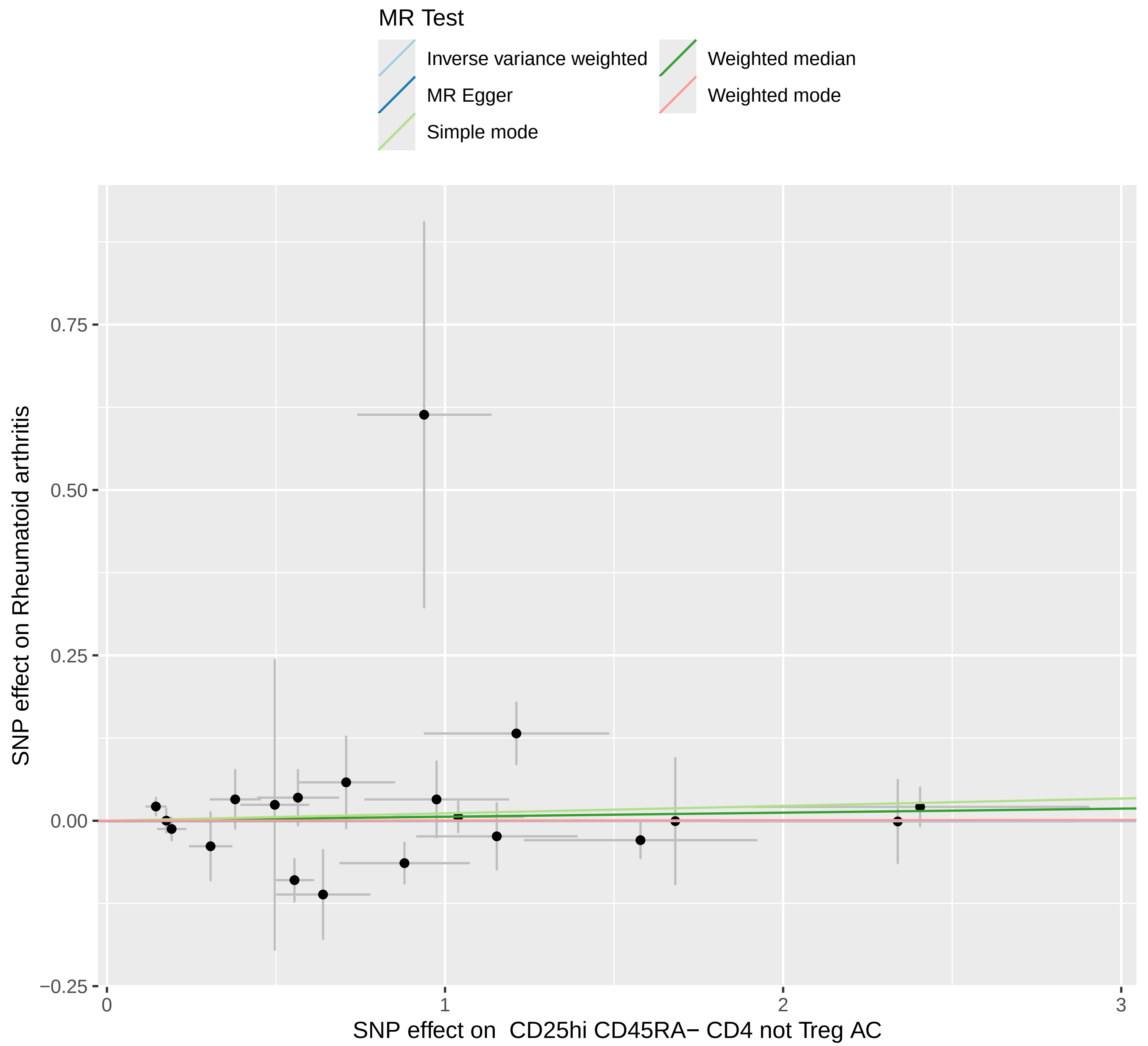
MR Test
Inverse variance weighted
MR Egger
Simple mode
Weighted median
Weighted mode
0.75
0.50
0.25
0.00
−0.25
0
1
2
3
SNP effect on Rheumatoid arthritis
SNP effect on CD25hi CD45RA− CD4 not Treg AC

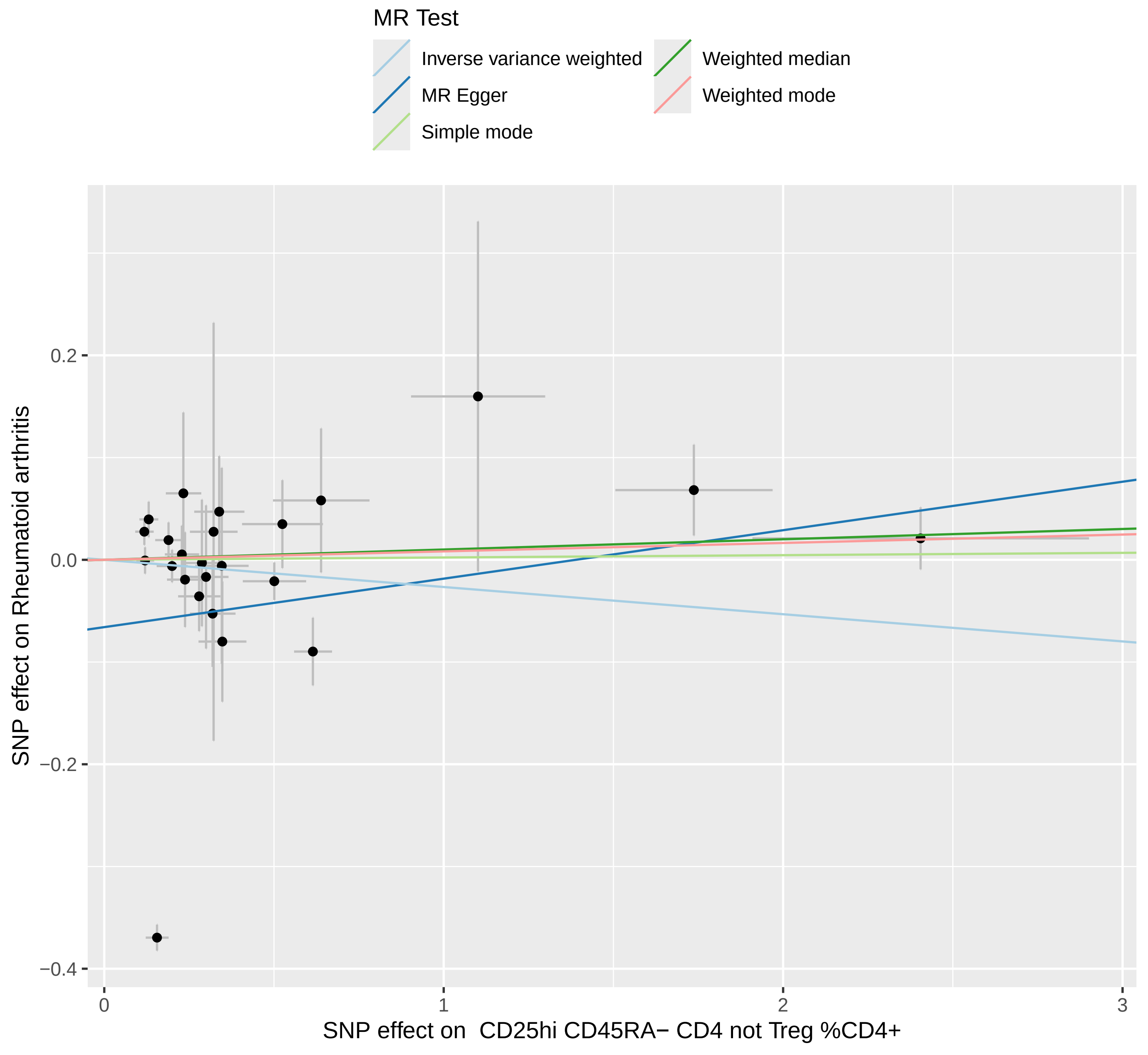
MR Test
Inverse variance weighted
MR Egger
Simple mode
Weighted median
Weighted mode
0.2
0.0
−0.2
−0.4
0
1
2
3
SNP effect on Rheumatoid arthritis
SNP effect on CD25hi CD45RA− CD4 not Treg %CD4+

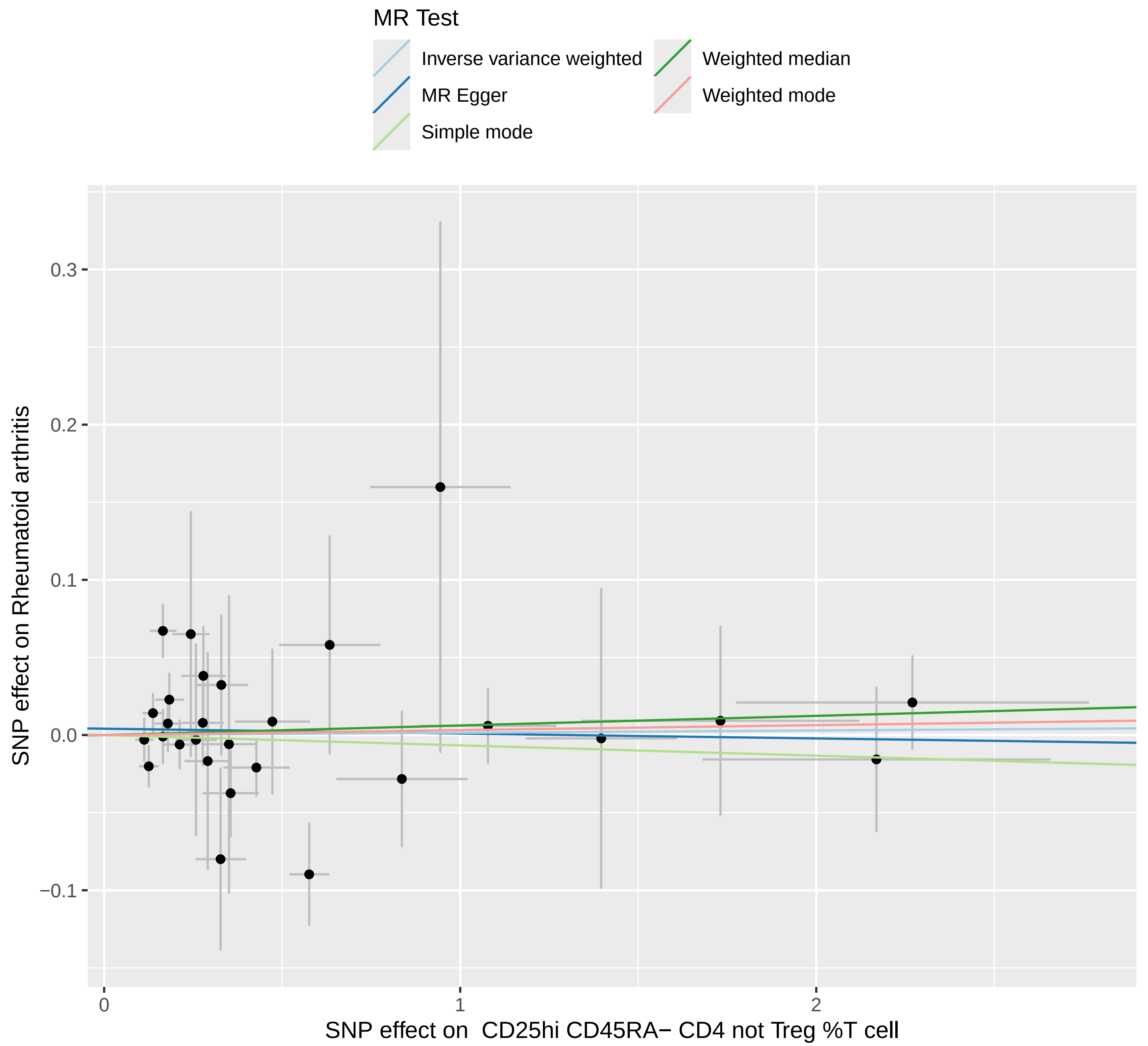

MR Test
Inverse variance weighted
MR Egger
Simple mode
Weighted median
Weighted mode
0.3
0.2
0.1
0.0
−0.1
0
1
2
SNP effect on Rheumatoid arthritis
SNP effect on CD25hi CD45RA− CD4 not Treg %T cell

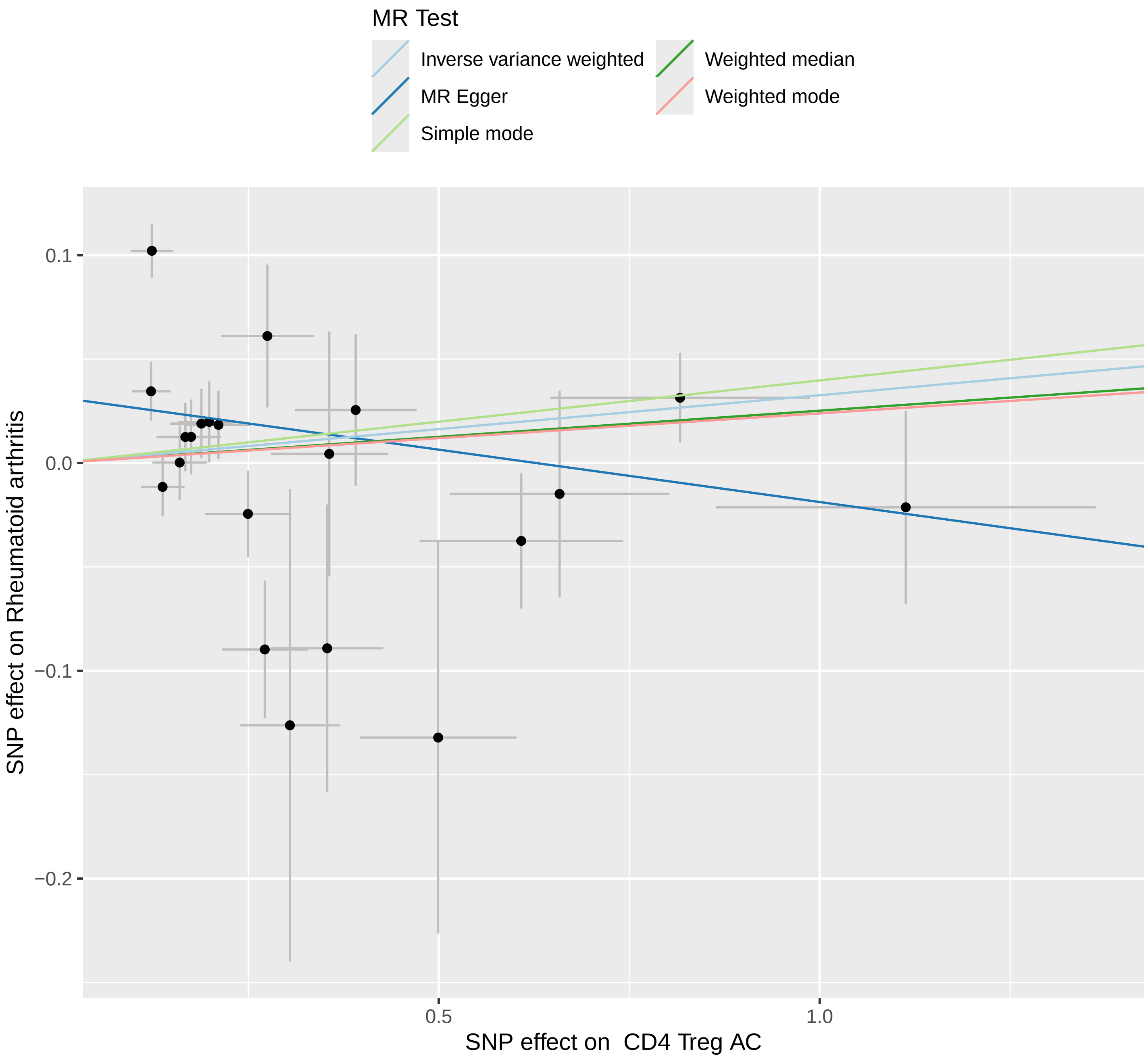

MR Test
Inverse variance weighted
MR Egger
Simple mode
Weighted median
Weighted mode
0.1
0.0
−0.1
−0.2
0.5
1.0
SNP effect on Rheumatoid arthritis
SNP effect on CD4 Treg AC

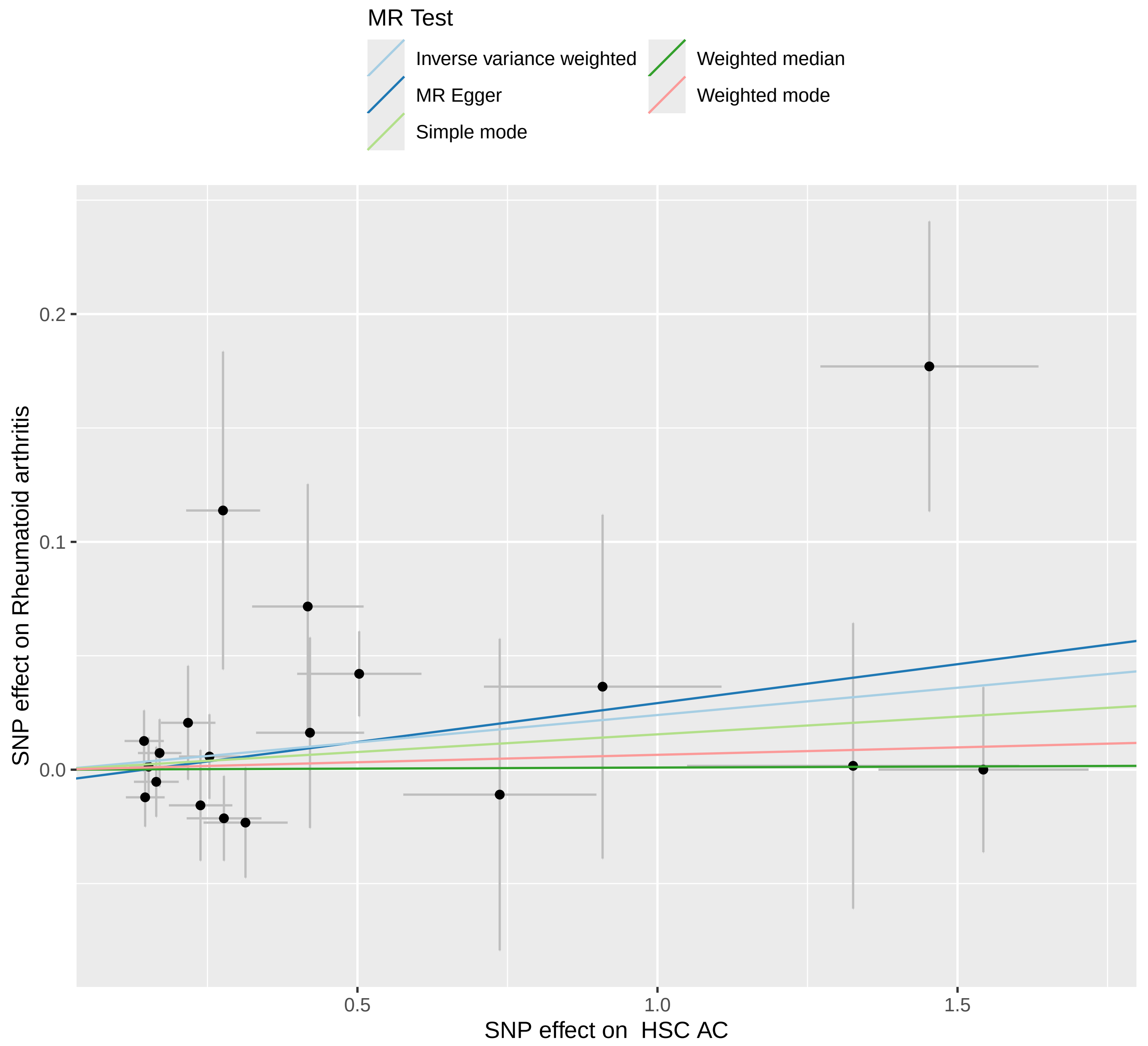

MR Test
Inverse variance weighted
MR Egger
Simple mode
Weighted median
Weighted mode
SNP effect on Rheumatoid arthritis
0.2
0.1
0.0
0.5
1.0
1.5
SNP effect on HSC AC

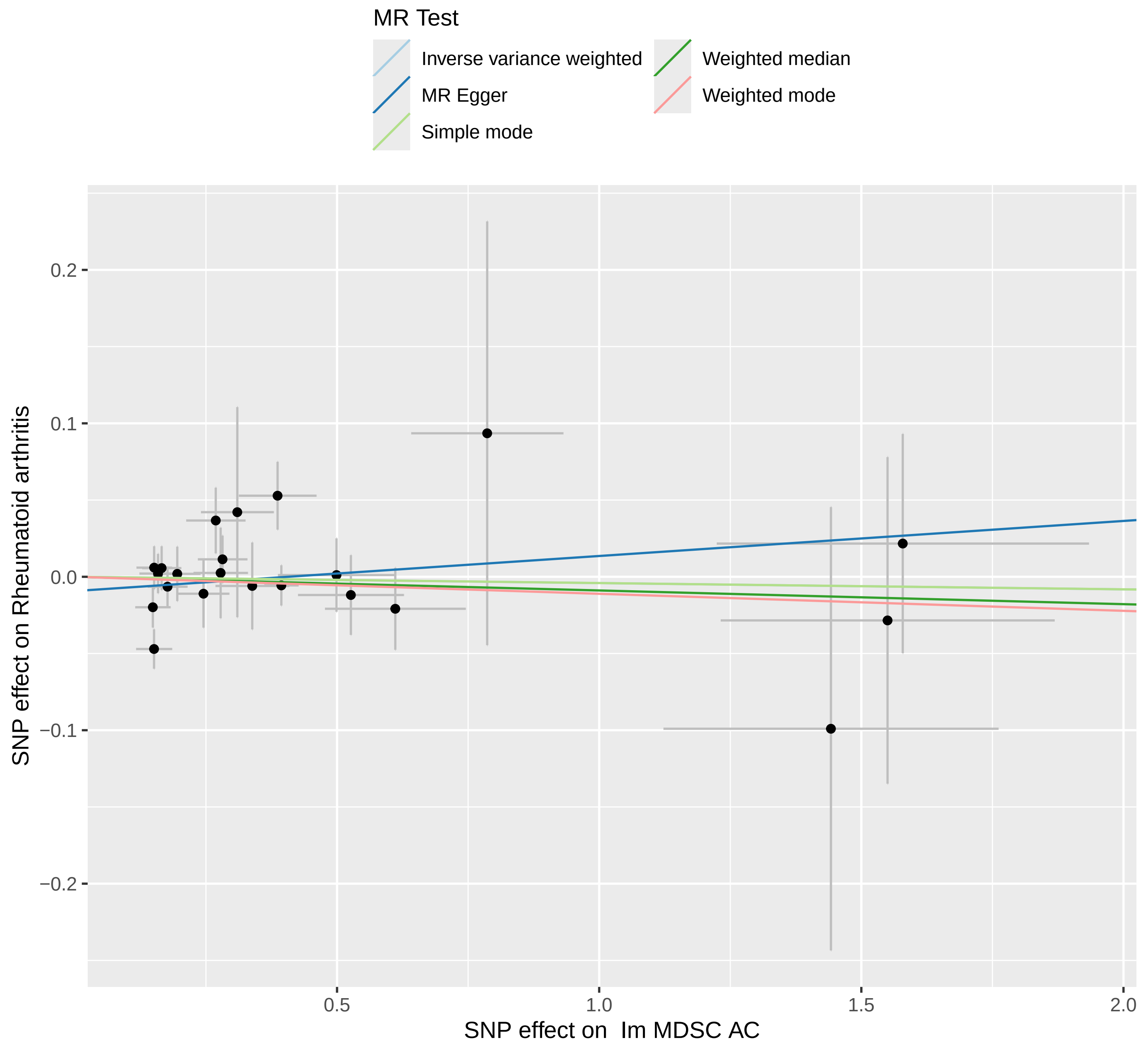

MR Test
Inverse variance weighted
MR Egger
Simple mode
Weighted median
Weighted mode
0.2
0.1
0.0
−0.1
−0.2
SNP effect on Rheumatoid arthritis
0.5
1.0
1.5
2.0
SNP effect on Im MDSC AC

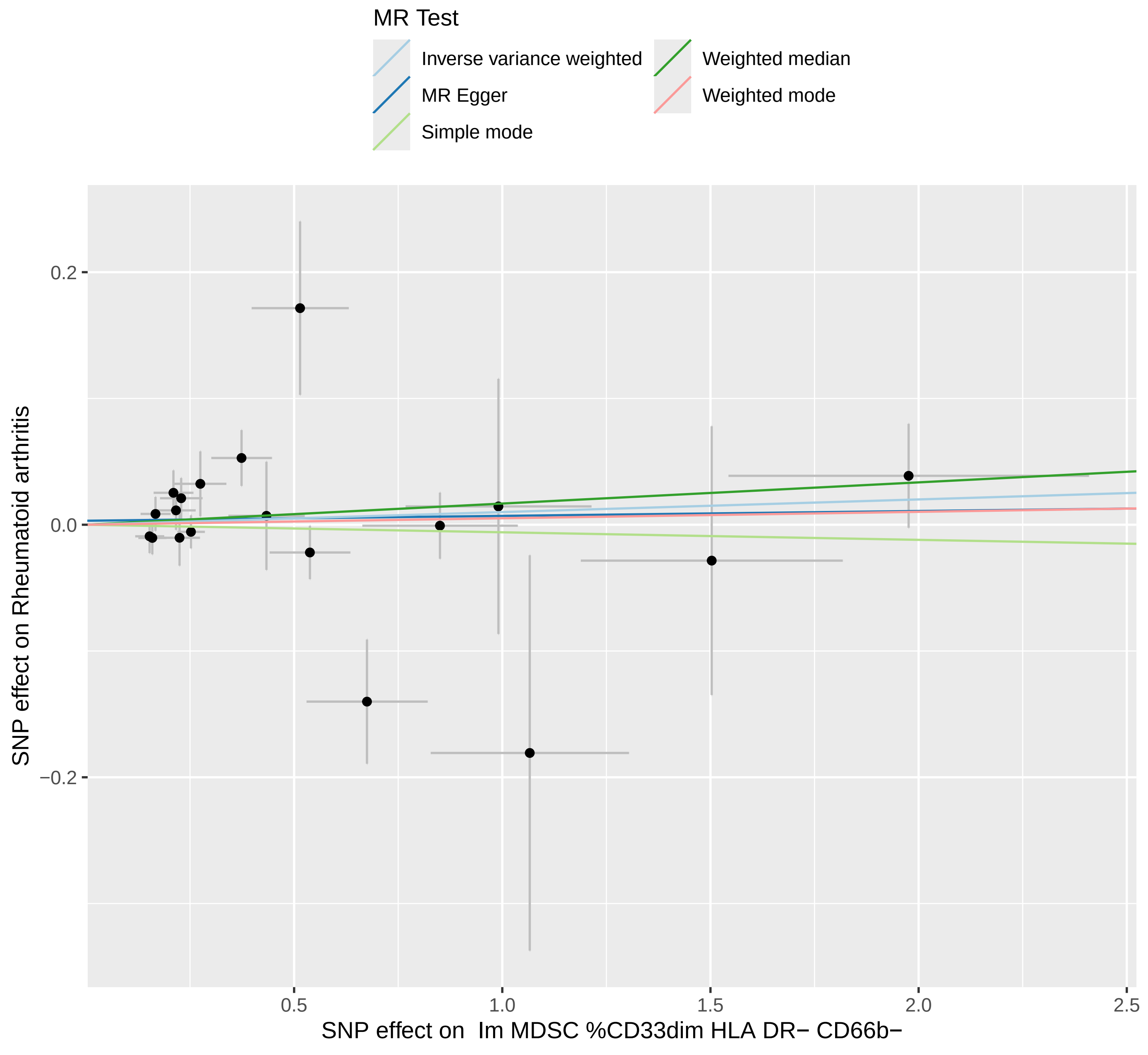

MR Test
Inverse variance weighted
MR Egger
Simple mode
Weighted median
Weighted mode
0.2
0.0
−0.2
0.5
1.0
1.5
2.0
2.5
SNP effect on Rheumatoid arthritis
SNP effect on Im MDSC %CD33dim HLA DR− CD66b−

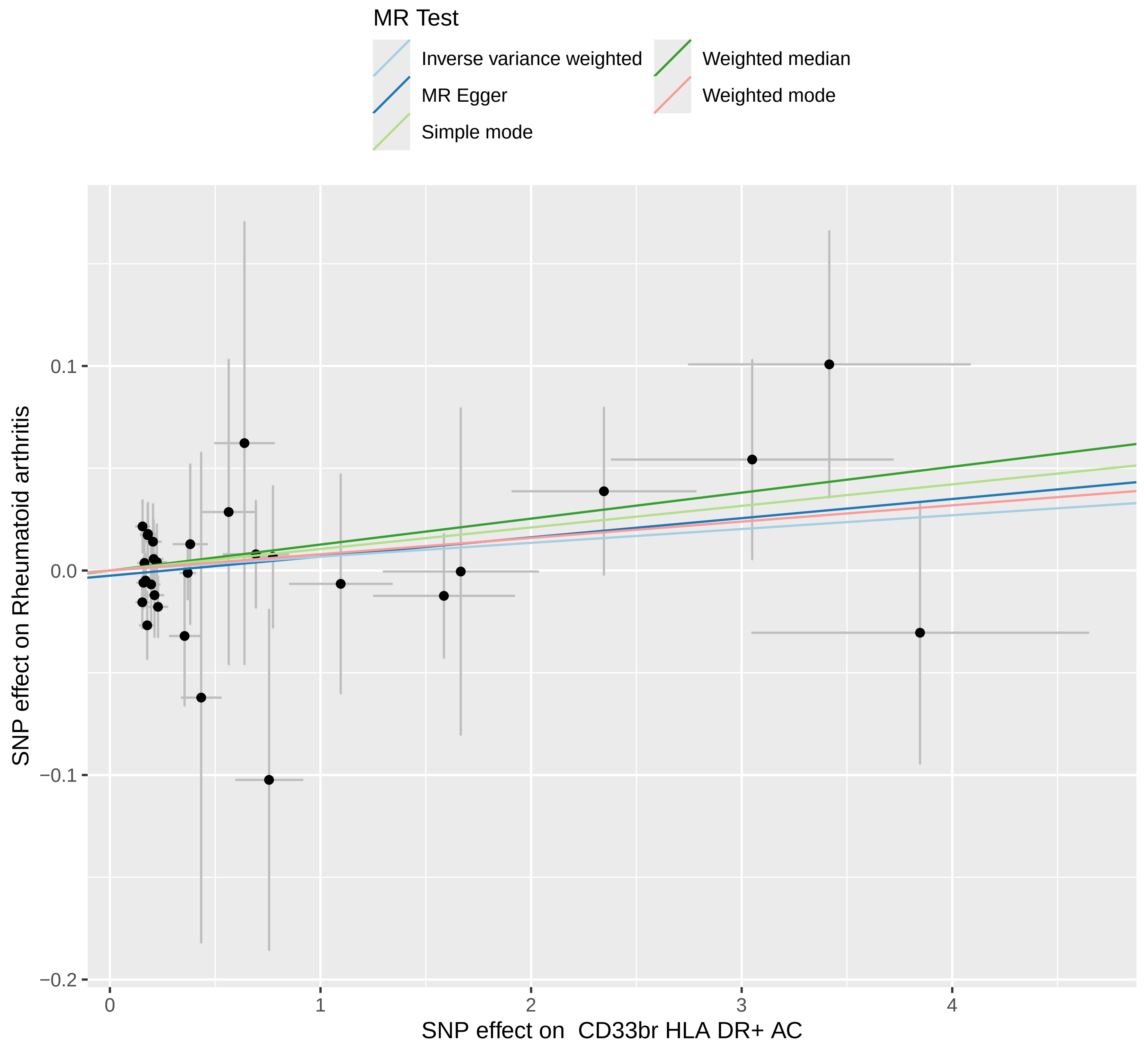

MR Test
Inverse variance weighted
MR Egger
Simple mode
Weighted median
Weighted mode
0.1
0.0
−0.1
−0.2
0
1
2
3
4
SNP effect on Rheumatoid arthritis
SNP effect on CD33br HLA DR+ AC

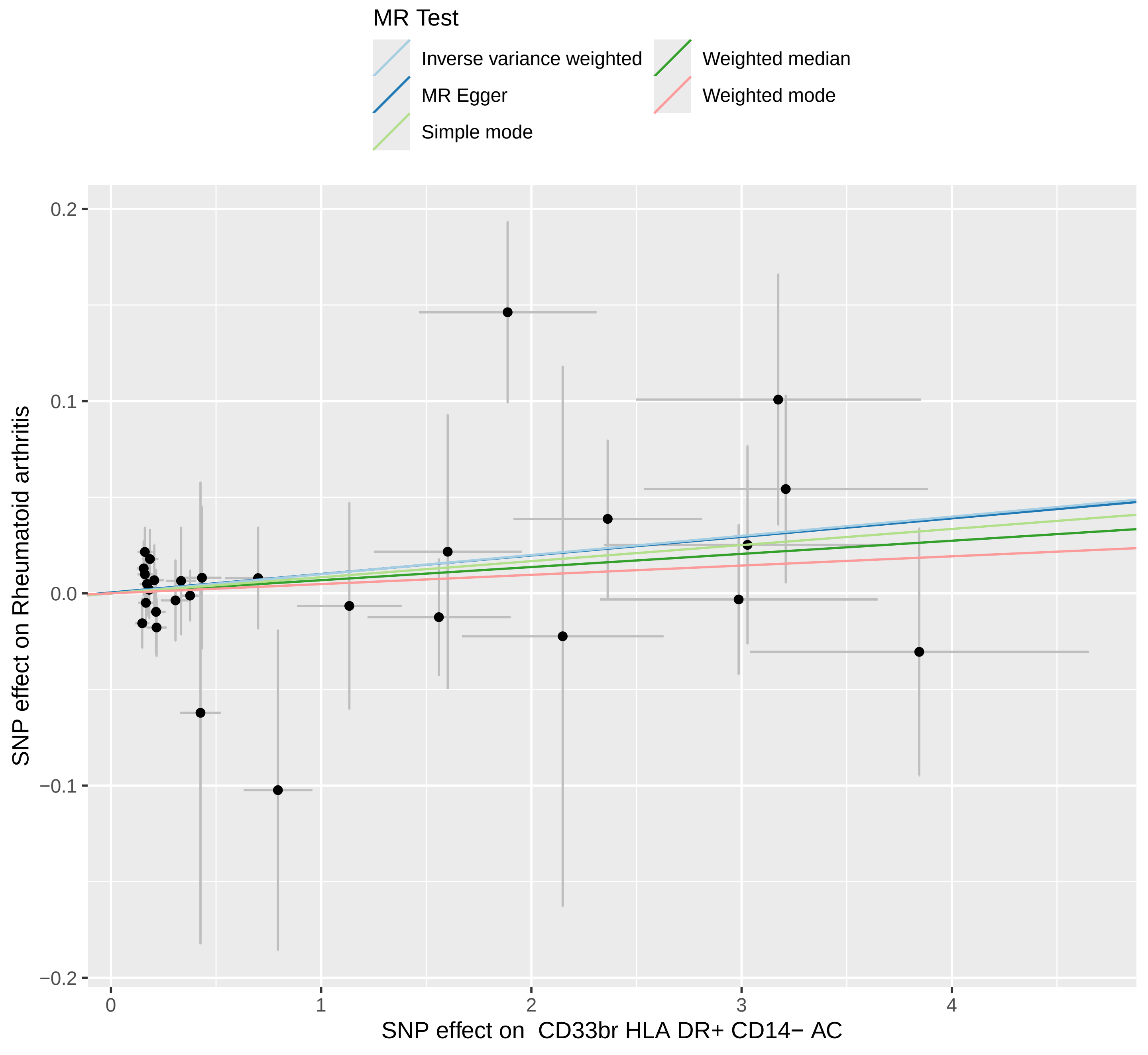
MR Test
Inverse variance weighted
MR Egger
Simple mode
Weighted median
Weighted mode
0.2
0.1
0.0
−0.1
−0.2
0
1
2
3
4
SNP effect on Rheumatoid arthritis
SNP effect on CD33br HLA DR+ CD14− AC

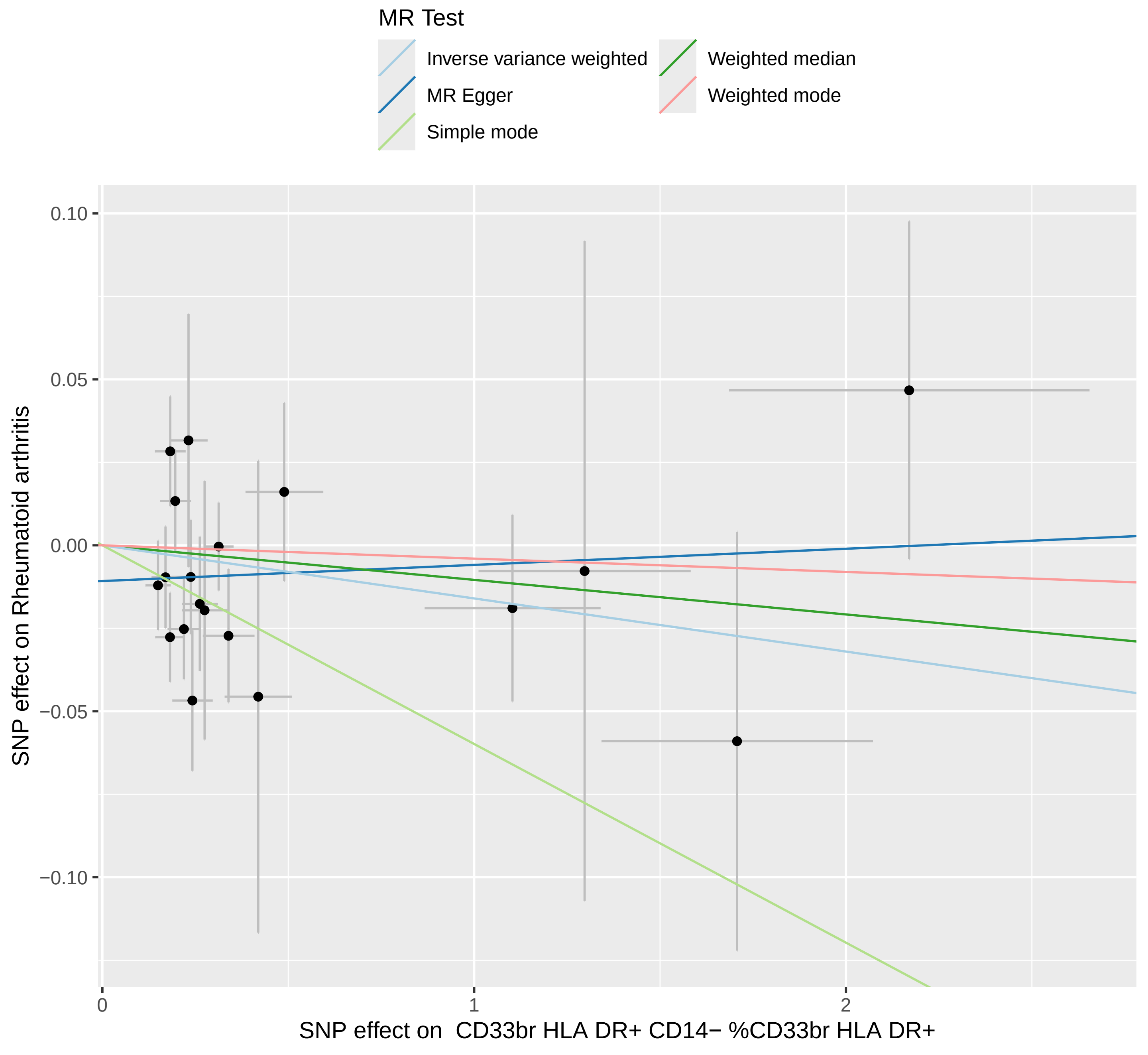

MR Test
Inverse variance weighted
MR Egger
Simple mode
Weighted median
Weighted mode
0.10
0.05
0.00
−0.05
−0.10
SNP effect on Rheumatoid arthritis
0
1
2
SNP effect on CD33br HLA DR+ CD14− %CD33br HLA DR+

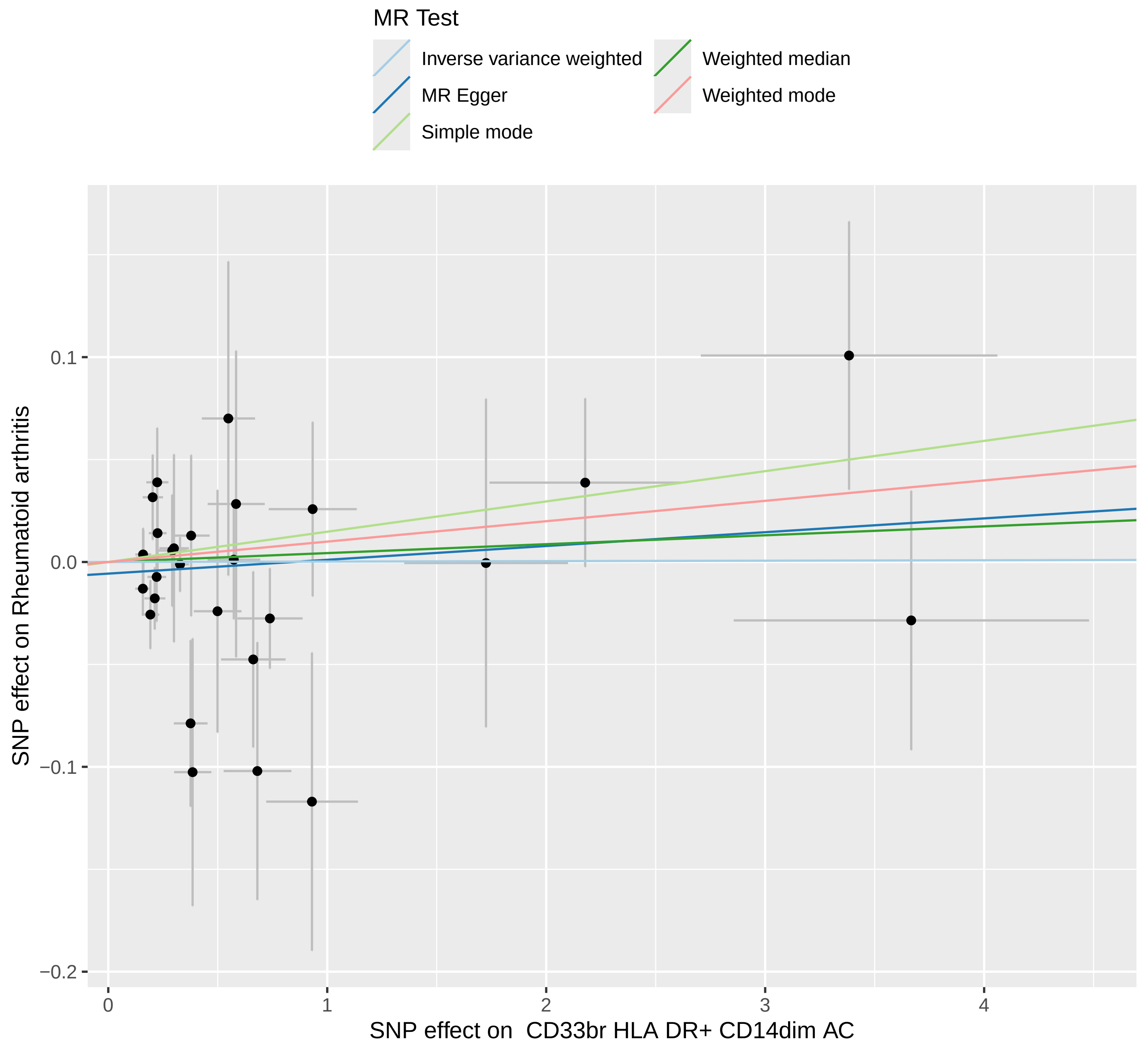
MR Test
Inverse variance weighted
MR Egger
Simple mode
Weighted median
Weighted mode
SNP effect on Rheumatoid arthritis
0.1
0.0
−0.1
−0.2
0
1
2
3
4
SNP effect on CD33br HLA DR+ CD14dim AC

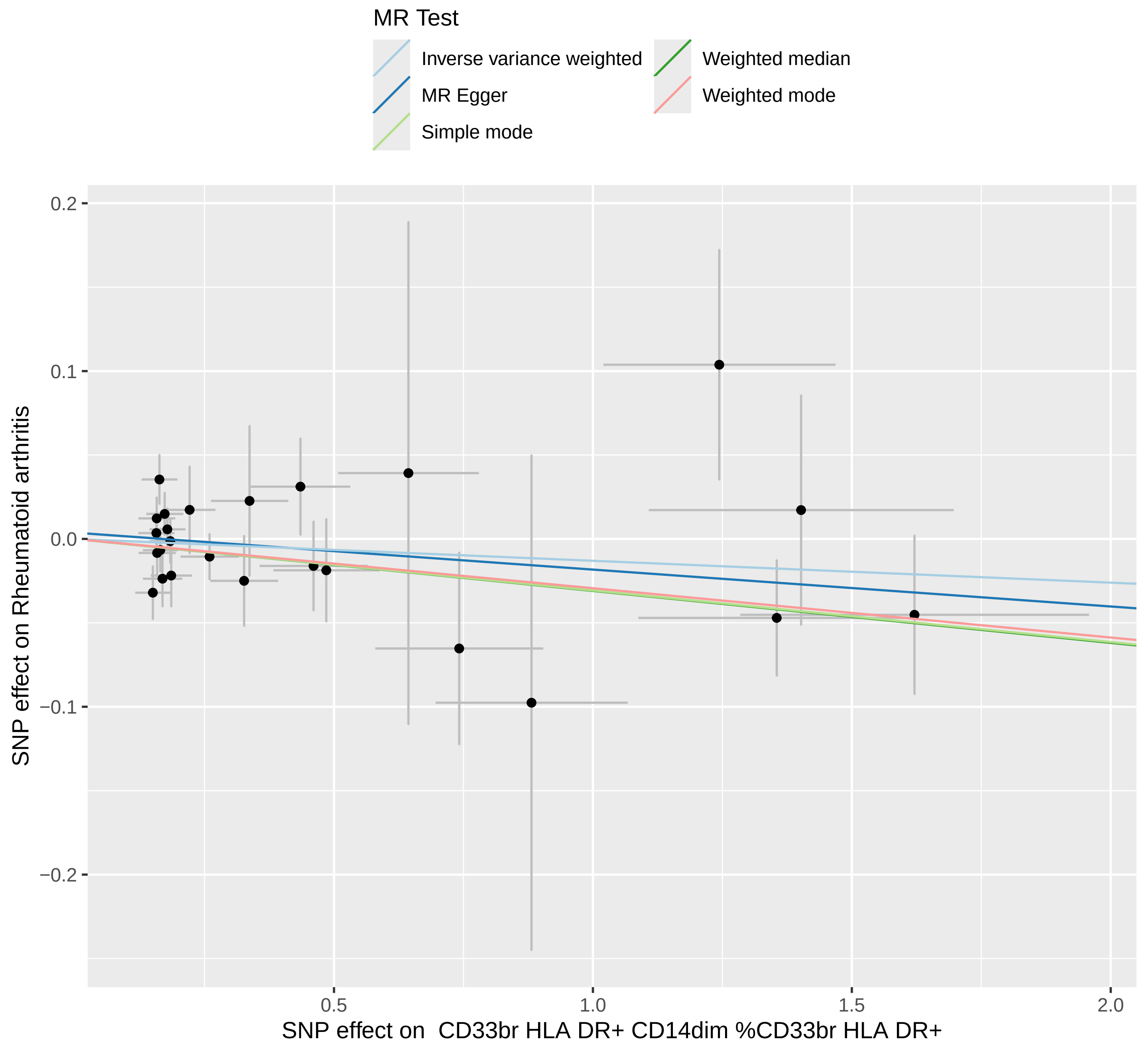

MR Test
Inverse variance weighted
MR Egger
Simple mode
Weighted median
Weighted mode
0.2
0.1
0.0
−0.1
−0.2
SNP effect on Rheumatoid arthritis
0.5
1.0
1.5
2.0
SNP effect on CD33br HLA DR+ CD14dim %CD33br HLA DR+

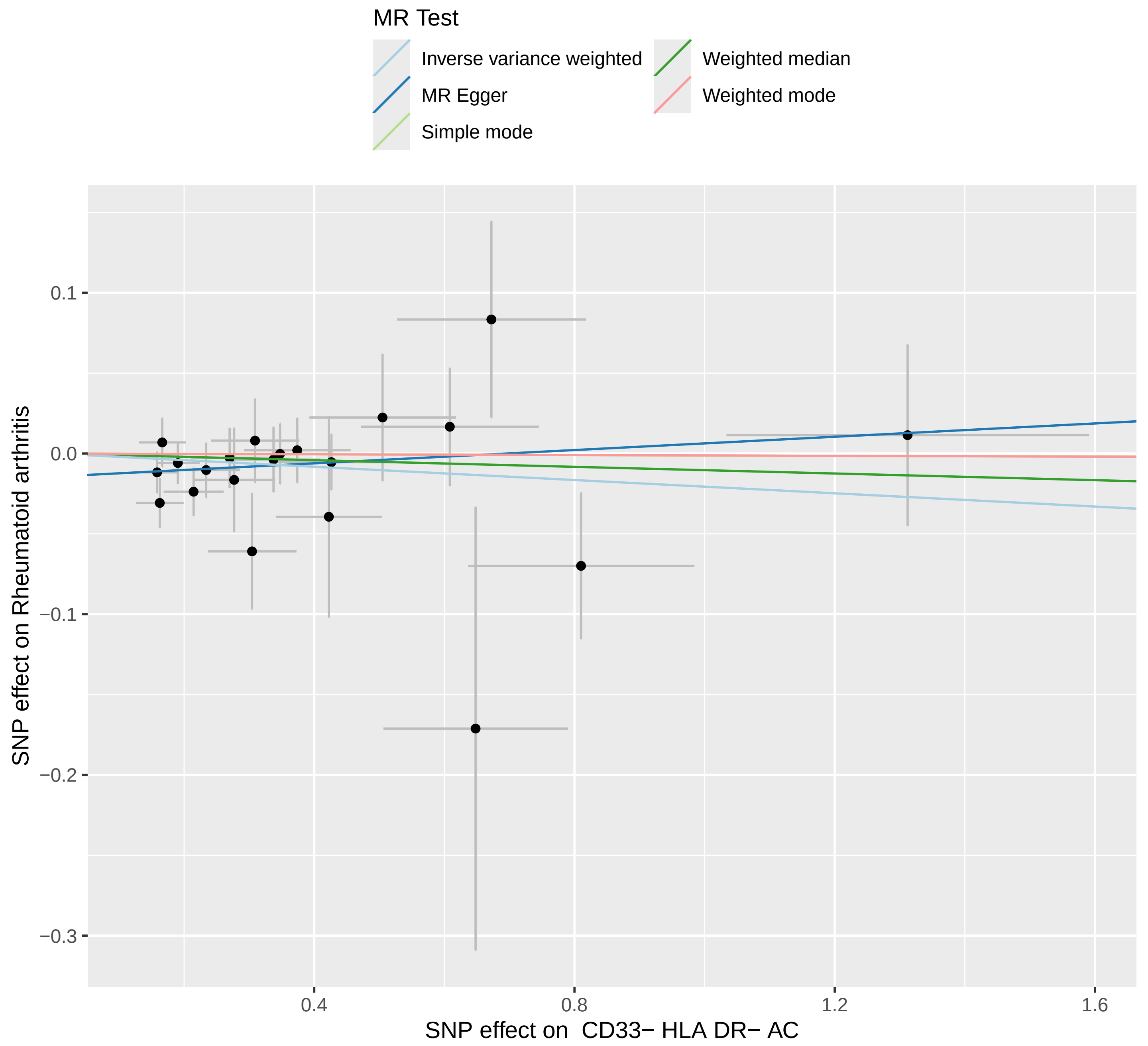

MR Test
Inverse variance weighted
MR Egger
Simple mode
Weighted median
Weighted mode
0.1
0.0
−0.1
−0.2
−0.3
SNP effect on Rheumatoid arthritis
0.4
0.8
1.2
1.6
SNP effect on CD33− HLA DR− AC

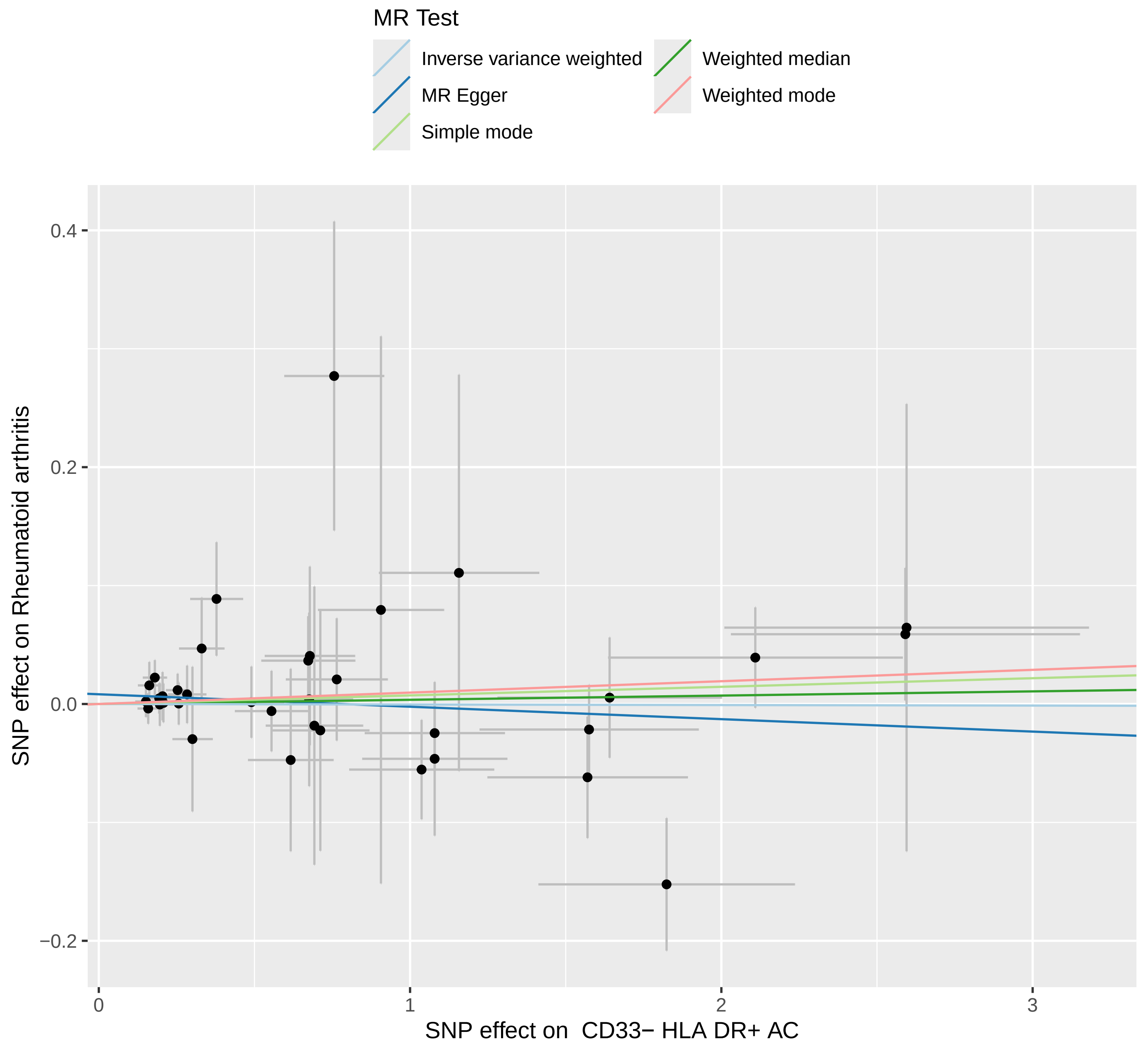

MR Test
Inverse variance weighted
MR Egger
Simple mode
Weighted median
Weighted mode
SNP effect on Rheumatoid arthritis
0.4
0.2
0.0
−0.2
0
1
2
3
SNP effect on CD33− HLA DR+ AC

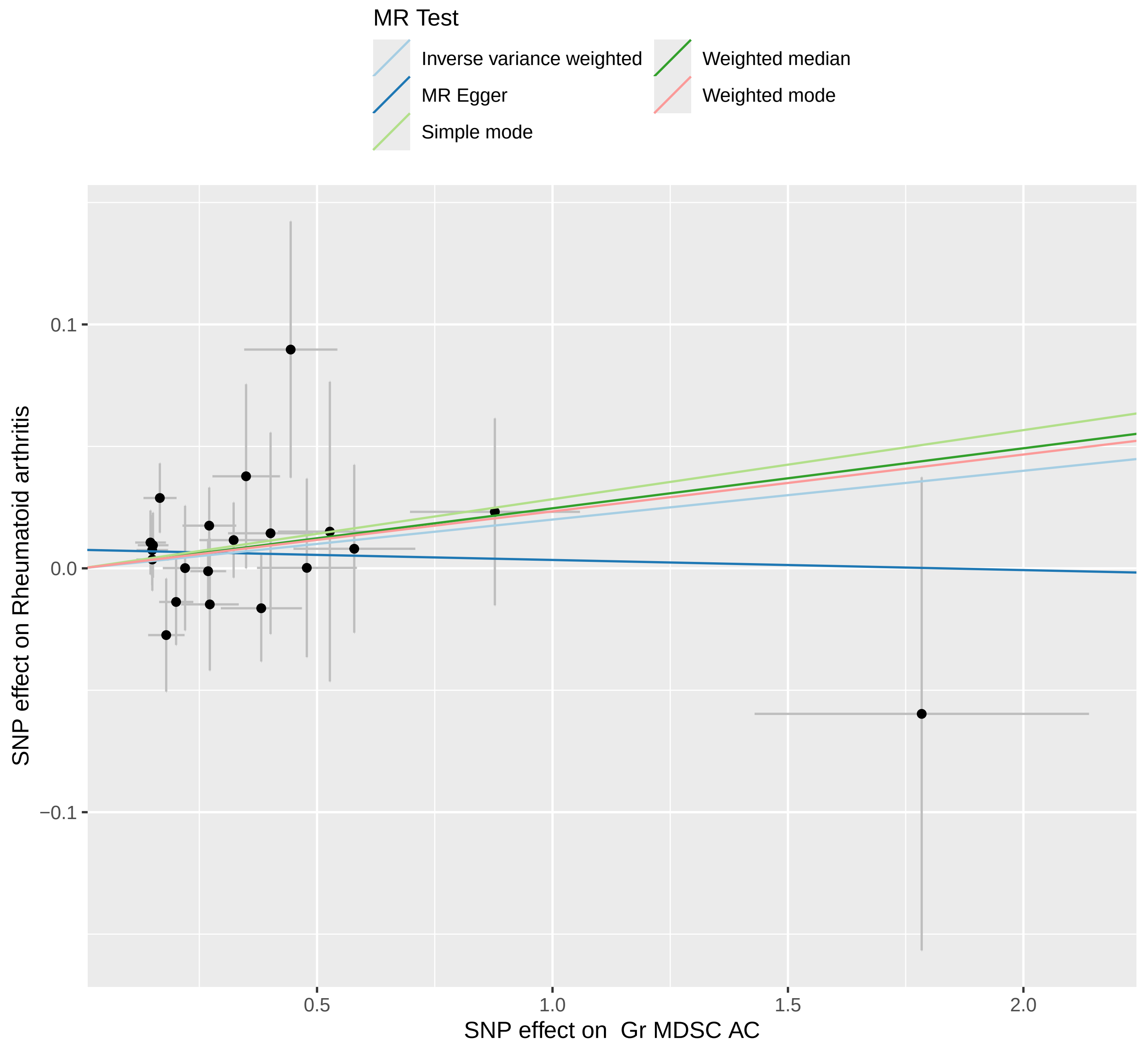
MR Test
Inverse variance weighted
MR Egger
Simple mode
Weighted median
Weighted mode
0.1
0.0
−0.1
0.5
1.0
1.5
2.0
SNP effect on Rheumatoid arthritis
SNP effect on Gr MDSC AC

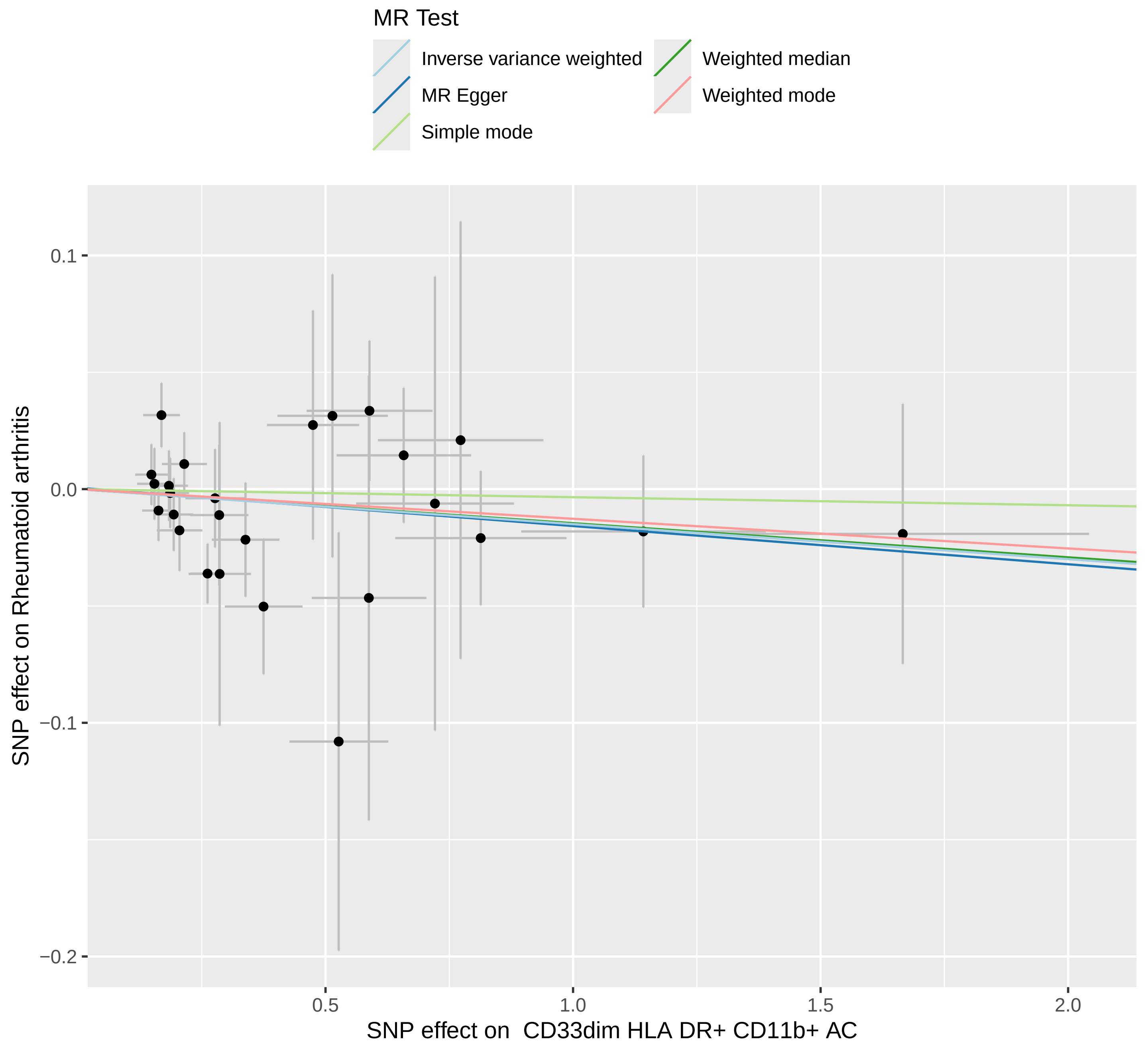
MR Test
Inverse variance weighted
MR Egger
Simple mode
Weighted median
Weighted mode
SNP effect on Rheumatoid arthritis
0.1
0.0
−0.1
−0.2
0.5
1.0
1.5
2.0
SNP effect on CD33dim HLA DR+ CD11b+ AC

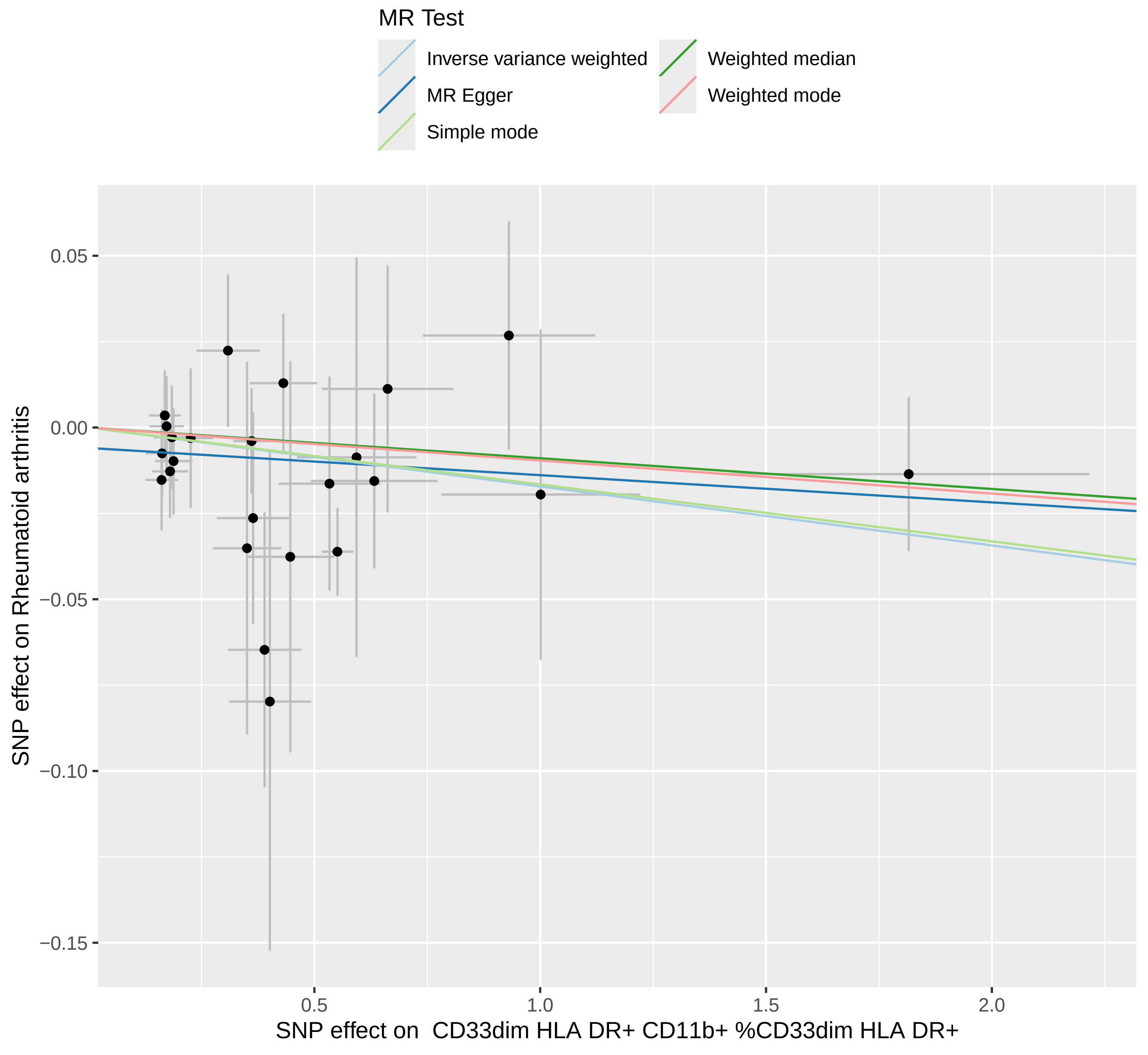
MR Test
Inverse variance weighted
MR Egger
Simple mode
Weighted median
Weighted mode
0.05
0.00
−0.05
−0.10
−0.15
0.5
1.0
1.5
2.0
SNP effect on Rheumatoid arthritis
SNP effect on CD33dim HLA DR+ CD11b+ %CD33dim HLA DR+

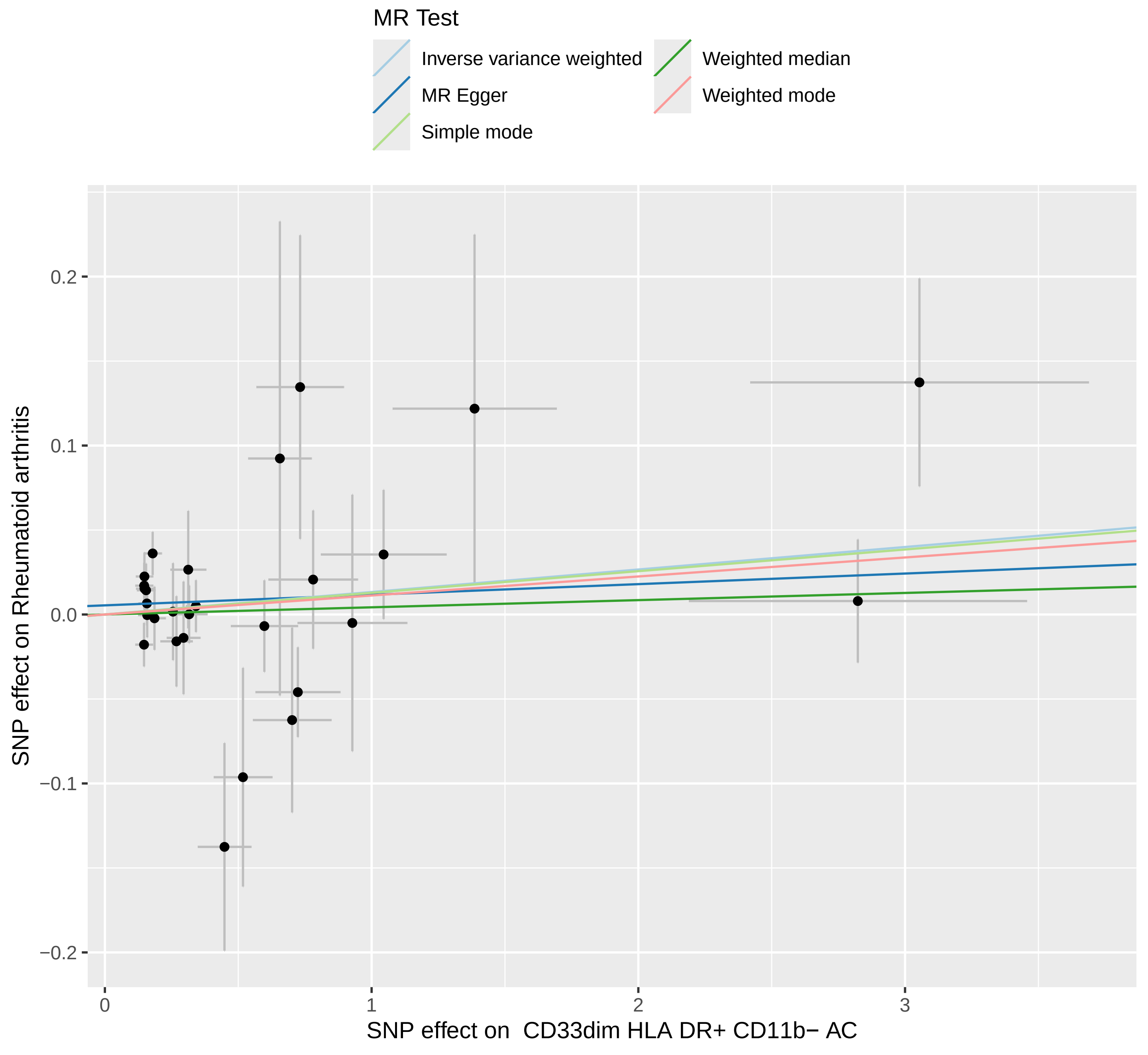

MR Test
Inverse variance weighted
MR Egger
Simple mode
Weighted median
Weighted mode
SNP effect on Rheumatoid arthritis
0.2
0.1
0.0
−0.1
−0.2
0
1
2
3
SNP effect on CD33dim HLA DR+ CD11b− AC

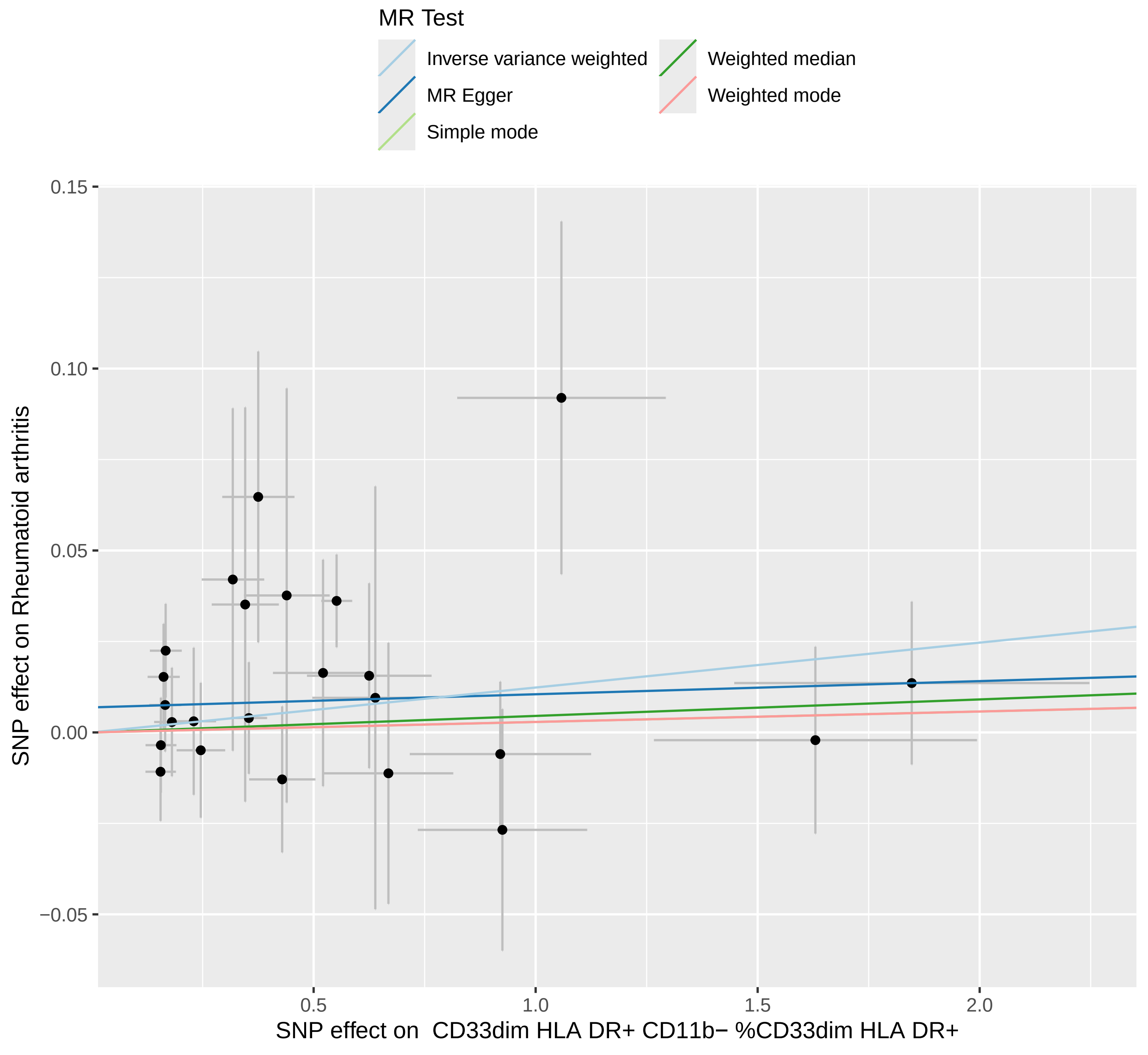
MR Test
Inverse variance weighted
MR Egger
Simple mode
Weighted median
Weighted mode
0.15
0.10
0.05
0.00
−0.05
0.5
1.0
1.5
2.0
SNP effect on Rheumatoid arthritis
SNP effect on CD33dim HLA DR+ CD11b− %CD33dim HLA DR+

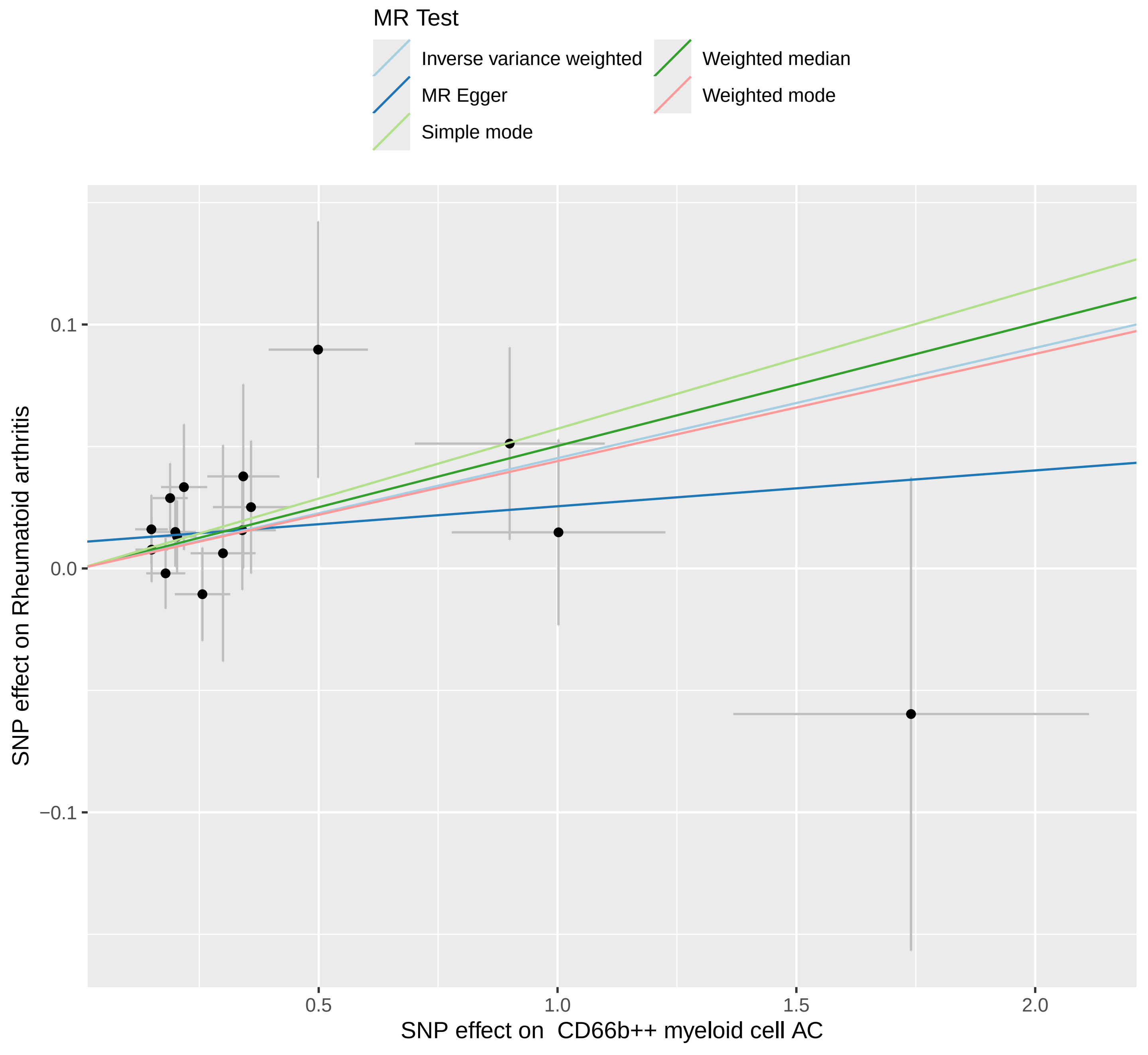

MR Test
Inverse variance weighted
MR Egger
Simple mode
Weighted median
Weighted mode
SNP effect on Rheumatoid arthritis
0.1
0.0
−0.1
0.5
1.0
1.5
2.0
SNP effect on CD66b++ myeloid cell AC

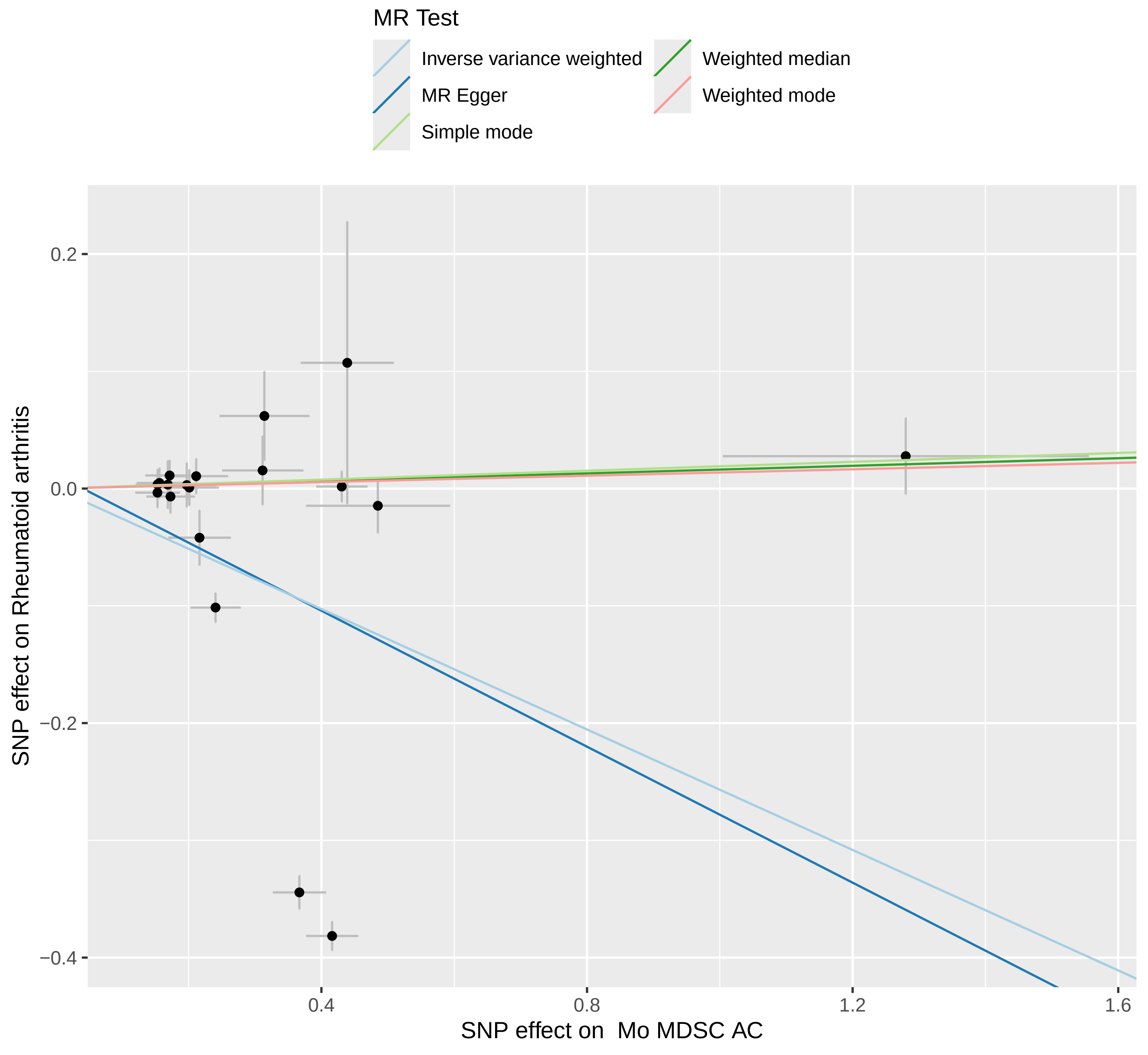
MR Test
Inverse variance weighted
MR Egger
Simple mode
Weighted median
Weighted mode
0.2
0.0
−0.2
−0.4
0.4
0.8
1.2
1.6
SNP effect on Rheumatoid arthritis
SNP effect on Mo MDSC AC

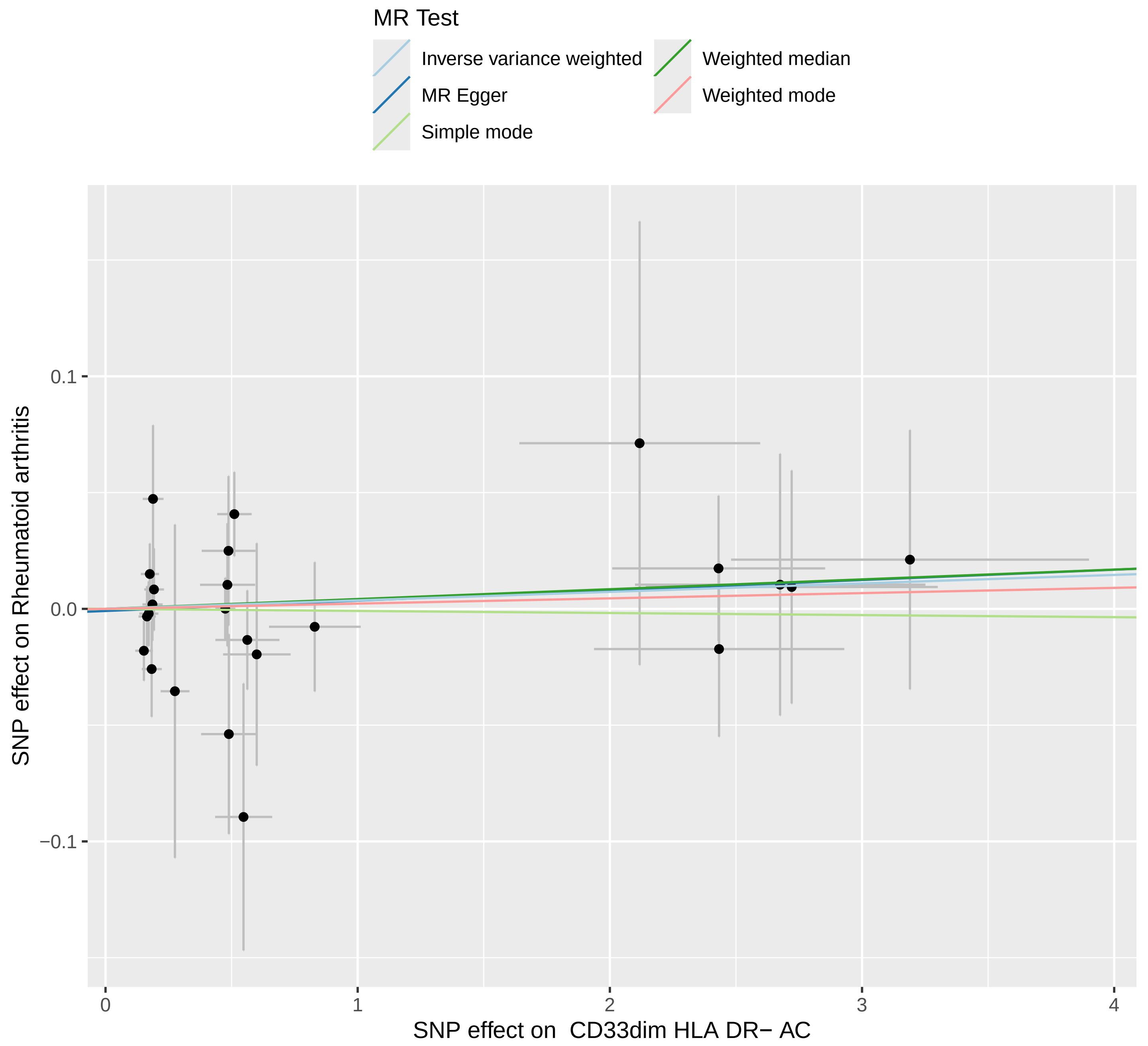

MR Test
Inverse variance weighted
MR Egger
Simple mode
Weighted median
Weighted mode
0.1
0.0
−0.1
SNP effect on Rheumatoid arthritis
0
1
2
3
4
SNP effect on CD33dim HLA DR− AC

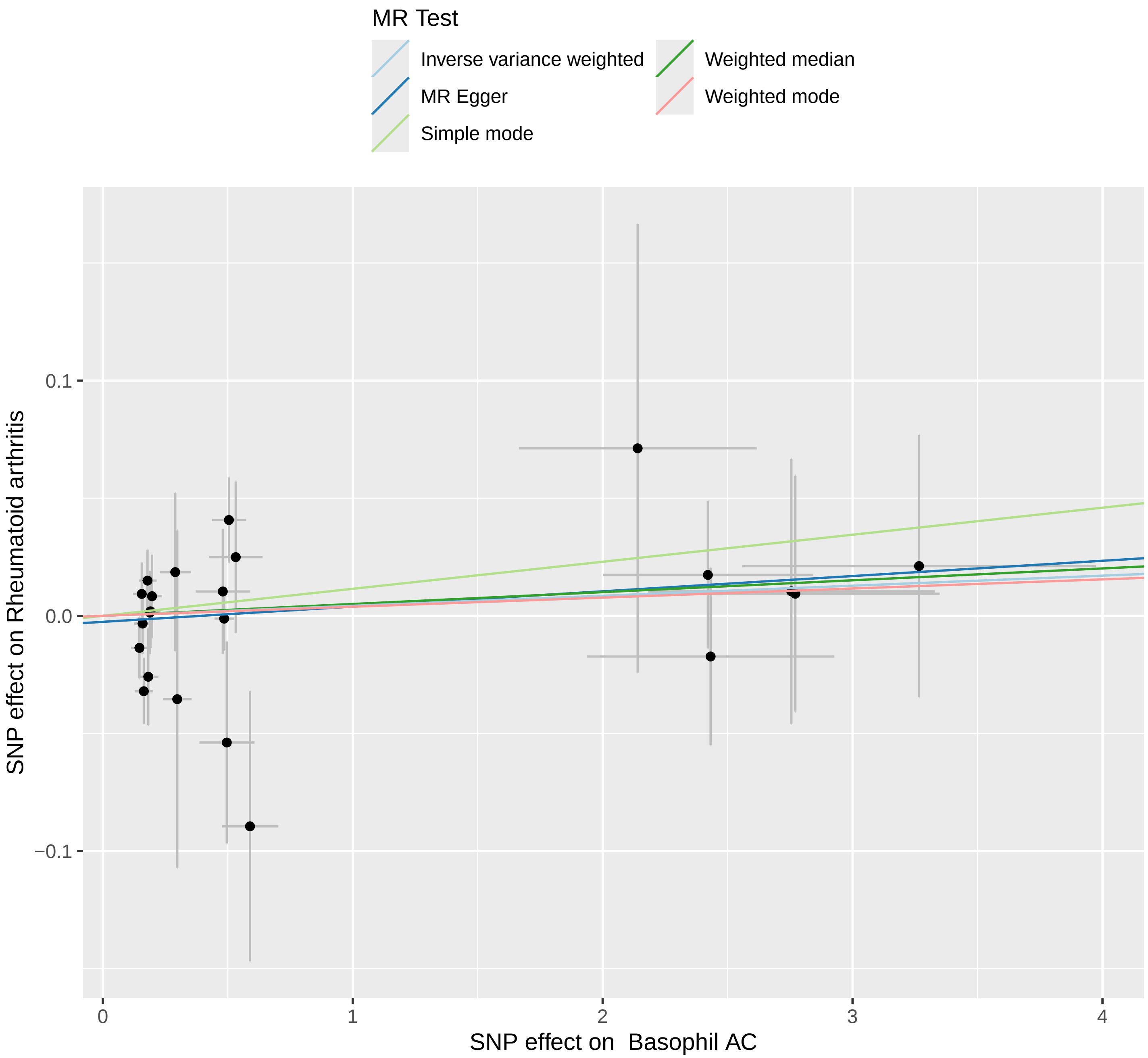
MR Test
Inverse variance weighted
MR Egger
Simple mode
Weighted median
Weighted mode
SNP effect on Rheumatoid arthritis
0.1
0.0
−0.1
0
1
2
3
4
SNP effect on Basophil AC

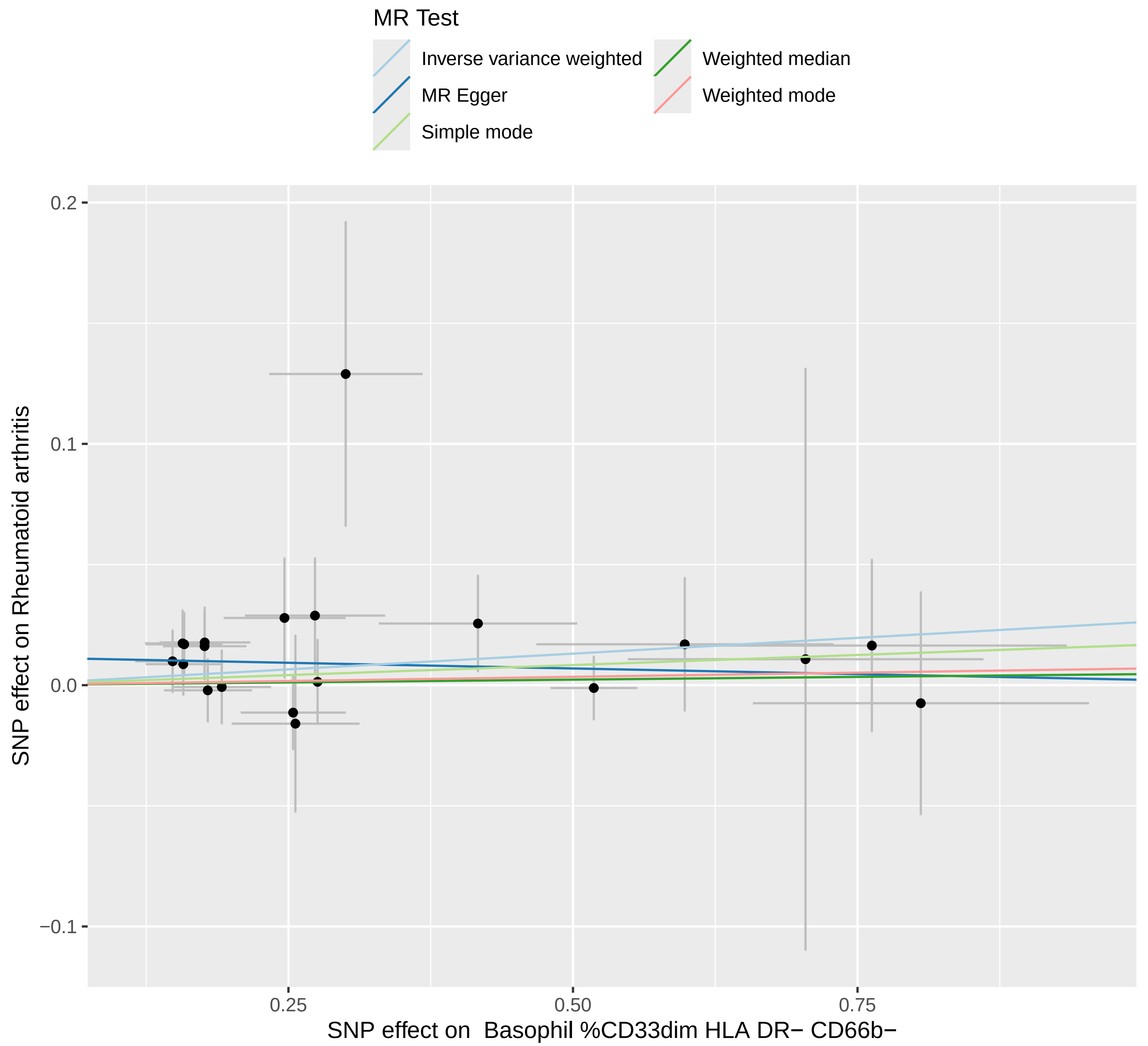
MR Test
Inverse variance weighted
MR Egger
Simple mode
Weighted median
Weighted mode
0.2
0.1
0.0
−0.1
0.25
0.50
0.75
SNP effect on Rheumatoid arthritis
SNP effect on Basophil %CD33dim HLA DR− CD66b−

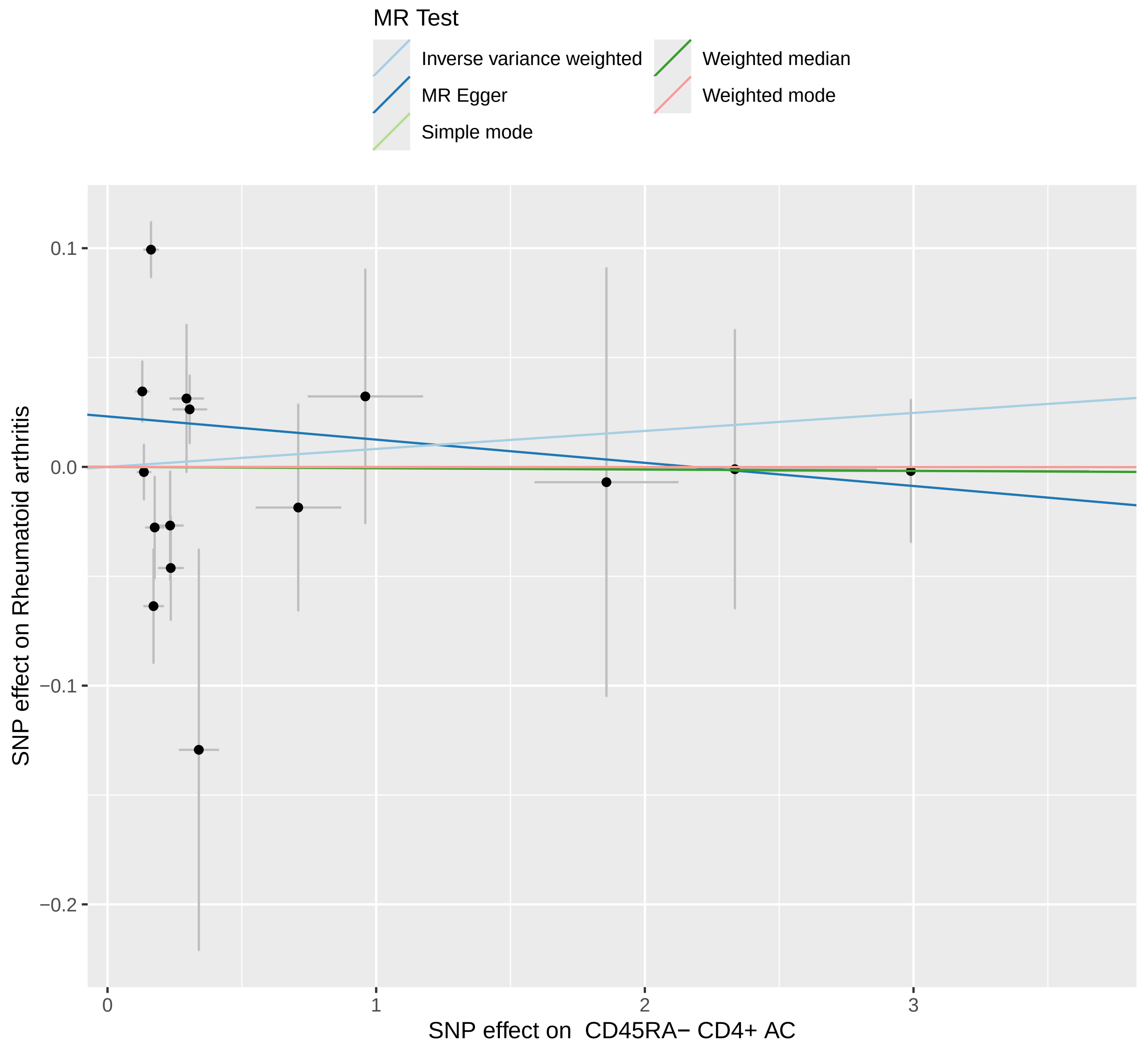
MR Test
Inverse variance weighted
MR Egger
Simple mode
Weighted median
Weighted mode
SNP effect on Rheumatoid arthritis
0.1
0.0
−0.1
−0.2
0
1
2
3
SNP effect on CD45RA− CD4+ AC

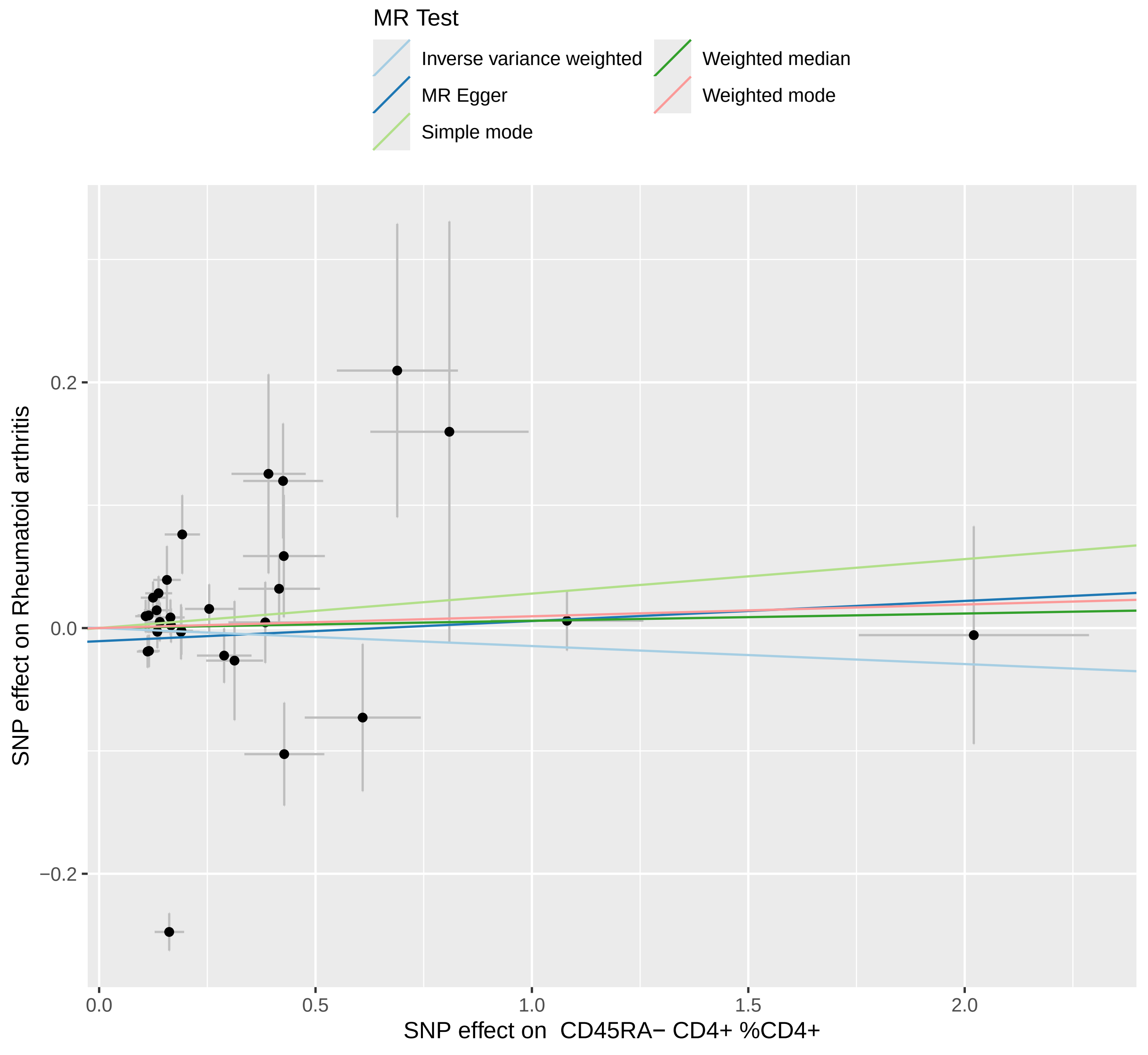

MR Test
Inverse variance weighted
MR Egger
Simple mode
Weighted median
Weighted mode
SNP effect on Rheumatoid arthritis
0.2
0.0
−0.2
0.0
0.5
1.0
1.5
2.0
SNP effect on CD45RA− CD4+ %CD4+

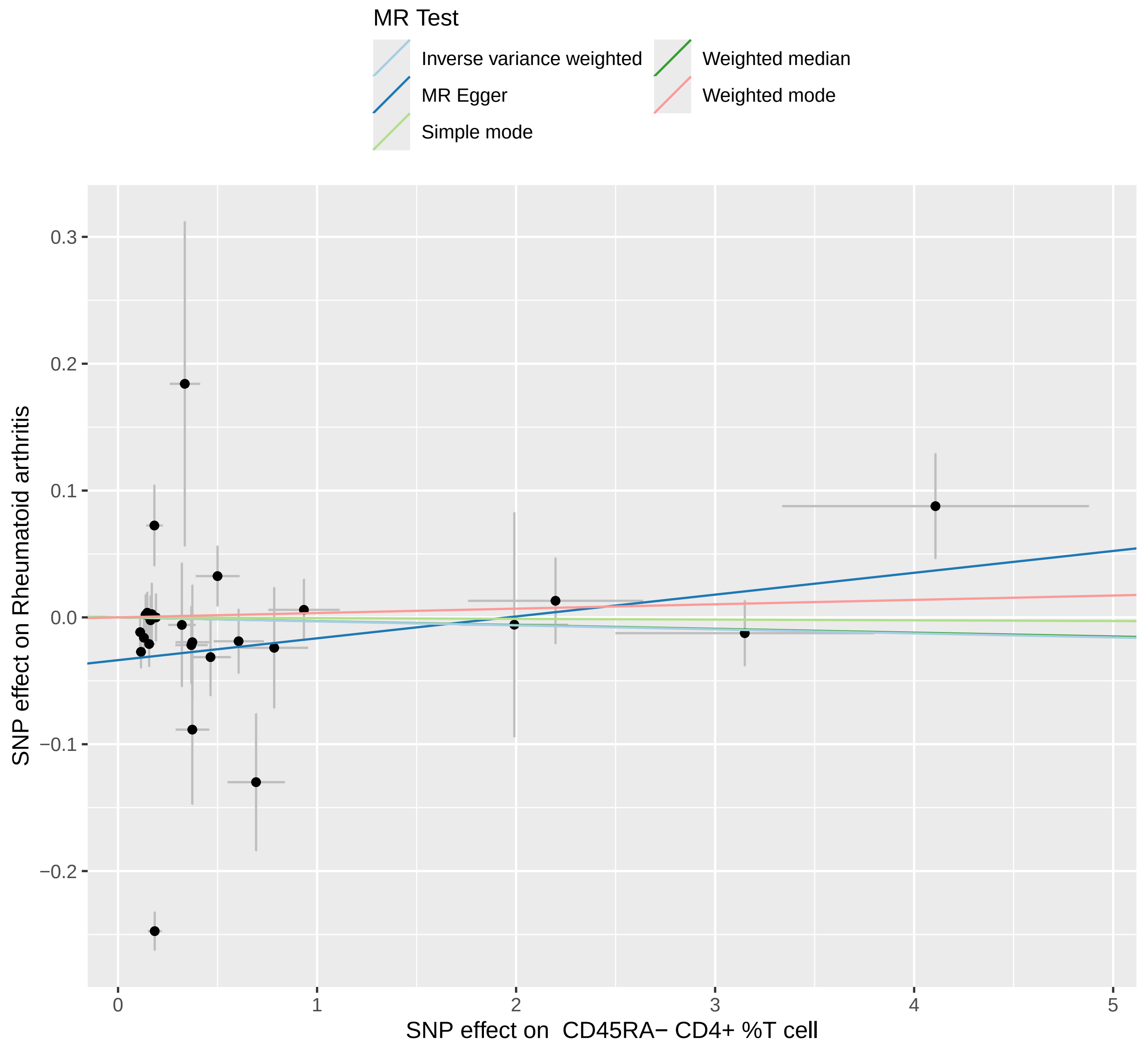
MR Test
Inverse variance weighted
MR Egger
Simple mode
Weighted median
Weighted mode
SNP effect on Rheumatoid arthritis
0.3
0.2
0.1
0.0
−0.1
−0.2
0
1
2
3
4
5
SNP effect on CD45RA− CD4+ %T cell

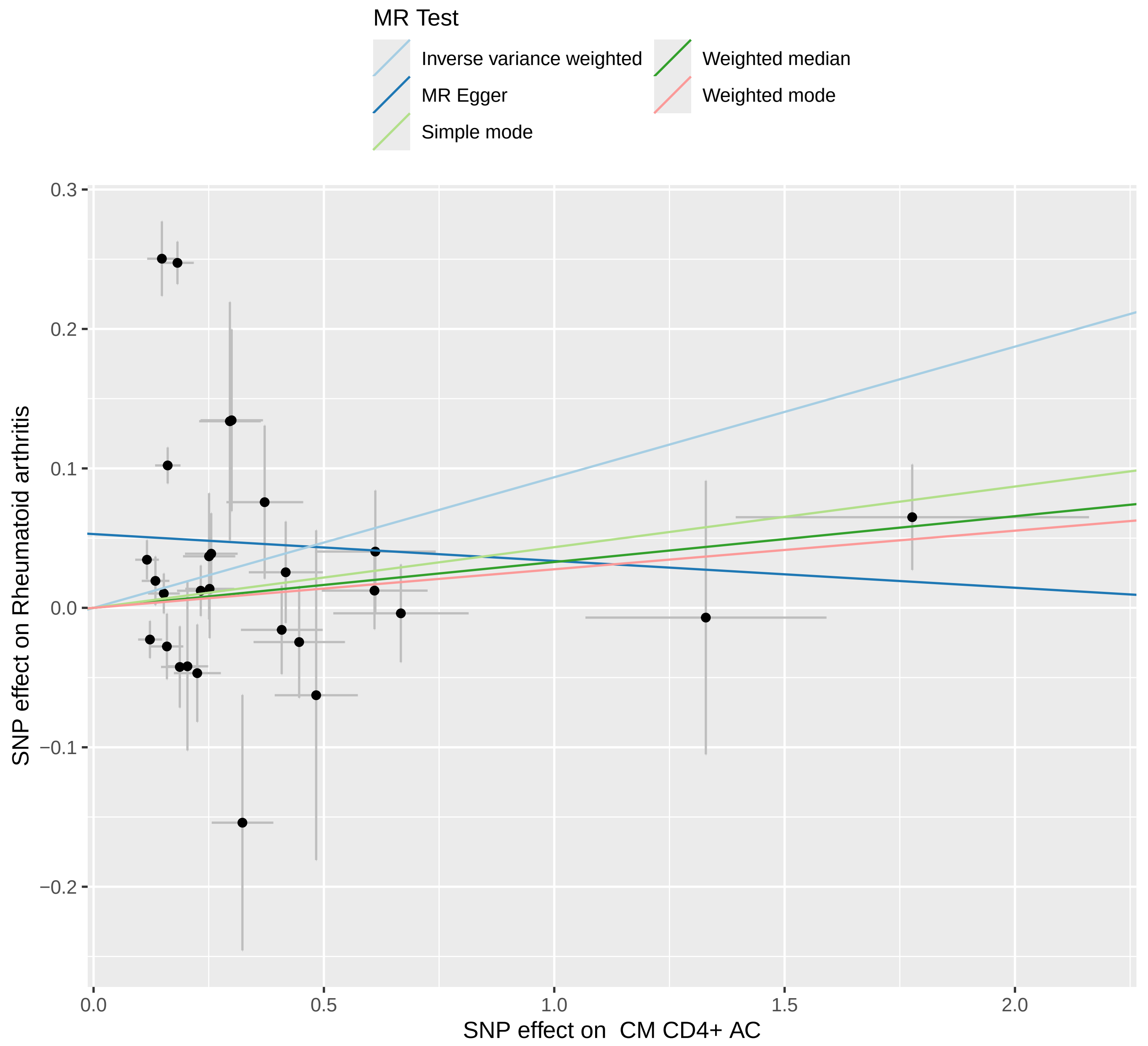

MR Test
Inverse variance weighted
MR Egger
Simple mode
Weighted median
Weighted mode
SNP effect on Rheumatoid arthritis
0.3
0.2
0.1
0.0
−0.1
−0.2
0.0
0.5
1.0
1.5
2.0
SNP effect on CM CD4+ AC

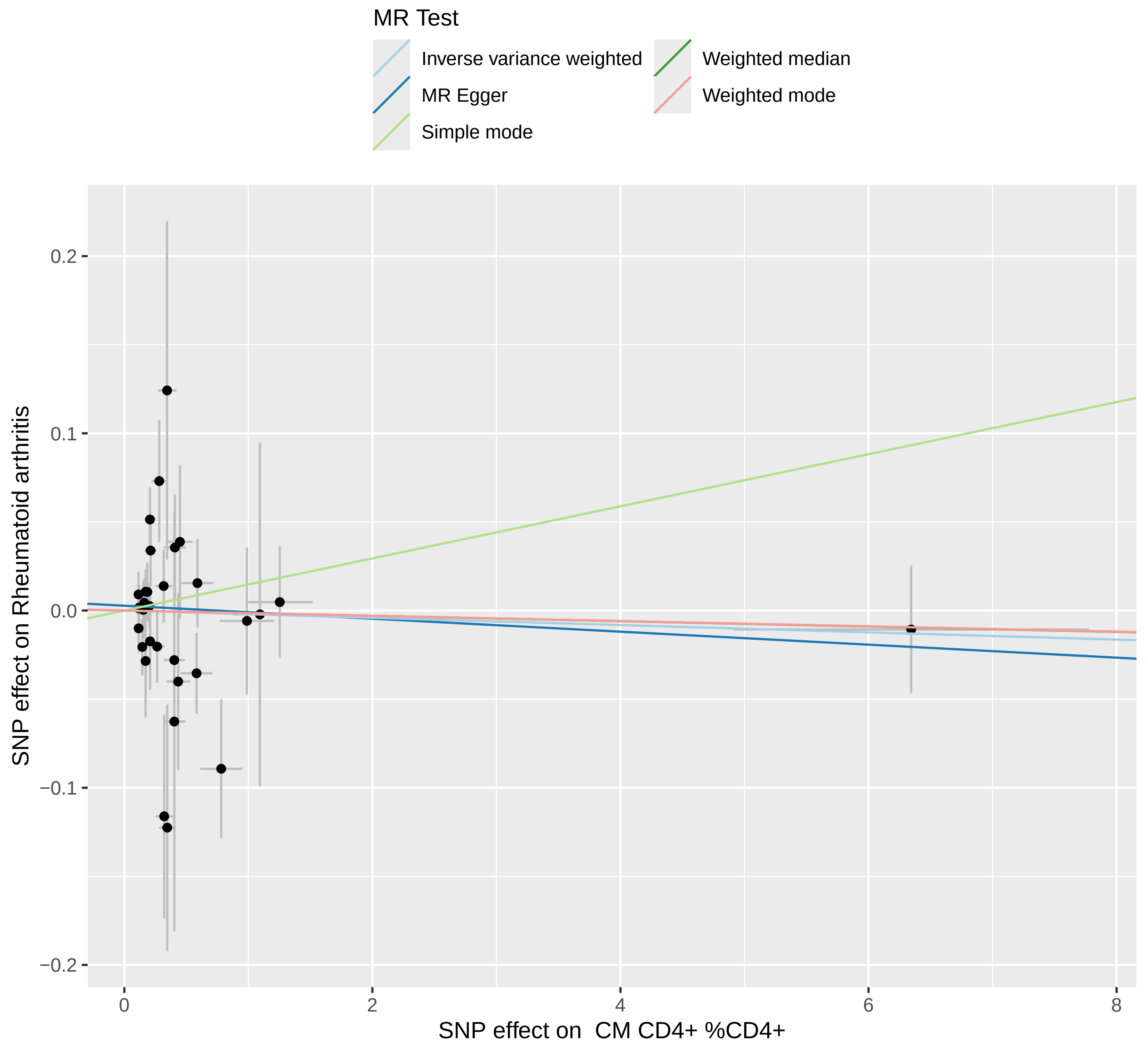
MR Test
Inverse variance weighted
MR Egger
Simple mode
Weighted median
Weighted mode
0.2
0.1
0.0
−0.1
−0.2
0
2
4
6
8
SNP effect on Rheumatoid arthritis
SNP effect on CM CD4+ %CD4+

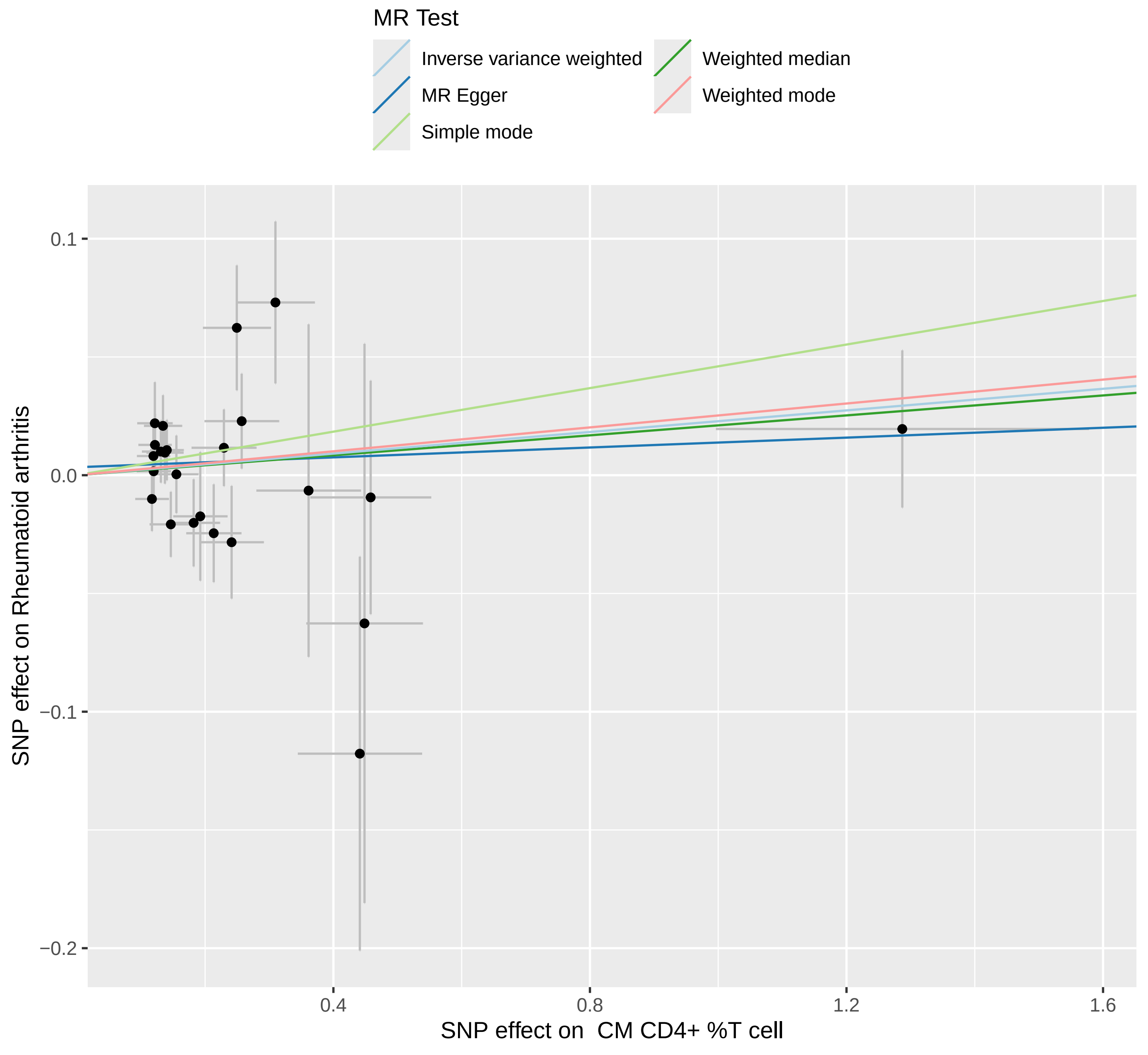

MR Test
Inverse variance weighted
MR Egger
Simple mode
Weighted median
Weighted mode
0.1
0.0
−0.1
−0.2
0.4
0.8
1.2
1.6
SNP effect on Rheumatoid arthritis
SNP effect on CM CD4+ %T cell

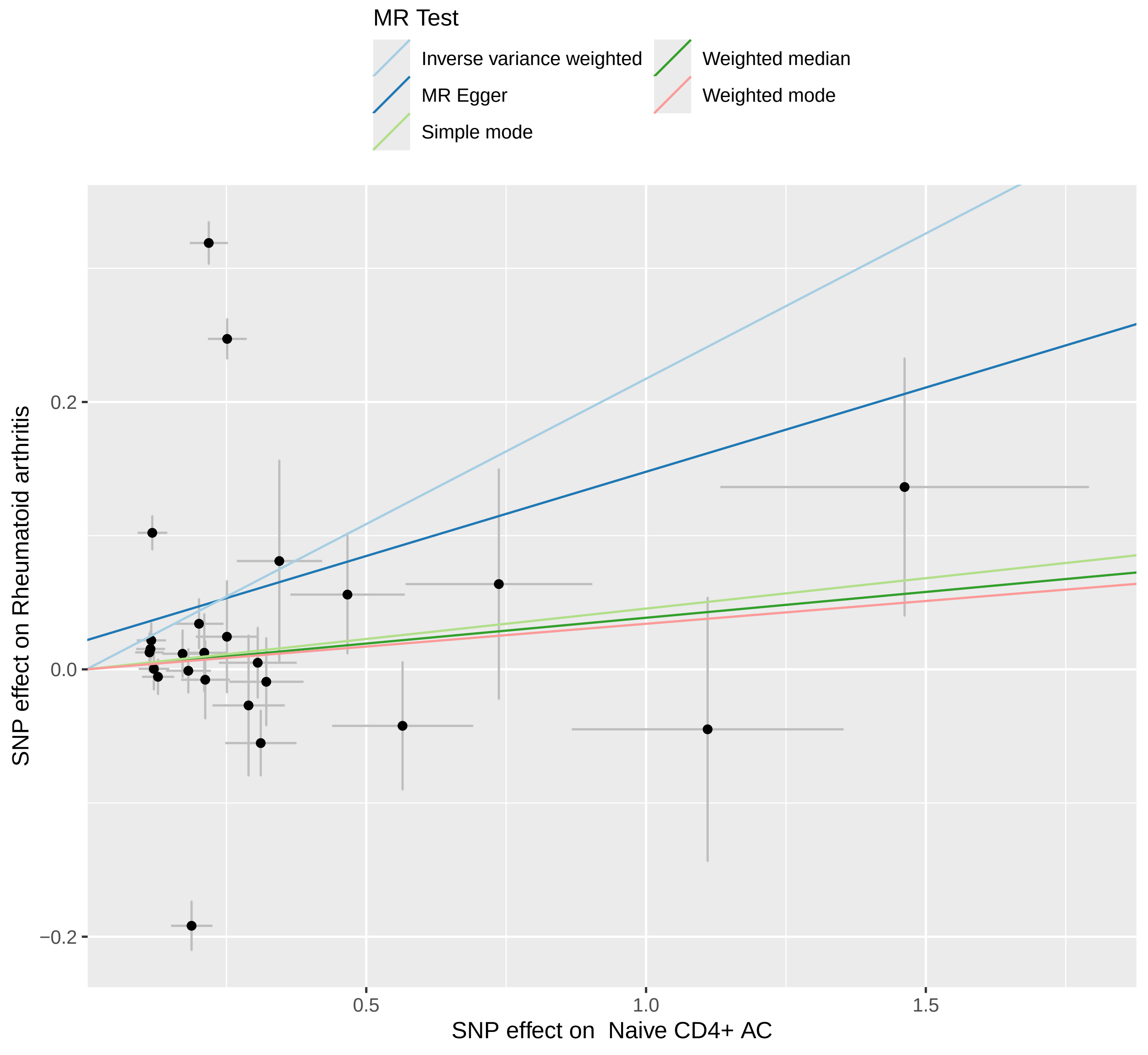
MR Test
Inverse variance weighted
MR Egger
Simple mode
Weighted median
Weighted mode
SNP effect on Rheumatoid arthritis
0.2
0.0
−0.2
0.5
1.0
1.5
SNP effect on Naive CD4+ AC

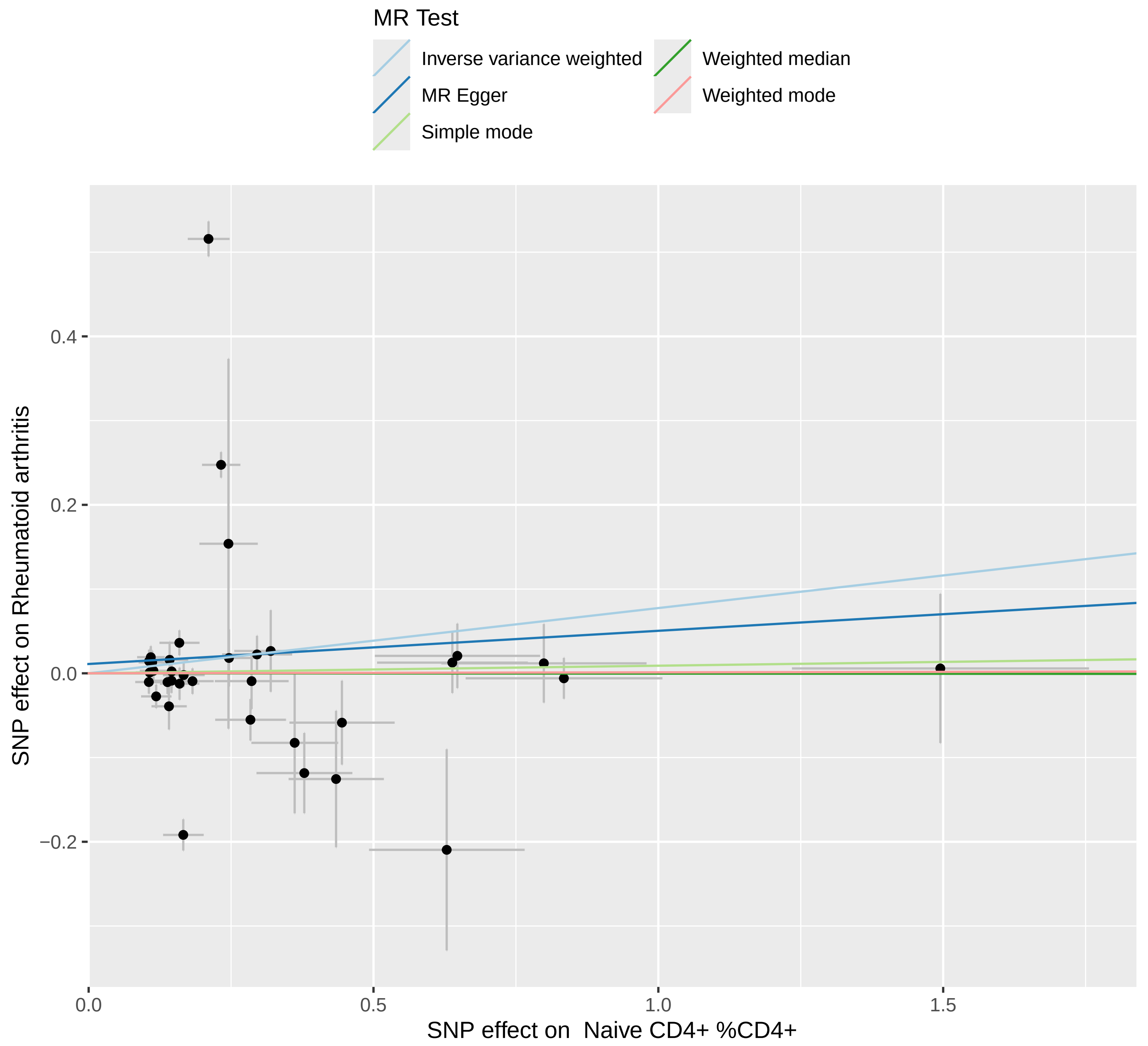

MR Test
Inverse variance weighted
MR Egger
Simple mode
Weighted median
Weighted mode
SNP effect on Rheumatoid arthritis
0.4
0.2
0.0
−0.2
0.0
0.5
1.0
1.5
SNP effect on Naive CD4+ %CD4+

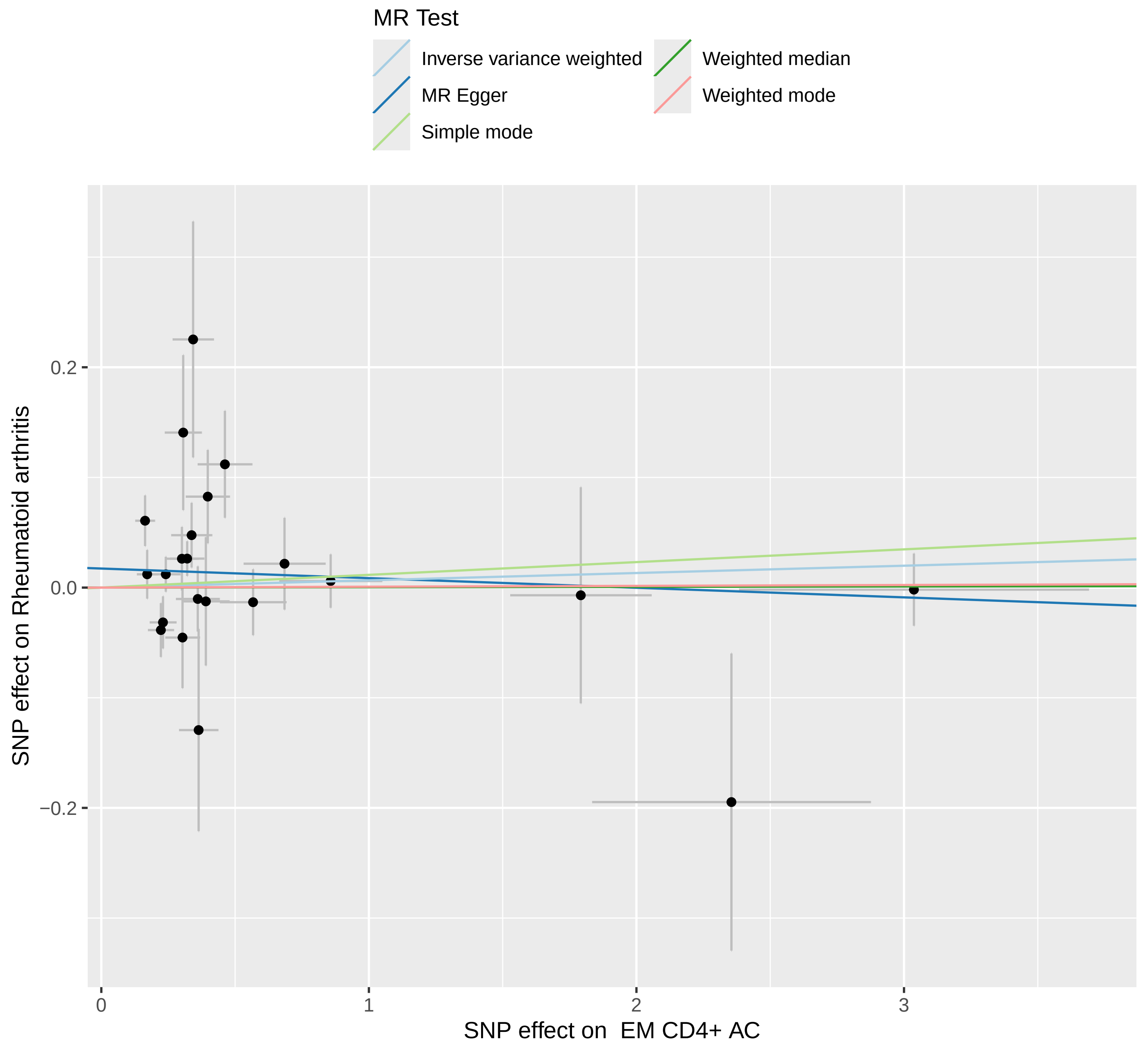
MR Test
Inverse variance weighted
MR Egger
Simple mode
Weighted median
Weighted mode
SNP effect on Rheumatoid arthritis
0.2
0.0
−0.2
0
1
2
3
SNP effect on EM CD4+ AC

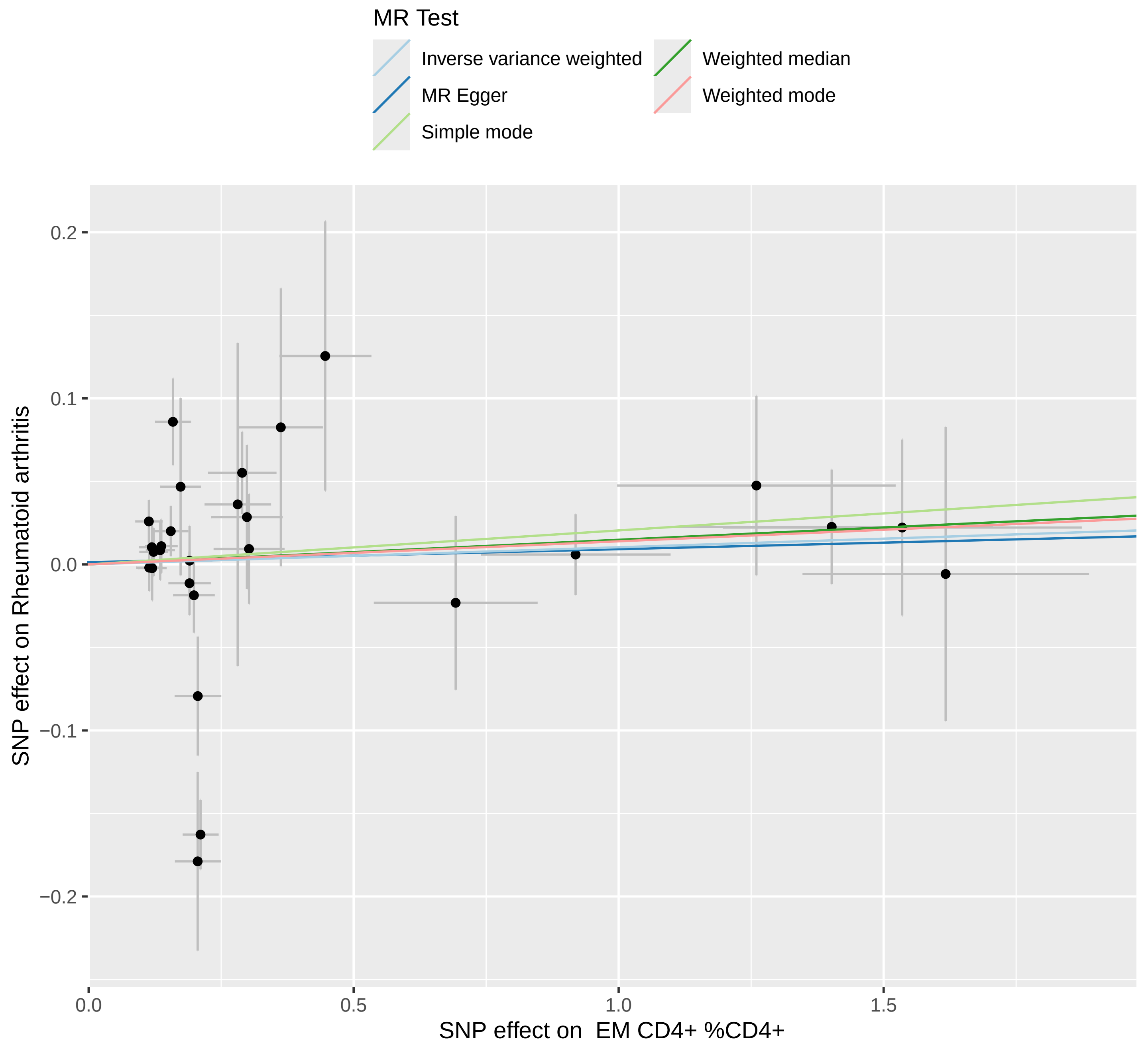
MR Test
Inverse variance weighted
MR Egger
Simple mode
Weighted median
Weighted mode
SNP effect on Rheumatoid arthritis
SNP effect on EM CD4+ %CD4+
0.2
0.1
0.0
−0.1
−0.2
0.0
0.5
1.0
1.5

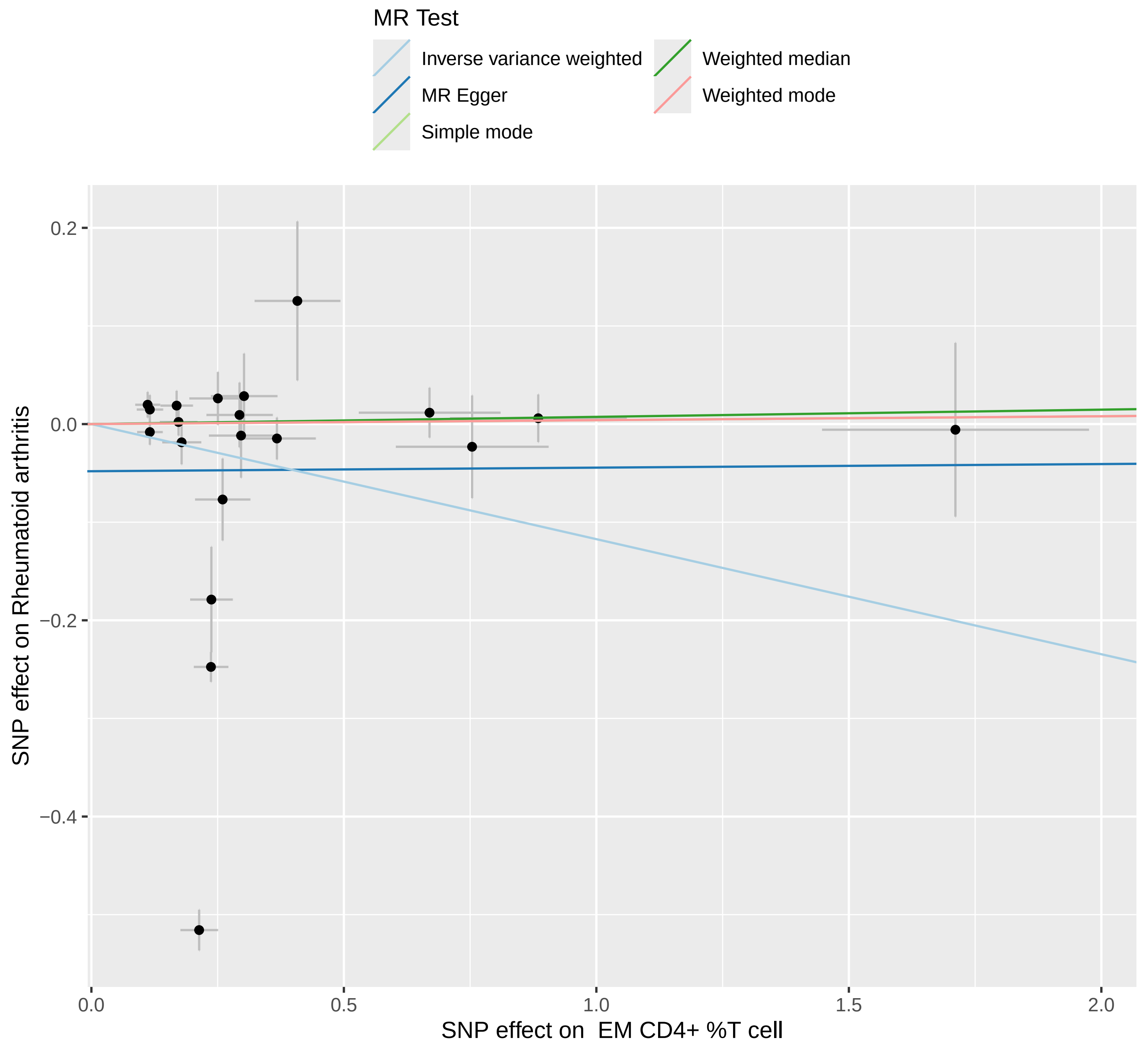

MR Test
Inverse variance weighted
MR Egger
Simple mode
Weighted median
Weighted mode
0.2
0.0
−0.2
−0.4
0.0
0.5
1.0
1.5
2.0
SNP effect on Rheumatoid arthritis
SNP effect on EM CD4+ %T cell

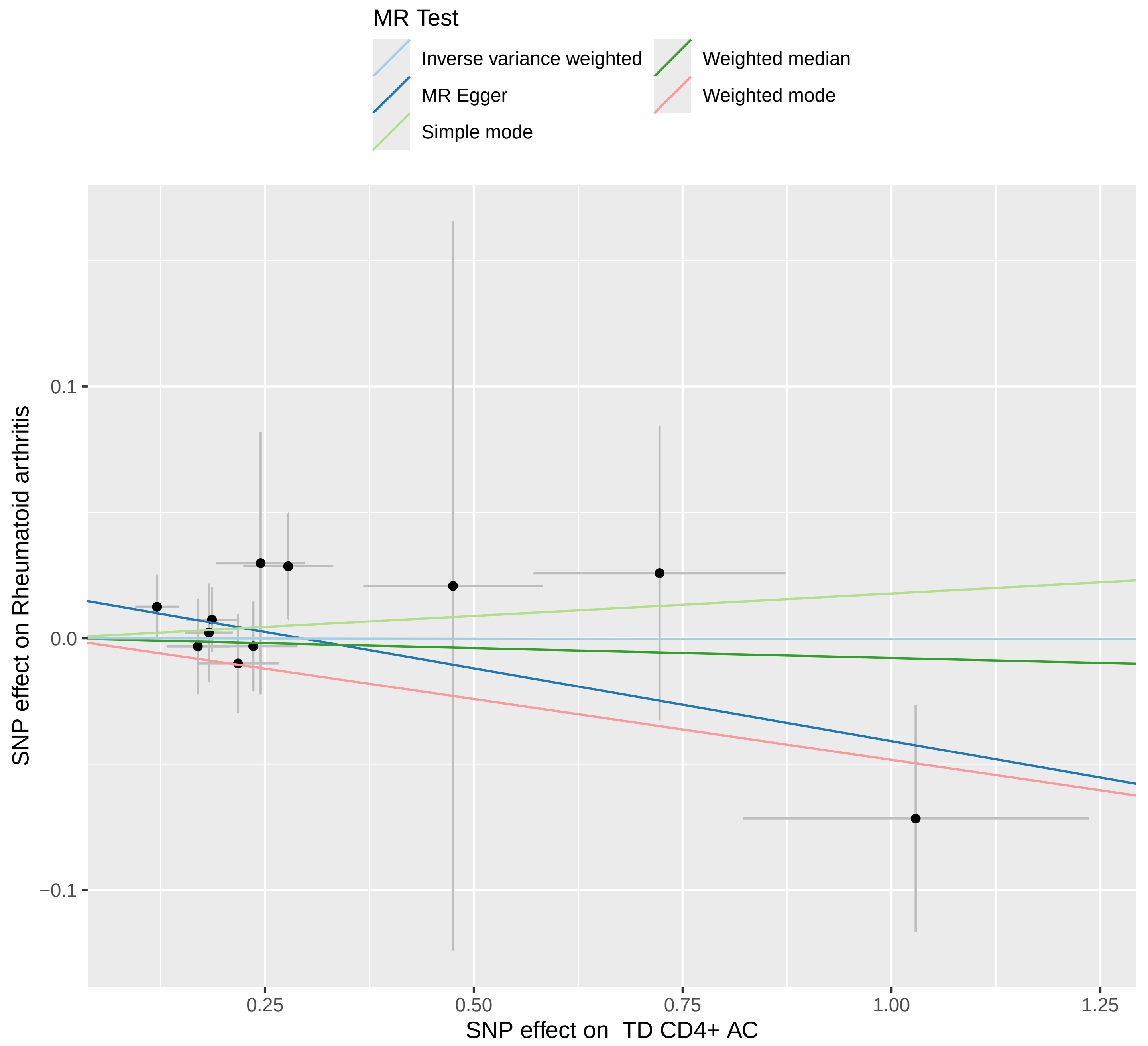
MR Test
Inverse variance weighted
MR Egger
Simple mode
Weighted median
Weighted mode
SNP effect on Rheumatoid arthritis
0.1
0.0
−0.1
0.25
0.50
0.75
1.00
1.25
SNP effect on TD CD4+ AC

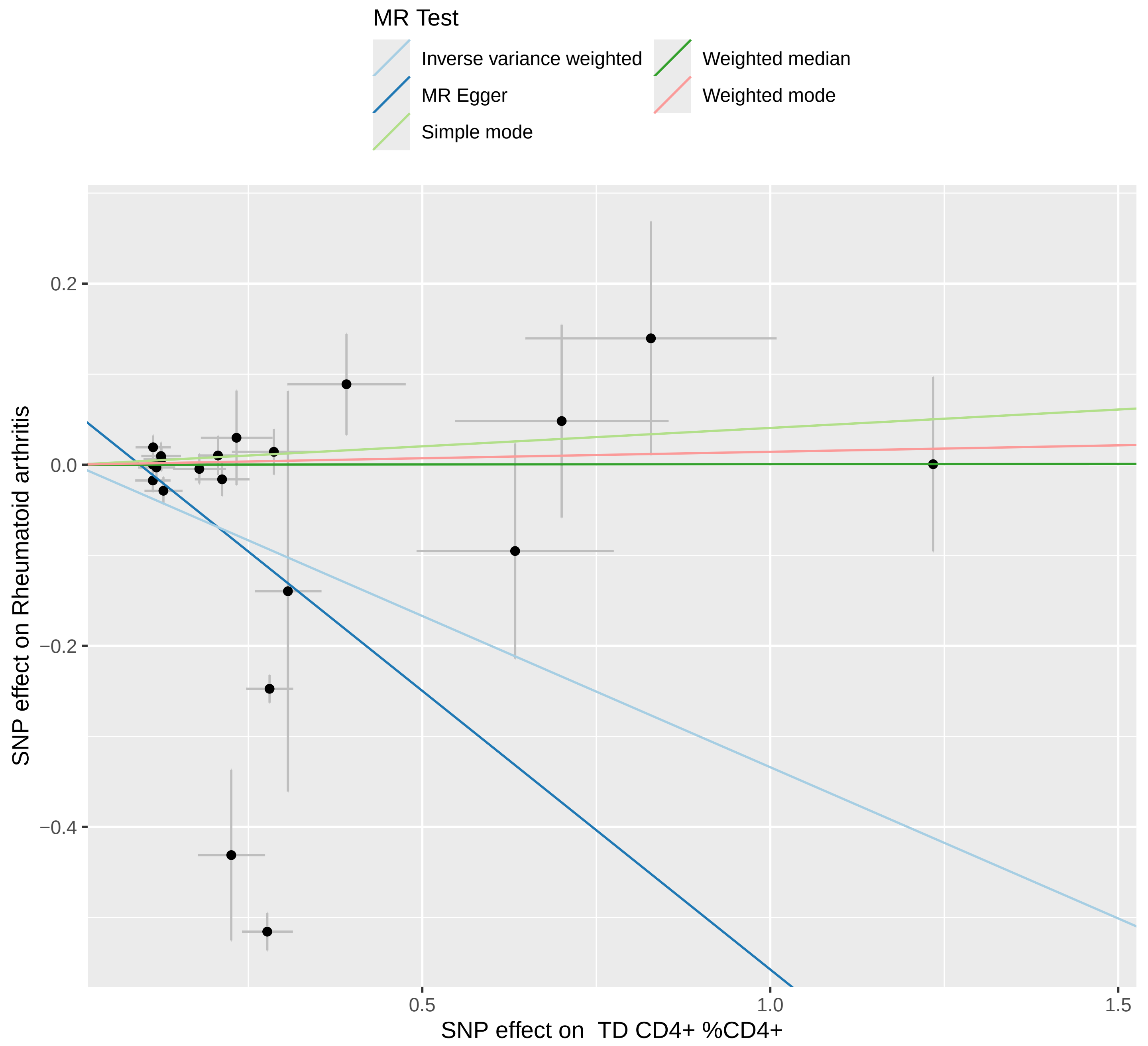

MR Test
Inverse variance weighted
MR Egger
Simple mode
Weighted median
Weighted mode
0.2
0.0
−0.2
−0.4
0.5
1.0
1.5
SNP effect on Rheumatoid arthritis
SNP effect on TD CD4+ %CD4+

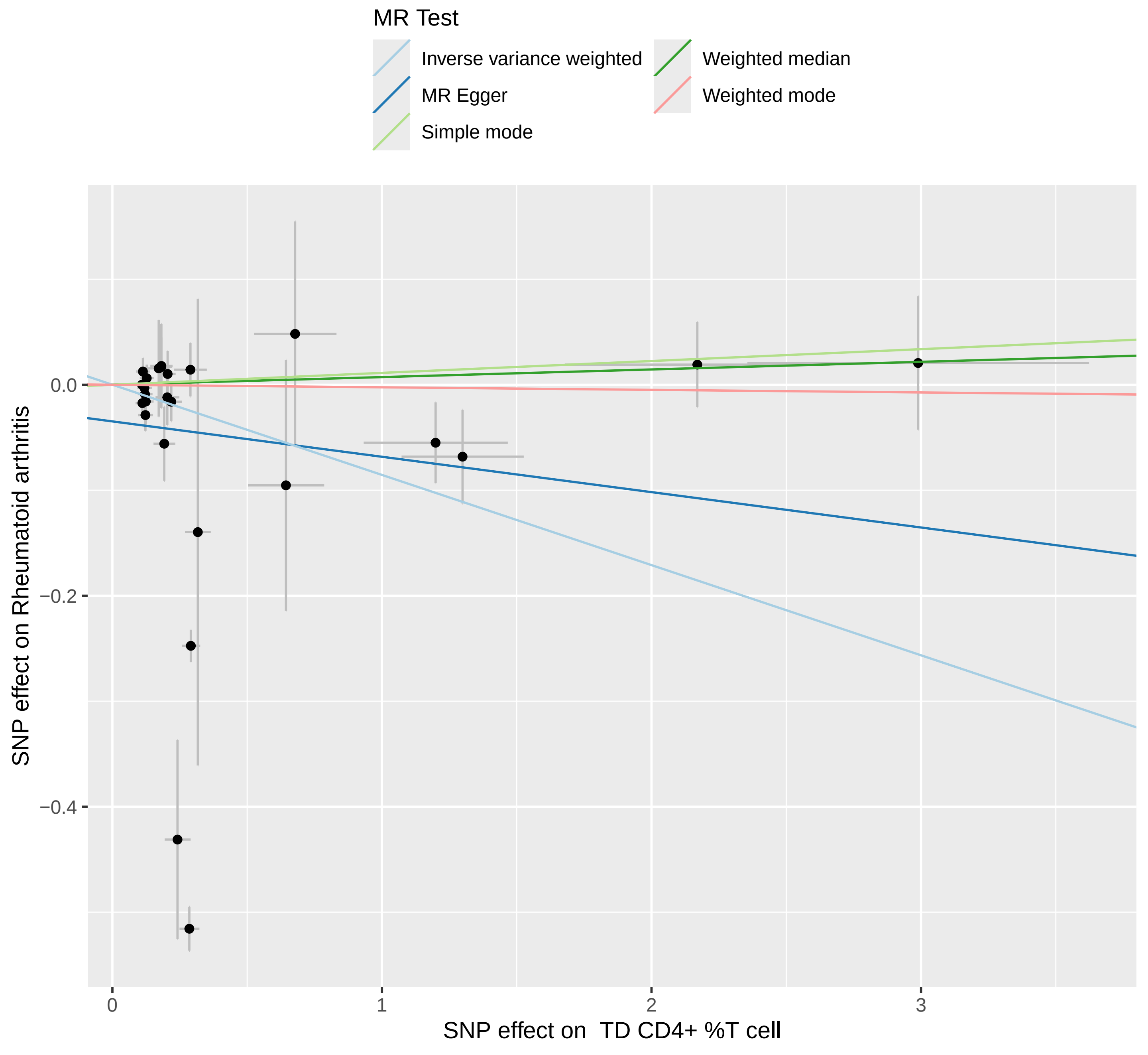

MR Test
Inverse variance weighted
MR Egger
Simple mode
Weighted median
Weighted mode
SNP effect on Rheumatoid arthritis
0.0
−0.2
−0.4
0
1
2
3
SNP effect on TD CD4+ %T cell

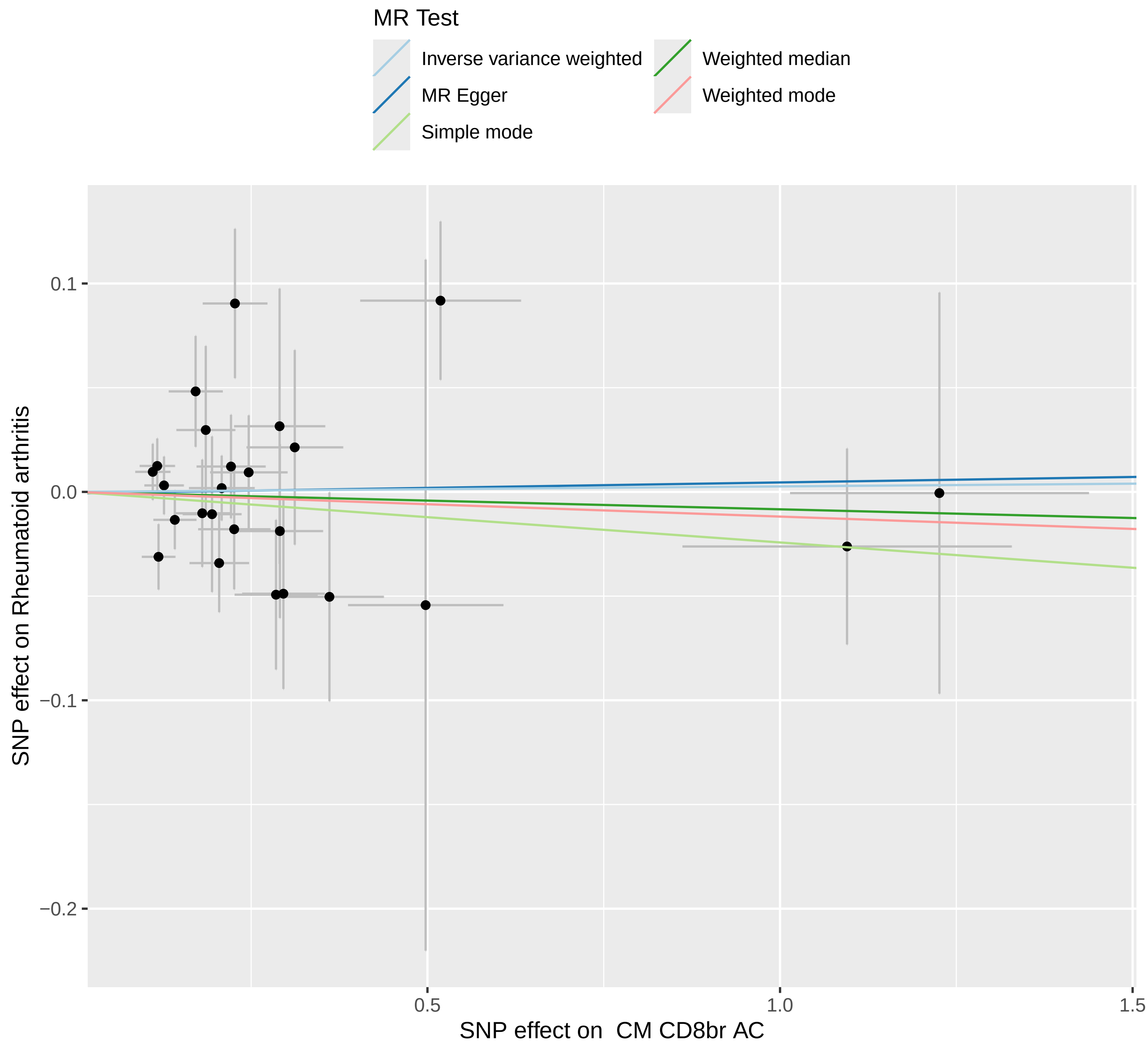

MR Test
Inverse variance weighted
MR Egger
Simple mode
Weighted median
Weighted mode
0.1
0.0
−0.1
−0.2
0.5
1.0
1.5
SNP effect on Rheumatoid arthritis
SNP effect on CM CD8br AC

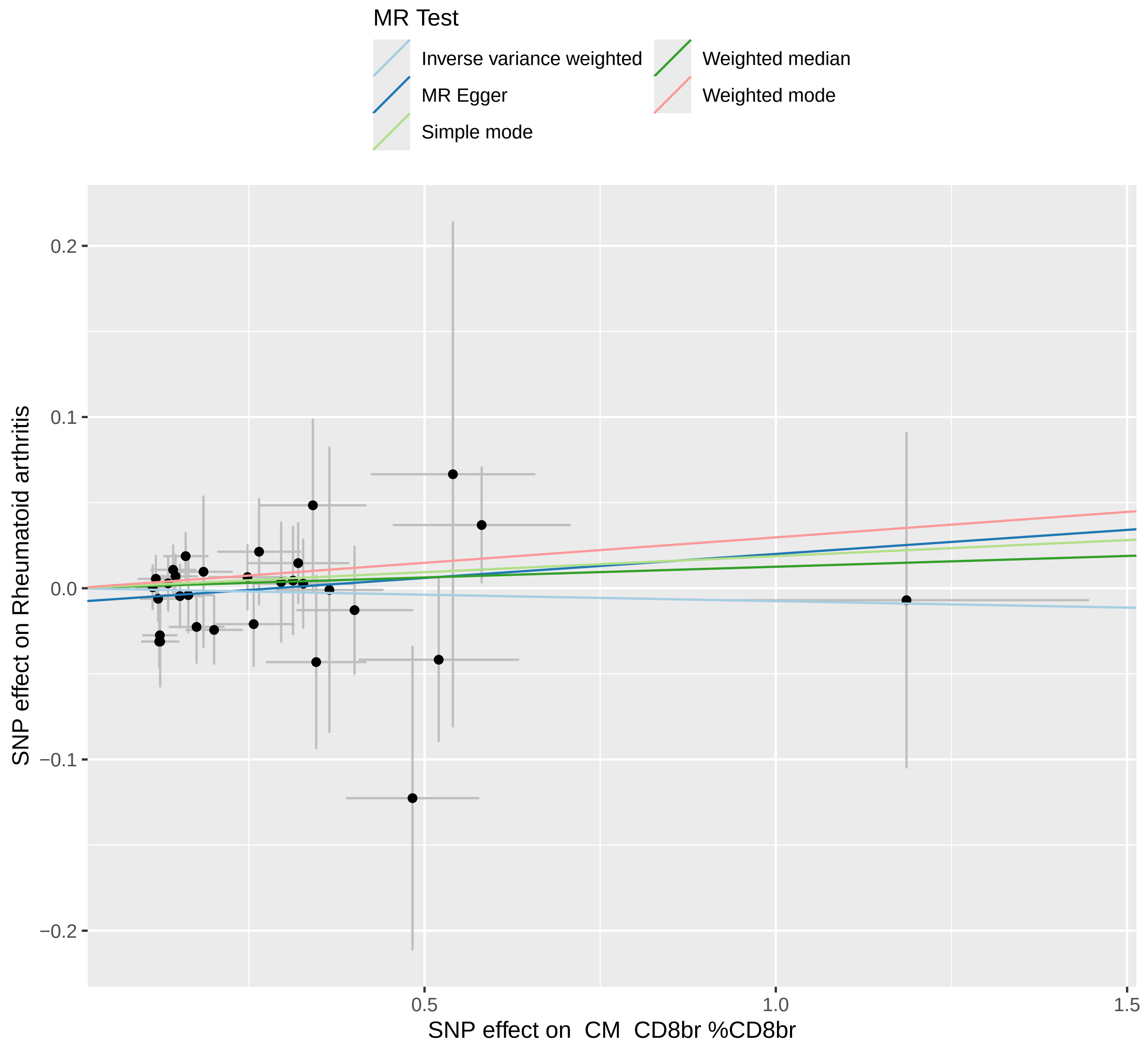
MR Test
Inverse variance weighted
MR Egger
Simple mode
Weighted median
Weighted mode
0.2
0.1
0.0
−0.1
−0.2
SNP effect on Rheumatoid arthritis
0.5
1.0
1.5
SNP effect on CM CD8br %CD8br

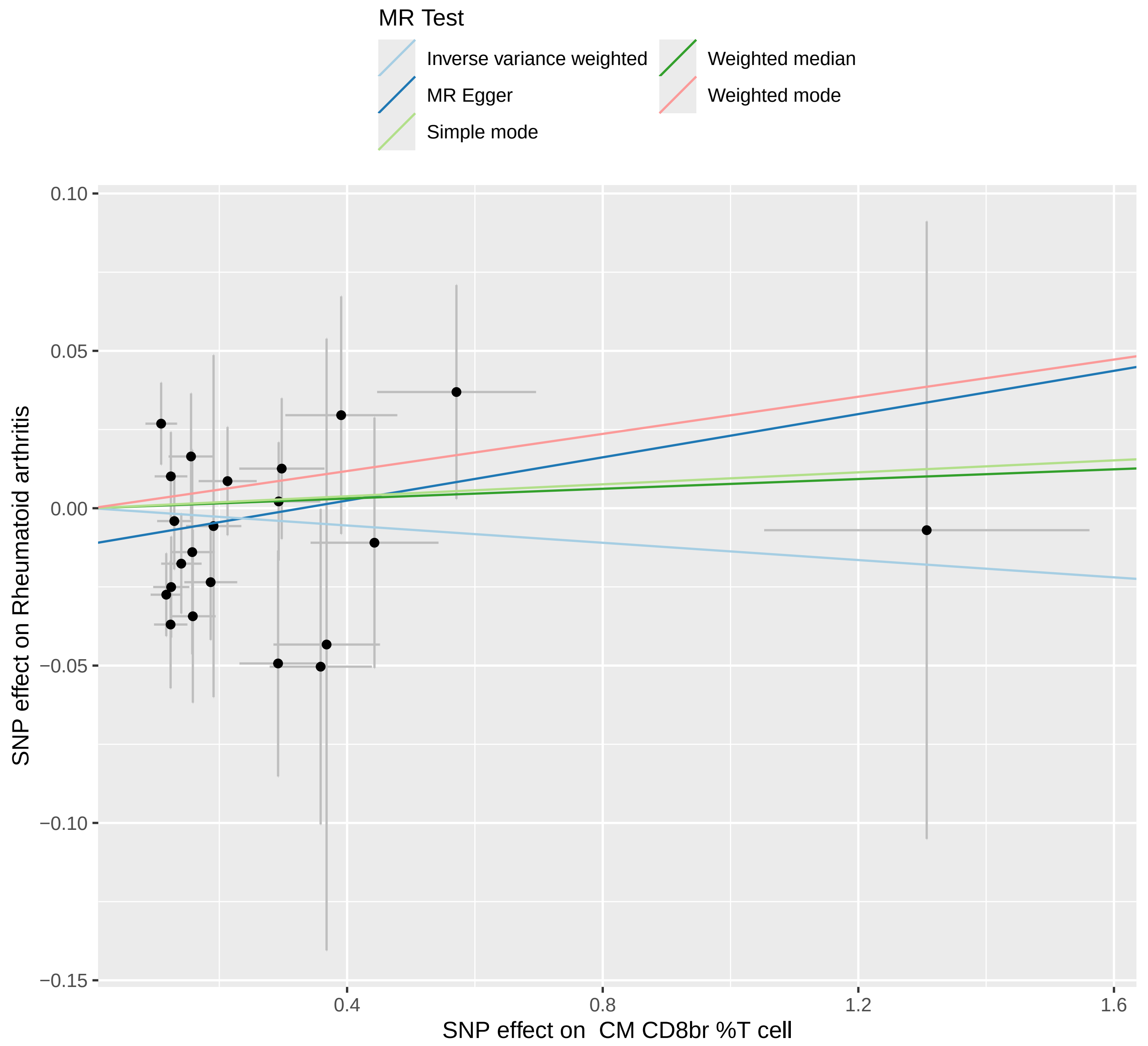
MR Test
Inverse variance weighted
MR Egger
Simple mode
Weighted median
Weighted mode
0.10
0.05
0.00
−0.05
−0.10
−0.15
SNP effect on Rheumatoid arthritis
0.4
0.8
1.2
1.6
SNP effect on CM CD8br %T cell

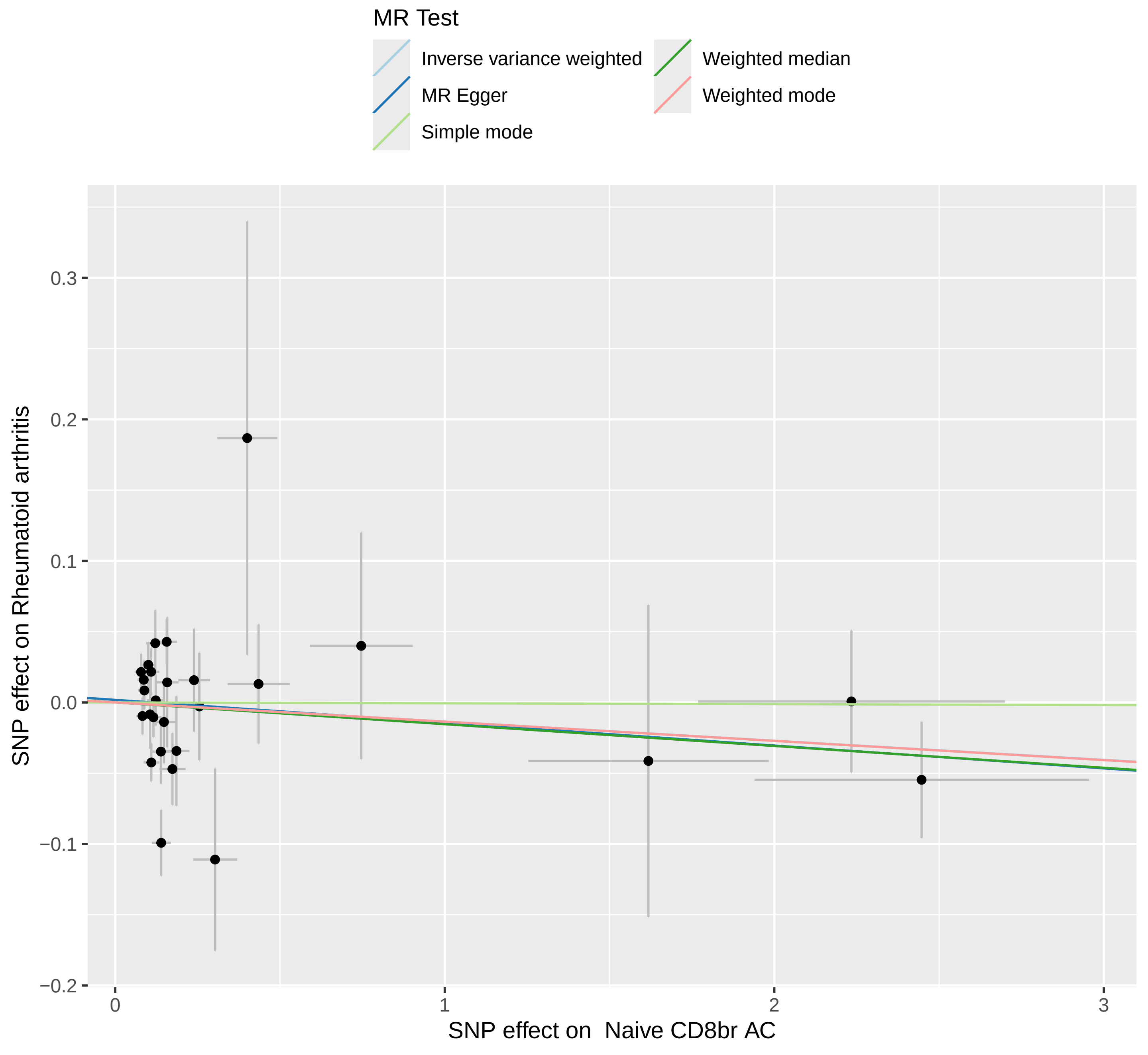
MR Test
Inverse variance weighted
MR Egger
Simple mode
Weighted median
Weighted mode
0.3
0.2
0.1
0.0
−0.1
−0.2
0
1
2
3
SNP effect on Rheumatoid arthritis
SNP effect on Naive CD8br AC

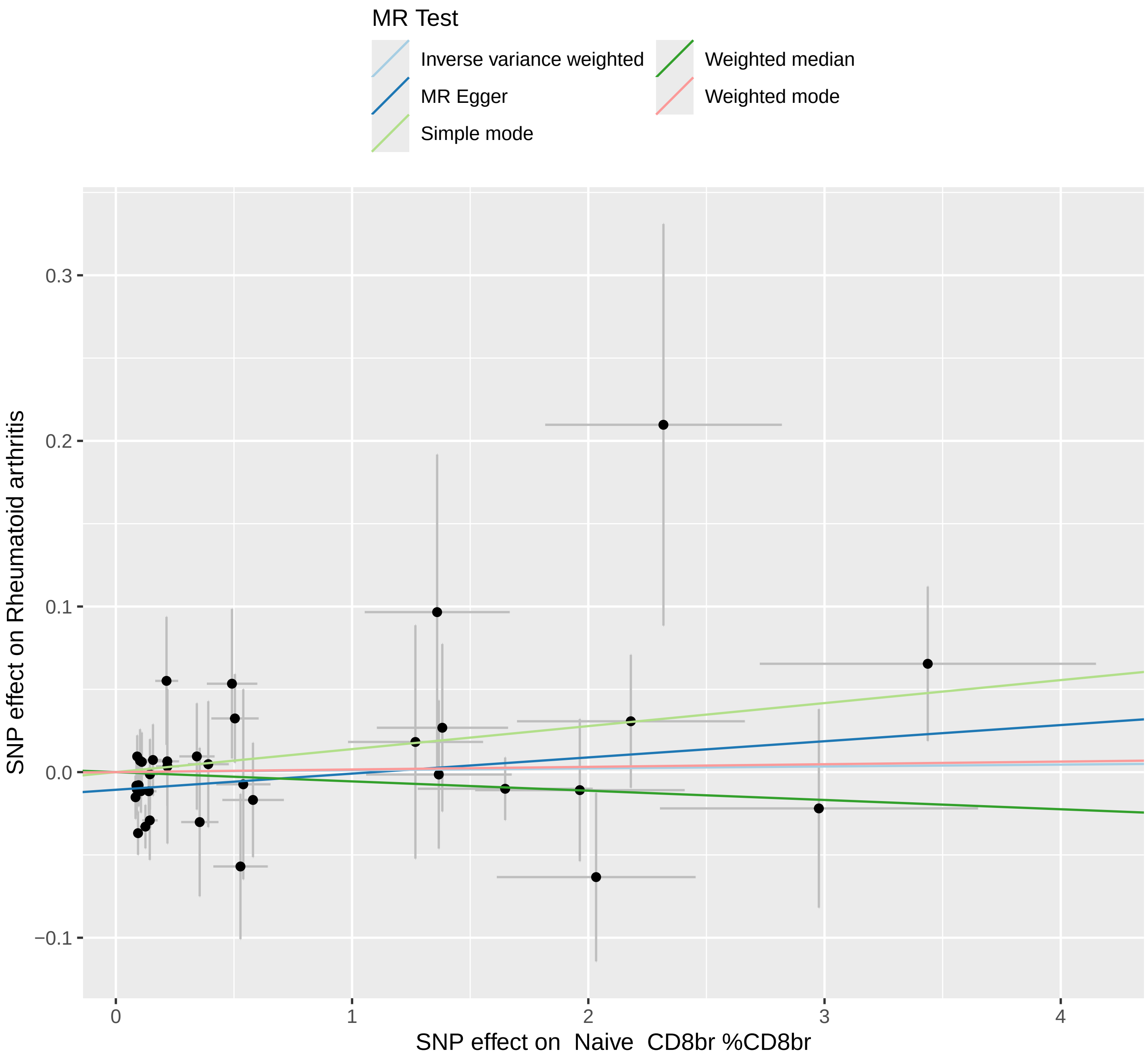
MR Test
Inverse variance weighted
MR Egger
Simple mode
Weighted median
Weighted mode
SNP effect on Rheumatoid arthritis
0.3
0.2
0.1
0.0
−0.1
0
1
2
3
4
SNP effect on Naive CD8br %CD8br

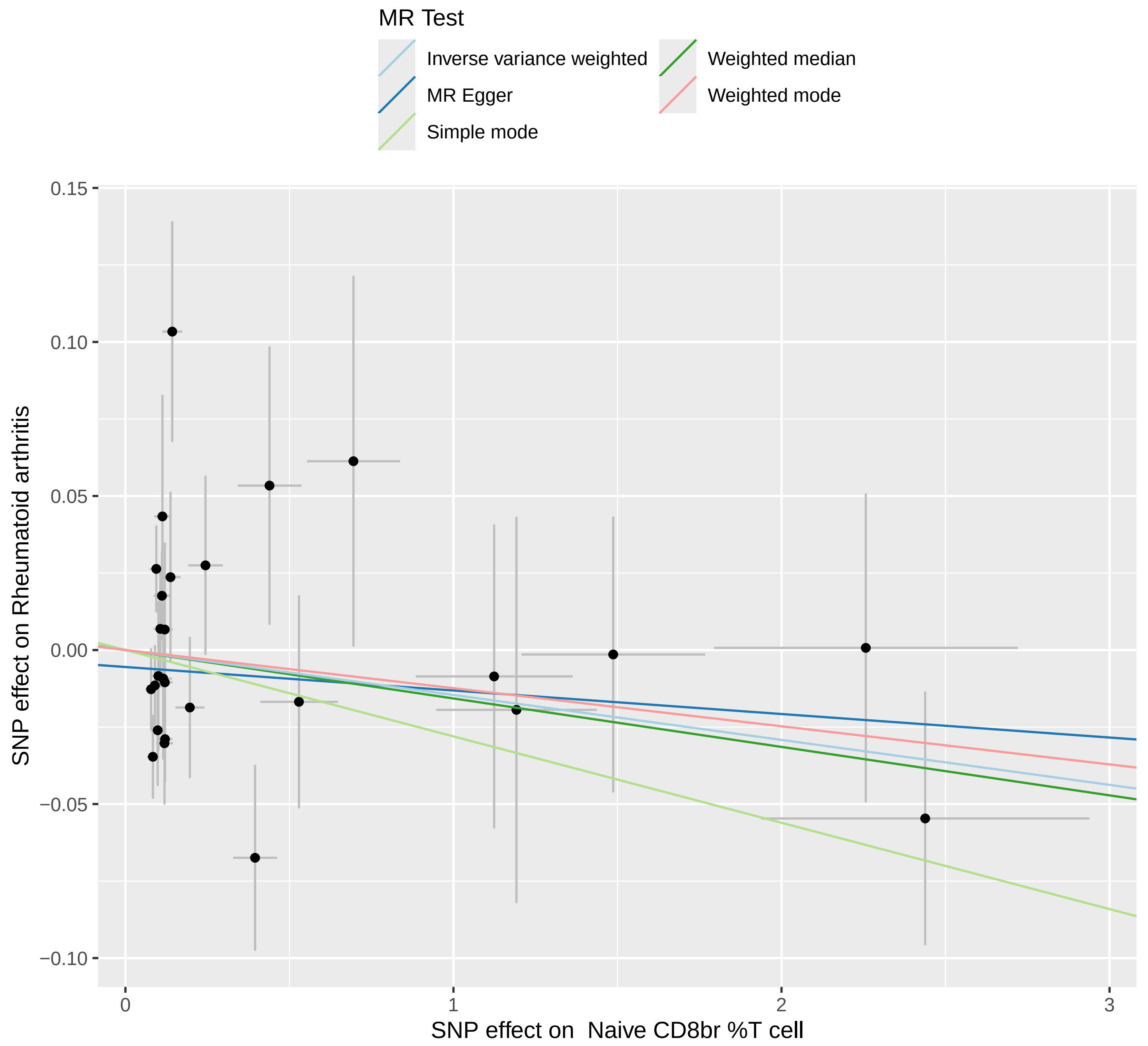
MR Test
Inverse variance weighted
MR Egger
Simple mode
Weighted median
Weighted mode
0.15
0.10
0.05
0.00
−0.05
−0.10
0
1
2
3
SNP effect on Rheumatoid arthritis
SNP effect on Naive CD8br %T cell

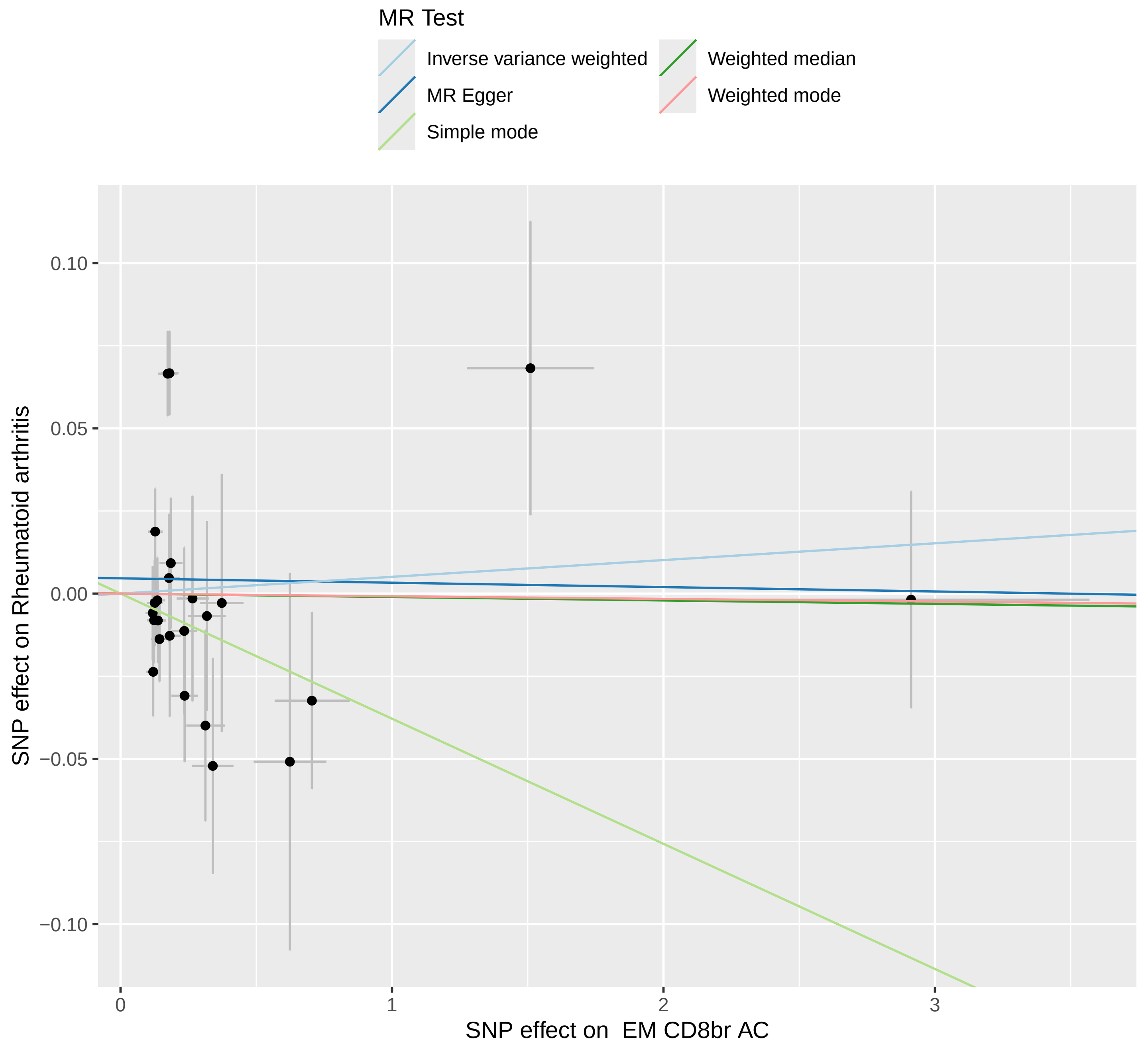

MR Test
Inverse variance weighted
MR Egger
Simple mode
Weighted median
Weighted mode
0.10
0.05
0.00
−0.05
−0.10
SNP effect on Rheumatoid arthritis
0
1
2
3
SNP effect on EM CD8br AC

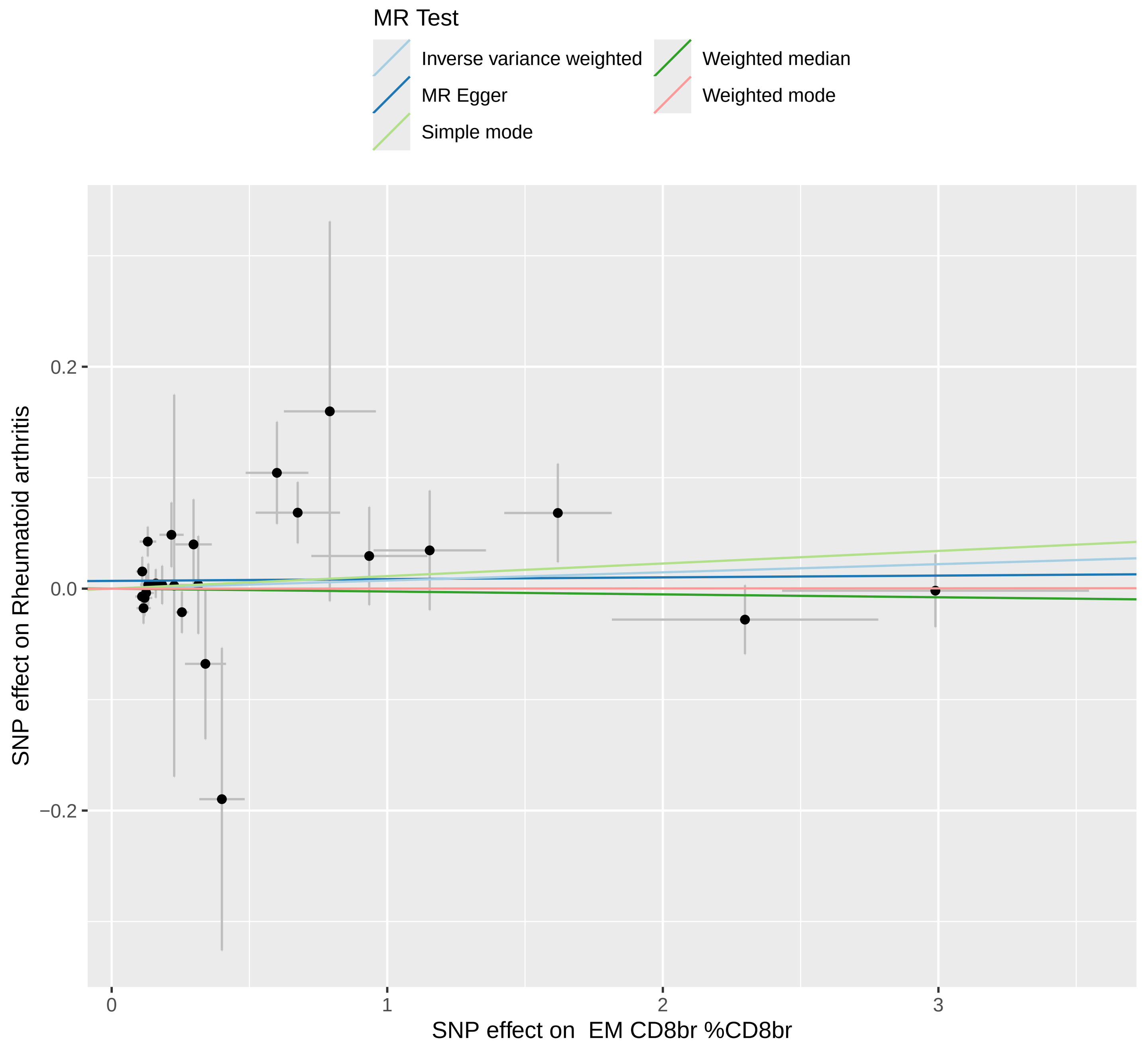
MR Test
Inverse variance weighted
MR Egger
Simple mode
Weighted median
Weighted mode
SNP effect on Rheumatoid arthritis
0.2
0.0
−0.2
0
1
2
3
SNP effect on EM CD8br %CD8br

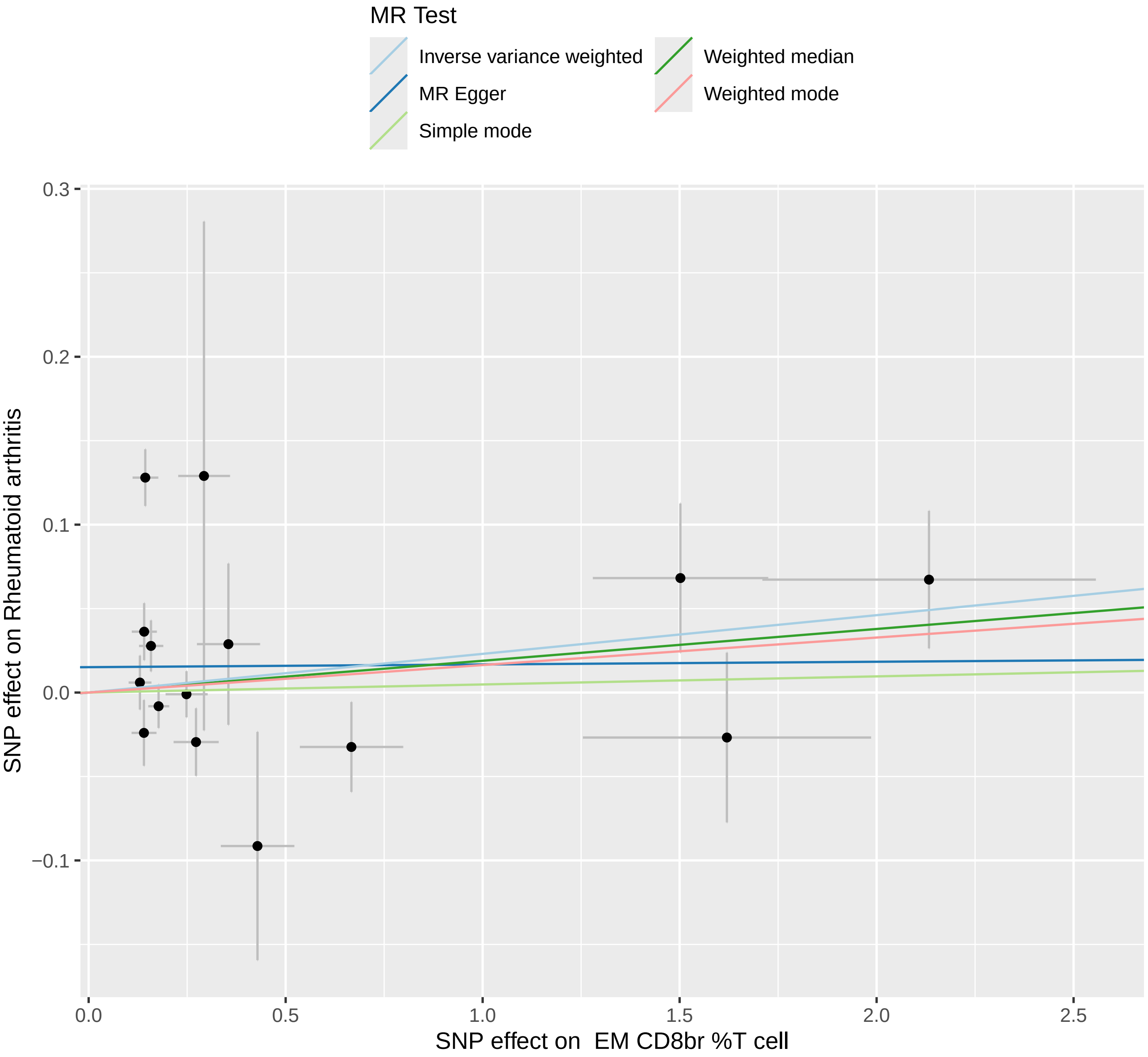

MR Test
Inverse variance weighted
Weighted median
MR Egger
Weighted mode
Simple mode
0.3
0.2
0.1
0.0
−0.1
SNP effect on Rheumatoid arthritis
0.0
0.5
1.0
1.5
2.0
2.5
SNP effect on EM CD8br %T cell

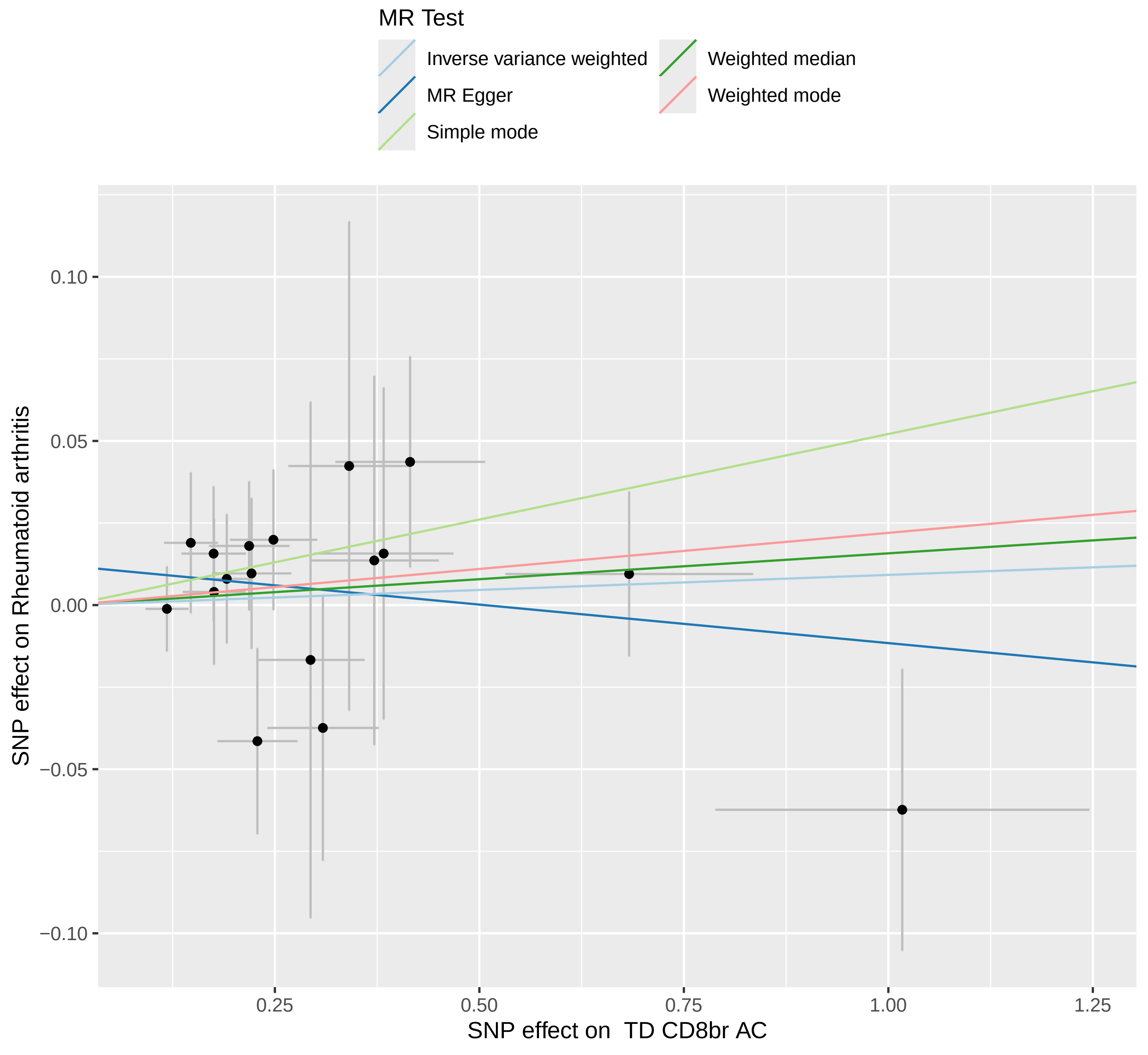
MR Test
Inverse variance weighted
MR Egger
Simple mode
Weighted median
Weighted mode
0.10
0.05
0.00
−0.05
−0.10
SNP effect on Rheumatoid arthritis
0.25
0.50
0.75
1.00
1.25
SNP effect on TD CD8br AC

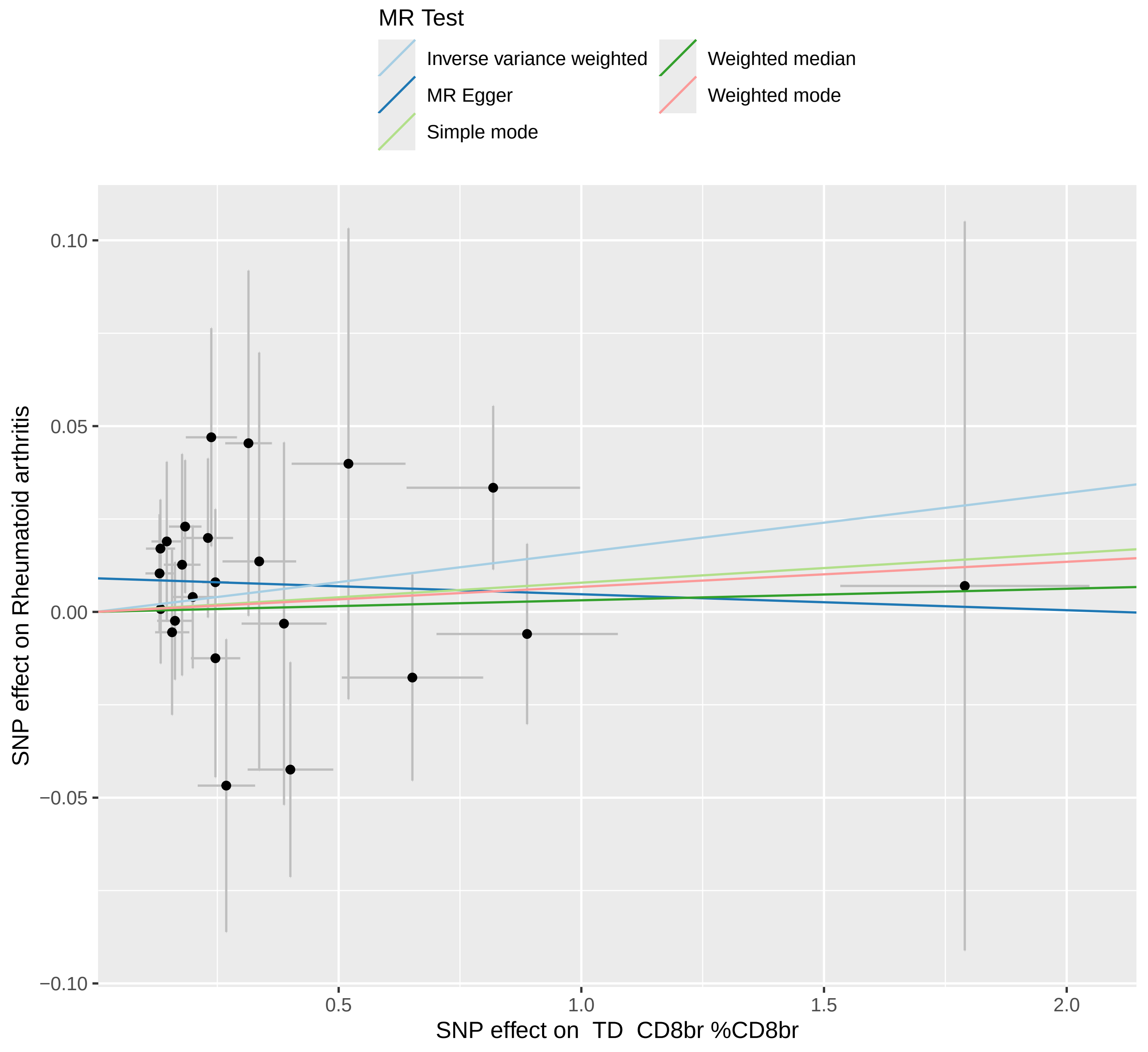

MR Test
Inverse variance weighted
MR Egger
Simple mode
Weighted median
Weighted mode
0.10
0.05
0.00
−0.05
−0.10
SNP effect on Rheumatoid arthritis
0.5
1.0
1.5
2.0
SNP effect on TD CD8br %CD8br

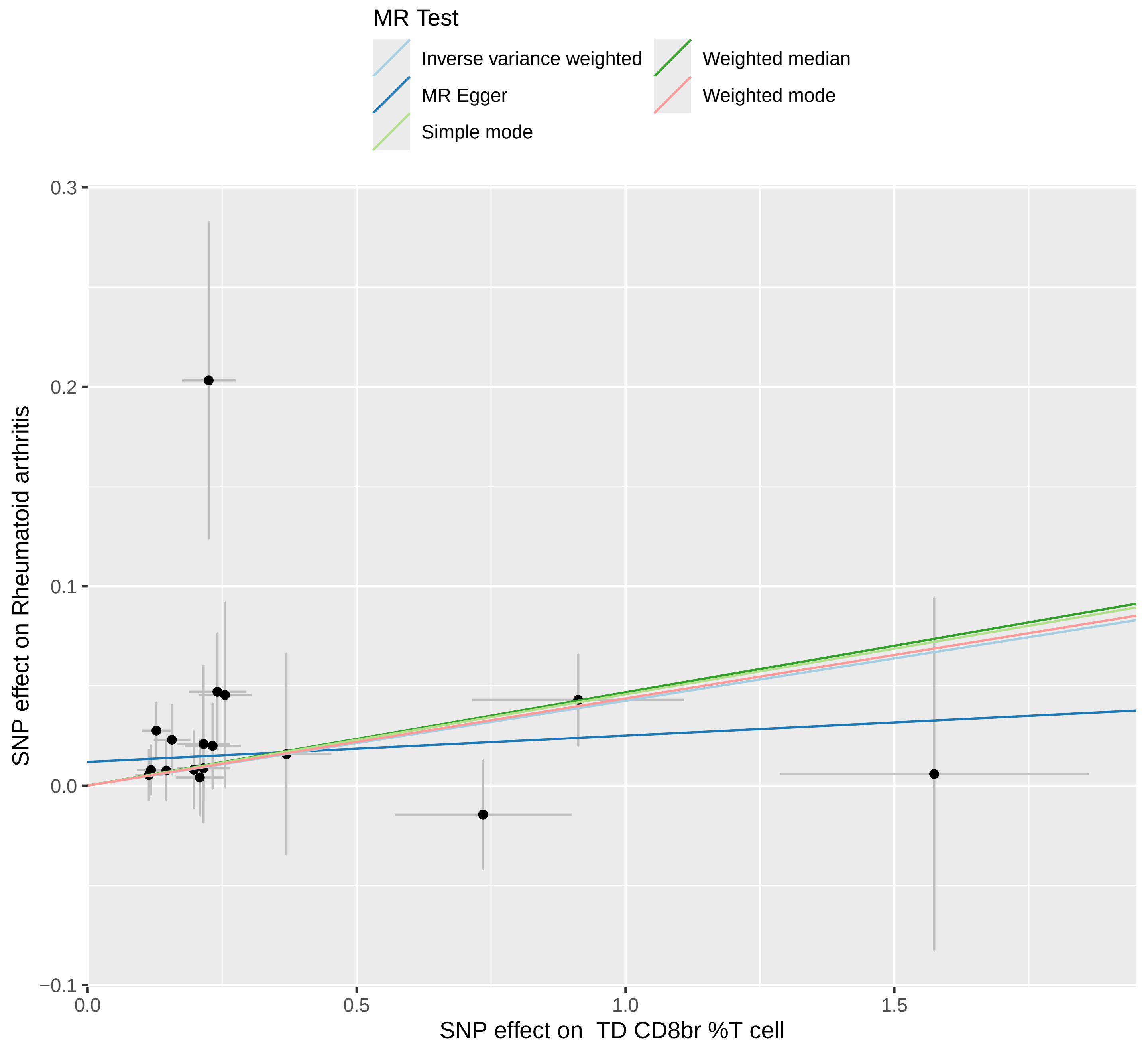
MR Test
Inverse variance weighted
MR Egger
Simple mode
Weighted median
Weighted mode
0.3
0.2
0.1
0.0
−0.1
0.0
0.5
1.0
1.5
SNP effect on Rheumatoid arthritis
SNP effect on TD CD8br %T cell

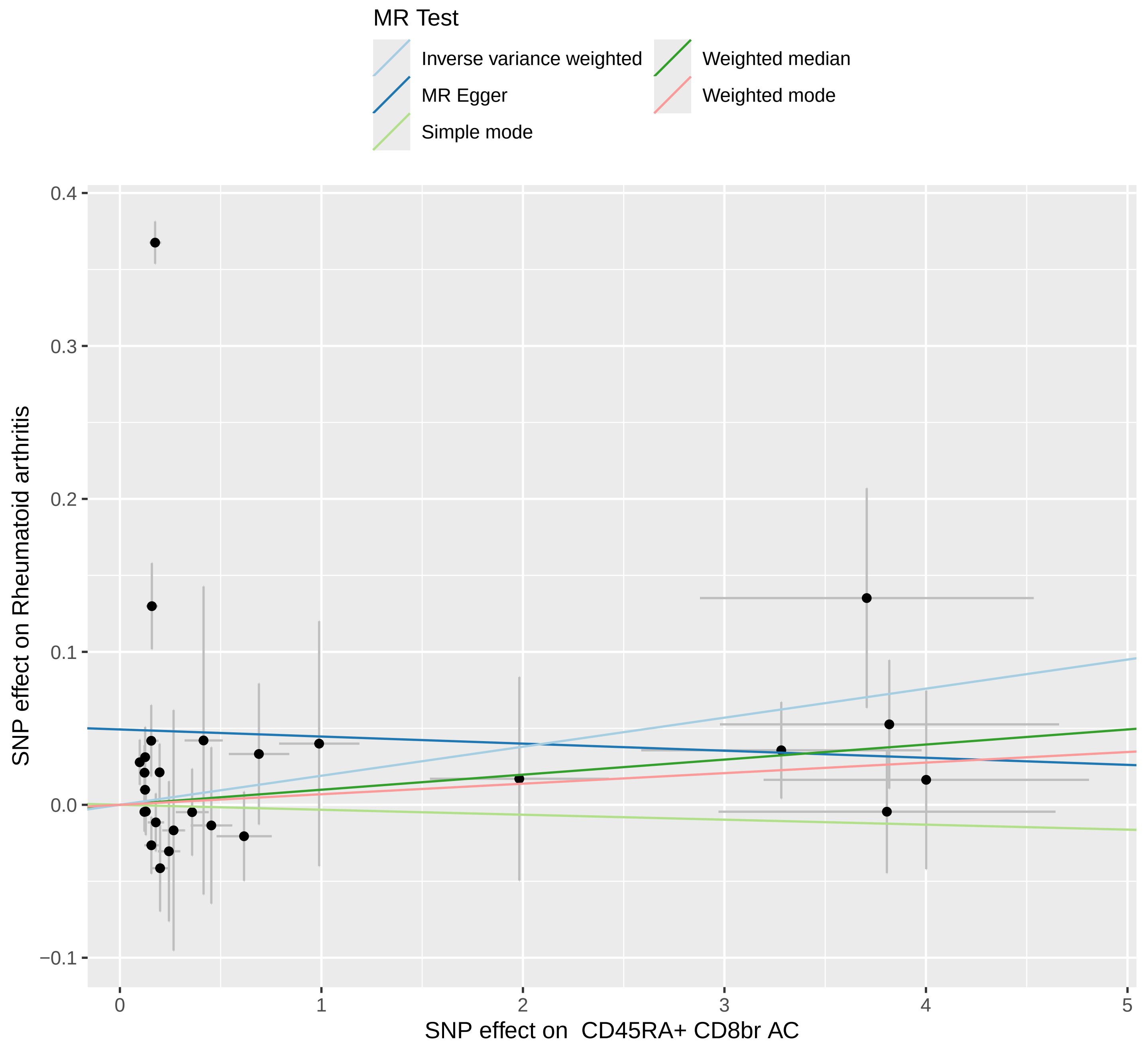
MR Test
Inverse variance weighted
MR Egger
Simple mode
Weighted median
Weighted mode
0.4
0.3
0.2
0.1
0.0
−0.1
SNP effect on Rheumatoid arthritis
0
1
2
3
4
5
SNP effect on CD45RA+ CD8br AC

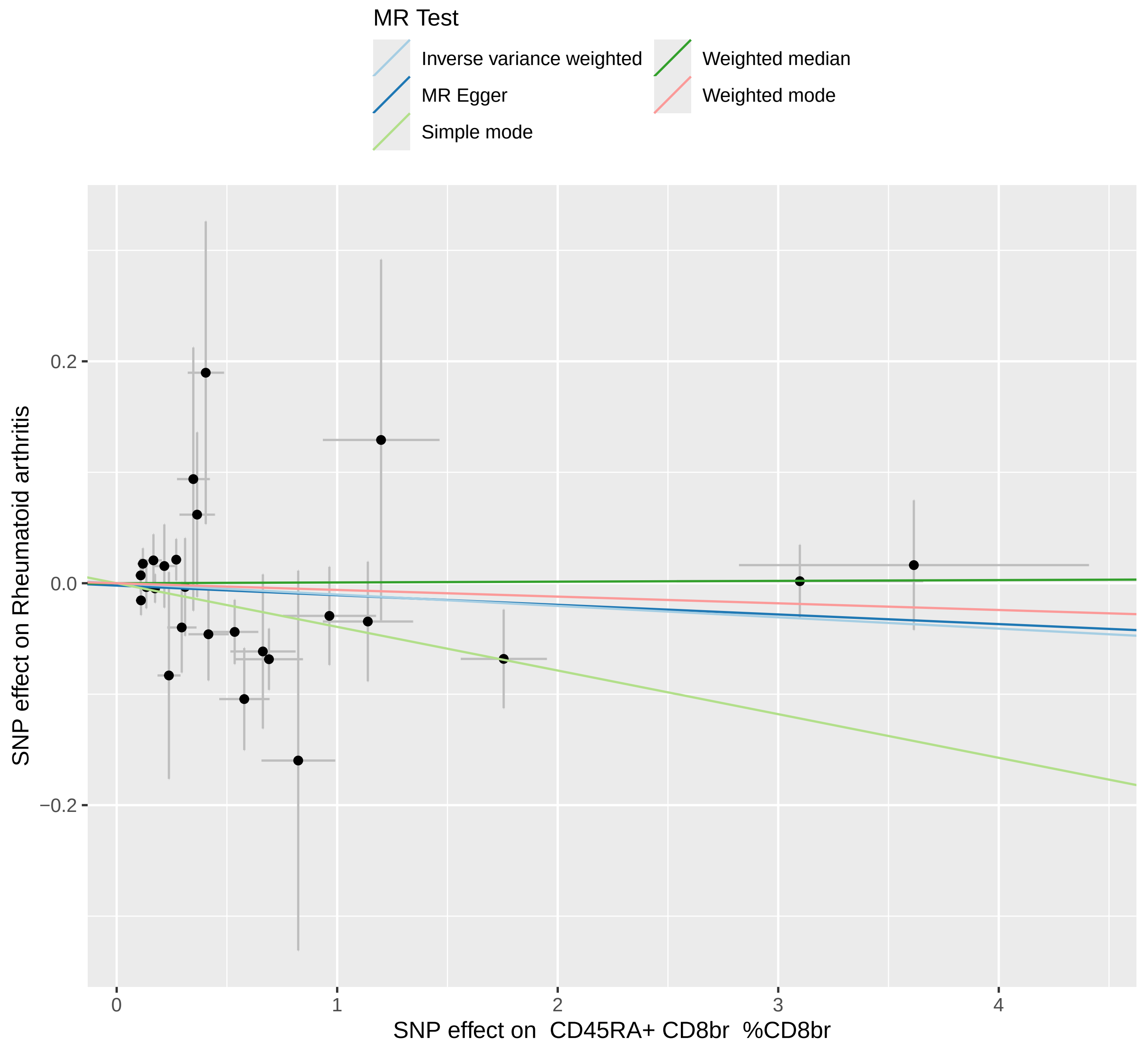
MR Test
Inverse variance weighted
MR Egger
Simple mode
Weighted median
Weighted mode
0.2
0.0
−0.2
SNP effect on Rheumatoid arthritis
0
1
2
3
4
SNP effect on CD45RA+ CD8br %CD8br

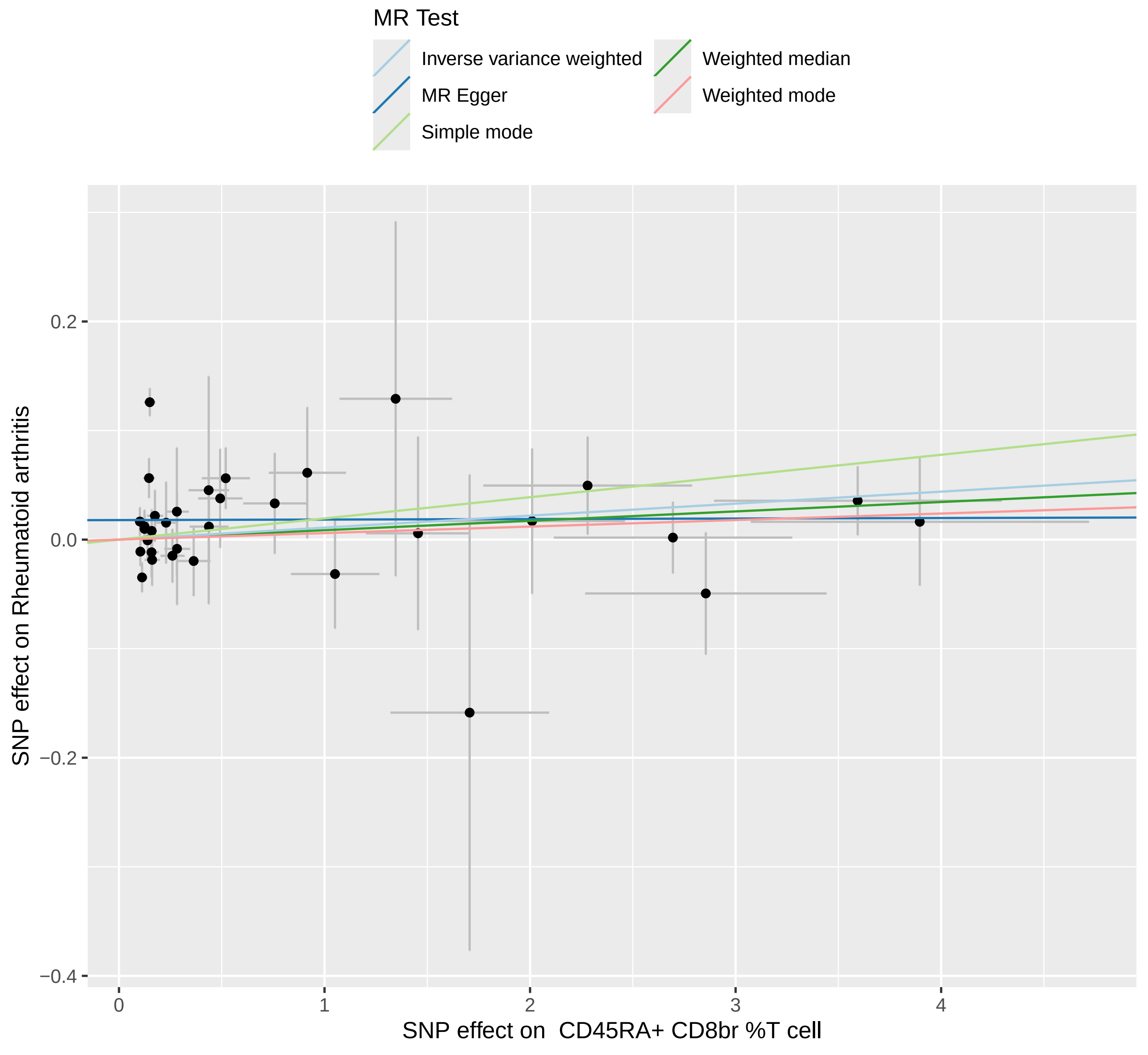

MR Test
Inverse variance weighted
MR Egger
Simple mode
Weighted median
Weighted mode
0.2
0.0
−0.2
−0.4
0
1
2
3
4
SNP effect on Rheumatoid arthritis
SNP effect on CD45RA+ CD8br %T cell

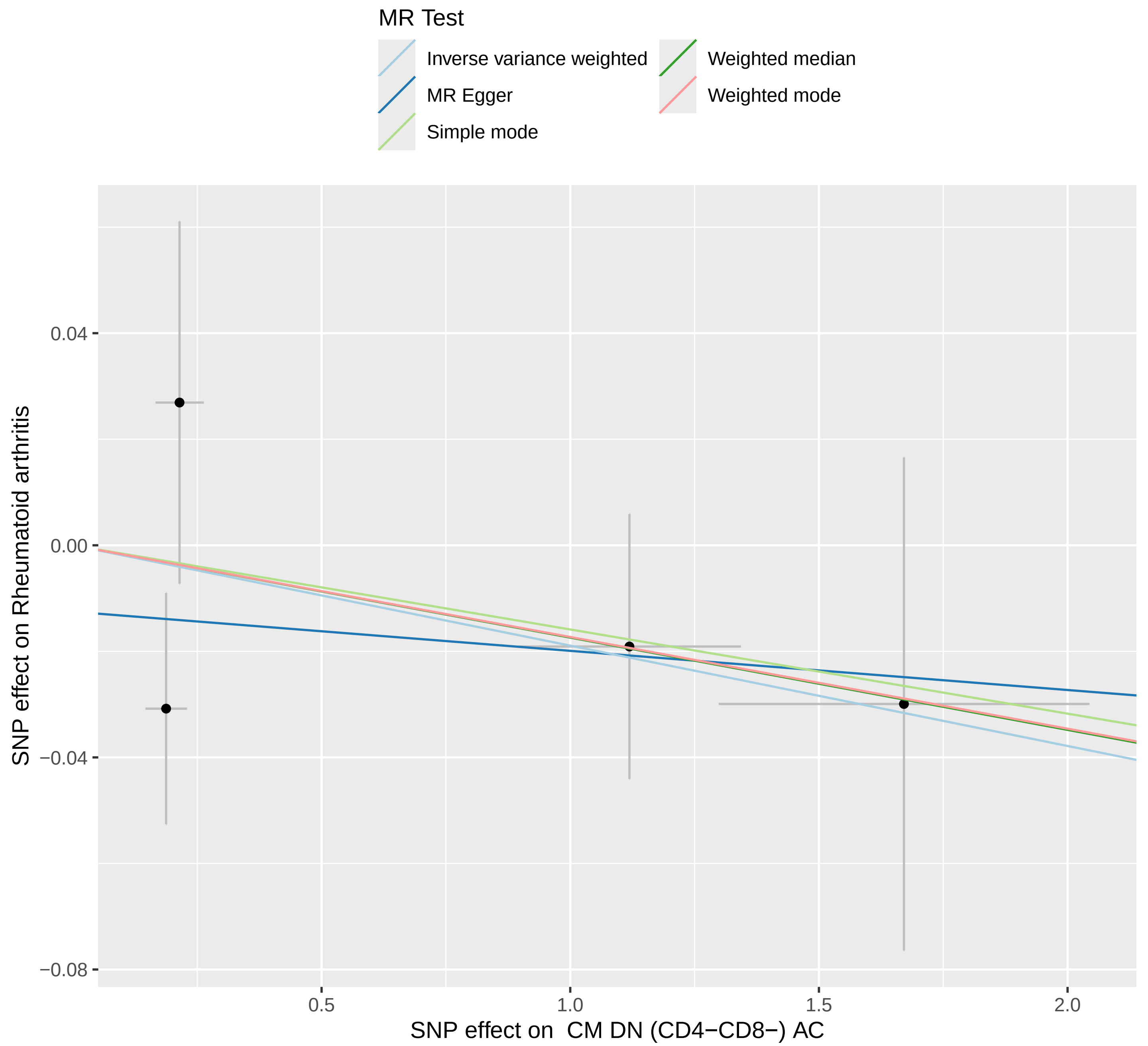
MR Test
Inverse variance weighted
MR Egger
Simple mode
Weighted median
Weighted mode
0.04
0.00
−0.04
−0.08
0.5
1.0
1.5
2.0
SNP effect on Rheumatoid arthritis
SNP effect on CM DN (CD4−CD8−) AC

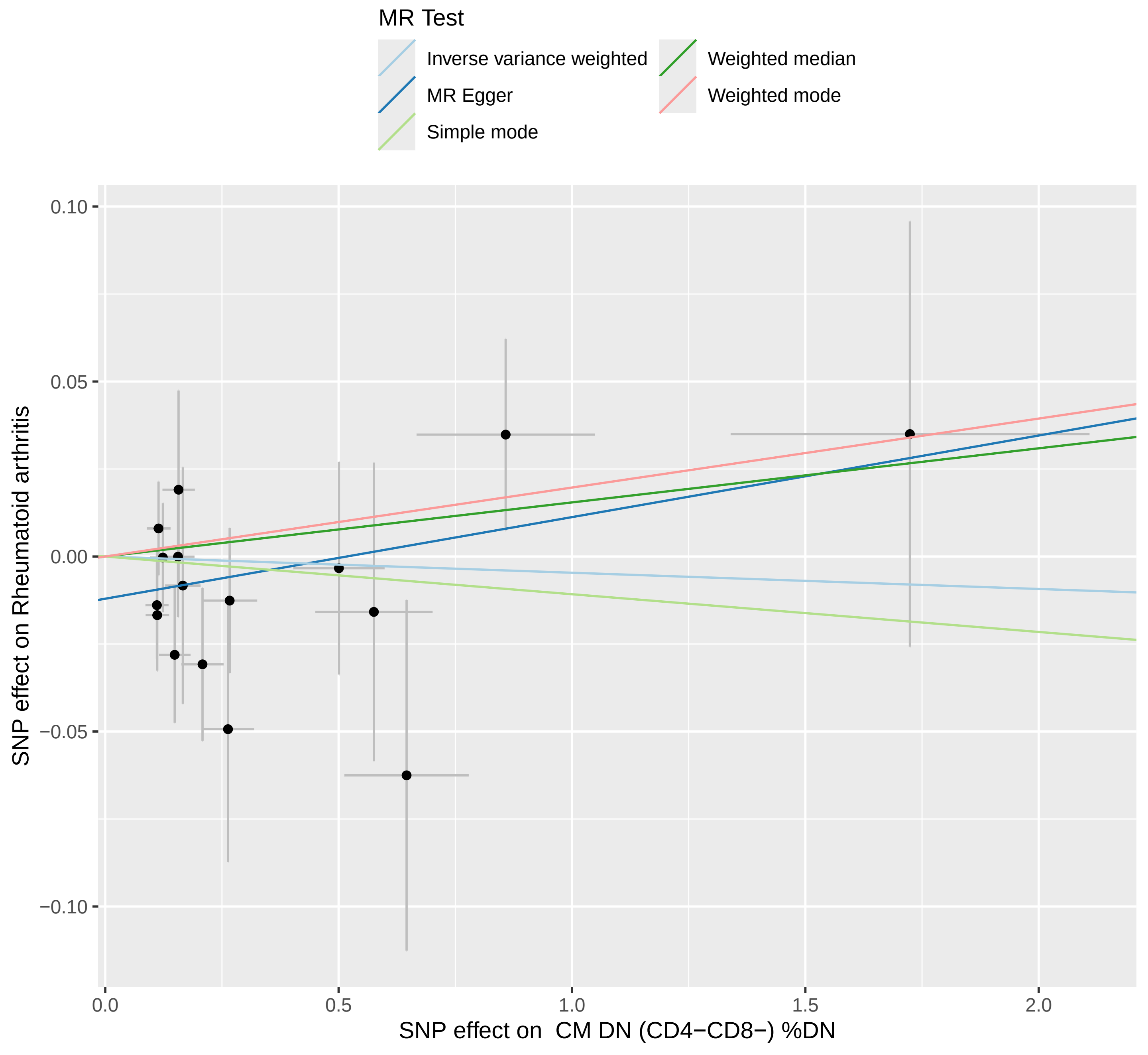

MR Test
Inverse variance weighted
MR Egger
Simple mode
Weighted median
Weighted mode
0.10
0.05
0.00
−0.05
−0.10
SNP effect on Rheumatoid arthritis
0.0
0.5
1.0
1.5
2.0
SNP effect on CM DN (CD4−CD8−) %DN

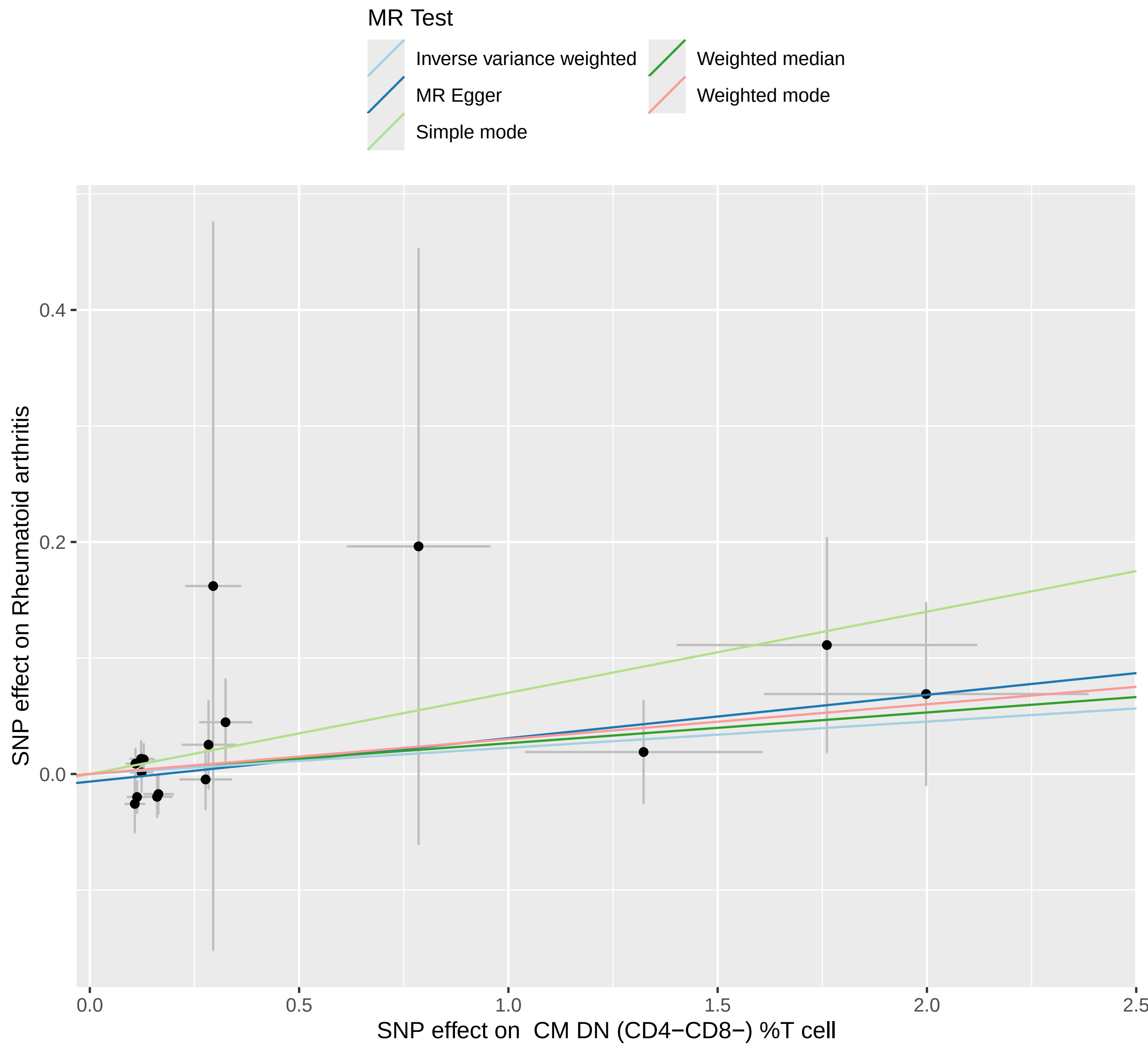

MR Test
Inverse variance weighted
MR Egger
Simple mode
Weighted median
Weighted mode
SNP effect on Rheumatoid arthritis
0.4
0.2
0.0
0.0
0.5
1.0
1.5
2.0
2.5
SNP effect on CM DN (CD4−CD8−) %T cell

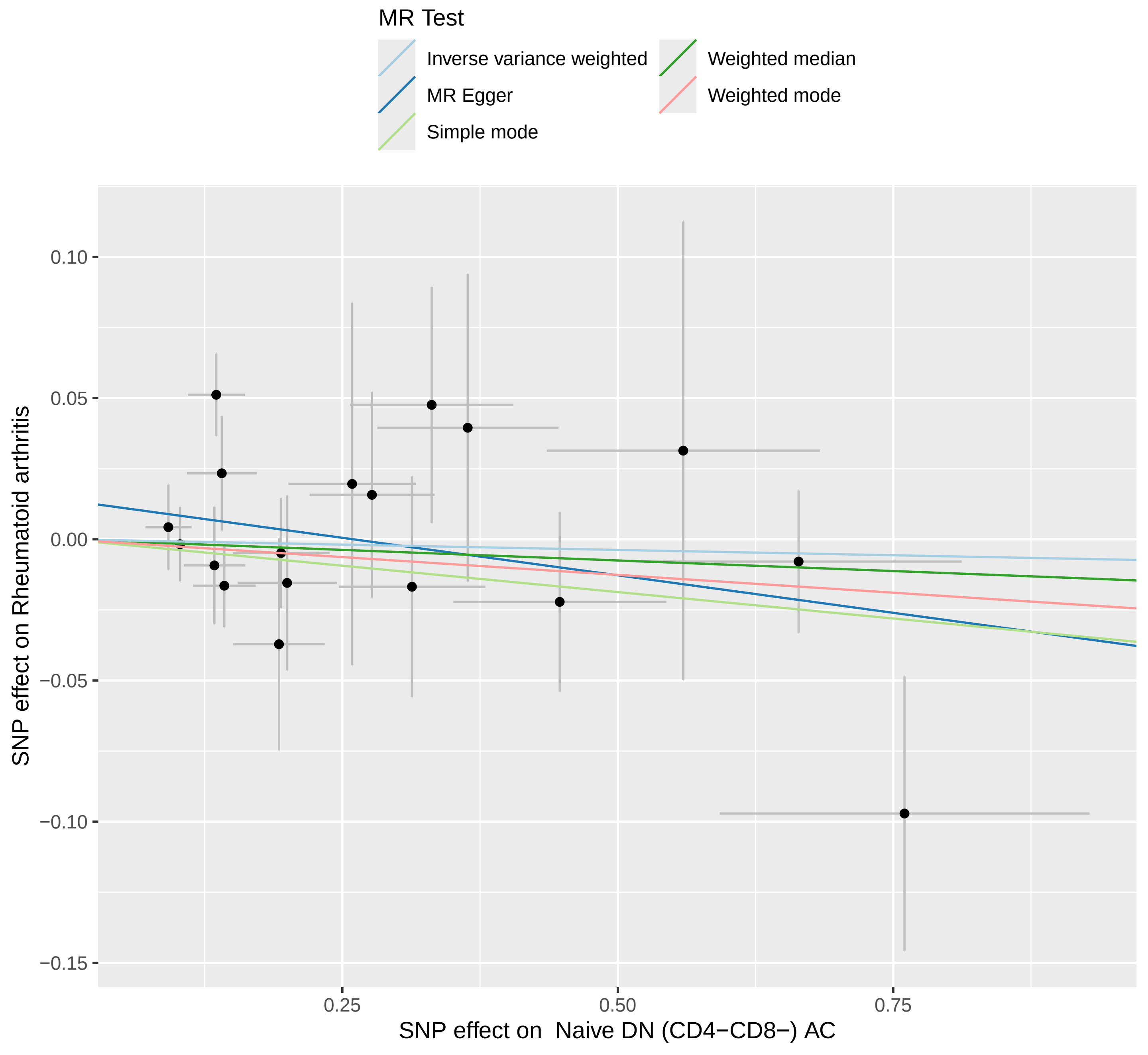
MR Test
Inverse variance weighted
MR Egger
Simple mode
Weighted median
Weighted mode
0.10
0.05
0.00
−0.05
−0.10
−0.15
SNP effect on Rheumatoid arthritis
0.25
0.50
0.75
SNP effect on Naive DN (CD4−CD8−) AC

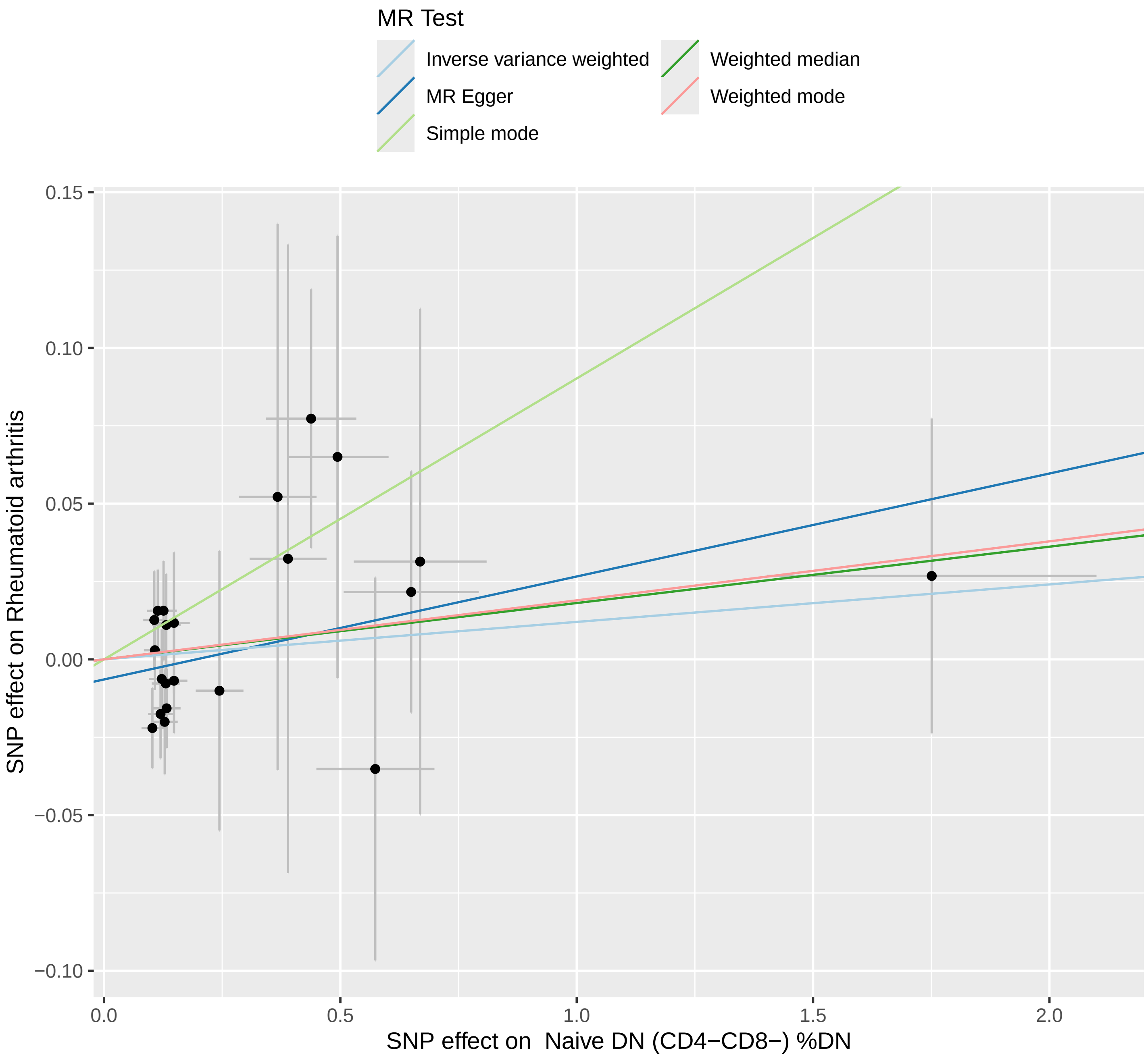

MR Test
Inverse variance weighted
MR Egger
Simple mode
Weighted median
Weighted mode
0.15
0.10
0.05
0.00
−0.05
−0.10
0.0
0.5
1.0
1.5
2.0
SNP effect on Rheumatoid arthritis
SNP effect on Naive DN (CD4−CD8−) %DN

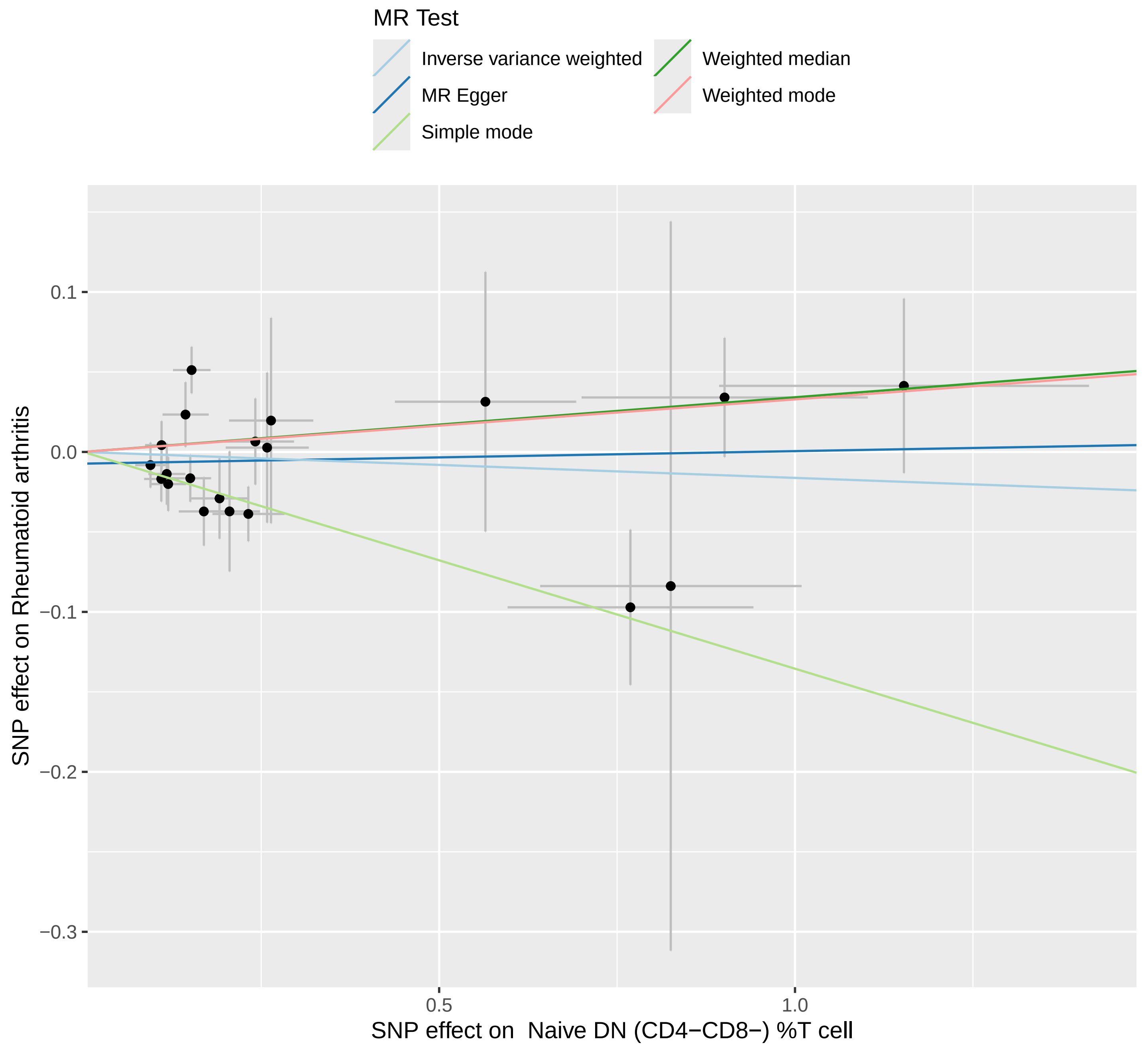

MR Test
Inverse variance weighted
MR Egger
Simple mode
Weighted median
Weighted mode
0.1
0.0
−0.1
−0.2
−0.3
0.5
1.0
SNP effect on Rheumatoid arthritis
SNP effect on Naive DN (CD4−CD8−) %T cell

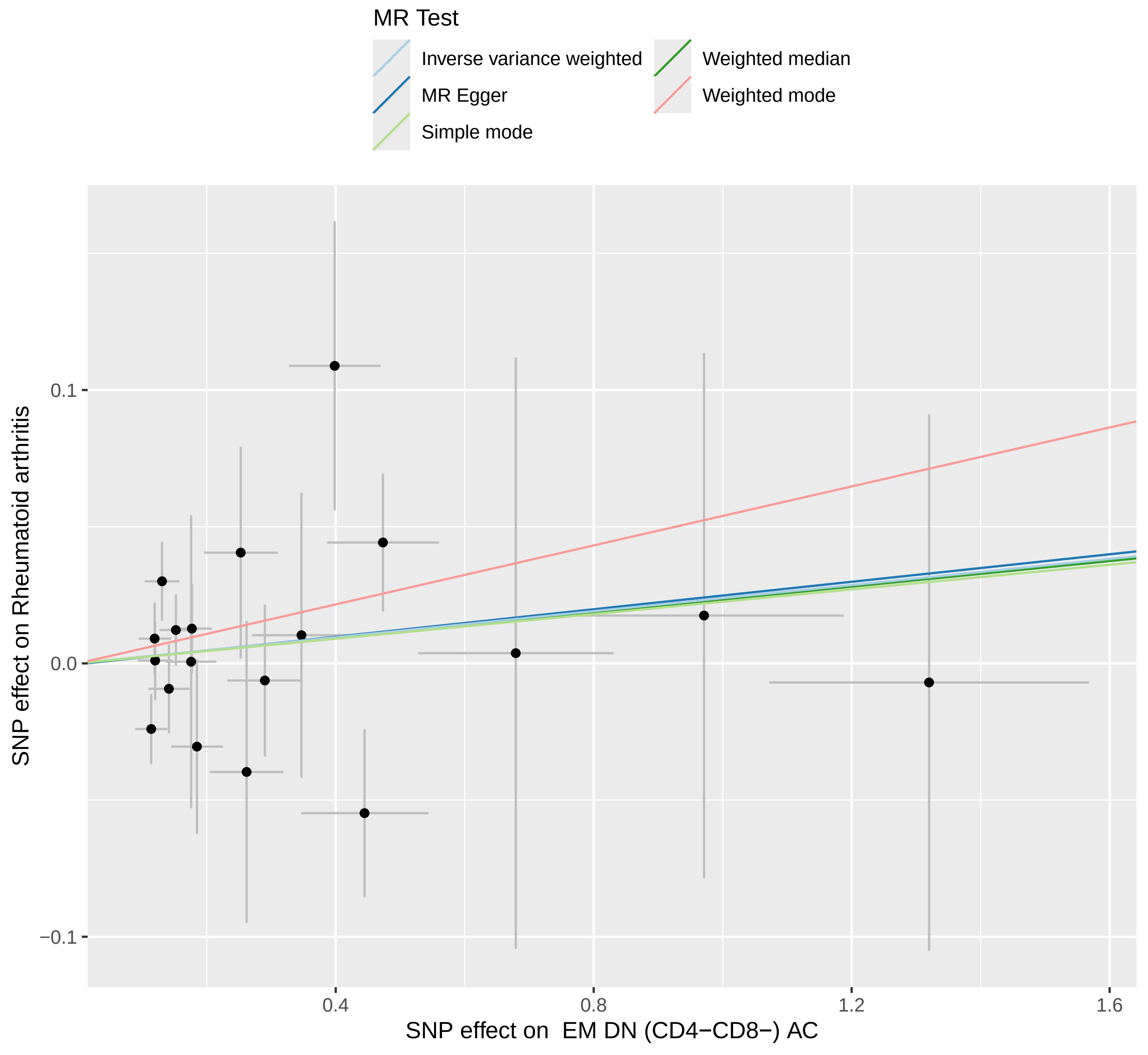
MR Test
Inverse variance weighted
MR Egger
Simple mode
Weighted median
Weighted mode
0.1
0.0
−0.1
0.4
0.8
1.2
1.6
SNP effect on Rheumatoid arthritis
SNP effect on EM DN (CD4−CD8−) AC

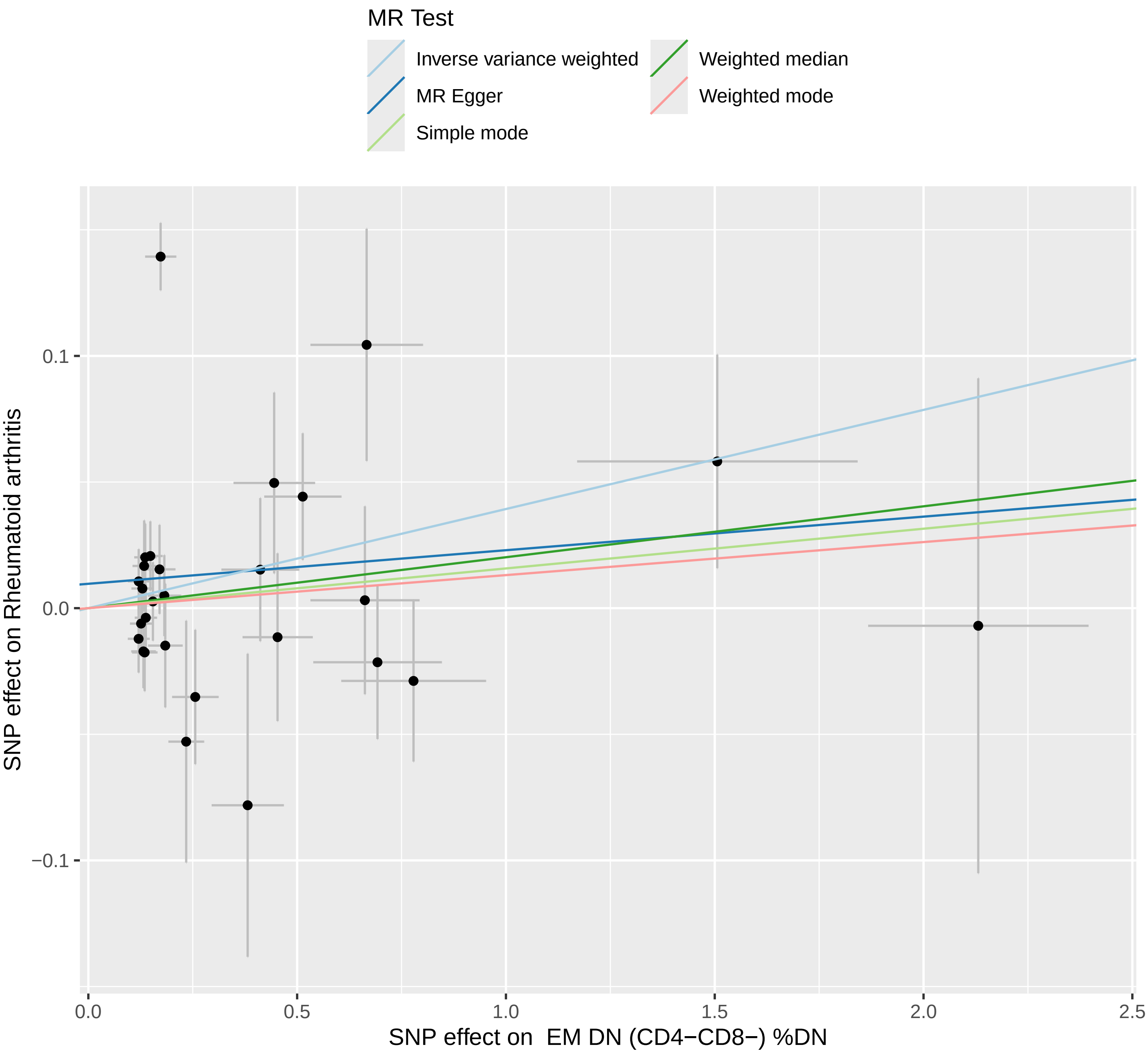
MR Test
Inverse variance weighted
MR Egger
Simple mode
Weighted median
Weighted mode
0.1
0.0
−0.1
0.0
0.5
1.0
1.5
2.0
2.5
SNP effect on Rheumatoid arthritis
SNP effect on EM DN (CD4−CD8−) %DN

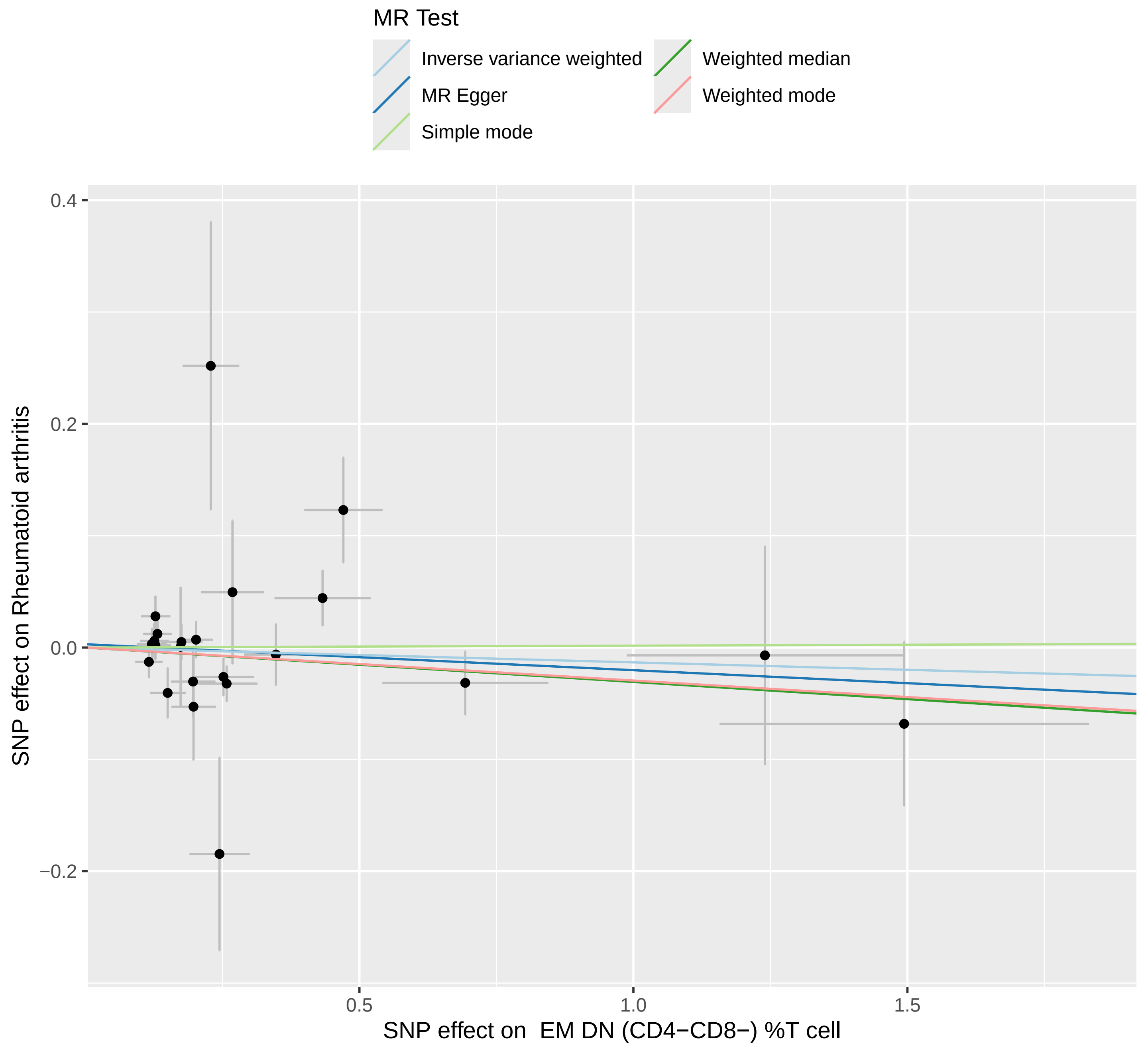

MR Test
Inverse variance weighted
MR Egger
Simple mode
Weighted median
Weighted mode
SNP effect on Rheumatoid arthritis
0.4
0.2
0.0
−0.2
0.5
1.0
1.5
SNP effect on EM DN (CD4−CD8−) %T cell

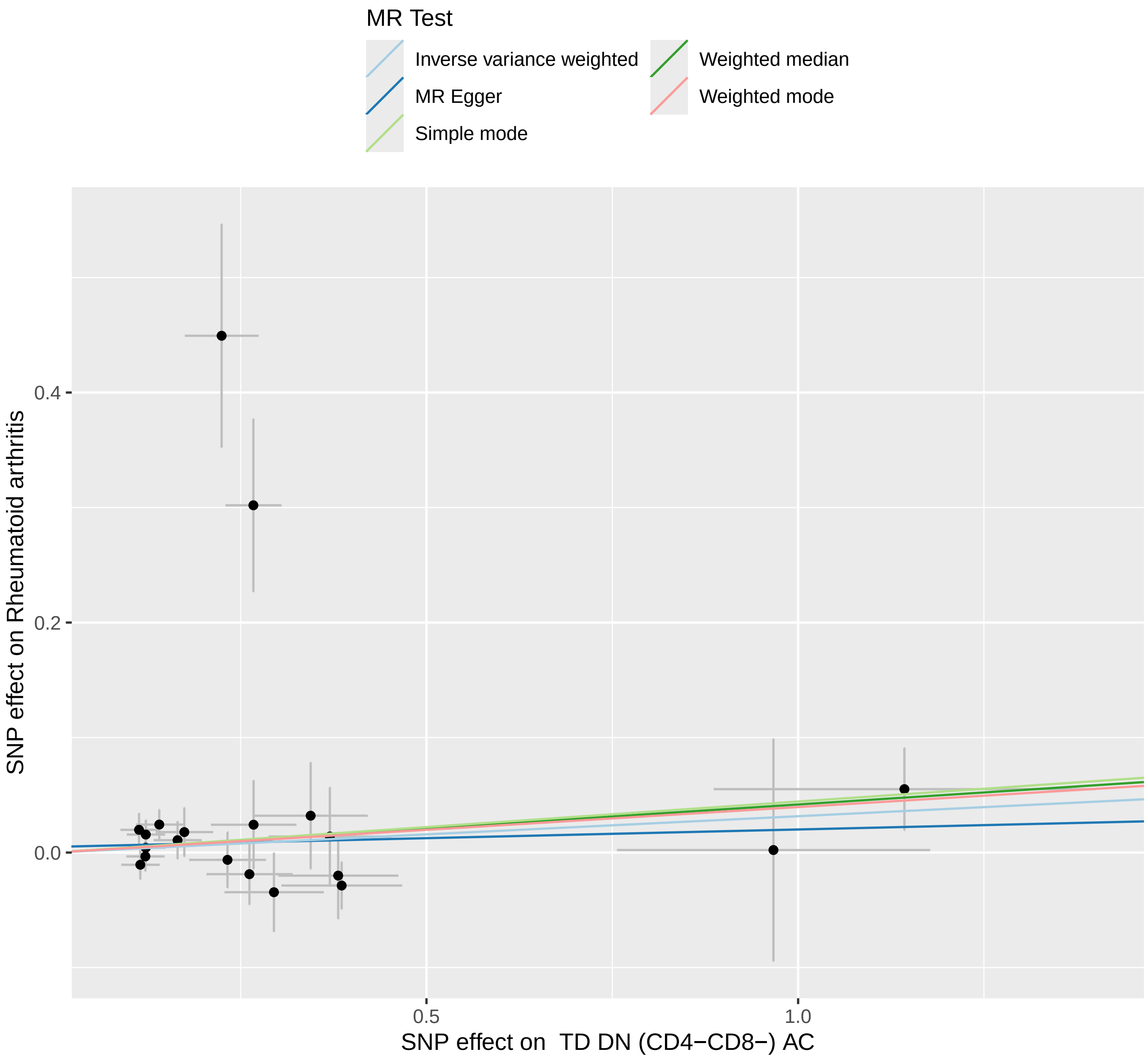

MR Test
Inverse variance weighted
MR Egger
Simple mode
Weighted median
Weighted mode
0.4
0.2
0.0
0.5
1.0
SNP effect on Rheumatoid arthritis
SNP effect on TD DN (CD4−CD8−) AC

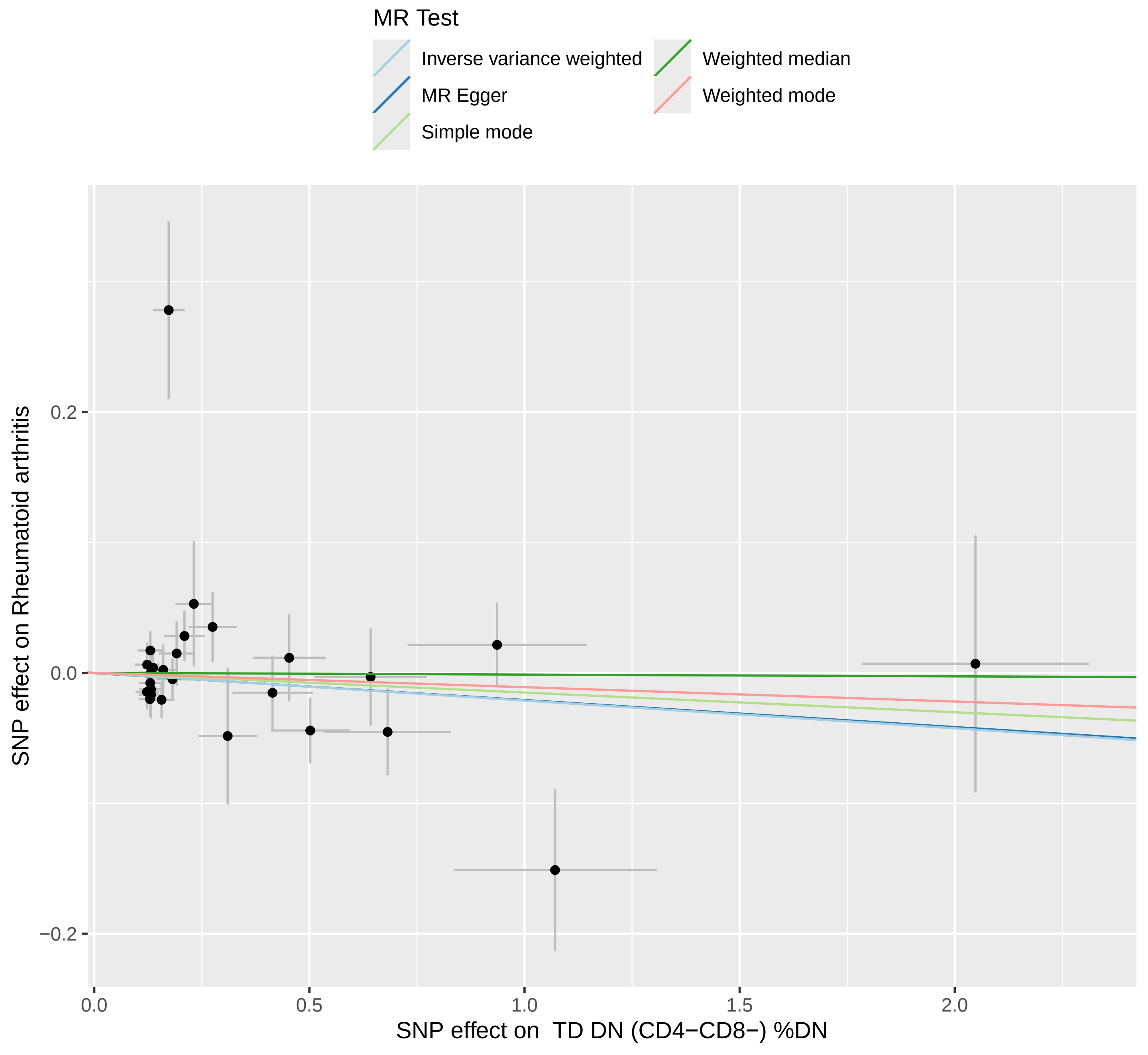

MR Test
Inverse variance weighted
MR Egger
Simple mode
Weighted median
Weighted mode
0.2
0.0
−0.2
0.0
0.5
1.0
1.5
2.0
SNP effect on Rheumatoid arthritis
SNP effect on TD DN (CD4−CD8−) %DN

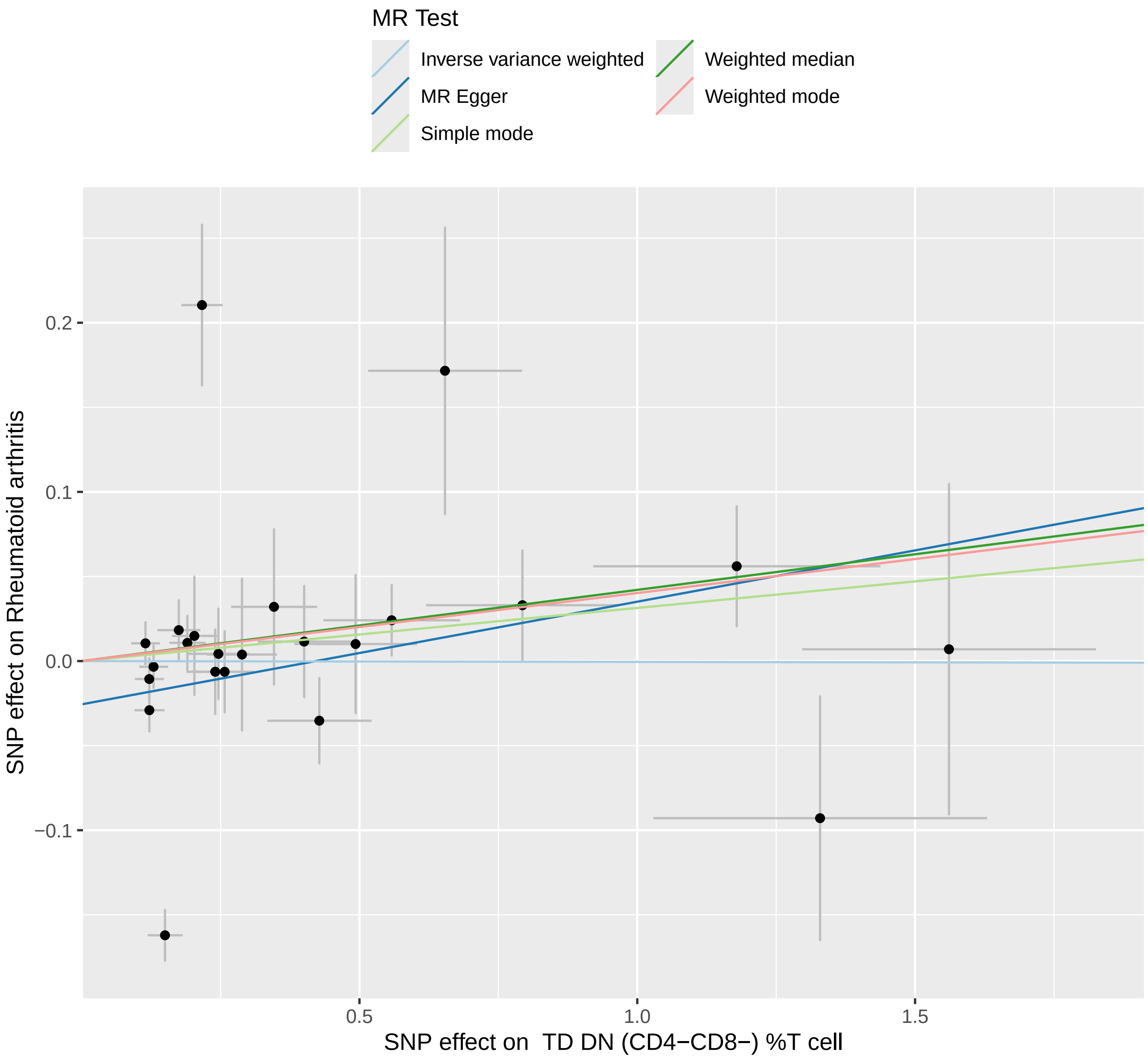

MR Test
Inverse variance weighted
MR Egger
Simple mode
Weighted median
Weighted mode
SNP effect on Rheumatoid arthritis
0.2
0.1
0.0
−0.1
0.5
1.0
1.5
SNP effect on TD DN (CD4−CD8−) %T cell

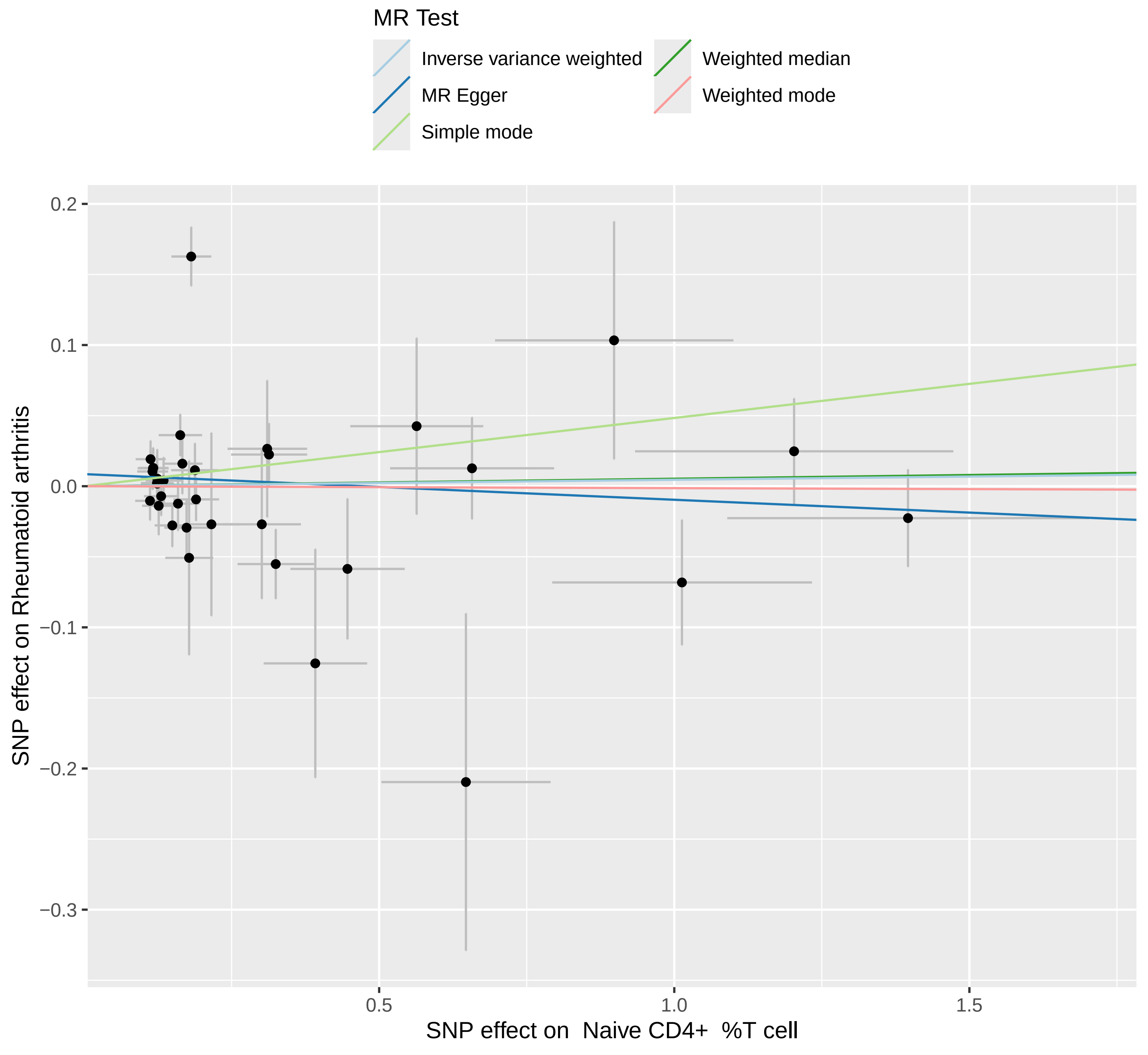
MR Test
Inverse variance weighted
Weighted median
MR Egger
Weighted mode
Simple mode
0.2
0.1
0.0
−0.1
−0.2
−0.3
0.5
1.0
1.5
SNP effect on Rheumatoid arthritis
SNP effect on Naive CD4+ %T cell

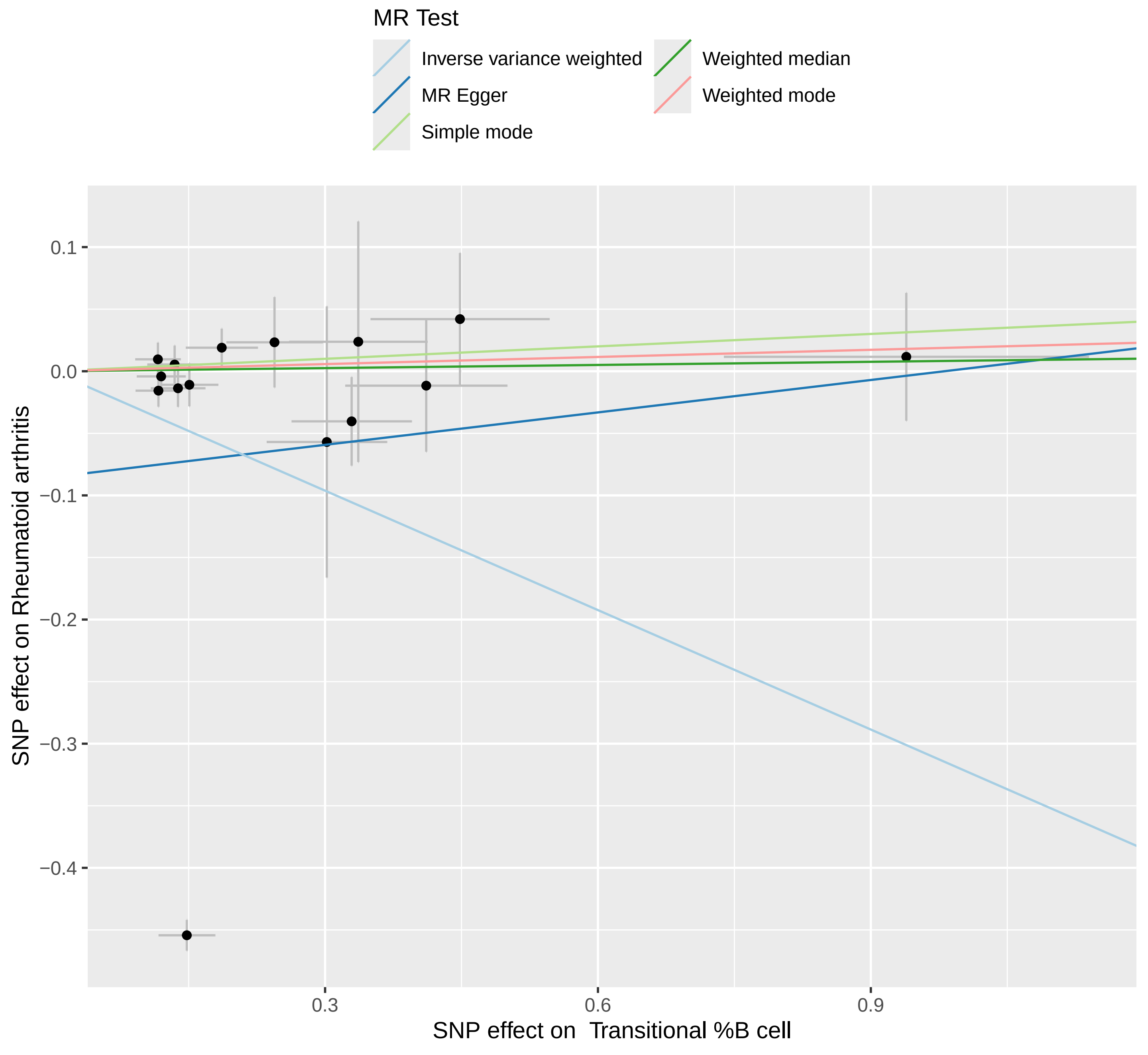

MR Test
Inverse variance weighted
MR Egger
Simple mode
Weighted median
Weighted mode
0.1
0.0
−0.1
−0.2
−0.3
−0.4
SNP effect on Rheumatoid arthritis
0.3
0.6
0.9
SNP effect on Transitional %B cell

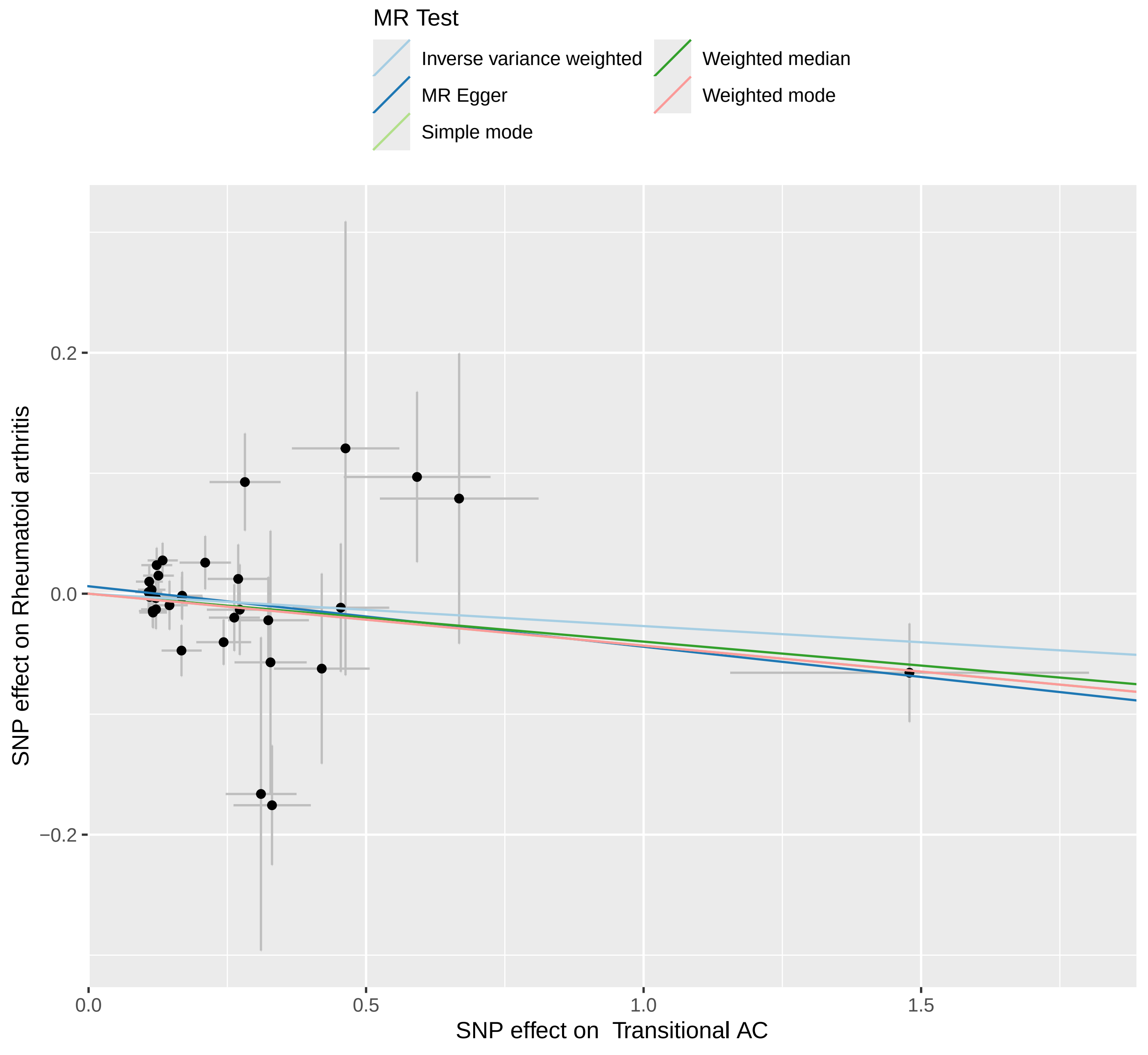

MR Test
Inverse variance weighted
MR Egger
Simple mode
Weighted median
Weighted mode
0.2
0.0
−0.2
0.0
0.5
1.0
1.5
SNP effect on Rheumatoid arthritis
SNP effect on Transitional AC

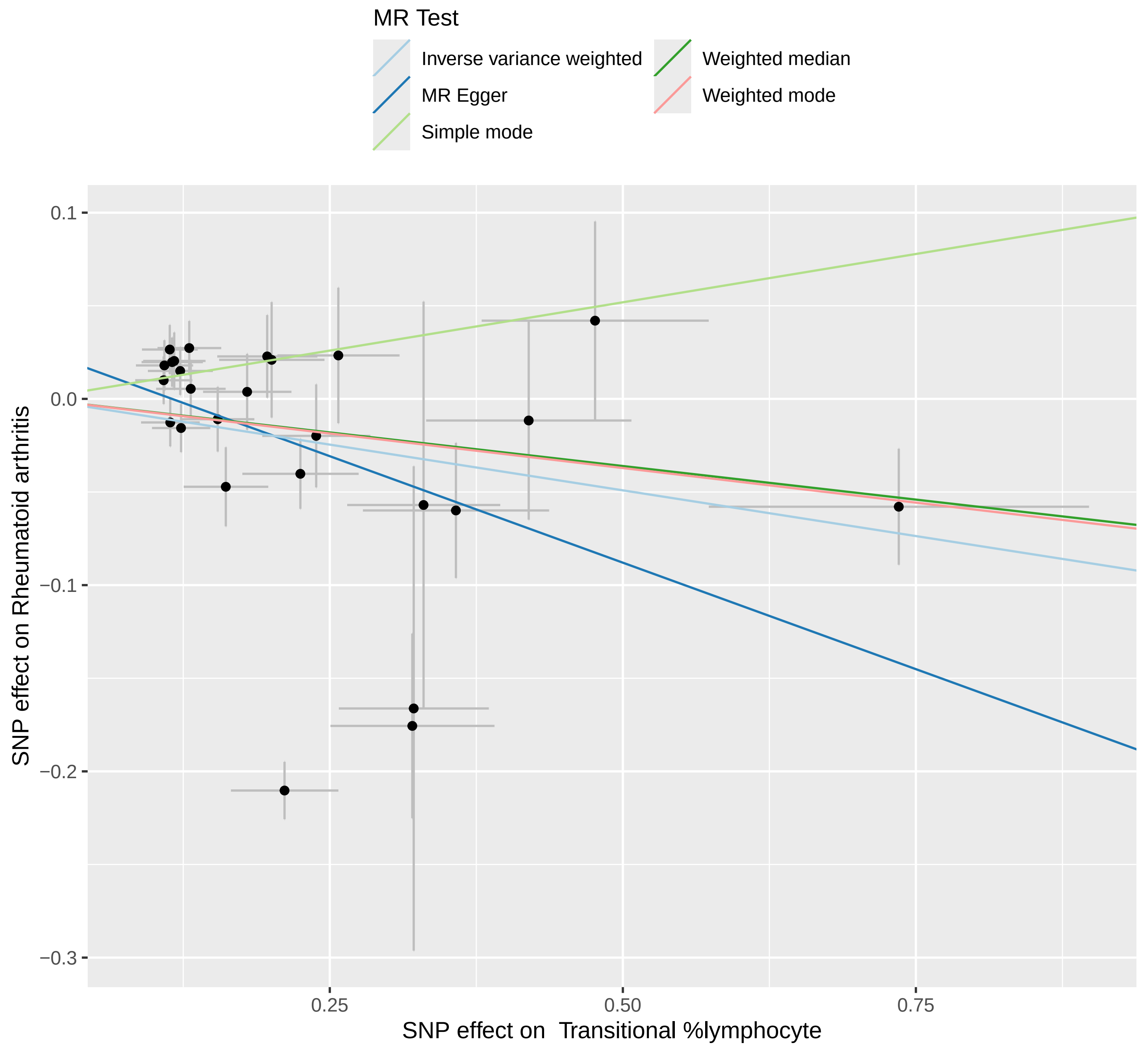

MR Test
Inverse variance weighted
MR Egger
Simple mode
Weighted median
Weighted mode
0.1
0.0
−0.1
−0.2
−0.3
SNP effect on Rheumatoid arthritis
0.25
0.50
0.75
SNP effect on Transitional %lymphocyte

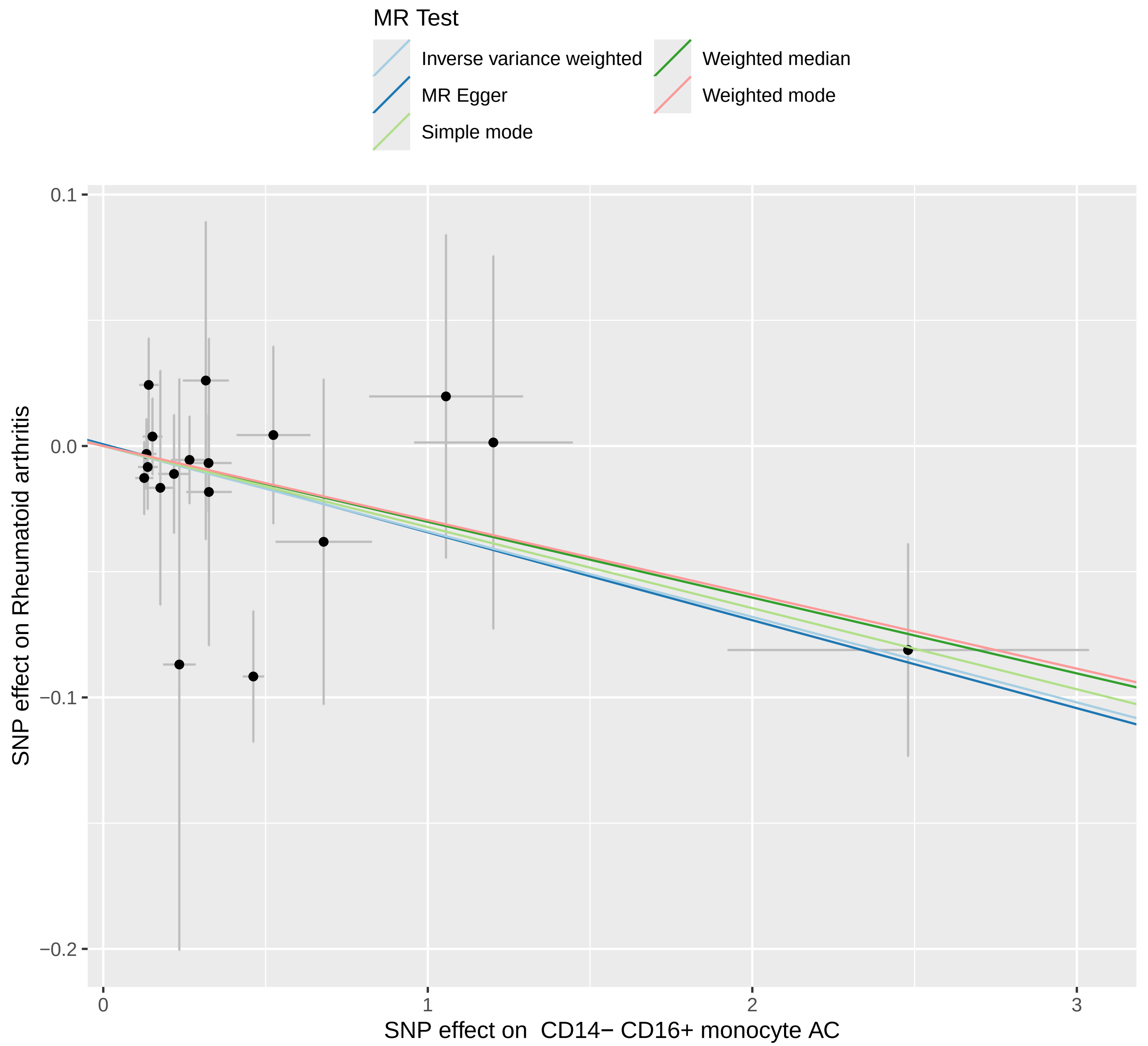
MR Test
Inverse variance weighted
MR Egger
Simple mode
Weighted median
Weighted mode
0.1
0.0
−0.1
−0.2
0
1
2
3
SNP effect on Rheumatoid arthritis
SNP effect on CD14− CD16+ monocyte AC

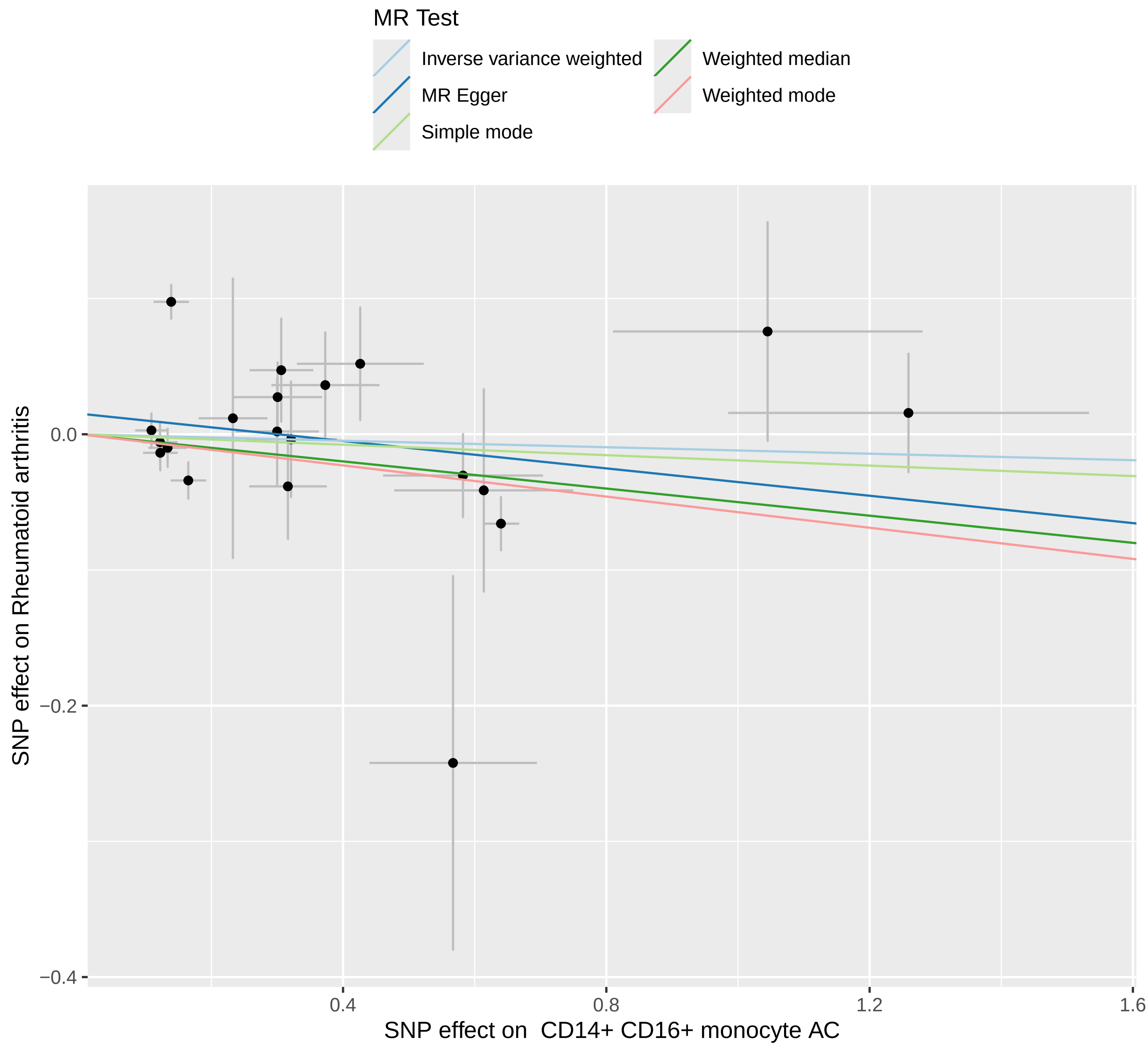

MR Test
Inverse variance weighted
MR Egger
Simple mode
Weighted median
Weighted mode
SNP effect on Rheumatoid arthritis
0.0
−0.2
−0.4
0.4
0.8
1.2
1.6
SNP effect on CD14+ CD16+ monocyte AC

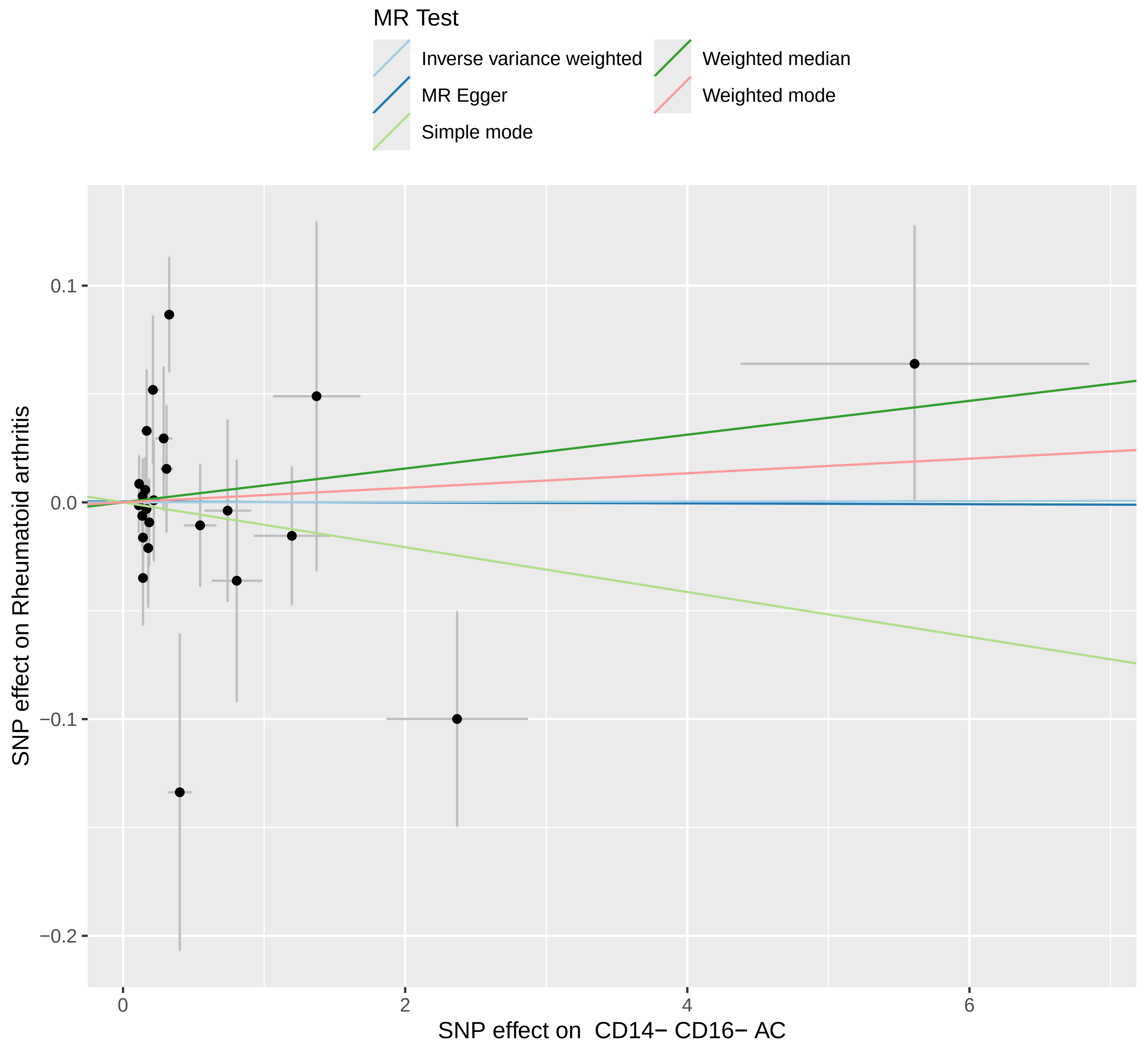

MR Test
Inverse variance weighted
MR Egger
Simple mode
Weighted median
Weighted mode
0.1
0.0
−0.1
−0.2
0
2
4
6
SNP effect on Rheumatoid arthritis
SNP effect on CD14− CD16− AC

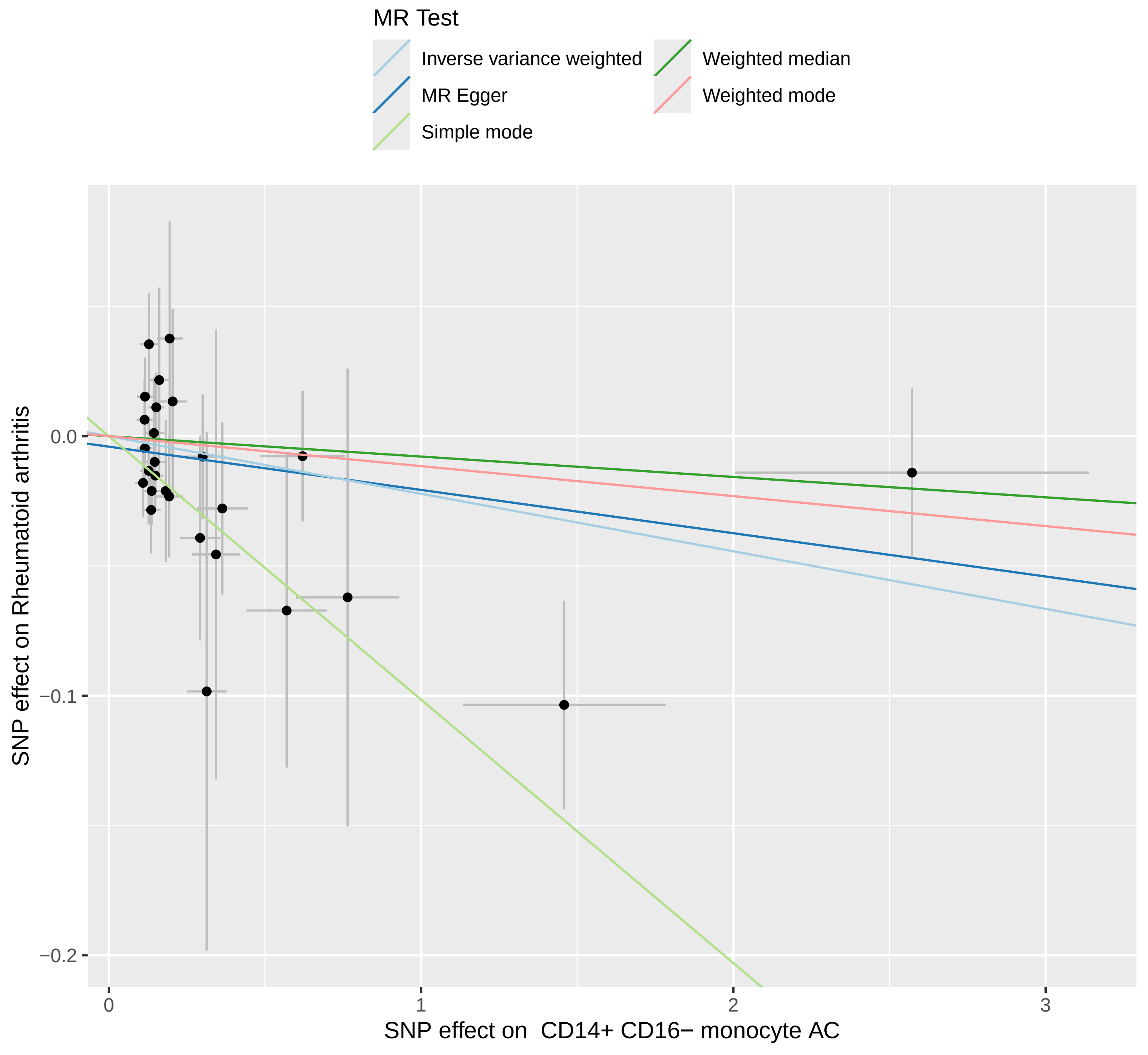

MR Test
Inverse variance weighted
MR Egger
Simple mode
Weighted median
Weighted mode
SNP effect on Rheumatoid arthritis
0.0
−0.1
−0.2
0
1
2
3
SNP effect on CD14+ CD16− monocyte AC

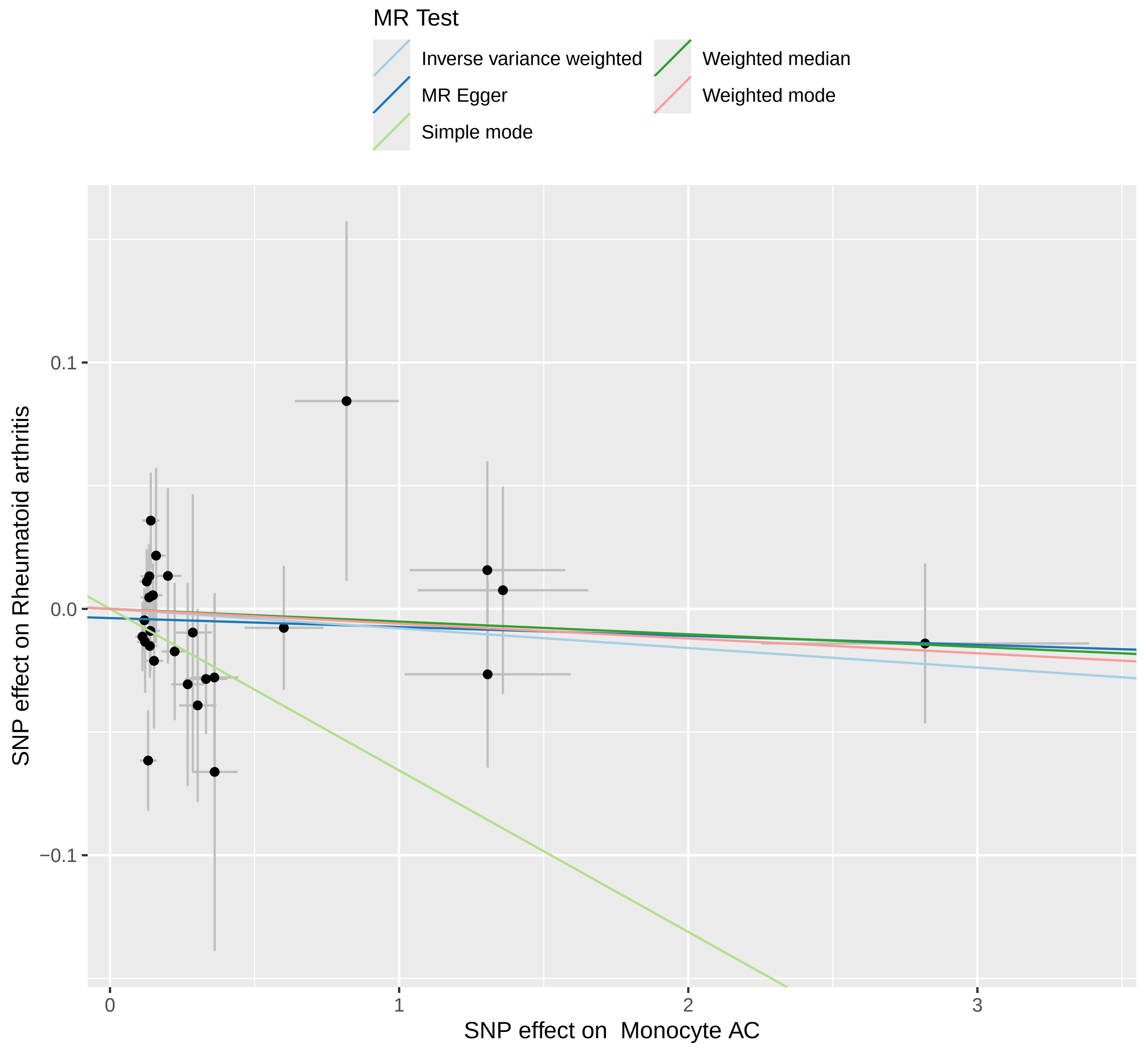
MR Test
Inverse variance weighted
MR Egger
Simple mode
Weighted median
Weighted mode
SNP effect on Rheumatoid arthritis
0.1
0.0
−0.1
0
1
2
3
SNP effect on Monocyte AC

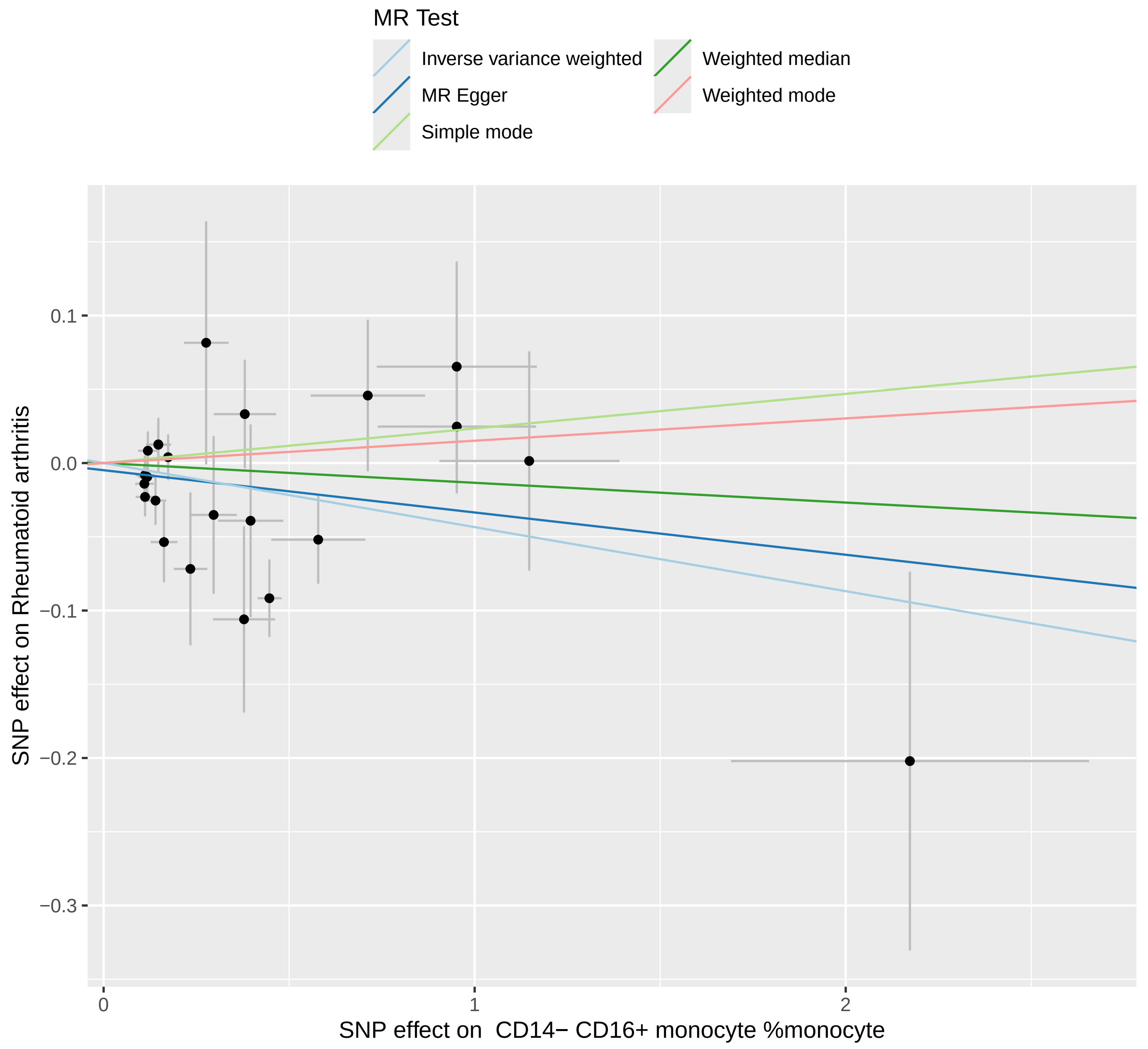

MR Test
Inverse variance weighted
MR Egger
Simple mode
Weighted median
Weighted mode
0.1
0.0
−0.1
−0.2
−0.3
0
1
2
SNP effect on Rheumatoid arthritis
SNP effect on CD14− CD16+ monocyte %monocyte

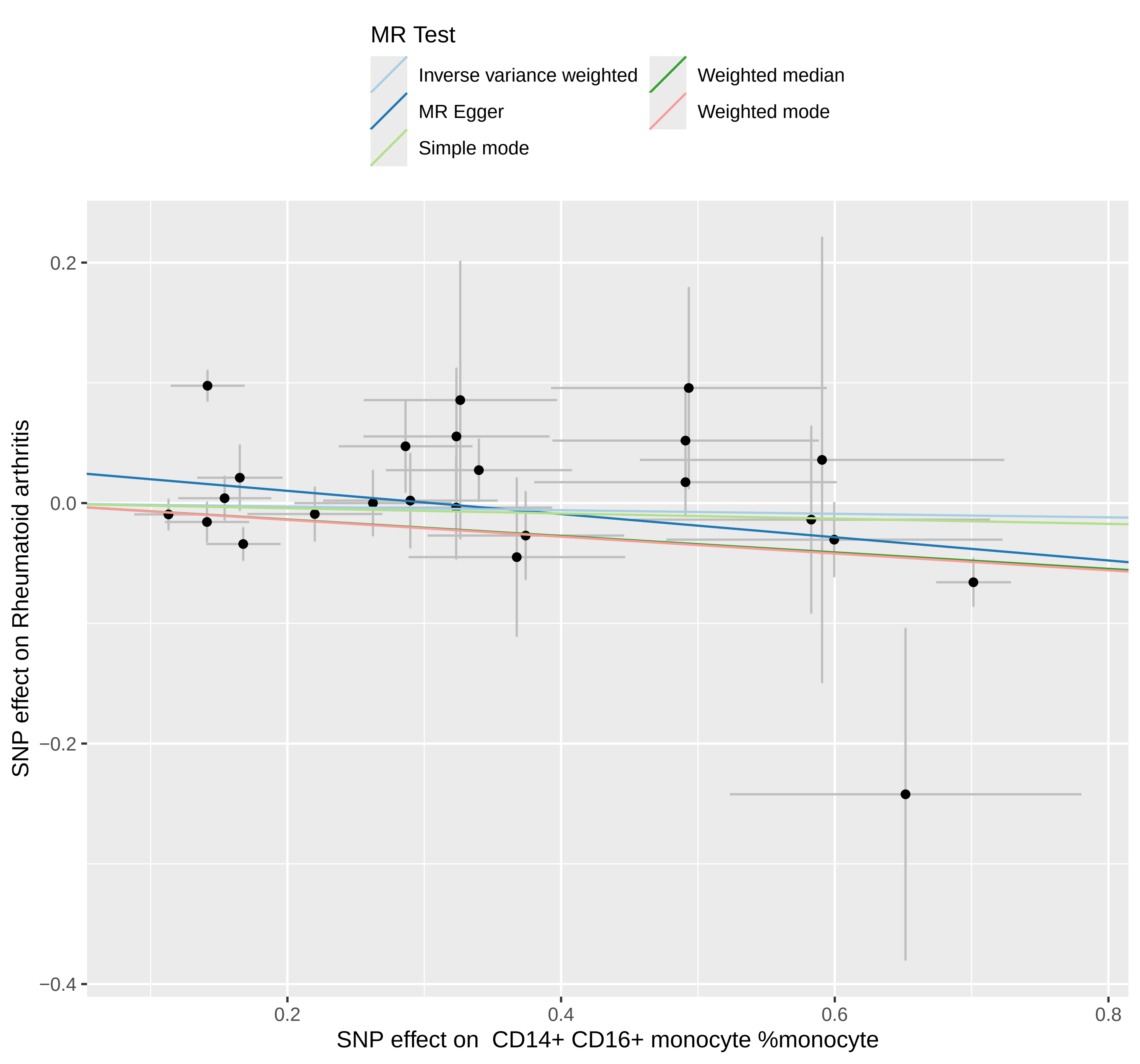

MR Test
Inverse variance weighted
MR Egger
Simple mode
Weighted median
Weighted mode
0.2
0.0
−0.2
−0.4
0.2
0.4
0.6
0.8
SNP effect on Rheumatoid arthritis
SNP effect on CD14+ CD16+ monocyte %monocyte

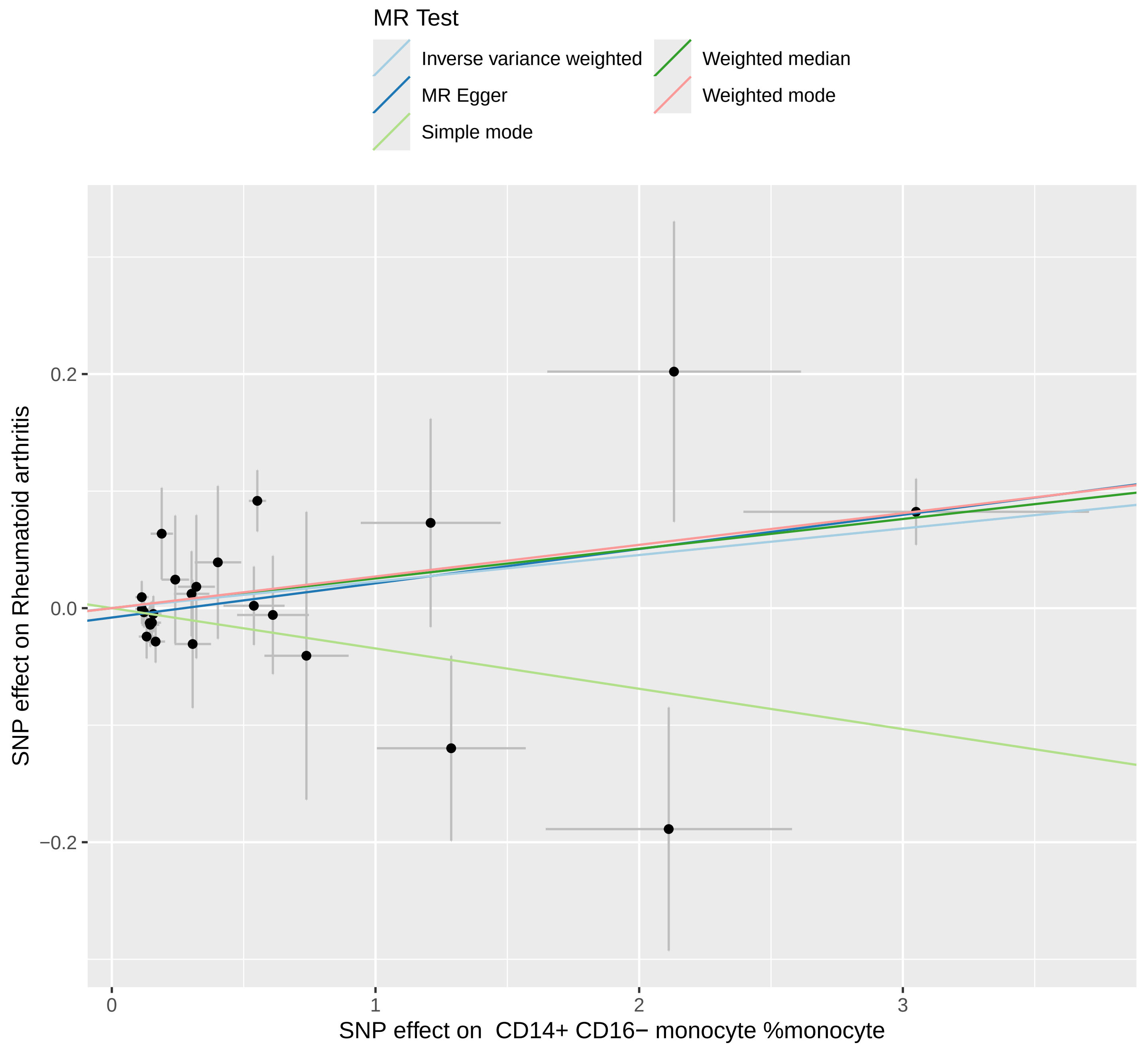

MR Test
Inverse variance weighted
MR Egger
Simple mode
Weighted median
Weighted mode
SNP effect on Rheumatoid arthritis
0.2
0.0
−0.2
0
1
2
3
SNP effect on CD14+ CD16− monocyte %monocyte

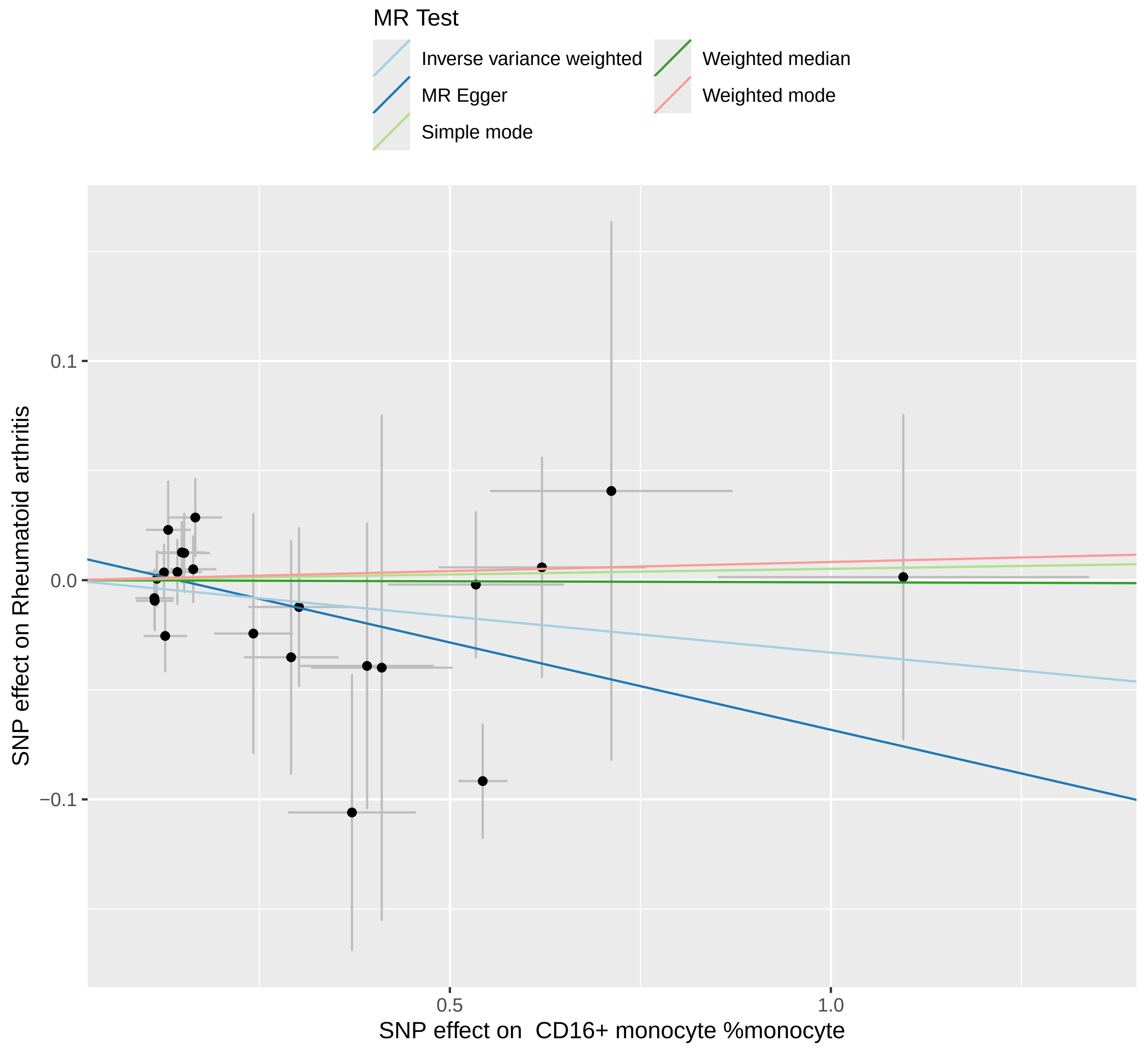
MR Test
Inverse variance weighted
MR Egger
Simple mode
Weighted median
Weighted mode
SNP effect on Rheumatoid arthritis
0.1
0.0
−0.1
0.5
1.0
SNP effect on CD16+ monocyte %monocyte

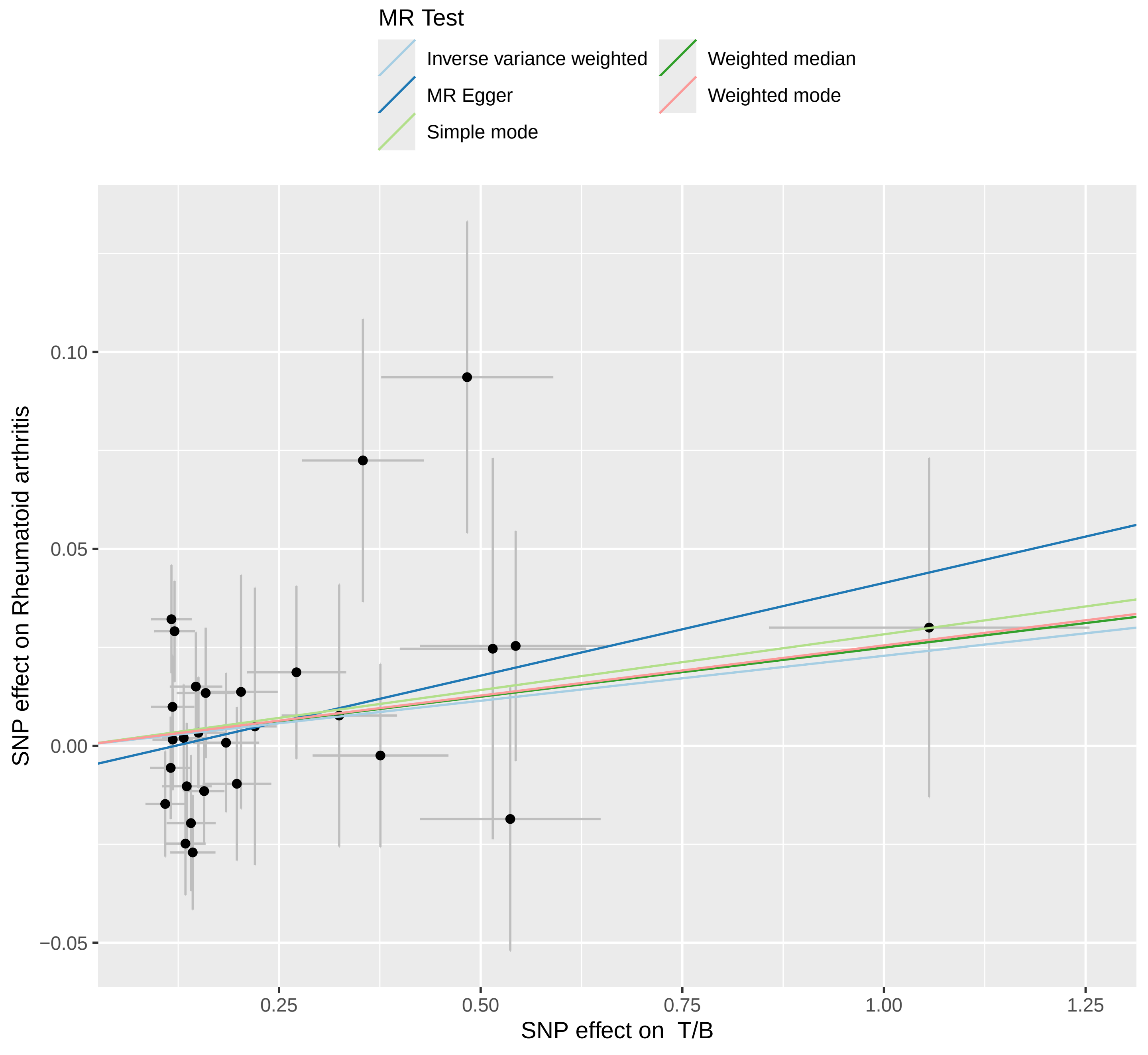

MR Test
Inverse variance weighted
MR Egger
Simple mode
Weighted median
Weighted mode
0.10
0.05
0.00
−0.05
0.25
0.50
0.75
1.00
1.25
SNP effect on Rheumatoid arthritis
SNP effect on T/B

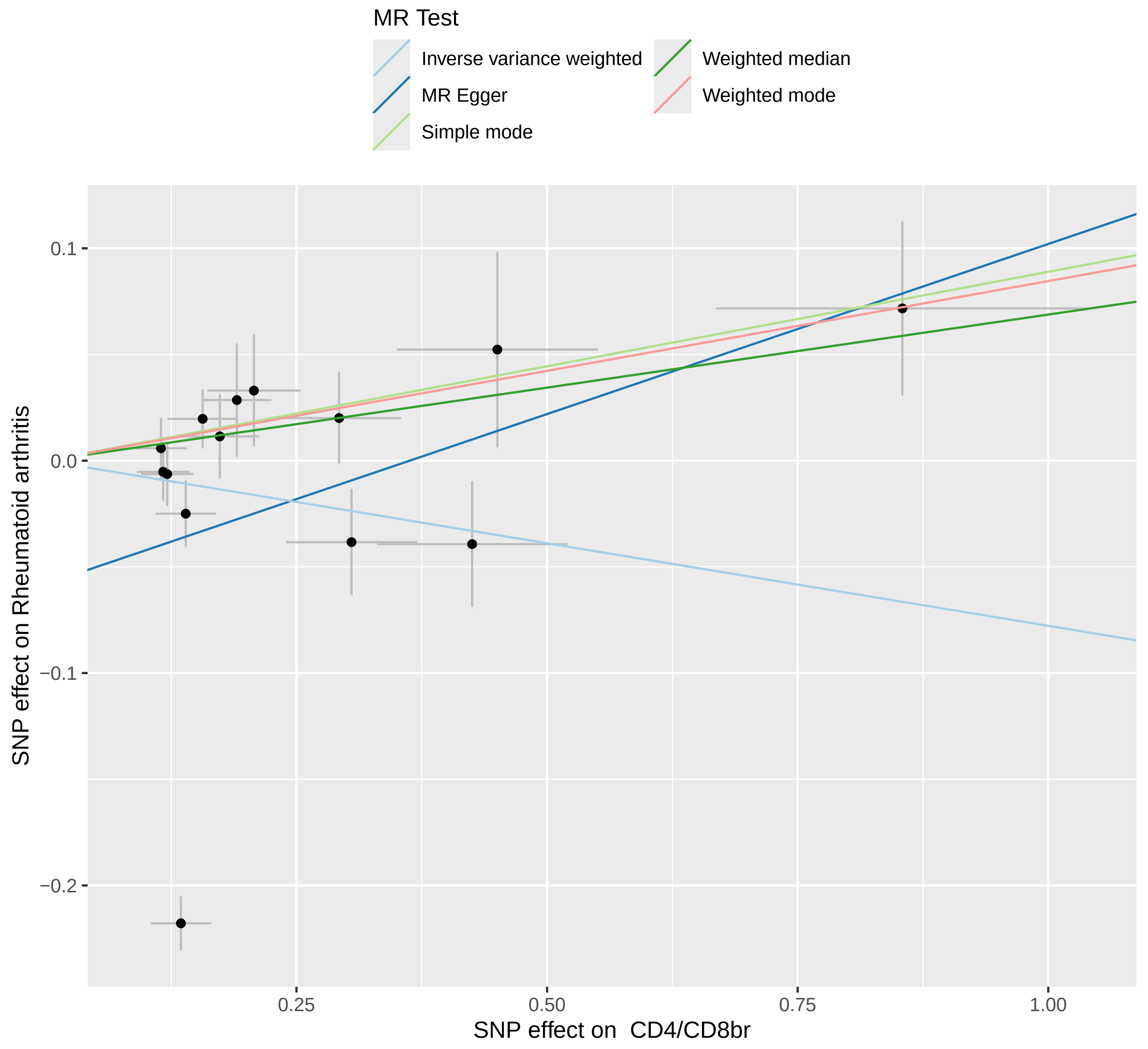
MR Test
Inverse variance weighted
MR Egger
Simple mode
Weighted median
Weighted mode
0.1
0.0
−0.1
−0.2
0.25
0.50
0.75
1.00
SNP effect on Rheumatoid arthritis
SNP effect on CD4/CD8br

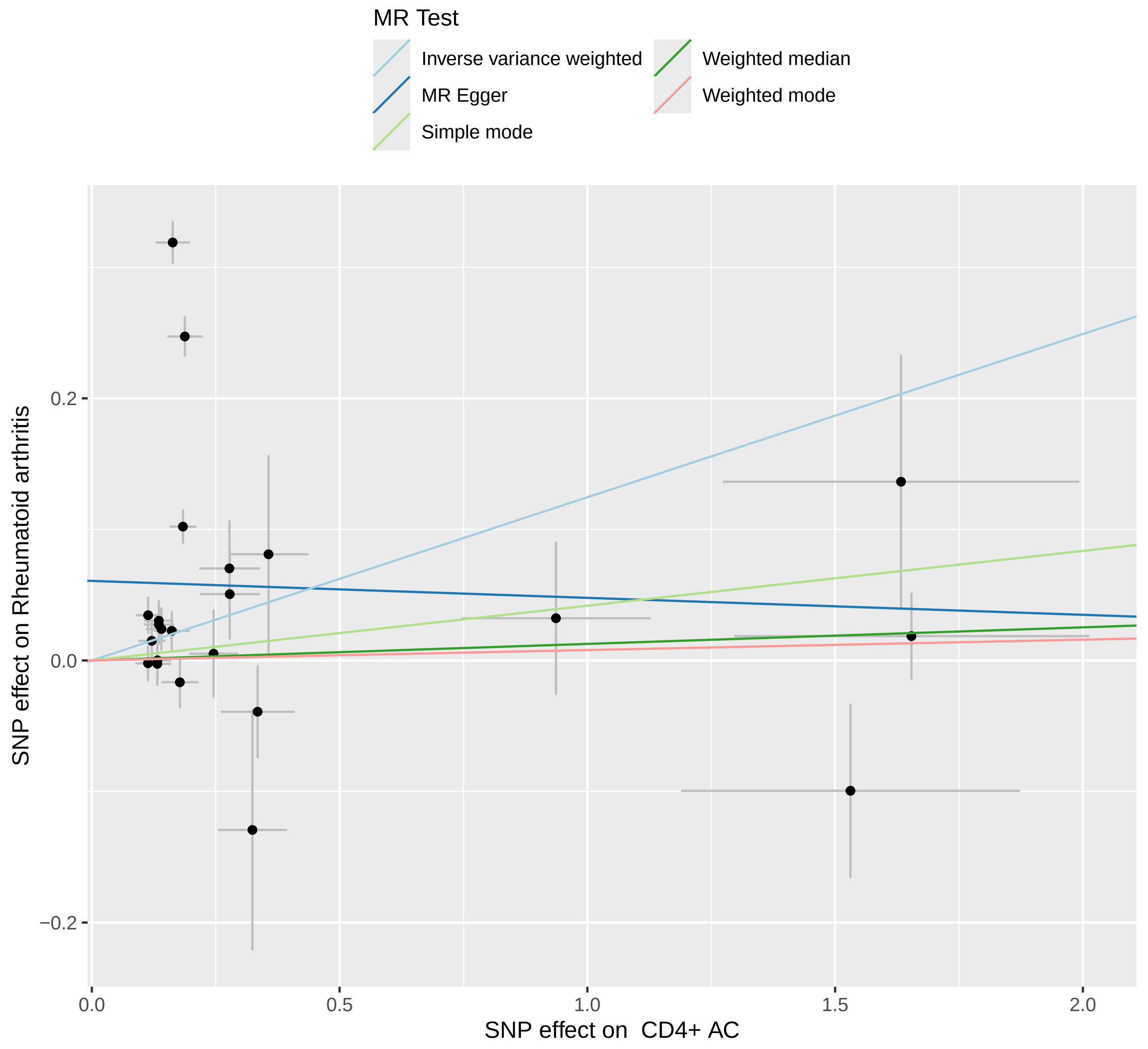

MR Test
Inverse variance weighted
MR Egger
Simple mode
Weighted median
Weighted mode
SNP effect on Rheumatoid arthritis
0.2
0.0
−0.2
0.0
0.5
1.0
1.5
2.0
SNP effect on CD4+ AC

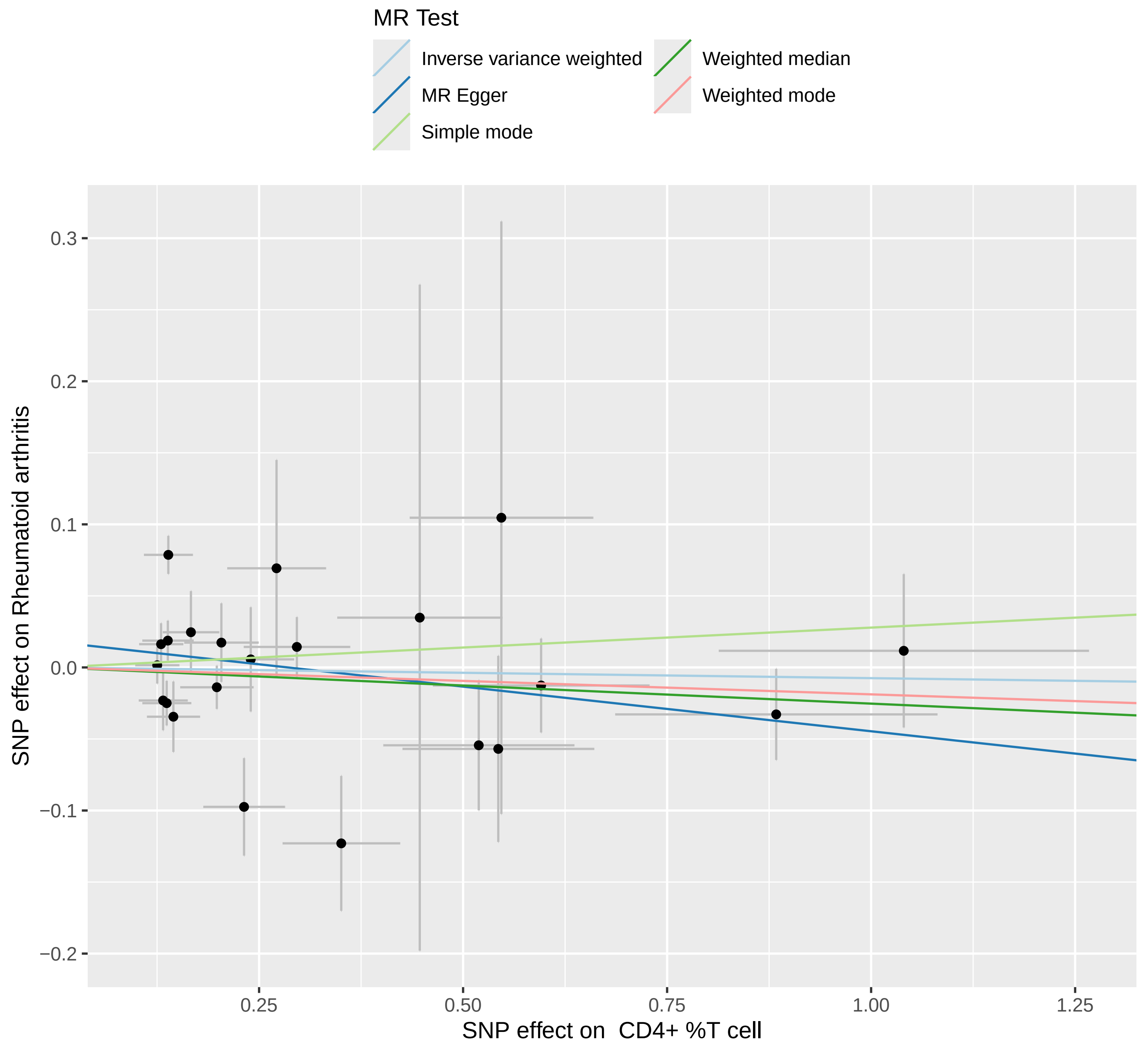

MR Test
Inverse variance weighted
MR Egger
Simple mode
Weighted median
Weighted mode
SNP effect on Rheumatoid arthritis
0.3
0.2
0.1
0.0
−0.1
−0.2
0.25
0.50
0.75
1.00
1.25
SNP effect on CD4+ %T cell

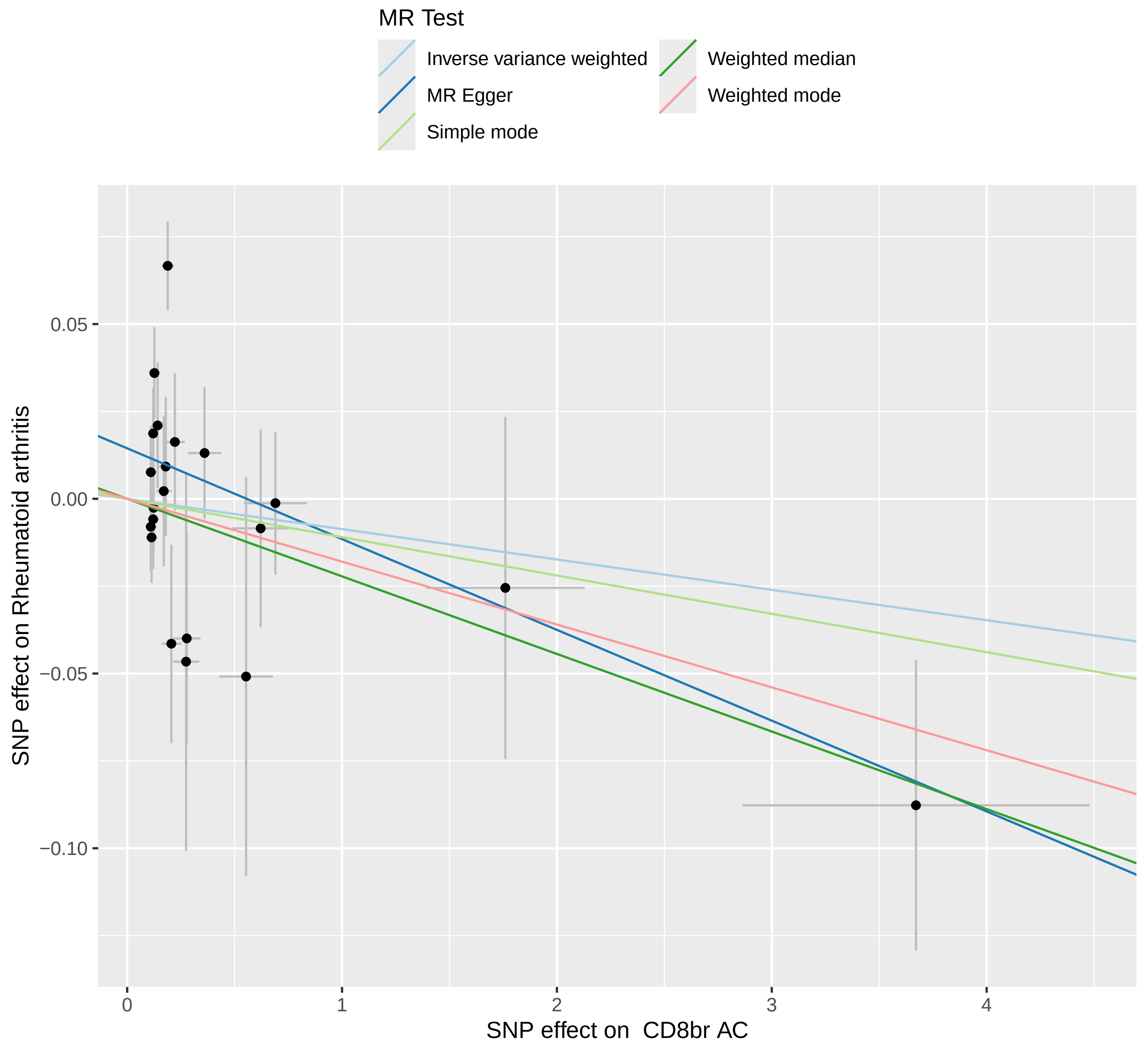
MR Test
Inverse variance weighted
MR Egger
Simple mode
Weighted median
Weighted mode
SNP effect on Rheumatoid arthritis
0.05
0.00
−0.05
−0.10
0
1
2
3
4
SNP effect on CD8br AC

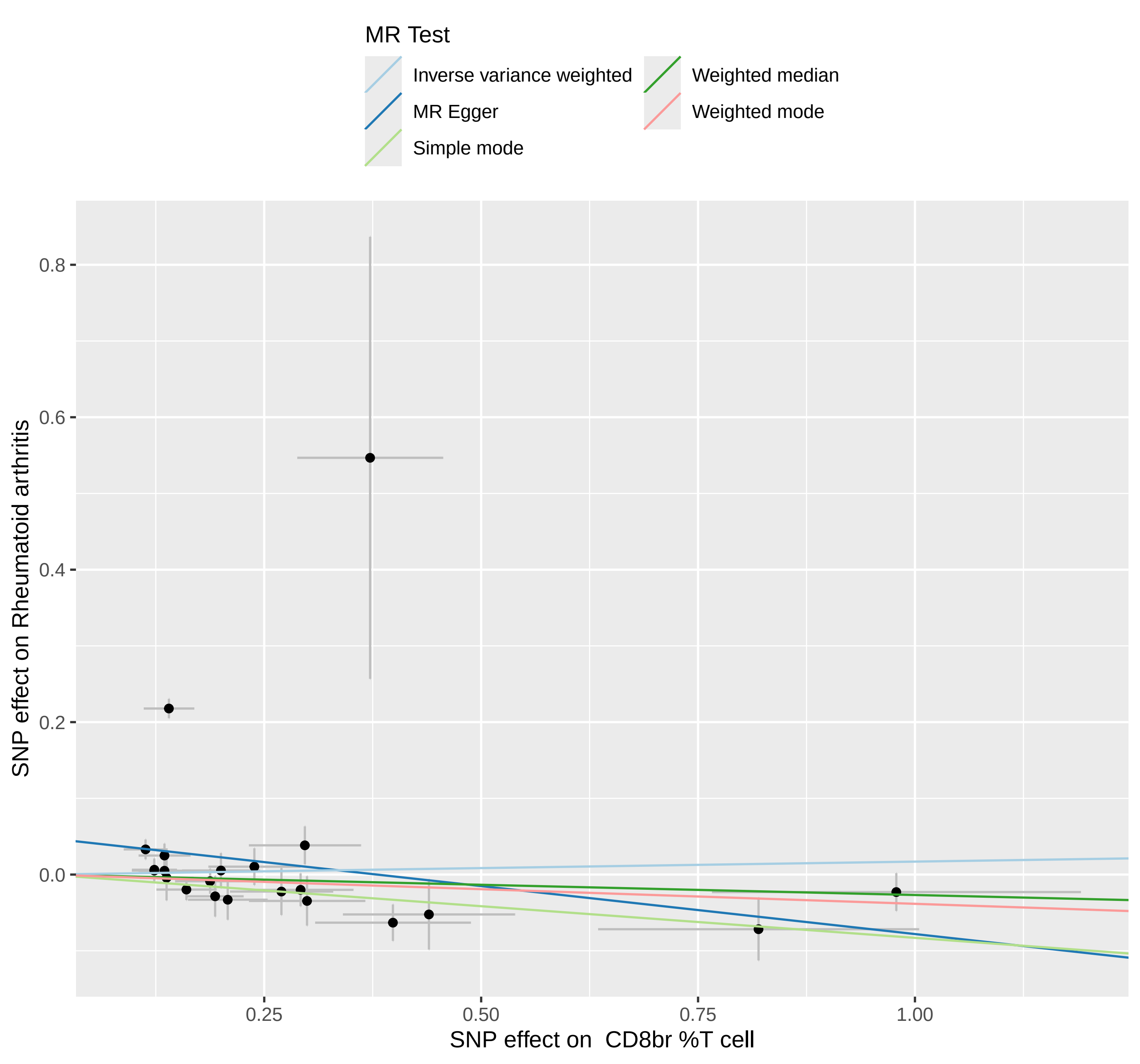
MR Test
Inverse variance weighted
MR Egger
Simple mode
Weighted median
Weighted mode
SNP effect on Rheumatoid arthritis
0.8
0.6
0.4
0.2
0.0
0.25
0.50
0.75
1.00
SNP effect on CD8br %T cell

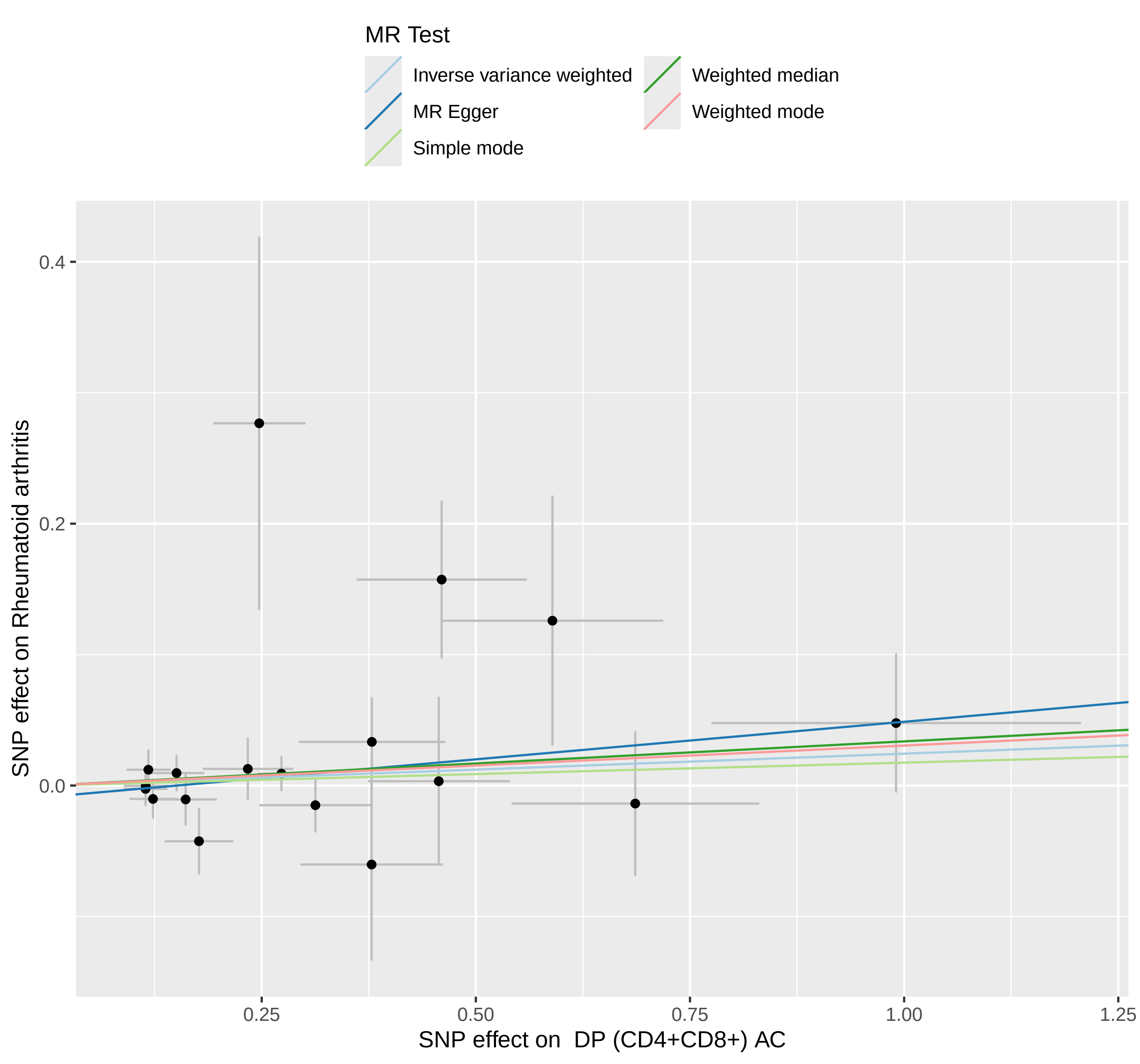
MR Test
Inverse variance weighted
MR Egger
Simple mode
Weighted median
Weighted mode
0.4
0.2
0.0
0.25
0.50
0.75
1.00
1.25
SNP effect on Rheumatoid arthritis
SNP effect on DP (CD4+CD8+) AC

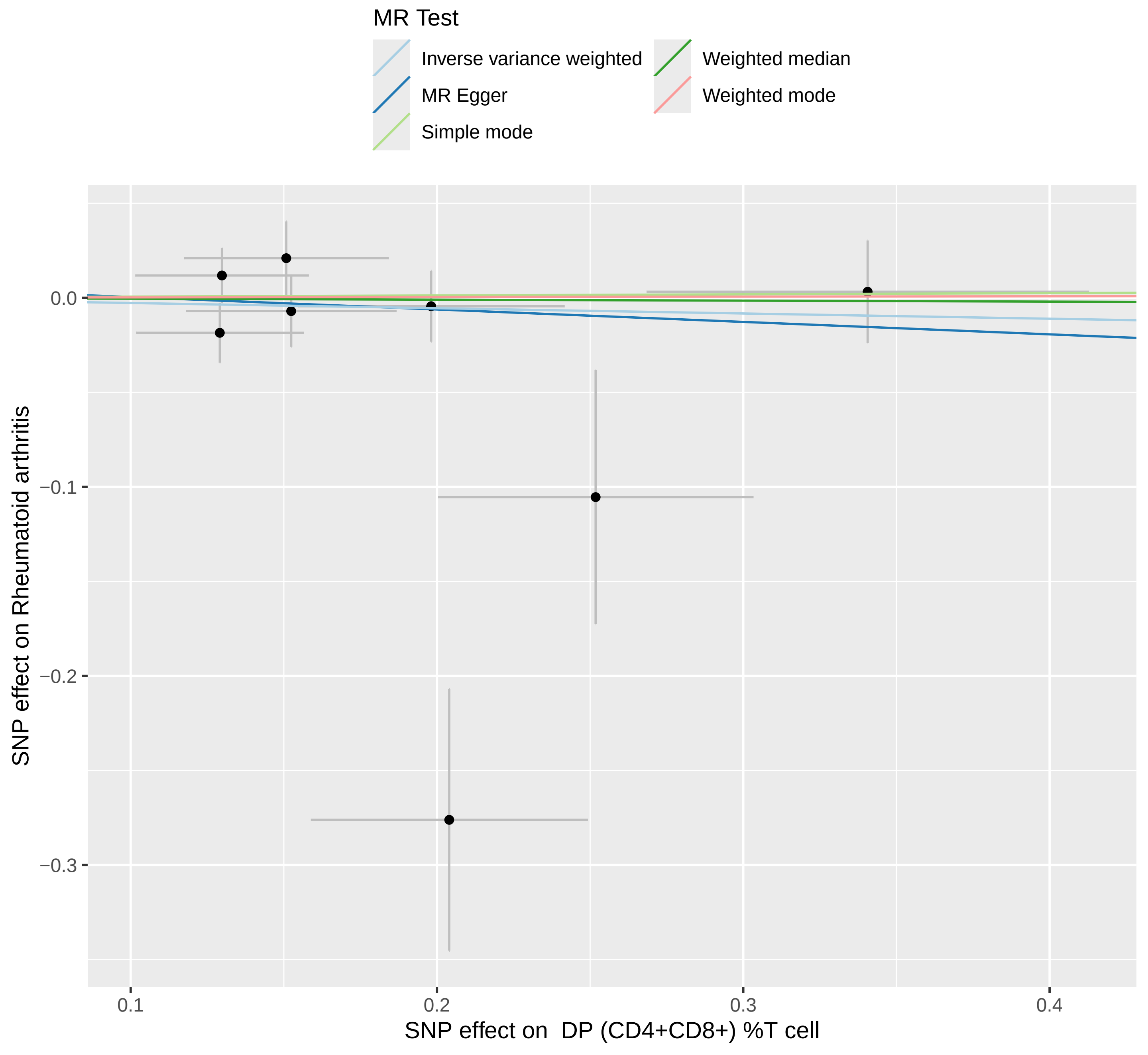

MR Test
Inverse variance weighted
MR Egger
Simple mode
Weighted median
Weighted mode
0.0
−0.1
−0.2
−0.3
0.1
0.2
0.3
0.4
SNP effect on Rheumatoid arthritis
SNP effect on DP (CD4+CD8+) %T cell

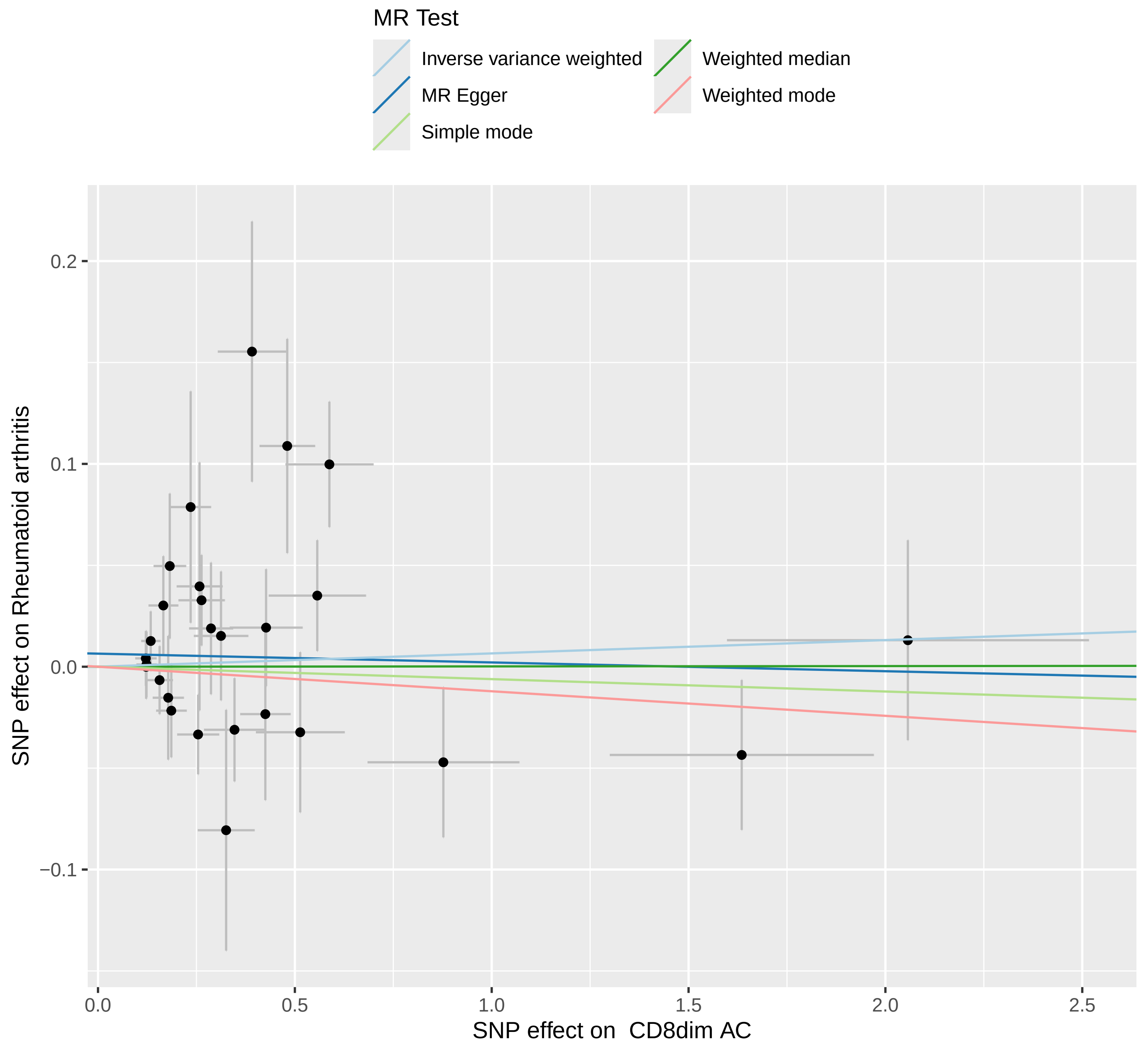
MR Test
Inverse variance weighted
MR Egger
Simple mode
Weighted median
Weighted mode
SNP effect on Rheumatoid arthritis
SNP effect on CD8dim AC
0.2
0.1
0.0
−0.1
0.0
0.5
1.0
1.5
2.0
2.5

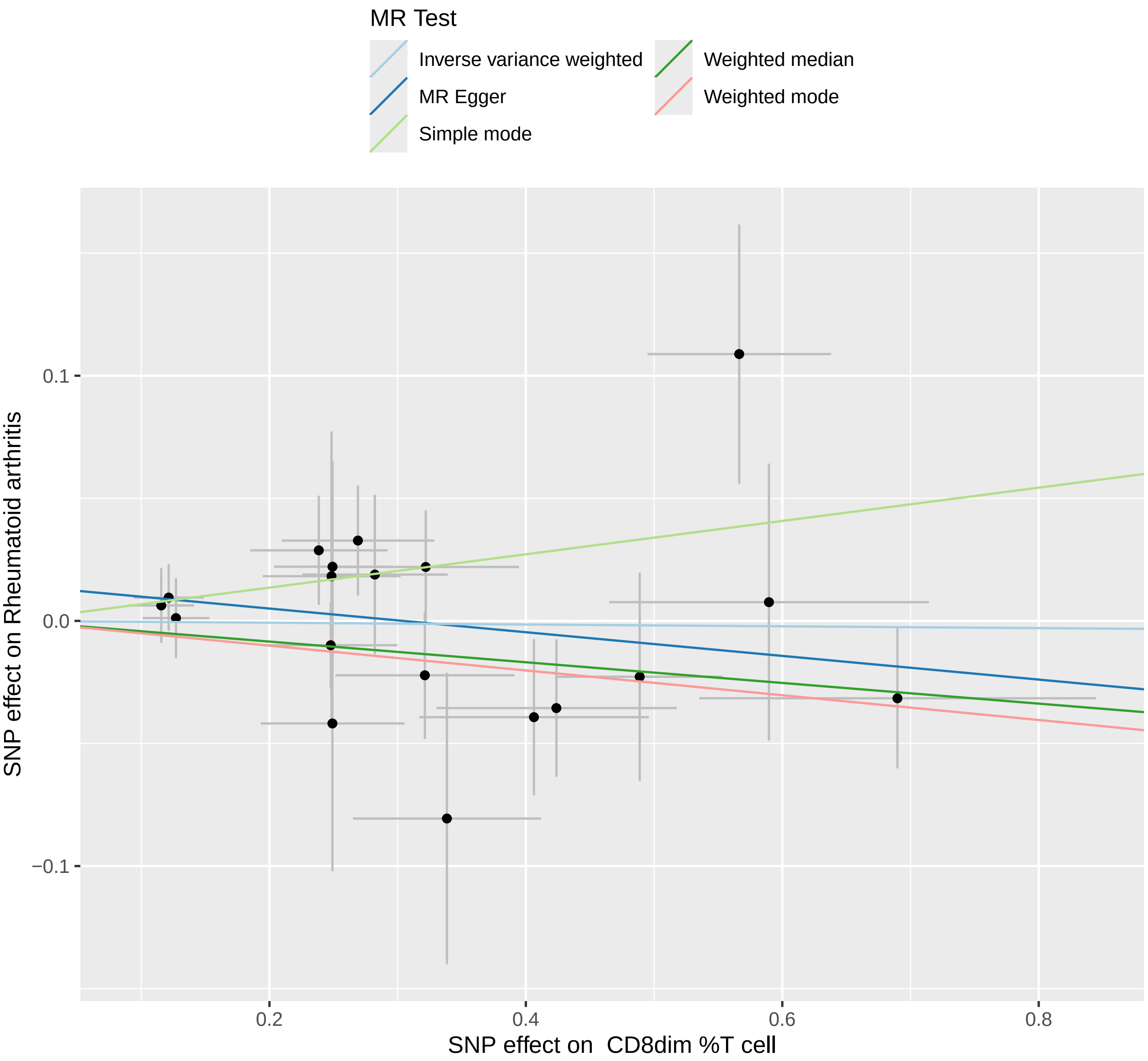
MR Test
Inverse variance weighted
MR Egger
Simple mode
Weighted median
Weighted mode
0.1
0.0
−0.1
SNP effect on Rheumatoid arthritis
0.2
0.4
0.6
0.8
SNP effect on CD8dim %T cell

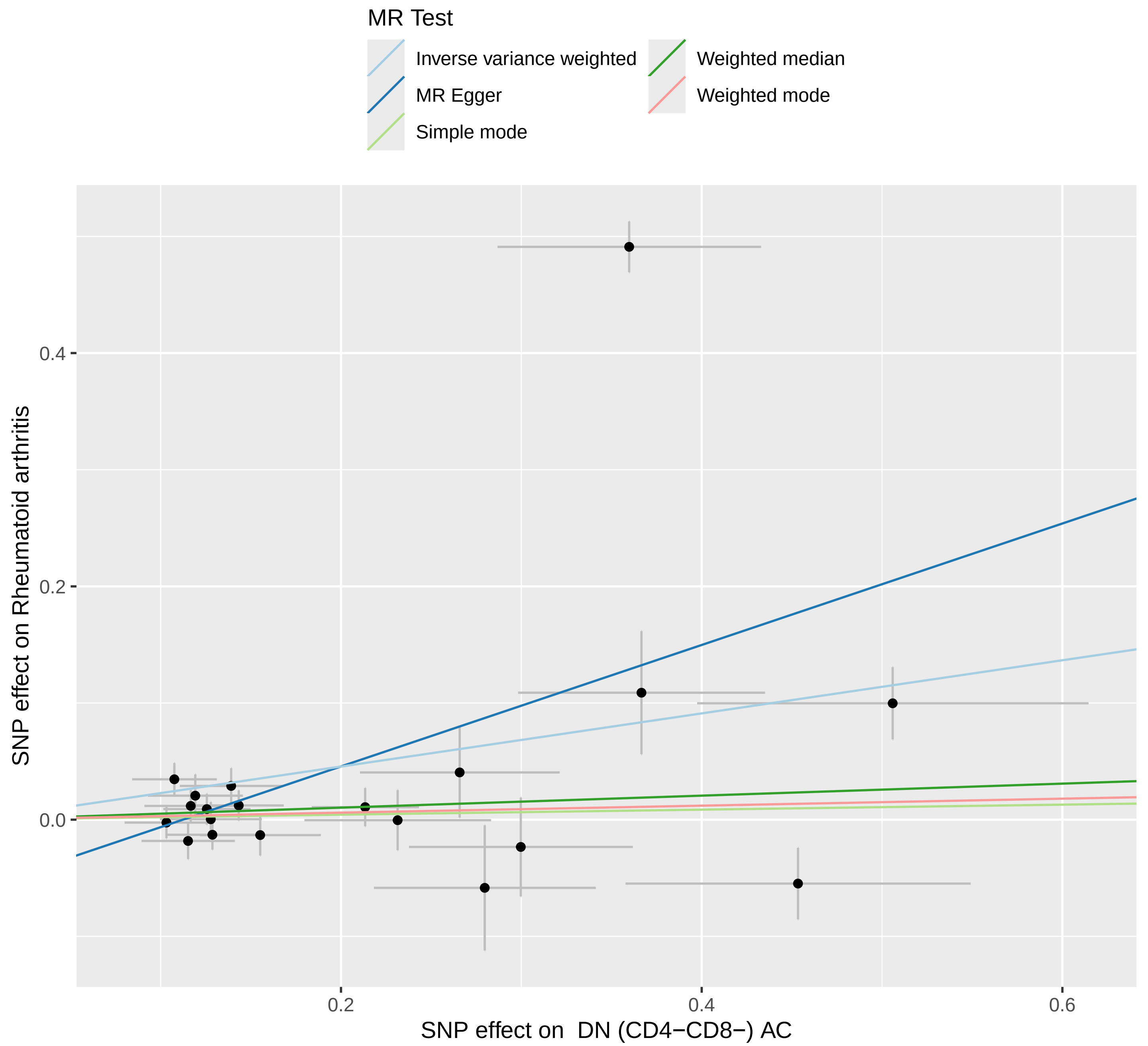
MR Test
Inverse variance weighted
MR Egger
Simple mode
Weighted median
Weighted mode
SNP effect on Rheumatoid arthritis
0.4
0.2
0.0
0.2
0.4
0.6
SNP effect on DN (CD4−CD8−) AC

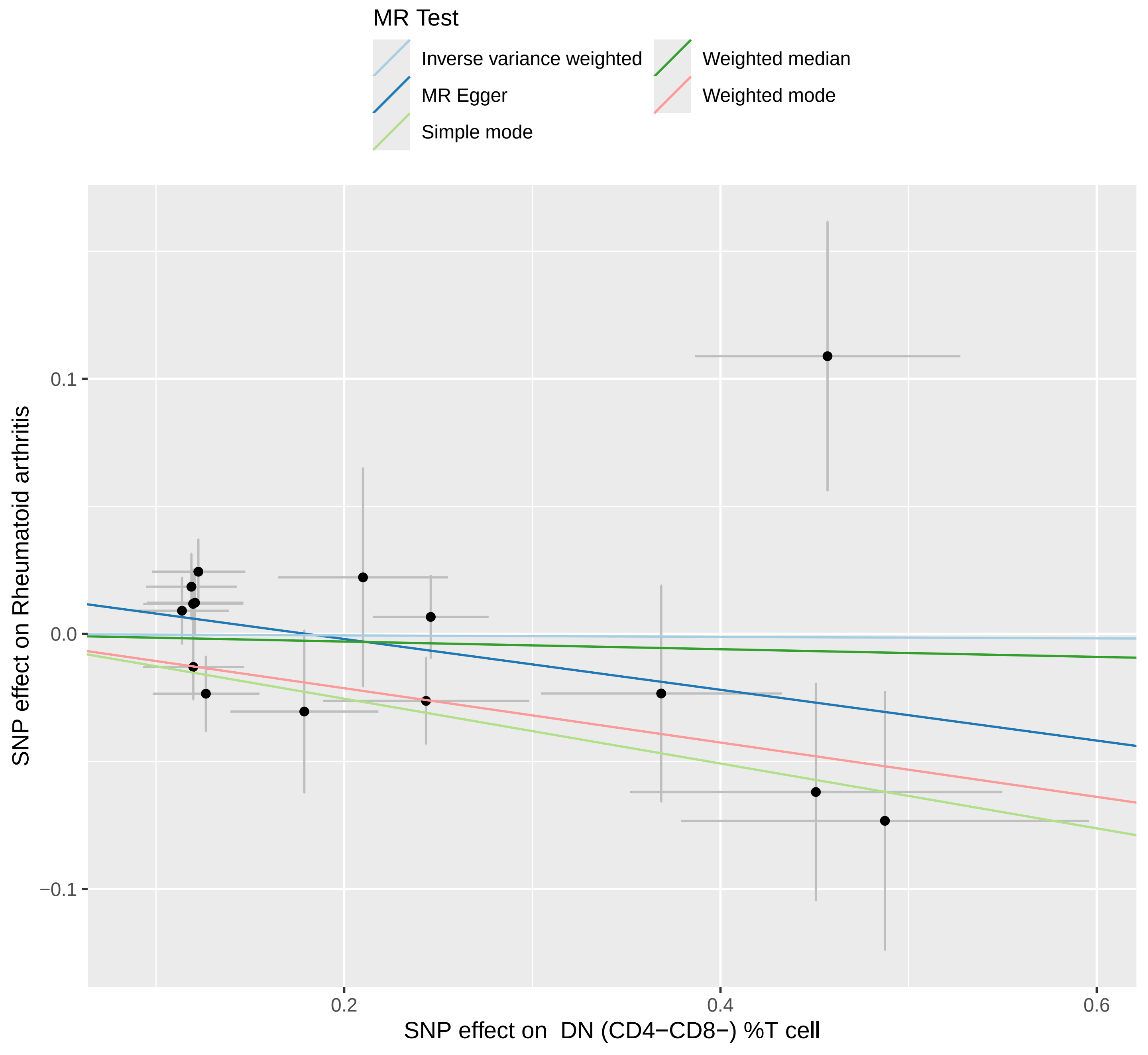
MR Test
Inverse variance weighted
MR Egger
Simple mode
Weighted median
Weighted mode
0.1
0.0
−0.1
0.2
0.4
0.6
SNP effect on Rheumatoid arthritis
SNP effect on DN (CD4−CD8−) %T cell

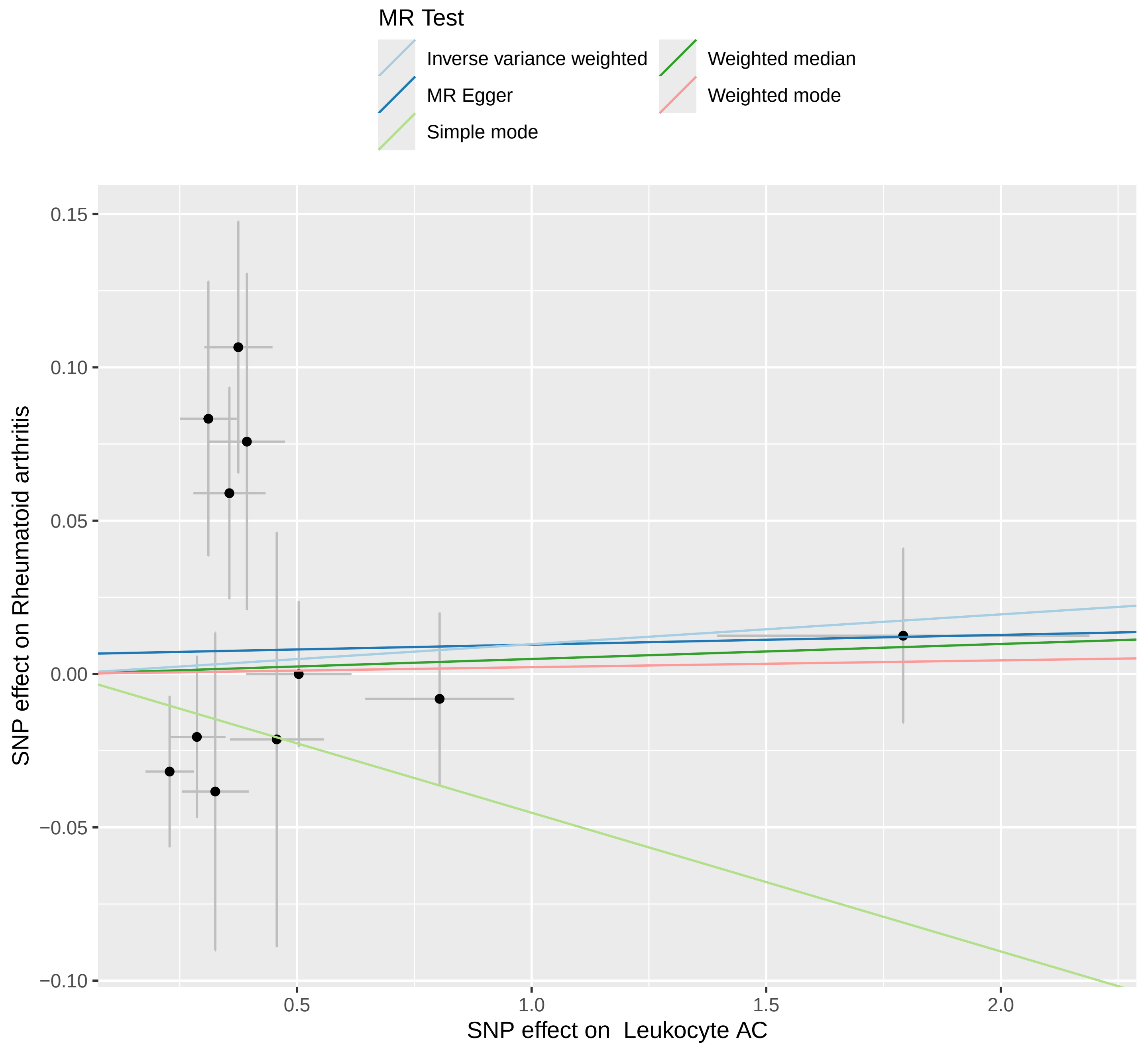
MR Test
Inverse variance weighted
MR Egger
Simple mode
Weighted median
Weighted mode
0.15
0.10
0.05
0.00
−0.05
−0.10
0.5
1.0
1.5
2.0
SNP effect on Rheumatoid arthritis
SNP effect on Leukocyte AC

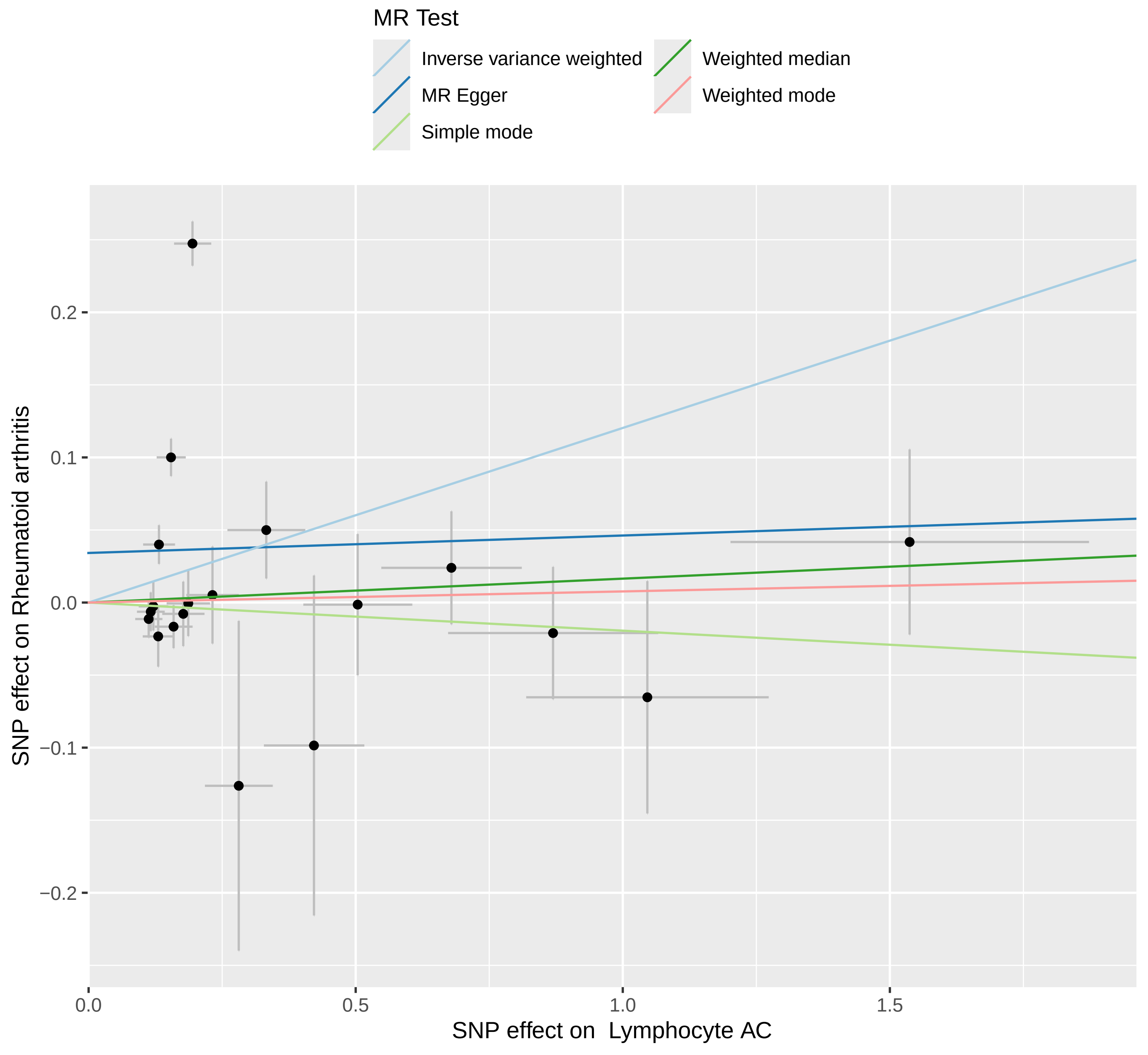
MR Test
Inverse variance weighted
MR Egger
Simple mode
Weighted median
Weighted mode
SNP effect on Rheumatoid arthritis
0.2
0.1
0.0
−0.1
−0.2
0.0
0.5
1.0
1.5
SNP effect on Lymphocyte AC

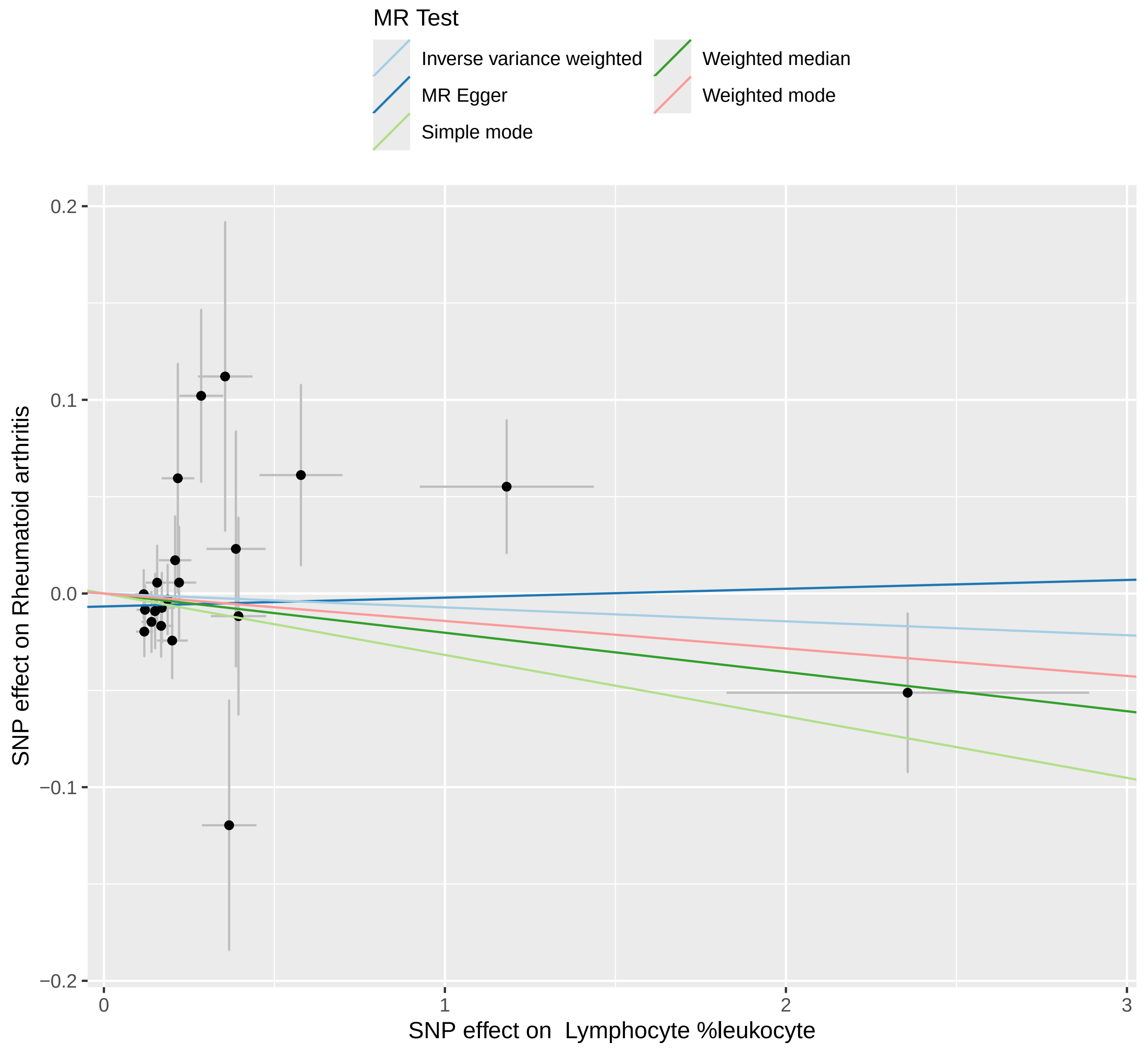
MR Test
Inverse variance weighted
MR Egger
Simple mode
Weighted median
Weighted mode
0.2
0.1
0.0
−0.1
−0.2
SNP effect on Rheumatoid arthritis
0
1
2
3
SNP effect on Lymphocyte %leukocyte

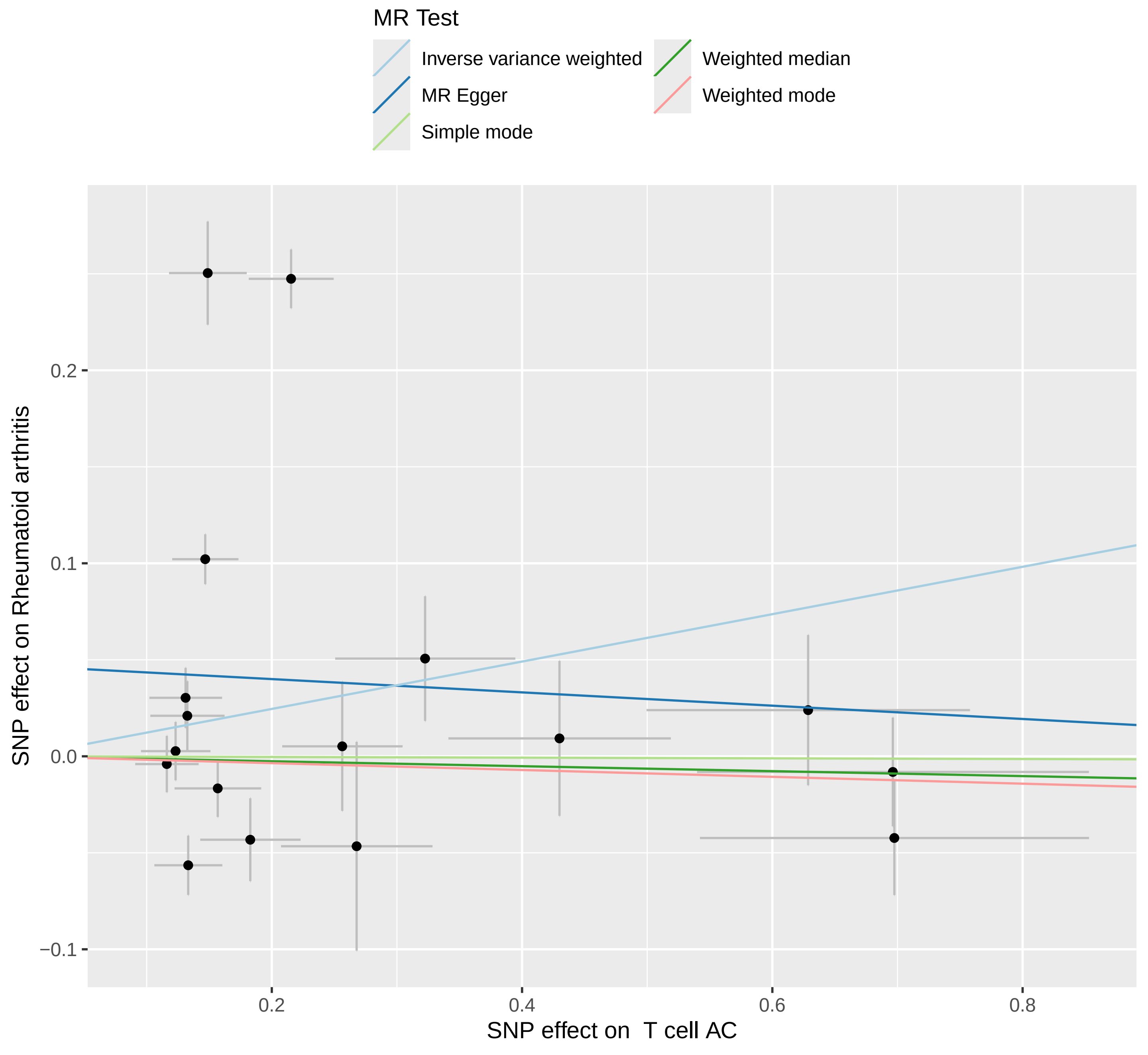
MR Test
Inverse variance weighted
MR Egger
Simple mode
Weighted median
Weighted mode
SNP effect on Rheumatoid arthritis
0.2
0.1
0.0
−0.1
0.2
0.4
0.6
0.8
SNP effect on T cell AC

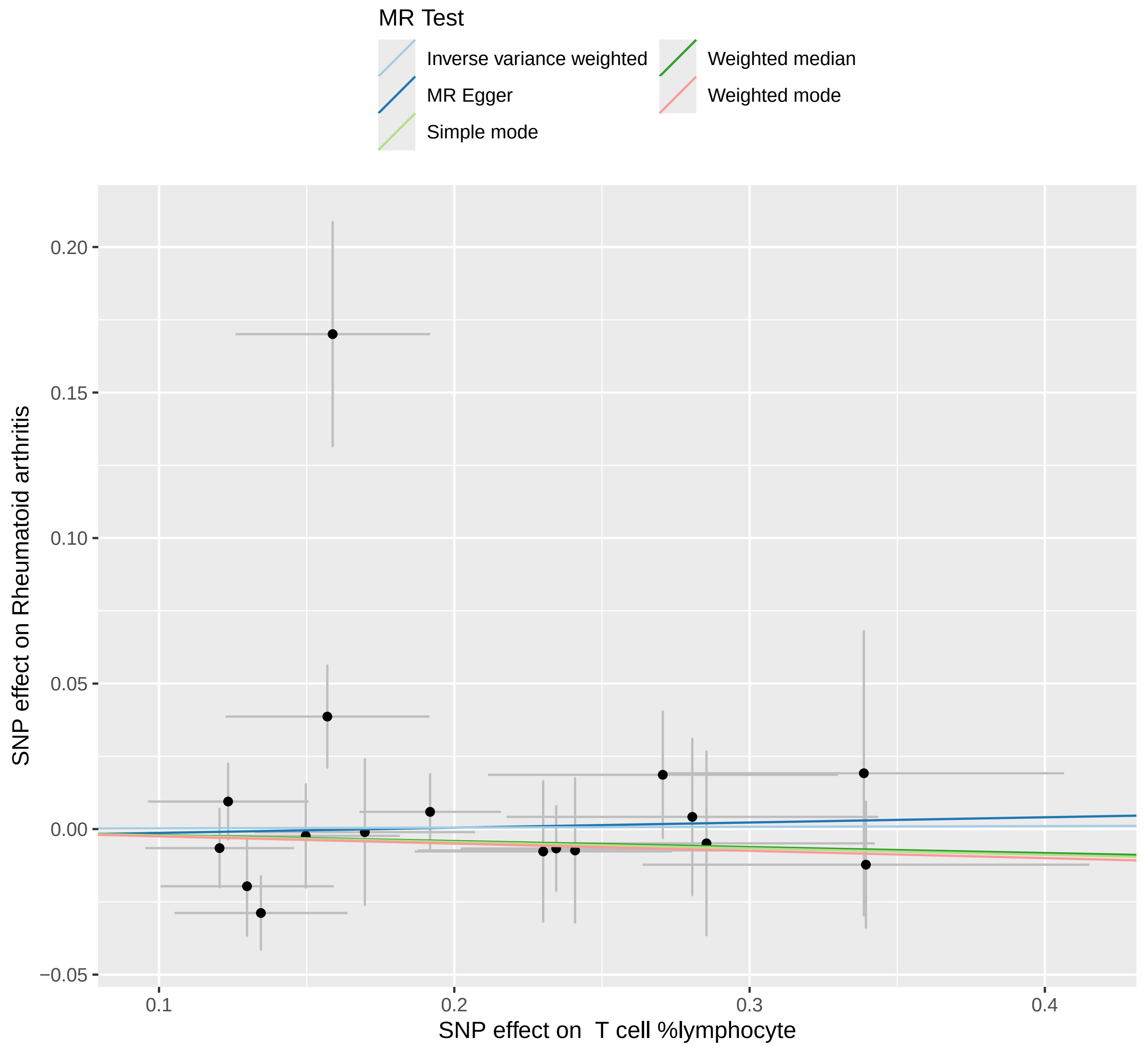
MR Test
Inverse variance weighted
MR Egger
Simple mode
Weighted median
Weighted mode
SNP effect on Rheumatoid arthritis
0.20
0.15
0.10
0.05
0.00
−0.05
0.1
0.2
0.3
0.4
SNP effect on T cell %lymphocyte

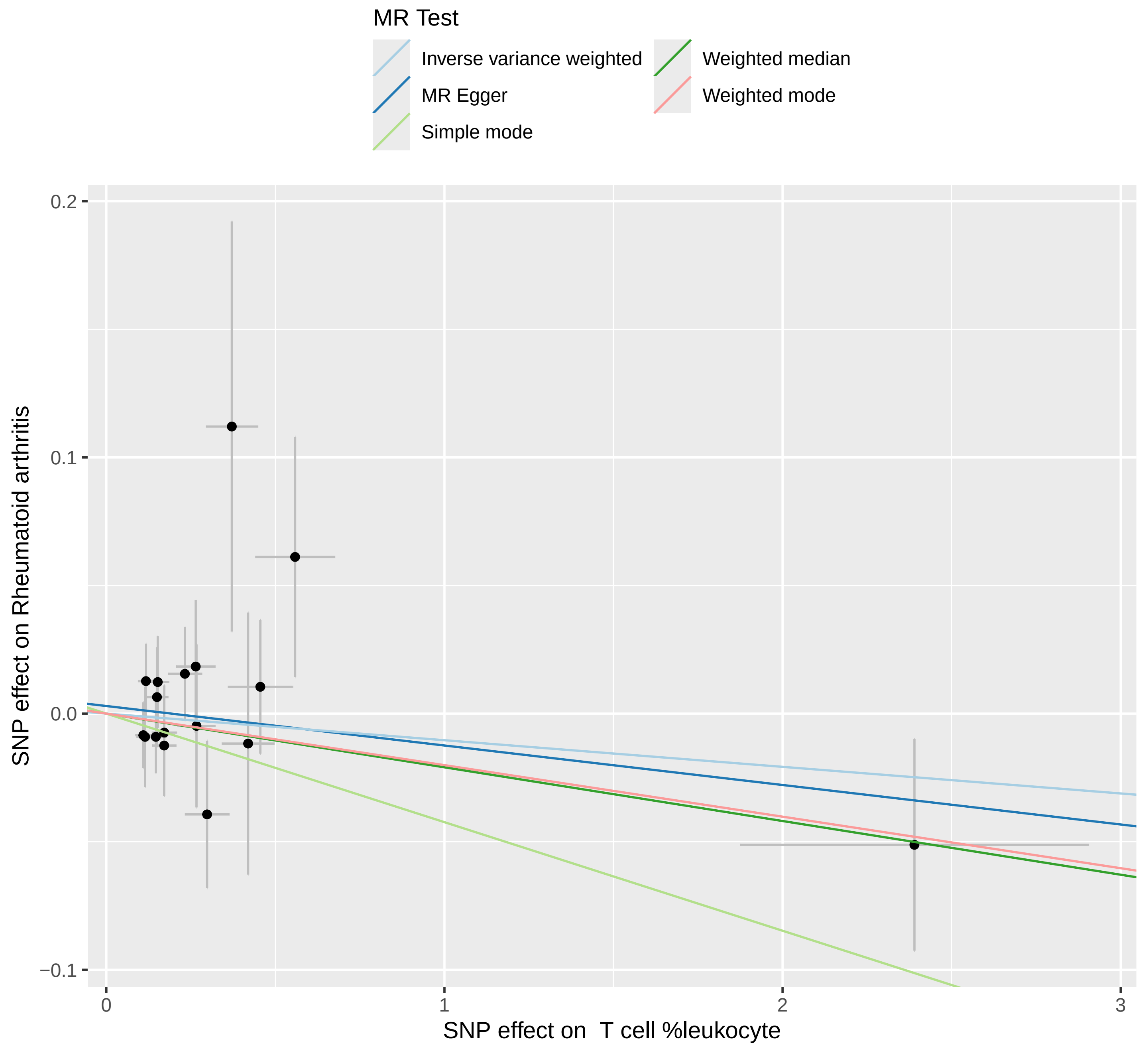
MR Test
Inverse variance weighted
MR Egger
Simple mode
Weighted median
Weighted mode
0.2
0.1
0.0
−0.1
0
1
2
3
SNP effect on Rheumatoid arthritis
SNP effect on T cell %leukocyte

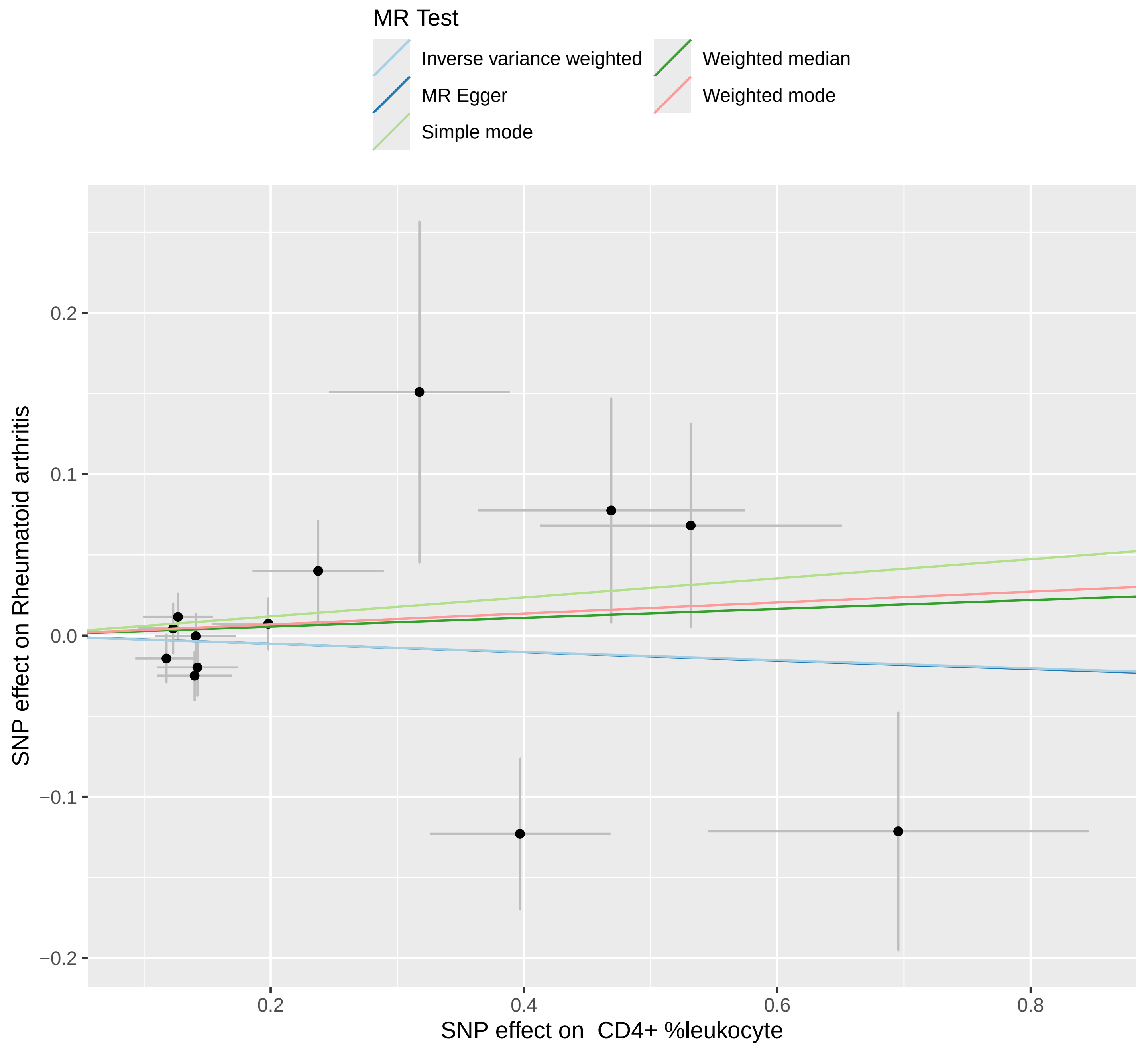
MR Test
Inverse variance weighted
MR Egger
Simple mode
Weighted median
Weighted mode
0.2
0.1
0.0
−0.1
−0.2
0.2
0.4
0.6
0.8
SNP effect on Rheumatoid arthritis
SNP effect on CD4+ %leukocyte

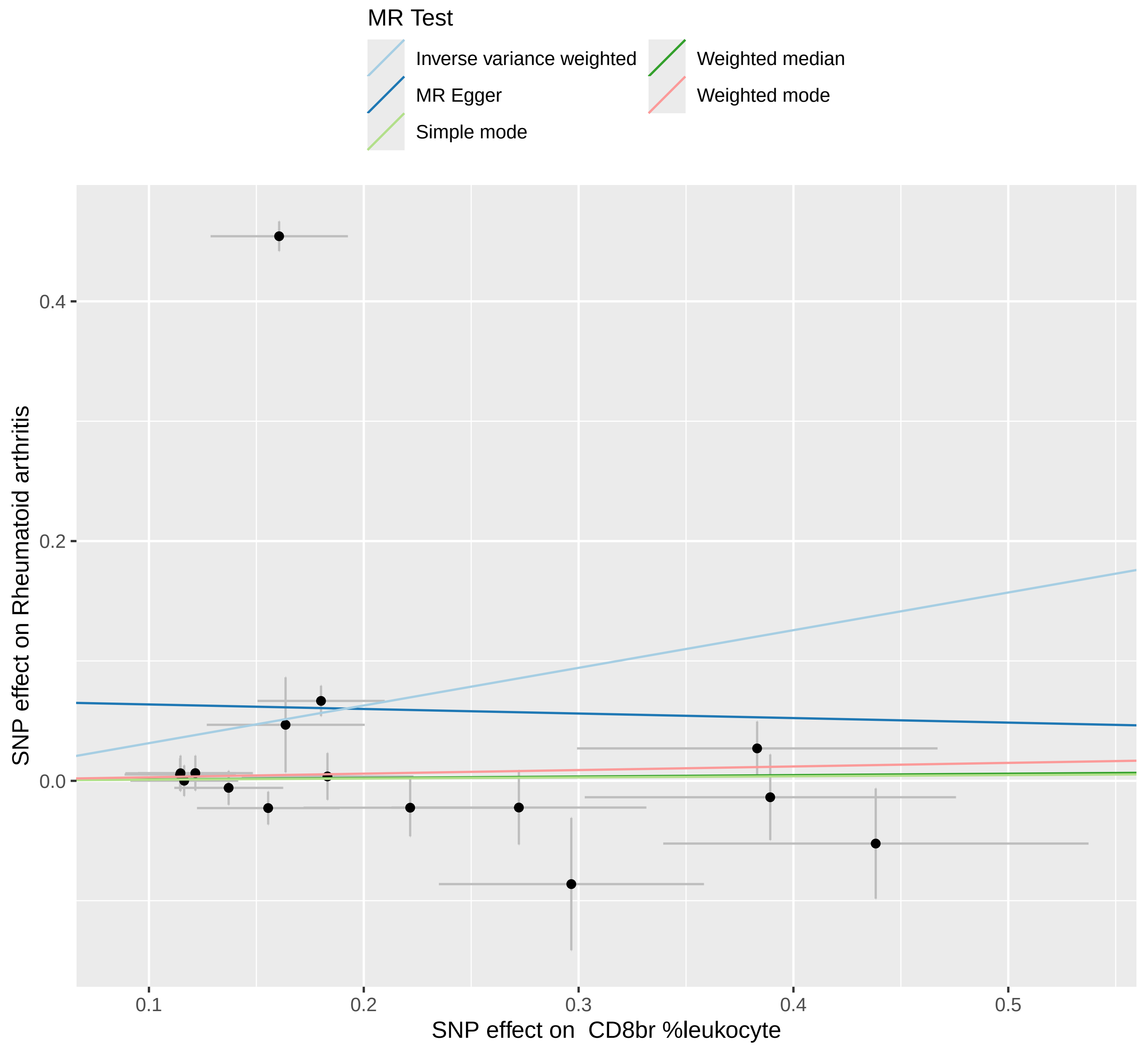

MR Test
Inverse variance weighted
MR Egger
Simple mode
Weighted median
Weighted mode
SNP effect on Rheumatoid arthritis
0.4
0.2
0.0
0.1
0.2
0.3
0.4
0.5
SNP effect on CD8br %leukocyte

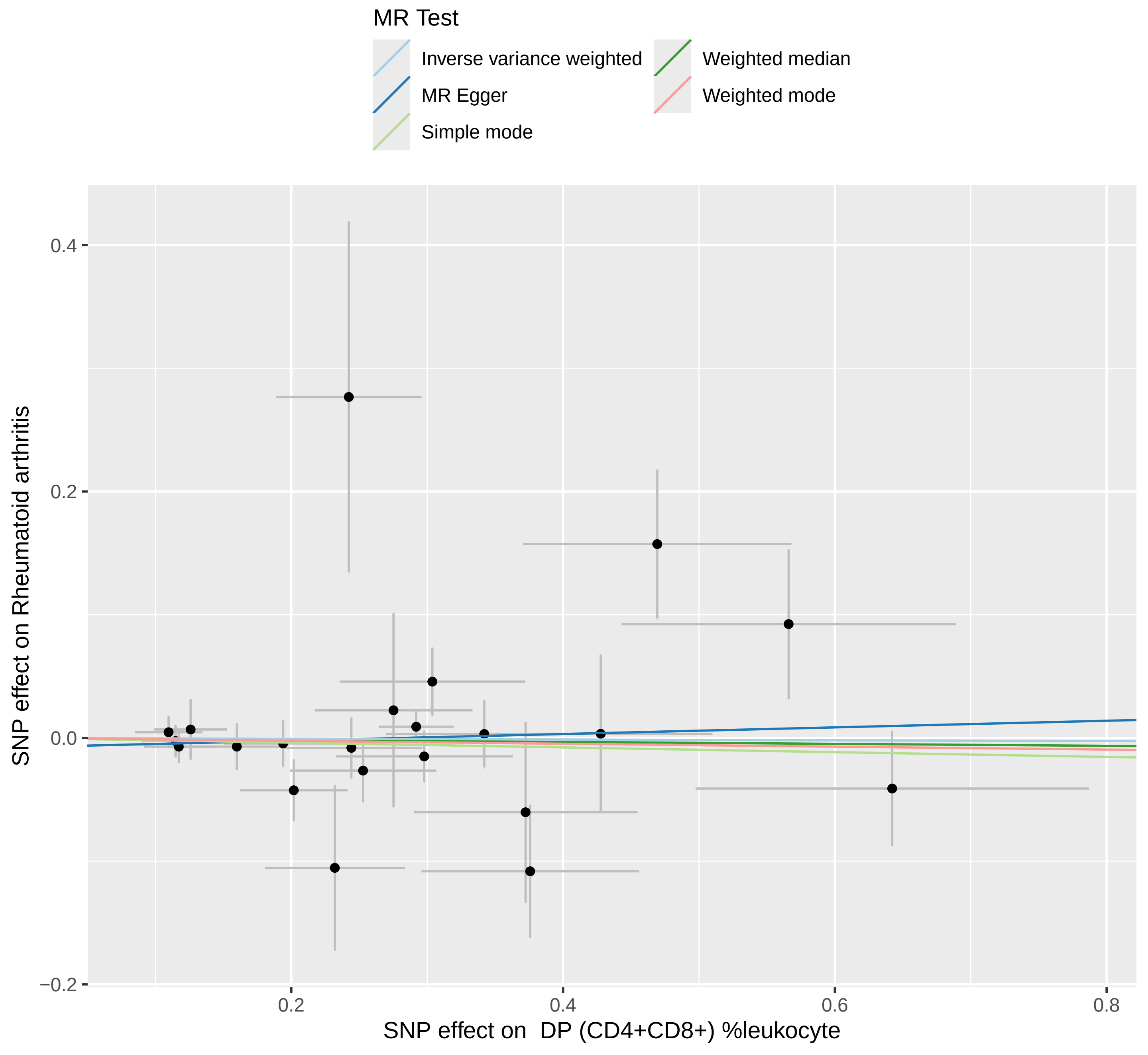
MR Test
Inverse variance weighted
Weighted median
MR Egger
Weighted mode
Simple mode
0.4
0.2
0.0
−0.2
0.2
0.4
0.6
0.8
SNP effect on Rheumatoid arthritis
SNP effect on DP (CD4+CD8+) %leukocyte

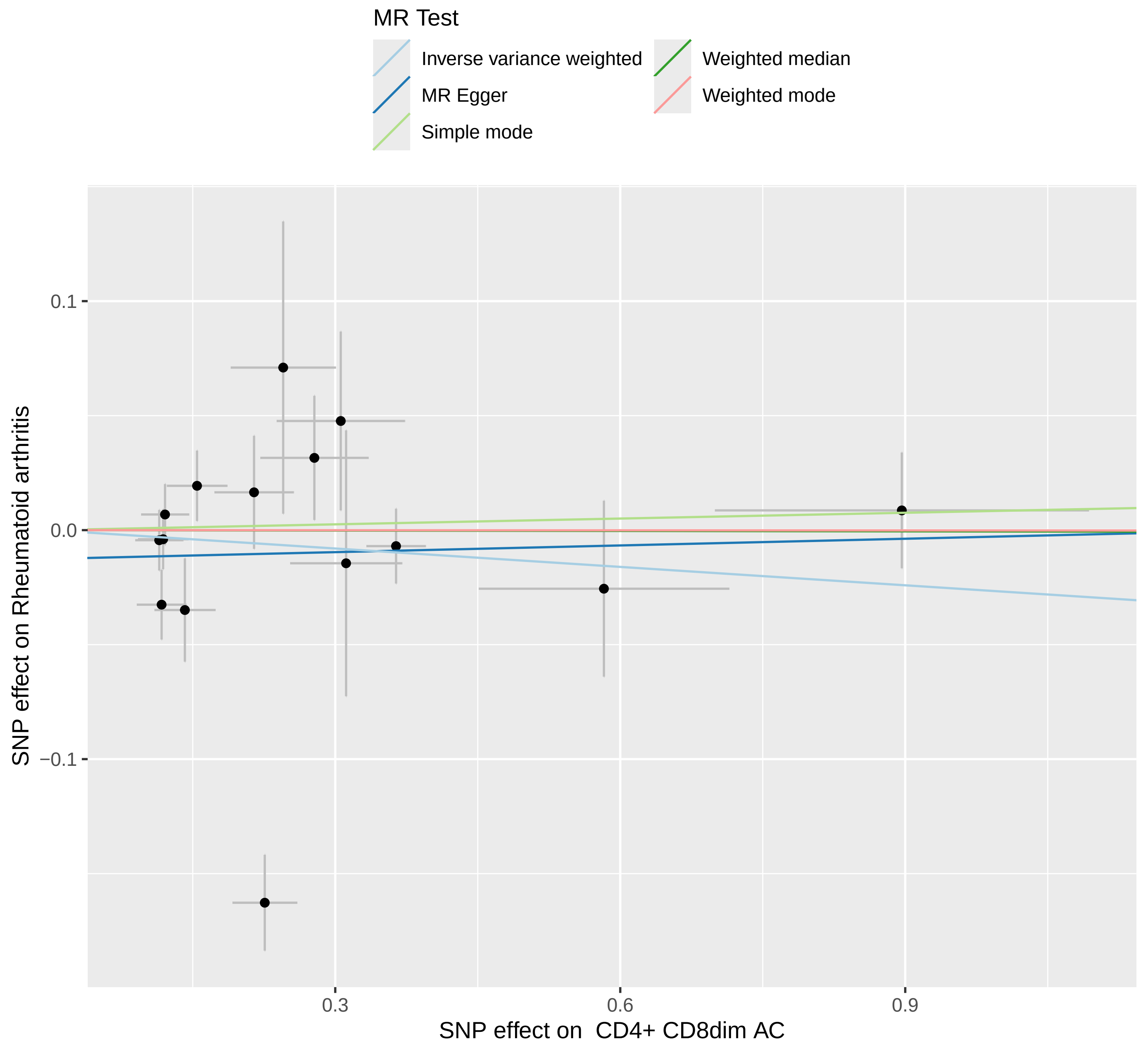
MR Test
Inverse variance weighted
MR Egger
Simple mode
Weighted median
Weighted mode
SNP effect on Rheumatoid arthritis
0.1
0.0
−0.1
0.3
0.6
0.9
SNP effect on CD4+ CD8dim AC

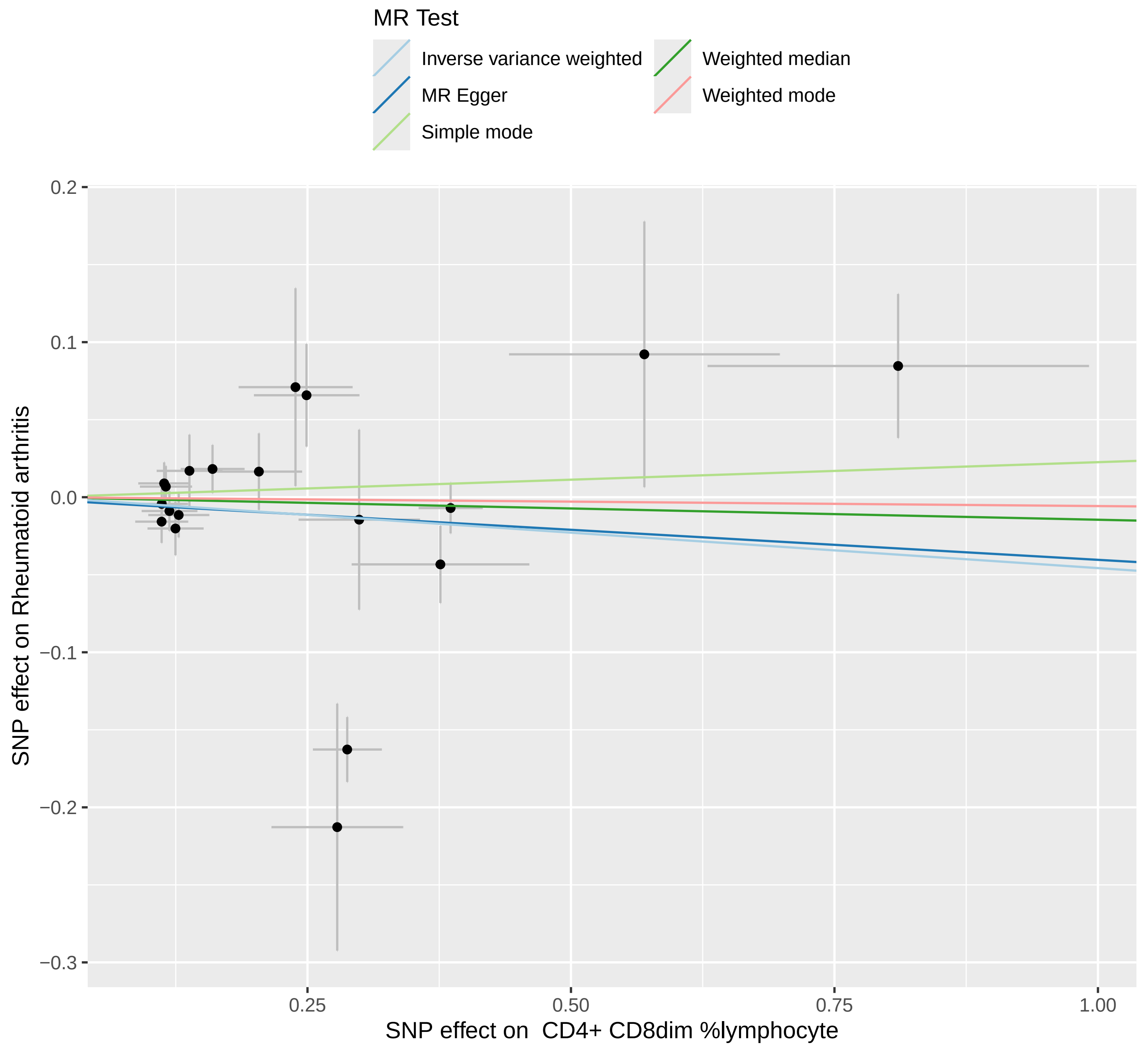
MR Test
Inverse variance weighted
MR Egger
Simple mode
Weighted median
Weighted mode
0.2
0.1
0.0
−0.1
−0.2
−0.3
0.25
0.50
0.75
1.00
SNP effect on Rheumatoid arthritis
SNP effect on CD4+ CD8dim %lymphocyte

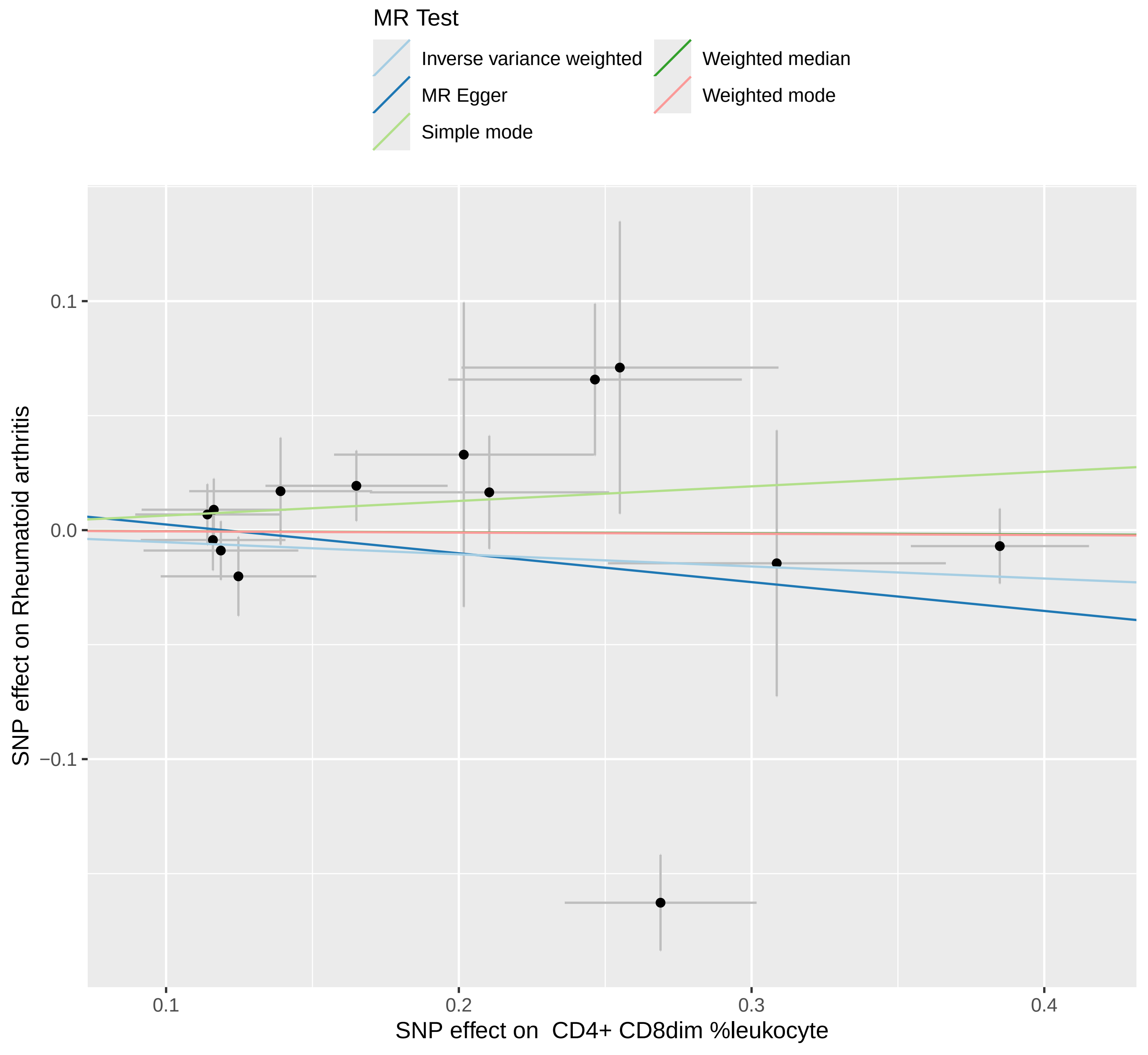
MR Test
Inverse variance weighted
MR Egger
Simple mode
Weighted median
Weighted mode
0.1
0.0
−0.1
0.1
0.2
0.3
0.4
SNP effect on Rheumatoid arthritis
SNP effect on CD4+ CD8dim %leukocyte

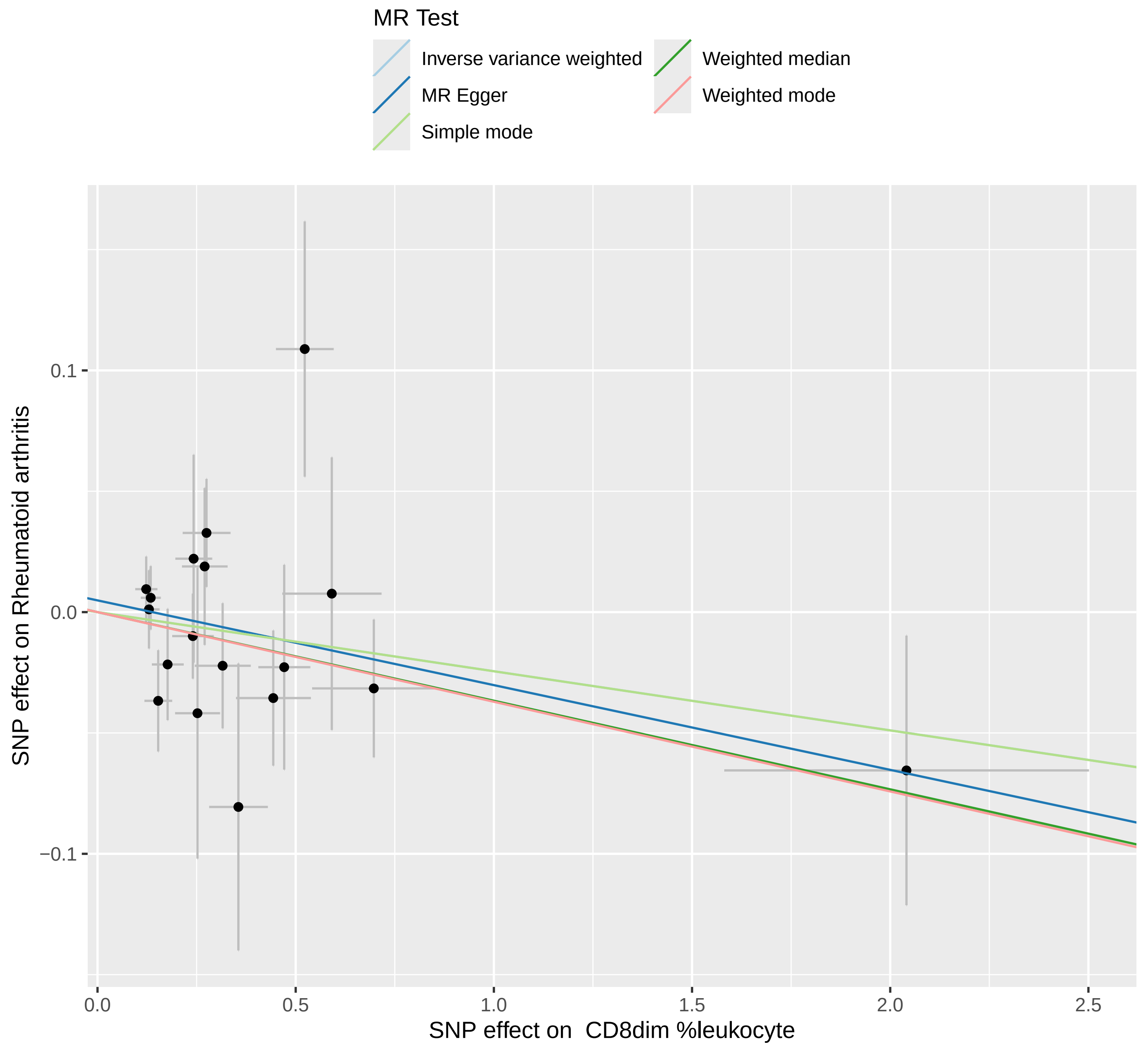
MR Test
Inverse variance weighted
MR Egger
Simple mode
Weighted median
Weighted mode
0.1
0.0
−0.1
0.0
0.5
1.0
1.5
2.0
2.5
SNP effect on Rheumatoid arthritis
SNP effect on CD8dim %leukocyte

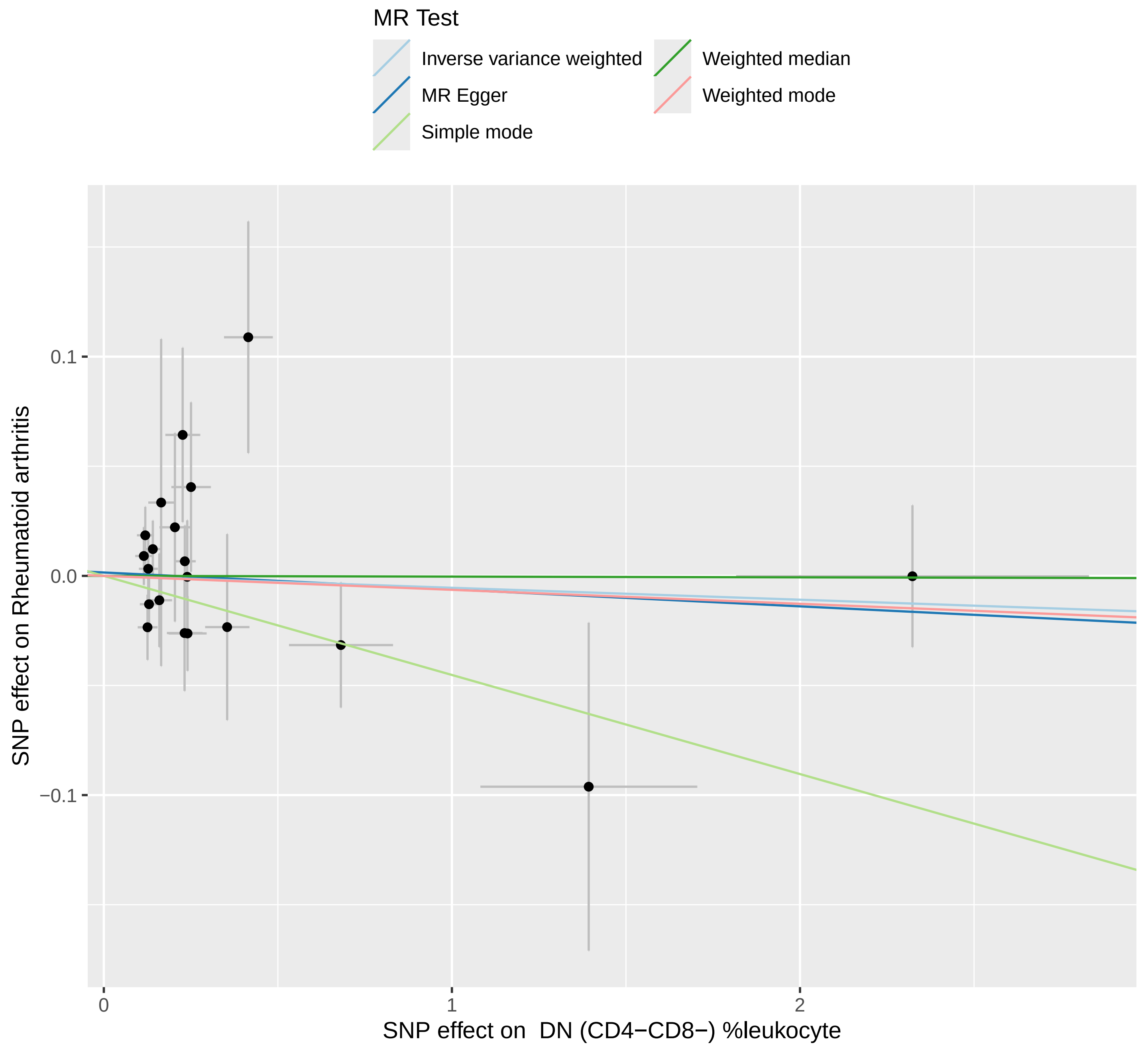

MR Test
Inverse variance weighted
MR Egger
Simple mode
Weighted median
Weighted mode
0.1
0.0
−0.1
0
1
2
SNP effect on Rheumatoid arthritis
SNP effect on DN (CD4−CD8−) %leukocyte

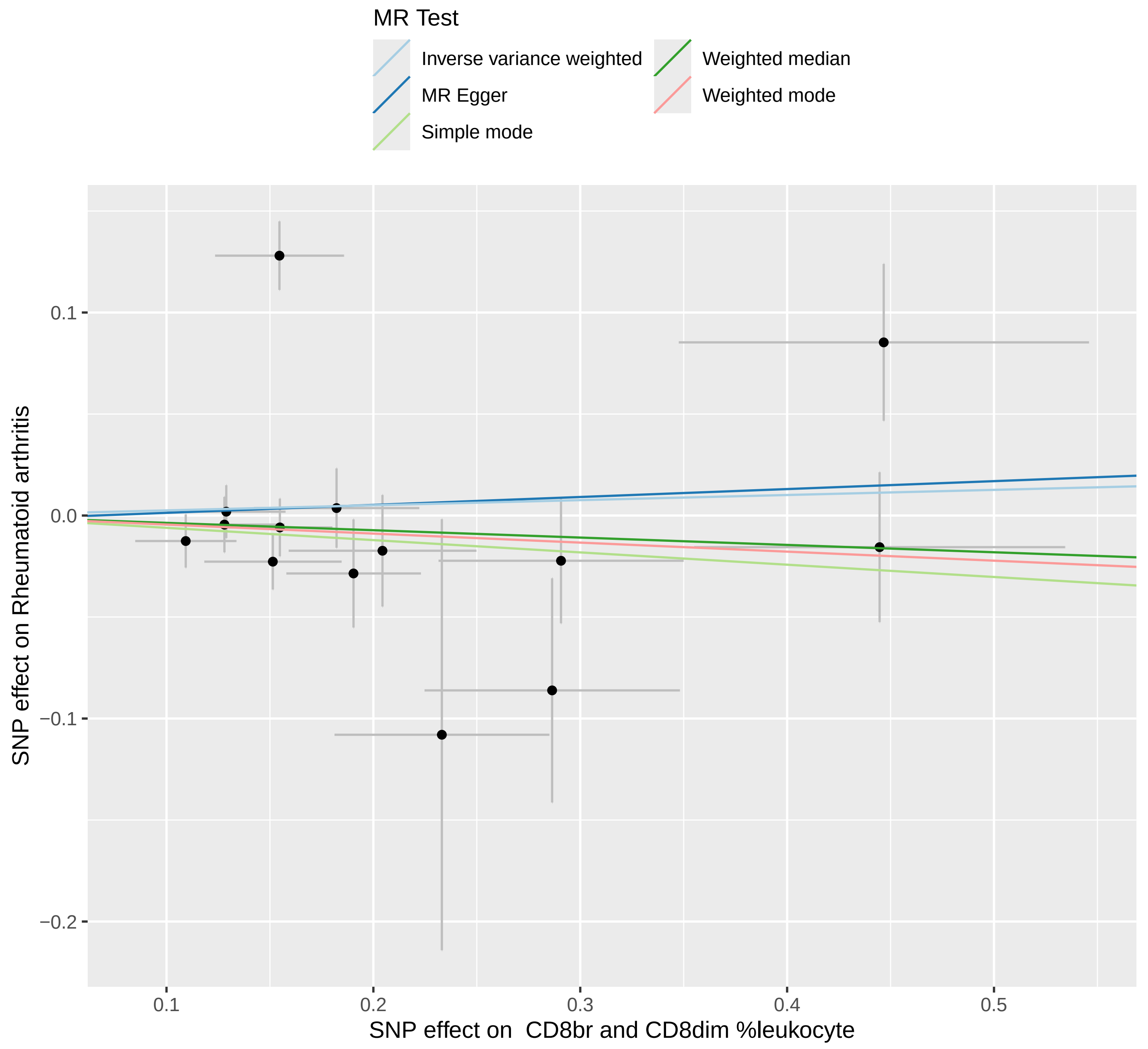

MR Test
Inverse variance weighted
MR Egger
Simple mode
Weighted median
Weighted mode
SNP effect on Rheumatoid arthritis
0.1
0.0
−0.1
−0.2
0.1
0.2
0.3
0.4
0.5
SNP effect on CD8br and CD8dim %leukocyte

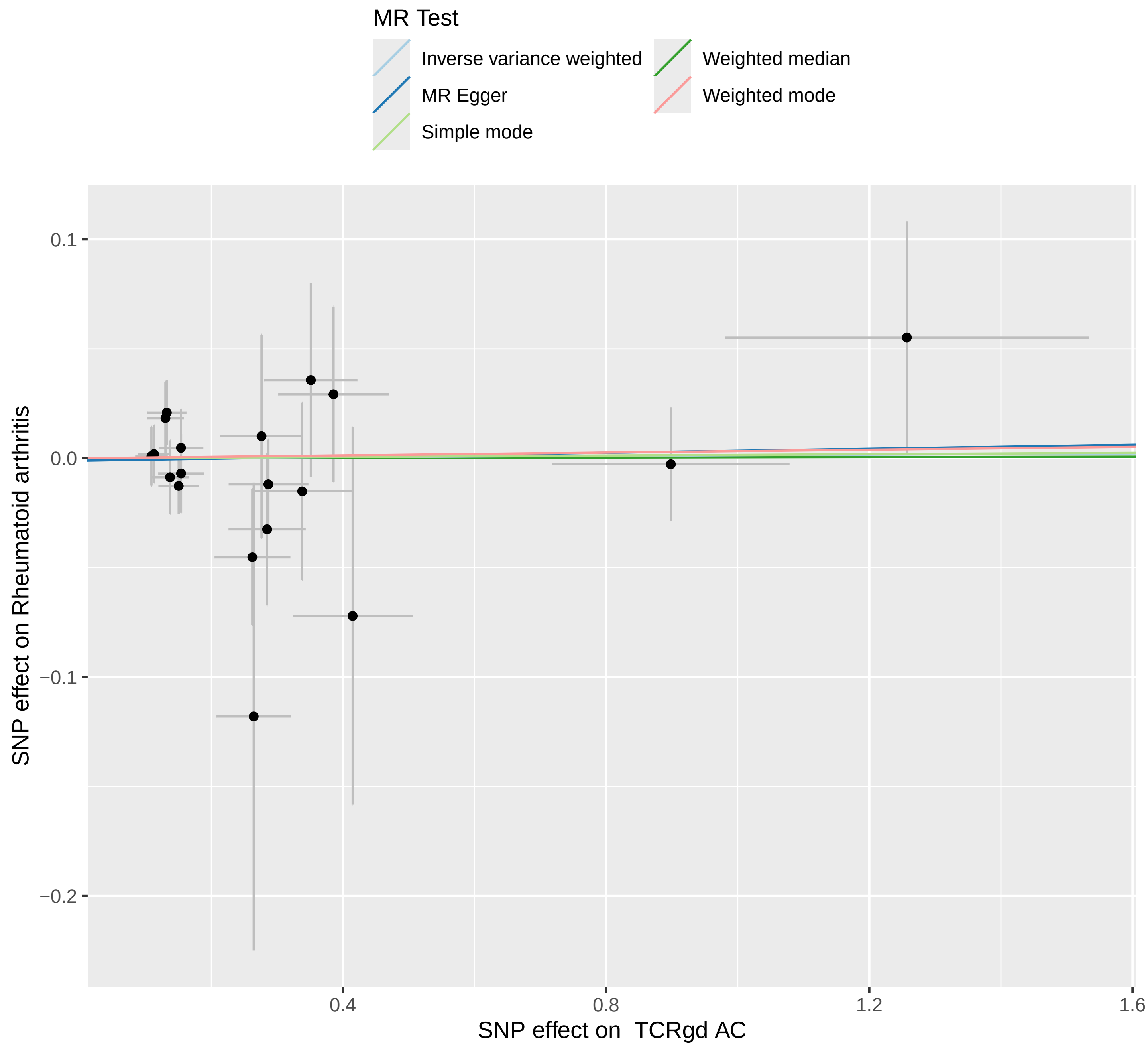
MR Test
Inverse variance weighted
MR Egger
Simple mode
Weighted median
Weighted mode
0.1
0.0
−0.1
−0.2
0.4
0.8
1.2
1.6
SNP effect on Rheumatoid arthritis
SNP effect on TCRgd AC

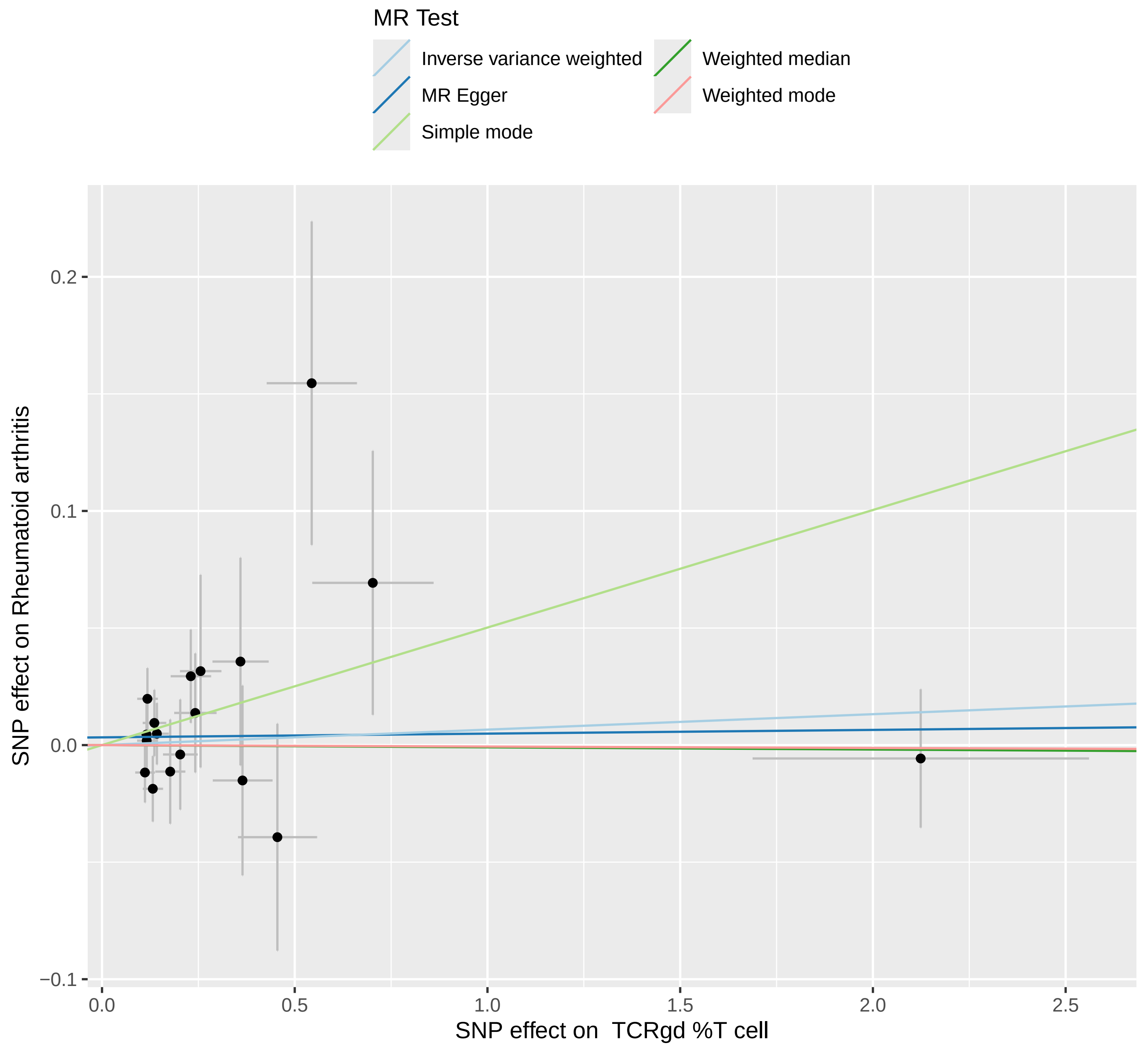

MR Test
Inverse variance weighted
Weighted median
MR Egger
Weighted mode
Simple mode
0.2
0.1
0.0
−0.1
0.0
0.5
1.0
1.5
2.0
2.5
SNP effect on Rheumatoid arthritis
SNP effect on TCRgd %T cell

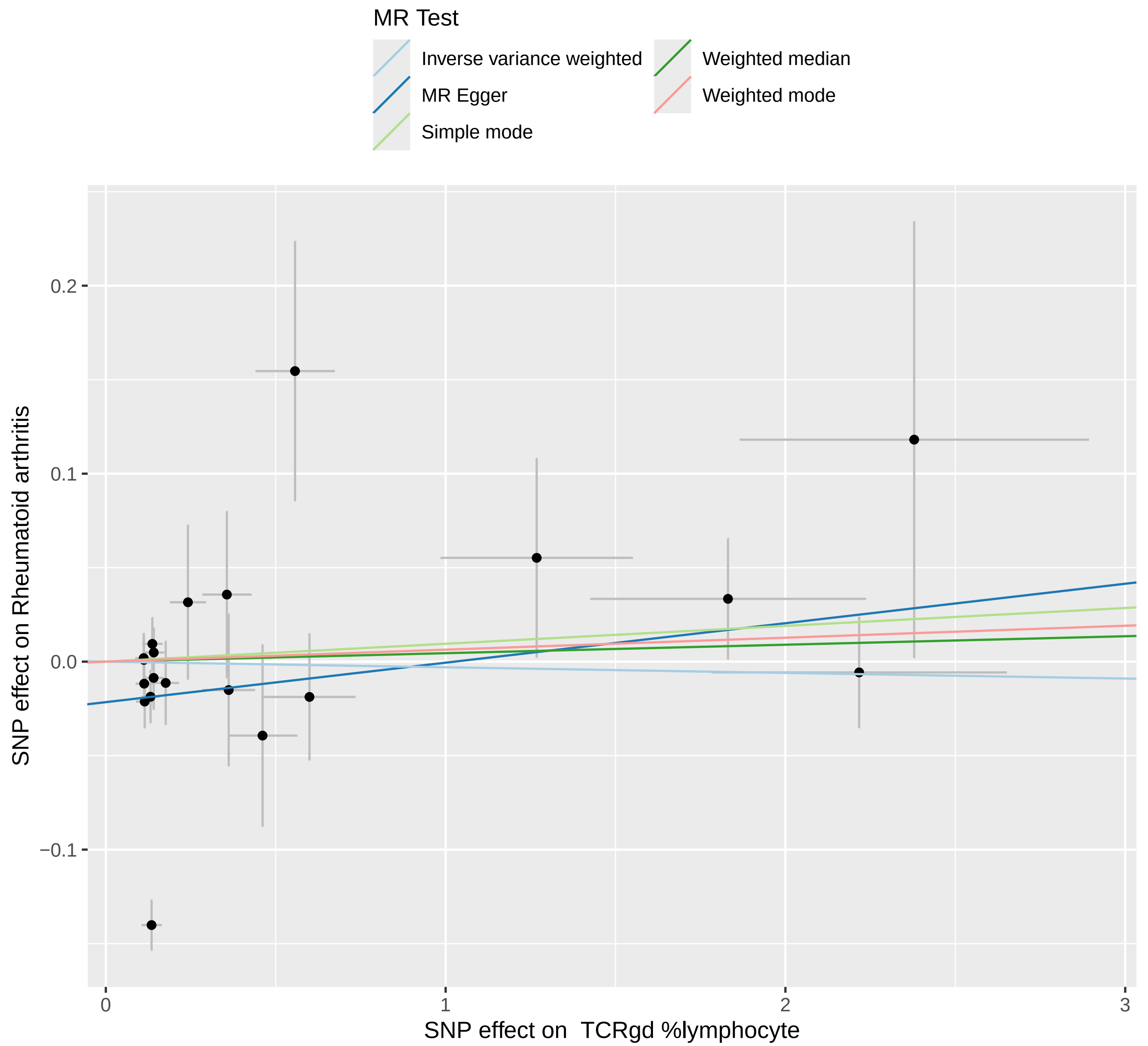
MR Test
Inverse variance weighted
MR Egger
Simple mode
Weighted median
Weighted mode
SNP effect on Rheumatoid arthritis
SNP effect on TCRgd %lymphocyte
0.2
0.1
0.0
−0.1
0
1
2
3

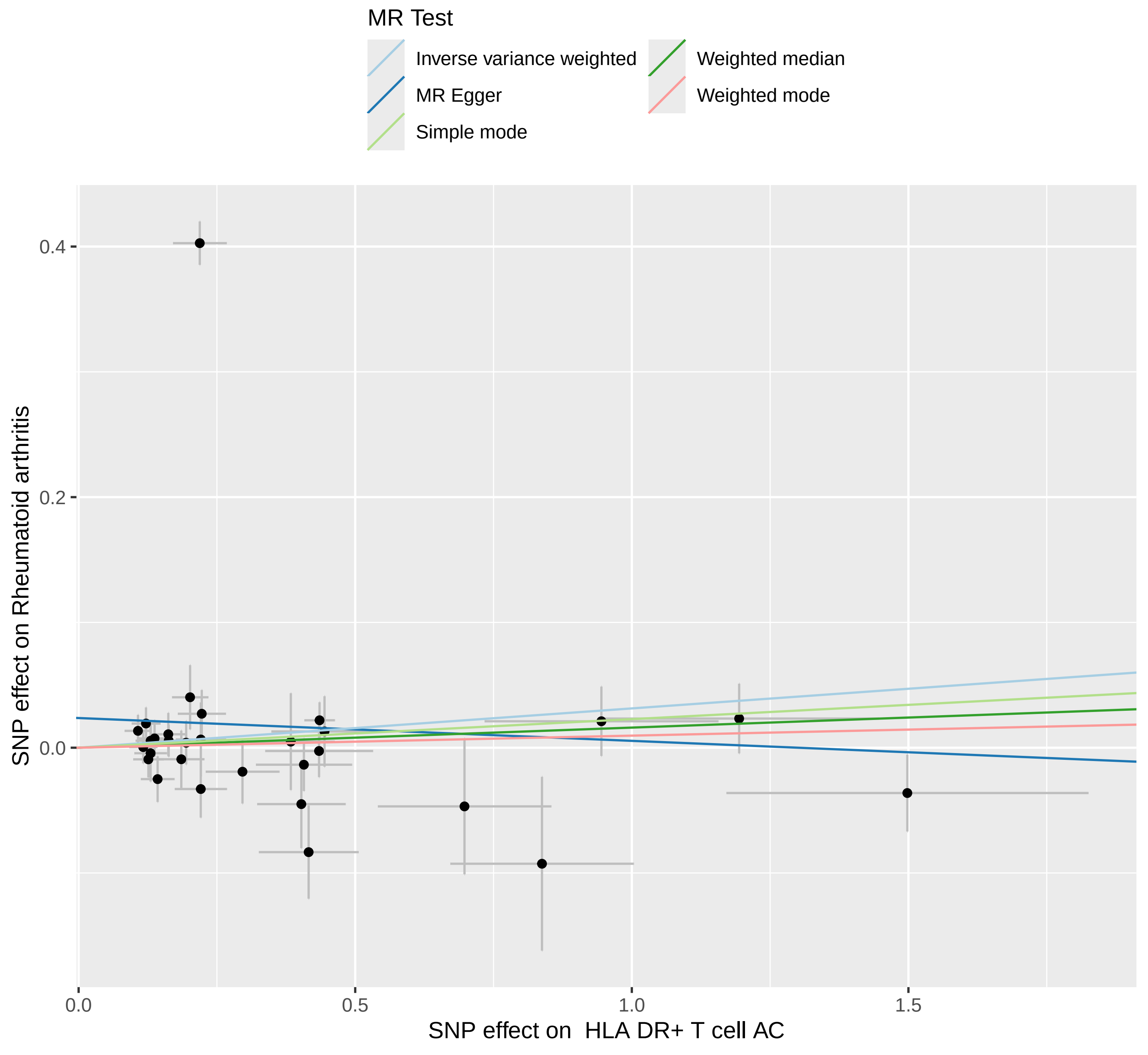
MR Test
Inverse variance weighted
MR Egger
Simple mode
Weighted median
Weighted mode
SNP effect on Rheumatoid arthritis
0.4
0.2
0.0
0.0
0.5
1.0
1.5
SNP effect on HLA DR+ T cell AC

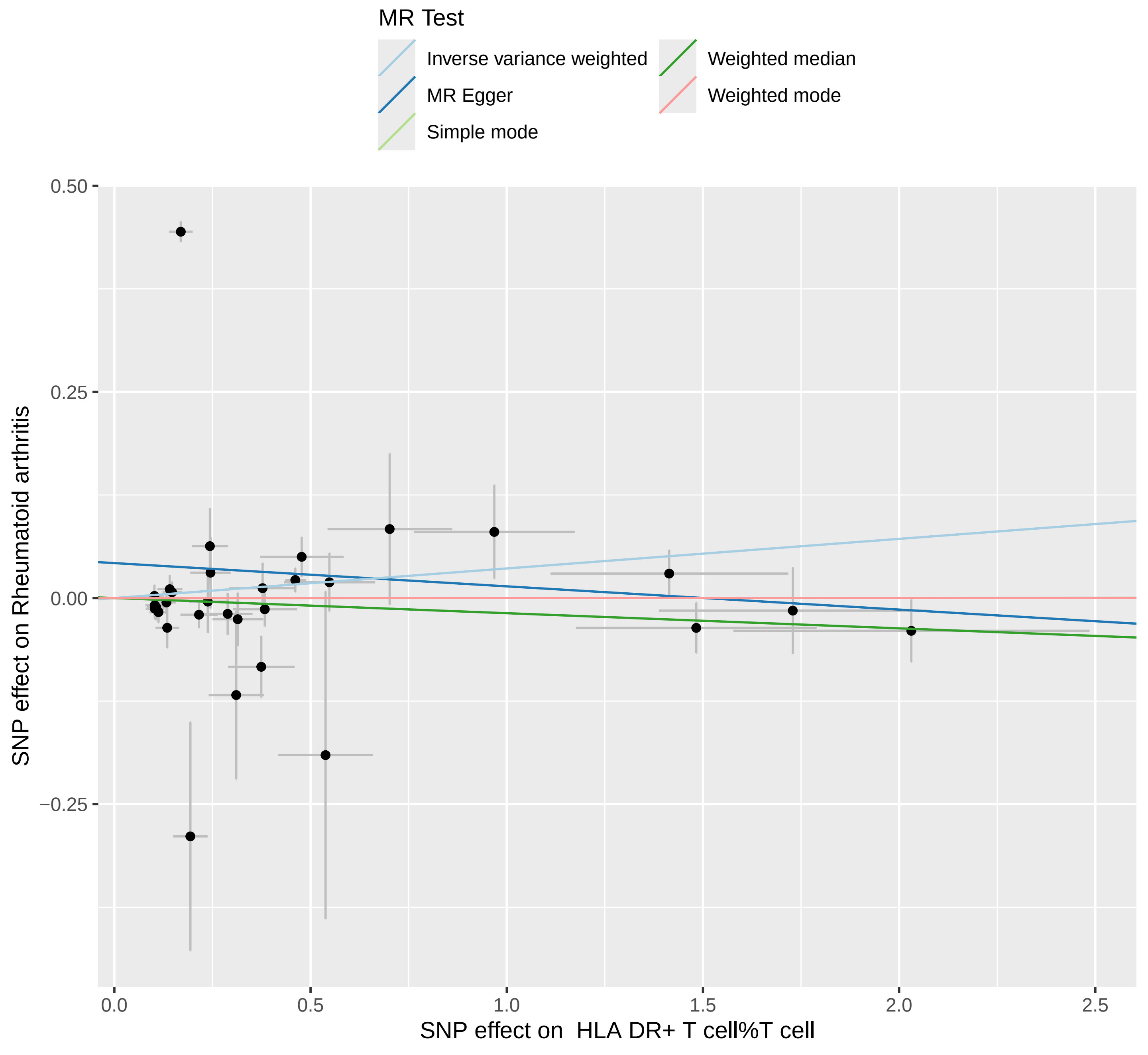
MR Test
Inverse variance weighted
MR Egger
Simple mode
Weighted median
Weighted mode
0.50
0.25
0.00
−0.25
SNP effect on Rheumatoid arthritis
0.0
0.5
1.0
1.5
2.0
2.5
SNP effect on HLA DR+ T cell%T cell

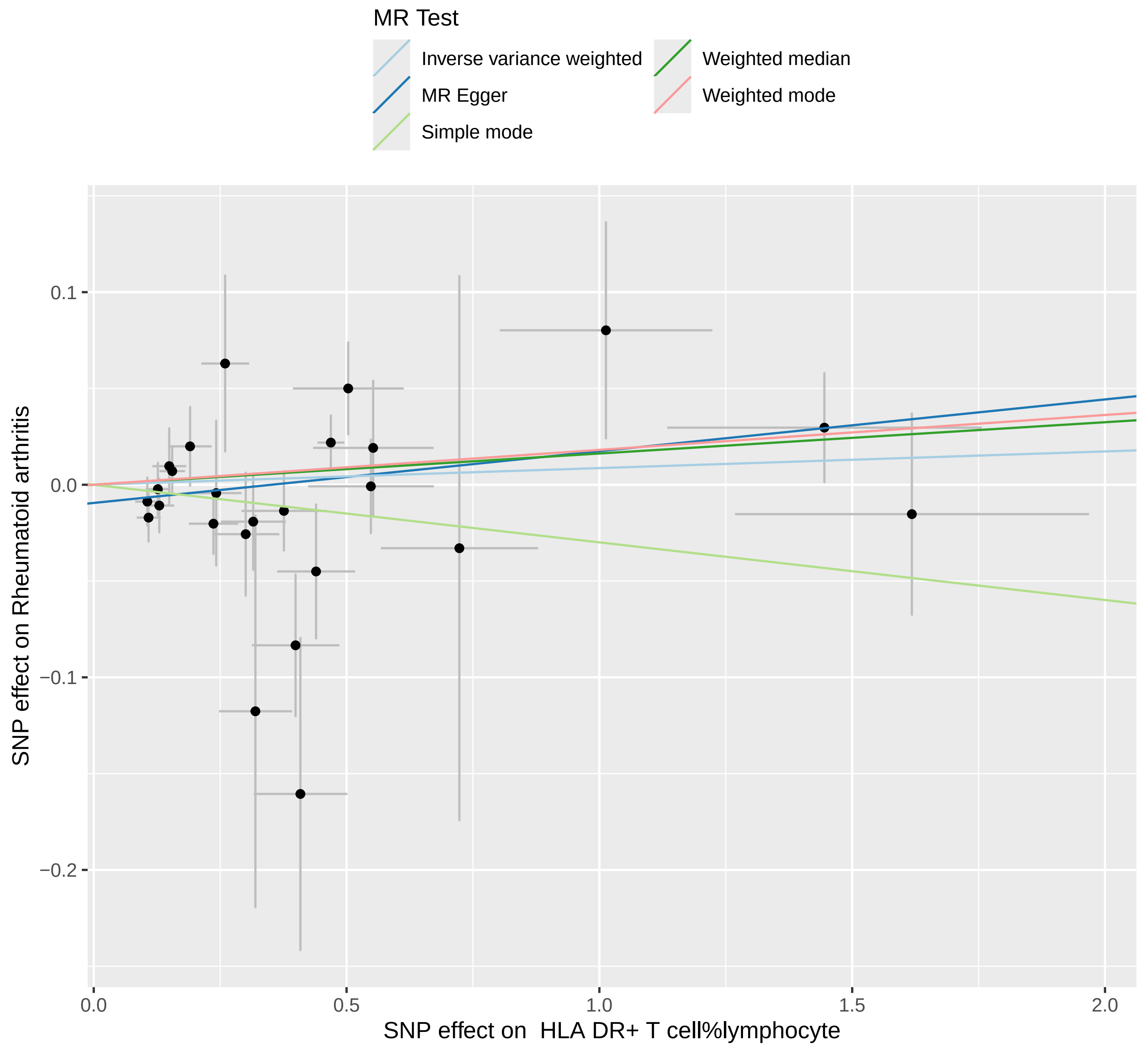
MR Test
Inverse variance weighted
MR Egger
Simple mode
Weighted median
Weighted mode
SNP effect on Rheumatoid arthritis
0.1
0.0
−0.1
−0.2
0.0
0.5
1.0
1.5
2.0
SNP effect on HLA DR+ T cell%lymphocyte

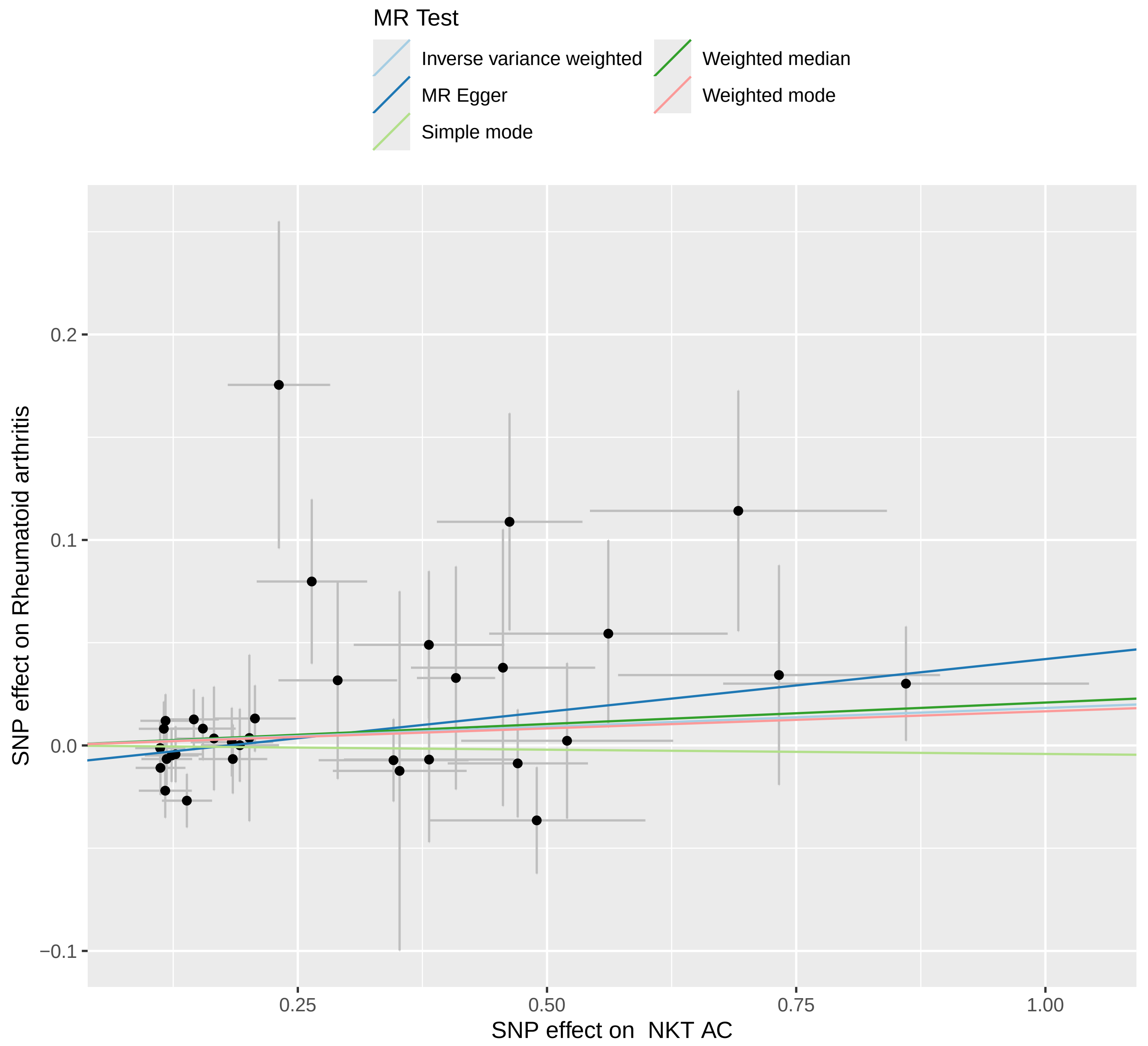

MR Test
Inverse variance weighted
MR Egger
Simple mode
Weighted median
Weighted mode
SNP effect on Rheumatoid arthritis
0.2
0.1
0.0
−0.1
0.25
0.50
0.75
1.00
SNP effect on NKT AC

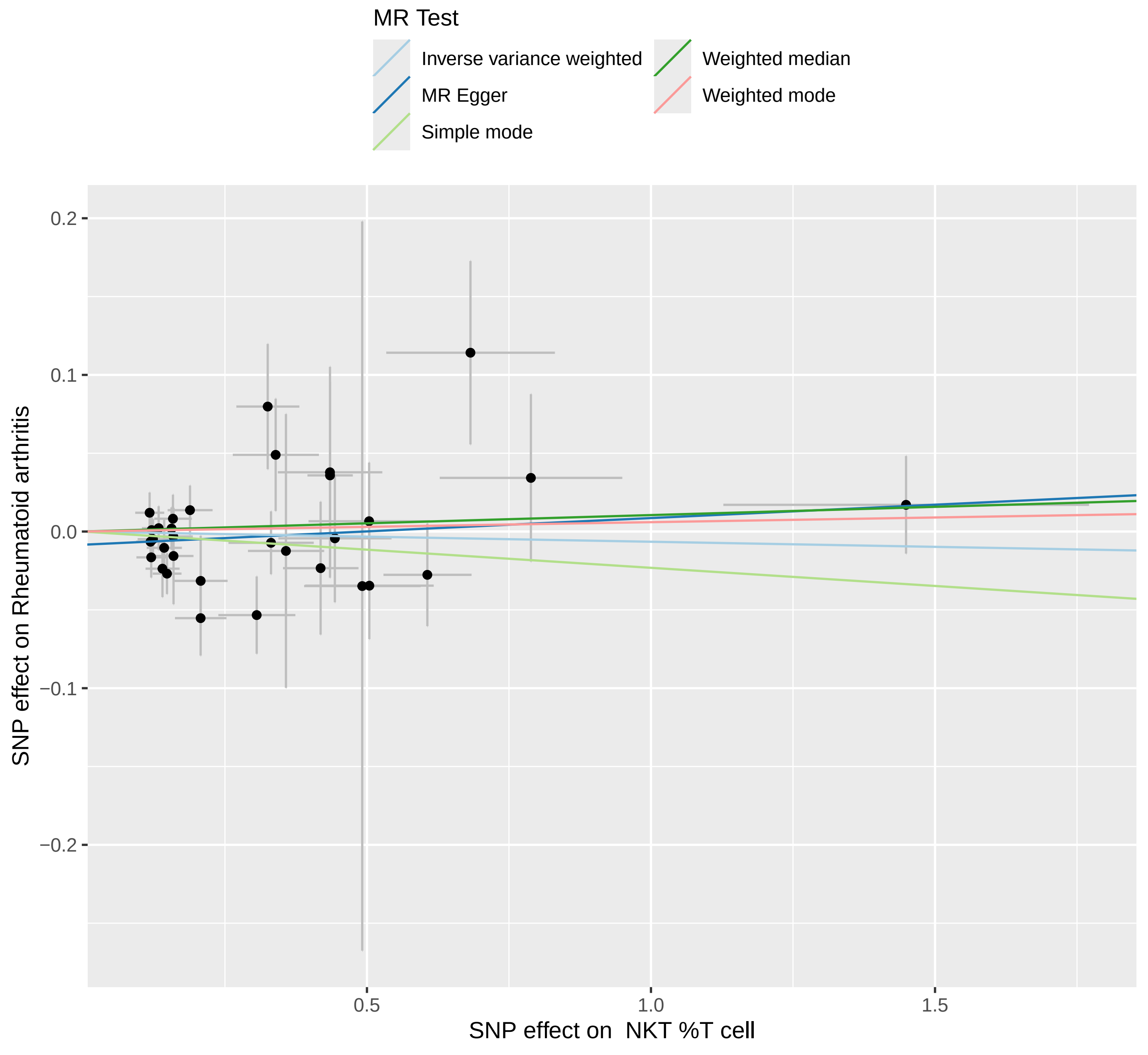

MR Test
Inverse variance weighted
MR Egger
Simple mode
Weighted median
Weighted mode
0.2
0.1
0.0
−0.1
−0.2
SNP effect on Rheumatoid arthritis
0.5
1.0
1.5
SNP effect on NKT %T cell

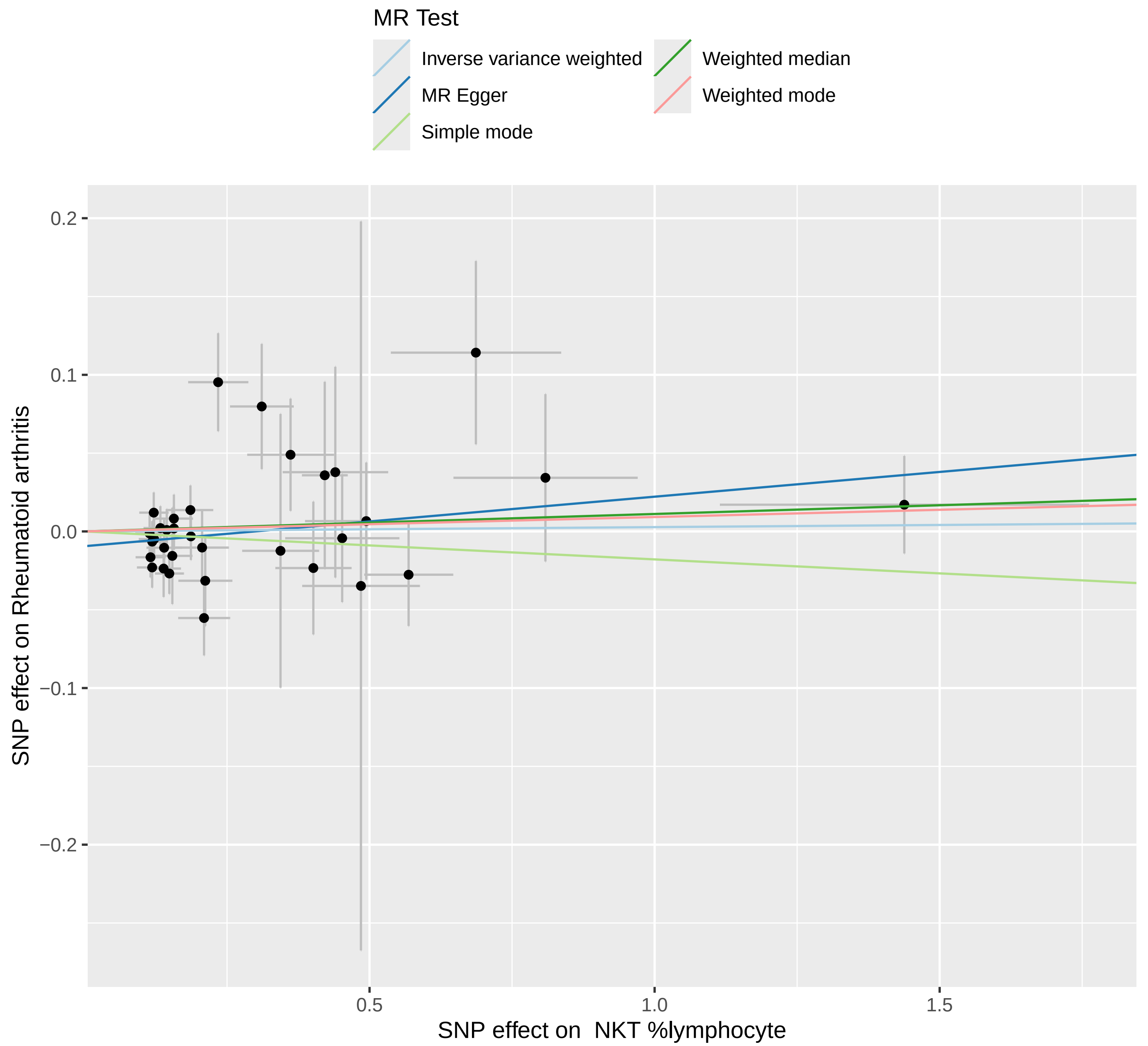
MR Test
Inverse variance weighted
MR Egger
Simple mode
Weighted median
Weighted mode
0.2
0.1
0.0
−0.1
−0.2
SNP effect on Rheumatoid arthritis
0.5
1.0
1.5
SNP effect on NKT %lymphocyte

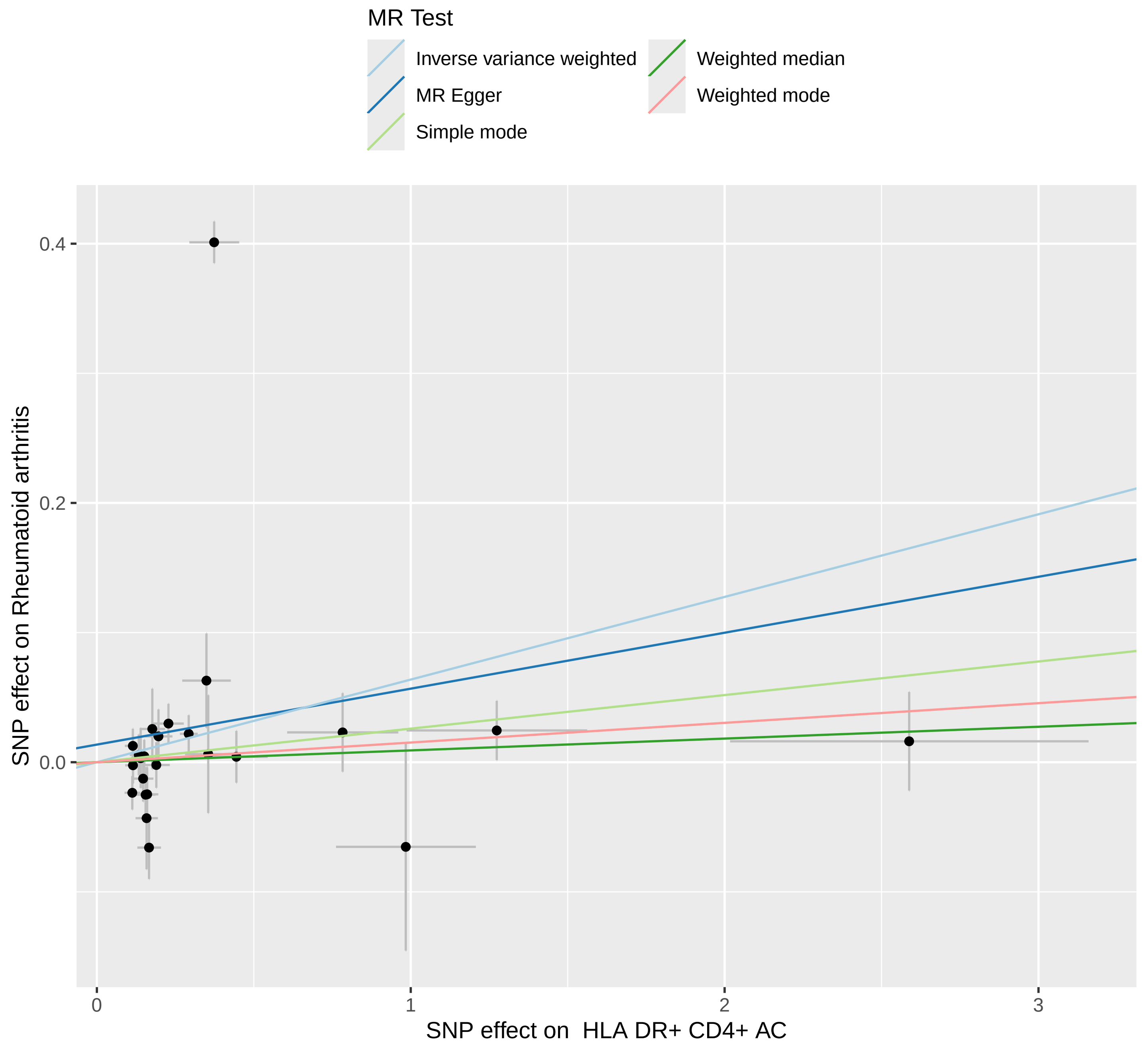
MR Test
Inverse variance weighted
MR Egger
Simple mode
Weighted median
Weighted mode
0.4
0.2
0.0
SNP effect on Rheumatoid arthritis
0
1
2
3
SNP effect on HLA DR+ CD4+ AC

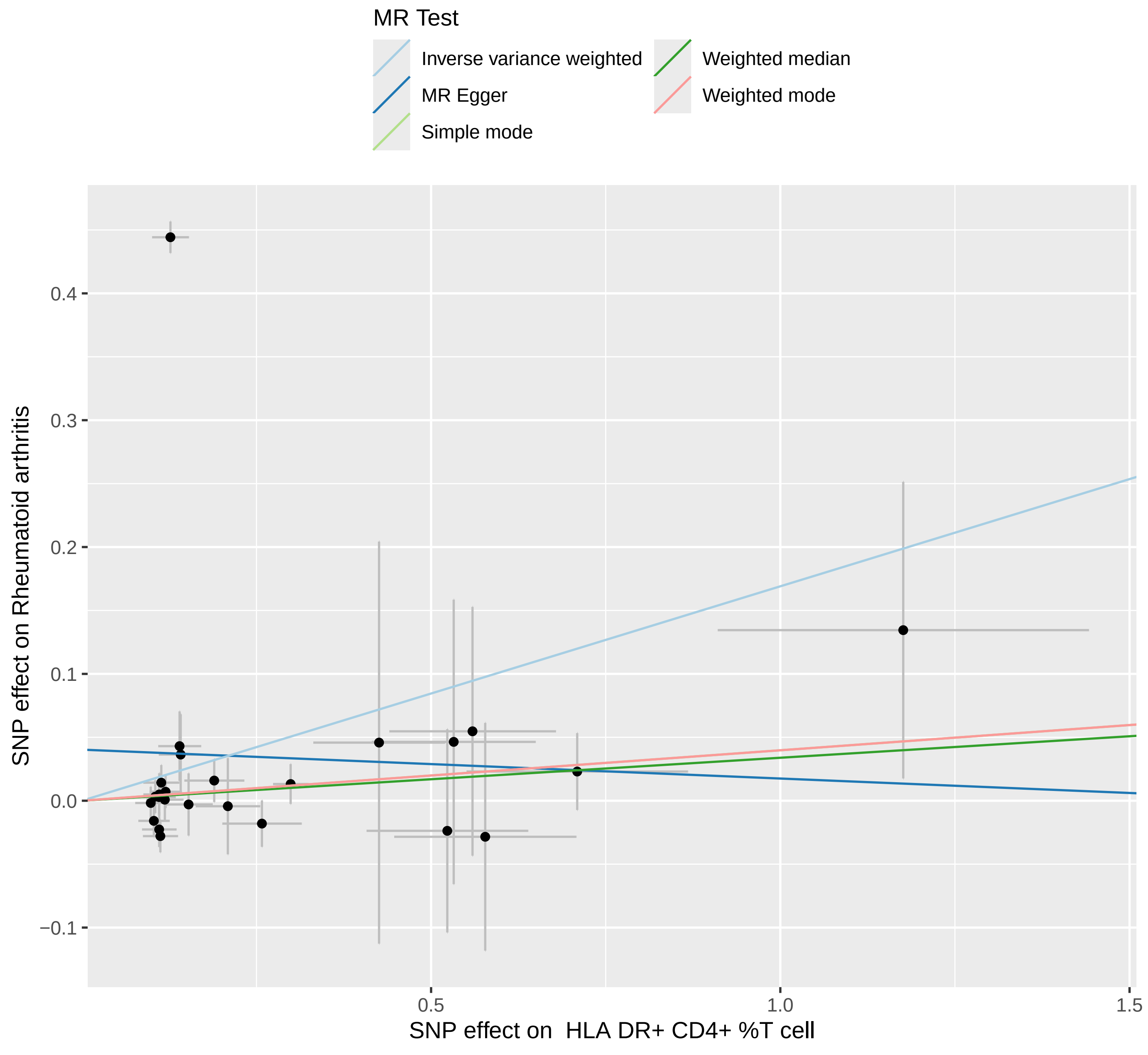
MR Test
Inverse variance weighted
MR Egger
Simple mode
Weighted median
Weighted mode
SNP effect on Rheumatoid arthritis
0.4
0.3
0.2
0.1
0.0
−0.1
0.5
1.0
1.5
SNP effect on HLA DR+ CD4+ %T cell

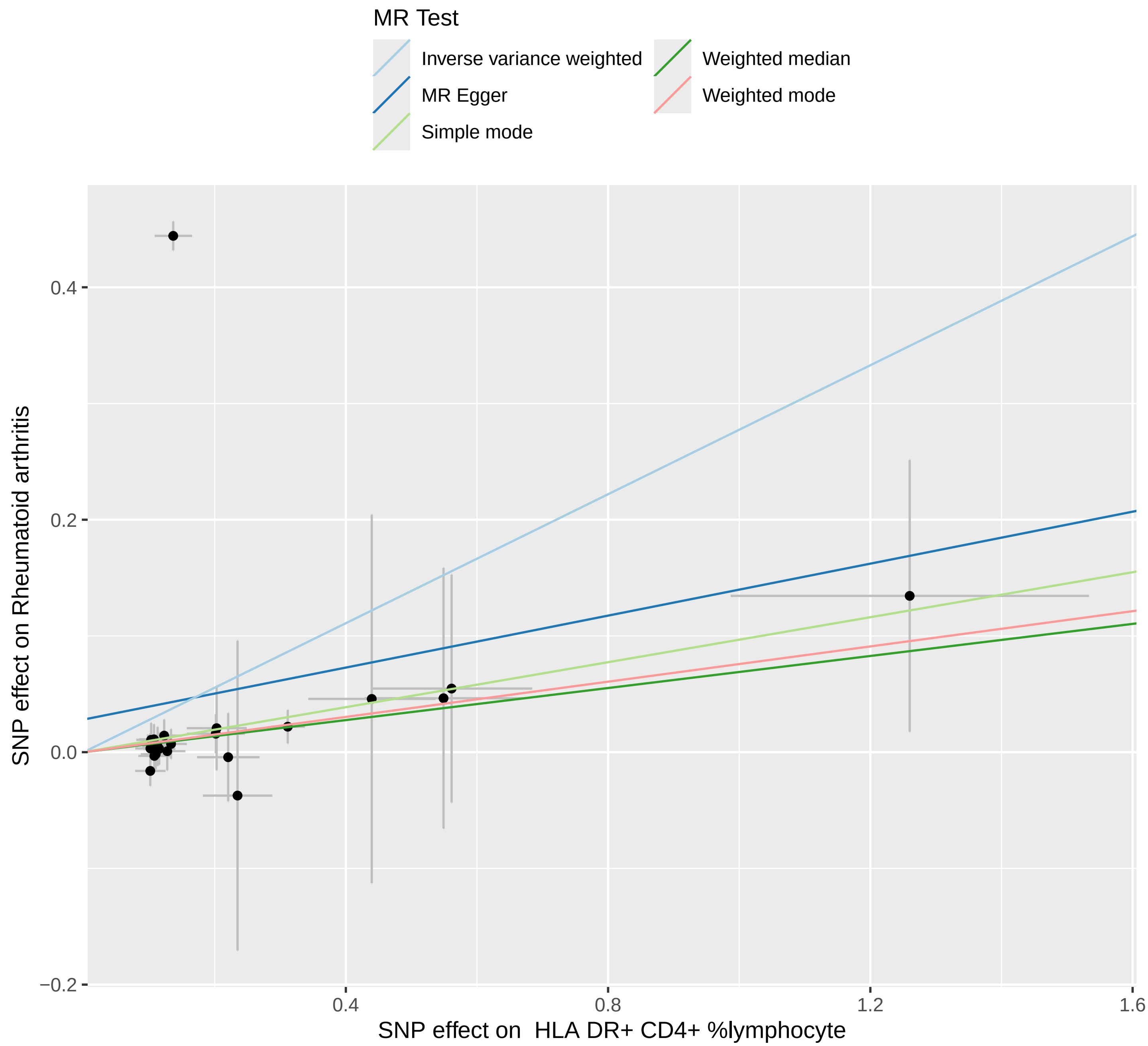
MR Test
Inverse variance weighted
MR Egger
Simple mode
Weighted median
Weighted mode
SNP effect on Rheumatoid arthritis
0.4
0.2
0.0
−0.2
0.4
0.8
1.2
1.6
SNP effect on HLA DR+ CD4+ %lymphocyte

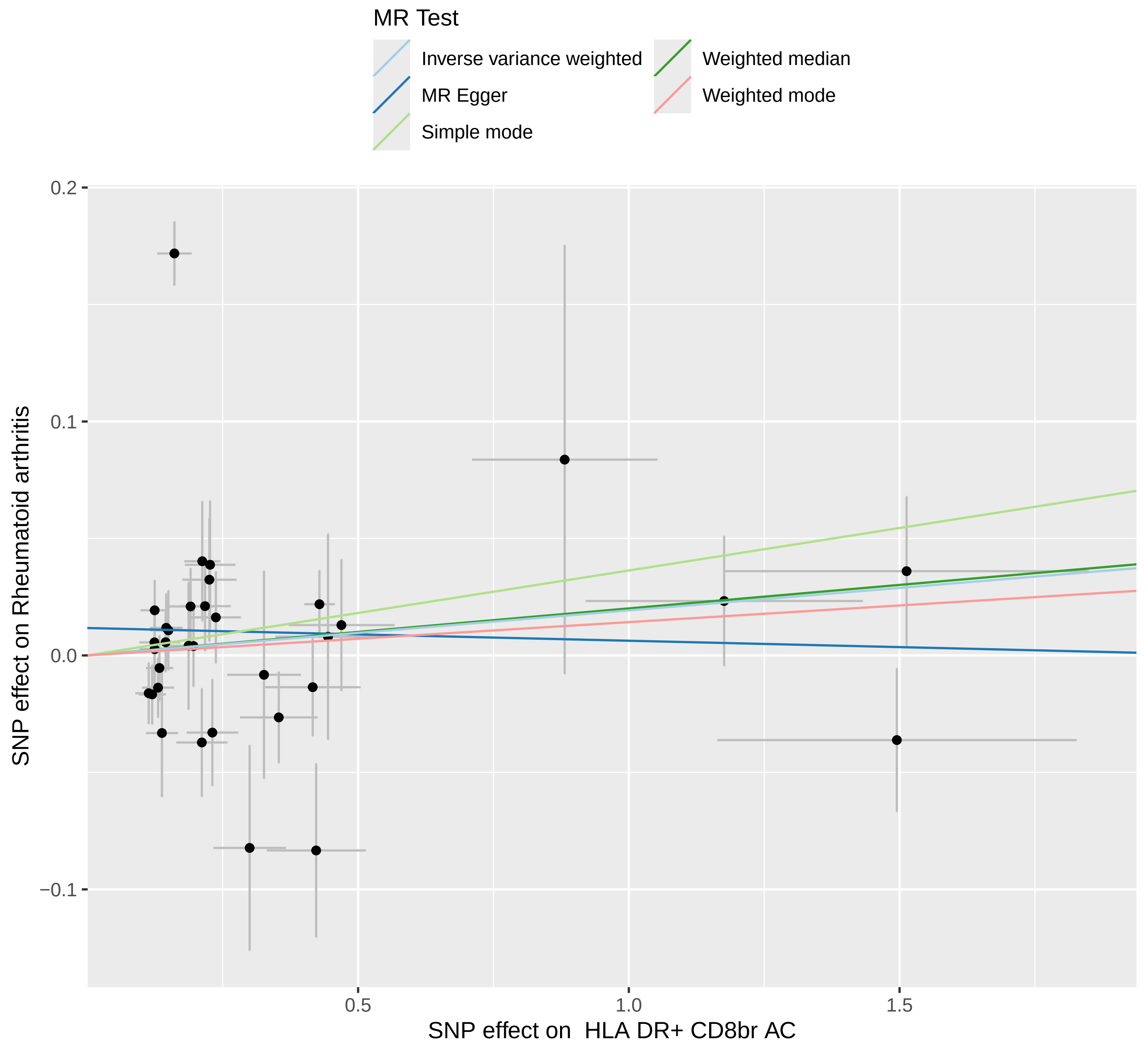
MR Test
Inverse variance weighted
MR Egger
Simple mode
Weighted median
Weighted mode
0.2
0.1
0.0
−0.1
0.5
1.0
1.5
SNP effect on Rheumatoid arthritis
SNP effect on HLA DR+ CD8br AC

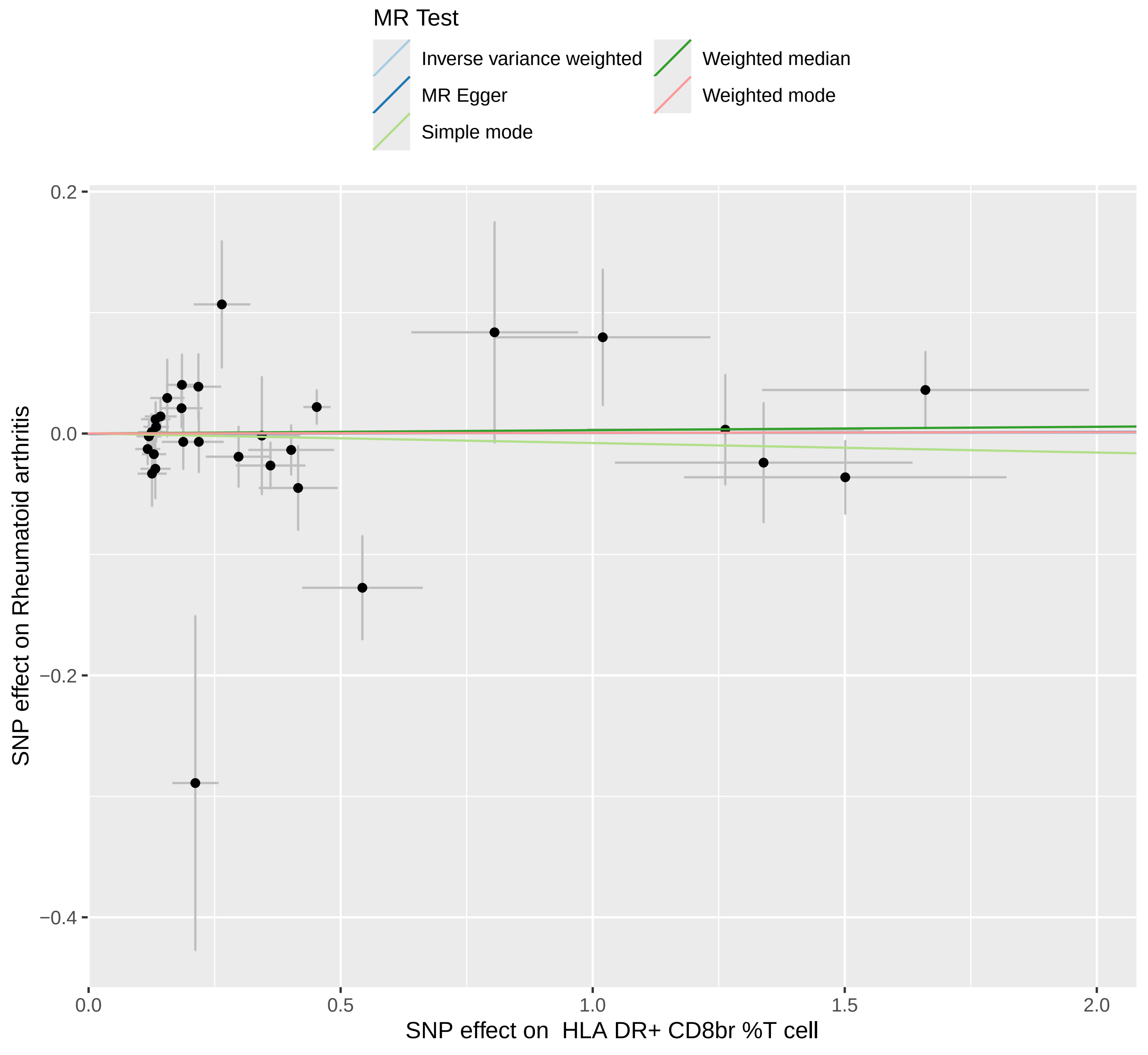

MR Test
Inverse variance weighted
MR Egger
Simple mode
Weighted median
Weighted mode
0.2
0.0
−0.2
−0.4
0.0
0.5
1.0
1.5
2.0
SNP effect on HLA DR+ CD8br %T cell
SNP effect on Rheumatoid arthritis

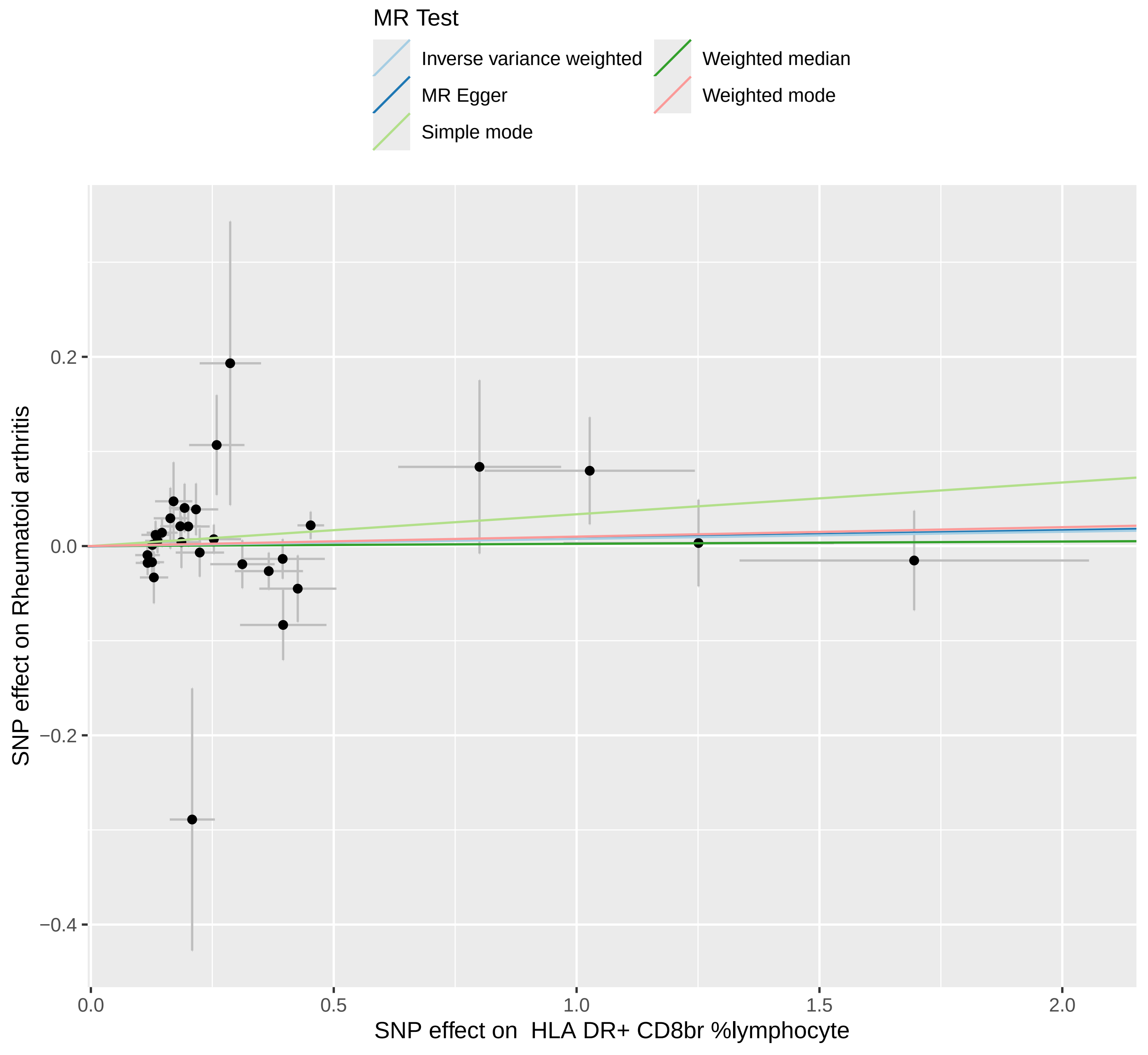
MR Test
Inverse variance weighted
MR Egger
Simple mode
Weighted median
Weighted mode
0.2
0.0
−0.2
−0.4
SNP effect on Rheumatoid arthritis
0.0
0.5
1.0
1.5
2.0
SNP effect on HLA DR+ CD8br %lymphocyte

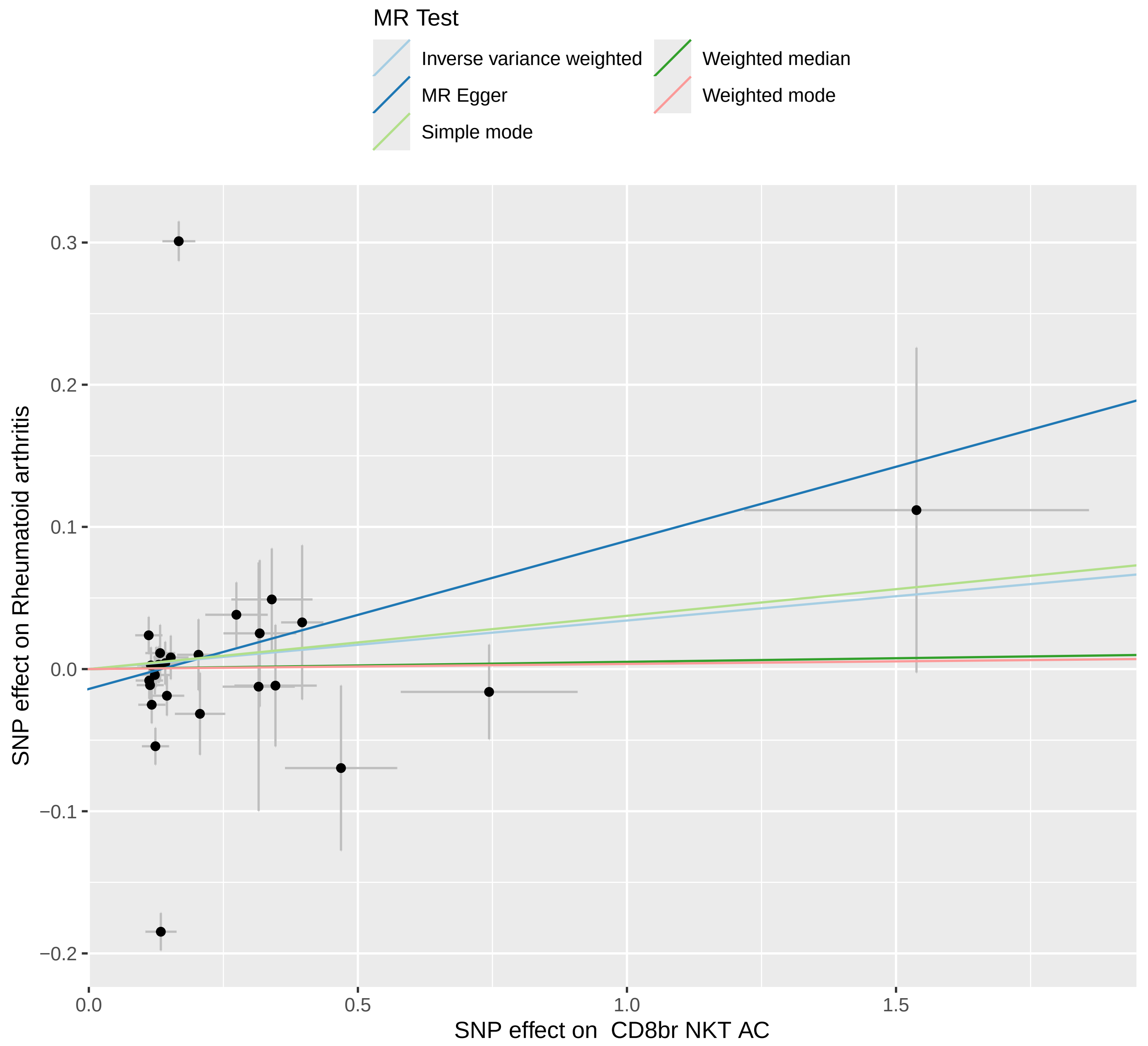

MR Test
Inverse variance weighted
MR Egger
Simple mode
Weighted median
Weighted mode
0.3
0.2
0.1
0.0
−0.1
−0.2
0.0
0.5
1.0
1.5
SNP effect on Rheumatoid arthritis
SNP effect on CD8br NKT AC

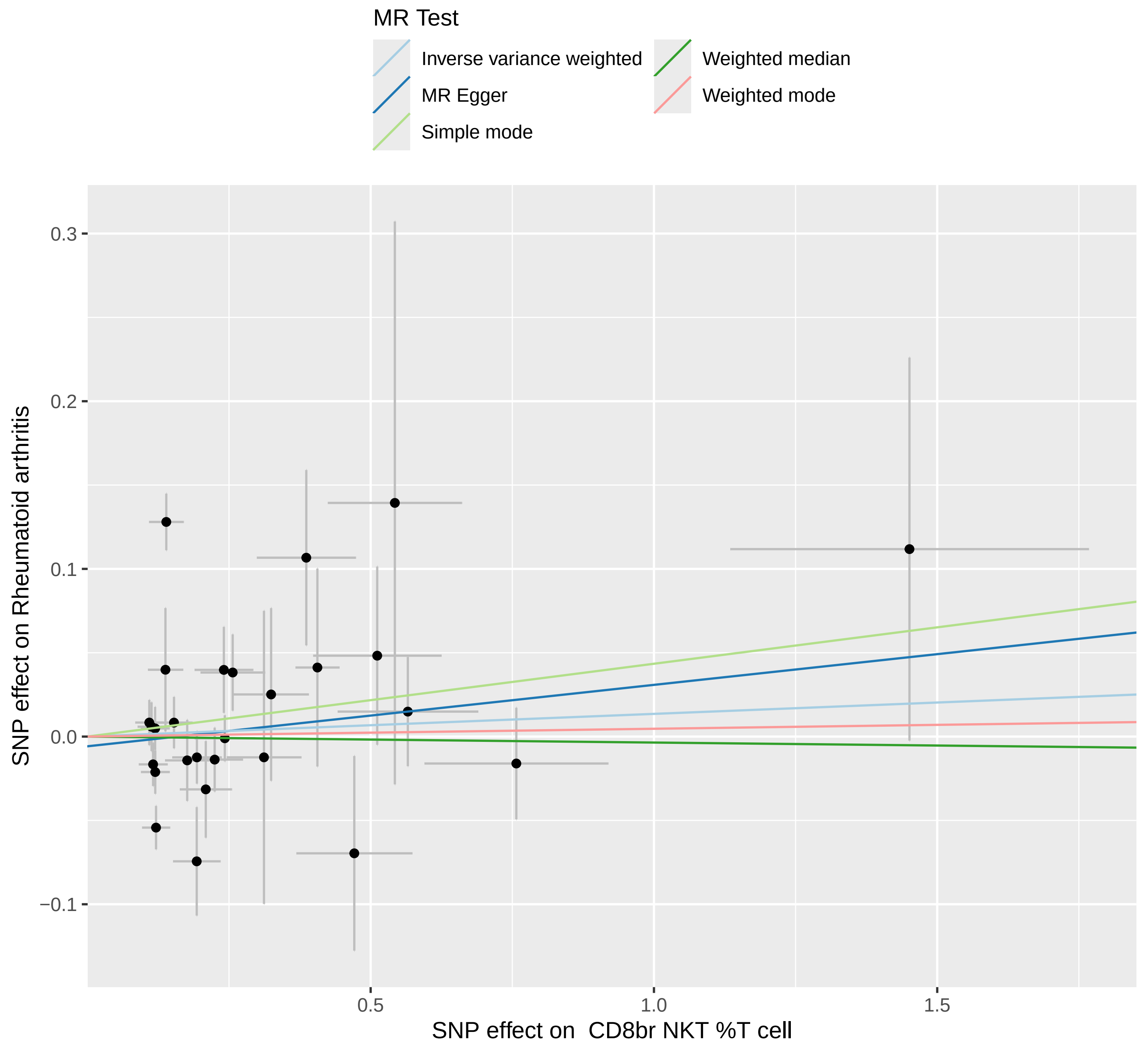

MR Test
Inverse variance weighted
MR Egger
Simple mode
Weighted median
Weighted mode
SNP effect on Rheumatoid arthritis
0.3
0.2
0.1
0.0
−0.1
0.5
1.0
1.5
SNP effect on CD8br NKT %T cell

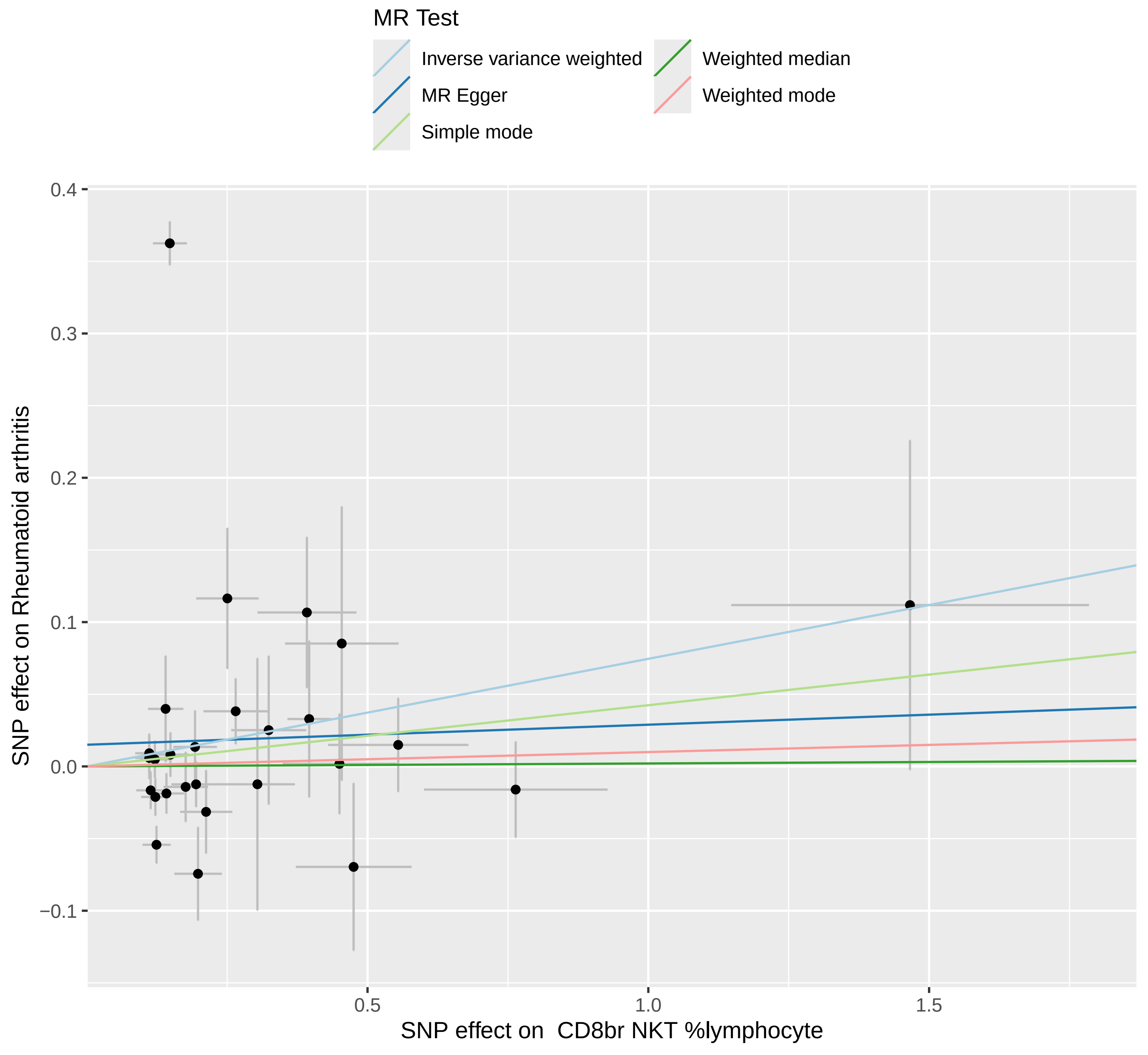

MR Test
Inverse variance weighted
MR Egger
Simple mode
Weighted median
Weighted mode
SNP effect on Rheumatoid arthritis
0.4
0.3
0.2
0.1
0.0
−0.1
0.5
1.0
1.5
SNP effect on CD8br NKT %lymphocyte

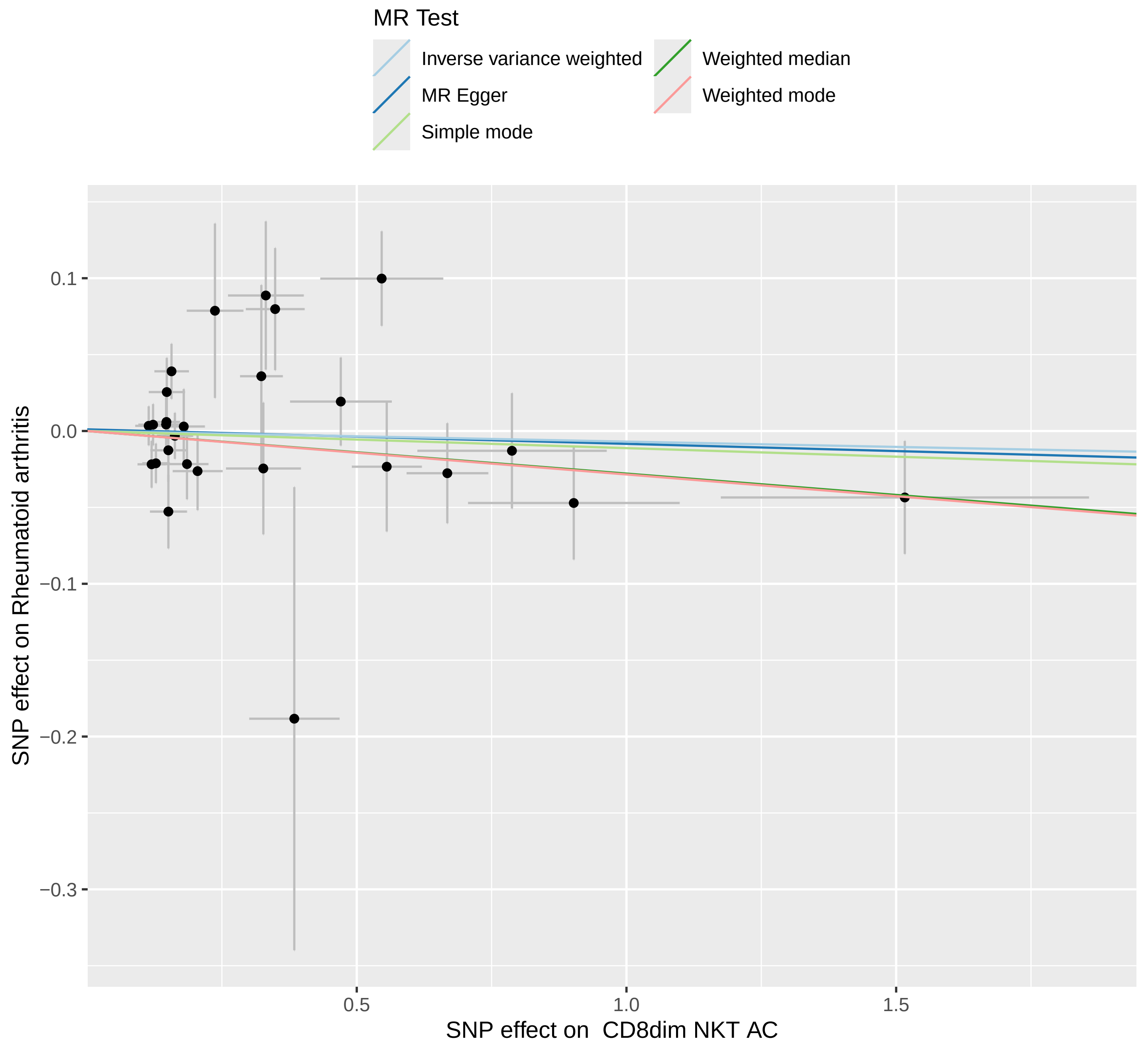
MR Test
Inverse variance weighted
MR Egger
Simple mode
Weighted median
Weighted mode
0.1
0.0
−0.1
−0.2
−0.3
0.5
1.0
1.5
SNP effect on Rheumatoid arthritis
SNP effect on CD8dim NKT AC

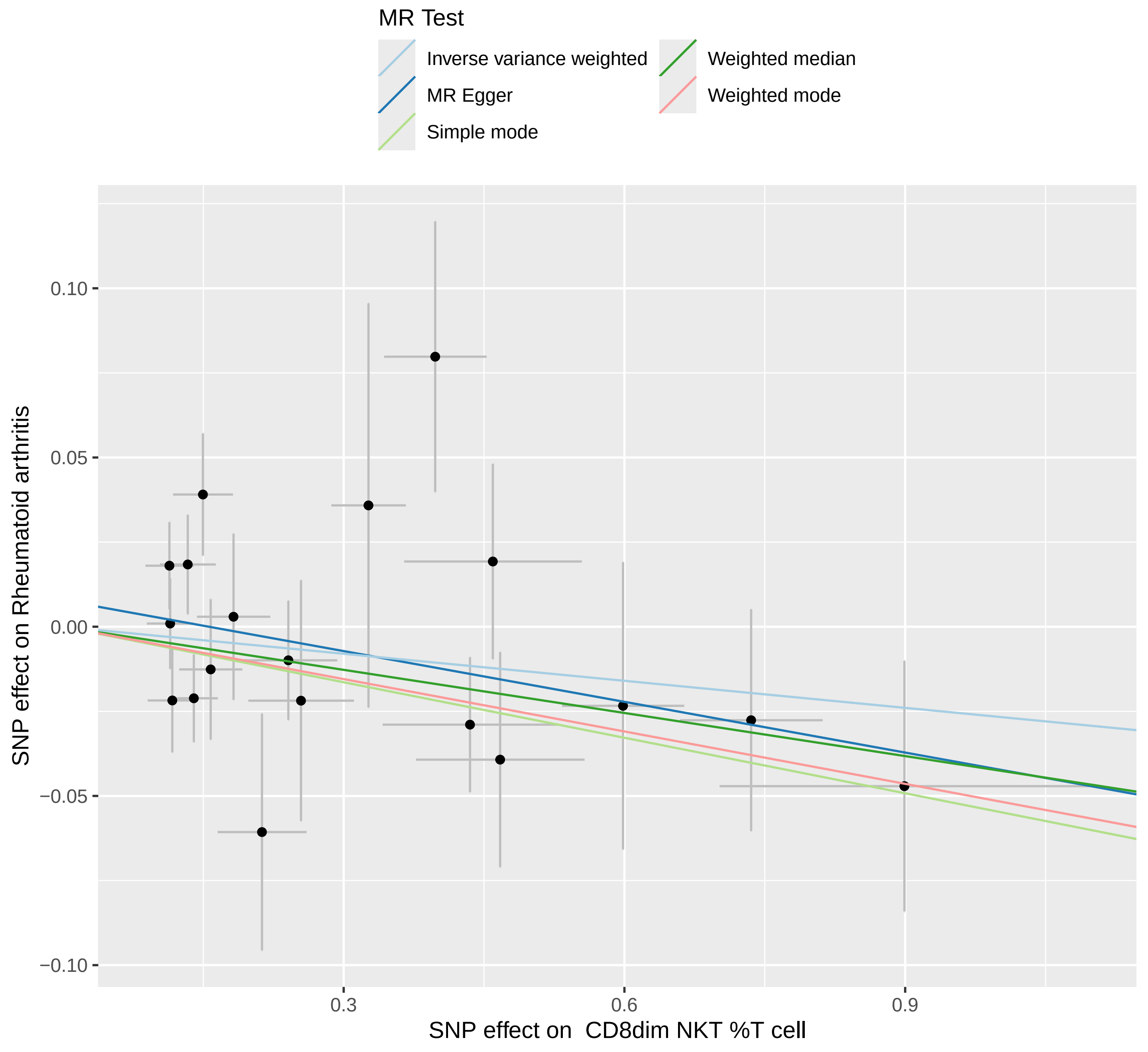
MR Test
Inverse variance weighted
MR Egger
Simple mode
Weighted median
Weighted mode
SNP effect on Rheumatoid arthritis
0.10
0.05
0.00
−0.05
−0.10
0.3
0.6
0.9
SNP effect on CD8dim NKT %T cell

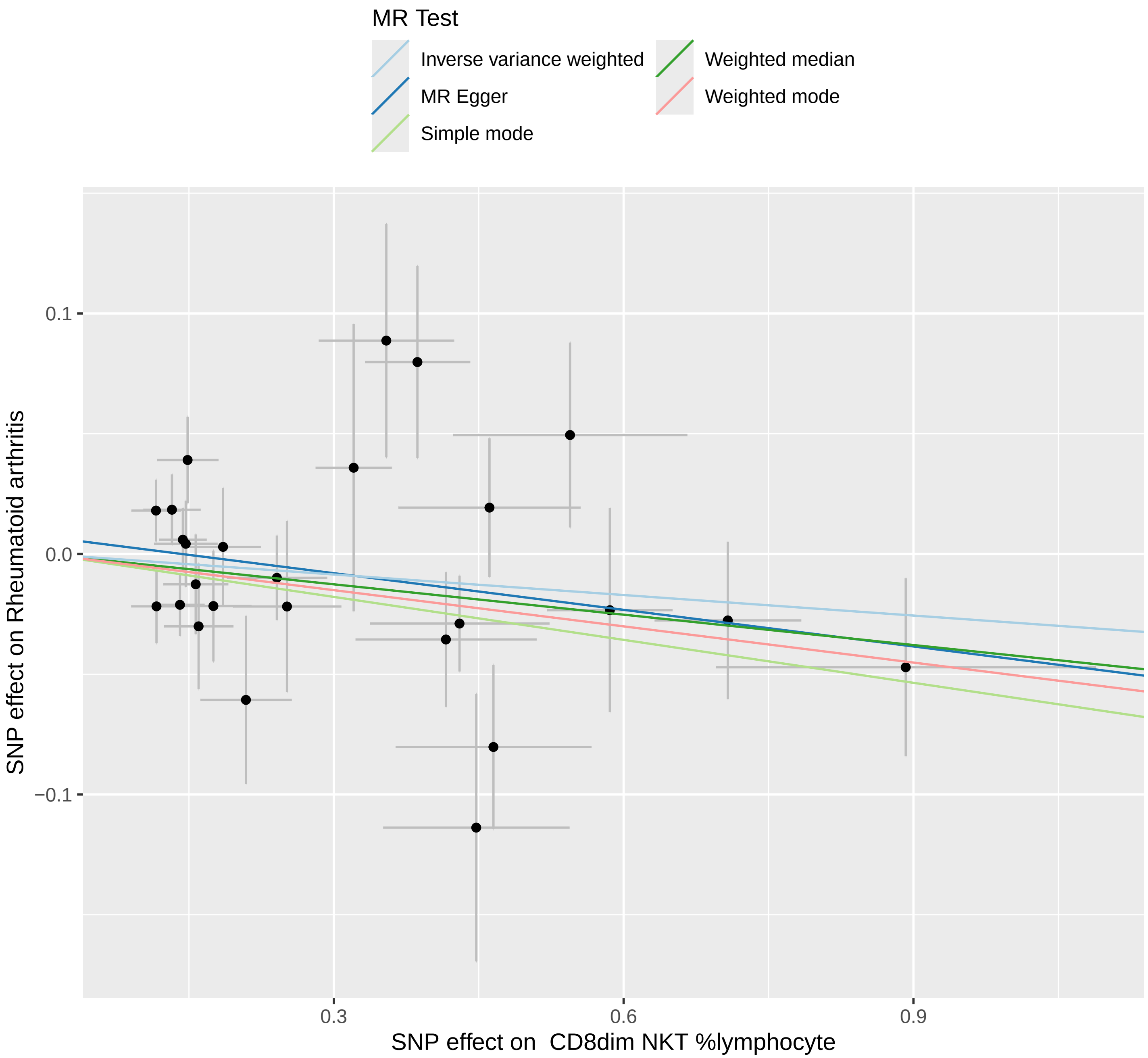

MR Test
Inverse variance weighted
MR Egger
Simple mode
Weighted median
Weighted mode
SNP effect on Rheumatoid arthritis
0.1
0.0
−0.1
0.3
0.6
0.9
SNP effect on CD8dim NKT %lymphocyte

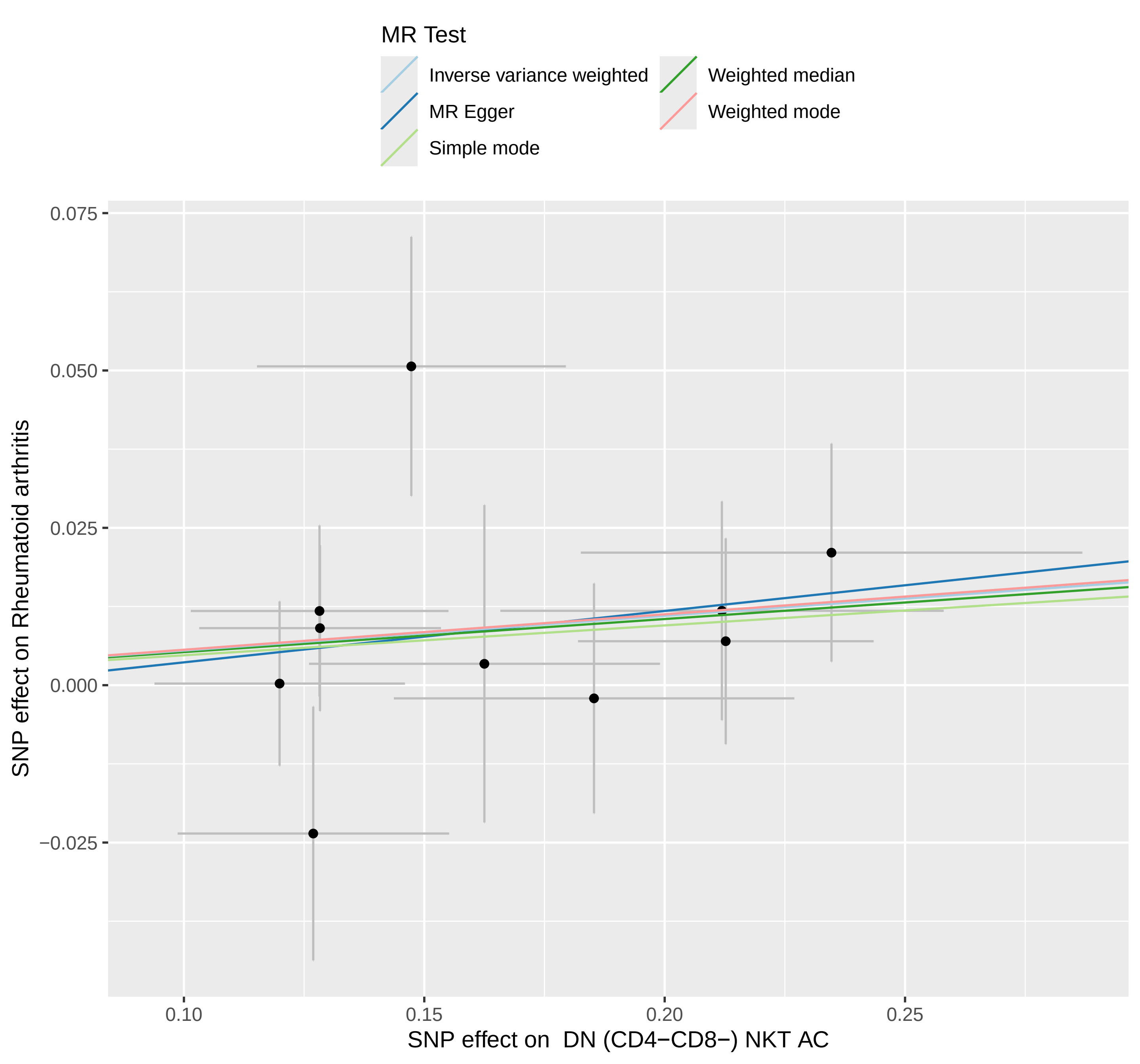

MR Test
Inverse variance weighted
MR Egger
Simple mode
Weighted median
Weighted mode
0.075
0.050
0.025
0.000
−0.025
0.10
0.15
0.20
0.25
SNP effect on Rheumatoid arthritis
SNP effect on DN (CD4−CD8−) NKT AC

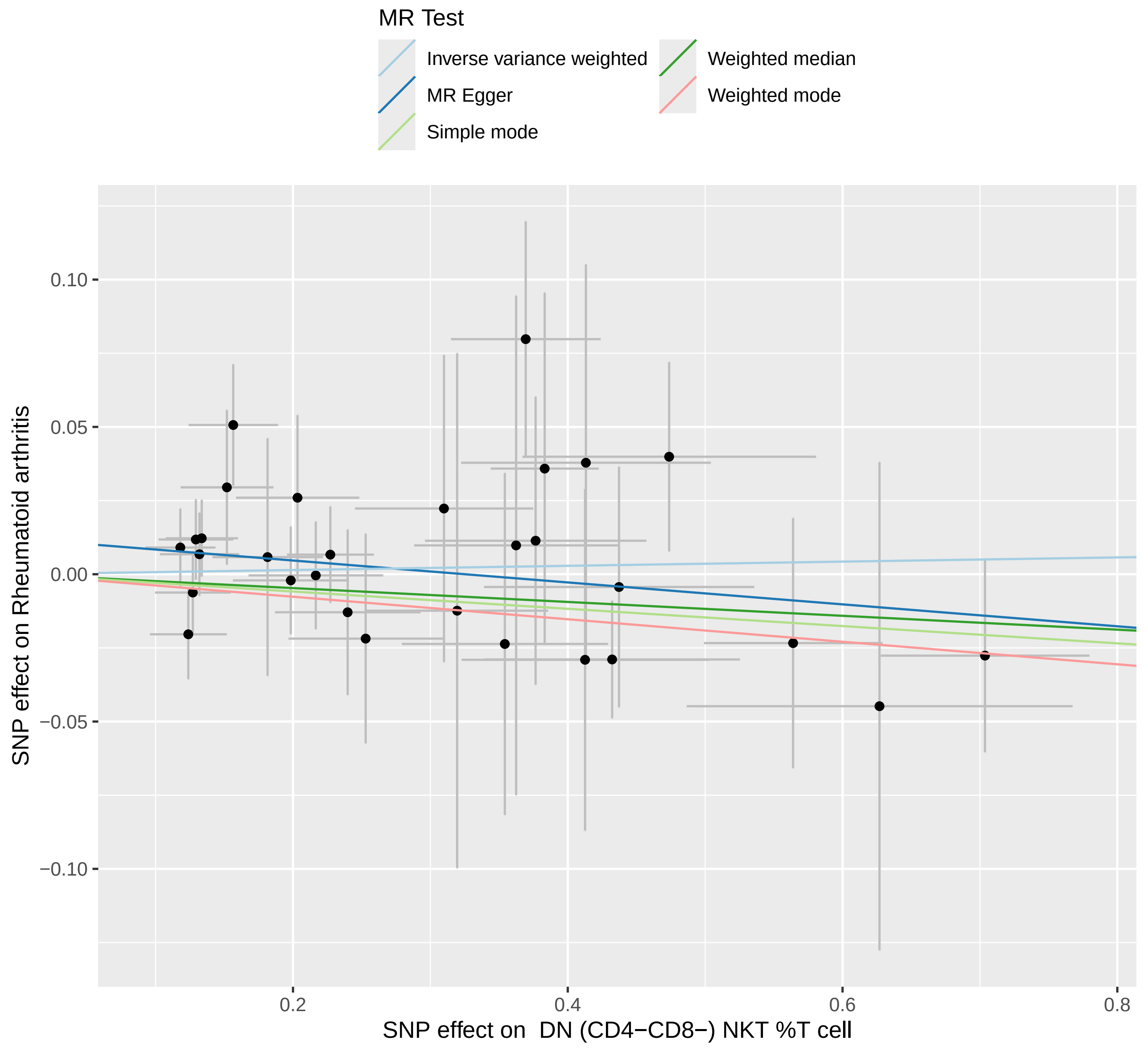
MR Test
Inverse variance weighted
MR Egger
Simple mode
Weighted median
Weighted mode
SNP effect on Rheumatoid arthritis
0.10
0.05
0.00
−0.05
−0.10
0.2
0.4
0.6
0.8
SNP effect on DN (CD4−CD8−) NKT %T cell

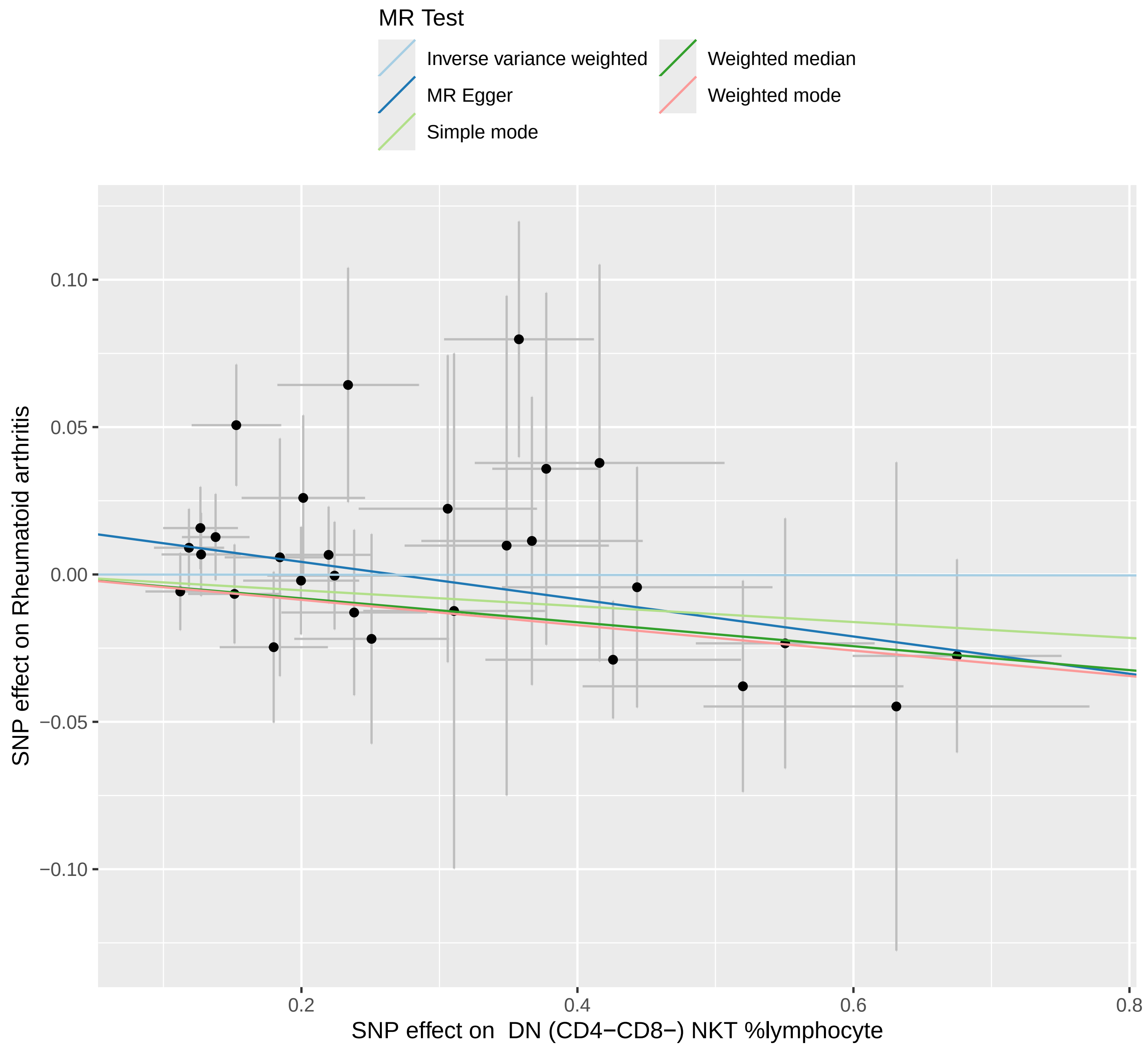

MR Test
Inverse variance weighted
MR Egger
Simple mode
Weighted median
Weighted mode
0.10
0.05
0.00
−0.05
−0.10
SNP effect on Rheumatoid arthritis
0.2
0.4
0.6
0.8
SNP effect on DN (CD4−CD8−) NKT %lymphocyte

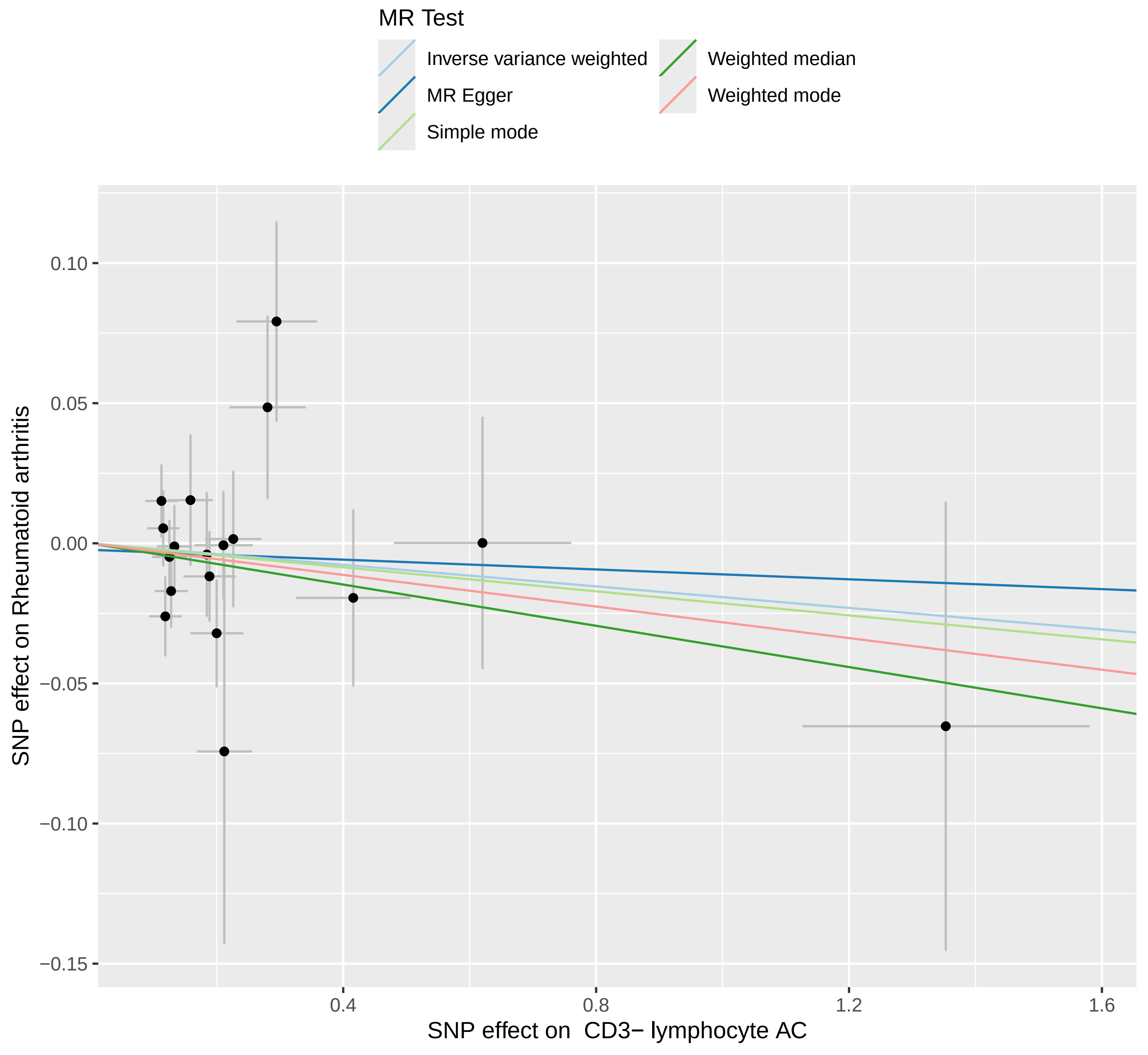

MR Test
Inverse variance weighted
MR Egger
Simple mode
Weighted median
Weighted mode
0.10
0.05
0.00
−0.05
−0.10
−0.15
0.4
0.8
1.2
1.6
SNP effect on Rheumatoid arthritis
SNP effect on CD3− lymphocyte AC

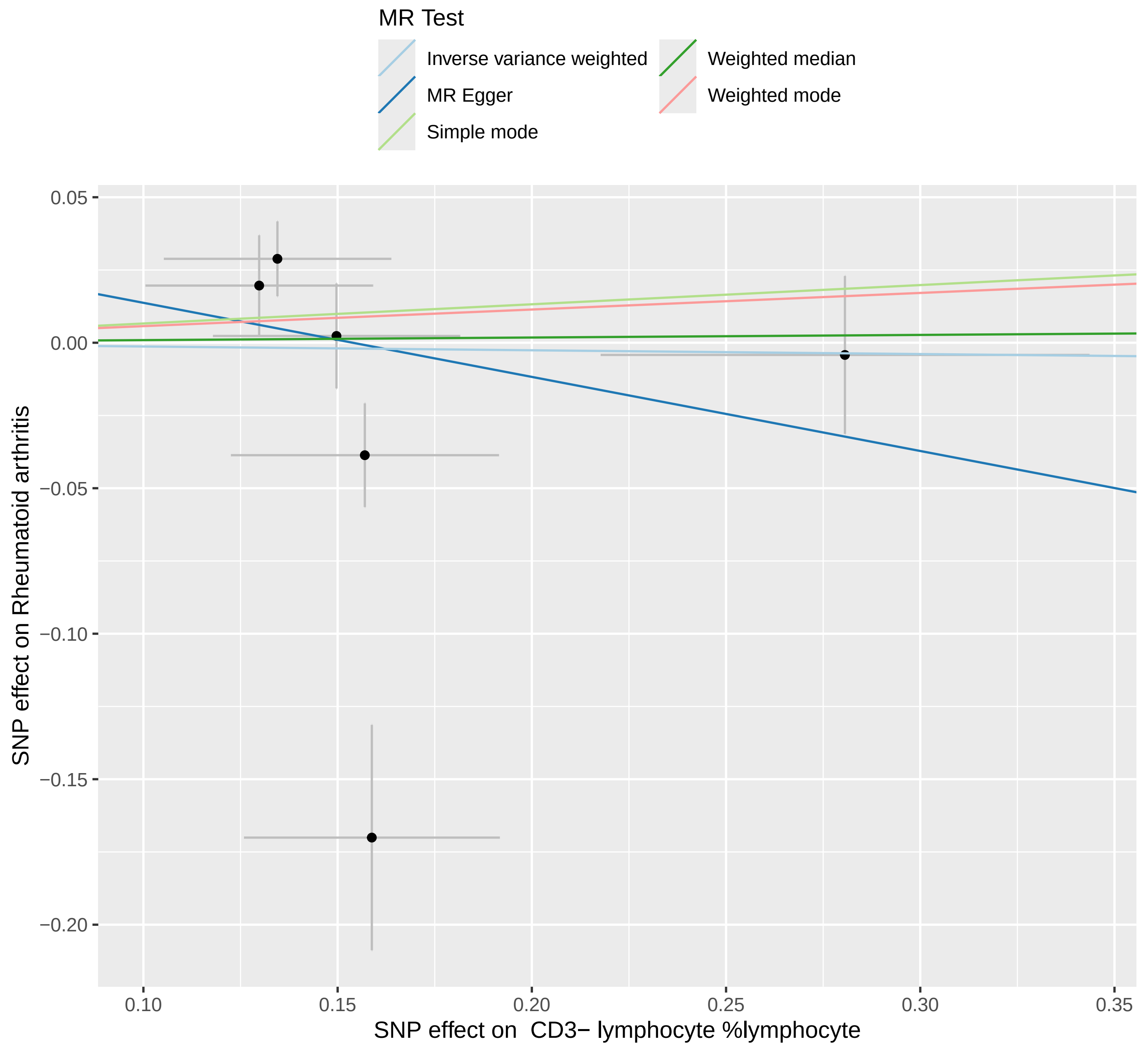

MR Test
Inverse variance weighted
MR Egger
Simple mode
Weighted median
Weighted mode
0.05
0.00
−0.05
−0.10
−0.15
−0.20
0.10
0.15
0.20
0.25
0.30
0.35
SNP effect on Rheumatoid arthritis
SNP effect on CD3− lymphocyte %lymphocyte

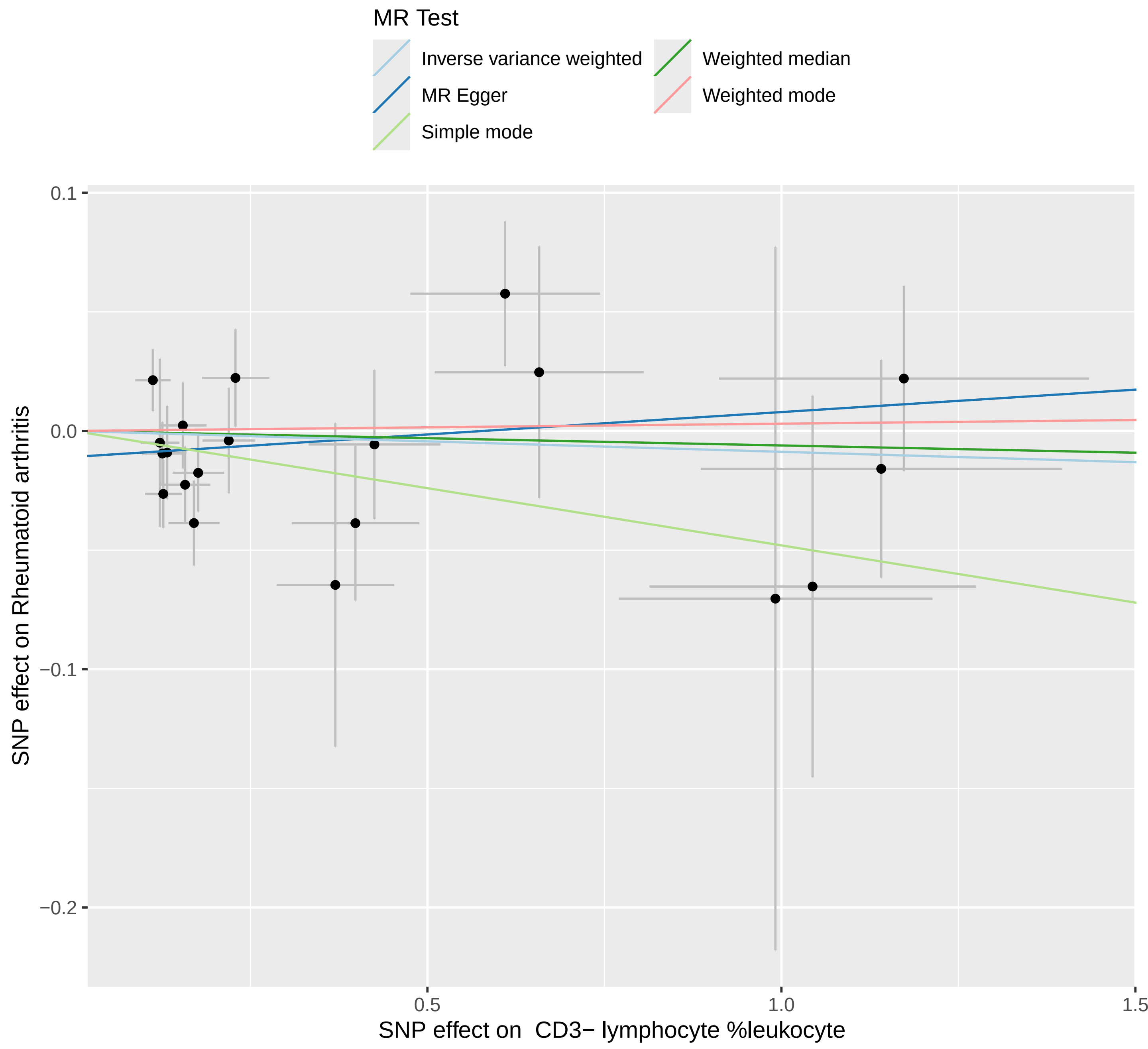
MR Test
Inverse variance weighted
MR Egger
Simple mode
Weighted median
Weighted mode
0.1
0.0
−0.1
−0.2
0.5
1.0
1.5
SNP effect on Rheumatoid arthritis
SNP effect on CD3− lymphocyte %leukocyte

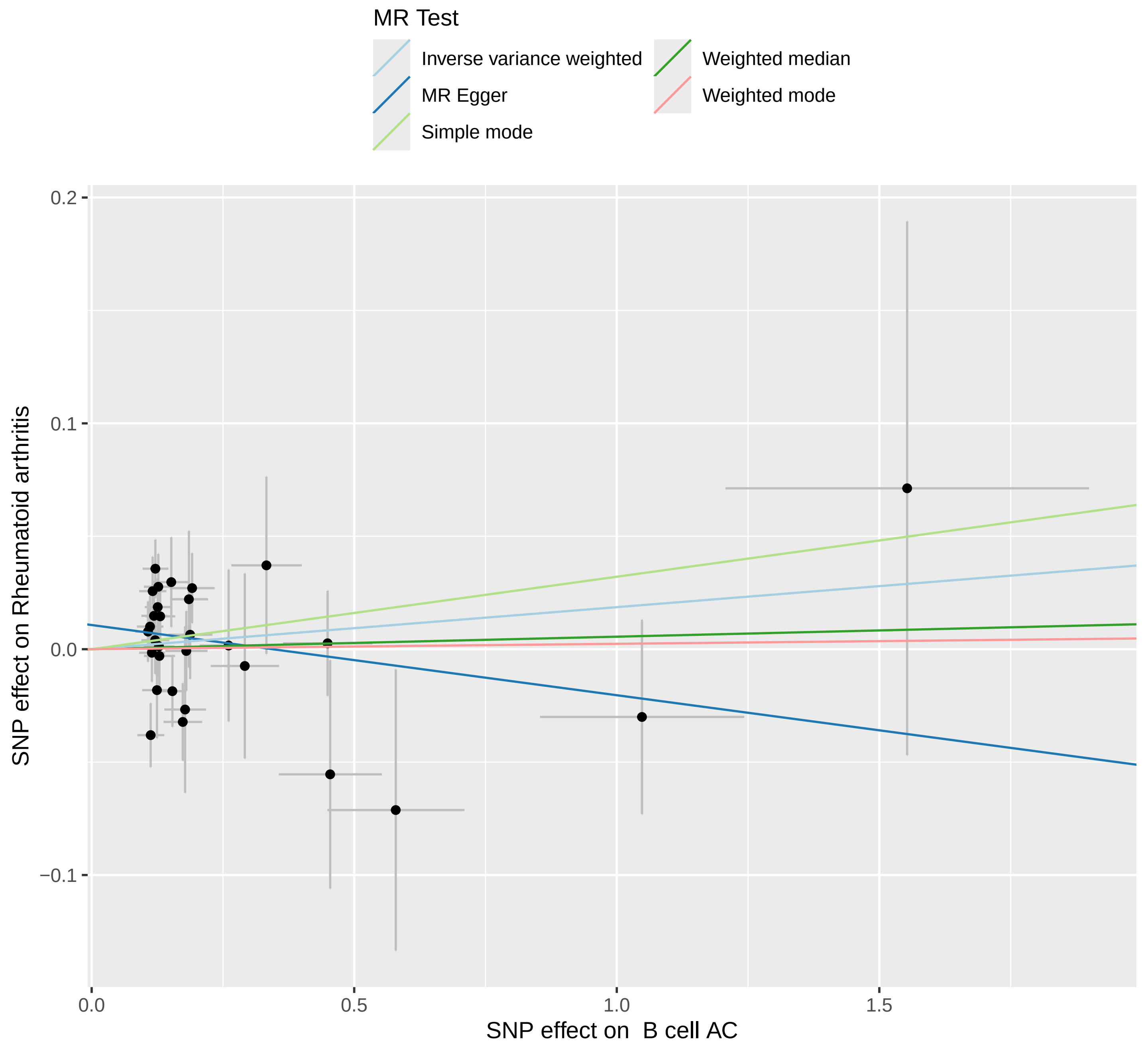
MR Test
Inverse variance weighted
MR Egger
Simple mode
Weighted median
Weighted mode
0.2
0.1
0.0
−0.1
SNP effect on Rheumatoid arthritis
0.0
0.5
1.0
1.5
SNP effect on B cell AC

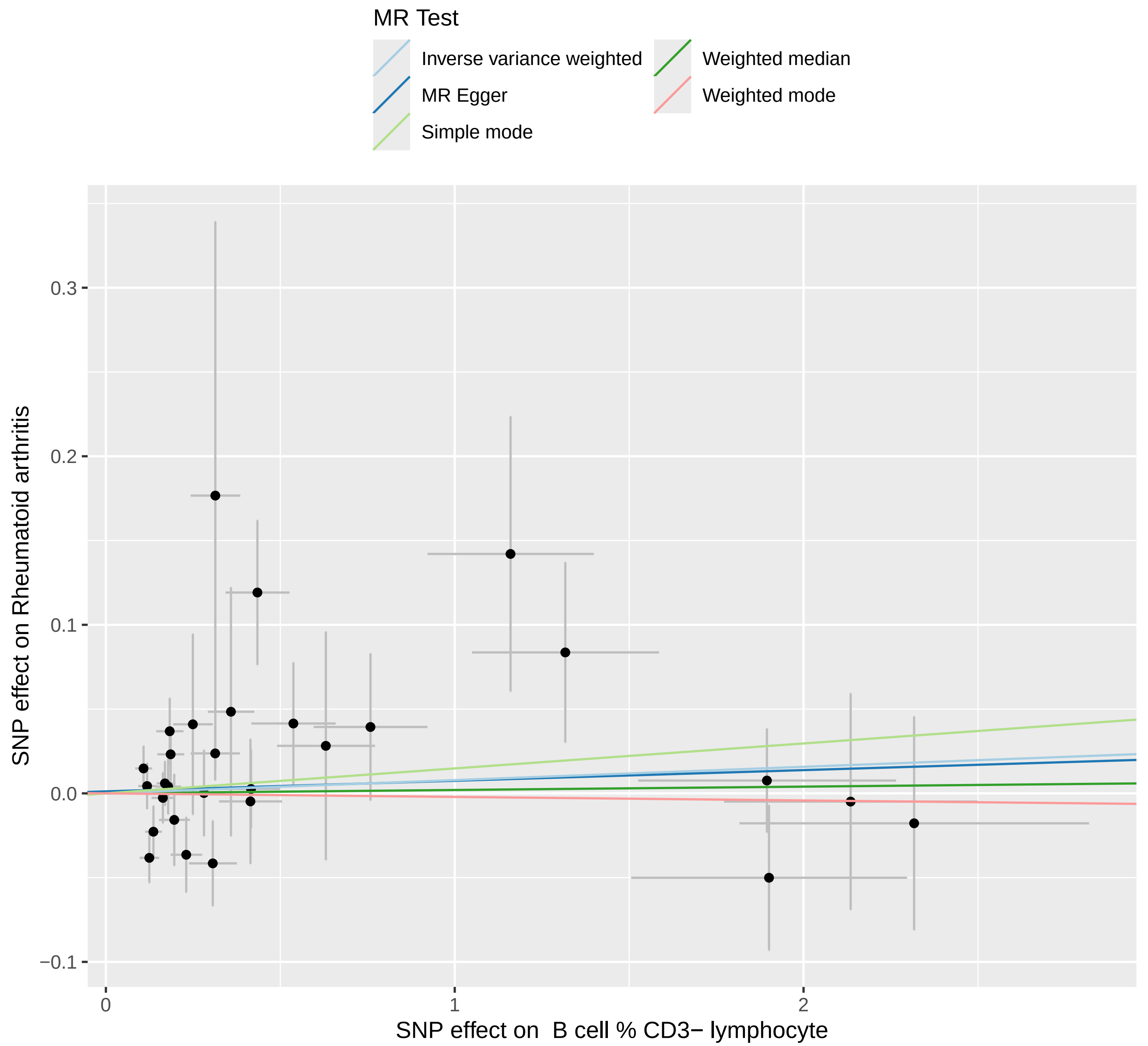

MR Test
Inverse variance weighted
MR Egger
Simple mode
Weighted median
Weighted mode
SNP effect on Rheumatoid arthritis
0.3
0.2
0.1
0.0
−0.1
0
1
2
SNP effect on B cell % CD3− lymphocyte

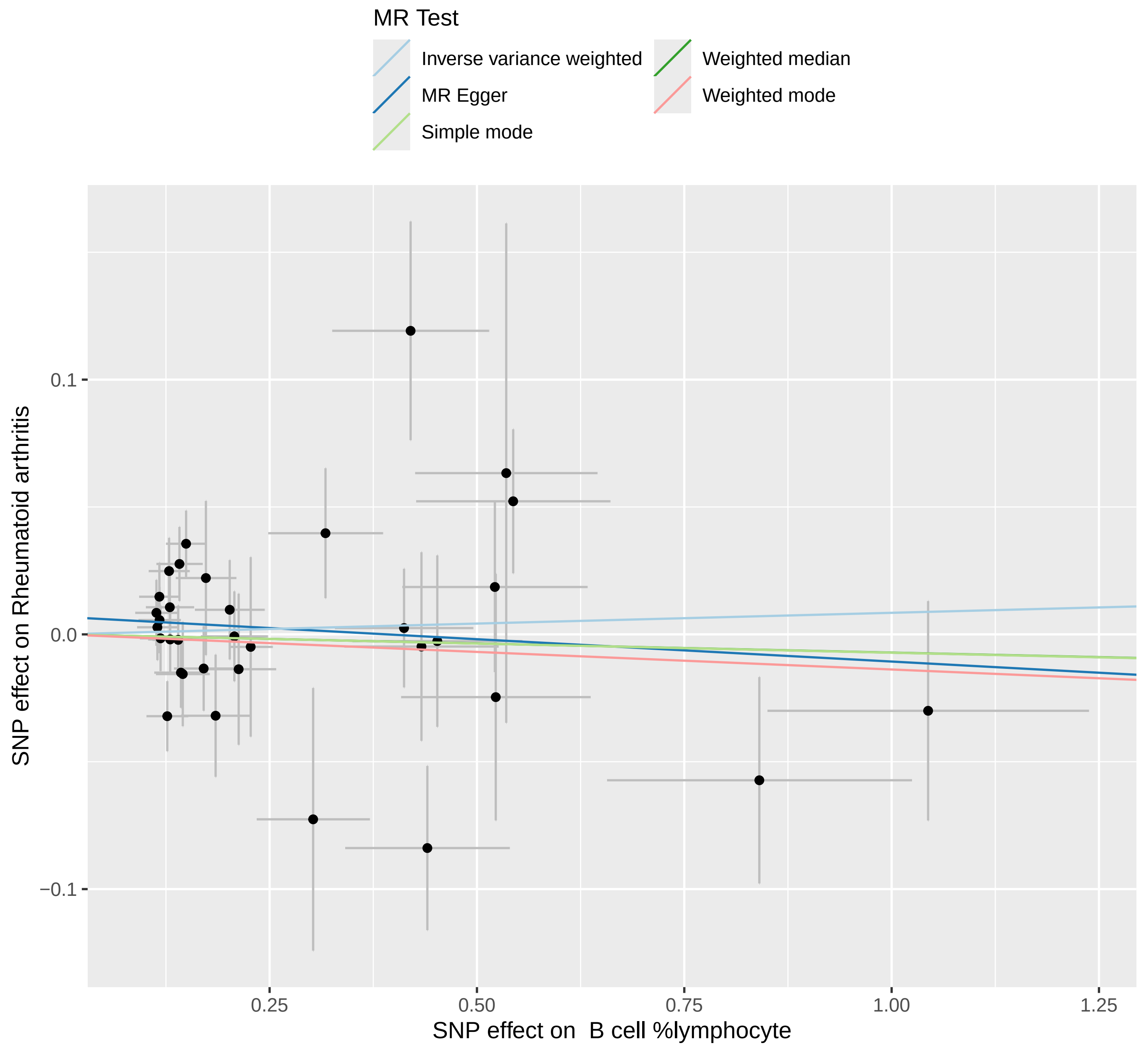

MR Test
Inverse variance weighted
MR Egger
Simple mode
Weighted median
Weighted mode
0.1
0.0
−0.1
0.25
0.50
0.75
1.00
1.25
SNP effect on Rheumatoid arthritis
SNP effect on B cell %lymphocyte

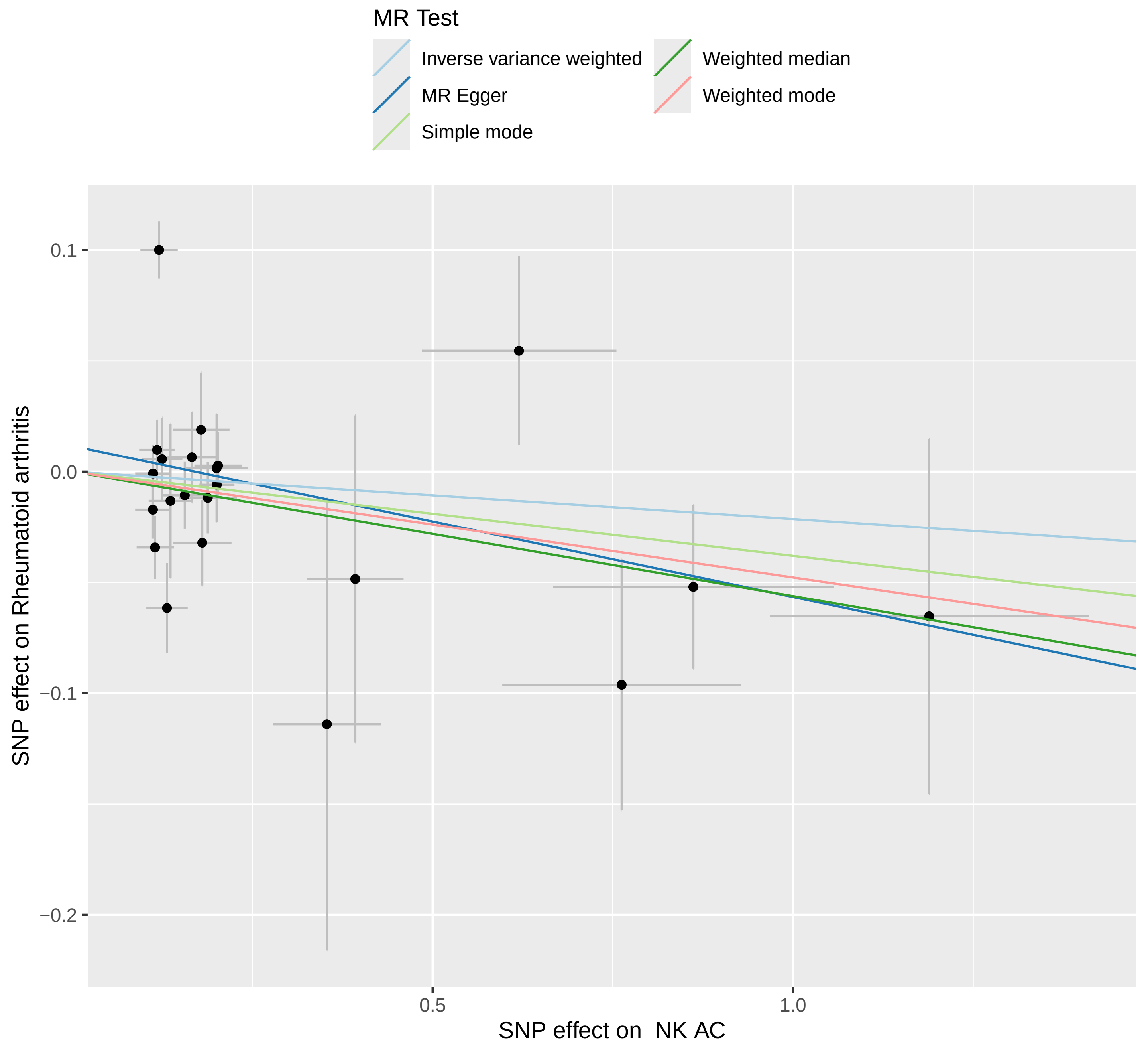
MR Test
Inverse variance weighted
MR Egger
Simple mode
Weighted median
Weighted mode
SNP effect on Rheumatoid arthritis
0.1
0.0
−0.1
−0.2
0.5
1.0
SNP effect on NK AC

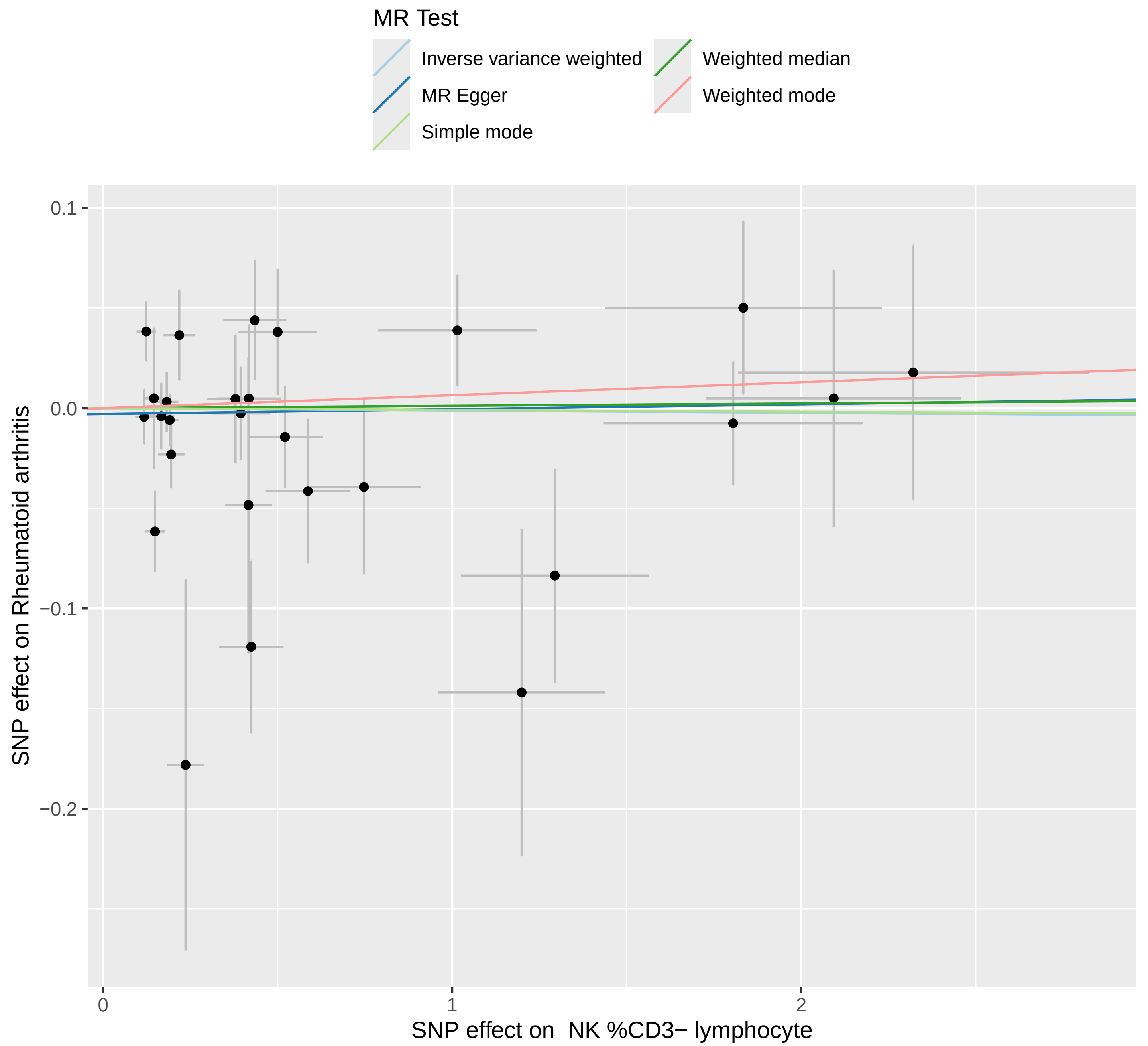
MR Test
Inverse variance weighted
MR Egger
Simple mode
Weighted median
Weighted mode
0.1
0.0
−0.1
−0.2
0
1
2
SNP effect on Rheumatoid arthritis
SNP effect on NK %CD3− lymphocyte

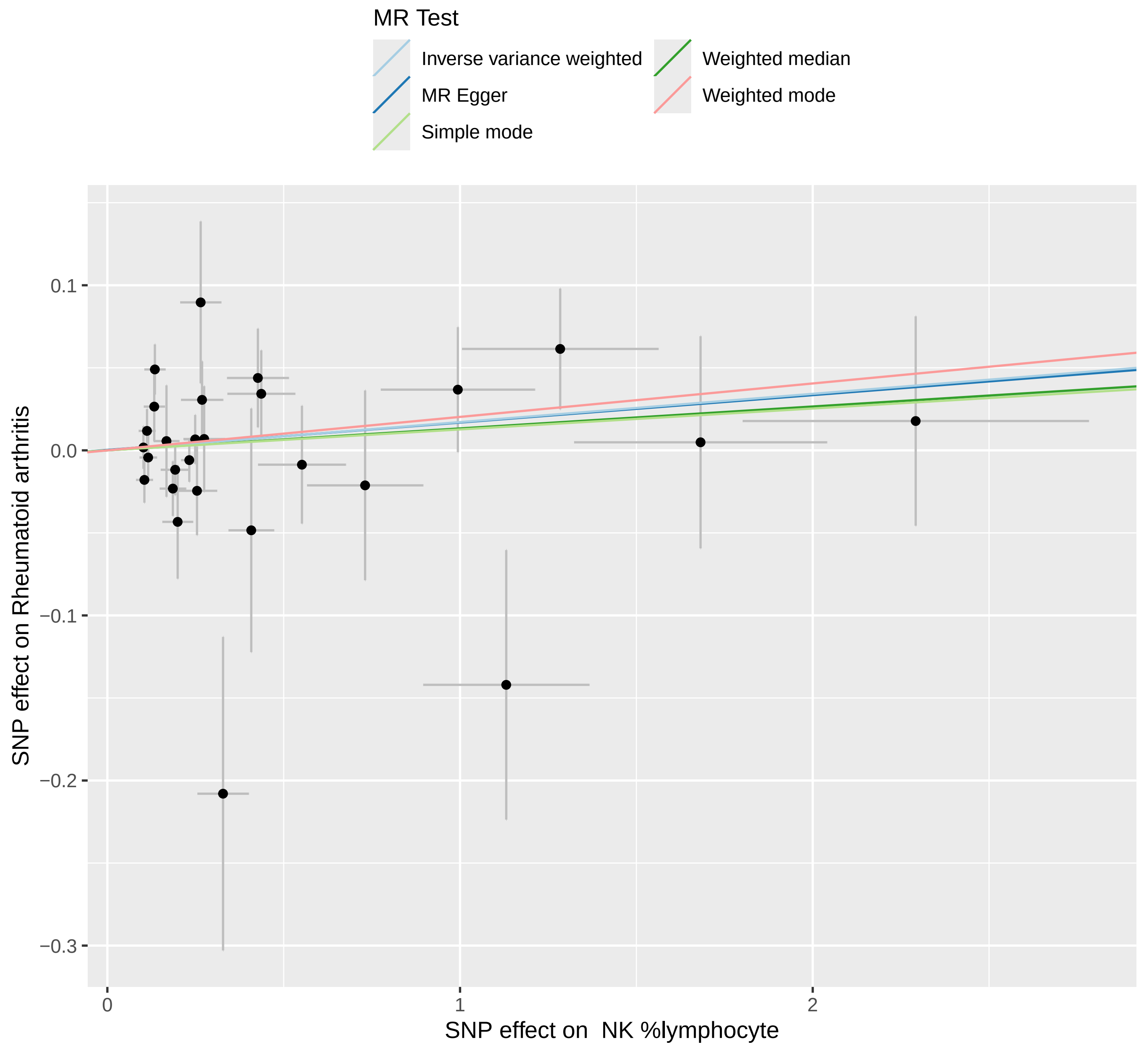
MR Test
Inverse variance weighted
MR Egger
Simple mode
Weighted median
Weighted mode
SNP effect on Rheumatoid arthritis
SNP effect on NK %lymphocyte
0.1
0.0
−0.1
−0.2
−0.3
0
1
2

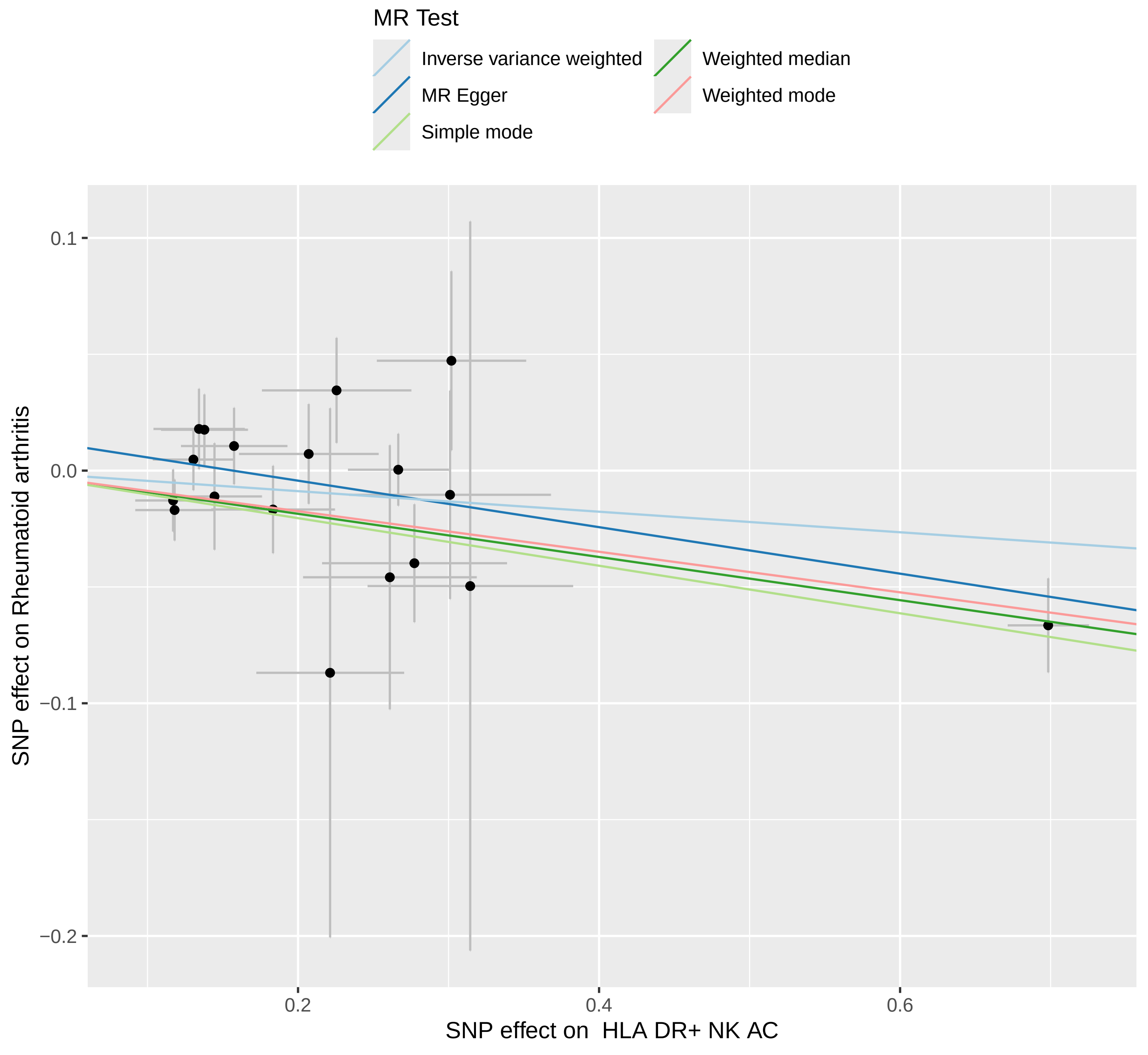
MR Test
Inverse variance weighted
MR Egger
Simple mode
Weighted median
Weighted mode
SNP effect on Rheumatoid arthritis
0.1
0.0
−0.1
−0.2
0.2
0.4
0.6
SNP effect on HLA DR+ NK AC

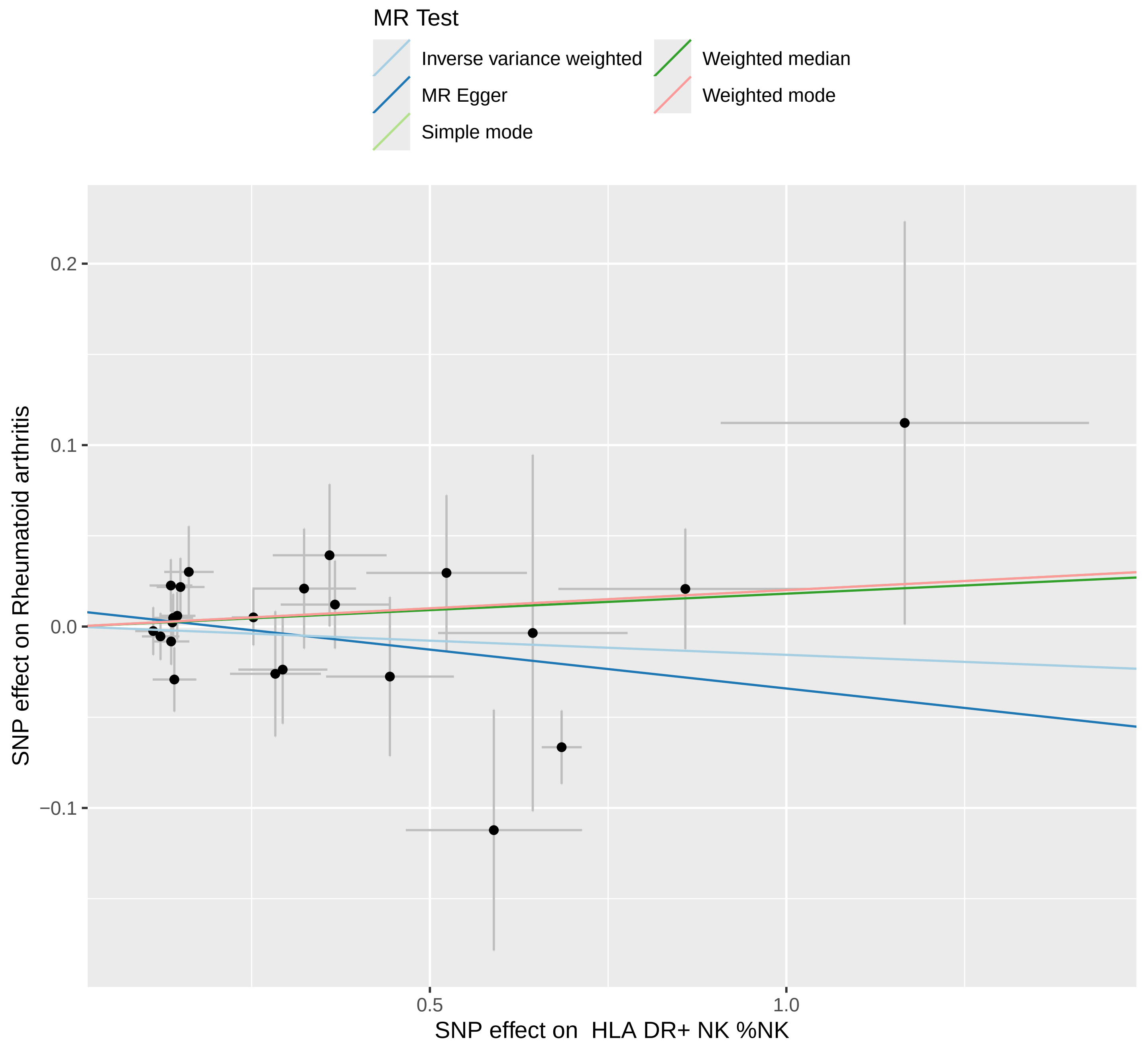
MR Test
Inverse variance weighted
MR Egger
Simple mode
Weighted median
Weighted mode
0.2
0.1
0.0
−0.1
0.5
1.0
SNP effect on Rheumatoid arthritis
SNP effect on HLA DR+ NK %NK

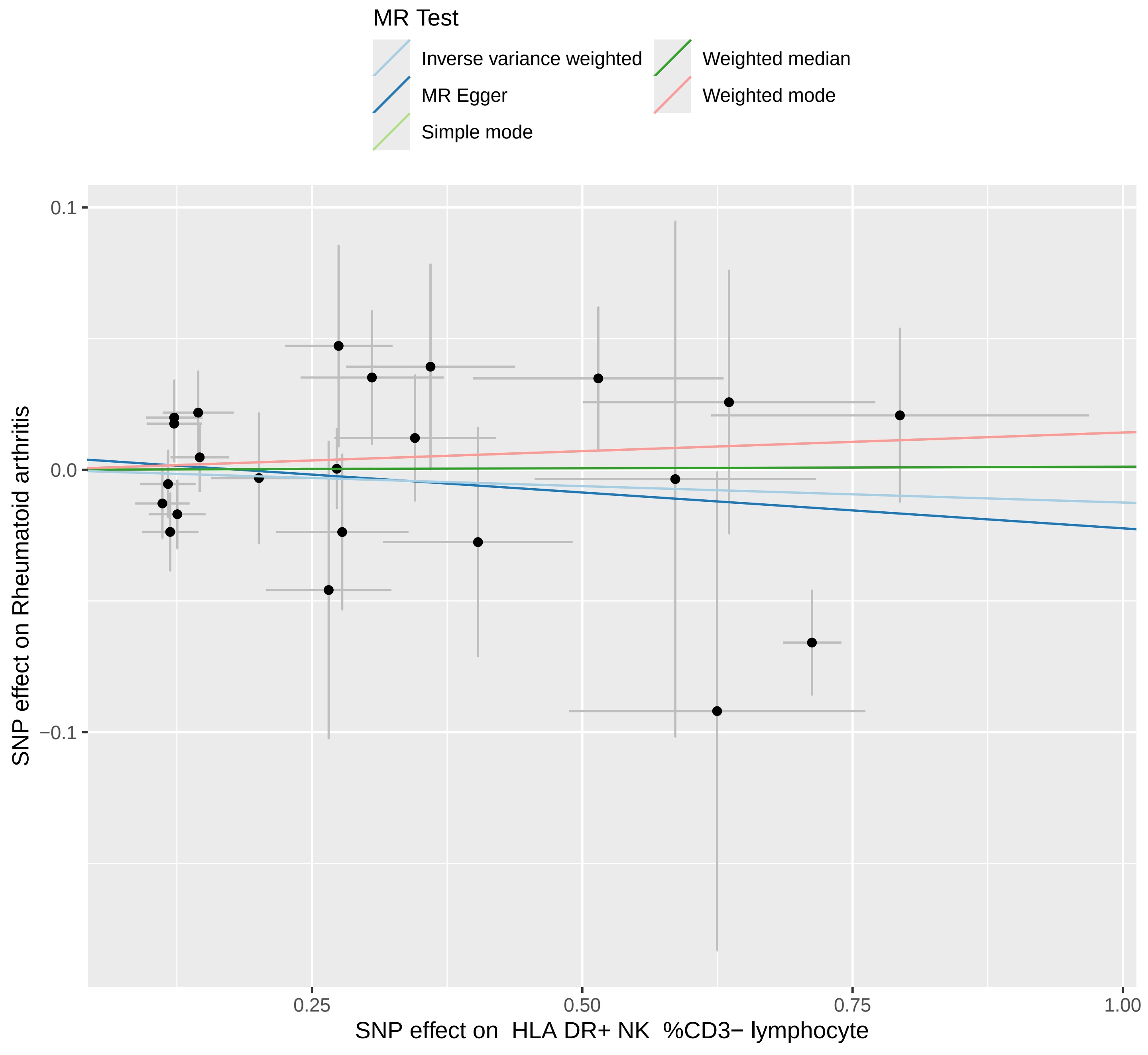
MR Test
Inverse variance weighted
MR Egger
Simple mode
Weighted median
Weighted mode
0.1
0.0
−0.1
SNP effect on Rheumatoid arthritis
0.25
0.50
0.75
1.00
SNP effect on HLA DR+ NK %CD3− lymphocyte

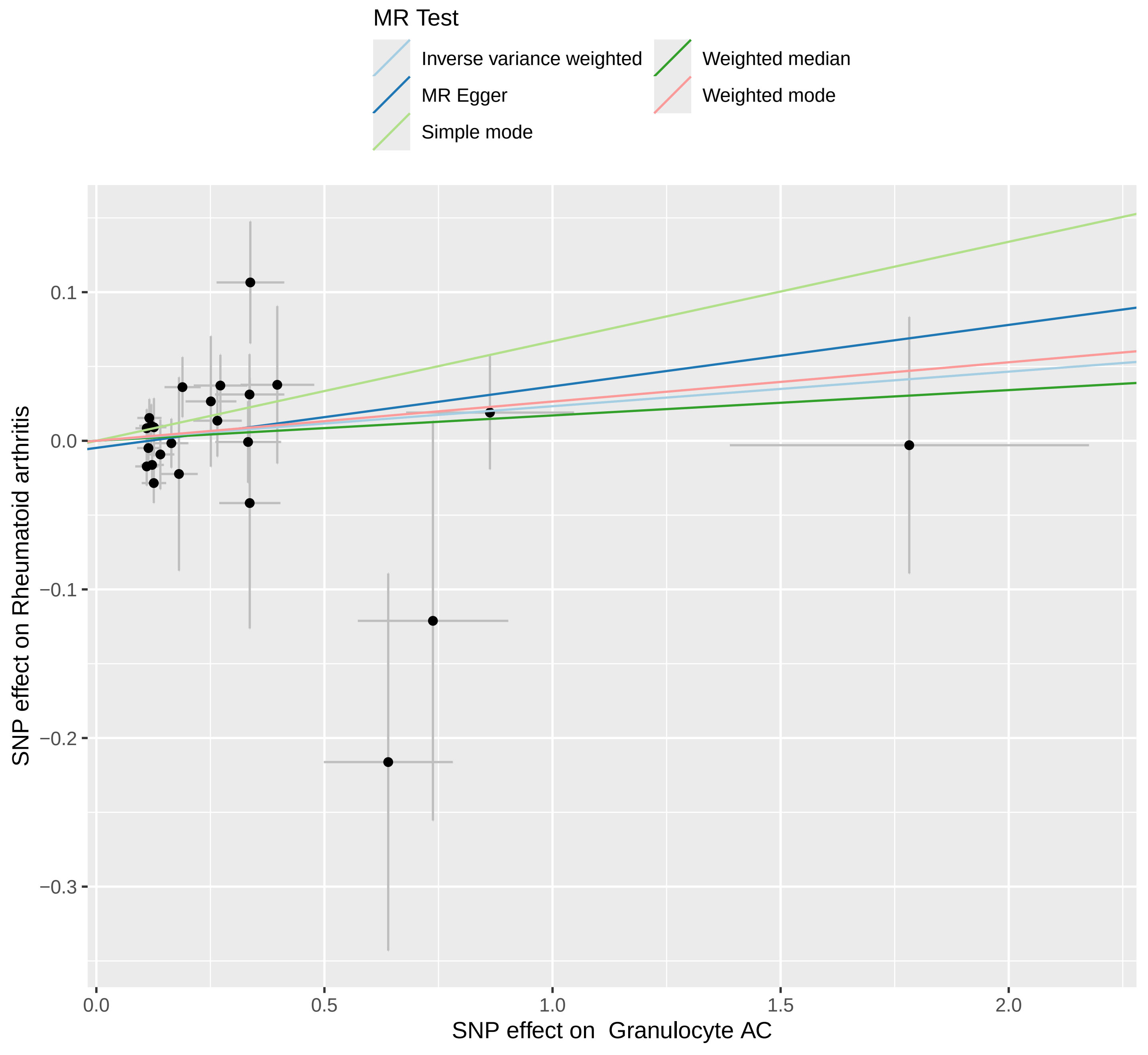

MR Test
Inverse variance weighted
MR Egger
Simple mode
Weighted median
Weighted mode
0.1
0.0
−0.1
−0.2
−0.3
SNP effect on Rheumatoid arthritis
0.0
0.5
1.0
1.5
2.0
SNP effect on Granulocyte AC

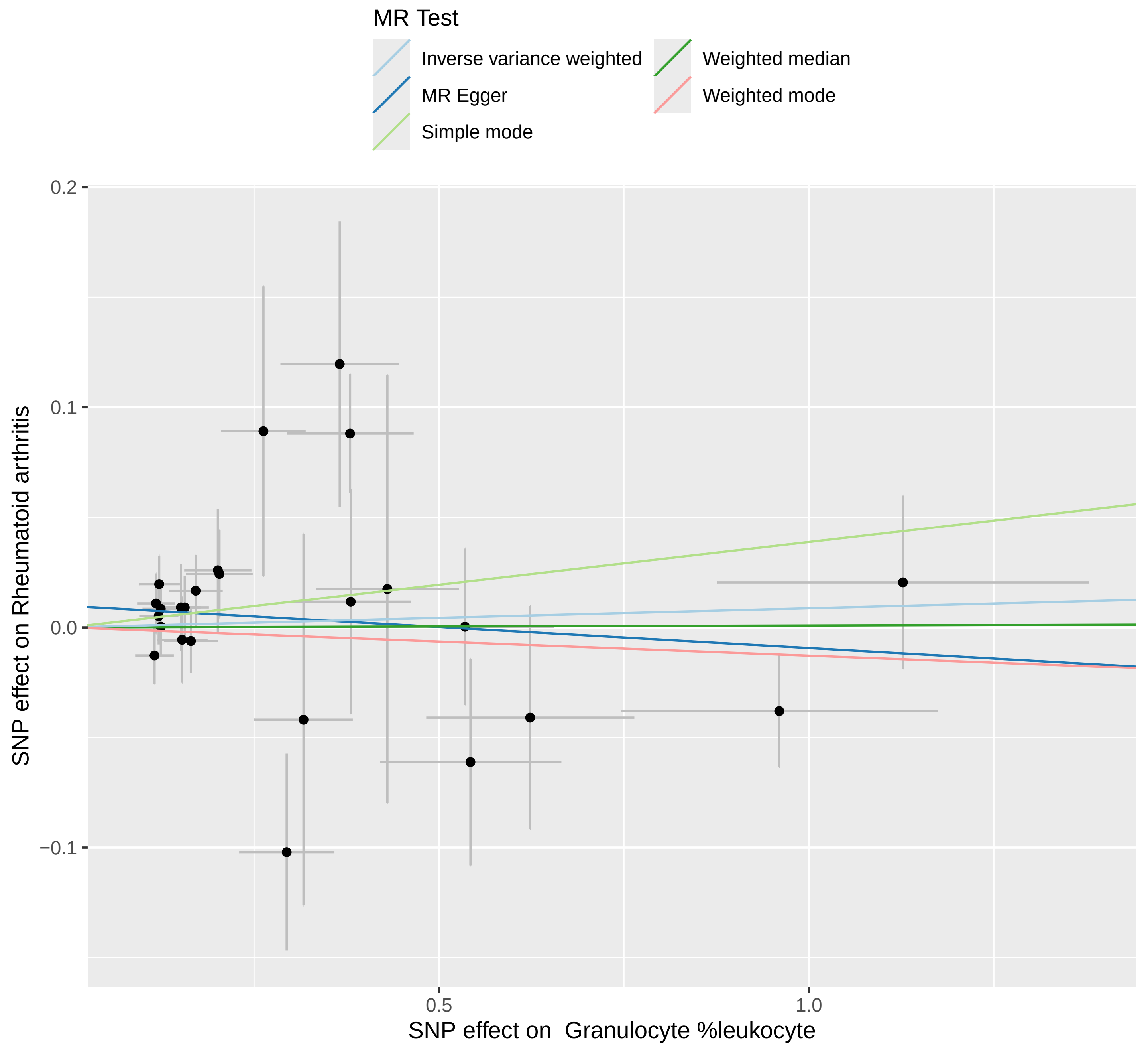
MR Test
Inverse variance weighted
MR Egger
Simple mode
Weighted median
Weighted mode
0.2
0.1
0.0
−0.1
0.5
1.0
SNP effect on Rheumatoid arthritis
SNP effect on Granulocyte %leukocyte

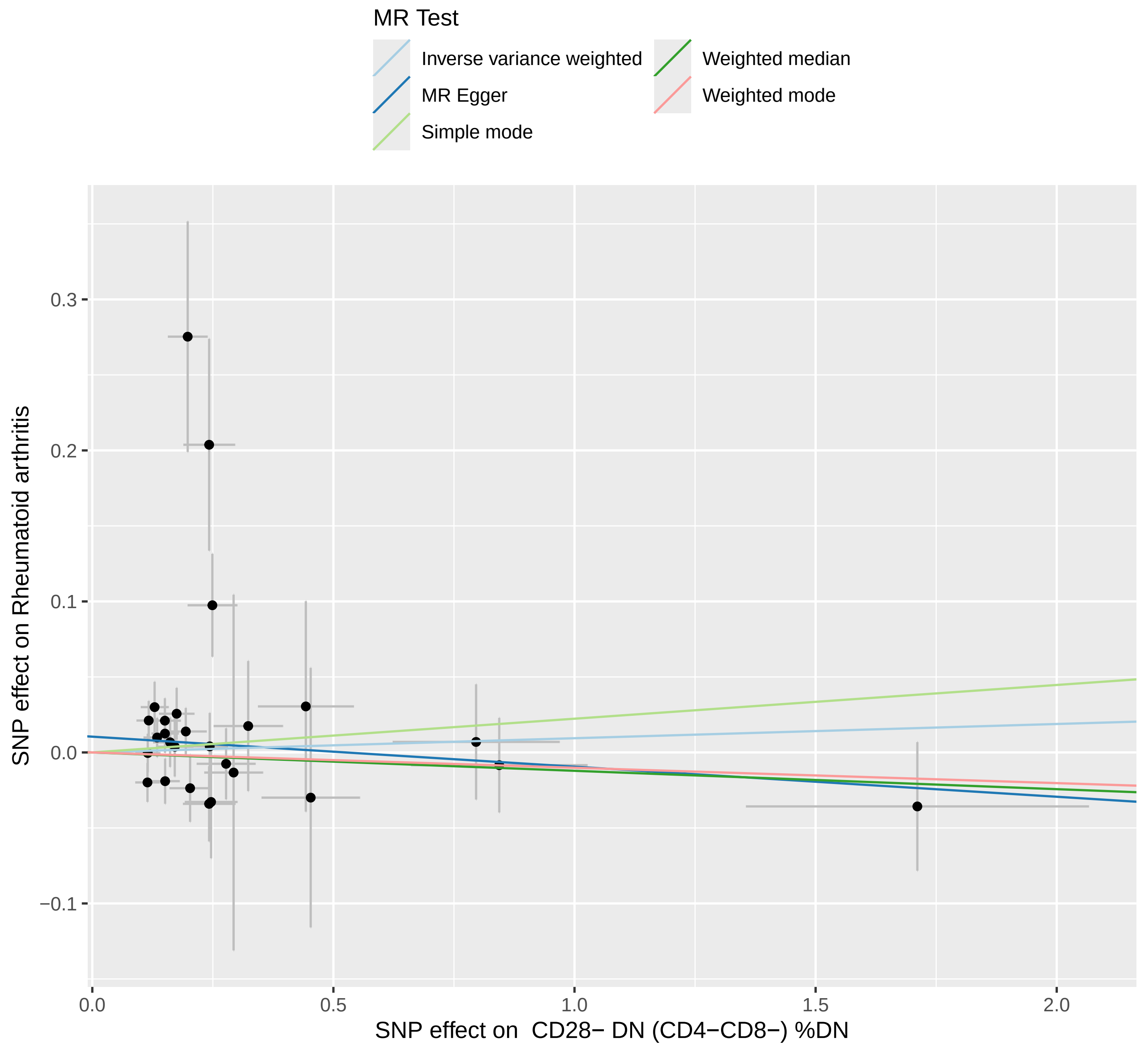

MR Test
Inverse variance weighted
MR Egger
Simple mode
Weighted median
Weighted mode
SNP effect on Rheumatoid arthritis
0.3
0.2
0.1
0.0
−0.1
0.0
0.5
1.0
1.5
2.0
SNP effect on CD28− DN (CD4−CD8−) %DN

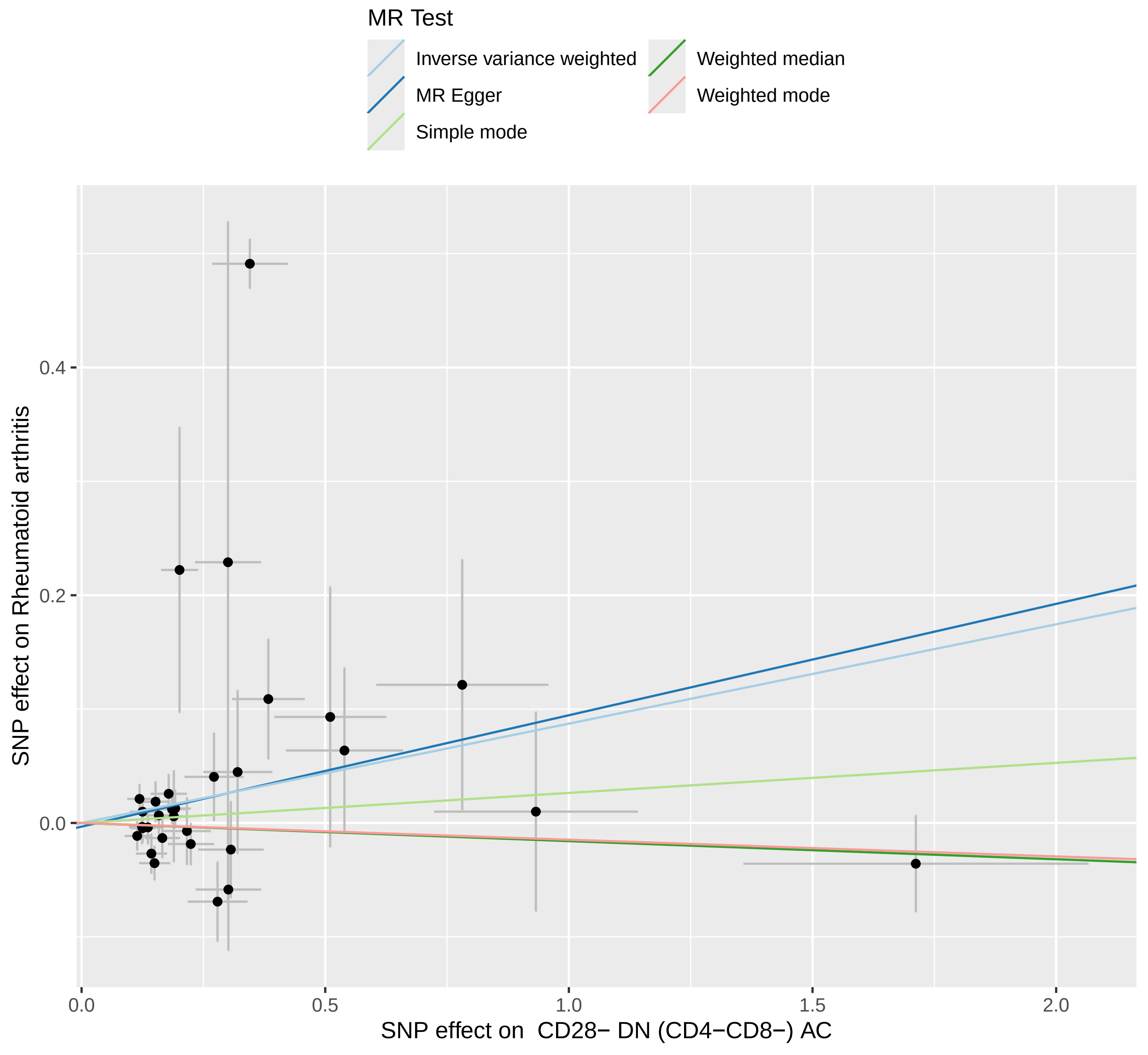

MR Test
Inverse variance weighted
MR Egger
Simple mode
Weighted median
Weighted mode
SNP effect on Rheumatoid arthritis
0.4
0.2
0.0
0.0
0.5
1.0
1.5
2.0
SNP effect on CD28− DN (CD4−CD8−) AC

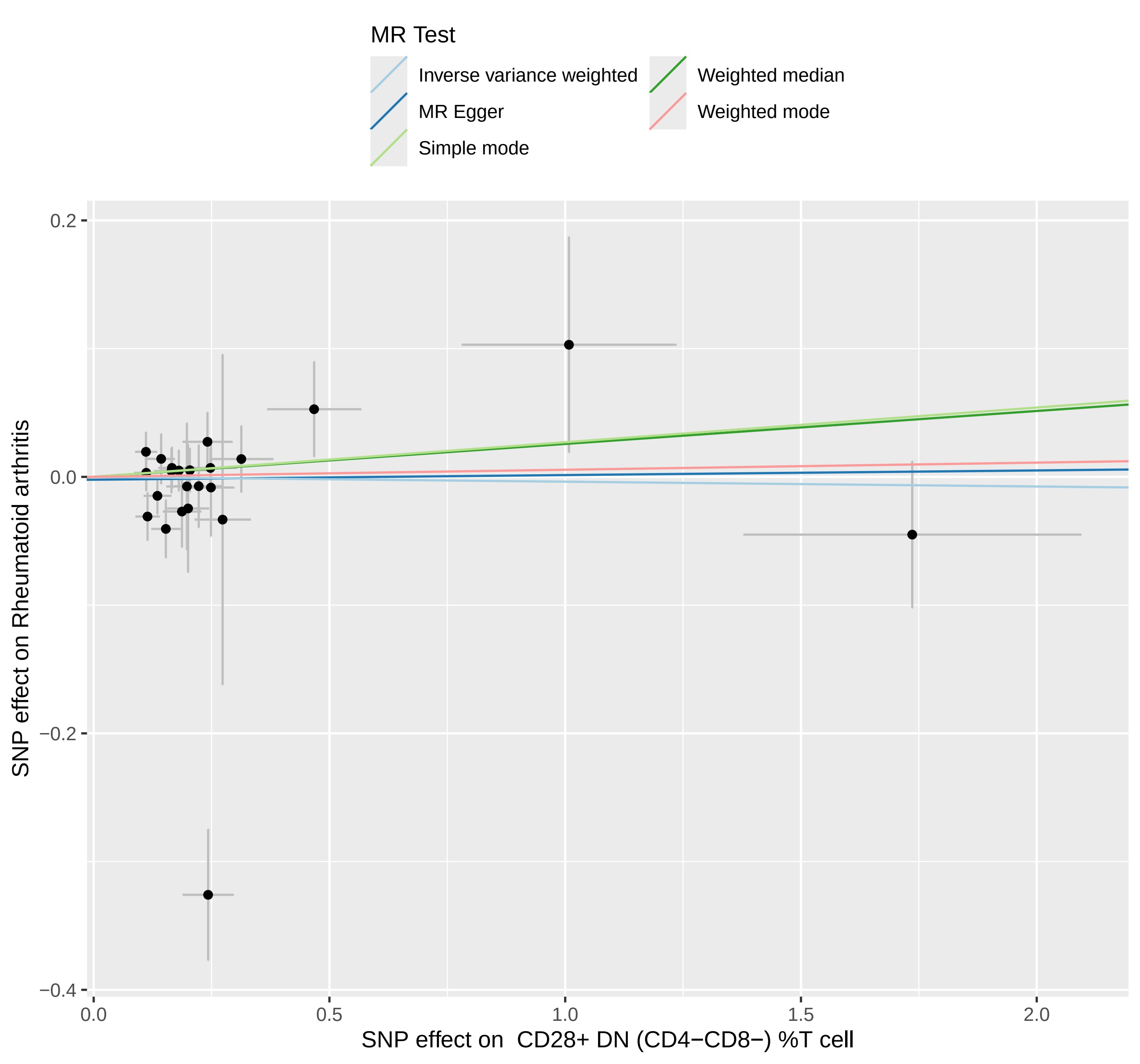

MR Test
Inverse variance weighted
MR Egger
Simple mode
Weighted median
Weighted mode
0.2
0.0
−0.2
−0.4
0.0
0.5
1.0
1.5
2.0
SNP effect on Rheumatoid arthritis
SNP effect on CD28+ DN (CD4−CD8−) %T cell

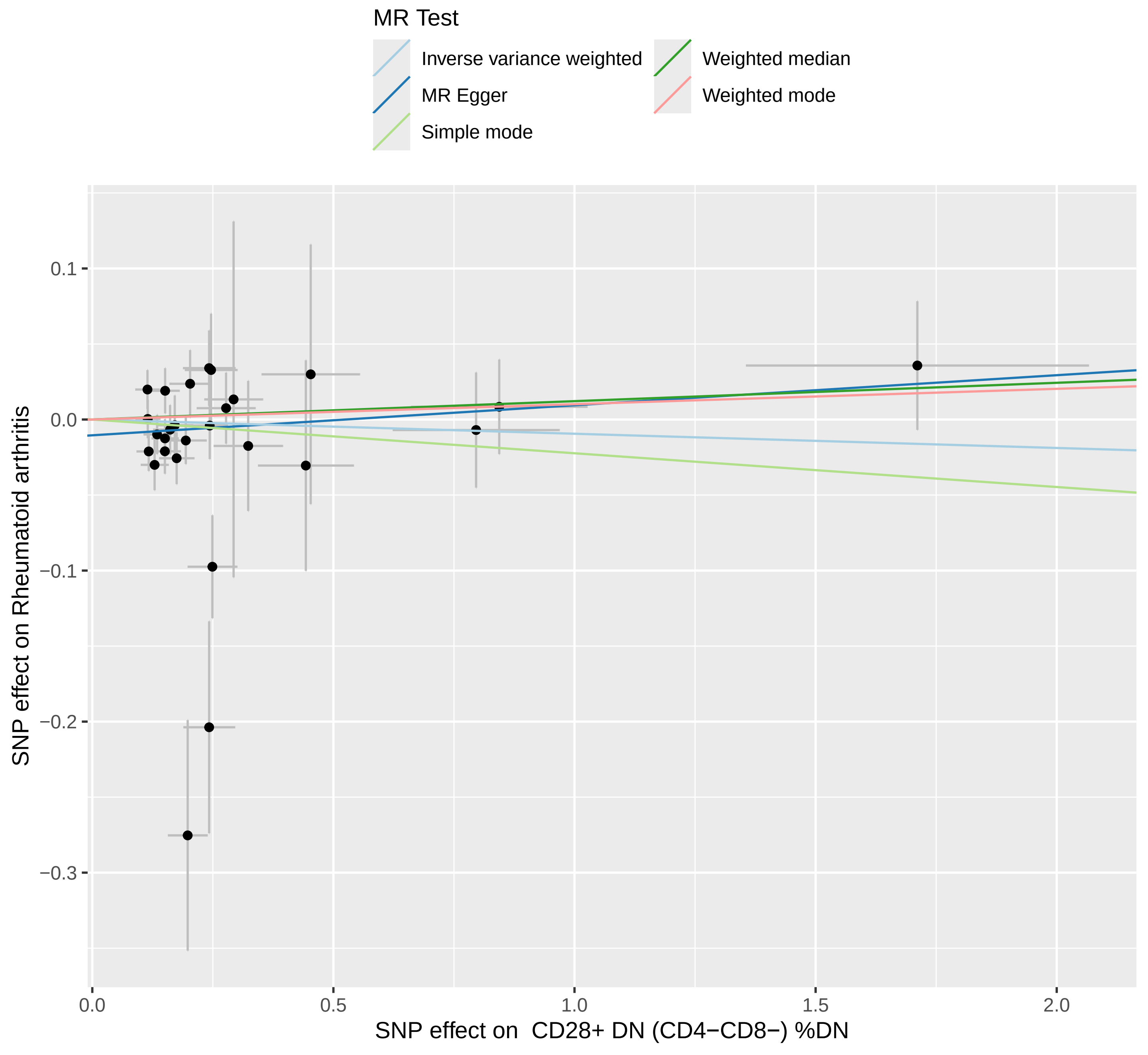
MR Test
Inverse variance weighted
MR Egger
Simple mode
Weighted median
Weighted mode
SNP effect on Rheumatoid arthritis
0.1
0.0
−0.1
−0.2
−0.3
0.0
0.5
1.0
1.5
2.0
SNP effect on CD28+ DN (CD4−CD8−) %DN

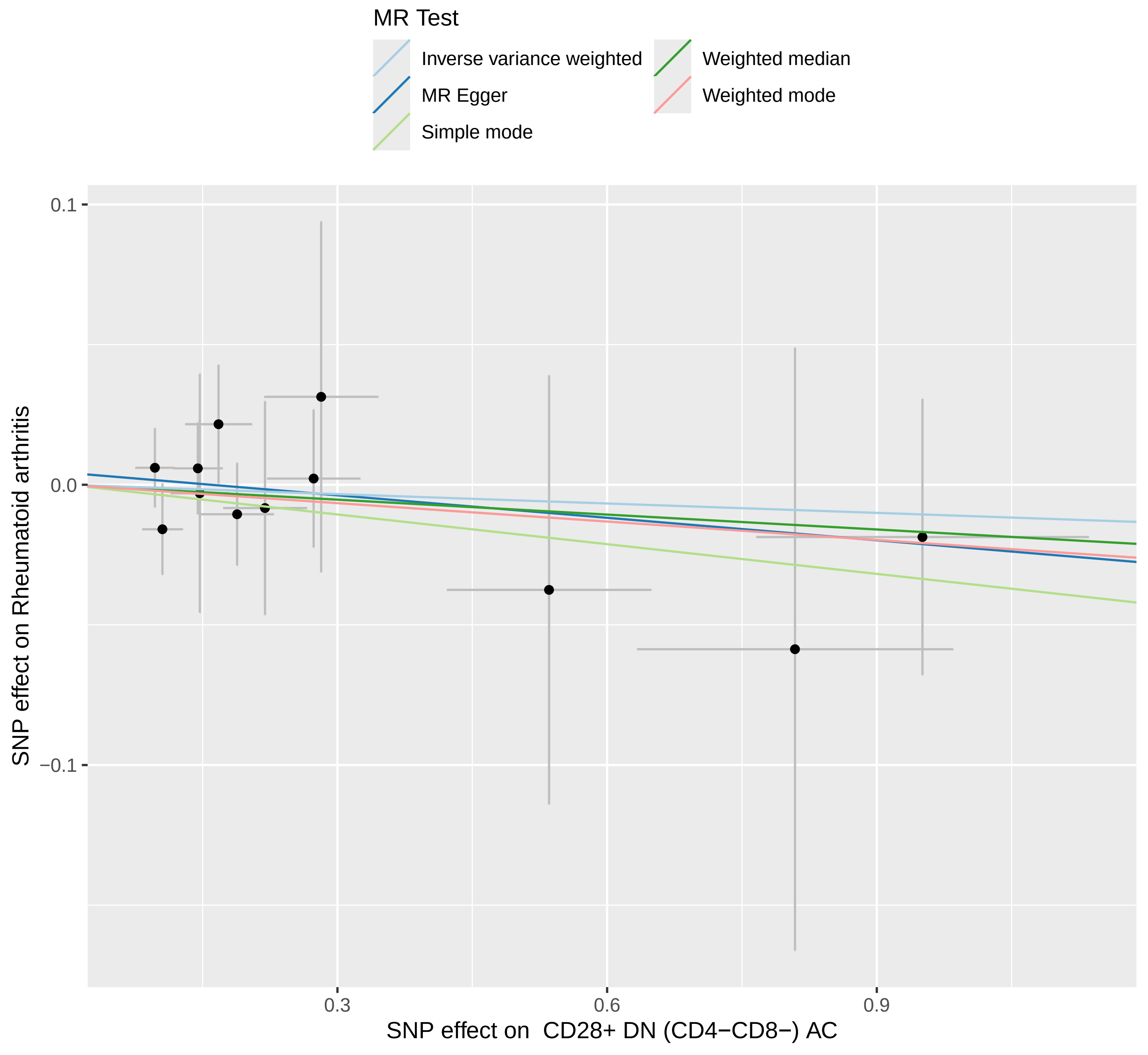
MR Test
Inverse variance weighted
MR Egger
Simple mode
Weighted median
Weighted mode
0.1
0.0
−0.1
0.3
0.6
0.9
SNP effect on Rheumatoid arthritis
SNP effect on CD28+ DN (CD4−CD8−) AC

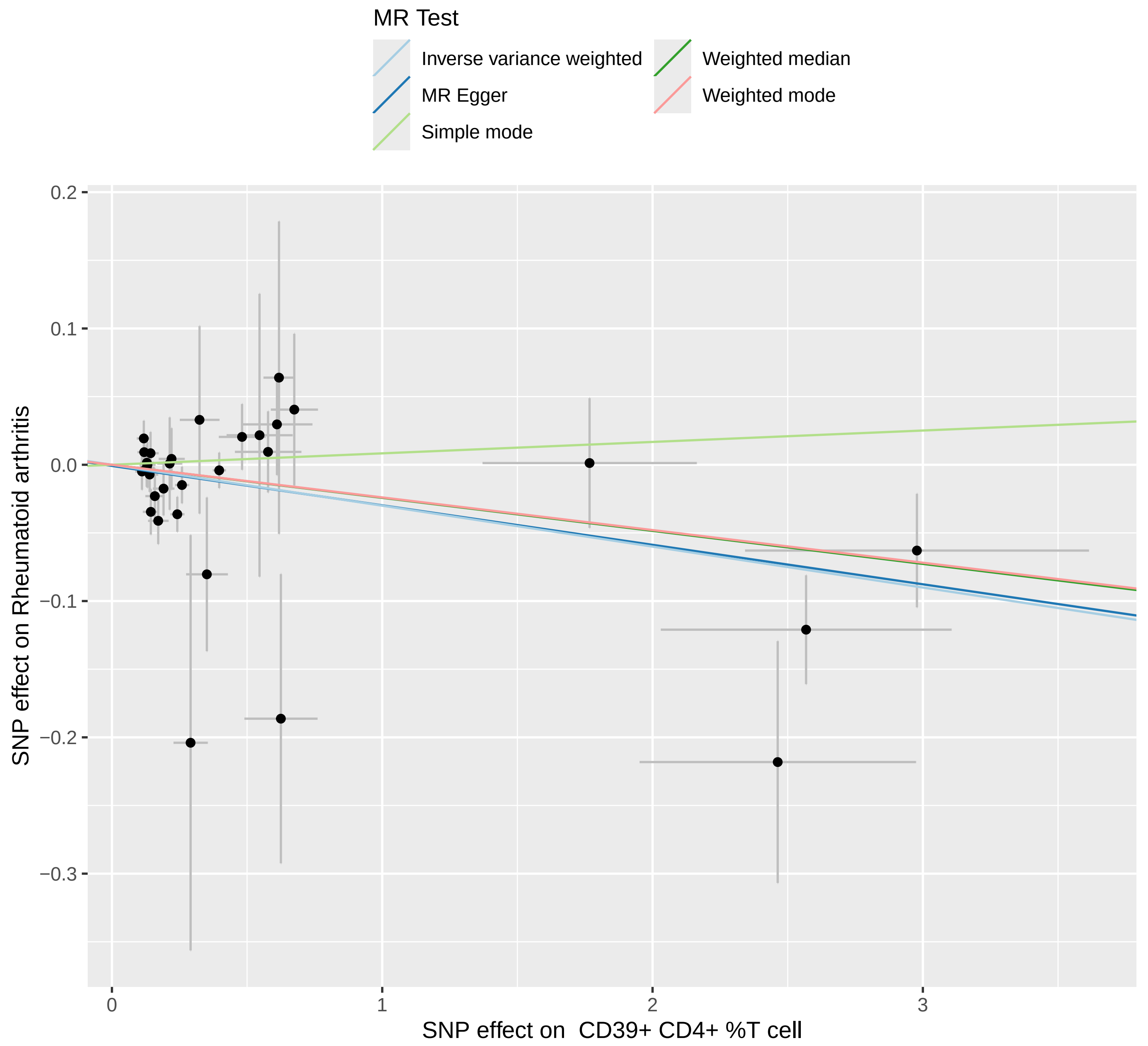
MR Test
Inverse variance weighted
MR Egger
Simple mode
Weighted median
Weighted mode
0.2
0.1
0.0
−0.1
−0.2
−0.3
0
1
2
3
SNP effect on Rheumatoid arthritis
SNP effect on CD39+ CD4+ %T cell

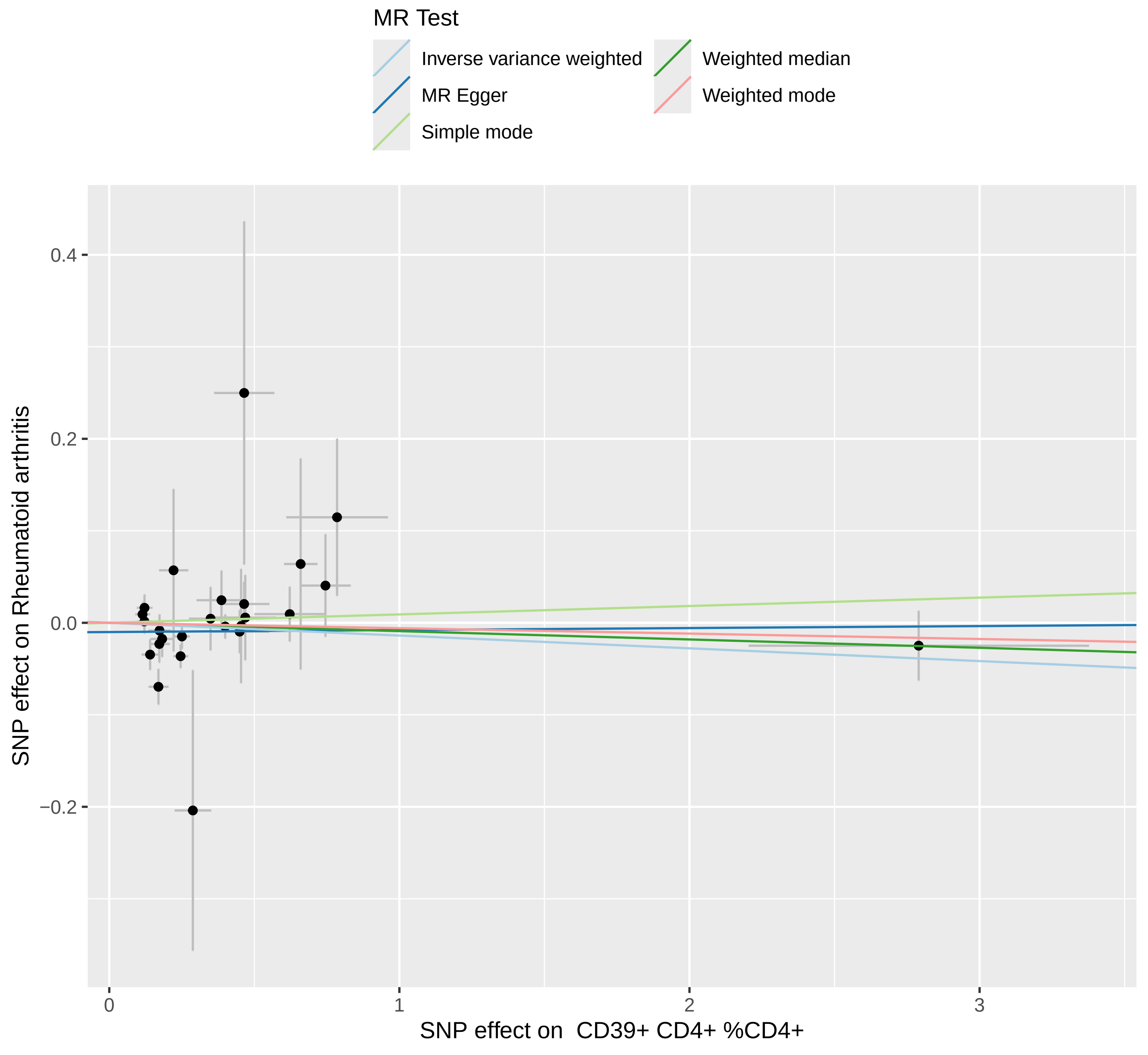
MR Test
Inverse variance weighted
MR Egger
Simple mode
Weighted median
Weighted mode
0.4
0.2
0.0
−0.2
SNP effect on Rheumatoid arthritis
0
1
2
3
SNP effect on CD39+ CD4+ %CD4+

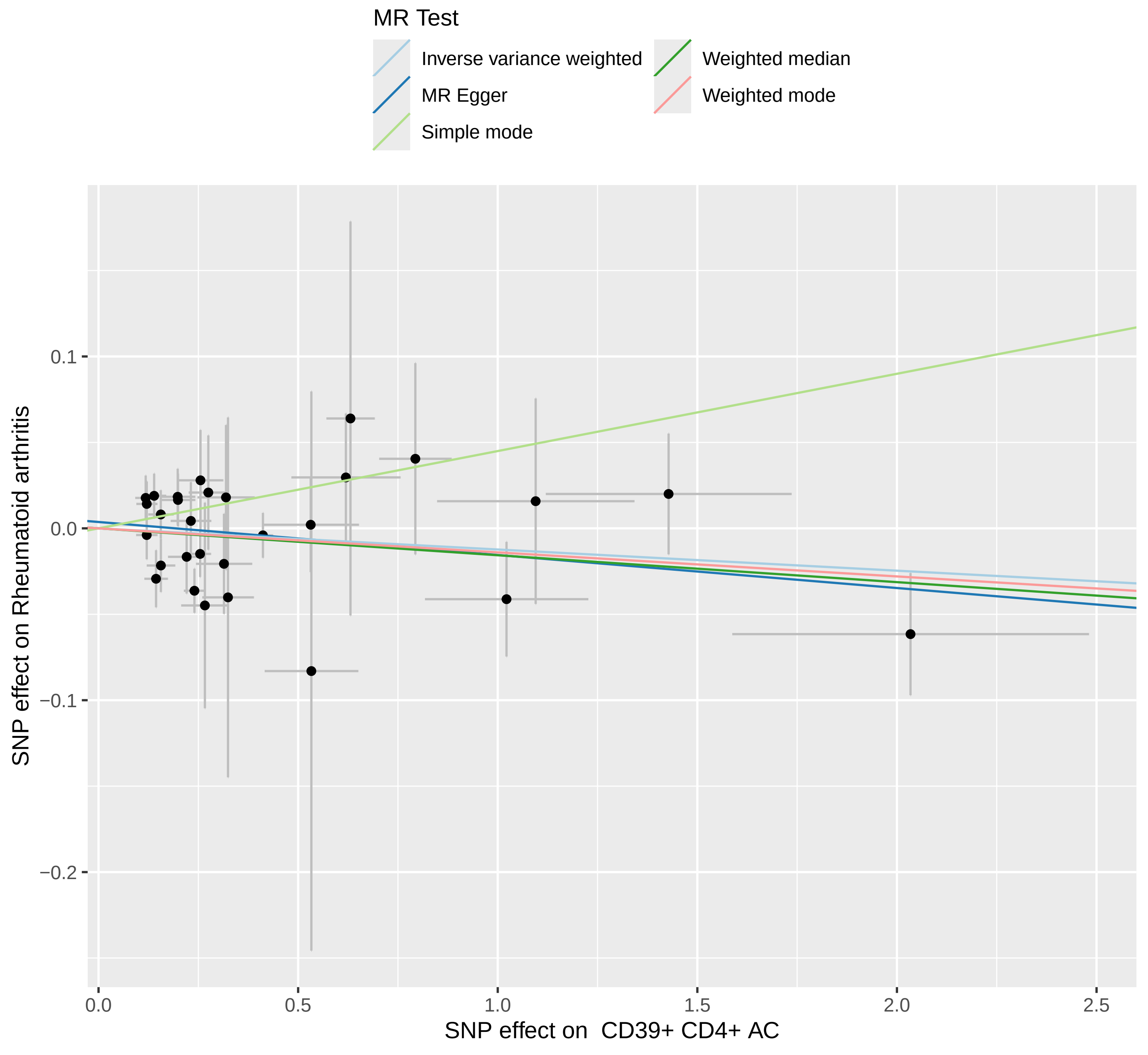

MR Test
Inverse variance weighted
MR Egger
Simple mode
Weighted median
Weighted mode
SNP effect on Rheumatoid arthritis
0.1
0.0
−0.1
−0.2
0.0
0.5
1.0
1.5
2.0
2.5
SNP effect on CD39+ CD4+ AC

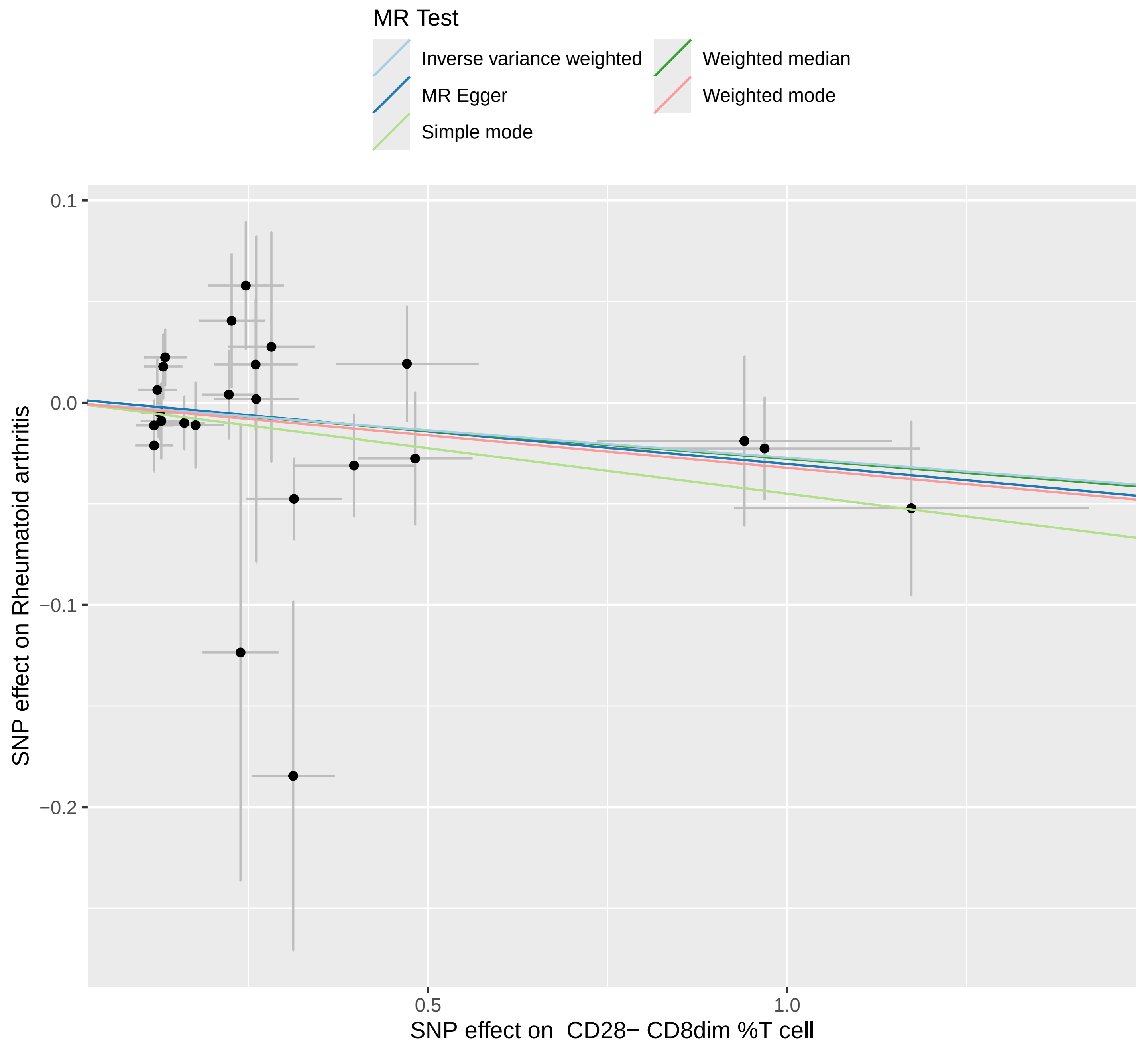

MR Test
Inverse variance weighted
MR Egger
Simple mode
Weighted median
Weighted mode
0.1
0.0
−0.1
−0.2
SNP effect on Rheumatoid arthritis
0.5
1.0
SNP effect on CD28− CD8dim %T cell

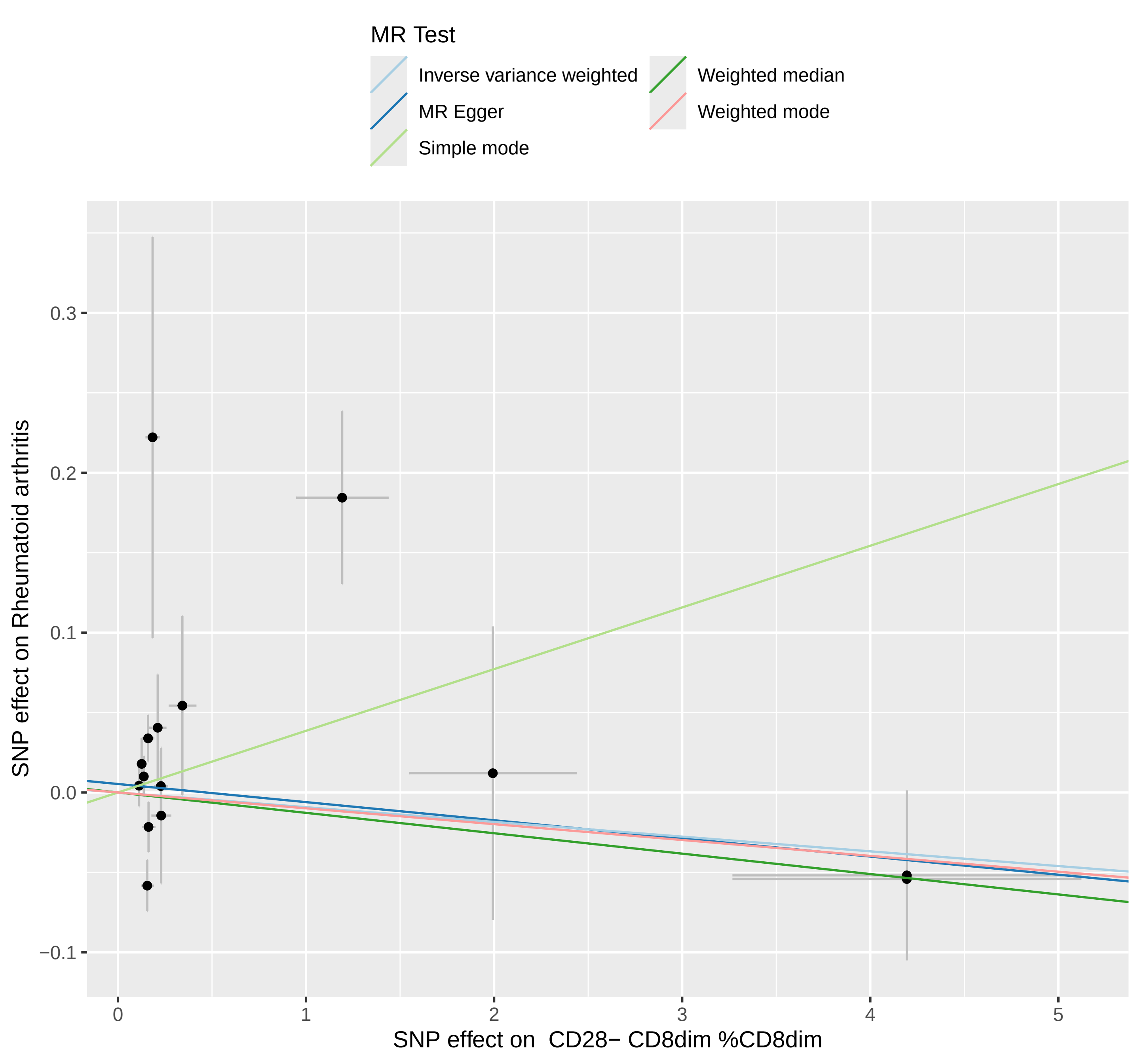

MR Test
Inverse variance weighted
MR Egger
Simple mode
Weighted median
Weighted mode
0.3
0.2
0.1
0.0
−0.1
0
1
2
3
4
5
SNP effect on Rheumatoid arthritis
SNP effect on CD28− CD8dim %CD8dim

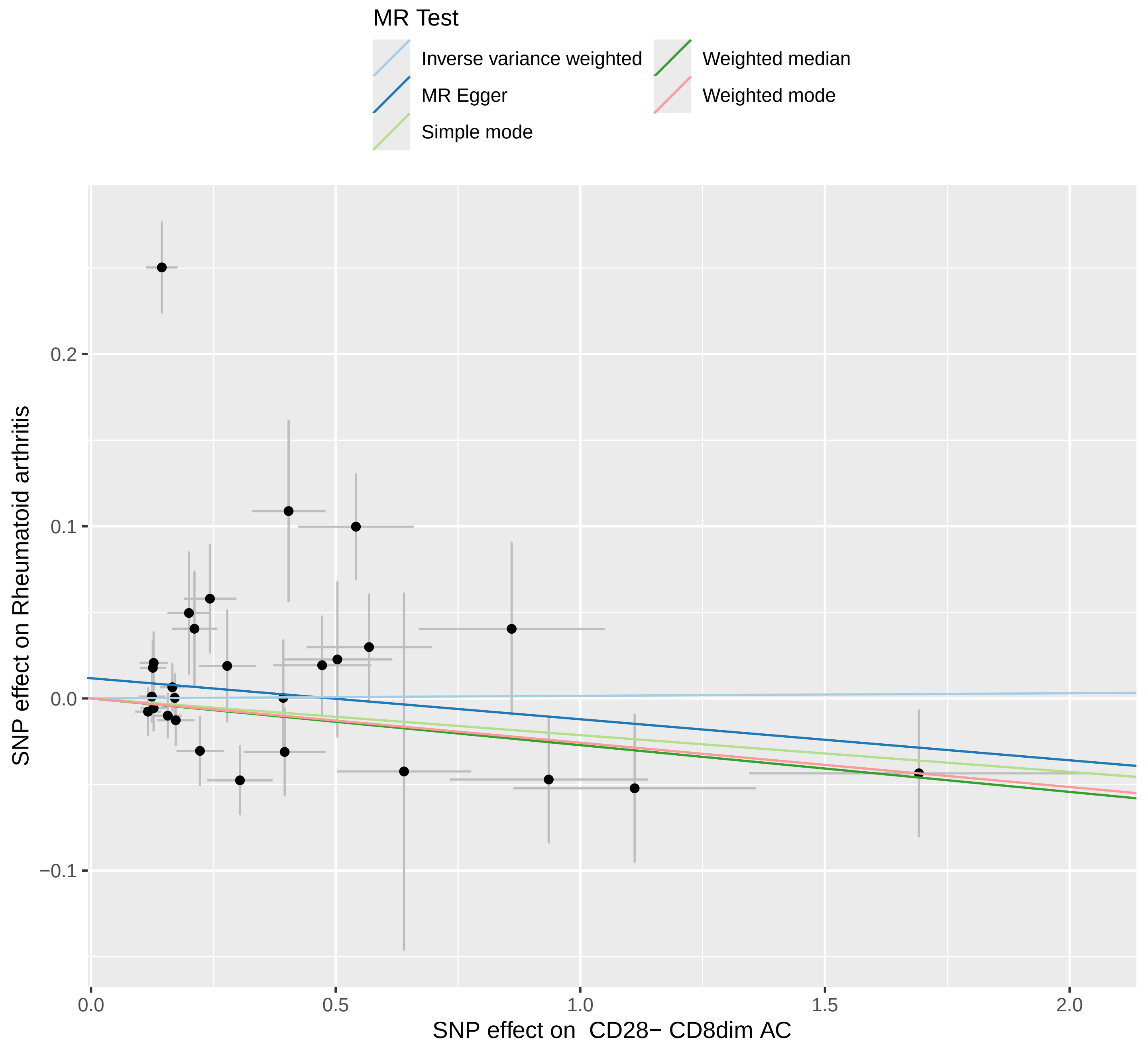
MR Test
Inverse variance weighted
MR Egger
Simple mode
Weighted median
Weighted mode
SNP effect on Rheumatoid arthritis
0.2
0.1
0.0
−0.1
0.0
0.5
1.0
1.5
2.0
SNP effect on CD28− CD8dim AC

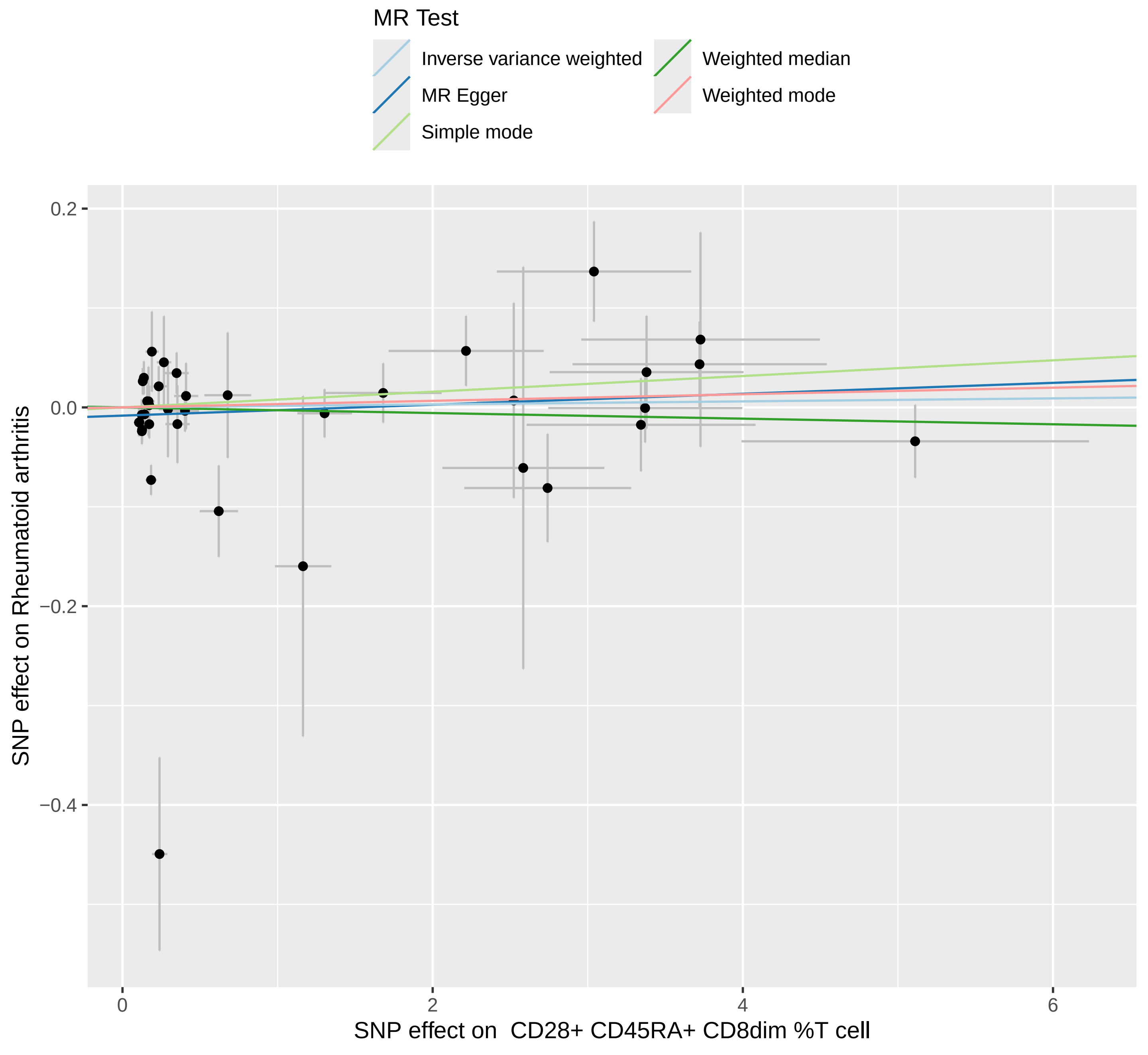

MR Test
Inverse variance weighted
MR Egger
Simple mode
Weighted median
Weighted mode
0.2
0.0
−0.2
−0.4
0
2
4
6
SNP effect on Rheumatoid arthritis
SNP effect on CD28+ CD45RA+ CD8dim %T cell

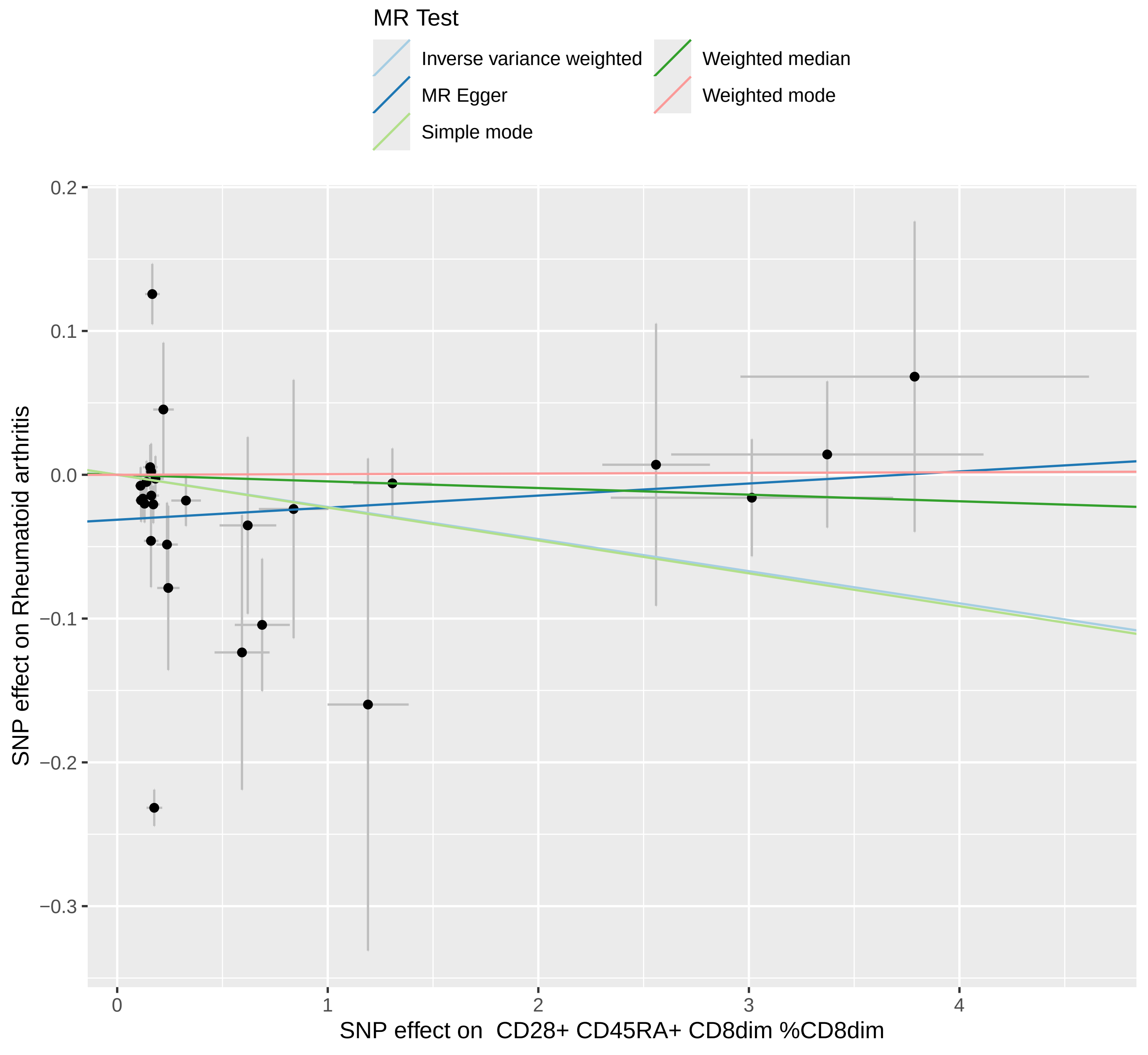
MR Test
Inverse variance weighted
MR Egger
Simple mode
Weighted median
Weighted mode
0.2
0.1
0.0
−0.1
−0.2
−0.3
SNP effect on Rheumatoid arthritis
0
1
2
3
4
SNP effect on CD28+ CD45RA+ CD8dim %CD8dim

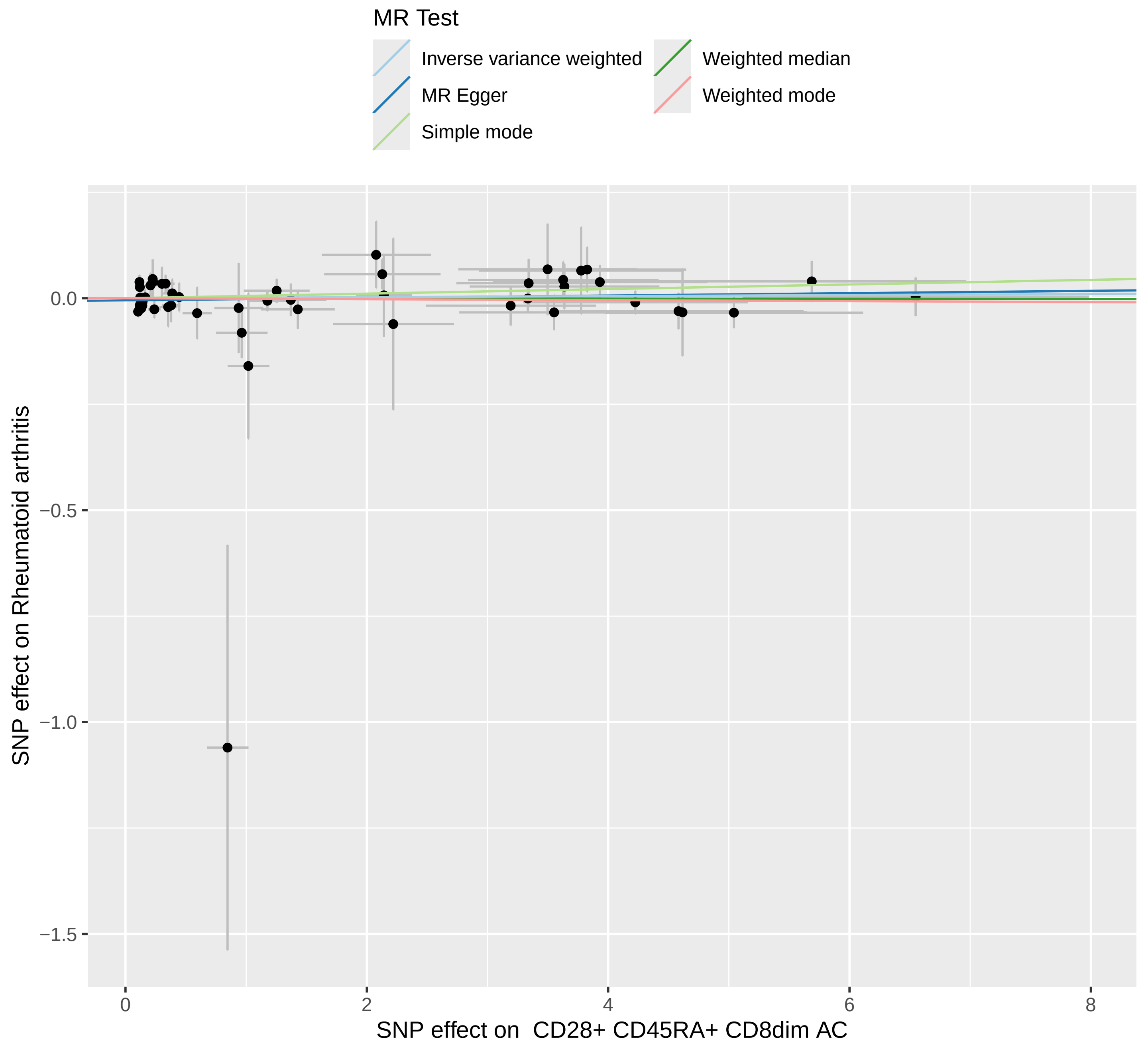

MR Test
Inverse variance weighted
MR Egger
Simple mode
Weighted median
Weighted mode
0.0
−0.5
−1.0
−1.5
0
2
4
6
8
SNP effect on Rheumatoid arthritis
SNP effect on CD28+ CD45RA+ CD8dim AC

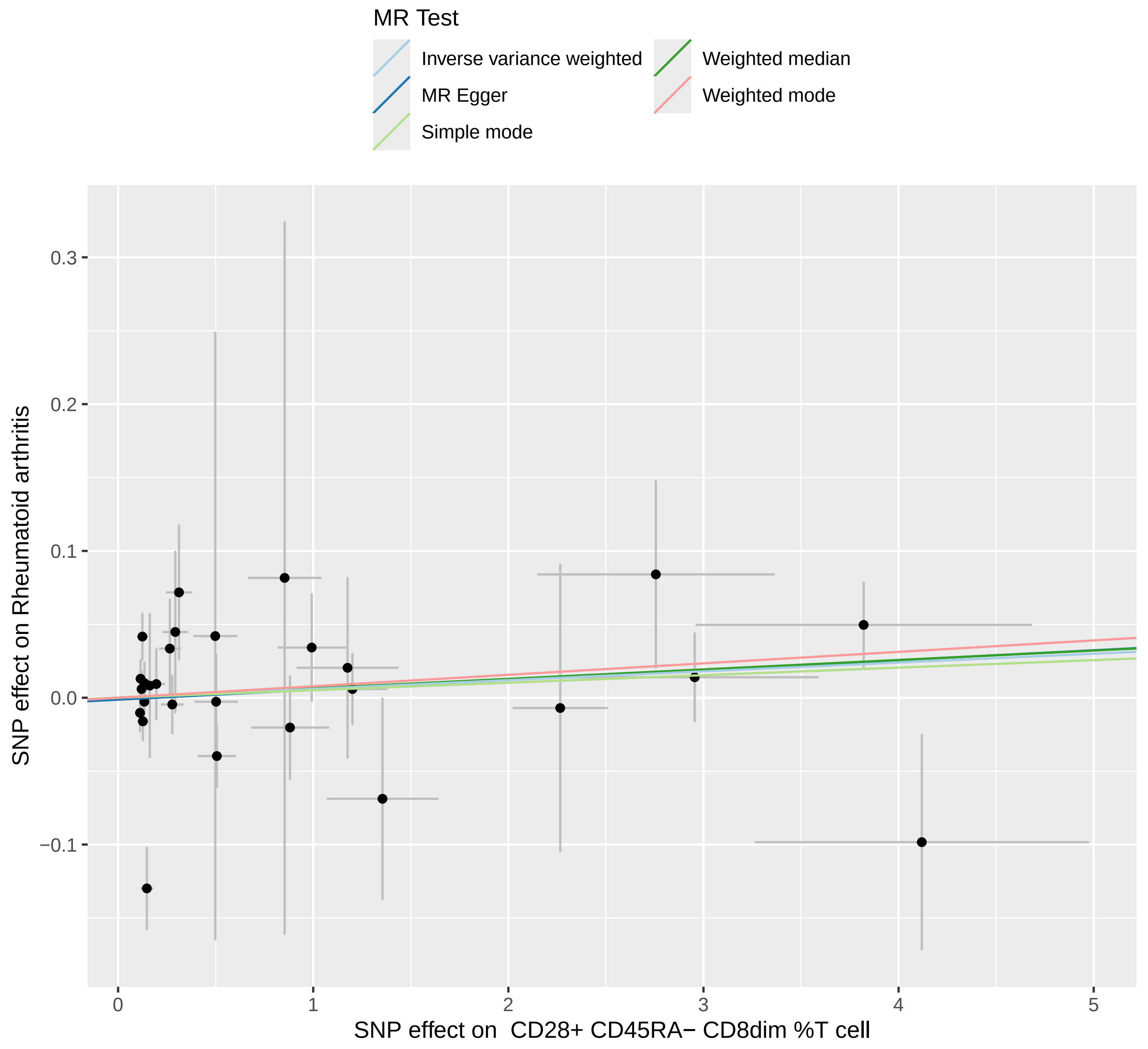
MR Test
Inverse variance weighted
MR Egger
Simple mode
Weighted median
Weighted mode
SNP effect on Rheumatoid arthritis
0.3
0.2
0.1
0.0
−0.1
0
1
2
3
4
5
SNP effect on CD28+ CD45RA− CD8dim %T cell

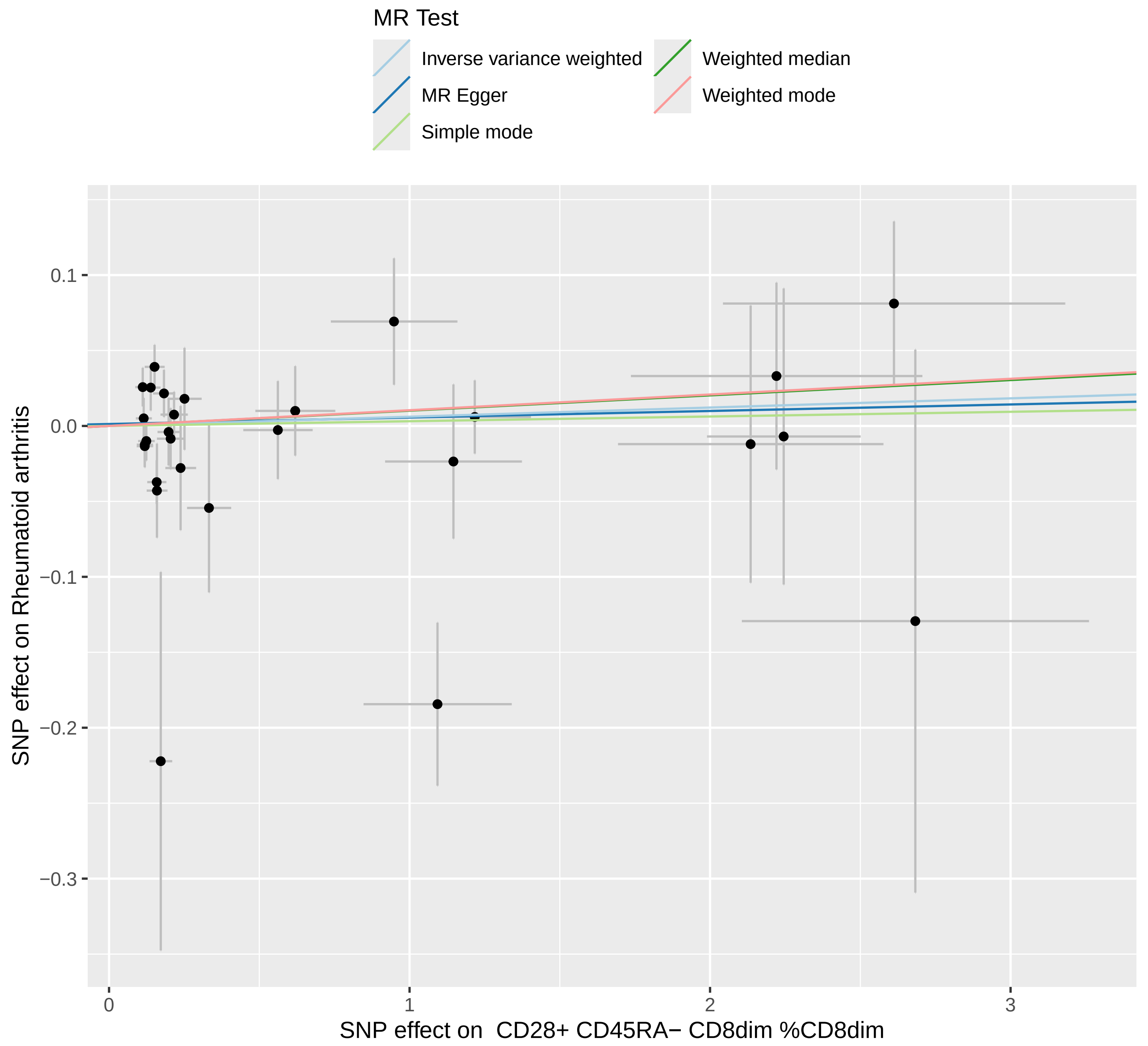

MR Test
Inverse variance weighted
MR Egger
Simple mode
Weighted median
Weighted mode
0.1
0.0
−0.1
−0.2
−0.3
0
1
2
3
SNP effect on Rheumatoid arthritis
SNP effect on CD28+ CD45RA− CD8dim %CD8dim

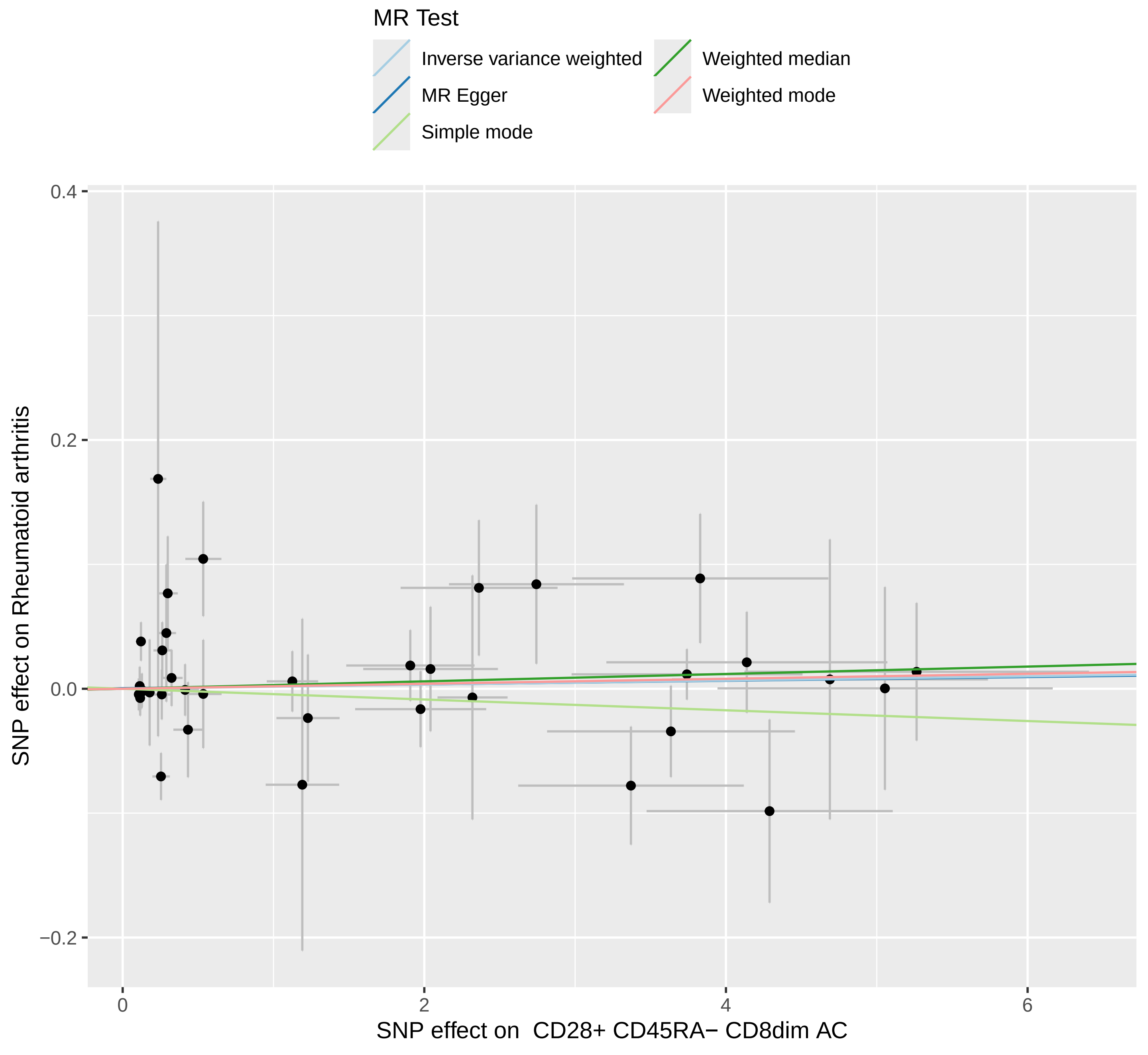
MR Test
Inverse variance weighted
MR Egger
Simple mode
Weighted median
Weighted mode
0.4
0.2
0.0
−0.2
SNP effect on Rheumatoid arthritis
0
2
4
6
SNP effect on CD28+ CD45RA− CD8dim AC

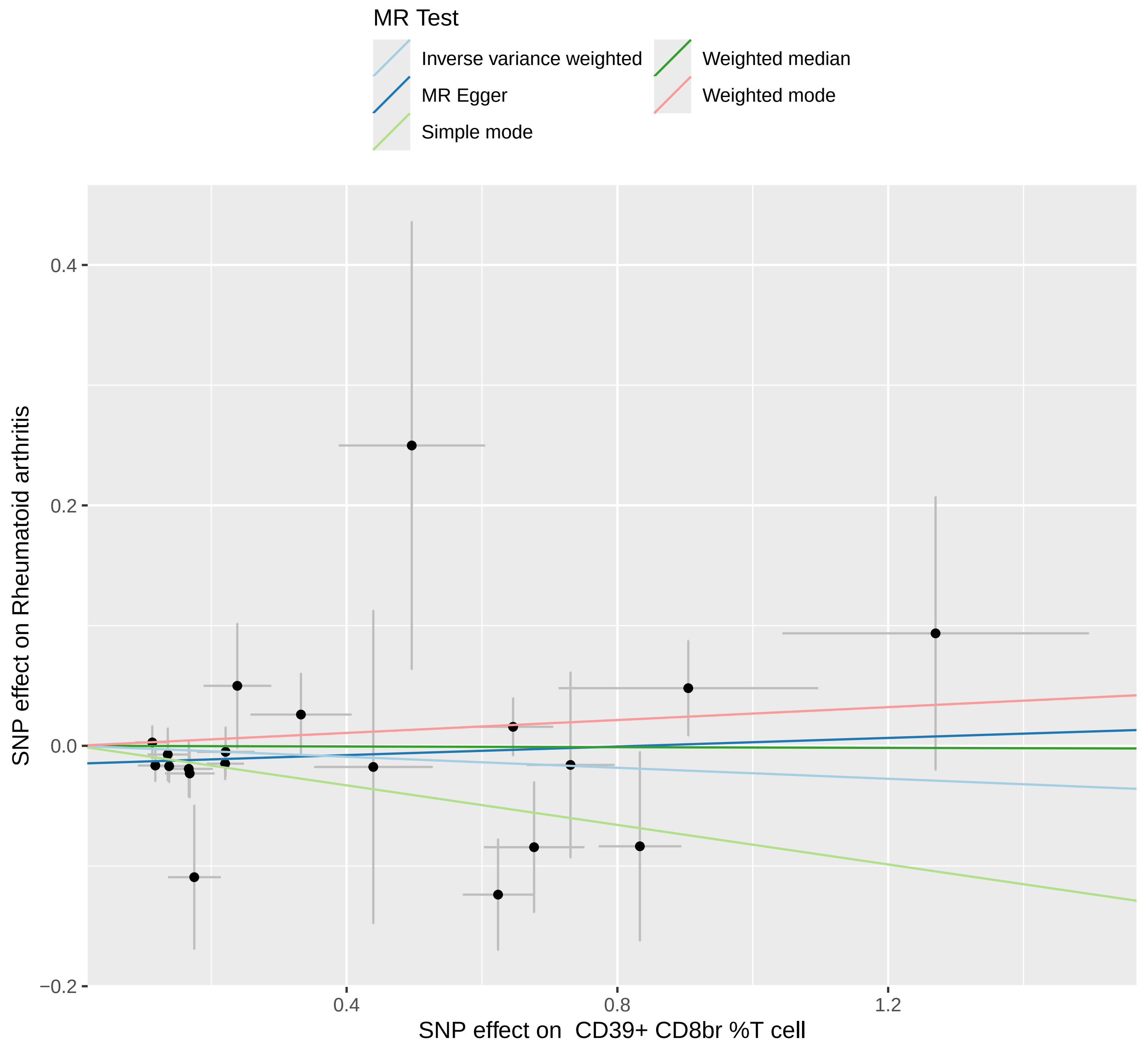
MR Test
Inverse variance weighted
MR Egger
Simple mode
Weighted median
Weighted mode
0.4
0.2
0.0
−0.2
0.4
0.8
1.2
SNP effect on Rheumatoid arthritis
SNP effect on CD39+ CD8br %T cell

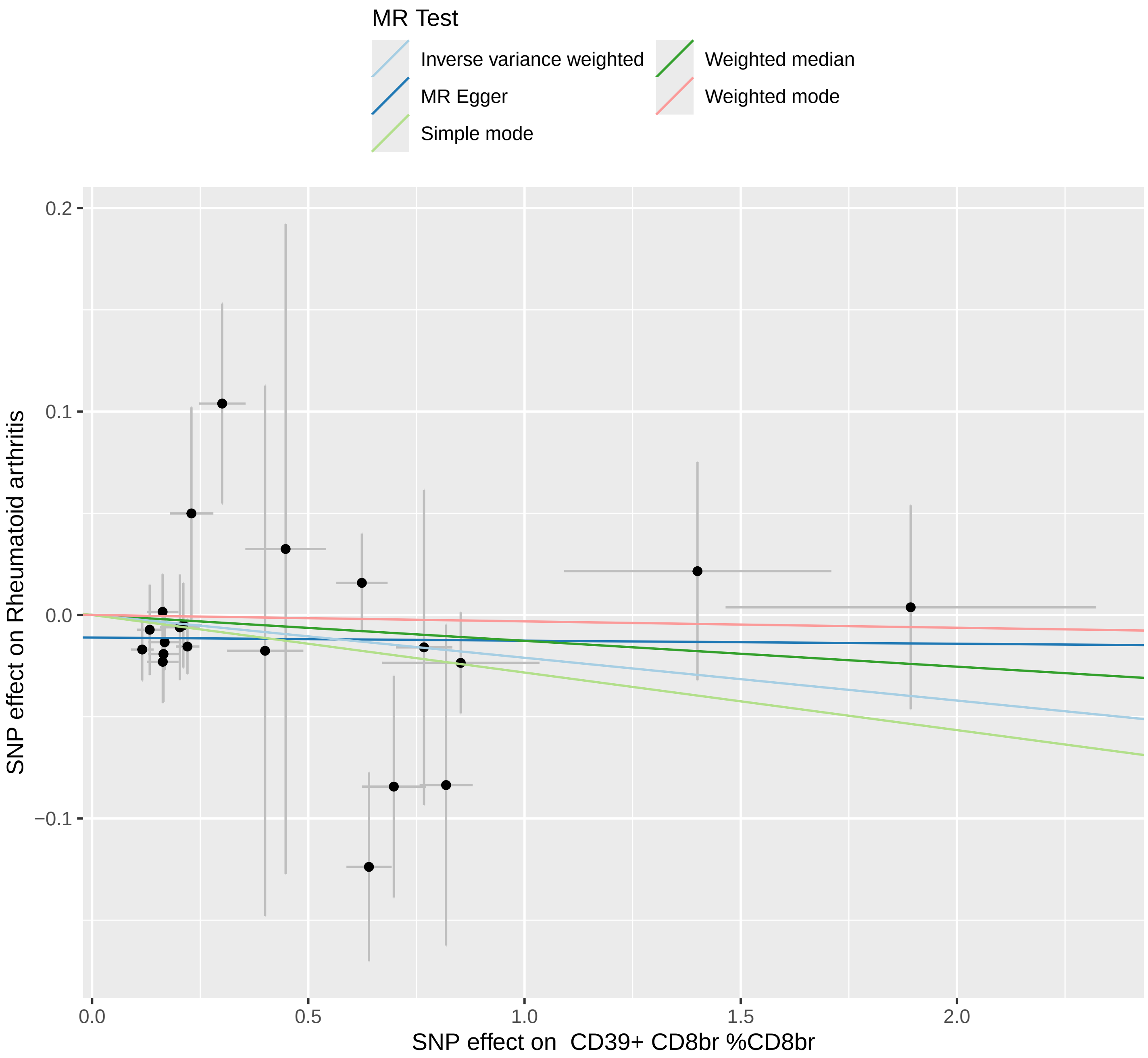

MR Test
Inverse variance weighted
MR Egger
Simple mode
Weighted median
Weighted mode
0.2
0.1
0.0
−0.1
0.0
0.5
1.0
1.5
2.0
SNP effect on Rheumatoid arthritis
SNP effect on CD39+ CD8br %CD8br

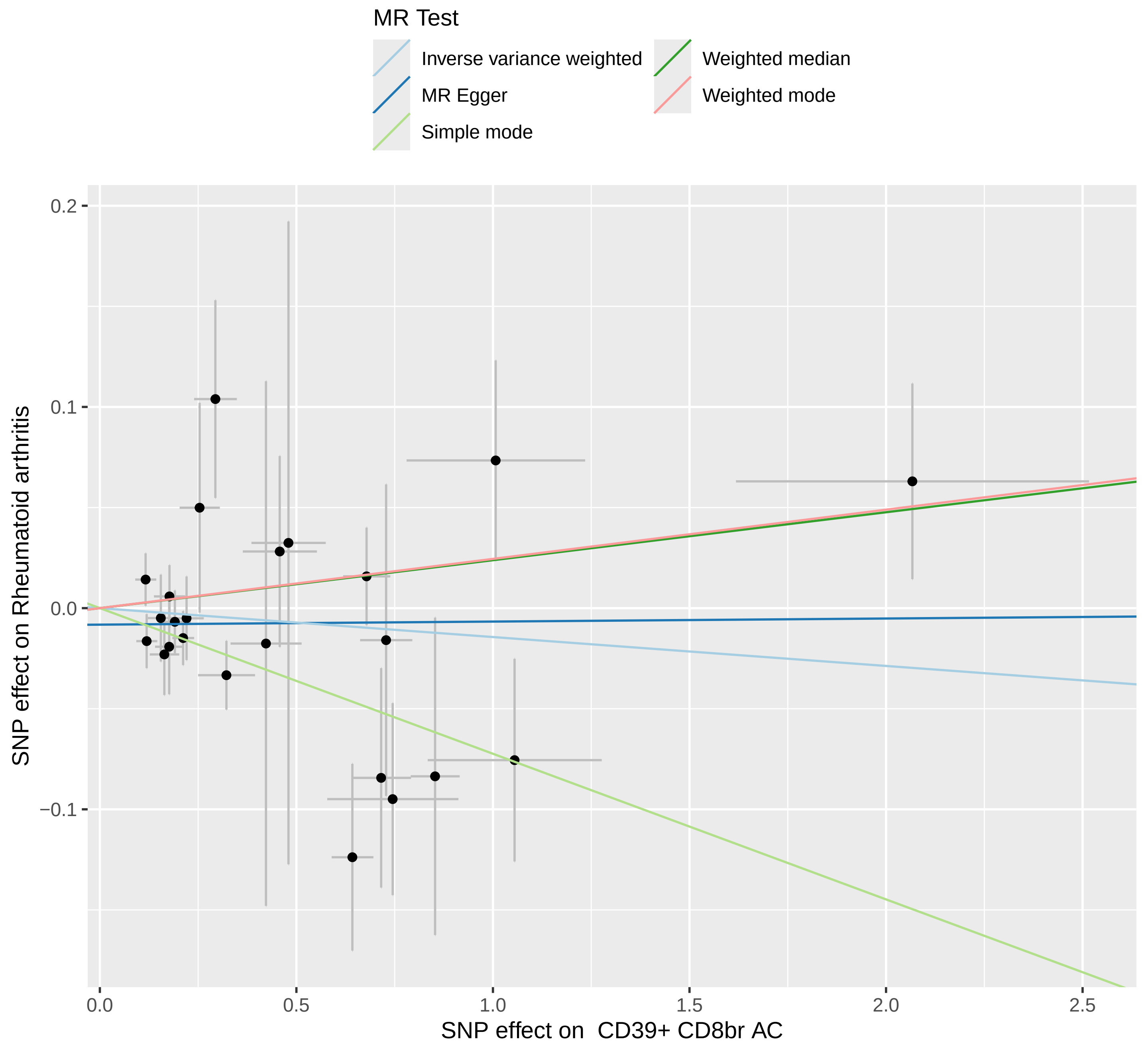
MR Test
Inverse variance weighted
MR Egger
Simple mode
Weighted median
Weighted mode
0.2
0.1
0.0
−0.1
SNP effect on Rheumatoid arthritis
0.0
0.5
1.0
1.5
2.0
2.5
SNP effect on CD39+ CD8br AC

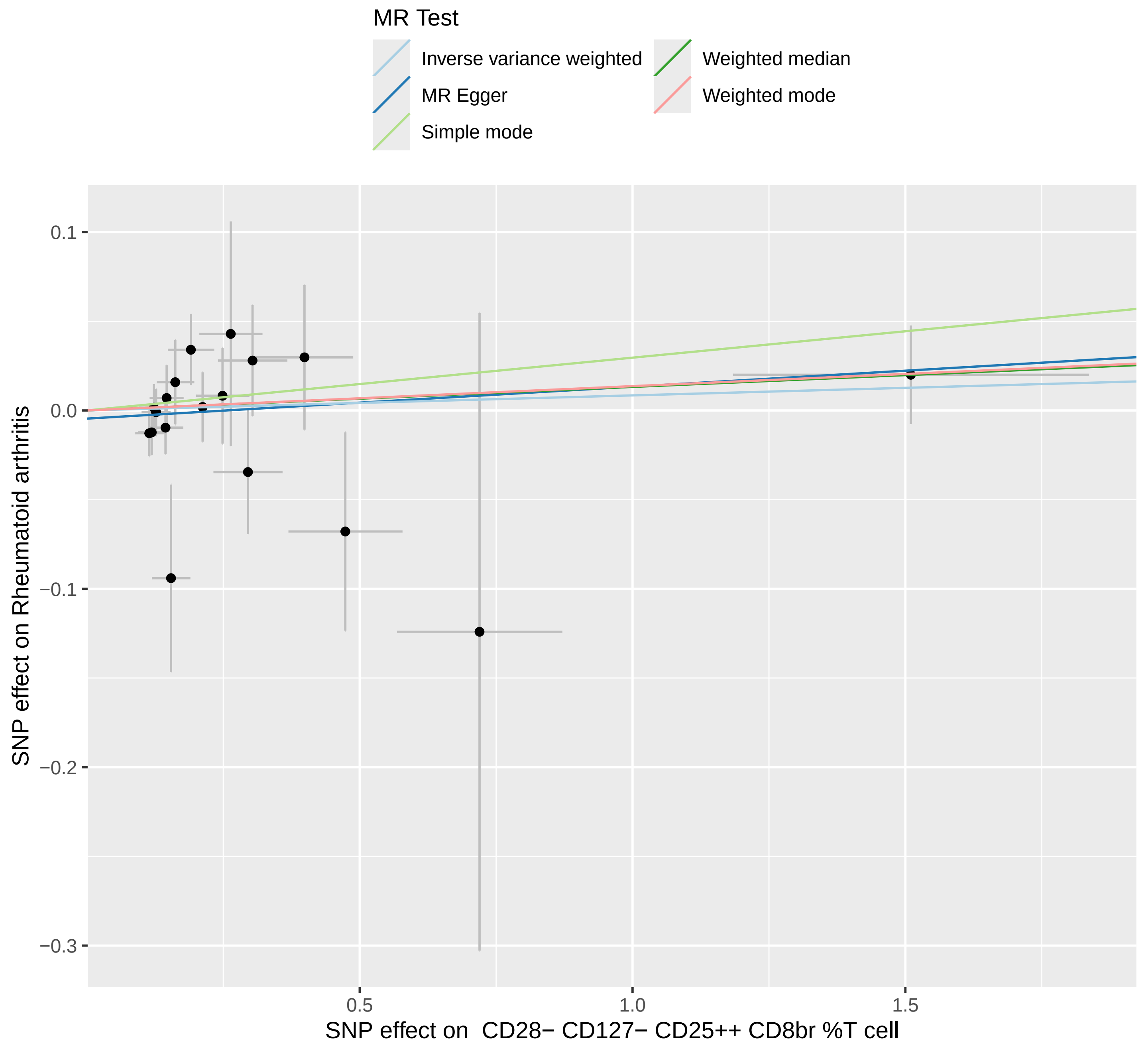
MR Test
Inverse variance weighted
MR Egger
Simple mode
Weighted median
Weighted mode
0.1
0.0
−0.1
−0.2
−0.3
0.5
1.0
1.5
SNP effect on Rheumatoid arthritis
SNP effect on CD28− CD127− CD25++ CD8br %T cell

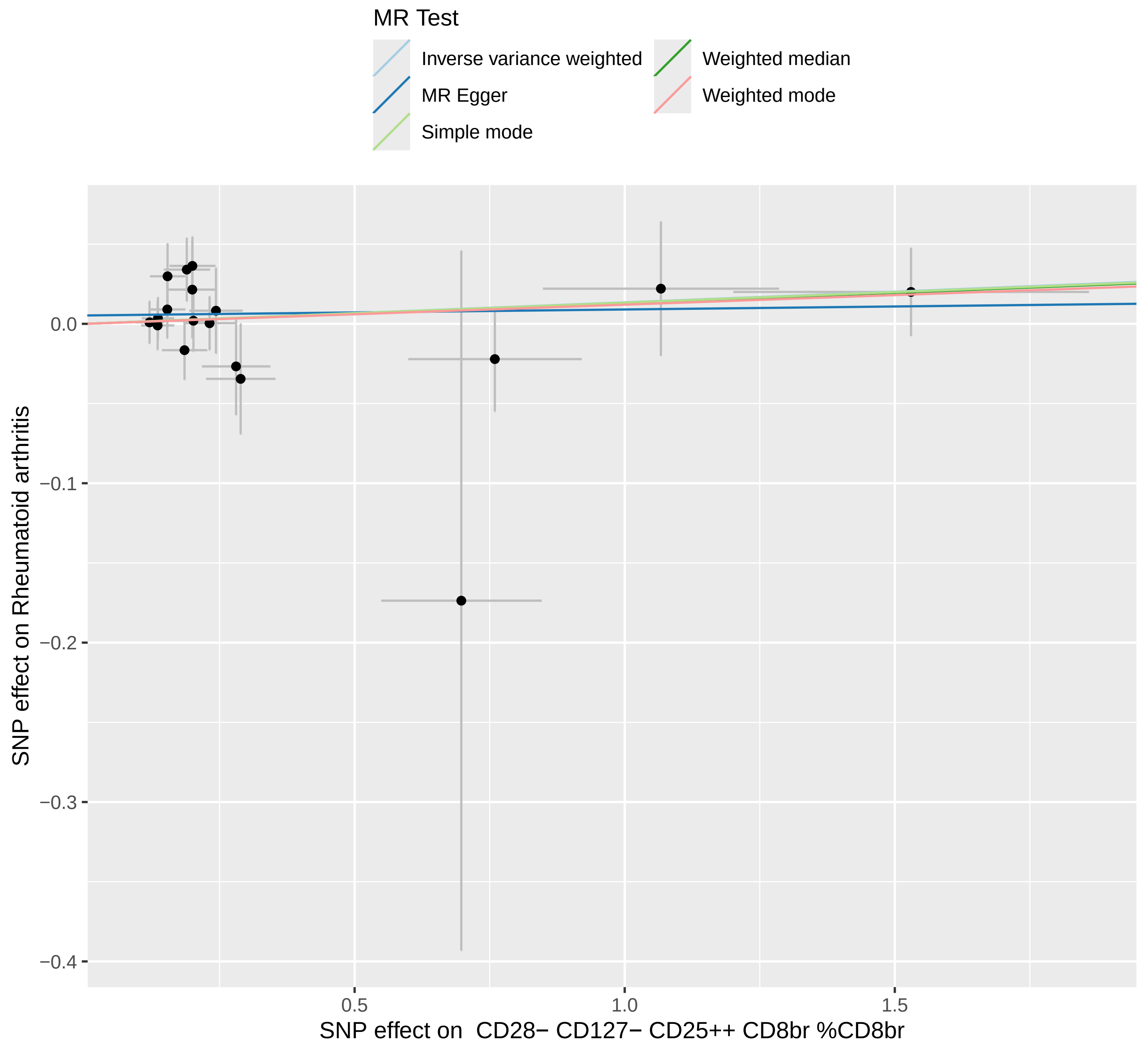

MR Test
Inverse variance weighted
MR Egger
Simple mode
Weighted median
Weighted mode
SNP effect on Rheumatoid arthritis
0.0
−0.1
−0.2
−0.3
−0.4
0.5
1.0
1.5
SNP effect on CD28− CD127− CD25++ CD8br %CD8br

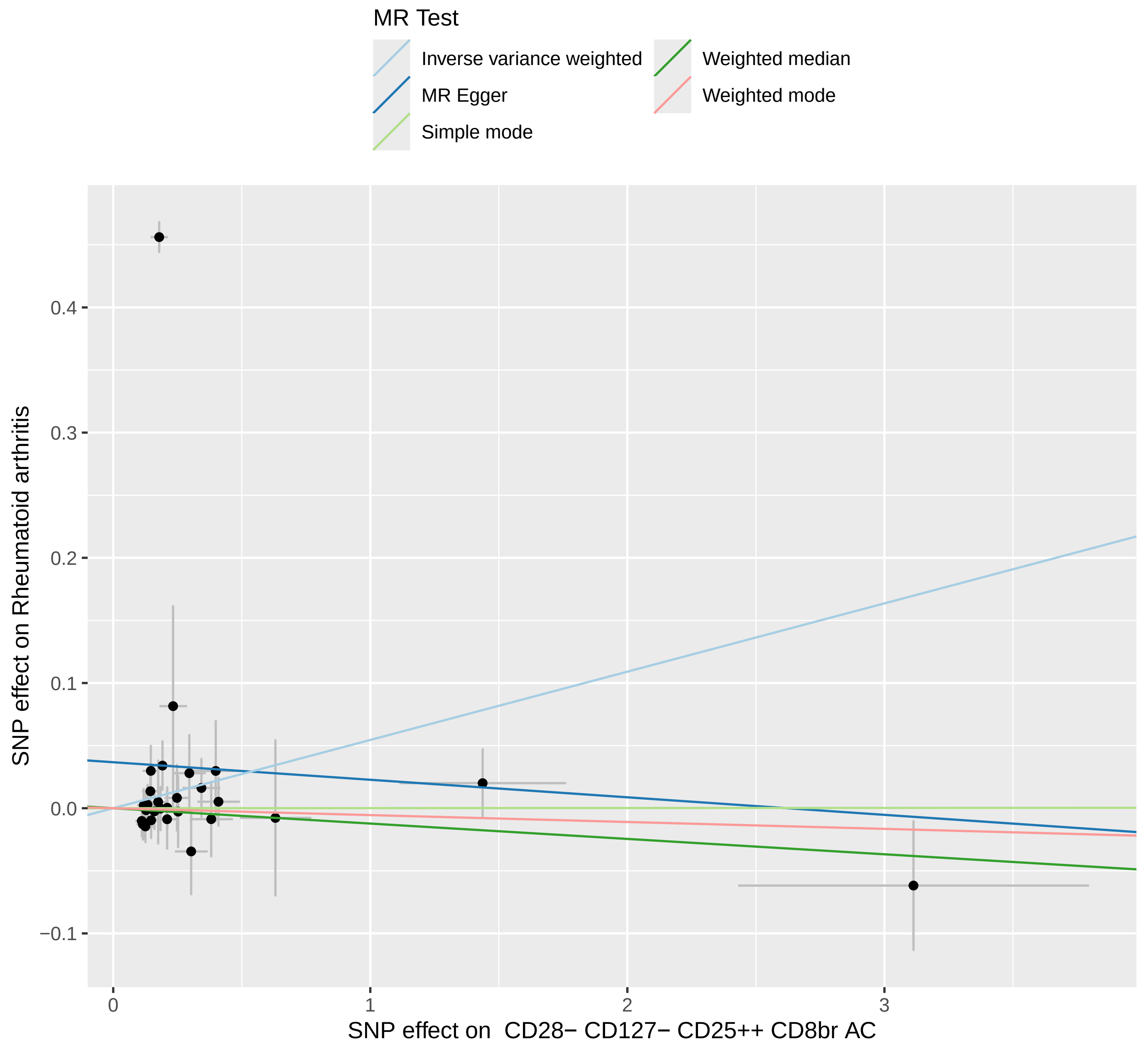
MR Test
Inverse variance weighted
MR Egger
Simple mode
Weighted median
Weighted mode
SNP effect on Rheumatoid arthritis
0.4
0.3
0.2
0.1
0.0
−0.1
0
1
2
3
SNP effect on CD28− CD127− CD25++ CD8br AC

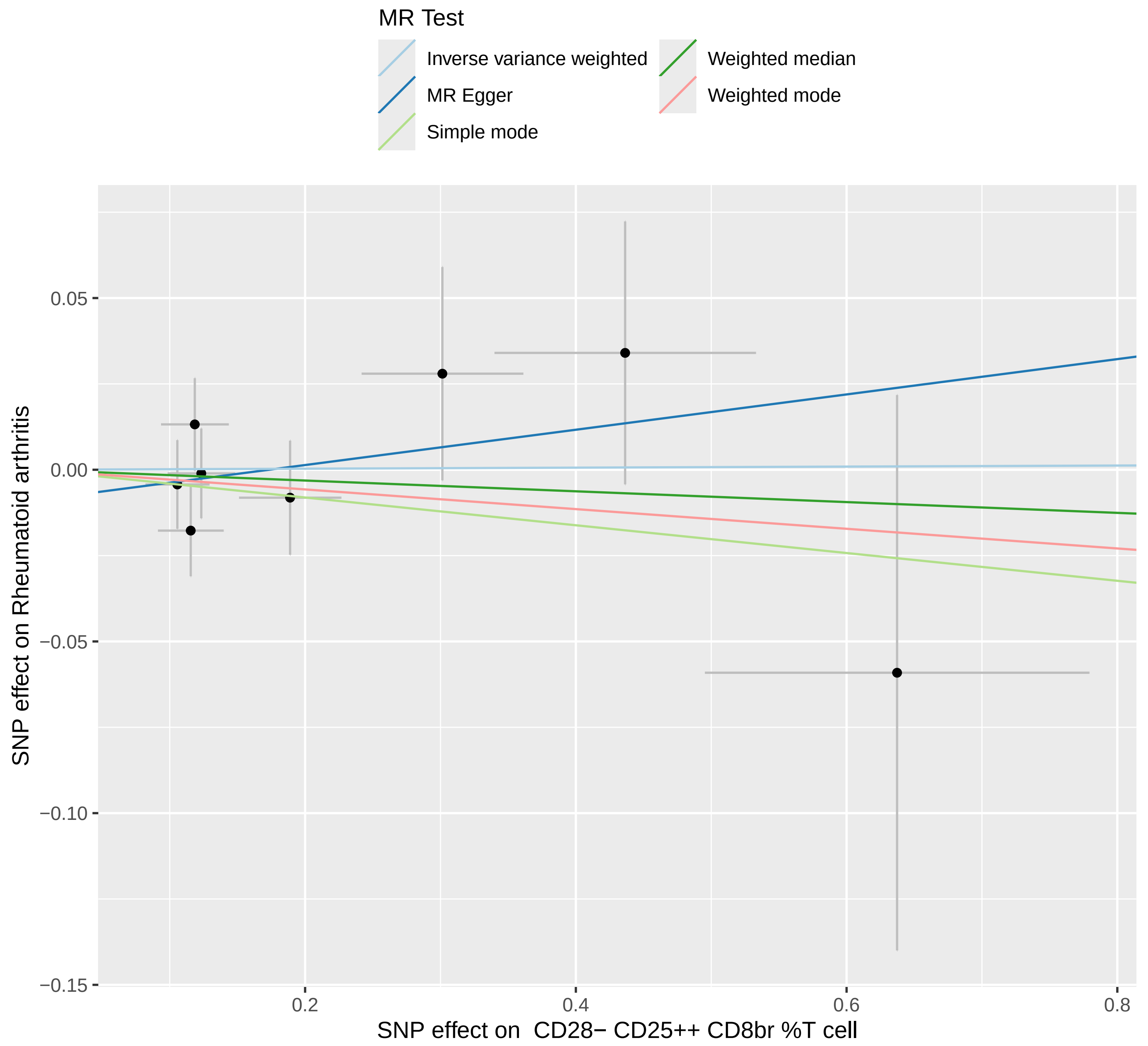
MR Test
Inverse variance weighted
MR Egger
Simple mode
Weighted median
Weighted mode
0.05
0.00
−0.05
−0.10
−0.15
0.2
0.4
0.6
0.8
SNP effect on Rheumatoid arthritis
SNP effect on CD28− CD25++ CD8br %T cell

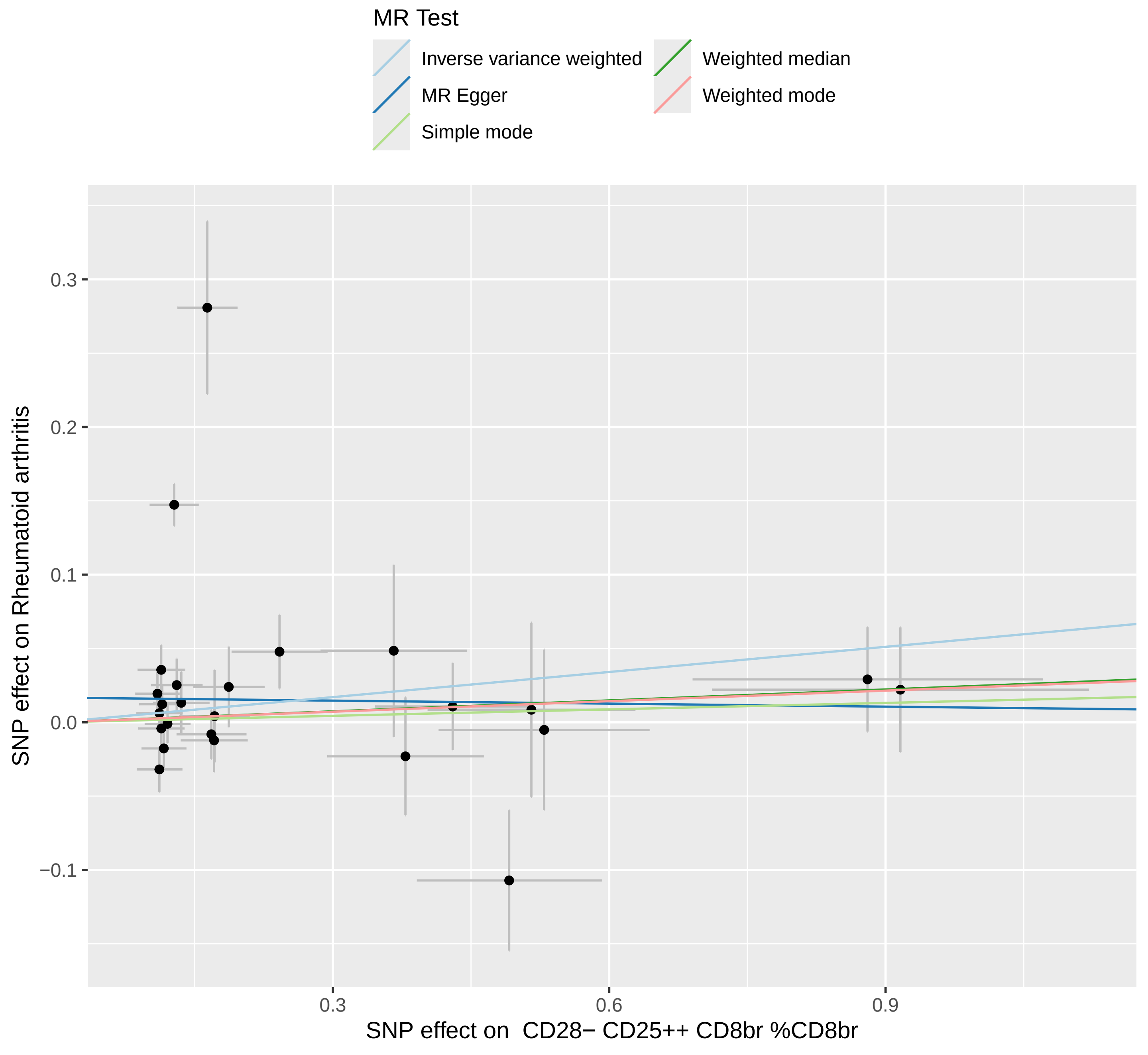
MR Test
Inverse variance weighted
MR Egger
Simple mode
Weighted median
Weighted mode
0.3
0.2
0.1
0.0
−0.1
0.3
0.6
0.9
SNP effect on Rheumatoid arthritis
SNP effect on CD28− CD25++ CD8br %CD8br

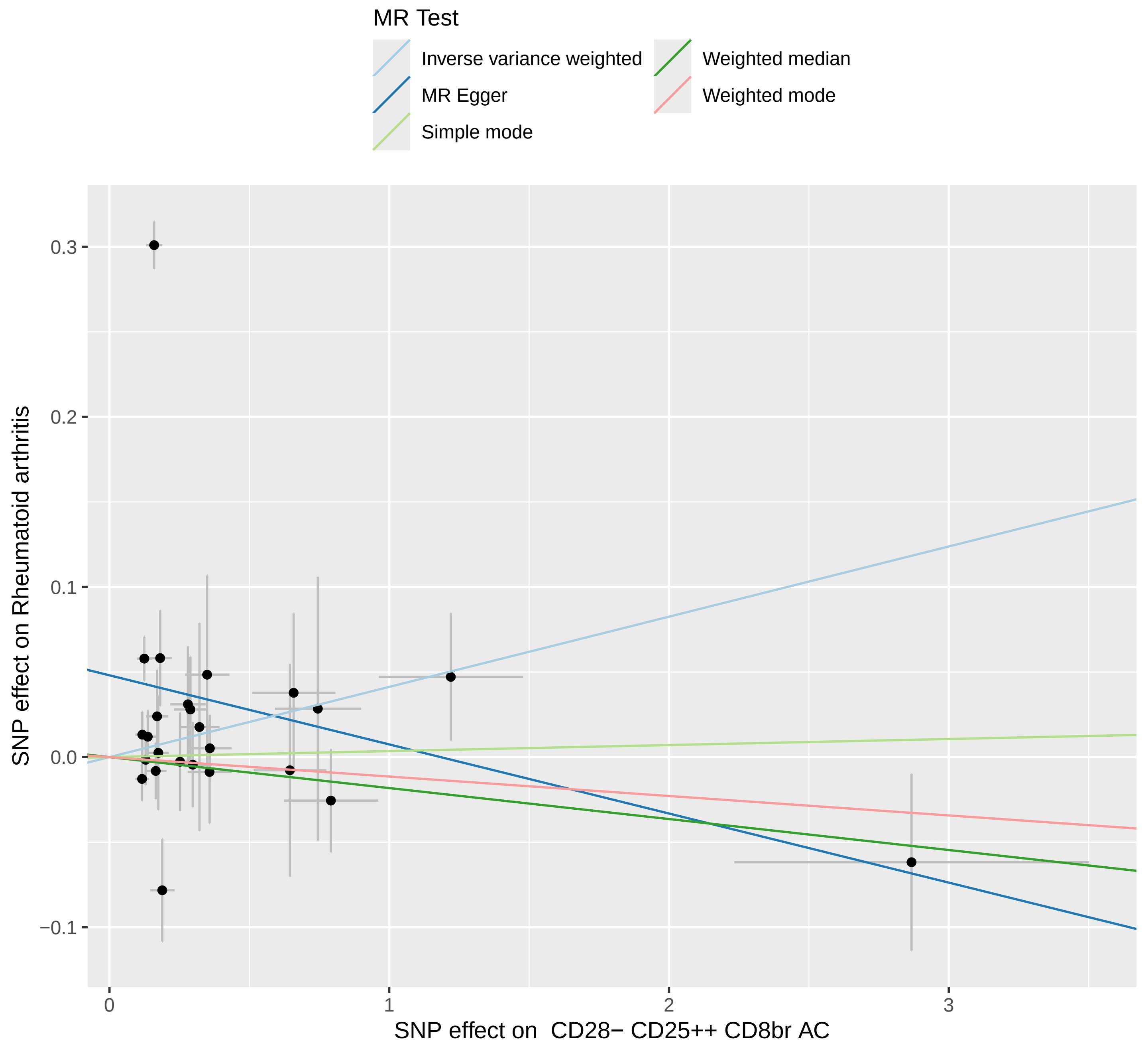

MR Test
Inverse variance weighted
Weighted median
MR Egger
Weighted mode
Simple mode
0.3
0.2
0.1
0.0
−0.1
0
1
2
3
SNP effect on Rheumatoid arthritis
SNP effect on CD28− CD25++ CD8br AC

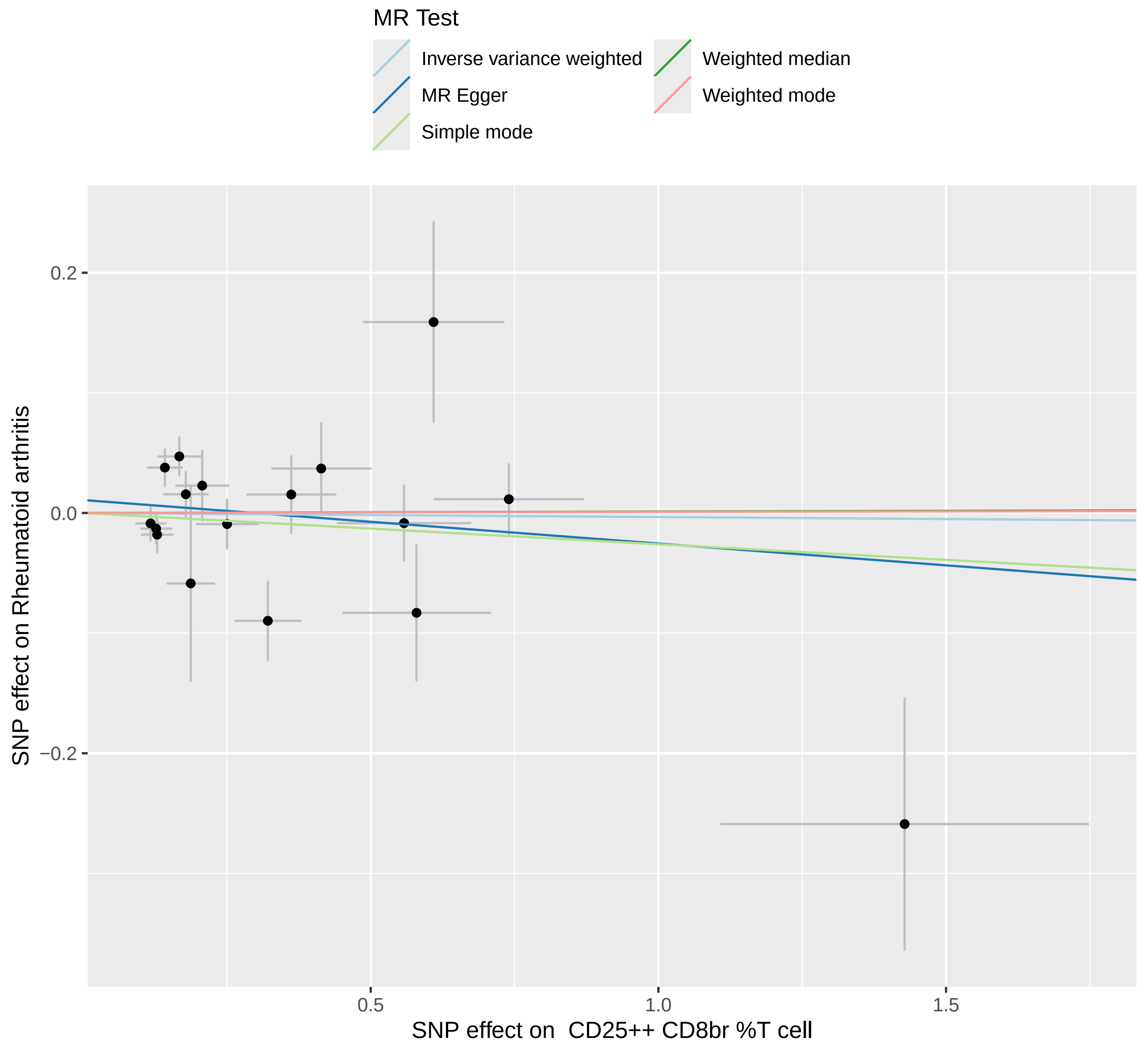

MR Test
Inverse variance weighted
Weighted median
MR Egger
Weighted mode
Simple mode
0.2
0.0
−0.2
0.5
1.0
1.5
SNP effect on Rheumatoid arthritis
SNP effect on CD25++ CD8br %T cell

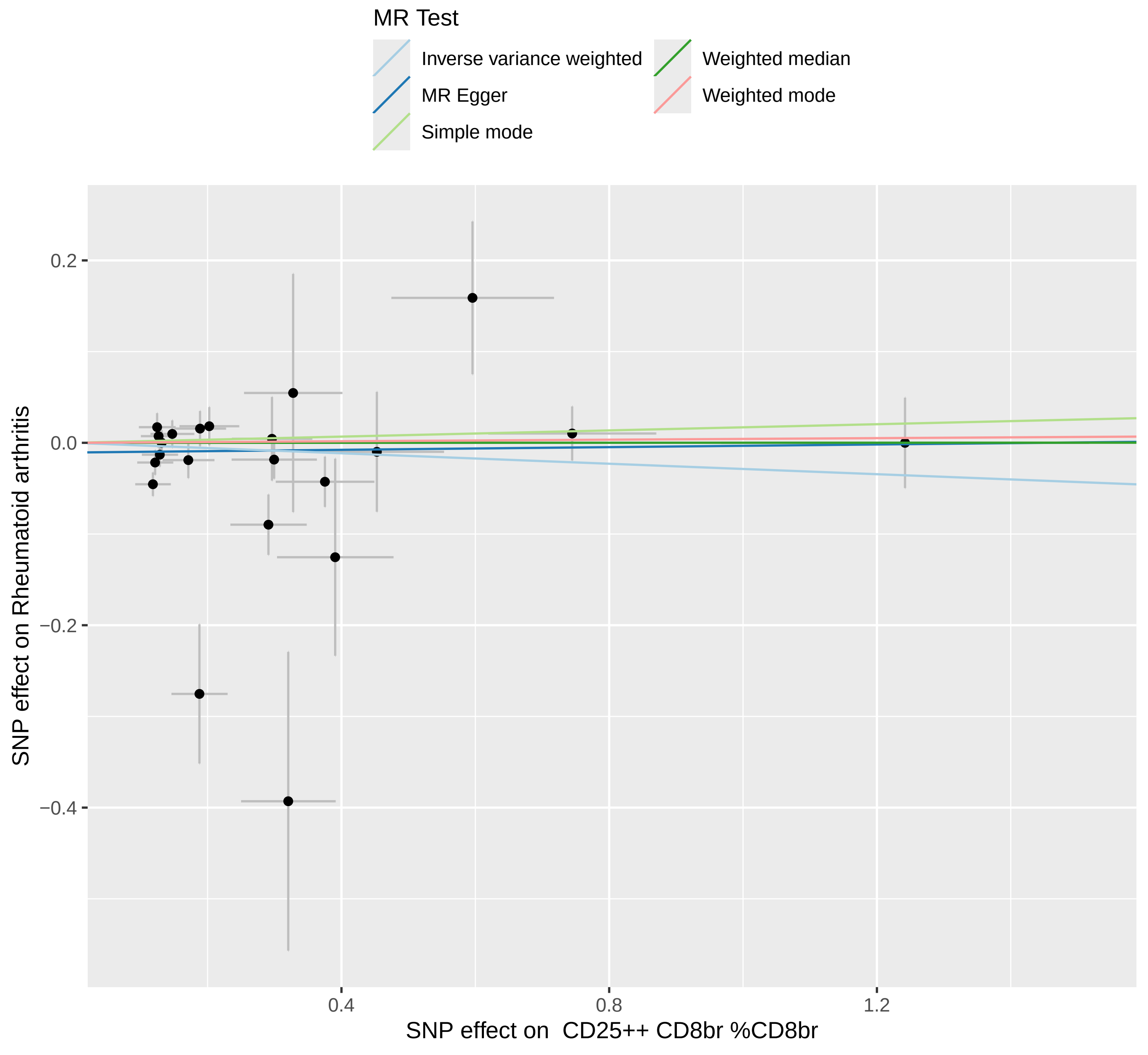

MR Test
Inverse variance weighted
MR Egger
Simple mode
Weighted median
Weighted mode
0.2
0.0
−0.2
−0.4
0.4
0.8
1.2
SNP effect on Rheumatoid arthritis
SNP effect on CD25++ CD8br %CD8br

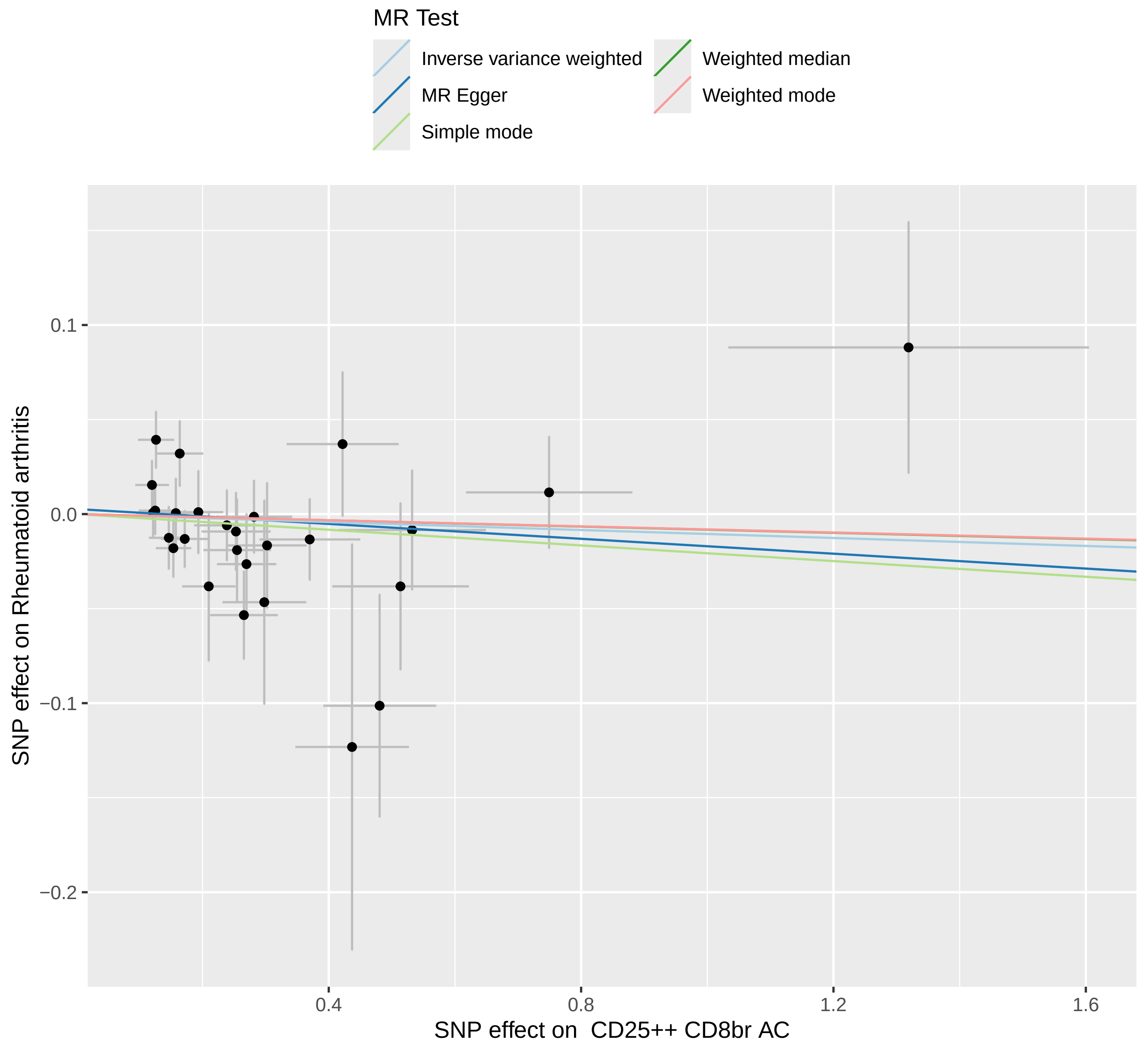
MR Test
Inverse variance weighted
MR Egger
Simple mode
Weighted median
Weighted mode
0.1
0.0
−0.1
−0.2
0.4
0.8
1.2
1.6
SNP effect on Rheumatoid arthritis
SNP effect on CD25++ CD8br AC

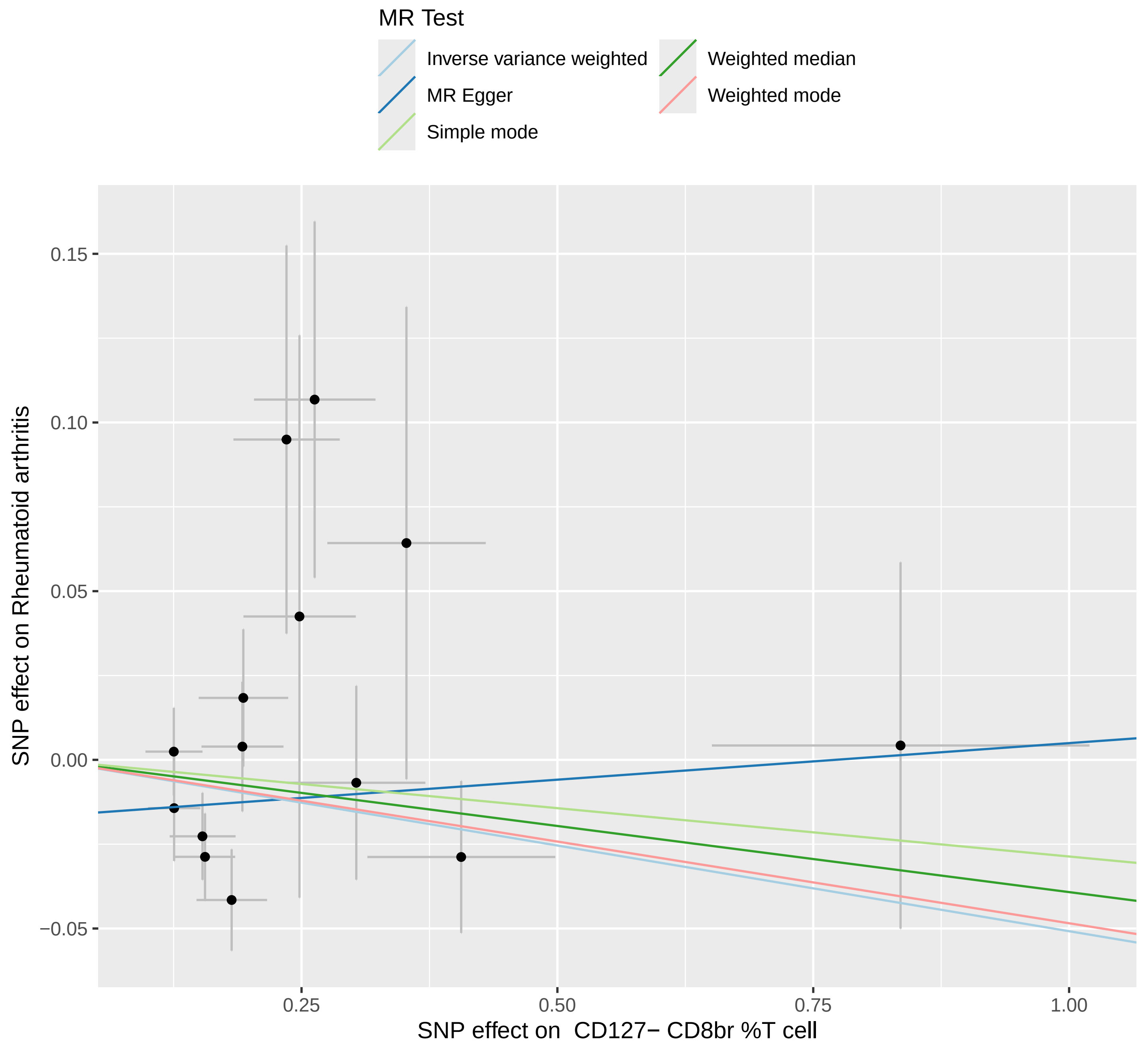
MR Test
Inverse variance weighted
MR Egger
Simple mode
Weighted median
Weighted mode
0.15
0.10
0.05
0.00
−0.05
0.25
0.50
0.75
1.00
SNP effect on Rheumatoid arthritis
SNP effect on CD127− CD8br %T cell

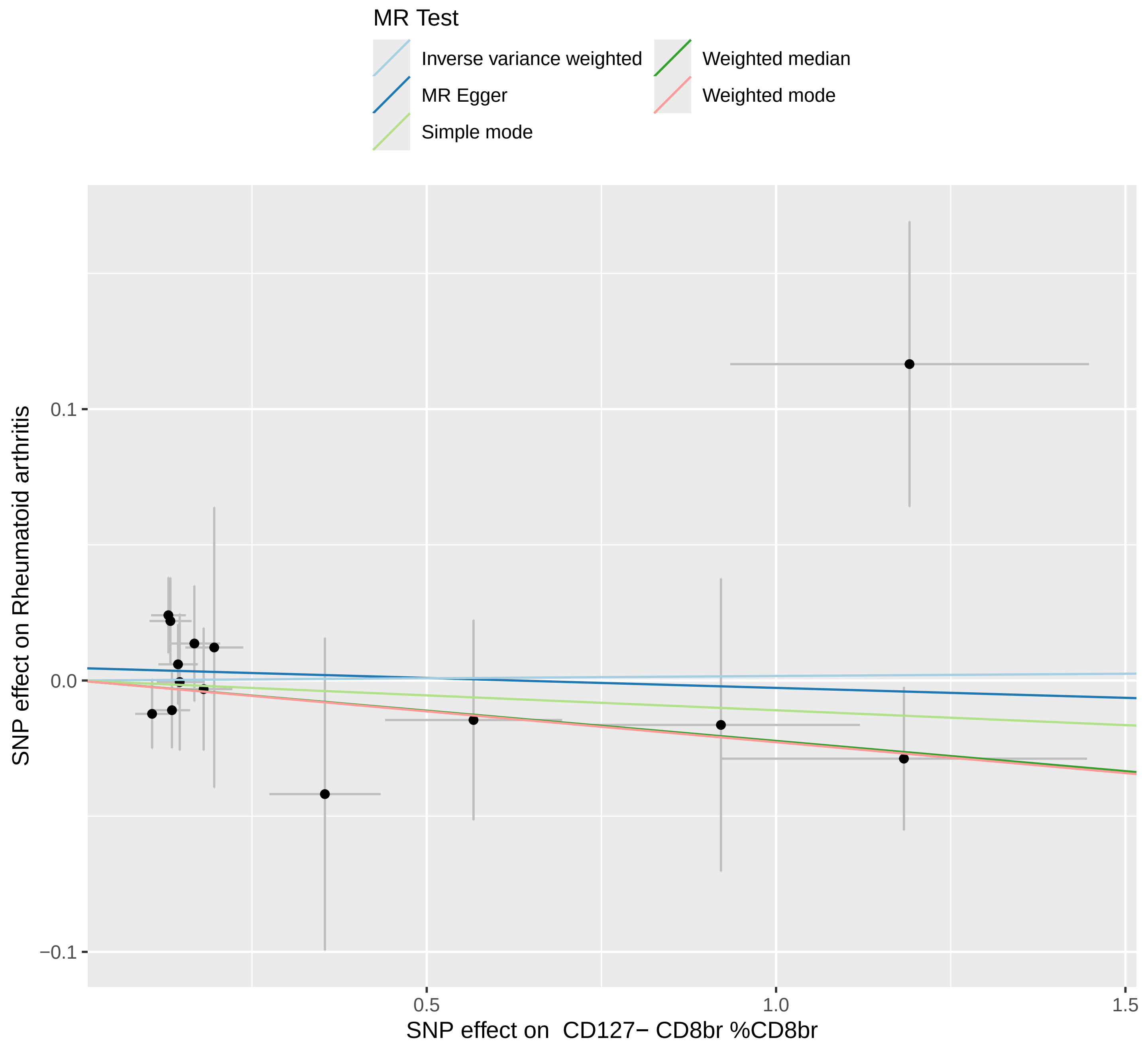
MR Test
Inverse variance weighted
MR Egger
Simple mode
Weighted median
Weighted mode
SNP effect on Rheumatoid arthritis
0.1
0.0
−0.1
0.5
1.0
1.5
SNP effect on CD127− CD8br %CD8br

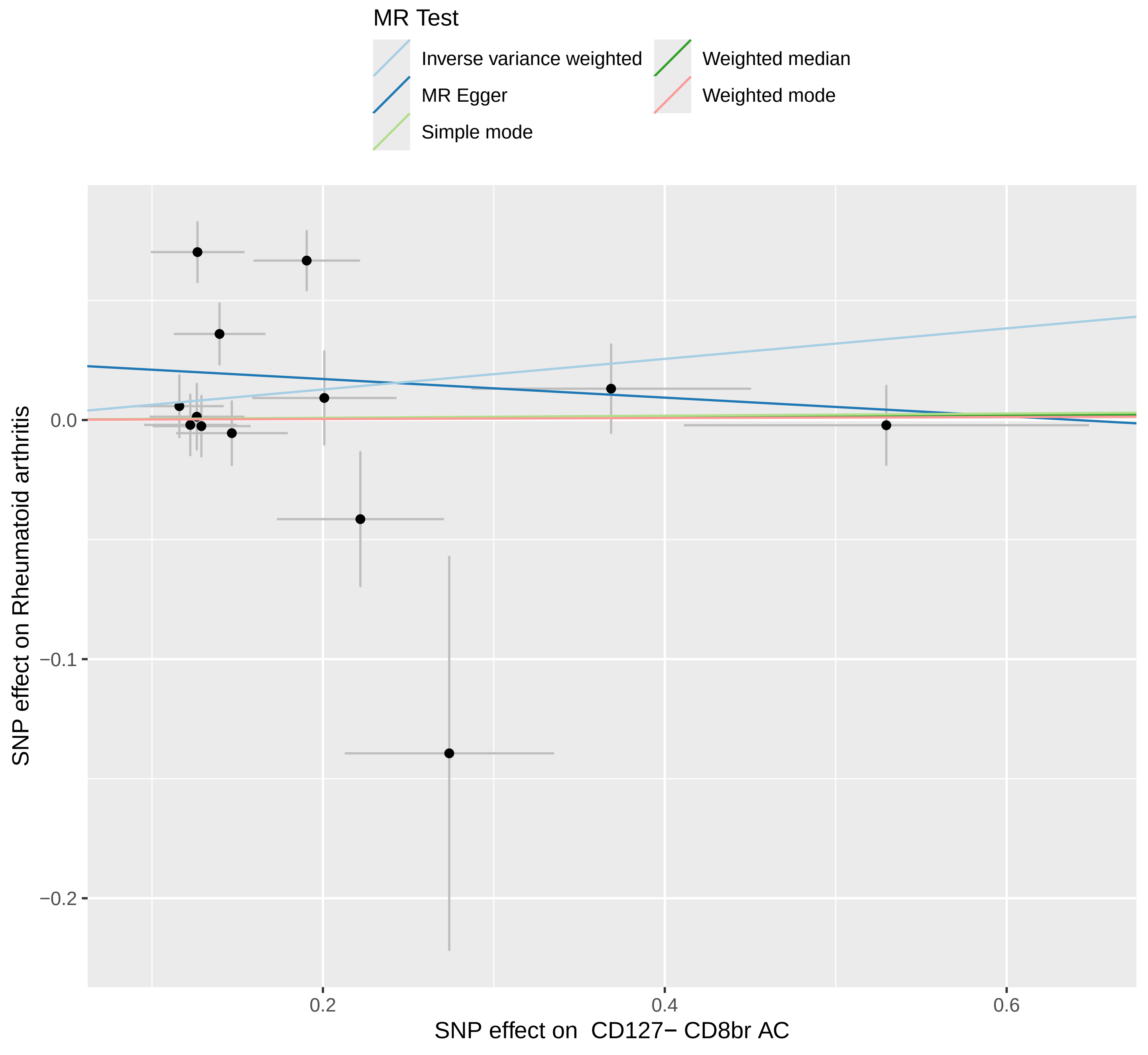
MR Test
Inverse variance weighted
MR Egger
Simple mode
Weighted median
Weighted mode
SNP effect on Rheumatoid arthritis
0.0
−0.1
−0.2
0.2
0.4
0.6
SNP effect on CD127− CD8br AC

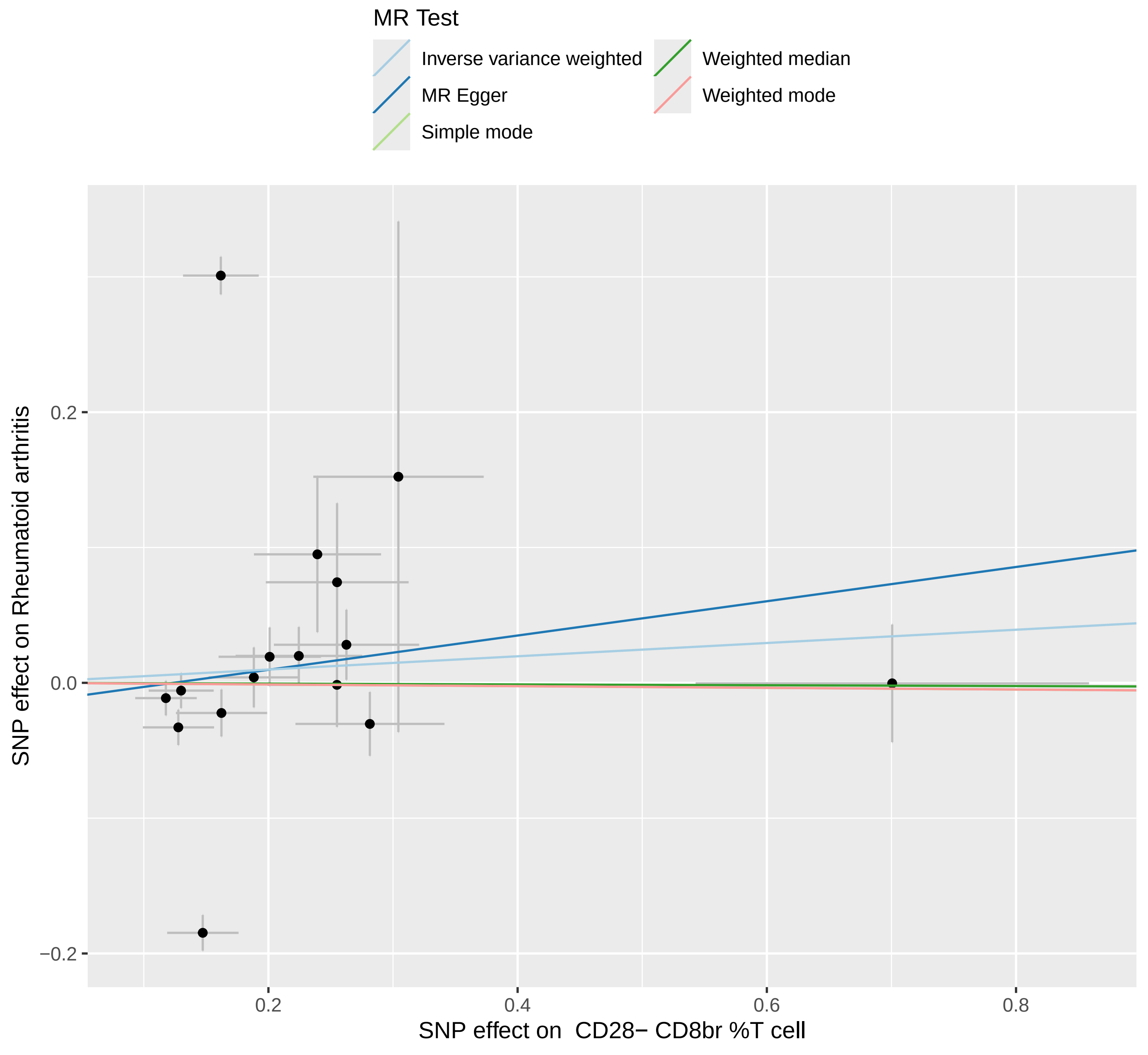
MR Test
Inverse variance weighted
MR Egger
Simple mode
Weighted median
Weighted mode
SNP effect on Rheumatoid arthritis
0.2
0.0
−0.2
0.2
0.4
0.6
0.8
SNP effect on CD28− CD8br %T cell

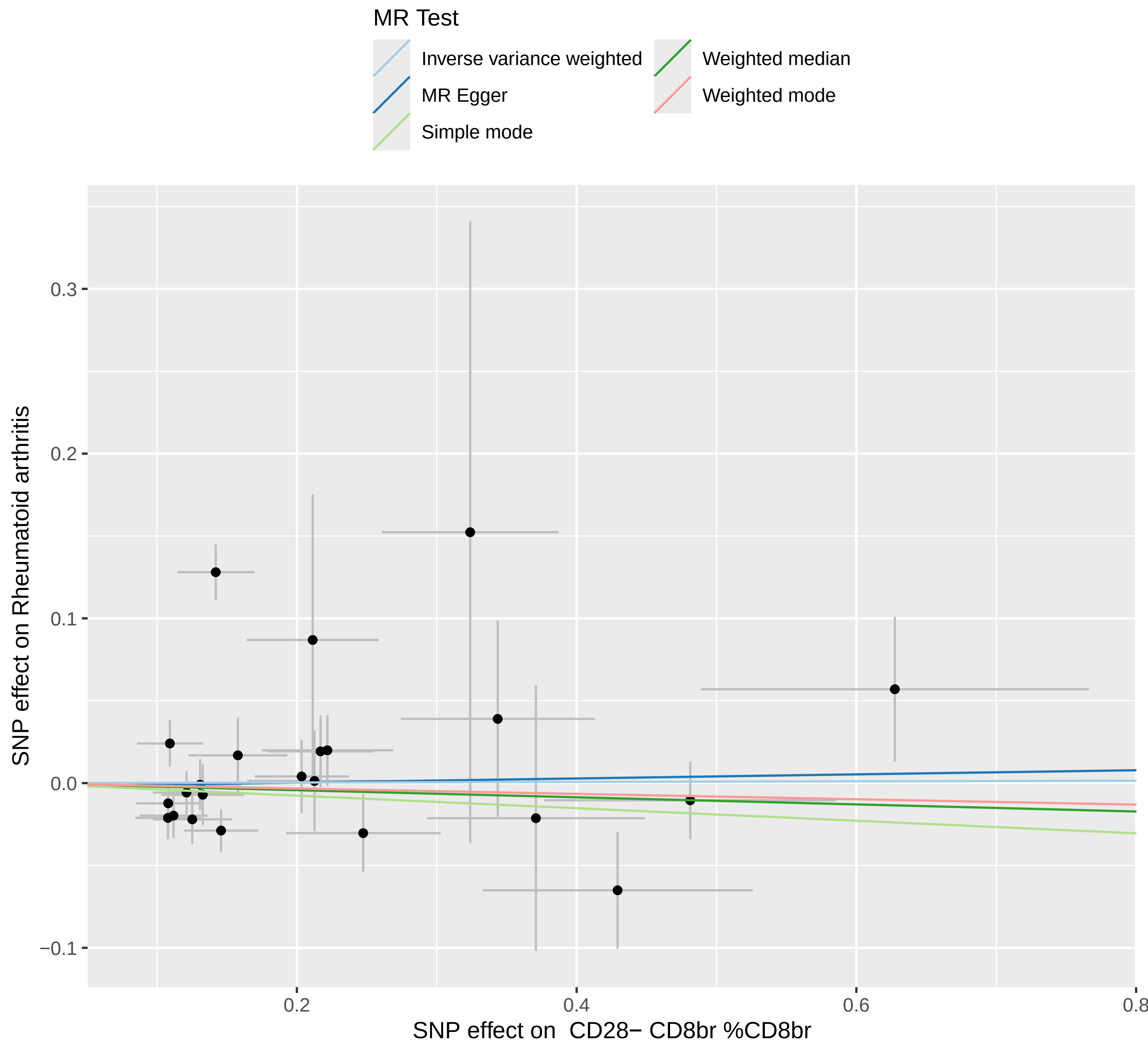

MR Test
Inverse variance weighted
MR Egger
Simple mode
Weighted median
Weighted mode
SNP effect on Rheumatoid arthritis
0.3
0.2
0.1
0.0
−0.1
0.2
0.4
0.6
0.8
SNP effect on CD28− CD8br %CD8br

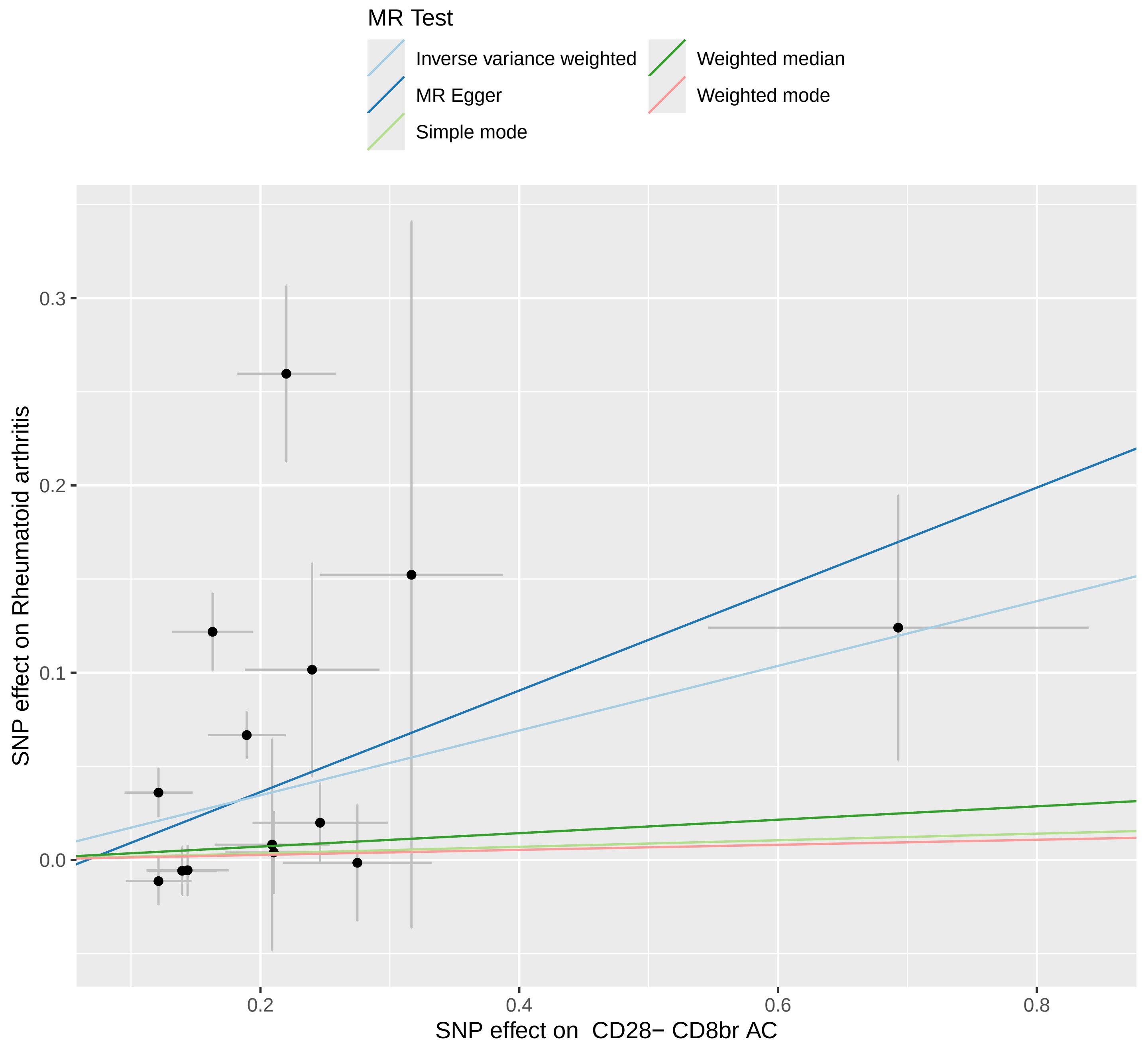

MR Test
Inverse variance weighted
MR Egger
Simple mode
Weighted median
Weighted mode
SNP effect on Rheumatoid arthritis
0.3
0.2
0.1
0.0
0.2
0.4
0.6
0.8
SNP effect on CD28− CD8br AC

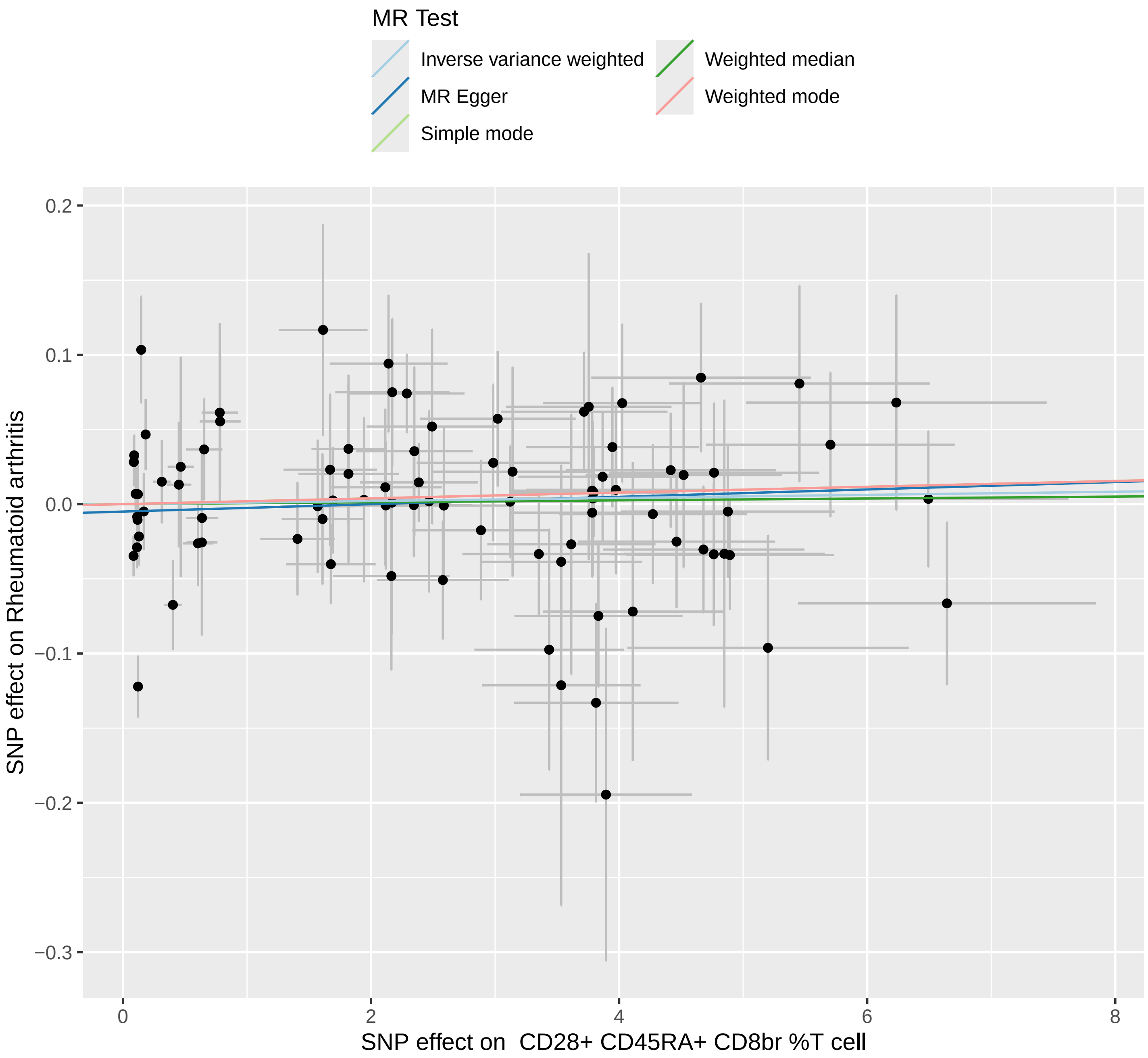

MR Test
Inverse variance weighted
MR Egger
Simple mode
Weighted median
Weighted mode
0.2
0.1
0.0
−0.1
−0.2
−0.3
SNP effect on Rheumatoid arthritis
0
2
4
6
8
SNP effect on CD28+ CD45RA+ CD8br %T cell

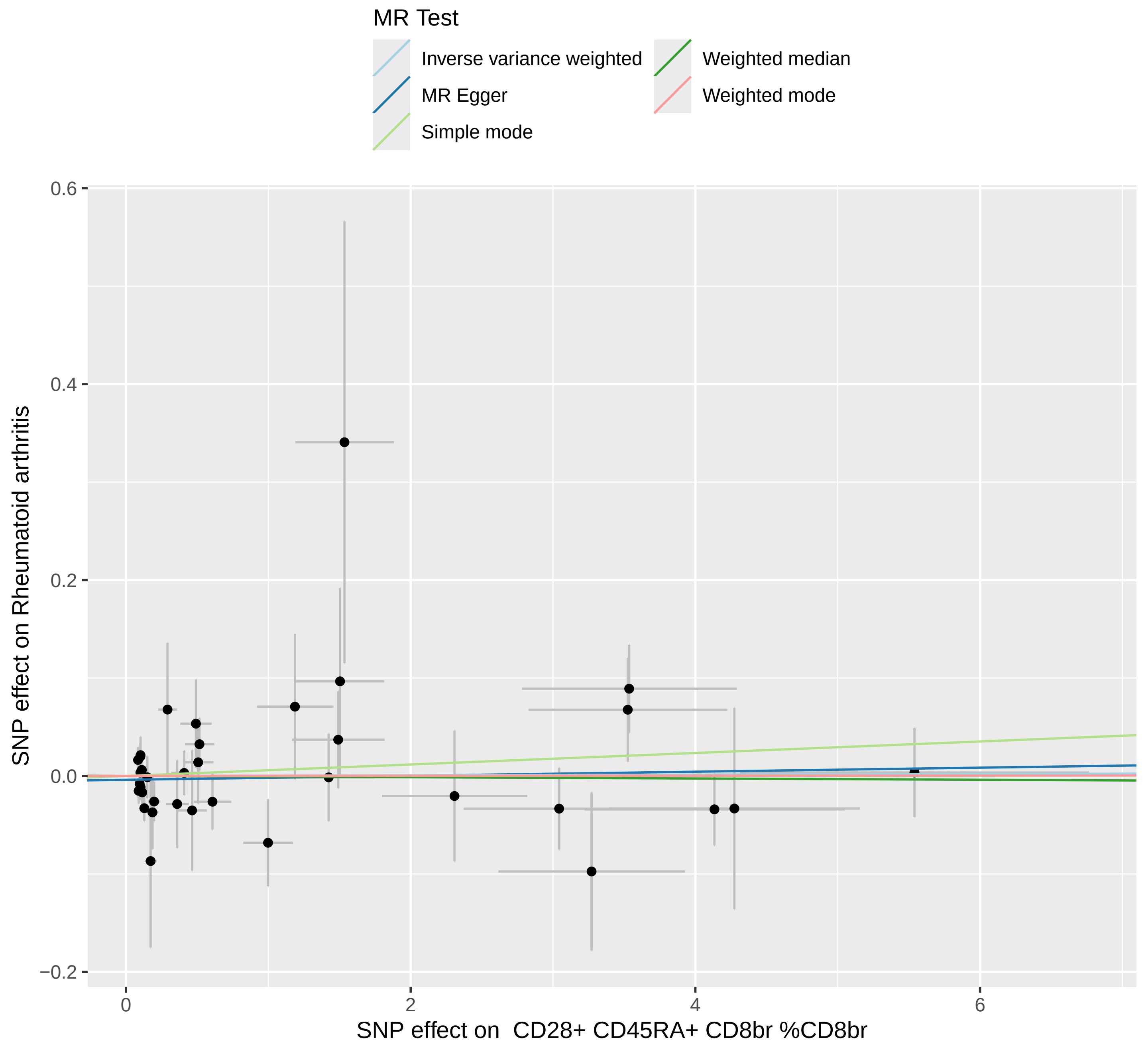
MR Test
Inverse variance weighted
MR Egger
Simple mode
Weighted median
Weighted mode
0.6
0.4
0.2
0.0
−0.2
0
2
4
6
SNP effect on Rheumatoid arthritis
SNP effect on CD28+ CD45RA+ CD8br %CD8br

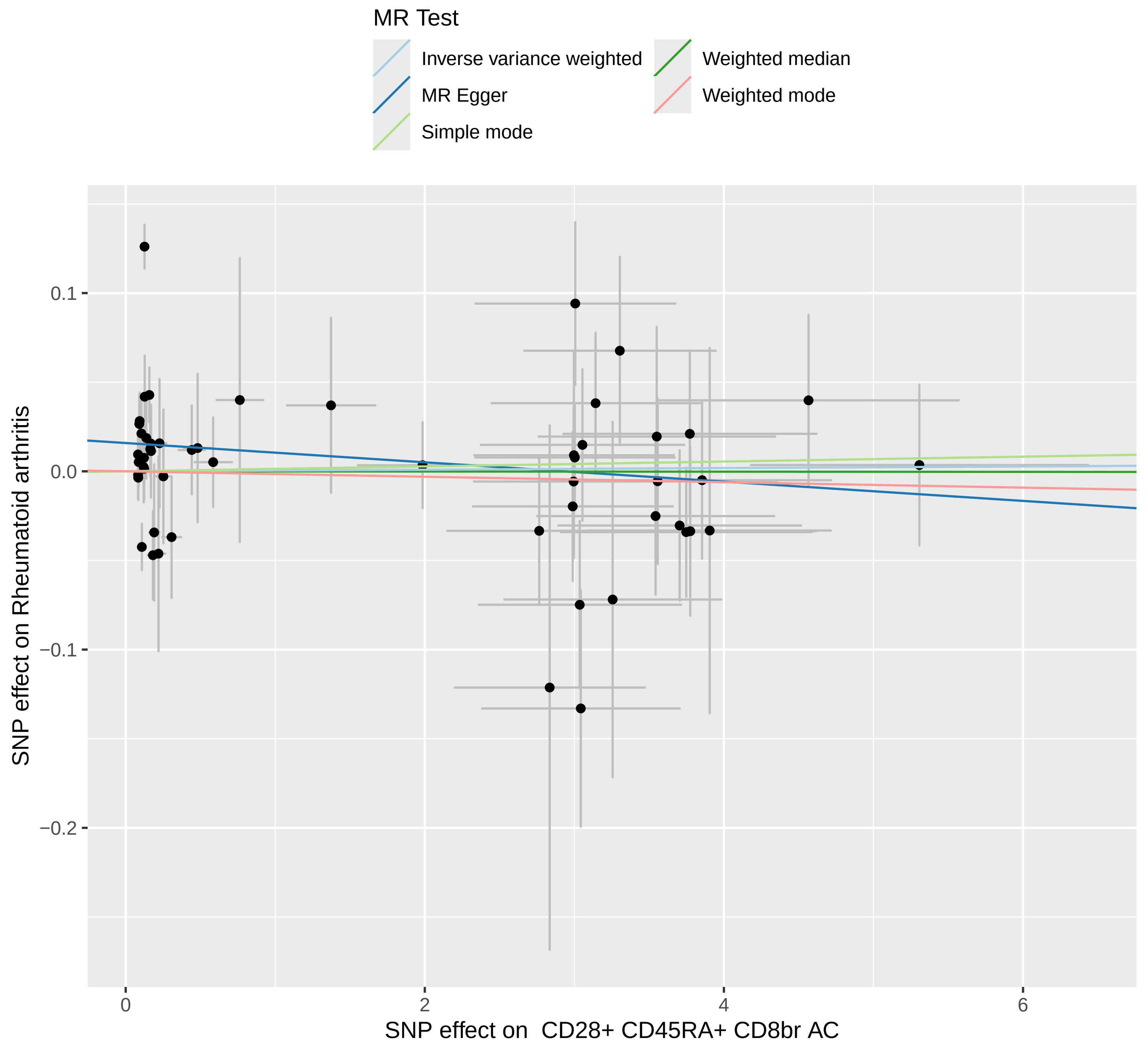
MR Test
Inverse variance weighted
MR Egger
Simple mode
Weighted median
Weighted mode
0.1
0.0
−0.1
−0.2
0
2
4
6
SNP effect on Rheumatoid arthritis
SNP effect on CD28+ CD45RA+ CD8br AC

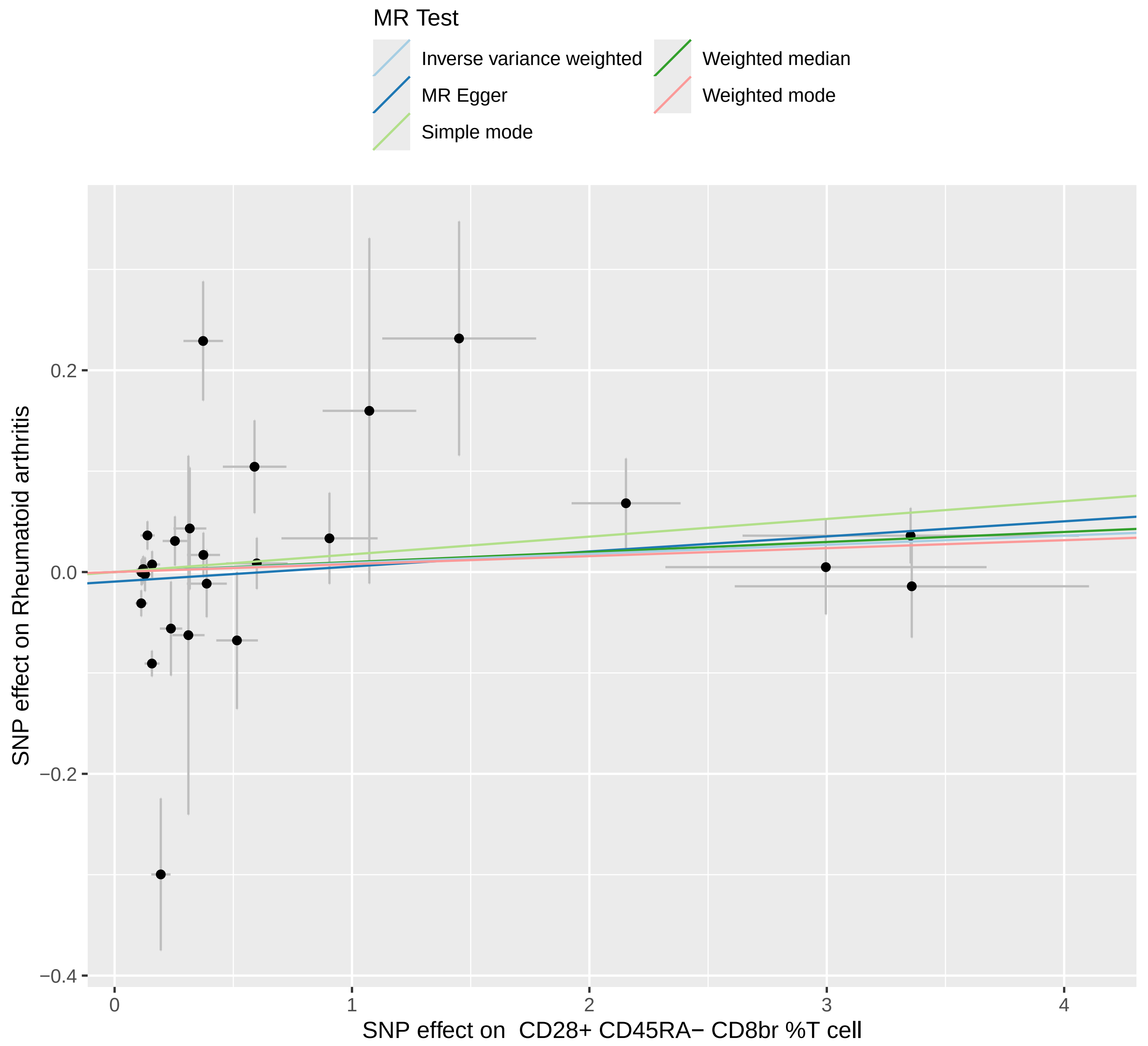

MR Test
Inverse variance weighted
MR Egger
Simple mode
Weighted median
Weighted mode
SNP effect on Rheumatoid arthritis
0.2
0.0
−0.2
−0.4
0
1
2
3
4
SNP effect on CD28+ CD45RA− CD8br %T cell

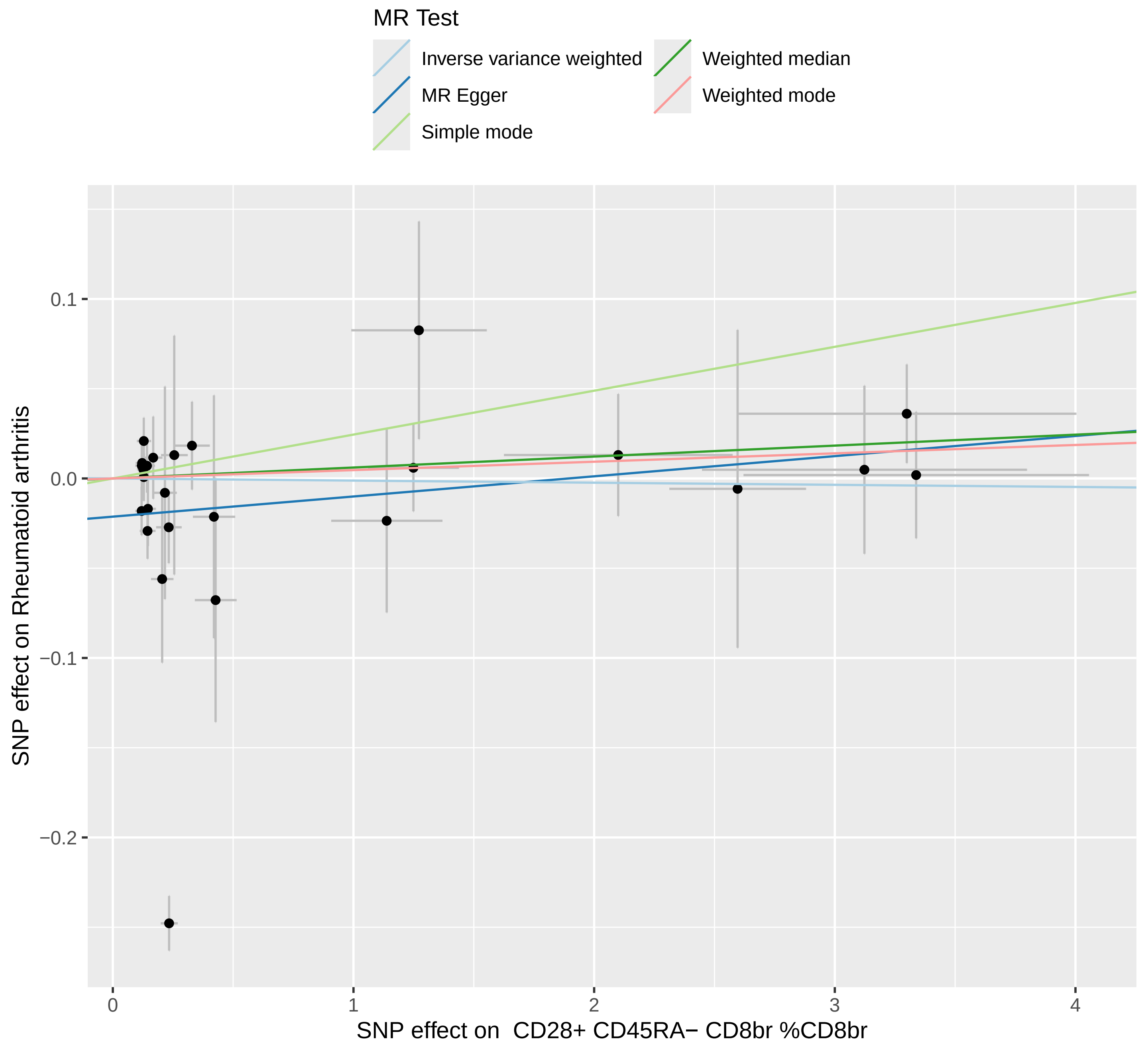
MR Test
Inverse variance weighted
MR Egger
Simple mode
Weighted median
Weighted mode
0.1
0.0
−0.1
−0.2
0
1
2
3
4
SNP effect on Rheumatoid arthritis
SNP effect on CD28+ CD45RA− CD8br %CD8br

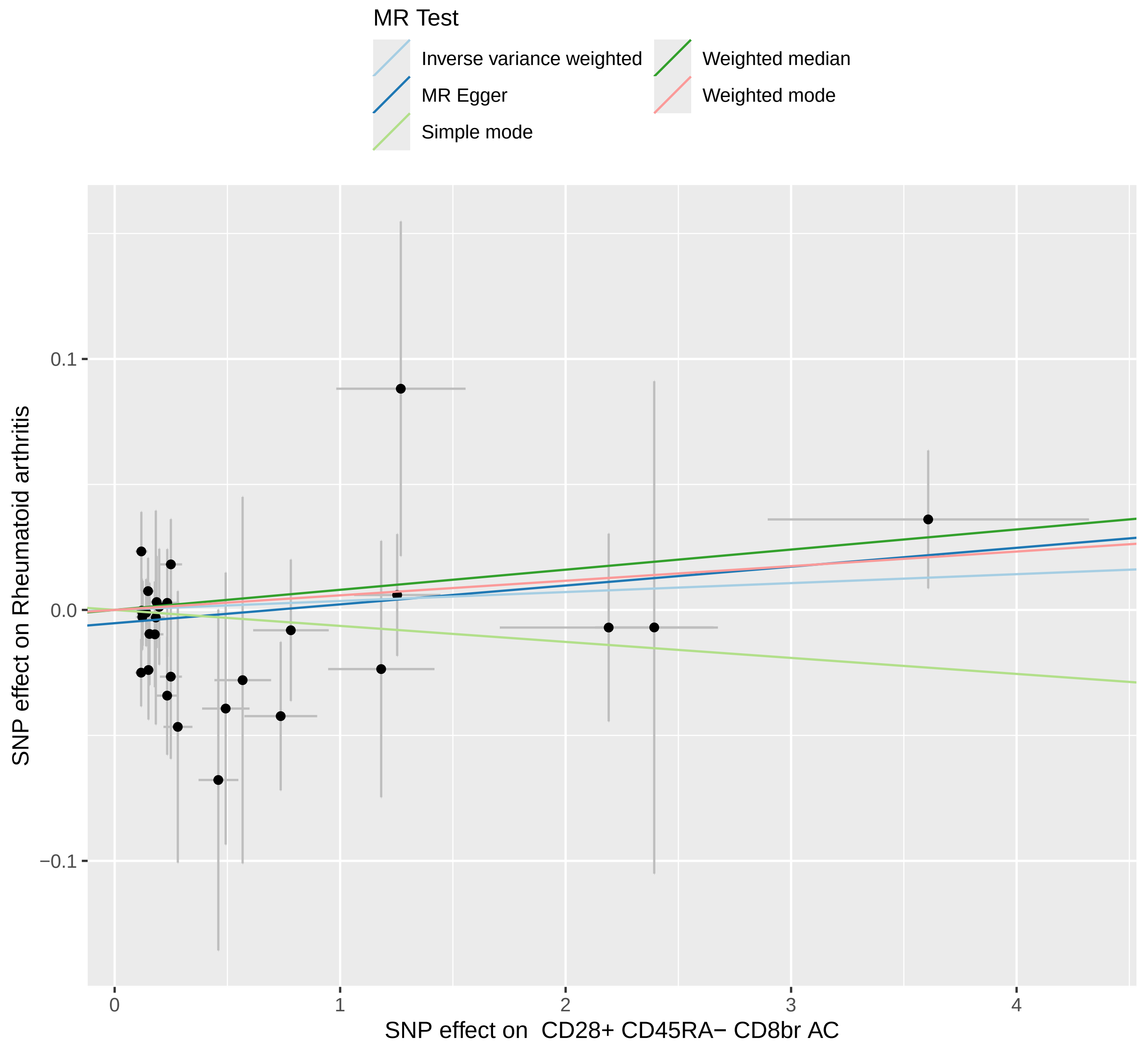

MR Test
Inverse variance weighted
MR Egger
Simple mode
Weighted median
Weighted mode
0.1
0.0
−0.1
0
1
2
3
4
SNP effect on Rheumatoid arthritis
SNP effect on CD28+ CD45RA− CD8br AC

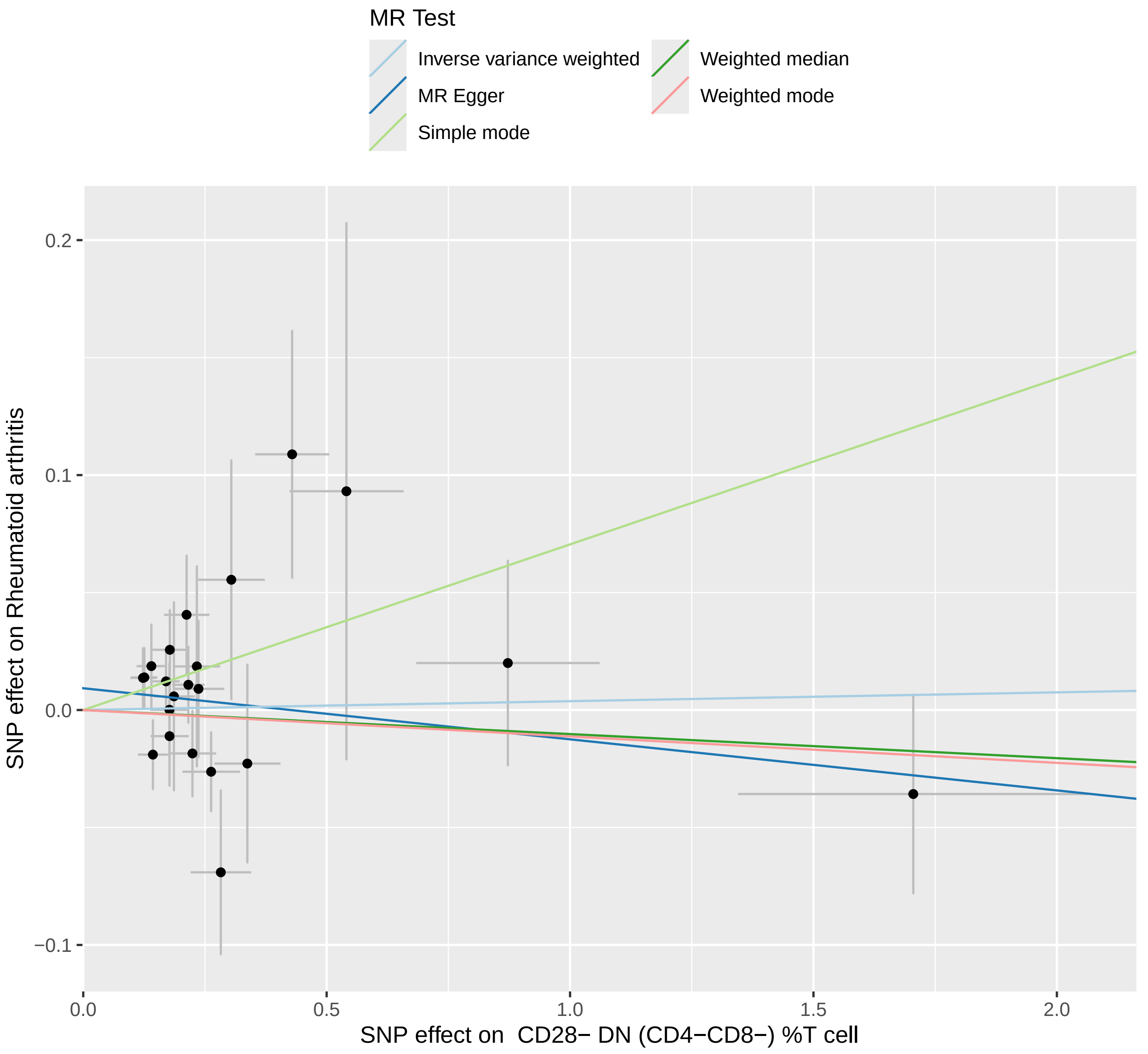

MR Test
Inverse variance weighted
MR Egger
Simple mode
Weighted median
Weighted mode
SNP effect on Rheumatoid arthritis
0.2
0.1
0.0
−0.1
0.0
0.5
1.0
1.5
2.0
SNP effect on CD28− DN (CD4−CD8−) %T cell

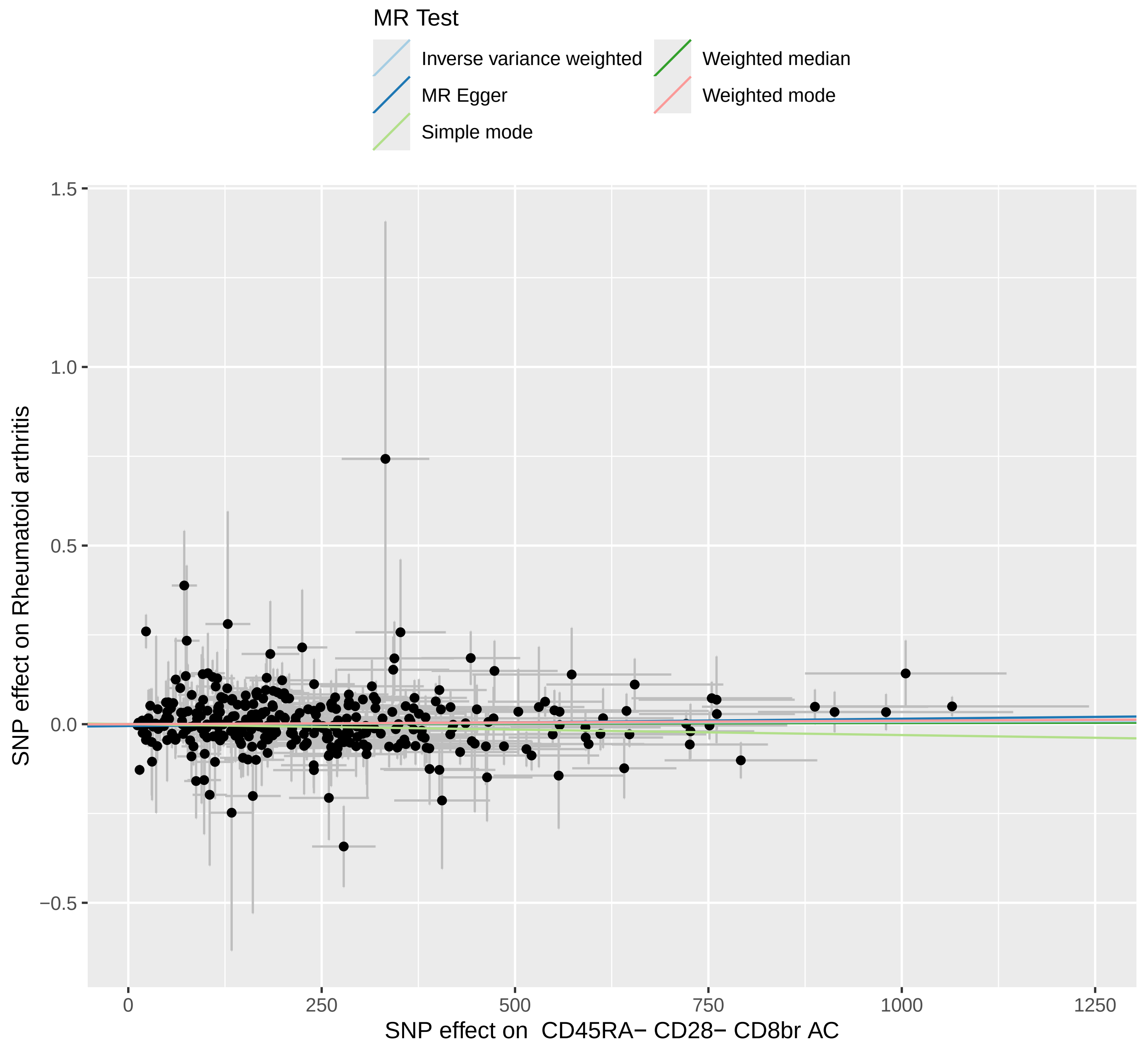
MR Test
Inverse variance weighted
MR Egger
Simple mode
Weighted median
Weighted mode
1.5
1.0
0.5
0.0
−0.5
SNP effect on Rheumatoid arthritis
0
250
500
750
1000
1250
SNP effect on CD45RA− CD28− CD8br AC

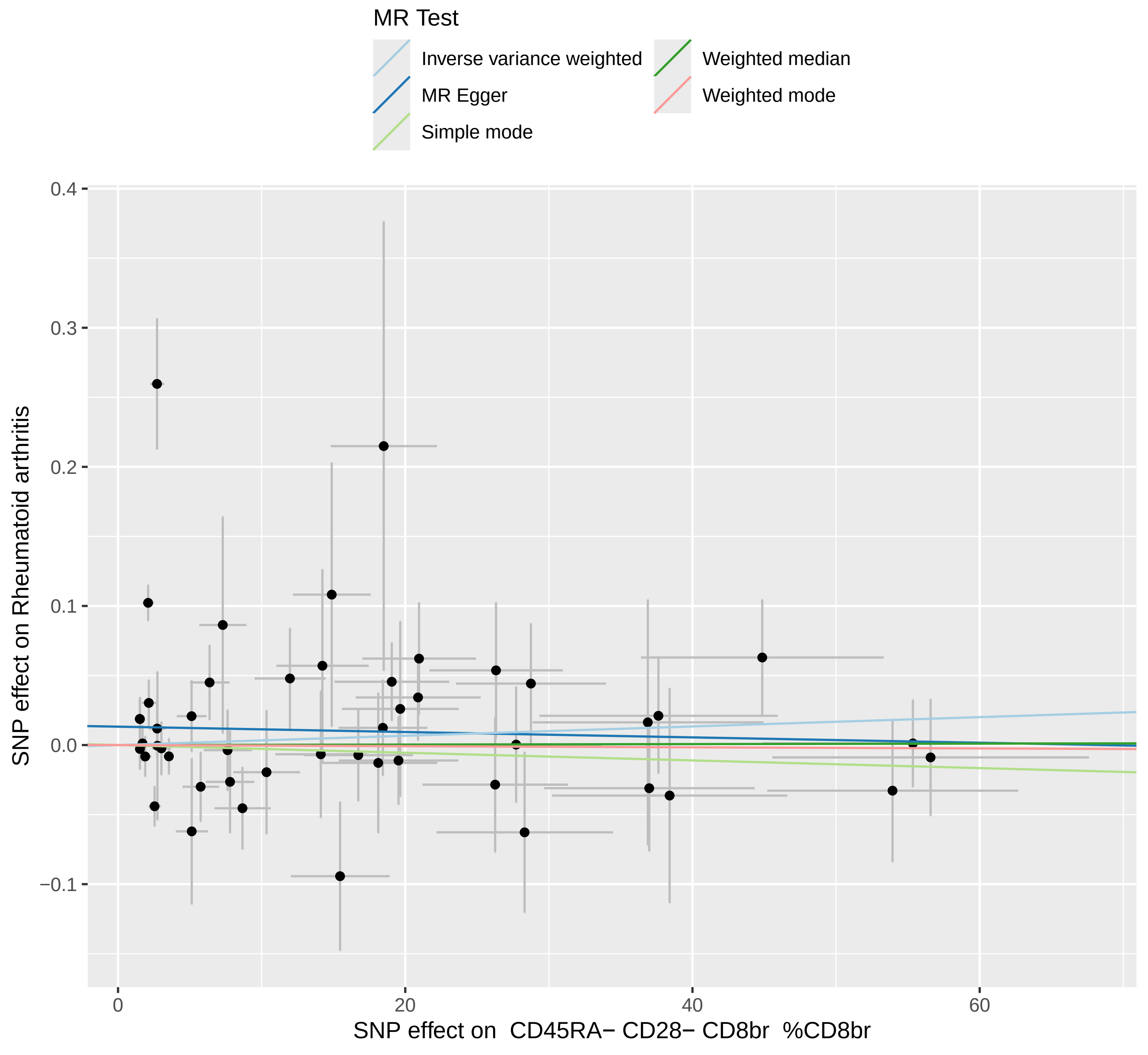
MR Test
Inverse variance weighted
MR Egger
Simple mode
Weighted median
Weighted mode
0.4
0.3
0.2
0.1
0.0
−0.1
SNP effect on Rheumatoid arthritis
0
20
40
60
SNP effect on CD45RA− CD28− CD8br %CD8br

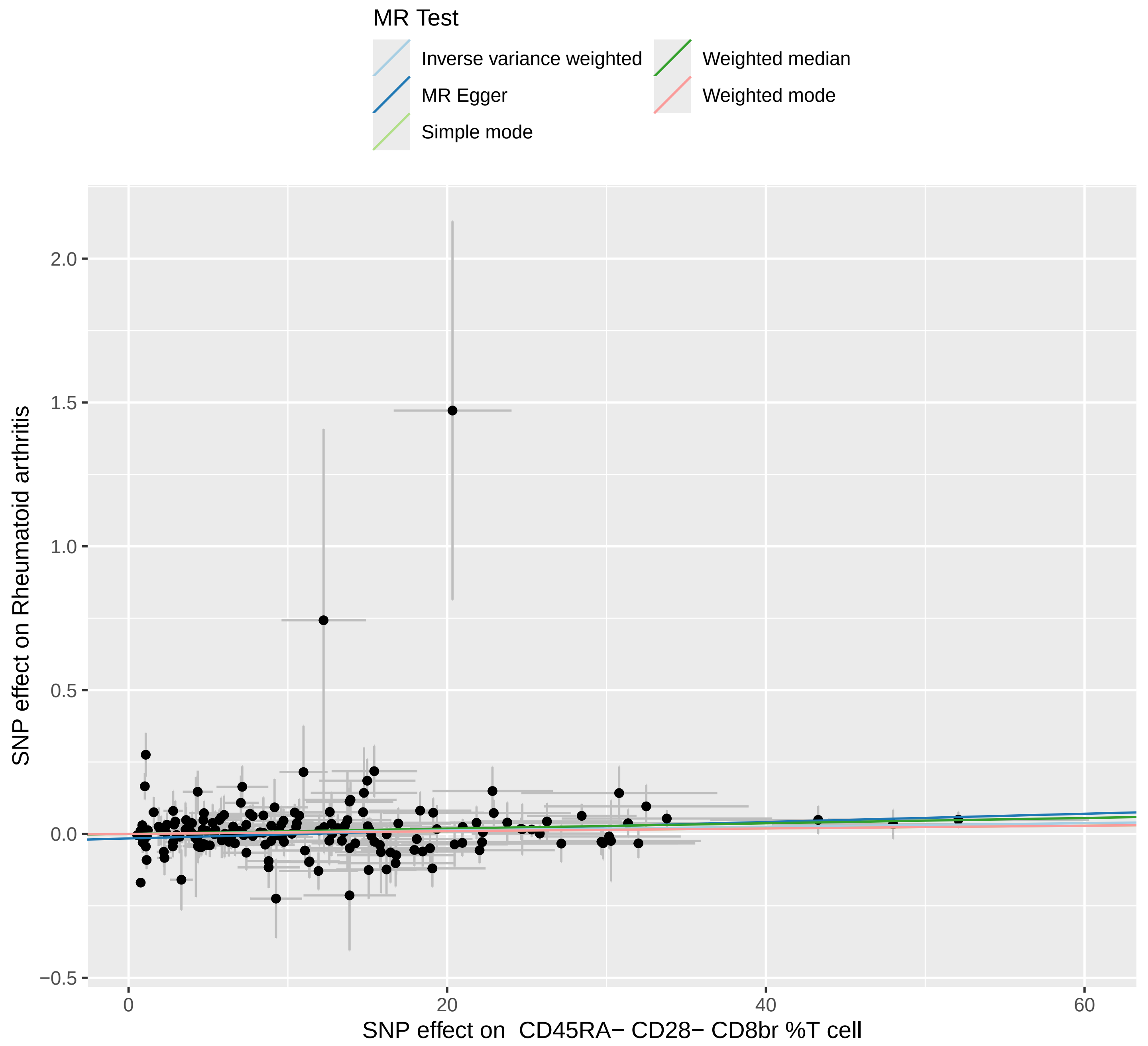
MR Test
Inverse variance weighted
MR Egger
Simple mode
Weighted median
Weighted mode
SNP effect on Rheumatoid arthritis
2.0
1.5
1.0
0.5
0.0
−0.5
0
20
40
60
SNP effect on CD45RA− CD28− CD8br %T cell

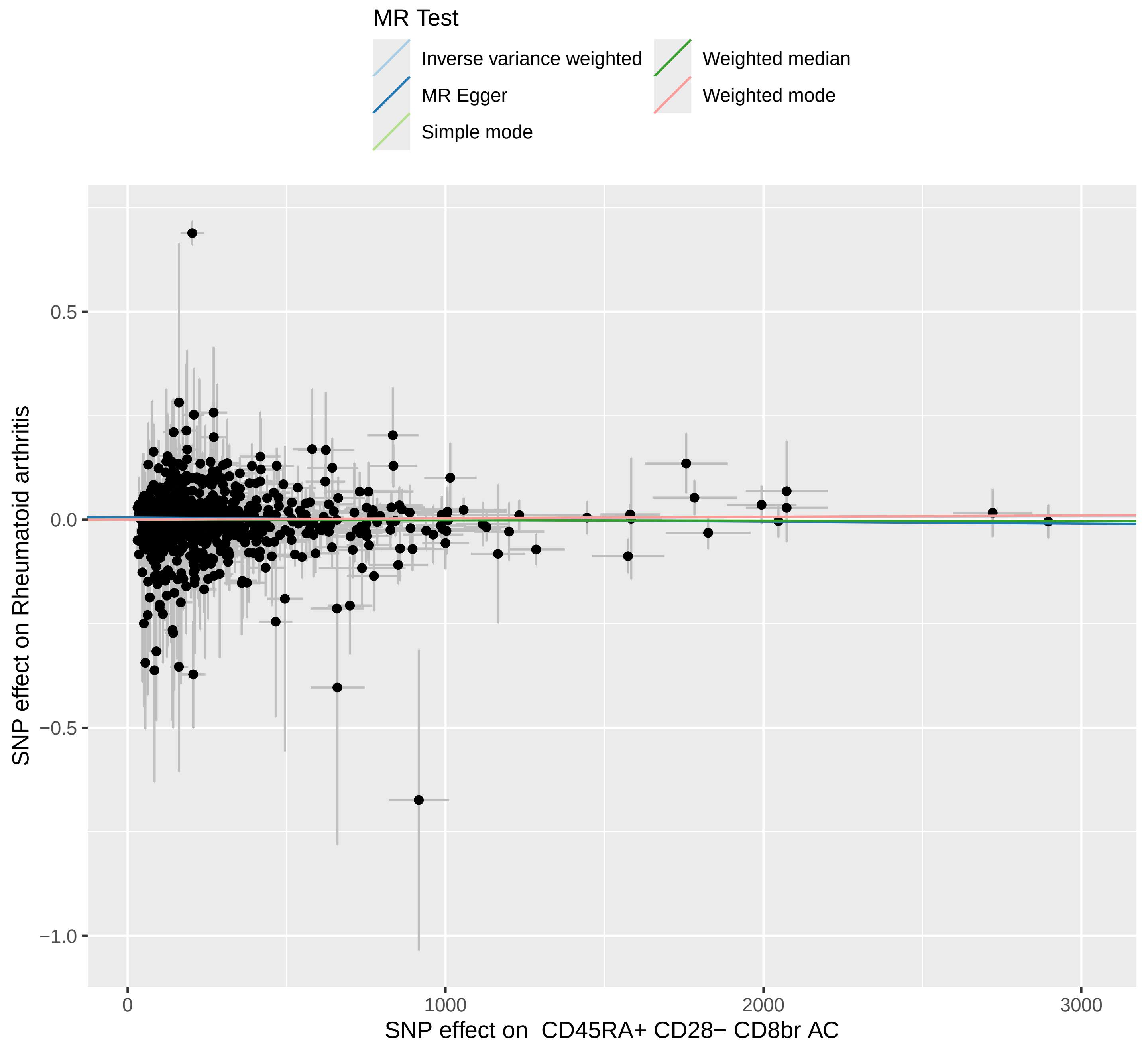

MR Test
Inverse variance weighted
MR Egger
Simple mode
Weighted median
Weighted mode
0.5
0.0
−0.5
−1.0
0
1000
2000
3000
SNP effect on Rheumatoid arthritis
SNP effect on CD45RA+ CD28− CD8br AC

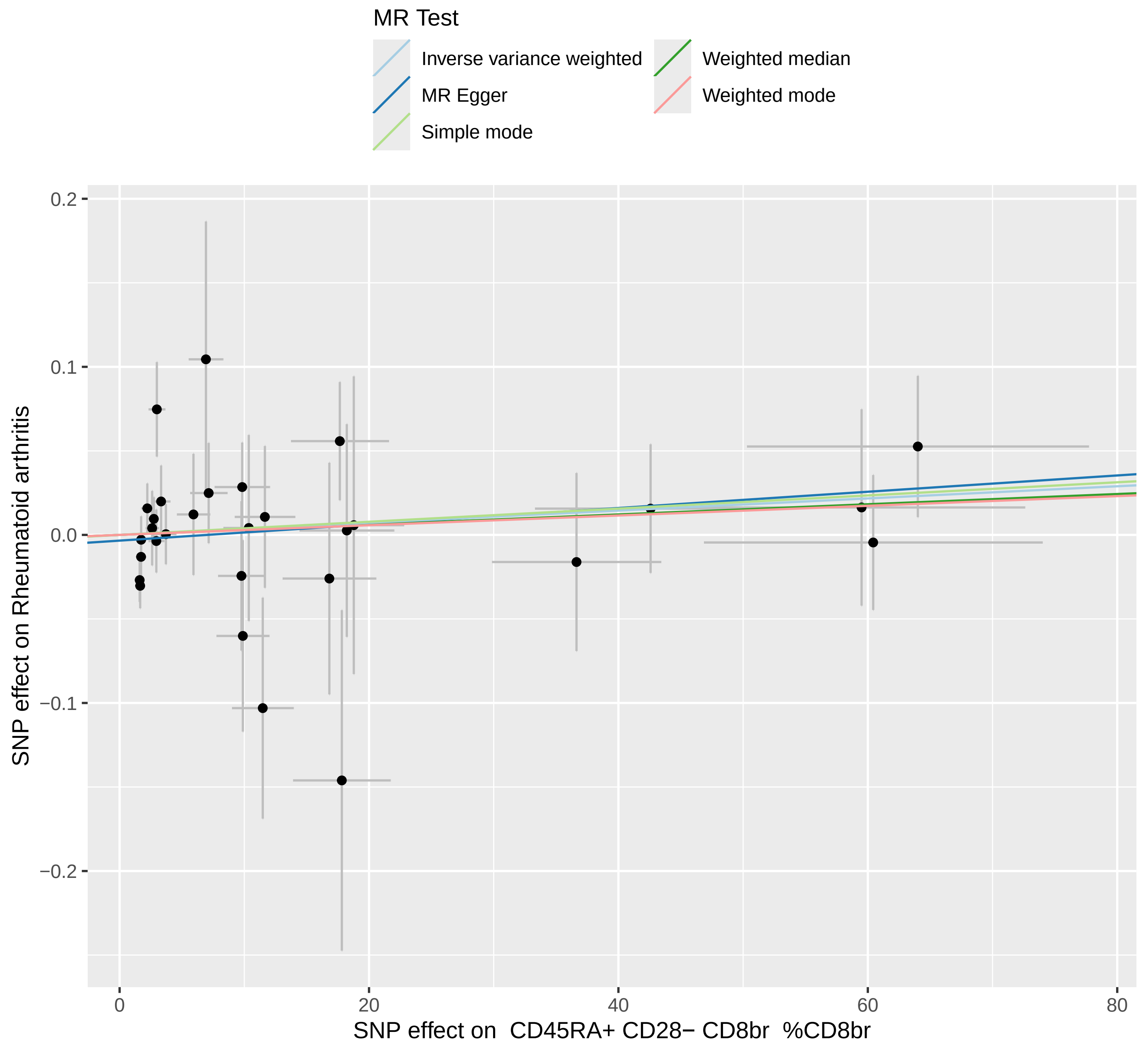

MR Test
Inverse variance weighted
MR Egger
Simple mode
Weighted median
Weighted mode
0.2
0.1
0.0
−0.1
−0.2
SNP effect on Rheumatoid arthritis
0
20
40
60
80
SNP effect on CD45RA+ CD28− CD8br %CD8br

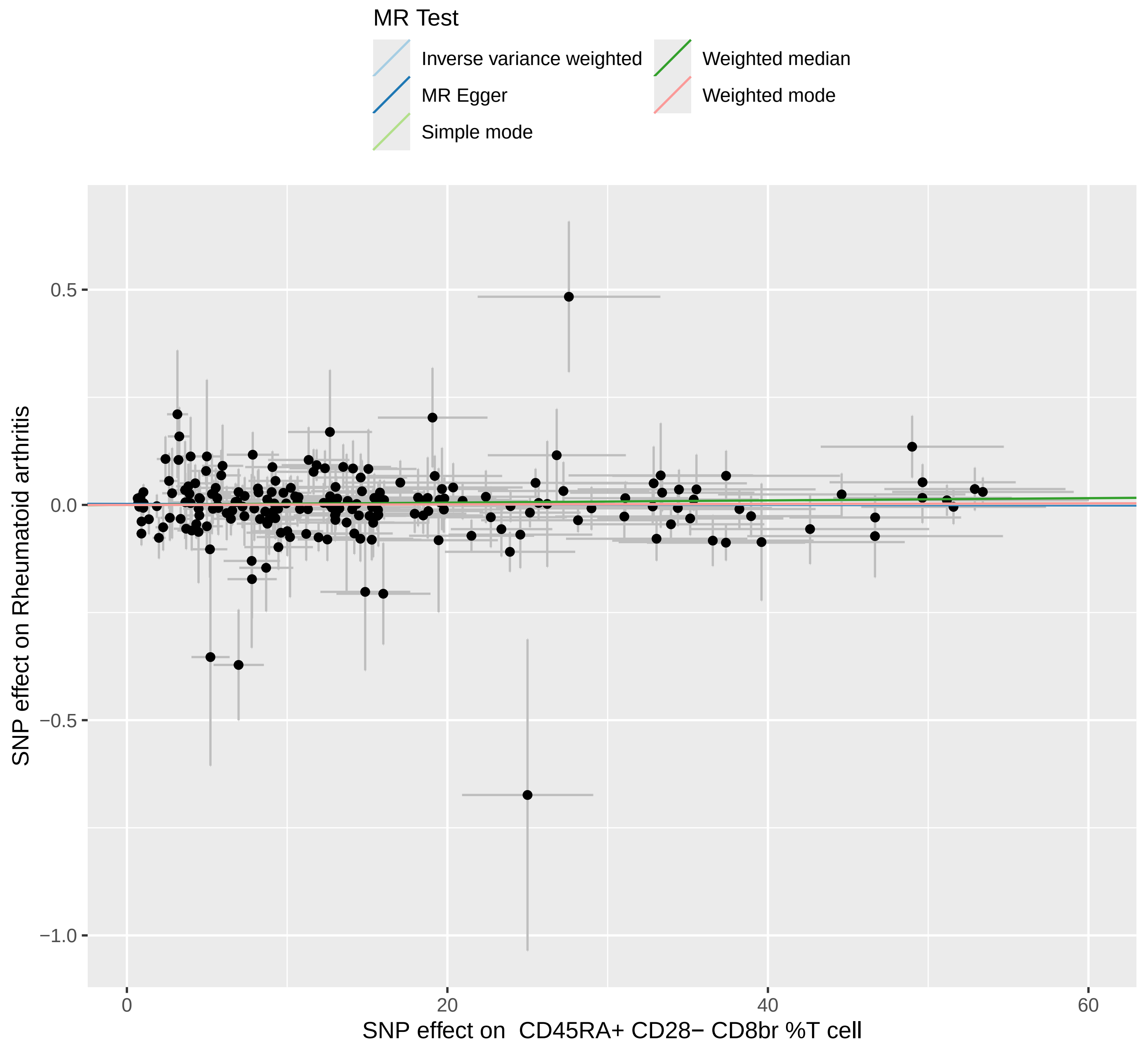

MR Test
Inverse variance weighted
MR Egger
Simple mode
Weighted median
Weighted mode
0.5
0.0
−0.5
−1.0
0
20
40
60
SNP effect on Rheumatoid arthritis
SNP effect on CD45RA+ CD28− CD8br %T cell

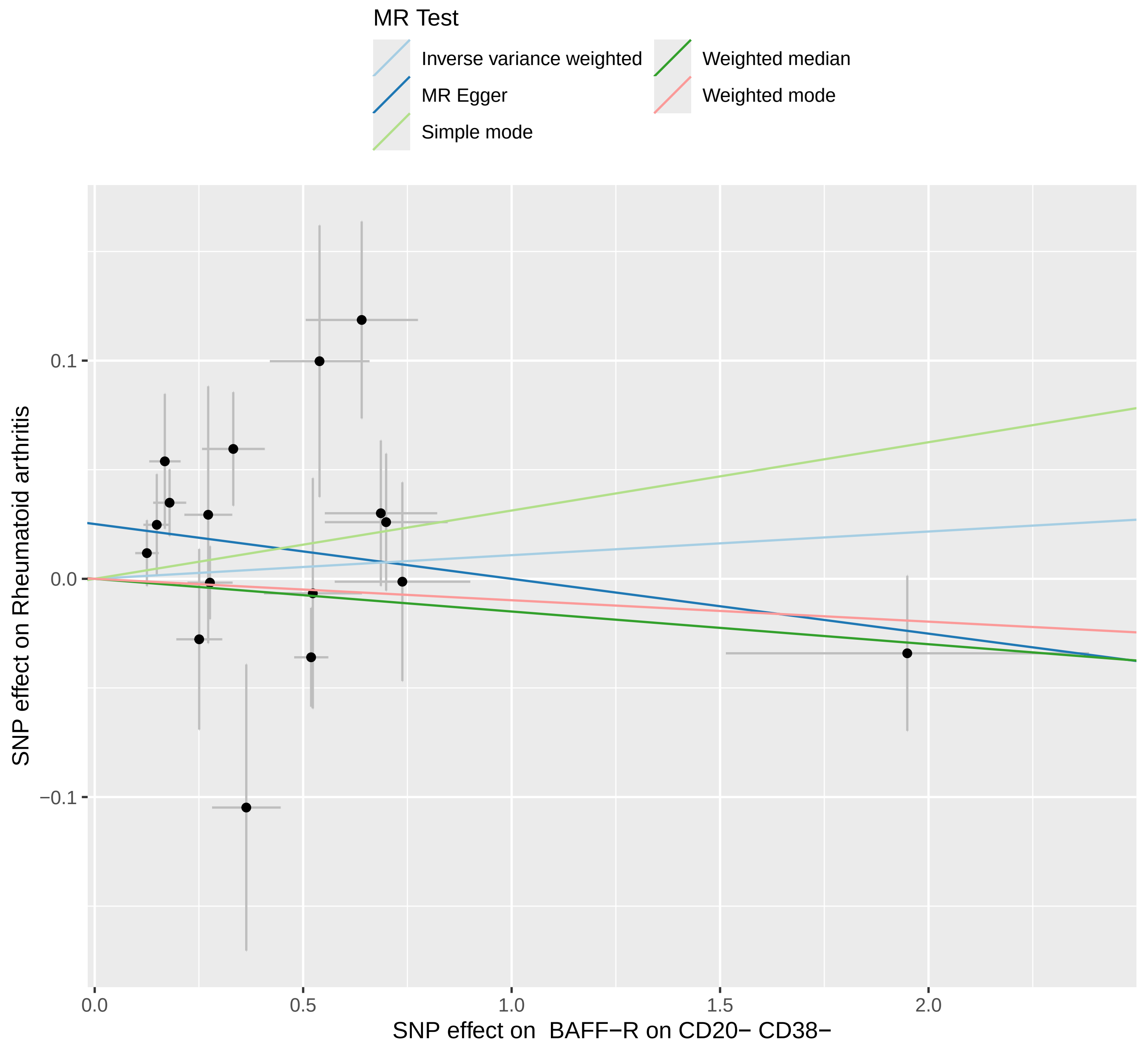

MR Test
Inverse variance weighted
MR Egger
Simple mode
Weighted median
Weighted mode
0.1
0.0
−0.1
SNP effect on Rheumatoid arthritis
0.0
0.5
1.0
1.5
2.0
SNP effect on BAFF−R on CD20− CD38−

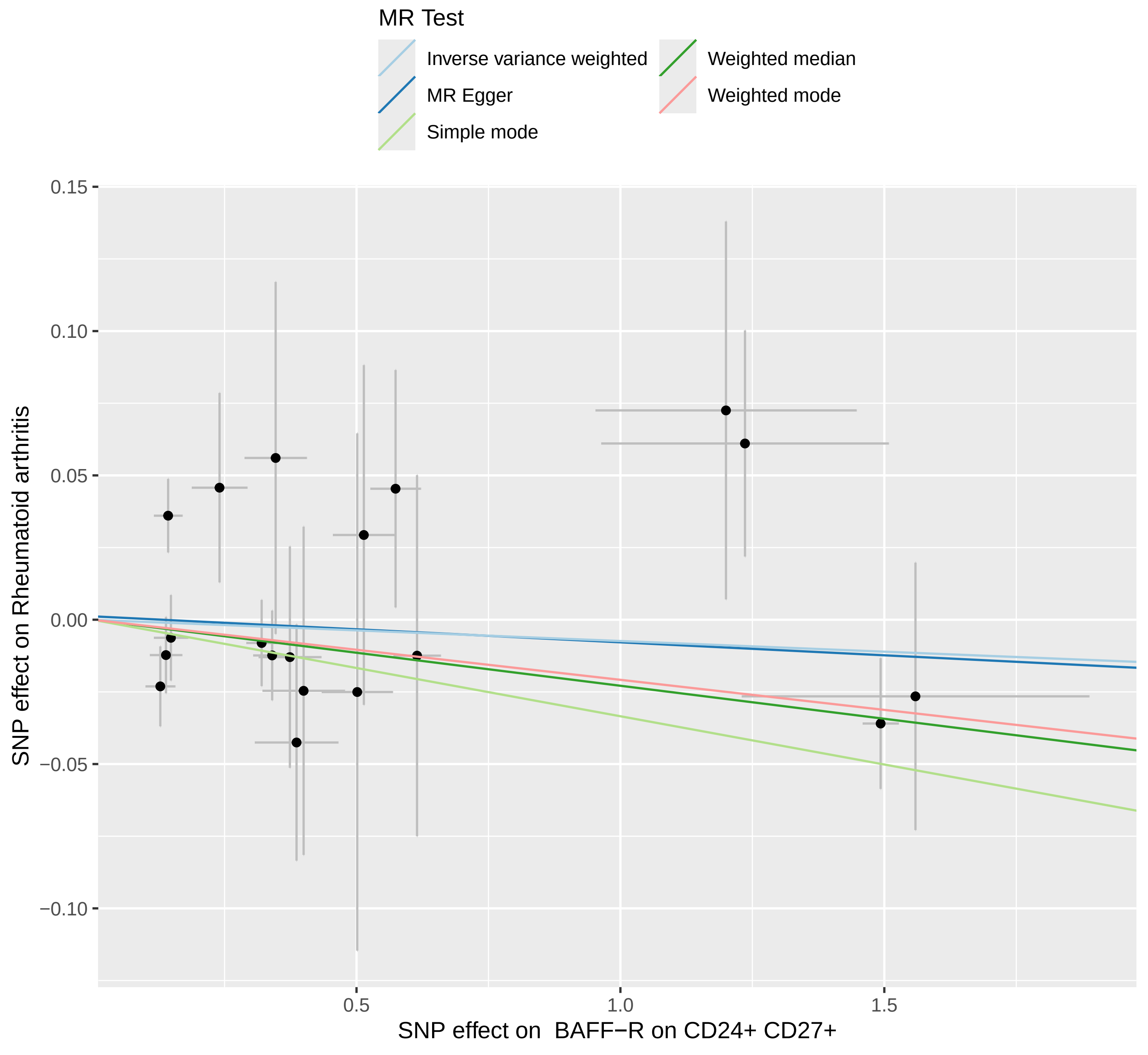
MR Test
Inverse variance weighted
MR Egger
Simple mode
Weighted median
Weighted mode
0.15
0.10
0.05
0.00
−0.05
−0.10
SNP effect on Rheumatoid arthritis
0.5
1.0
1.5
SNP effect on BAFF−R on CD24+ CD27+

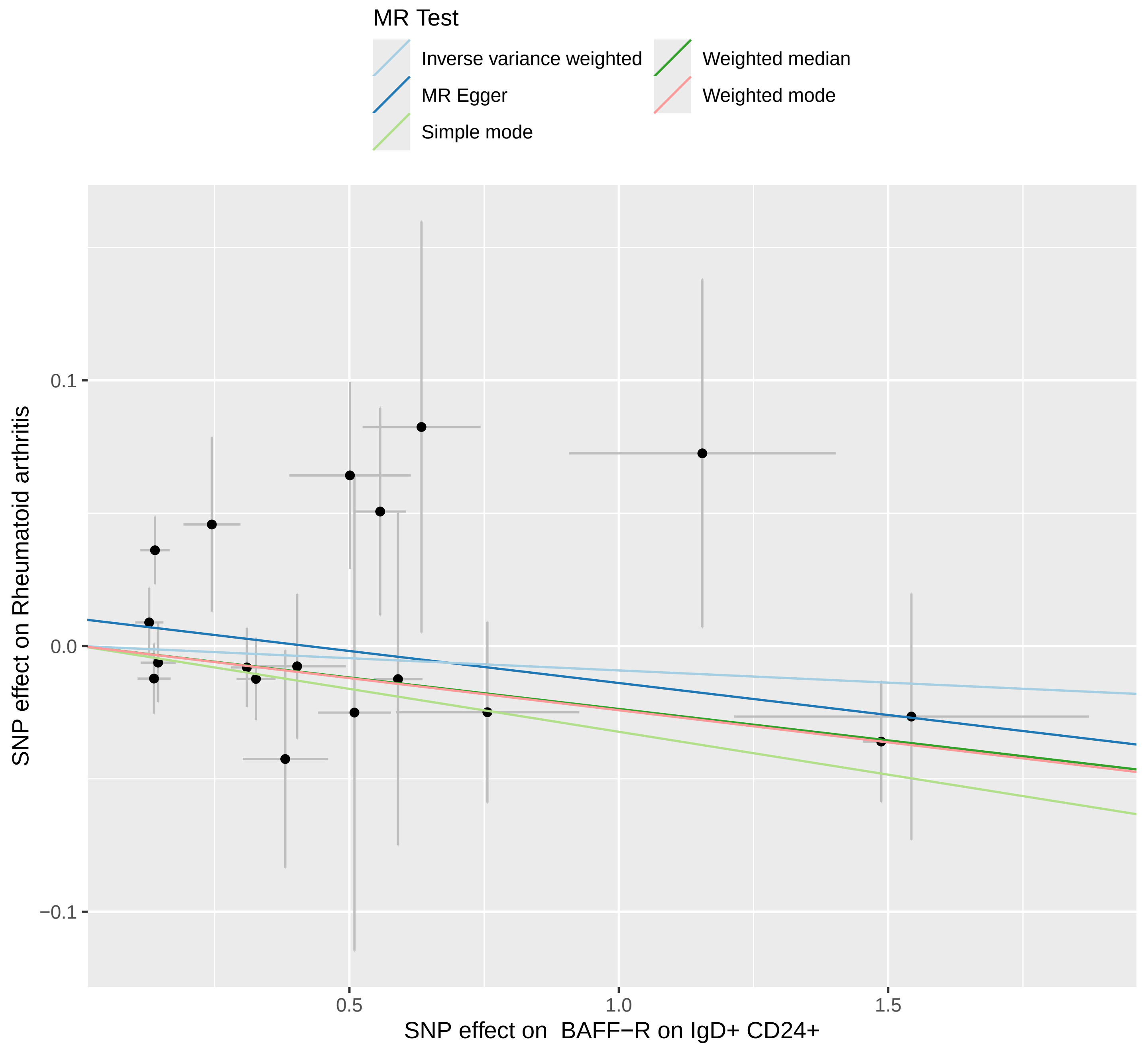
MR Test
Inverse variance weighted
MR Egger
Simple mode
Weighted median
Weighted mode
0.1
0.0
−0.1
0.5
1.0
1.5
SNP effect on Rheumatoid arthritis
SNP effect on BAFF−R on IgD+ CD24+

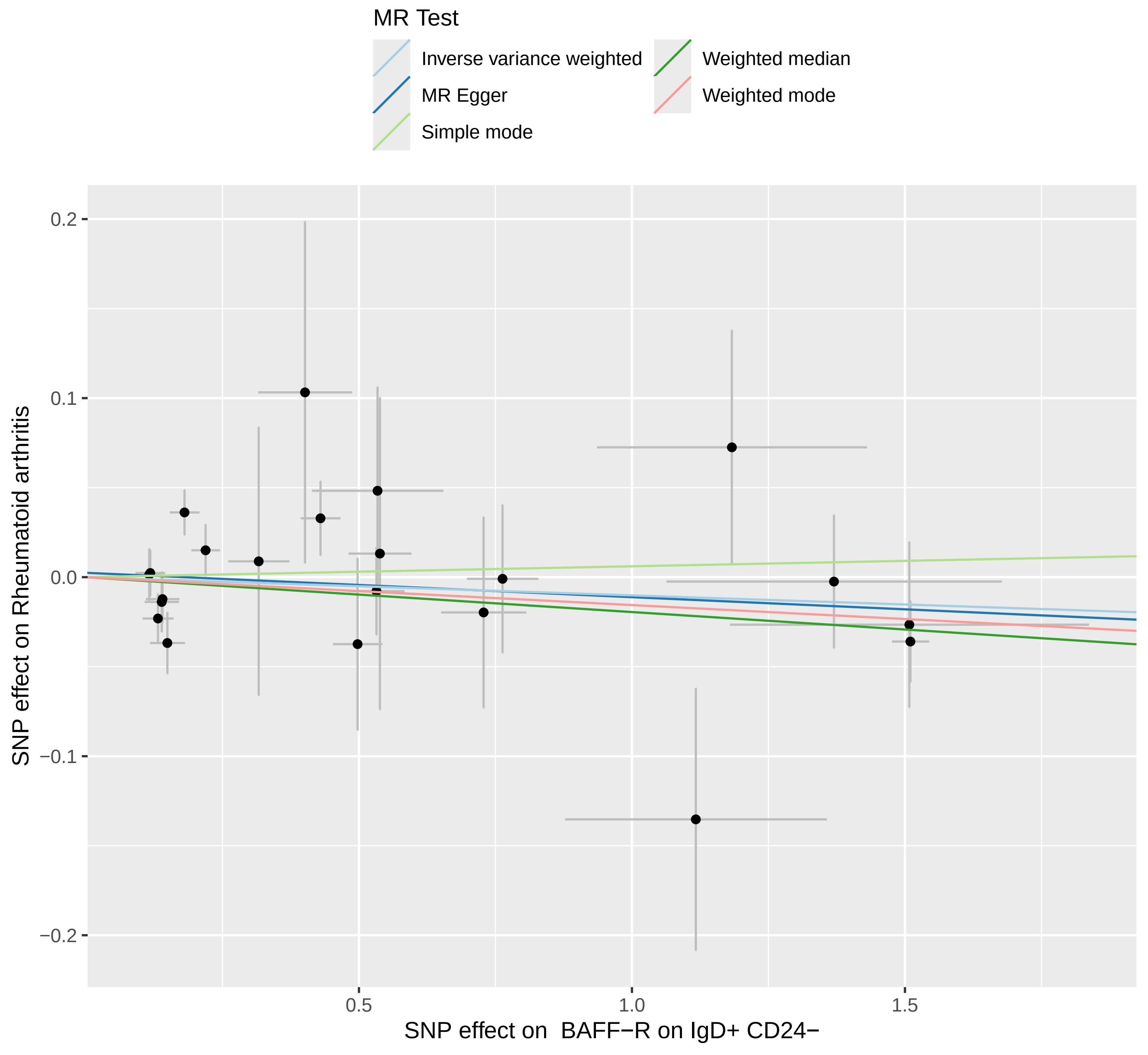
MR Test
Inverse variance weighted
MR Egger
Simple mode
Weighted median
Weighted mode
0.2
0.1
0.0
−0.1
−0.2
0.5
1.0
1.5
SNP effect on Rheumatoid arthritis
SNP effect on BAFF−R on IgD+ CD24−

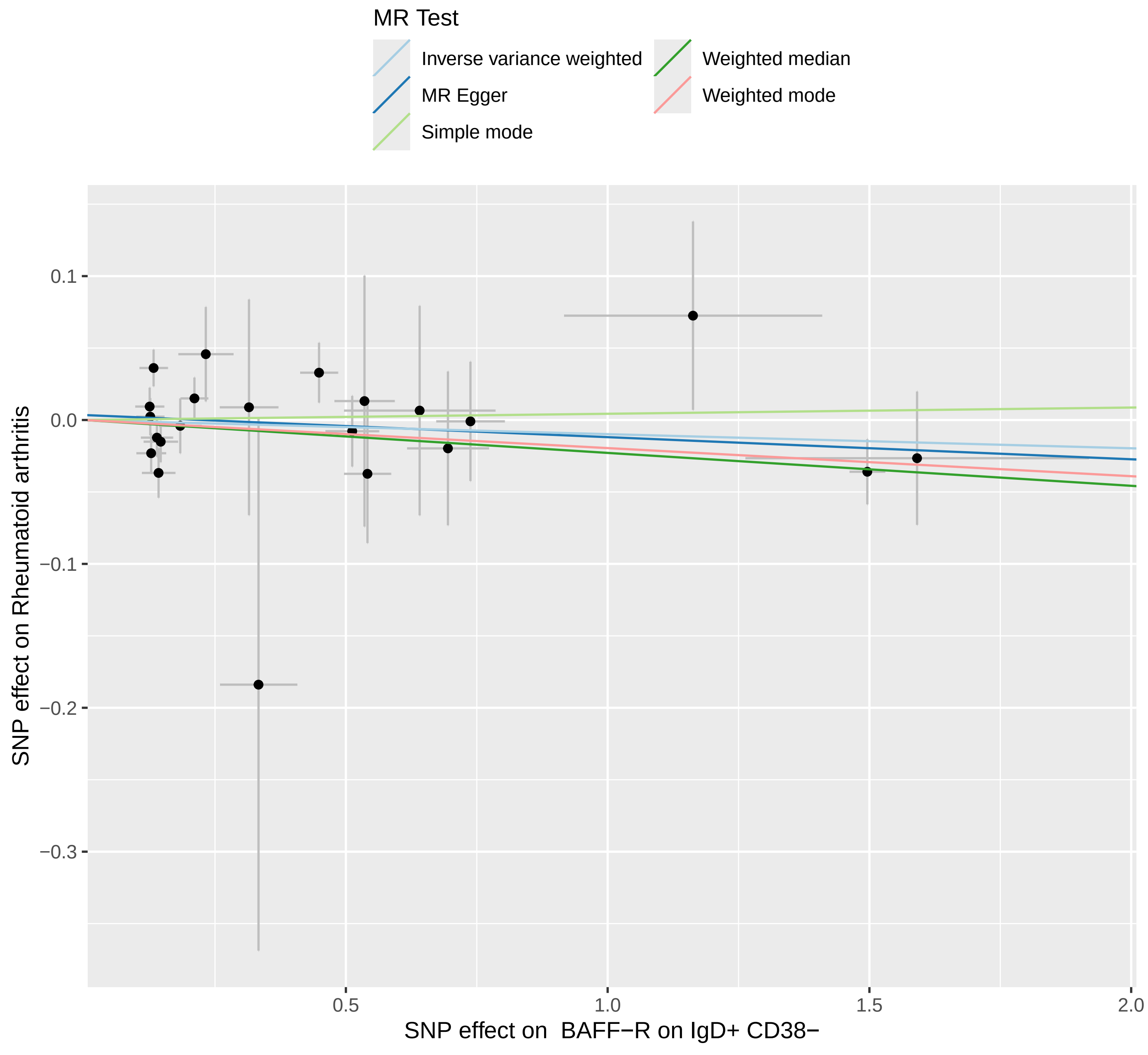

MR Test
Inverse variance weighted
MR Egger
Simple mode
Weighted median
Weighted mode
0.1
0.0
−0.1
−0.2
−0.3
SNP effect on Rheumatoid arthritis
0.5
1.0
1.5
2.0
SNP effect on BAFF−R on IgD+ CD38−

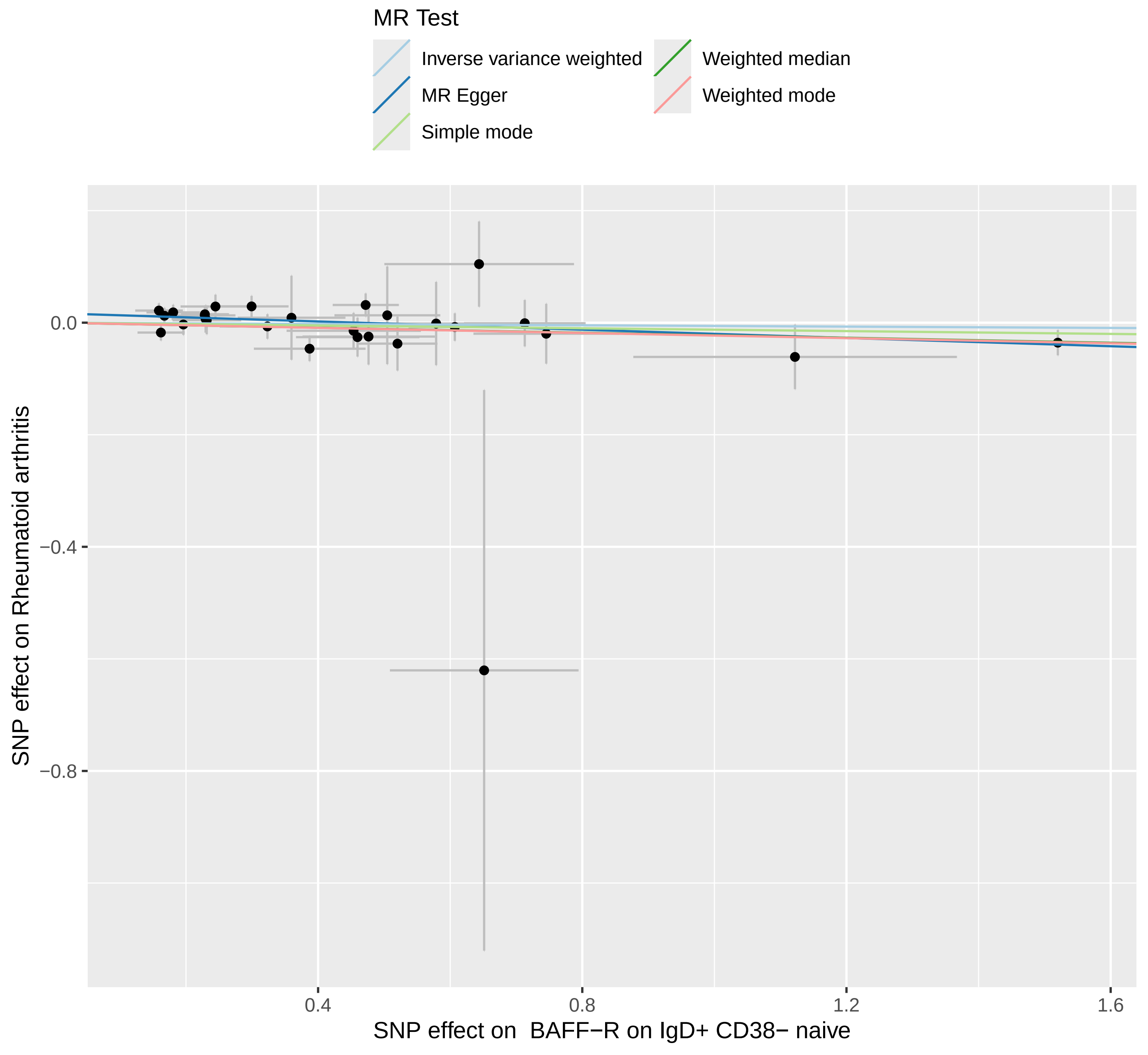

MR Test
Inverse variance weighted
MR Egger
Simple mode
Weighted median
Weighted mode
0.0
−0.4
−0.8
0.4
0.8
1.2
1.6
SNP effect on Rheumatoid arthritis
SNP effect on BAFF−R on IgD+ CD38− naive

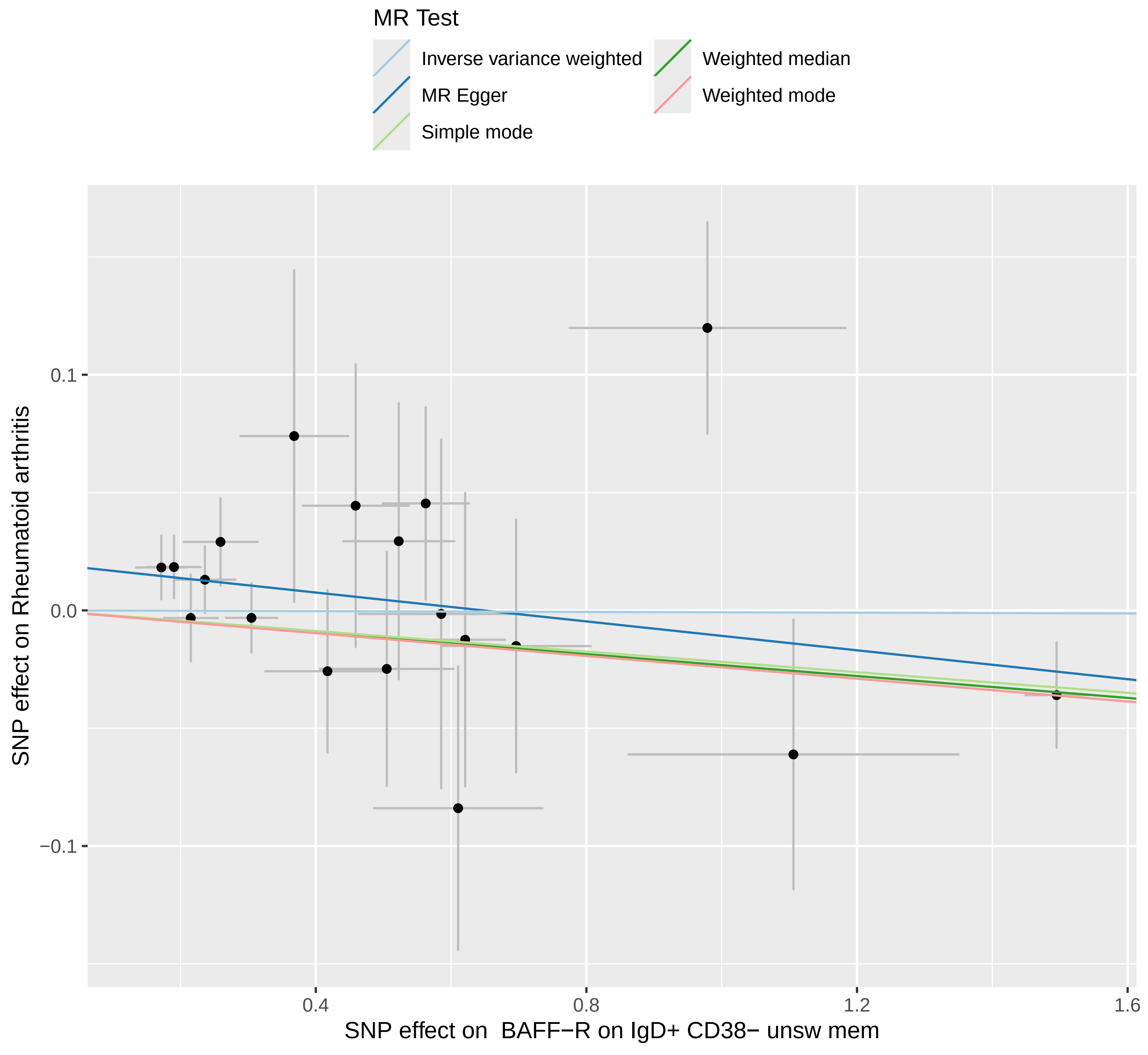

MR Test
Inverse variance weighted
MR Egger
Simple mode
Weighted median
Weighted mode
0.1
0.0
−0.1
0.4
0.8
1.2
1.6
SNP effect on Rheumatoid arthritis
SNP effect on BAFF−R on IgD+ CD38− unsw mem

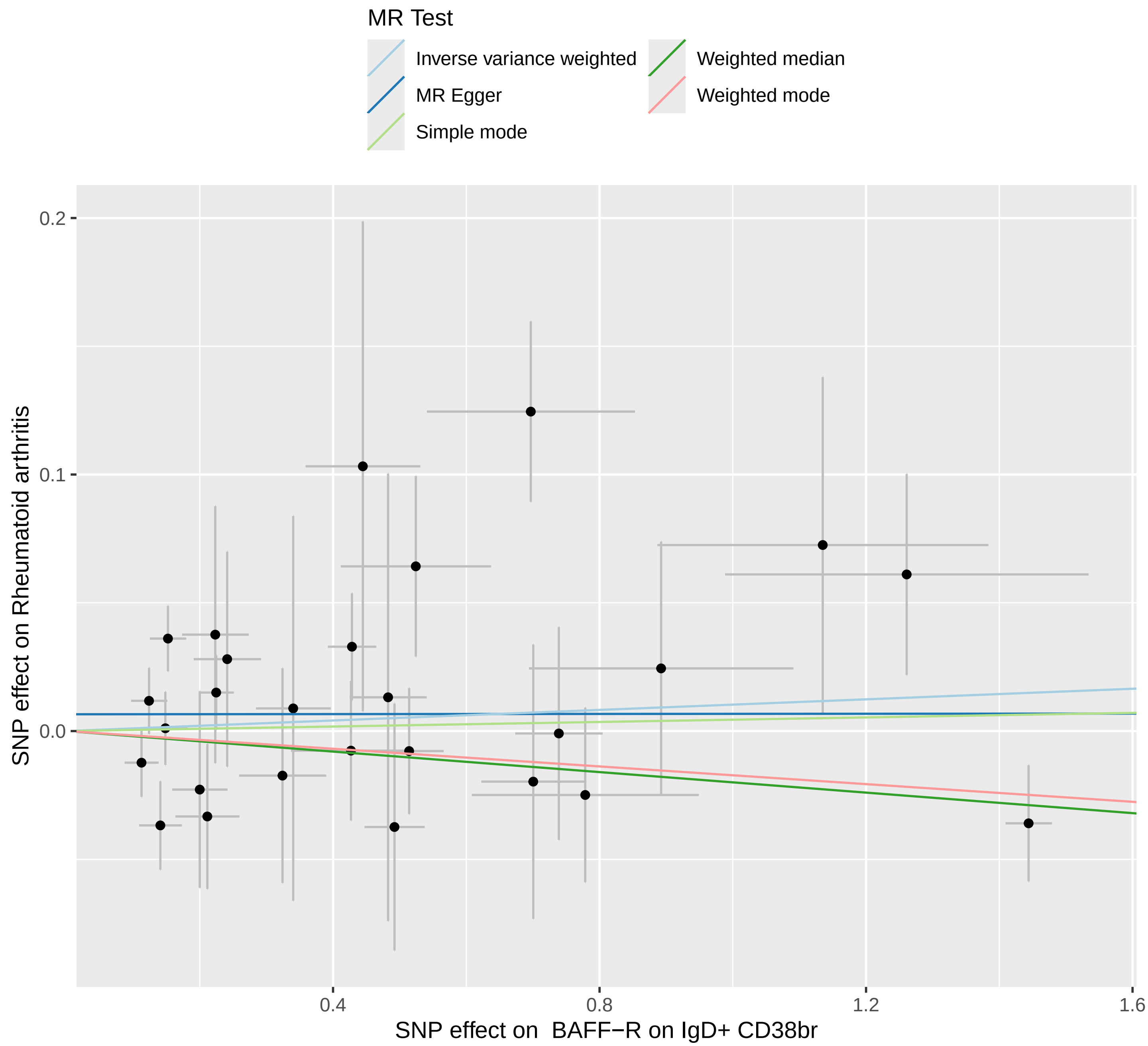
MR Test
Inverse variance weighted
MR Egger
Simple mode
Weighted median
Weighted mode
0.2
0.1
0.0
0.4
0.8
1.2
1.6
SNP effect on Rheumatoid arthritis
SNP effect on BAFF−R on IgD+ CD38br

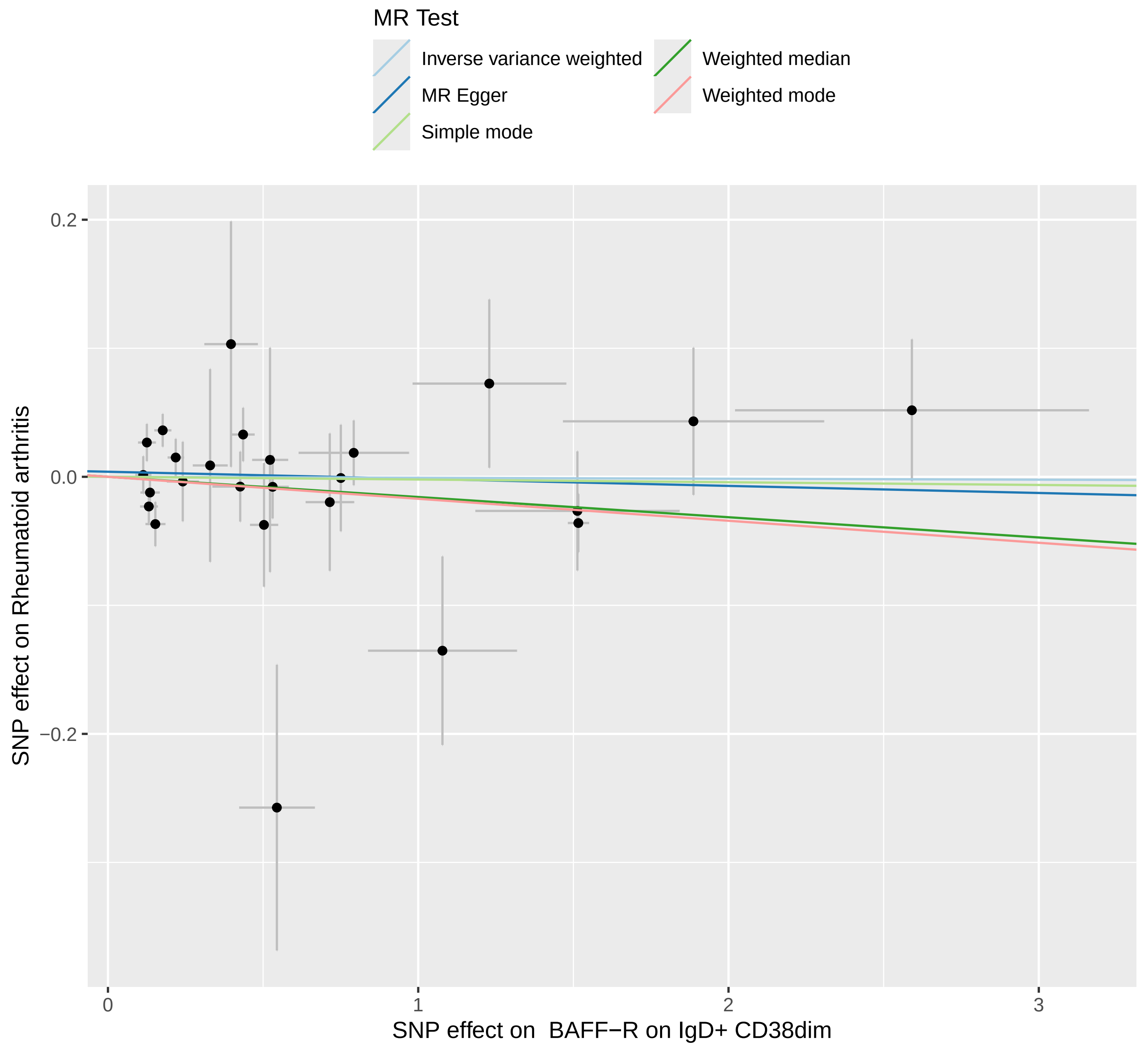

MR Test
Inverse variance weighted
MR Egger
Simple mode
Weighted median
Weighted mode
0.2
0.0
−0.2
0
1
2
3
SNP effect on Rheumatoid arthritis
SNP effect on BAFF−R on IgD+ CD38dim

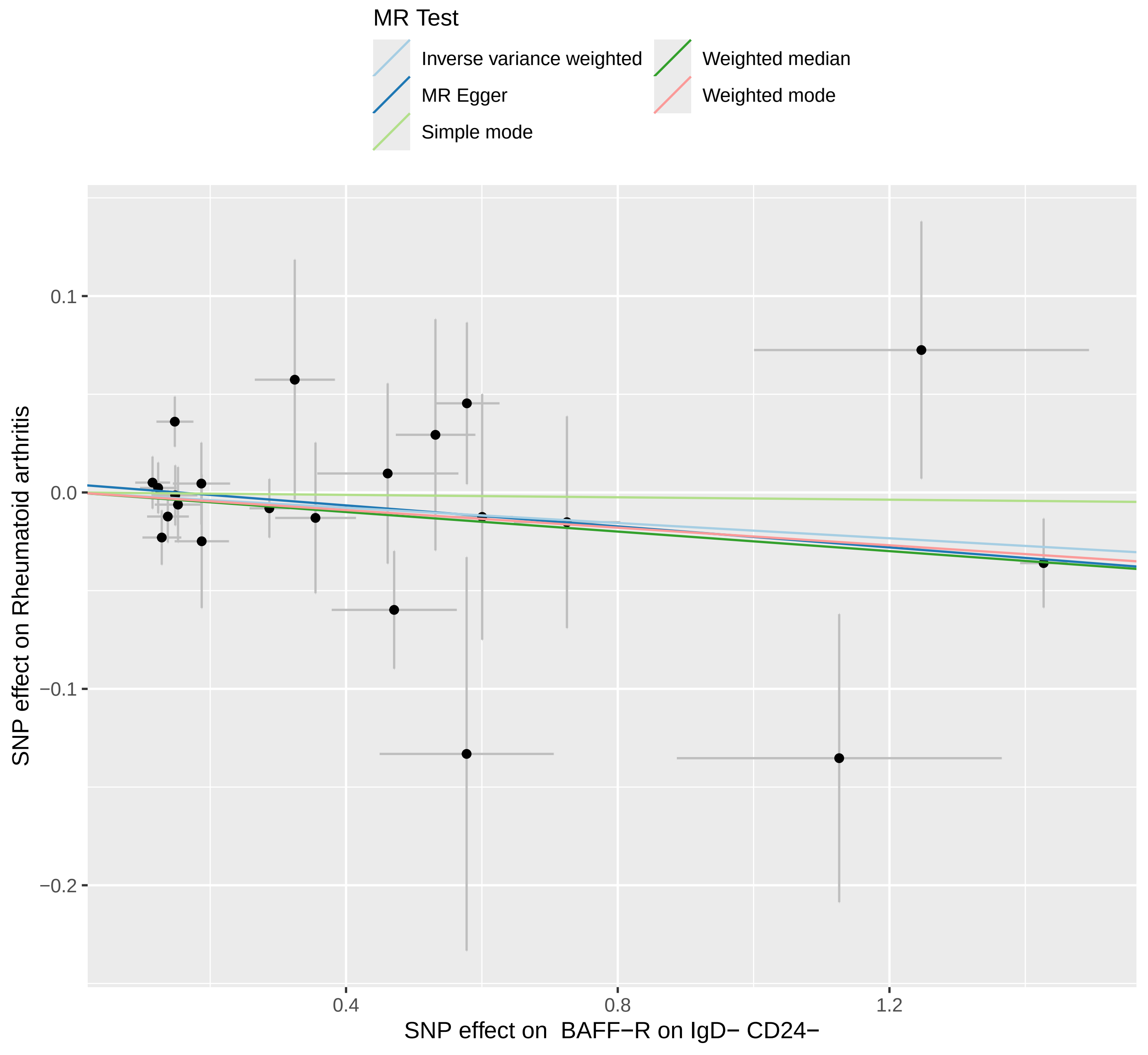
MR Test
Inverse variance weighted
MR Egger
Simple mode
Weighted median
Weighted mode
0.1
0.0
−0.1
−0.2
0.4
0.8
1.2
SNP effect on Rheumatoid arthritis
SNP effect on BAFF−R on IgD− CD24−

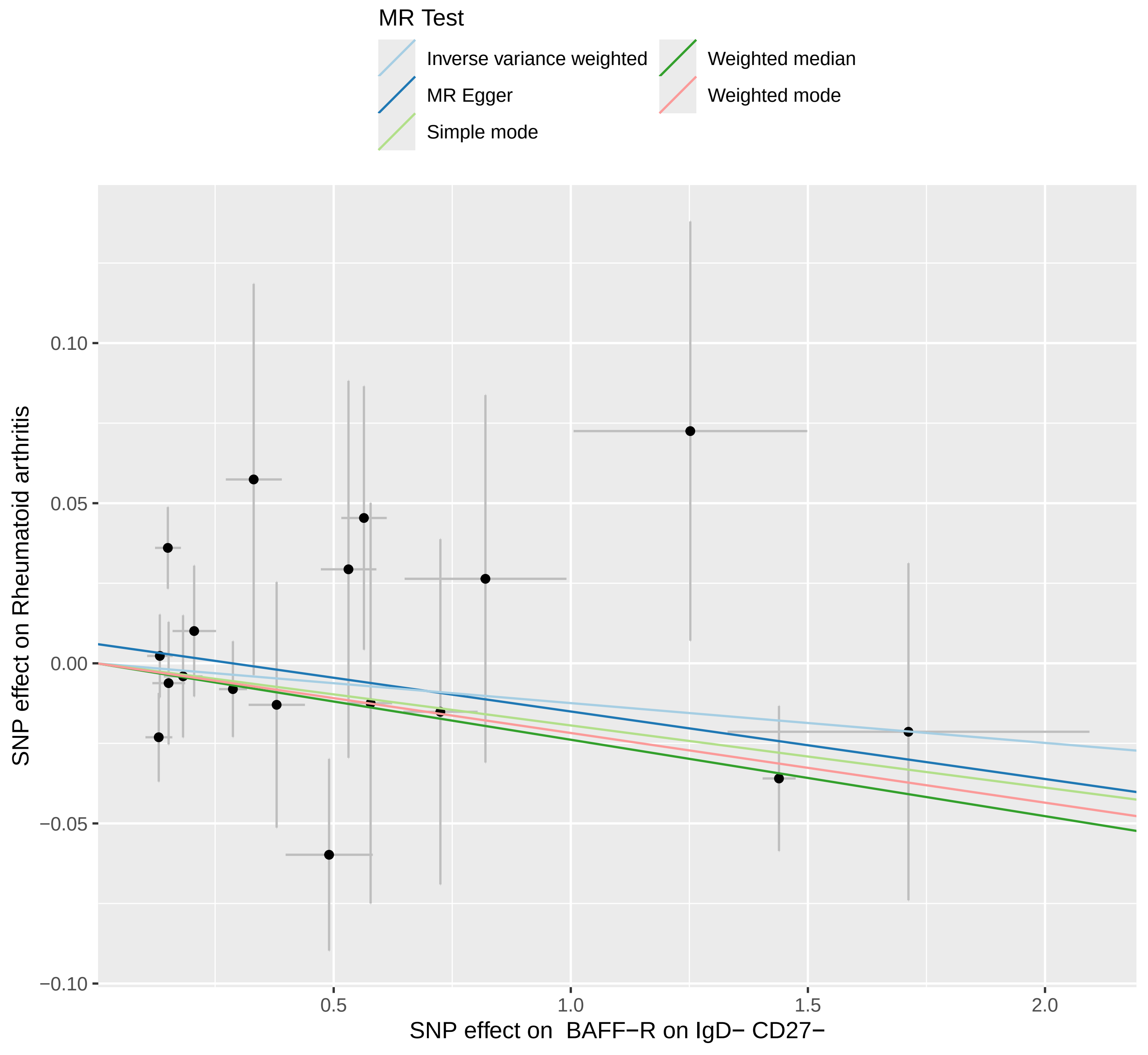
MR Test
Inverse variance weighted
MR Egger
Simple mode
Weighted median
Weighted mode
0.10
0.05
0.00
−0.05
−0.10
0.5
1.0
1.5
2.0
SNP effect on Rheumatoid arthritis
SNP effect on BAFF−R on IgD− CD27−

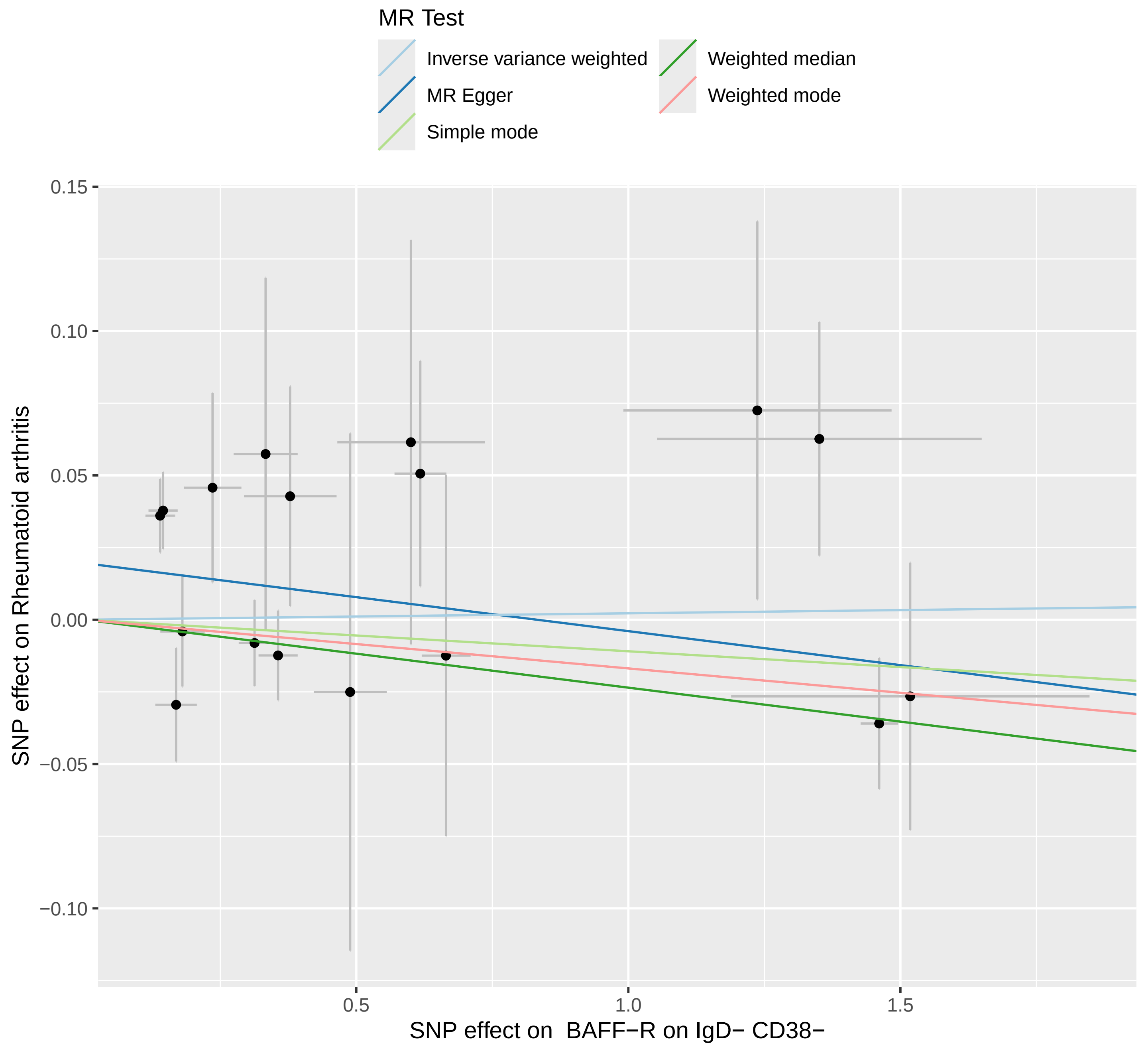

MR Test
Inverse variance weighted
MR Egger
Simple mode
Weighted median
Weighted mode
0.15
0.10
0.05
0.00
−0.05
−0.10
SNP effect on Rheumatoid arthritis
0.5
1.0
1.5
SNP effect on BAFF−R on IgD− CD38−

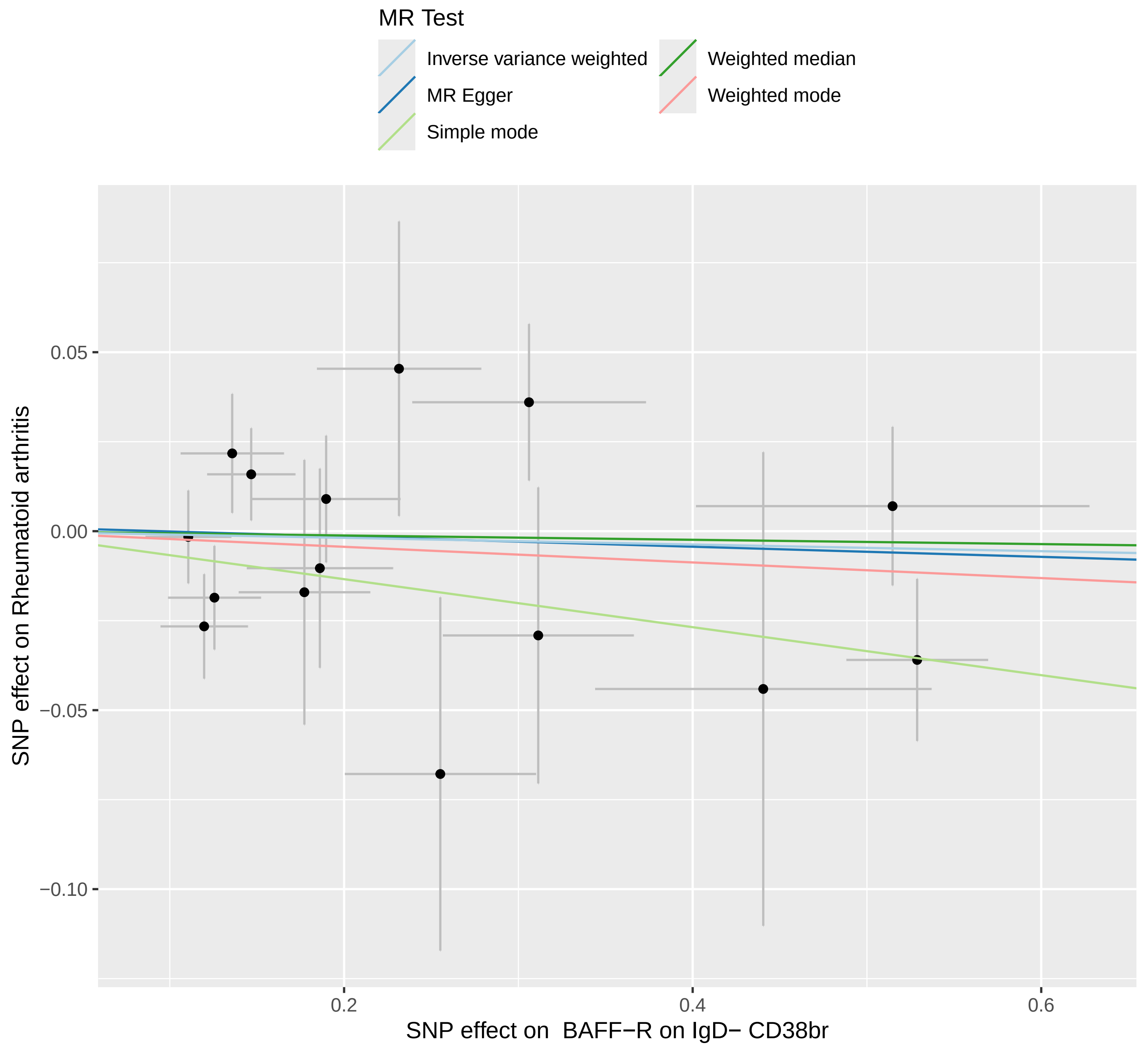

MR Test
Inverse variance weighted
MR Egger
Simple mode
Weighted median
Weighted mode
0.05
0.00
−0.05
−0.10
0.2
0.4
0.6
SNP effect on Rheumatoid arthritis
SNP effect on BAFF−R on IgD− CD38br

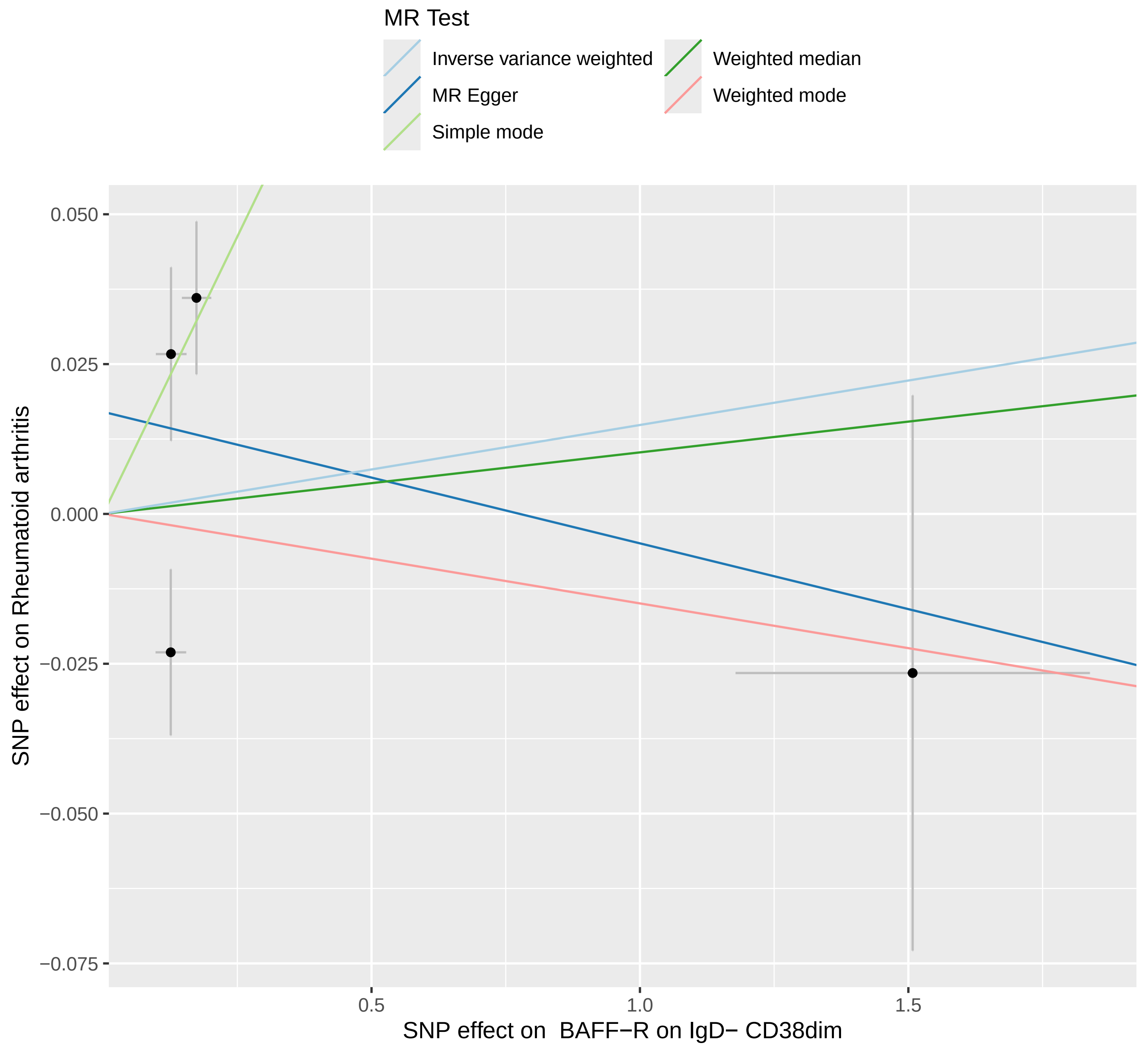

MR Test
Inverse variance weighted
MR Egger
Simple mode
Weighted median
Weighted mode
0.050
0.025
0.000
−0.025
−0.050
−0.075
0.5
1.0
1.5
SNP effect on Rheumatoid arthritis
SNP effect on BAFF−R on IgD− CD38dim

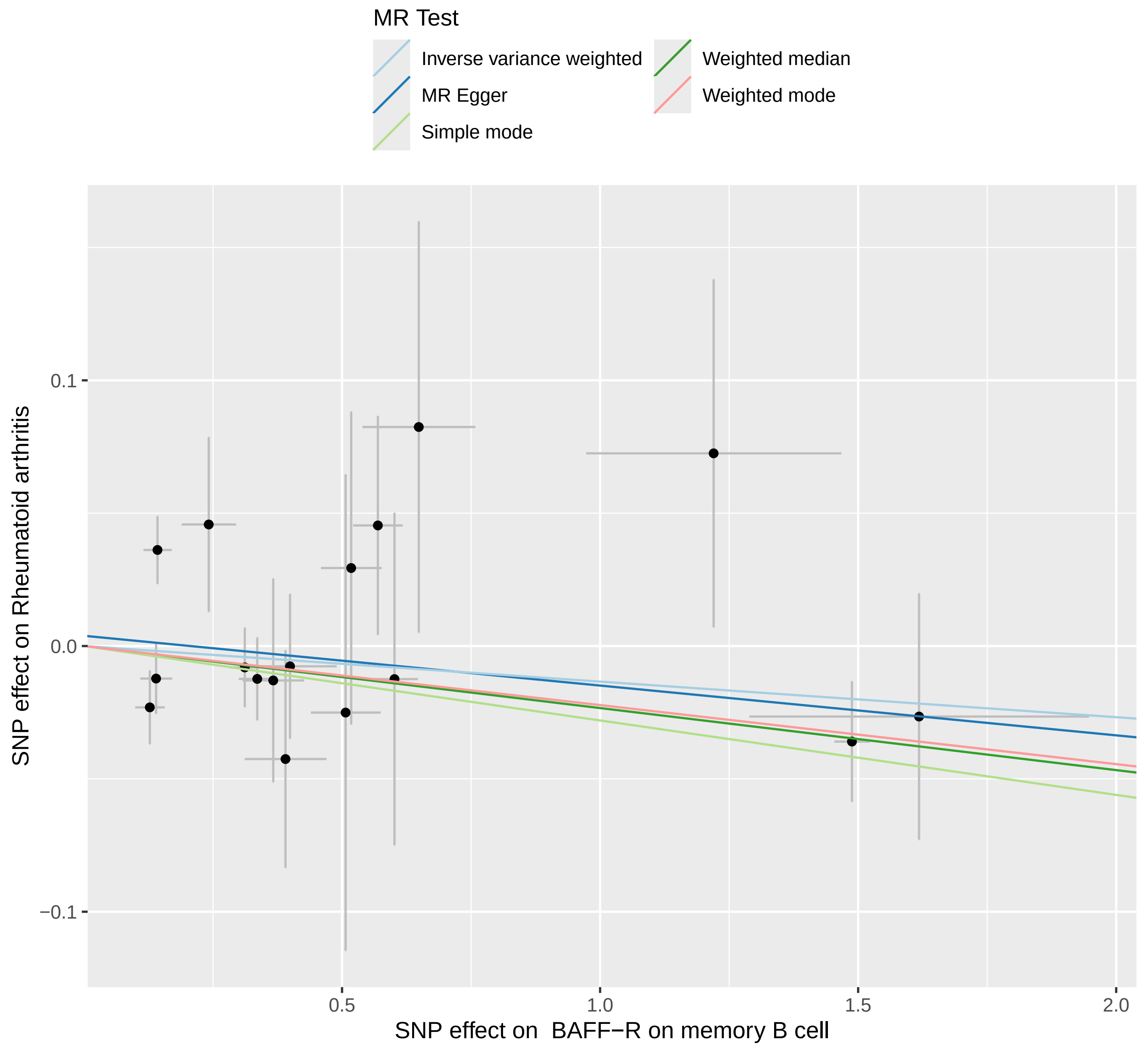
MR Test
Inverse variance weighted
MR Egger
Simple mode
Weighted median
Weighted mode
0.1
0.0
−0.1
SNP effect on Rheumatoid arthritis
0.5
1.0
1.5
2.0
SNP effect on BAFF−R on memory B cell

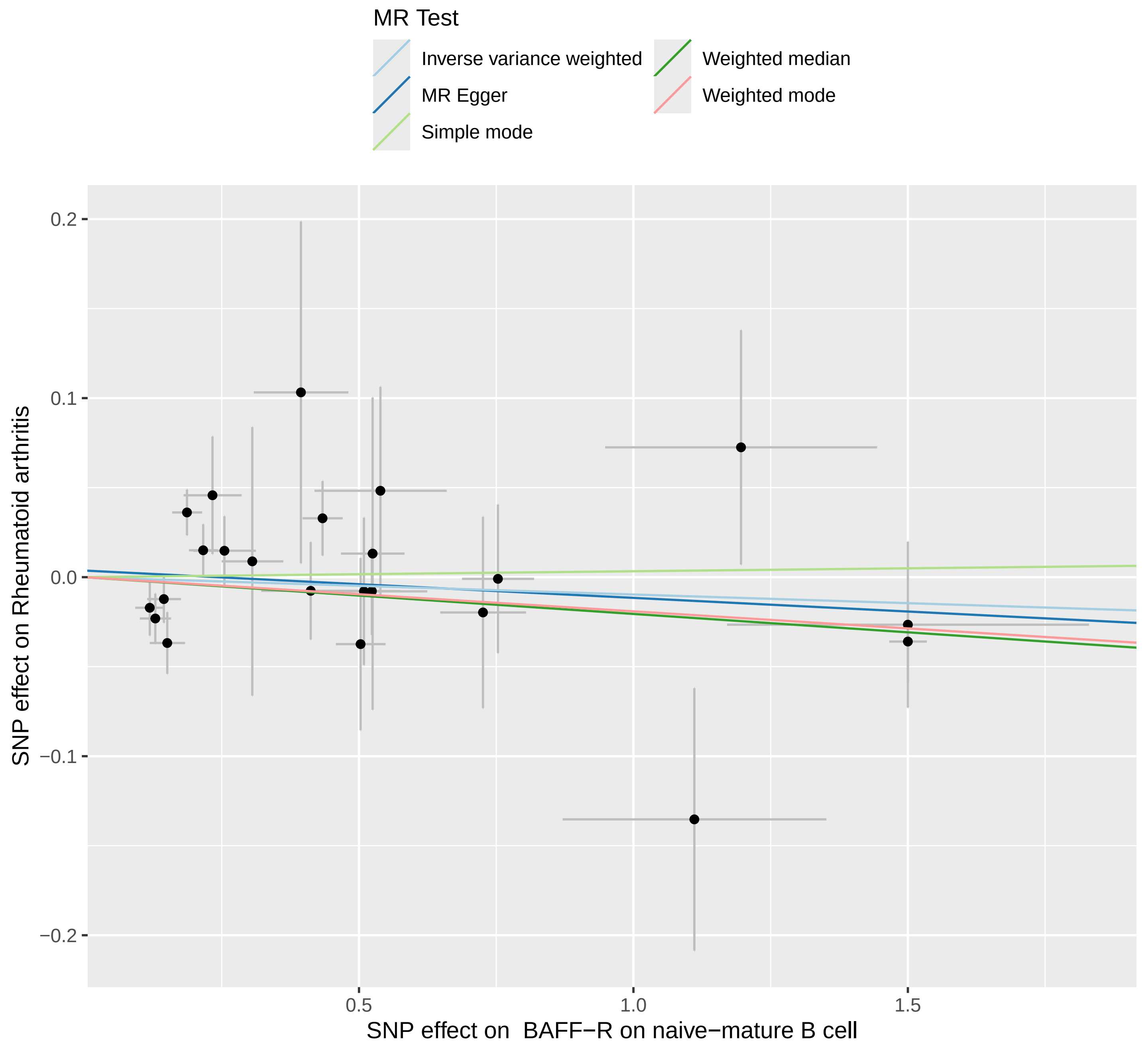
MR Test
Inverse variance weighted
MR Egger
Simple mode
Weighted median
Weighted mode
0.2
0.1
0.0
−0.1
−0.2
0.5
1.0
1.5
SNP effect on Rheumatoid arthritis
SNP effect on BAFF−R on naive−mature B cell

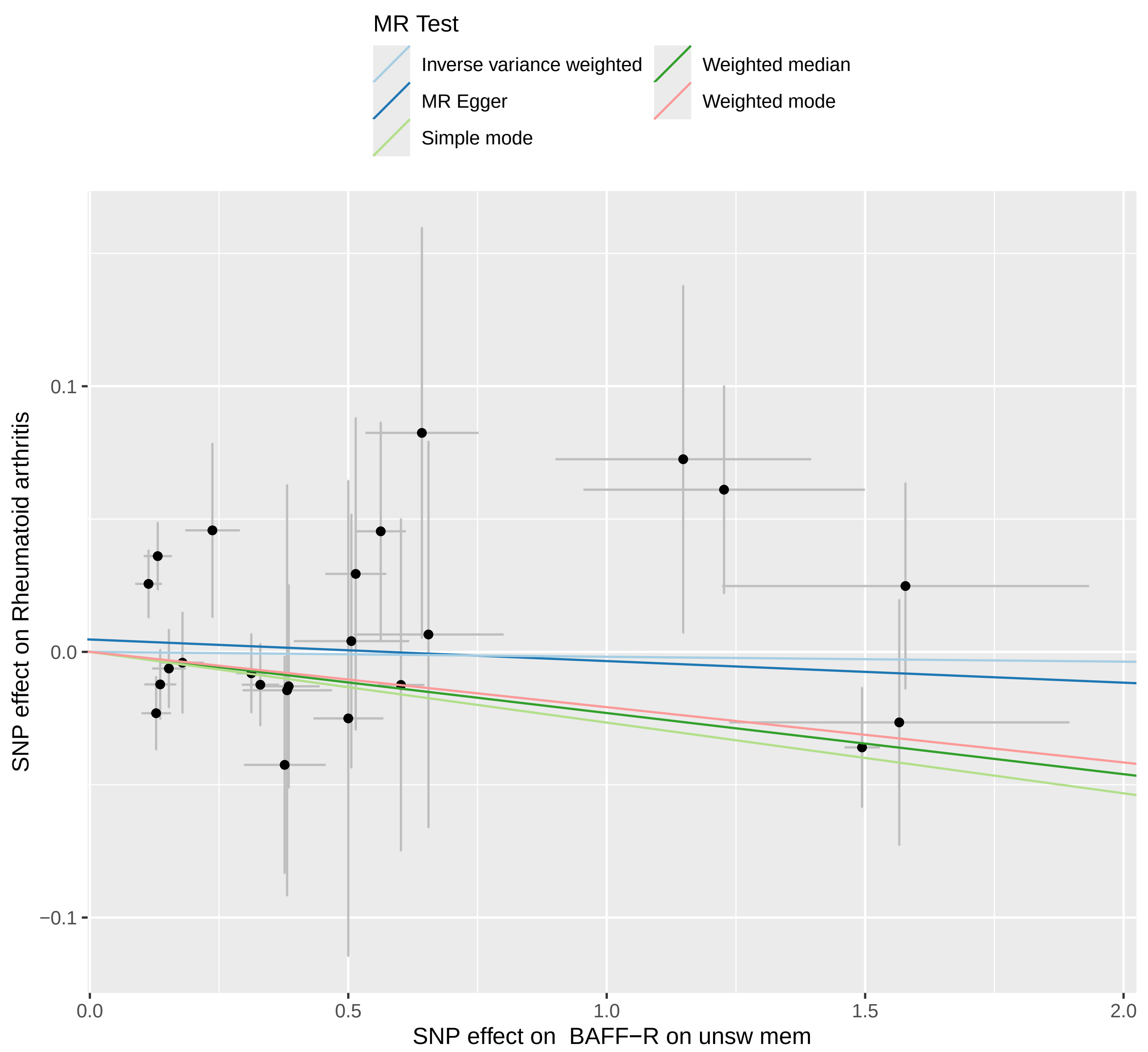
MR Test
Inverse variance weighted
MR Egger
Simple mode
Weighted median
Weighted mode
0.1
0.0
−0.1
SNP effect on Rheumatoid arthritis
0.0
0.5
1.0
1.5
2.0
SNP effect on BAFF−R on unsw mem

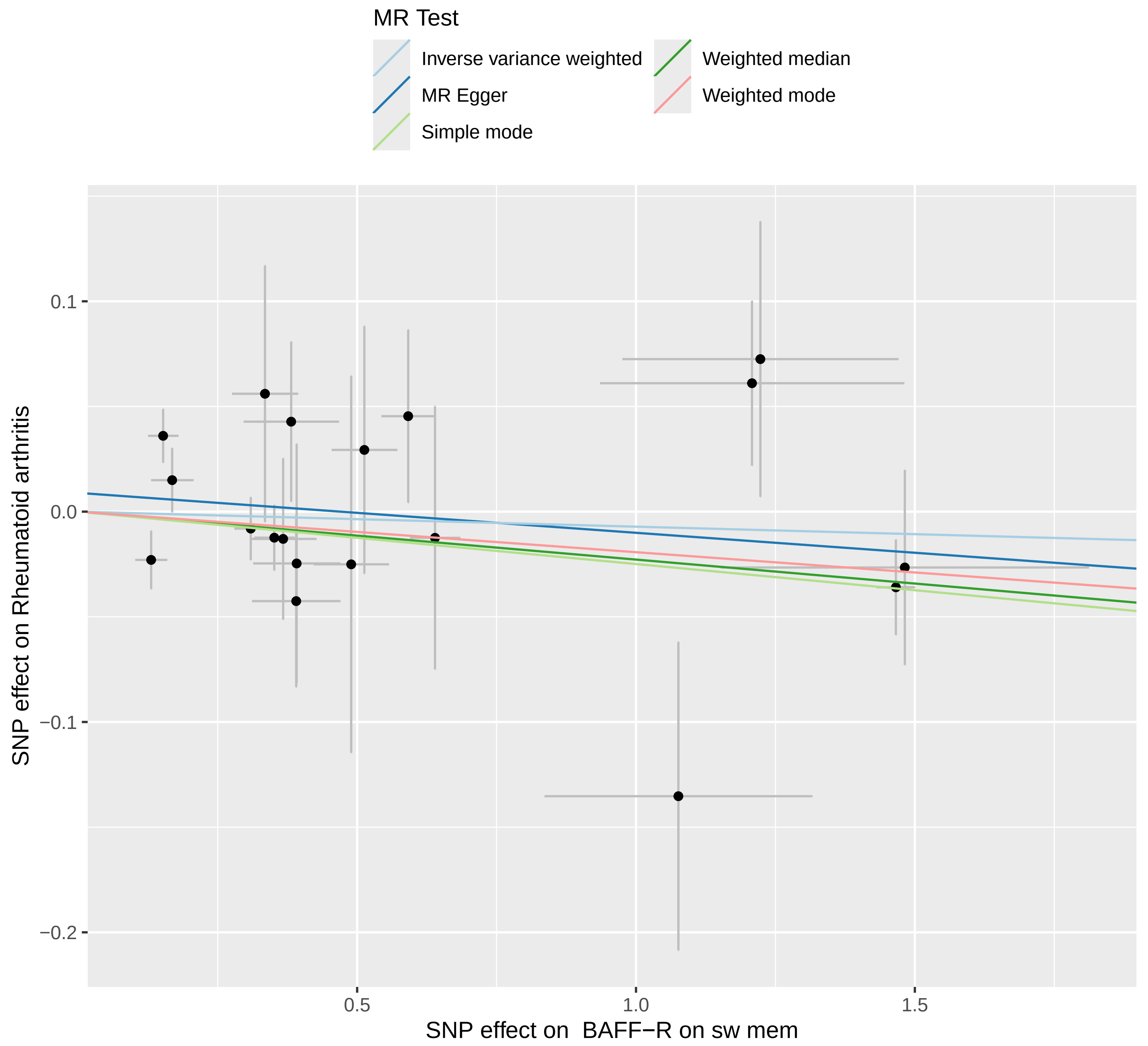

MR Test
Inverse variance weighted
MR Egger
Simple mode
Weighted median
Weighted mode
0.1
0.0
−0.1
−0.2
0.5
1.0
1.5
SNP effect on Rheumatoid arthritis
SNP effect on BAFF−R on sw mem

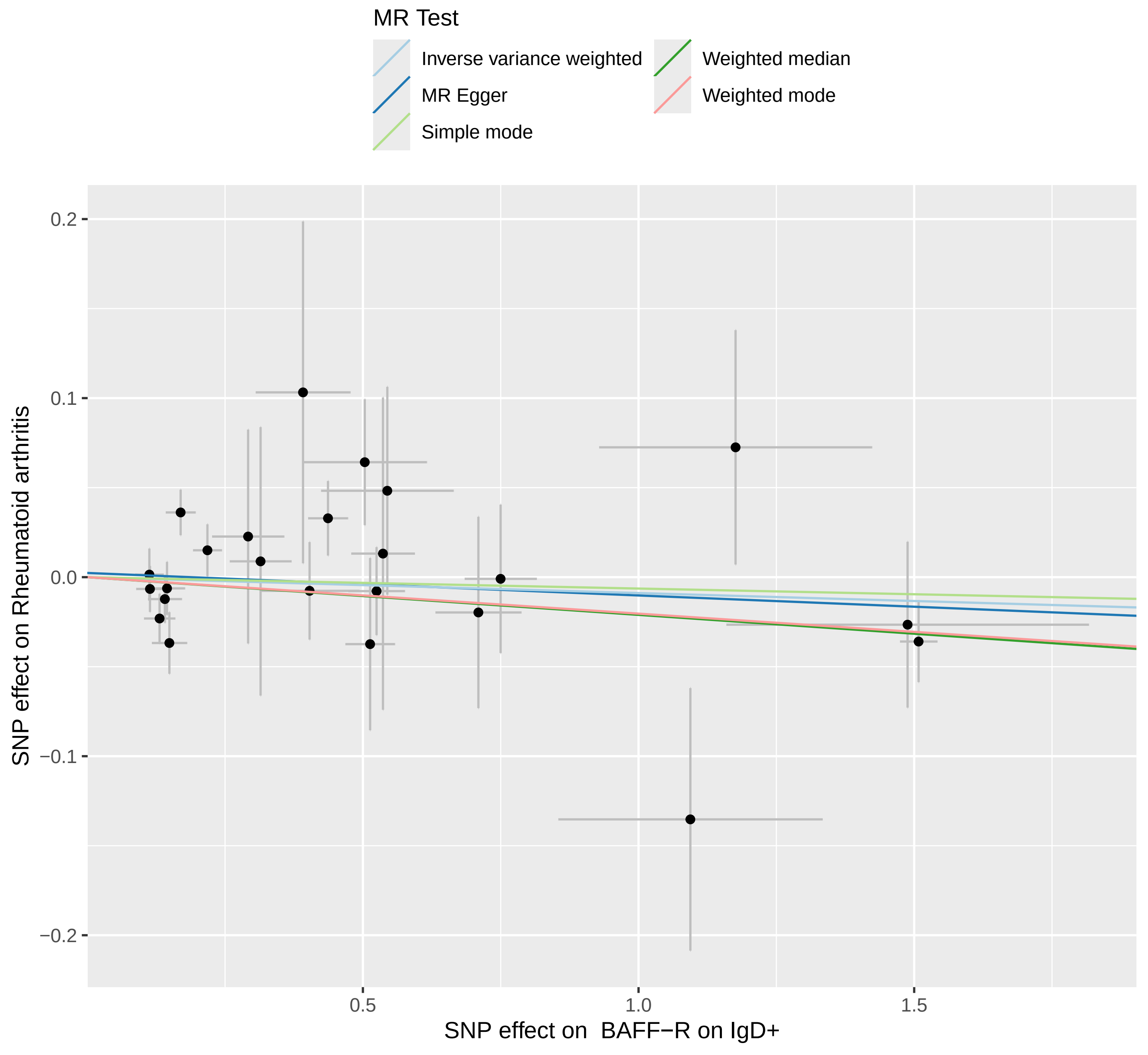

MR Test
Inverse variance weighted
MR Egger
Simple mode
Weighted median
Weighted mode
0.2
0.1
0.0
−0.1
−0.2
0.5
1.0
1.5
SNP effect on Rheumatoid arthritis
SNP effect on BAFF−R on IgD+

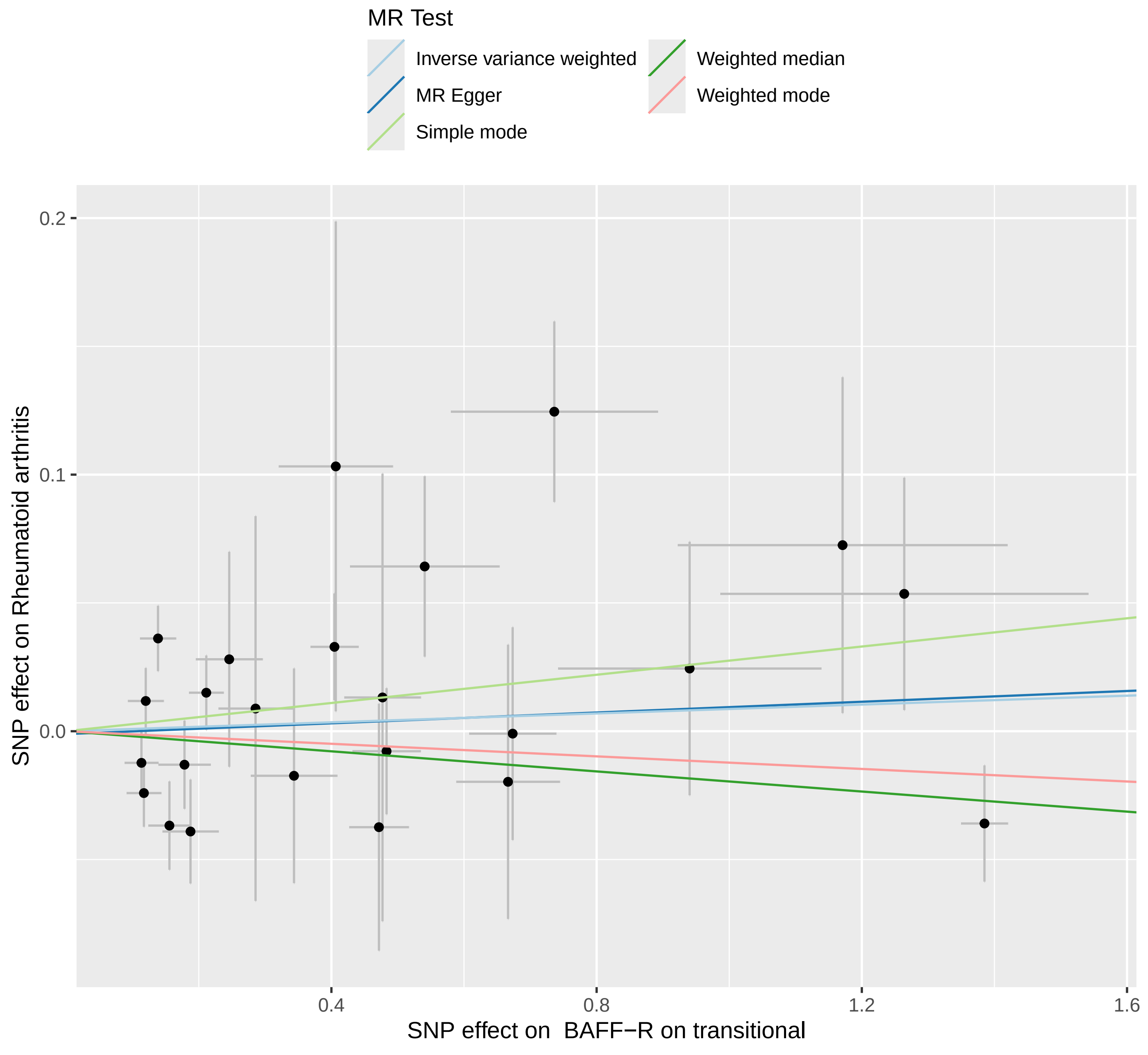

MR Test
Inverse variance weighted
MR Egger
Simple mode
Weighted median
Weighted mode
SNP effect on Rheumatoid arthritis
0.2
0.1
0.0
0.4
0.8
1.2
1.6
SNP effect on BAFF−R on transitional

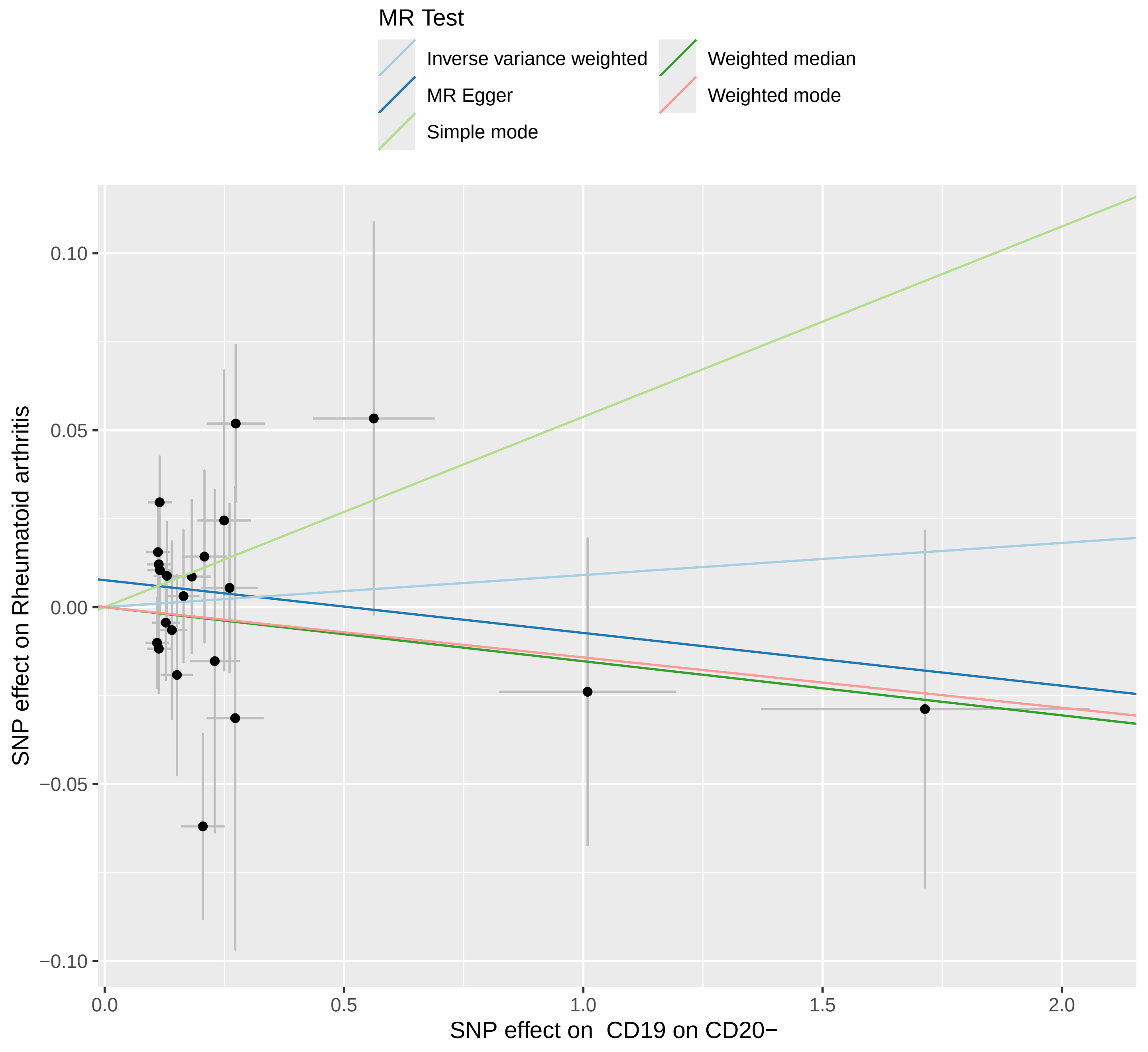
MR Test
Inverse variance weighted
MR Egger
Simple mode
Weighted median
Weighted mode
0.10
0.05
0.00
−0.05
−0.10
SNP effect on Rheumatoid arthritis
0.0
0.5
1.0
1.5
2.0
SNP effect on CD19 on CD20−

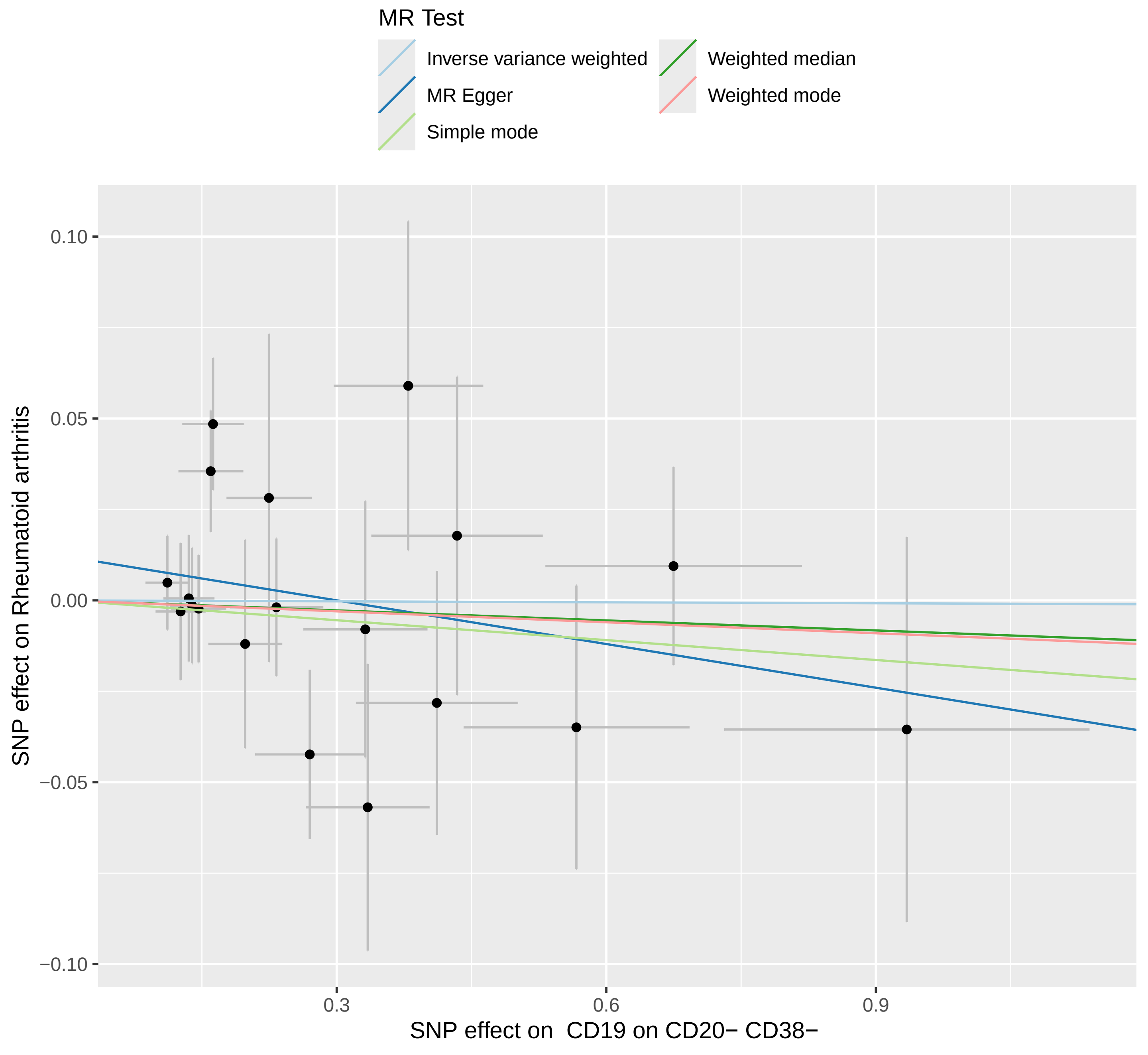

MR Test
Inverse variance weighted
MR Egger
Simple mode
Weighted median
Weighted mode
SNP effect on Rheumatoid arthritis
0.10
0.05
0.00
−0.05
−0.10
0.3
0.6
0.9
SNP effect on CD19 on CD20− CD38−

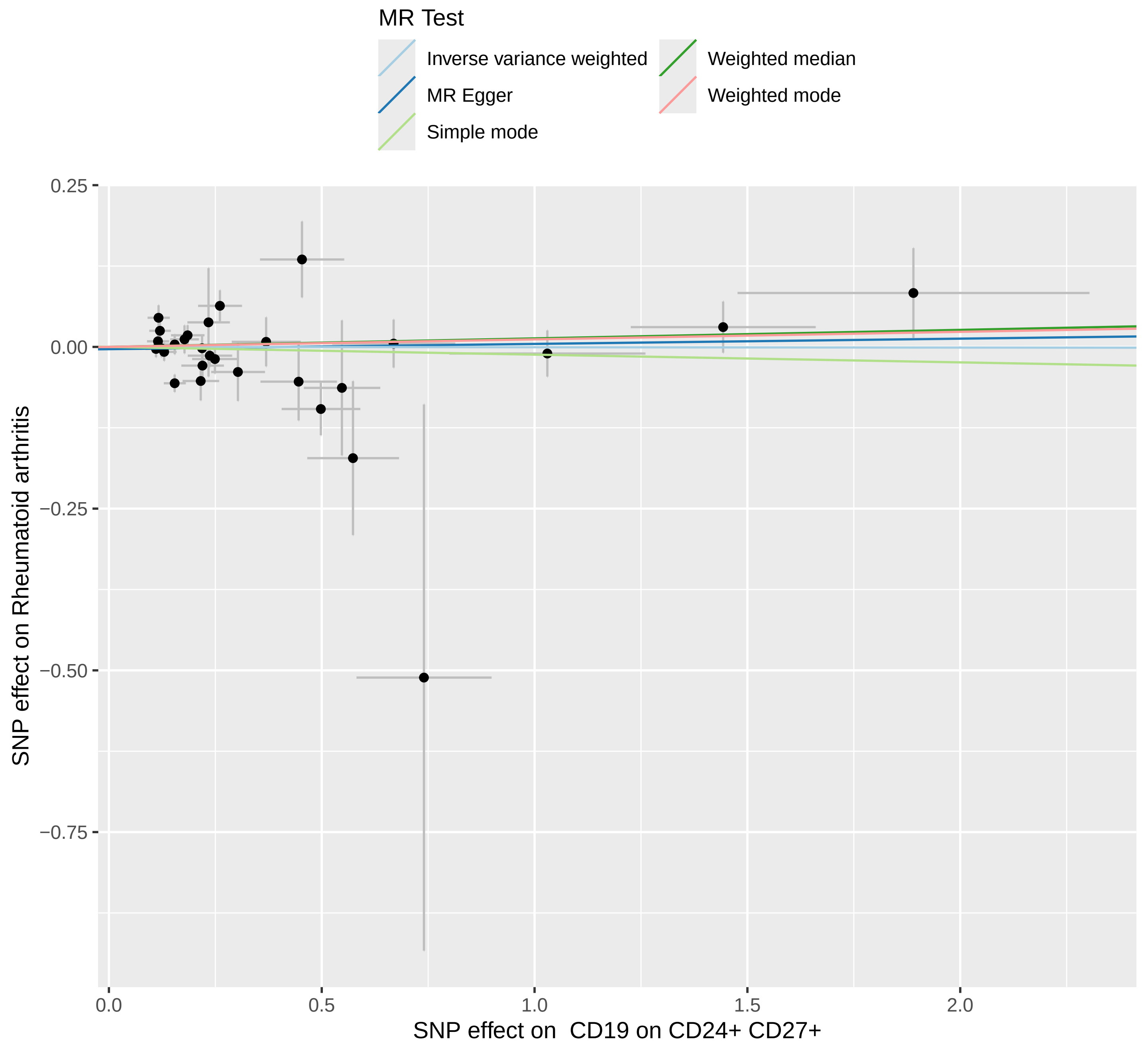

MR Test
Inverse variance weighted
MR Egger
Simple mode
Weighted median
Weighted mode
0.25
0.00
−0.25
−0.50
−0.75
SNP effect on Rheumatoid arthritis
0.0
0.5
1.0
1.5
2.0
SNP effect on CD19 on CD24+ CD27+

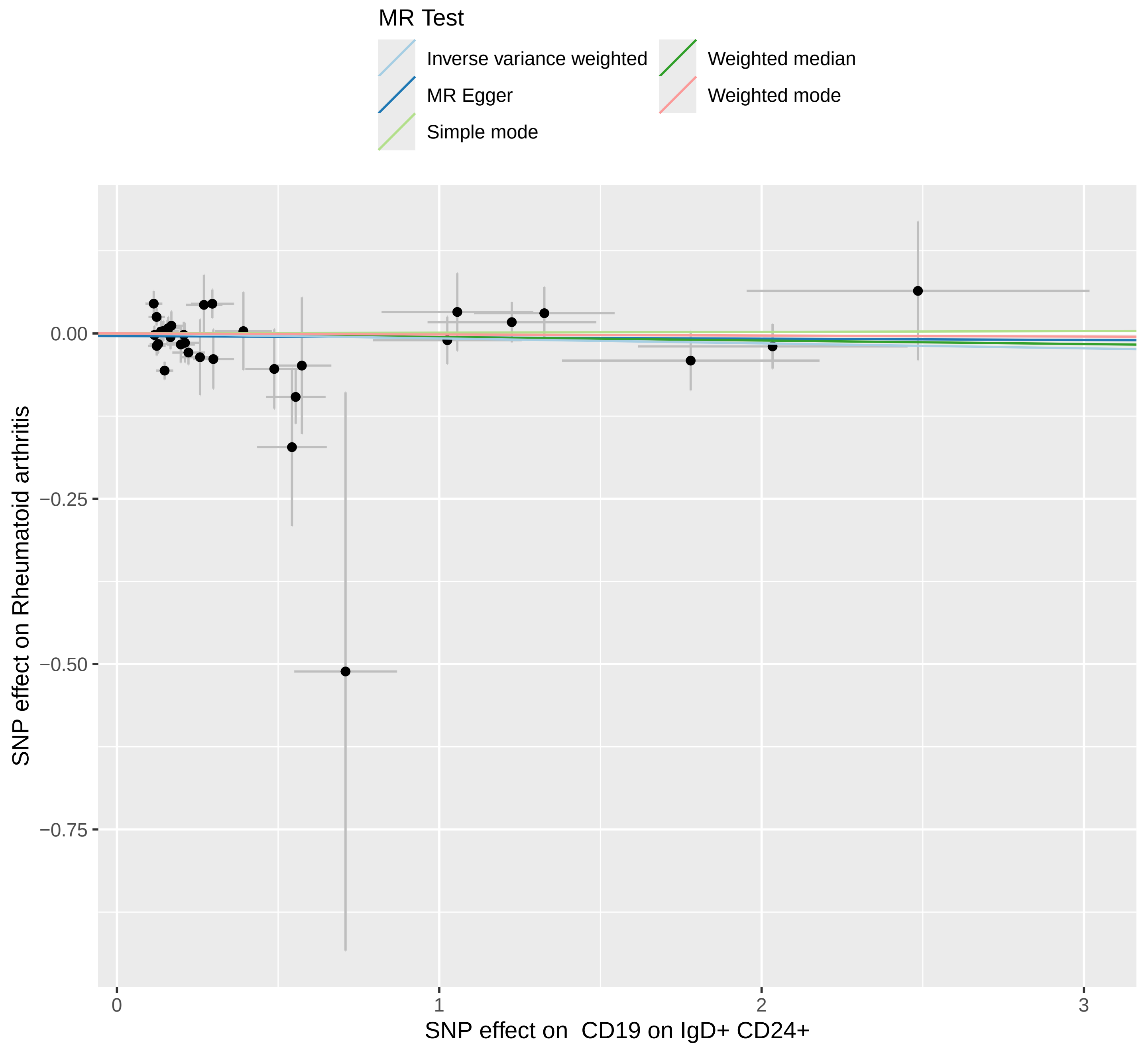

MR Test
Inverse variance weighted
MR Egger
Simple mode
Weighted median
Weighted mode
SNP effect on Rheumatoid arthritis
0.00
−0.25
−0.50
−0.75
0
1
2
3
SNP effect on CD19 on IgD+ CD24+

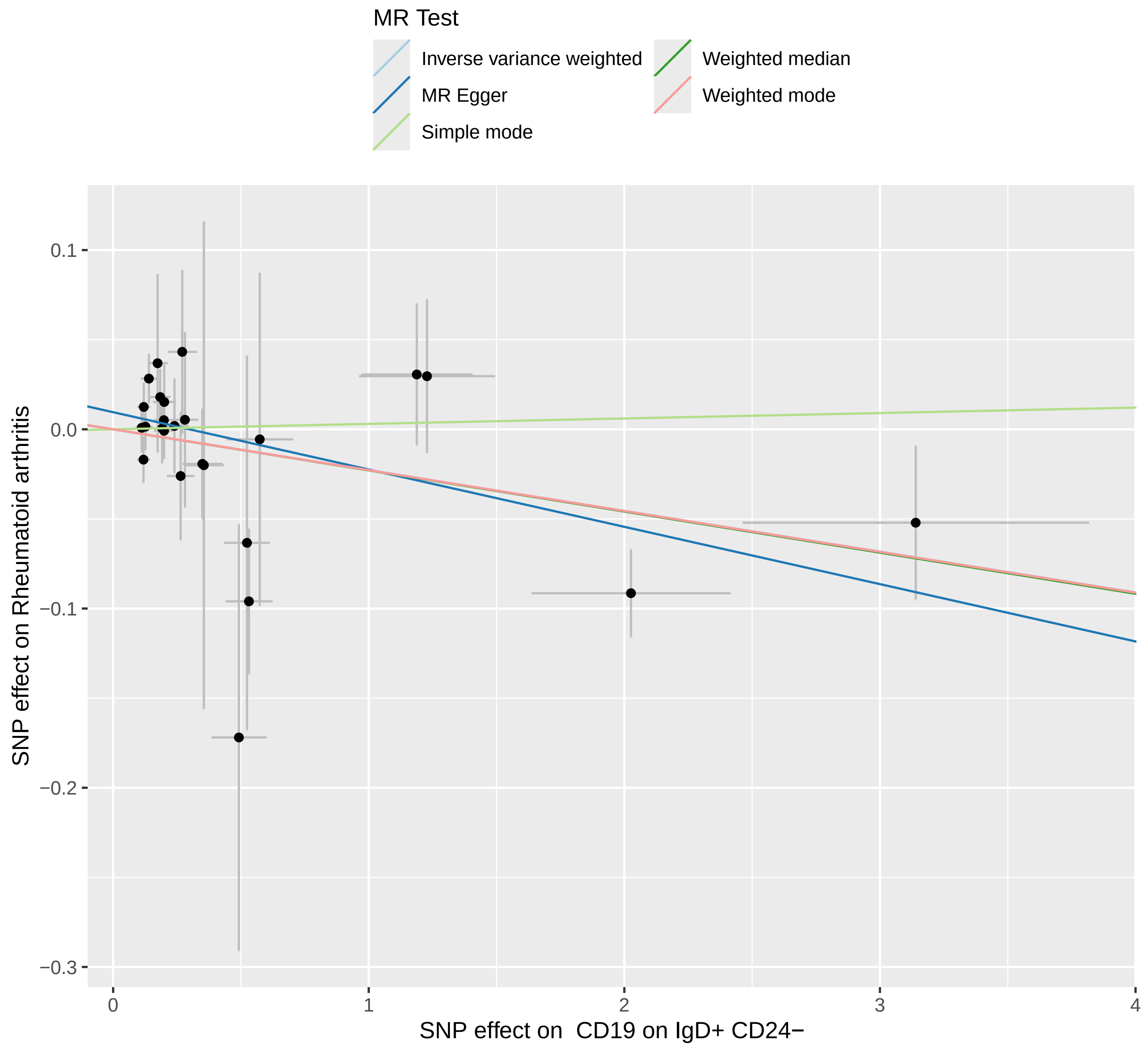
MR Test
Inverse variance weighted
MR Egger
Simple mode
Weighted median
Weighted mode
SNP effect on Rheumatoid arthritis
0.1
0.0
−0.1
−0.2
−0.3
0
1
2
3
4
SNP effect on CD19 on IgD+ CD24−

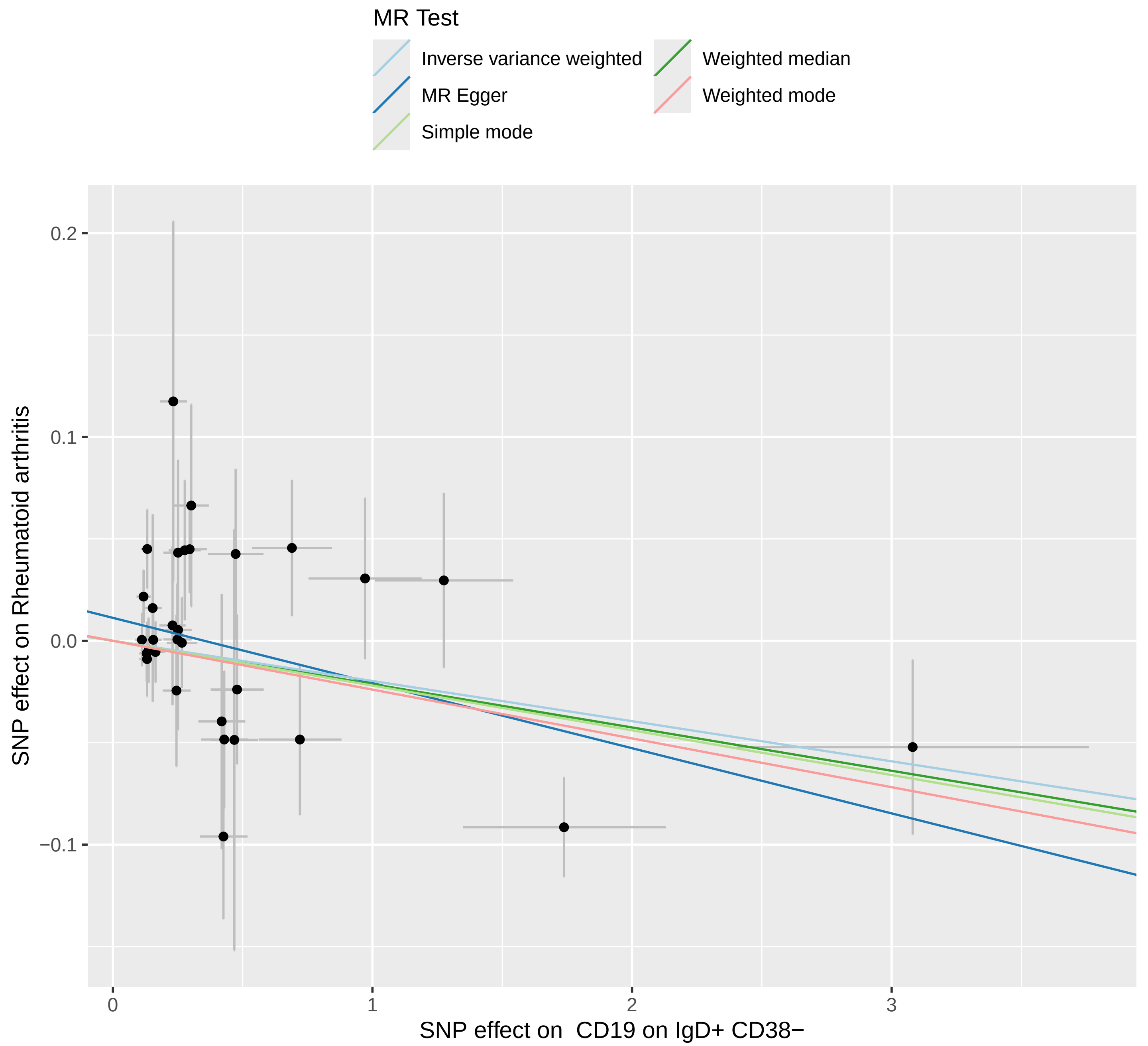
MR Test
Inverse variance weighted
MR Egger
Simple mode
Weighted median
Weighted mode
SNP effect on Rheumatoid arthritis
0.2
0.1
0.0
−0.1
0
1
2
3
SNP effect on CD19 on IgD+ CD38−

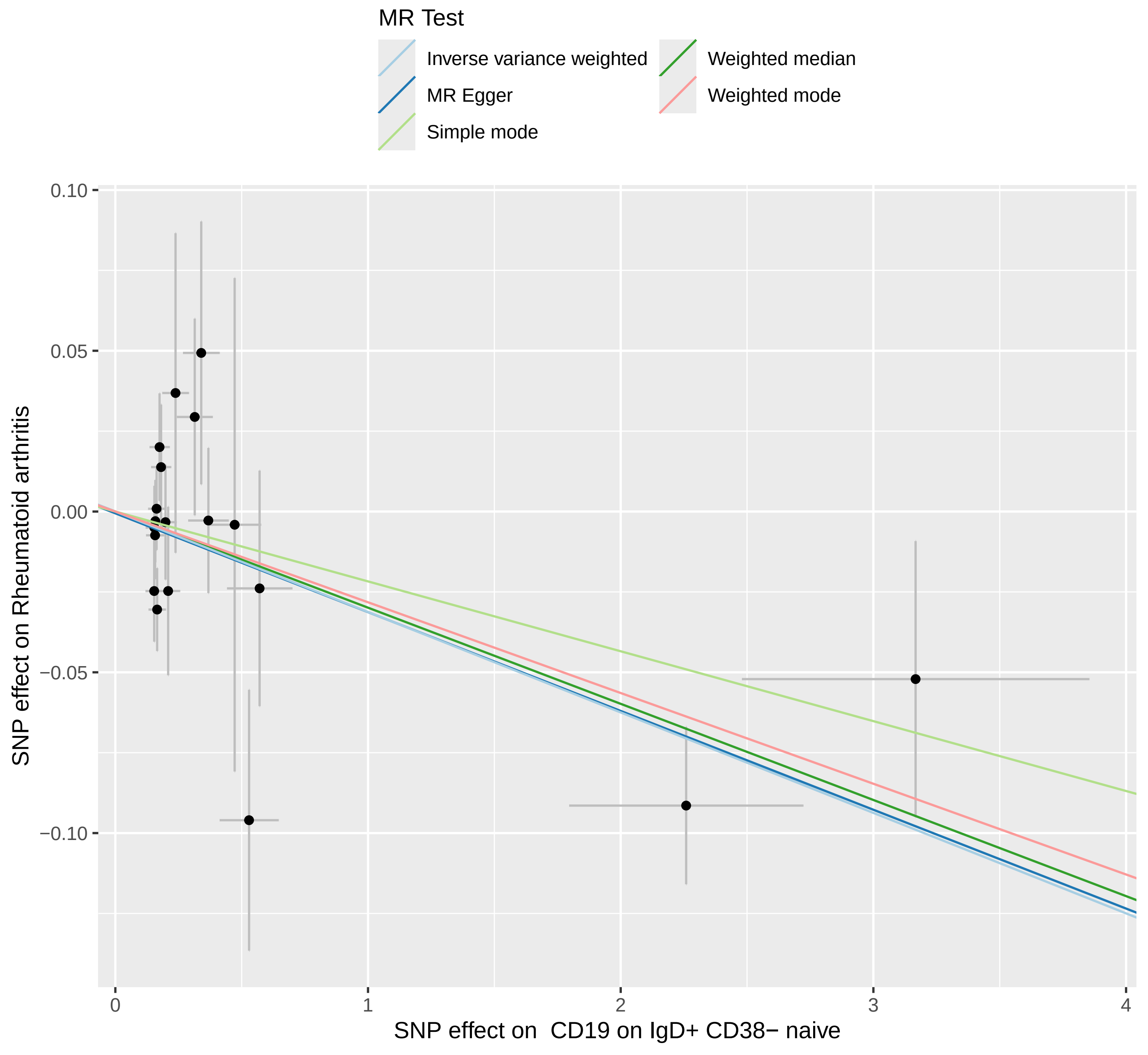

MR Test
Inverse variance weighted
MR Egger
Simple mode
Weighted median
Weighted mode
0.10
0.05
0.00
−0.05
−0.10
SNP effect on Rheumatoid arthritis
0
1
2
3
4
SNP effect on CD19 on IgD+ CD38− naive

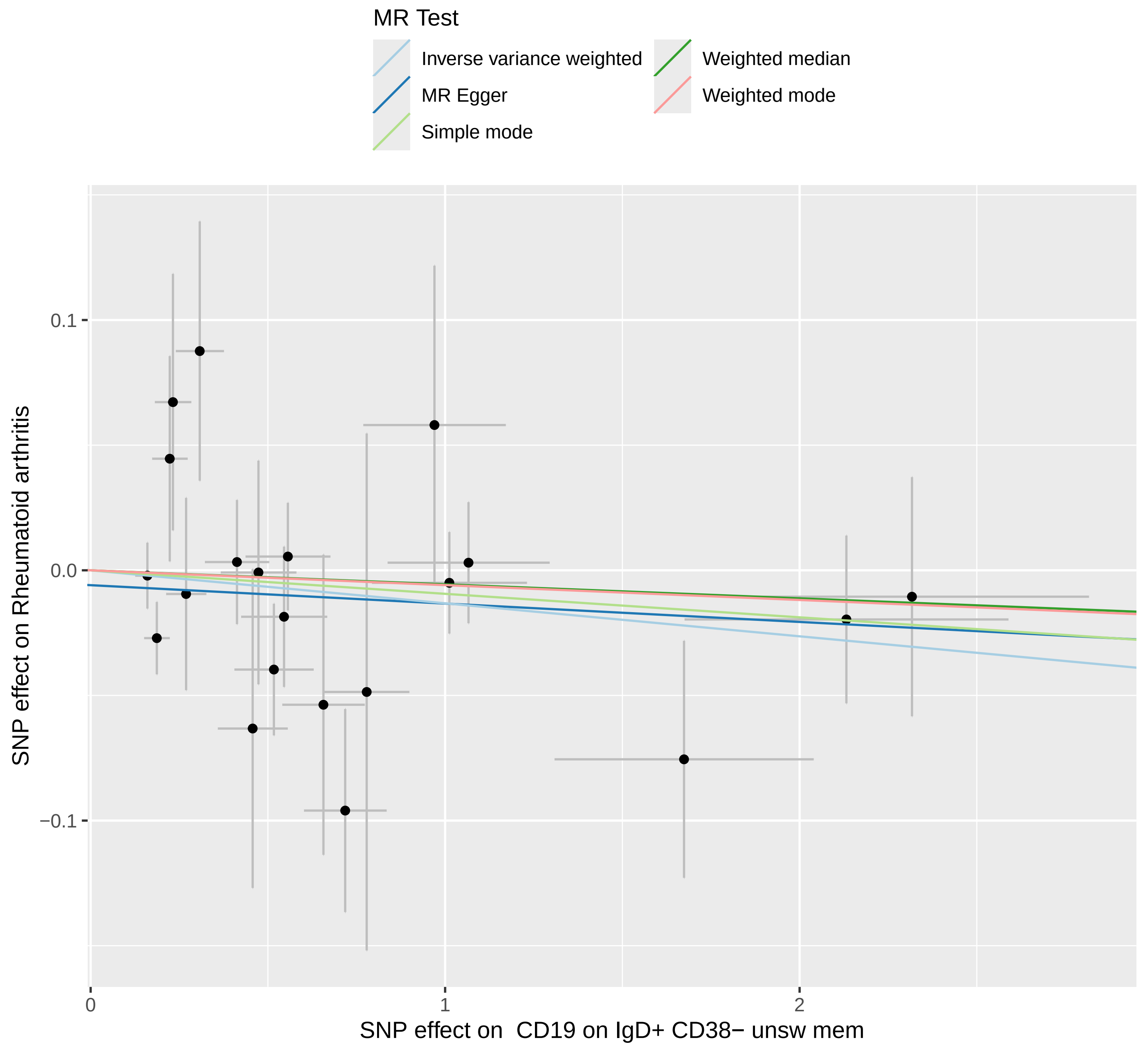

MR Test
Inverse variance weighted
MR Egger
Simple mode
Weighted median
Weighted mode
0.1
0.0
−0.1
0
1
2
SNP effect on Rheumatoid arthritis
SNP effect on CD19 on IgD+ CD38− unsw mem

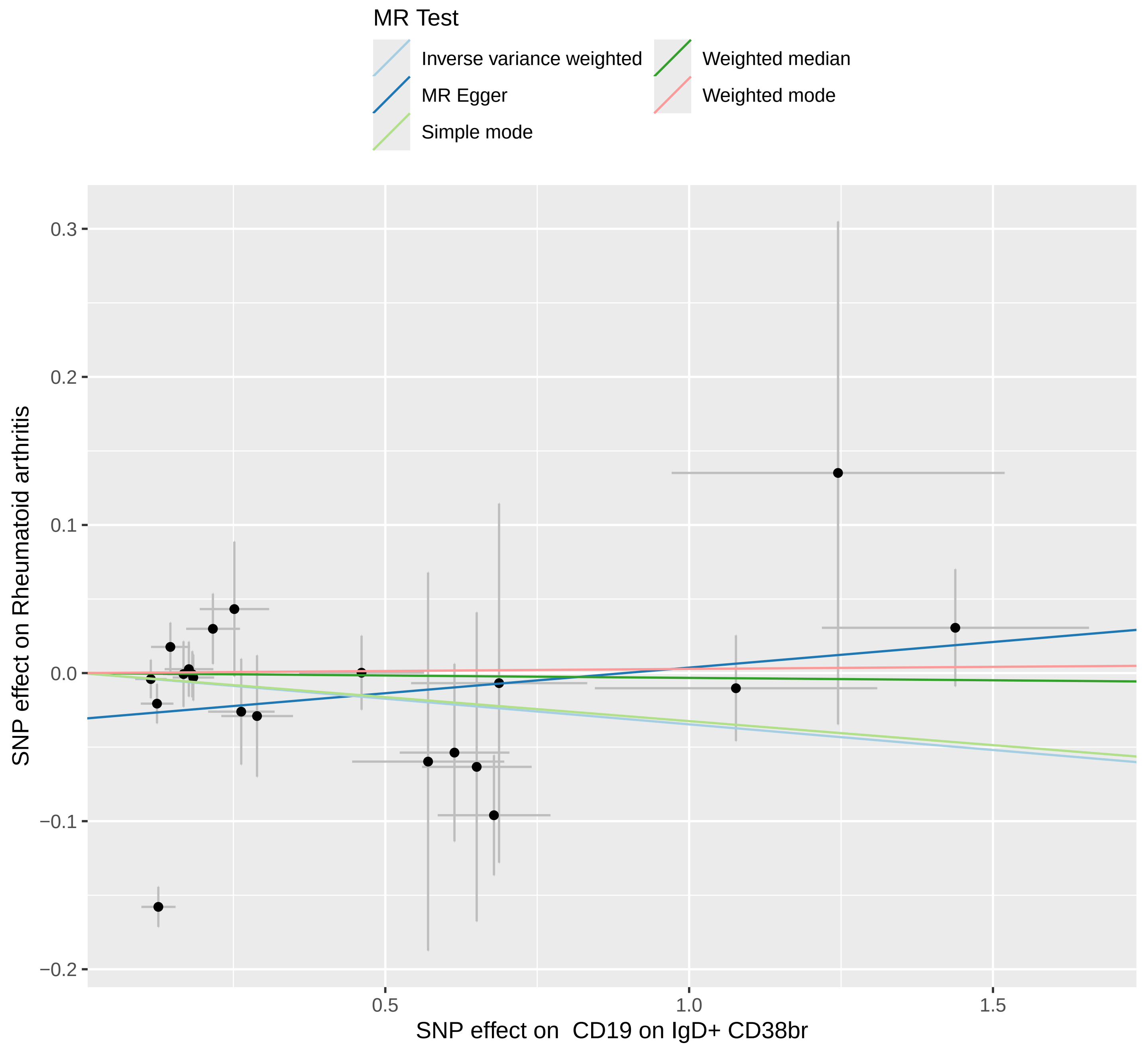

MR Test
Inverse variance weighted
MR Egger
Simple mode
Weighted median
Weighted mode
SNP effect on Rheumatoid arthritis
0.3
0.2
0.1
0.0
−0.1
−0.2
0.5
1.0
1.5
SNP effect on CD19 on IgD+ CD38br

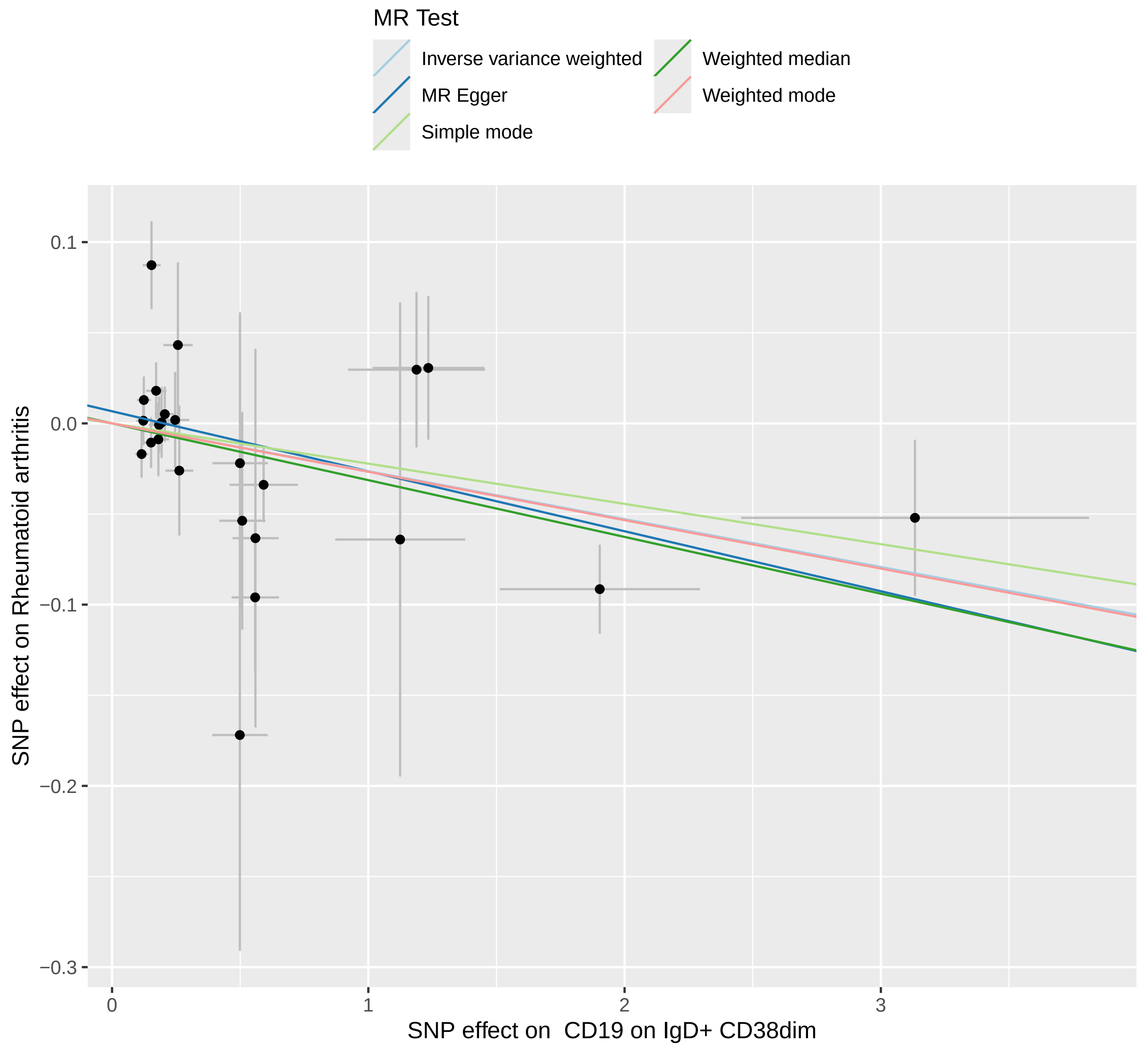

MR Test
Inverse variance weighted
MR Egger
Simple mode
Weighted median
Weighted mode
0.1
0.0
−0.1
−0.2
−0.3
0
1
2
3
SNP effect on Rheumatoid arthritis
SNP effect on CD19 on IgD+ CD38dim

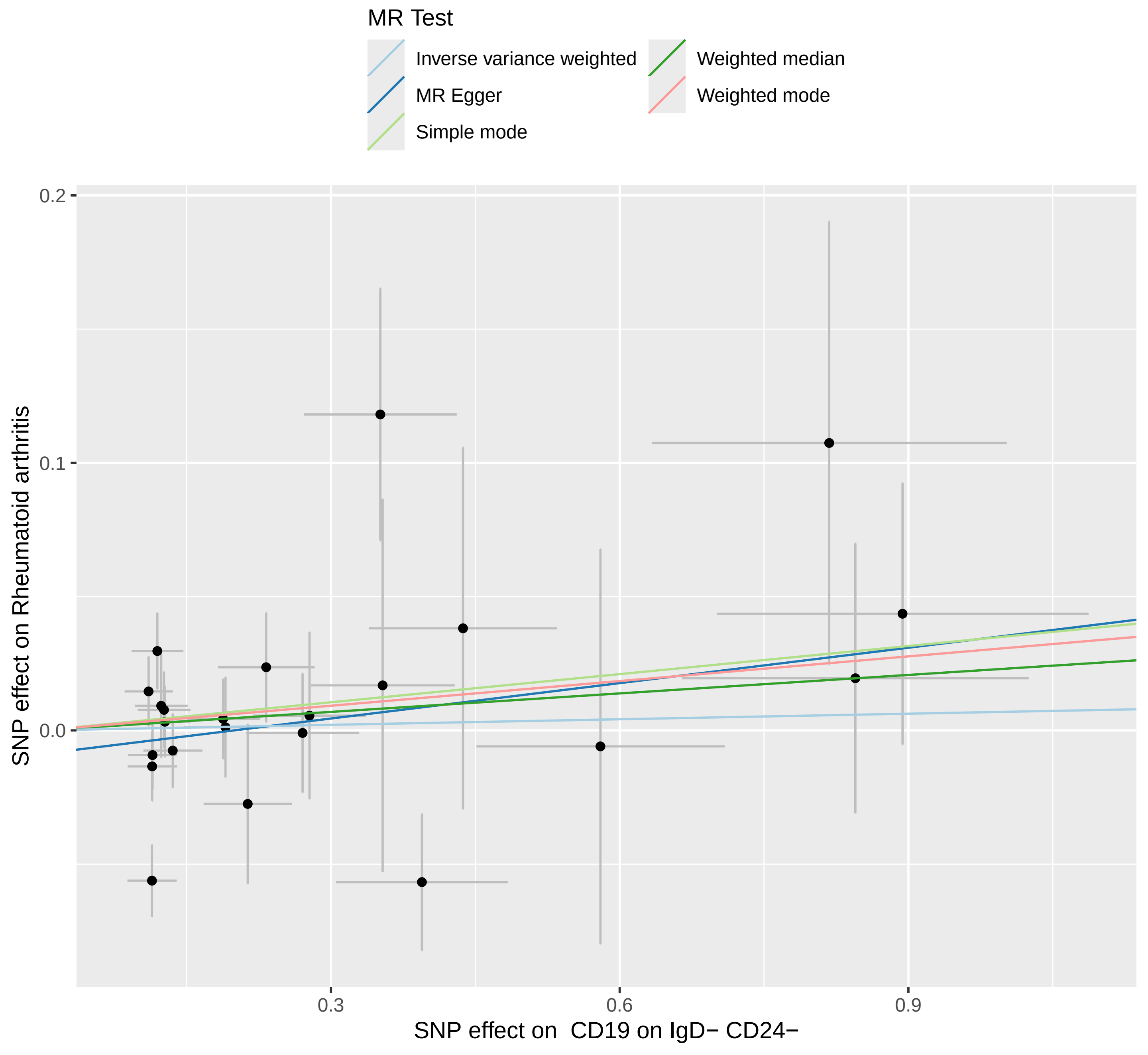
MR Test
Inverse variance weighted
MR Egger
Simple mode
Weighted median
Weighted mode
0.2
0.1
0.0
0.3
0.6
0.9
SNP effect on Rheumatoid arthritis
SNP effect on CD19 on IgD− CD24−

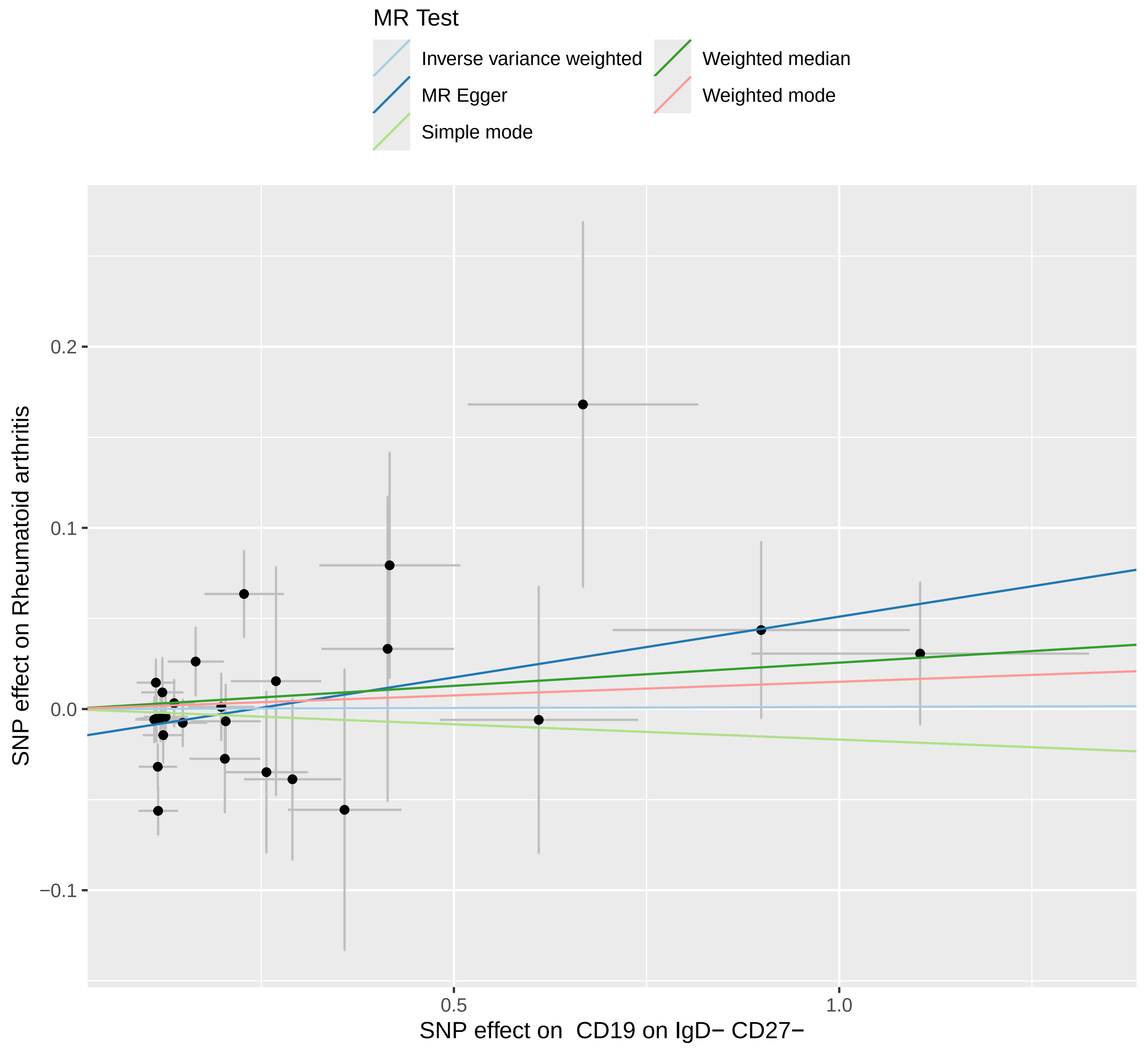
MR Test
Inverse variance weighted
MR Egger
Simple mode
Weighted median
Weighted mode
0.2
0.1
0.0
−0.1
0.5
1.0
SNP effect on Rheumatoid arthritis
SNP effect on CD19 on IgD− CD27−

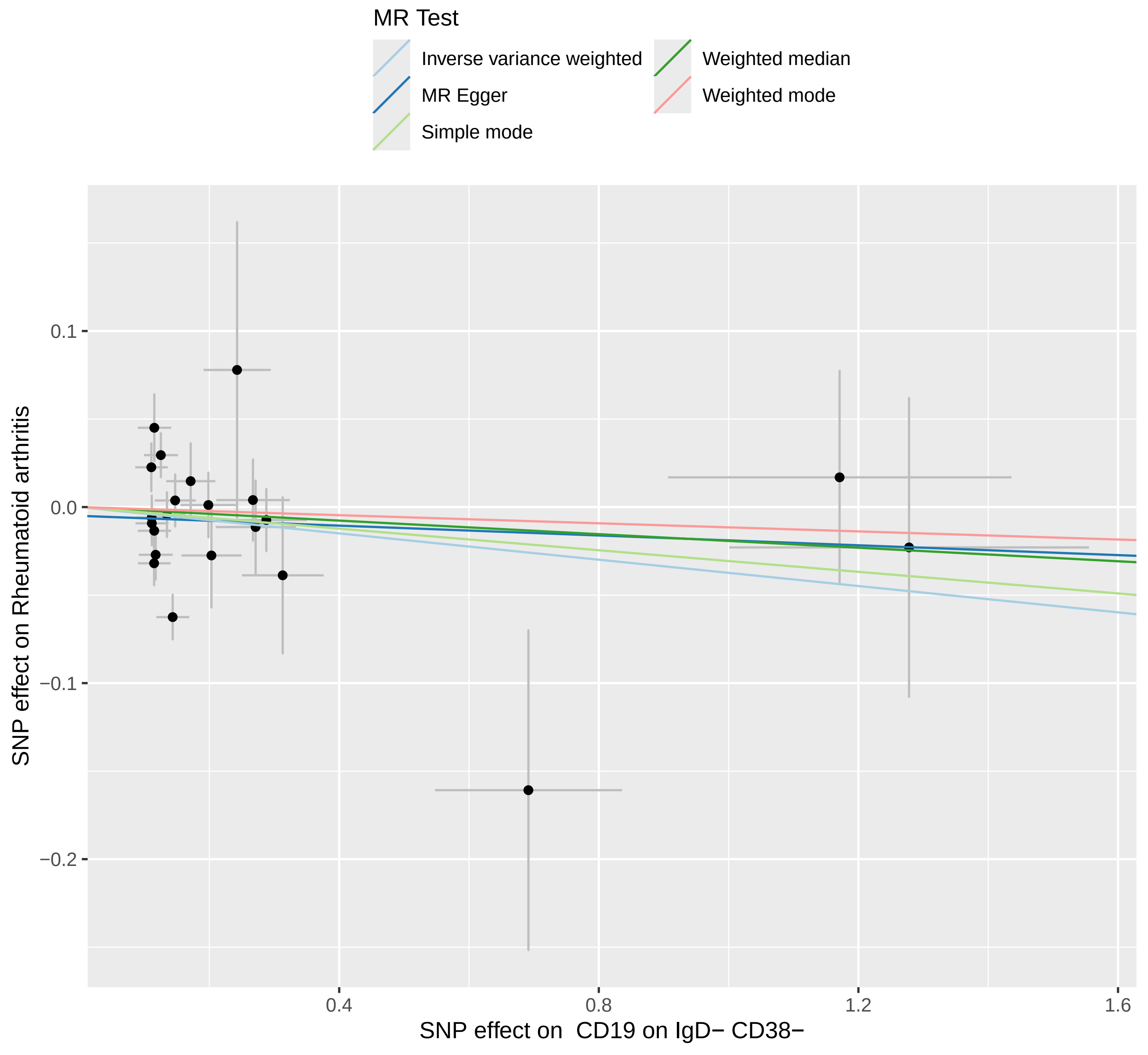

MR Test
Inverse variance weighted
MR Egger
Simple mode
Weighted median
Weighted mode
0.1
0.0
−0.1
−0.2
0.4
0.8
1.2
1.6
SNP effect on Rheumatoid arthritis
SNP effect on CD19 on IgD− CD38−

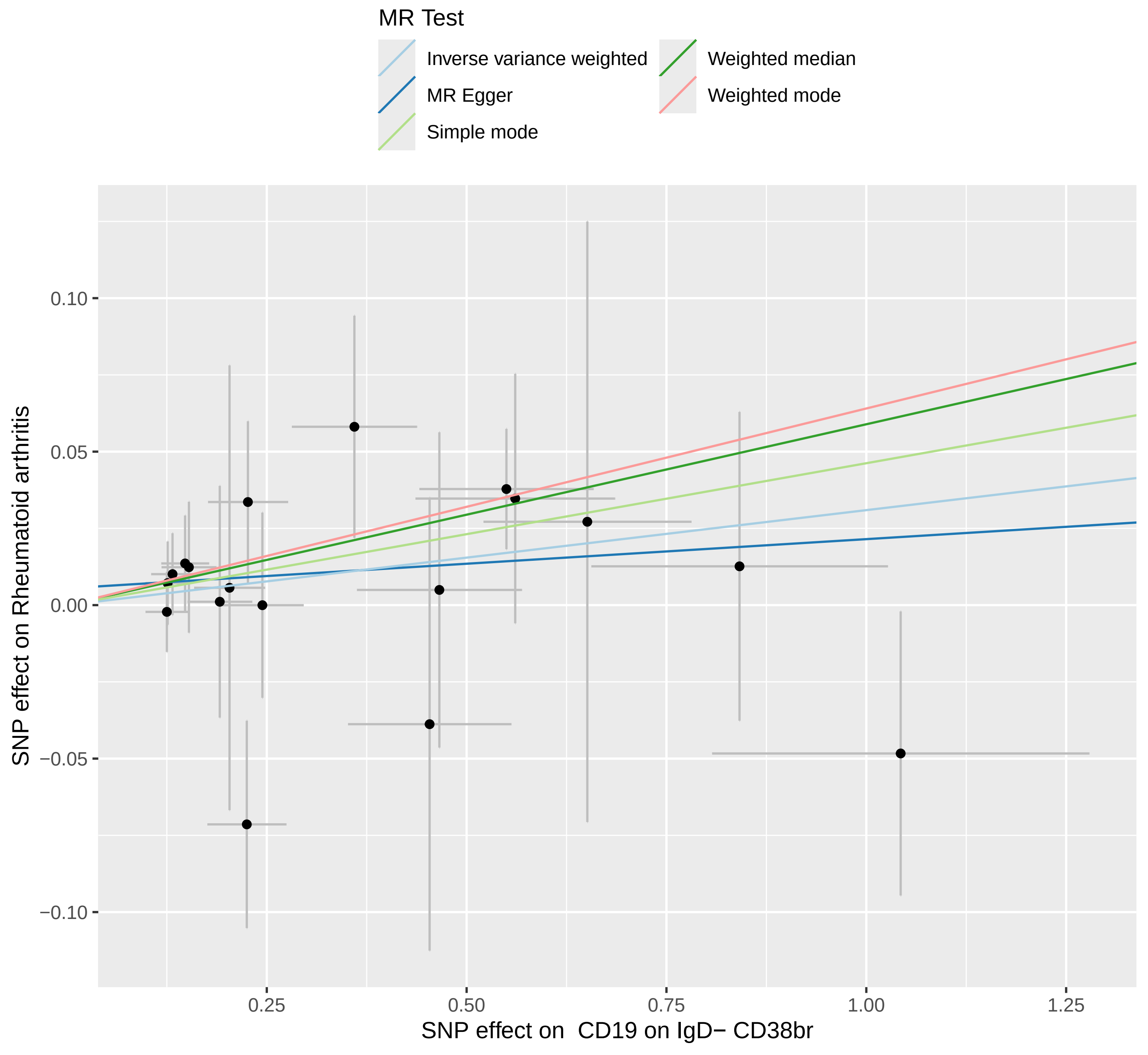

MR Test
Inverse variance weighted
MR Egger
Simple mode
Weighted median
Weighted mode
SNP effect on Rheumatoid arthritis
0.10
0.05
0.00
−0.05
−0.10
0.25
0.50
0.75
1.00
1.25
SNP effect on CD19 on IgD− CD38br

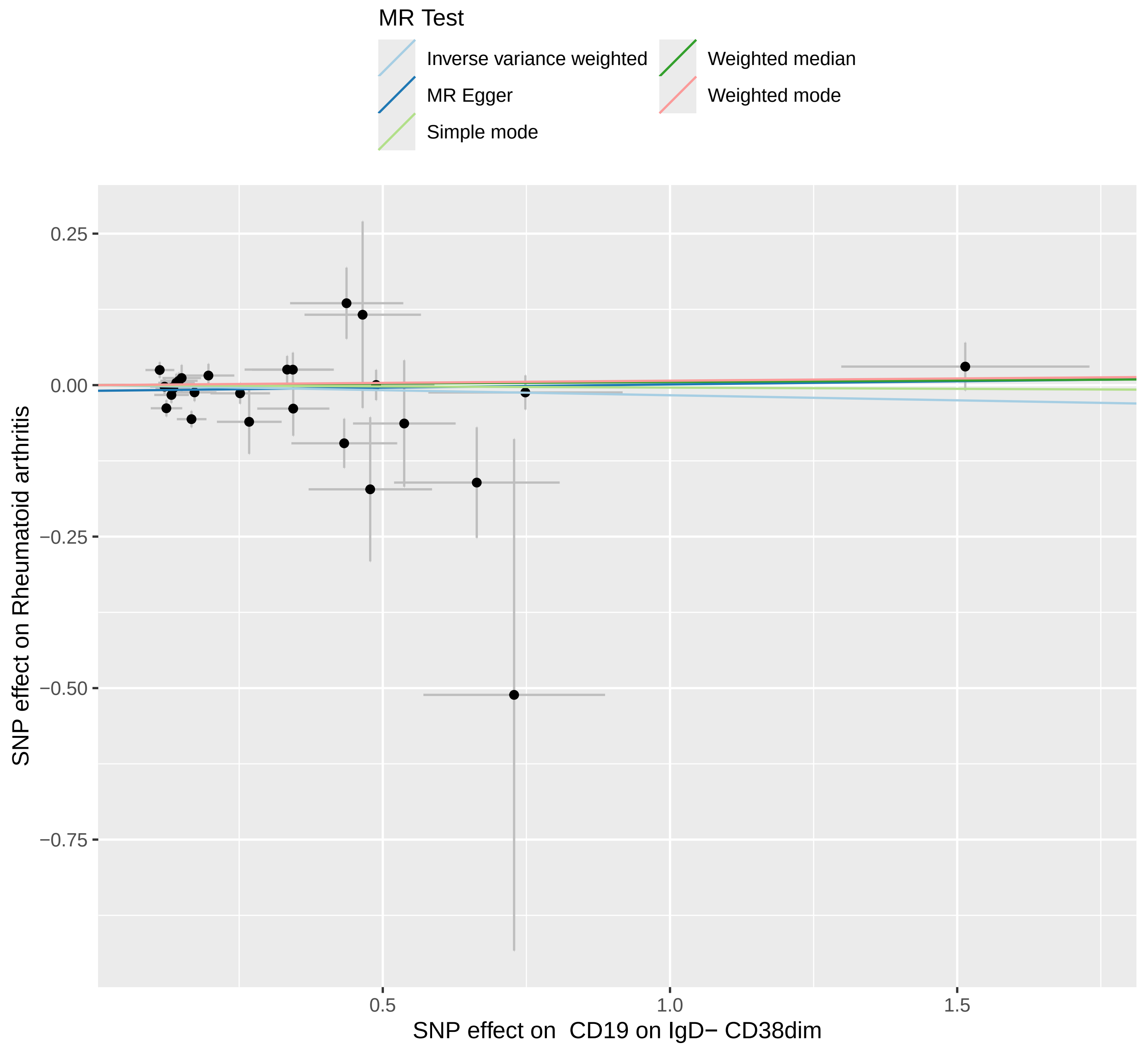

MR Test
Inverse variance weighted
MR Egger
Simple mode
Weighted median
Weighted mode
0.25
0.00
−0.25
−0.50
−0.75
0.5
1.0
1.5
SNP effect on Rheumatoid arthritis
SNP effect on CD19 on IgD− CD38dim

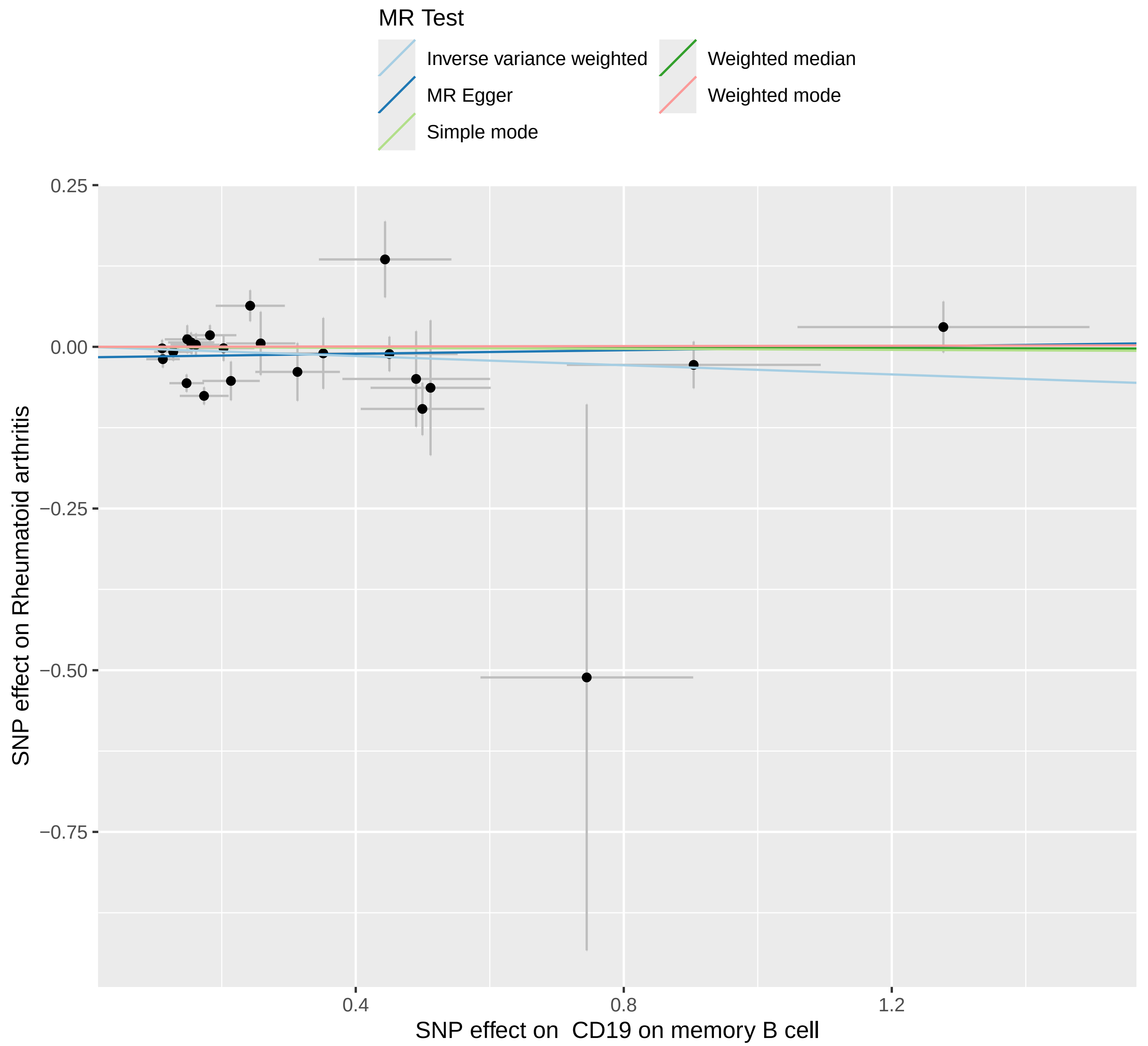
MR Test
Inverse variance weighted
MR Egger
Simple mode
Weighted median
Weighted mode
0.25
0.00
−0.25
−0.50
−0.75
0.4
0.8
1.2
SNP effect on Rheumatoid arthritis
SNP effect on CD19 on memory B cell

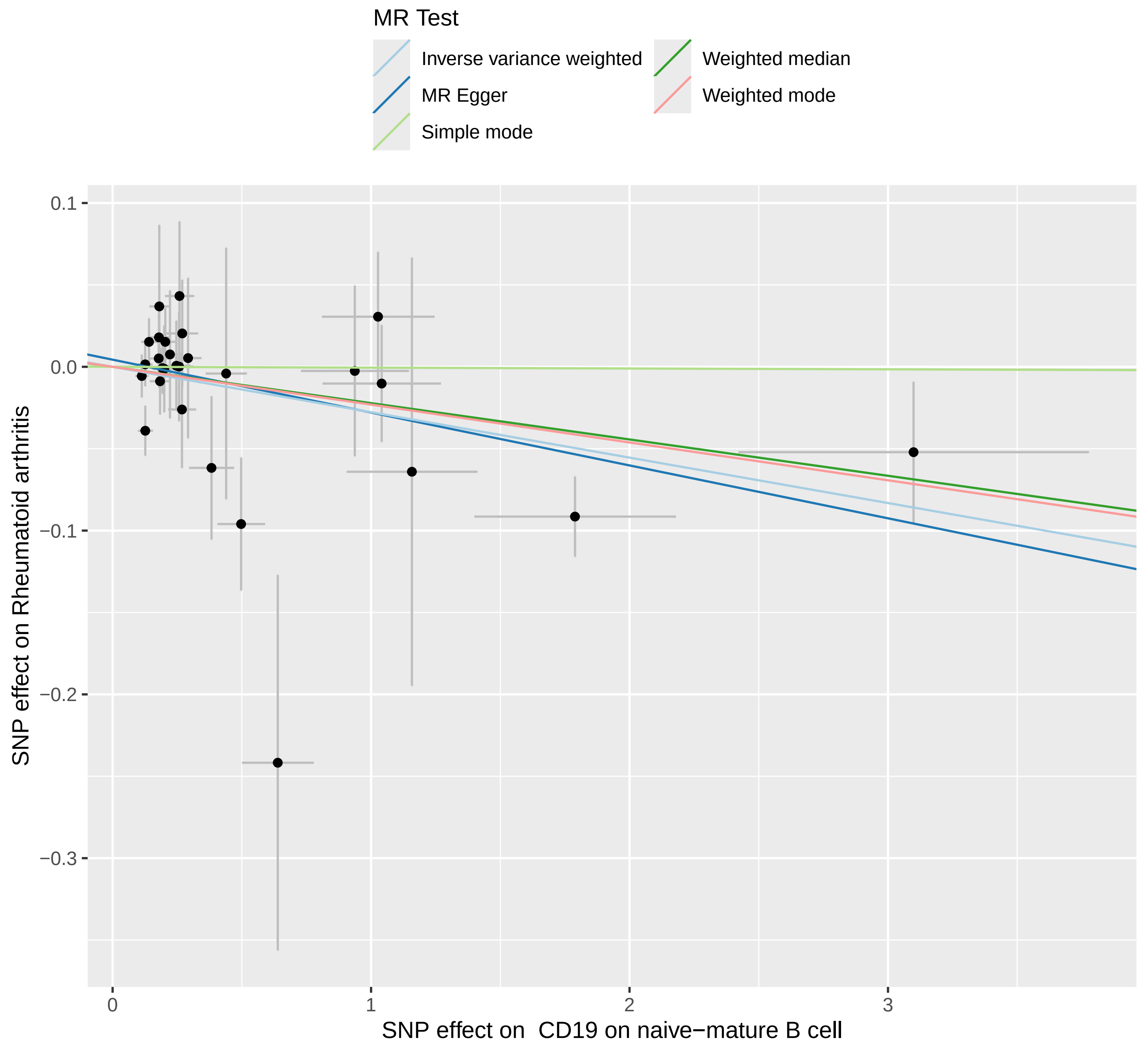

MR Test
Inverse variance weighted
MR Egger
Simple mode
Weighted median
Weighted mode
0.1
0.0
−0.1
−0.2
−0.3
0
1
2
3
SNP effect on Rheumatoid arthritis
SNP effect on CD19 on naive−mature B cell

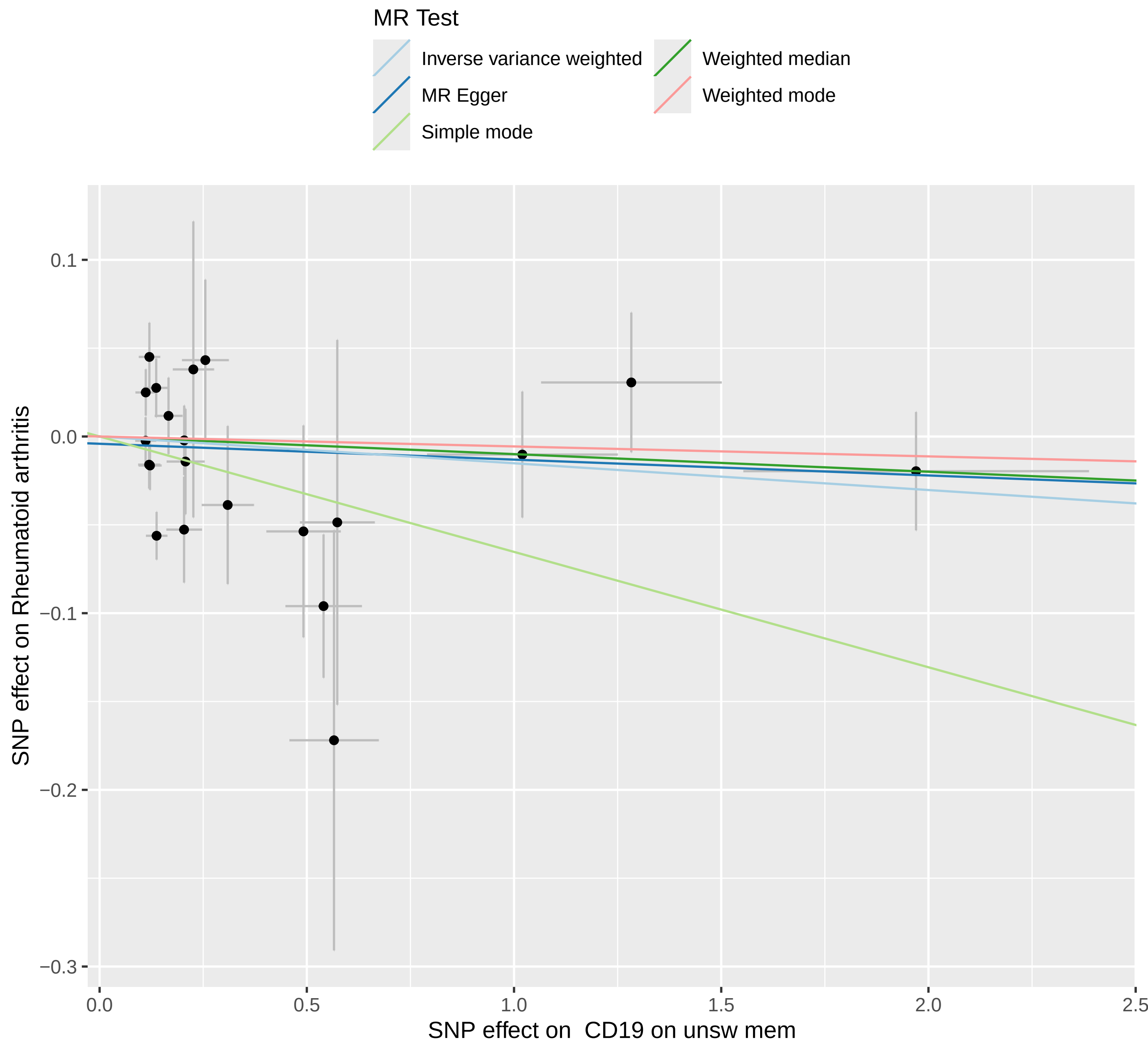

MR Test
Inverse variance weighted
MR Egger
Simple mode
Weighted median
Weighted mode
SNP effect on Rheumatoid arthritis
0.1
0.0
−0.1
−0.2
−0.3
0.0
0.5
1.0
1.5
2.0
2.5
SNP effect on CD19 on unsw mem

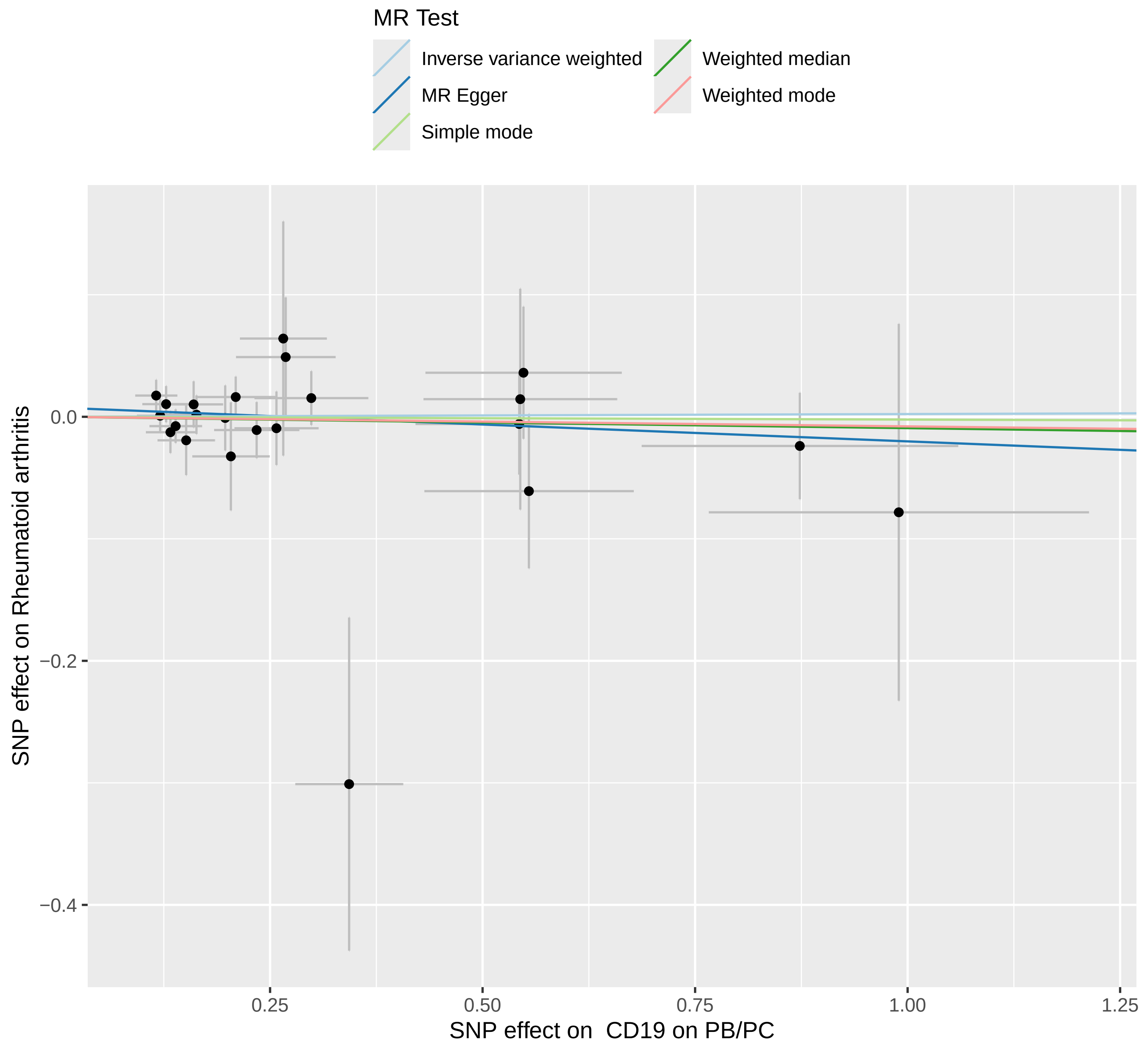

MR Test
Inverse variance weighted
MR Egger
Simple mode
Weighted median
Weighted mode
SNP effect on Rheumatoid arthritis
0.0
−0.2
−0.4
0.25
0.50
0.75
1.00
1.25
SNP effect on CD19 on PB/PC

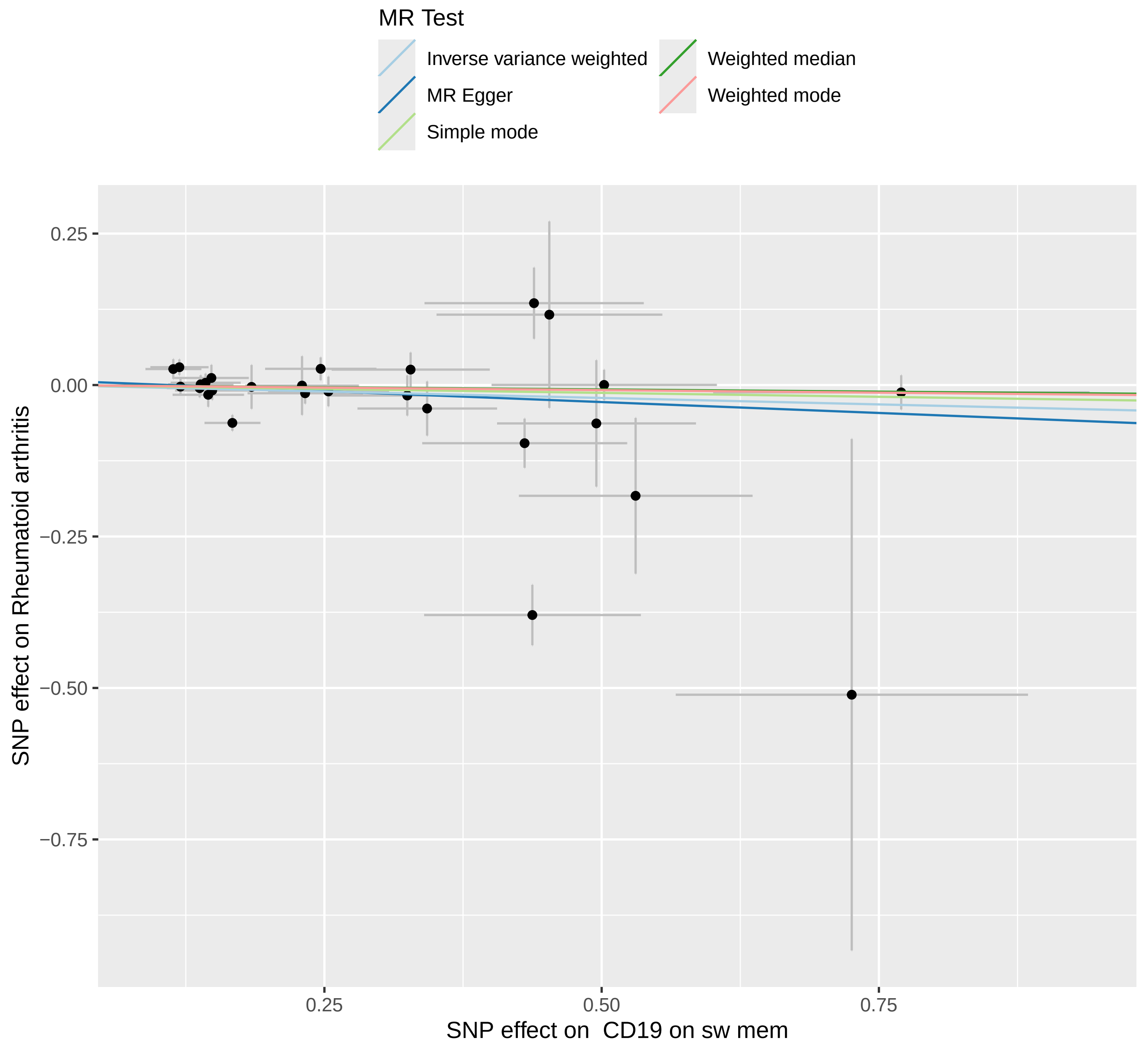

MR Test
Inverse variance weighted
MR Egger
Simple mode
Weighted median
Weighted mode
0.25
0.00
−0.25
−0.50
−0.75
SNP effect on Rheumatoid arthritis
0.25
0.50
0.75
SNP effect on CD19 on sw mem

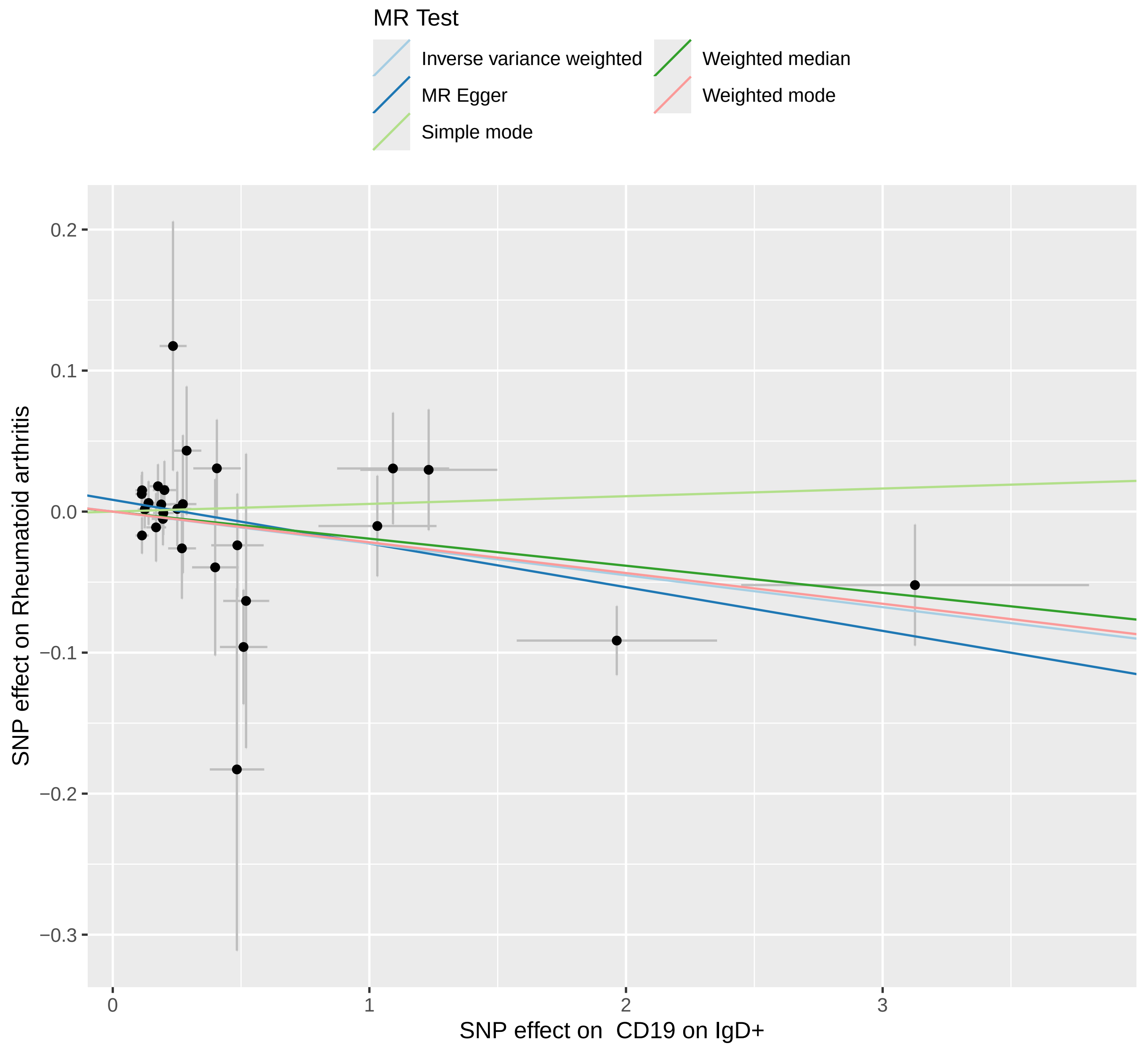

MR Test
Inverse variance weighted
MR Egger
Simple mode
Weighted median
Weighted mode
0.2
0.1
0.0
−0.1
−0.2
−0.3
0
1
2
3
SNP effect on Rheumatoid arthritis
SNP effect on CD19 on IgD+

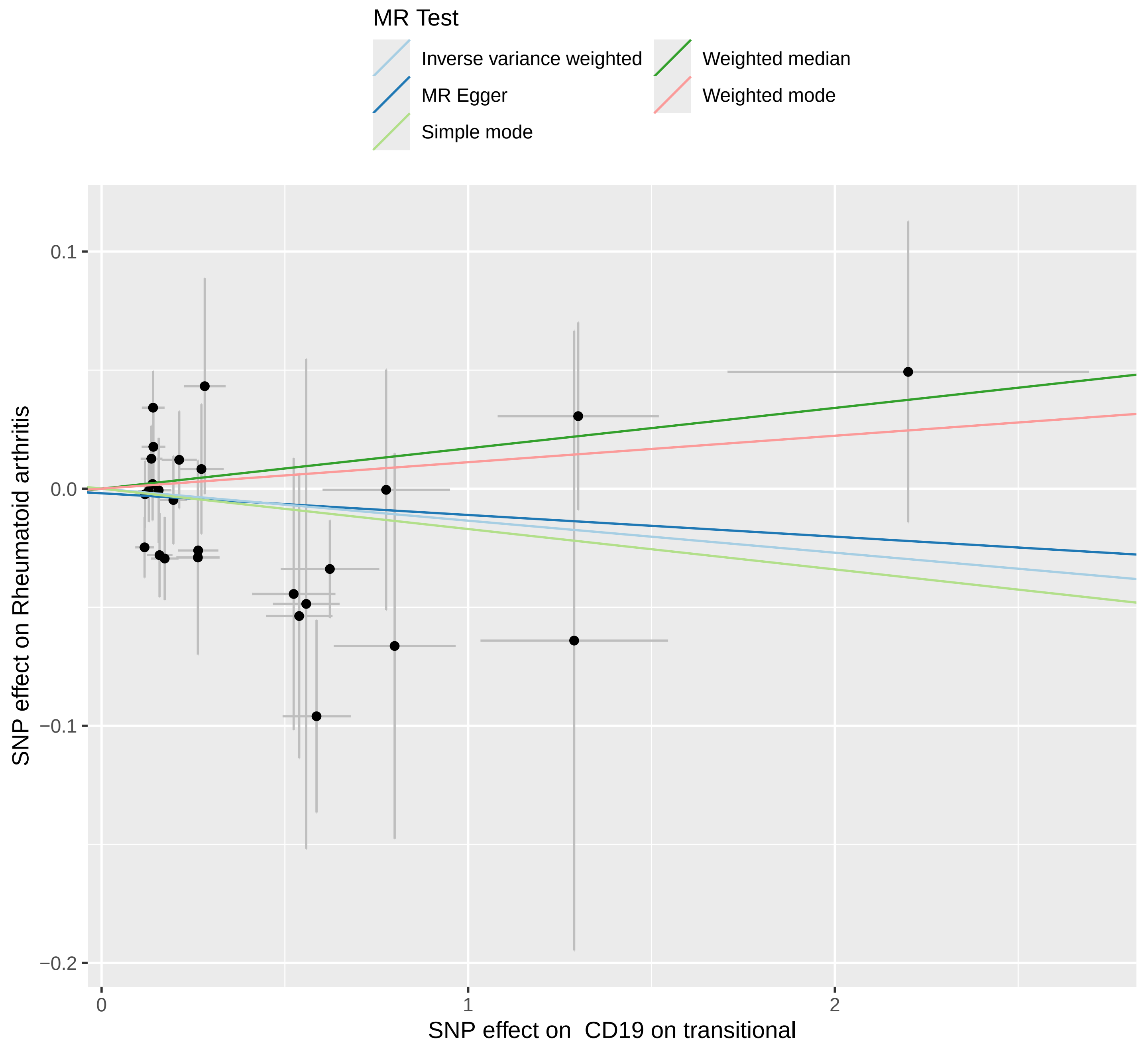

MR Test
Inverse variance weighted
MR Egger
Simple mode
Weighted median
Weighted mode
0.1
0.0
−0.1
−0.2
SNP effect on Rheumatoid arthritis
0
1
2
SNP effect on CD19 on transitional

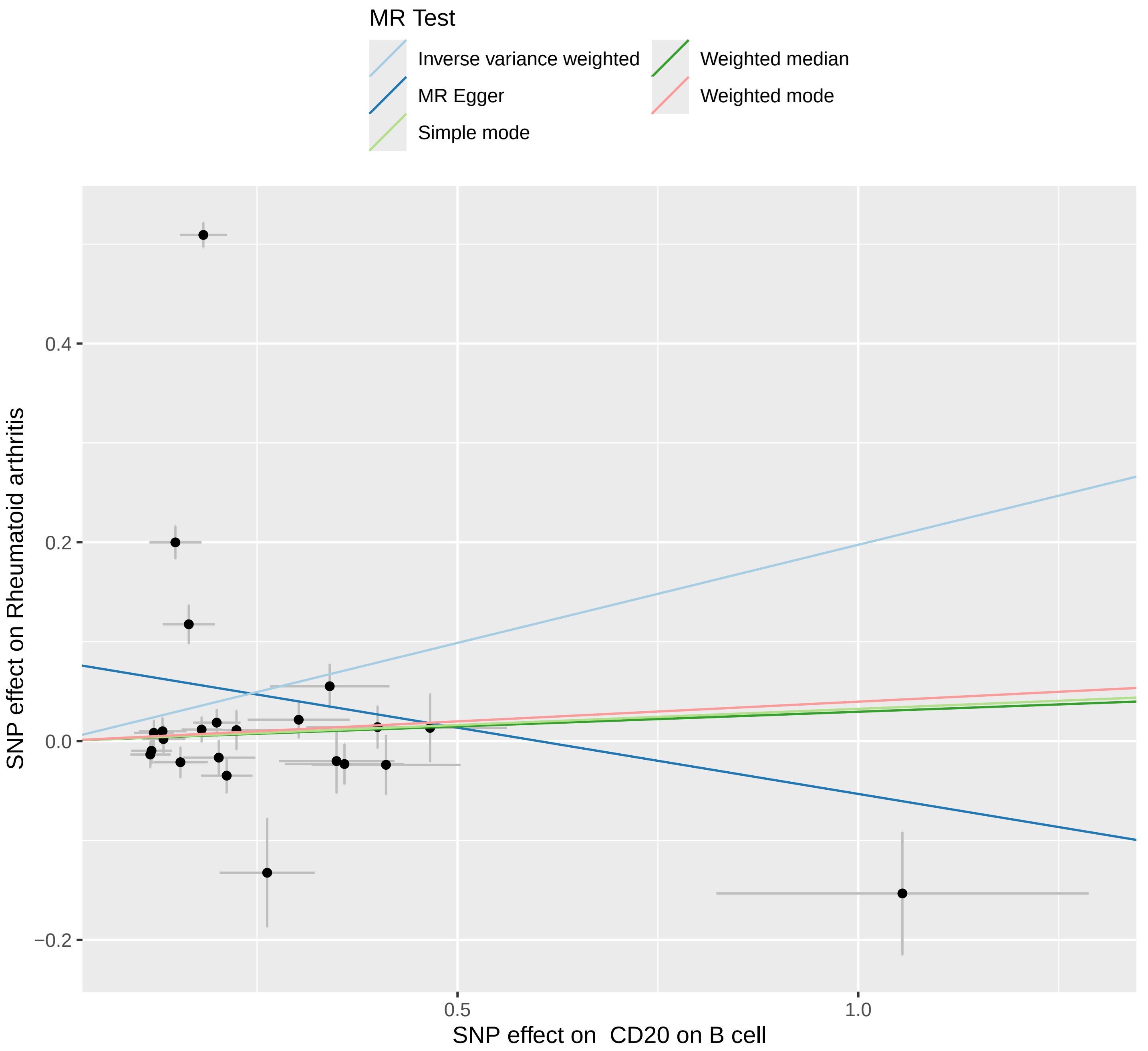

MR Test
Inverse variance weighted
MR Egger
Simple mode
Weighted median
Weighted mode
0.4
0.2
0.0
−0.2
0.5
1.0
SNP effect on Rheumatoid arthritis
SNP effect on CD20 on B cell

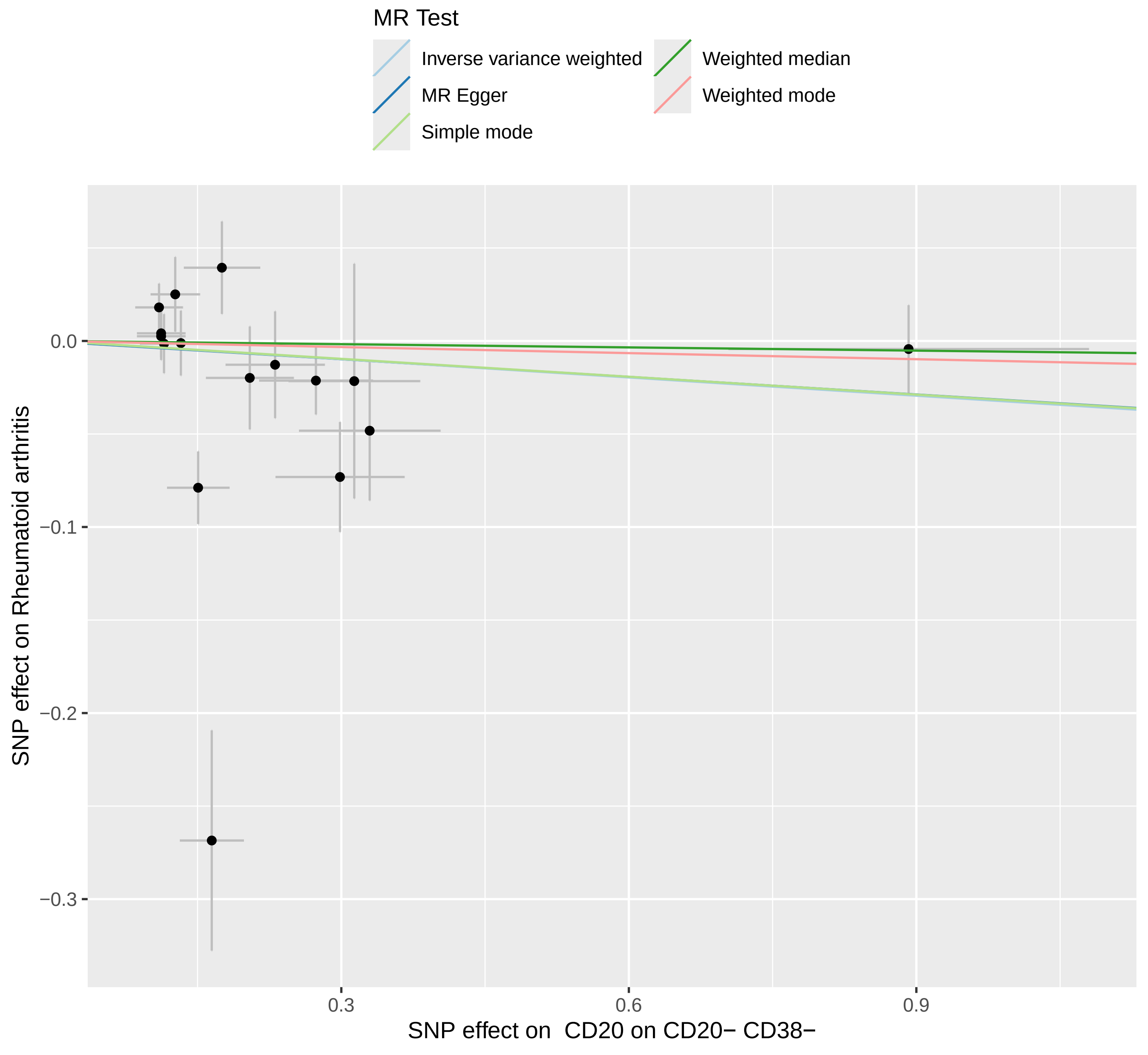
MR Test
Inverse variance weighted
MR Egger
Simple mode
Weighted median
Weighted mode
SNP effect on Rheumatoid arthritis
0.0
−0.1
−0.2
−0.3
0.3
0.6
0.9
SNP effect on CD20 on CD20− CD38−

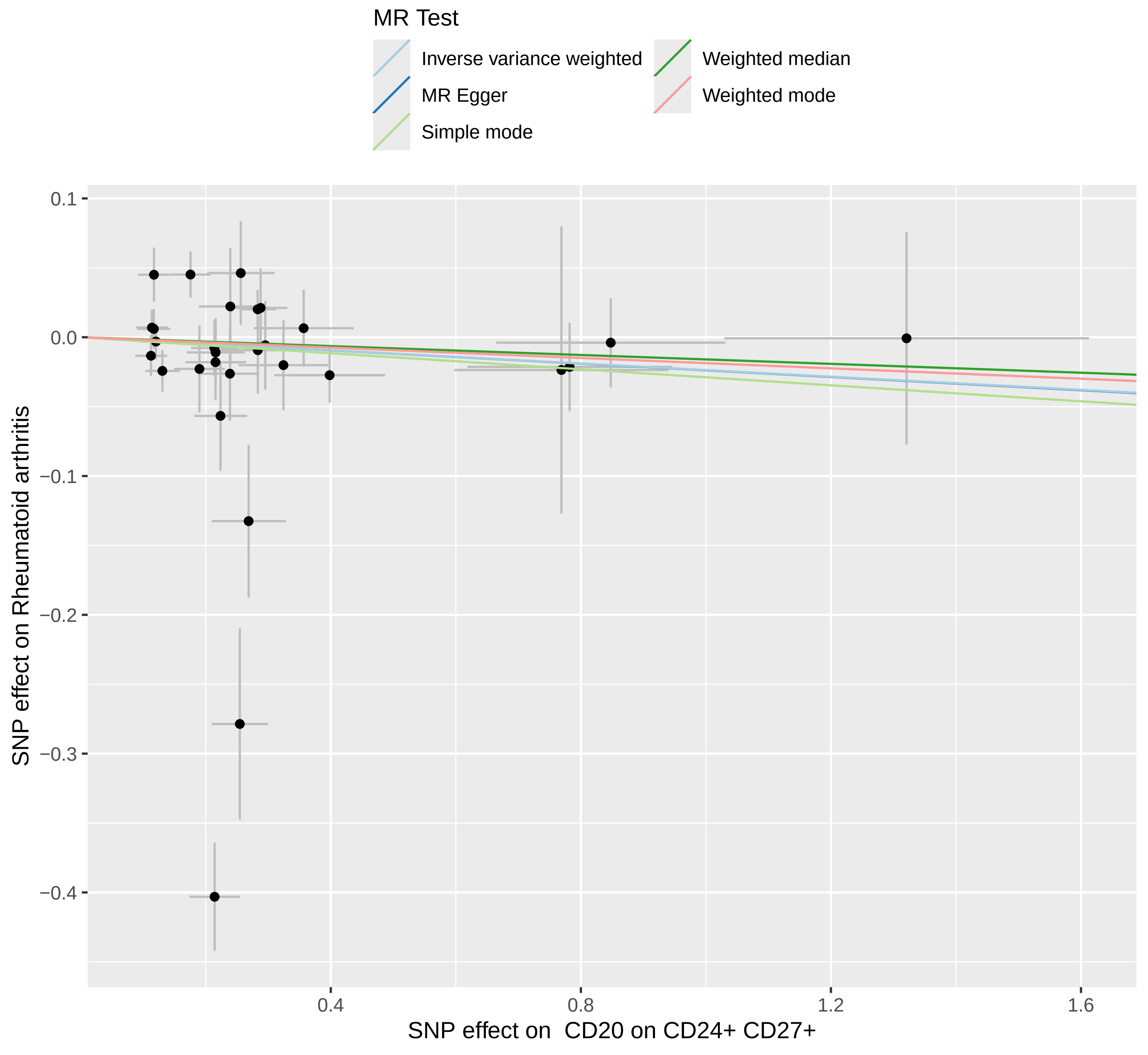
MR Test
Inverse variance weighted
MR Egger
Simple mode
Weighted median
Weighted mode
0.1
0.0
−0.1
−0.2
−0.3
−0.4
0.4
0.8
1.2
1.6
SNP effect on Rheumatoid arthritis
SNP effect on CD20 on CD24+ CD27+

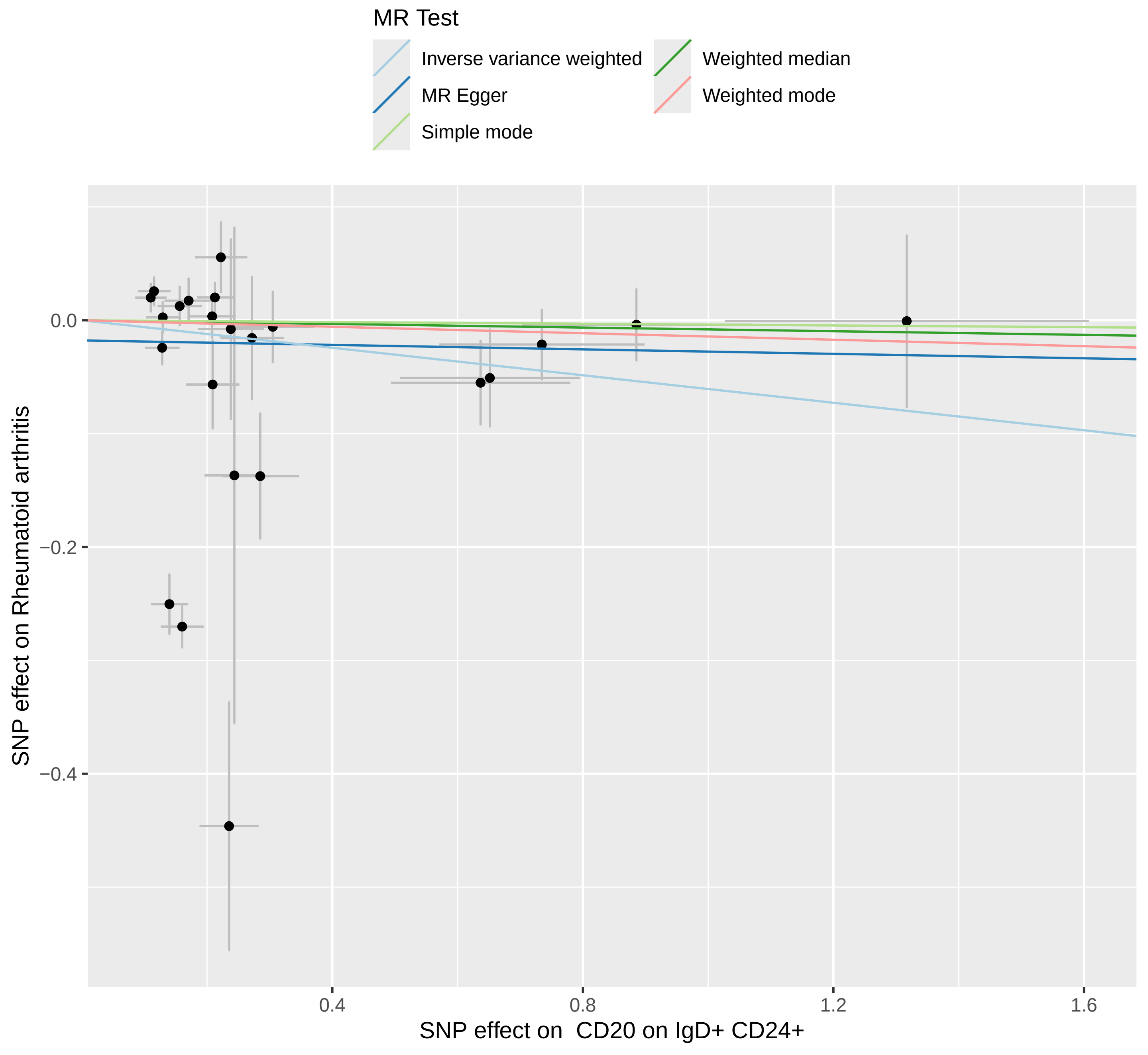

MR Test
Inverse variance weighted
MR Egger
Simple mode
Weighted median
Weighted mode
0.0
−0.2
−0.4
0.4
0.8
1.2
1.6
SNP effect on Rheumatoid arthritis
SNP effect on CD20 on IgD+ CD24+

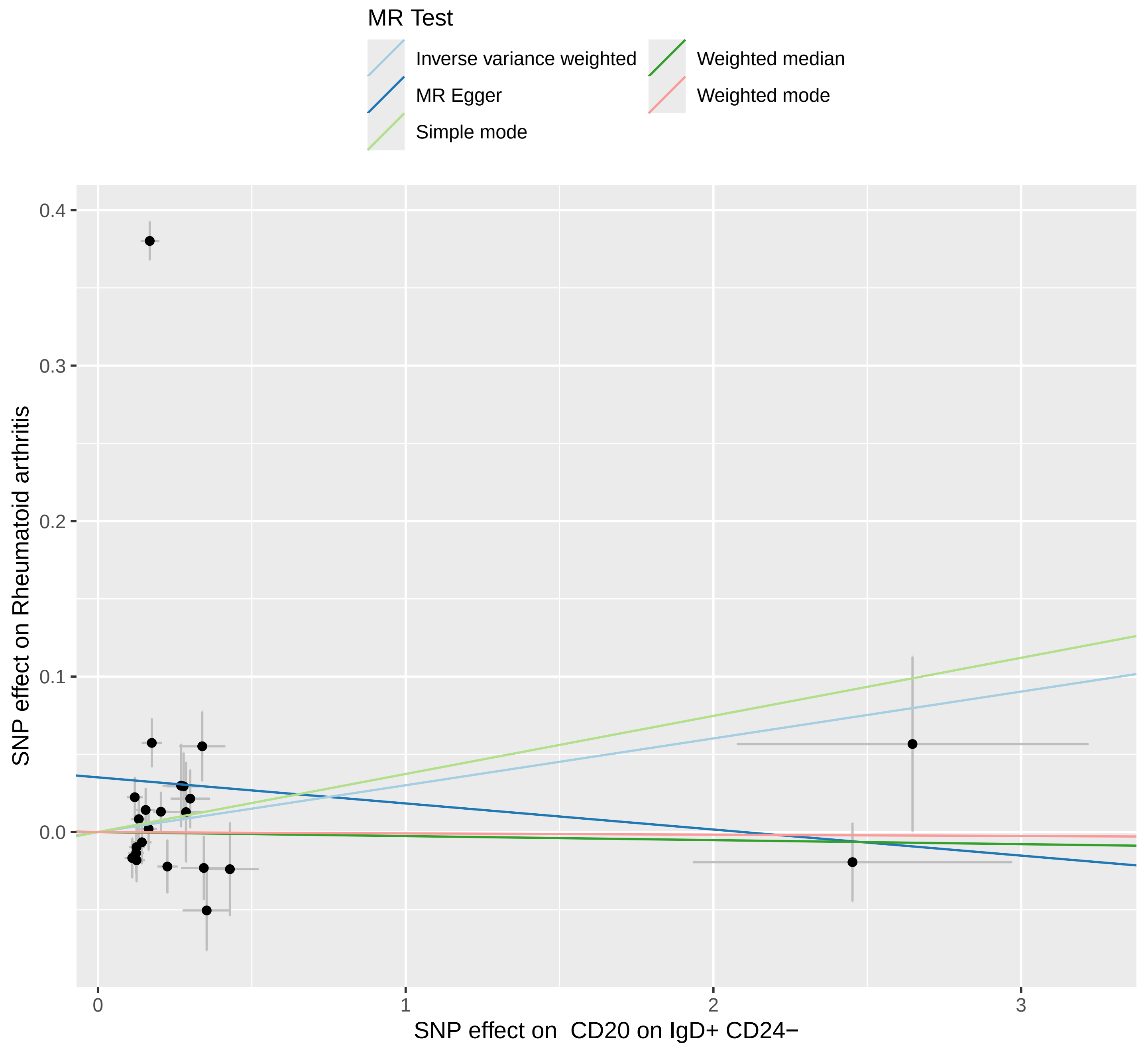

MR Test
Inverse variance weighted
MR Egger
Simple mode
Weighted median
Weighted mode
SNP effect on Rheumatoid arthritis
0.4
0.3
0.2
0.1
0.0
0
1
2
3
SNP effect on CD20 on IgD+ CD24−

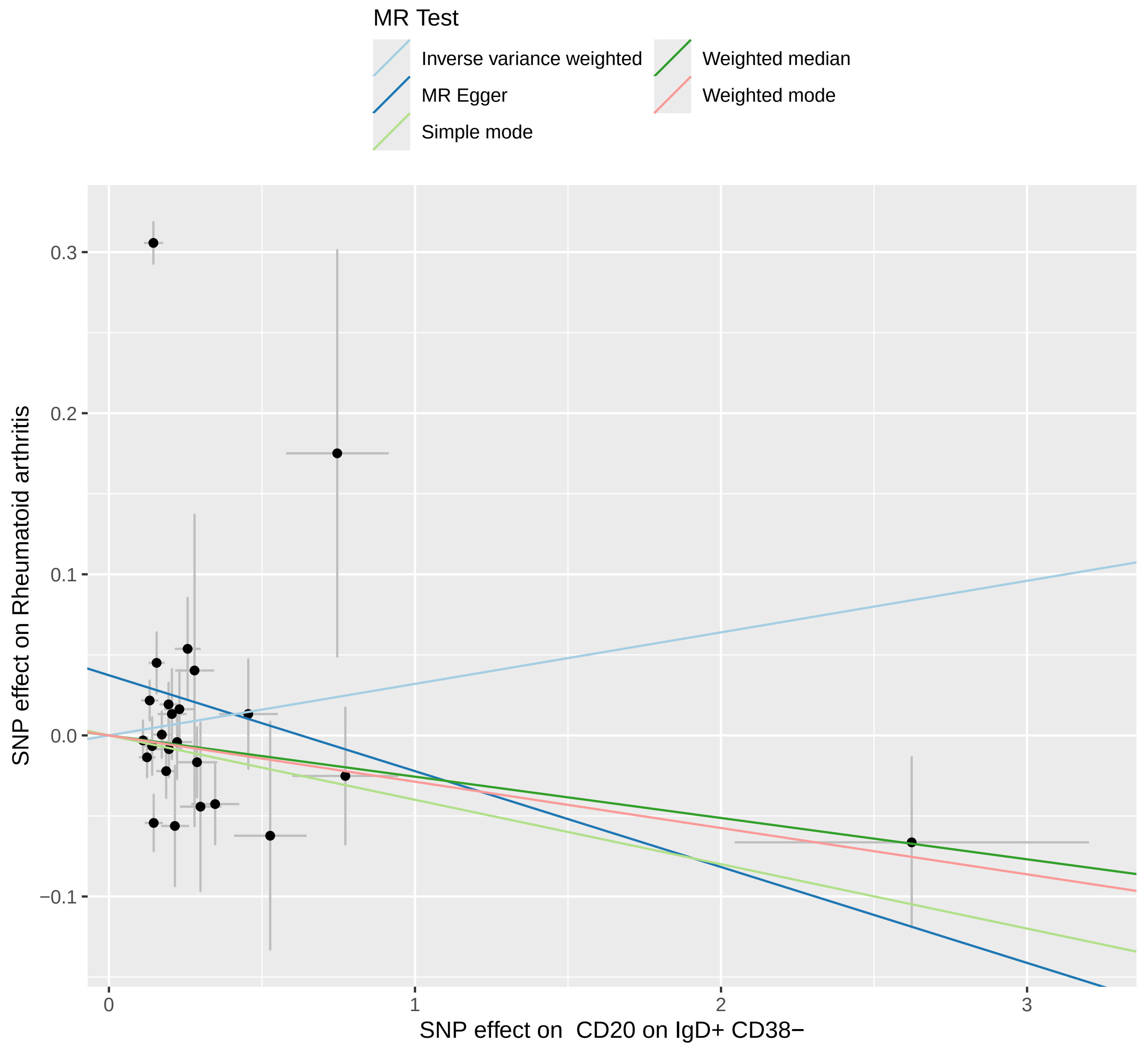

MR Test
Inverse variance weighted
MR Egger
Simple mode
Weighted median
Weighted mode
SNP effect on Rheumatoid arthritis
0.3
0.2
0.1
0.0
−0.1
0
1
2
3
SNP effect on CD20 on IgD+ CD38−

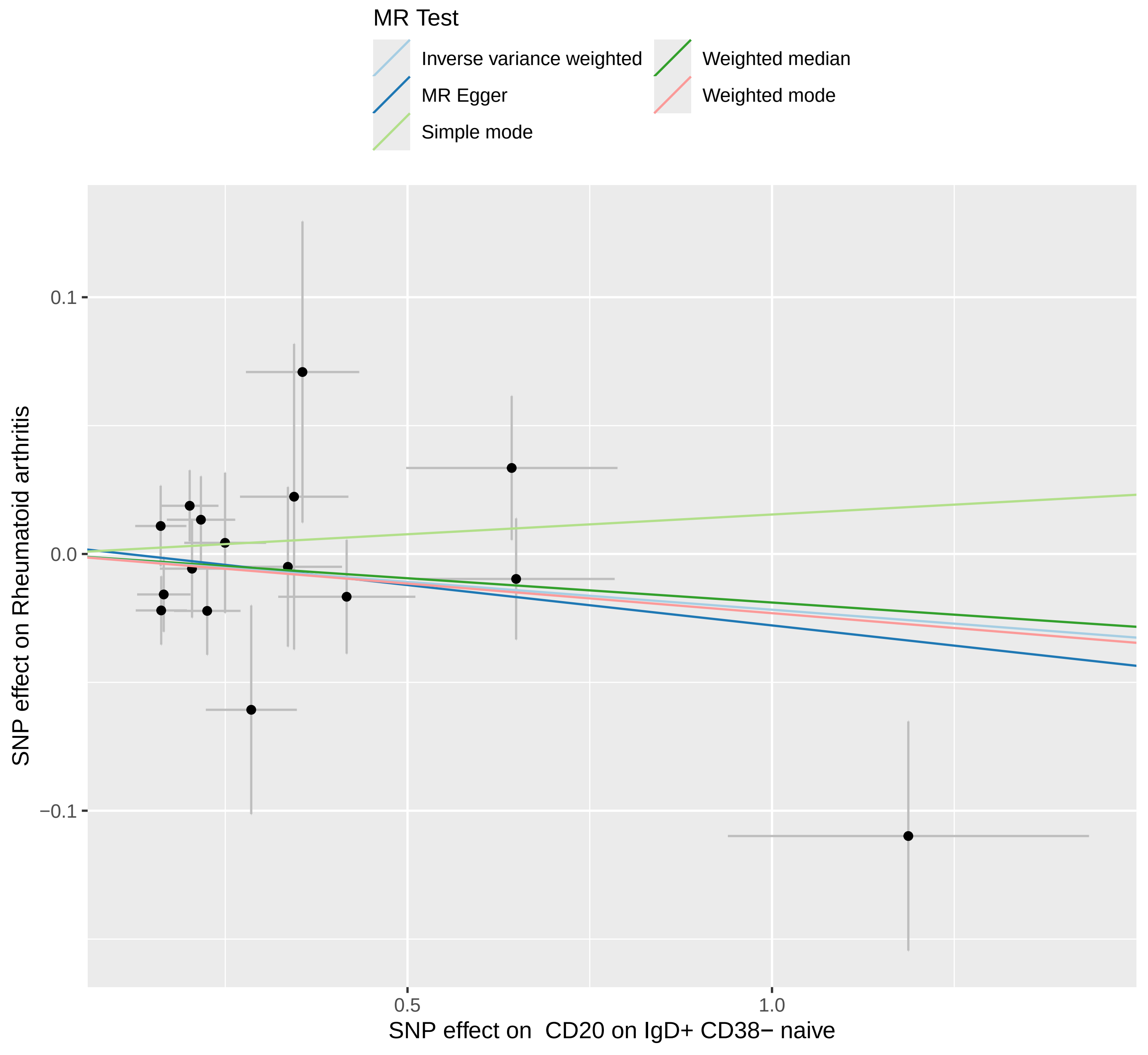

MR Test
Inverse variance weighted
MR Egger
Simple mode
Weighted median
Weighted mode
0.1
0.0
−0.1
0.5
1.0
SNP effect on Rheumatoid arthritis
SNP effect on CD20 on IgD+ CD38− naive

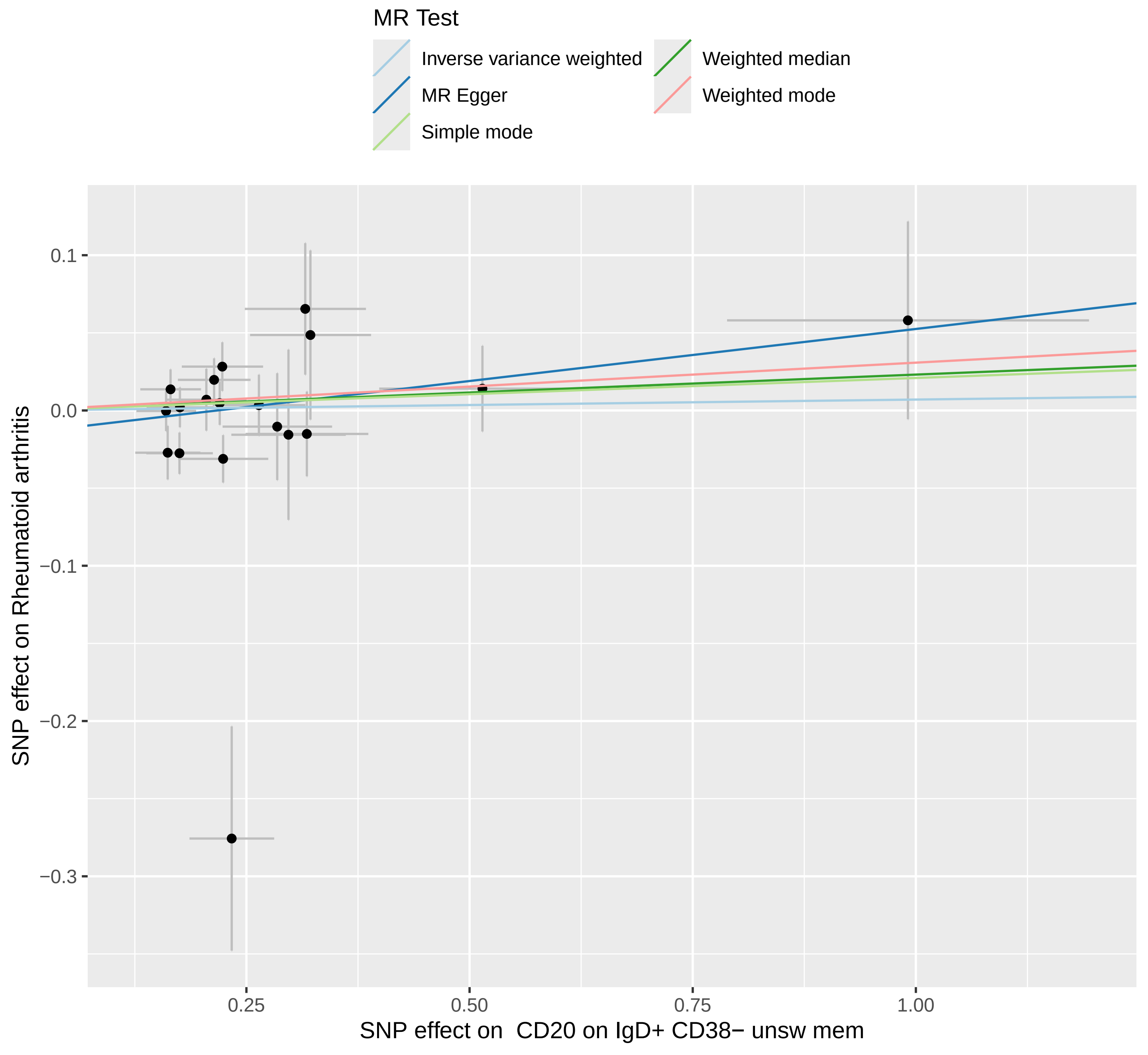

MR Test
Inverse variance weighted
MR Egger
Simple mode
Weighted median
Weighted mode
0.1
0.0
−0.1
−0.2
−0.3
0.25
0.50
0.75
1.00
SNP effect on Rheumatoid arthritis
SNP effect on CD20 on IgD+ CD38− unsw mem

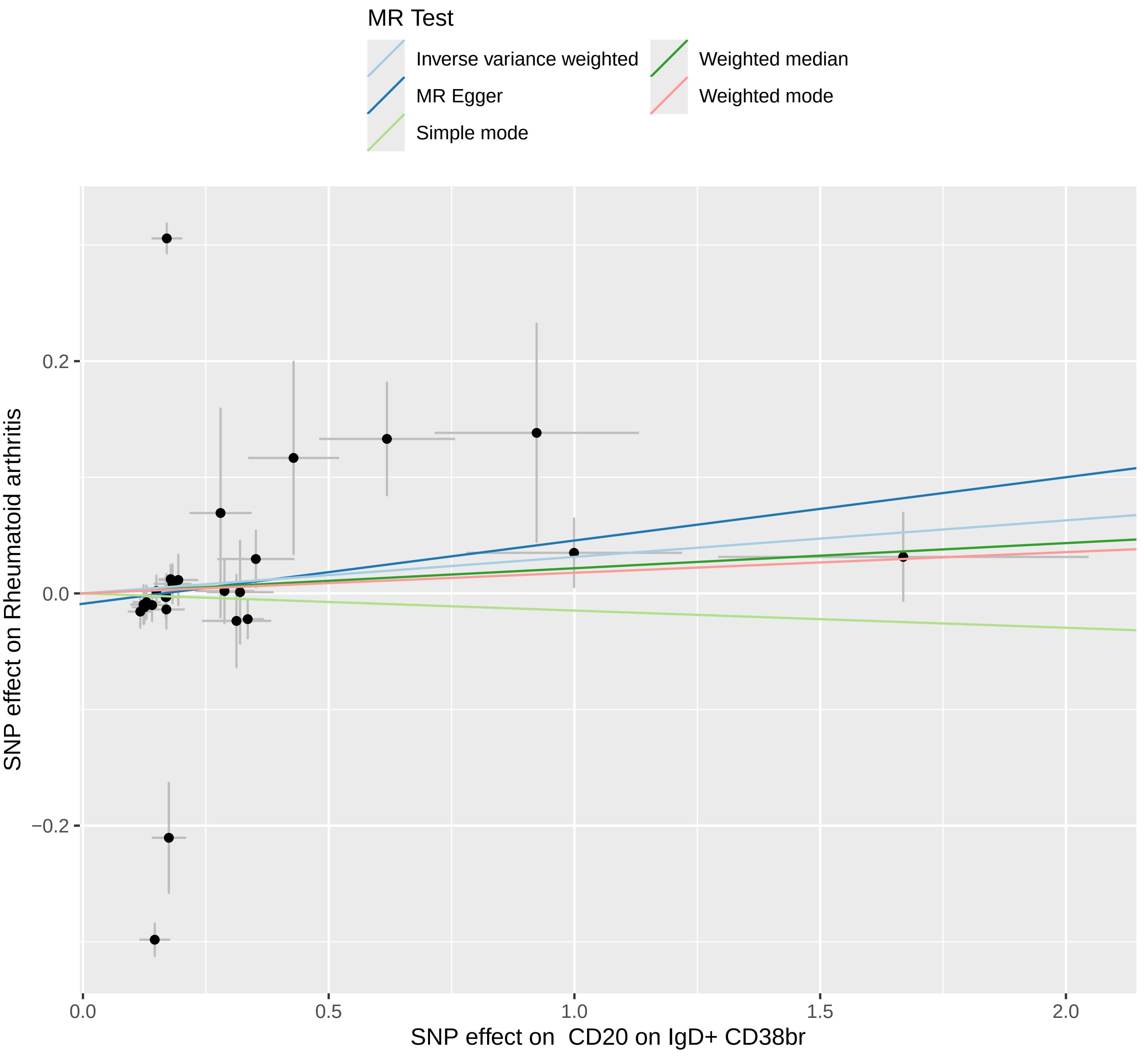

MR Test
Inverse variance weighted
MR Egger
Simple mode
Weighted median
Weighted mode
SNP effect on Rheumatoid arthritis
0.2
0.0
−0.2
0.0
0.5
1.0
1.5
2.0
SNP effect on CD20 on IgD+ CD38br

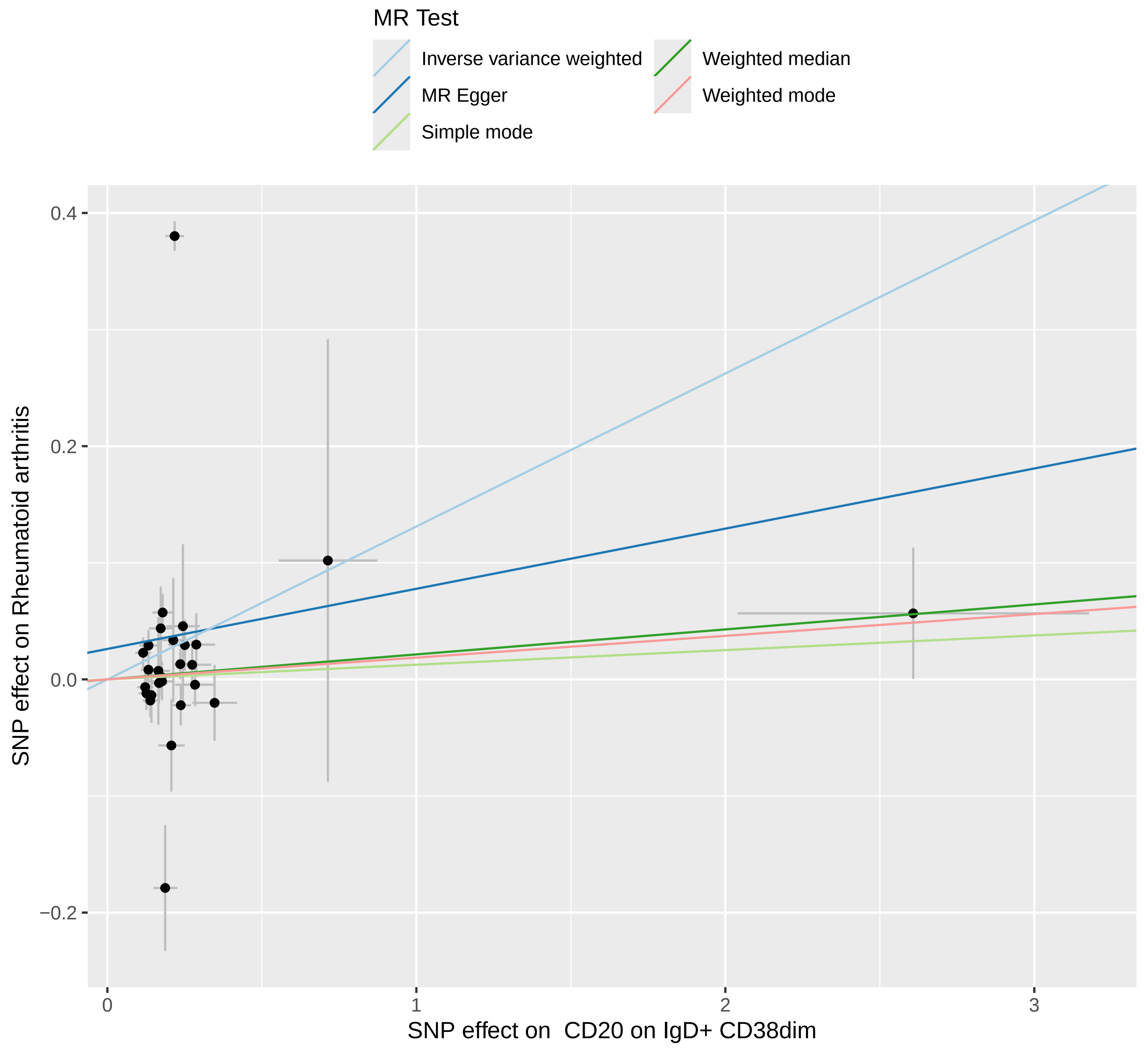
MR Test
Inverse variance weighted
MR Egger
Simple mode
Weighted median
Weighted mode
SNP effect on Rheumatoid arthritis
SNP effect on CD20 on IgD+ CD38dim
0.4
0.2
0.0
−0.2
0
1
2
3

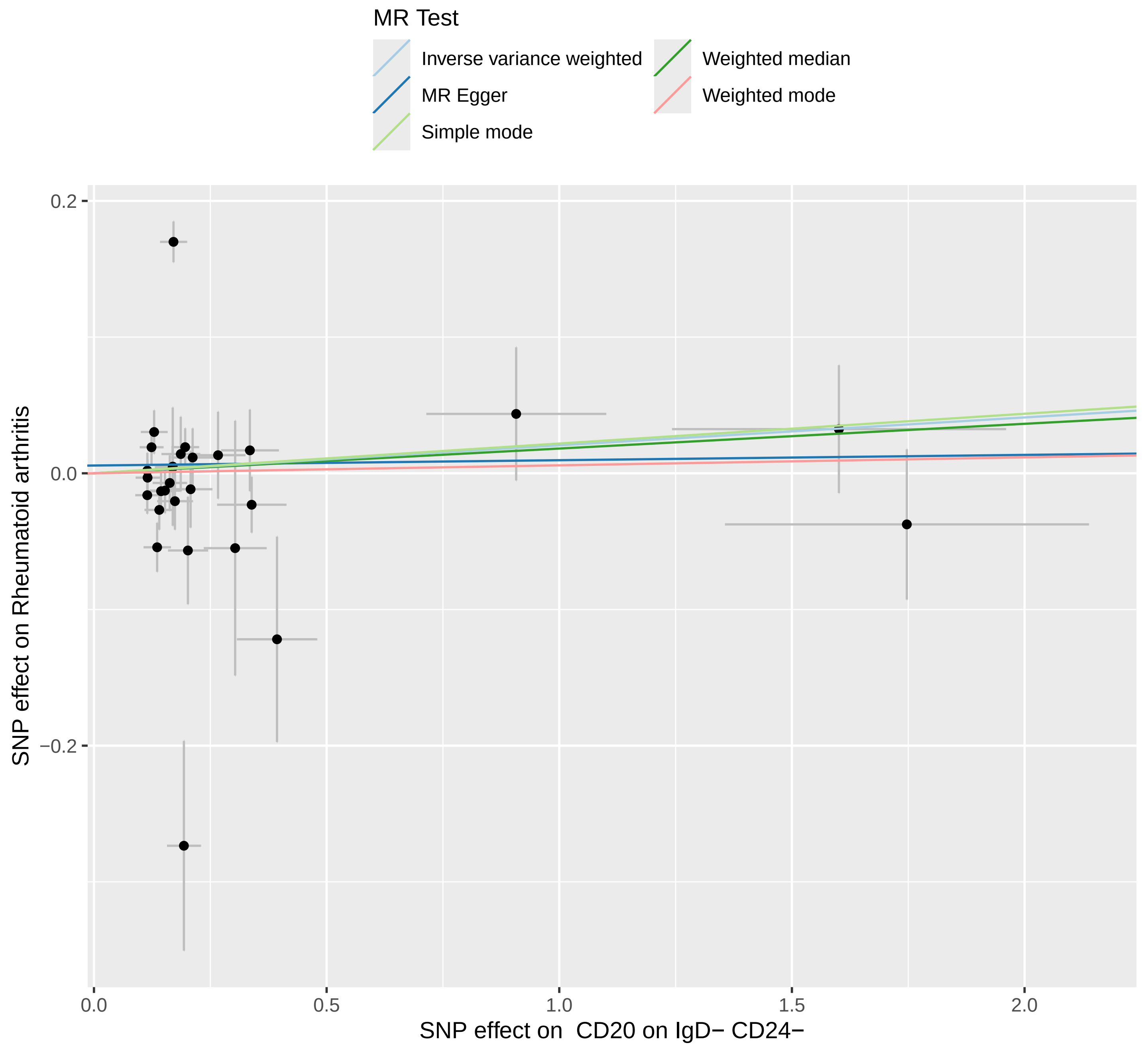
MR Test
Inverse variance weighted
MR Egger
Simple mode
Weighted median
Weighted mode
0.2
0.0
−0.2
0.0
0.5
1.0
1.5
2.0
SNP effect on Rheumatoid arthritis
SNP effect on CD20 on IgD− CD24−

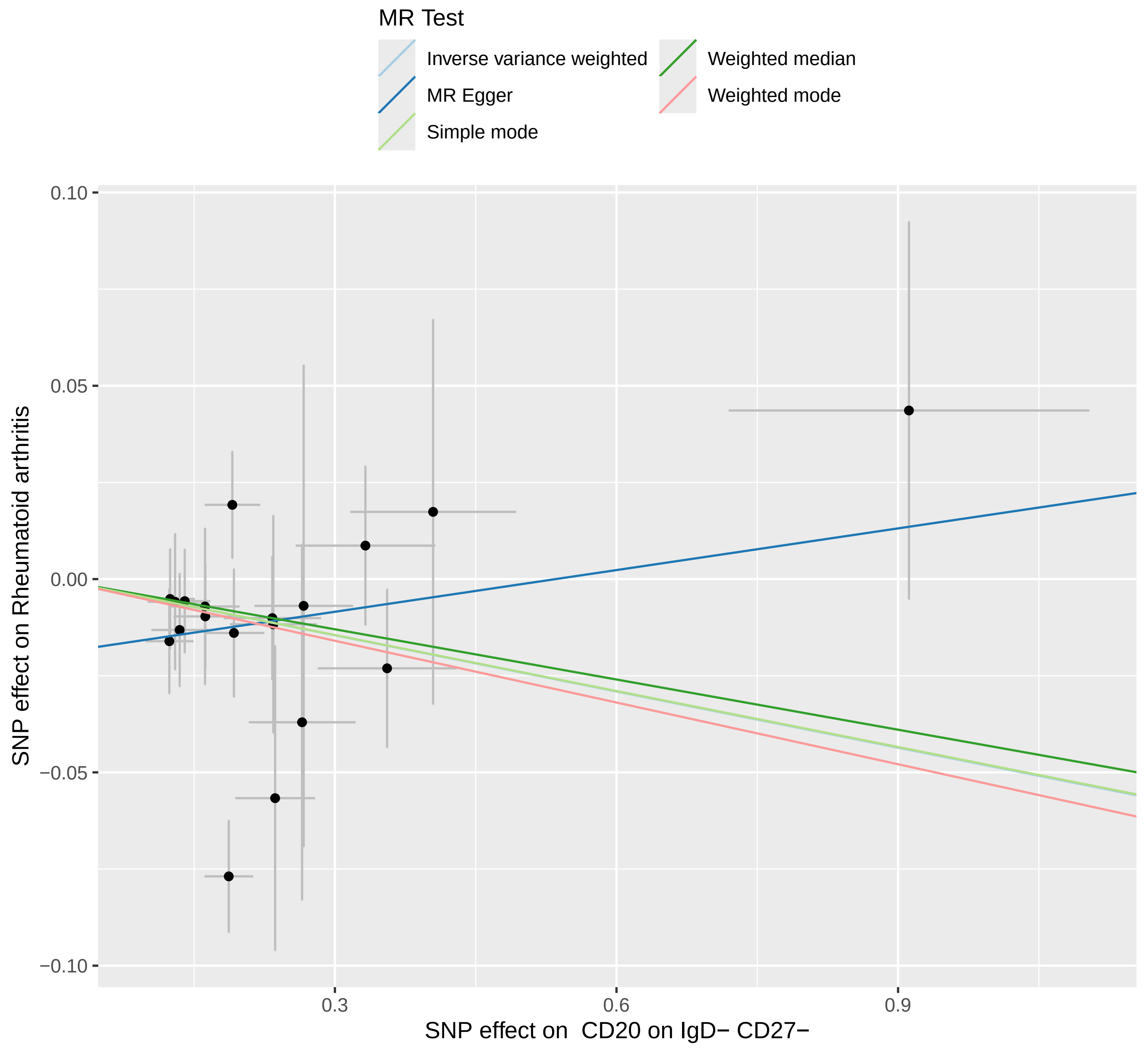

MR Test
Inverse variance weighted
MR Egger
Simple mode
Weighted median
Weighted mode
0.10
0.05
0.00
−0.05
−0.10
0.3
0.6
0.9
SNP effect on Rheumatoid arthritis
SNP effect on CD20 on IgD− CD27−

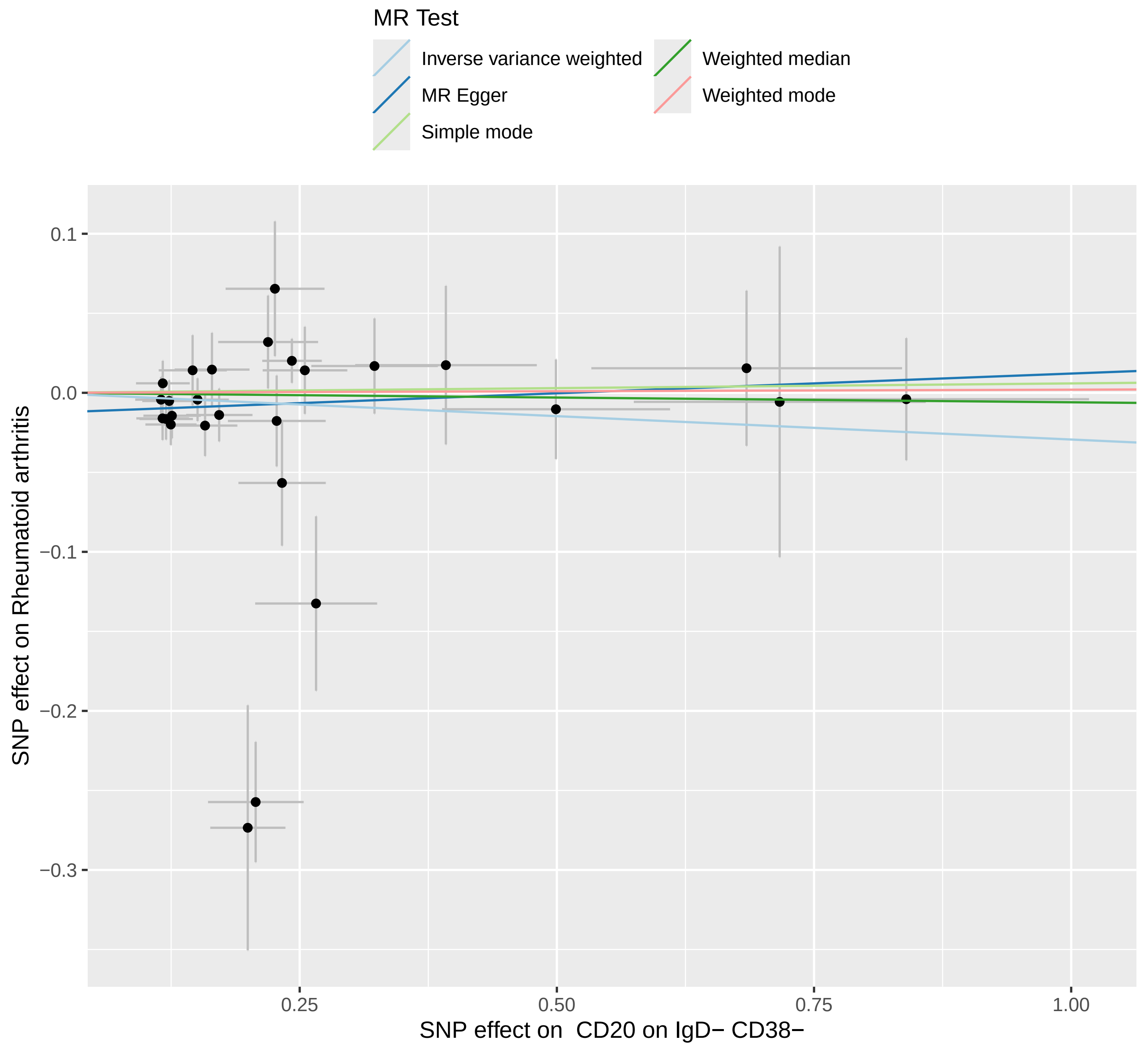

MR Test
Inverse variance weighted
MR Egger
Simple mode
Weighted median
Weighted mode
0.1
0.0
−0.1
−0.2
−0.3
0.25
0.50
0.75
1.00
SNP effect on Rheumatoid arthritis
SNP effect on CD20 on IgD− CD38−

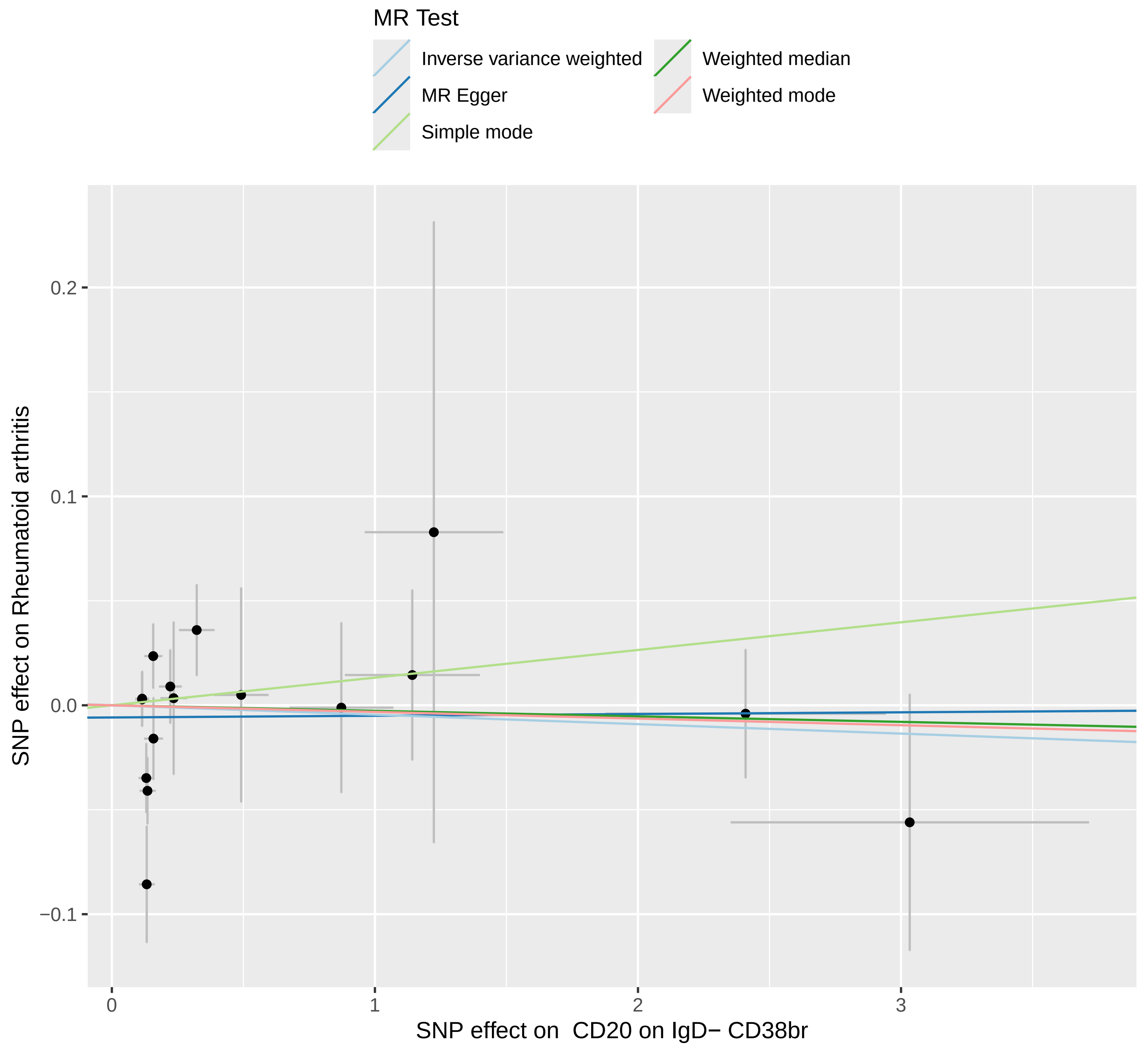

MR Test
Inverse variance weighted
MR Egger
Simple mode
Weighted median
Weighted mode
0.2
0.1
0.0
−0.1
0
1
2
3
SNP effect on Rheumatoid arthritis
SNP effect on CD20 on IgD− CD38br

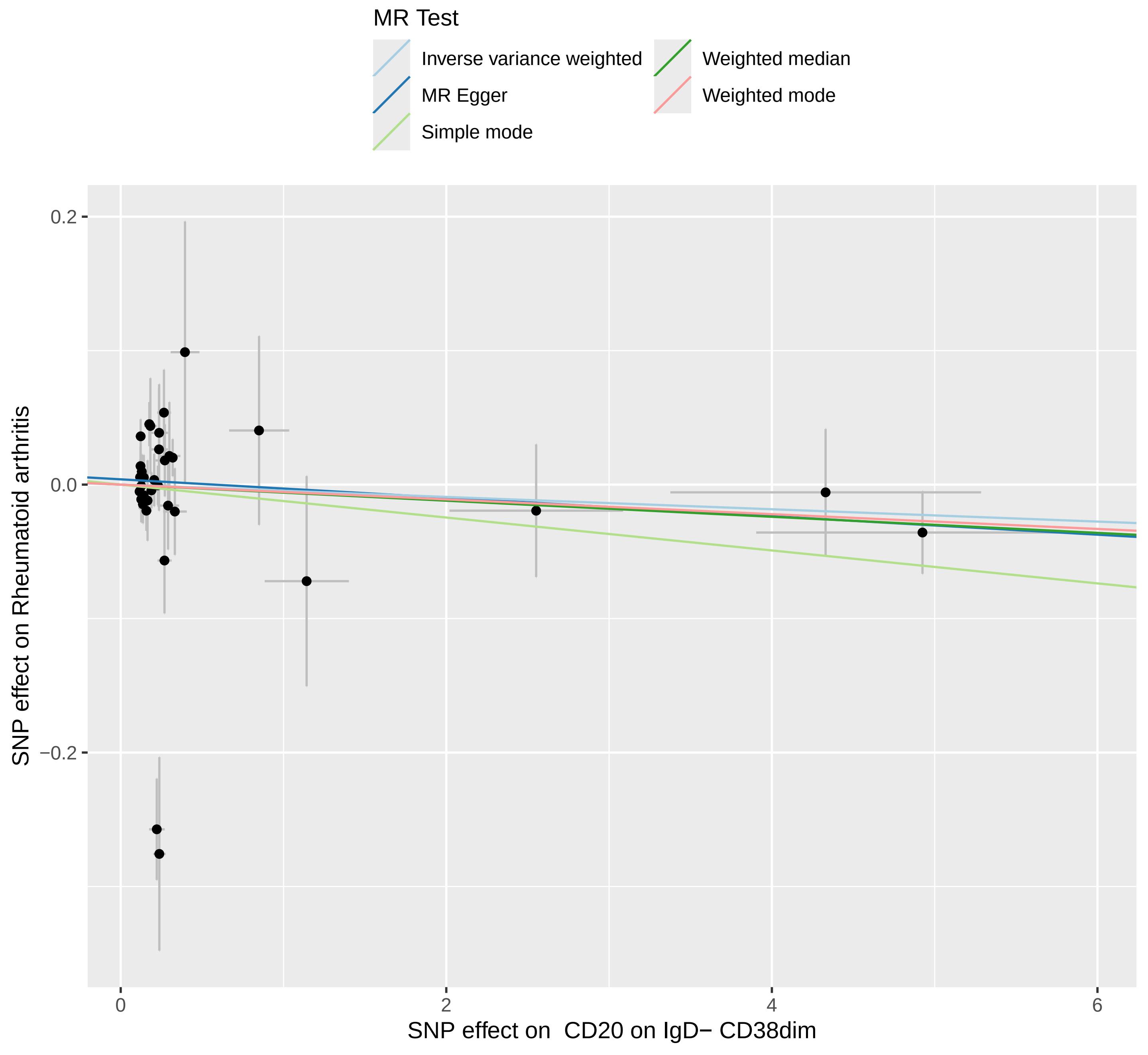
MR Test
Inverse variance weighted
MR Egger
Simple mode
Weighted median
Weighted mode
0.2
0.0
−0.2
0
2
4
6
SNP effect on Rheumatoid arthritis
SNP effect on CD20 on IgD− CD38dim

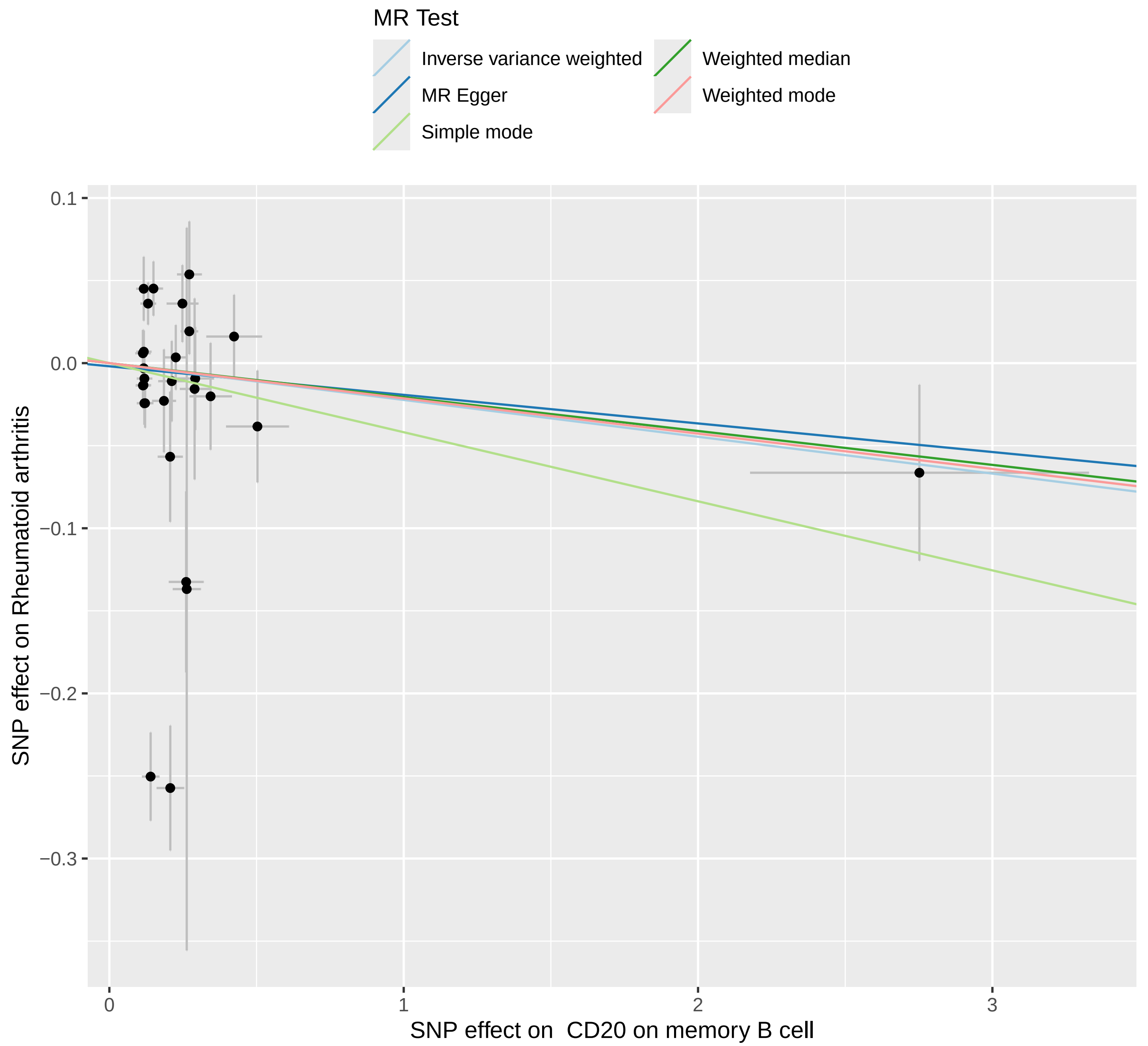

MR Test
Inverse variance weighted
MR Egger
Simple mode
Weighted median
Weighted mode
0.1
0.0
−0.1
−0.2
−0.3
0
1
2
3
SNP effect on Rheumatoid arthritis
SNP effect on CD20 on memory B cell

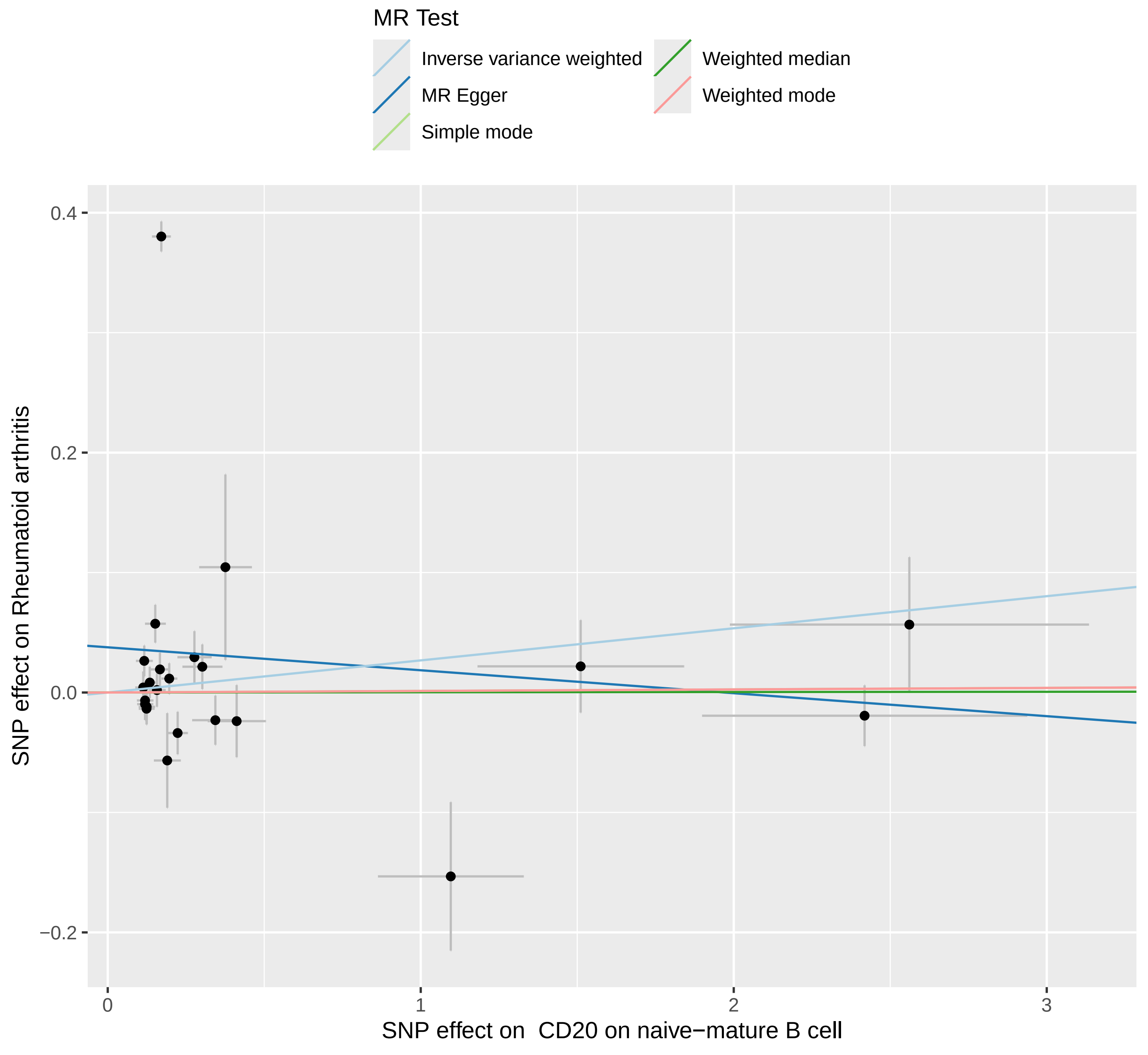

MR Test
Inverse variance weighted
Weighted median
MR Egger
Weighted mode
Simple mode
0.4
0.2
0.0
−0.2
0
1
2
3
SNP effect on Rheumatoid arthritis
SNP effect on CD20 on naive−mature B cell

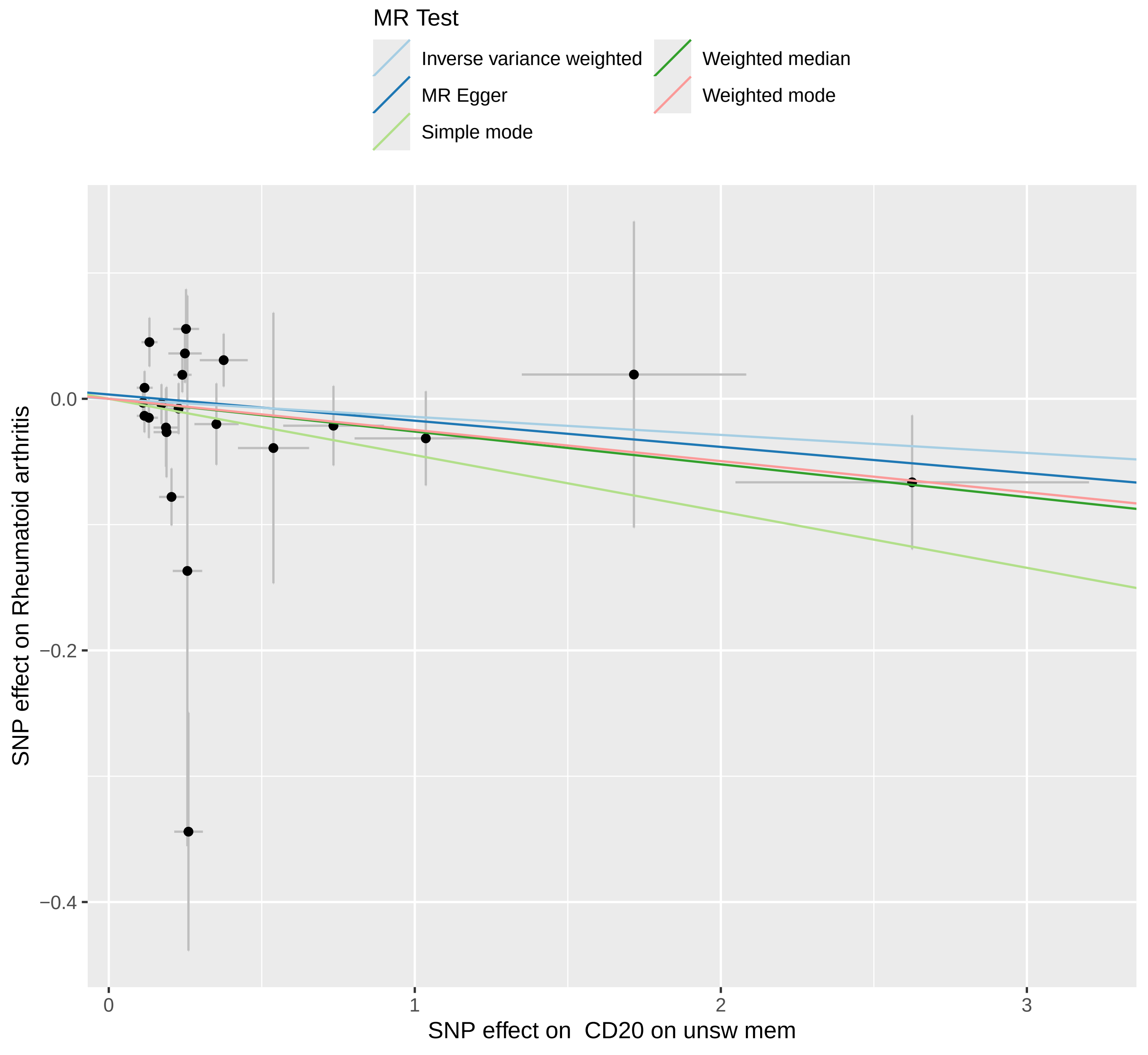

MR Test
Inverse variance weighted
MR Egger
Simple mode
Weighted median
Weighted mode
SNP effect on Rheumatoid arthritis
0.0
−0.2
−0.4
0
1
2
3
SNP effect on CD20 on unsw mem

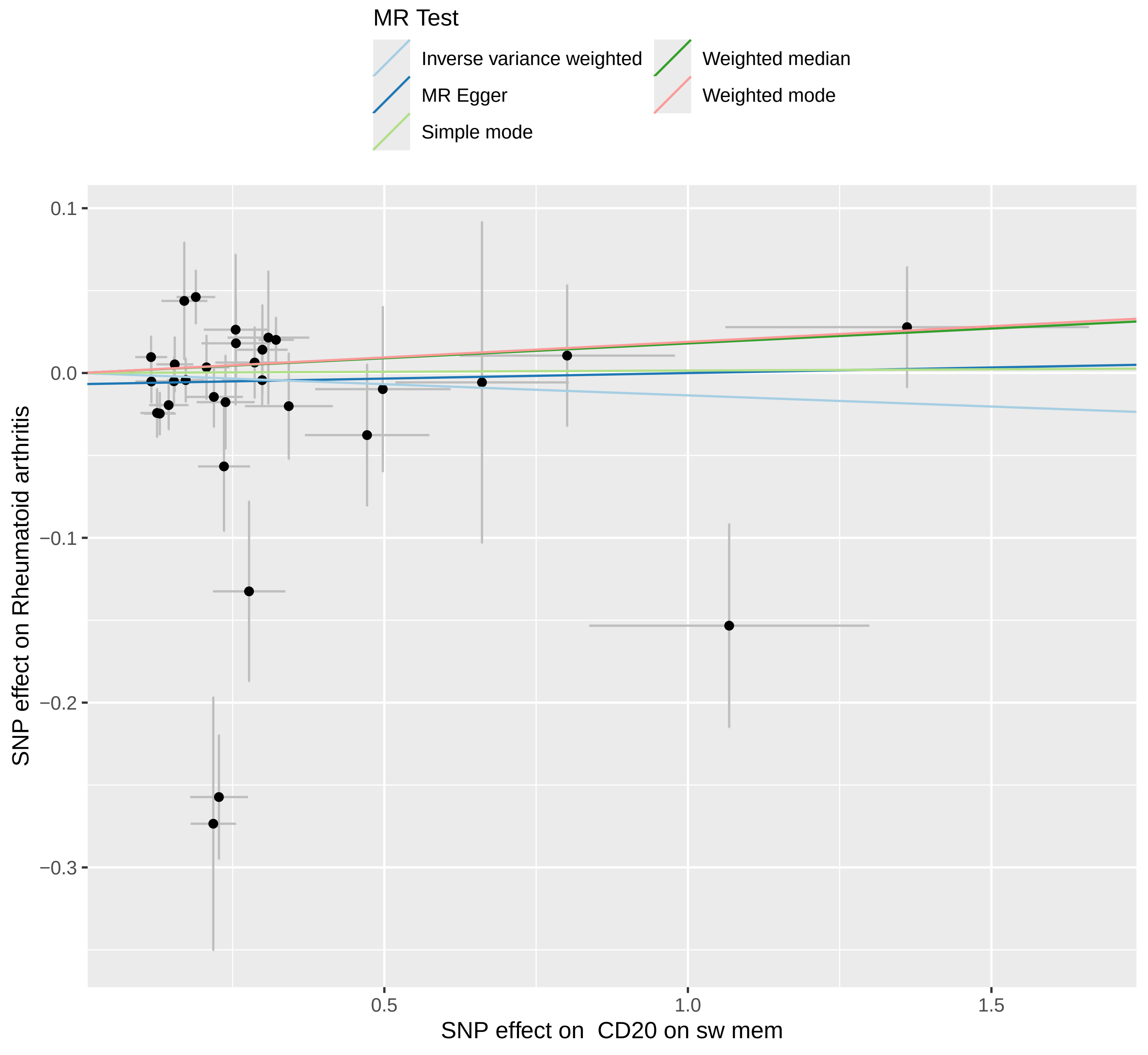
MR Test
Inverse variance weighted
MR Egger
Simple mode
Weighted median
Weighted mode
0.1
0.0
−0.1
−0.2
−0.3
0.5
1.0
1.5
SNP effect on Rheumatoid arthritis
SNP effect on CD20 on sw mem

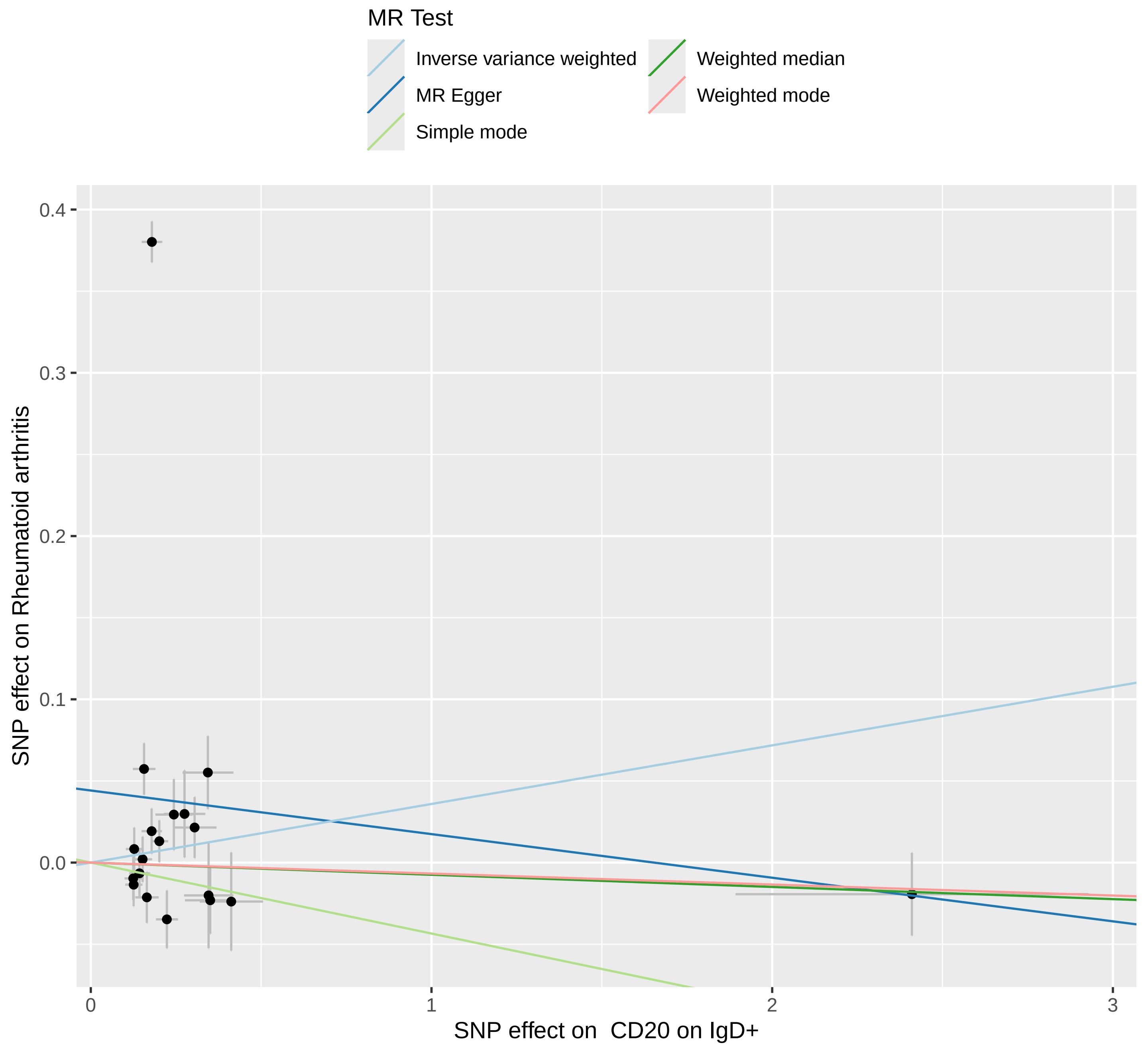
MR Test
Inverse variance weighted
MR Egger
Simple mode
Weighted median
Weighted mode
SNP effect on Rheumatoid arthritis
SNP effect on CD20 on IgD+
0.4
0.3
0.2
0.1
0.0
0
1
2
3

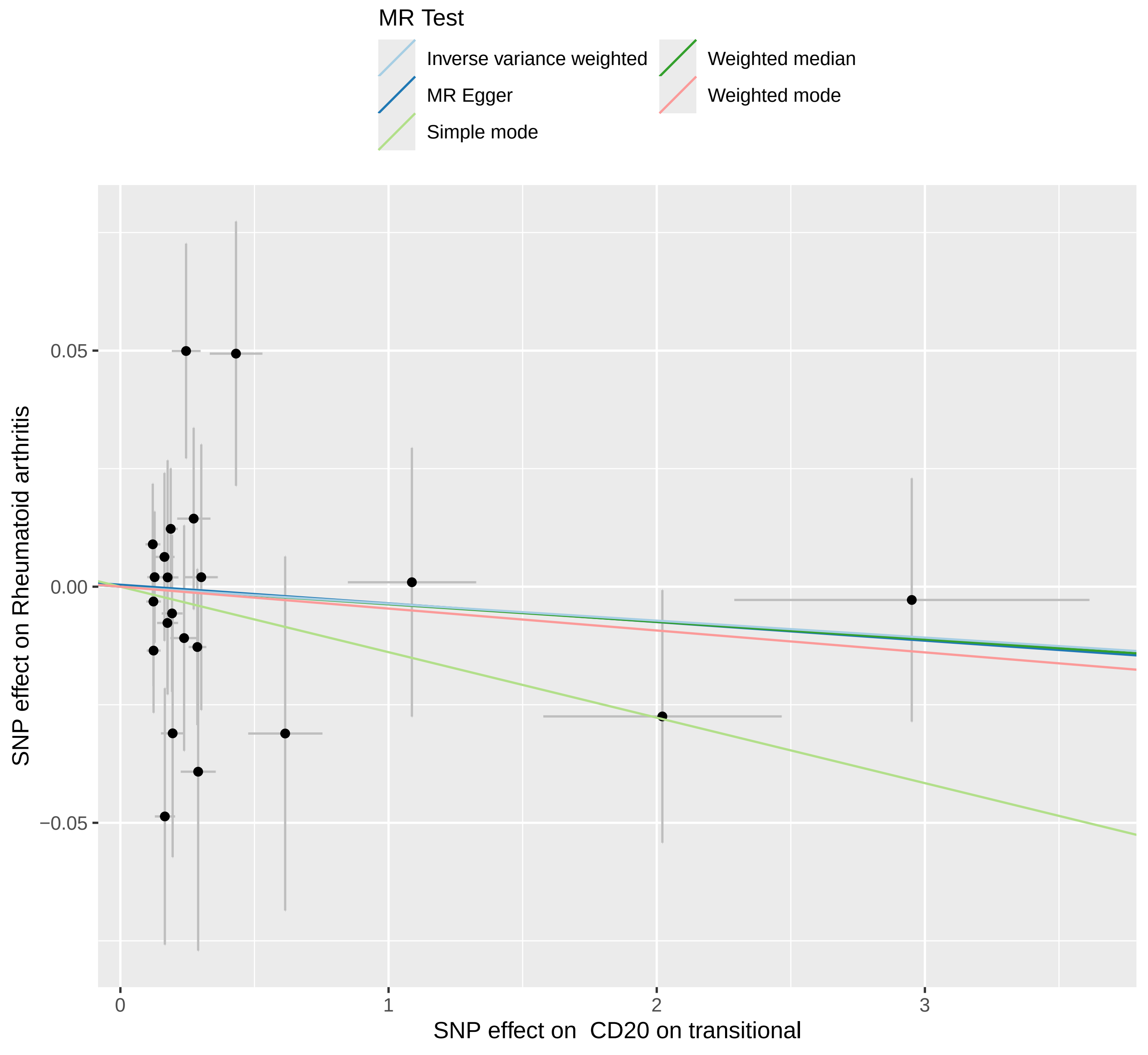
MR Test
Inverse variance weighted
MR Egger
Simple mode
Weighted median
Weighted mode
0.05
0.00
−0.05
0
1
2
3
SNP effect on Rheumatoid arthritis
SNP effect on CD20 on transitional

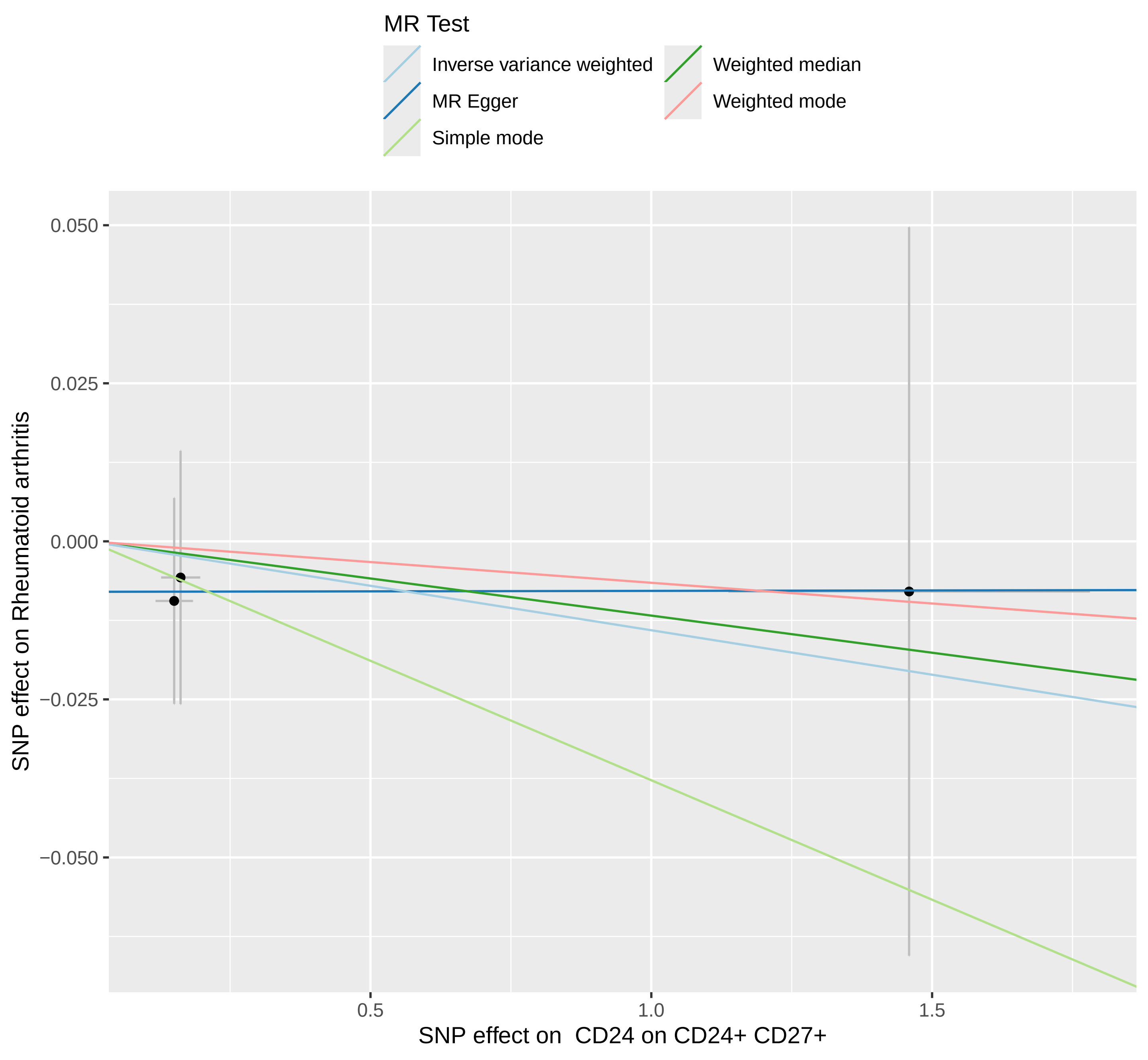
MR Test
Inverse variance weighted
MR Egger
Simple mode
Weighted median
Weighted mode
0.050
0.025
0.000
−0.025
−0.050
SNP effect on Rheumatoid arthritis
0.5
1.0
1.5
SNP effect on CD24 on CD24+ CD27+

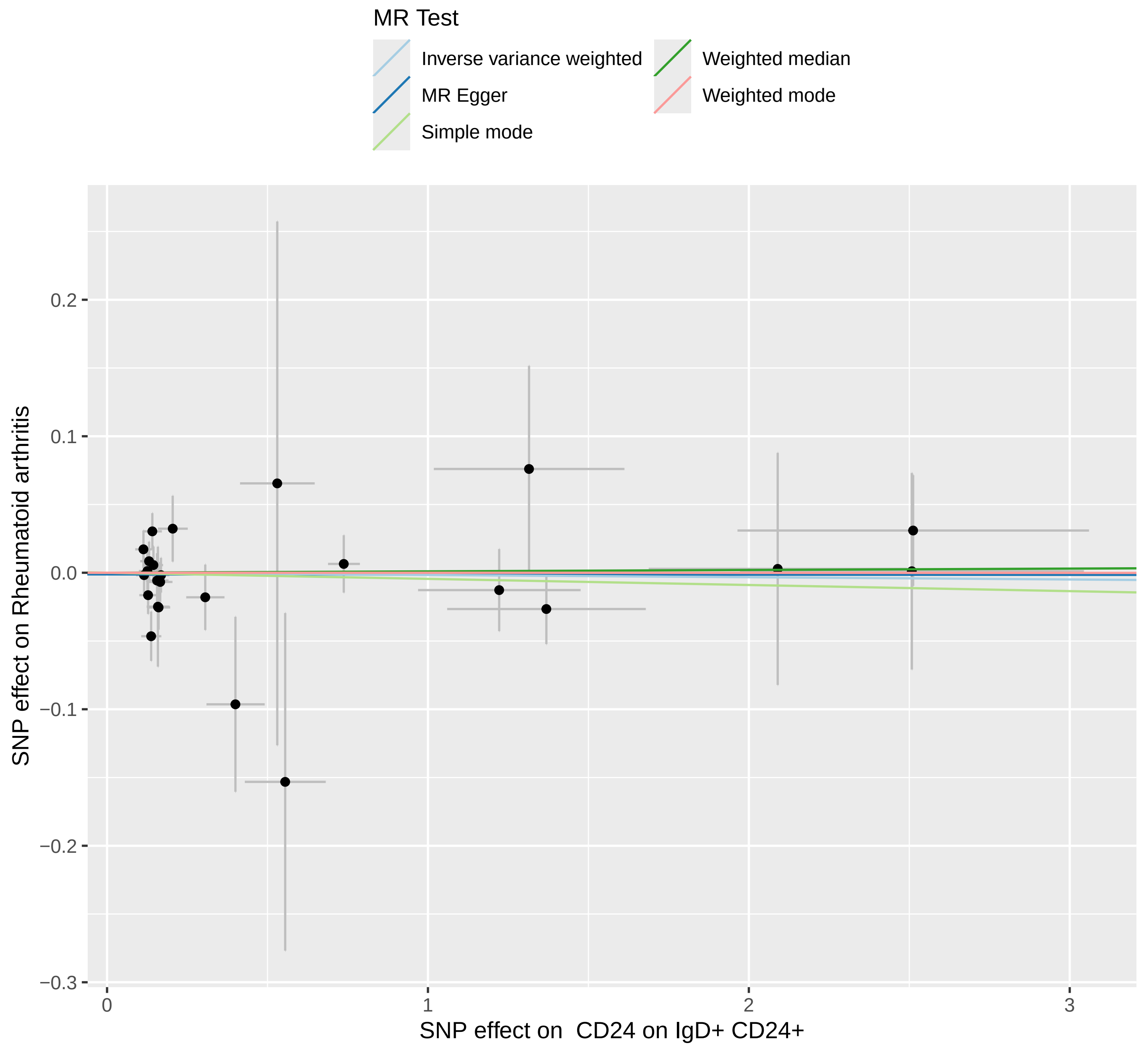
MR Test
Inverse variance weighted
MR Egger
Simple mode
Weighted median
Weighted mode
SNP effect on Rheumatoid arthritis
0.2
0.1
0.0
−0.1
−0.2
−0.3
0
1
2
3
SNP effect on CD24 on IgD+ CD24+

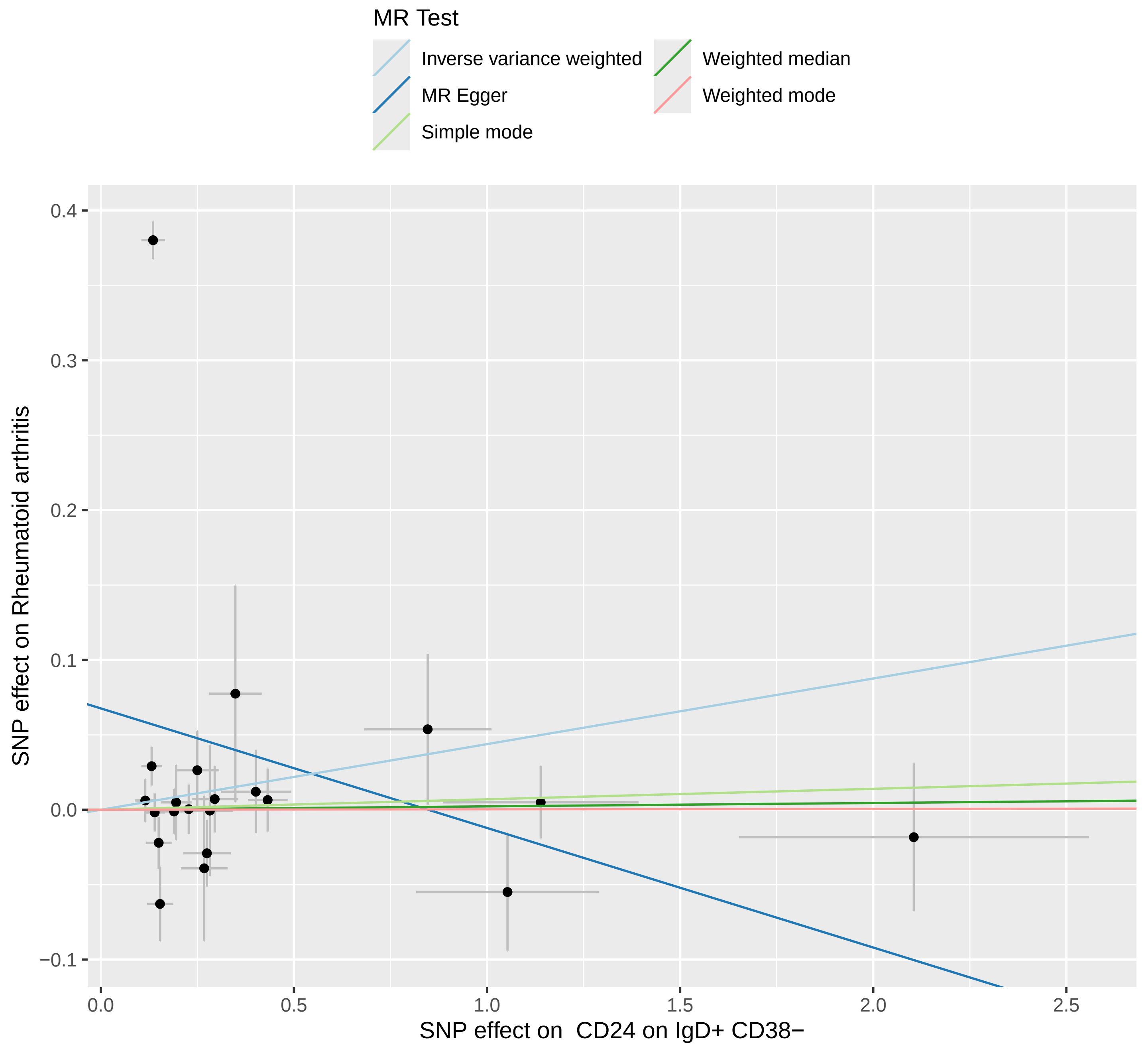
MR Test
Inverse variance weighted
MR Egger
Simple mode
Weighted median
Weighted mode
SNP effect on Rheumatoid arthritis
0.4
0.3
0.2
0.1
0.0
−0.1
0.0
0.5
1.0
1.5
2.0
2.5
SNP effect on CD24 on IgD+ CD38−

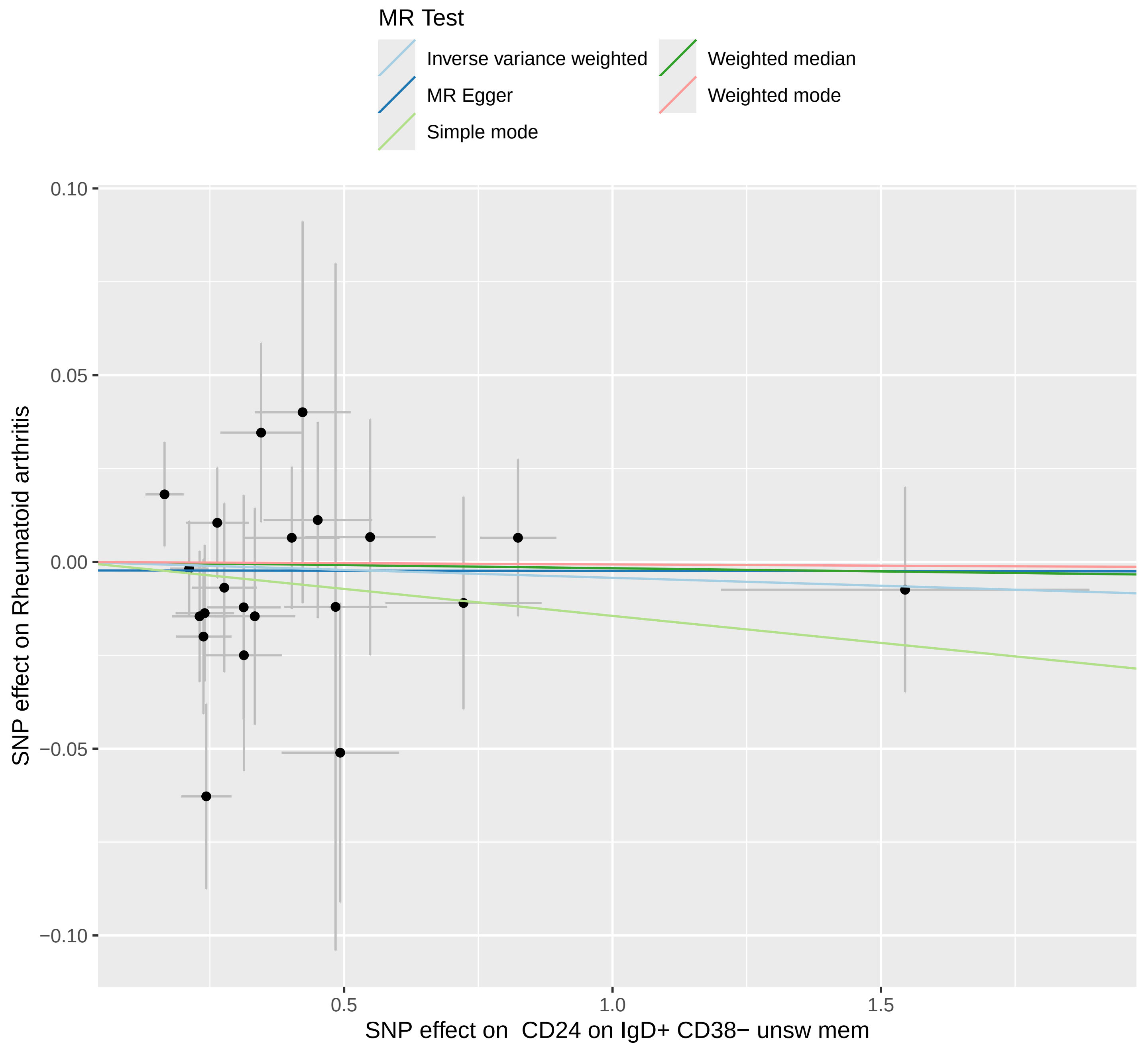
MR Test
Inverse variance weighted
MR Egger
Simple mode
Weighted median
Weighted mode
0.10
0.05
0.00
−0.05
−0.10
0.5
1.0
1.5
SNP effect on Rheumatoid arthritis
SNP effect on CD24 on IgD+ CD38− unsw mem

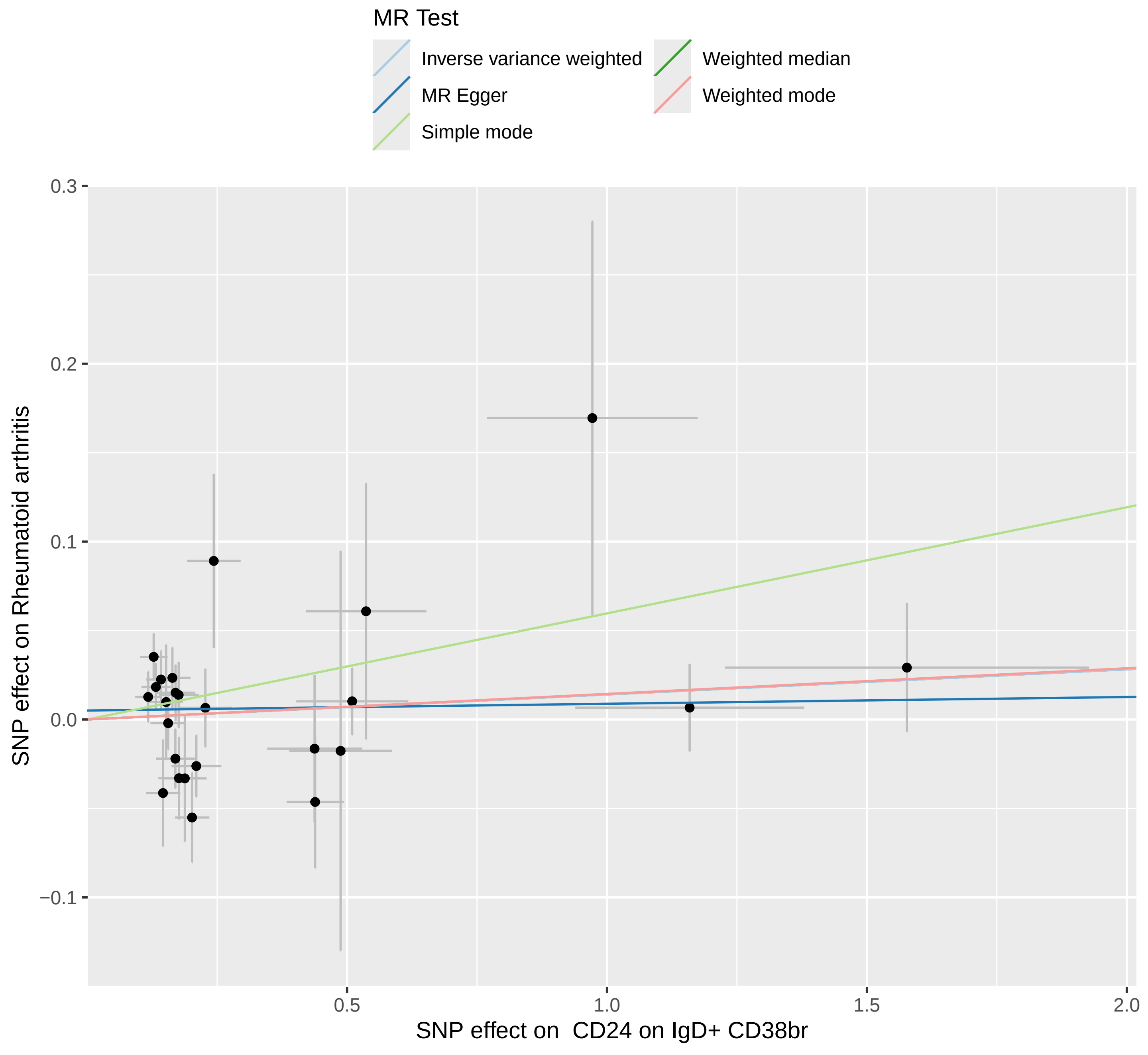

MR Test
Inverse variance weighted
MR Egger
Simple mode
Weighted median
Weighted mode
0.3
0.2
0.1
0.0
−0.1
SNP effect on Rheumatoid arthritis
0.5
1.0
1.5
2.0
SNP effect on CD24 on IgD+ CD38br

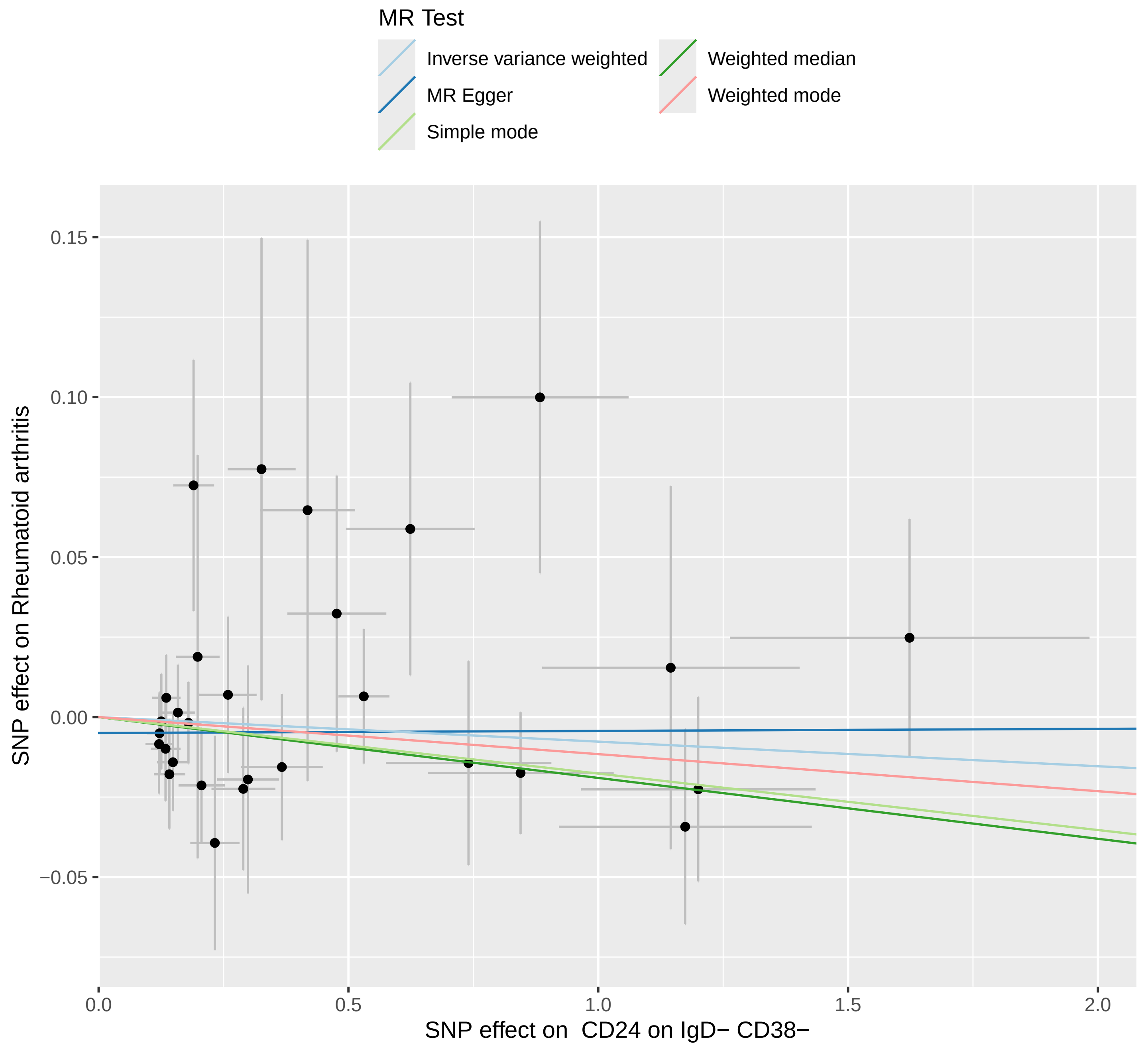
MR Test
Inverse variance weighted
MR Egger
Simple mode
Weighted median
Weighted mode
0.15
0.10
0.05
0.00
−0.05
0.0
0.5
1.0
1.5
2.0
SNP effect on Rheumatoid arthritis
SNP effect on CD24 on IgD− CD38−

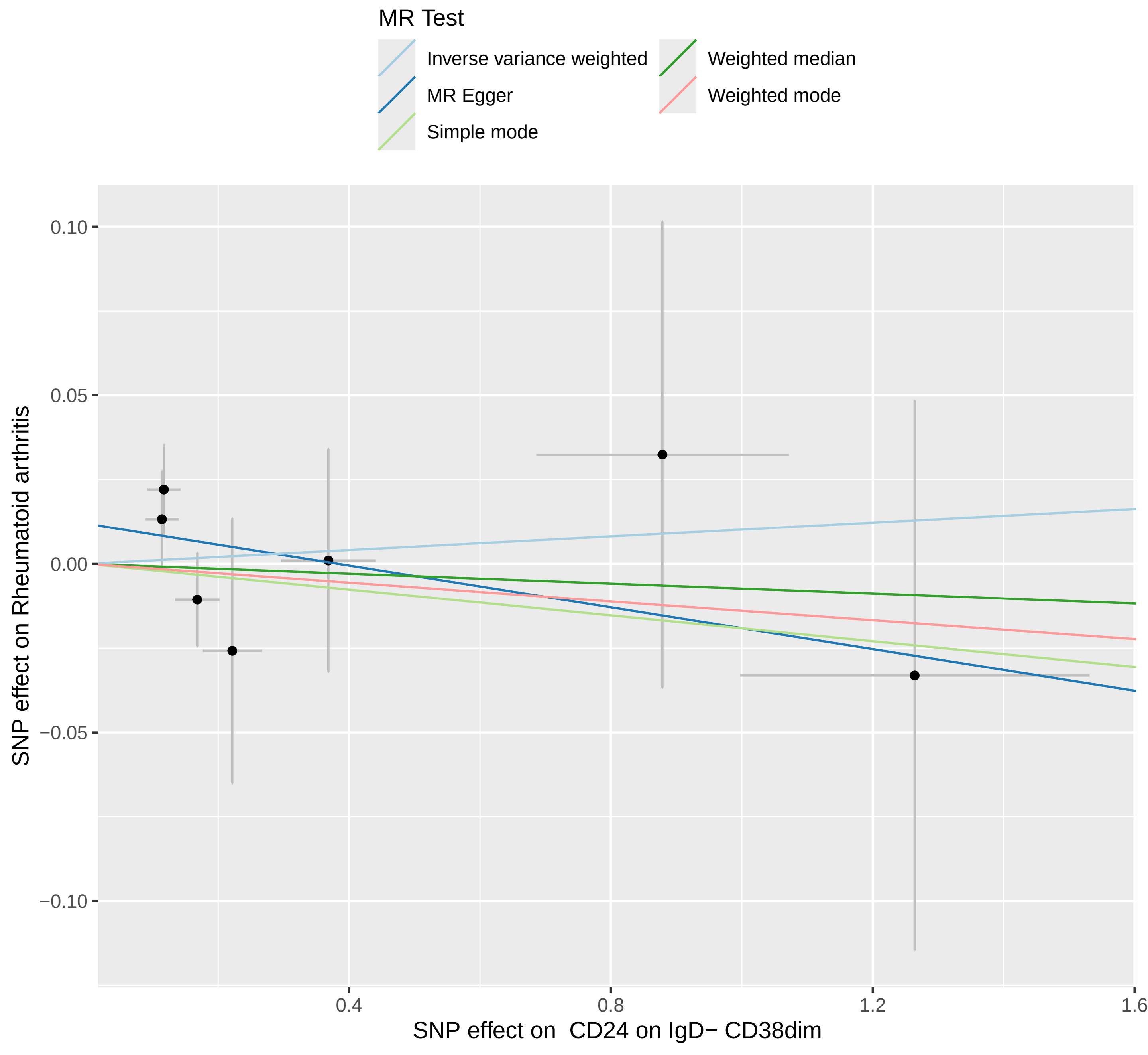

MR Test
Inverse variance weighted
MR Egger
Simple mode
Weighted median
Weighted mode
0.10
0.05
0.00
−0.05
−0.10
0.4
0.8
1.2
1.6
SNP effect on Rheumatoid arthritis
SNP effect on CD24 on IgD− CD38dim

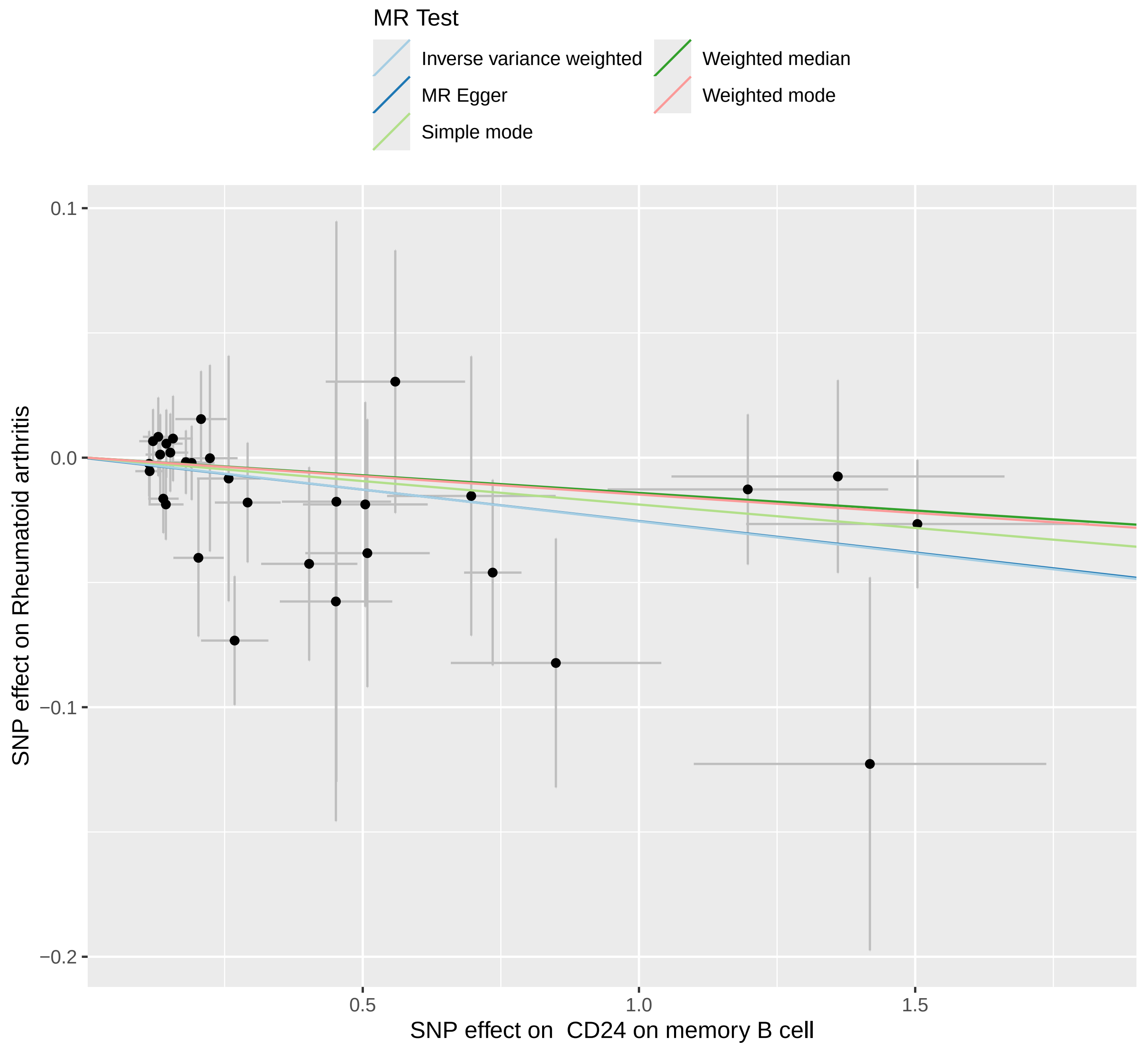

MR Test
Inverse variance weighted
MR Egger
Simple mode
Weighted median
Weighted mode
0.1
0.0
−0.1
−0.2
0.5
1.0
1.5
SNP effect on Rheumatoid arthritis
SNP effect on CD24 on memory B cell

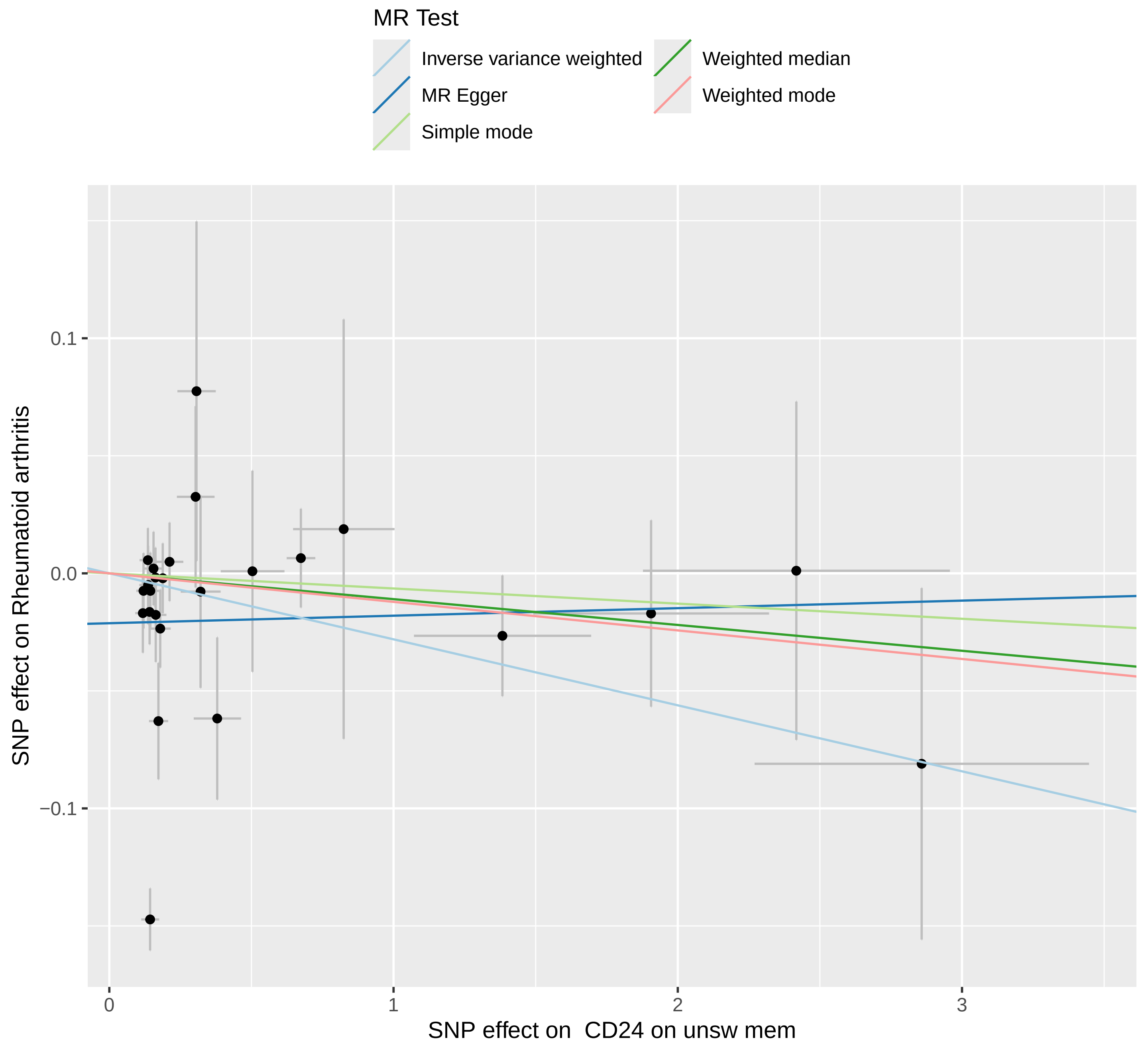
MR Test
Inverse variance weighted
MR Egger
Simple mode
Weighted median
Weighted mode
0.1
0.0
−0.1
0
1
2
3
SNP effect on Rheumatoid arthritis
SNP effect on CD24 on unsw mem

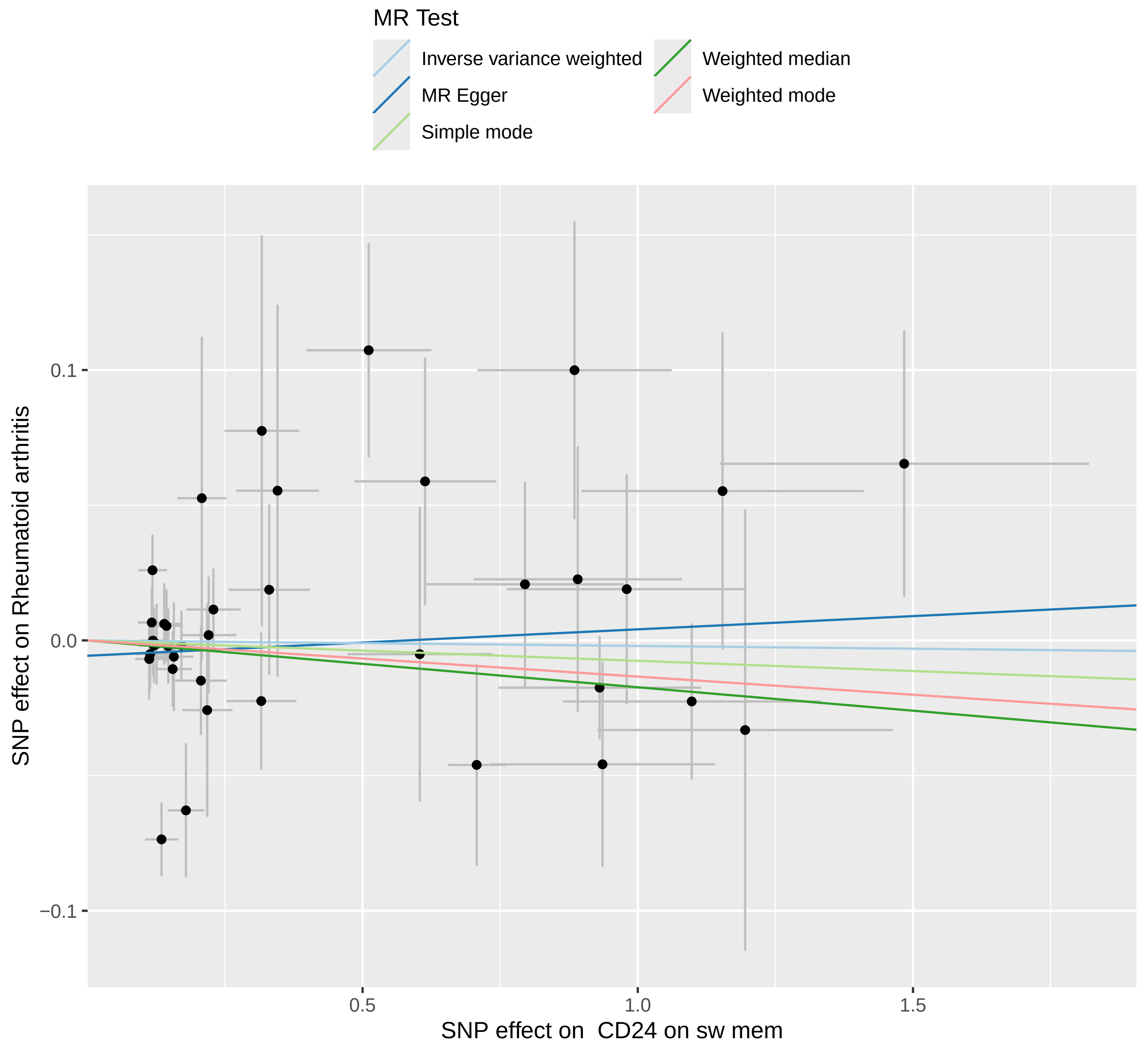
MR Test
Inverse variance weighted
MR Egger
Simple mode
Weighted median
Weighted mode
0.1
0.0
−0.1
0.5
1.0
1.5
SNP effect on Rheumatoid arthritis
SNP effect on CD24 on sw mem

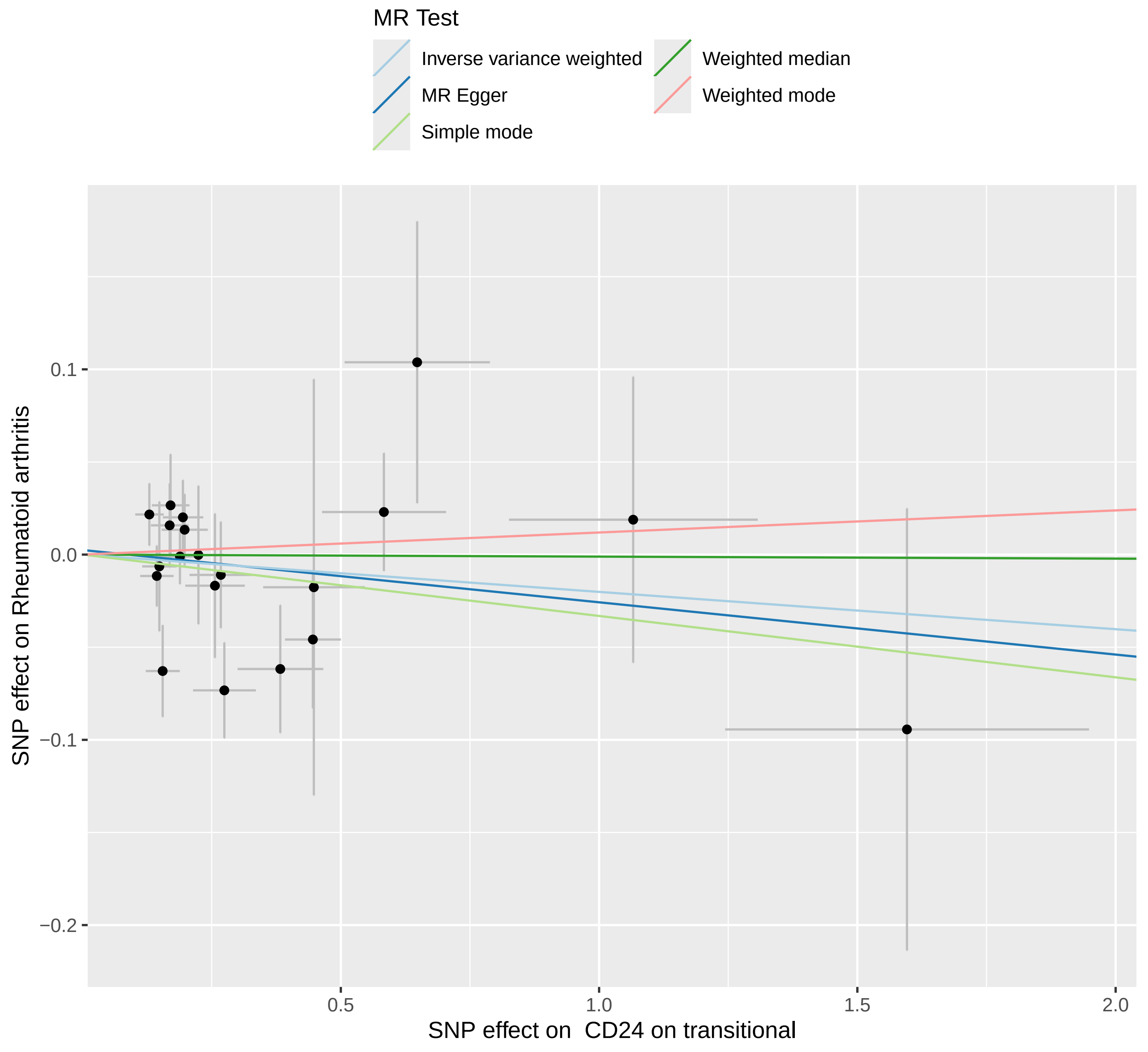

MR Test
Inverse variance weighted
MR Egger
Simple mode
Weighted median
Weighted mode
0.1
0.0
−0.1
−0.2
SNP effect on Rheumatoid arthritis
0.5
1.0
1.5
2.0
SNP effect on CD24 on transitional

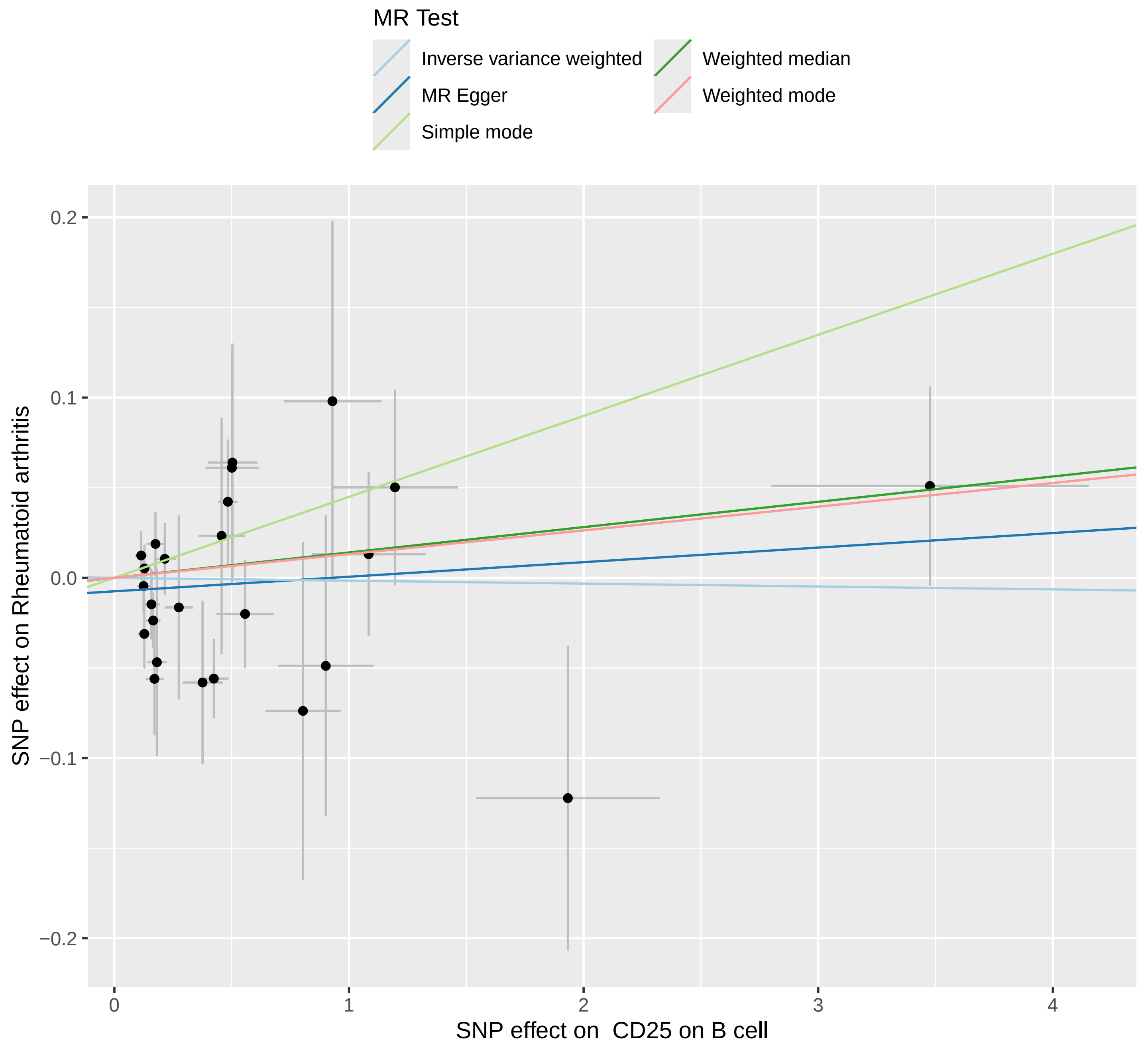

MR Test
Inverse variance weighted
MR Egger
Simple mode
Weighted median
Weighted mode
0.2
0.1
0.0
−0.1
−0.2
0
1
2
3
4
SNP effect on Rheumatoid arthritis
SNP effect on CD25 on B cell

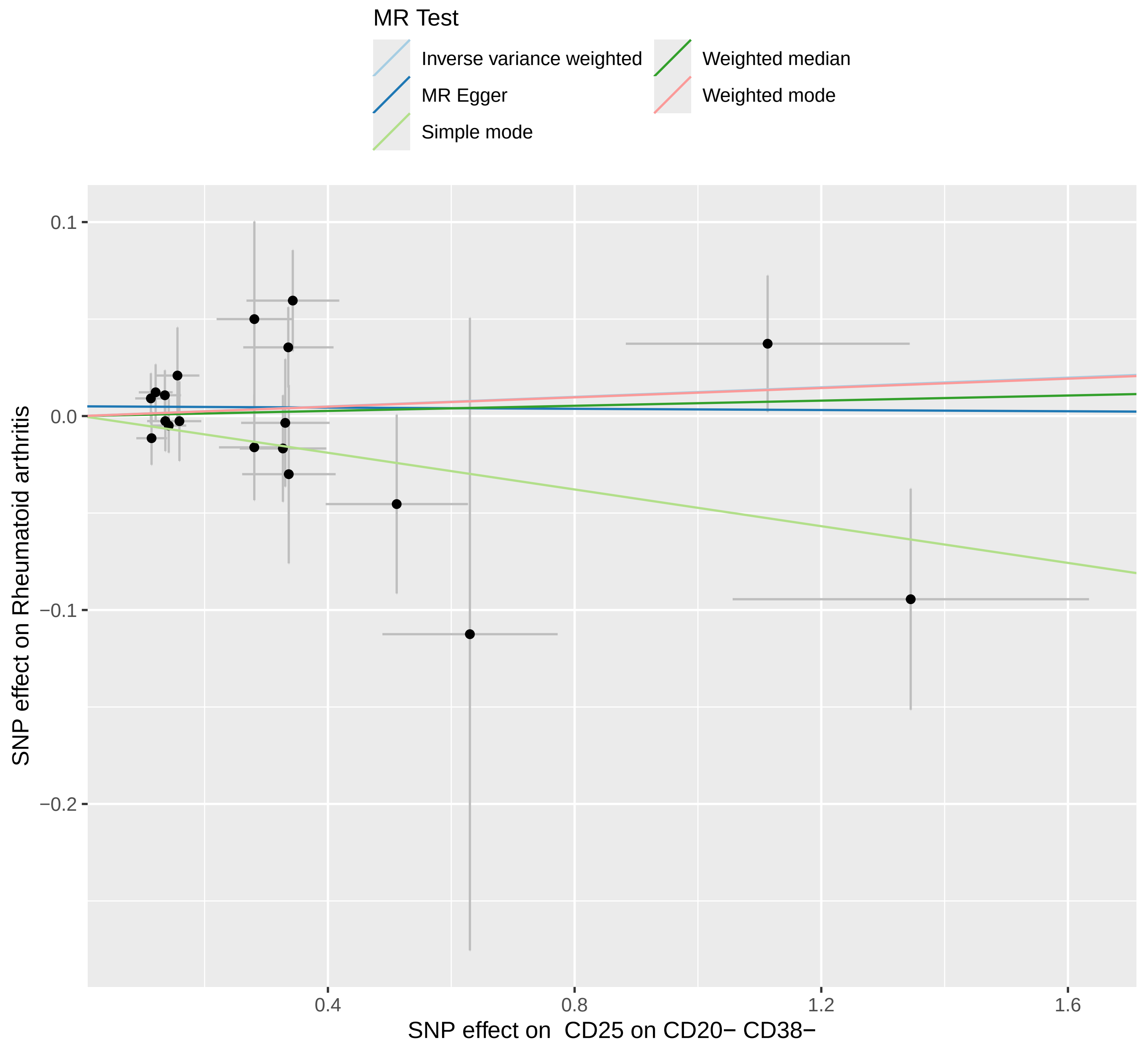

MR Test
Inverse variance weighted
MR Egger
Simple mode
Weighted median
Weighted mode
0.1
0.0
−0.1
−0.2
0.4
0.8
1.2
1.6
SNP effect on Rheumatoid arthritis
SNP effect on CD25 on CD20− CD38−

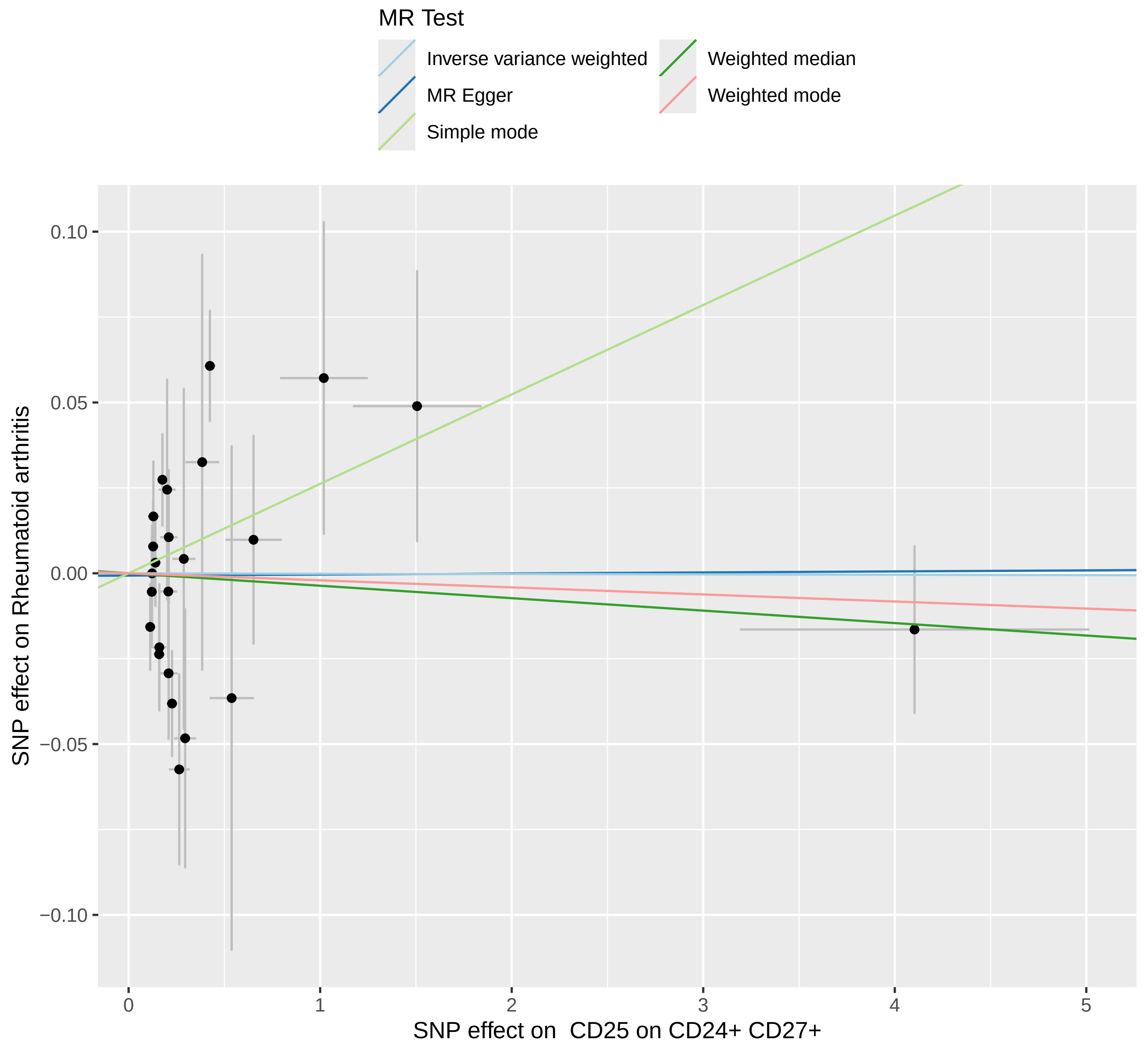
MR Test
Inverse variance weighted
MR Egger
Simple mode
Weighted median
Weighted mode
SNP effect on Rheumatoid arthritis
0.10
0.05
0.00
−0.05
−0.10
0
1
2
3
4
5
SNP effect on CD25 on CD24+ CD27+

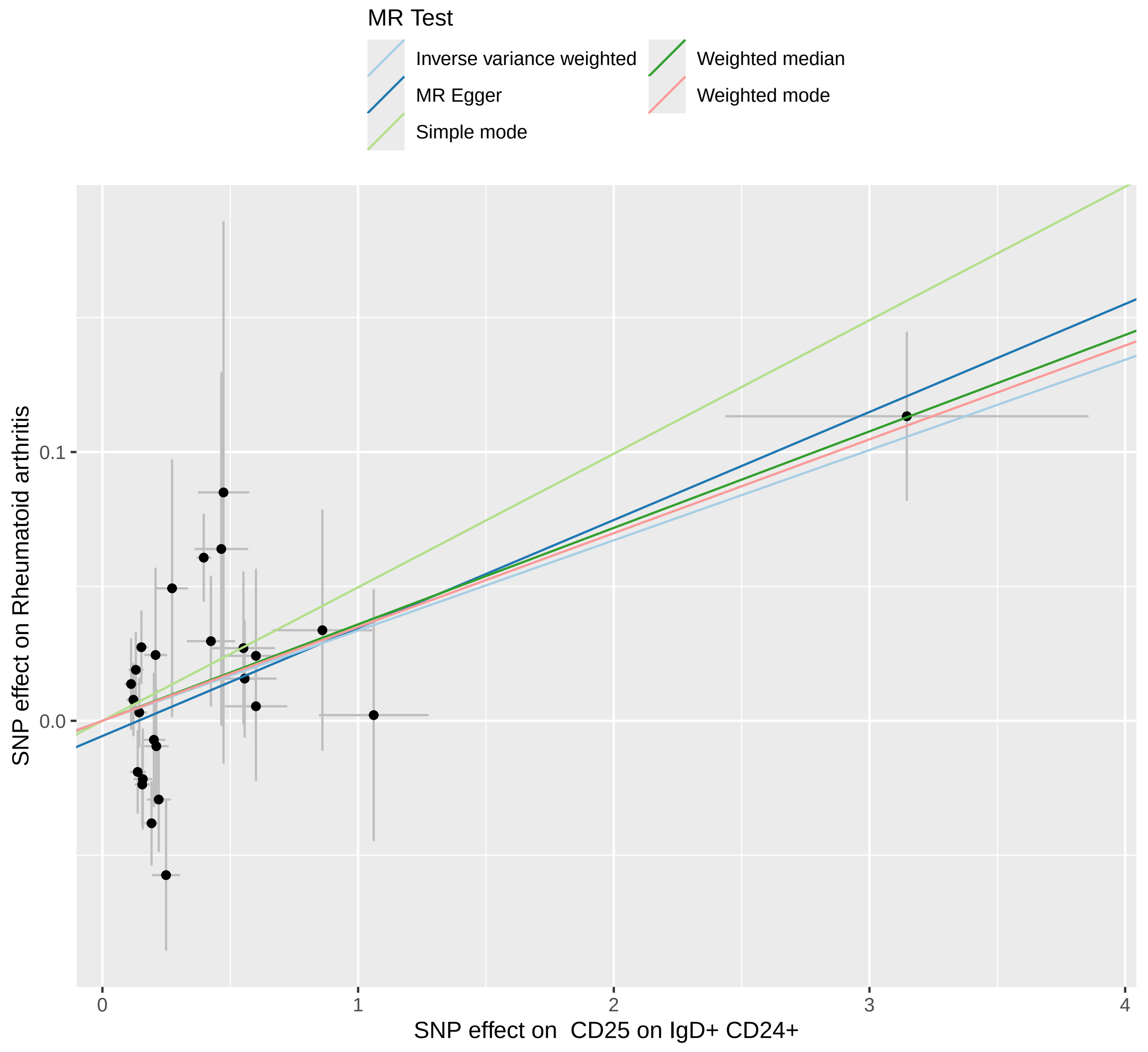

MR Test
Inverse variance weighted
MR Egger
Simple mode
Weighted median
Weighted mode
SNP effect on Rheumatoid arthritis
0.1
0.0
0
1
2
3
4
SNP effect on CD25 on IgD+ CD24+

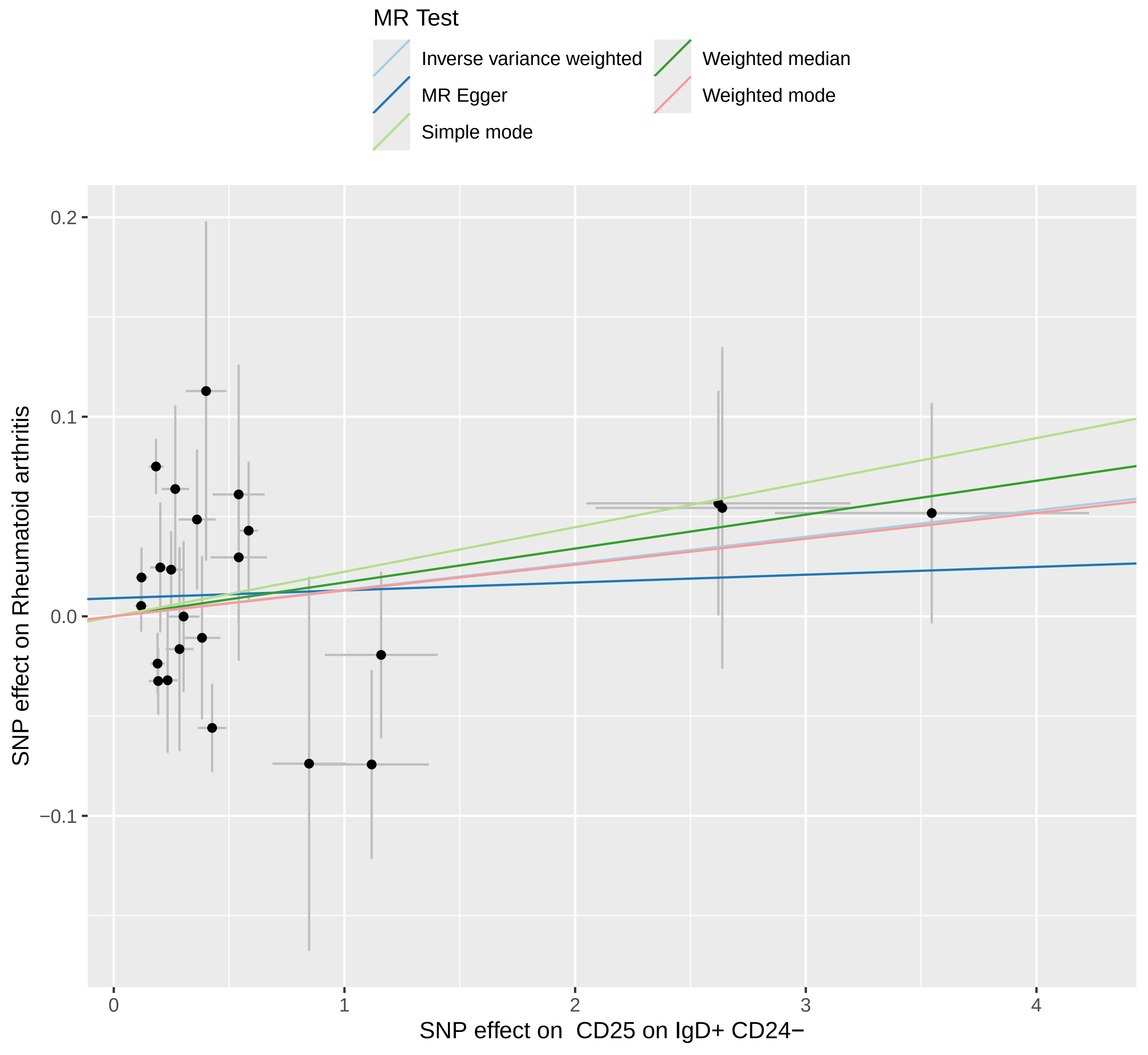

MR Test
Inverse variance weighted
MR Egger
Simple mode
Weighted median
Weighted mode
0.2
0.1
0.0
−0.1
0
1
2
3
4
SNP effect on Rheumatoid arthritis
SNP effect on CD25 on IgD+ CD24−

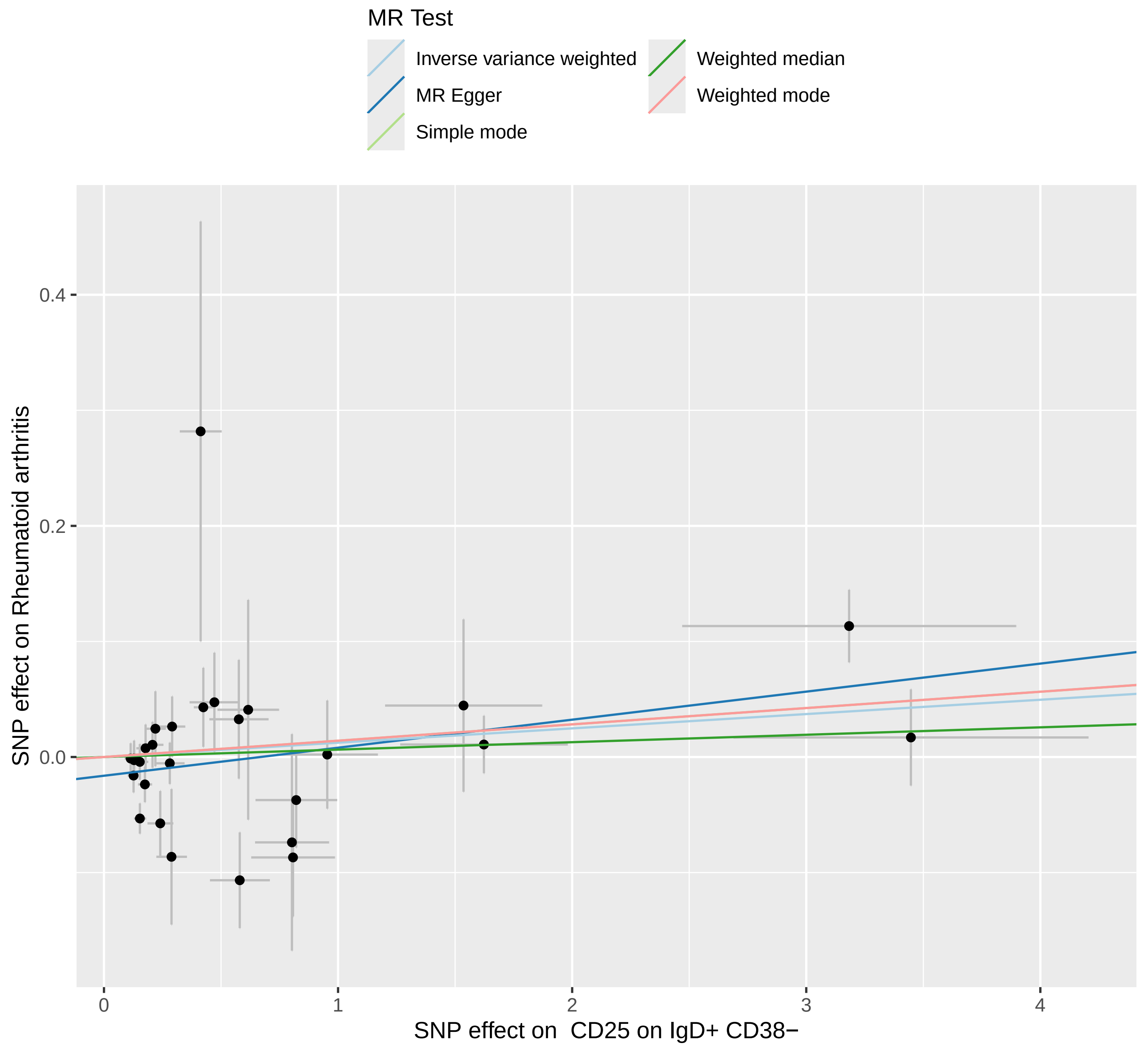
MR Test
Inverse variance weighted
MR Egger
Simple mode
Weighted median
Weighted mode
SNP effect on Rheumatoid arthritis
0.4
0.2
0.0
0
1
2
3
4
SNP effect on CD25 on IgD+ CD38−

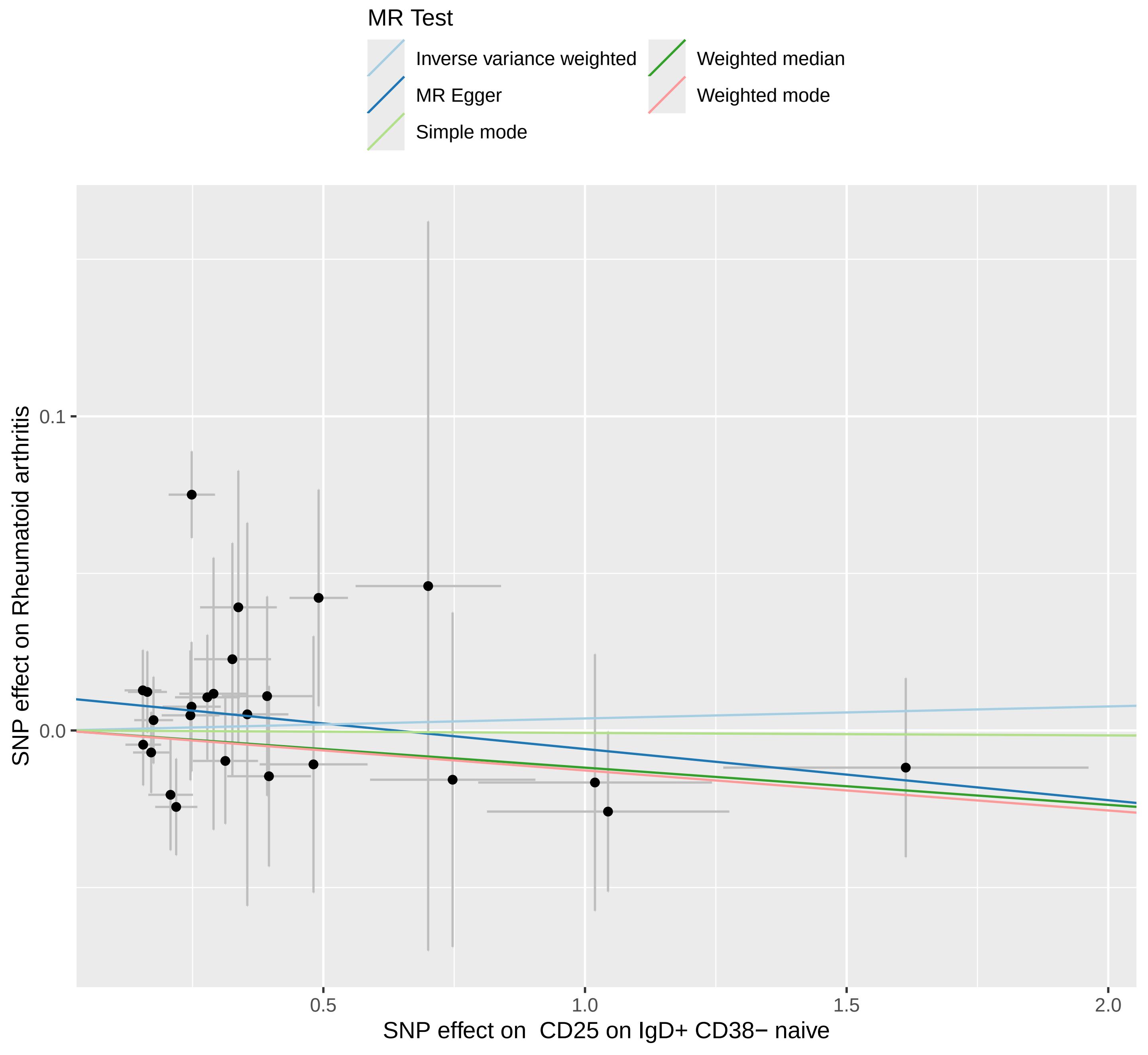
MR Test
Inverse variance weighted
MR Egger
Simple mode
Weighted median
Weighted mode
SNP effect on Rheumatoid arthritis
0.1
0.0
0.5
1.0
1.5
2.0
SNP effect on CD25 on IgD+ CD38− naive

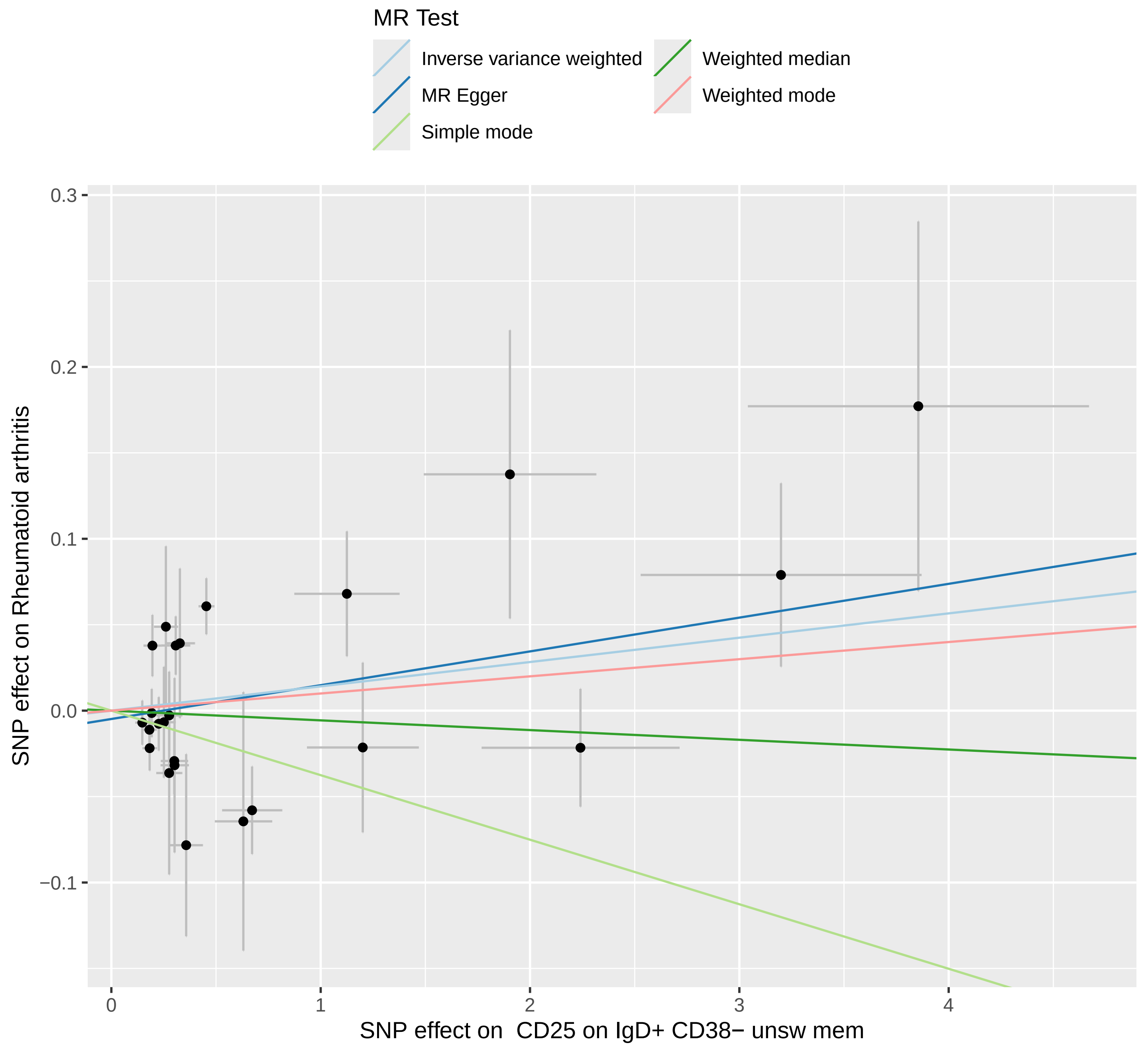

MR Test
Inverse variance weighted
MR Egger
Simple mode
Weighted median
Weighted mode
0.3
0.2
0.1
0.0
−0.1
SNP effect on Rheumatoid arthritis
0
1
2
3
4
SNP effect on CD25 on IgD+ CD38− unsw mem

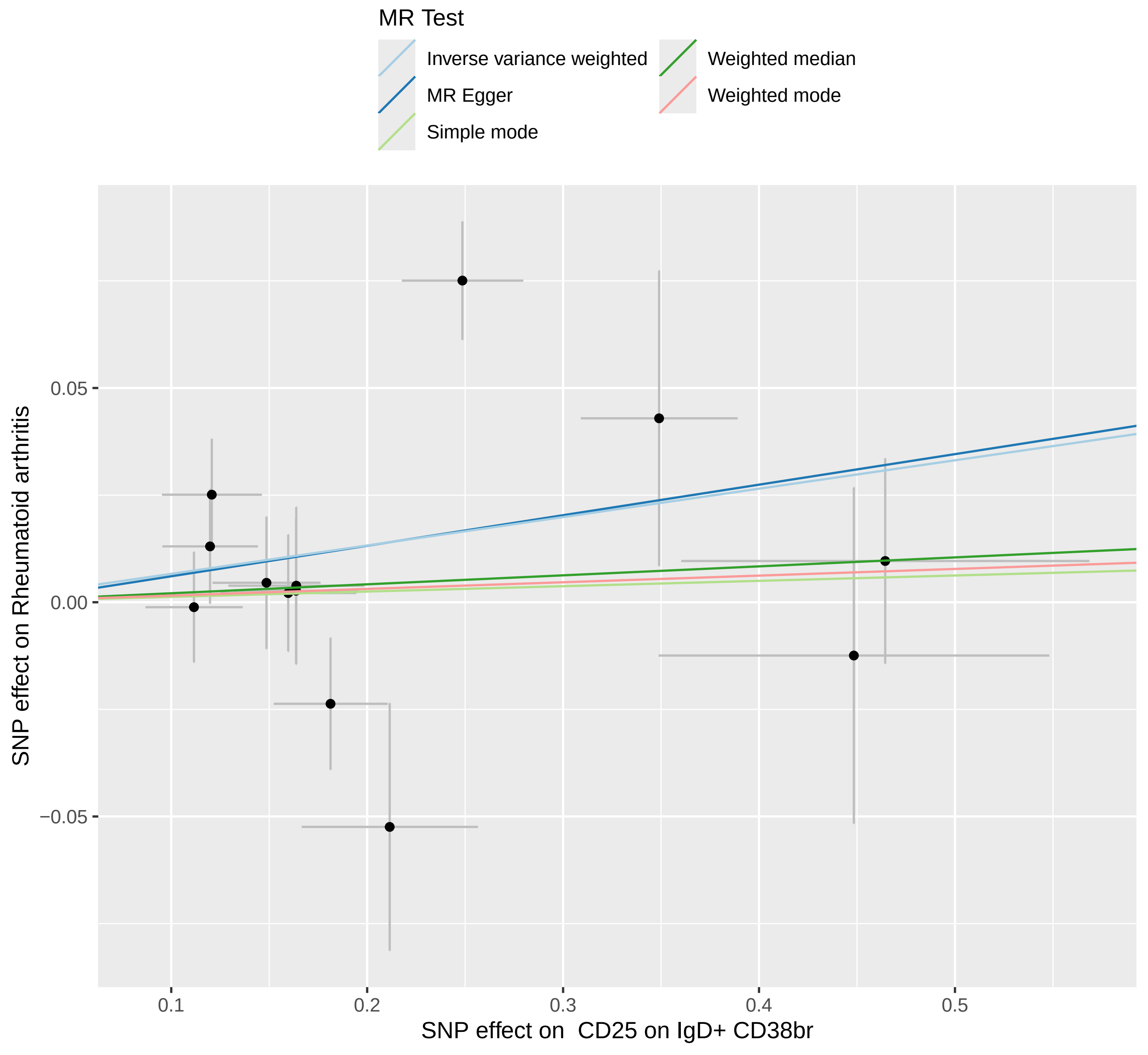

MR Test
Inverse variance weighted
MR Egger
Simple mode
Weighted median
Weighted mode
SNP effect on Rheumatoid arthritis
0.05
0.00
−0.05
0.1
0.2
0.3
0.4
0.5
SNP effect on CD25 on IgD+ CD38br

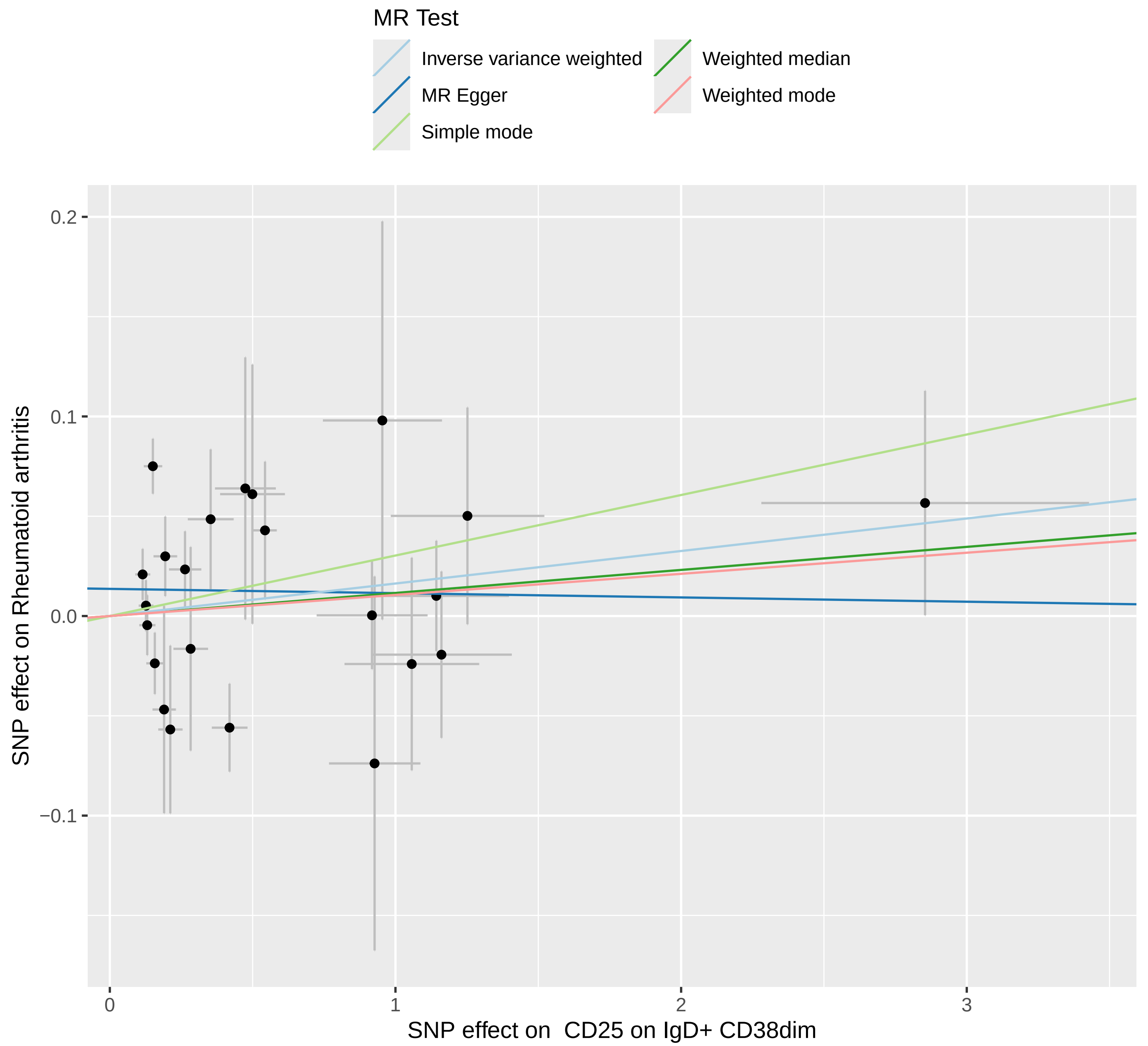
MR Test
Inverse variance weighted
MR Egger
Simple mode
Weighted median
Weighted mode
0.2
0.1
0.0
−0.1
SNP effect on Rheumatoid arthritis
0
1
2
3
SNP effect on CD25 on IgD+ CD38dim

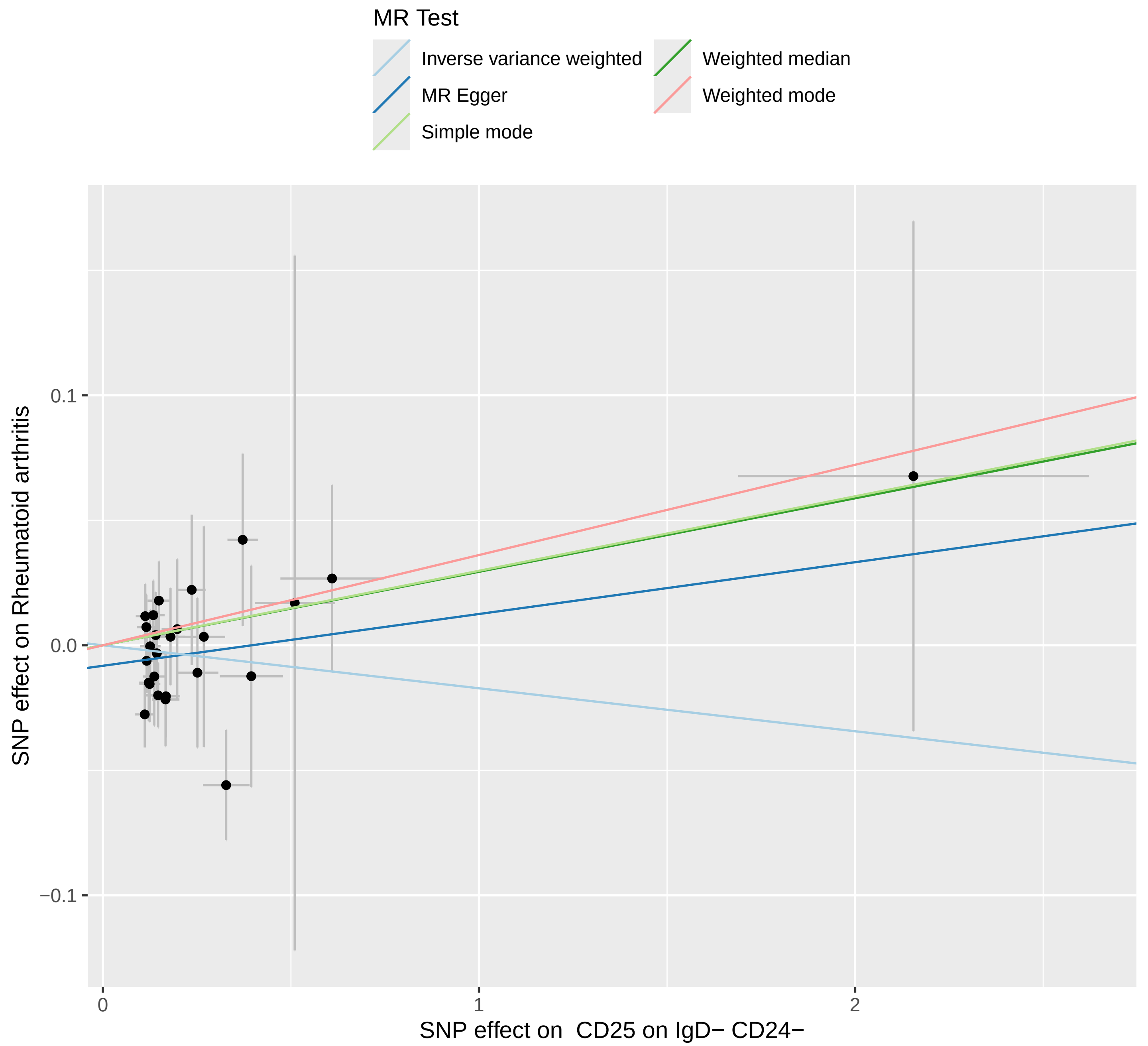
MR Test
Inverse variance weighted
MR Egger
Simple mode
Weighted median
Weighted mode
0.1
0.0
−0.1
0
1
2
SNP effect on Rheumatoid arthritis
SNP effect on CD25 on IgD− CD24−

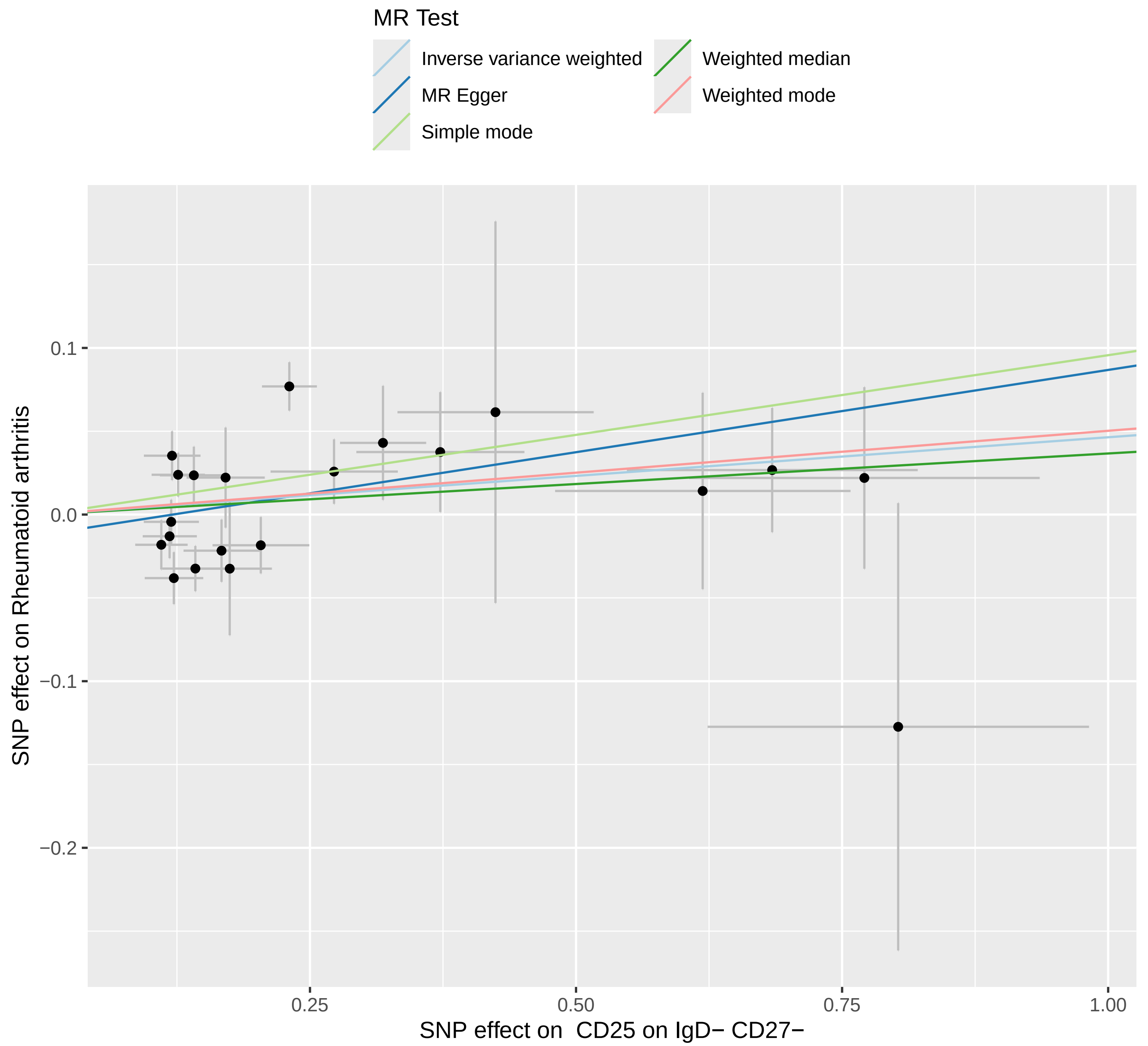

MR Test
Inverse variance weighted
MR Egger
Simple mode
Weighted median
Weighted mode
0.1
0.0
−0.1
−0.2
0.25
0.50
0.75
1.00
SNP effect on Rheumatoid arthritis
SNP effect on CD25 on IgD− CD27−

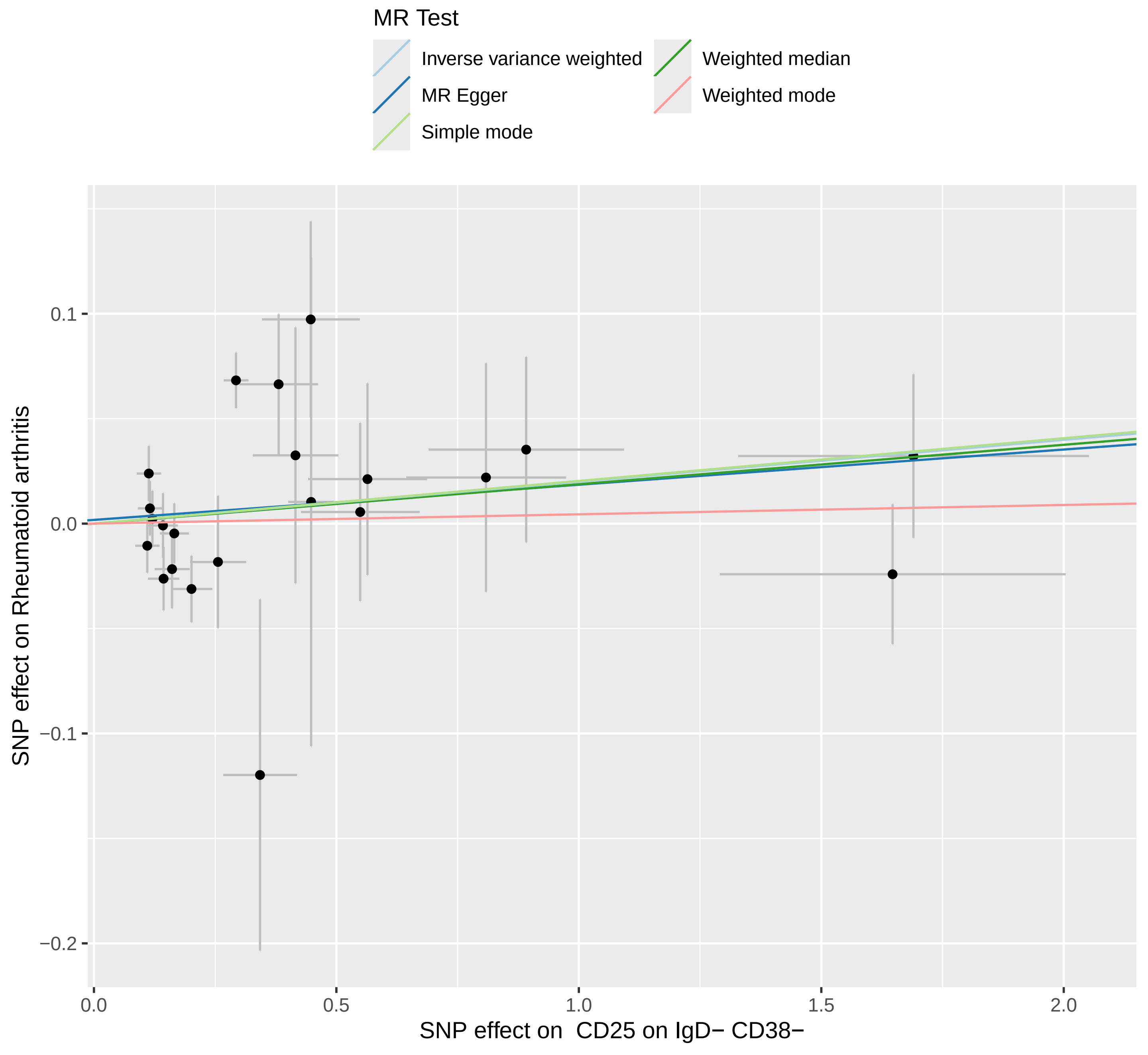

MR Test
Inverse variance weighted
MR Egger
Simple mode
Weighted median
Weighted mode
0.1
0.0
−0.1
−0.2
SNP effect on Rheumatoid arthritis
0.0
0.5
1.0
1.5
2.0
SNP effect on CD25 on IgD− CD38−

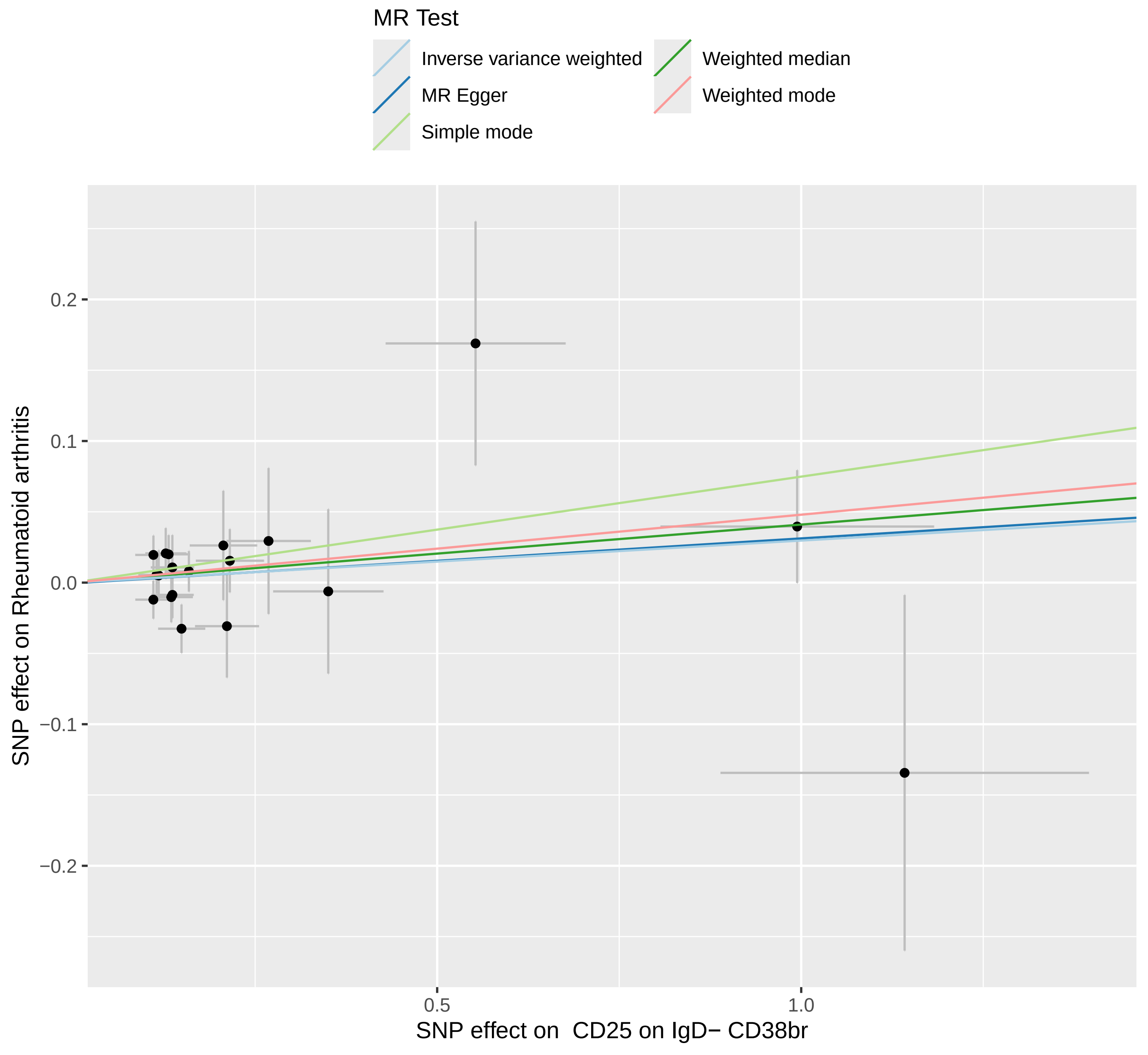
MR Test
Inverse variance weighted
MR Egger
Simple mode
Weighted median
Weighted mode
SNP effect on Rheumatoid arthritis
0.2
0.1
0.0
−0.1
−0.2
0.5
1.0
SNP effect on CD25 on IgD− CD38br

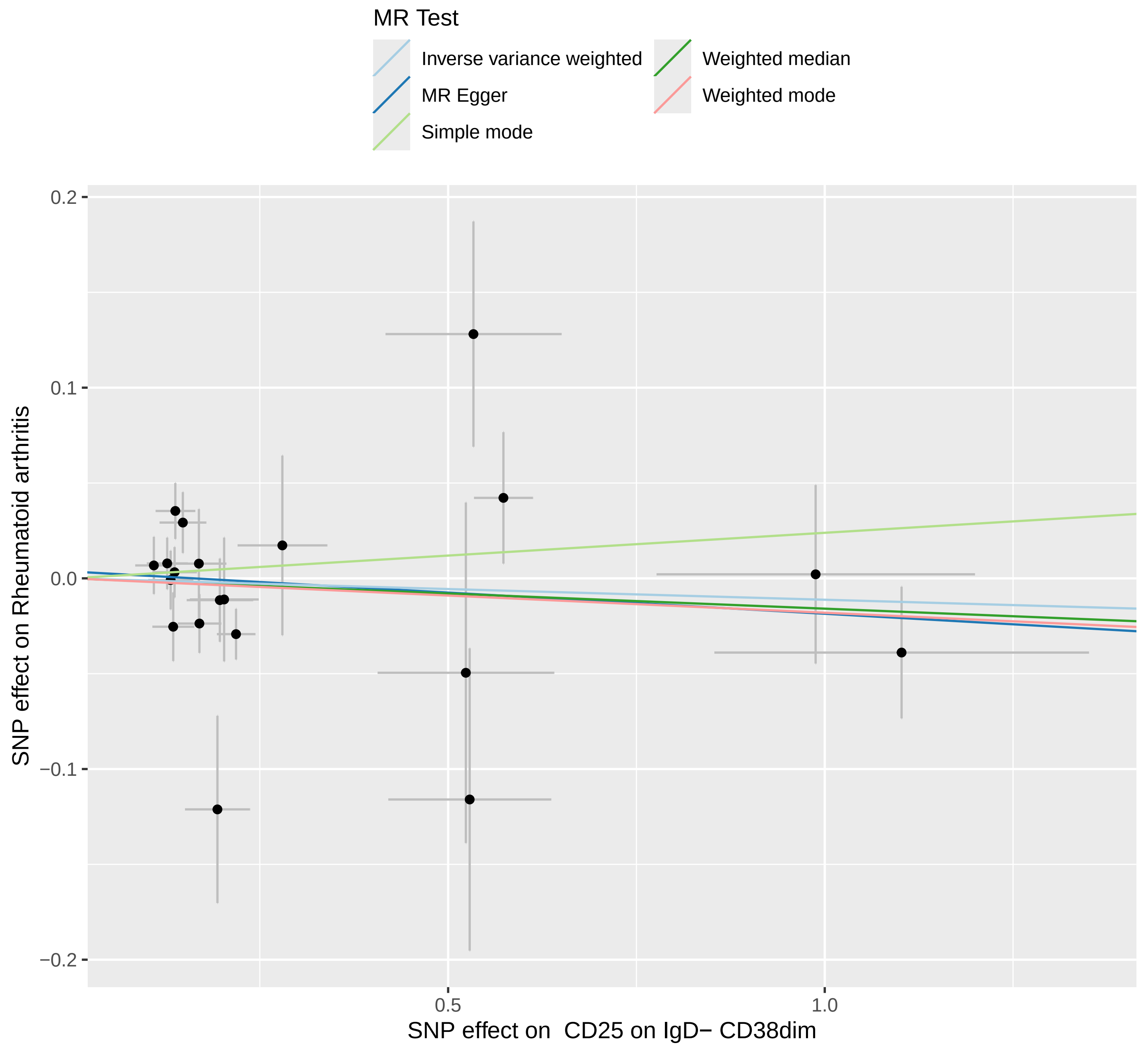

MR Test
Inverse variance weighted
MR Egger
Simple mode
Weighted median
Weighted mode
0.2
0.1
0.0
−0.1
−0.2
SNP effect on Rheumatoid arthritis
0.5
1.0
SNP effect on CD25 on IgD− CD38dim

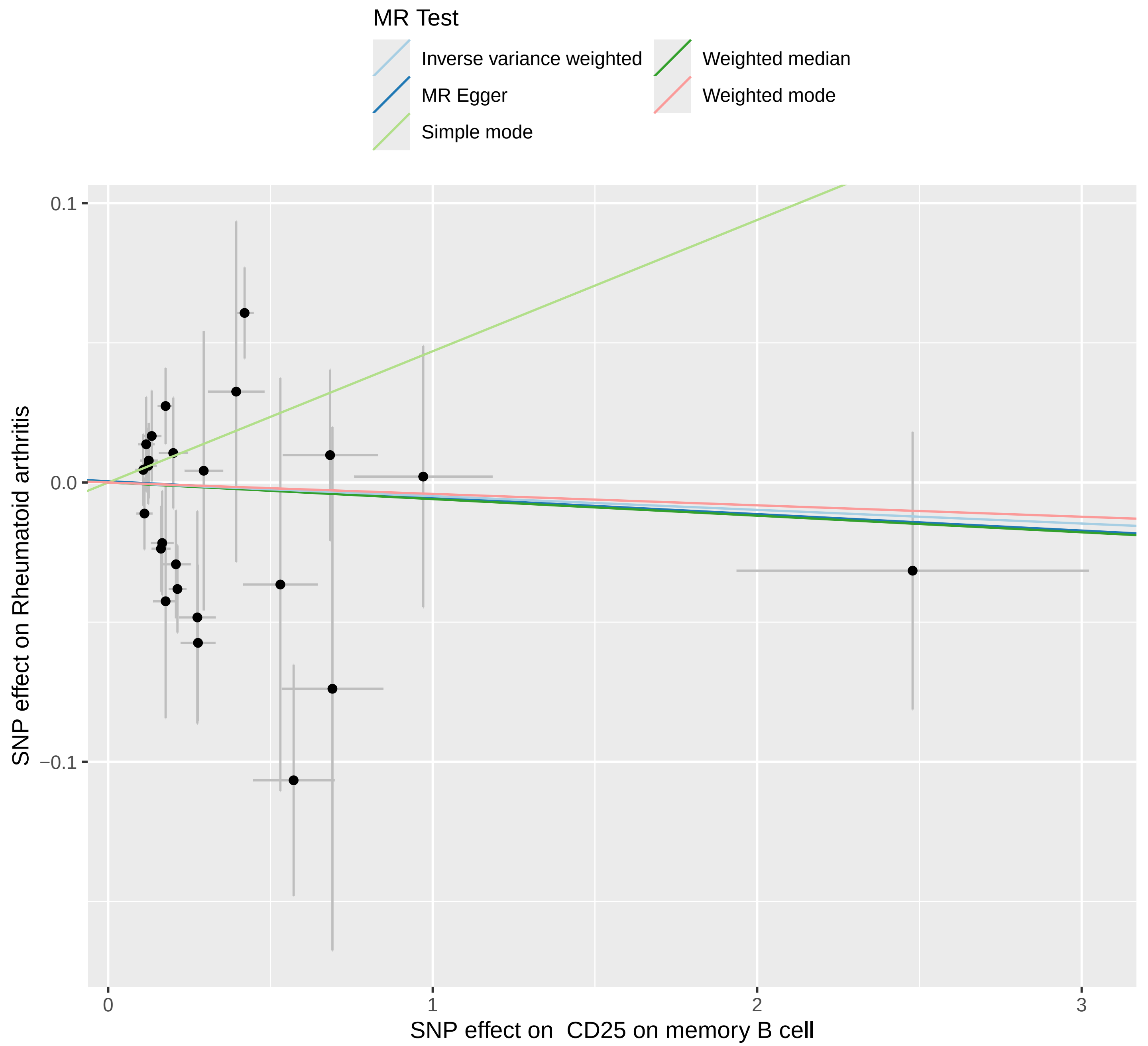
MR Test
Inverse variance weighted
MR Egger
Simple mode
Weighted median
Weighted mode
0.1
0.0
−0.1
0
1
2
3
SNP effect on Rheumatoid arthritis
SNP effect on CD25 on memory B cell

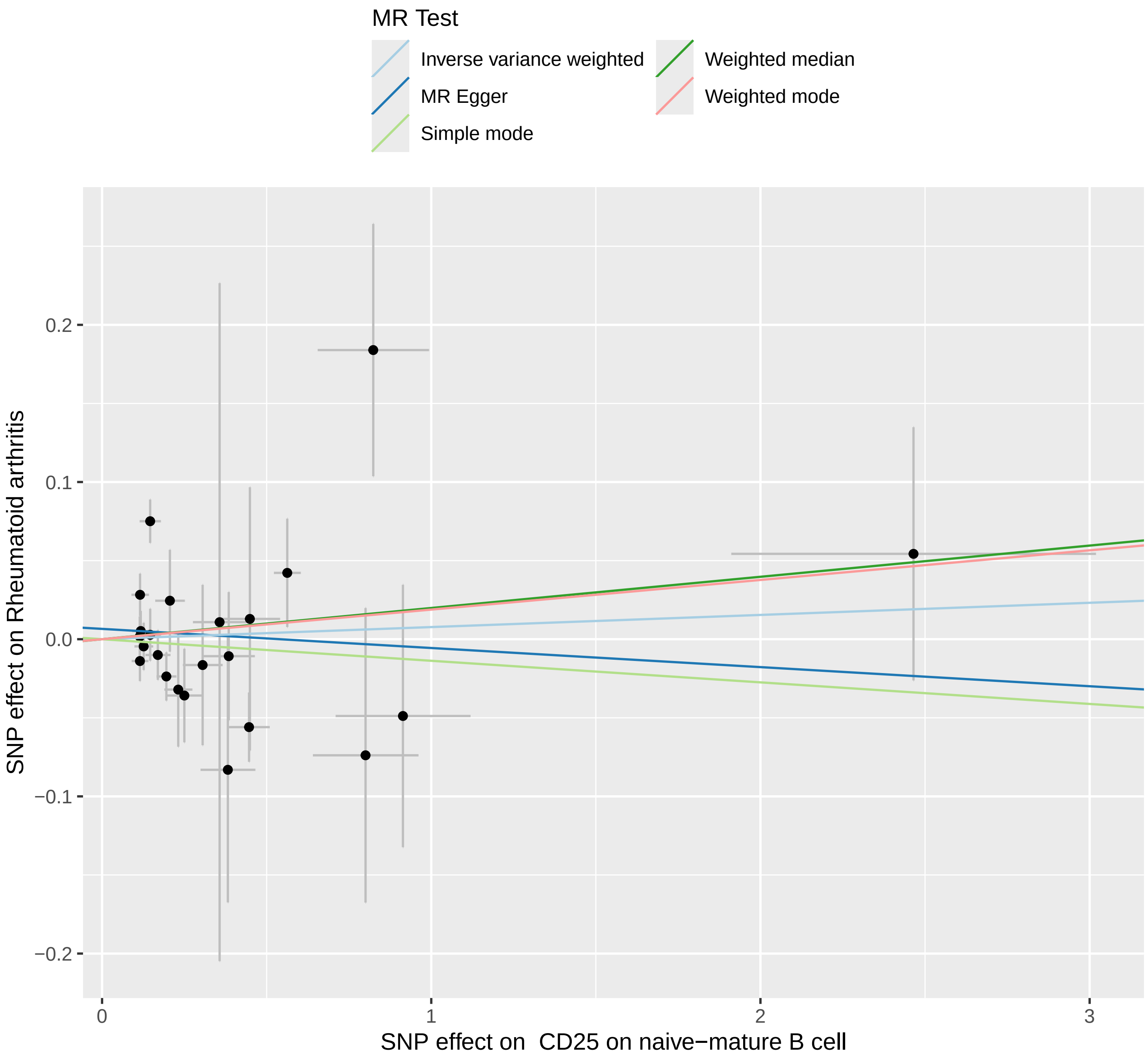

MR Test
Inverse variance weighted
MR Egger
Simple mode
Weighted median
Weighted mode
SNP effect on Rheumatoid arthritis
0.2
0.1
0.0
−0.1
−0.2
0
1
2
3
SNP effect on CD25 on naive−mature B cell

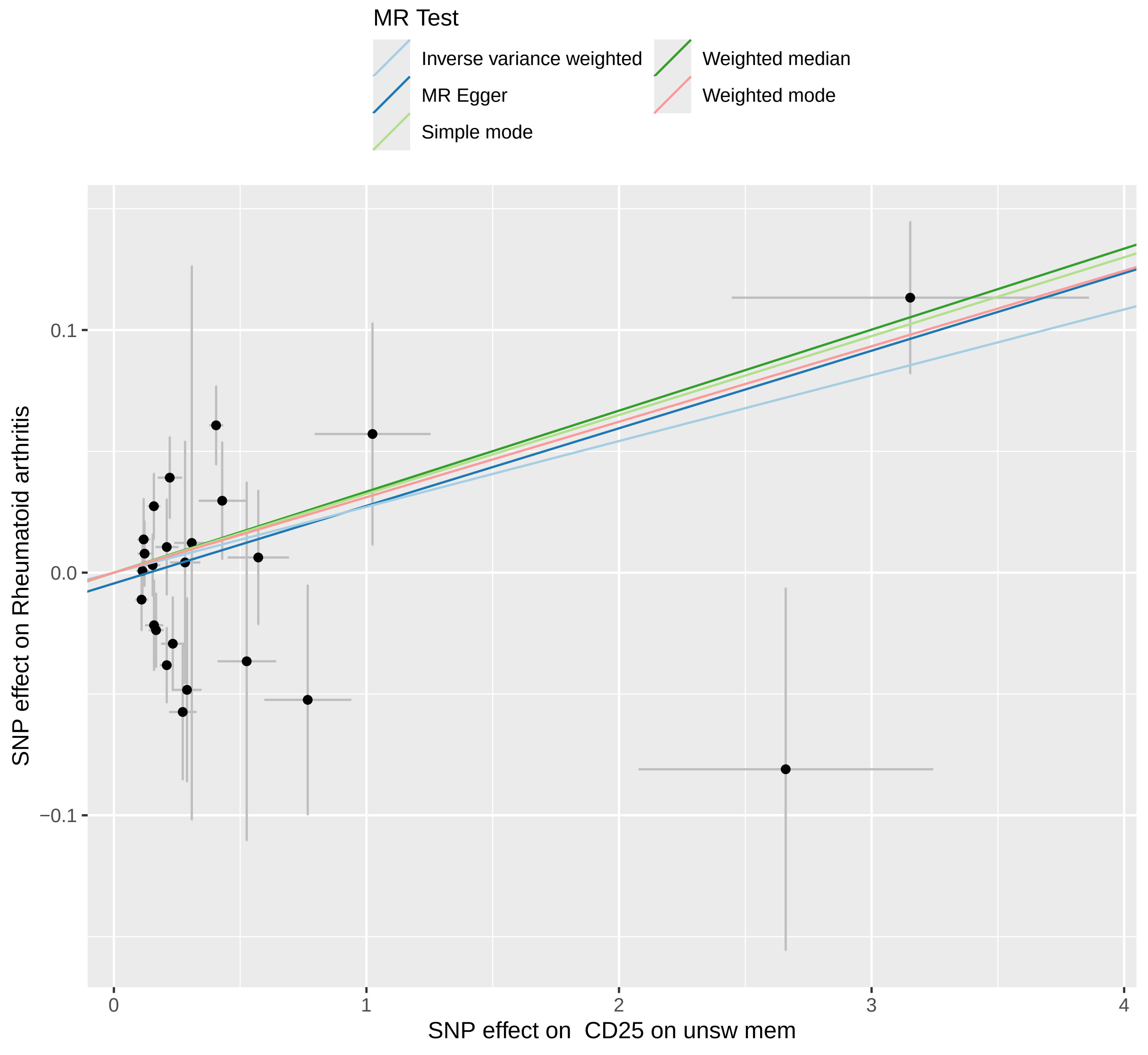

MR Test
Inverse variance weighted
MR Egger
Simple mode
Weighted median
Weighted mode
0.1
0.0
−0.1
0
1
2
3
4
SNP effect on Rheumatoid arthritis
SNP effect on CD25 on unsw mem

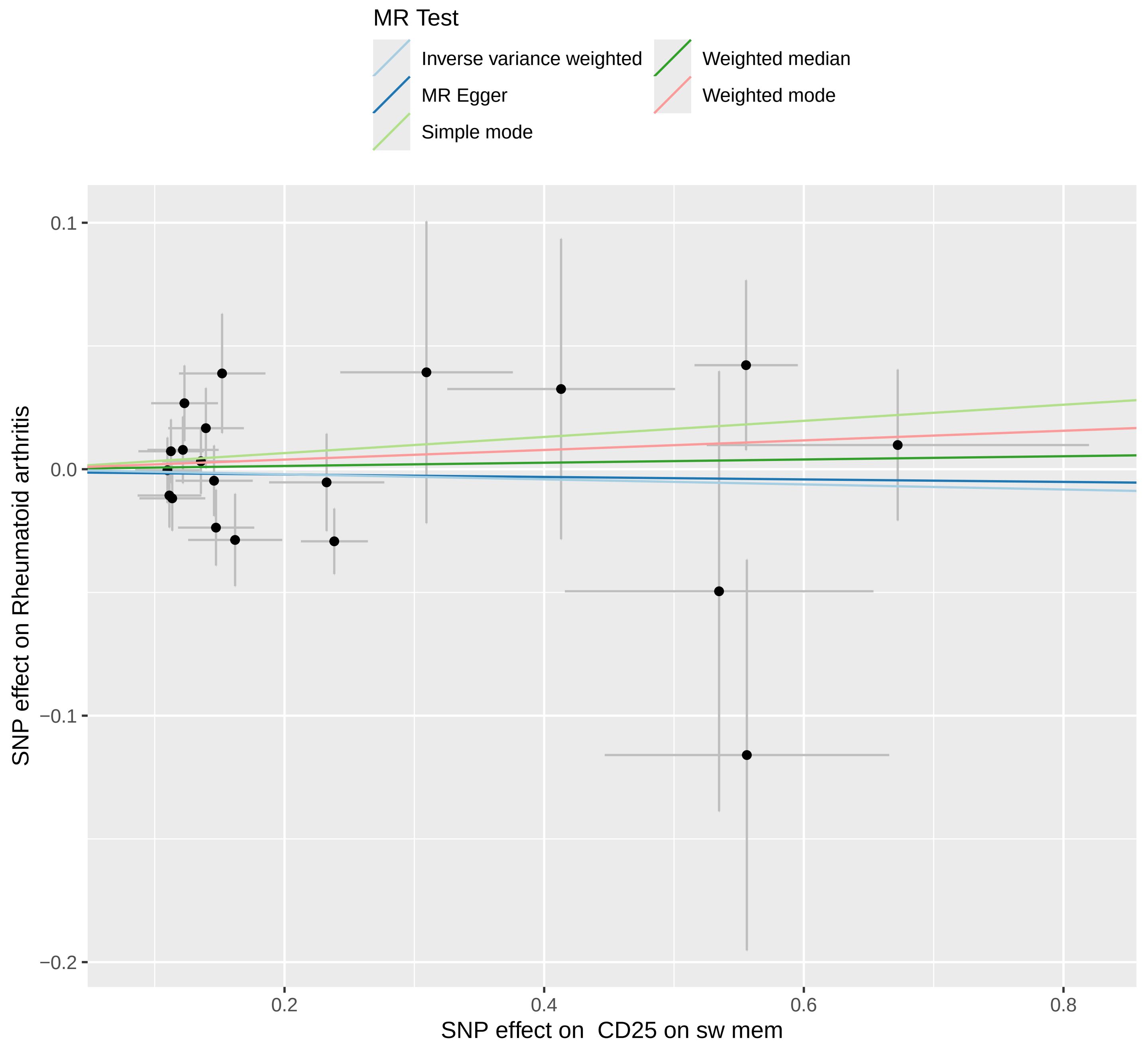

MR Test
Inverse variance weighted
MR Egger
Simple mode
Weighted median
Weighted mode
0.1
0.0
−0.1
−0.2
0.2
0.4
0.6
0.8
SNP effect on Rheumatoid arthritis
SNP effect on CD25 on sw mem

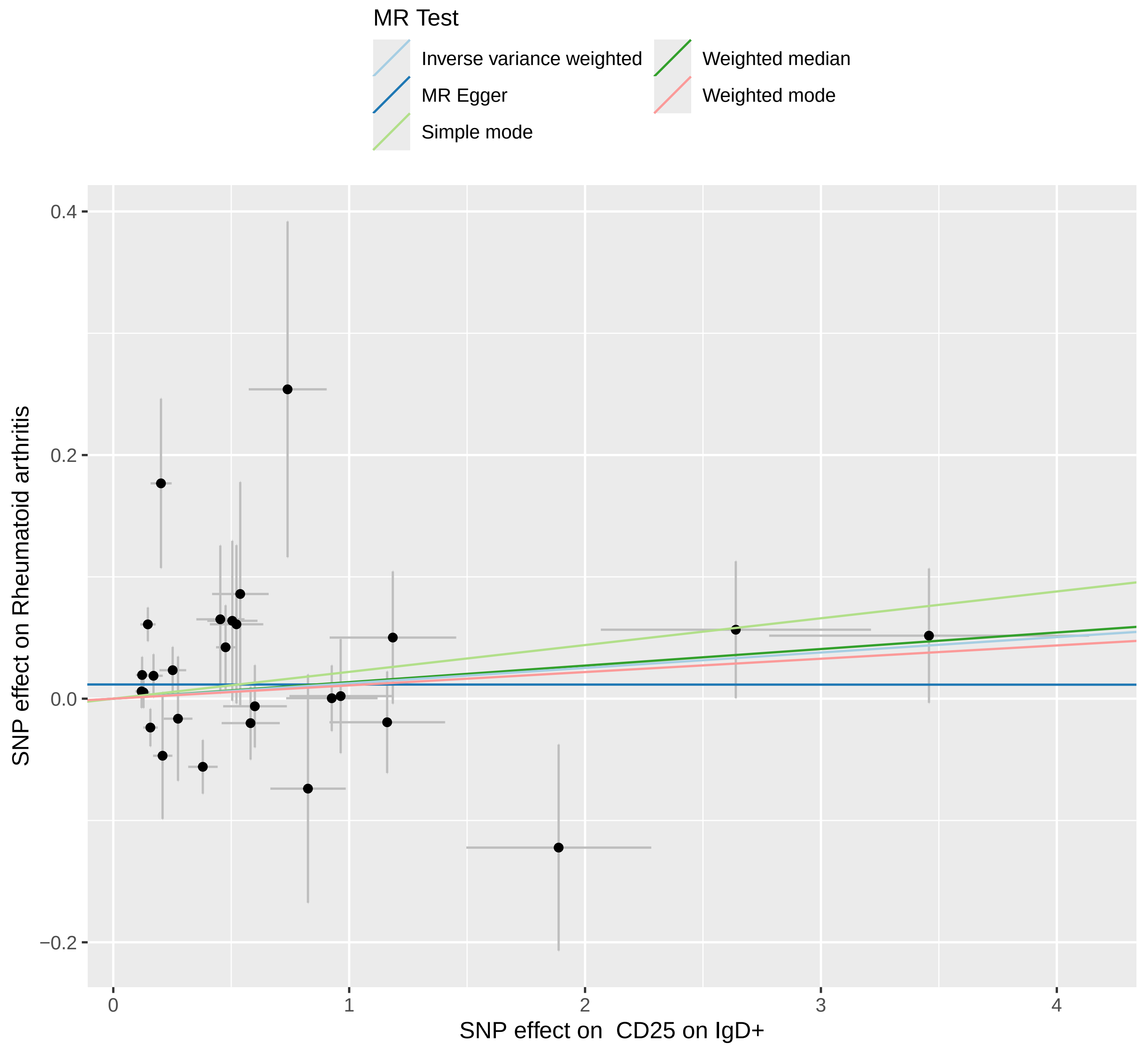

MR Test
Inverse variance weighted
MR Egger
Simple mode
Weighted median
Weighted mode
0.4
0.2
0.0
−0.2
0
1
2
3
4
SNP effect on Rheumatoid arthritis
SNP effect on CD25 on IgD+

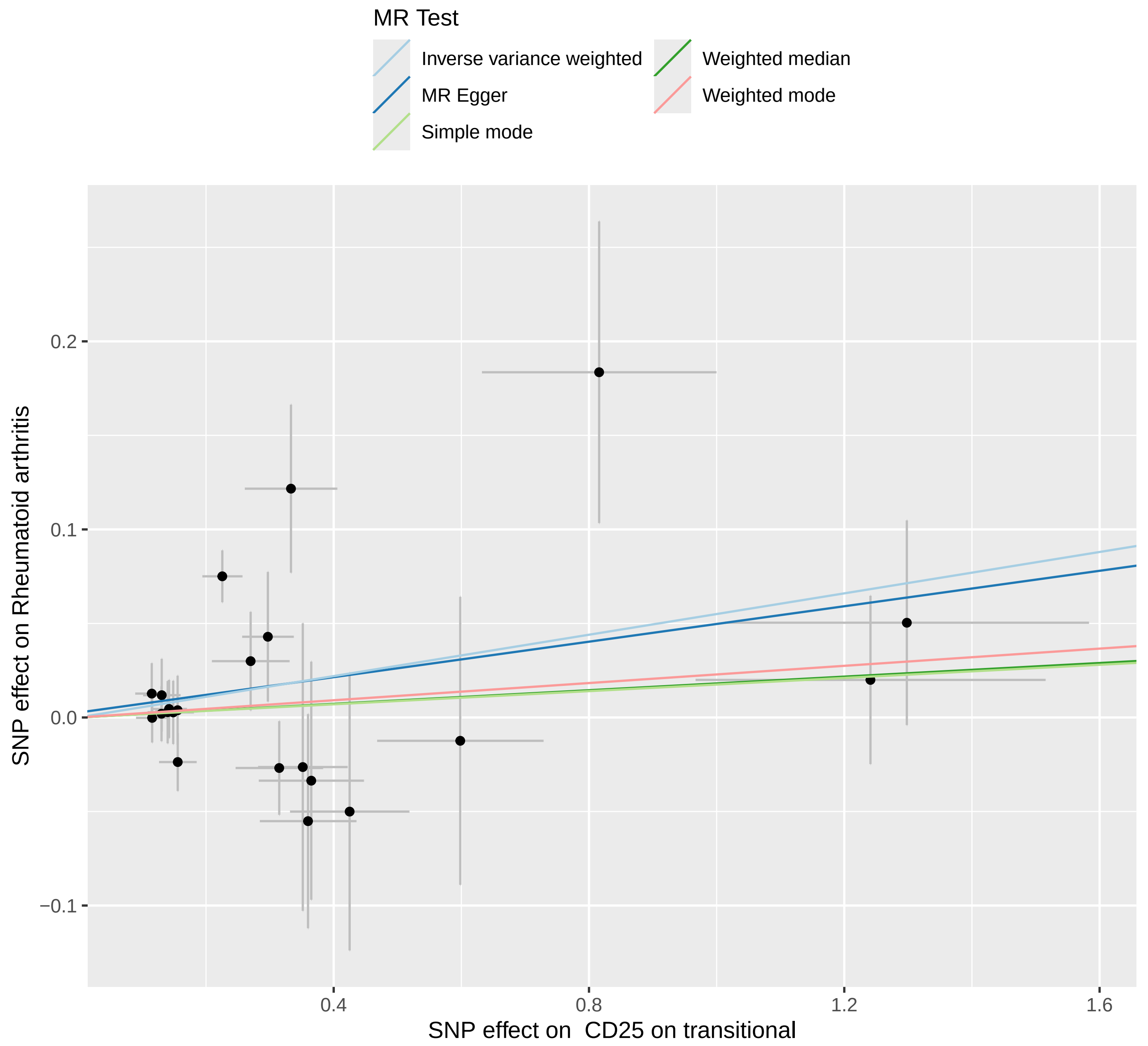
MR Test
Inverse variance weighted
MR Egger
Simple mode
Weighted median
Weighted mode
0.2
0.1
0.0
−0.1
0.4
0.8
1.2
1.6
SNP effect on Rheumatoid arthritis
SNP effect on CD25 on transitional

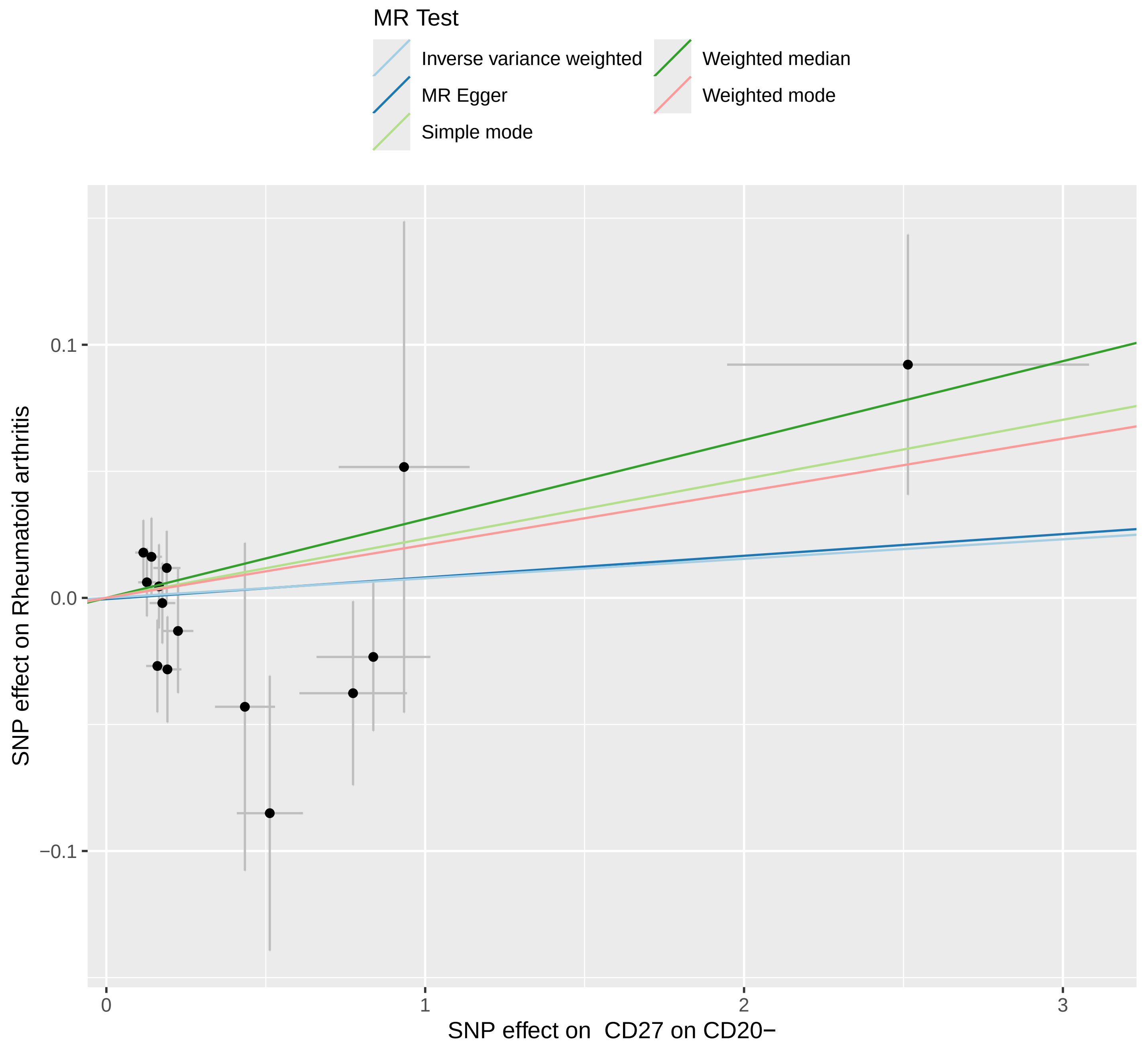

MR Test
Inverse variance weighted
MR Egger
Simple mode
Weighted median
Weighted mode
SNP effect on Rheumatoid arthritis
0.1
0.0
−0.1
0
1
2
3
SNP effect on CD27 on CD20−

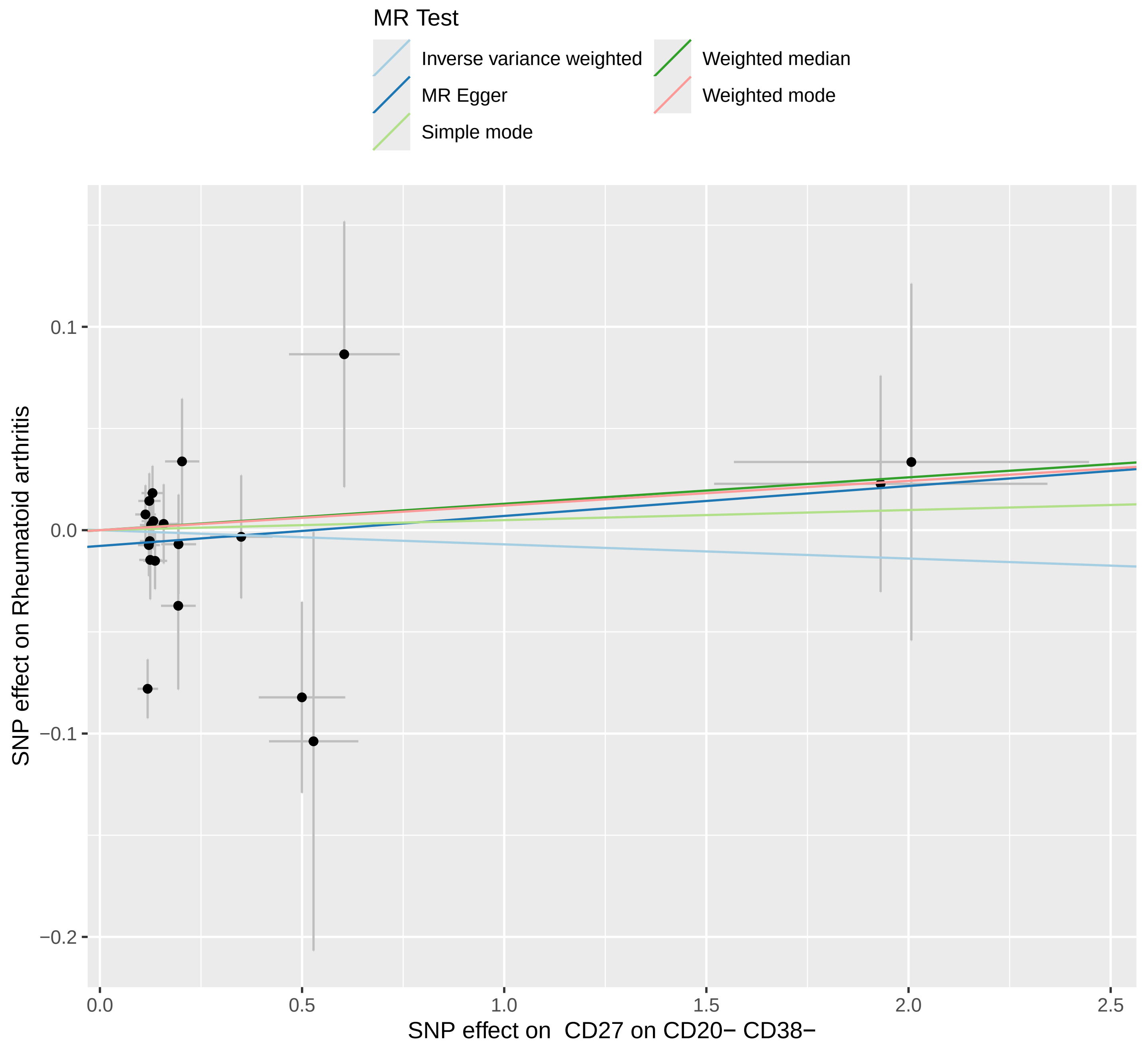
MR Test
Inverse variance weighted
MR Egger
Simple mode
Weighted median
Weighted mode
SNP effect on Rheumatoid arthritis
0.1
0.0
−0.1
−0.2
0.0
0.5
1.0
1.5
2.0
2.5
SNP effect on CD27 on CD20− CD38−

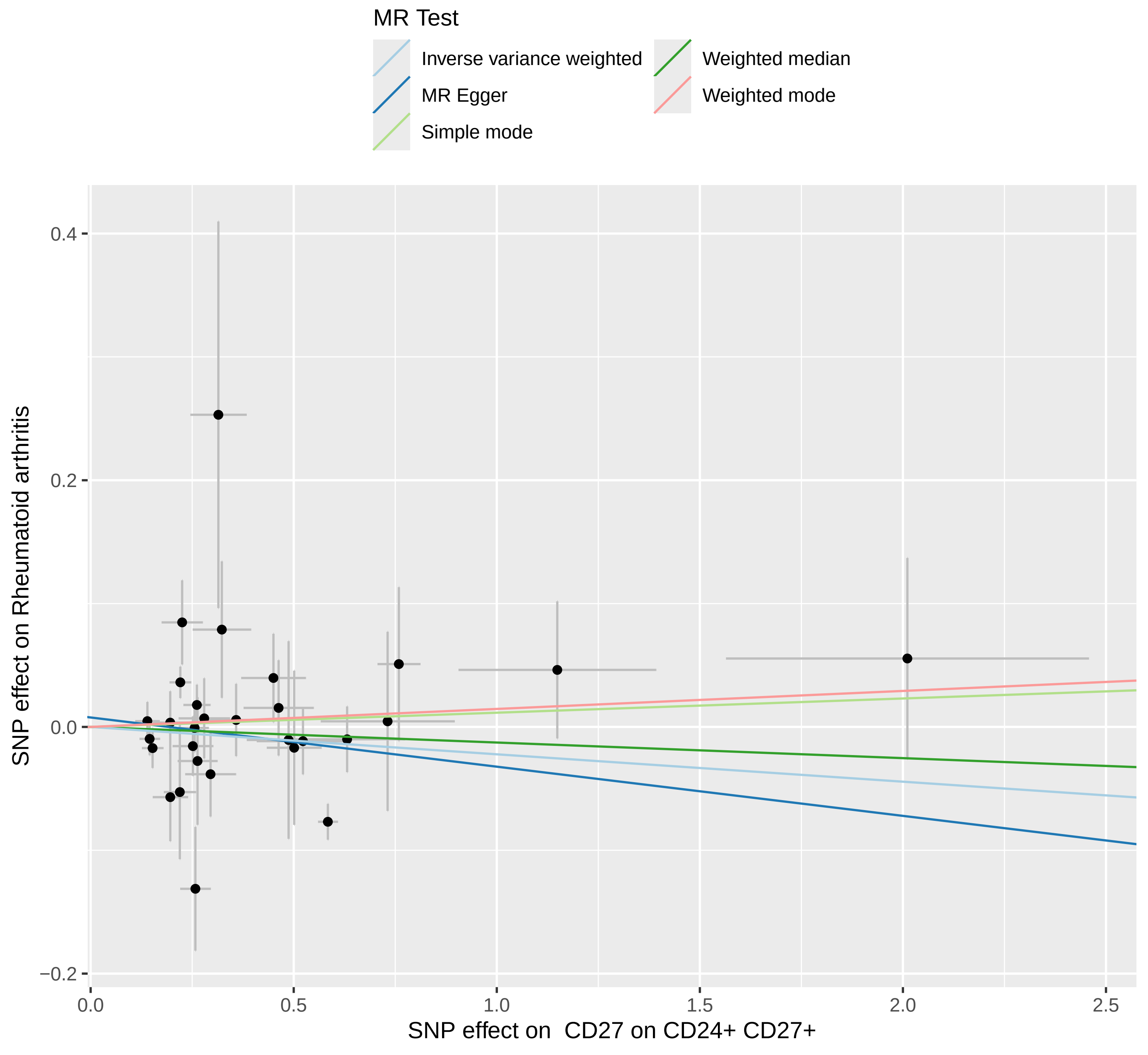

MR Test
Inverse variance weighted
MR Egger
Simple mode
Weighted median
Weighted mode
0.4
0.2
0.0
−0.2
0.0
0.5
1.0
1.5
2.0
2.5
SNP effect on Rheumatoid arthritis
SNP effect on CD27 on CD24+ CD27+

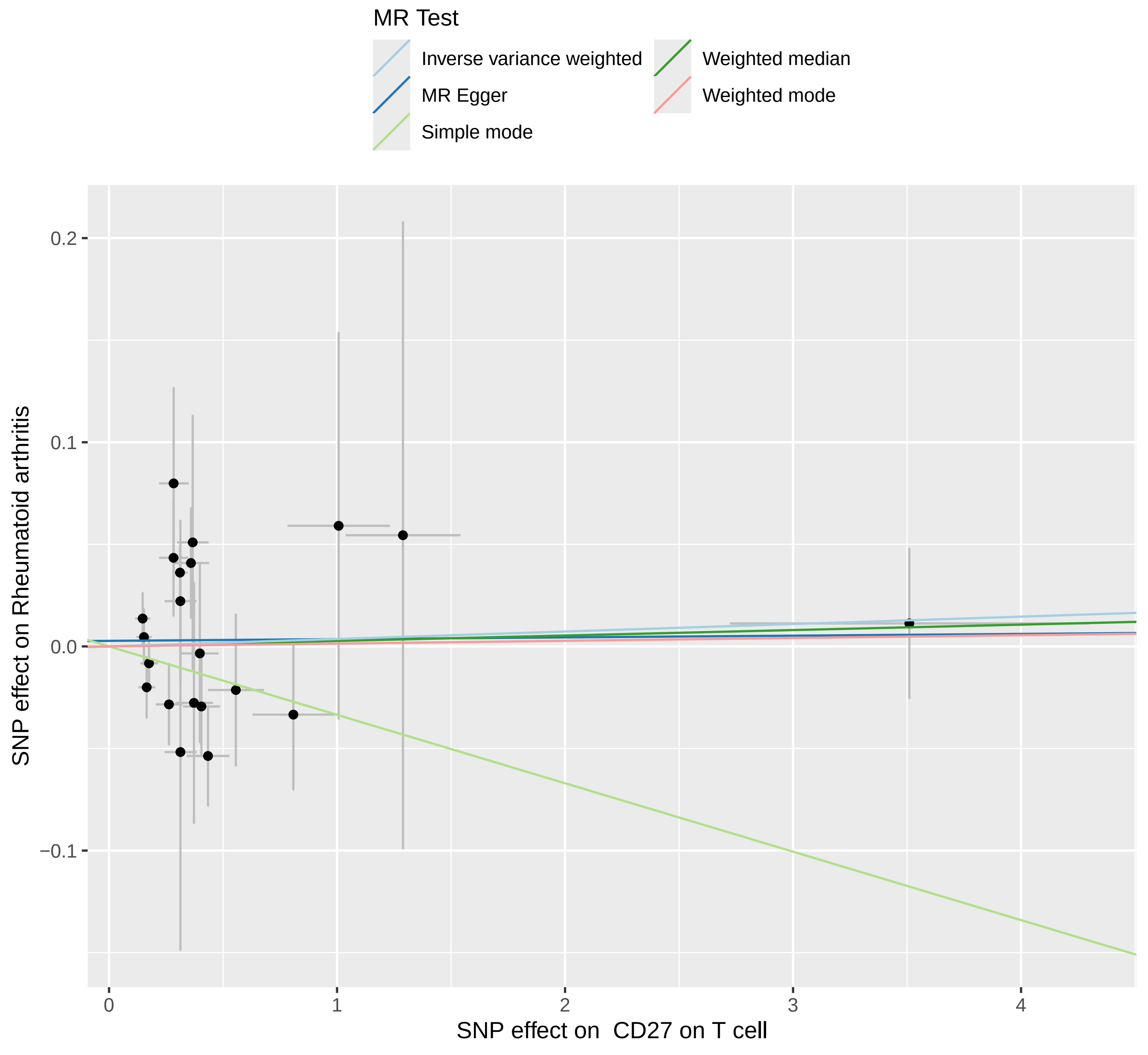

MR Test
Inverse variance weighted
MR Egger
Simple mode
Weighted median
Weighted mode
0.2
0.1
0.0
−0.1
0
1
2
3
4
SNP effect on Rheumatoid arthritis
SNP effect on CD27 on T cell

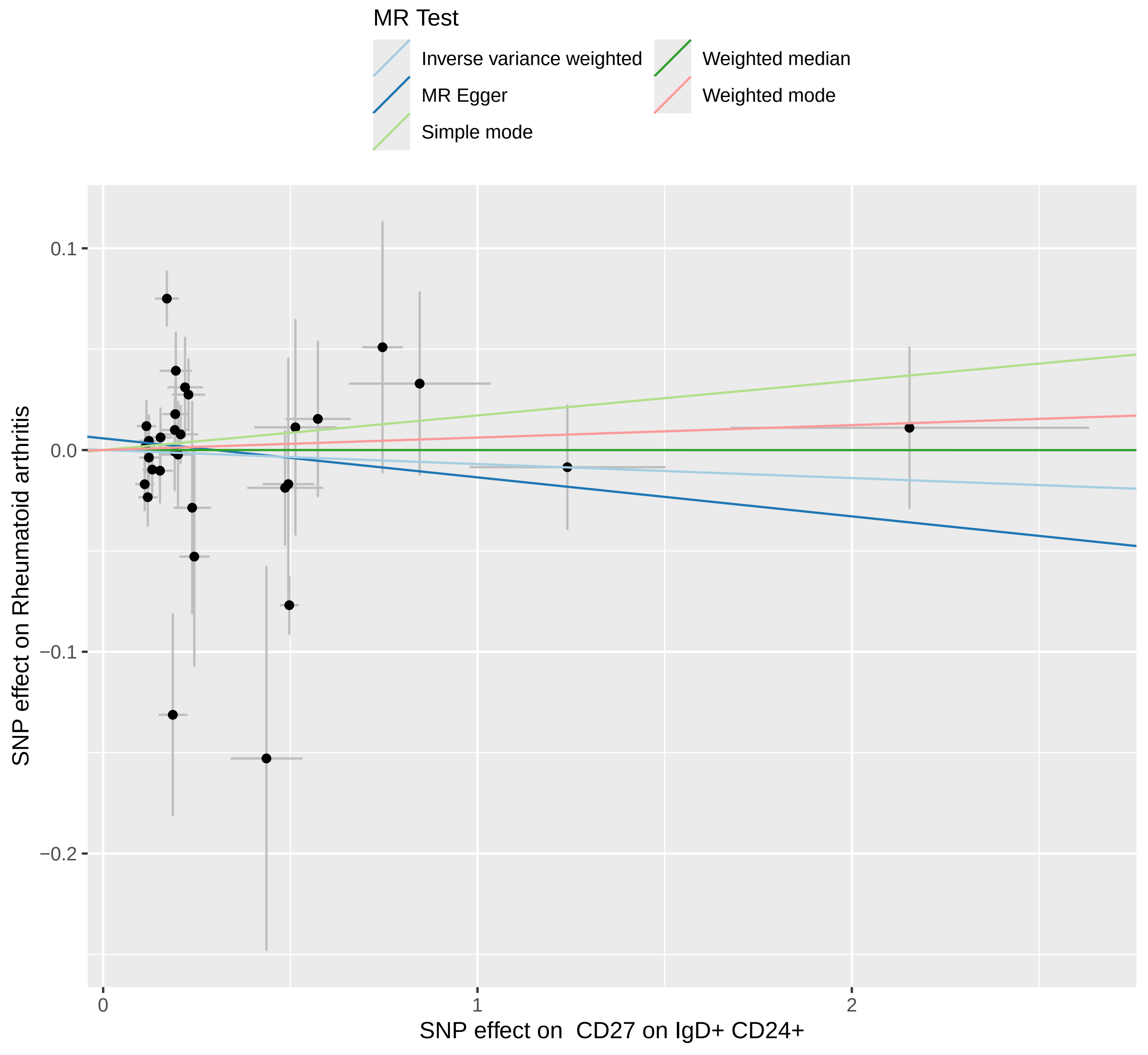

MR Test
Inverse variance weighted
MR Egger
Simple mode
Weighted median
Weighted mode
0.1
0.0
−0.1
−0.2
SNP effect on Rheumatoid arthritis
0
1
2
SNP effect on CD27 on IgD+ CD24+

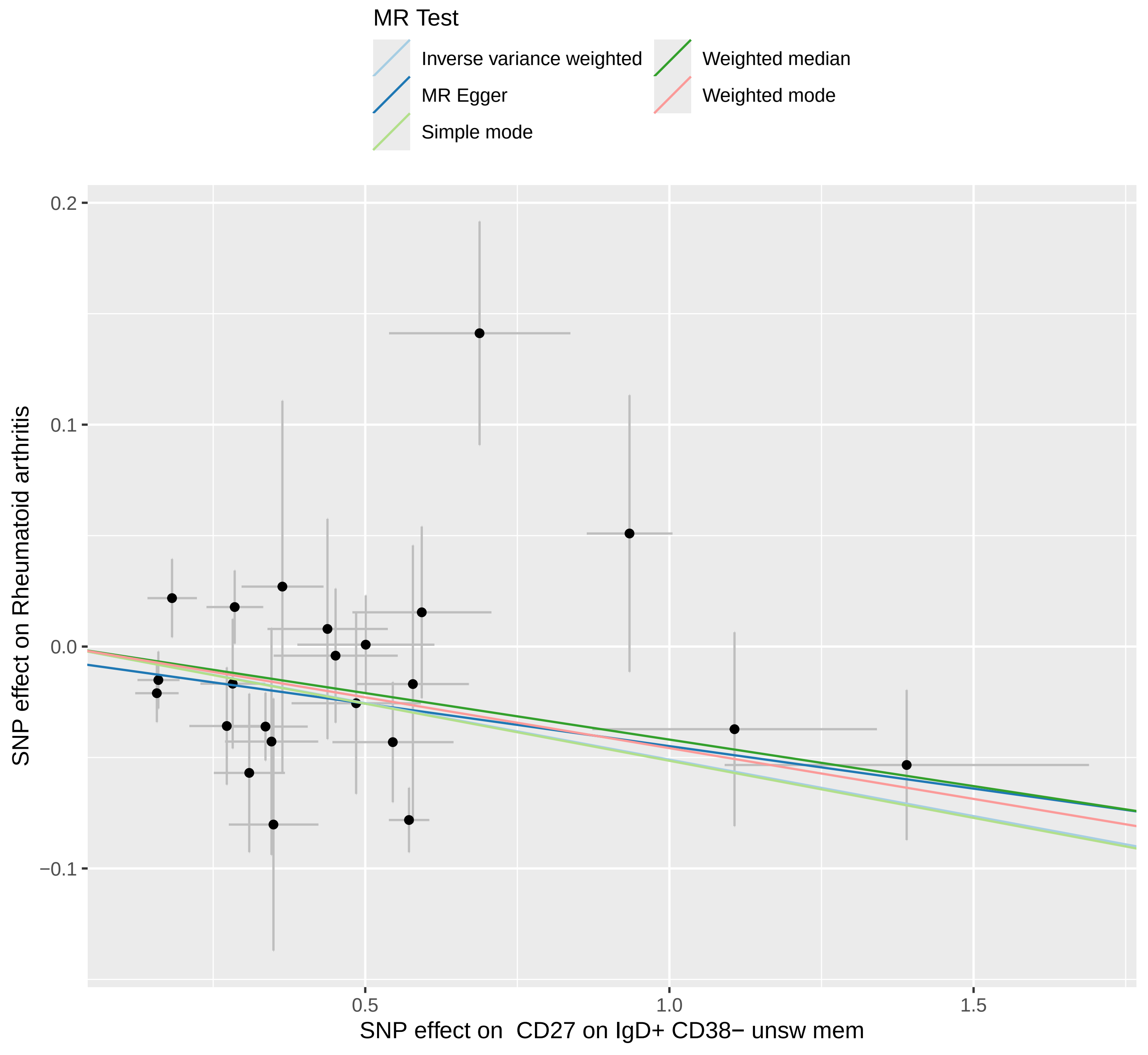
MR Test
Inverse variance weighted
MR Egger
Simple mode
Weighted median
Weighted mode
0.2
0.1
0.0
−0.1
0.5
1.0
1.5
SNP effect on Rheumatoid arthritis
SNP effect on CD27 on IgD+ CD38− unsw mem

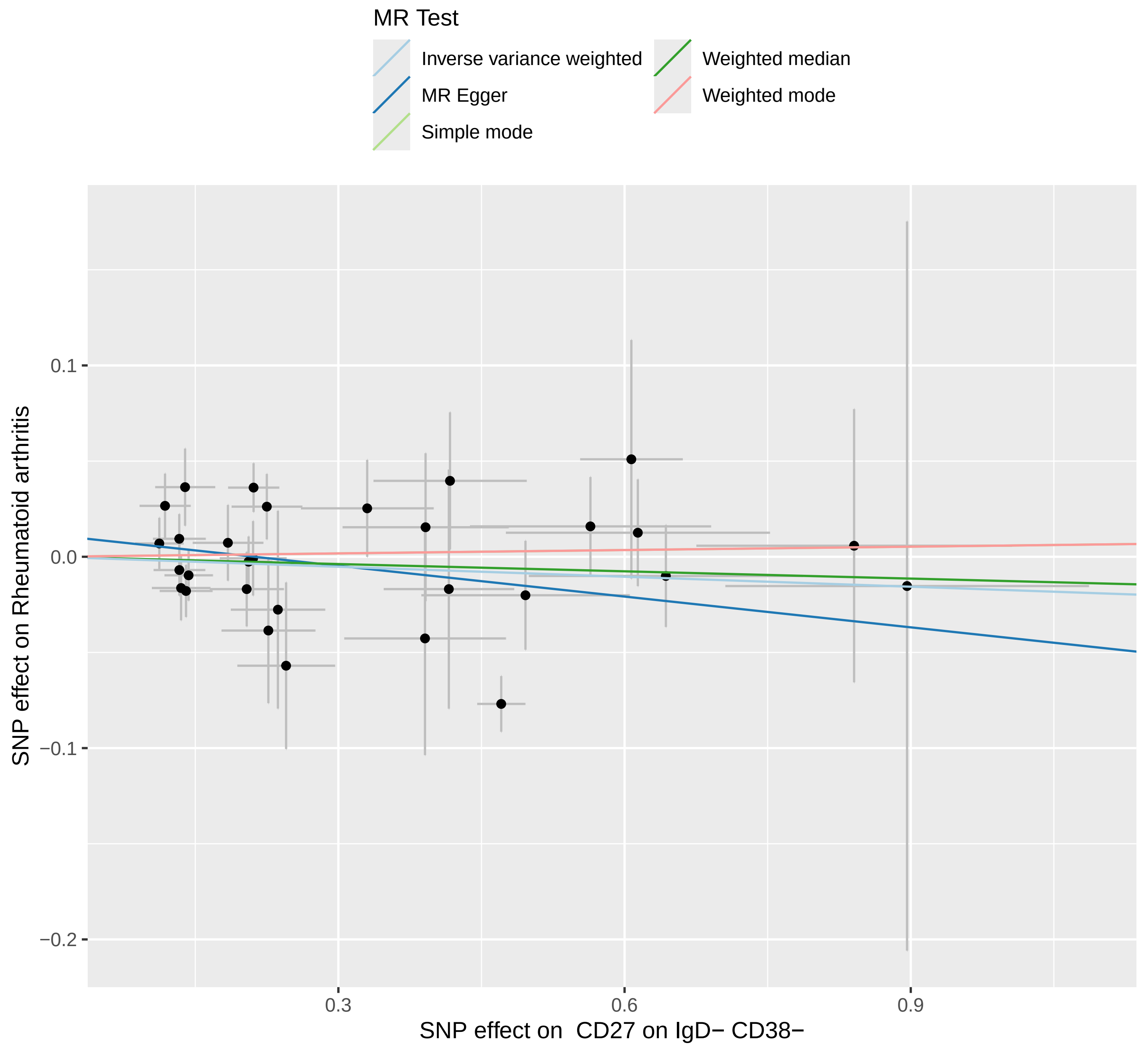

MR Test
Inverse variance weighted
MR Egger
Simple mode
Weighted median
Weighted mode
0.1
0.0
−0.1
−0.2
SNP effect on Rheumatoid arthritis
0.3
0.6
0.9
SNP effect on CD27 on IgD− CD38−

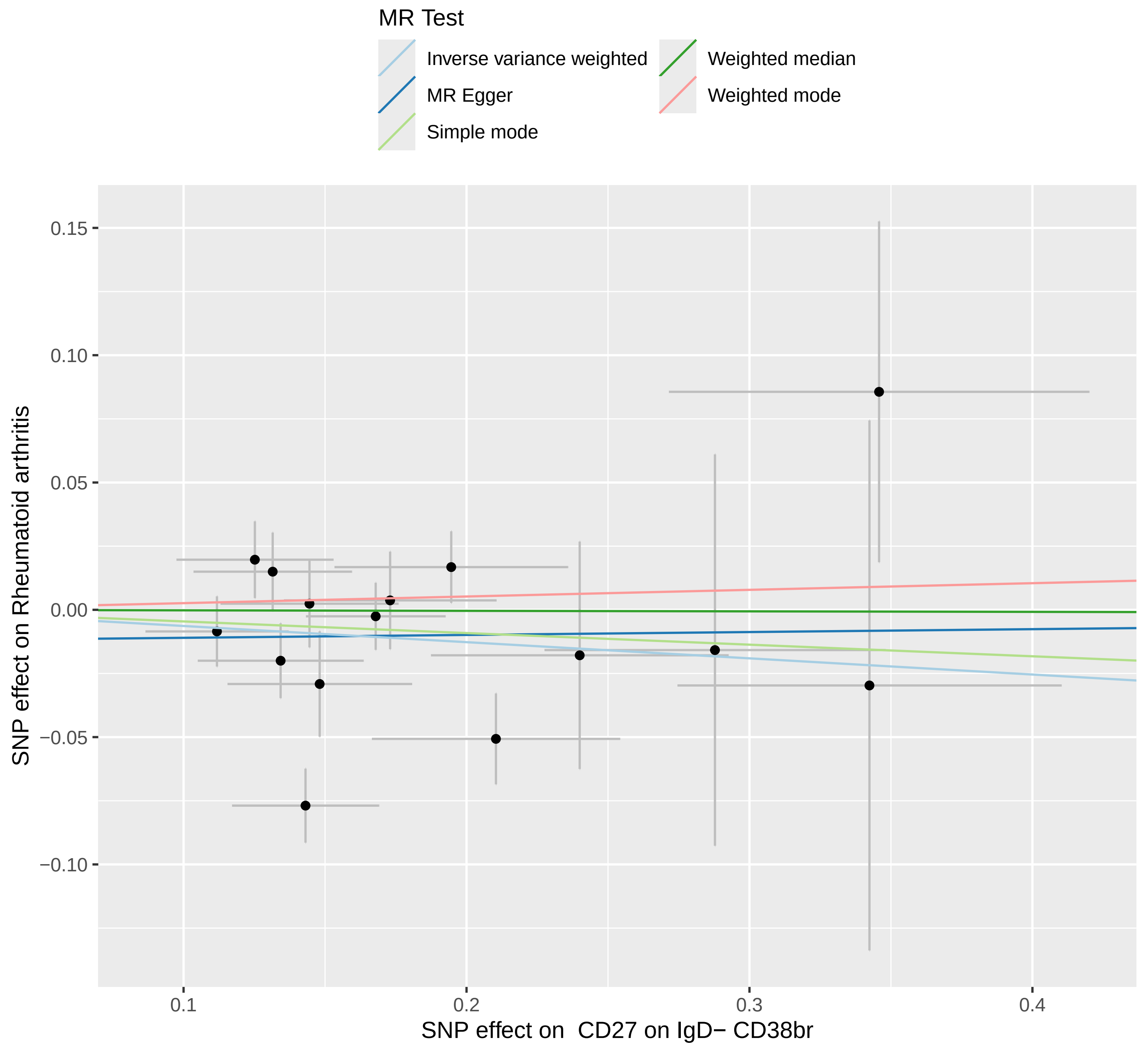

MR Test
Inverse variance weighted
MR Egger
Simple mode
Weighted median
Weighted mode
0.15
0.10
0.05
0.00
−0.05
−0.10
SNP effect on Rheumatoid arthritis
0.1
0.2
0.3
0.4
SNP effect on CD27 on IgD− CD38br

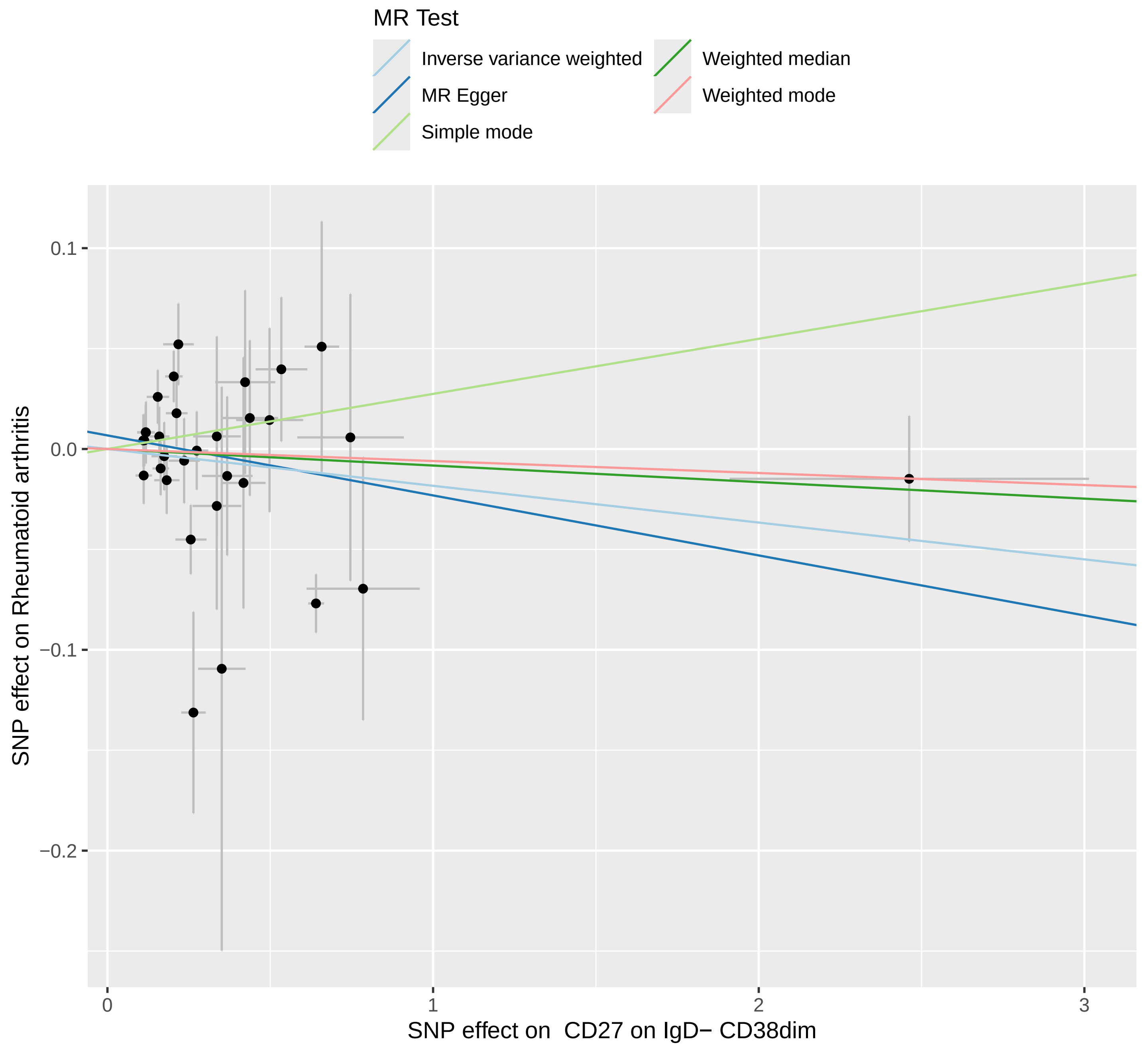
MR Test
Inverse variance weighted
MR Egger
Simple mode
Weighted median
Weighted mode
0.1
0.0
−0.1
−0.2
SNP effect on Rheumatoid arthritis
0
1
2
3
SNP effect on CD27 on IgD− CD38dim

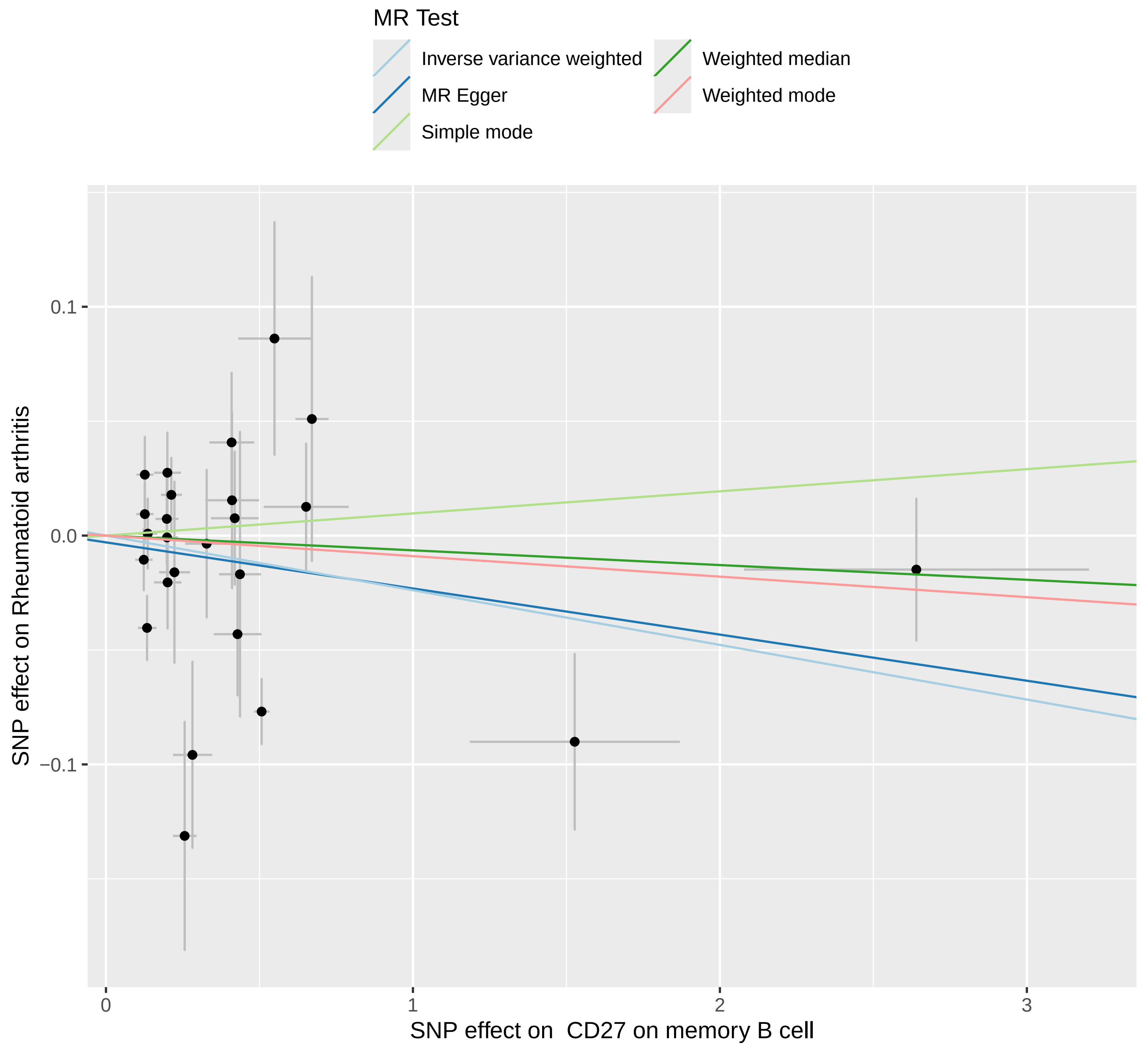
MR Test
Inverse variance weighted
MR Egger
Simple mode
Weighted median
Weighted mode
0.1
0.0
−0.1
0
1
2
3
SNP effect on Rheumatoid arthritis
SNP effect on CD27 on memory B cell

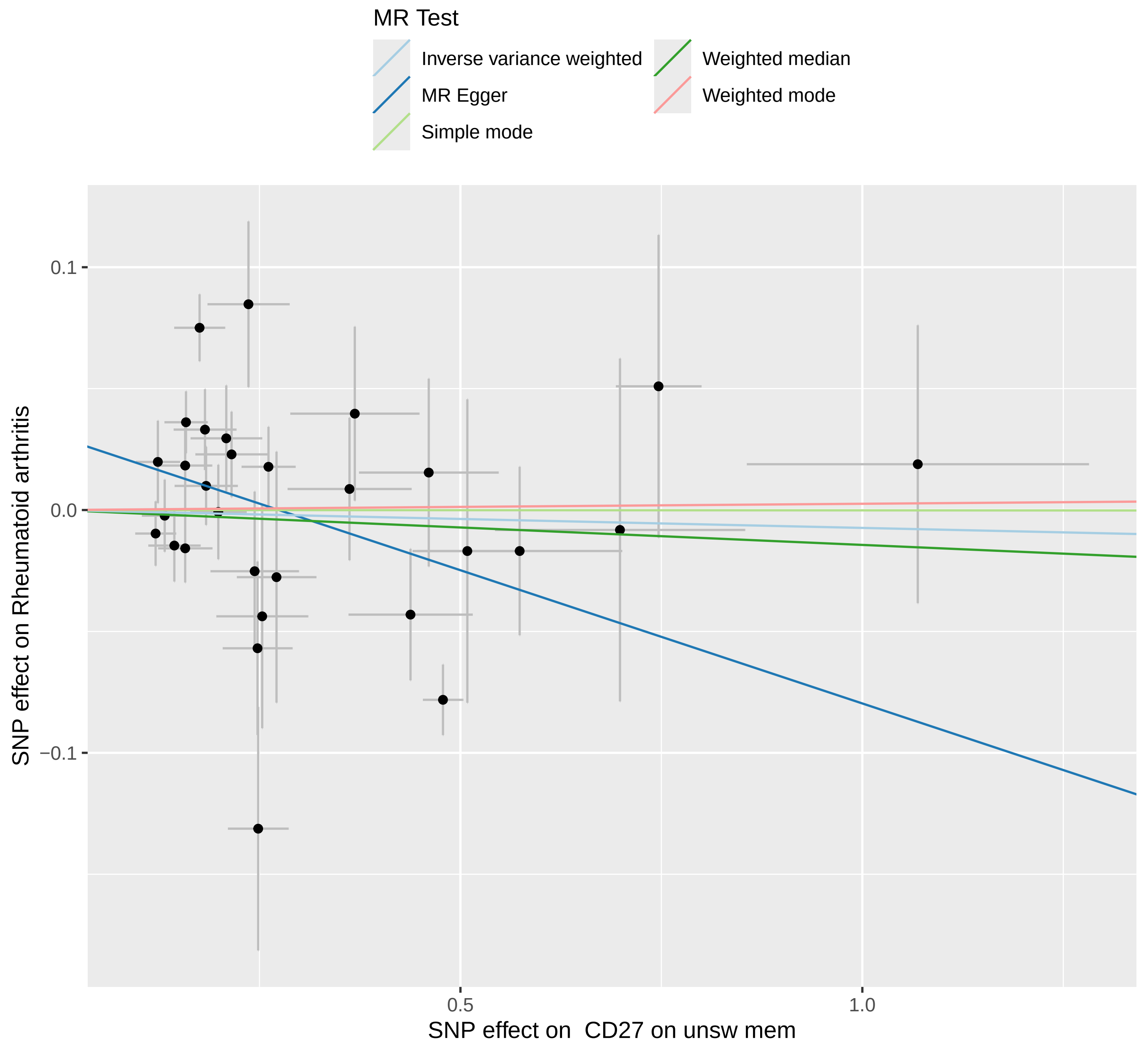
MR Test
Inverse variance weighted
MR Egger
Simple mode
Weighted median
Weighted mode
SNP effect on Rheumatoid arthritis
0.1
0.0
−0.1
0.5
1.0
SNP effect on CD27 on unsw mem

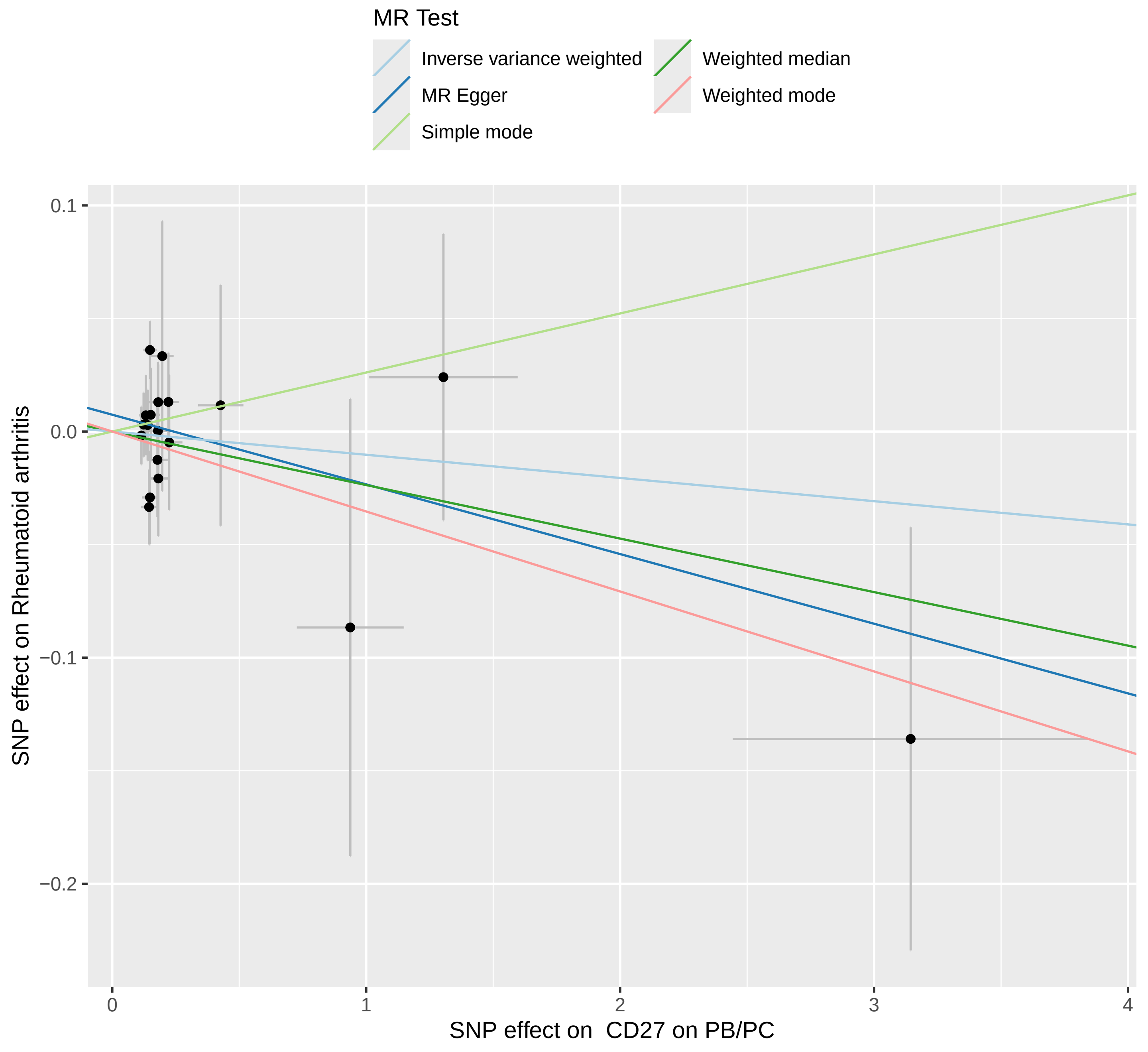
MR Test
Inverse variance weighted
MR Egger
Simple mode
Weighted median
Weighted mode
0.1
0.0
−0.1
−0.2
0
1
2
3
4
SNP effect on Rheumatoid arthritis
SNP effect on CD27 on PB/PC

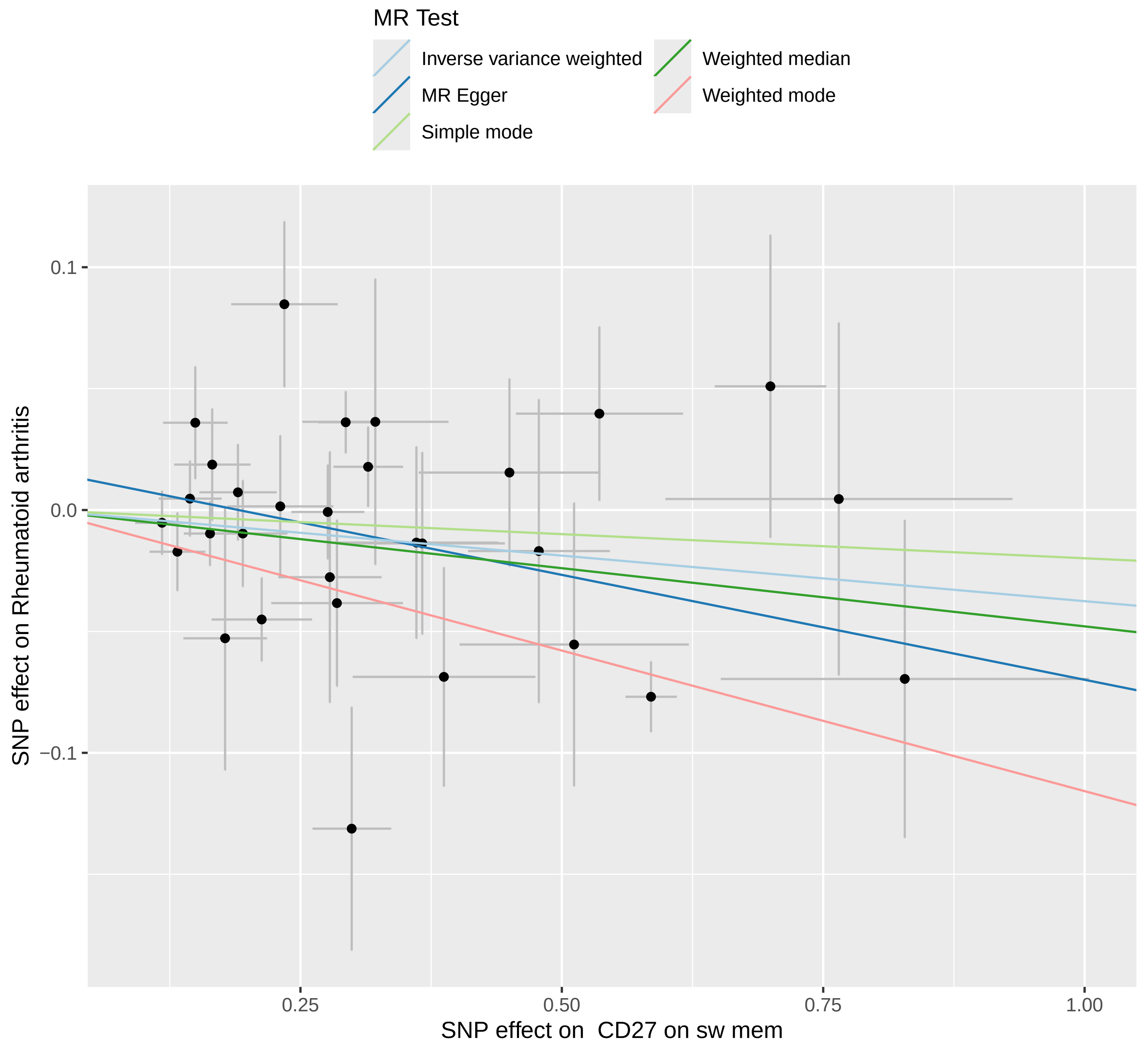

MR Test
Inverse variance weighted
MR Egger
Simple mode
Weighted median
Weighted mode
0.1
0.0
−0.1
0.25
0.50
0.75
1.00
SNP effect on Rheumatoid arthritis
SNP effect on CD27 on sw mem

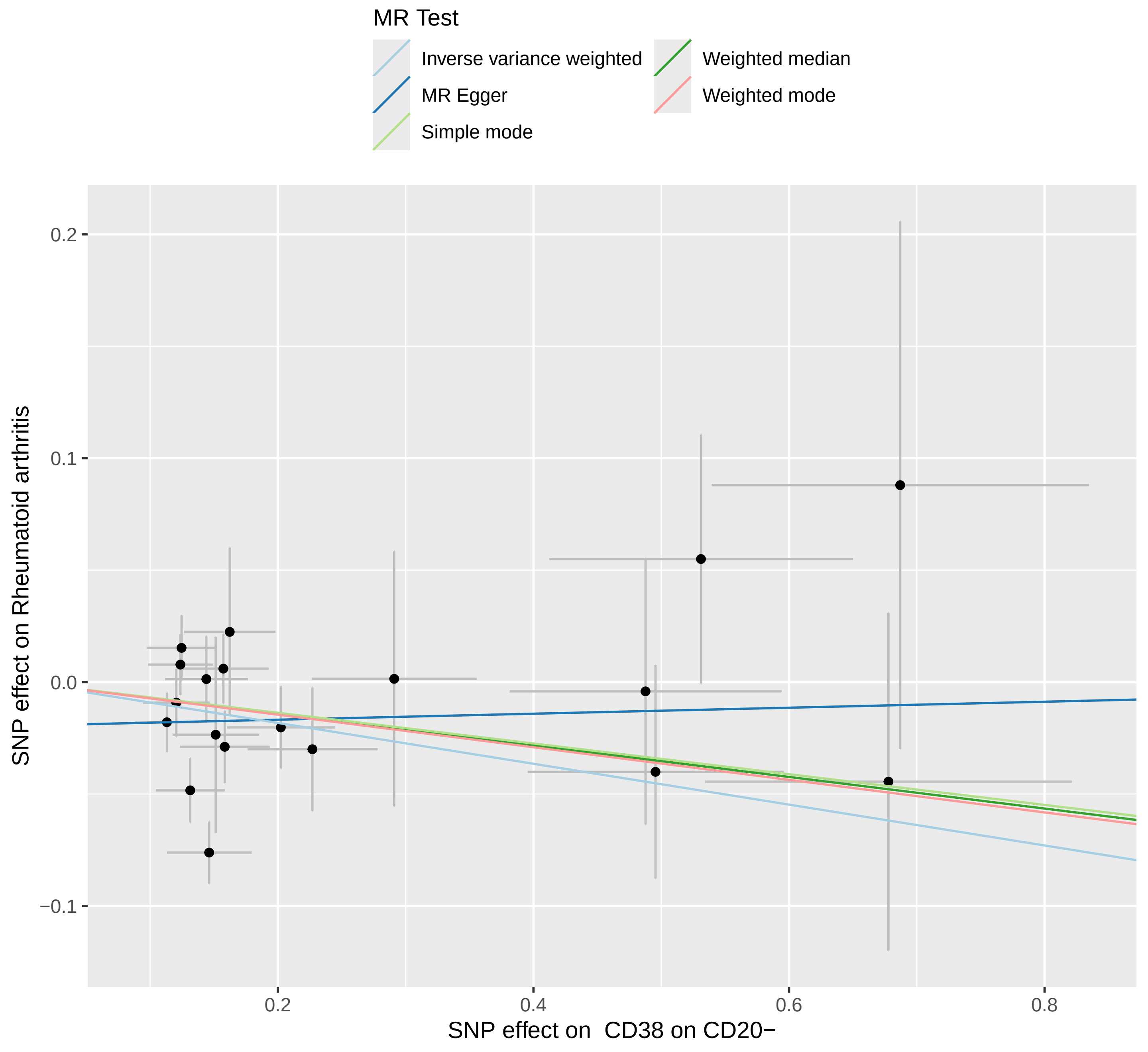

MR Test
Inverse variance weighted
MR Egger
Simple mode
Weighted median
Weighted mode
0.2
0.1
0.0
−0.1
SNP effect on Rheumatoid arthritis
0.2
0.4
0.6
0.8
SNP effect on CD38 on CD20−

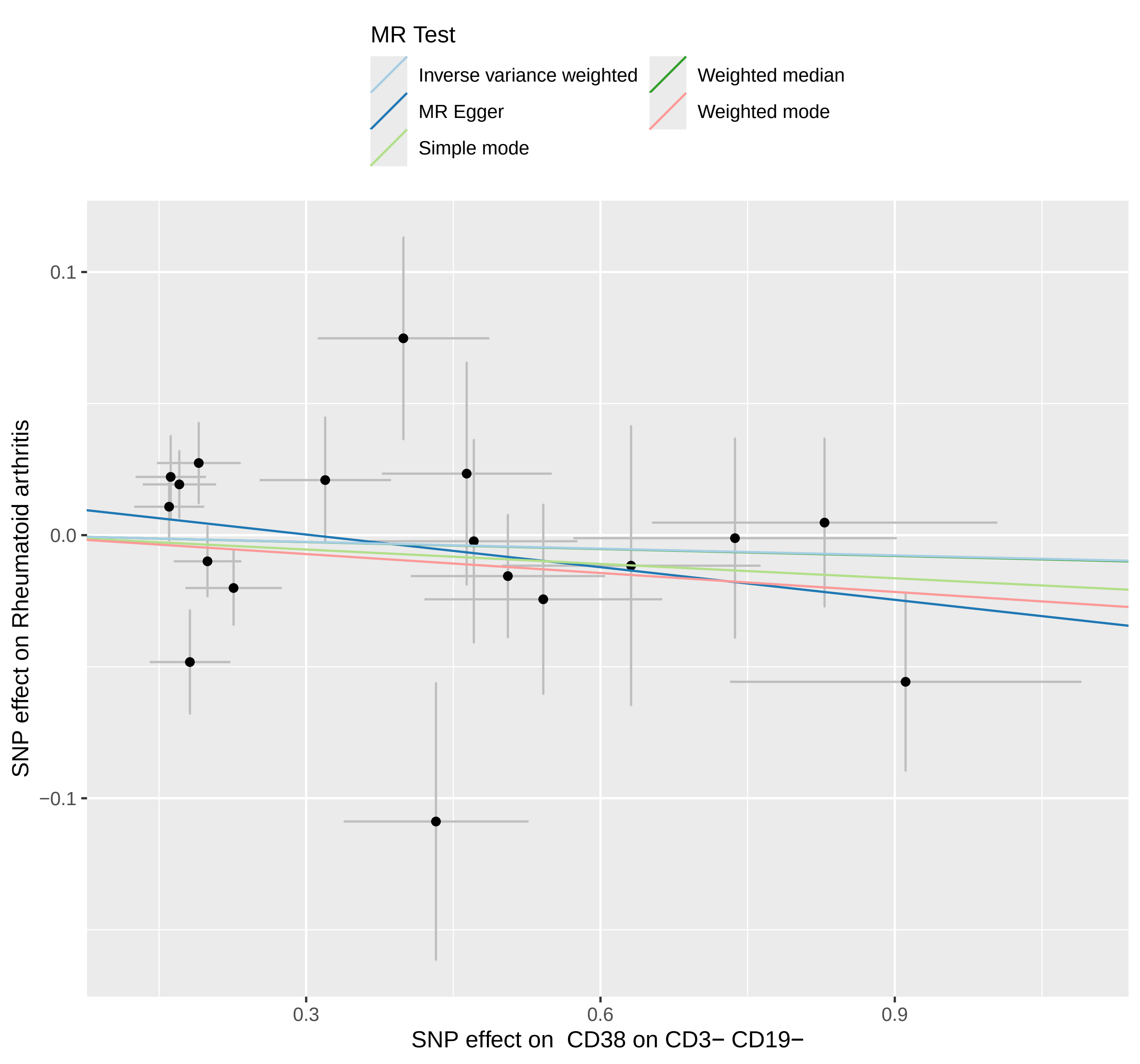
MR Test
Inverse variance weighted
MR Egger
Simple mode
Weighted median
Weighted mode
0.1
0.0
−0.1
0.3
0.6
0.9
SNP effect on Rheumatoid arthritis
SNP effect on CD38 on CD3− CD19−

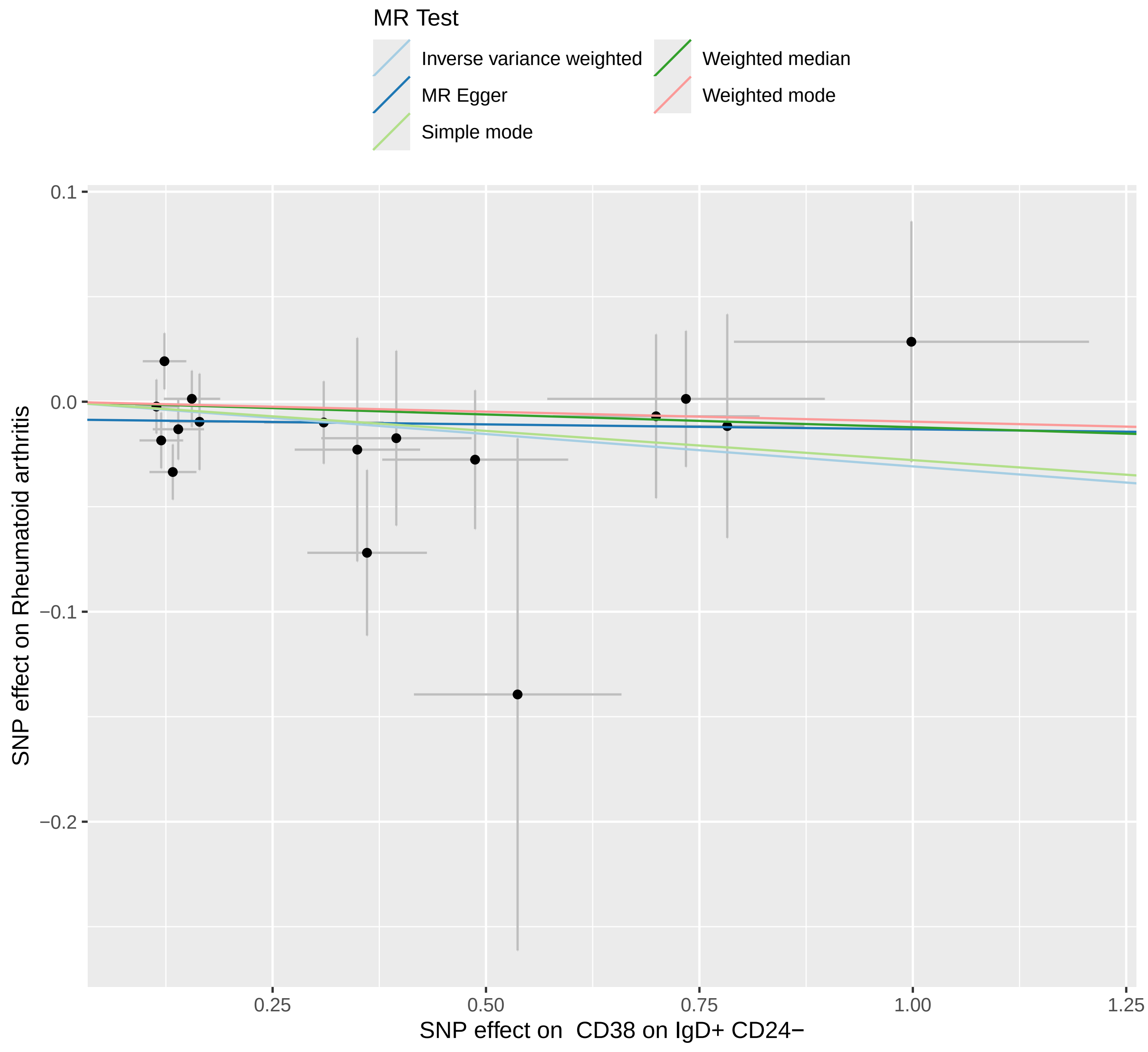

MR Test
Inverse variance weighted
MR Egger
Simple mode
Weighted median
Weighted mode
0.1
0.0
−0.1
−0.2
SNP effect on Rheumatoid arthritis
0.25
0.50
0.75
1.00
1.25
SNP effect on CD38 on IgD+ CD24−

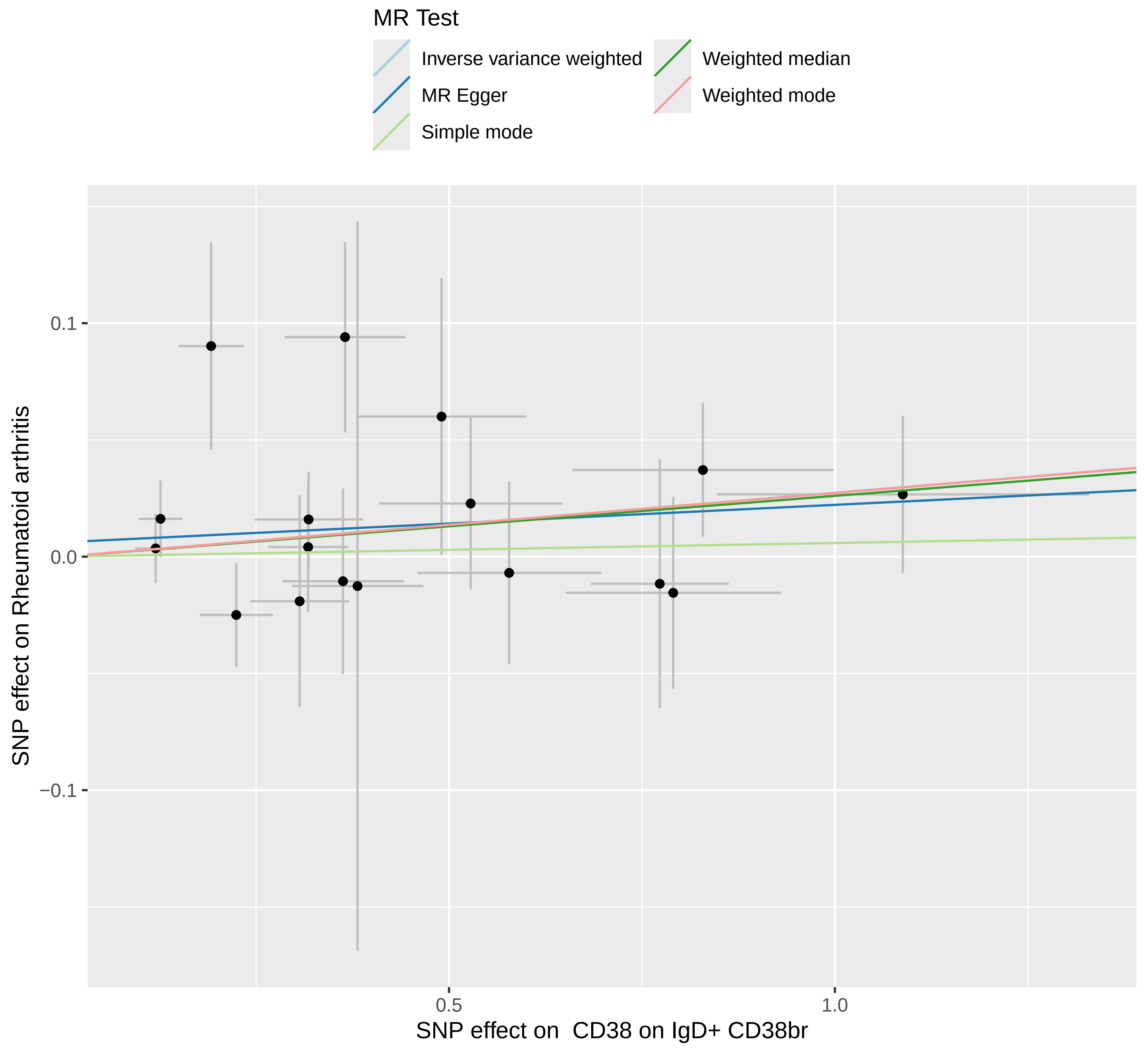

MR Test
Inverse variance weighted
MR Egger
Simple mode
Weighted median
Weighted mode
0.1
0.0
−0.1
0.5
1.0
SNP effect on Rheumatoid arthritis
SNP effect on CD38 on IgD+ CD38br

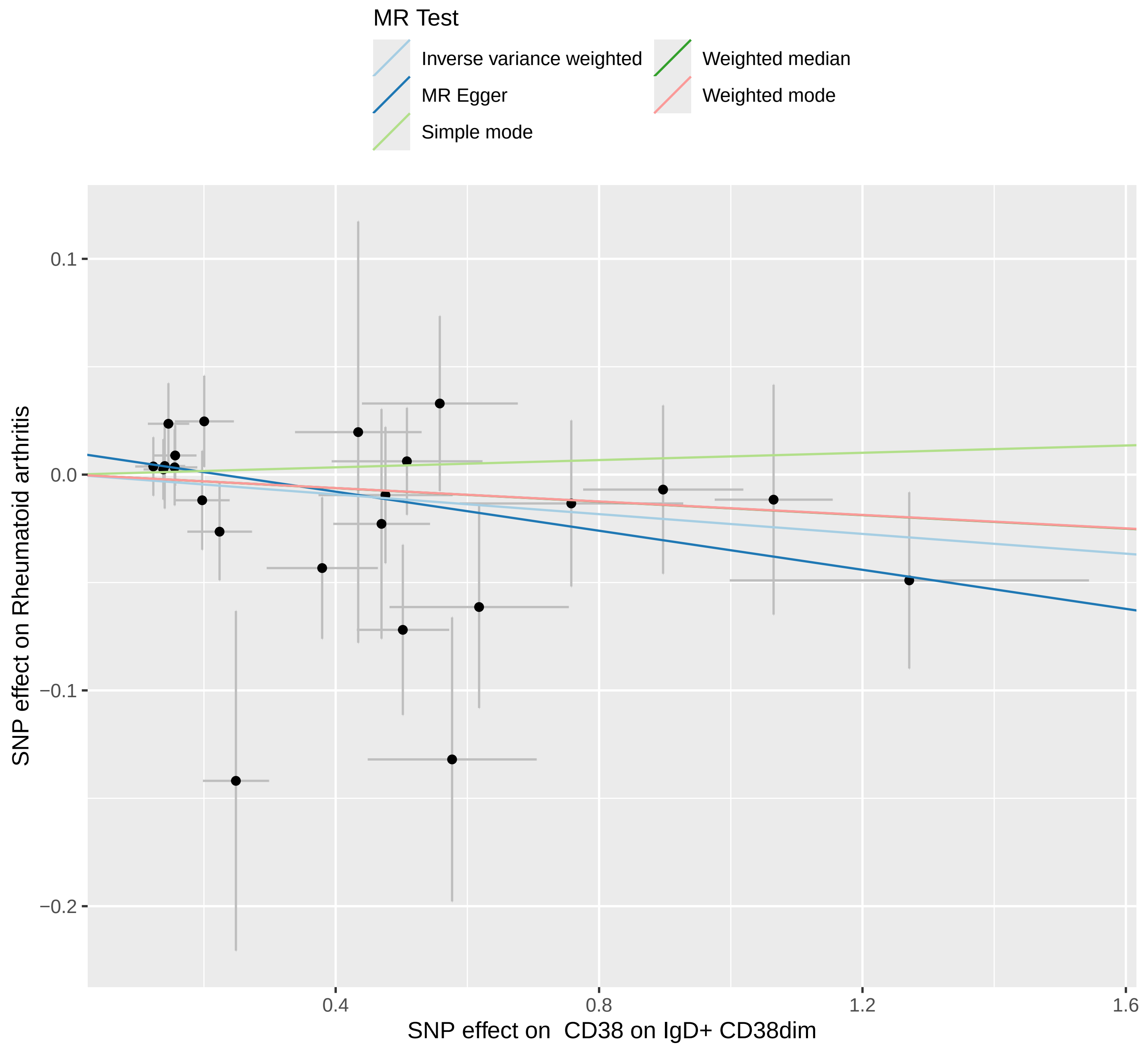
MR Test
Inverse variance weighted
MR Egger
Simple mode
Weighted median
Weighted mode
0.1
0.0
−0.1
−0.2
0.4
0.8
1.2
1.6
SNP effect on Rheumatoid arthritis
SNP effect on CD38 on IgD+ CD38dim

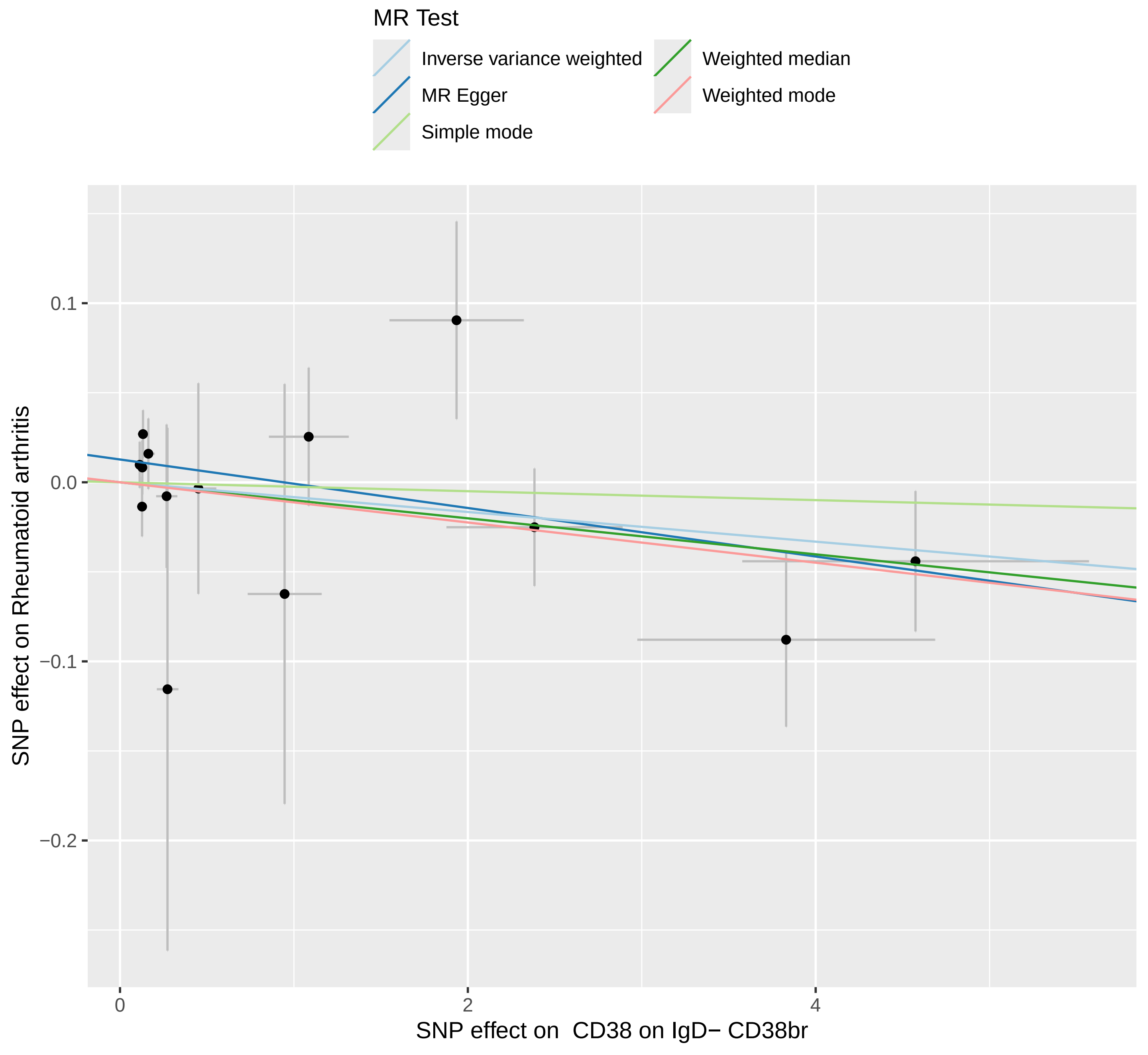
MR Test
Inverse variance weighted
MR Egger
Simple mode
Weighted median
Weighted mode
SNP effect on Rheumatoid arthritis
0.1
0.0
−0.1
−0.2
0
2
4
SNP effect on CD38 on IgD− CD38br

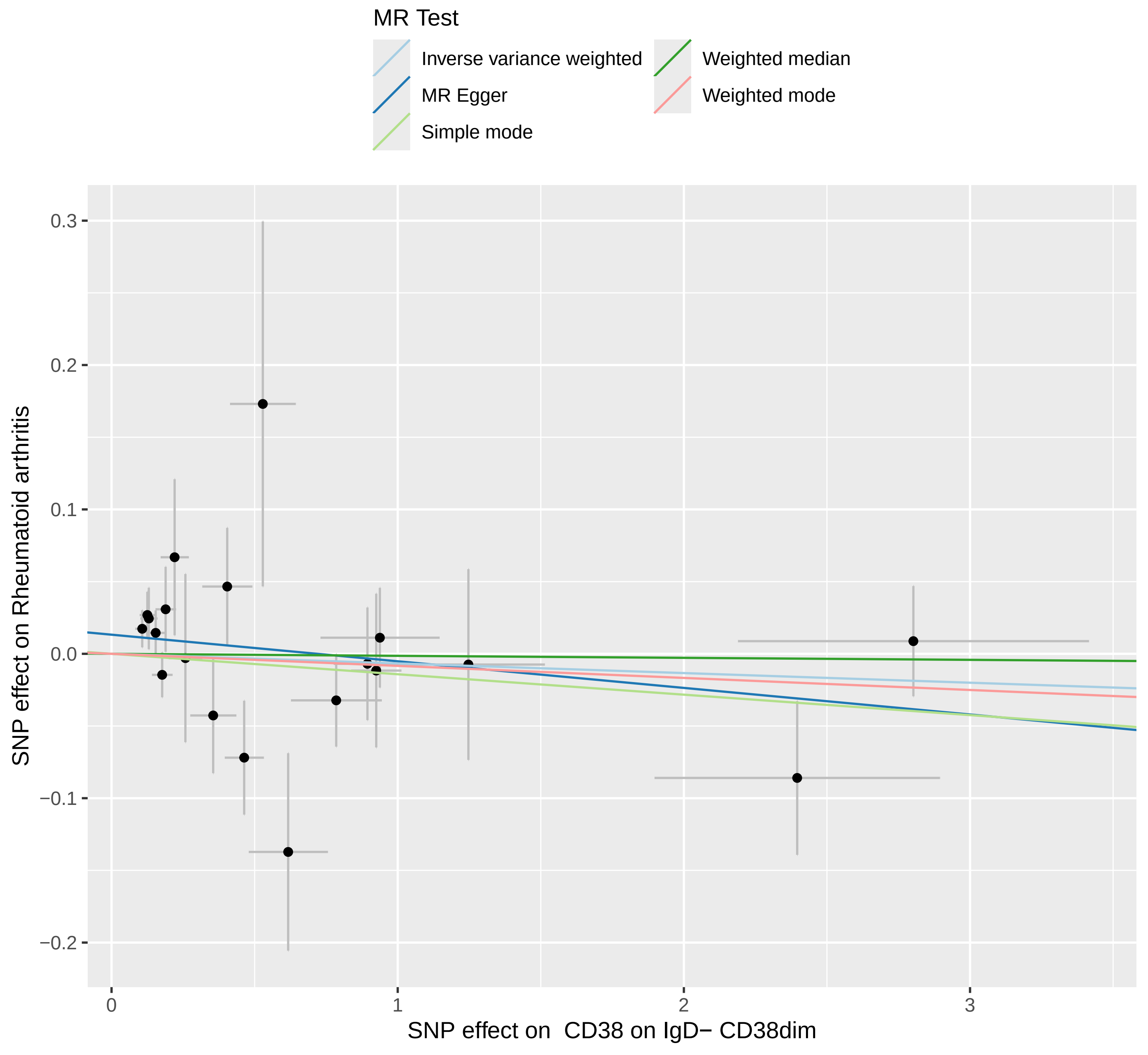
MR Test
Inverse variance weighted
MR Egger
Simple mode
Weighted median
Weighted mode
SNP effect on Rheumatoid arthritis
0.3
0.2
0.1
0.0
−0.1
−0.2
0
1
2
3
SNP effect on CD38 on IgD− CD38dim

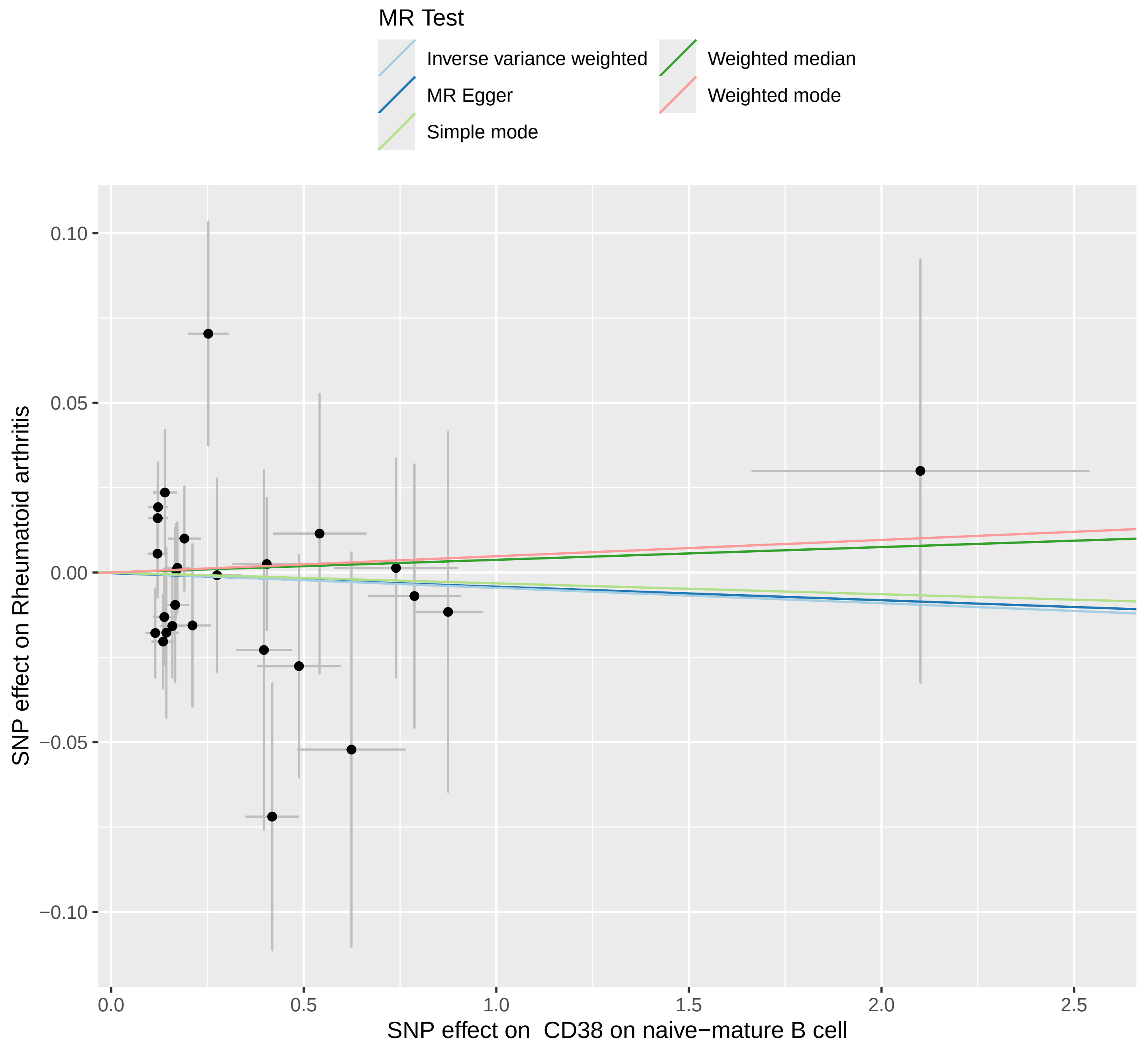

MR Test
Inverse variance weighted
MR Egger
Simple mode
Weighted median
Weighted mode
SNP effect on Rheumatoid arthritis
0.10
0.05
0.00
−0.05
−0.10
0.0
0.5
1.0
1.5
2.0
2.5
SNP effect on CD38 on naive−mature B cell

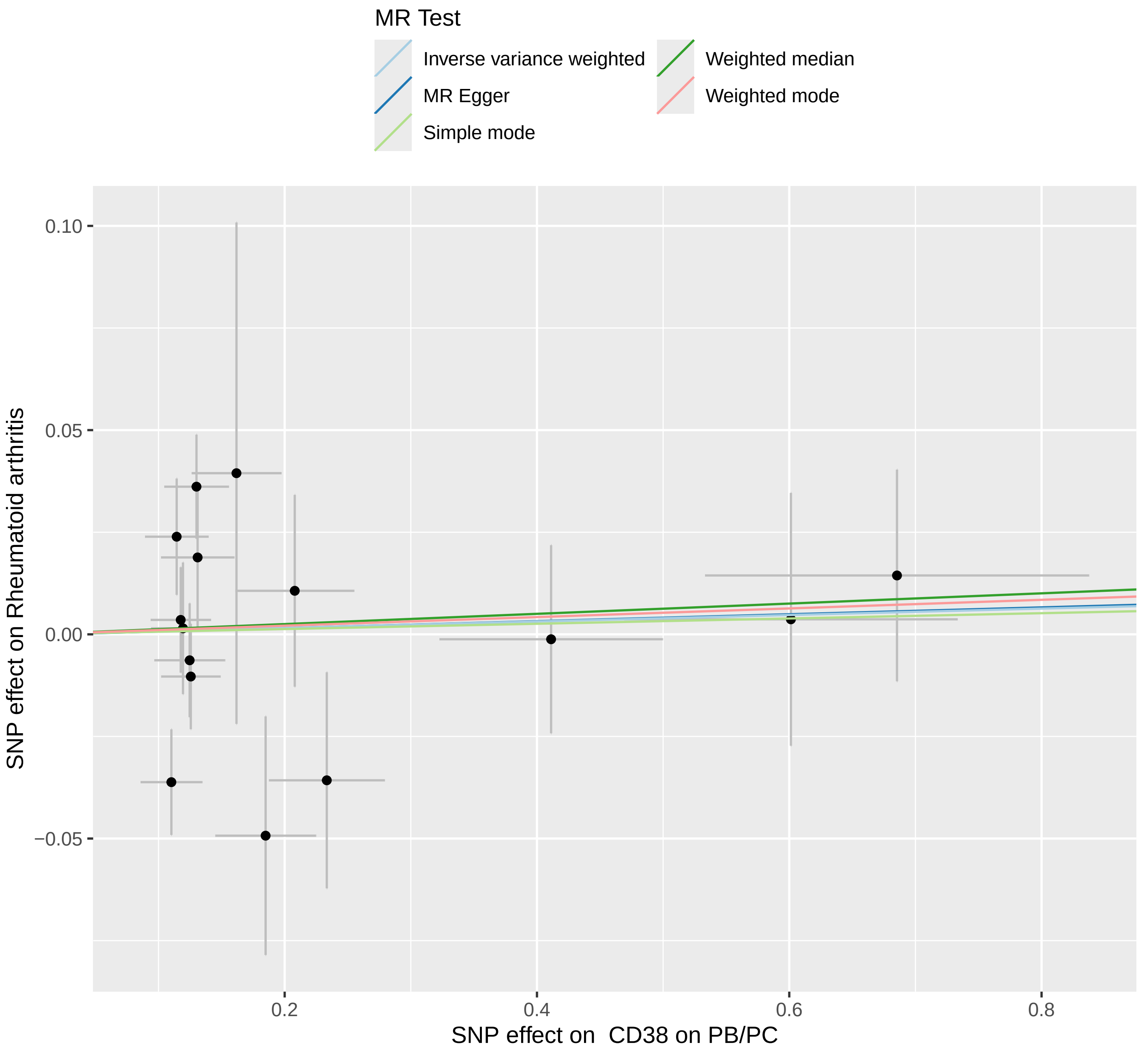
MR Test
Inverse variance weighted
MR Egger
Simple mode
Weighted median
Weighted mode
SNP effect on Rheumatoid arthritis
0.10
0.05
0.00
−0.05
0.2
0.4
0.6
0.8
SNP effect on CD38 on PB/PC

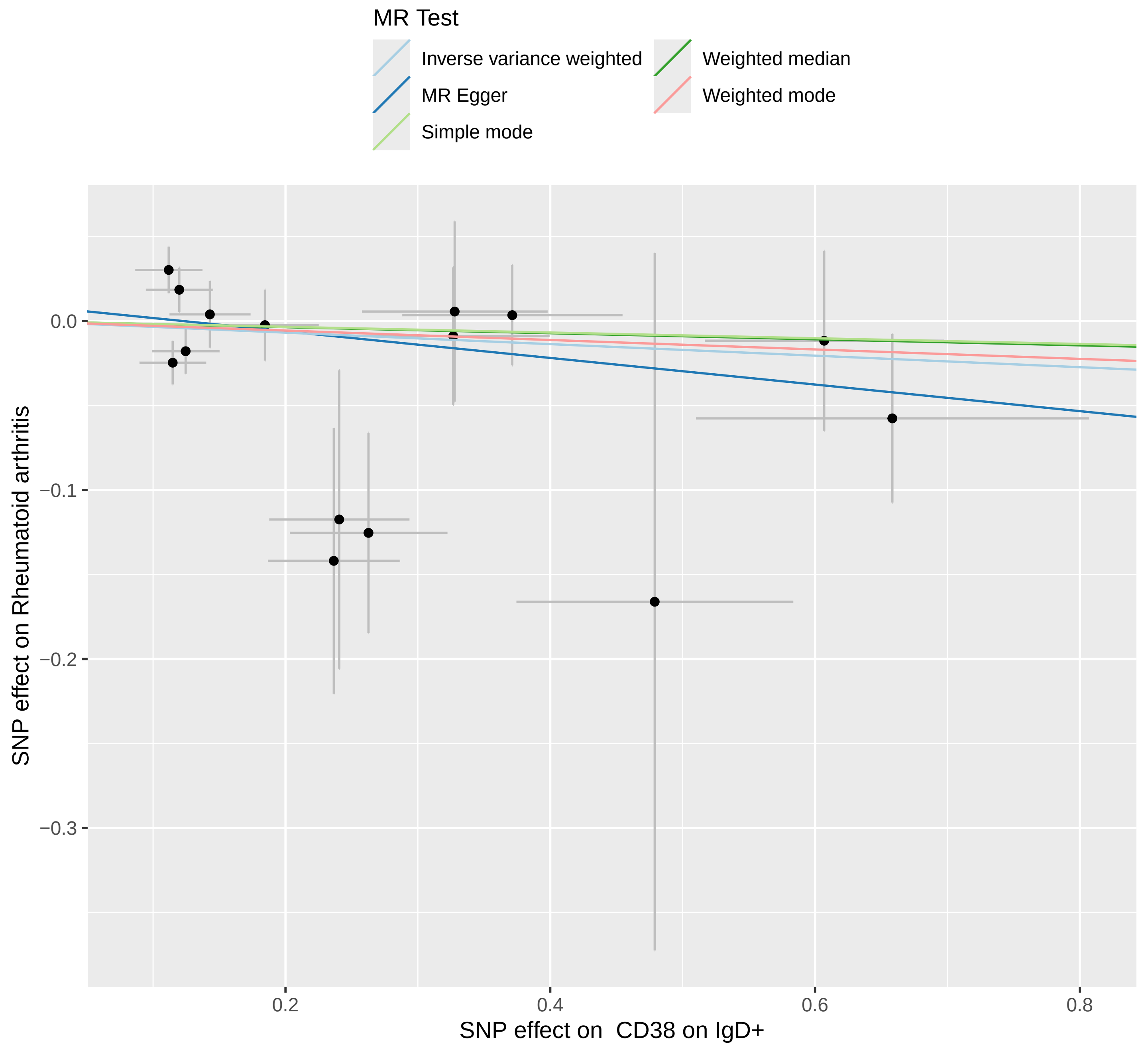

MR Test
Inverse variance weighted
MR Egger
Simple mode
Weighted median
Weighted mode
0.0
−0.1
−0.2
−0.3
0.2
0.4
0.6
0.8
SNP effect on Rheumatoid arthritis
SNP effect on CD38 on IgD+

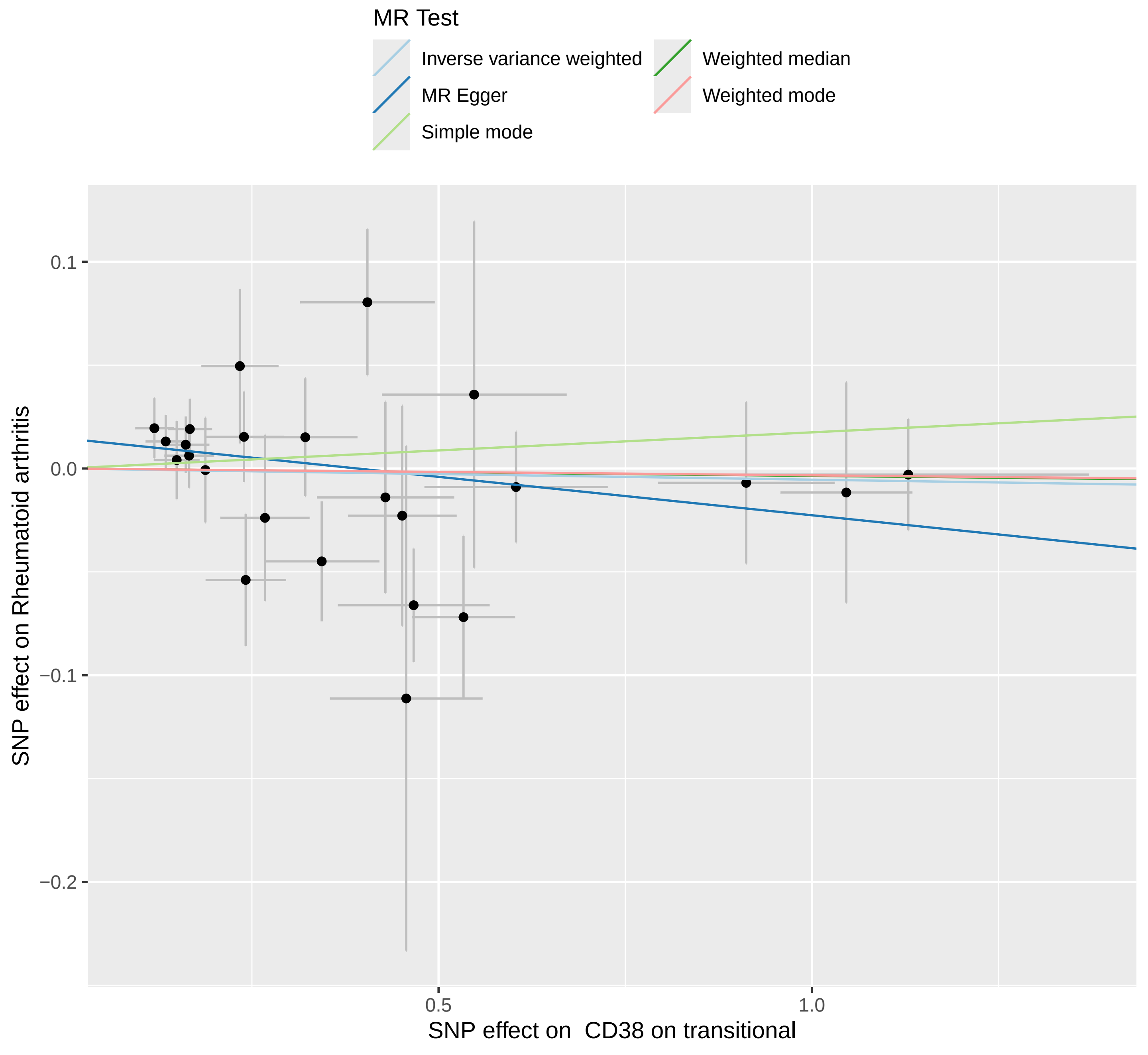
MR Test
Inverse variance weighted
MR Egger
Simple mode
Weighted median
Weighted mode
0.1
0.0
−0.1
−0.2
0.5
1.0
SNP effect on Rheumatoid arthritis
SNP effect on CD38 on transitional

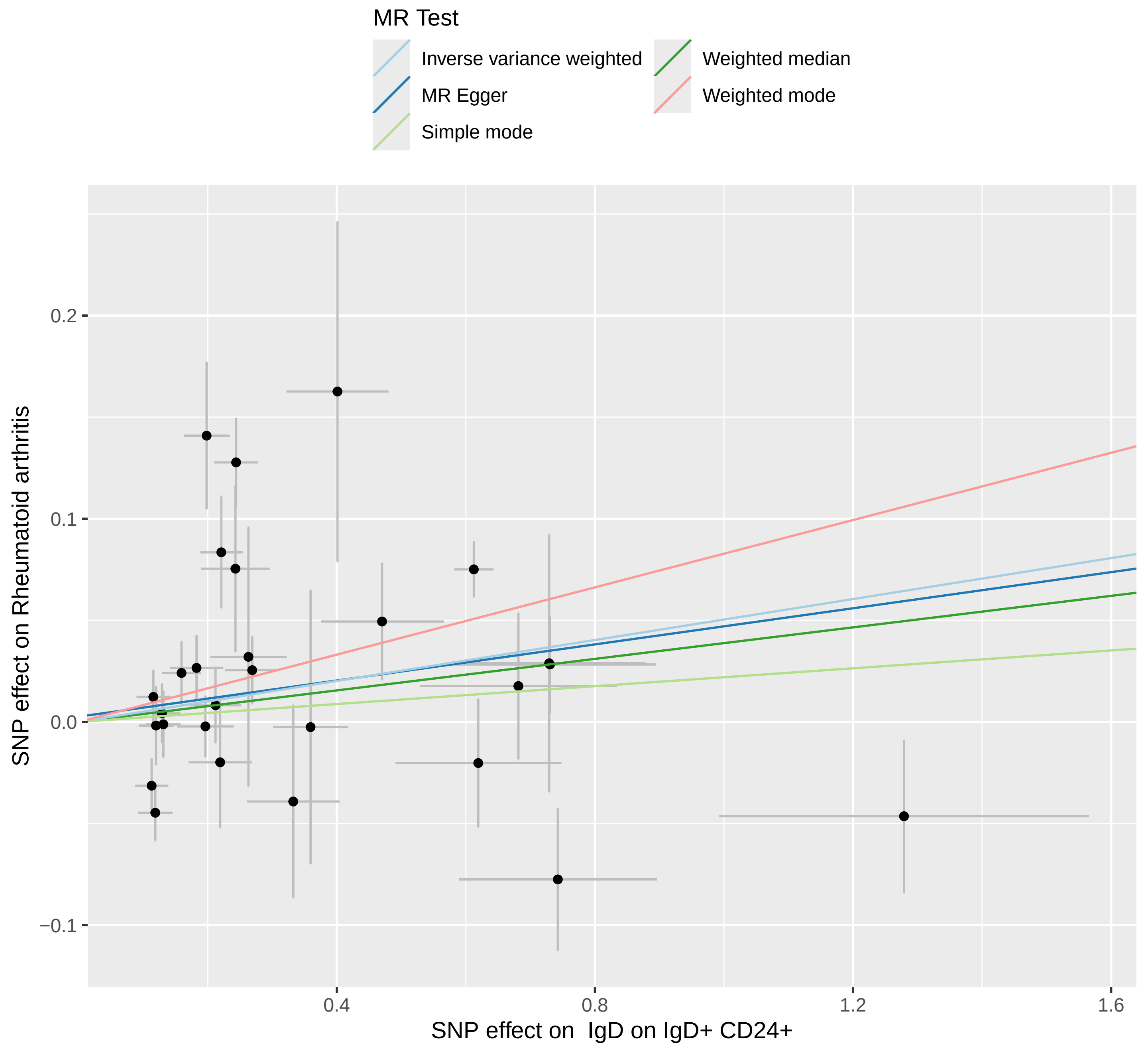

MR Test
Inverse variance weighted
MR Egger
Simple mode
Weighted median
Weighted mode
0.2
0.1
0.0
−0.1
0.4
0.8
1.2
1.6
SNP effect on Rheumatoid arthritis
SNP effect on IgD on IgD+ CD24+

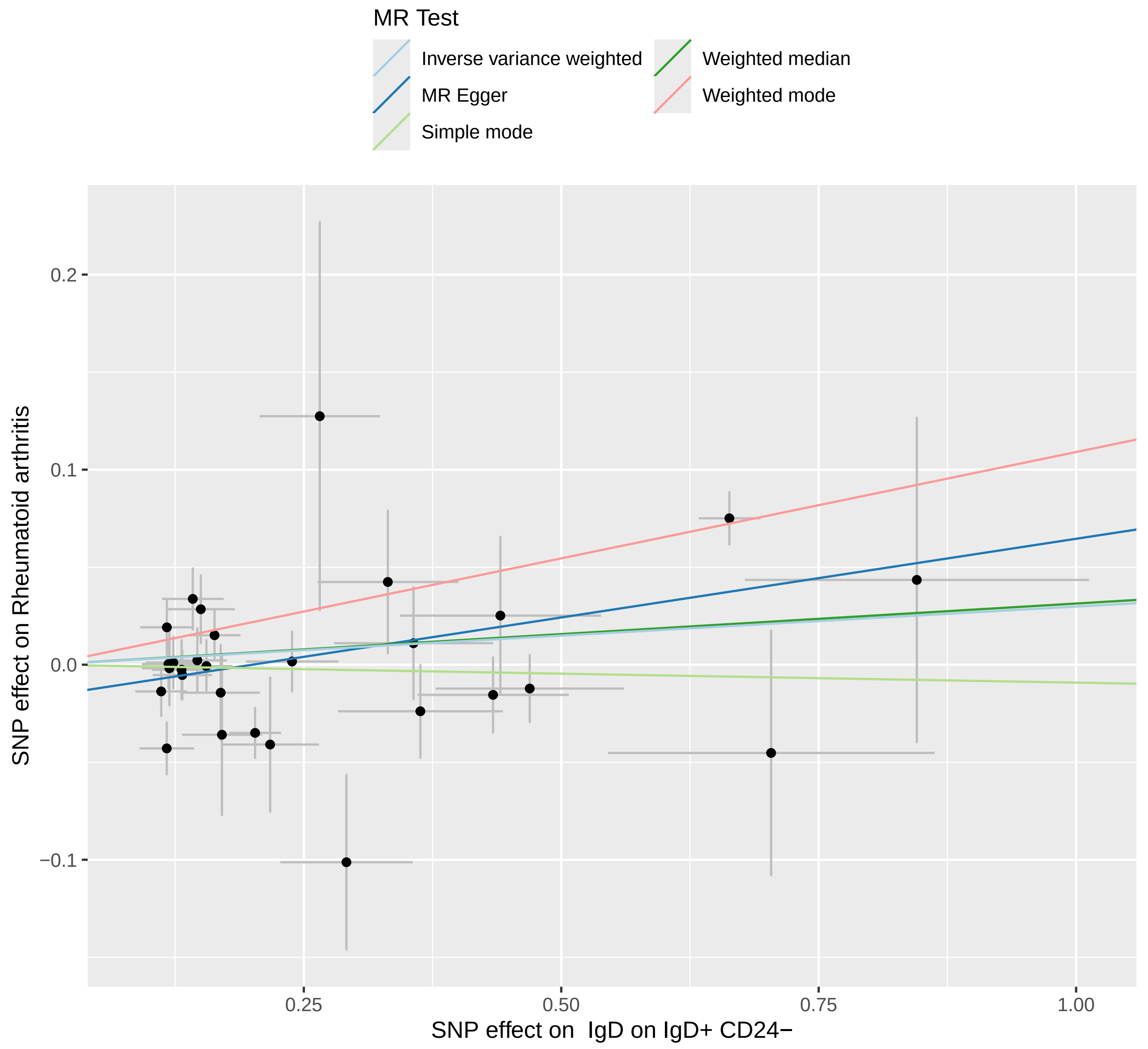
MR Test
Inverse variance weighted
MR Egger
Simple mode
Weighted median
Weighted mode
SNP effect on Rheumatoid arthritis
0.2
0.1
0.0
−0.1
0.25
0.50
0.75
1.00
SNP effect on IgD on IgD+ CD24−

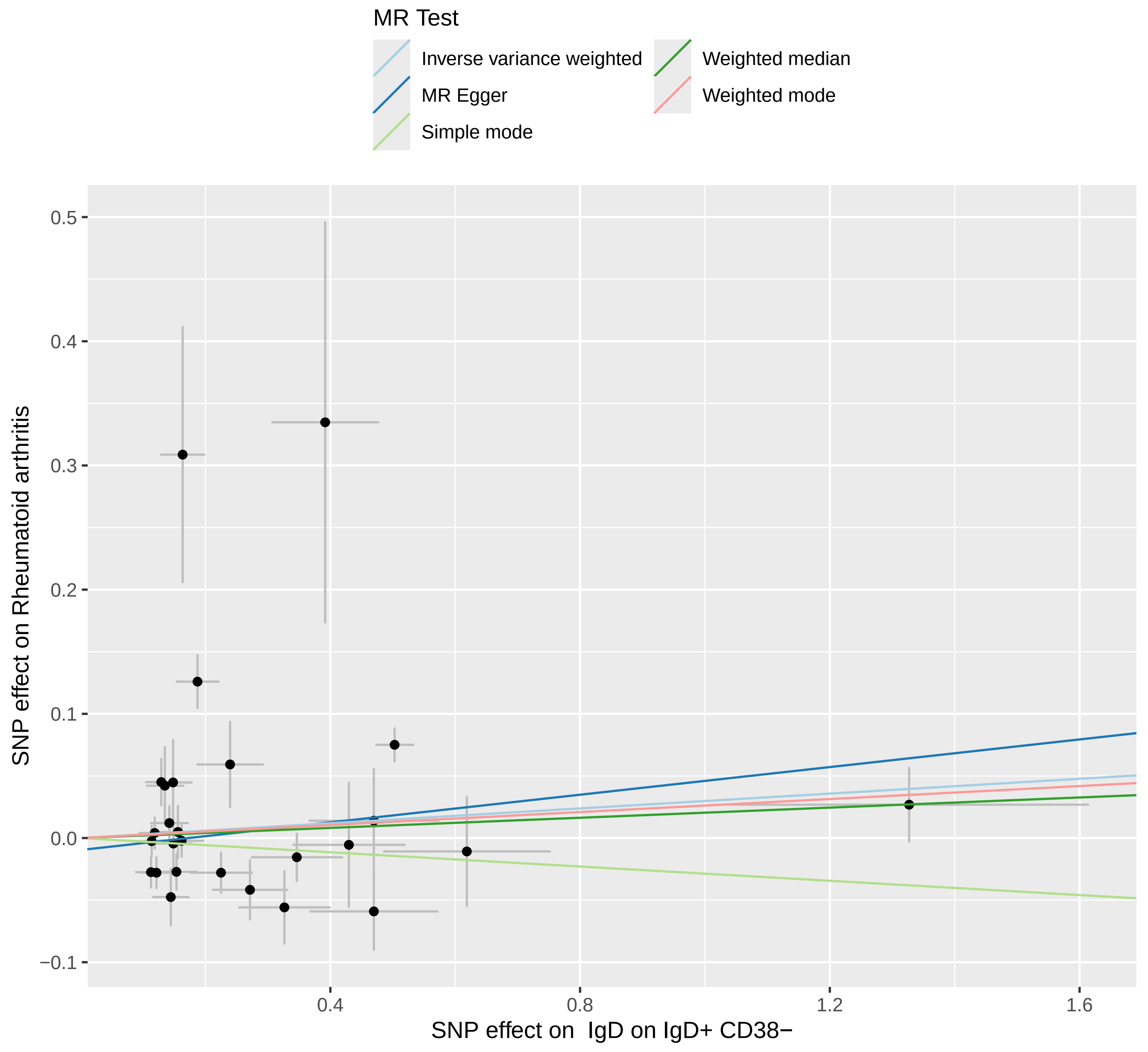

MR Test
Inverse variance weighted
MR Egger
Simple mode
Weighted median
Weighted mode
SNP effect on Rheumatoid arthritis
0.5
0.4
0.3
0.2
0.1
0.0
−0.1
0.4
0.8
1.2
1.6
SNP effect on IgD on IgD+ CD38−

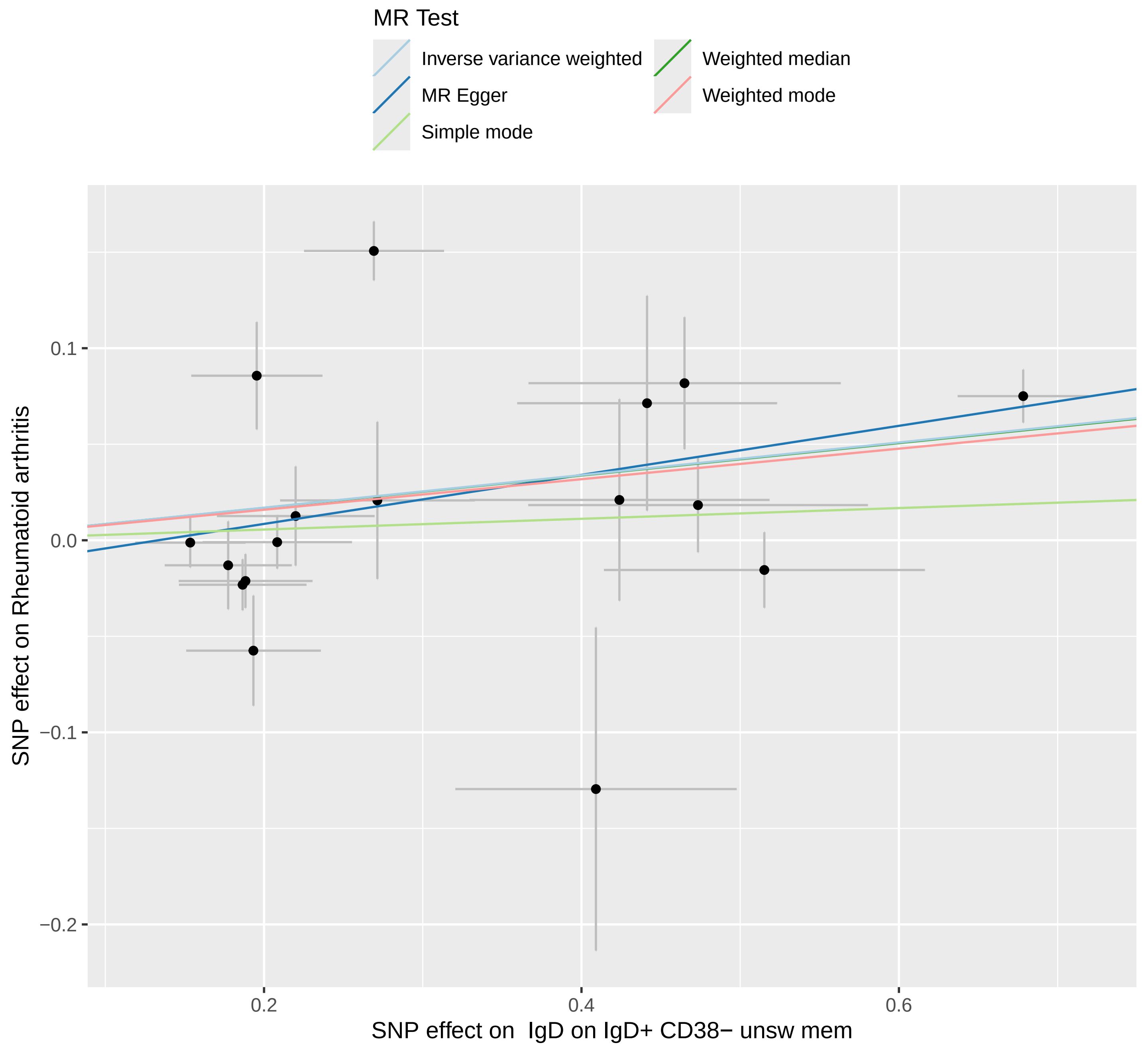

MR Test
Inverse variance weighted
MR Egger
Simple mode
Weighted median
Weighted mode
0.1
0.0
−0.1
−0.2
0.2
0.4
0.6
SNP effect on Rheumatoid arthritis
SNP effect on IgD on IgD+ CD38− unsw mem

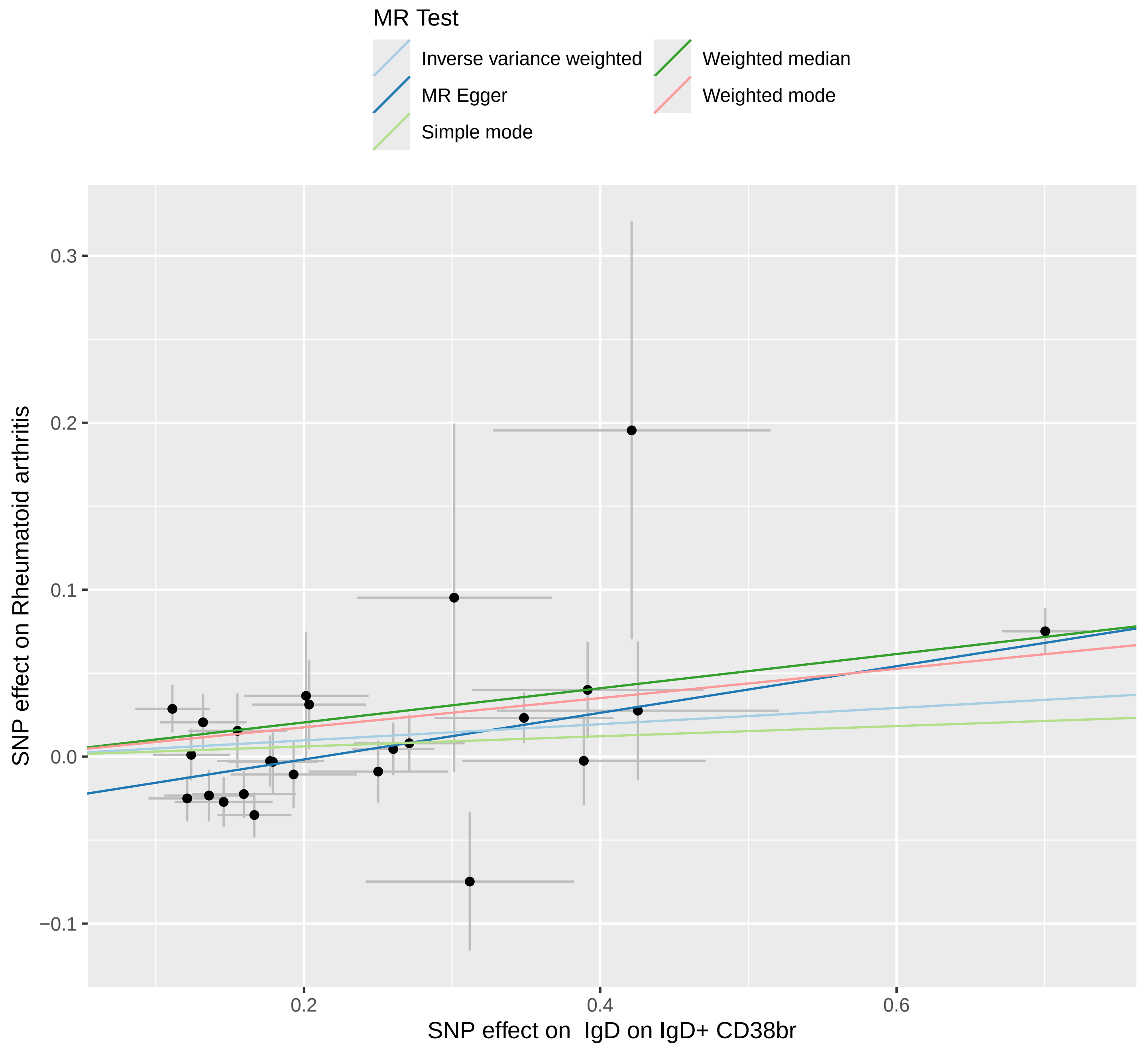

MR Test
Inverse variance weighted
MR Egger
Simple mode
Weighted median
Weighted mode
SNP effect on Rheumatoid arthritis
0.3
0.2
0.1
0.0
−0.1
0.2
0.4
0.6
SNP effect on IgD on IgD+ CD38br

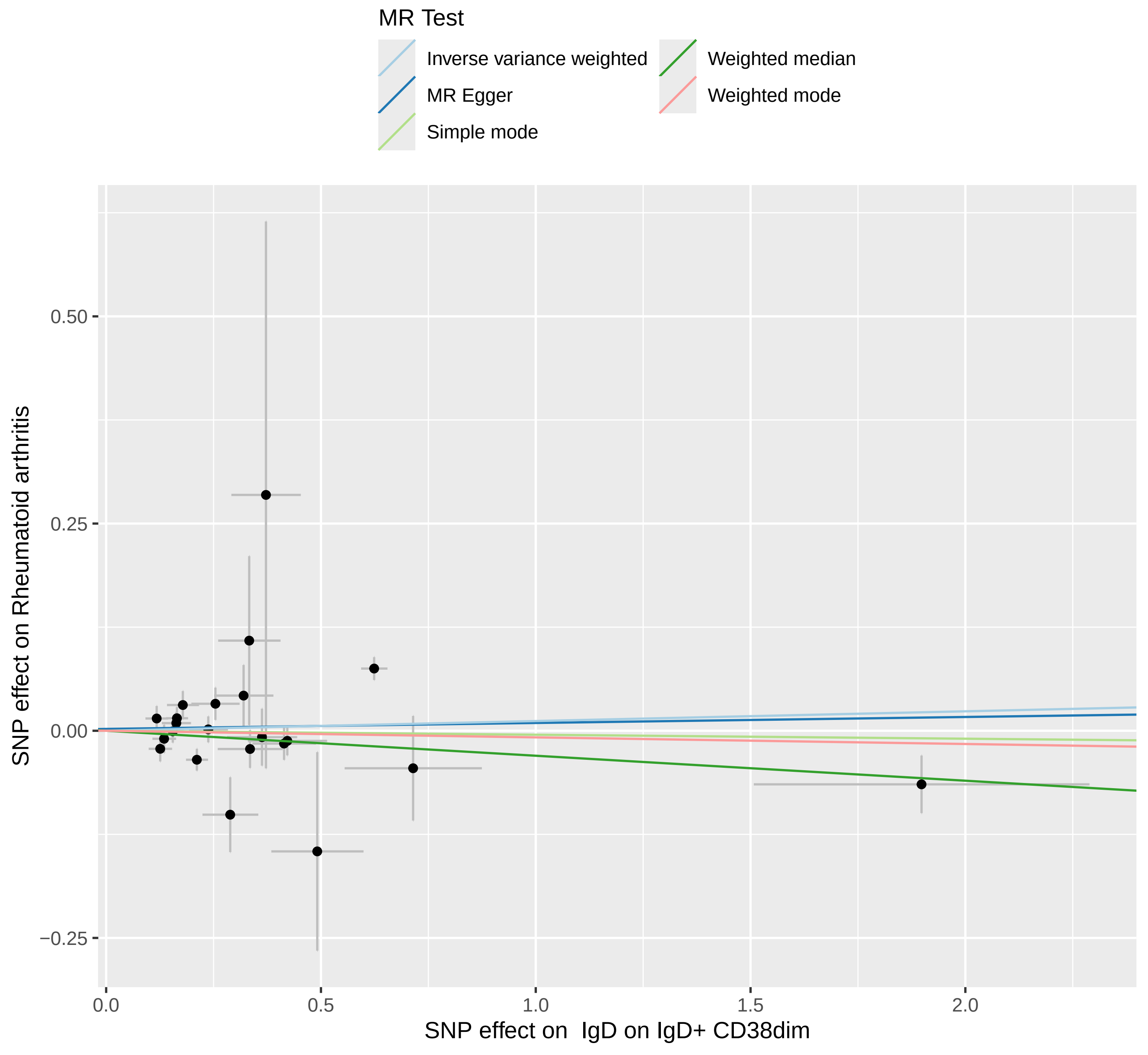

MR Test
Inverse variance weighted
MR Egger
Simple mode
Weighted median
Weighted mode
0.50
0.25
0.00
−0.25
0.0
0.5
1.0
1.5
2.0
SNP effect on Rheumatoid arthritis
SNP effect on IgD on IgD+ CD38dim

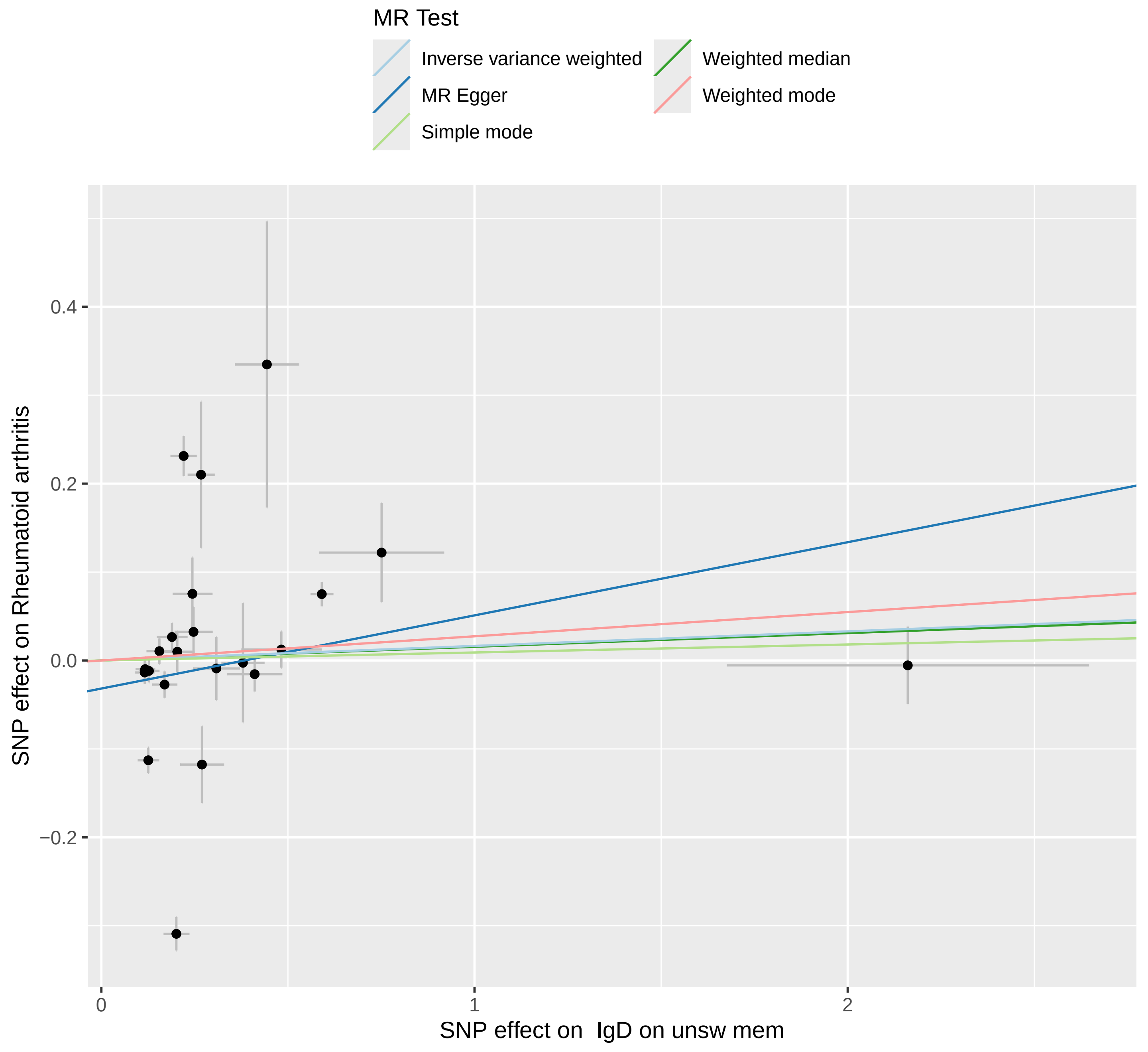

MR Test
Inverse variance weighted
MR Egger
Simple mode
Weighted median
Weighted mode
0.4
0.2
0.0
−0.2
SNP effect on Rheumatoid arthritis
0
1
2
SNP effect on IgD on unsw mem

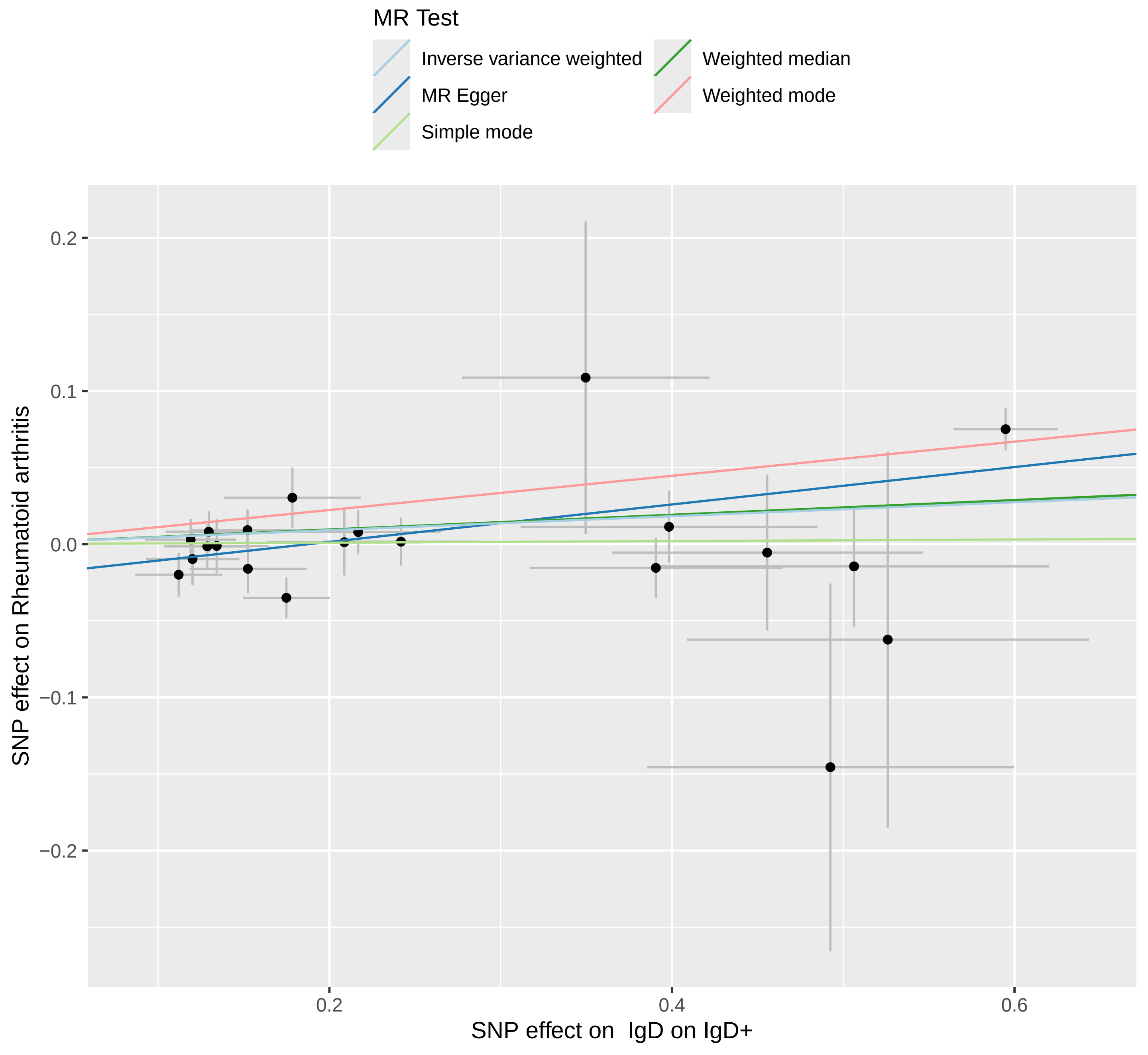
MR Test
Inverse variance weighted
MR Egger
Simple mode
Weighted median
Weighted mode
SNP effect on Rheumatoid arthritis
0.2
0.1
0.0
−0.1
−0.2
0.2
0.4
0.6
SNP effect on IgD on IgD+

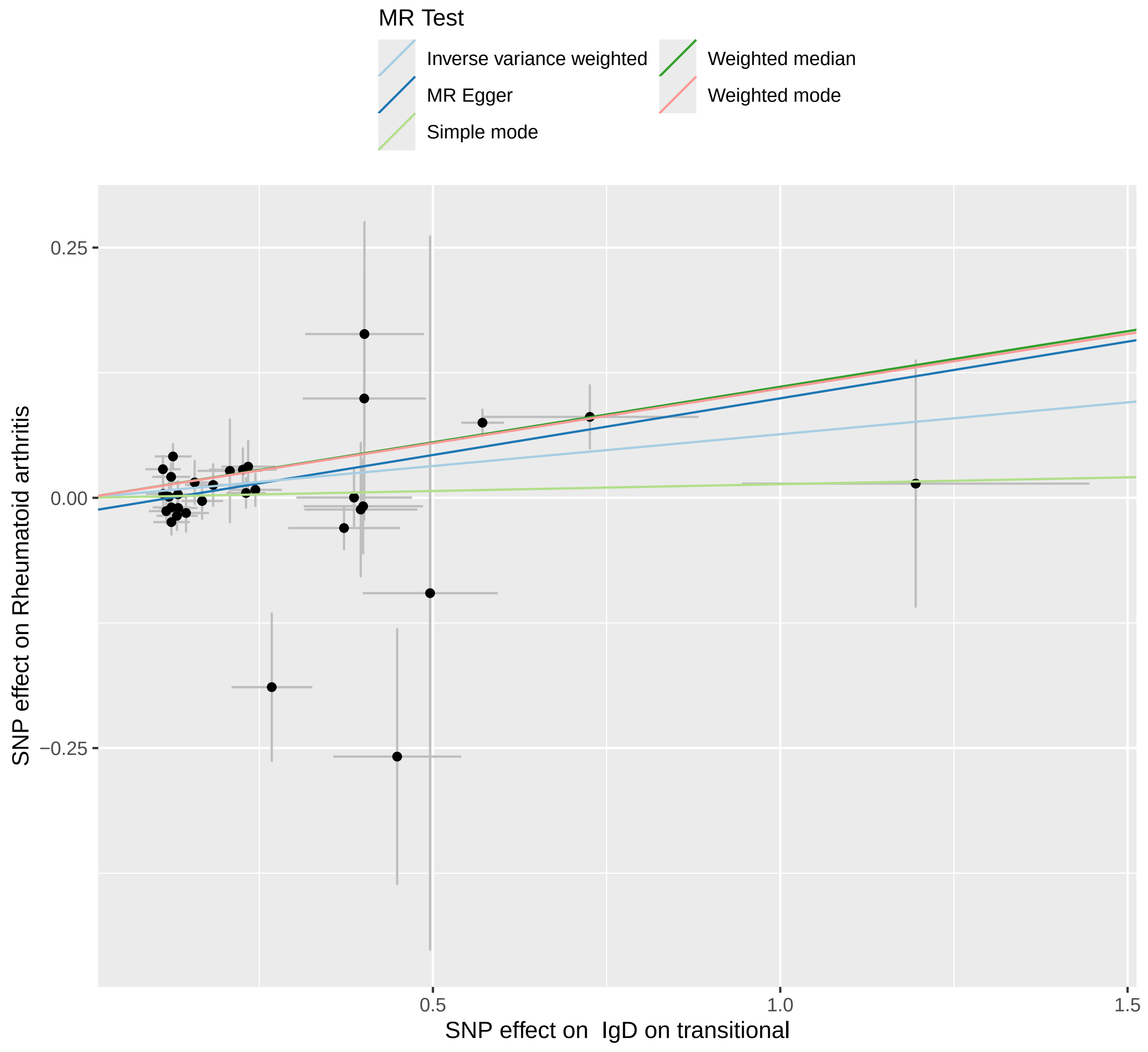

MR Test
Inverse variance weighted
MR Egger
Simple mode
Weighted median
Weighted mode
0.25
0.00
−0.25
0.5
1.0
1.5
SNP effect on Rheumatoid arthritis
SNP effect on IgD on transitional

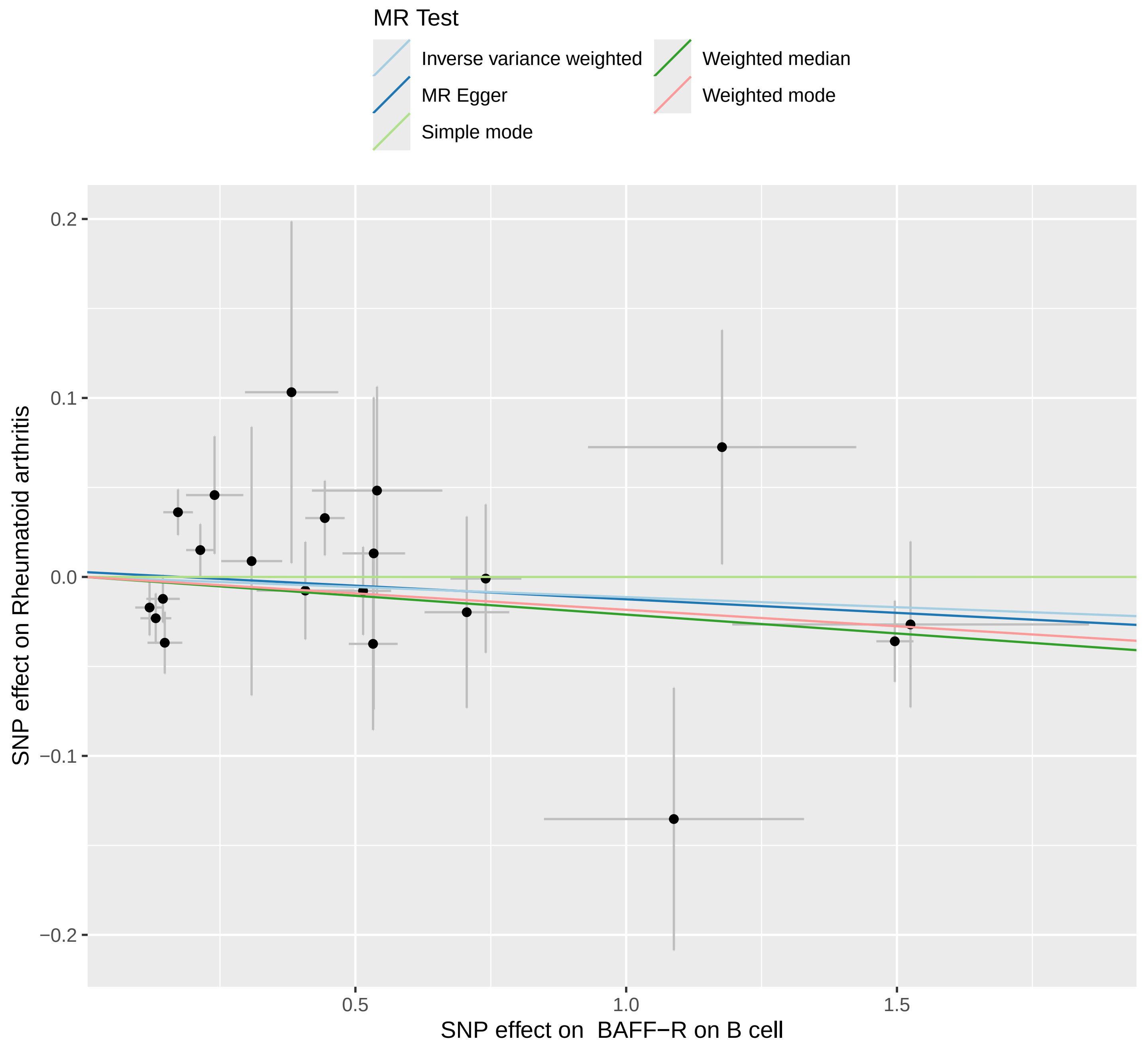
MR Test
Inverse variance weighted
MR Egger
Simple mode
Weighted median
Weighted mode
0.2
0.1
0.0
−0.1
−0.2
0.5
1.0
1.5
SNP effect on Rheumatoid arthritis
SNP effect on BAFF−R on B cell

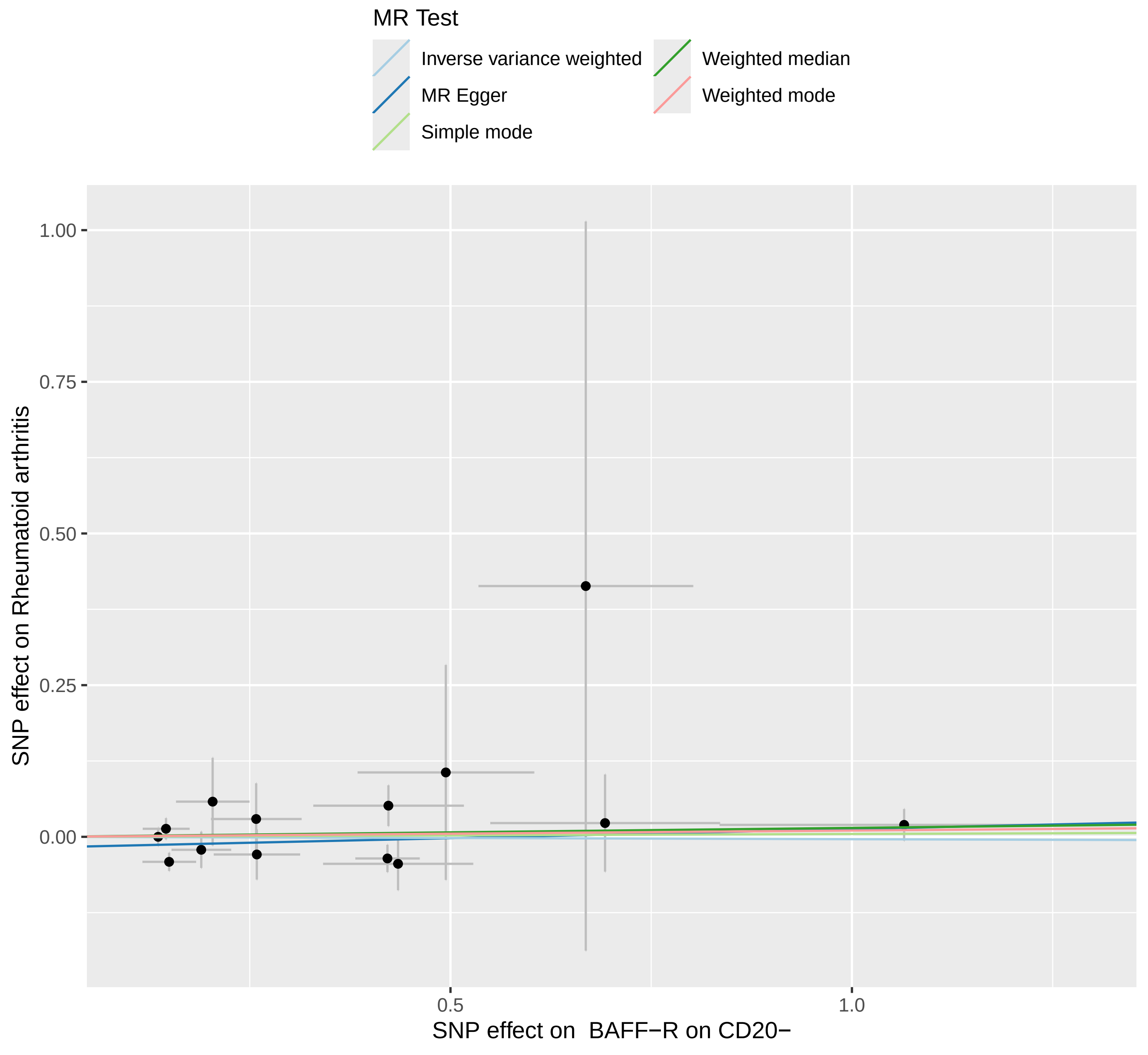
MR Test
Inverse variance weighted
MR Egger
Simple mode
Weighted median
Weighted mode
1.00
0.75
0.50
0.25
0.00
SNP effect on Rheumatoid arthritis
0.5
1.0
SNP effect on BAFF−R on CD20−

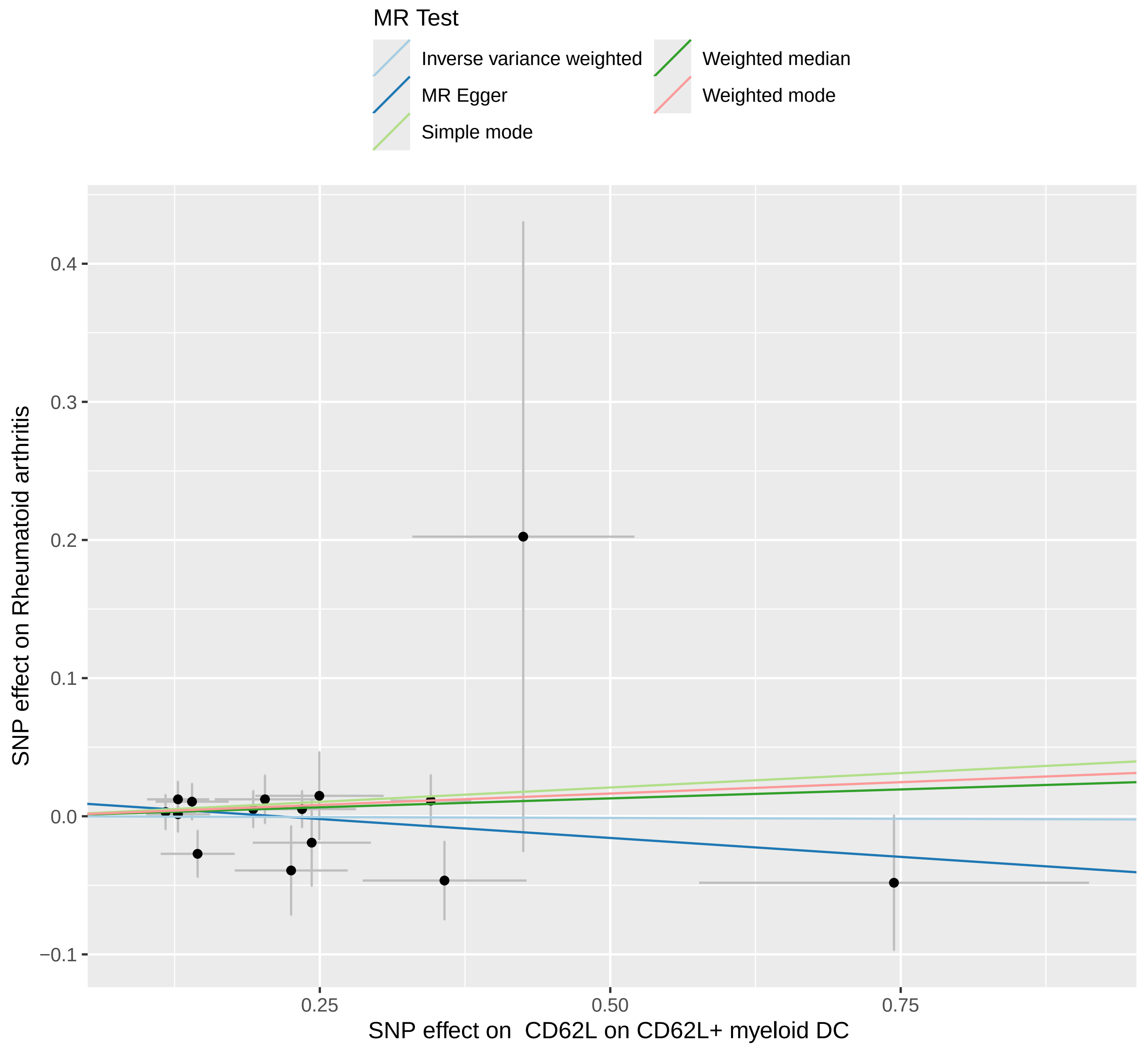
MR Test
Inverse variance weighted
MR Egger
Simple mode
Weighted median
Weighted mode
0.4
0.3
0.2
0.1
0.0
−0.1
0.25
0.50
0.75
SNP effect on Rheumatoid arthritis
SNP effect on CD62L on CD62L+ myeloid DC

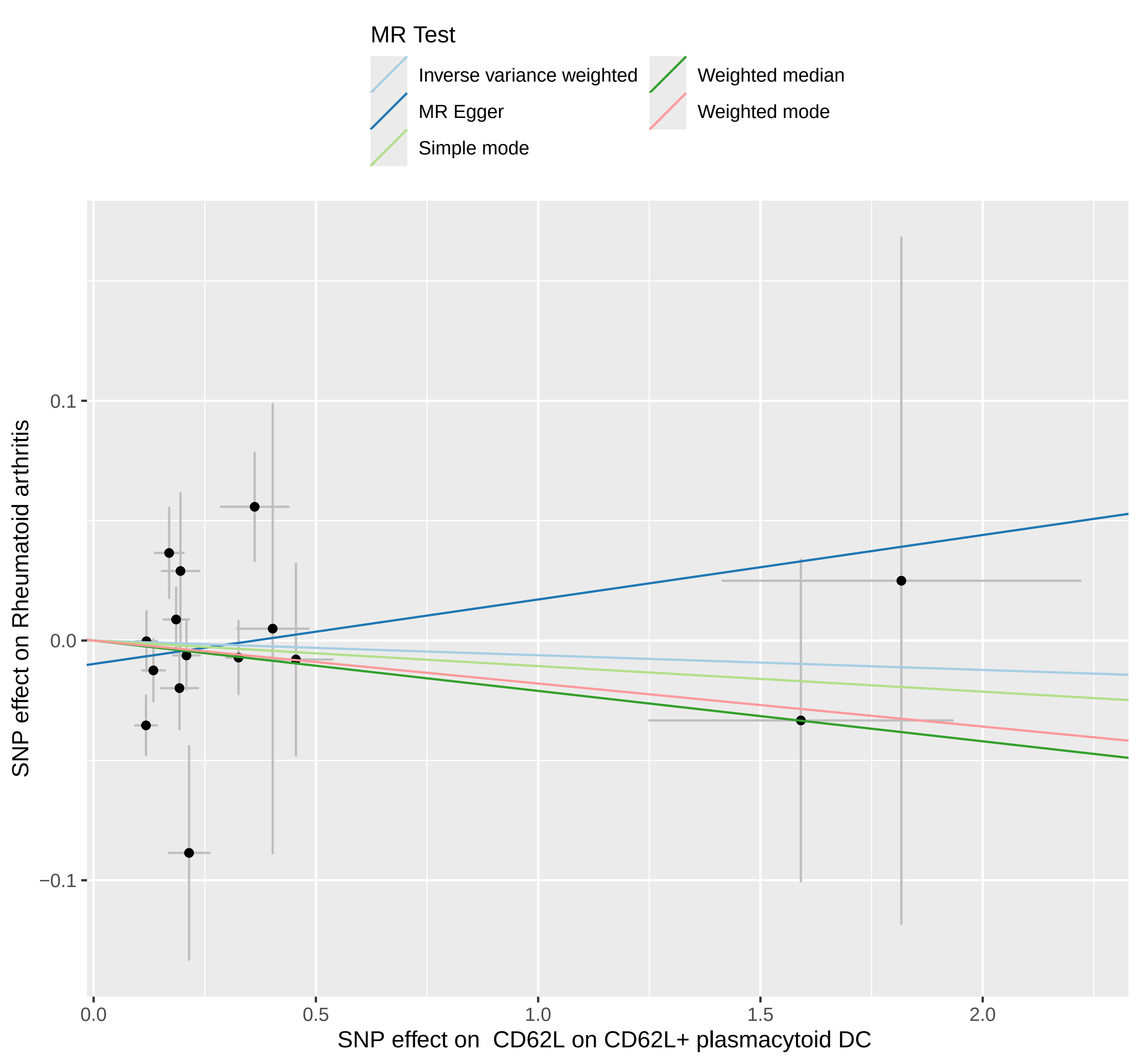
MR Test
Inverse variance weighted
MR Egger
Simple mode
Weighted median
Weighted mode
0.1
0.0
−0.1
0.0
0.5
1.0
1.5
2.0
SNP effect on Rheumatoid arthritis
SNP effect on CD62L on CD62L+ plasmacytoid DC

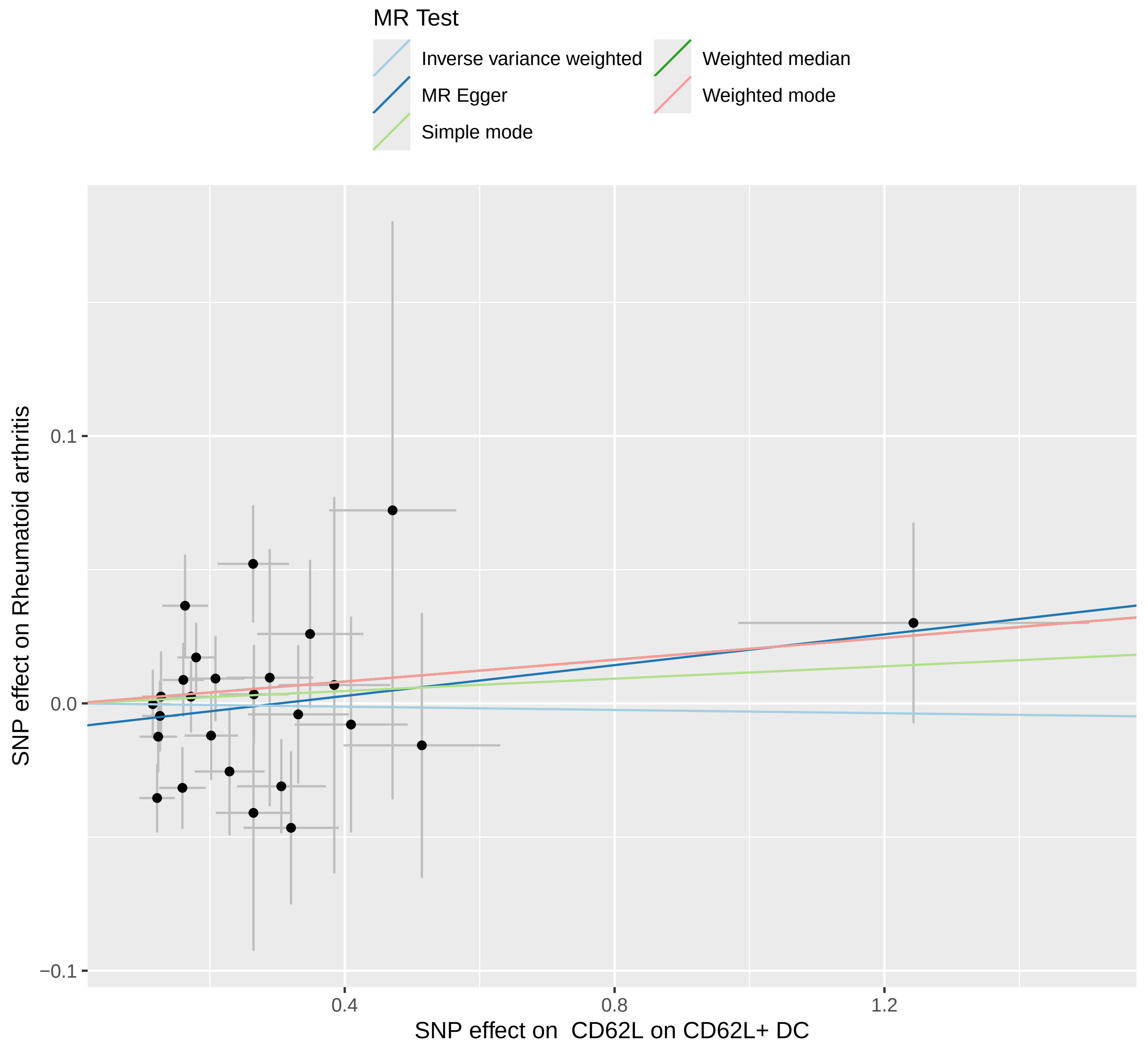
MR Test
Inverse variance weighted
MR Egger
Simple mode
Weighted median
Weighted mode
0.1
0.0
−0.1
0.4
0.8
1.2
SNP effect on Rheumatoid arthritis
SNP effect on CD62L on CD62L+ DC

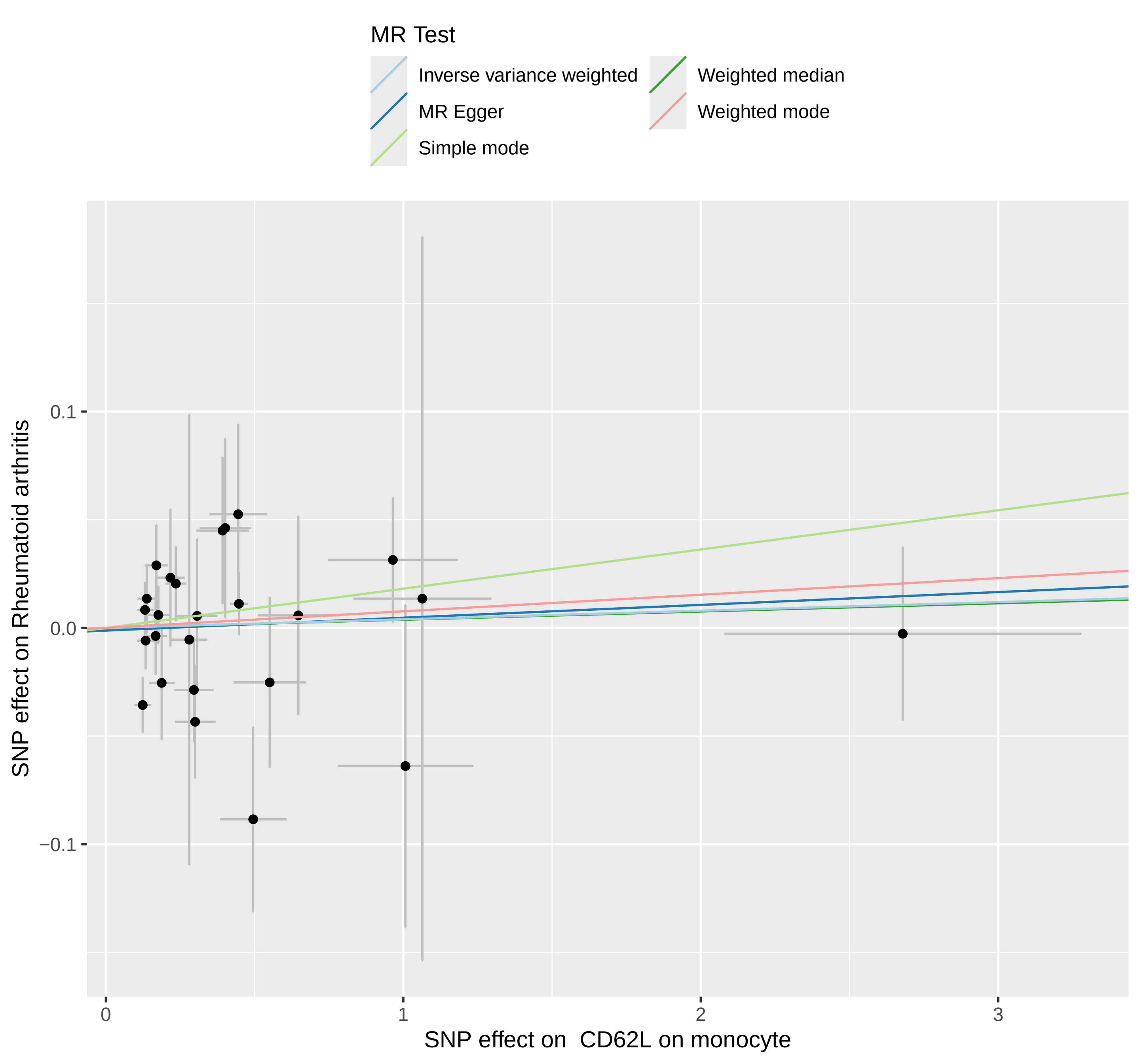
MR Test
Inverse variance weighted
MR Egger
Simple mode
Weighted median
Weighted mode
0.1
0.0
−0.1
0
1
2
3
SNP effect on Rheumatoid arthritis
SNP effect on CD62L on monocyte

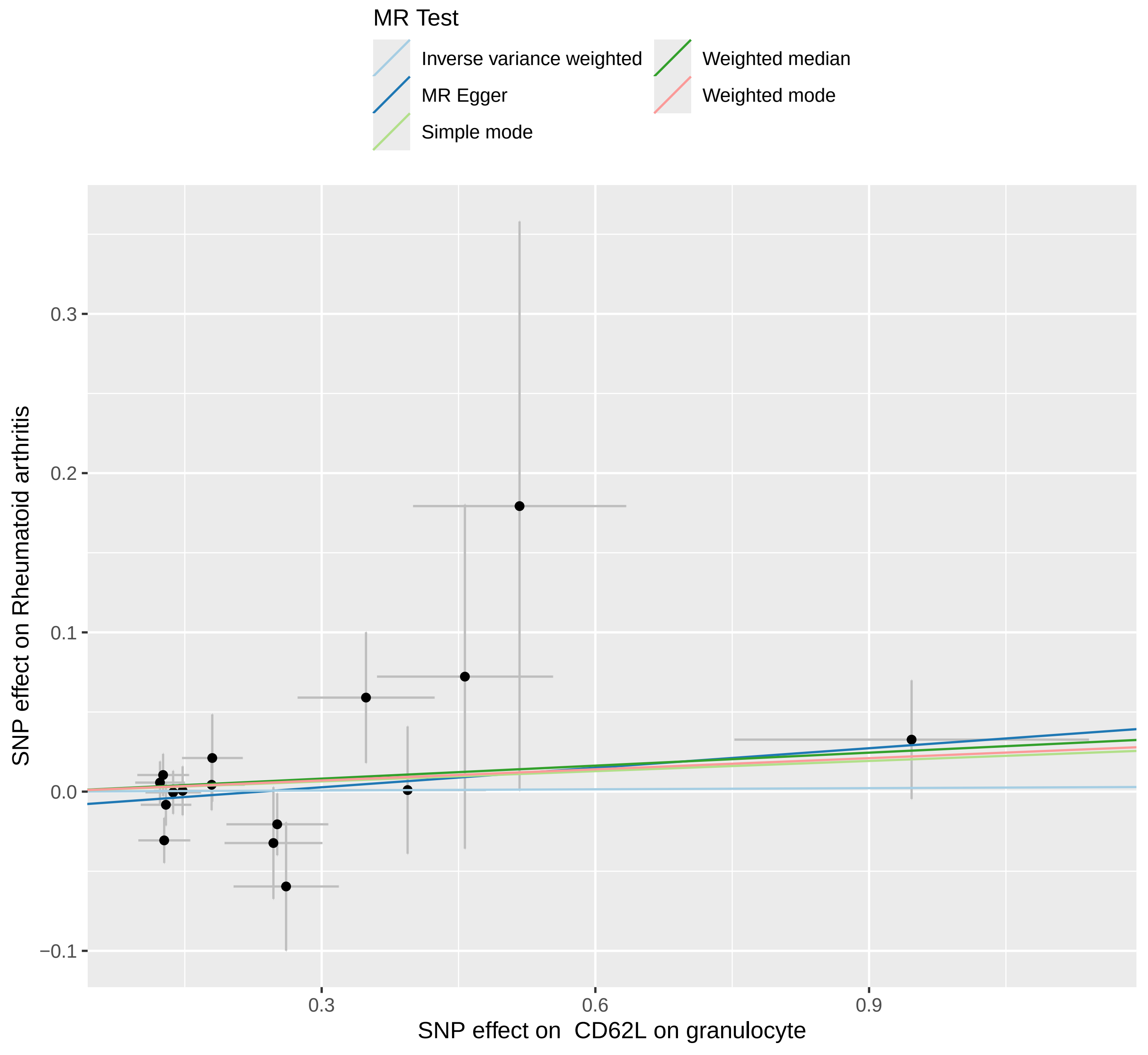
MR Test
Inverse variance weighted
MR Egger
Simple mode
Weighted median
Weighted mode
SNP effect on Rheumatoid arthritis
0.3
0.2
0.1
0.0
−0.1
0.3
0.6
0.9
SNP effect on CD62L on granulocyte

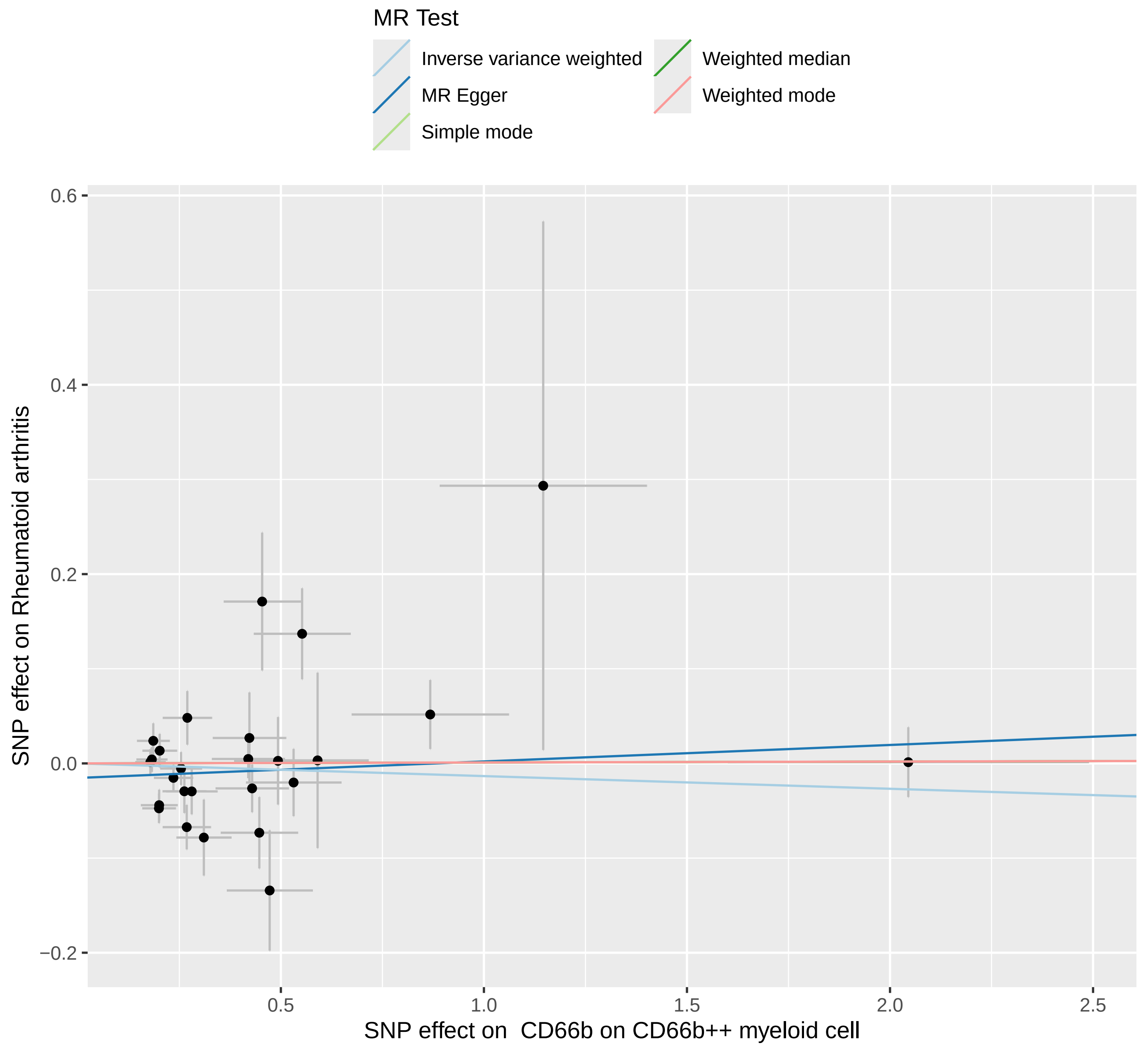
MR Test
Inverse variance weighted
MR Egger
Simple mode
Weighted median
Weighted mode
0.6
0.4
0.2
0.0
−0.2
0.5
1.0
1.5
2.0
2.5
SNP effect on Rheumatoid arthritis
SNP effect on CD66b on CD66b++ myeloid cell

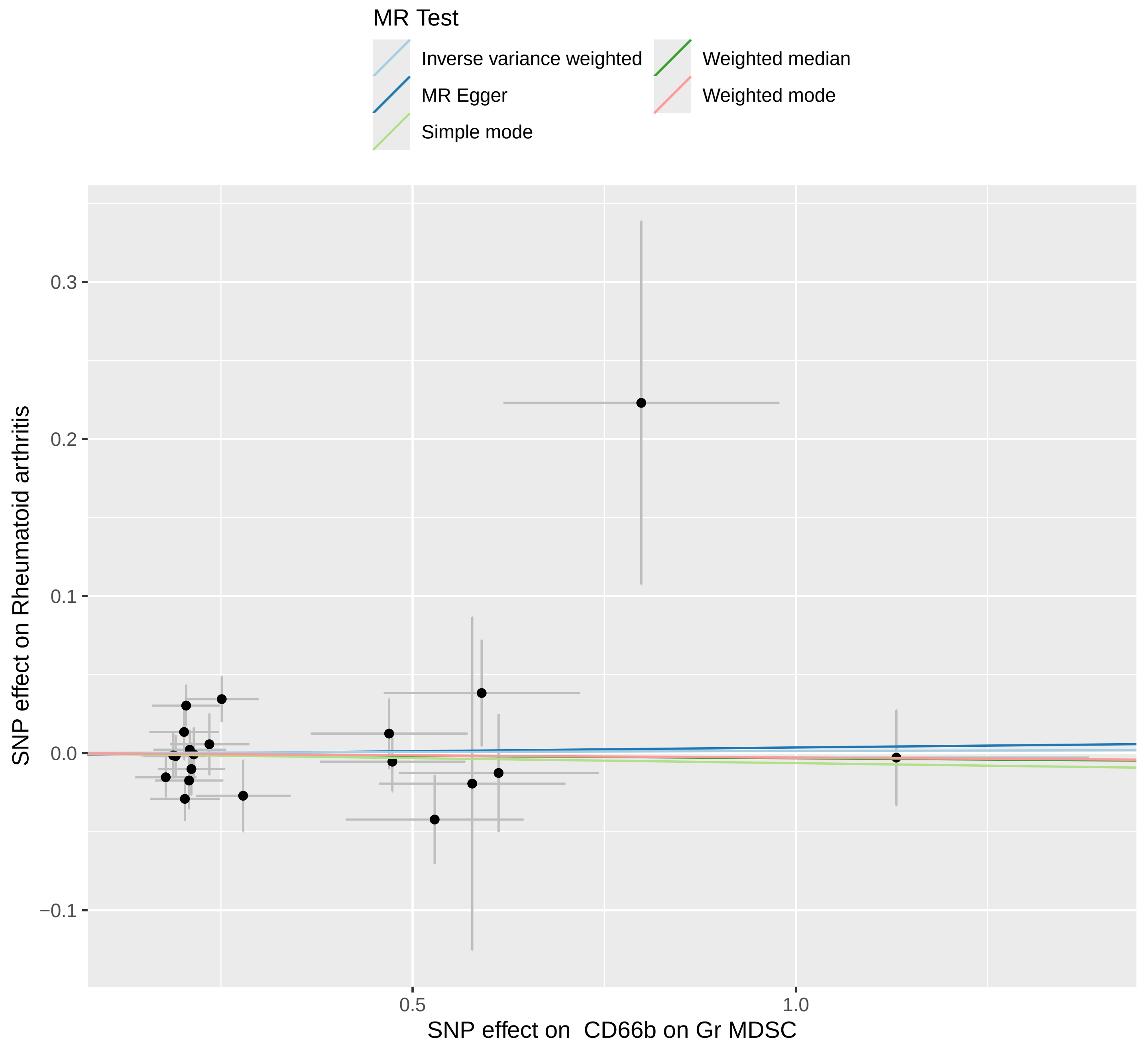

MR Test
Inverse variance weighted
MR Egger
Simple mode
Weighted median
Weighted mode
0.3
0.2
0.1
0.0
−0.1
0.5
1.0
SNP effect on Rheumatoid arthritis
SNP effect on CD66b on Gr MDSC

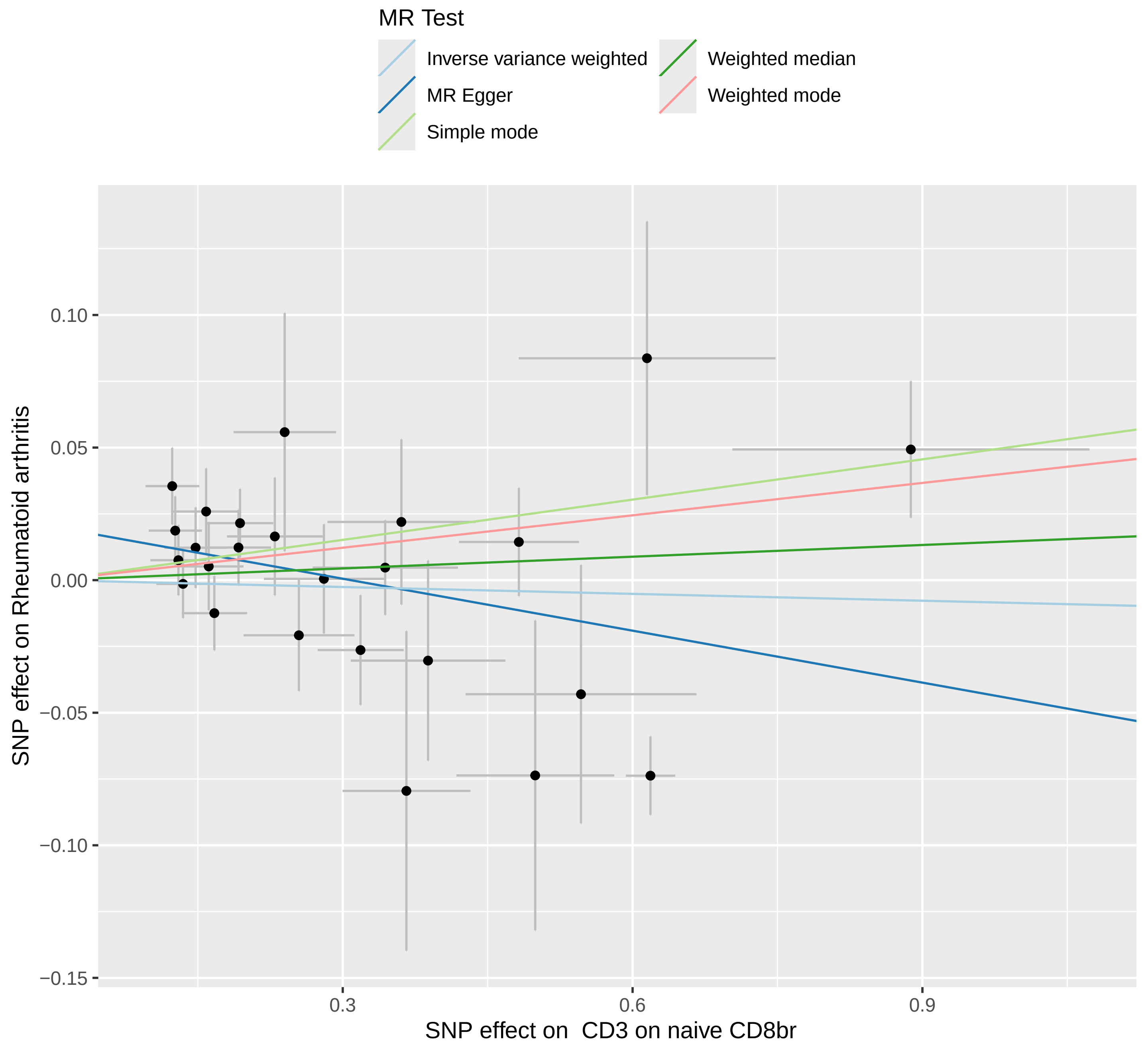
MR Test
Inverse variance weighted
MR Egger
Simple mode
Weighted median
Weighted mode
SNP effect on Rheumatoid arthritis
0.10
0.05
0.00
−0.05
−0.10
−0.15
0.3
0.6
0.9
SNP effect on CD3 on naive CD8br

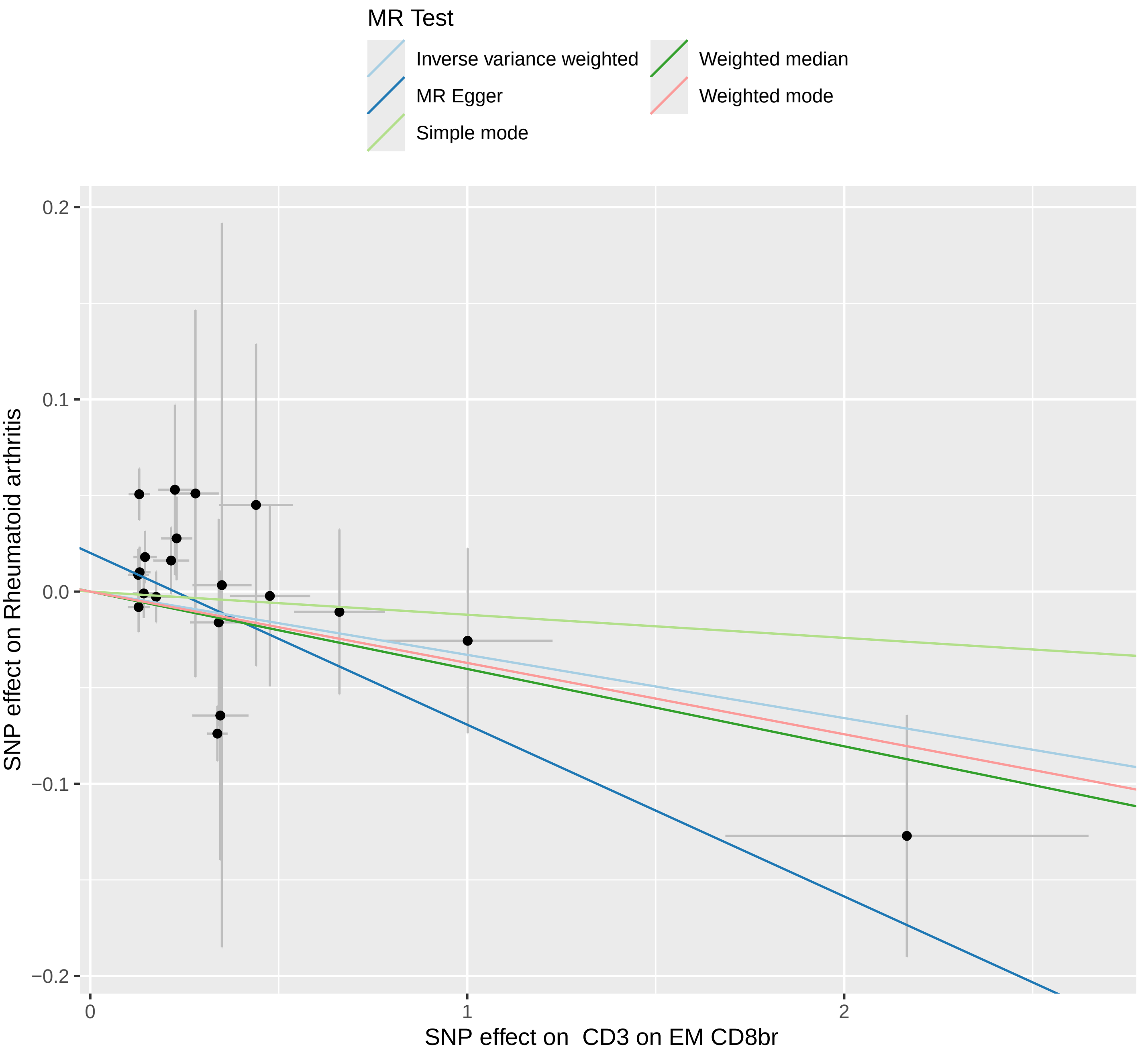
MR Test
Inverse variance weighted
MR Egger
Simple mode
Weighted median
Weighted mode
0.2
0.1
0.0
−0.1
−0.2
0
1
2
SNP effect on Rheumatoid arthritis
SNP effect on CD3 on EM CD8br

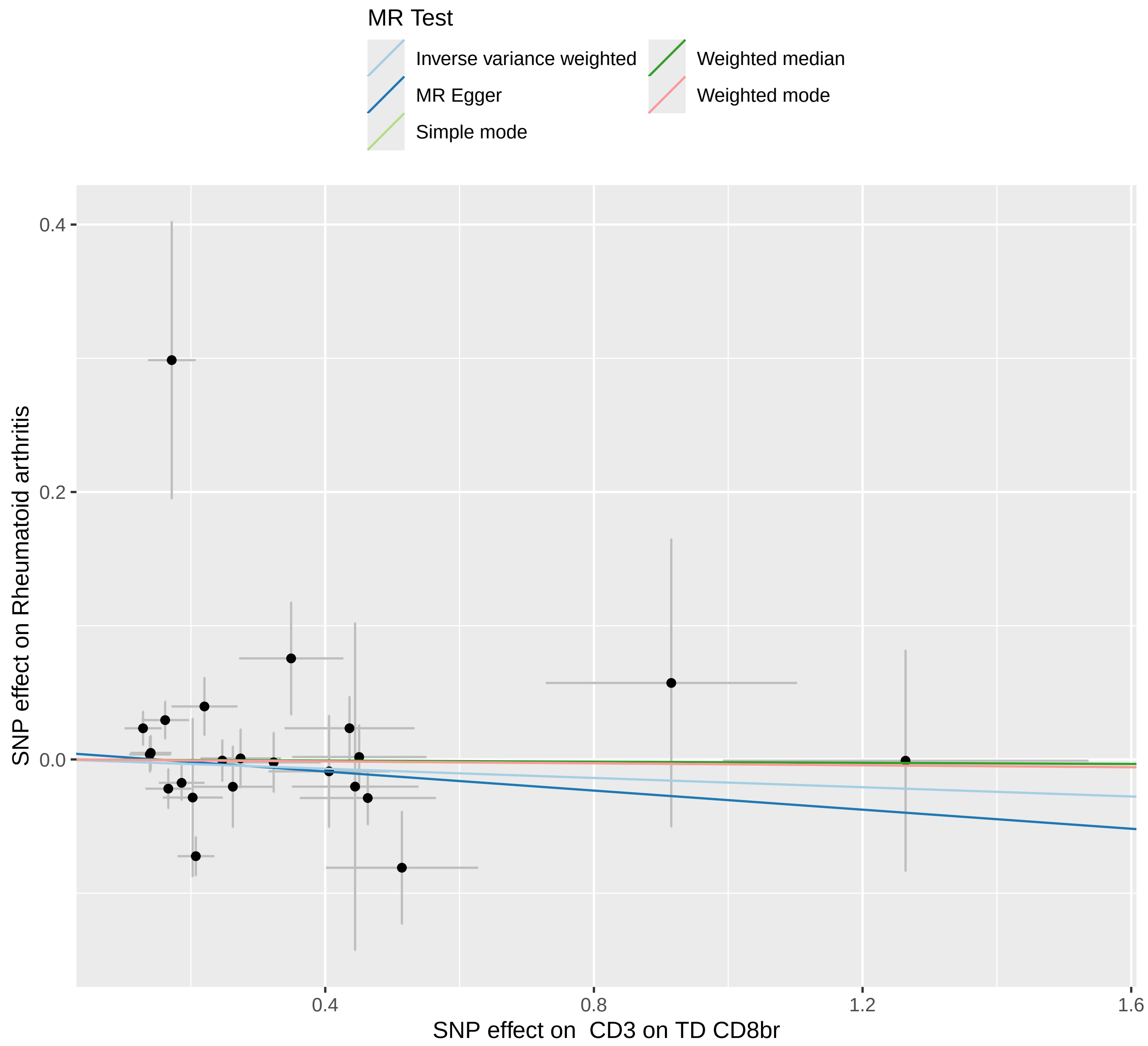

MR Test
Inverse variance weighted
MR Egger
Simple mode
Weighted median
Weighted mode
0.4
0.2
0.0
0.4
0.8
1.2
1.6
SNP effect on Rheumatoid arthritis
SNP effect on CD3 on TD CD8br

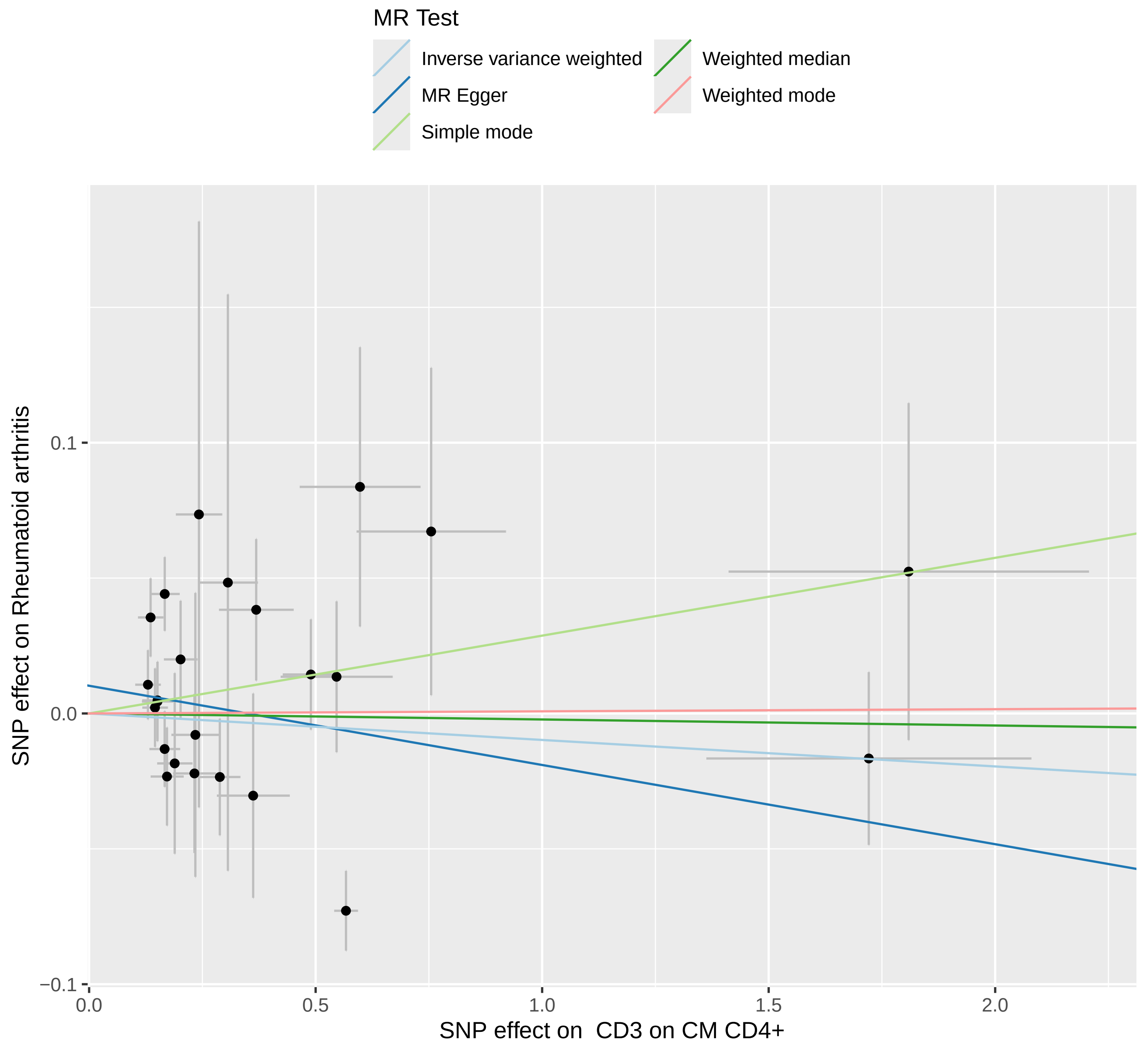

MR Test
Inverse variance weighted
MR Egger
Simple mode
Weighted median
Weighted mode
SNP effect on Rheumatoid arthritis
0.1
0.0
−0.1
0.0
0.5
1.0
1.5
2.0
SNP effect on CD3 on CM CD4+

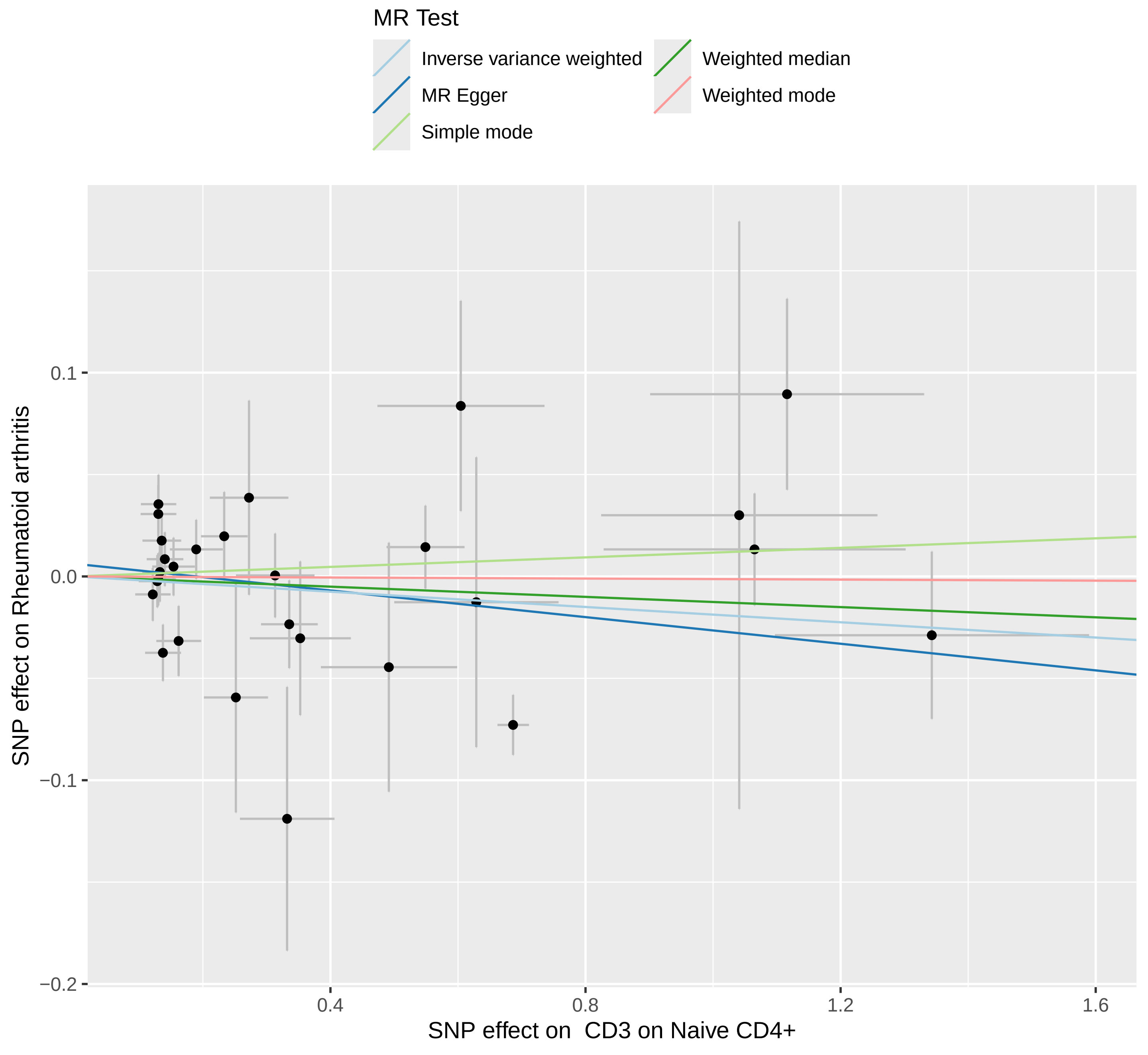
MR Test
Inverse variance weighted
MR Egger
Simple mode
Weighted median
Weighted mode
SNP effect on Rheumatoid arthritis
0.1
0.0
−0.1
−0.2
0.4
0.8
1.2
1.6
SNP effect on CD3 on Naive CD4+

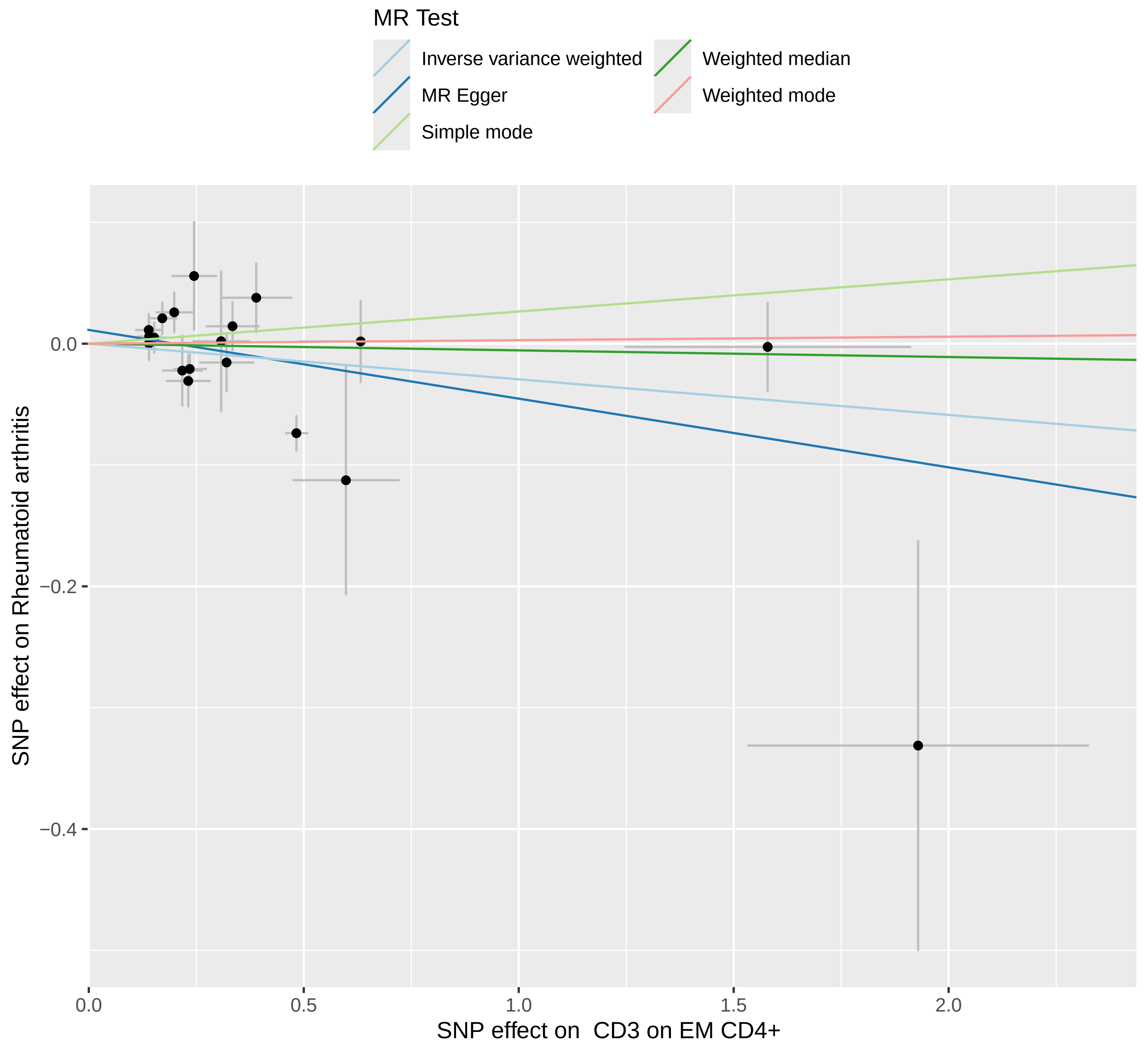
MR Test
Inverse variance weighted
MR Egger
Simple mode
Weighted median
Weighted mode
SNP effect on Rheumatoid arthritis
0.0
−0.2
−0.4
0.0
0.5
1.0
1.5
2.0
SNP effect on CD3 on EM CD4+

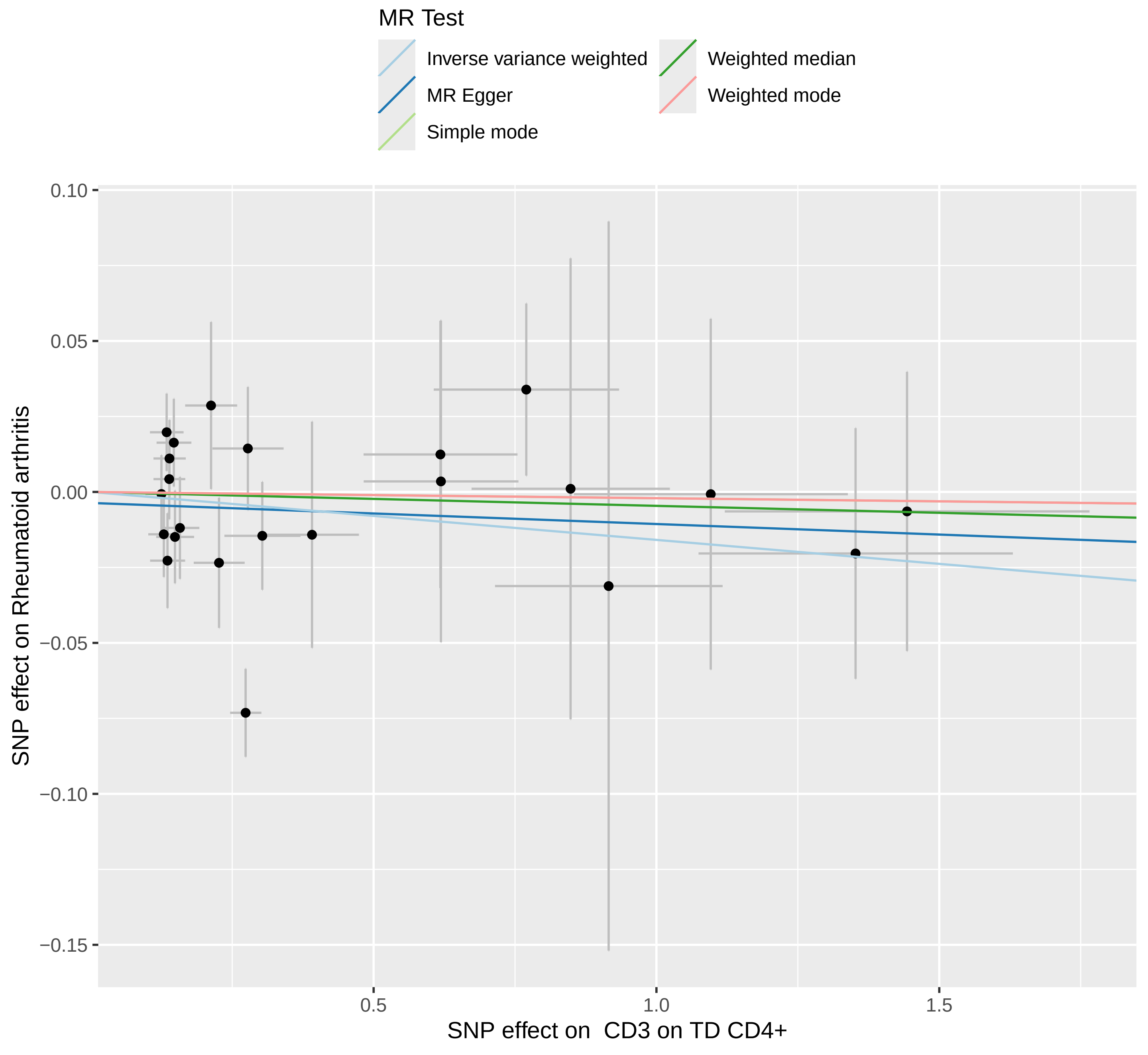
MR Test
Inverse variance weighted
MR Egger
Simple mode
Weighted median
Weighted mode
0.10
0.05
0.00
−0.05
−0.10
−0.15
SNP effect on Rheumatoid arthritis
0.5
1.0
1.5
SNP effect on CD3 on TD CD4+

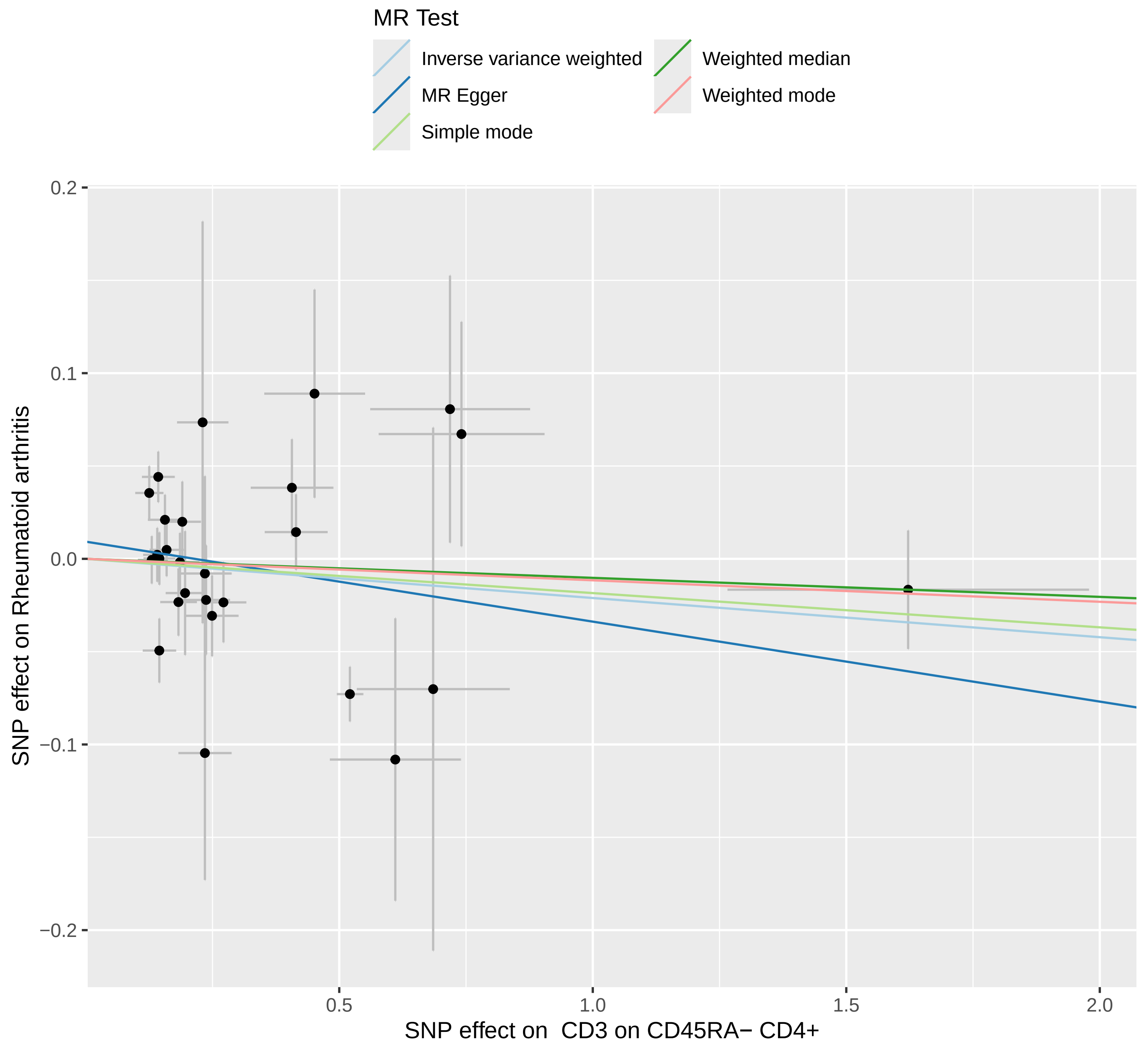

MR Test
Inverse variance weighted
MR Egger
Simple mode
Weighted median
Weighted mode
0.2
0.1
0.0
−0.1
−0.2
0.5
1.0
1.5
2.0
SNP effect on Rheumatoid arthritis
SNP effect on CD3 on CD45RA− CD4+

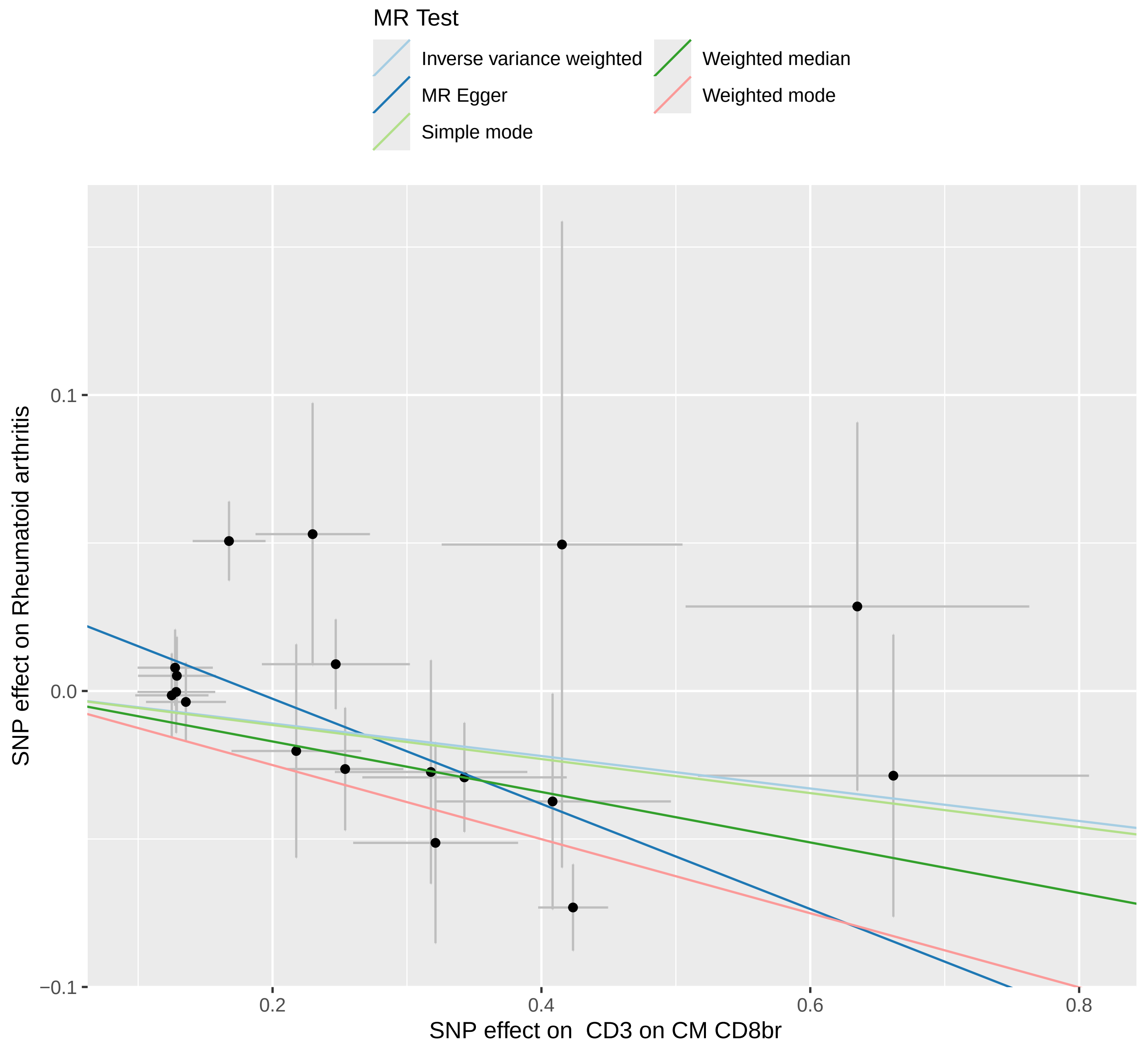

MR Test
Inverse variance weighted
MR Egger
Simple mode
Weighted median
Weighted mode
0.1
0.0
−0.1
0.2
0.4
0.6
0.8
SNP effect on Rheumatoid arthritis
SNP effect on CD3 on CM CD8br

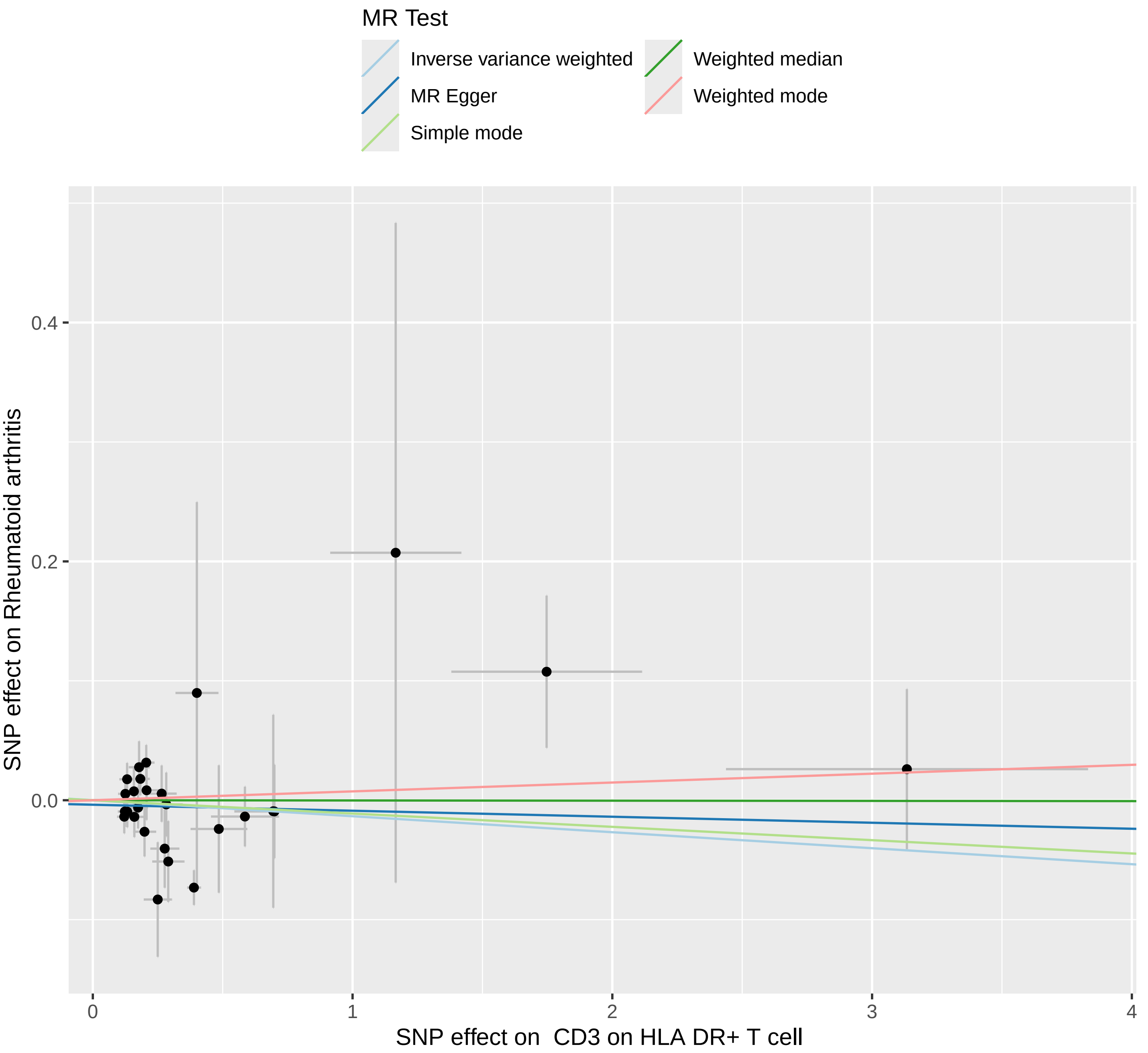
MR Test
Inverse variance weighted
MR Egger
Simple mode
Weighted median
Weighted mode
SNP effect on Rheumatoid arthritis
0.4
0.2
0.0
0
1
2
3
4
SNP effect on CD3 on HLA DR+ T cell

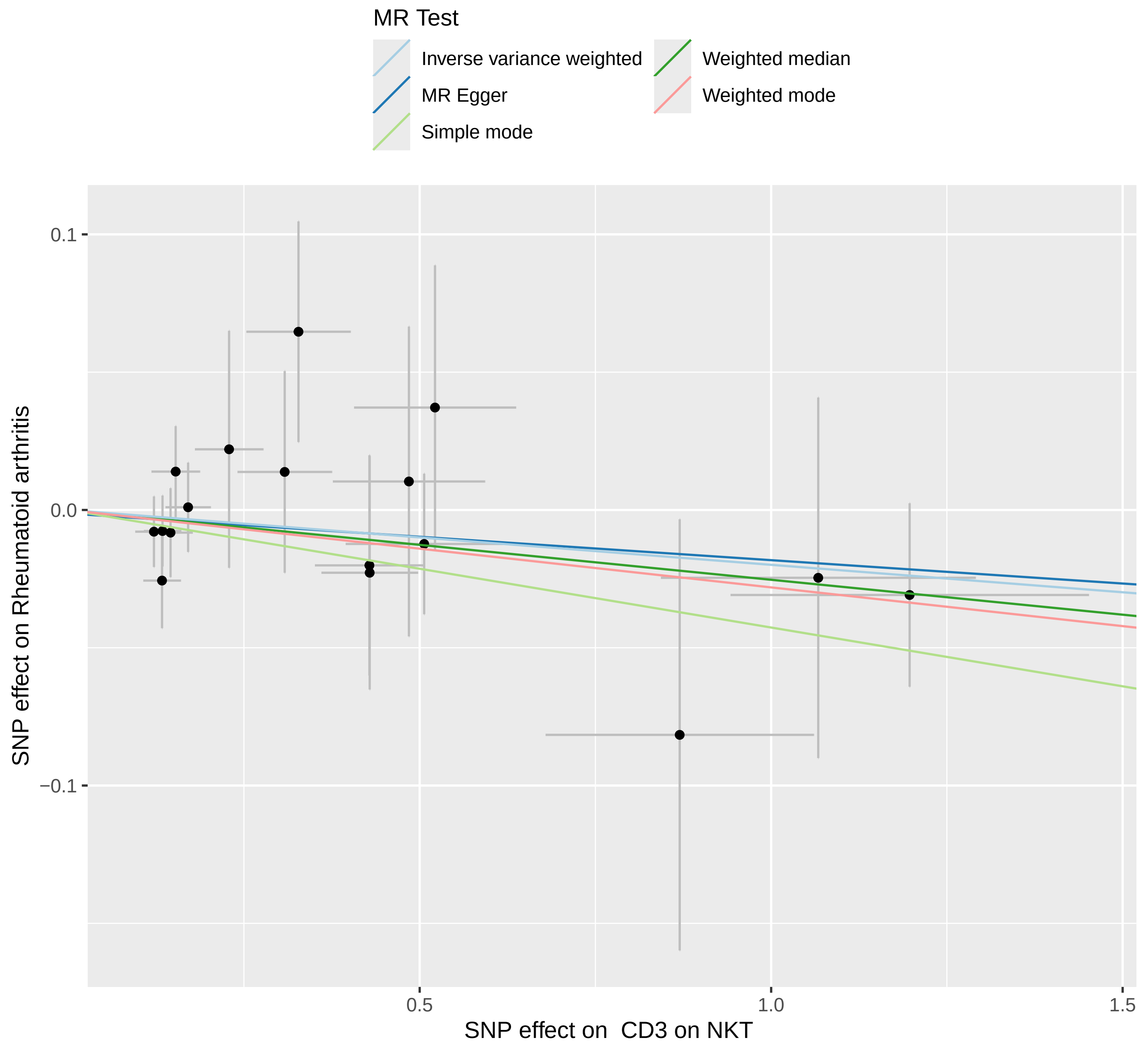
MR Test
Inverse variance weighted
MR Egger
Simple mode
Weighted median
Weighted mode
0.1
0.0
−0.1
SNP effect on Rheumatoid arthritis
0.5
1.0
1.5
SNP effect on CD3 on NKT

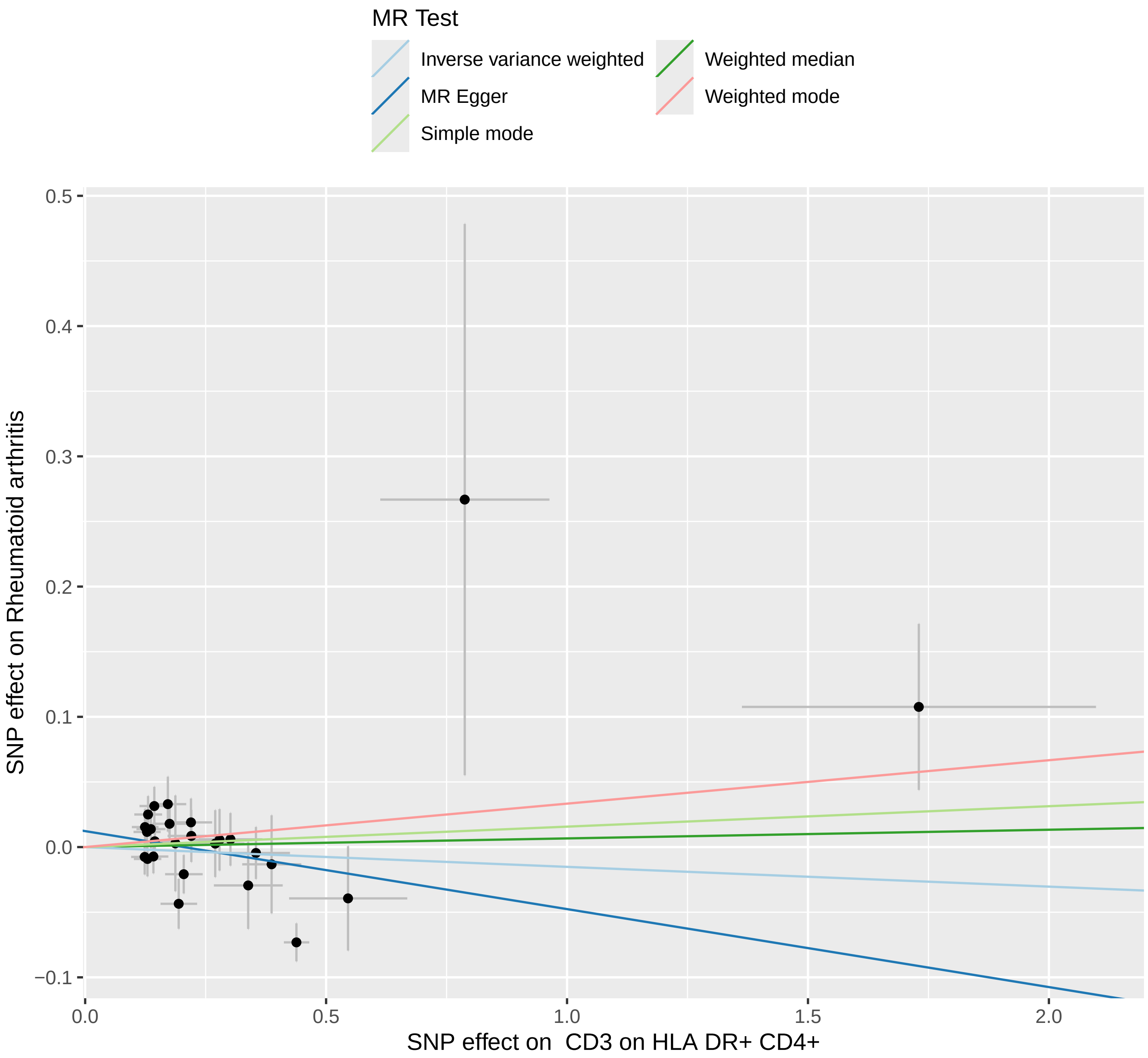
MR Test
Inverse variance weighted
MR Egger
Simple mode
Weighted median
Weighted mode
SNP effect on Rheumatoid arthritis
SNP effect on CD3 on HLA DR+ CD4+
0.5
0.4
0.3
0.2
0.1
0.0
−0.1
0.0
0.5
1.0
1.5
2.0

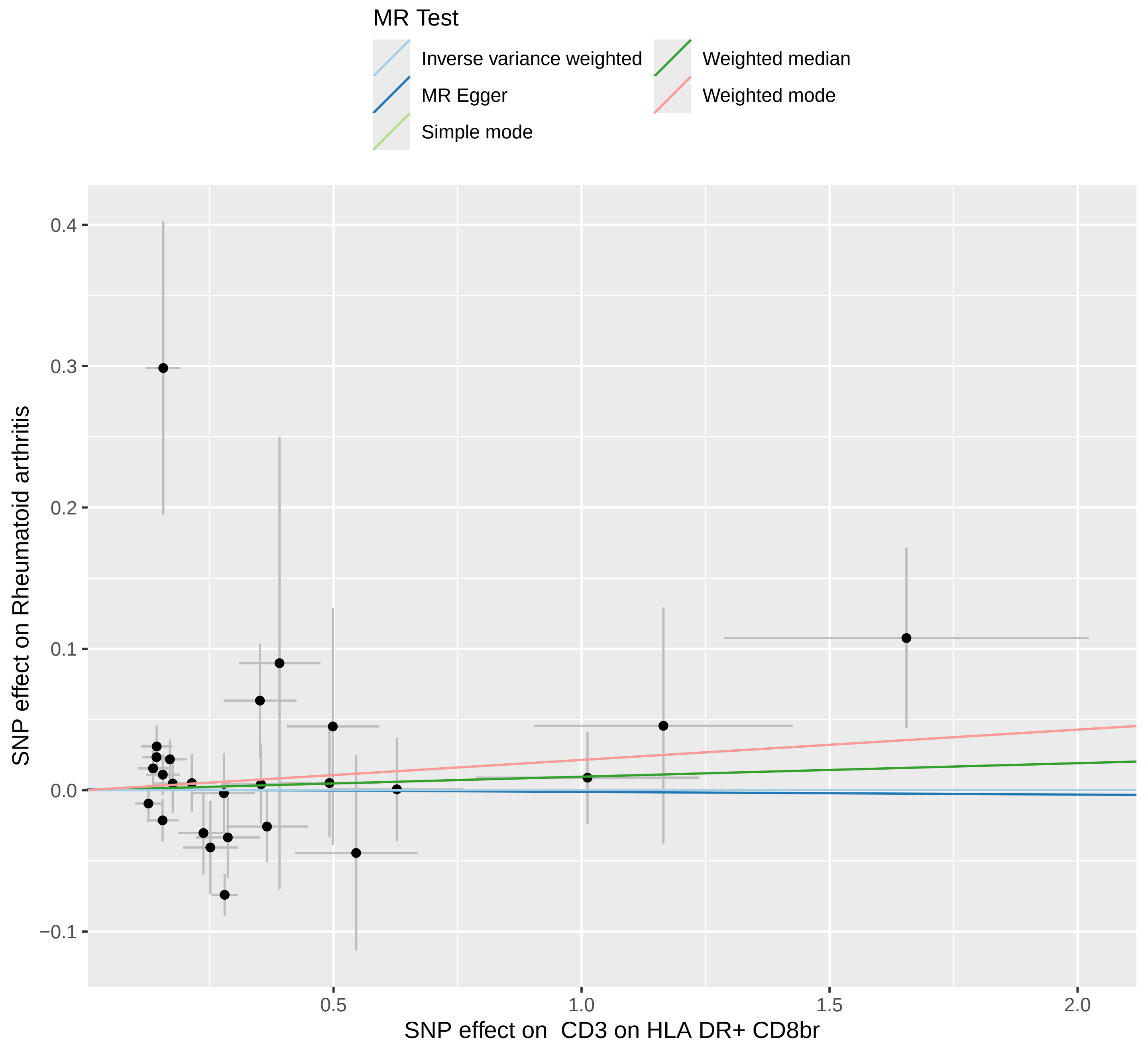

MR Test
Inverse variance weighted
MR Egger
Simple mode
Weighted median
Weighted mode
SNP effect on Rheumatoid arthritis
0.4
0.3
0.2
0.1
0.0
−0.1
0.5
1.0
1.5
2.0
SNP effect on CD3 on HLA DR+ CD8br

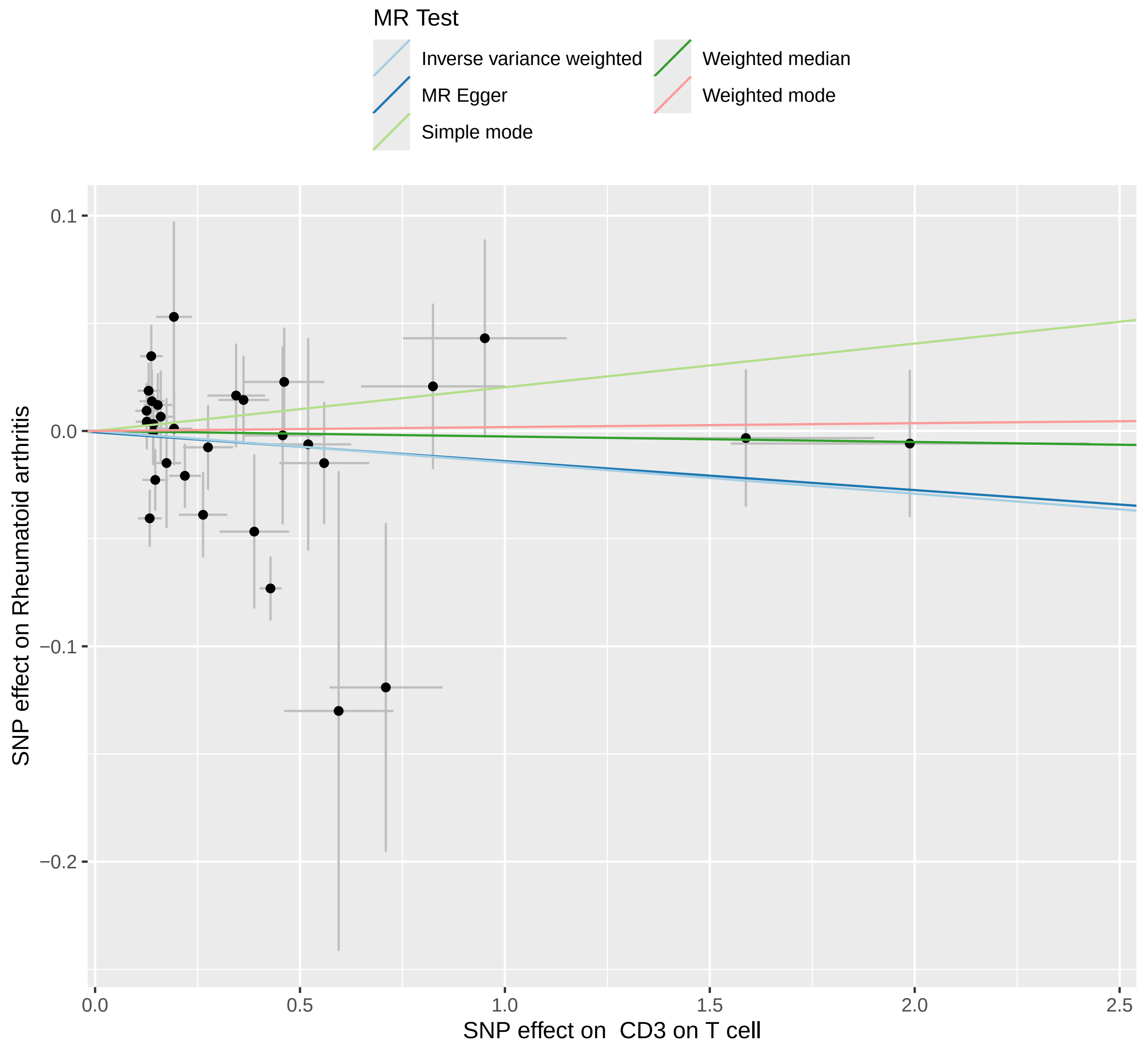
MR Test
Inverse variance weighted
MR Egger
Simple mode
Weighted median
Weighted mode
0.1
0.0
−0.1
−0.2
0.0
0.5
1.0
1.5
2.0
2.5
SNP effect on Rheumatoid arthritis
SNP effect on CD3 on T cell

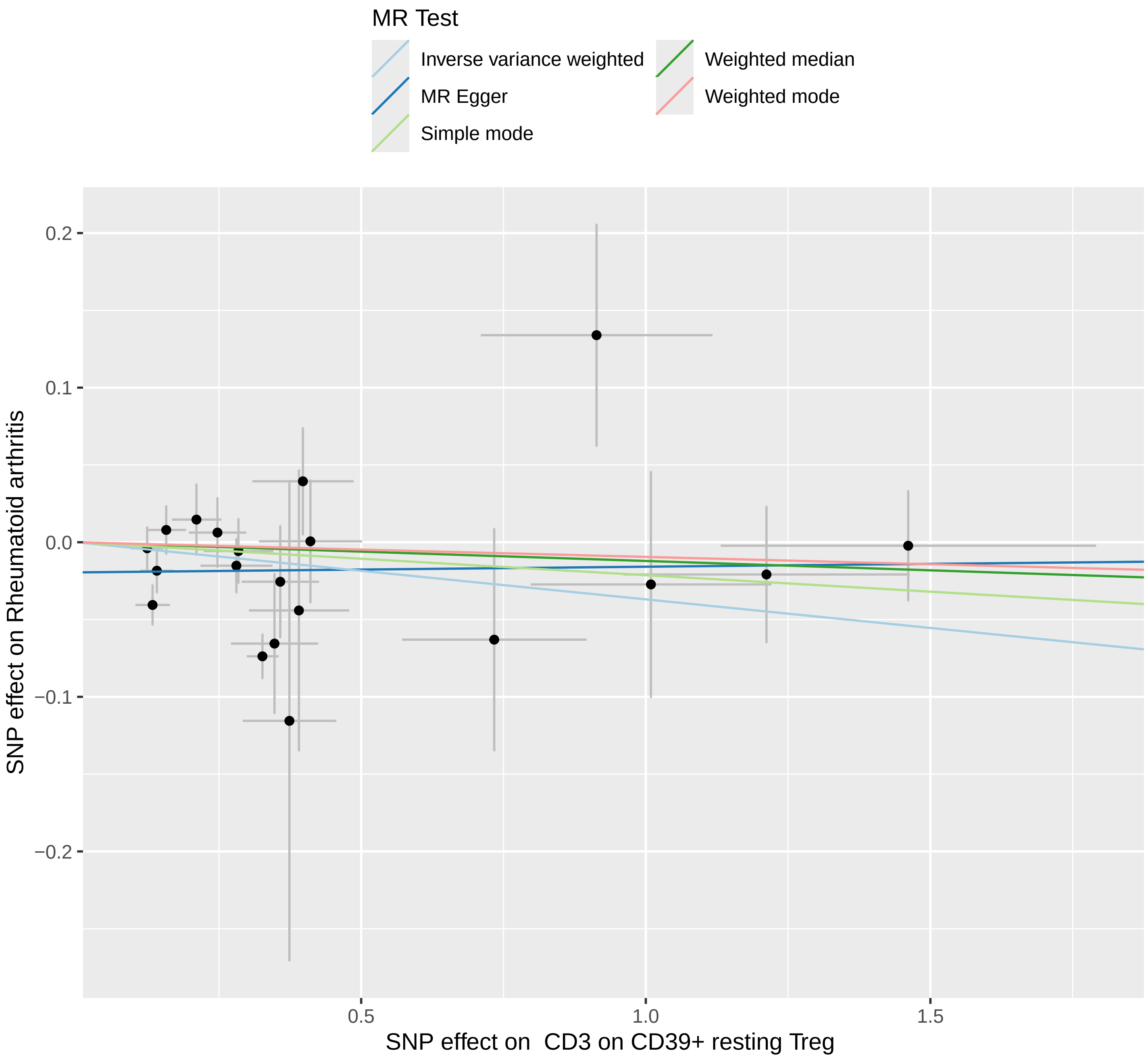

MR Test
Inverse variance weighted
MR Egger
Simple mode
Weighted median
Weighted mode
0.2
0.1
0.0
−0.1
−0.2
0.5
1.0
1.5
SNP effect on Rheumatoid arthritis
SNP effect on CD3 on CD39+ resting Treg

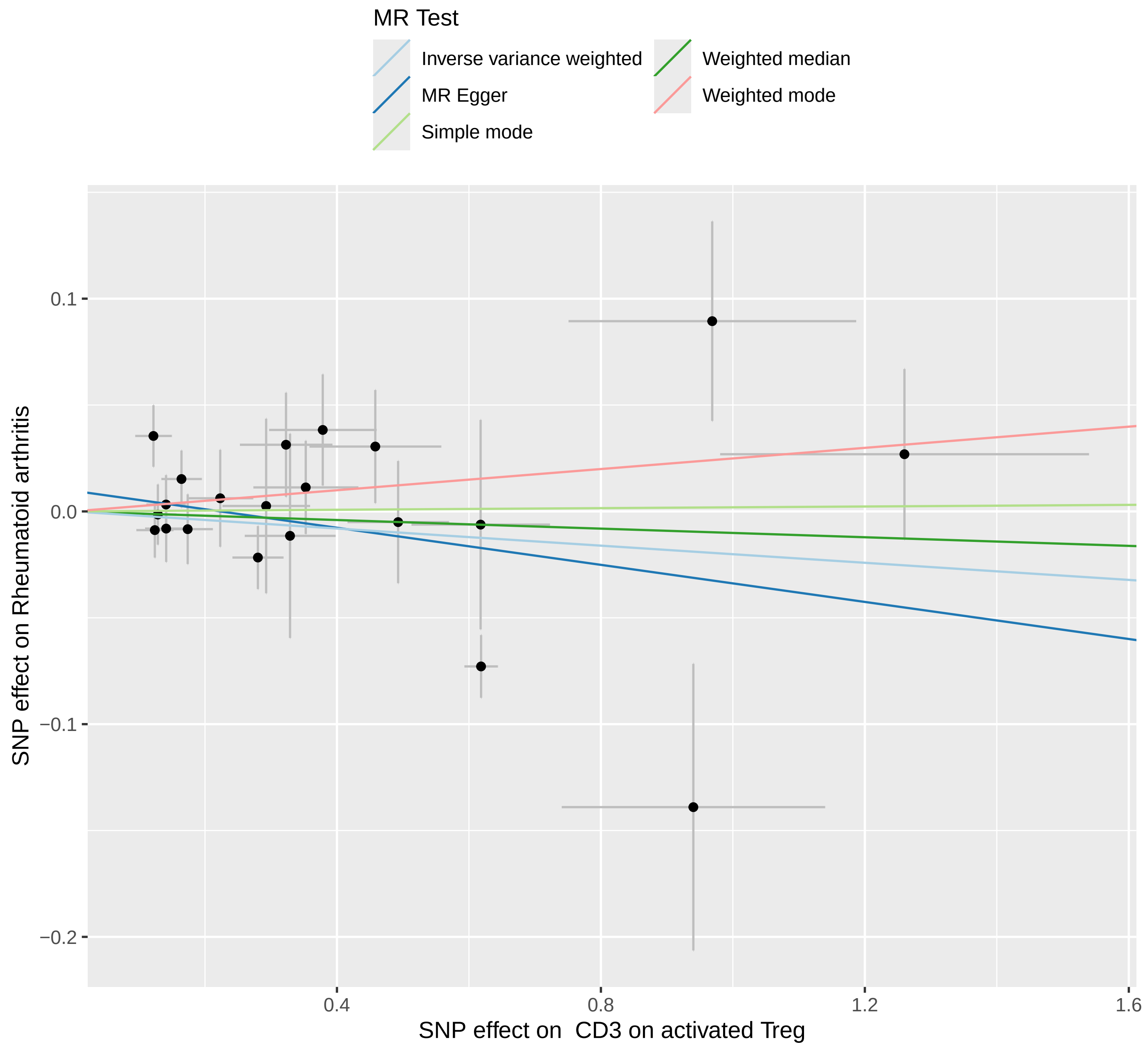

MR Test
Inverse variance weighted
MR Egger
Simple mode
Weighted median
Weighted mode
SNP effect on Rheumatoid arthritis
0.1
0.0
−0.1
−0.2
0.4
0.8
1.2
1.6
SNP effect on CD3 on activated Treg

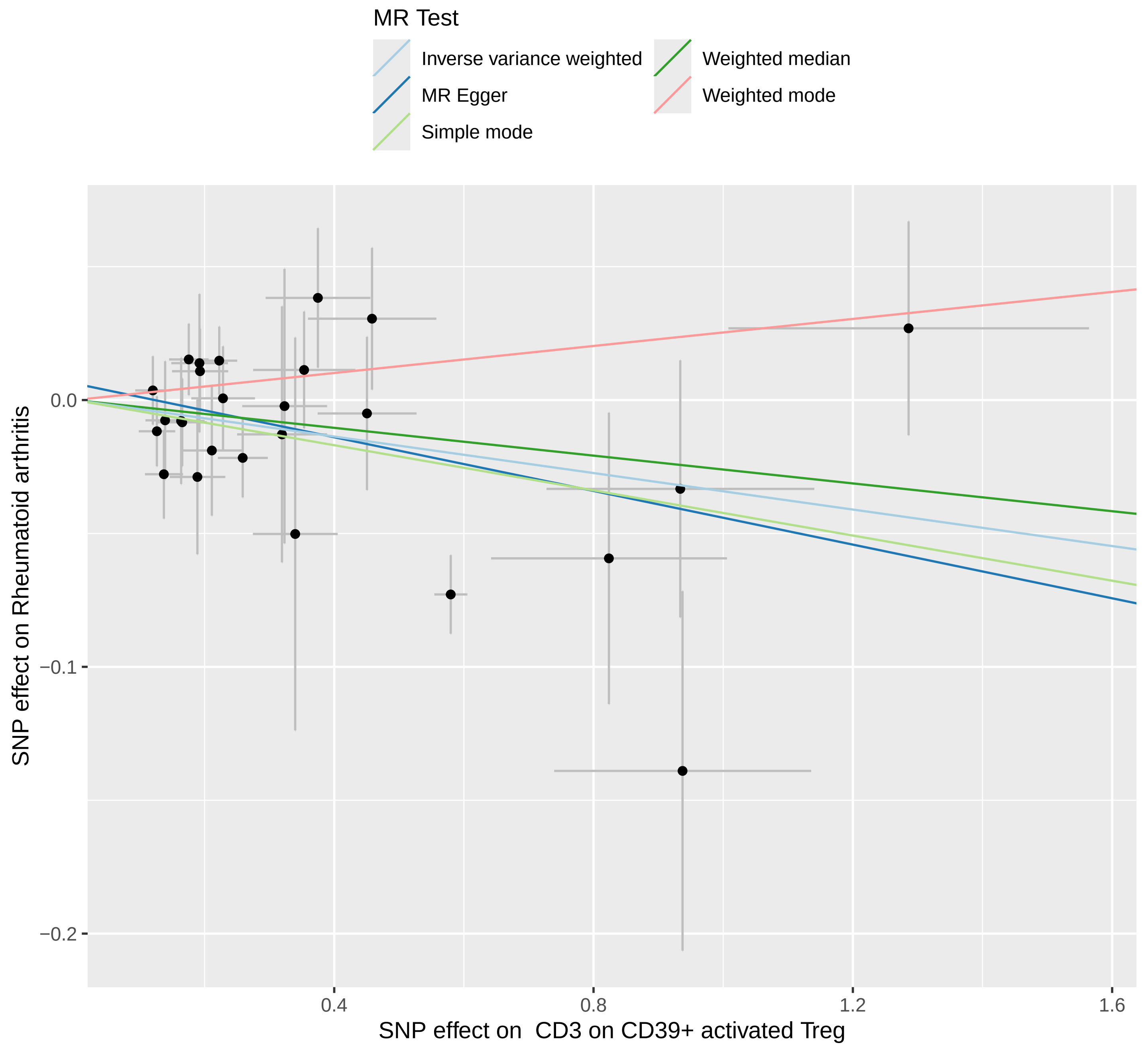
MR Test
Inverse variance weighted
MR Egger
Simple mode
Weighted median
Weighted mode
SNP effect on Rheumatoid arthritis
0.0
−0.1
−0.2
0.4
0.8
1.2
1.6
SNP effect on CD3 on CD39+ activated Treg

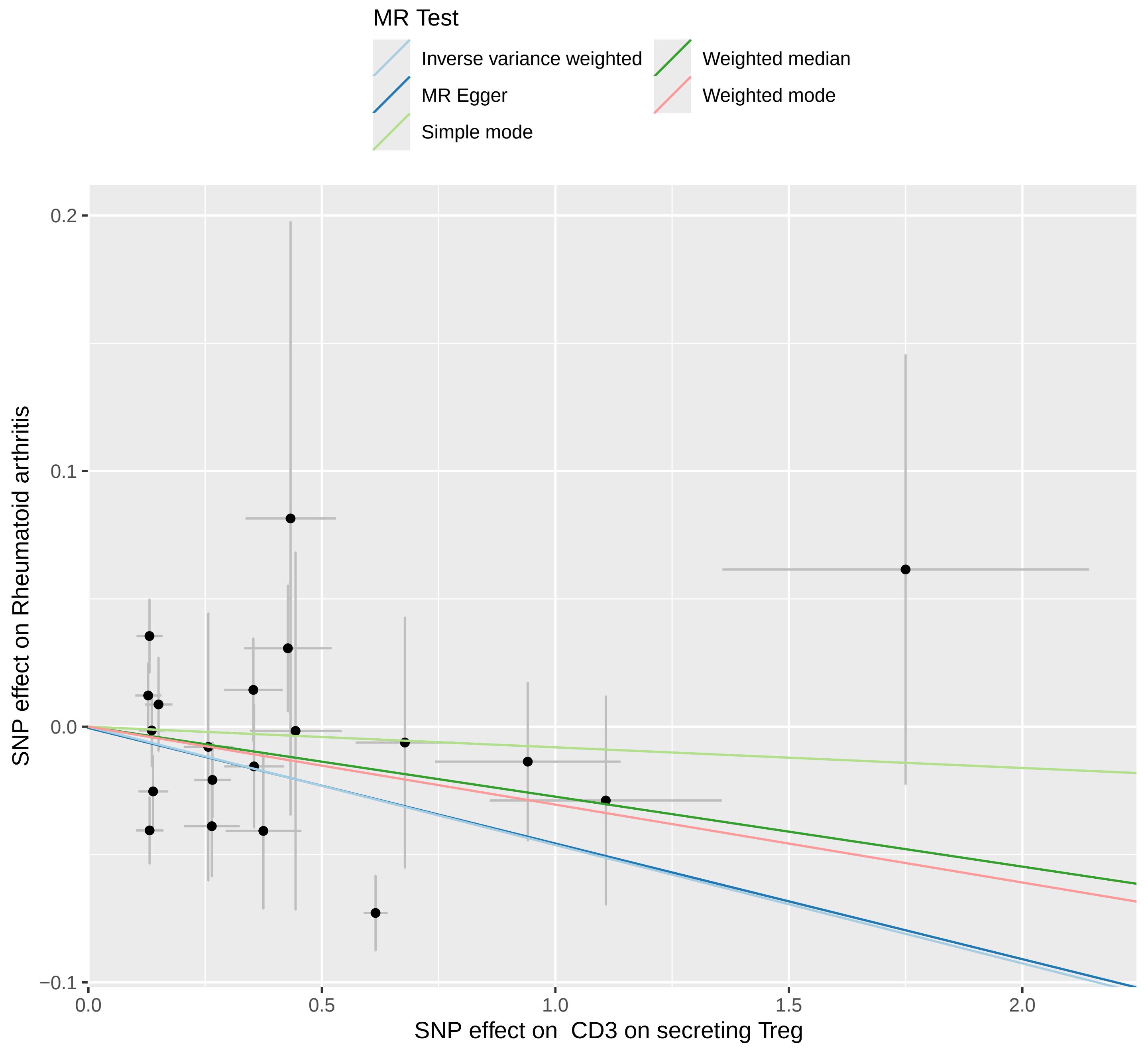

MR Test
Inverse variance weighted
MR Egger
Simple mode
Weighted median
Weighted mode
0.2
0.1
0.0
−0.1
0.0
0.5
1.0
1.5
2.0
SNP effect on Rheumatoid arthritis
SNP effect on CD3 on secreting Treg

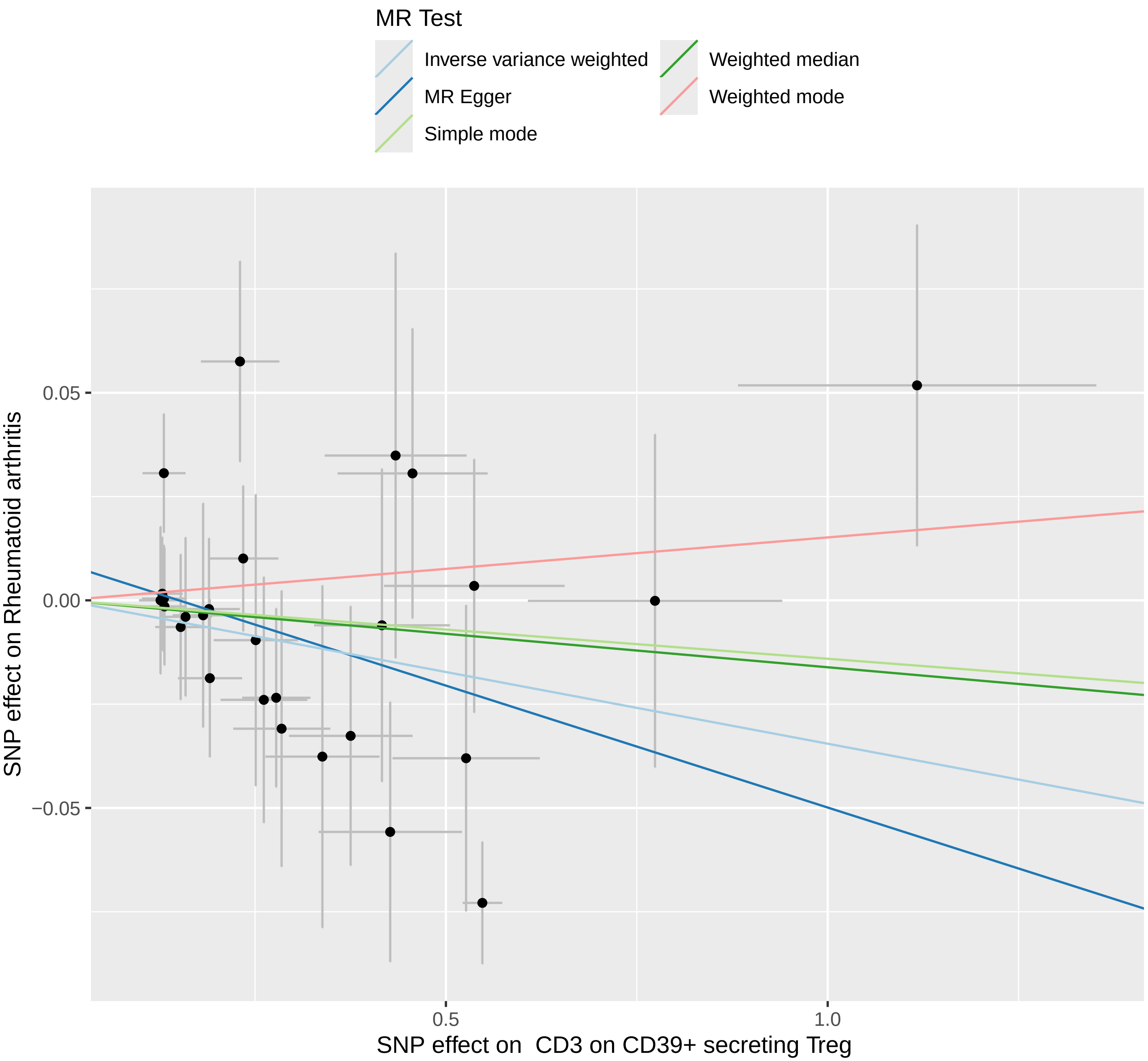
MR Test
Inverse variance weighted
MR Egger
Simple mode
Weighted median
Weighted mode
0.05
0.00
−0.05
0.5
1.0
SNP effect on Rheumatoid arthritis
SNP effect on CD3 on CD39+ secreting Treg

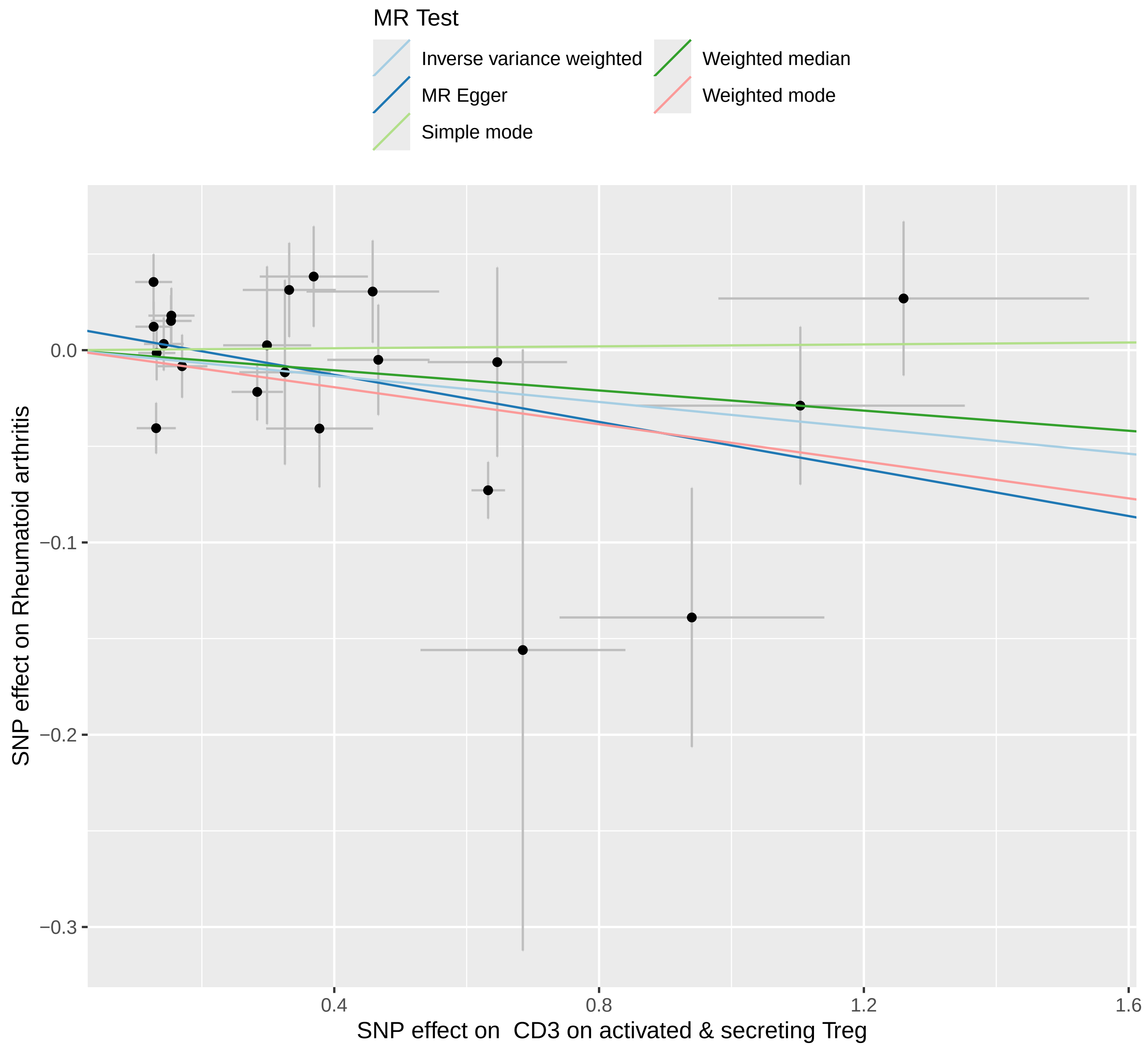
MR Test
Inverse variance weighted
MR Egger
Simple mode
Weighted median
Weighted mode
0.0
−0.1
−0.2
−0.3
0.4
0.8
1.2
1.6
SNP effect on Rheumatoid arthritis
SNP effect on CD3 on activated & secreting Treg

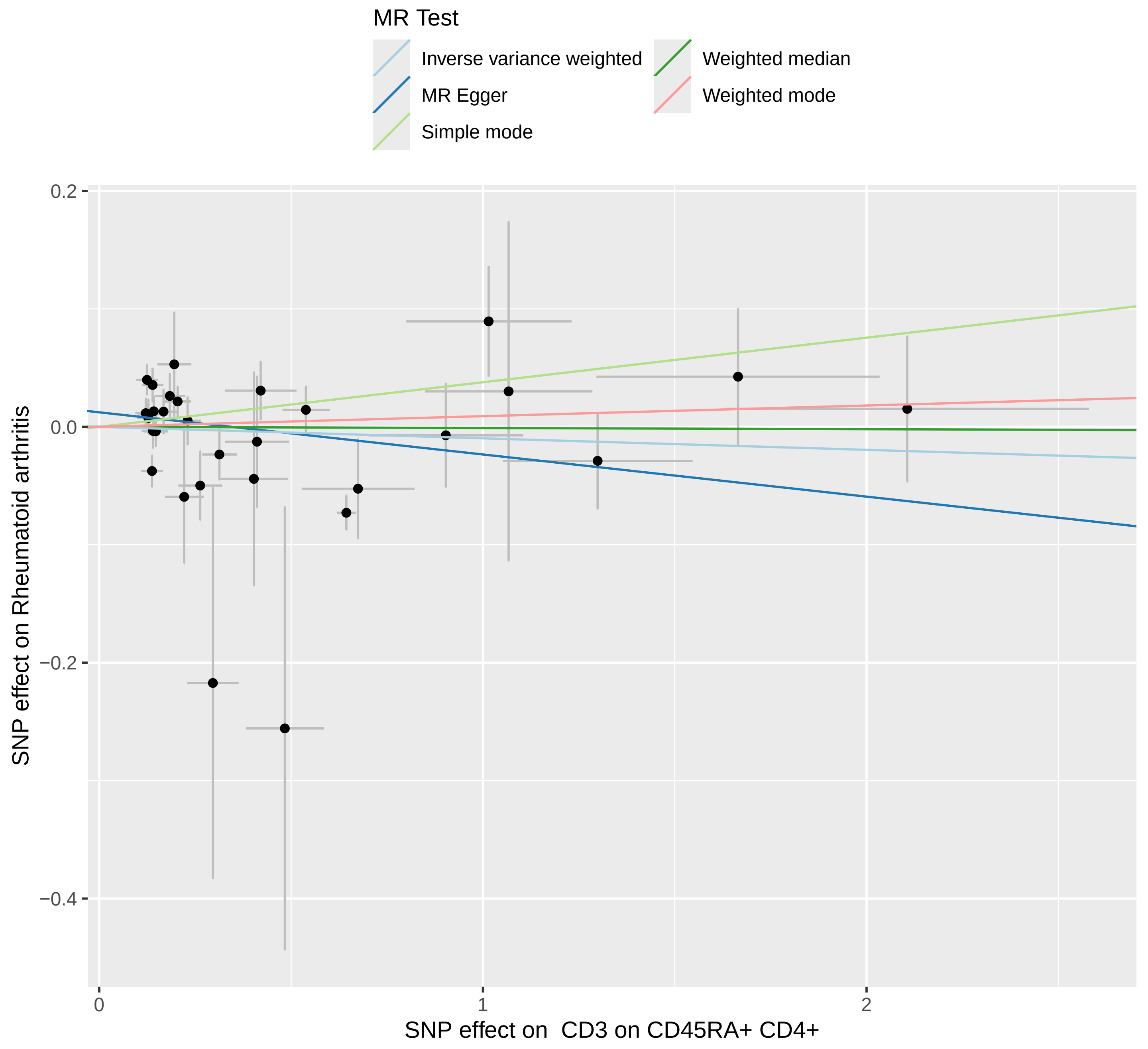
MR Test
Inverse variance weighted
MR Egger
Simple mode
Weighted median
Weighted mode
0.2
0.0
−0.2
−0.4
0
1
2
SNP effect on Rheumatoid arthritis
SNP effect on CD3 on CD45RA+ CD4+

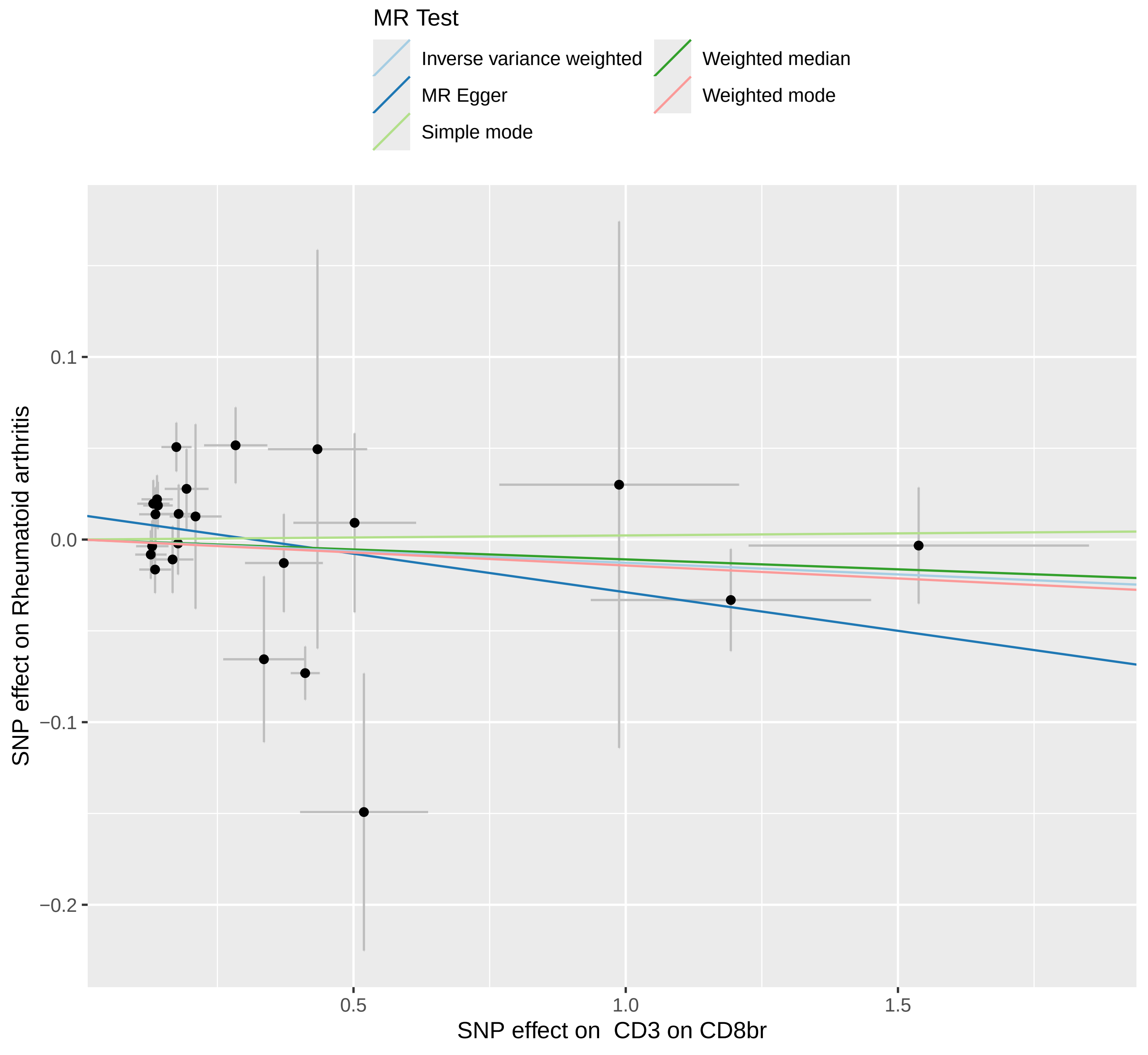

MR Test
Inverse variance weighted
MR Egger
Simple mode
Weighted median
Weighted mode
0.1
0.0
−0.1
−0.2
SNP effect on Rheumatoid arthritis
0.5
1.0
1.5
SNP effect on CD3 on CD8br

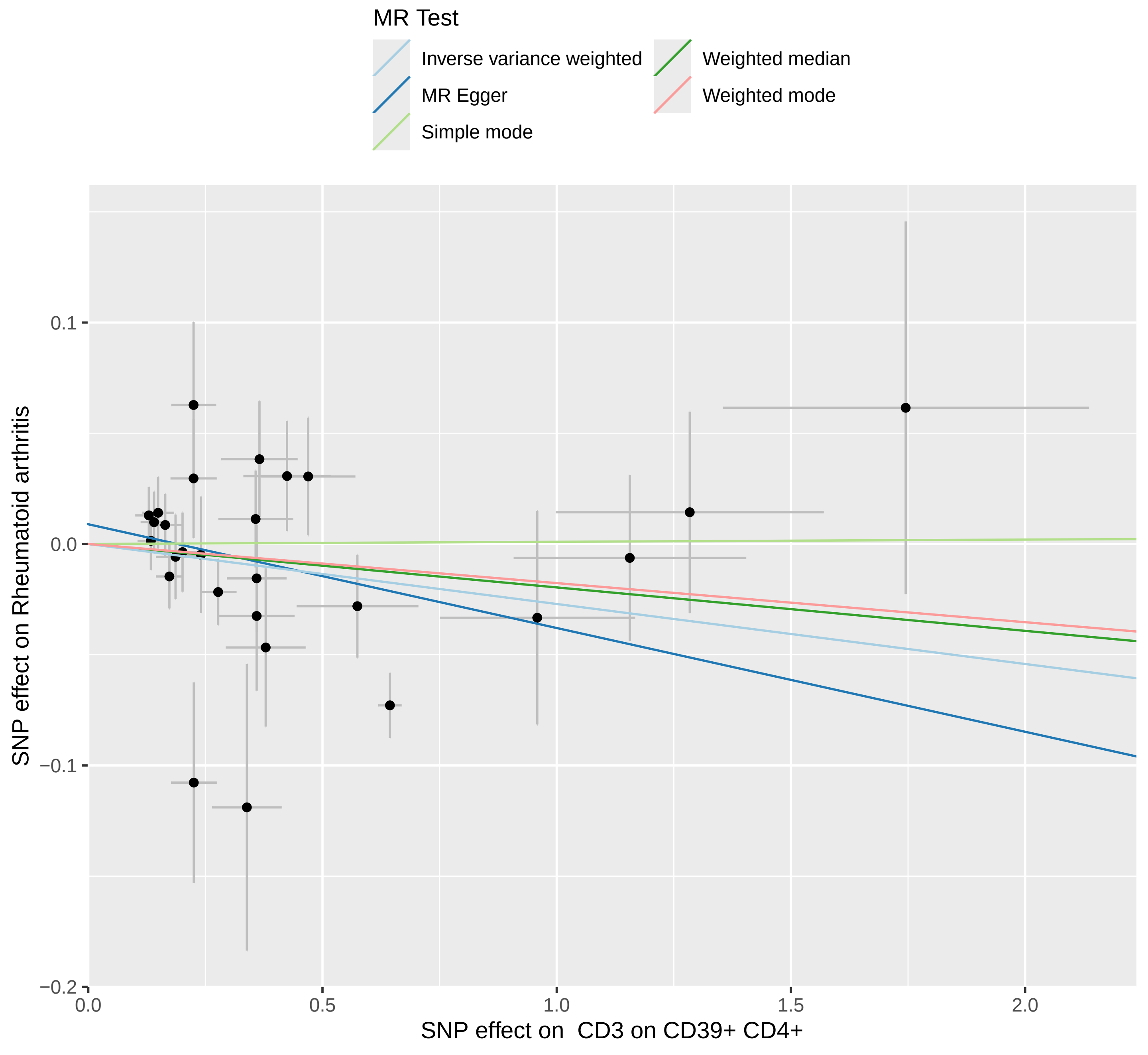
MR Test
Inverse variance weighted
MR Egger
Simple mode
Weighted median
Weighted mode
SNP effect on Rheumatoid arthritis
0.1
0.0
−0.1
−0.2
0.0
0.5
1.0
1.5
2.0
SNP effect on CD3 on CD39+ CD4+

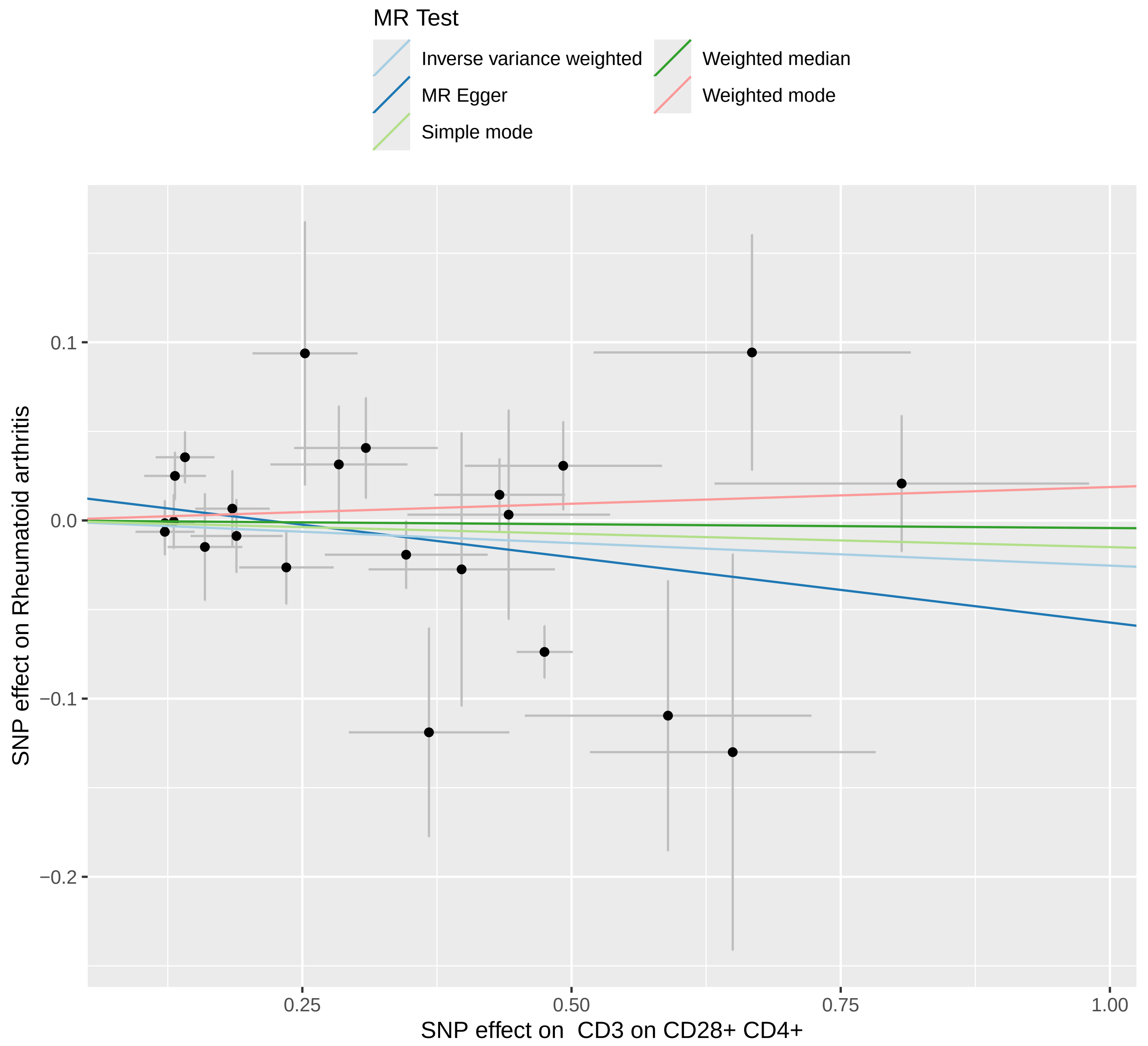

MR Test
Inverse variance weighted
MR Egger
Simple mode
Weighted median
Weighted mode
0.1
0.0
−0.1
−0.2
SNP effect on Rheumatoid arthritis
0.25
0.50
0.75
1.00
SNP effect on CD3 on CD28+ CD4+

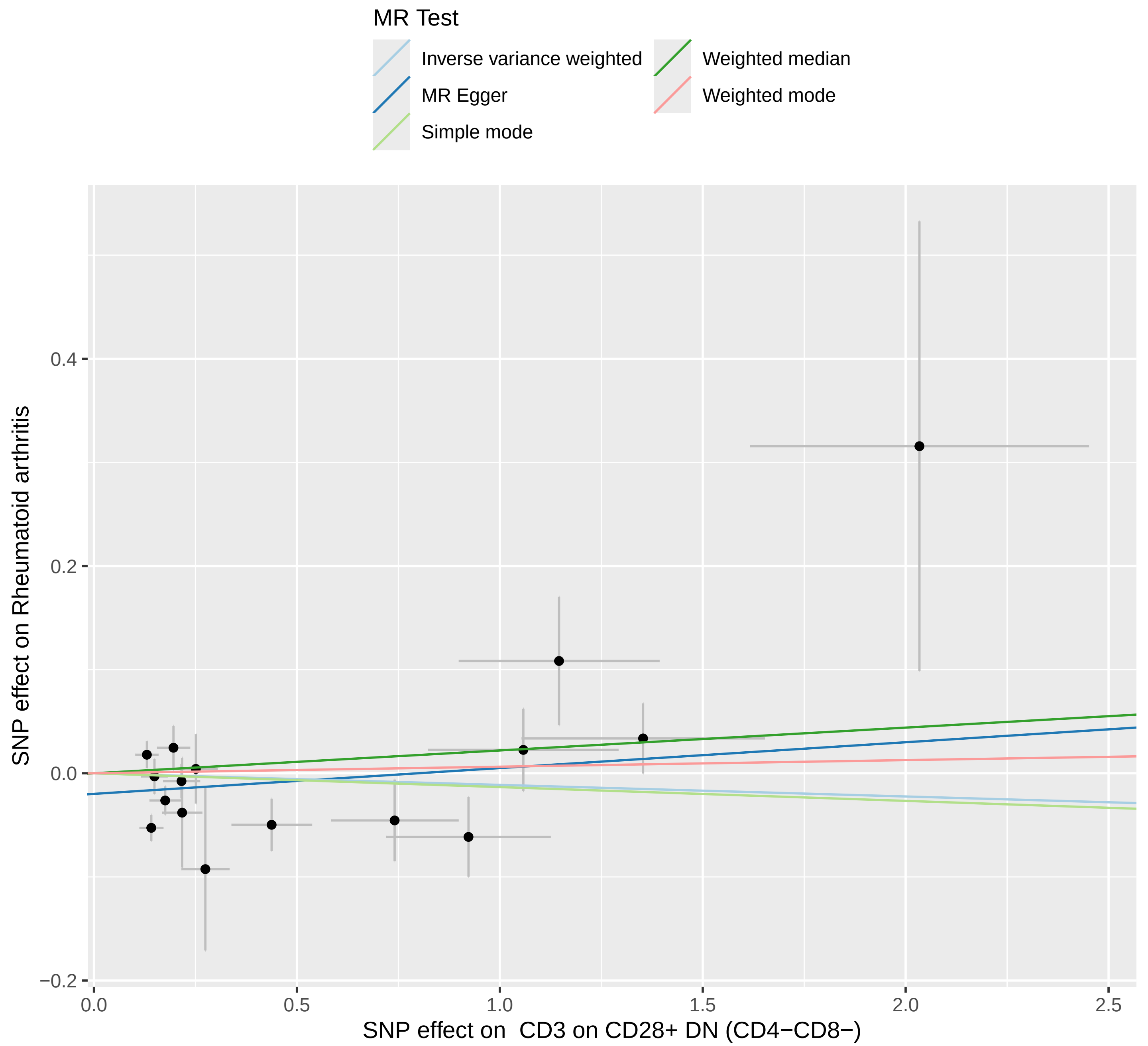

MR Test
Inverse variance weighted
MR Egger
Simple mode
Weighted median
Weighted mode
SNP effect on Rheumatoid arthritis
0.4
0.2
0.0
−0.2
0.0
0.5
1.0
1.5
2.0
2.5
SNP effect on CD3 on CD28+ DN (CD4−CD8−)

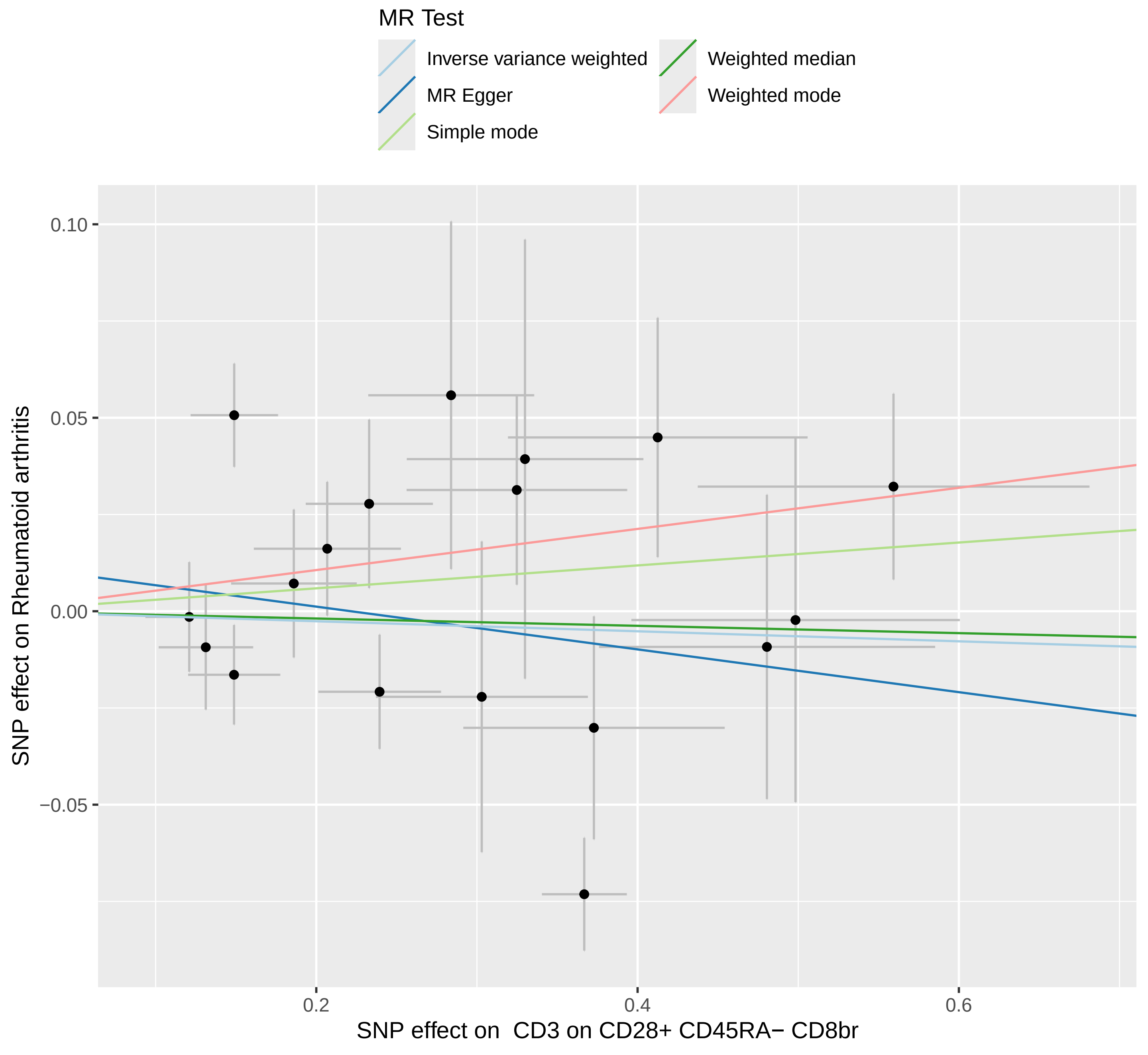

MR Test
Inverse variance weighted
MR Egger
Simple mode
Weighted median
Weighted mode
0.10
0.05
0.00
−0.05
SNP effect on Rheumatoid arthritis
0.2
0.4
0.6
SNP effect on CD3 on CD28+ CD45RA− CD8br

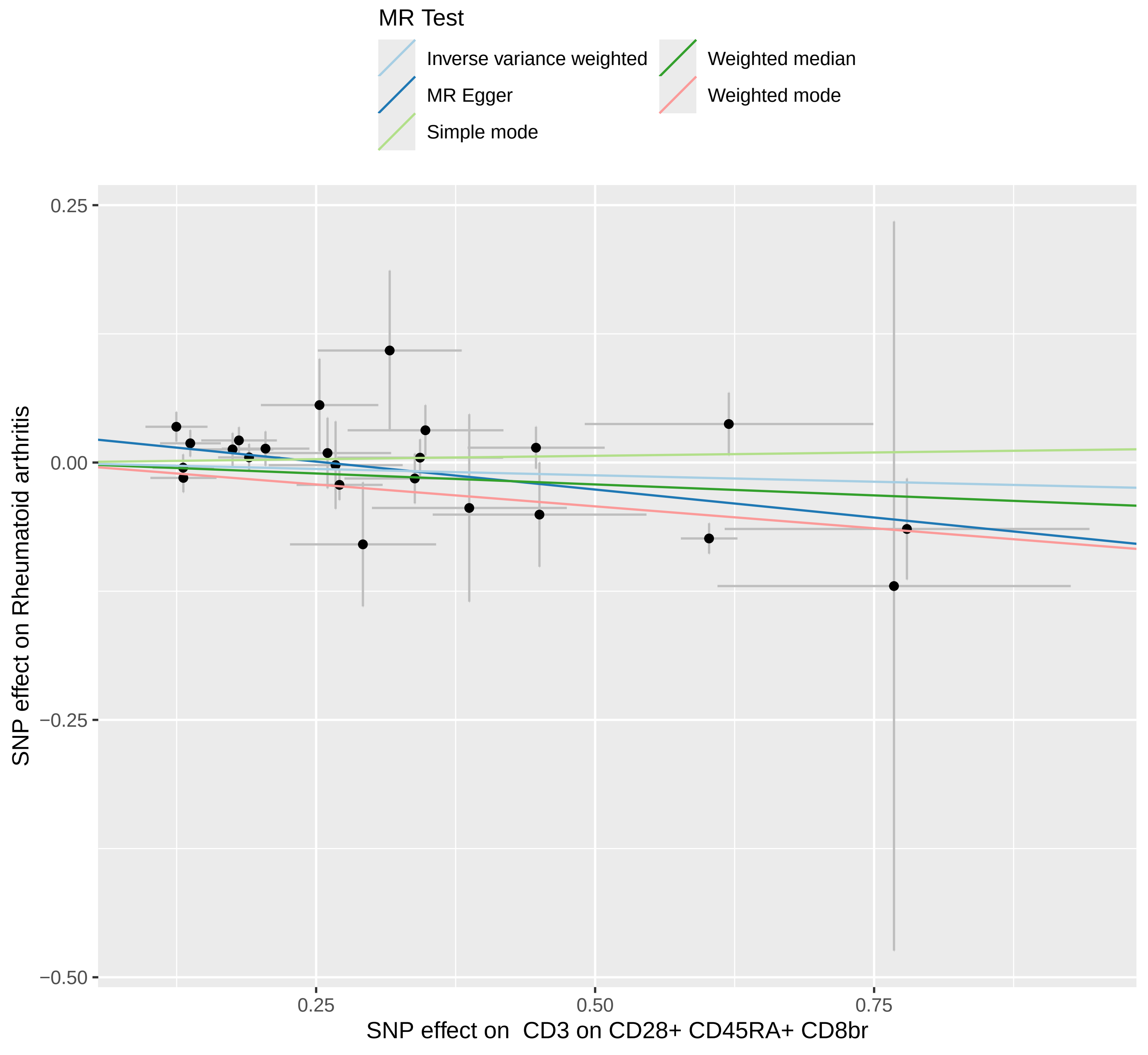
MR Test
Inverse variance weighted
MR Egger
Simple mode
Weighted median
Weighted mode
0.25
0.00
−0.25
−0.50
0.25
0.50
0.75
SNP effect on Rheumatoid arthritis
SNP effect on CD3 on CD28+ CD45RA+ CD8br

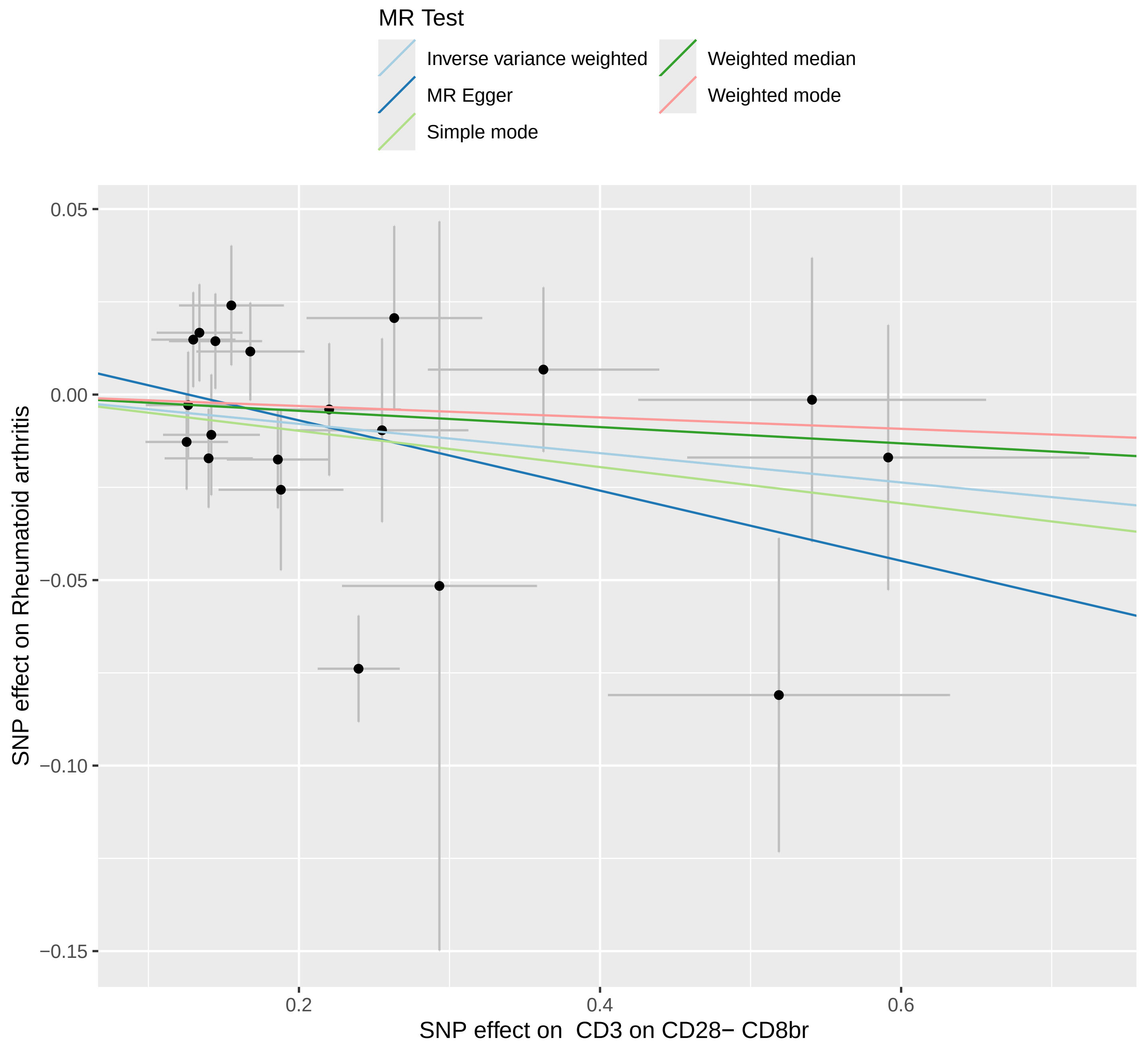
MR Test
Inverse variance weighted
MR Egger
Simple mode
Weighted median
Weighted mode
0.05
0.00
−0.05
−0.10
−0.15
0.2
0.4
0.6
SNP effect on Rheumatoid arthritis
SNP effect on CD3 on CD28− CD8br

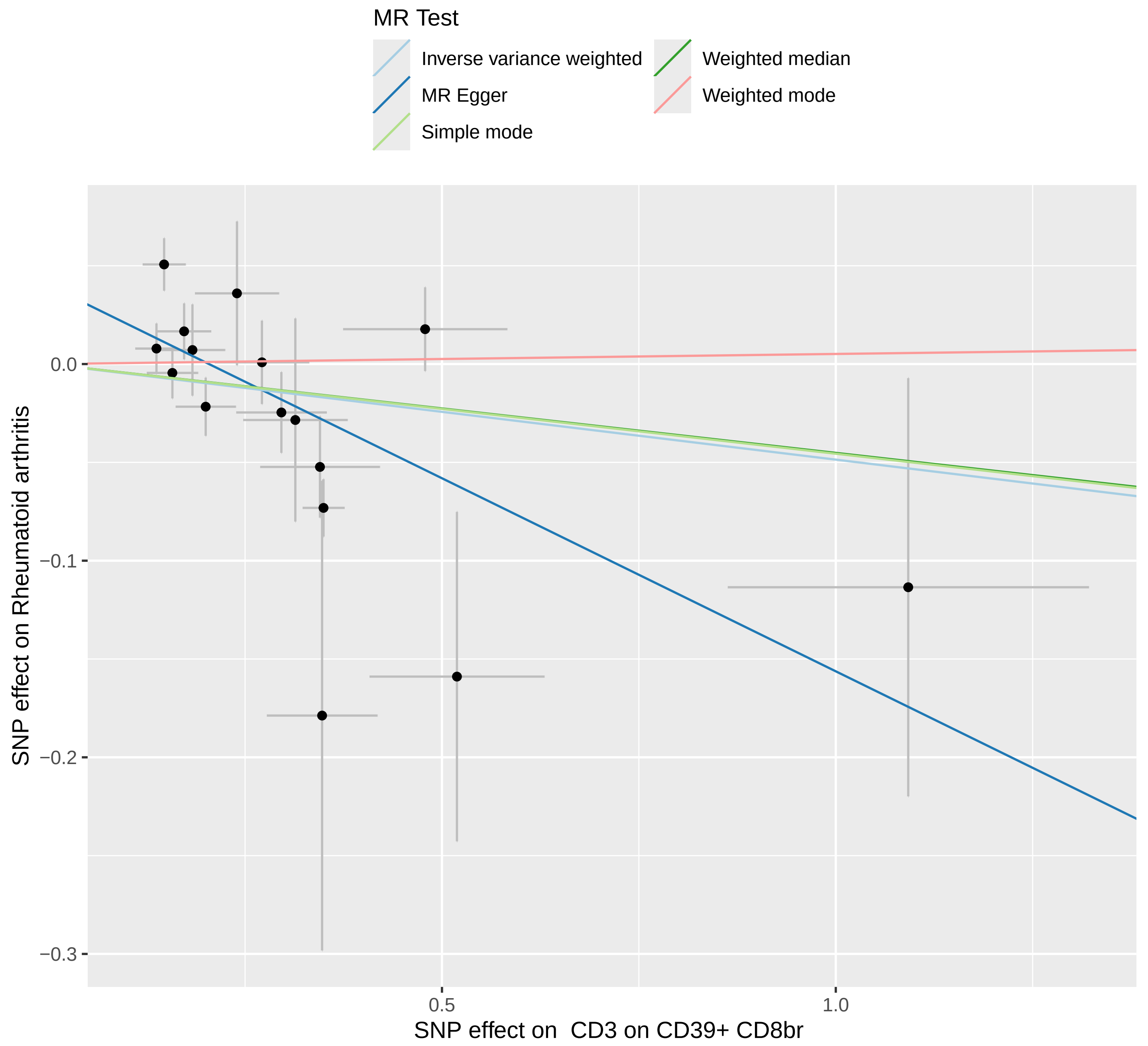

MR Test
Inverse variance weighted
MR Egger
Simple mode
Weighted median
Weighted mode
0.0
−0.1
−0.2
−0.3
0.5
1.0
SNP effect on Rheumatoid arthritis
SNP effect on CD3 on CD39+ CD8br

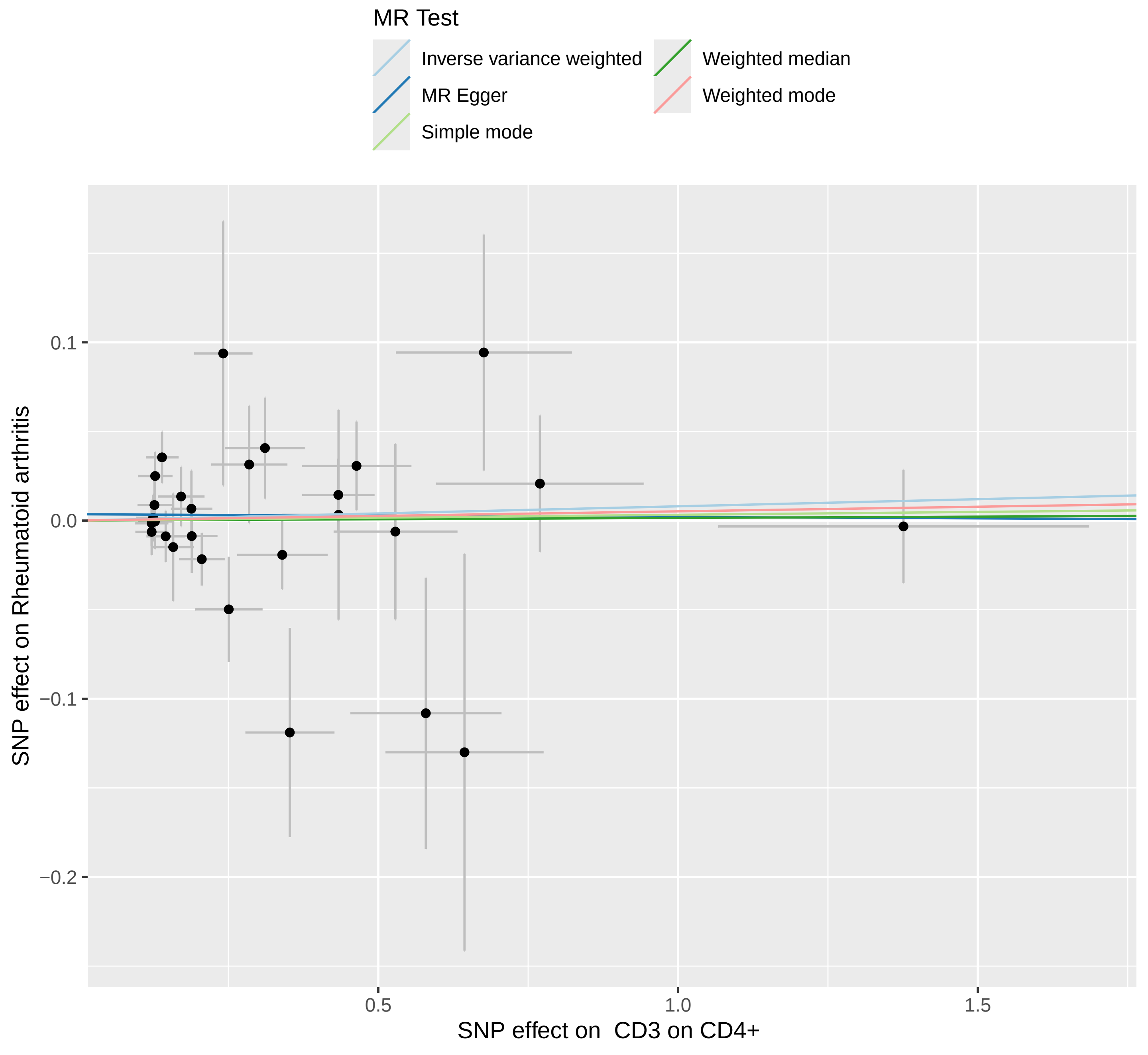
MR Test
Inverse variance weighted
MR Egger
Simple mode
Weighted median
Weighted mode
0.1
0.0
−0.1
−0.2
0.5
1.0
1.5
SNP effect on Rheumatoid arthritis
SNP effect on CD3 on CD4+

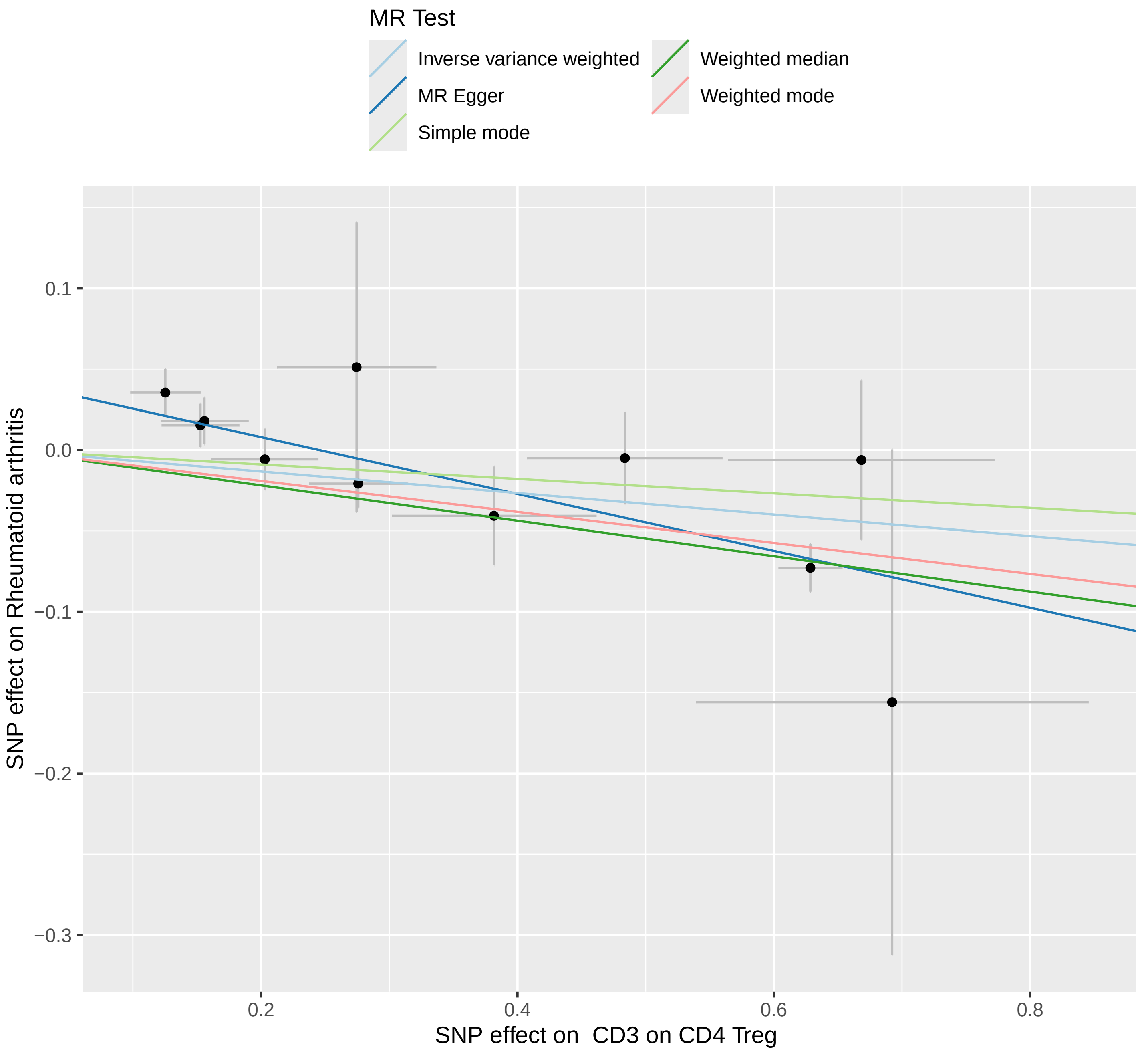
MR Test
Inverse variance weighted
MR Egger
Simple mode
Weighted median
Weighted mode
0.1
0.0
−0.1
−0.2
−0.3
0.2
0.4
0.6
0.8
SNP effect on Rheumatoid arthritis
SNP effect on CD3 on CD4 Treg

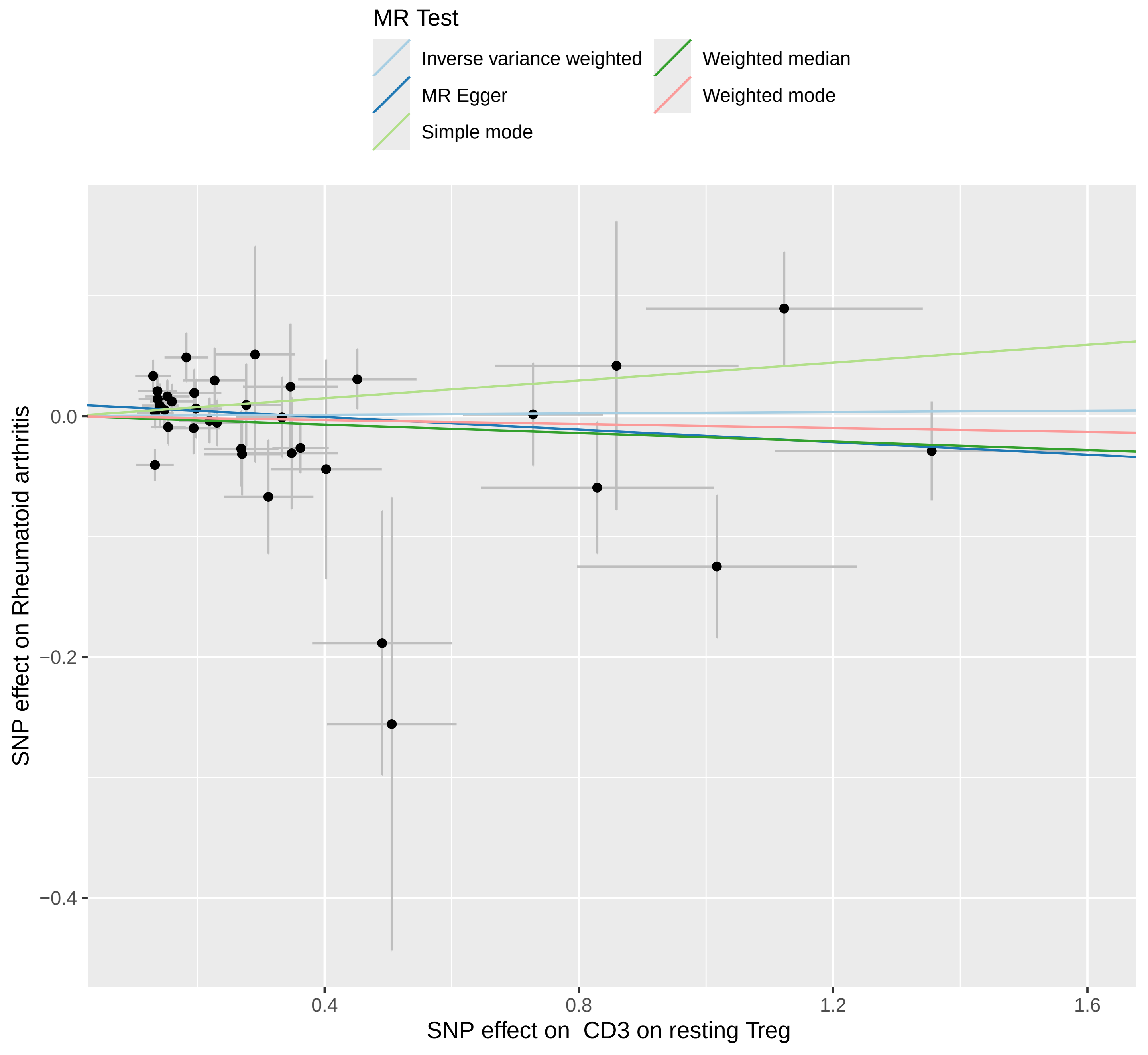

MR Test
Inverse variance weighted
MR Egger
Simple mode
Weighted median
Weighted mode
0.0
−0.2
−0.4
0.4
0.8
1.2
1.6
SNP effect on Rheumatoid arthritis
SNP effect on CD3 on resting Treg

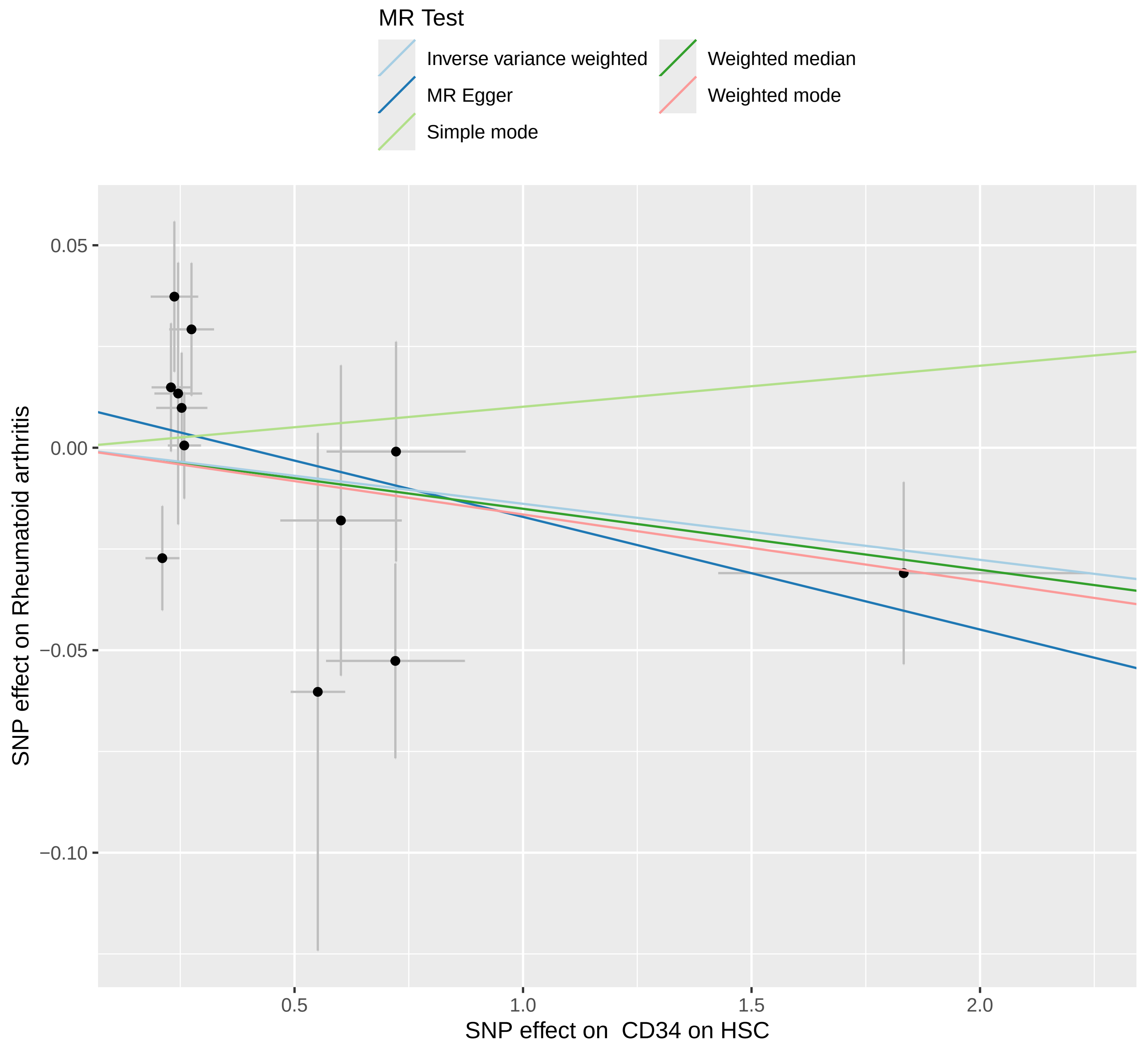
MR Test
Inverse variance weighted
MR Egger
Simple mode
Weighted median
Weighted mode
0.05
0.00
−0.05
−0.10
0.5
1.0
1.5
2.0
SNP effect on Rheumatoid arthritis
SNP effect on CD34 on HSC

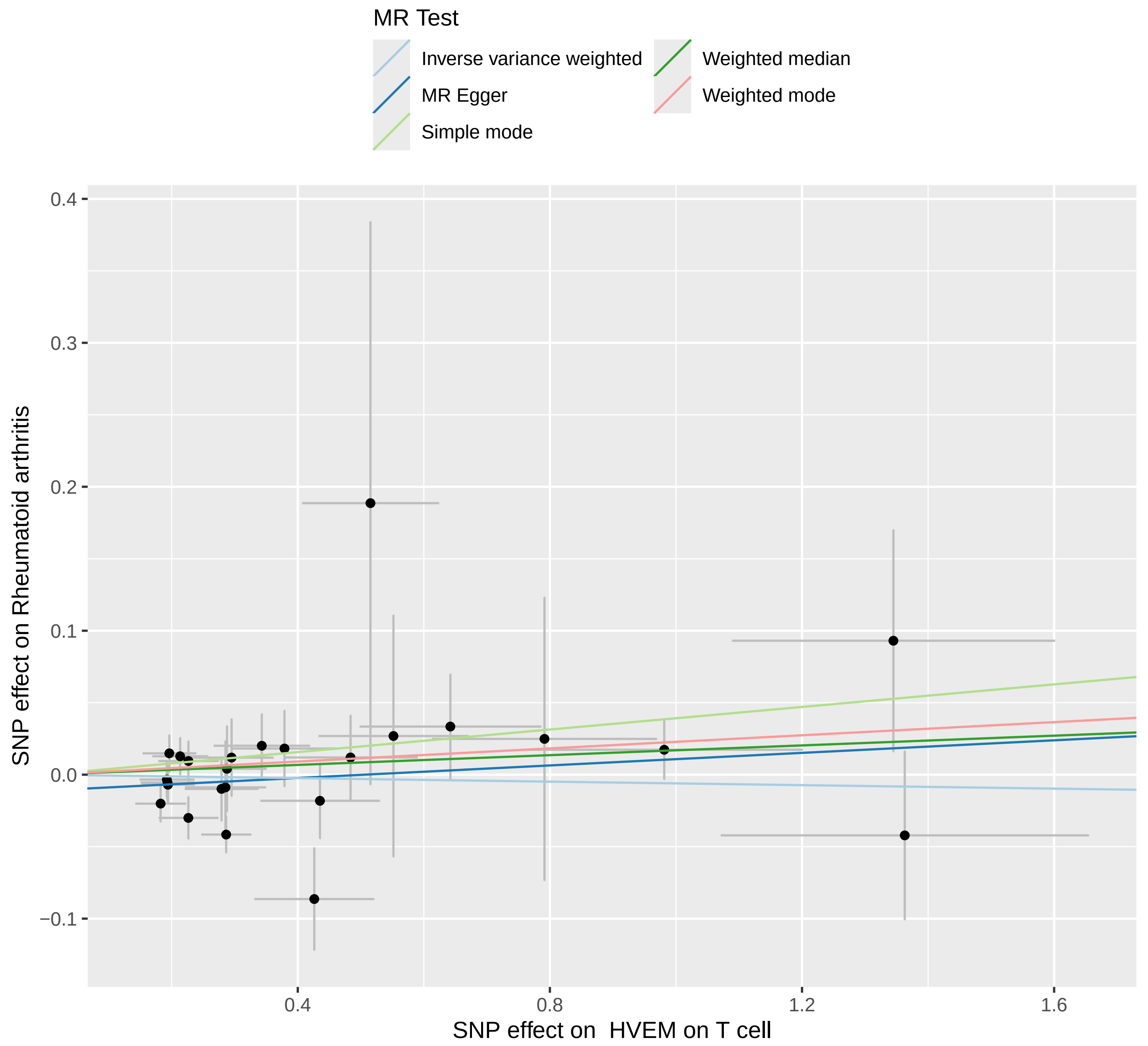
MR Test
Inverse variance weighted
MR Egger
Simple mode
Weighted median
Weighted mode
0.4
0.3
0.2
0.1
0.0
−0.1
SNP effect on Rheumatoid arthritis
0.4
0.8
1.2
1.6
SNP effect on HVEM on T cell

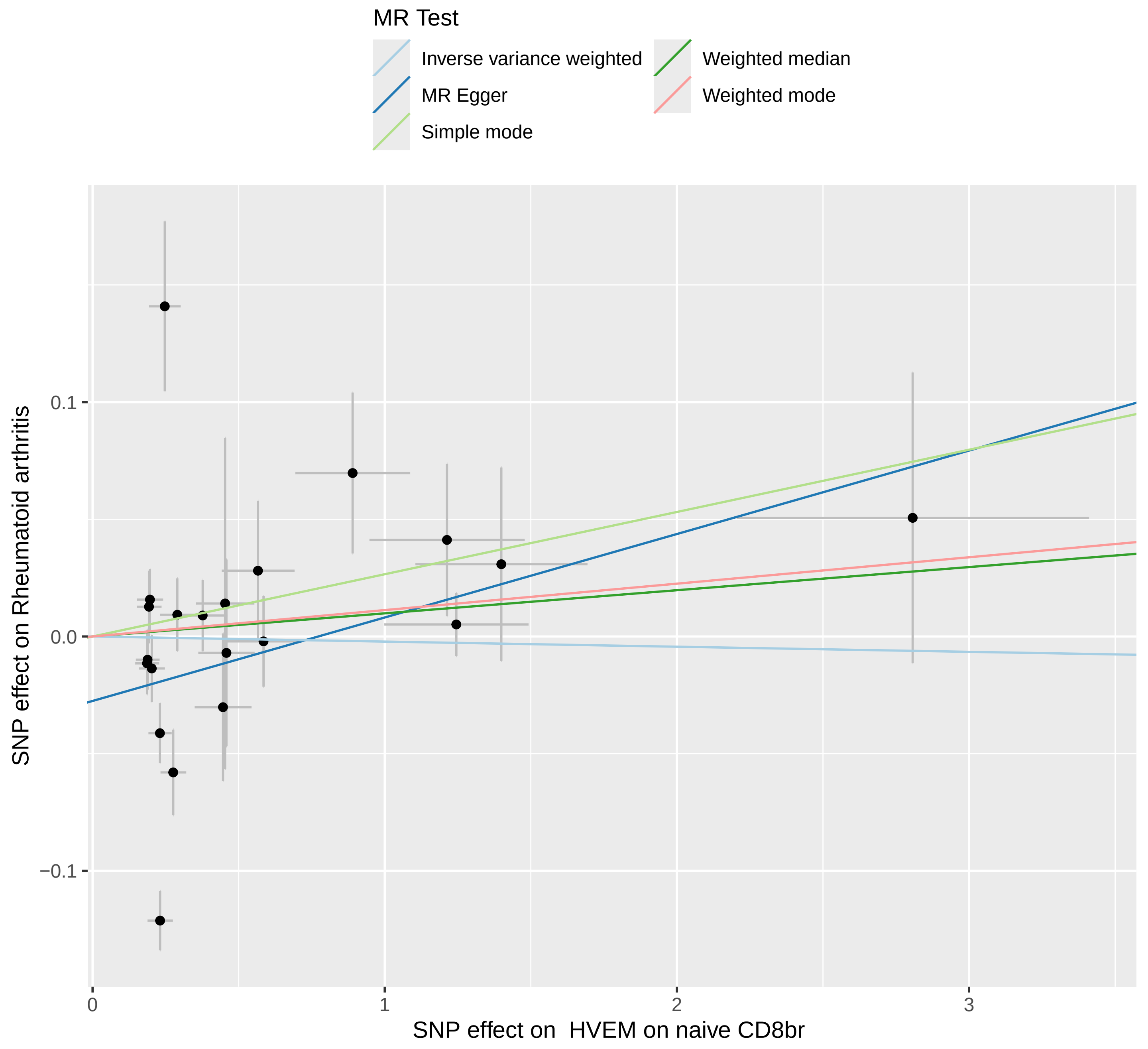
MR Test
Inverse variance weighted
MR Egger
Simple mode
Weighted median
Weighted mode
0.1
0.0
−0.1
0
1
2
3
SNP effect on Rheumatoid arthritis
SNP effect on HVEM on naive CD8br

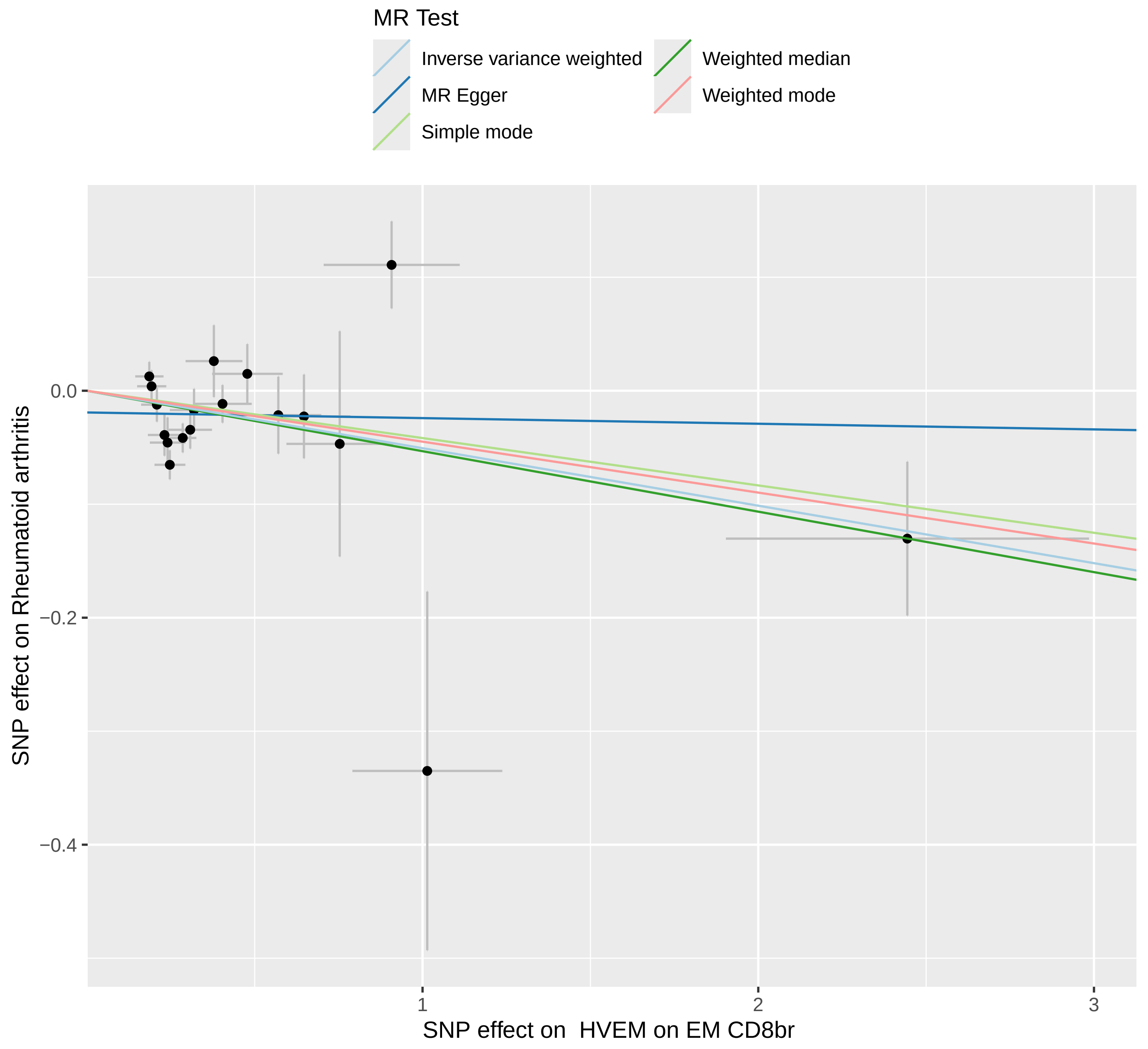

MR Test
Inverse variance weighted
MR Egger
Simple mode
Weighted median
Weighted mode
SNP effect on Rheumatoid arthritis
0.0
−0.2
−0.4
1
2
3
SNP effect on HVEM on EM CD8br

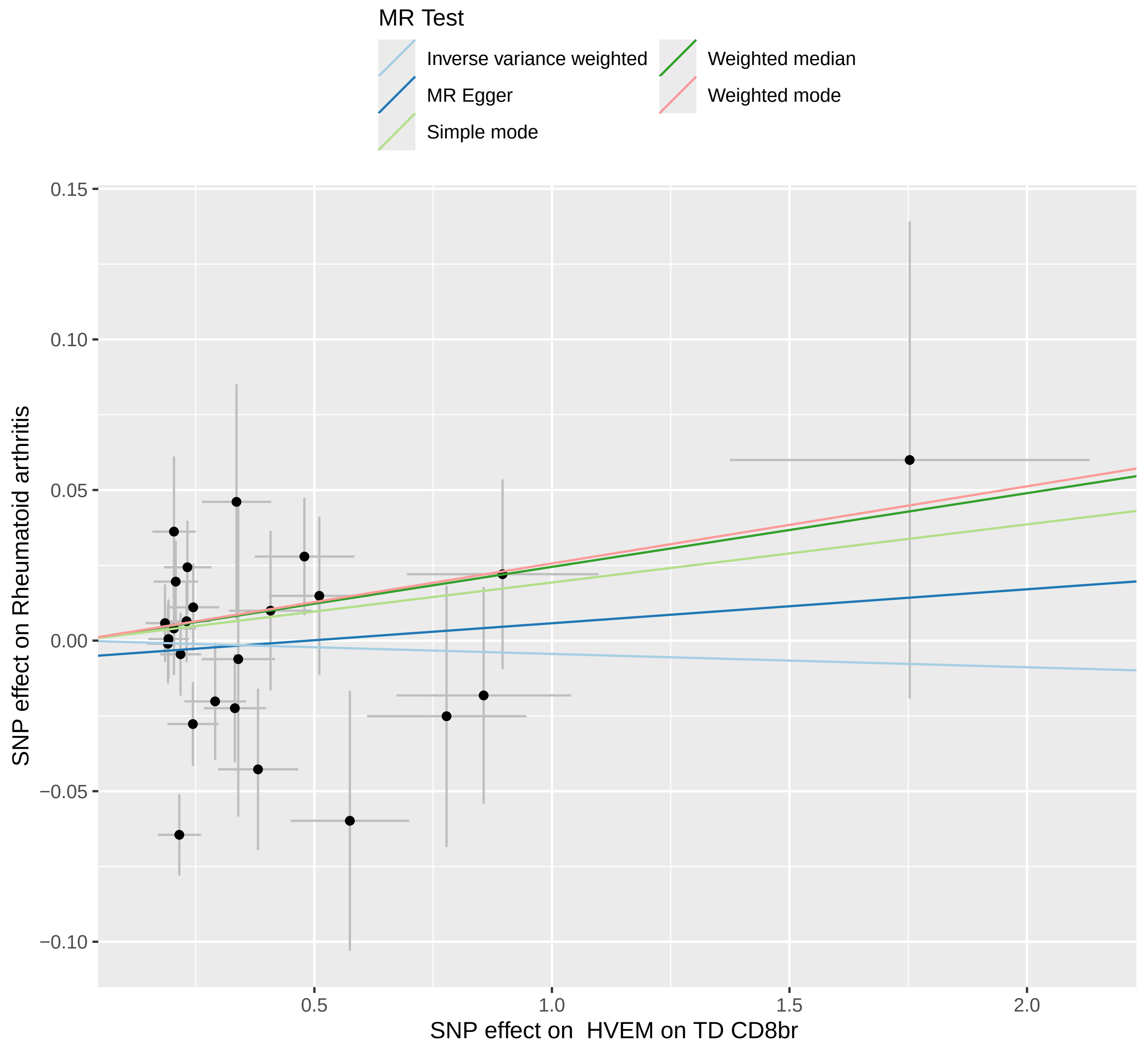
MR Test
Inverse variance weighted
MR Egger
Simple mode
Weighted median
Weighted mode
0.15
0.10
0.05
0.00
−0.05
−0.10
0.5
1.0
1.5
2.0
SNP effect on Rheumatoid arthritis
SNP effect on HVEM on TD CD8br

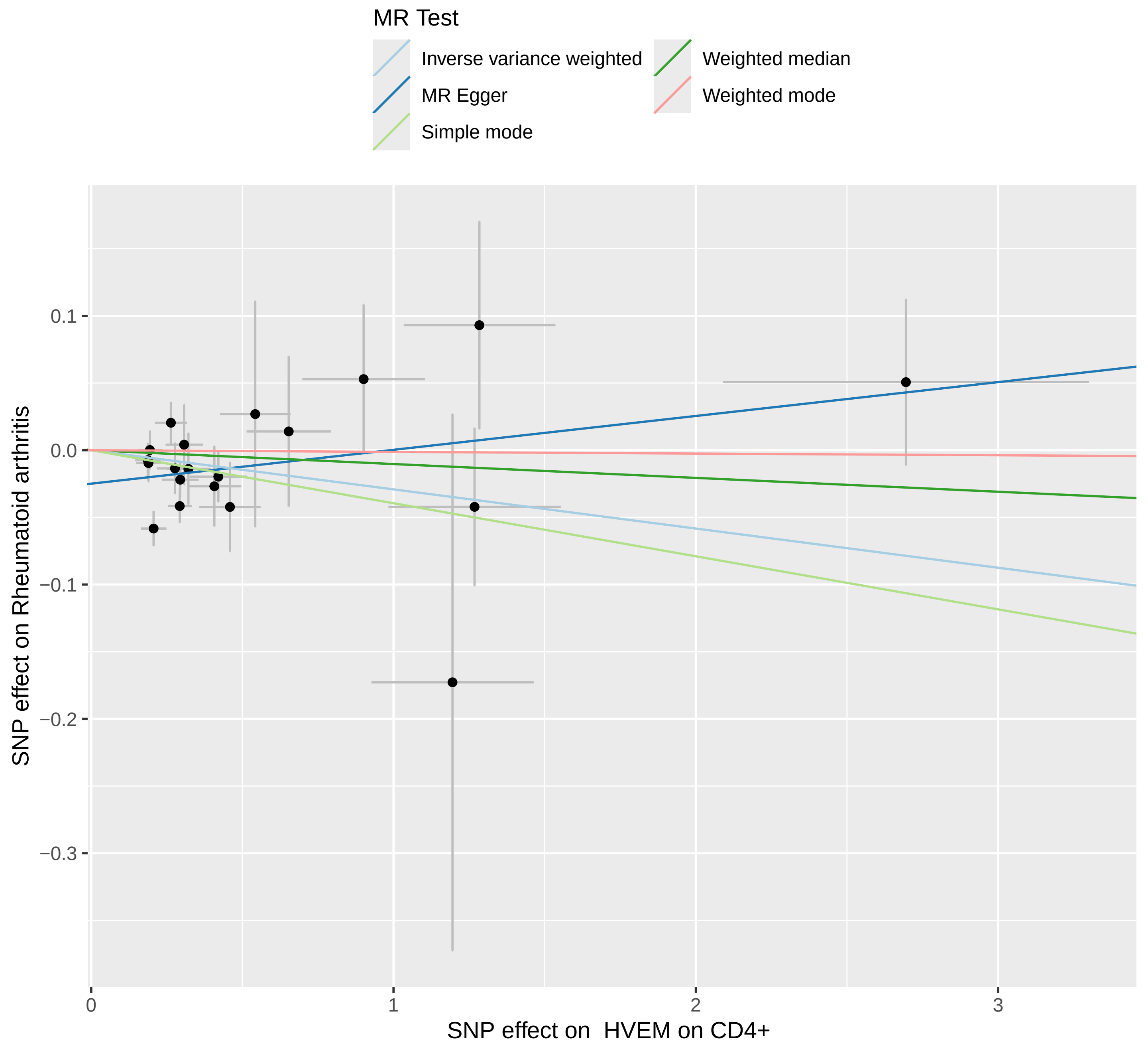
MR Test
Inverse variance weighted
MR Egger
Simple mode
Weighted median
Weighted mode
0.1
0.0
−0.1
−0.2
−0.3
SNP effect on Rheumatoid arthritis
0
1
2
3
SNP effect on HVEM on CD4+

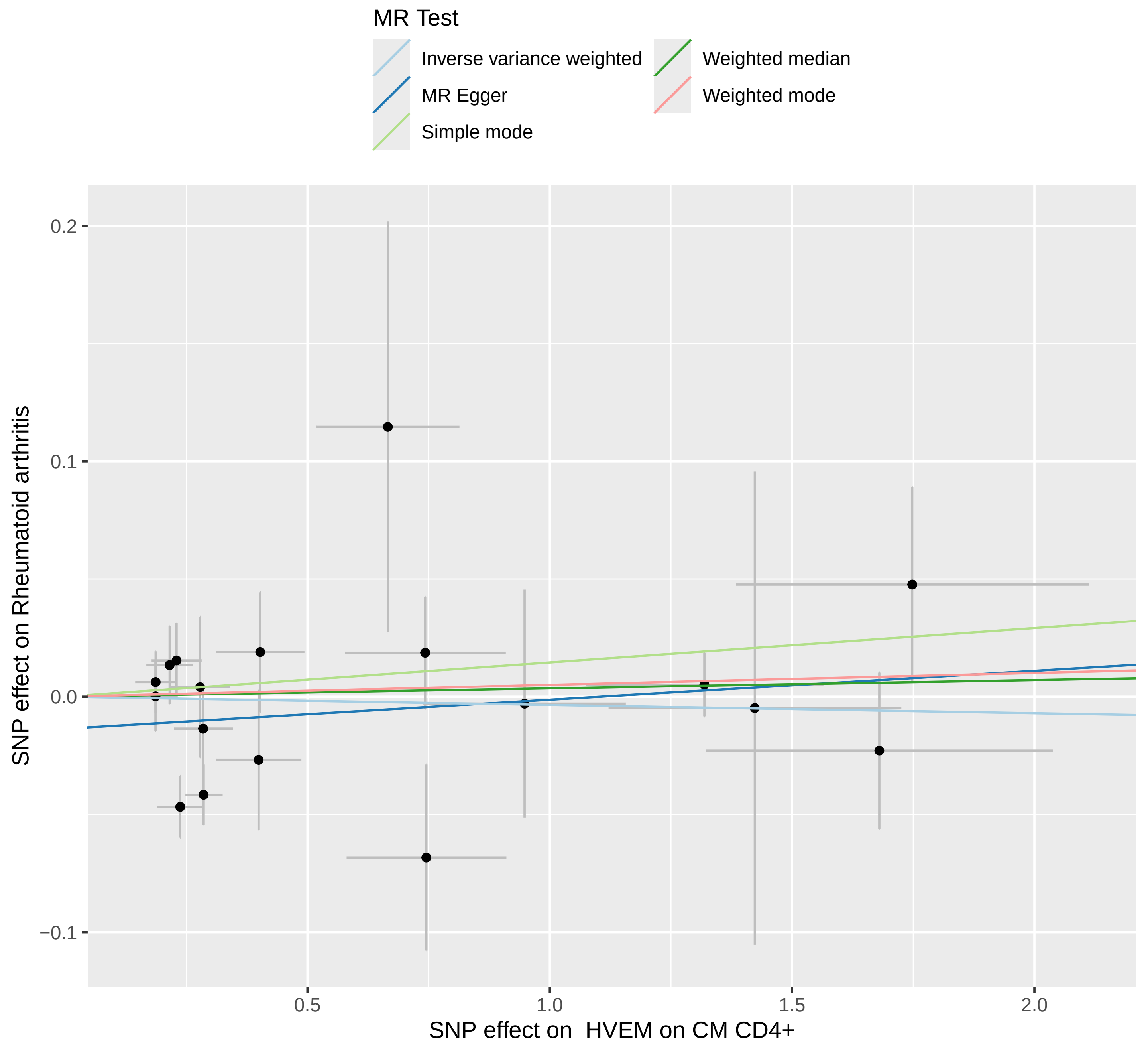

MR Test
Inverse variance weighted
MR Egger
Simple mode
Weighted median
Weighted mode
0.2
0.1
0.0
−0.1
0.5
1.0
1.5
2.0
SNP effect on Rheumatoid arthritis
SNP effect on HVEM on CM CD4+

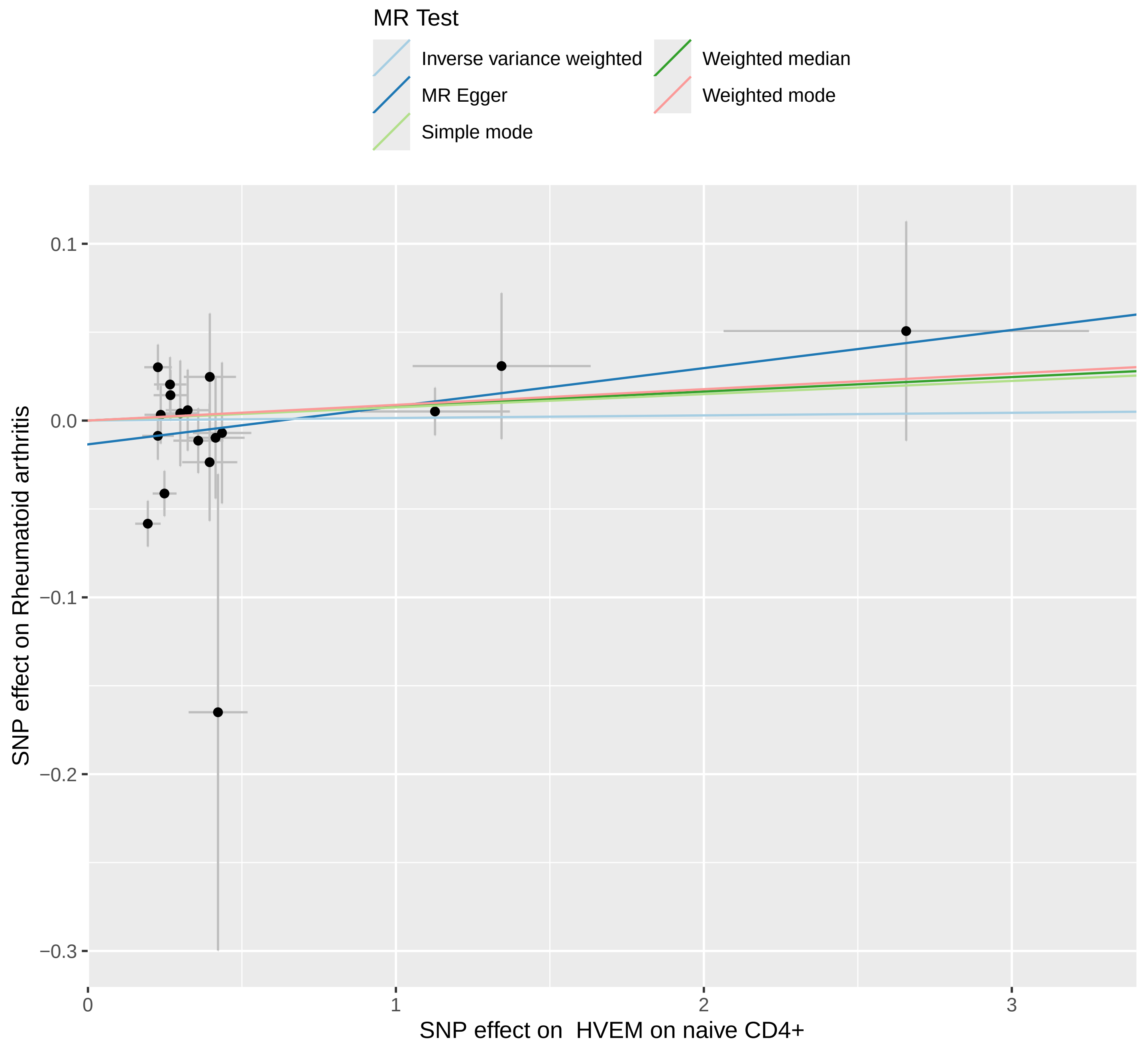

MR Test
Inverse variance weighted
MR Egger
Simple mode
Weighted median
Weighted mode
0.1
0.0
−0.1
−0.2
−0.3
SNP effect on Rheumatoid arthritis
0
1
2
3
SNP effect on HVEM on naive CD4+

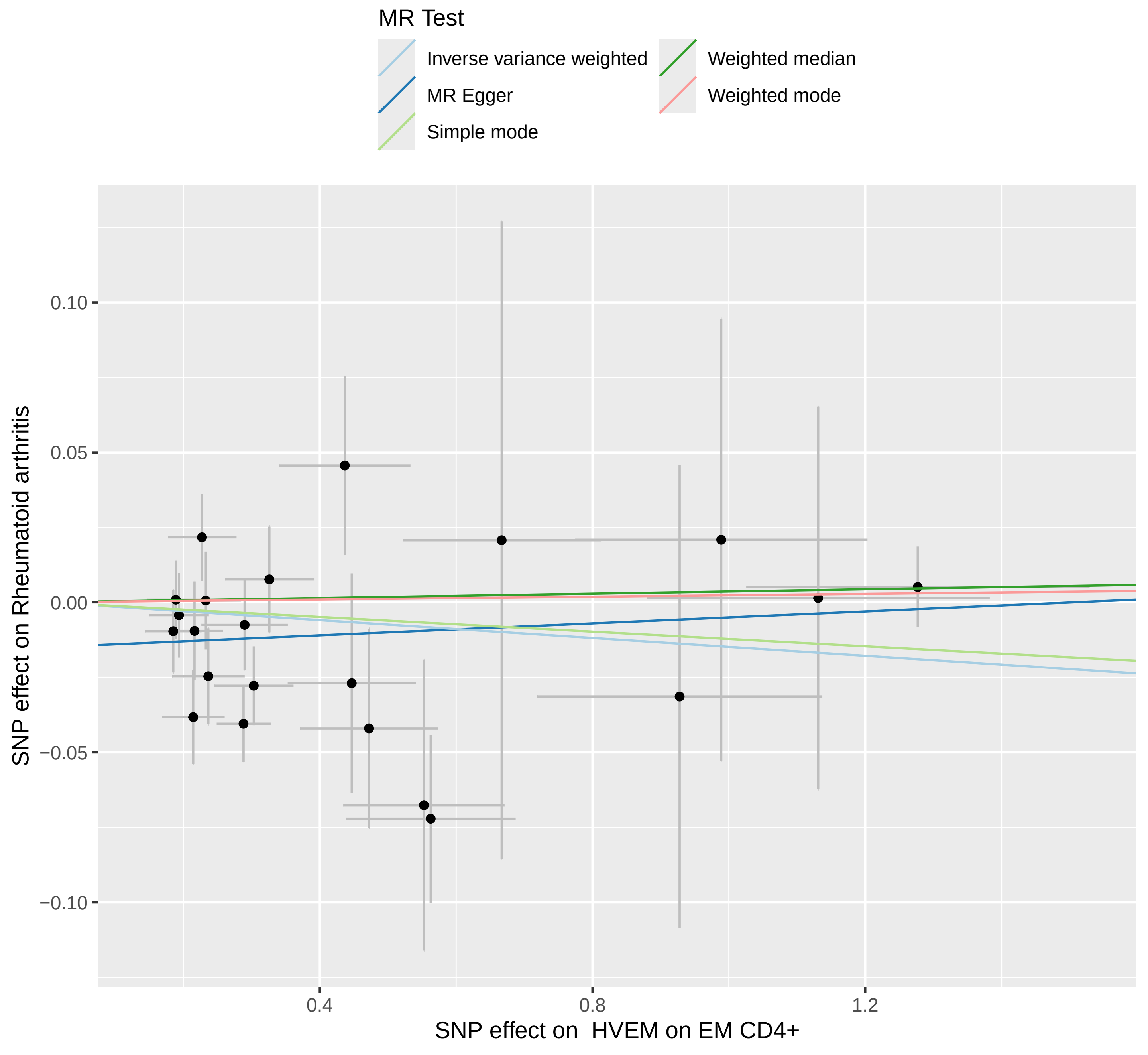
MR Test
Inverse variance weighted
MR Egger
Simple mode
Weighted median
Weighted mode
SNP effect on Rheumatoid arthritis
0.10
0.05
0.00
−0.05
−0.10
0.4
0.8
1.2
SNP effect on HVEM on EM CD4+

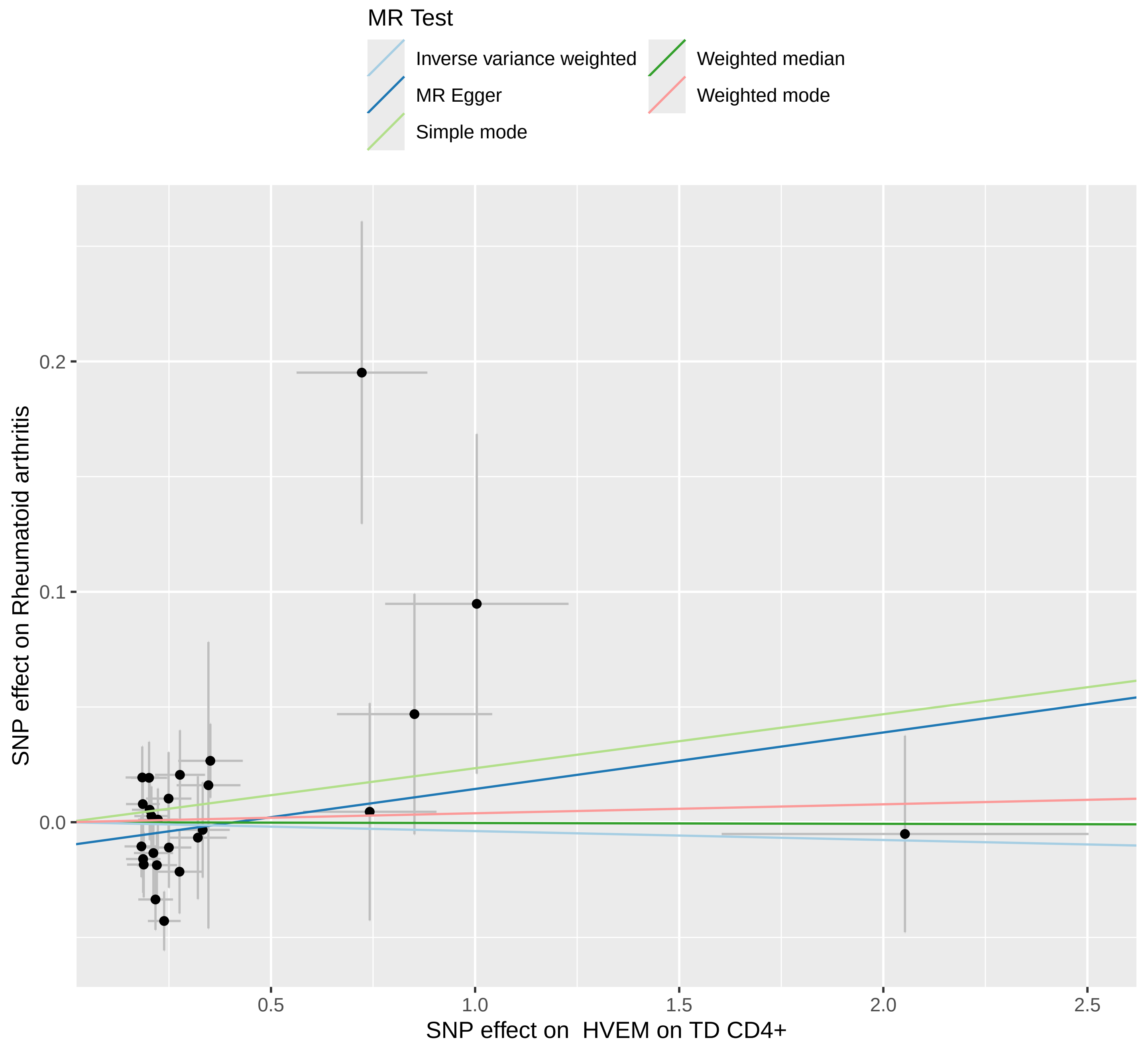

MR Test
Inverse variance weighted
MR Egger
Simple mode
Weighted median
Weighted mode
0.2
0.1
0.0
0.5
1.0
1.5
2.0
2.5
SNP effect on Rheumatoid arthritis
SNP effect on HVEM on TD CD4+

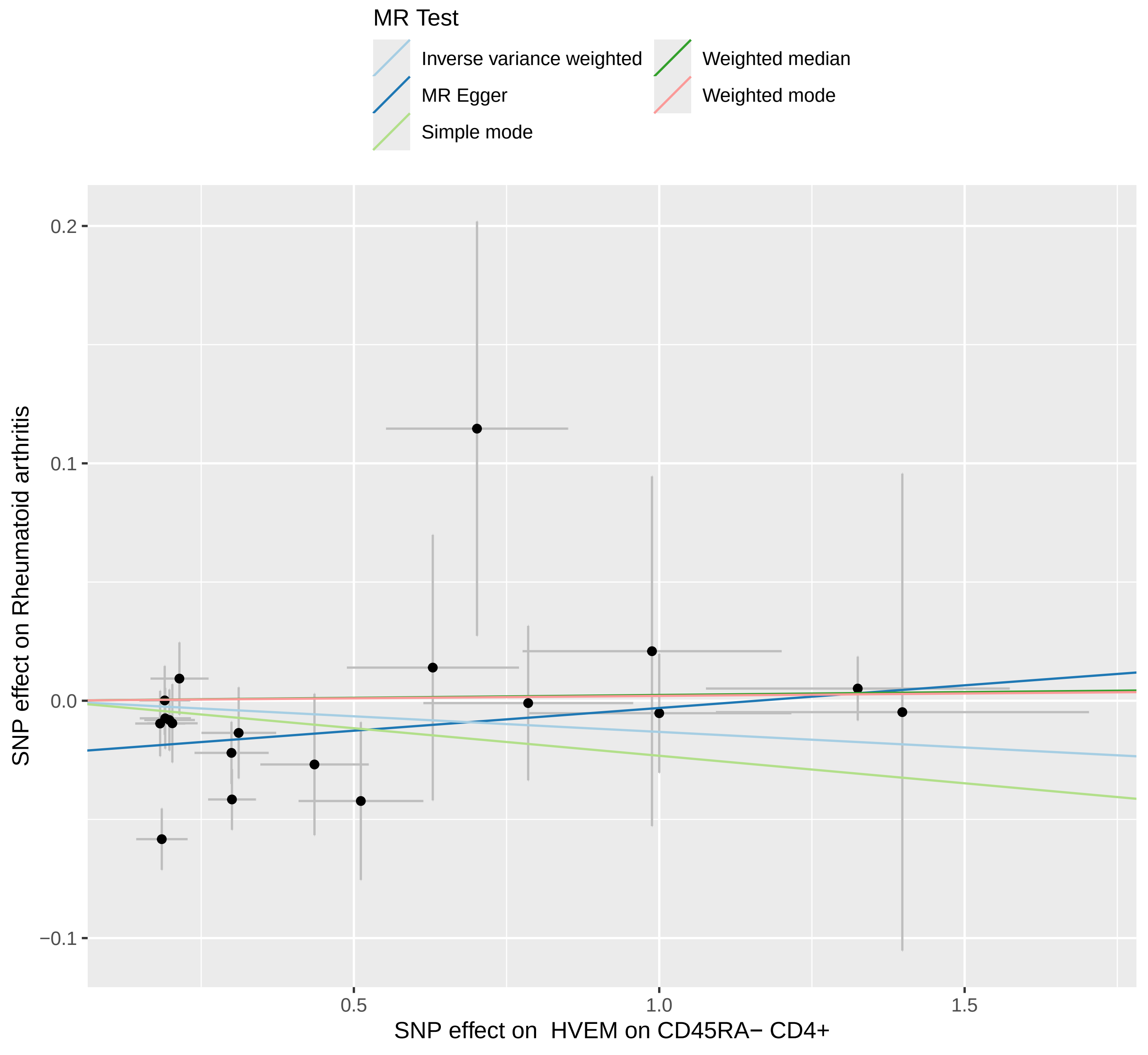

MR Test
Inverse variance weighted
MR Egger
Simple mode
Weighted median
Weighted mode
0.2
0.1
0.0
−0.1
0.5
1.0
1.5
SNP effect on Rheumatoid arthritis
SNP effect on HVEM on CD45RA− CD4+

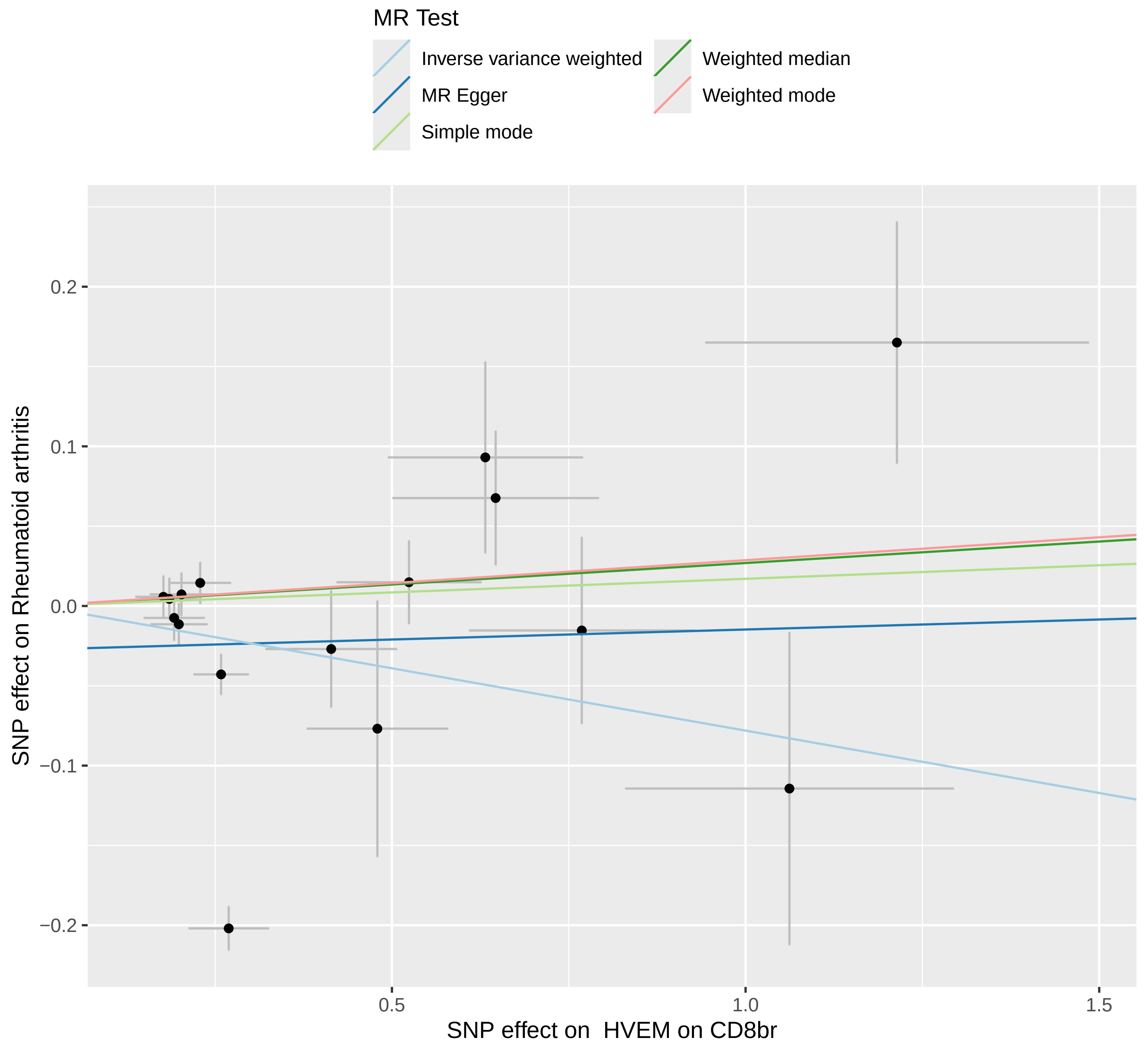
MR Test
Inverse variance weighted
MR Egger
Simple mode
Weighted median
Weighted mode
SNP effect on Rheumatoid arthritis
0.2
0.1
0.0
−0.1
−0.2
0.5
1.0
1.5
SNP effect on HVEM on CD8br

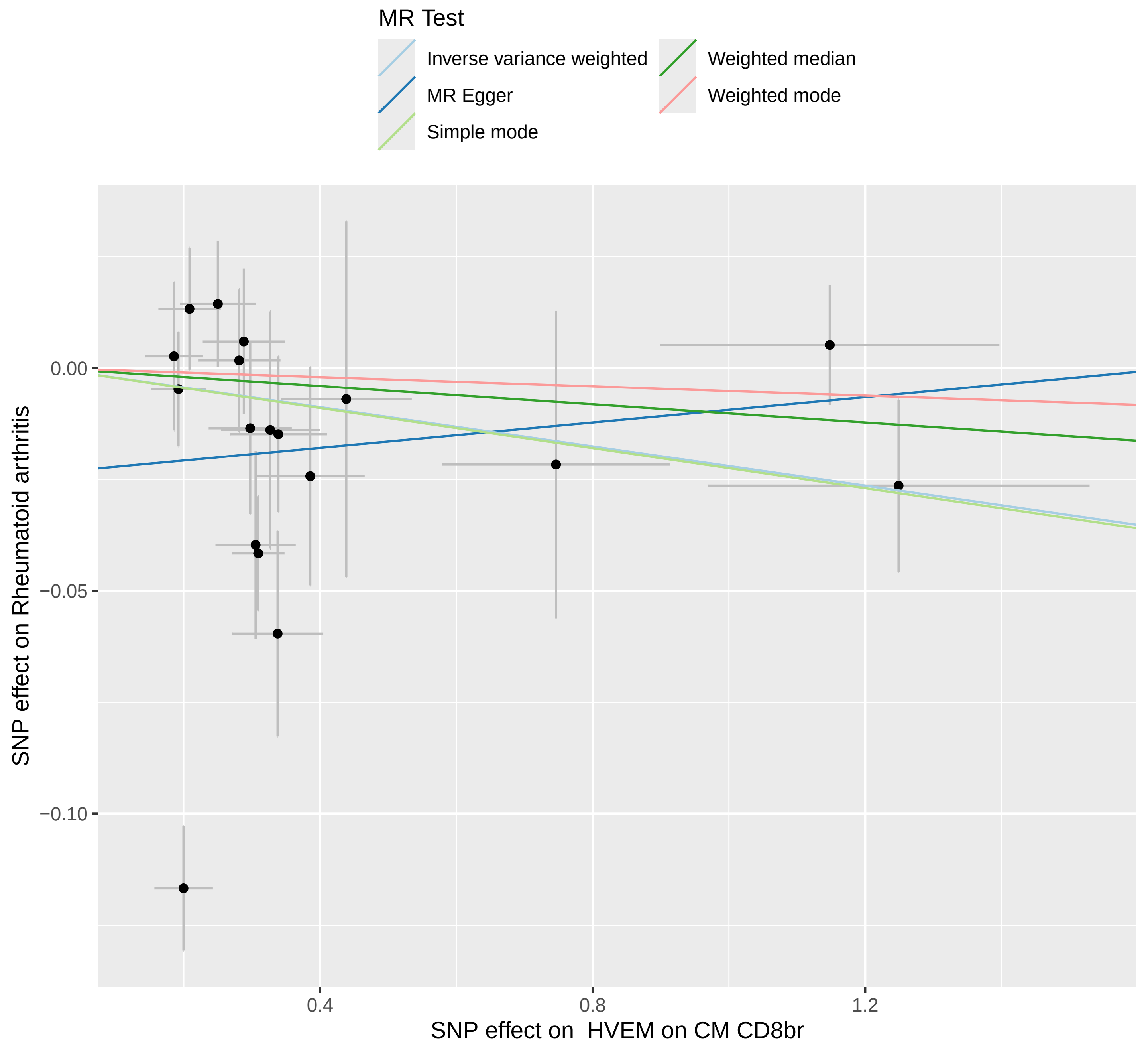
MR Test
Inverse variance weighted
MR Egger
Simple mode
Weighted median
Weighted mode
0.00
−0.05
−0.10
0.4
0.8
1.2
SNP effect on HVEM on CM CD8br
SNP effect on Rheumatoid arthritis

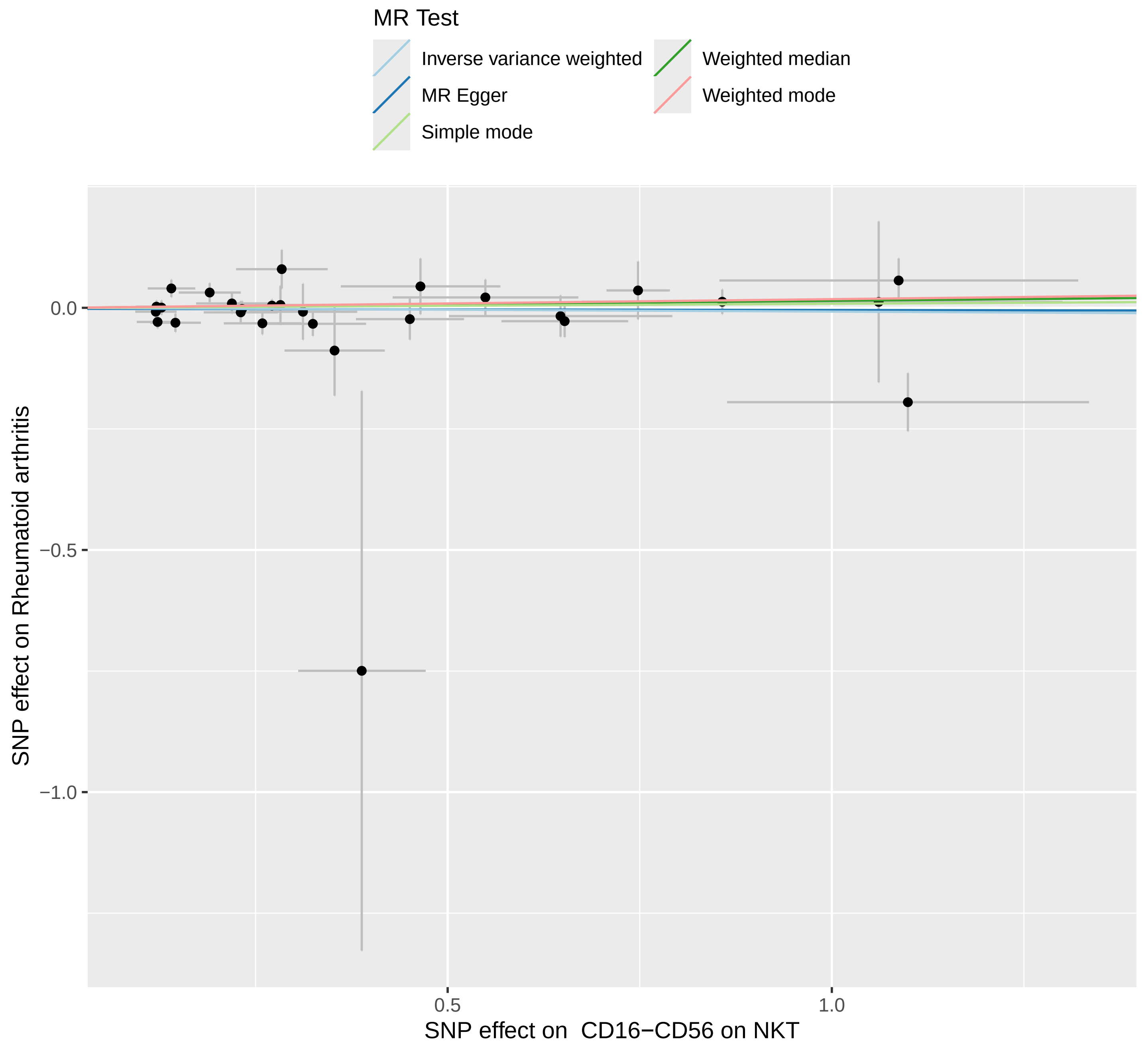

MR Test
Inverse variance weighted
MR Egger
Simple mode
Weighted median
Weighted mode
SNP effect on Rheumatoid arthritis
0.0
−0.5
−1.0
0.5
1.0
SNP effect on CD16−CD56 on NKT

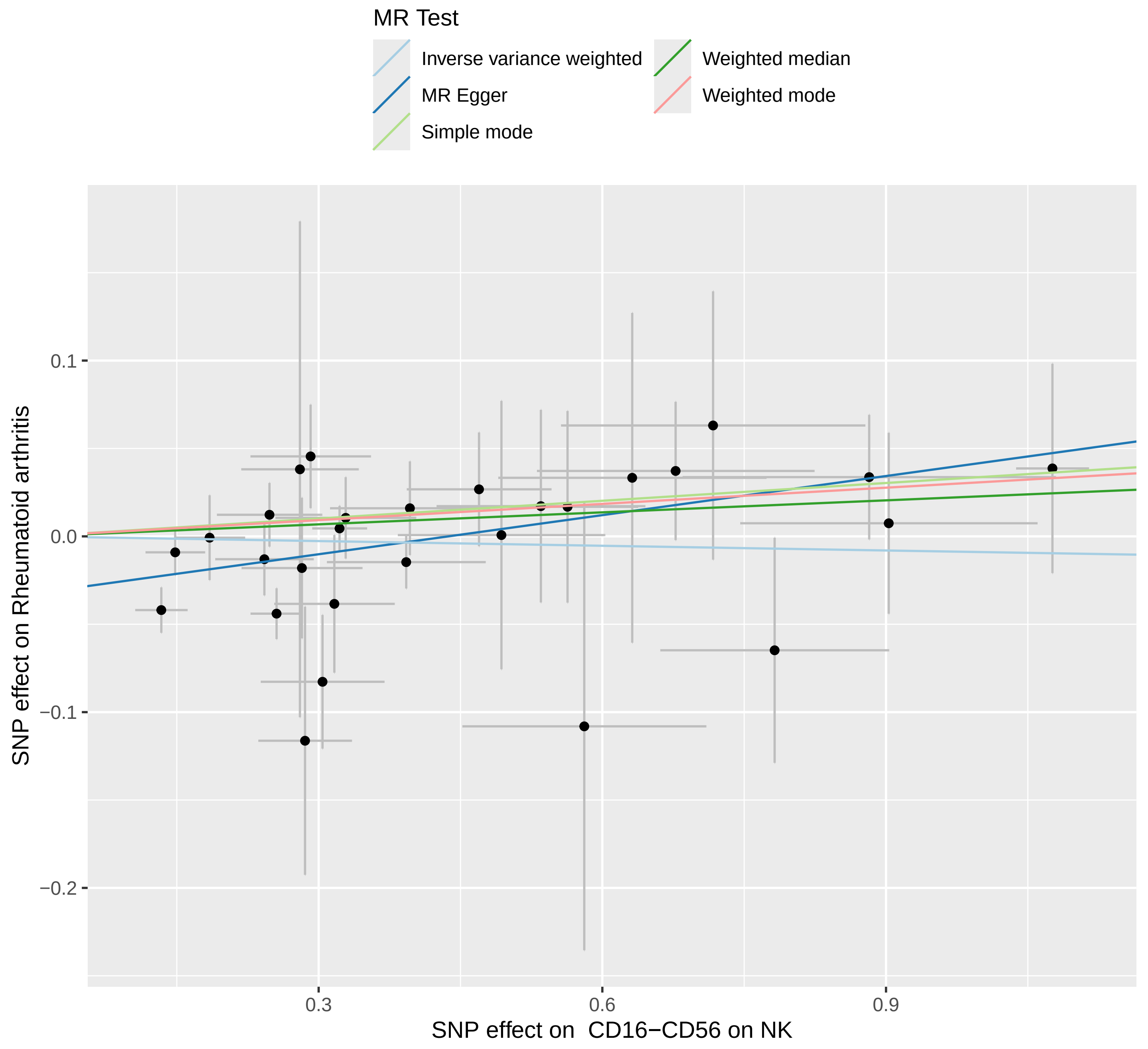
MR Test
Inverse variance weighted
MR Egger
Simple mode
Weighted median
Weighted mode
0.1
0.0
−0.1
−0.2
SNP effect on Rheumatoid arthritis
0.3
0.6
0.9
SNP effect on CD16−CD56 on NK

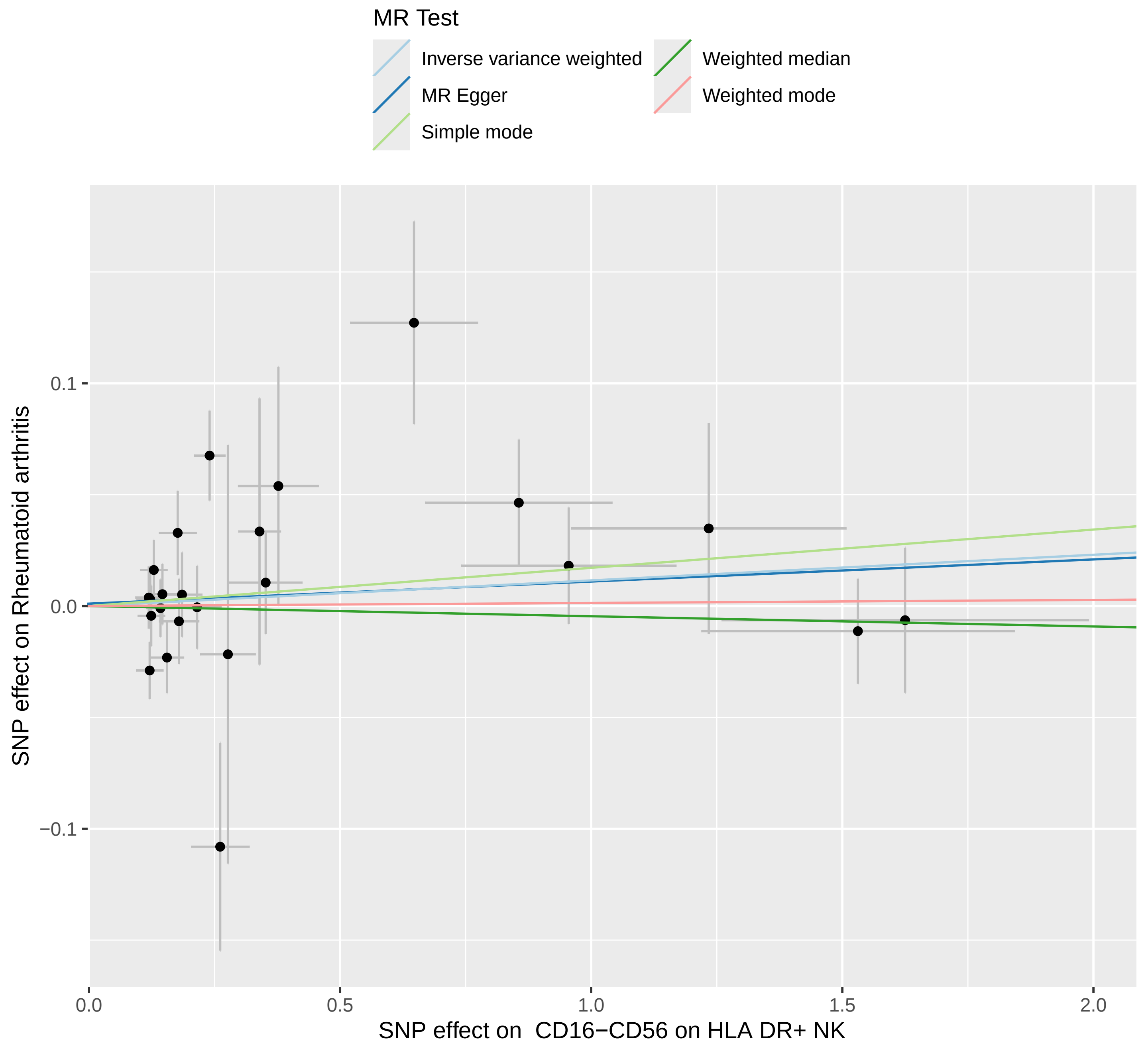
MR Test
Inverse variance weighted
MR Egger
Simple mode
Weighted median
Weighted mode
0.1
0.0
−0.1
SNP effect on Rheumatoid arthritis
0.0
0.5
1.0
1.5
2.0
SNP effect on CD16−CD56 on HLA DR+ NK

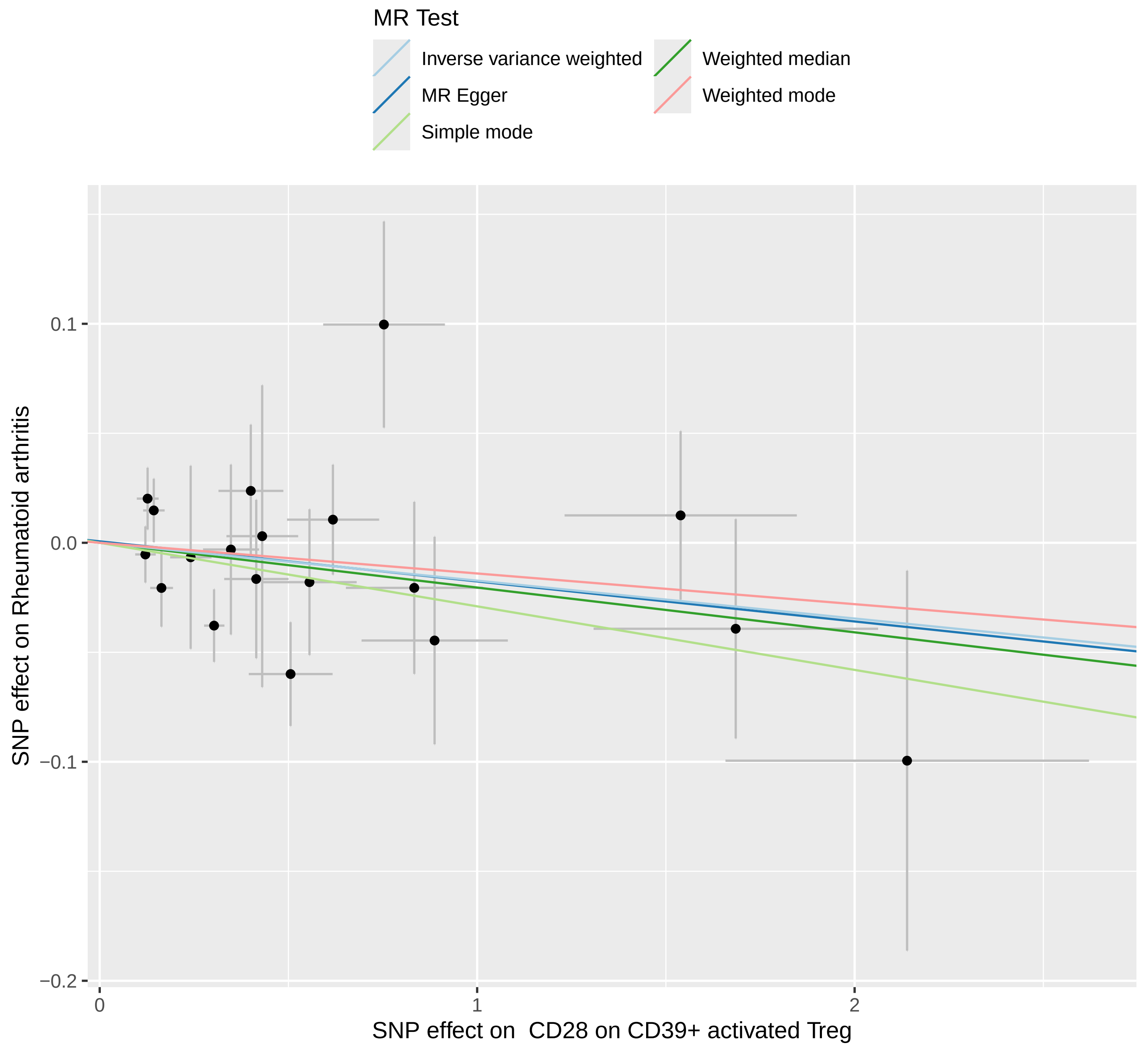
MR Test
Inverse variance weighted
MR Egger
Simple mode
Weighted median
Weighted mode
0.1
0.0
−0.1
−0.2
0
1
2
SNP effect on Rheumatoid arthritis
SNP effect on CD28 on CD39+ activated Treg

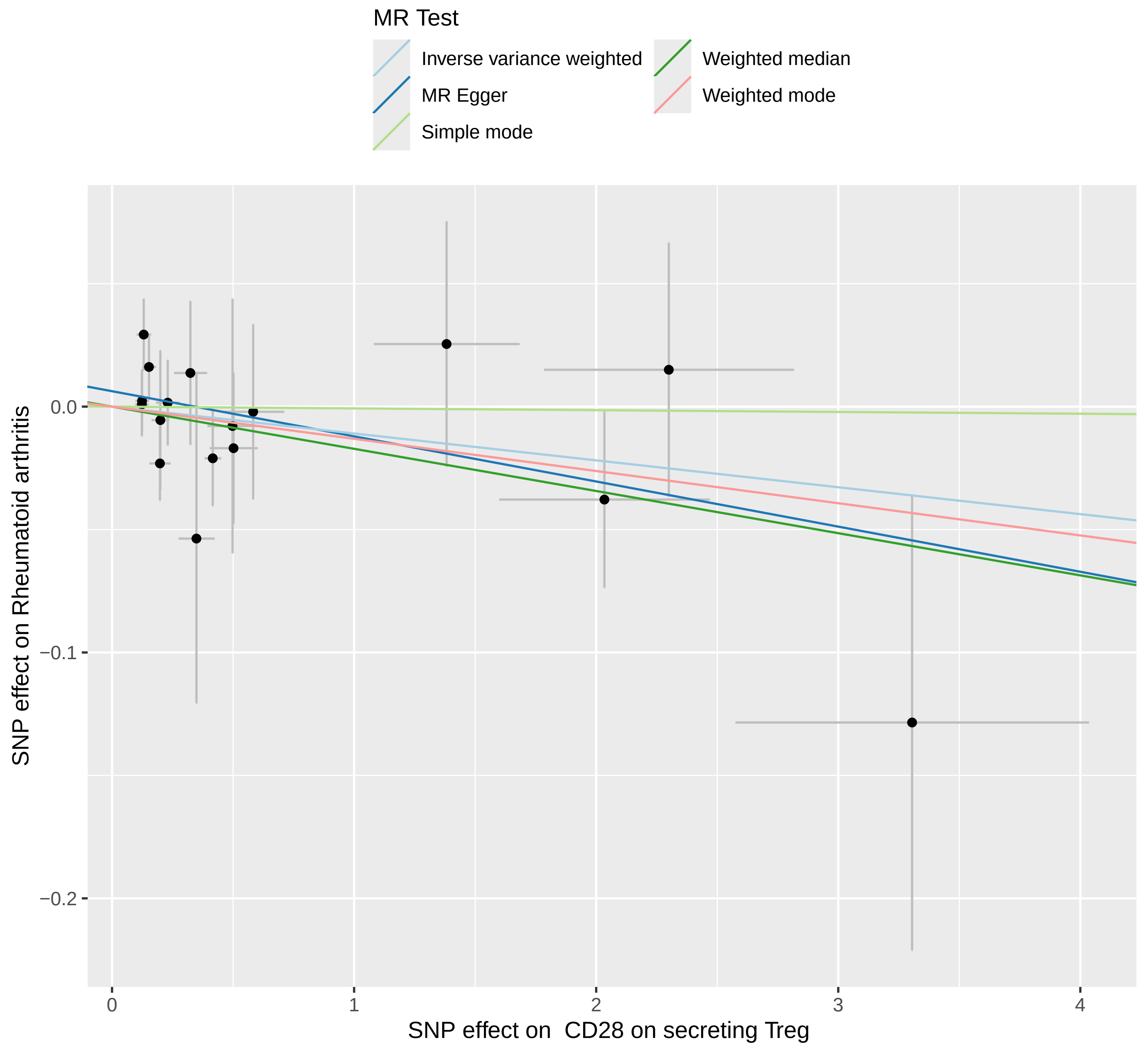
MR Test
Inverse variance weighted
MR Egger
Simple mode
Weighted median
Weighted mode
SNP effect on Rheumatoid arthritis
0.0
−0.1
−0.2
0
1
2
3
4
SNP effect on CD28 on secreting Treg

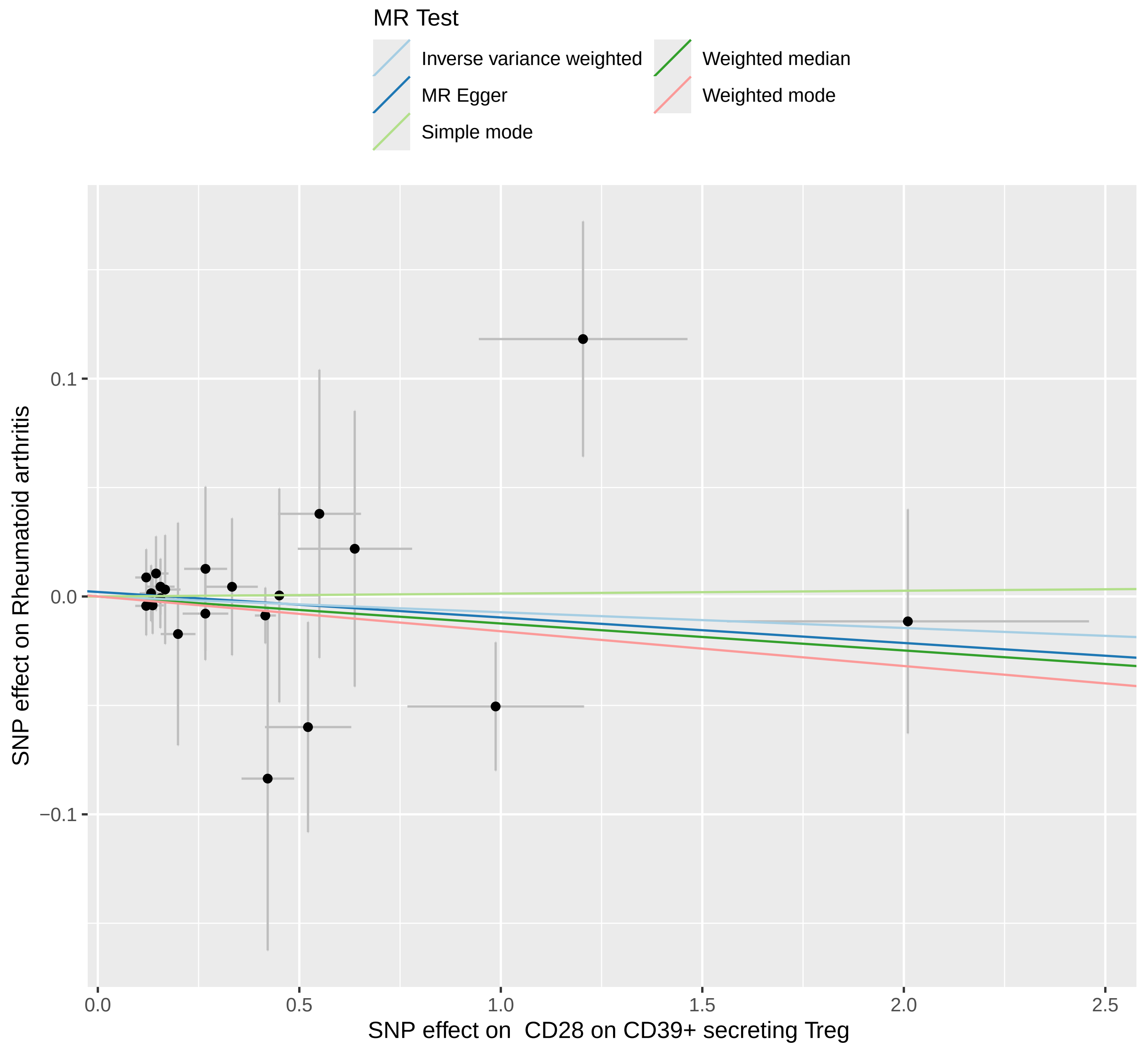
MR Test
Inverse variance weighted
MR Egger
Simple mode
Weighted median
Weighted mode
0.1
0.0
−0.1
0.0
0.5
1.0
1.5
2.0
2.5
SNP effect on Rheumatoid arthritis
SNP effect on CD28 on CD39+ secreting Treg

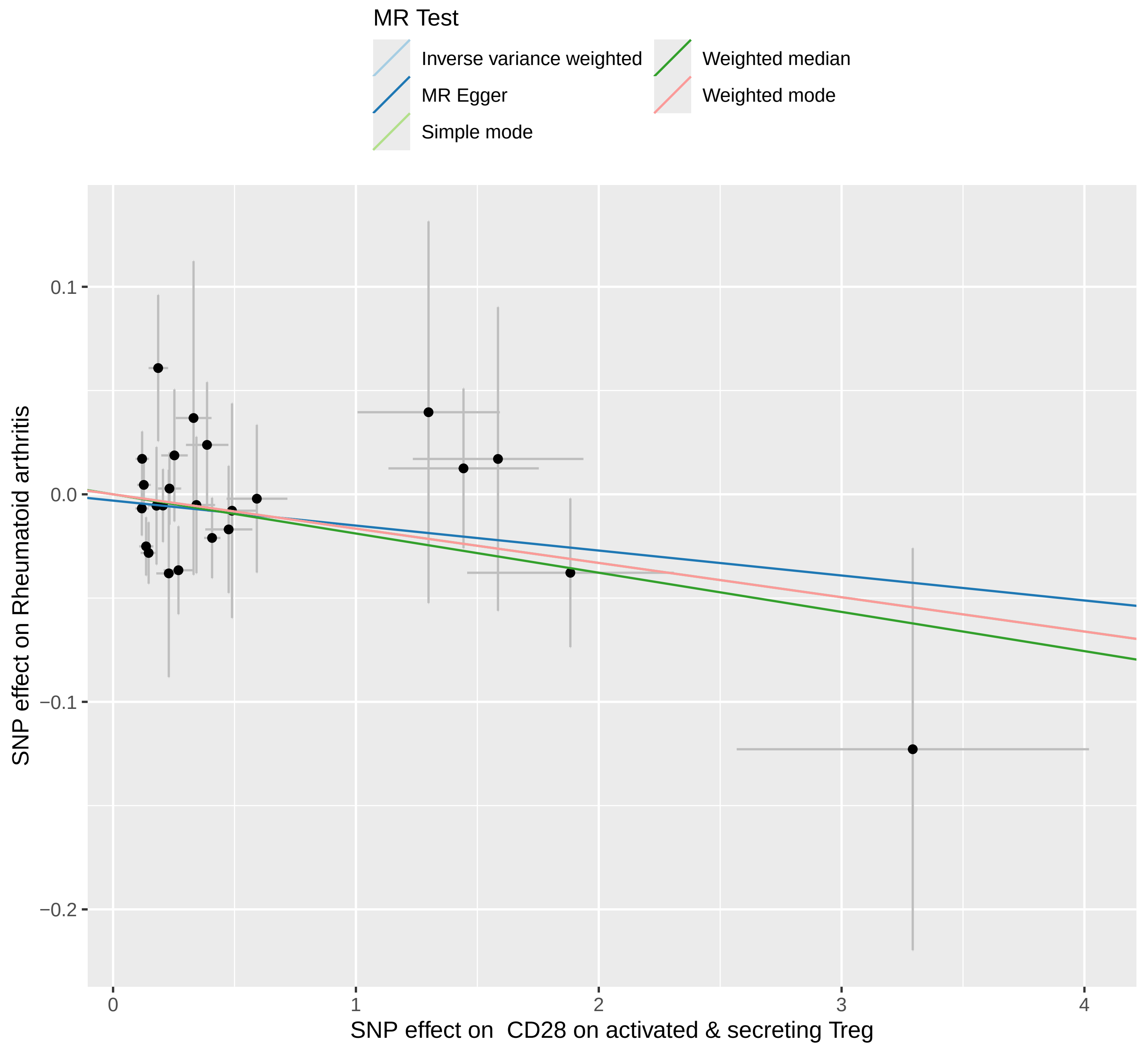

MR Test
Inverse variance weighted
MR Egger
Simple mode
Weighted median
Weighted mode
0.1
0.0
−0.1
−0.2
0
1
2
3
4
SNP effect on Rheumatoid arthritis
SNP effect on CD28 on activated & secreting Treg

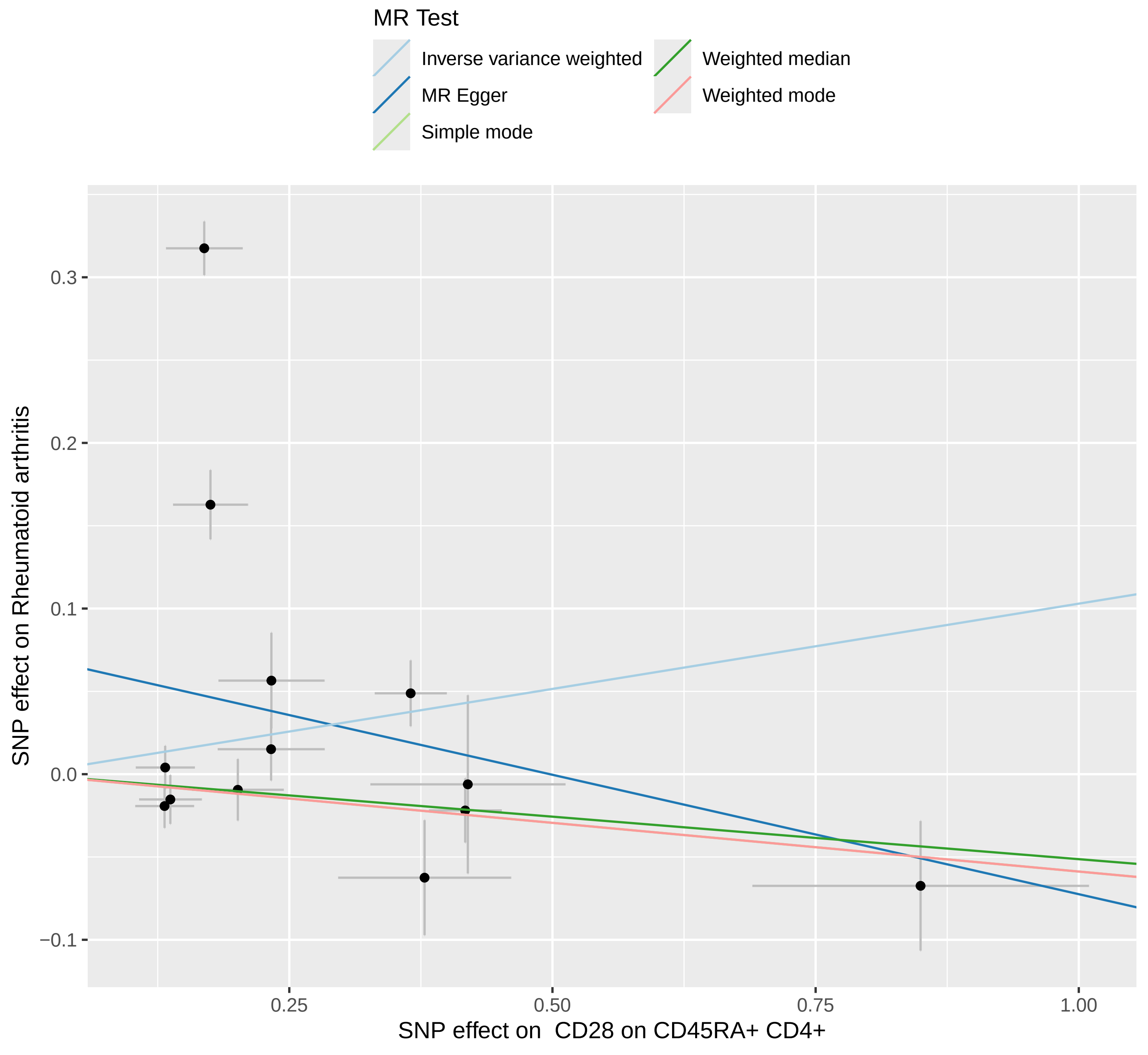

MR Test
Inverse variance weighted
MR Egger
Simple mode
Weighted median
Weighted mode
0.3
0.2
0.1
0.0
−0.1
0.25
0.50
0.75
1.00
SNP effect on Rheumatoid arthritis
SNP effect on CD28 on CD45RA+ CD4+

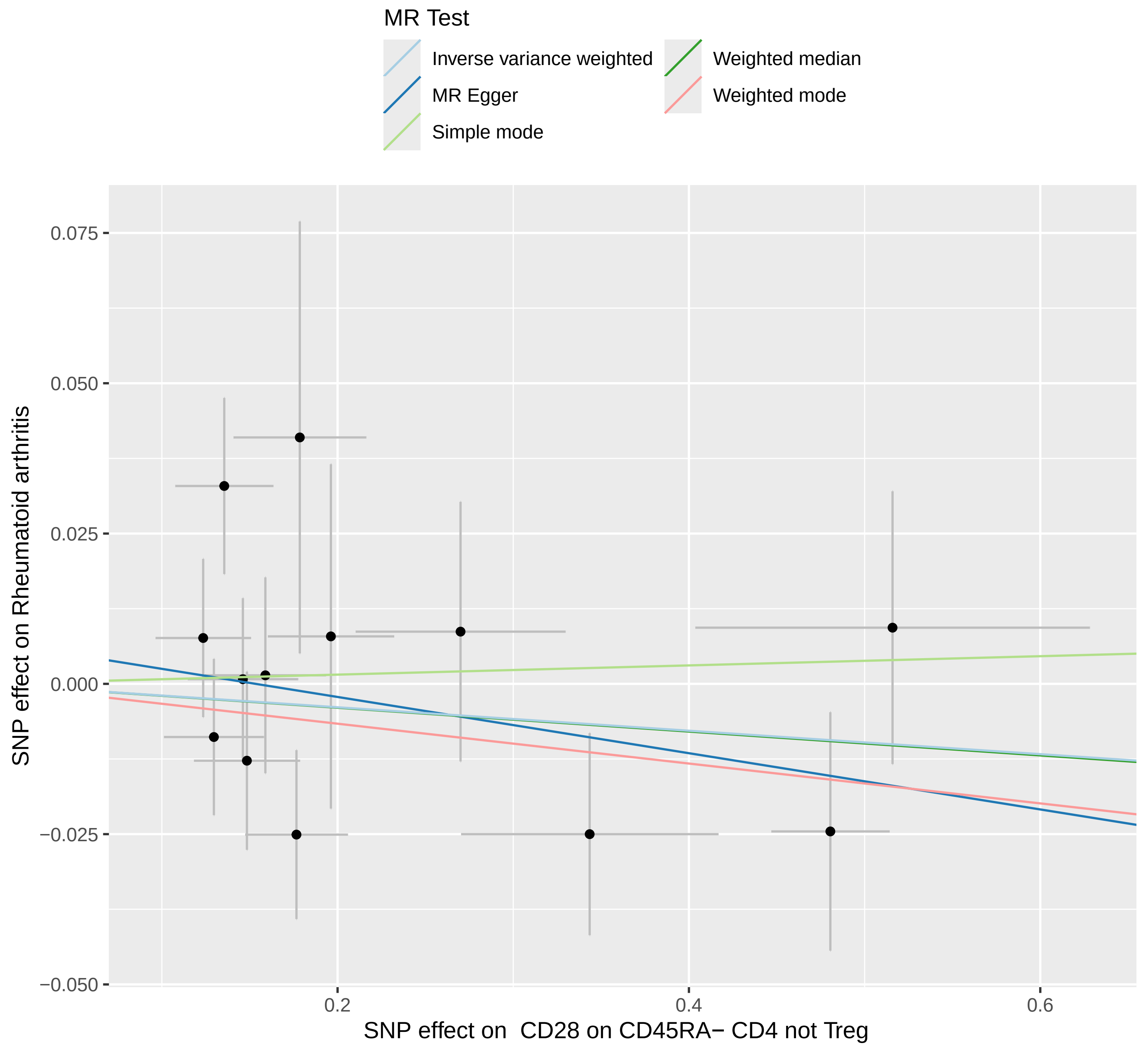
MR Test
Inverse variance weighted
MR Egger
Simple mode
Weighted median
Weighted mode
0.075
0.050
0.025
0.000
−0.025
−0.050
0.2
0.4
0.6
SNP effect on Rheumatoid arthritis
SNP effect on CD28 on CD45RA− CD4 not Treg

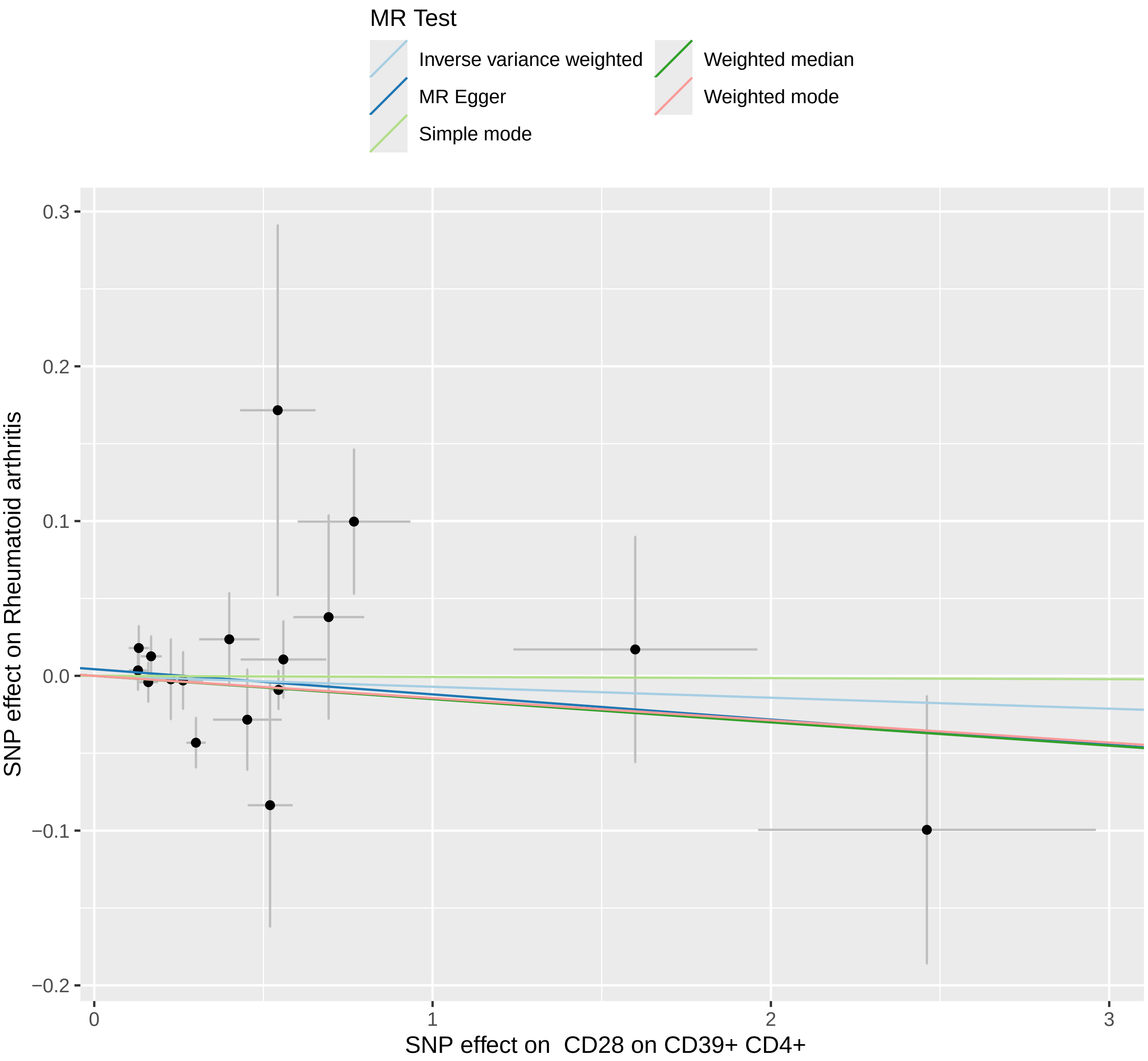
MR Test
Inverse variance weighted
MR Egger
Simple mode
Weighted median
Weighted mode
SNP effect on Rheumatoid arthritis
0.3
0.2
0.1
0.0
−0.1
−0.2
0
1
2
3
SNP effect on CD28 on CD39+ CD4+

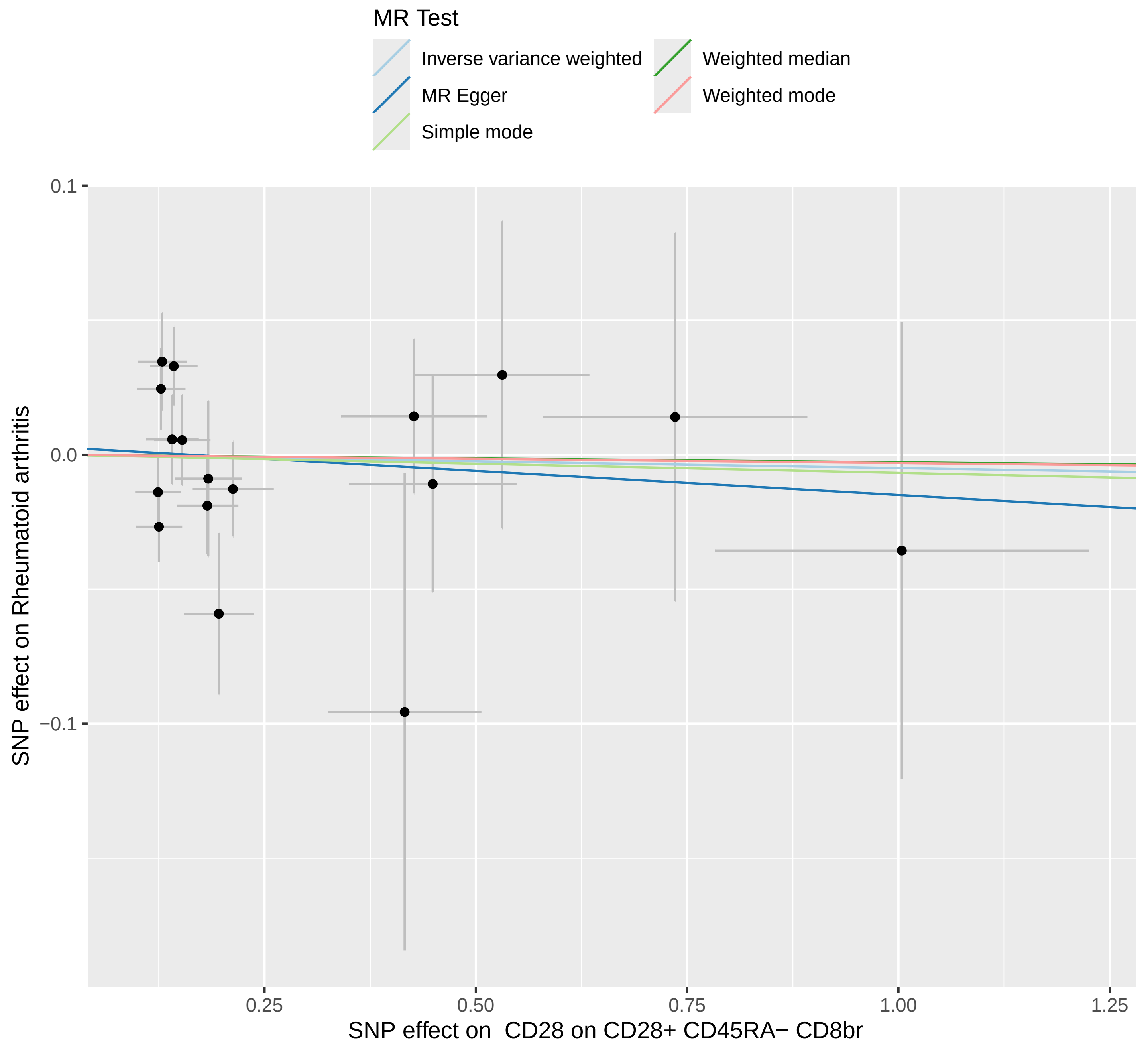

MR Test
Inverse variance weighted
MR Egger
Simple mode
Weighted median
Weighted mode
0.1
0.0
−0.1
SNP effect on Rheumatoid arthritis
0.25
0.50
0.75
1.00
1.25
SNP effect on CD28 on CD28+ CD45RA− CD8br

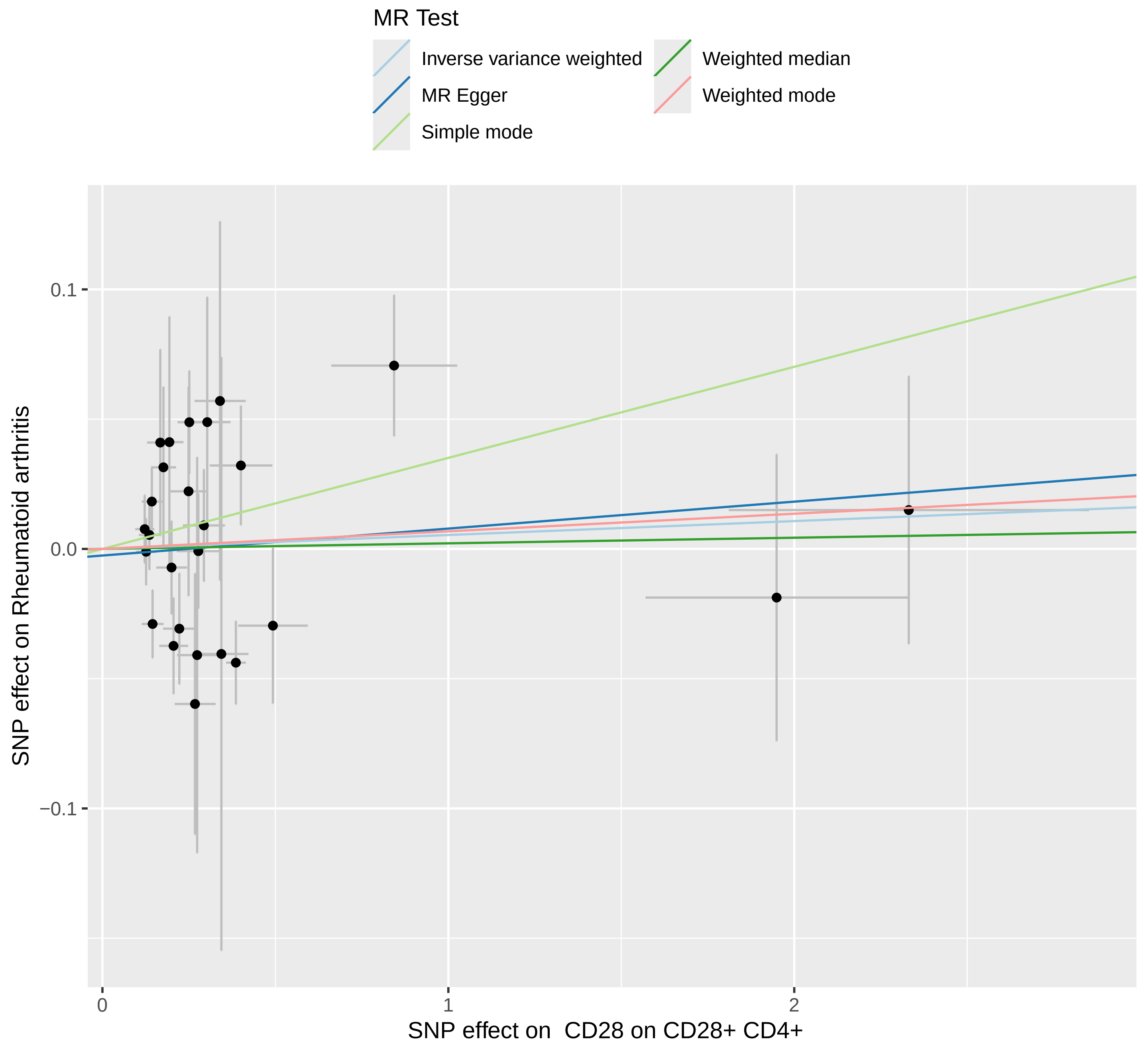
MR Test
Inverse variance weighted
MR Egger
Simple mode
Weighted median
Weighted mode
0.1
0.0
−0.1
0
1
2
SNP effect on Rheumatoid arthritis
SNP effect on CD28 on CD28+ CD4+

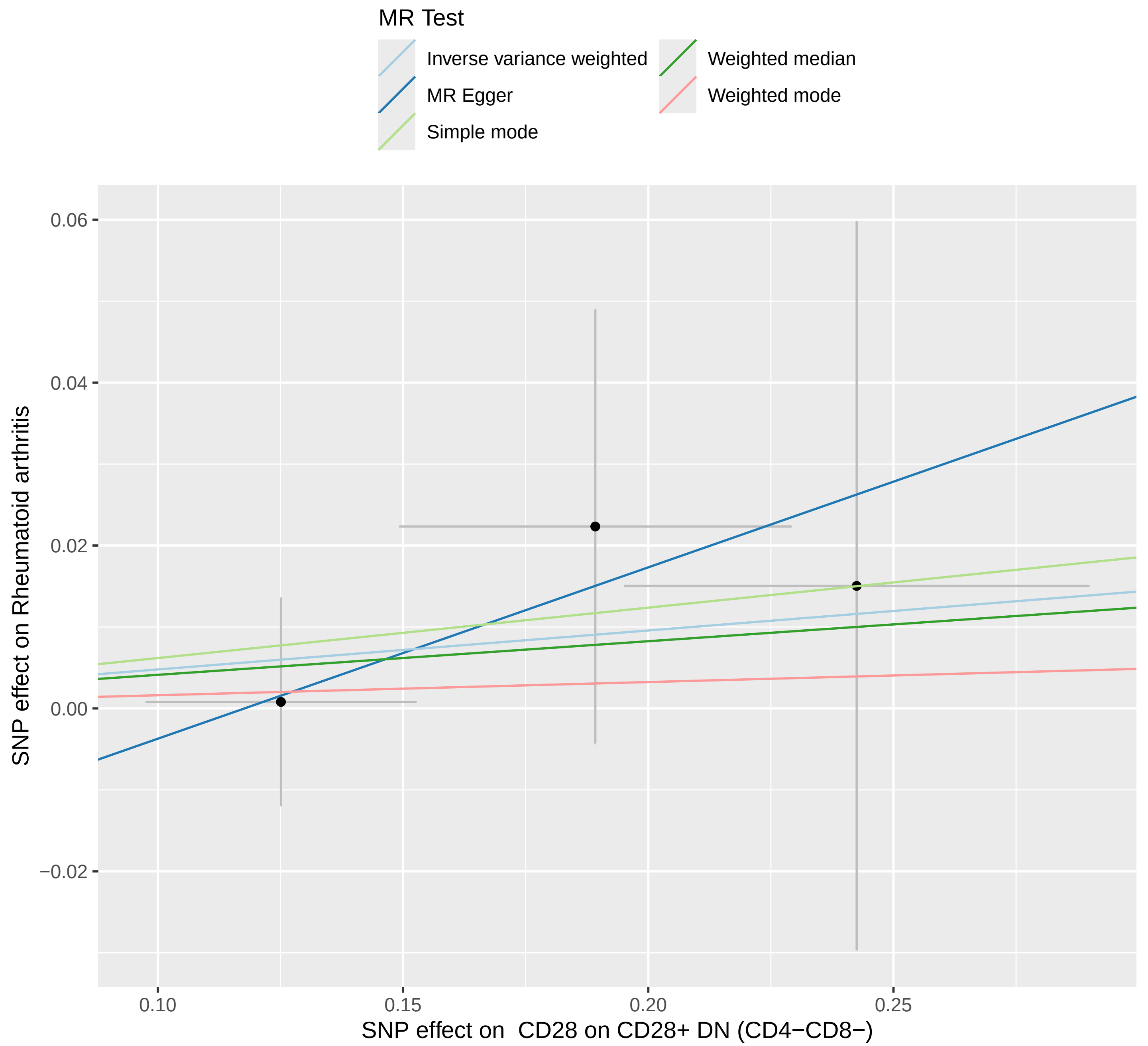
MR Test
Inverse variance weighted
MR Egger
Simple mode
Weighted median
Weighted mode
SNP effect on Rheumatoid arthritis
0.06
0.04
0.02
0.00
−0.02
0.10
0.15
0.20
0.25
SNP effect on CD28 on CD28+ DN (CD4−CD8−)

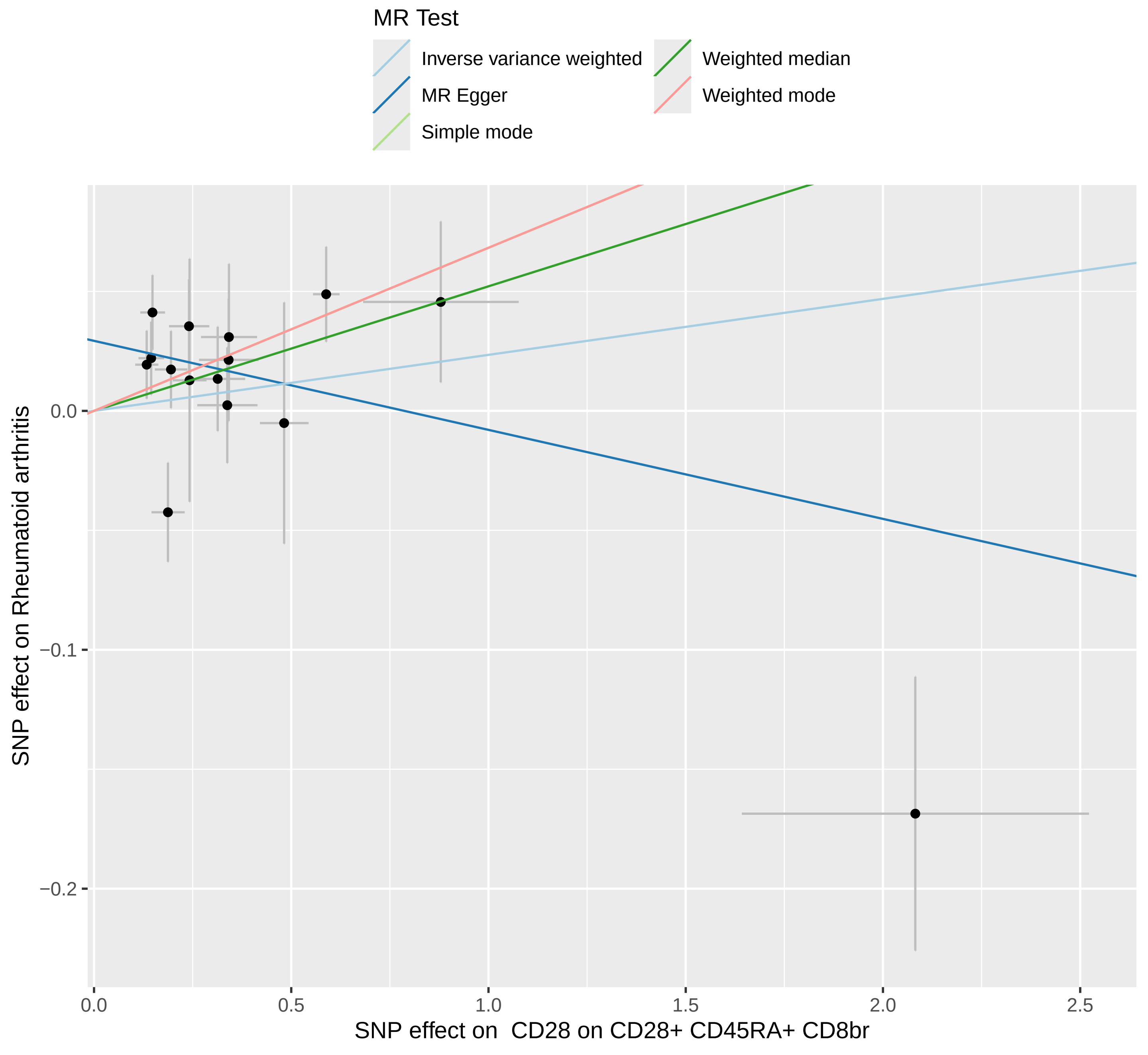
MR Test
Inverse variance weighted
MR Egger
Simple mode
Weighted median
Weighted mode
SNP effect on Rheumatoid arthritis
0.0
−0.1
−0.2
0.0
0.5
1.0
1.5
2.0
2.5
SNP effect on CD28 on CD28+ CD45RA+ CD8br

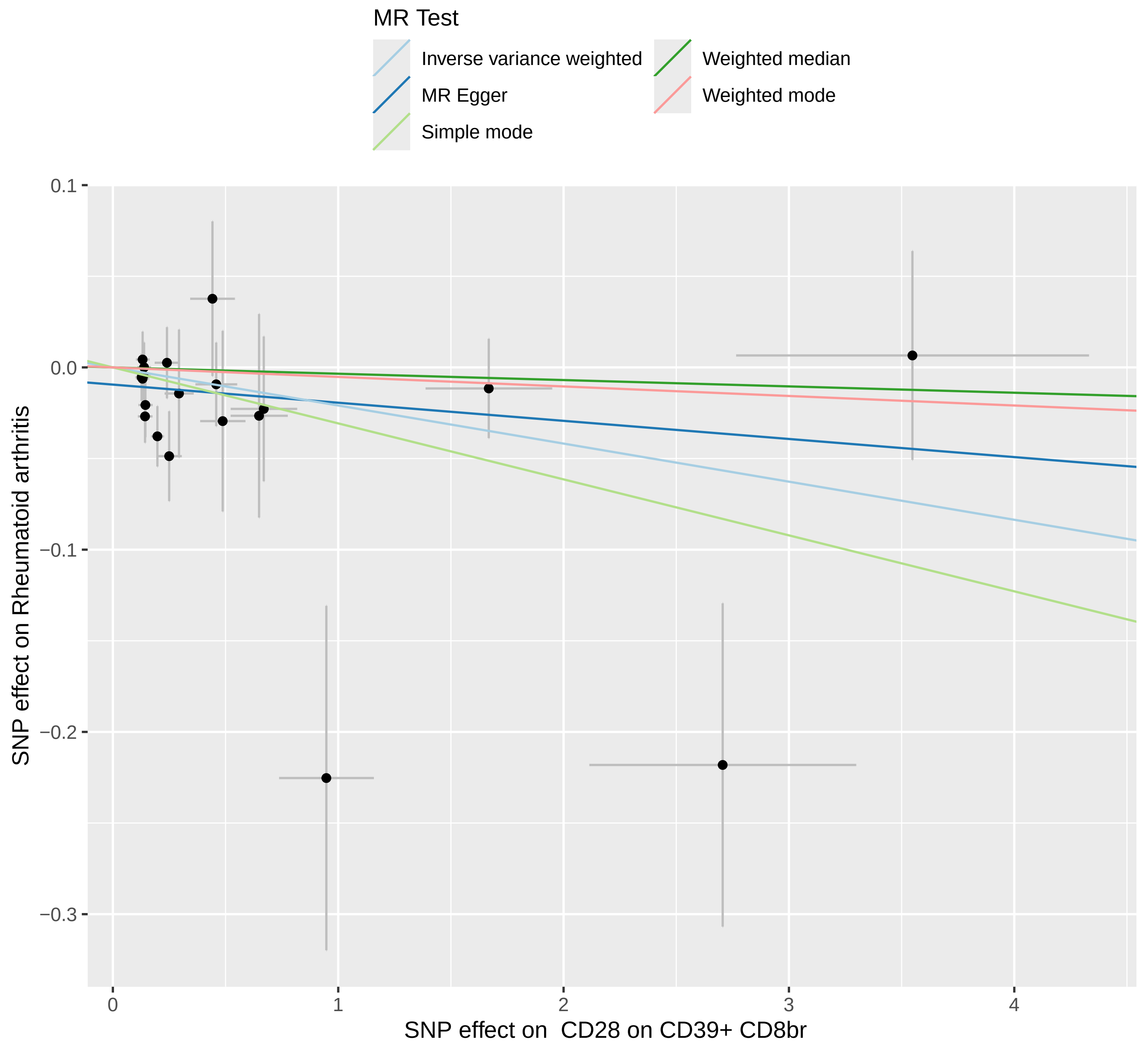
MR Test
Inverse variance weighted
MR Egger
Simple mode
Weighted median
Weighted mode
0.1
0.0
−0.1
−0.2
−0.3
SNP effect on Rheumatoid arthritis
0
1
2
3
4
SNP effect on CD28 on CD39+ CD8br

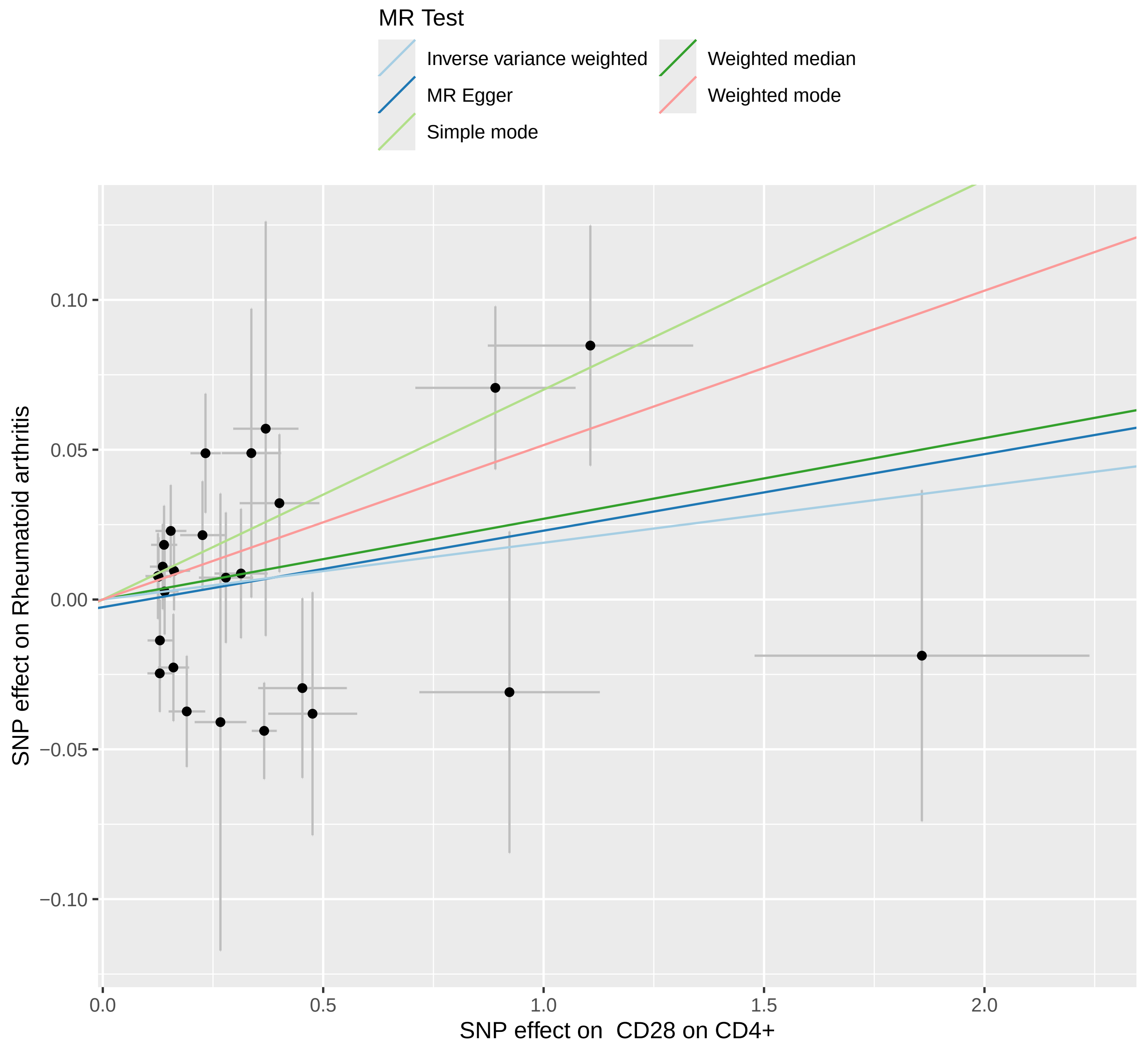

MR Test
Inverse variance weighted
MR Egger
Simple mode
Weighted median
Weighted mode
0.10
0.05
0.00
−0.05
−0.10
0.0
0.5
1.0
1.5
2.0
SNP effect on Rheumatoid arthritis
SNP effect on CD28 on CD4+

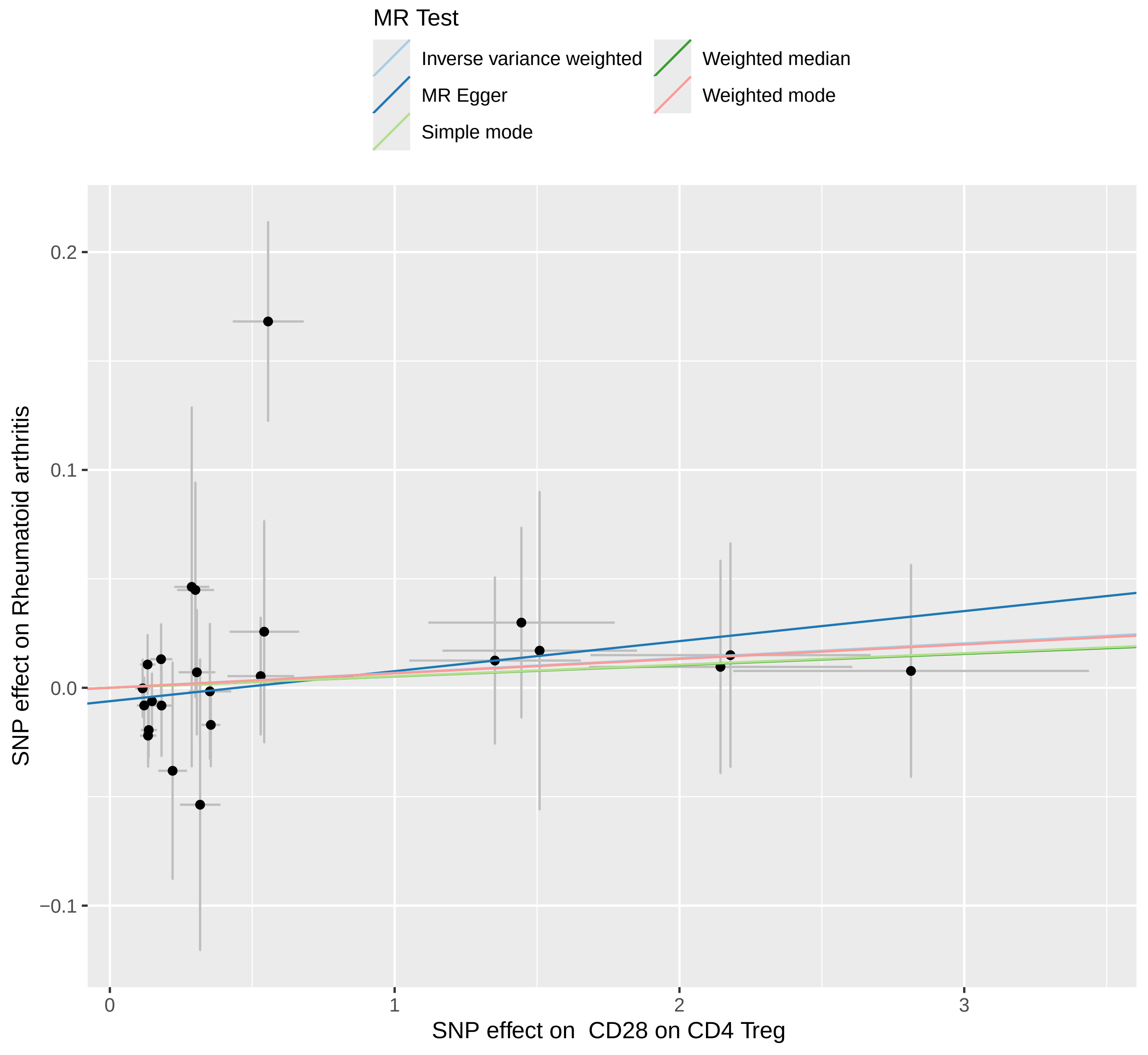

MR Test
Inverse variance weighted
MR Egger
Simple mode
Weighted median
Weighted mode
SNP effect on Rheumatoid arthritis
0.2
0.1
0.0
−0.1
0
1
2
3
SNP effect on CD28 on CD4 Treg

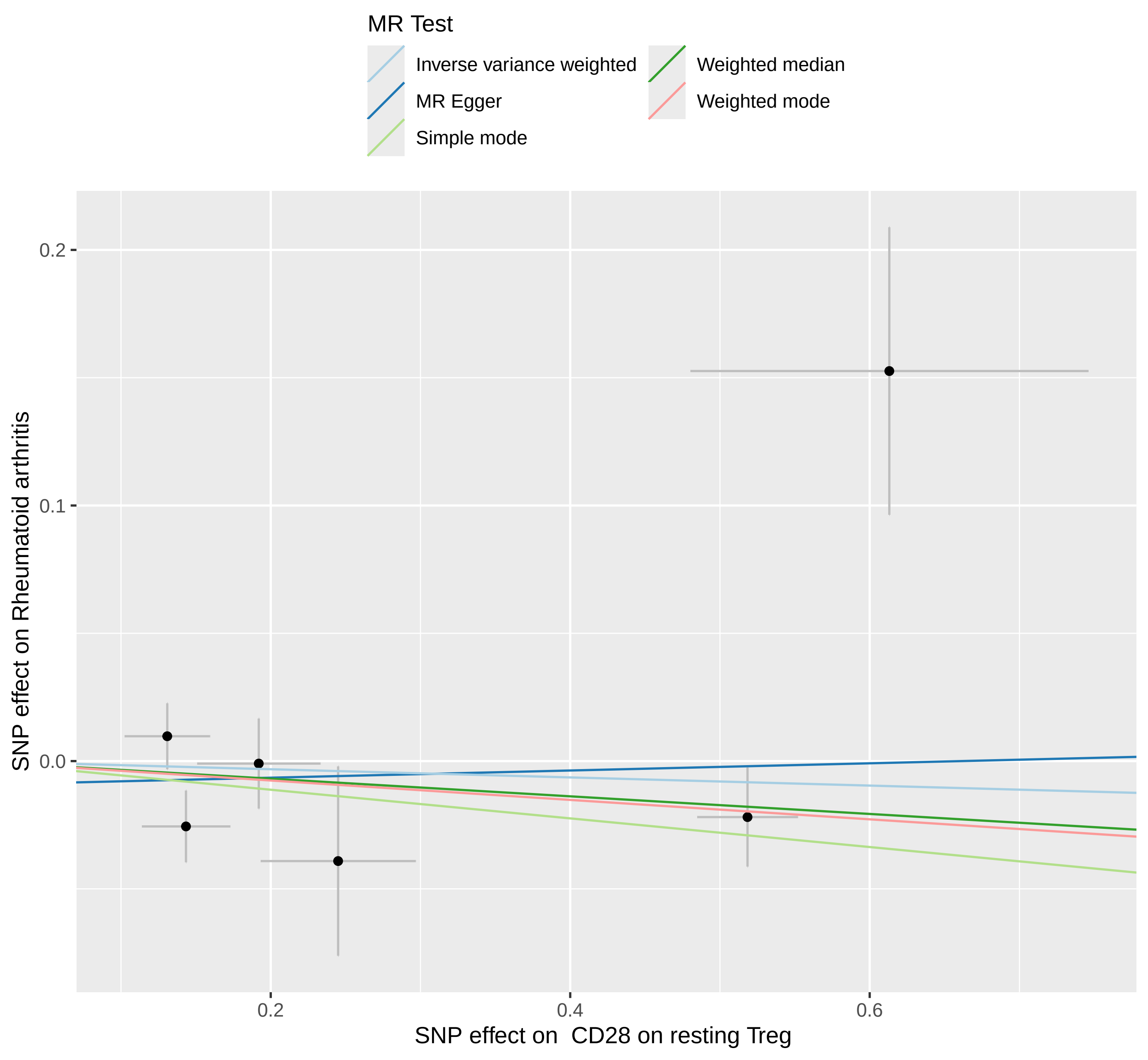

MR Test
Inverse variance weighted
MR Egger
Simple mode
Weighted median
Weighted mode
0.2
0.1
0.0
0.2
0.4
0.6
SNP effect on Rheumatoid arthritis
SNP effect on CD28 on resting Treg

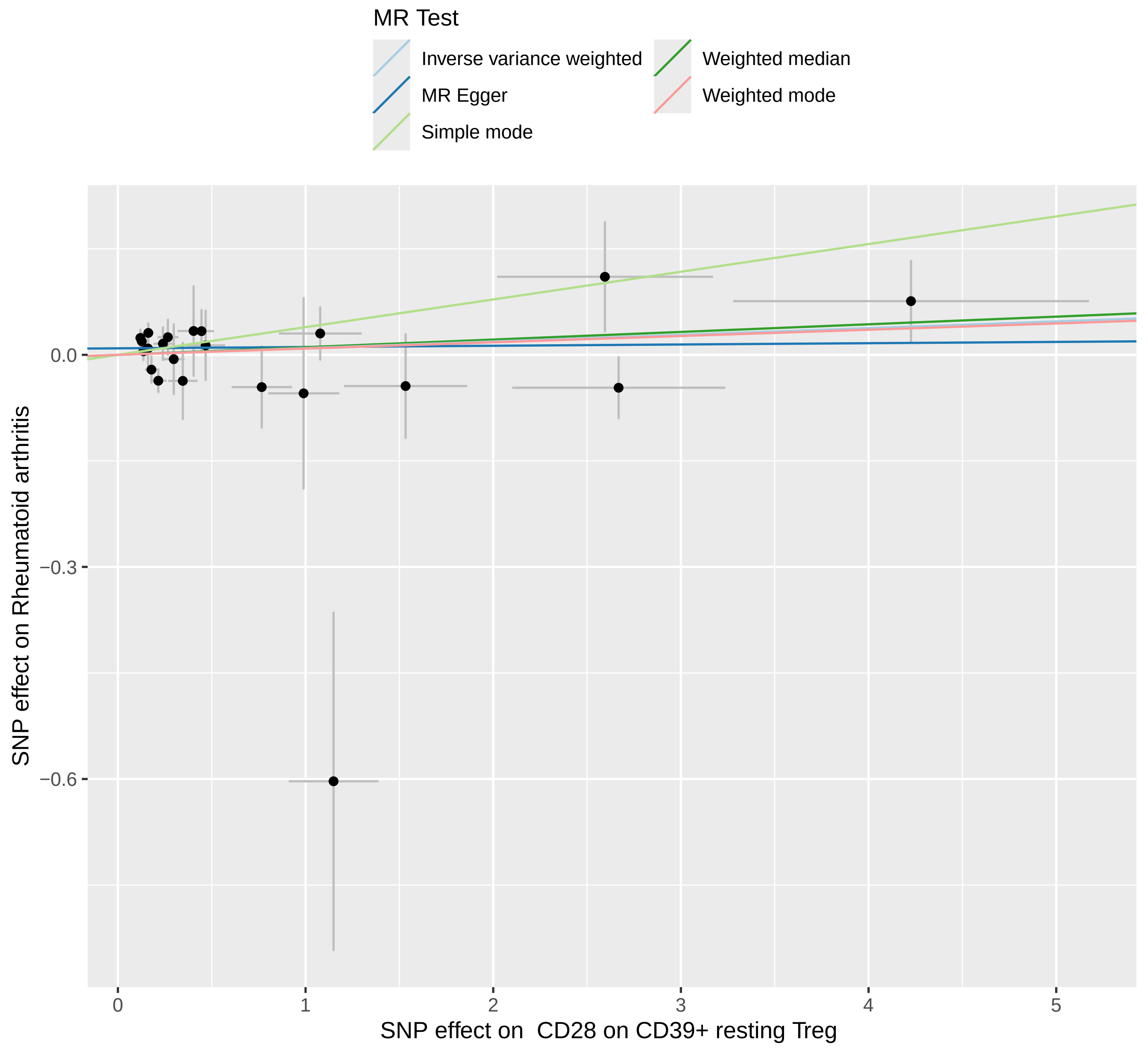

MR Test
Inverse variance weighted
MR Egger
Simple mode
Weighted median
Weighted mode
SNP effect on Rheumatoid arthritis
0.0
−0.3
−0.6
0
1
2
3
4
5
SNP effect on CD28 on CD39+ resting Treg

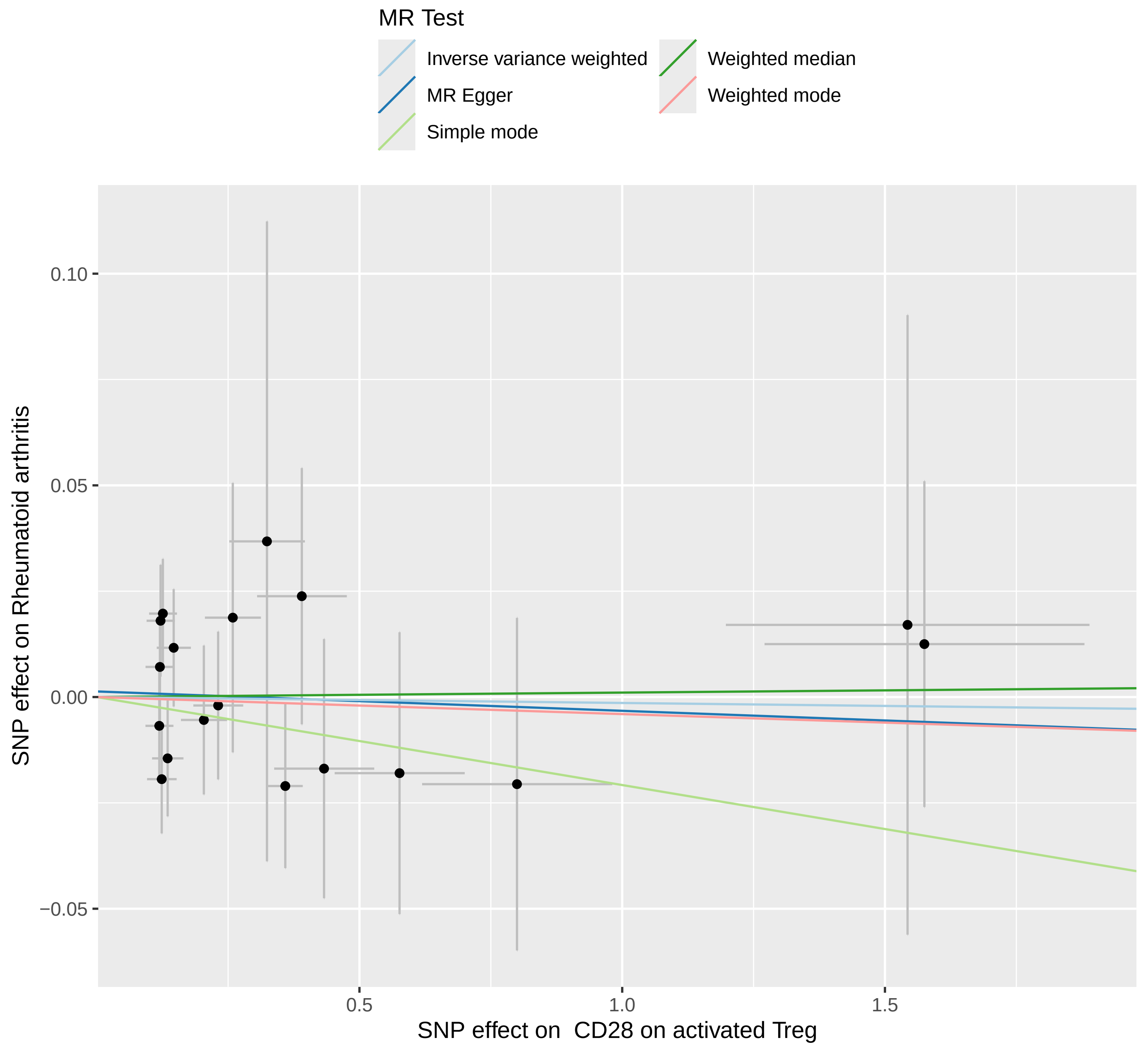
MR Test
Inverse variance weighted
MR Egger
Simple mode
Weighted median
Weighted mode
SNP effect on Rheumatoid arthritis
0.10
0.05
0.00
−0.05
0.5
1.0
1.5
SNP effect on CD28 on activated Treg

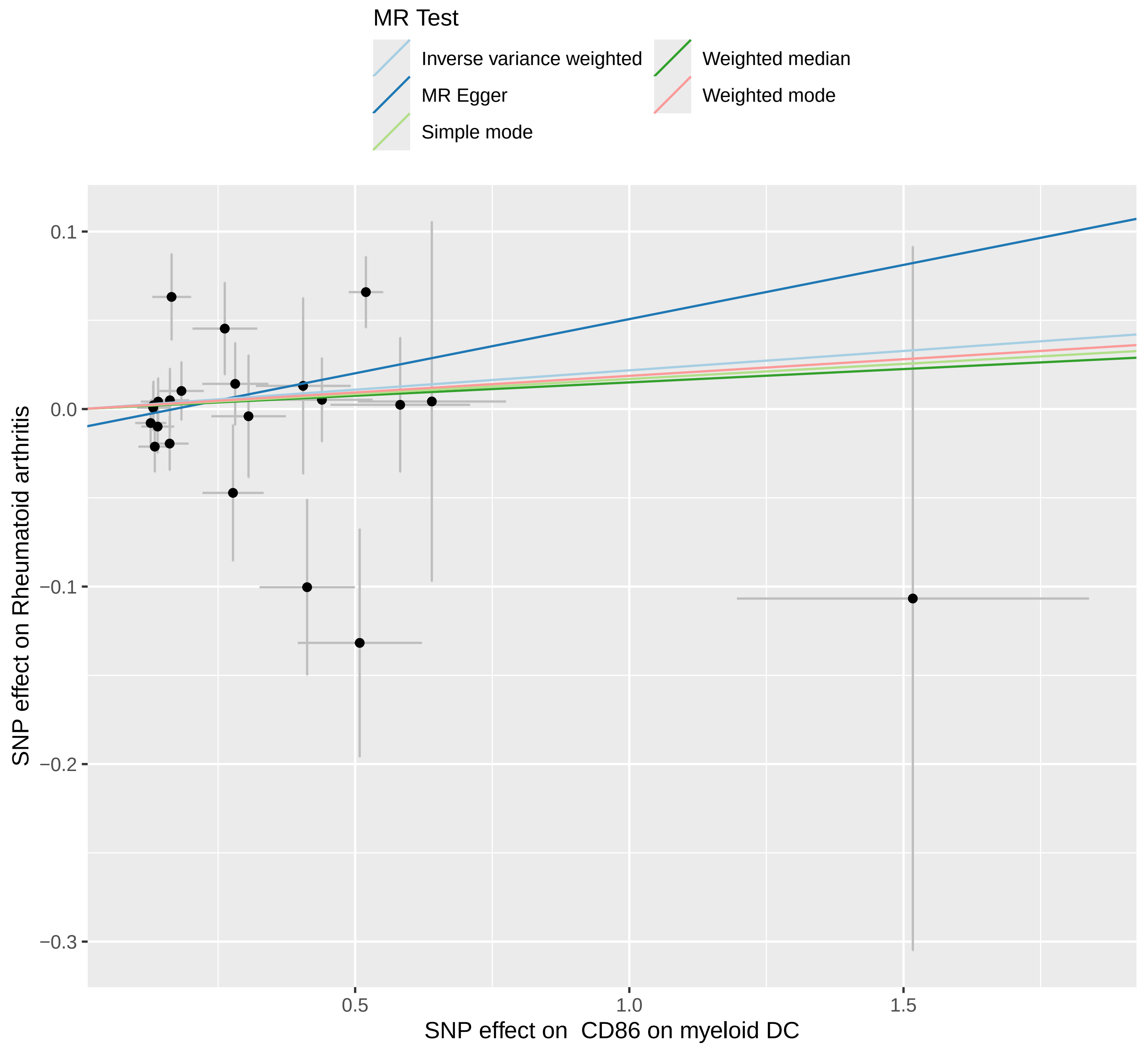

MR Test
Inverse variance weighted
MR Egger
Simple mode
Weighted median
Weighted mode
0.1
0.0
−0.1
−0.2
−0.3
0.5
1.0
1.5
SNP effect on Rheumatoid arthritis
SNP effect on CD86 on myeloid DC

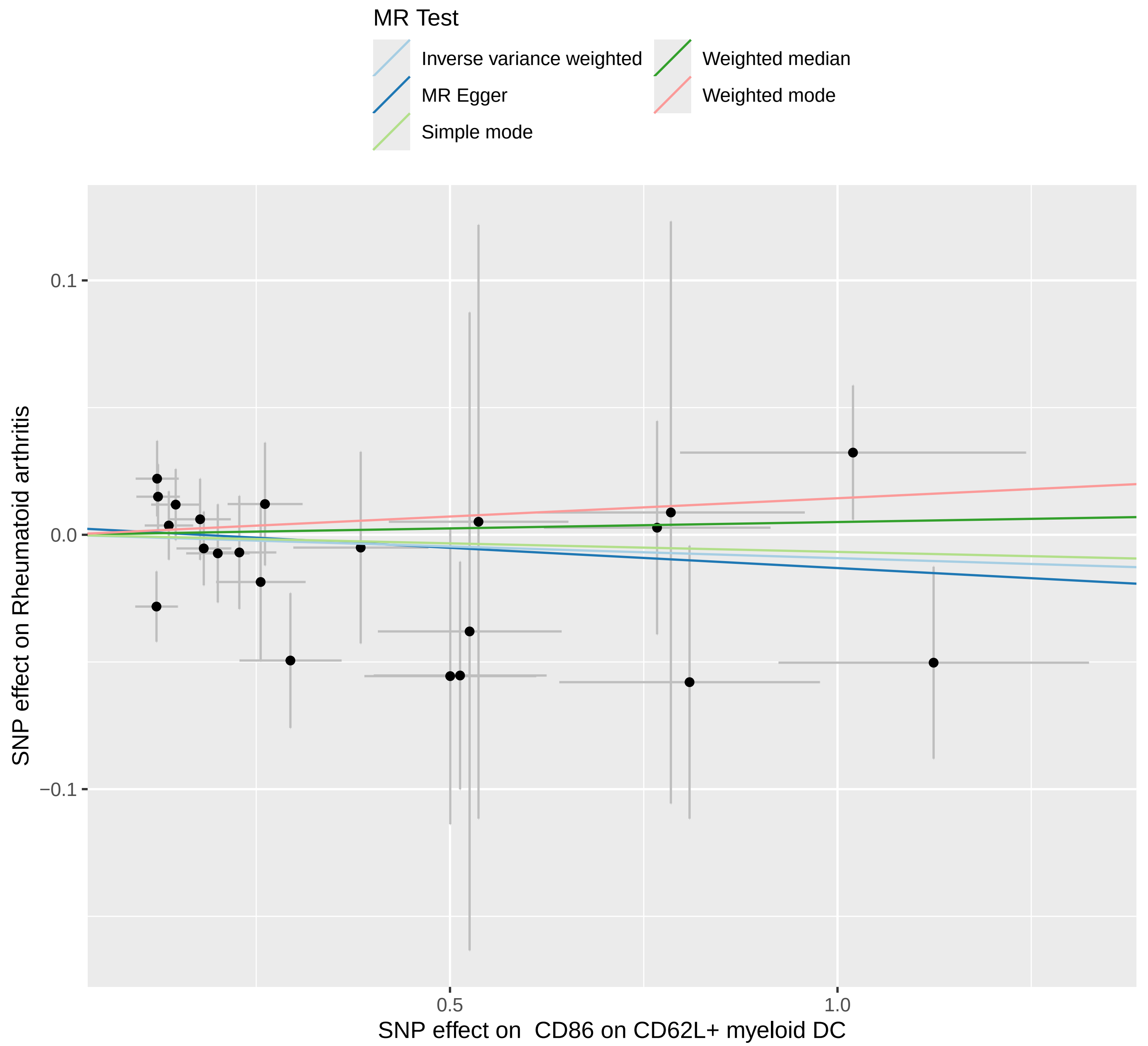
MR Test
Inverse variance weighted
MR Egger
Simple mode
Weighted median
Weighted mode
0.1
0.0
−0.1
0.5
1.0
SNP effect on Rheumatoid arthritis
SNP effect on CD86 on CD62L+ myeloid DC

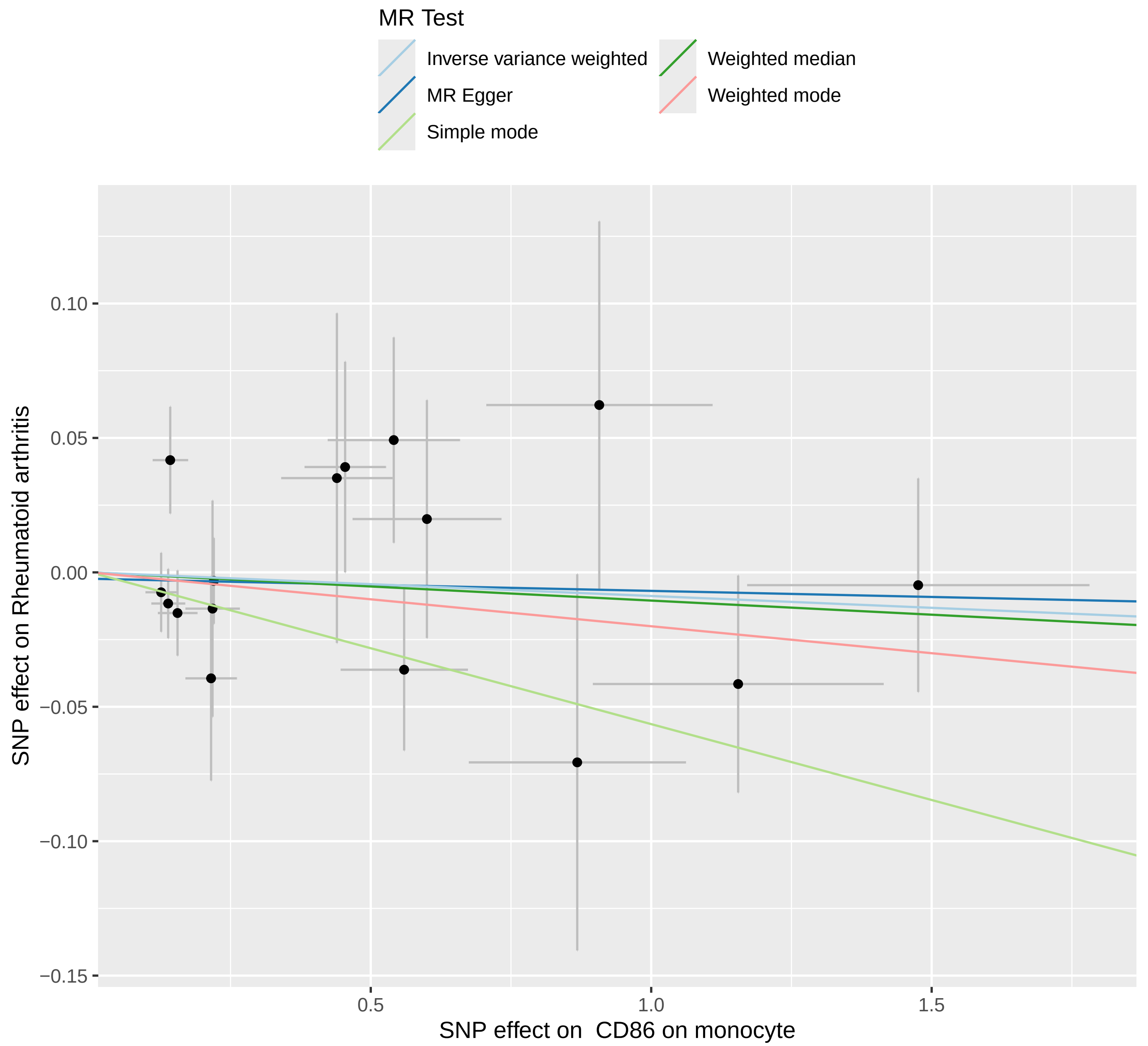
MR Test
Inverse variance weighted
MR Egger
Simple mode
Weighted median
Weighted mode
0.10
0.05
0.00
−0.05
−0.10
−0.15
0.5
1.0
1.5
SNP effect on Rheumatoid arthritis
SNP effect on CD86 on monocyte

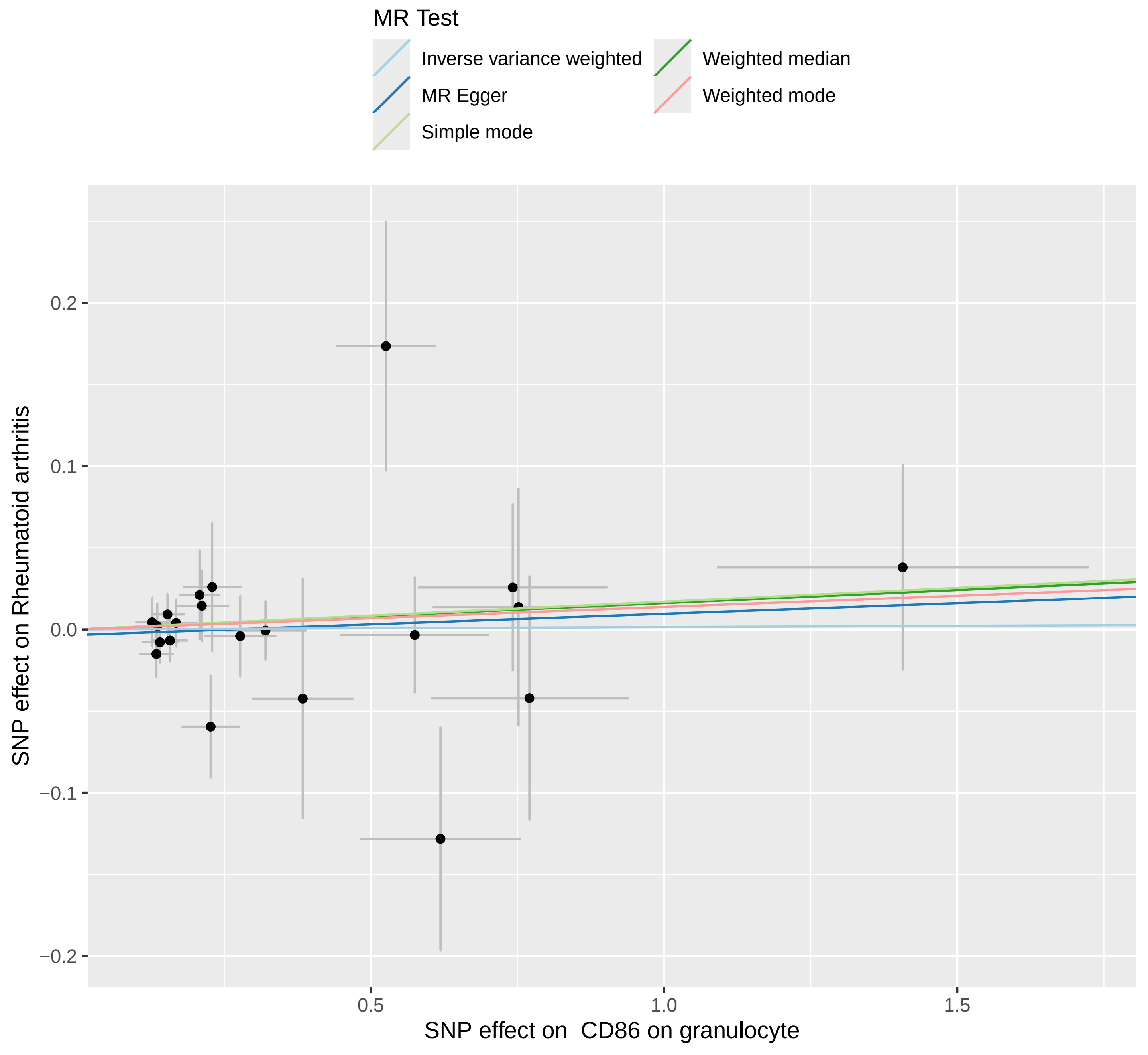
MR Test
Inverse variance weighted
MR Egger
Simple mode
Weighted median
Weighted mode
0.2
0.1
0.0
−0.1
−0.2
SNP effect on Rheumatoid arthritis
0.5
1.0
1.5
SNP effect on CD86 on granulocyte

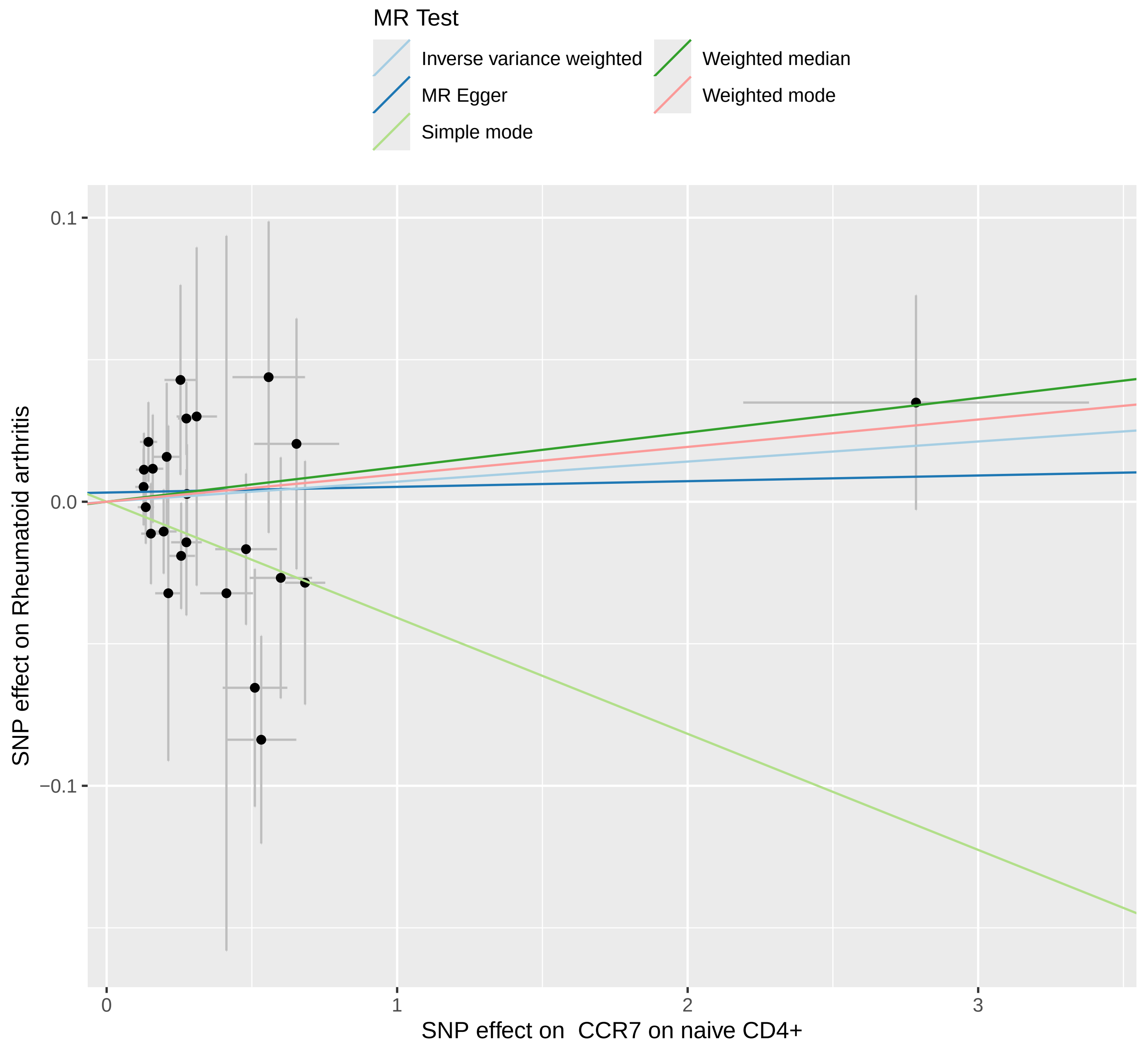
MR Test
Inverse variance weighted
MR Egger
Simple mode
Weighted median
Weighted mode
0.1
0.0
−0.1
0
1
2
3
SNP effect on Rheumatoid arthritis
SNP effect on CCR7 on naive CD4+

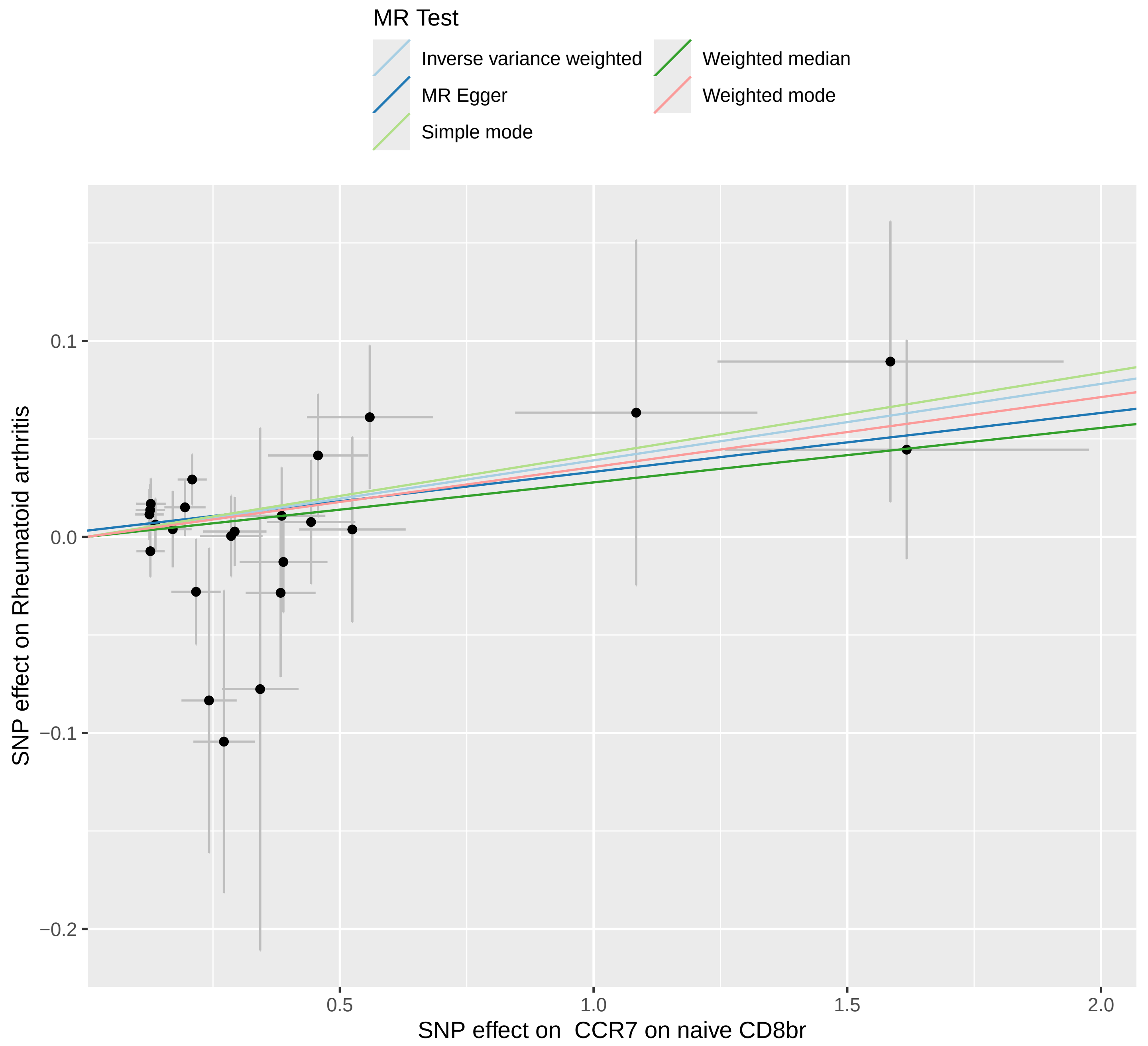
MR Test
Inverse variance weighted
MR Egger
Simple mode
Weighted median
Weighted mode
SNP effect on Rheumatoid arthritis
0.1
0.0
−0.1
−0.2
0.5
1.0
1.5
2.0
SNP effect on CCR7 on naive CD8br

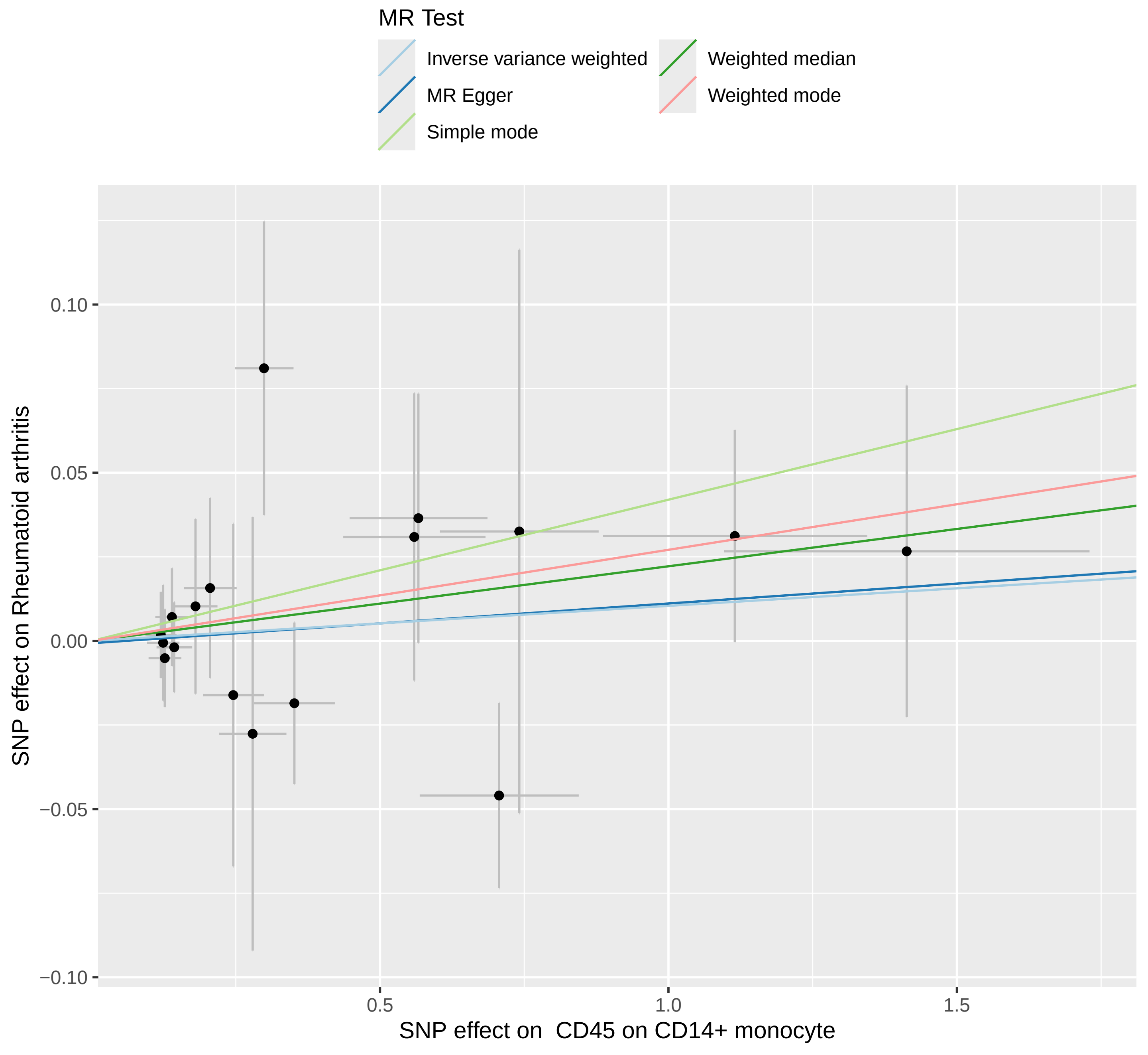
MR Test
Inverse variance weighted
MR Egger
Simple mode
Weighted median
Weighted mode
0.10
0.05
0.00
−0.05
−0.10
SNP effect on Rheumatoid arthritis
0.5
1.0
1.5
SNP effect on CD45 on CD14+ monocyte

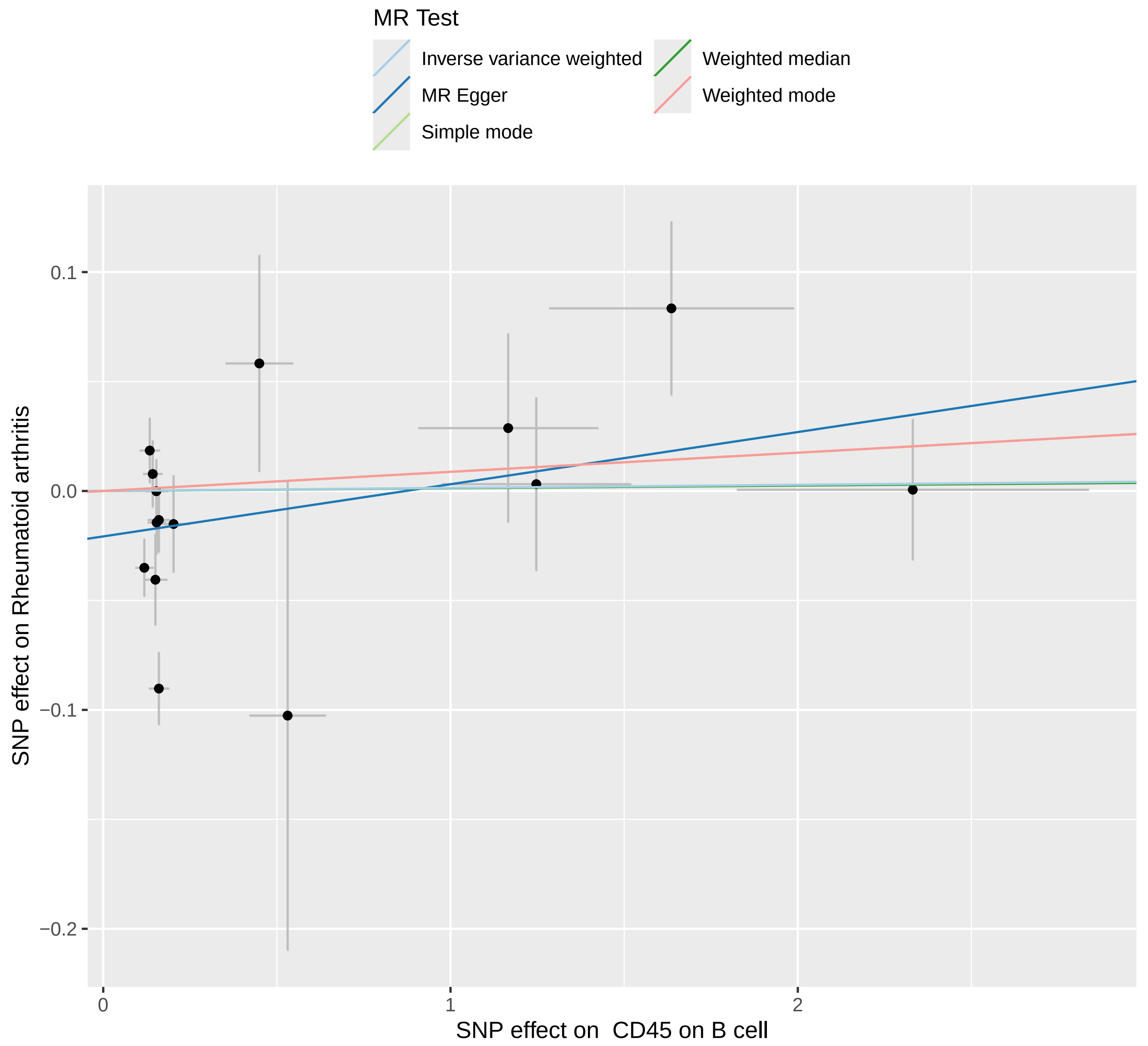

MR Test
Inverse variance weighted
MR Egger
Simple mode
Weighted median
Weighted mode
0.1
0.0
−0.1
−0.2
0
1
2
SNP effect on Rheumatoid arthritis
SNP effect on CD45 on B cell

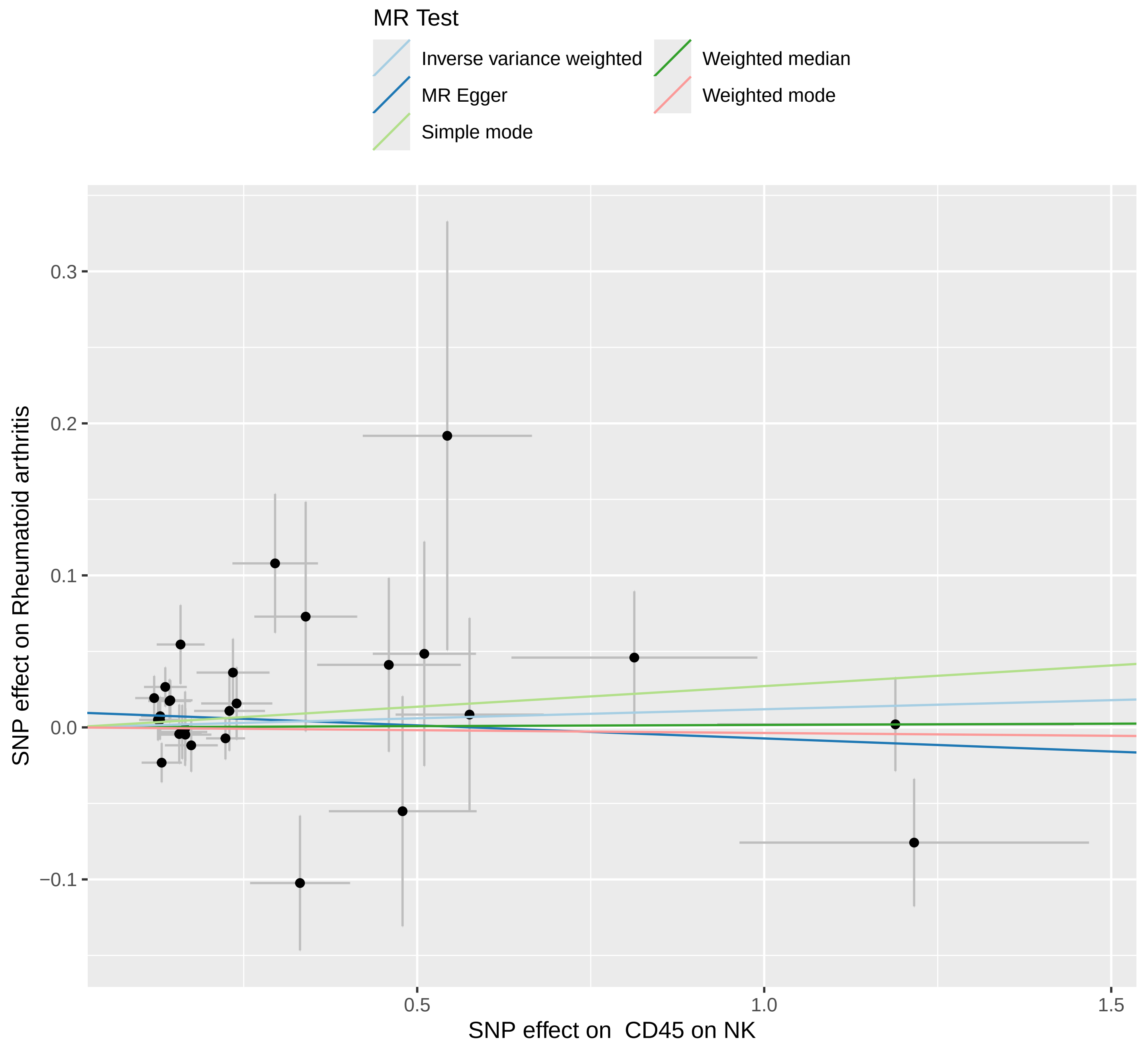

MR Test
Inverse variance weighted
MR Egger
Simple mode
Weighted median
Weighted mode
SNP effect on Rheumatoid arthritis
0.3
0.2
0.1
0.0
−0.1
0.5
1.0
1.5
SNP effect on CD45 on NK

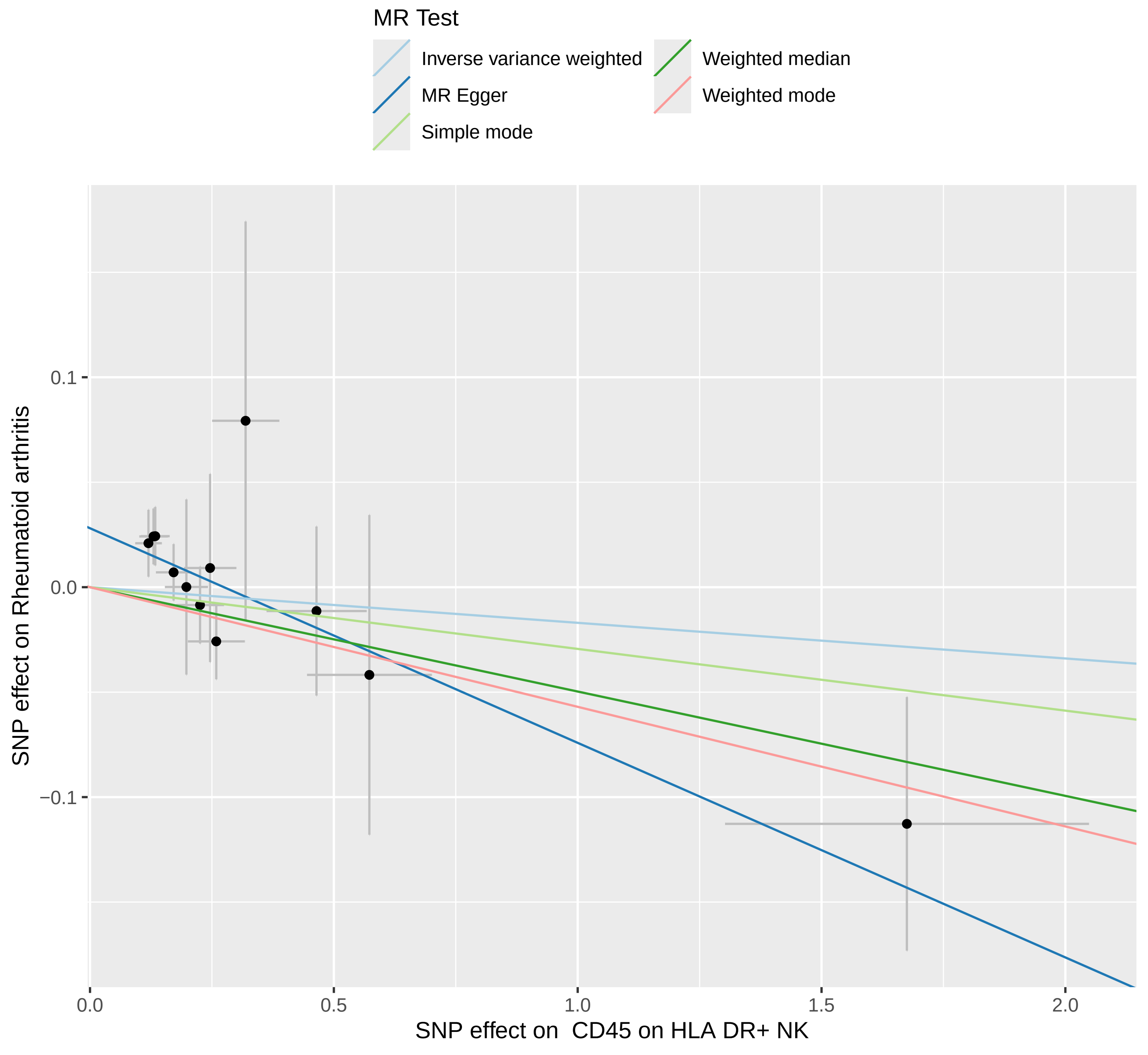

MR Test
Inverse variance weighted
MR Egger
Simple mode
Weighted median
Weighted mode
SNP effect on Rheumatoid arthritis
0.1
0.0
−0.1
0.0
0.5
1.0
1.5
2.0
SNP effect on CD45 on HLA DR+ NK

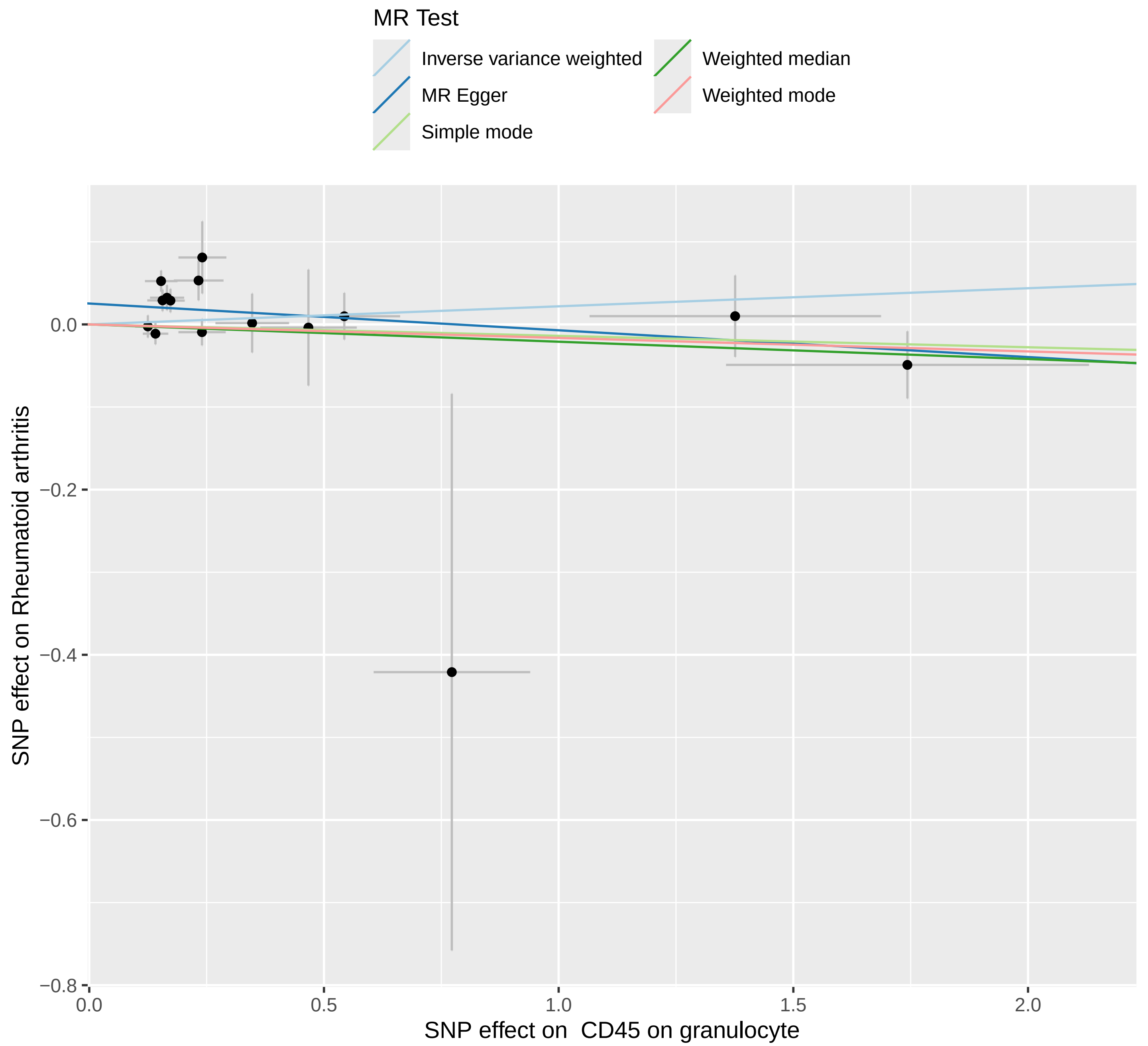

MR Test
Inverse variance weighted
MR Egger
Simple mode
Weighted median
Weighted mode
SNP effect on Rheumatoid arthritis
0.0
−0.2
−0.4
−0.6
−0.8
0.0
0.5
1.0
1.5
2.0
SNP effect on CD45 on granulocyte

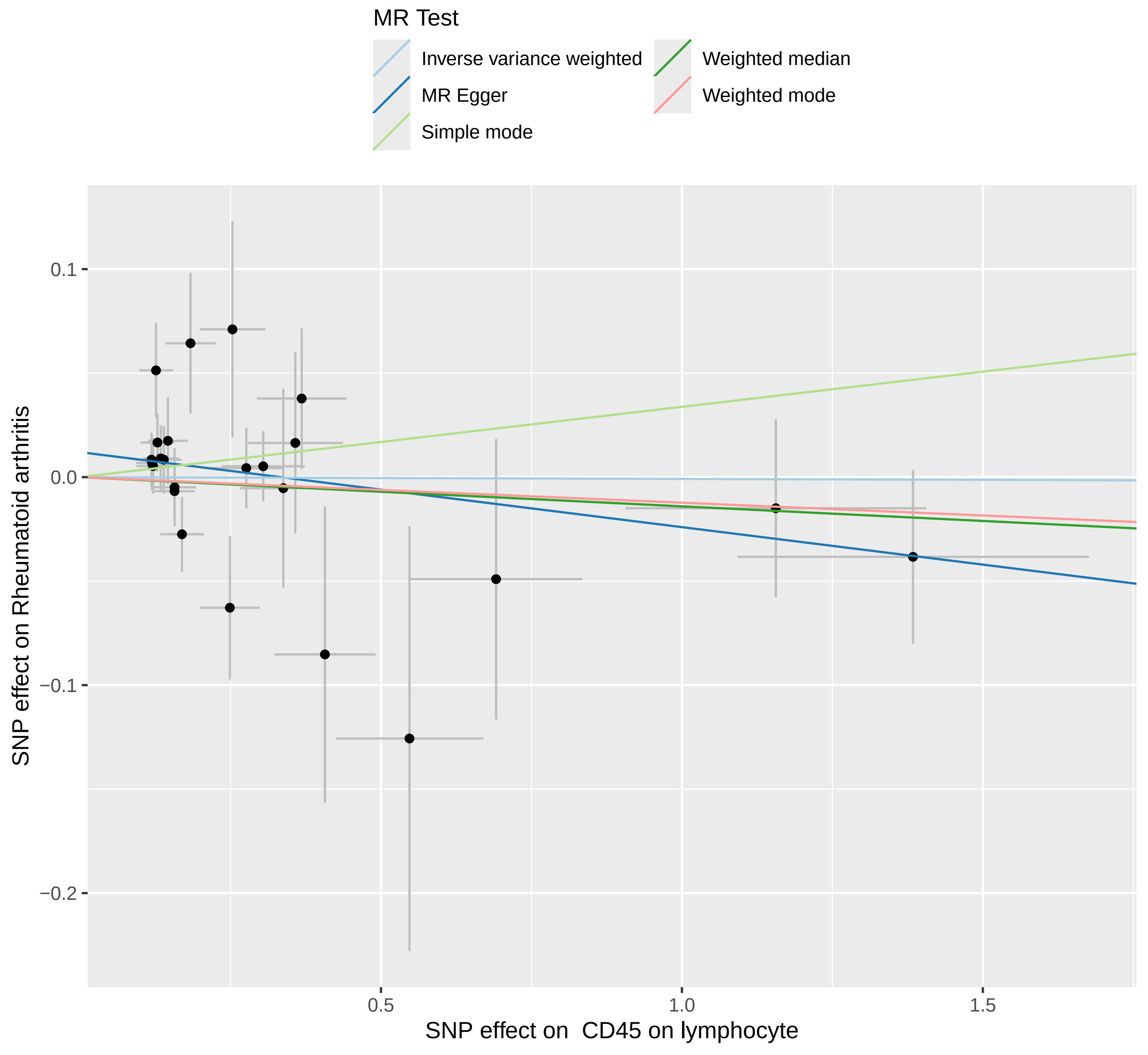
MR Test
Inverse variance weighted
MR Egger
Simple mode
Weighted median
Weighted mode
0.1
0.0
−0.1
−0.2
SNP effect on Rheumatoid arthritis
0.5
1.0
1.5
SNP effect on CD45 on lymphocyte

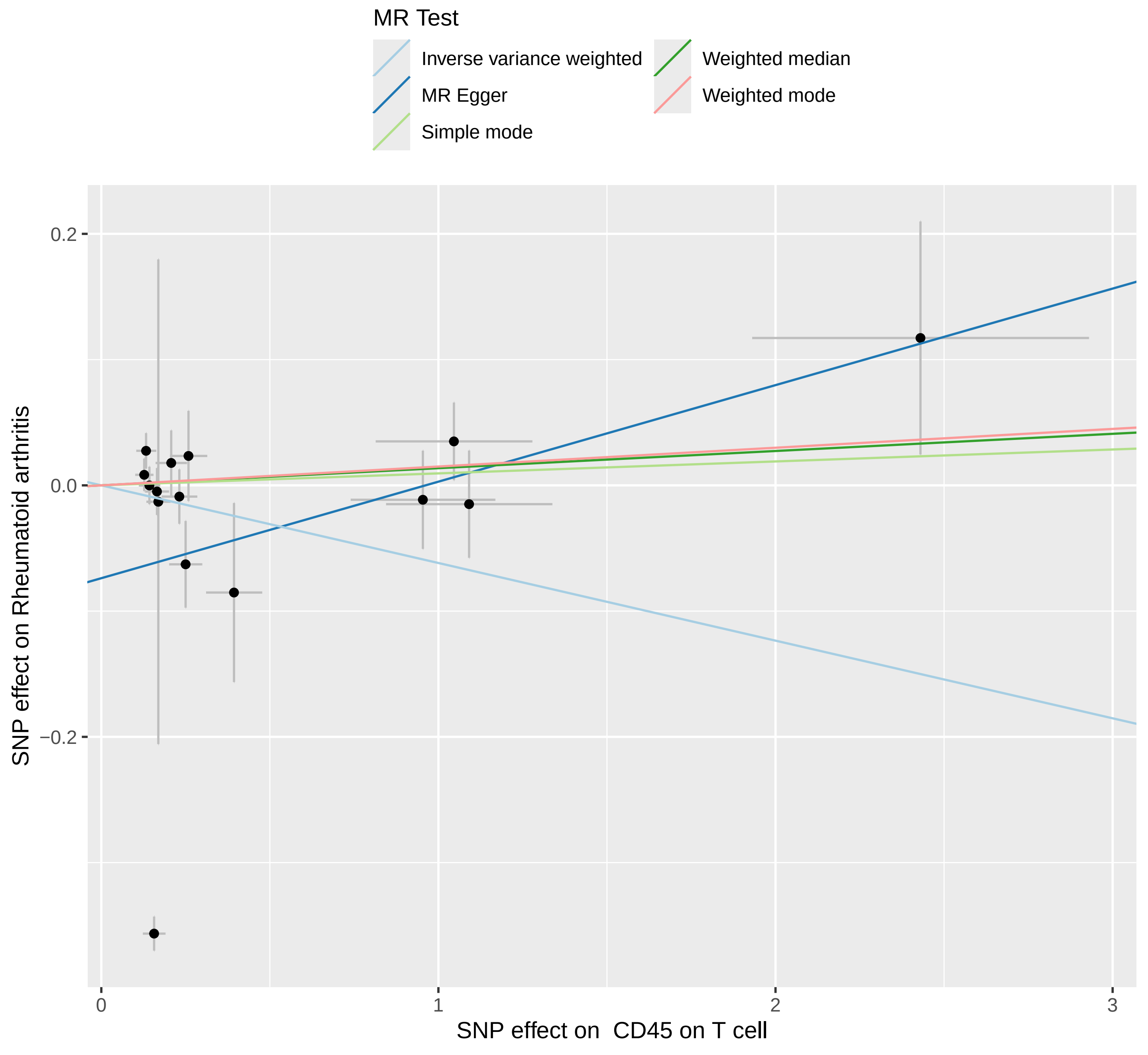
MR Test
Inverse variance weighted
MR Egger
Simple mode
Weighted median
Weighted mode
0.2
0.0
−0.2
0
1
2
3
SNP effect on Rheumatoid arthritis
SNP effect on CD45 on T cell

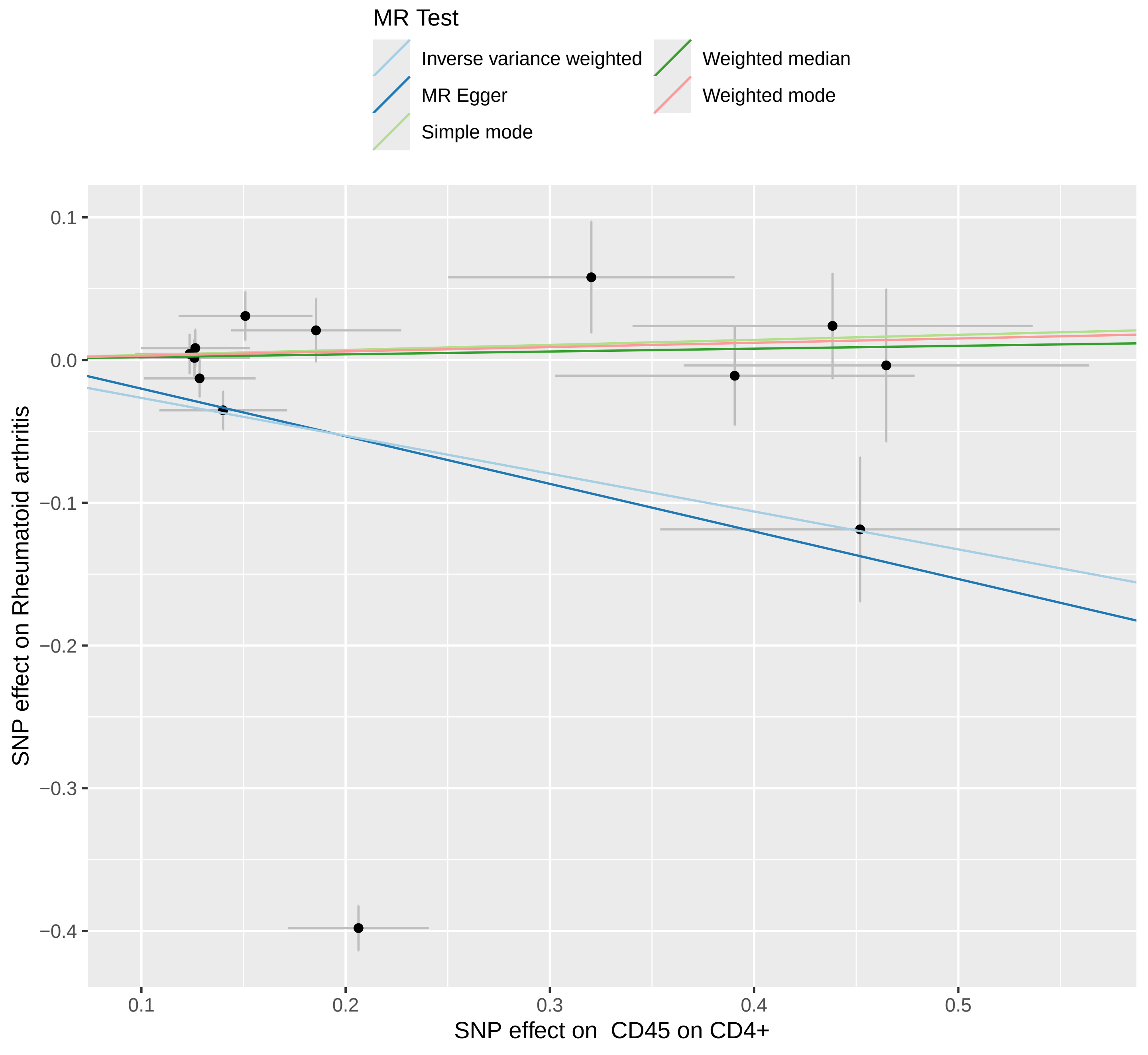
MR Test
Inverse variance weighted
MR Egger
Simple mode
Weighted median
Weighted mode
SNP effect on Rheumatoid arthritis
SNP effect on CD45 on CD4+
0.1
0.0
−0.1
−0.2
−0.3
−0.4
0.1
0.2
0.3
0.4
0.5

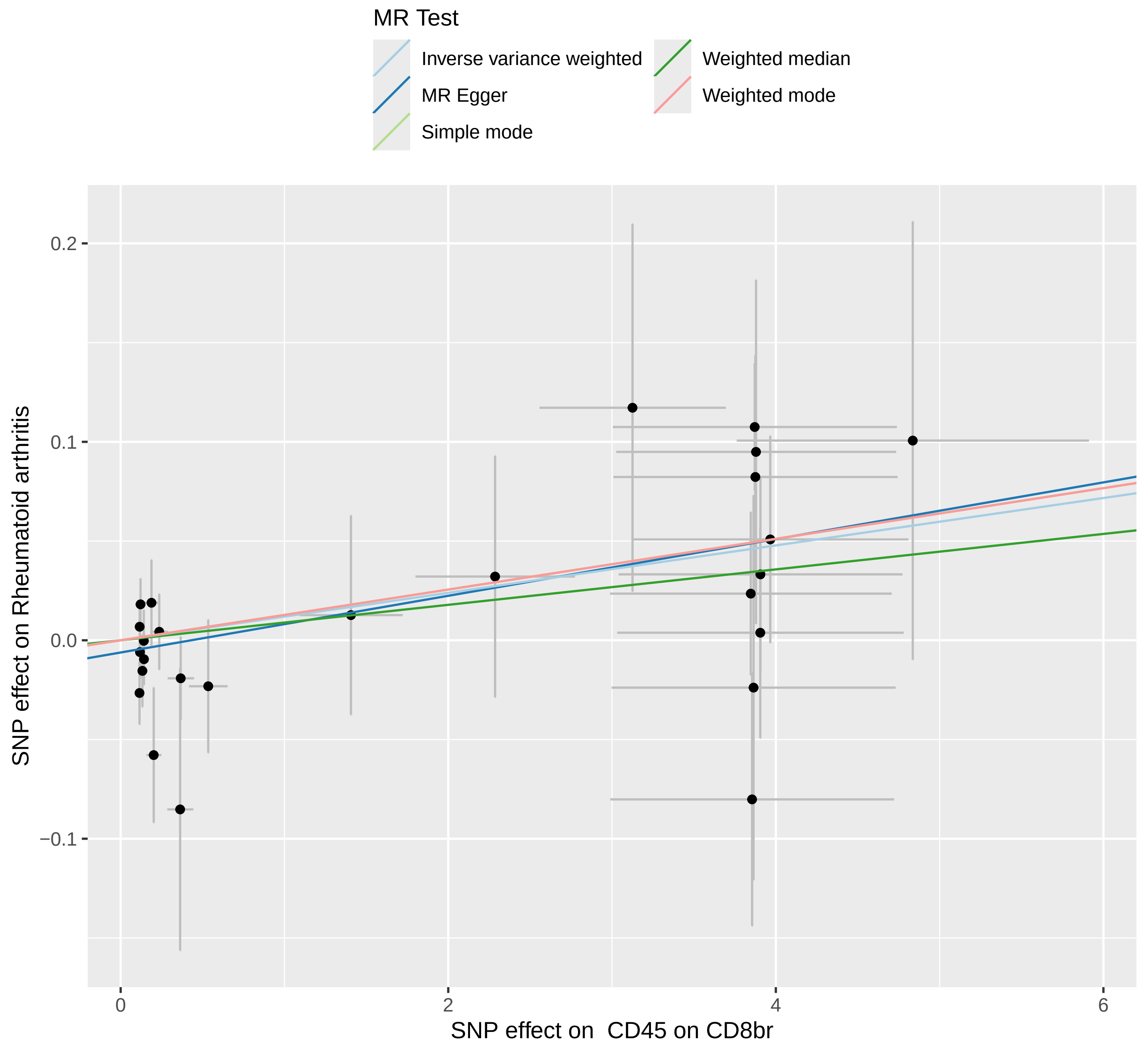

MR Test
Inverse variance weighted
MR Egger
Simple mode
Weighted median
Weighted mode
0.2
0.1
0.0
−0.1
0
2
4
6
SNP effect on Rheumatoid arthritis
SNP effect on CD45 on CD8br

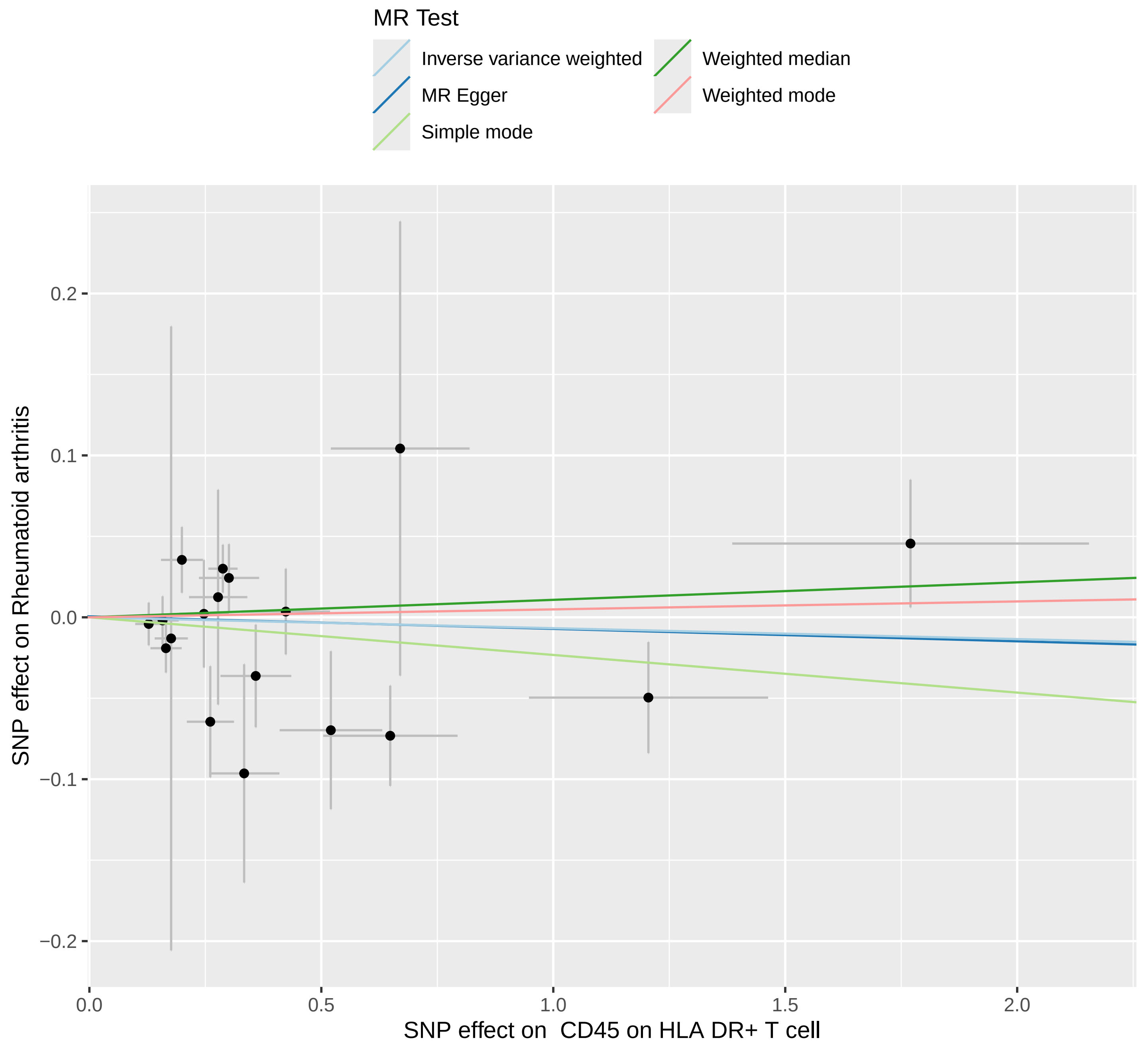

MR Test
Inverse variance weighted
MR Egger
Simple mode
Weighted median
Weighted mode
0.2
0.1
0.0
−0.1
−0.2
SNP effect on Rheumatoid arthritis
0.0
0.5
1.0
1.5
2.0
SNP effect on CD45 on HLA DR+ T cell

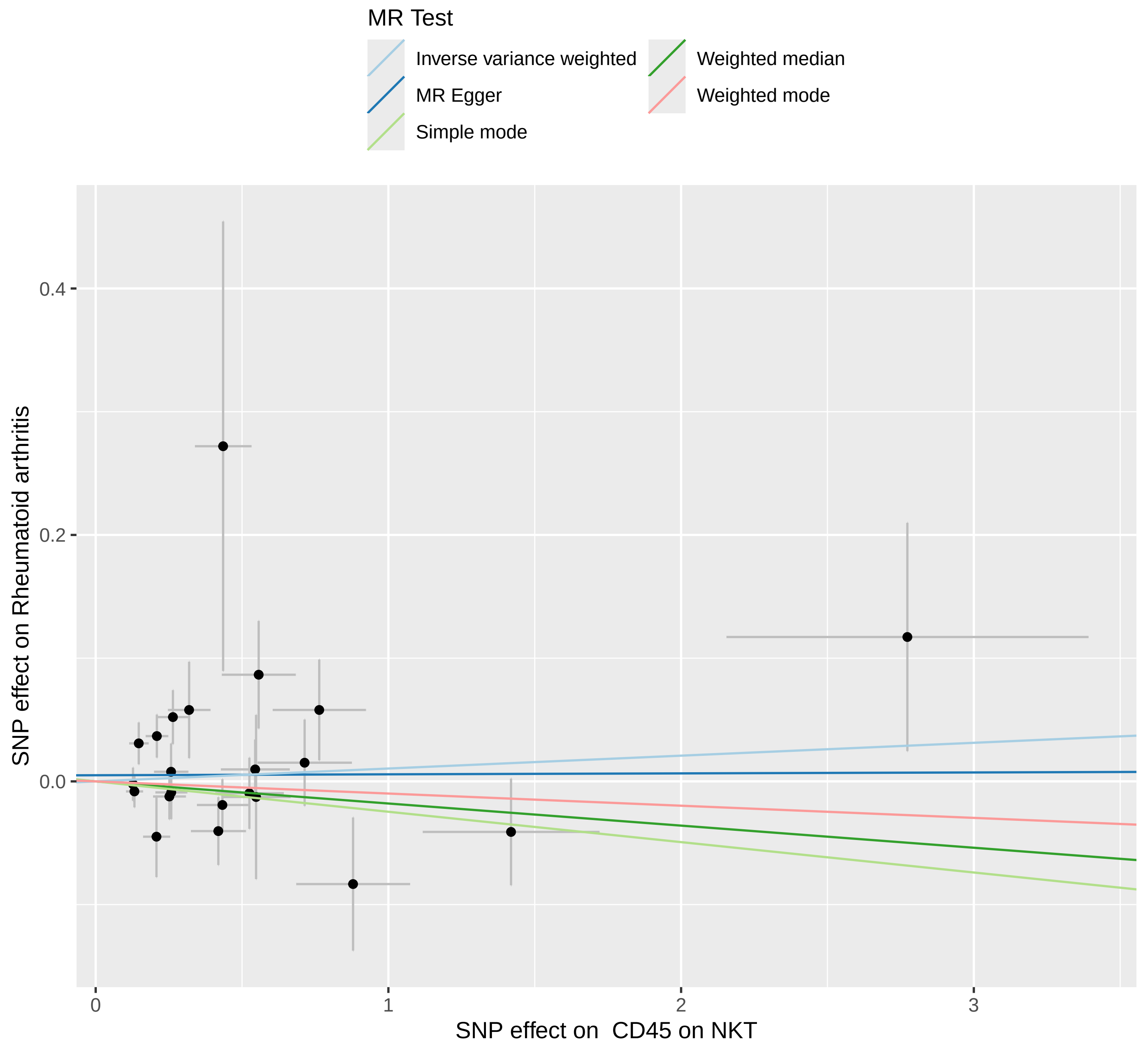

MR Test
Inverse variance weighted
MR Egger
Simple mode
Weighted median
Weighted mode
SNP effect on Rheumatoid arthritis
0.4
0.2
0.0
0
1
2
3
SNP effect on CD45 on NKT

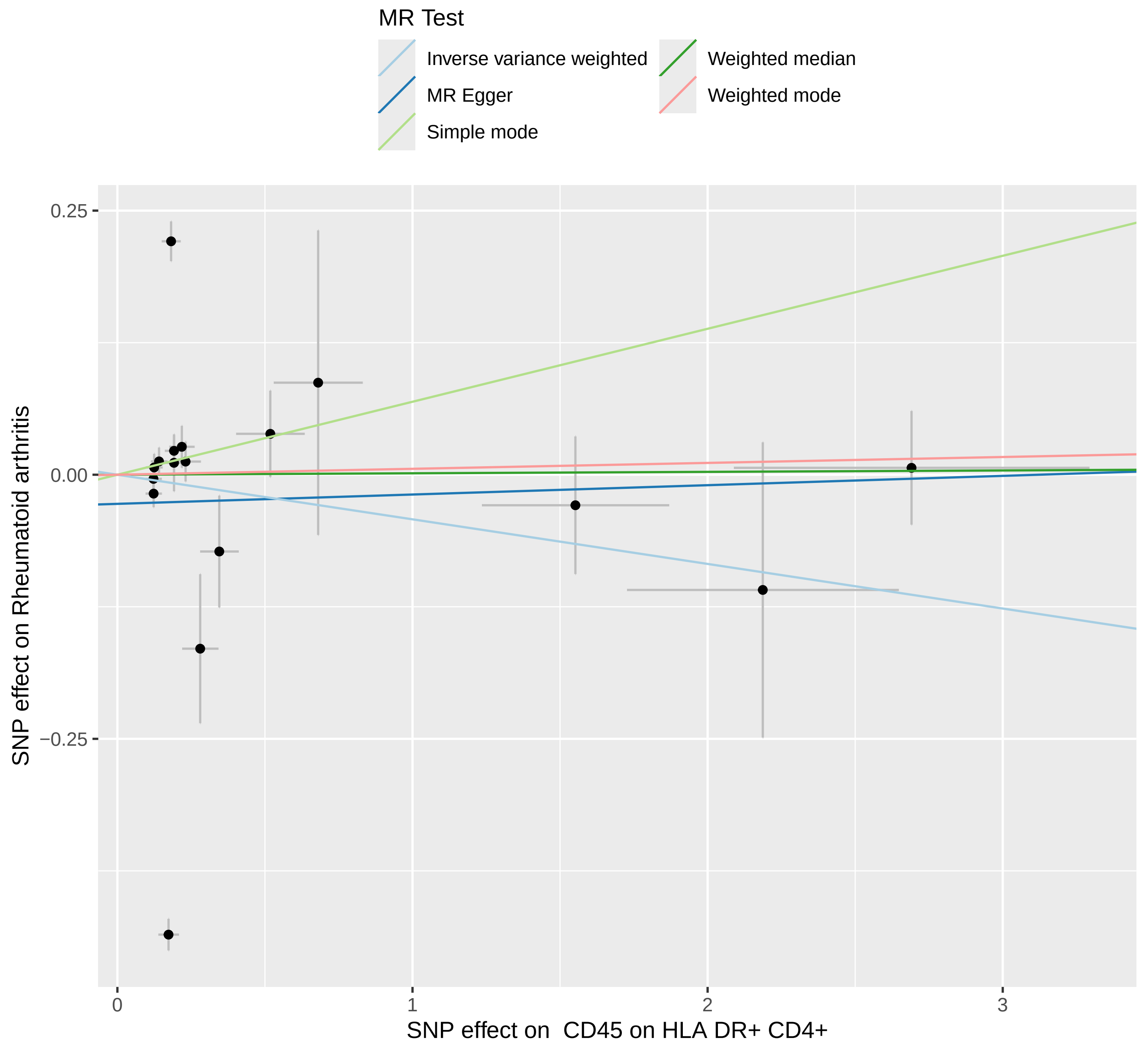
MR Test
Inverse variance weighted
MR Egger
Simple mode
Weighted median
Weighted mode
0.25
0.00
−0.25
0
1
2
3
SNP effect on Rheumatoid arthritis
SNP effect on CD45 on HLA DR+ CD4+

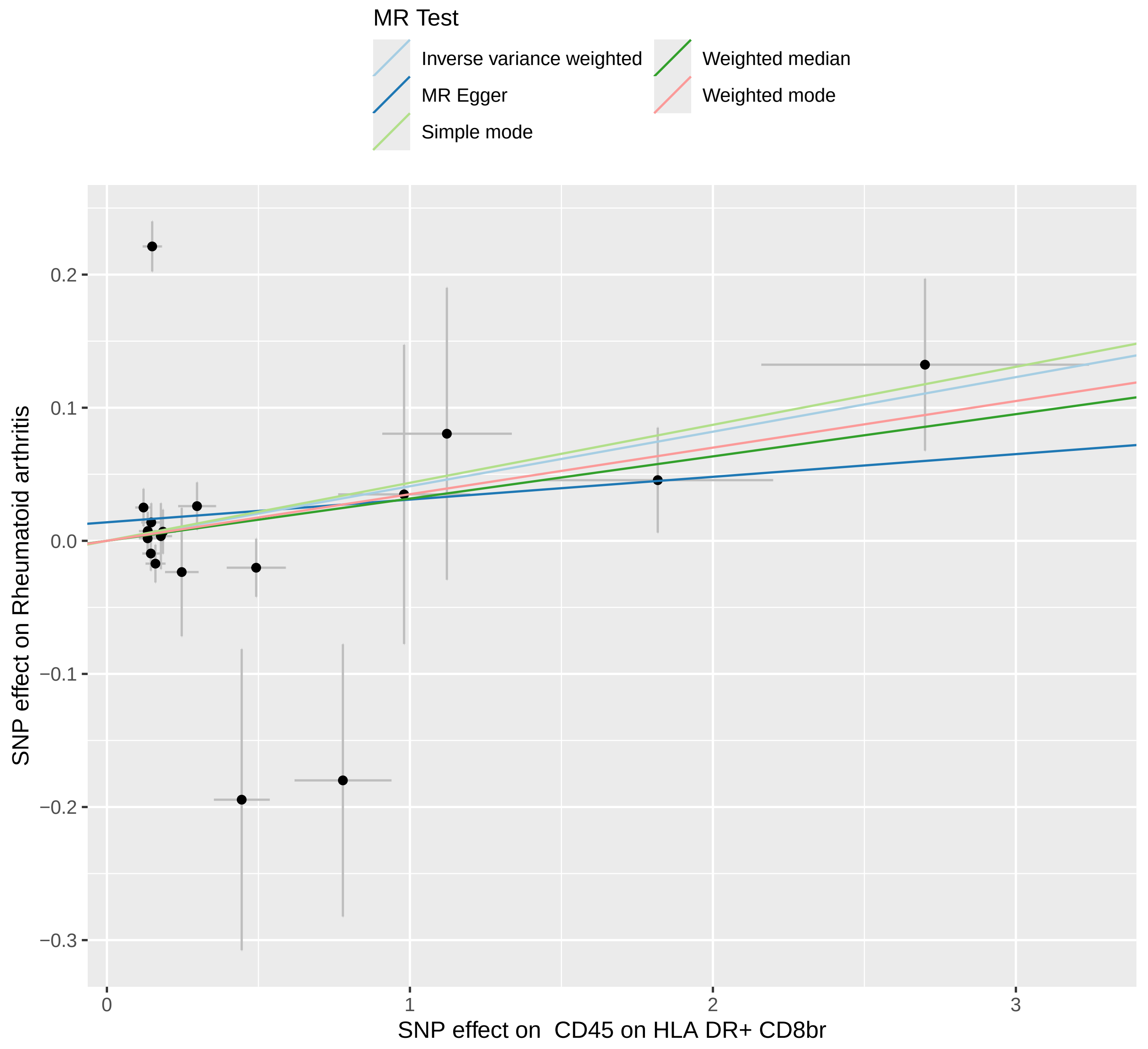
MR Test
Inverse variance weighted
MR Egger
Simple mode
Weighted median
Weighted mode
SNP effect on Rheumatoid arthritis
0.2
0.1
0.0
−0.1
−0.2
−0.3
0
1
2
3
SNP effect on CD45 on HLA DR+ CD8br

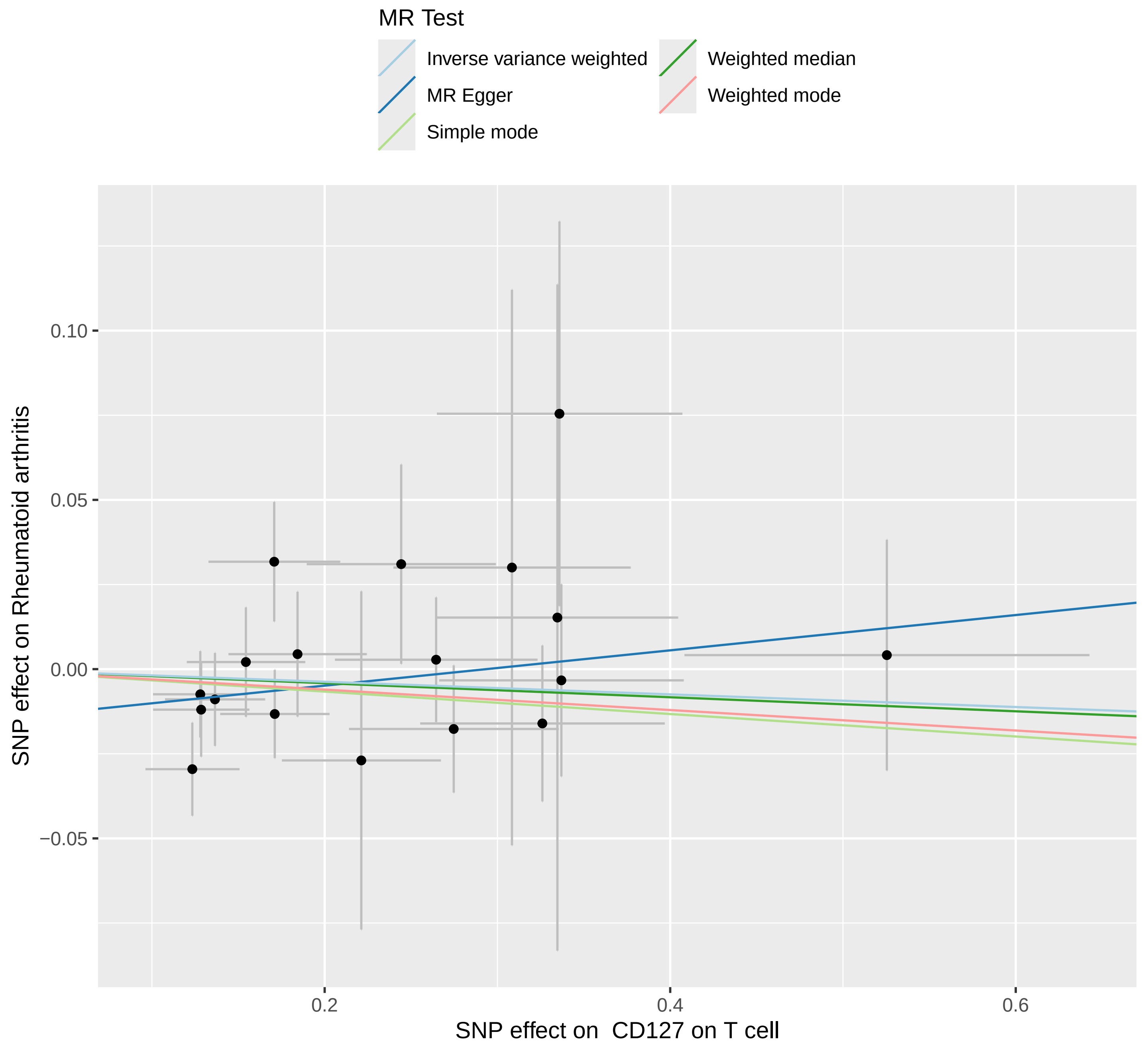

MR Test
Inverse variance weighted
MR Egger
Simple mode
Weighted median
Weighted mode
0.10
0.05
0.00
−0.05
0.2
0.4
0.6
SNP effect on Rheumatoid arthritis
SNP effect on CD127 on T cell

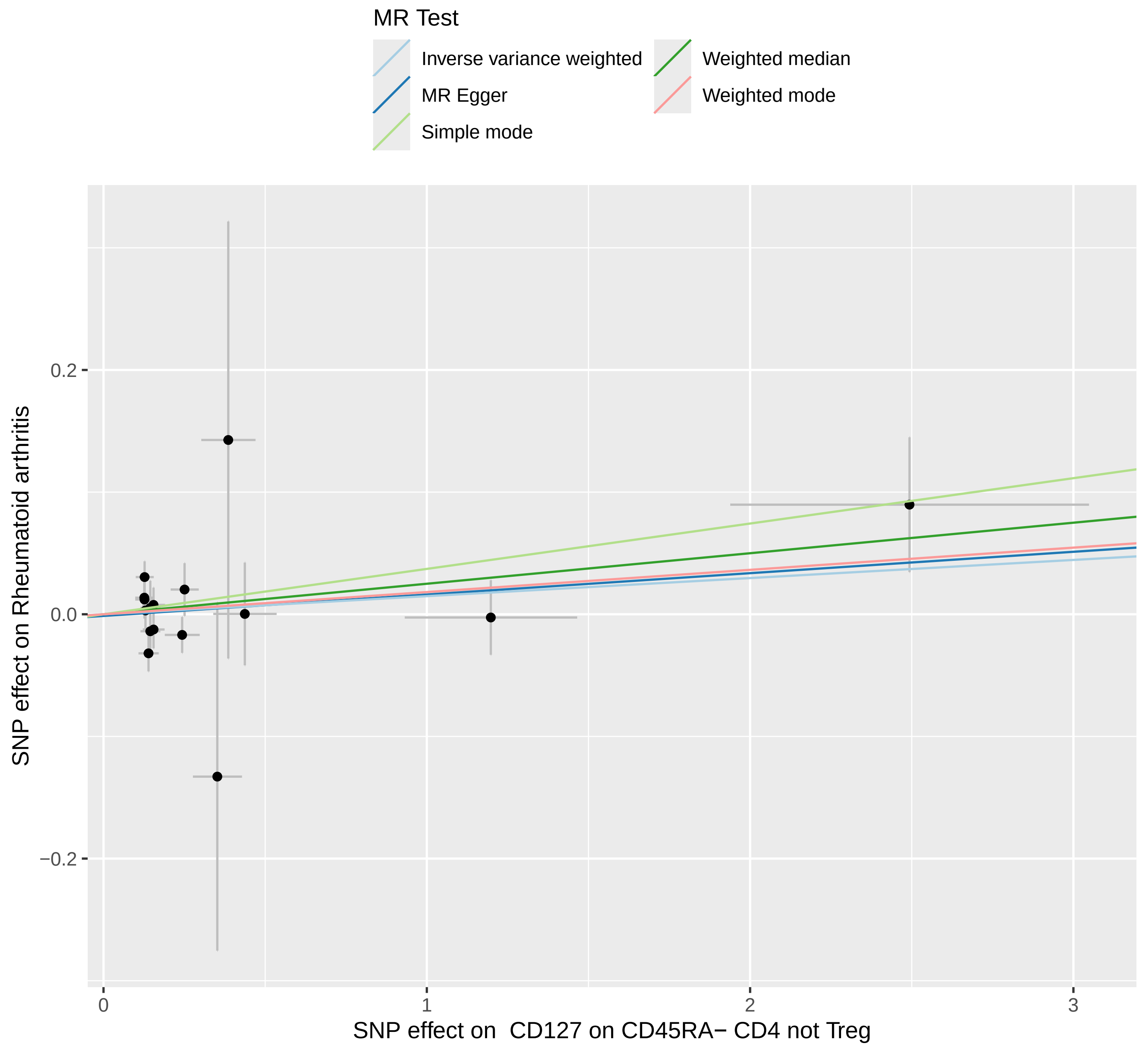

MR Test
Inverse variance weighted
MR Egger
Simple mode
Weighted median
Weighted mode
0.2
0.0
−0.2
0
1
2
3
SNP effect on Rheumatoid arthritis
SNP effect on CD127 on CD45RA− CD4 not Treg

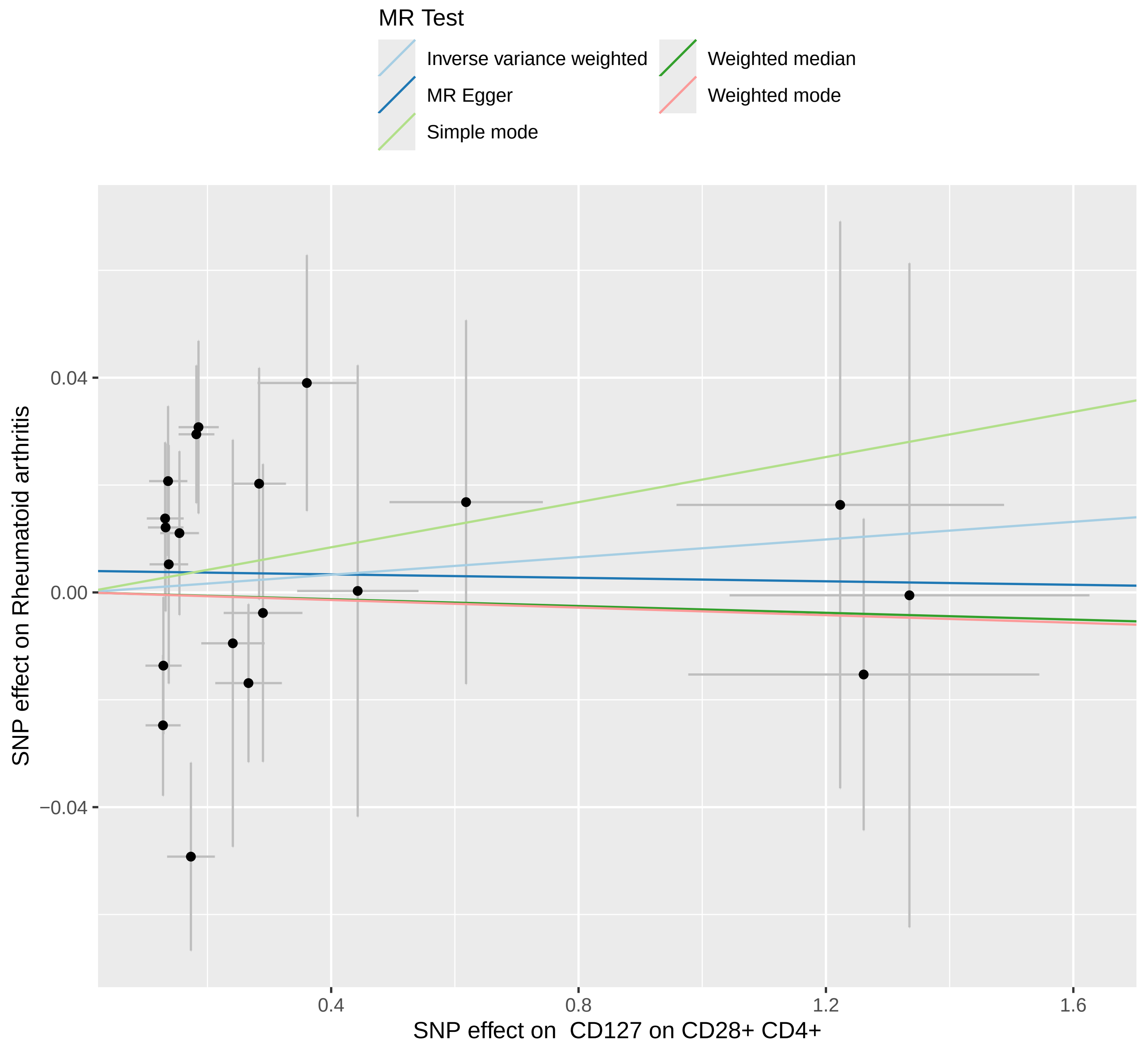
MR Test
Inverse variance weighted
MR Egger
Simple mode
Weighted median
Weighted mode
0.04
0.00
−0.04
0.4
0.8
1.2
1.6
SNP effect on Rheumatoid arthritis
SNP effect on CD127 on CD28+ CD4+

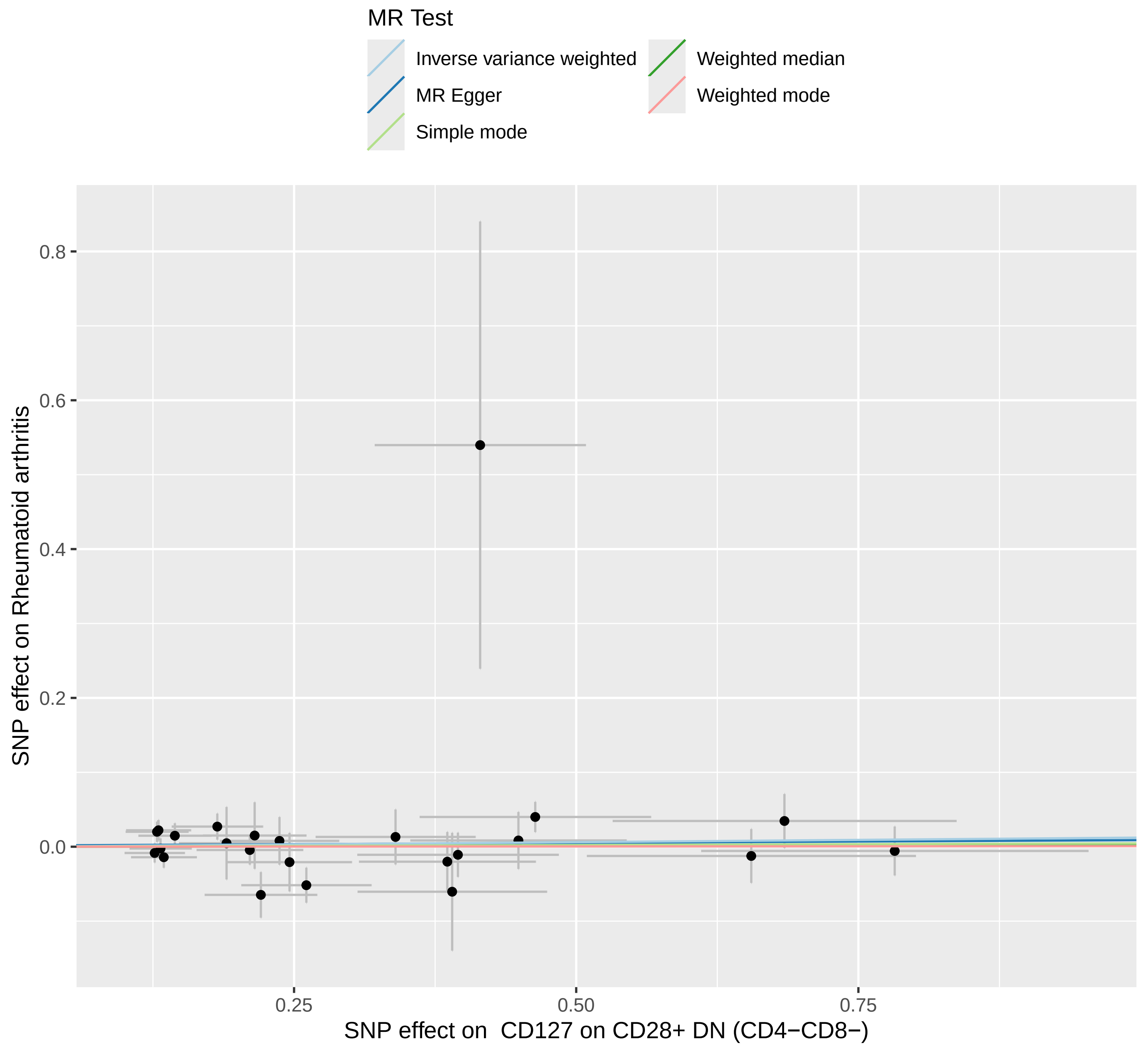

MR Test
Inverse variance weighted
MR Egger
Simple mode
Weighted median
Weighted mode
0.8
0.6
0.4
0.2
0.0
SNP effect on Rheumatoid arthritis
0.25
0.50
0.75
SNP effect on CD127 on CD28+ DN (CD4−CD8−)

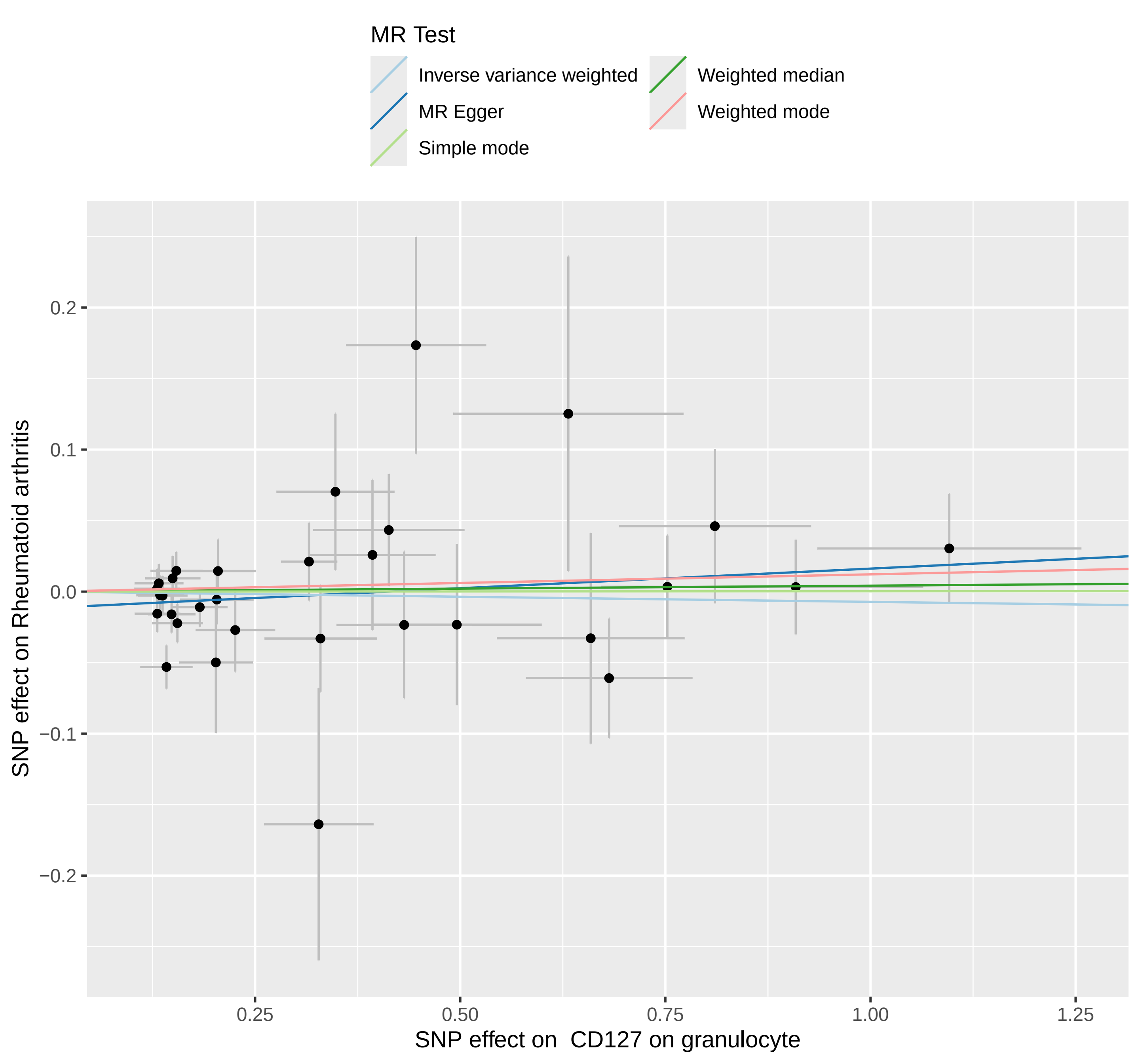

MR Test
Inverse variance weighted
MR Egger
Simple mode
Weighted median
Weighted mode
SNP effect on Rheumatoid arthritis
0.2
0.1
0.0
−0.1
−0.2
0.25
0.50
0.75
1.00
1.25
SNP effect on CD127 on granulocyte

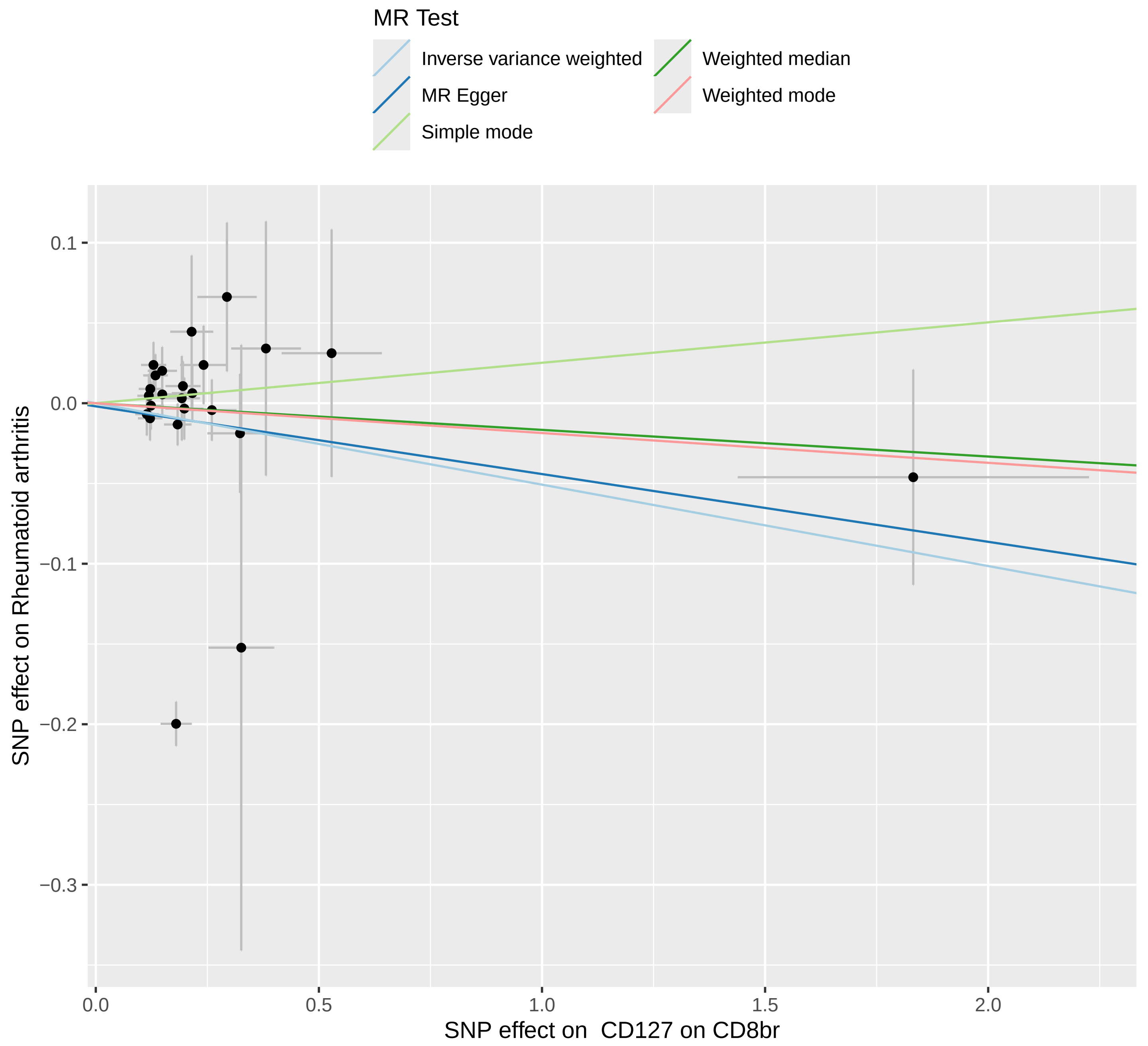

MR Test
Inverse variance weighted
MR Egger
Simple mode
Weighted median
Weighted mode
0.1
0.0
−0.1
−0.2
−0.3
SNP effect on Rheumatoid arthritis
0.0
0.5
1.0
1.5
2.0
SNP effect on CD127 on CD8br

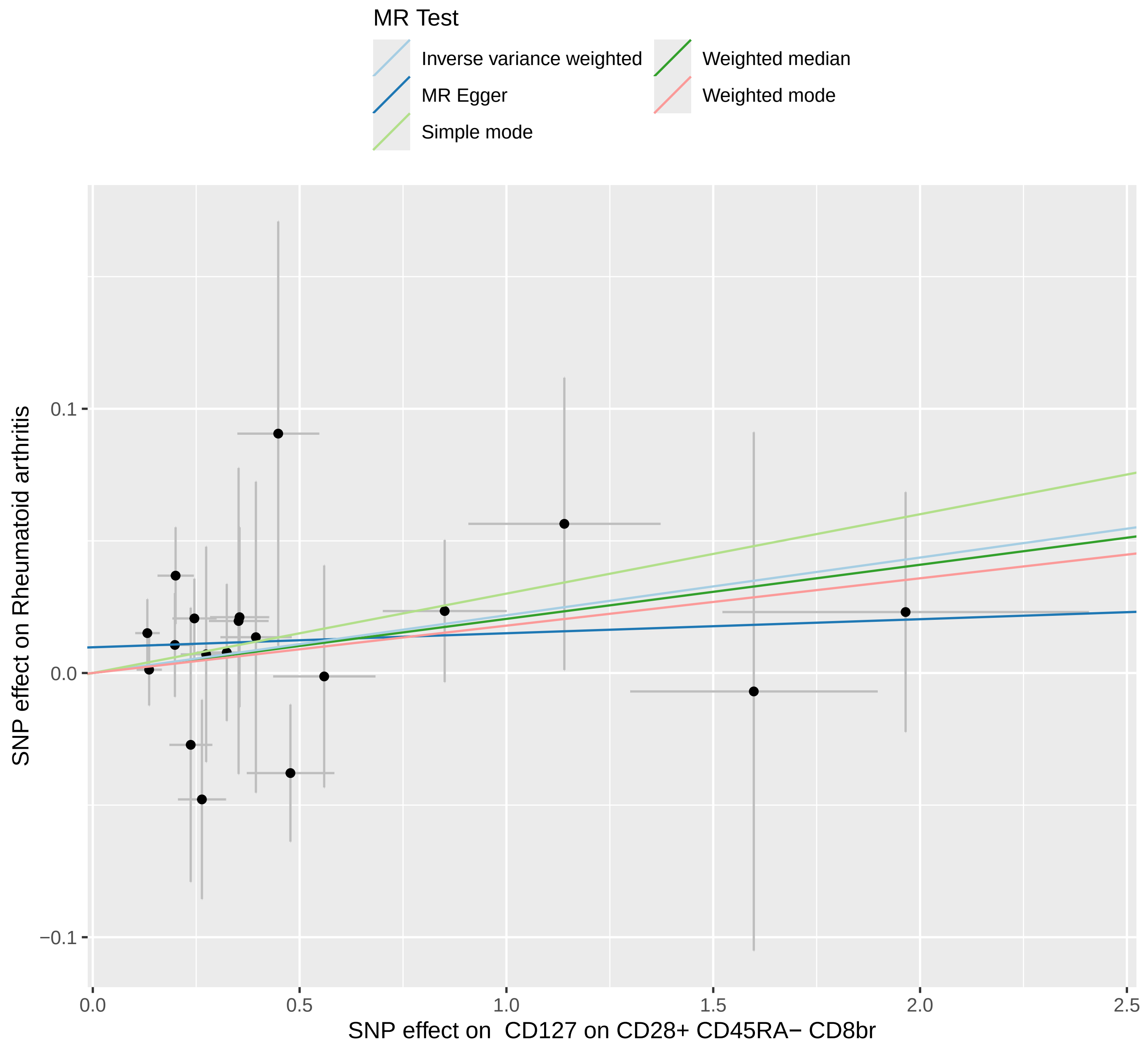
MR Test
Inverse variance weighted
MR Egger
Simple mode
Weighted median
Weighted mode
0.1
0.0
−0.1
SNP effect on Rheumatoid arthritis
0.0
0.5
1.0
1.5
2.0
2.5
SNP effect on CD127 on CD28+ CD45RA− CD8br

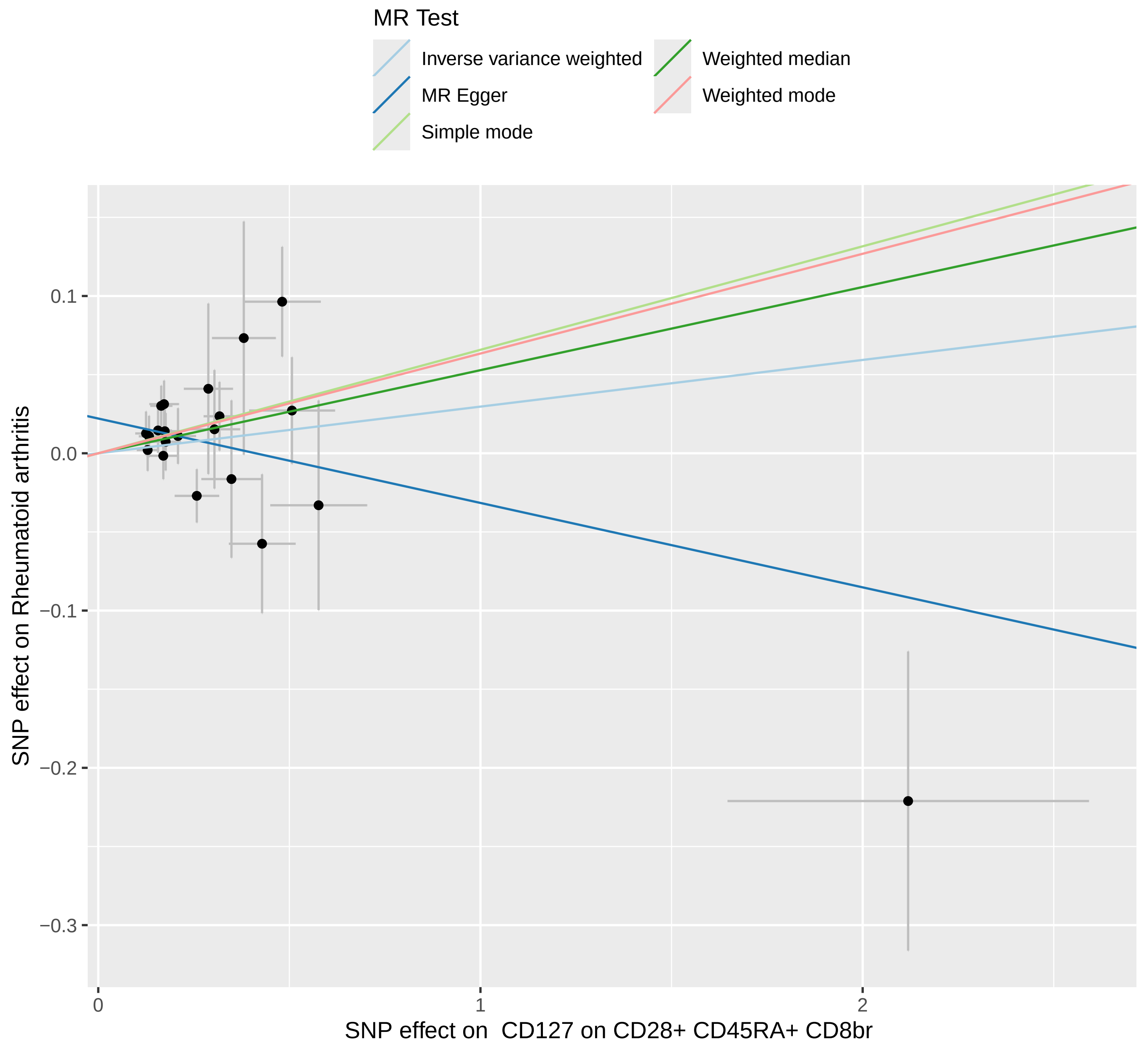

MR Test
Inverse variance weighted
MR Egger
Simple mode
Weighted median
Weighted mode
0.1
0.0
−0.1
−0.2
−0.3
0
1
2
SNP effect on Rheumatoid arthritis
SNP effect on CD127 on CD28+ CD45RA+ CD8br

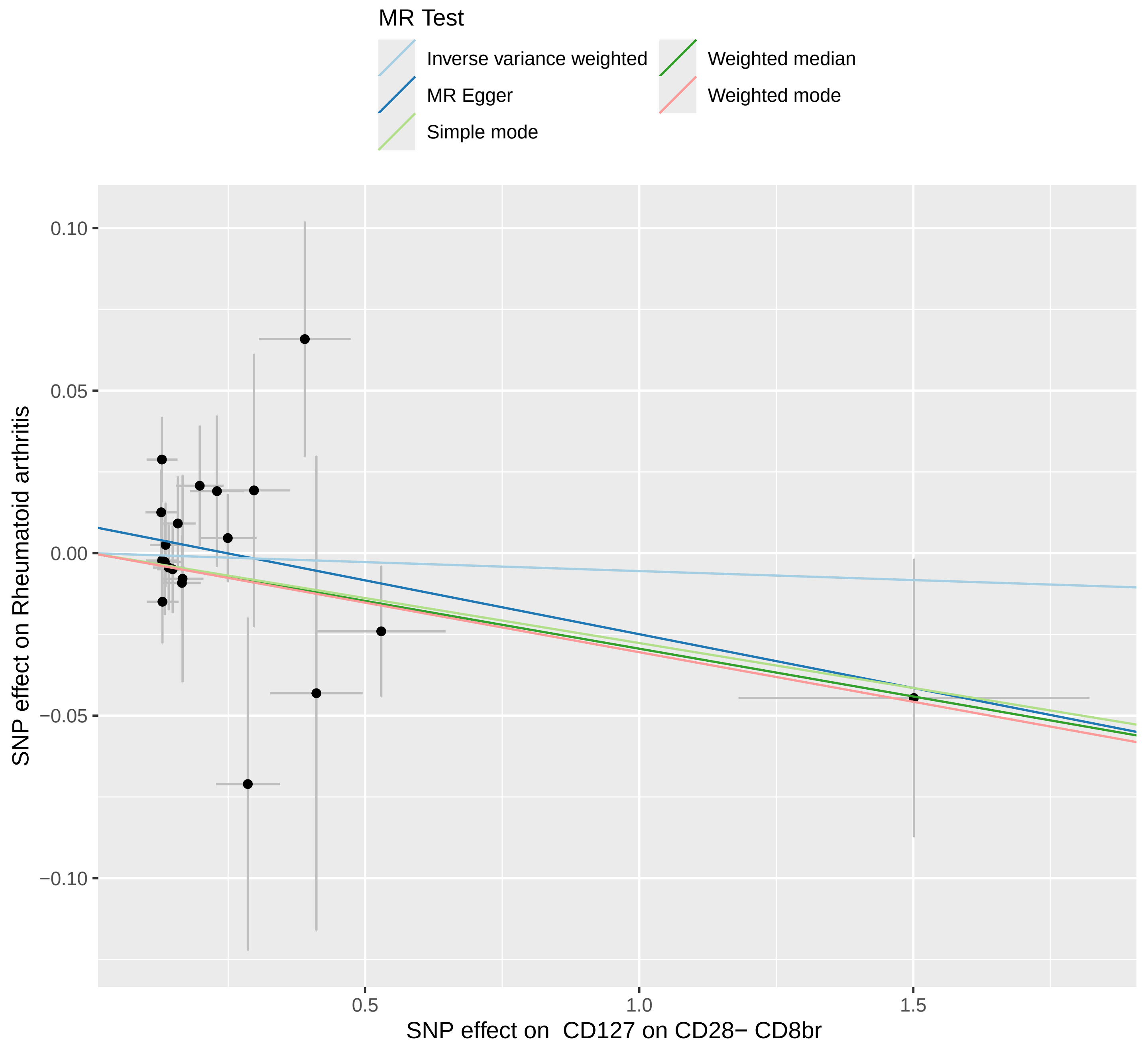

MR Test
Inverse variance weighted
MR Egger
Simple mode
Weighted median
Weighted mode
0.10
0.05
0.00
−0.05
−0.10
0.5
1.0
1.5
SNP effect on Rheumatoid arthritis
SNP effect on CD127 on CD28− CD8br

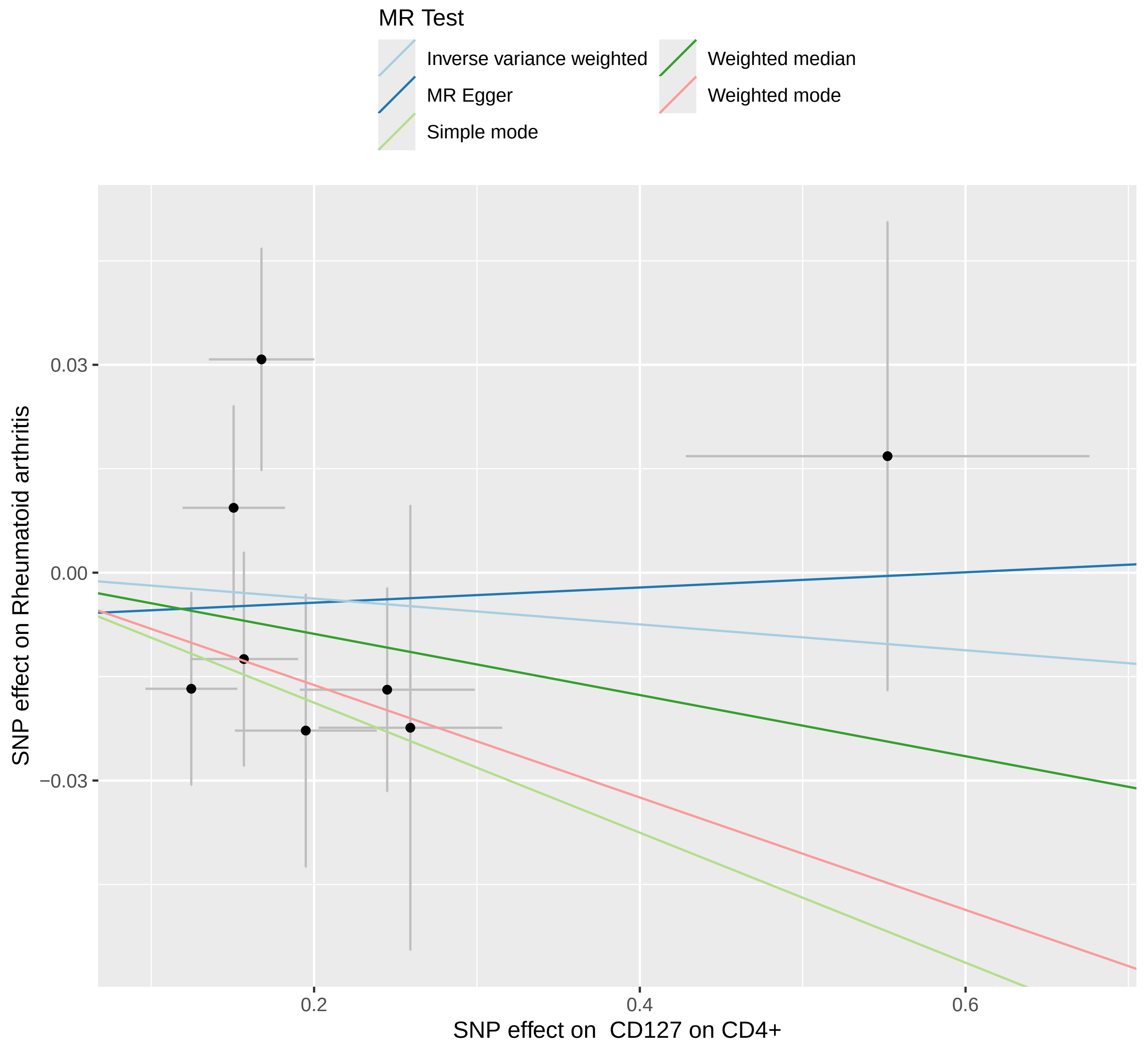
MR Test
Inverse variance weighted
MR Egger
Simple mode
Weighted median
Weighted mode
0.03
0.00
−0.03
0.2
0.4
0.6
SNP effect on Rheumatoid arthritis
SNP effect on CD127 on CD4+

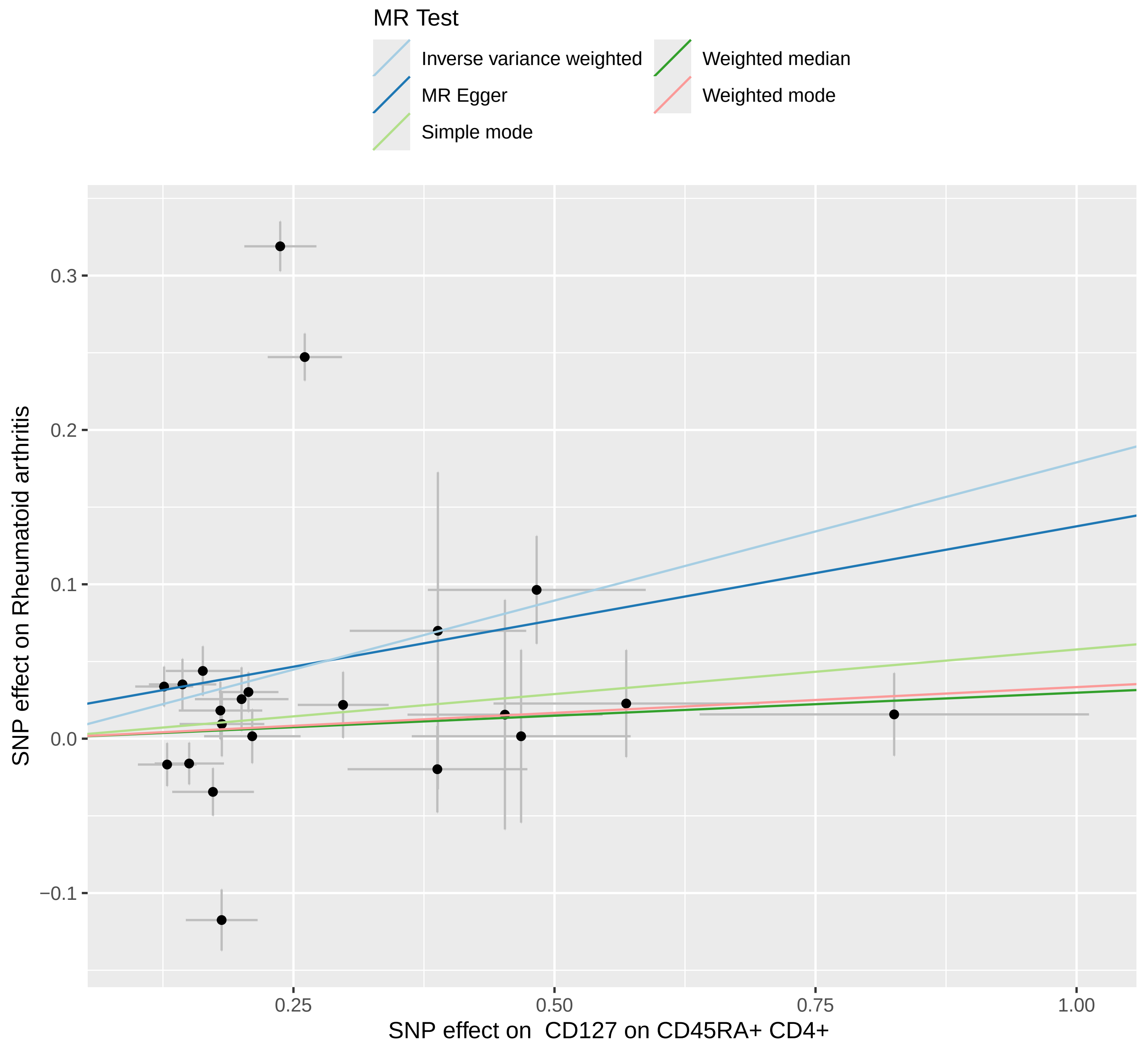

MR Test
Inverse variance weighted
MR Egger
Simple mode
Weighted median
Weighted mode
0.3
0.2
0.1
0.0
−0.1
0.25
0.50
0.75
1.00
SNP effect on Rheumatoid arthritis
SNP effect on CD127 on CD45RA+ CD4+

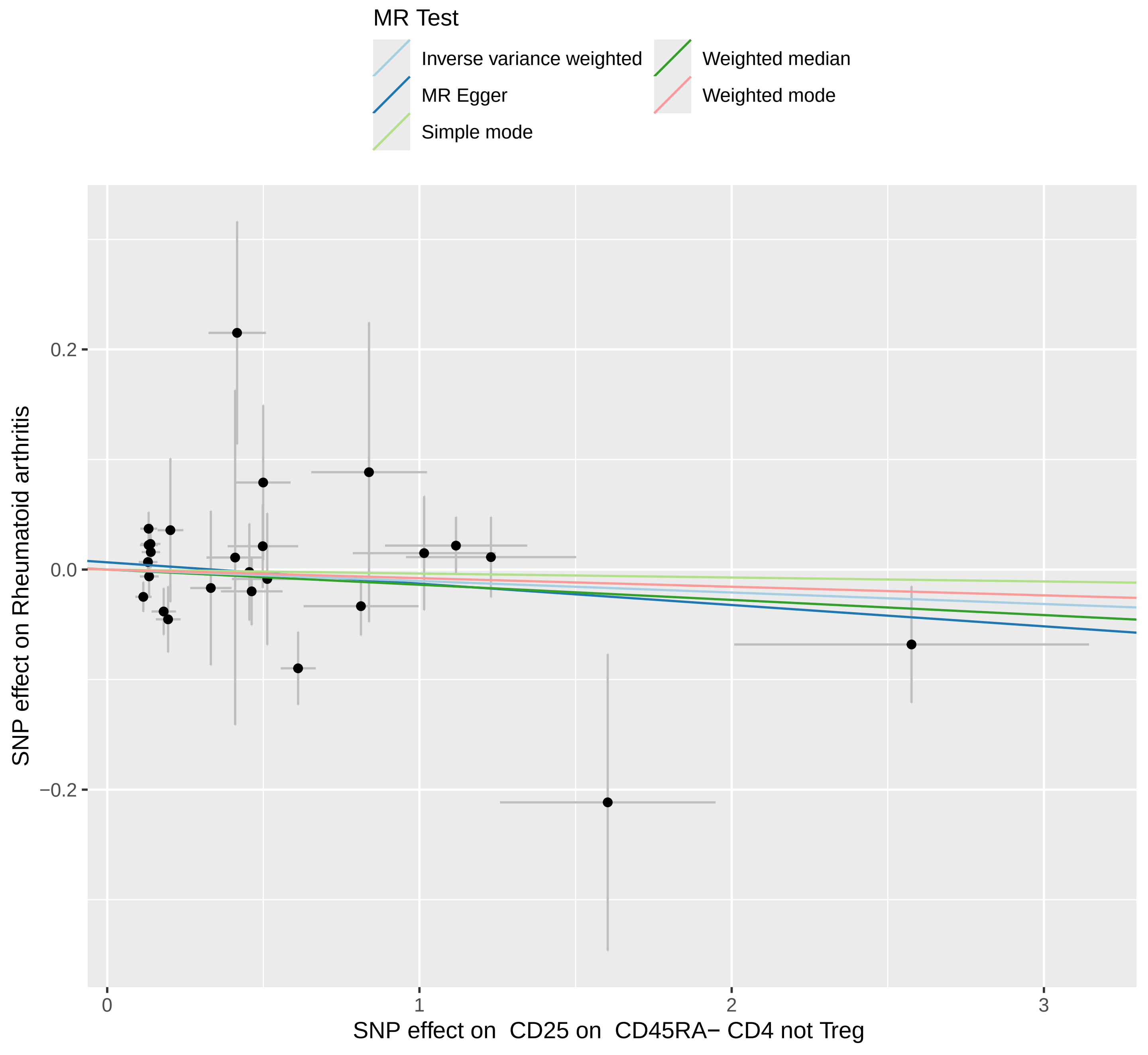
MR Test
Inverse variance weighted
MR Egger
Simple mode
Weighted median
Weighted mode
0.2
0.0
−0.2
0
1
2
3
SNP effect on Rheumatoid arthritis
SNP effect on CD25 on CD45RA− CD4 not Treg

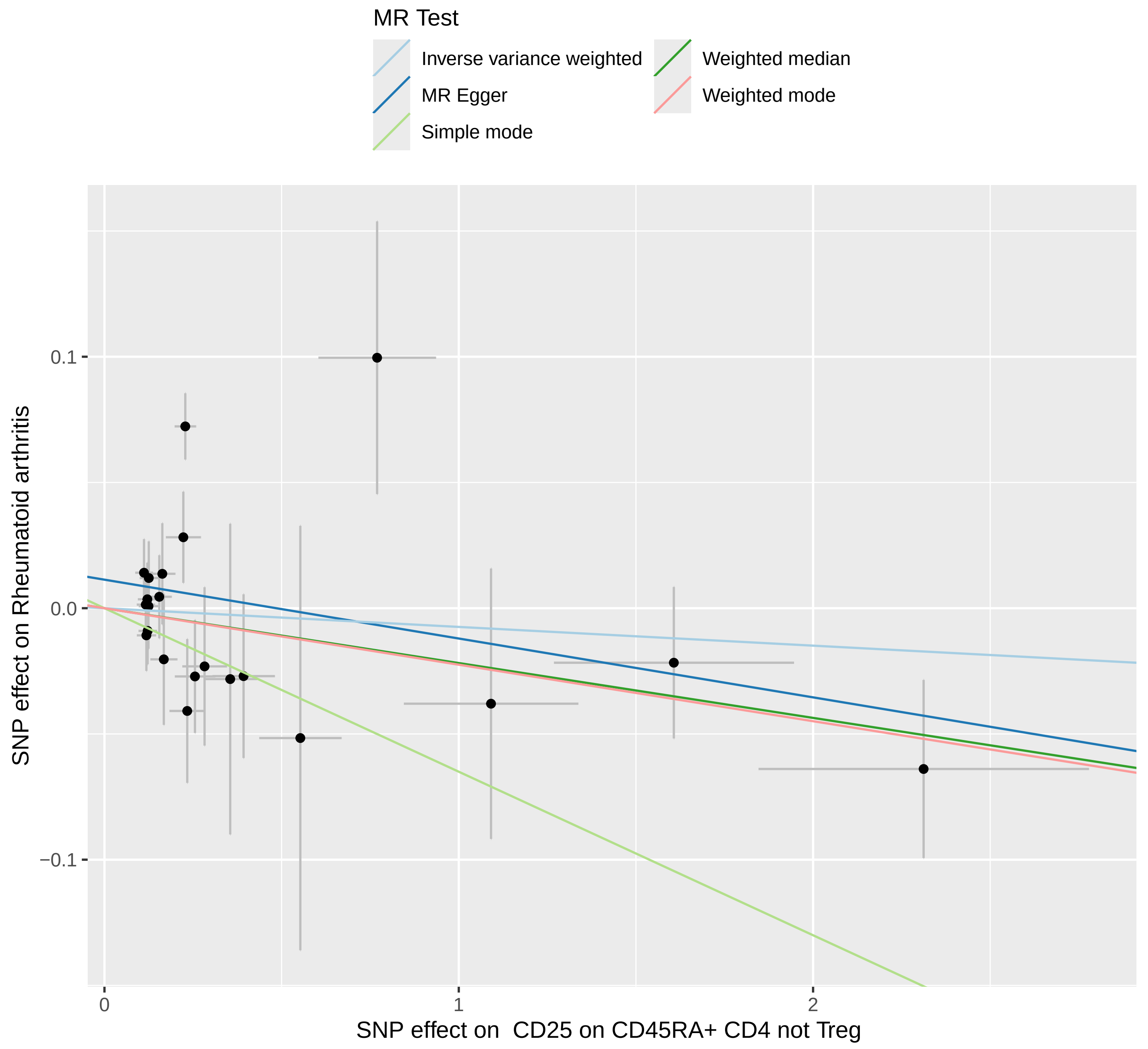
MR Test
Inverse variance weighted
MR Egger
Simple mode
Weighted median
Weighted mode
0.1
0.0
−0.1
0
1
2
SNP effect on Rheumatoid arthritis
SNP effect on CD25 on CD45RA+ CD4 not Treg

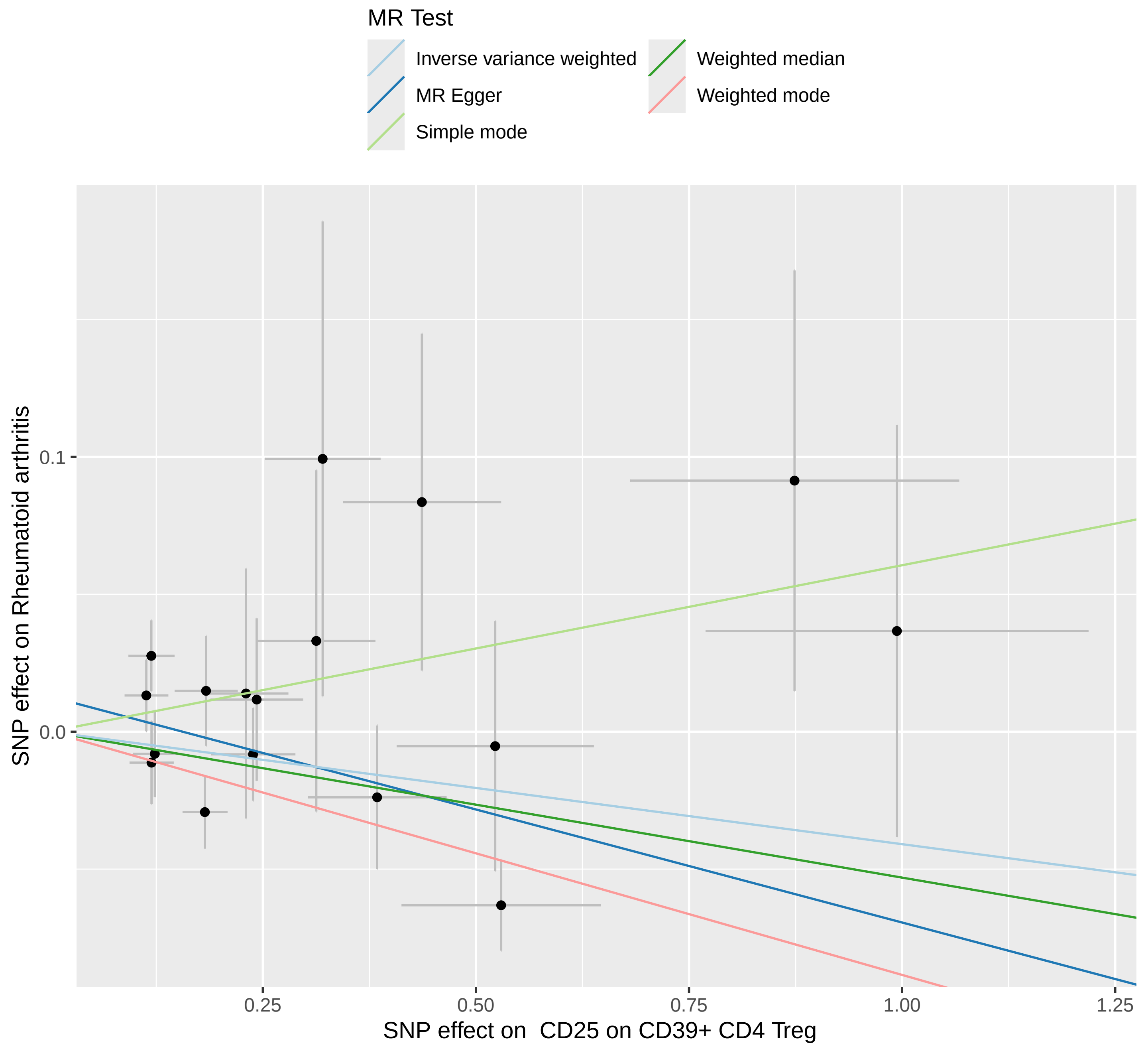
MR Test
Inverse variance weighted
MR Egger
Simple mode
Weighted median
Weighted mode
SNP effect on Rheumatoid arthritis
0.1
0.0
0.25
0.50
0.75
1.00
1.25
SNP effect on CD25 on CD39+ CD4 Treg

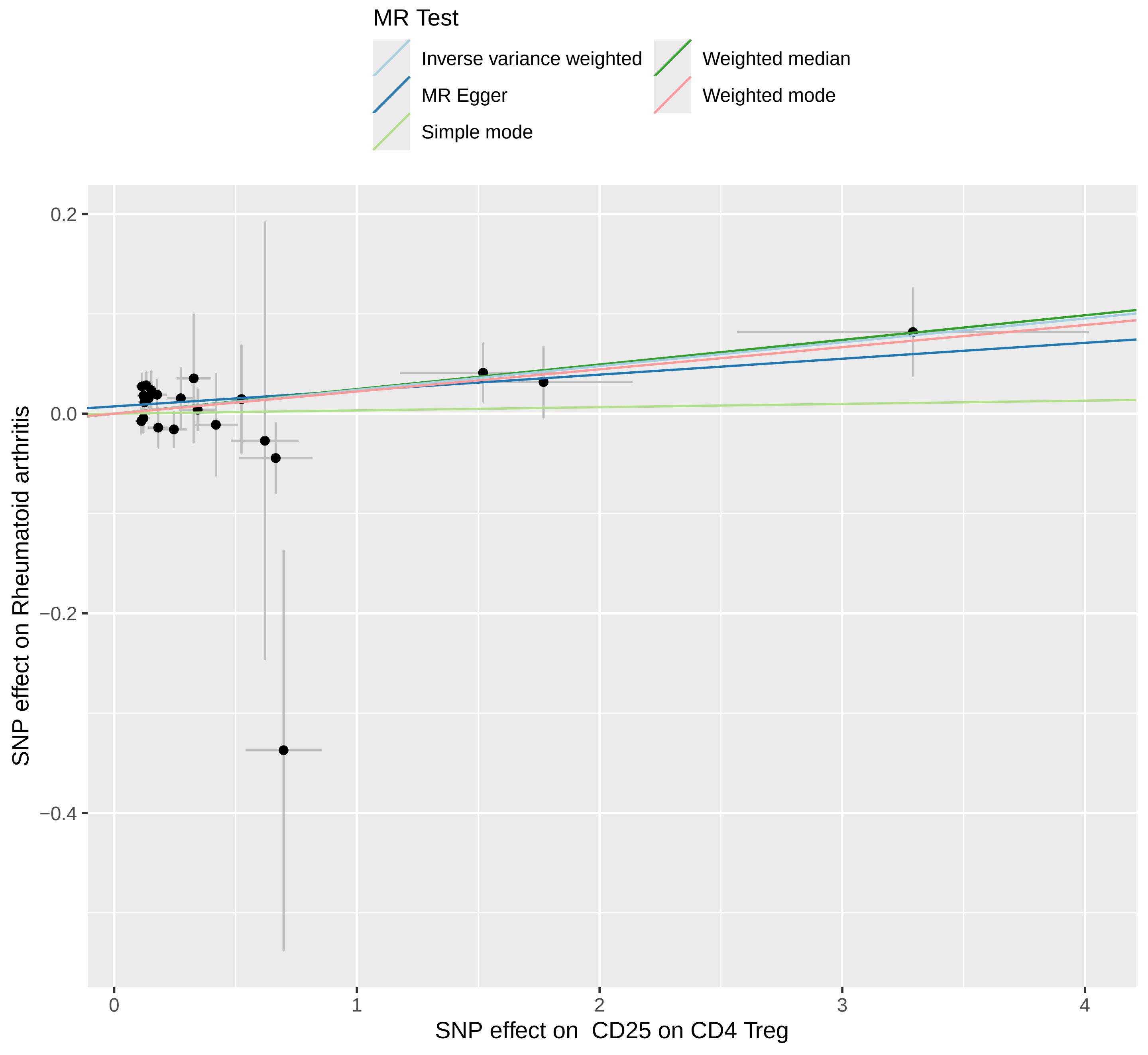

MR Test
Inverse variance weighted
MR Egger
Simple mode
Weighted median
Weighted mode
0.2
0.0
−0.2
−0.4
0
1
2
3
4
SNP effect on Rheumatoid arthritis
SNP effect on CD25 on CD4 Treg

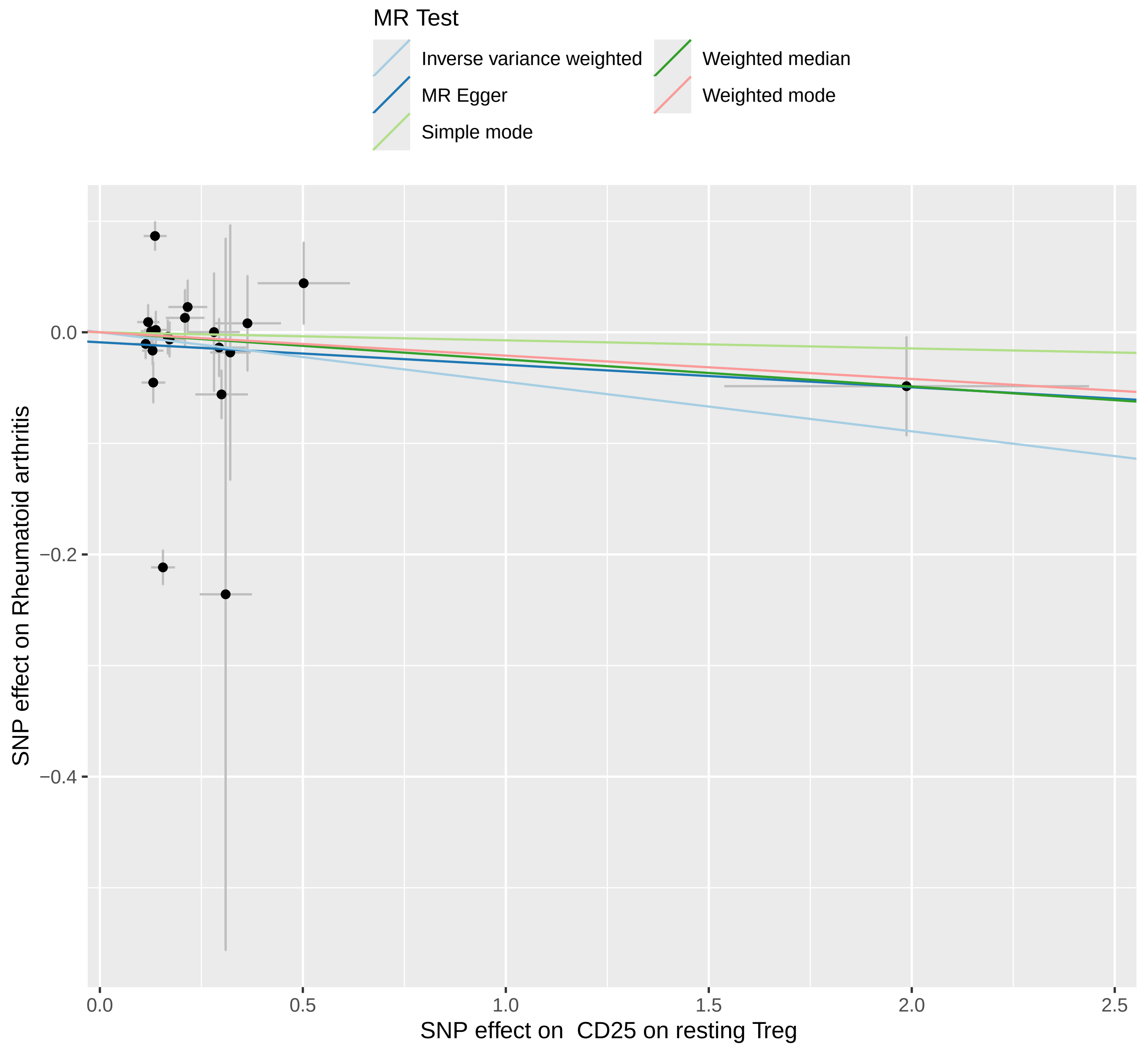

MR Test
Inverse variance weighted
MR Egger
Simple mode
Weighted median
Weighted mode
0.0
−0.2
−0.4
SNP effect on Rheumatoid arthritis
0.0
0.5
1.0
1.5
2.0
2.5
SNP effect on CD25 on resting Treg

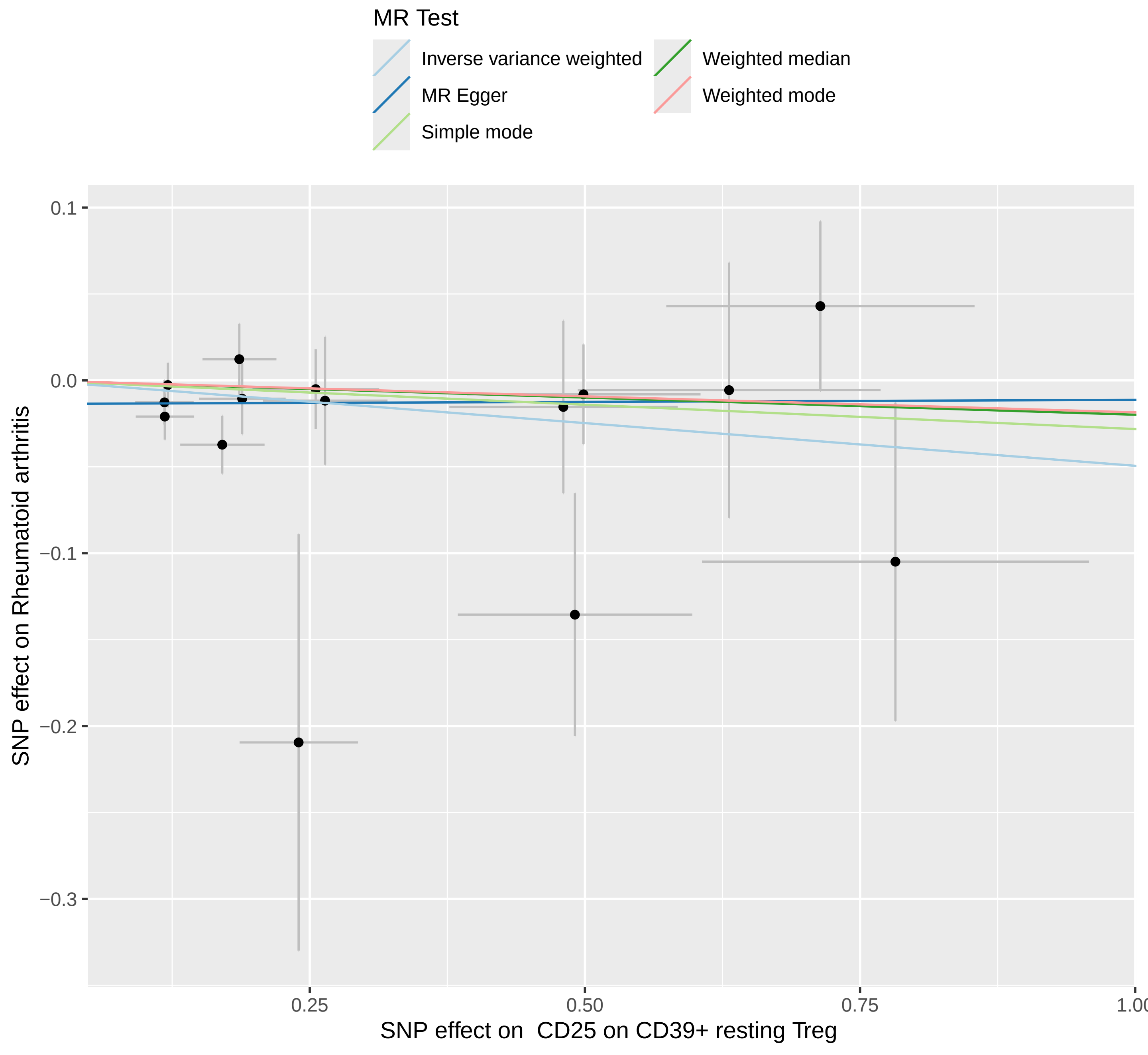
MR Test
Inverse variance weighted
MR Egger
Simple mode
Weighted median
Weighted mode
0.1
0.0
−0.1
−0.2
−0.3
0.25
0.50
0.75
SNP effect on Rheumatoid arthritis
SNP effect on CD25 on CD39+ resting Treg

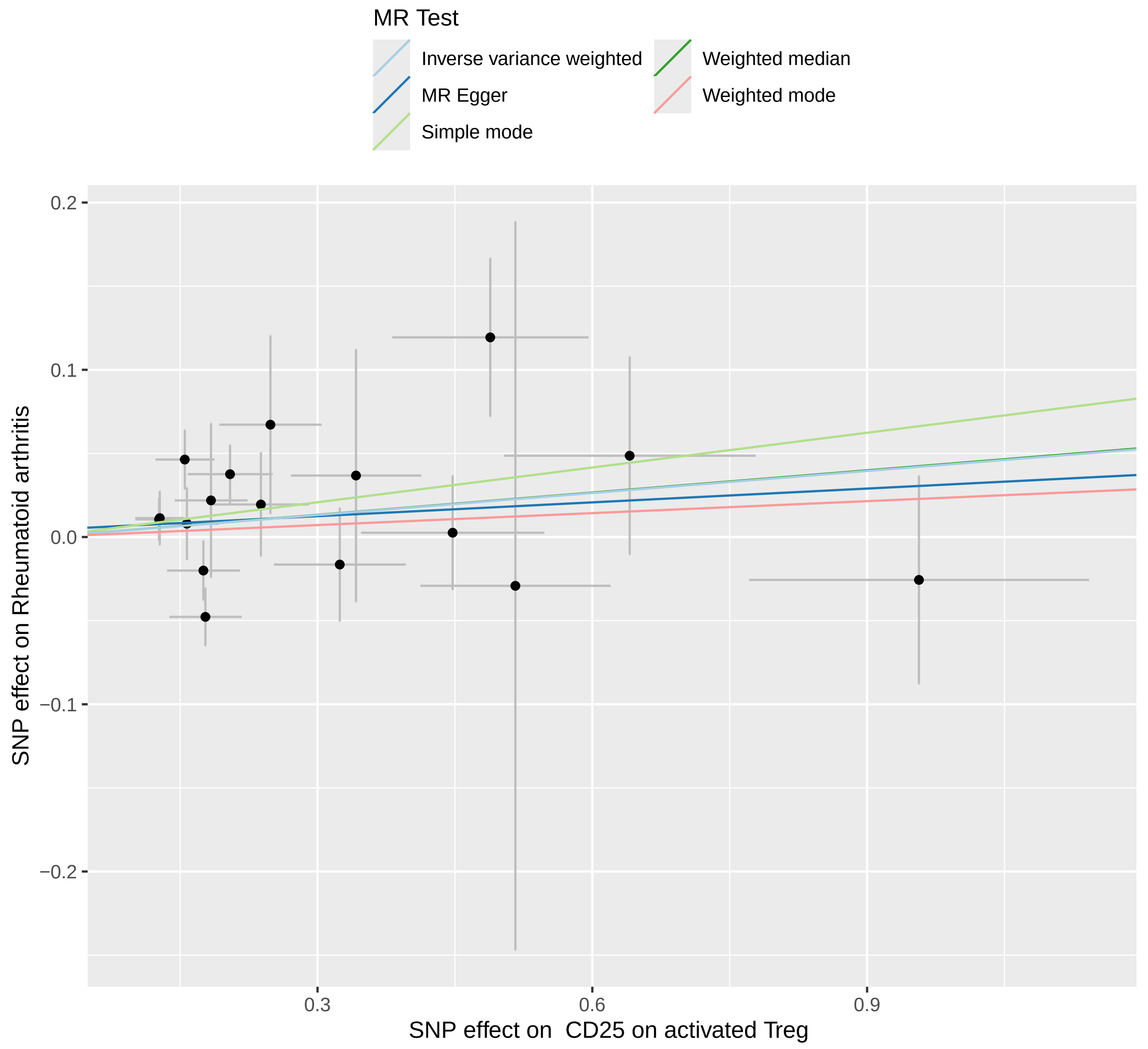
MR Test
Inverse variance weighted
MR Egger
Simple mode
Weighted median
Weighted mode
0.2
0.1
0.0
−0.1
−0.2
0.3
0.6
0.9
SNP effect on Rheumatoid arthritis
SNP effect on CD25 on activated Treg

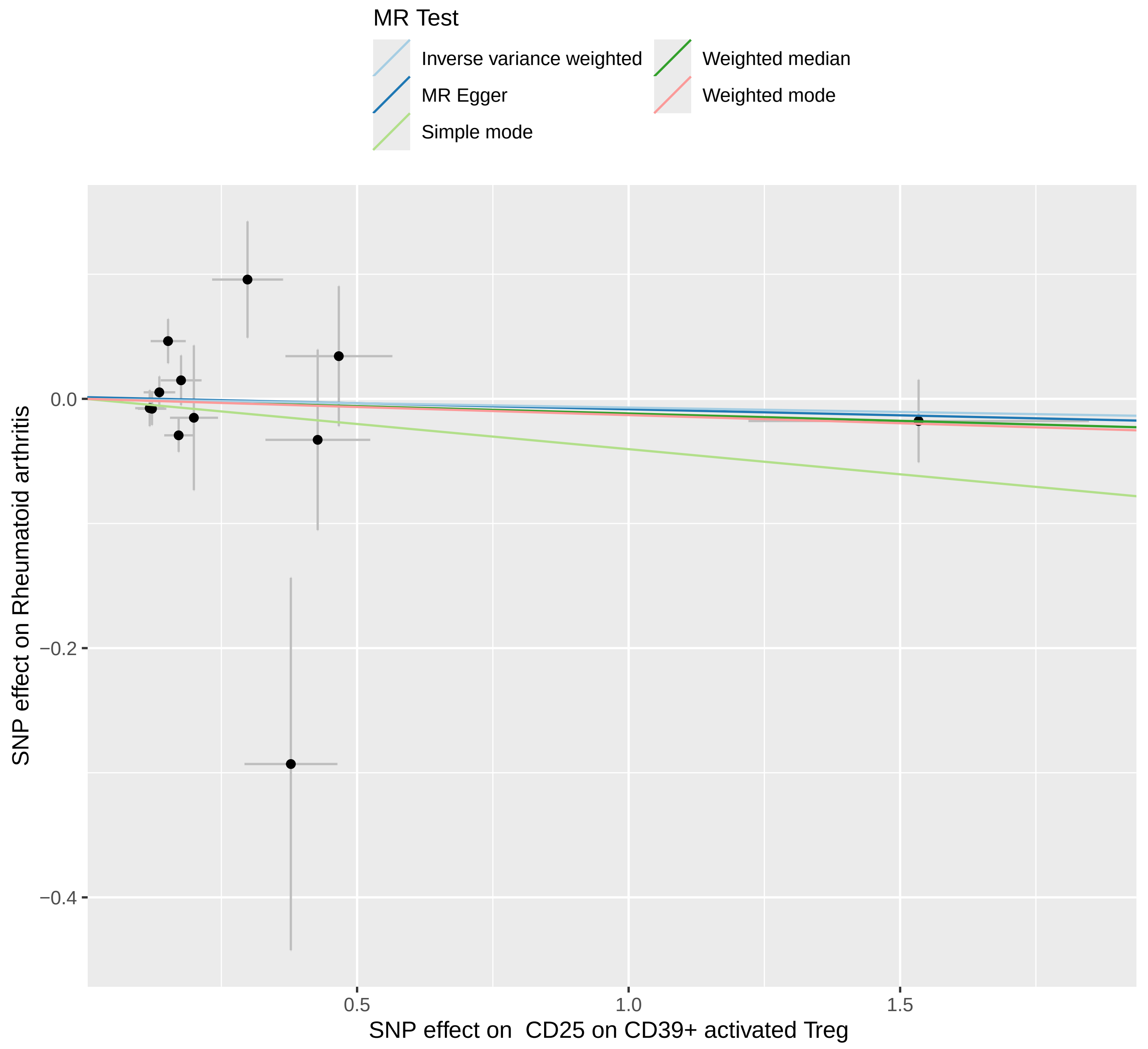

MR Test
Inverse variance weighted
MR Egger
Simple mode
Weighted median
Weighted mode
0.0
−0.2
−0.4
SNP effect on Rheumatoid arthritis
0.5
1.0
1.5
SNP effect on CD25 on CD39+ activated Treg

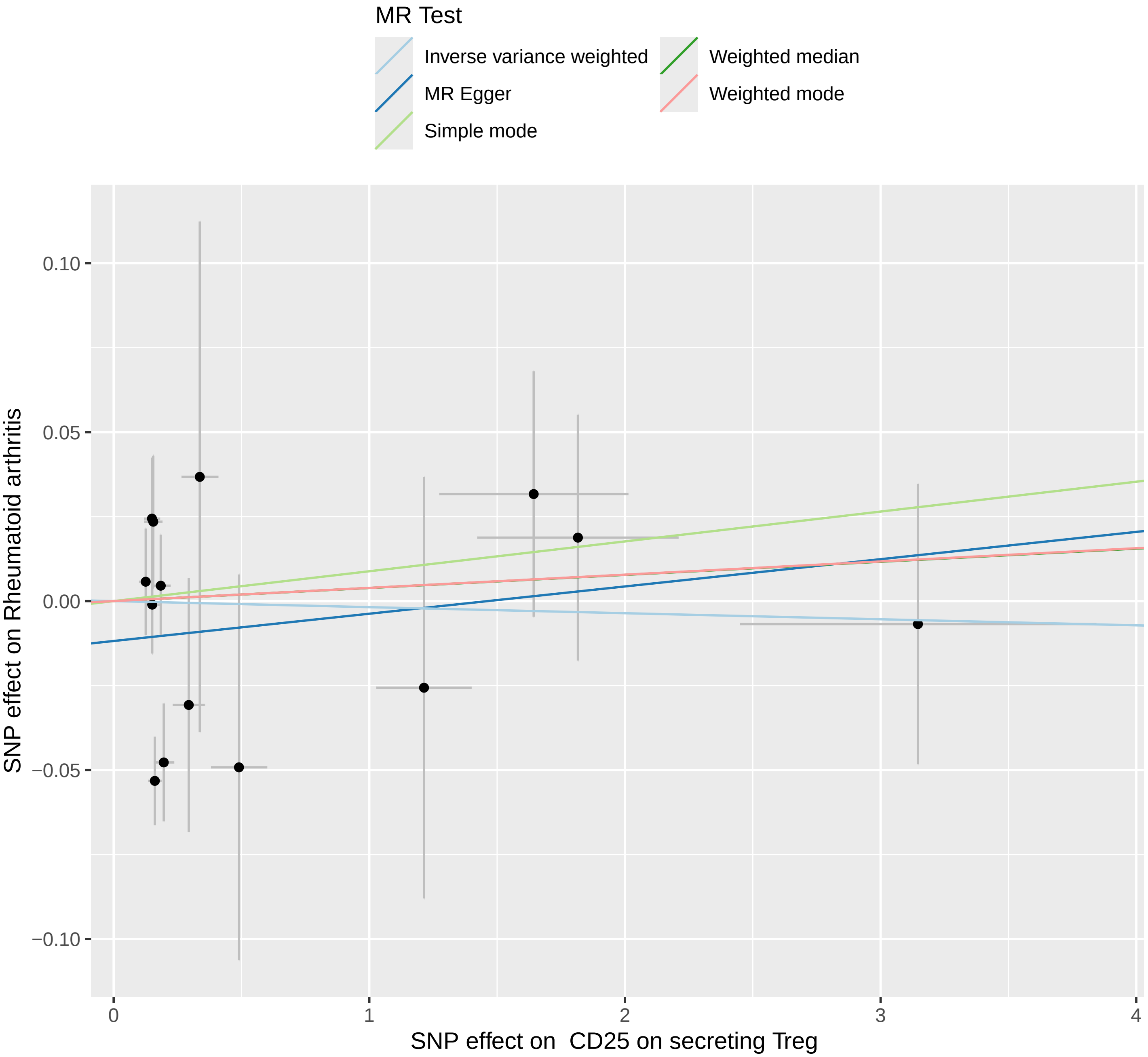
MR Test
Inverse variance weighted
MR Egger
Simple mode
Weighted median
Weighted mode
0.10
0.05
0.00
−0.05
−0.10
0
1
2
3
4
SNP effect on Rheumatoid arthritis
SNP effect on CD25 on secreting Treg

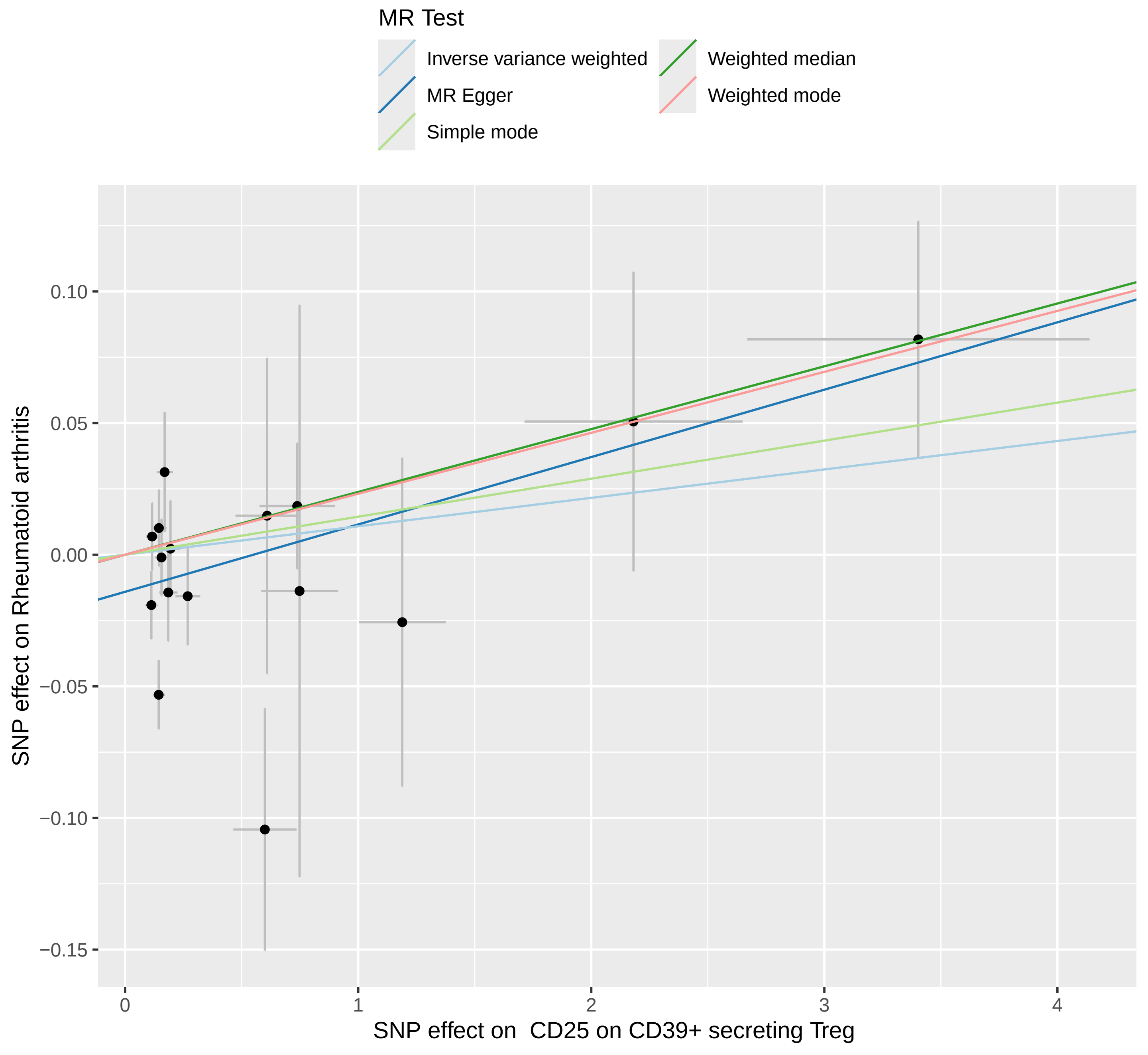

MR Test
Inverse variance weighted
MR Egger
Simple mode
Weighted median
Weighted mode
0.10
0.05
0.00
−0.05
−0.10
−0.15
0
1
2
3
4
SNP effect on Rheumatoid arthritis
SNP effect on CD25 on CD39+ secreting Treg

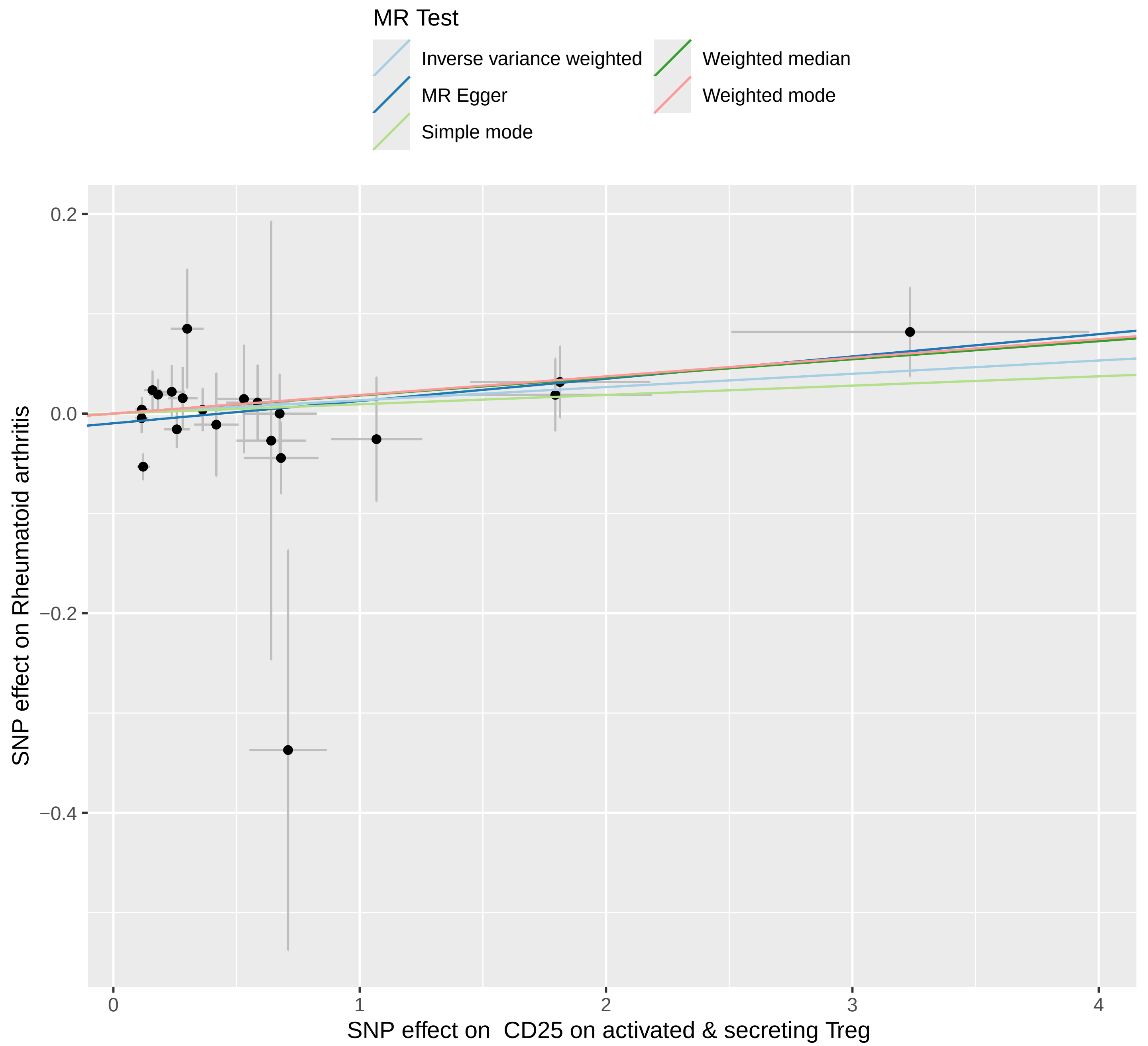

MR Test
Inverse variance weighted
MR Egger
Simple mode
Weighted median
Weighted mode
0.2
0.0
−0.2
−0.4
0
1
2
3
4
SNP effect on Rheumatoid arthritis
SNP effect on CD25 on activated & secreting Treg

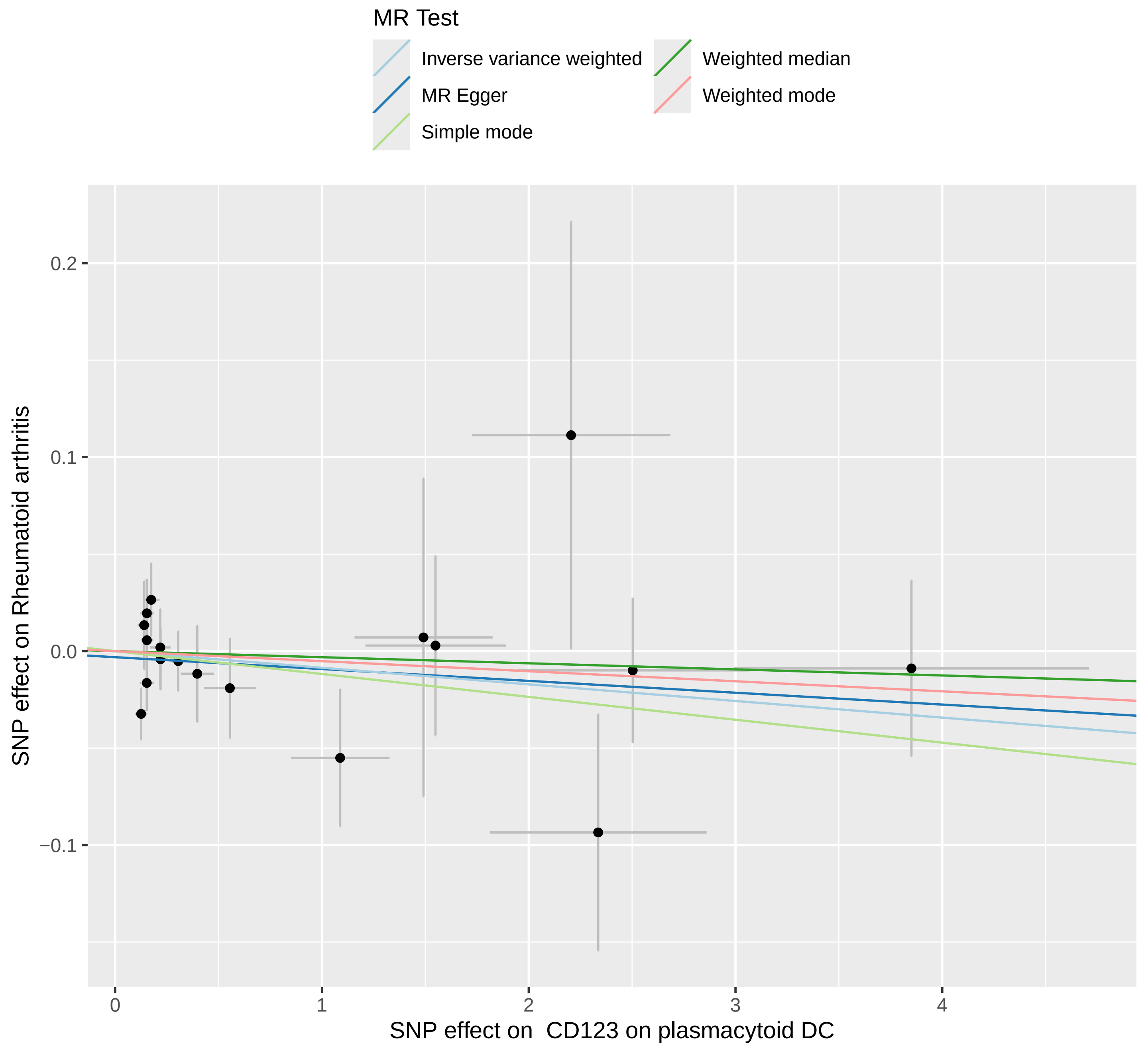
MR Test
Inverse variance weighted
MR Egger
Simple mode
Weighted median
Weighted mode
0.2
0.1
0.0
−0.1
0
1
2
3
4
SNP effect on Rheumatoid arthritis
SNP effect on CD123 on plasmacytoid DC

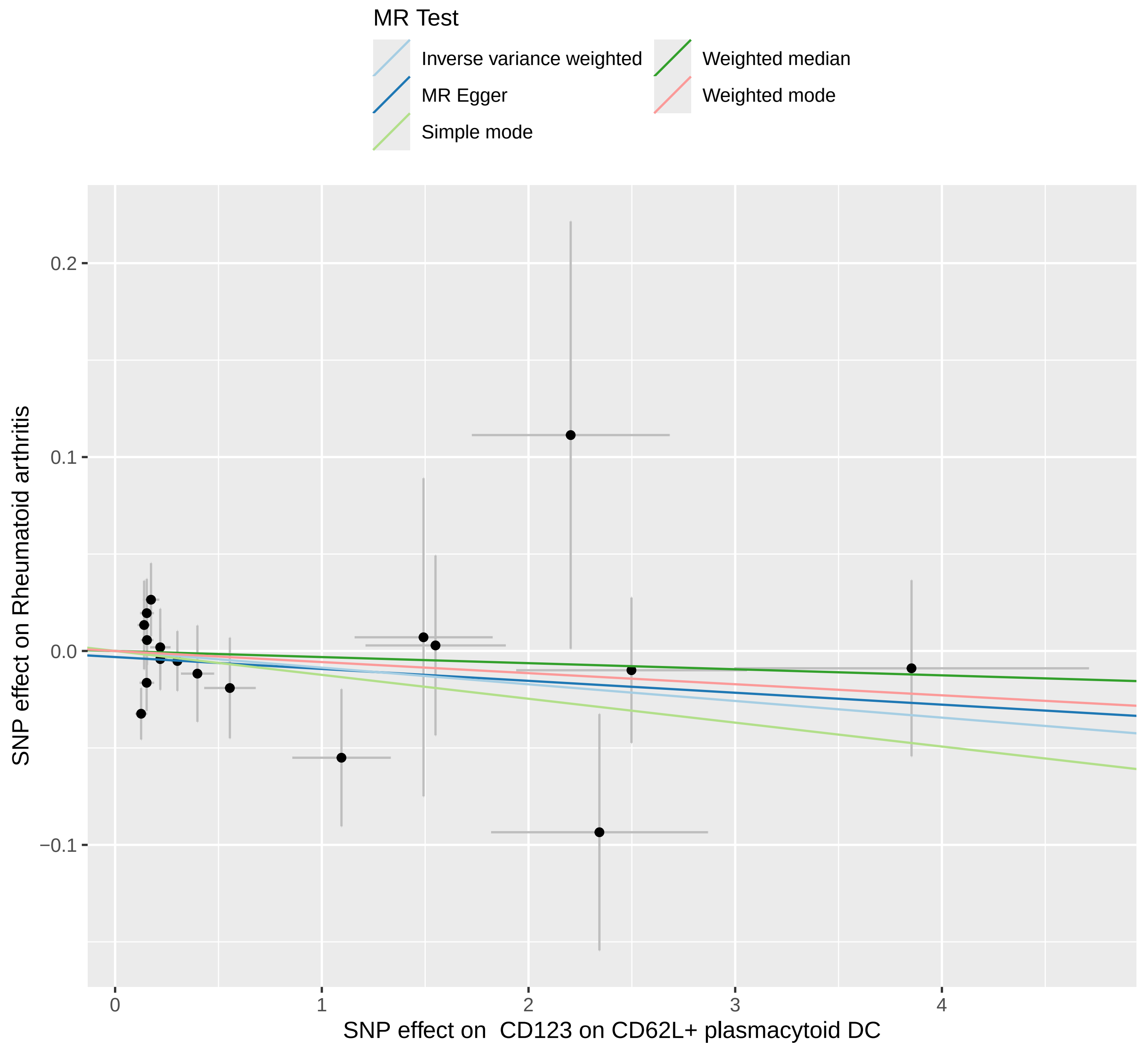
MR Test
Inverse variance weighted
MR Egger
Simple mode
Weighted median
Weighted mode
SNP effect on Rheumatoid arthritis
0.2
0.1
0.0
−0.1
0
1
2
3
4
SNP effect on CD123 on CD62L+ plasmacytoid DC

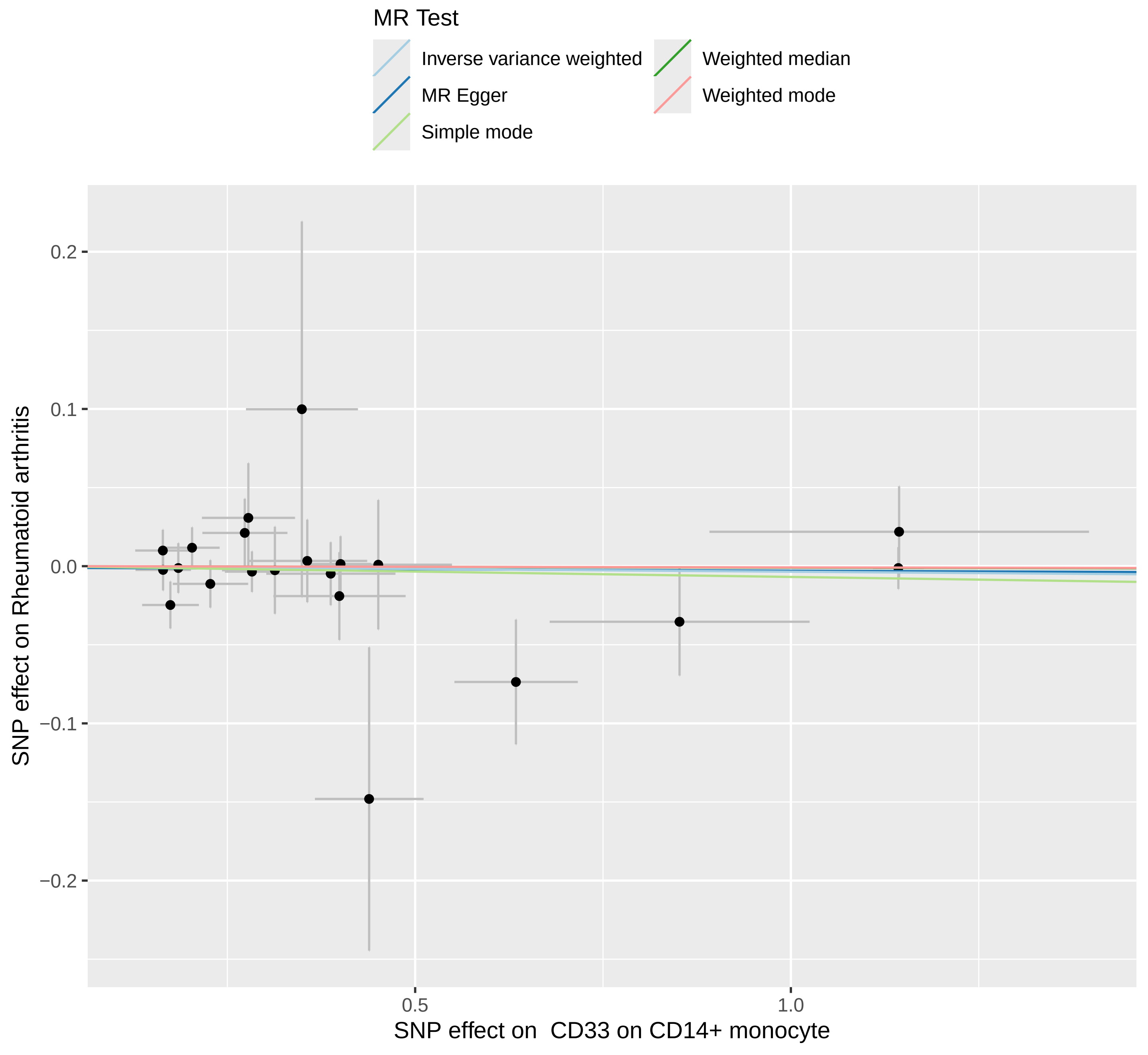

MR Test
Inverse variance weighted
MR Egger
Simple mode
Weighted median
Weighted mode
0.2
0.1
0.0
−0.1
−0.2
SNP effect on Rheumatoid arthritis
0.5
1.0
SNP effect on CD33 on CD14+ monocyte

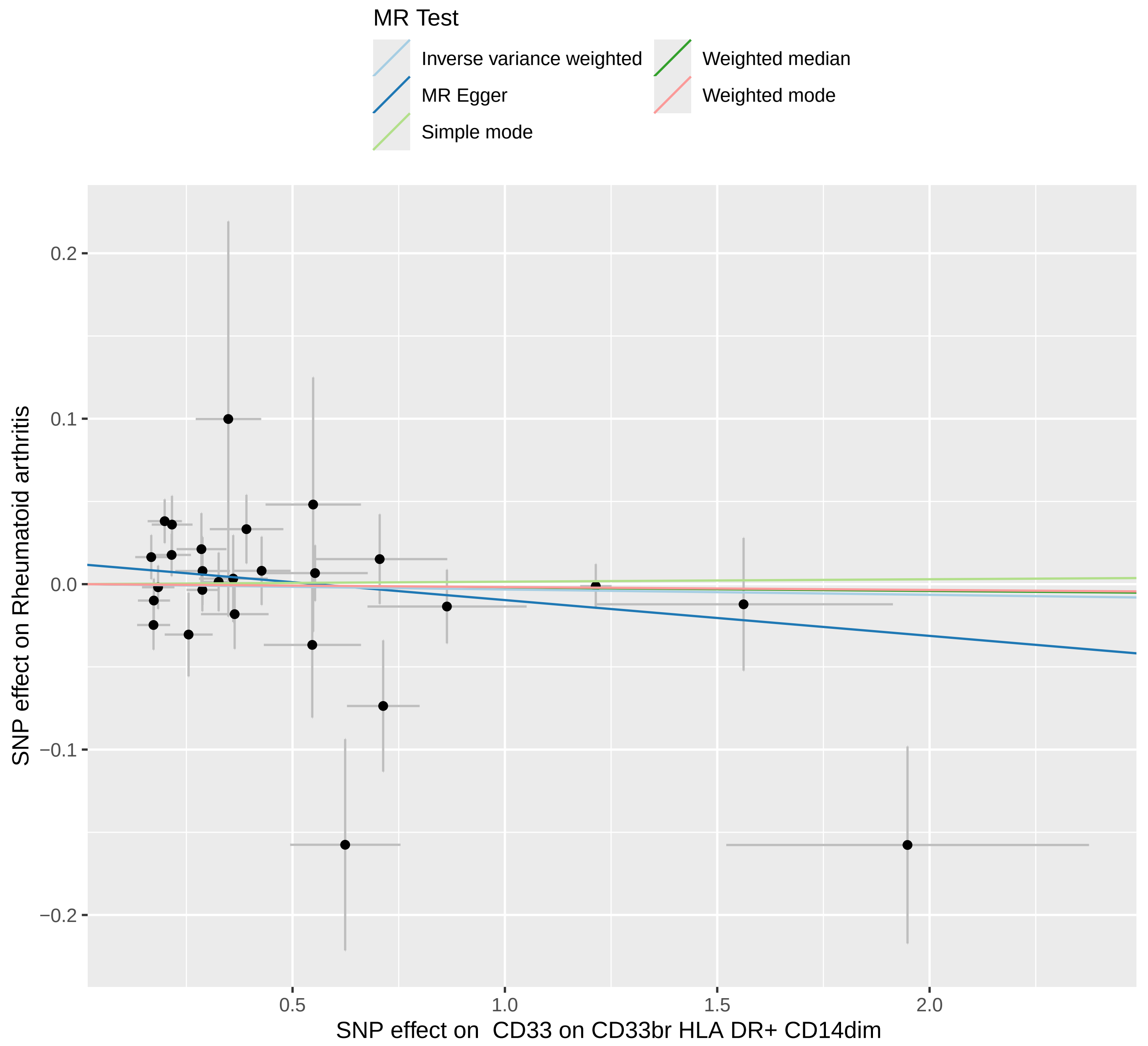

MR Test
Inverse variance weighted
MR Egger
Simple mode
Weighted median
Weighted mode
SNP effect on Rheumatoid arthritis
0.2
0.1
0.0
−0.1
−0.2
0.5
1.0
1.5
2.0
SNP effect on CD33 on CD33br HLA DR+ CD14dim

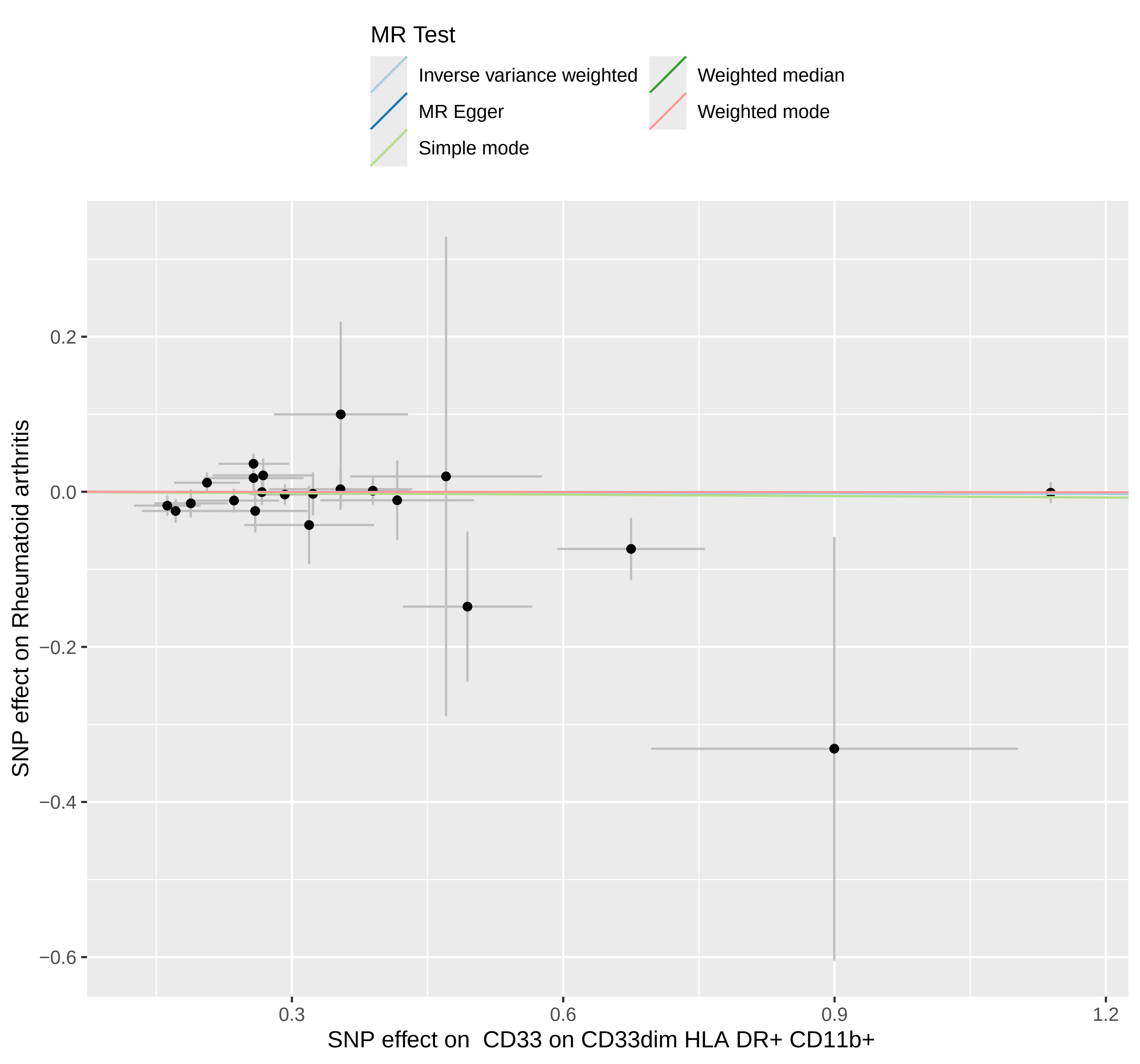
MR Test
Inverse variance weighted
MR Egger
Simple mode
Weighted median
Weighted mode
SNP effect on Rheumatoid arthritis
0.2
0.0
−0.2
−0.4
−0.6
0.3
0.6
0.9
1.2
SNP effect on CD33 on CD33dim HLA DR+ CD11b+

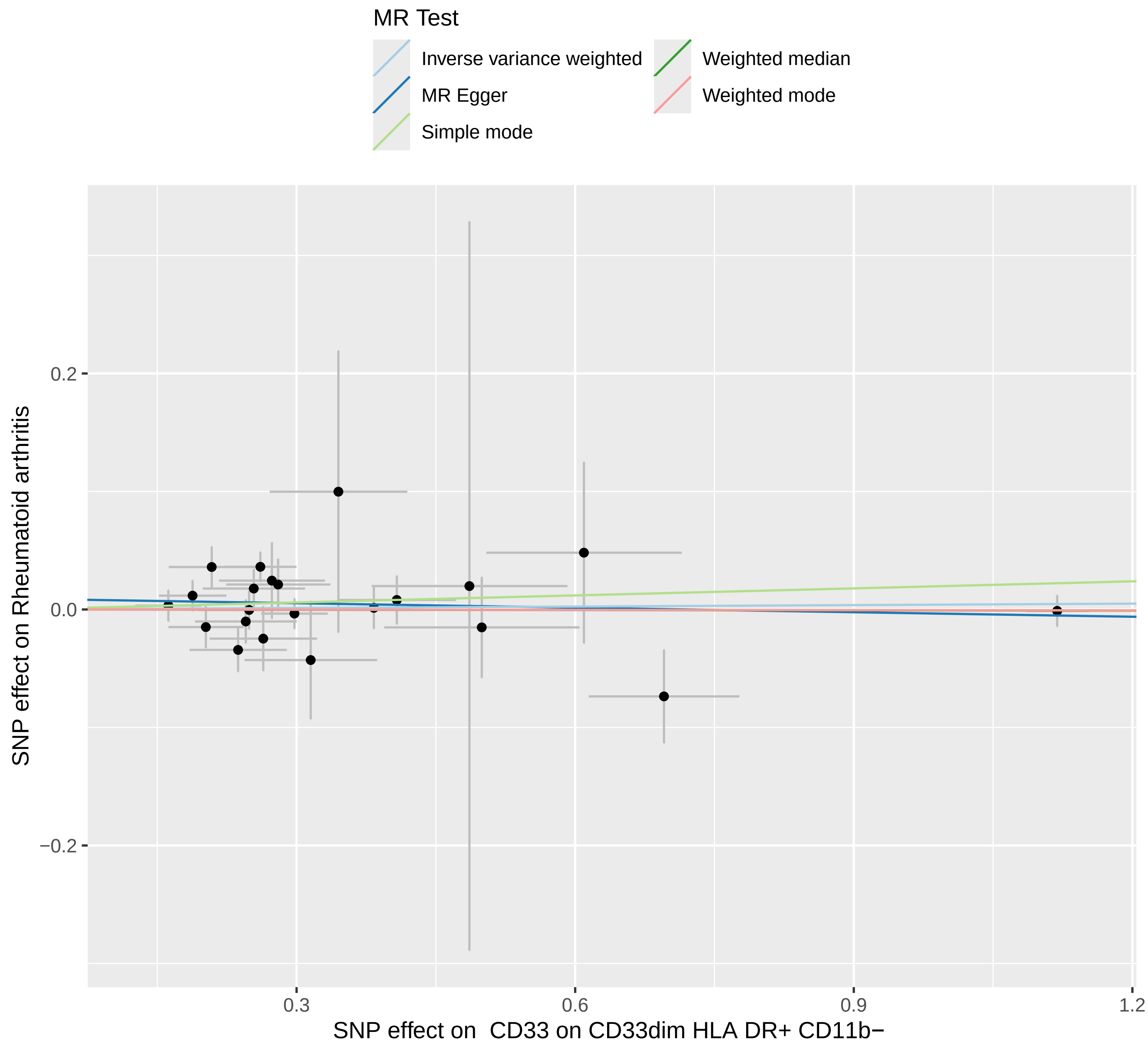
MR Test
Inverse variance weighted
MR Egger
Simple mode
Weighted median
Weighted mode
0.2
0.0
−0.2
0.3
0.6
0.9
1.2
SNP effect on Rheumatoid arthritis
SNP effect on CD33 on CD33dim HLA DR+ CD11b−

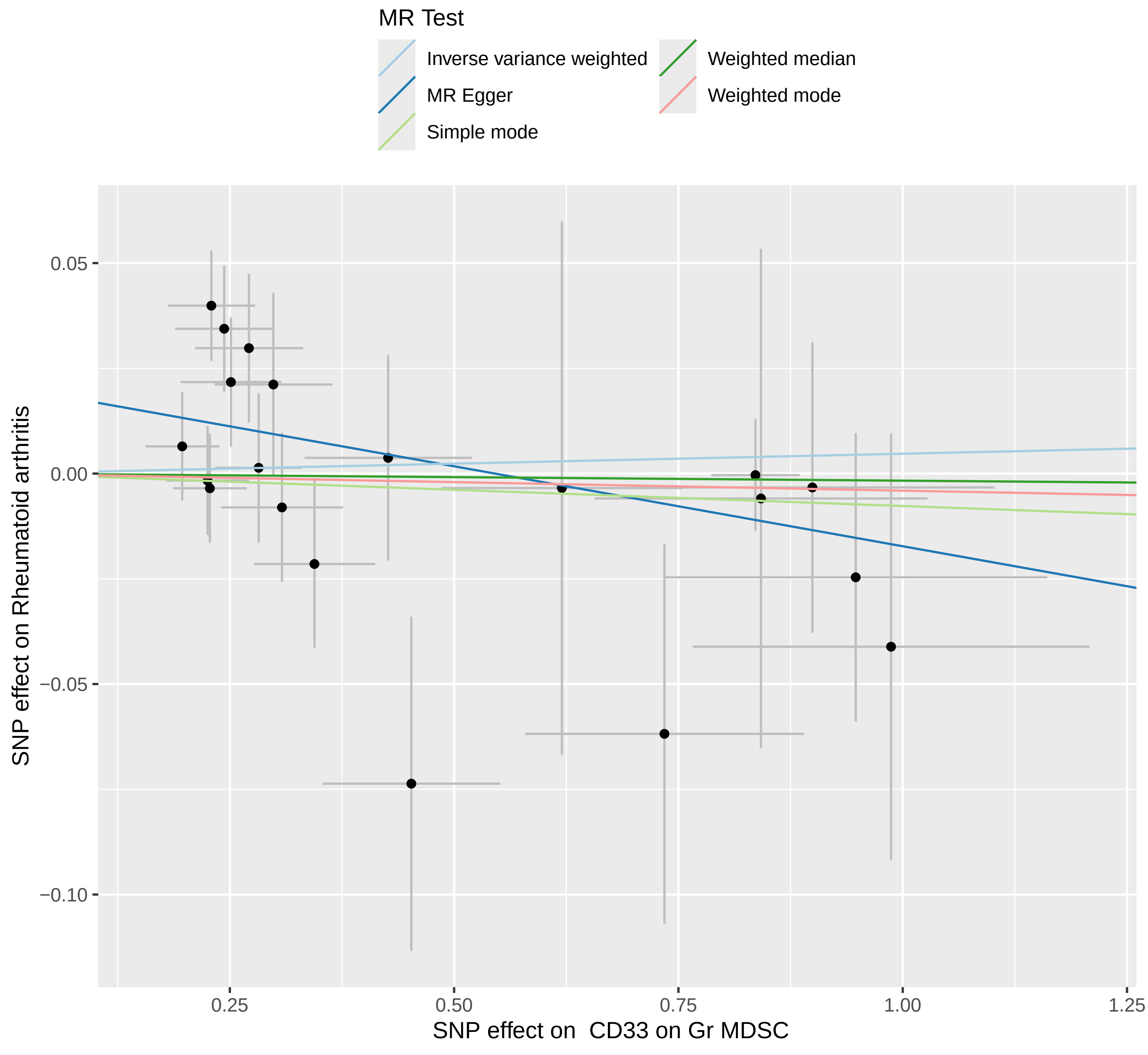
MR Test
Inverse variance weighted
MR Egger
Simple mode
Weighted median
Weighted mode
0.05
0.00
−0.05
−0.10
0.25
0.50
0.75
1.00
1.25
SNP effect on Rheumatoid arthritis
SNP effect on CD33 on Gr MDSC

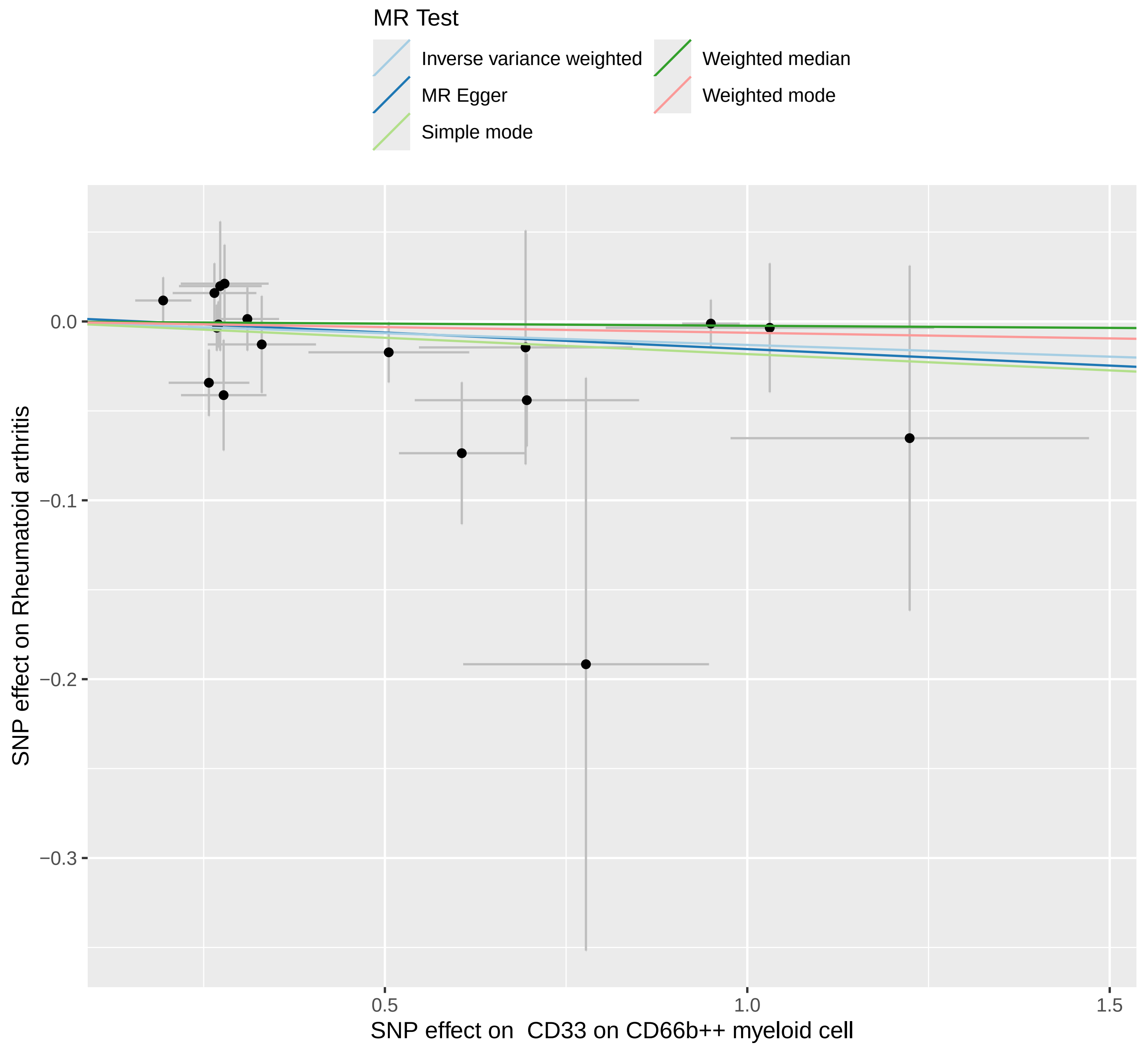
MR Test
Inverse variance weighted
MR Egger
Simple mode
Weighted median
Weighted mode
SNP effect on Rheumatoid arthritis
0.0
−0.1
−0.2
−0.3
0.5
1.0
1.5
SNP effect on CD33 on CD66b++ myeloid cell

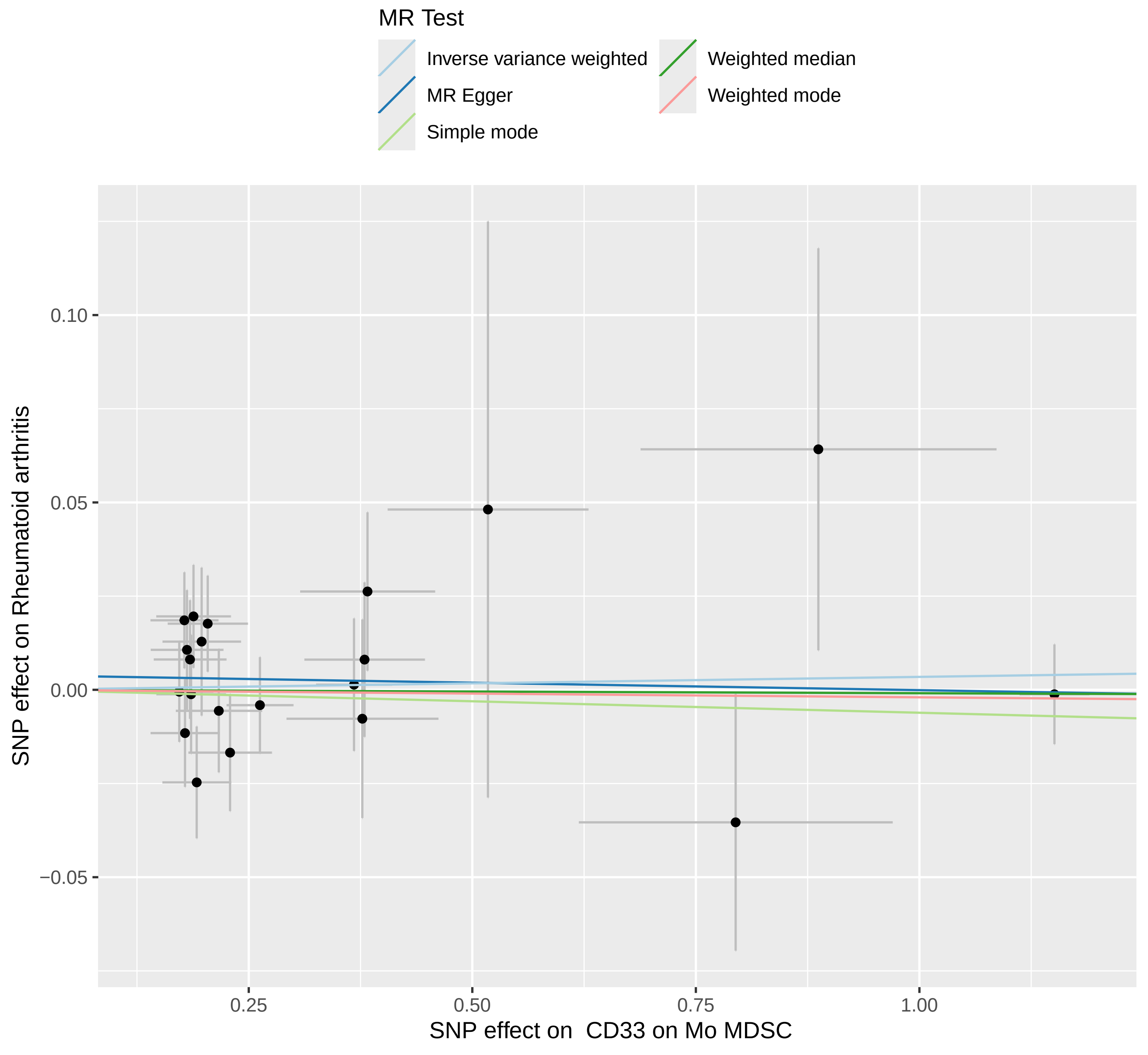

MR Test
Inverse variance weighted
MR Egger
Simple mode
Weighted median
Weighted mode
0.10
0.05
0.00
−0.05
SNP effect on Rheumatoid arthritis
0.25
0.50
0.75
1.00
SNP effect on CD33 on Mo MDSC

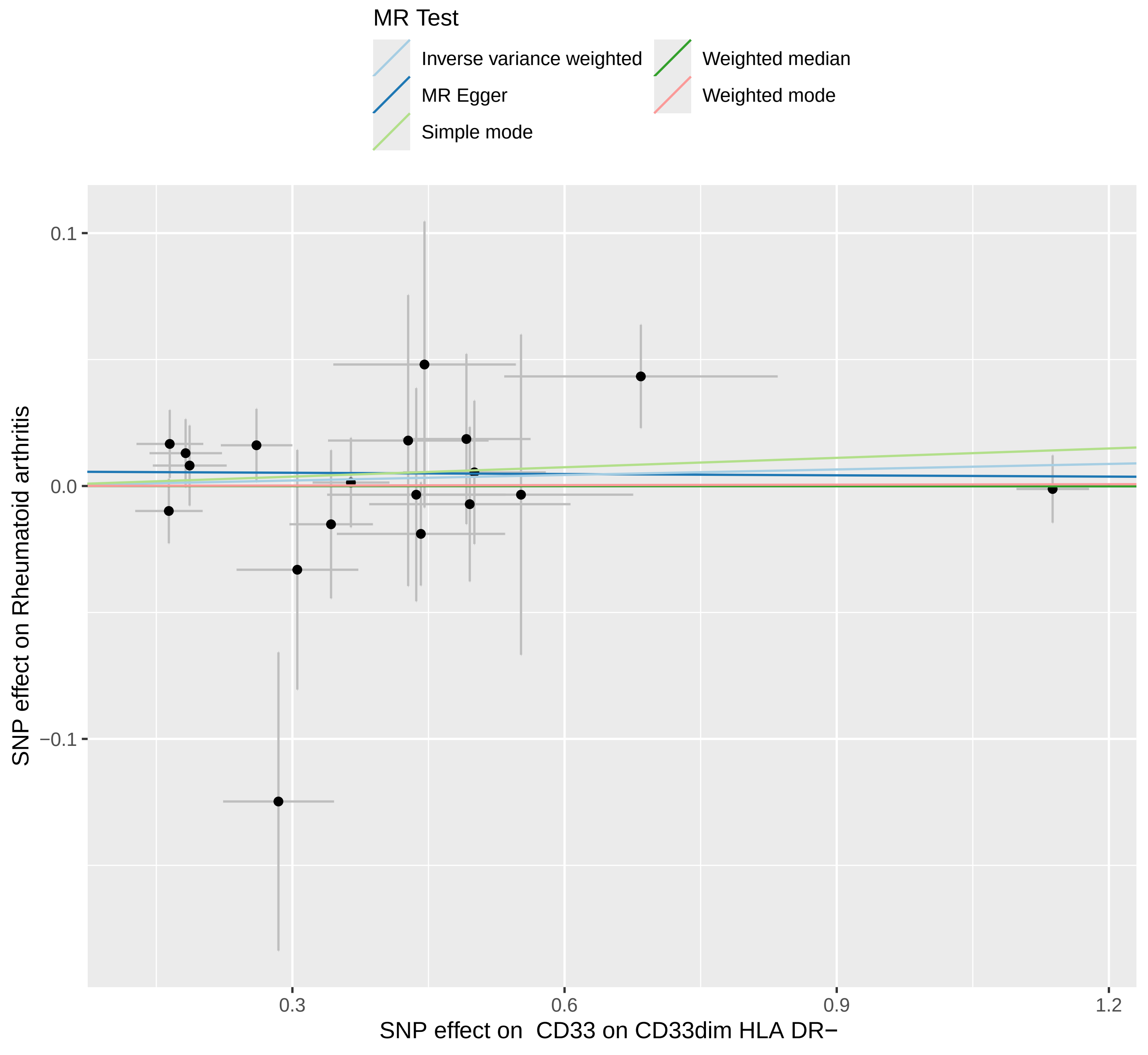

MR Test
Inverse variance weighted
MR Egger
Simple mode
Weighted median
Weighted mode
0.1
0.0
−0.1
0.3
0.6
0.9
1.2
SNP effect on Rheumatoid arthritis
SNP effect on CD33 on CD33dim HLA DR−

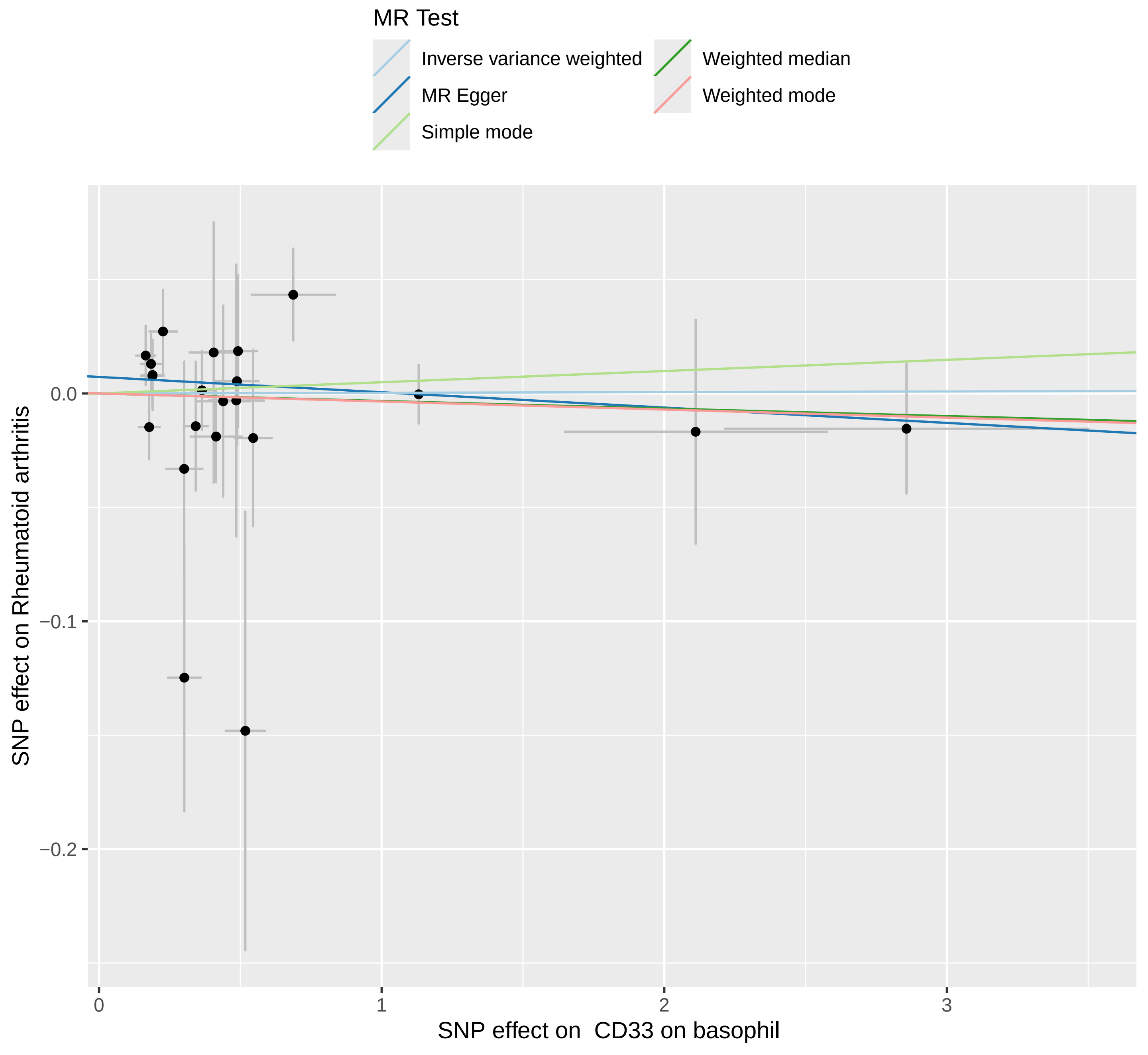
MR Test
Inverse variance weighted
MR Egger
Simple mode
Weighted median
Weighted mode
SNP effect on Rheumatoid arthritis
0.0
−0.1
−0.2
0
1
2
3
SNP effect on CD33 on basophil

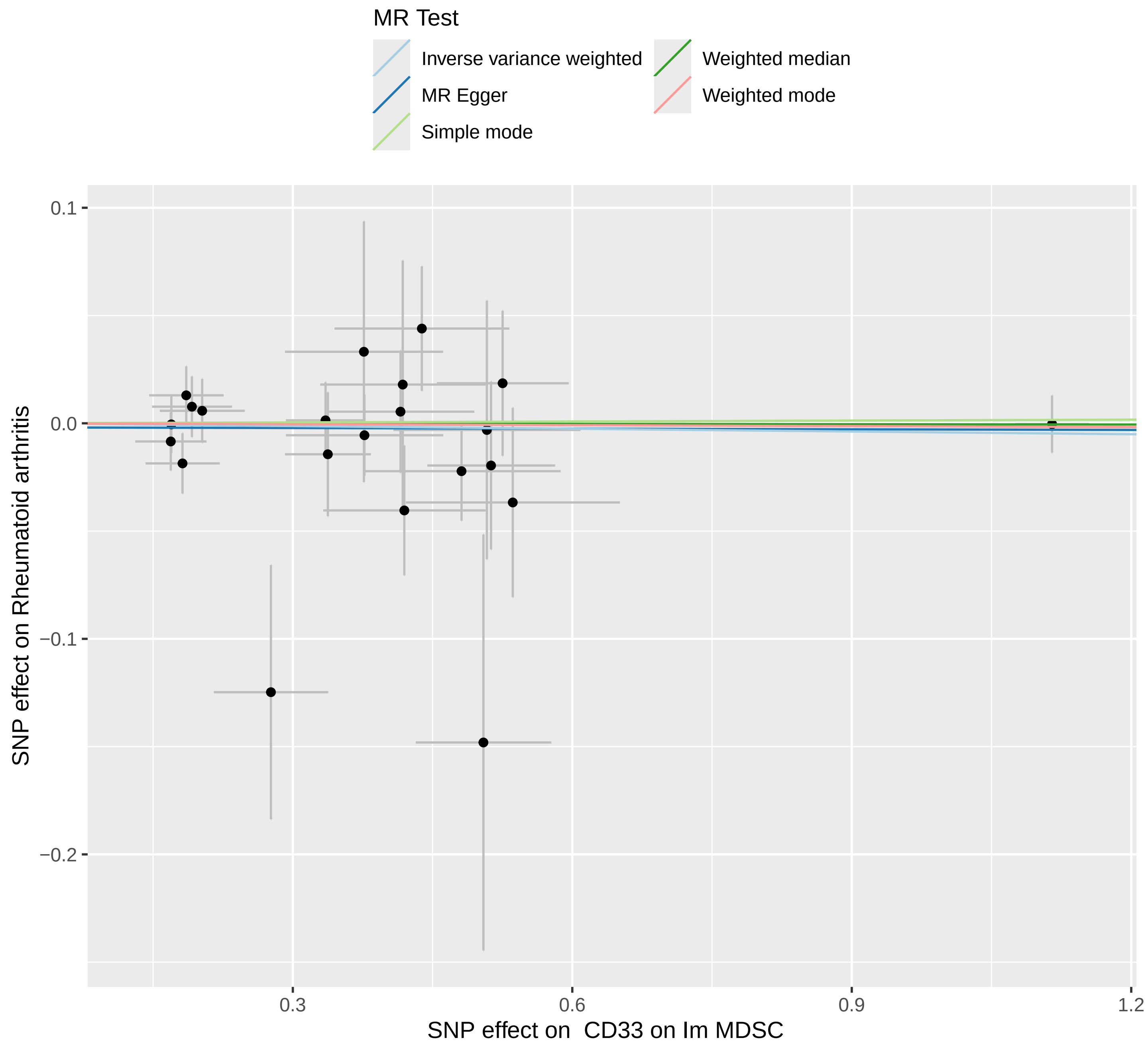

MR Test
Inverse variance weighted
MR Egger
Simple mode
Weighted median
Weighted mode
0.1
0.0
−0.1
−0.2
0.3
0.6
0.9
1.2
SNP effect on Rheumatoid arthritis
SNP effect on CD33 on Im MDSC

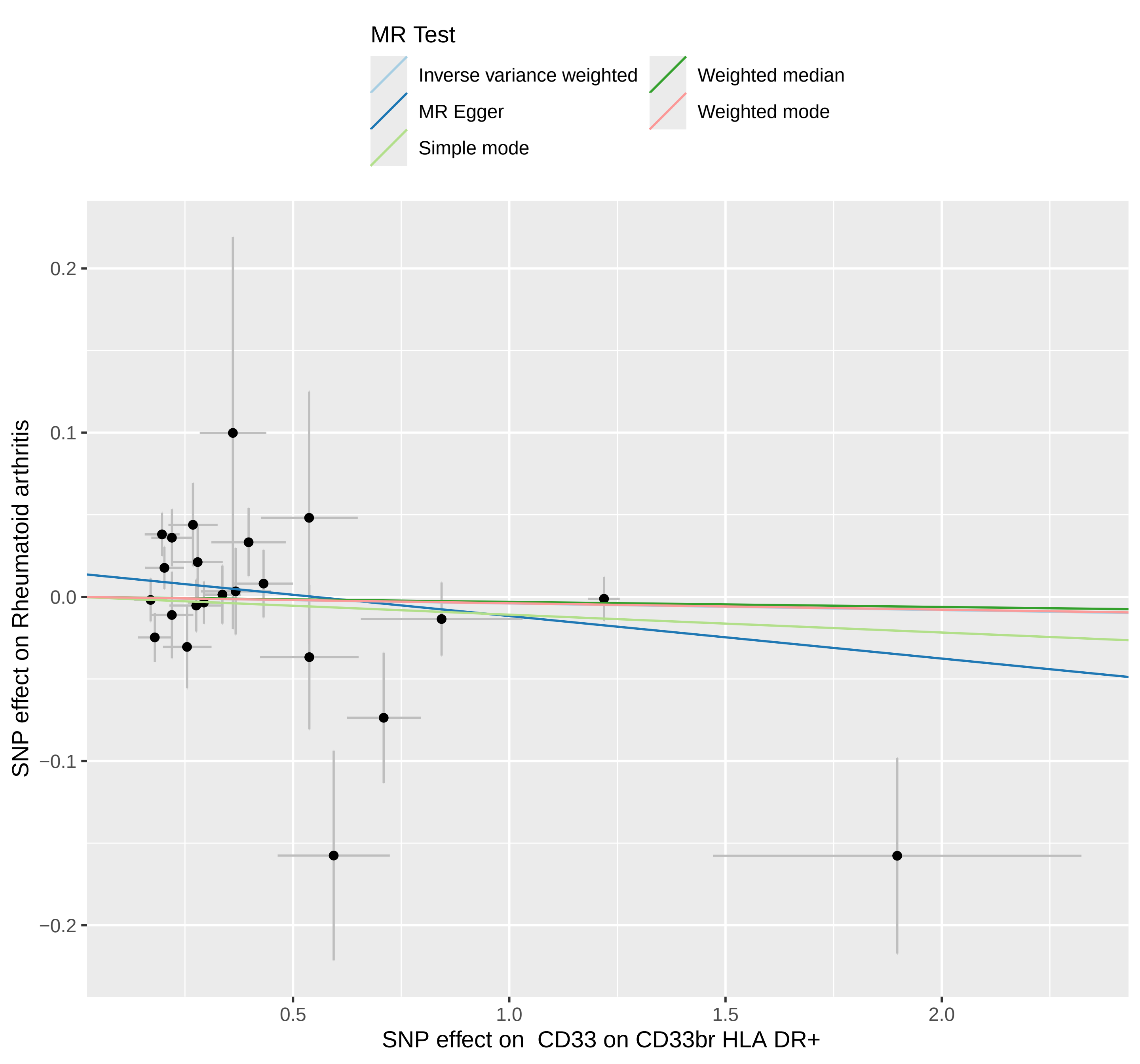

MR Test
Inverse variance weighted
MR Egger
Simple mode
Weighted median
Weighted mode
0.2
0.1
0.0
−0.1
−0.2
SNP effect on Rheumatoid arthritis
0.5
1.0
1.5
2.0
SNP effect on CD33 on CD33br HLA DR+

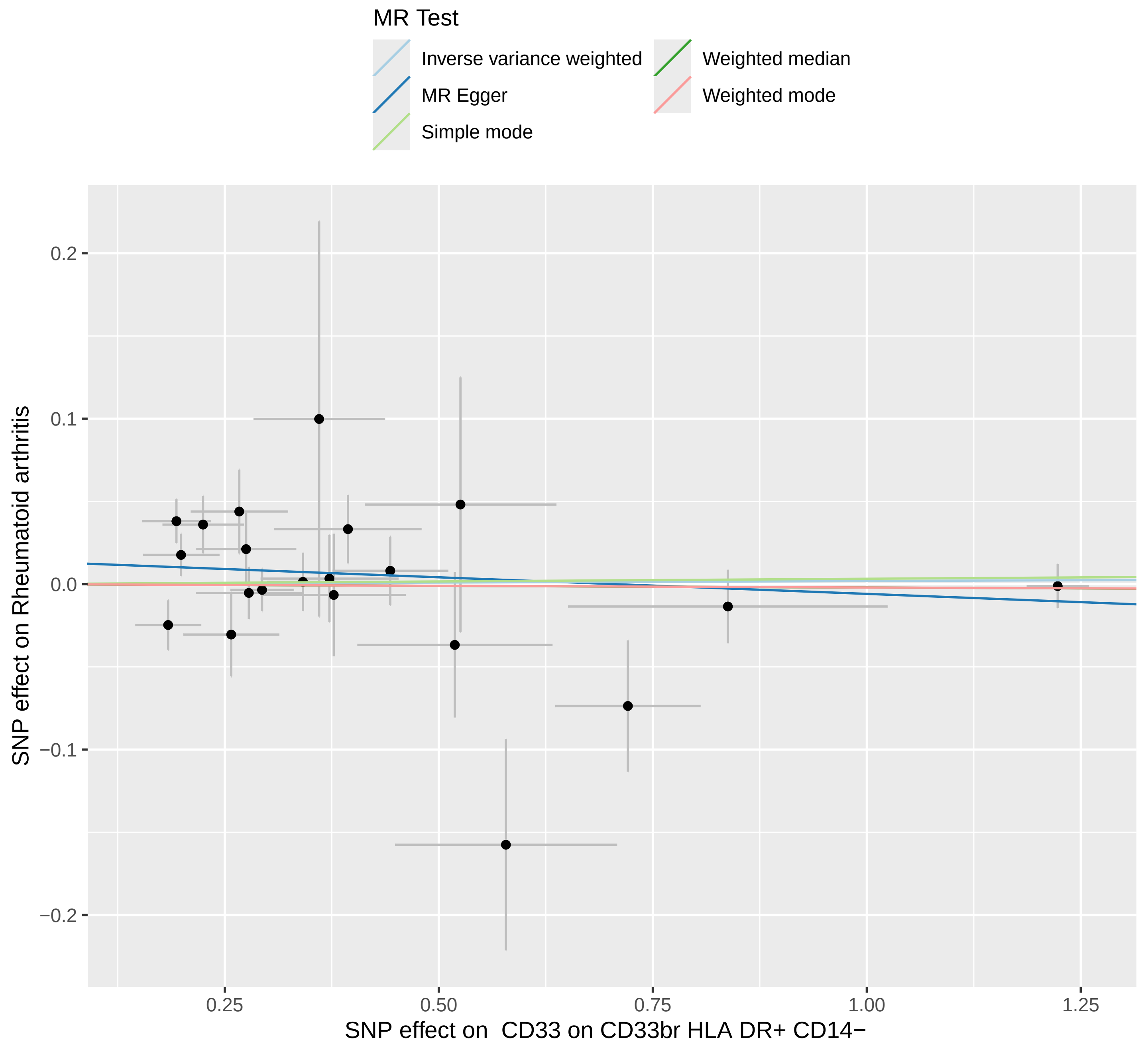
MR Test
Inverse variance weighted
MR Egger
Simple mode
Weighted median
Weighted mode
0.2
0.1
0.0
−0.1
−0.2
0.25
0.50
0.75
1.00
1.25
SNP effect on Rheumatoid arthritis
SNP effect on CD33 on CD33br HLA DR+ CD14−

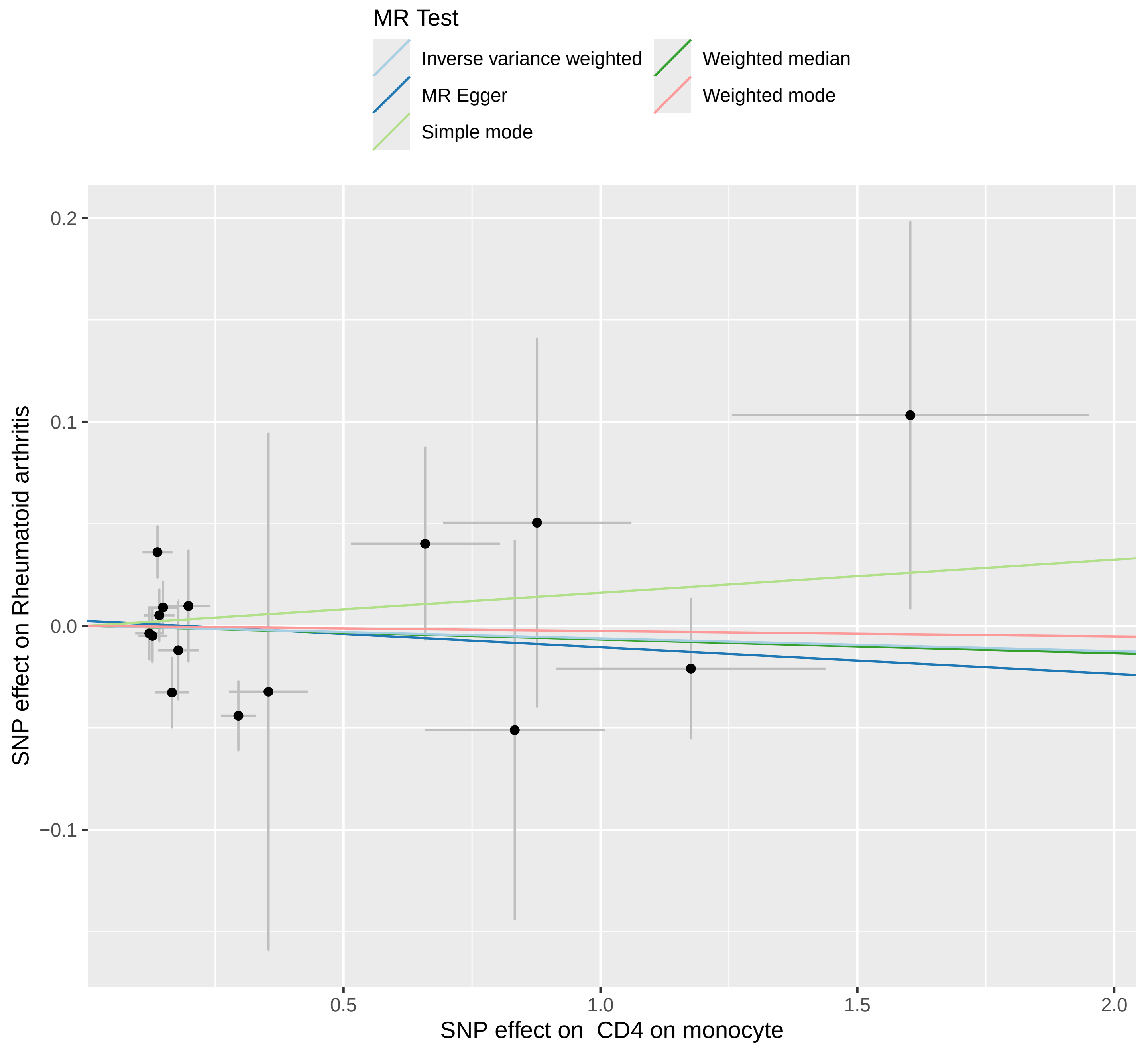
MR Test
Inverse variance weighted
MR Egger
Simple mode
Weighted median
Weighted mode
0.2
0.1
0.0
−0.1
0.5
1.0
1.5
2.0
SNP effect on Rheumatoid arthritis
SNP effect on CD4 on monocyte

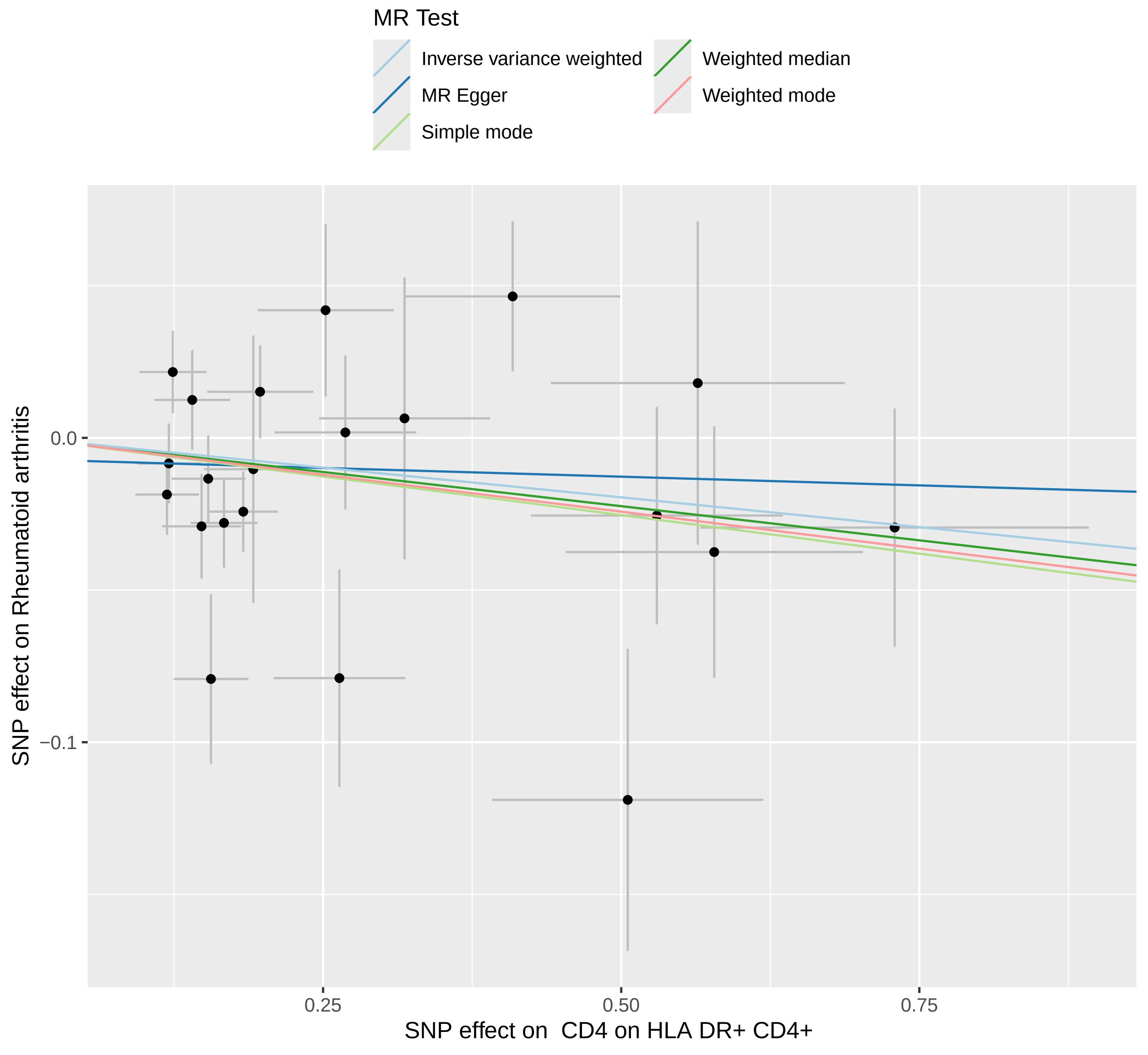

MR Test
Inverse variance weighted
MR Egger
Simple mode
Weighted median
Weighted mode
0.0
−0.1
0.25
0.50
0.75
SNP effect on Rheumatoid arthritis
SNP effect on CD4 on HLA DR+ CD4+

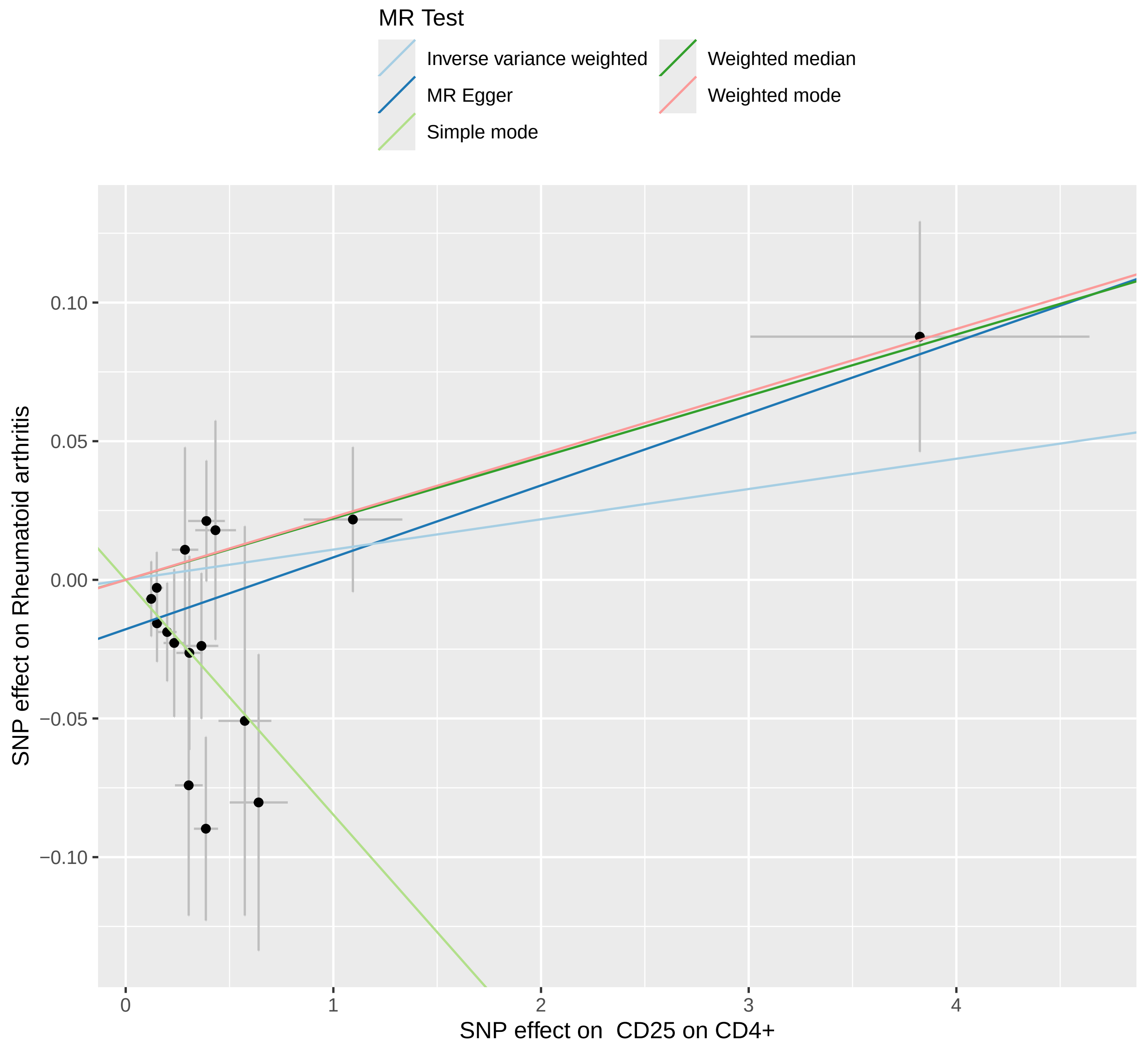

MR Test
Inverse variance weighted
MR Egger
Simple mode
Weighted median
Weighted mode
SNP effect on Rheumatoid arthritis
0.10
0.05
0.00
−0.05
−0.10
0
1
2
3
4
SNP effect on CD25 on CD4+

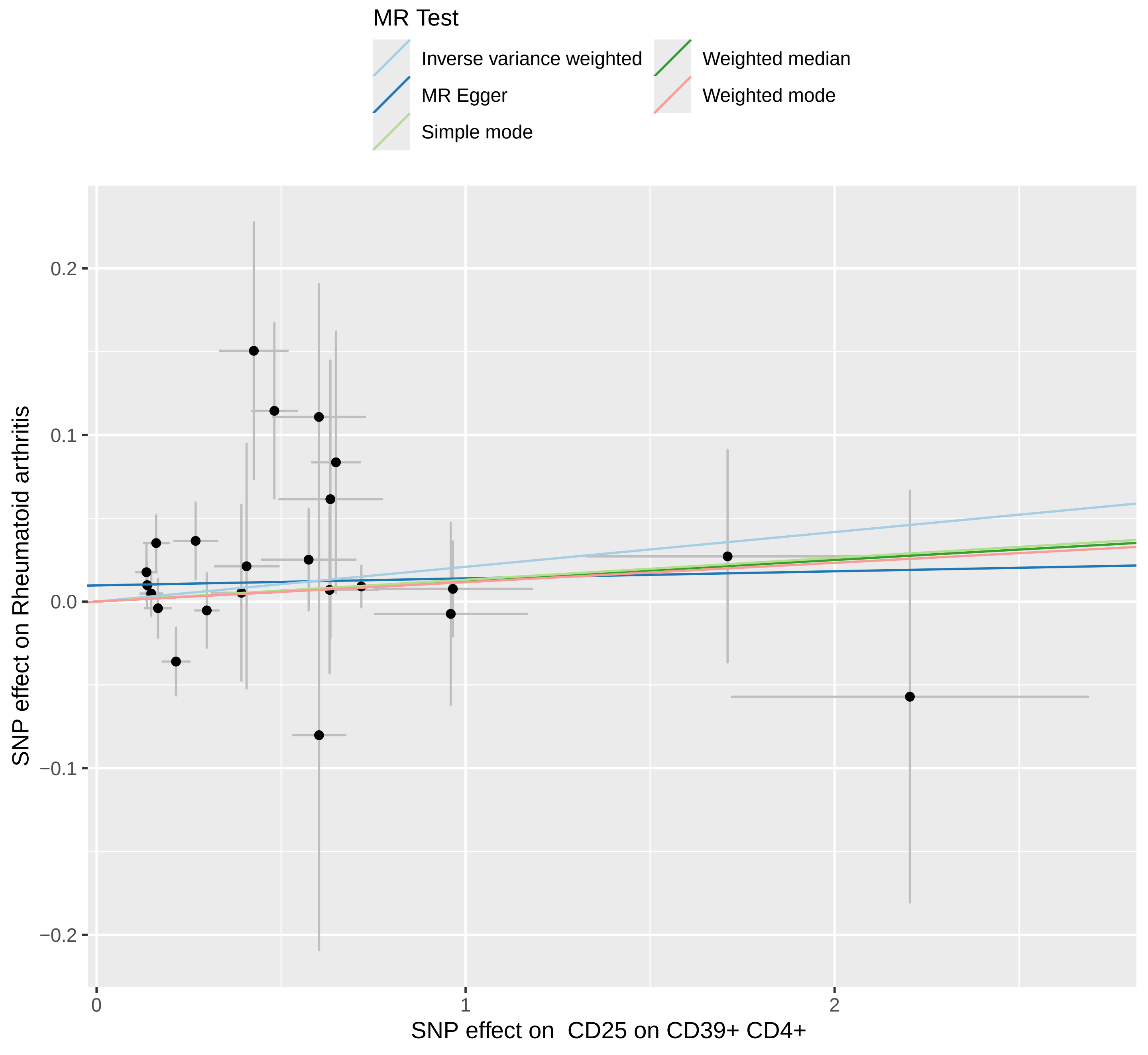
MR Test
Inverse variance weighted
MR Egger
Simple mode
Weighted median
Weighted mode
0.2
0.1
0.0
−0.1
−0.2
0
1
2
SNP effect on Rheumatoid arthritis
SNP effect on CD25 on CD39+ CD4+

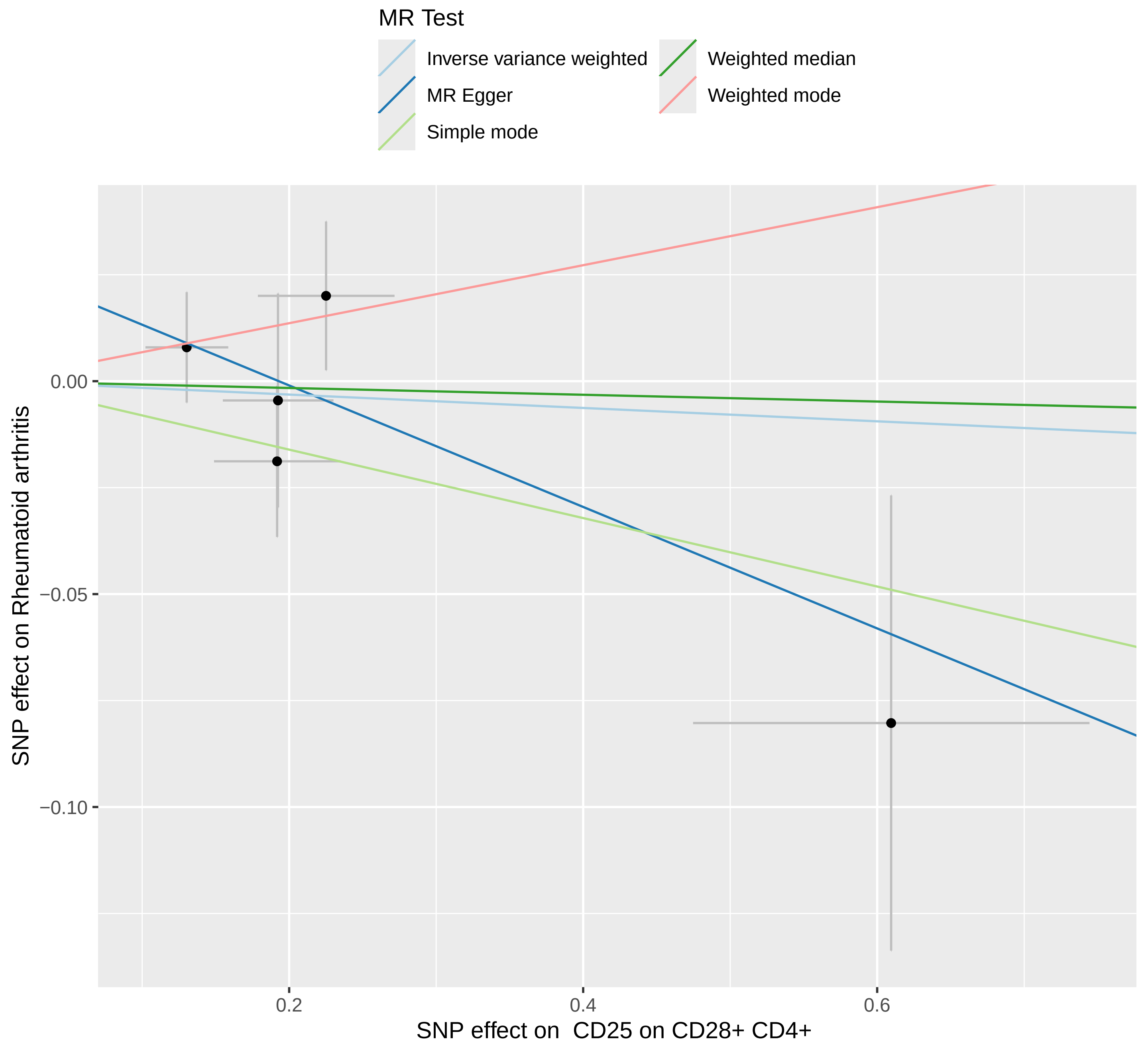
MR Test
Inverse variance weighted
MR Egger
Simple mode
Weighted median
Weighted mode
0.00
−0.05
−0.10
0.2
0.4
0.6
SNP effect on Rheumatoid arthritis
SNP effect on CD25 on CD28+ CD4+

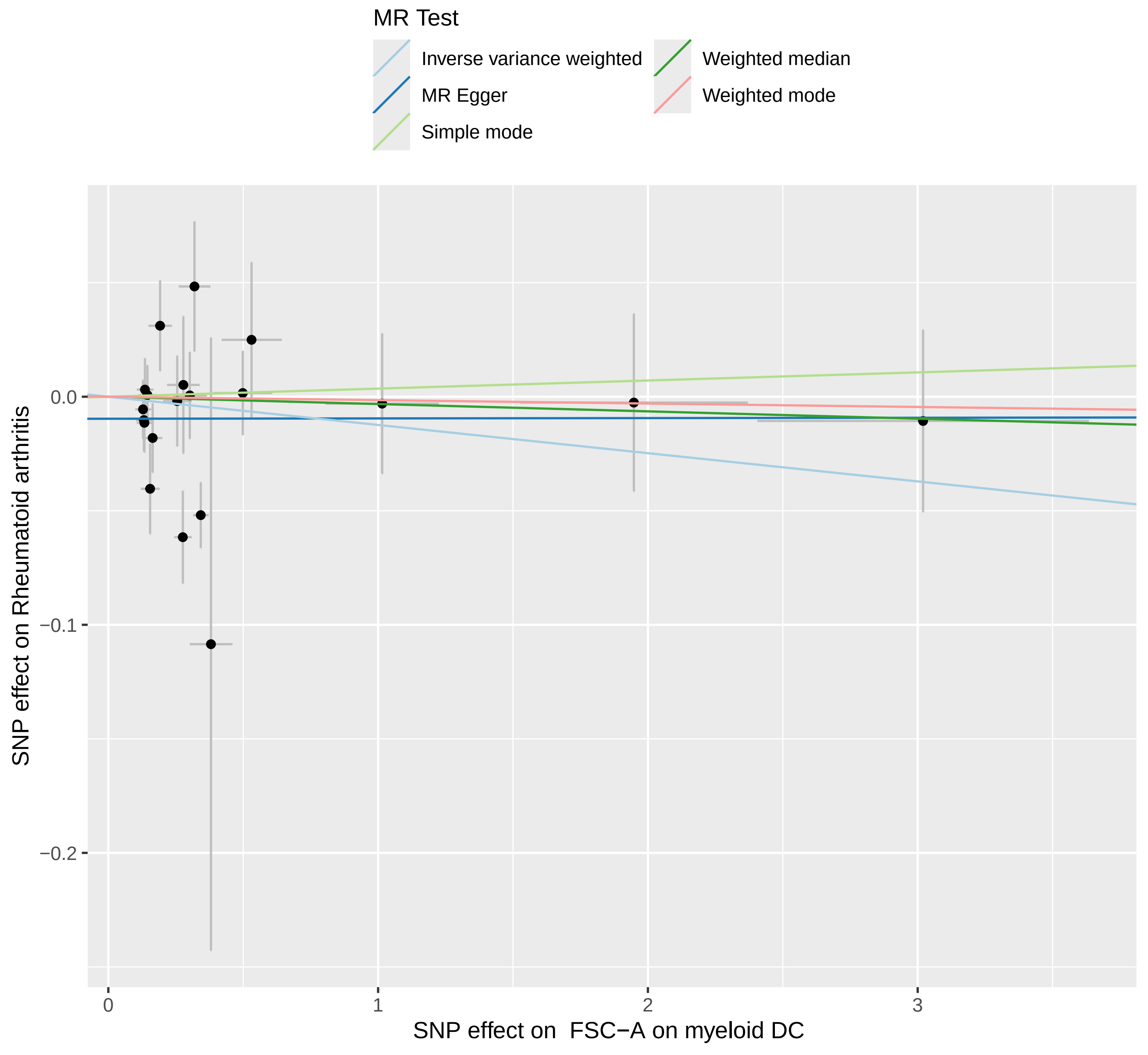
MR Test
Inverse variance weighted
MR Egger
Simple mode
Weighted median
Weighted mode
SNP effect on Rheumatoid arthritis
0.0
−0.1
−0.2
0
1
2
3
SNP effect on FSC−A on myeloid DC

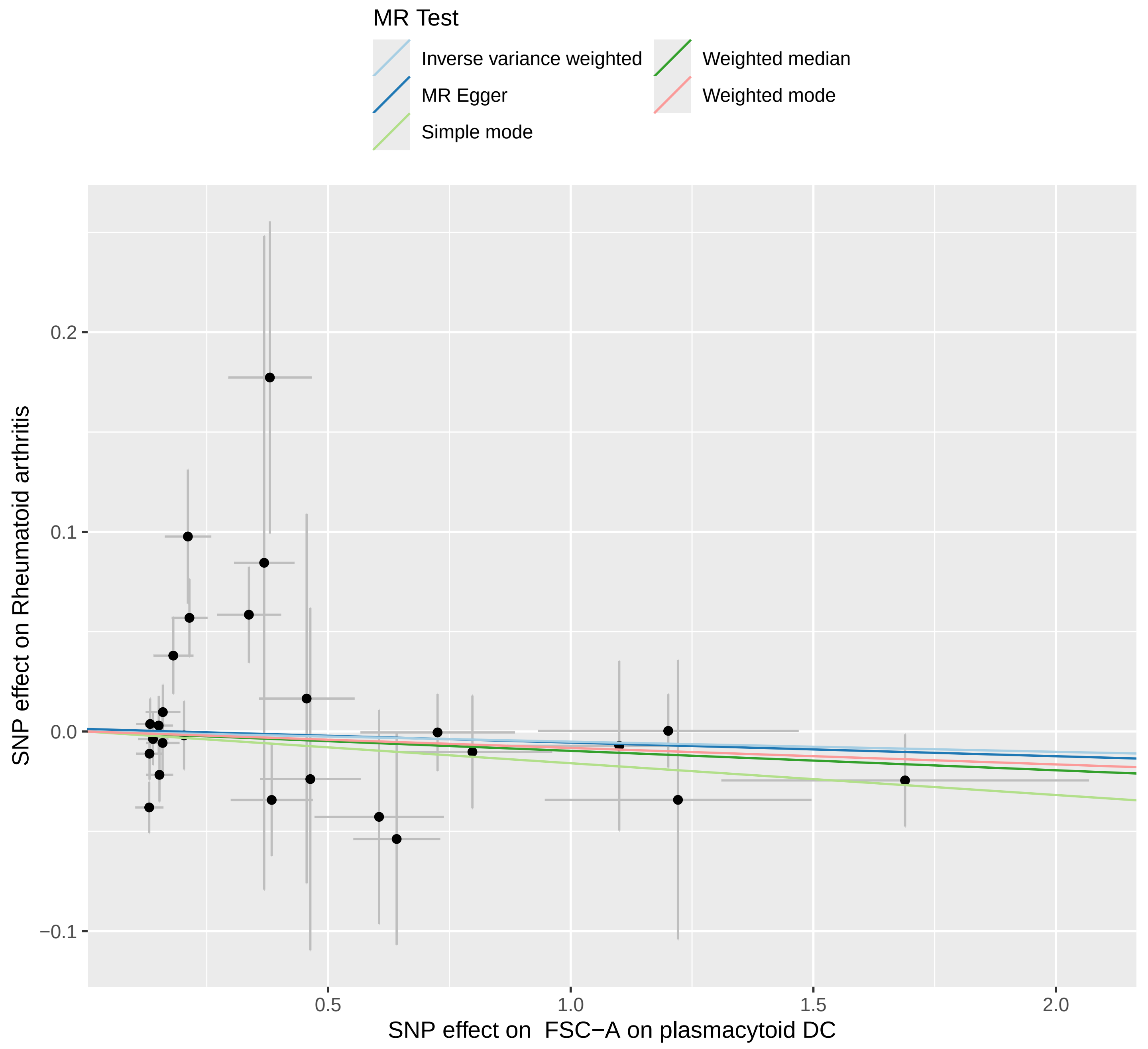

MR Test
Inverse variance weighted
MR Egger
Simple mode
Weighted median
Weighted mode
SNP effect on Rheumatoid arthritis
0.2
0.1
0.0
−0.1
0.5
1.0
1.5
2.0
SNP effect on FSC−A on plasmacytoid DC

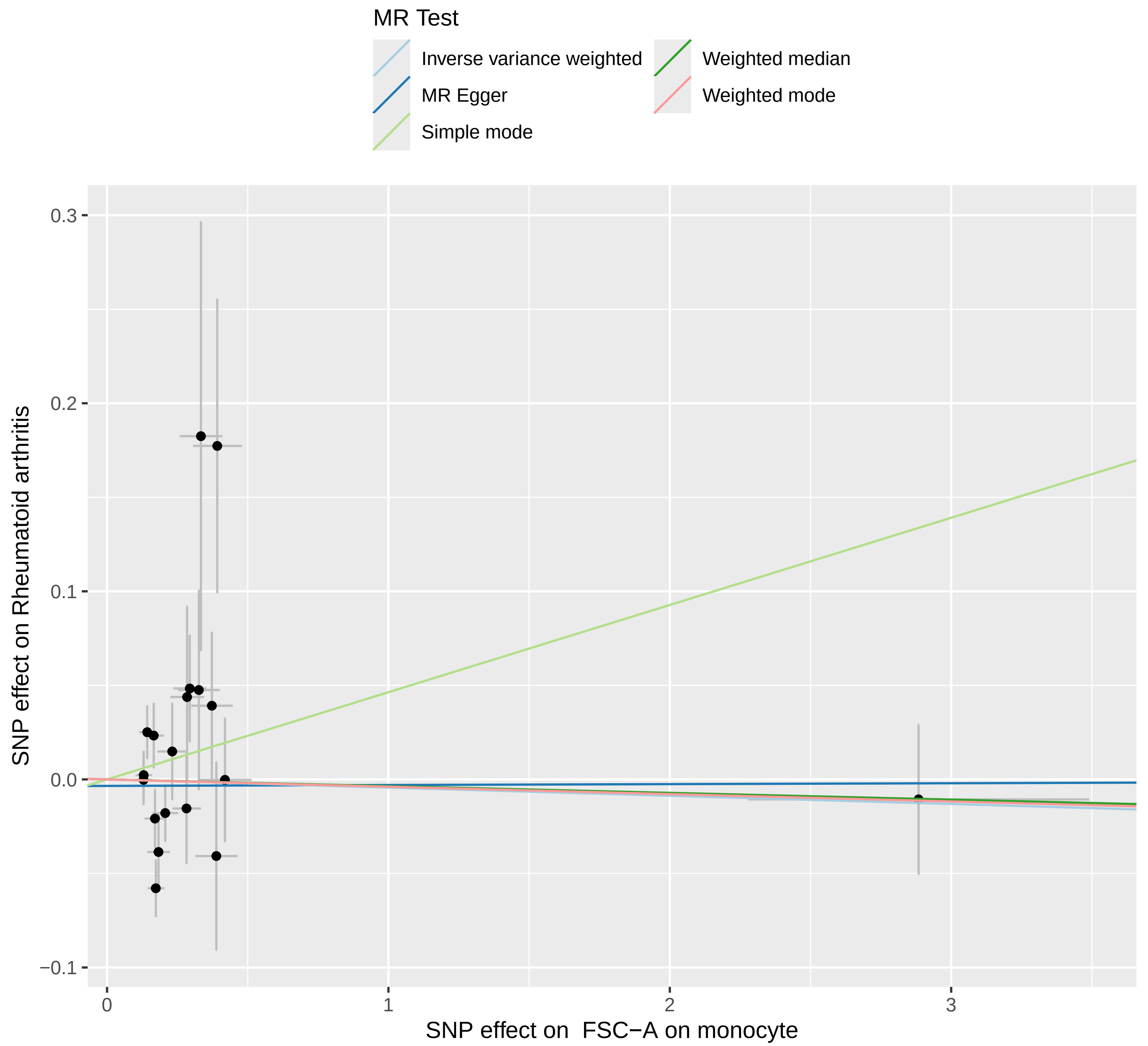
MR Test
Inverse variance weighted
MR Egger
Simple mode
Weighted median
Weighted mode
0.3
0.2
0.1
0.0
−0.1
0
1
2
3
SNP effect on Rheumatoid arthritis
SNP effect on FSC−A on monocyte

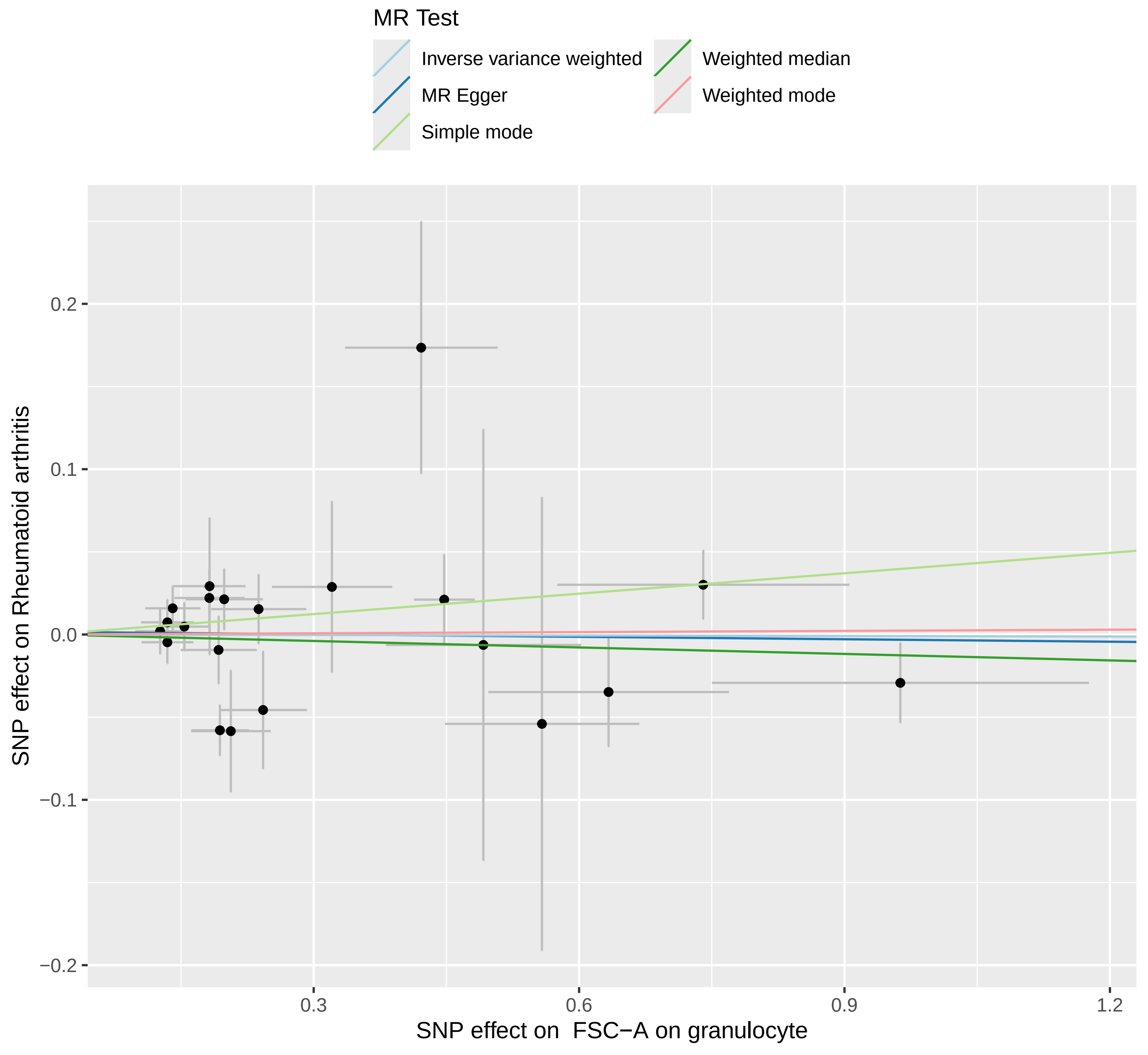
MR Test
Inverse variance weighted
MR Egger
Simple mode
Weighted median
Weighted mode
SNP effect on Rheumatoid arthritis
0.2
0.1
0.0
−0.1
−0.2
0.3
0.6
0.9
1.2
SNP effect on FSC−A on granulocyte

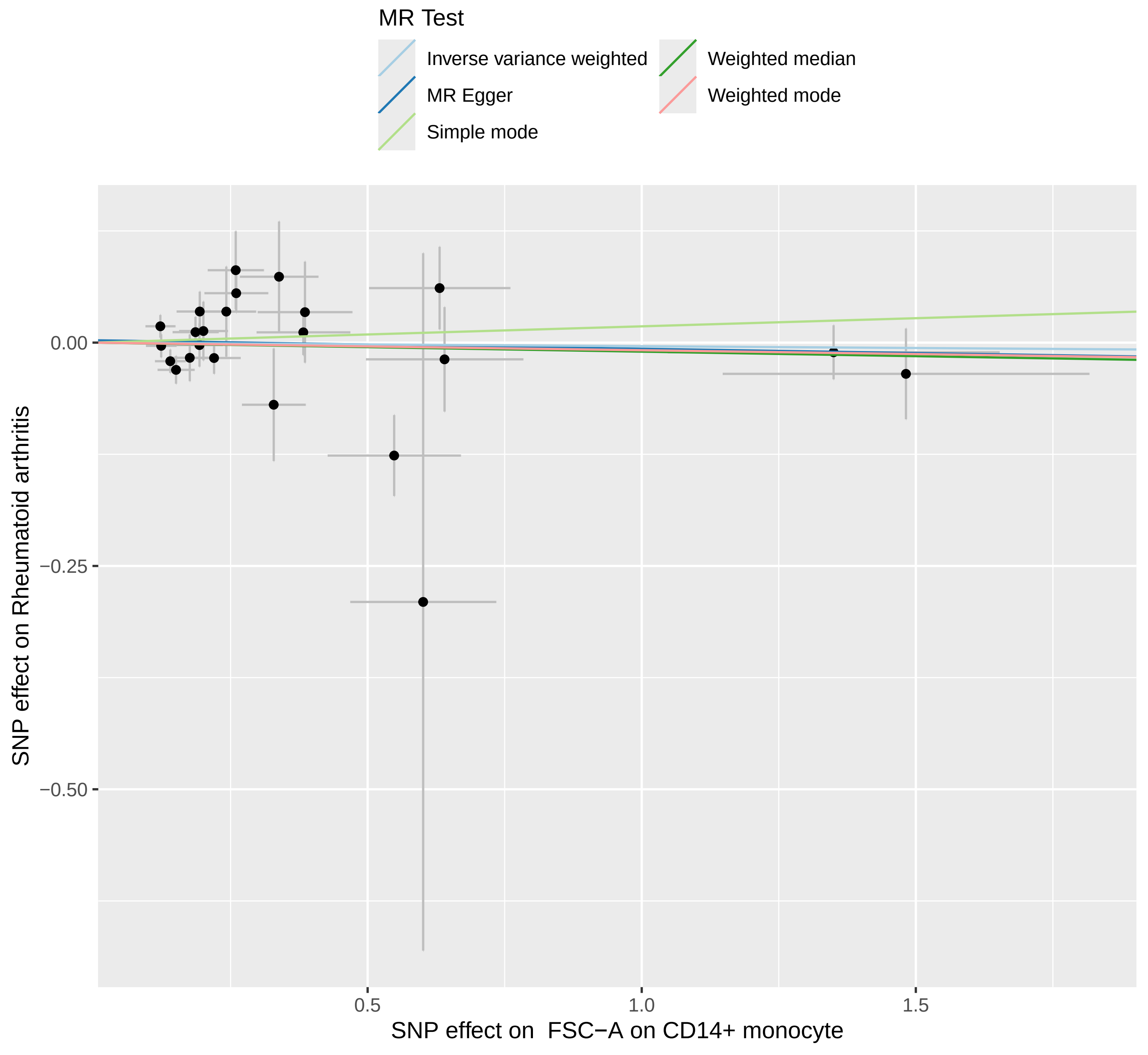
MR Test
Inverse variance weighted
MR Egger
Simple mode
Weighted median
Weighted mode
0.00
−0.25
−0.50
0.5
1.0
1.5
SNP effect on Rheumatoid arthritis
SNP effect on FSC−A on CD14+ monocyte

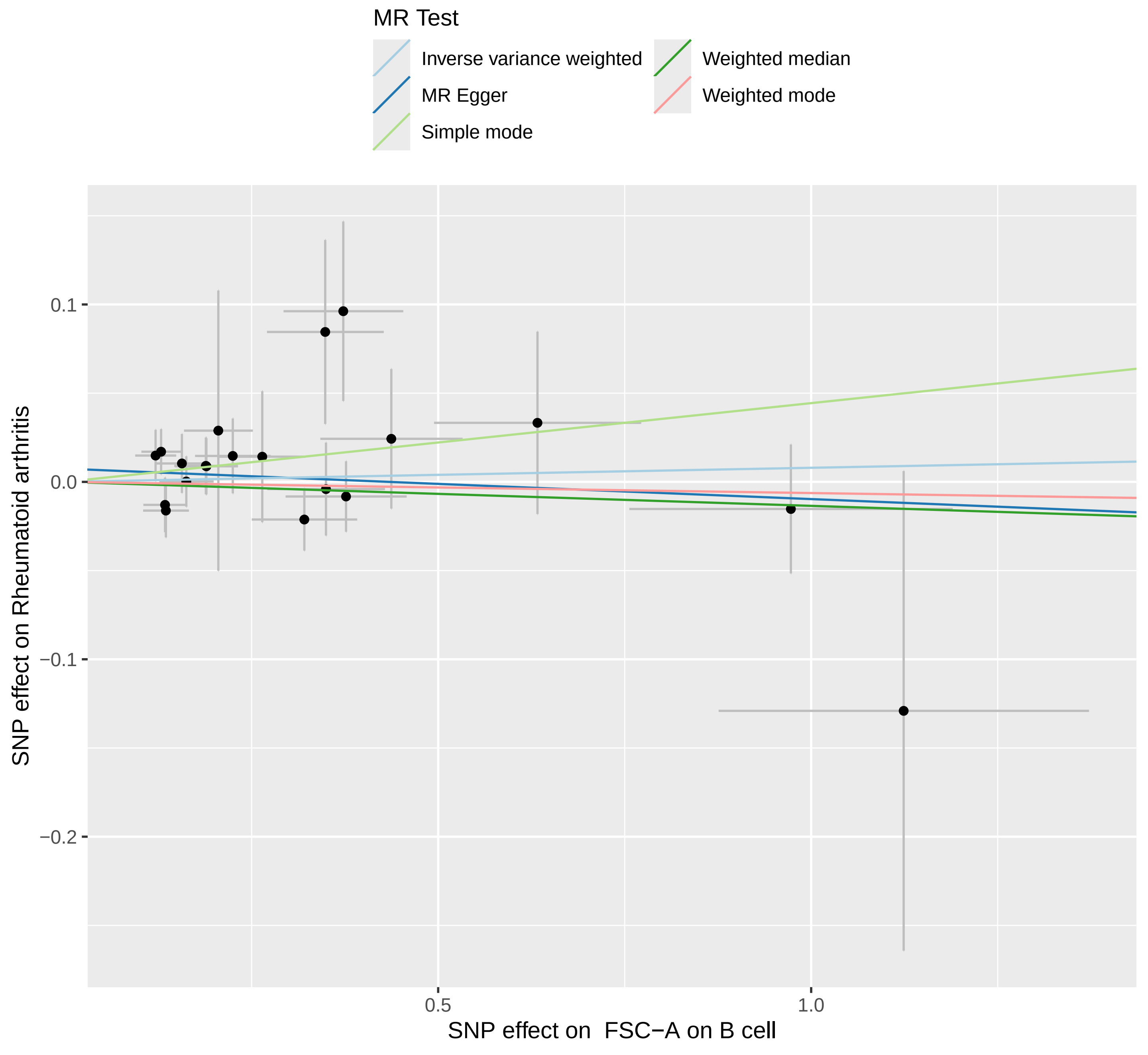

MR Test
Inverse variance weighted
MR Egger
Simple mode
Weighted median
Weighted mode
0.1
0.0
−0.1
−0.2
SNP effect on Rheumatoid arthritis
0.5
1.0
SNP effect on FSC−A on B cell

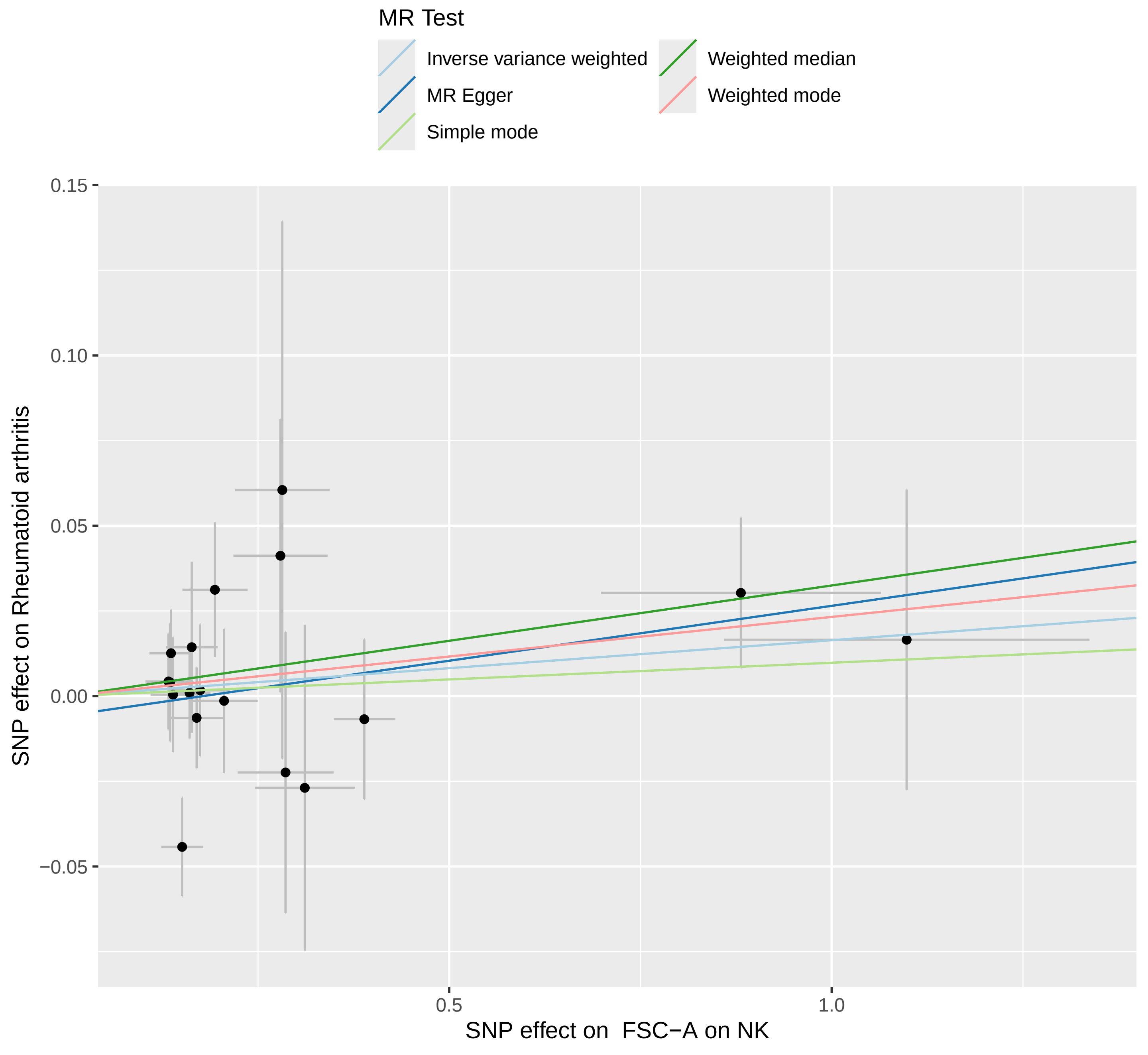
MR Test
Inverse variance weighted
MR Egger
Simple mode
Weighted median
Weighted mode
0.15
0.10
0.05
0.00
−0.05
0.5
1.0
SNP effect on Rheumatoid arthritis
SNP effect on FSC−A on NK

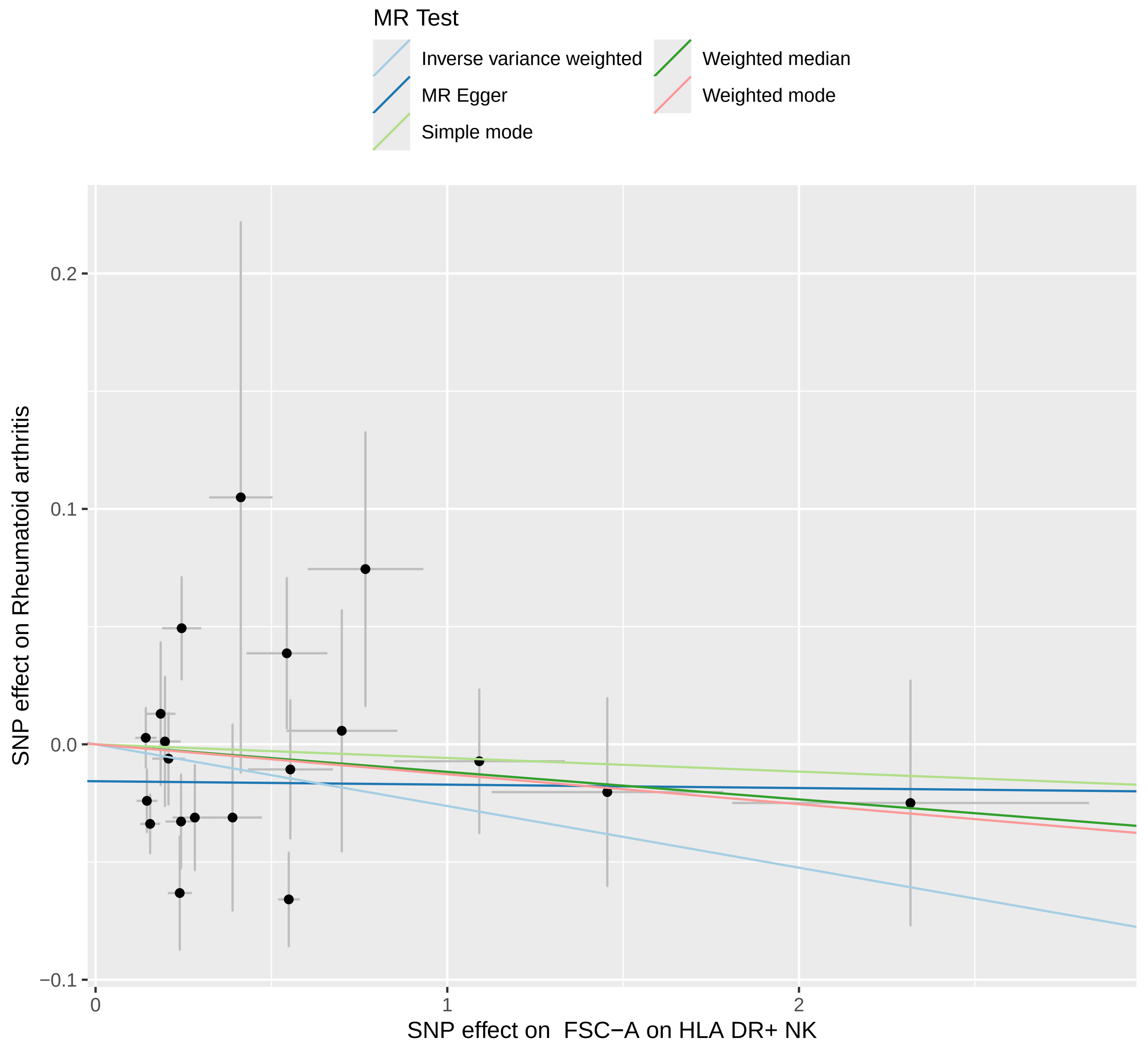
MR Test
Inverse variance weighted
MR Egger
Simple mode
Weighted median
Weighted mode
SNP effect on Rheumatoid arthritis
0.2
0.1
0.0
−0.1
0
1
2
SNP effect on FSC−A on HLA DR+ NK

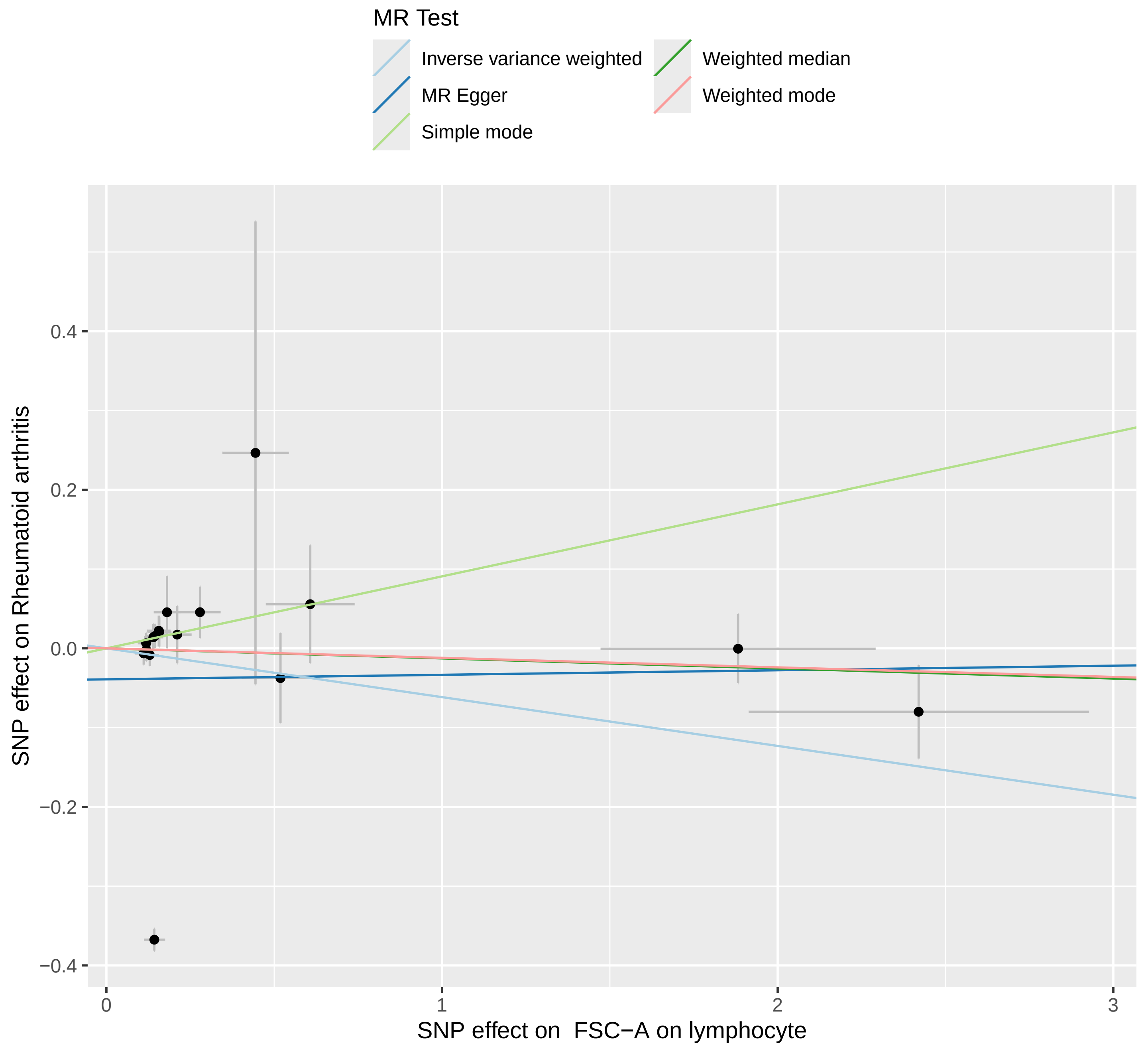
MR Test
Inverse variance weighted
MR Egger
Simple mode
Weighted median
Weighted mode
SNP effect on Rheumatoid arthritis
0.4
0.2
0.0
−0.2
−0.4
0
1
2
3
SNP effect on FSC−A on lymphocyte

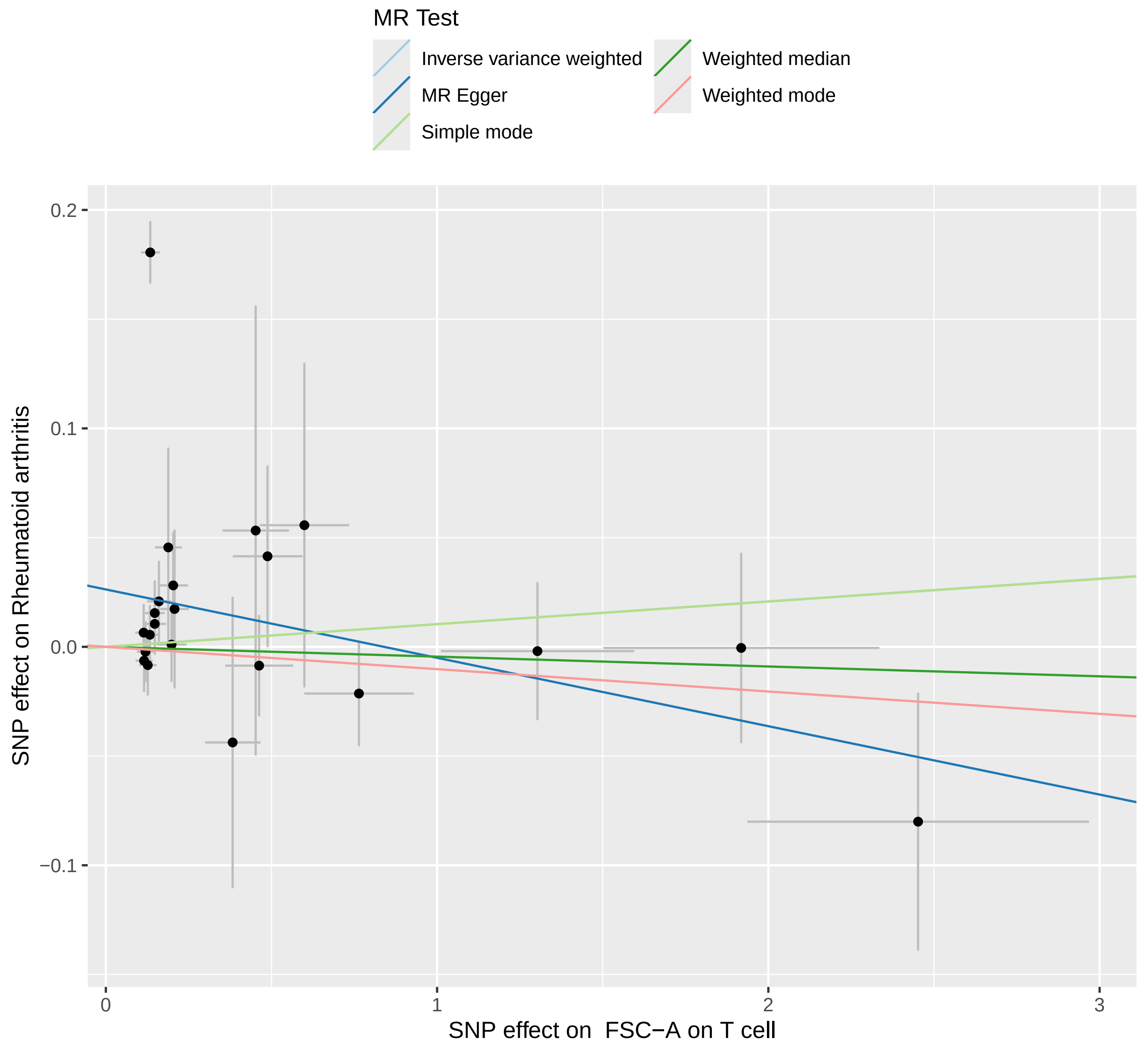
MR Test
Inverse variance weighted
MR Egger
Simple mode
Weighted median
Weighted mode
0.2
0.1
0.0
−0.1
SNP effect on Rheumatoid arthritis
0
1
2
3
SNP effect on FSC−A on T cell

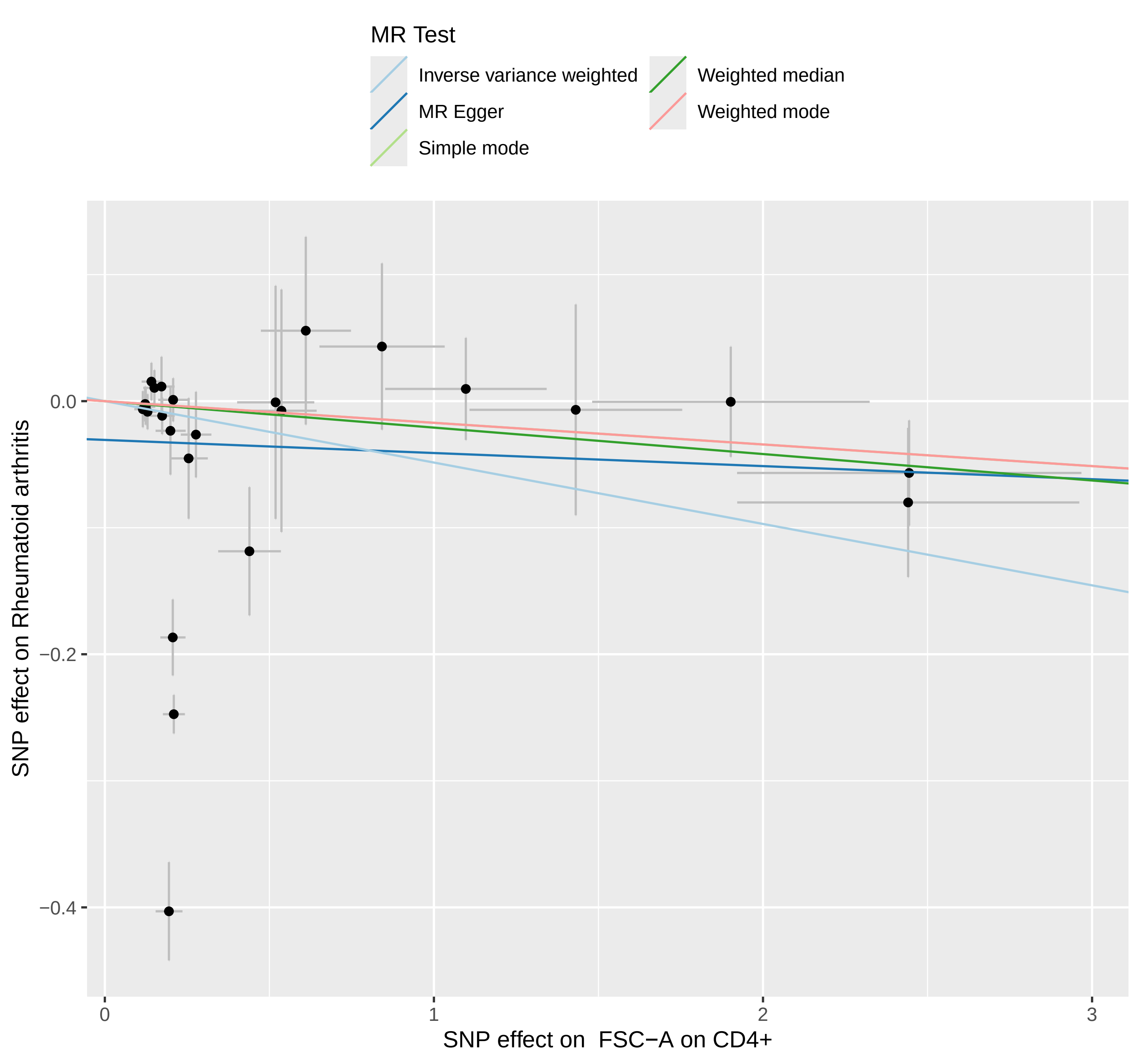
MR Test
Inverse variance weighted
MR Egger
Simple mode
Weighted median
Weighted mode
SNP effect on Rheumatoid arthritis
0.0
−0.2
−0.4
0
1
2
3
SNP effect on FSC−A on CD4+

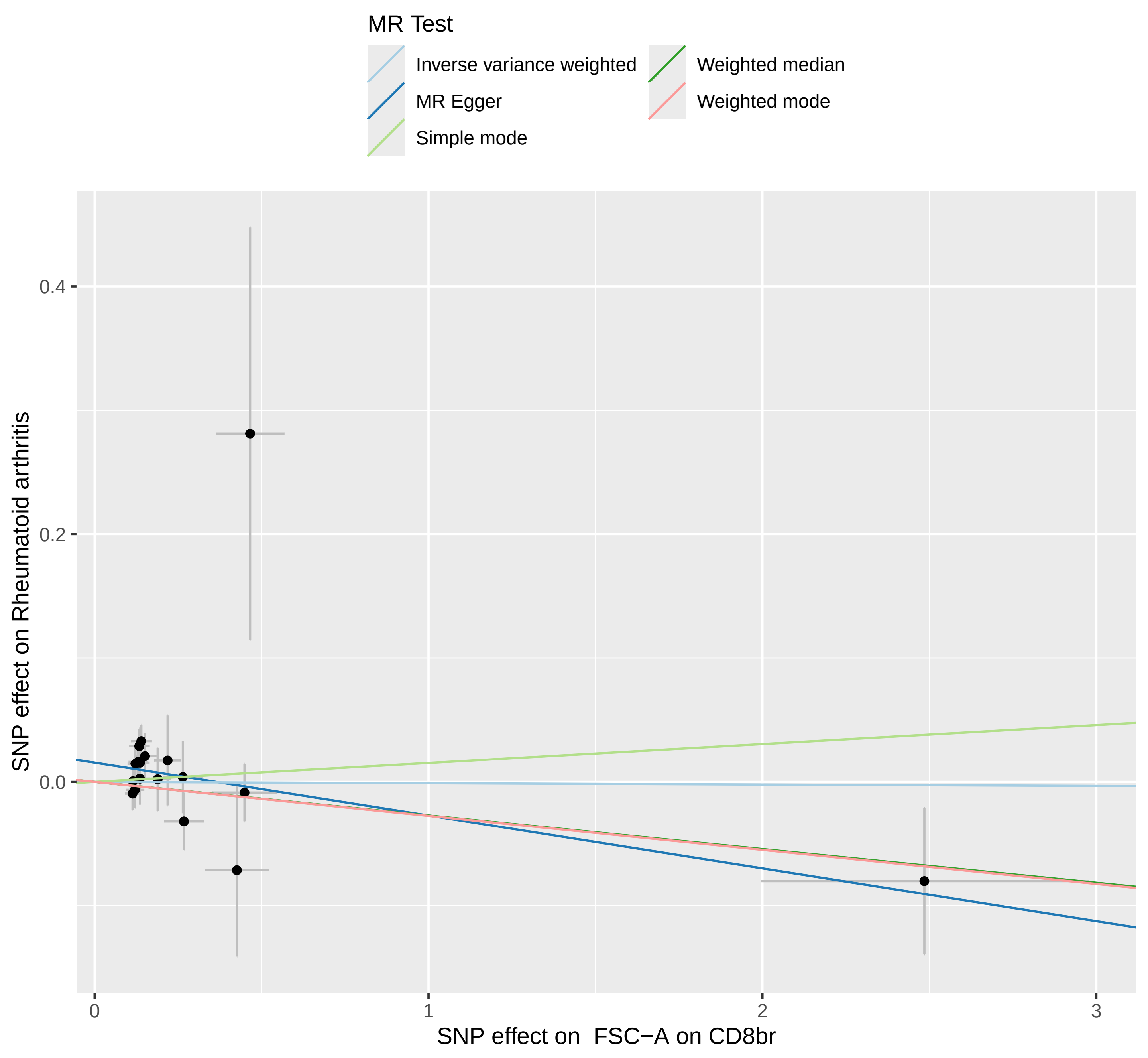

MR Test
Inverse variance weighted
MR Egger
Simple mode
Weighted median
Weighted mode
SNP effect on Rheumatoid arthritis
0.4
0.2
0.0
0
1
2
3
SNP effect on FSC−A on CD8br

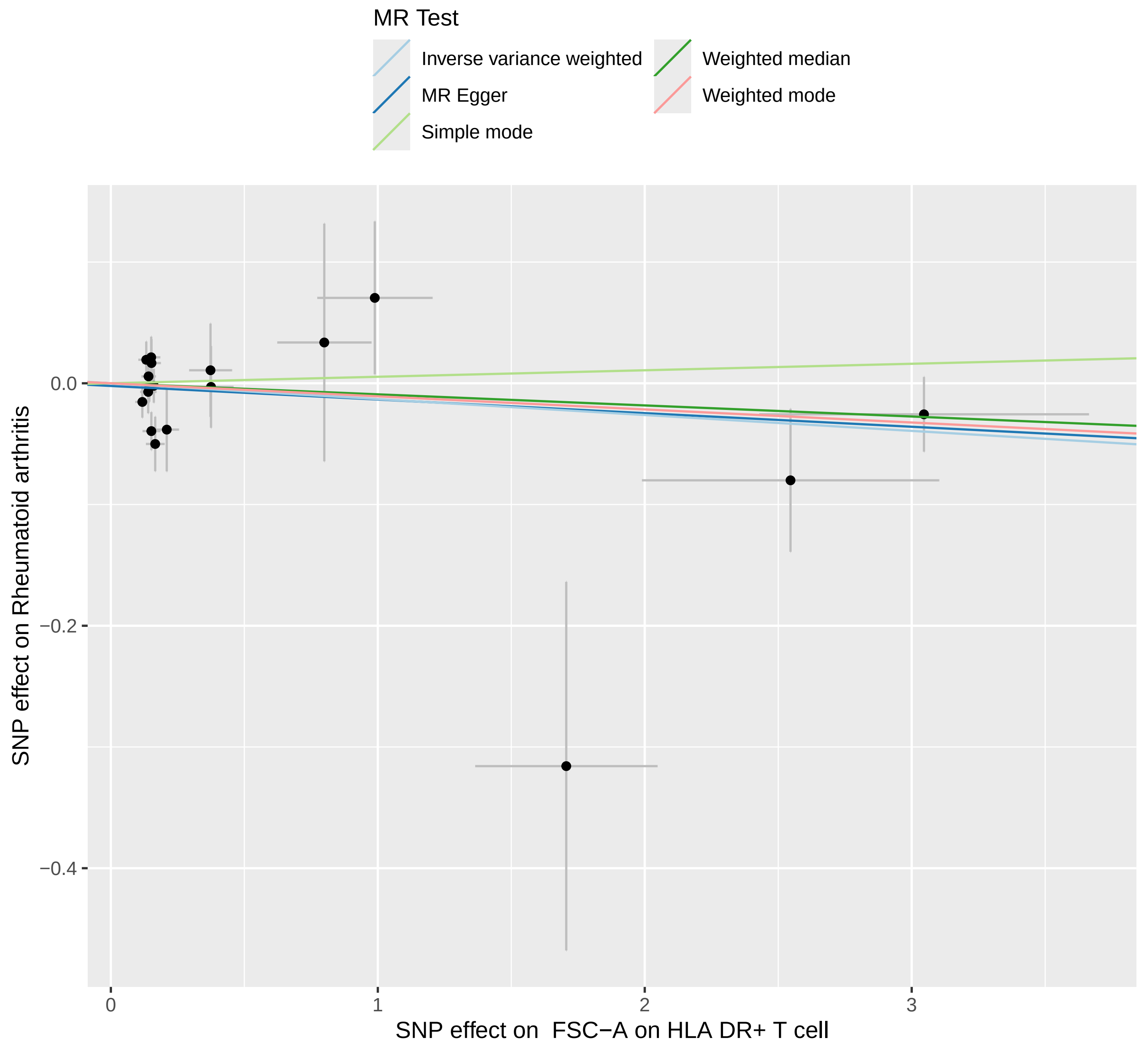

MR Test
Inverse variance weighted
MR Egger
Simple mode
Weighted median
Weighted mode
0.0
−0.2
−0.4
SNP effect on Rheumatoid arthritis
0
1
2
3
SNP effect on FSC−A on HLA DR+ T cell

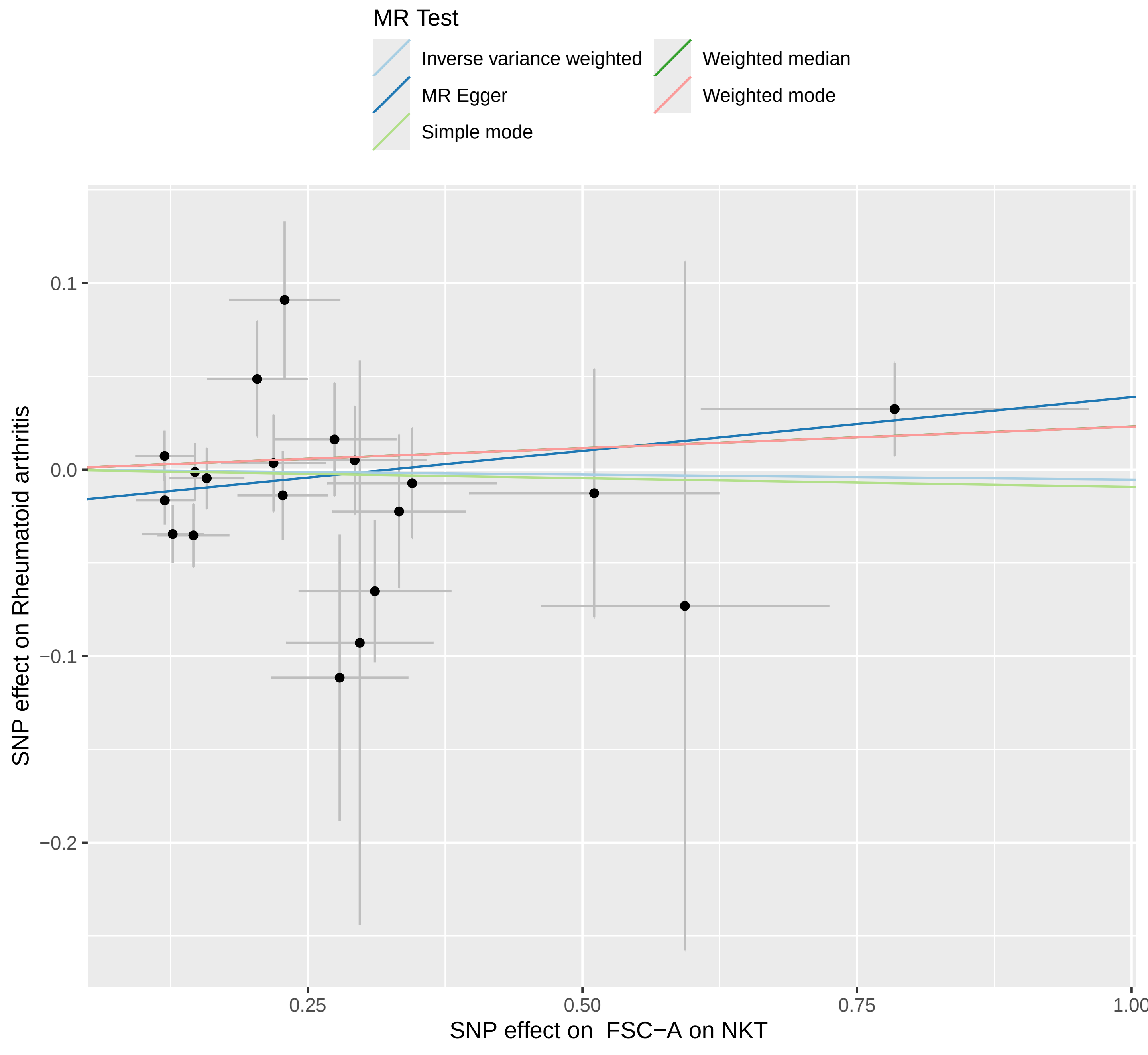
MR Test
Inverse variance weighted
MR Egger
Simple mode
Weighted median
Weighted mode
0.1
0.0
−0.1
−0.2
0.25
0.50
0.75
1.00
SNP effect on Rheumatoid arthritis
SNP effect on FSC−A on NKT

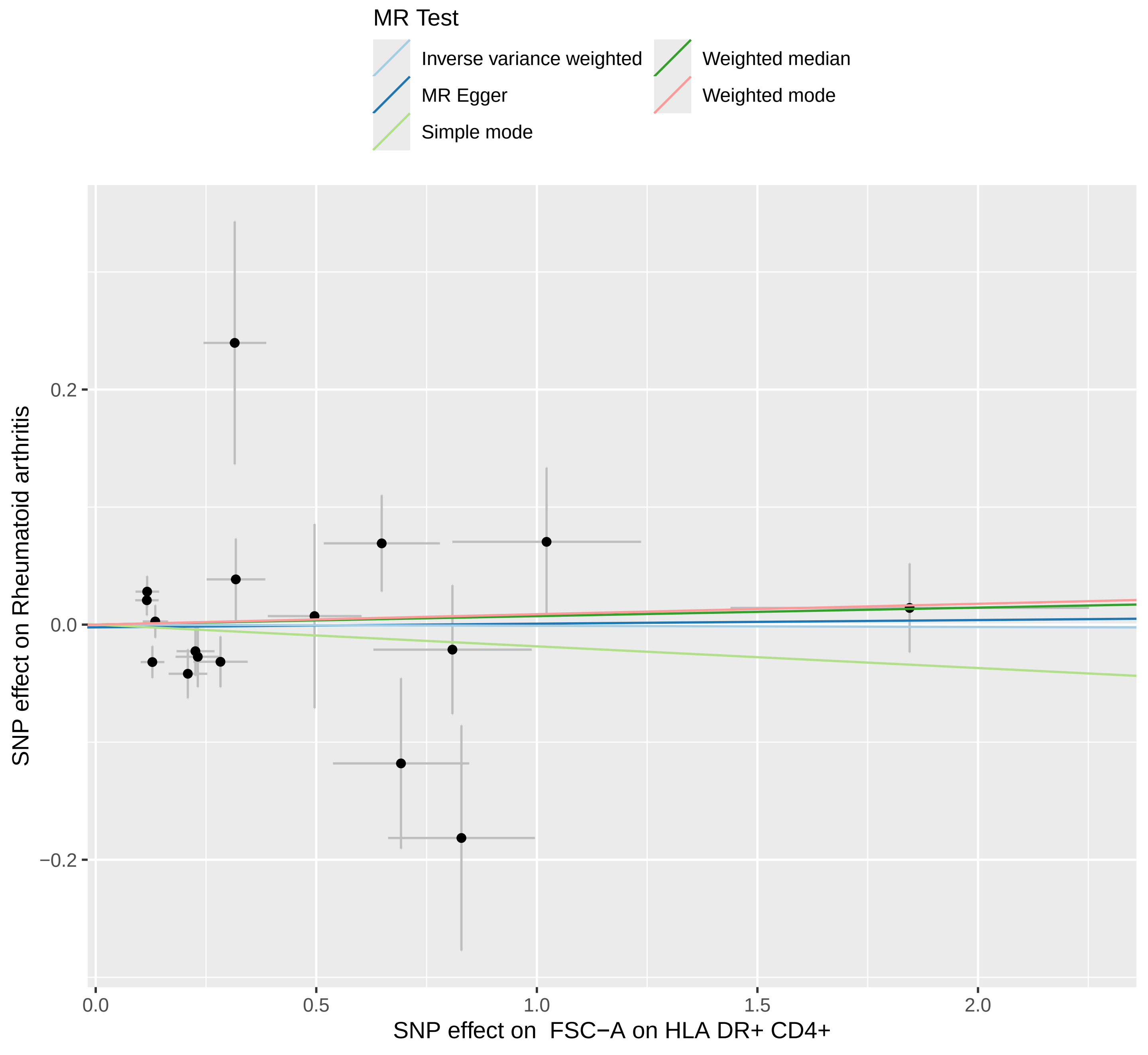

MR Test
Inverse variance weighted
MR Egger
Simple mode
Weighted median
Weighted mode
0.2
0.0
−0.2
SNP effect on Rheumatoid arthritis
0.0
0.5
1.0
1.5
2.0
SNP effect on FSC−A on HLA DR+ CD4+

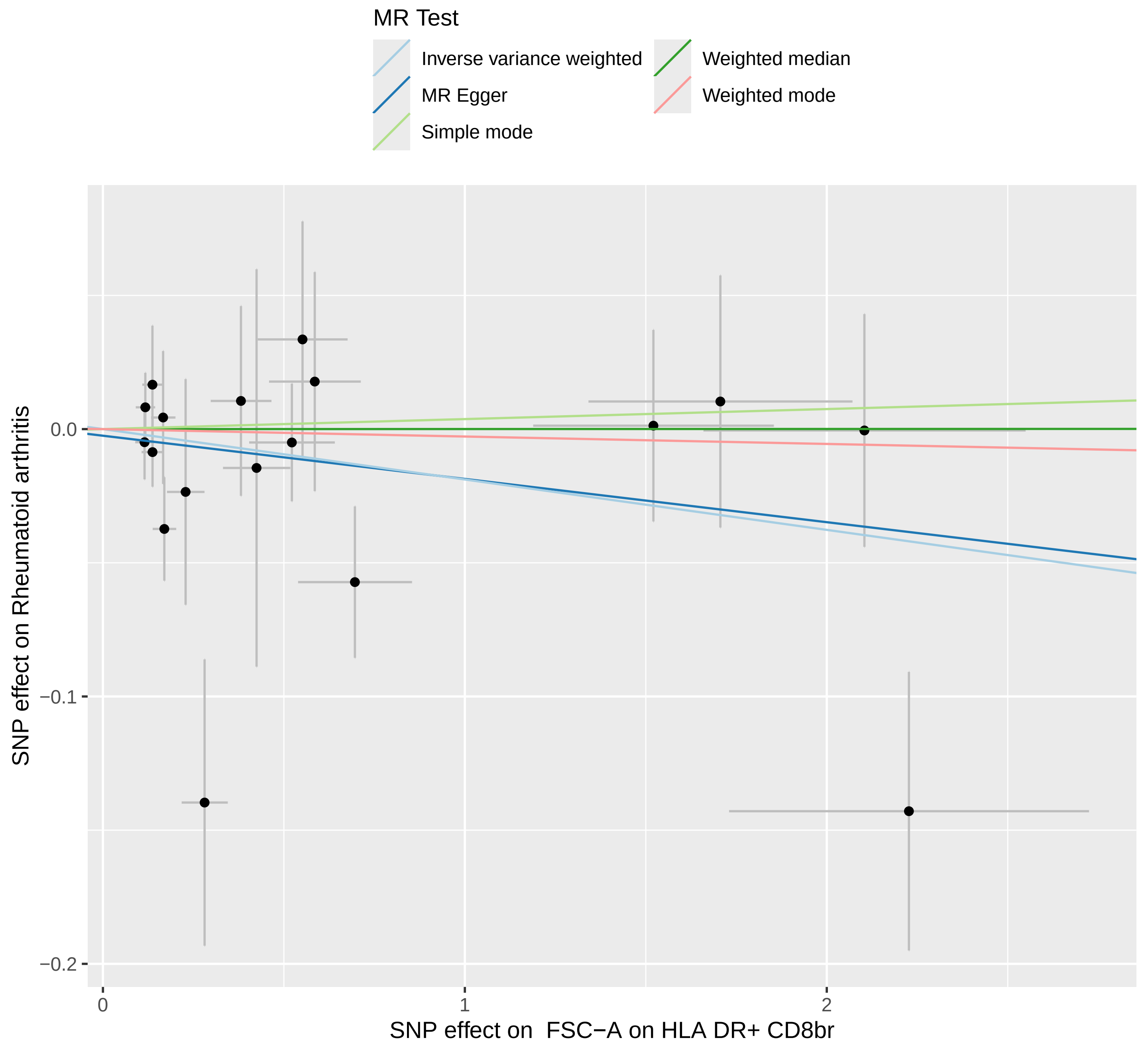
MR Test
Inverse variance weighted
MR Egger
Simple mode
Weighted median
Weighted mode
0.0
−0.1
−0.2
0
1
2
SNP effect on Rheumatoid arthritis
SNP effect on FSC−A on HLA DR+ CD8br

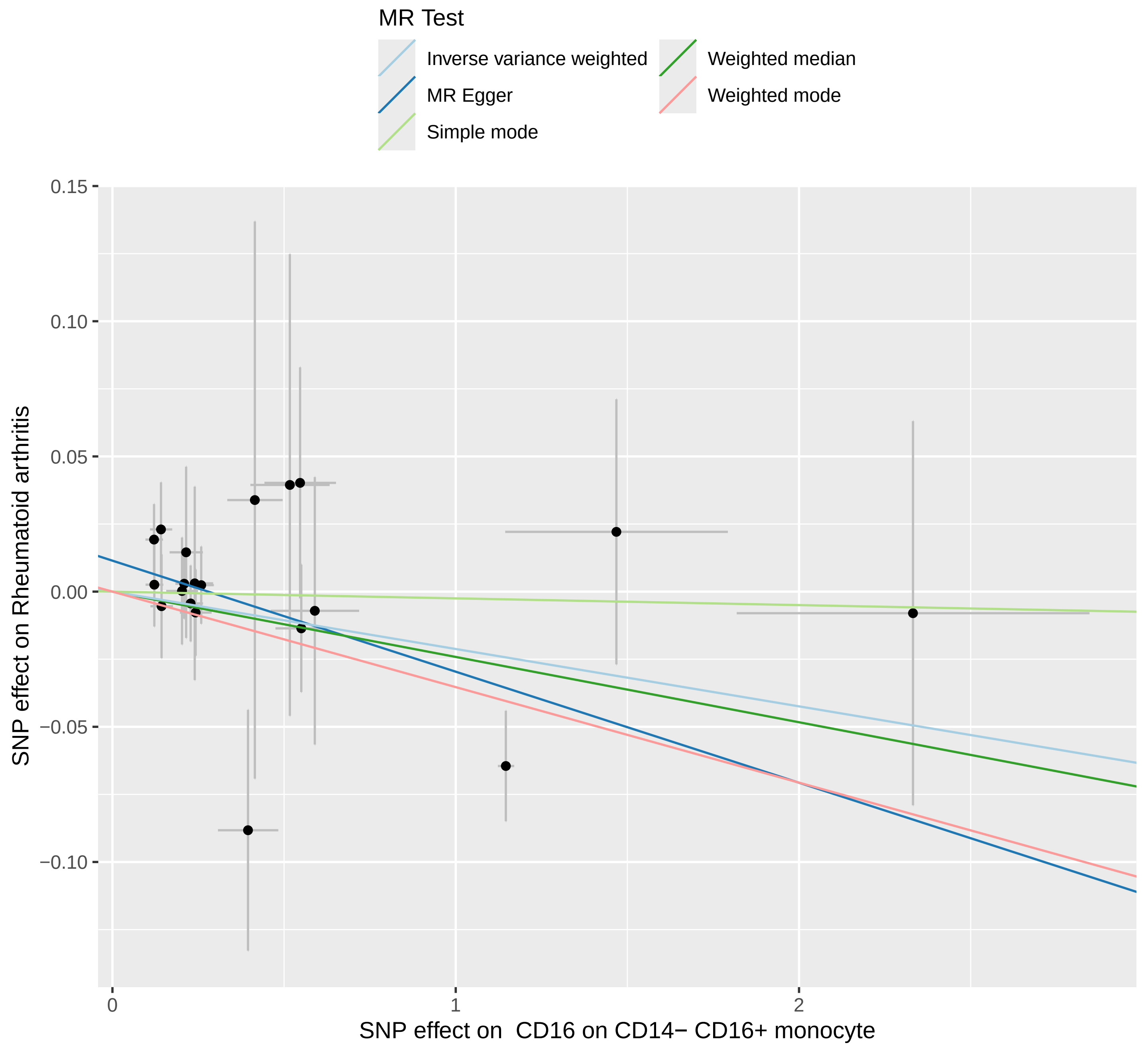

MR Test
Inverse variance weighted
MR Egger
Simple mode
Weighted median
Weighted mode
0.15
0.10
0.05
0.00
−0.05
−0.10
SNP effect on Rheumatoid arthritis
0
1
2
SNP effect on CD16 on CD14− CD16+ monocyte

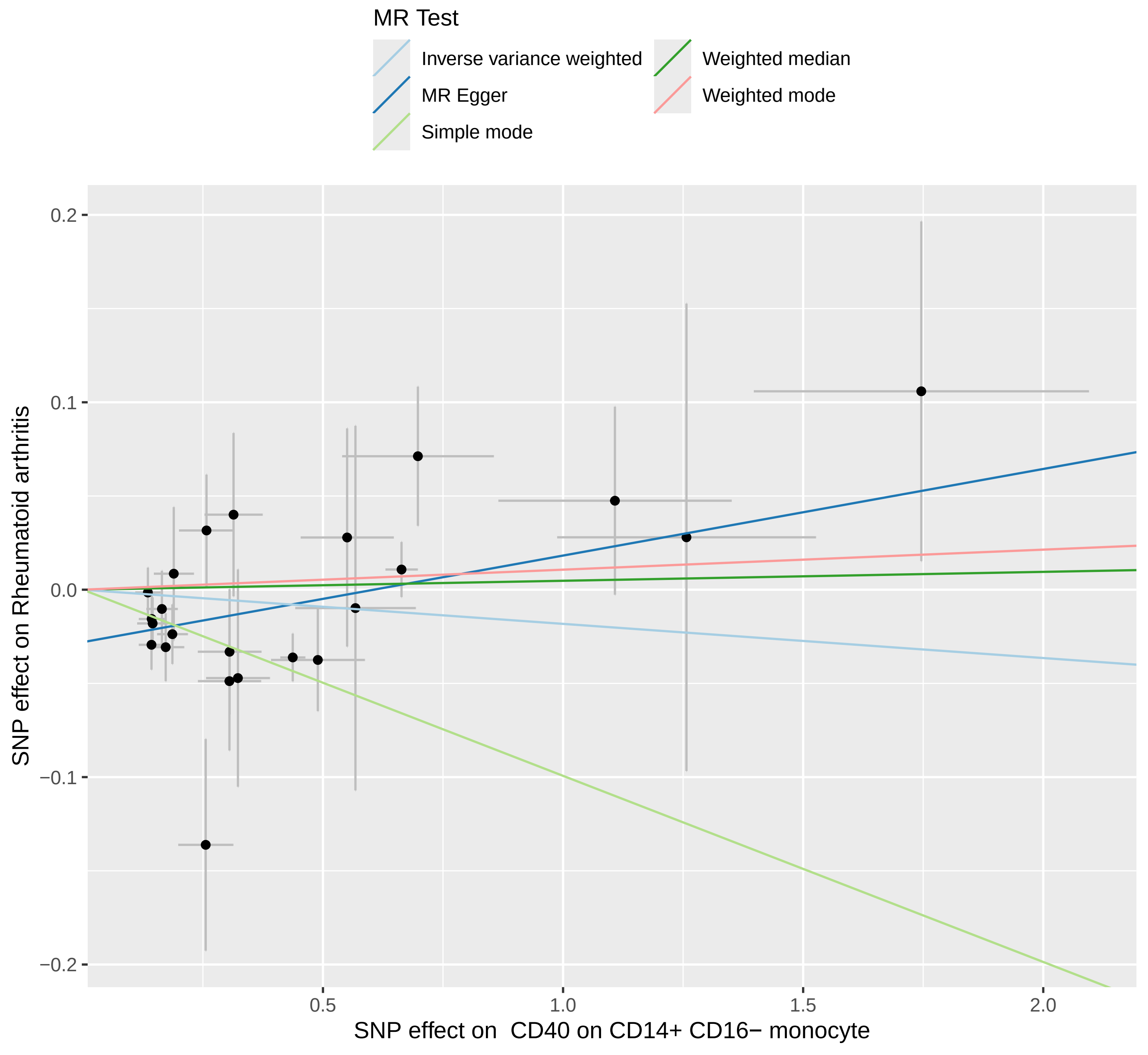

MR Test
Inverse variance weighted
MR Egger
Simple mode
Weighted median
Weighted mode
SNP effect on Rheumatoid arthritis
0.2
0.1
0.0
−0.1
−0.2
0.5
1.0
1.5
2.0
SNP effect on CD40 on CD14+ CD16− monocyte

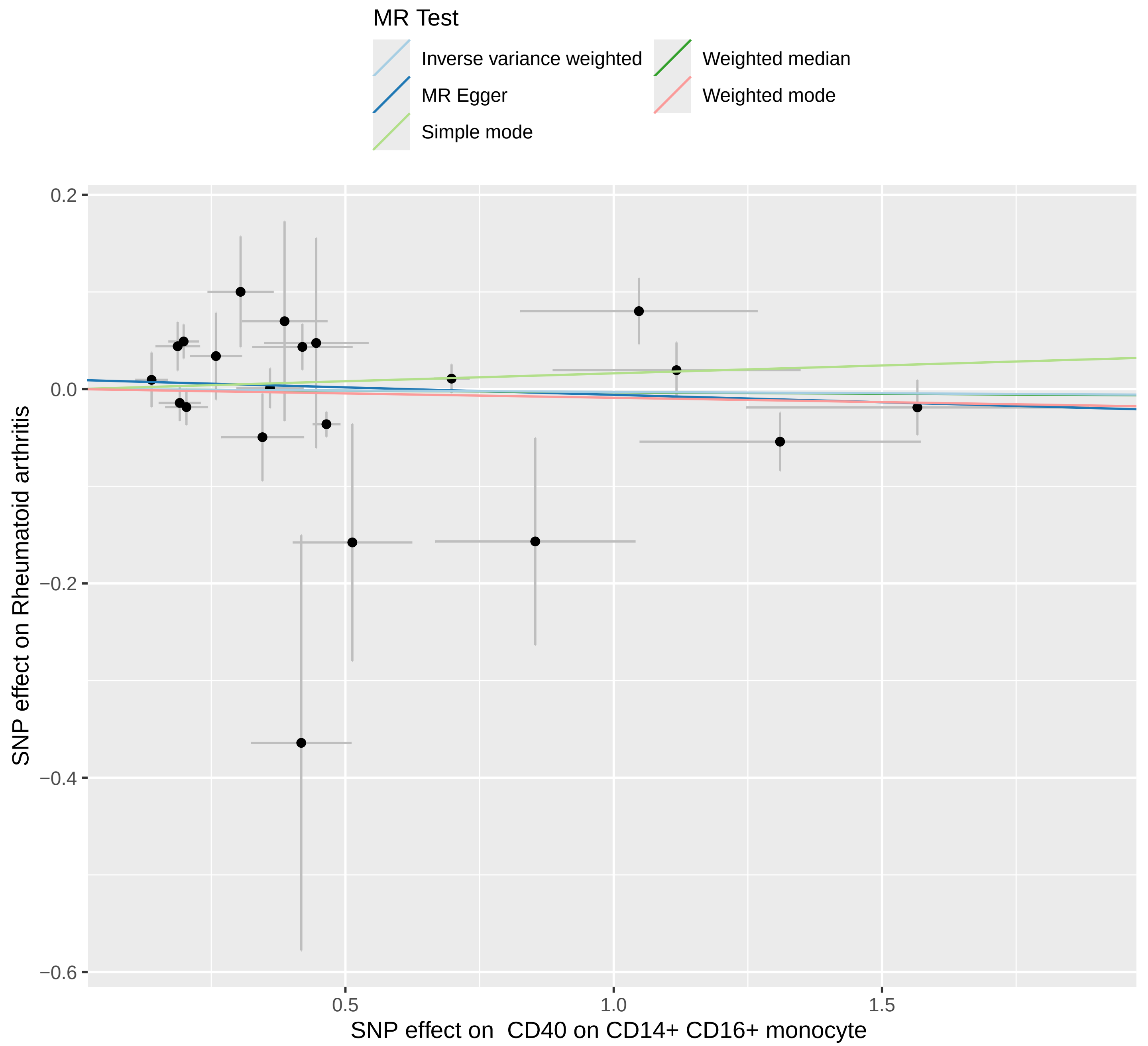
MR Test
Inverse variance weighted
MR Egger
Simple mode
Weighted median
Weighted mode
0.2
0.0
−0.2
−0.4
−0.6
0.5
1.0
1.5
SNP effect on Rheumatoid arthritis
SNP effect on CD40 on CD14+ CD16+ monocyte

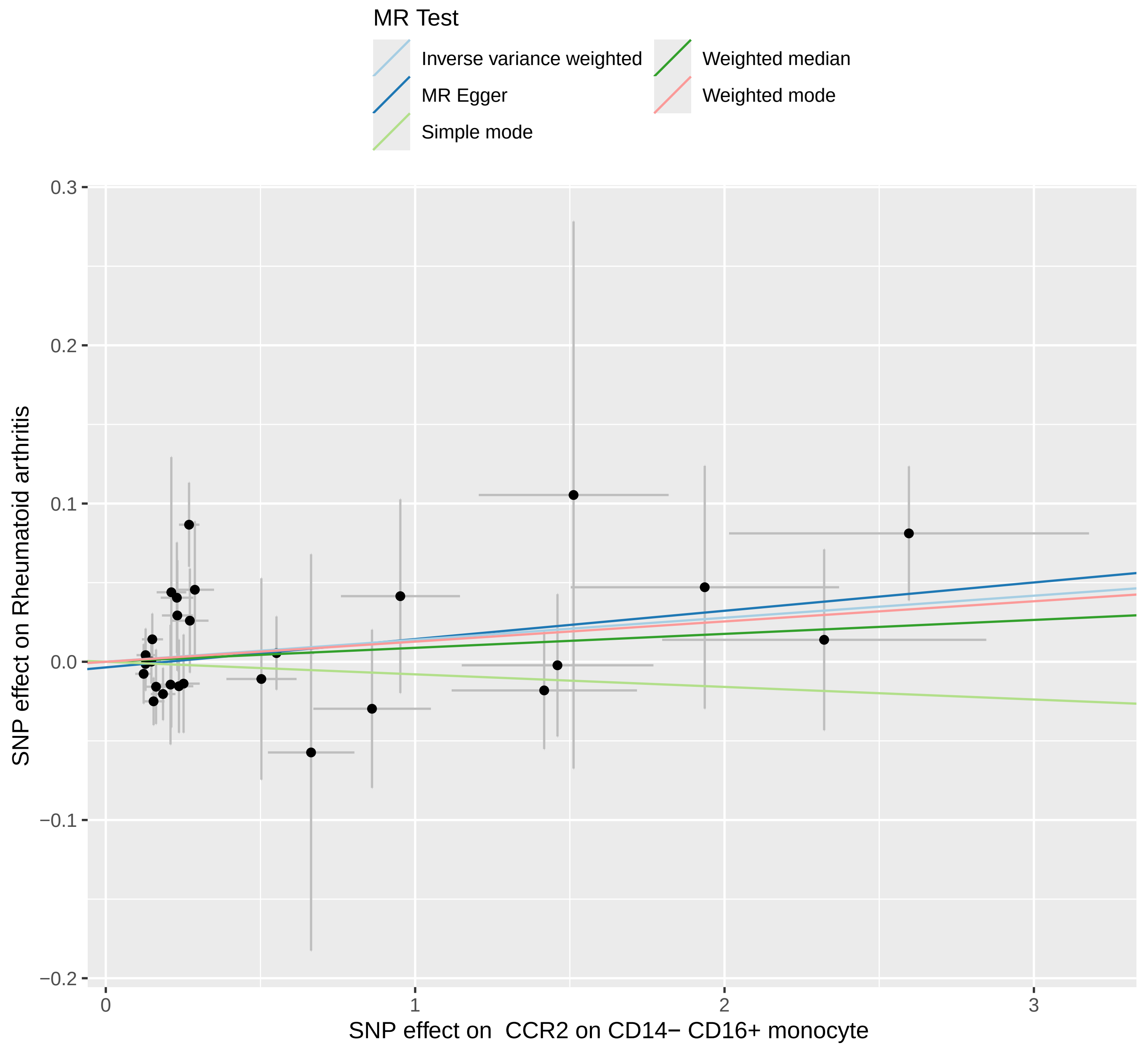

MR Test
Inverse variance weighted
MR Egger
Simple mode
Weighted median
Weighted mode
0.3
0.2
0.1
0.0
−0.1
−0.2
0
1
2
3
SNP effect on Rheumatoid arthritis
SNP effect on CCR2 on CD14− CD16+ monocyte

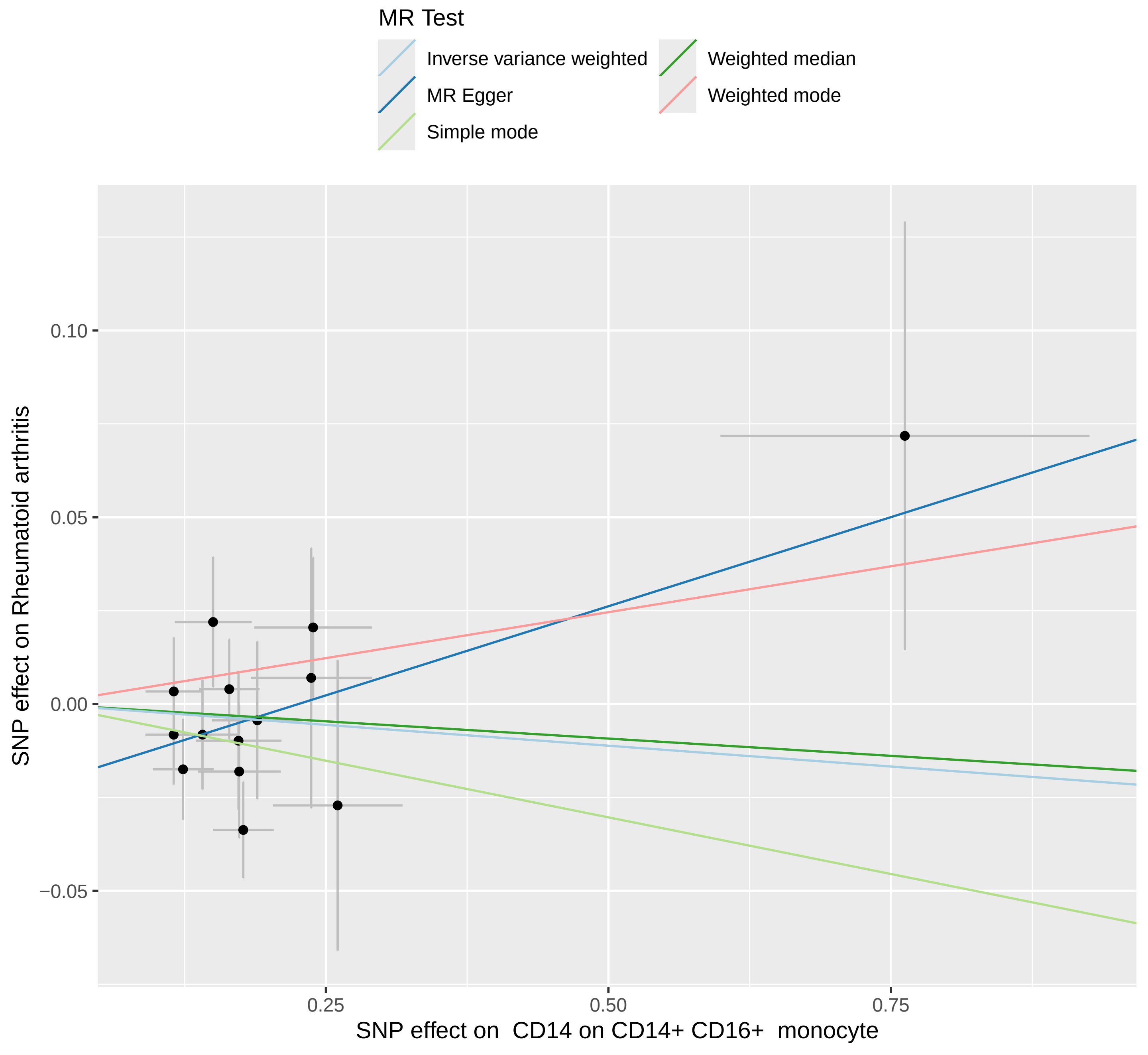

MR Test
Inverse variance weighted
MR Egger
Simple mode
Weighted median
Weighted mode
0.10
0.05
0.00
−0.05
SNP effect on Rheumatoid arthritis
0.25
0.50
0.75
SNP effect on CD14 on CD14+ CD16+ monocyte

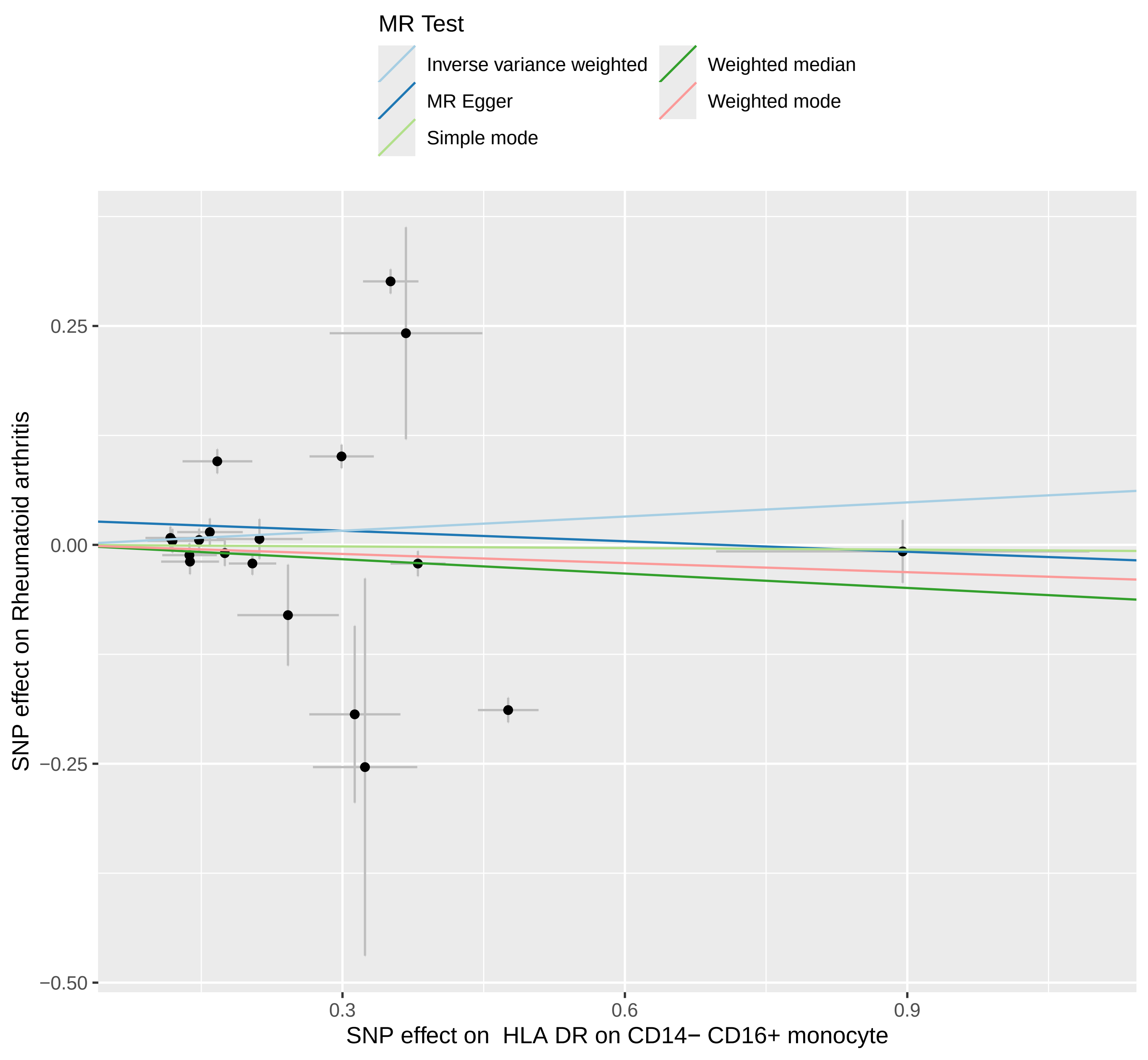

MR Test
Inverse variance weighted
MR Egger
Simple mode
Weighted median
Weighted mode
0.25
0.00
−0.25
−0.50
0.3
0.6
0.9
SNP effect on Rheumatoid arthritis
SNP effect on HLA DR on CD14− CD16+ monocyte

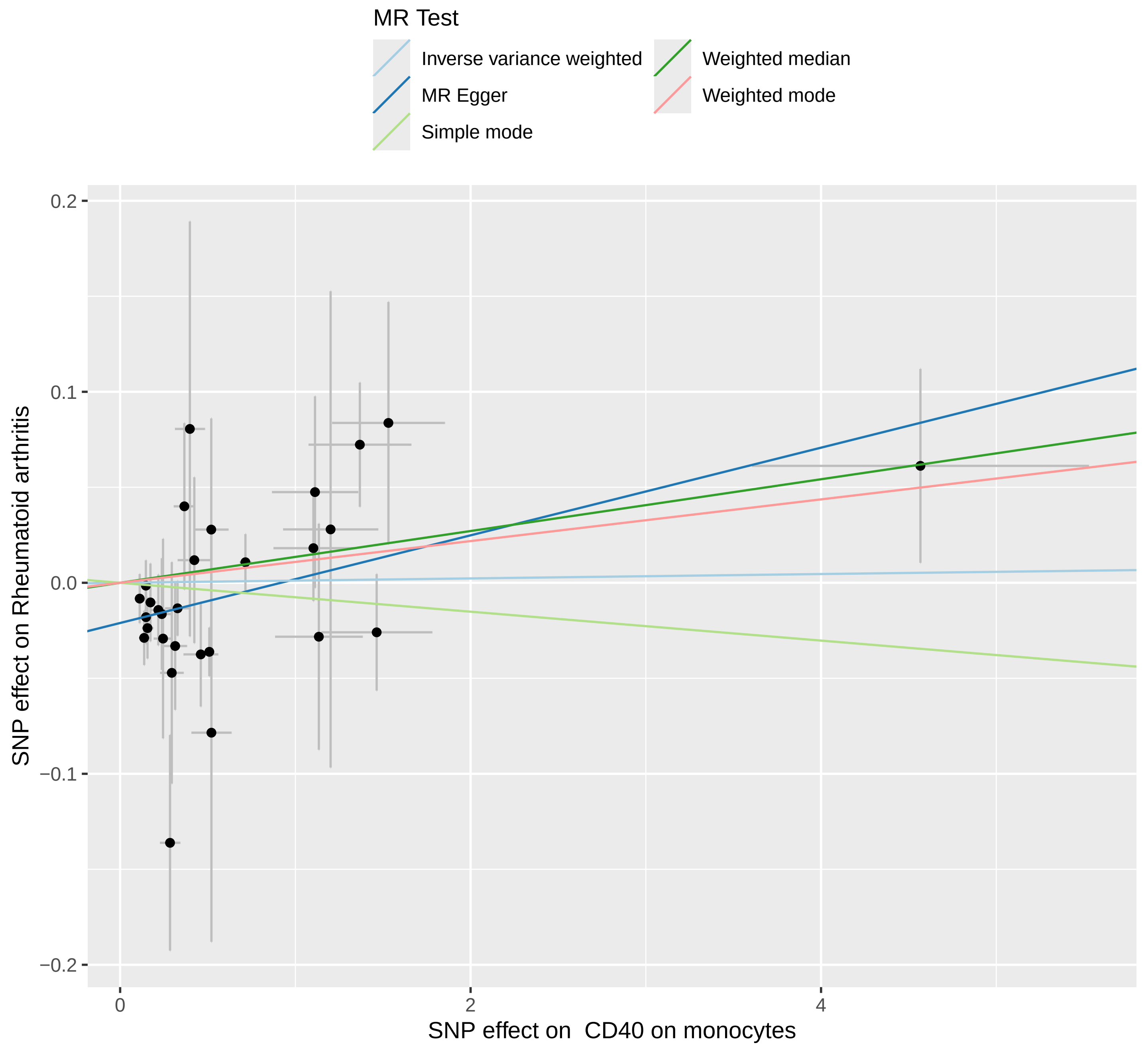

MR Test
Inverse variance weighted
MR Egger
Simple mode
Weighted median
Weighted mode
0.2
0.1
0.0
−0.1
−0.2
SNP effect on Rheumatoid arthritis
0
2
4
SNP effect on CD40 on monocytes

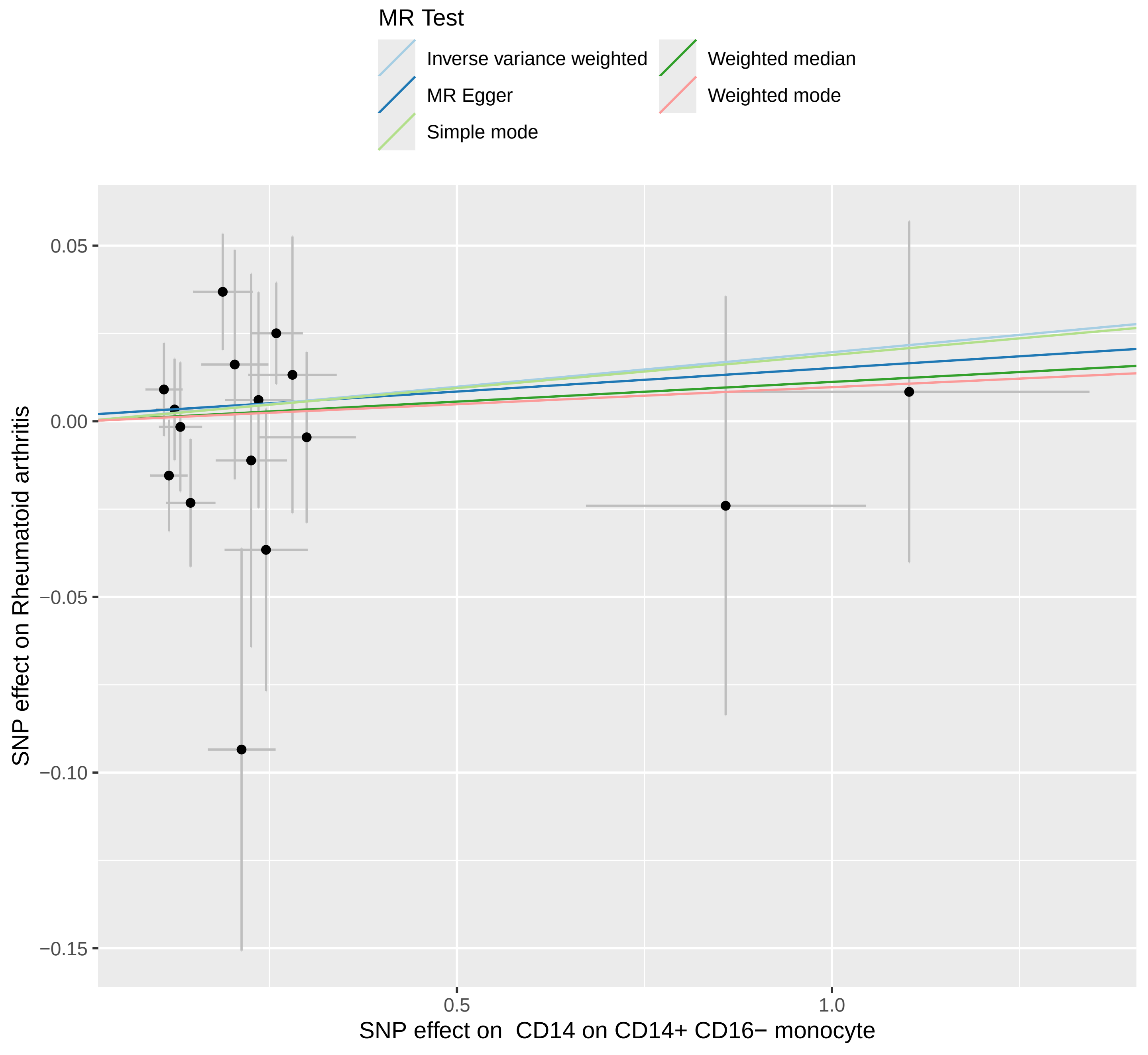
MR Test
Inverse variance weighted
MR Egger
Simple mode
Weighted median
Weighted mode
0.05
0.00
−0.05
−0.10
−0.15
0.5
1.0
SNP effect on Rheumatoid arthritis
SNP effect on CD14 on CD14+ CD16− monocyte

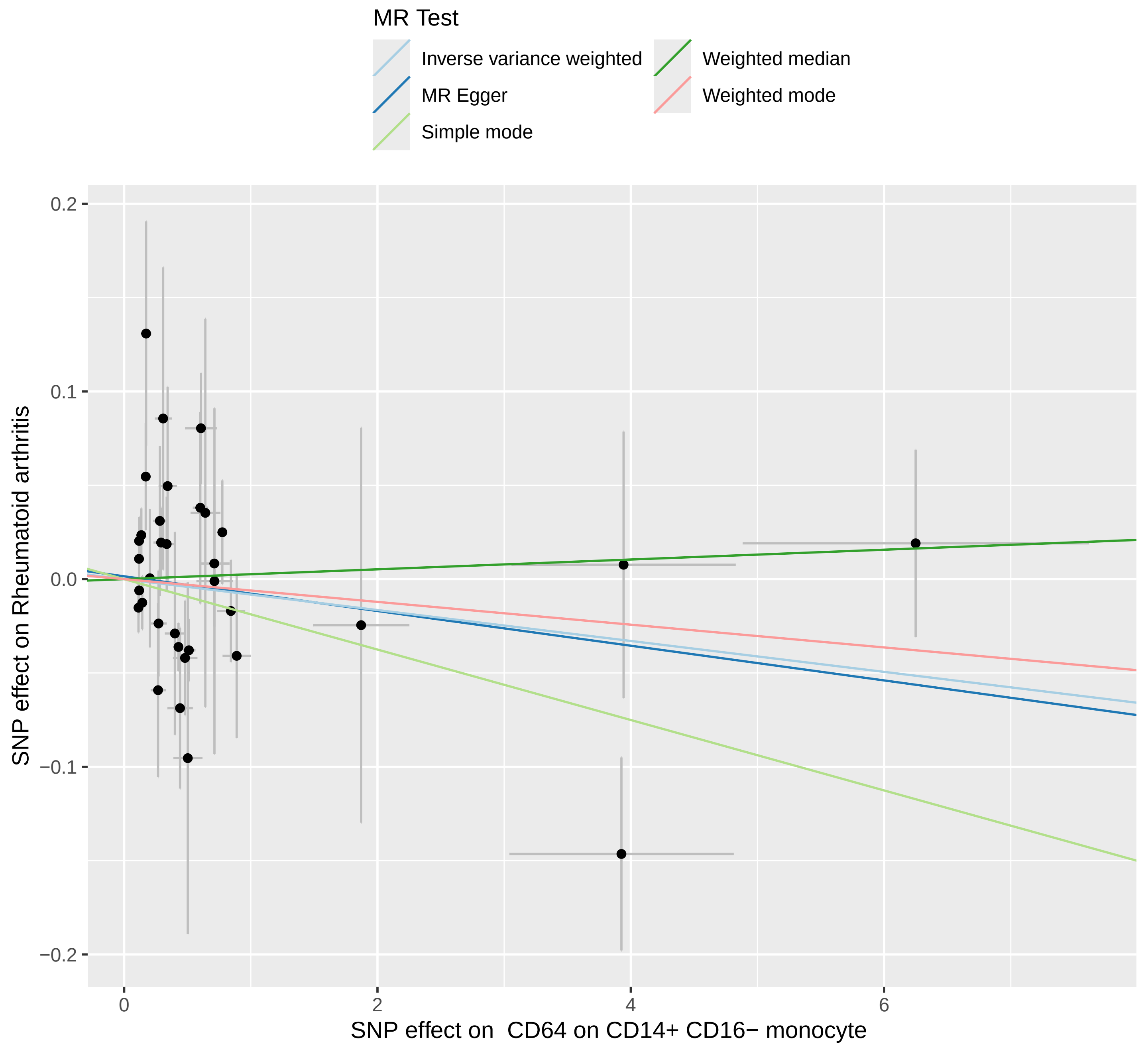
MR Test
Inverse variance weighted
MR Egger
Simple mode
Weighted median
Weighted mode
0.2
0.1
0.0
−0.1
−0.2
0
2
4
6
SNP effect on Rheumatoid arthritis
SNP effect on CD64 on CD14+ CD16− monocyte

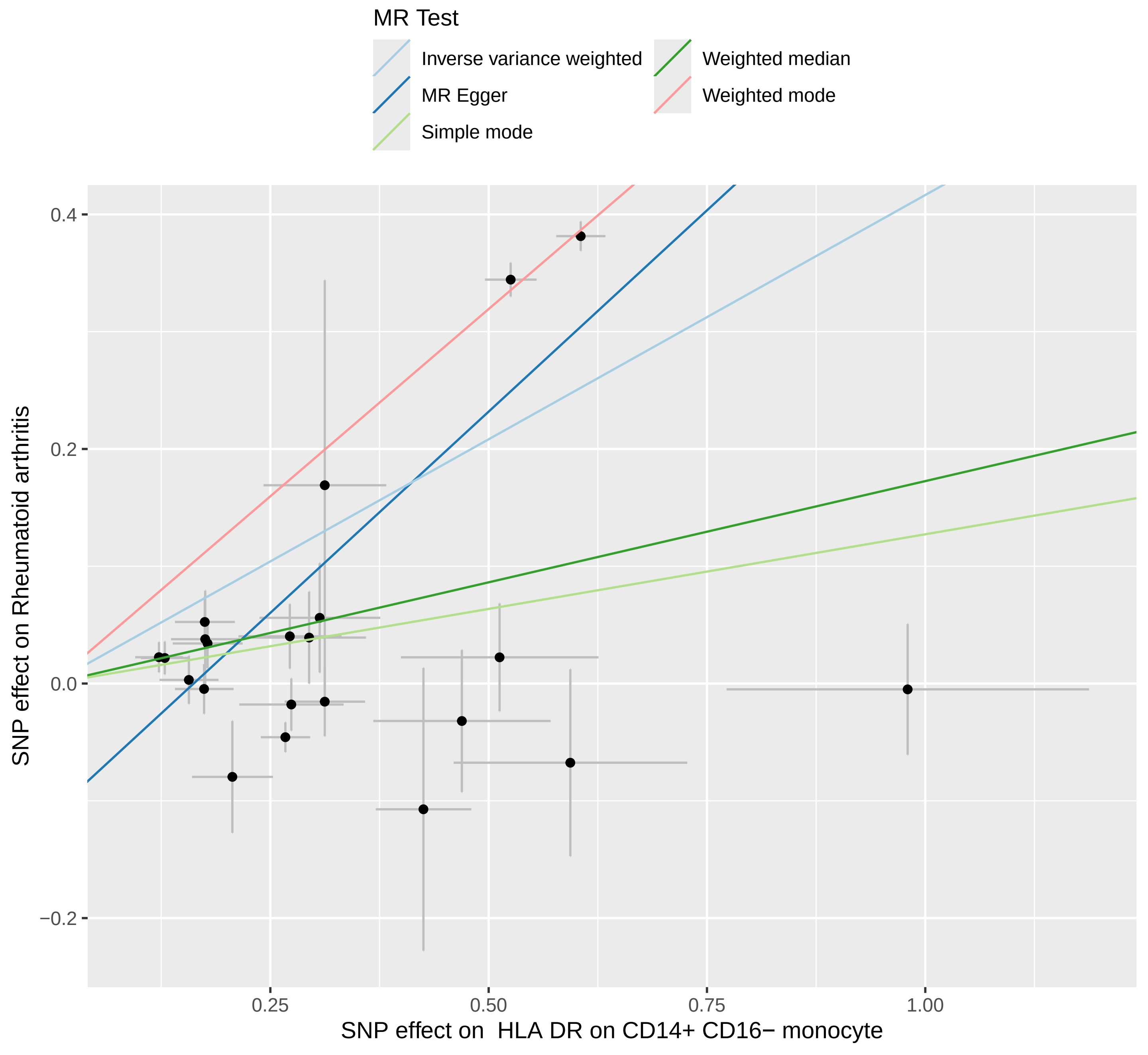

MR Test
Inverse variance weighted
MR Egger
Simple mode
Weighted median
Weighted mode
SNP effect on Rheumatoid arthritis
0.4
0.2
0.0
−0.2
0.25
0.50
0.75
1.00
SNP effect on HLA DR on CD14+ CD16− monocyte

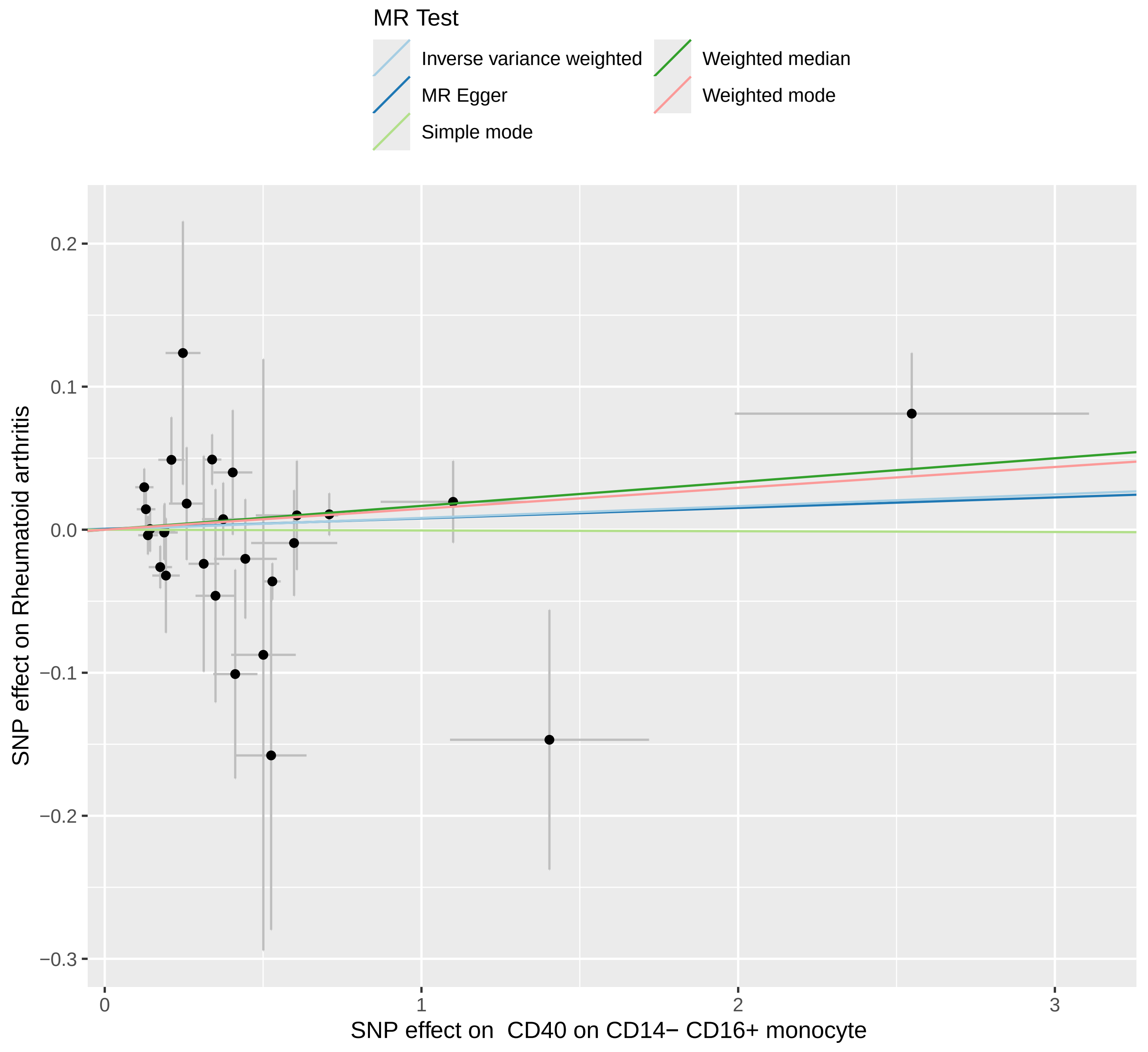

MR Test
Inverse variance weighted
Weighted median
MR Egger
Weighted mode
Simple mode
SNP effect on Rheumatoid arthritis
0.2
0.1
0.0
−0.1
−0.2
−0.3
0
1
2
3
SNP effect on CD40 on CD14− CD16+ monocyte

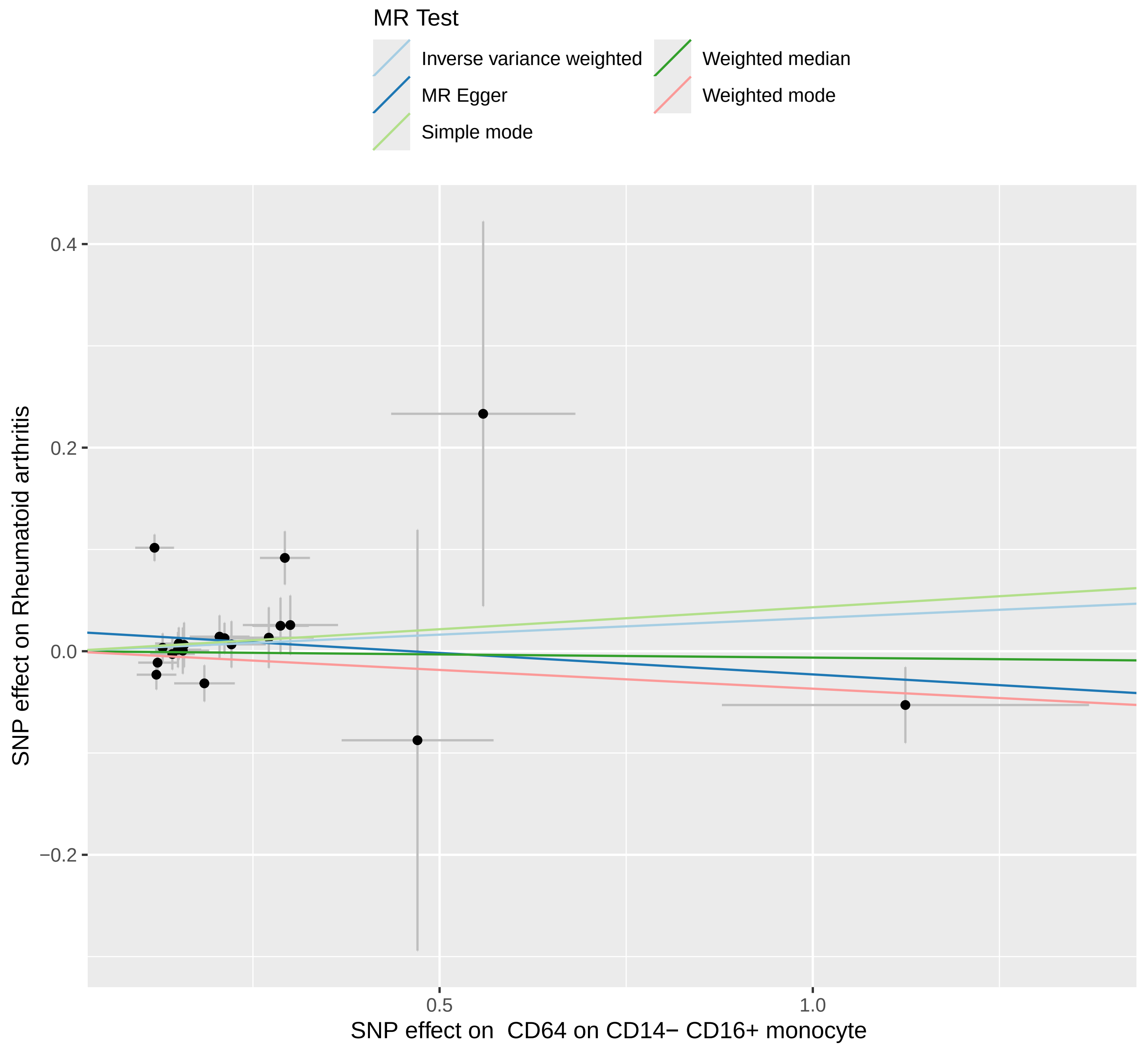

MR Test
Inverse variance weighted
MR Egger
Simple mode
Weighted median
Weighted mode
0.4
0.2
0.0
−0.2
0.5
1.0
SNP effect on Rheumatoid arthritis
SNP effect on CD64 on CD14− CD16+ monocyte

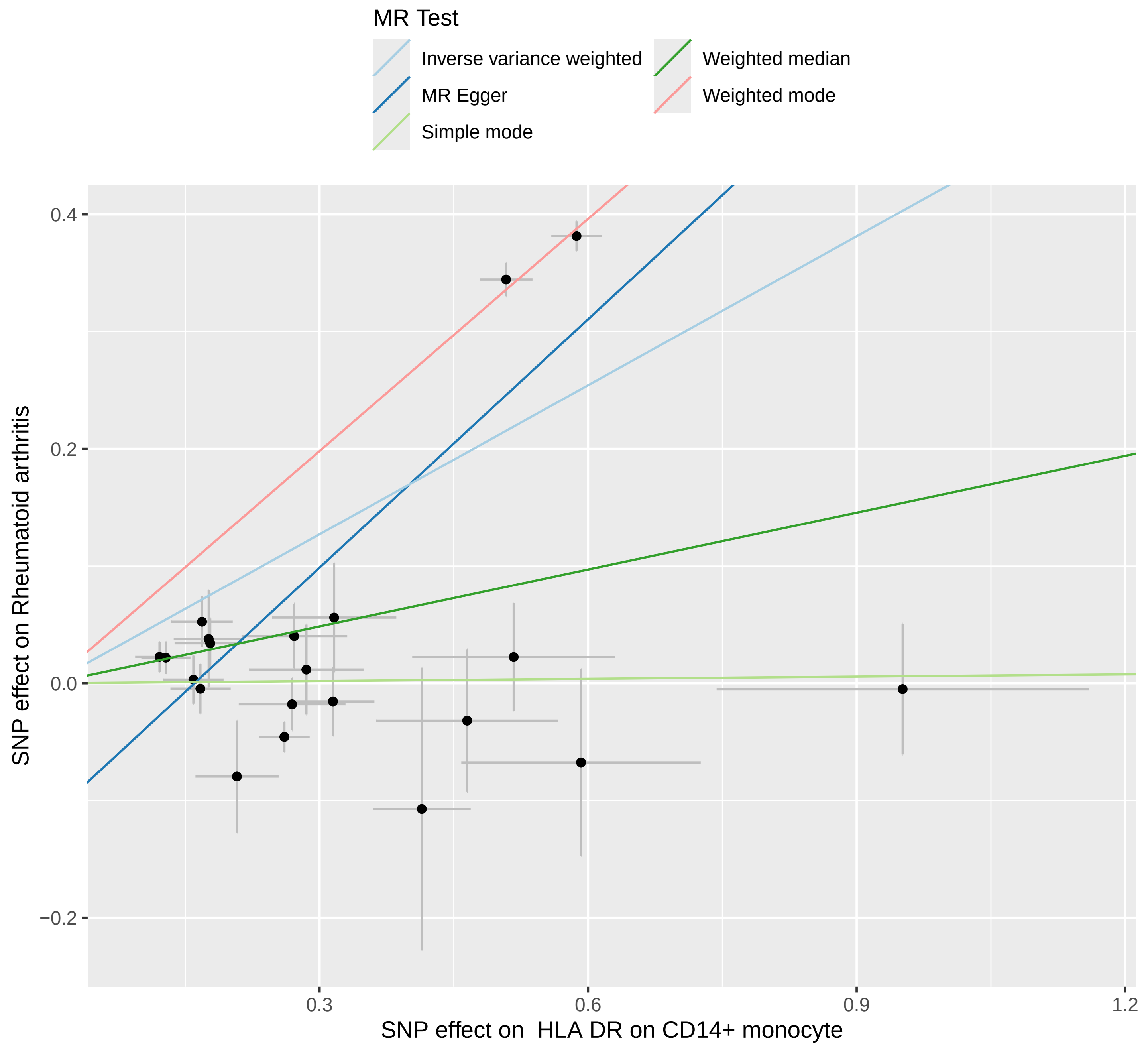

MR Test
Inverse variance weighted
MR Egger
Simple mode
Weighted median
Weighted mode
0.4
0.2
0.0
−0.2
0.3
0.6
0.9
1.2
SNP effect on Rheumatoid arthritis
SNP effect on HLA DR on CD14+ monocyte

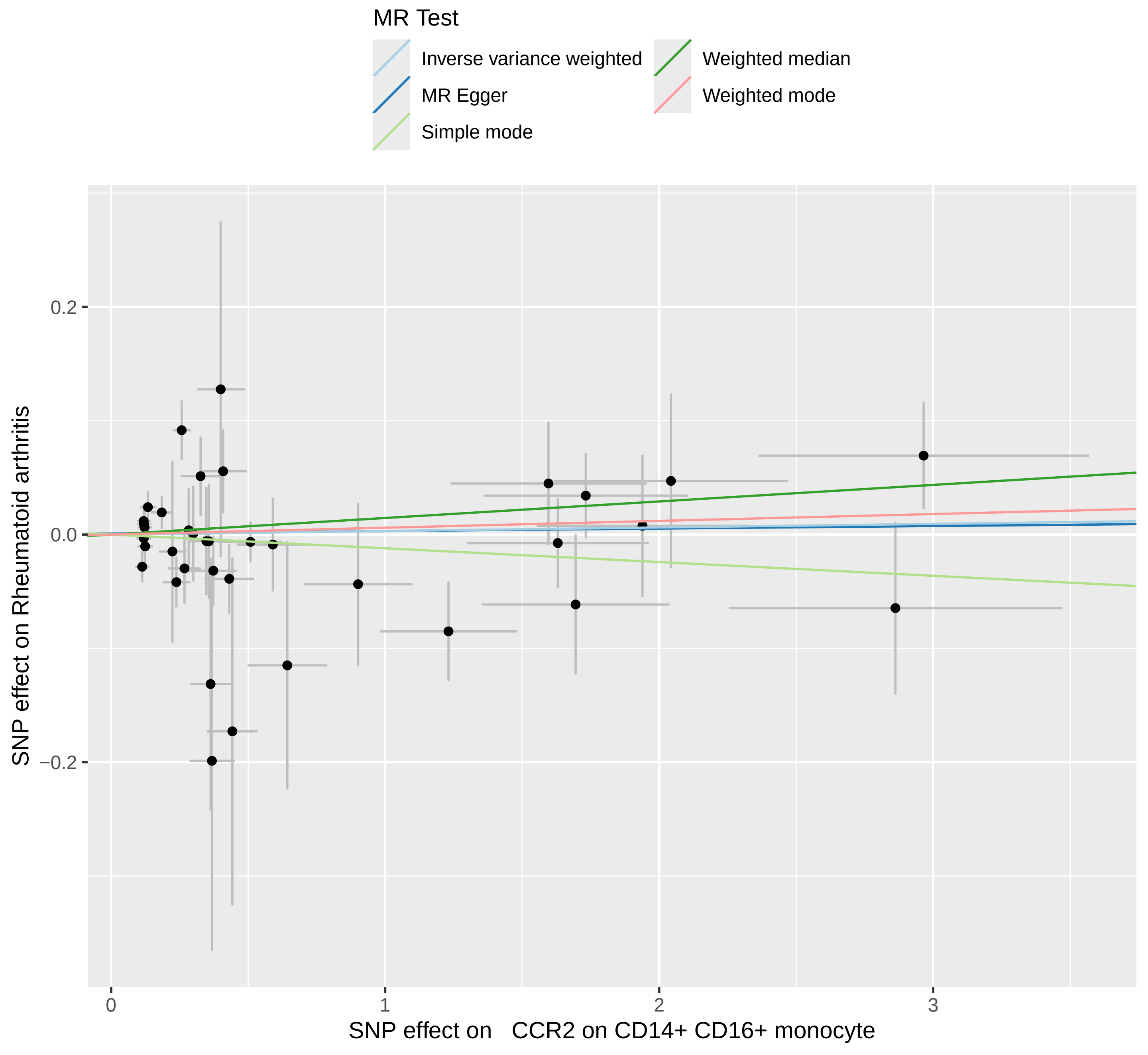

MR Test
Inverse variance weighted
MR Egger
Simple mode
Weighted median
Weighted mode
0.2
0.0
−0.2
0
1
2
3
SNP effect on Rheumatoid arthritis
SNP effect on CCR2 on CD14+ CD16+ monocyte

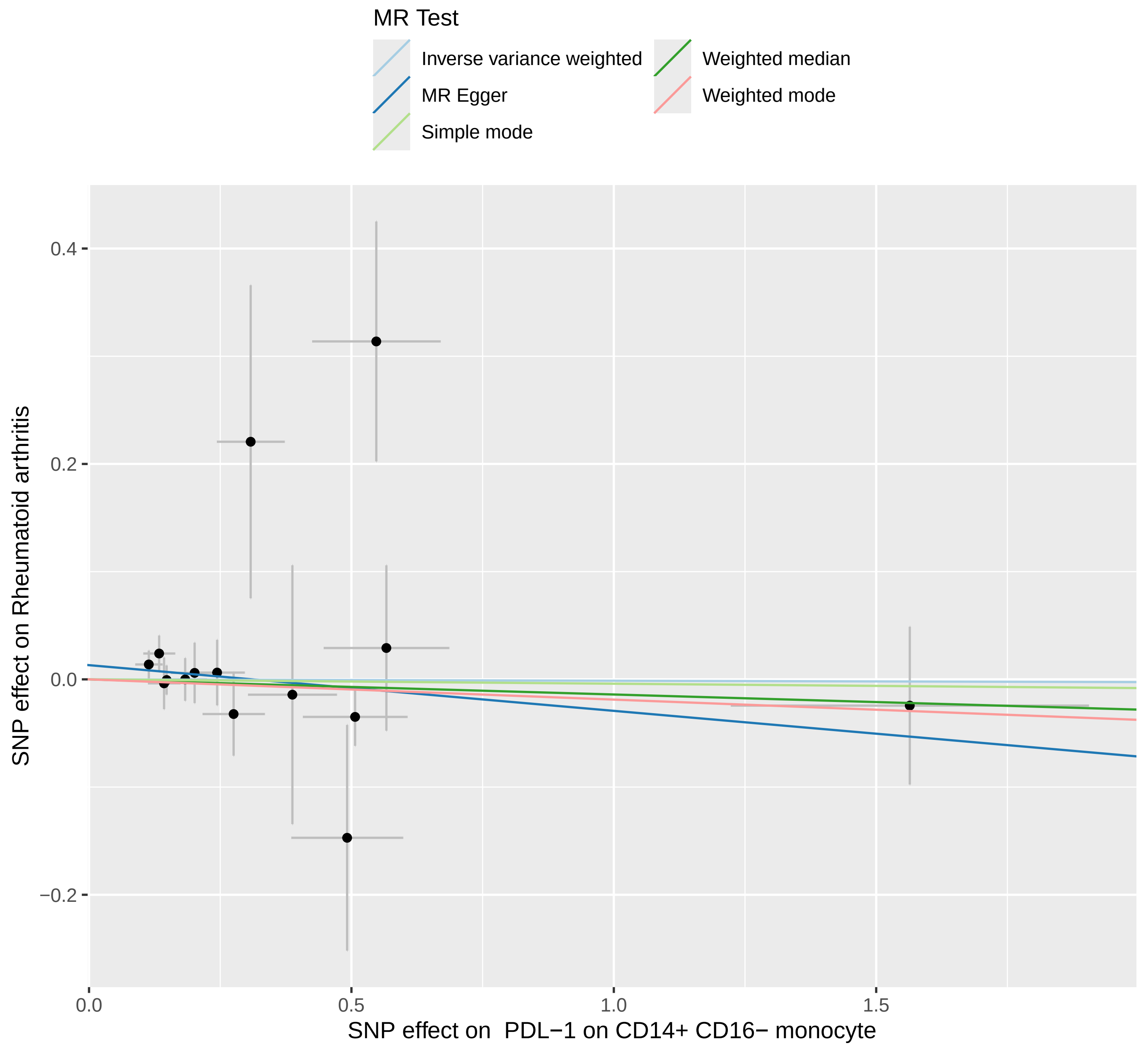

MR Test
Inverse variance weighted
MR Egger
Simple mode
Weighted median
Weighted mode
0.4
0.2
0.0
−0.2
0.0
0.5
1.0
1.5
SNP effect on Rheumatoid arthritis
SNP effect on PDL−1 on CD14+ CD16− monocyte

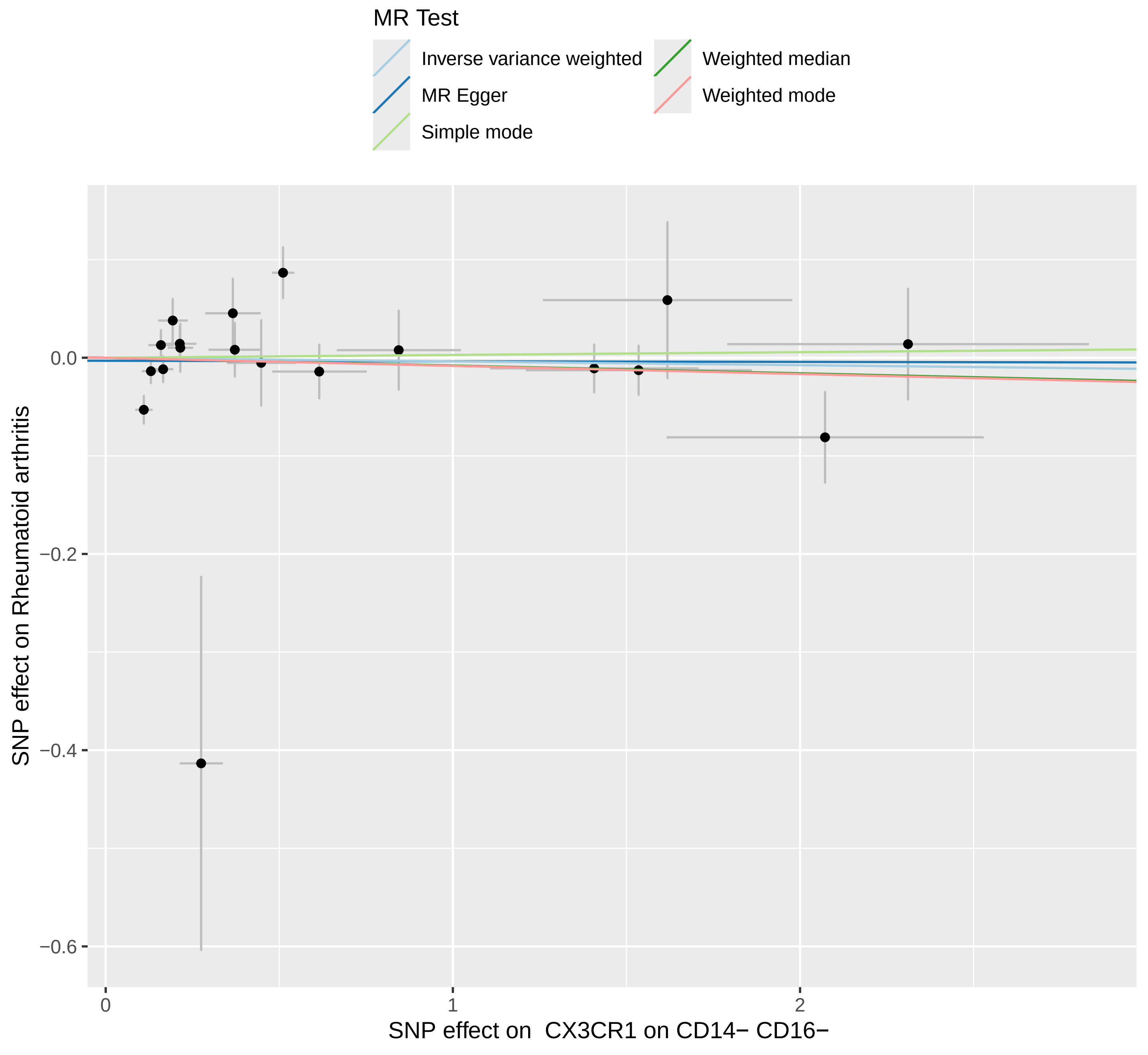
MR Test
Inverse variance weighted
MR Egger
Simple mode
Weighted median
Weighted mode
SNP effect on Rheumatoid arthritis
0.0
−0.2
−0.4
−0.6
0
1
2
SNP effect on CX3CR1 on CD14− CD16−

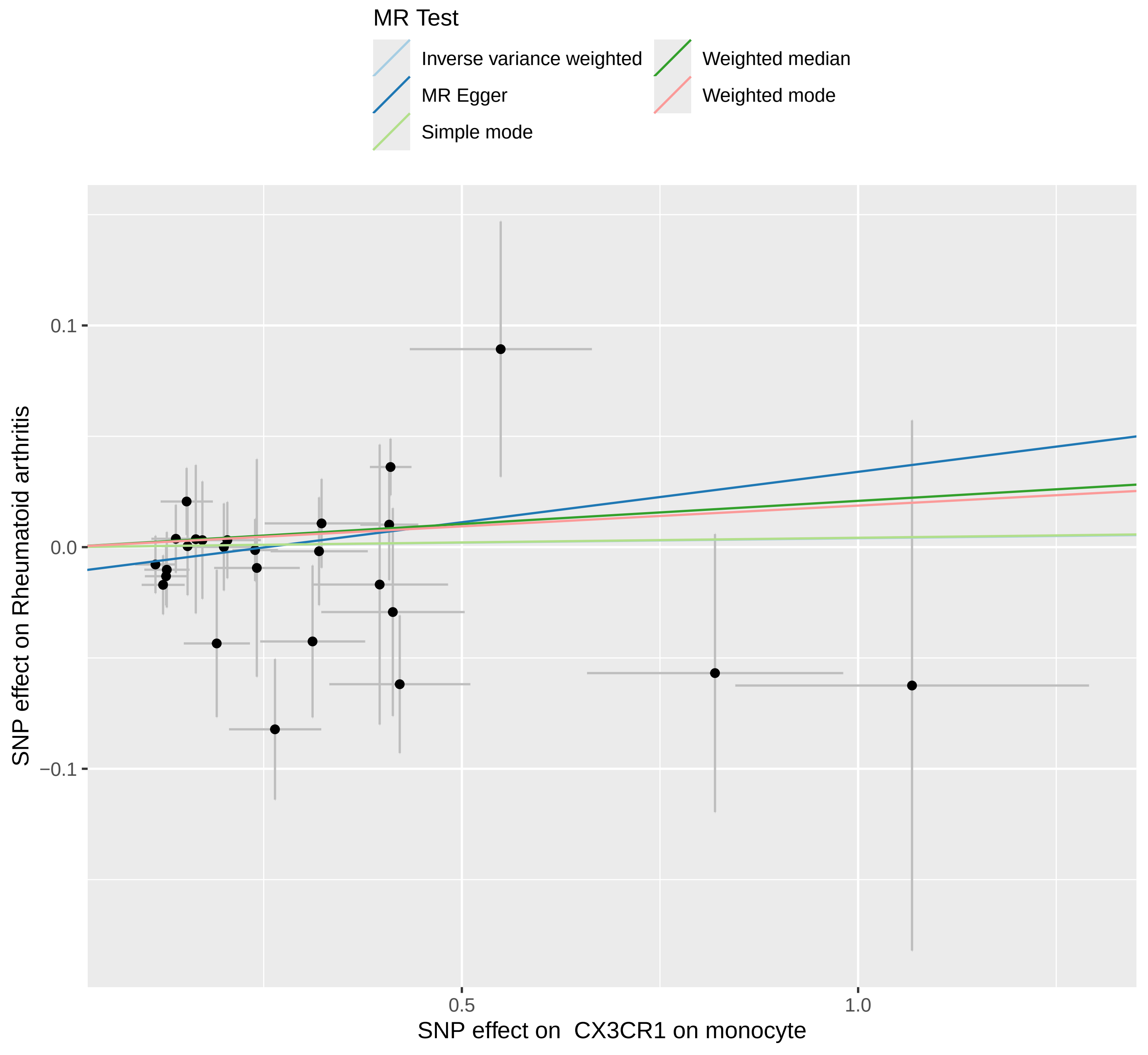

MR Test
Inverse variance weighted
MR Egger
Simple mode
Weighted median
Weighted mode
0.1
0.0
−0.1
0.5
1.0
SNP effect on Rheumatoid arthritis
SNP effect on CX3CR1 on monocyte

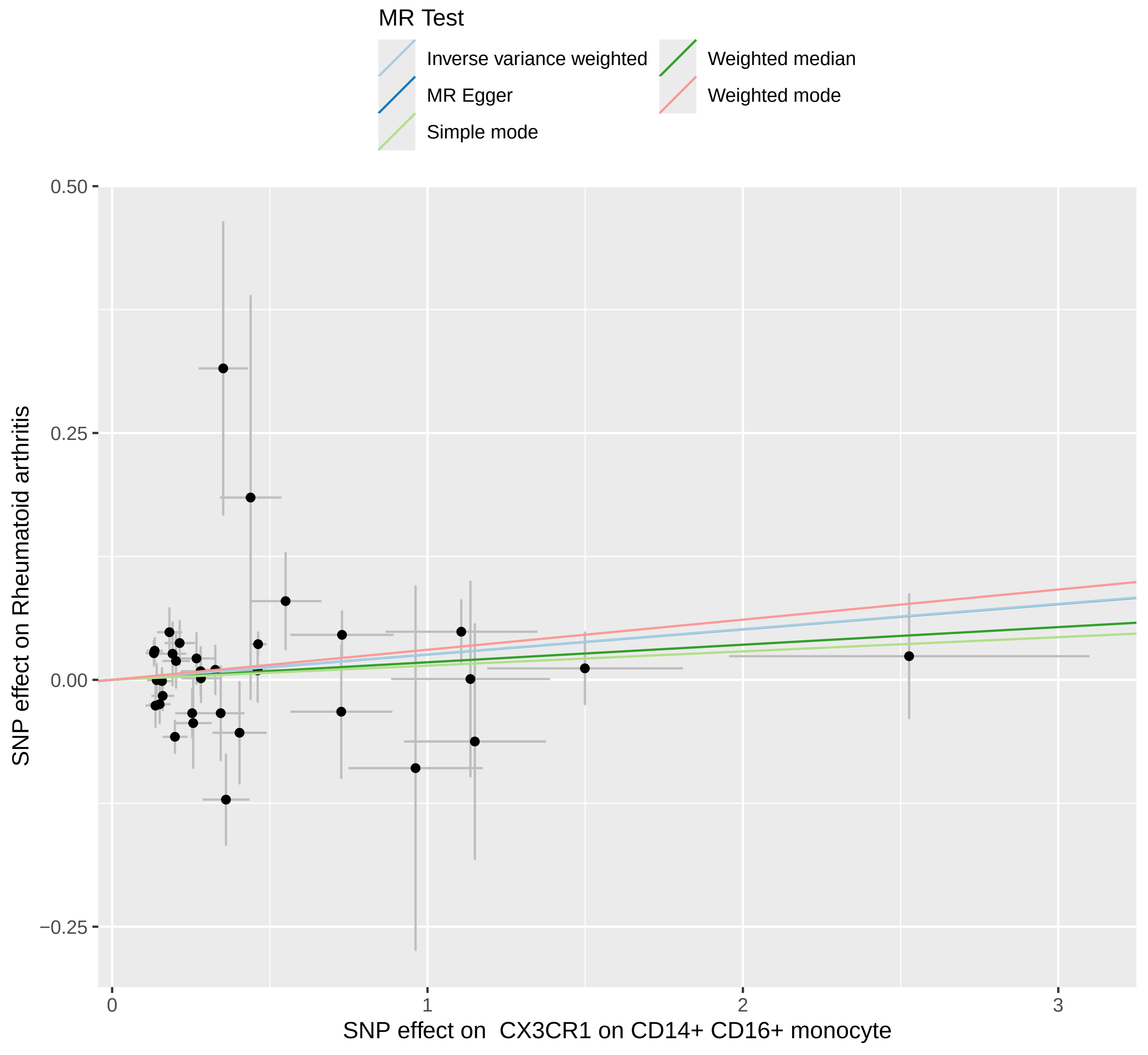

MR Test
Inverse variance weighted
MR Egger
Simple mode
Weighted median
Weighted mode
0.50
0.25
0.00
−0.25
0
1
2
3
SNP effect on Rheumatoid arthritis
SNP effect on CX3CR1 on CD14+ CD16+ monocyte

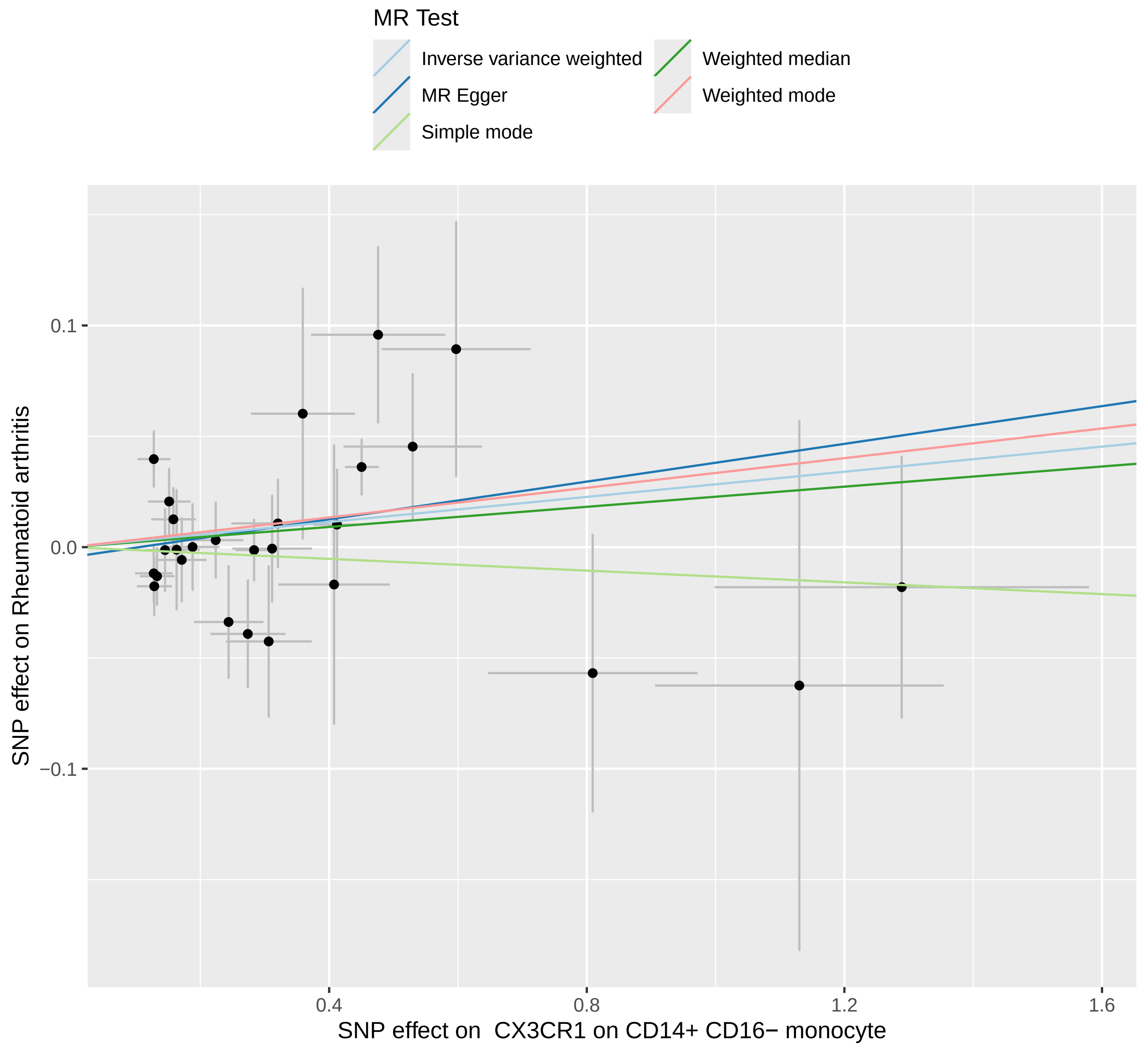

MR Test
Inverse variance weighted
MR Egger
Simple mode
Weighted median
Weighted mode
0.1
0.0
−0.1
0.4
0.8
1.2
1.6
SNP effect on Rheumatoid arthritis
SNP effect on CX3CR1 on CD14+ CD16− monocyte

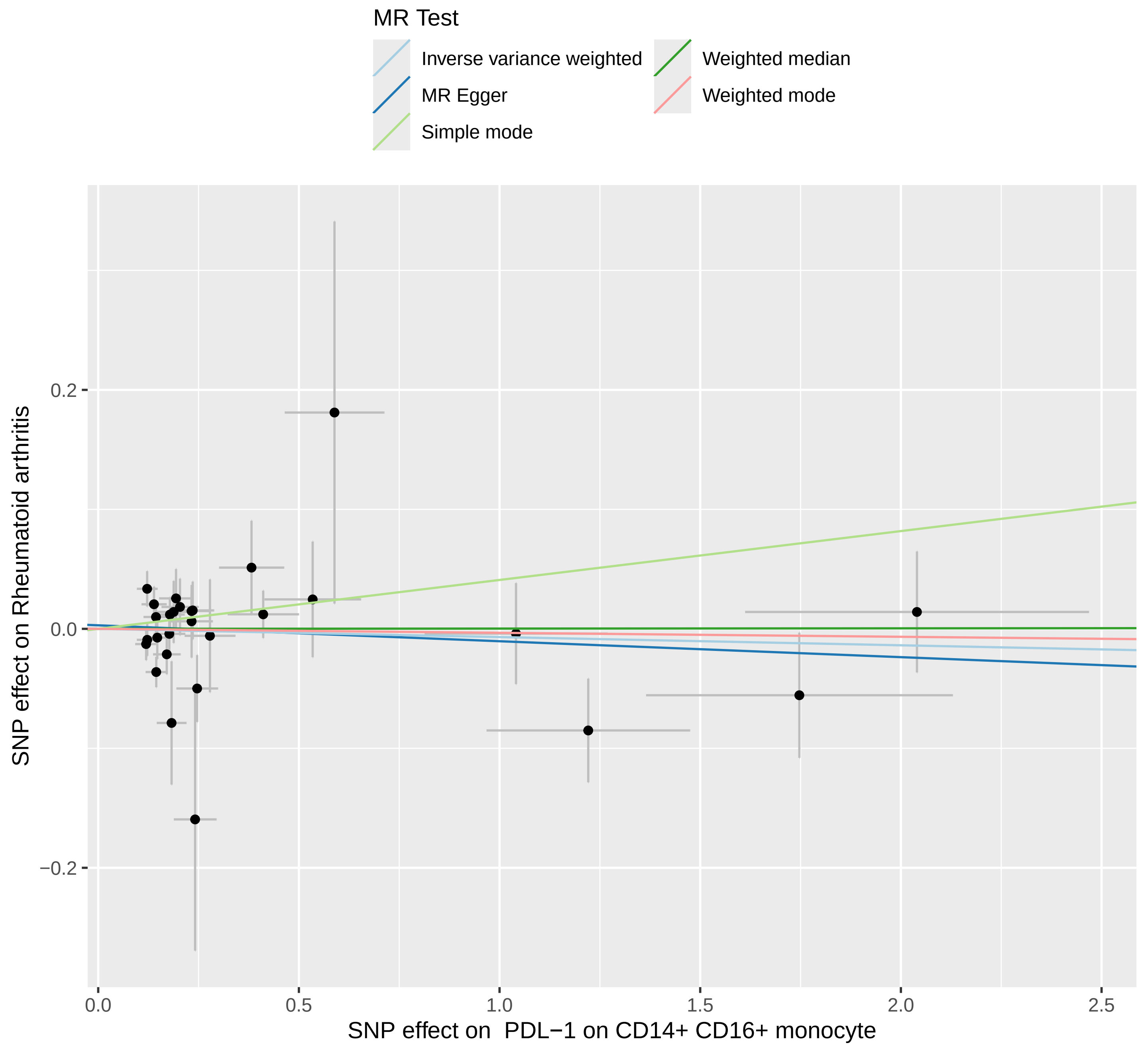
MR Test
Inverse variance weighted
MR Egger
Simple mode
Weighted median
Weighted mode
0.2
0.0
−0.2
0.0
0.5
1.0
1.5
2.0
2.5
SNP effect on Rheumatoid arthritis
SNP effect on PDL−1 on CD14+ CD16+ monocyte

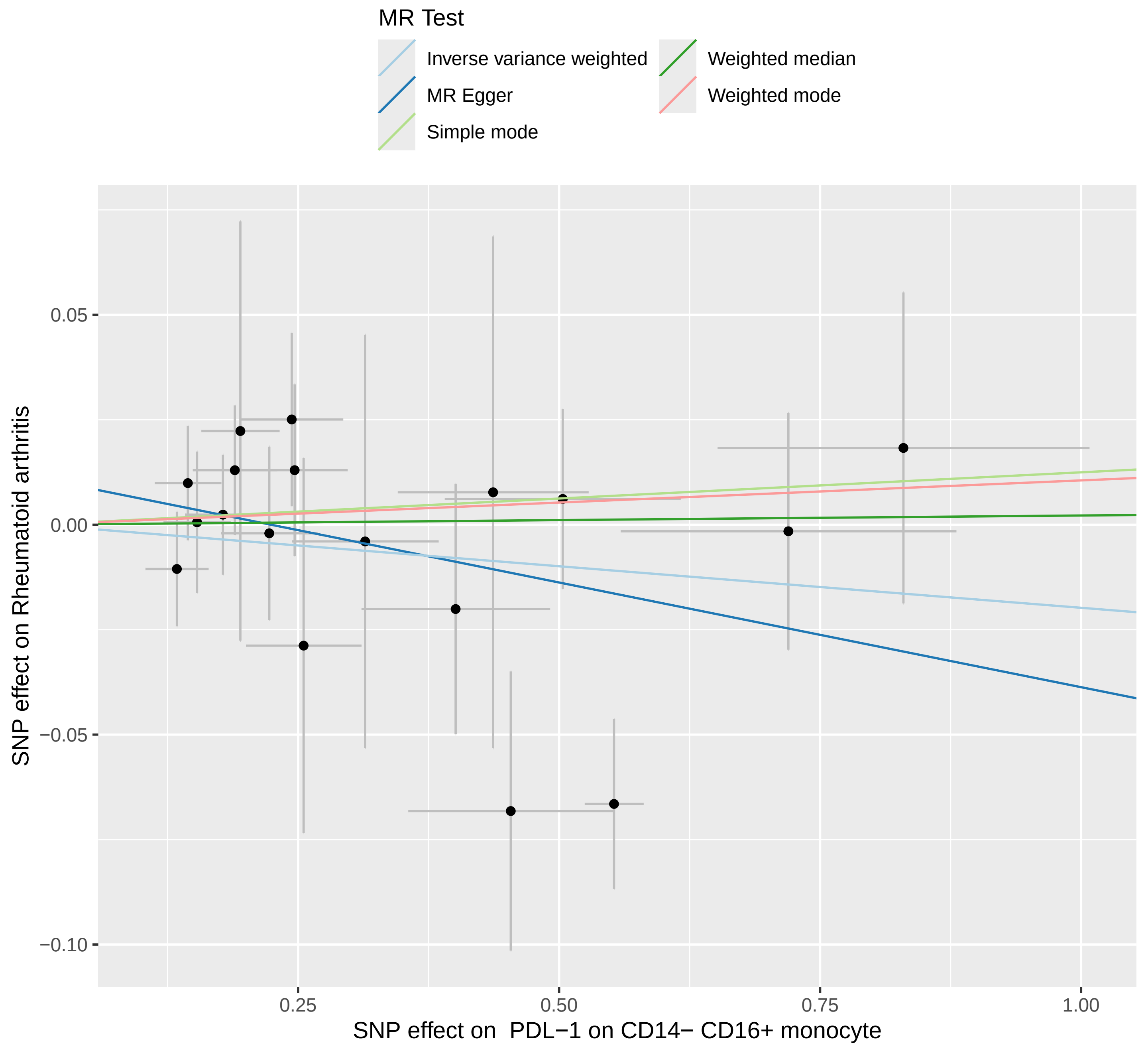
MR Test
Inverse variance weighted
MR Egger
Simple mode
Weighted median
Weighted mode
0.05
0.00
−0.05
−0.10
0.25
0.50
0.75
1.00
SNP effect on Rheumatoid arthritis
SNP effect on PDL−1 on CD14− CD16+ monocyte

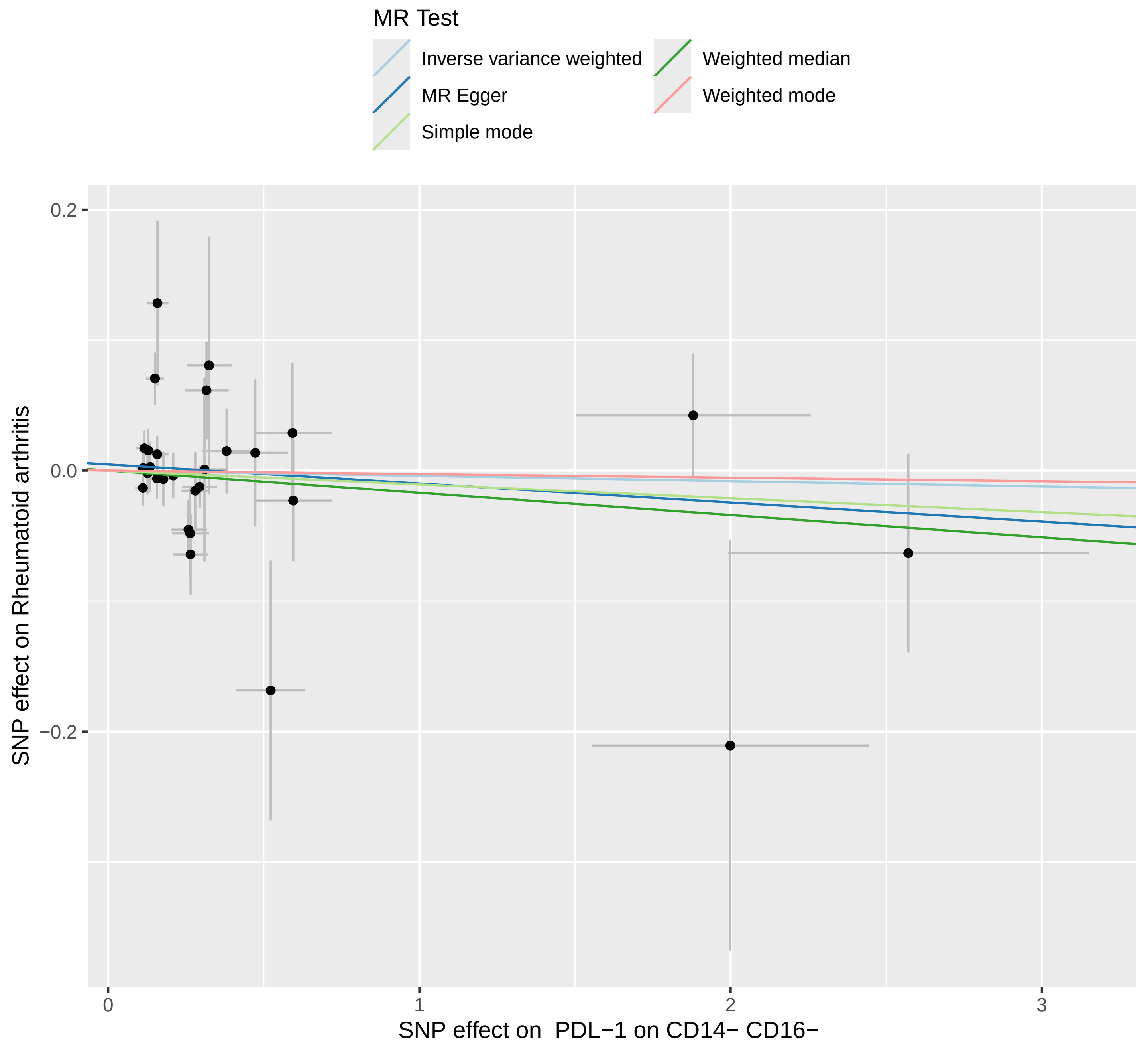
MR Test
Inverse variance weighted
MR Egger
Simple mode
Weighted median
Weighted mode
0.2
0.0
−0.2
0
1
2
3
SNP effect on Rheumatoid arthritis
SNP effect on PDL−1 on CD14− CD16−

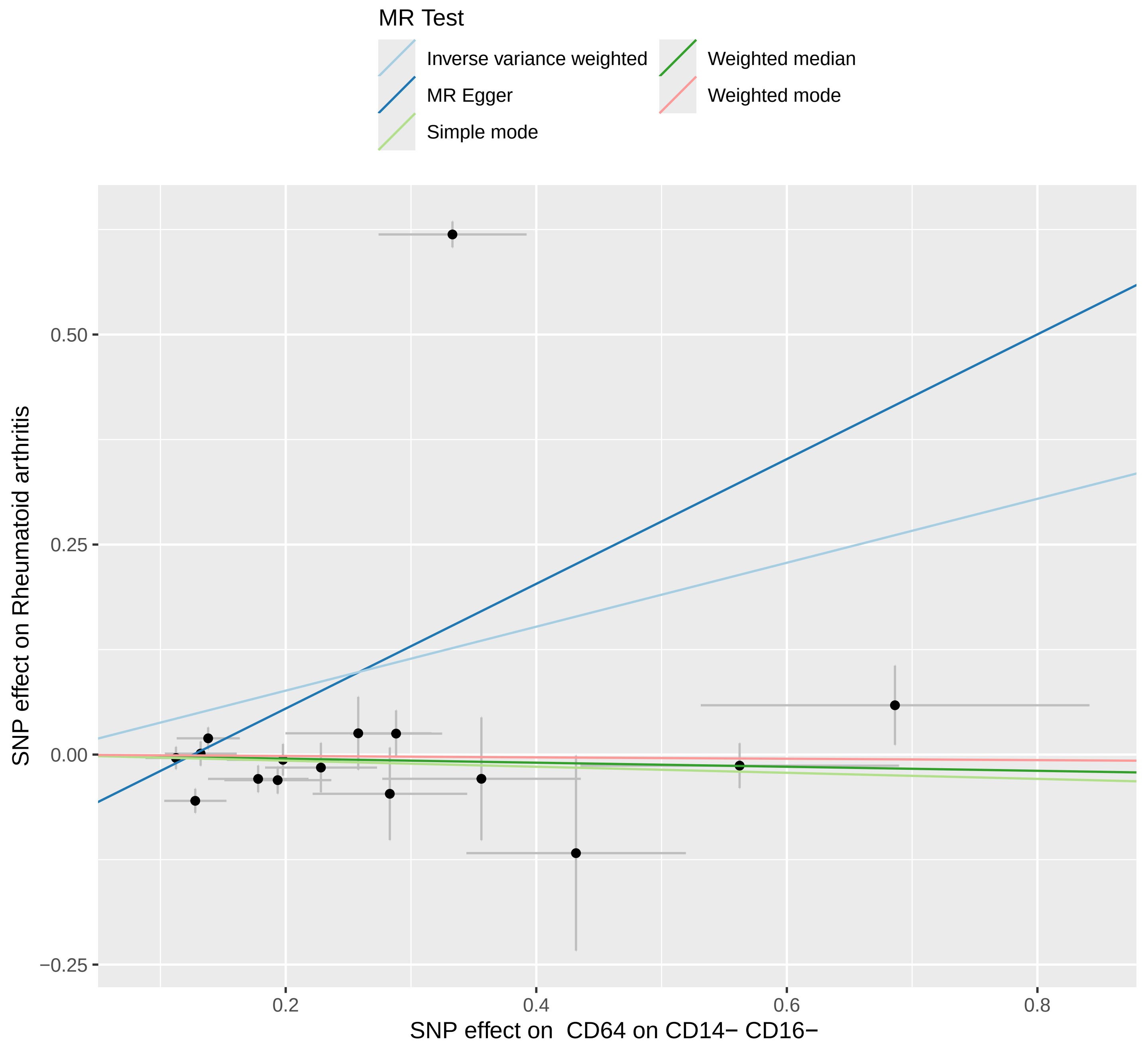
MR Test
Inverse variance weighted
MR Egger
Simple mode
Weighted median
Weighted mode
0.50
0.25
0.00
−0.25
0.2
0.4
0.6
0.8
SNP effect on Rheumatoid arthritis
SNP effect on CD64 on CD14− CD16−

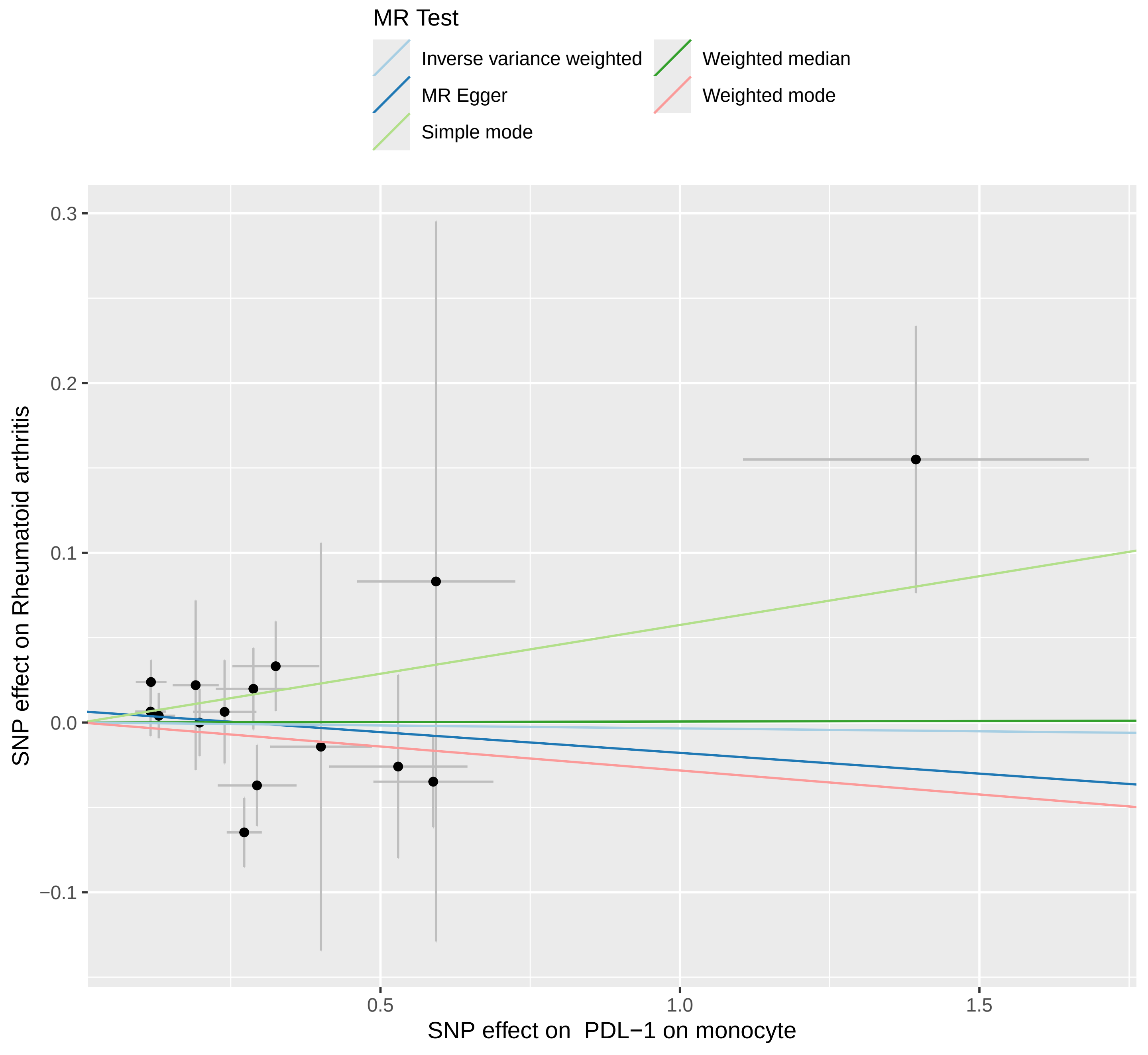
MR Test
Inverse variance weighted
Weighted median
MR Egger
Weighted mode
Simple mode
0.3
0.2
0.1
0.0
−0.1
0.5
1.0
1.5
SNP effect on Rheumatoid arthritis
SNP effect on PDL−1 on monocyte

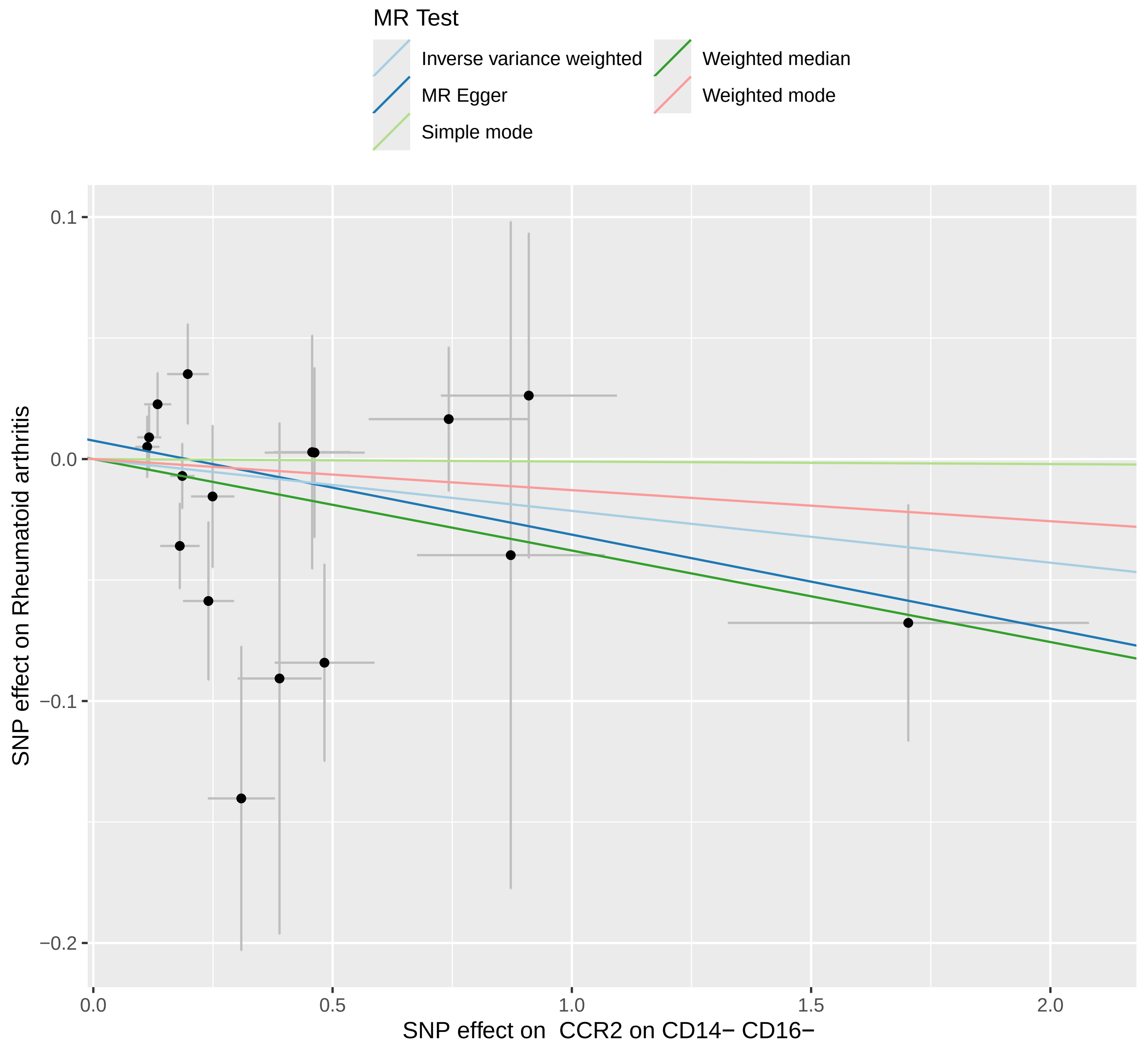
MR Test
Inverse variance weighted
MR Egger
Simple mode
Weighted median
Weighted mode
0.1
0.0
−0.1
−0.2
0.0
0.5
1.0
1.5
2.0
SNP effect on Rheumatoid arthritis
SNP effect on CCR2 on CD14− CD16−

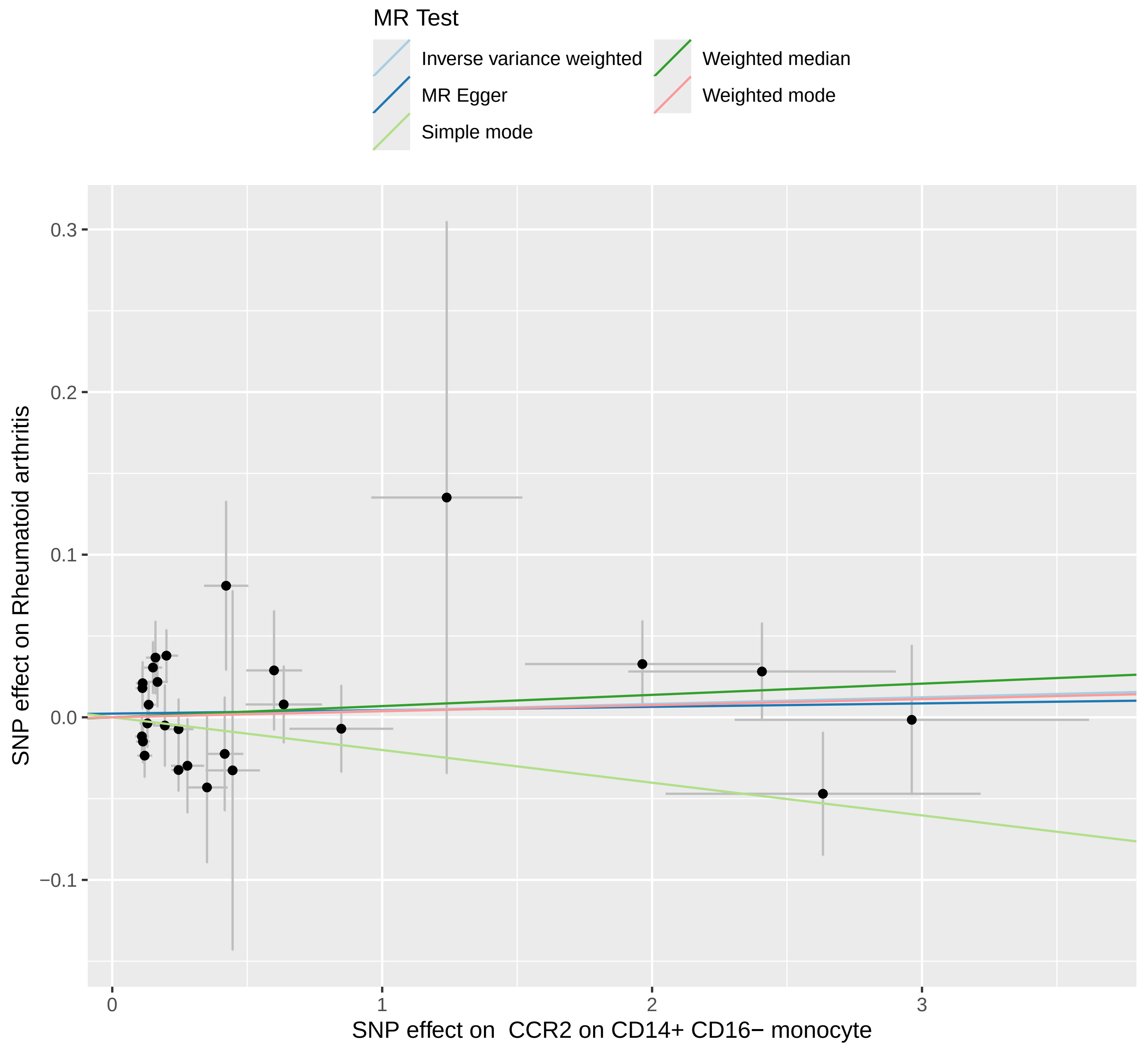
MR Test
Inverse variance weighted
MR Egger
Simple mode
Weighted median
Weighted mode
SNP effect on Rheumatoid arthritis
0.3
0.2
0.1
0.0
−0.1
0
1
2
3
SNP effect on CCR2 on CD14+ CD16− monocyte

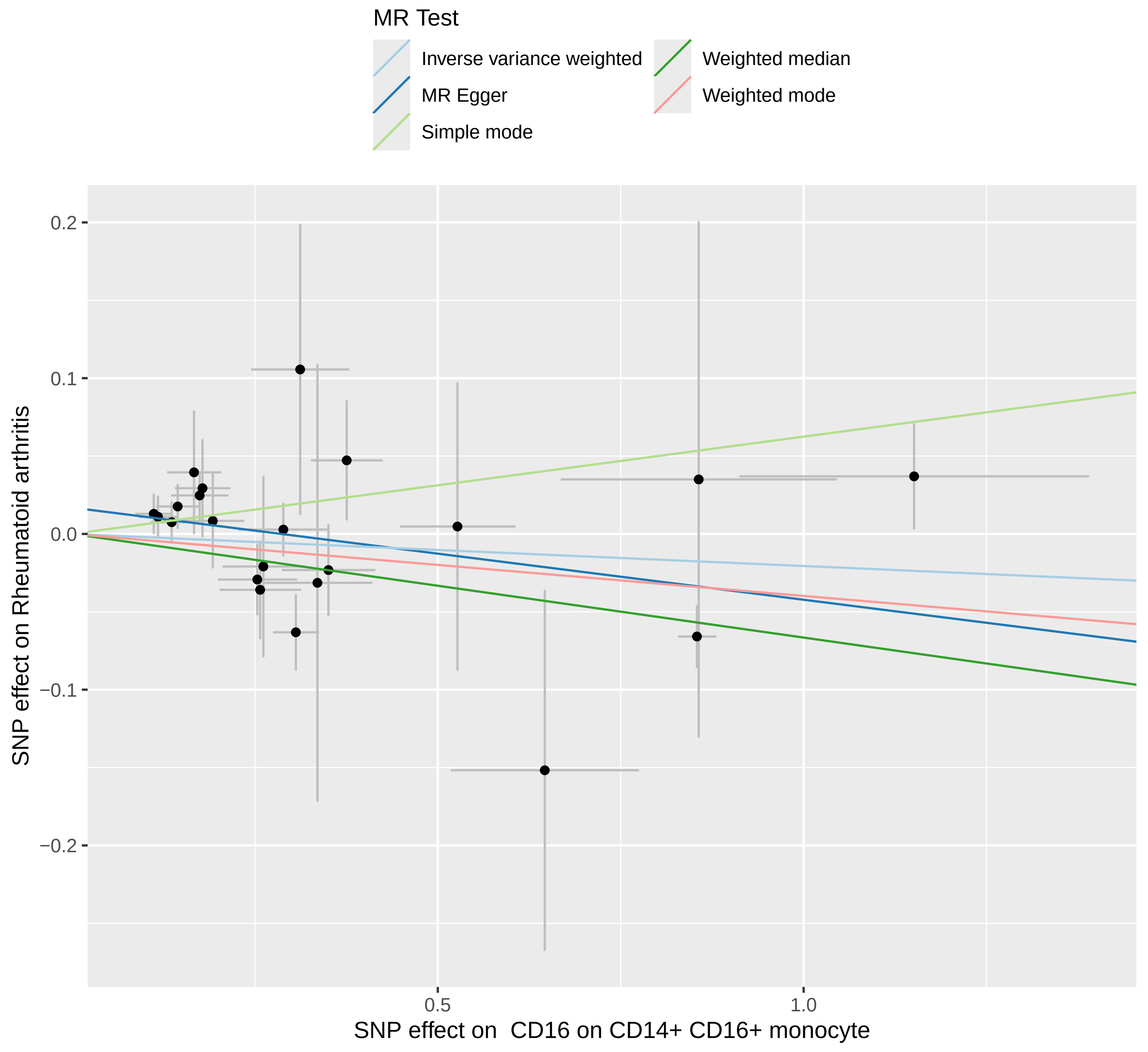
MR Test
Inverse variance weighted
MR Egger
Simple mode
Weighted median
Weighted mode
0.2
0.1
0.0
−0.1
−0.2
0.5
1.0
SNP effect on Rheumatoid arthritis
SNP effect on CD16 on CD14+ CD16+ monocyte

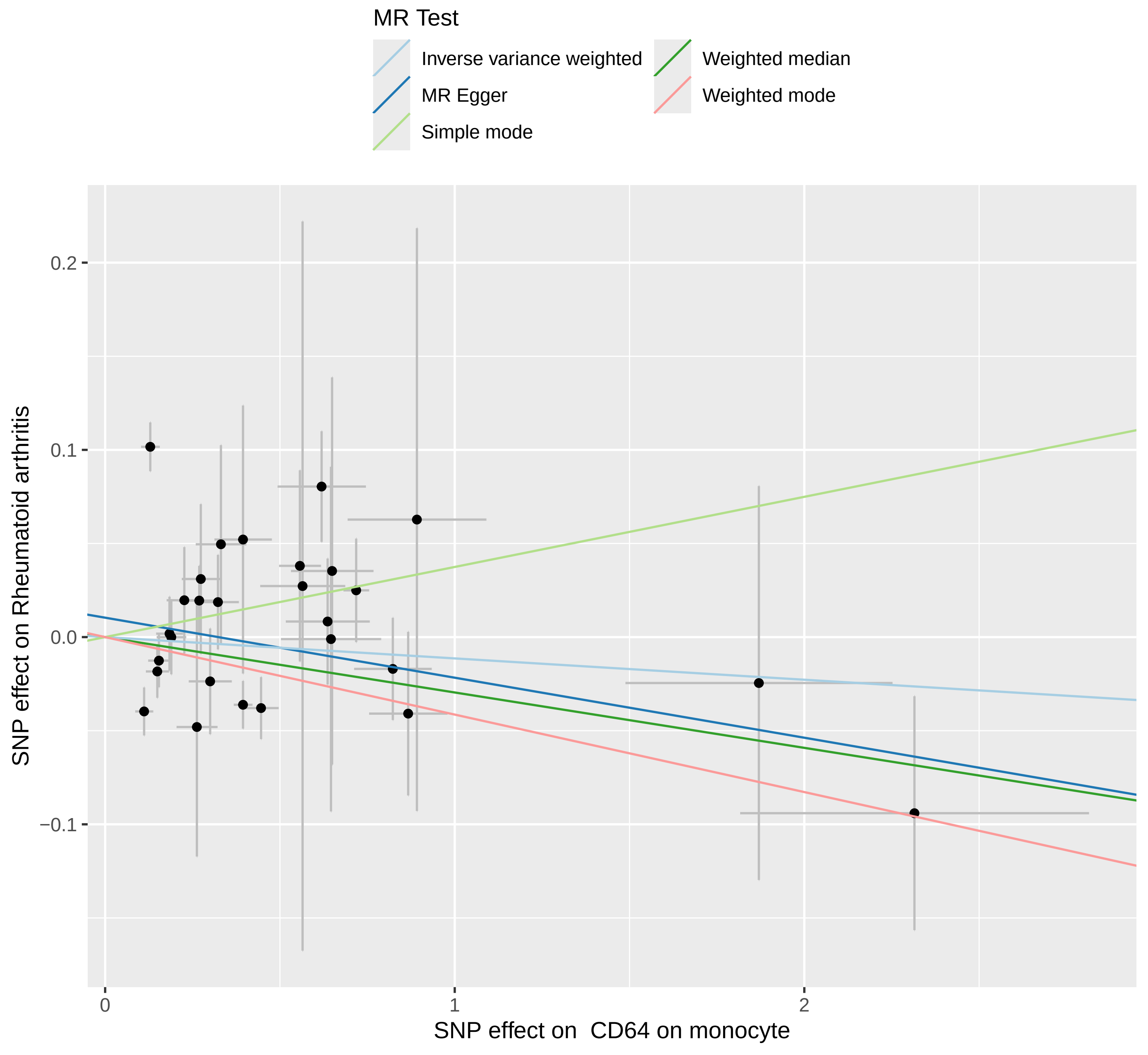

MR Test
Inverse variance weighted
Weighted median
MR Egger
Weighted mode
Simple mode
SNP effect on Rheumatoid arthritis
0.2
0.1
0.0
−0.1
0
1
2
SNP effect on CD64 on monocyte

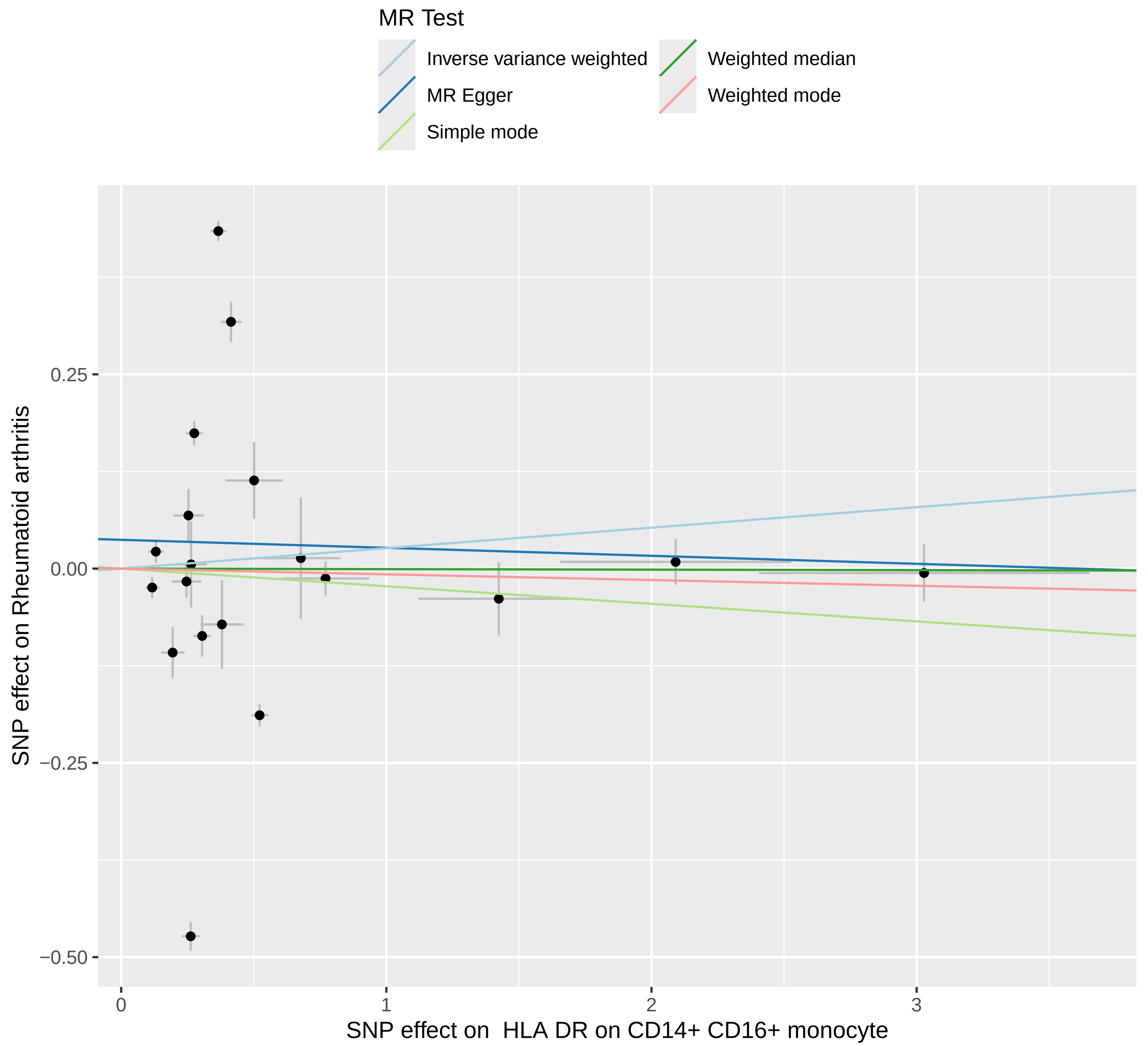

MR Test
Inverse variance weighted
MR Egger
Simple mode
Weighted median
Weighted mode
0.25
0.00
−0.25
−0.50
0
1
2
3
SNP effect on Rheumatoid arthritis
SNP effect on HLA DR on CD14+ CD16+ monocyte

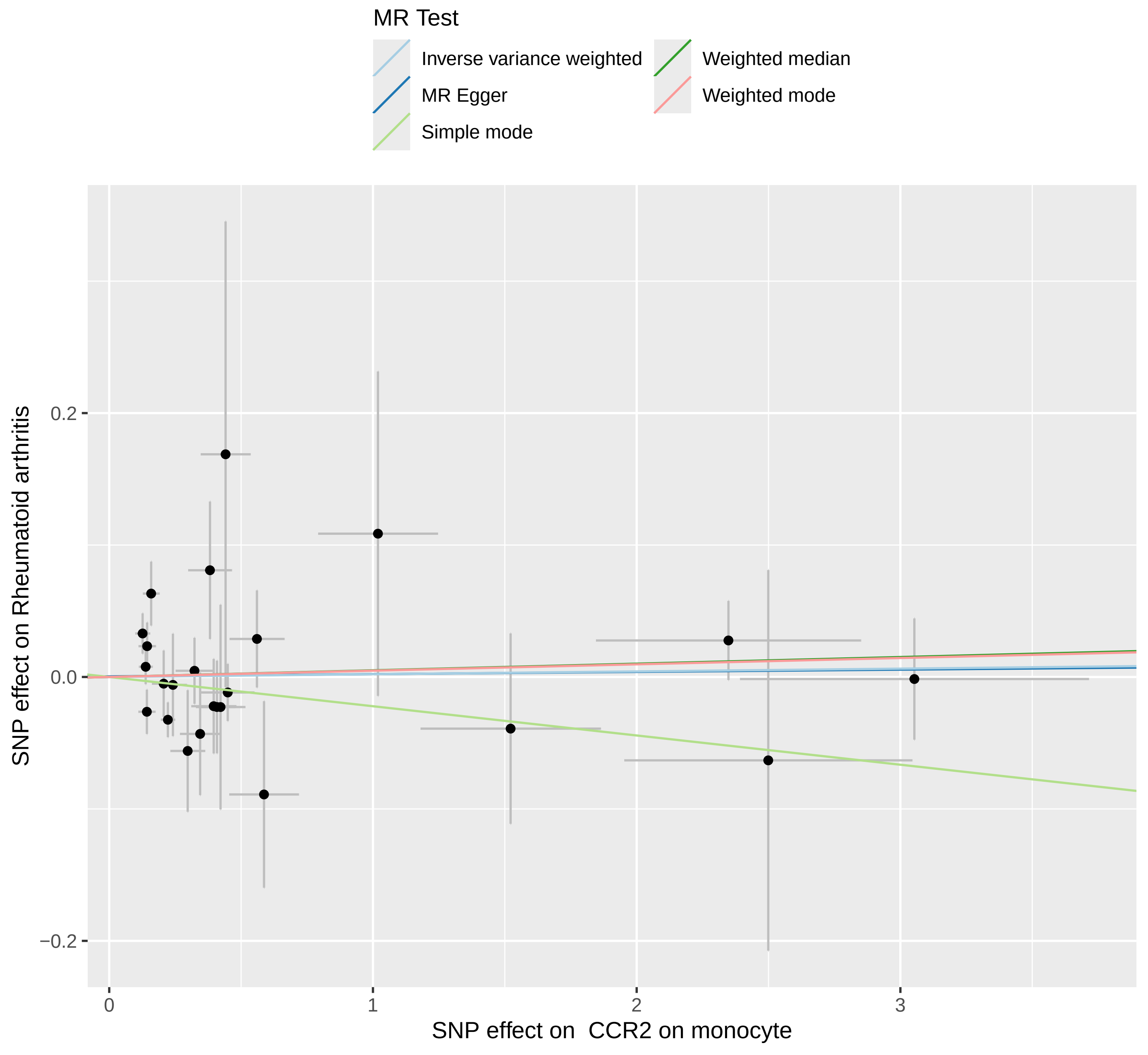

MR Test
Inverse variance weighted
MR Egger
Simple mode
Weighted median
Weighted mode
0.2
0.0
−0.2
0
1
2
3
SNP effect on Rheumatoid arthritis
SNP effect on CCR2 on monocyte

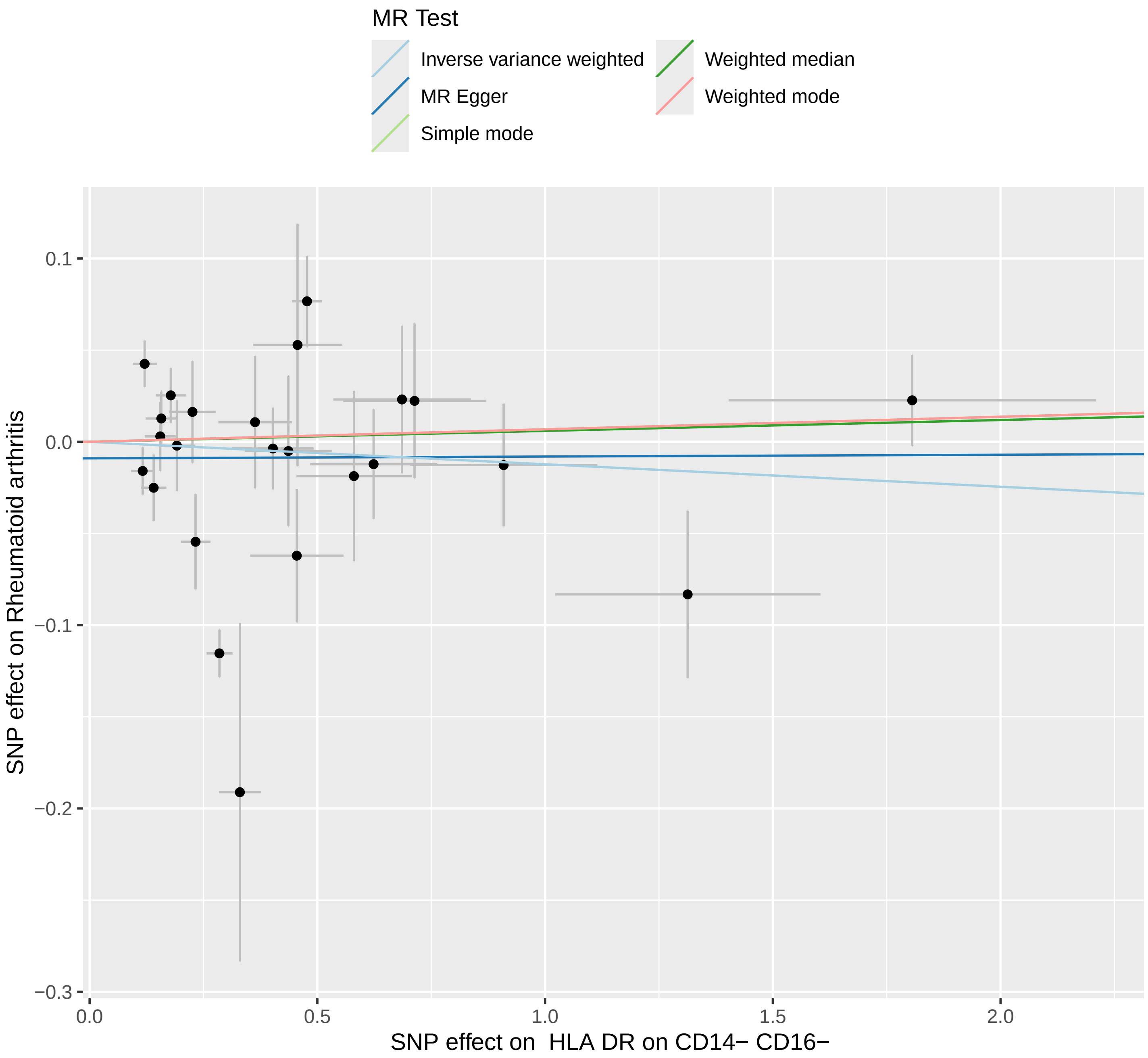

MR Test
Inverse variance weighted
MR Egger
Simple mode
Weighted median
Weighted mode
SNP effect on Rheumatoid arthritis
0.1
0.0
−0.1
−0.2
−0.3
0.0
0.5
1.0
1.5
2.0
SNP effect on HLA DR on CD14− CD16−

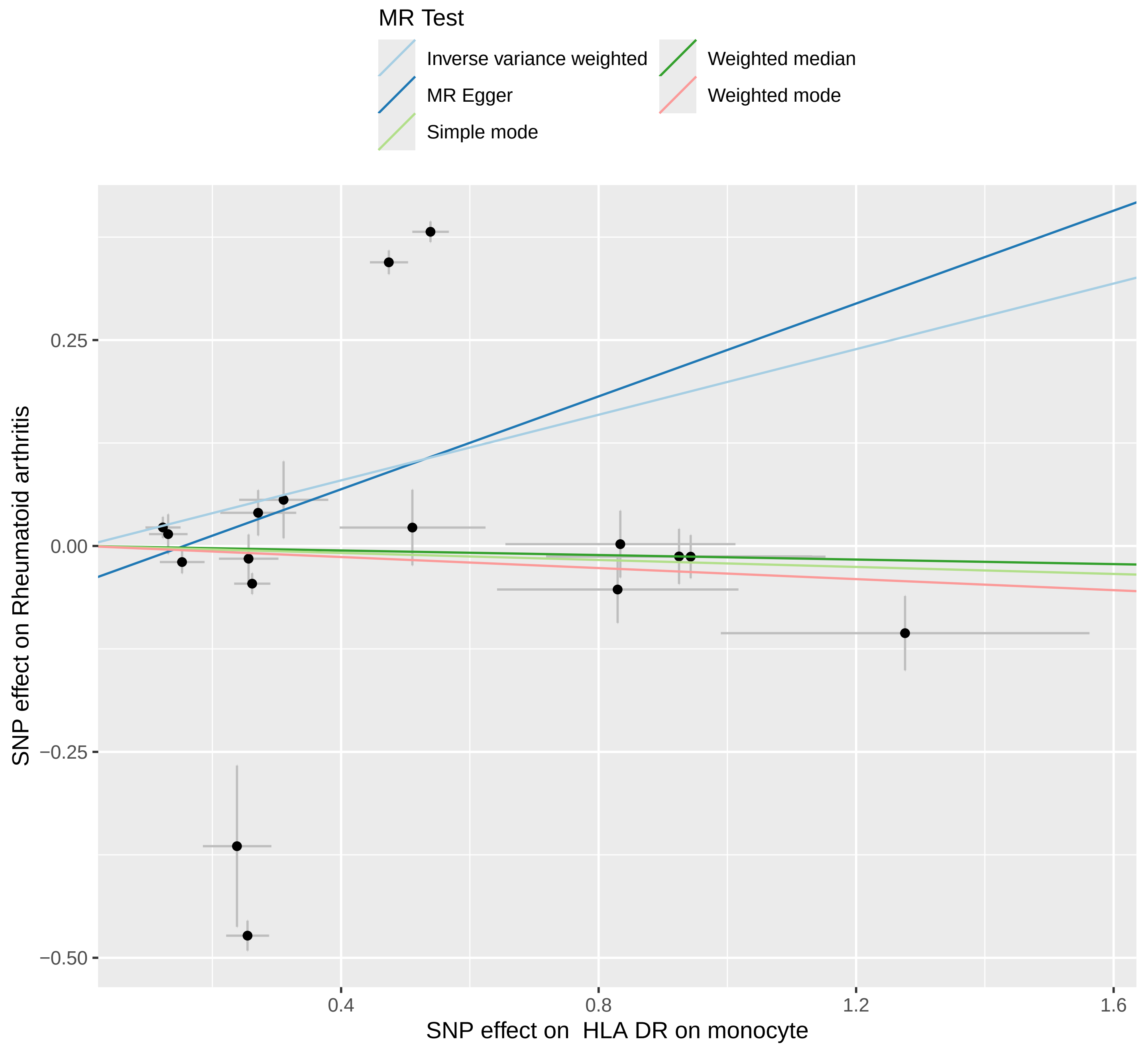

MR Test
Inverse variance weighted
MR Egger
Simple mode
Weighted median
Weighted mode
0.25
0.00
−0.25
−0.50
0.4
0.8
1.2
1.6
SNP effect on Rheumatoid arthritis
SNP effect on HLA DR on monocyte

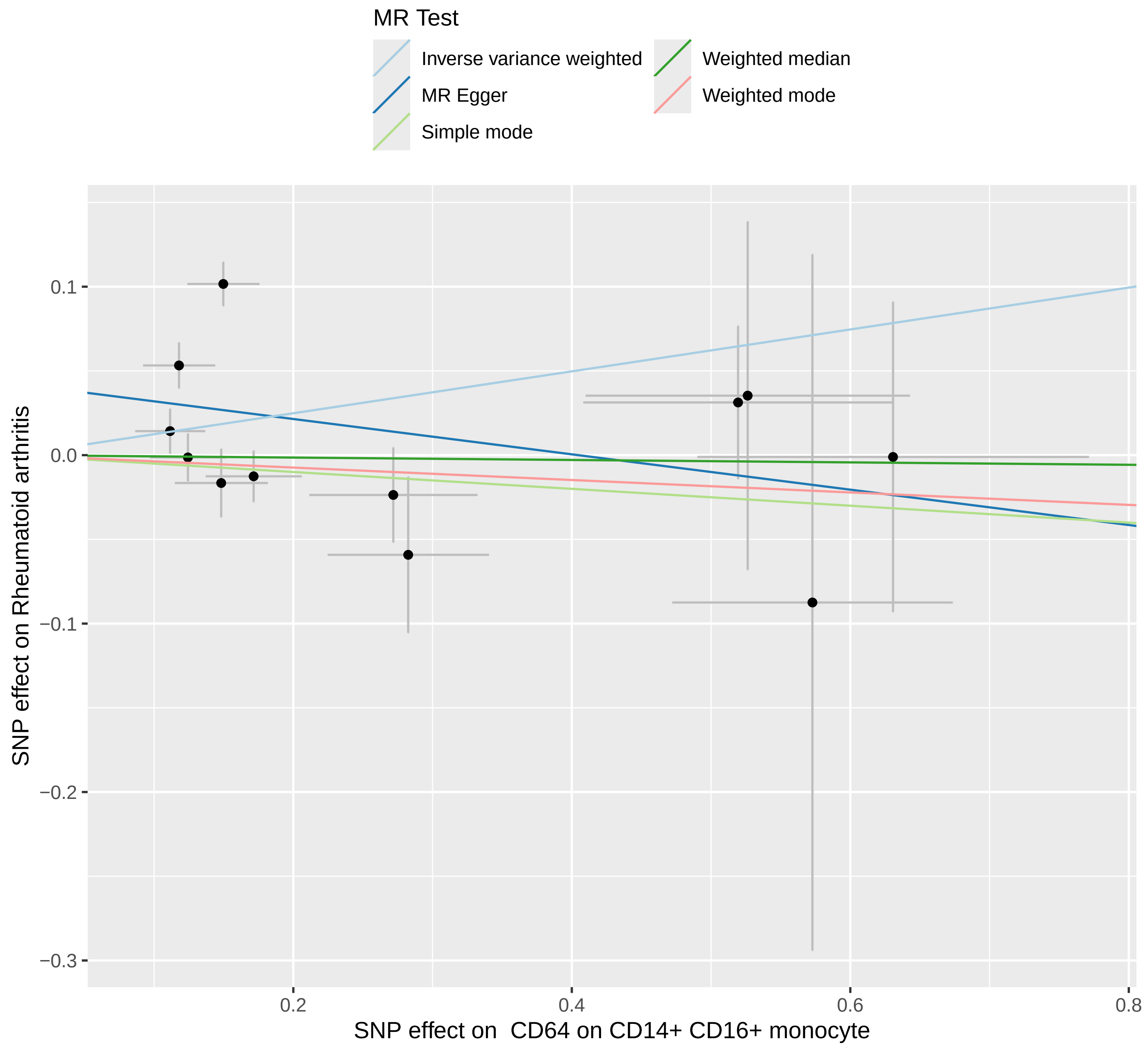
MR Test
Inverse variance weighted
MR Egger
Simple mode
Weighted median
Weighted mode
0.1
0.0
−0.1
−0.2
−0.3
0.2
0.4
0.6
0.8
SNP effect on Rheumatoid arthritis
SNP effect on CD64 on CD14+ CD16+ monocyte

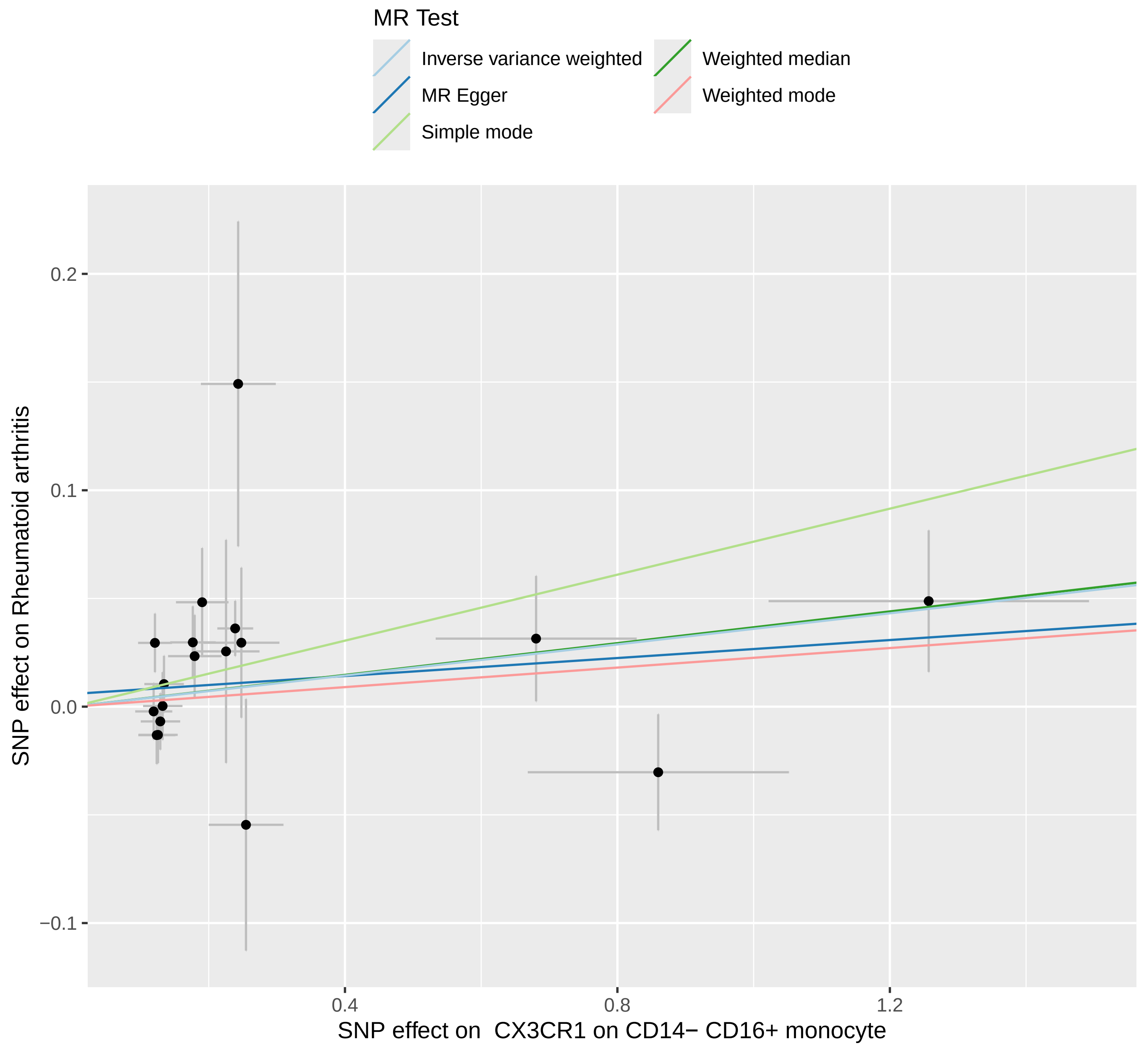
MR Test
Inverse variance weighted
MR Egger
Simple mode
Weighted median
Weighted mode
0.2
0.1
0.0
−0.1
0.4
0.8
1.2
SNP effect on Rheumatoid arthritis
SNP effect on CX3CR1 on CD14− CD16+ monocyte

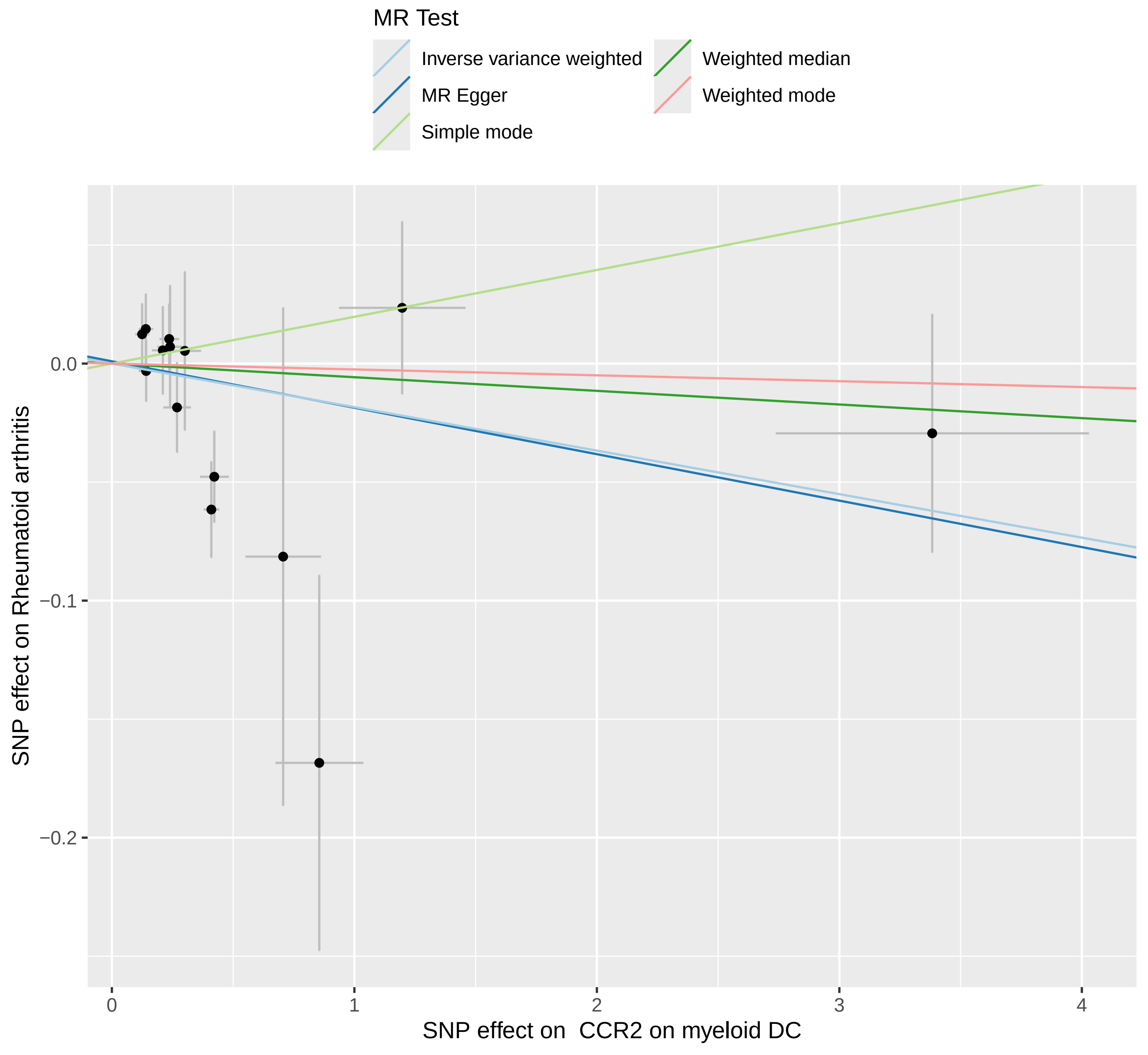

MR Test
Inverse variance weighted
MR Egger
Simple mode
Weighted median
Weighted mode
SNP effect on Rheumatoid arthritis
0.0
−0.1
−0.2
0
1
2
3
4
SNP effect on CCR2 on myeloid DC

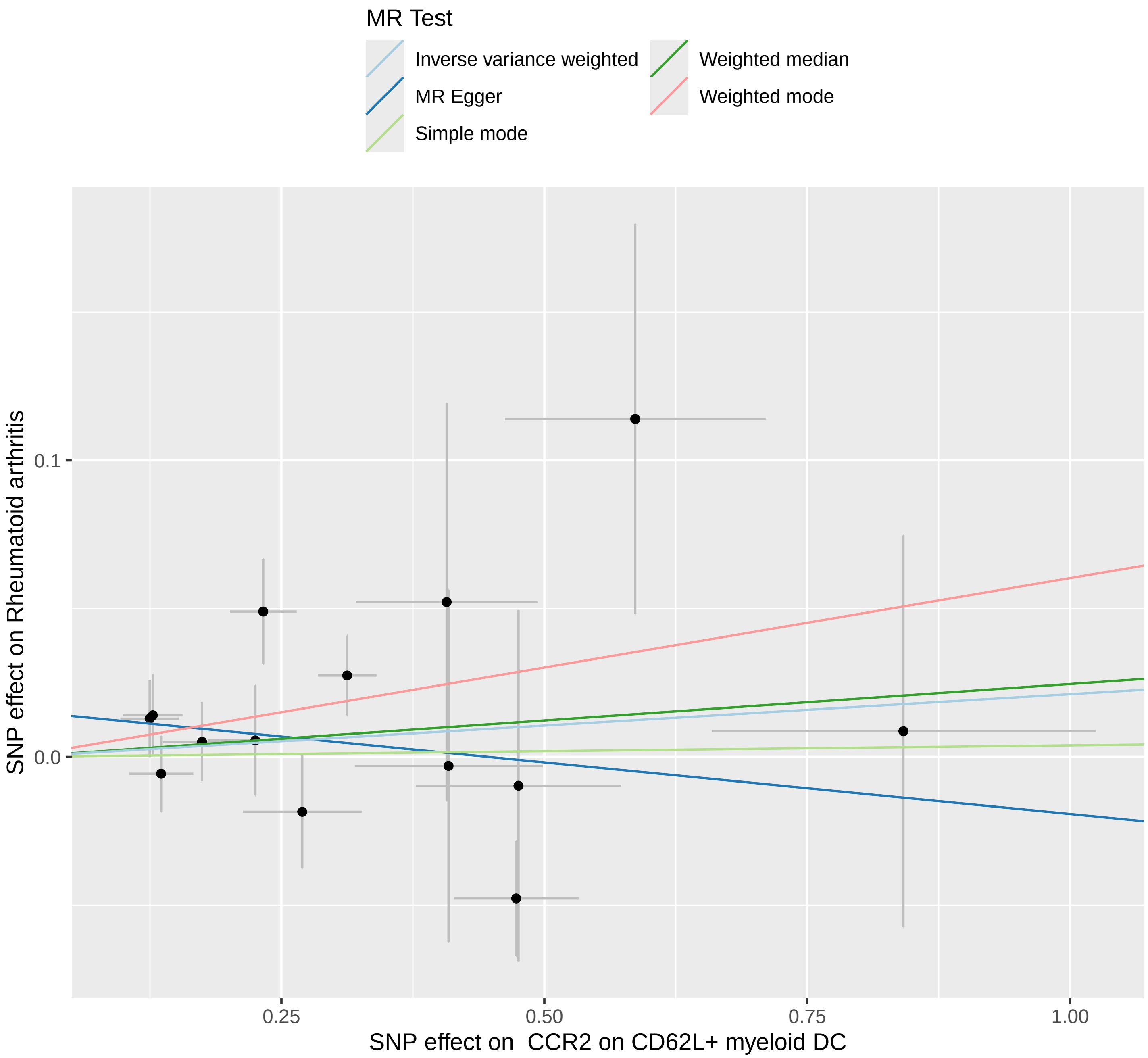
MR Test
Inverse variance weighted
MR Egger
Simple mode
Weighted median
Weighted mode
SNP effect on Rheumatoid arthritis
0.1
0.0
0.25
0.50
0.75
1.00
SNP effect on CCR2 on CD62L+ myeloid DC

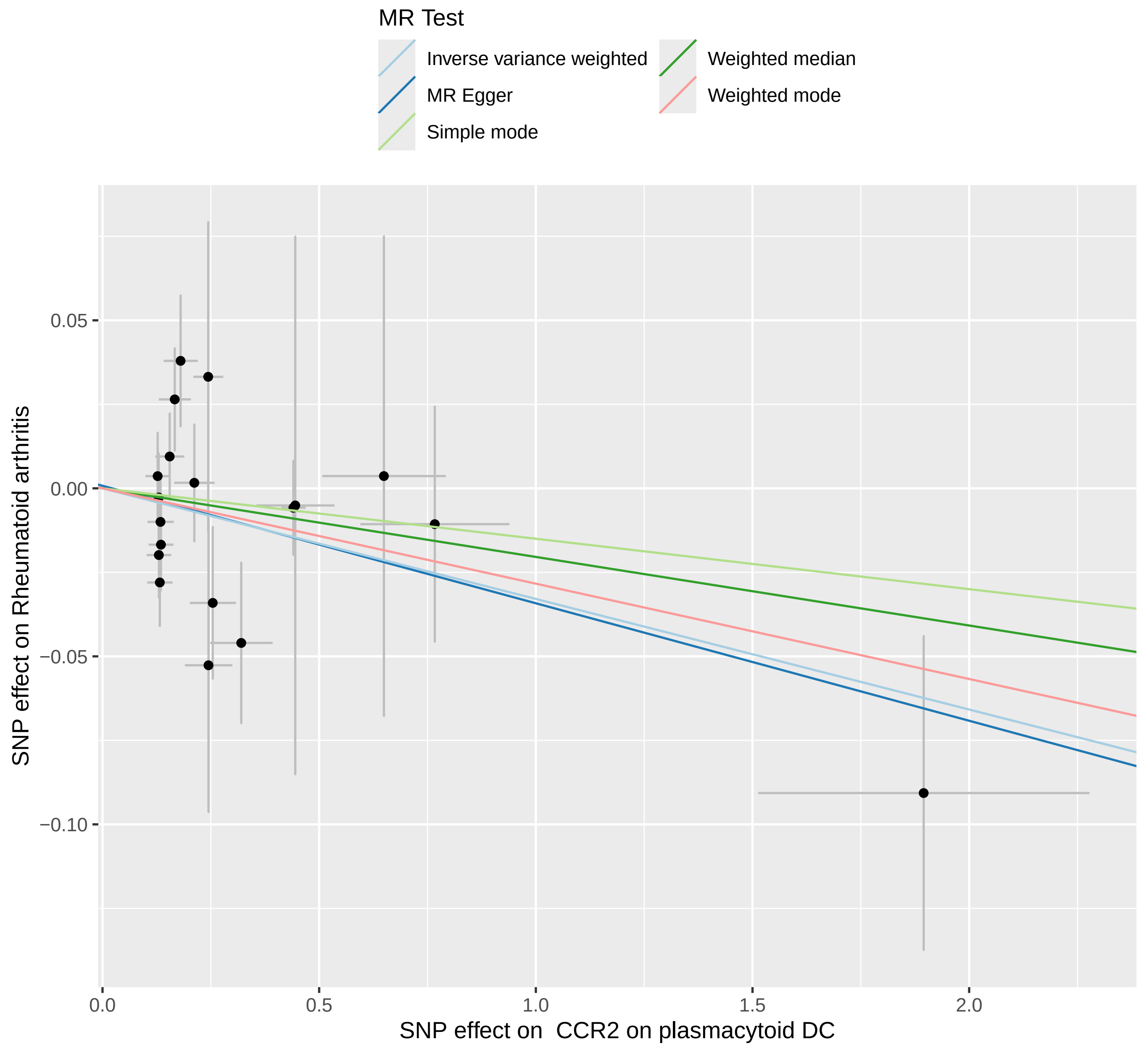
MR Test
Inverse variance weighted
MR Egger
Simple mode
Weighted median
Weighted mode
0.05
0.00
−0.05
−0.10
0.0
0.5
1.0
1.5
2.0
SNP effect on Rheumatoid arthritis
SNP effect on CCR2 on plasmacytoid DC

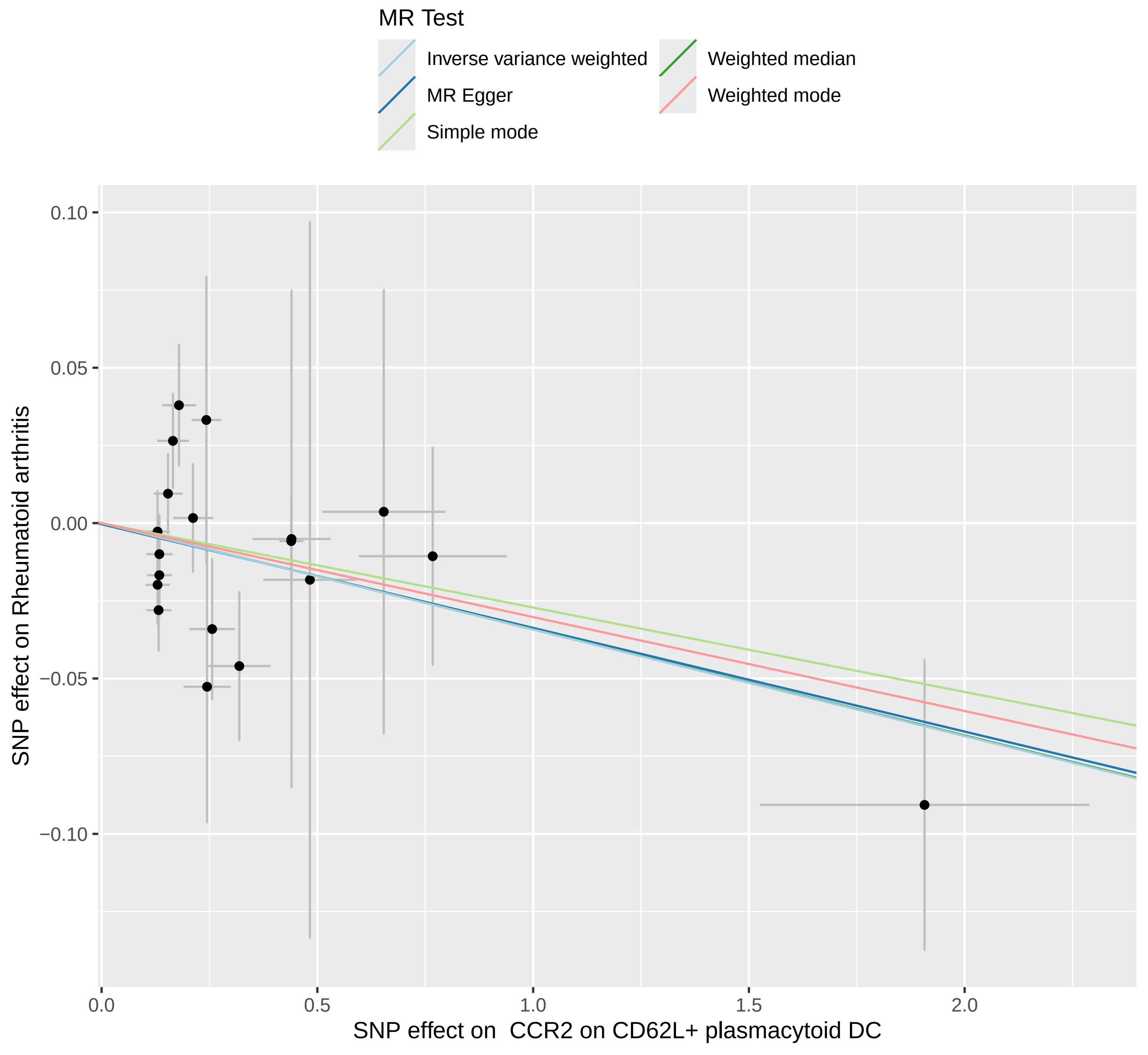
MR Test
Inverse variance weighted
MR Egger
Simple mode
Weighted median
Weighted mode
0.10
0.05
0.00
−0.05
−0.10
0.0
0.5
1.0
1.5
2.0
SNP effect on Rheumatoid arthritis
SNP effect on CCR2 on CD62L+ plasmacytoid DC

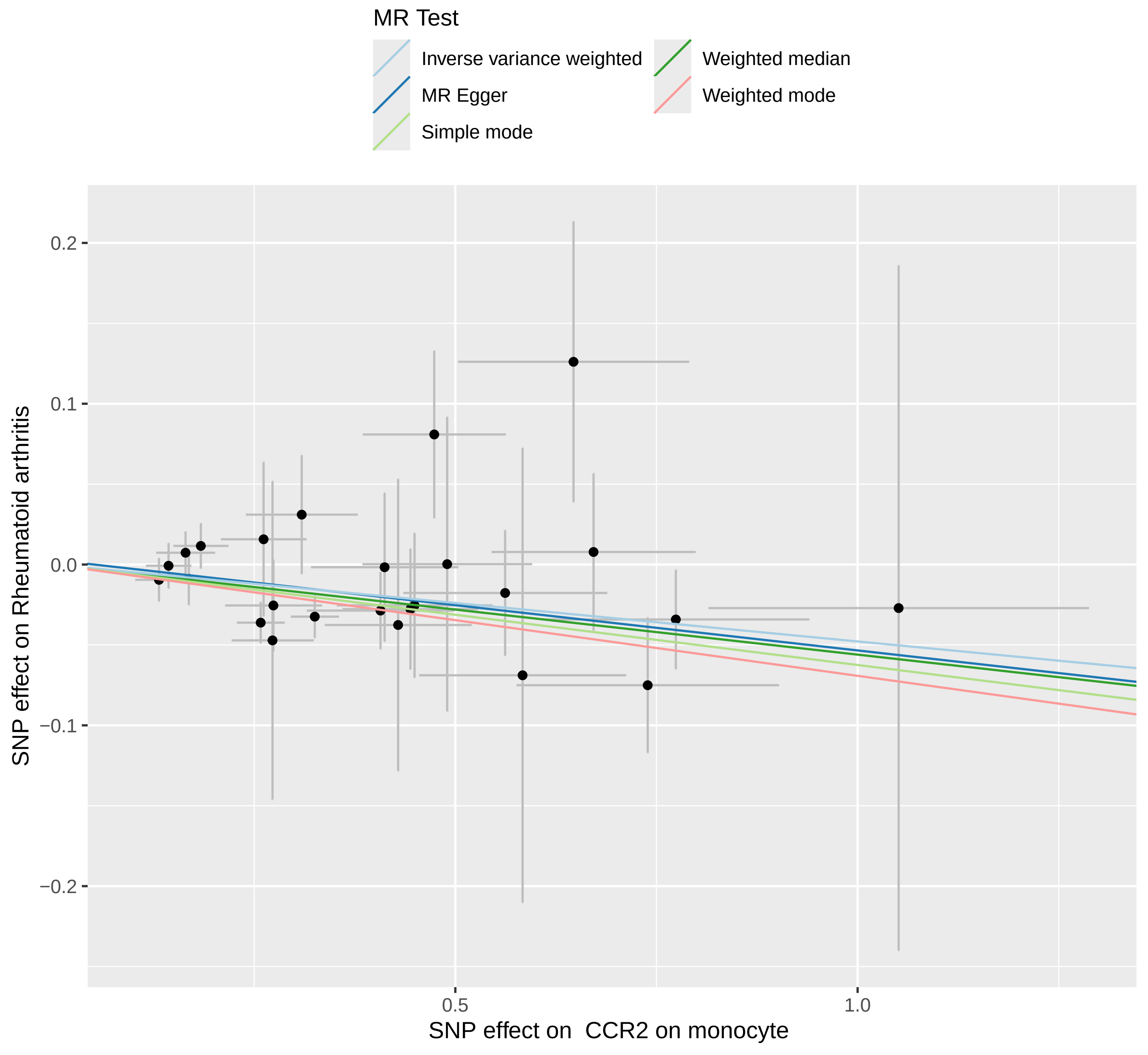

MR Test
Inverse variance weighted
MR Egger
Simple mode
Weighted median
Weighted mode
0.2
0.1
0.0
−0.1
−0.2
SNP effect on Rheumatoid arthritis
0.5
1.0
SNP effect on CCR2 on monocyte

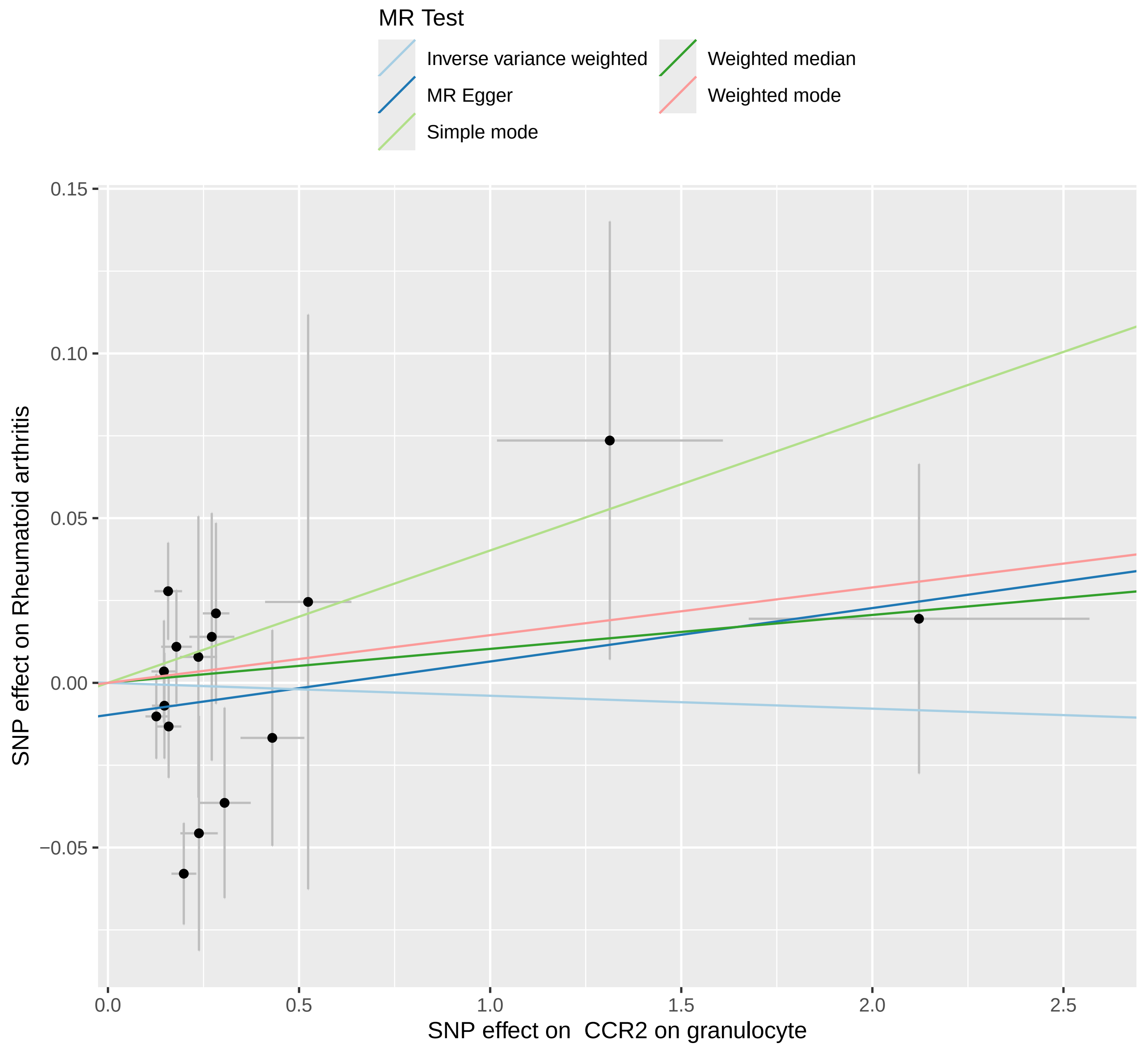
MR Test
Inverse variance weighted
MR Egger
Simple mode
Weighted median
Weighted mode
0.15
0.10
0.05
0.00
−0.05
0.0
0.5
1.0
1.5
2.0
2.5
SNP effect on Rheumatoid arthritis
SNP effect on CCR2 on granulocyte

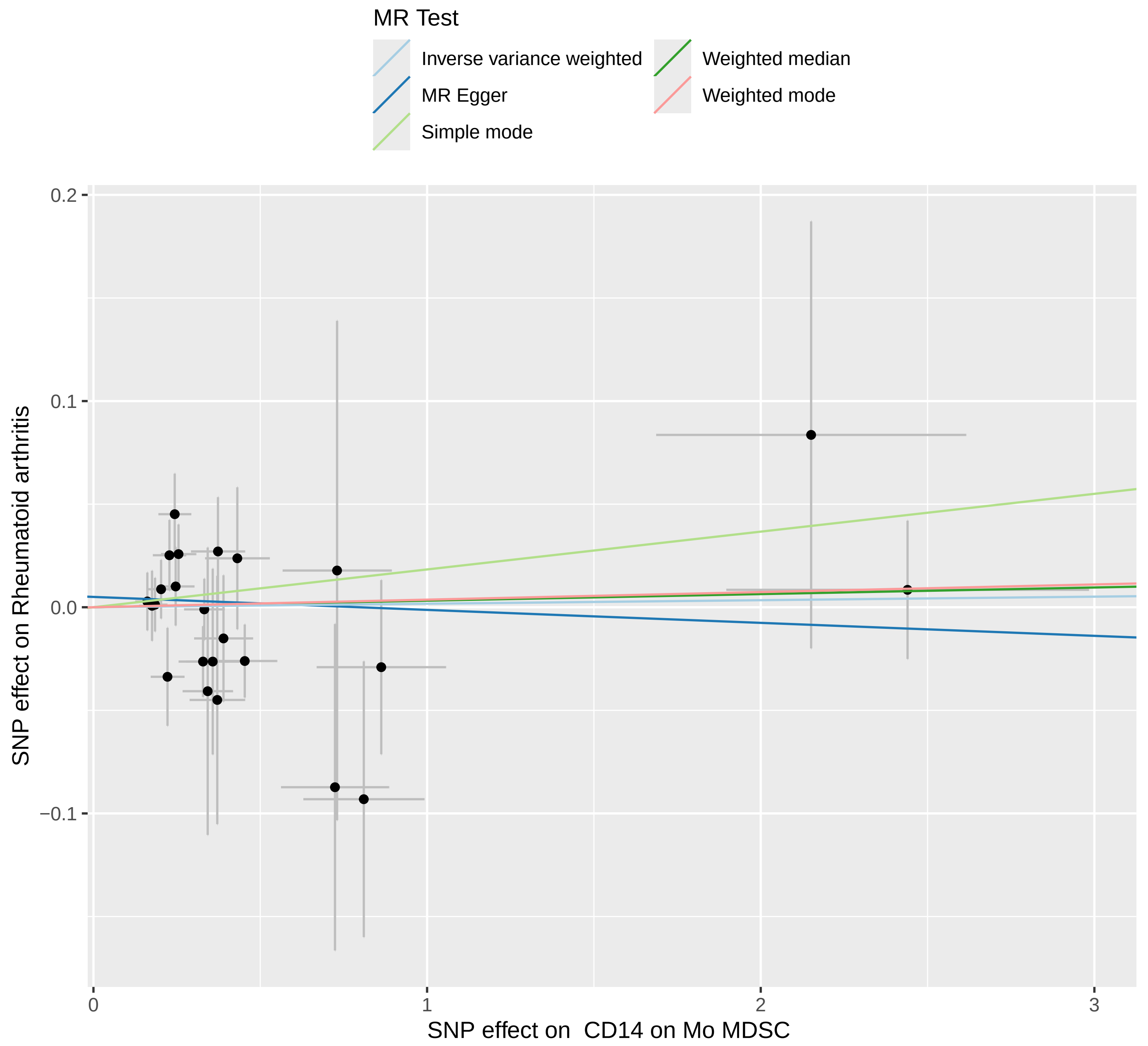
MR Test
Inverse variance weighted
MR Egger
Simple mode
Weighted median
Weighted mode
SNP effect on Rheumatoid arthritis
SNP effect on CD14 on Mo MDSC
0.2
0.1
0.0
−0.1
0
1
2
3

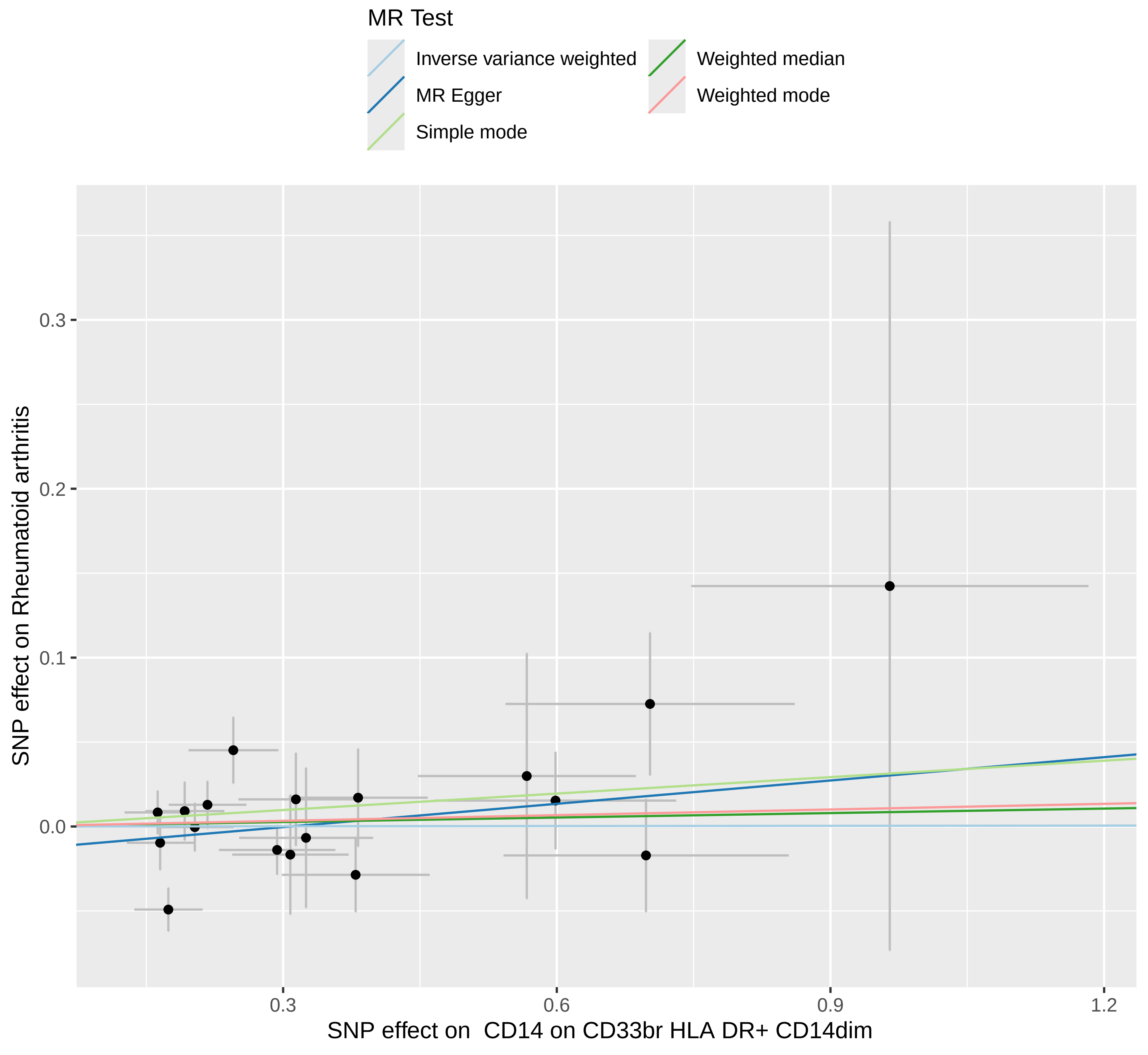

MR Test
Inverse variance weighted
MR Egger
Simple mode
Weighted median
Weighted mode
0.3
0.2
0.1
0.0
SNP effect on Rheumatoid arthritis
0.3
0.6
0.9
1.2
SNP effect on CD14 on CD33br HLA DR+ CD14dim

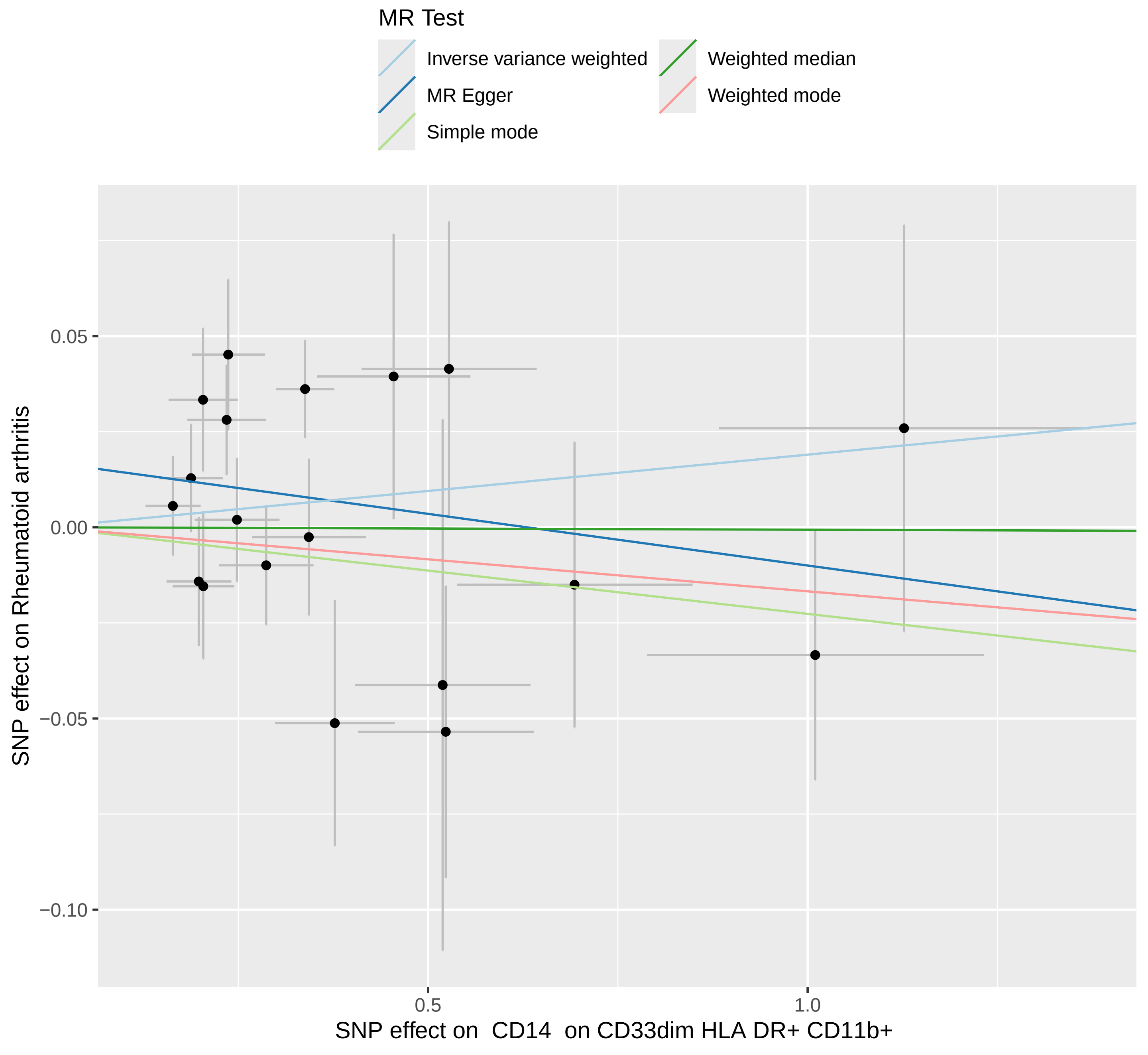
MR Test
Inverse variance weighted
MR Egger
Simple mode
Weighted median
Weighted mode
0.05
0.00
−0.05
−0.10
0.5
1.0
SNP effect on Rheumatoid arthritis
SNP effect on CD14 on CD33dim HLA DR+ CD11b+

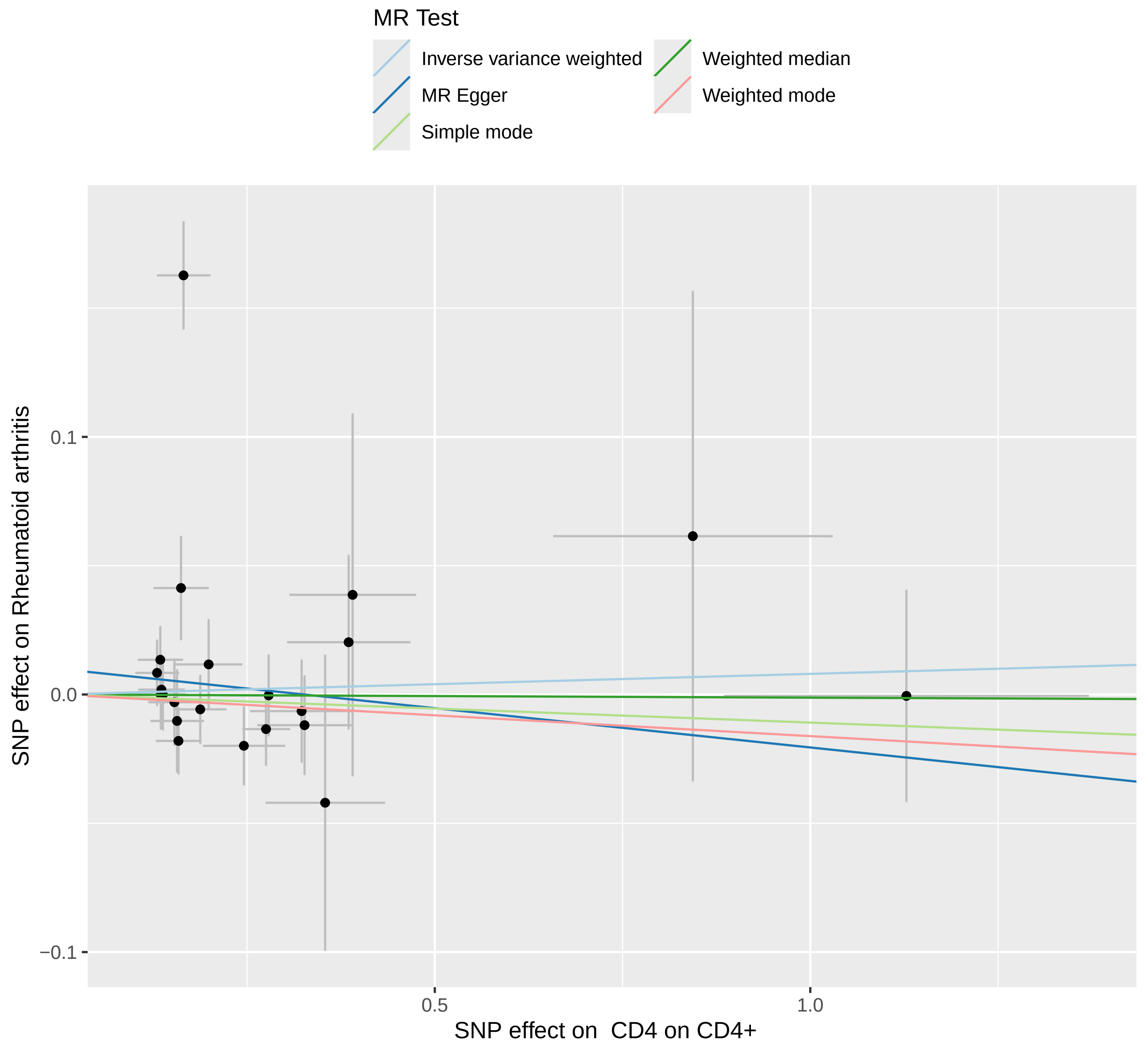
MR Test
Inverse variance weighted
MR Egger
Simple mode
Weighted median
Weighted mode
0.1
0.0
−0.1
0.5
1.0
SNP effect on Rheumatoid arthritis
SNP effect on CD4 on CD4+

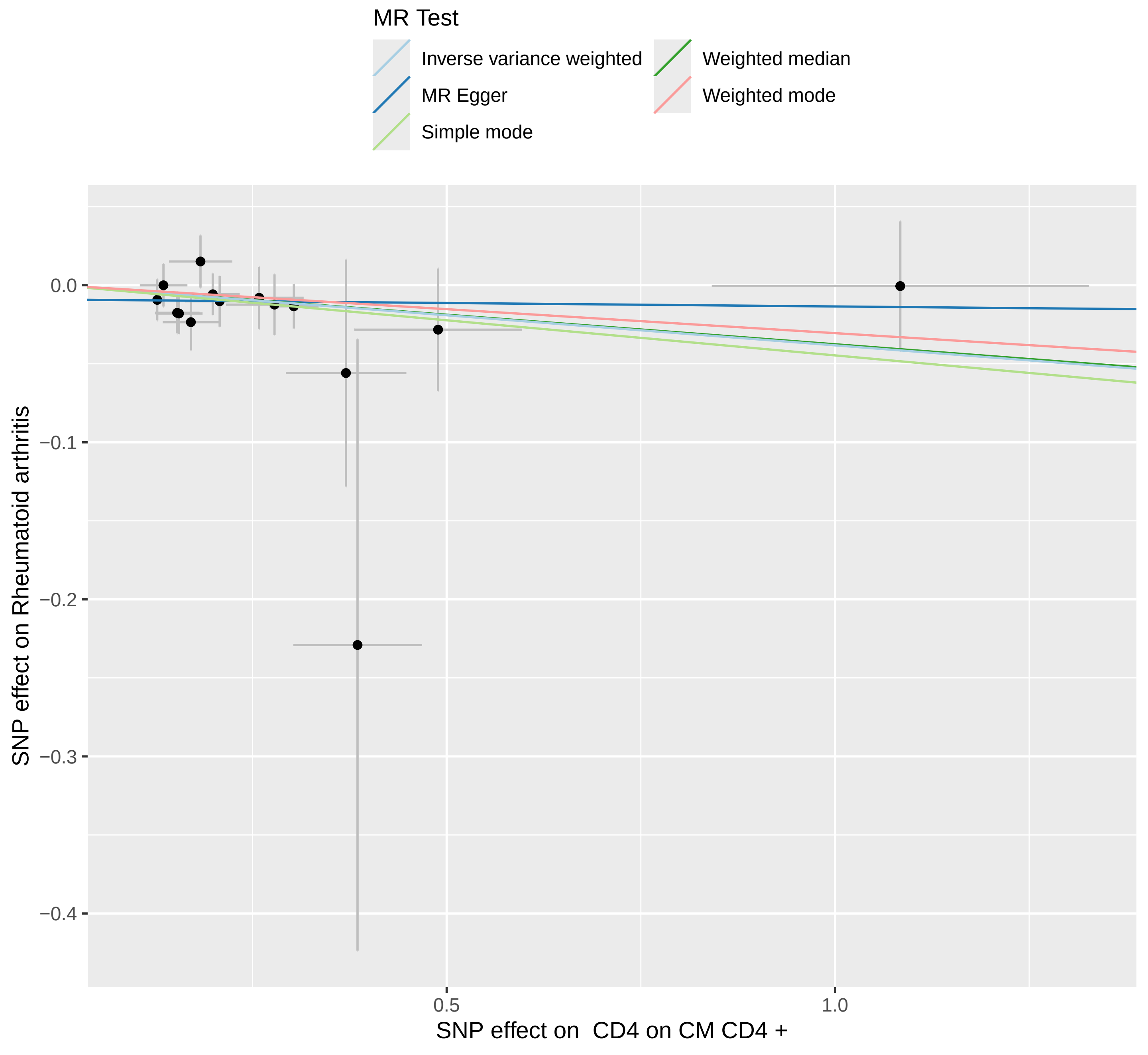
MR Test
Inverse variance weighted
MR Egger
Simple mode
Weighted median
Weighted mode
0.0
−0.1
−0.2
−0.3
−0.4
0.5
1.0
SNP effect on Rheumatoid arthritis
SNP effect on CD4 on CM CD4 +

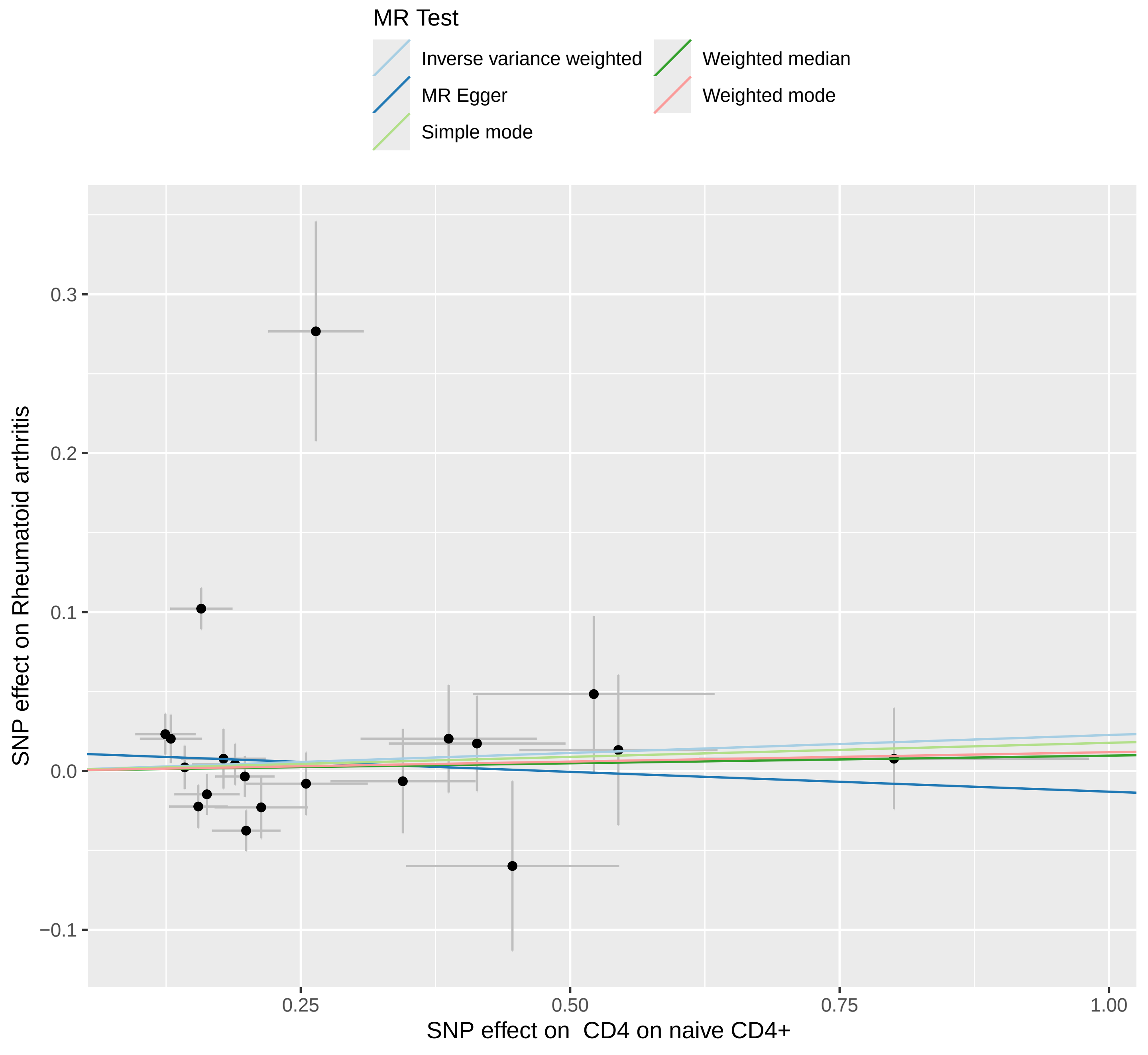

MR Test
Inverse variance weighted
MR Egger
Simple mode
Weighted median
Weighted mode
0.3
0.2
0.1
0.0
−0.1
SNP effect on Rheumatoid arthritis
0.25
0.50
0.75
1.00
SNP effect on CD4 on naive CD4+

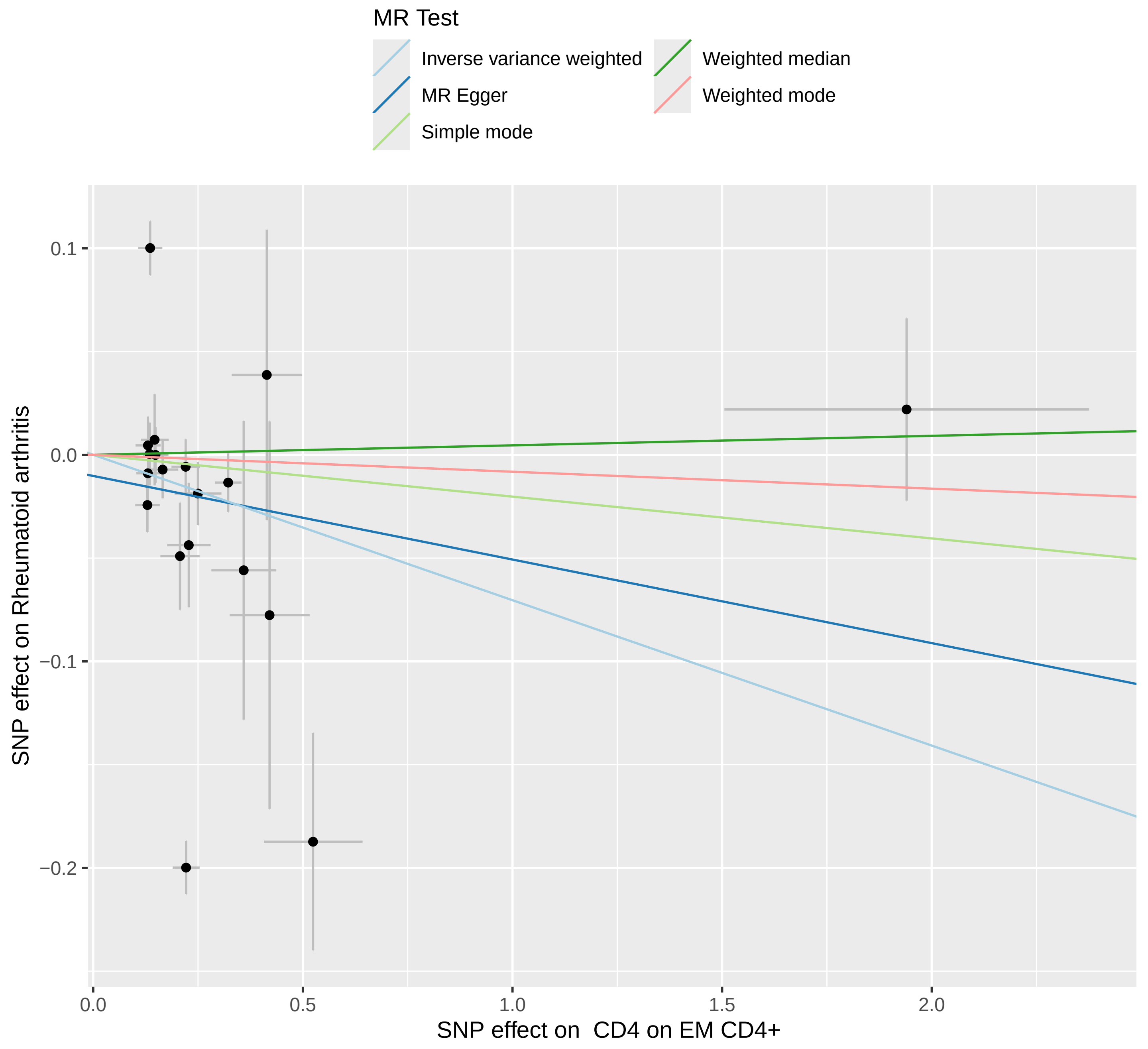
MR Test
Inverse variance weighted
MR Egger
Simple mode
Weighted median
Weighted mode
0.1
0.0
−0.1
−0.2
0.0
0.5
1.0
1.5
2.0
SNP effect on Rheumatoid arthritis
SNP effect on CD4 on EM CD4+

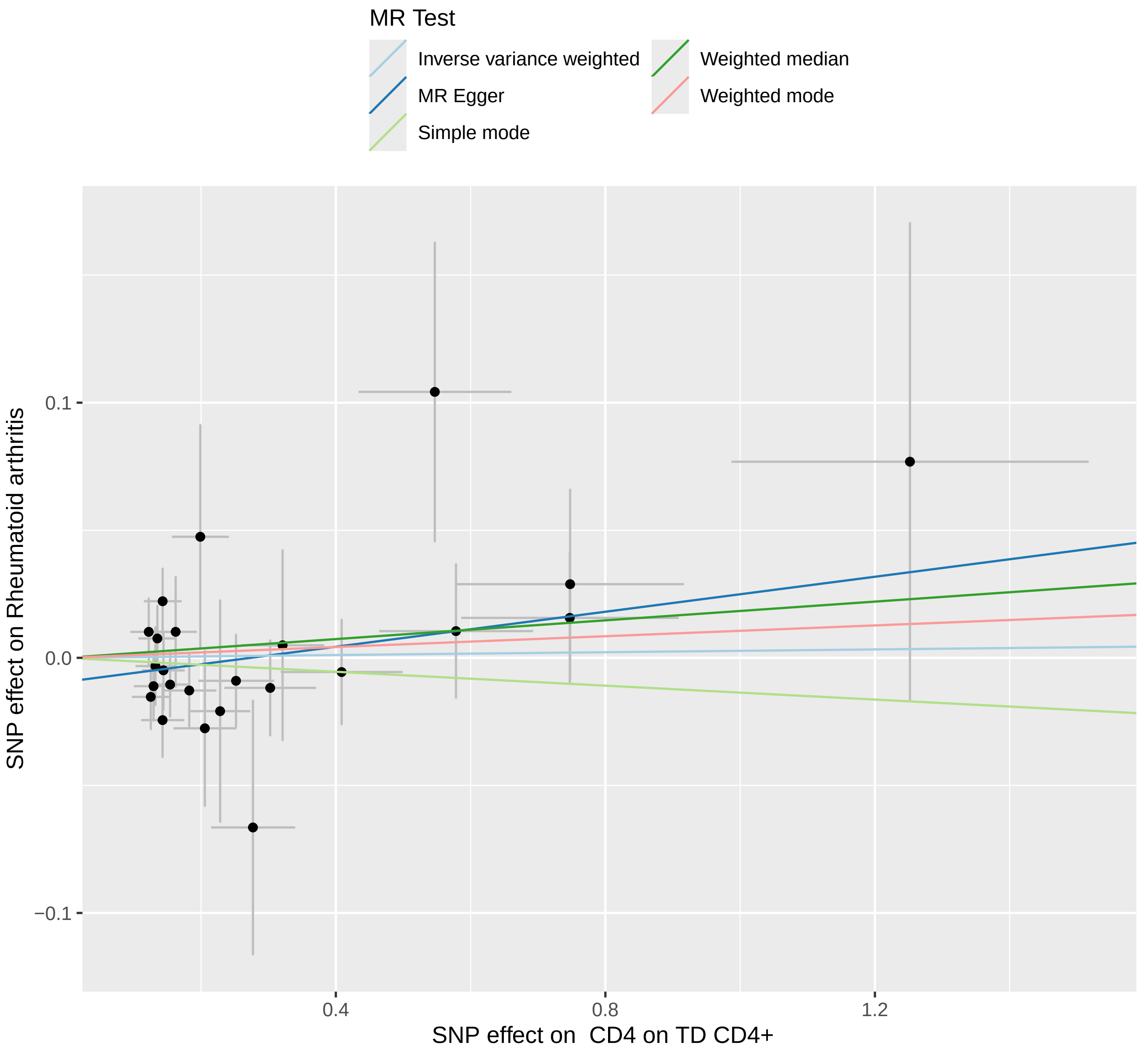
MR Test
Inverse variance weighted
MR Egger
Simple mode
Weighted median
Weighted mode
0.1
0.0
−0.1
0.4
0.8
1.2
SNP effect on Rheumatoid arthritis
SNP effect on CD4 on TD CD4+

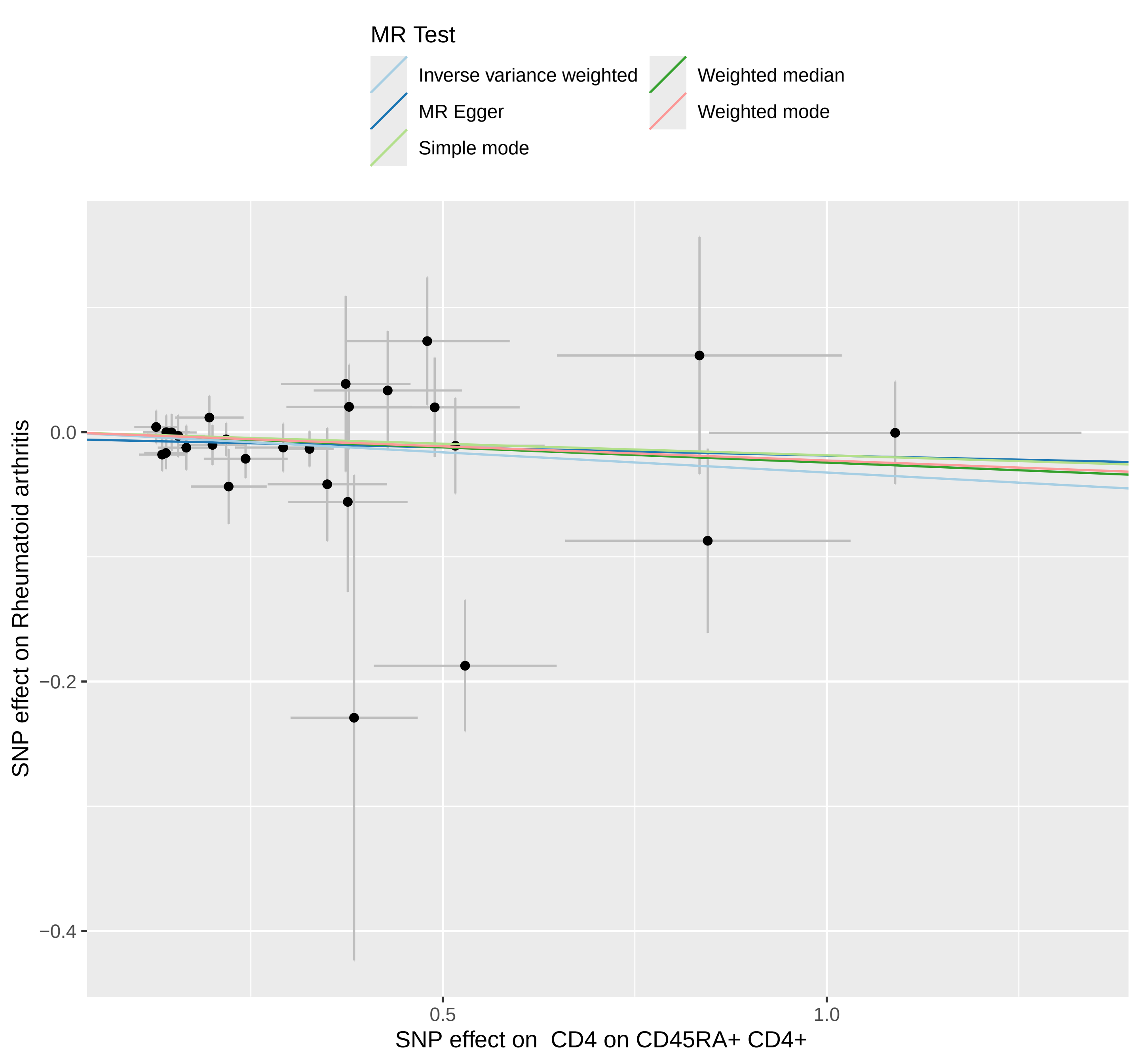
MR Test
Inverse variance weighted
MR Egger
Simple mode
Weighted median
Weighted mode
SNP effect on Rheumatoid arthritis
0.0
−0.2
−0.4
0.5
1.0
SNP effect on CD4 on CD45RA+ CD4+

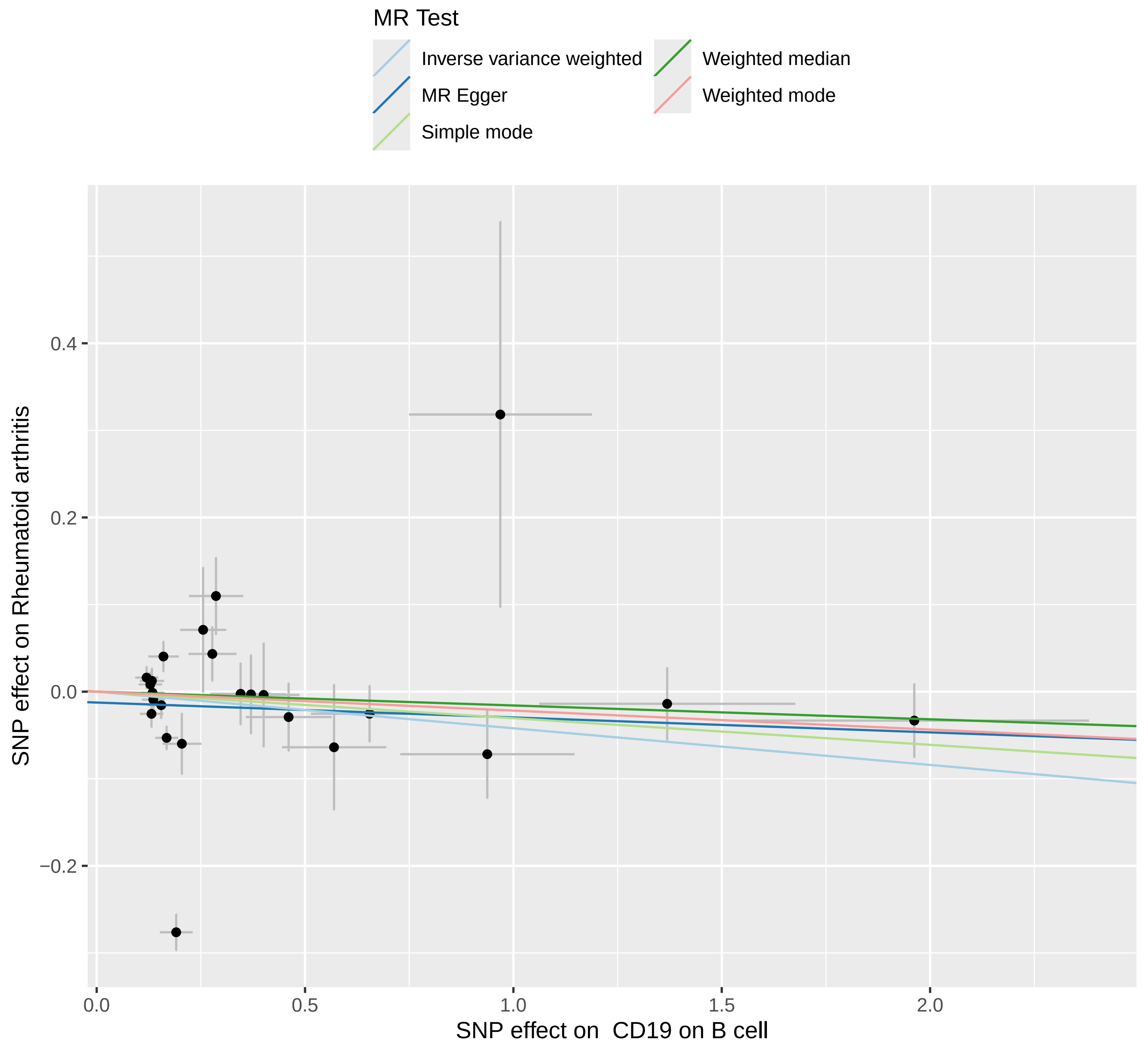

MR Test
Inverse variance weighted
MR Egger
Simple mode
Weighted median
Weighted mode
0.4
0.2
0.0
−0.2
SNP effect on Rheumatoid arthritis
0.0
0.5
1.0
1.5
2.0
SNP effect on CD19 on B cell

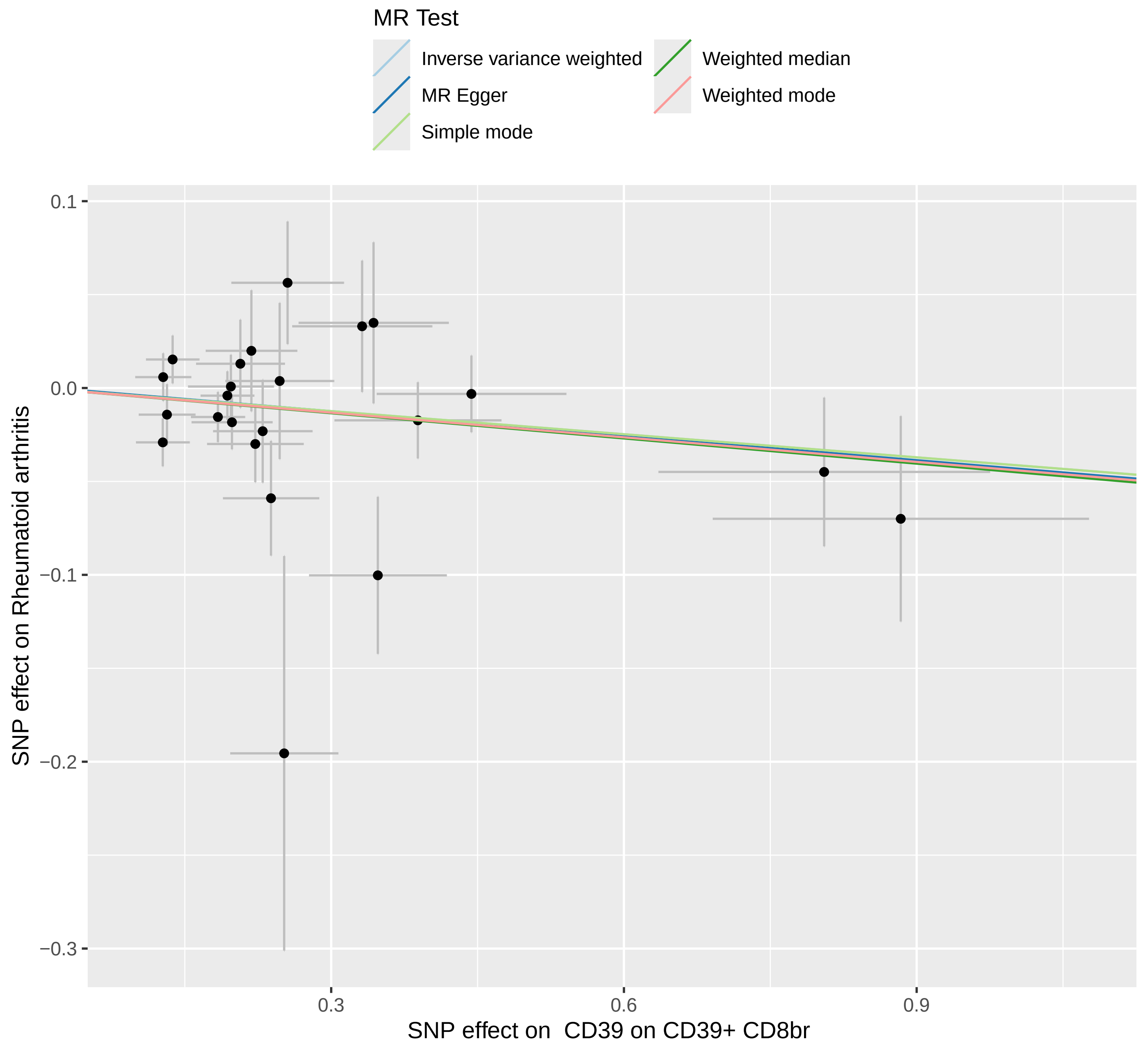
MR Test
Inverse variance weighted
MR Egger
Simple mode
Weighted median
Weighted mode
0.1
0.0
−0.1
−0.2
−0.3
SNP effect on Rheumatoid arthritis
0.3
0.6
0.9
SNP effect on CD39 on CD39+ CD8br

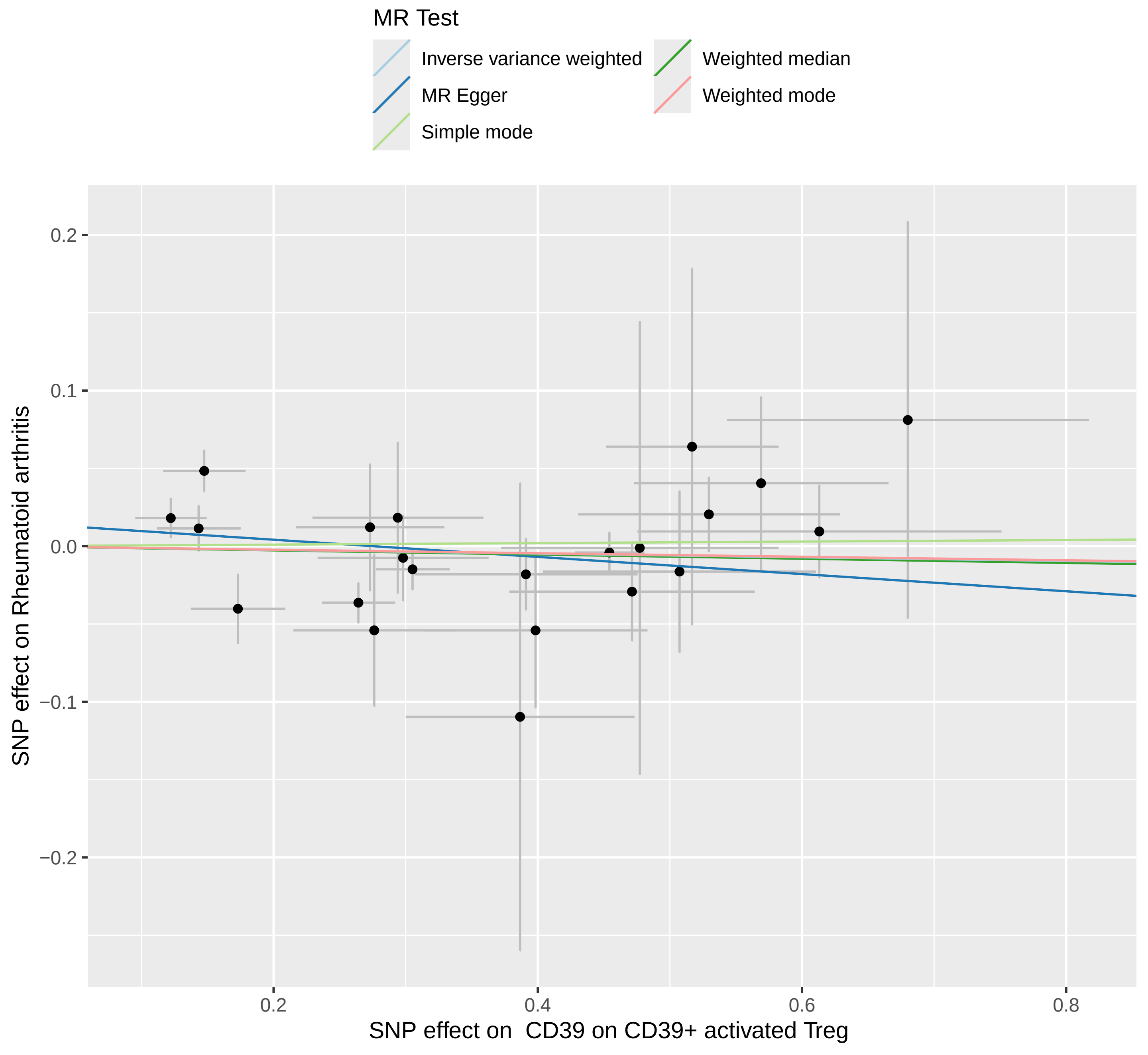

MR Test
Inverse variance weighted
MR Egger
Simple mode
Weighted median
Weighted mode
SNP effect on Rheumatoid arthritis
0.2
0.1
0.0
−0.1
−0.2
0.2
0.4
0.6
0.8
SNP effect on CD39 on CD39+ activated Treg

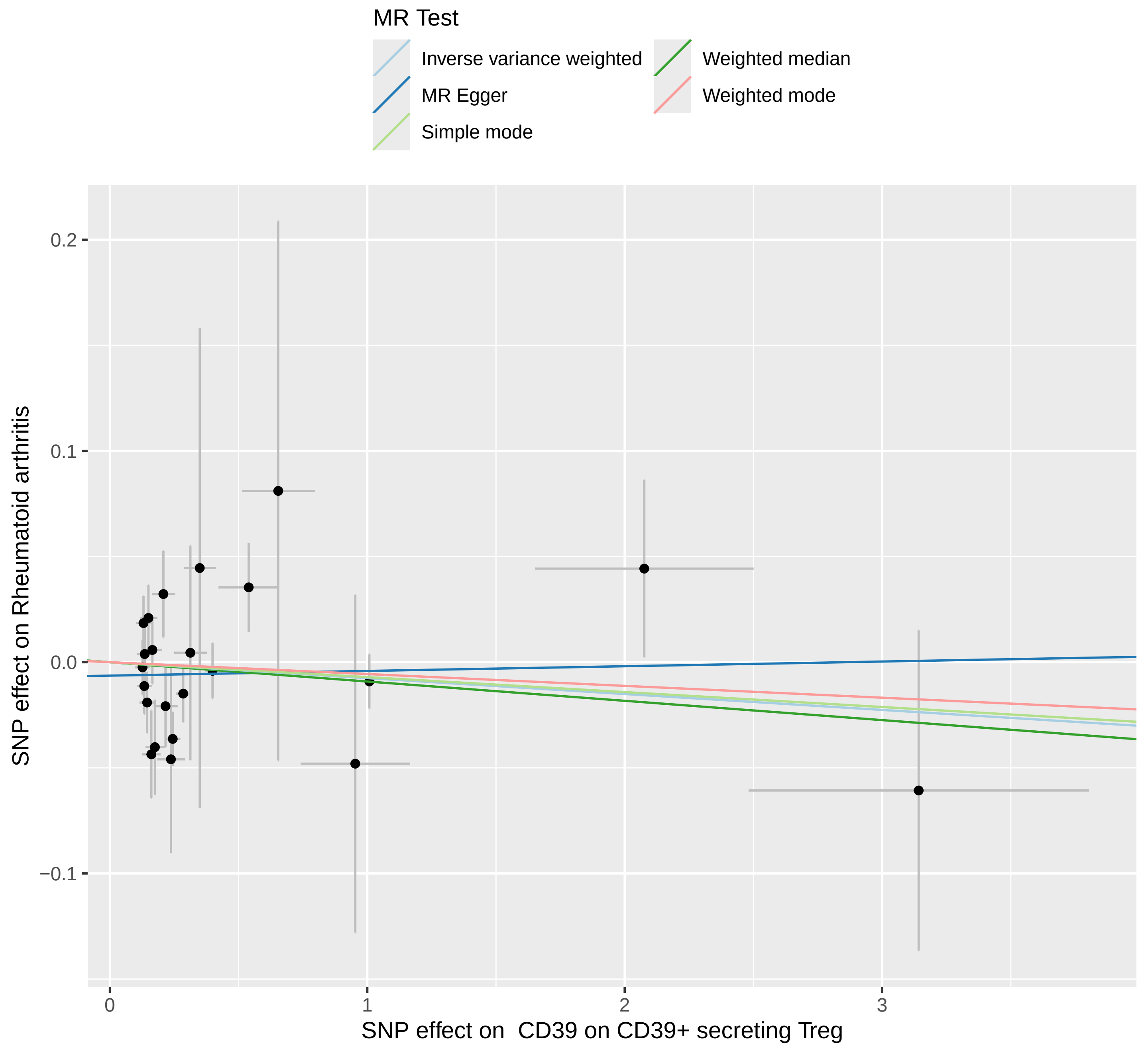
MR Test
Inverse variance weighted
MR Egger
Simple mode
Weighted median
Weighted mode
0.2
0.1
0.0
−0.1
SNP effect on Rheumatoid arthritis
0
1
2
3
SNP effect on CD39 on CD39+ secreting Treg

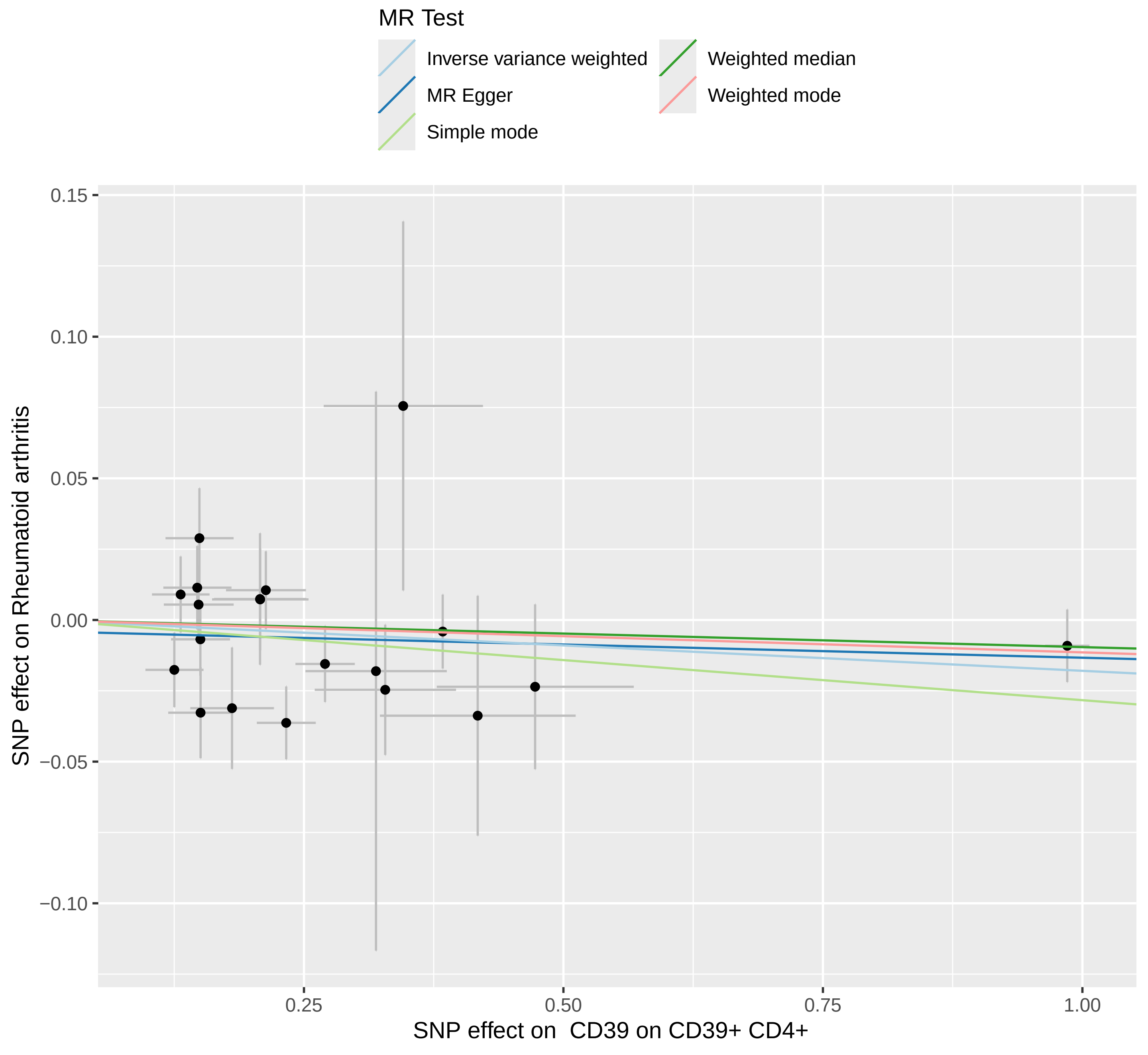

MR Test
Inverse variance weighted
MR Egger
Simple mode
Weighted median
Weighted mode
0.15
0.10
0.05
0.00
−0.05
−0.10
SNP effect on Rheumatoid arthritis
0.25
0.50
0.75
1.00
SNP effect on CD39 on CD39+ CD4+

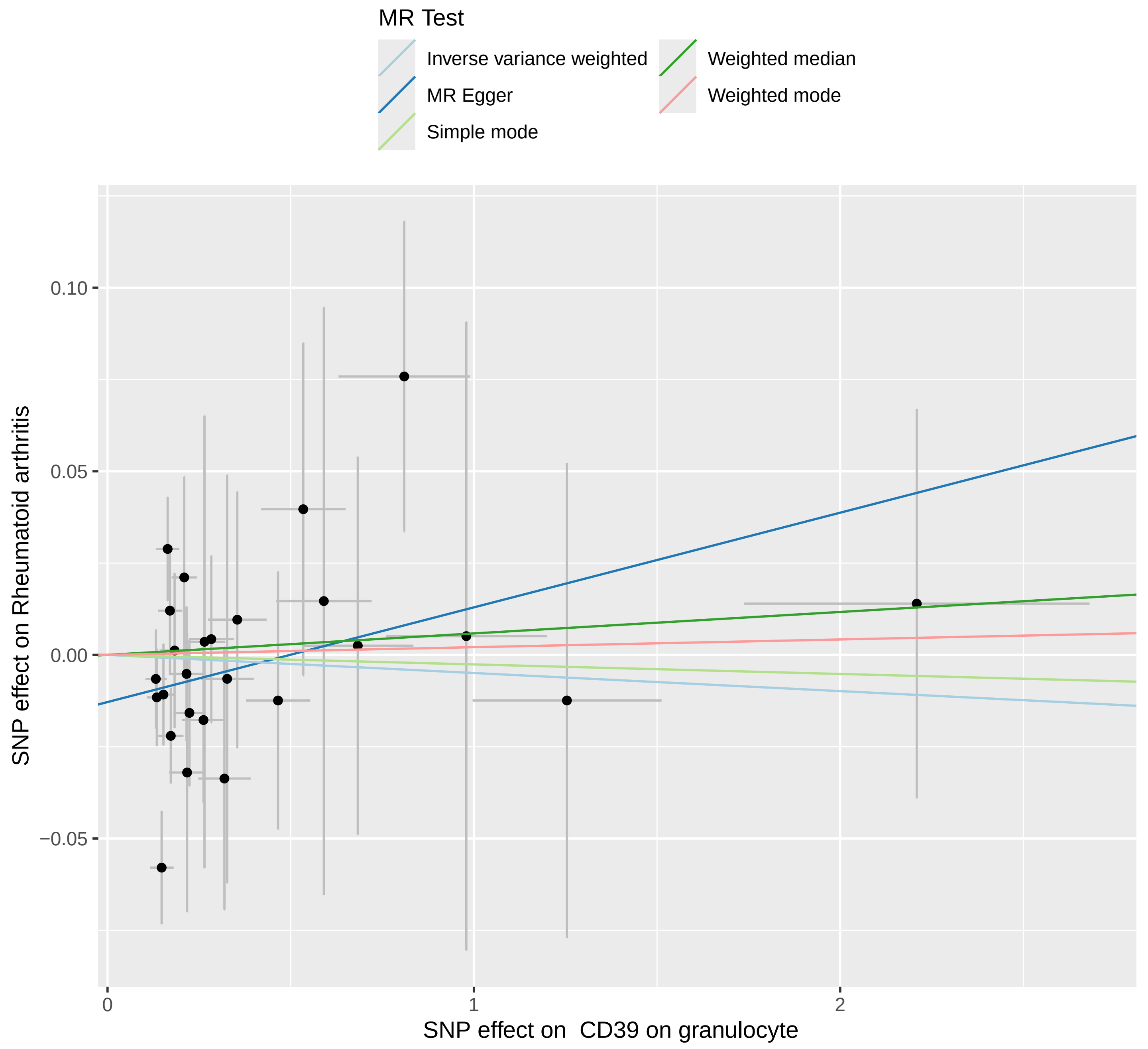

MR Test
Inverse variance weighted
MR Egger
Simple mode
Weighted median
Weighted mode
0.10
0.05
0.00
−0.05
SNP effect on Rheumatoid arthritis
0
1
2
SNP effect on CD39 on granulocyte

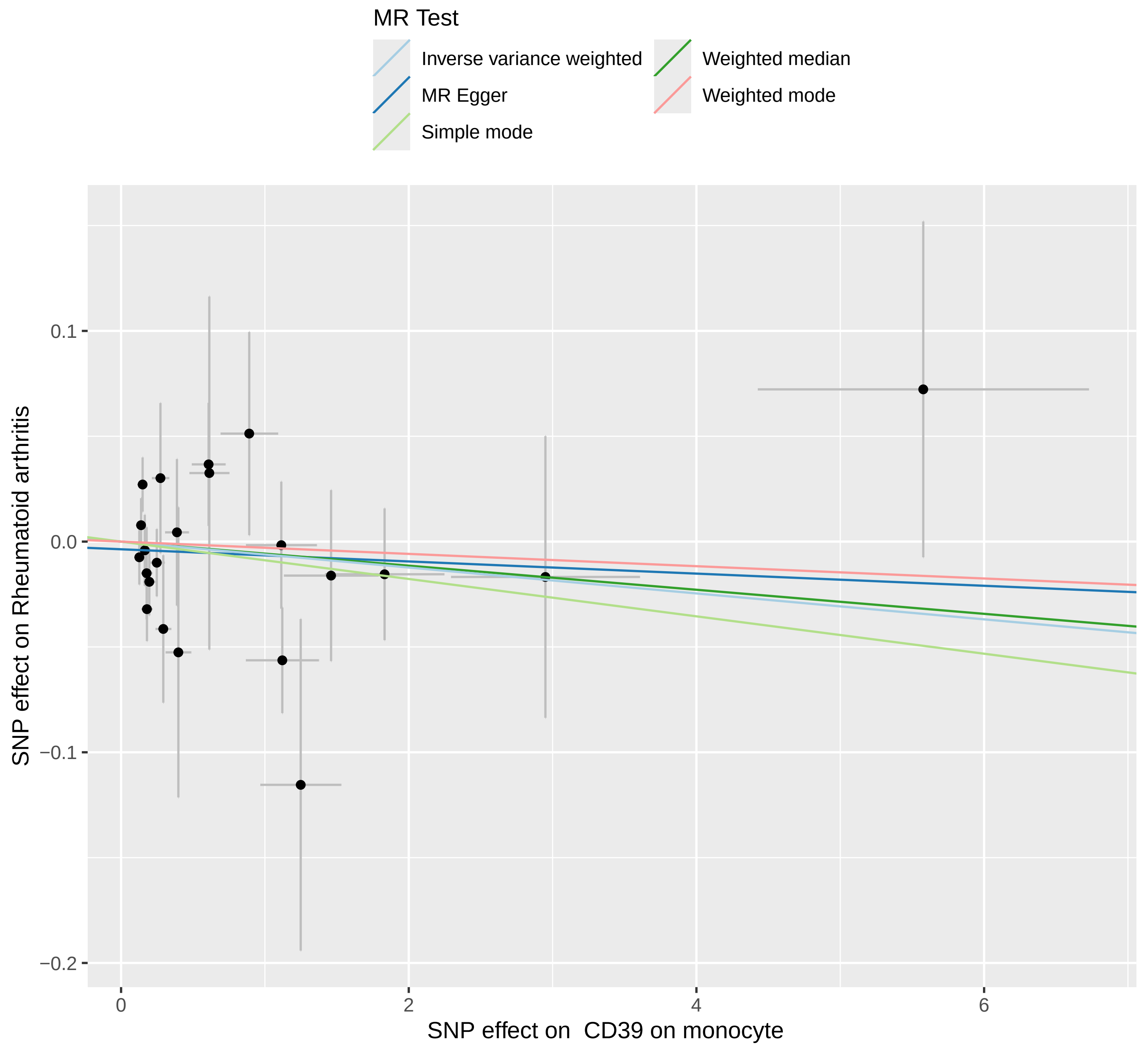
MR Test
Inverse variance weighted
MR Egger
Simple mode
Weighted median
Weighted mode
0.1
0.0
−0.1
−0.2
0
2
4
6
SNP effect on Rheumatoid arthritis
SNP effect on CD39 on monocyte

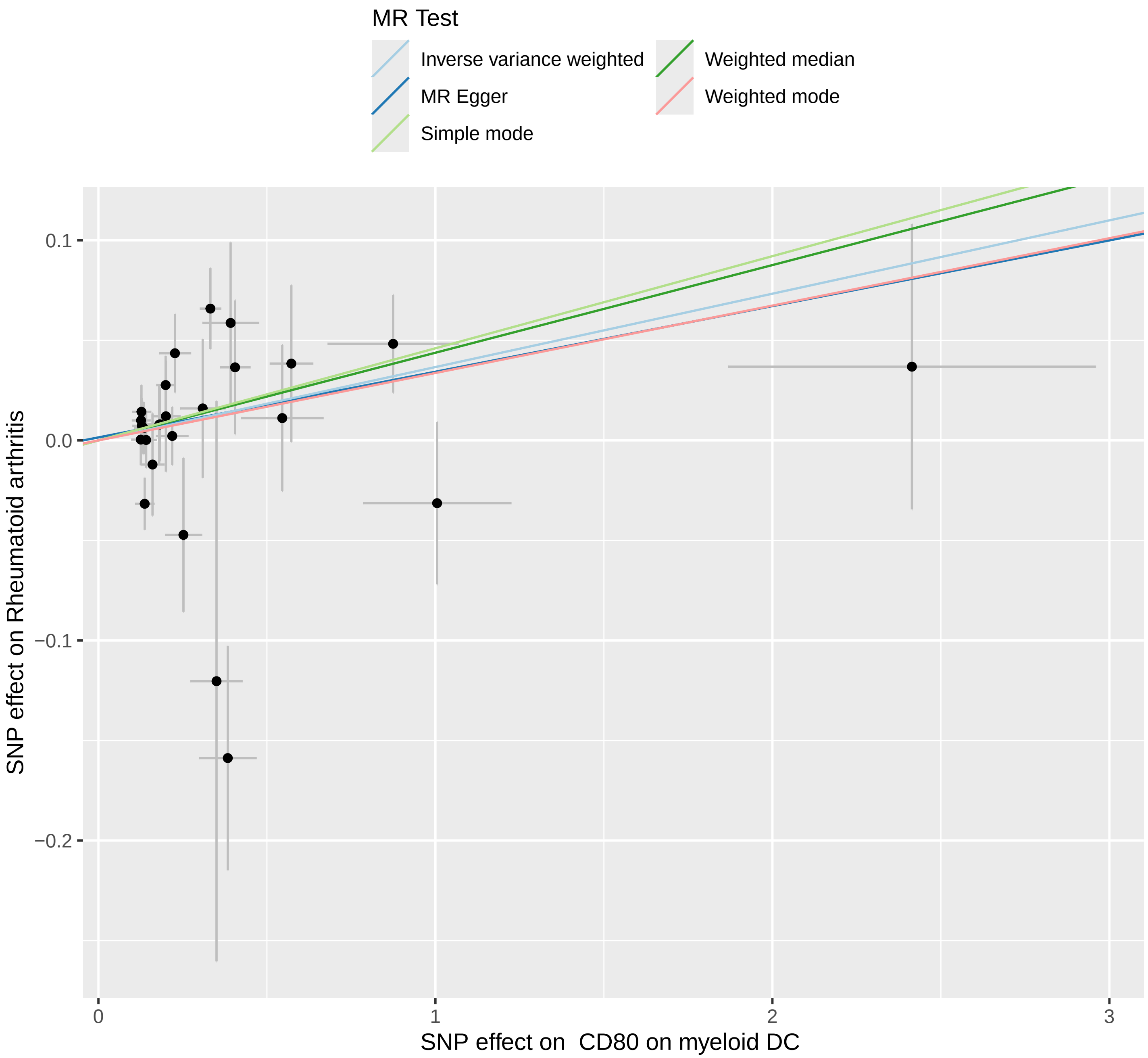
MR Test
Inverse variance weighted
MR Egger
Simple mode
Weighted median
Weighted mode
0.1
0.0
−0.1
−0.2
SNP effect on Rheumatoid arthritis
0
1
2
3
SNP effect on CD80 on myeloid DC

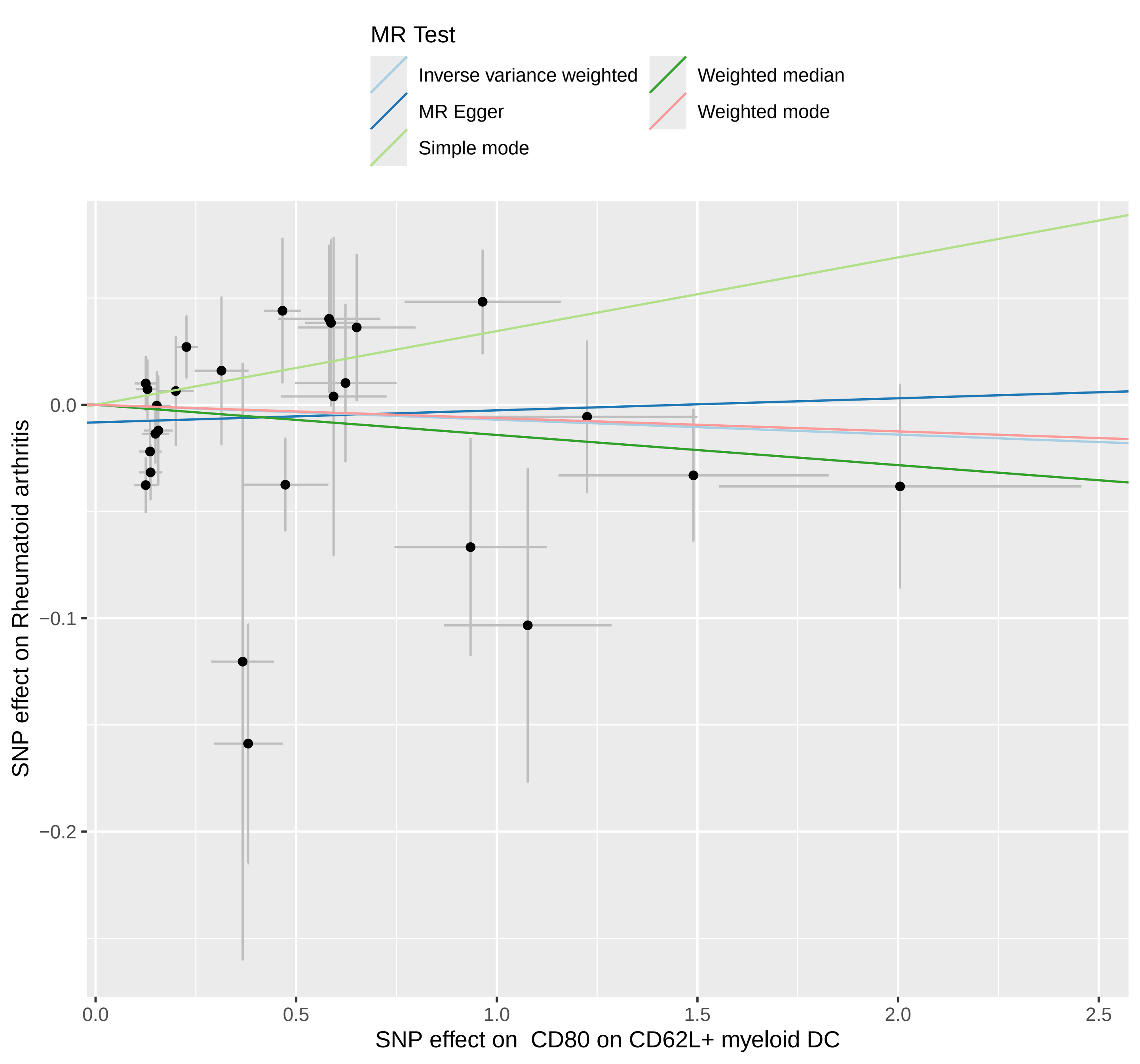

MR Test
Inverse variance weighted
MR Egger
Simple mode
Weighted median
Weighted mode
SNP effect on Rheumatoid arthritis
0.0
−0.1
−0.2
0.0
0.5
1.0
1.5
2.0
2.5
SNP effect on CD80 on CD62L+ myeloid DC

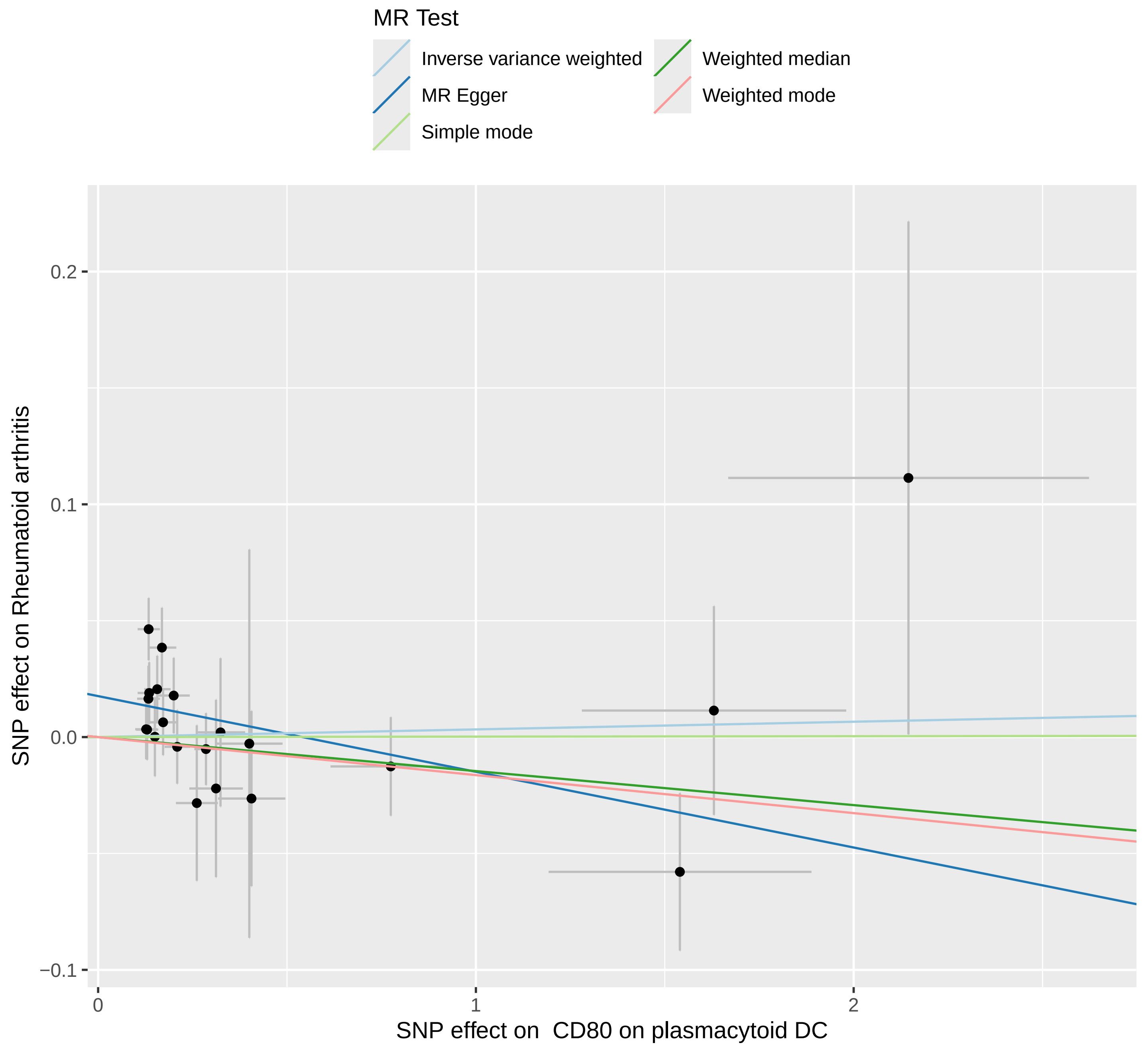
MR Test
Inverse variance weighted
MR Egger
Simple mode
Weighted median
Weighted mode
SNP effect on Rheumatoid arthritis
0.2
0.1
0.0
−0.1
0
1
2
SNP effect on CD80 on plasmacytoid DC

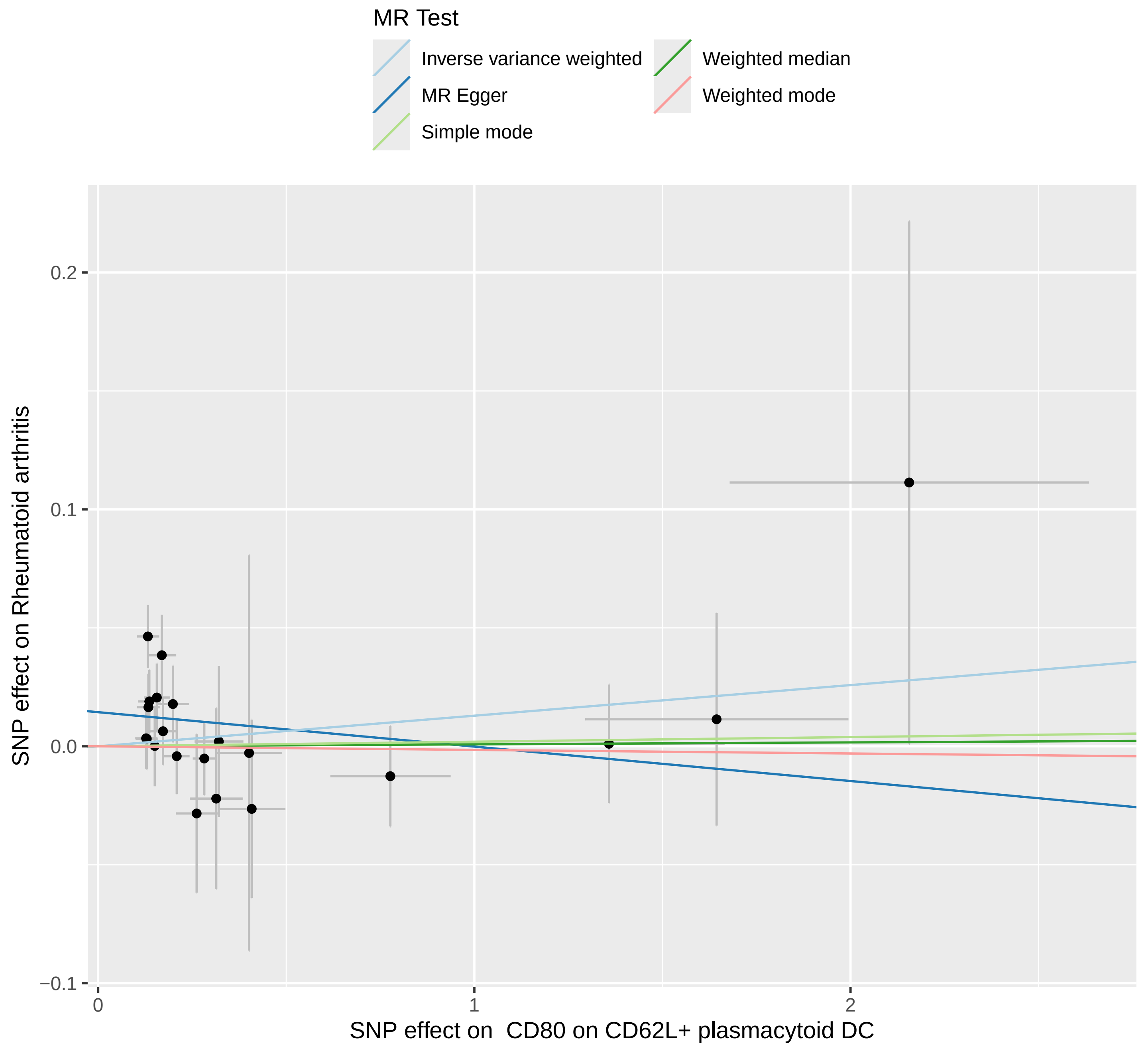

MR Test
Inverse variance weighted
MR Egger
Simple mode
Weighted median
Weighted mode
0.2
0.1
0.0
−0.1
0
1
2
SNP effect on Rheumatoid arthritis
SNP effect on CD80 on CD62L+ plasmacytoid DC

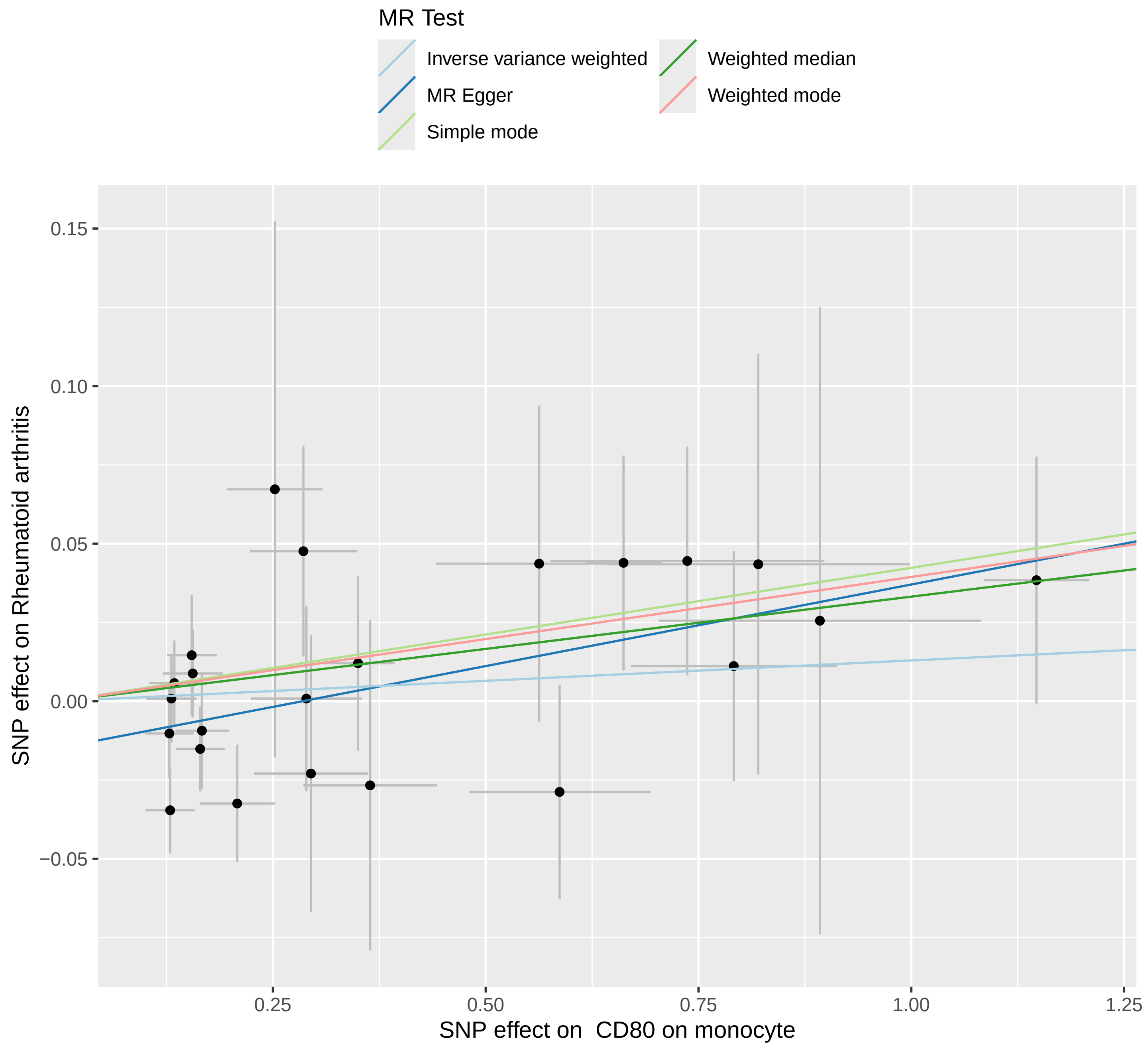
MR Test
Inverse variance weighted
MR Egger
Simple mode
Weighted median
Weighted mode
0.15
0.10
0.05
0.00
−0.05
SNP effect on Rheumatoid arthritis
0.25
0.50
0.75
1.00
1.25
SNP effect on CD80 on monocyte

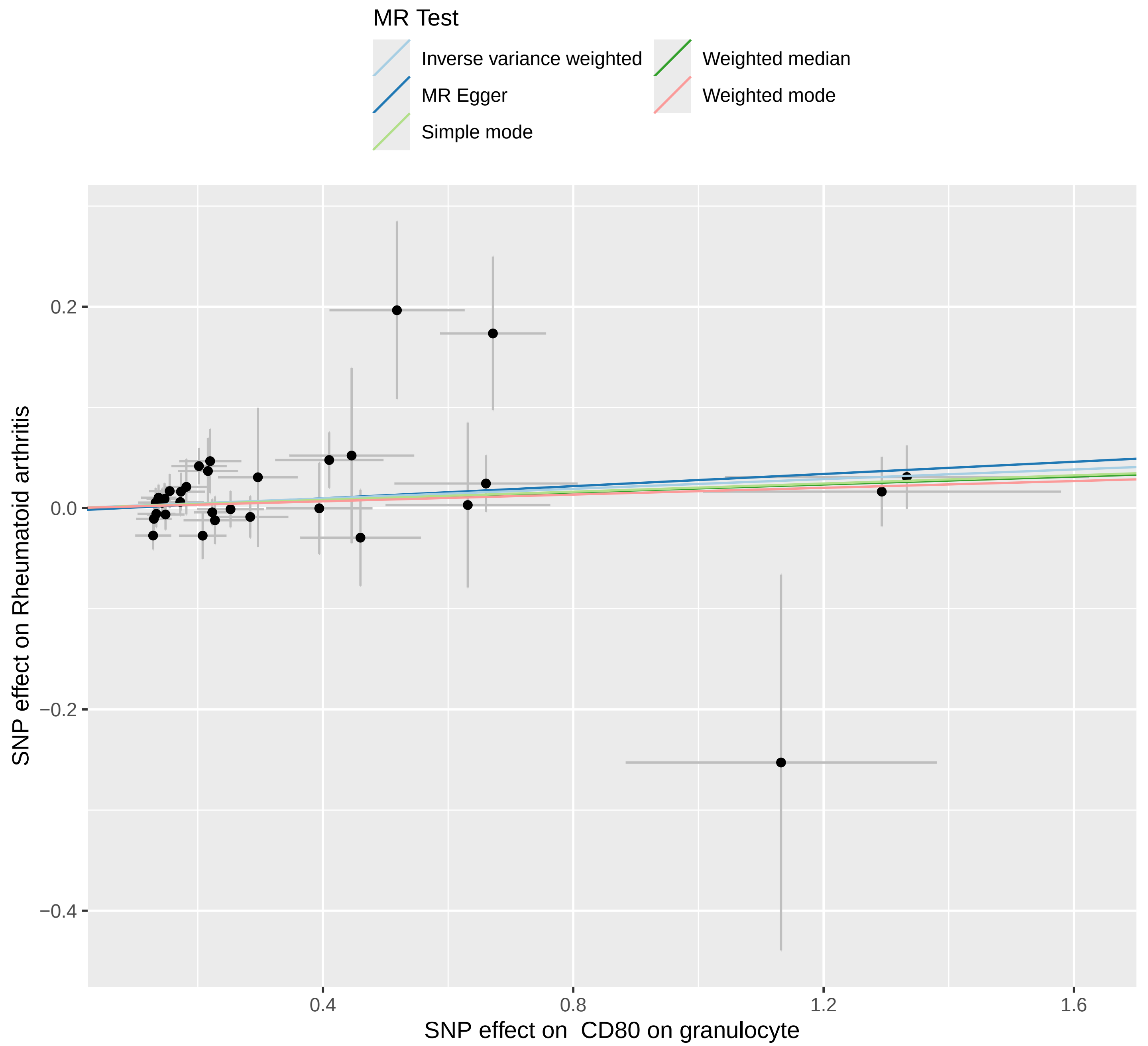
MR Test
Inverse variance weighted
MR Egger
Simple mode
Weighted median
Weighted mode
SNP effect on Rheumatoid arthritis
0.2
0.0
−0.2
−0.4
0.4
0.8
1.2
1.6
SNP effect on CD80 on granulocyte

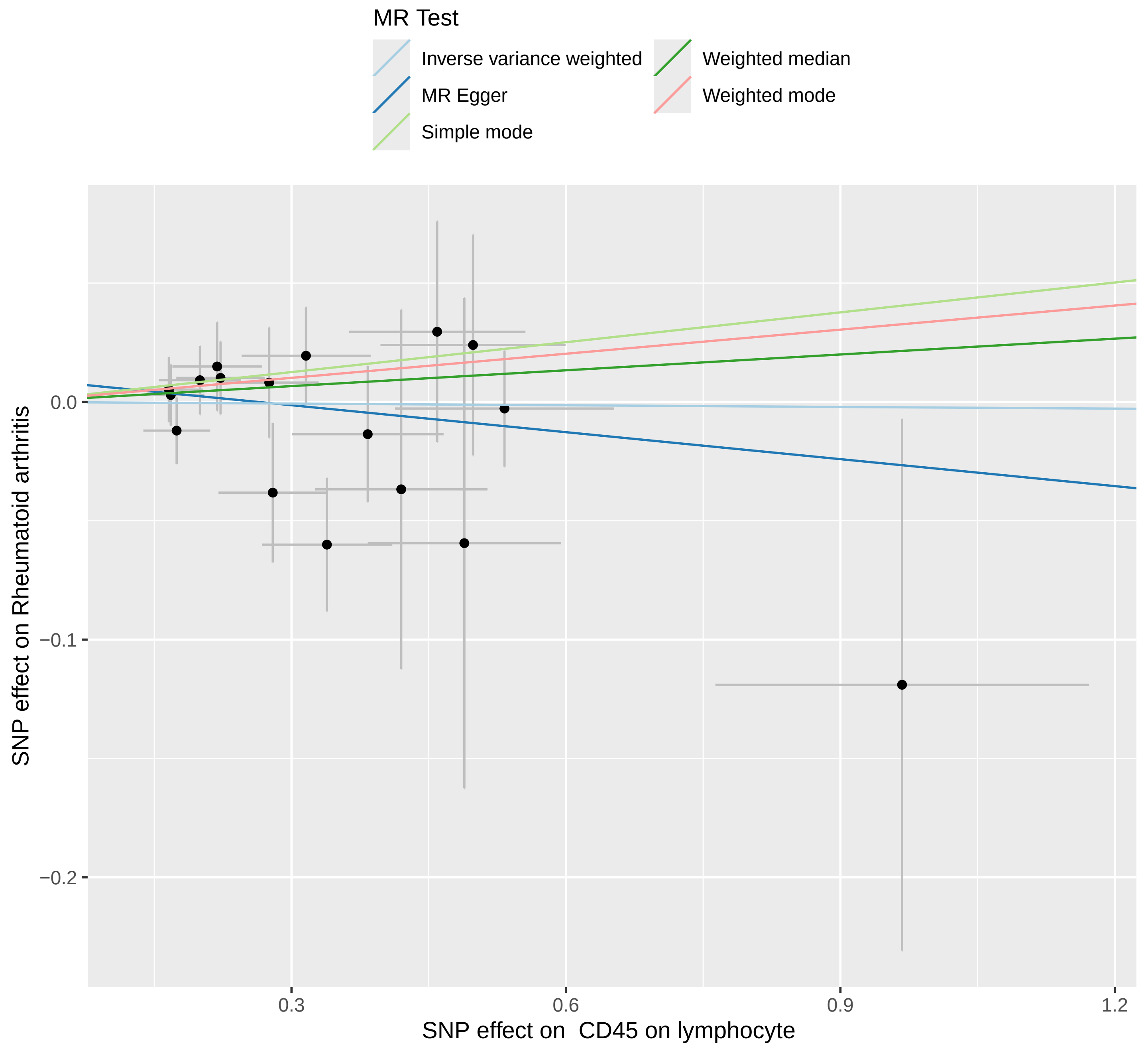
MR Test
Inverse variance weighted
MR Egger
Simple mode
Weighted median
Weighted mode
0.0
−0.1
−0.2
0.3
0.6
0.9
1.2
SNP effect on Rheumatoid arthritis
SNP effect on CD45 on lymphocyte

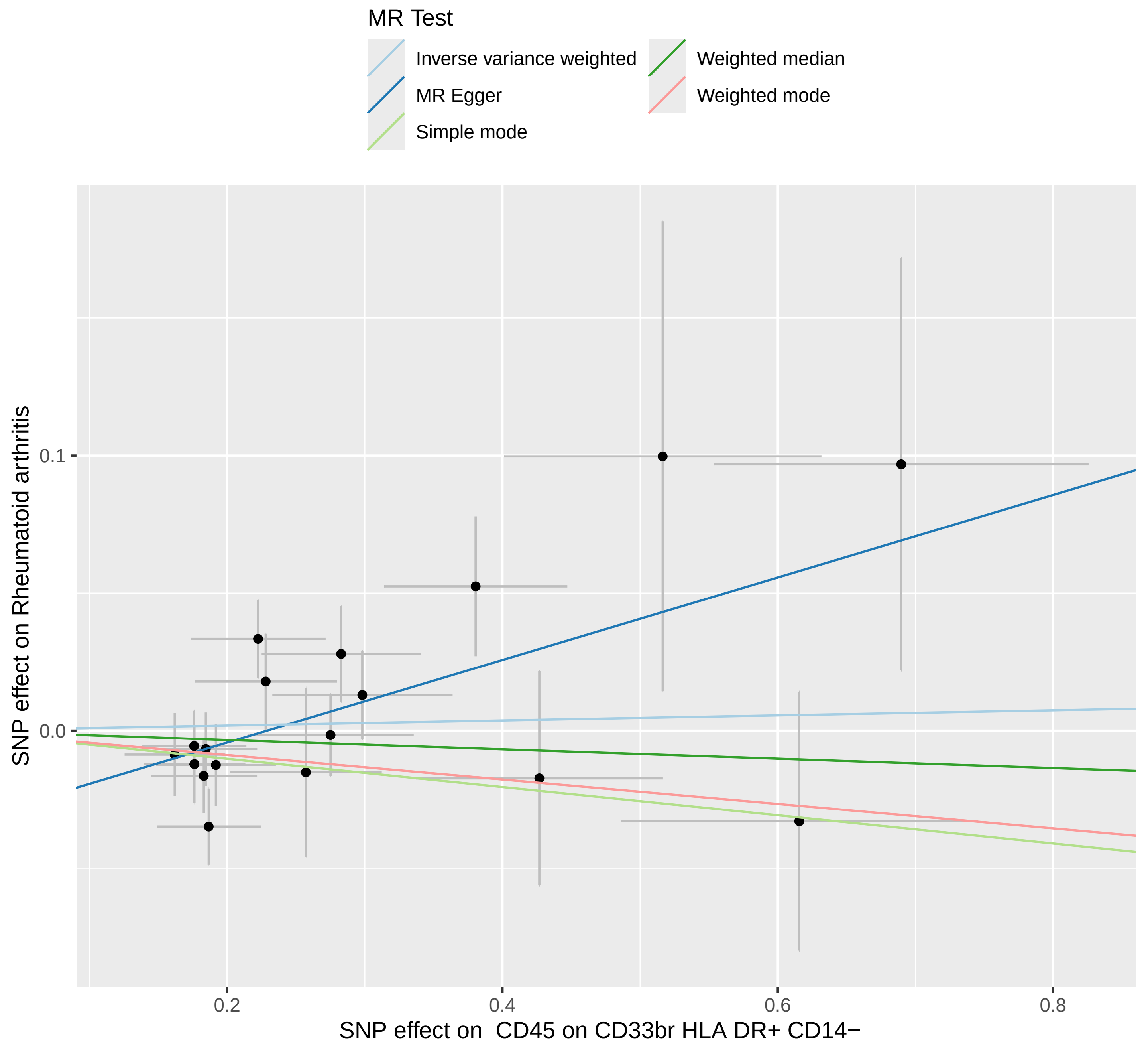

MR Test
Inverse variance weighted
MR Egger
Simple mode
Weighted median
Weighted mode
0.1
0.0
0.2
0.4
0.6
0.8
SNP effect on Rheumatoid arthritis
SNP effect on CD45 on CD33br HLA DR+ CD14−

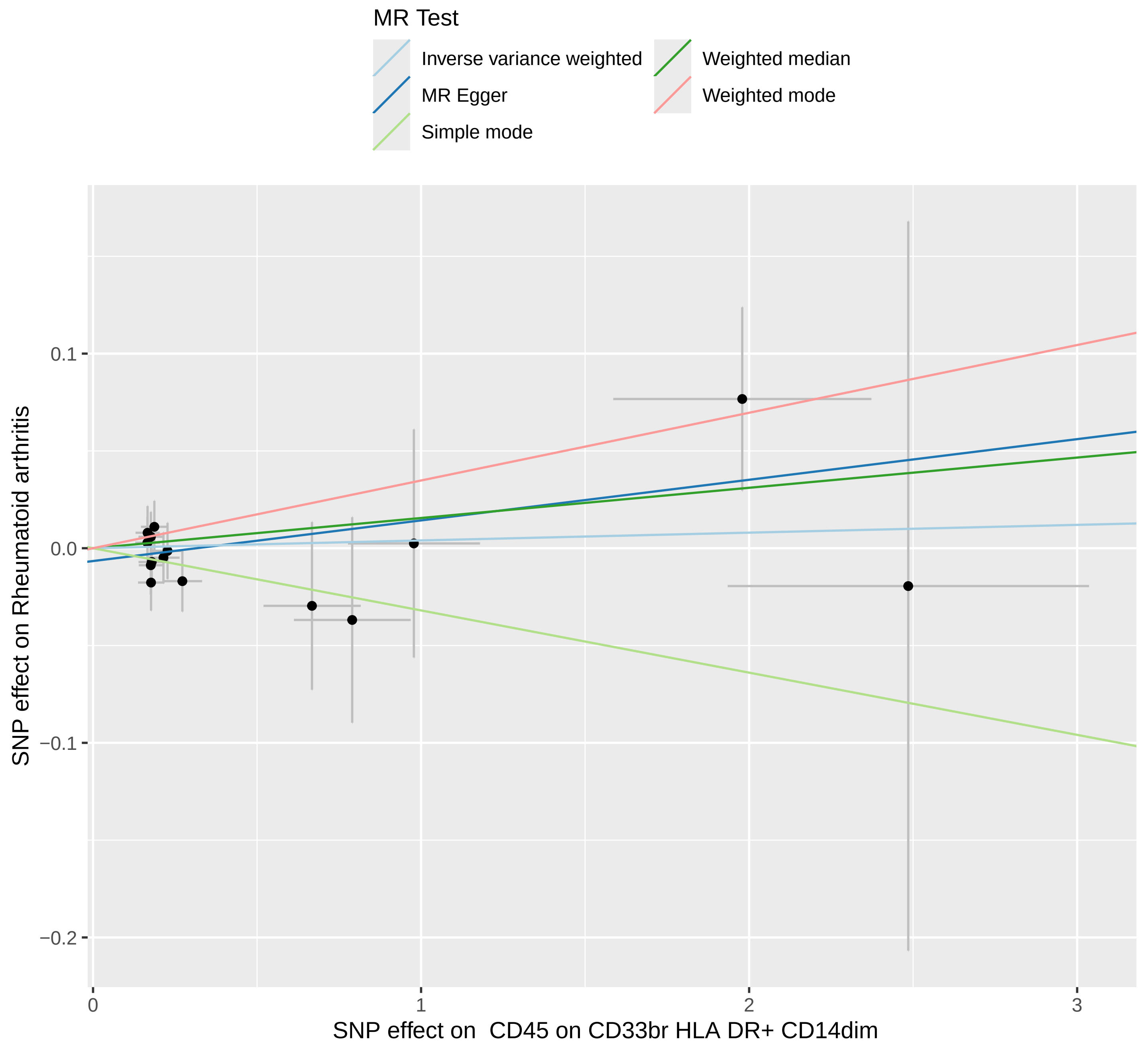

MR Test
Inverse variance weighted
MR Egger
Simple mode
Weighted median
Weighted mode
SNP effect on Rheumatoid arthritis
0.1
0.0
−0.1
−0.2
0
1
2
3
SNP effect on CD45 on CD33br HLA DR+ CD14dim

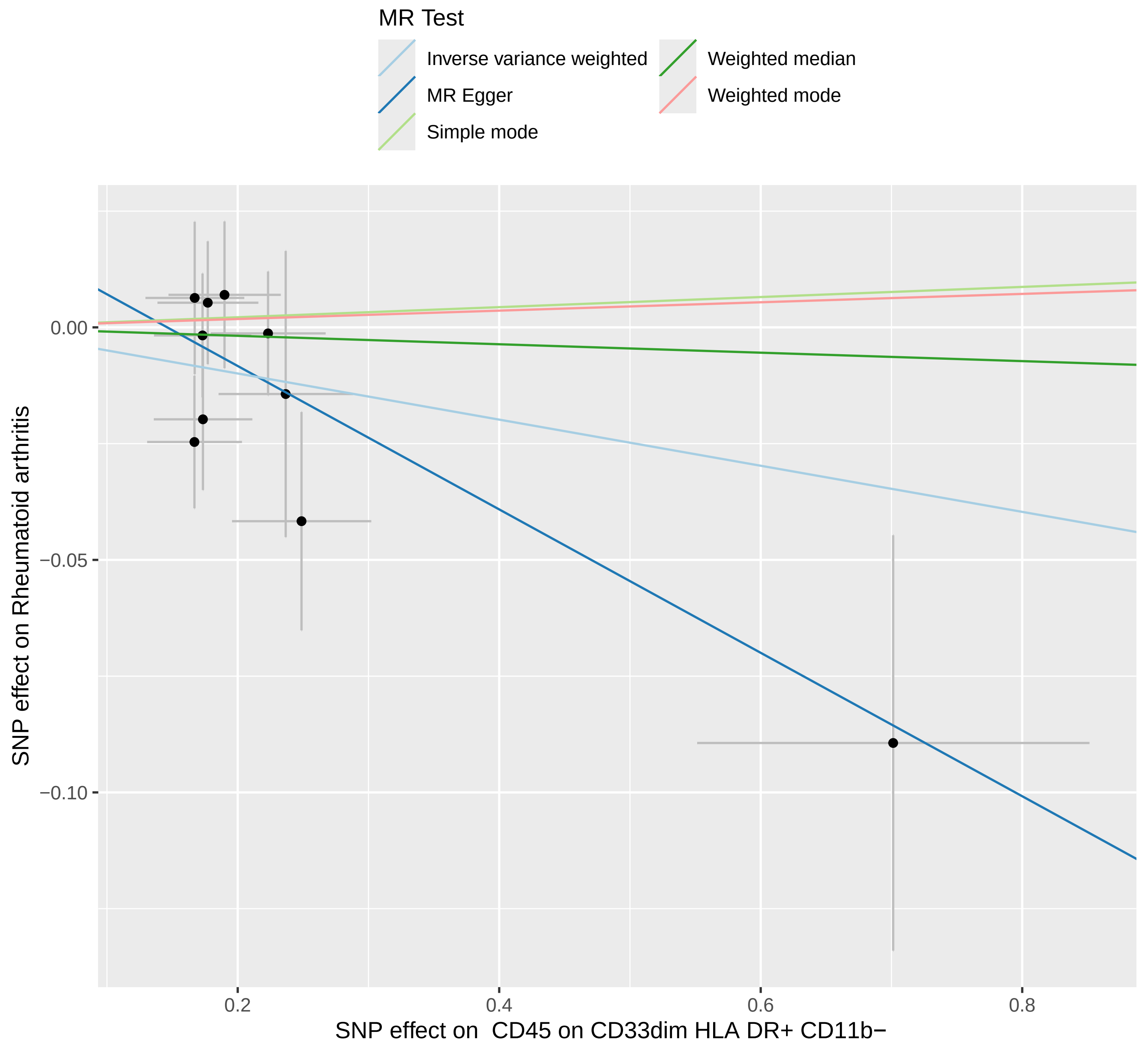

MR Test
Inverse variance weighted
MR Egger
Simple mode
Weighted median
Weighted mode
0.00
−0.05
−0.10
0.2
0.4
0.6
0.8
SNP effect on Rheumatoid arthritis
SNP effect on CD45 on CD33dim HLA DR+ CD11b−

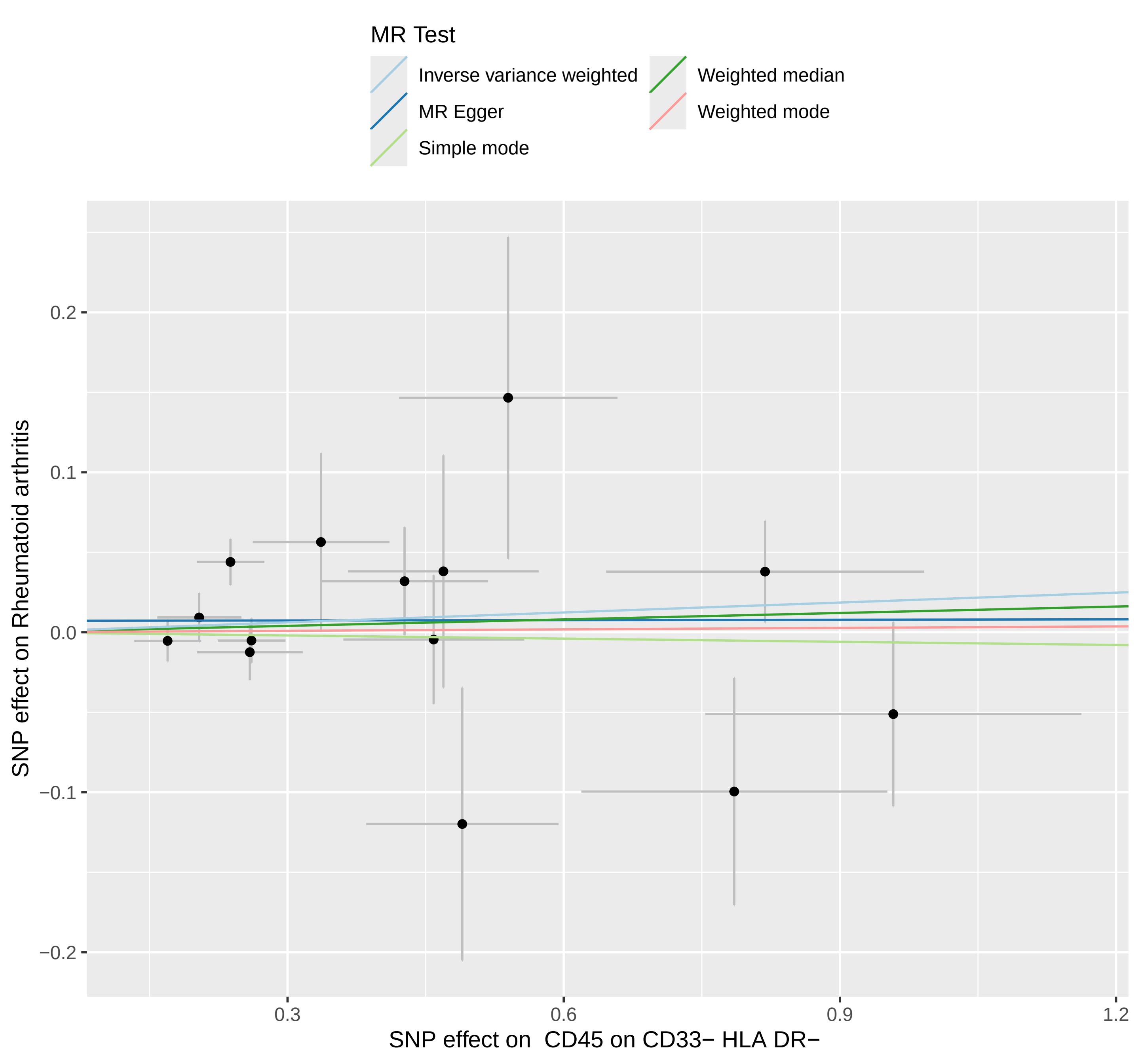

MR Test
Inverse variance weighted
MR Egger
Simple mode
Weighted median
Weighted mode
0.2
0.1
0.0
−0.1
−0.2
0.3
0.6
0.9
1.2
SNP effect on Rheumatoid arthritis
SNP effect on CD45 on CD33− HLA DR−

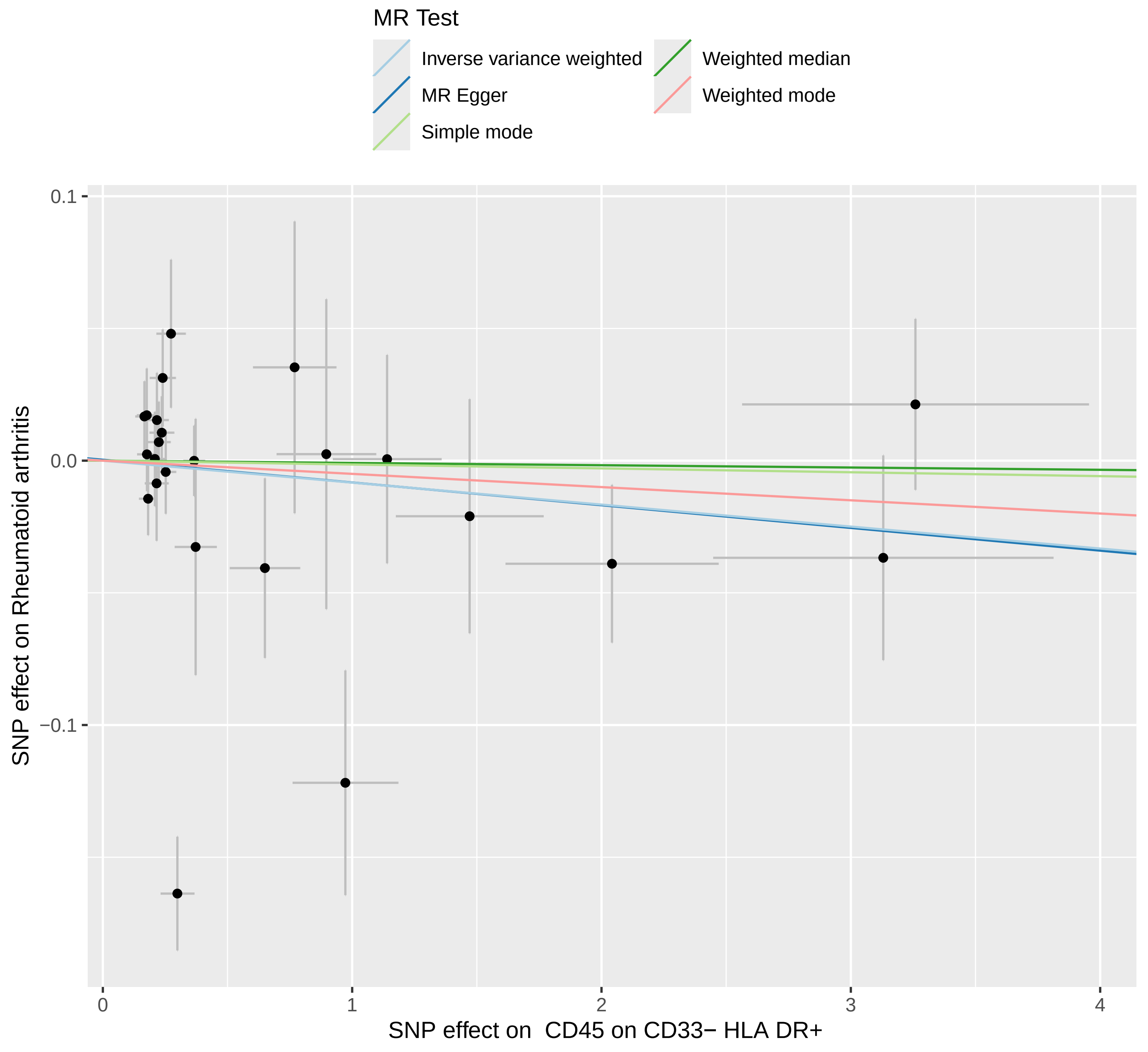

MR Test
Inverse variance weighted
MR Egger
Simple mode
Weighted median
Weighted mode
0.1
0.0
−0.1
0
1
2
3
4
SNP effect on Rheumatoid arthritis
SNP effect on CD45 on CD33− HLA DR+

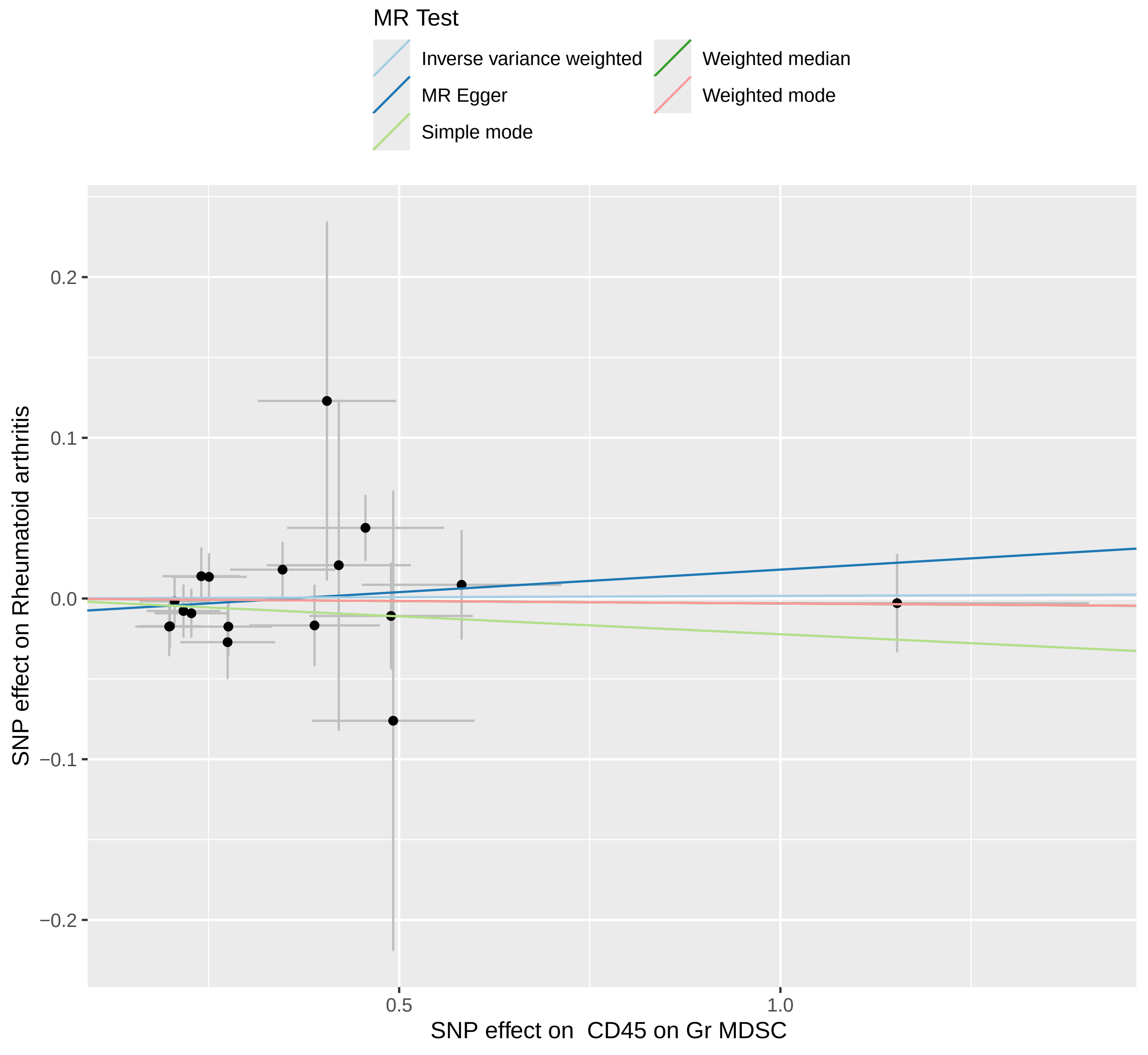

MR Test
Inverse variance weighted
MR Egger
Simple mode
Weighted median
Weighted mode
0.2
0.1
0.0
−0.1
−0.2
SNP effect on Rheumatoid arthritis
0.5
1.0
SNP effect on CD45 on Gr MDSC

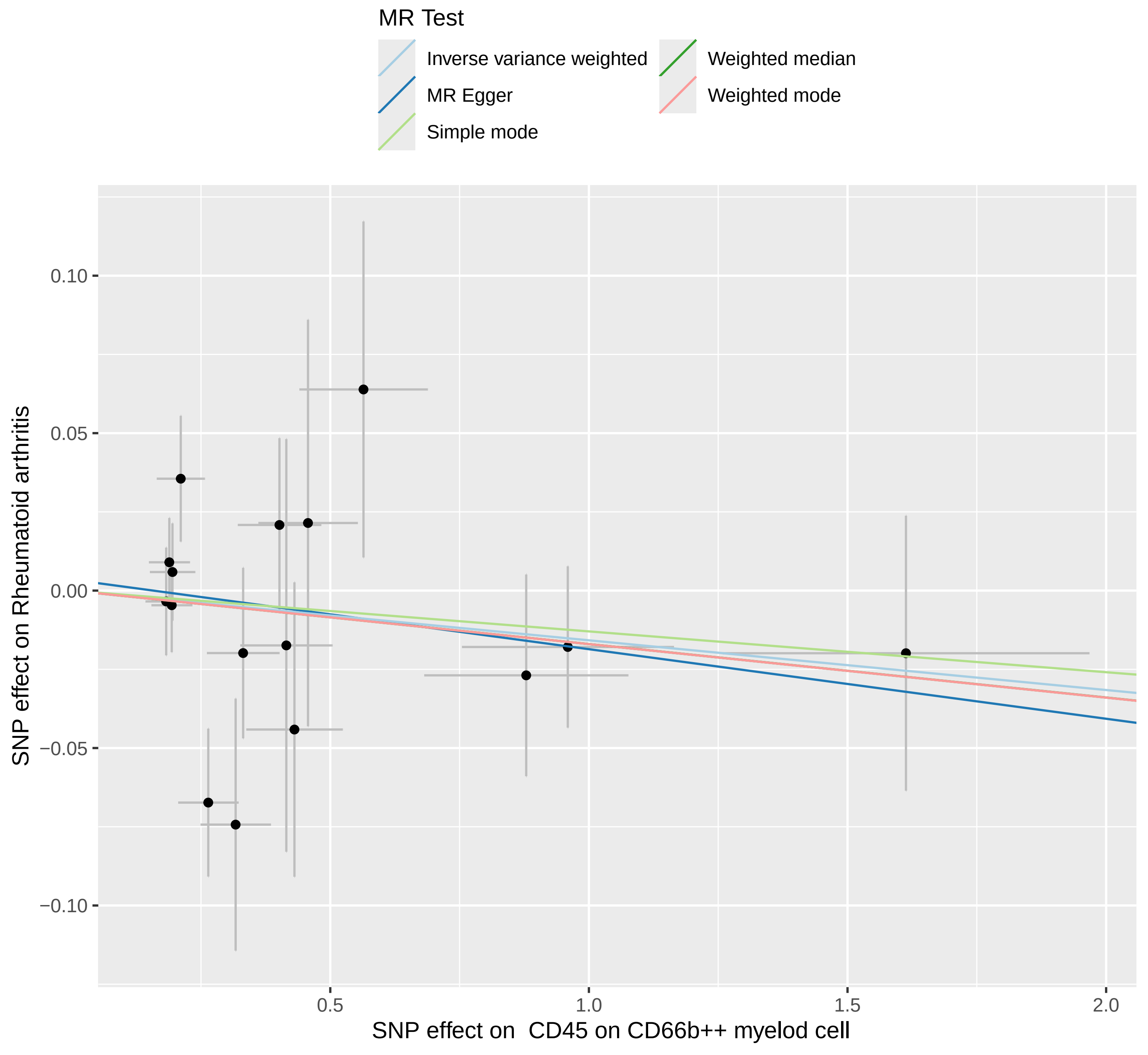
MR Test
Inverse variance weighted
MR Egger
Simple mode
Weighted median
Weighted mode
0.10
0.05
0.00
−0.05
−0.10
SNP effect on Rheumatoid arthritis
0.5
1.0
1.5
2.0
SNP effect on CD45 on CD66b++ myelod cell

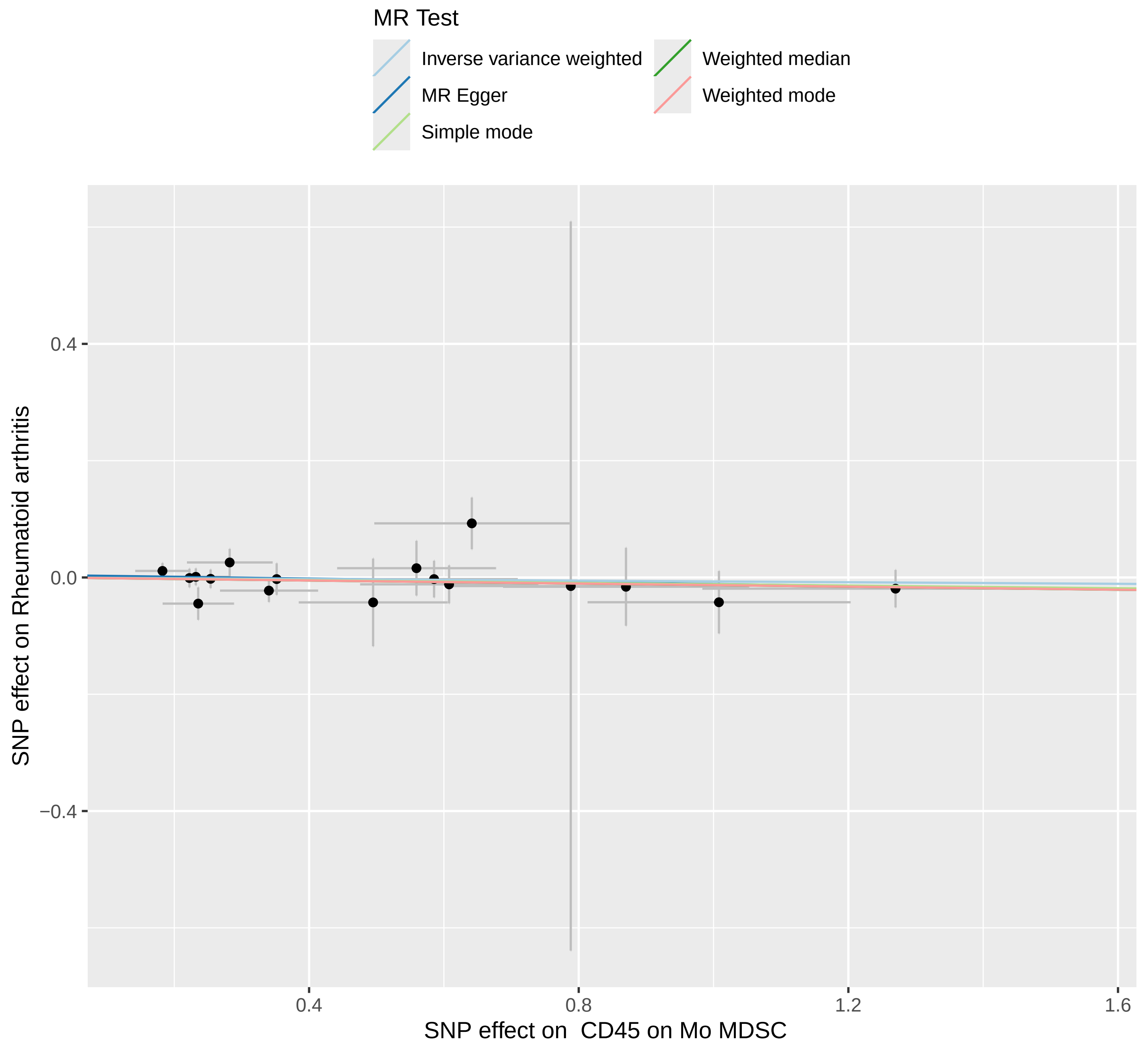

MR Test
Inverse variance weighted
MR Egger
Simple mode
Weighted median
Weighted mode
0.4
0.0
−0.4
SNP effect on Rheumatoid arthritis
0.4
0.8
1.2
1.6
SNP effect on CD45 on Mo MDSC

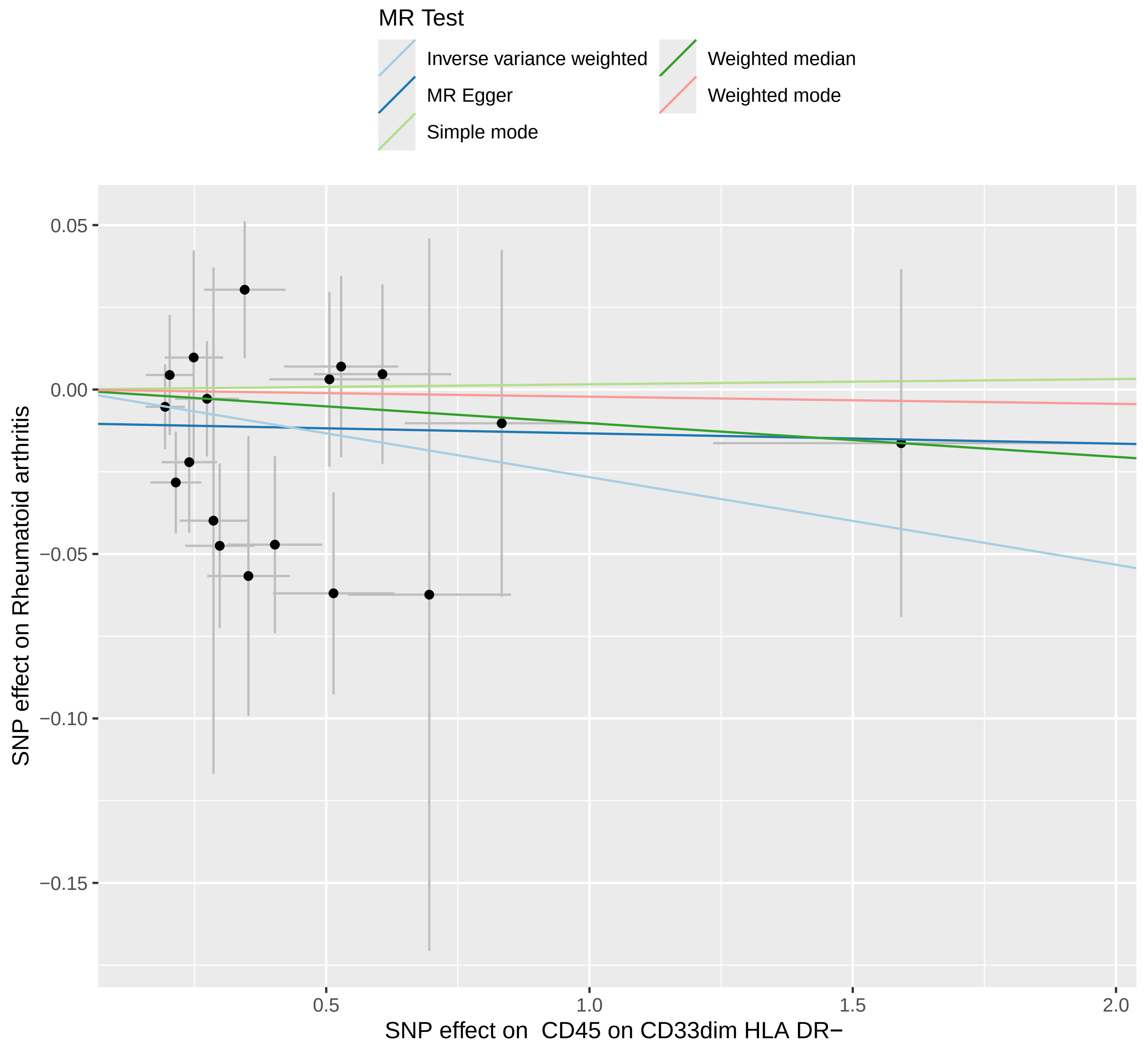
MR Test
Inverse variance weighted
MR Egger
Simple mode
Weighted median
Weighted mode
0.05
0.00
−0.05
−0.10
−0.15
0.5
1.0
1.5
2.0
SNP effect on Rheumatoid arthritis
SNP effect on CD45 on CD33dim HLA DR−

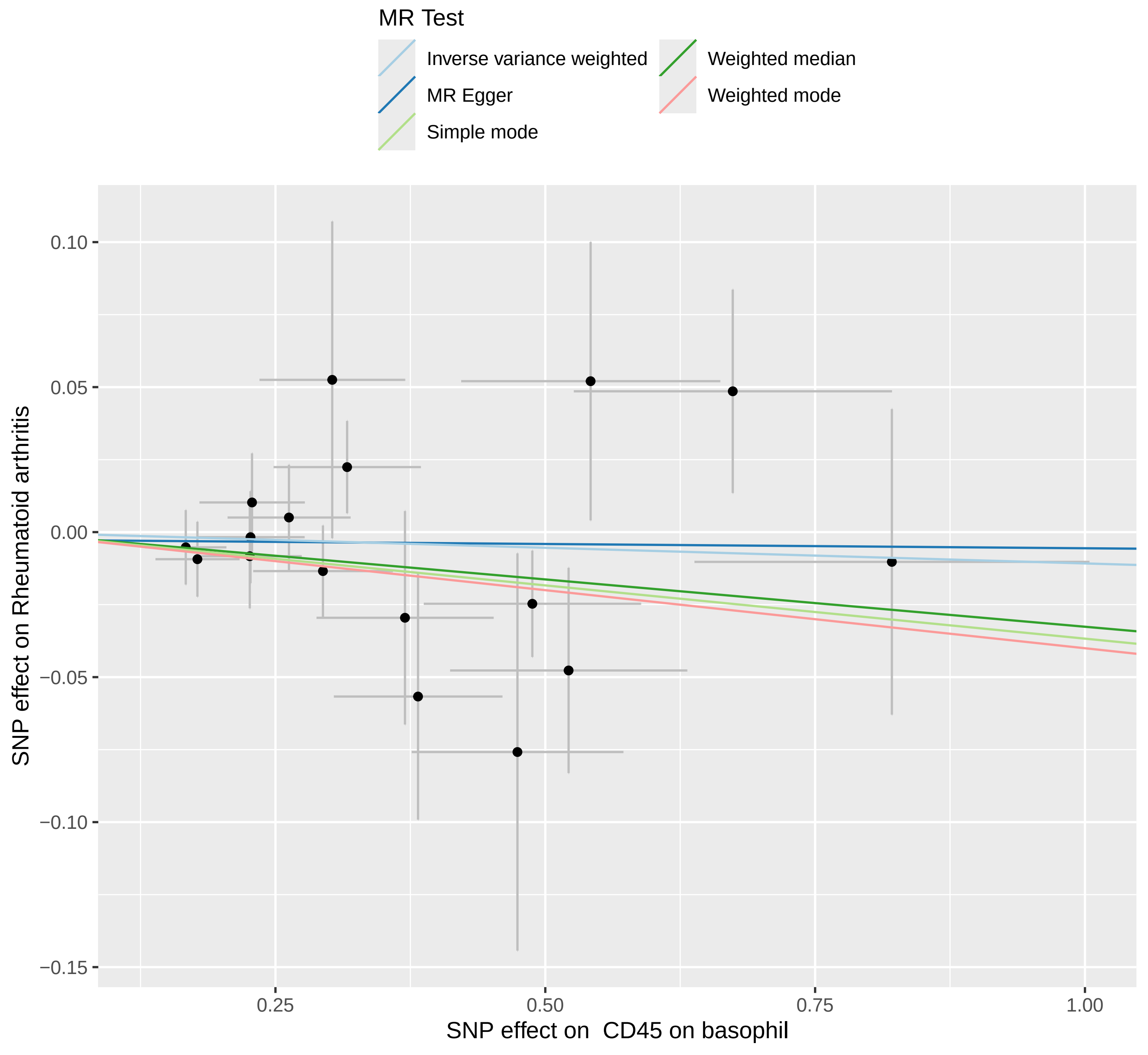
MR Test
Inverse variance weighted
MR Egger
Simple mode
Weighted median
Weighted mode
0.10
0.05
0.00
−0.05
−0.10
−0.15
0.25
0.50
0.75
1.00
SNP effect on Rheumatoid arthritis
SNP effect on CD45 on basophil

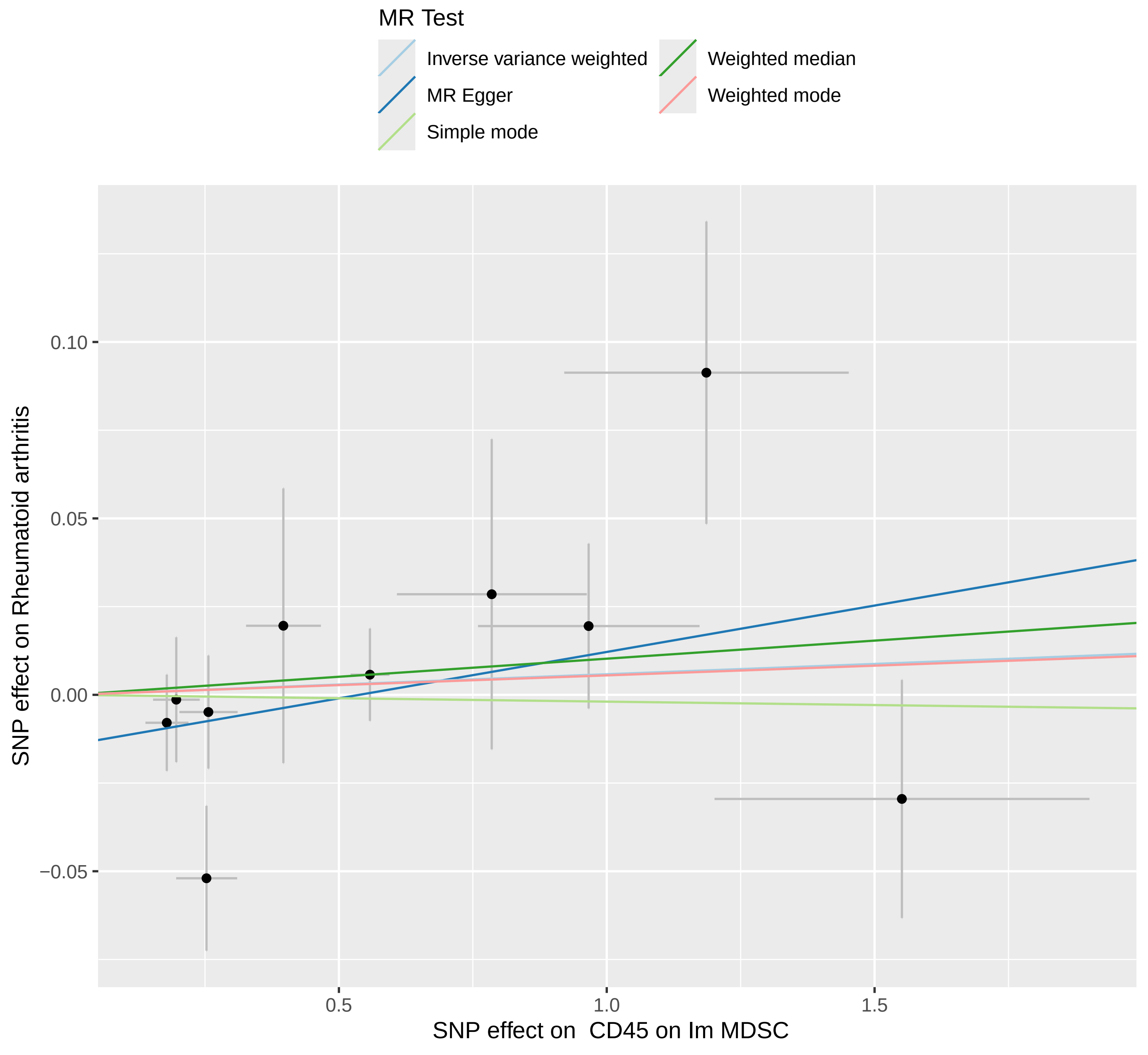

MR Test
Inverse variance weighted
MR Egger
Simple mode
Weighted median
Weighted mode
0.10
0.05
0.00
−0.05
0.5
1.0
1.5
SNP effect on Rheumatoid arthritis
SNP effect on CD45 on Im MDSC

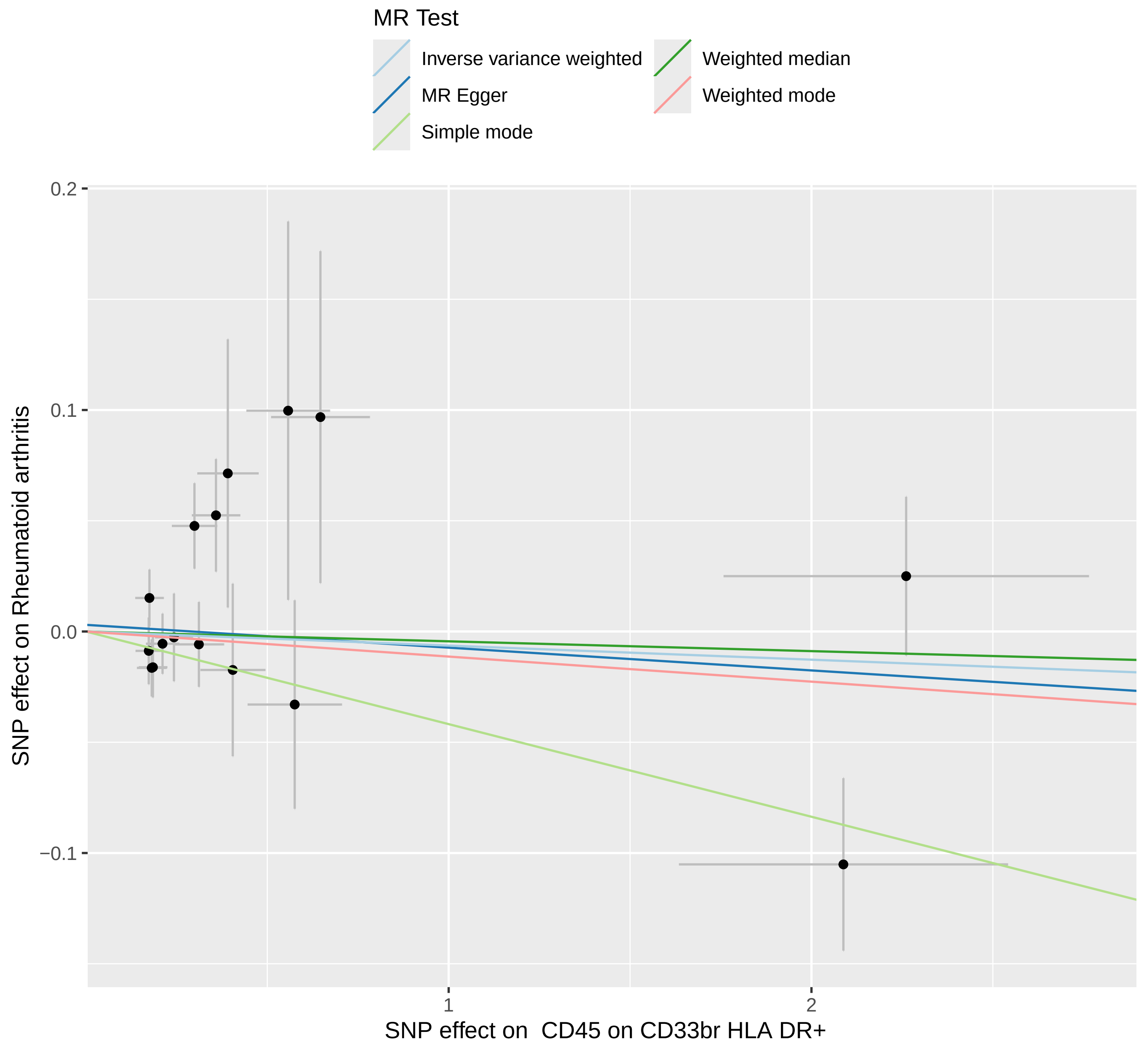

MR Test
Inverse variance weighted
MR Egger
Simple mode
Weighted median
Weighted mode
0.2
0.1
0.0
−0.1
SNP effect on Rheumatoid arthritis
1
2
SNP effect on CD45 on CD33br HLA DR+

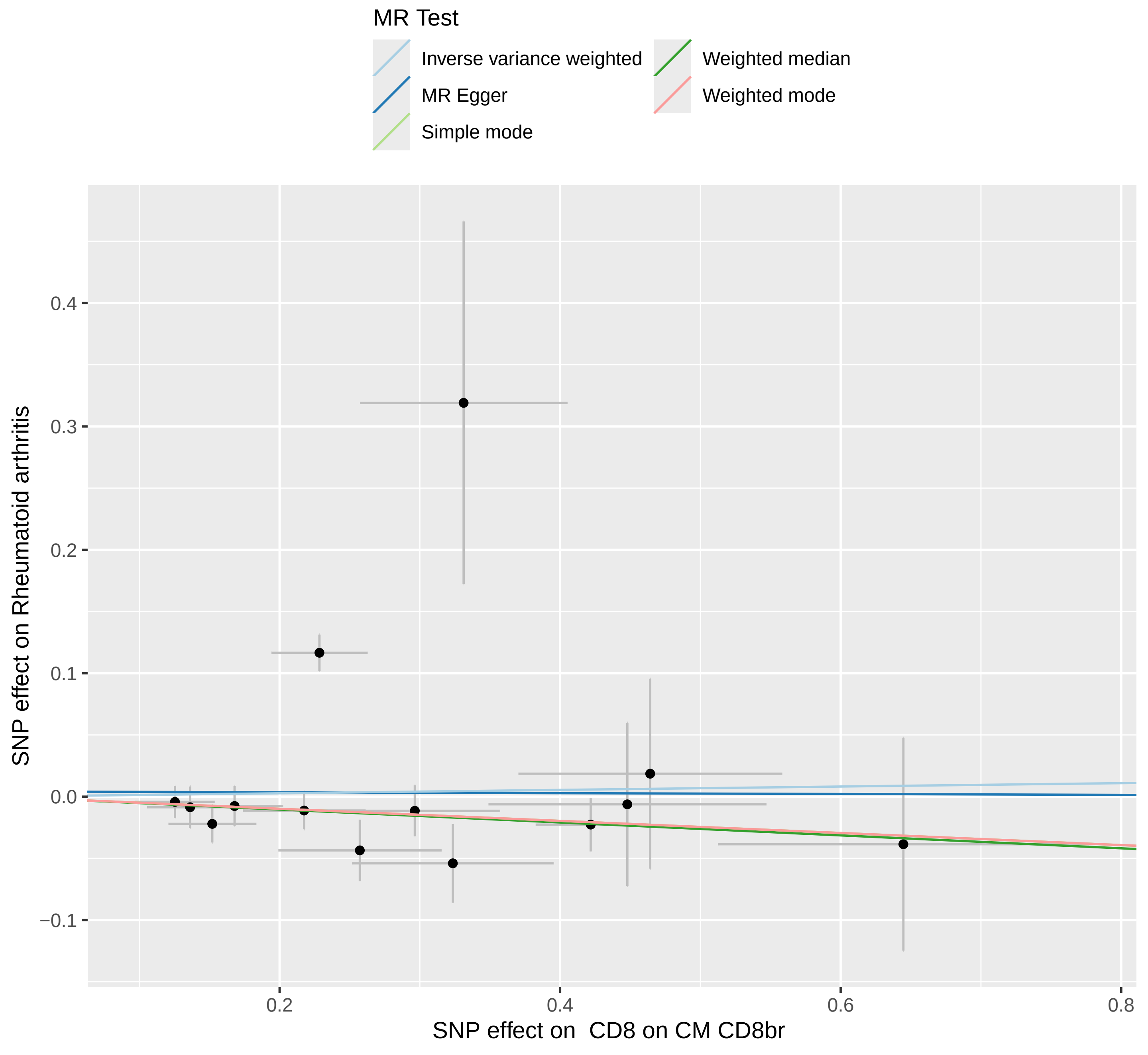
MR Test
Inverse variance weighted
MR Egger
Simple mode
Weighted median
Weighted mode
0.4
0.3
0.2
0.1
0.0
−0.1
0.2
0.4
0.6
0.8
SNP effect on Rheumatoid arthritis
SNP effect on CD8 on CM CD8br

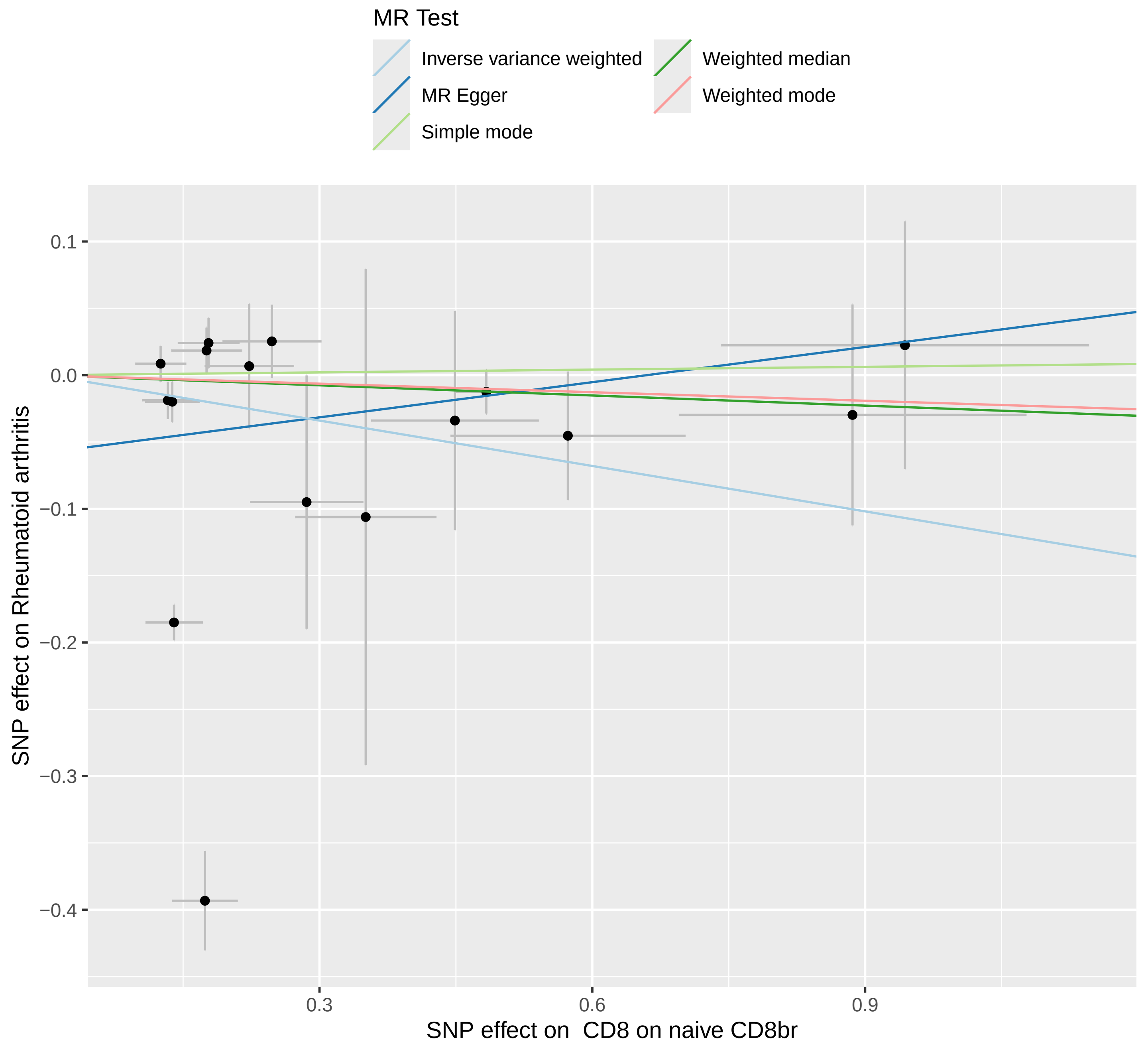
MR Test
Inverse variance weighted
MR Egger
Simple mode
Weighted median
Weighted mode
0.1
0.0
−0.1
−0.2
−0.3
−0.4
0.3
0.6
0.9
SNP effect on Rheumatoid arthritis
SNP effect on CD8 on naive CD8br

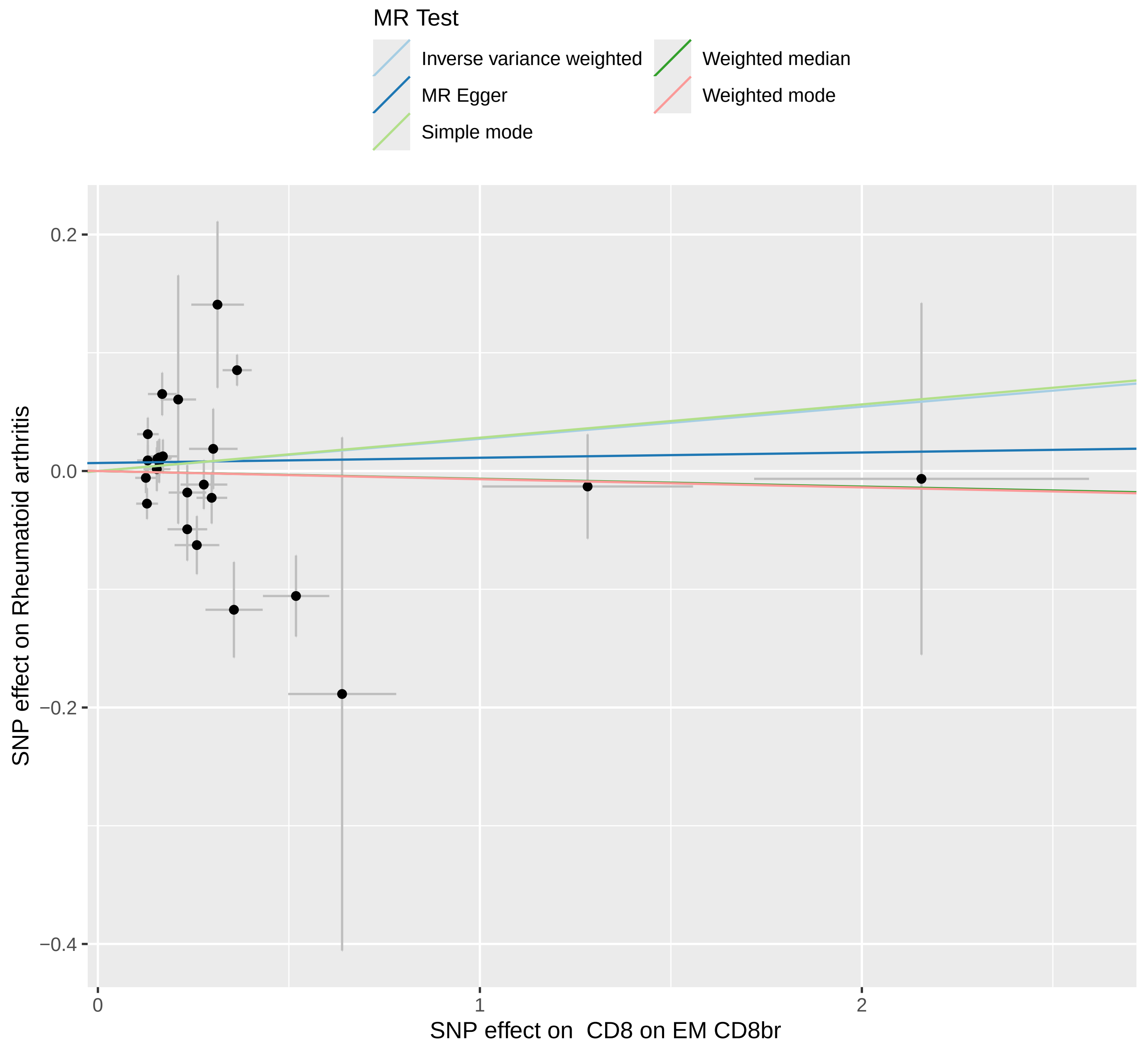
MR Test
Inverse variance weighted
MR Egger
Simple mode
Weighted median
Weighted mode
0.2
0.0
−0.2
−0.4
0
1
2
SNP effect on Rheumatoid arthritis
SNP effect on CD8 on EM CD8br

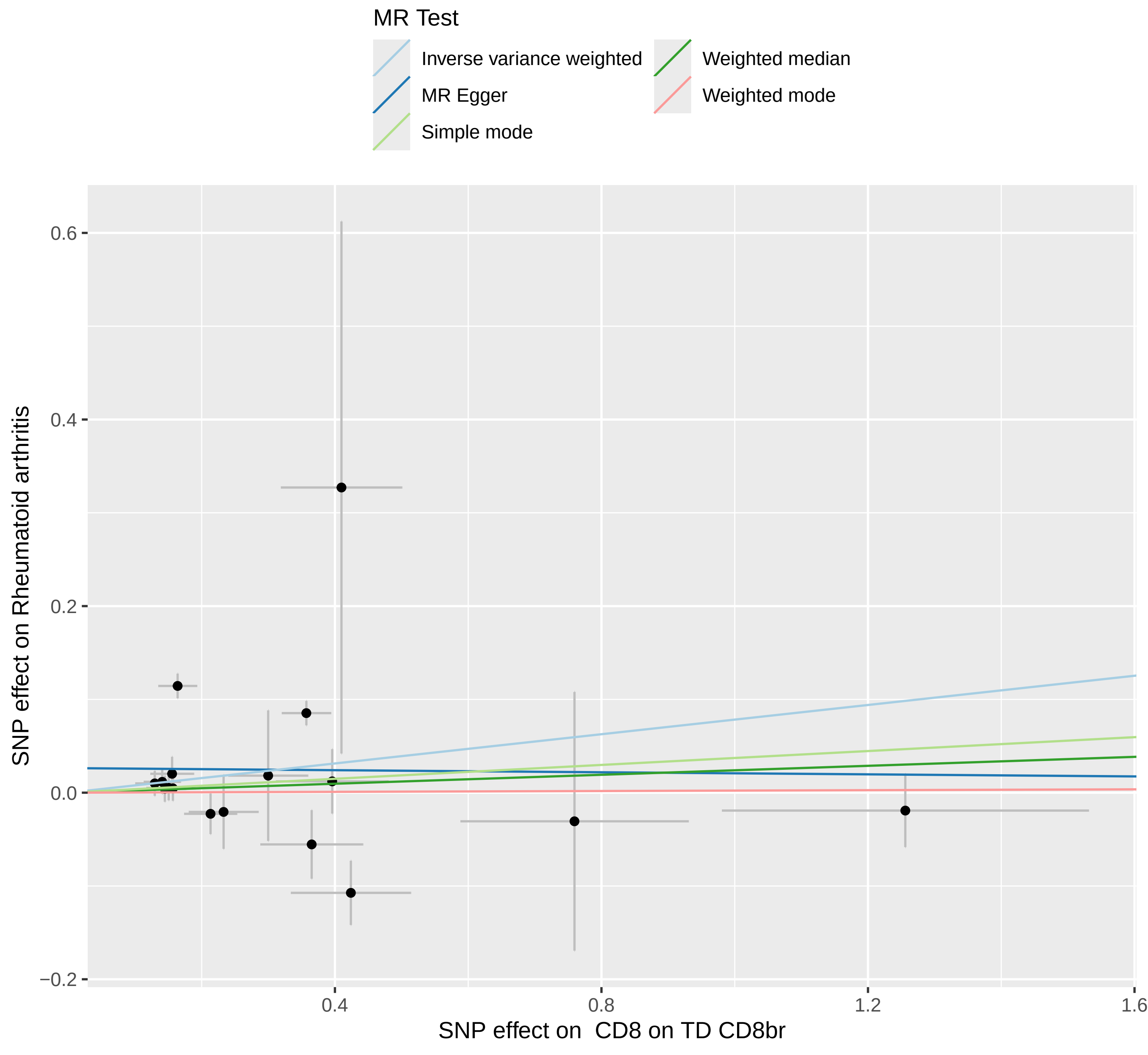

MR Test
Inverse variance weighted
MR Egger
Simple mode
Weighted median
Weighted mode
0.6
0.4
0.2
0.0
−0.2
0.4
0.8
1.2
1.6
SNP effect on Rheumatoid arthritis
SNP effect on CD8 on TD CD8br

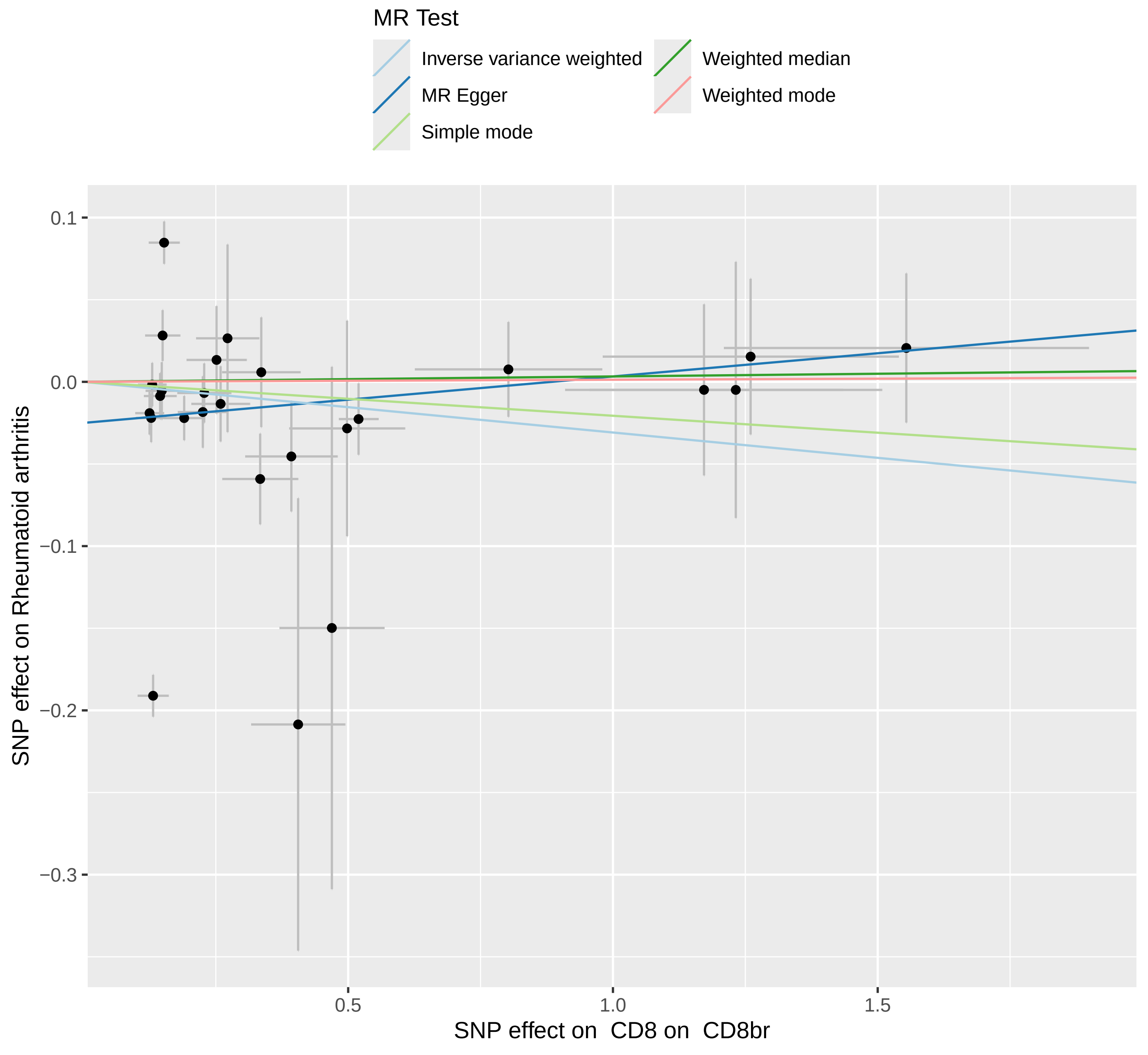
MR Test
Inverse variance weighted
MR Egger
Simple mode
Weighted median
Weighted mode
0.1
0.0
−0.1
−0.2
−0.3
0.5
1.0
1.5
SNP effect on Rheumatoid arthritis
SNP effect on CD8 on CD8br

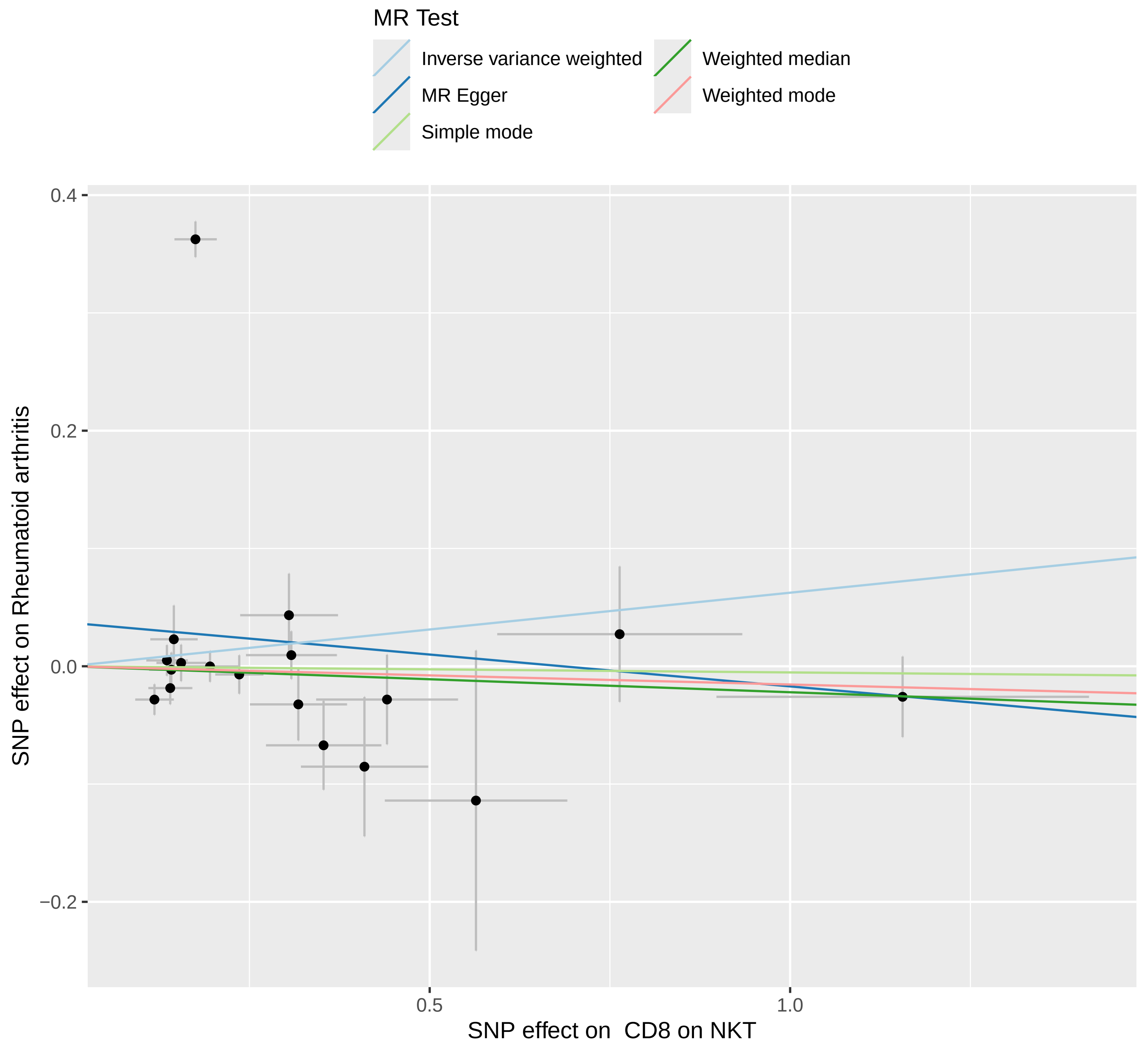
MR Test
Inverse variance weighted
MR Egger
Simple mode
Weighted median
Weighted mode
0.4
0.2
0.0
−0.2
0.5
1.0
SNP effect on Rheumatoid arthritis
SNP effect on CD8 on NKT

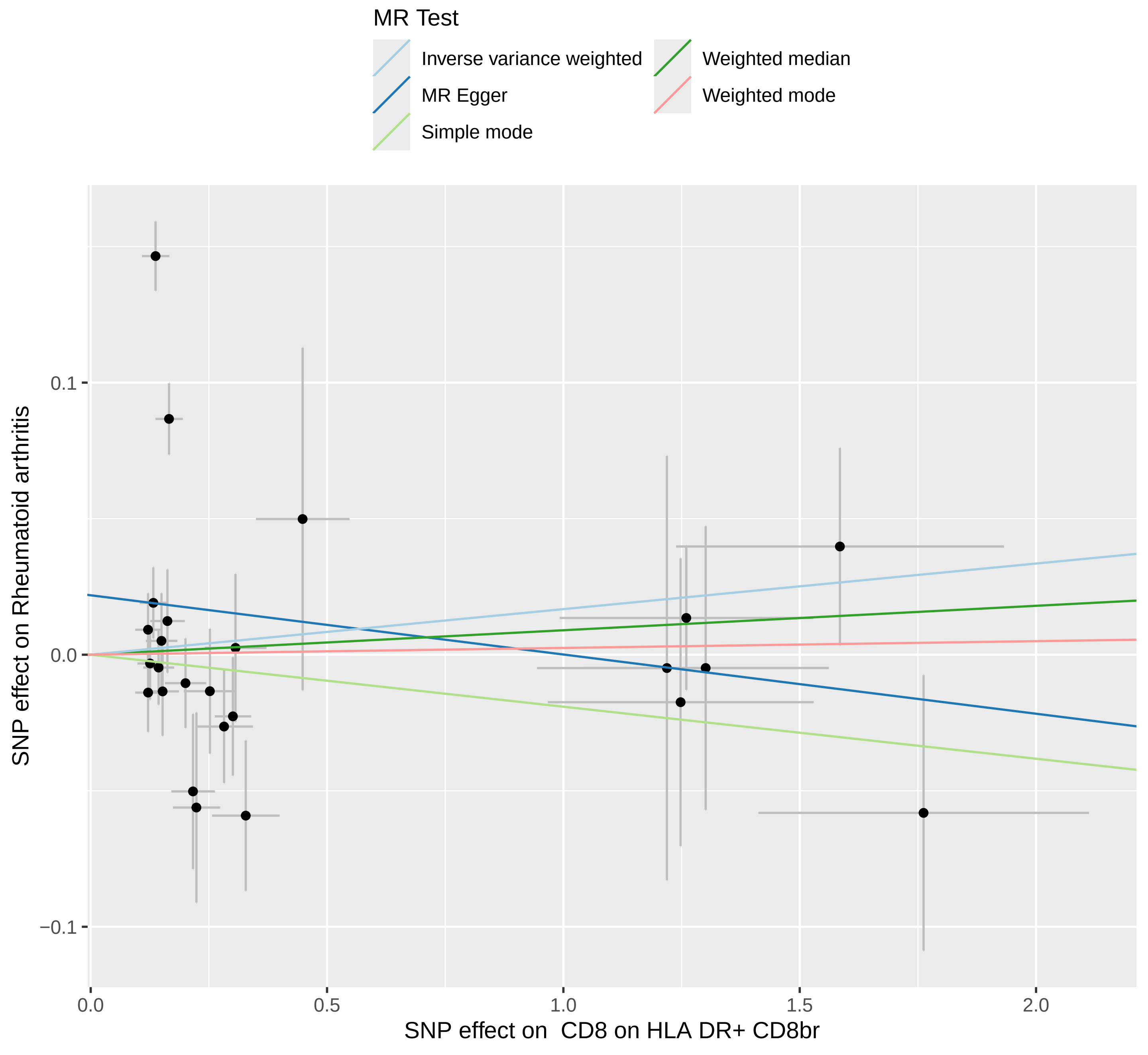
MR Test
Inverse variance weighted
MR Egger
Simple mode
Weighted median
Weighted mode
SNP effect on Rheumatoid arthritis
0.1
0.0
−0.1
0.0
0.5
1.0
1.5
2.0
SNP effect on CD8 on HLA DR+ CD8br

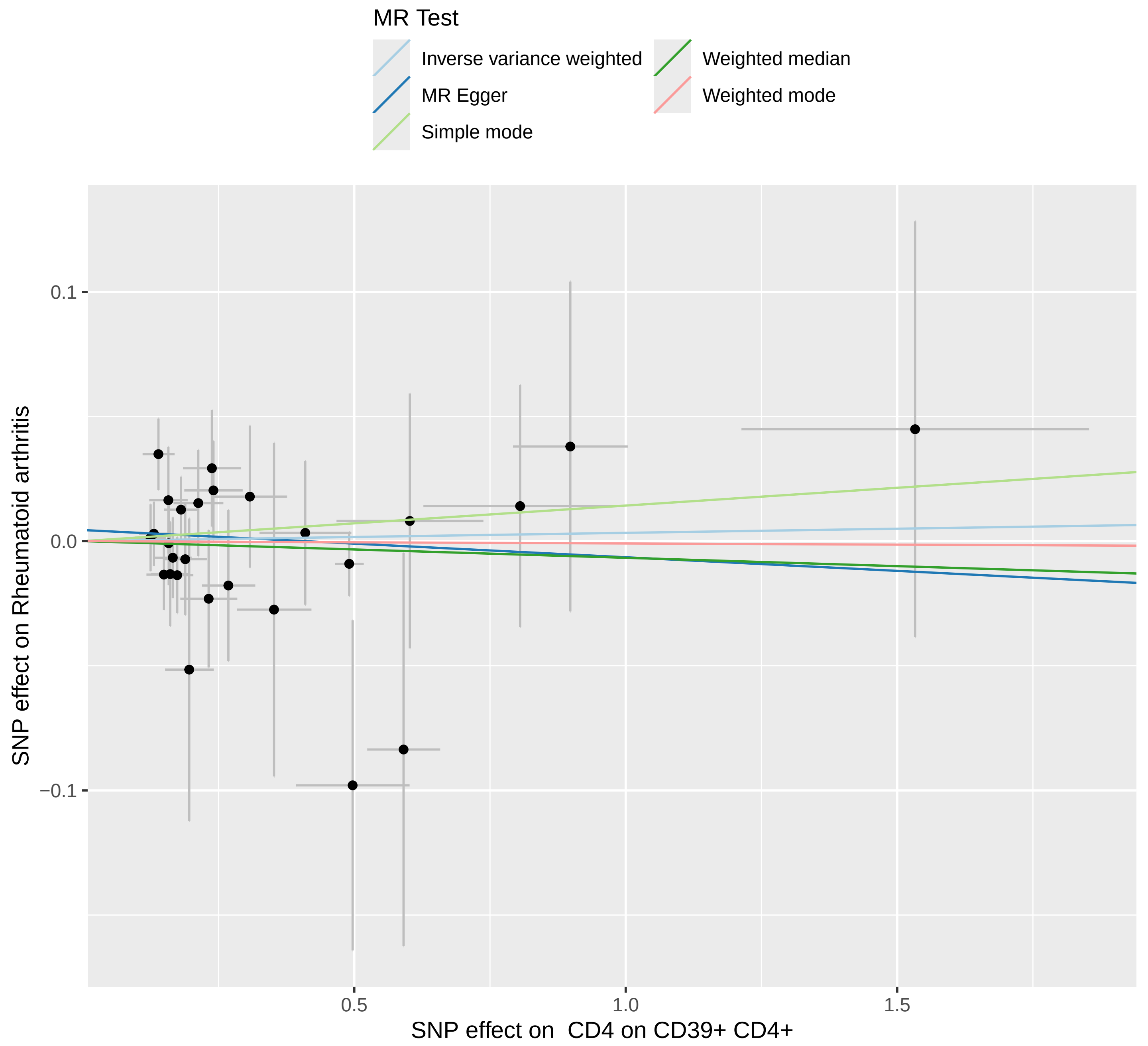
MR Test
Inverse variance weighted
MR Egger
Simple mode
Weighted median
Weighted mode
0.1
0.0
−0.1
0.5
1.0
1.5
SNP effect on Rheumatoid arthritis
SNP effect on CD4 on CD39+ CD4+

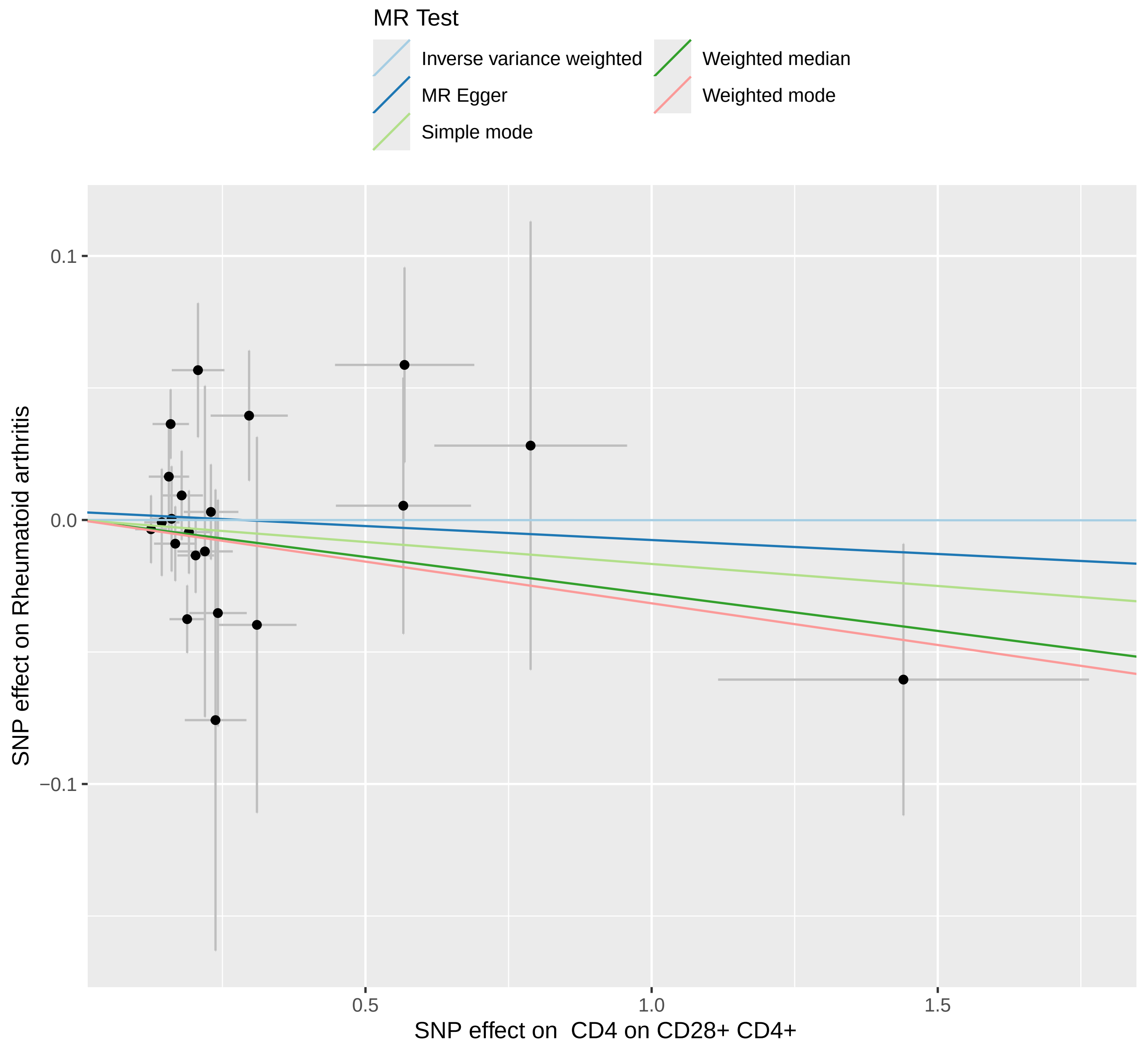

MR Test
Inverse variance weighted
MR Egger
Simple mode
Weighted median
Weighted mode
0.1
0.0
−0.1
0.5
1.0
1.5
SNP effect on Rheumatoid arthritis
SNP effect on CD4 on CD28+ CD4+

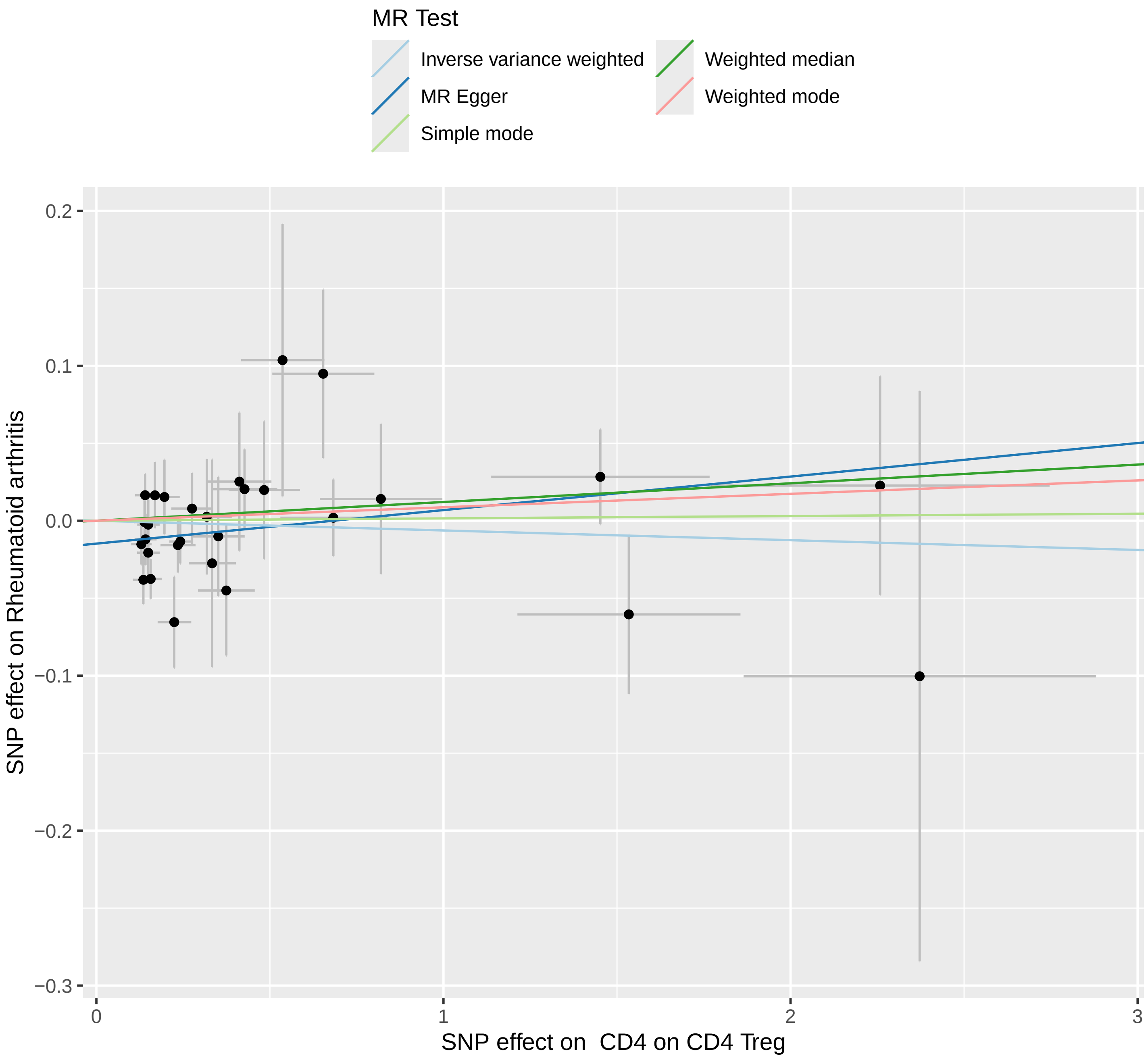
MR Test
Inverse variance weighted
MR Egger
Simple mode
Weighted median
Weighted mode
0.2
0.1
0.0
−0.1
−0.2
−0.3
0
1
2
3
SNP effect on Rheumatoid arthritis
SNP effect on CD4 on CD4 Treg

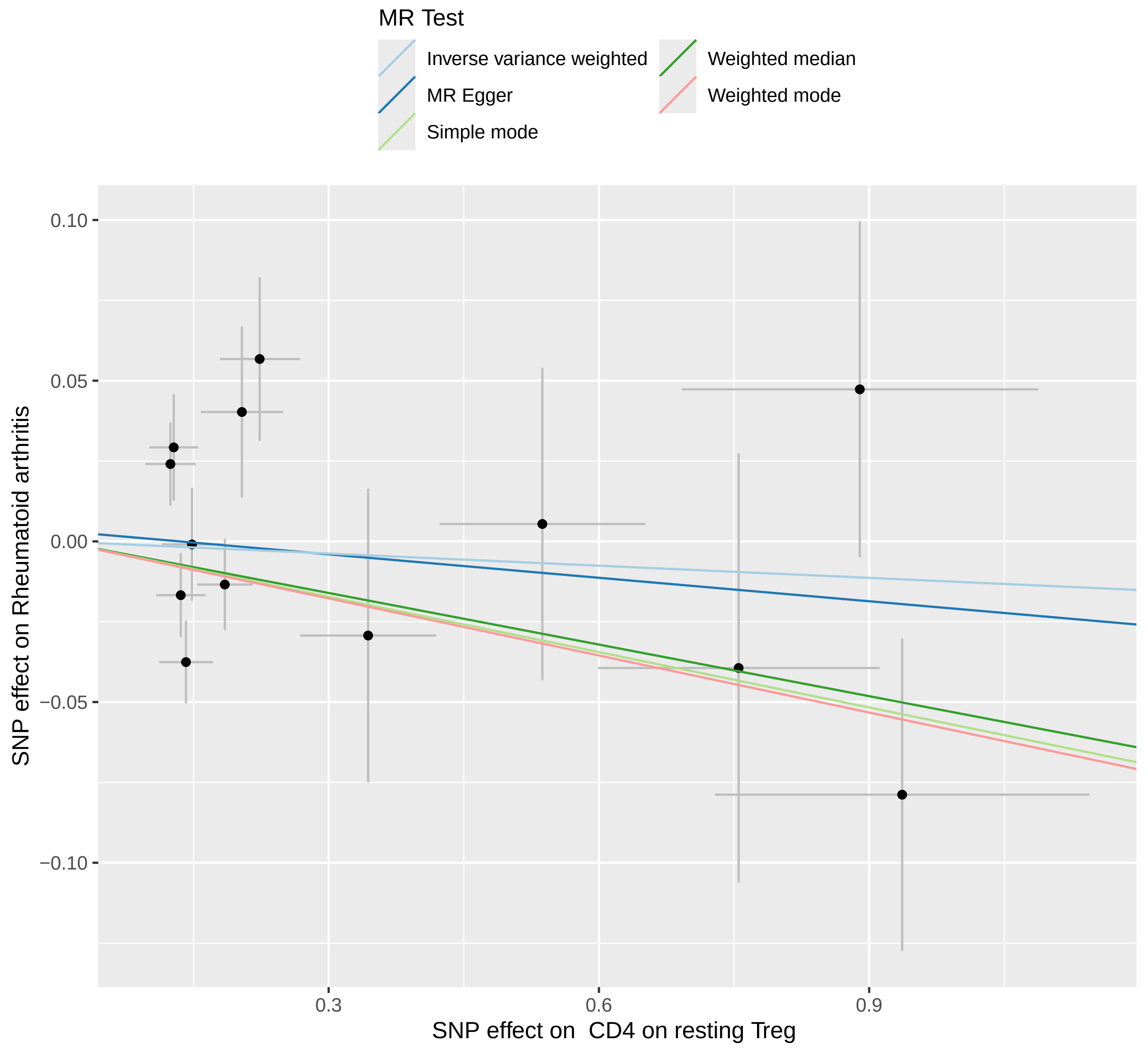
MR Test
Inverse variance weighted
MR Egger
Simple mode
Weighted median
Weighted mode
0.10
0.05
0.00
−0.05
−0.10
0.3
0.6
0.9
SNP effect on Rheumatoid arthritis
SNP effect on CD4 on resting Treg

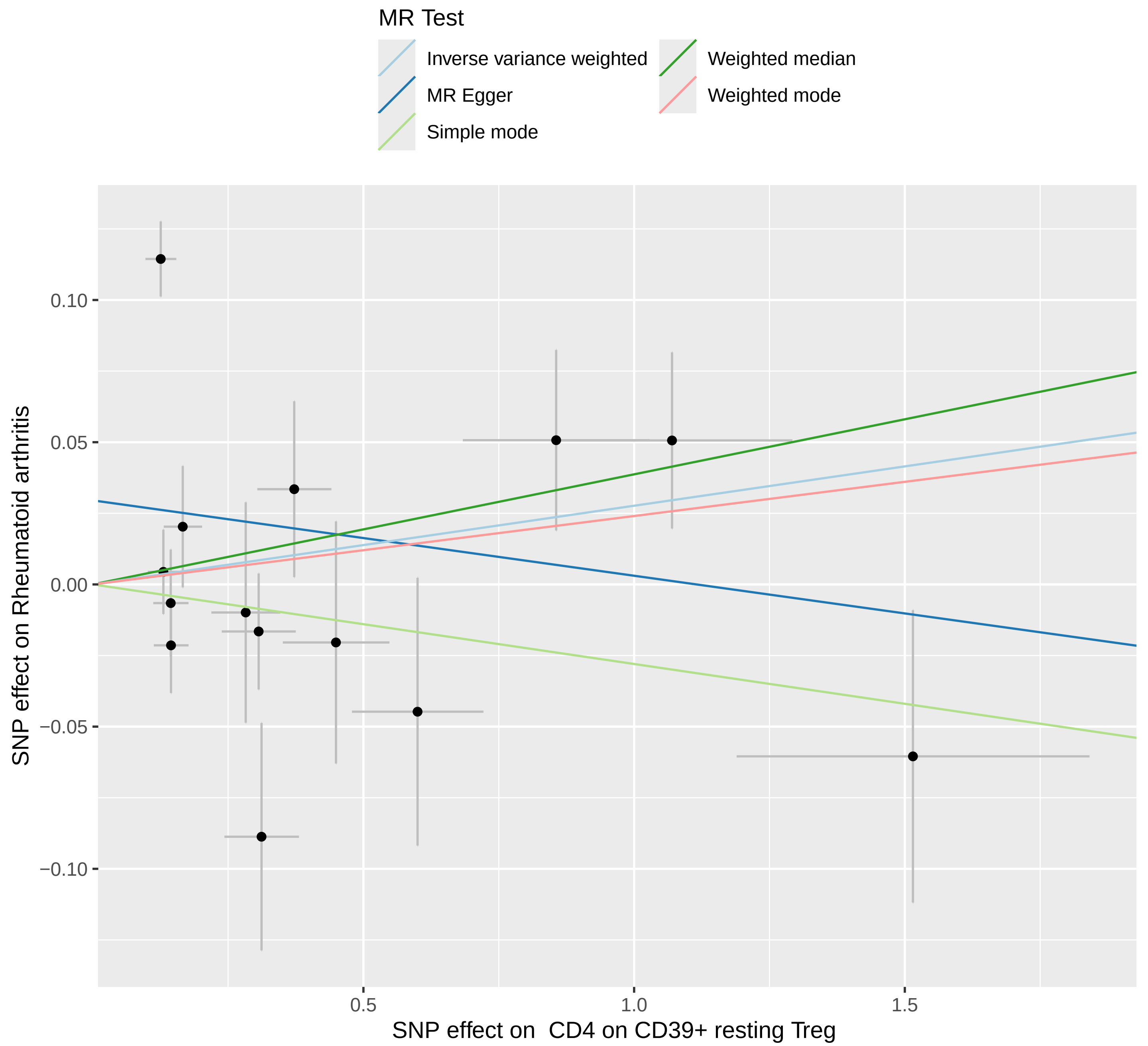
MR Test
Inverse variance weighted
MR Egger
Simple mode
Weighted median
Weighted mode
0.10
0.05
0.00
−0.05
−0.10
0.5
1.0
1.5
SNP effect on Rheumatoid arthritis
SNP effect on CD4 on CD39+ resting Treg

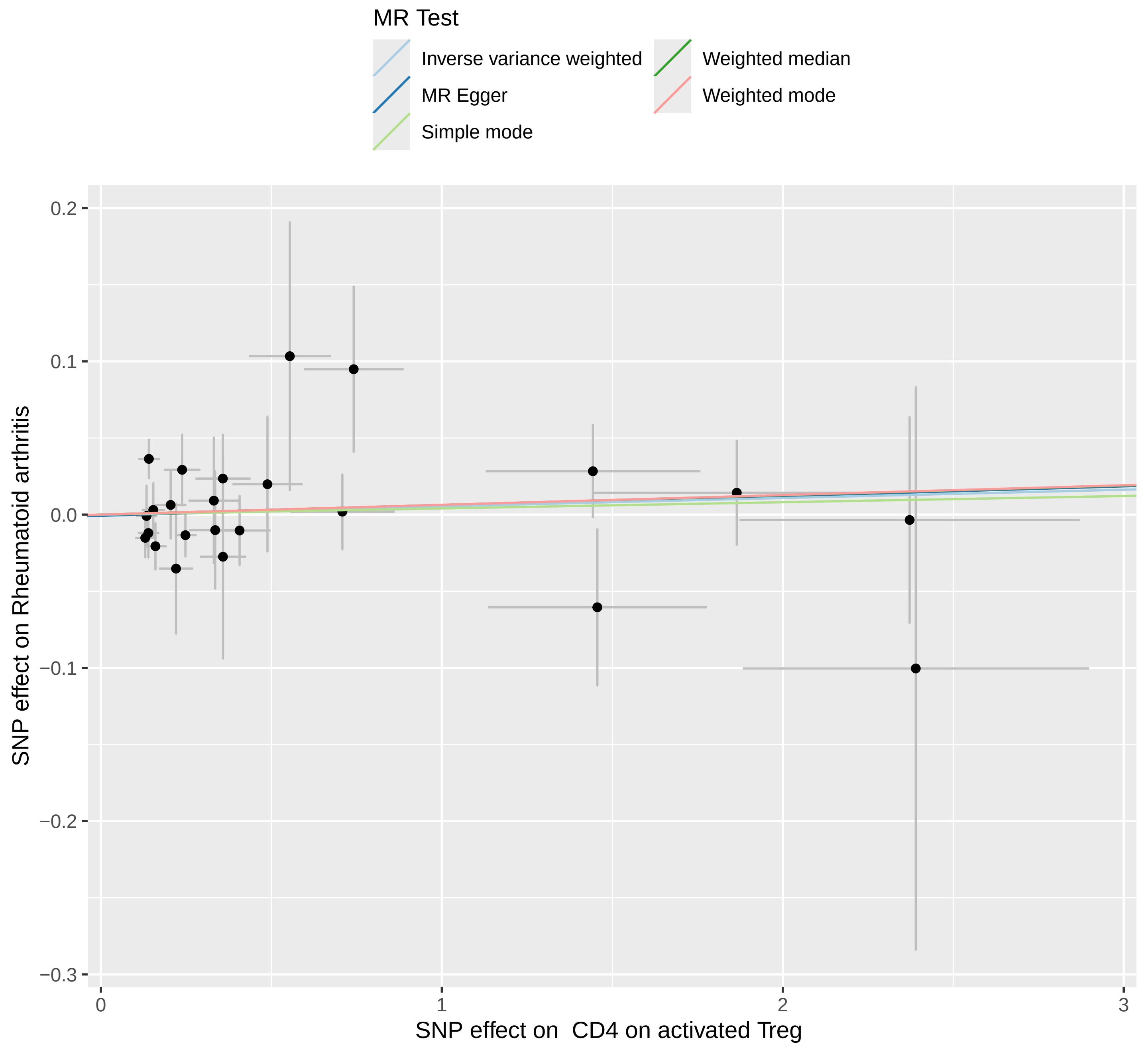

MR Test
Inverse variance weighted
MR Egger
Simple mode
Weighted median
Weighted mode
0.2
0.1
0.0
−0.1
−0.2
−0.3
0
1
2
3
SNP effect on Rheumatoid arthritis
SNP effect on CD4 on activated Treg

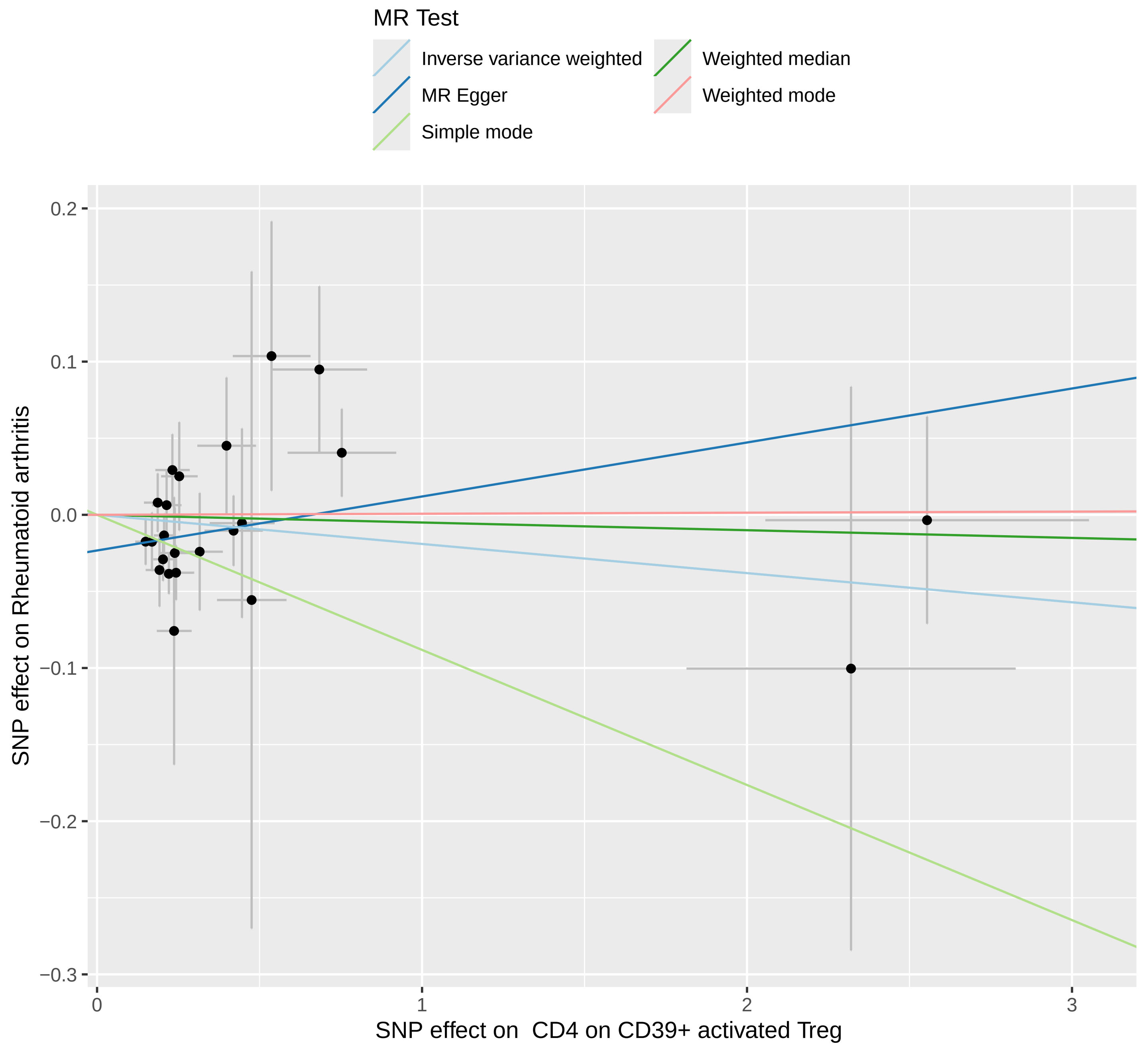

MR Test
Inverse variance weighted
MR Egger
Simple mode
Weighted median
Weighted mode
0.2
0.1
0.0
−0.1
−0.2
−0.3
0
1
2
3
SNP effect on Rheumatoid arthritis
SNP effect on CD4 on CD39+ activated Treg

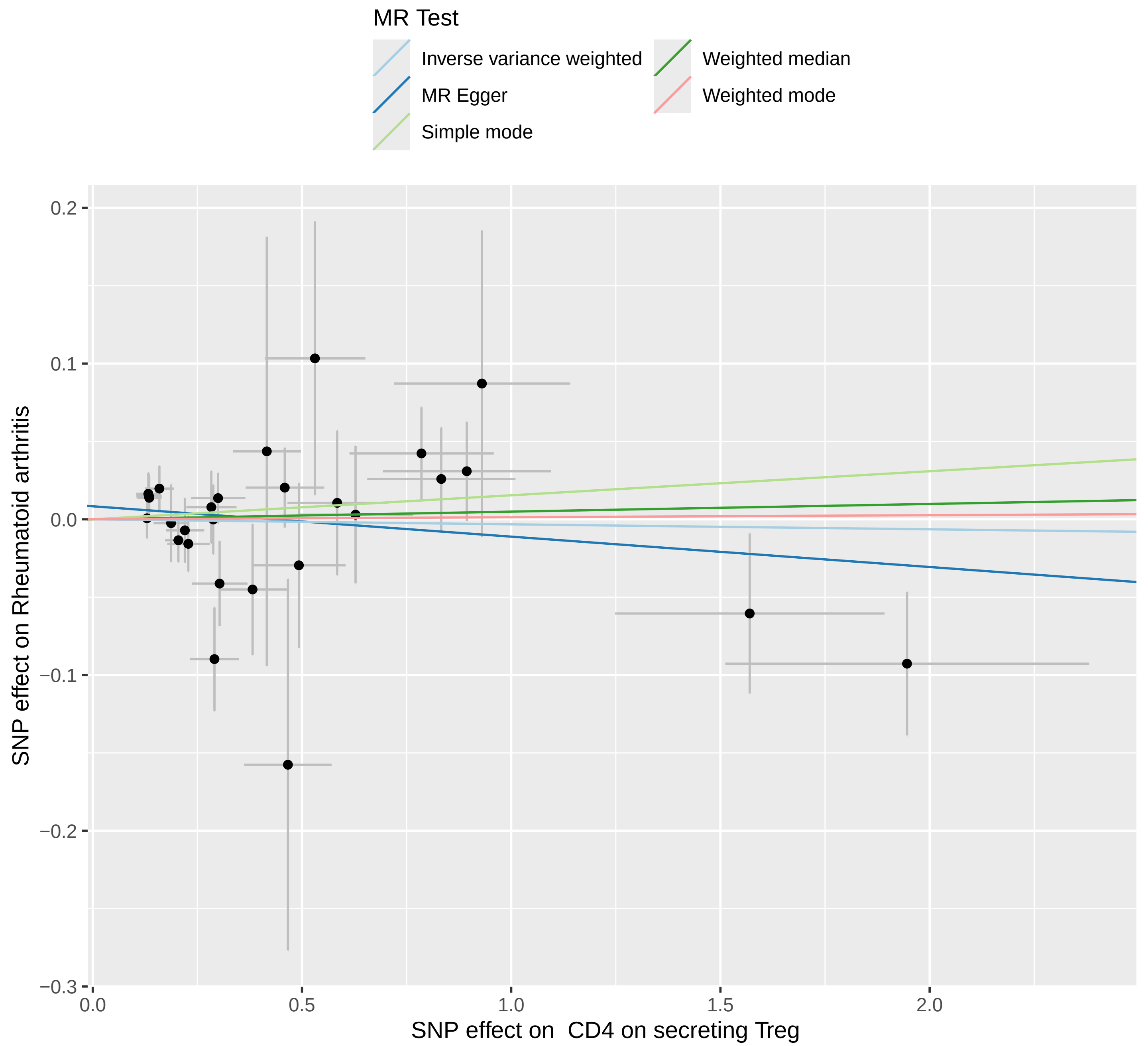

MR Test
Inverse variance weighted
MR Egger
Simple mode
Weighted median
Weighted mode
0.2
0.1
0.0
−0.1
−0.2
−0.3
0.0
0.5
1.0
1.5
2.0
SNP effect on Rheumatoid arthritis
SNP effect on CD4 on secreting Treg

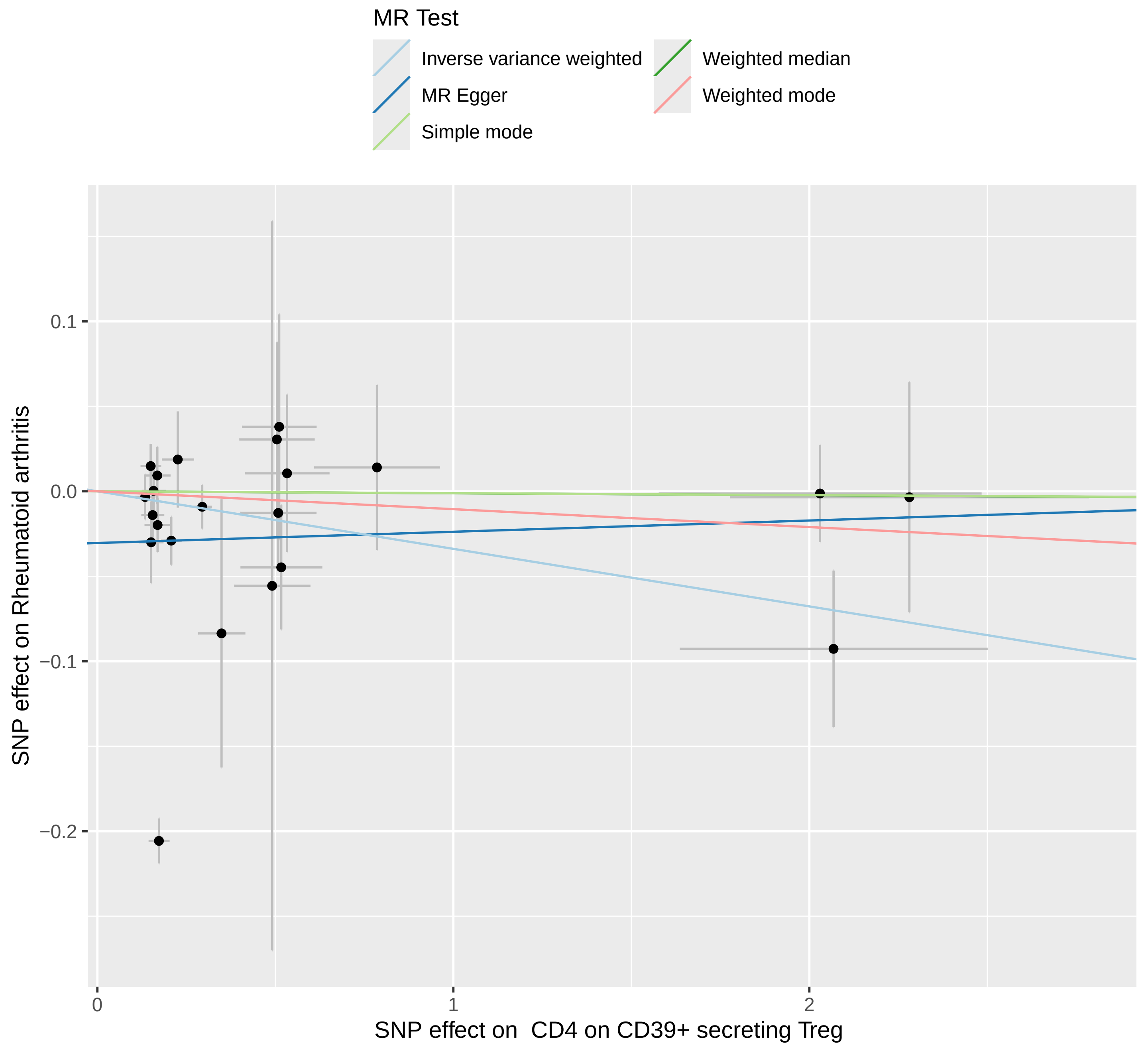

MR Test
Inverse variance weighted
MR Egger
Simple mode
Weighted median
Weighted mode
0.1
0.0
−0.1
−0.2
0
1
2
SNP effect on Rheumatoid arthritis
SNP effect on CD4 on CD39+ secreting Treg

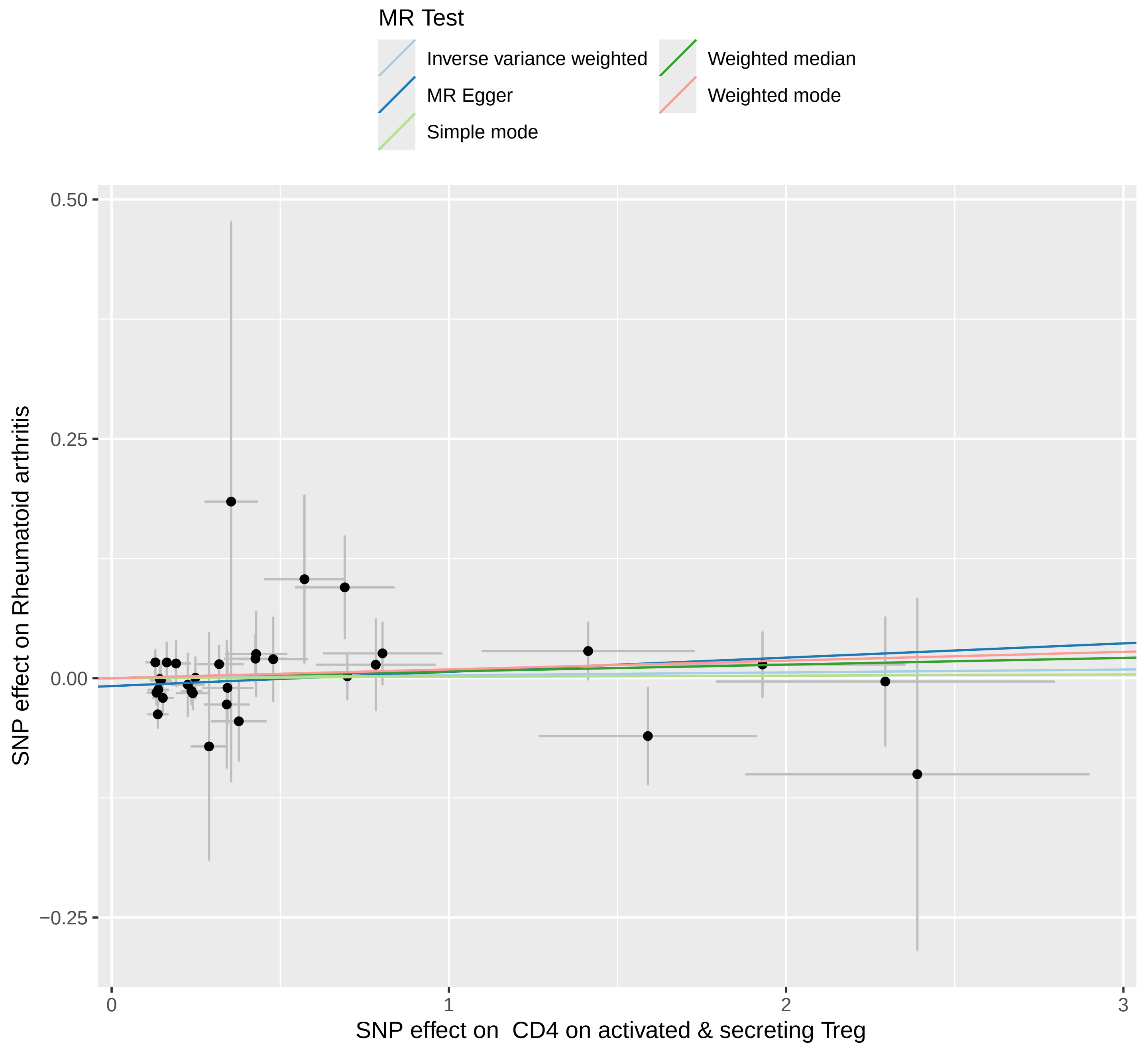

MR Test
Inverse variance weighted
MR Egger
Simple mode
Weighted median
Weighted mode
0.50
0.25
0.00
−0.25
SNP effect on Rheumatoid arthritis
0
1
2
3
SNP effect on CD4 on activated & secreting Treg

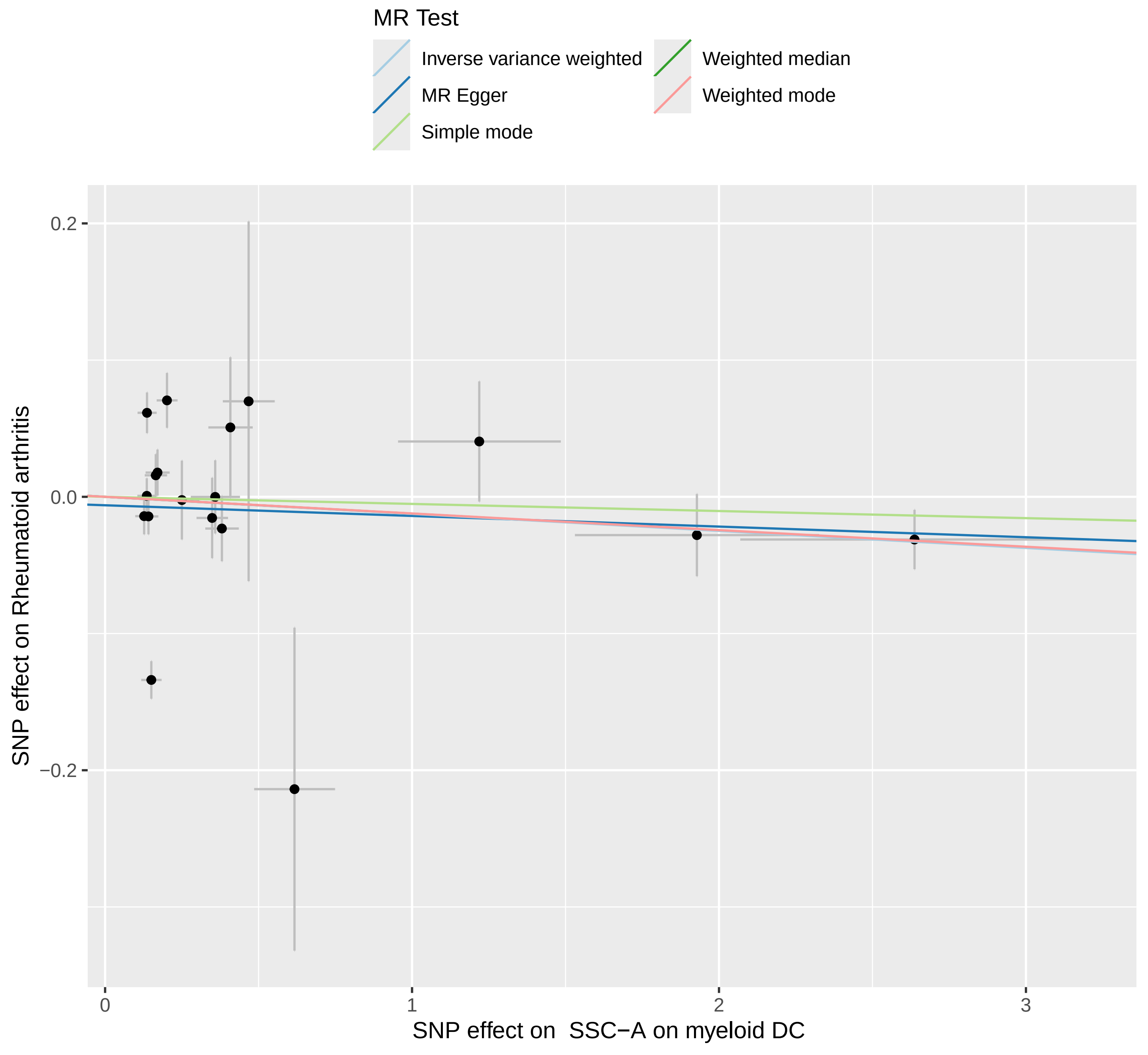
MR Test
Inverse variance weighted
MR Egger
Simple mode
Weighted median
Weighted mode
0.2
0.0
−0.2
0
1
2
3
SNP effect on Rheumatoid arthritis
SNP effect on SSC−A on myeloid DC

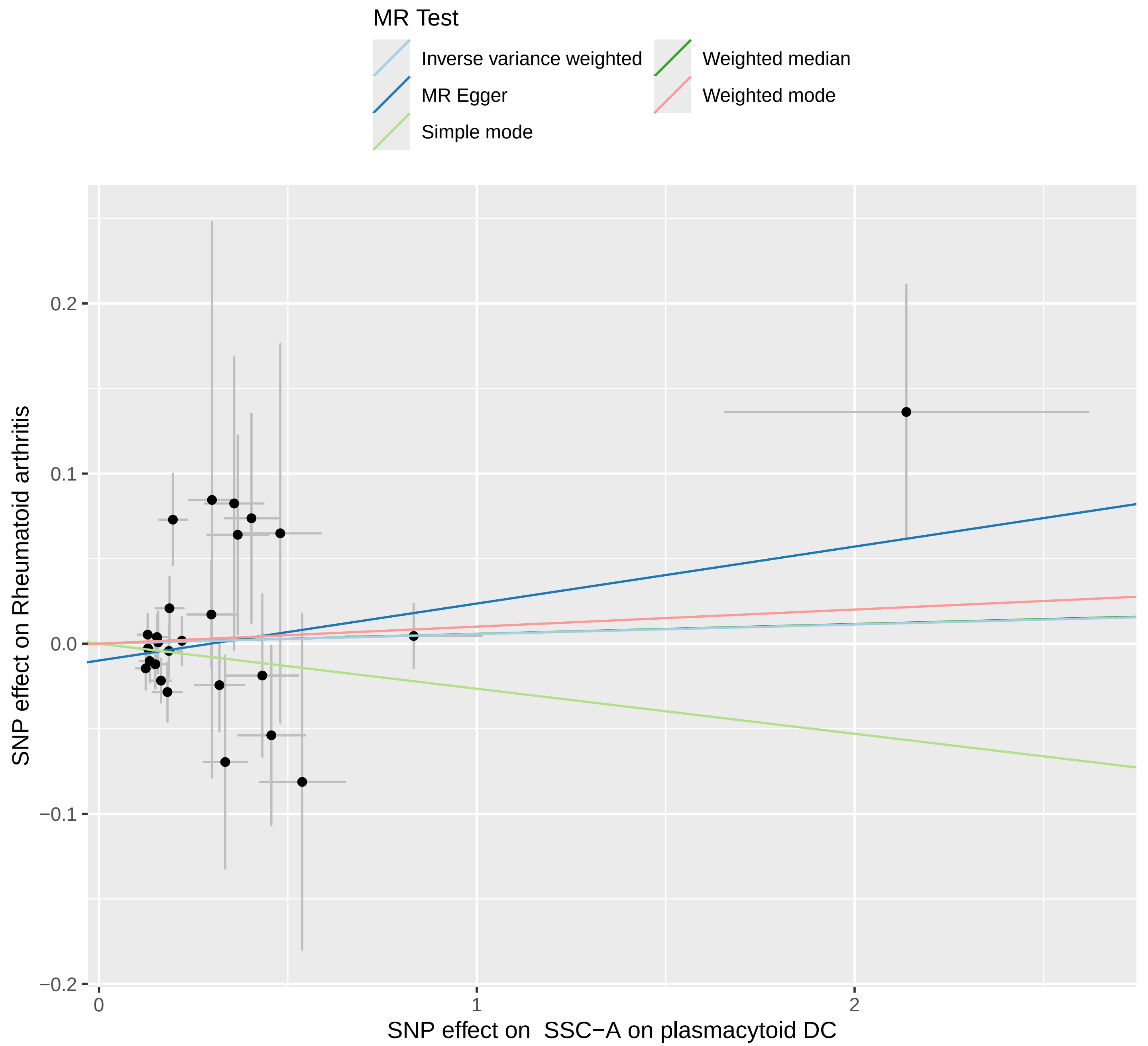
MR Test
Inverse variance weighted
MR Egger
Simple mode
Weighted median
Weighted mode
SNP effect on Rheumatoid arthritis
0.2
0.1
0.0
−0.1
−0.2
0
1
2
SNP effect on SSC−A on plasmacytoid DC

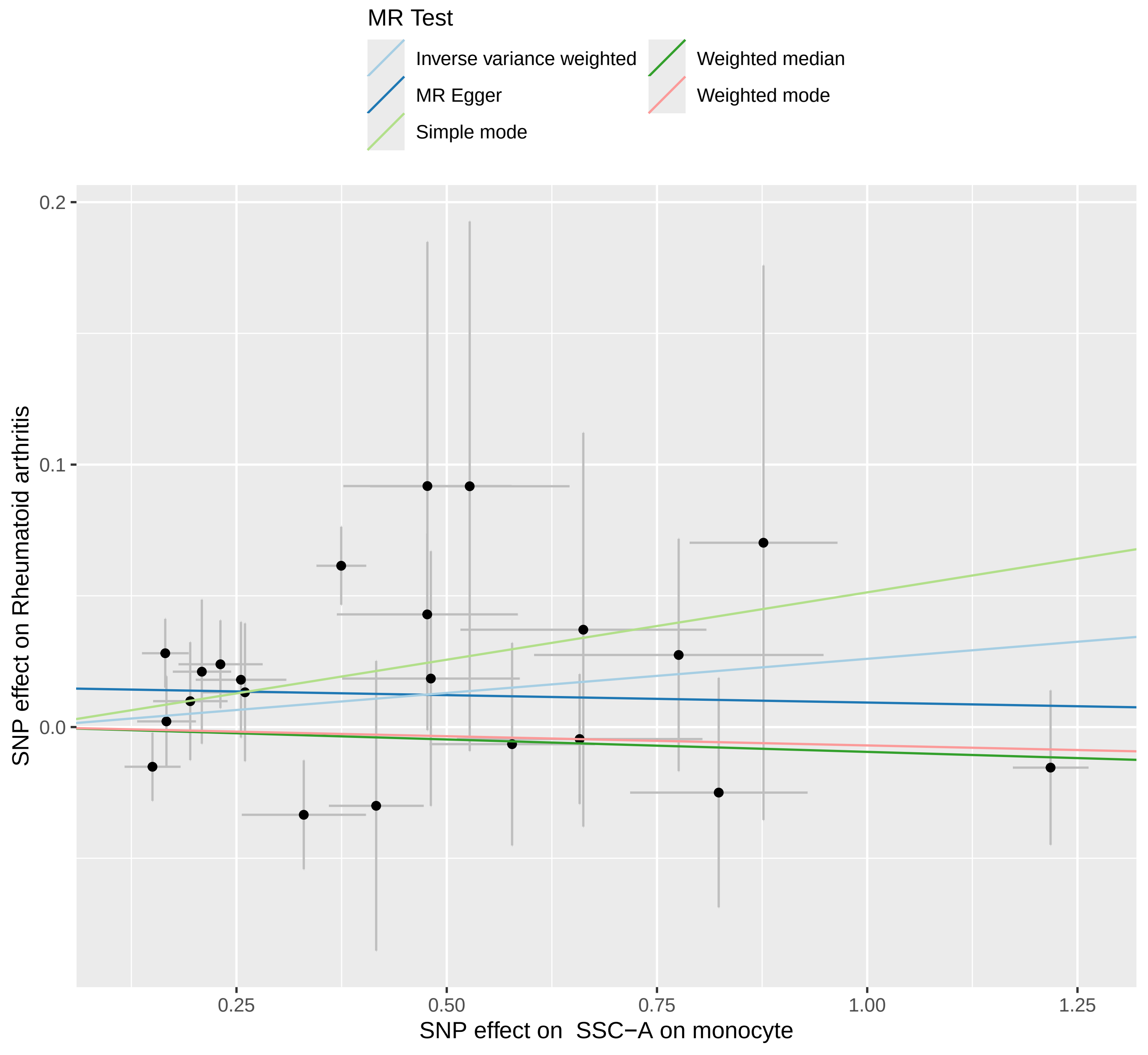

MR Test
Inverse variance weighted
MR Egger
Simple mode
Weighted median
Weighted mode
0.2
0.1
0.0
SNP effect on Rheumatoid arthritis
0.25
0.50
0.75
1.00
1.25
SNP effect on SSC−A on monocyte

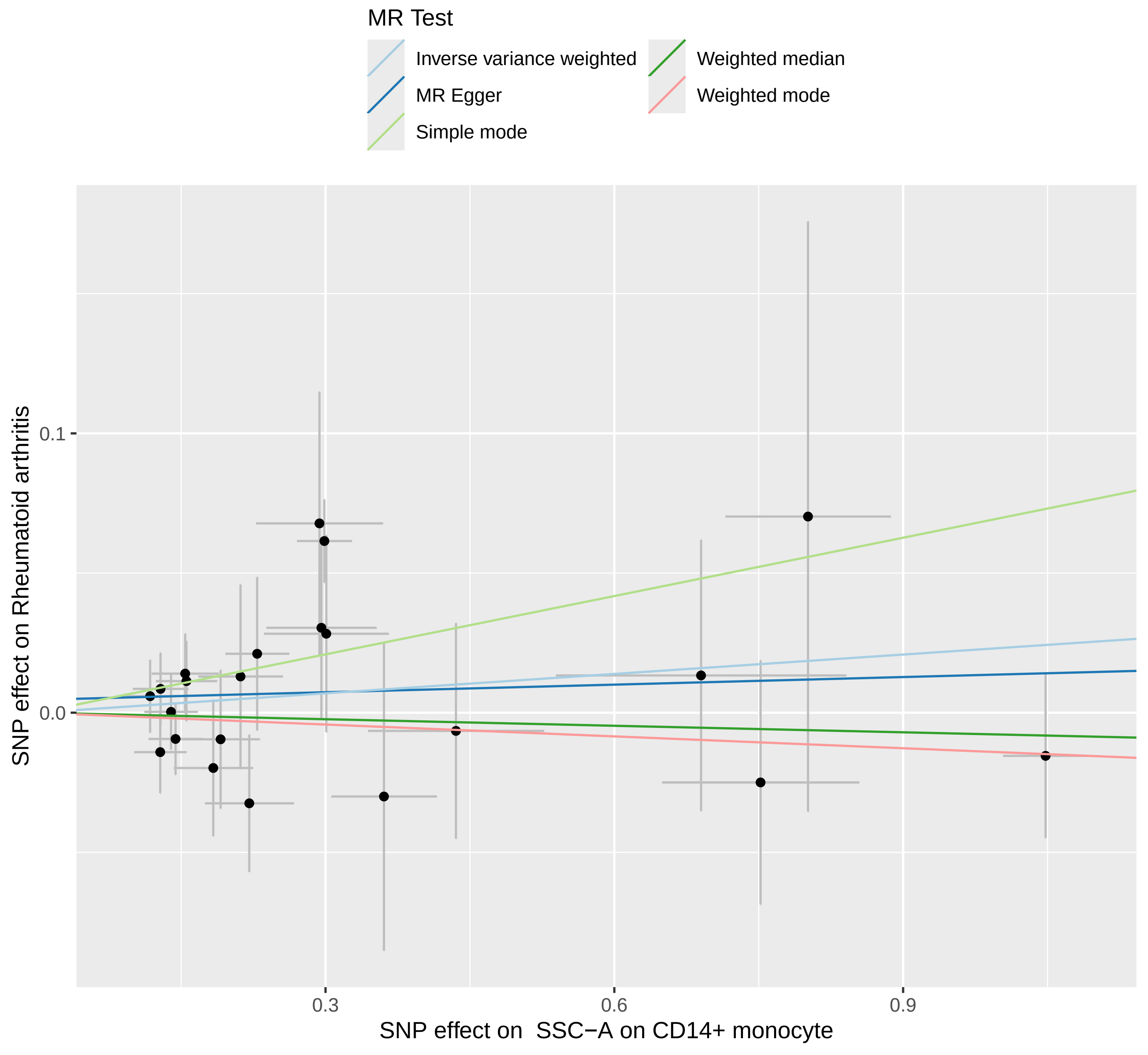

MR Test
Inverse variance weighted
MR Egger
Simple mode
Weighted median
Weighted mode
0.1
0.0
0.3
0.6
0.9
SNP effect on Rheumatoid arthritis
SNP effect on SSC−A on CD14+ monocyte

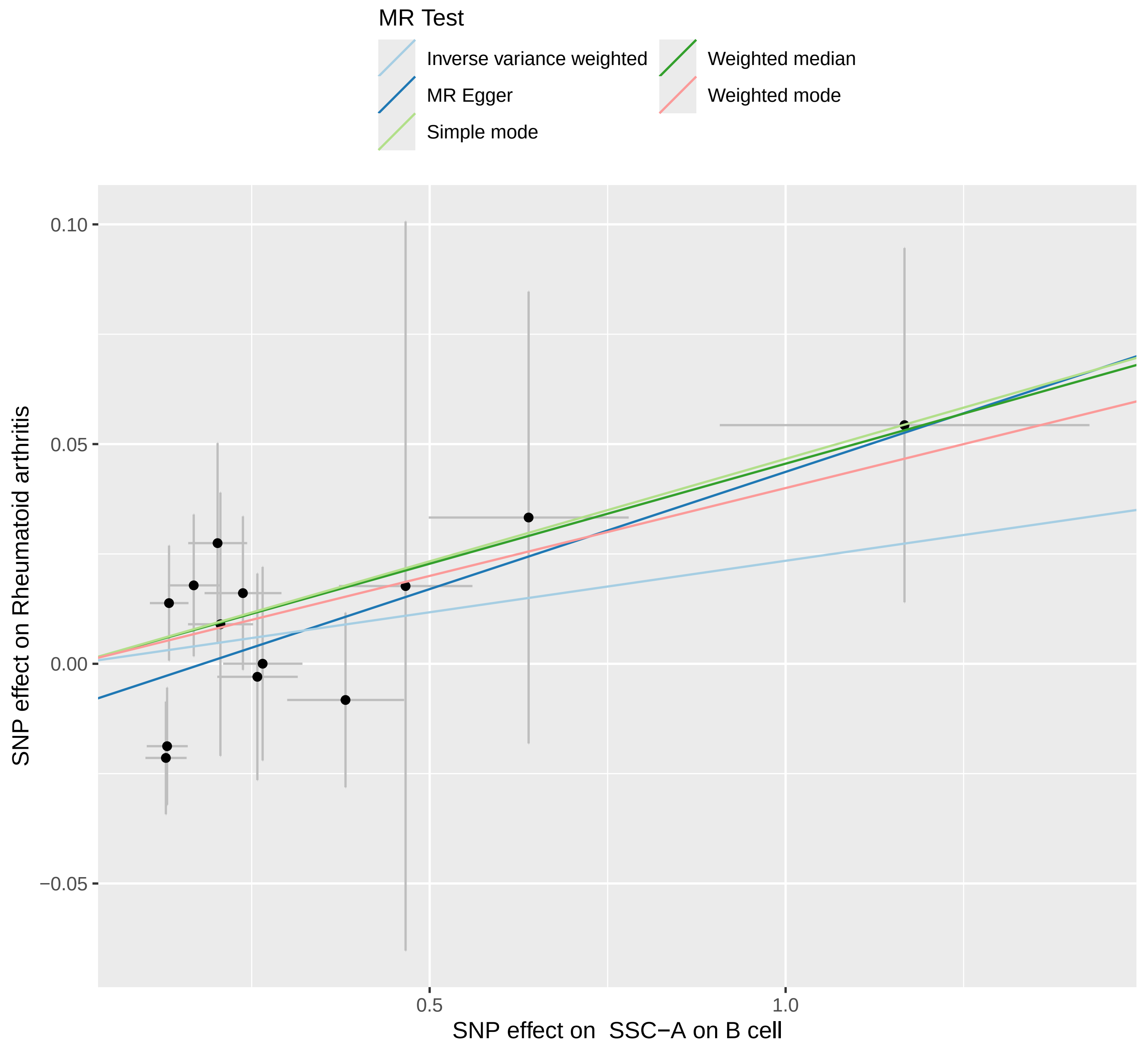

MR Test
Inverse variance weighted
MR Egger
Simple mode
Weighted median
Weighted mode
0.10
0.05
0.00
−0.05
0.5
1.0
SNP effect on SSC−A on B cell
SNP effect on Rheumatoid arthritis

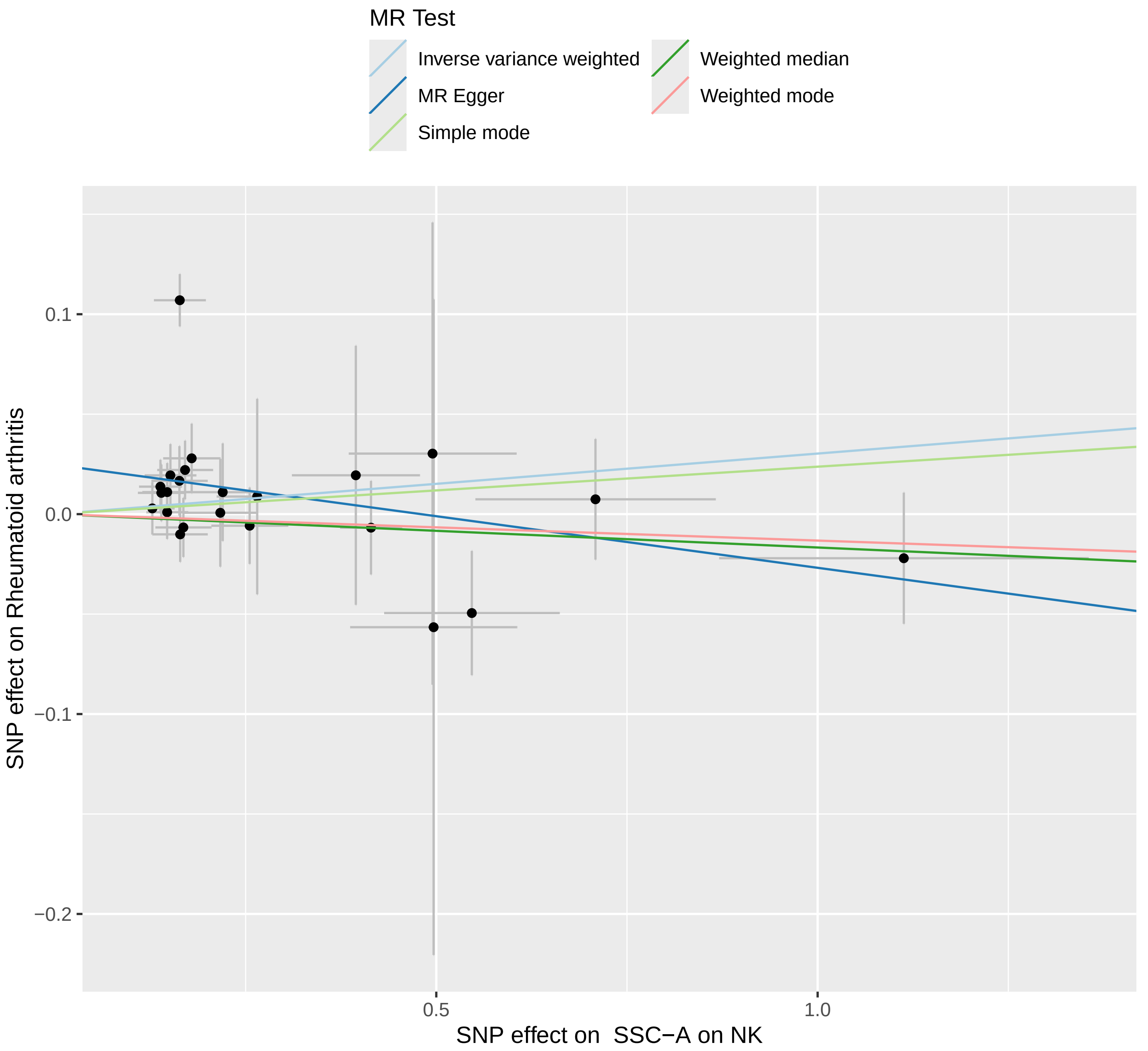
MR Test
Inverse variance weighted
MR Egger
Simple mode
Weighted median
Weighted mode
0.1
0.0
−0.1
−0.2
0.5
1.0
SNP effect on Rheumatoid arthritis
SNP effect on SSC−A on NK

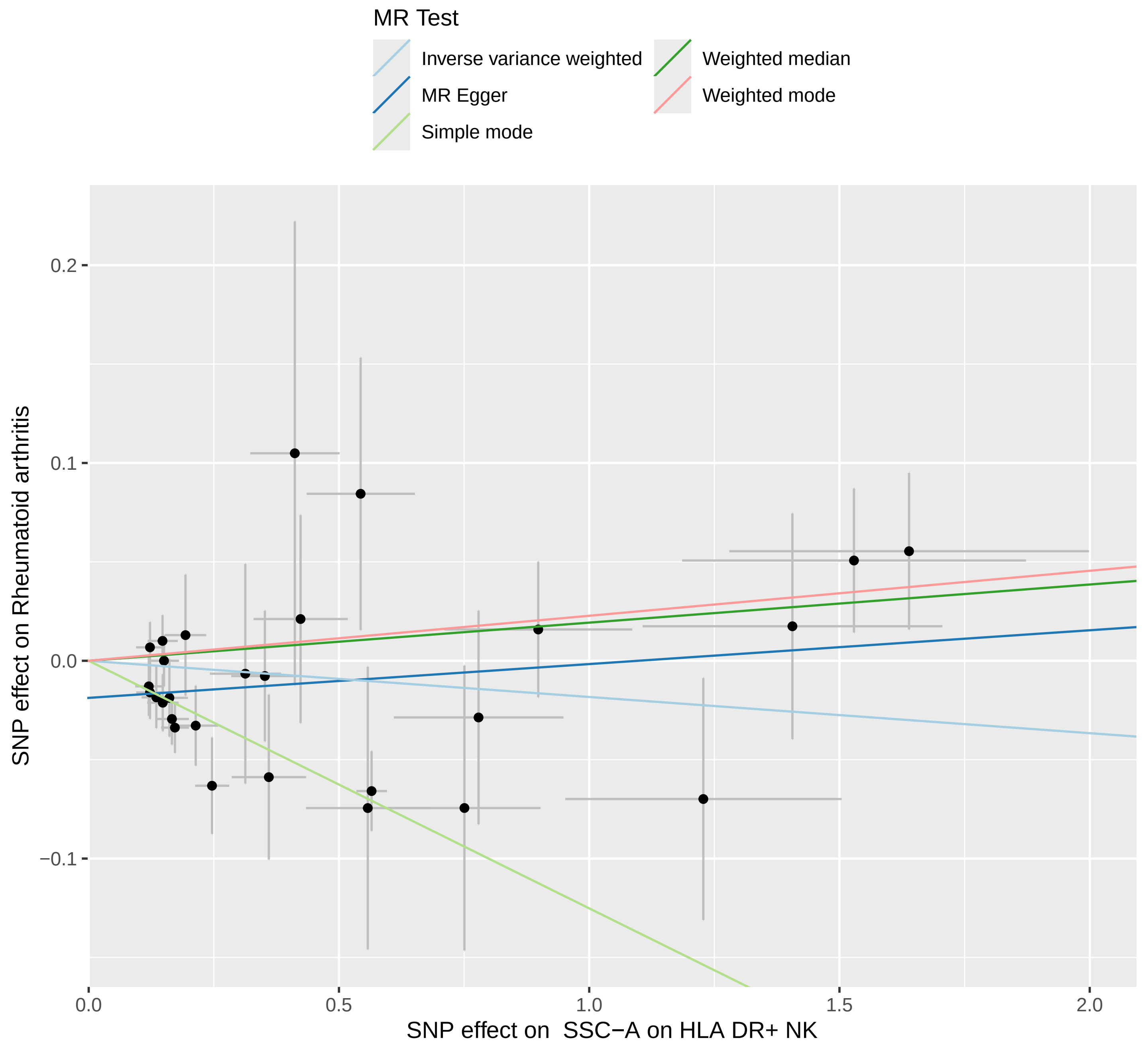

MR Test
Inverse variance weighted
MR Egger
Simple mode
Weighted median
Weighted mode
SNP effect on Rheumatoid arthritis
0.2
0.1
0.0
−0.1
0.0
0.5
1.0
1.5
2.0
SNP effect on SSC−A on HLA DR+ NK

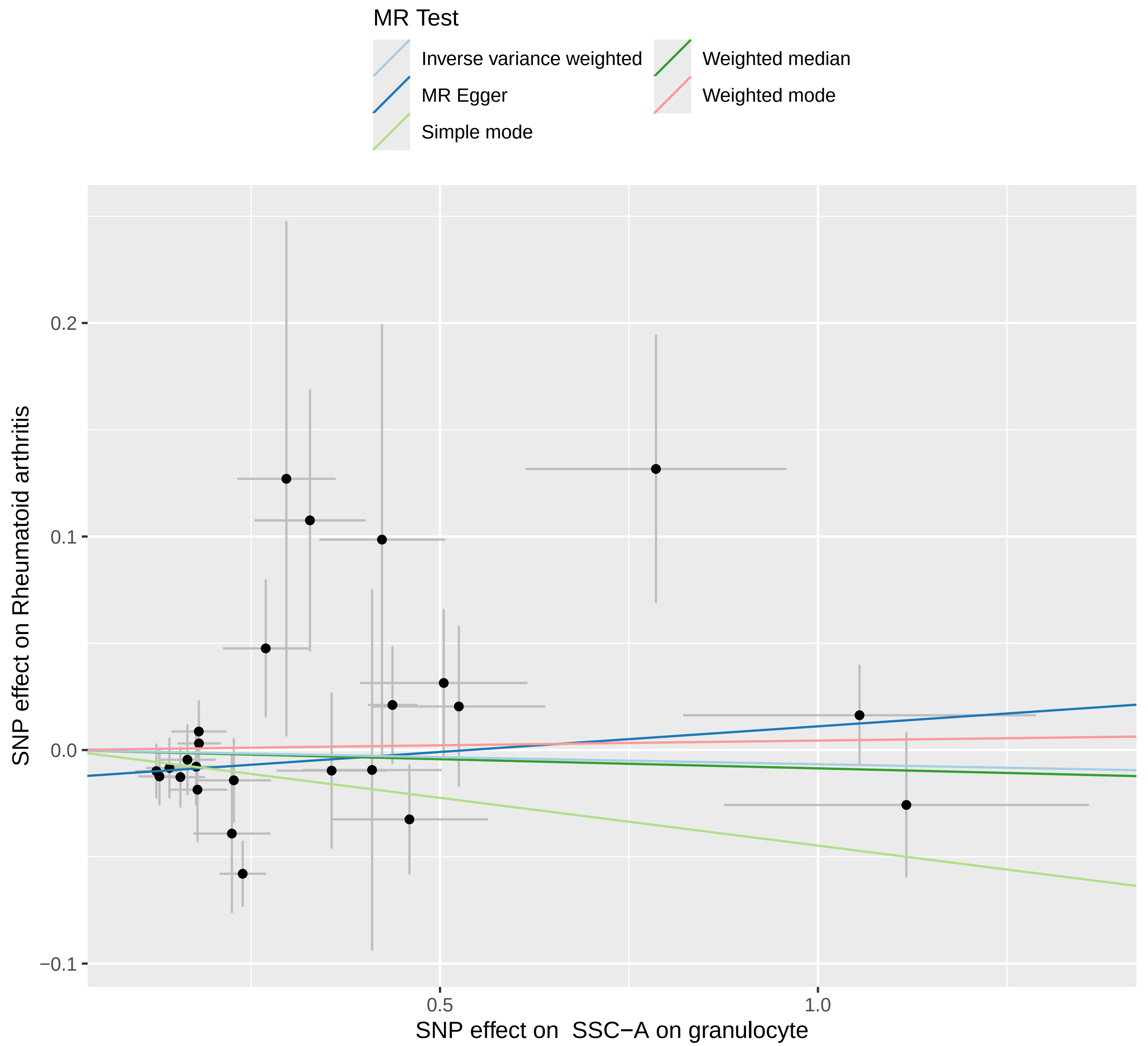
MR Test
Inverse variance weighted
MR Egger
Simple mode
Weighted median
Weighted mode
0.2
0.1
0.0
−0.1
0.5
1.0
SNP effect on Rheumatoid arthritis
SNP effect on SSC−A on granulocyte

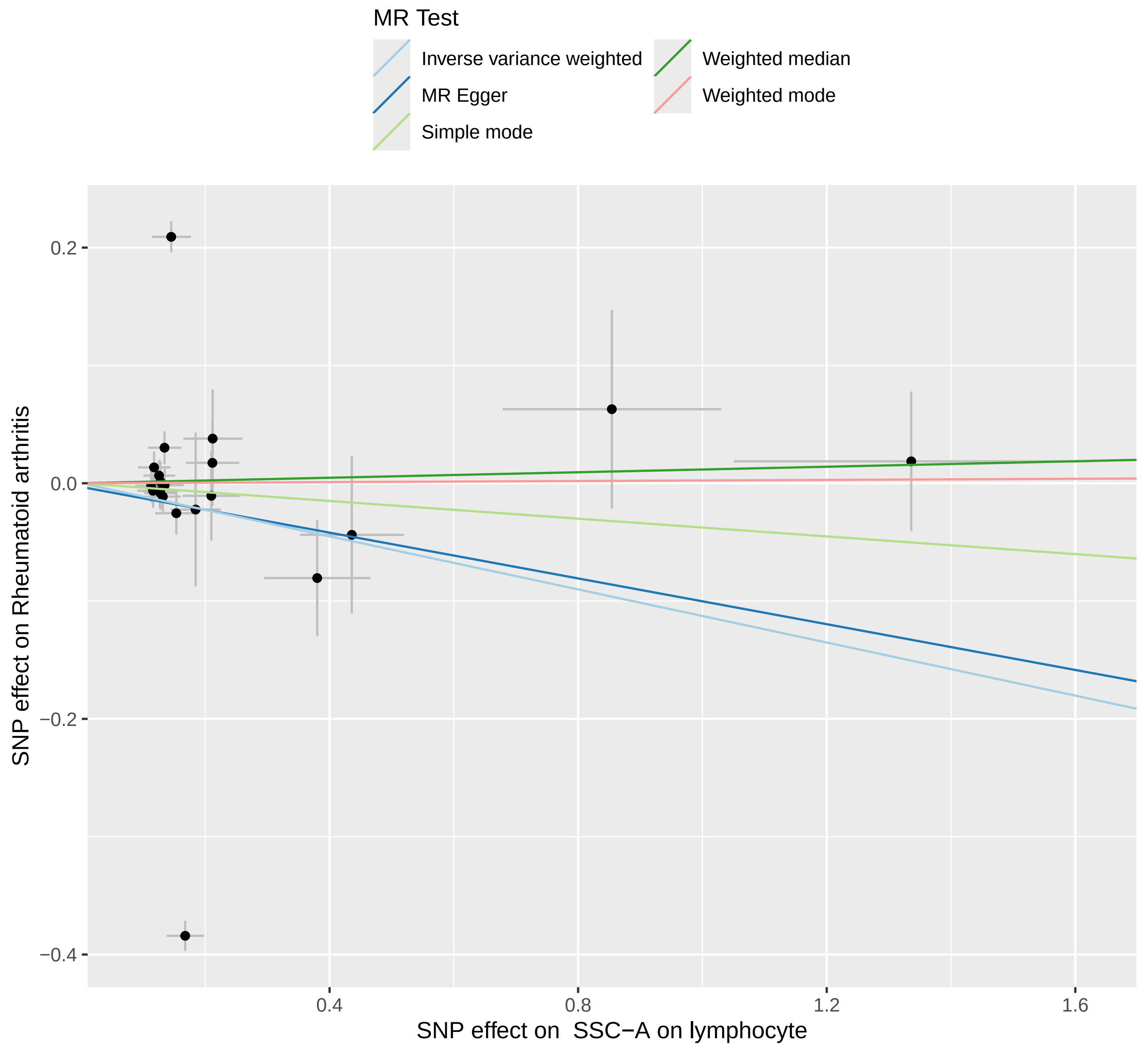

MR Test
Inverse variance weighted
MR Egger
Simple mode
Weighted median
Weighted mode
0.2
0.0
−0.2
−0.4
SNP effect on Rheumatoid arthritis
0.4
0.8
1.2
1.6
SNP effect on SSC−A on lymphocyte

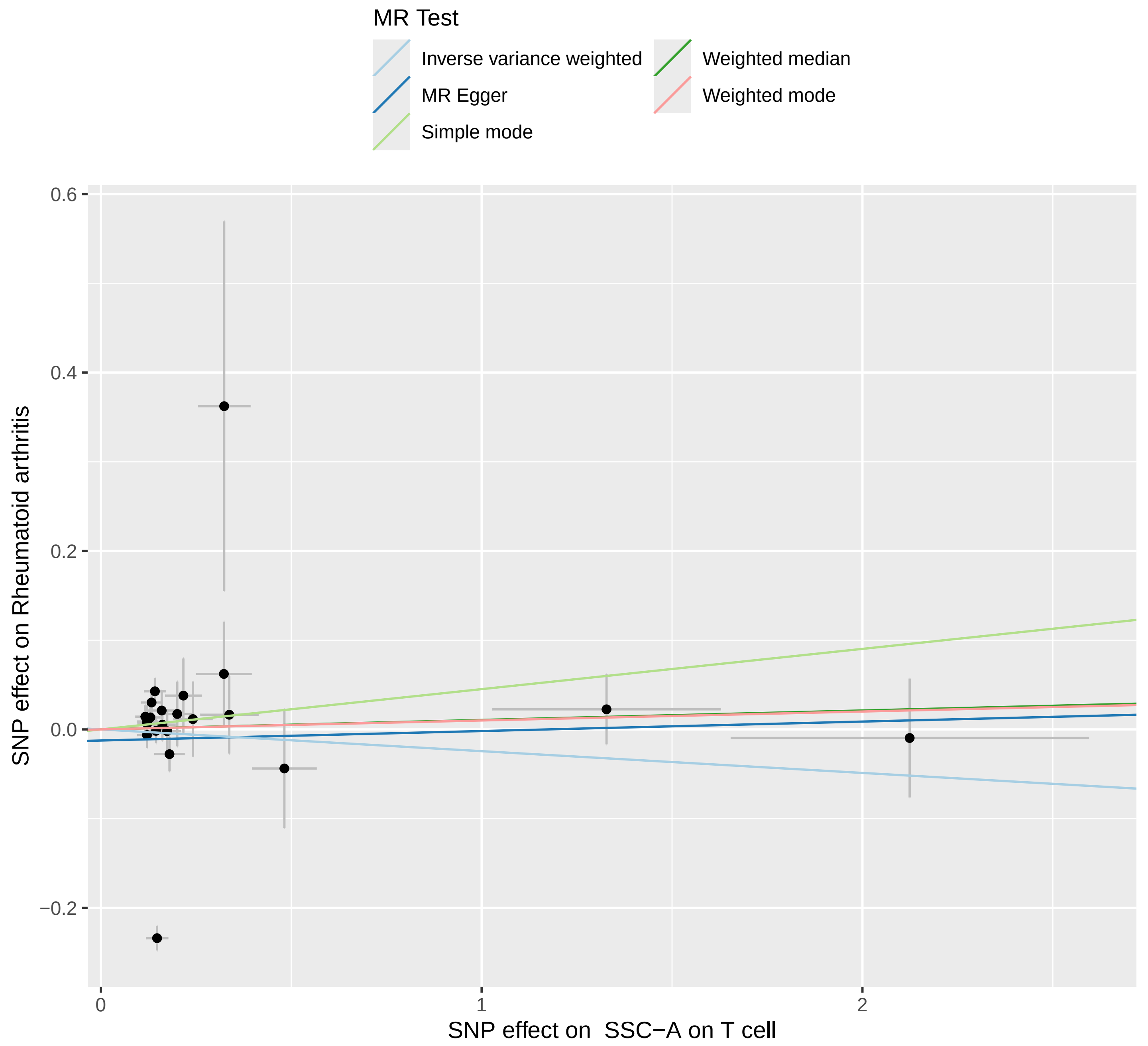
MR Test
Inverse variance weighted
MR Egger
Simple mode
Weighted median
Weighted mode
0.6
0.4
0.2
0.0
−0.2
0
1
2
SNP effect on Rheumatoid arthritis
SNP effect on SSC−A on T cell

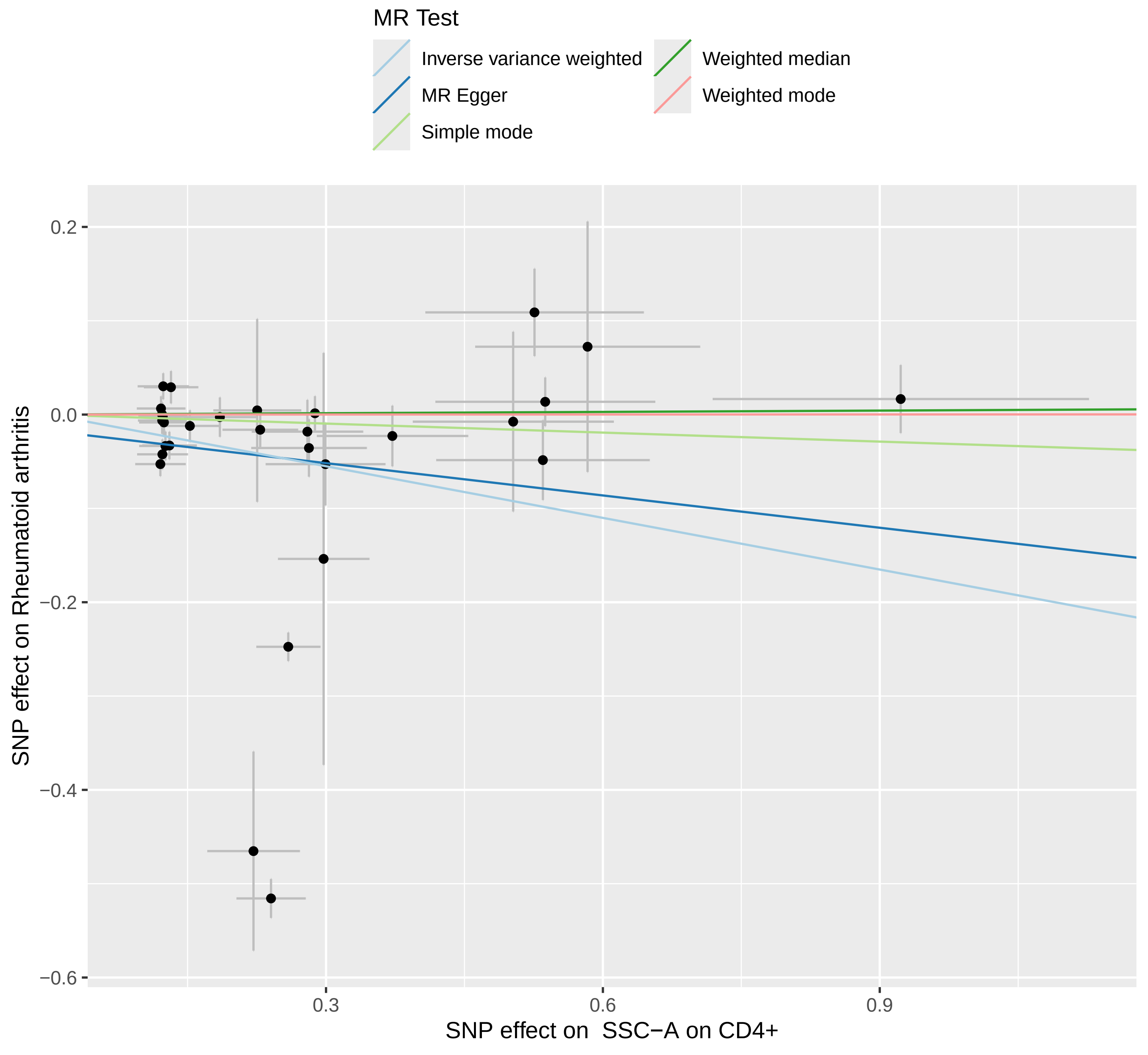
MR Test
Inverse variance weighted
MR Egger
Simple mode
Weighted median
Weighted mode
0.2
0.0
−0.2
−0.4
−0.6
SNP effect on Rheumatoid arthritis
0.3
0.6
0.9
SNP effect on SSC−A on CD4+

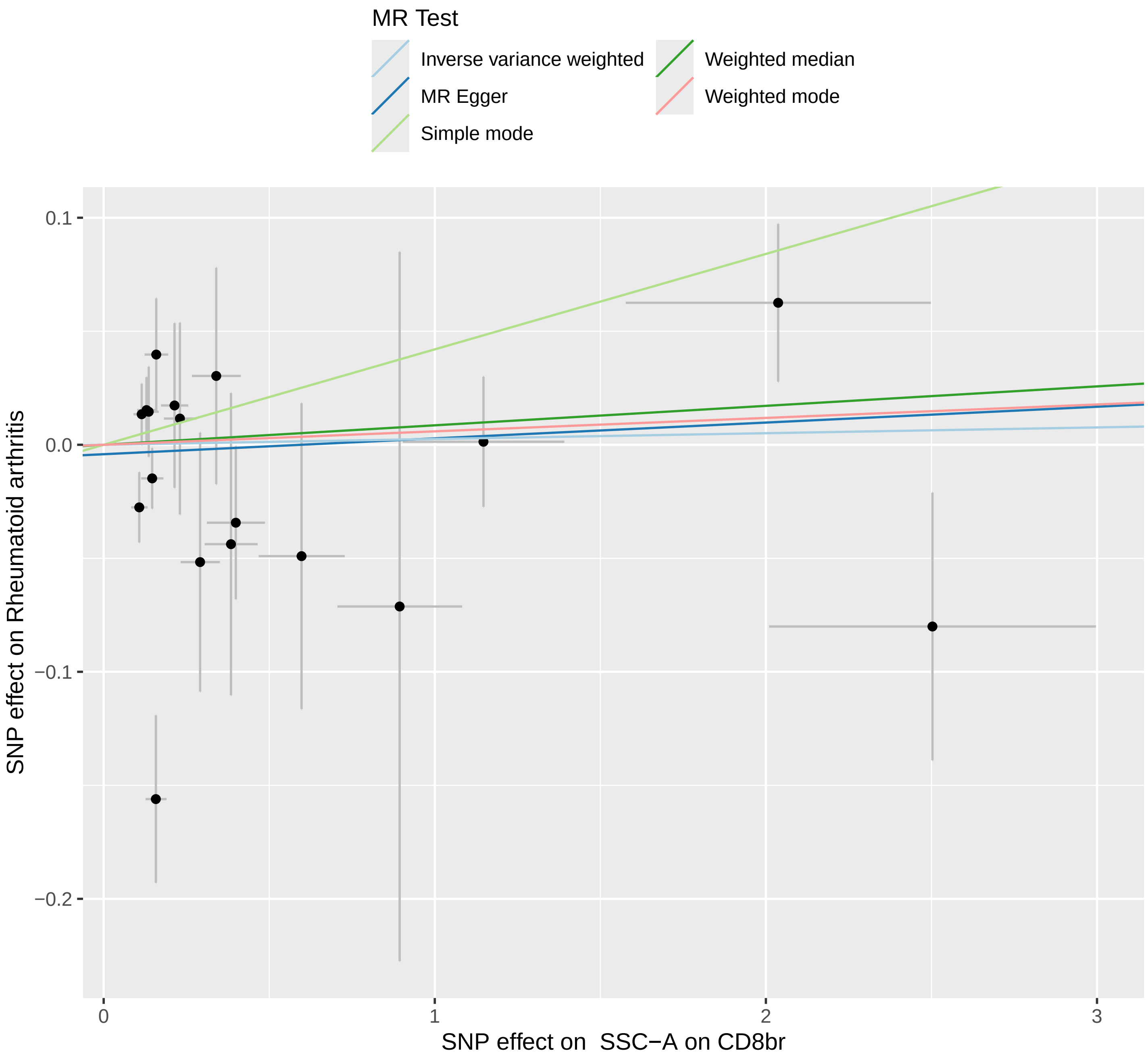
MR Test
Inverse variance weighted
MR Egger
Simple mode
Weighted median
Weighted mode
0.1
0.0
−0.1
−0.2
0
1
2
3
SNP effect on Rheumatoid arthritis
SNP effect on SSC−A on CD8br

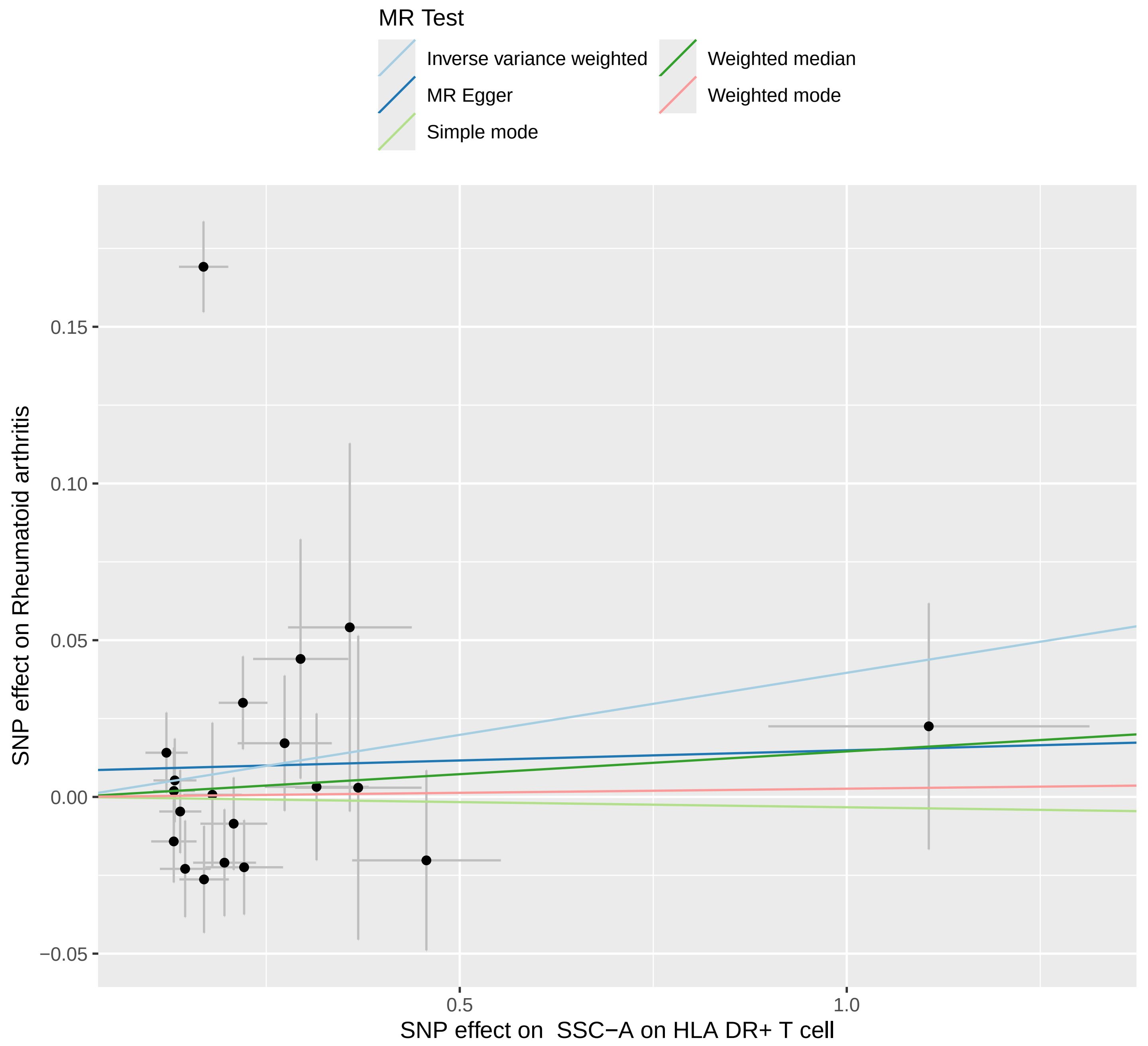

MR Test
Inverse variance weighted
MR Egger
Simple mode
Weighted median
Weighted mode
SNP effect on Rheumatoid arthritis
0.15
0.10
0.05
0.00
−0.05
0.5
1.0
SNP effect on SSC−A on HLA DR+ T cell

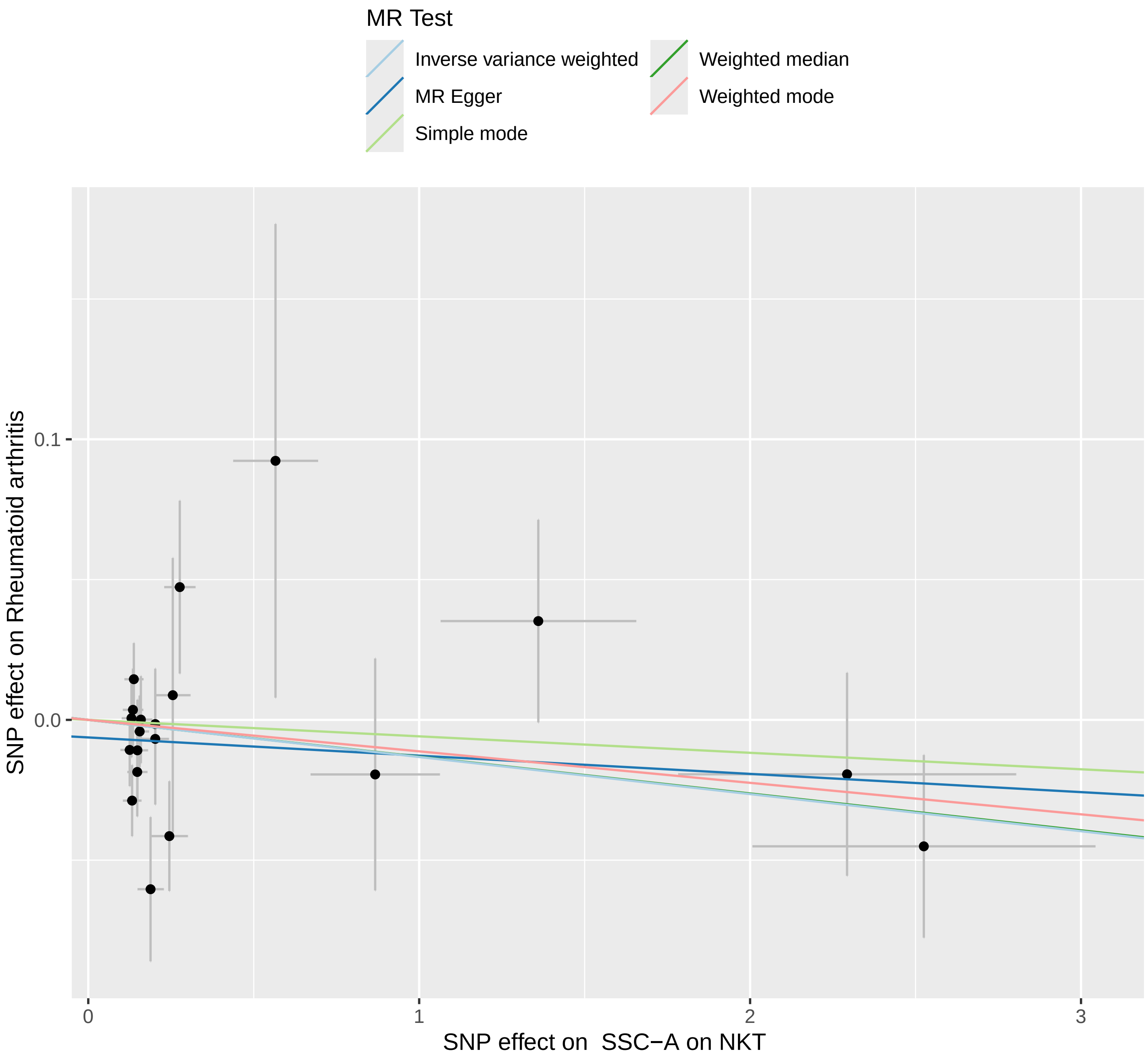
MR Test
Inverse variance weighted
MR Egger
Simple mode
Weighted median
Weighted mode
0.1
0.0
0
1
2
3
SNP effect on Rheumatoid arthritis
SNP effect on SSC−A on NKT

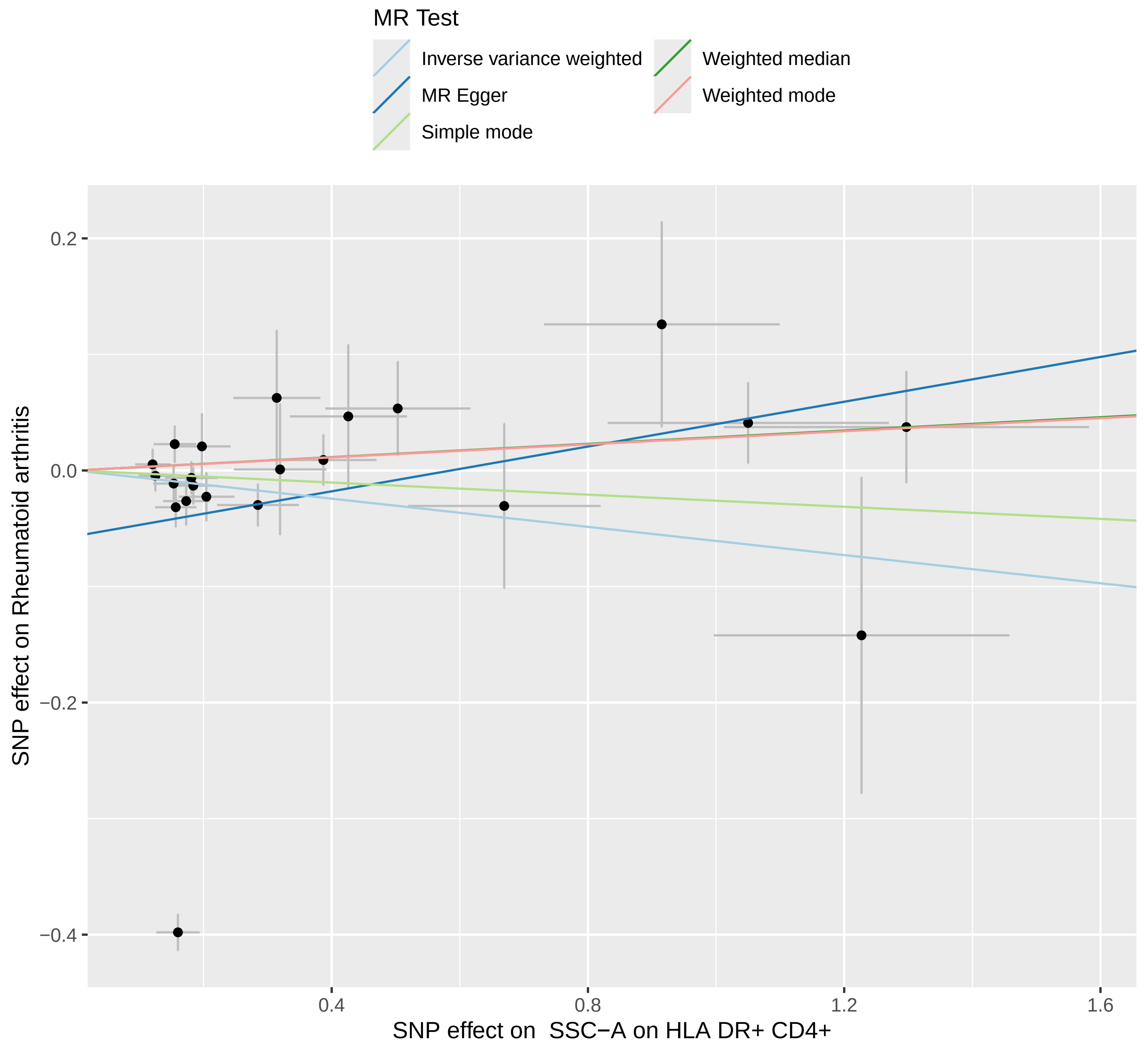

MR Test
Inverse variance weighted
MR Egger
Simple mode
Weighted median
Weighted mode
0.2
0.0
−0.2
−0.4
0.4
0.8
1.2
1.6
SNP effect on Rheumatoid arthritis
SNP effect on SSC−A on HLA DR+ CD4+

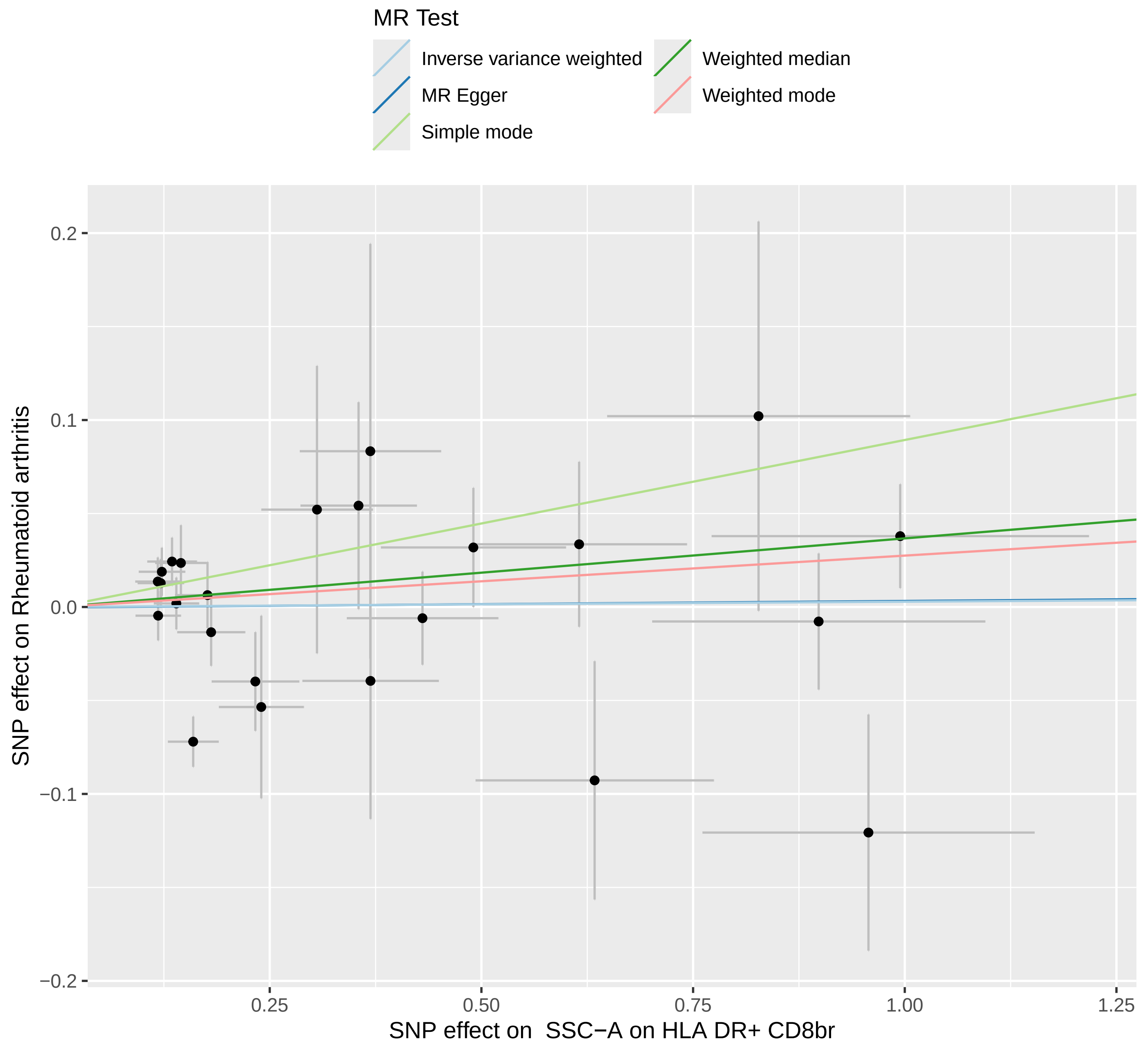
MR Test
Inverse variance weighted
MR Egger
Simple mode
Weighted median
Weighted mode
SNP effect on Rheumatoid arthritis
0.2
0.1
0.0
−0.1
−0.2
0.25
0.50
0.75
1.00
1.25
SNP effect on SSC−A on HLA DR+ CD8br

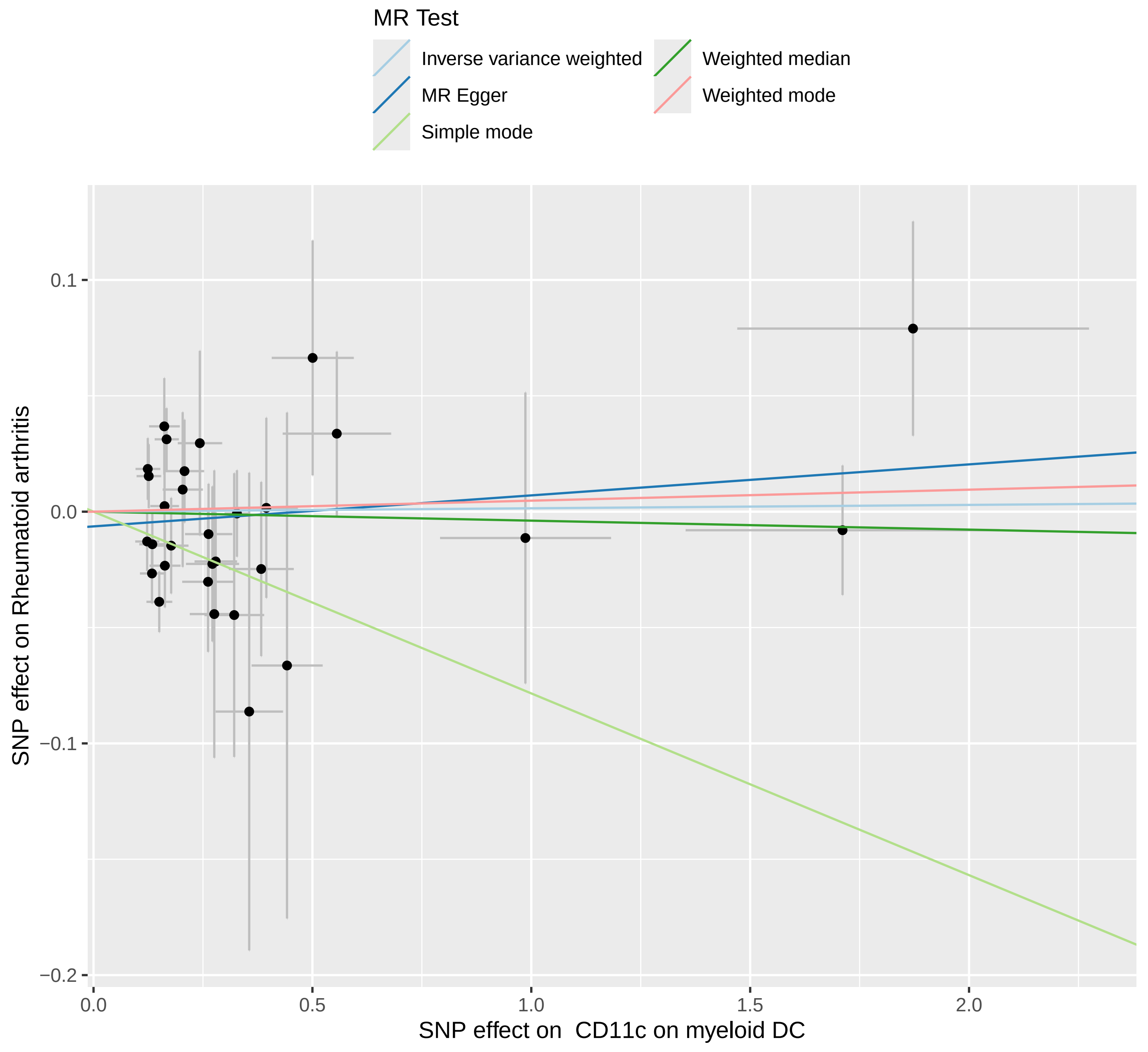
MR Test
Inverse variance weighted
MR Egger
Simple mode
Weighted median
Weighted mode
SNP effect on Rheumatoid arthritis
0.1
0.0
−0.1
−0.2
0.0
0.5
1.0
1.5
2.0
SNP effect on CD11c on myeloid DC

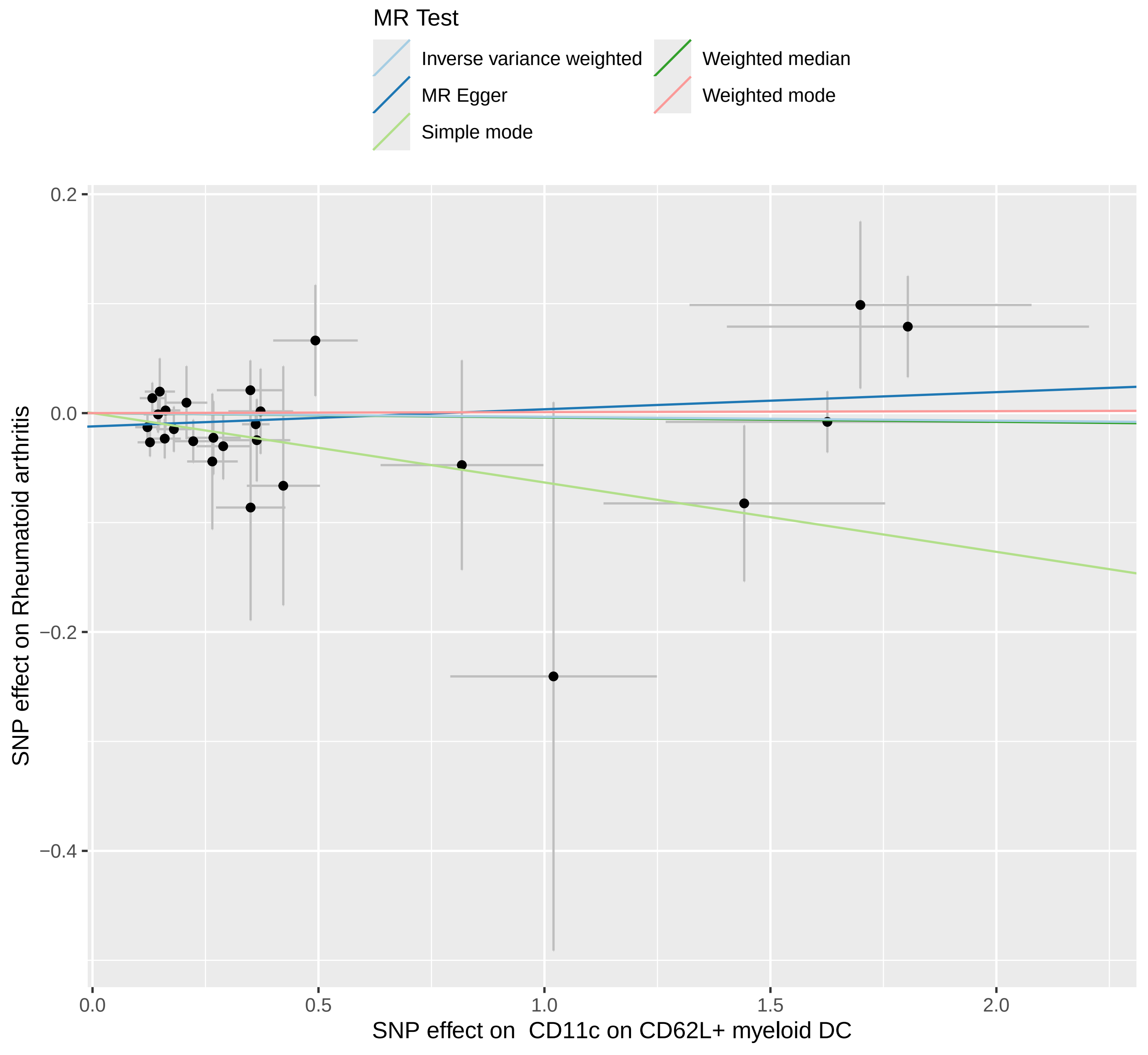
MR Test
Inverse variance weighted
MR Egger
Simple mode
Weighted median
Weighted mode
0.2
0.0
−0.2
−0.4
SNP effect on Rheumatoid arthritis
0.0
0.5
1.0
1.5
2.0
SNP effect on CD11c on CD62L+ myeloid DC

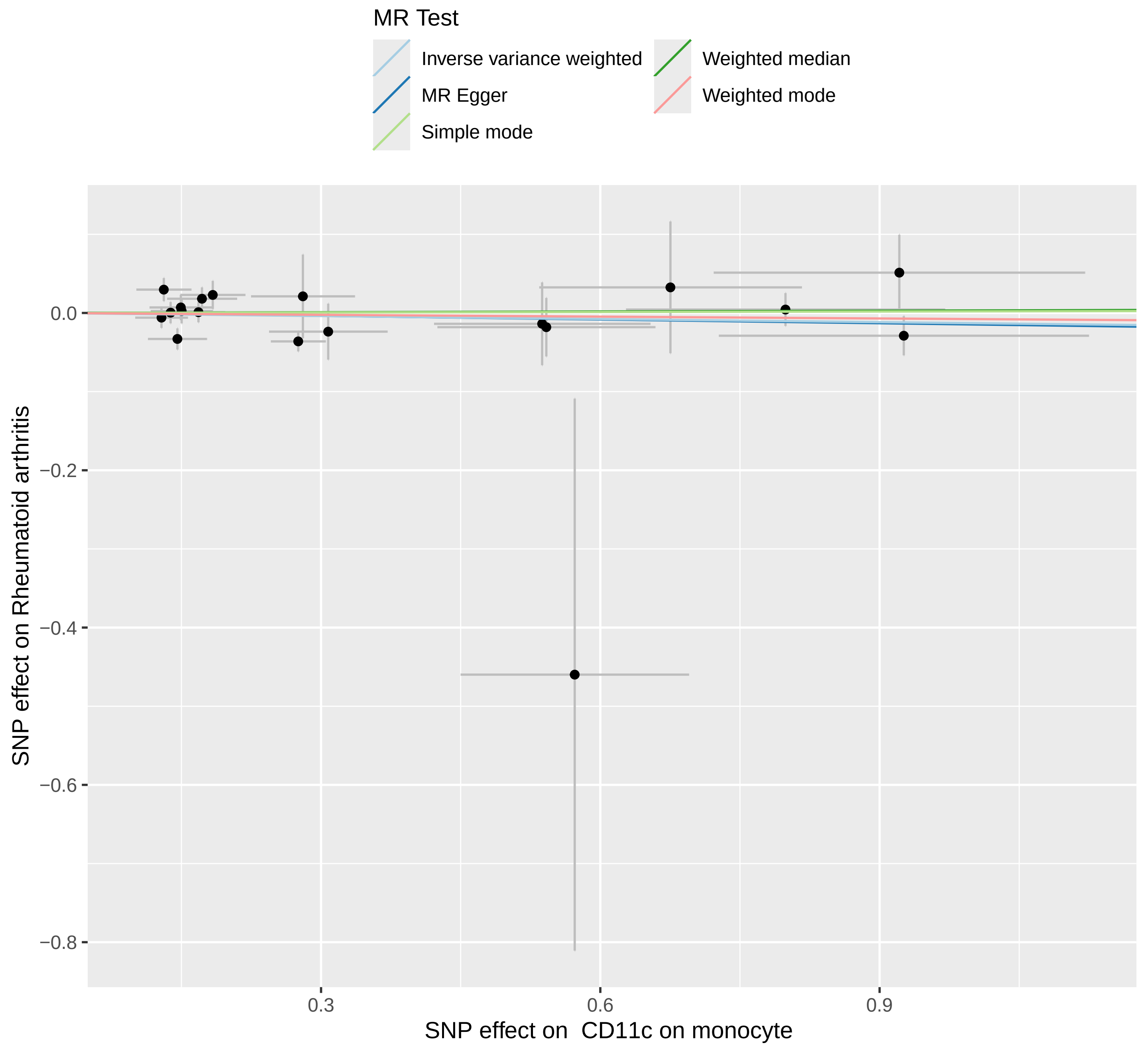

MR Test
Inverse variance weighted
MR Egger
Simple mode
Weighted median
Weighted mode
0.0
−0.2
−0.4
−0.6
−0.8
0.3
0.6
0.9
SNP effect on Rheumatoid arthritis
SNP effect on CD11c on monocyte

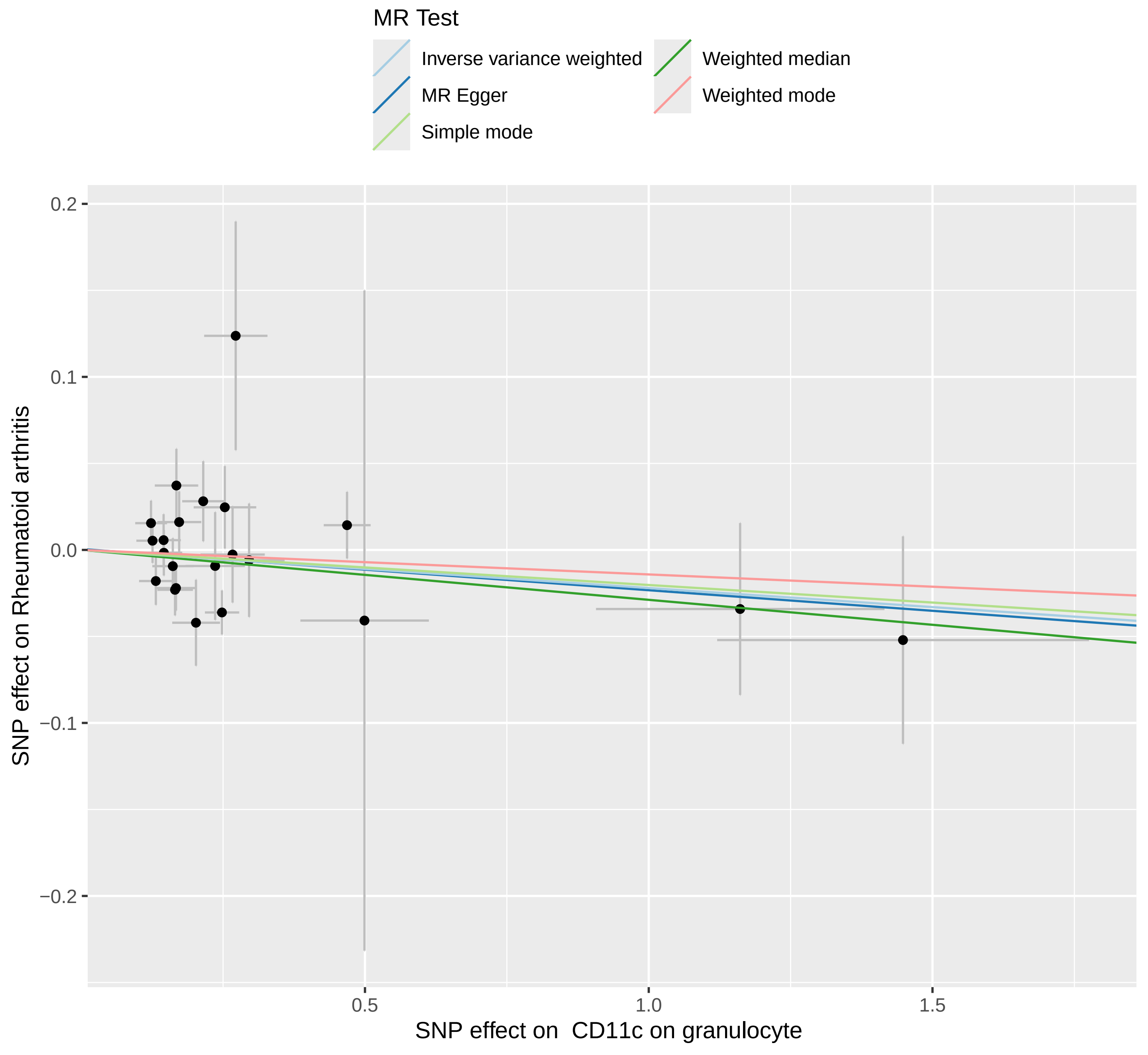
MR Test
Inverse variance weighted
MR Egger
Simple mode
Weighted median
Weighted mode
0.2
0.1
0.0
−0.1
−0.2
0.5
1.0
1.5
SNP effect on Rheumatoid arthritis
SNP effect on CD11c on granulocyte

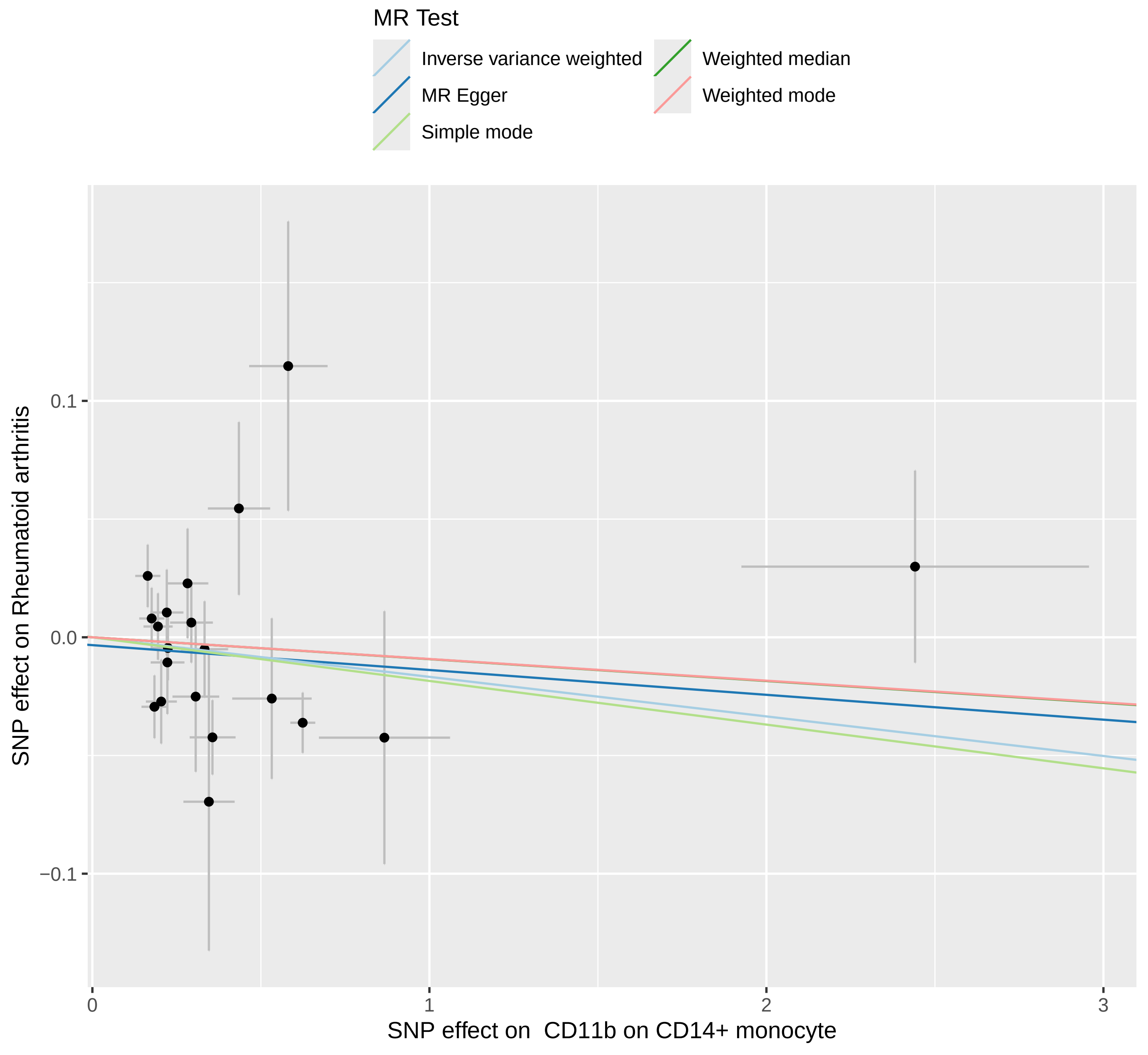

MR Test
Inverse variance weighted
MR Egger
Simple mode
Weighted median
Weighted mode
SNP effect on Rheumatoid arthritis
0.1
0.0
−0.1
0
1
2
3
SNP effect on CD11b on CD14+ monocyte

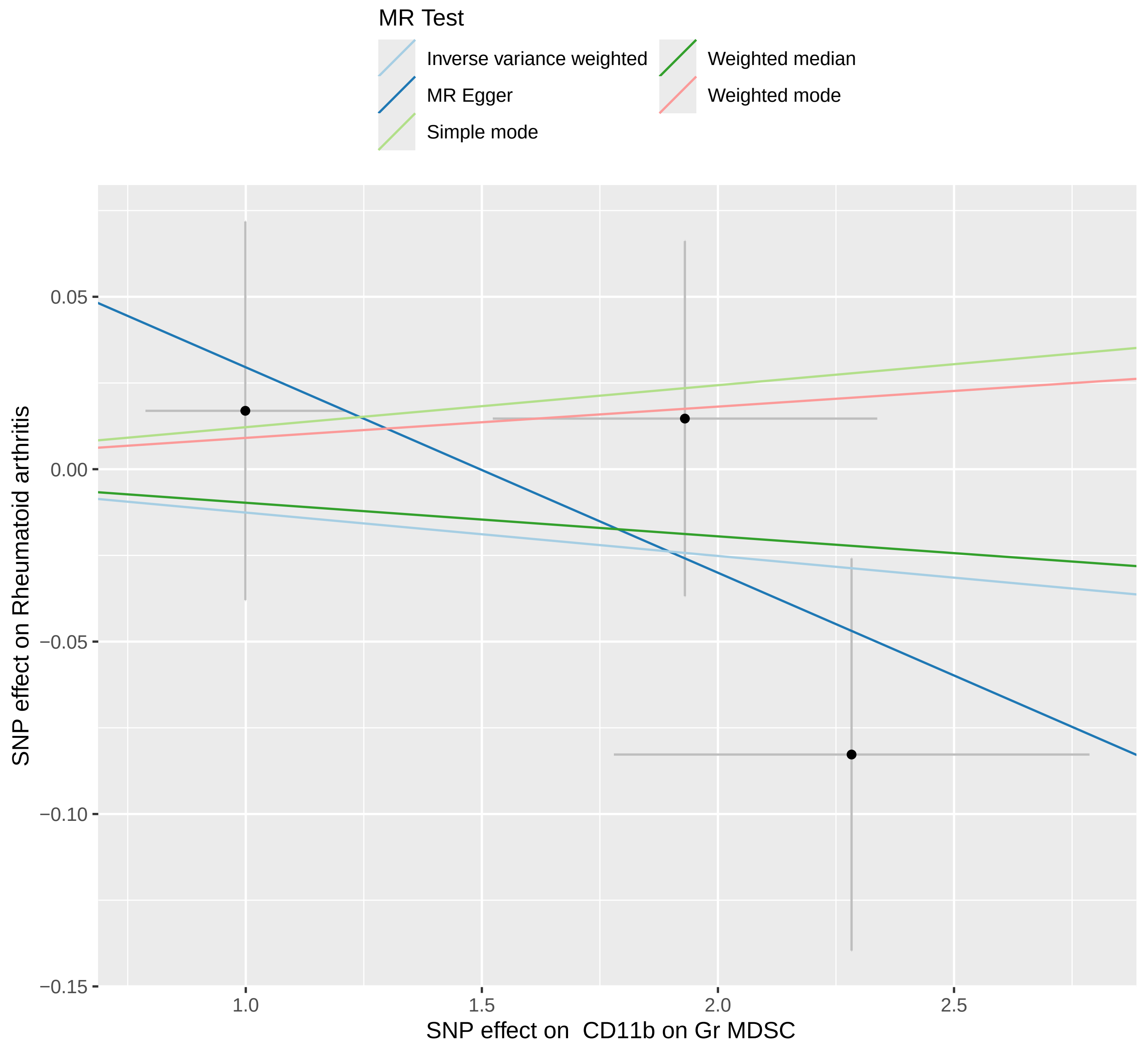

MR Test
Inverse variance weighted
MR Egger
Simple mode
Weighted median
Weighted mode
0.05
0.00
−0.05
−0.10
−0.15
1.0
1.5
2.0
2.5
SNP effect on Rheumatoid arthritis
SNP effect on CD11b on Gr MDSC

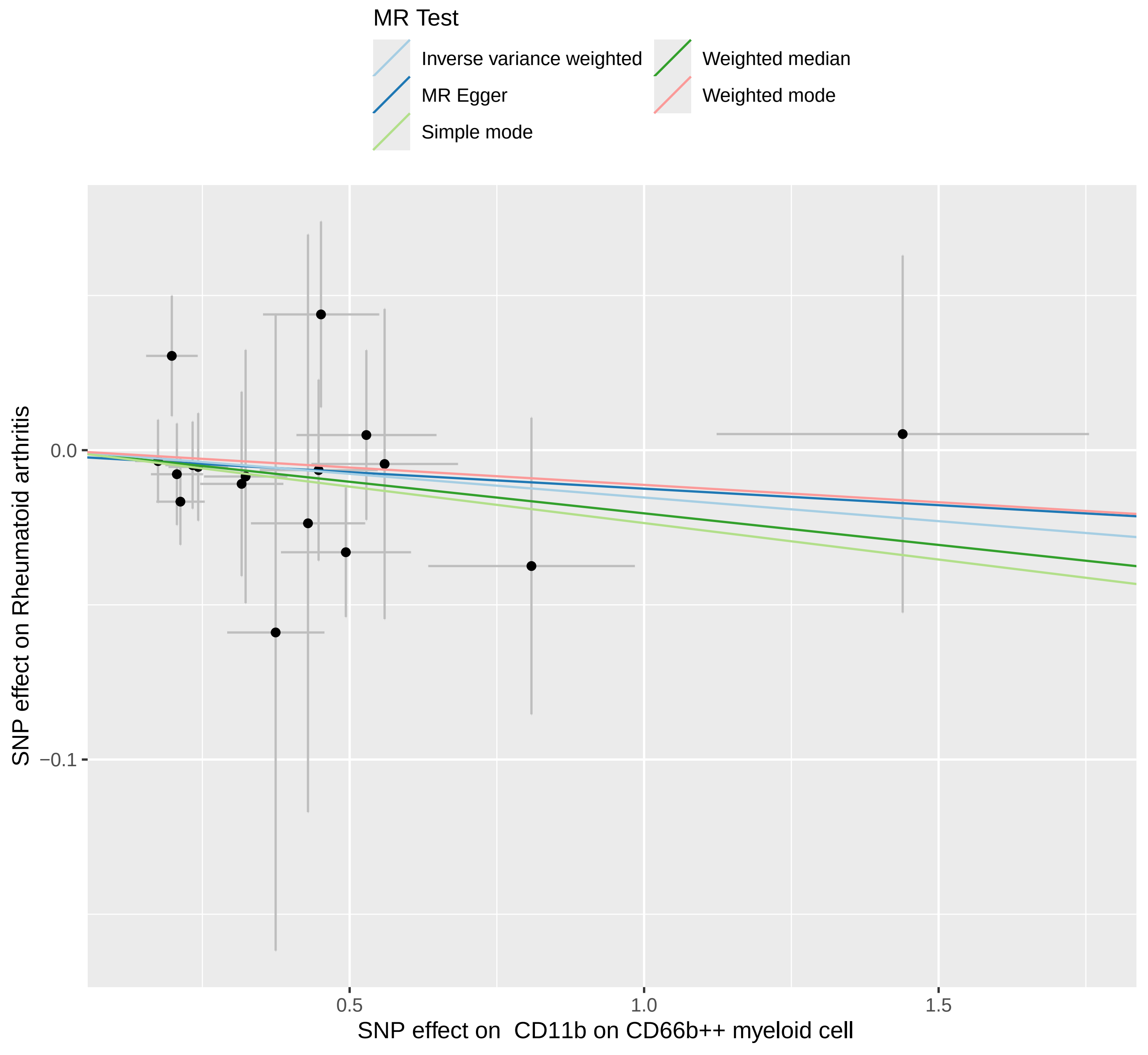
MR Test
Inverse variance weighted
MR Egger
Simple mode
Weighted median
Weighted mode
SNP effect on Rheumatoid arthritis
0.0
−0.1
0.5
1.0
1.5
SNP effect on CD11b on CD66b++ myeloid cell

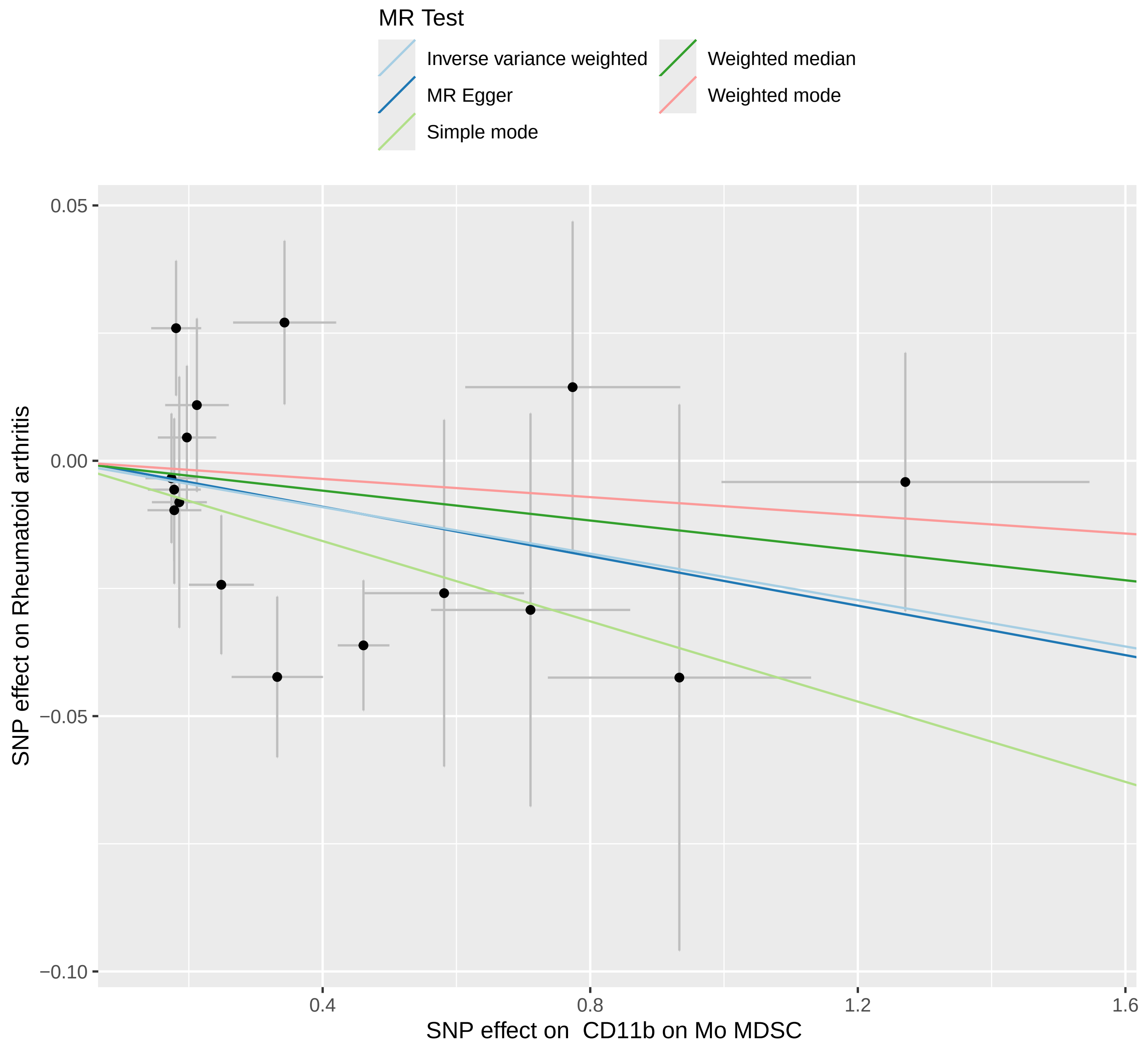

MR Test
Inverse variance weighted
MR Egger
Simple mode
Weighted median
Weighted mode
0.05
0.00
−0.05
−0.10
0.4
0.8
1.2
1.6
SNP effect on CD11b on Mo MDSC
SNP effect on Rheumatoid arthritis

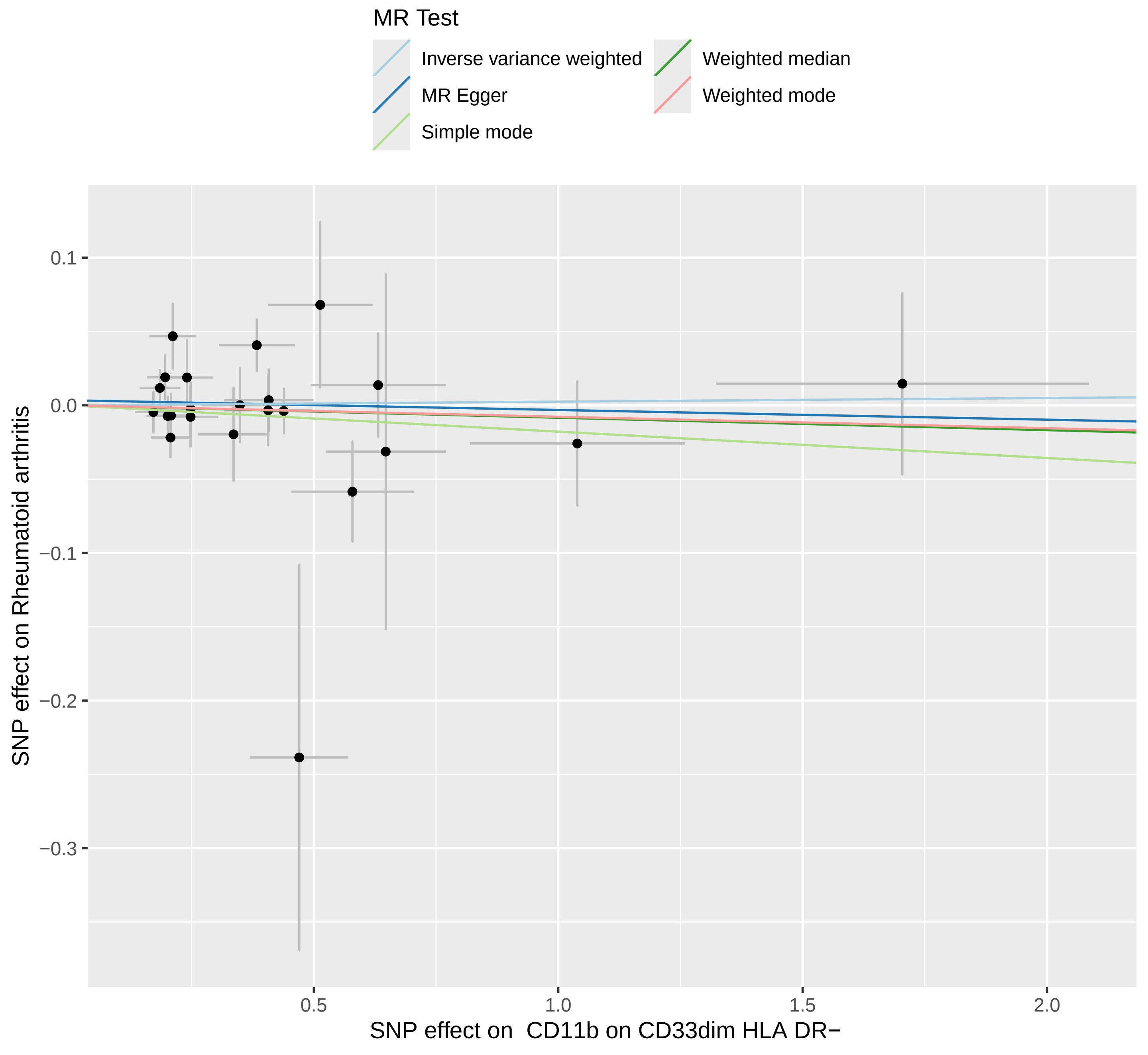
MR Test
Inverse variance weighted
MR Egger
Simple mode
Weighted median
Weighted mode
0.1
0.0
−0.1
−0.2
−0.3
0.5
1.0
1.5
2.0
SNP effect on Rheumatoid arthritis
SNP effect on CD11b on CD33dim HLA DR−

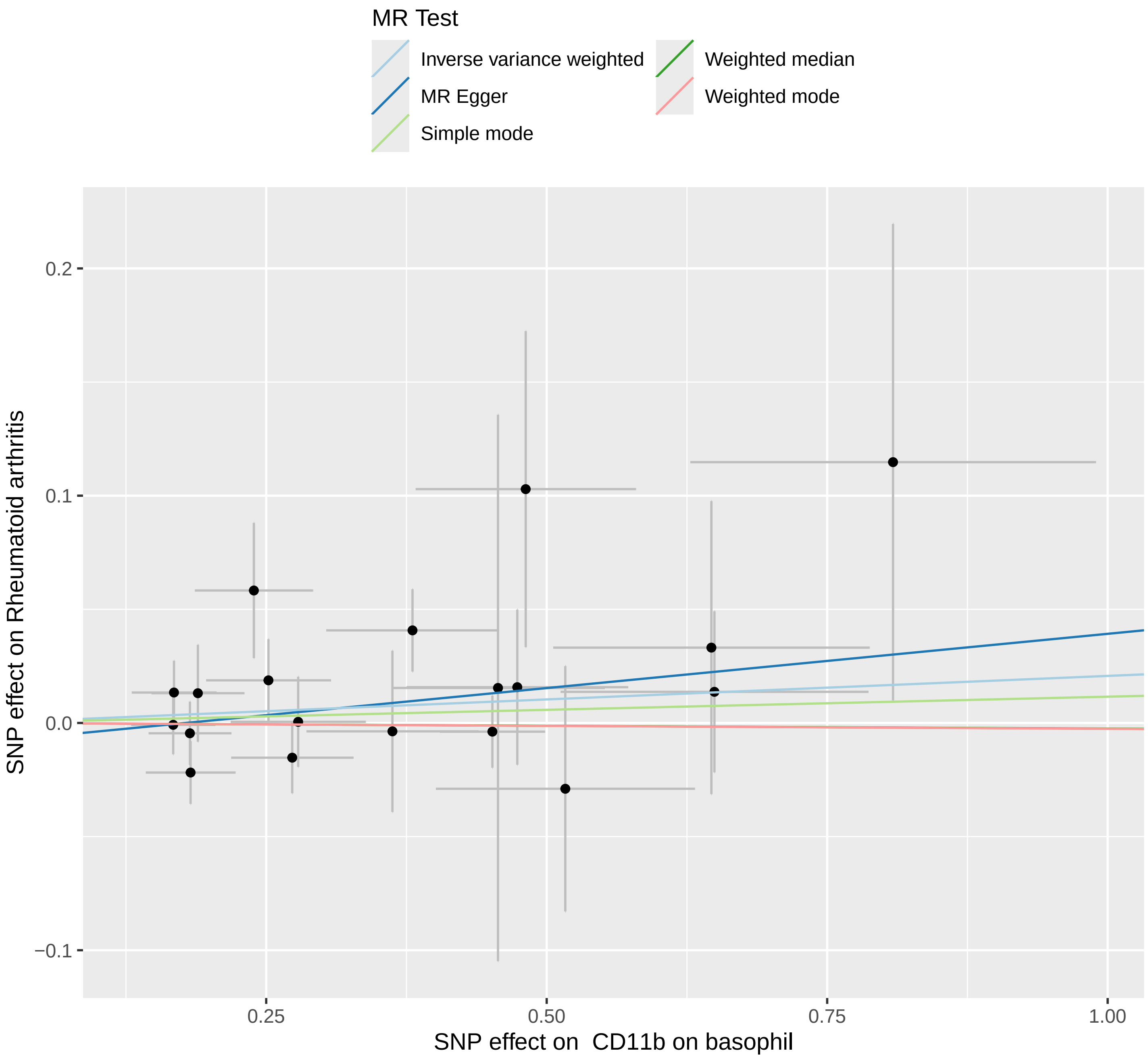
MR Test
Inverse variance weighted
MR Egger
Simple mode
Weighted median
Weighted mode
0.2
0.1
0.0
−0.1
0.25
0.50
0.75
1.00
SNP effect on Rheumatoid arthritis
SNP effect on CD11b on basophil

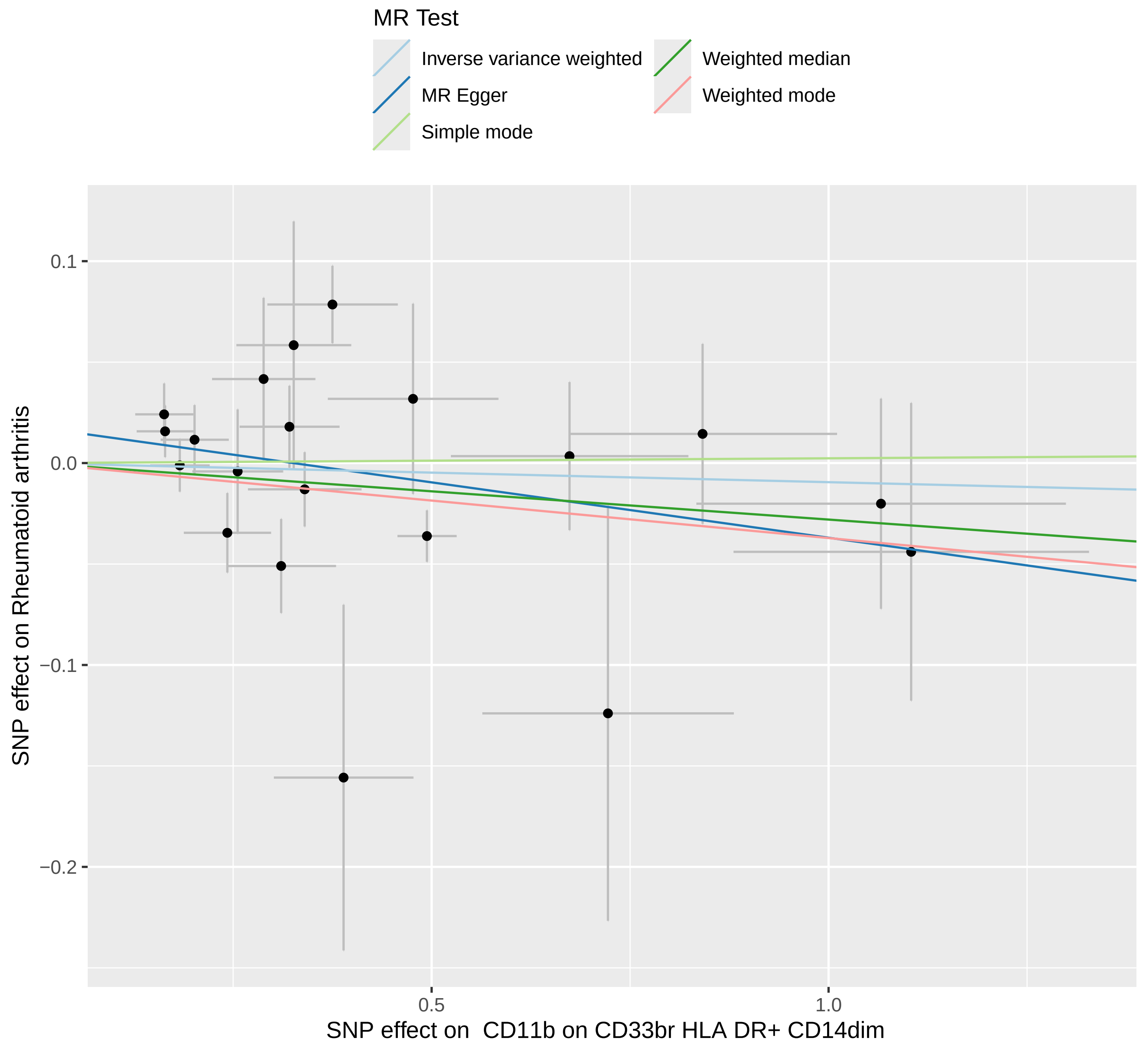

MR Test
Inverse variance weighted
MR Egger
Simple mode
Weighted median
Weighted mode
0.1
0.0
−0.1
−0.2
0.5
1.0
SNP effect on Rheumatoid arthritis
SNP effect on CD11b on CD33br HLA DR+ CD14dim

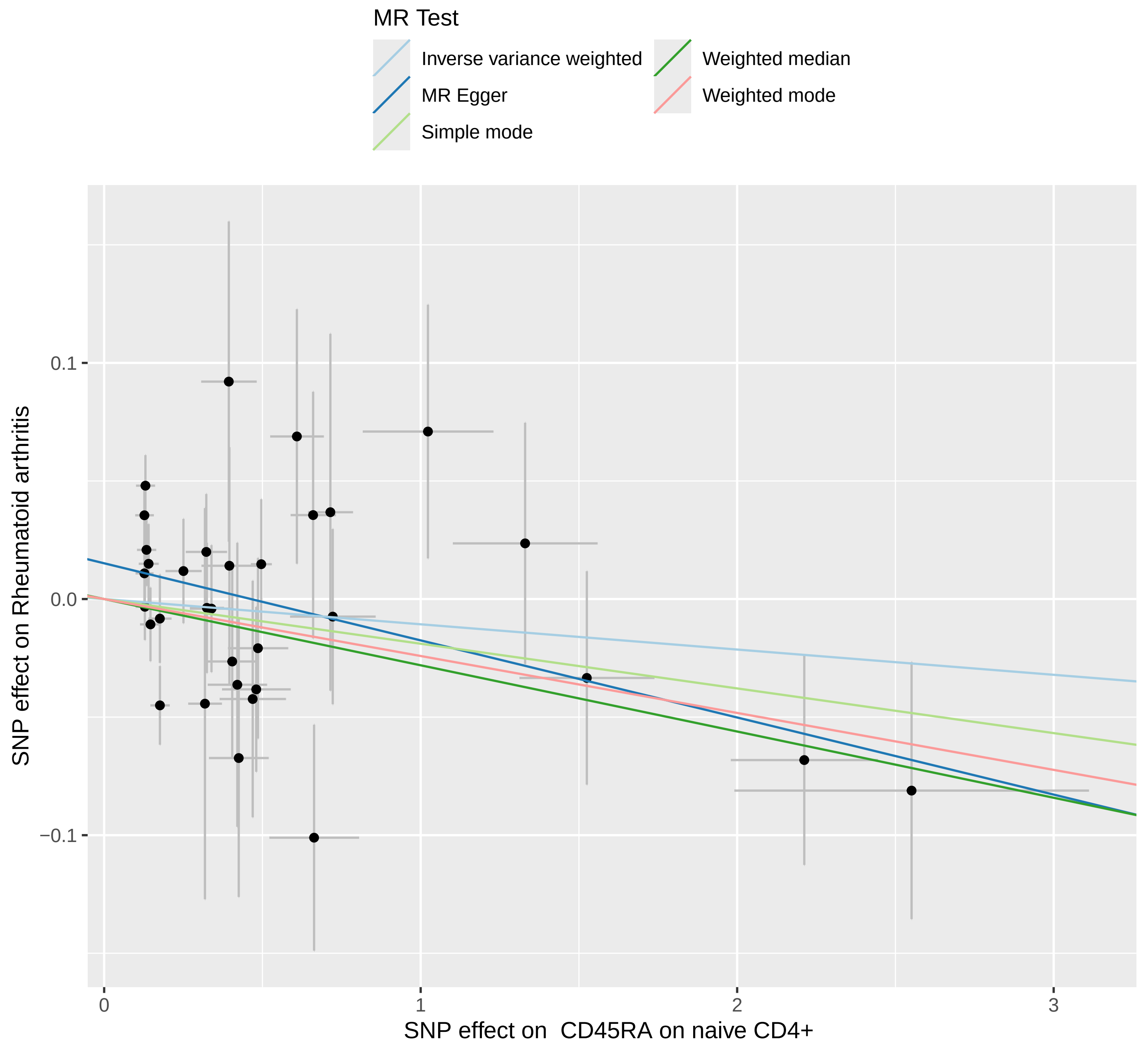

MR Test
Inverse variance weighted
MR Egger
Simple mode
Weighted median
Weighted mode
0.1
0.0
−0.1
SNP effect on Rheumatoid arthritis
0
1
2
3
SNP effect on CD45RA on naive CD4+

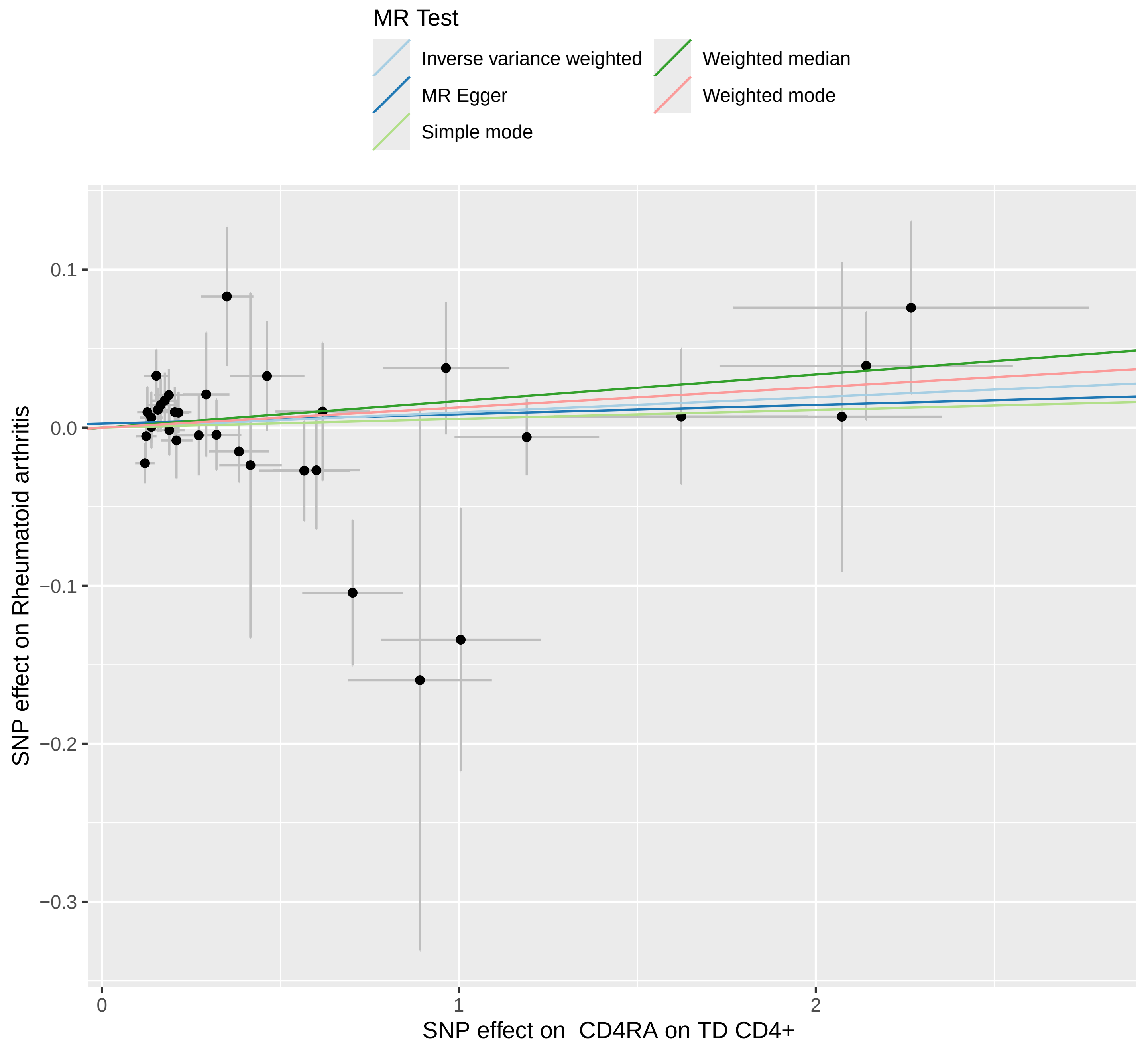

MR Test
Inverse variance weighted
MR Egger
Simple mode
Weighted median
Weighted mode
0.1
0.0
−0.1
−0.2
−0.3
0
1
2
SNP effect on Rheumatoid arthritis
SNP effect on CD4RA on TD CD4+

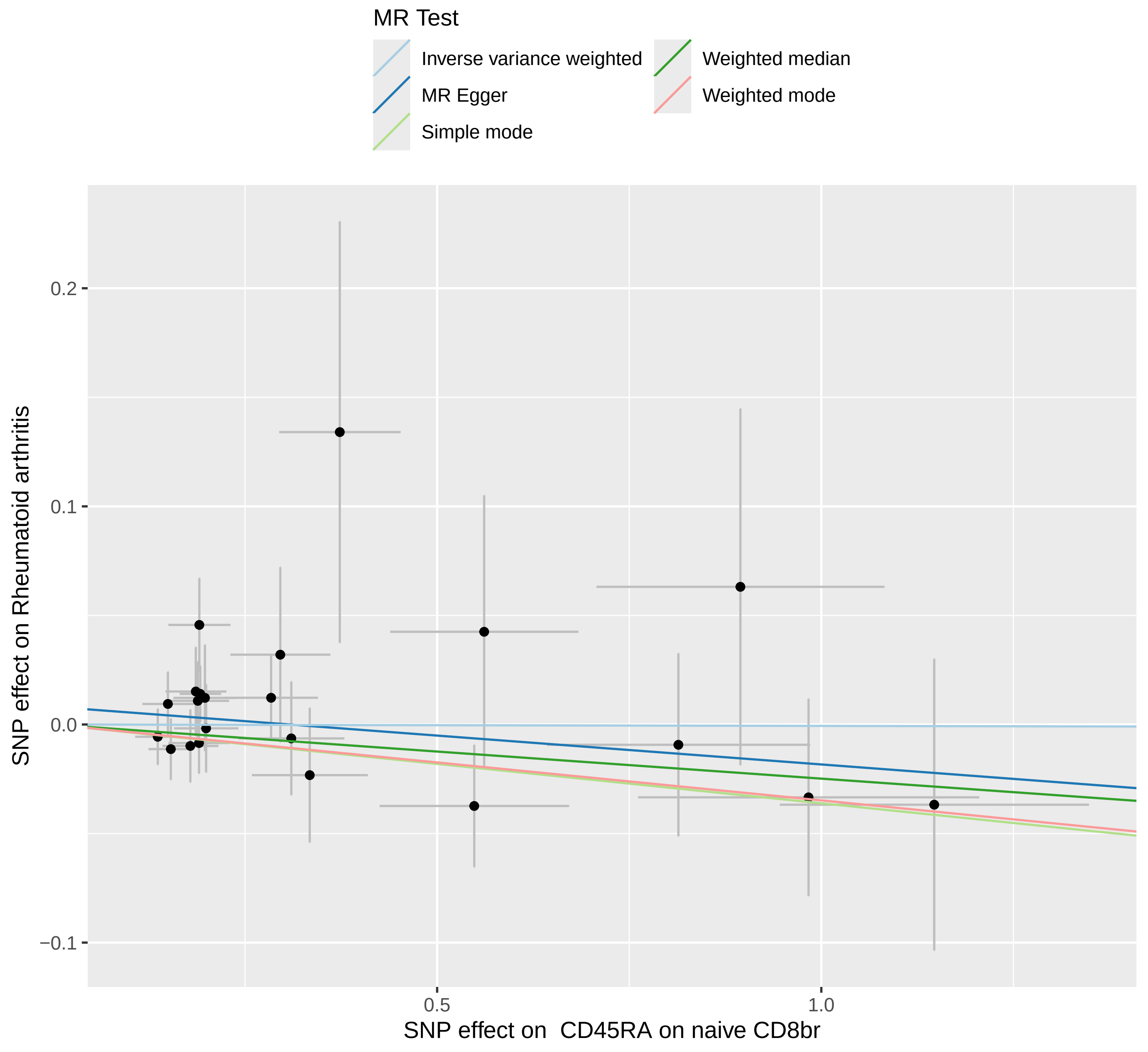
MR Test
Inverse variance weighted
MR Egger
Simple mode
Weighted median
Weighted mode
0.2
0.1
0.0
−0.1
0.5
1.0
SNP effect on Rheumatoid arthritis
SNP effect on CD45RA on naive CD8br

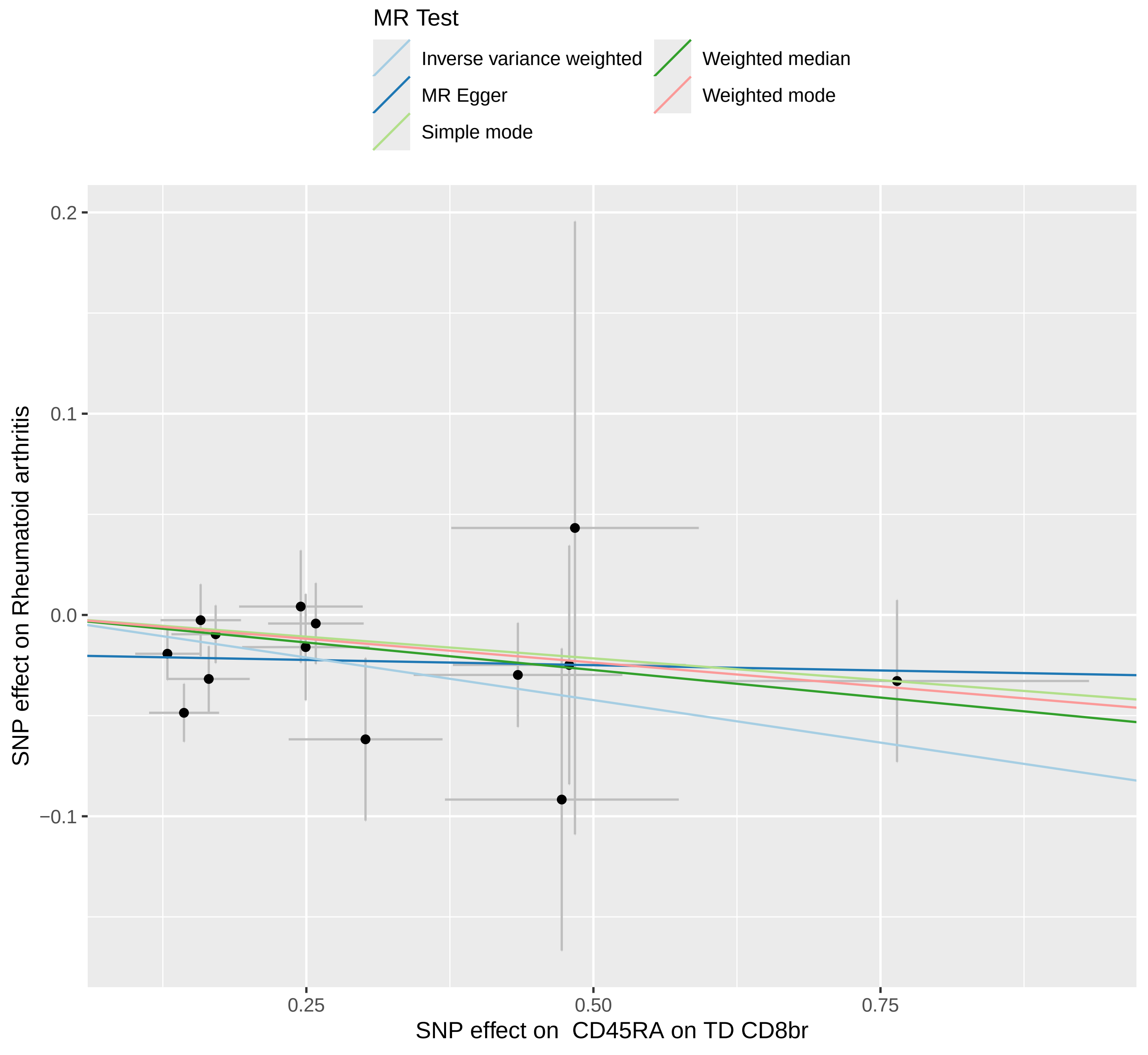
MR Test
Inverse variance weighted
MR Egger
Simple mode
Weighted median
Weighted mode
0.2
0.1
0.0
−0.1
0.25
0.50
0.75
SNP effect on Rheumatoid arthritis
SNP effect on CD45RA on TD CD8br

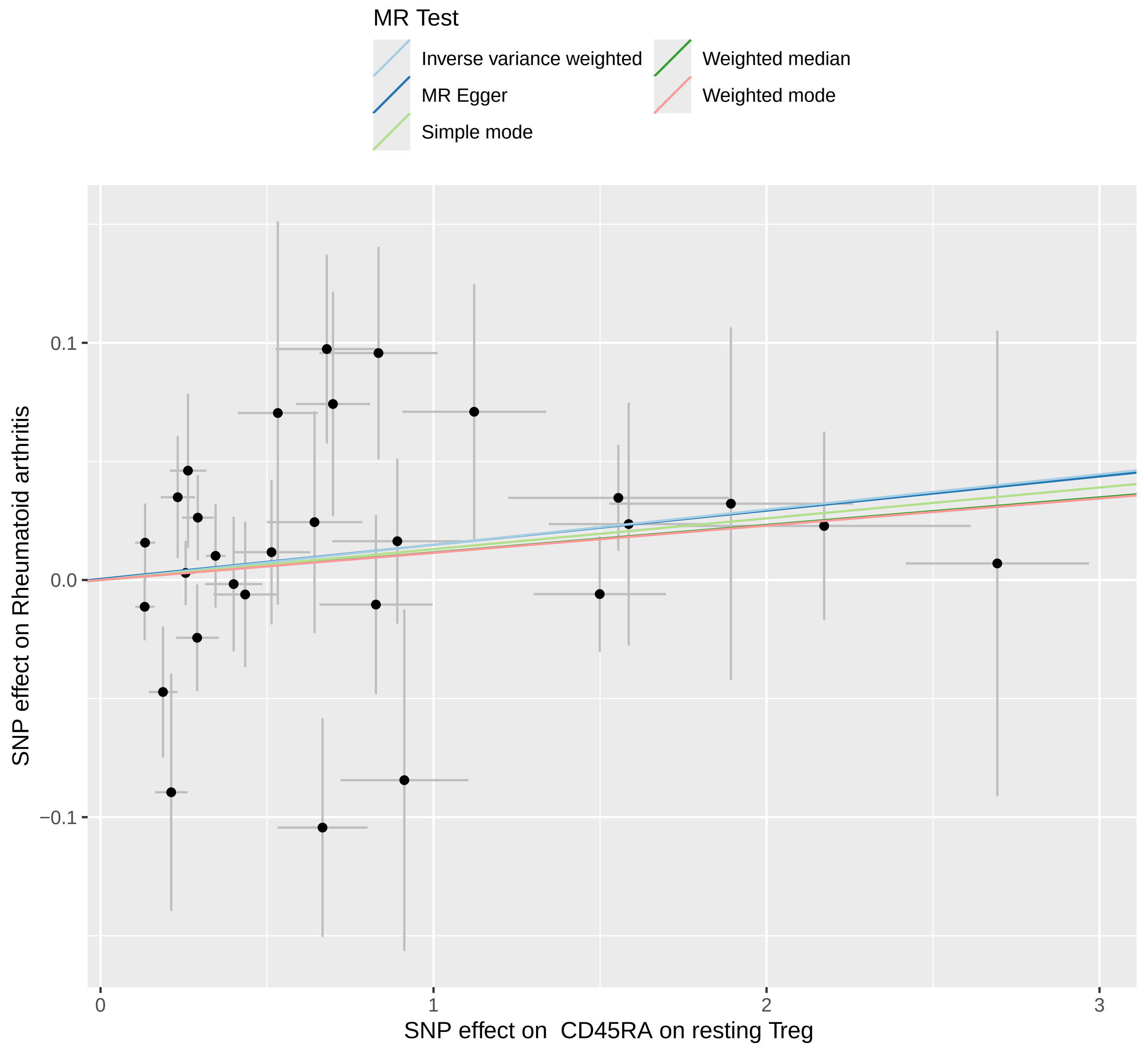
MR Test
Inverse variance weighted
MR Egger
Simple mode
Weighted median
Weighted mode
0.1
0.0
−0.1
0
1
2
3
SNP effect on Rheumatoid arthritis
SNP effect on CD45RA on resting Treg

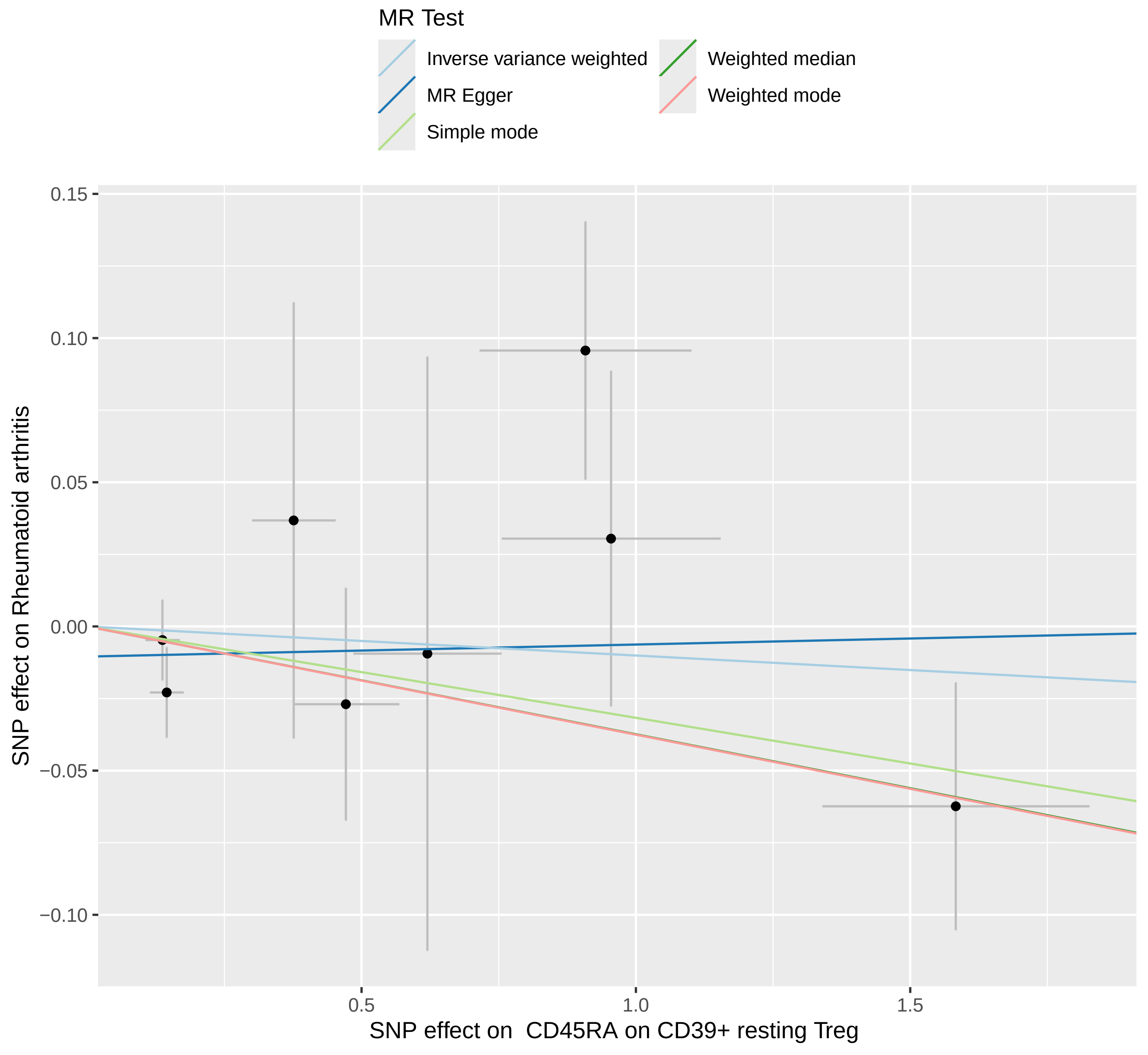
MR Test
Inverse variance weighted
MR Egger
Simple mode
Weighted median
Weighted mode
0.15
0.10
0.05
0.00
−0.05
−0.10
SNP effect on Rheumatoid arthritis
0.5
1.0
1.5
SNP effect on CD45RA on CD39+ resting Treg

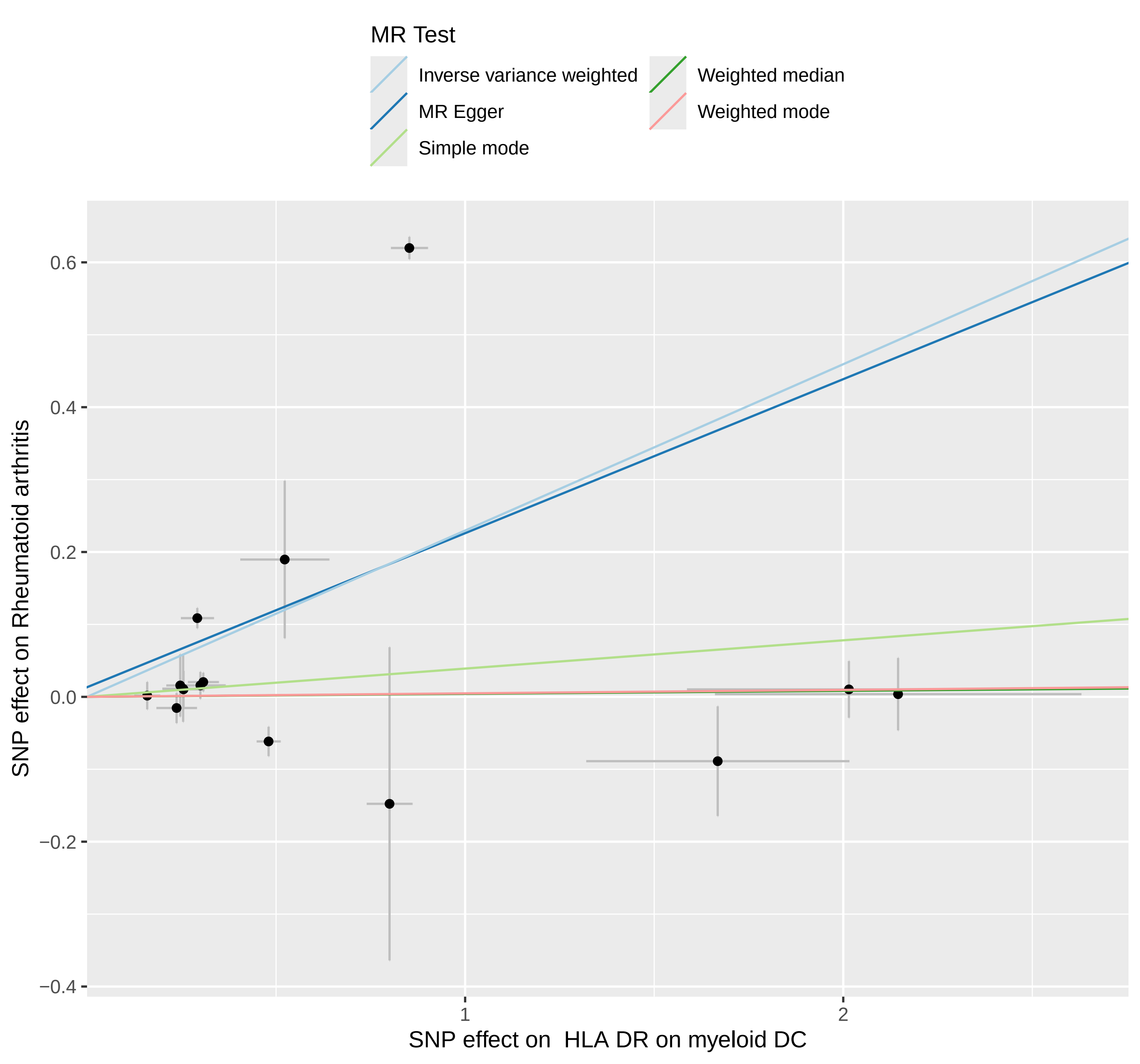
MR Test
Inverse variance weighted
MR Egger
Simple mode
Weighted median
Weighted mode
SNP effect on Rheumatoid arthritis
0.6
0.4
0.2
0.0
−0.2
−0.4
1
2
SNP effect on HLA DR on myeloid DC

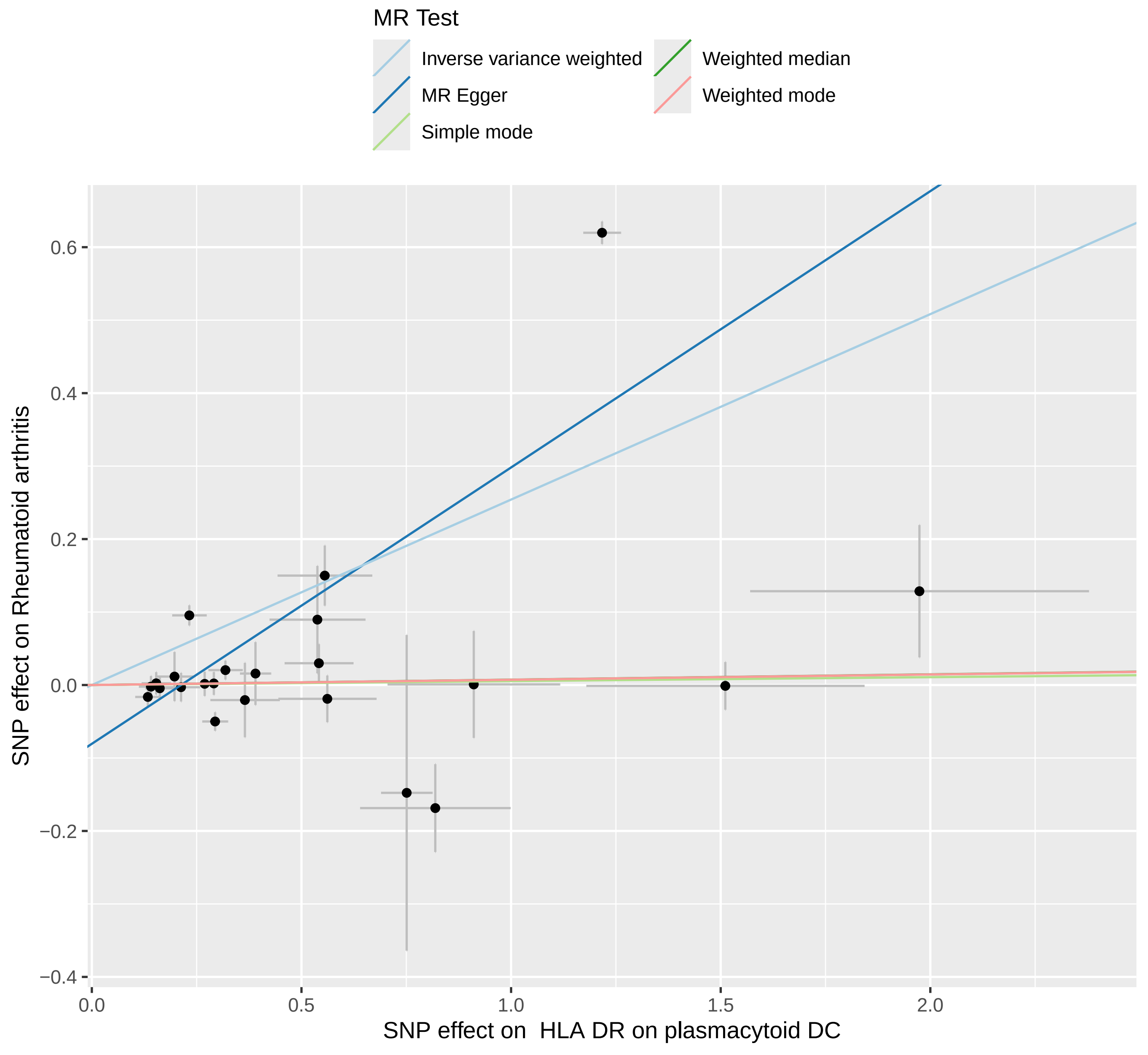
MR Test
Inverse variance weighted
MR Egger
Simple mode
Weighted median
Weighted mode
SNP effect on Rheumatoid arthritis
SNP effect on HLA DR on plasmacytoid DC
0.6
0.4
0.2
0.0
−0.2
−0.4
0.0
0.5
1.0
1.5
2.0

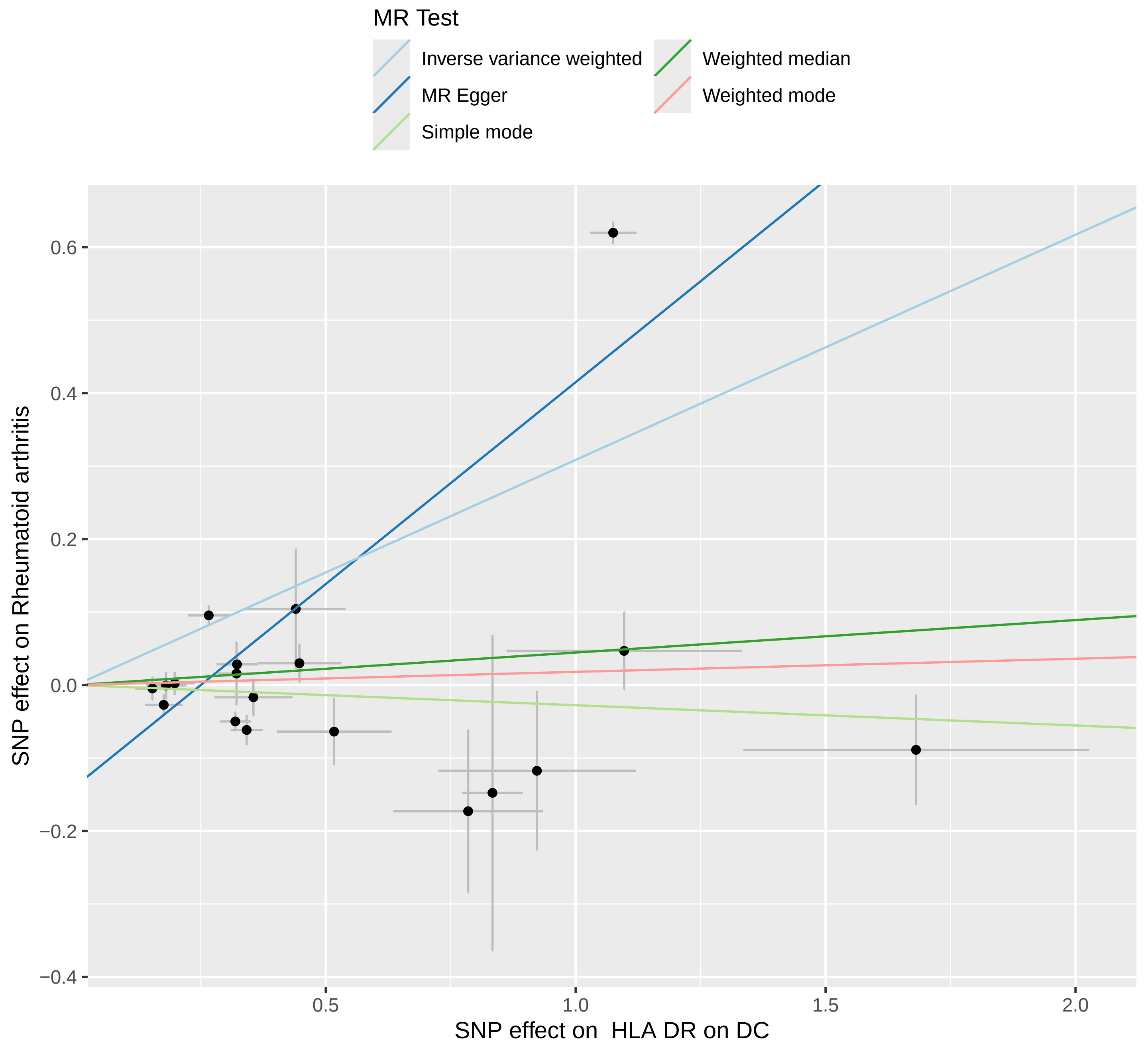
MR Test
Inverse variance weighted
MR Egger
Simple mode
Weighted median
Weighted mode
SNP effect on Rheumatoid arthritis
SNP effect on HLA DR on DC
0.6
0.4
0.2
0.0
−0.2
−0.4
0.5
1.0
1.5
2.0

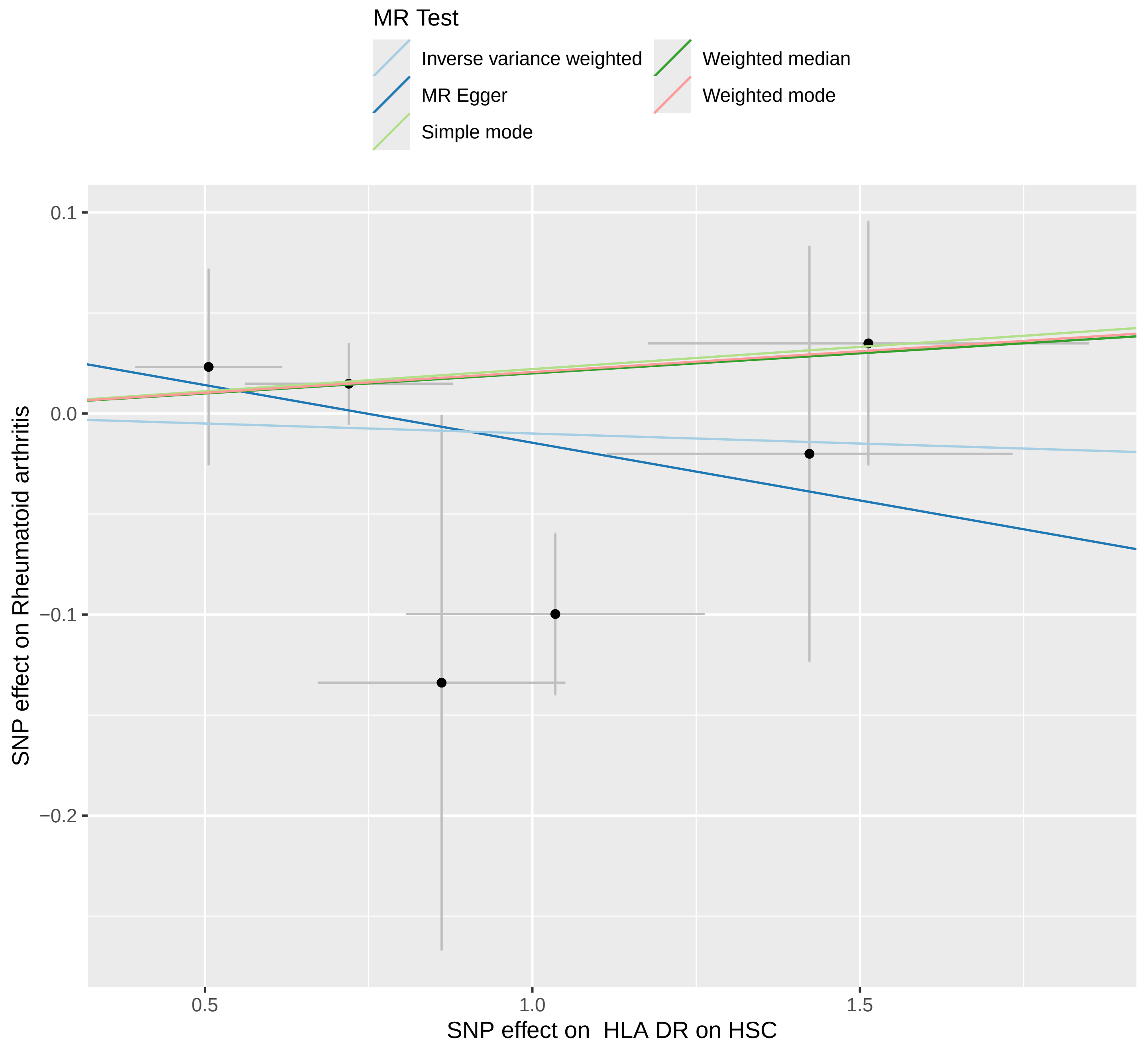
MR Test
Inverse variance weighted
MR Egger
Simple mode
Weighted median
Weighted mode
0.1
0.0
−0.1
−0.2
0.5
1.0
1.5
SNP effect on Rheumatoid arthritis
SNP effect on HLA DR on HSC

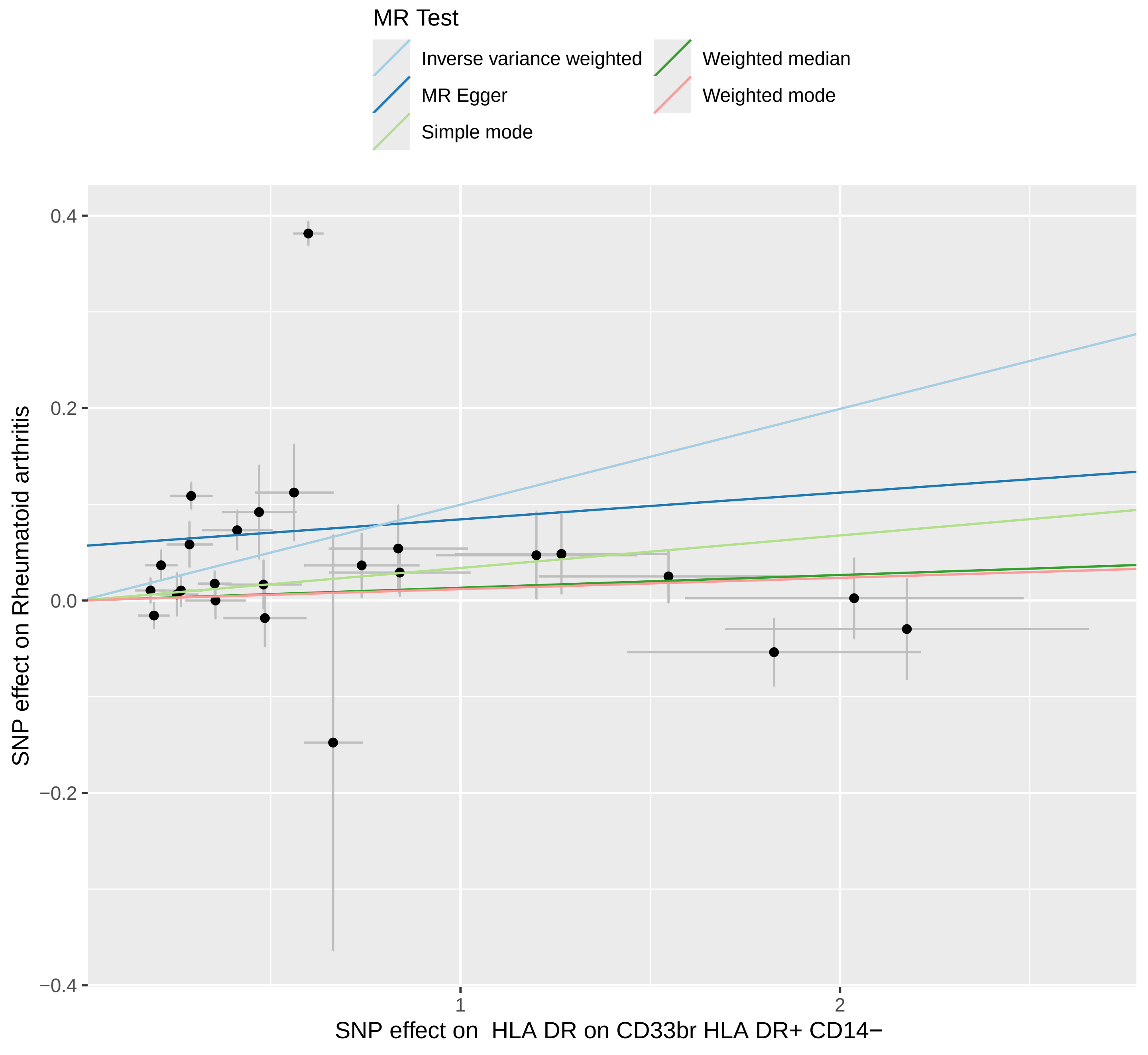
MR Test
Inverse variance weighted
MR Egger
Simple mode
Weighted median
Weighted mode
0.4
0.2
0.0
−0.2
−0.4
1
2
SNP effect on Rheumatoid arthritis
SNP effect on HLA DR on CD33br HLA DR+ CD14−

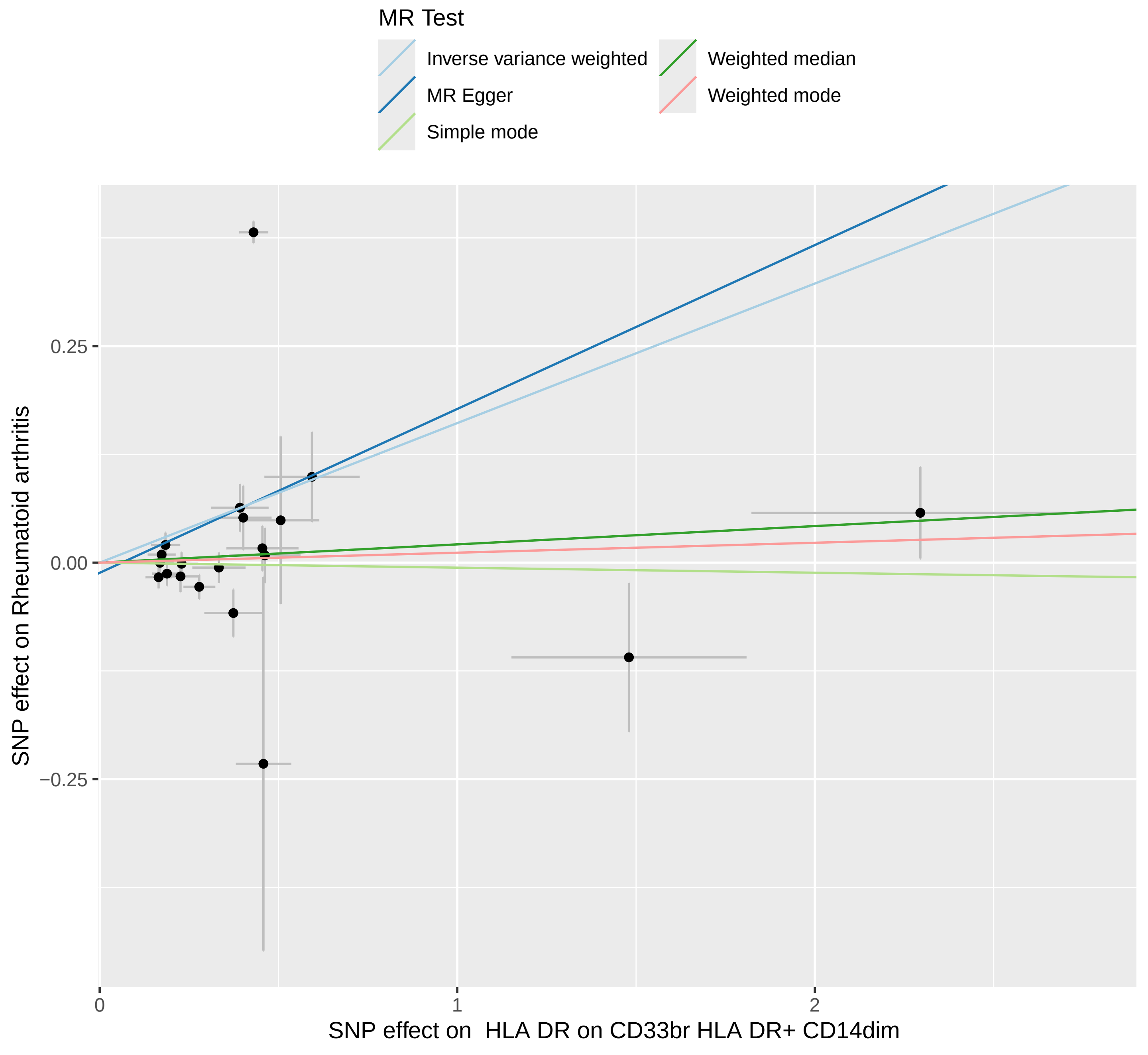
MR Test
Inverse variance weighted
MR Egger
Simple mode
Weighted median
Weighted mode
0.25
0.00
−0.25
0
1
2
SNP effect on Rheumatoid arthritis
SNP effect on HLA DR on CD33br HLA DR+ CD14dim

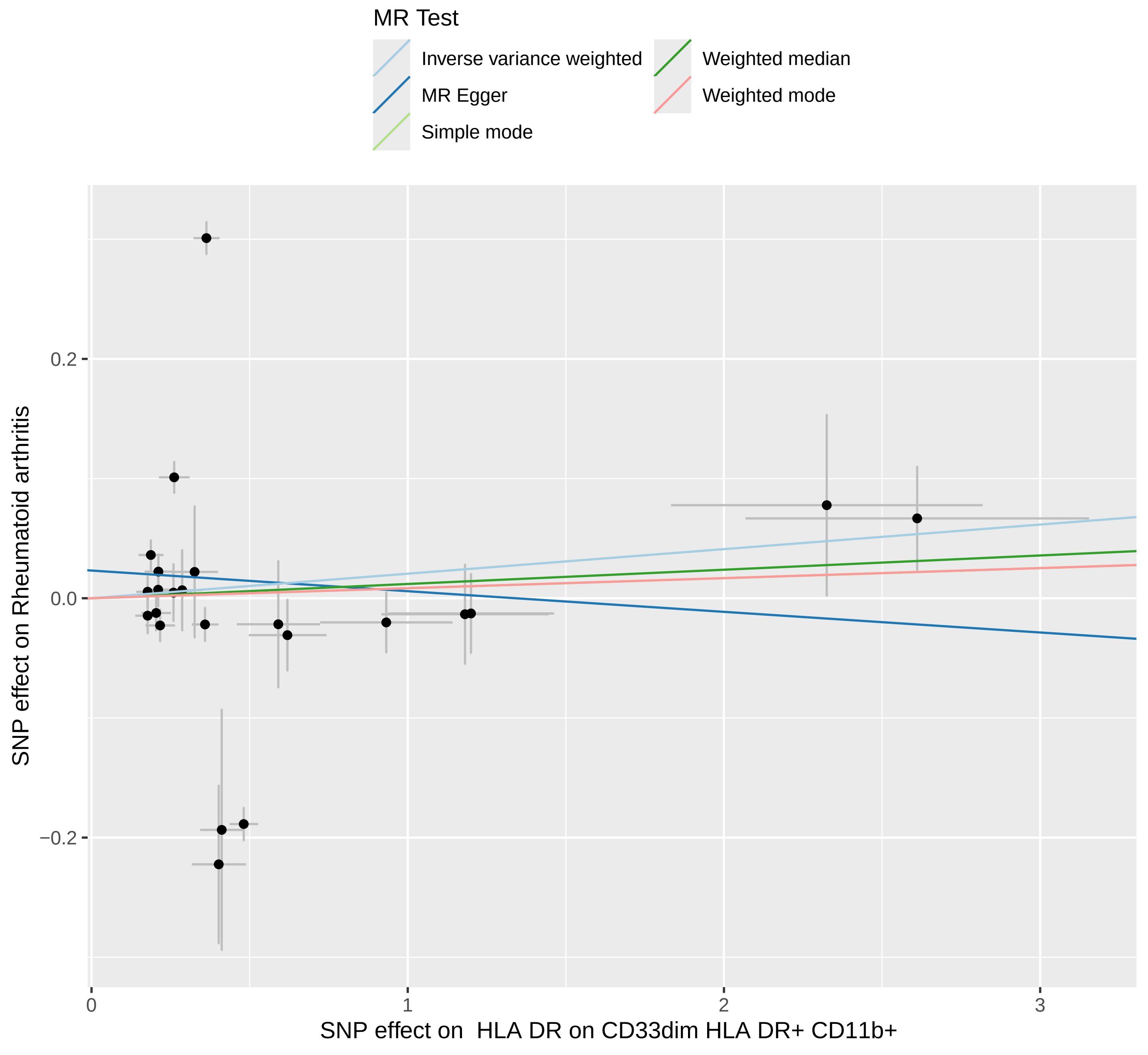

MR Test
Inverse variance weighted
MR Egger
Simple mode
Weighted median
Weighted mode
0.2
0.0
−0.2
0
1
2
3
SNP effect on Rheumatoid arthritis
SNP effect on HLA DR on CD33dim HLA DR+ CD11b+

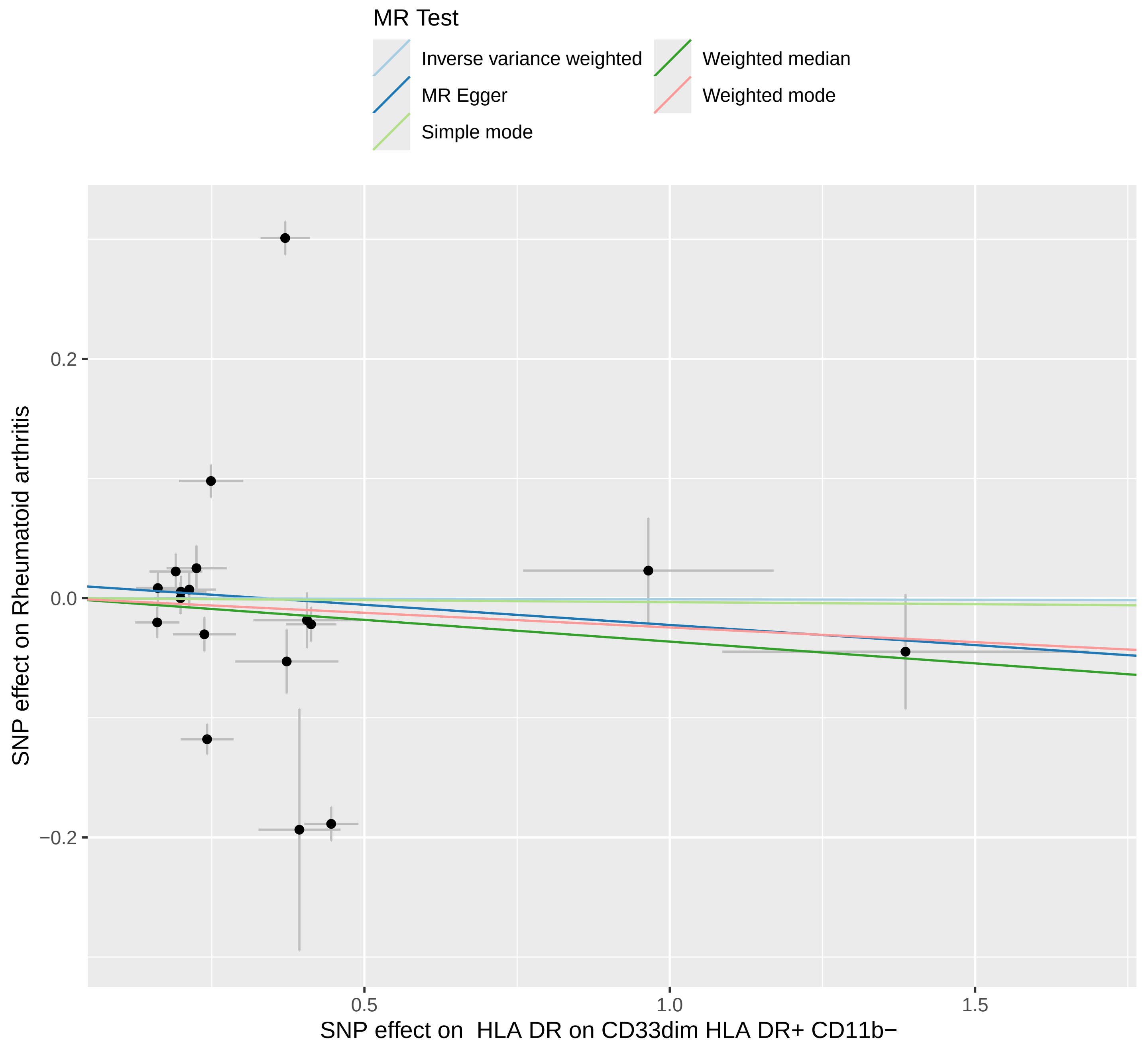
MR Test
Inverse variance weighted
MR Egger
Simple mode
Weighted median
Weighted mode
0.2
0.0
−0.2
SNP effect on Rheumatoid arthritis
0.5
1.0
1.5
SNP effect on HLA DR on CD33dim HLA DR+ CD11b−

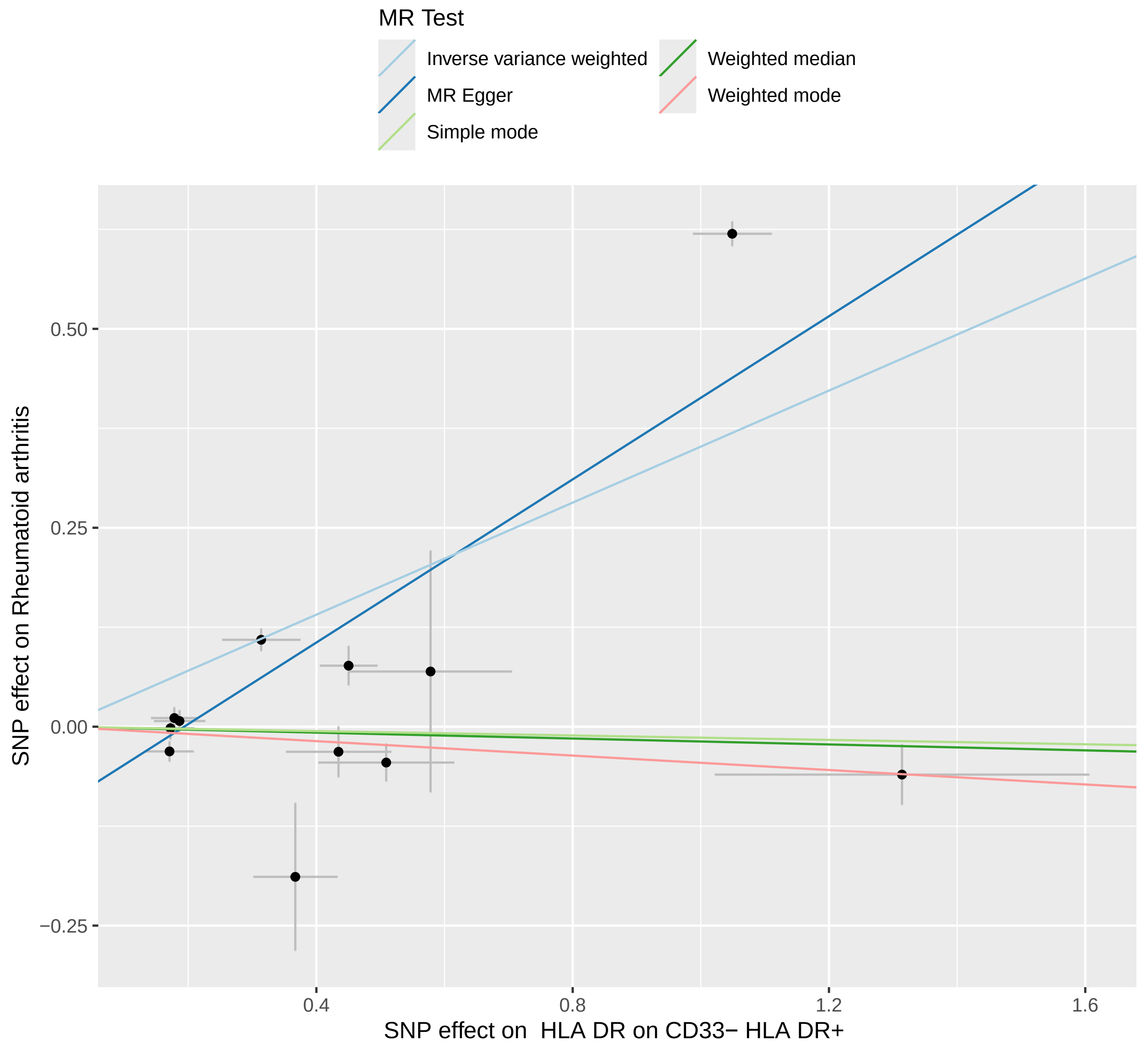

MR Test
Inverse variance weighted
MR Egger
Simple mode
Weighted median
Weighted mode
SNP effect on Rheumatoid arthritis
0.50
0.25
0.00
−0.25
0.4
0.8
1.2
1.6
SNP effect on HLA DR on CD33− HLA DR+

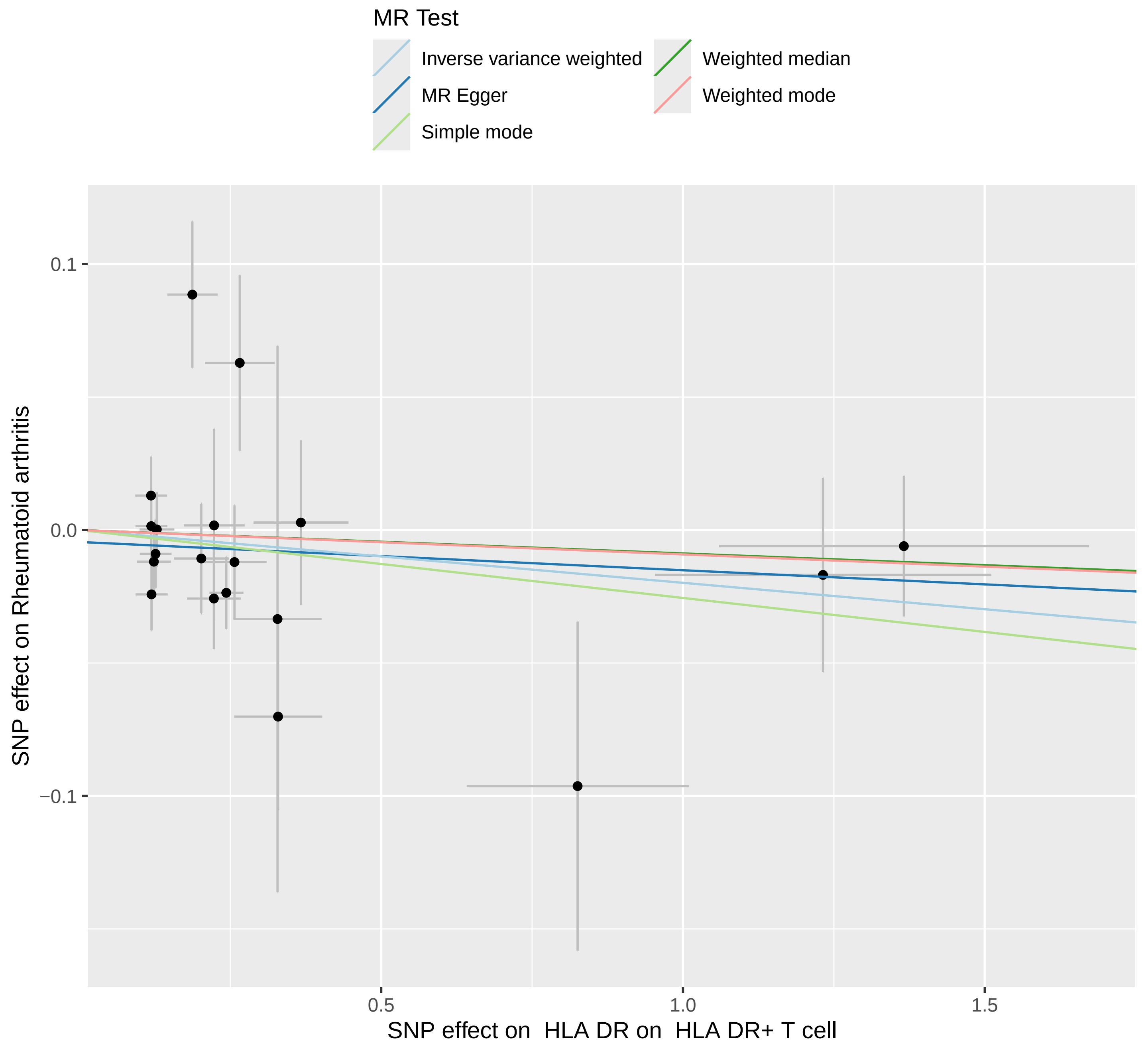

MR Test
Inverse variance weighted
MR Egger
Simple mode
Weighted median
Weighted mode
SNP effect on Rheumatoid arthritis
0.1
0.0
−0.1
0.5
1.0
1.5
SNP effect on HLA DR on HLA DR+ T cell

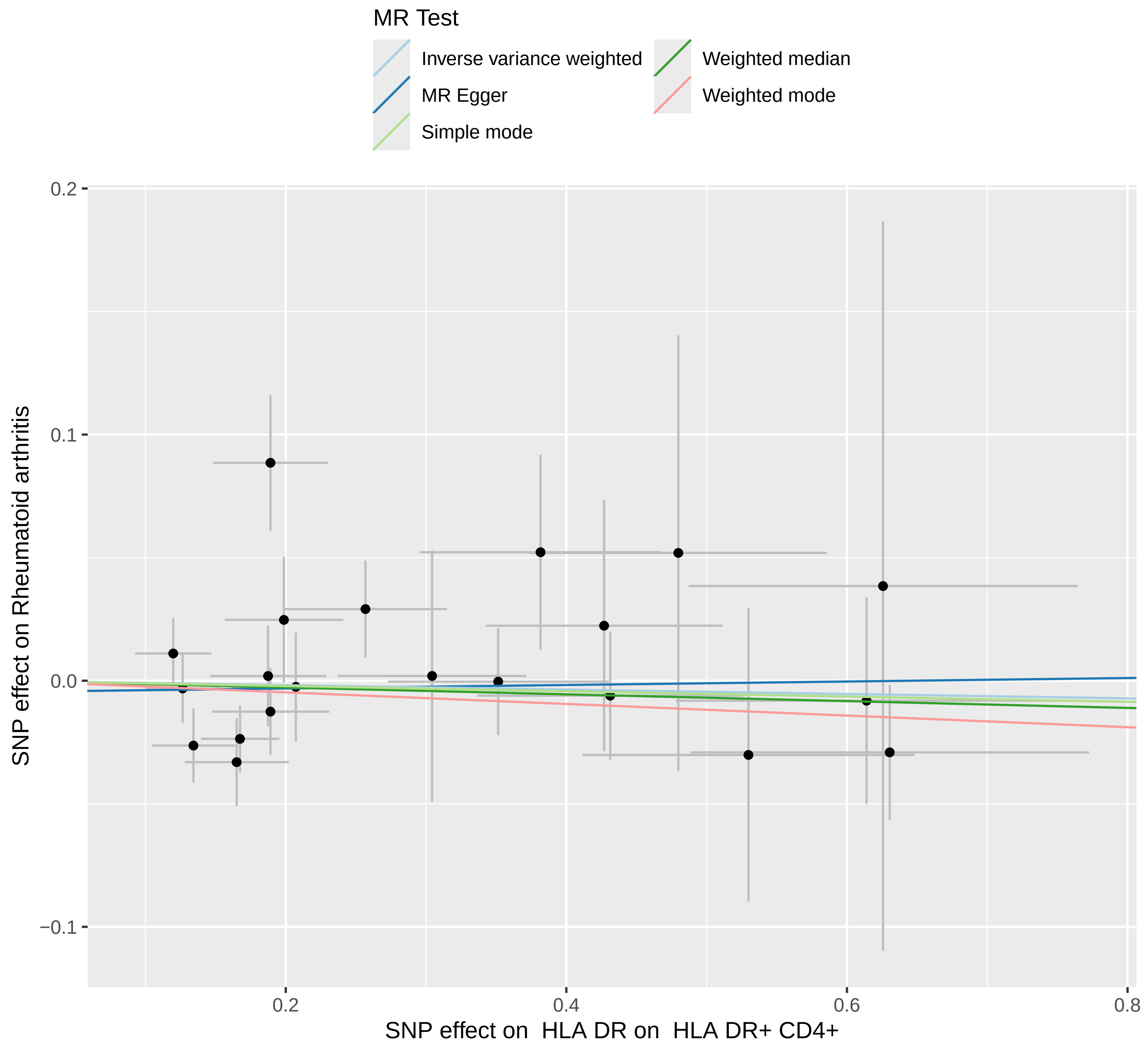

MR Test
Inverse variance weighted
MR Egger
Simple mode
Weighted median
Weighted mode
0.2
0.1
0.0
−0.1
SNP effect on Rheumatoid arthritis
0.2
0.4
0.6
0.8
SNP effect on HLA DR on HLA DR+ CD4+

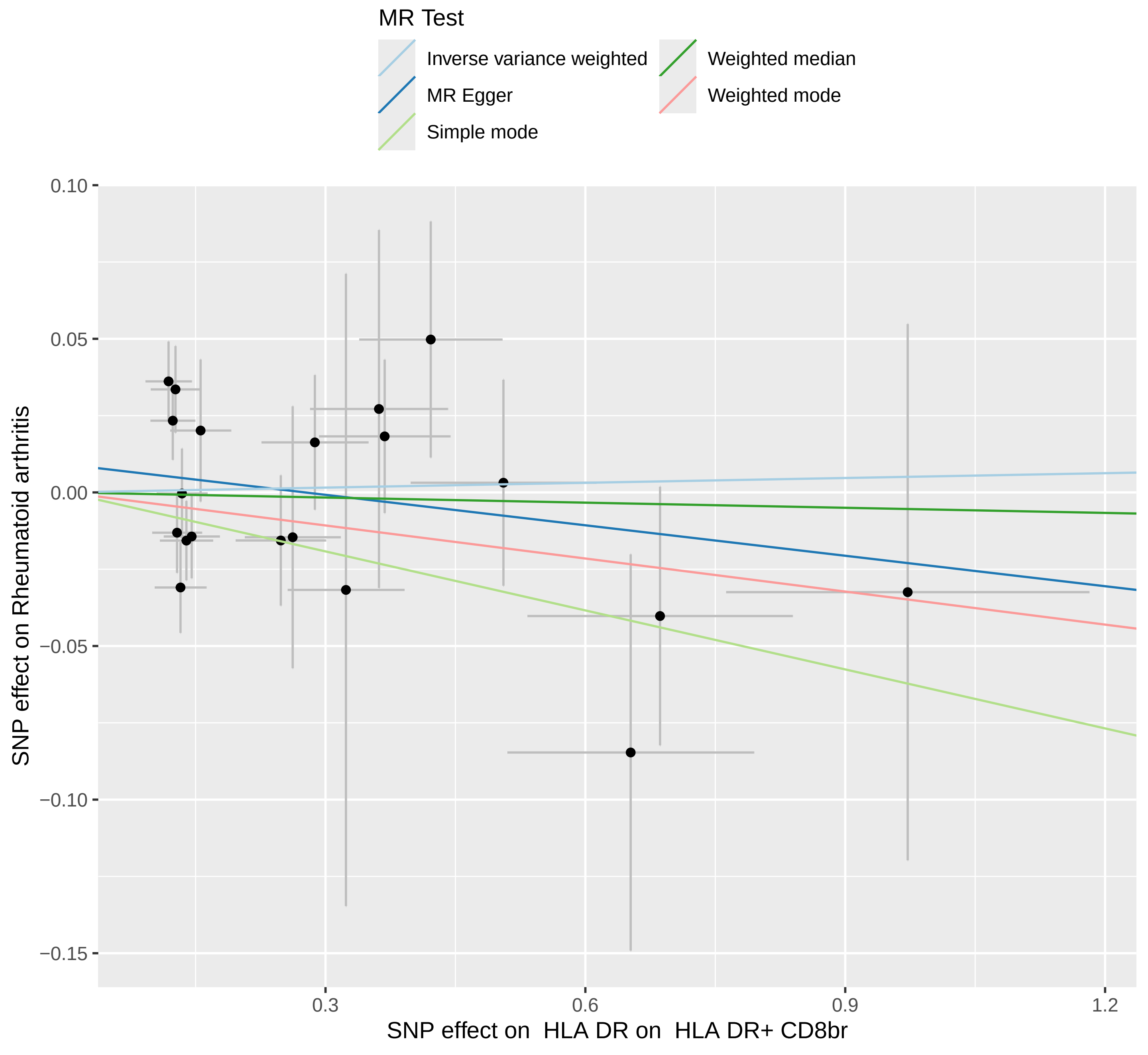

MR Test
Inverse variance weighted
MR Egger
Simple mode
Weighted median
Weighted mode
0.10
0.05
0.00
−0.05
−0.10
−0.15
SNP effect on Rheumatoid arthritis
0.3
0.6
0.9
1.2
SNP effect on HLA DR on HLA DR+ CD8br

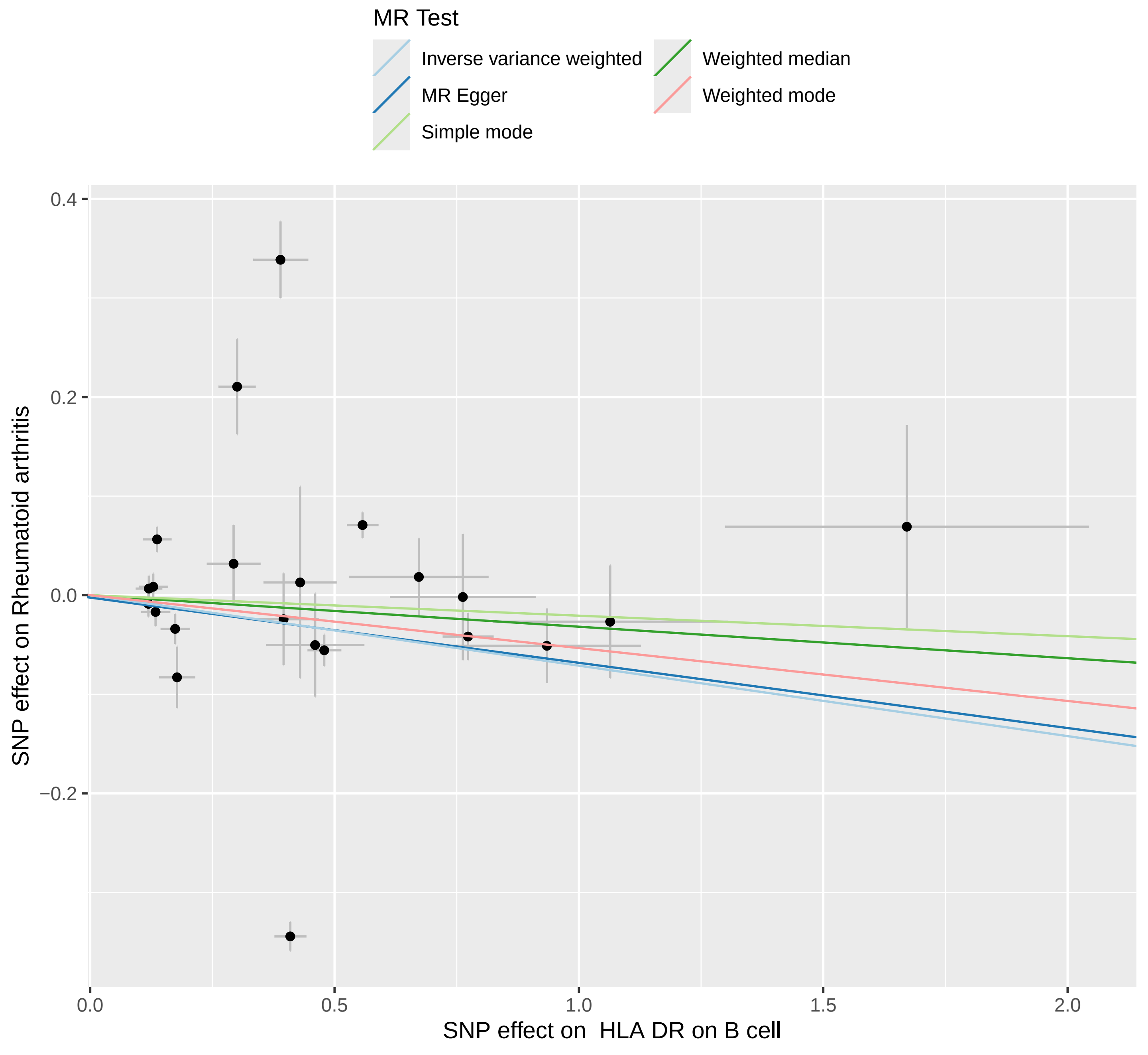

MR Test
Inverse variance weighted
MR Egger
Simple mode
Weighted median
Weighted mode
SNP effect on Rheumatoid arthritis
0.4
0.2
0.0
−0.2
0.0
0.5
1.0
1.5
2.0
SNP effect on HLA DR on B cell

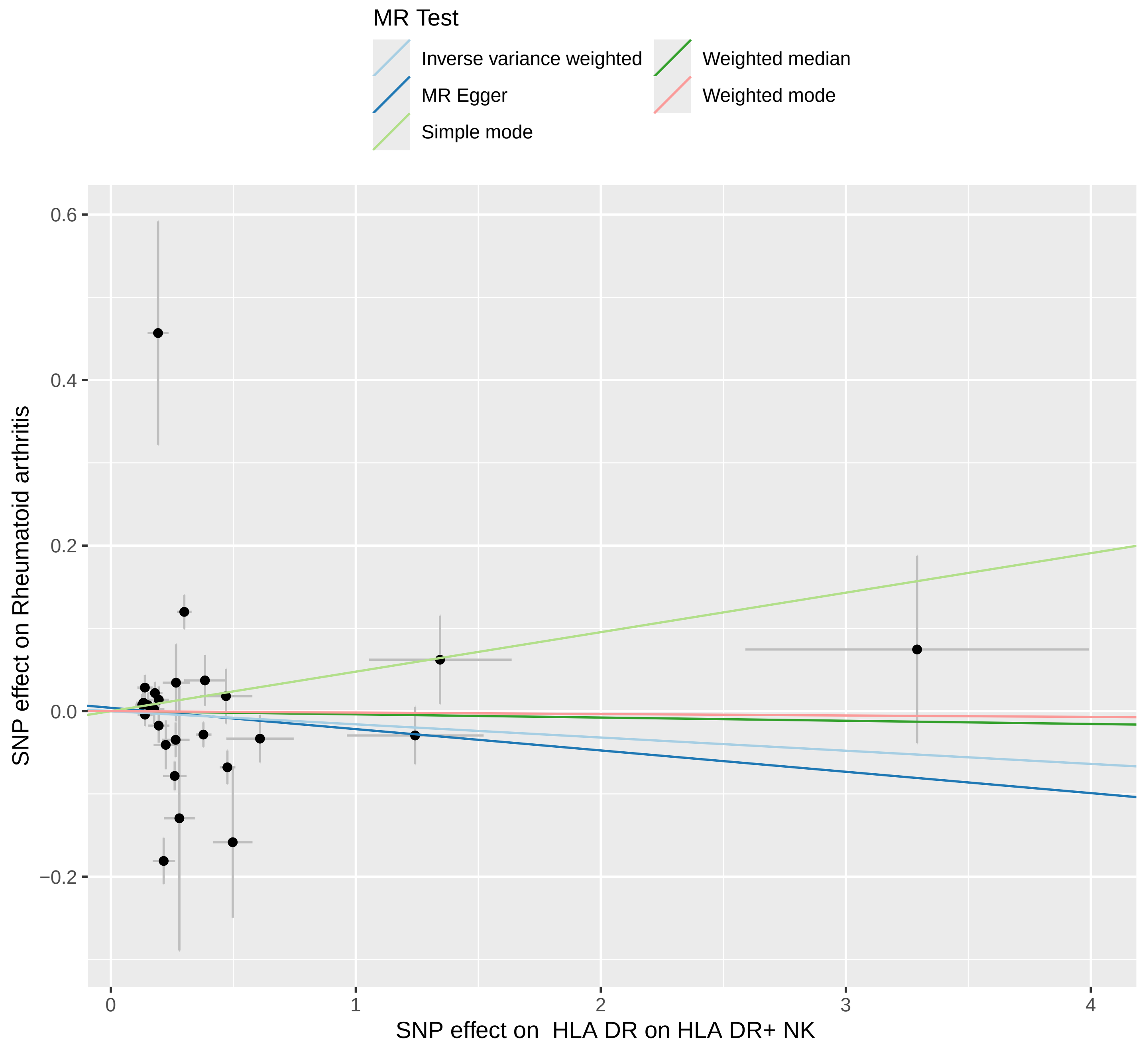
MR Test
Inverse variance weighted
MR Egger
Simple mode
Weighted median
Weighted mode
0.6
0.4
0.2
0.0
−0.2
0
1
2
3
4
SNP effect on Rheumatoid arthritis
SNP effect on HLA DR on HLA DR+ NK

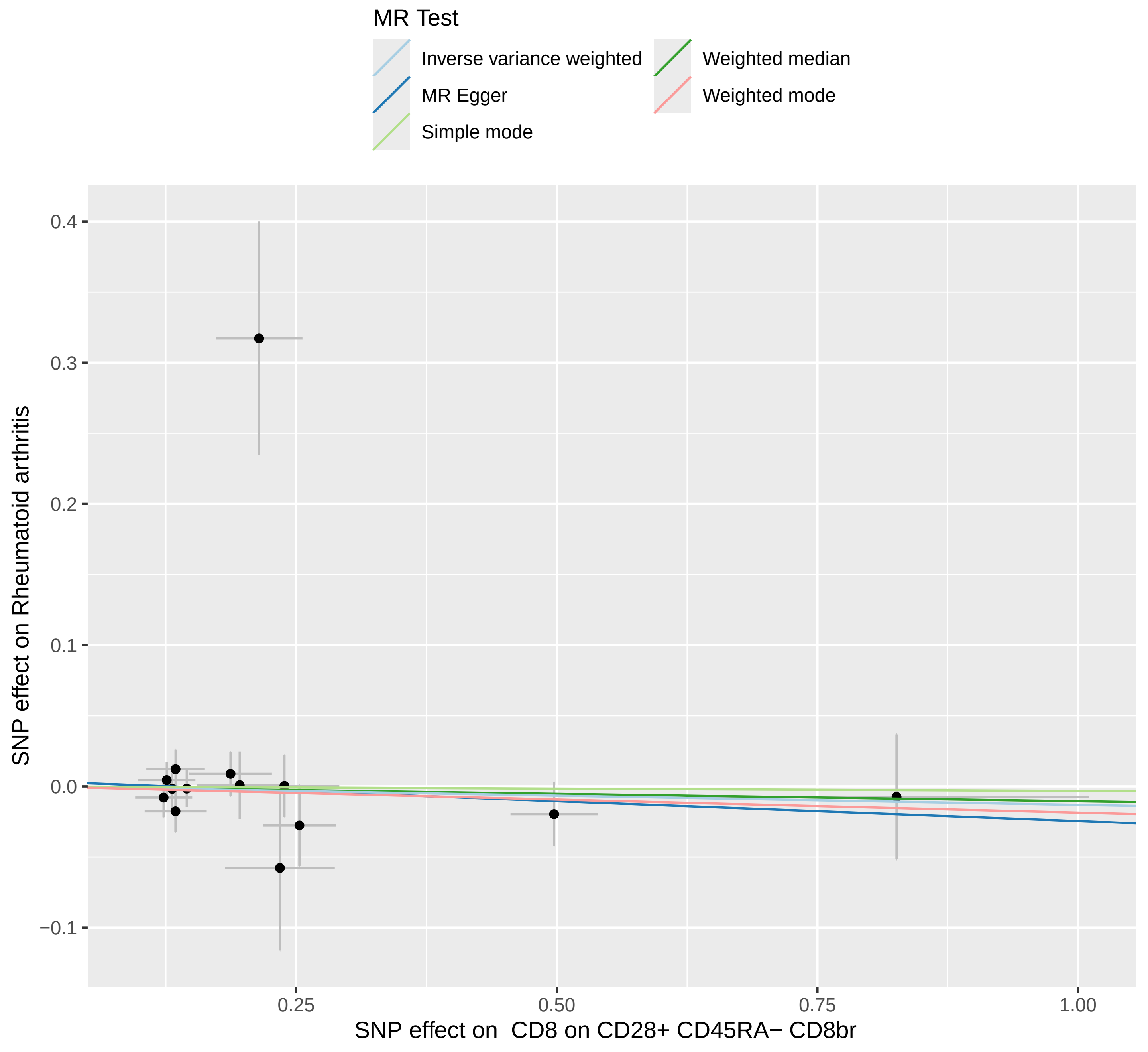
MR Test
Inverse variance weighted
MR Egger
Simple mode
Weighted median
Weighted mode
SNP effect on Rheumatoid arthritis
0.4
0.3
0.2
0.1
0.0
−0.1
0.25
0.50
0.75
1.00
SNP effect on CD8 on CD28+ CD45RA− CD8br

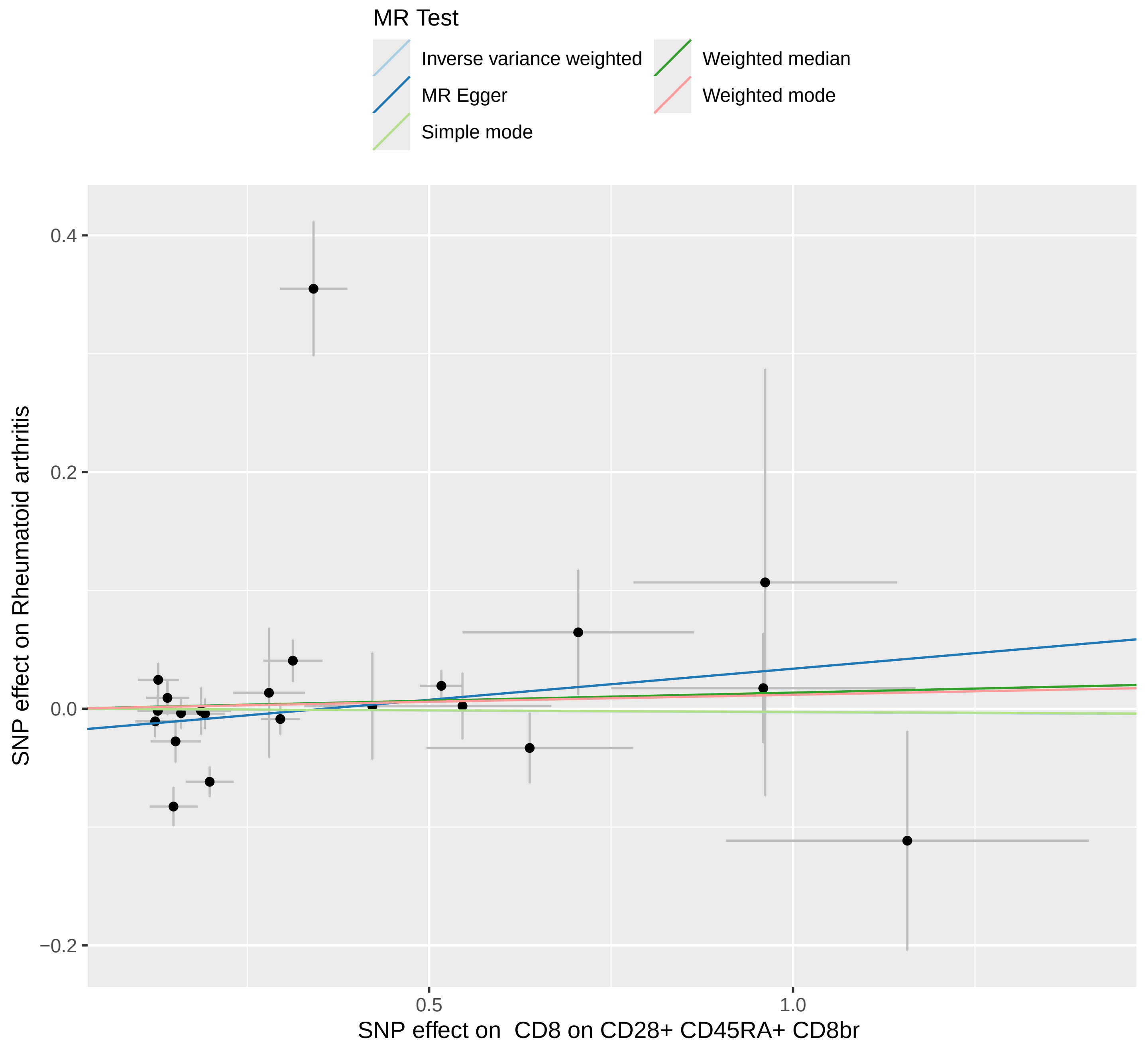
MR Test
Inverse variance weighted
MR Egger
Simple mode
Weighted median
Weighted mode
0.4
0.2
0.0
−0.2
0.5
1.0
SNP effect on Rheumatoid arthritis
SNP effect on CD8 on CD28+ CD45RA+ CD8br

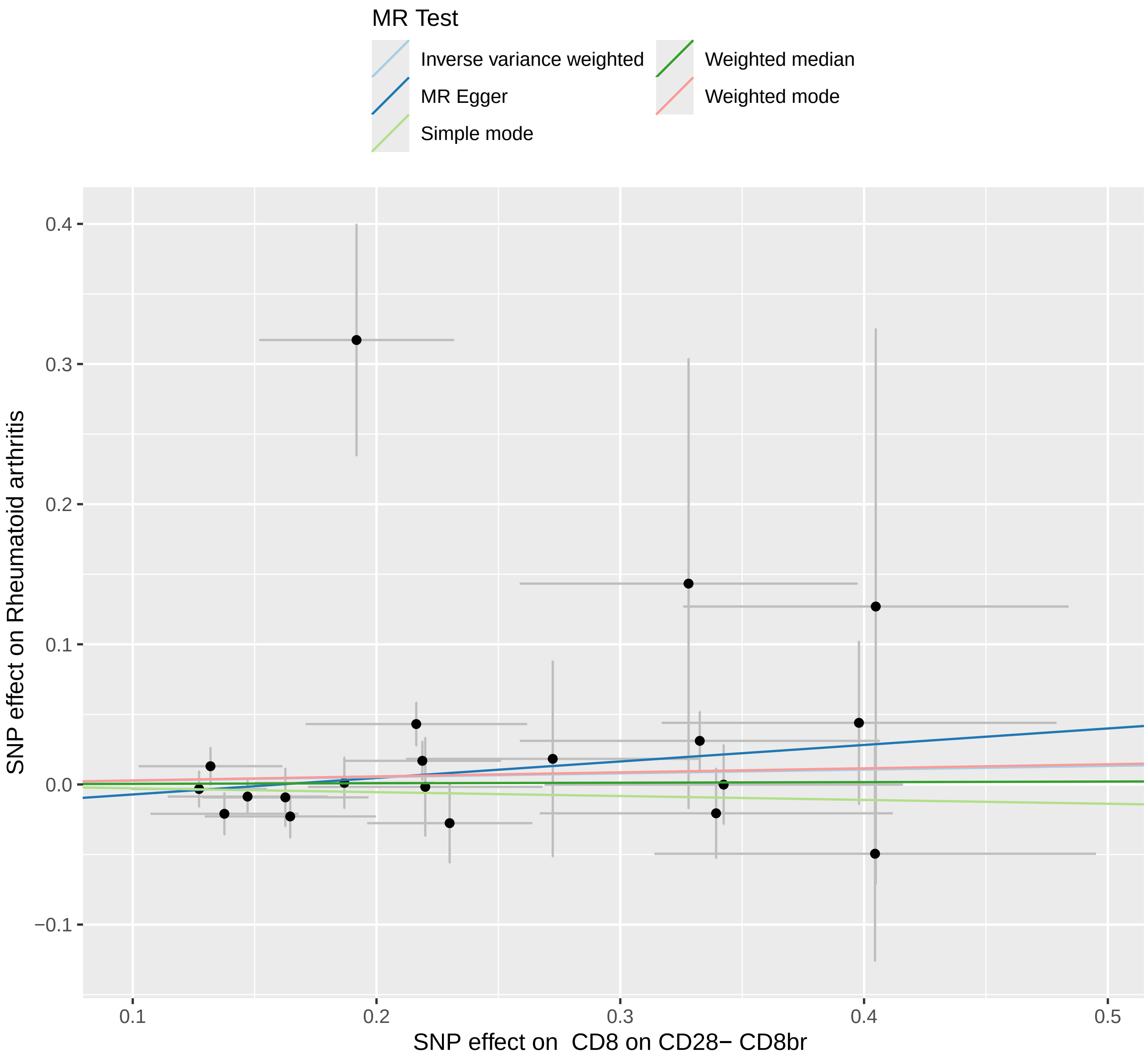
MR Test
Inverse variance weighted
MR Egger
Simple mode
Weighted median
Weighted mode
SNP effect on Rheumatoid arthritis
0.4
0.3
0.2
0.1
0.0
−0.1
0.1
0.2
0.3
0.4
0.5
SNP effect on CD8 on CD28− CD8br

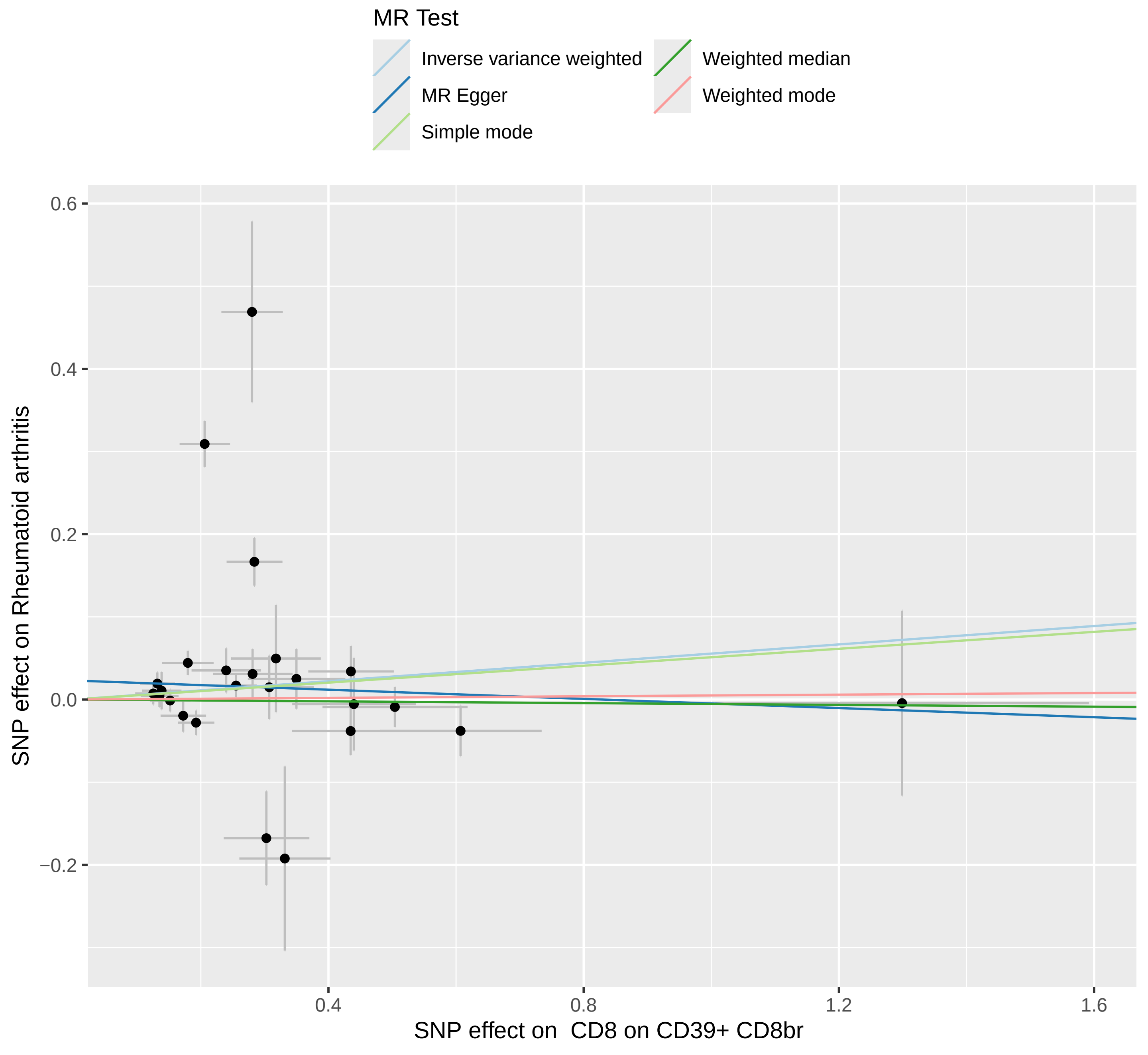

MR Test
Inverse variance weighted
MR Egger
Simple mode
Weighted median
Weighted mode
0.6
0.4
0.2
0.0
−0.2
SNP effect on Rheumatoid arthritis
0.4
0.8
1.2
1.6
SNP effect on CD8 on CD39+ CD8br

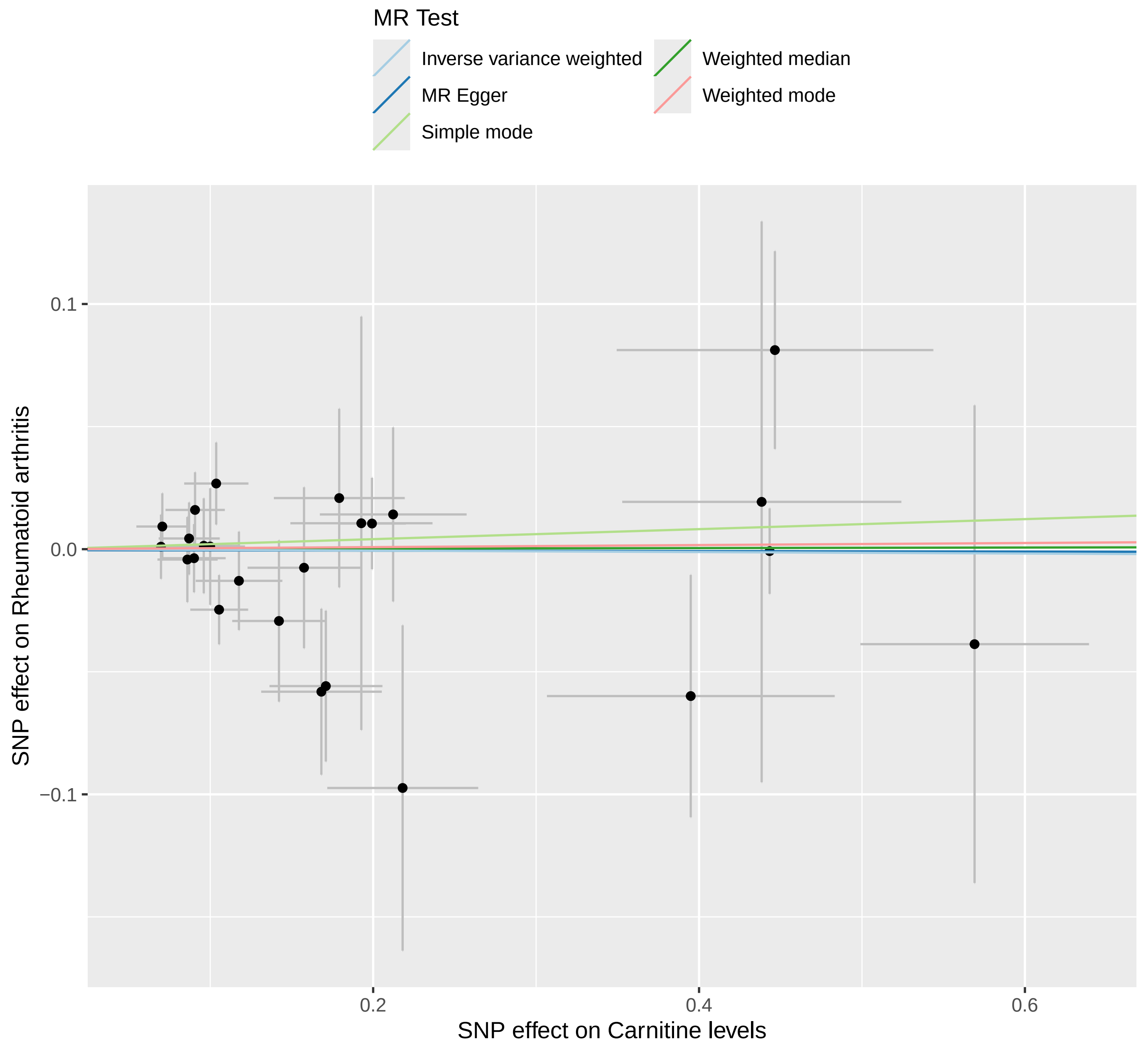
MR Test
Inverse variance weighted
MR Egger
Simple mode
Weighted median
Weighted mode
0.1
0.0
−0.1
0.2
0.4
0.6
SNP effect on Rheumatoid arthritis
SNP effect on Carnitine levels

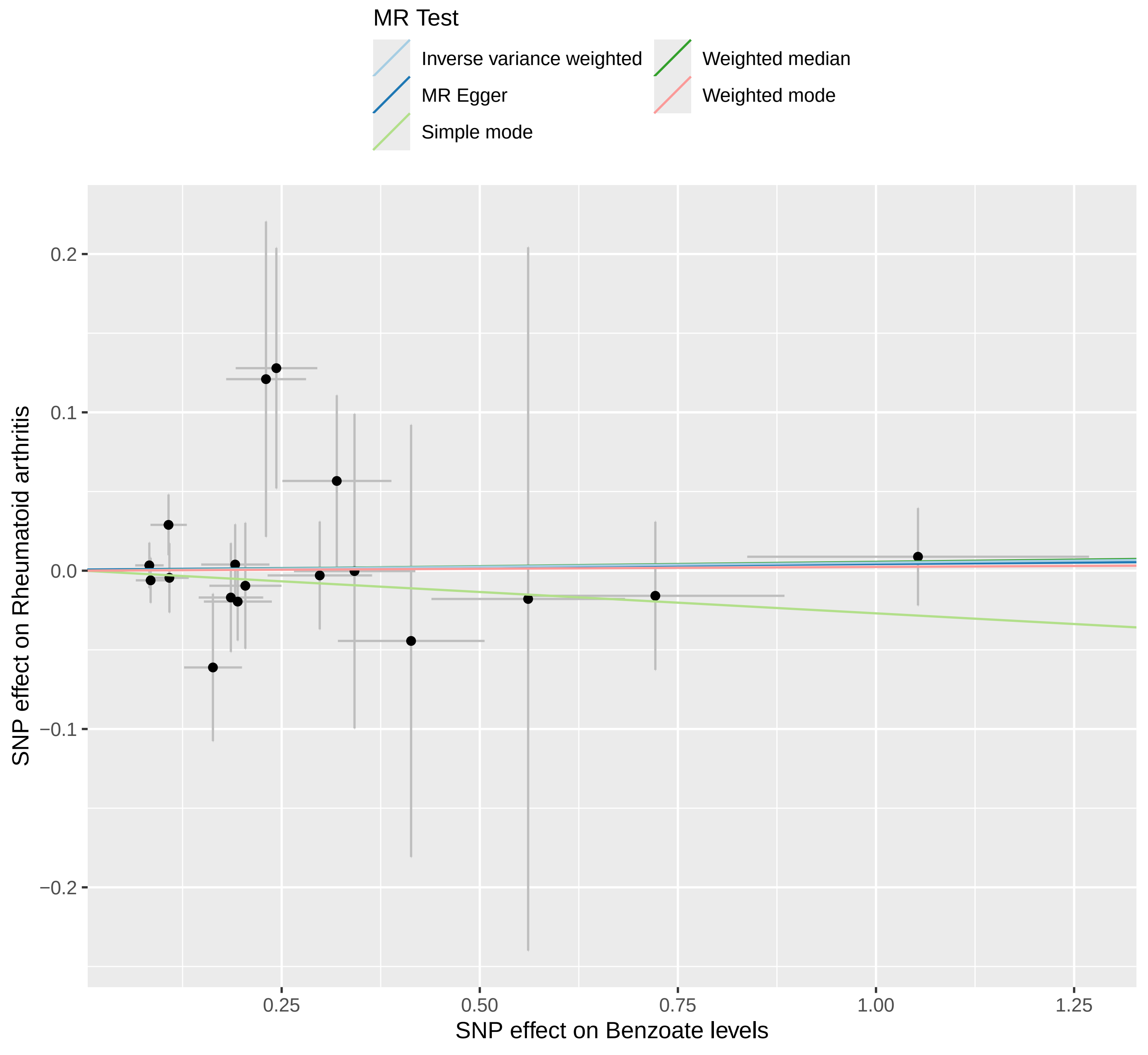

MR Test
Inverse variance weighted
MR Egger
Simple mode
Weighted median
Weighted mode
0.2
0.1
0.0
−0.1
−0.2
0.25
0.50
0.75
1.00
1.25
SNP effect on Rheumatoid arthritis
SNP effect on Benzoate levels

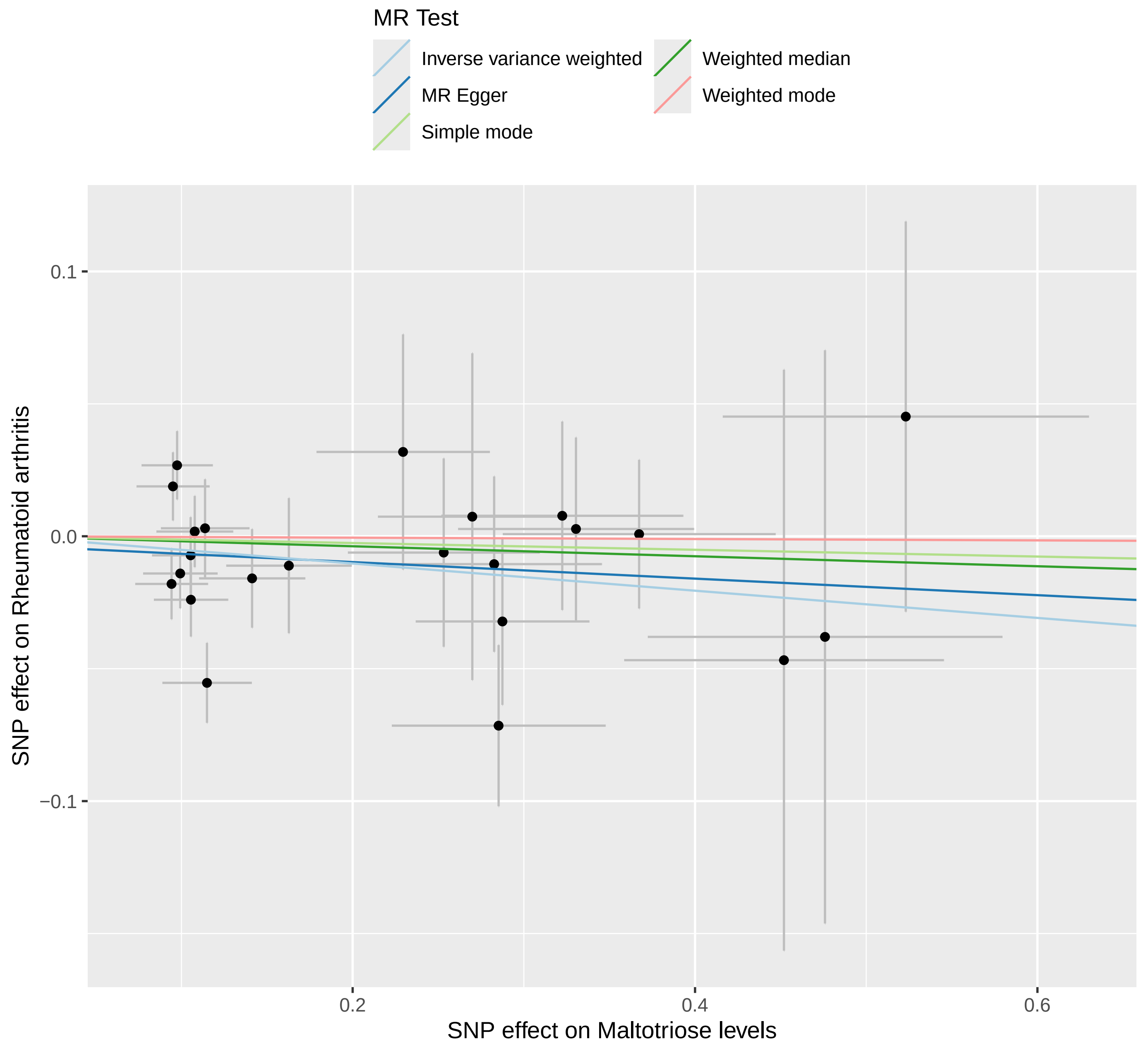

MR Test
Inverse variance weighted
MR Egger
Simple mode
Weighted median
Weighted mode
0.1
0.0
−0.1
SNP effect on Rheumatoid arthritis
0.2
0.4
0.6
SNP effect on Maltotriose levels

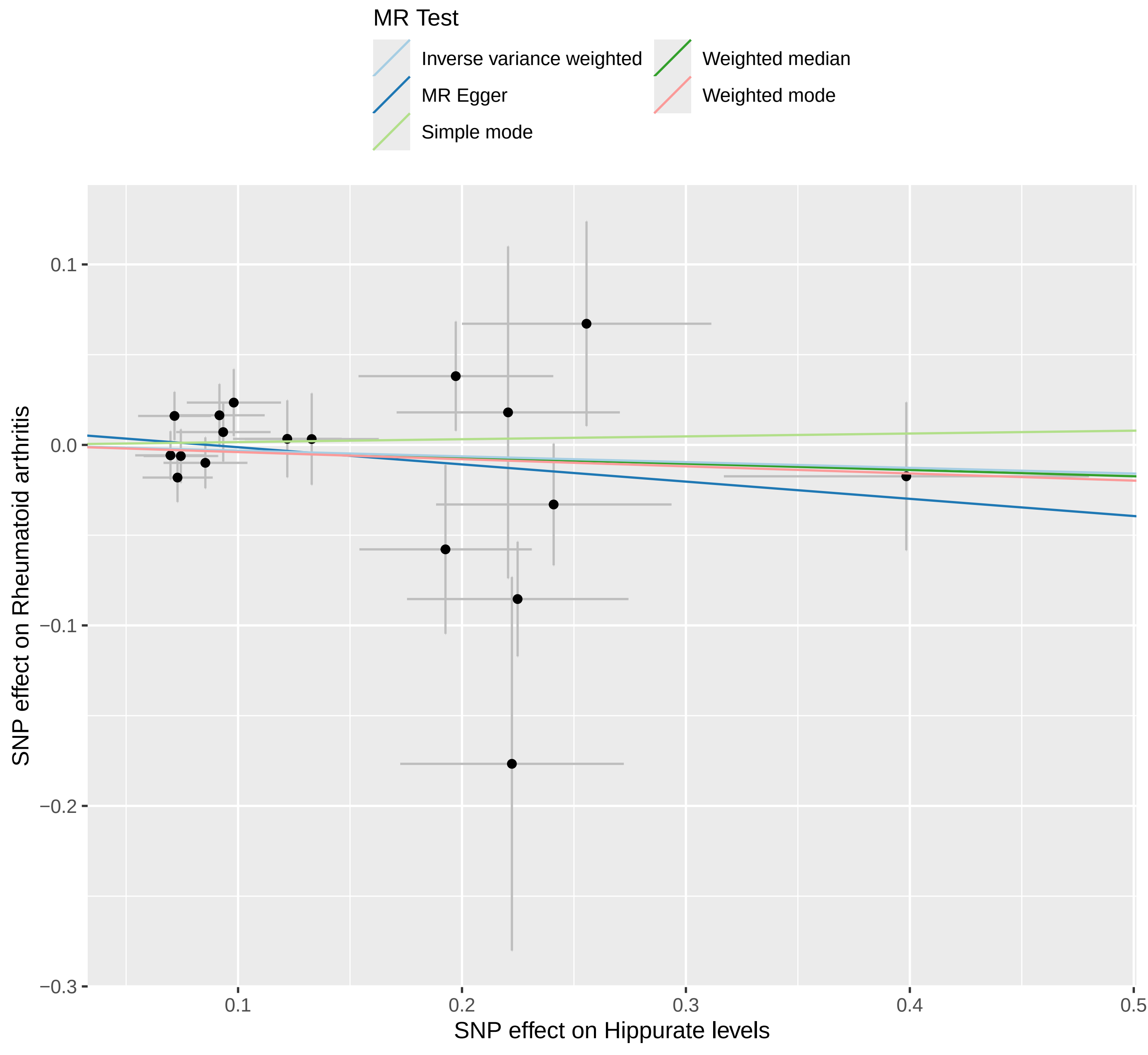
MR Test
Inverse variance weighted
MR Egger
Simple mode
Weighted median
Weighted mode
0.1
0.0
−0.1
−0.2
−0.3
0.1
0.2
0.3
0.4
0.5
SNP effect on Rheumatoid arthritis
SNP effect on Hippurate levels

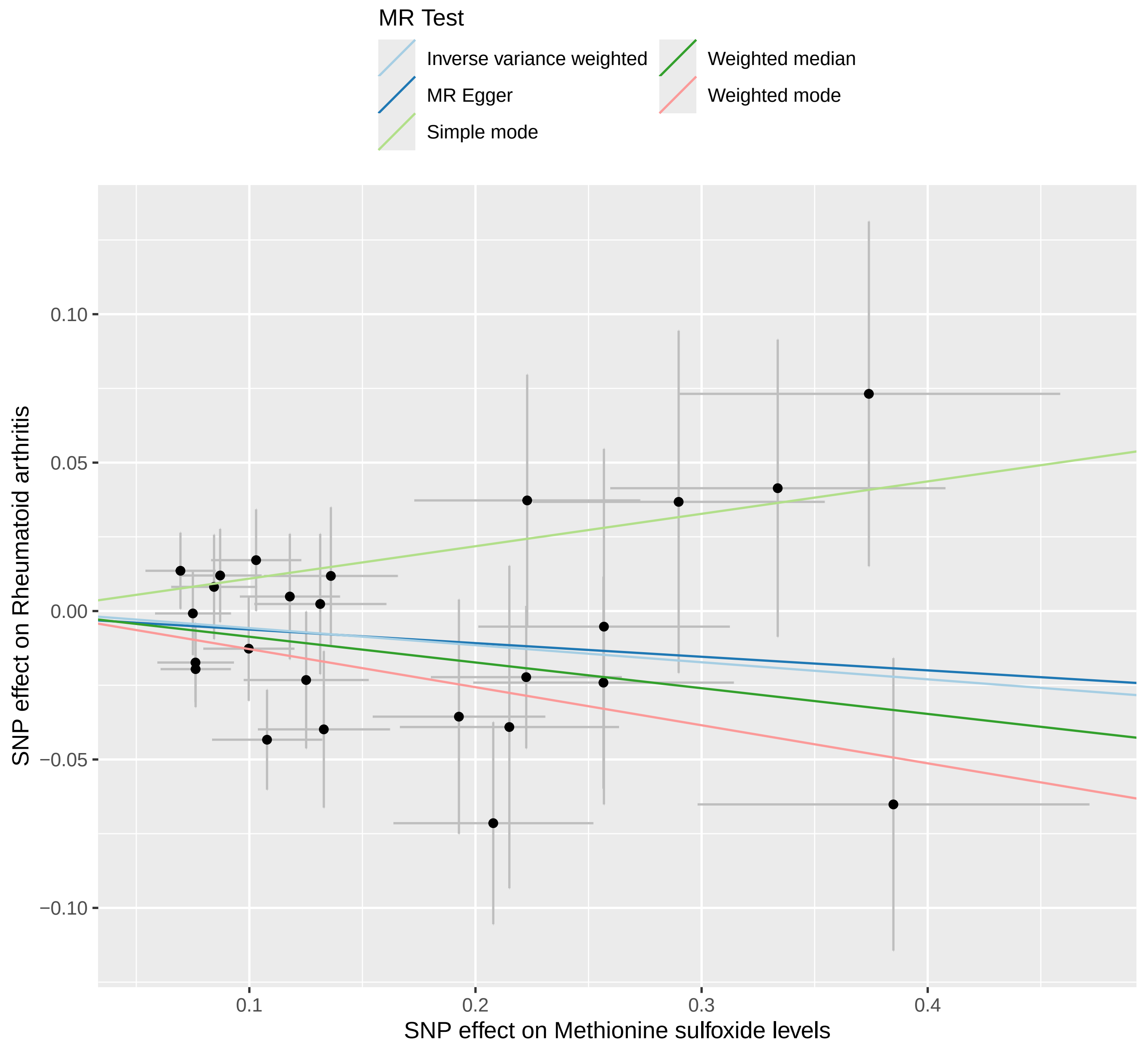
MR Test
Inverse variance weighted
MR Egger
Simple mode
Weighted median
Weighted mode
SNP effect on Rheumatoid arthritis
0.10
0.05
0.00
−0.05
−0.10
0.1
0.2
0.3
0.4
SNP effect on Methionine sulfoxide levels

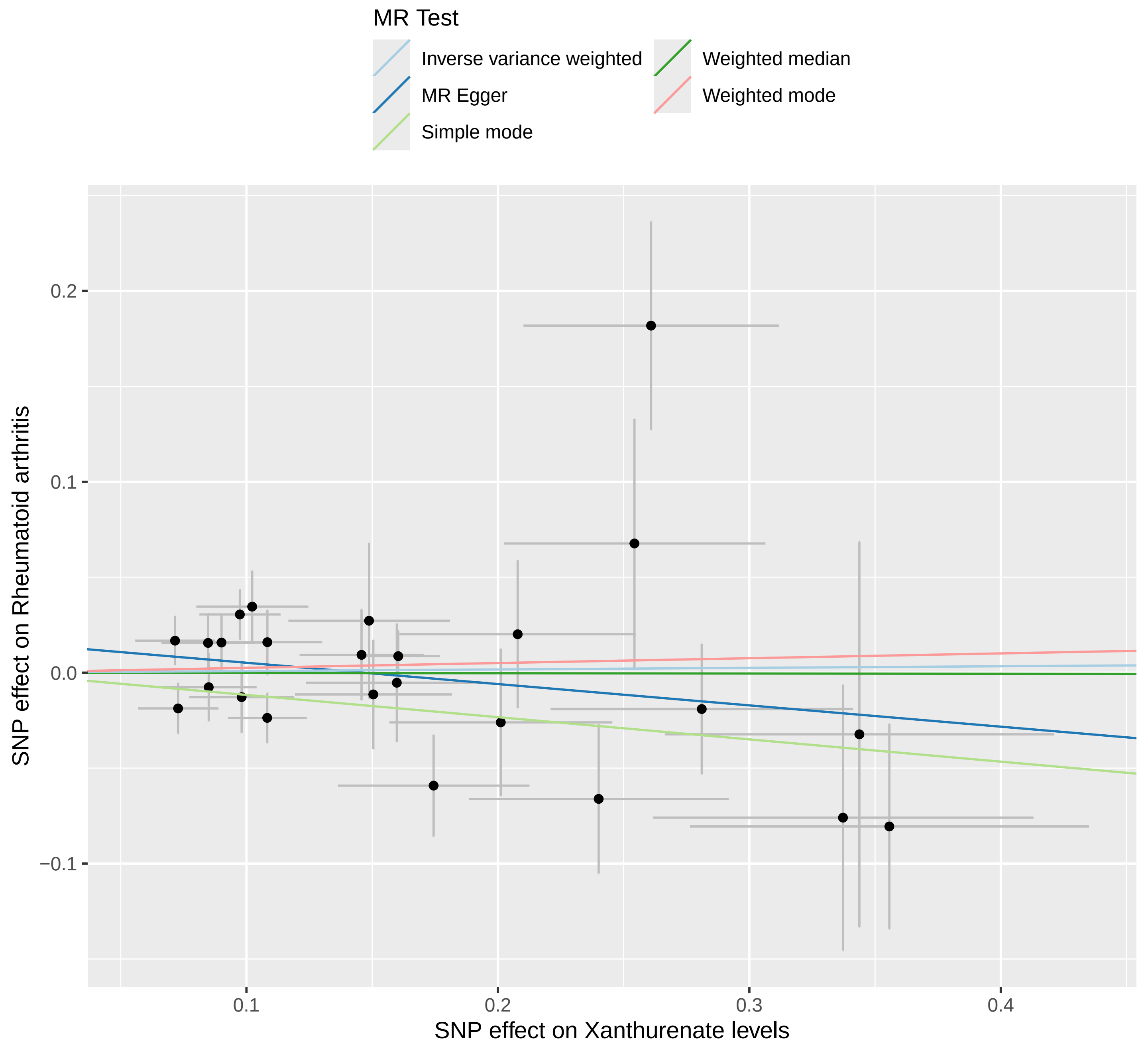
MR Test
Inverse variance weighted
MR Egger
Simple mode
Weighted median
Weighted mode
SNP effect on Rheumatoid arthritis
0.2
0.1
0.0
−0.1
0.1
0.2
0.3
0.4
SNP effect on Xanthurenate levels

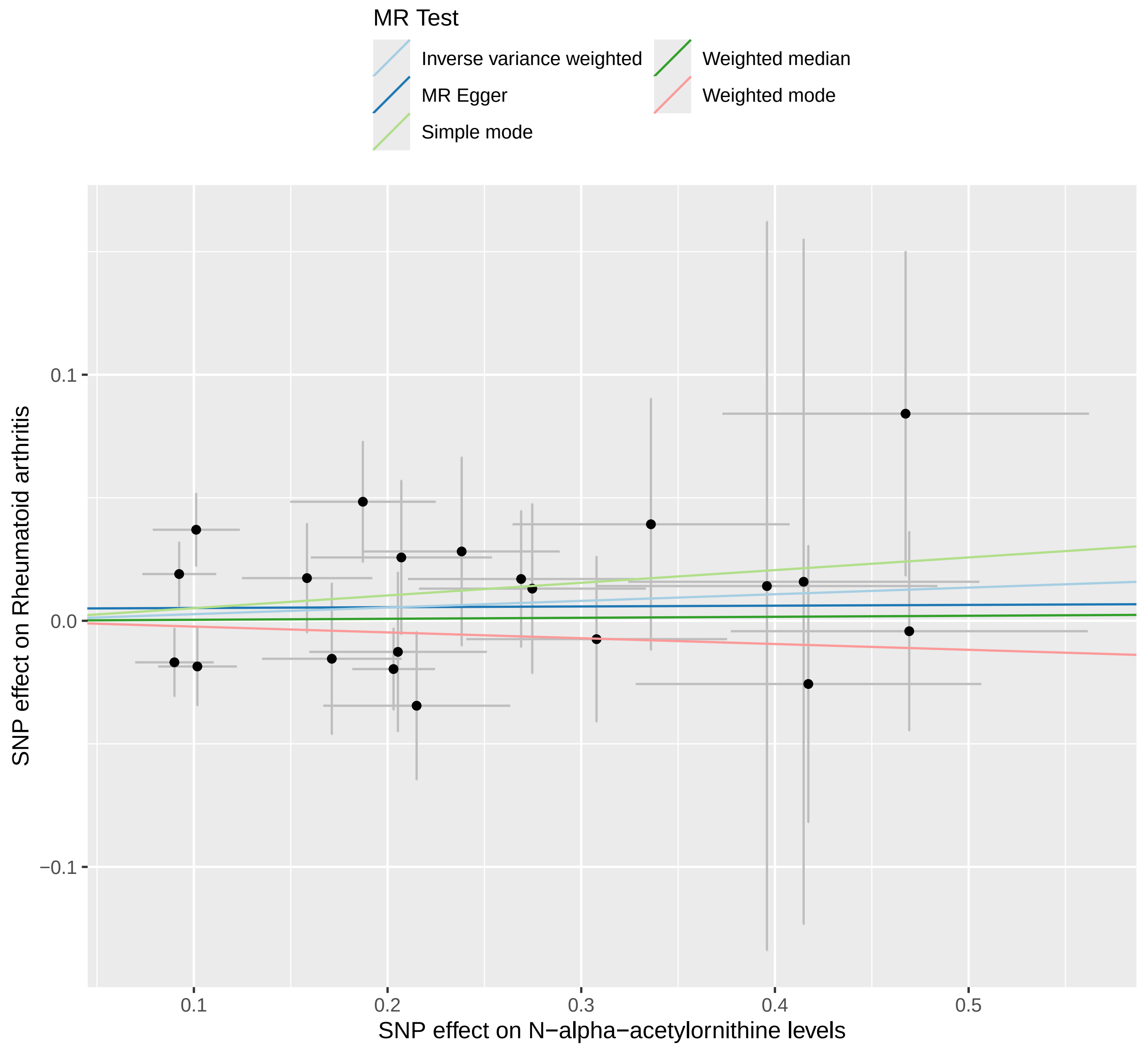
MR Test
Inverse variance weighted
MR Egger
Simple mode
Weighted median
Weighted mode
0.1
0.0
−0.1
SNP effect on Rheumatoid arthritis
0.1
0.2
0.3
0.4
0.5
SNP effect on N−alpha−acetylornithine levels

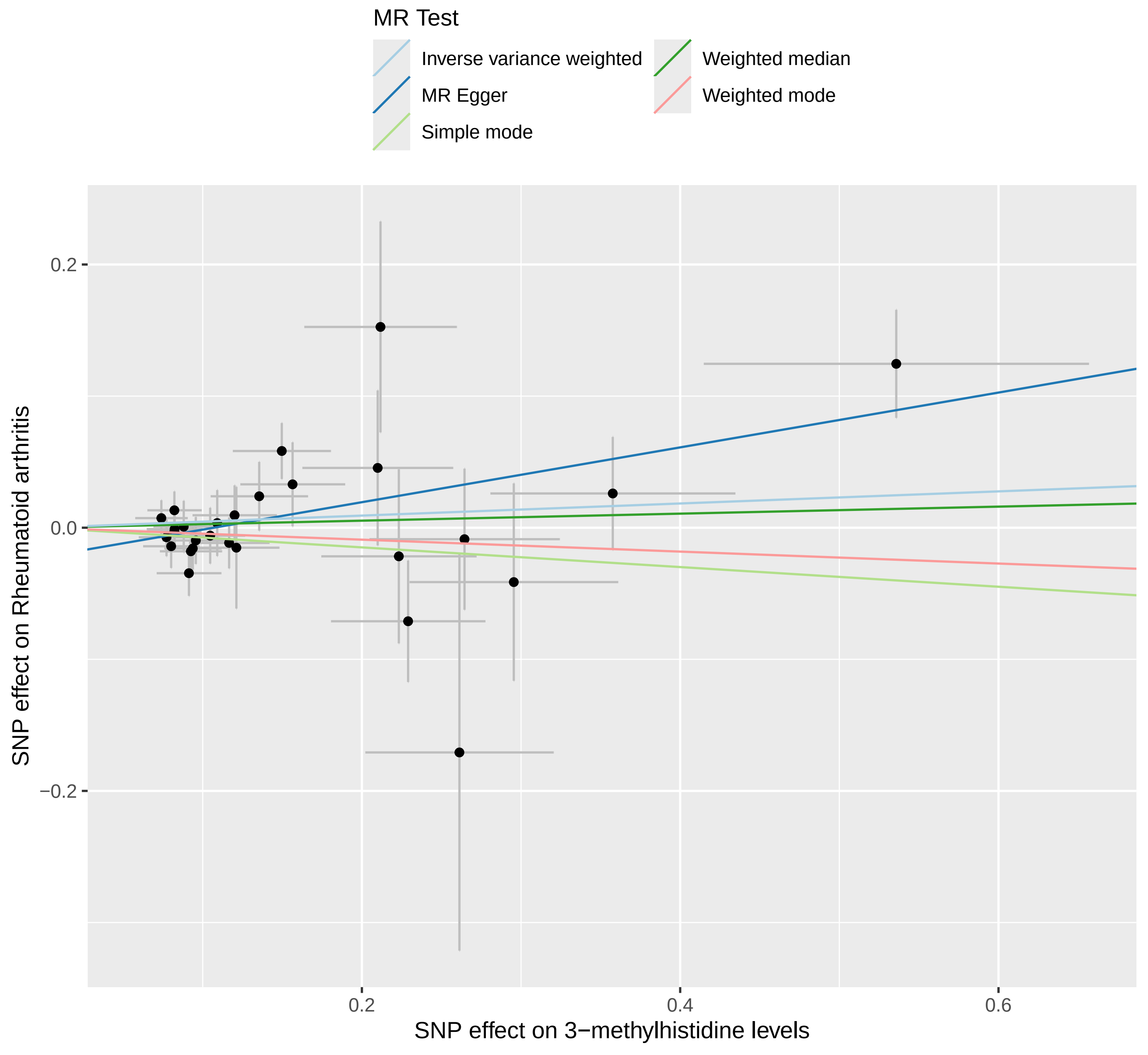
MR Test
Inverse variance weighted
MR Egger
Simple mode
Weighted median
Weighted mode
0.2
0.0
−0.2
0.2
0.4
0.6
SNP effect on Rheumatoid arthritis
SNP effect on 3−methylhistidine levels

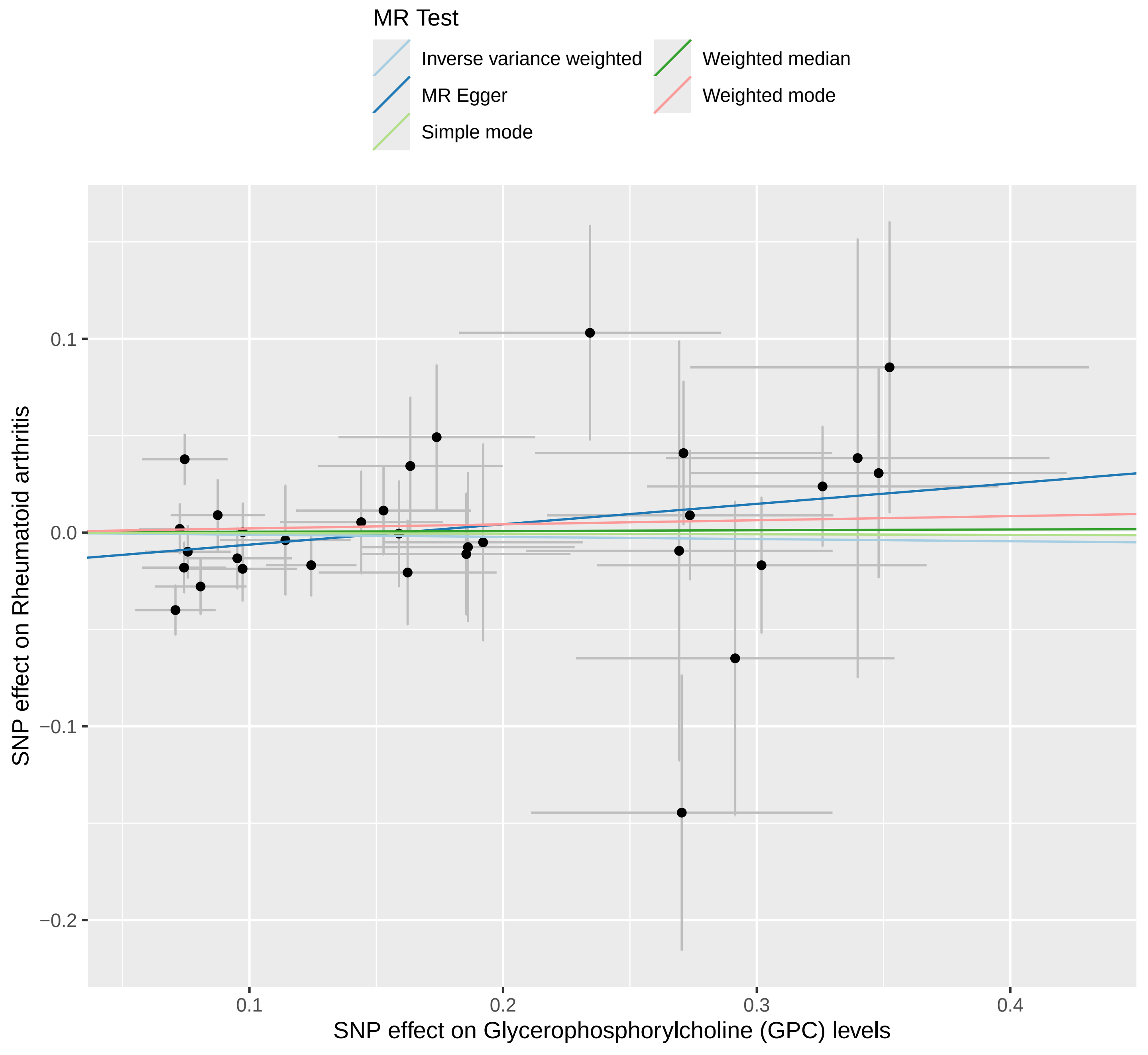

MR Test
Inverse variance weighted
MR Egger
Simple mode
Weighted median
Weighted mode
0.1
0.0
−0.1
−0.2
SNP effect on Rheumatoid arthritis
0.1
0.2
0.3
0.4
SNP effect on Glycerophosphorylcholine (GPC) levels

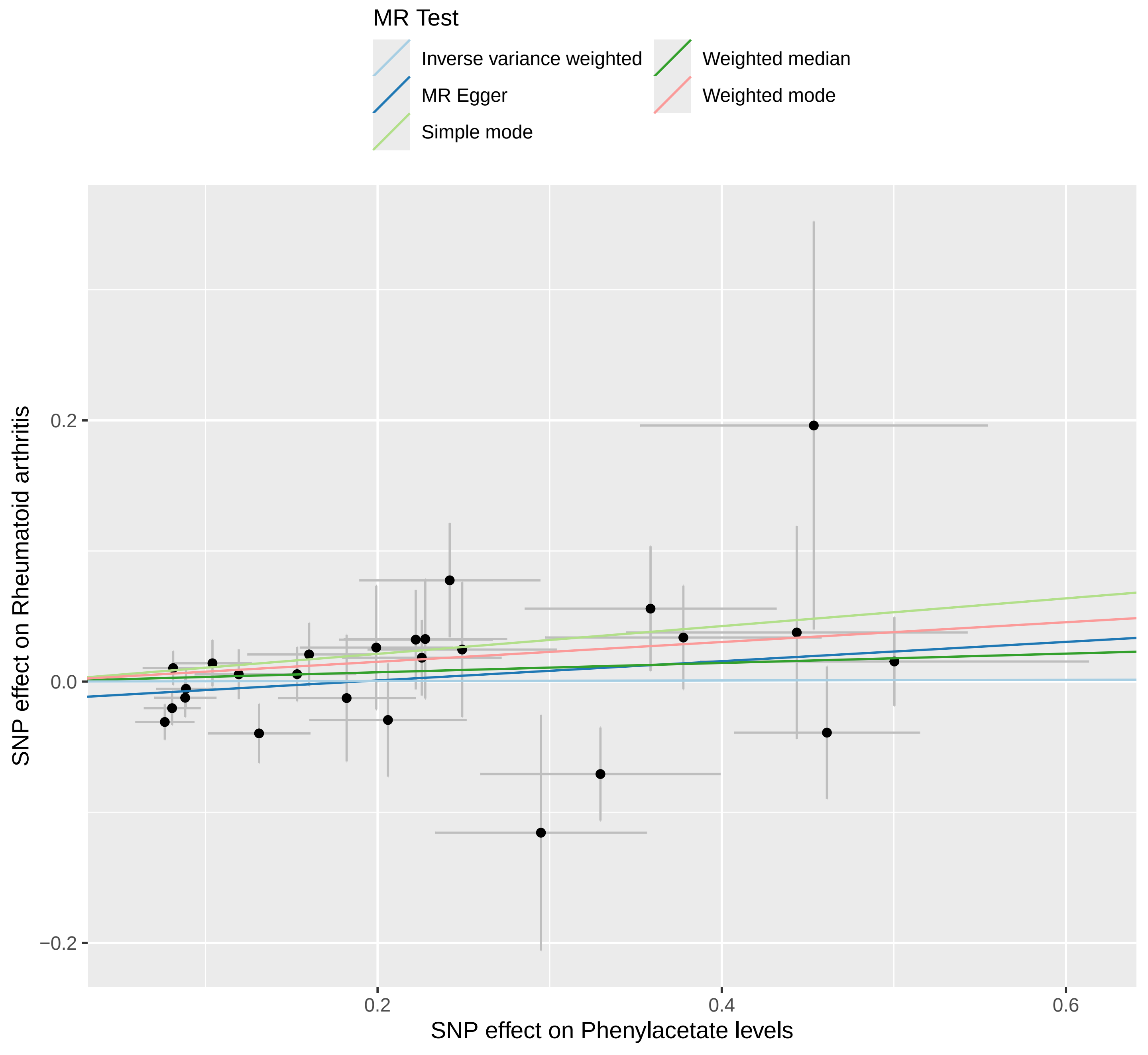
MR Test
Inverse variance weighted
MR Egger
Simple mode
Weighted median
Weighted mode
SNP effect on Rheumatoid arthritis
0.2
0.0
−0.2
0.2
0.4
0.6
SNP effect on Phenylacetate levels

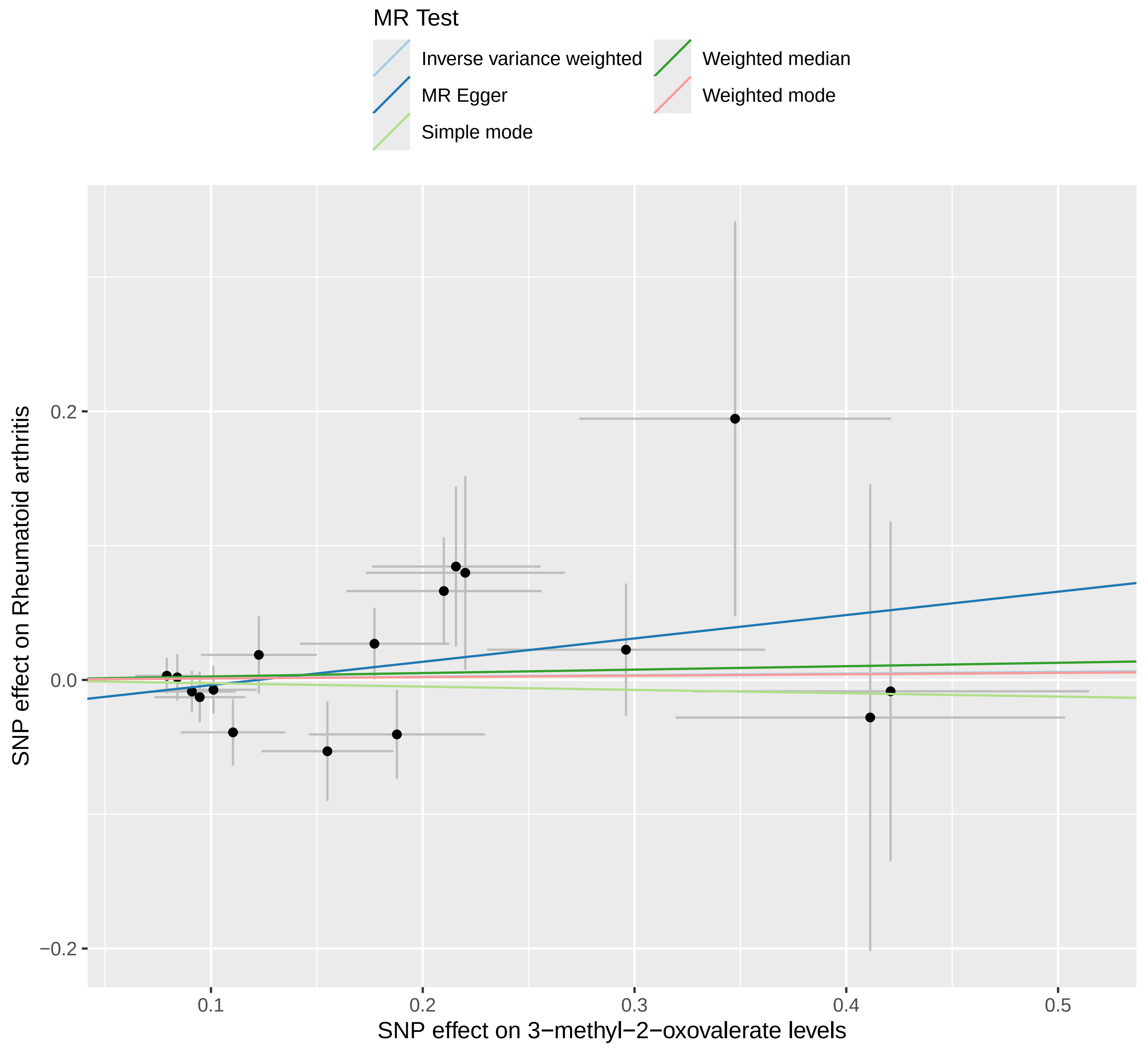
MR Test
Inverse variance weighted
MR Egger
Simple mode
Weighted median
Weighted mode
0.2
0.0
−0.2
0.1
0.2
0.3
0.4
0.5
SNP effect on Rheumatoid arthritis
SNP effect on 3−methyl−2−oxovalerate levels

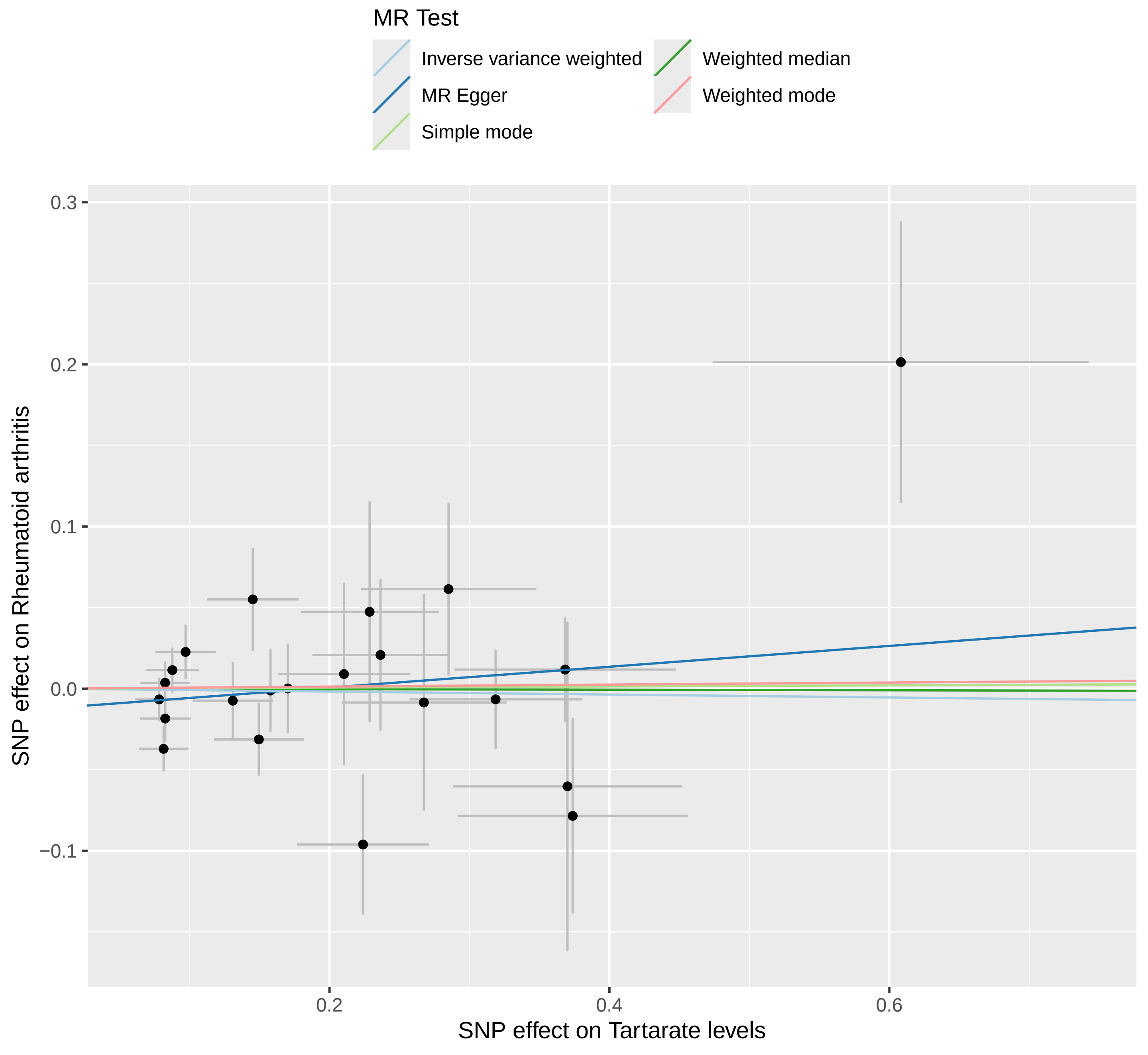
MR Test
Inverse variance weighted
MR Egger
Simple mode
Weighted median
Weighted mode
0.3
0.2
0.1
0.0
−0.1
SNP effect on Rheumatoid arthritis
0.2
0.4
0.6
SNP effect on Tartarate levels

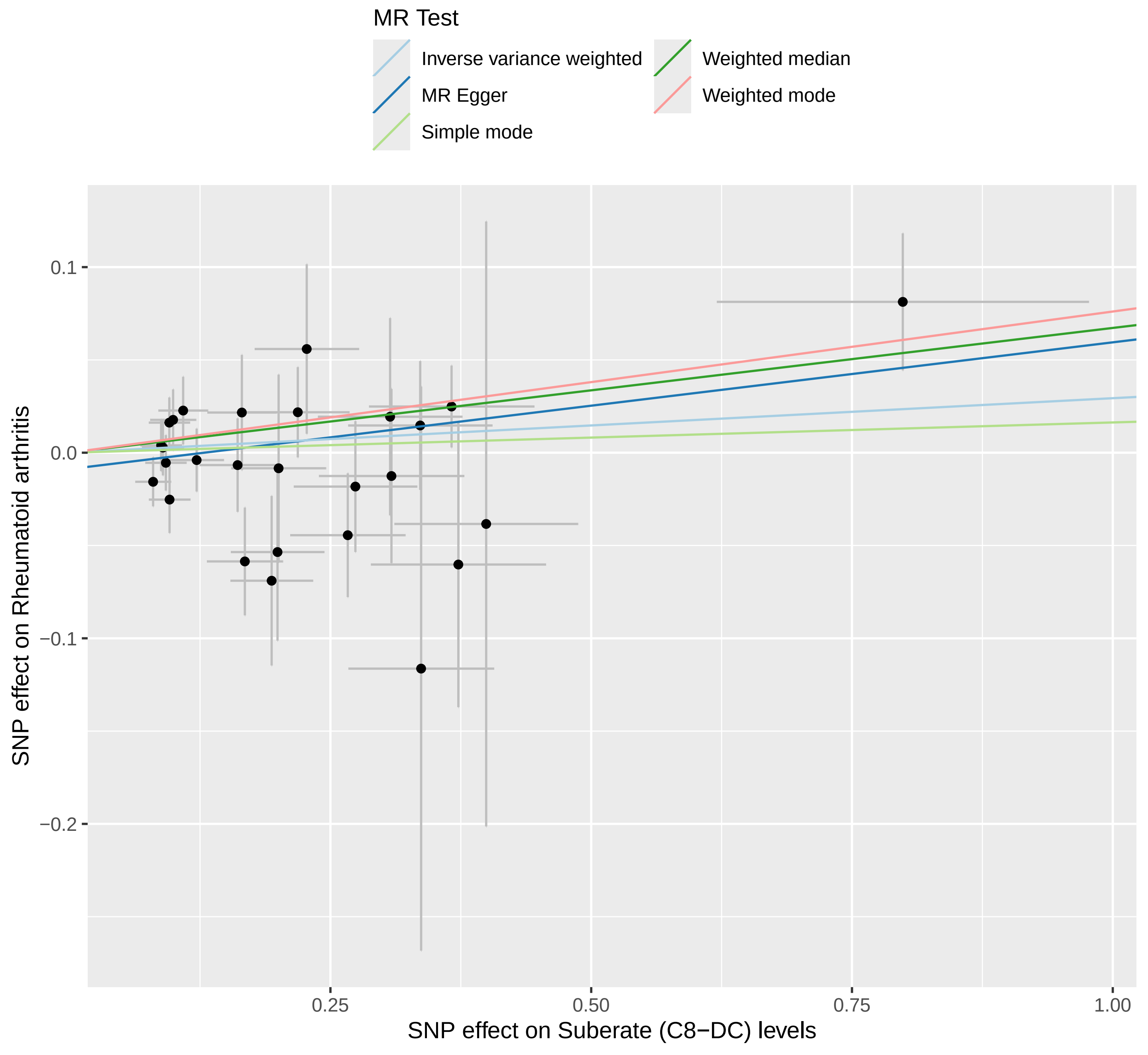
MR Test
Inverse variance weighted
MR Egger
Simple mode
Weighted median
Weighted mode
0.1
0.0
−0.1
−0.2
0.25
0.50
0.75
1.00
SNP effect on Rheumatoid arthritis
SNP effect on Suberate (C8−DC) levels

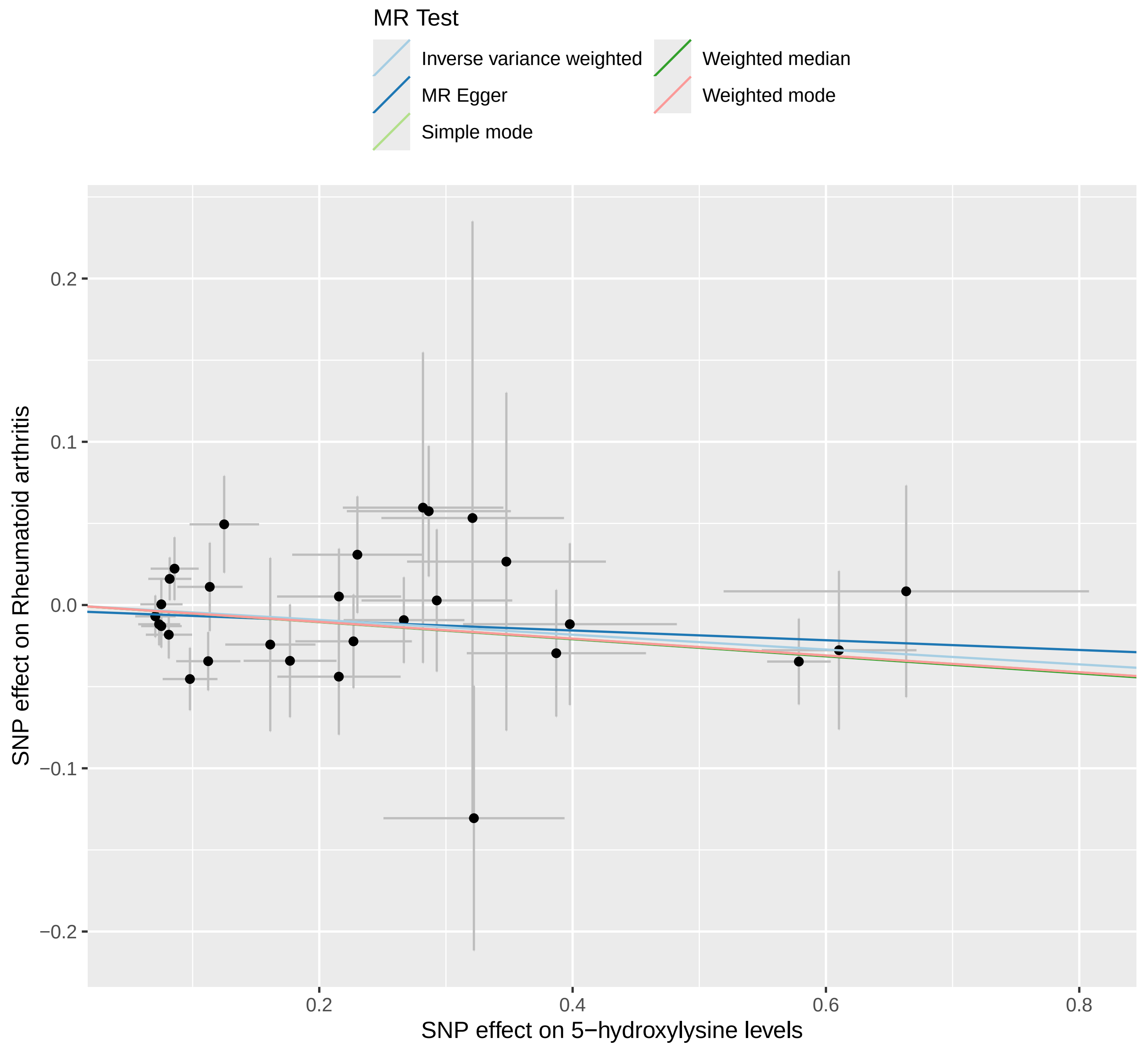
MR Test
Inverse variance weighted
MR Egger
Simple mode
Weighted median
Weighted mode
0.2
0.1
0.0
−0.1
−0.2
0.2
0.4
0.6
0.8
SNP effect on Rheumatoid arthritis
SNP effect on 5−hydroxylysine levels

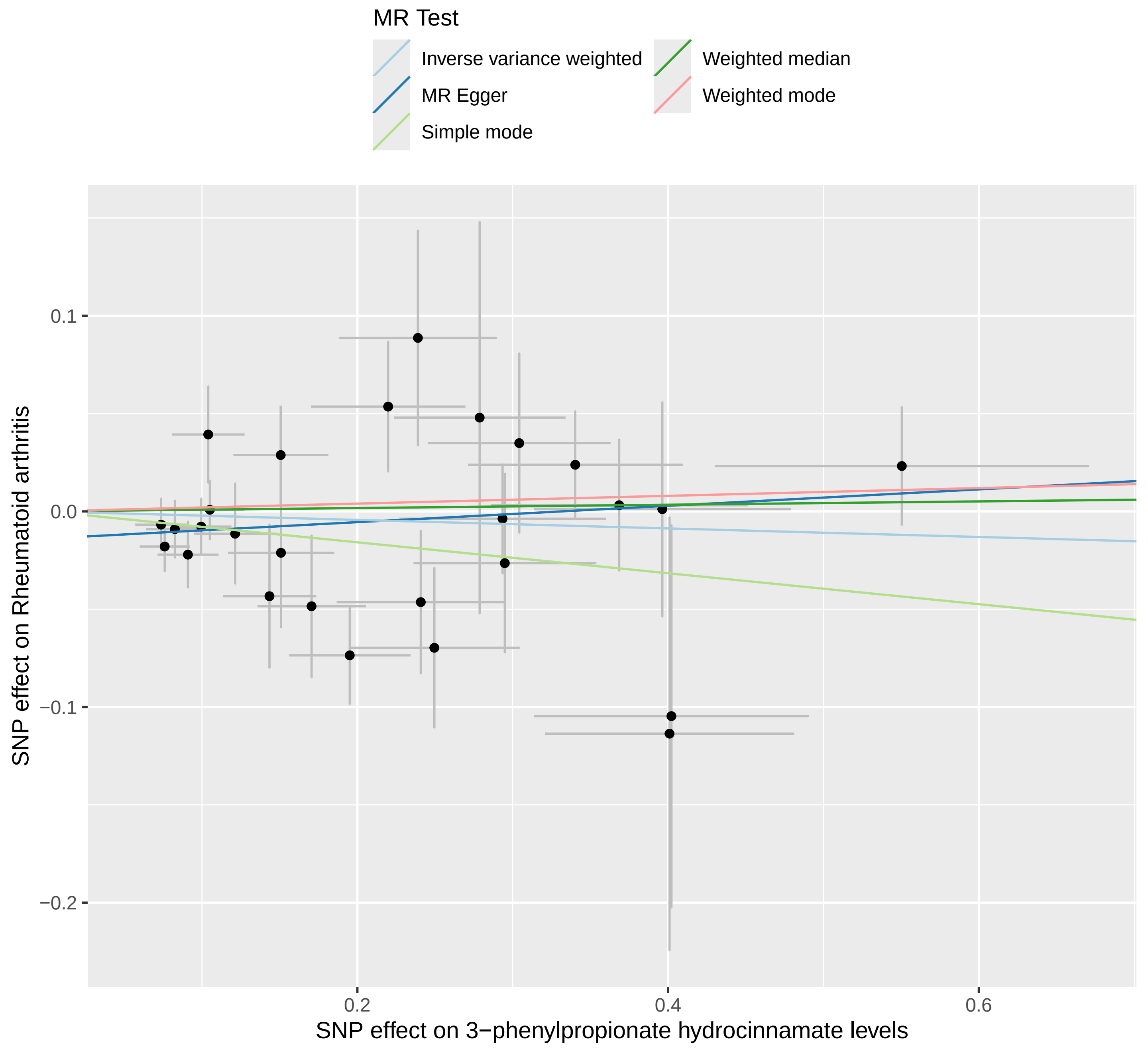

MR Test
Inverse variance weighted
MR Egger
Simple mode
Weighted median
Weighted mode
0.1
0.0
−0.1
−0.2
0.2
0.4
0.6
SNP effect on Rheumatoid arthritis
SNP effect on 3−phenylpropionate hydrocinnamate levels

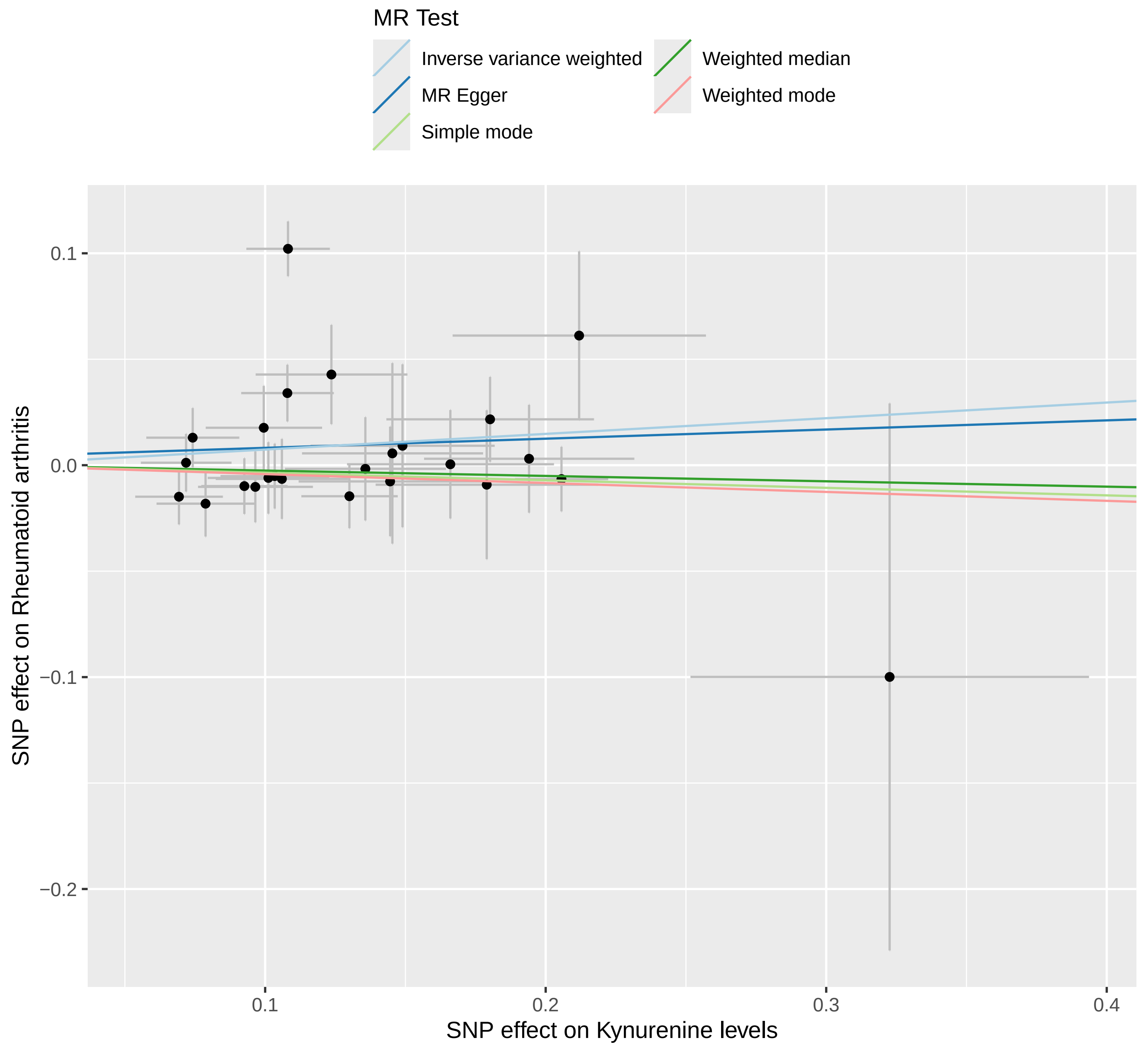

MR Test
Inverse variance weighted
MR Egger
Simple mode
Weighted median
Weighted mode
0.1
0.0
−0.1
−0.2
SNP effect on Rheumatoid arthritis
0.1
0.2
0.3
0.4
SNP effect on Kynurenine levels

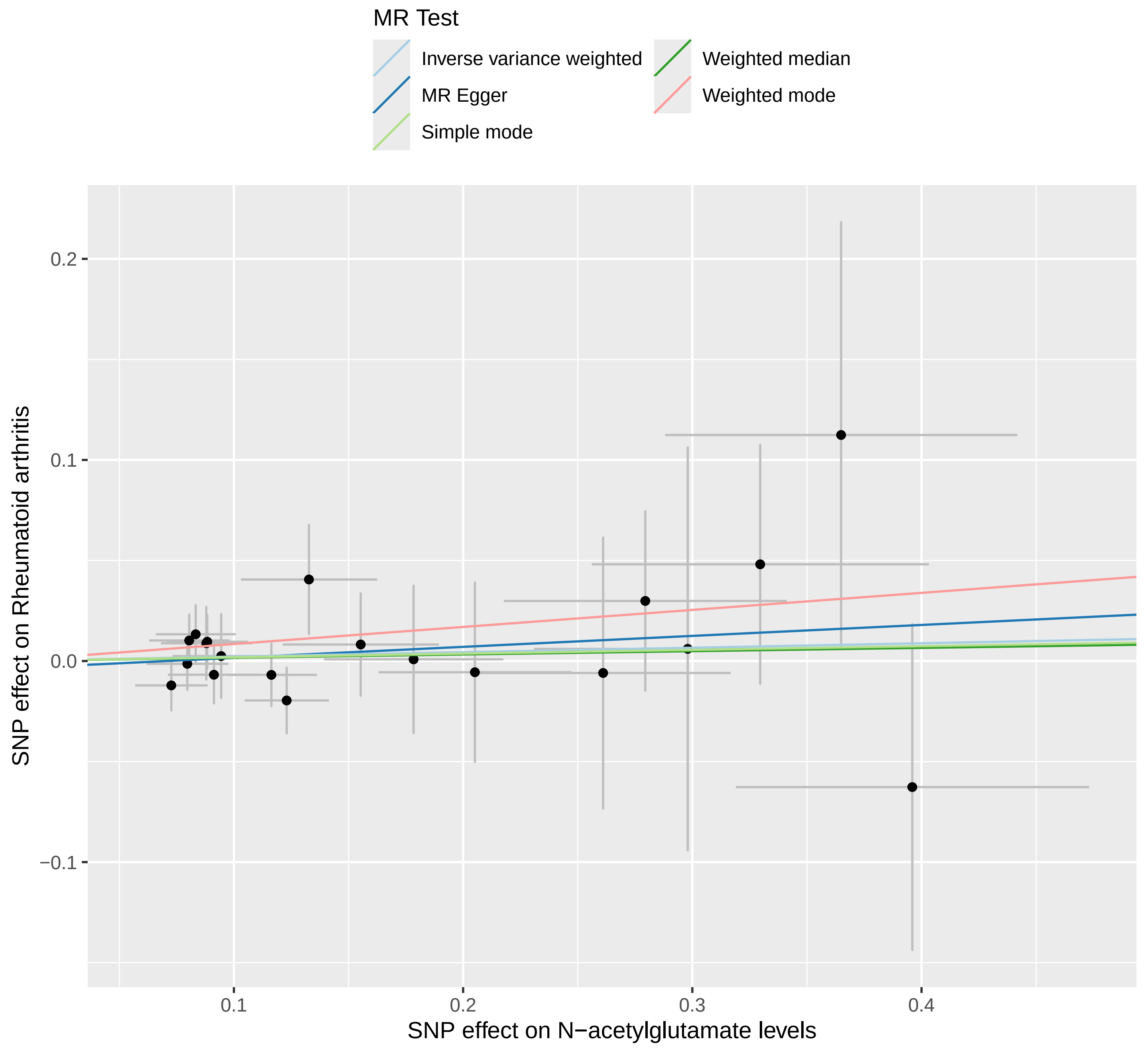
MR Test
Inverse variance weighted
MR Egger
Simple mode
Weighted median
Weighted mode
0.2
0.1
0.0
−0.1
0.1
0.2
0.3
0.4
SNP effect on Rheumatoid arthritis
SNP effect on N−acetylglutamate levels

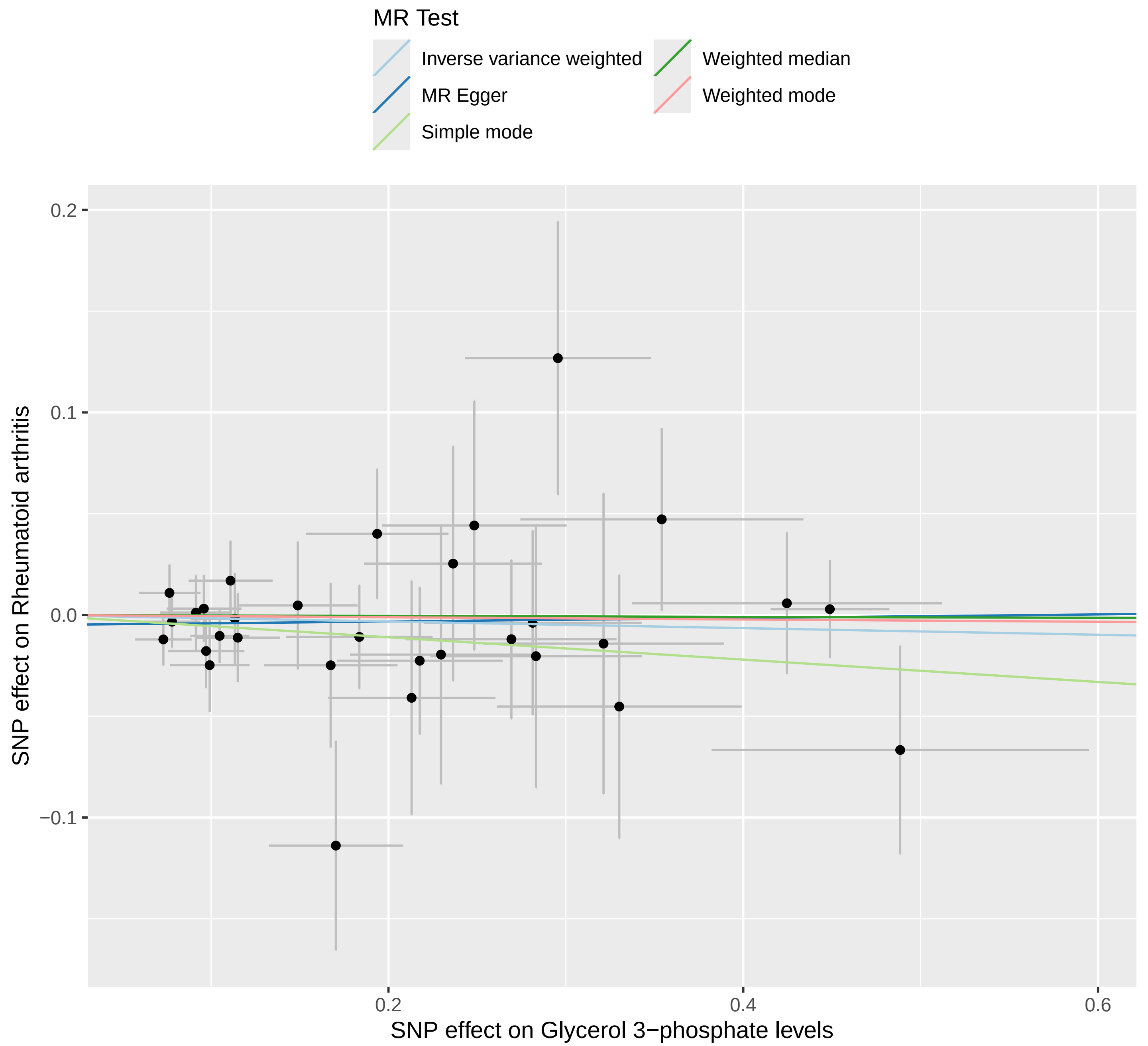

MR Test
Inverse variance weighted
MR Egger
Simple mode
Weighted median
Weighted mode
0.2
0.1
0.0
−0.1
SNP effect on Rheumatoid arthritis
0.2
0.4
0.6
SNP effect on Glycerol 3−phosphate levels

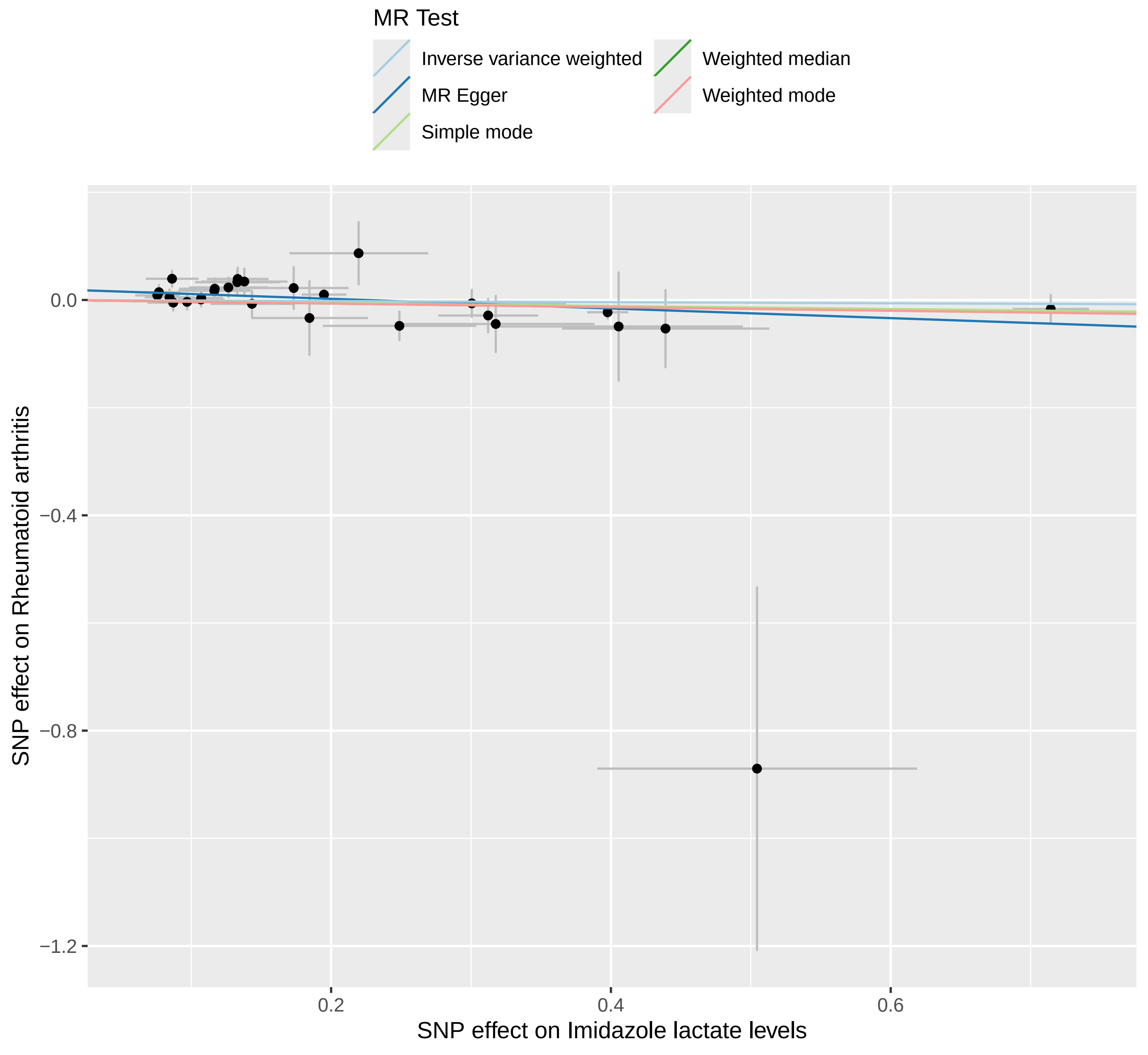
MR Test
Inverse variance weighted
MR Egger
Simple mode
Weighted median
Weighted mode
0.0
−0.4
−0.8
−1.2
0.2
0.4
0.6
SNP effect on Rheumatoid arthritis
SNP effect on Imidazole lactate levels

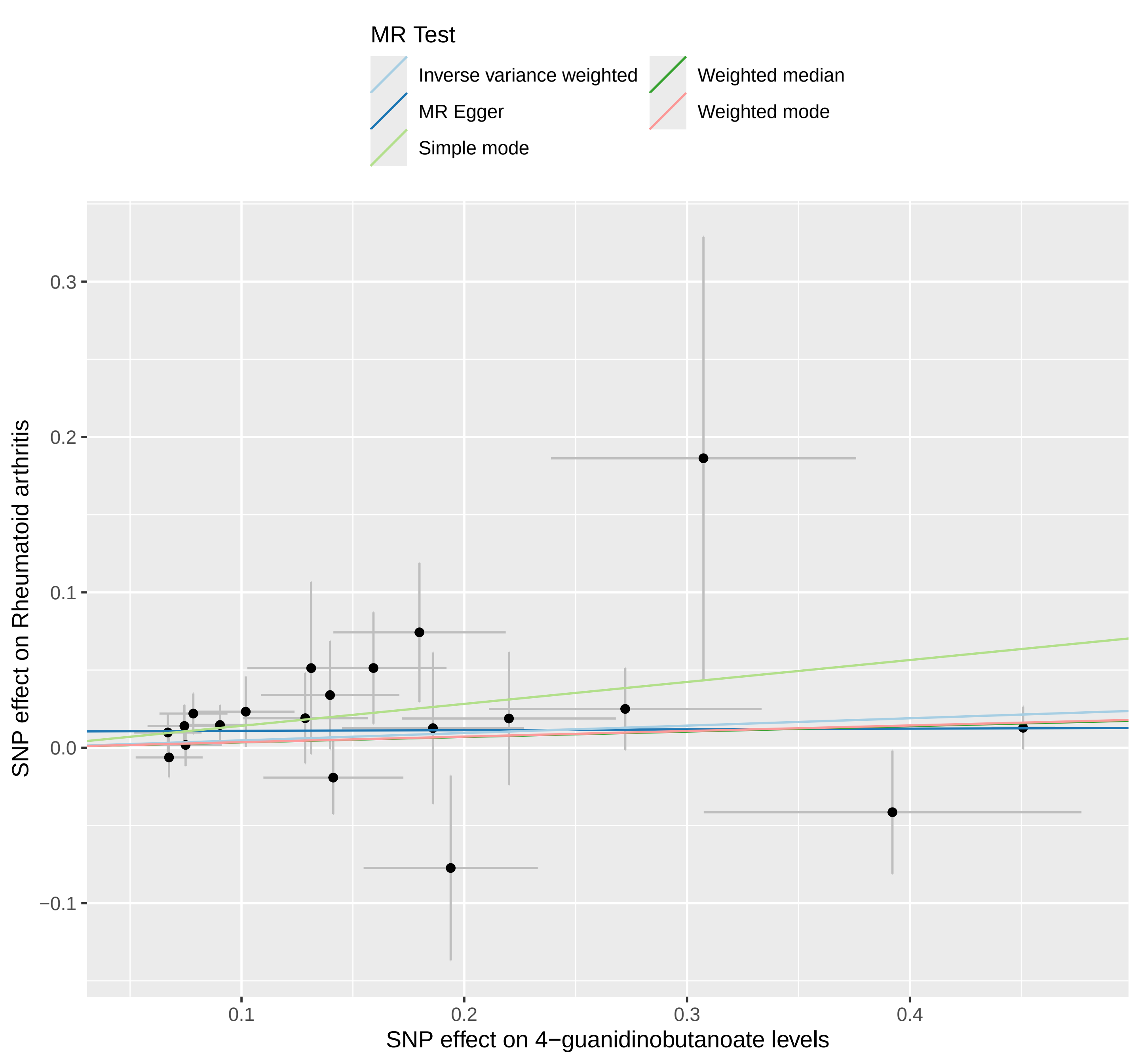

MR Test
Inverse variance weighted
MR Egger
Simple mode
Weighted median
Weighted mode
0.3
0.2
0.1
0.0
−0.1
0.1
0.2
0.3
0.4
SNP effect on Rheumatoid arthritis
SNP effect on 4−guanidinobutanoate levels

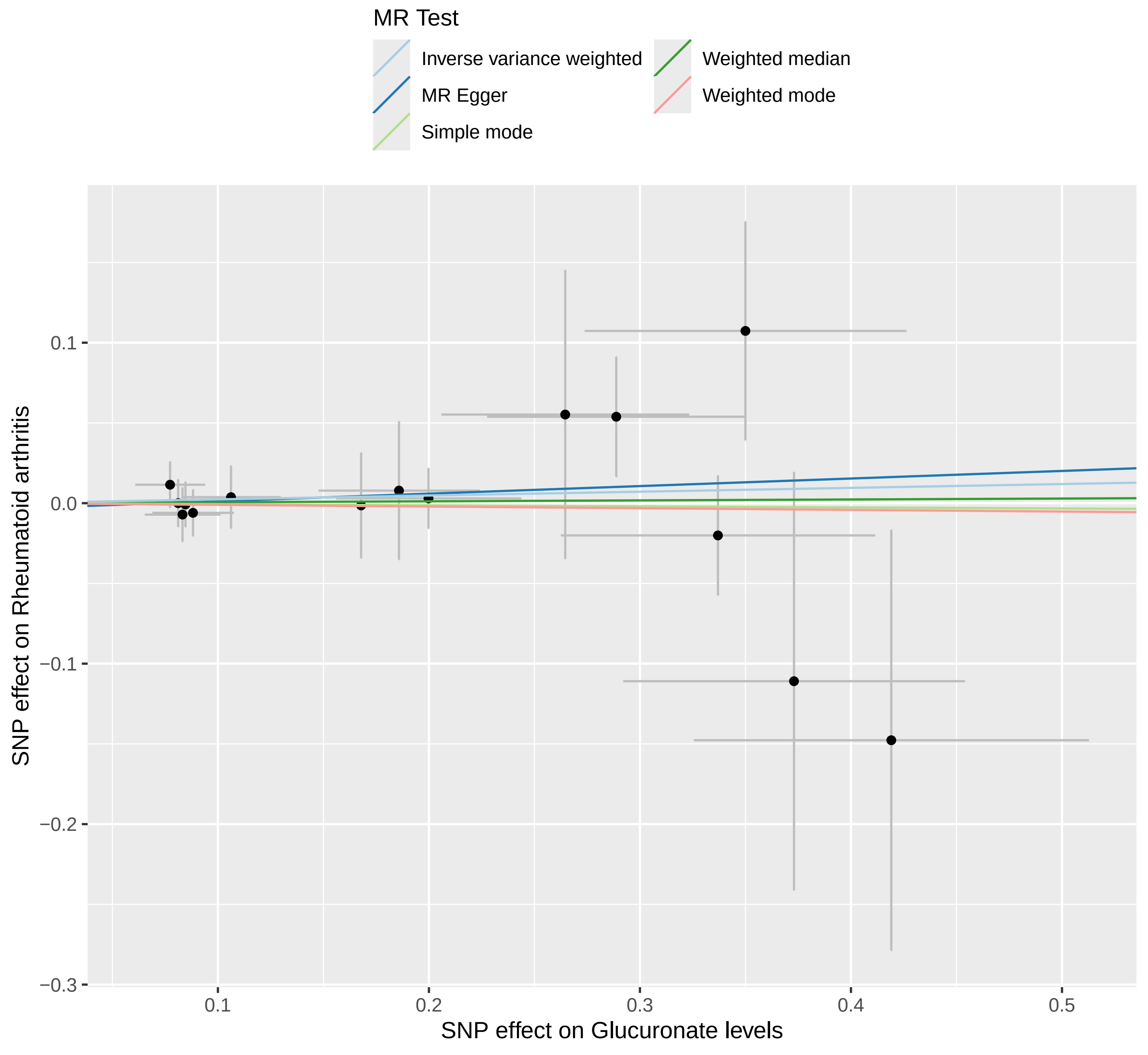
MR Test
Inverse variance weighted
MR Egger
Simple mode
Weighted median
Weighted mode
SNP effect on Rheumatoid arthritis
0.1
0.0
−0.1
−0.2
−0.3
0.1
0.2
0.3
0.4
0.5
SNP effect on Glucuronate levels

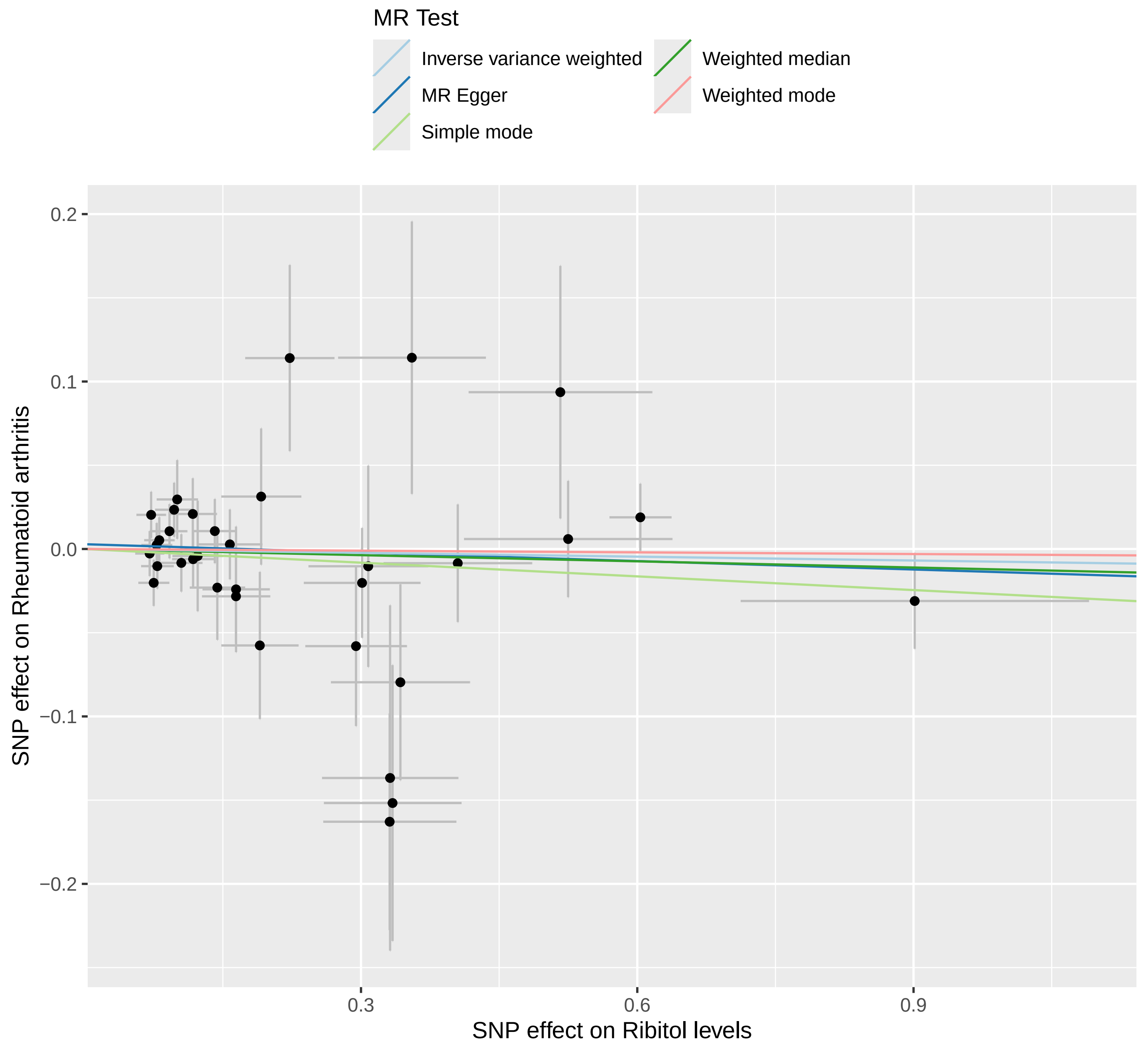

MR Test
Inverse variance weighted
MR Egger
Simple mode
Weighted median
Weighted mode
0.2
0.1
0.0
−0.1
−0.2
0.3
0.6
0.9
SNP effect on Rheumatoid arthritis
SNP effect on Ribitol levels

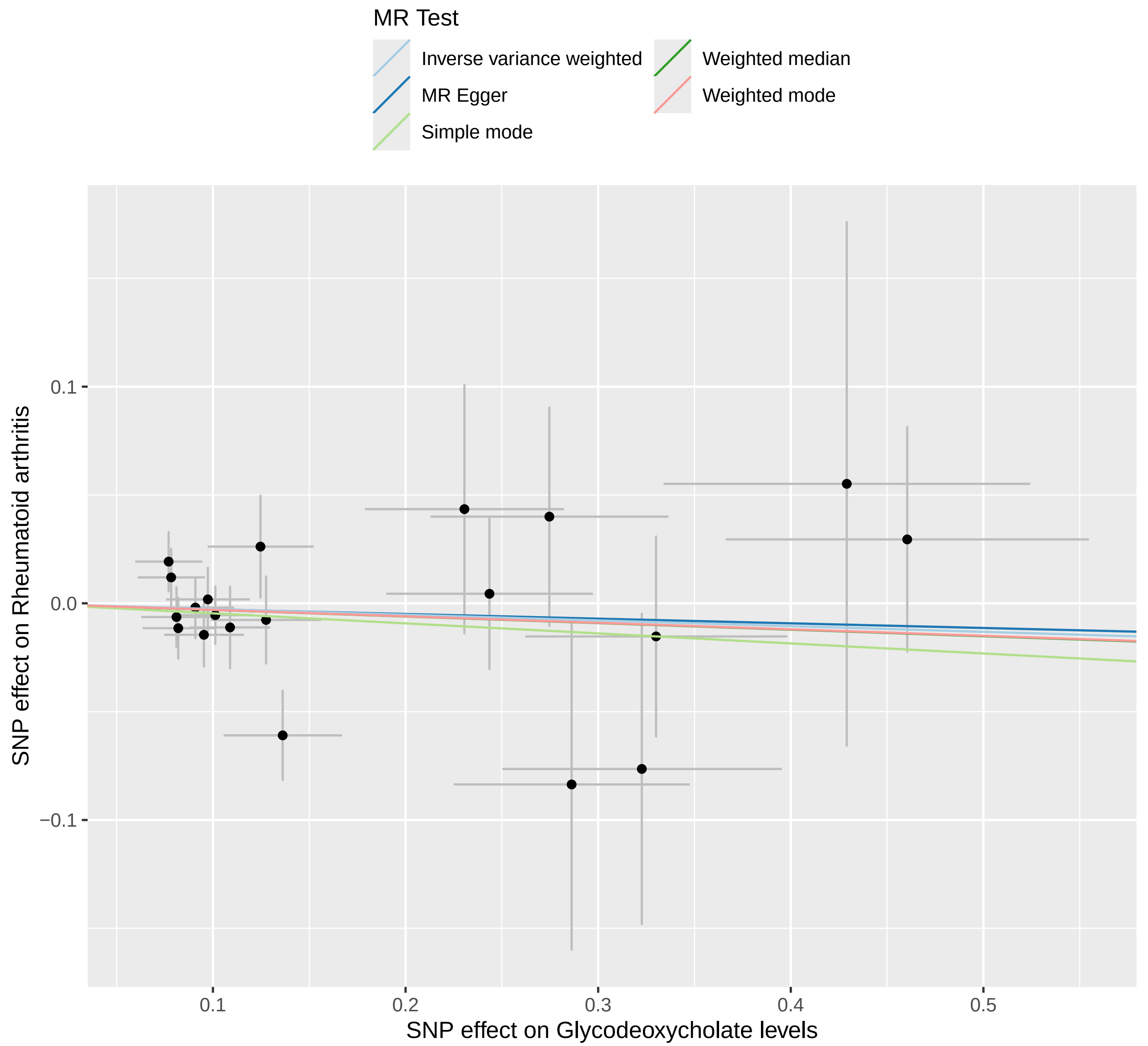
MR Test
Inverse variance weighted
MR Egger
Simple mode
Weighted median
Weighted mode
0.1
0.0
−0.1
0.1
0.2
0.3
0.4
0.5
SNP effect on Rheumatoid arthritis
SNP effect on Glycodeoxycholate levels

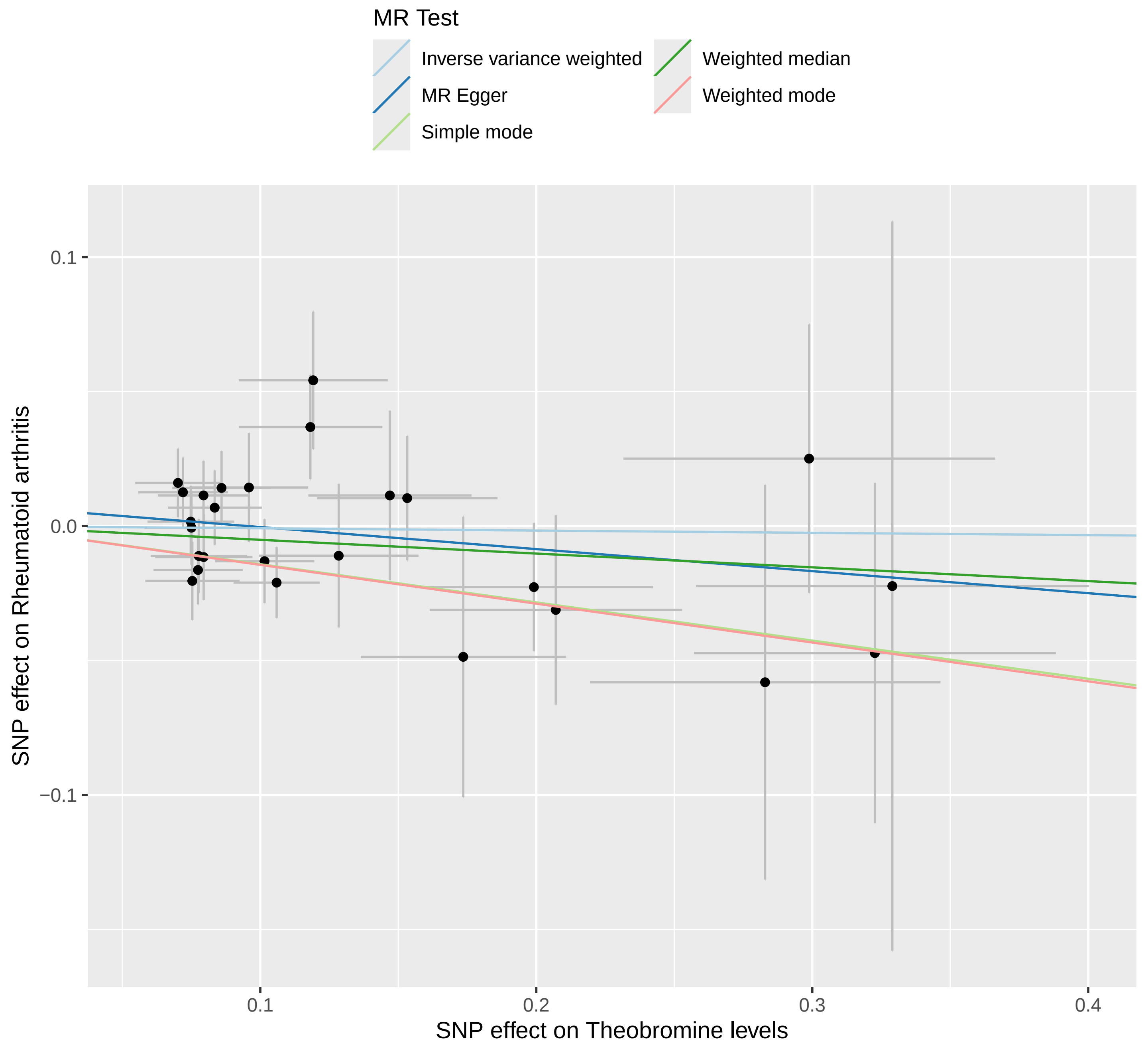
MR Test
Inverse variance weighted
MR Egger
Simple mode
Weighted median
Weighted mode
0.1
0.0
−0.1
0.1
0.2
0.3
0.4
SNP effect on Rheumatoid arthritis
SNP effect on Theobromine levels

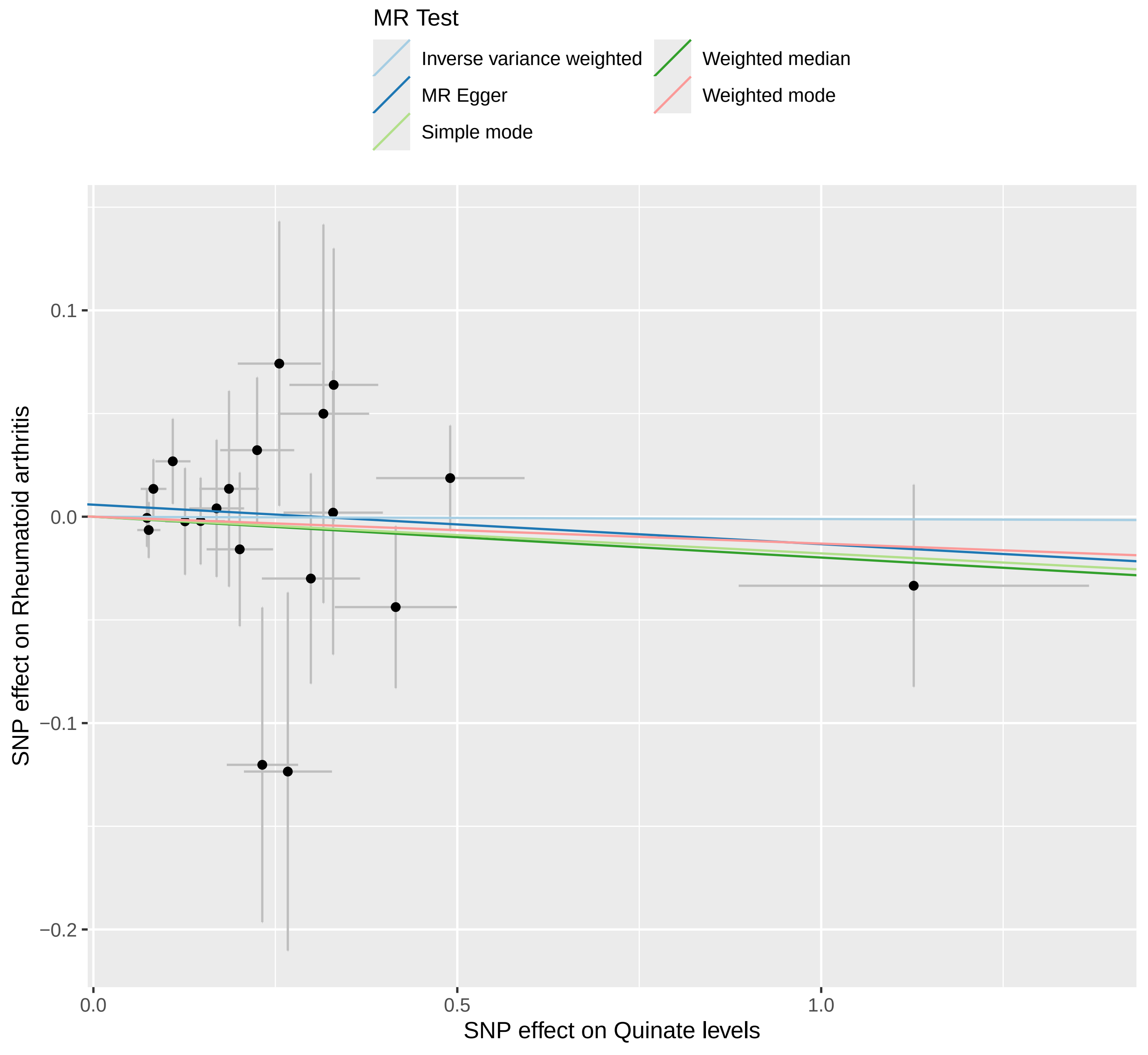

MR Test
Inverse variance weighted
MR Egger
Simple mode
Weighted median
Weighted mode
SNP effect on Rheumatoid arthritis
0.1
0.0
−0.1
−0.2
0.0
0.5
1.0
SNP effect on Quinate levels

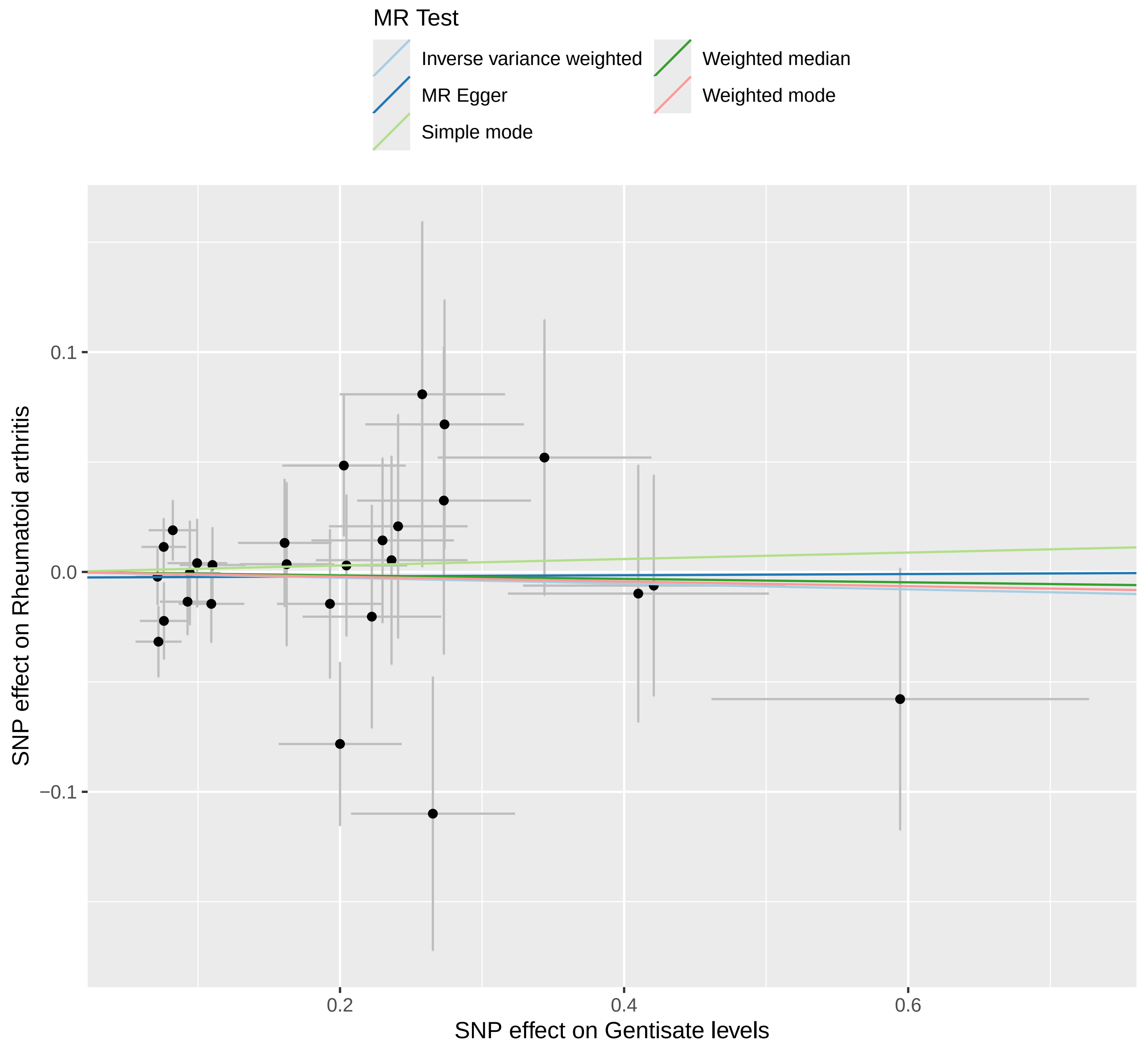
MR Test
Inverse variance weighted
MR Egger
Simple mode
Weighted median
Weighted mode
0.1
0.0
−0.1
SNP effect on Rheumatoid arthritis
0.2
0.4
0.6
SNP effect on Gentisate levels

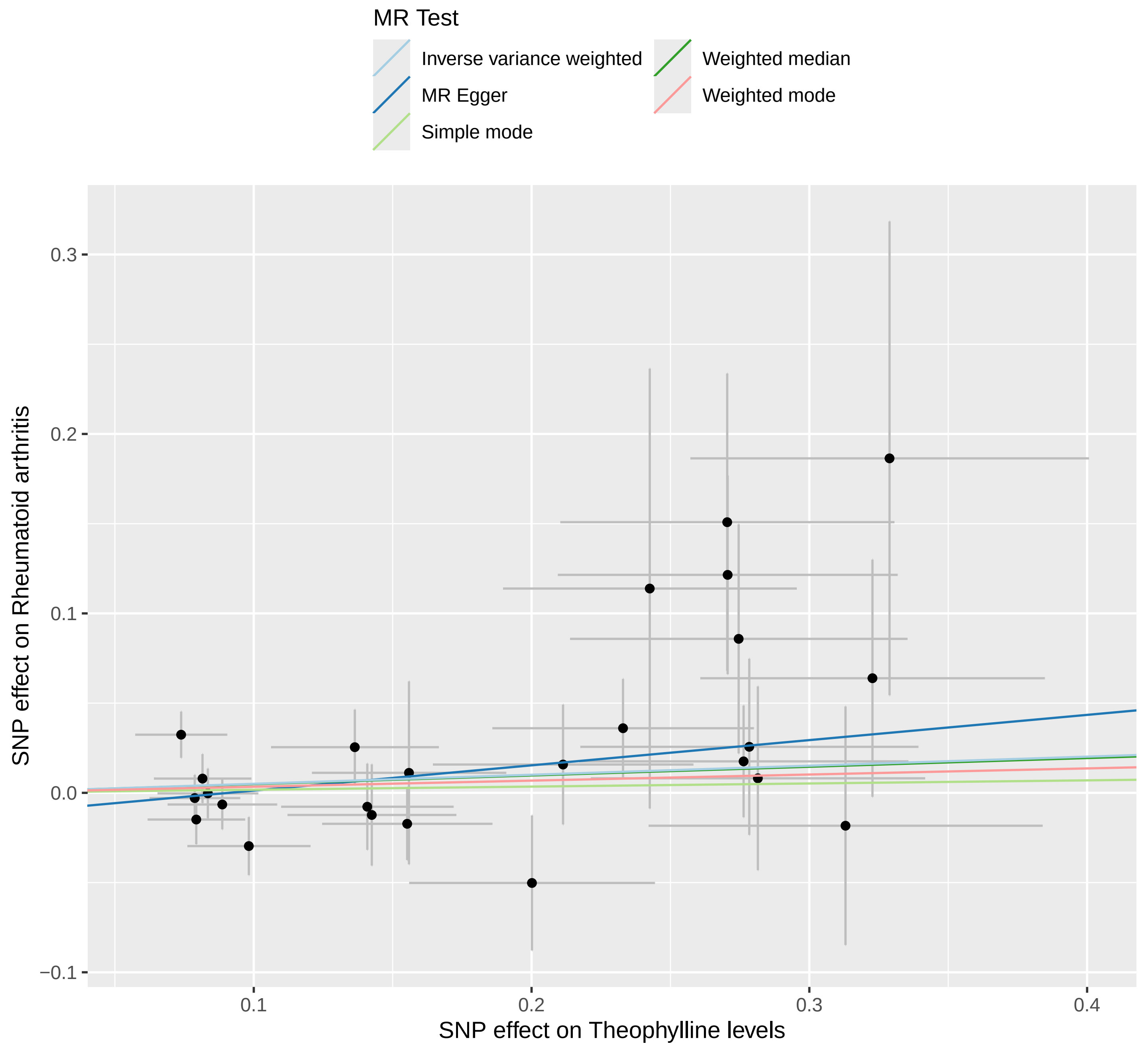
MR Test
Inverse variance weighted
MR Egger
Simple mode
Weighted median
Weighted mode
SNP effect on Rheumatoid arthritis
0.3
0.2
0.1
0.0
−0.1
0.1
0.2
0.3
0.4
SNP effect on Theophylline levels

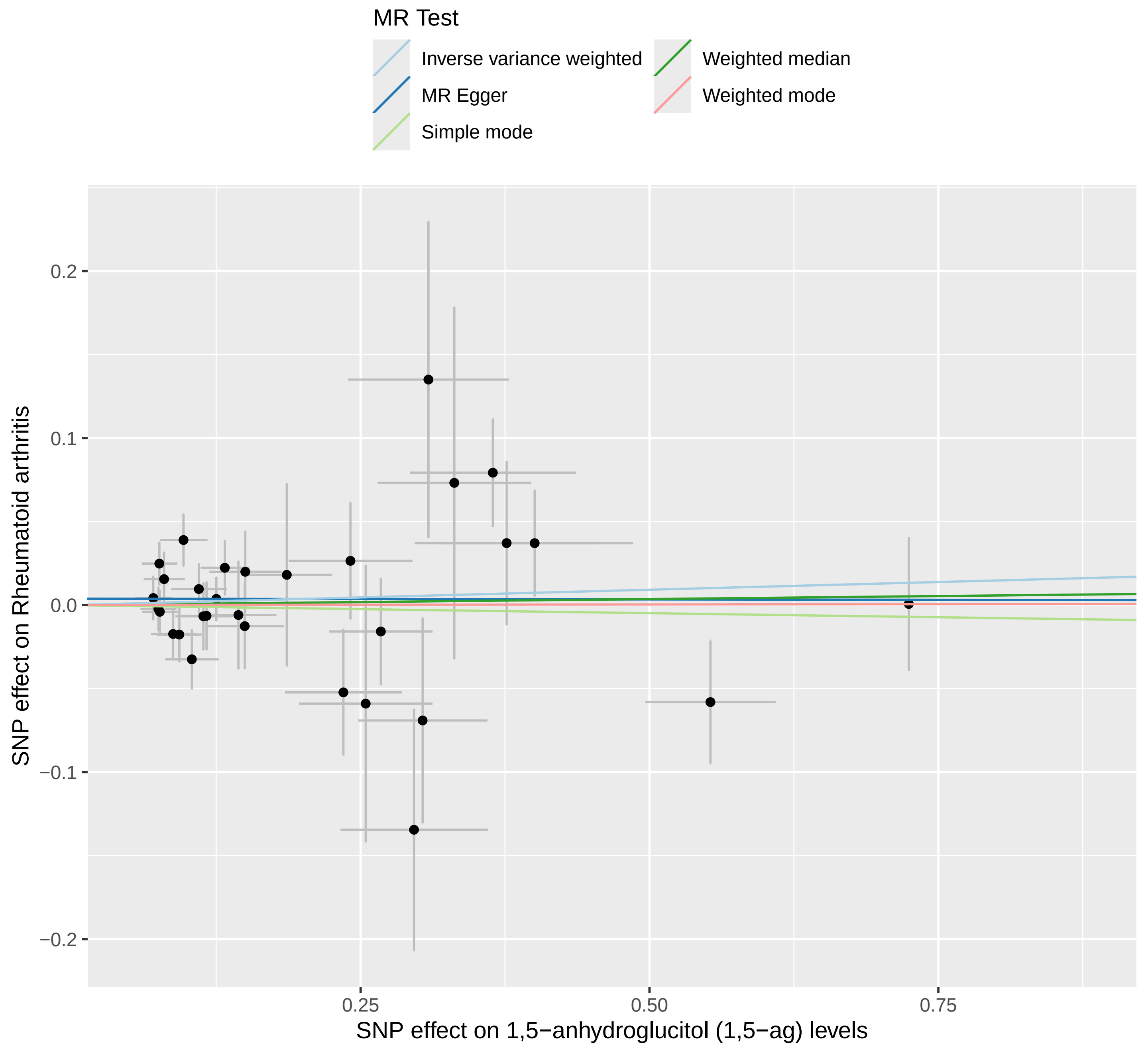
MR Test
Inverse variance weighted
MR Egger
Simple mode
Weighted median
Weighted mode
SNP effect on Rheumatoid arthritis
0.2
0.1
0.0
−0.1
−0.2
0.25
0.50
0.75
SNP effect on 1,5−anhydroglucitol (1,5−ag) levels

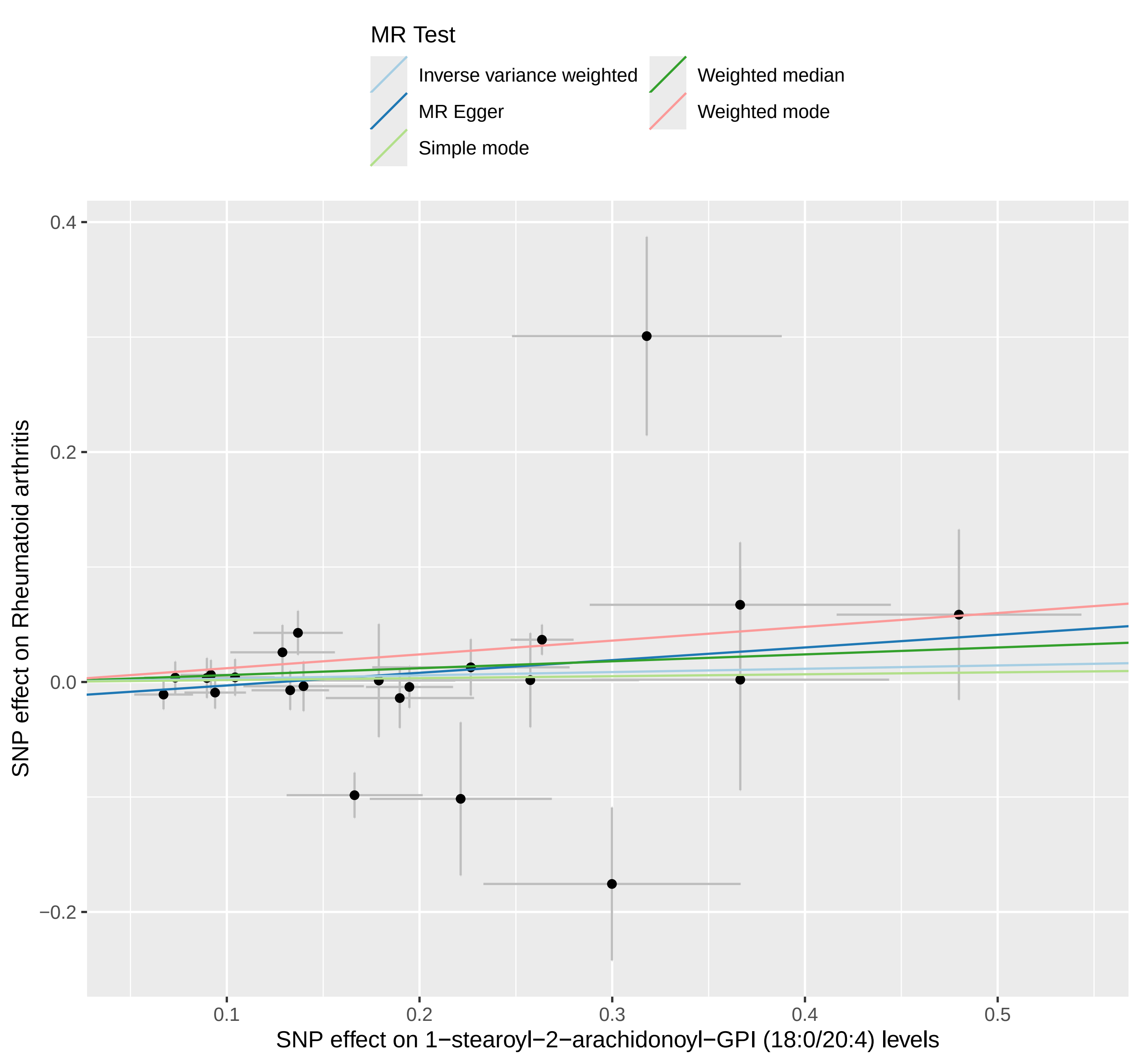

MR Test
Inverse variance weighted
MR Egger
Simple mode
Weighted median
Weighted mode
SNP effect on Rheumatoid arthritis
0.4
0.2
0.0
−0.2
0.1
0.2
0.3
0.4
0.5
SNP effect on 1−stearoyl−2−arachidonoyl−GPI (18:0/20:4) levels

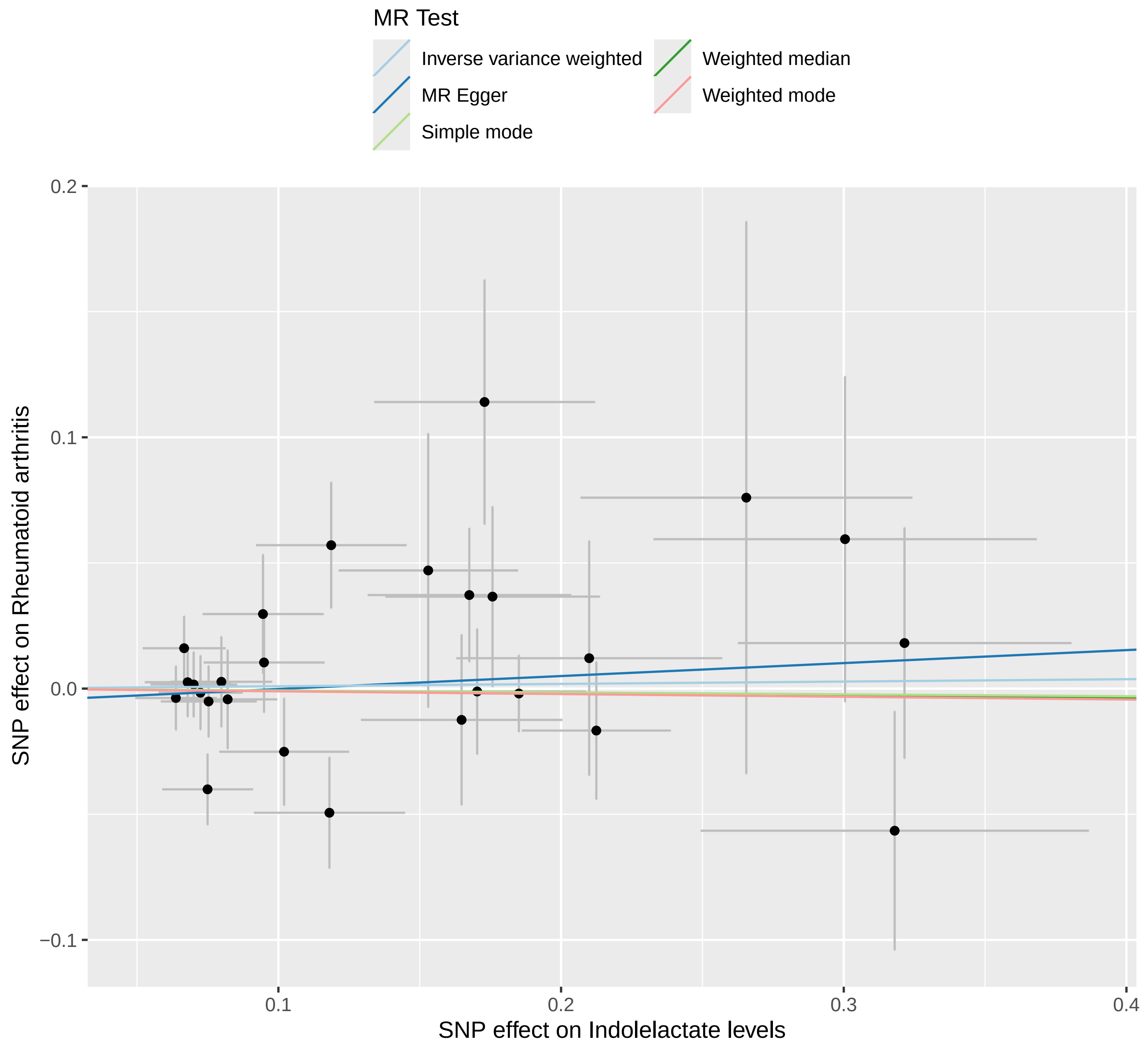

MR Test
Inverse variance weighted
MR Egger
Simple mode
Weighted median
Weighted mode
0.2
0.1
0.0
−0.1
0.1
0.2
0.3
0.4
SNP effect on Indolelactate levels
SNP effect on Rheumatoid arthritis

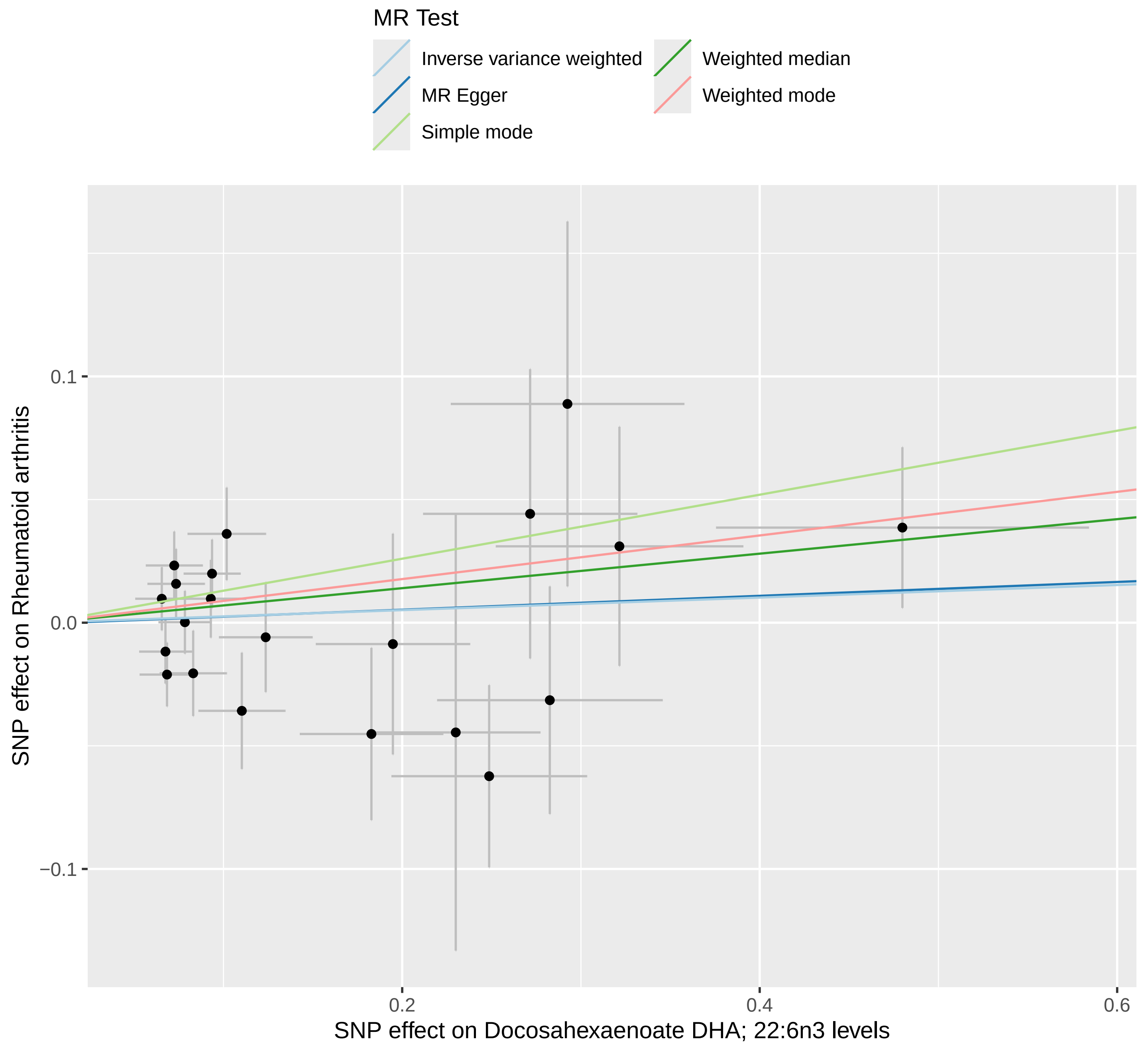
MR Test
Inverse variance weighted
MR Egger
Simple mode
Weighted median
Weighted mode
0.1
0.0
−0.1
0.2
0.4
0.6
SNP effect on Rheumatoid arthritis
SNP effect on Docosahexaenoate DHA; 22:6n3 levels

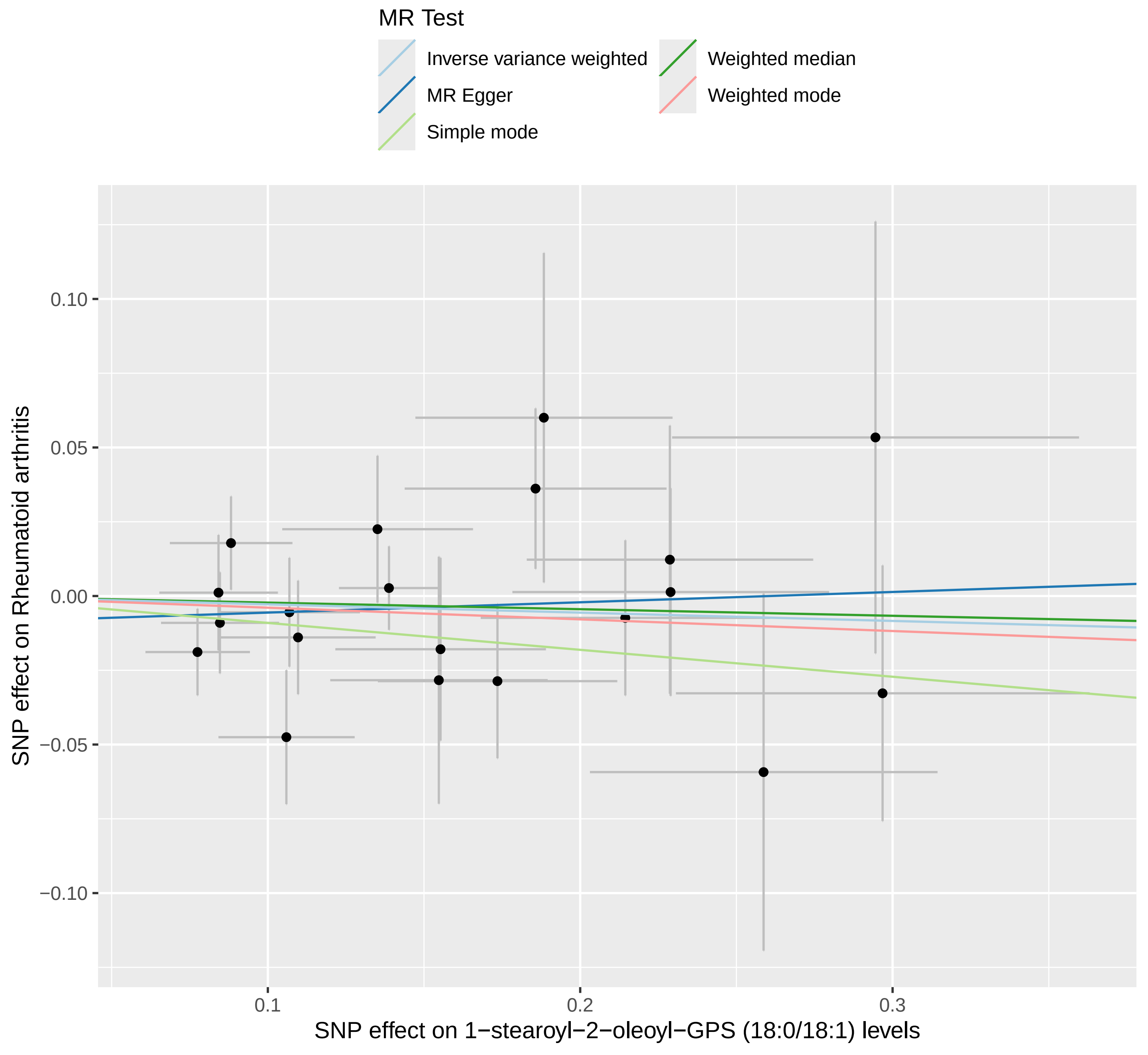

MR Test
Inverse variance weighted
MR Egger
Simple mode
Weighted median
Weighted mode
SNP effect on Rheumatoid arthritis
0.10
0.05
0.00
−0.05
−0.10
0.1
0.2
0.3
SNP effect on 1−stearoyl−2−oleoyl−GPS (18:0/18:1) levels

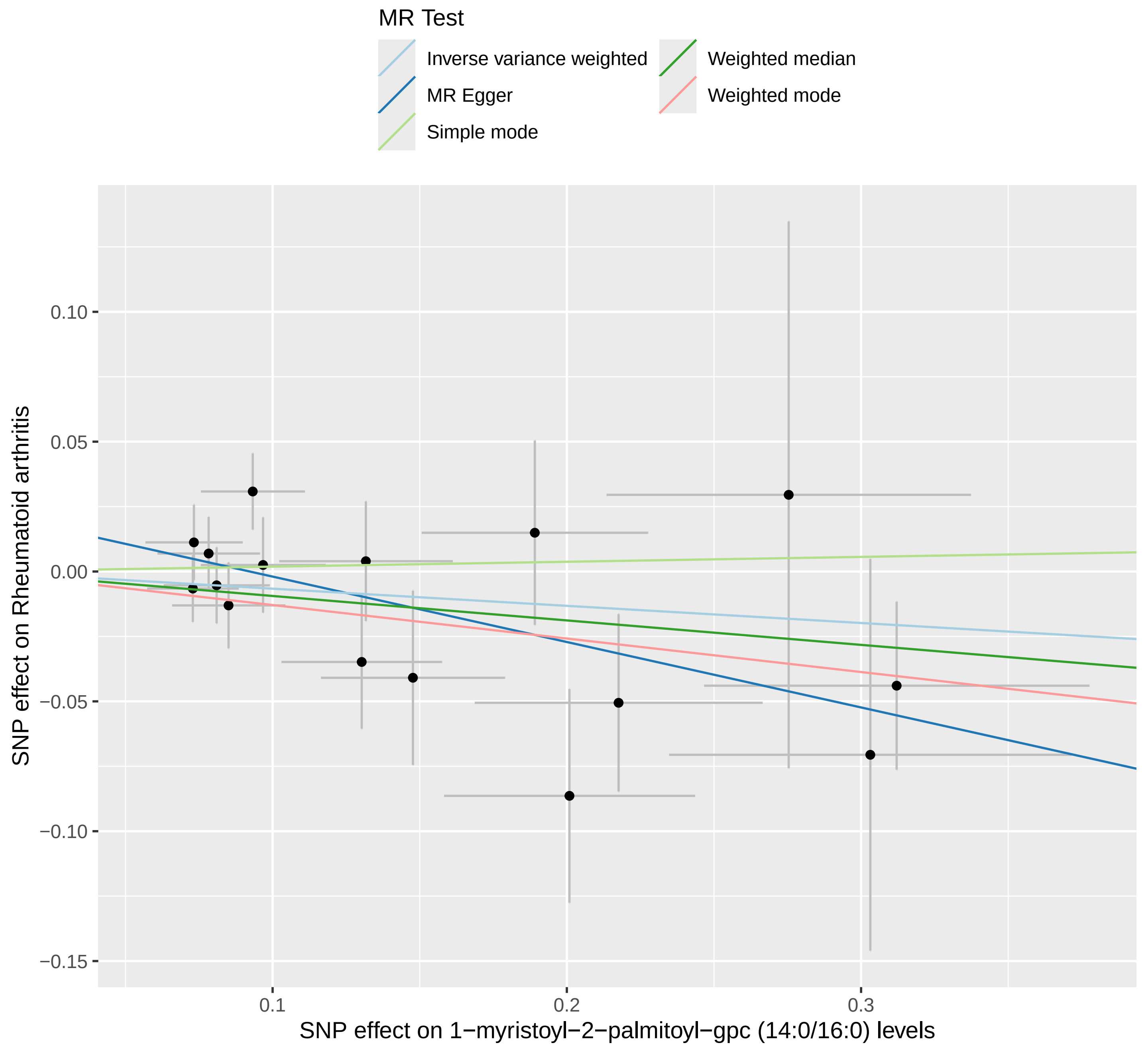

MR Test
Inverse variance weighted
MR Egger
Simple mode
Weighted median
Weighted mode
0.10
0.05
0.00
−0.05
−0.10
−0.15
0.1
0.2
0.3
SNP effect on Rheumatoid arthritis
SNP effect on 1−myristoyl−2−palmitoyl−gpc (14:0/16:0) levels

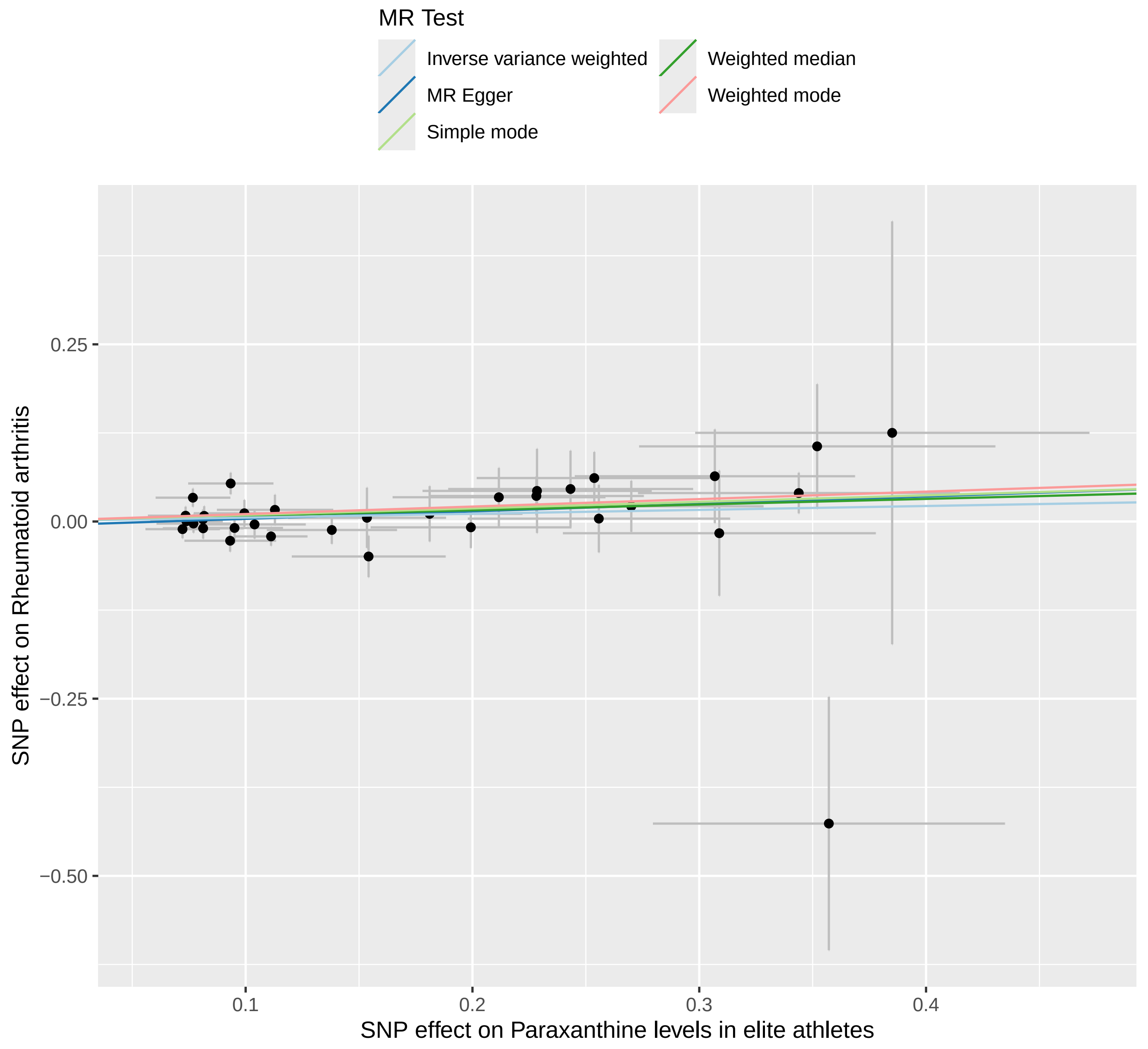

MR Test
Inverse variance weighted
MR Egger
Simple mode
Weighted median
Weighted mode
SNP effect on Rheumatoid arthritis
0.25
0.00
−0.25
−0.50
0.1
0.2
0.3
0.4
SNP effect on Paraxanthine levels in elite athletes

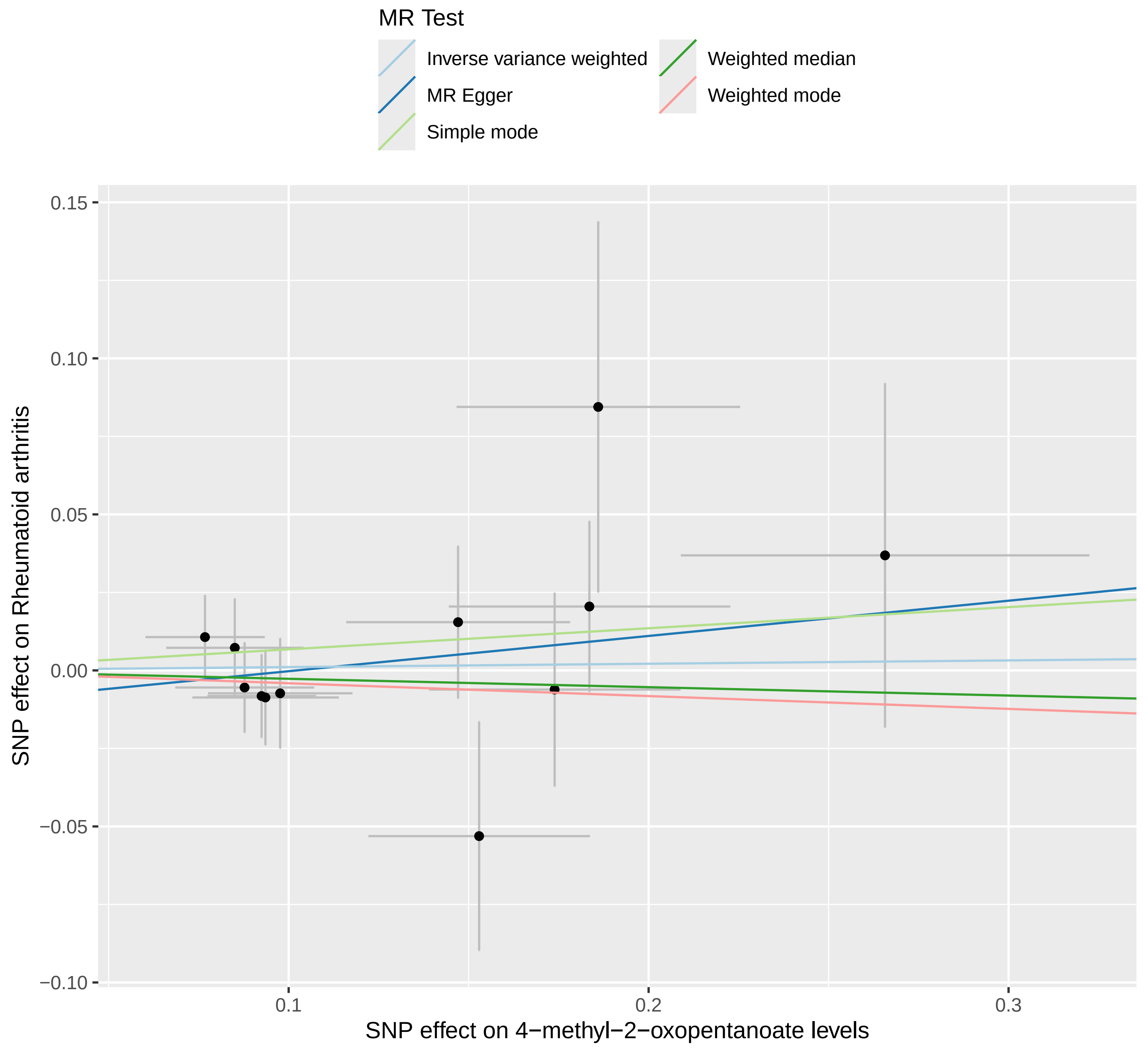
MR Test
Inverse variance weighted
MR Egger
Simple mode
Weighted median
Weighted mode
0.15
0.10
0.05
0.00
−0.05
−0.10
0.1
0.2
0.3
SNP effect on Rheumatoid arthritis
SNP effect on 4−methyl−2−oxopentanoate levels

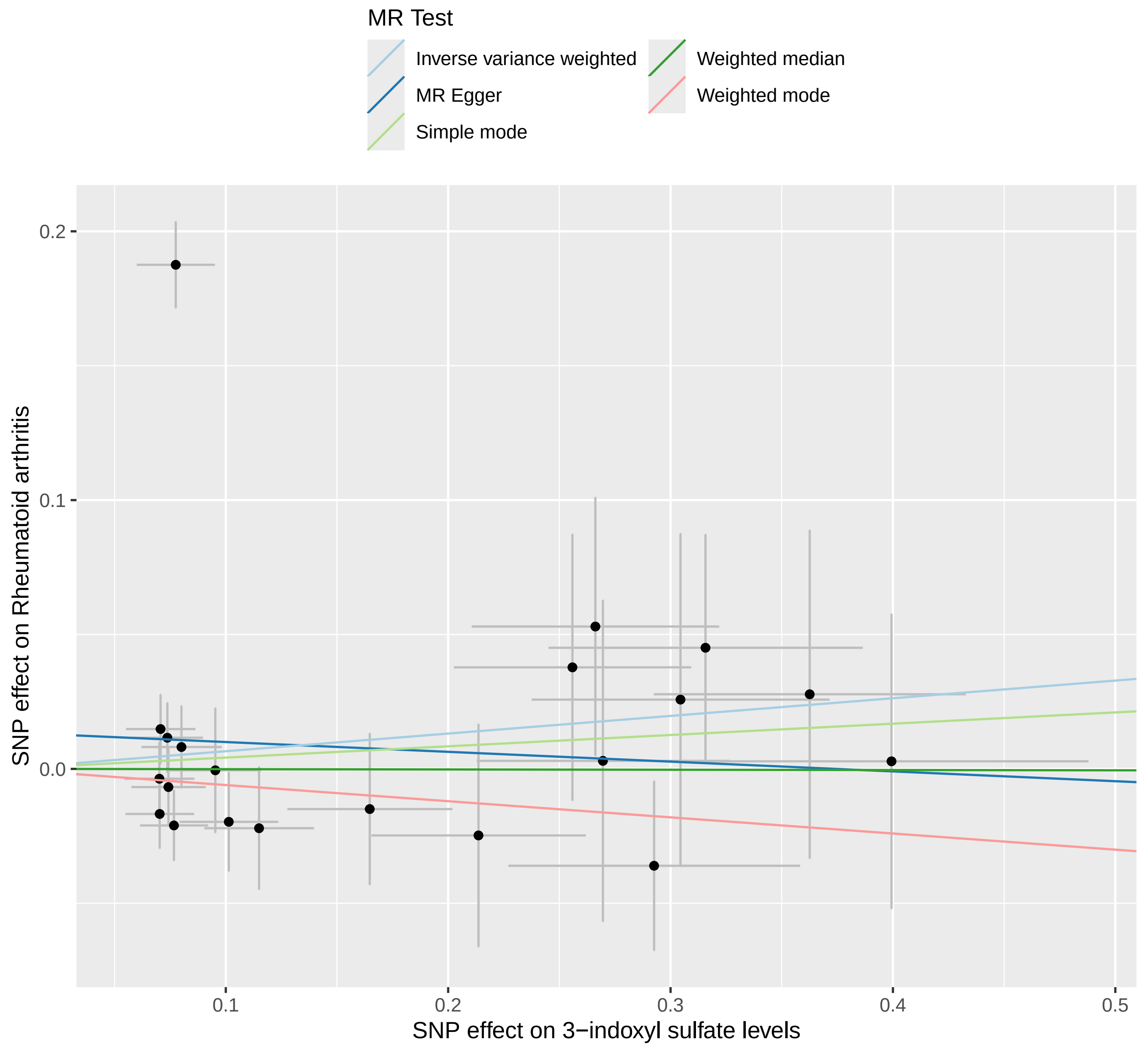

MR Test
Inverse variance weighted
MR Egger
Simple mode
Weighted median
Weighted mode
0.2
0.1
0.0
0.1
0.2
0.3
0.4
0.5
SNP effect on Rheumatoid arthritis
SNP effect on 3−indoxyl sulfate levels

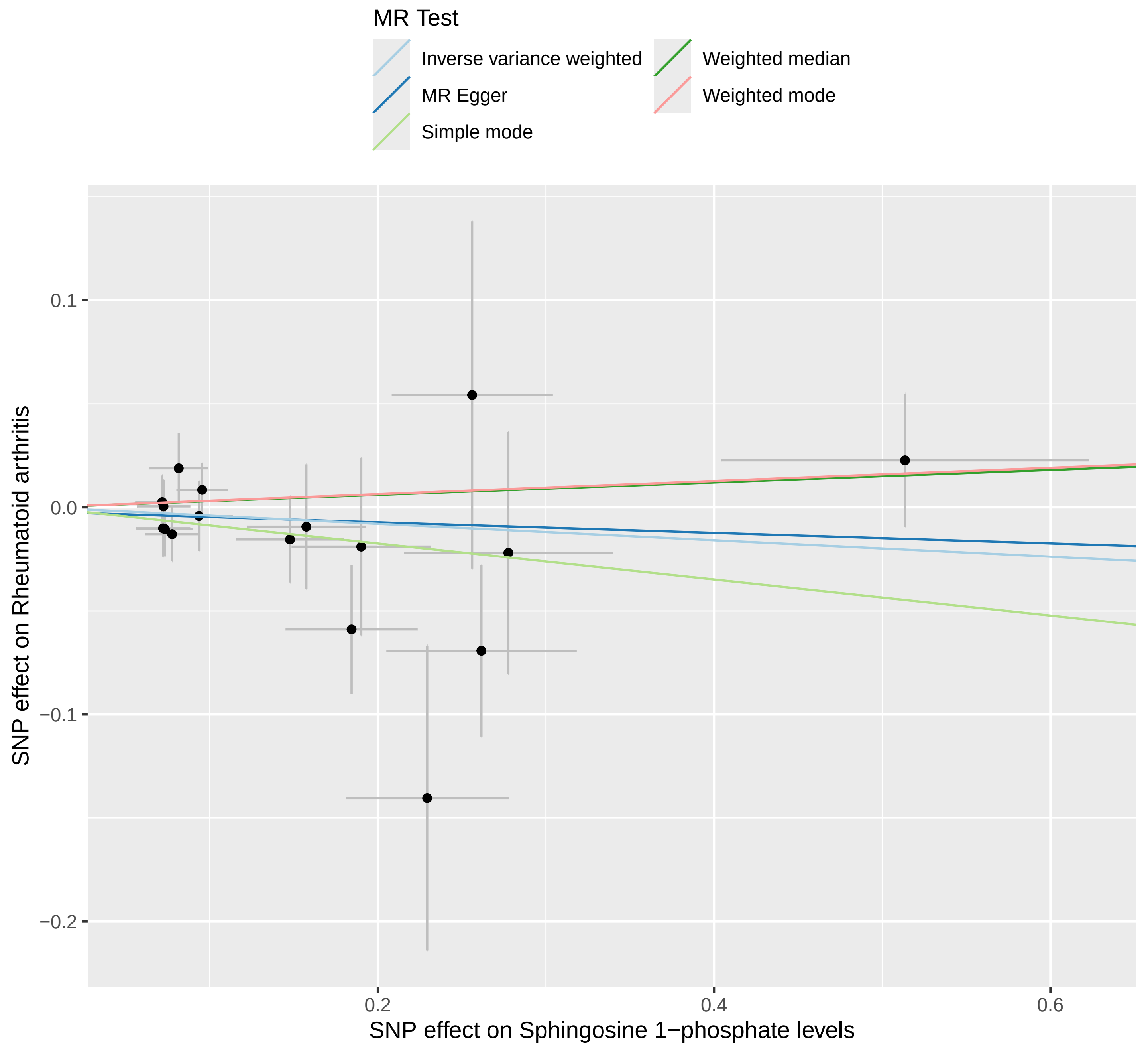
MR Test
Inverse variance weighted
MR Egger
Simple mode
Weighted median
Weighted mode
SNP effect on Rheumatoid arthritis
0.1
0.0
−0.1
−0.2
0.2
0.4
0.6
SNP effect on Sphingosine 1−phosphate levels

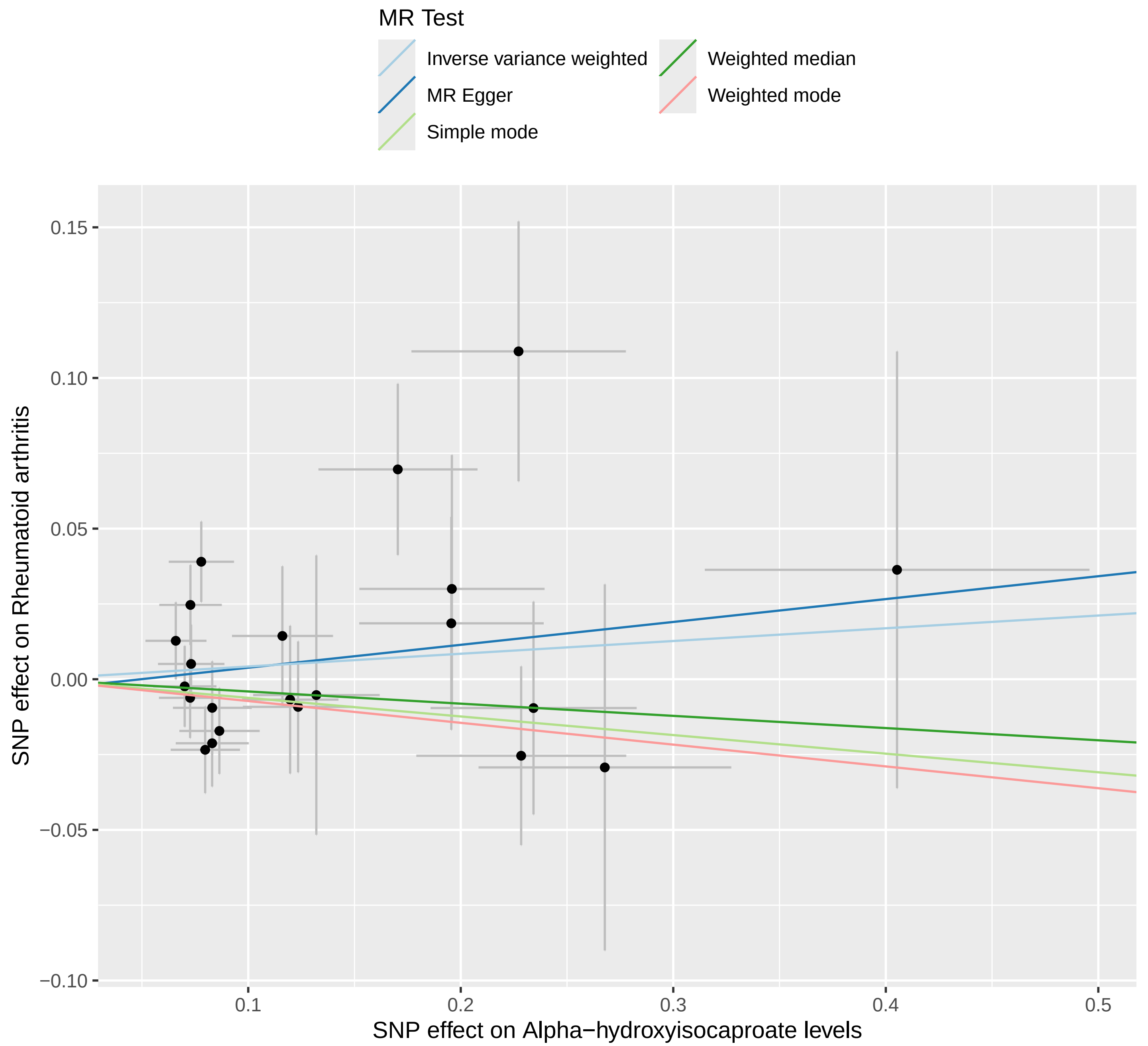
MR Test
Inverse variance weighted
MR Egger
Simple mode
Weighted median
Weighted mode
SNP effect on Rheumatoid arthritis
SNP effect on Alpha−hydroxyisocaproate levels
0.15
0.10
0.05
0.00
−0.05
−0.10
0.1
0.2
0.3
0.4
0.5

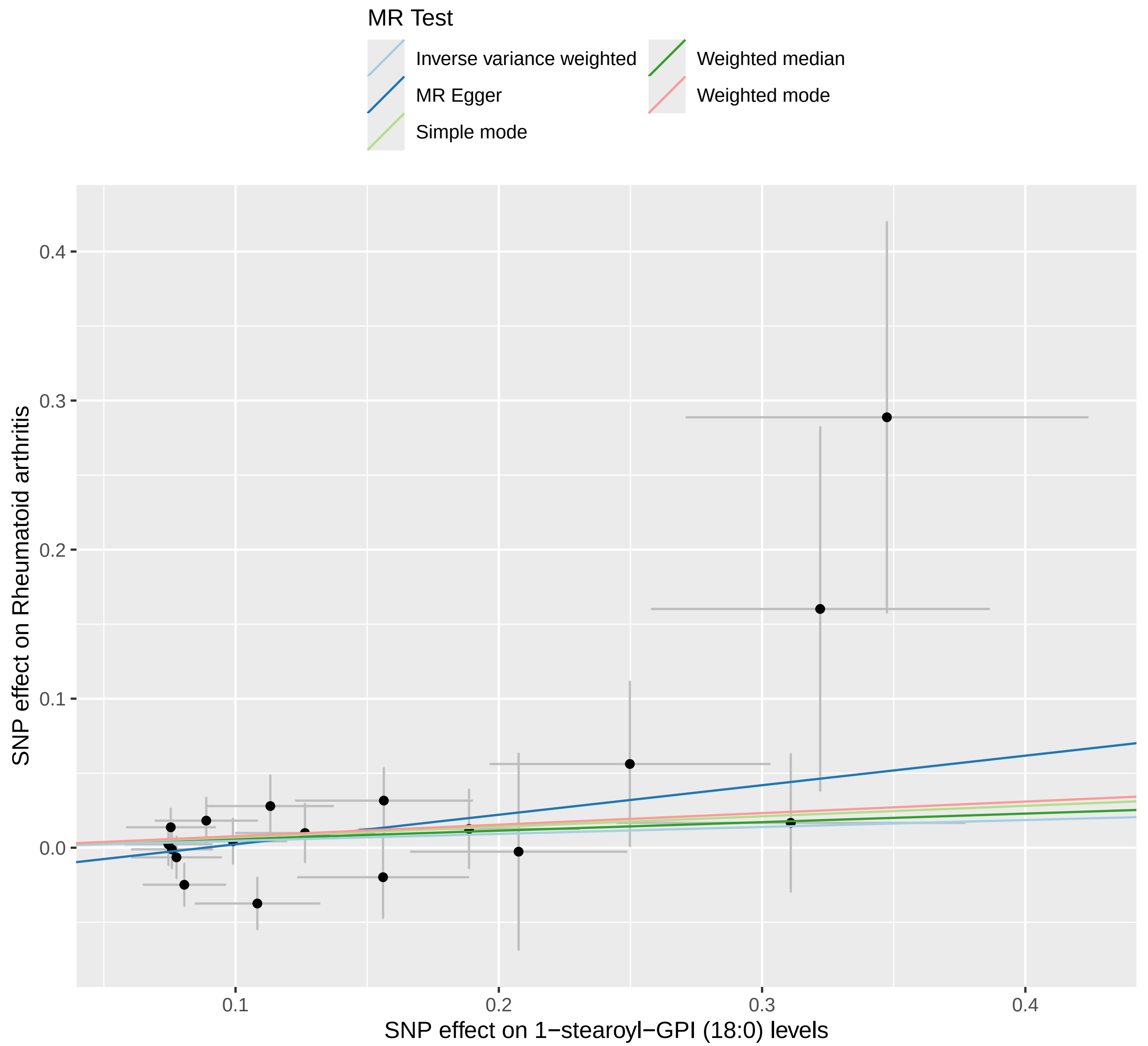
MR Test
Inverse variance weighted
MR Egger
Simple mode
Weighted median
Weighted mode
SNP effect on Rheumatoid arthritis
0.0
0.1
0.2
0.3
0.4
0.1
0.2
0.3
0.4
SNP effect on 1−stearoyl−GPI (18:0) levels

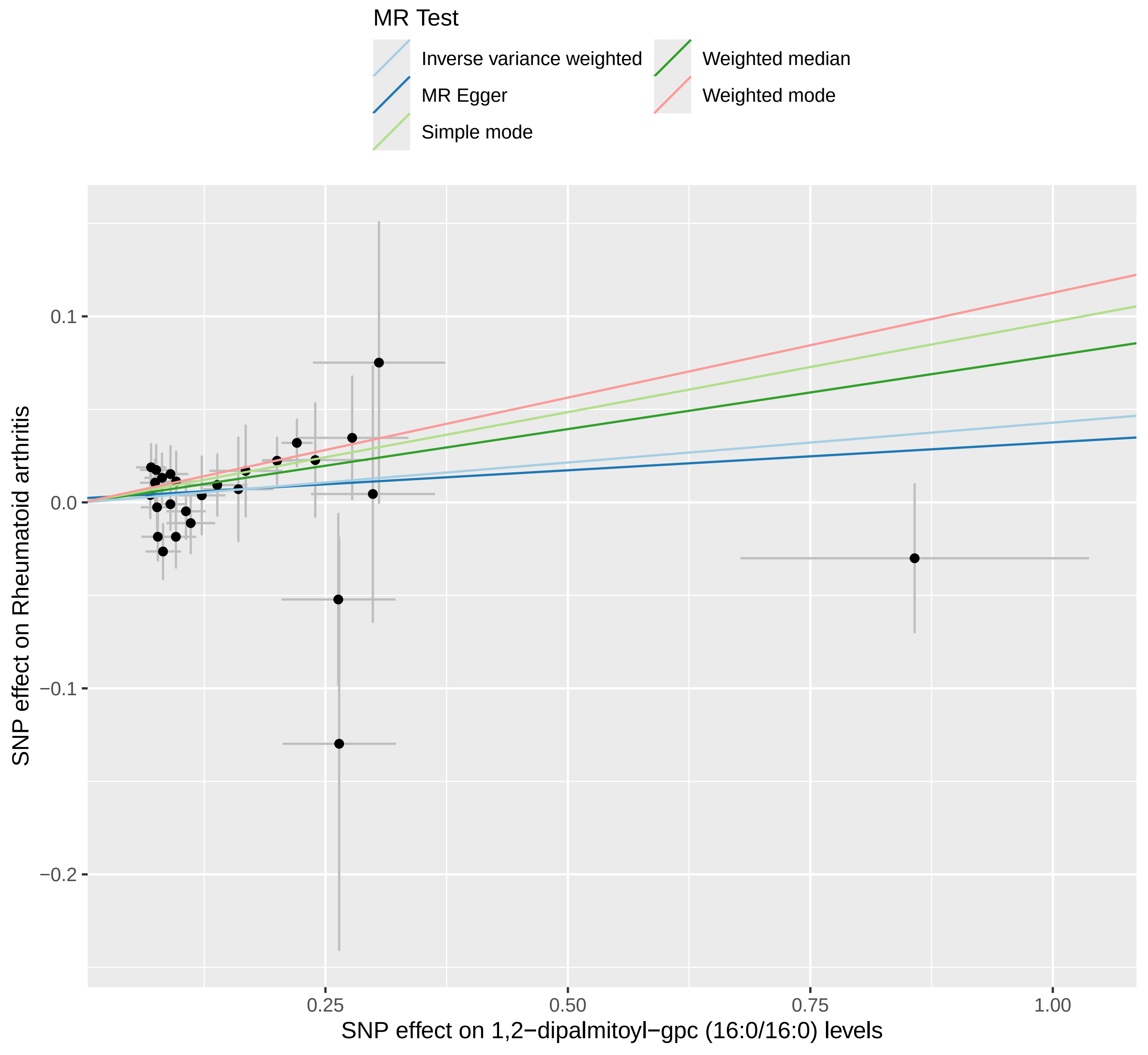
MR Test
Inverse variance weighted
MR Egger
Simple mode
Weighted median
Weighted mode
0.1
0.0
−0.1
−0.2
0.25
0.50
0.75
1.00
SNP effect on Rheumatoid arthritis
SNP effect on 1,2−dipalmitoyl−gpc (16:0/16:0) levels

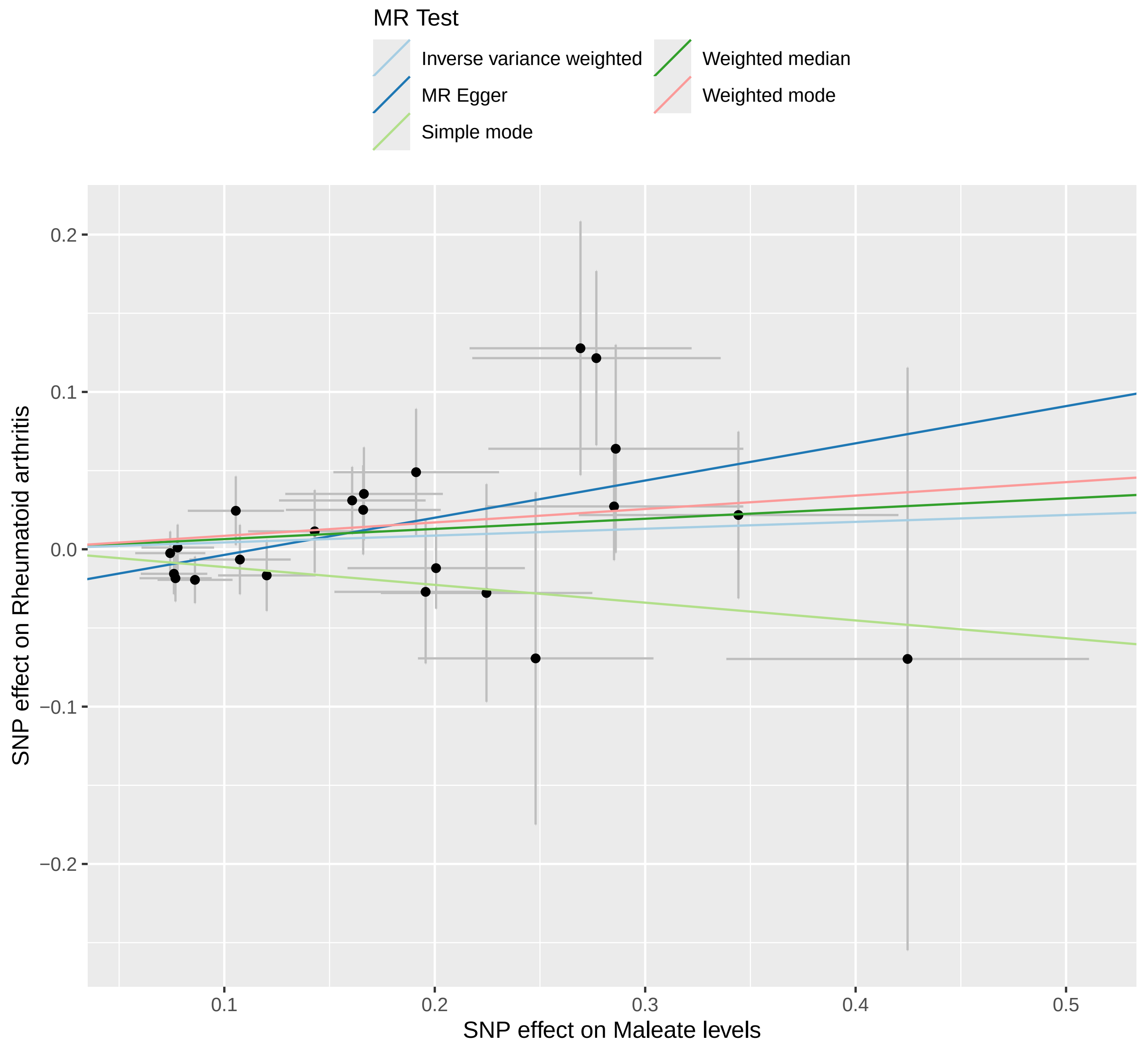
MR Test
Inverse variance weighted
MR Egger
Simple mode
Weighted median
Weighted mode
0.2
0.1
0.0
−0.1
−0.2
SNP effect on Rheumatoid arthritis
0.1
0.2
0.3
0.4
0.5
SNP effect on Maleate levels

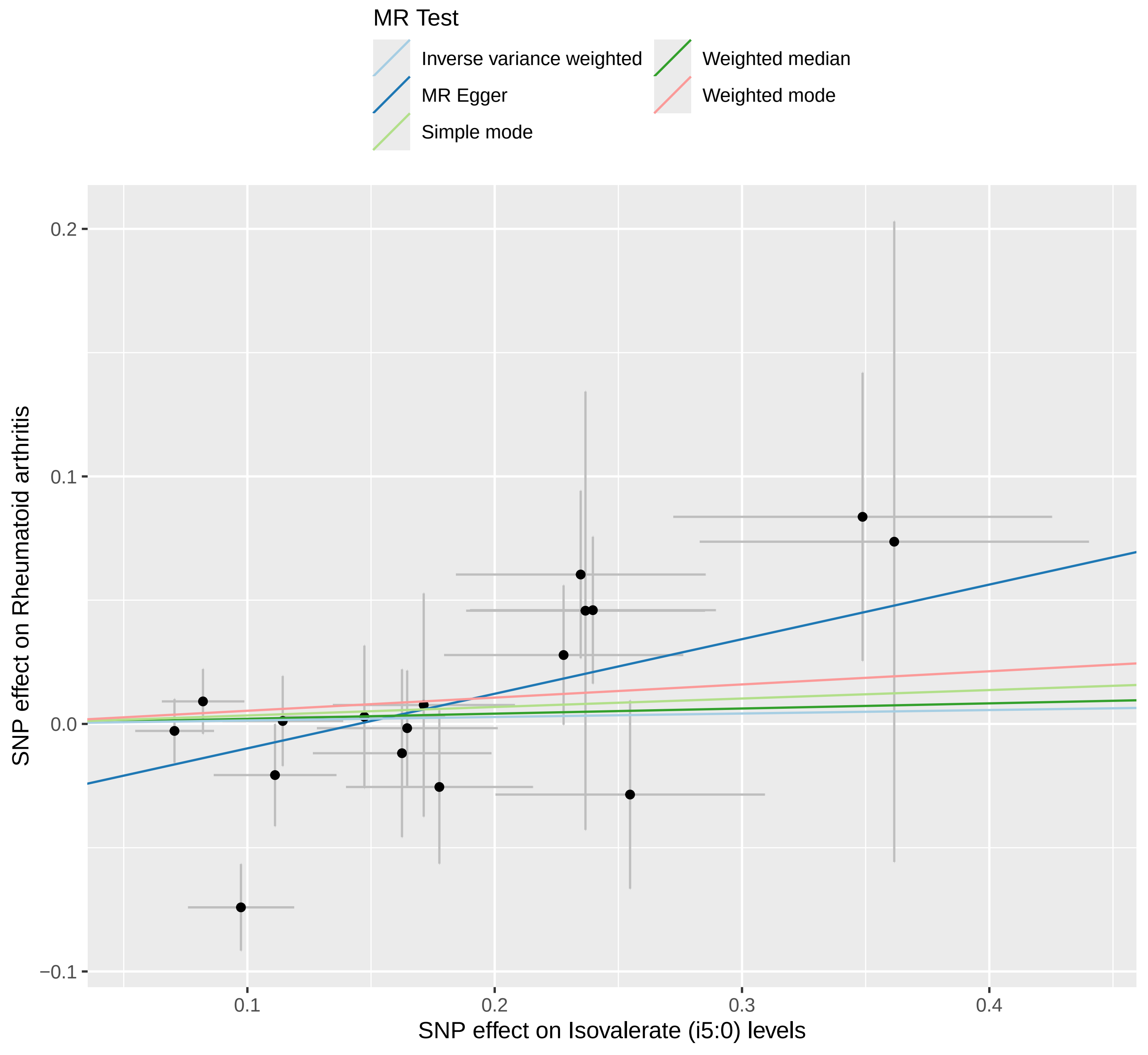
MR Test
Inverse variance weighted
MR Egger
Simple mode
Weighted median
Weighted mode
0.2
0.1
0.0
−0.1
0.1
0.2
0.3
0.4
SNP effect on Rheumatoid arthritis
SNP effect on Isovalerate (i5:0) levels

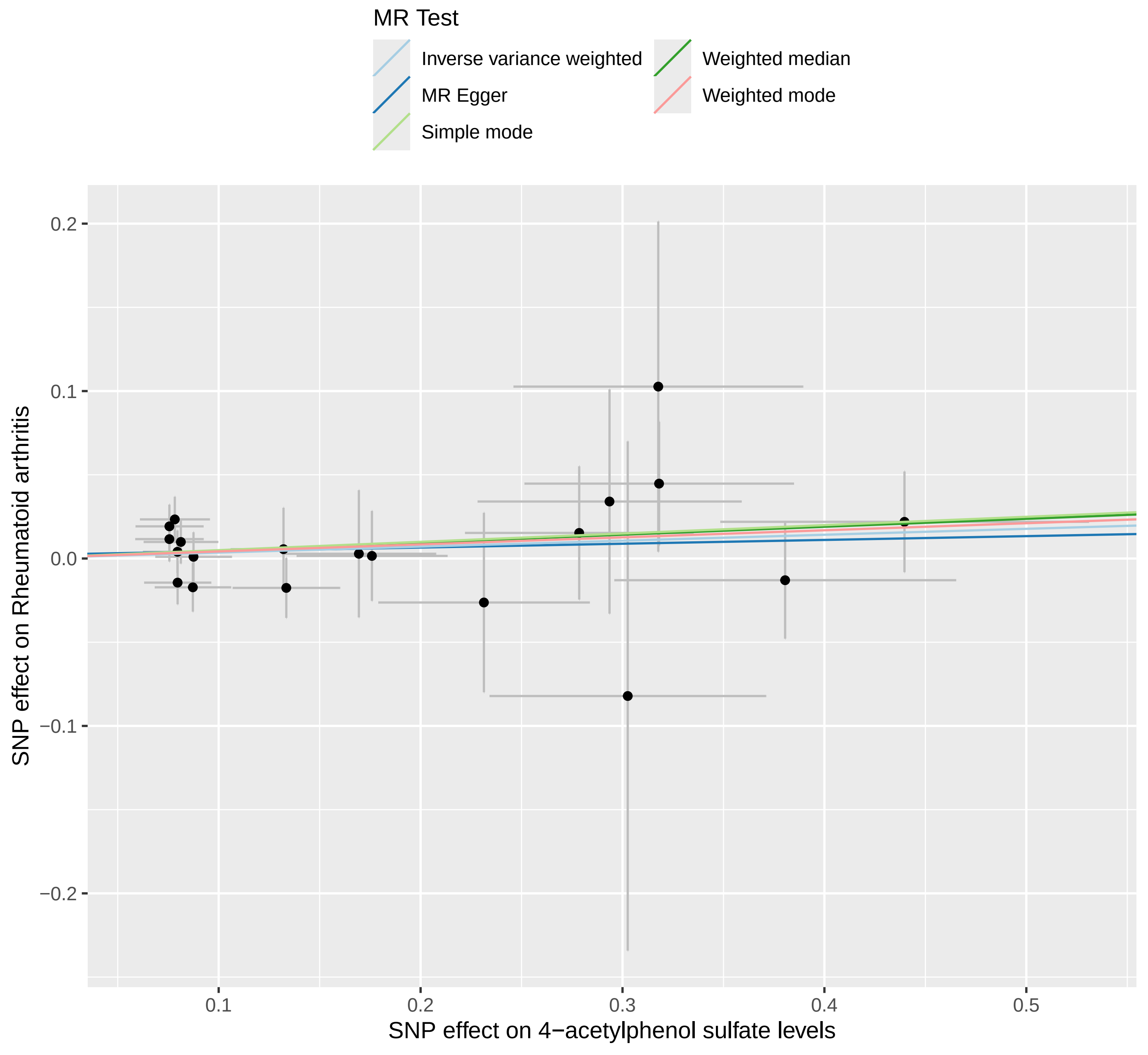

MR Test
Inverse variance weighted
MR Egger
Simple mode
Weighted median
Weighted mode
0.2
0.1
0.0
−0.1
−0.2
SNP effect on Rheumatoid arthritis
0.1
0.2
0.3
0.4
0.5
SNP effect on 4−acetylphenol sulfate levels

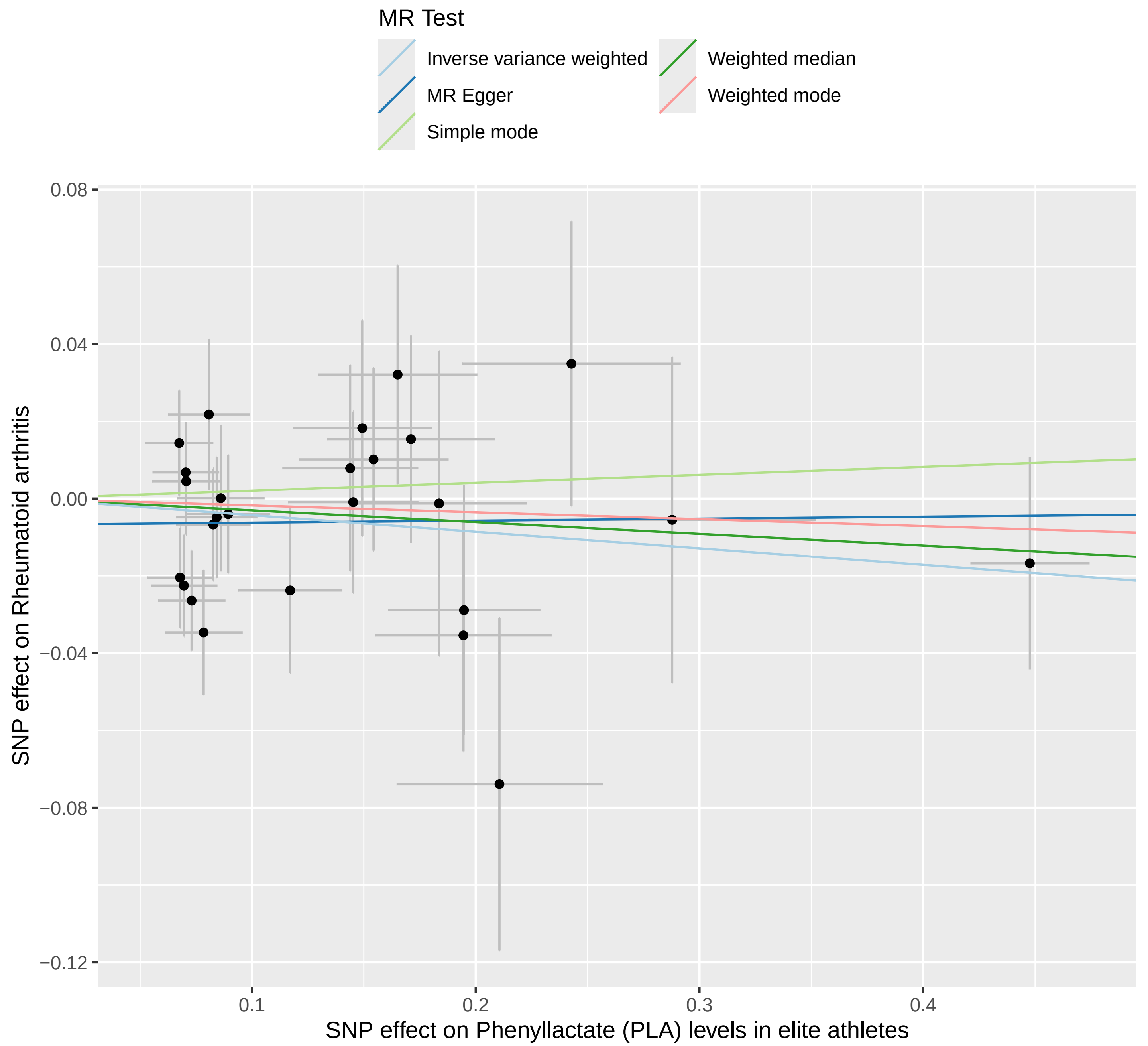

MR Test
Inverse variance weighted
MR Egger
Simple mode
Weighted median
Weighted mode
0.08
0.04
0.00
−0.04
−0.08
−0.12
SNP effect on Rheumatoid arthritis
0.1
0.2
0.3
0.4
SNP effect on Phenyllactate (PLA) levels in elite athletes

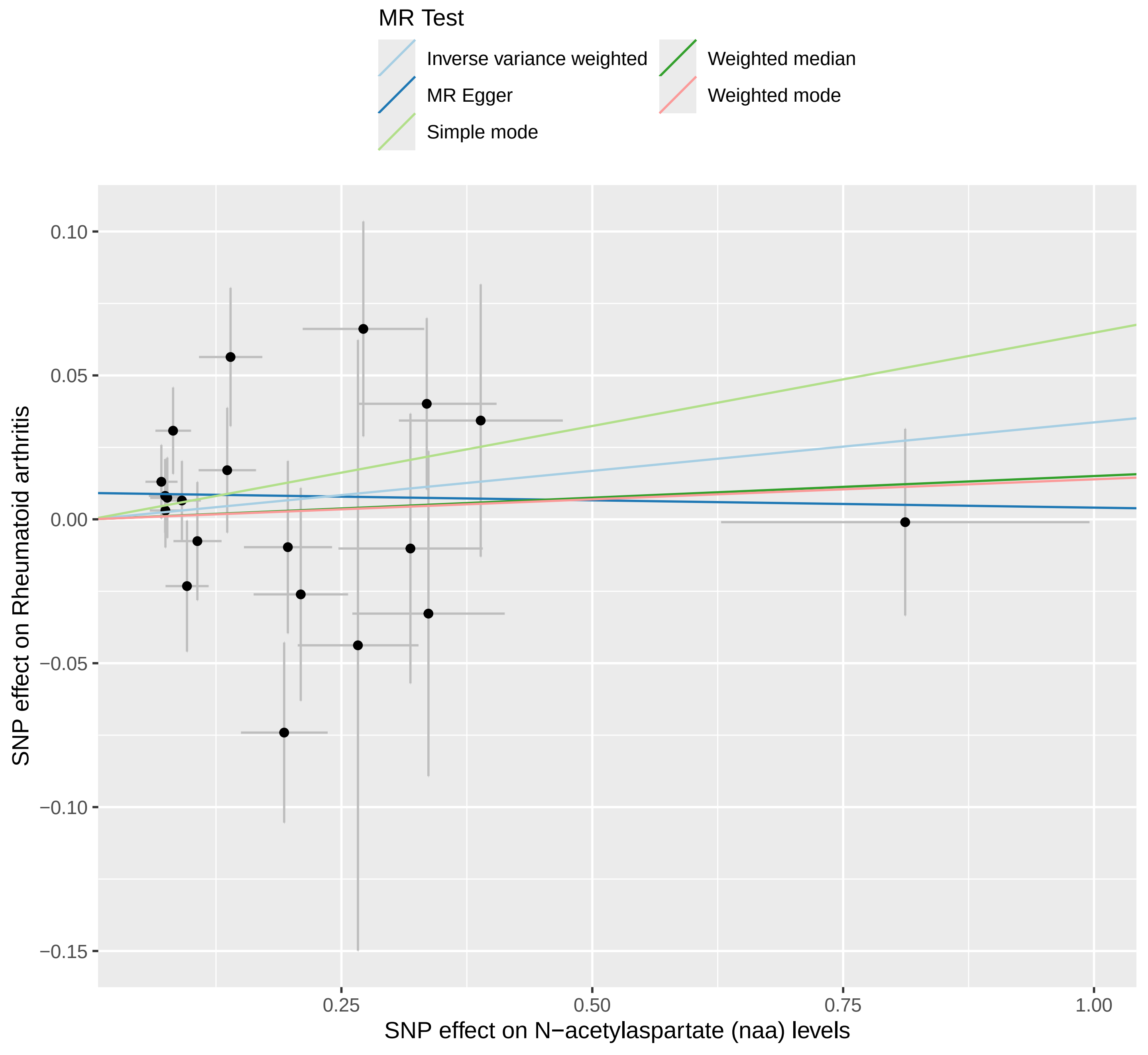

MR Test
Inverse variance weighted
MR Egger
Simple mode
Weighted median
Weighted mode
0.10
0.05
0.00
−0.05
−0.10
−0.15
0.25
0.50
0.75
1.00
SNP effect on Rheumatoid arthritis
SNP effect on N−acetylaspartate (naa) levels

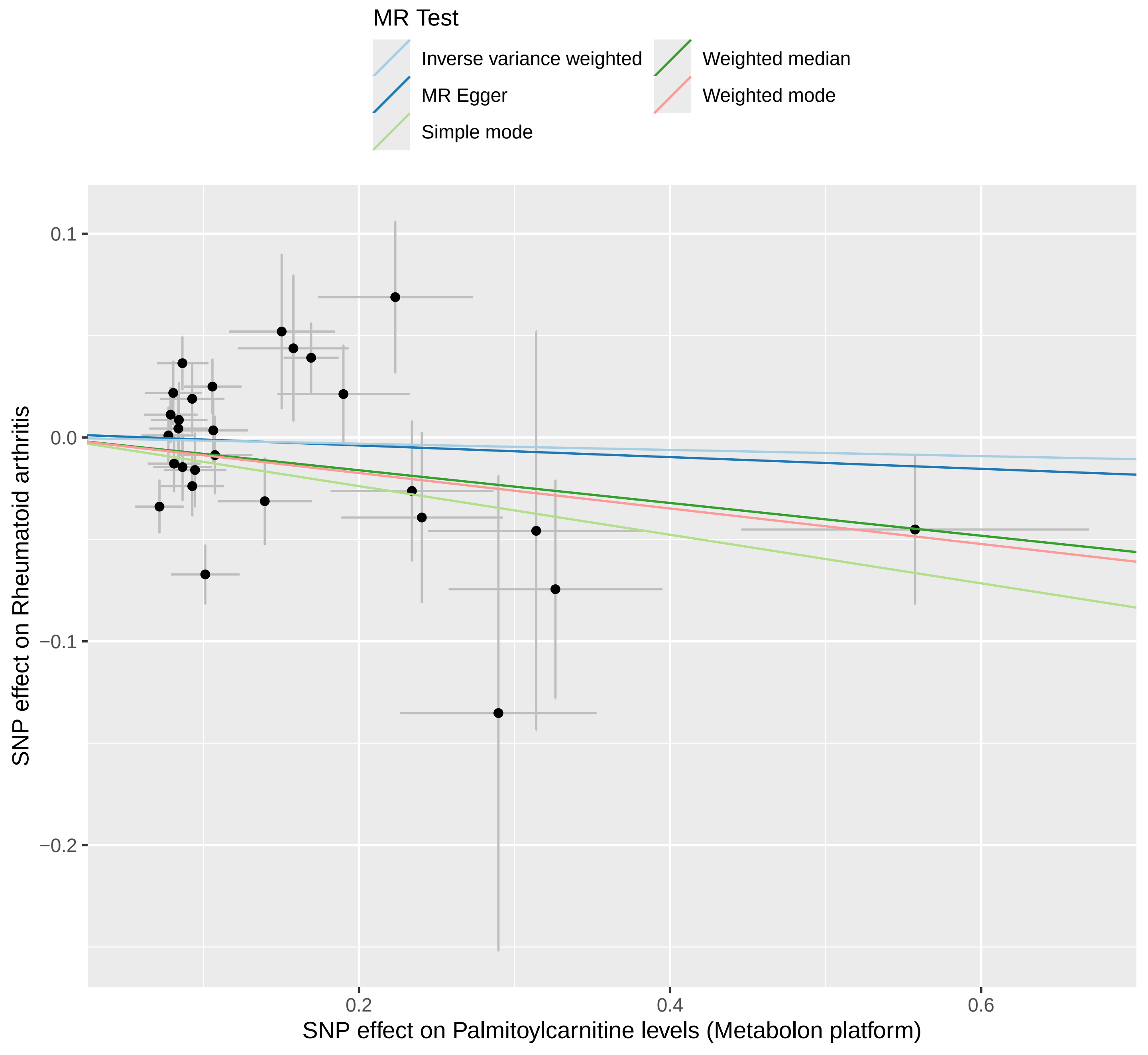
MR Test
Inverse variance weighted
MR Egger
Simple mode
Weighted median
Weighted mode
0.1
0.0
−0.1
−0.2
0.2
0.4
0.6
SNP effect on Rheumatoid arthritis
SNP effect on Palmitoylcarnitine levels (Metabolon platform)

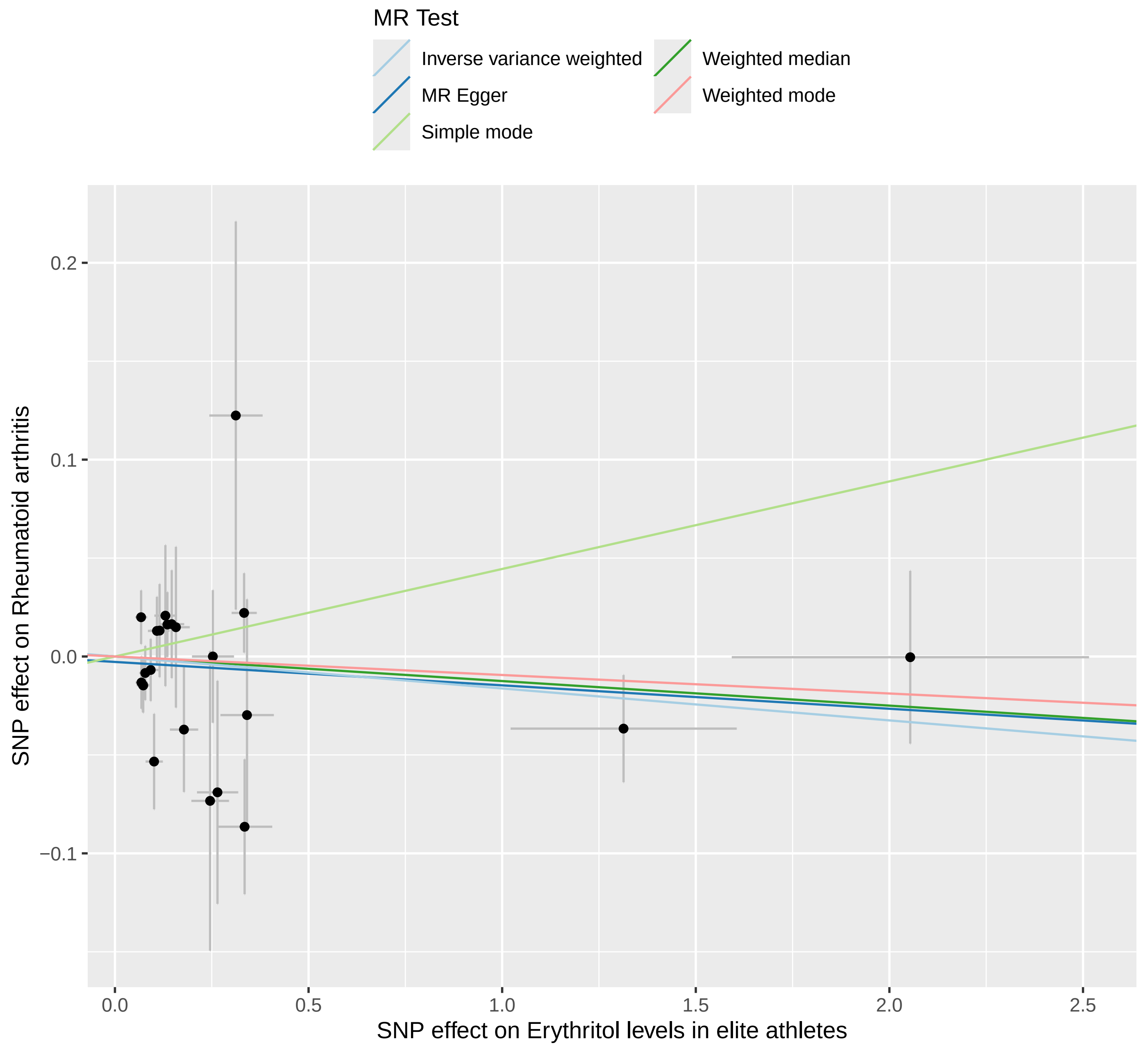

MR Test
Inverse variance weighted
MR Egger
Simple mode
Weighted median
Weighted mode
0.2
0.1
0.0
−0.1
0.0
0.5
1.0
1.5
2.0
2.5
SNP effect on Rheumatoid arthritis
SNP effect on Erythritol levels in elite athletes

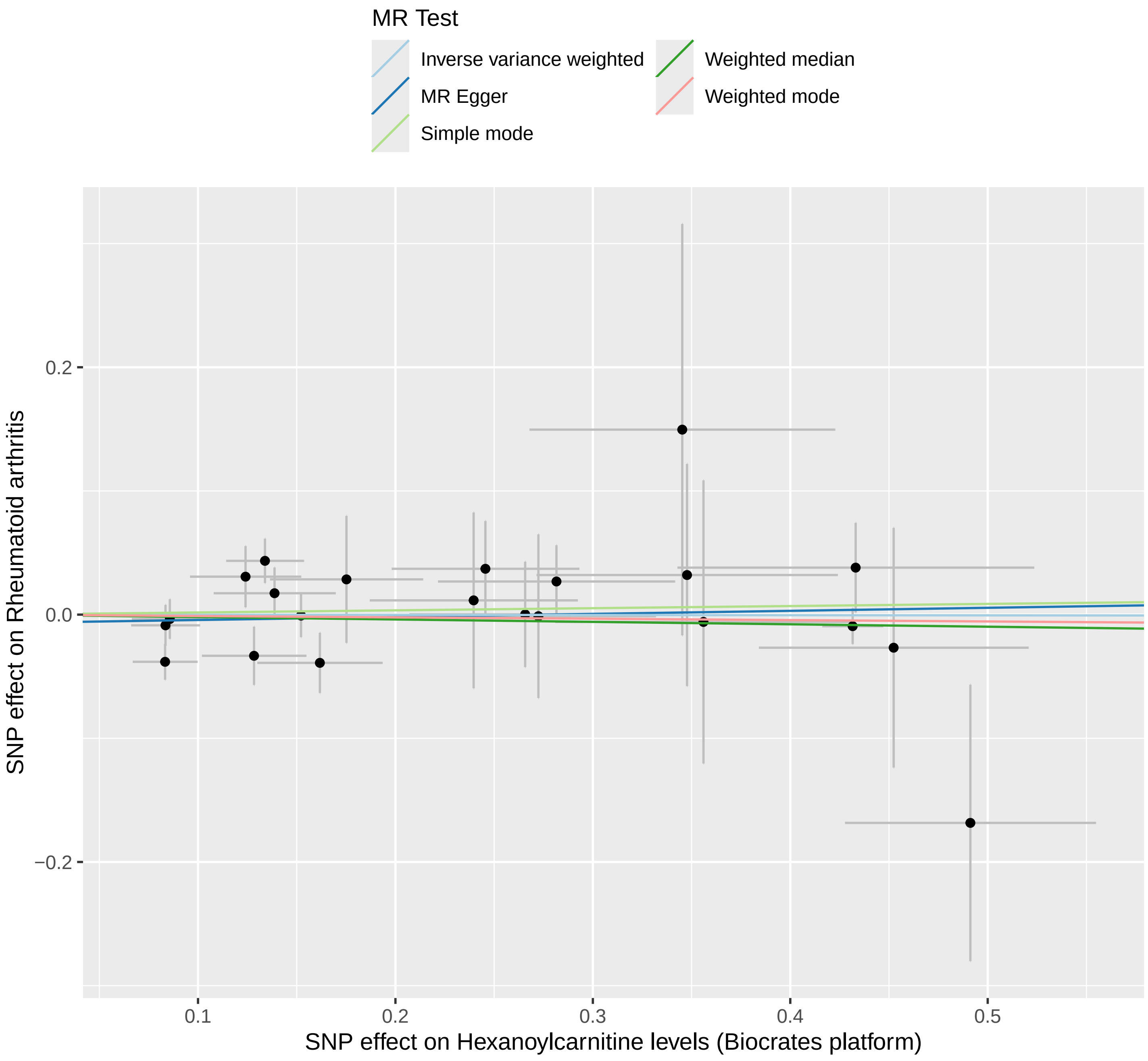

MR Test
Inverse variance weighted
MR Egger
Simple mode
Weighted median
Weighted mode
0.2
0.0
−0.2
0.1
0.2
0.3
0.4
0.5
SNP effect on Rheumatoid arthritis
SNP effect on Hexanoylcarnitine levels (Biocrates platform)

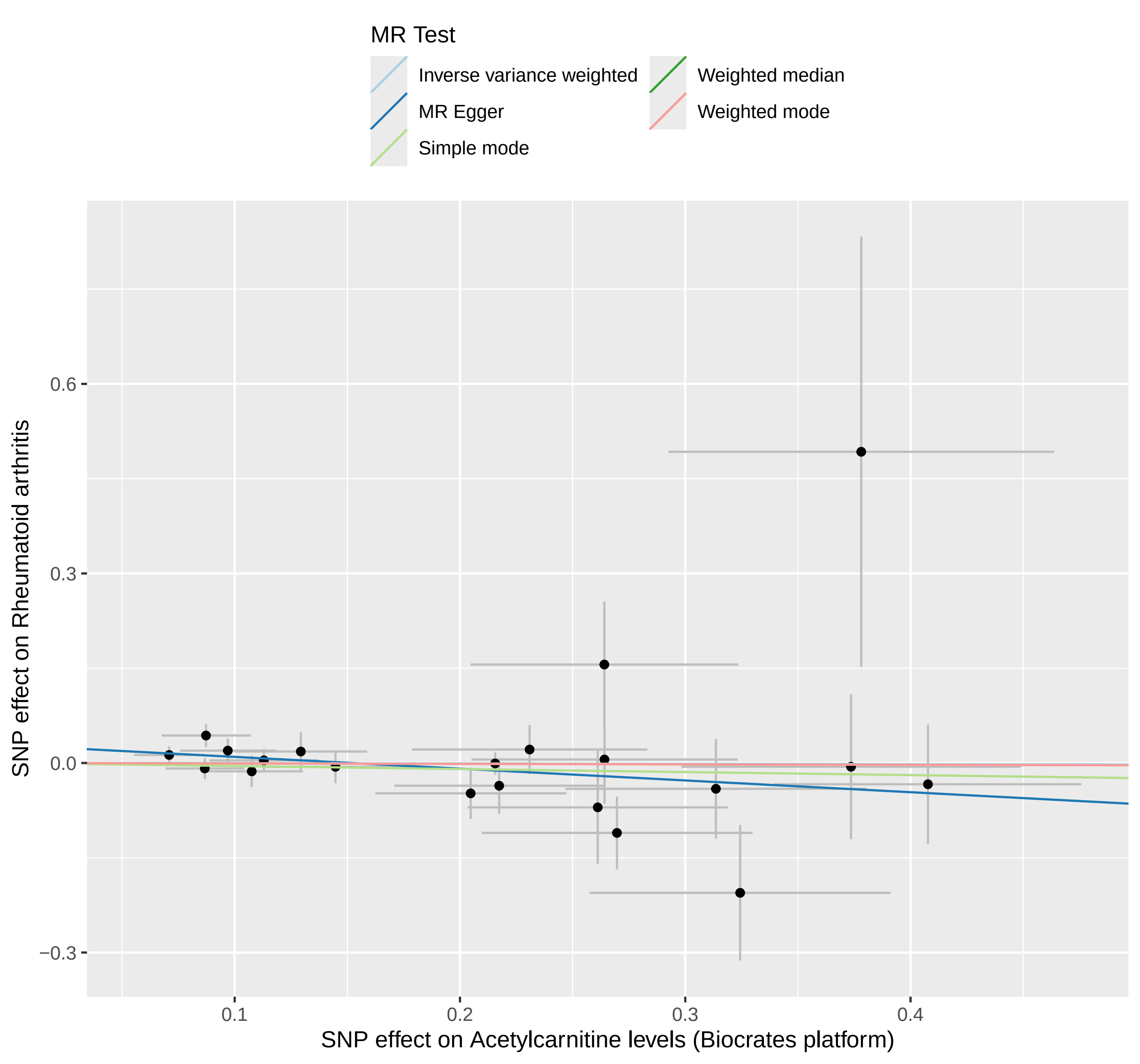

MR Test
Inverse variance weighted
MR Egger
Simple mode
Weighted median
Weighted mode
0.6
0.3
0.0
−0.3
0.1
0.2
0.3
0.4
SNP effect on Rheumatoid arthritis
SNP effect on Acetylcarnitine levels (Biocrates platform)

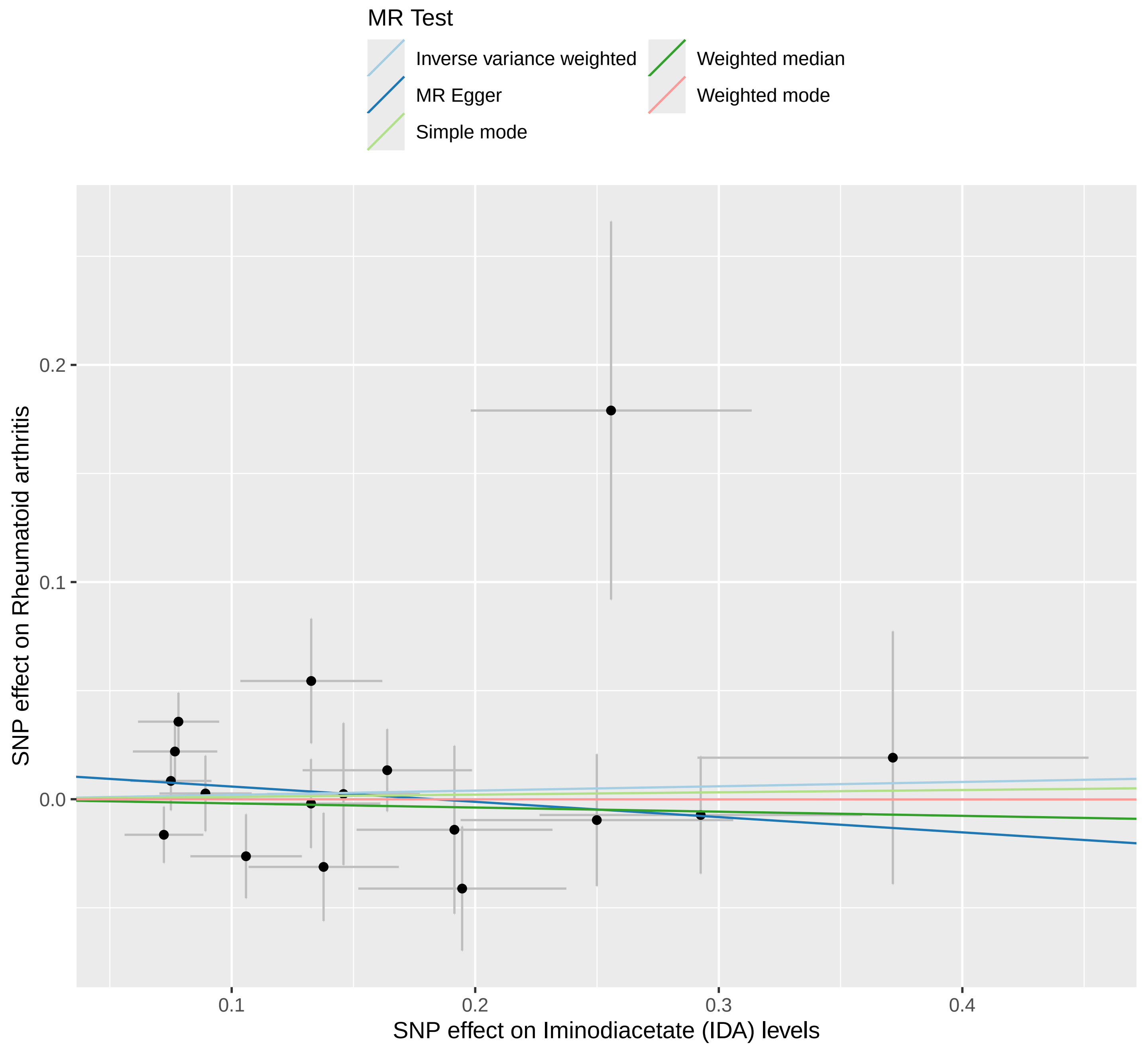
MR Test
Inverse variance weighted
MR Egger
Simple mode
Weighted median
Weighted mode
0.2
0.1
0.0
0.1
0.2
0.3
0.4
SNP effect on Rheumatoid arthritis
SNP effect on Iminodiacetate (IDA) levels

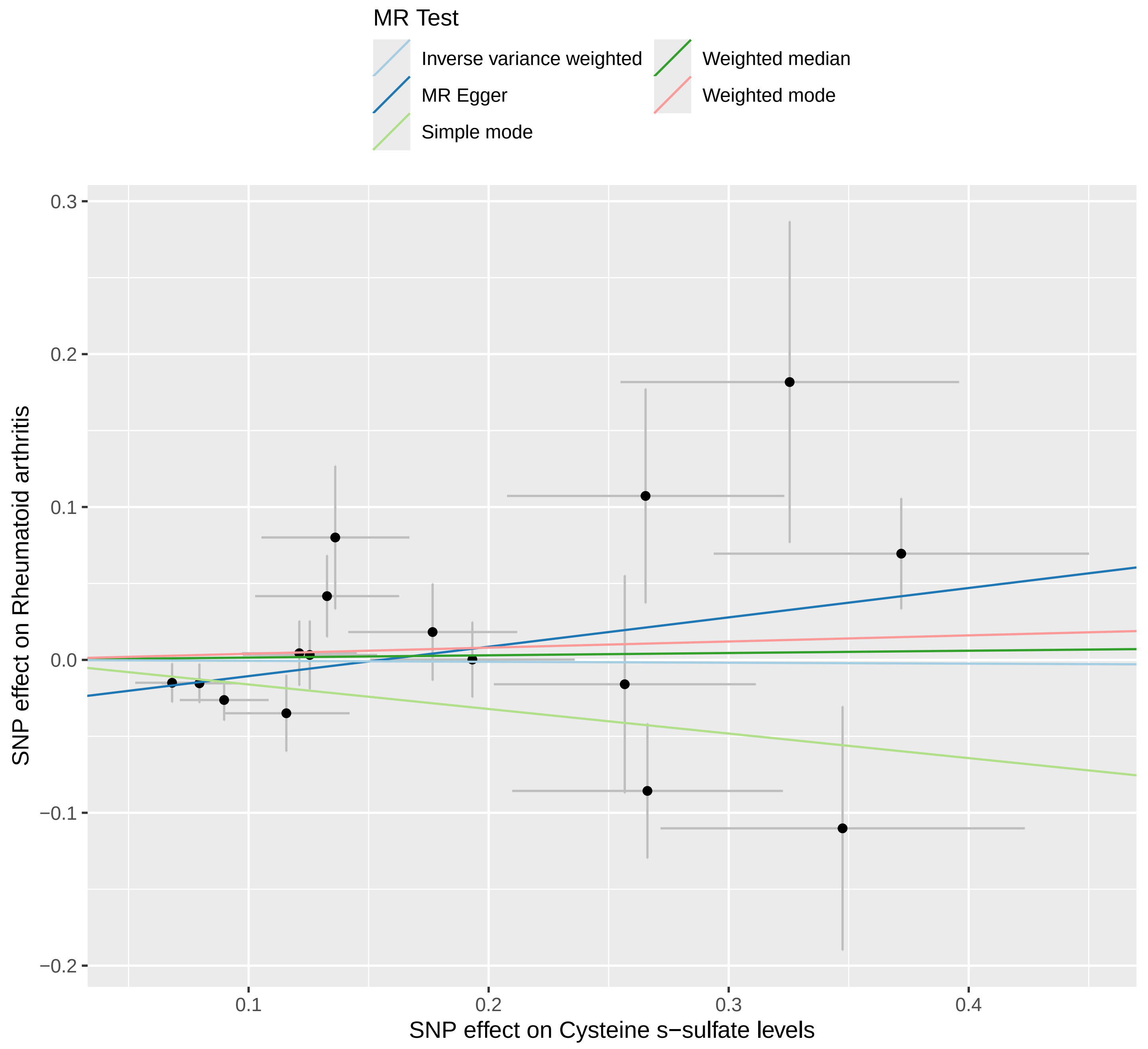
MR Test
Inverse variance weighted
MR Egger
Simple mode
Weighted median
Weighted mode
0.3
0.2
0.1
0.0
−0.1
−0.2
SNP effect on Rheumatoid arthritis
0.1
0.2
0.3
0.4
SNP effect on Cysteine s−sulfate levels

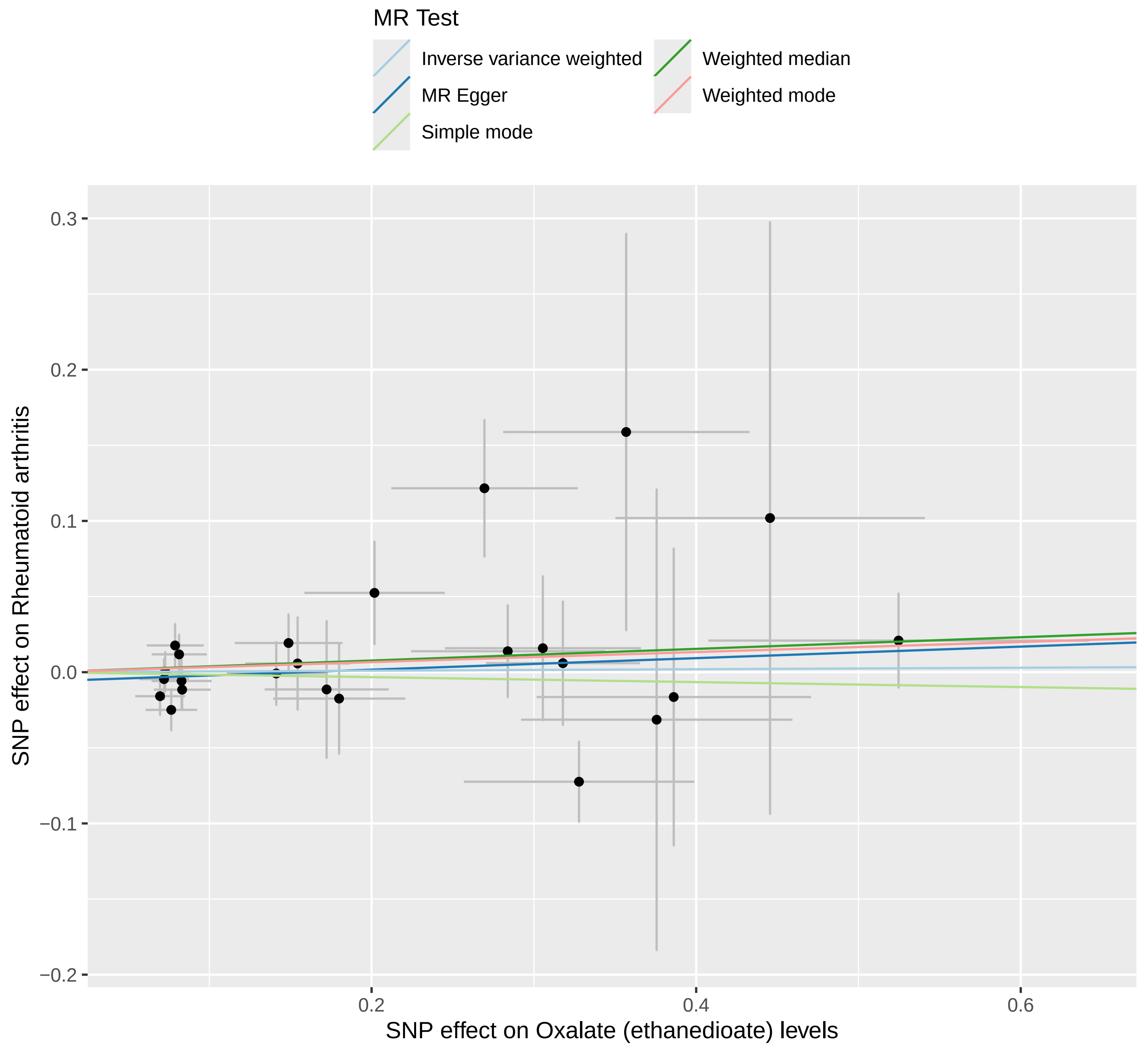
MR Test
Inverse variance weighted
MR Egger
Simple mode
Weighted median
Weighted mode
SNP effect on Rheumatoid arthritis
0.3
0.2
0.1
0.0
−0.1
−0.2
0.2
0.4
0.6
SNP effect on Oxalate (ethanedioate) levels

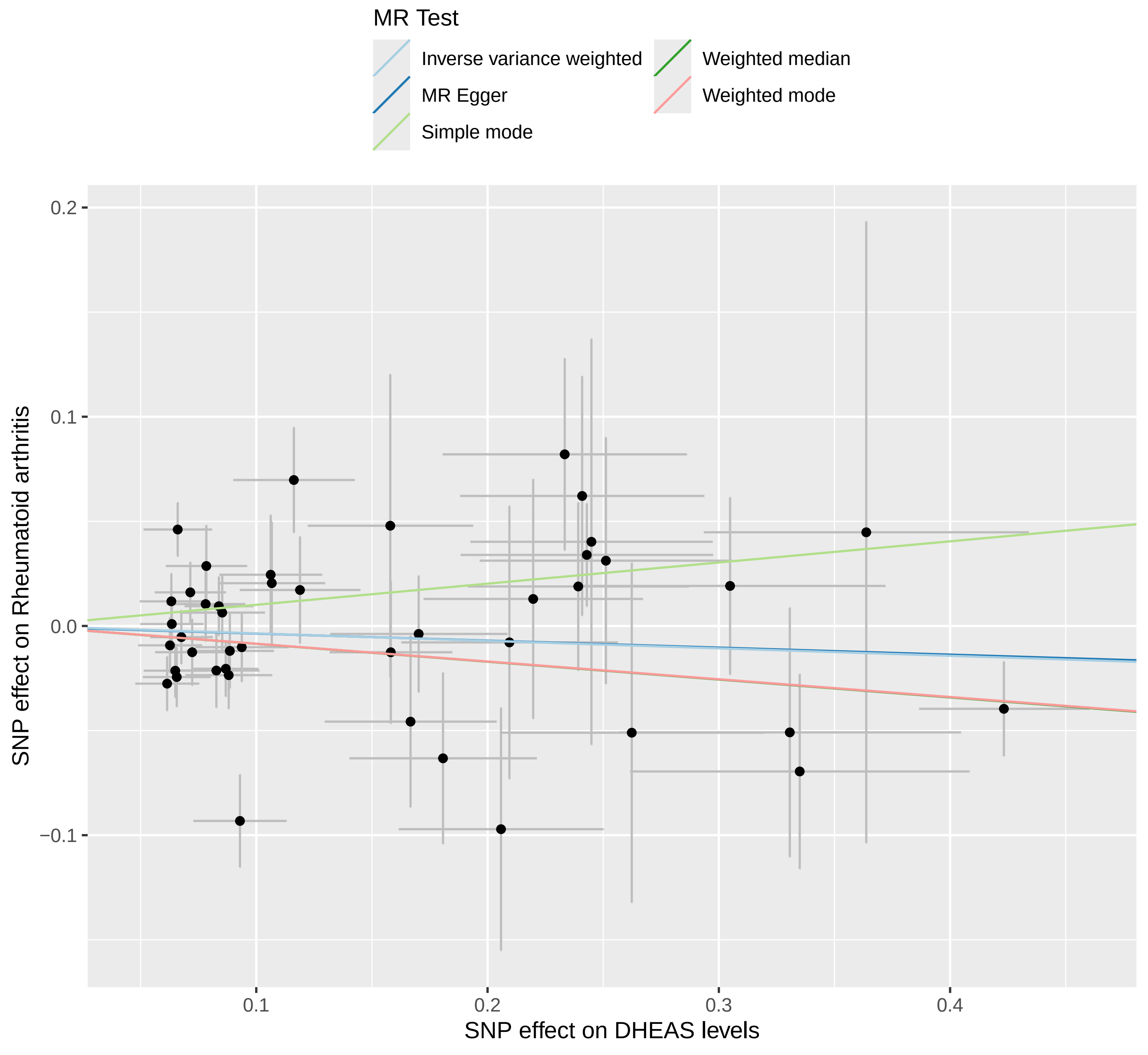
MR Test
Inverse variance weighted
MR Egger
Simple mode
Weighted median
Weighted mode
0.2
0.1
0.0
−0.1
0.1
0.2
0.3
0.4
SNP effect on Rheumatoid arthritis
SNP effect on DHEAS levels

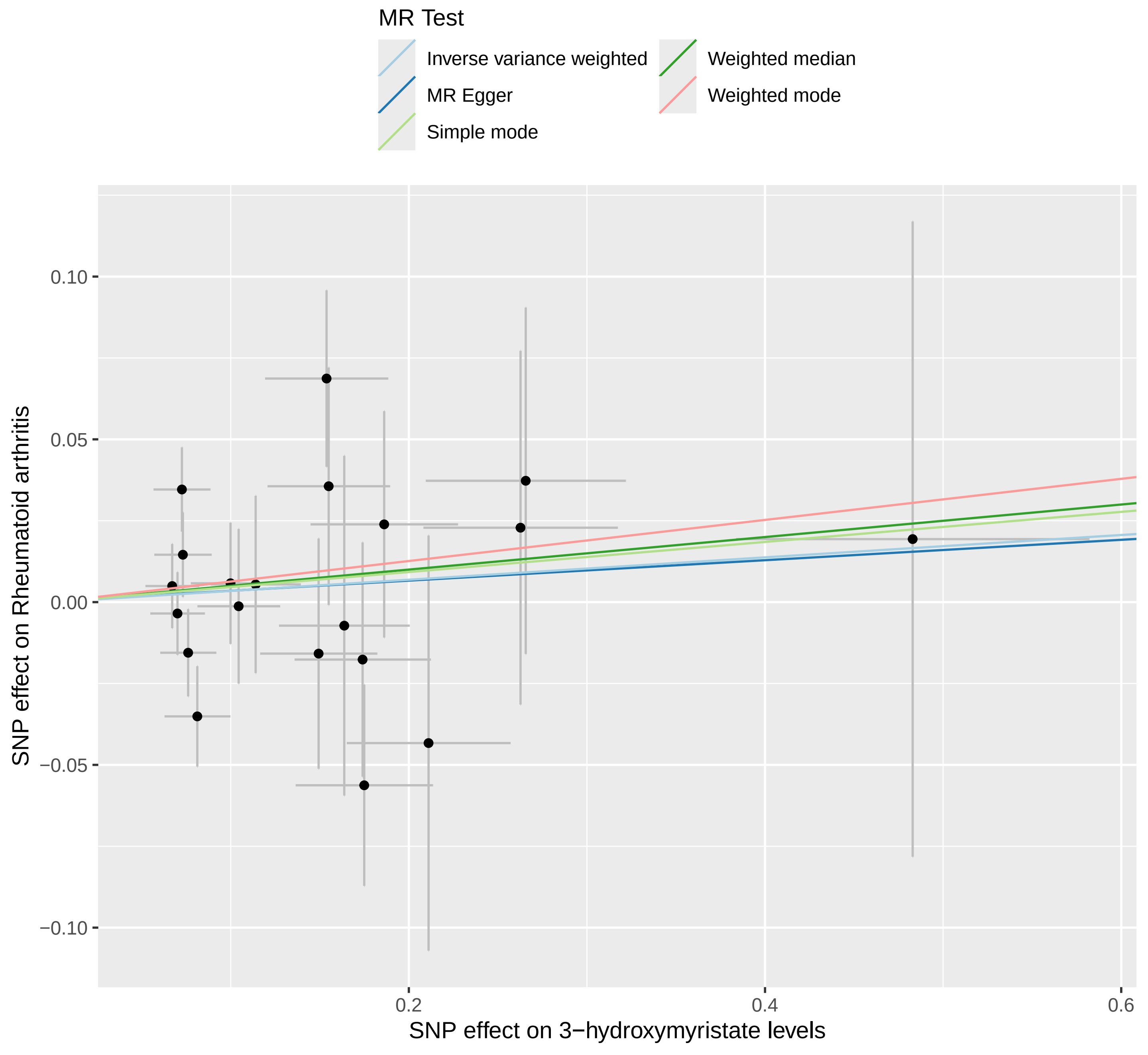
MR Test
Inverse variance weighted
MR Egger
Simple mode
Weighted median
Weighted mode
SNP effect on Rheumatoid arthritis
0.10
0.05
0.00
−0.05
−0.10
0.2
0.4
0.6
SNP effect on 3−hydroxymyristate levels

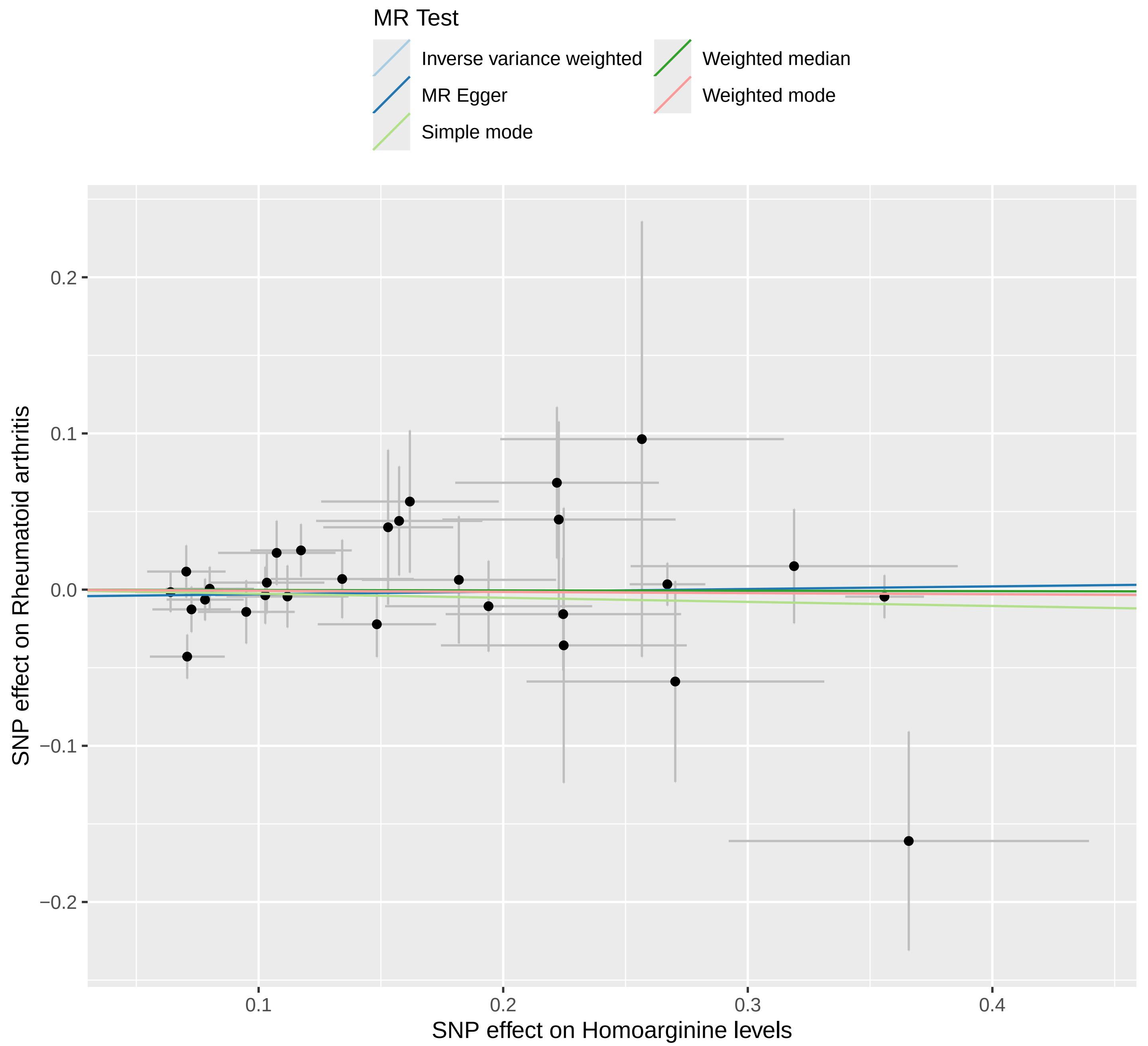

MR Test
Inverse variance weighted
MR Egger
Simple mode
Weighted median
Weighted mode
SNP effect on Rheumatoid arthritis
0.2
0.1
0.0
−0.1
−0.2
0.1
0.2
0.3
0.4
SNP effect on Homoarginine levels

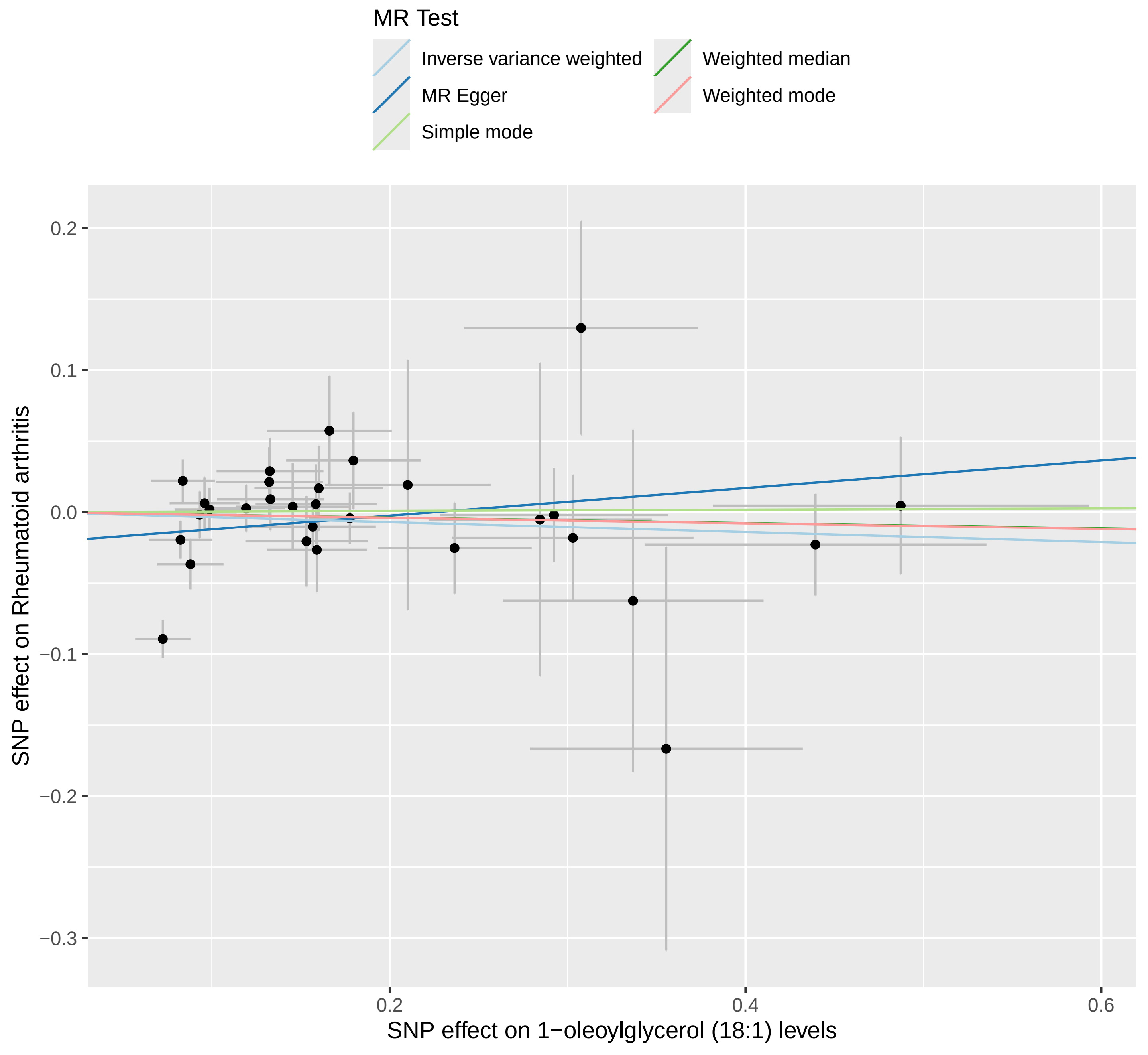

MR Test
Inverse variance weighted
MR Egger
Simple mode
Weighted median
Weighted mode
0.2
0.1
0.0
−0.1
−0.2
−0.3
0.2
0.4
0.6
SNP effect on Rheumatoid arthritis
SNP effect on 1−oleoylglycerol (18:1) levels

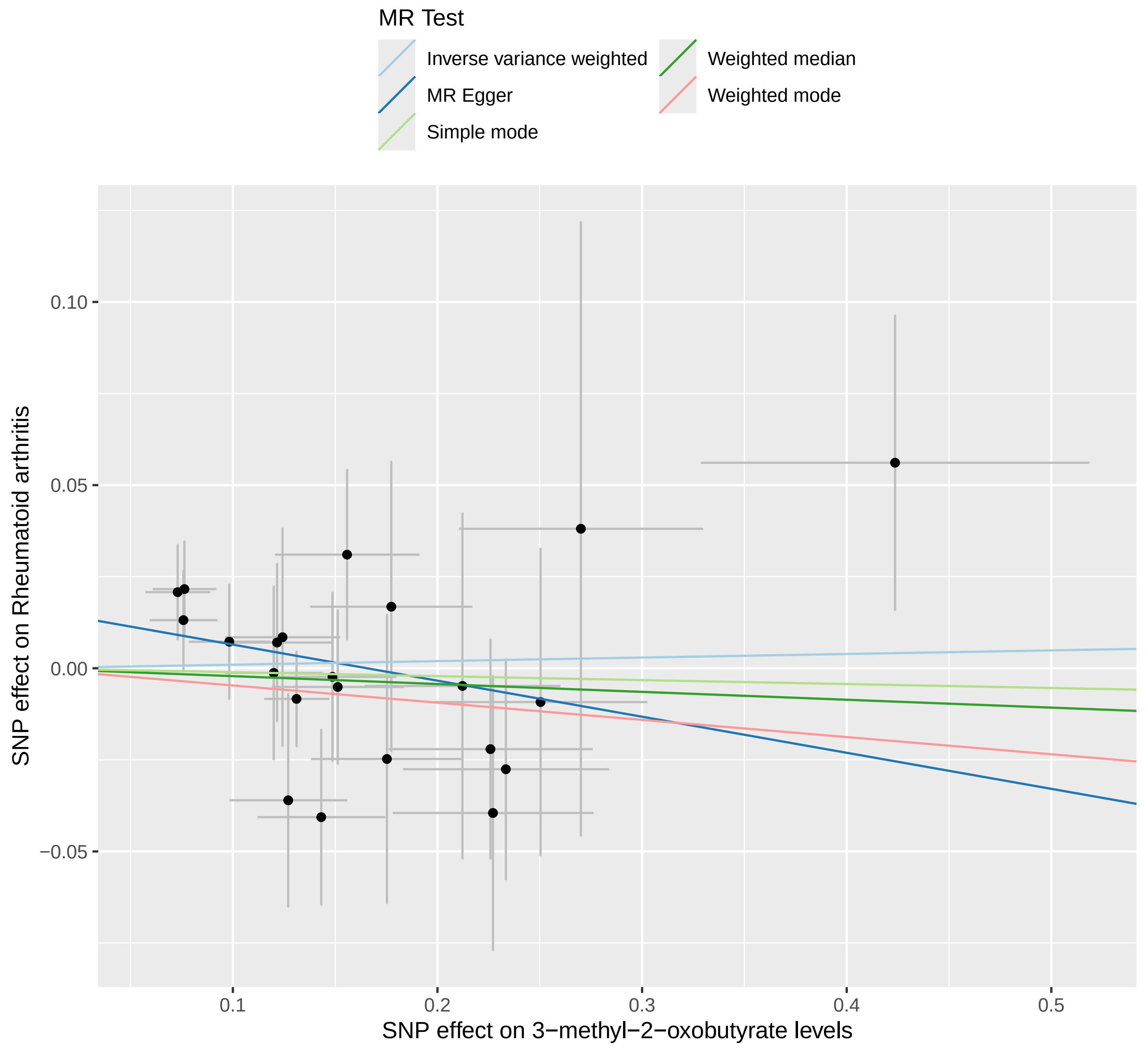

MR Test
Inverse variance weighted
MR Egger
Simple mode
Weighted median
Weighted mode
0.10
0.05
0.00
−0.05
SNP effect on Rheumatoid arthritis
0.1
0.2
0.3
0.4
0.5
SNP effect on 3−methyl−2−oxobutyrate levels

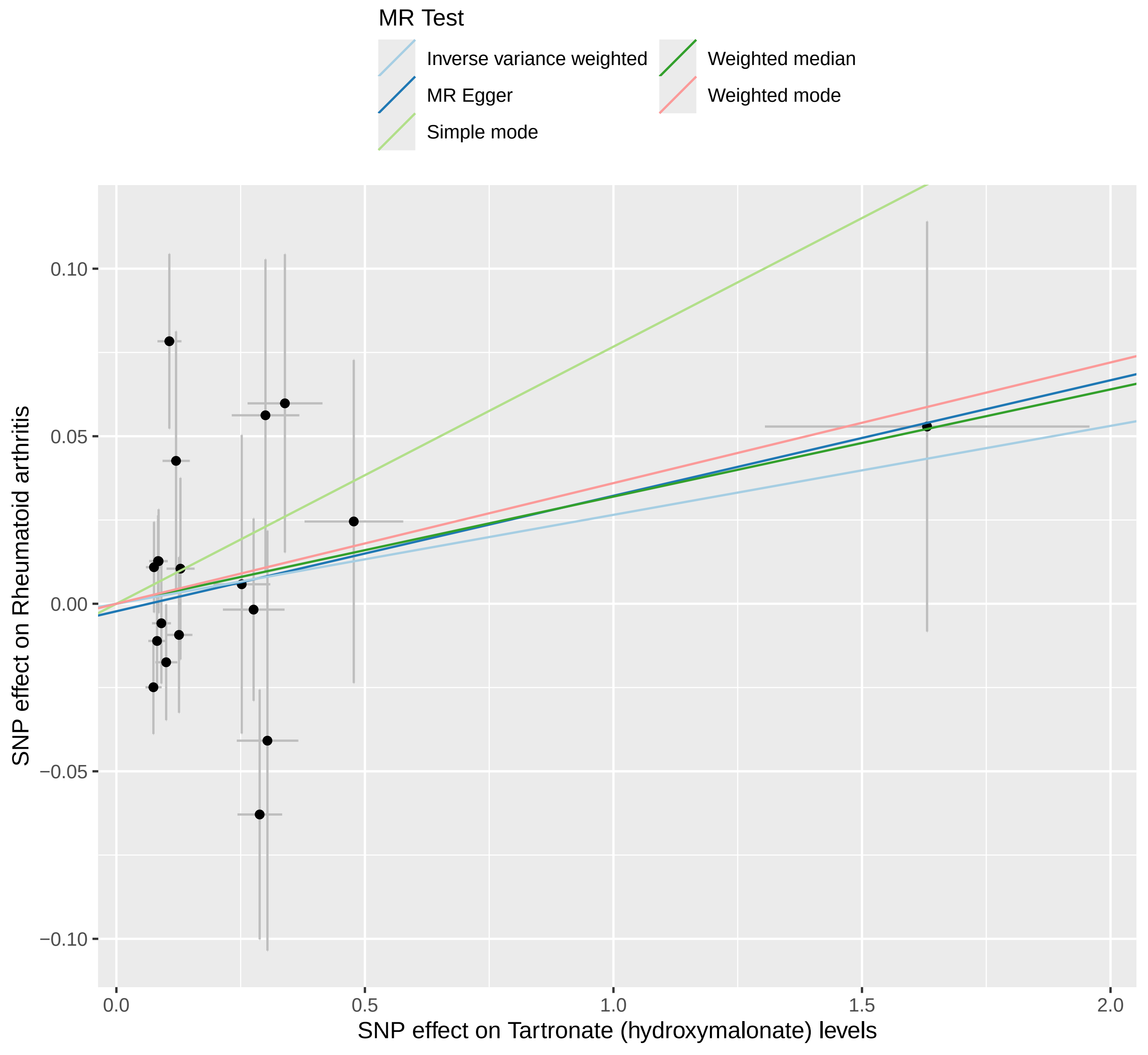
MR Test
Inverse variance weighted
MR Egger
Simple mode
Weighted median
Weighted mode
SNP effect on Rheumatoid arthritis
0.10
0.05
0.00
−0.05
−0.10
0.0
0.5
1.0
1.5
2.0
SNP effect on Tartronate (hydroxymalonate) levels

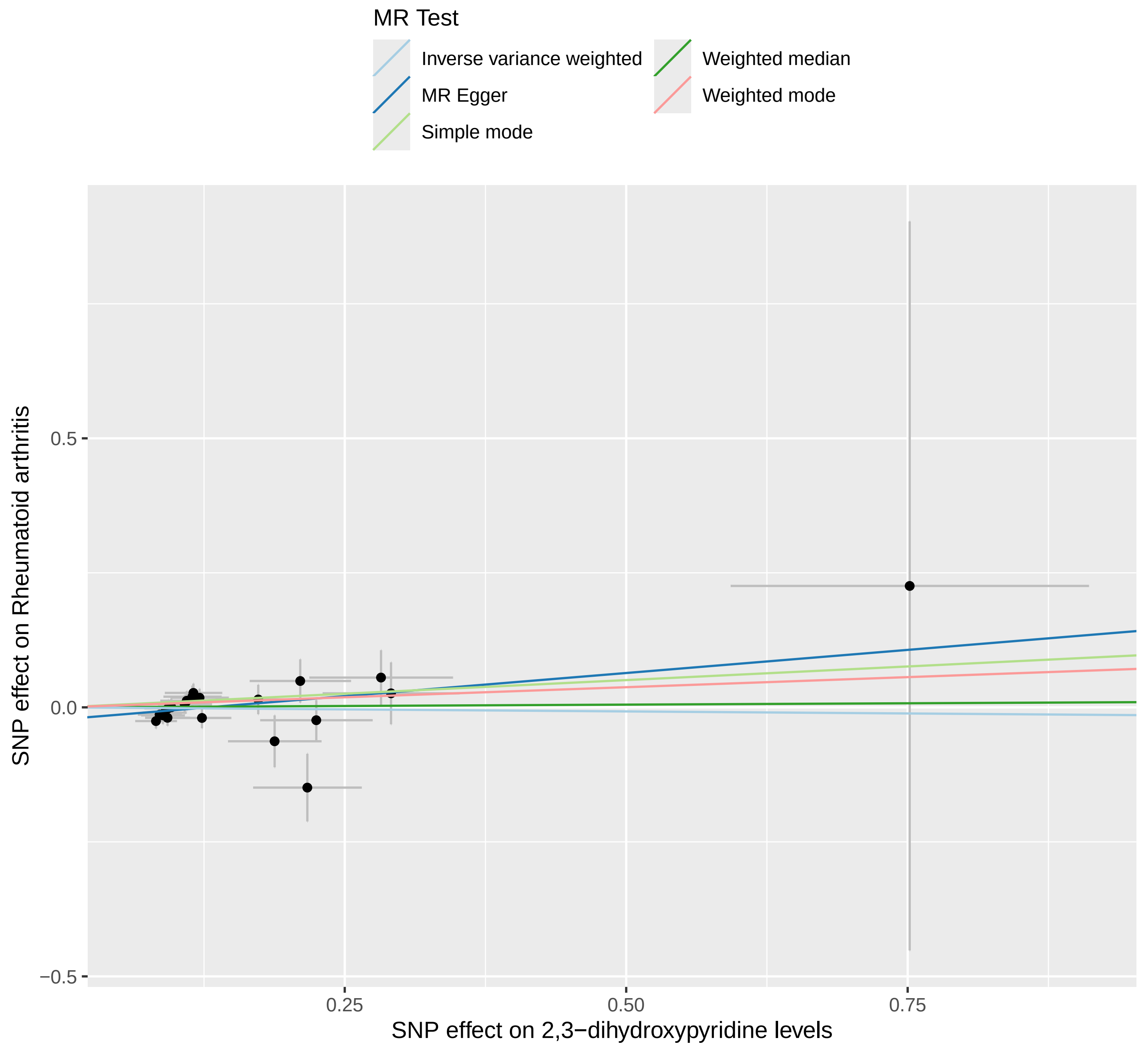
MR Test
Inverse variance weighted
MR Egger
Simple mode
Weighted median
Weighted mode
SNP effect on Rheumatoid arthritis
0.5
0.0
−0.5
0.25
0.50
0.75
SNP effect on 2,3−dihydroxypyridine levels

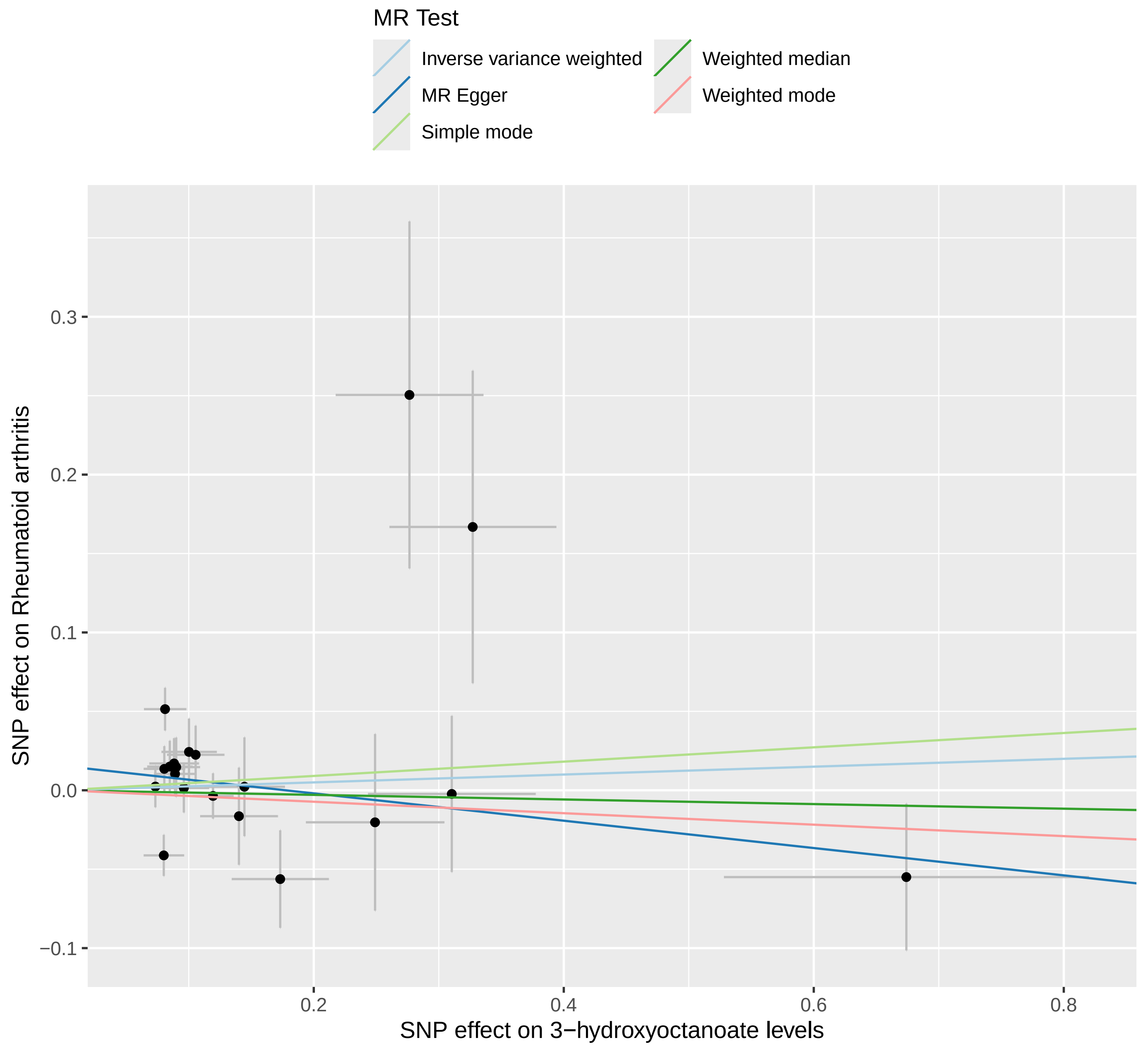

MR Test
Inverse variance weighted
MR Egger
Simple mode
Weighted median
Weighted mode
0.3
0.2
0.1
0.0
−0.1
0.2
0.4
0.6
0.8
SNP effect on Rheumatoid arthritis
SNP effect on 3−hydroxyoctanoate levels

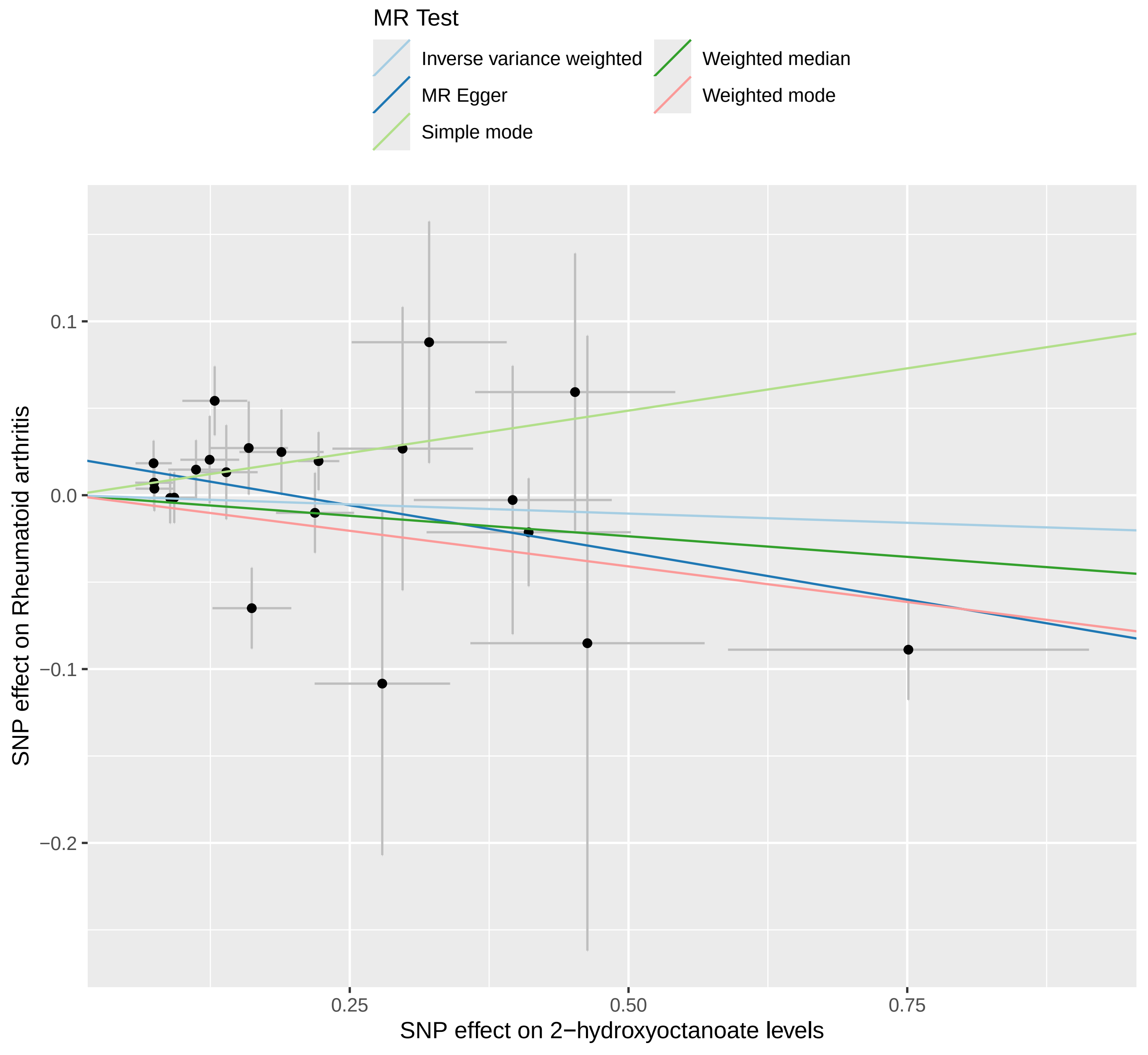
MR Test
Inverse variance weighted
MR Egger
Simple mode
Weighted median
Weighted mode
0.1
0.0
−0.1
−0.2
SNP effect on Rheumatoid arthritis
0.25
0.50
0.75
SNP effect on 2−hydroxyoctanoate levels

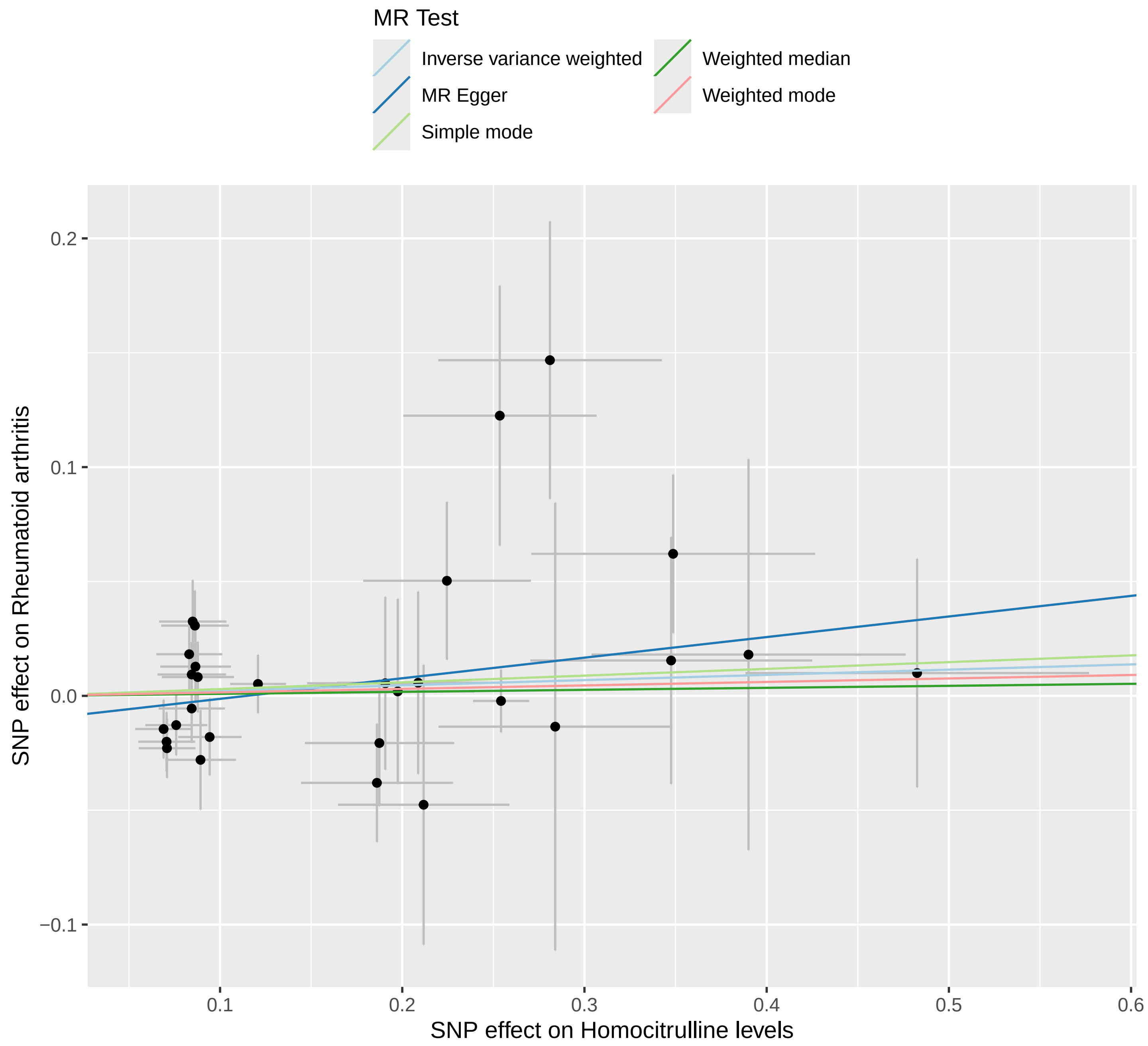
MR Test
Inverse variance weighted
MR Egger
Simple mode
Weighted median
Weighted mode
0.2
0.1
0.0
−0.1
0.1
0.2
0.3
0.4
0.5
0.6
SNP effect on Rheumatoid arthritis
SNP effect on Homocitrulline levels

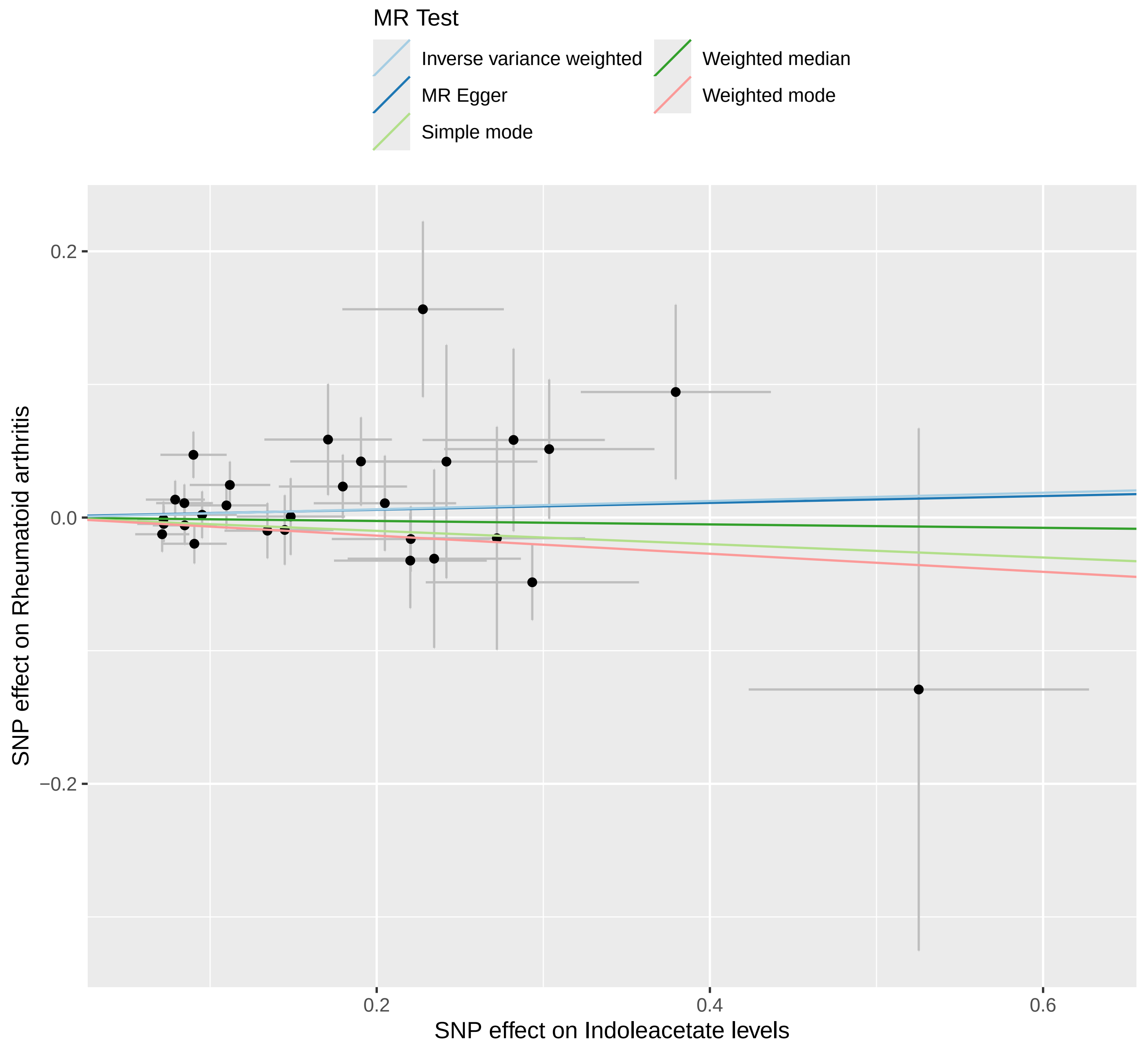

MR Test
Inverse variance weighted
MR Egger
Simple mode
Weighted median
Weighted mode
0.2
0.0
−0.2
0.2
0.4
0.6
SNP effect on Rheumatoid arthritis
SNP effect on Indoleacetate levels

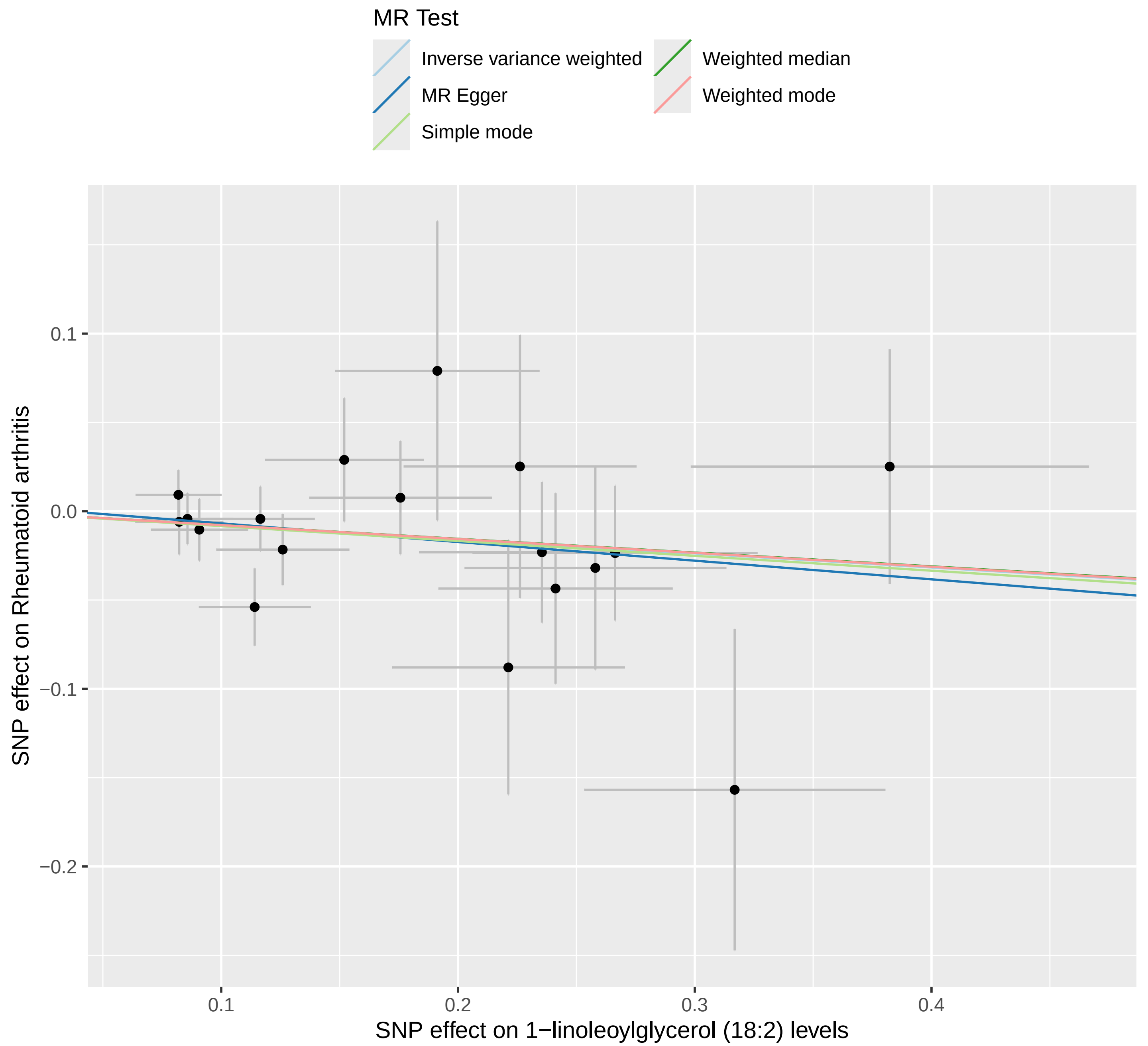
MR Test
Inverse variance weighted
MR Egger
Simple mode
Weighted median
Weighted mode
0.1
0.0
−0.1
−0.2
0.1
0.2
0.3
0.4
SNP effect on Rheumatoid arthritis
SNP effect on 1−linoleoylglycerol (18:2) levels

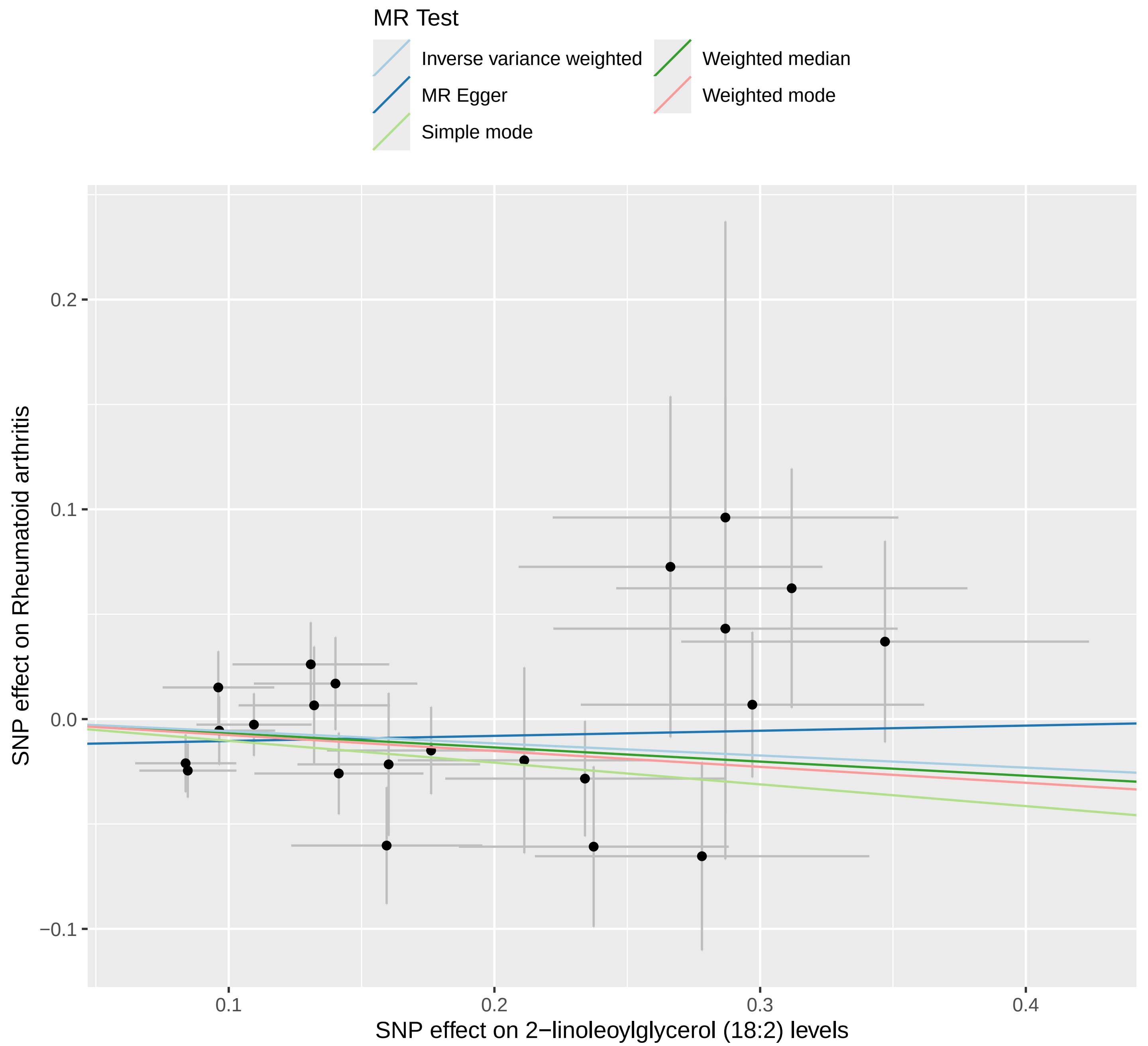

MR Test
Inverse variance weighted
MR Egger
Simple mode
Weighted median
Weighted mode
SNP effect on Rheumatoid arthritis
0.2
0.1
0.0
−0.1
0.1
0.2
0.3
0.4
SNP effect on 2−linoleoylglycerol (18:2) levels

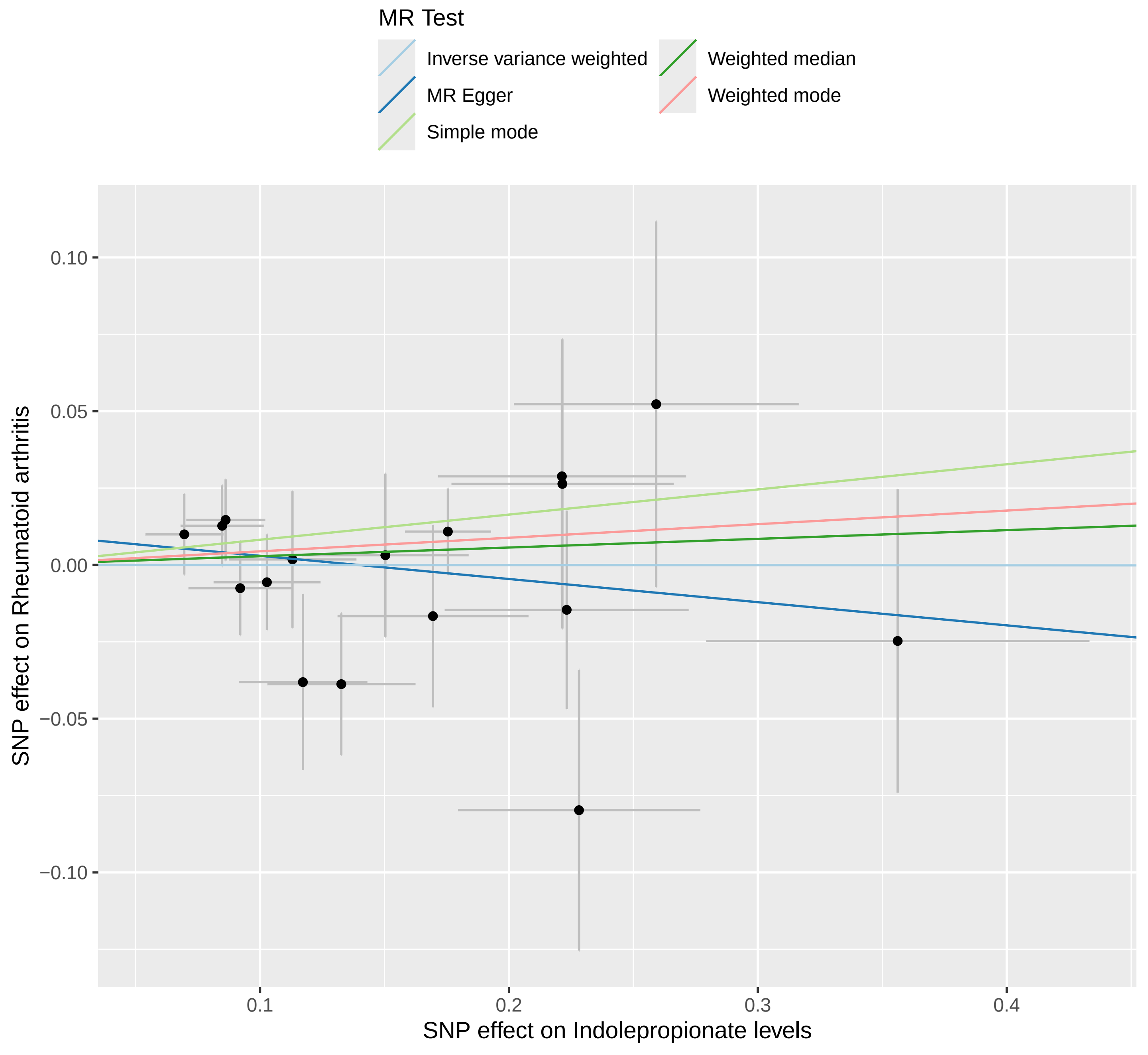

MR Test
Inverse variance weighted
MR Egger
Simple mode
Weighted median
Weighted mode
0.10
0.05
0.00
−0.05
−0.10
SNP effect on Rheumatoid arthritis
0.1
0.2
0.3
0.4
SNP effect on Indolepropionate levels

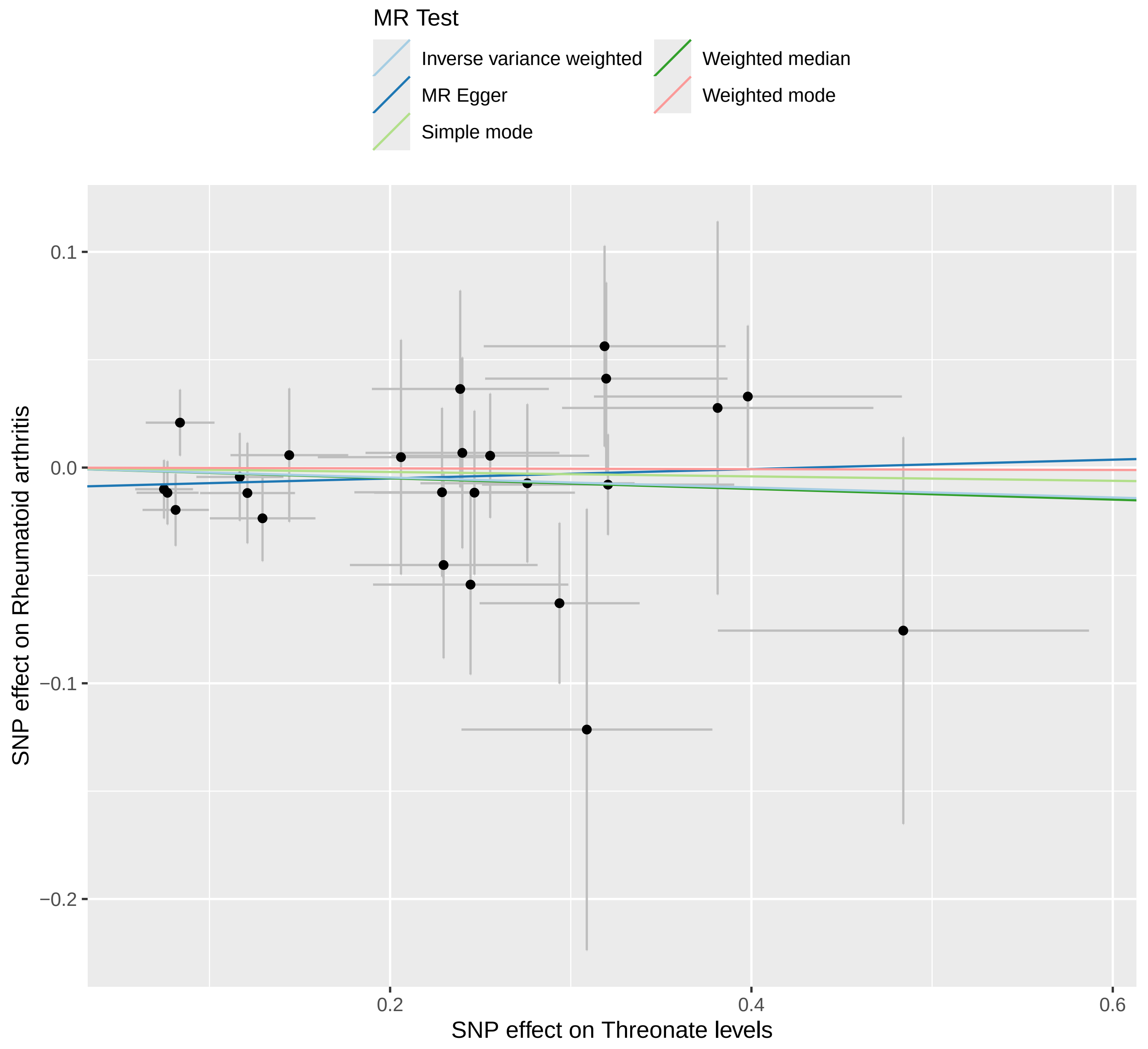
MR Test
Inverse variance weighted
MR Egger
Simple mode
Weighted median
Weighted mode
SNP effect on Rheumatoid arthritis
SNP effect on Threonate levels
0.1
0.0
−0.1
−0.2
0.2
0.4
0.6

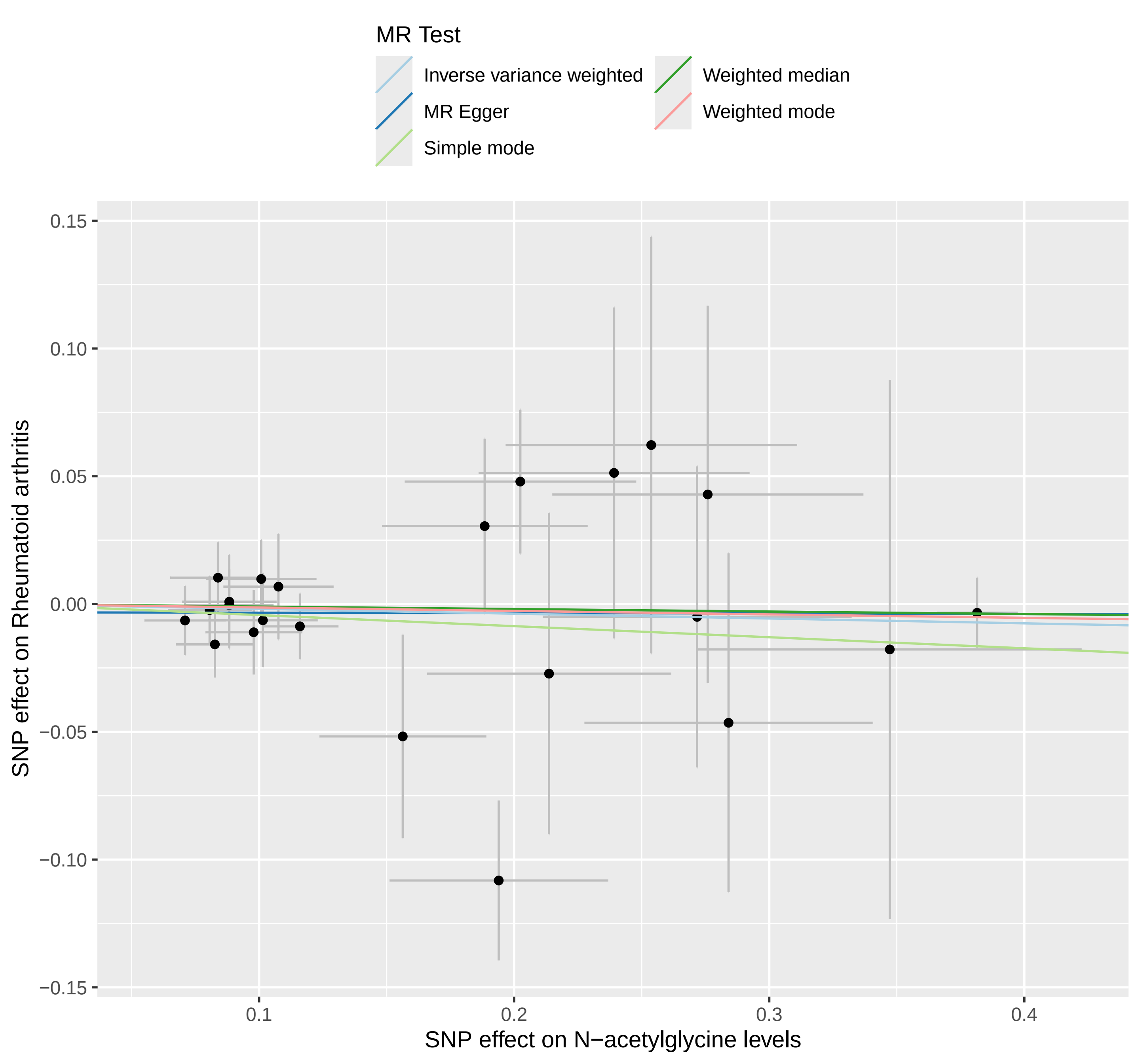
MR Test
Inverse variance weighted
MR Egger
Simple mode
Weighted median
Weighted mode
0.15
0.10
0.05
0.00
−0.05
−0.10
−0.15
0.1
0.2
0.3
0.4
SNP effect on Rheumatoid arthritis
SNP effect on N−acetylglycine levels

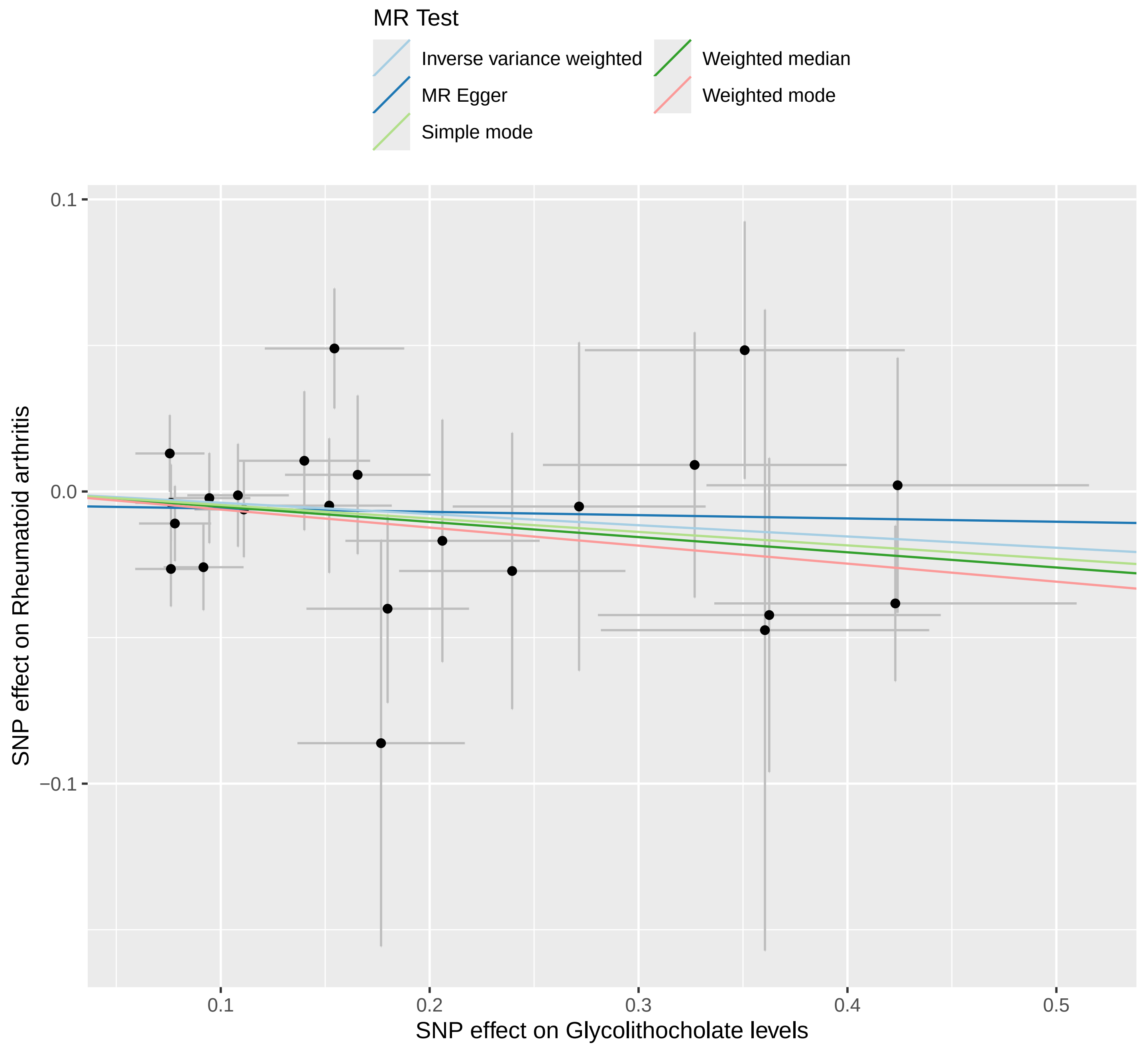
MR Test
Inverse variance weighted
MR Egger
Simple mode
Weighted median
Weighted mode
0.1
0.0
−0.1
SNP effect on Rheumatoid arthritis
0.1
0.2
0.3
0.4
0.5
SNP effect on Glycolithocholate levels

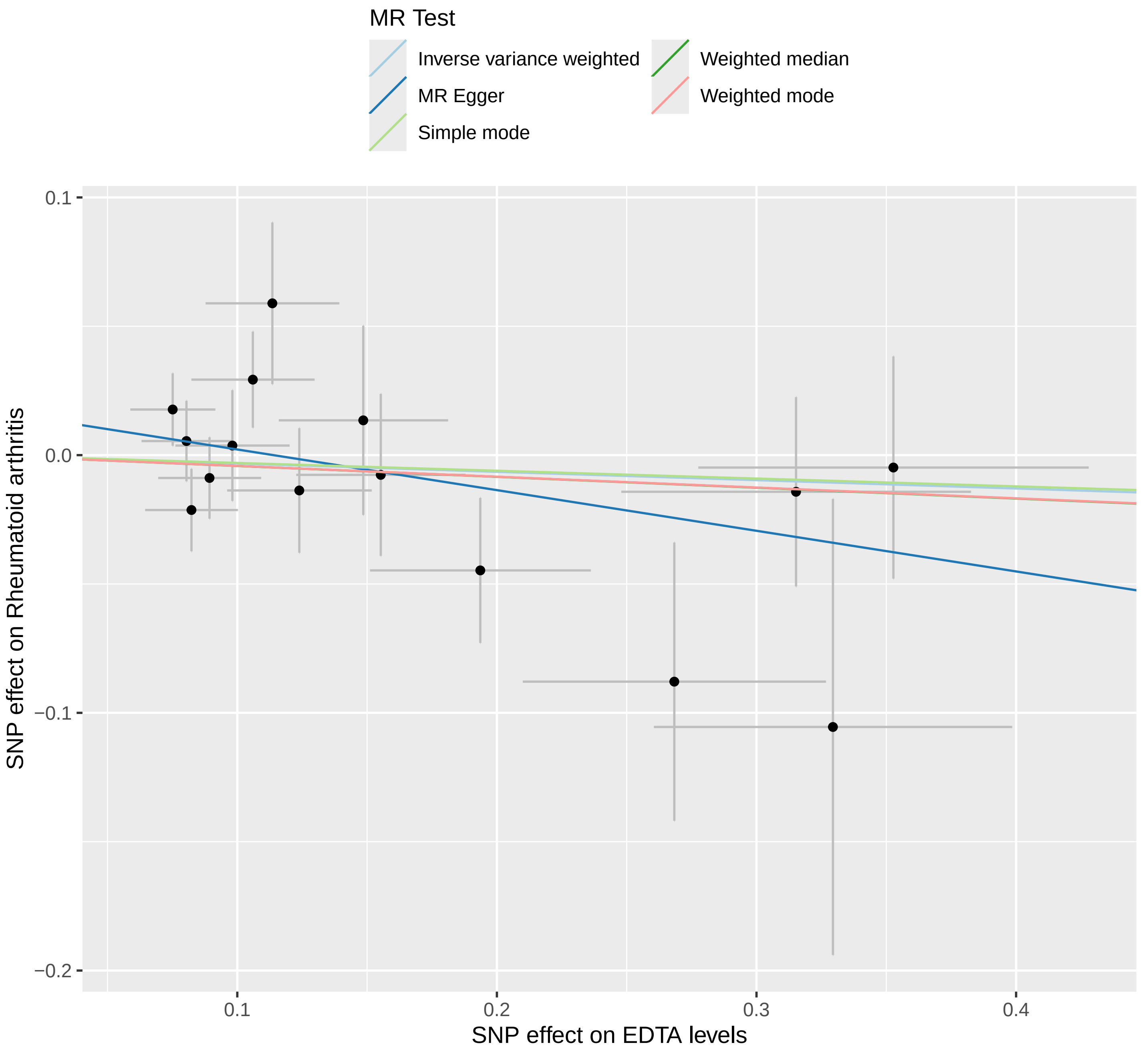
MR Test
Inverse variance weighted
MR Egger
Simple mode
Weighted median
Weighted mode
0.1
0.0
−0.1
−0.2
SNP effect on Rheumatoid arthritis
0.1
0.2
0.3
0.4
SNP effect on EDTA levels

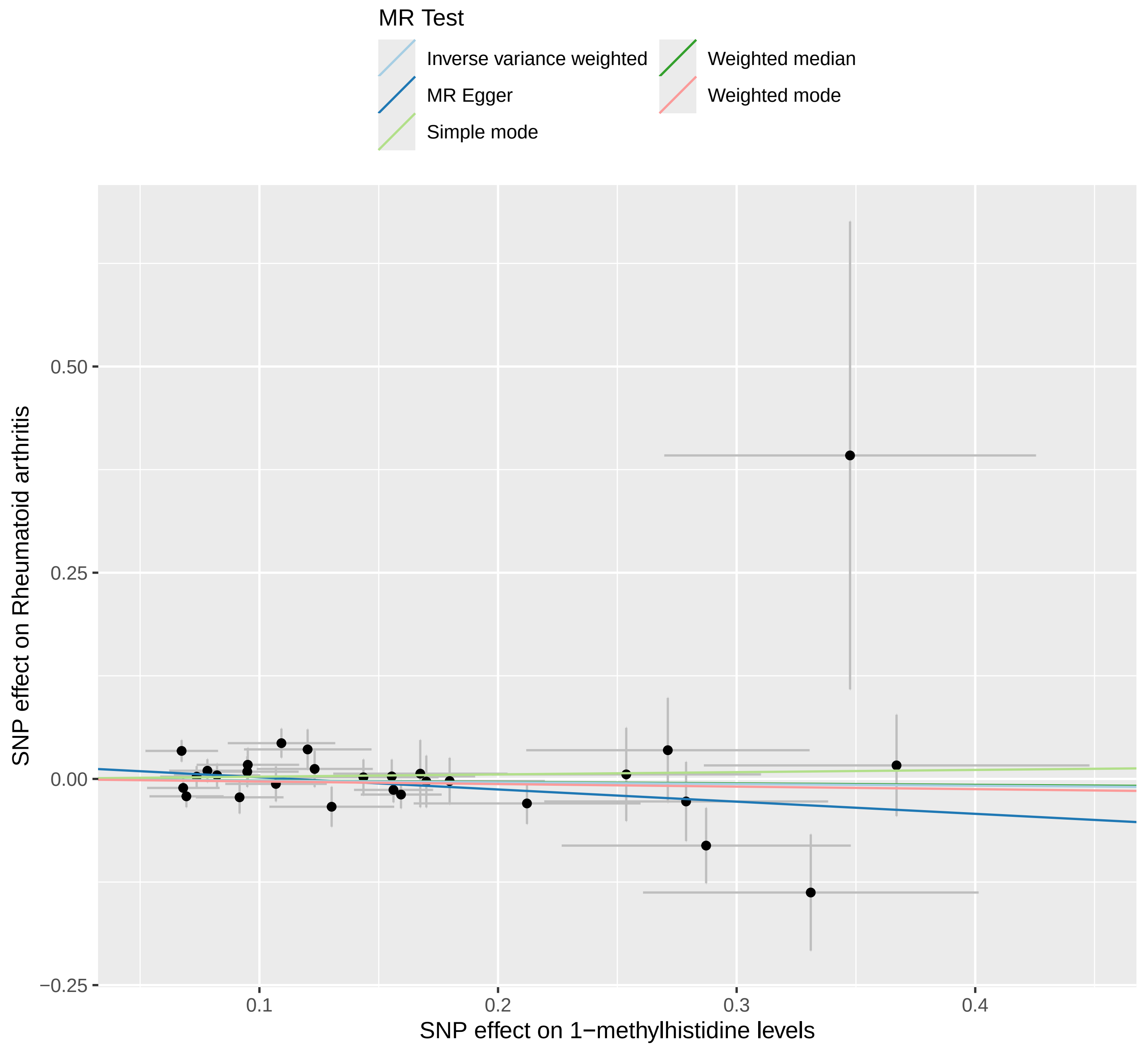
MR Test
Inverse variance weighted
MR Egger
Simple mode
Weighted median
Weighted mode
SNP effect on Rheumatoid arthritis
0.50
0.25
0.00
−0.25
0.1
0.2
0.3
0.4
SNP effect on 1−methylhistidine levels

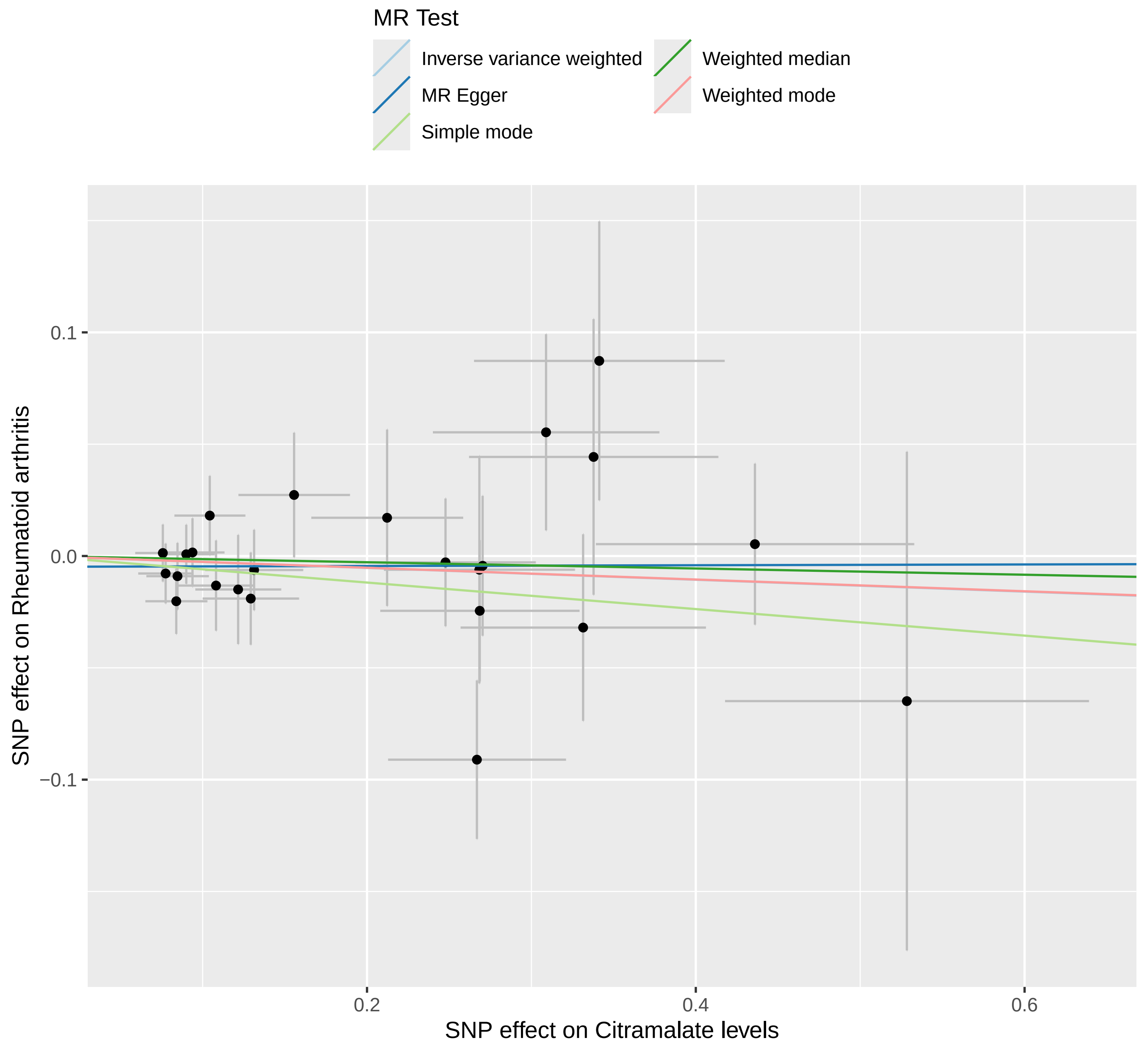
MR Test
Inverse variance weighted
MR Egger
Simple mode
Weighted median
Weighted mode
0.1
0.0
−0.1
0.2
0.4
0.6
SNP effect on Rheumatoid arthritis
SNP effect on Citramalate levels

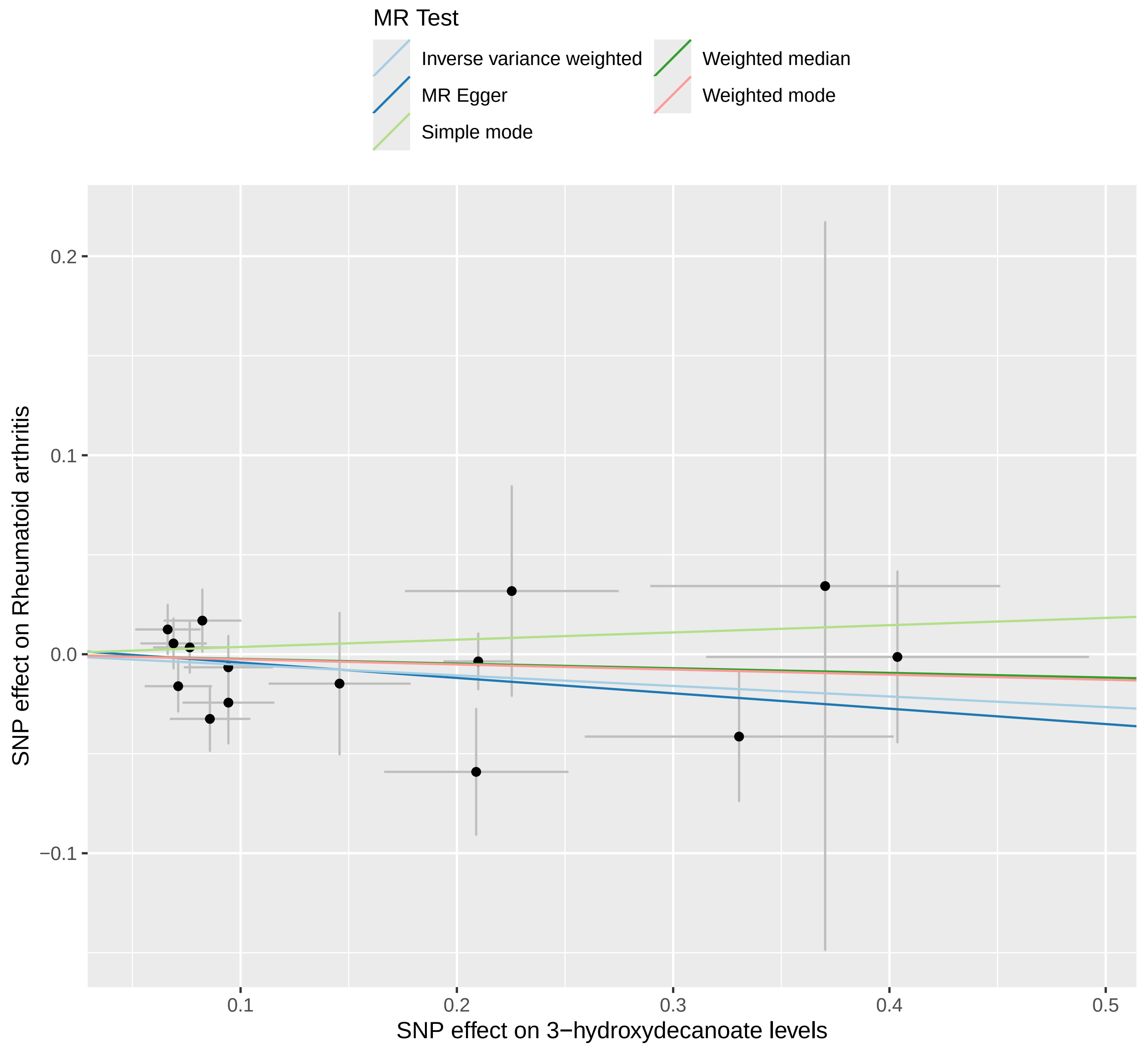

MR Test
Inverse variance weighted
MR Egger
Simple mode
Weighted median
Weighted mode
0.2
0.1
0.0
−0.1
SNP effect on Rheumatoid arthritis
0.1
0.2
0.3
0.4
0.5
SNP effect on 3−hydroxydecanoate levels

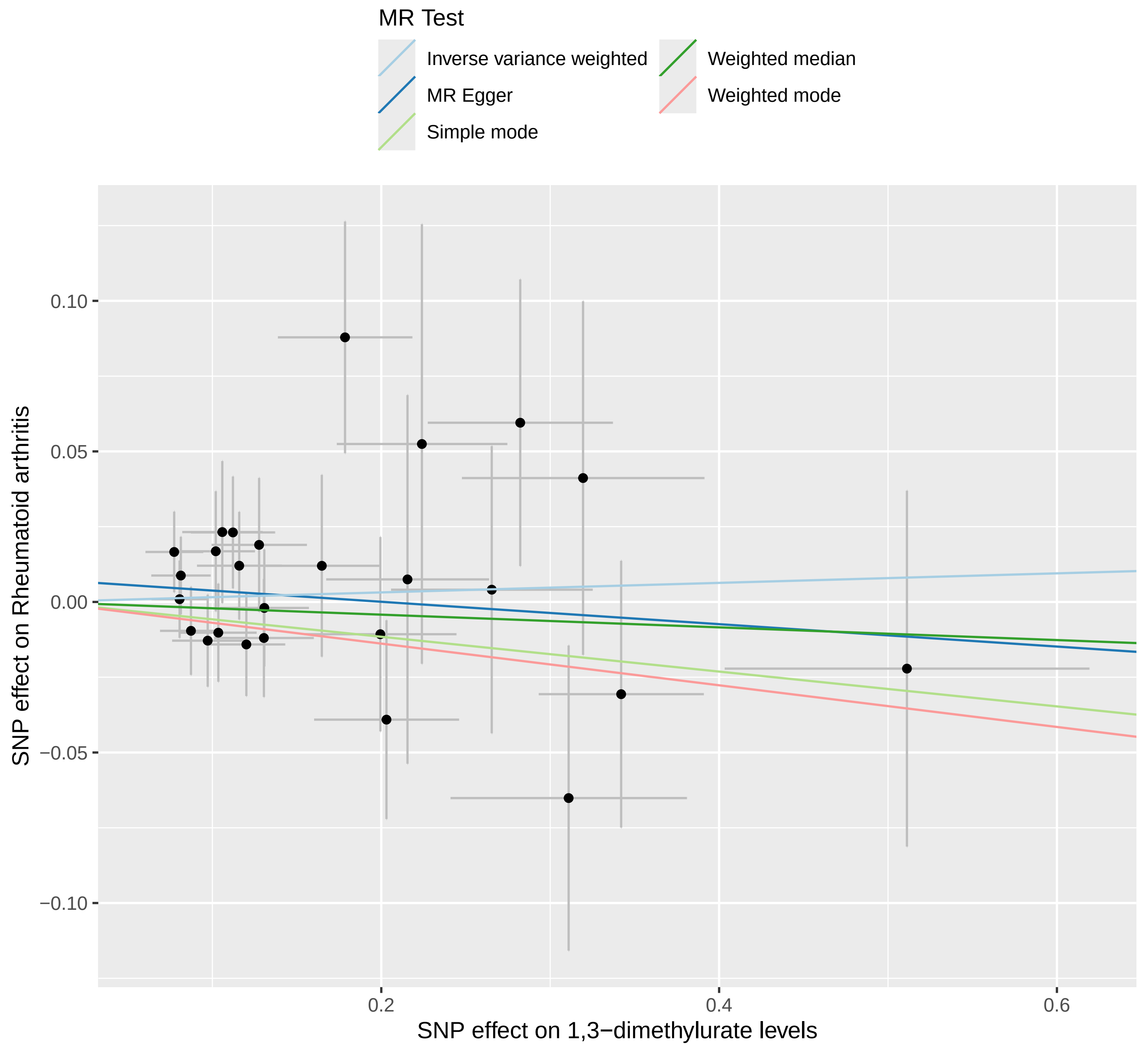
MR Test
Inverse variance weighted
MR Egger
Simple mode
Weighted median
Weighted mode
0.10
0.05
0.00
−0.05
−0.10
SNP effect on Rheumatoid arthritis
0.2
0.4
0.6
SNP effect on 1,3−dimethylurate levels

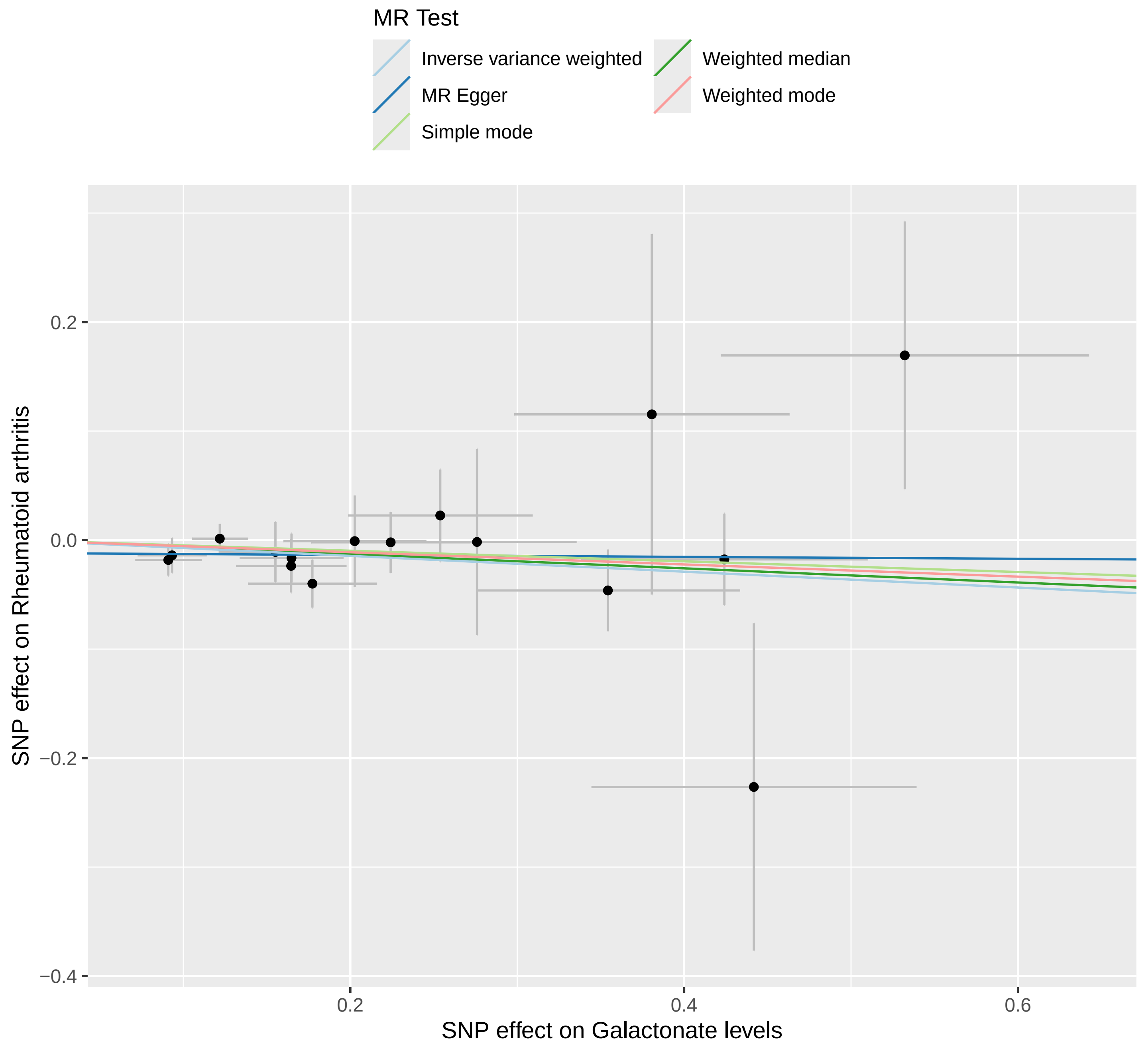
MR Test
Inverse variance weighted
MR Egger
Simple mode
Weighted median
Weighted mode
0.2
0.0
−0.2
−0.4
0.2
0.4
0.6
SNP effect on Rheumatoid arthritis
SNP effect on Galactonate levels

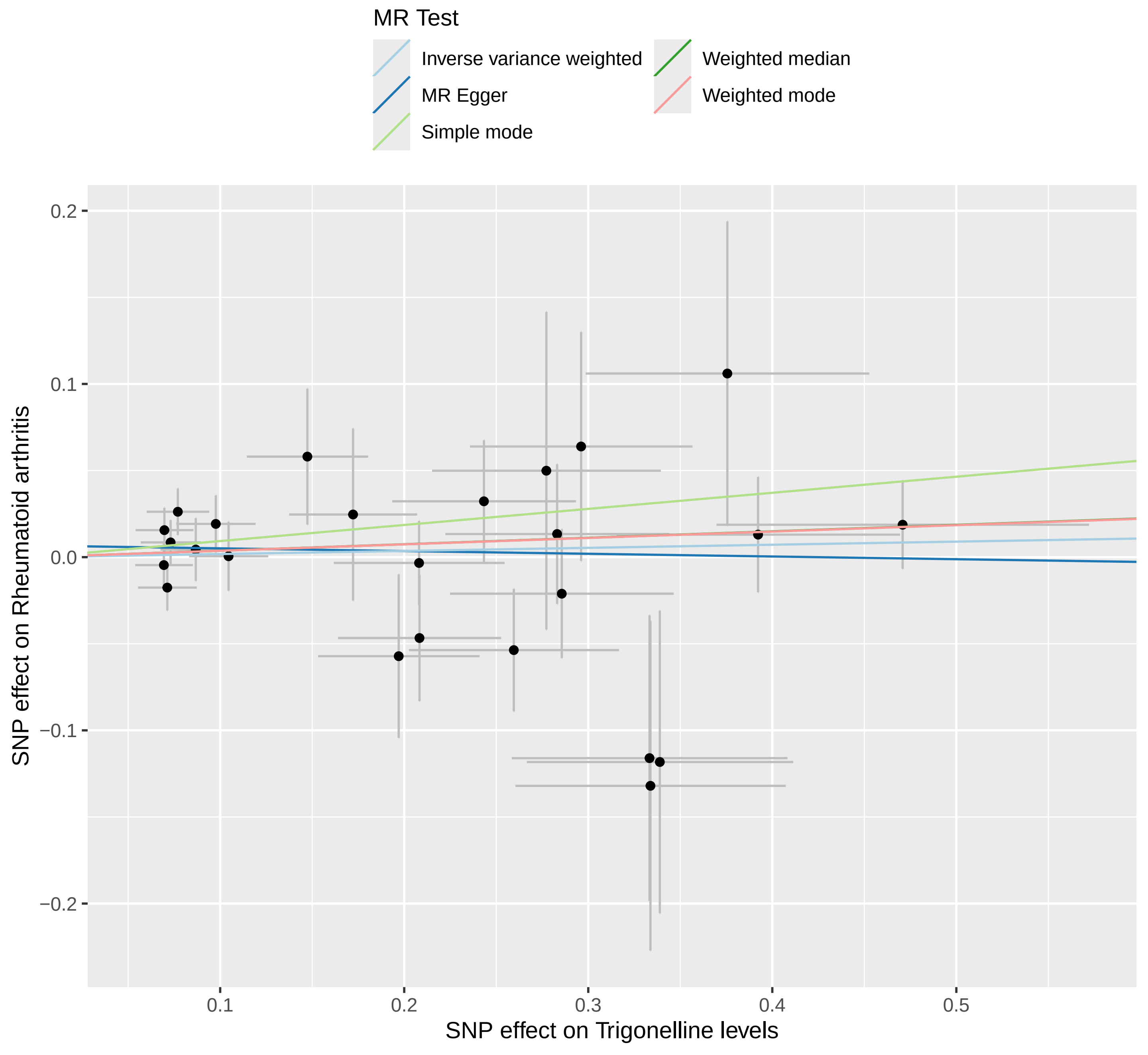

MR Test
Inverse variance weighted
MR Egger
Simple mode
Weighted median
Weighted mode
0.2
0.1
0.0
−0.1
−0.2
SNP effect on Rheumatoid arthritis
0.1
0.2
0.3
0.4
0.5
SNP effect on Trigonelline levels

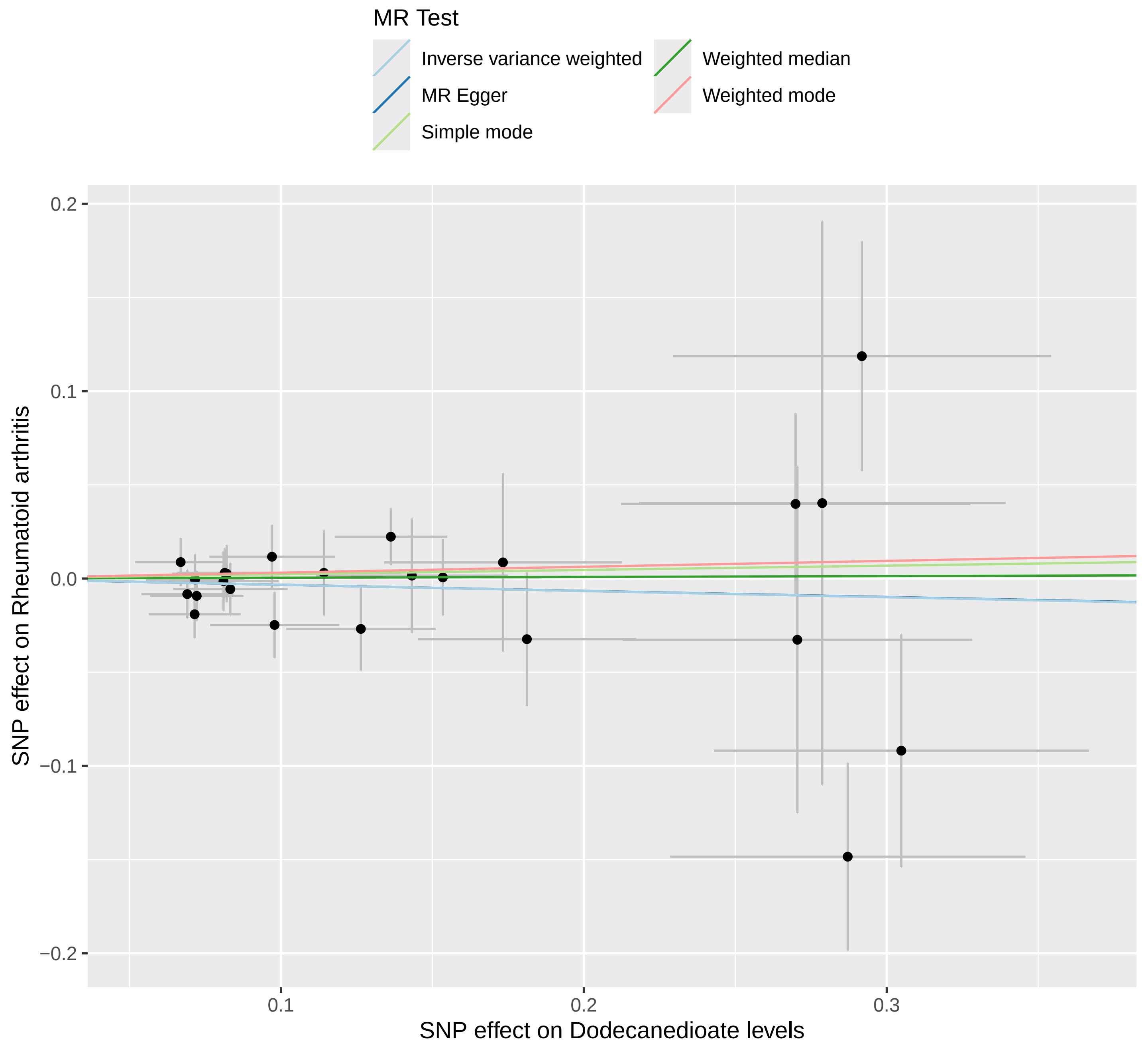

MR Test
Inverse variance weighted
MR Egger
Simple mode
Weighted median
Weighted mode
0.2
0.1
0.0
−0.1
−0.2
SNP effect on Rheumatoid arthritis
0.1
0.2
0.3
SNP effect on Dodecanedioate levels

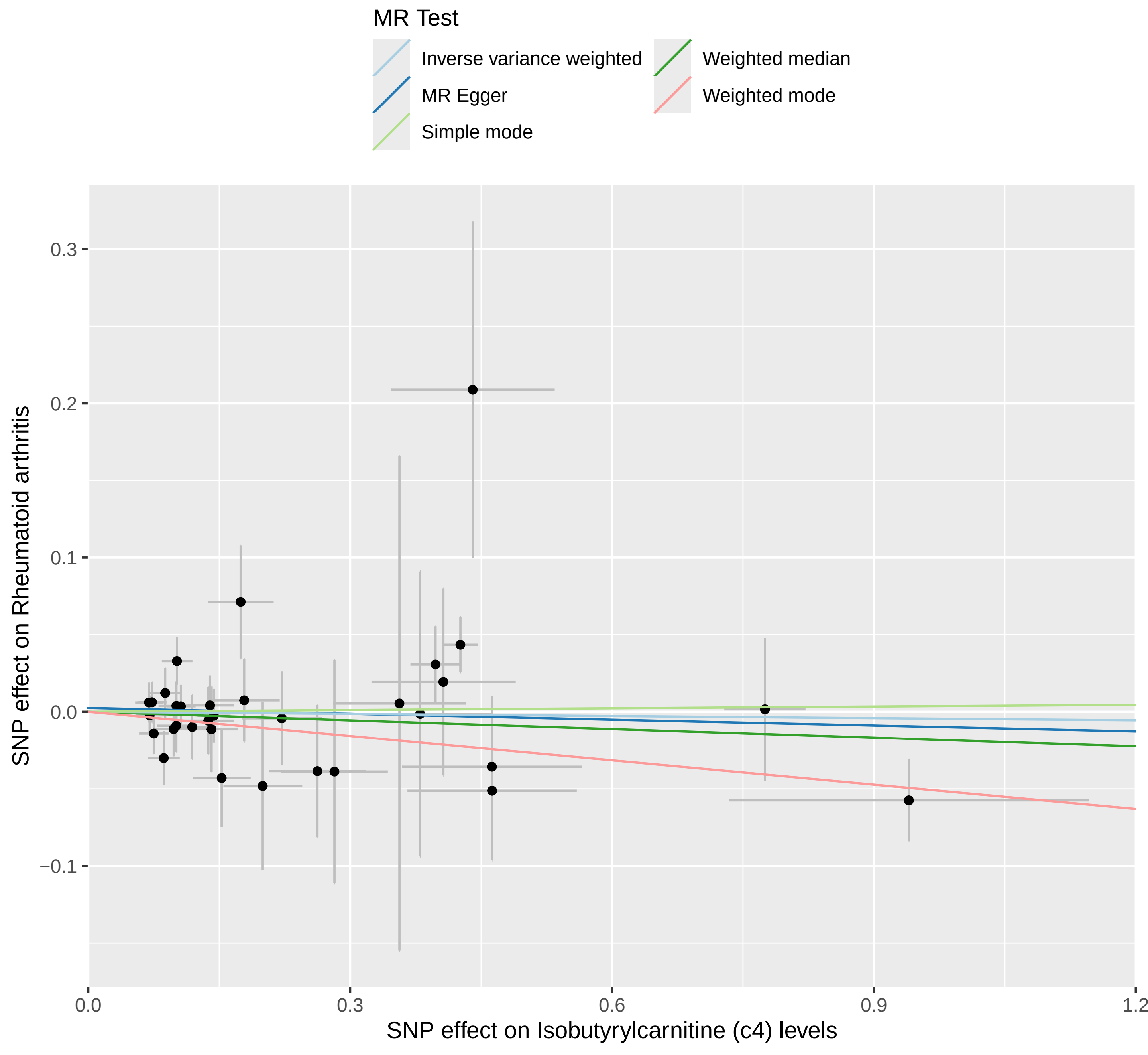

MR Test
Inverse variance weighted
MR Egger
Simple mode
Weighted median
Weighted mode
0.3
0.2
0.1
0.0
−0.1
0.0
0.3
0.6
0.9
1.2
SNP effect on Rheumatoid arthritis
SNP effect on Isobutyrylcarnitine (c4) levels

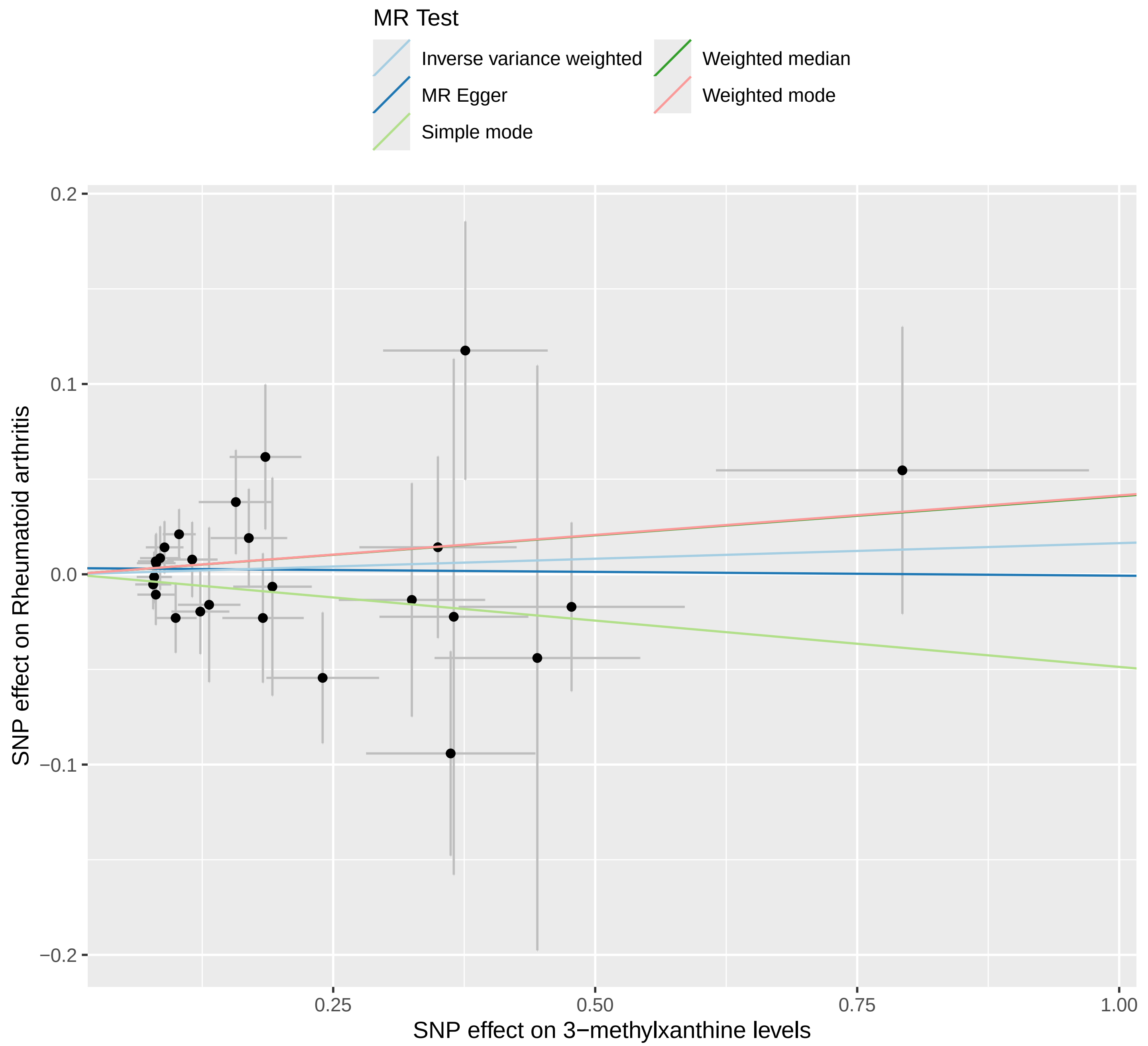
MR Test
Inverse variance weighted
MR Egger
Simple mode
Weighted median
Weighted mode
SNP effect on Rheumatoid arthritis
0.2
0.1
0.0
−0.1
−0.2
0.25
0.50
0.75
1.00
SNP effect on 3−methylxanthine levels

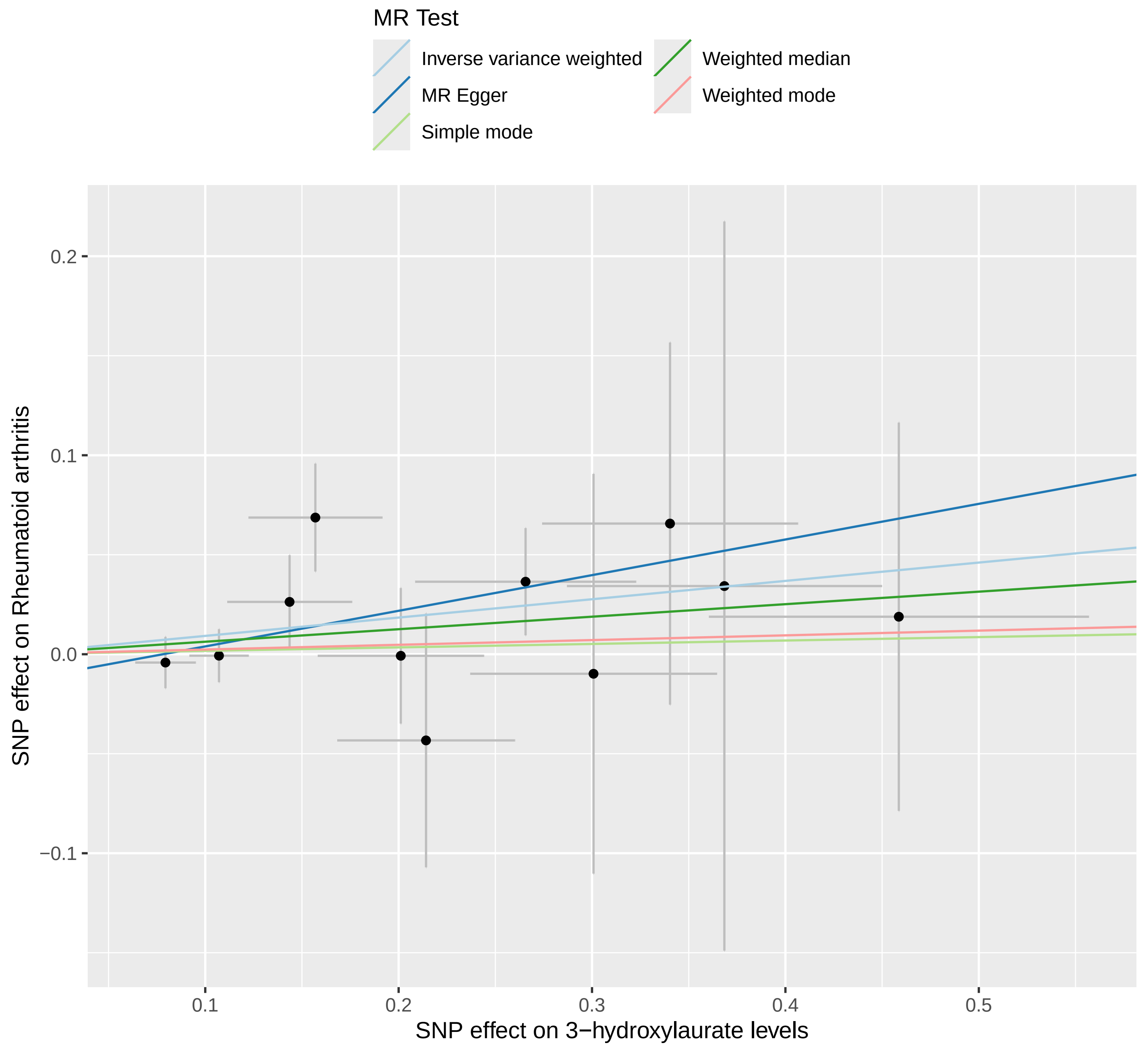

MR Test
Inverse variance weighted
MR Egger
Simple mode
Weighted median
Weighted mode
SNP effect on Rheumatoid arthritis
0.2
0.1
0.0
−0.1
0.1
0.2
0.3
0.4
0.5
SNP effect on 3−hydroxylaurate levels

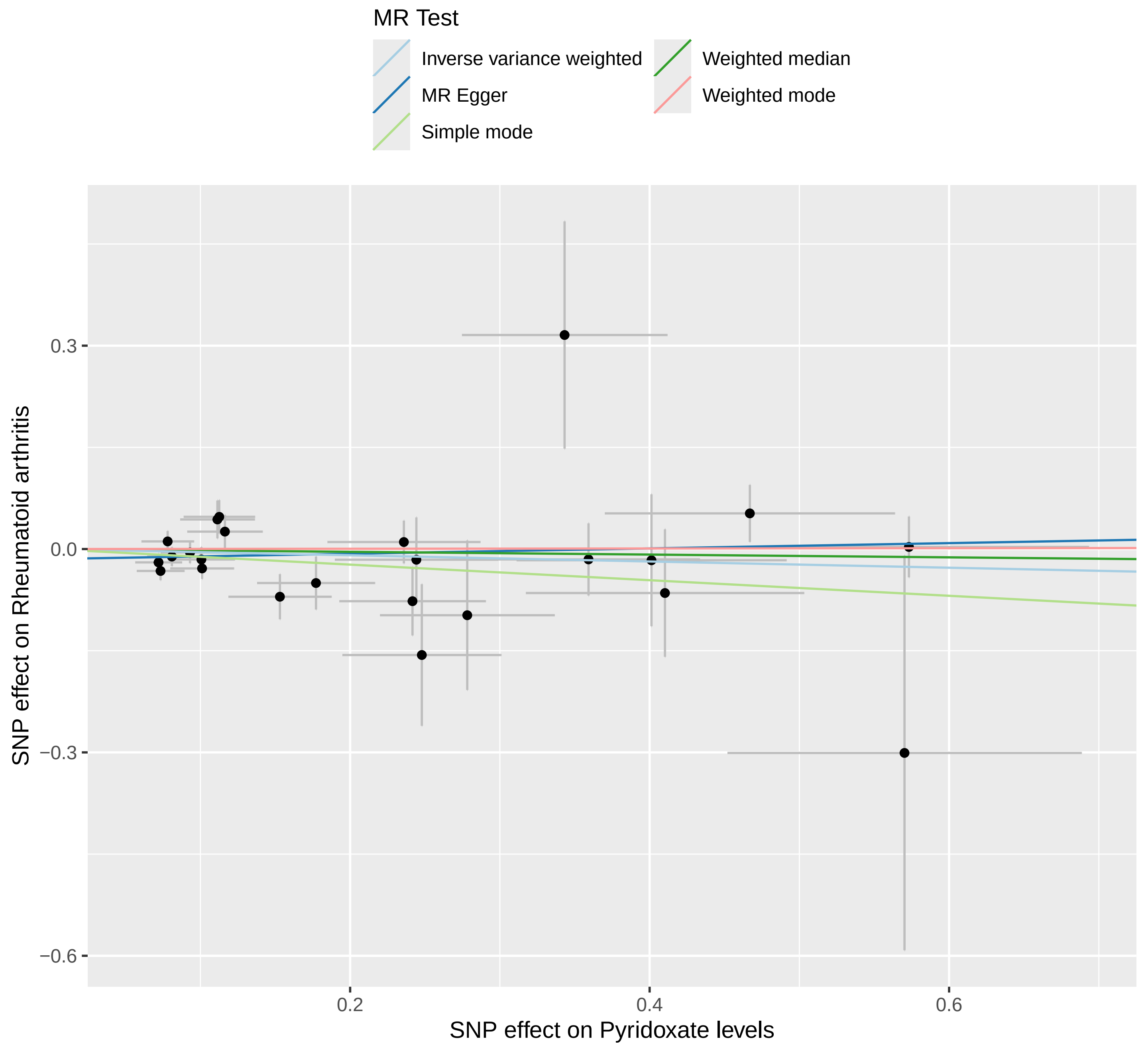
MR Test
Inverse variance weighted
MR Egger
Simple mode
Weighted median
Weighted mode
0.3
0.0
−0.3
−0.6
0.2
0.4
0.6
SNP effect on Rheumatoid arthritis
SNP effect on Pyridoxate levels

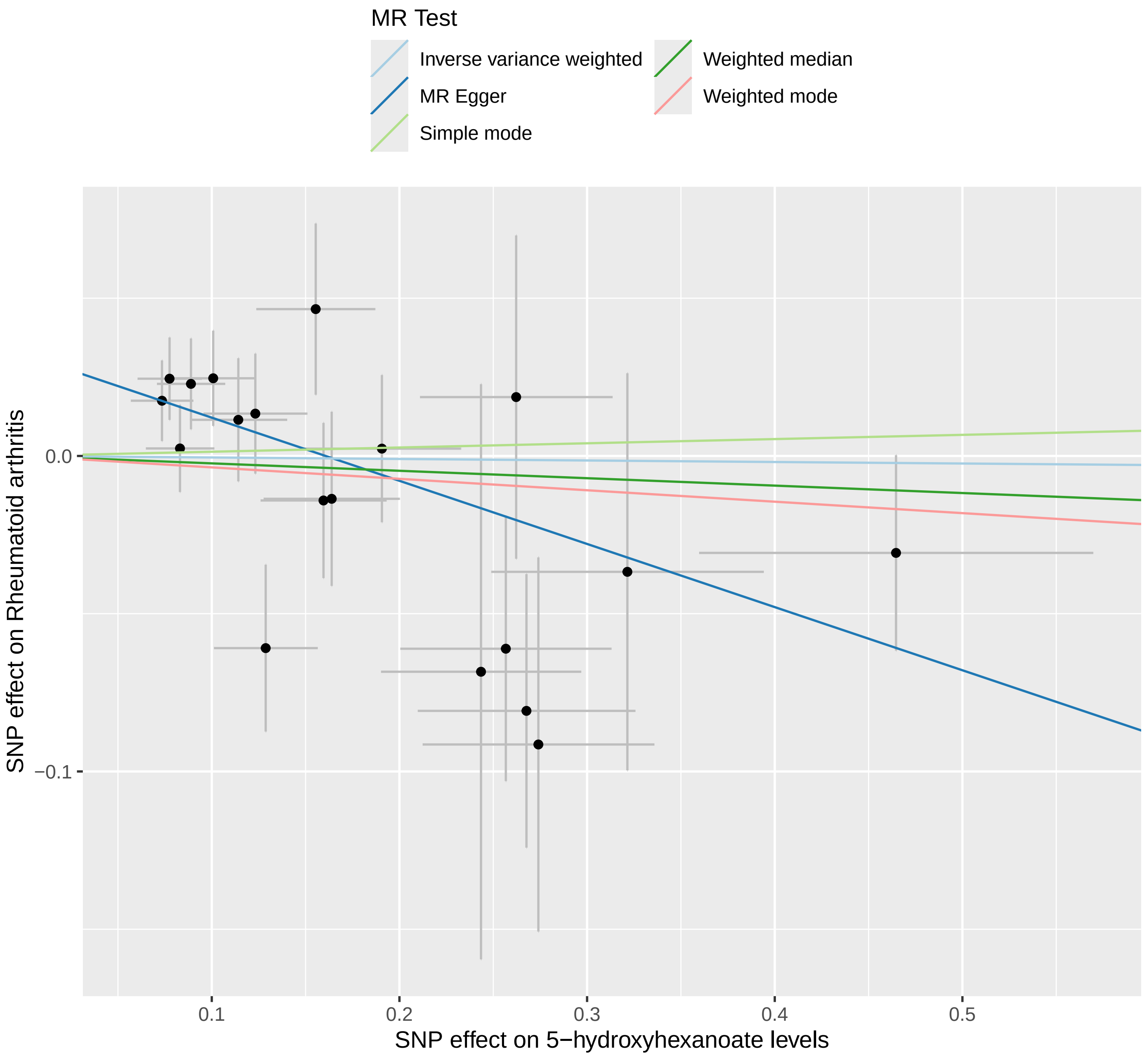
MR Test
Inverse variance weighted
MR Egger
Simple mode
Weighted median
Weighted mode
0.0
−0.1
0.1
0.2
0.3
0.4
0.5
SNP effect on Rheumatoid arthritis
SNP effect on 5−hydroxyhexanoate levels

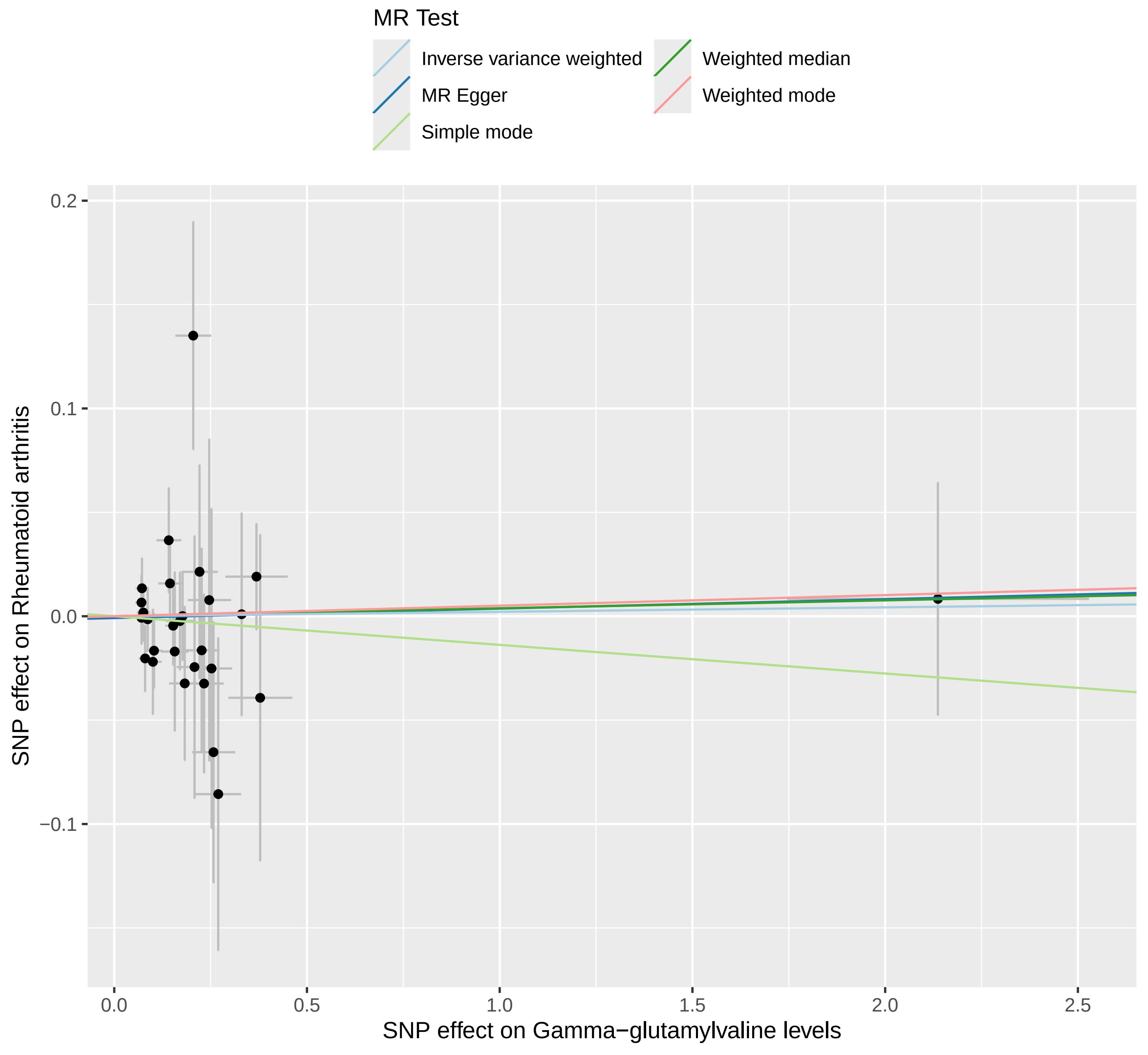

MR Test
Inverse variance weighted
MR Egger
Simple mode
Weighted median
Weighted mode
0.2
0.1
0.0
−0.1
0.0
0.5
1.0
1.5
2.0
2.5
SNP effect on Rheumatoid arthritis
SNP effect on Gamma−glutamylvaline levels

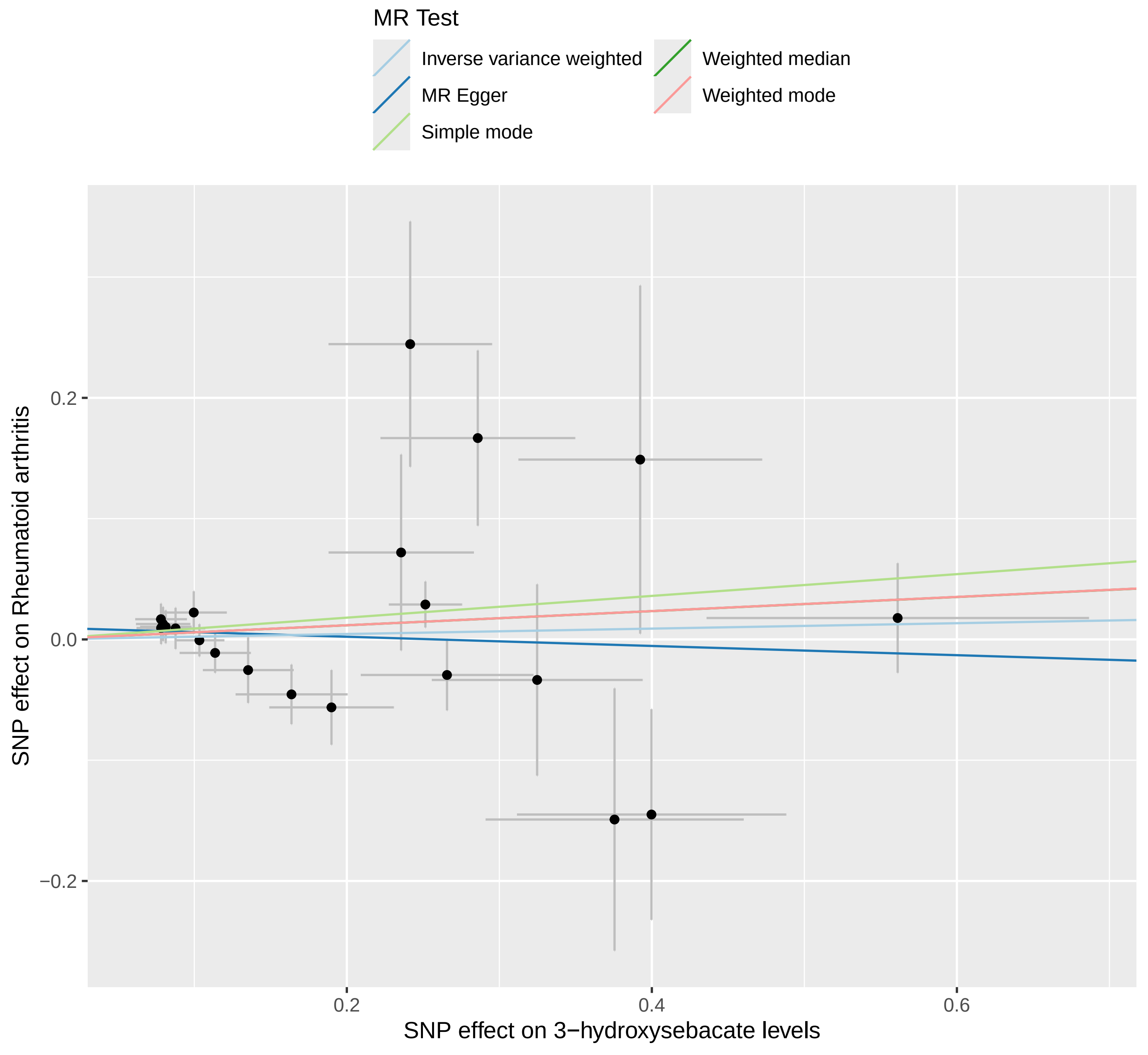
MR Test
Inverse variance weighted
MR Egger
Simple mode
Weighted median
Weighted mode
0.2
0.0
−0.2
SNP effect on Rheumatoid arthritis
0.2
0.4
0.6
SNP effect on 3−hydroxysebacate levels

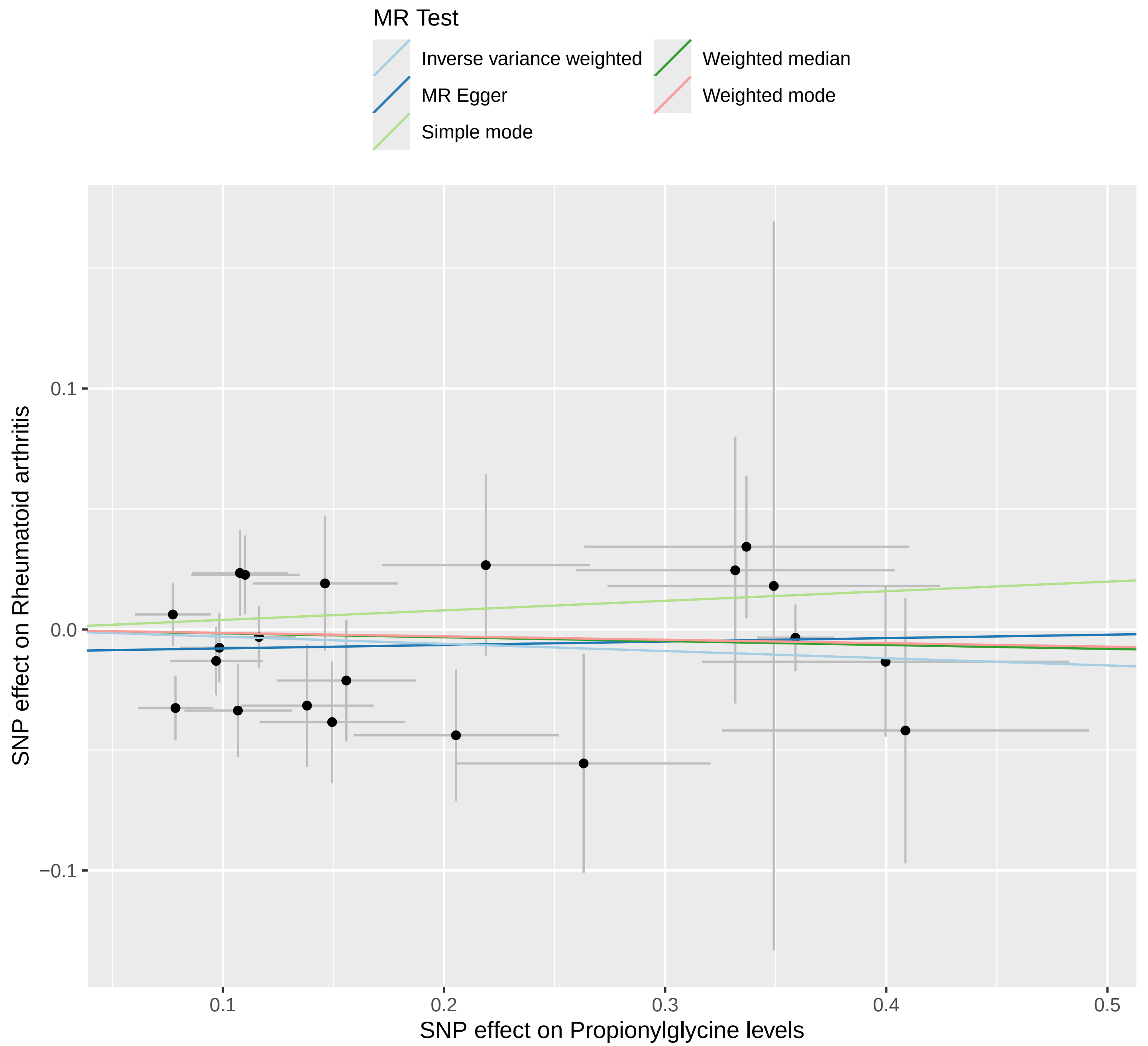

MR Test
Inverse variance weighted
MR Egger
Simple mode
Weighted median
Weighted mode
0.1
0.0
−0.1
SNP effect on Rheumatoid arthritis
0.1
0.2
0.3
0.4
0.5
SNP effect on Propionylglycine levels

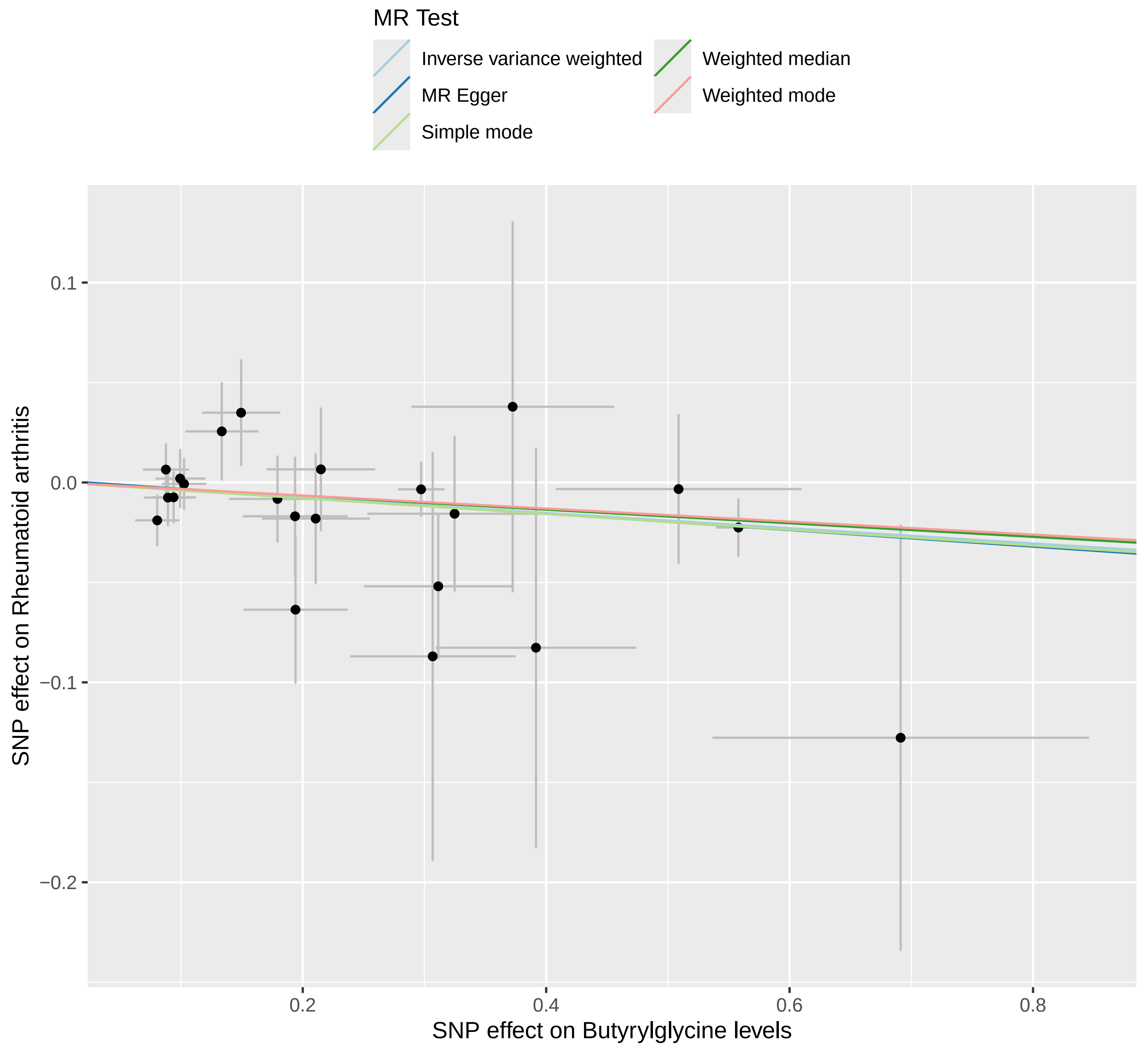
MR Test
Inverse variance weighted
MR Egger
Simple mode
Weighted median
Weighted mode
0.1
0.0
−0.1
−0.2
SNP effect on Rheumatoid arthritis
0.2
0.4
0.6
0.8
SNP effect on Butyrylglycine levels

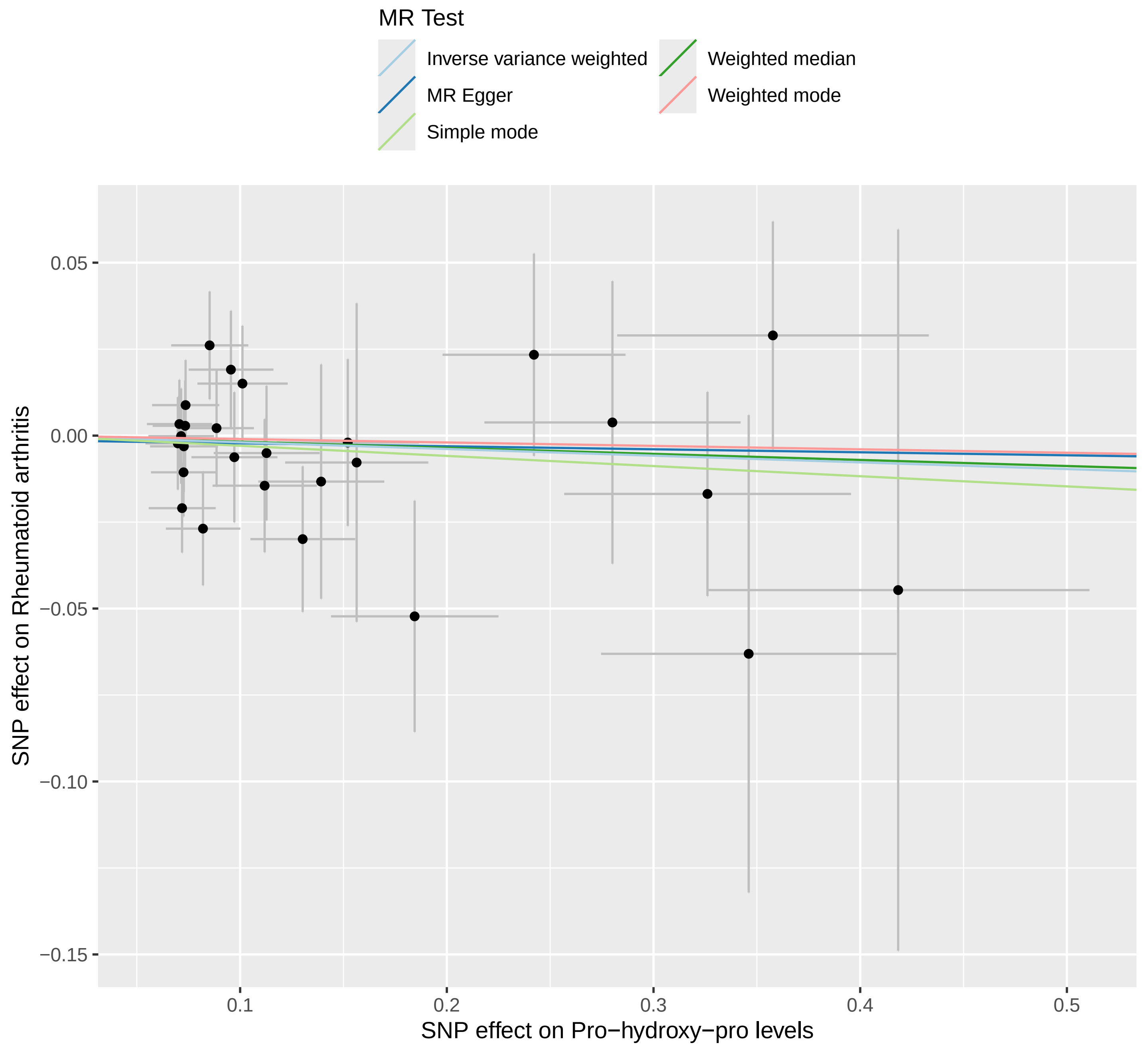

MR Test
Inverse variance weighted
MR Egger
Simple mode
Weighted median
Weighted mode
0.05
0.00
−0.05
−0.10
−0.15
SNP effect on Rheumatoid arthritis
0.1
0.2
0.3
0.4
0.5
SNP effect on Pro−hydroxy−pro levels

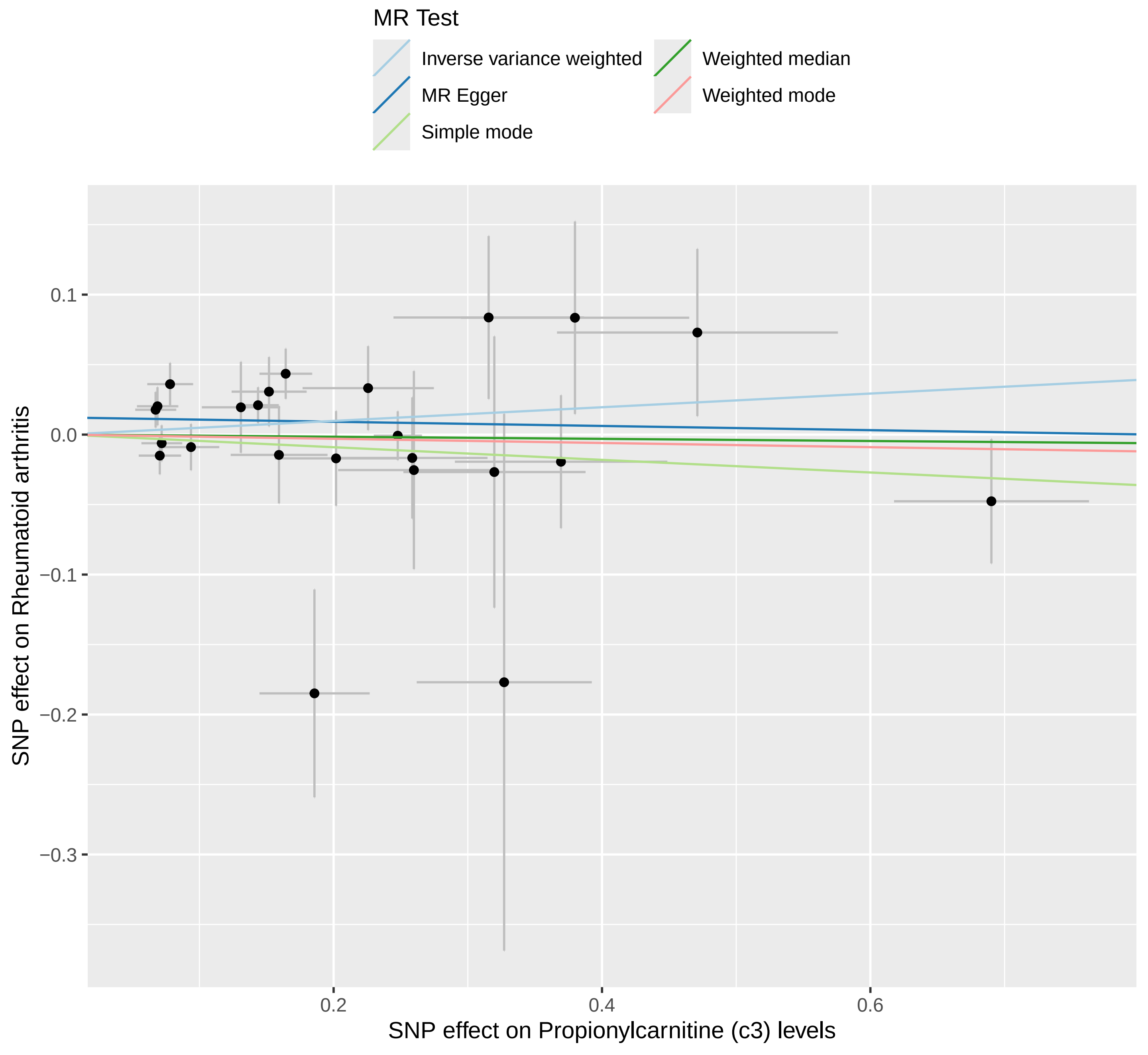
MR Test
Inverse variance weighted
MR Egger
Simple mode
Weighted median
Weighted mode
0.1
0.0
−0.1
−0.2
−0.3
SNP effect on Rheumatoid arthritis
0.2
0.4
0.6
SNP effect on Propionylcarnitine (c3) levels

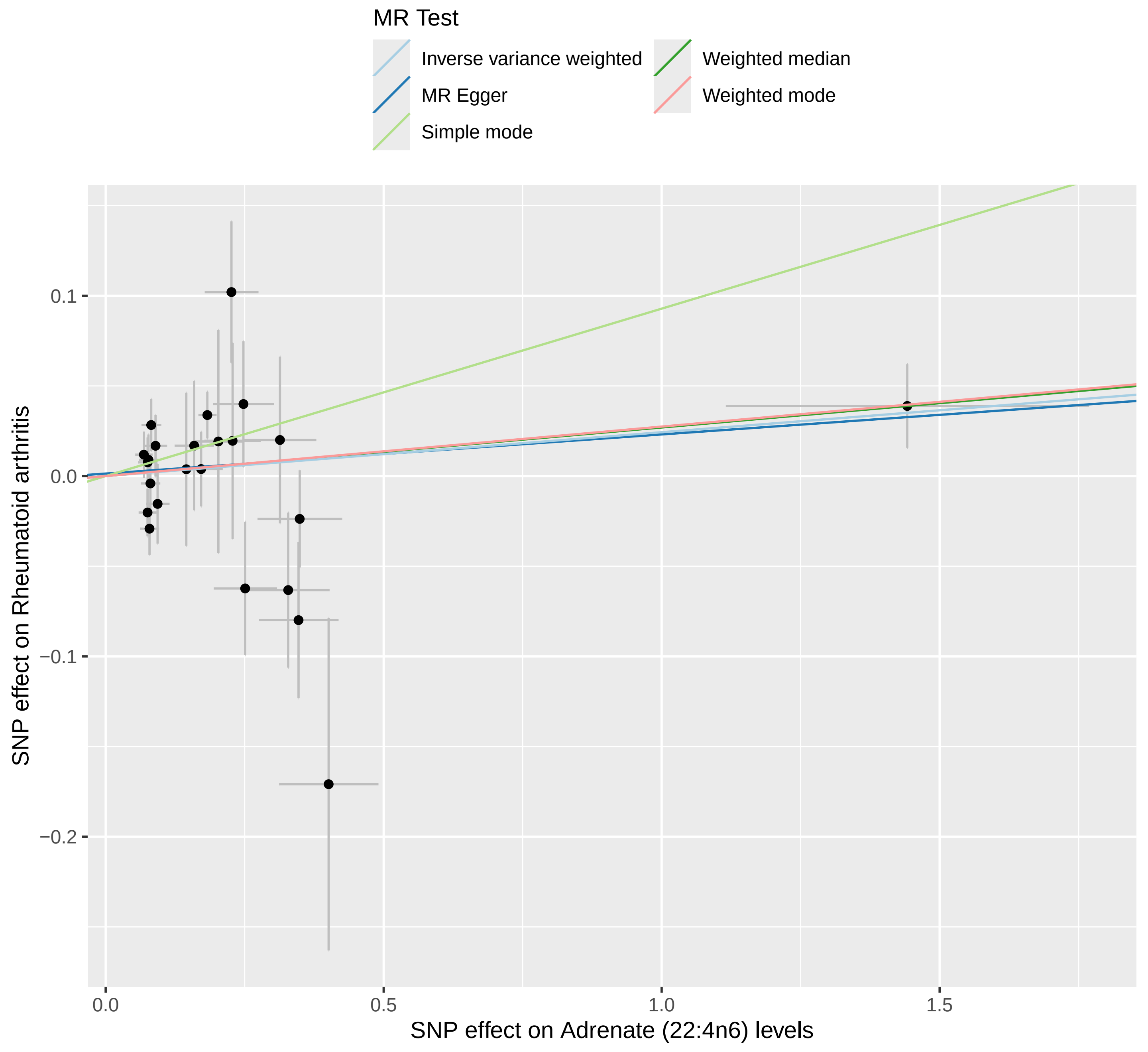

MR Test
Inverse variance weighted
MR Egger
Simple mode
Weighted median
Weighted mode
SNP effect on Rheumatoid arthritis
0.1
0.0
−0.1
−0.2
0.0
0.5
1.0
1.5
SNP effect on Adrenate (22:4n6) levels

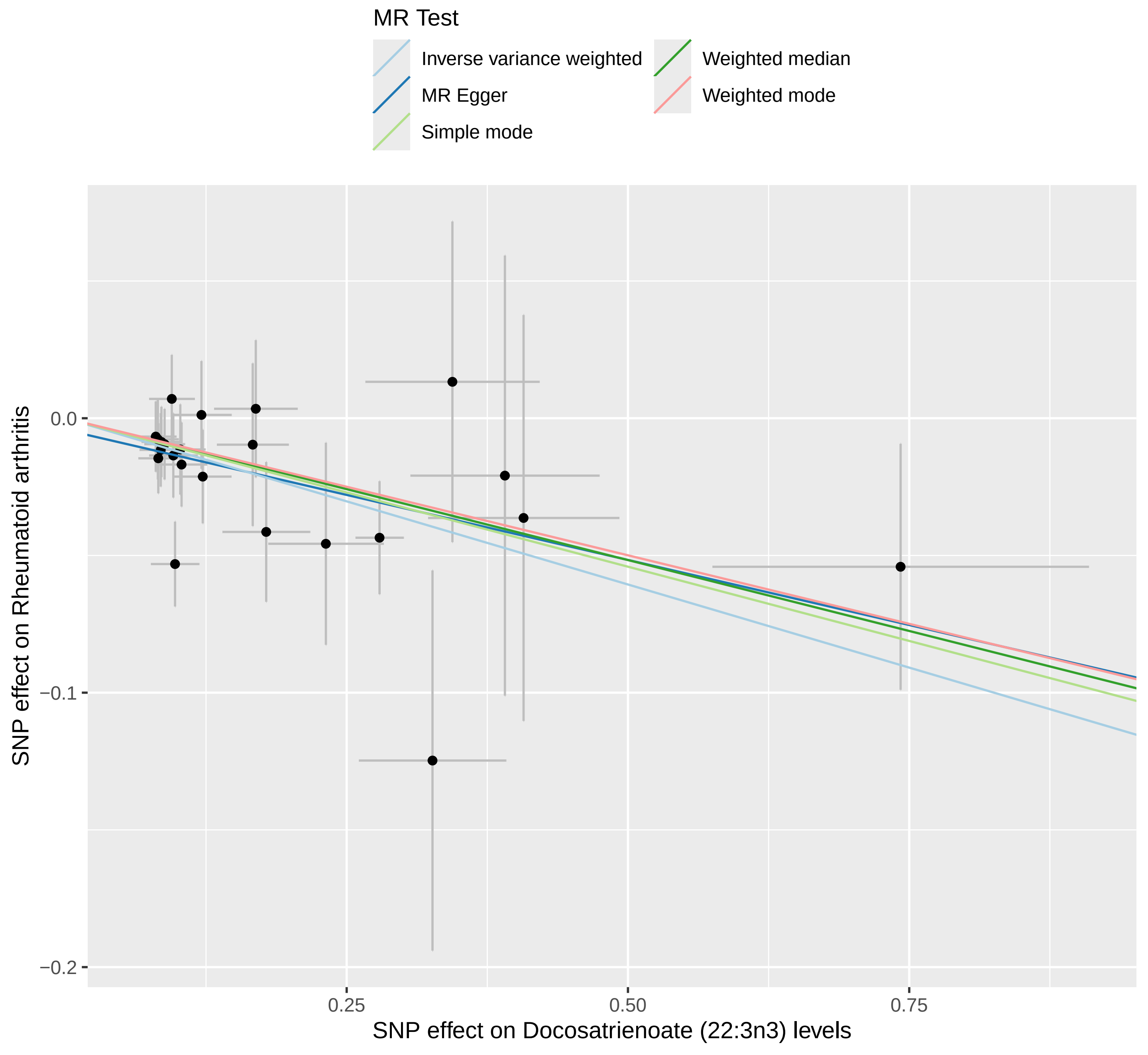

MR Test
Inverse variance weighted
MR Egger
Simple mode
Weighted median
Weighted mode
0.0
−0.1
−0.2
0.25
0.50
0.75
SNP effect on Rheumatoid arthritis
SNP effect on Docosatrienoate (22:3n3) levels

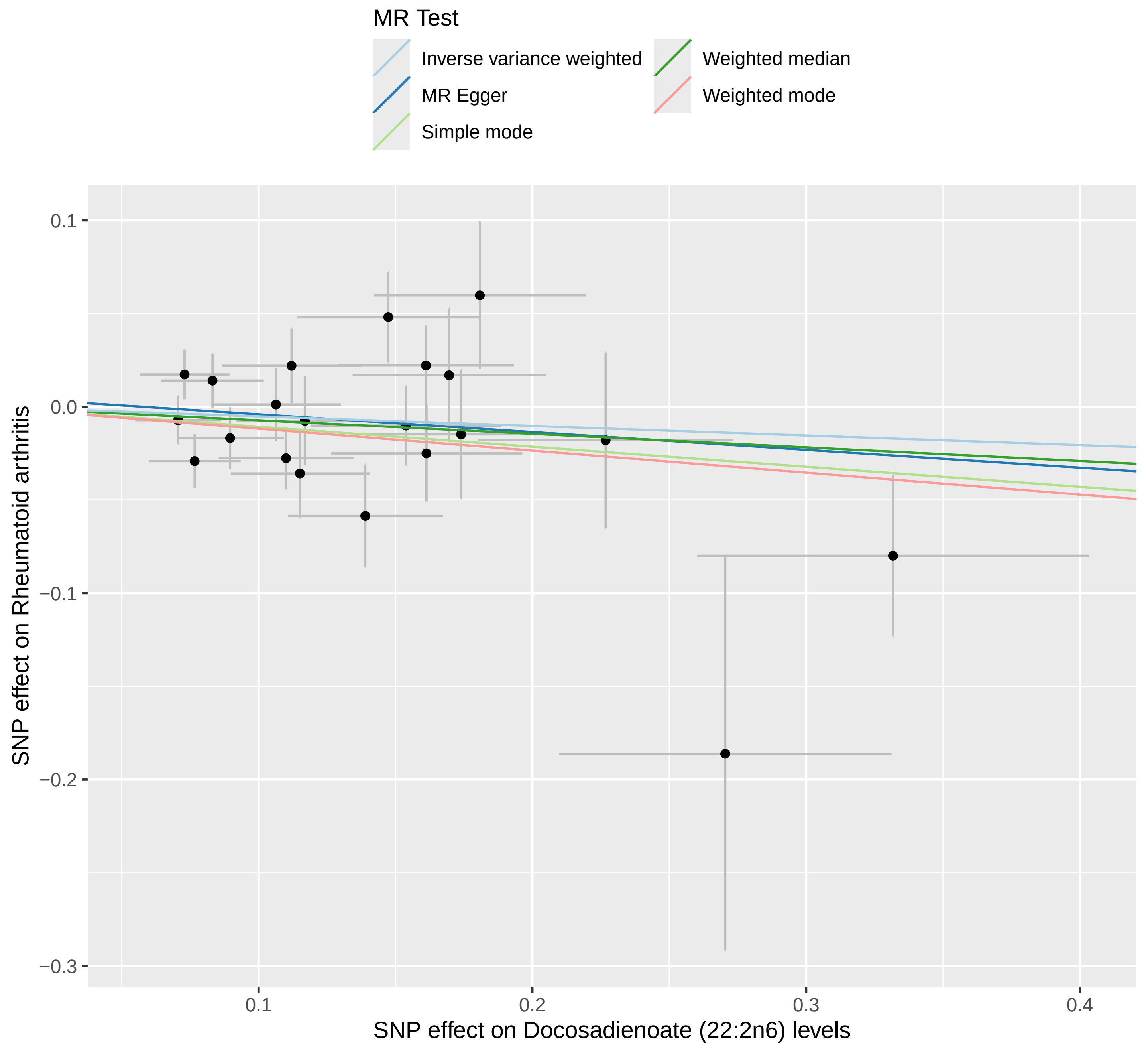
MR Test
Inverse variance weighted
MR Egger
Simple mode
Weighted median
Weighted mode
0.1
0.0
−0.1
−0.2
−0.3
SNP effect on Rheumatoid arthritis
0.1
0.2
0.3
0.4
SNP effect on Docosadienoate (22:2n6) levels

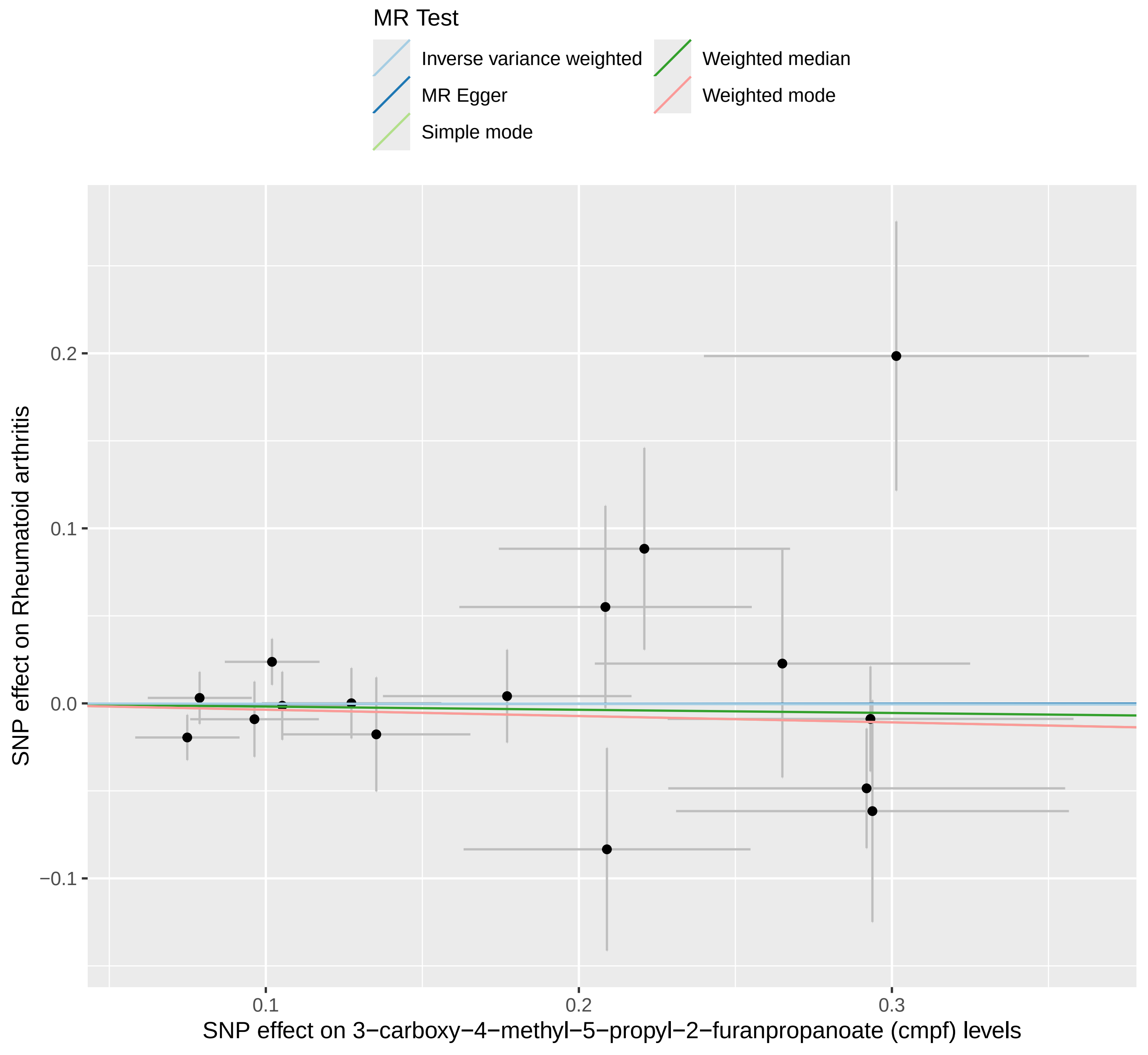

MR Test
Inverse variance weighted
MR Egger
Simple mode
Weighted median
Weighted mode
0.2
0.1
0.0
−0.1
0.1
0.2
0.3
SNP effect on Rheumatoid arthritis
SNP effect on 3−carboxy−4−methyl−5−propyl−2−furanpropanoate (cmpf) levels

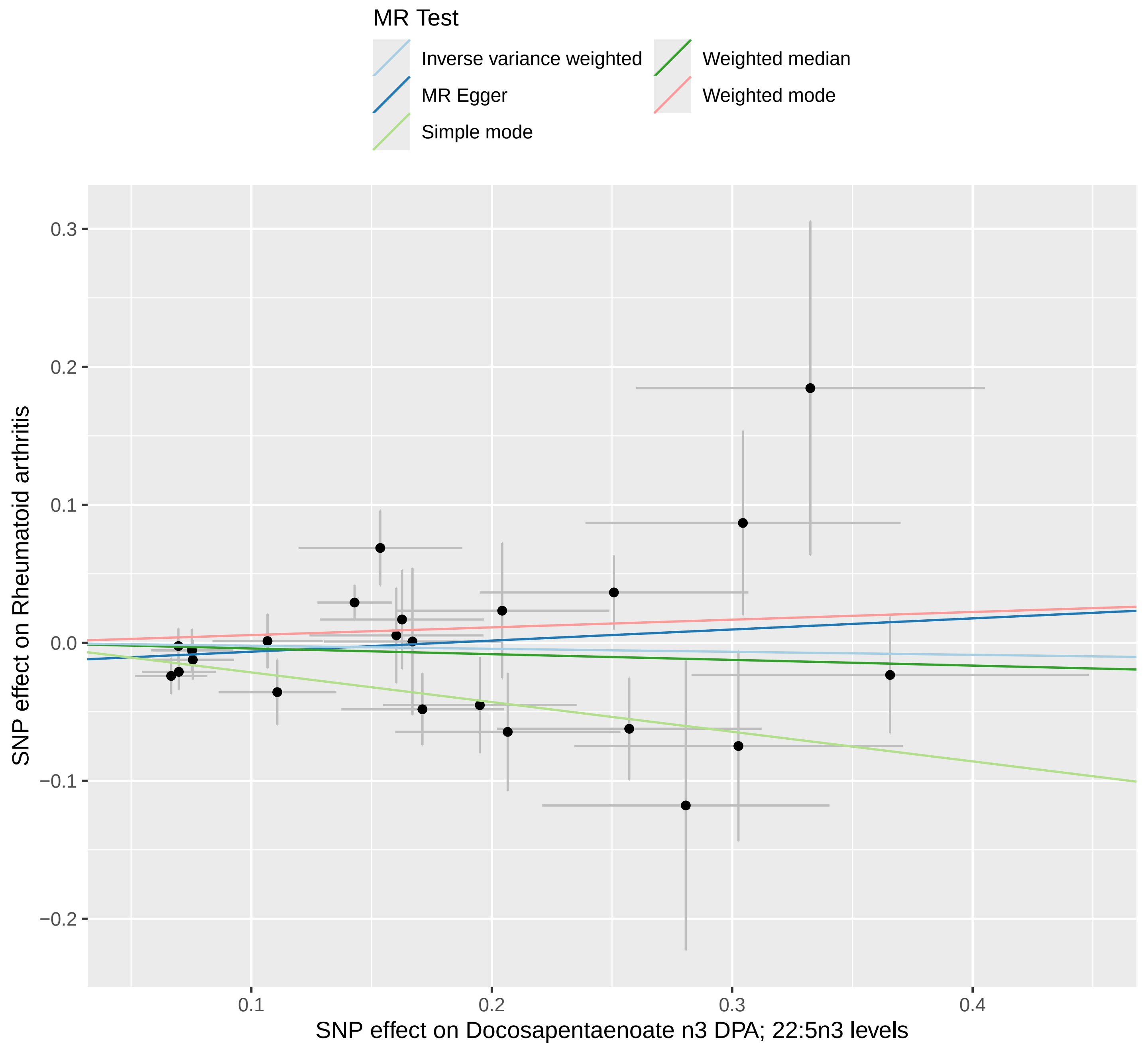
MR Test
Inverse variance weighted
MR Egger
Simple mode
Weighted median
Weighted mode
0.3
0.2
0.1
0.0
−0.1
−0.2
SNP effect on Rheumatoid arthritis
0.1
0.2
0.3
0.4
SNP effect on Docosapentaenoate n3 DPA; 22:5n3 levels

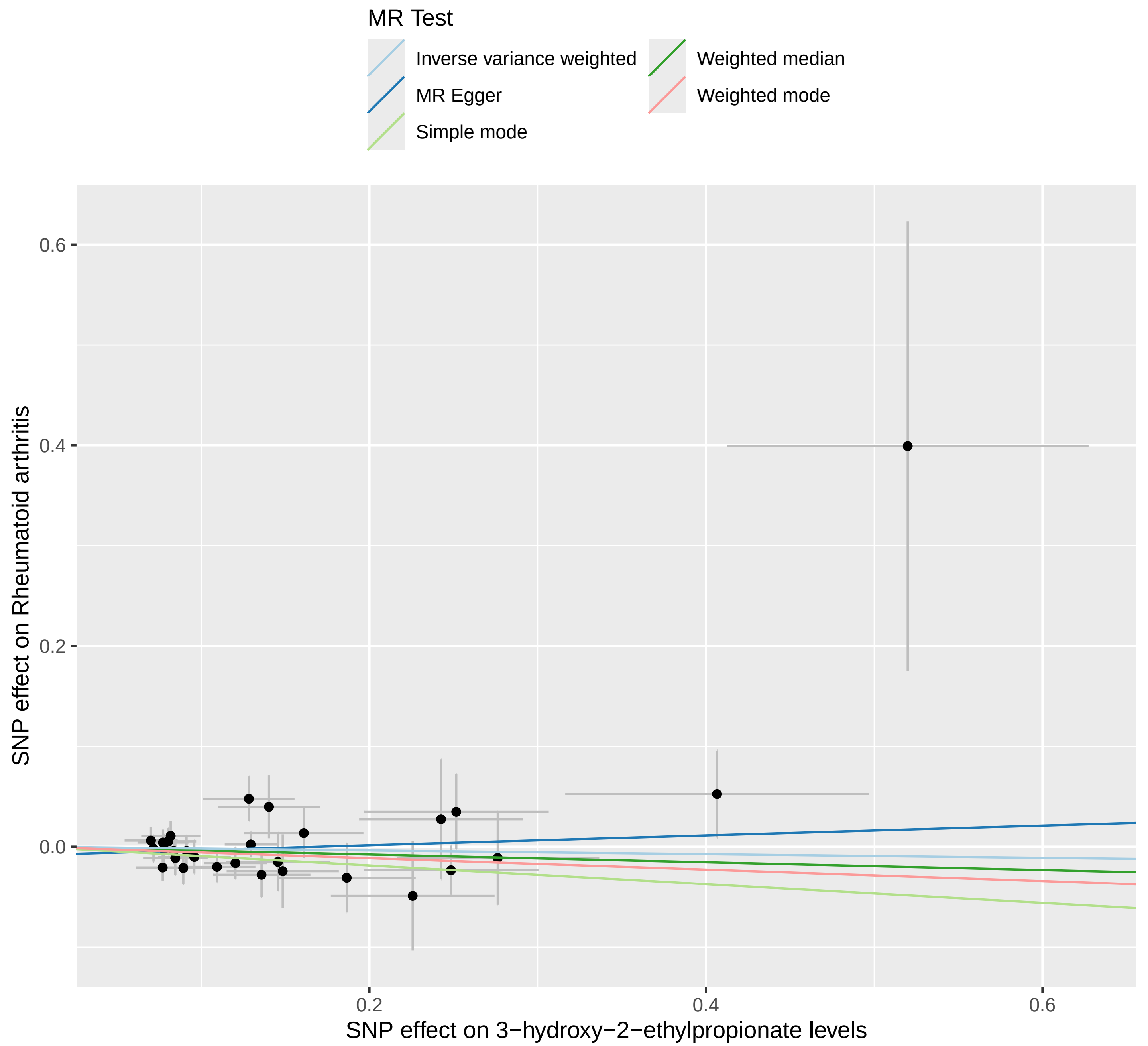
MR Test
Inverse variance weighted
MR Egger
Simple mode
Weighted median
Weighted mode
SNP effect on Rheumatoid arthritis
0.6
0.4
0.2
0.0
0.2
0.4
0.6
SNP effect on 3−hydroxy−2−ethylpropionate levels

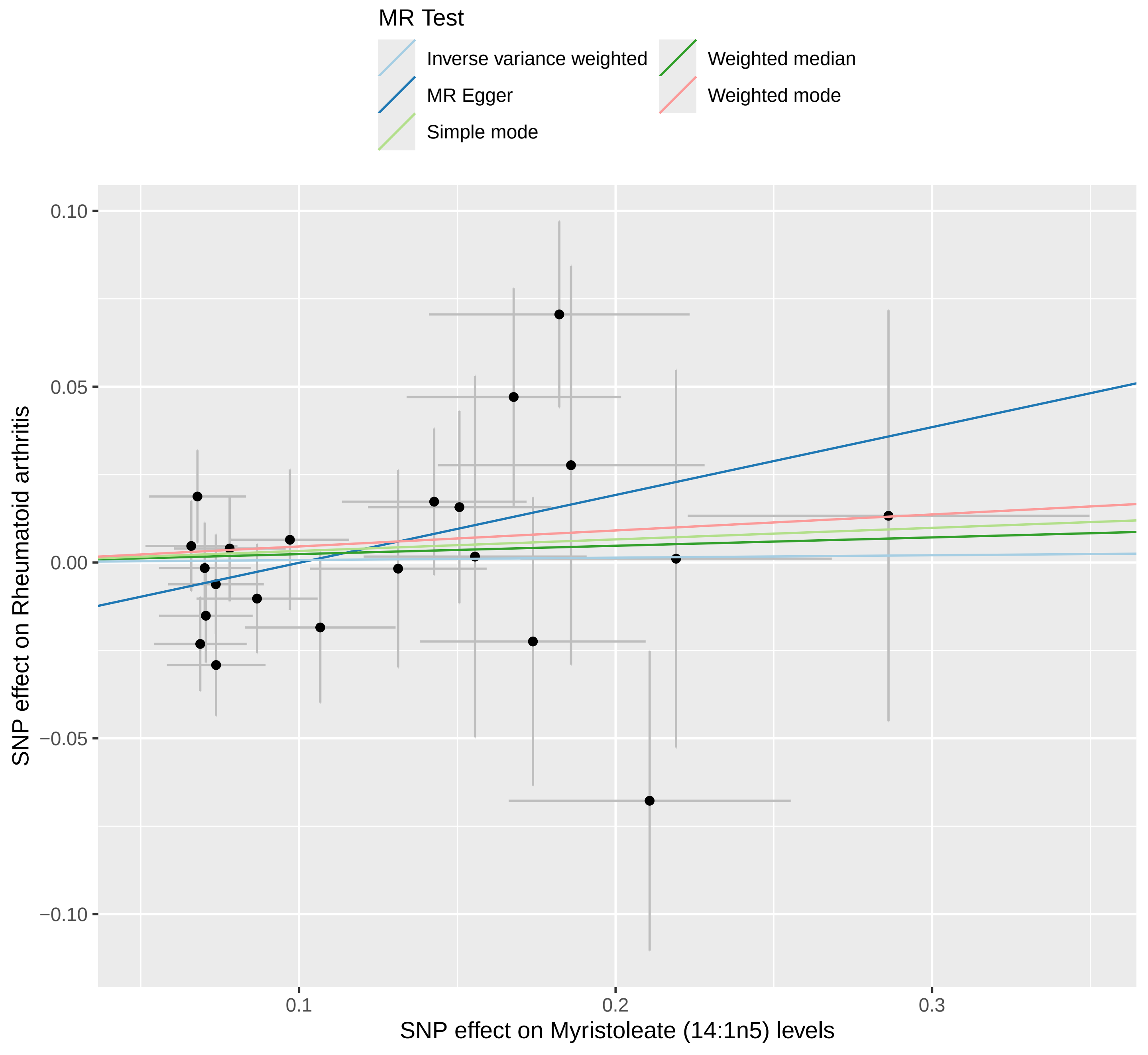

MR Test
Inverse variance weighted
MR Egger
Simple mode
Weighted median
Weighted mode
0.10
0.05
0.00
−0.05
−0.10
0.1
0.2
0.3
SNP effect on Rheumatoid arthritis
SNP effect on Myristoleate (14:1n5) levels

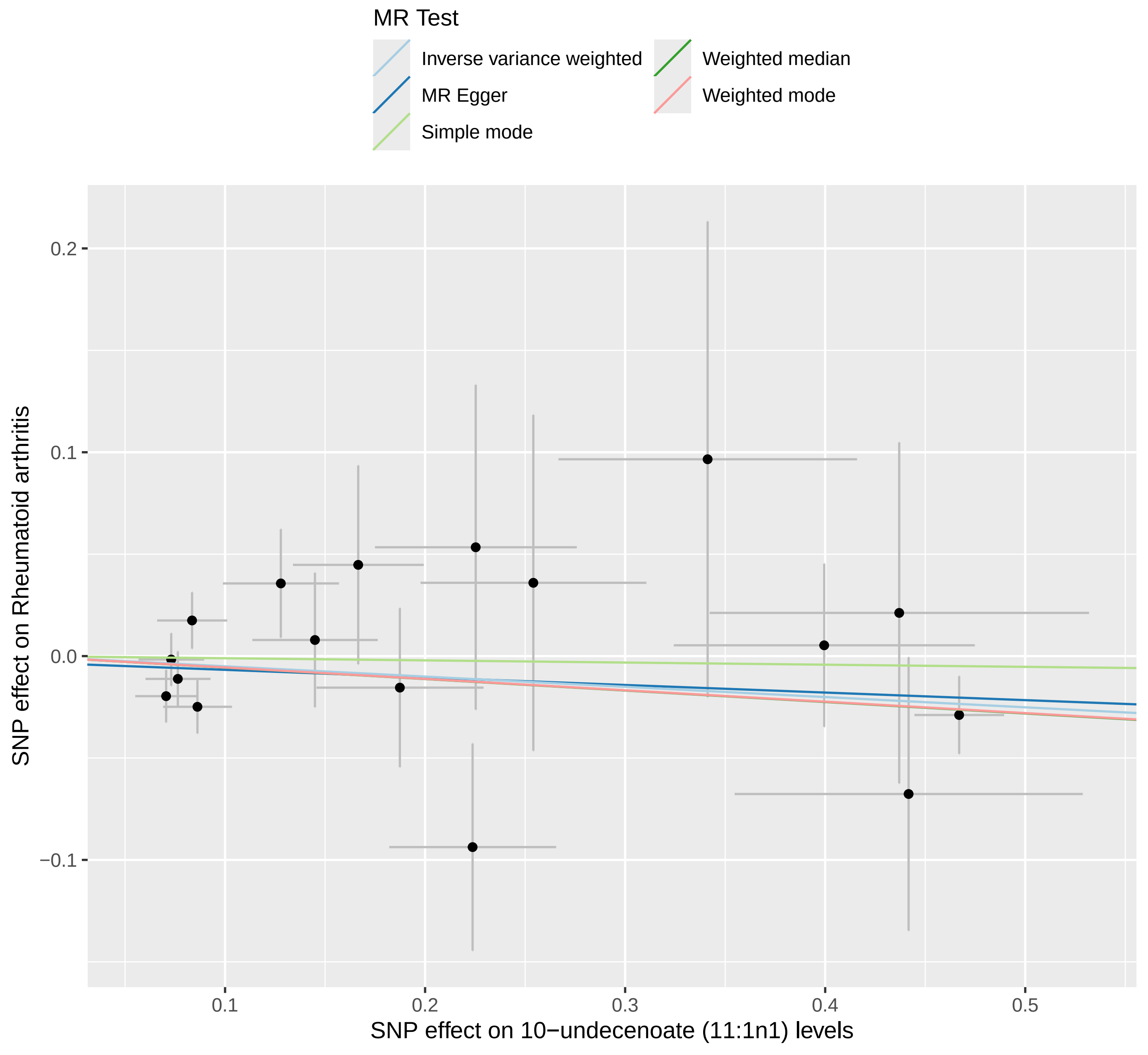
MR Test
Inverse variance weighted
MR Egger
Simple mode
Weighted median
Weighted mode
SNP effect on Rheumatoid arthritis
0.2
0.1
0.0
−0.1
0.1
0.2
0.3
0.4
0.5
SNP effect on 10−undecenoate (11:1n1) levels

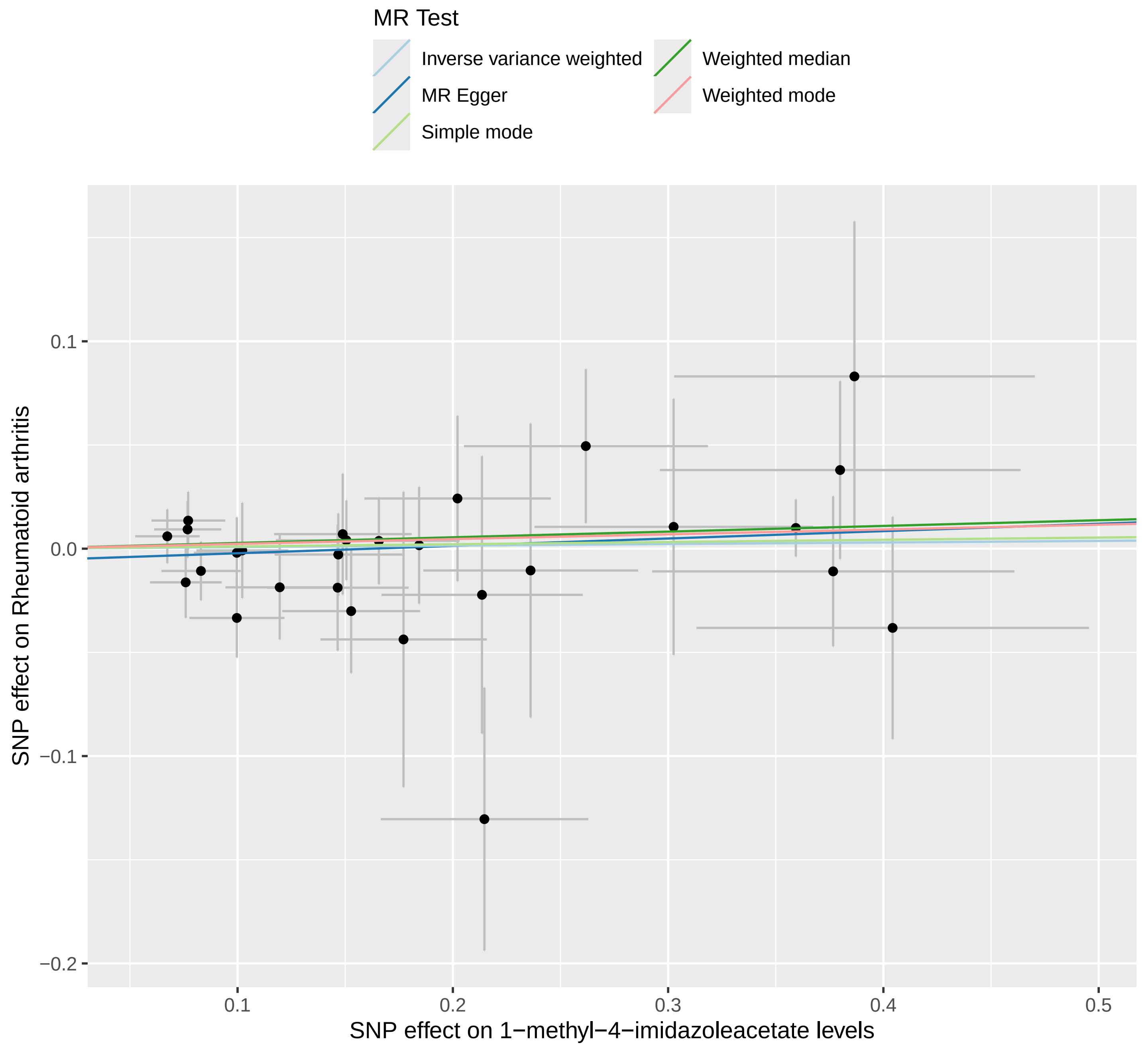

MR Test
Inverse variance weighted
MR Egger
Simple mode
Weighted median
Weighted mode
0.1
0.0
−0.1
−0.2
0.1
0.2
0.3
0.4
0.5
SNP effect on Rheumatoid arthritis
SNP effect on 1−methyl−4−imidazoleacetate levels

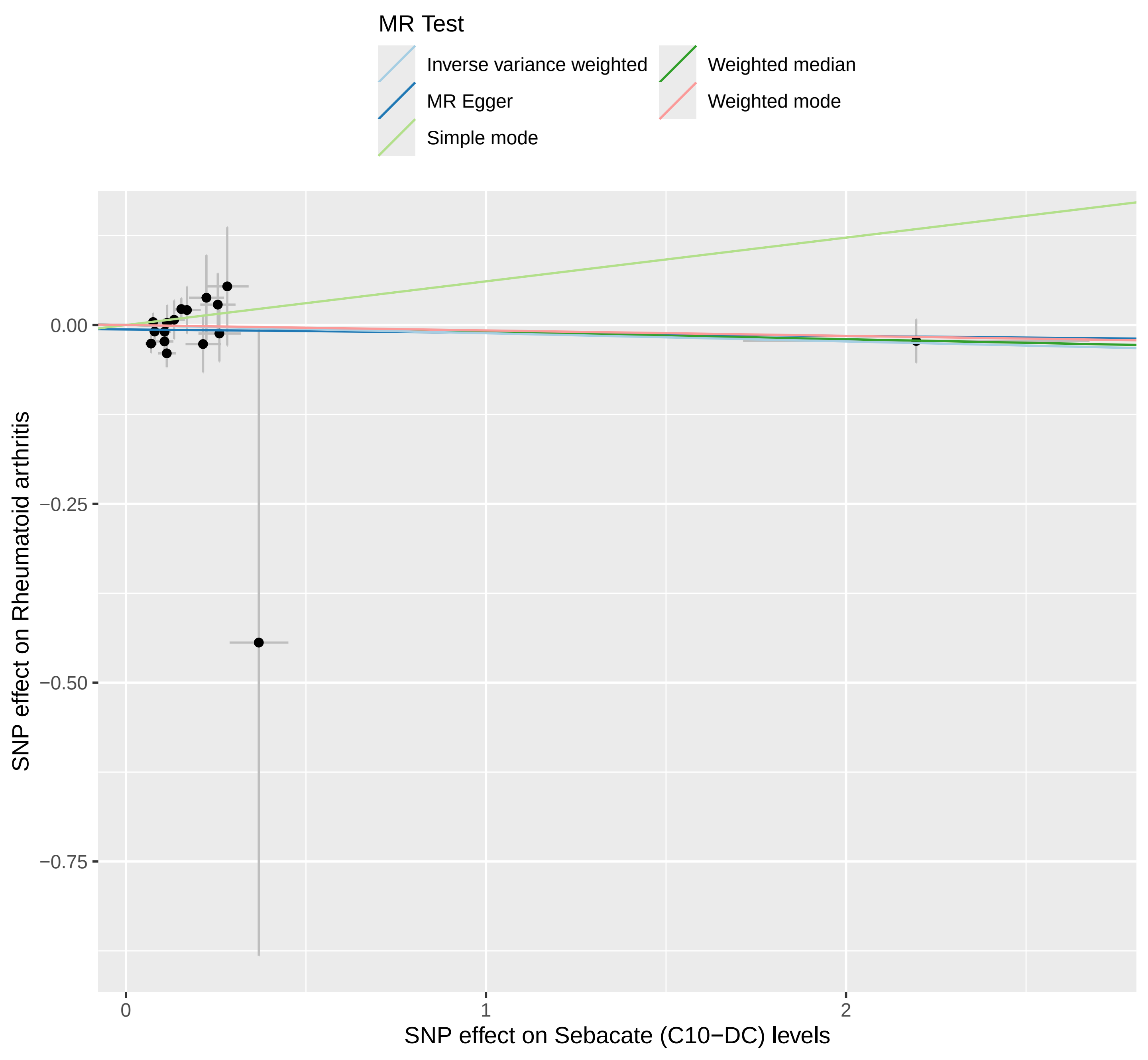

MR Test
Inverse variance weighted
MR Egger
Simple mode
Weighted median
Weighted mode
0.00
−0.25
−0.50
−0.75
0
1
2
SNP effect on Rheumatoid arthritis
SNP effect on Sebacate (C10−DC) levels

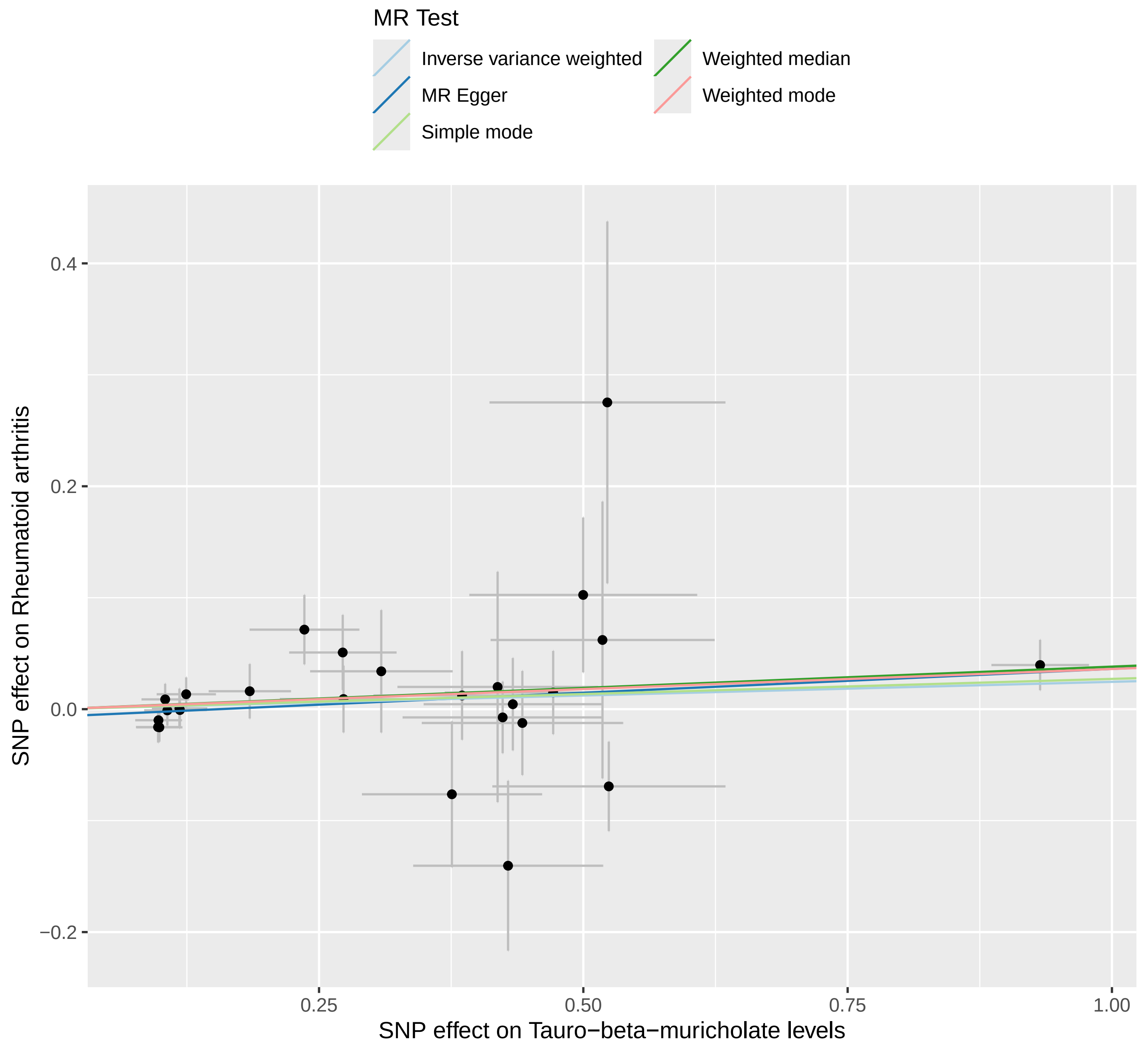

MR Test
Inverse variance weighted
MR Egger
Simple mode
Weighted median
Weighted mode
0.4
0.2
0.0
−0.2
0.25
0.50
0.75
1.00
SNP effect on Rheumatoid arthritis
SNP effect on Tauro−beta−muricholate levels

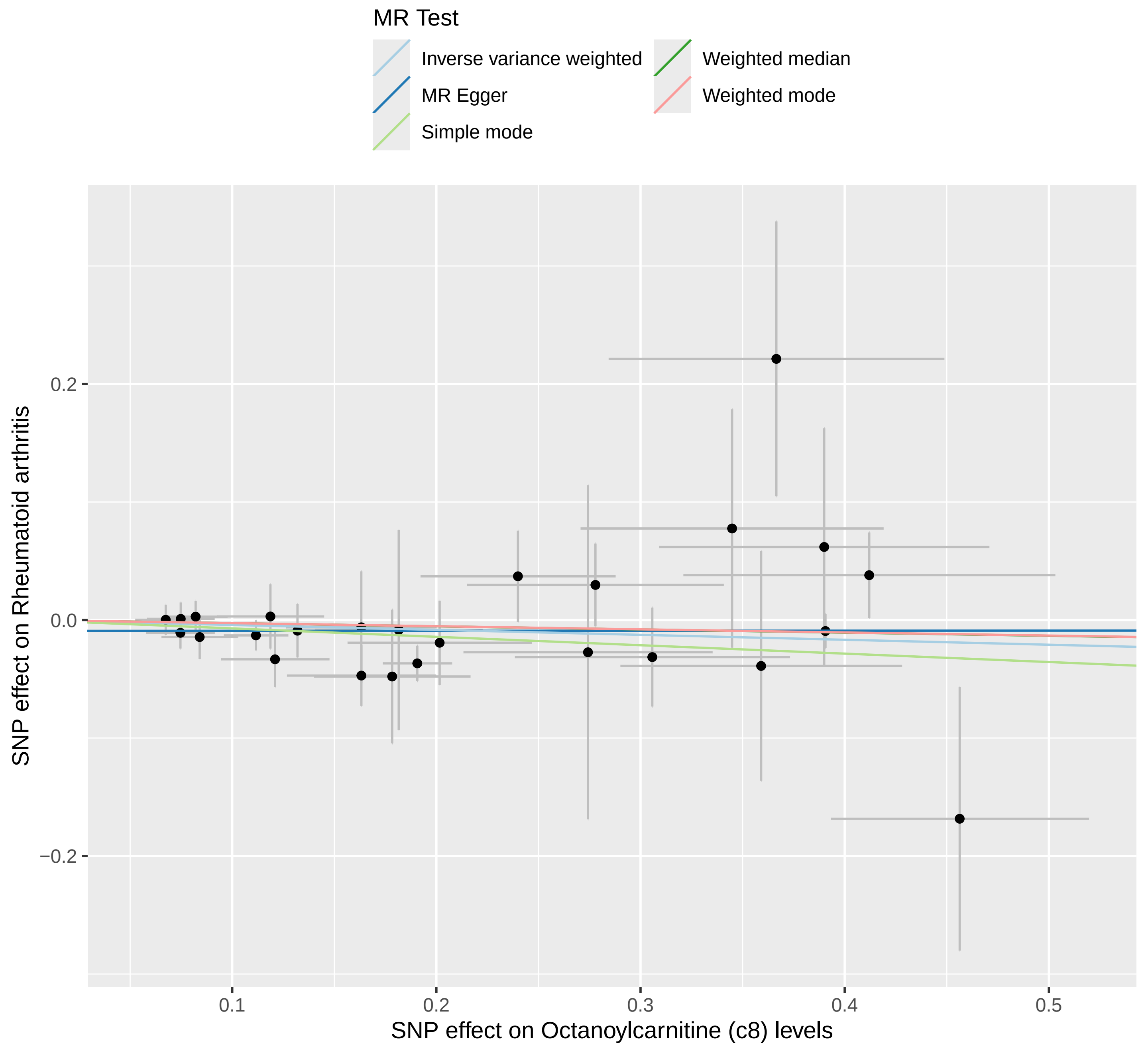
MR Test
Inverse variance weighted
MR Egger
Simple mode
Weighted median
Weighted mode
0.2
0.0
−0.2
SNP effect on Rheumatoid arthritis
0.1
0.2
0.3
0.4
0.5
SNP effect on Octanoylcarnitine (c8) levels

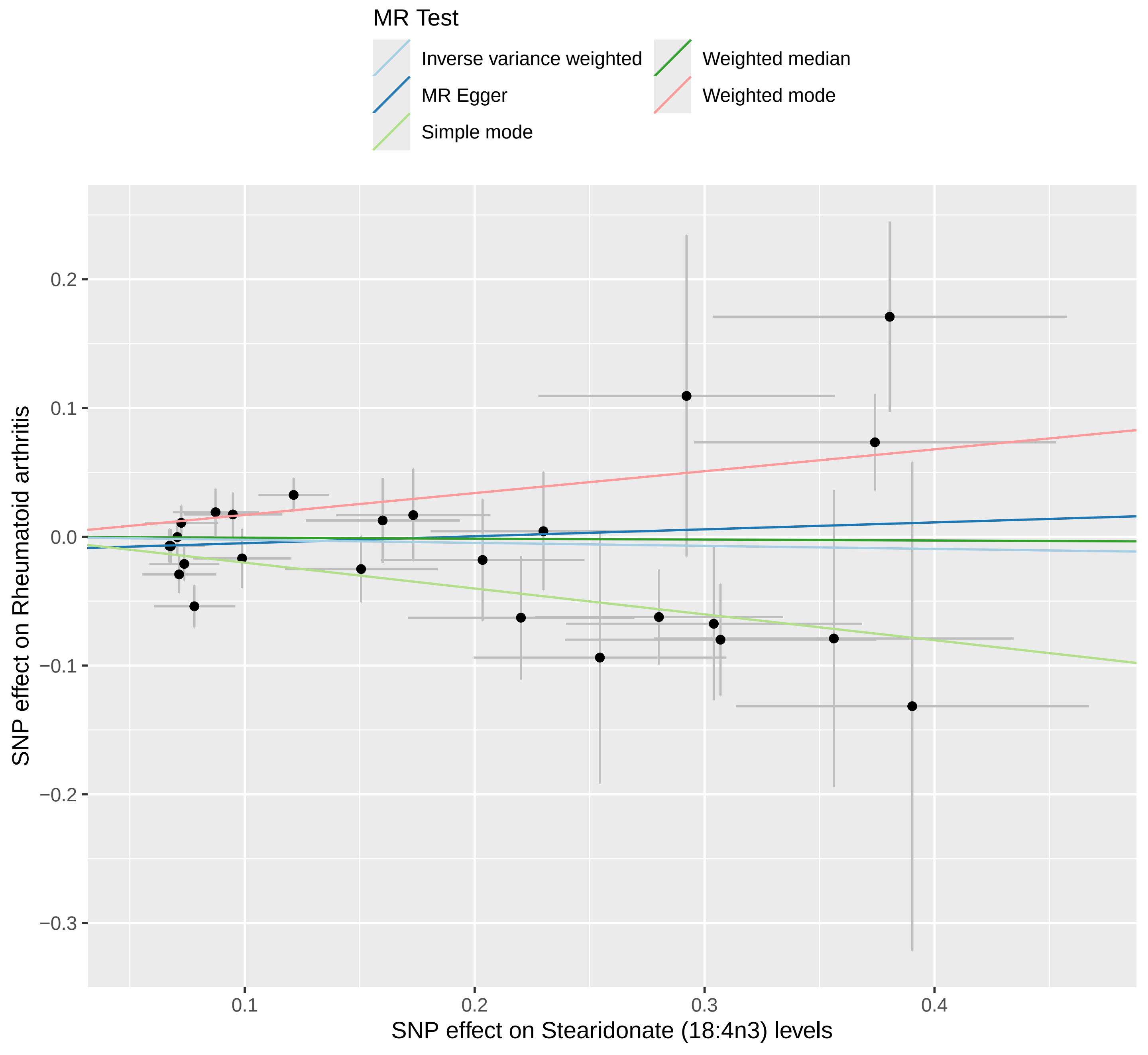
MR Test
Inverse variance weighted
MR Egger
Simple mode
Weighted median
Weighted mode
0.2
0.1
0.0
−0.1
−0.2
−0.3
SNP effect on Rheumatoid arthritis
0.1
0.2
0.3
0.4
SNP effect on Stearidonate (18:4n3) levels

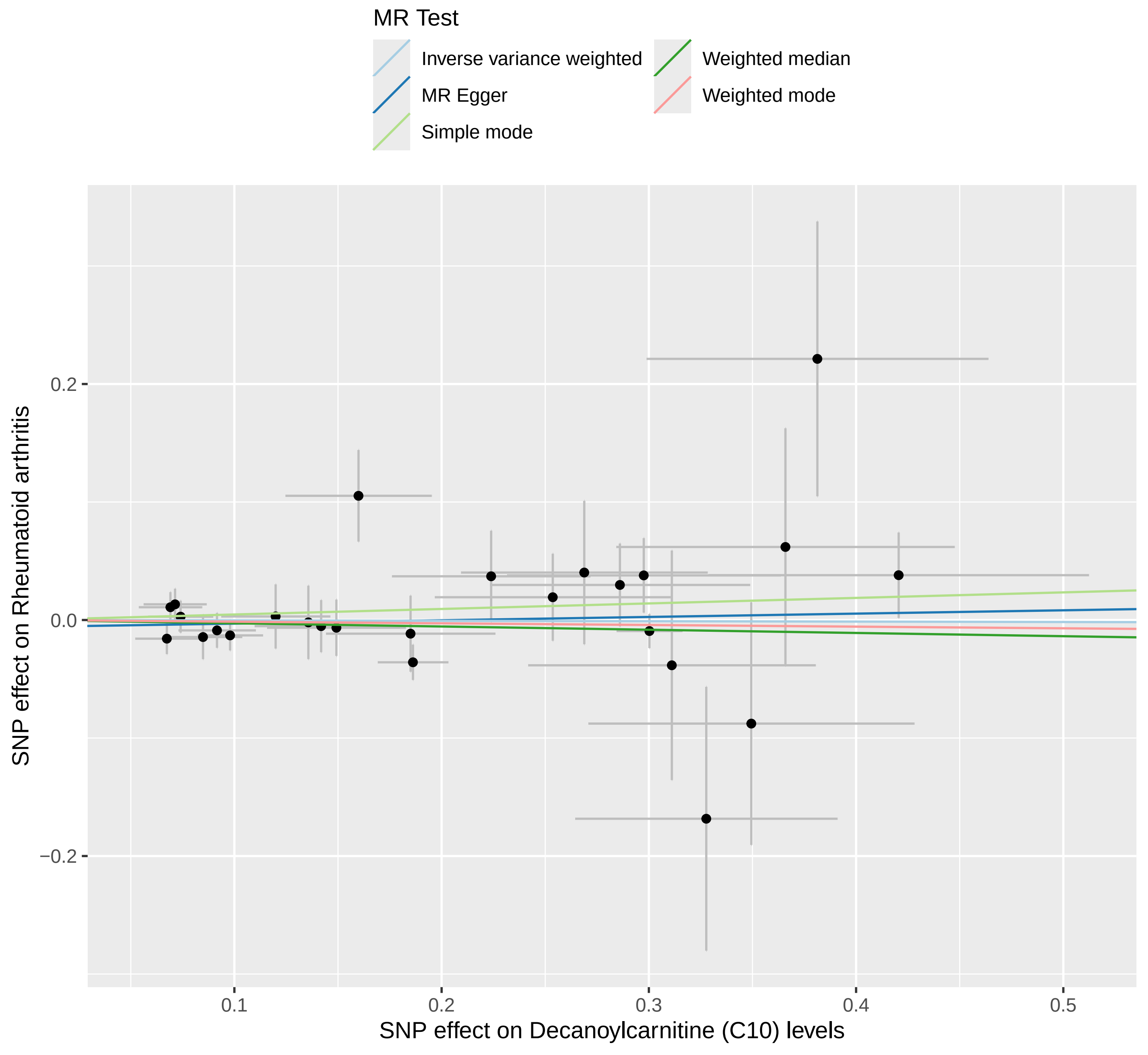
MR Test
Inverse variance weighted
MR Egger
Simple mode
Weighted median
Weighted mode
SNP effect on Rheumatoid arthritis
0.2
0.0
−0.2
0.1
0.2
0.3
0.4
0.5
SNP effect on Decanoylcarnitine (C10) levels

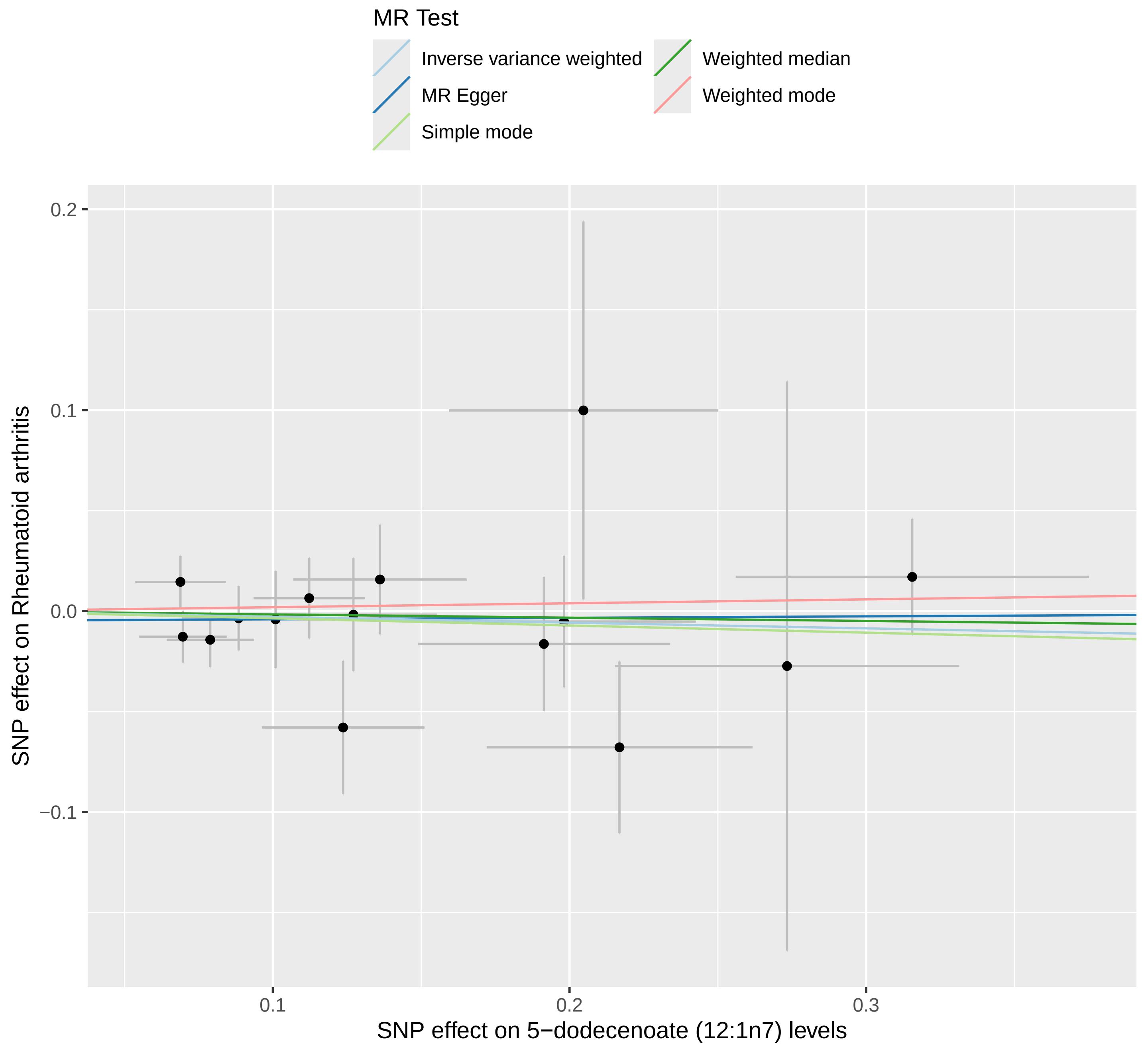
MR Test
Inverse variance weighted
MR Egger
Simple mode
Weighted median
Weighted mode
0.2
0.1
0.0
−0.1
0.1
0.2
0.3
SNP effect on Rheumatoid arthritis
SNP effect on 5−dodecenoate (12:1n7) levels

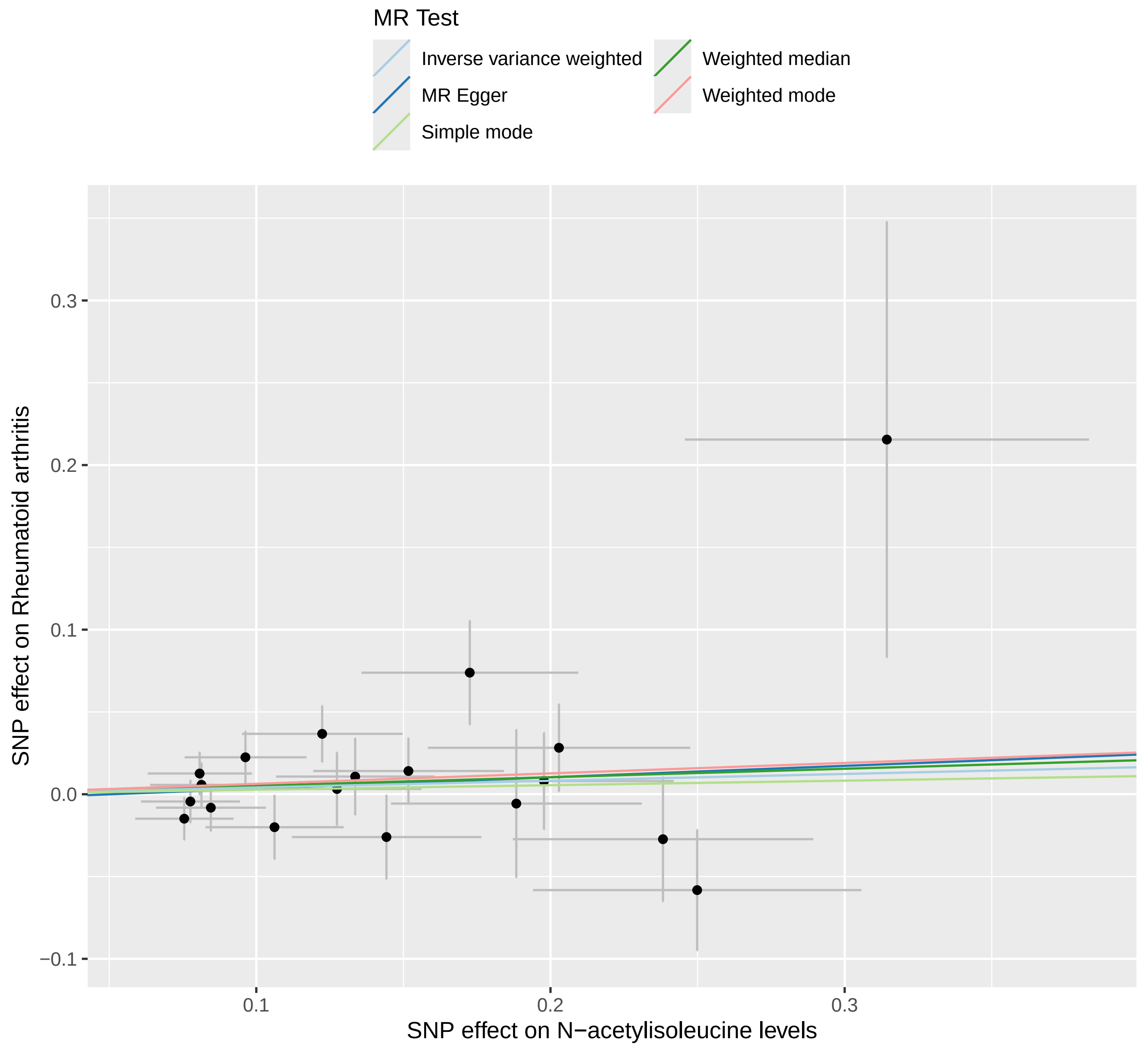
MR Test
Inverse variance weighted
MR Egger
Simple mode
Weighted median
Weighted mode
0.3
0.2
0.1
0.0
−0.1
0.1
0.2
0.3
SNP effect on Rheumatoid arthritis
SNP effect on N−acetylisoleucine levels

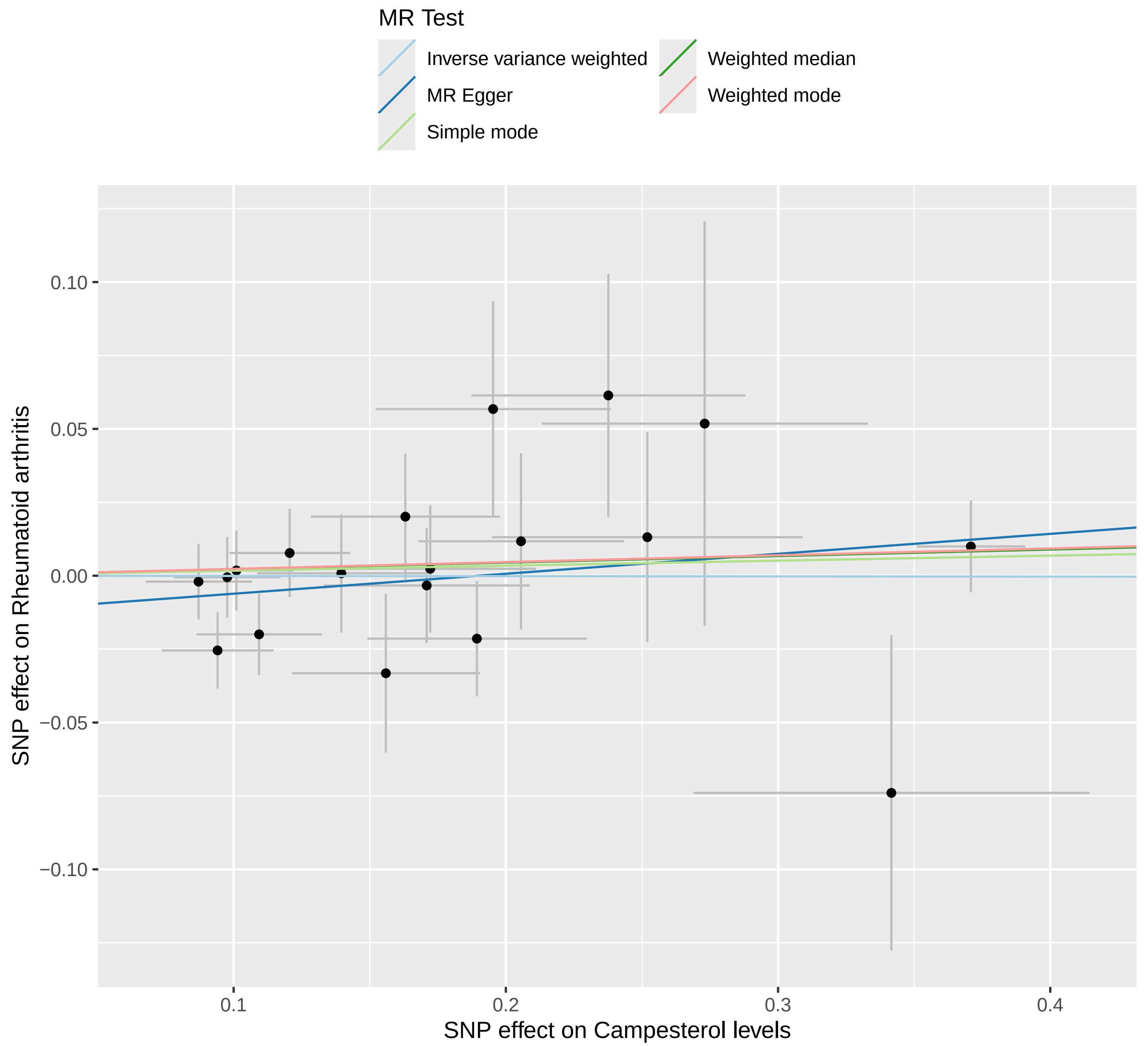
MR Test
Inverse variance weighted
MR Egger
Simple mode
Weighted median
Weighted mode
SNP effect on Rheumatoid arthritis
0.10
0.05
0.00
−0.05
−0.10
0.1
0.2
0.3
0.4
SNP effect on Campesterol levels

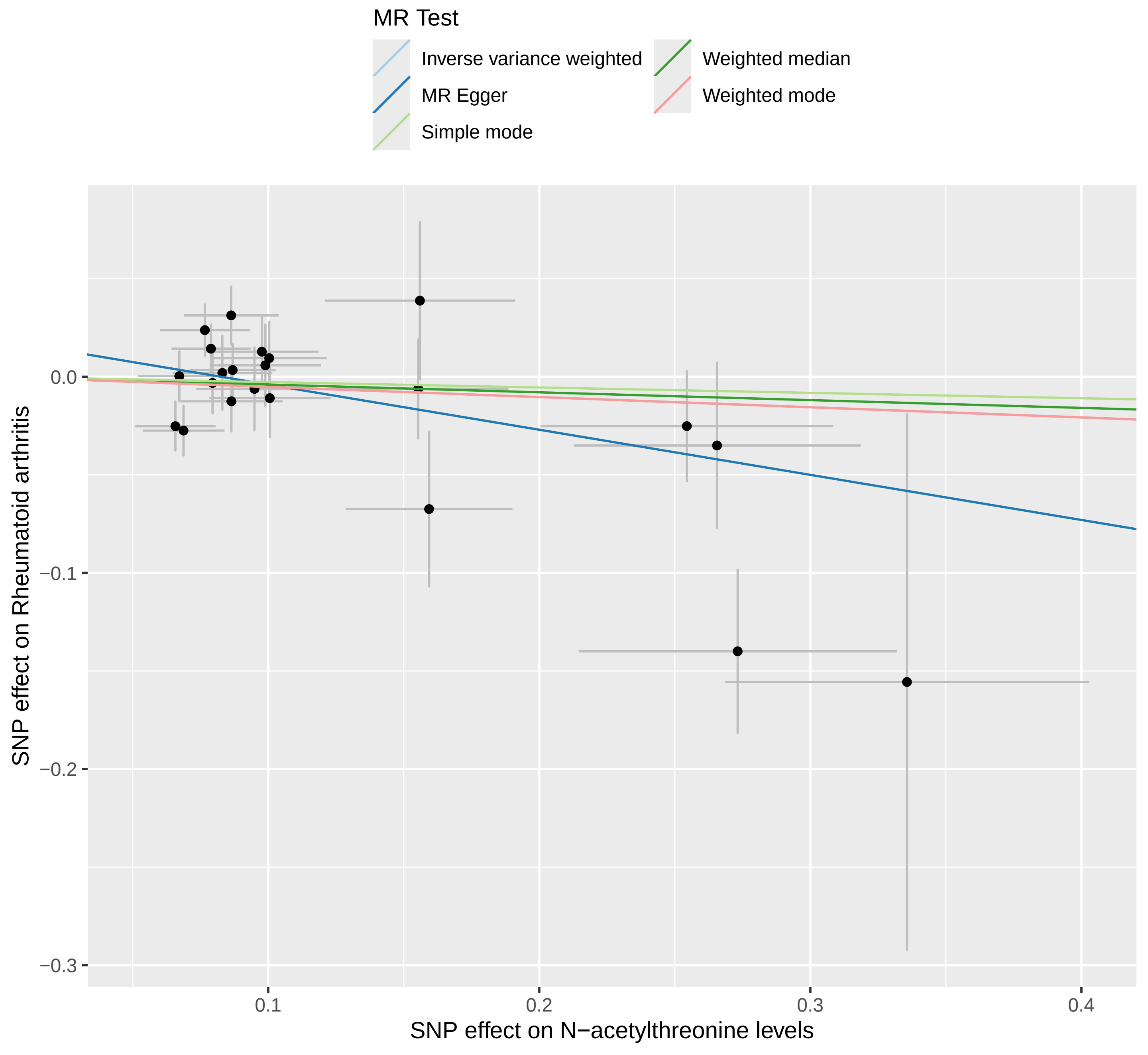

MR Test
Inverse variance weighted
MR Egger
Simple mode
Weighted median
Weighted mode
0.0
−0.1
−0.2
−0.3
0.1
0.2
0.3
0.4
SNP effect on Rheumatoid arthritis
SNP effect on N−acetylthreonine levels

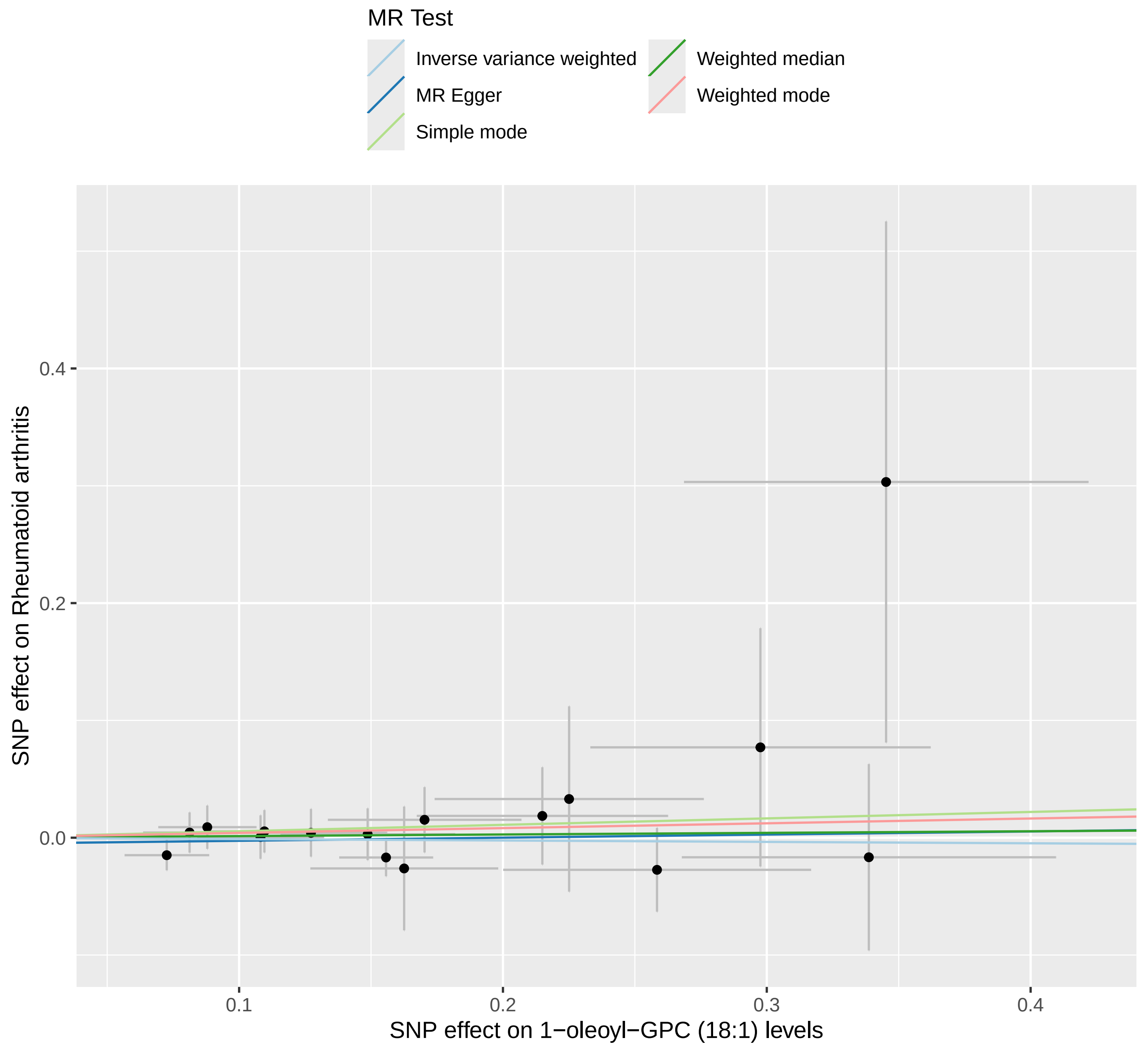
MR Test
Inverse variance weighted
MR Egger
Simple mode
Weighted median
Weighted mode
0.4
0.2
0.0
0.1
0.2
0.3
0.4
SNP effect on Rheumatoid arthritis
SNP effect on 1−oleoyl−GPC (18:1) levels

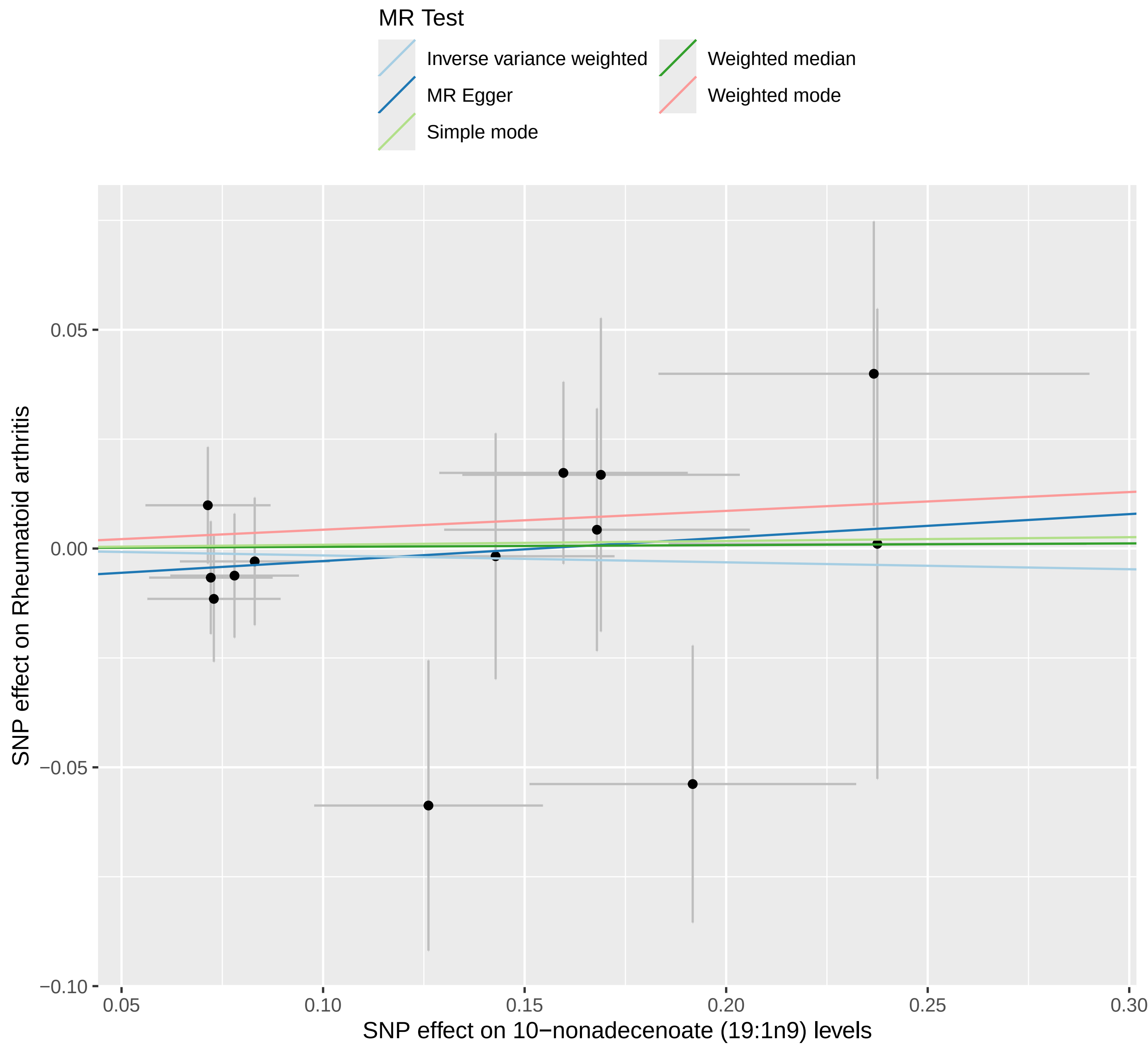

MR Test
Inverse variance weighted
MR Egger
Simple mode
Weighted median
Weighted mode
SNP effect on Rheumatoid arthritis
0.05
0.00
−0.05
−0.10
0.05
0.10
0.15
0.20
0.25
0.30
SNP effect on 10−nonadecenoate (19:1n9) levels

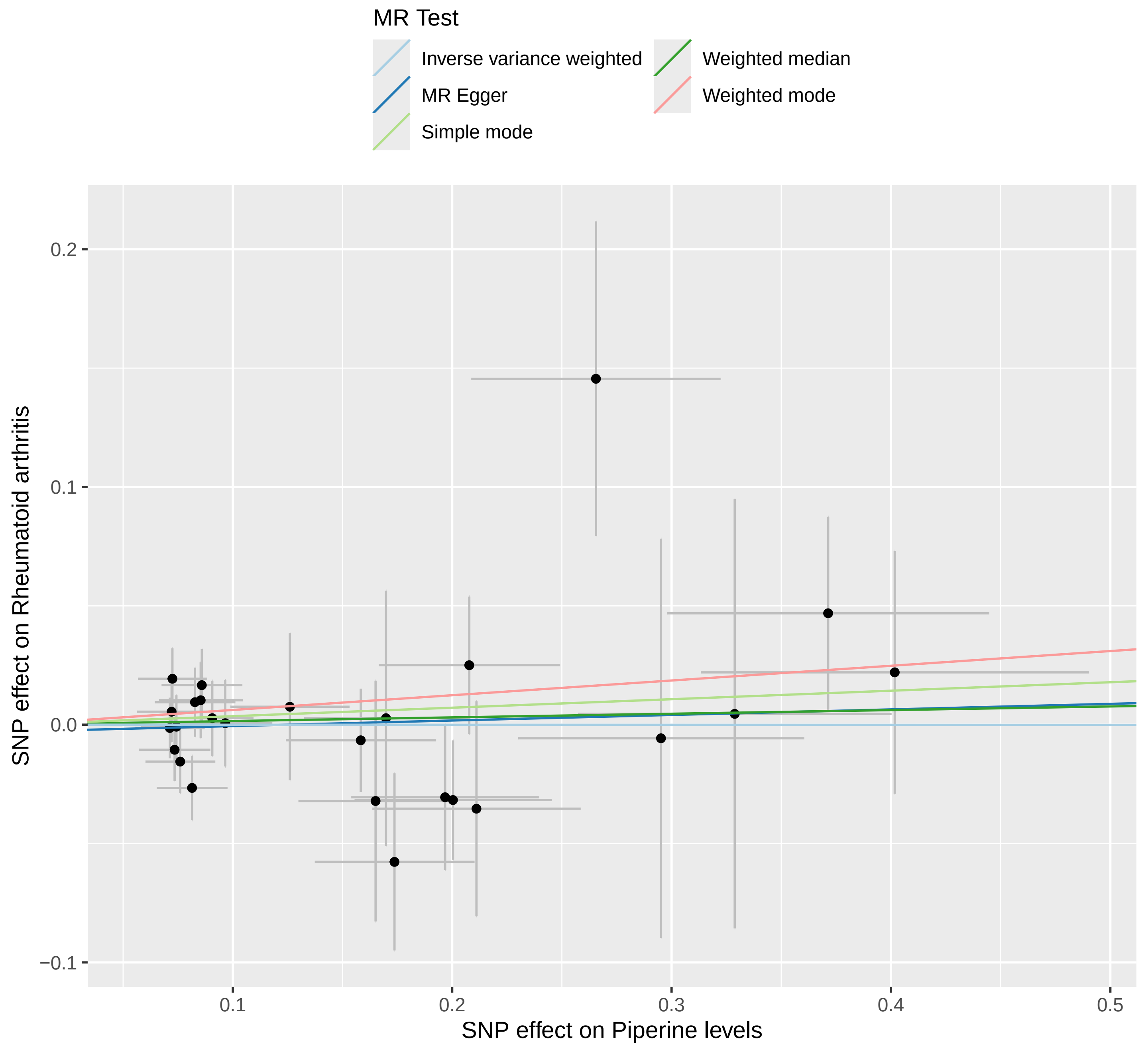
MR Test
Inverse variance weighted
MR Egger
Simple mode
Weighted median
Weighted mode
0.2
0.1
0.0
−0.1
0.1
0.2
0.3
0.4
0.5
SNP effect on Rheumatoid arthritis
SNP effect on Piperine levels

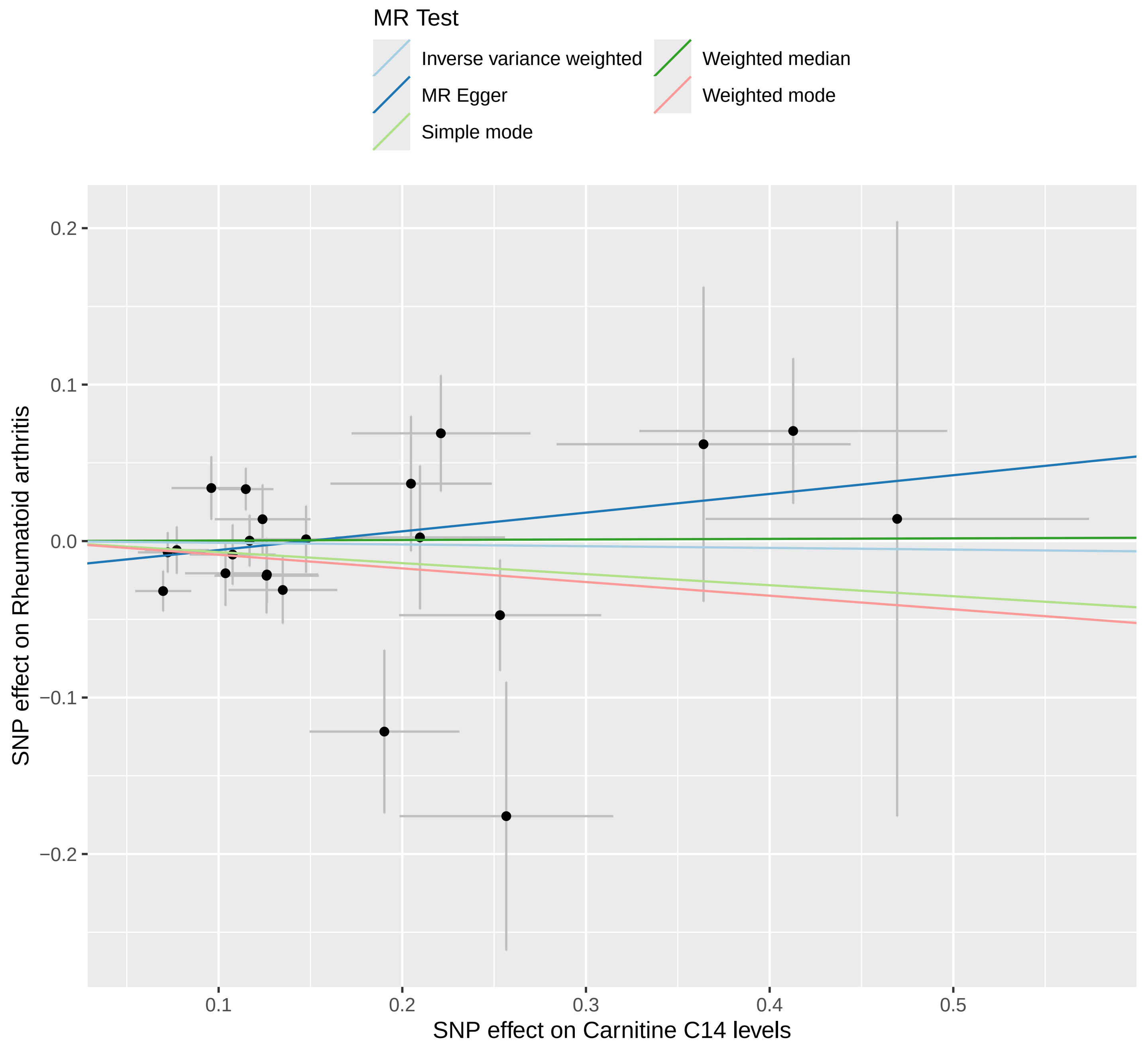

MR Test
Inverse variance weighted
MR Egger
Simple mode
Weighted median
Weighted mode
0.2
0.1
0.0
−0.1
−0.2
SNP effect on Rheumatoid arthritis
0.1
0.2
0.3
0.4
0.5
SNP effect on Carnitine C14 levels

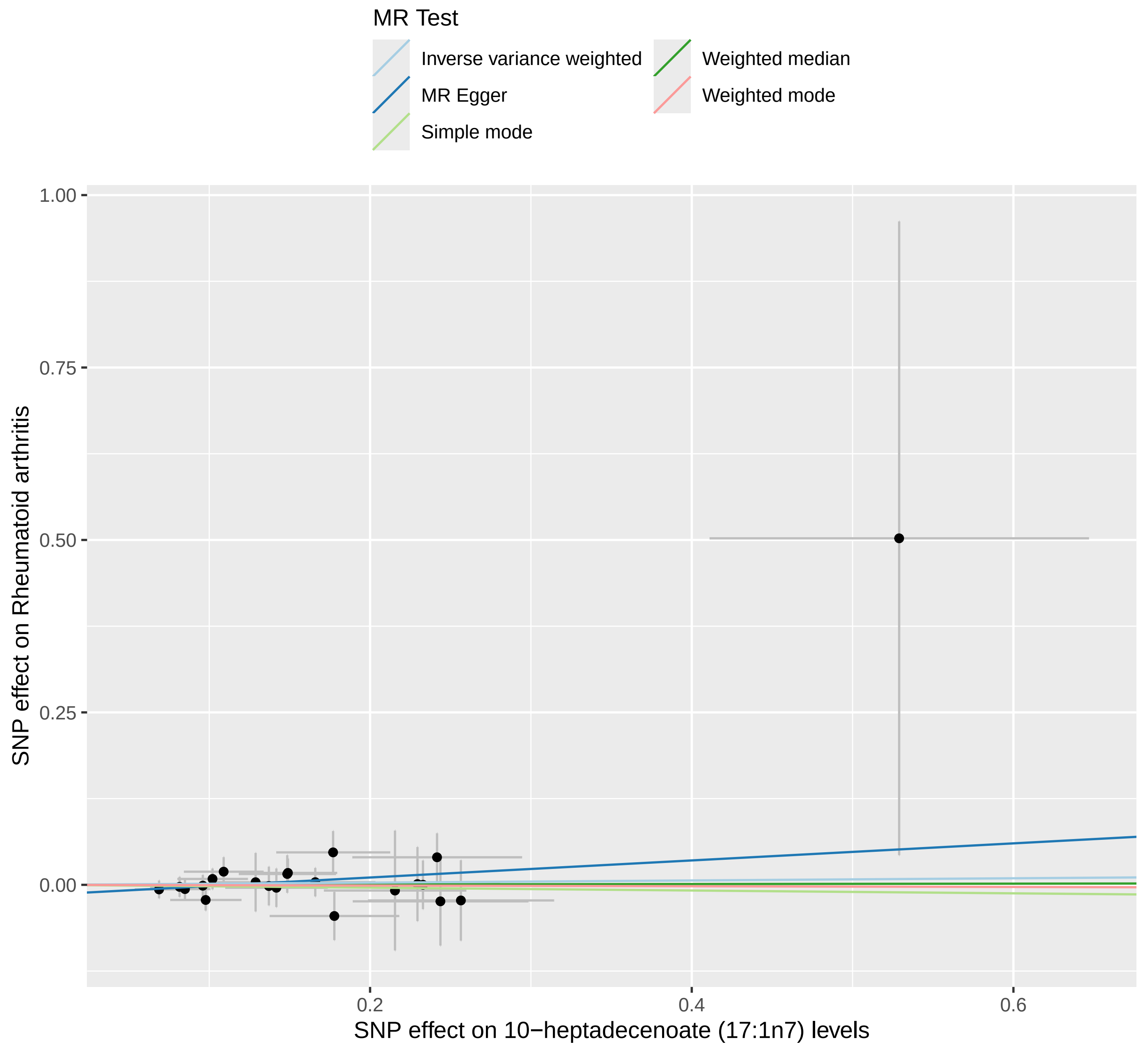
MR Test
Inverse variance weighted
MR Egger
Simple mode
Weighted median
Weighted mode
1.00
0.75
0.50
0.25
0.00
SNP effect on Rheumatoid arthritis
0.2
0.4
0.6
SNP effect on 10−heptadecenoate (17:1n7) levels

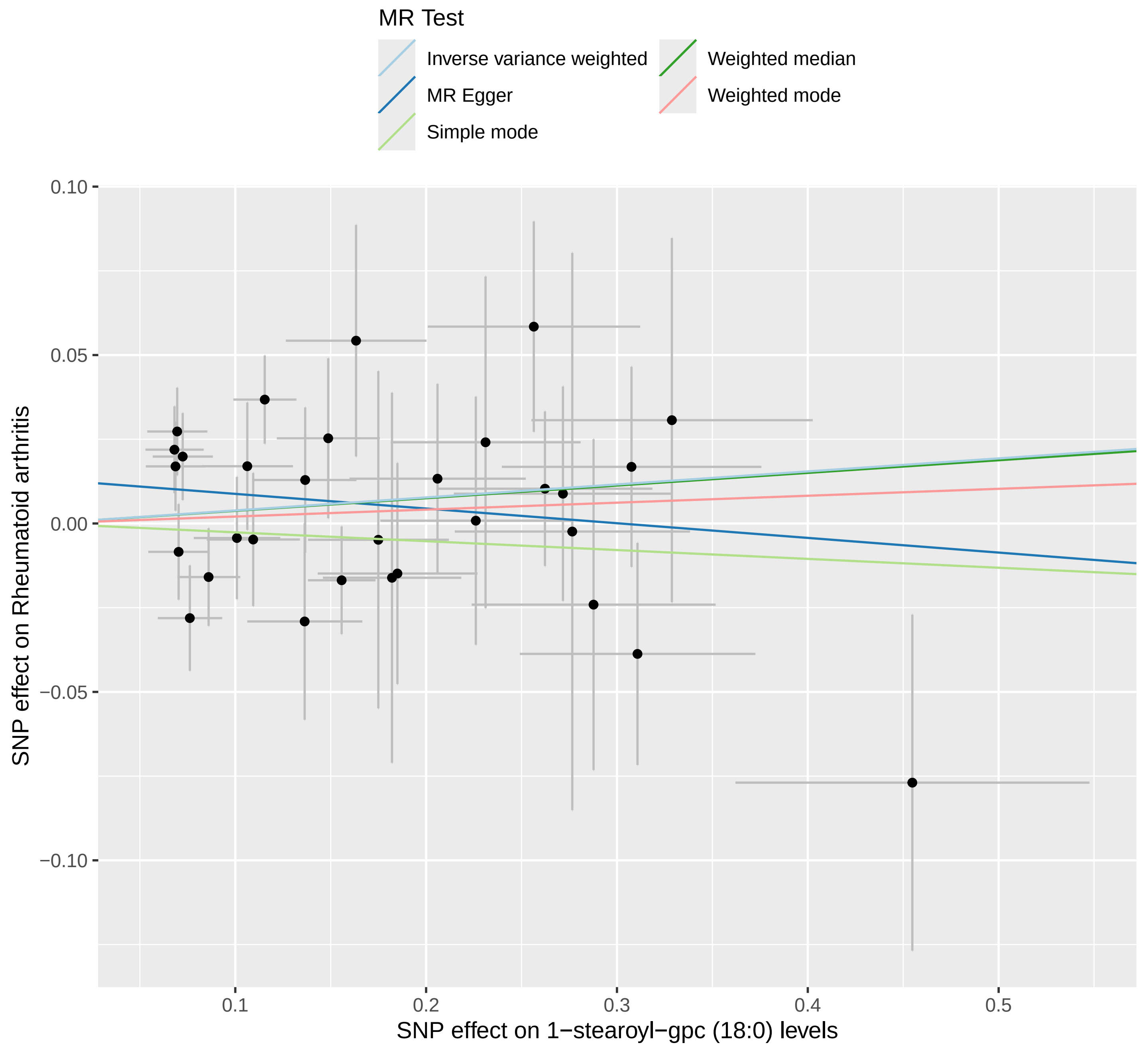

MR Test
Inverse variance weighted
MR Egger
Simple mode
Weighted median
Weighted mode
0.10
0.05
0.00
−0.05
−0.10
0.1
0.2
0.3
0.4
0.5
SNP effect on Rheumatoid arthritis
SNP effect on 1−stearoyl−gpc (18:0) levels

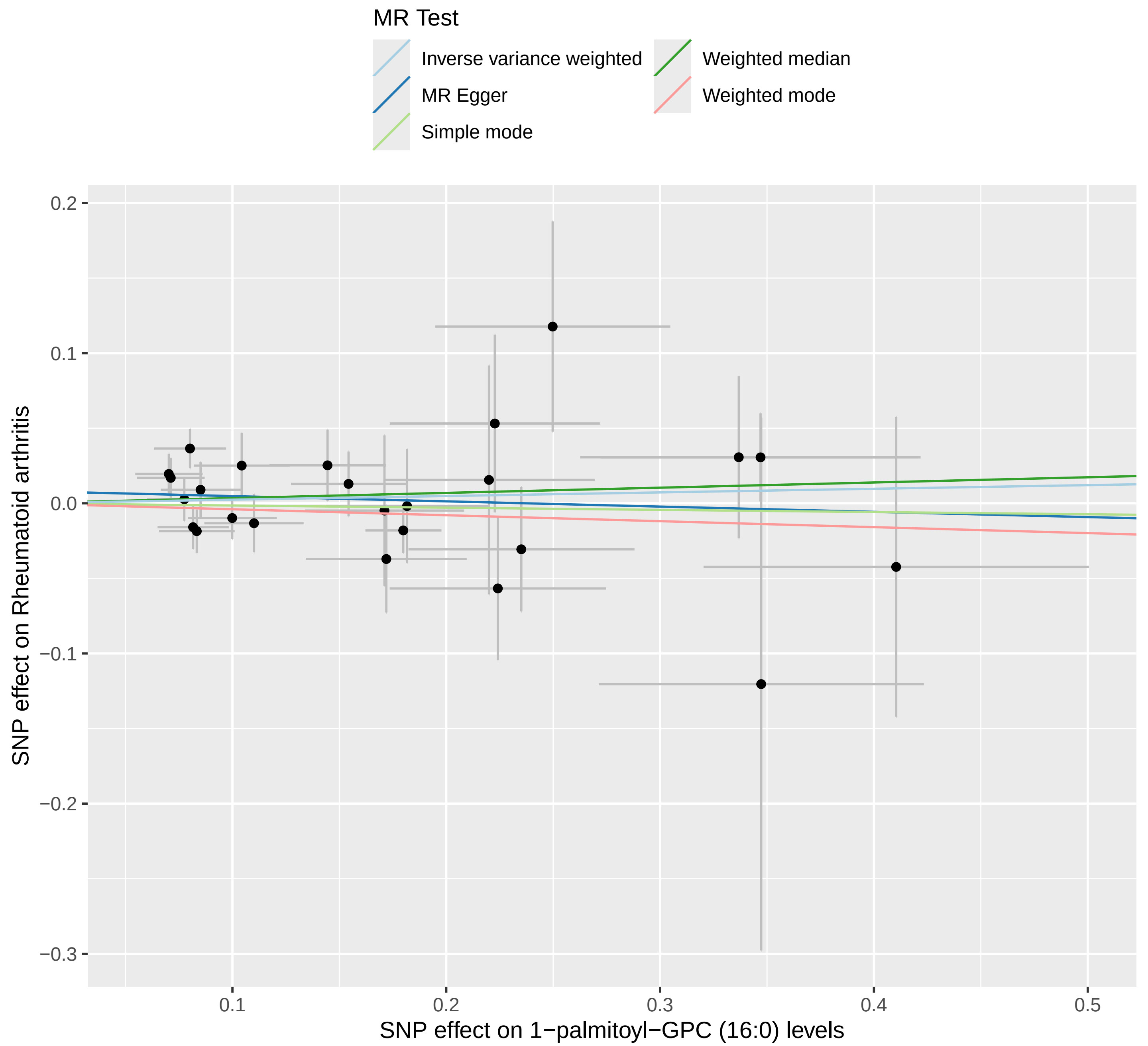

MR Test
Inverse variance weighted
MR Egger
Simple mode
Weighted median
Weighted mode
SNP effect on Rheumatoid arthritis
SNP effect on 1−palmitoyl−GPC (16:0) levels
0.2
0.1
0.0
−0.1
−0.2
−0.3
0.1
0.2
0.3
0.4
0.5

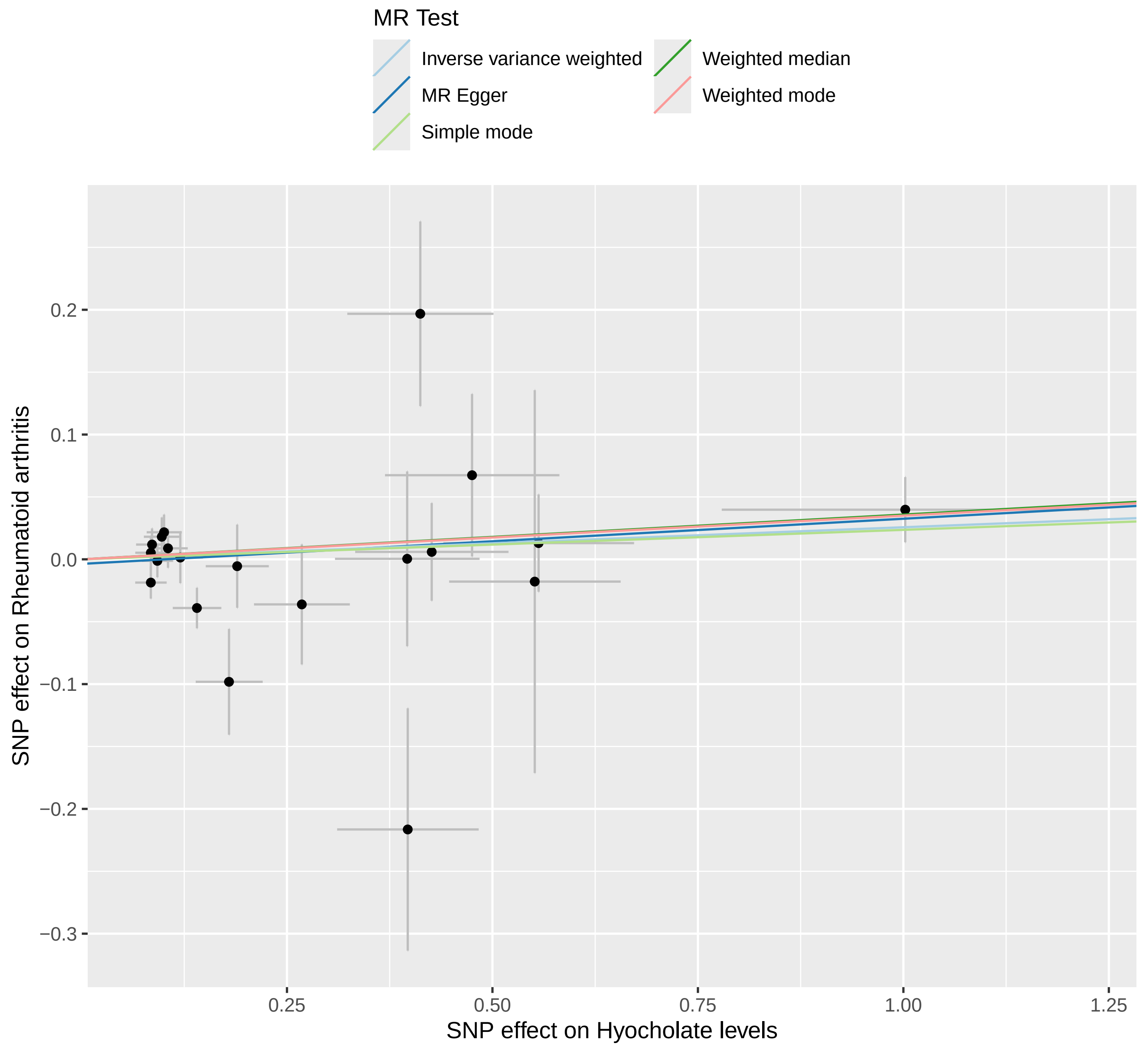

MR Test
Inverse variance weighted
MR Egger
Simple mode
Weighted median
Weighted mode
SNP effect on Rheumatoid arthritis
0.2
0.1
0.0
−0.1
−0.2
−0.3
0.25
0.50
0.75
1.00
1.25
SNP effect on Hyocholate levels

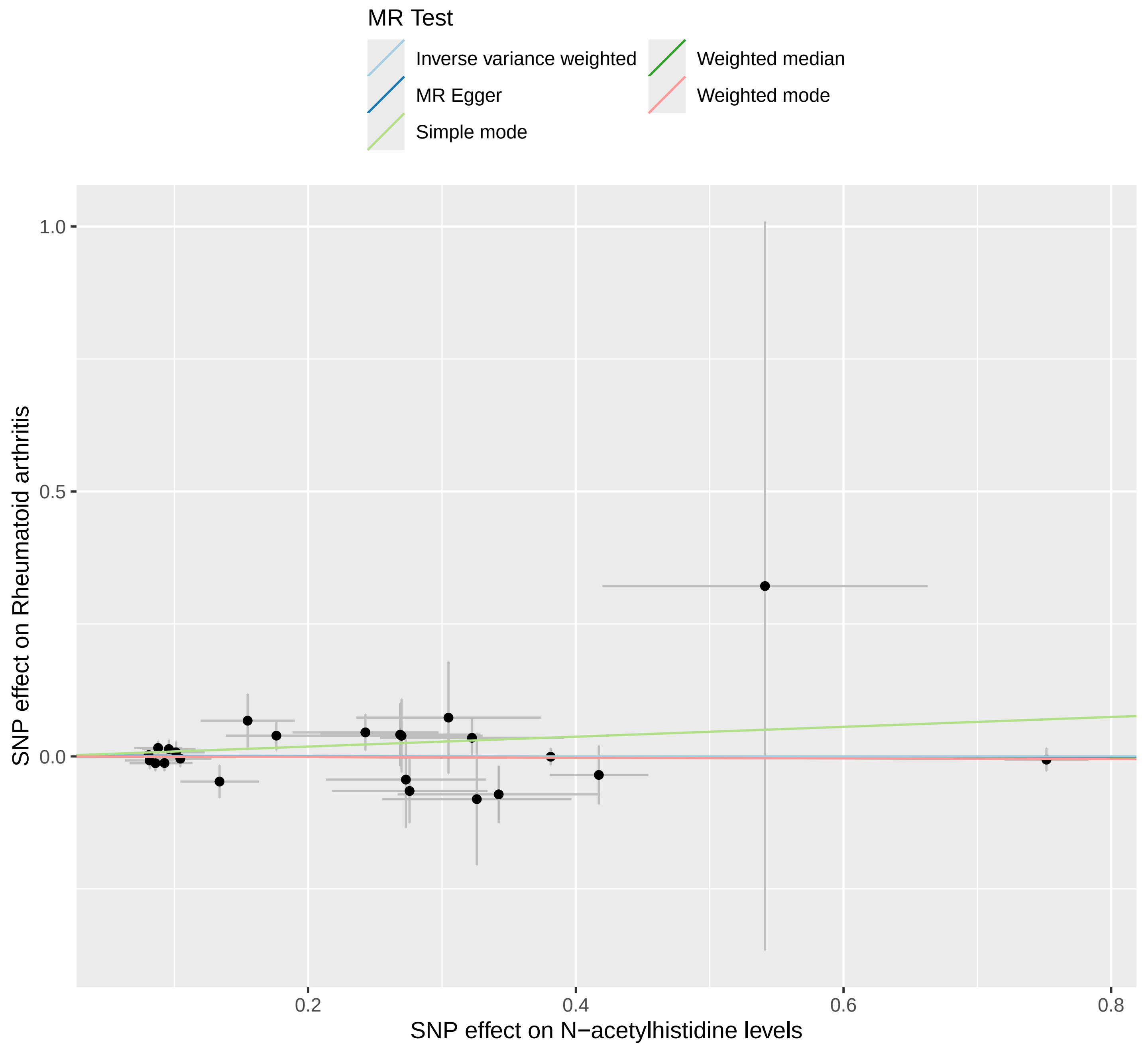
MR Test
Inverse variance weighted
MR Egger
Simple mode
Weighted median
Weighted mode
1.0
0.5
0.0
0.2
0.4
0.6
0.8
SNP effect on Rheumatoid arthritis
SNP effect on N−acetylhistidine levels

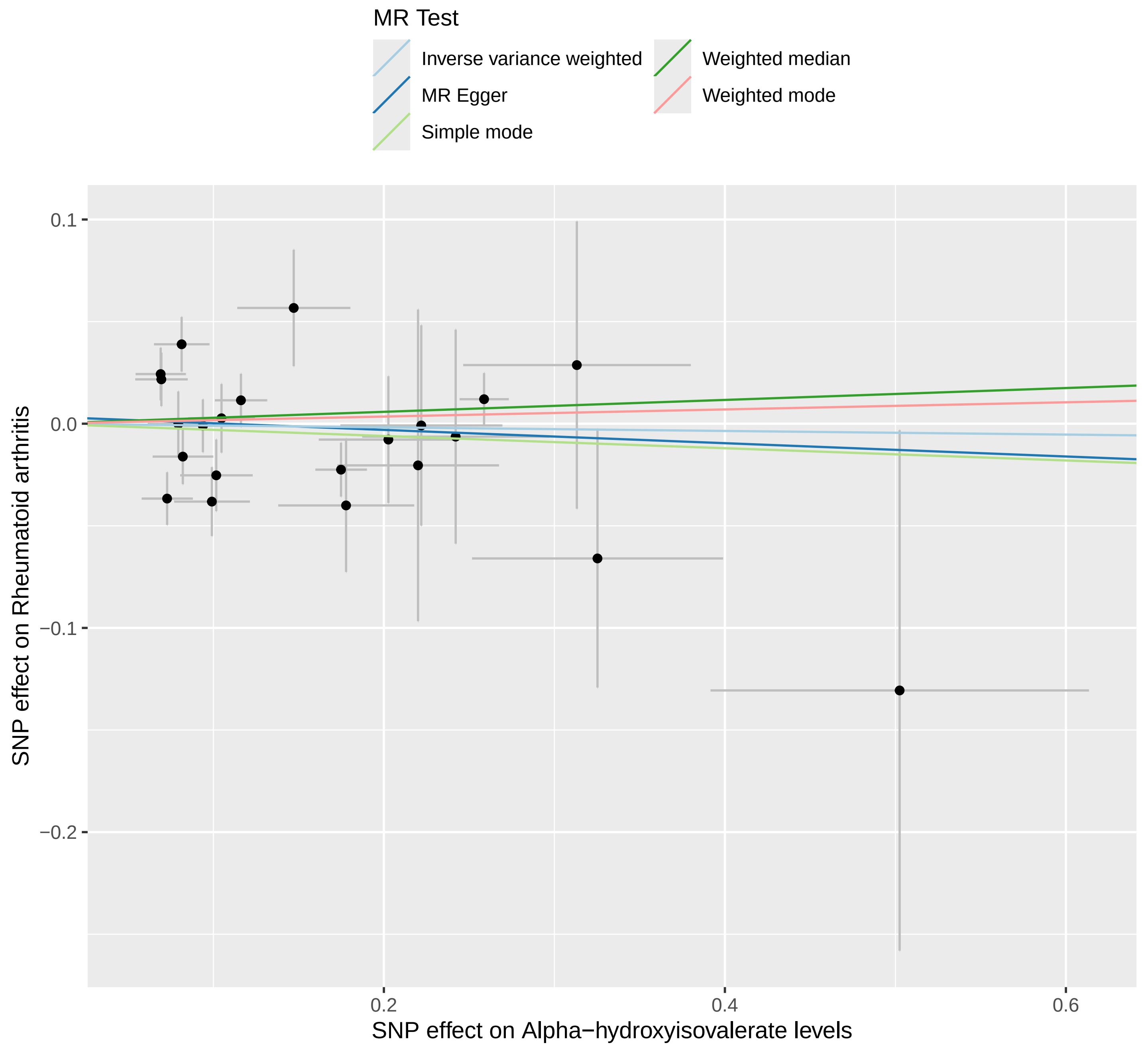
MR Test
Inverse variance weighted
MR Egger
Simple mode
Weighted median
Weighted mode
0.1
0.0
−0.1
−0.2
0.2
0.4
0.6
SNP effect on Rheumatoid arthritis
SNP effect on Alpha−hydroxyisovalerate levels

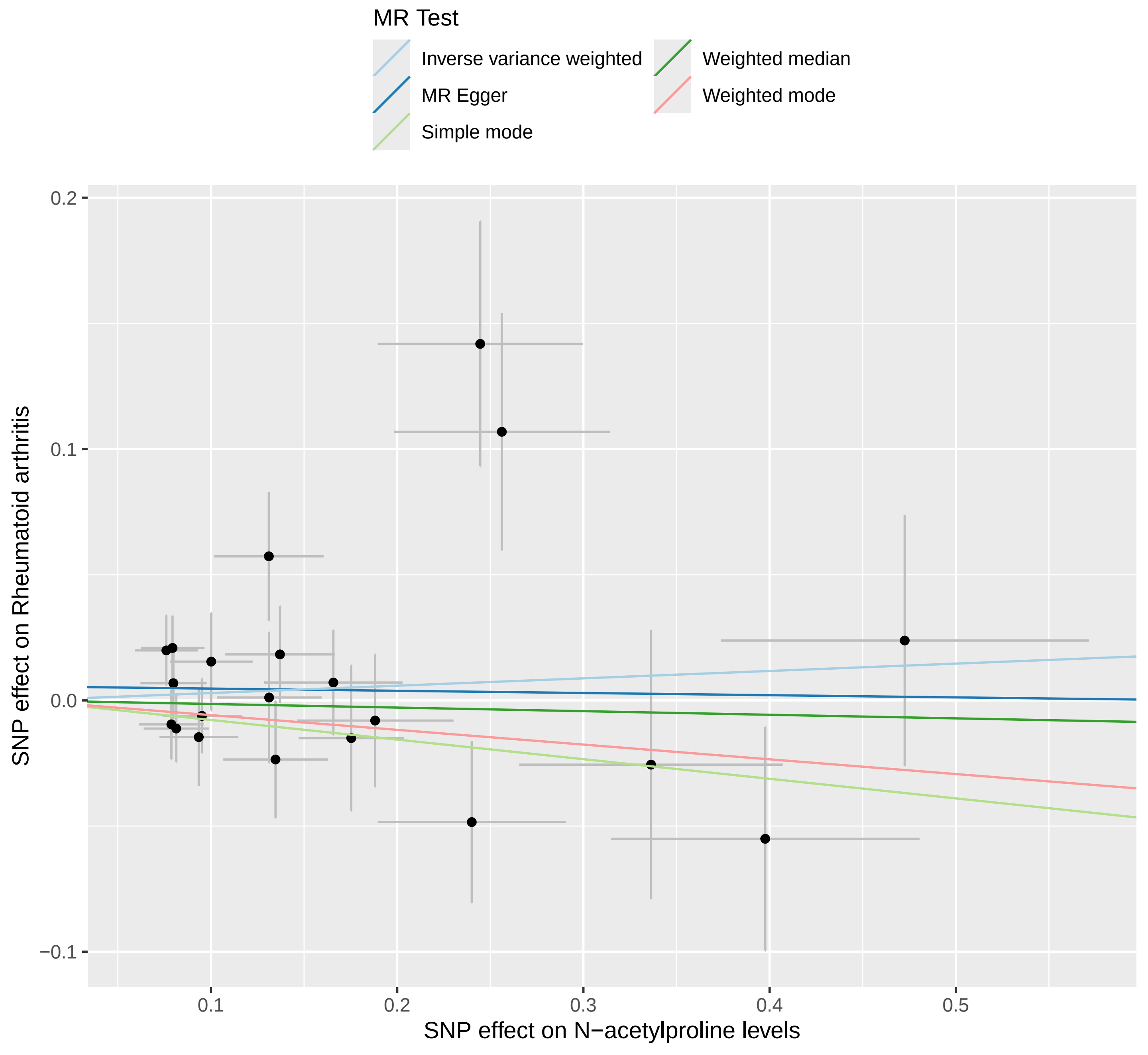
MR Test
Inverse variance weighted
MR Egger
Simple mode
Weighted median
Weighted mode
0.2
0.1
0.0
−0.1
0.1
0.2
0.3
0.4
0.5
SNP effect on Rheumatoid arthritis
SNP effect on N−acetylproline levels

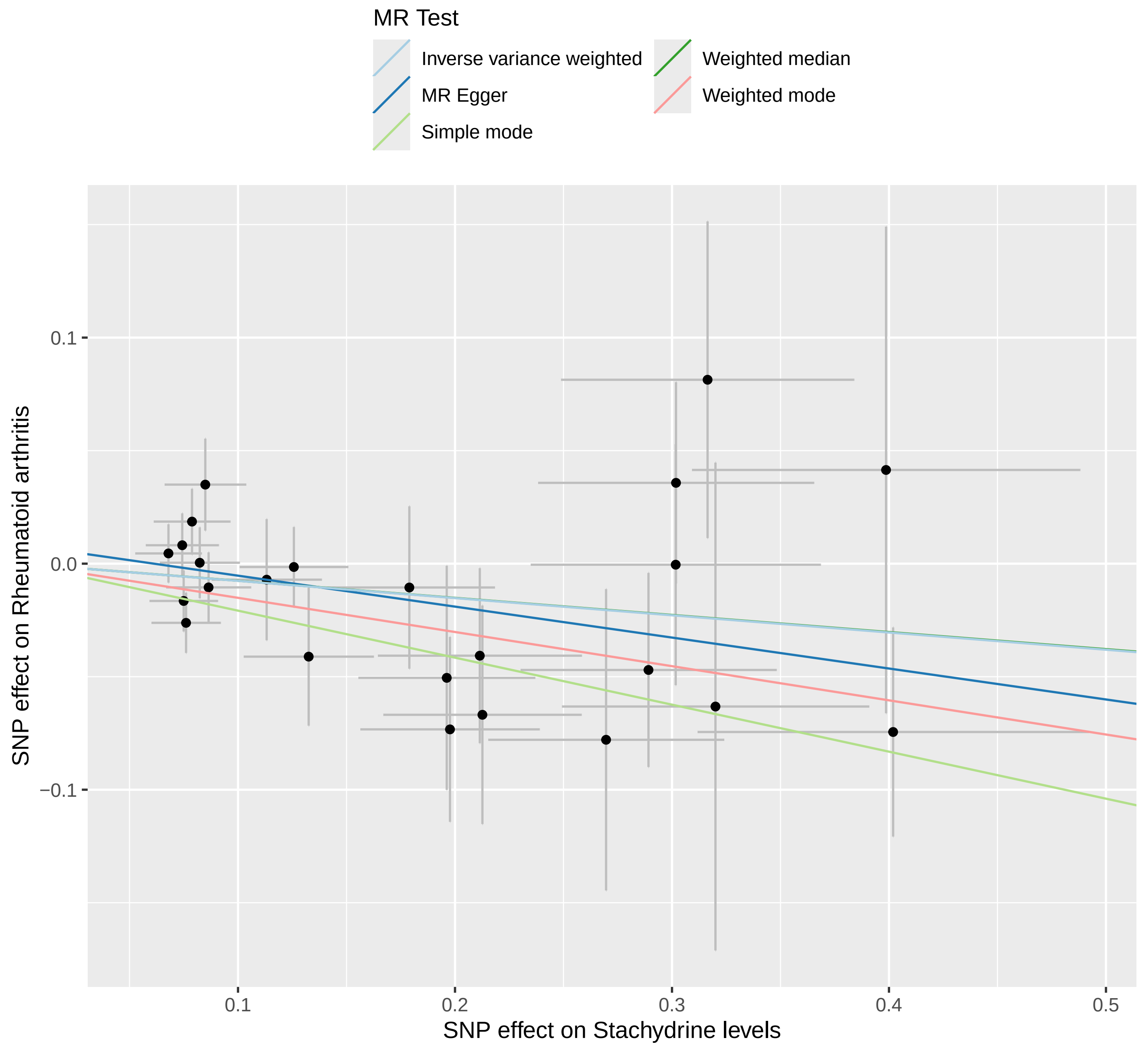

MR Test
Inverse variance weighted
MR Egger
Simple mode
Weighted median
Weighted mode
0.1
0.0
−0.1
SNP effect on Rheumatoid arthritis
0.1
0.2
0.3
0.4
0.5
SNP effect on Stachydrine levels

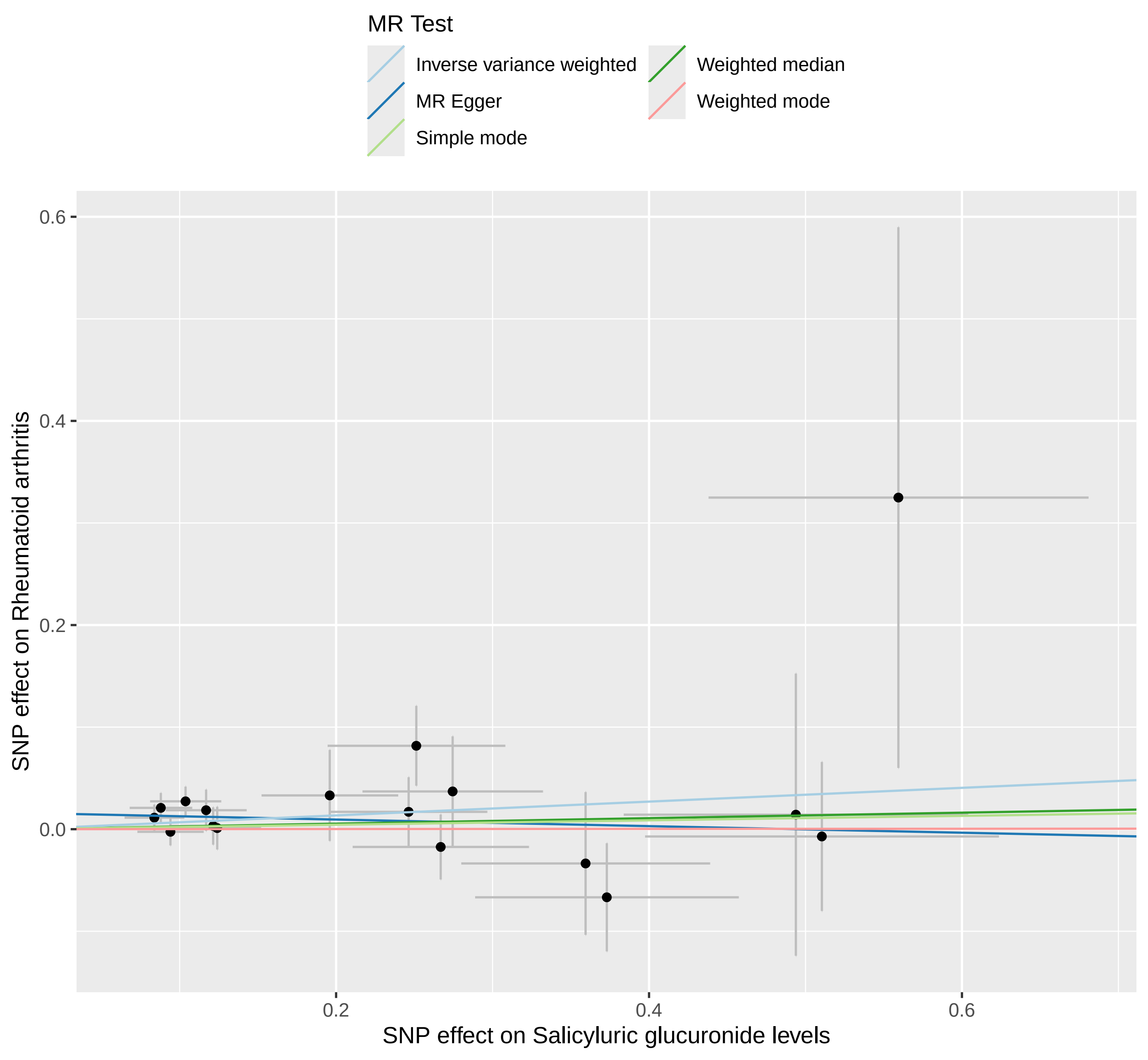

MR Test
Inverse variance weighted
MR Egger
Simple mode
Weighted median
Weighted mode
0.6
0.4
0.2
0.0
SNP effect on Rheumatoid arthritis
0.2
0.4
0.6
SNP effect on Salicyluric glucuronide levels

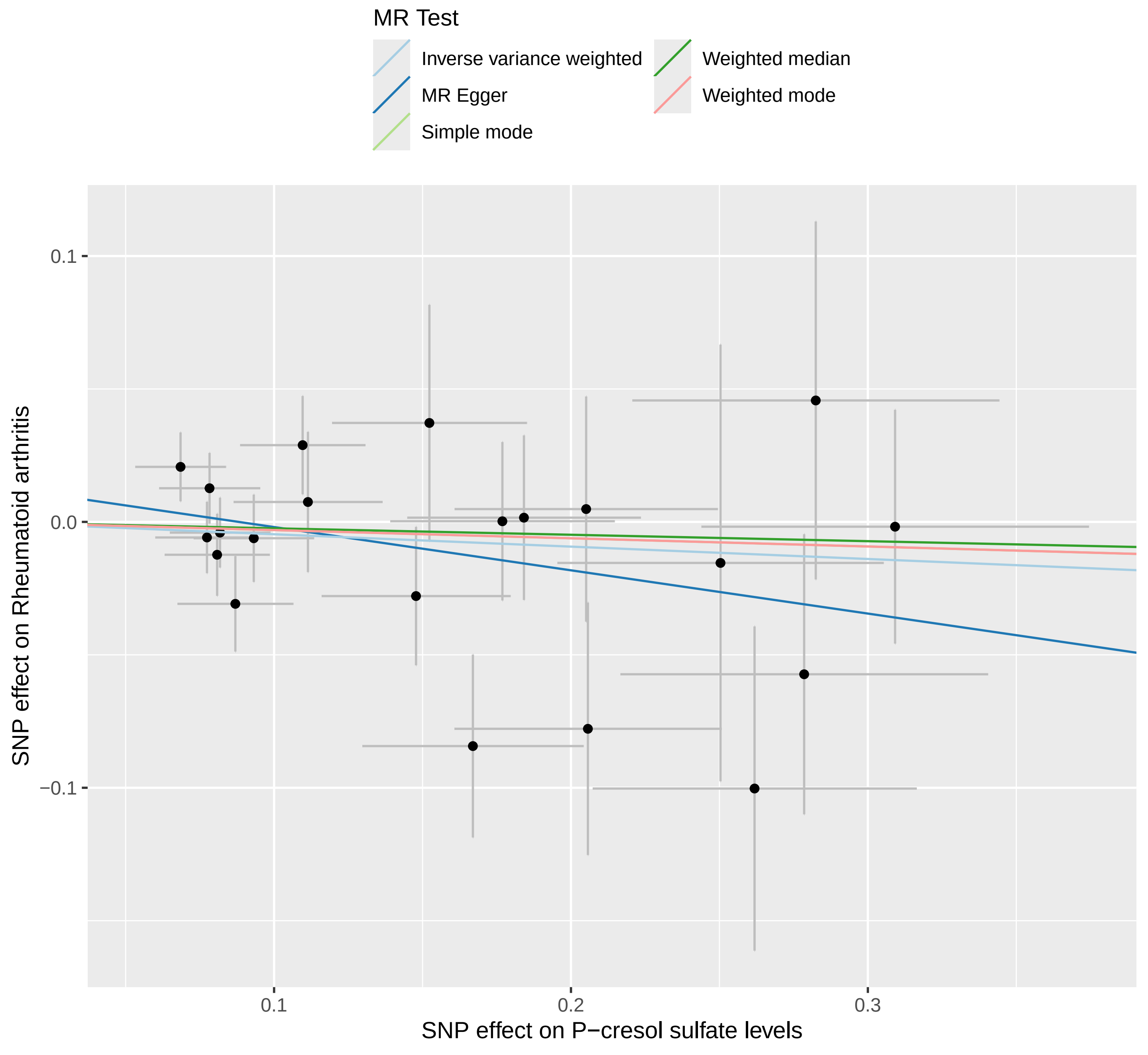

MR Test
Inverse variance weighted
MR Egger
Simple mode
Weighted median
Weighted mode
0.1
0.0
−0.1
SNP effect on Rheumatoid arthritis
0.1
0.2
0.3
SNP effect on P−cresol sulfate levels

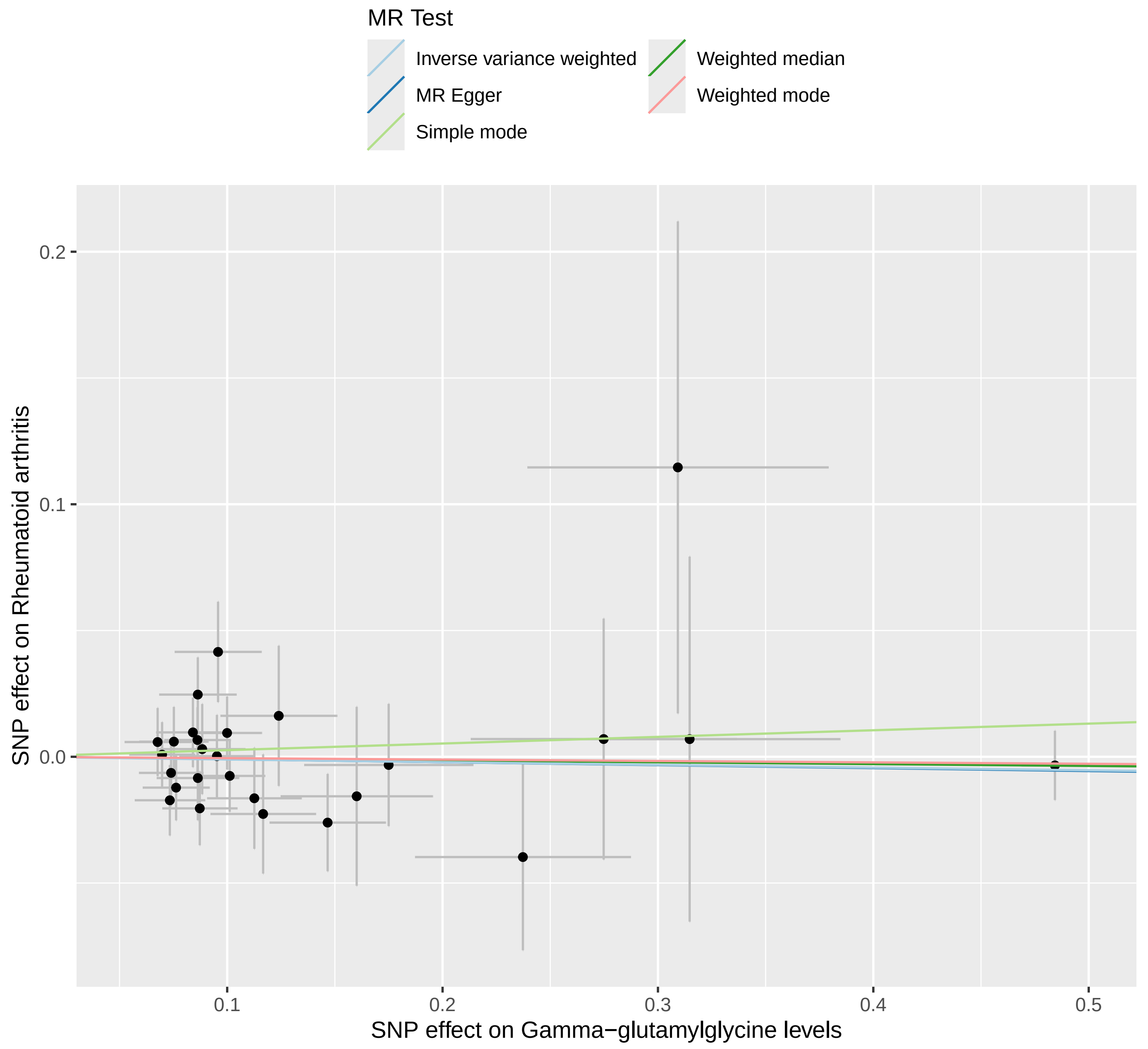
MR Test
Inverse variance weighted
MR Egger
Simple mode
Weighted median
Weighted mode
0.2
0.1
0.0
0.1
0.2
0.3
0.4
0.5
SNP effect on Rheumatoid arthritis
SNP effect on Gamma−glutamylglycine levels

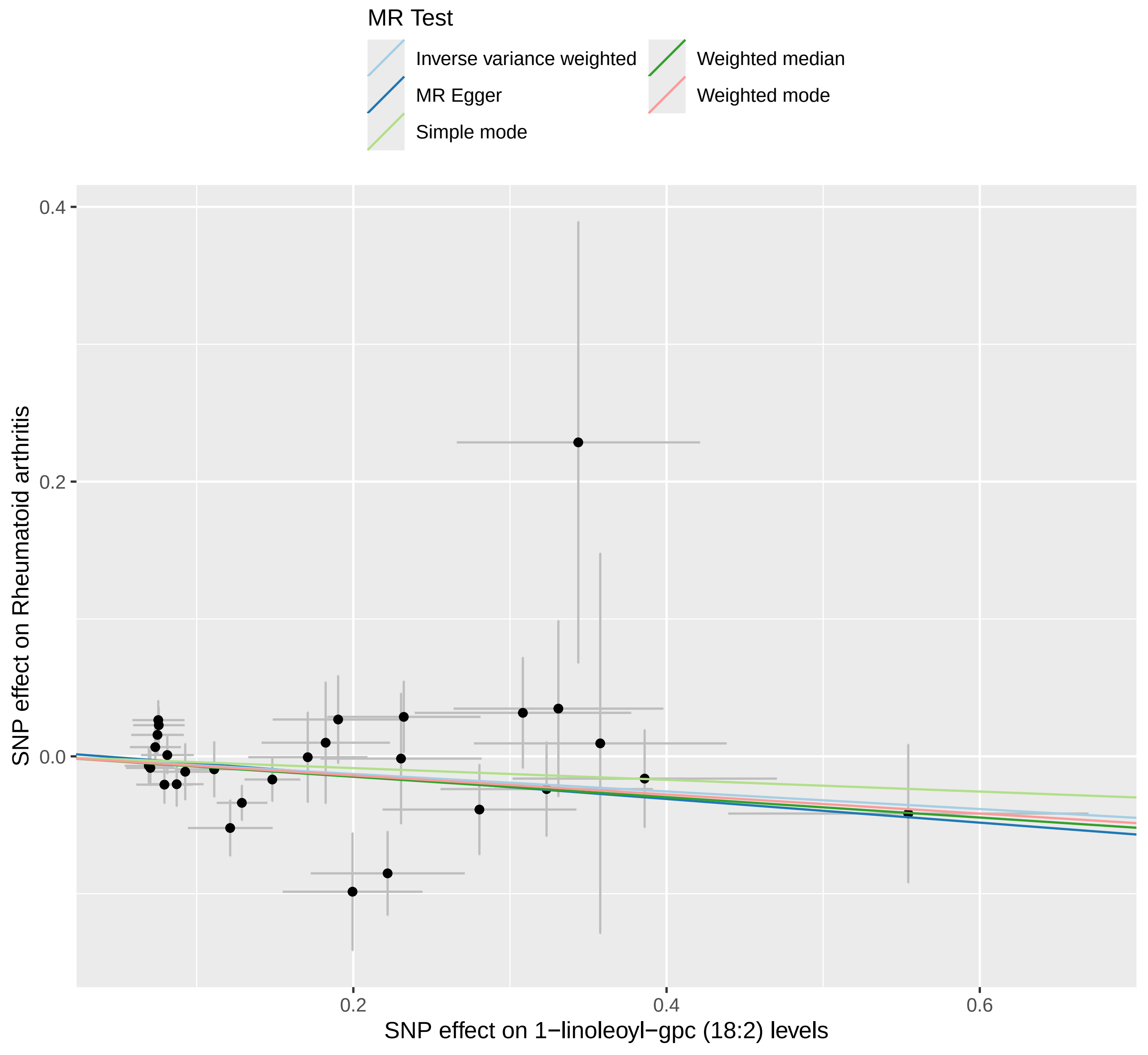
MR Test
Inverse variance weighted
MR Egger
Simple mode
Weighted median
Weighted mode
SNP effect on Rheumatoid arthritis
0.4
0.2
0.0
0.2
0.4
0.6
SNP effect on 1−linoleoyl−gpc (18:2) levels

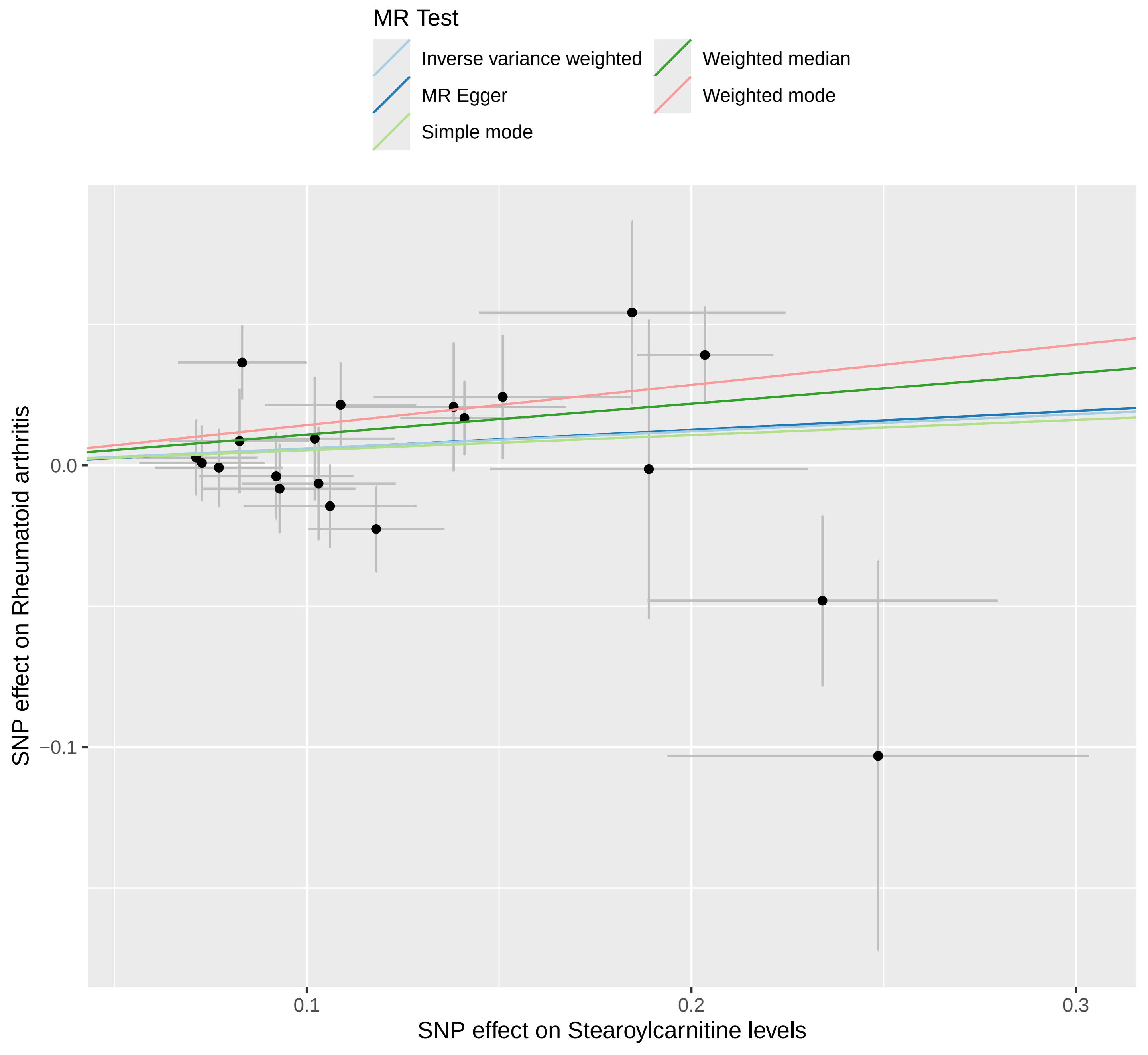

MR Test
Inverse variance weighted
MR Egger
Simple mode
Weighted median
Weighted mode
SNP effect on Rheumatoid arthritis
0.0
−0.1
0.1
0.2
0.3
SNP effect on Stearoylcarnitine levels

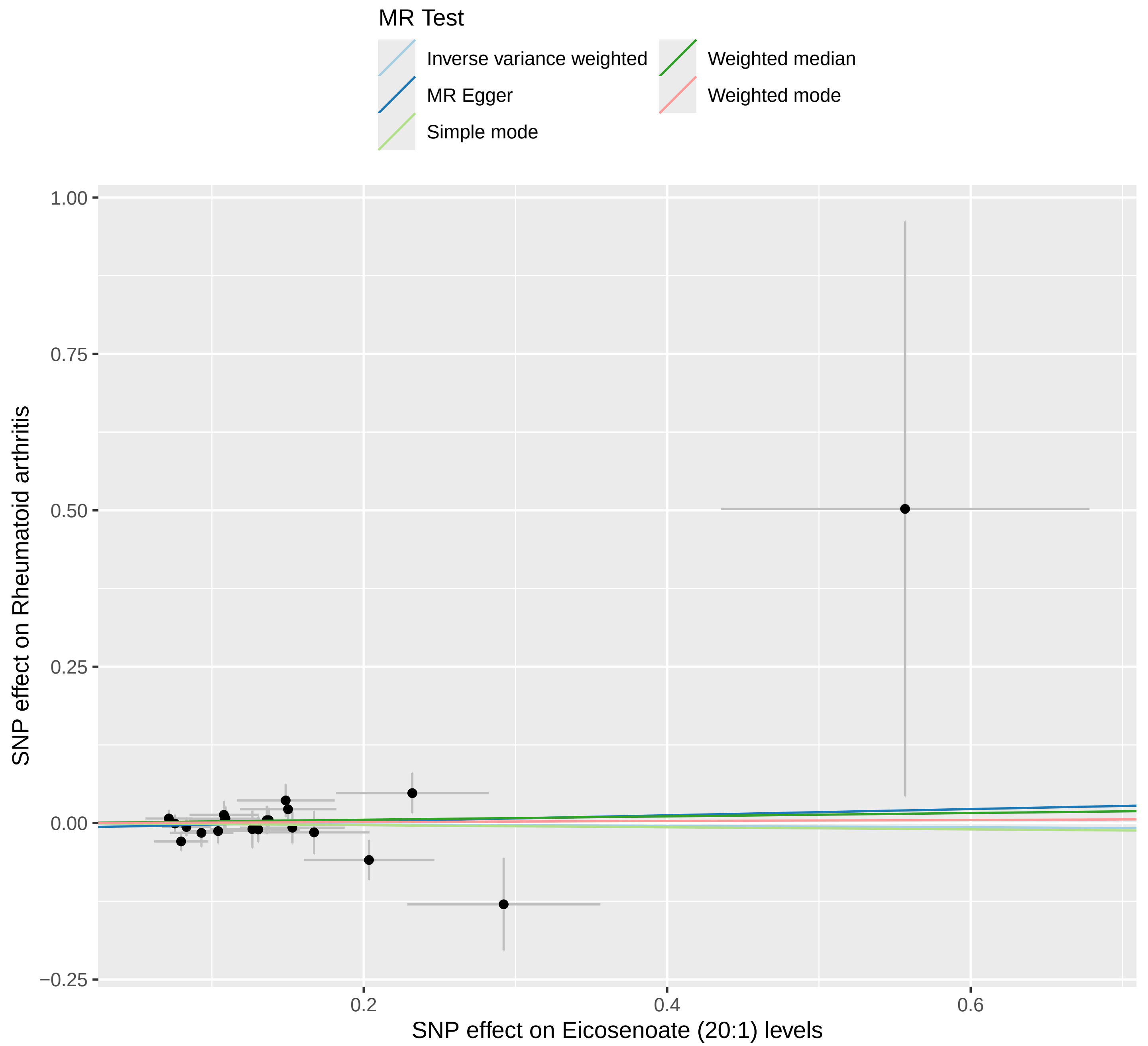
MR Test
Inverse variance weighted
MR Egger
Simple mode
Weighted median
Weighted mode
1.00
0.75
0.50
0.25
0.00
−0.25
0.2
0.4
0.6
SNP effect on Rheumatoid arthritis
SNP effect on Eicosenoate (20:1) levels

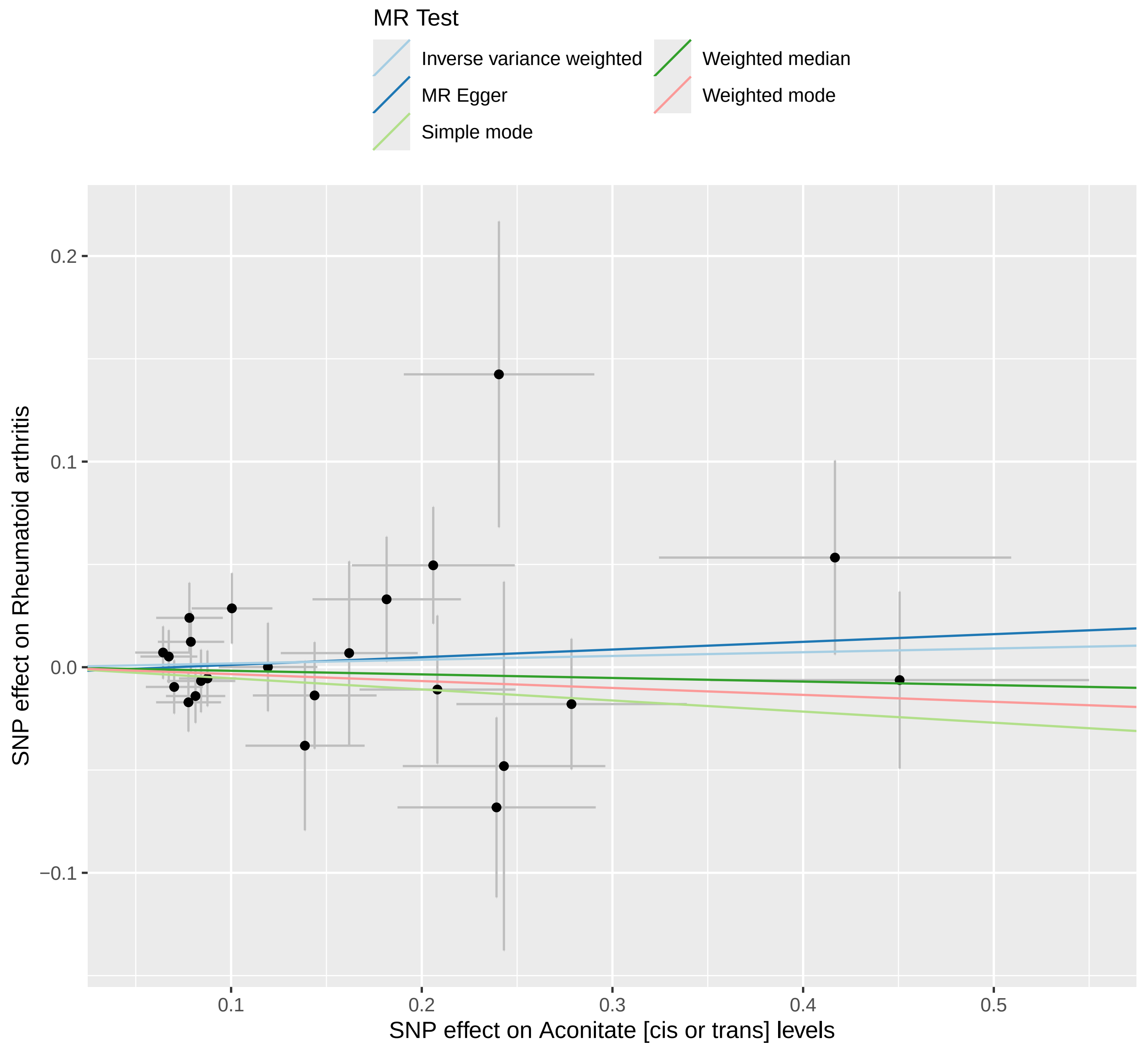

MR Test
Inverse variance weighted
MR Egger
Simple mode
Weighted median
Weighted mode
0.2
0.1
0.0
−0.1
SNP effect on Rheumatoid arthritis
0.1
0.2
0.3
0.4
0.5
SNP effect on Aconitate [cis or trans] levels

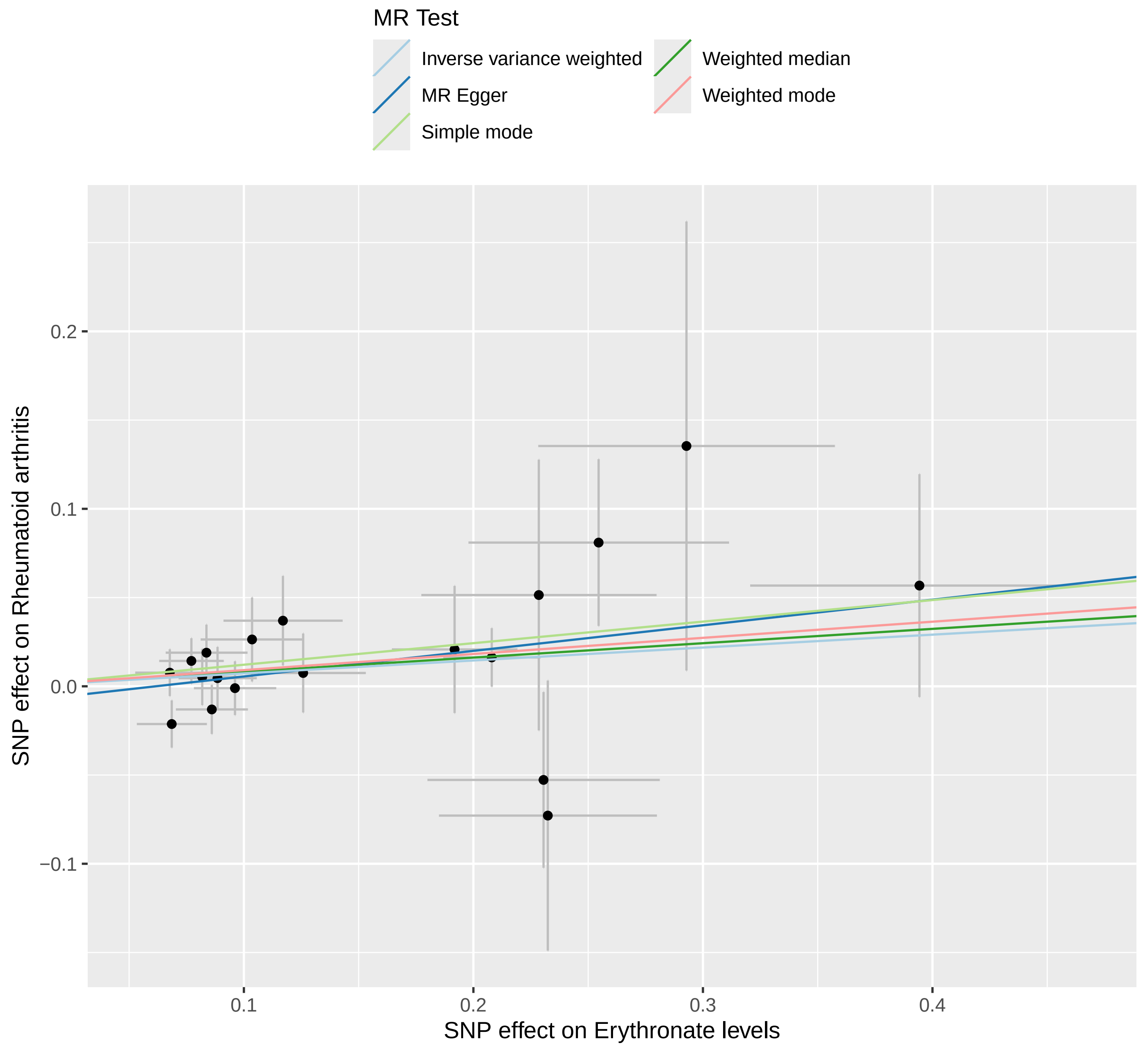
MR Test
Inverse variance weighted
MR Egger
Simple mode
Weighted median
Weighted mode
0.2
0.1
0.0
−0.1
SNP effect on Rheumatoid arthritis
0.1
0.2
0.3
0.4
SNP effect on Erythronate levels

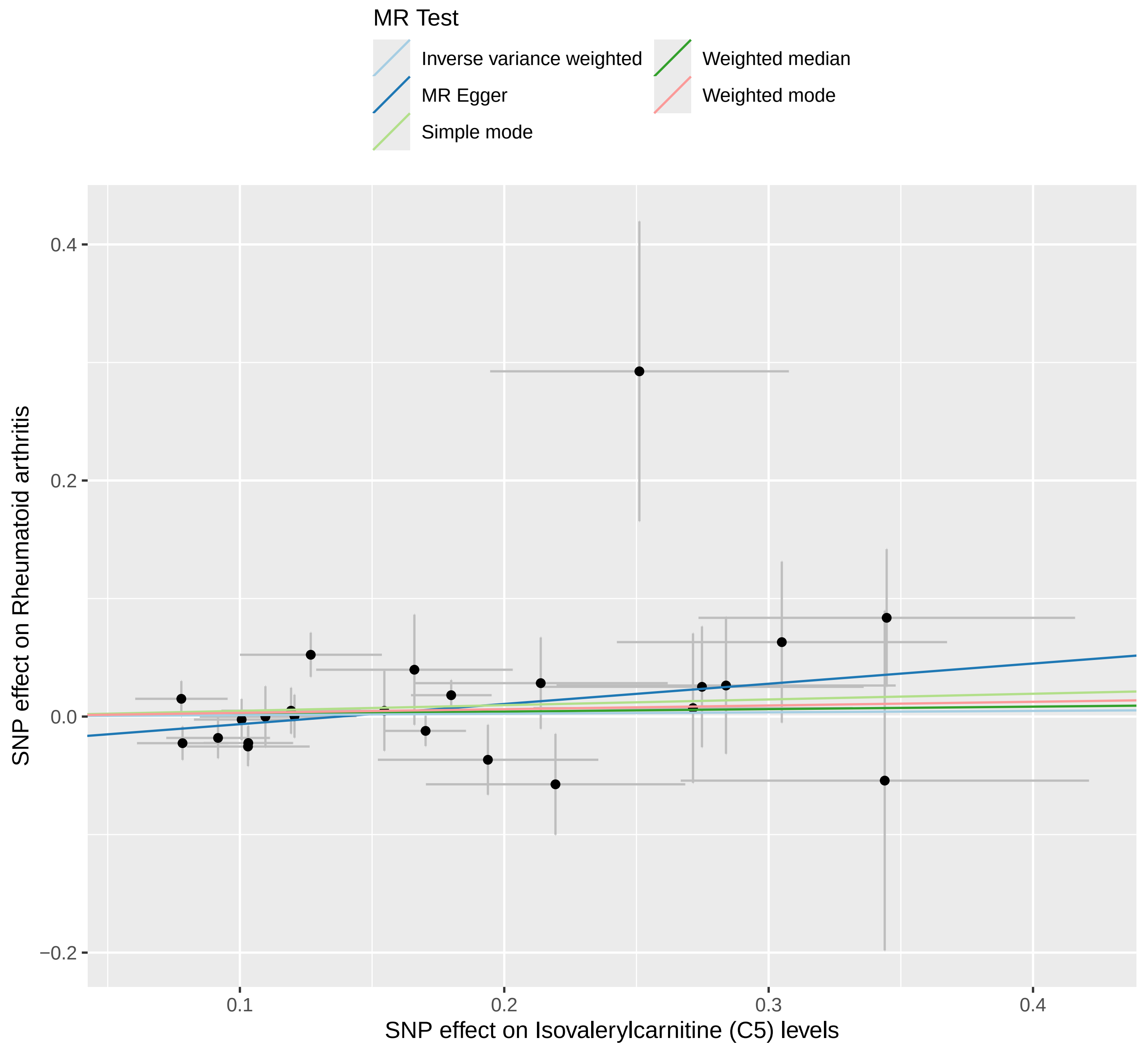

MR Test
Inverse variance weighted
MR Egger
Simple mode
Weighted median
Weighted mode
0.4
0.2
0.0
−0.2
0.1
0.2
0.3
0.4
SNP effect on Rheumatoid arthritis
SNP effect on Isovalerylcarnitine (C5) levels

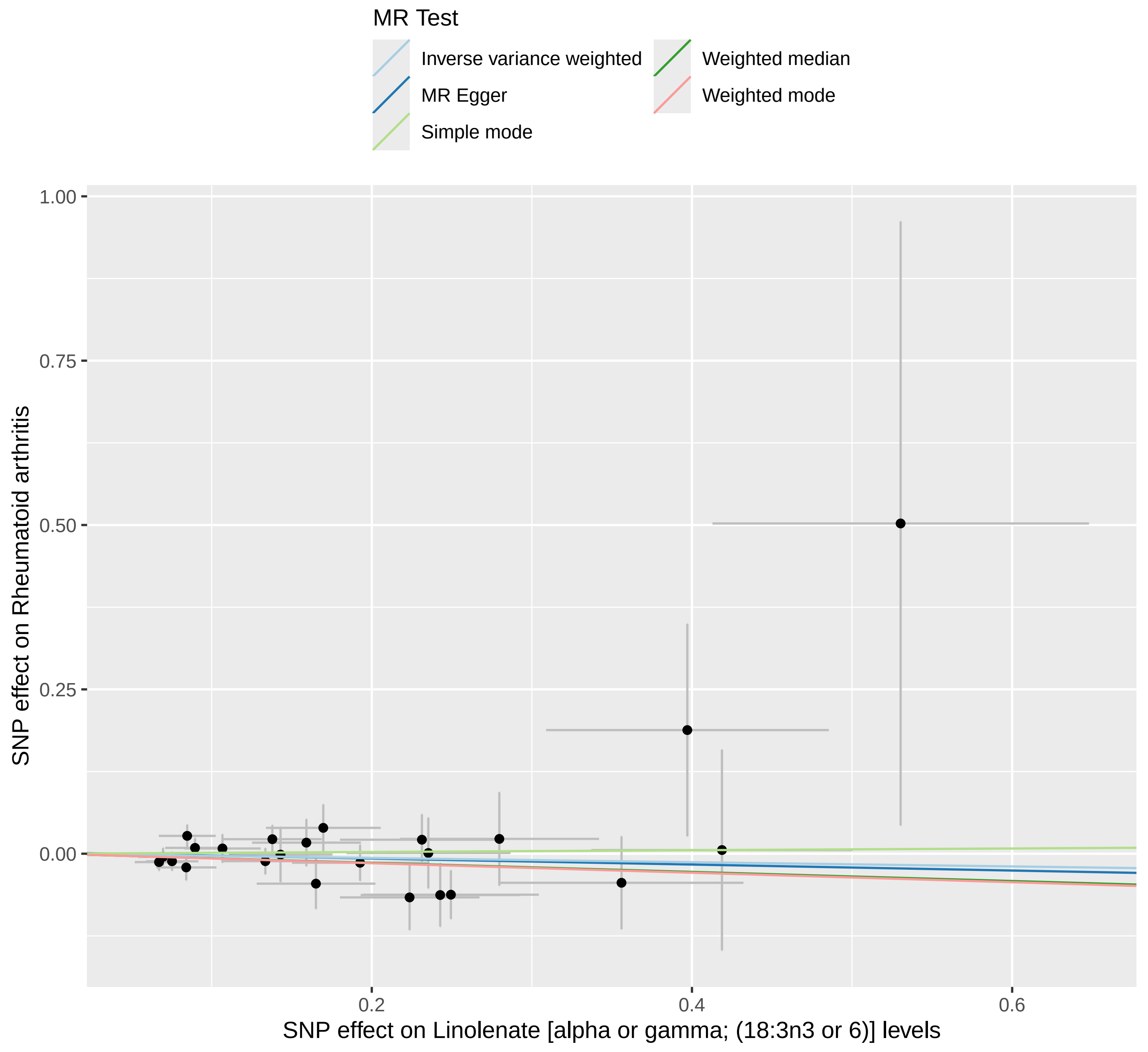

MR Test
Inverse variance weighted
MR Egger
Simple mode
Weighted median
Weighted mode
1.00
0.75
0.50
0.25
0.00
0.2
0.4
0.6
SNP effect on Rheumatoid arthritis
SNP effect on Linolenate [alpha or gamma; (18:3n3 or 6)] levels

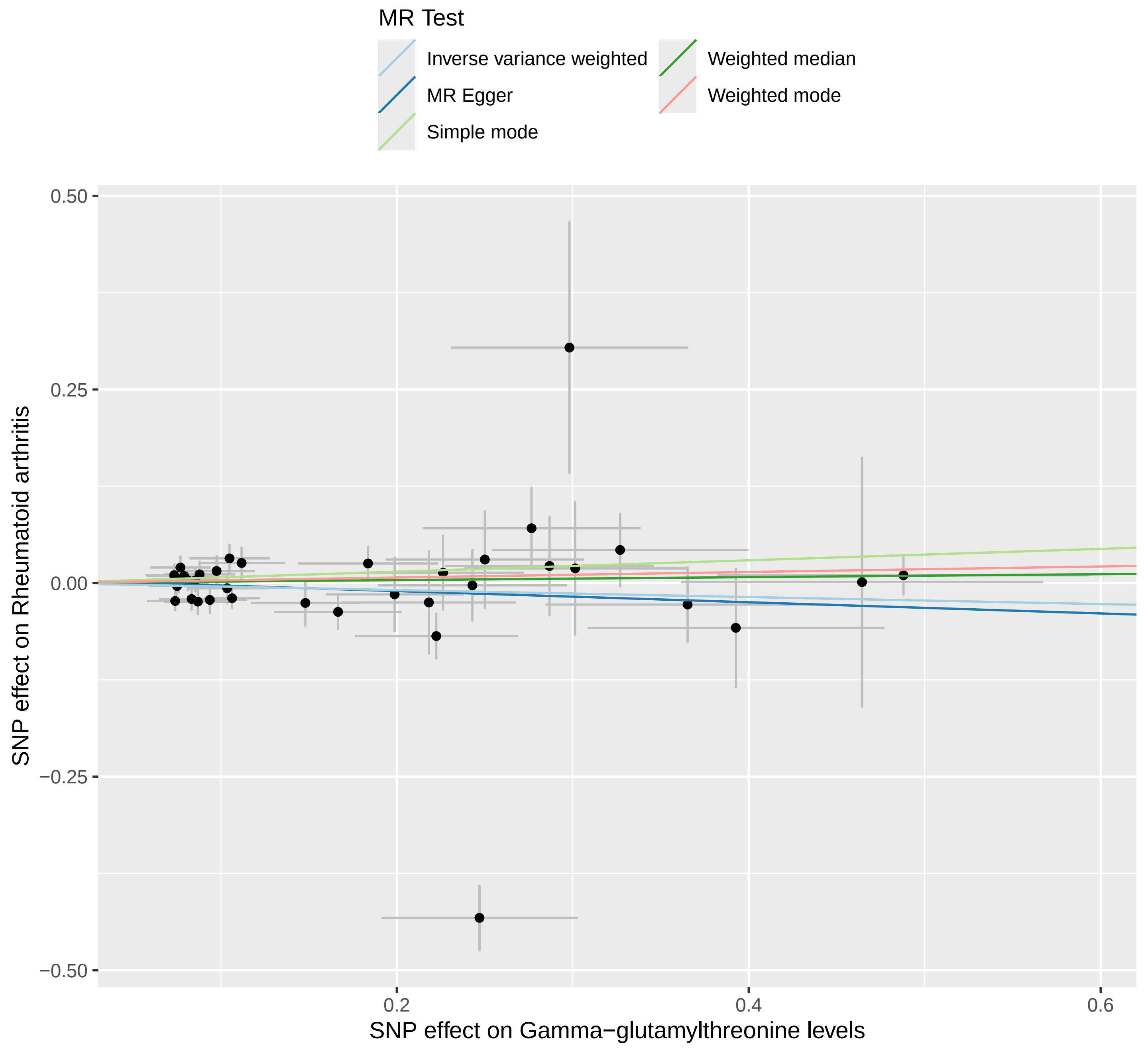
MR Test
Inverse variance weighted
MR Egger
Simple mode
Weighted median
Weighted mode
SNP effect on Rheumatoid arthritis
0.50
0.25
0.00
−0.25
−0.50
0.2
0.4
0.6
SNP effect on Gamma−glutamylthreonine levels

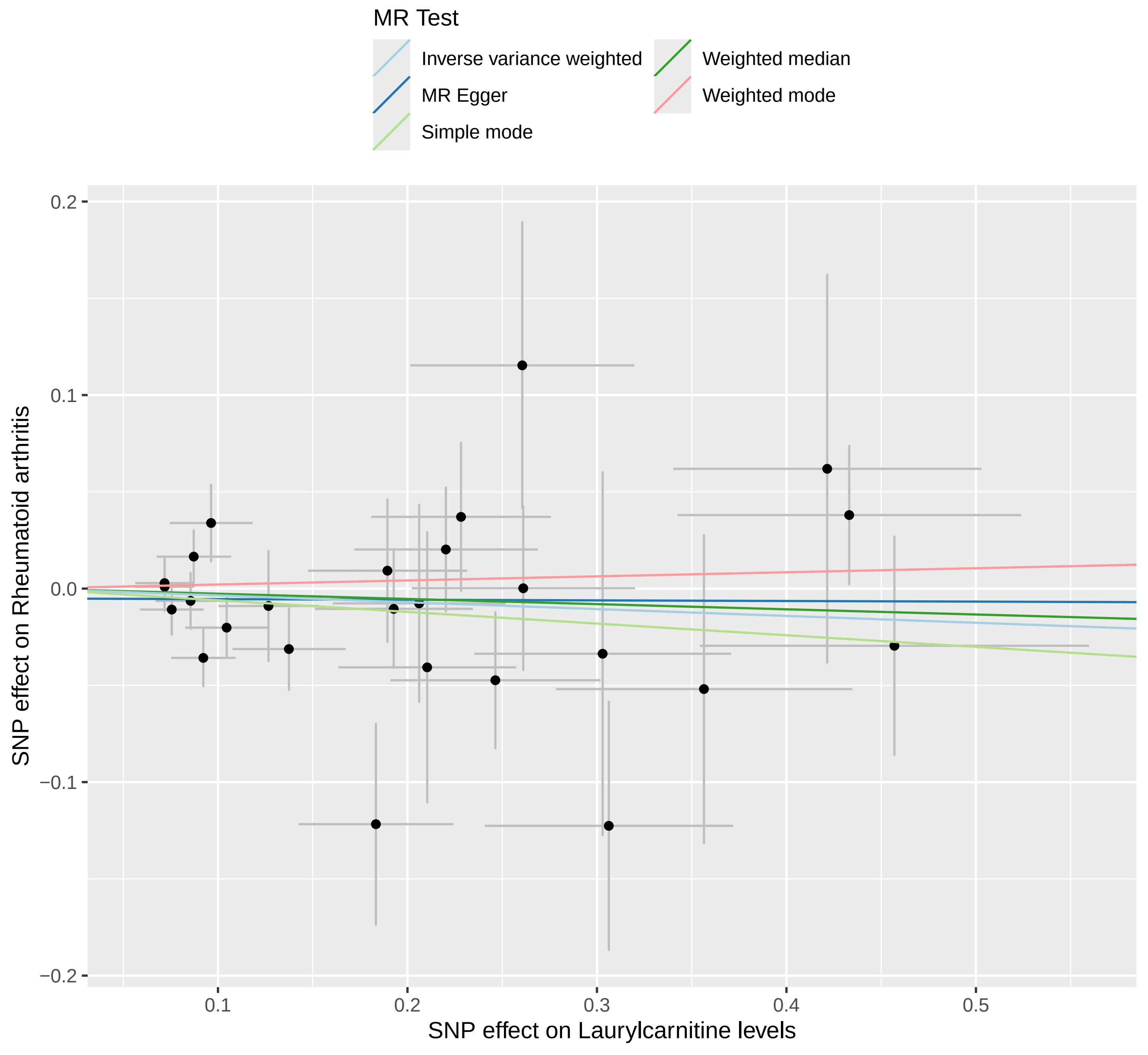
MR Test
Inverse variance weighted
MR Egger
Simple mode
Weighted median
Weighted mode
0.2
0.1
0.0
−0.1
−0.2
0.1
0.2
0.3
0.4
0.5
SNP effect on Laurylcarnitine levels
SNP effect on Rheumatoid arthritis

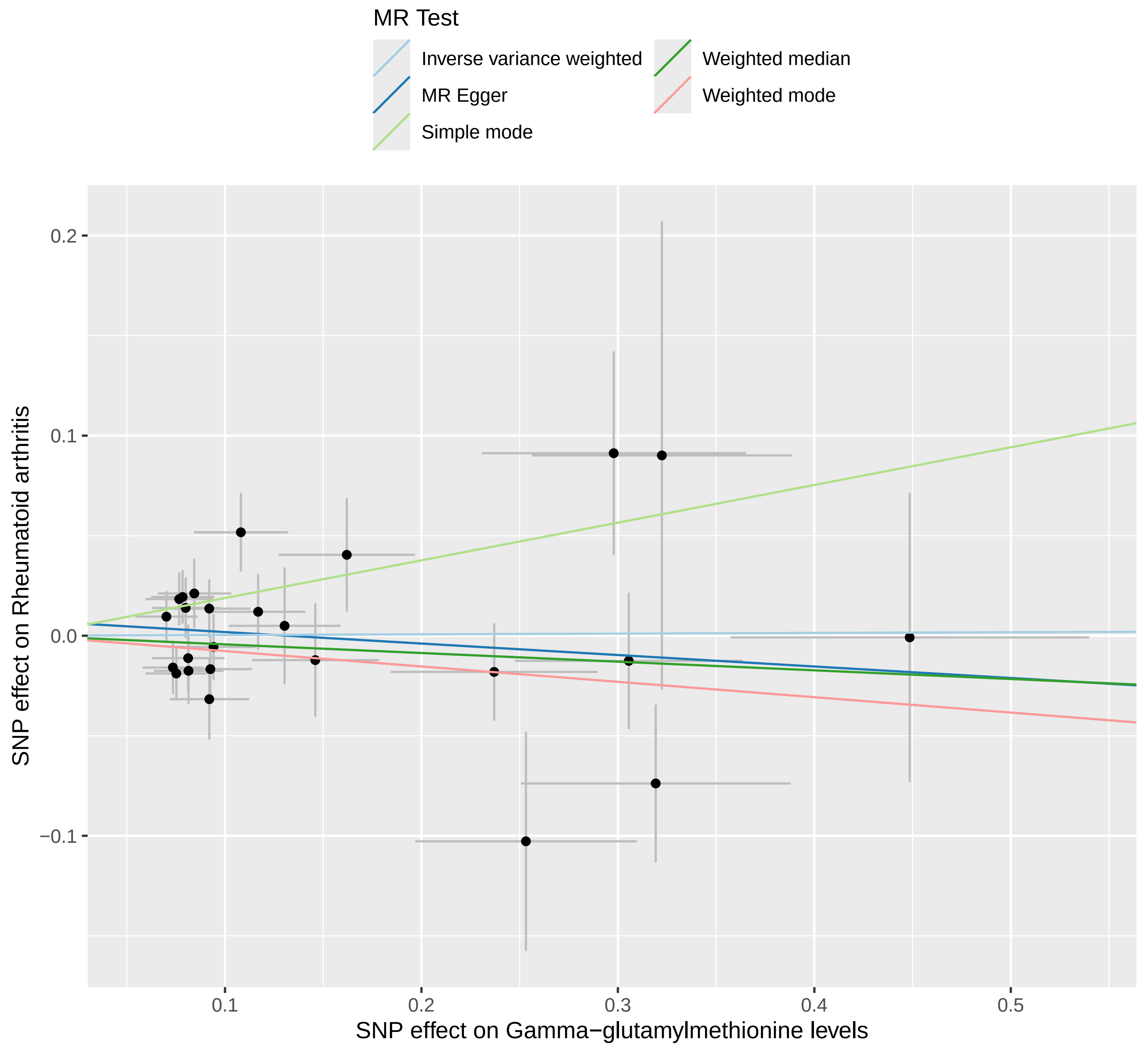

MR Test
Inverse variance weighted
Weighted median
MR Egger
Weighted mode
Simple mode
0.2
0.1
0.0
−0.1
0.1
0.2
0.3
0.4
0.5
SNP effect on Rheumatoid arthritis
SNP effect on Gamma−glutamylmethionine levels

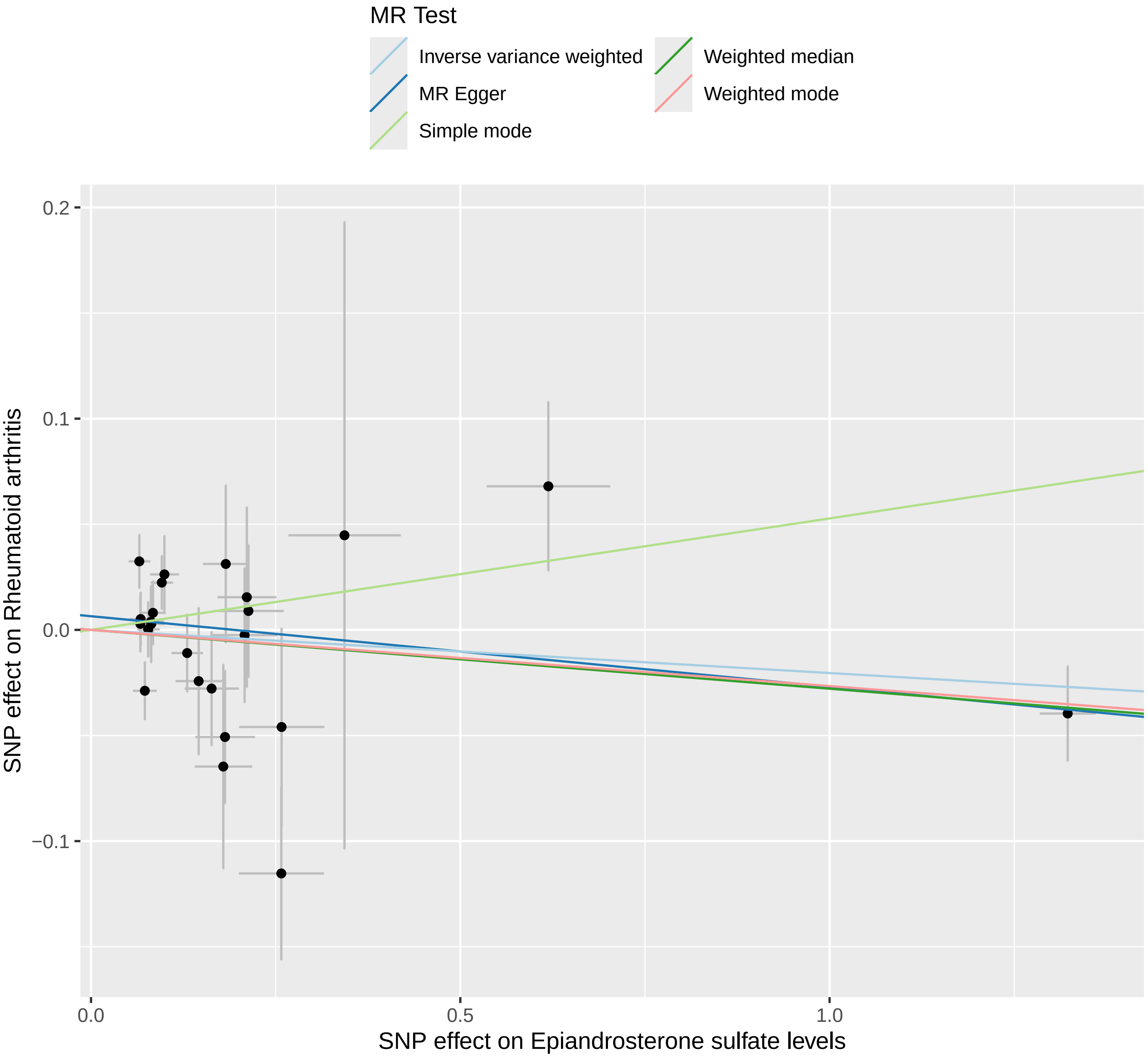
MR Test
Inverse variance weighted
MR Egger
Simple mode
Weighted median
Weighted mode
0.2
0.1
0.0
−0.1
0.0
0.5
1.0
SNP effect on Rheumatoid arthritis
SNP effect on Epiandrosterone sulfate levels

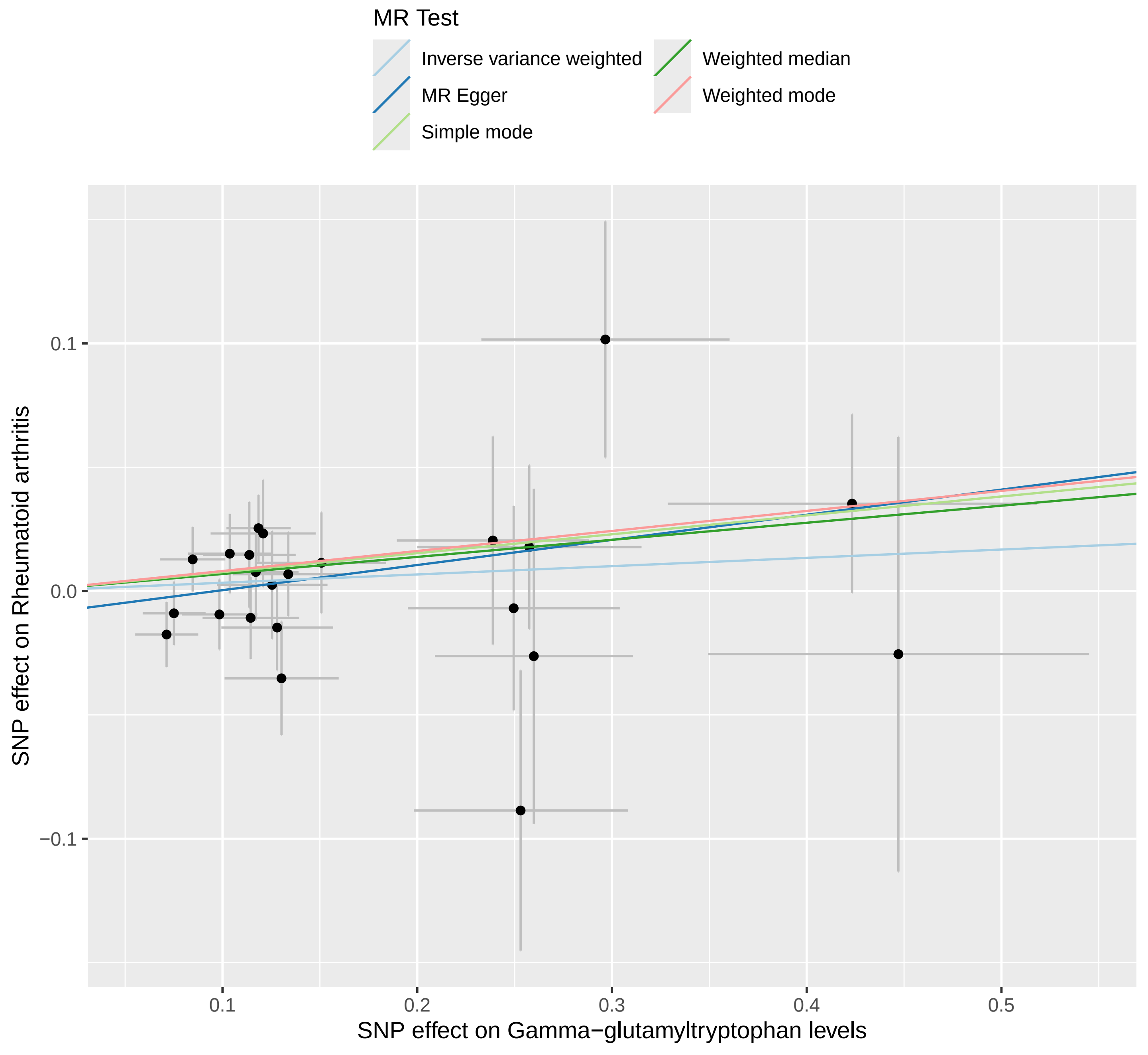
MR Test
Inverse variance weighted
MR Egger
Simple mode
Weighted median
Weighted mode
0.1
0.0
−0.1
SNP effect on Rheumatoid arthritis
0.1
0.2
0.3
0.4
0.5
SNP effect on Gamma−glutamyltryptophan levels

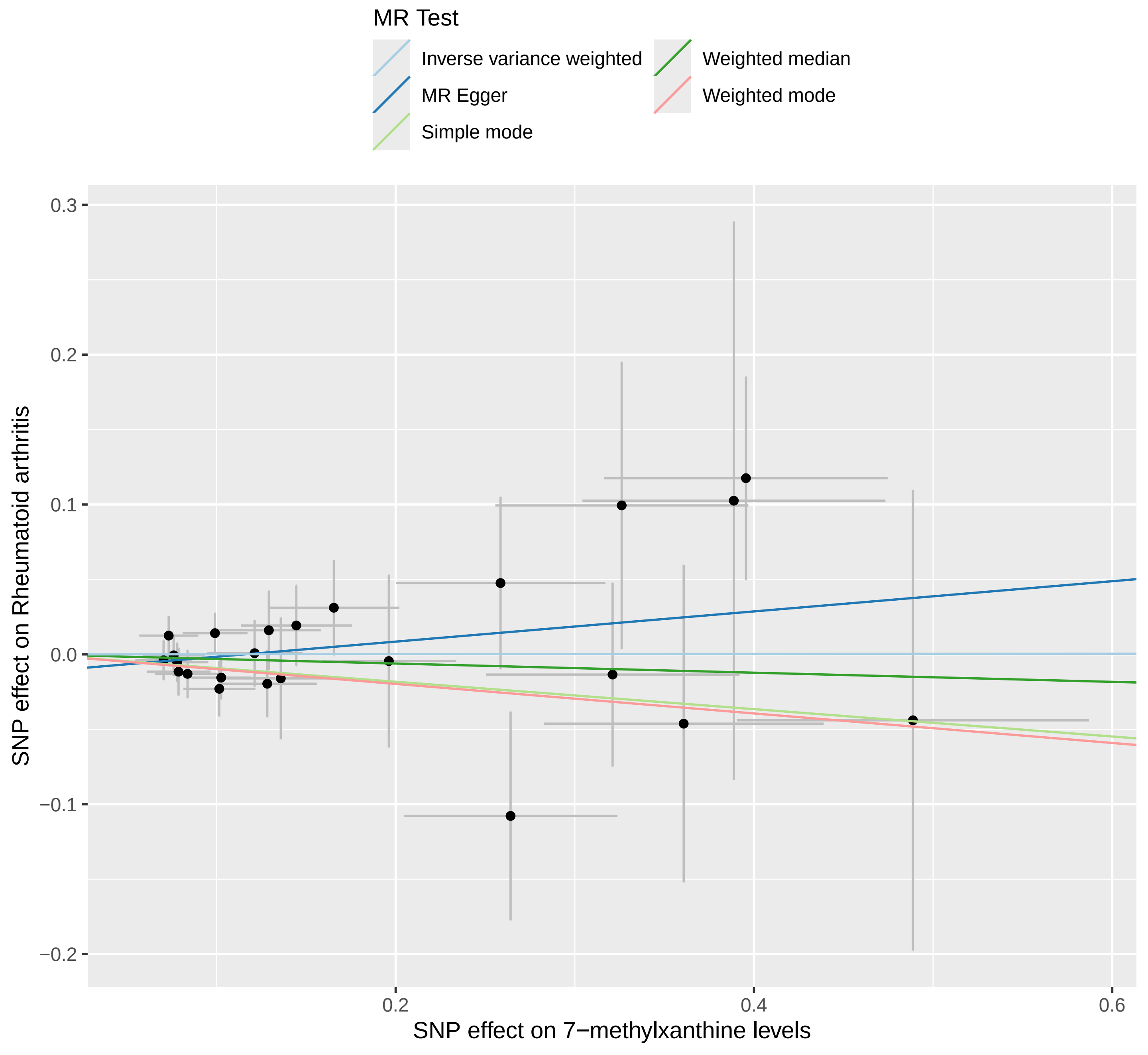

MR Test
Inverse variance weighted
MR Egger
Simple mode
Weighted median
Weighted mode
0.3
0.2
0.1
0.0
−0.1
−0.2
SNP effect on Rheumatoid arthritis
0.2
0.4
0.6
SNP effect on 7−methylxanthine levels

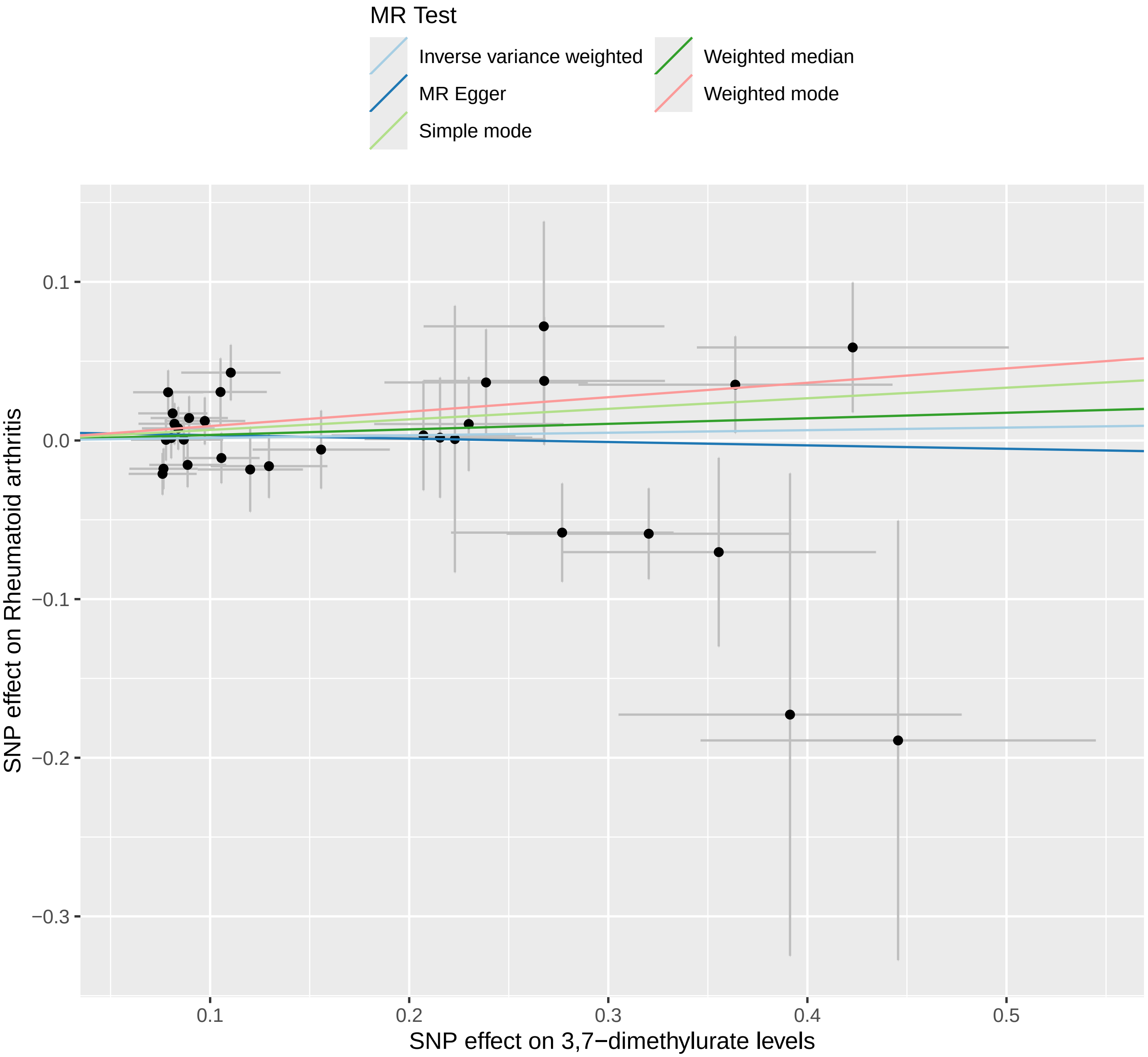
MR Test
Inverse variance weighted
MR Egger
Simple mode
Weighted median
Weighted mode
SNP effect on Rheumatoid arthritis
0.1
0.0
−0.1
−0.2
−0.3
0.1
0.2
0.3
0.4
0.5
SNP effect on 3,7−dimethylurate levels

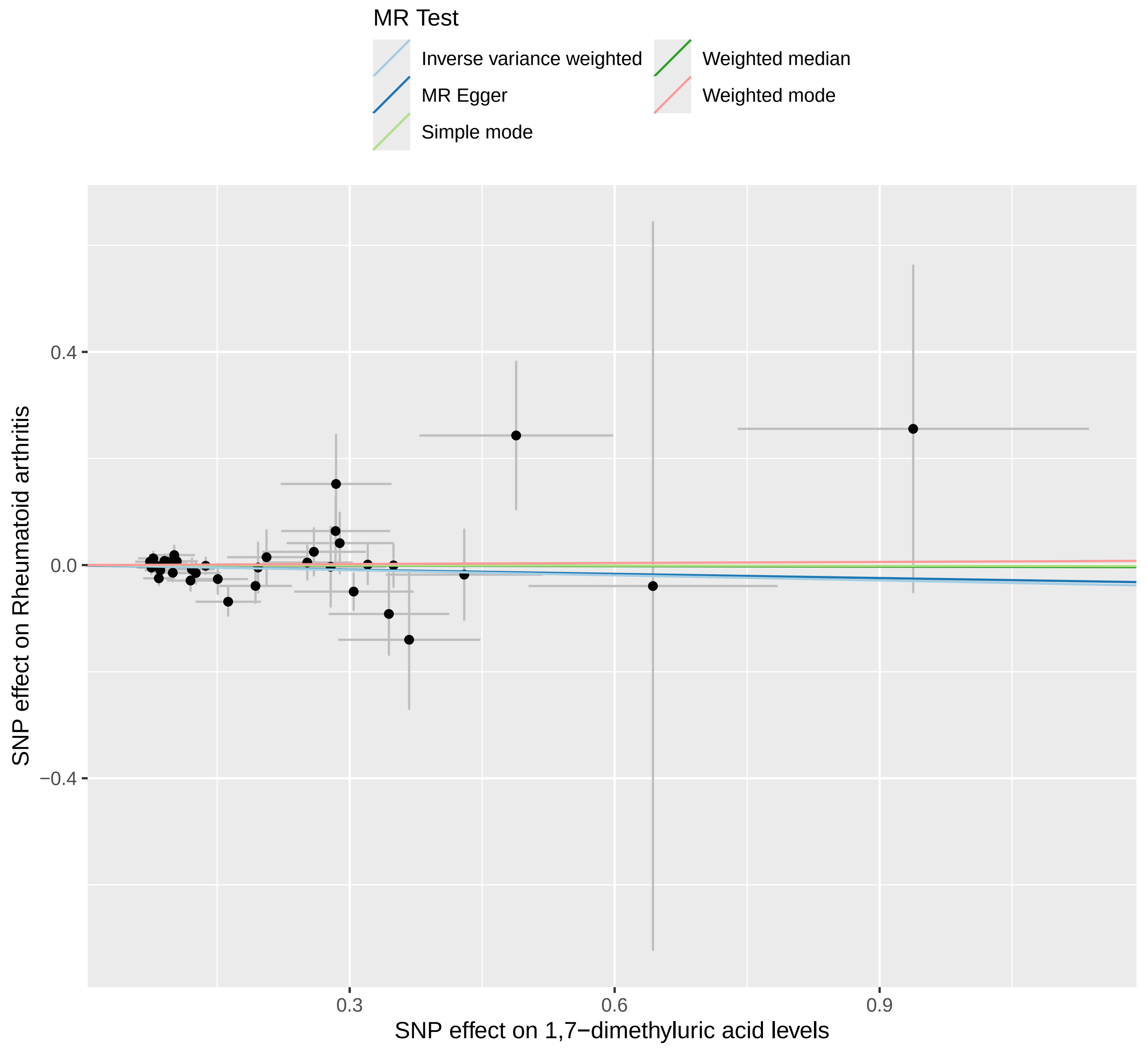
MR Test
Inverse variance weighted
MR Egger
Simple mode
Weighted median
Weighted mode
0.4
0.0
−0.4
0.3
0.6
0.9
SNP effect on Rheumatoid arthritis
SNP effect on 1,7−dimethyluric acid levels

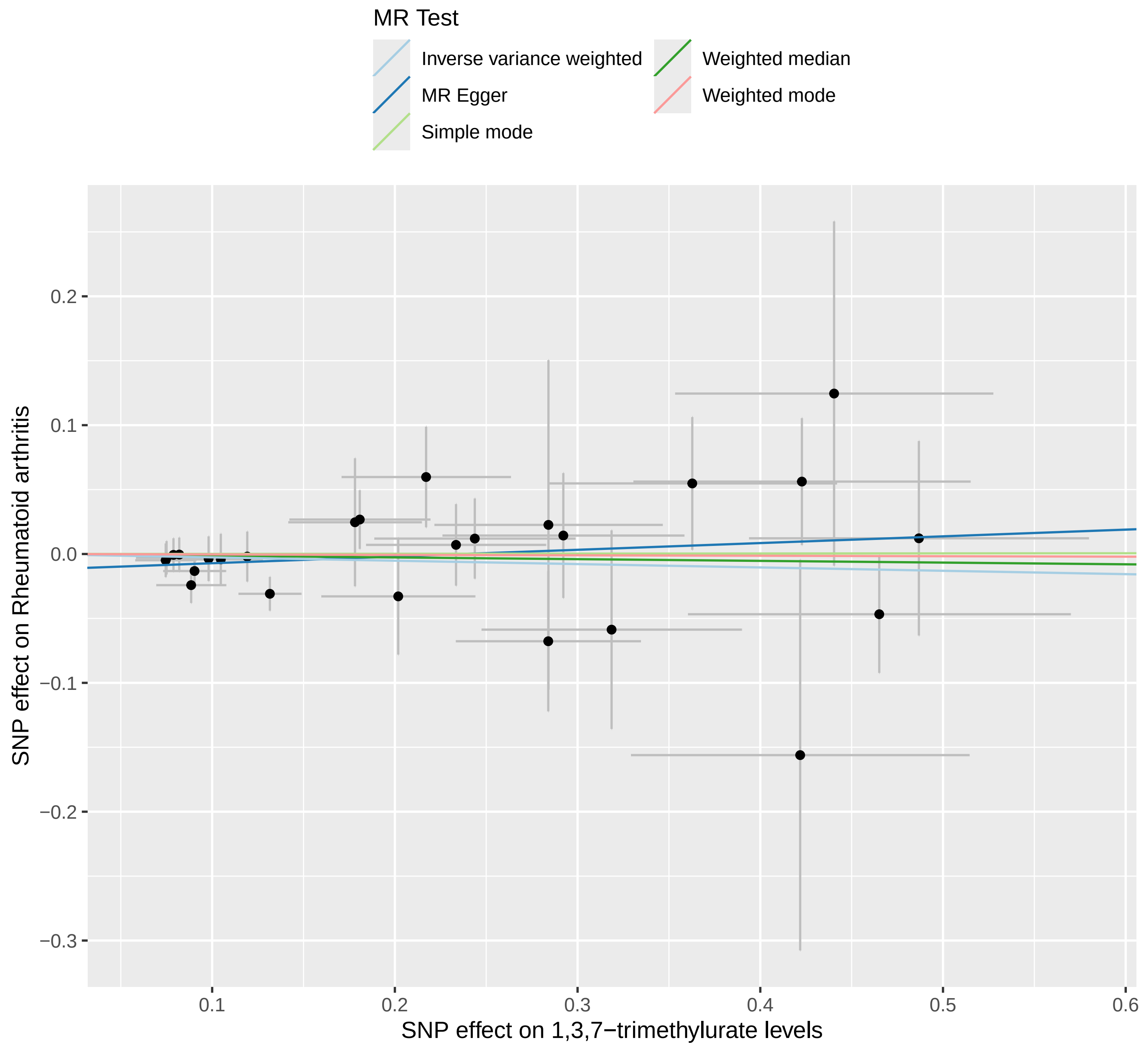

MR Test
Inverse variance weighted
MR Egger
Simple mode
Weighted median
Weighted mode
0.2
0.1
0.0
−0.1
−0.2
−0.3
0.1
0.2
0.3
0.4
0.5
0.6
SNP effect on Rheumatoid arthritis
SNP effect on 1,3,7−trimethylurate levels

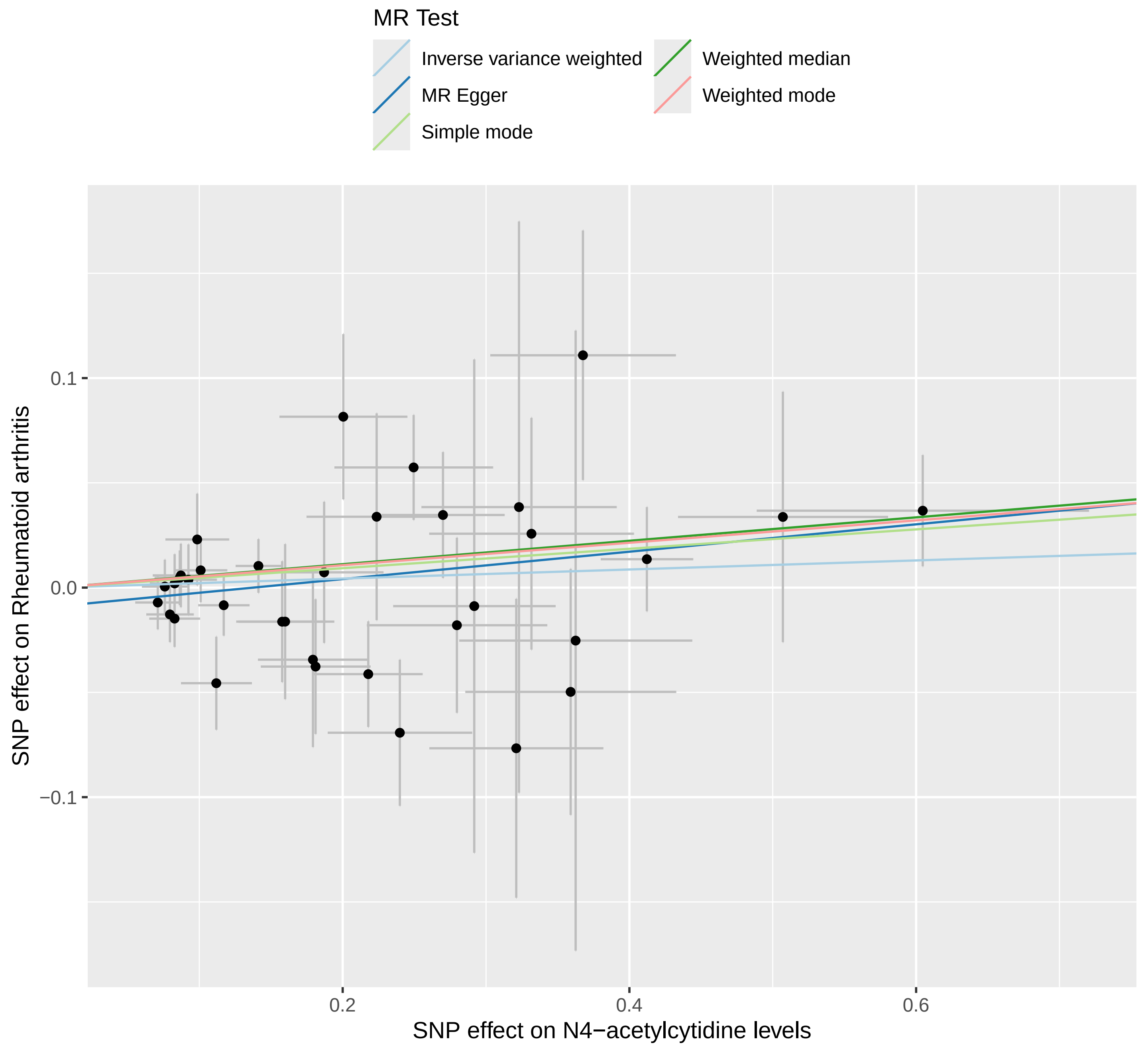
MR Test
Inverse variance weighted
MR Egger
Simple mode
Weighted median
Weighted mode
0.1
0.0
−0.1
0.2
0.4
0.6
SNP effect on Rheumatoid arthritis
SNP effect on N4−acetylcytidine levels

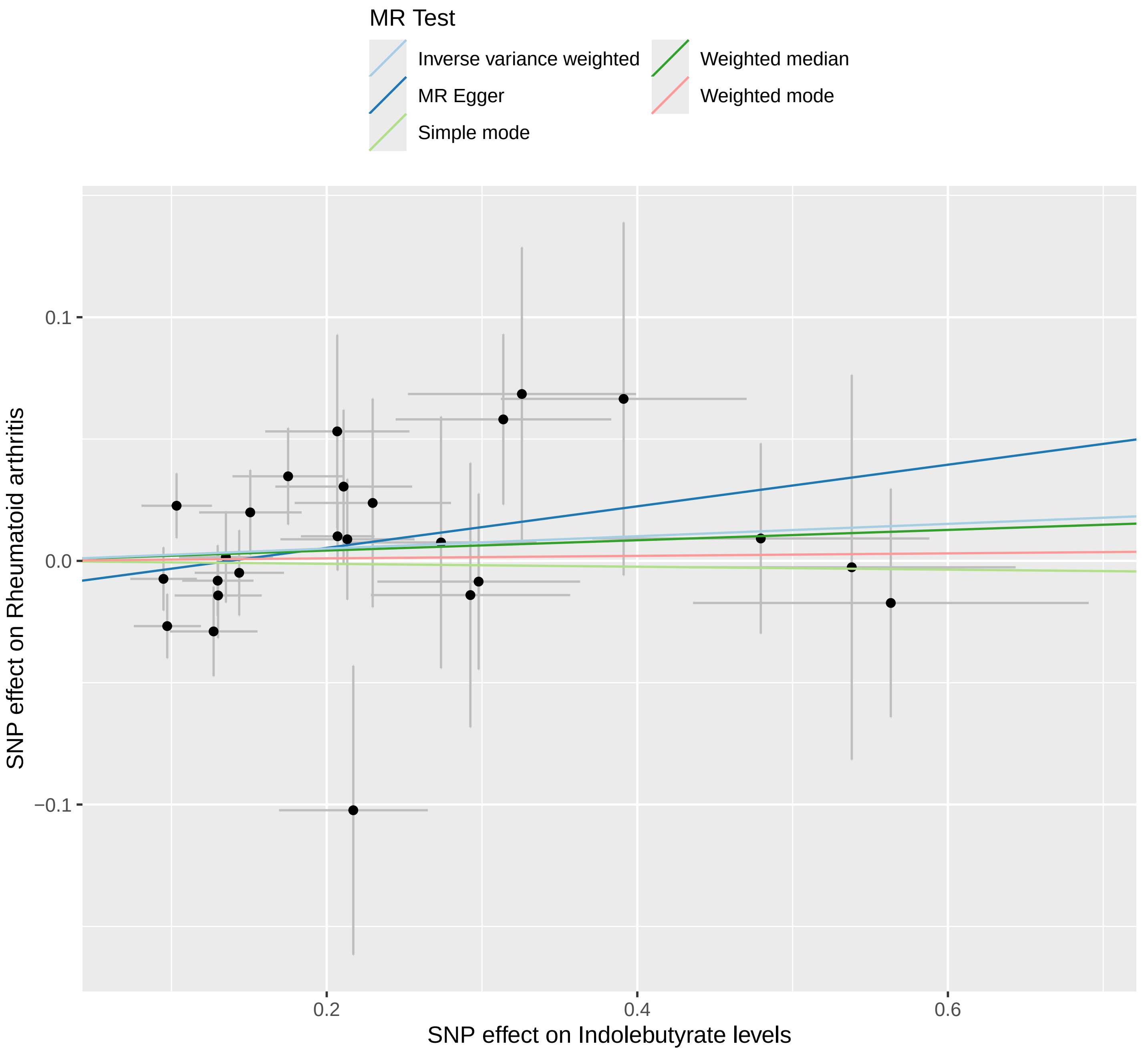

MR Test
Inverse variance weighted
MR Egger
Simple mode
Weighted median
Weighted mode
0.1
0.0
−0.1
0.2
0.4
0.6
SNP effect on Rheumatoid arthritis
SNP effect on Indolebutyrate levels

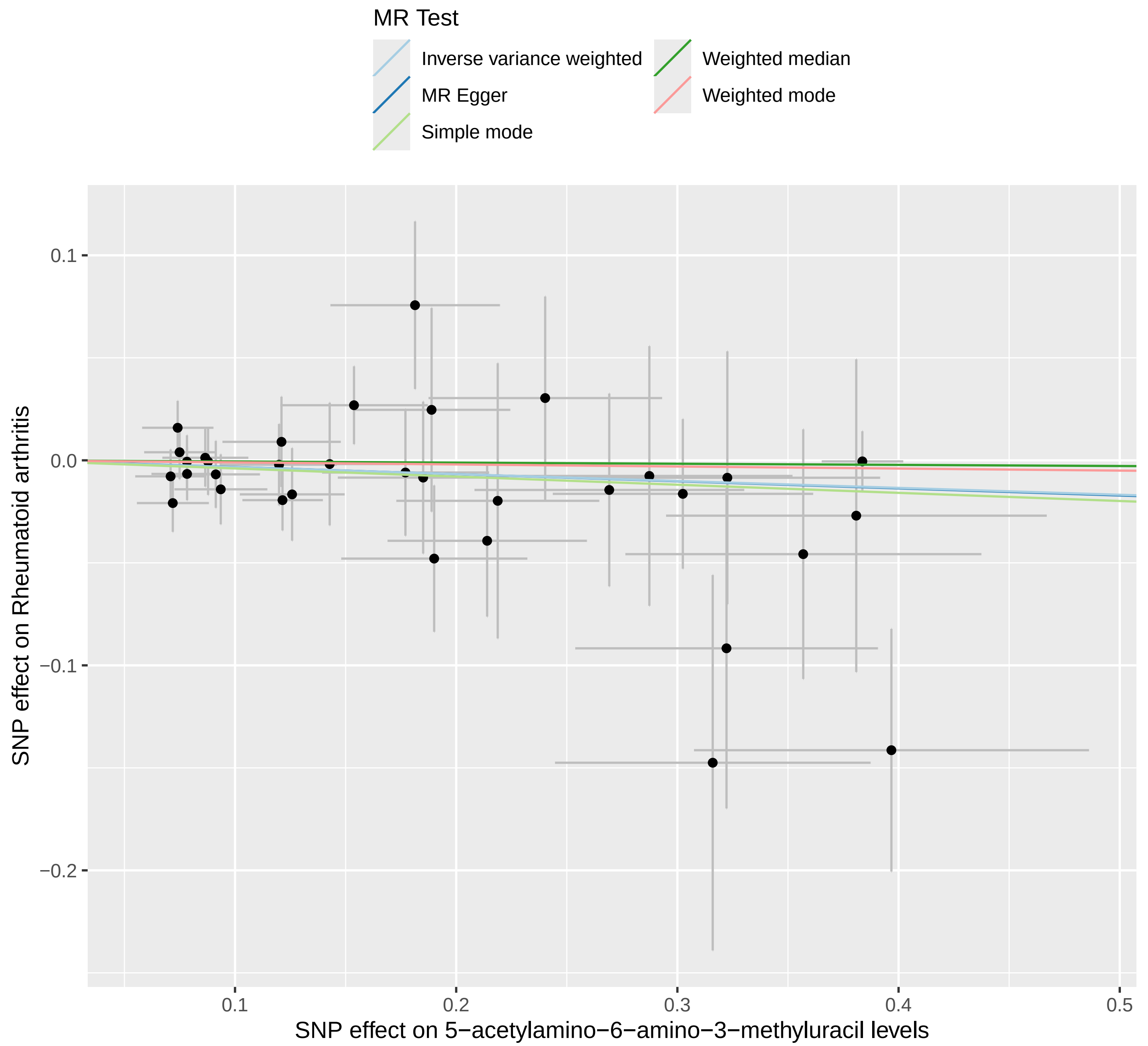
MR Test
Inverse variance weighted
MR Egger
Simple mode
Weighted median
Weighted mode
SNP effect on Rheumatoid arthritis
0.1
0.0
−0.1
−0.2
0.1
0.2
0.3
0.4
0.5
SNP effect on 5−acetylamino−6−amino−3−methyluracil levels

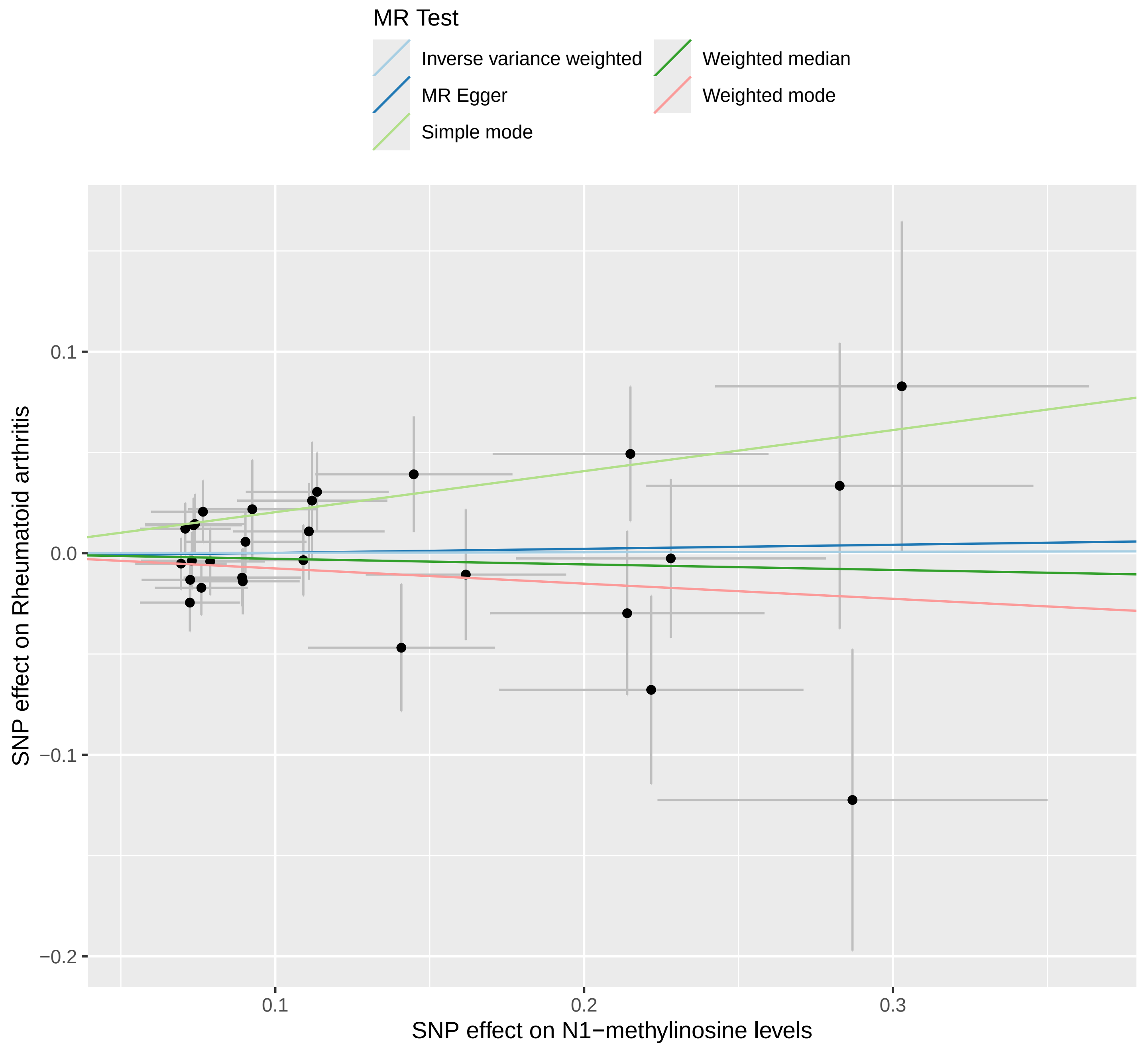

MR Test
Inverse variance weighted
MR Egger
Simple mode
Weighted median
Weighted mode
0.1
0.0
−0.1
−0.2
0.1
0.2
0.3
SNP effect on Rheumatoid arthritis
SNP effect on N1−methylinosine levels

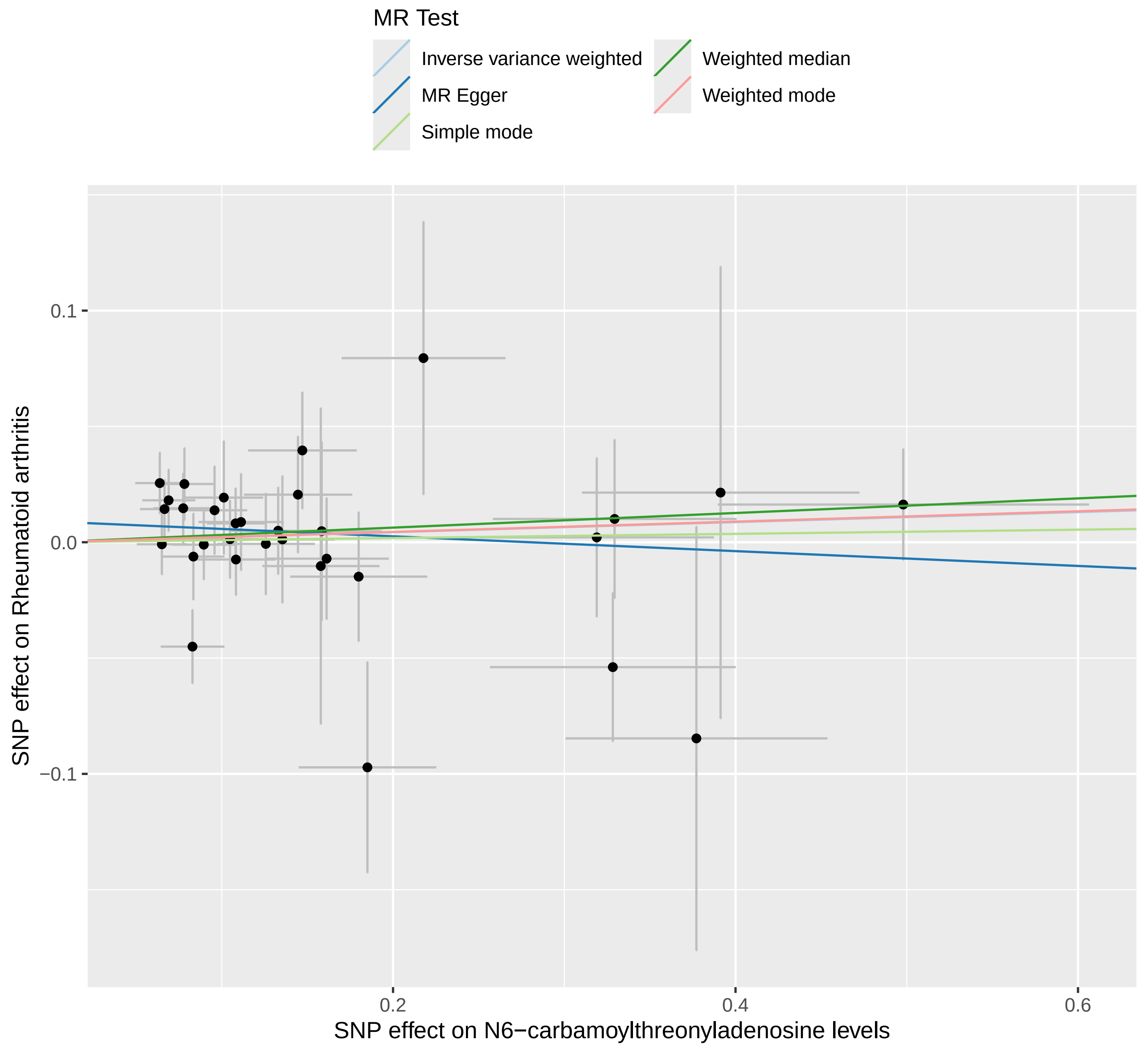

MR Test
Inverse variance weighted
MR Egger
Simple mode
Weighted median
Weighted mode
0.1
0.0
−0.1
0.2
0.4
0.6
SNP effect on Rheumatoid arthritis
SNP effect on N6−carbamoylthreonyladenosine levels

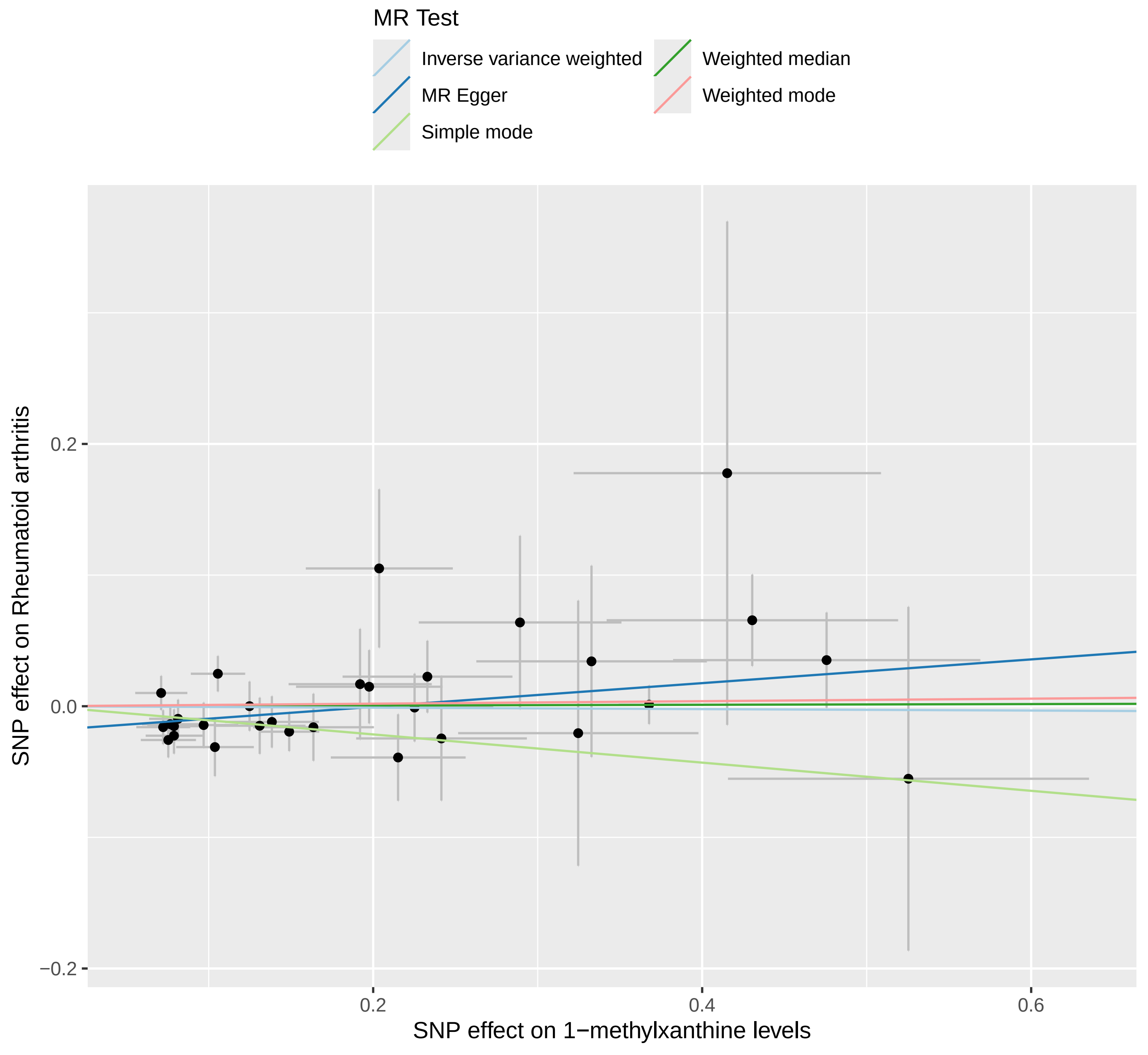

MR Test
Inverse variance weighted
MR Egger
Simple mode
Weighted median
Weighted mode
0.2
0.0
−0.2
0.2
0.4
0.6
SNP effect on Rheumatoid arthritis
SNP effect on 1−methylxanthine levels

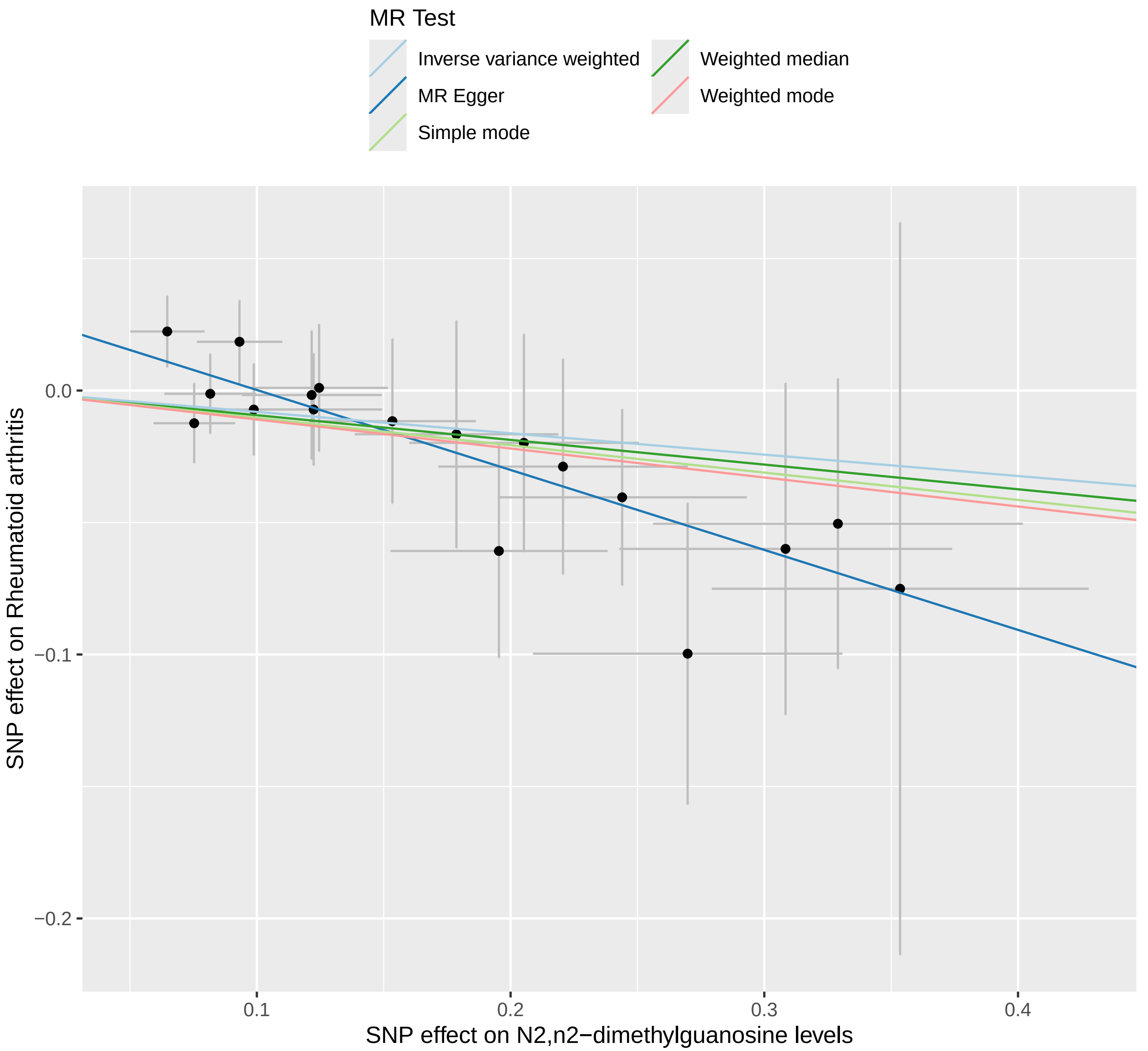
MR Test
Inverse variance weighted
MR Egger
Simple mode
Weighted median
Weighted mode
0.0
−0.1
−0.2
0.1
0.2
0.3
0.4
SNP effect on Rheumatoid arthritis
SNP effect on N2,n2−dimethylguanosine levels

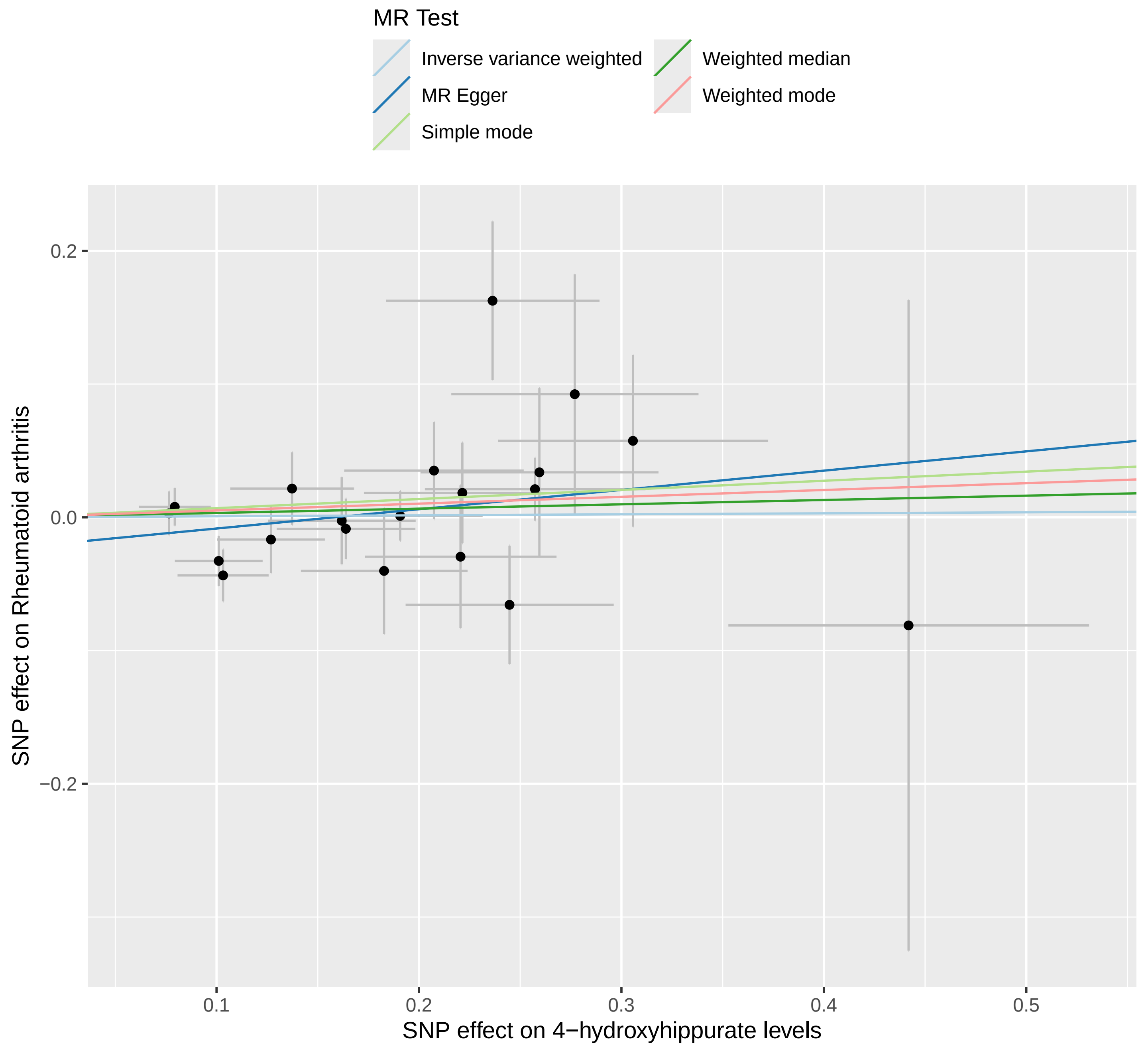

MR Test
Inverse variance weighted
MR Egger
Simple mode
Weighted median
Weighted mode
0.2
0.0
−0.2
0.1
0.2
0.3
0.4
0.5
SNP effect on Rheumatoid arthritis
SNP effect on 4−hydroxyhippurate levels

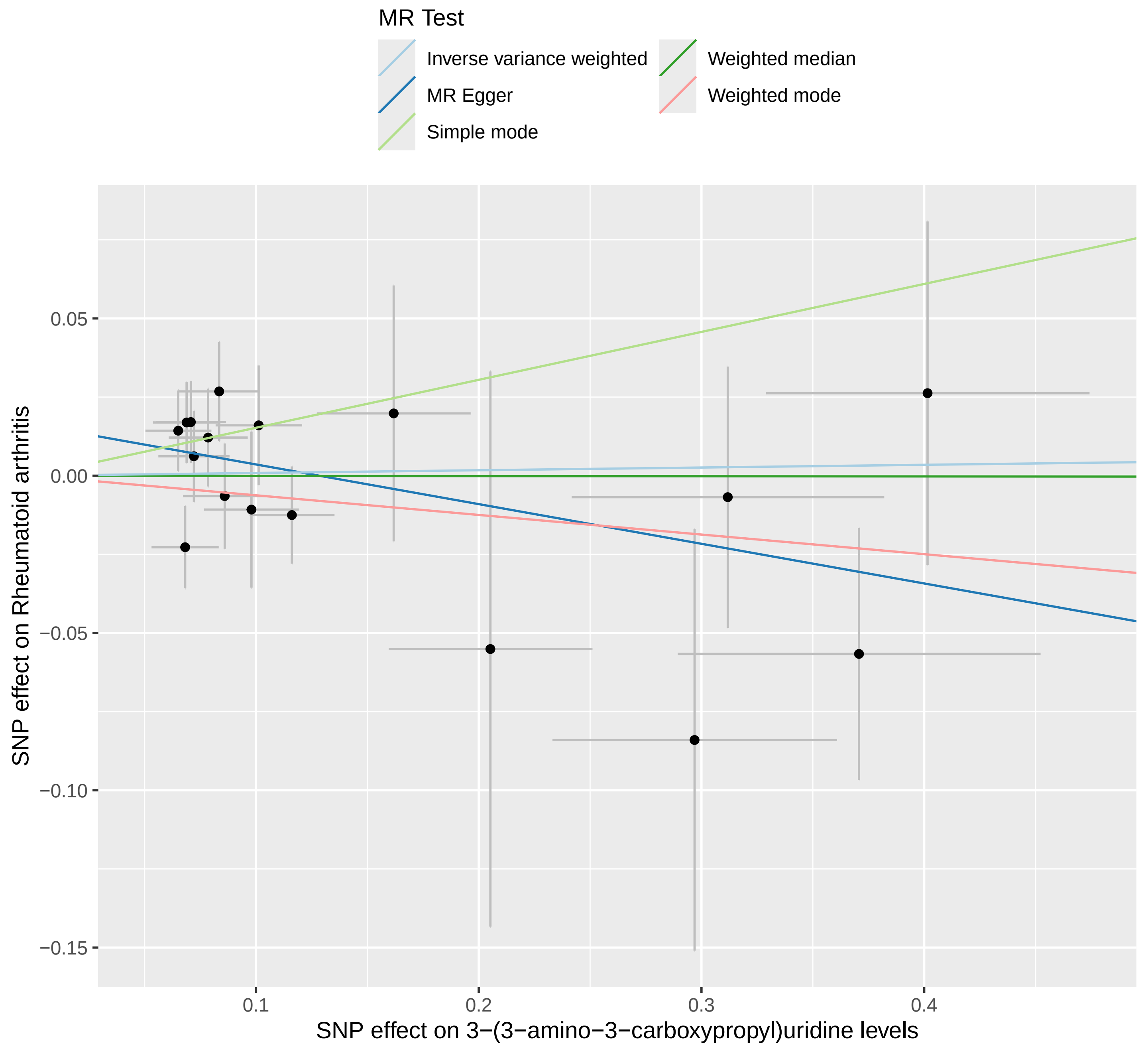

MR Test
Inverse variance weighted
MR Egger
Simple mode
Weighted median
Weighted mode
0.05
0.00
−0.05
−0.10
−0.15
0.1
0.2
0.3
0.4
SNP effect on Rheumatoid arthritis
SNP effect on 3−(3−amino−3−carboxypropyl)uridine levels

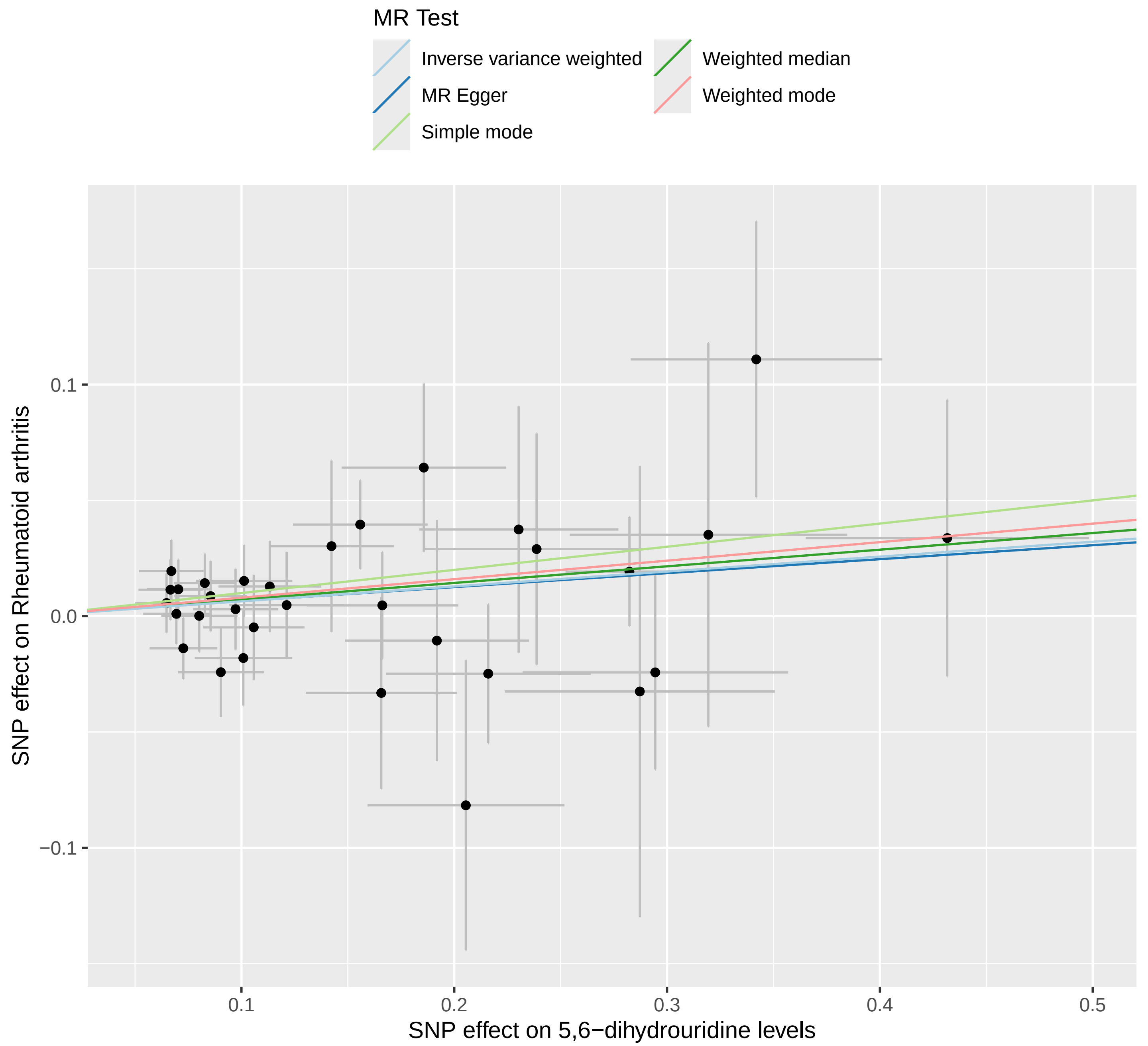

MR Test
Inverse variance weighted
MR Egger
Simple mode
Weighted median
Weighted mode
SNP effect on Rheumatoid arthritis
0.1
0.0
−0.1
0.1
0.2
0.3
0.4
0.5
SNP effect on 5,6−dihydrouridine levels

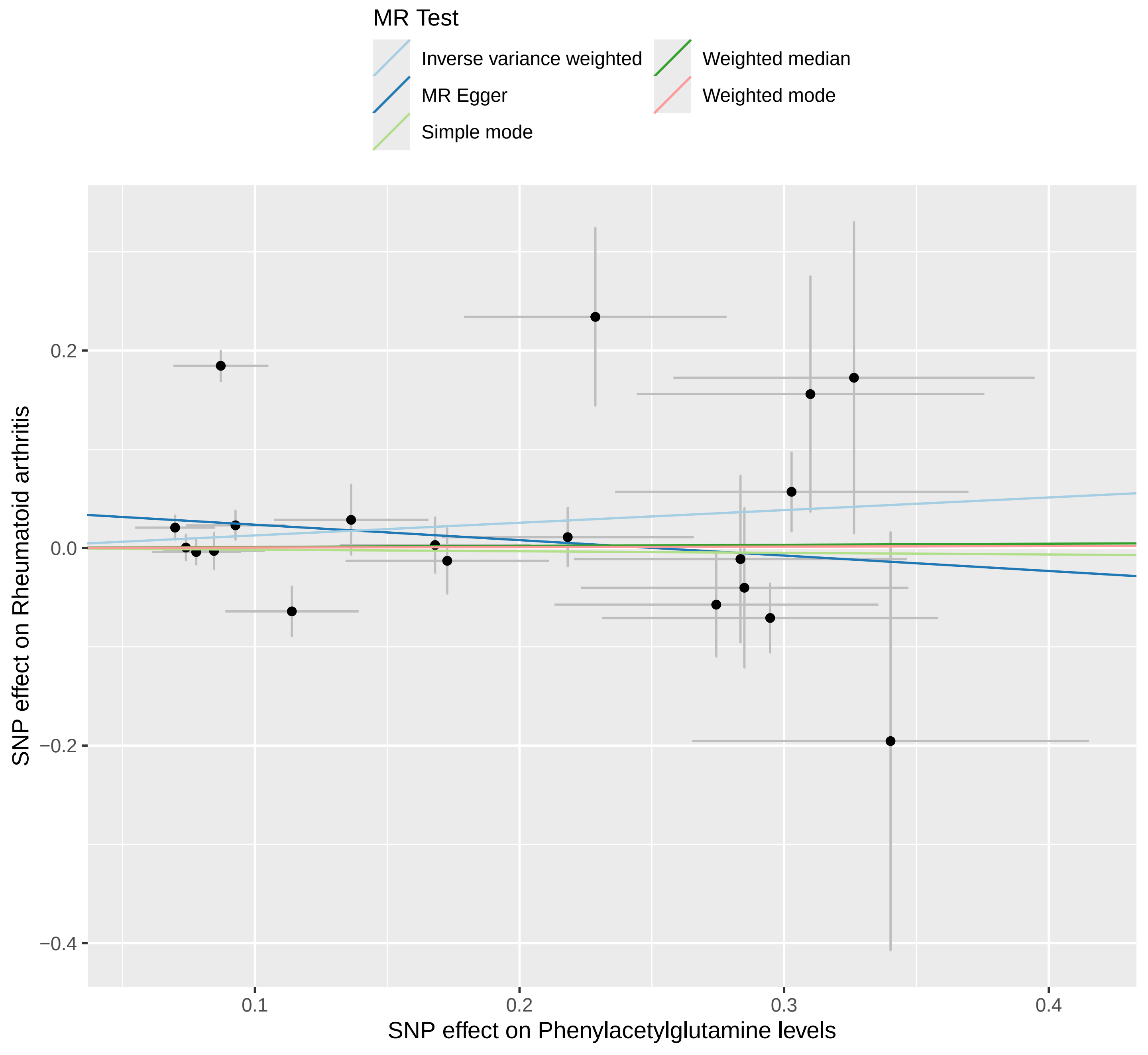
MR Test
Inverse variance weighted
MR Egger
Simple mode
Weighted median
Weighted mode
0.2
0.0
−0.2
−0.4
SNP effect on Rheumatoid arthritis
0.1
0.2
0.3
0.4
SNP effect on Phenylacetylglutamine levels

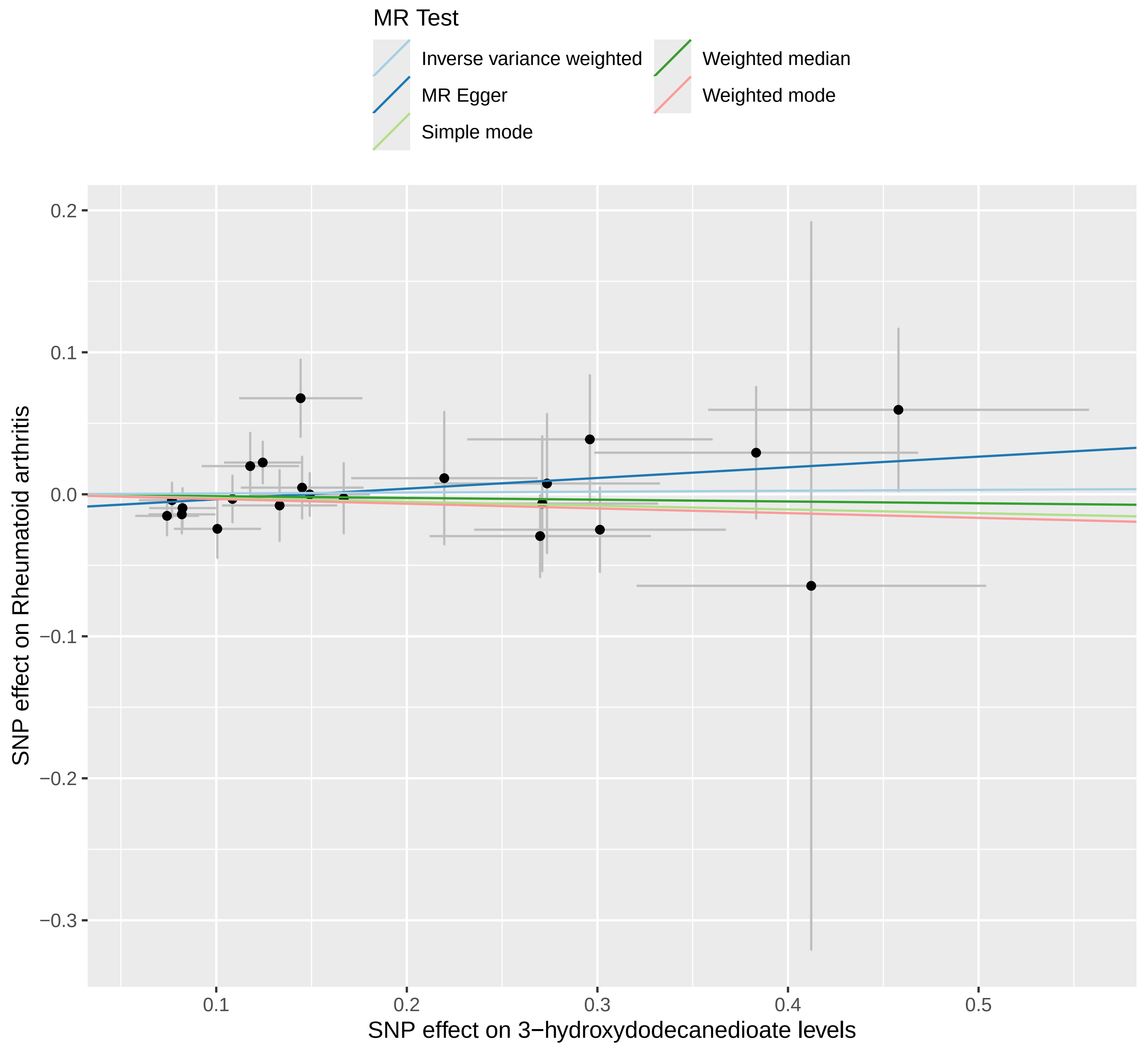
MR Test
Inverse variance weighted
MR Egger
Simple mode
Weighted median
Weighted mode
0.2
0.1
0.0
−0.1
−0.2
−0.3
SNP effect on Rheumatoid arthritis
0.1
0.2
0.3
0.4
0.5
SNP effect on 3−hydroxydodecanedioate levels

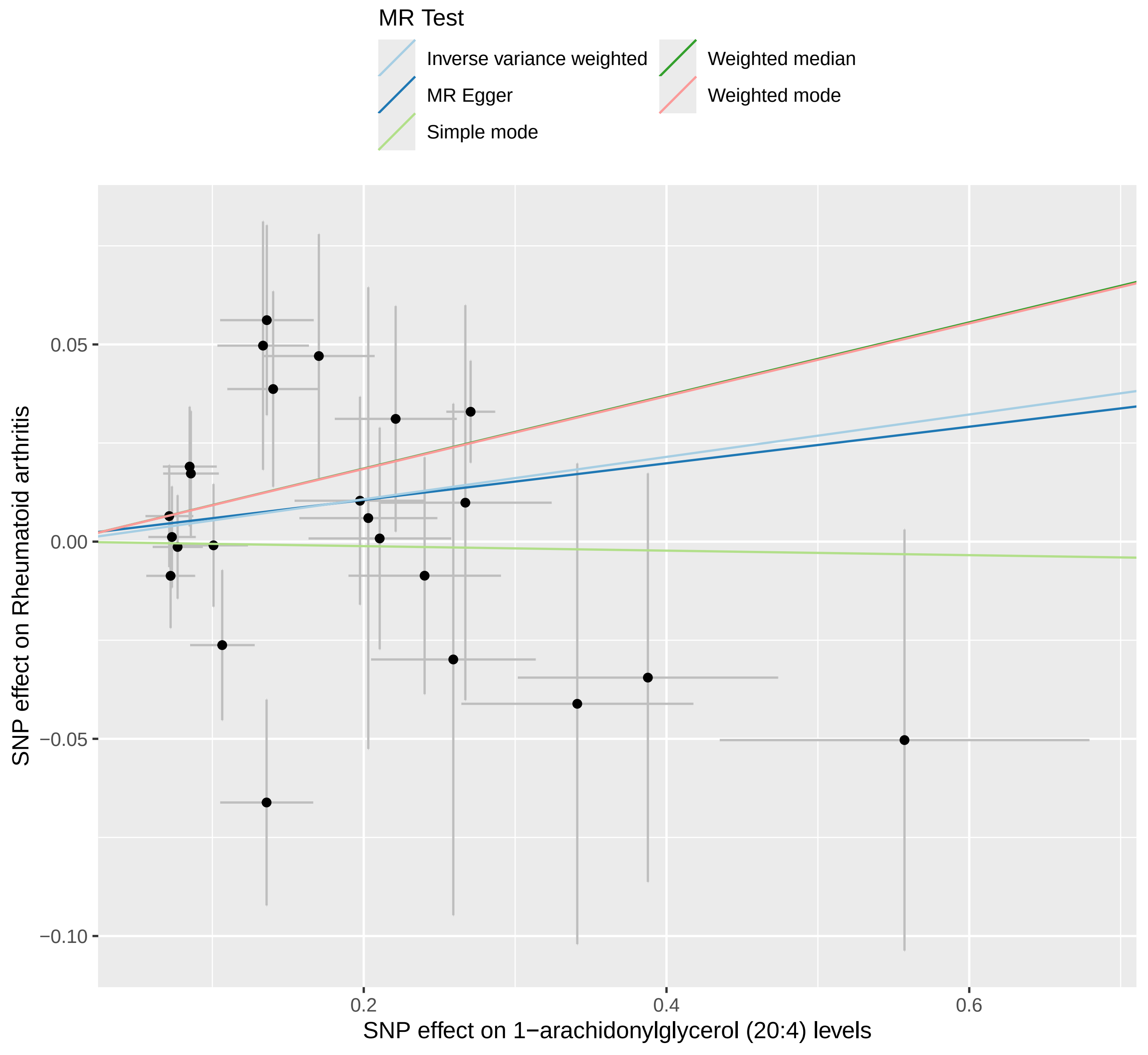

MR Test
Inverse variance weighted
MR Egger
Simple mode
Weighted median
Weighted mode
0.05
0.00
−0.05
−0.10
0.2
0.4
0.6
SNP effect on Rheumatoid arthritis
SNP effect on 1−arachidonylglycerol (20:4) levels

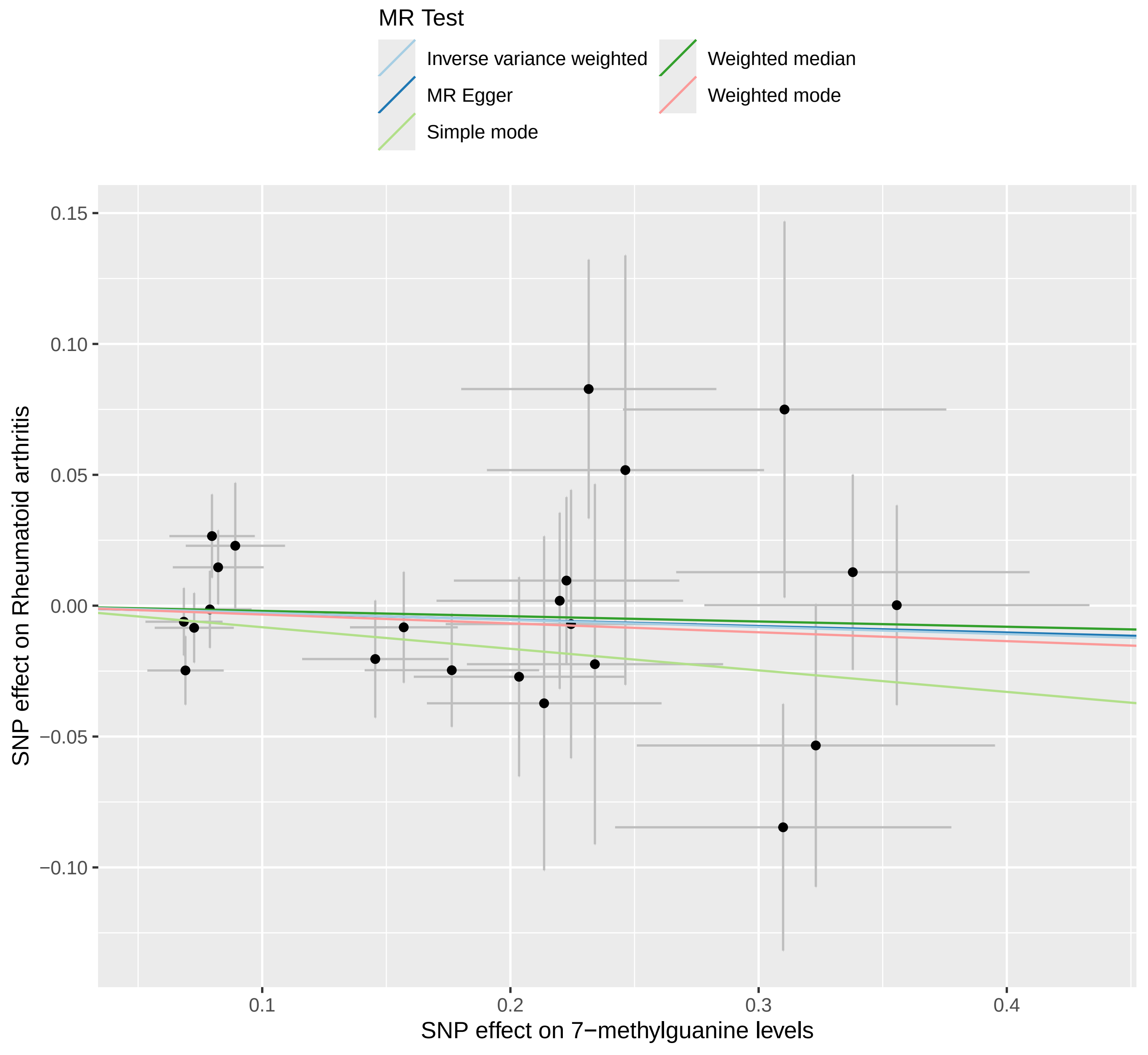
MR Test
Inverse variance weighted
MR Egger
Simple mode
Weighted median
Weighted mode
0.15
0.10
0.05
0.00
−0.05
−0.10
SNP effect on Rheumatoid arthritis
0.1
0.2
0.3
0.4
SNP effect on 7−methylguanine levels

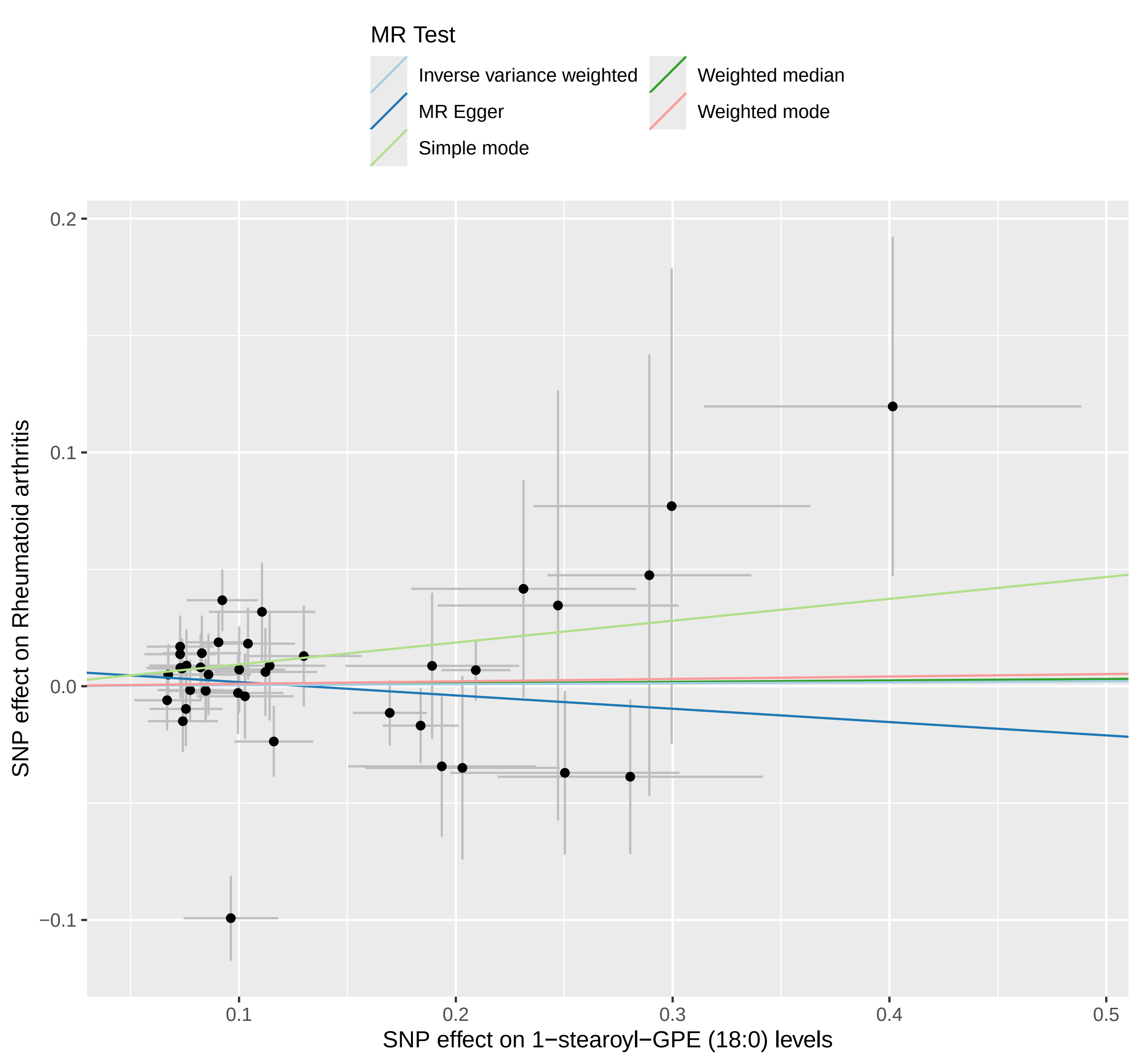
MR Test
Inverse variance weighted
MR Egger
Simple mode
Weighted median
Weighted mode
0.2
0.1
0.0
−0.1
SNP effect on Rheumatoid arthritis
0.1
0.2
0.3
0.4
0.5
SNP effect on 1−stearoyl−GPE (18:0) levels

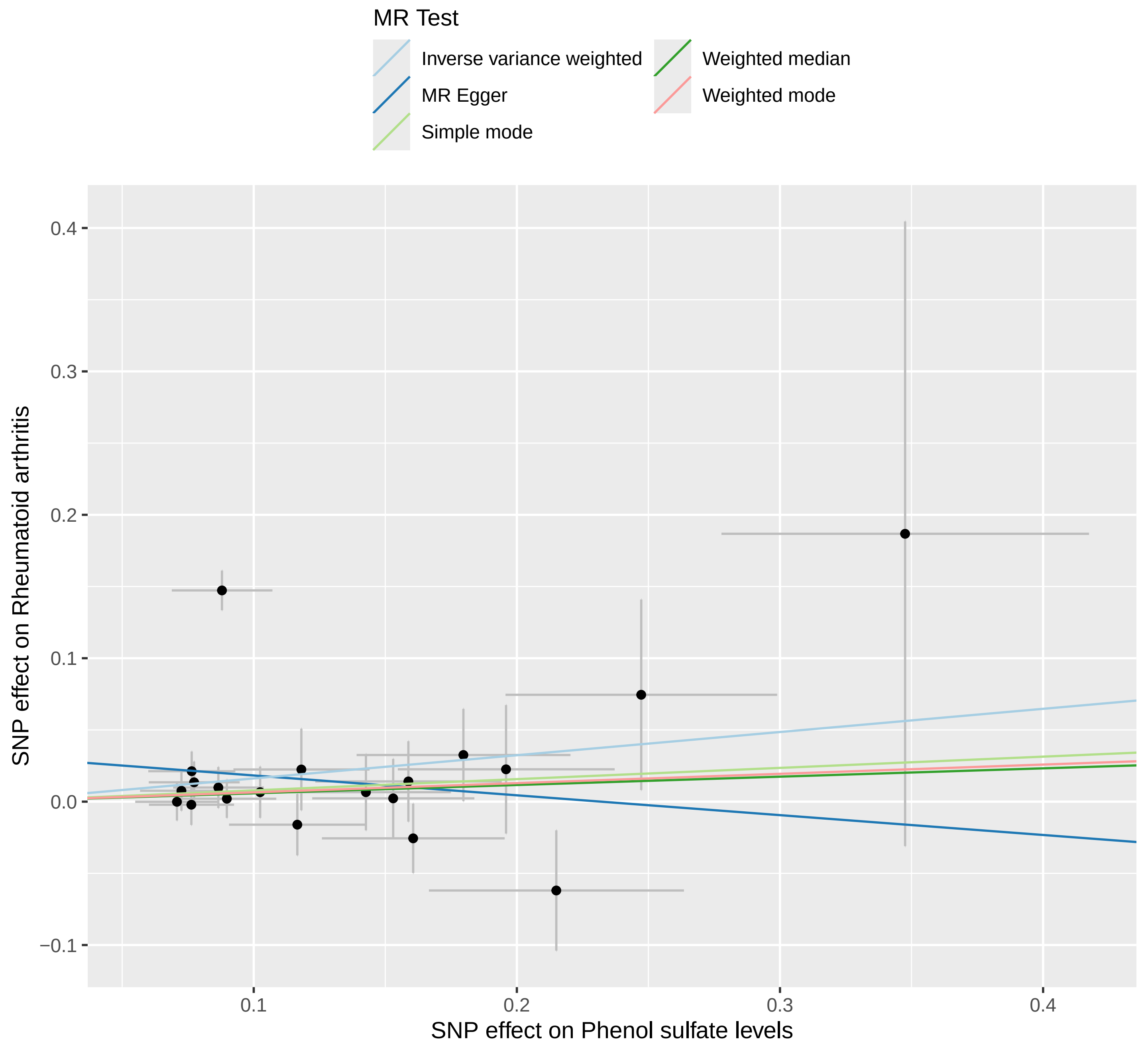
MR Test
Inverse variance weighted
MR Egger
Simple mode
Weighted median
Weighted mode
0.4
0.3
0.2
0.1
0.0
−0.1
0.1
0.2
0.3
0.4
SNP effect on Rheumatoid arthritis
SNP effect on Phenol sulfate levels

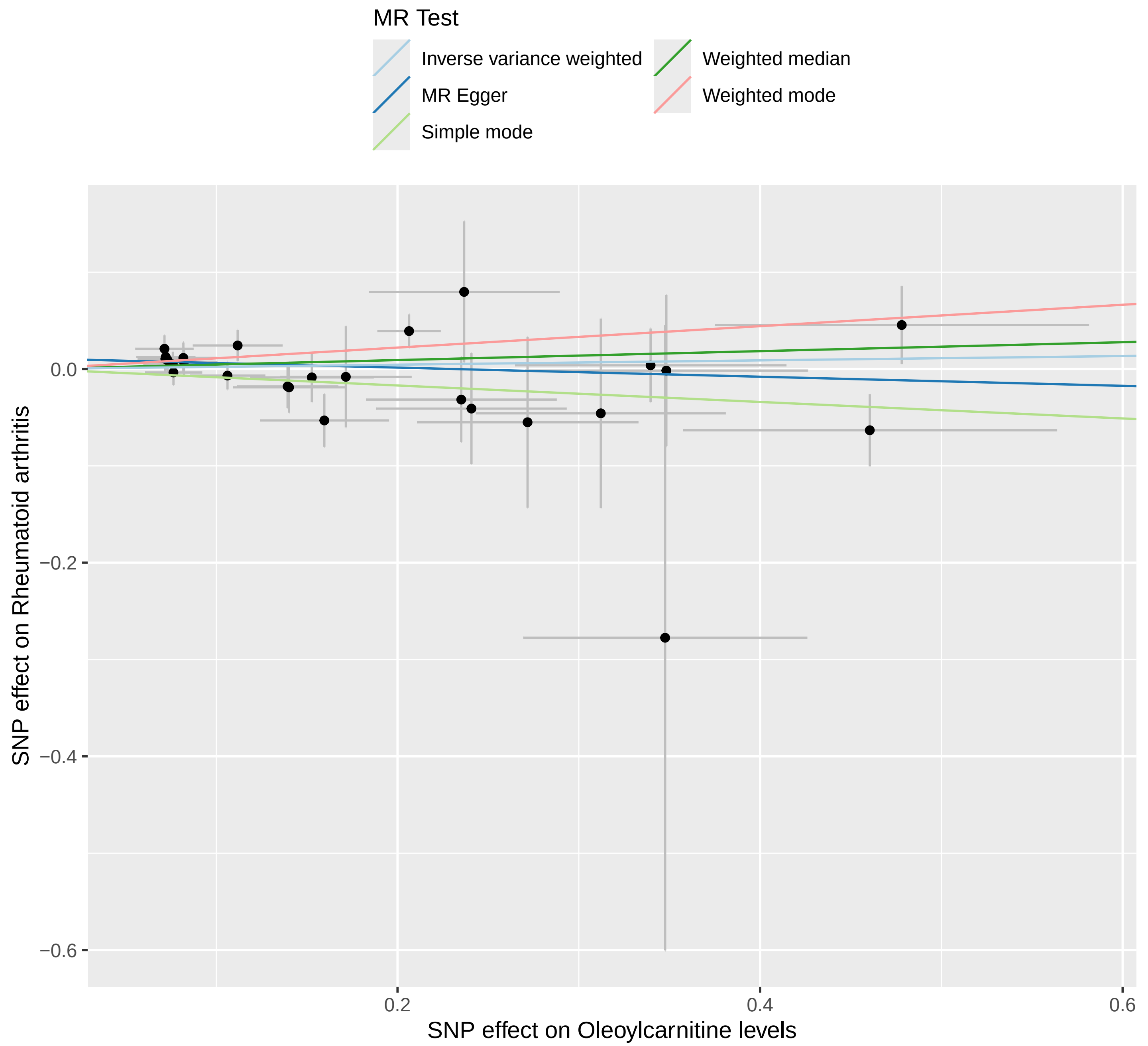

MR Test
Inverse variance weighted
MR Egger
Simple mode
Weighted median
Weighted mode
0.0
−0.2
−0.4
−0.6
0.2
0.4
0.6
SNP effect on Rheumatoid arthritis
SNP effect on Oleoylcarnitine levels

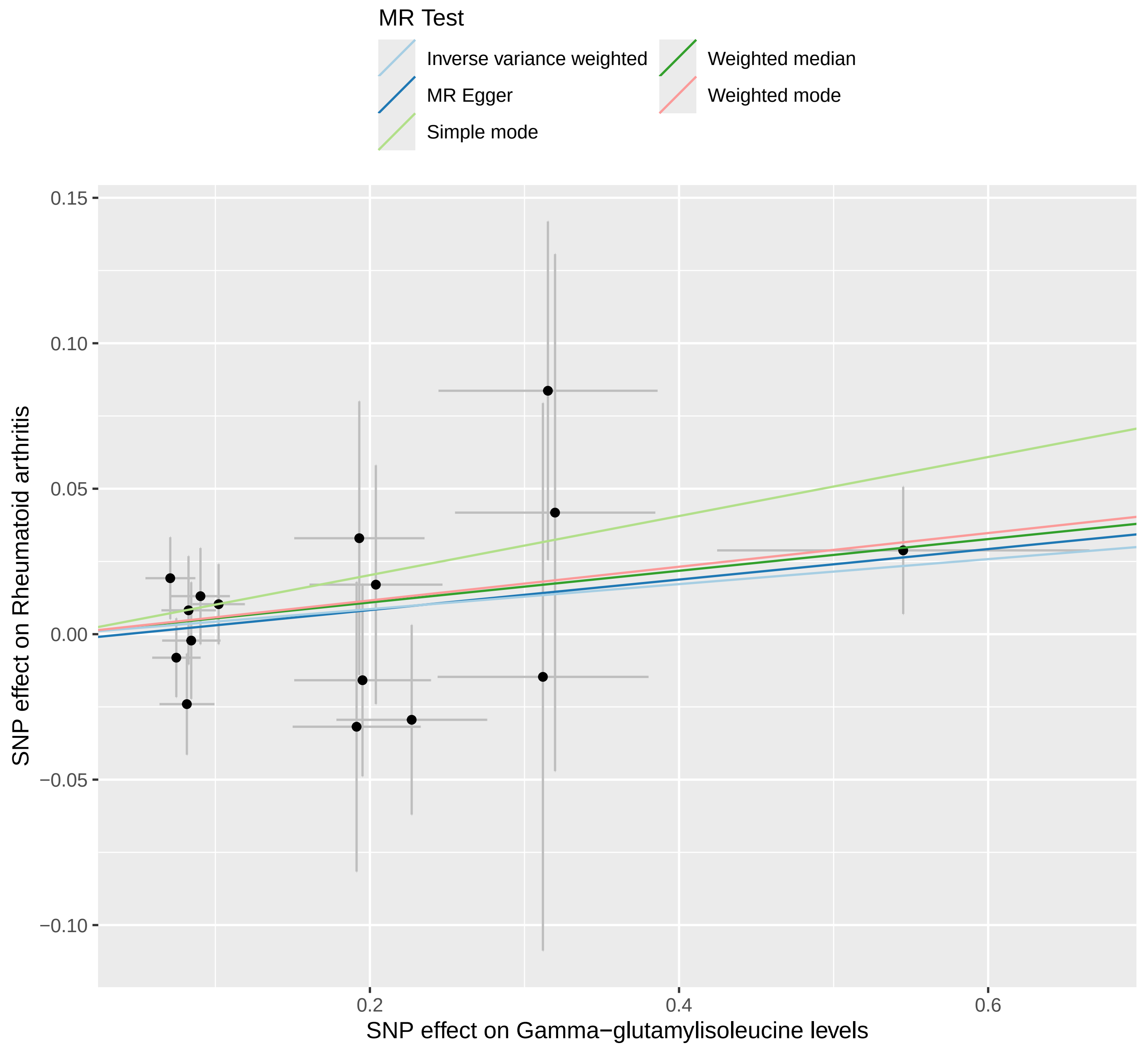
MR Test
Inverse variance weighted
MR Egger
Simple mode
Weighted median
Weighted mode
0.15
0.10
0.05
0.00
−0.05
−0.10
0.2
0.4
0.6
SNP effect on Rheumatoid arthritis
SNP effect on Gamma−glutamylisoleucine levels

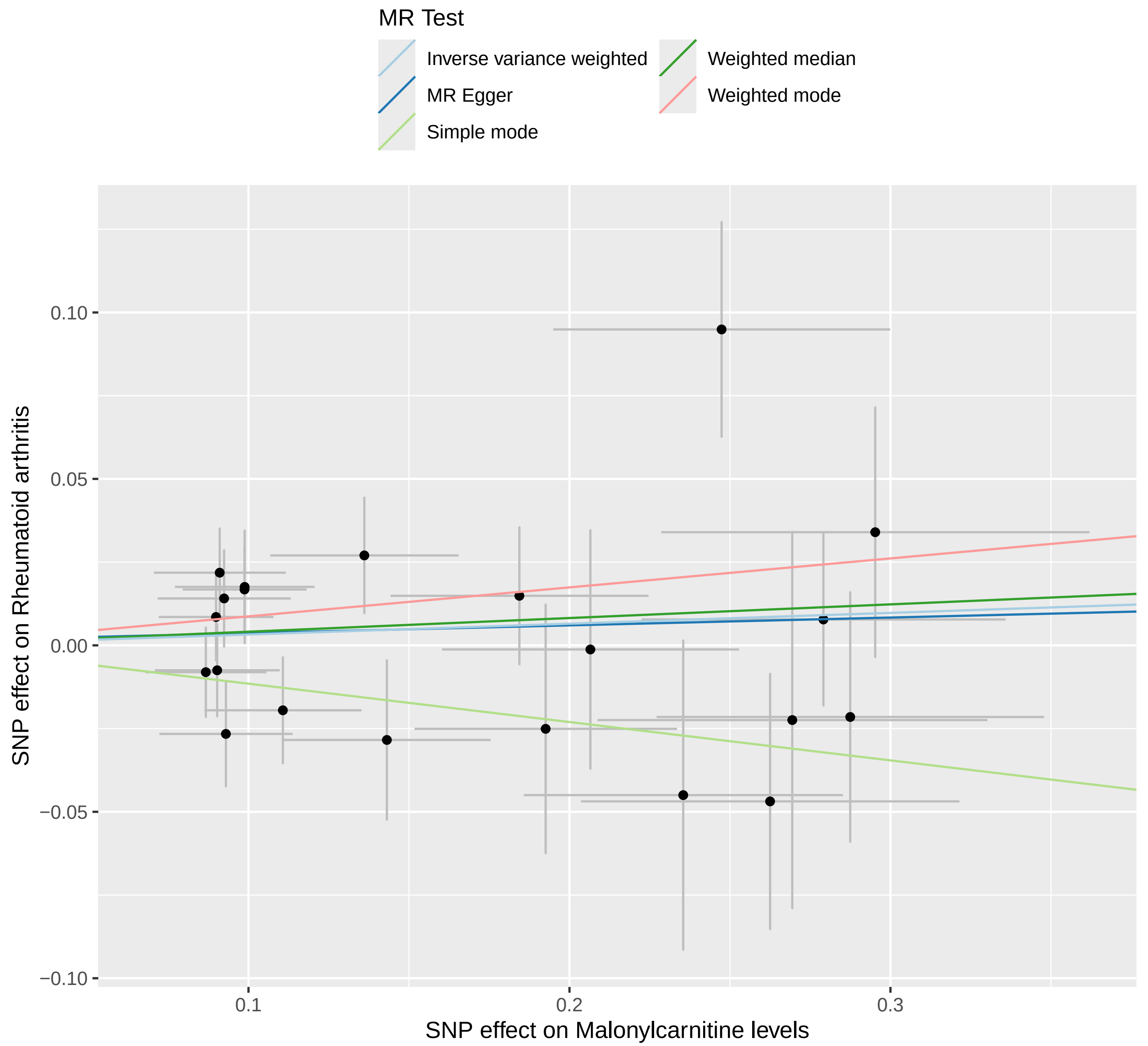

MR Test
Inverse variance weighted
MR Egger
Simple mode
Weighted median
Weighted mode
0.10
0.05
0.00
−0.05
−0.10
SNP effect on Rheumatoid arthritis
0.1
0.2
0.3
SNP effect on Malonylcarnitine levels

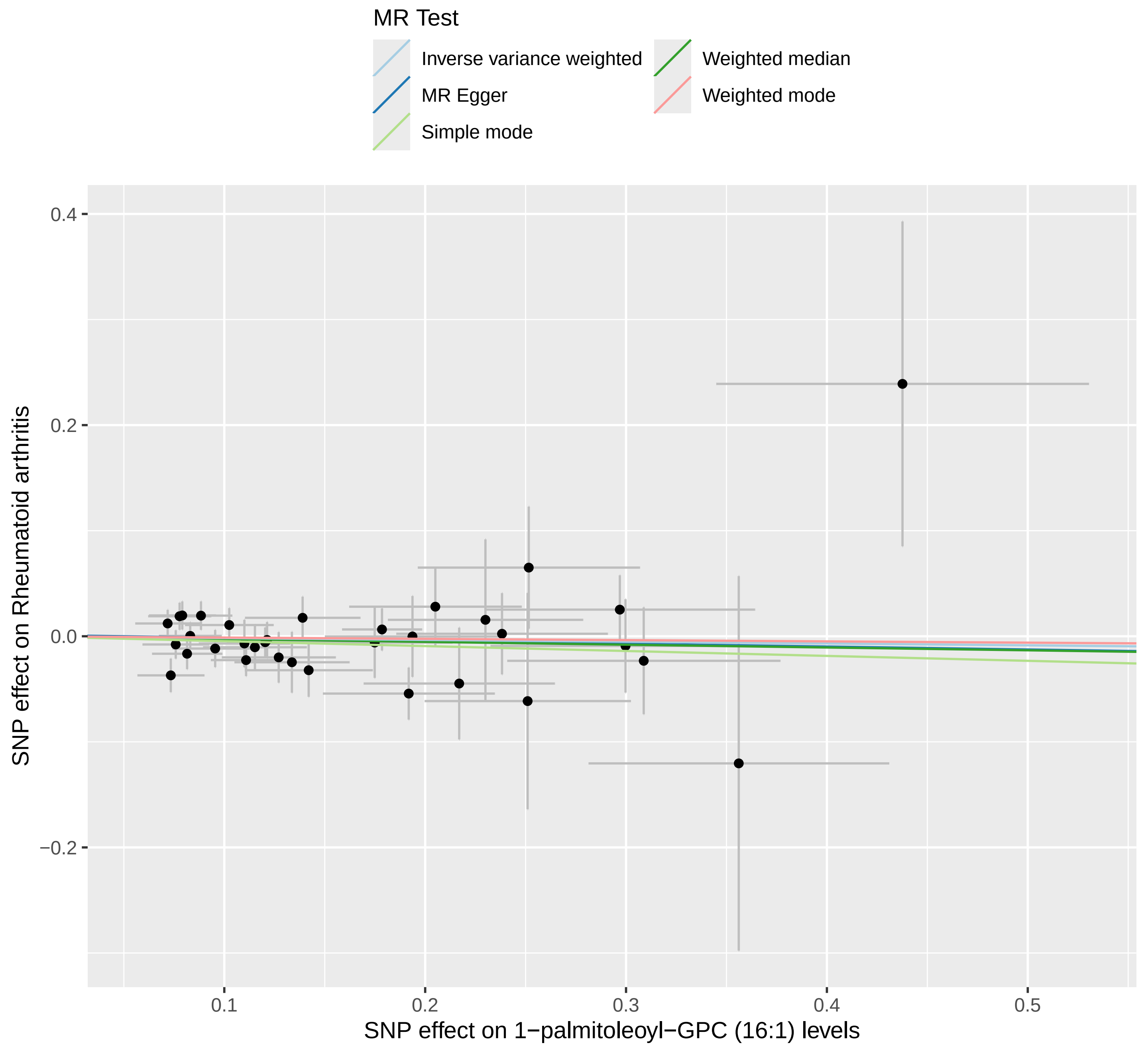

MR Test
Inverse variance weighted
MR Egger
Simple mode
Weighted median
Weighted mode
0.4
0.2
0.0
−0.2
SNP effect on Rheumatoid arthritis
0.1
0.2
0.3
0.4
0.5
SNP effect on 1−palmitoleoyl−GPC (16:1) levels

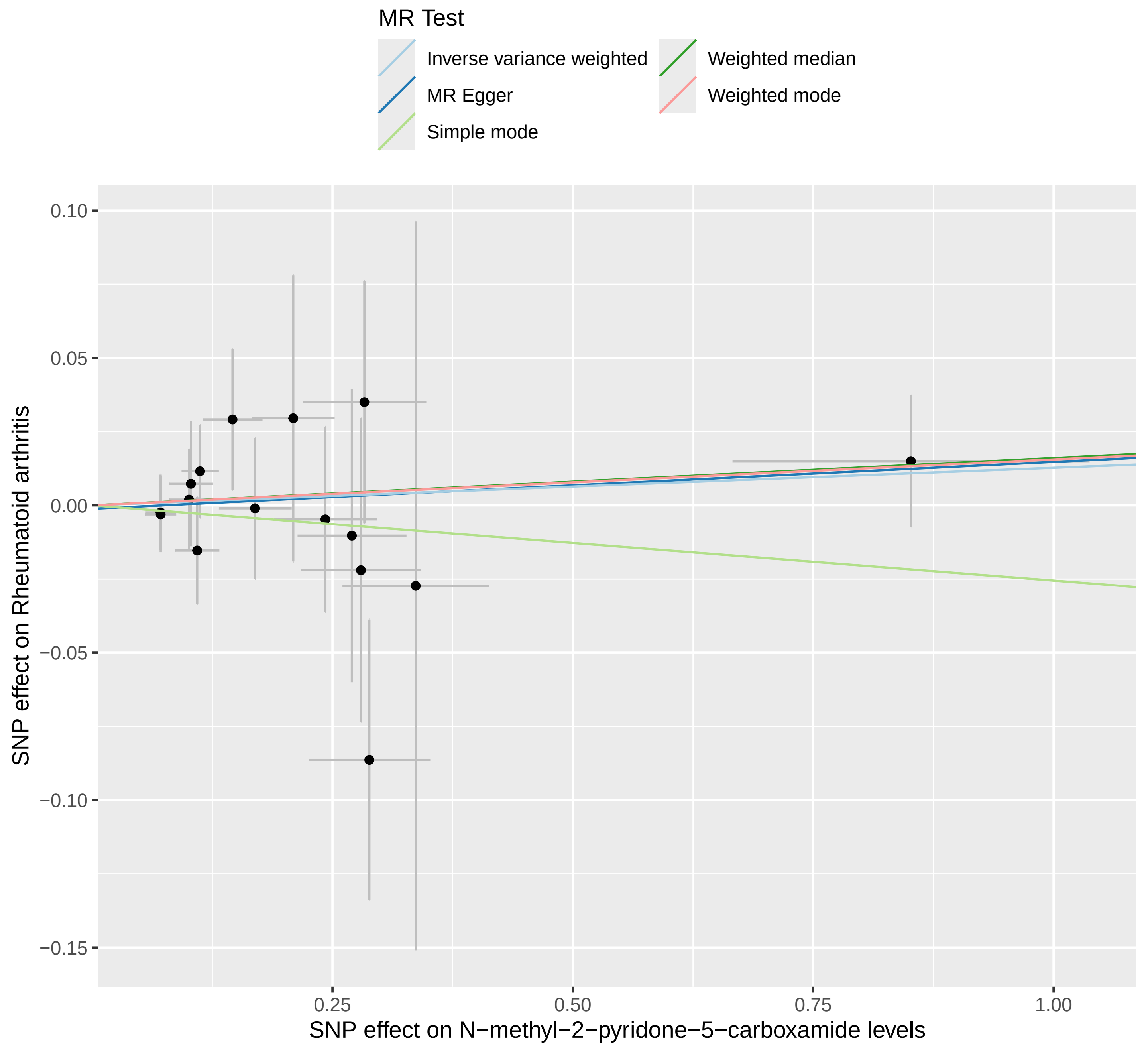
MR Test
Inverse variance weighted
MR Egger
Simple mode
Weighted median
Weighted mode
0.10
0.05
0.00
−0.05
−0.10
−0.15
SNP effect on Rheumatoid arthritis
0.25
0.50
0.75
1.00
SNP effect on N−methyl−2−pyridone−5−carboxamide levels

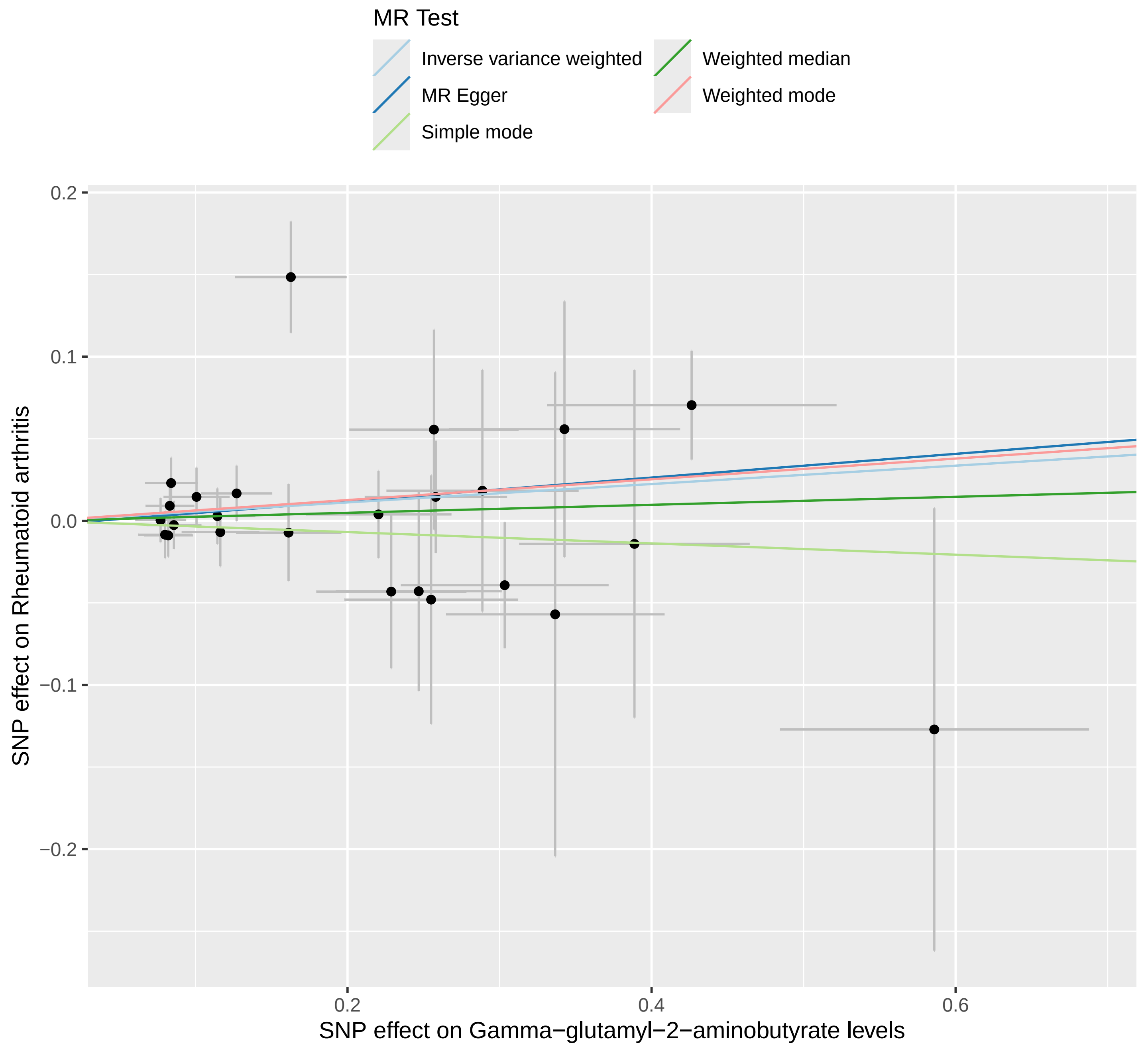
MR Test
Inverse variance weighted
MR Egger
Simple mode
Weighted median
Weighted mode
0.2
0.1
0.0
−0.1
−0.2
SNP effect on Rheumatoid arthritis
0.2
0.4
0.6
SNP effect on Gamma−glutamyl−2−aminobutyrate levels

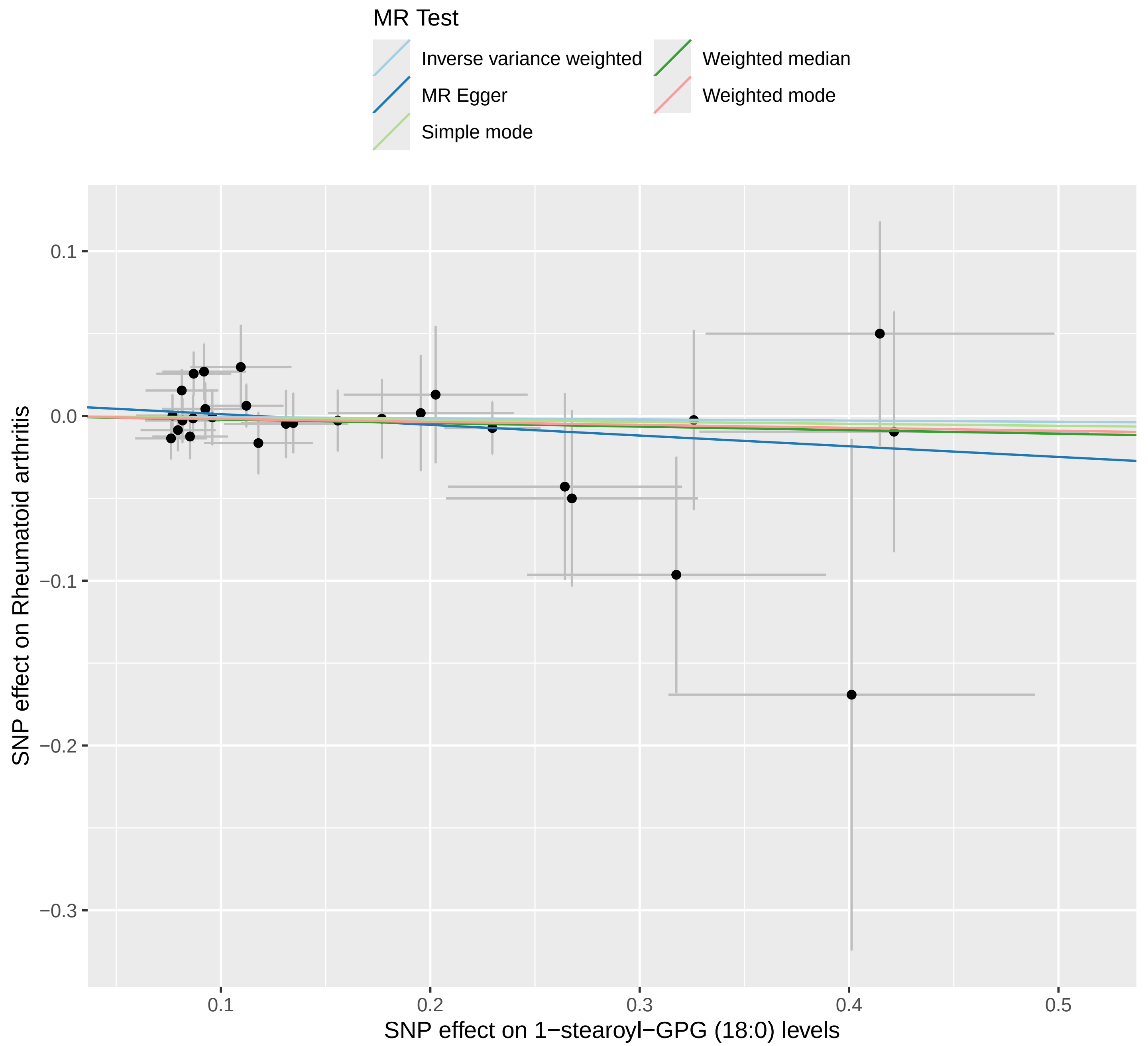
MR Test
Inverse variance weighted
MR Egger
Simple mode
Weighted median
Weighted mode
0.1
0.0
−0.1
−0.2
−0.3
0.1
0.2
0.3
0.4
0.5
SNP effect on Rheumatoid arthritis
SNP effect on 1−stearoyl−GPG (18:0) levels

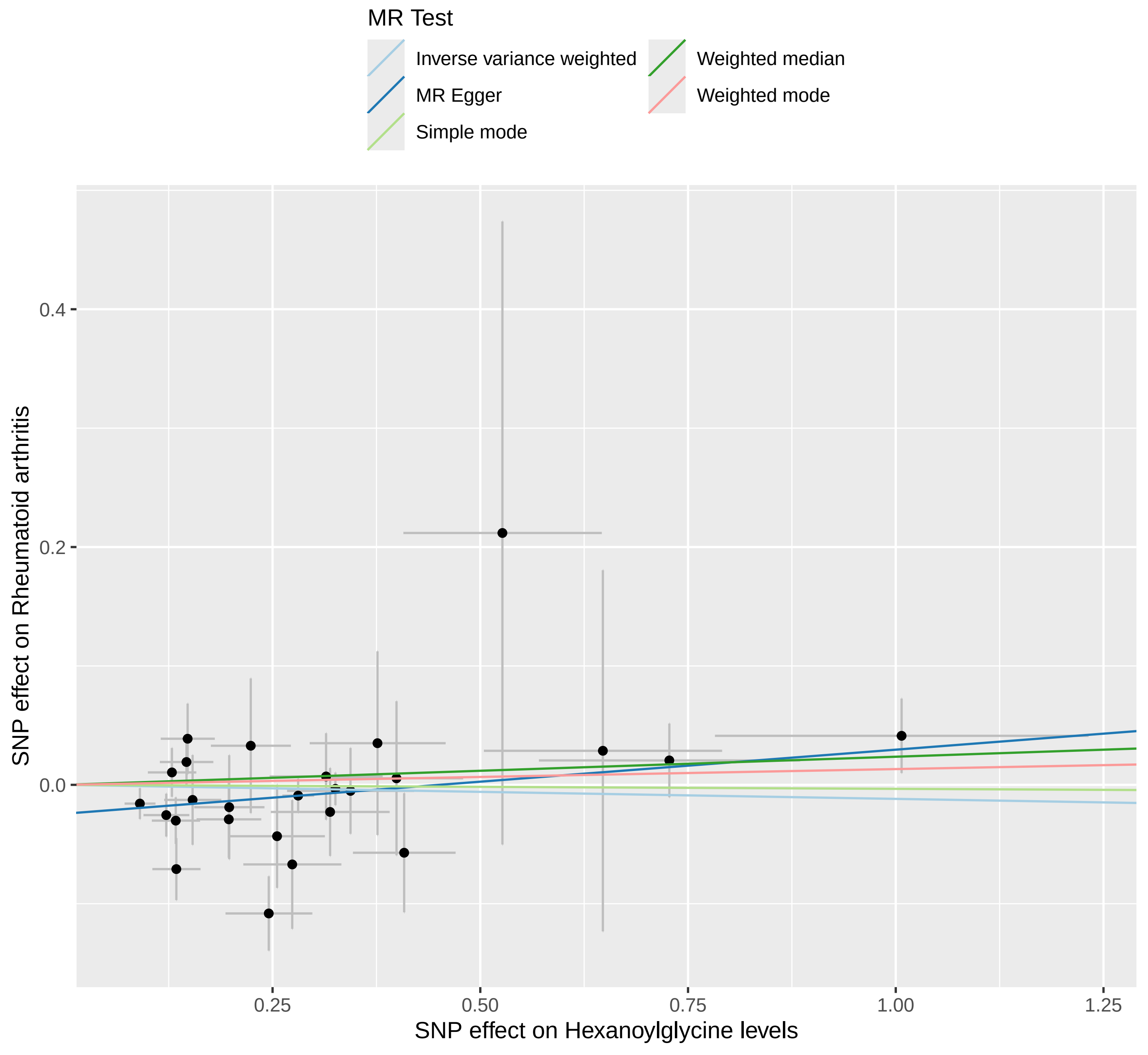

MR Test
Inverse variance weighted
MR Egger
Simple mode
Weighted median
Weighted mode
0.4
0.2
0.0
0.25
0.50
0.75
1.00
1.25
SNP effect on Rheumatoid arthritis
SNP effect on Hexanoylglycine levels

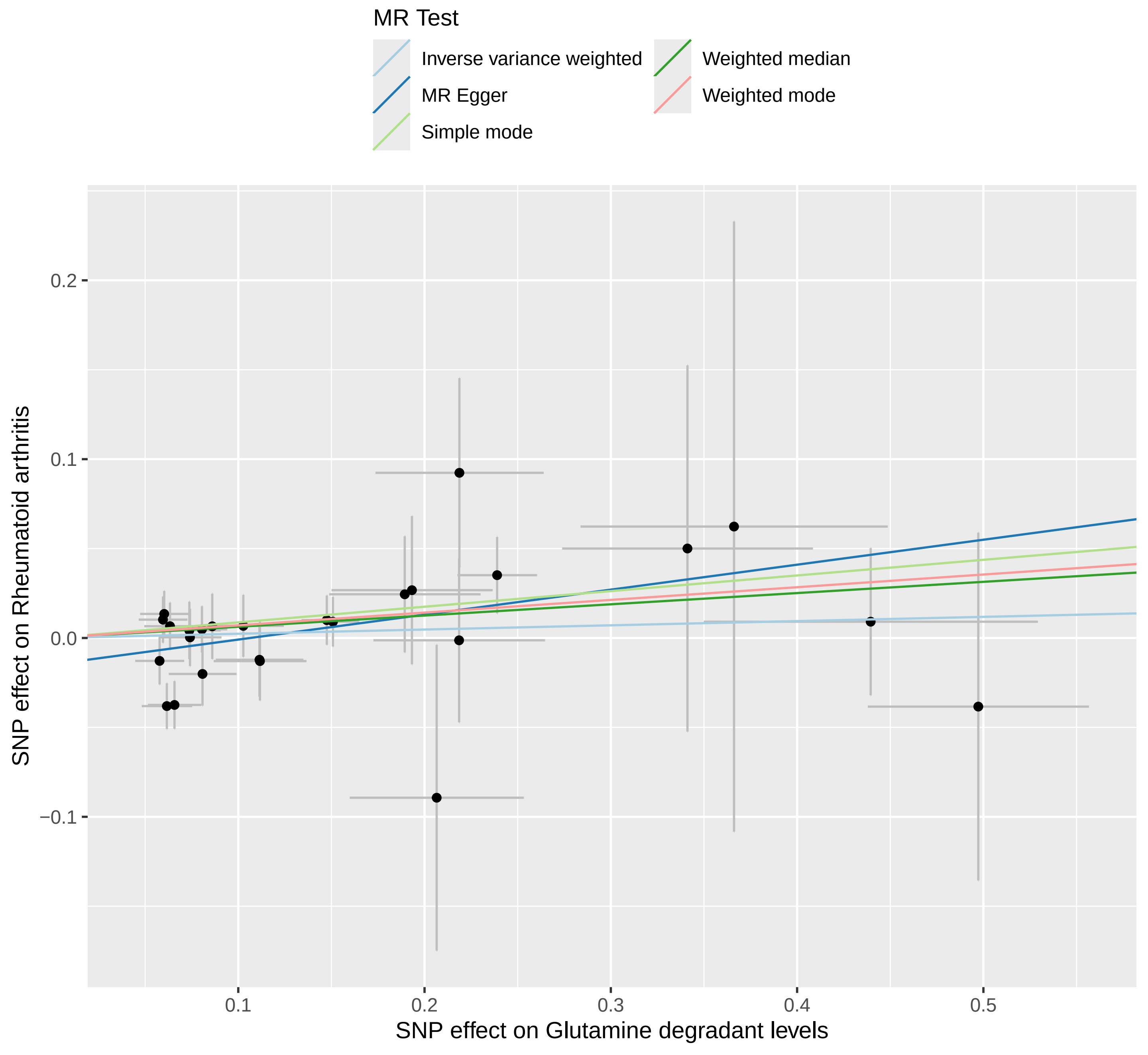
MR Test
Inverse variance weighted
MR Egger
Simple mode
Weighted median
Weighted mode
SNP effect on Rheumatoid arthritis
0.2
0.1
0.0
−0.1
0.1
0.2
0.3
0.4
0.5
SNP effect on Glutamine degradant levels

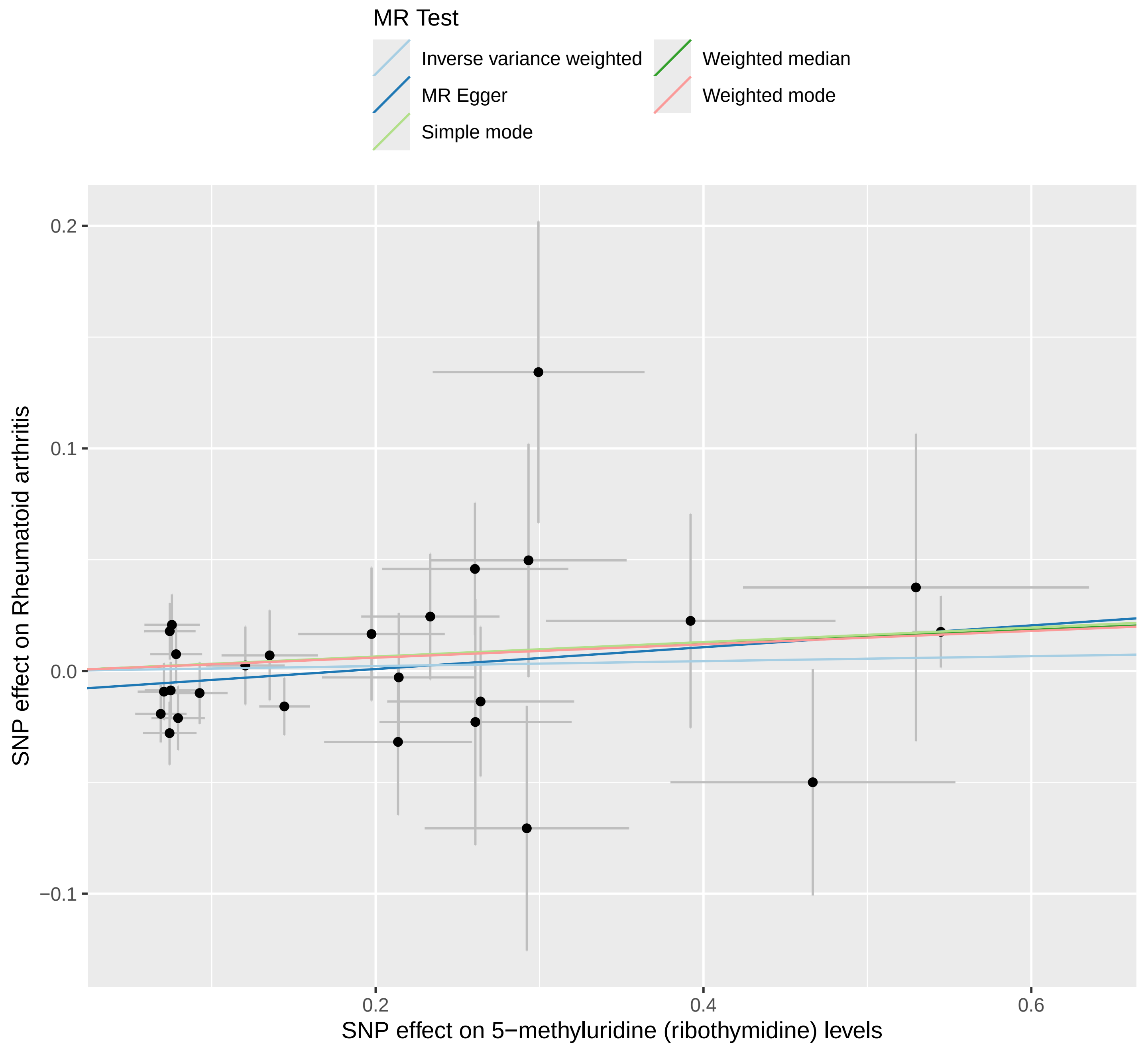

MR Test
Inverse variance weighted
MR Egger
Simple mode
Weighted median
Weighted mode
0.2
0.1
0.0
−0.1
SNP effect on Rheumatoid arthritis
0.2
0.4
0.6
SNP effect on 5−methyluridine (ribothymidine) levels

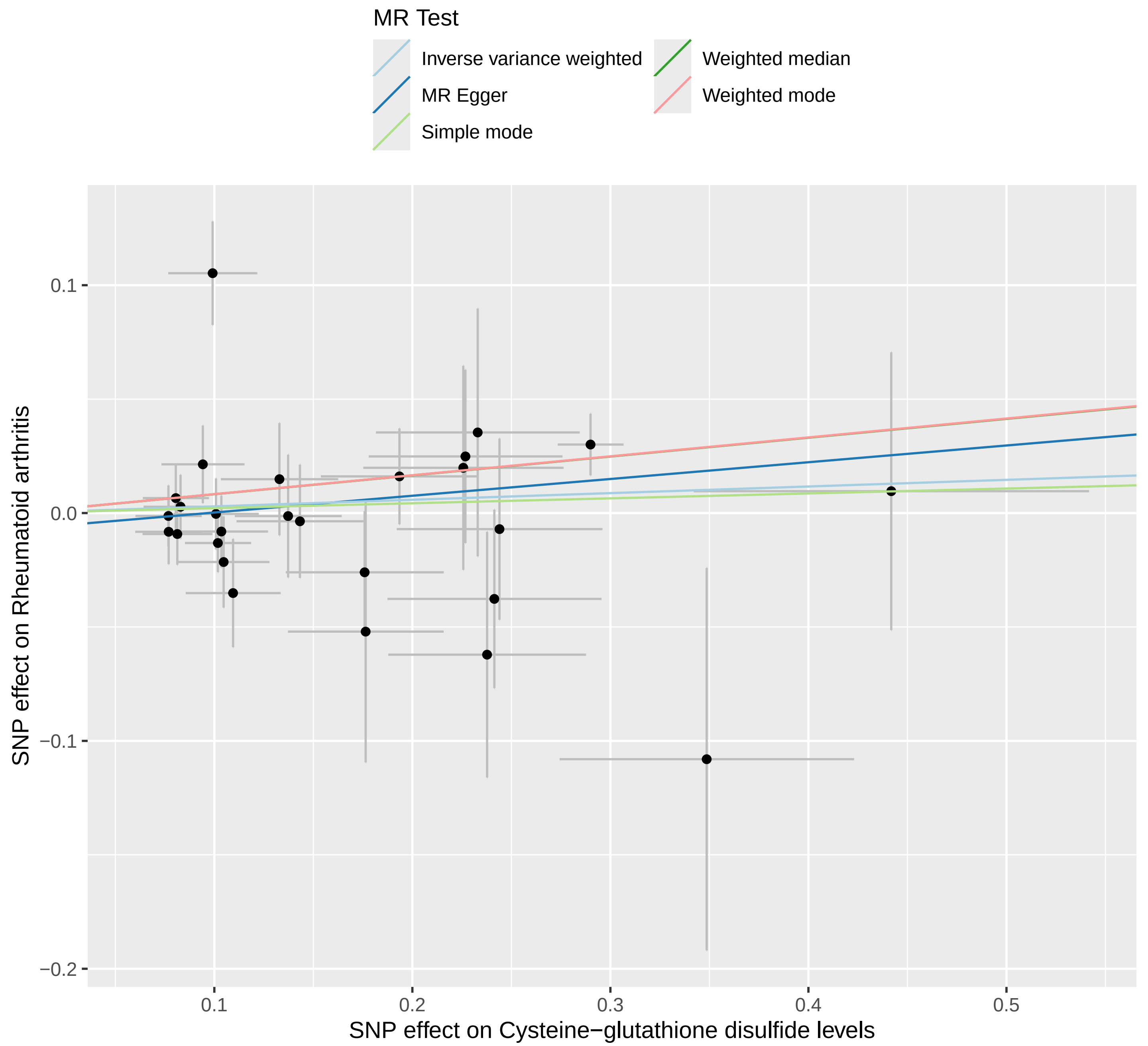
MR Test
Inverse variance weighted
MR Egger
Simple mode
Weighted median
Weighted mode
SNP effect on Rheumatoid arthritis
0.1
0.0
−0.1
−0.2
0.1
0.2
0.3
0.4
0.5
SNP effect on Cysteine−glutathione disulfide levels

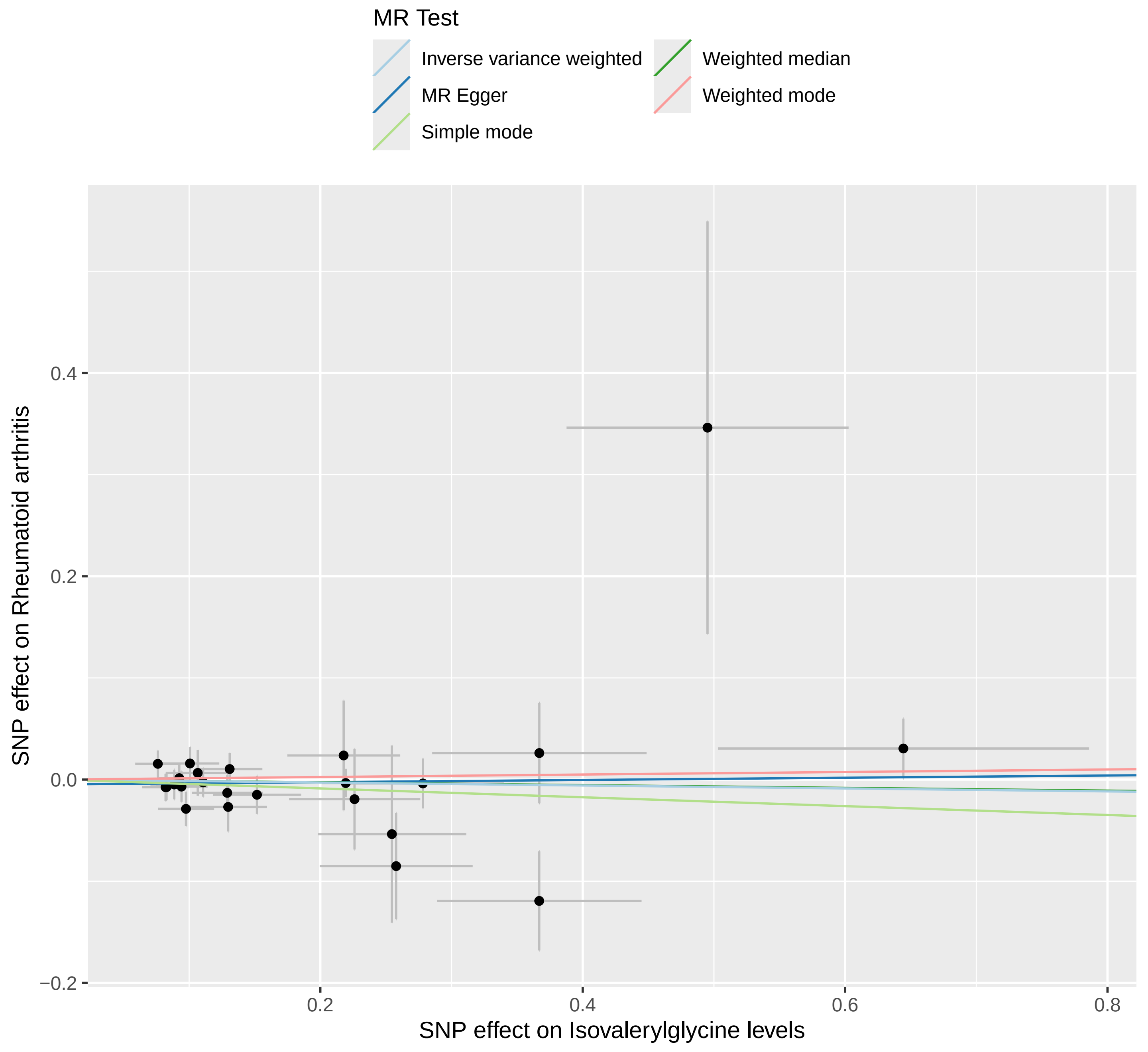
MR Test
Inverse variance weighted
MR Egger
Simple mode
Weighted median
Weighted mode
SNP effect on Rheumatoid arthritis
0.4
0.2
0.0
−0.2
0.2
0.4
0.6
0.8
SNP effect on Isovalerylglycine levels

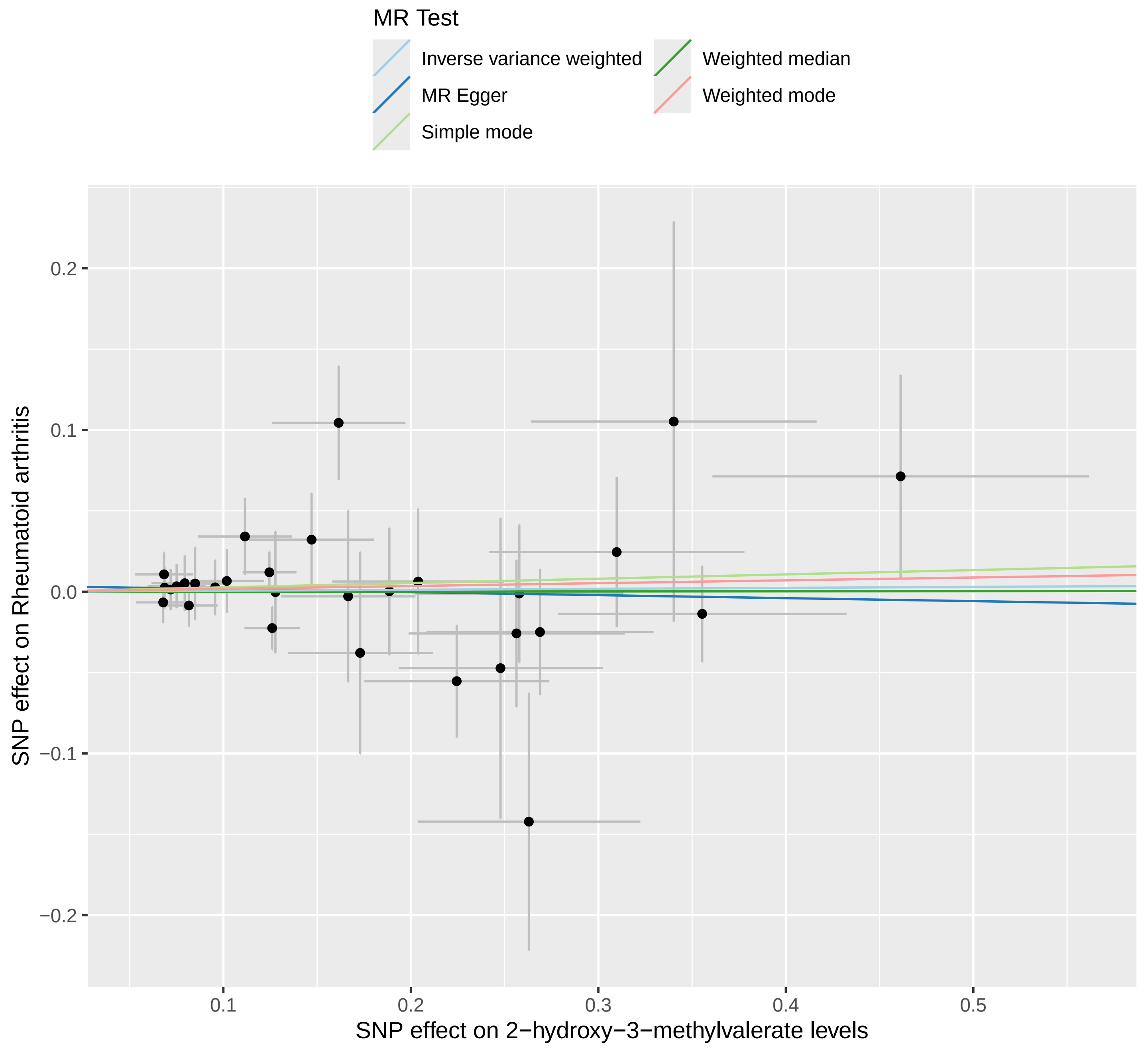
MR Test
Inverse variance weighted
MR Egger
Simple mode
Weighted median
Weighted mode
SNP effect on Rheumatoid arthritis
0.2
0.1
0.0
−0.1
−0.2
0.1
0.2
0.3
0.4
0.5
SNP effect on 2−hydroxy−3−methylvalerate levels

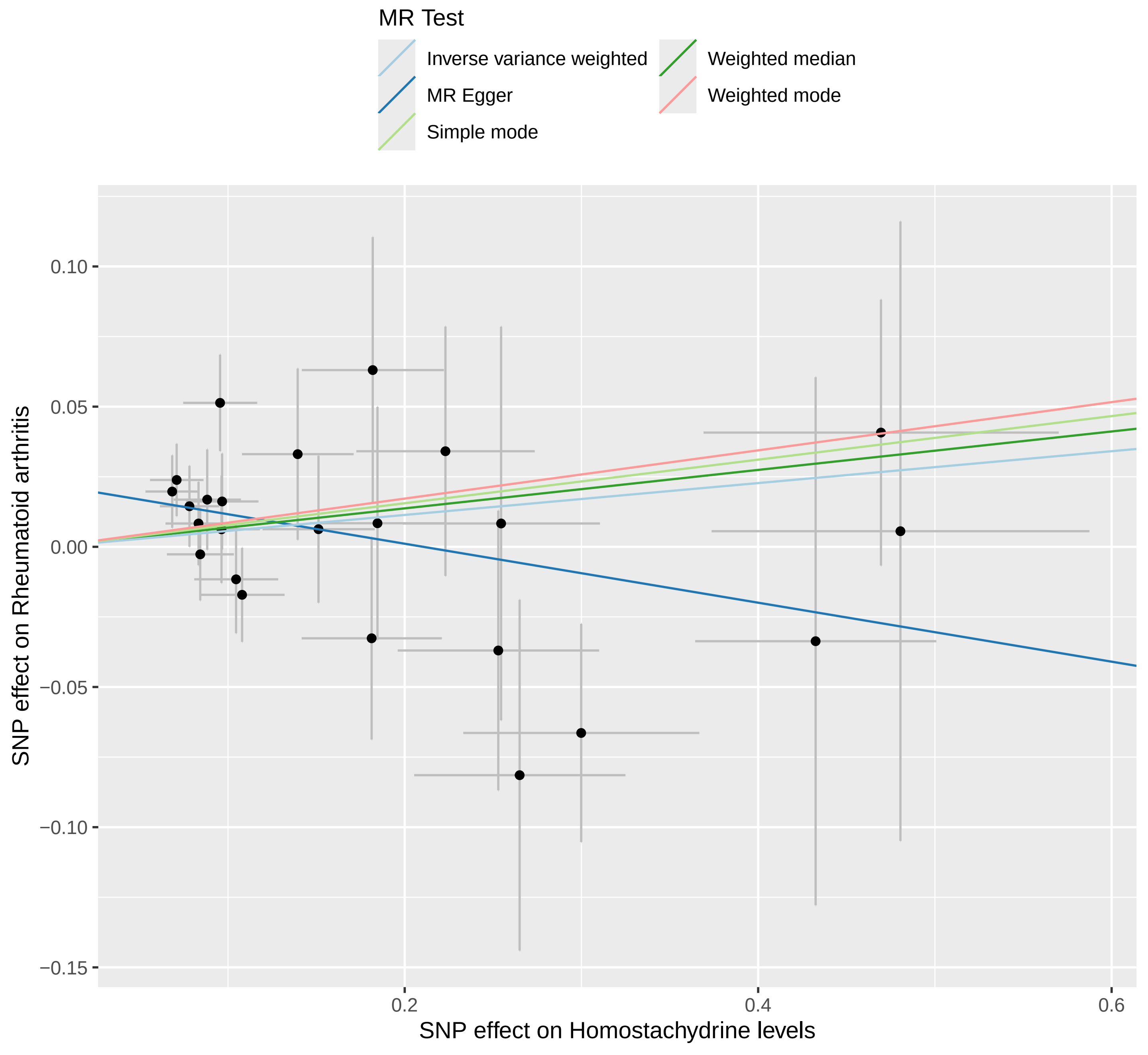
MR Test
Inverse variance weighted
MR Egger
Simple mode
Weighted median
Weighted mode
0.10
0.05
0.00
−0.05
−0.10
−0.15
SNP effect on Rheumatoid arthritis
0.2
0.4
0.6
SNP effect on Homostachydrine levels

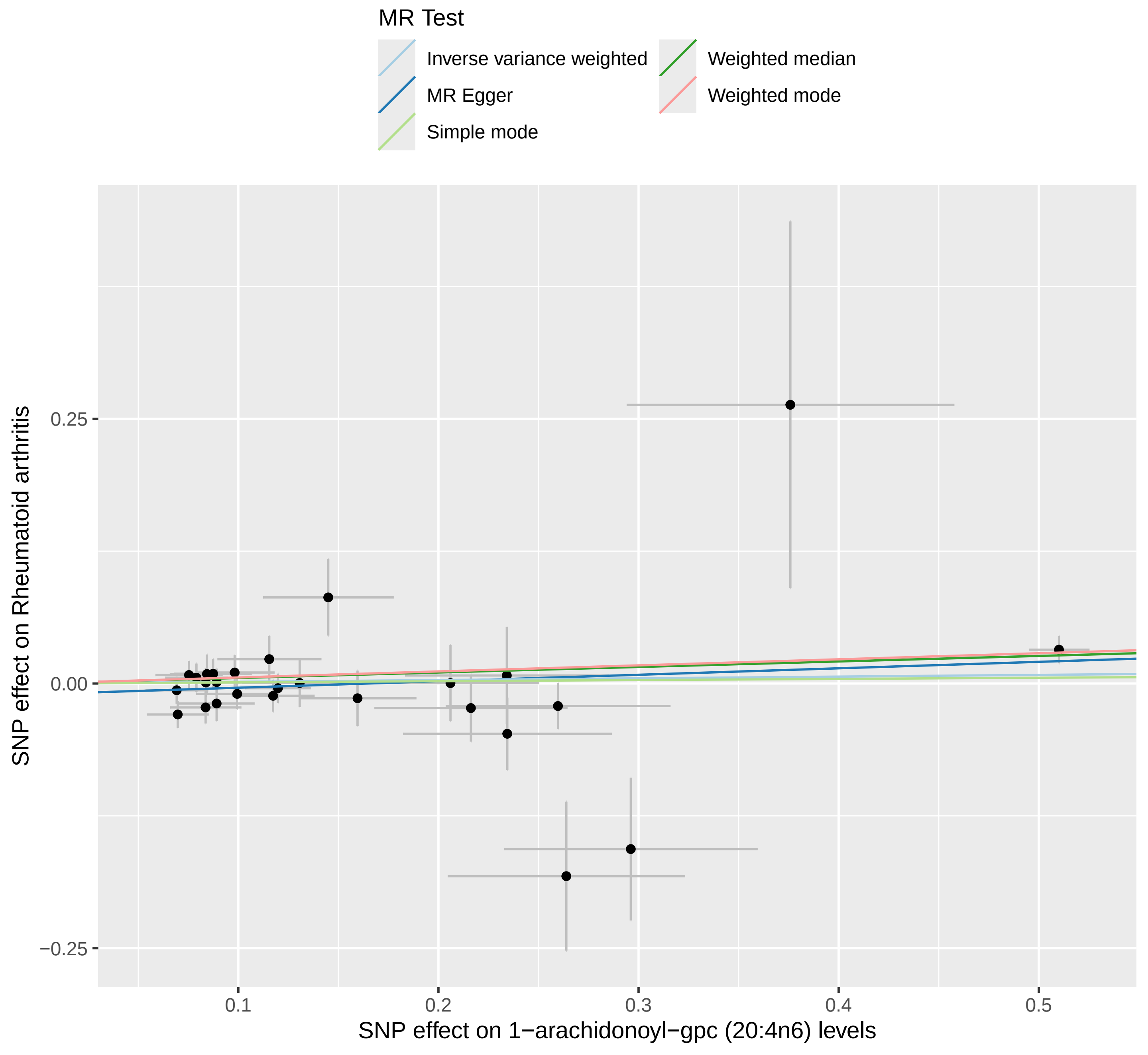

MR Test
Inverse variance weighted
MR Egger
Simple mode
Weighted median
Weighted mode
0.25
0.00
−0.25
0.1
0.2
0.3
0.4
0.5
SNP effect on Rheumatoid arthritis
SNP effect on 1−arachidonoyl−gpc (20:4n6) levels

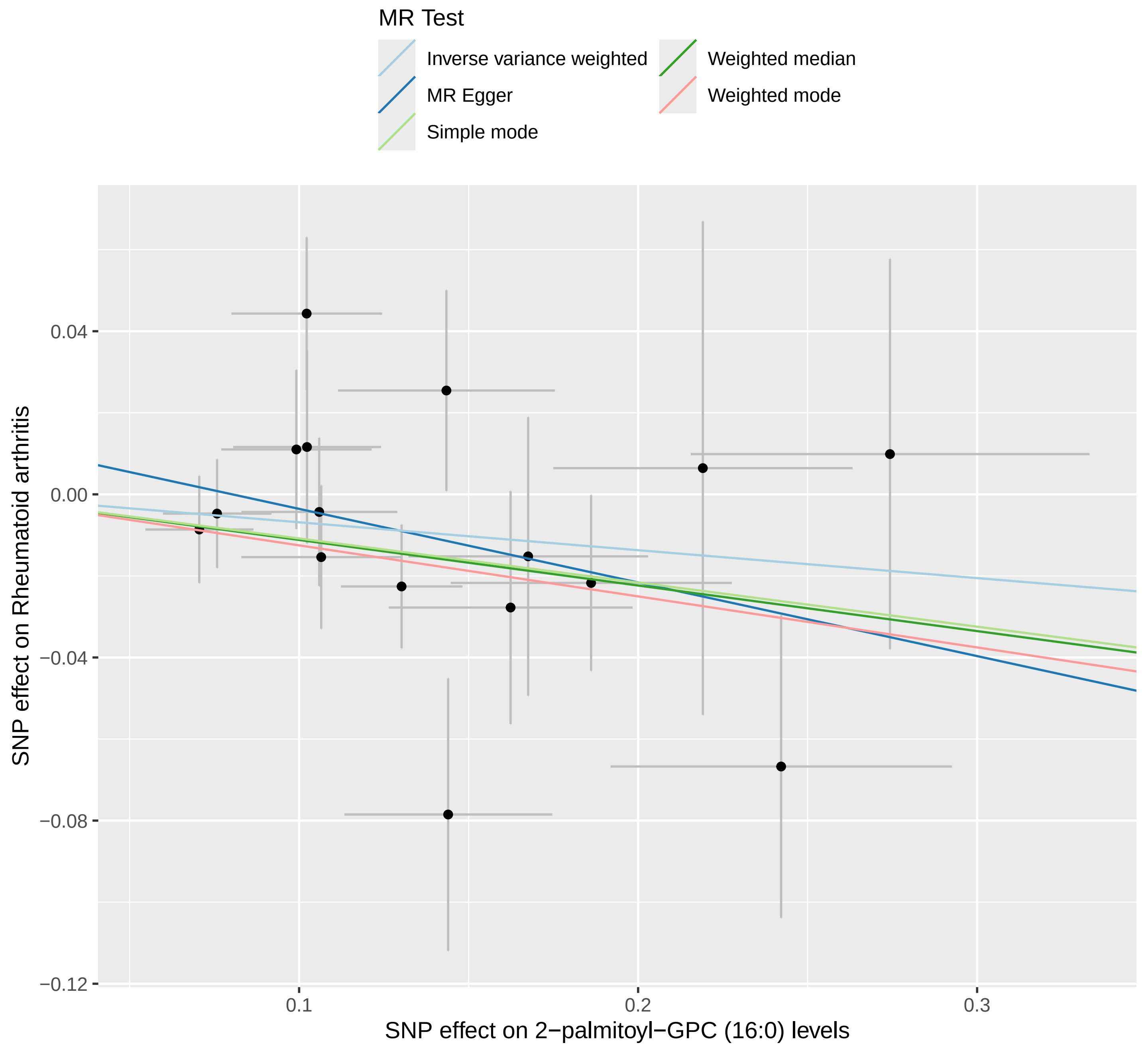
MR Test
Inverse variance weighted
MR Egger
Simple mode
Weighted median
Weighted mode
0.04
0.00
−0.04
−0.08
−0.12
SNP effect on Rheumatoid arthritis
0.1
0.2
0.3
SNP effect on 2−palmitoyl−GPC (16:0) levels

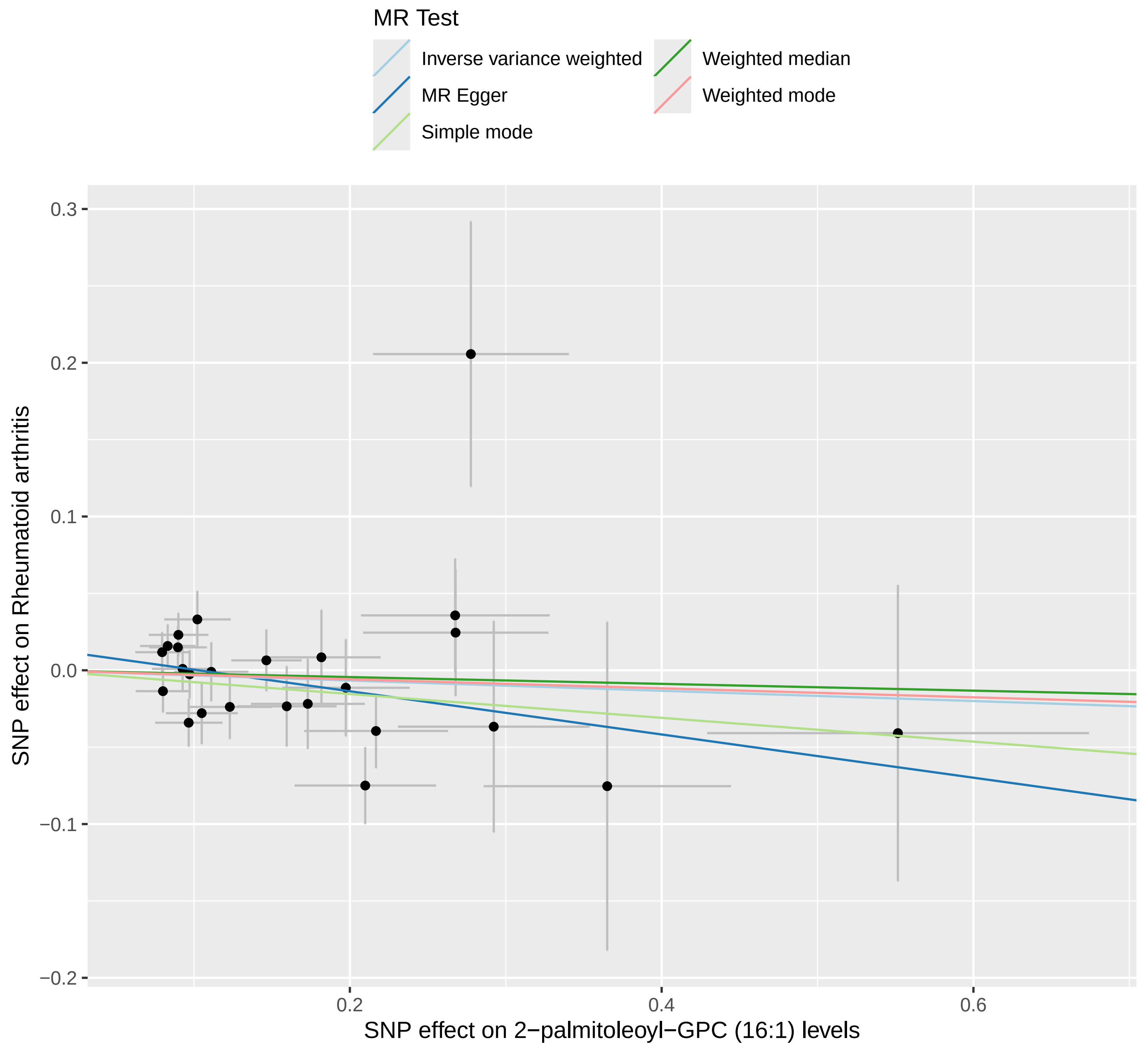
MR Test
Inverse variance weighted
MR Egger
Simple mode
Weighted median
Weighted mode
0.3
0.2
0.1
0.0
−0.1
−0.2
0.2
0.4
0.6
SNP effect on Rheumatoid arthritis
SNP effect on 2−palmitoleoyl−GPC (16:1) levels

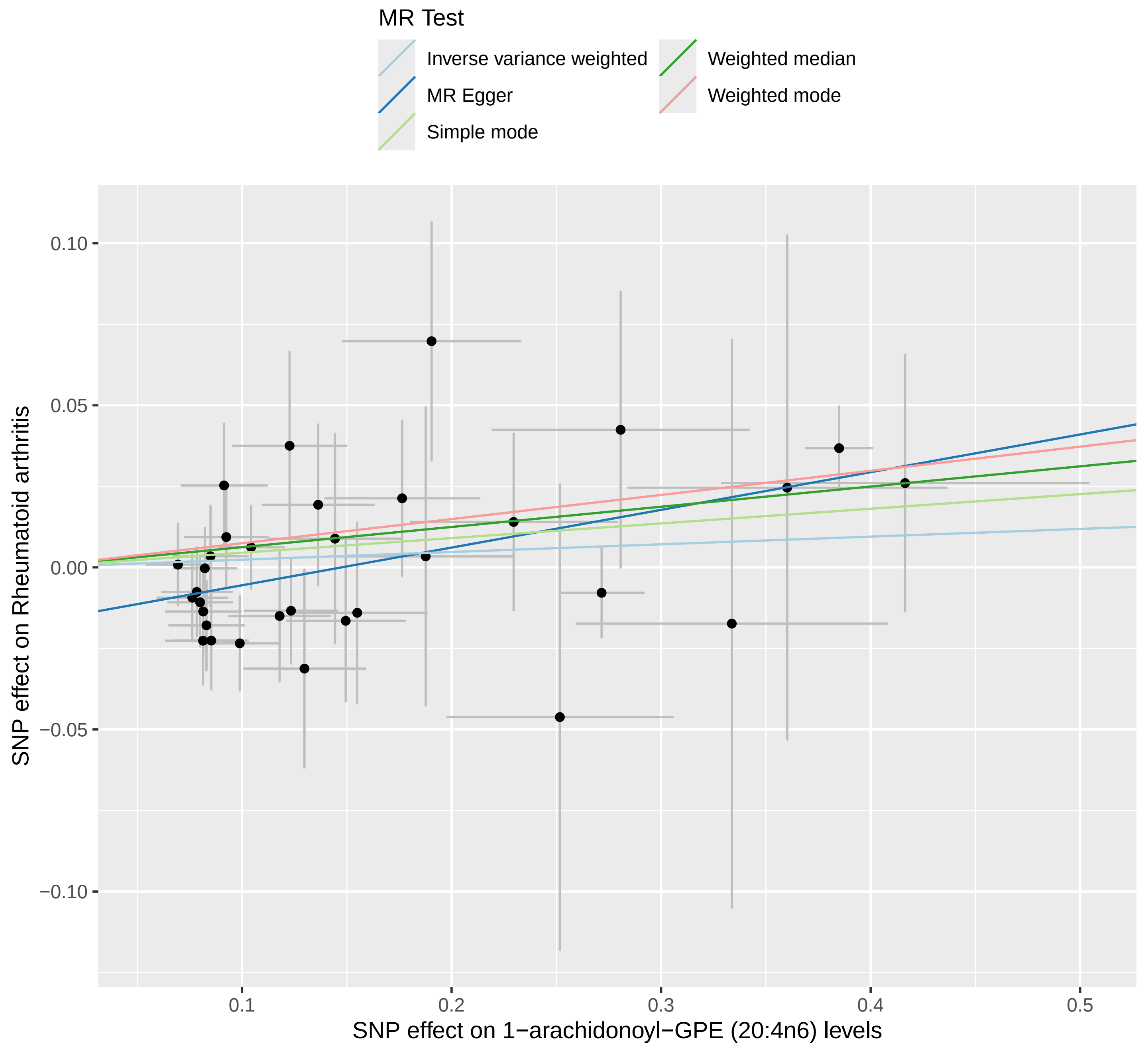
MR Test
Inverse variance weighted
MR Egger
Simple mode
Weighted median
Weighted mode
0.10
0.05
0.00
−0.05
−0.10
0.1
0.2
0.3
0.4
0.5
SNP effect on Rheumatoid arthritis
SNP effect on 1−arachidonoyl−GPE (20:4n6) levels

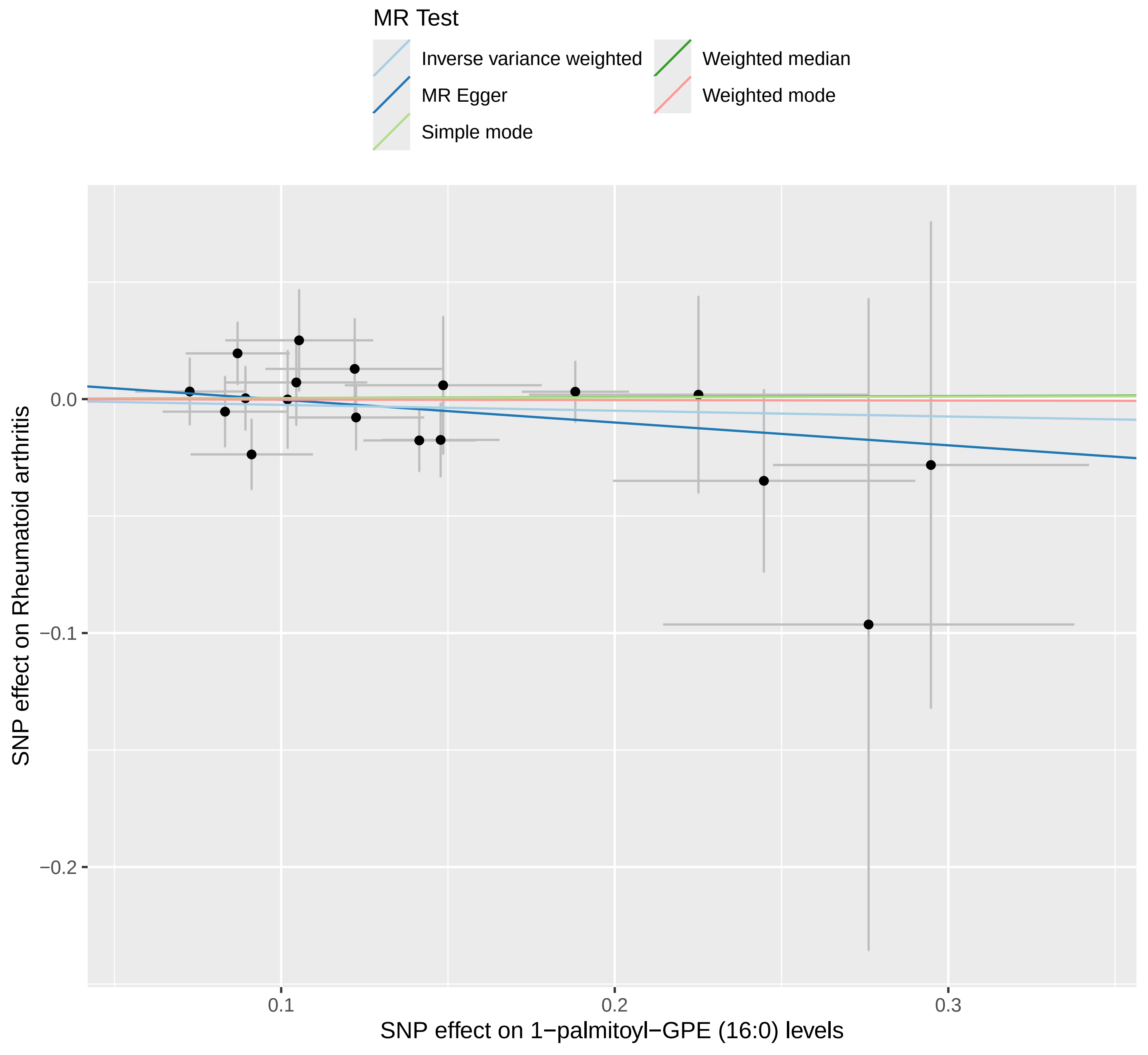
MR Test
Inverse variance weighted
MR Egger
Simple mode
Weighted median
Weighted mode
SNP effect on Rheumatoid arthritis
0.0
−0.1
−0.2
0.1
0.2
0.3
SNP effect on 1−palmitoyl−GPE (16:0) levels

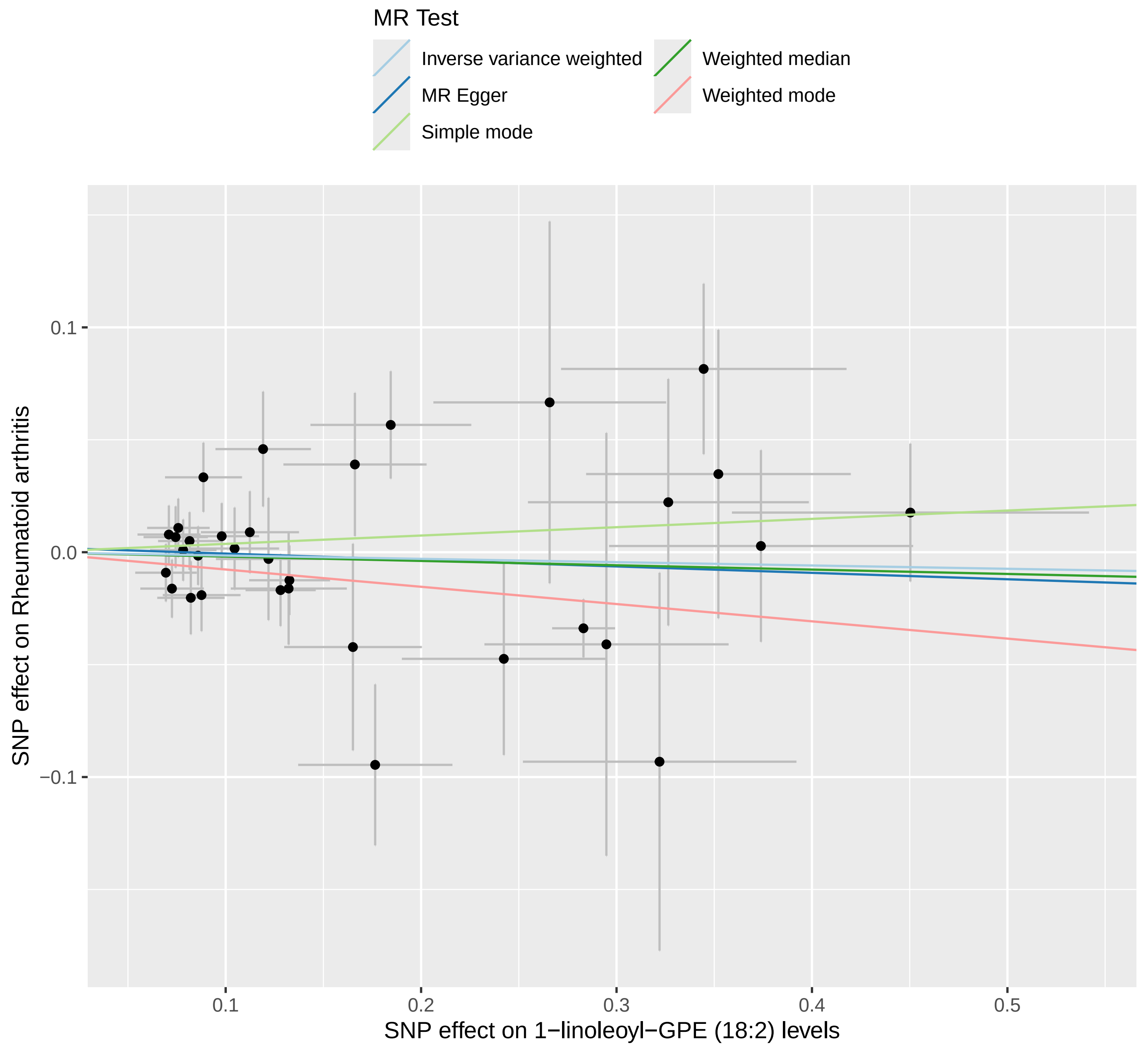
MR Test
Inverse variance weighted
MR Egger
Simple mode
Weighted median
Weighted mode
0.1
0.0
−0.1
SNP effect on Rheumatoid arthritis
0.1
0.2
0.3
0.4
0.5
SNP effect on 1−linoleoyl−GPE (18:2) levels

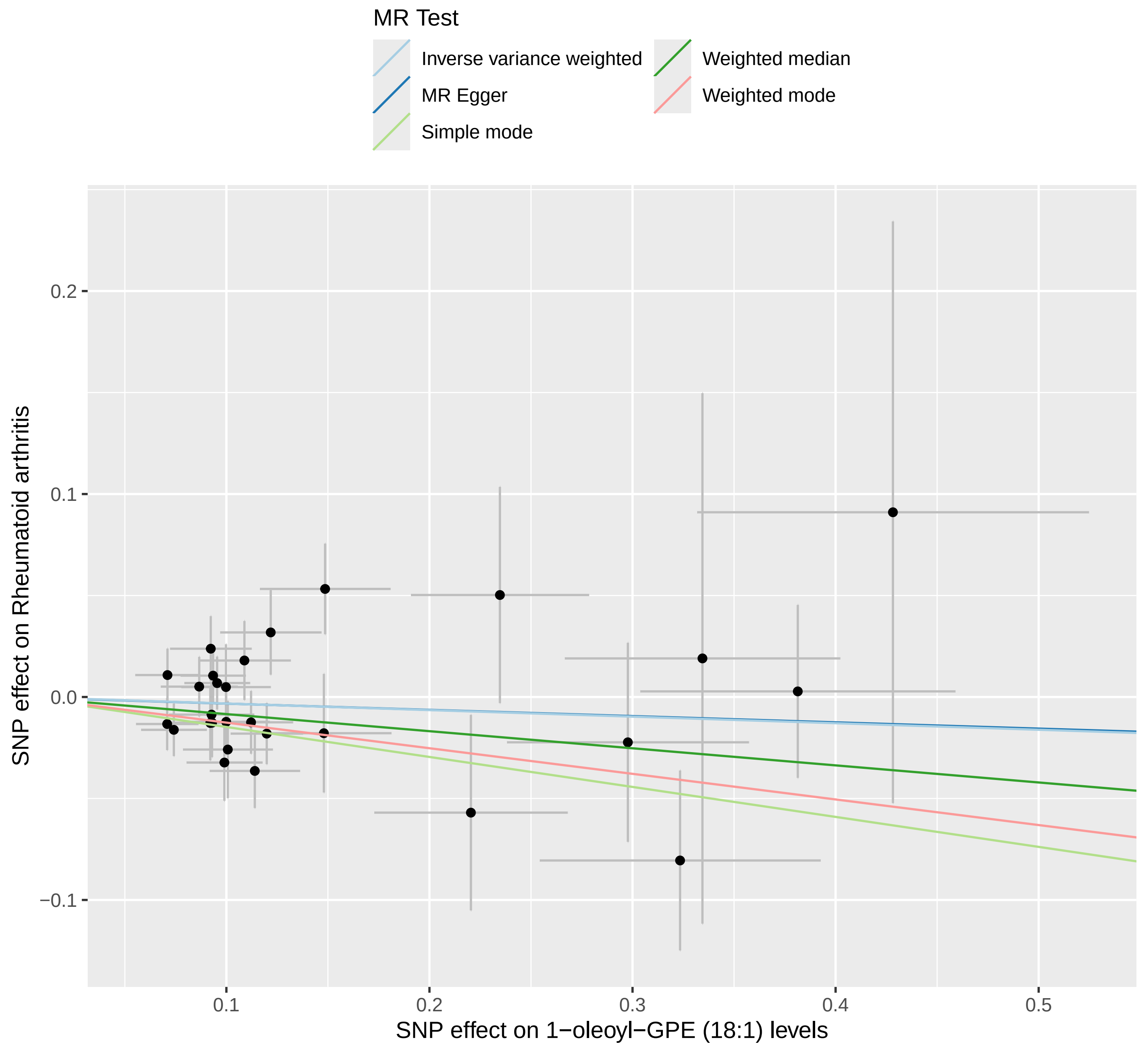
MR Test
Inverse variance weighted
MR Egger
Simple mode
Weighted median
Weighted mode
0.2
0.1
0.0
−0.1
SNP effect on Rheumatoid arthritis
0.1
0.2
0.3
0.4
0.5
SNP effect on 1−oleoyl−GPE (18:1) levels

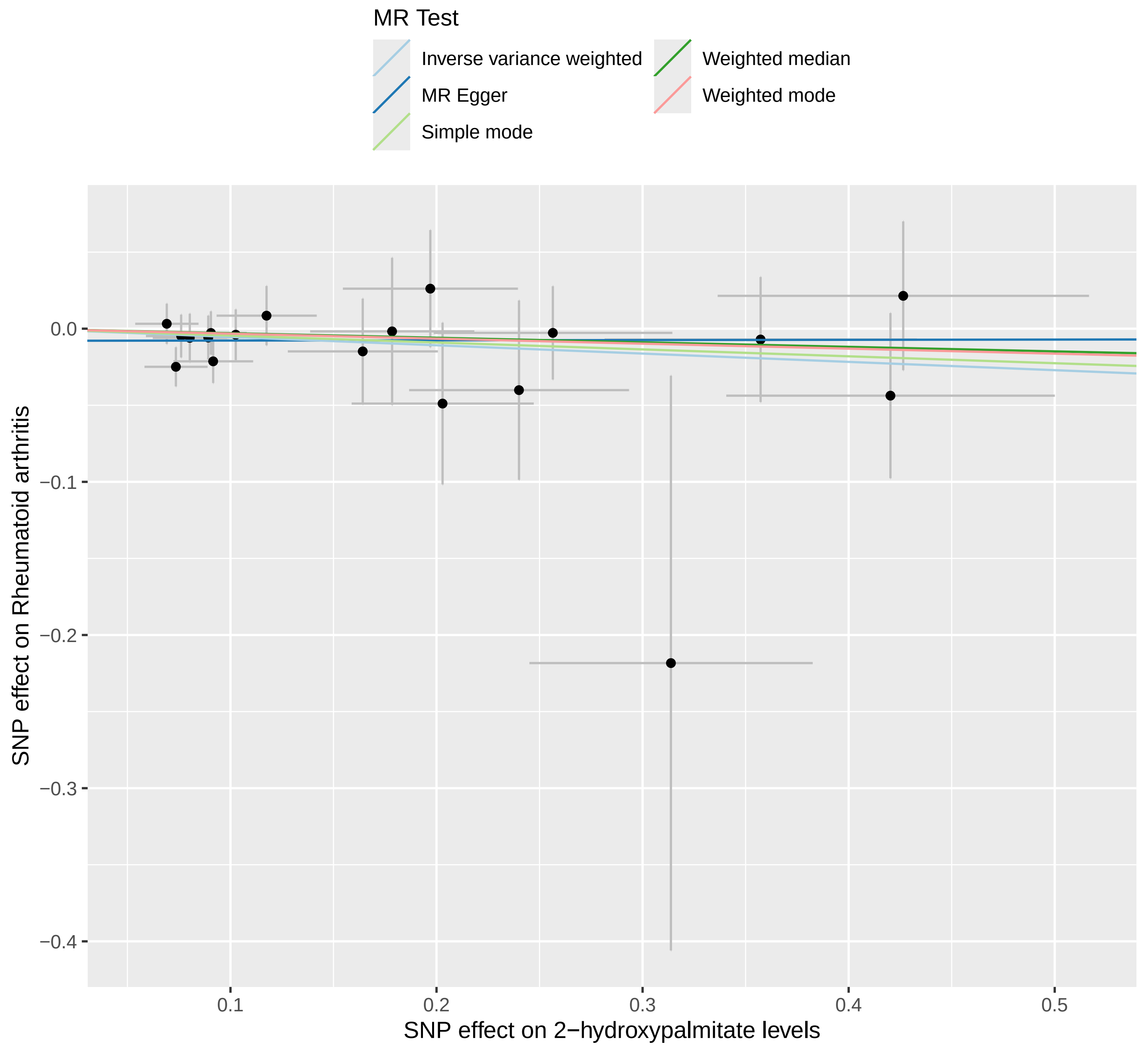

MR Test
Inverse variance weighted
MR Egger
Simple mode
Weighted median
Weighted mode
0.0
−0.1
−0.2
−0.3
−0.4
SNP effect on Rheumatoid arthritis
0.1
0.2
0.3
0.4
0.5
SNP effect on 2−hydroxypalmitate levels

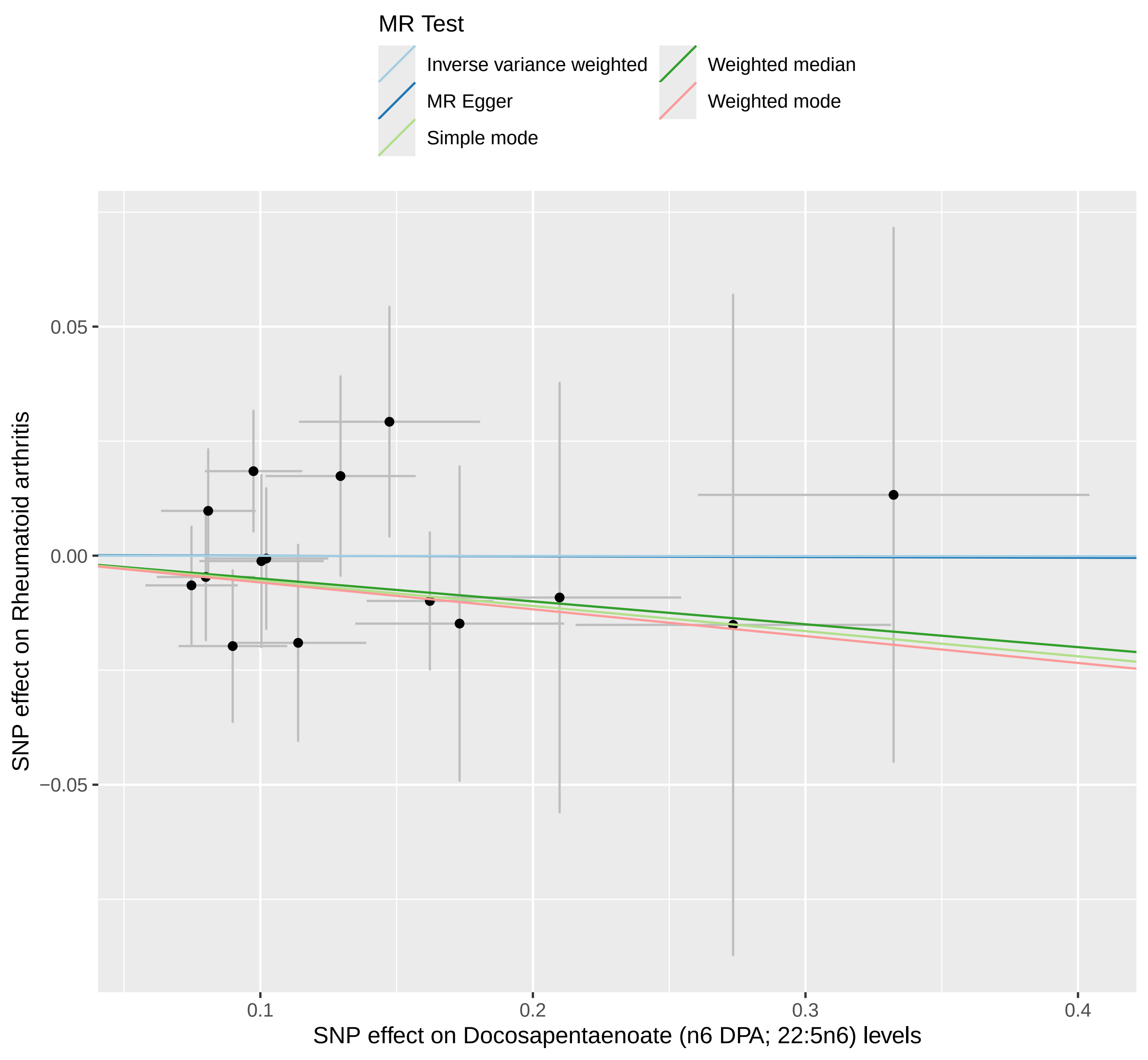
MR Test
Inverse variance weighted
MR Egger
Simple mode
Weighted median
Weighted mode
0.05
0.00
−0.05
0.1
0.2
0.3
0.4
SNP effect on Rheumatoid arthritis
SNP effect on Docosapentaenoate (n6 DPA; 22:5n6) levels

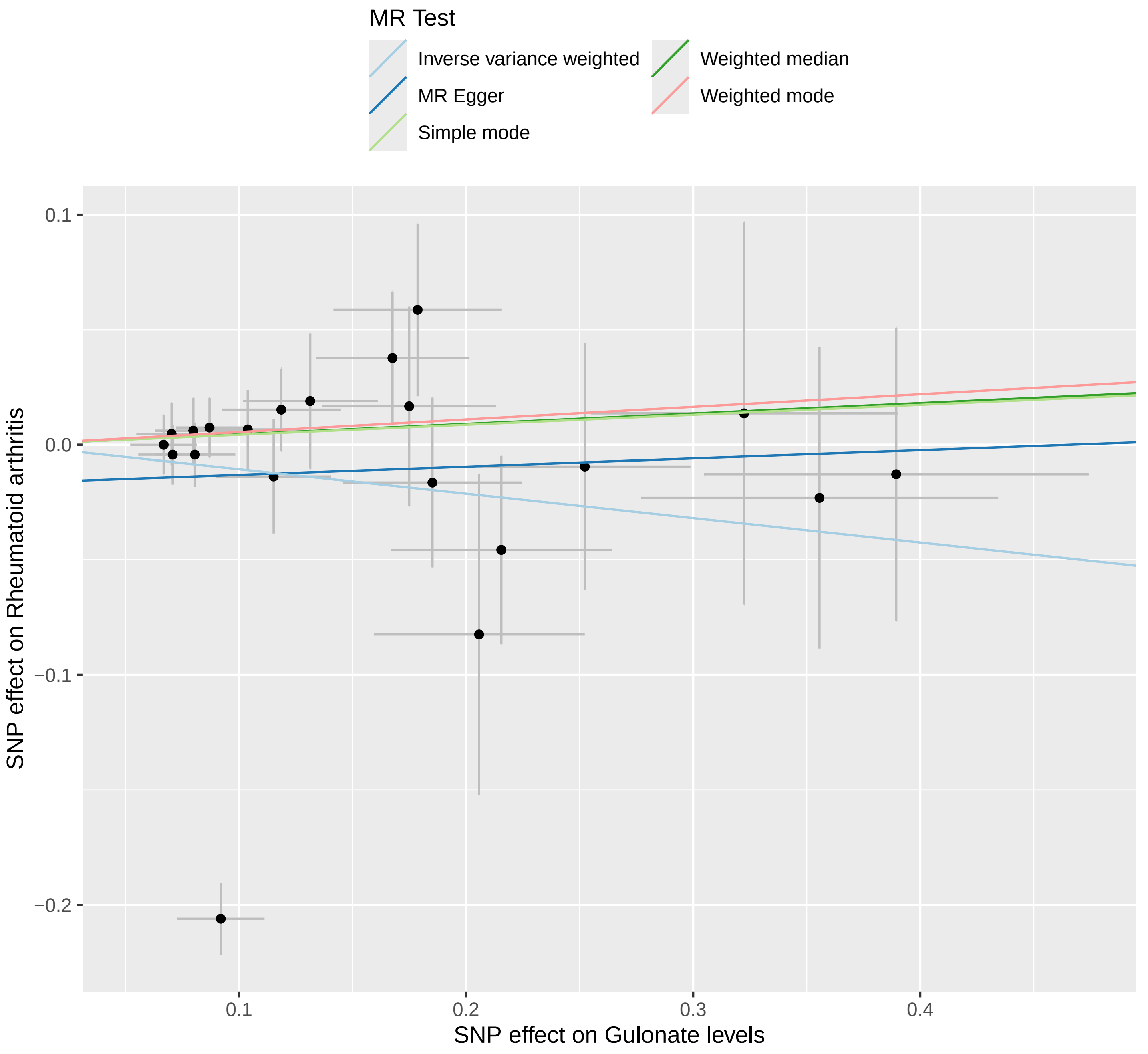

MR Test
Inverse variance weighted
Weighted median
MR Egger
Weighted mode
Simple mode
0.1
0.0
−0.1
−0.2
0.1
0.2
0.3
0.4
SNP effect on Rheumatoid arthritis
SNP effect on Gulonate levels

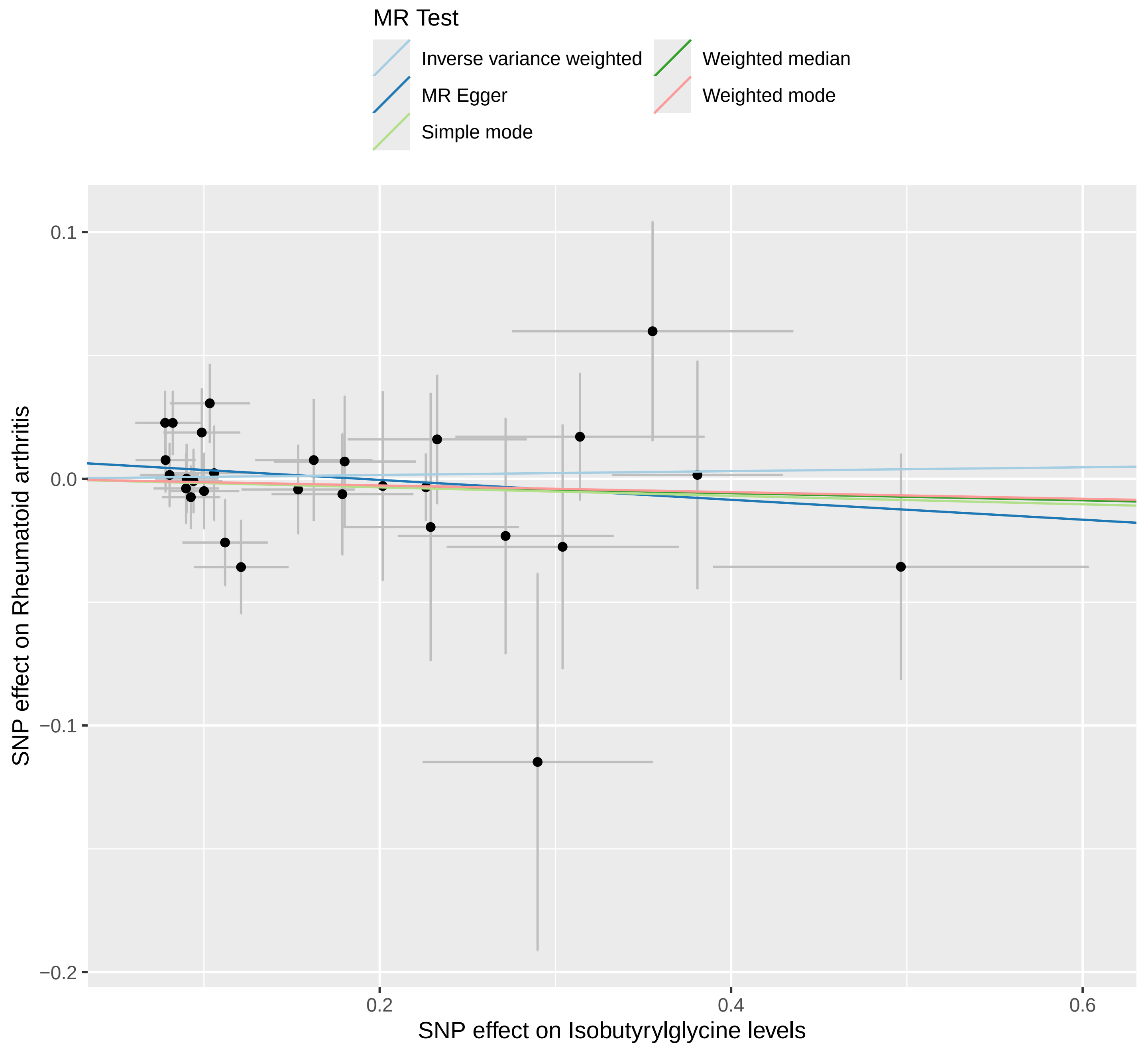

MR Test
Inverse variance weighted
MR Egger
Simple mode
Weighted median
Weighted mode
0.1
0.0
−0.1
−0.2
0.2
0.4
0.6
SNP effect on Rheumatoid arthritis
SNP effect on Isobutyrylglycine levels

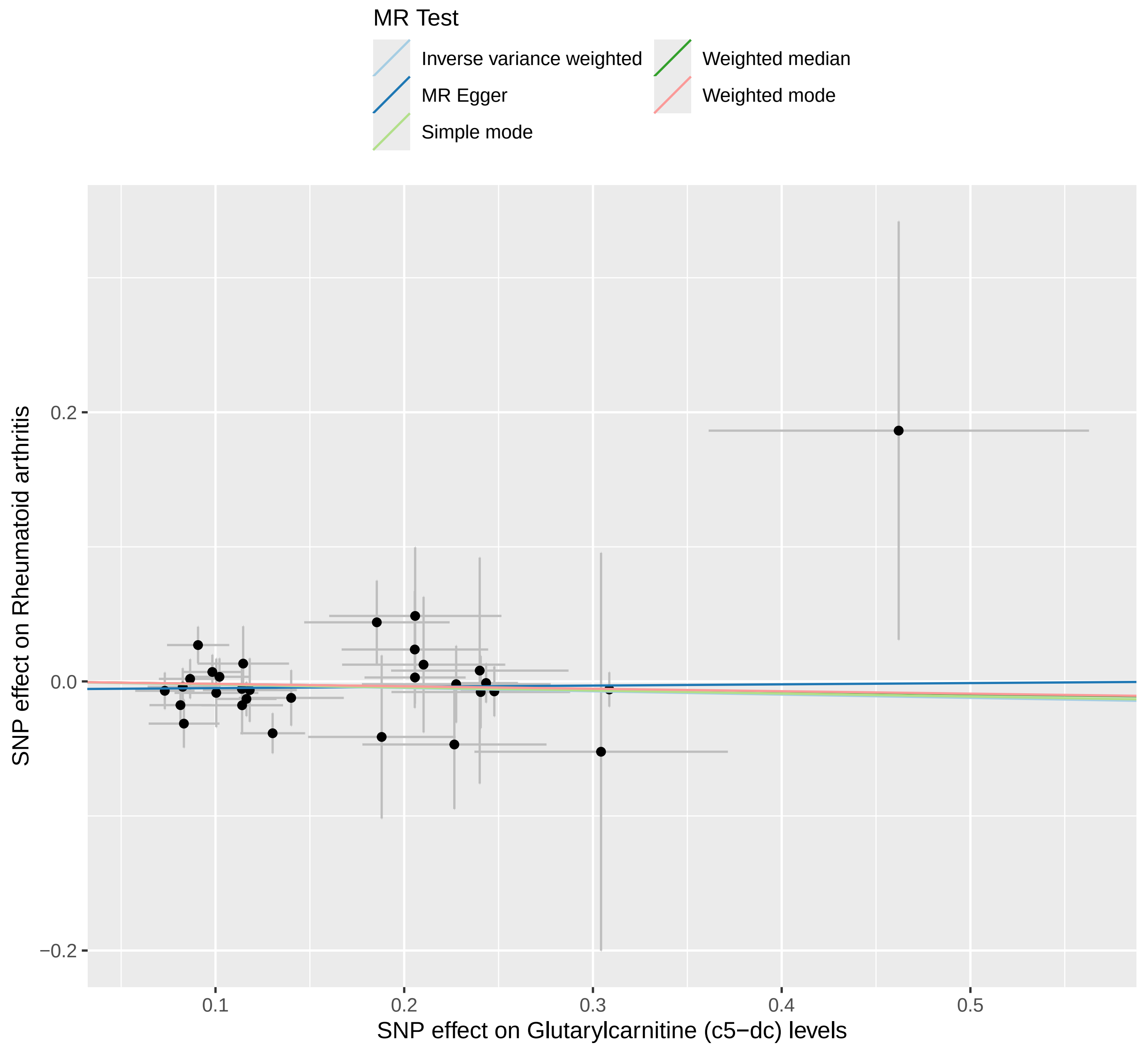
MR Test
Inverse variance weighted
MR Egger
Simple mode
Weighted median
Weighted mode
SNP effect on Rheumatoid arthritis
0.2
0.0
−0.2
0.1
0.2
0.3
0.4
0.5
SNP effect on Glutarylcarnitine (c5−dc) levels

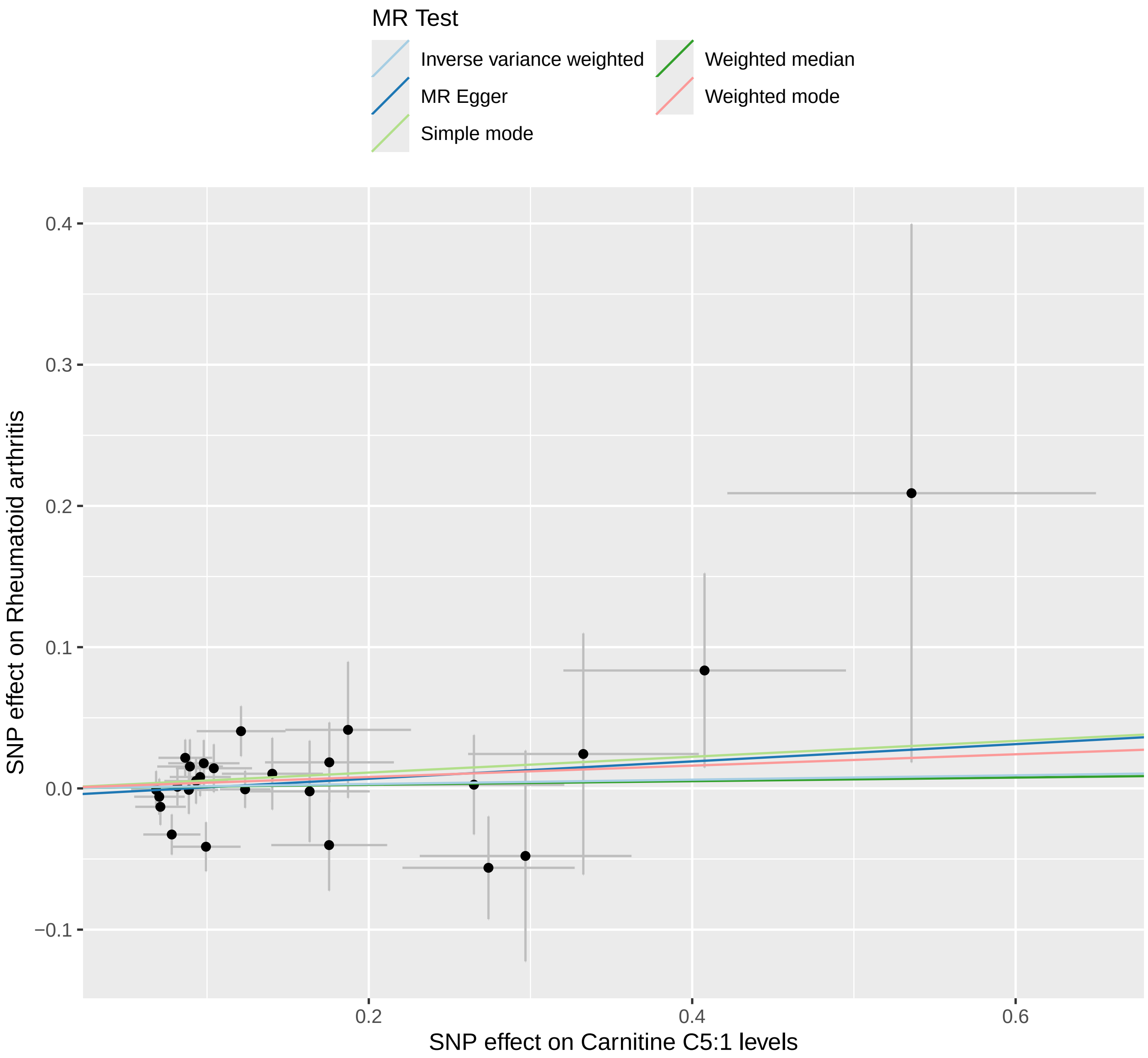

MR Test
Inverse variance weighted
MR Egger
Simple mode
Weighted median
Weighted mode
SNP effect on Rheumatoid arthritis
0.4
0.3
0.2
0.1
0.0
−0.1
0.2
0.4
0.6
SNP effect on Carnitine C5:1 levels

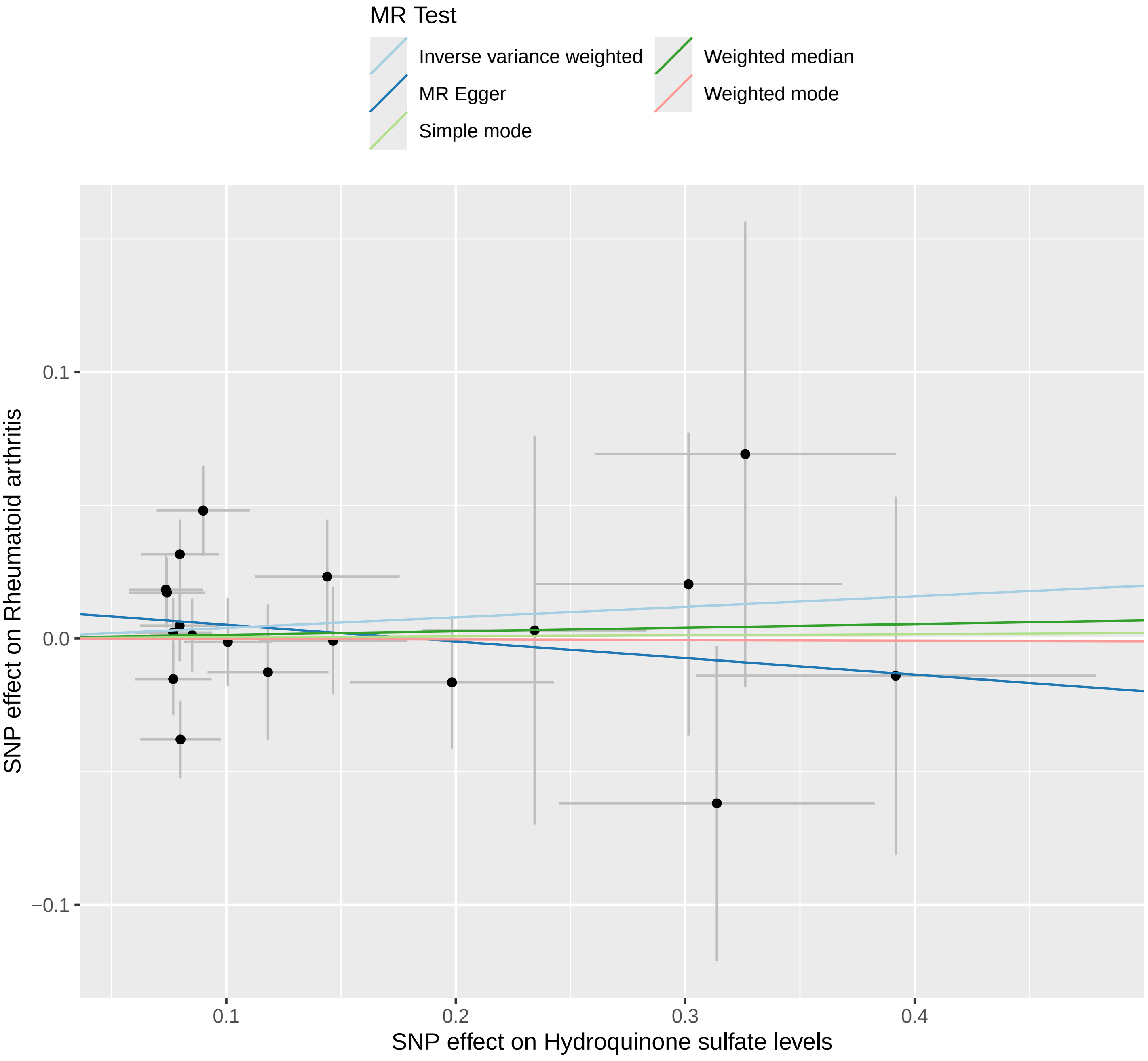
MR Test
Inverse variance weighted
MR Egger
Simple mode
Weighted median
Weighted mode
SNP effect on Rheumatoid arthritis
0.1
0.0
−0.1
0.1
0.2
0.3
0.4
SNP effect on Hydroquinone sulfate levels

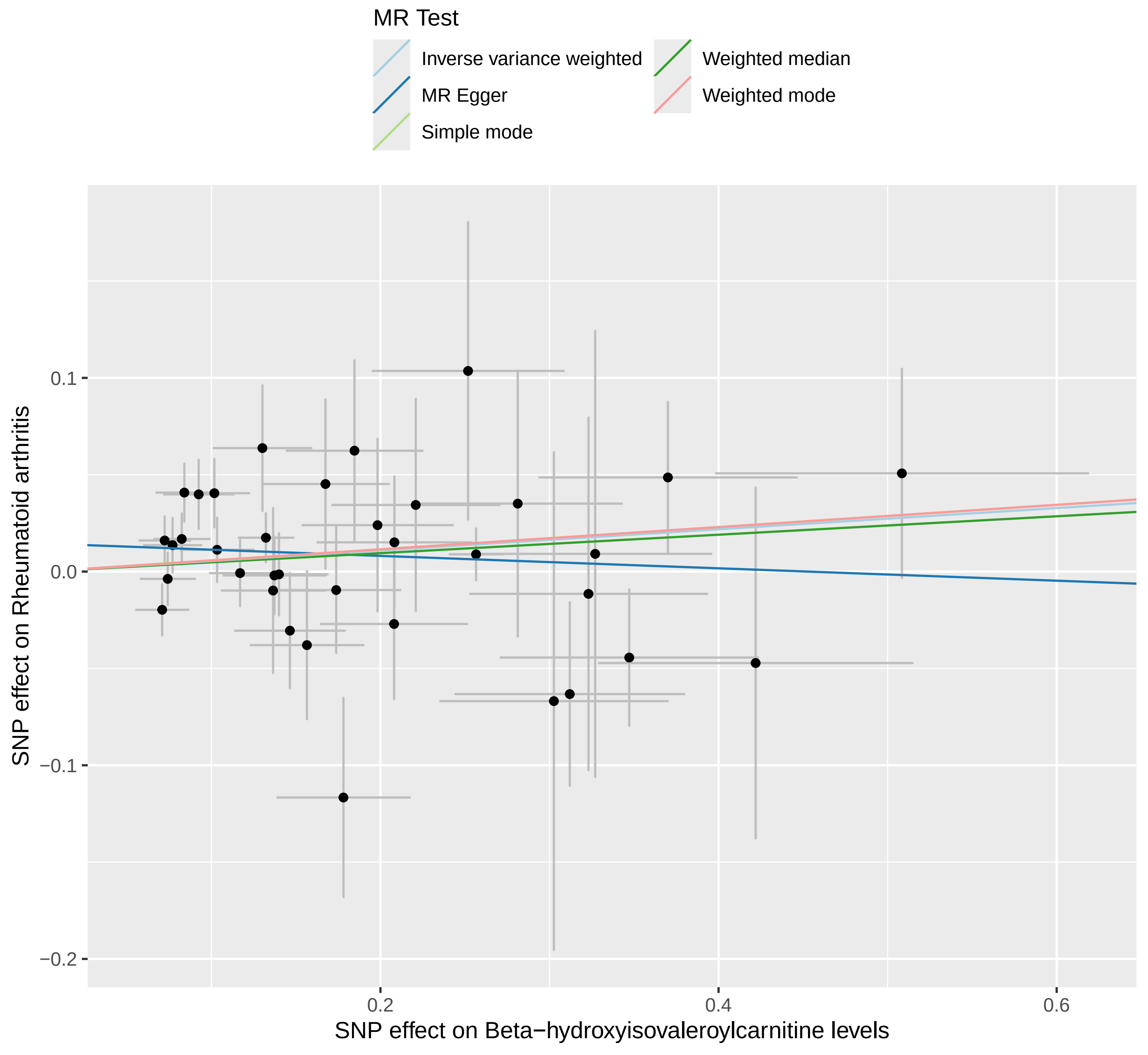
MR Test
Inverse variance weighted
MR Egger
Simple mode
Weighted median
Weighted mode
0.1
0.0
−0.1
−0.2
0.2
0.4
0.6
SNP effect on Rheumatoid arthritis
SNP effect on Beta−hydroxyisovaleroylcarnitine levels

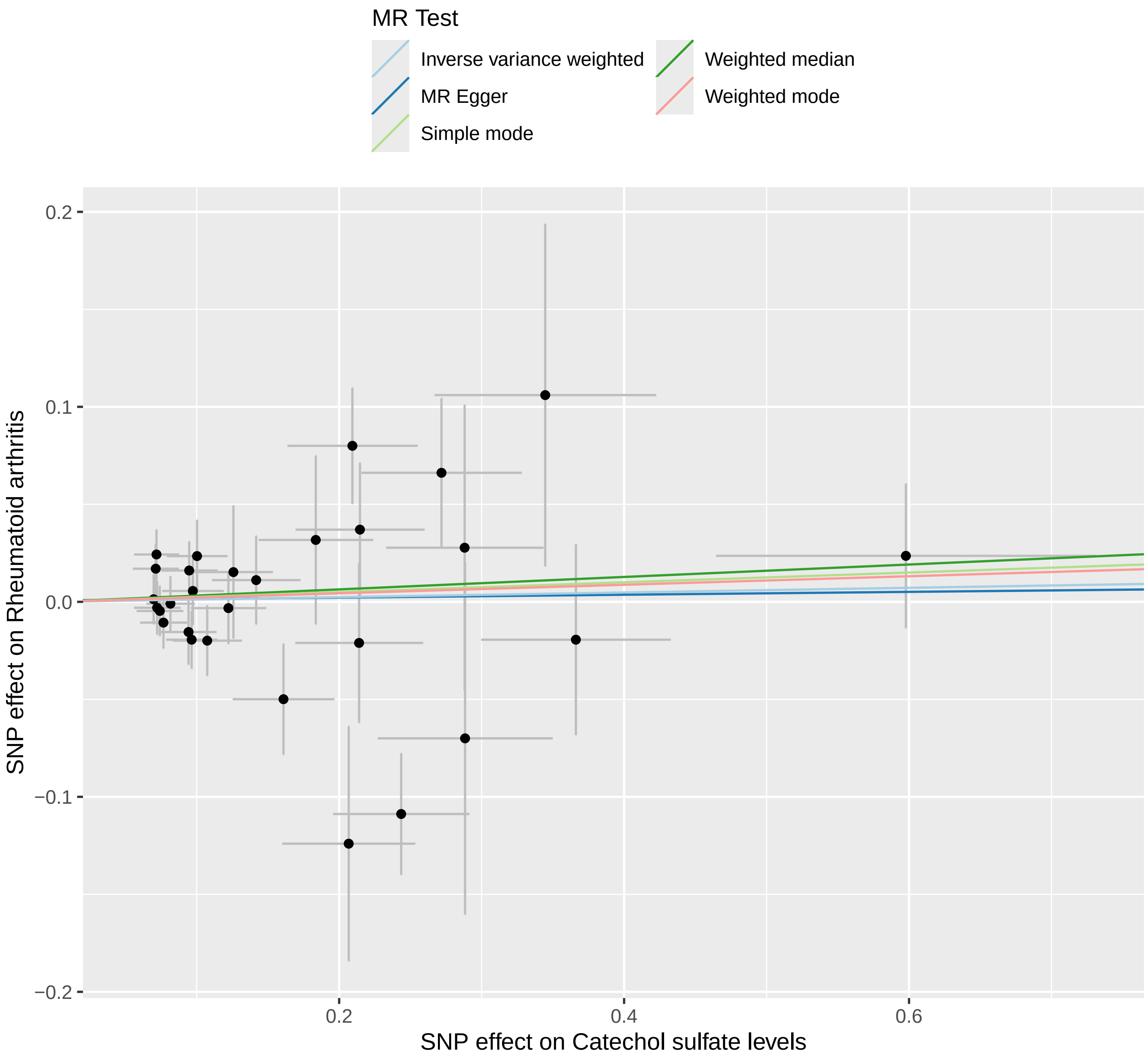
MR Test
Inverse variance weighted
MR Egger
Simple mode
Weighted median
Weighted mode
0.2
0.1
0.0
−0.1
−0.2
0.2
0.4
0.6
SNP effect on Rheumatoid arthritis
SNP effect on Catechol sulfate levels

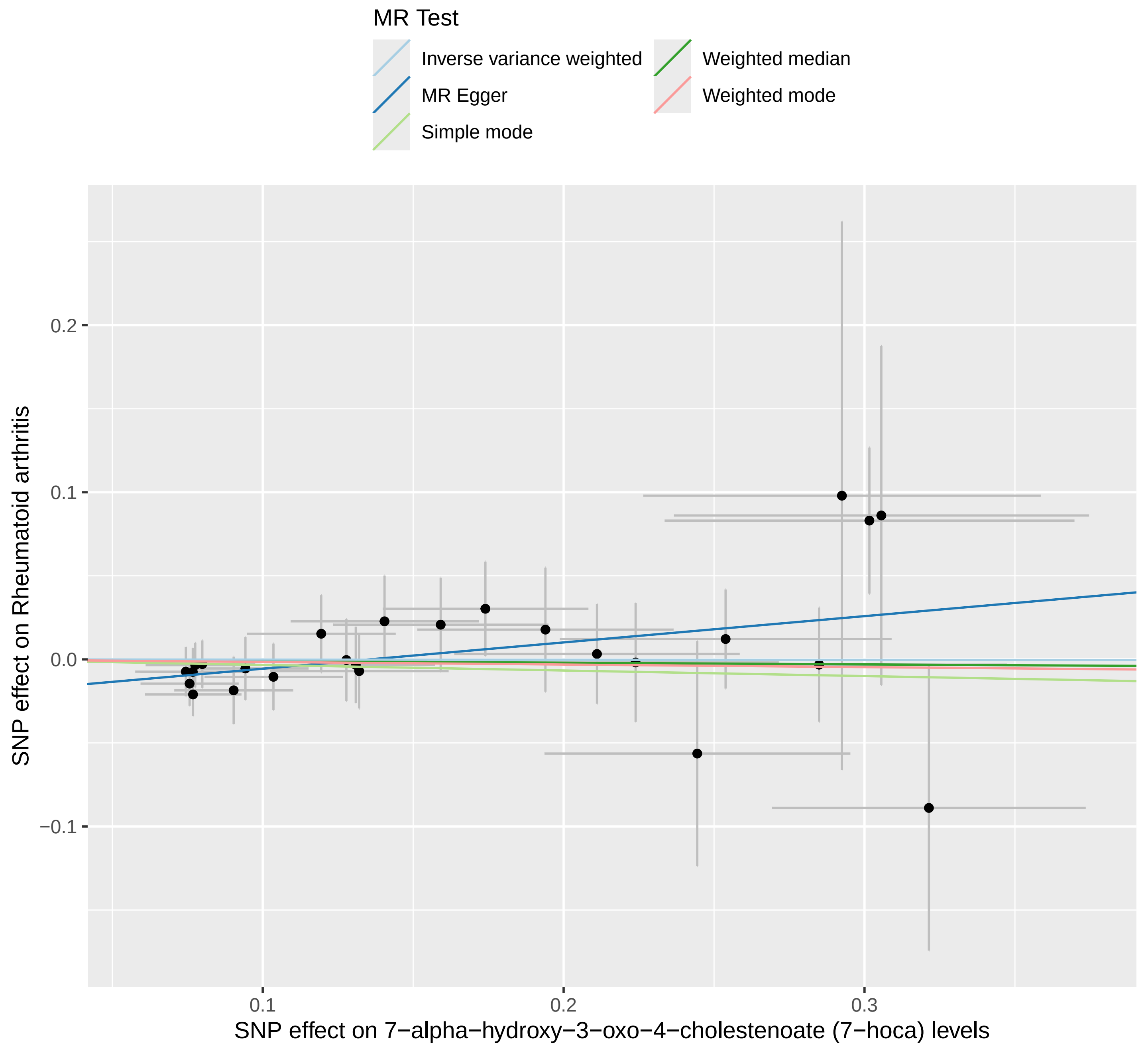
MR Test
Inverse variance weighted
MR Egger
Simple mode
Weighted median
Weighted mode
0.2
0.1
0.0
−0.1
0.1
0.2
0.3
SNP effect on Rheumatoid arthritis
SNP effect on 7−alpha−hydroxy−3−oxo−4−cholestenoate (7−hoca) levels

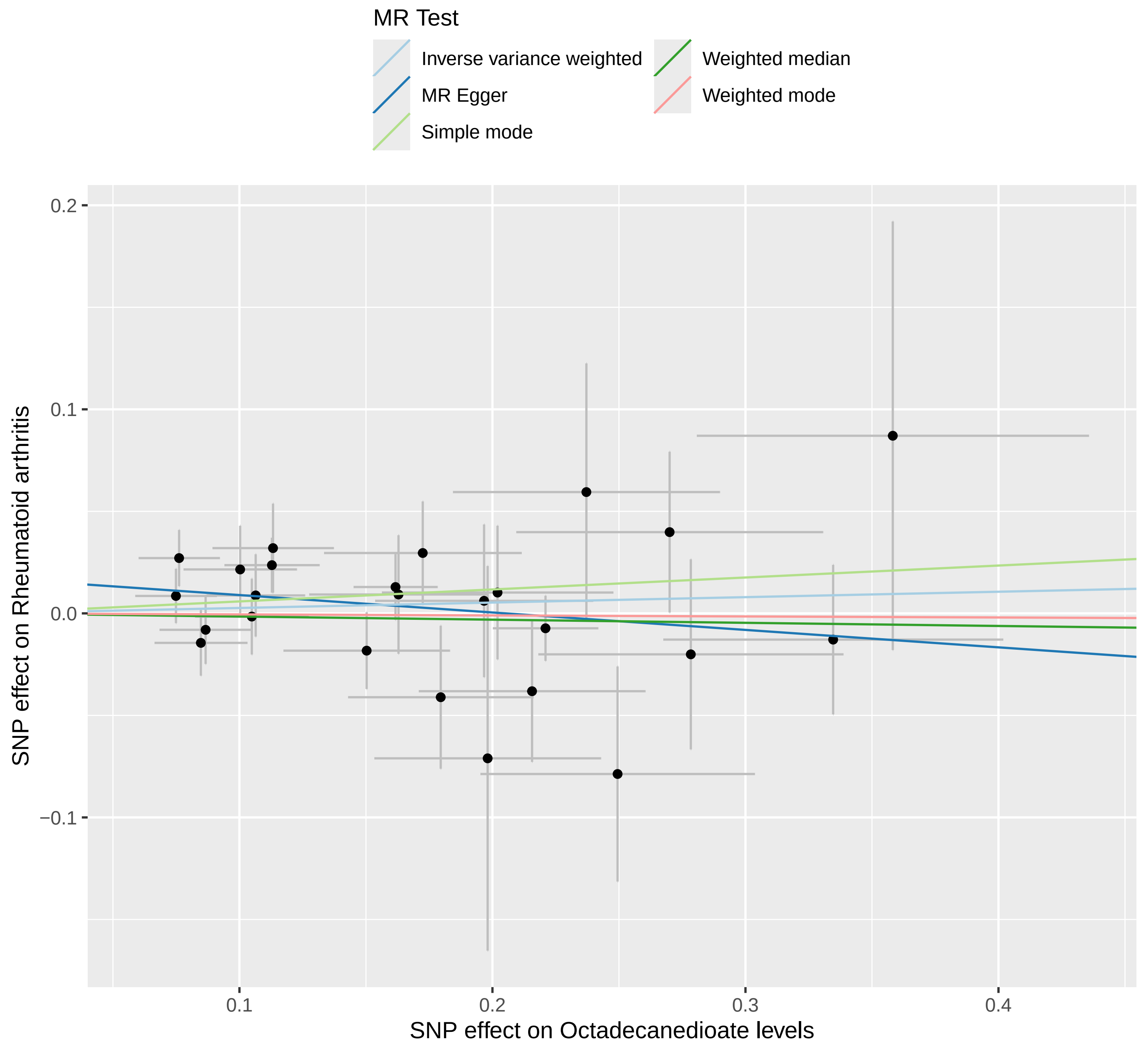
MR Test
Inverse variance weighted
MR Egger
Simple mode
Weighted median
Weighted mode
0.2
0.1
0.0
−0.1
0.1
0.2
0.3
0.4
SNP effect on Rheumatoid arthritis
SNP effect on Octadecanedioate levels

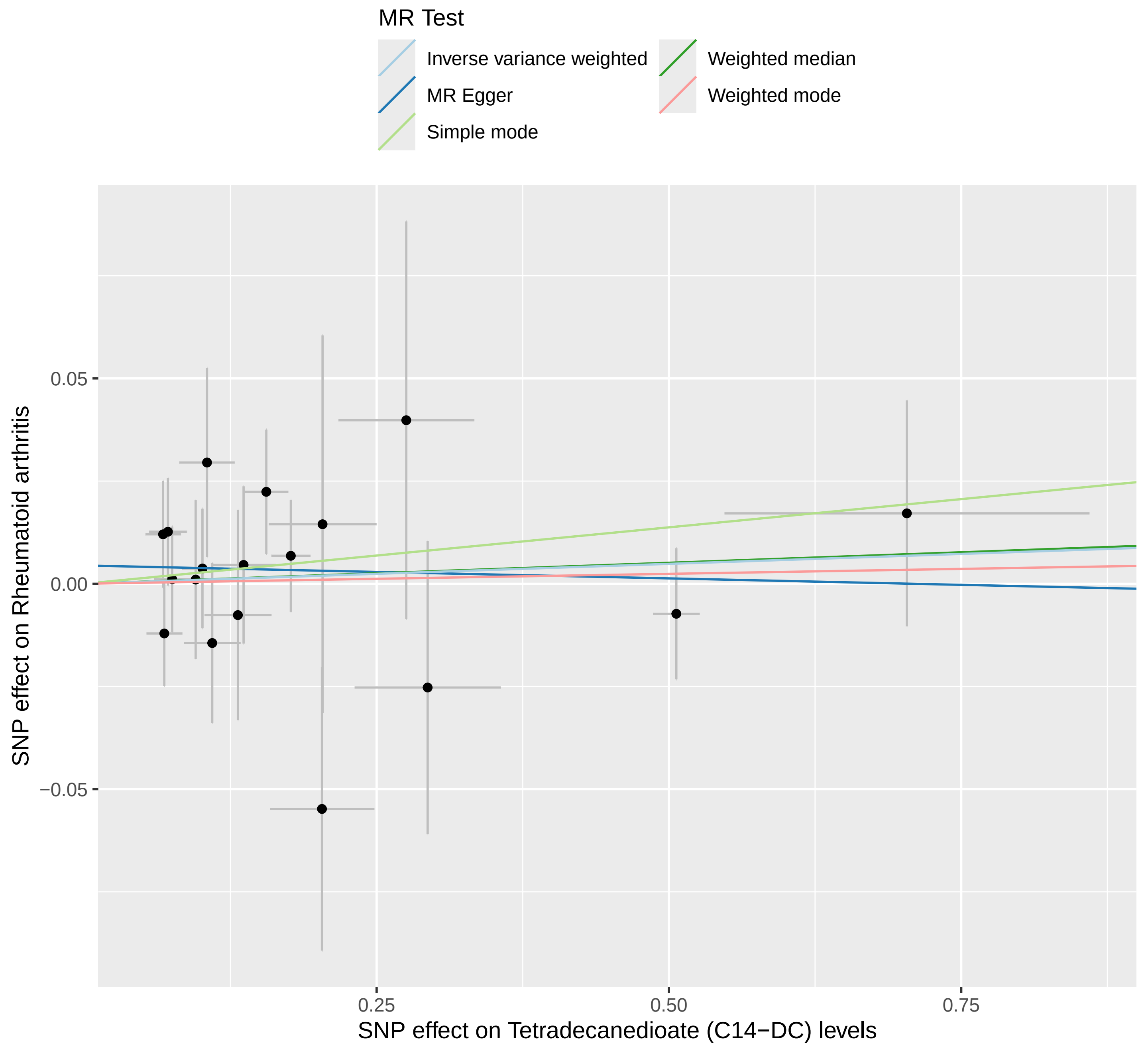
MR Test
Inverse variance weighted
MR Egger
Simple mode
Weighted median
Weighted mode
SNP effect on Rheumatoid arthritis
0.05
0.00
−0.05
0.25
0.50
0.75
SNP effect on Tetradecanedioate (C14−DC) levels

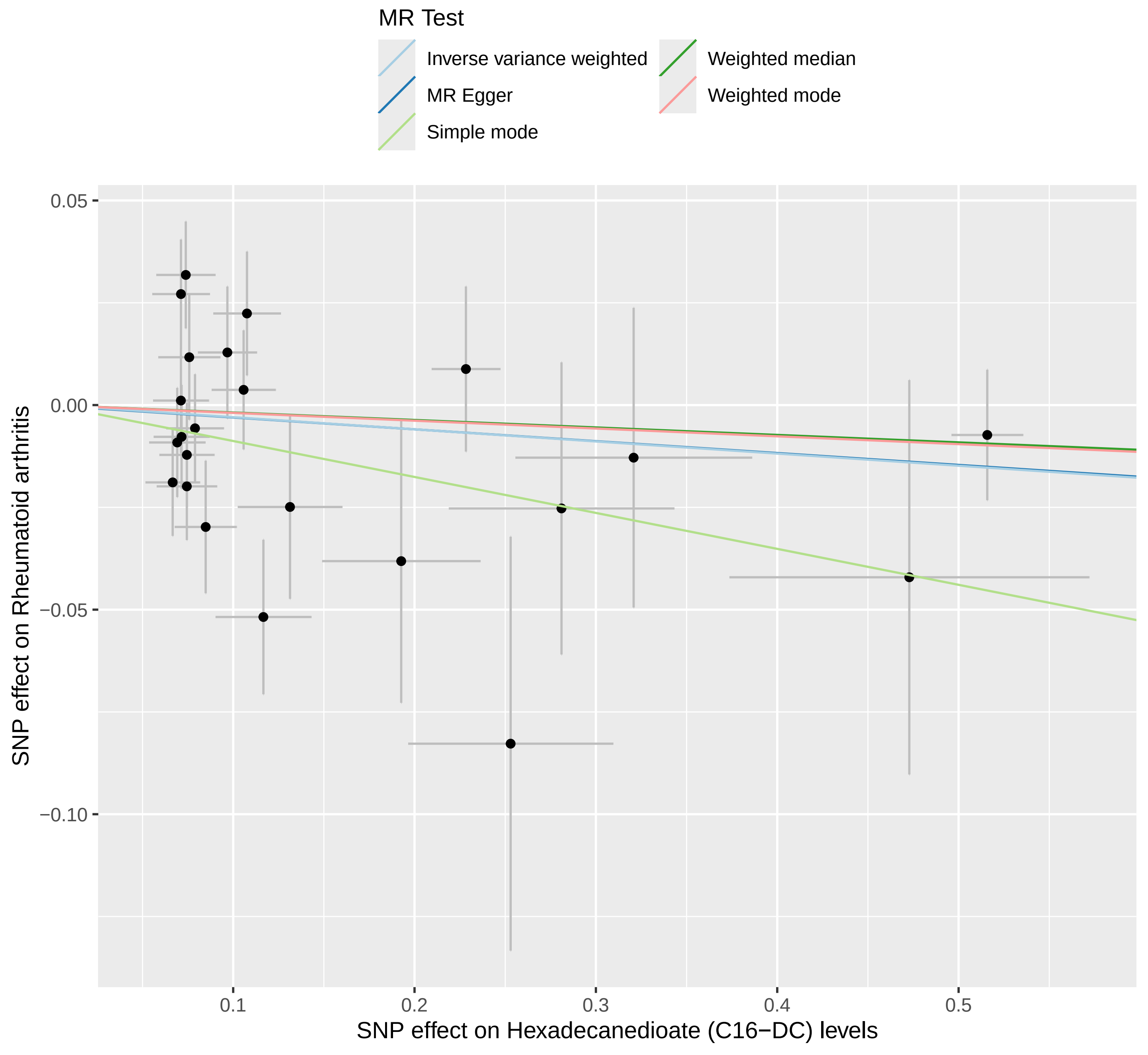
MR Test
Inverse variance weighted
MR Egger
Simple mode
Weighted median
Weighted mode
0.05
0.00
−0.05
−0.10
0.1
0.2
0.3
0.4
0.5
SNP effect on Rheumatoid arthritis
SNP effect on Hexadecanedioate (C16−DC) levels

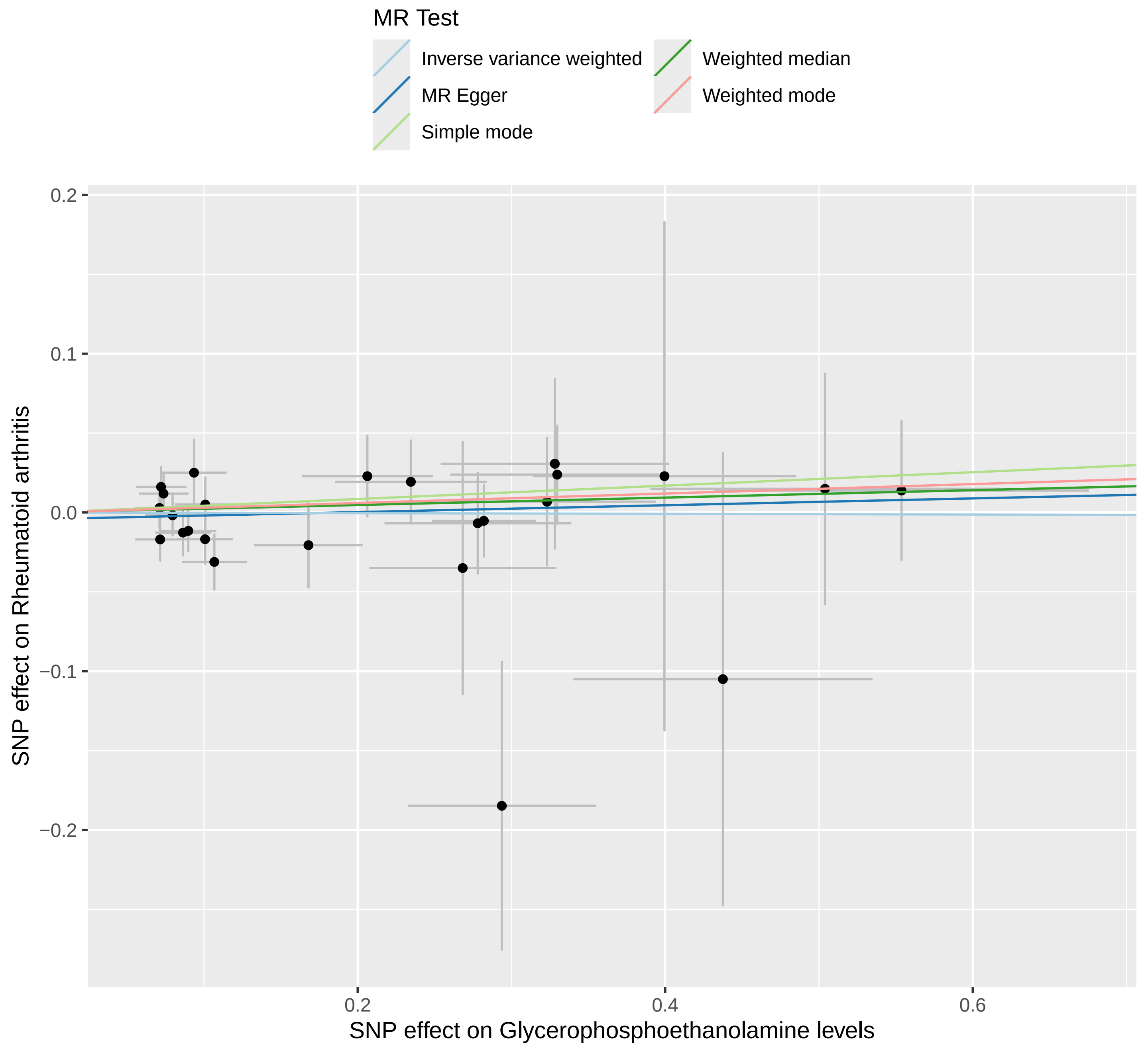
MR Test
Inverse variance weighted
MR Egger
Simple mode
Weighted median
Weighted mode
0.2
0.1
0.0
−0.1
−0.2
SNP effect on Rheumatoid arthritis
0.2
0.4
0.6
SNP effect on Glycerophosphoethanolamine levels

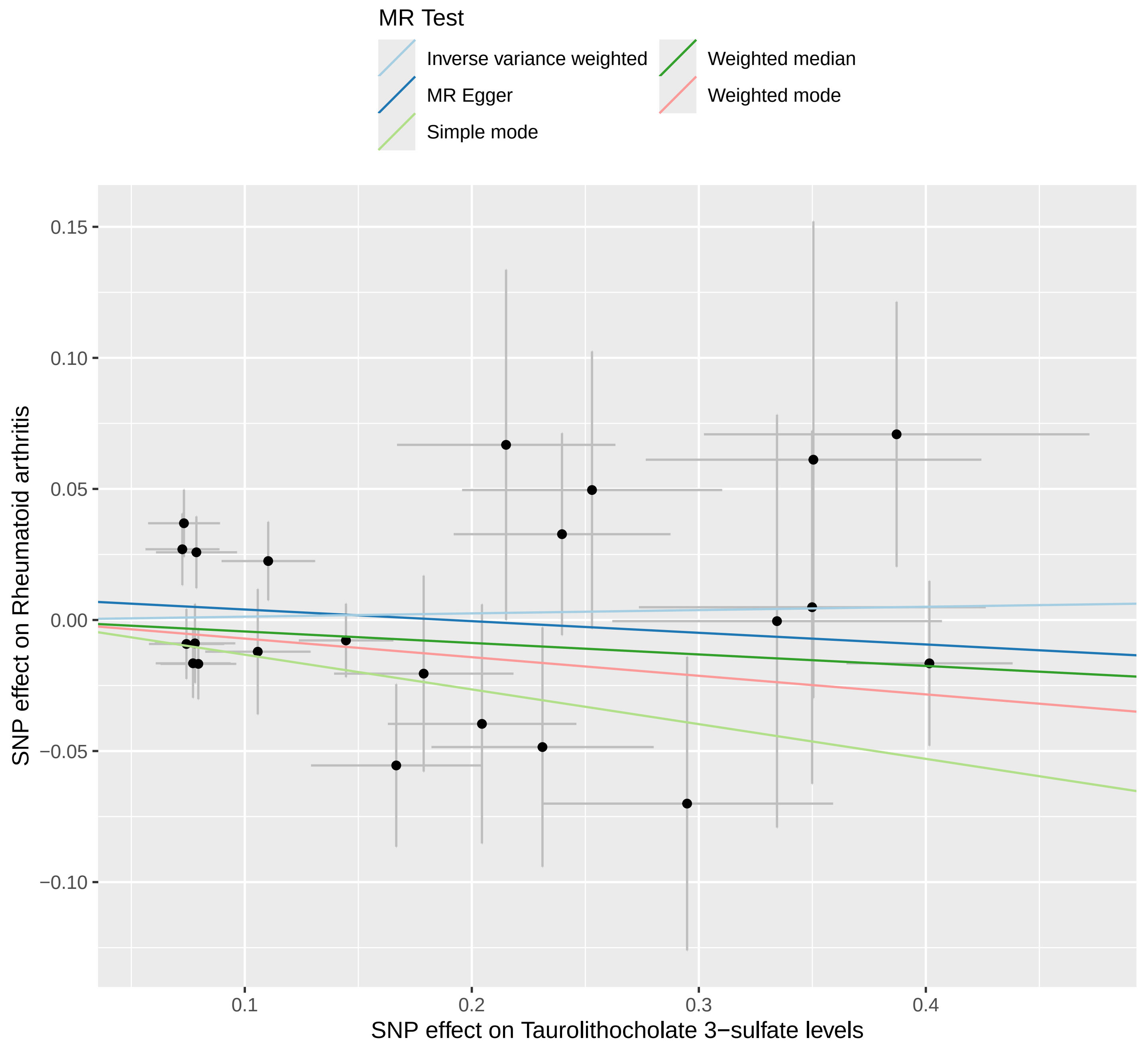
MR Test
Inverse variance weighted
MR Egger
Simple mode
Weighted median
Weighted mode
SNP effect on Rheumatoid arthritis
0.15
0.10
0.05
0.00
−0.05
−0.10
0.1
0.2
0.3
0.4
SNP effect on Taurolithocholate 3−sulfate levels

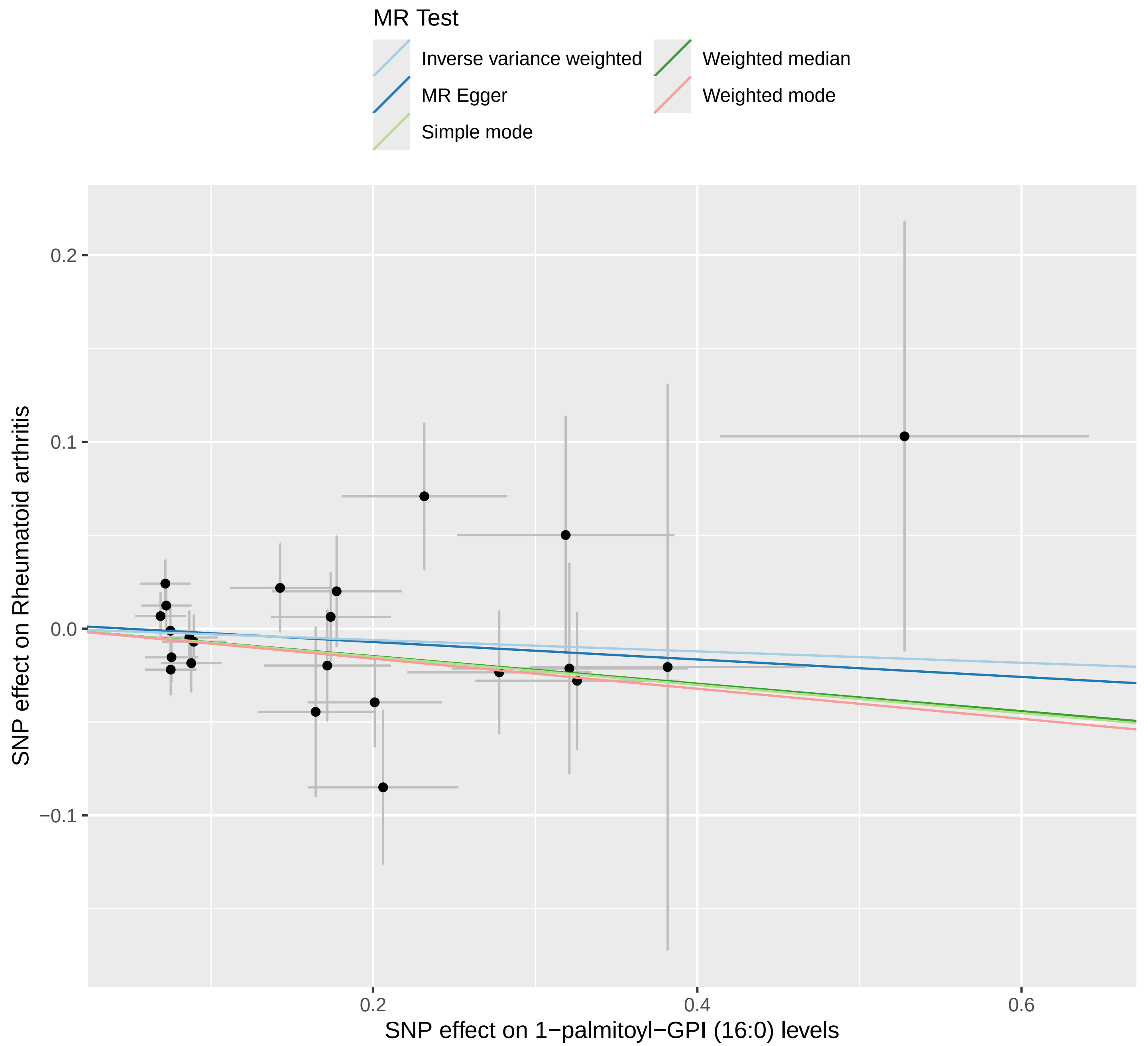
MR Test
Inverse variance weighted
MR Egger
Simple mode
Weighted median
Weighted mode
0.2
0.1
0.0
−0.1
SNP effect on Rheumatoid arthritis
0.2
0.4
0.6
SNP effect on 1−palmitoyl−GPI (16:0) levels

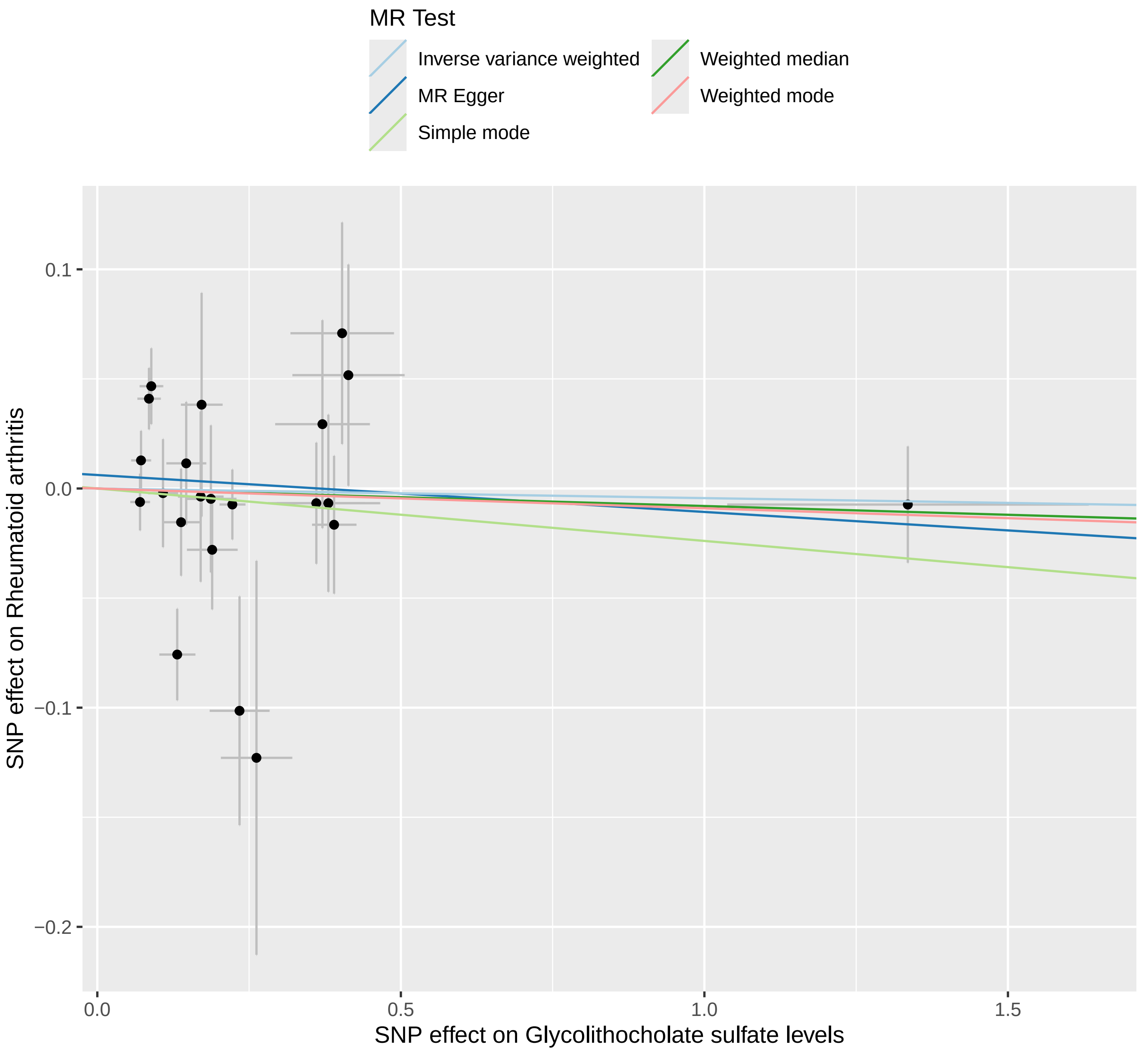

MR Test
Inverse variance weighted
MR Egger
Simple mode
Weighted median
Weighted mode
0.1
0.0
−0.1
−0.2
SNP effect on Rheumatoid arthritis
0.0
0.5
1.0
1.5
SNP effect on Glycolithocholate sulfate levels

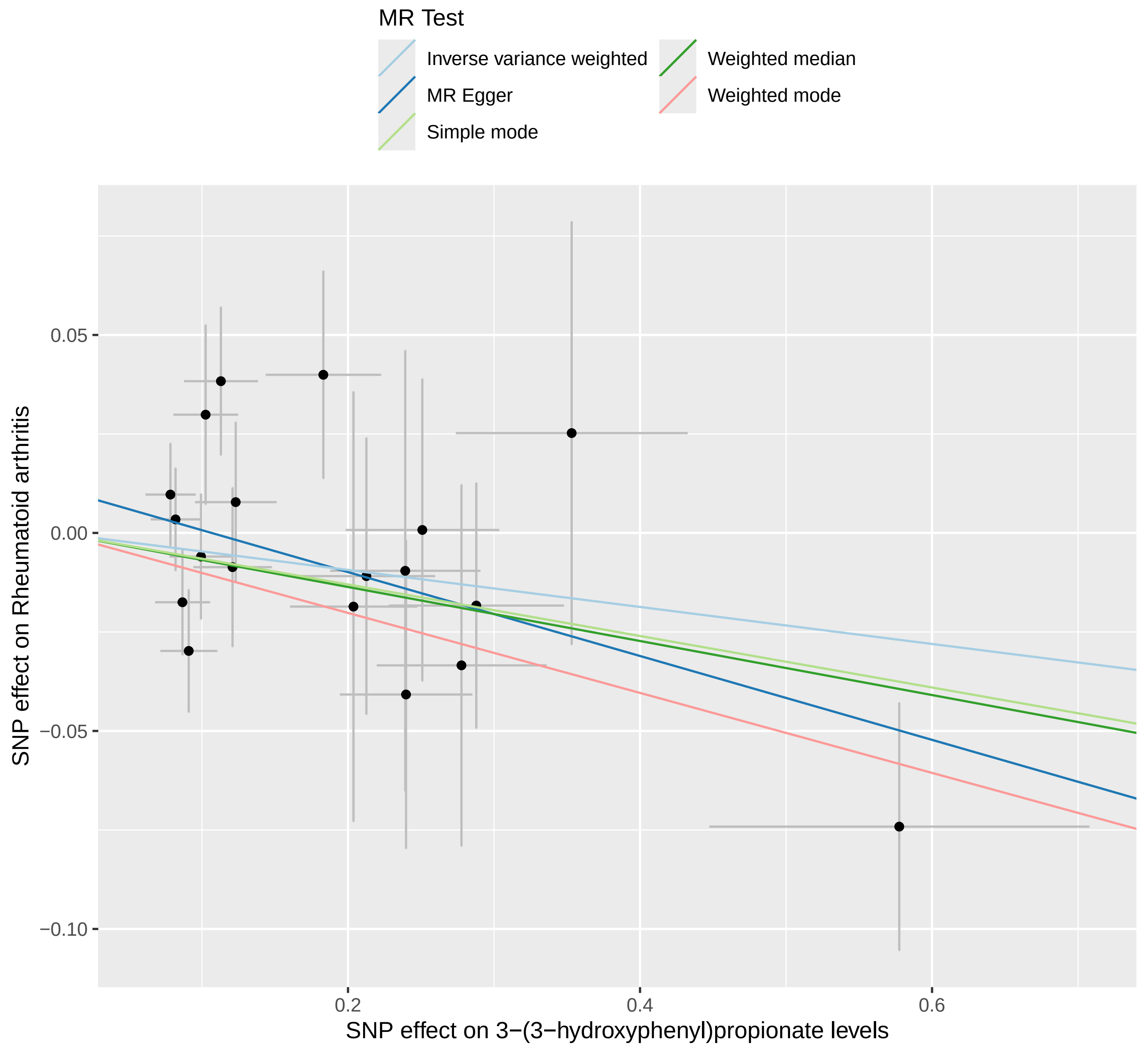
MR Test
Inverse variance weighted
MR Egger
Simple mode
Weighted median
Weighted mode
0.05
0.00
−0.05
−0.10
0.2
0.4
0.6
SNP effect on Rheumatoid arthritis
SNP effect on 3−(3−hydroxyphenyl)propionate levels

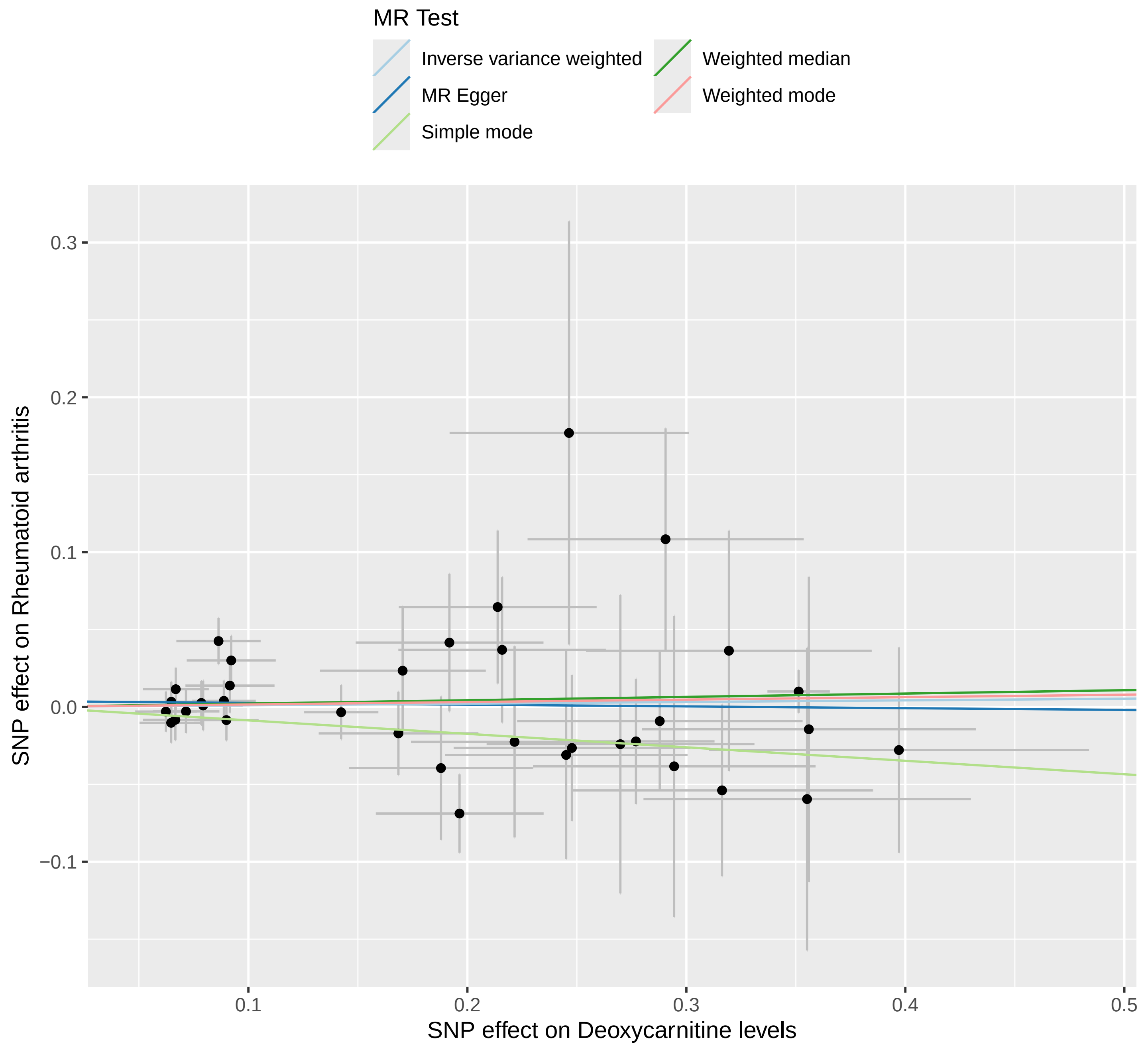

MR Test
Inverse variance weighted
MR Egger
Simple mode
Weighted median
Weighted mode
0.3
0.2
0.1
0.0
−0.1
SNP effect on Rheumatoid arthritis
0.1
0.2
0.3
0.4
0.5
SNP effect on Deoxycarnitine levels

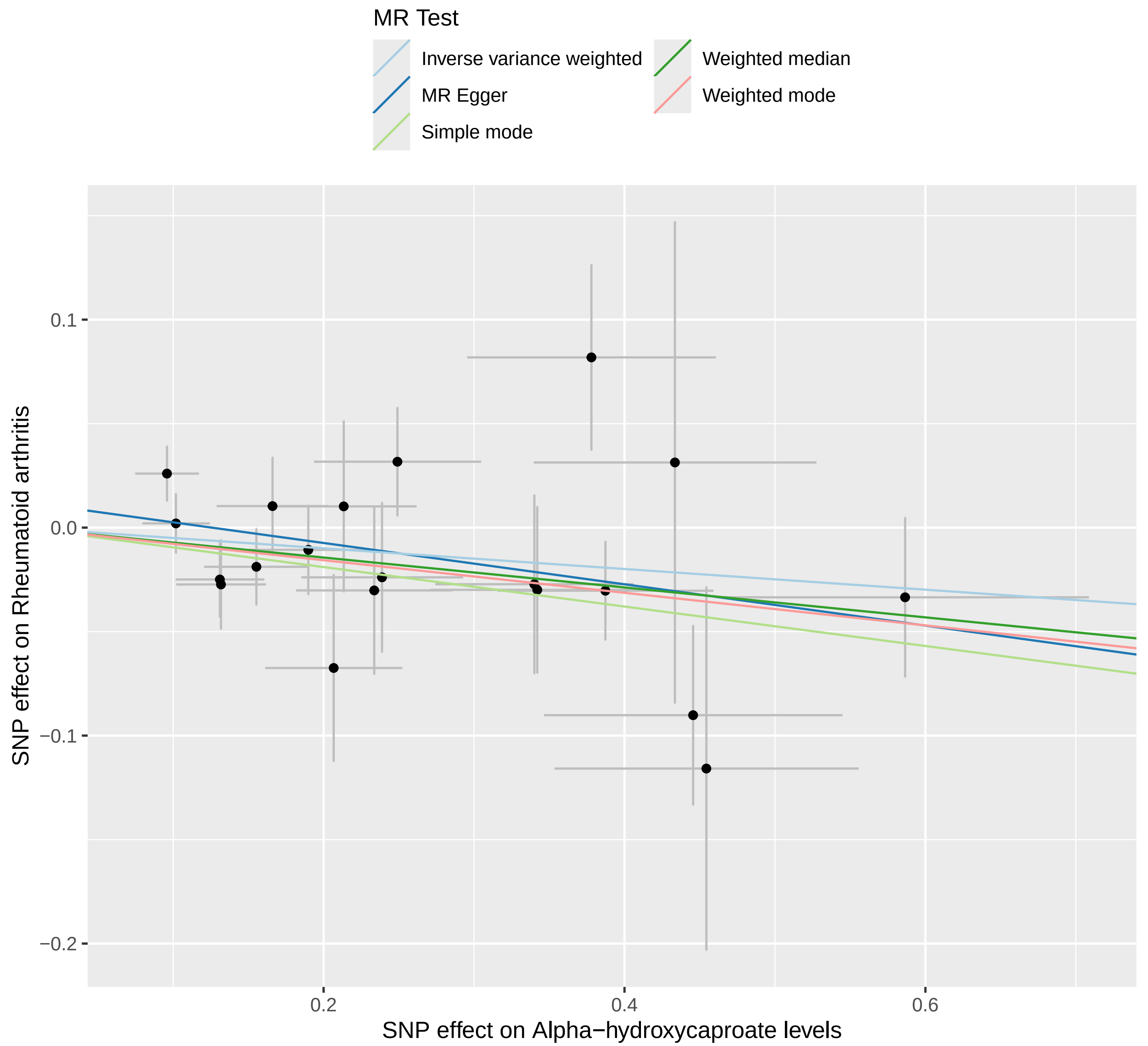
MR Test
Inverse variance weighted
MR Egger
Simple mode
Weighted median
Weighted mode
0.1
0.0
−0.1
−0.2
SNP effect on Rheumatoid arthritis
0.2
0.4
0.6
SNP effect on Alpha−hydroxycaproate levels

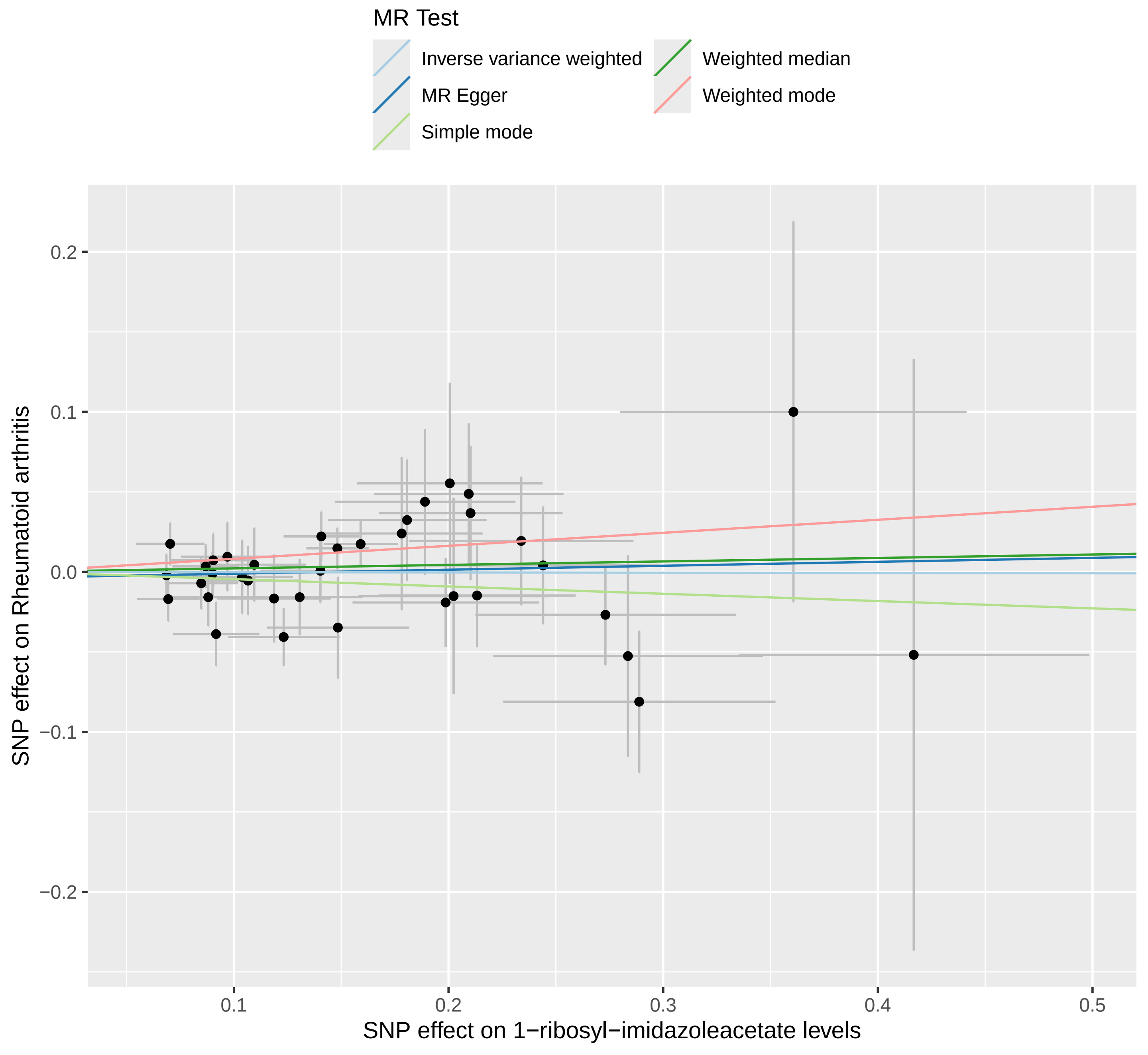

MR Test
Inverse variance weighted
MR Egger
Simple mode
Weighted median
Weighted mode
0.2
0.1
0.0
−0.1
−0.2
0.1
0.2
0.3
0.4
0.5
SNP effect on Rheumatoid arthritis
SNP effect on 1−ribosyl−imidazoleacetate levels

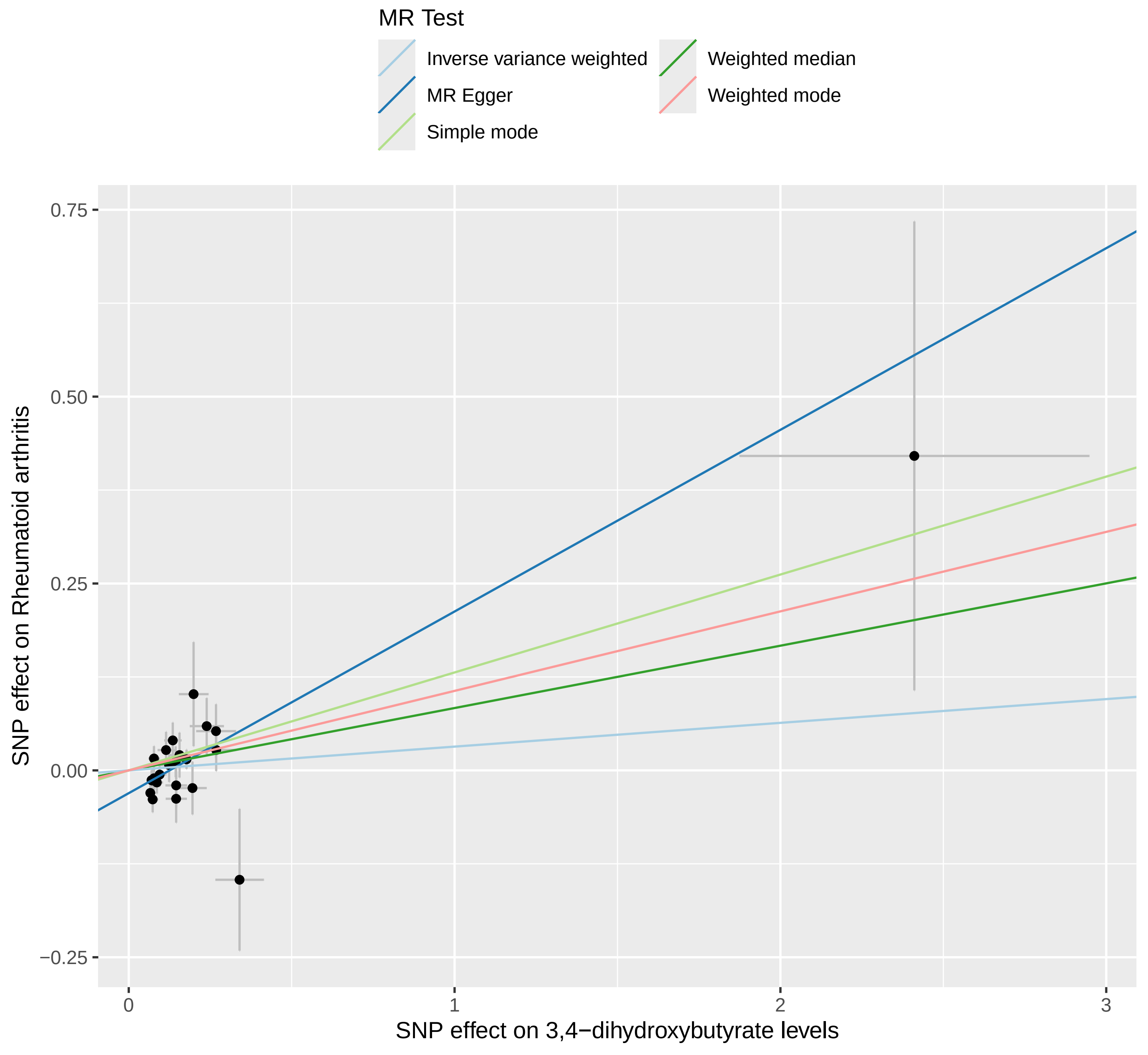

MR Test
Inverse variance weighted
MR Egger
Simple mode
Weighted median
Weighted mode
SNP effect on Rheumatoid arthritis
0.75
0.50
0.25
0.00
−0.25
0
1
2
3
SNP effect on 3,4−dihydroxybutyrate levels

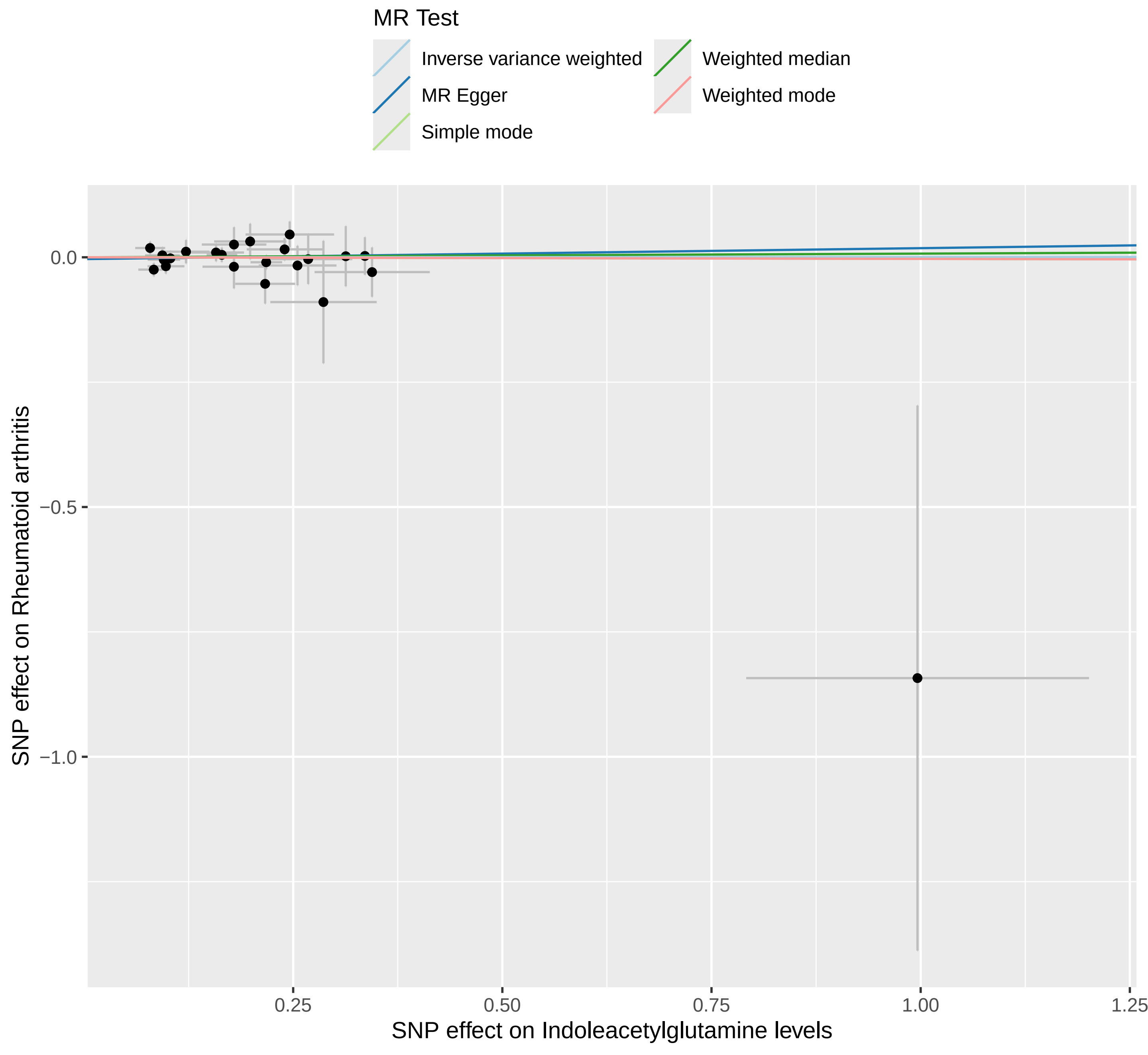
MR Test
Inverse variance weighted
MR Egger
Simple mode
Weighted median
Weighted mode
0.0
−0.5
−1.0
SNP effect on Rheumatoid arthritis
0.25
0.50
0.75
1.00
1.25
SNP effect on Indoleacetylglutamine levels

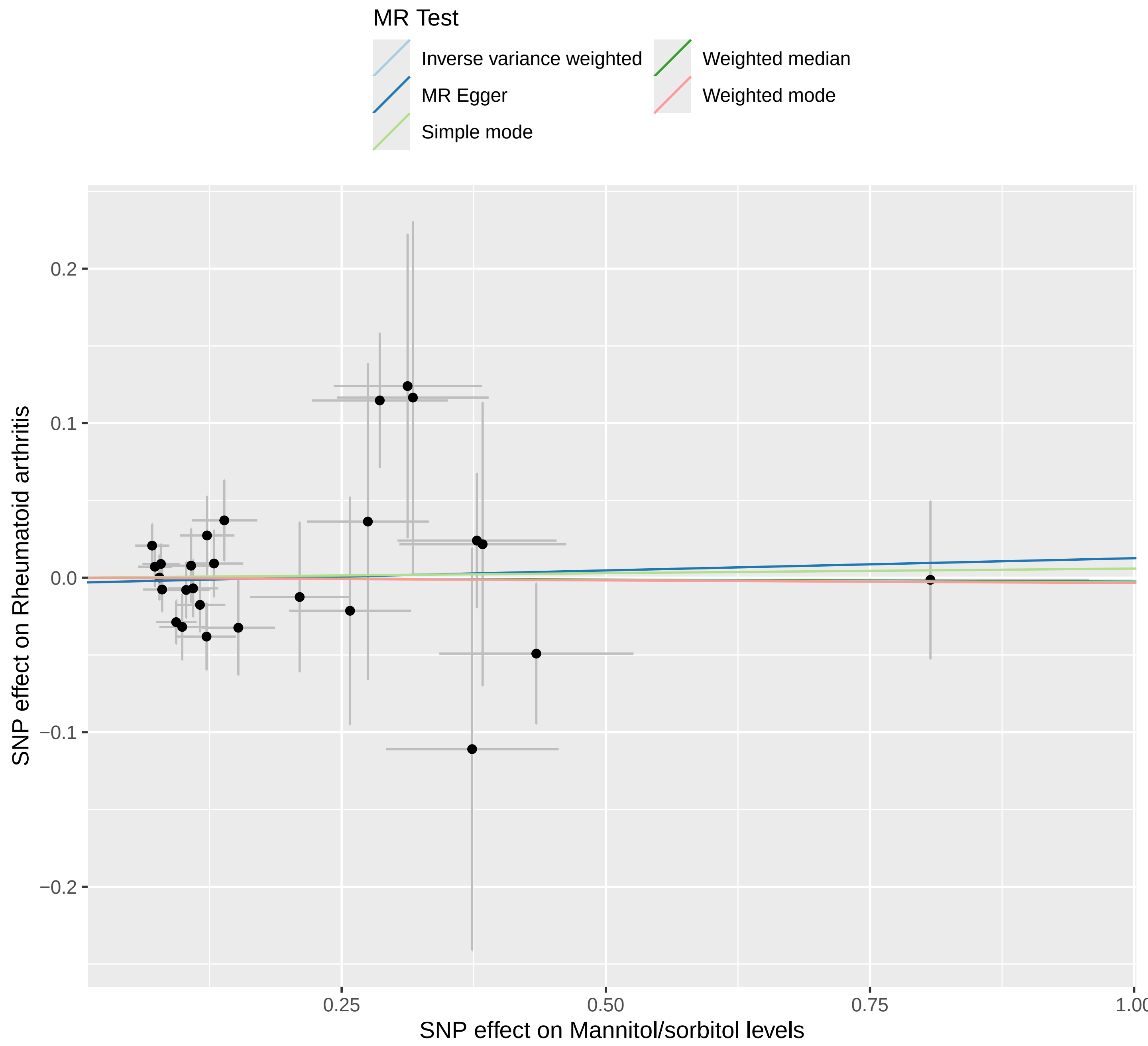
MR Test
Inverse variance weighted
MR Egger
Simple mode
Weighted median
Weighted mode
0.2
0.1
0.0
−0.1
−0.2
0.25
0.50
0.75
1.00
SNP effect on Rheumatoid arthritis
SNP effect on Mannitol/sorbitol levels

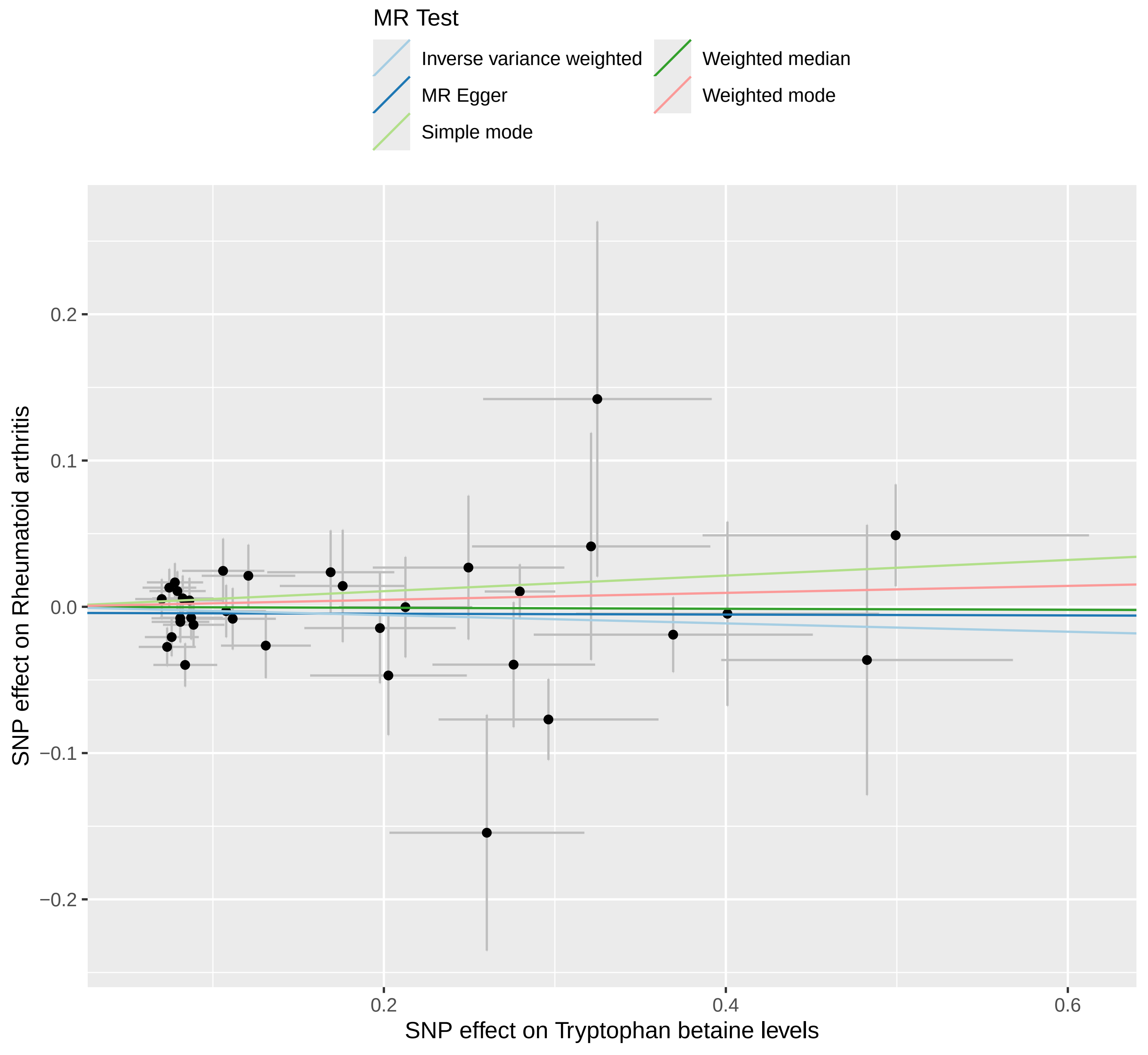

MR Test
Inverse variance weighted
MR Egger
Simple mode
Weighted median
Weighted mode
0.2
0.1
0.0
−0.1
−0.2
SNP effect on Rheumatoid arthritis
0.2
0.4
0.6
SNP effect on Tryptophan betaine levels

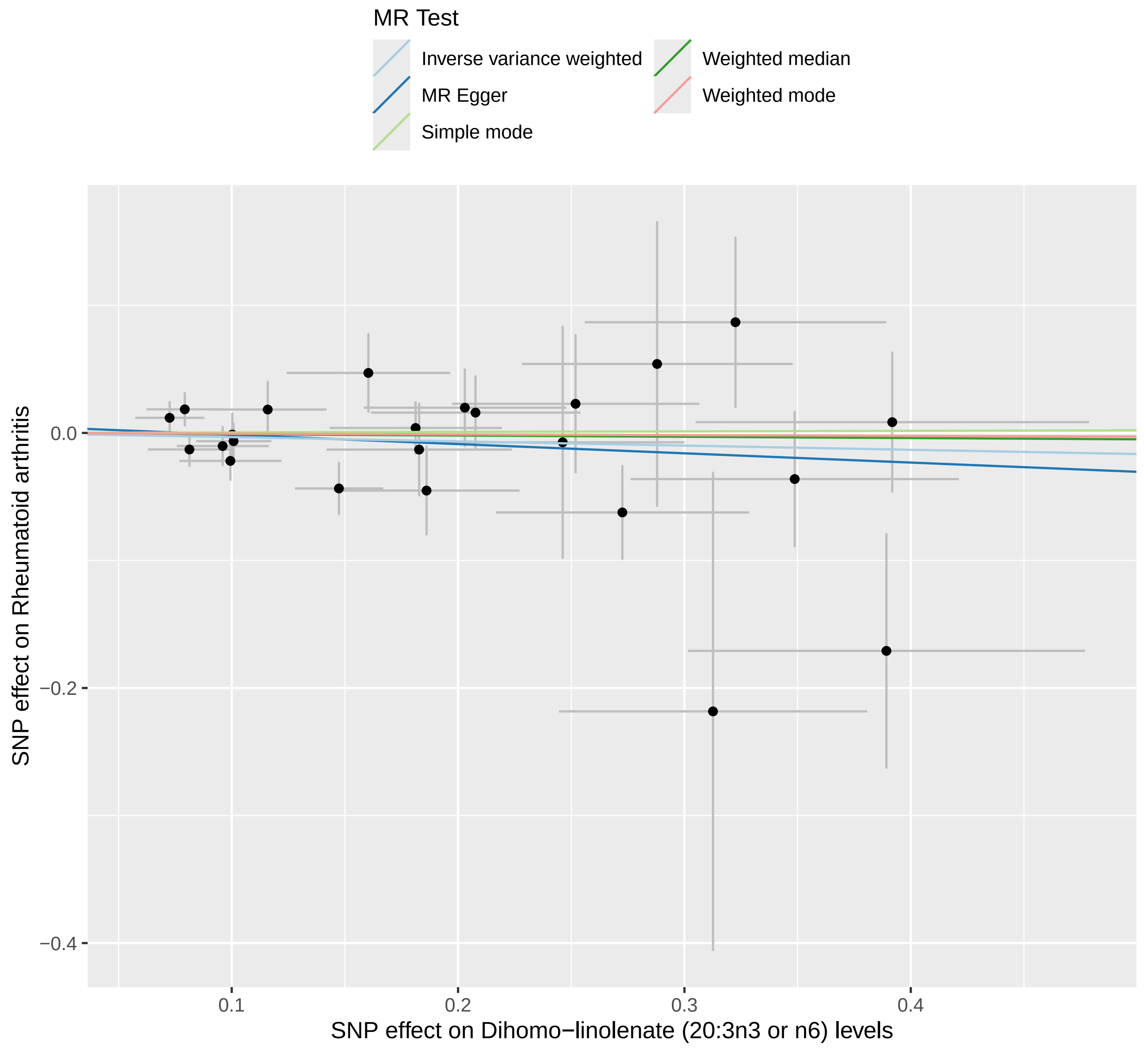

MR Test
Inverse variance weighted
MR Egger
Simple mode
Weighted median
Weighted mode
0.0
−0.2
−0.4
0.1
0.2
0.3
0.4
SNP effect on Rheumatoid arthritis
SNP effect on Dihomo−linolenate (20:3n3 or n6) levels

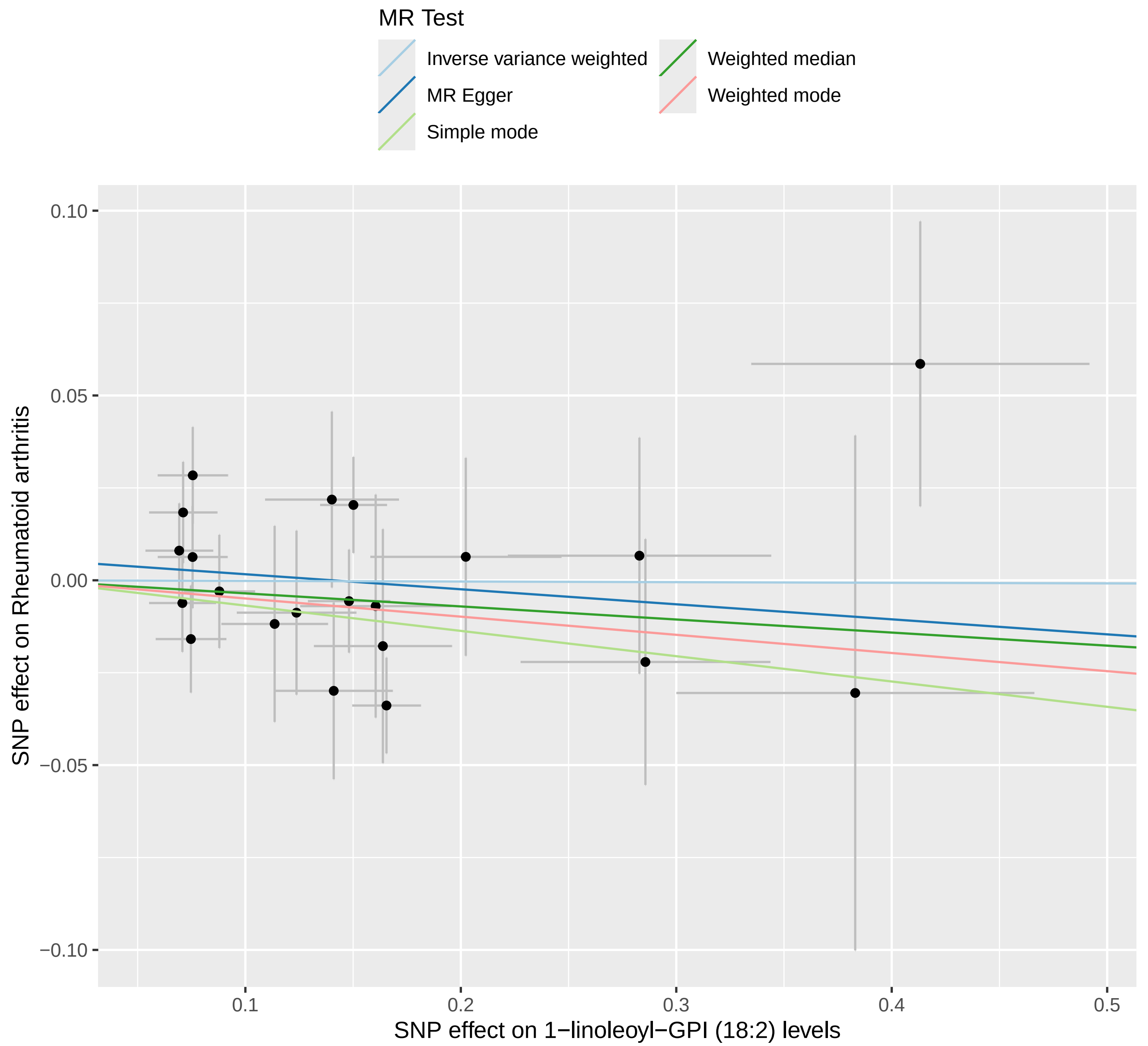
MR Test
Inverse variance weighted
MR Egger
Simple mode
Weighted median
Weighted mode
0.10
0.05
0.00
−0.05
−0.10
0.1
0.2
0.3
0.4
0.5
SNP effect on Rheumatoid arthritis
SNP effect on 1−linoleoyl−GPI (18:2) levels

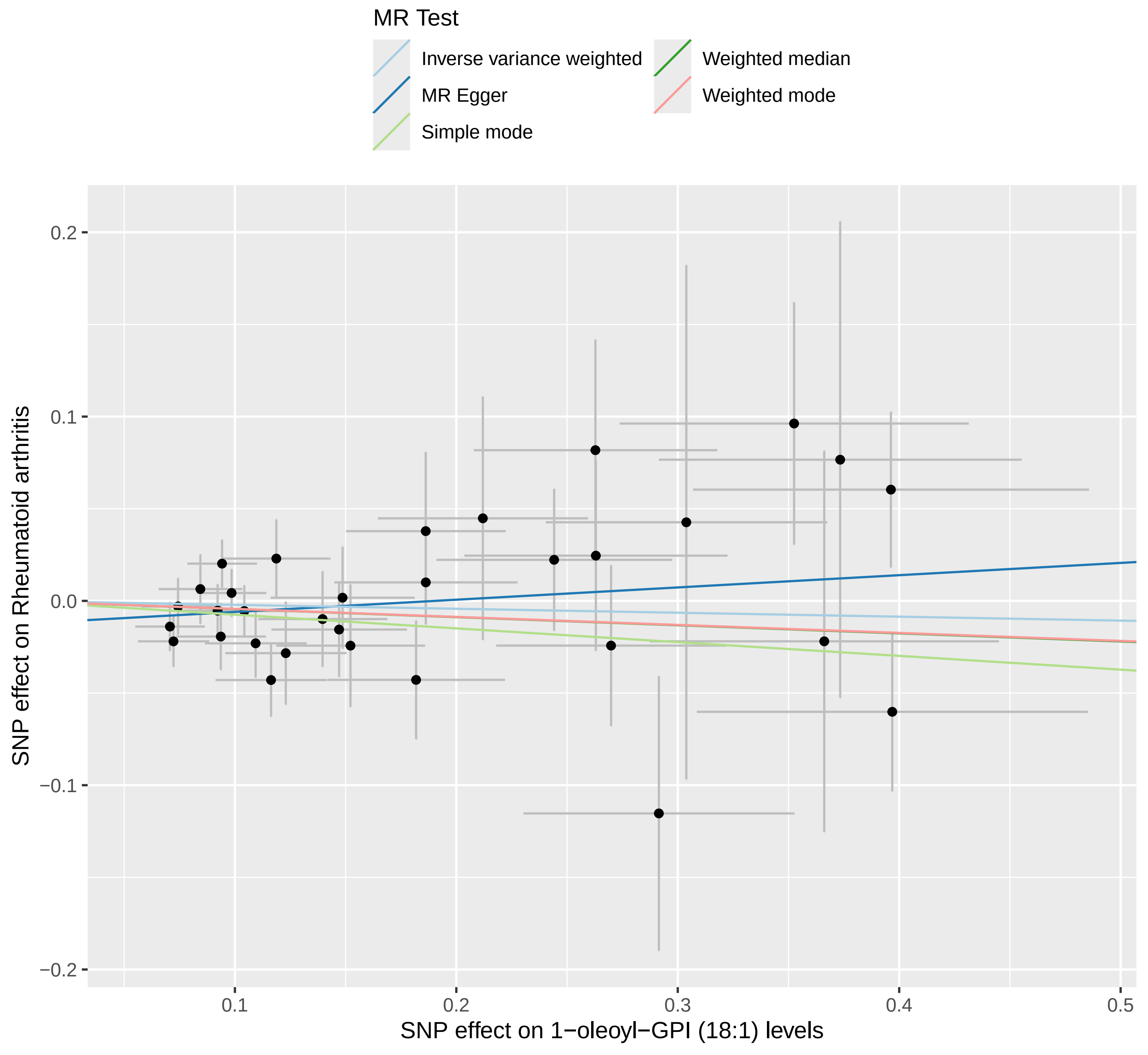
MR Test
Inverse variance weighted
MR Egger
Simple mode
Weighted median
Weighted mode
SNP effect on Rheumatoid arthritis
0.2
0.1
0.0
−0.1
−0.2
0.1
0.2
0.3
0.4
0.5
SNP effect on 1−oleoyl−GPI (18:1) levels

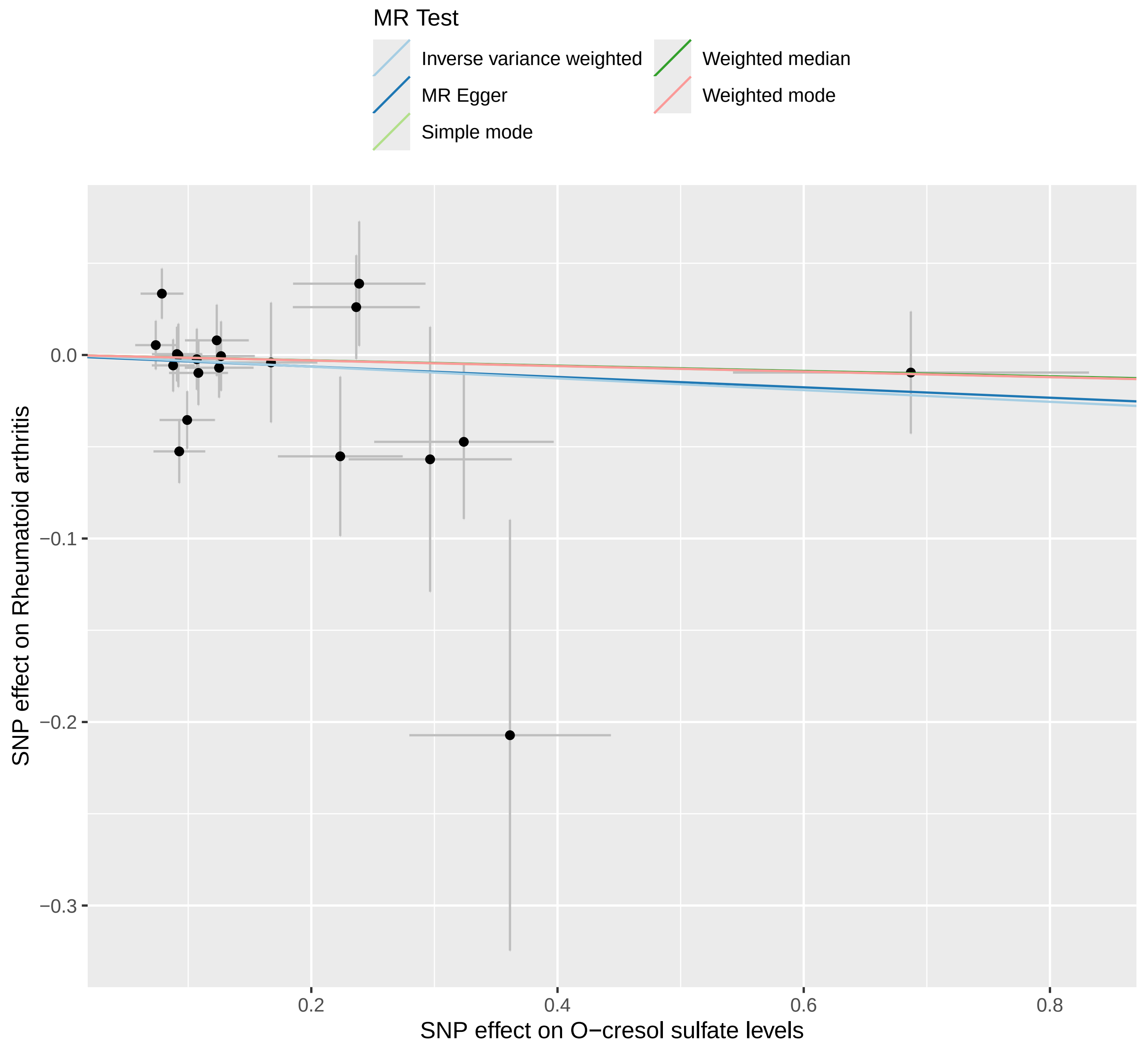

MR Test
Inverse variance weighted
MR Egger
Simple mode
Weighted median
Weighted mode
SNP effect on Rheumatoid arthritis
0.0
−0.1
−0.2
−0.3
0.2
0.4
0.6
0.8
SNP effect on O−cresol sulfate levels

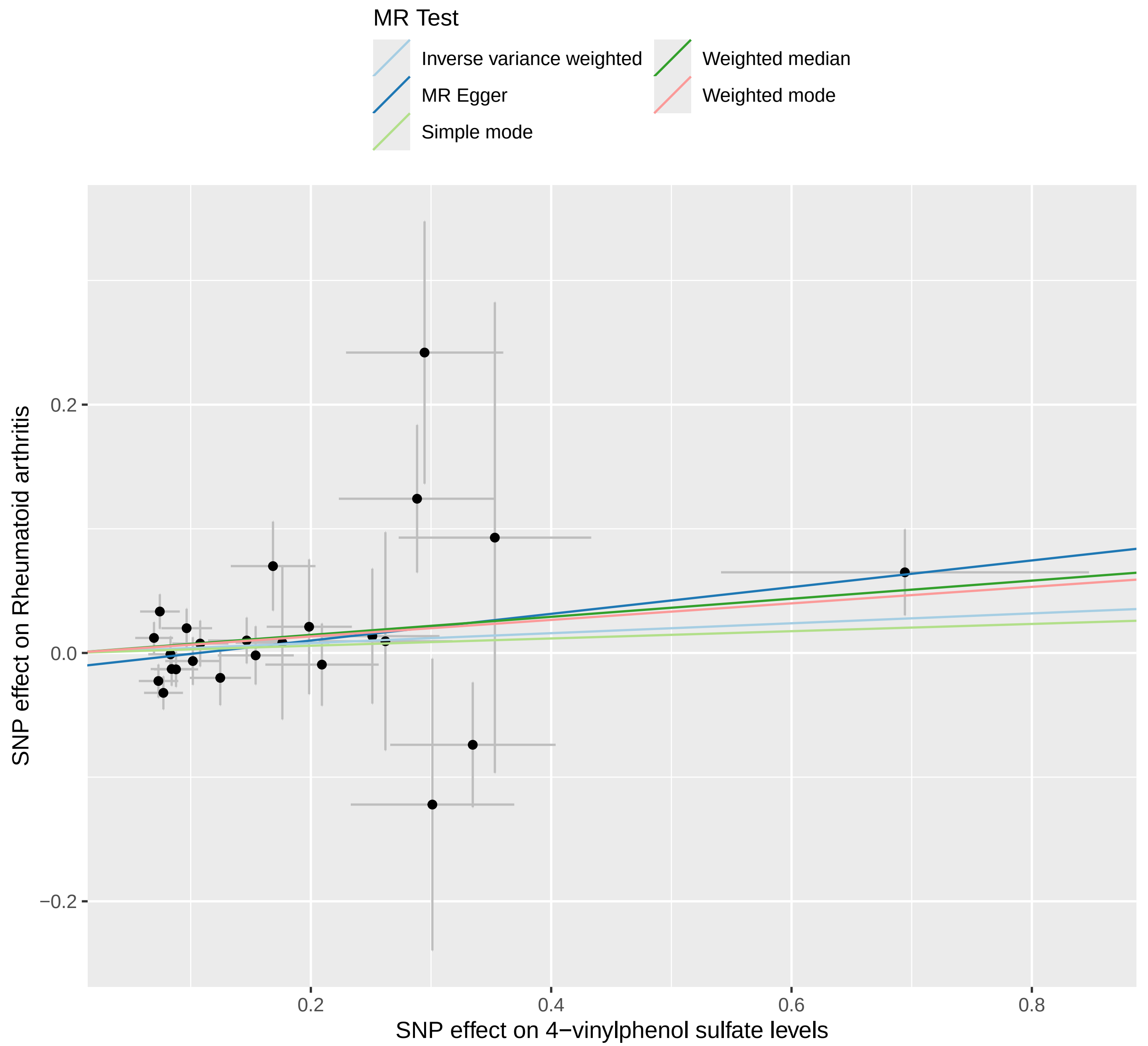
MR Test
Inverse variance weighted
MR Egger
Simple mode
Weighted median
Weighted mode
SNP effect on Rheumatoid arthritis
0.2
0.0
−0.2
0.2
0.4
0.6
0.8
SNP effect on 4−vinylphenol sulfate levels

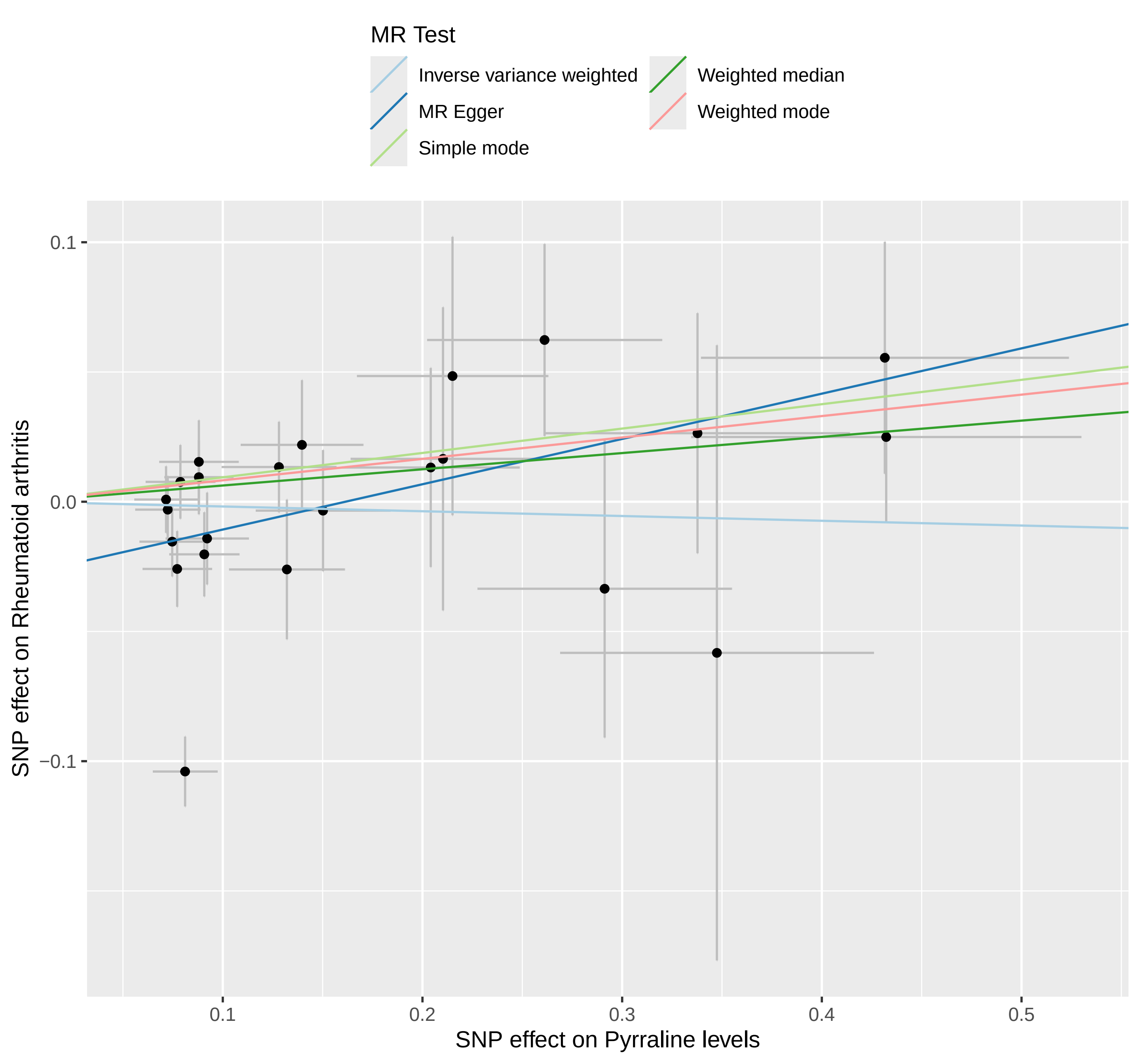
MR Test
Inverse variance weighted
MR Egger
Simple mode
Weighted median
Weighted mode
0.1
0.0
−0.1
SNP effect on Rheumatoid arthritis
0.1
0.2
0.3
0.4
0.5
SNP effect on Pyrraline levels

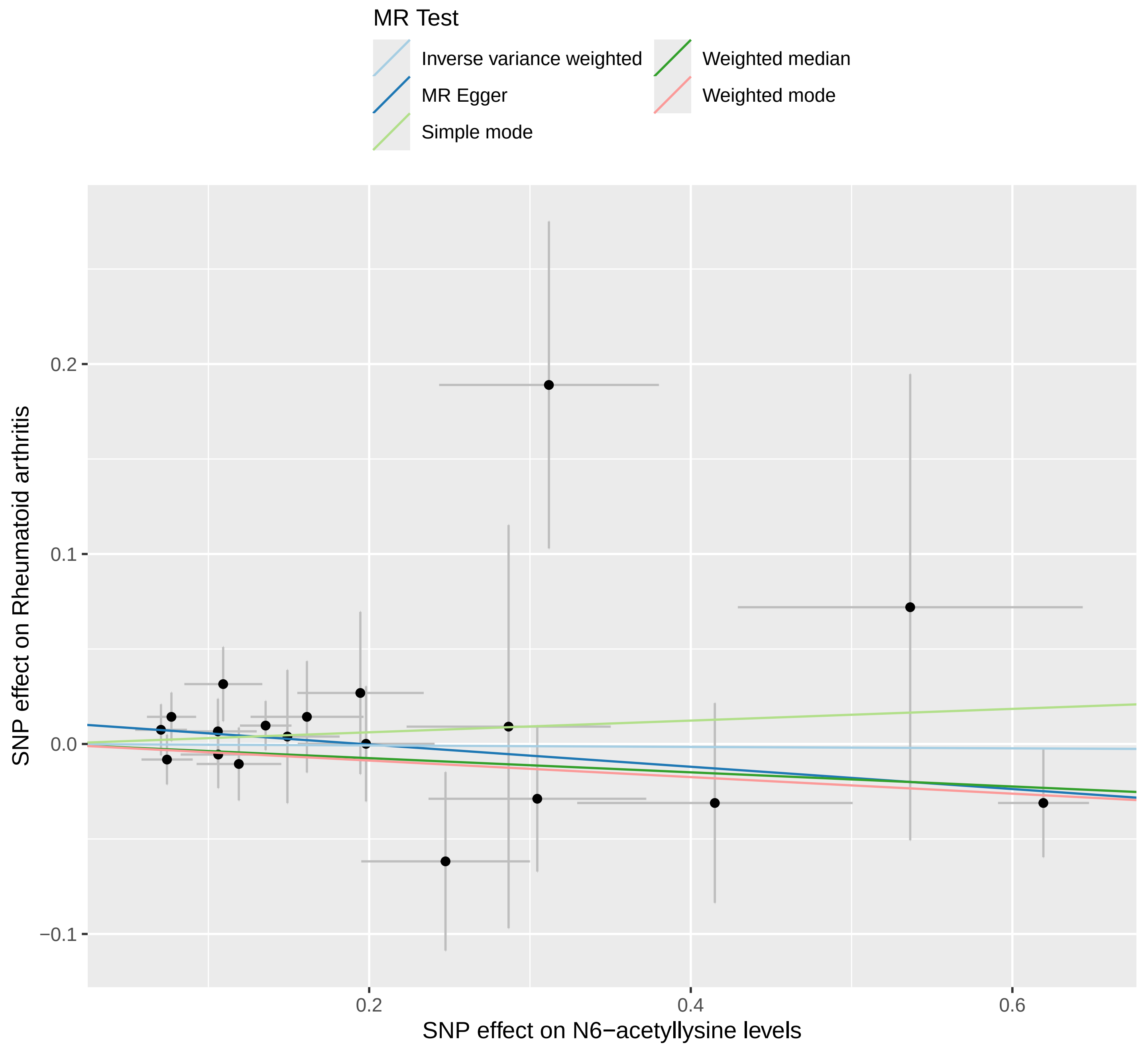
MR Test
Inverse variance weighted
MR Egger
Simple mode
Weighted median
Weighted mode
SNP effect on Rheumatoid arthritis
0.2
0.1
0.0
−0.1
0.2
0.4
0.6
SNP effect on N6−acetyllysine levels

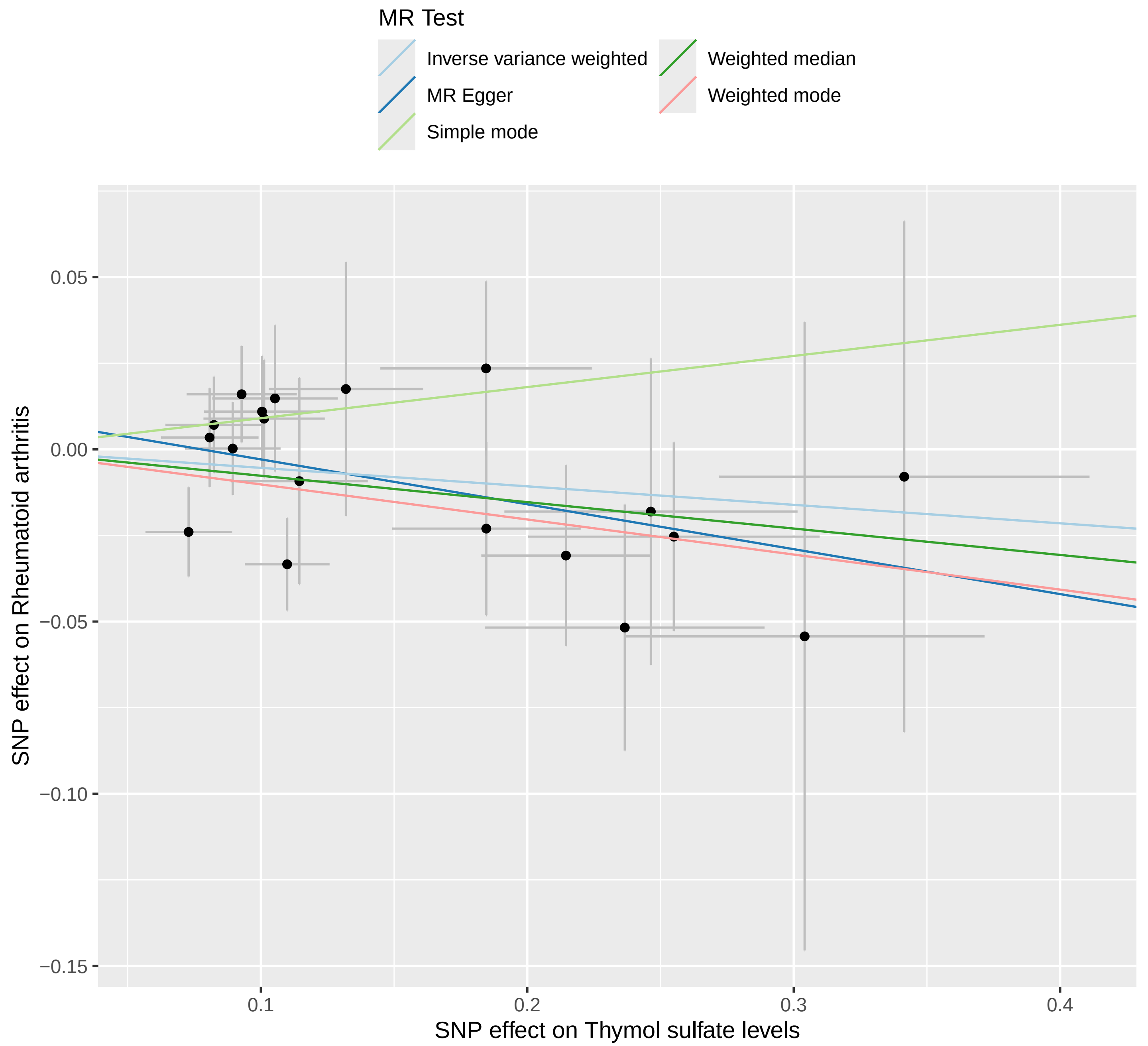

MR Test
Inverse variance weighted
MR Egger
Simple mode
Weighted median
Weighted mode
0.05
0.00
−0.05
−0.10
−0.15
0.1
0.2
0.3
0.4
SNP effect on Rheumatoid arthritis
SNP effect on Thymol sulfate levels

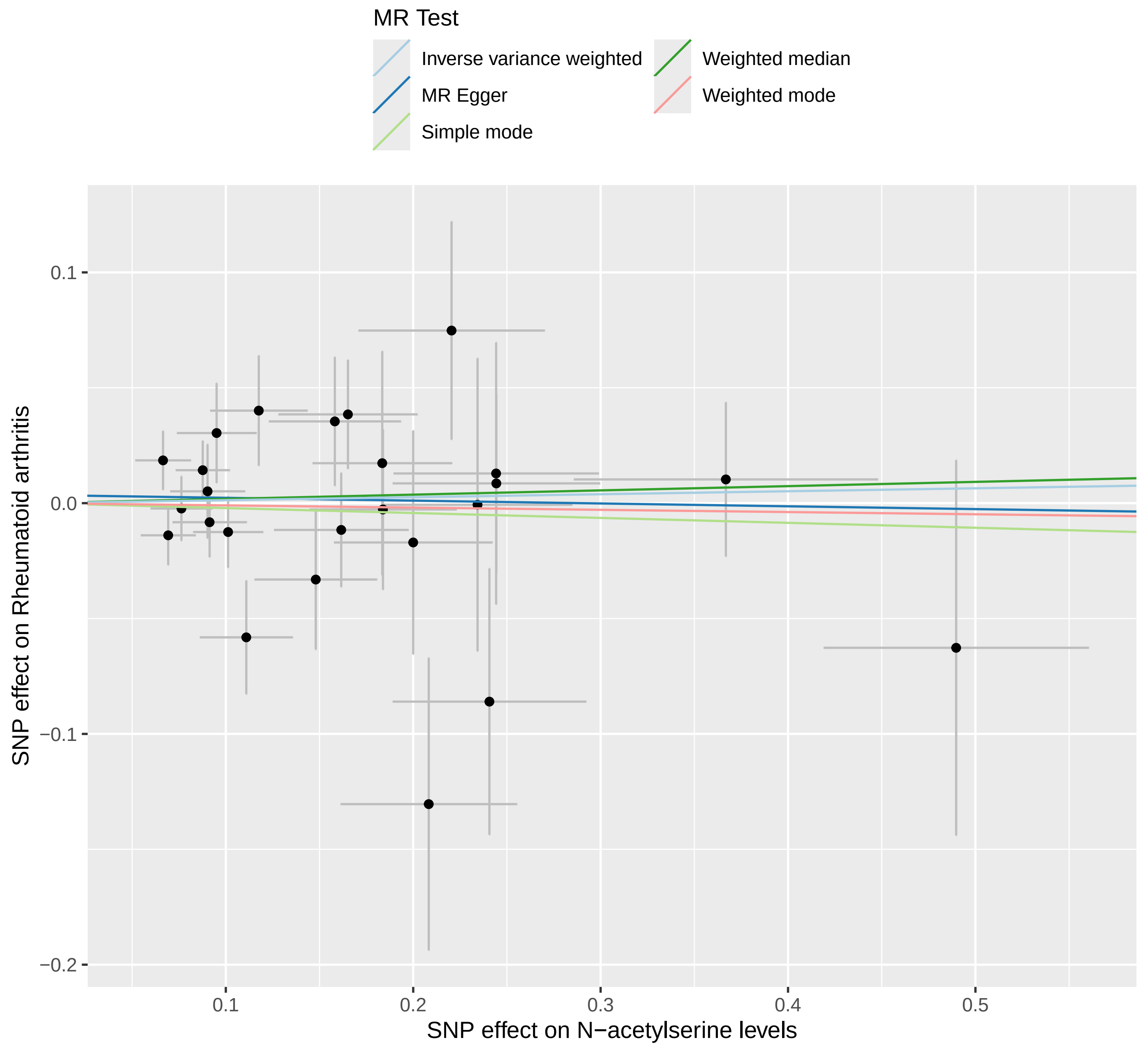
MR Test
Inverse variance weighted
MR Egger
Simple mode
Weighted median
Weighted mode
0.1
0.0
−0.1
−0.2
SNP effect on Rheumatoid arthritis
0.1
0.2
0.3
0.4
0.5
SNP effect on N−acetylserine levels

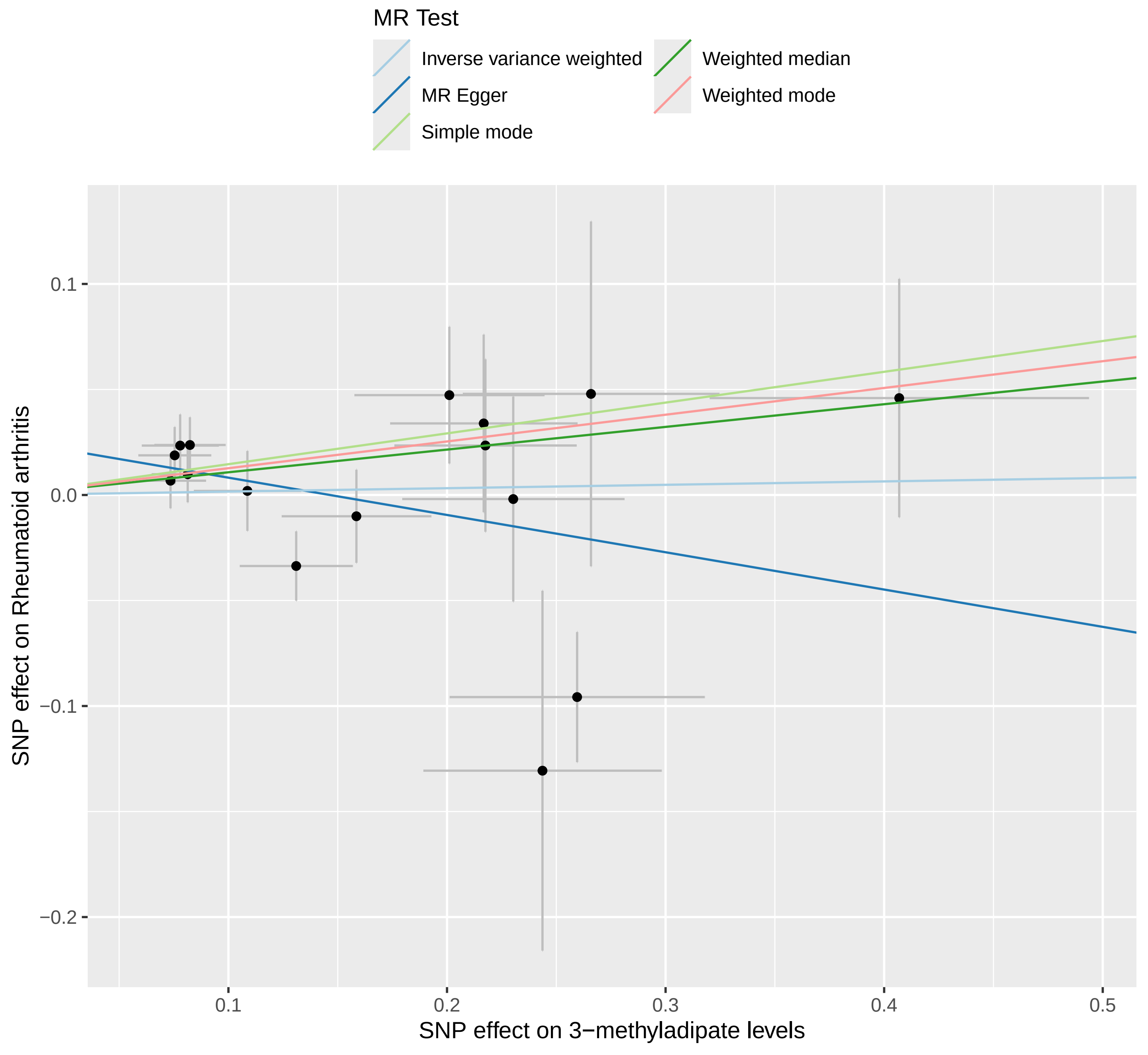
MR Test
Inverse variance weighted
MR Egger
Simple mode
Weighted median
Weighted mode
0.1
0.0
−0.1
−0.2
SNP effect on Rheumatoid arthritis
0.1
0.2
0.3
0.4
0.5
SNP effect on 3−methyladipate levels

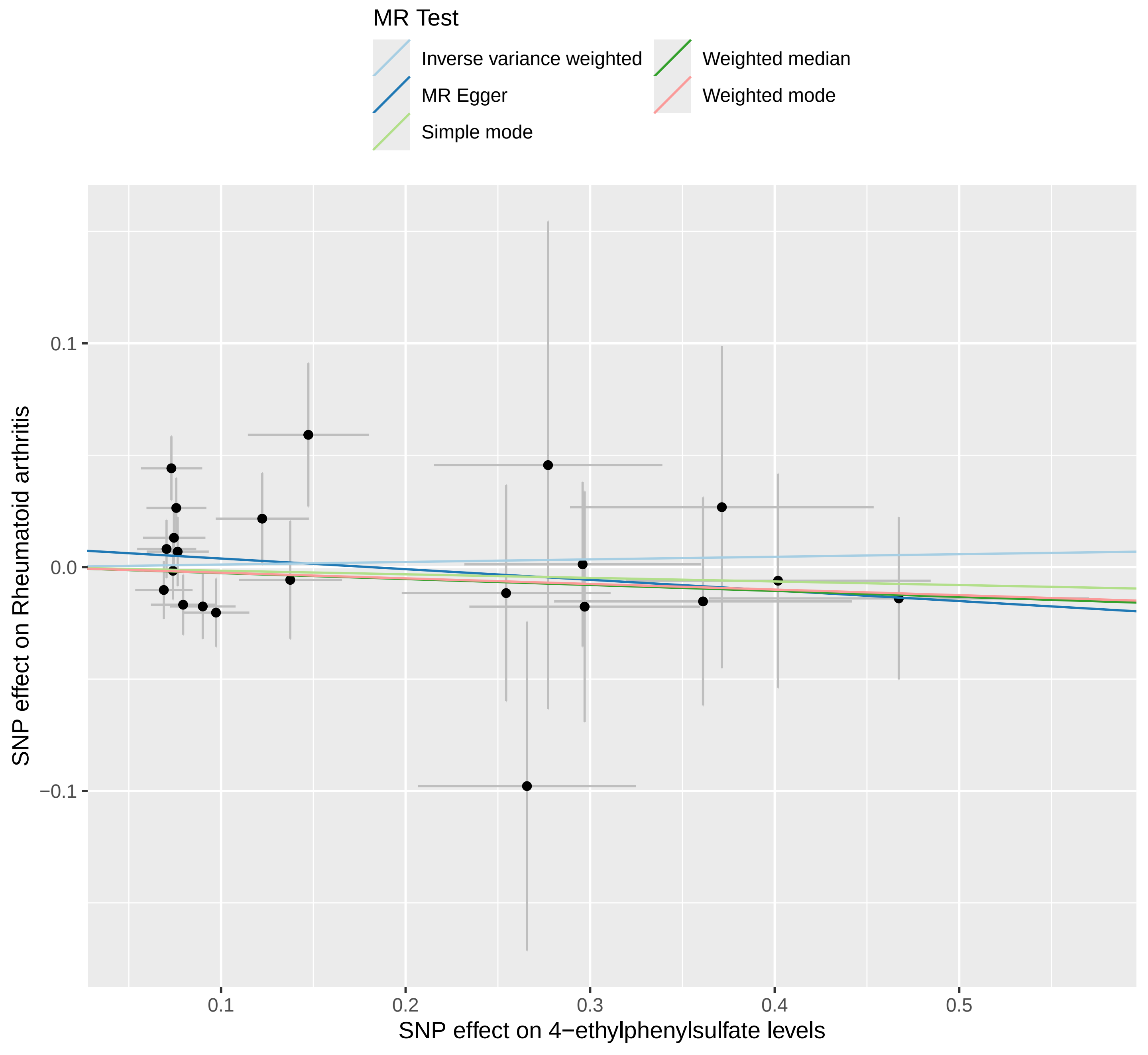
MR Test
Inverse variance weighted
MR Egger
Simple mode
Weighted median
Weighted mode
0.1
0.0
−0.1
SNP effect on Rheumatoid arthritis
0.1
0.2
0.3
0.4
0.5
SNP effect on 4−ethylphenylsulfate levels

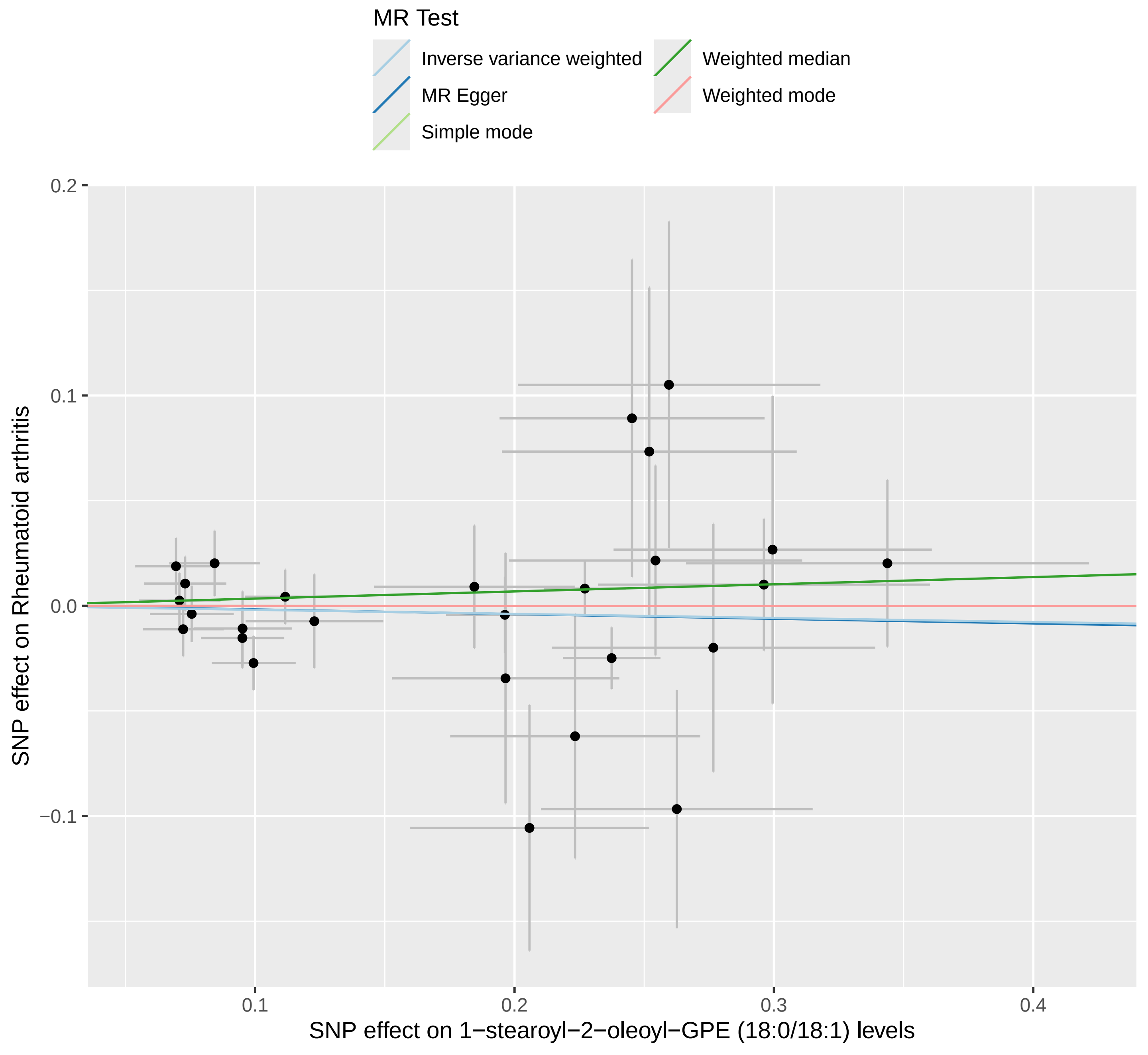
MR Test
Inverse variance weighted
MR Egger
Simple mode
Weighted median
Weighted mode
0.2
0.1
0.0
−0.1
0.1
0.2
0.3
0.4
SNP effect on Rheumatoid arthritis
SNP effect on 1−stearoyl−2−oleoyl−GPE (18:0/18:1) levels

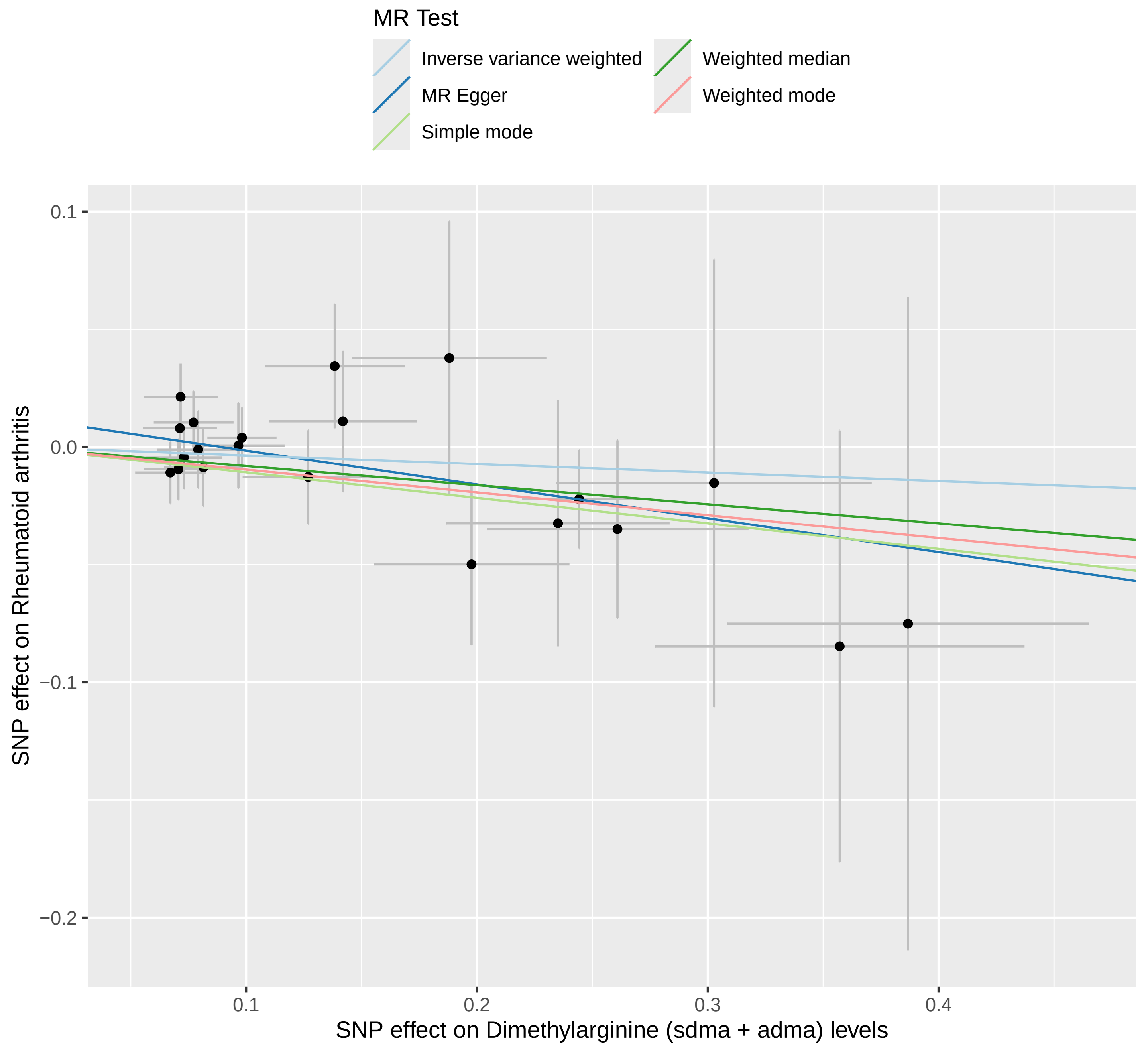

MR Test
Inverse variance weighted
MR Egger
Simple mode
Weighted median
Weighted mode
0.1
0.0
−0.1
−0.2
0.1
0.2
0.3
0.4
SNP effect on Rheumatoid arthritis
SNP effect on Dimethylarginine (sdma + adma) levels

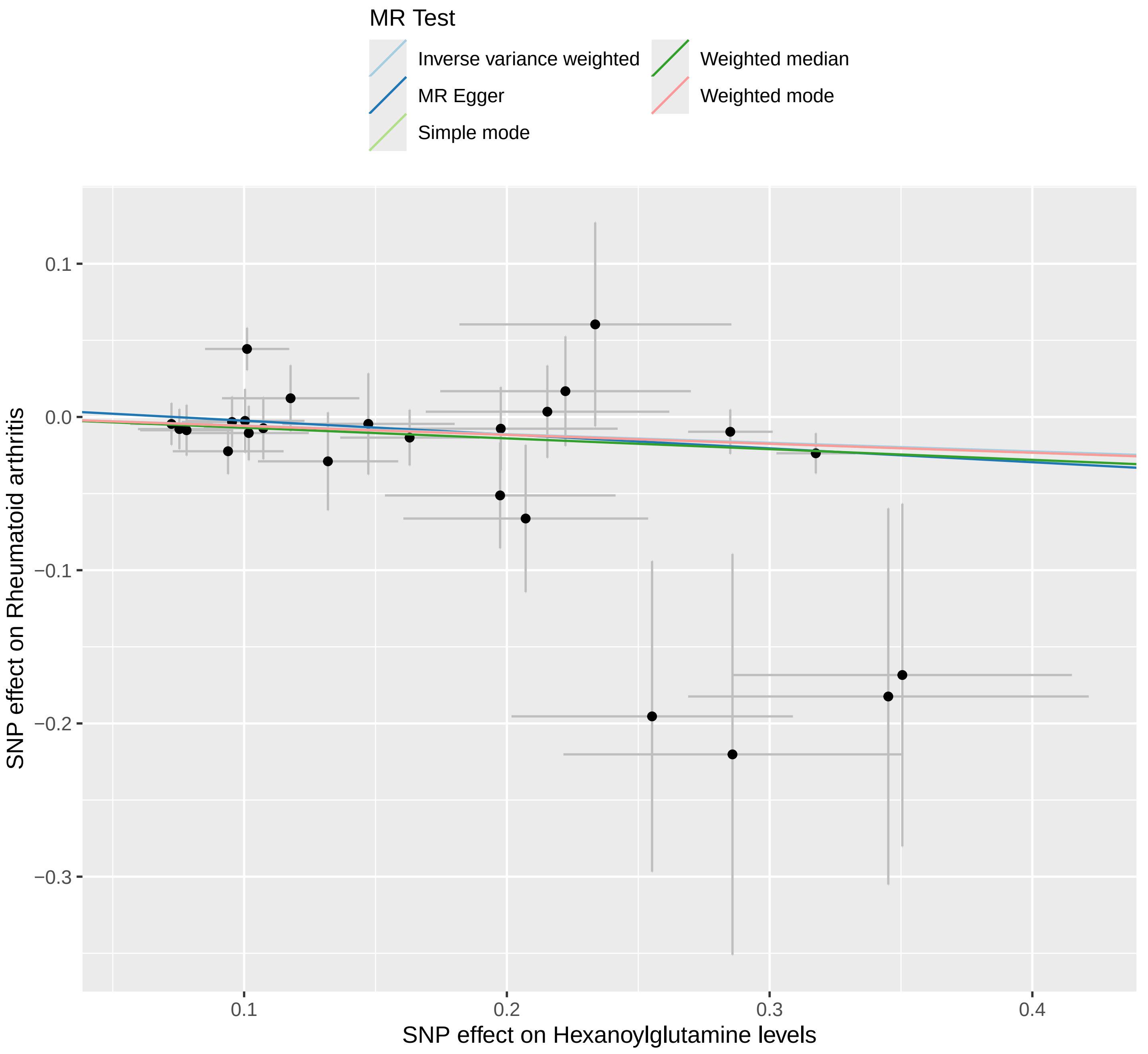
MR Test
Inverse variance weighted
MR Egger
Simple mode
Weighted median
Weighted mode
SNP effect on Rheumatoid arthritis
0.1
0.0
−0.1
−0.2
−0.3
0.1
0.2
0.3
0.4
SNP effect on Hexanoylglutamine levels

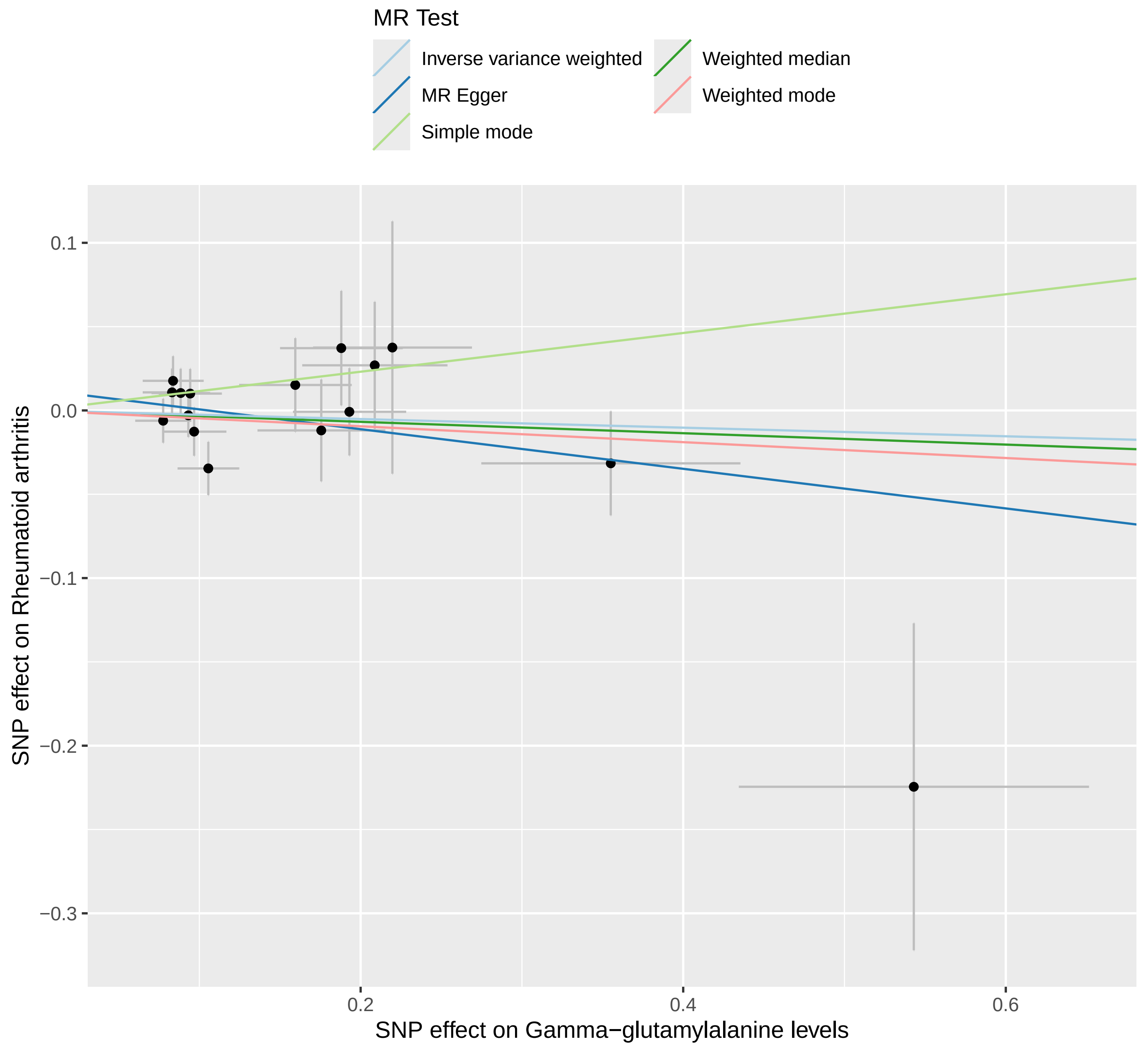

MR Test
Inverse variance weighted
MR Egger
Simple mode
Weighted median
Weighted mode
0.1
0.0
−0.1
−0.2
−0.3
SNP effect on Rheumatoid arthritis
0.2
0.4
0.6
SNP effect on Gamma−glutamylalanine levels

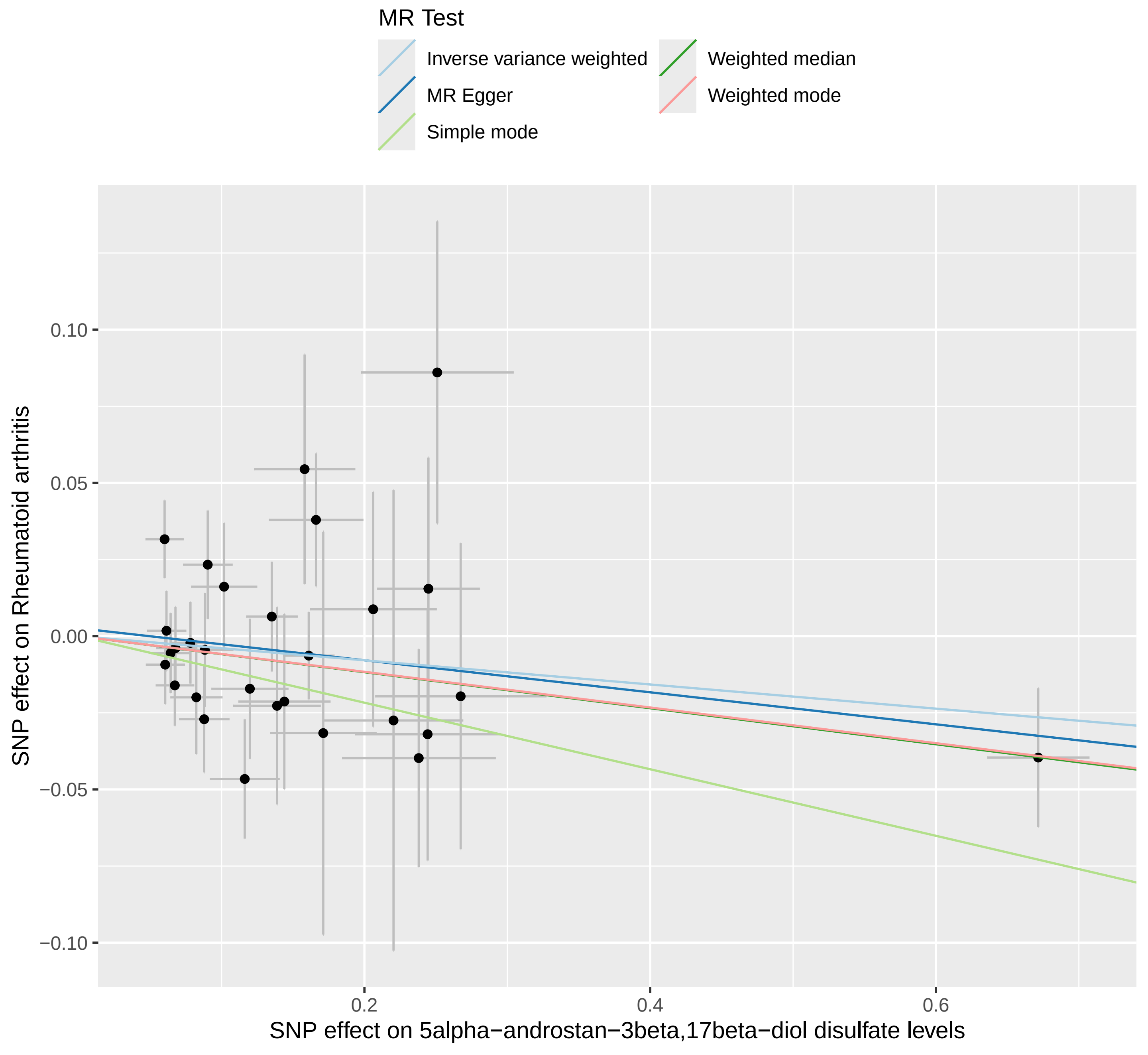
MR Test
Inverse variance weighted
MR Egger
Simple mode
Weighted median
Weighted mode
0.10
0.05
0.00
−0.05
−0.10
0.2
0.4
0.6
SNP effect on Rheumatoid arthritis
SNP effect on 5alpha−androstan−3beta,17beta−diol disulfate levels

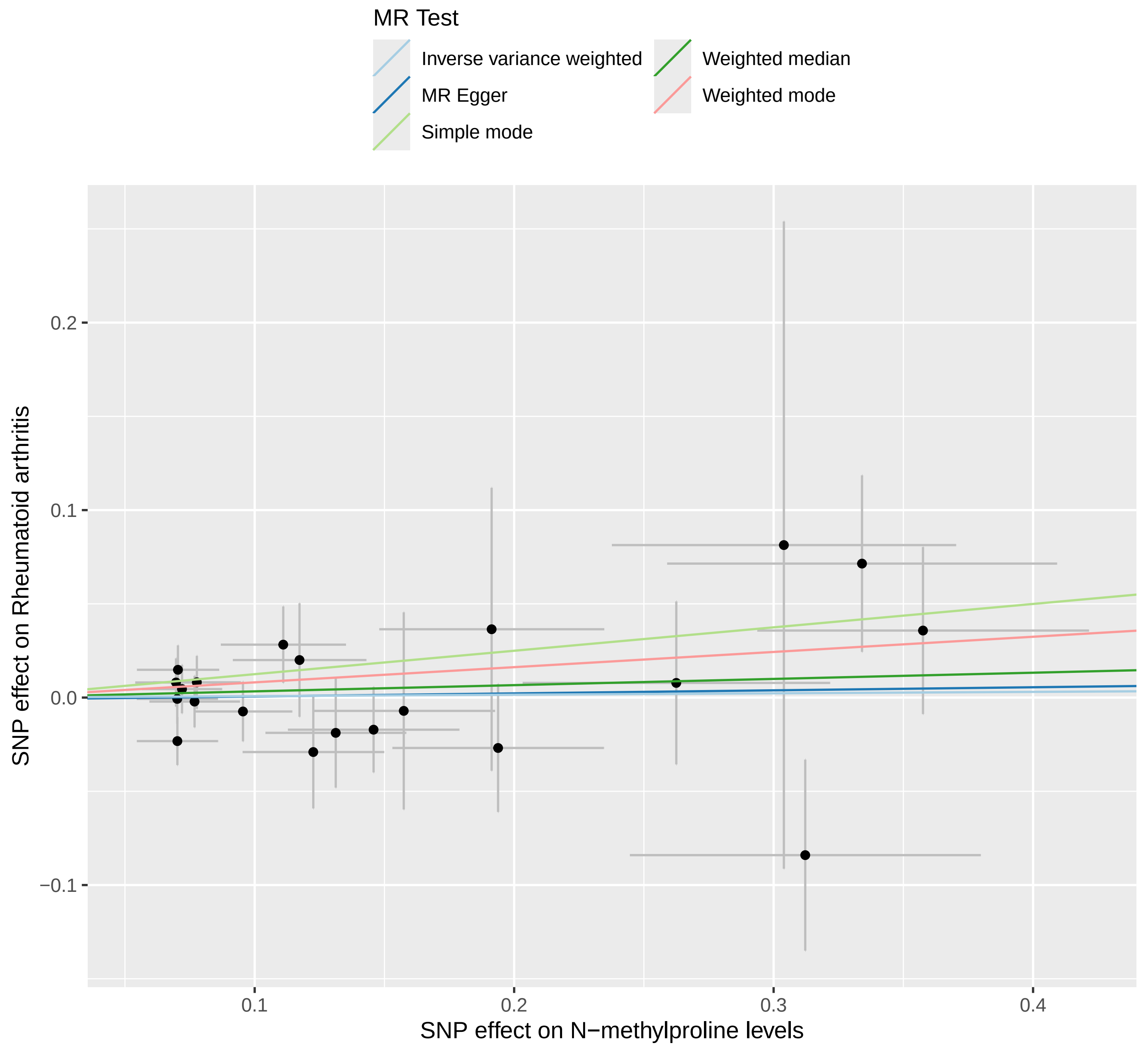
MR Test
Inverse variance weighted
MR Egger
Simple mode
Weighted median
Weighted mode
0.2
0.1
0.0
−0.1
SNP effect on Rheumatoid arthritis
0.1
0.2
0.3
0.4
SNP effect on N−methylproline levels

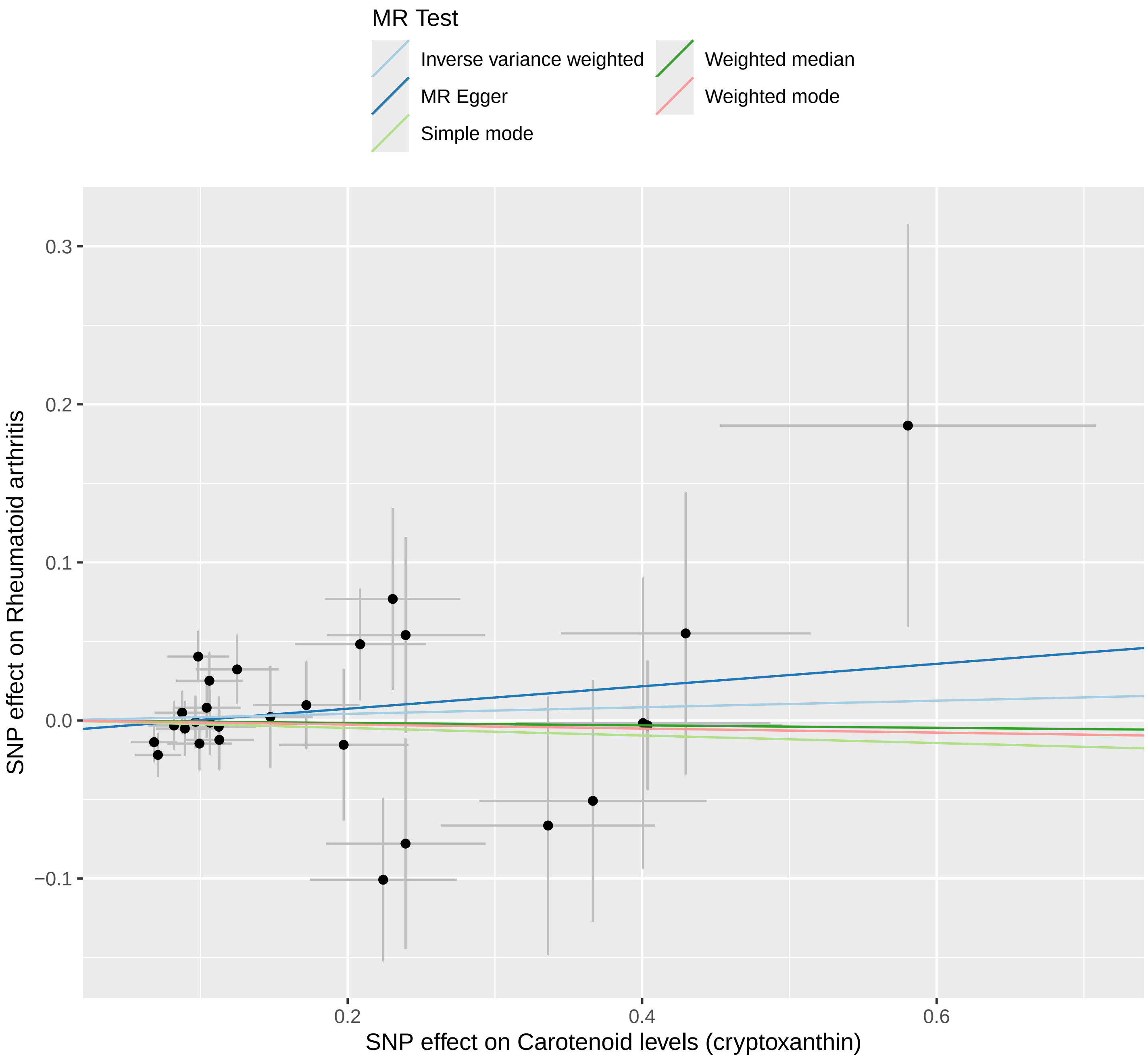
MR Test
Inverse variance weighted
MR Egger
Simple mode
Weighted median
Weighted mode
0.3
0.2
0.1
0.0
−0.1
0.2
0.4
0.6
SNP effect on Rheumatoid arthritis
SNP effect on Carotenoid levels (cryptoxanthin)

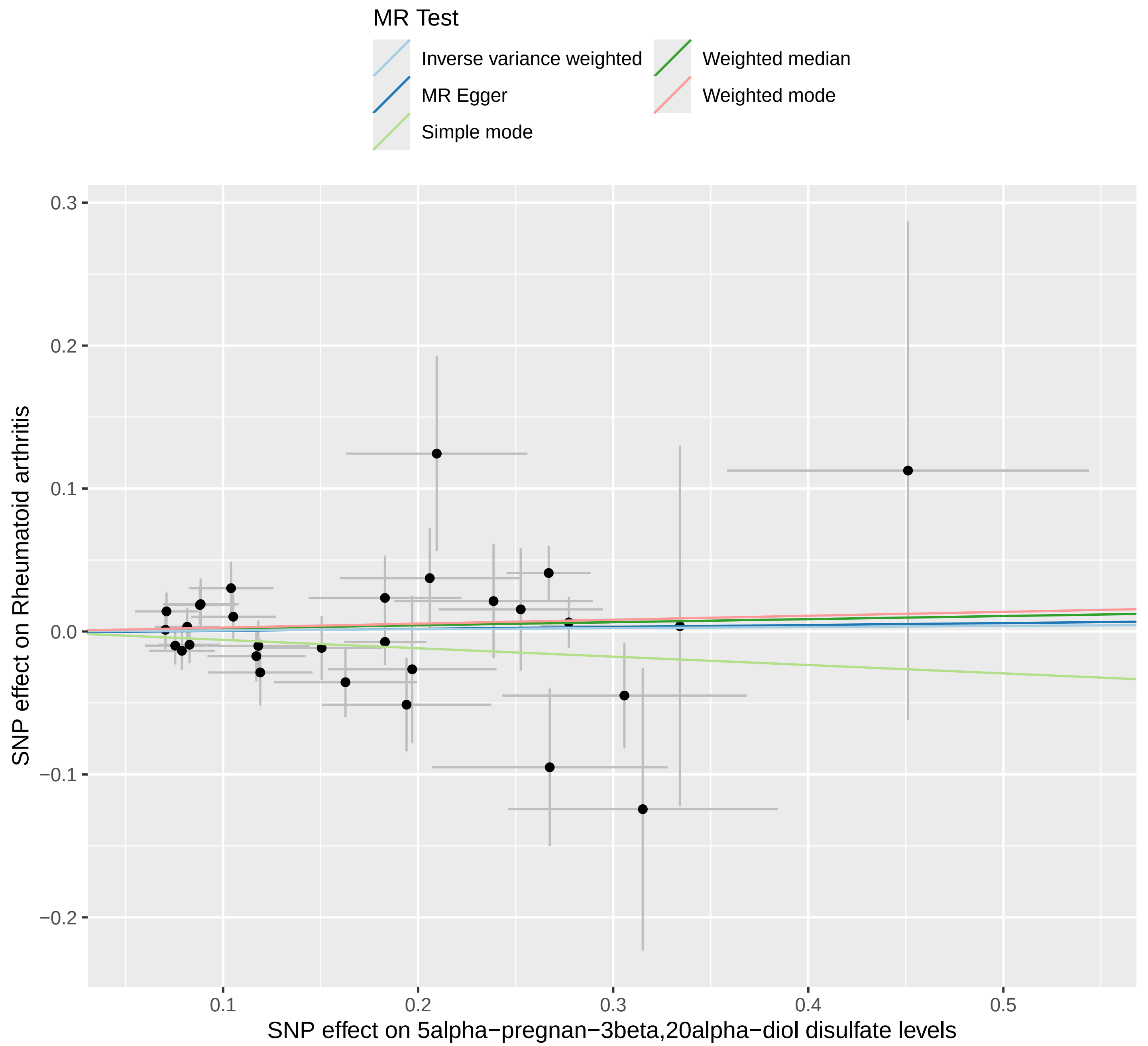
MR Test
Inverse variance weighted
MR Egger
Simple mode
Weighted median
Weighted mode
SNP effect on Rheumatoid arthritis
0.3
0.2
0.1
0.0
−0.1
−0.2
0.1
0.2
0.3
0.4
0.5
SNP effect on 5alpha−pregnan−3beta,20alpha−diol disulfate levels

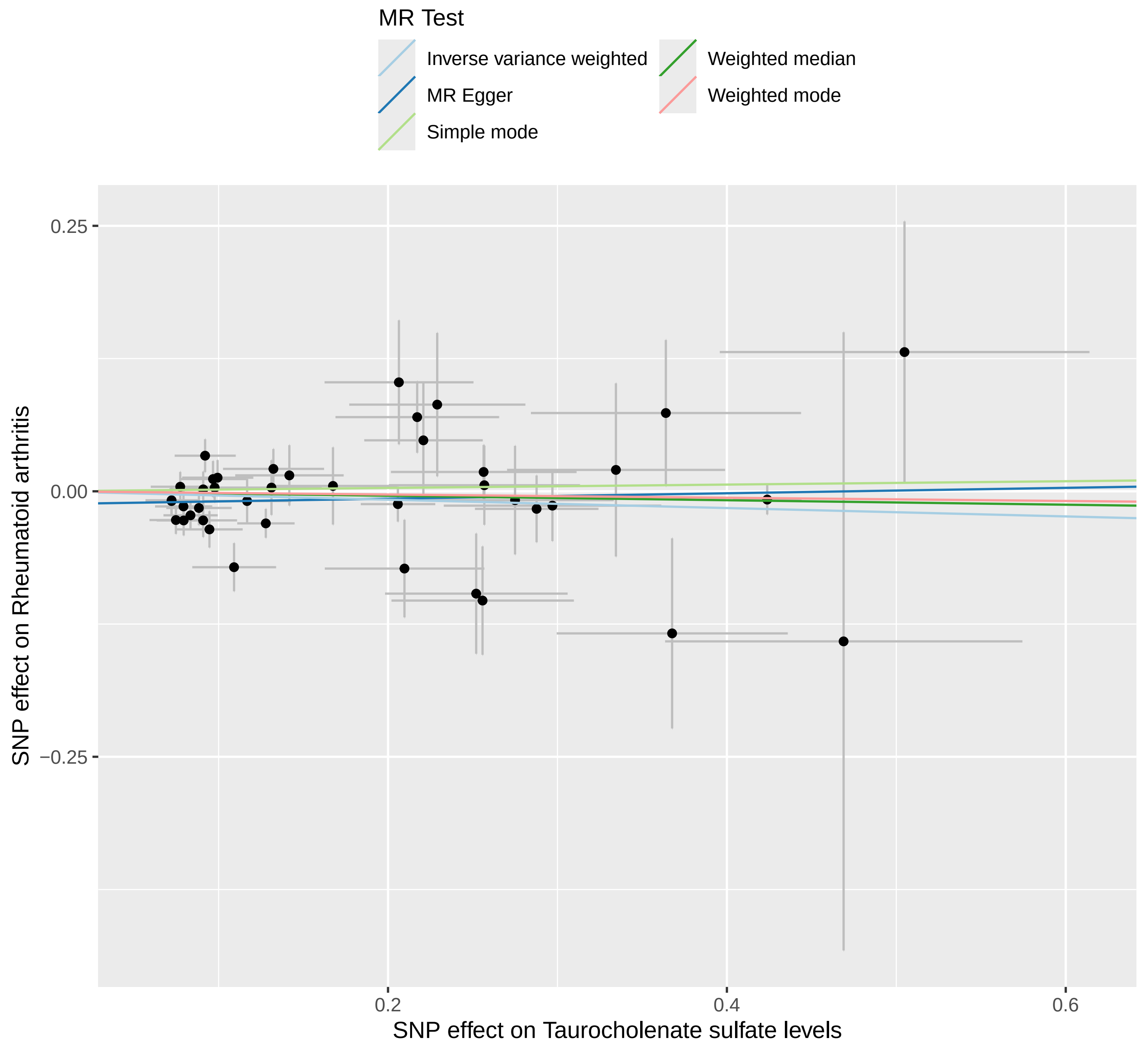
MR Test
Inverse variance weighted
MR Egger
Simple mode
Weighted median
Weighted mode
0.25
0.00
−0.25
0.2
0.4
0.6
SNP effect on Rheumatoid arthritis
SNP effect on Taurocholenate sulfate levels

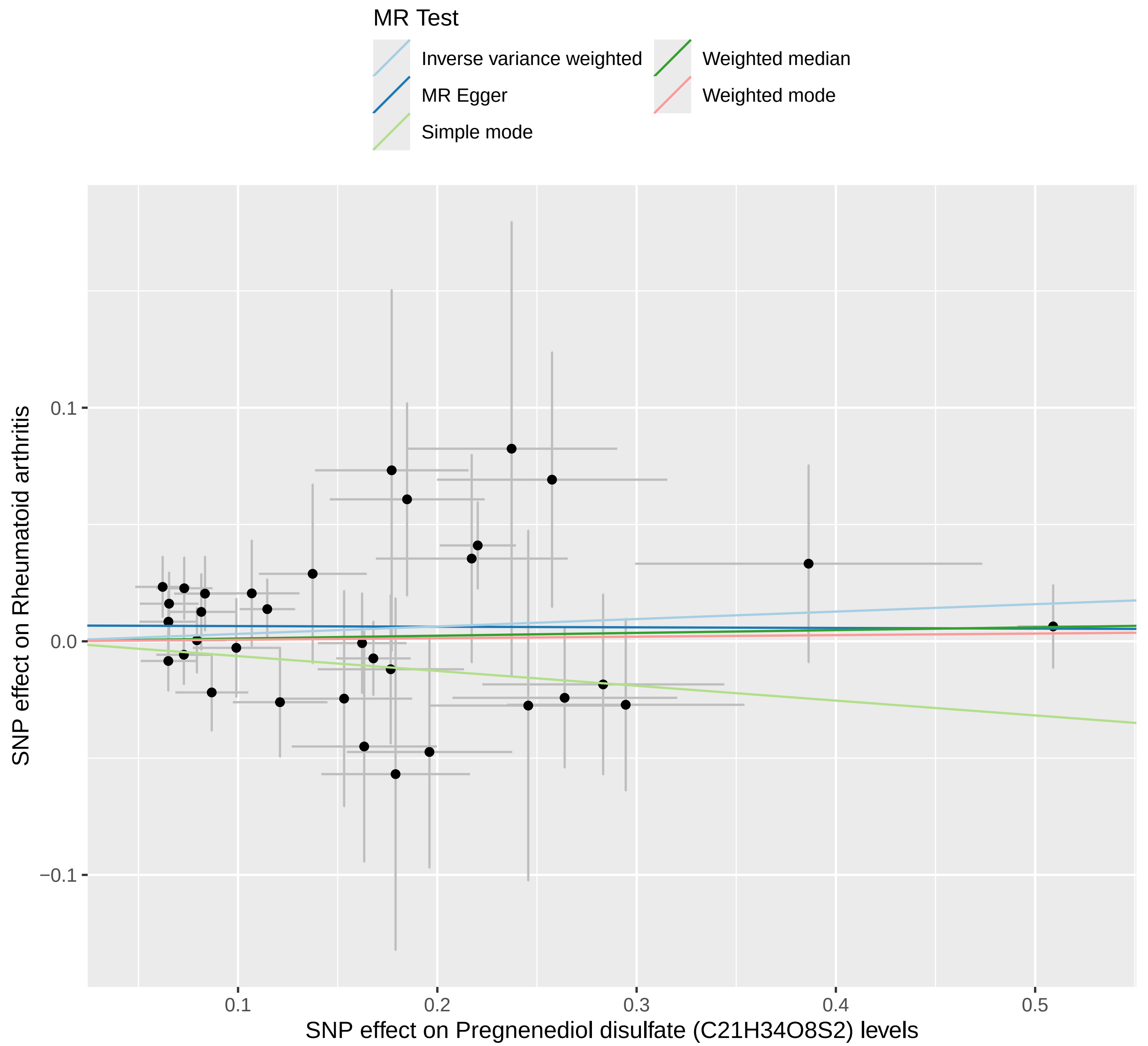
MR Test
Inverse variance weighted
MR Egger
Simple mode
Weighted median
Weighted mode
SNP effect on Rheumatoid arthritis
0.1
0.0
−0.1
0.1
0.2
0.3
0.4
0.5
SNP effect on Pregnenediol disulfate (C21H34O8S2) levels

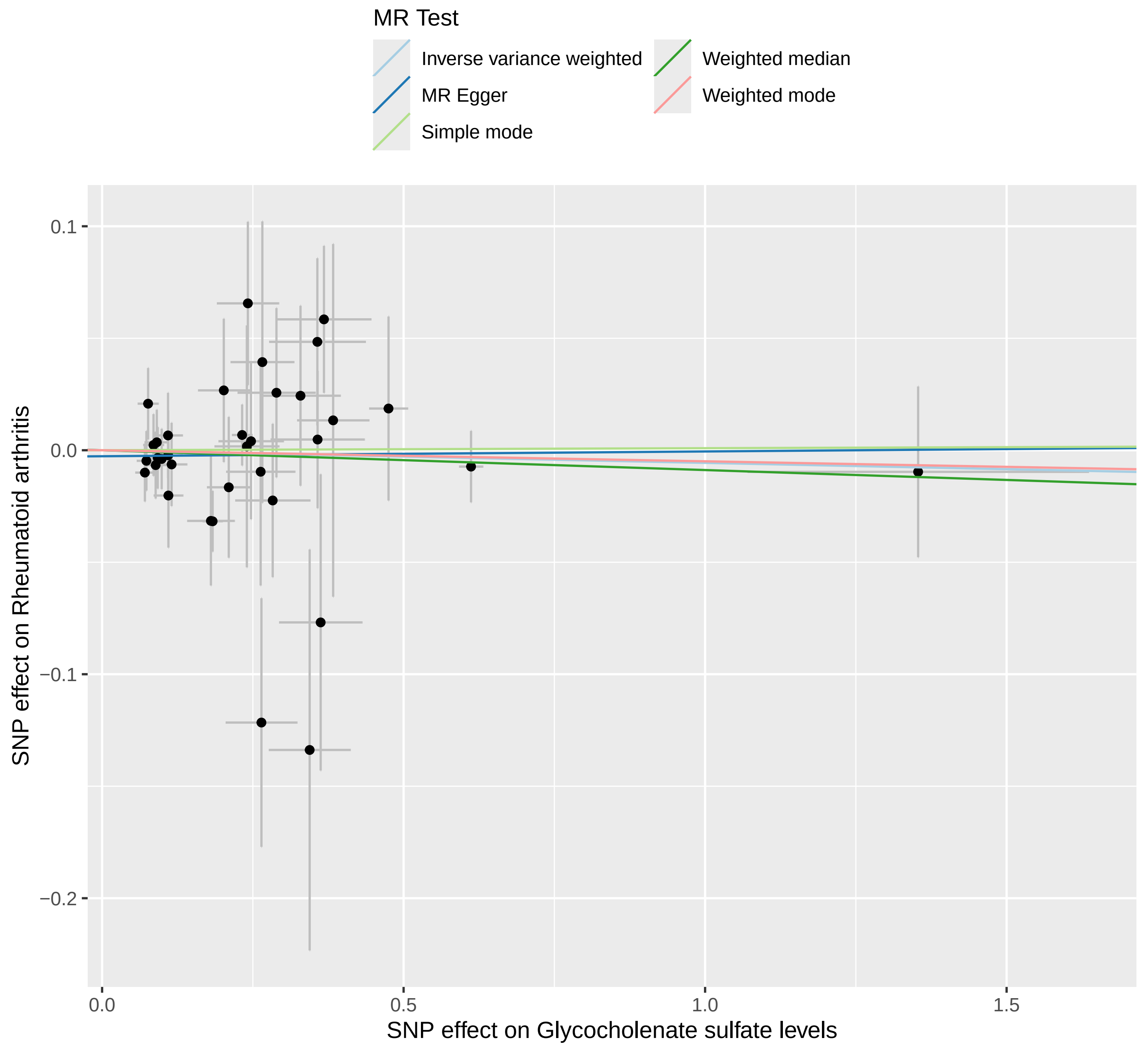

MR Test
Inverse variance weighted
MR Egger
Simple mode
Weighted median
Weighted mode
0.1
0.0
−0.1
−0.2
0.0
0.5
1.0
1.5
SNP effect on Rheumatoid arthritis
SNP effect on Glycocholenate sulfate levels

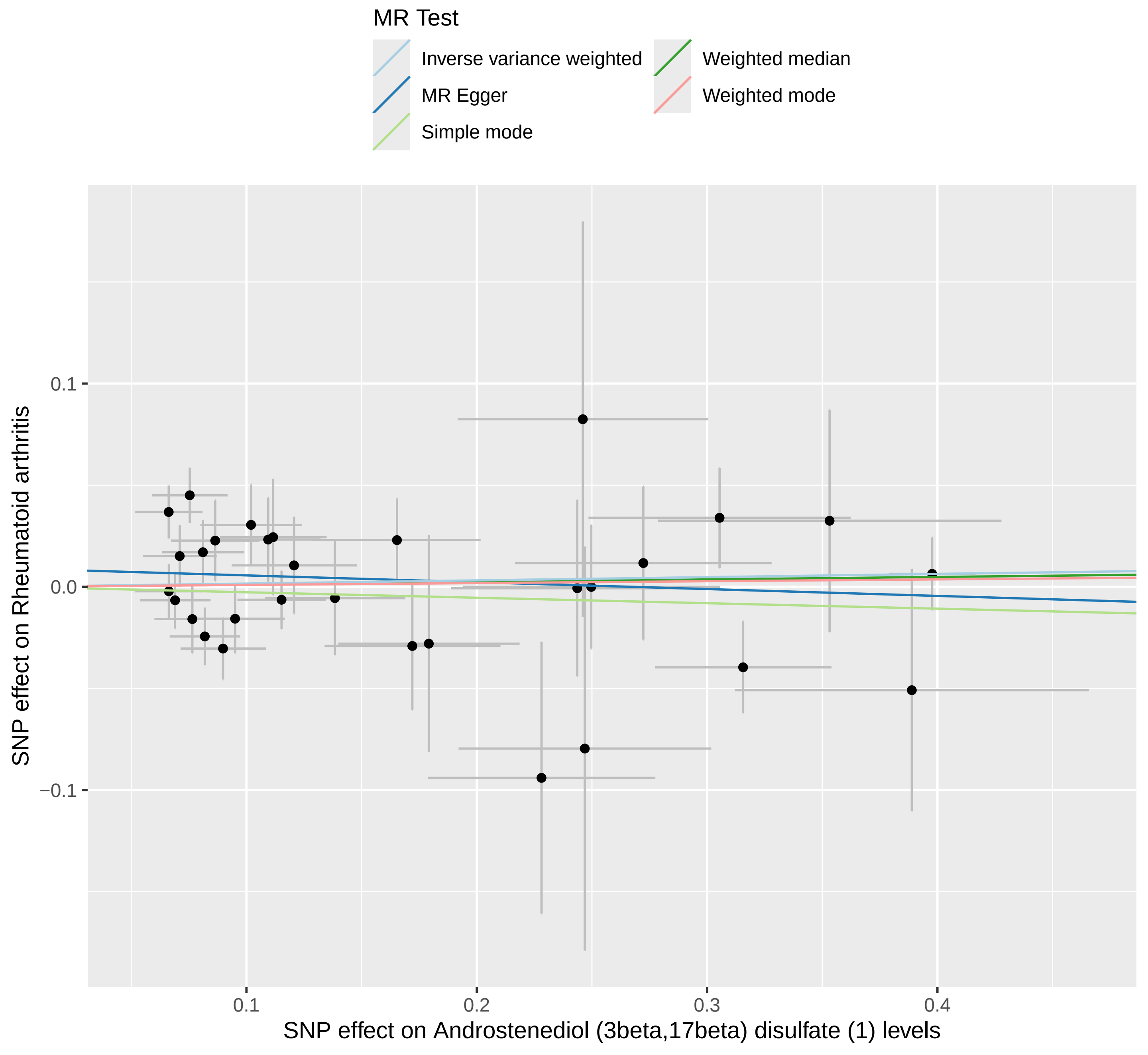

MR Test
Inverse variance weighted
MR Egger
Simple mode
Weighted median
Weighted mode
SNP effect on Rheumatoid arthritis
0.1
0.0
−0.1
0.1
0.2
0.3
0.4
SNP effect on Androstenediol (3beta,17beta) disulfate (1) levels

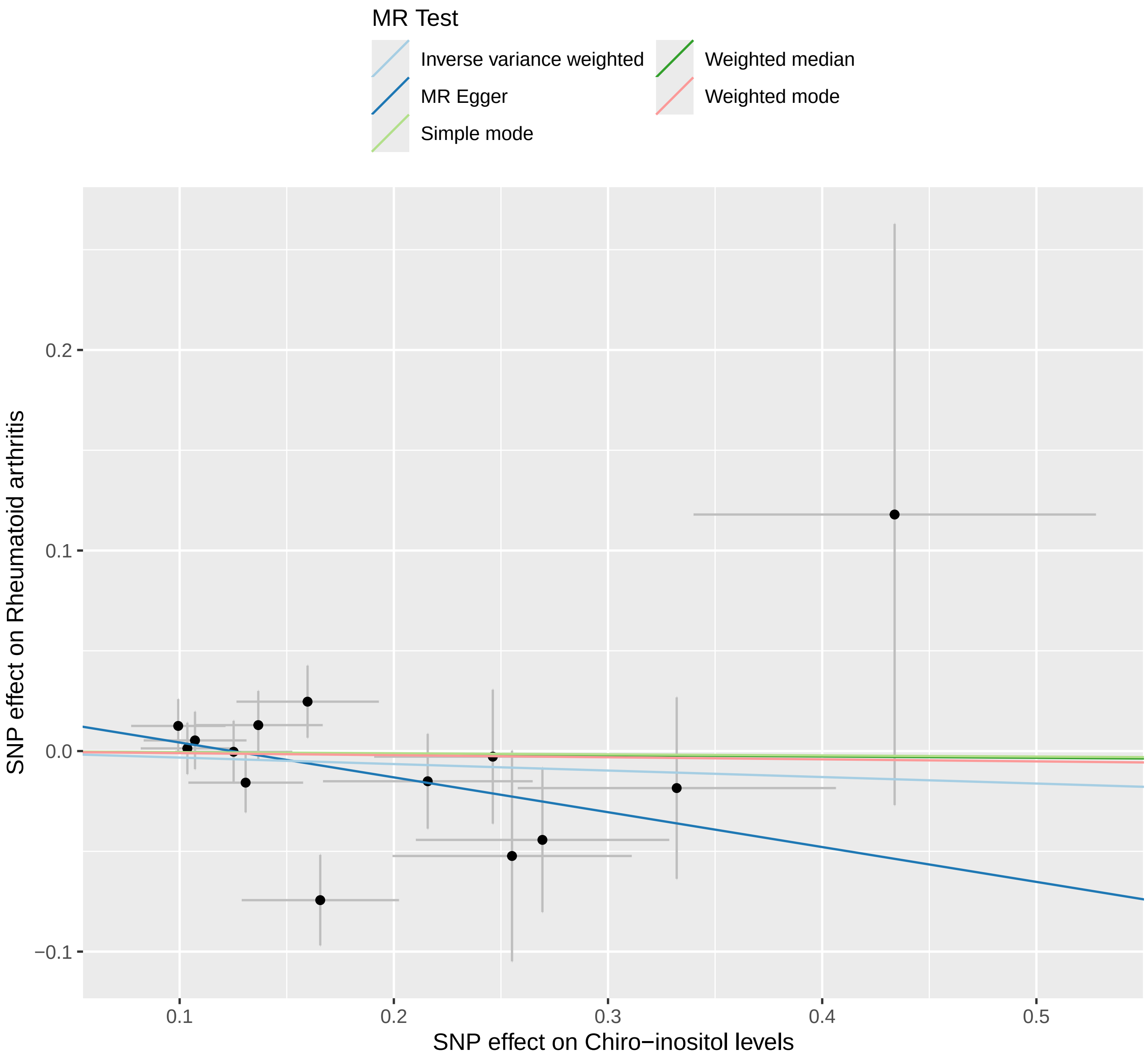
MR Test
Inverse variance weighted
MR Egger
Simple mode
Weighted median
Weighted mode
0.2
0.1
0.0
−0.1
0.1
0.2
0.3
0.4
0.5
SNP effect on Rheumatoid arthritis
SNP effect on Chiro−inositol levels

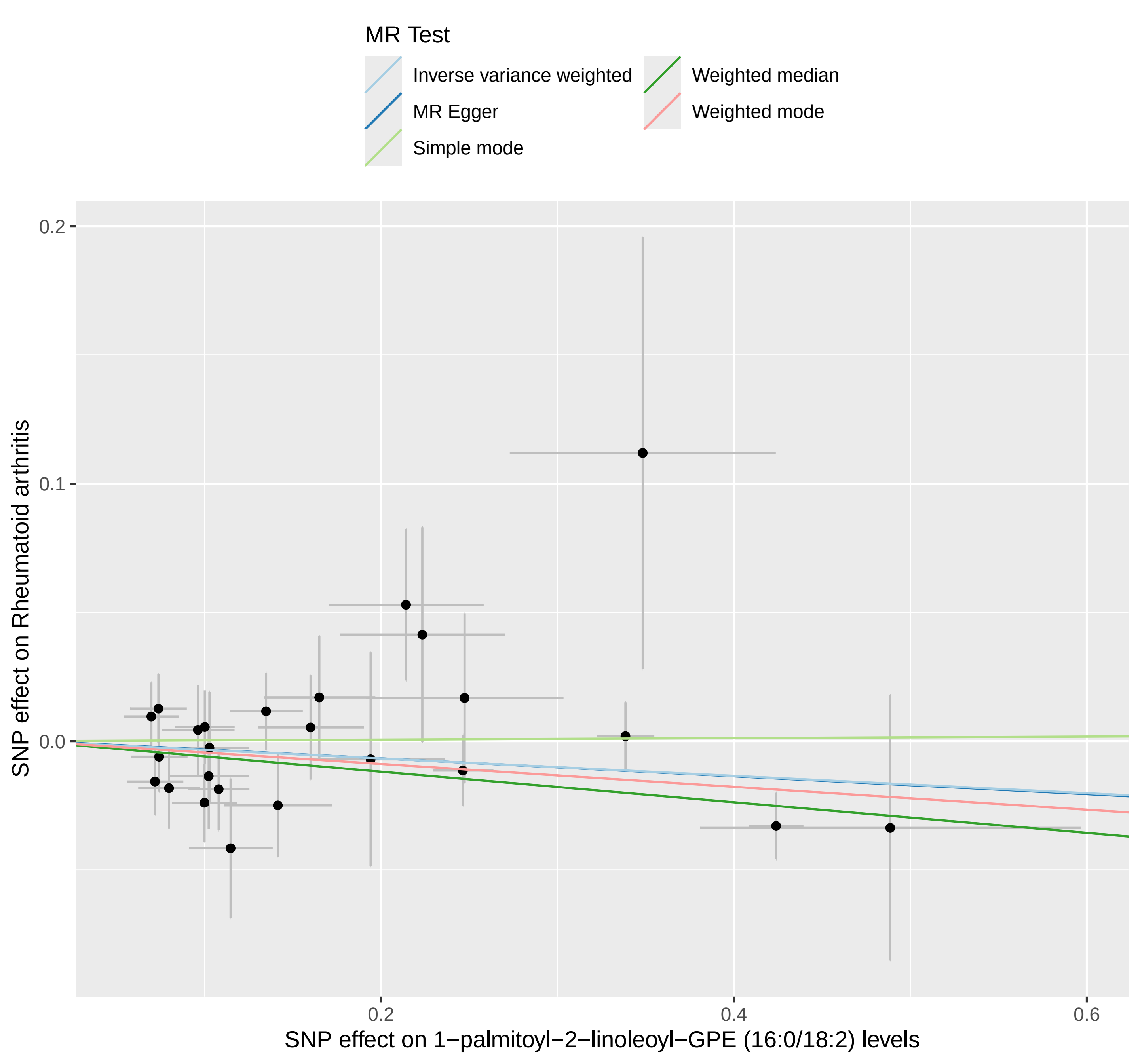
MR Test
Inverse variance weighted
MR Egger
Simple mode
Weighted median
Weighted mode
SNP effect on Rheumatoid arthritis
0.2
0.1
0.0
0.2
0.4
0.6
SNP effect on 1−palmitoyl−2−linoleoyl−GPE (16:0/18:2) levels

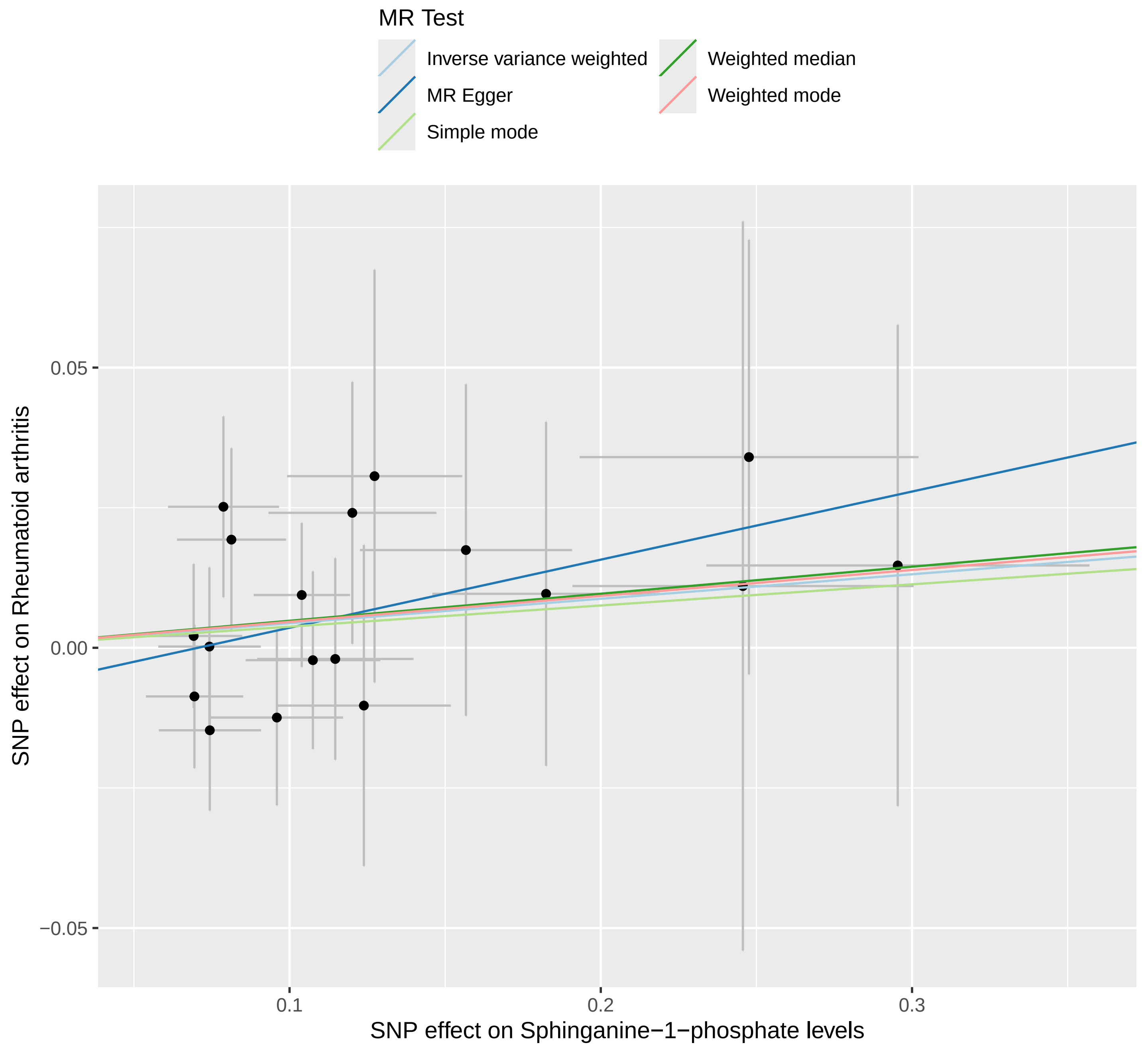

MR Test
Inverse variance weighted
MR Egger
Simple mode
Weighted median
Weighted mode
SNP effect on Rheumatoid arthritis
0.05
0.00
−0.05
0.1
0.2
0.3
SNP effect on Sphinganine−1−phosphate levels

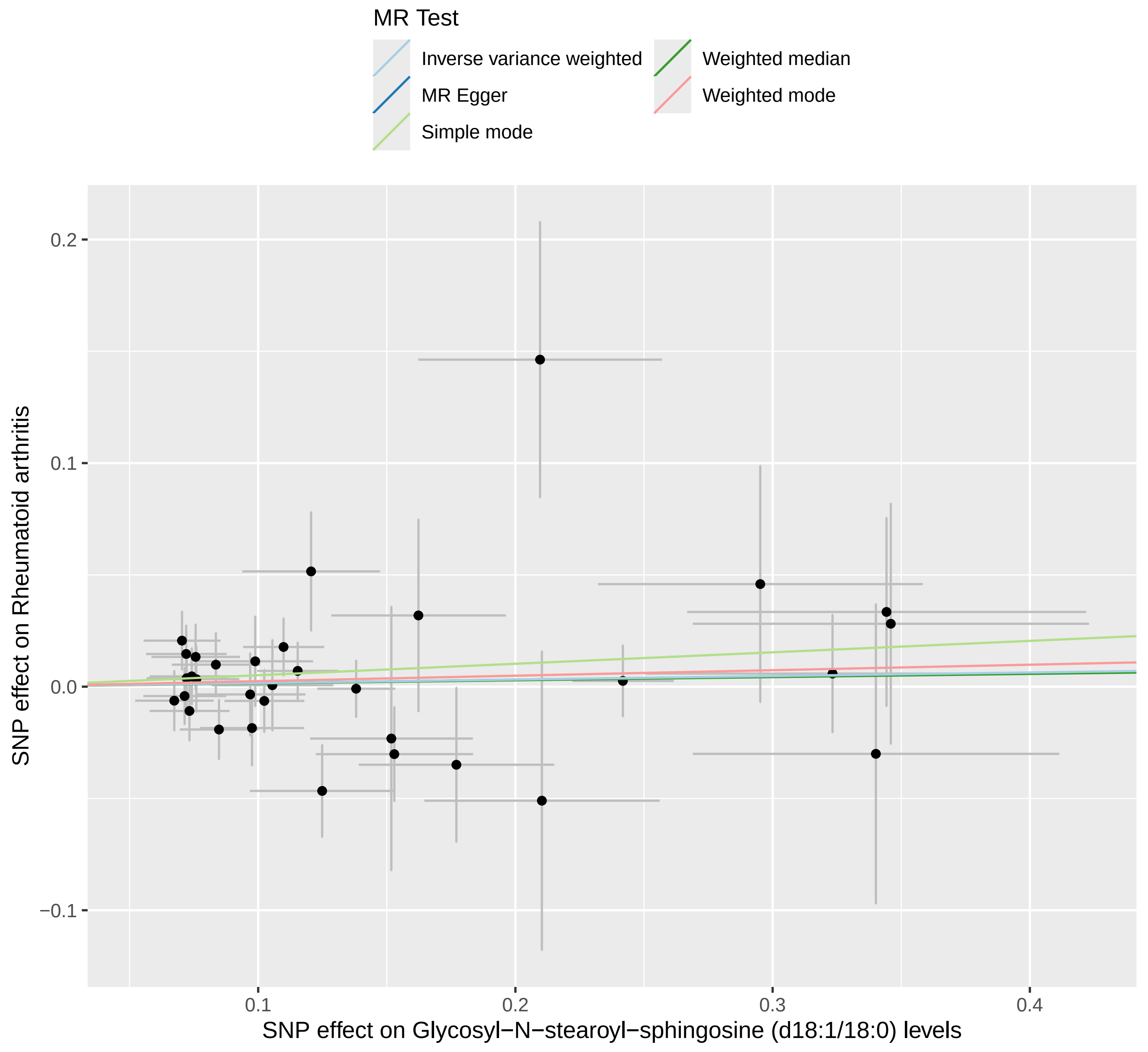
MR Test
Inverse variance weighted
MR Egger
Simple mode
Weighted median
Weighted mode
SNP effect on Rheumatoid arthritis
0.2
0.1
0.0
−0.1
0.1
0.2
0.3
0.4
SNP effect on Glycosyl−N−stearoyl−sphingosine (d18:1/18:0) levels

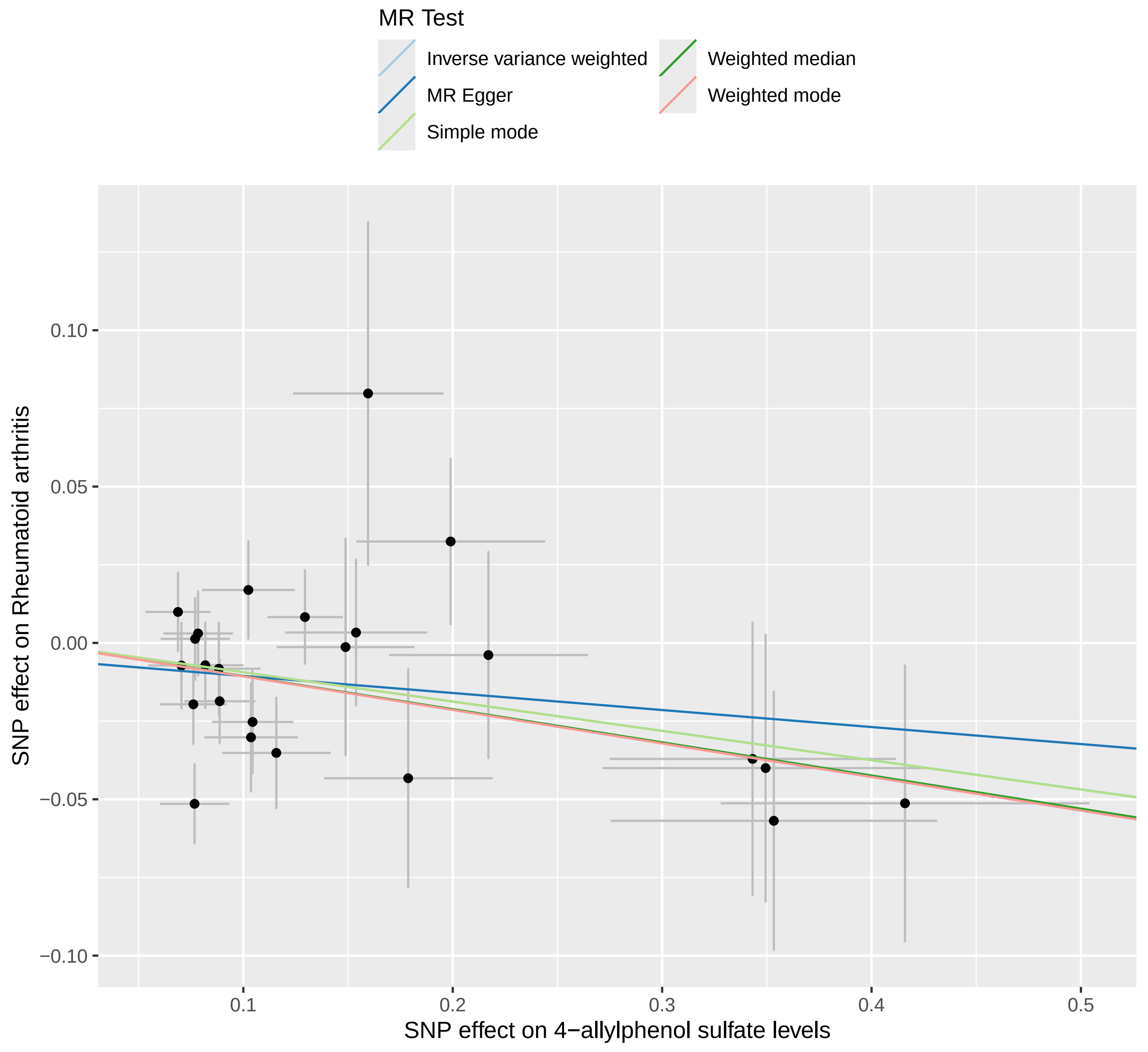

MR Test
Inverse variance weighted
MR Egger
Simple mode
Weighted median
Weighted mode
0.10
0.05
0.00
−0.05
−0.10
SNP effect on Rheumatoid arthritis
0.1
0.2
0.3
0.4
0.5
SNP effect on 4−allylphenol sulfate levels

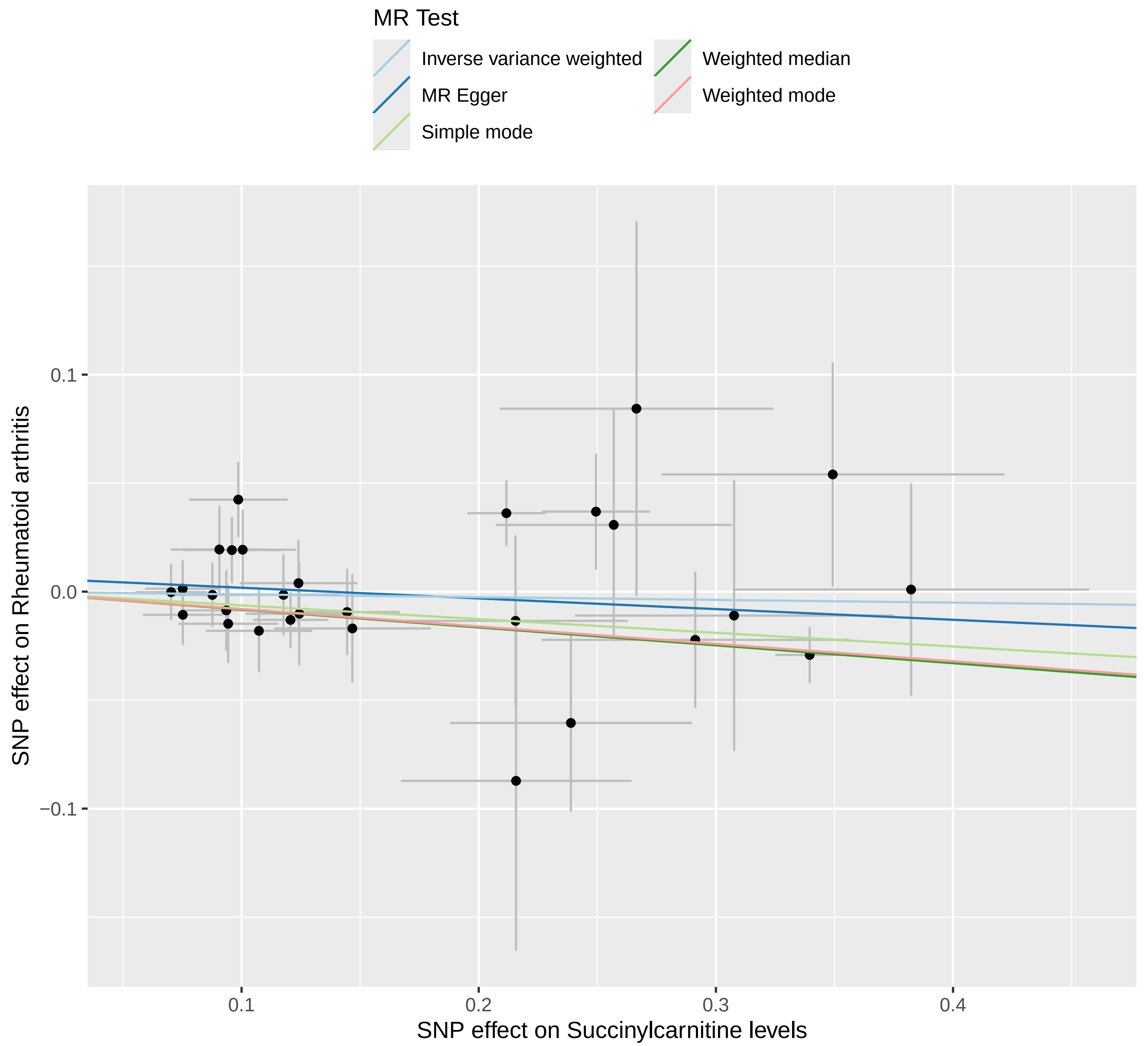

MR Test
Inverse variance weighted
MR Egger
Simple mode
Weighted median
Weighted mode
SNP effect on Rheumatoid arthritis
0.1
0.0
−0.1
0.1
0.2
0.3
0.4
SNP effect on Succinylcarnitine levels

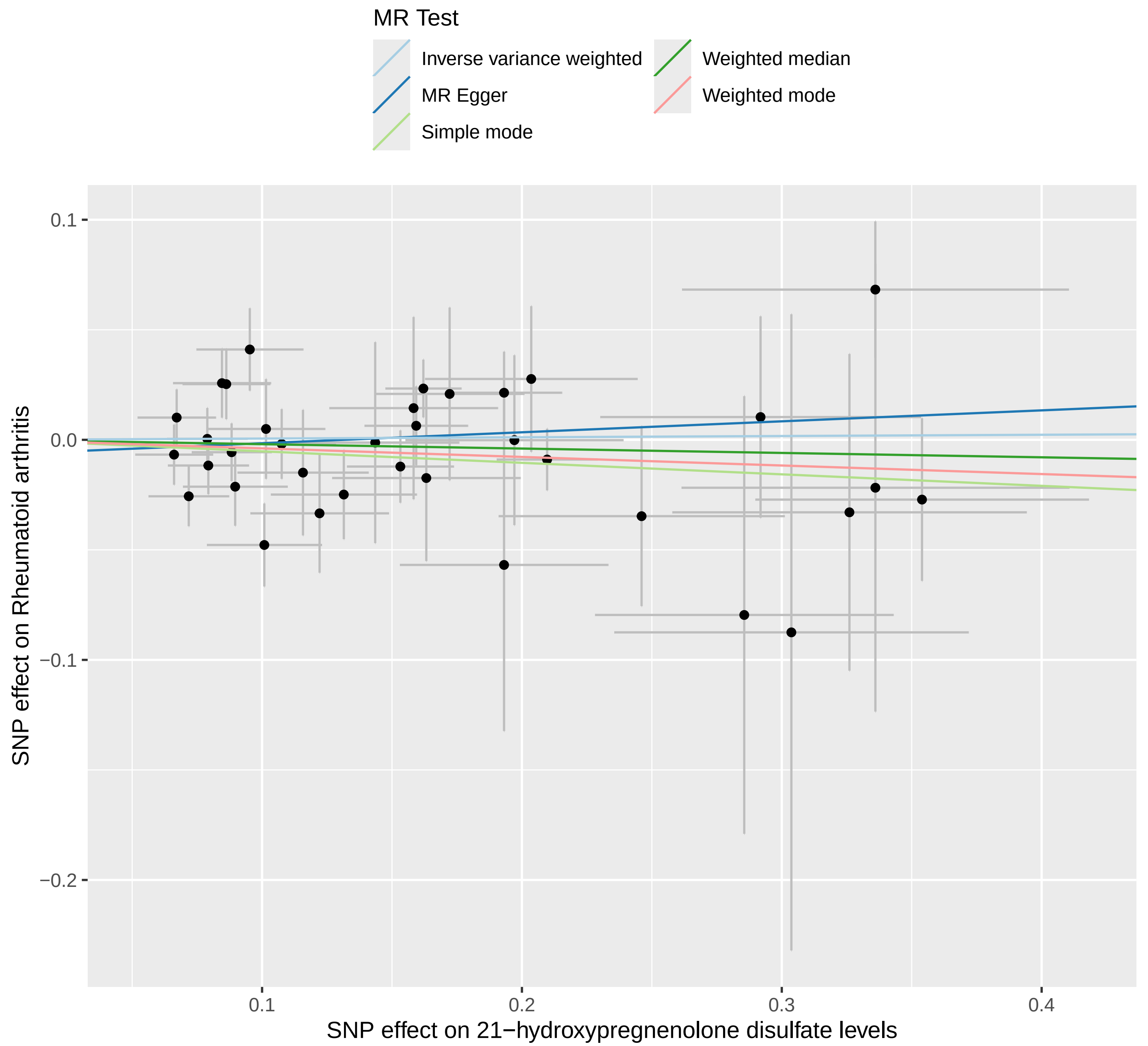
MR Test
Inverse variance weighted
MR Egger
Simple mode
Weighted median
Weighted mode
0.1
0.0
−0.1
−0.2
SNP effect on Rheumatoid arthritis
0.1
0.2
0.3
0.4
SNP effect on 21−hydroxypregnenolone disulfate levels

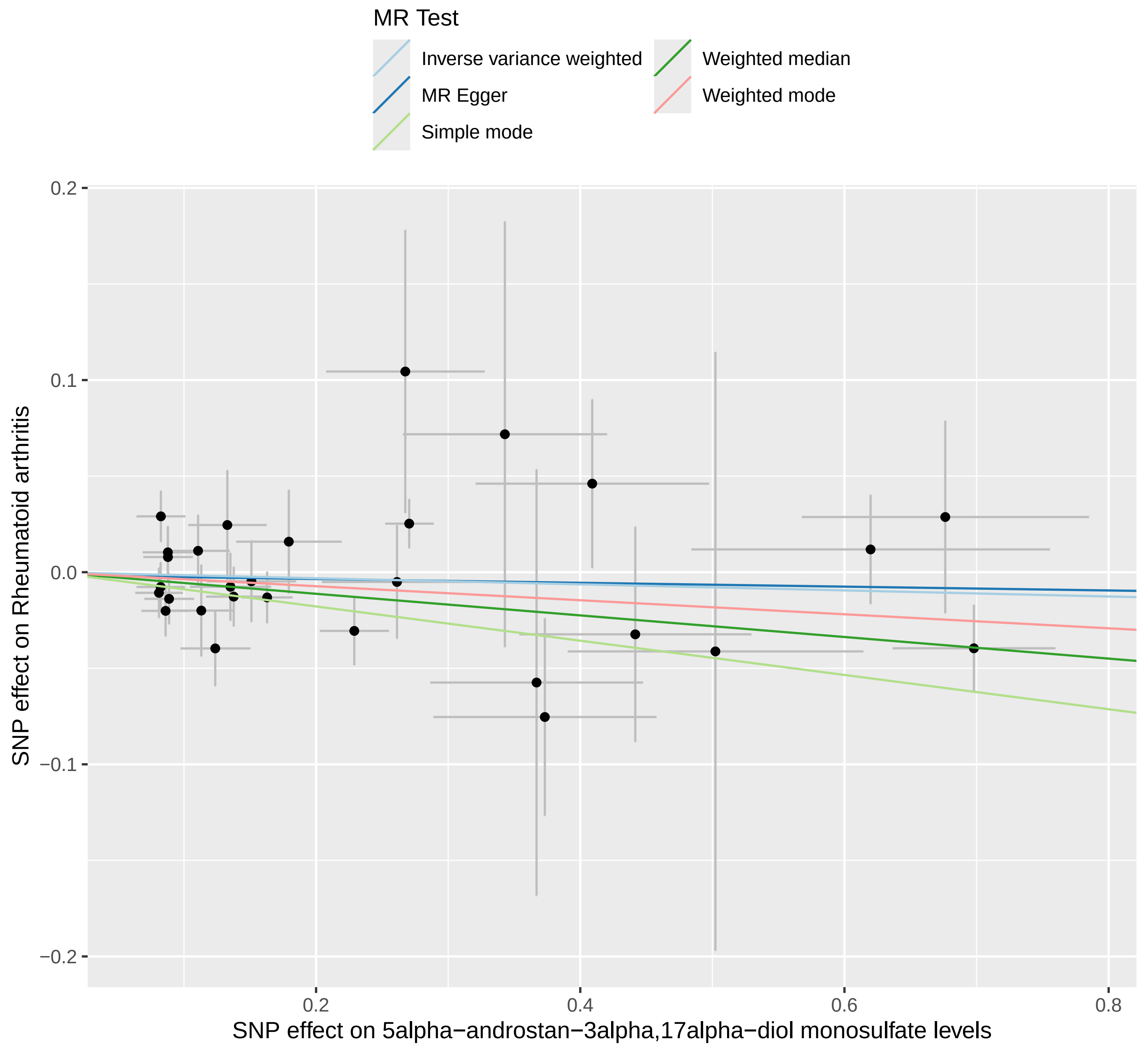
MR Test
Inverse variance weighted
MR Egger
Simple mode
Weighted median
Weighted mode
0.2
0.1
0.0
−0.1
−0.2
SNP effect on Rheumatoid arthritis
0.2
0.4
0.6
0.8
SNP effect on 5alpha−androstan−3alpha,17alpha−diol monosulfate levels

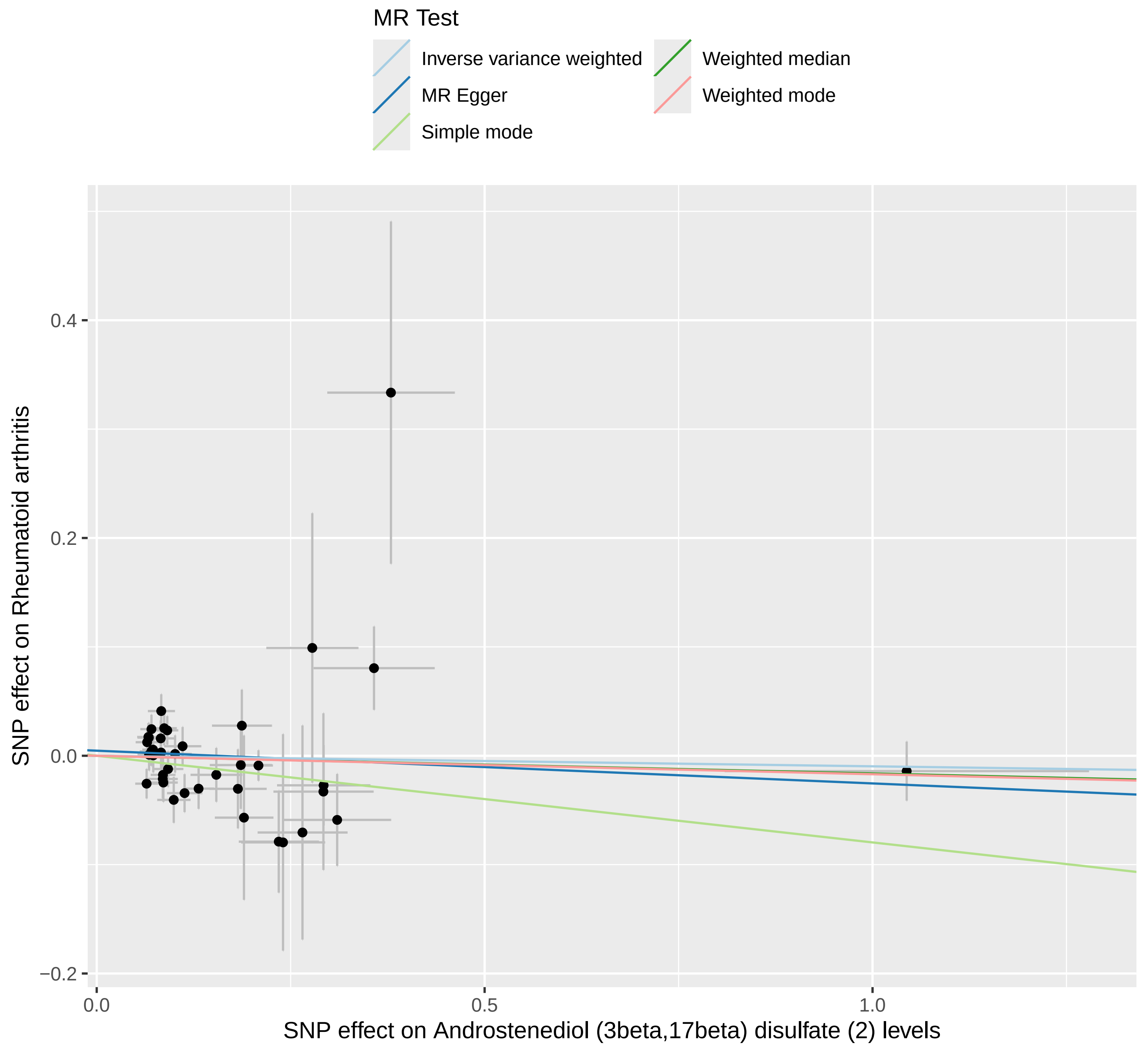
MR Test
Inverse variance weighted
MR Egger
Simple mode
Weighted median
Weighted mode
0.4
0.2
0.0
−0.2
0.0
0.5
1.0
SNP effect on Rheumatoid arthritis
SNP effect on Androstenediol (3beta,17beta) disulfate (2) levels

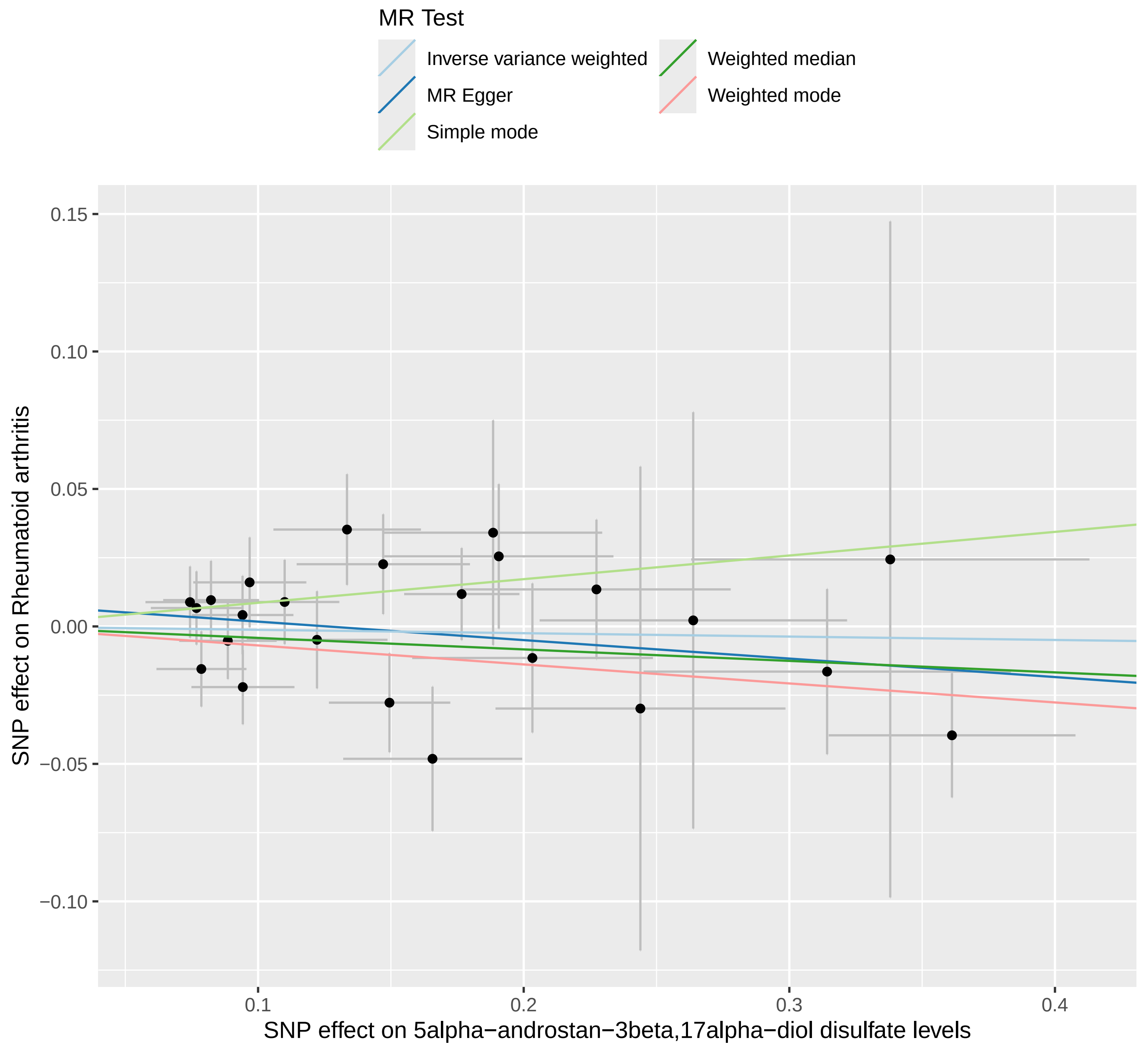

MR Test
Inverse variance weighted
MR Egger
Simple mode
Weighted median
Weighted mode
SNP effect on Rheumatoid arthritis
SNP effect on 5alpha−androstan−3beta,17alpha−diol disulfate levels
0.15
0.10
0.05
0.00
−0.05
−0.10
0.1
0.2
0.3
0.4

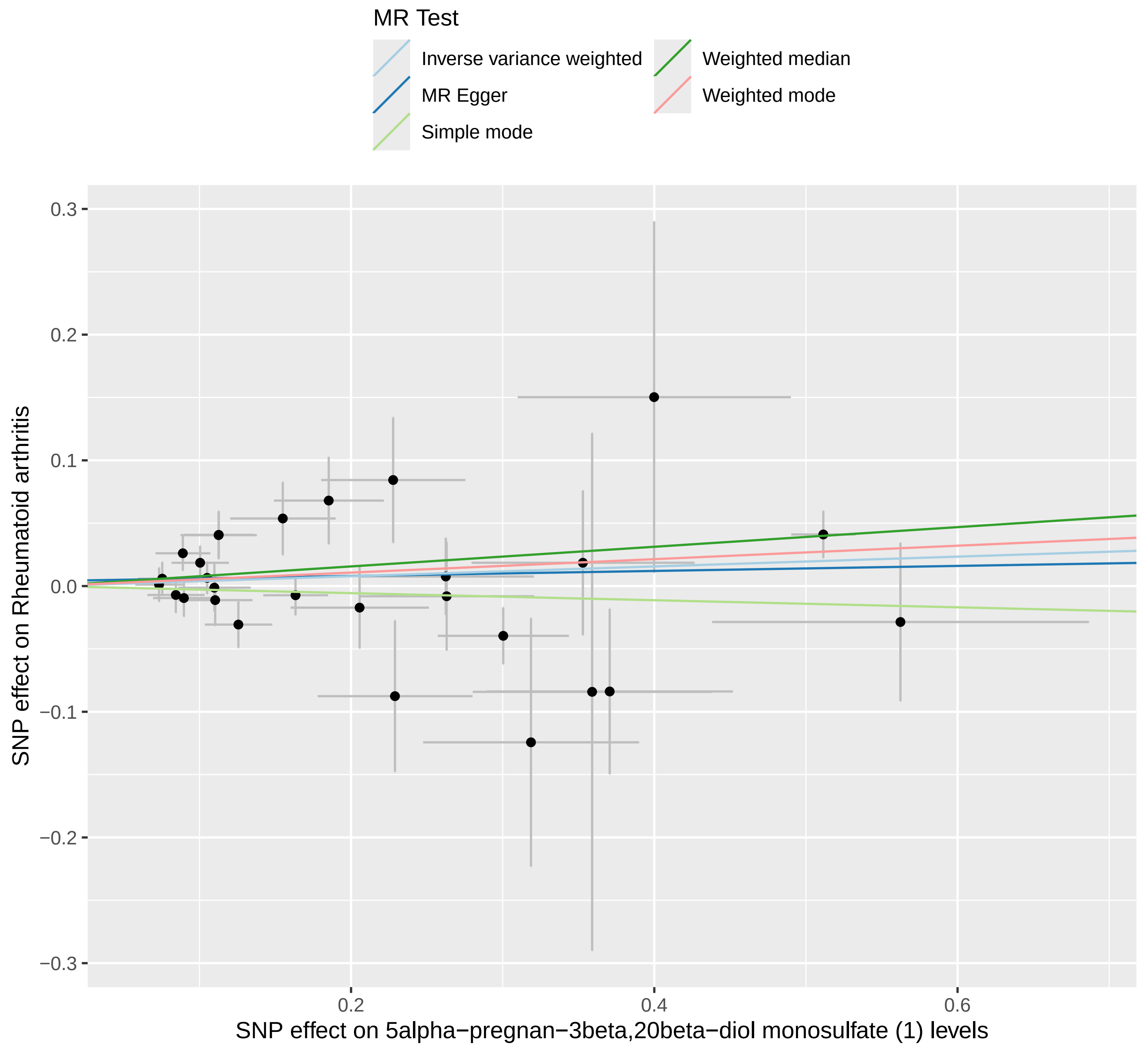
MR Test
Inverse variance weighted
MR Egger
Simple mode
Weighted median
Weighted mode
0.3
0.2
0.1
0.0
−0.1
−0.2
−0.3
SNP effect on Rheumatoid arthritis
0.2
0.4
0.6
SNP effect on 5alpha−pregnan−3beta,20beta−diol monosulfate (1) levels

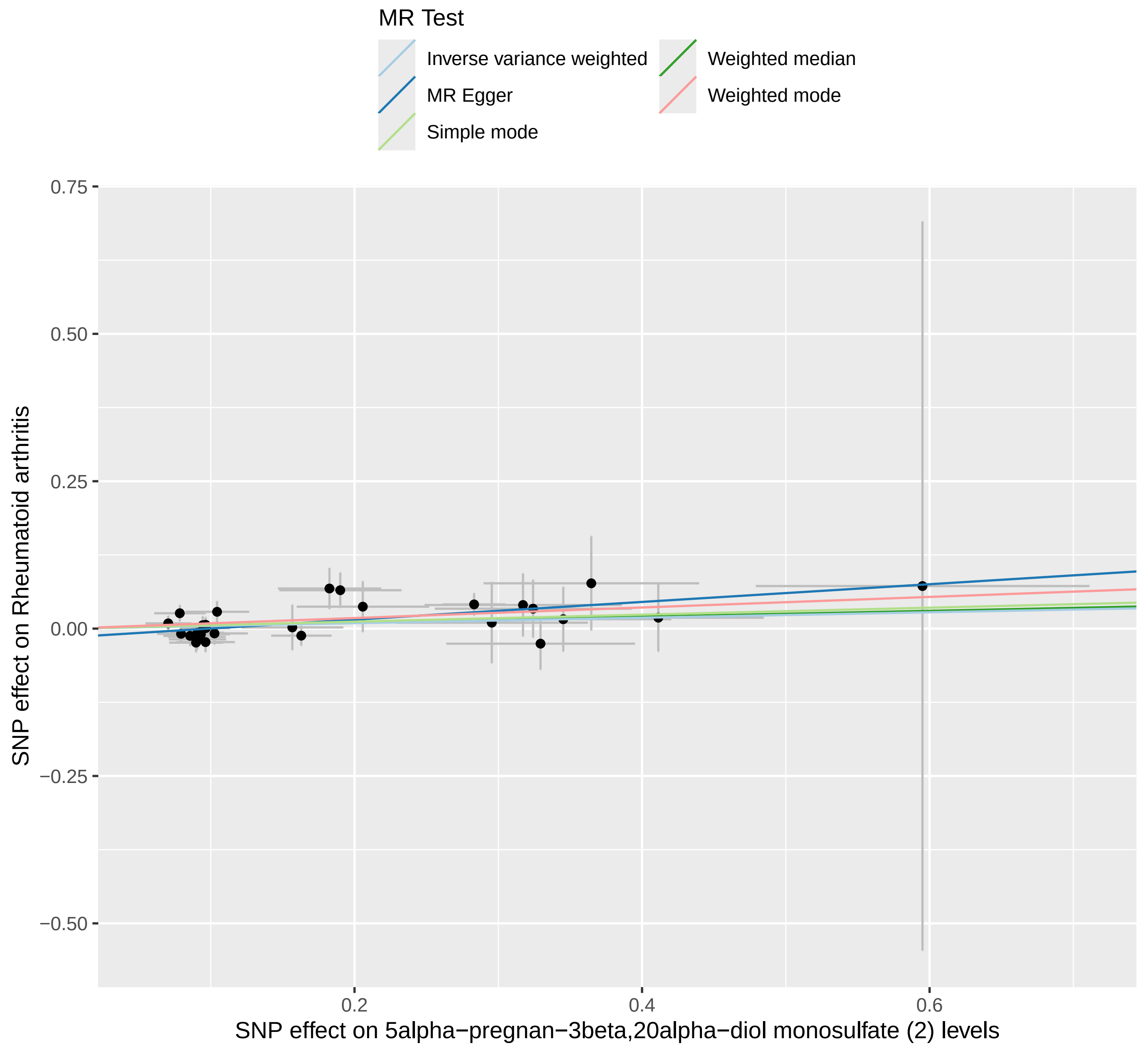
MR Test
Inverse variance weighted
MR Egger
Simple mode
Weighted median
Weighted mode
0.75
0.50
0.25
0.00
−0.25
−0.50
SNP effect on Rheumatoid arthritis
0.2
0.4
0.6
SNP effect on 5alpha−pregnan−3beta,20alpha−diol monosulfate (2) levels

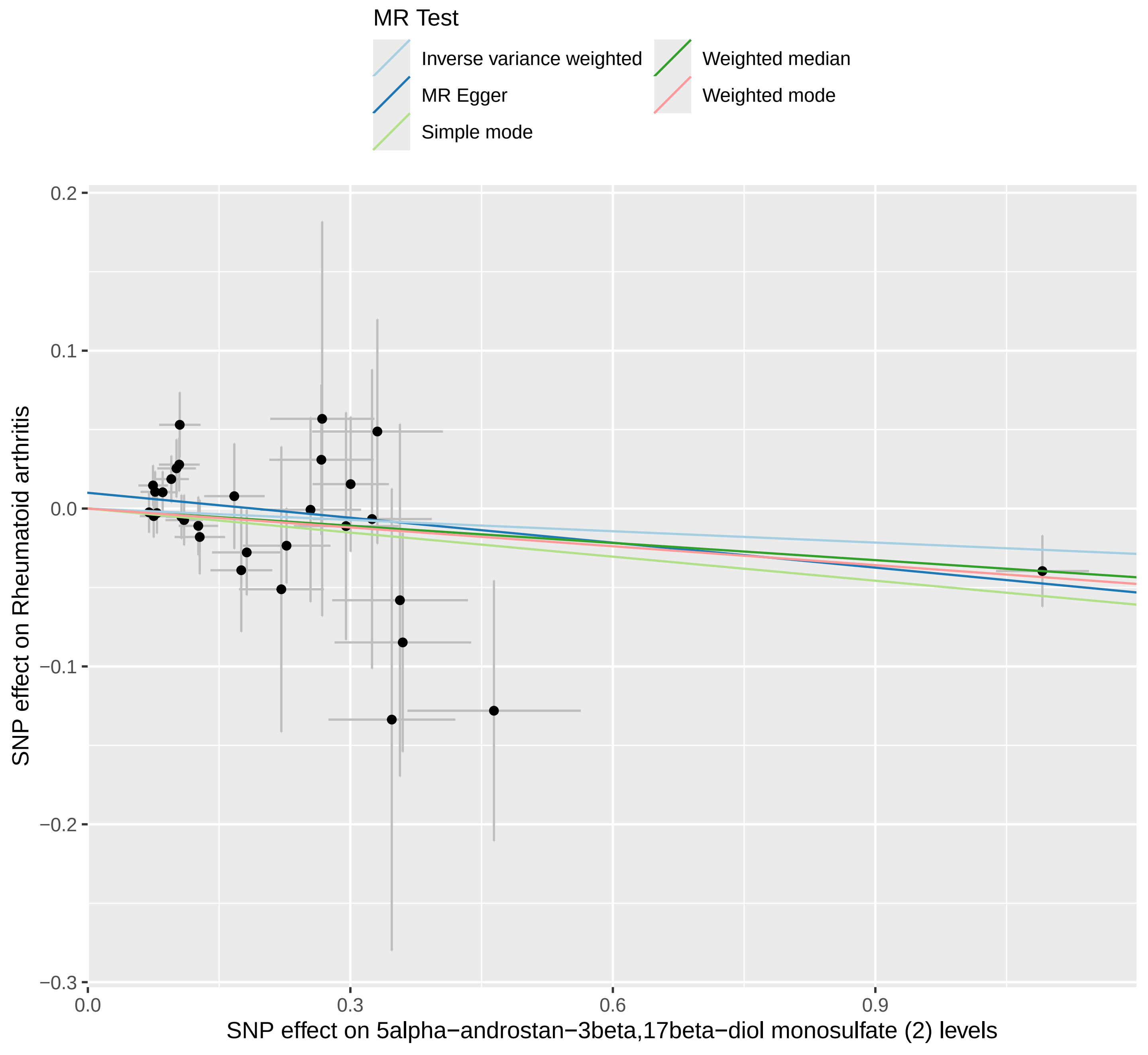

MR Test
Inverse variance weighted
MR Egger
Simple mode
Weighted median
Weighted mode
SNP effect on Rheumatoid arthritis
SNP effect on 5alpha−androstan−3beta,17beta−diol monosulfate (2) levels
0.2
0.1
0.0
−0.1
−0.2
−0.3
0.0
0.3
0.6
0.9

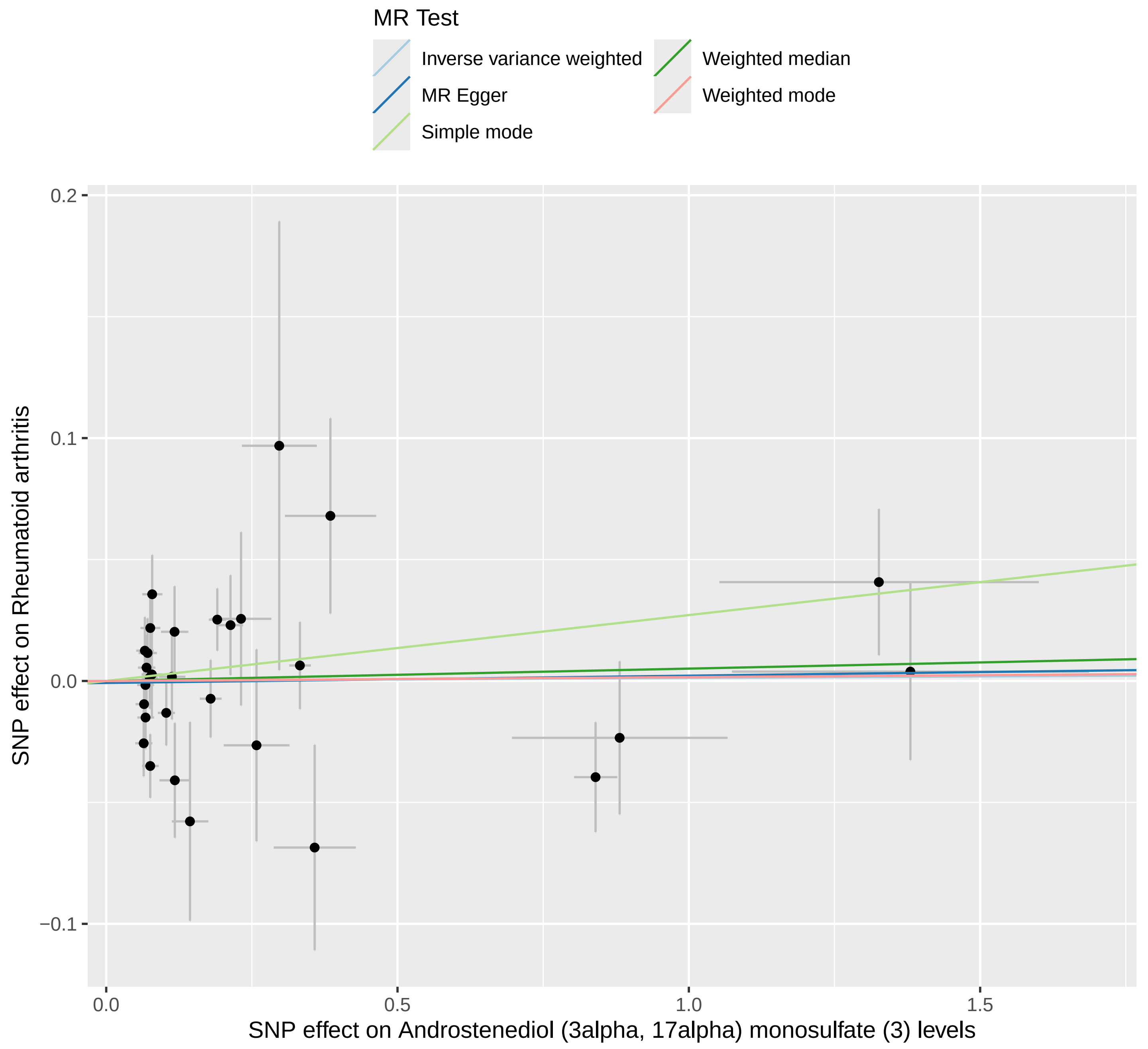
MR Test
Inverse variance weighted
MR Egger
Simple mode
Weighted median
Weighted mode
0.2
0.1
0.0
−0.1
0.0
0.5
1.0
1.5
SNP effect on Rheumatoid arthritis
SNP effect on Androstenediol (3alpha, 17alpha) monosulfate (3) levels

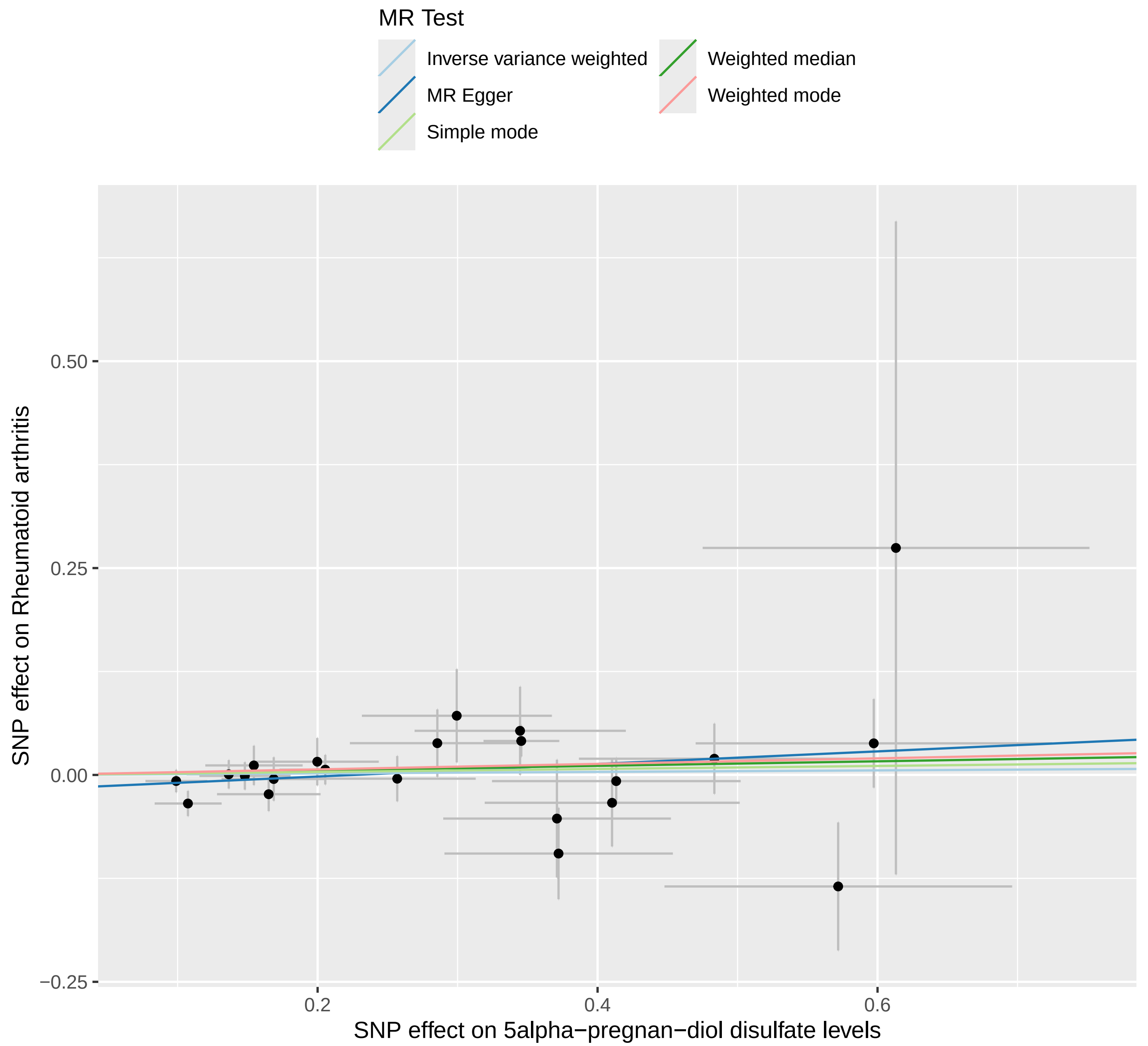

MR Test
Inverse variance weighted
MR Egger
Simple mode
Weighted median
Weighted mode
0.50
0.25
0.00
−0.25
0.2
0.4
0.6
SNP effect on Rheumatoid arthritis
SNP effect on 5alpha−pregnan−diol disulfate levels

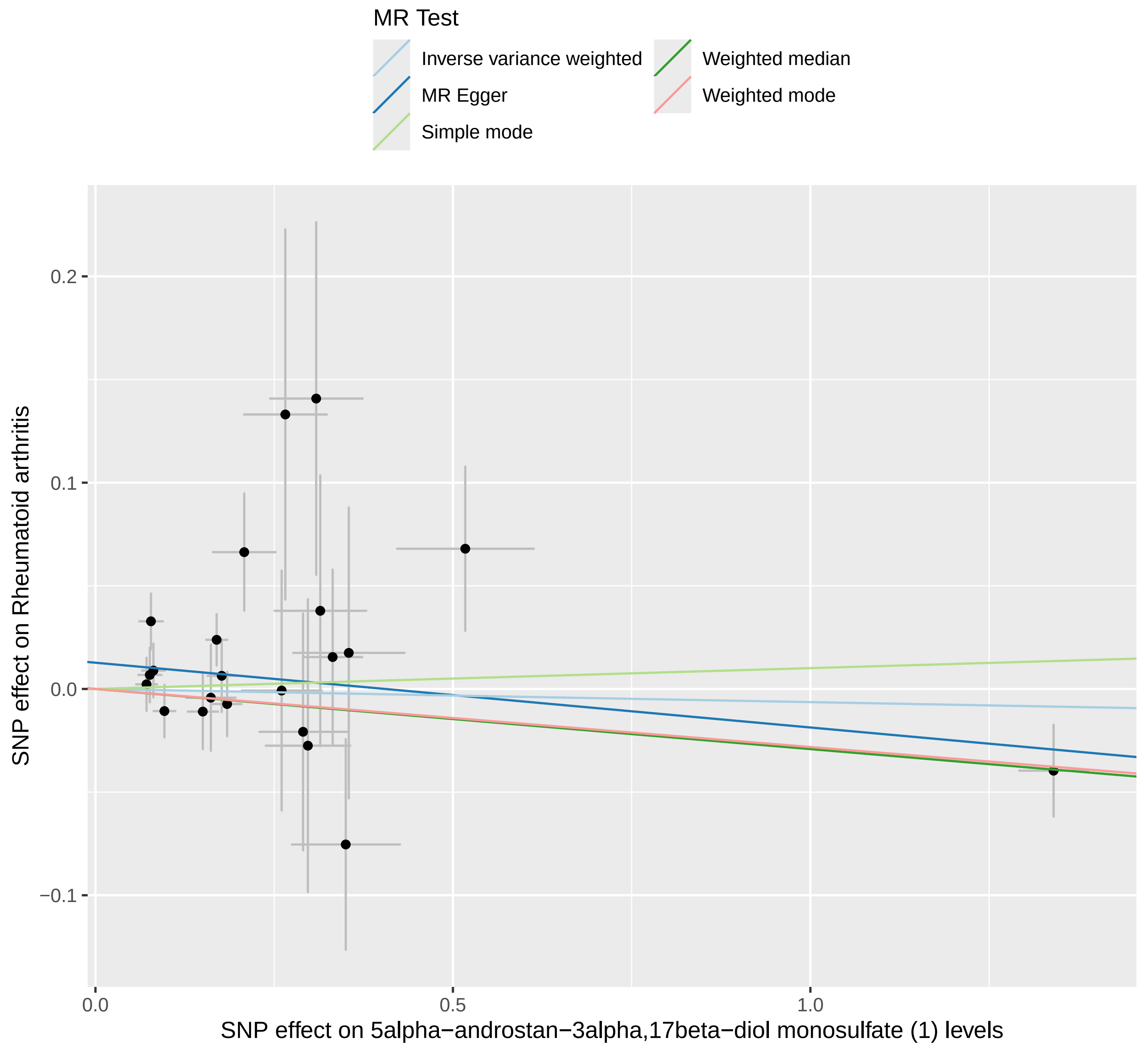

MR Test
Inverse variance weighted
MR Egger
Simple mode
Weighted median
Weighted mode
SNP effect on Rheumatoid arthritis
0.2
0.1
0.0
−0.1
0.0
0.5
1.0
SNP effect on 5alpha−androstan−3alpha,17beta−diol monosulfate (1) levels

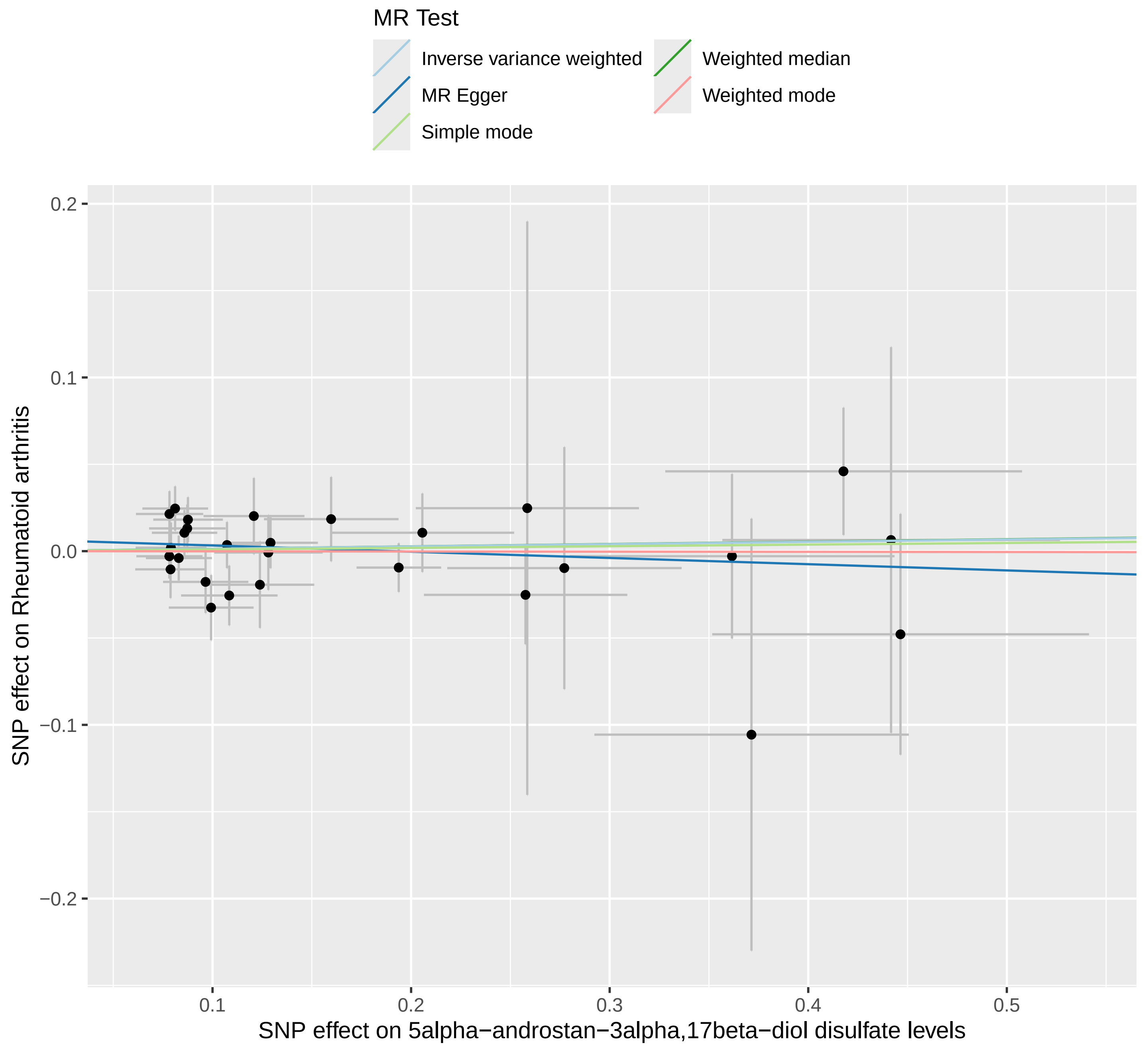
MR Test
Inverse variance weighted
MR Egger
Simple mode
Weighted median
Weighted mode
0.2
0.1
0.0
−0.1
−0.2
SNP effect on Rheumatoid arthritis
0.1
0.2
0.3
0.4
0.5
SNP effect on 5alpha−androstan−3alpha,17beta−diol disulfate levels

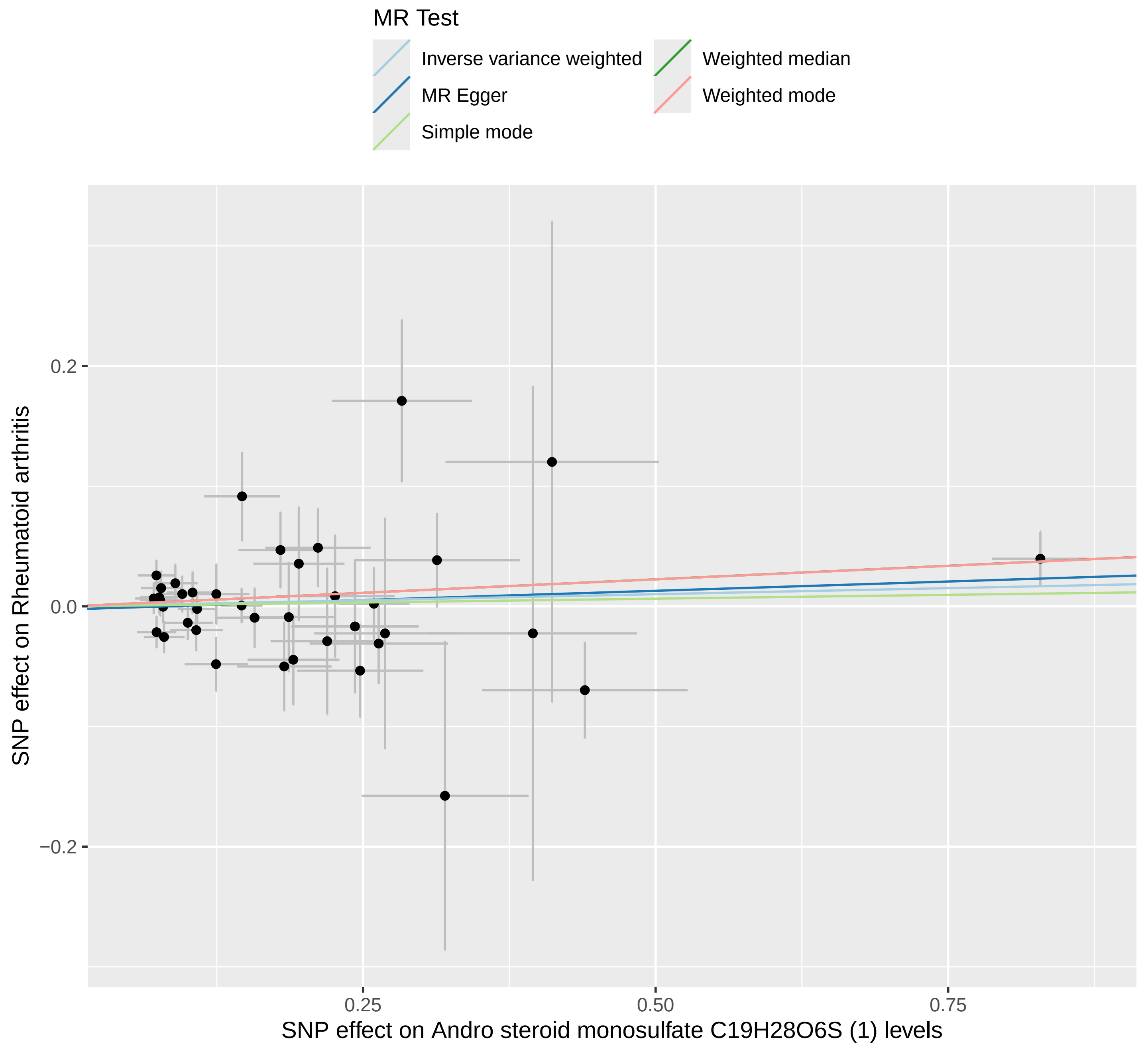

MR Test
Inverse variance weighted
MR Egger
Simple mode
Weighted median
Weighted mode
SNP effect on Rheumatoid arthritis
0.2
0.0
−0.2
0.25
0.50
0.75
SNP effect on Andro steroid monosulfate C19H28O6S (1) levels

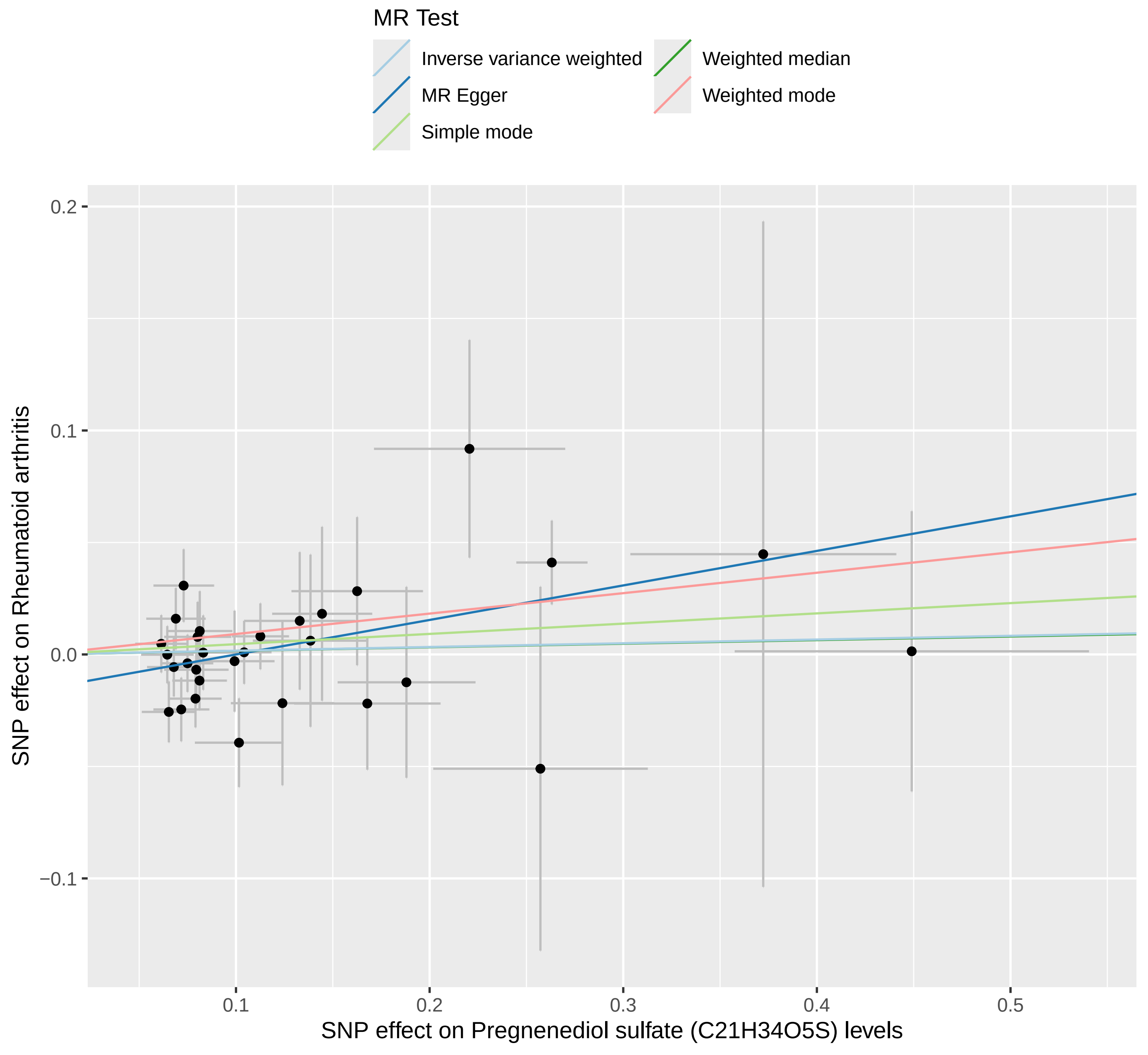
MR Test
Inverse variance weighted
MR Egger
Simple mode
Weighted median
Weighted mode
0.2
0.1
0.0
−0.1
0.1
0.2
0.3
0.4
0.5
SNP effect on Rheumatoid arthritis
SNP effect on Pregnenediol sulfate (C21H34O5S) levels

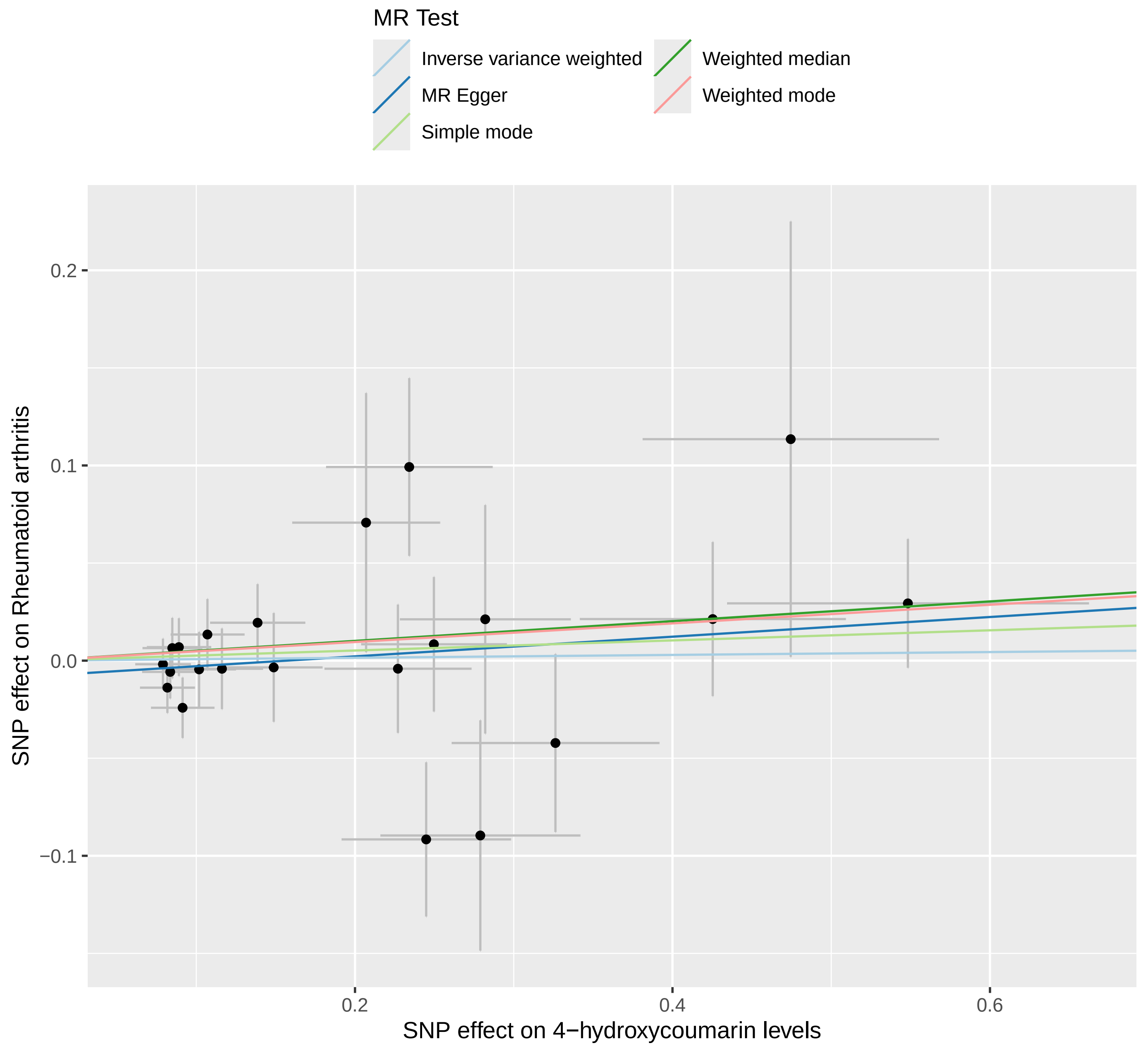

MR Test
Inverse variance weighted
MR Egger
Simple mode
Weighted median
Weighted mode
0.2
0.1
0.0
−0.1
SNP effect on Rheumatoid arthritis
0.2
0.4
0.6
SNP effect on 4−hydroxycoumarin levels

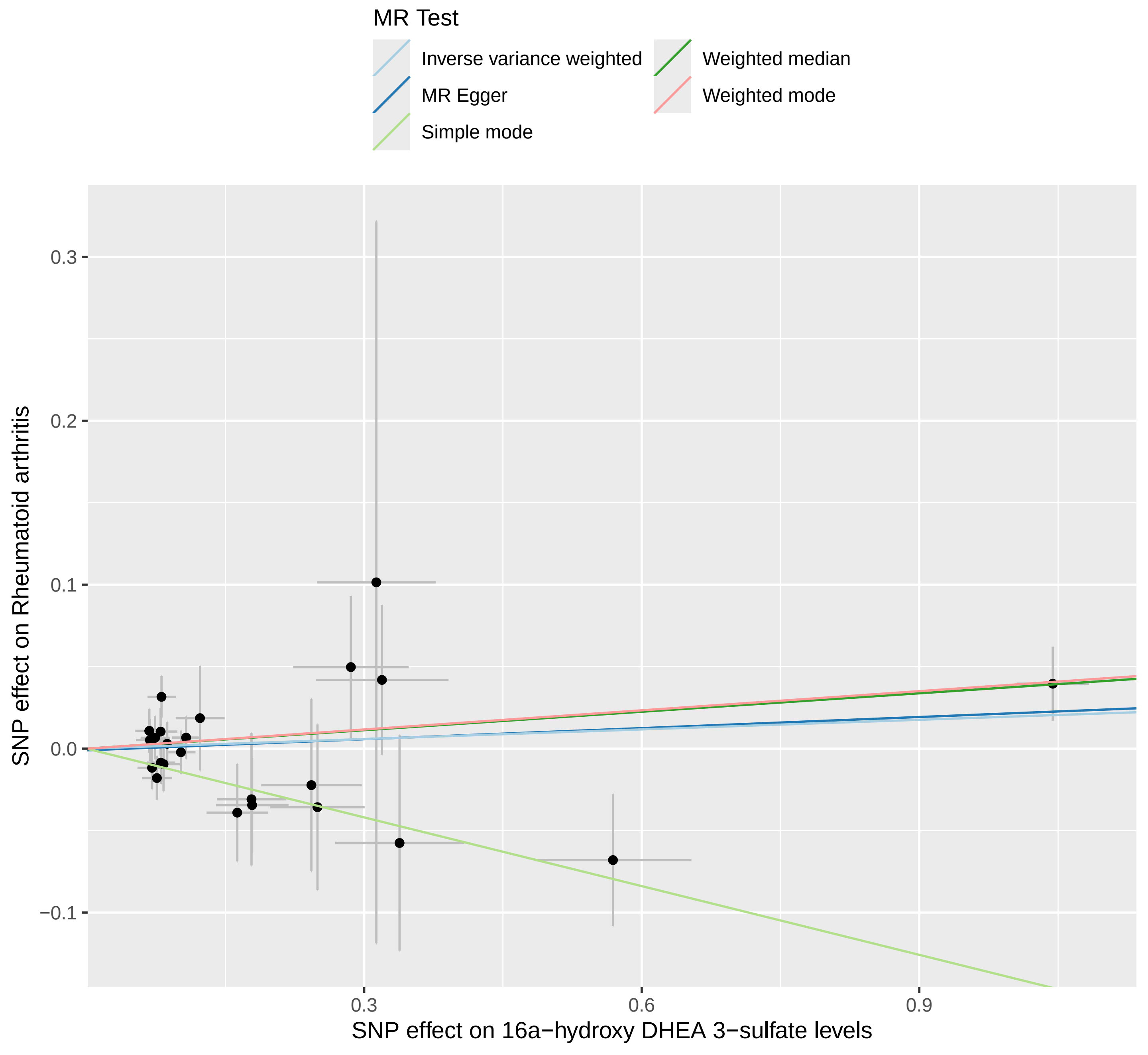

MR Test
Inverse variance weighted
MR Egger
Simple mode
Weighted median
Weighted mode
0.3
0.2
0.1
0.0
−0.1
0.3
0.6
0.9
SNP effect on Rheumatoid arthritis
SNP effect on 16a−hydroxy DHEA 3−sulfate levels

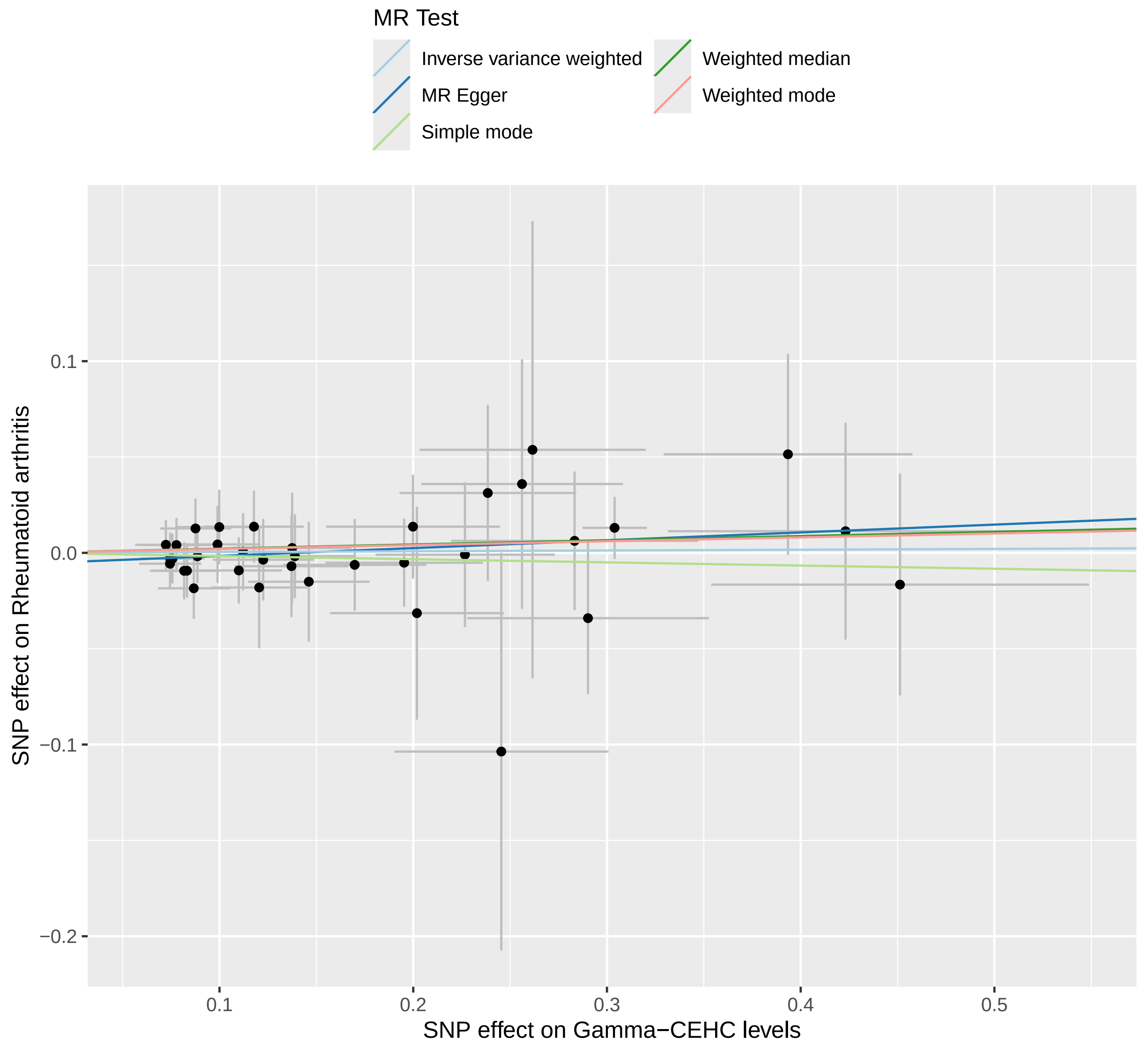

MR Test
Inverse variance weighted
MR Egger
Simple mode
Weighted median
Weighted mode
0.1
0.0
−0.1
−0.2
SNP effect on Rheumatoid arthritis
0.1
0.2
0.3
0.4
0.5
SNP effect on Gamma−CEHC levels

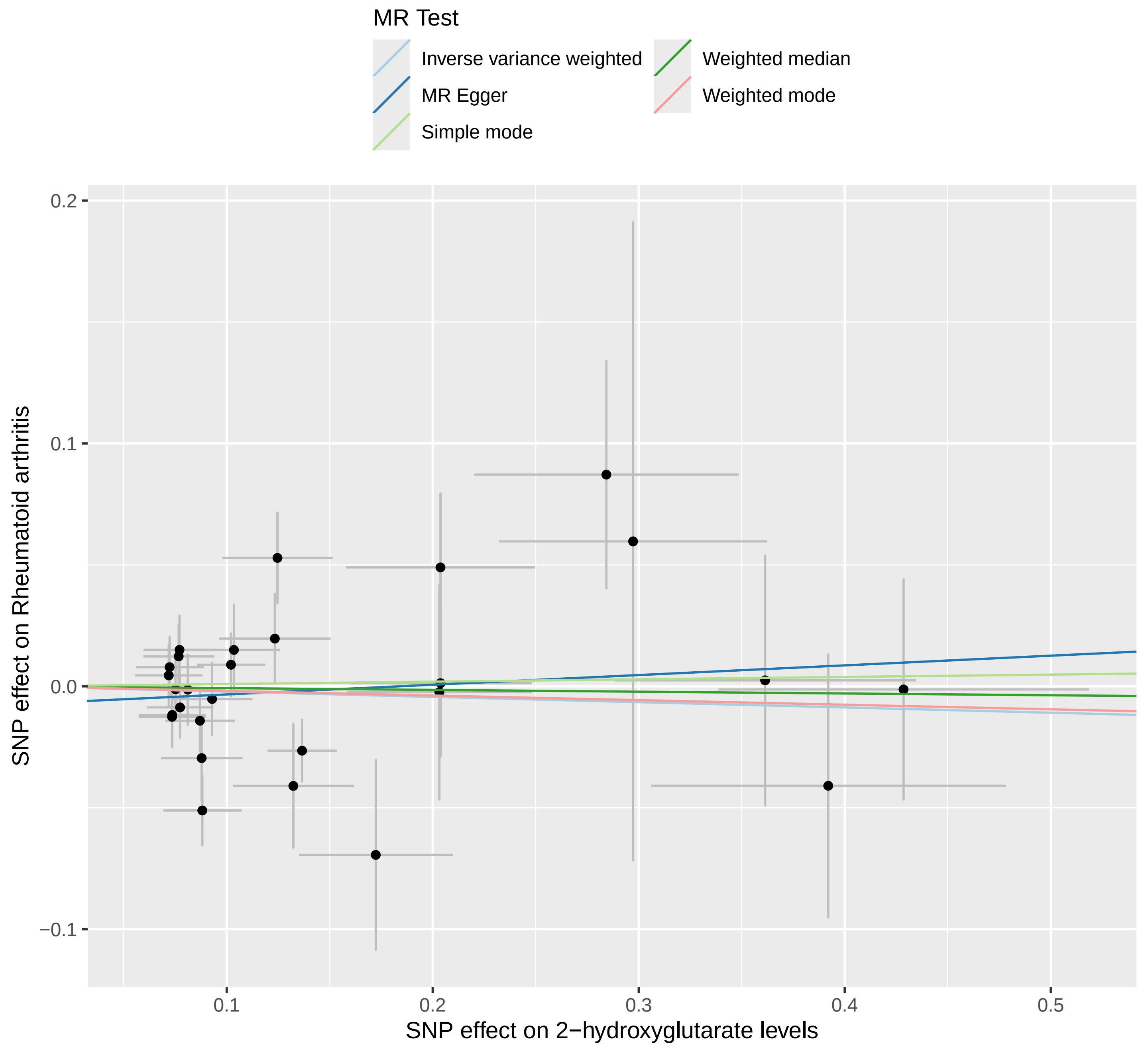
MR Test
Inverse variance weighted
MR Egger
Simple mode
Weighted median
Weighted mode
0.2
0.1
0.0
−0.1
SNP effect on Rheumatoid arthritis
0.1
0.2
0.3
0.4
0.5
SNP effect on 2−hydroxyglutarate levels

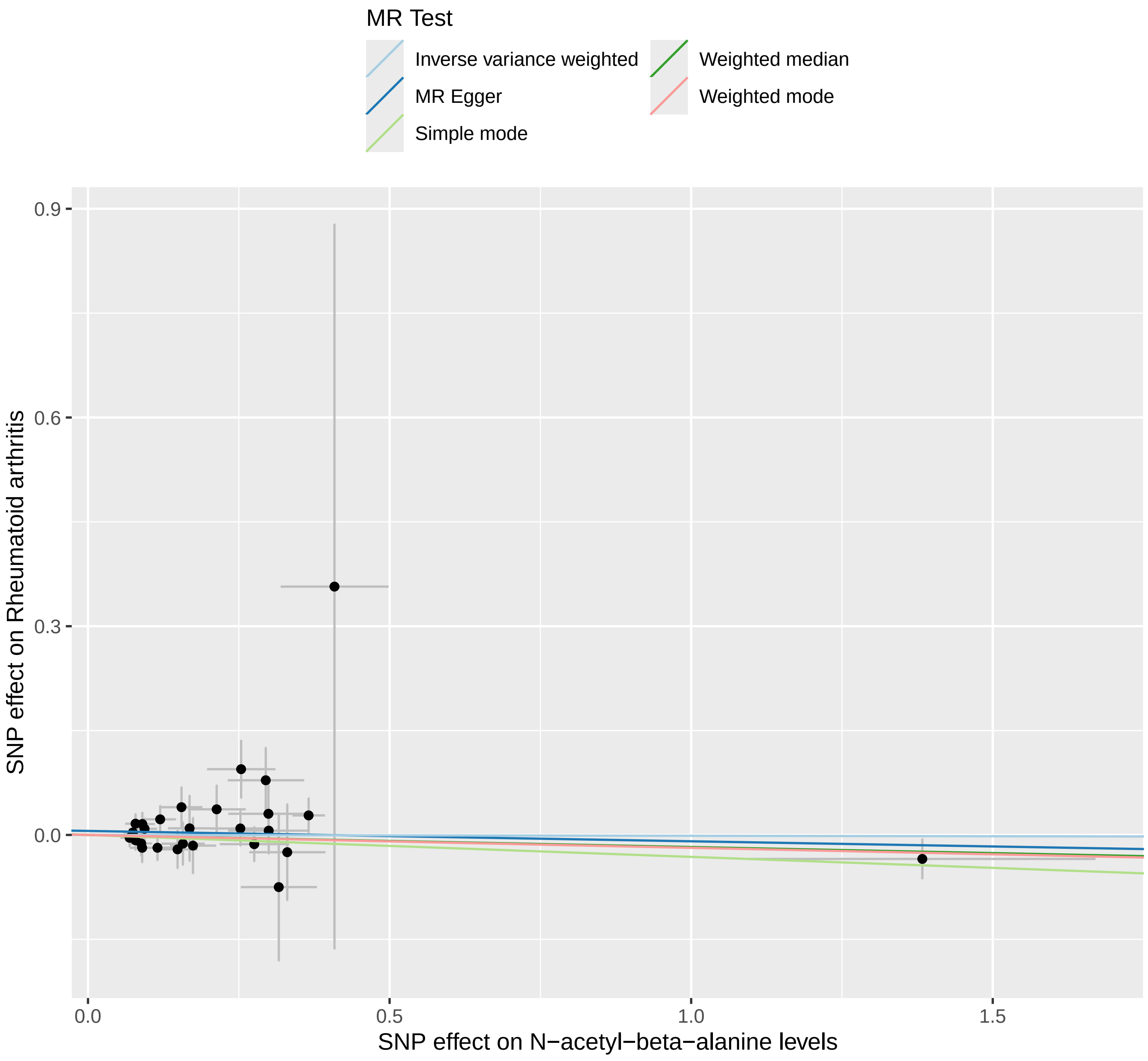
MR Test
Inverse variance weighted
MR Egger
Simple mode
Weighted median
Weighted mode
0.9
0.6
0.3
0.0
0.0
0.5
1.0
1.5
SNP effect on Rheumatoid arthritis
SNP effect on N−acetyl−beta−alanine levels

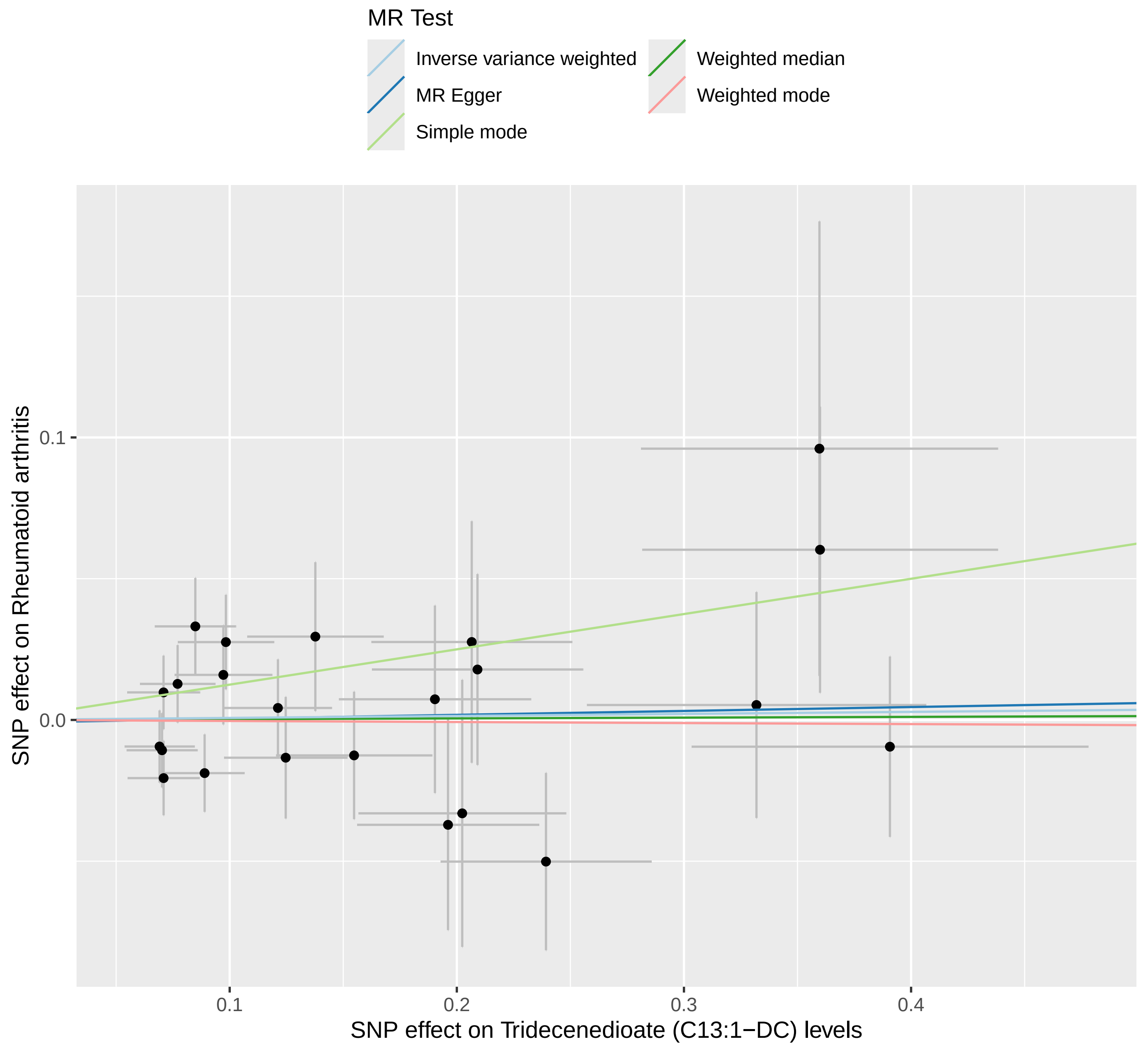
MR Test
Inverse variance weighted
MR Egger
Simple mode
Weighted median
Weighted mode
0.1
0.0
0.1
0.2
0.3
0.4
SNP effect on Rheumatoid arthritis
SNP effect on Tridecenedioate (C13:1−DC) levels

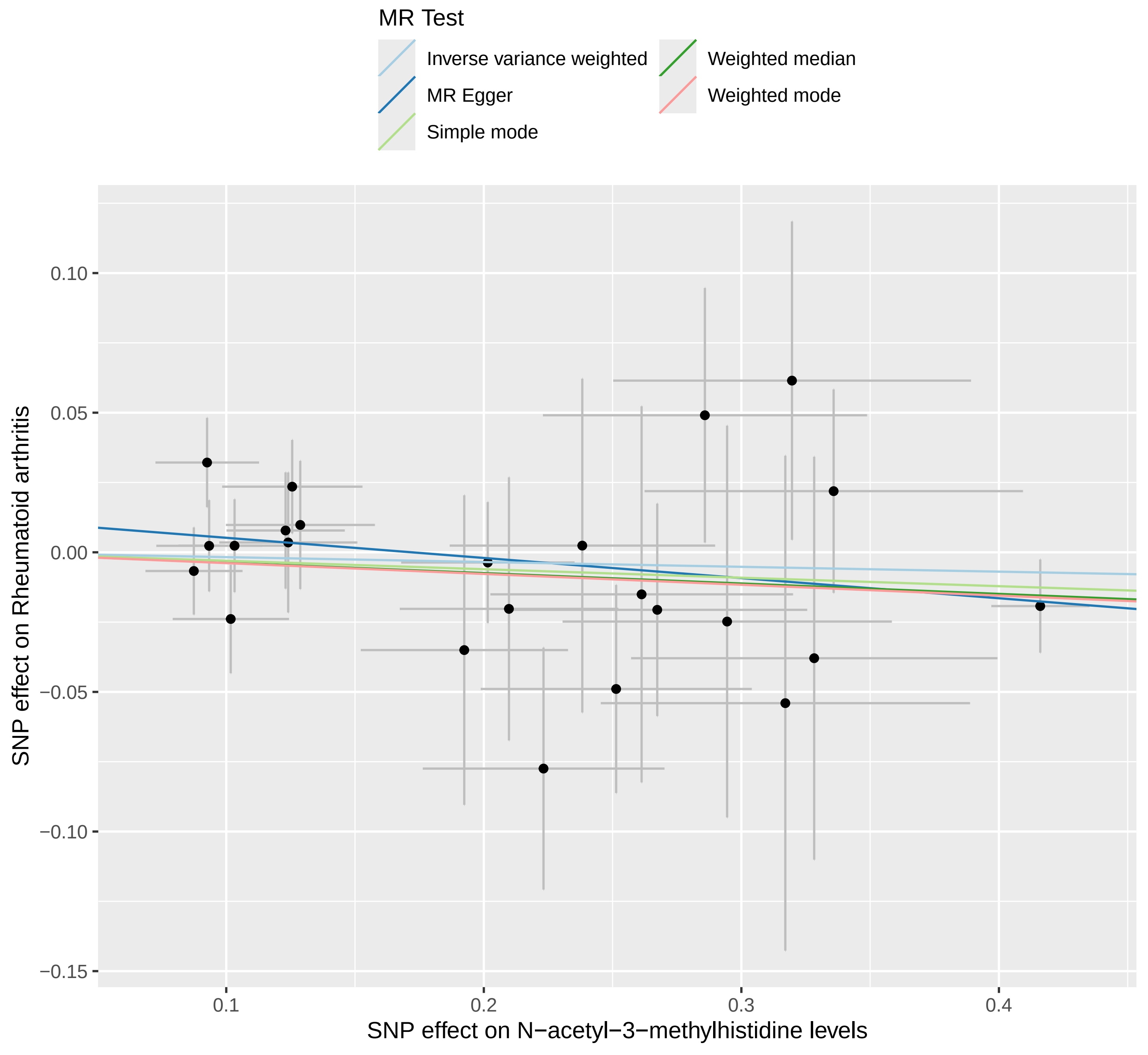
MR Test
Inverse variance weighted
MR Egger
Simple mode
Weighted median
Weighted mode
SNP effect on Rheumatoid arthritis
0.10
0.05
0.00
−0.05
−0.10
−0.15
0.1
0.2
0.3
0.4
SNP effect on N−acetyl−3−methylhistidine levels

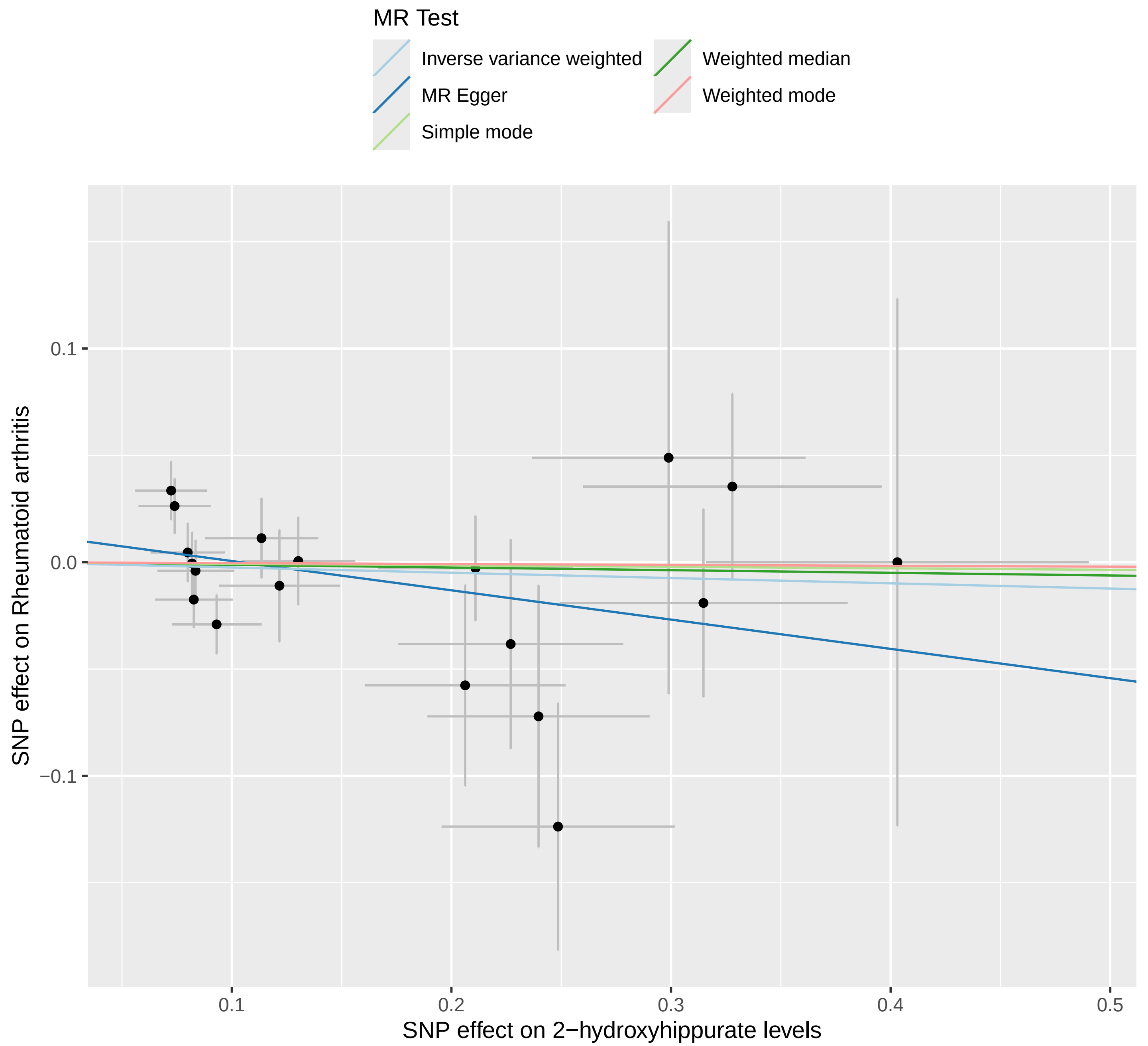
MR Test
Inverse variance weighted
MR Egger
Simple mode
Weighted median
Weighted mode
0.1
0.0
−0.1
SNP effect on Rheumatoid arthritis
0.1
0.2
0.3
0.4
0.5
SNP effect on 2−hydroxyhippurate levels

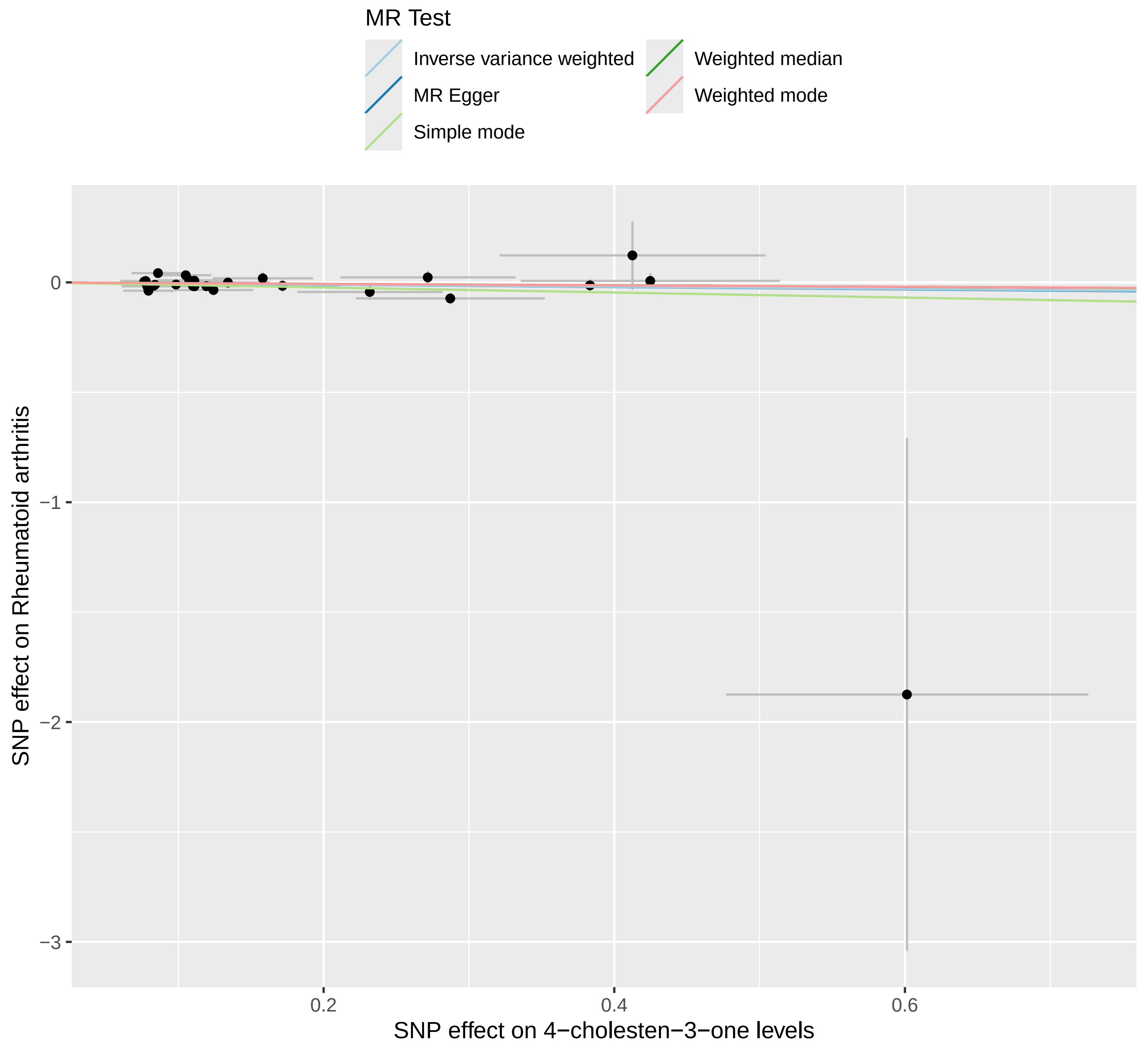
MR Test
Inverse variance weighted
MR Egger
Simple mode
Weighted median
Weighted mode
0
−1
−2
−3
0.2
0.4
0.6
SNP effect on Rheumatoid arthritis
SNP effect on 4−cholesten−3−one levels

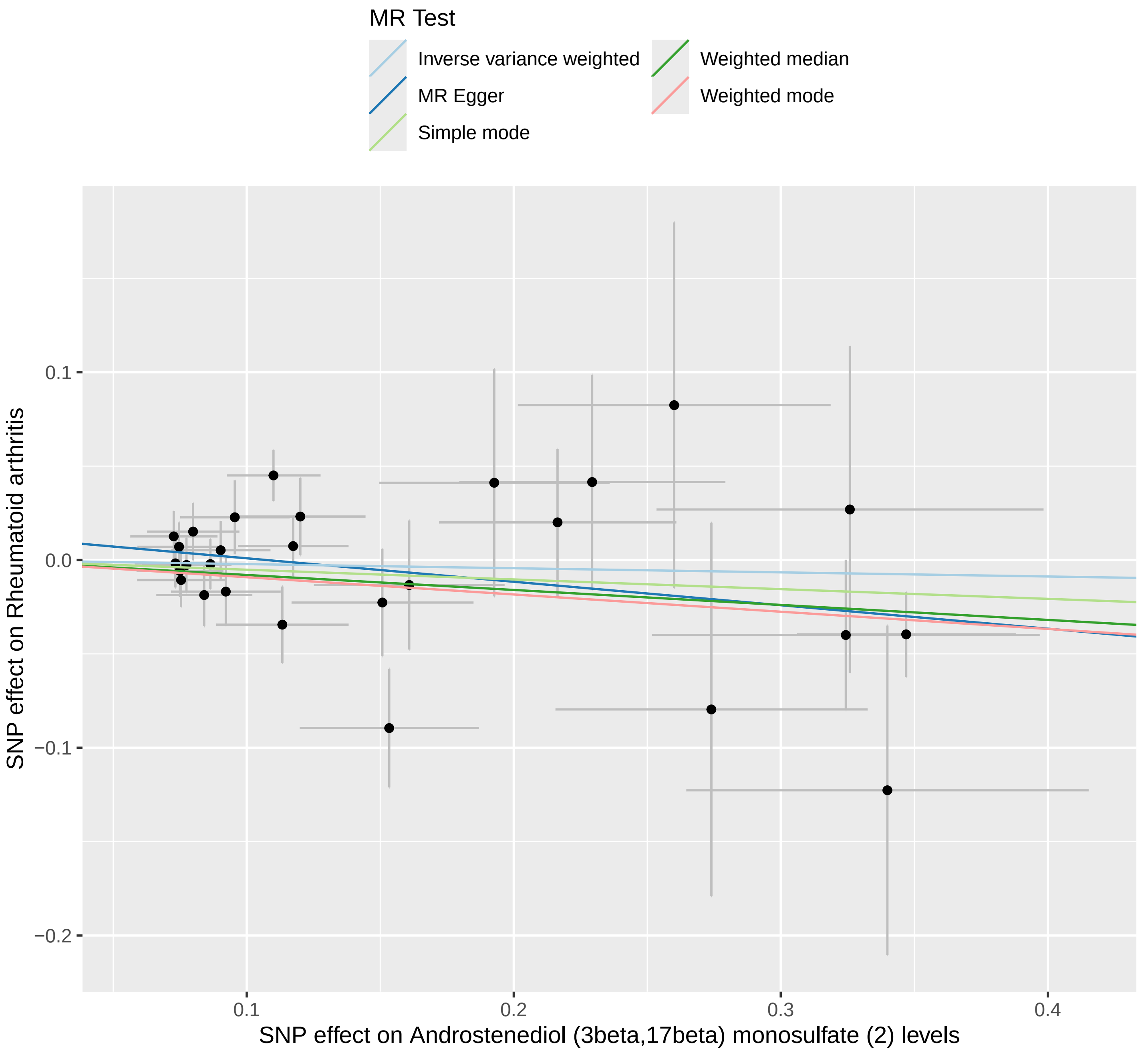

MR Test
Inverse variance weighted
MR Egger
Simple mode
Weighted median
Weighted mode
0.1
0.0
−0.1
−0.2
SNP effect on Rheumatoid arthritis
0.1
0.2
0.3
0.4
SNP effect on Androstenediol (3beta,17beta) monosulfate (2) levels

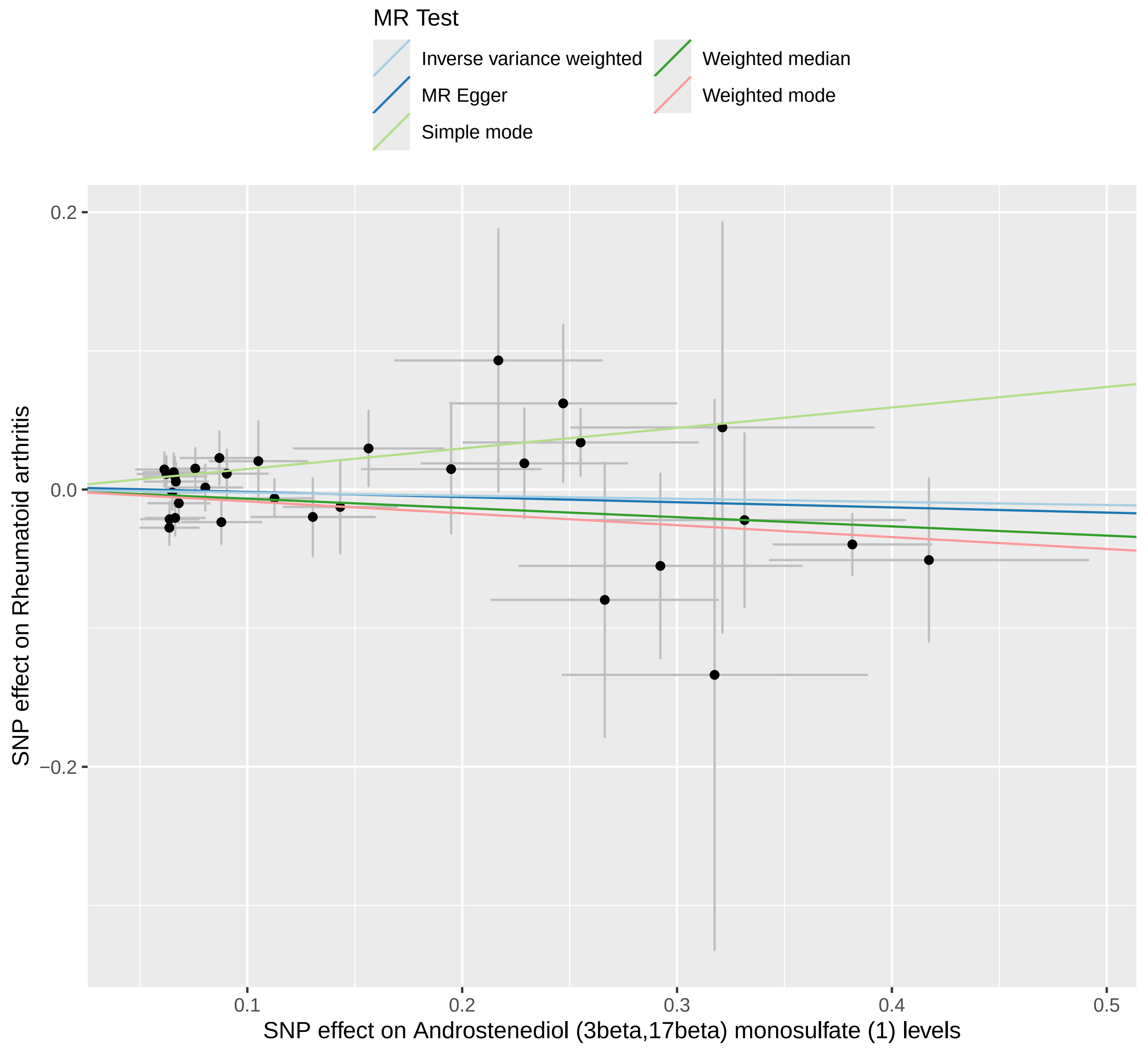
MR Test
Inverse variance weighted
MR Egger
Simple mode
Weighted median
Weighted mode
0.2
0.0
−0.2
0.1
0.2
0.3
0.4
0.5
SNP effect on Rheumatoid arthritis
SNP effect on Androstenediol (3beta,17beta) monosulfate (1) levels

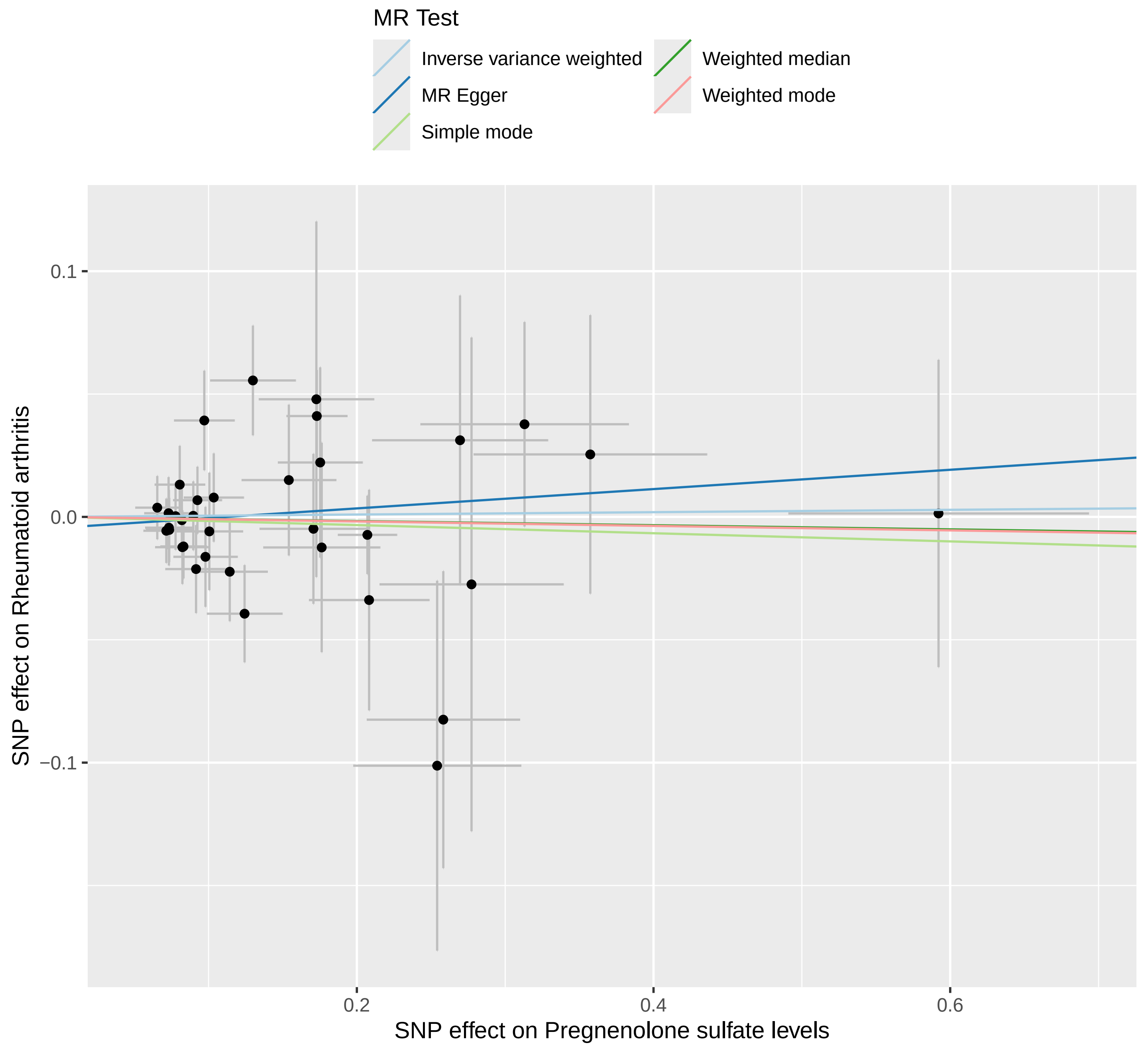

MR Test
Inverse variance weighted
MR Egger
Simple mode
Weighted median
Weighted mode
0.1
0.0
−0.1
0.2
0.4
0.6
SNP effect on Rheumatoid arthritis
SNP effect on Pregnenolone sulfate levels

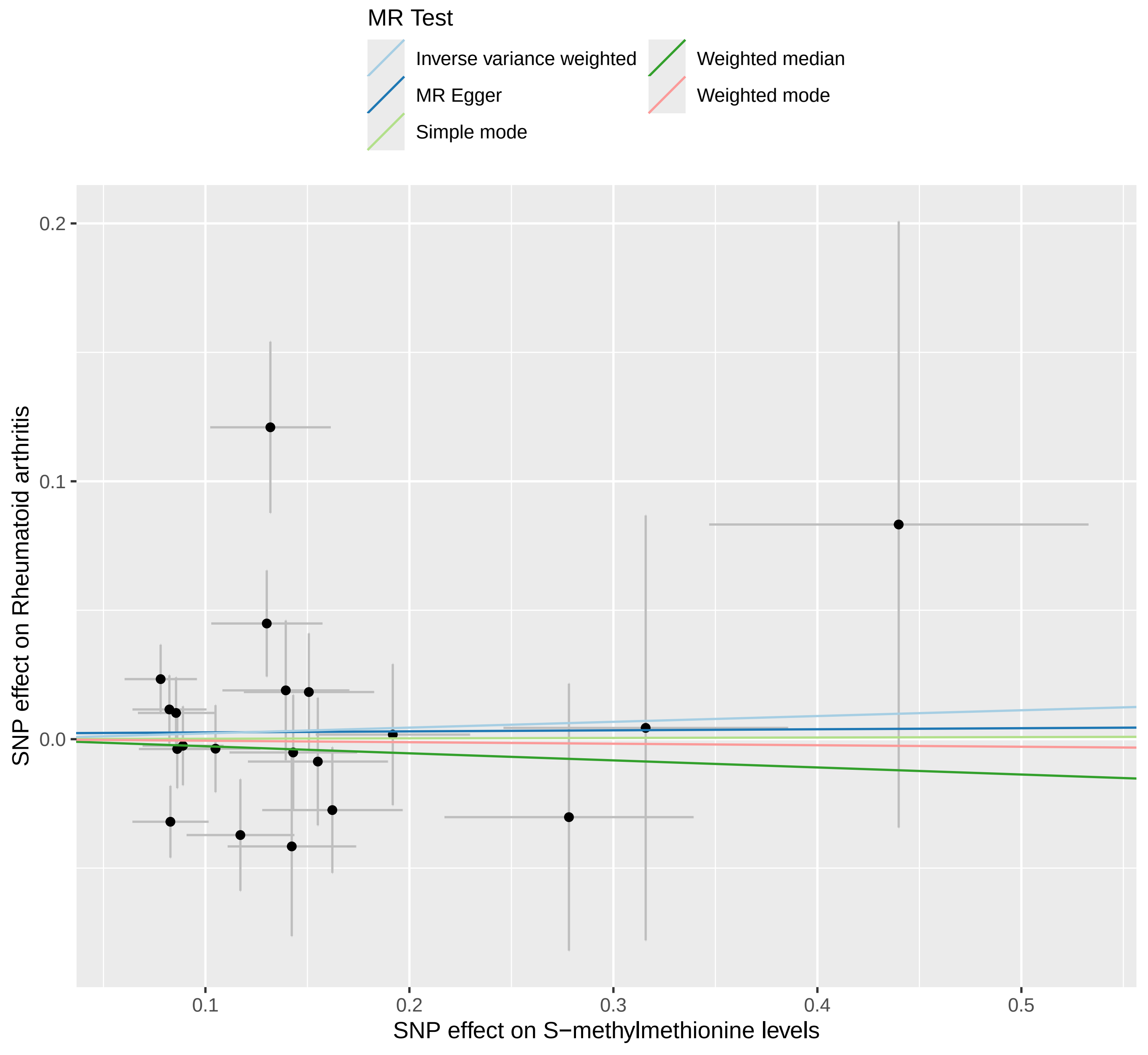

MR Test
Inverse variance weighted
MR Egger
Simple mode
Weighted median
Weighted mode
0.2
0.1
0.0
0.1
0.2
0.3
0.4
0.5
SNP effect on Rheumatoid arthritis
SNP effect on S−methylmethionine levels

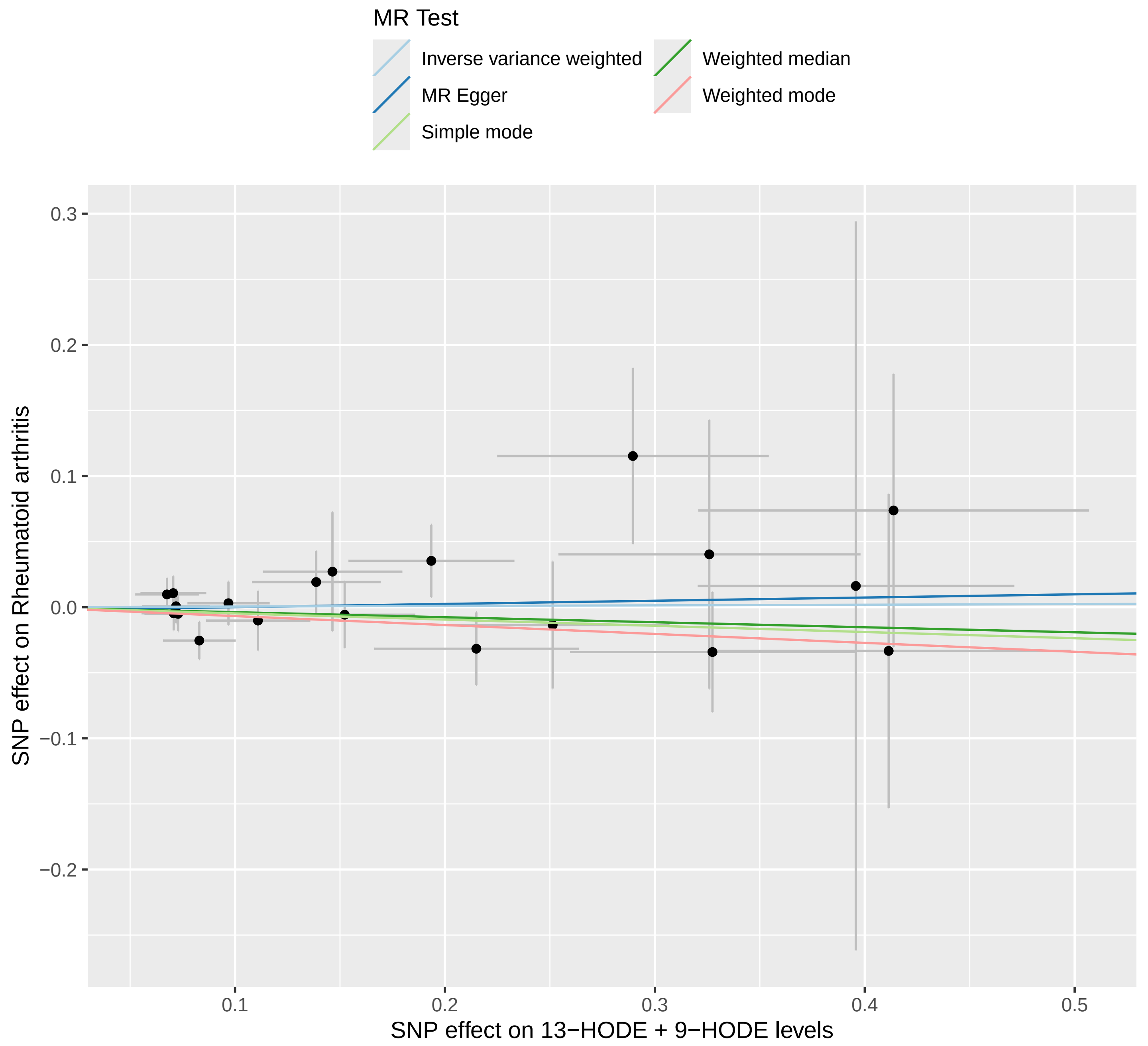
MR Test
Inverse variance weighted
MR Egger
Simple mode
Weighted median
Weighted mode
SNP effect on Rheumatoid arthritis
0.3
0.2
0.1
0.0
−0.1
−0.2
0.1
0.2
0.3
0.4
0.5
SNP effect on 13−HODE + 9−HODE levels

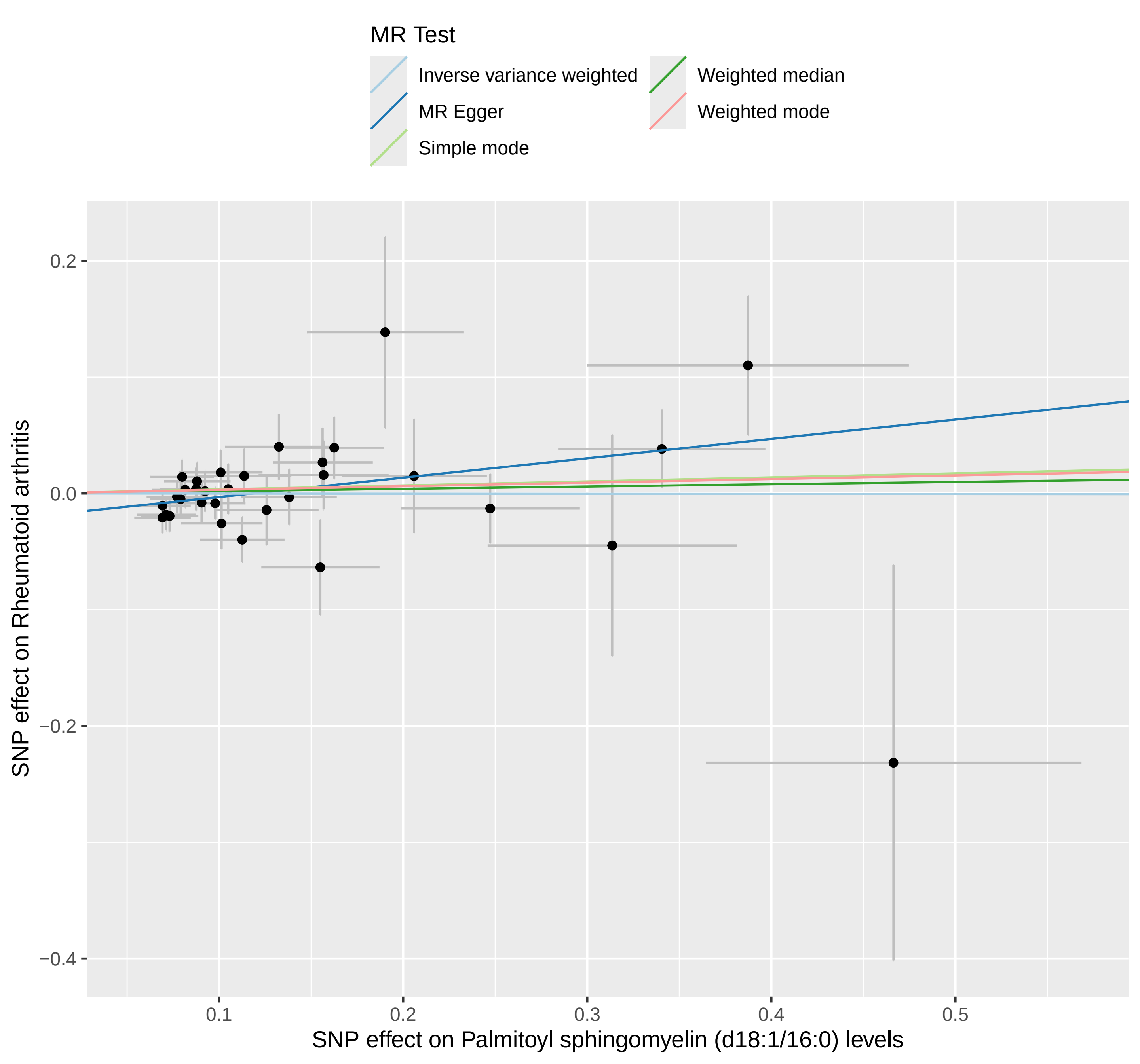
MR Test
Inverse variance weighted
MR Egger
Simple mode
Weighted median
Weighted mode
0.2
0.0
−0.2
−0.4
0.1
0.2
0.3
0.4
0.5
SNP effect on Rheumatoid arthritis
SNP effect on Palmitoyl sphingomyelin (d18:1/16:0) levels

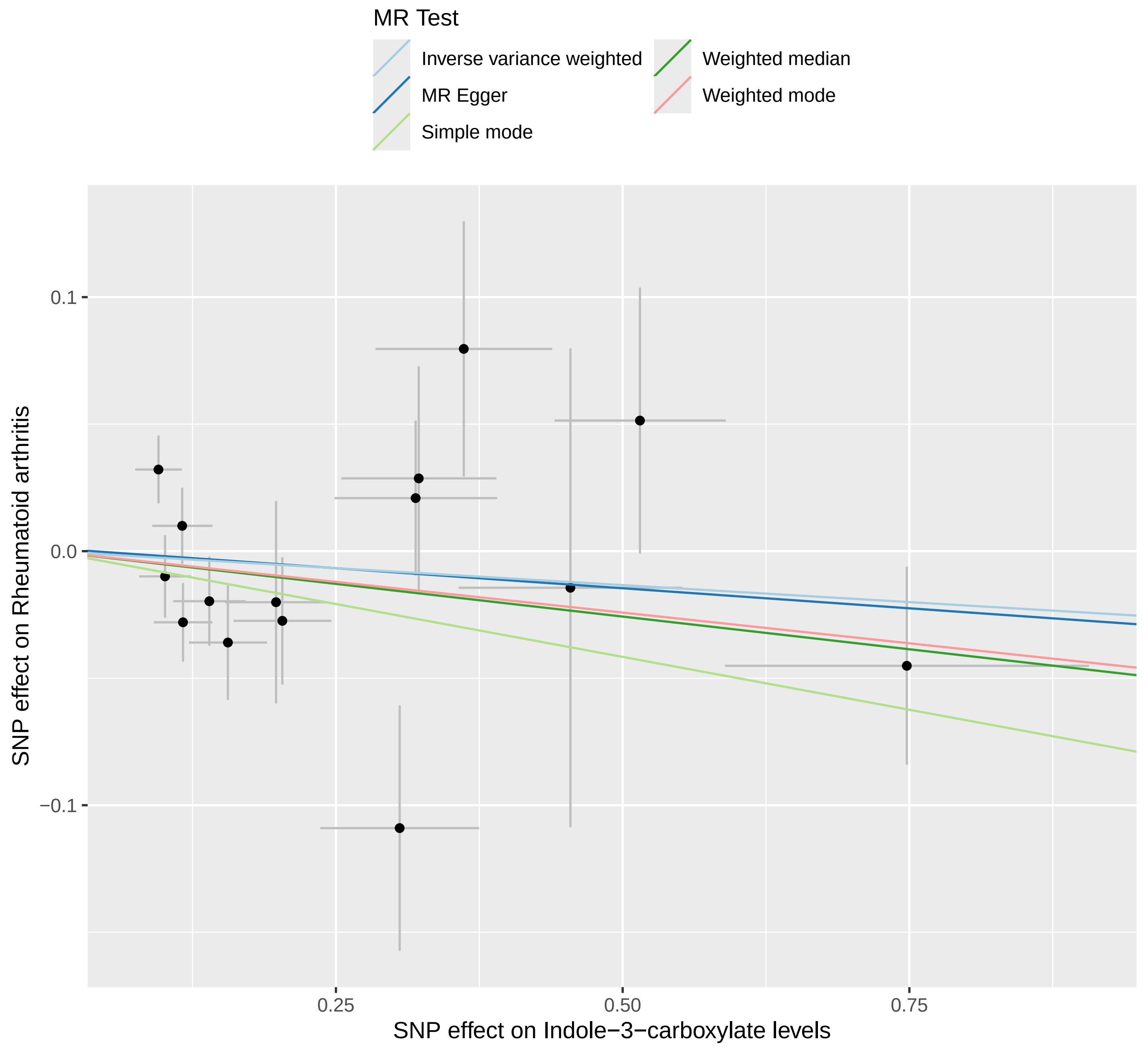

MR Test
Inverse variance weighted
MR Egger
Simple mode
Weighted median
Weighted mode
SNP effect on Rheumatoid arthritis
0.1
0.0
−0.1
0.25
0.50
0.75
SNP effect on Indole−3−carboxylate levels

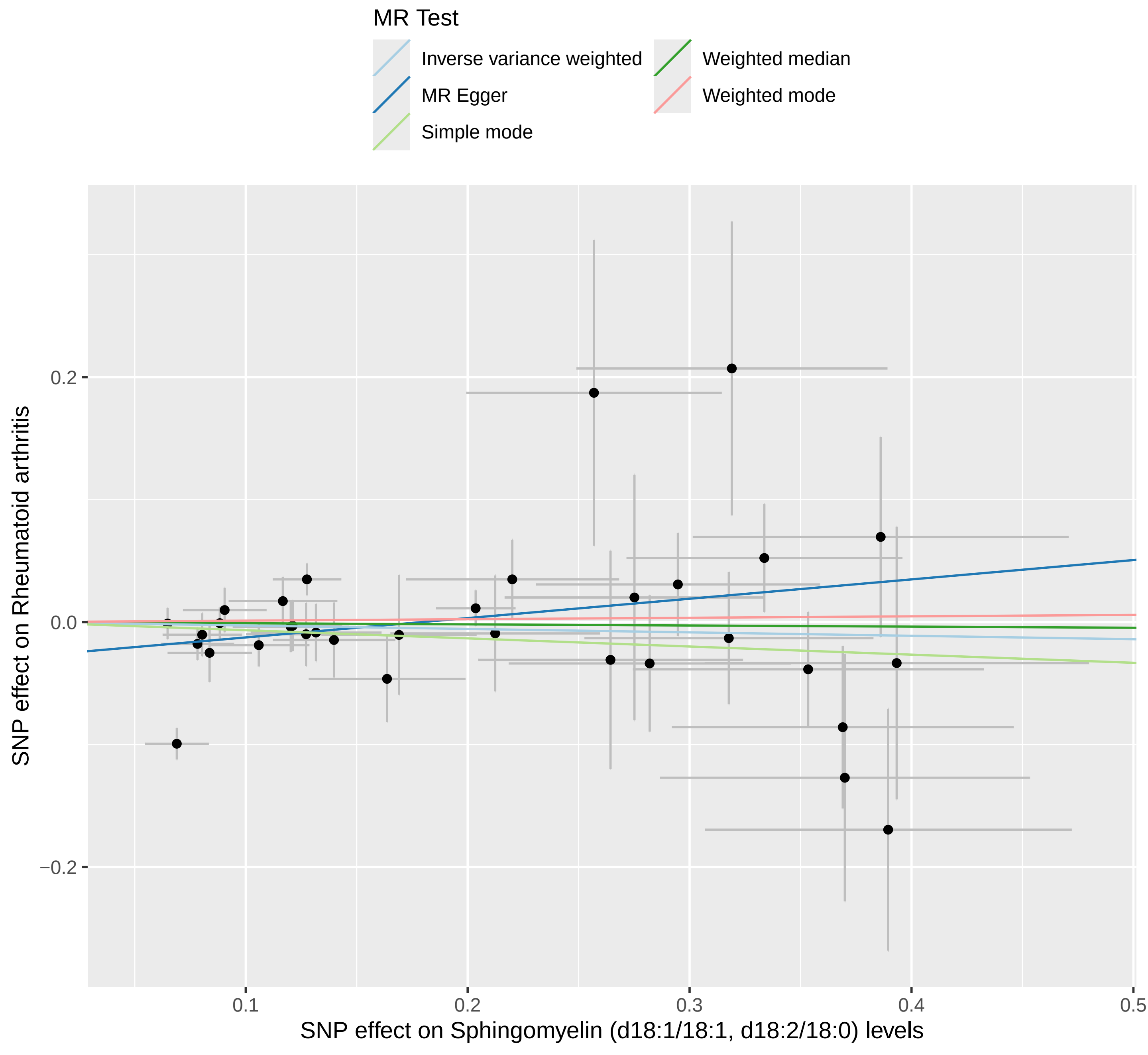
MR Test
Inverse variance weighted
MR Egger
Simple mode
Weighted median
Weighted mode
SNP effect on Rheumatoid arthritis
0.2
0.0
−0.2
0.1
0.2
0.3
0.4
0.5
SNP effect on Sphingomyelin (d18:1/18:1, d18:2/18:0) levels

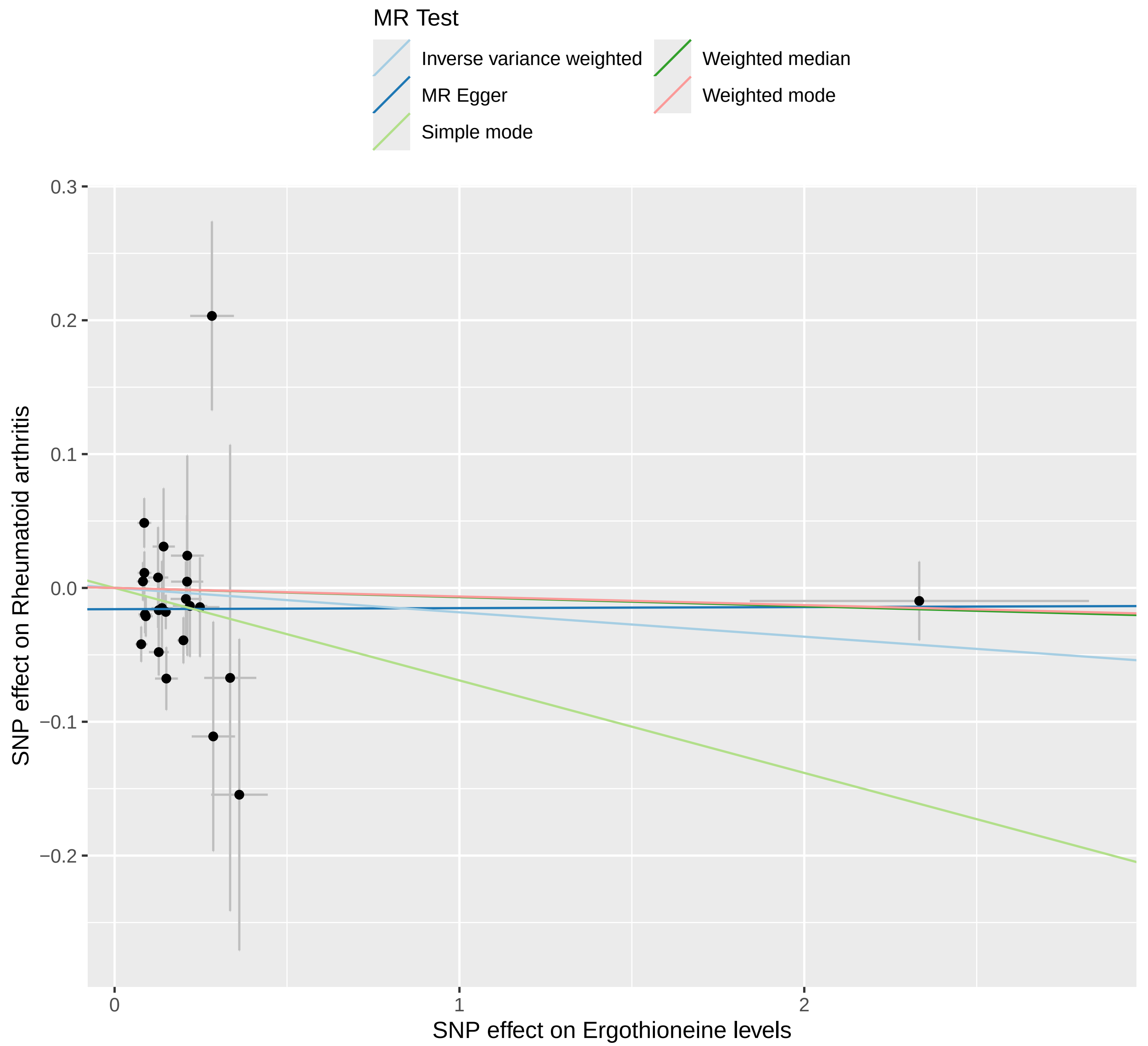
MR Test
Inverse variance weighted
MR Egger
Simple mode
Weighted median
Weighted mode
0.3
0.2
0.1
0.0
−0.1
−0.2
0
1
2
SNP effect on Rheumatoid arthritis
SNP effect on Ergothioneine levels

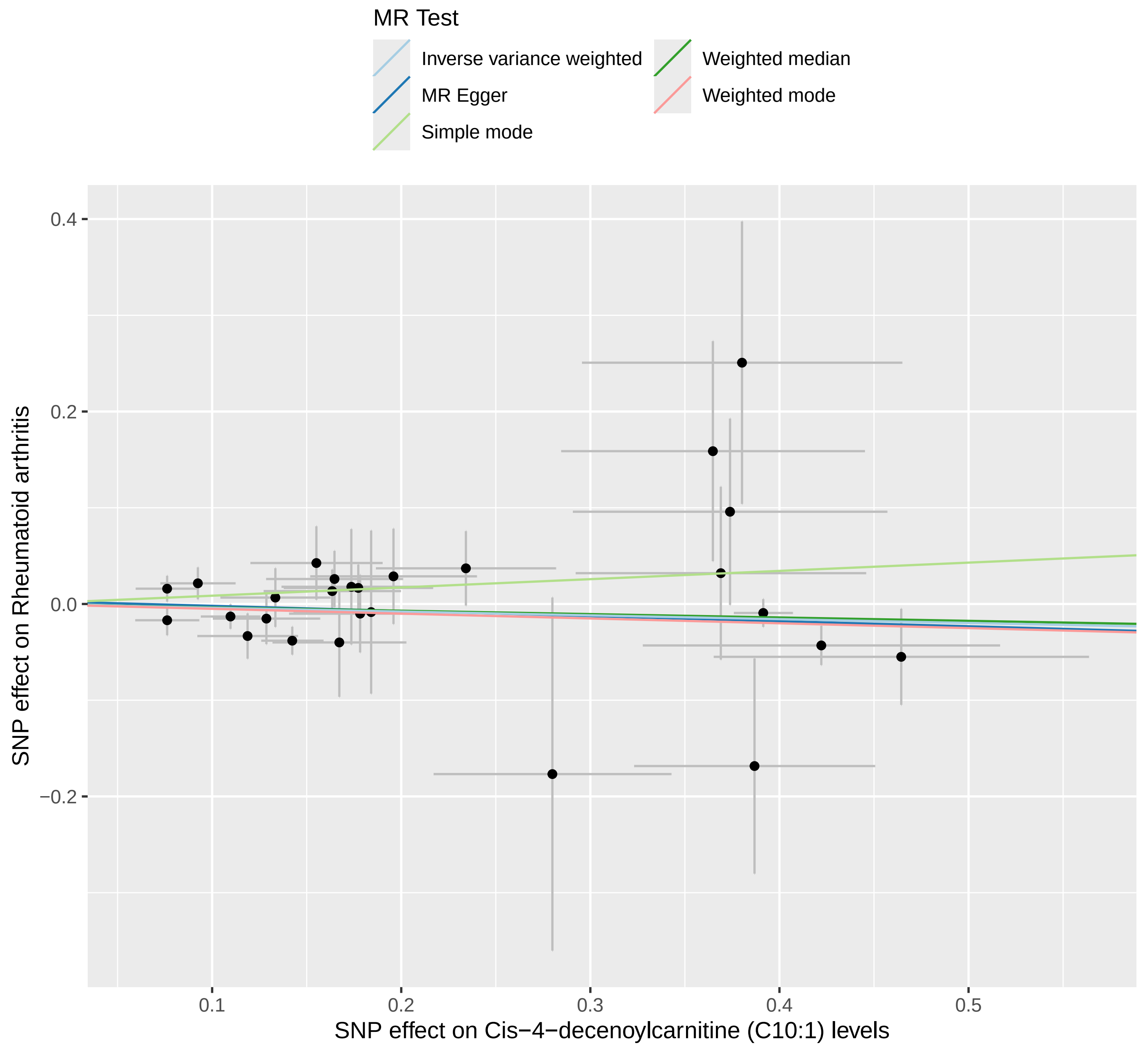
MR Test
Inverse variance weighted
MR Egger
Simple mode
Weighted median
Weighted mode
0.4
0.2
0.0
−0.2
0.1
0.2
0.3
0.4
0.5
SNP effect on Rheumatoid arthritis
SNP effect on Cis−4−decenoylcarnitine (C10:1) levels

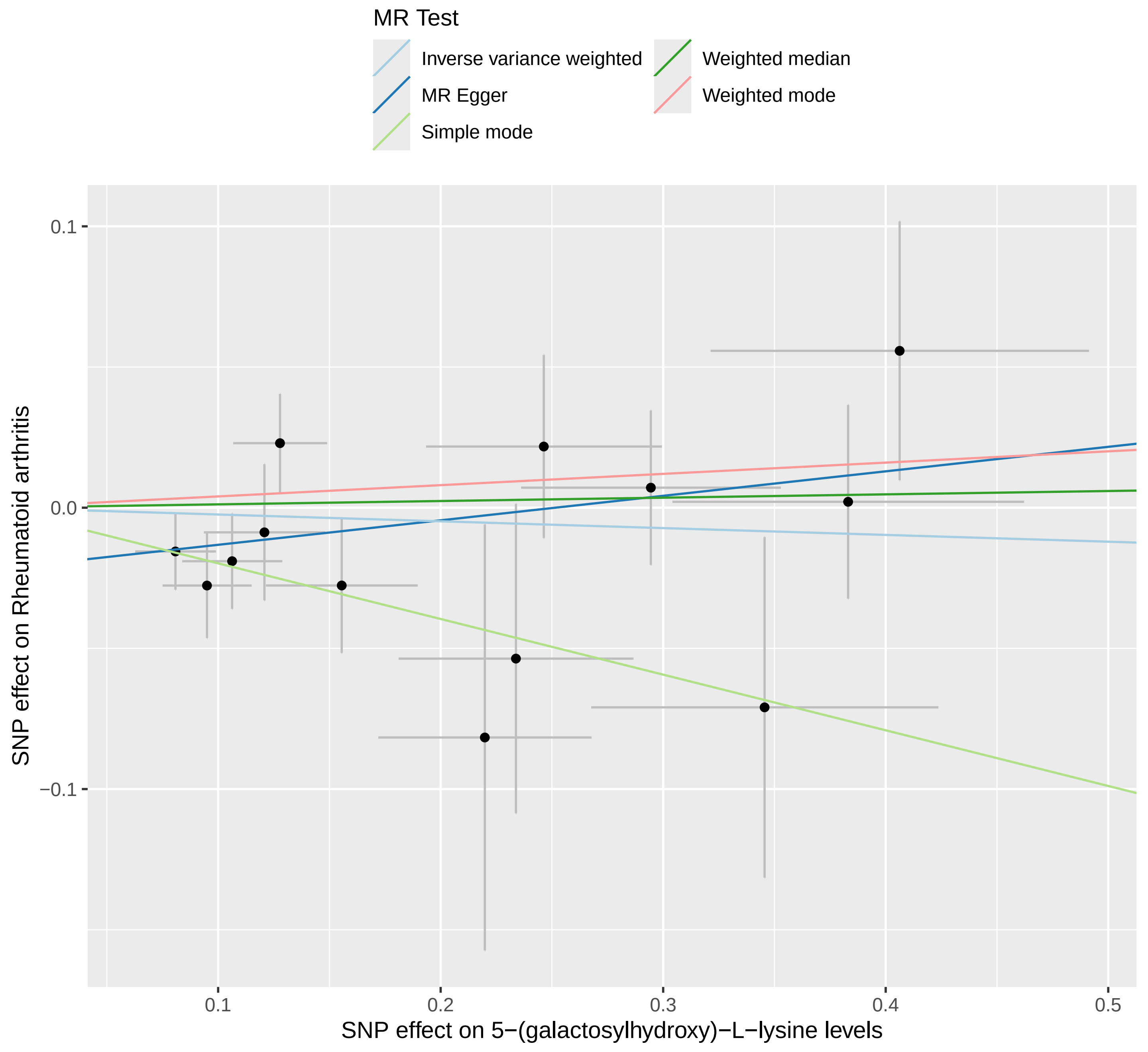

MR Test
Inverse variance weighted
MR Egger
Simple mode
Weighted median
Weighted mode
0.1
0.0
−0.1
SNP effect on Rheumatoid arthritis
0.1
0.2
0.3
0.4
0.5
SNP effect on 5−(galactosylhydroxy)−L−lysine levels

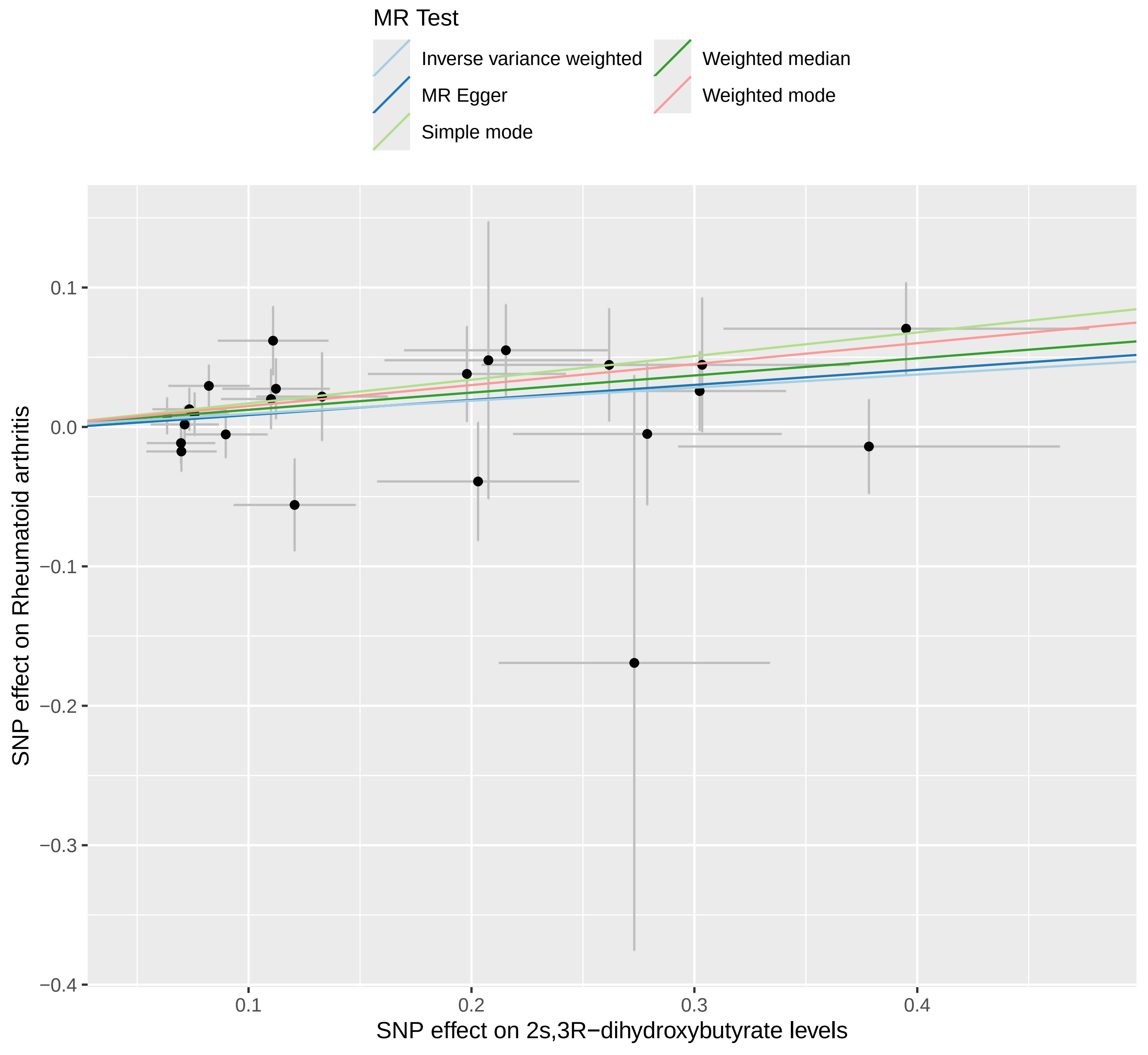

MR Test
Inverse variance weighted
MR Egger
Simple mode
Weighted median
Weighted mode
0.1
0.0
−0.1
−0.2
−0.3
−0.4
0.1
0.2
0.3
0.4
SNP effect on Rheumatoid arthritis
SNP effect on 2s,3R−dihydroxybutyrate levels

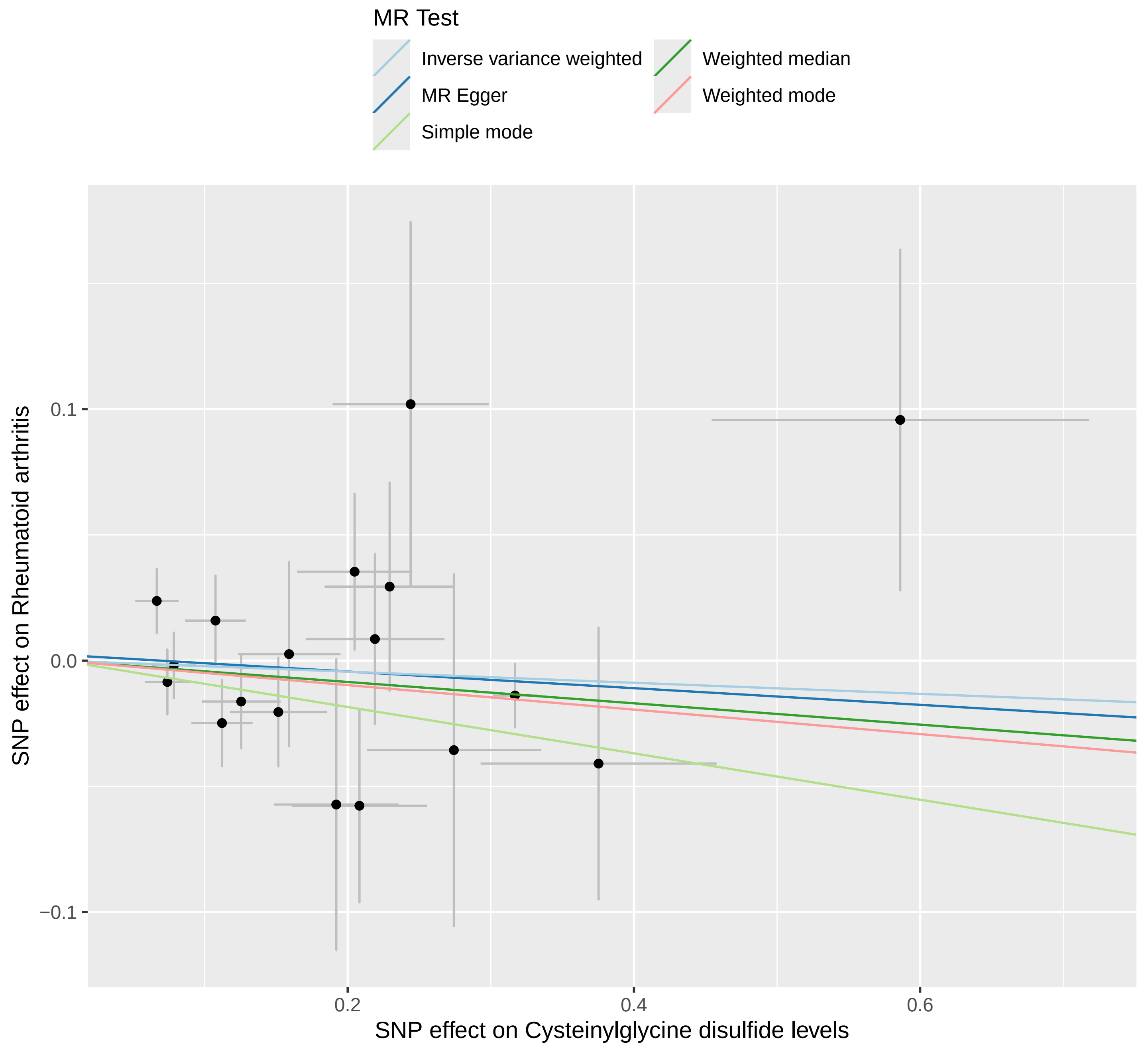

MR Test
Inverse variance weighted
MR Egger
Simple mode
Weighted median
Weighted mode
SNP effect on Rheumatoid arthritis
0.1
0.0
−0.1
0.2
0.4
0.6
SNP effect on Cysteinylglycine disulfide levels

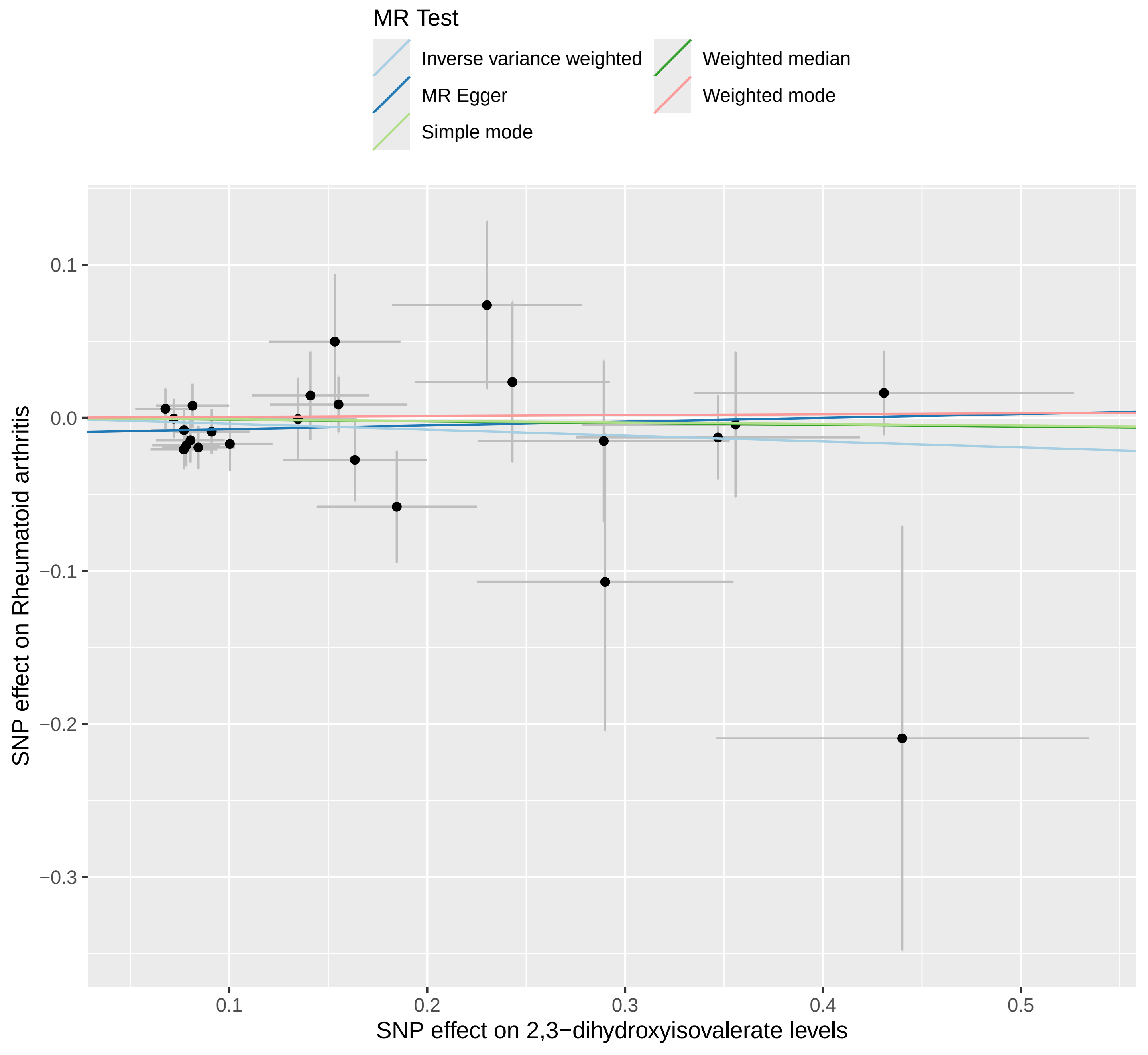

MR Test
Inverse variance weighted
MR Egger
Simple mode
Weighted median
Weighted mode
SNP effect on Rheumatoid arthritis
0.1
0.0
−0.1
−0.2
−0.3
0.1
0.2
0.3
0.4
0.5
SNP effect on 2,3−dihydroxyisovalerate levels

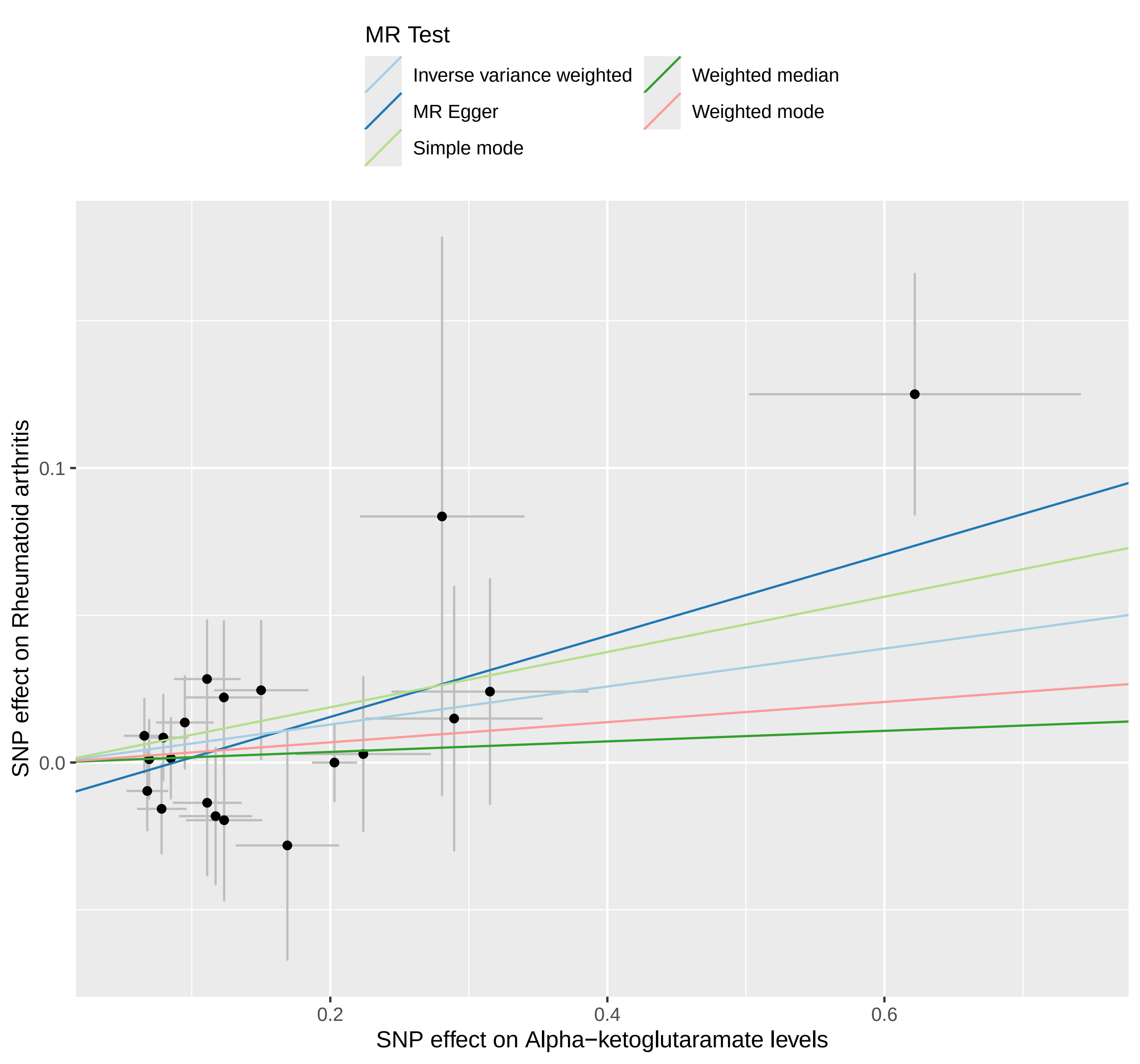
MR Test
Inverse variance weighted
MR Egger
Simple mode
Weighted median
Weighted mode
0.1
0.0
0.2
0.4
0.6
SNP effect on Rheumatoid arthritis
SNP effect on Alpha−ketoglutaramate levels

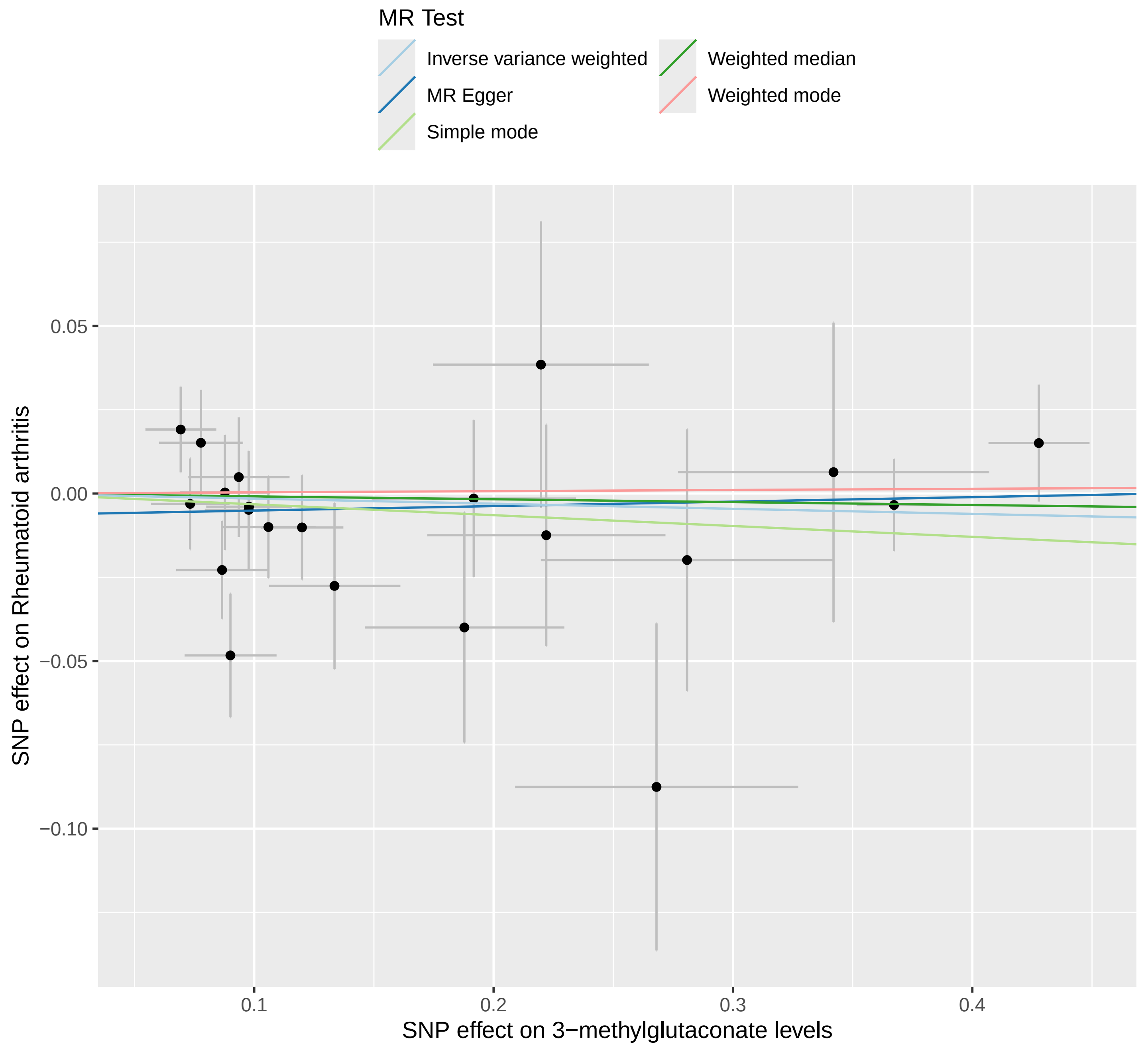

MR Test
Inverse variance weighted
MR Egger
Simple mode
Weighted median
Weighted mode
0.05
0.00
−0.05
−0.10
0.1
0.2
0.3
0.4
SNP effect on Rheumatoid arthritis
SNP effect on 3−methylglutaconate levels

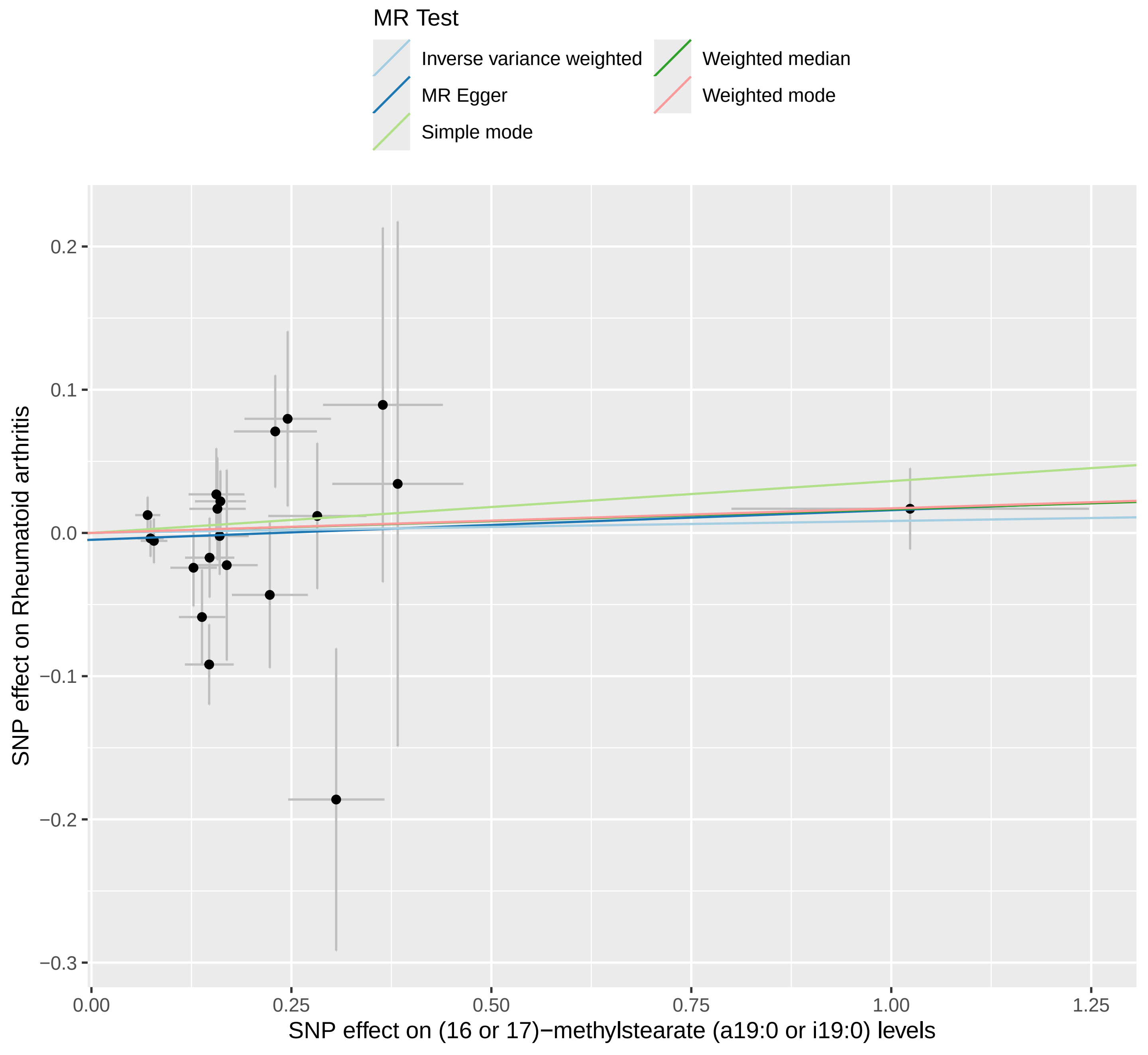
MR Test
Inverse variance weighted
MR Egger
Simple mode
Weighted median
Weighted mode
SNP effect on Rheumatoid arthritis
0.2
0.1
0.0
−0.1
−0.2
−0.3
0.00
0.25
0.50
0.75
1.00
1.25
SNP effect on (16 or 17)−methylstearate (a19:0 or i19:0) levels

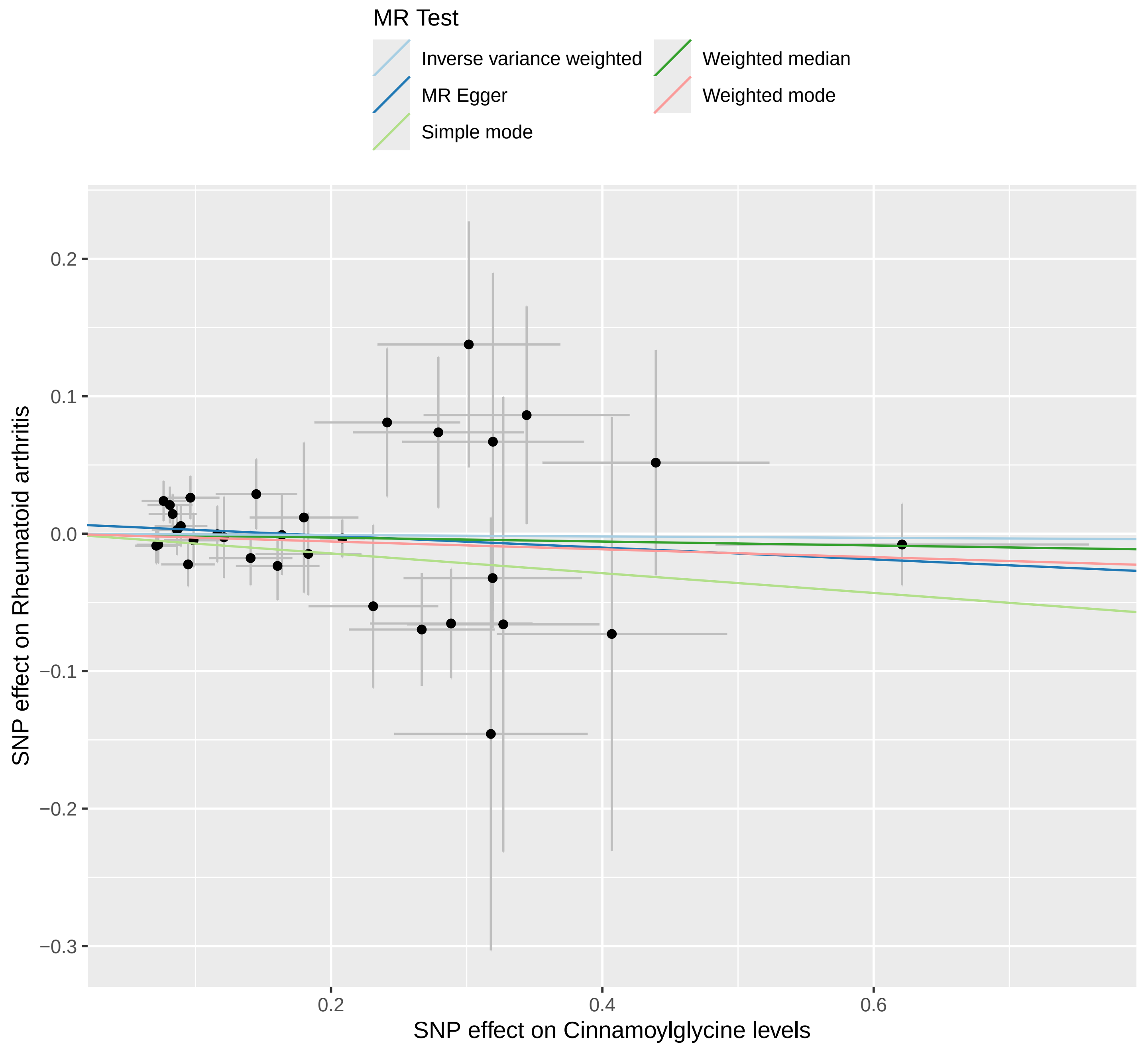

MR Test
Inverse variance weighted
MR Egger
Simple mode
Weighted median
Weighted mode
0.2
0.1
0.0
−0.1
−0.2
−0.3
0.2
0.4
0.6
SNP effect on Rheumatoid arthritis
SNP effect on Cinnamoylglycine levels

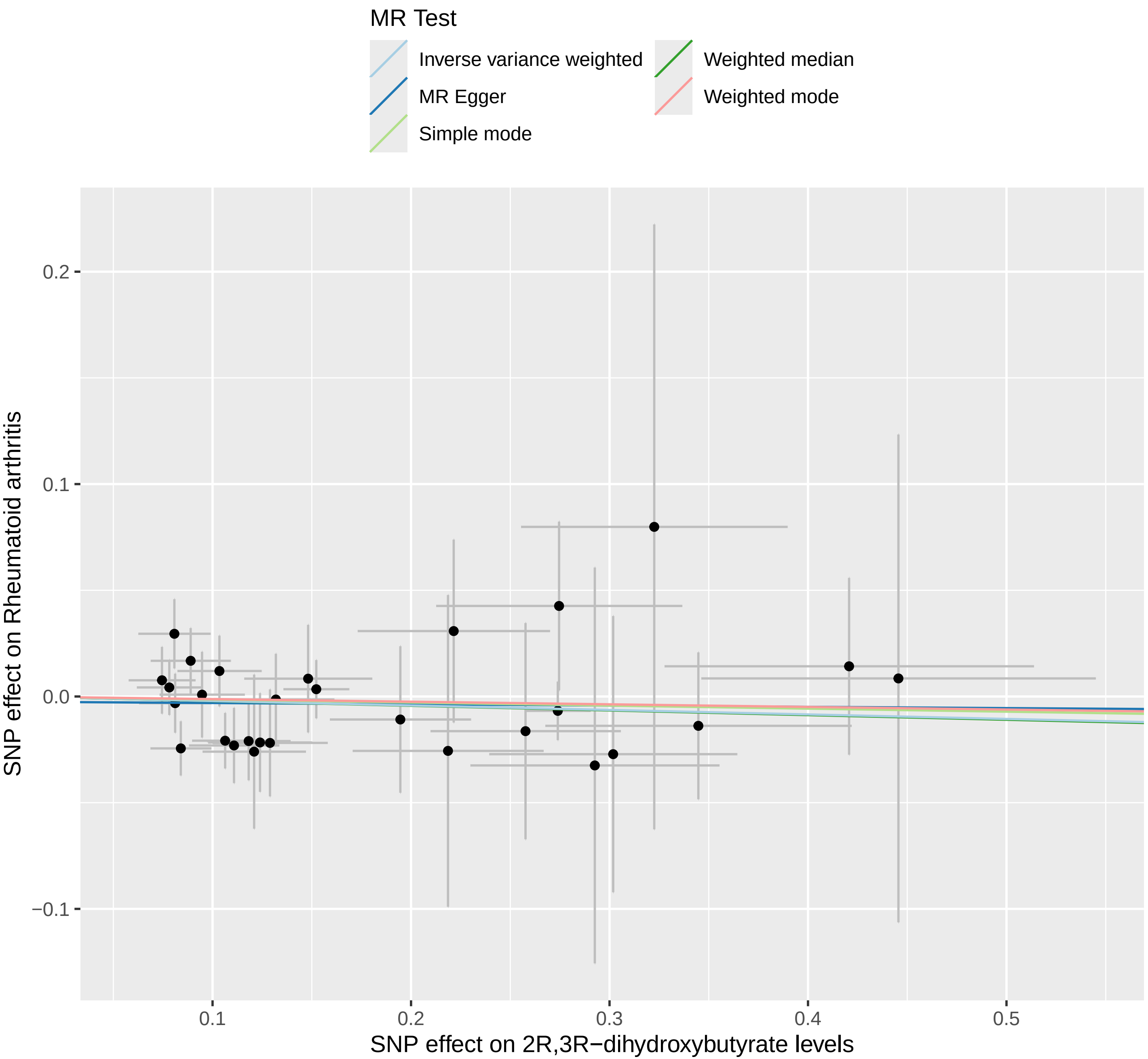

MR Test
Inverse variance weighted
MR Egger
Simple mode
Weighted median
Weighted mode
0.2
0.1
0.0
−0.1
SNP effect on Rheumatoid arthritis
0.1
0.2
0.3
0.4
0.5
SNP effect on 2R,3R−dihydroxybutyrate levels

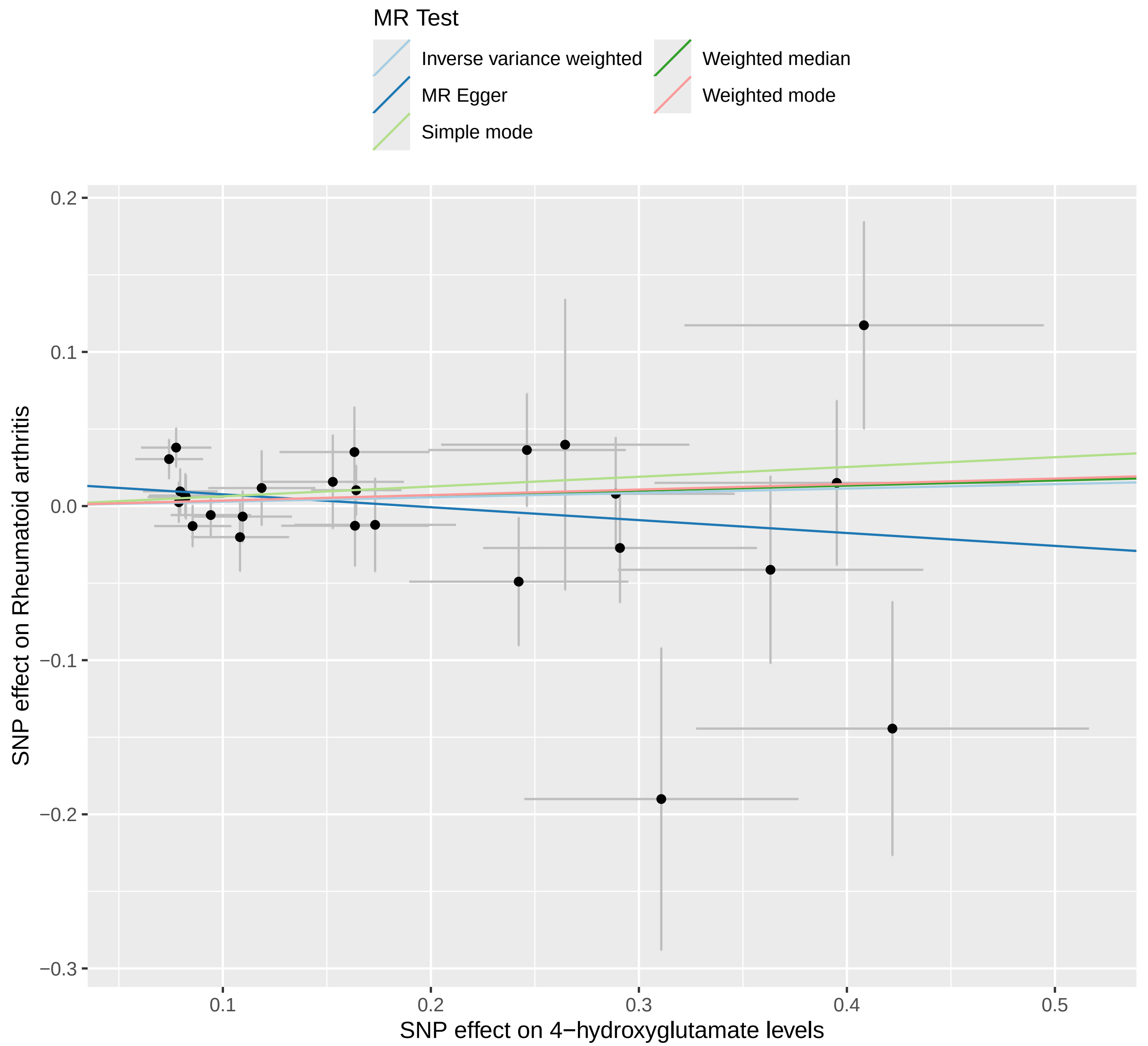

MR Test
Inverse variance weighted
MR Egger
Simple mode
Weighted median
Weighted mode
0.2
0.1
0.0
−0.1
−0.2
−0.3
0.1
0.2
0.3
0.4
0.5
SNP effect on Rheumatoid arthritis
SNP effect on 4−hydroxyglutamate levels

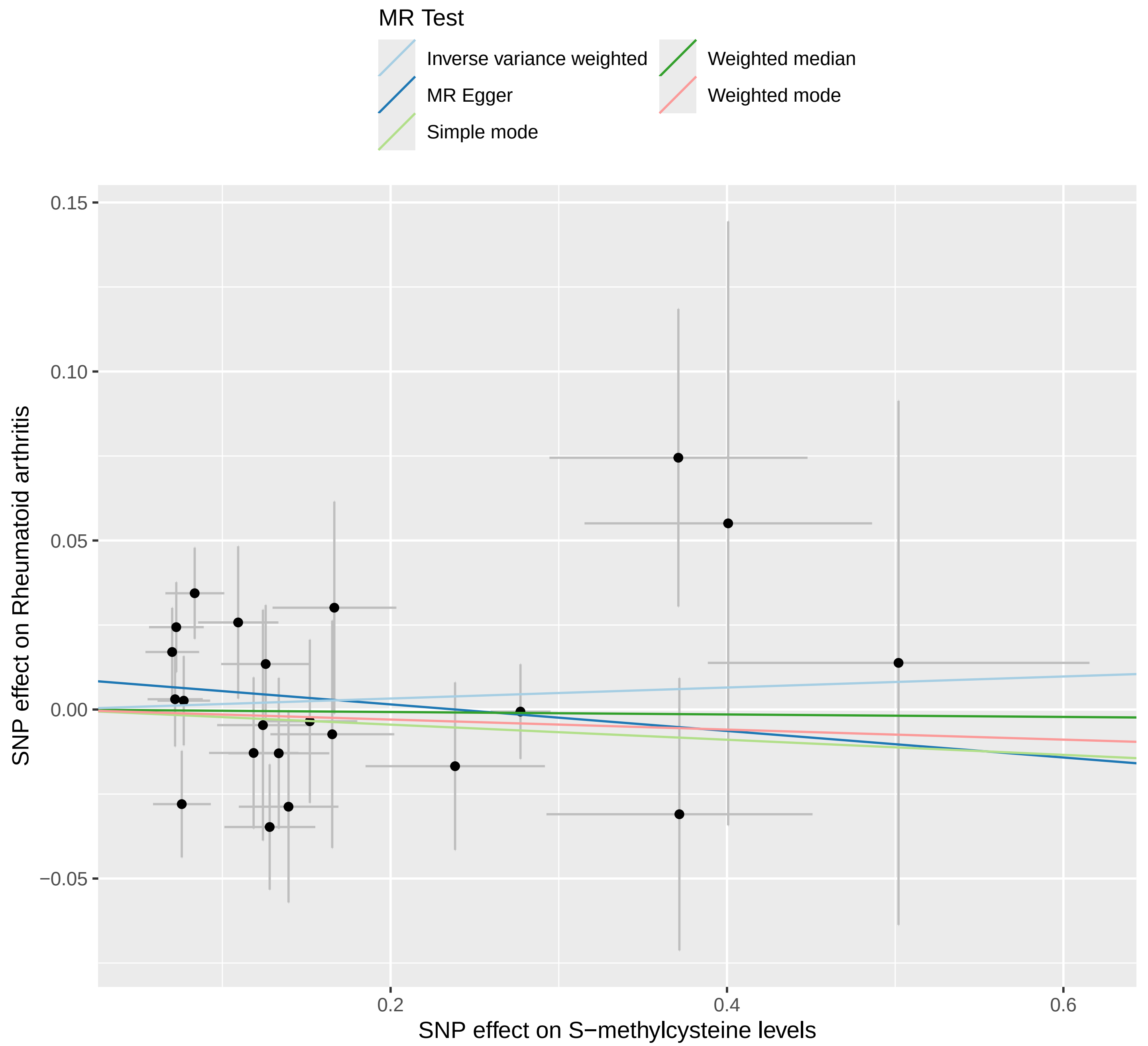

MR Test
Inverse variance weighted
MR Egger
Simple mode
Weighted median
Weighted mode
0.15
0.10
0.05
0.00
−0.05
0.2
0.4
0.6
SNP effect on Rheumatoid arthritis
SNP effect on S−methylcysteine levels

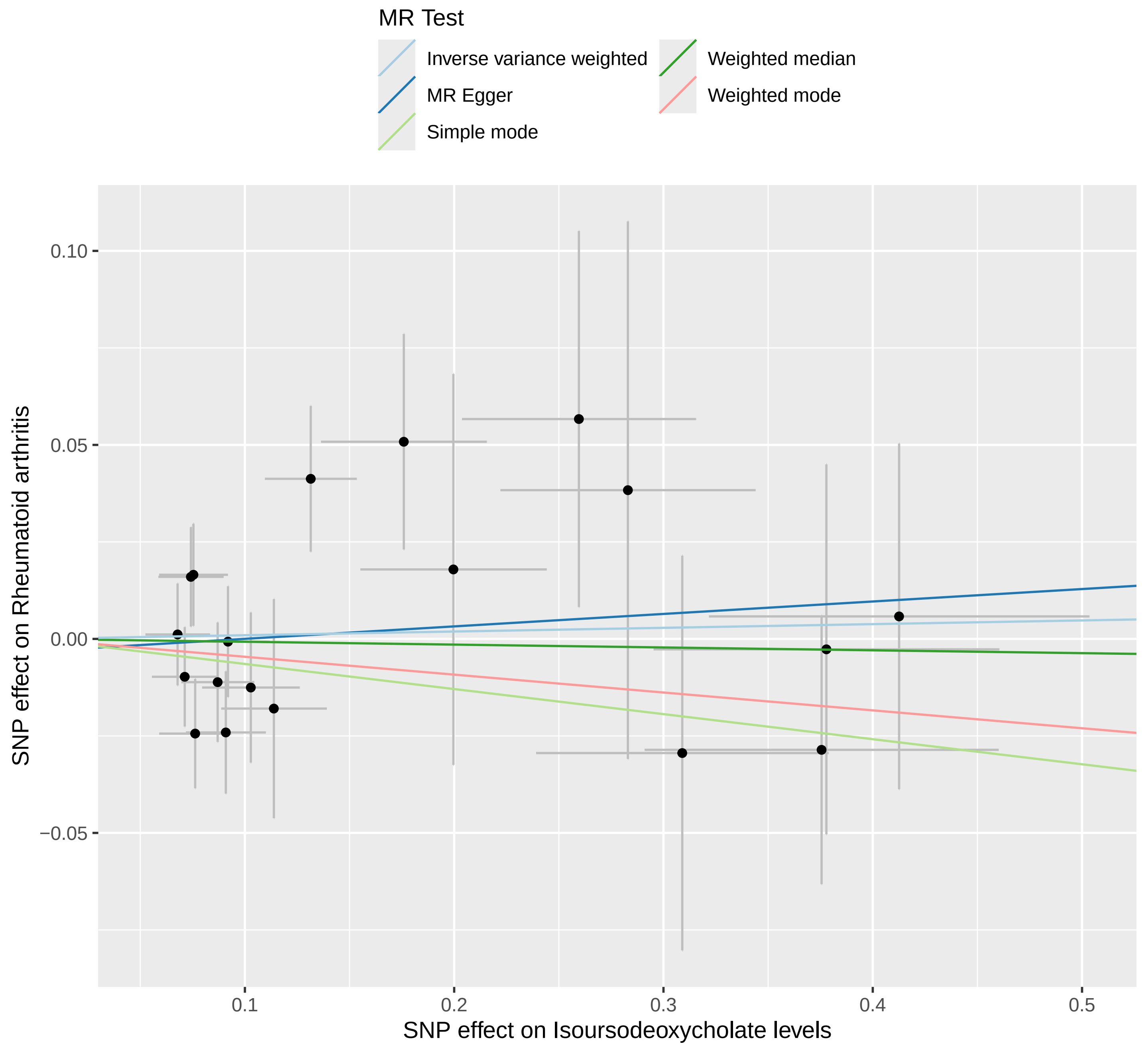

MR Test
Inverse variance weighted
MR Egger
Simple mode
Weighted median
Weighted mode
0.10
0.05
0.00
−0.05
SNP effect on Rheumatoid arthritis
0.1
0.2
0.3
0.4
0.5
SNP effect on Isoursodeoxycholate levels

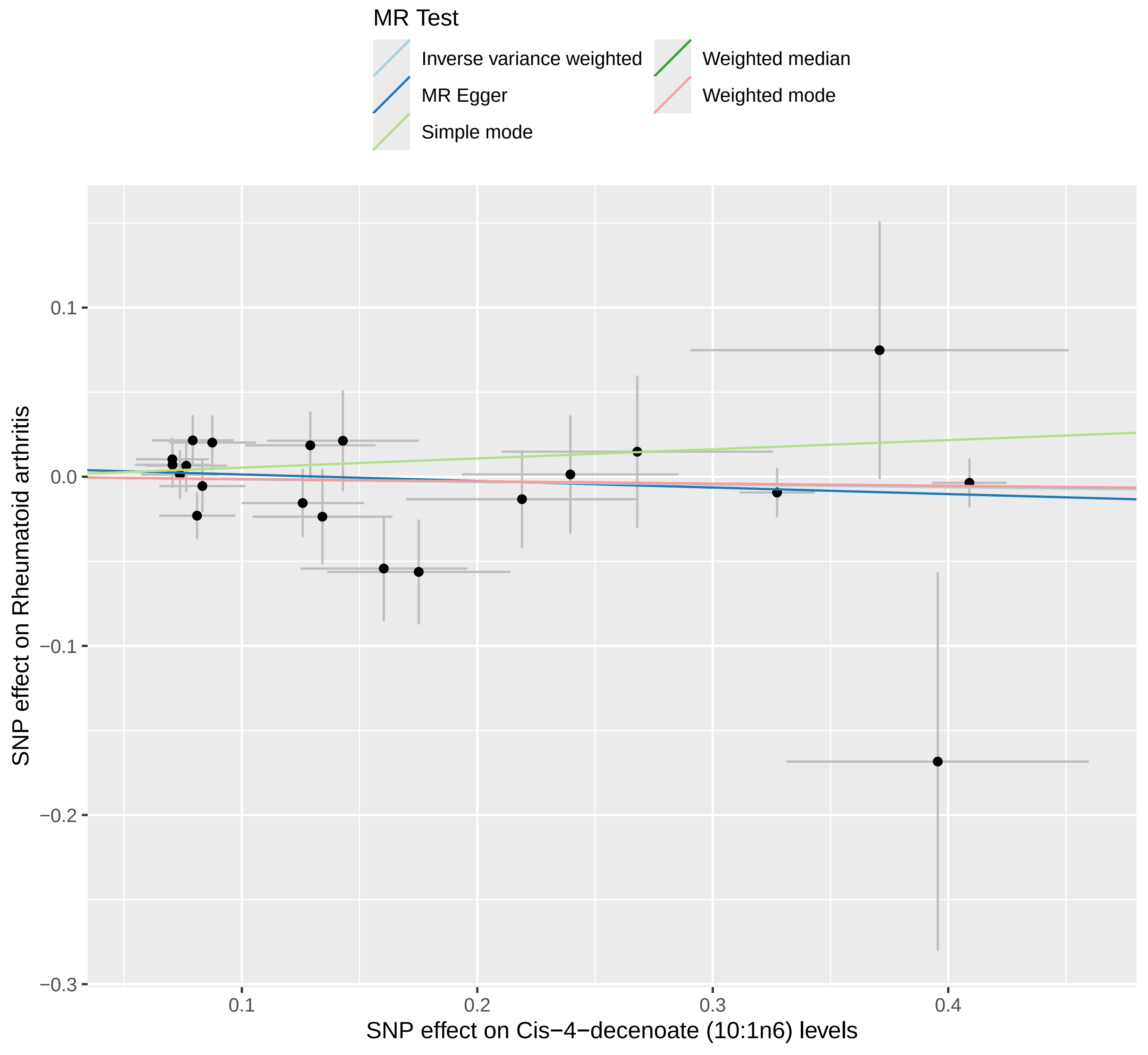

MR Test
Inverse variance weighted
MR Egger
Simple mode
Weighted median
Weighted mode
SNP effect on Rheumatoid arthritis
0.1
0.0
−0.1
−0.2
−0.3
0.1
0.2
0.3
0.4
SNP effect on Cis−4−decenoate (10:1n6) levels

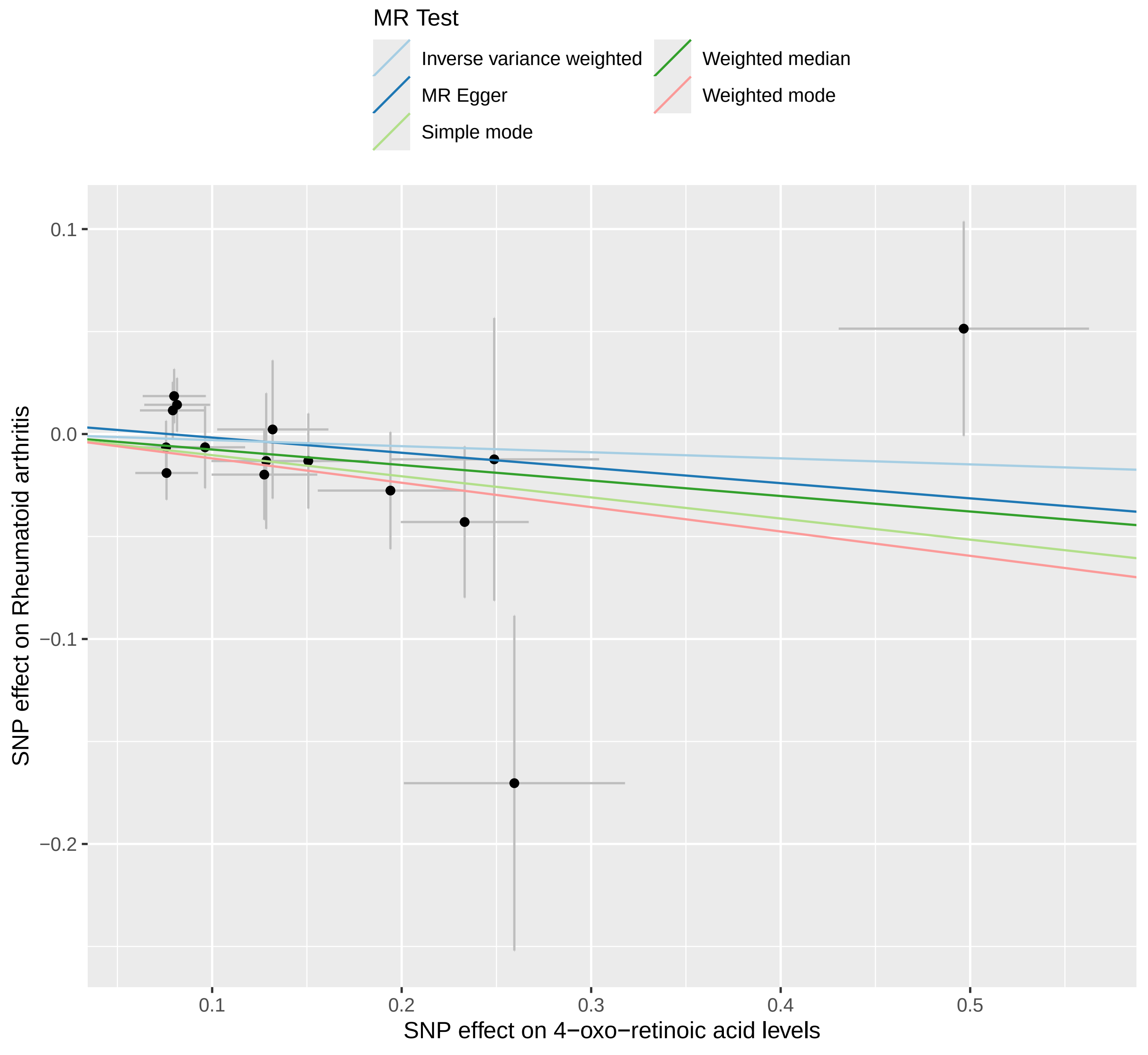
MR Test
Inverse variance weighted
MR Egger
Simple mode
Weighted median
Weighted mode
SNP effect on Rheumatoid arthritis
0.1
0.0
−0.1
−0.2
0.1
0.2
0.3
0.4
0.5
SNP effect on 4−oxo−retinoic acid levels

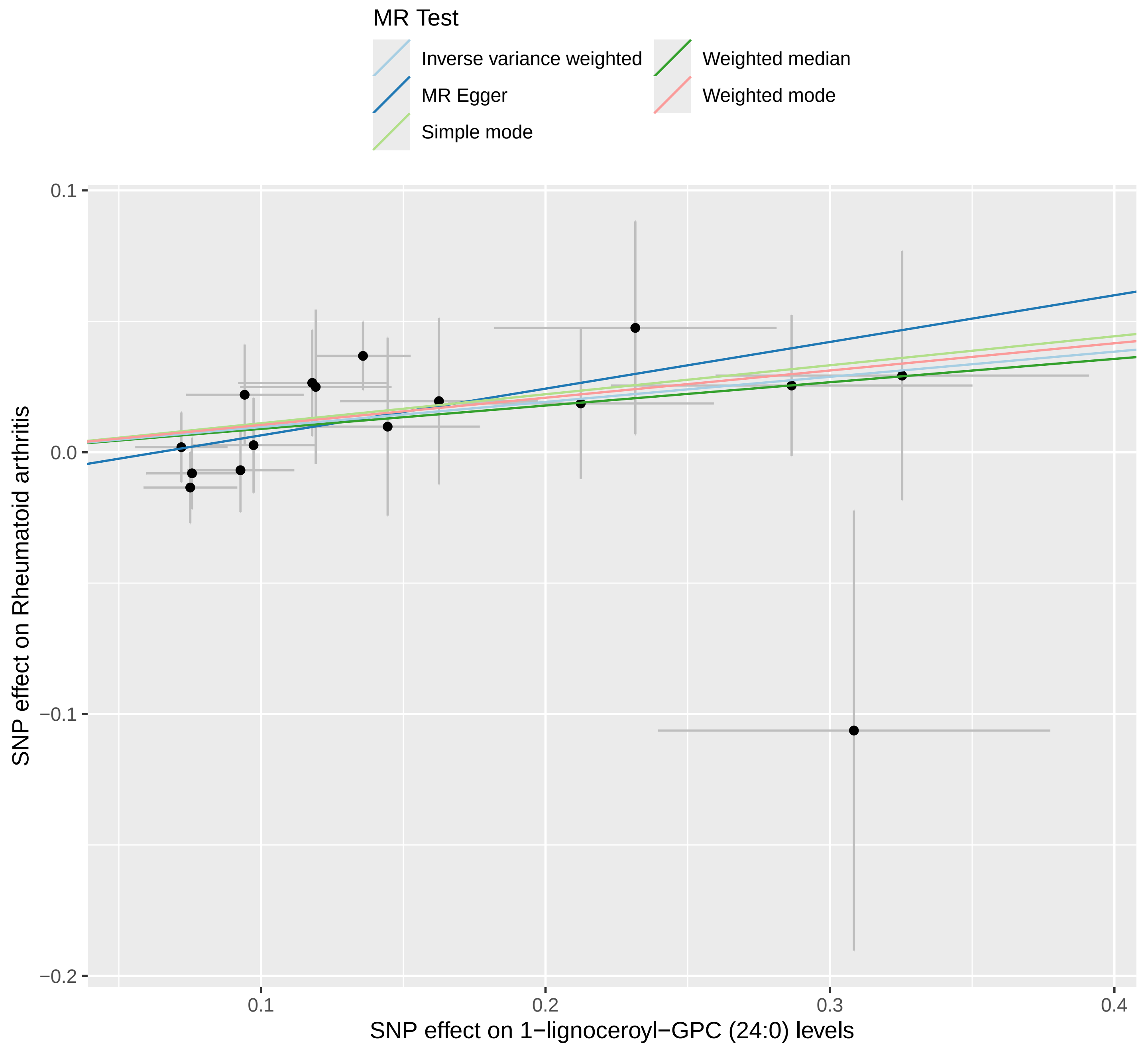

MR Test
Inverse variance weighted
MR Egger
Simple mode
Weighted median
Weighted mode
0.1
0.0
−0.1
−0.2
0.1
0.2
0.3
0.4
SNP effect on Rheumatoid arthritis
SNP effect on 1−lignoceroyl−GPC (24:0) levels

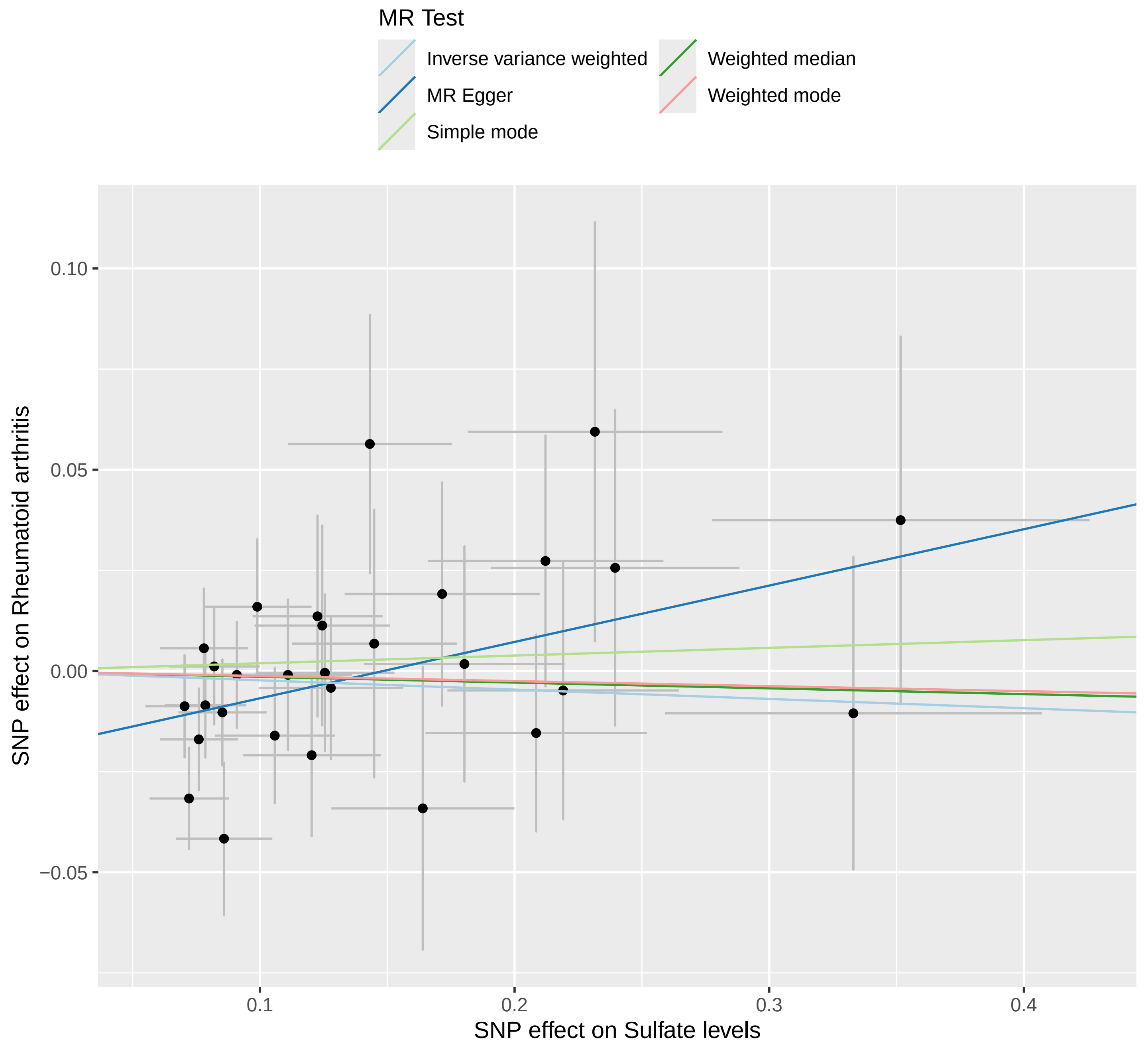

MR Test
Inverse variance weighted
MR Egger
Simple mode
Weighted median
Weighted mode
0.10
0.05
0.00
−0.05
0.1
0.2
0.3
0.4
SNP effect on Rheumatoid arthritis
SNP effect on Sulfate levels

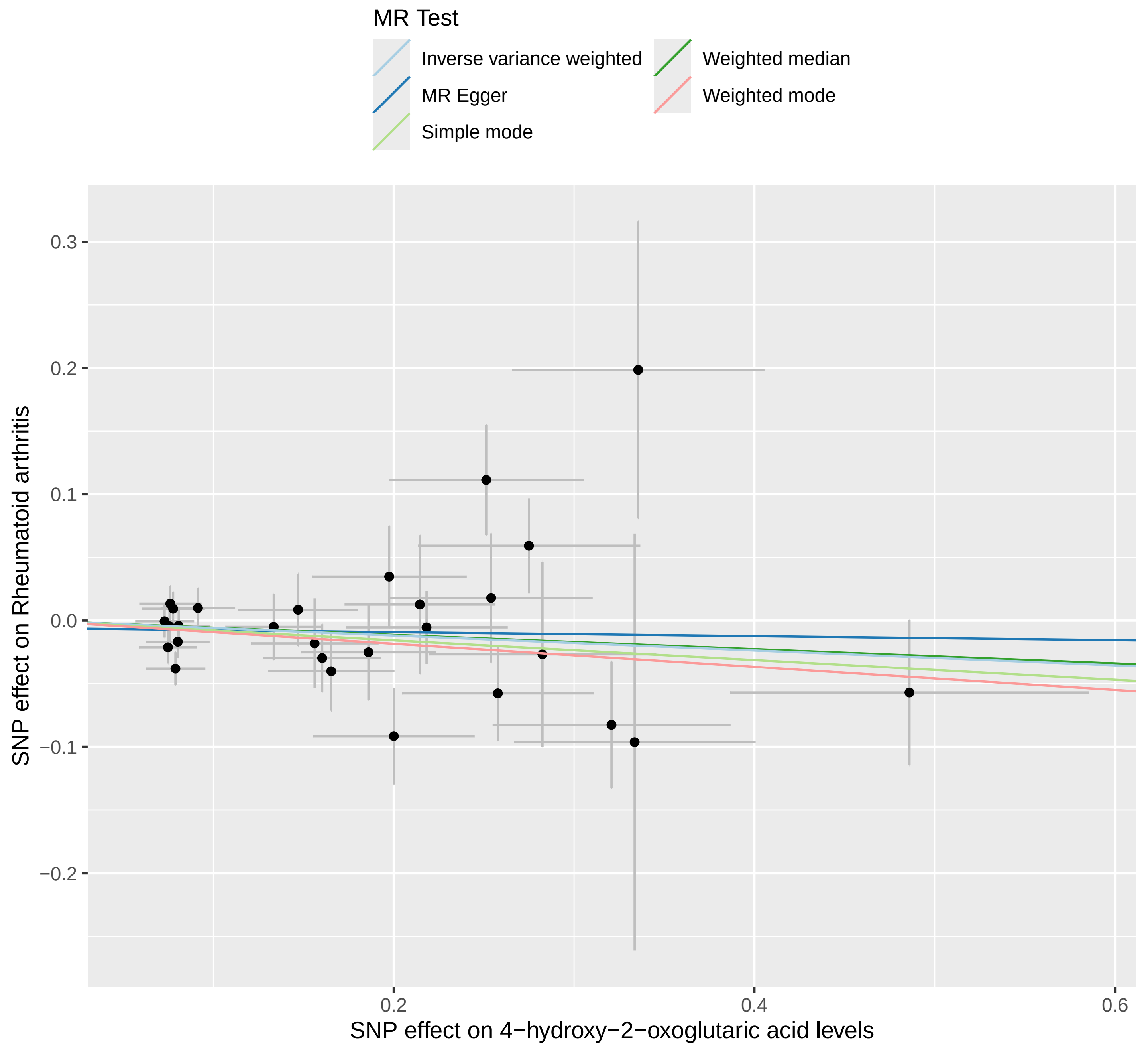
MR Test
Inverse variance weighted
MR Egger
Simple mode
Weighted median
Weighted mode
SNP effect on Rheumatoid arthritis
0.3
0.2
0.1
0.0
−0.1
−0.2
0.2
0.4
0.6
SNP effect on 4−hydroxy−2−oxoglutaric acid levels

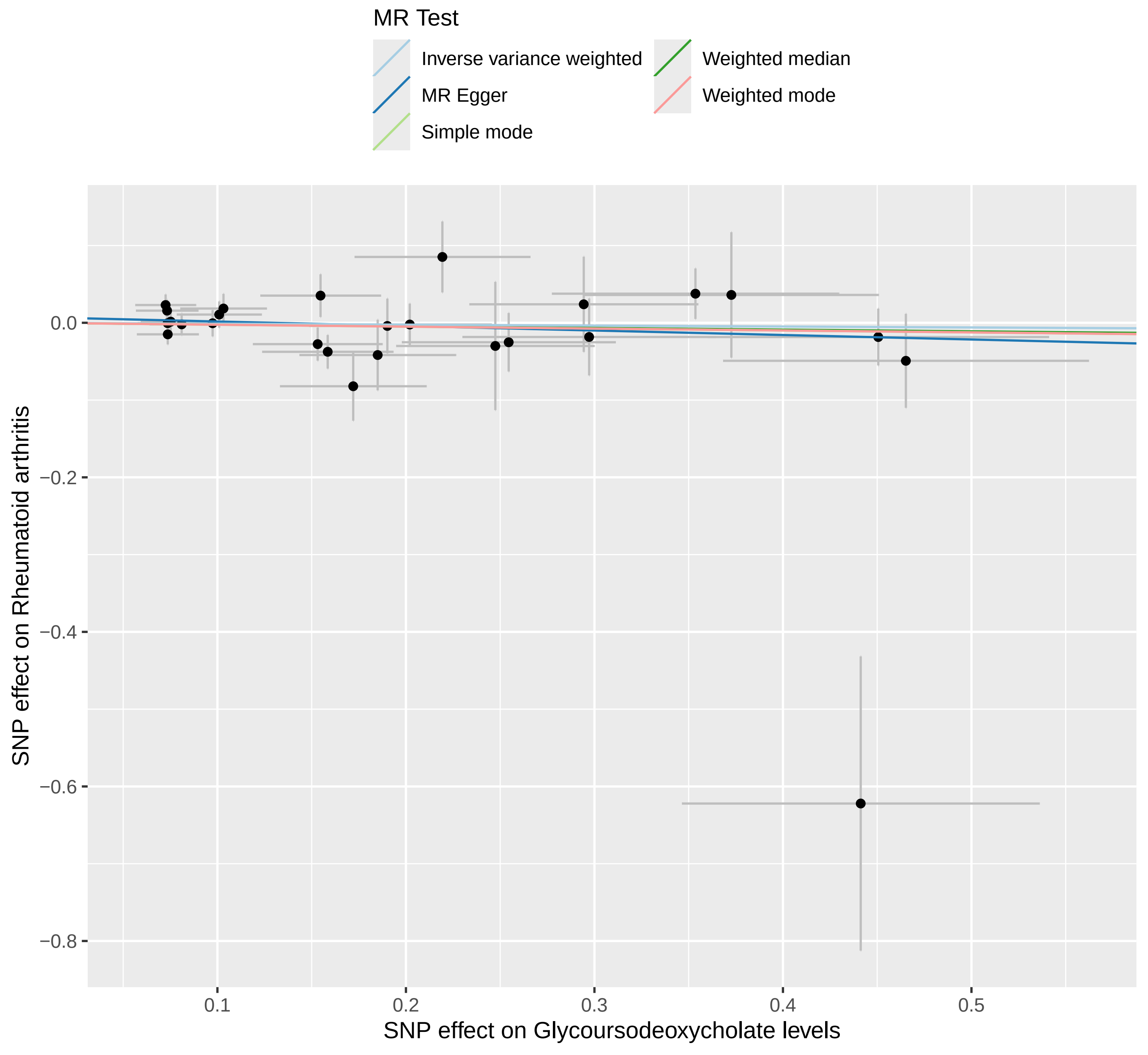

MR Test
Inverse variance weighted
MR Egger
Simple mode
Weighted median
Weighted mode
0.0
−0.2
−0.4
−0.6
−0.8
0.1
0.2
0.3
0.4
0.5
SNP effect on Rheumatoid arthritis
SNP effect on Glycoursodeoxycholate levels

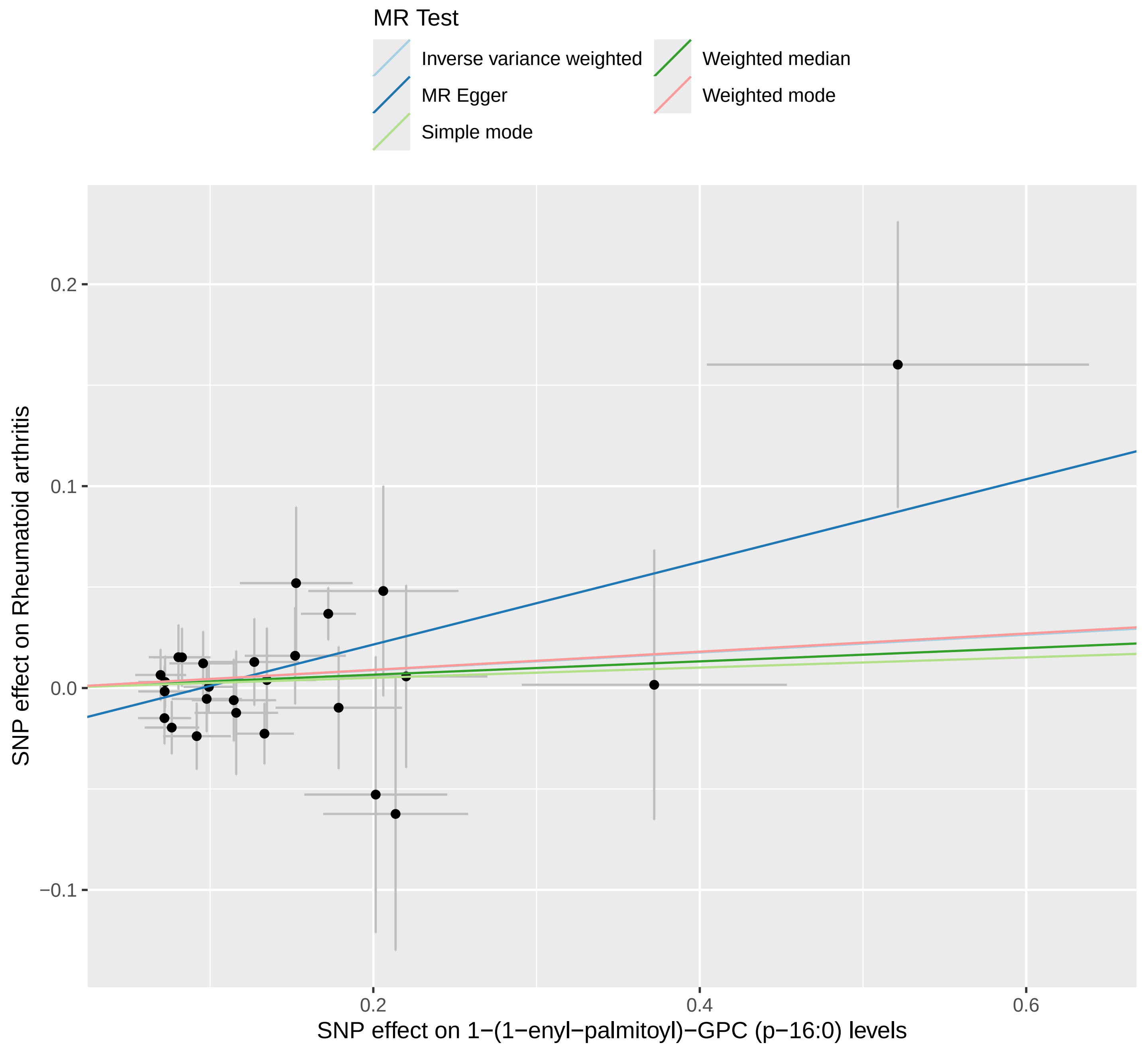

MR Test
Inverse variance weighted
MR Egger
Simple mode
Weighted median
Weighted mode
0.2
0.1
0.0
−0.1
0.2
0.4
0.6
SNP effect on Rheumatoid arthritis
SNP effect on 1−(1−enyl−palmitoyl)−GPC (p−16:0) levels

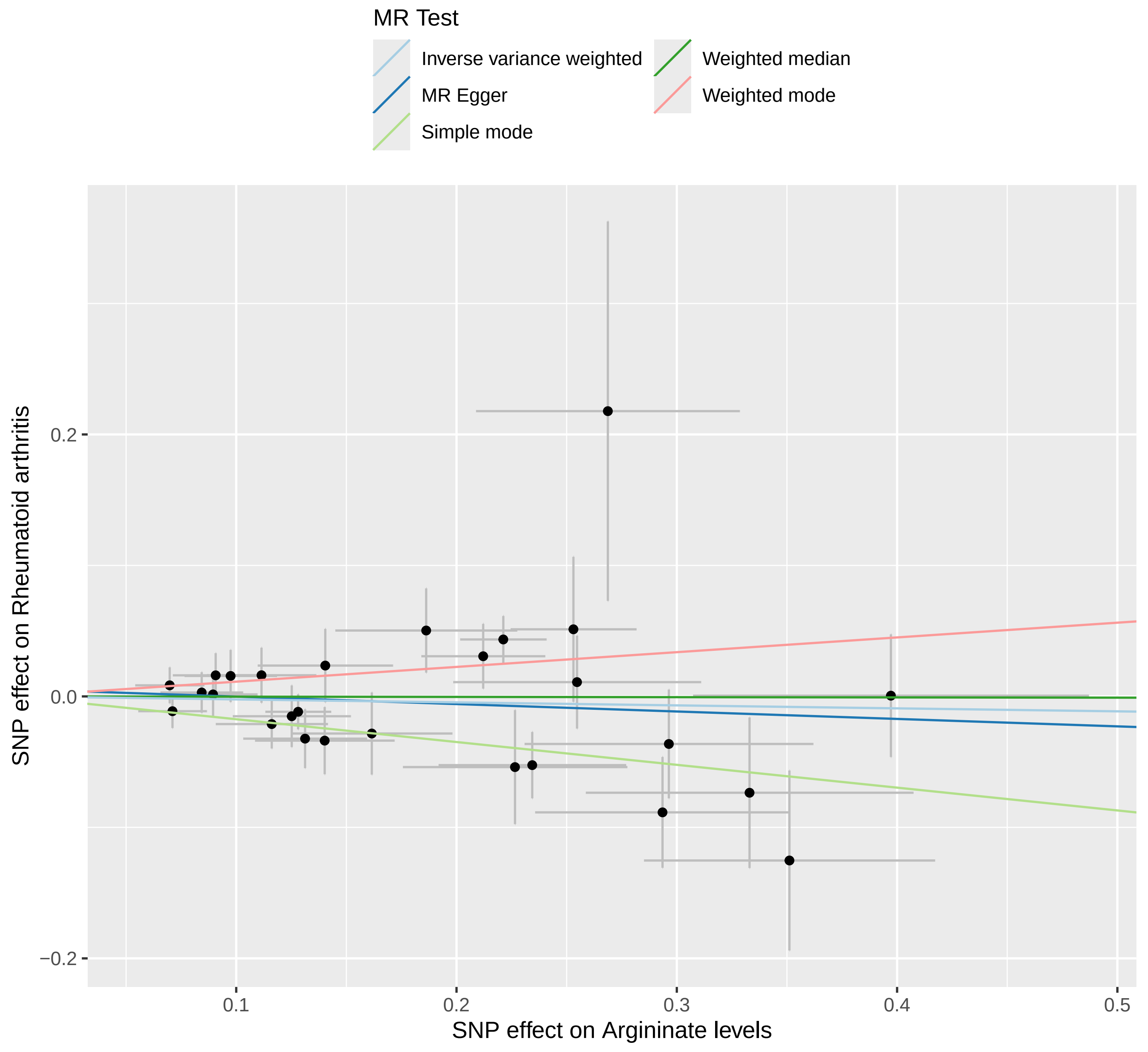

MR Test
Inverse variance weighted
MR Egger
Simple mode
Weighted median
Weighted mode
0.2
0.0
−0.2
0.1
0.2
0.3
0.4
0.5
SNP effect on Arginate levels
SNP effect on Rheumatoid arthritis

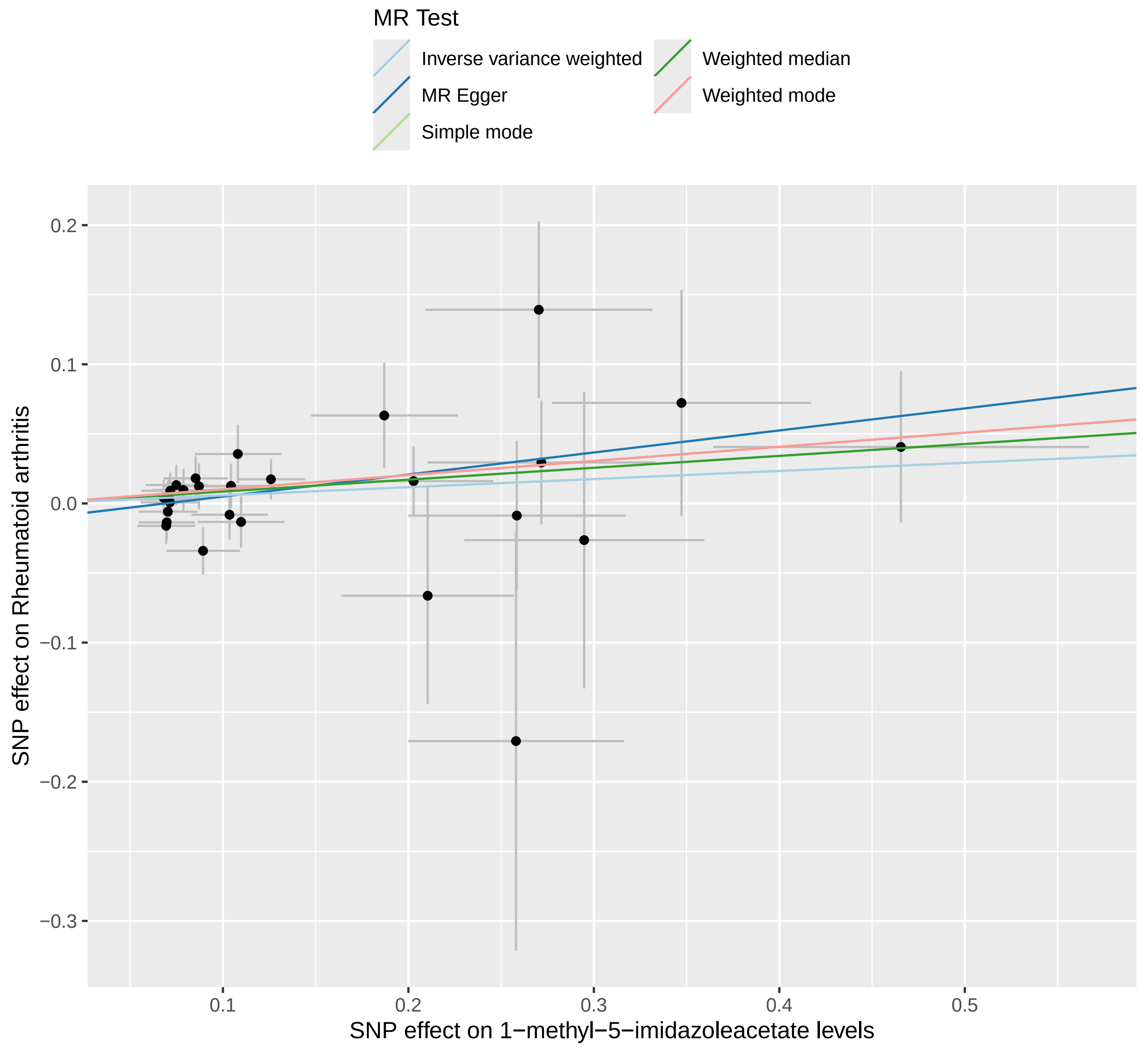

MR Test
Inverse variance weighted
MR Egger
Simple mode
Weighted median
Weighted mode
0.2
0.1
0.0
−0.1
−0.2
−0.3
SNP effect on Rheumatoid arthritis
0.1
0.2
0.3
0.4
0.5
SNP effect on 1−methyl−5−imidazoleacetate levels

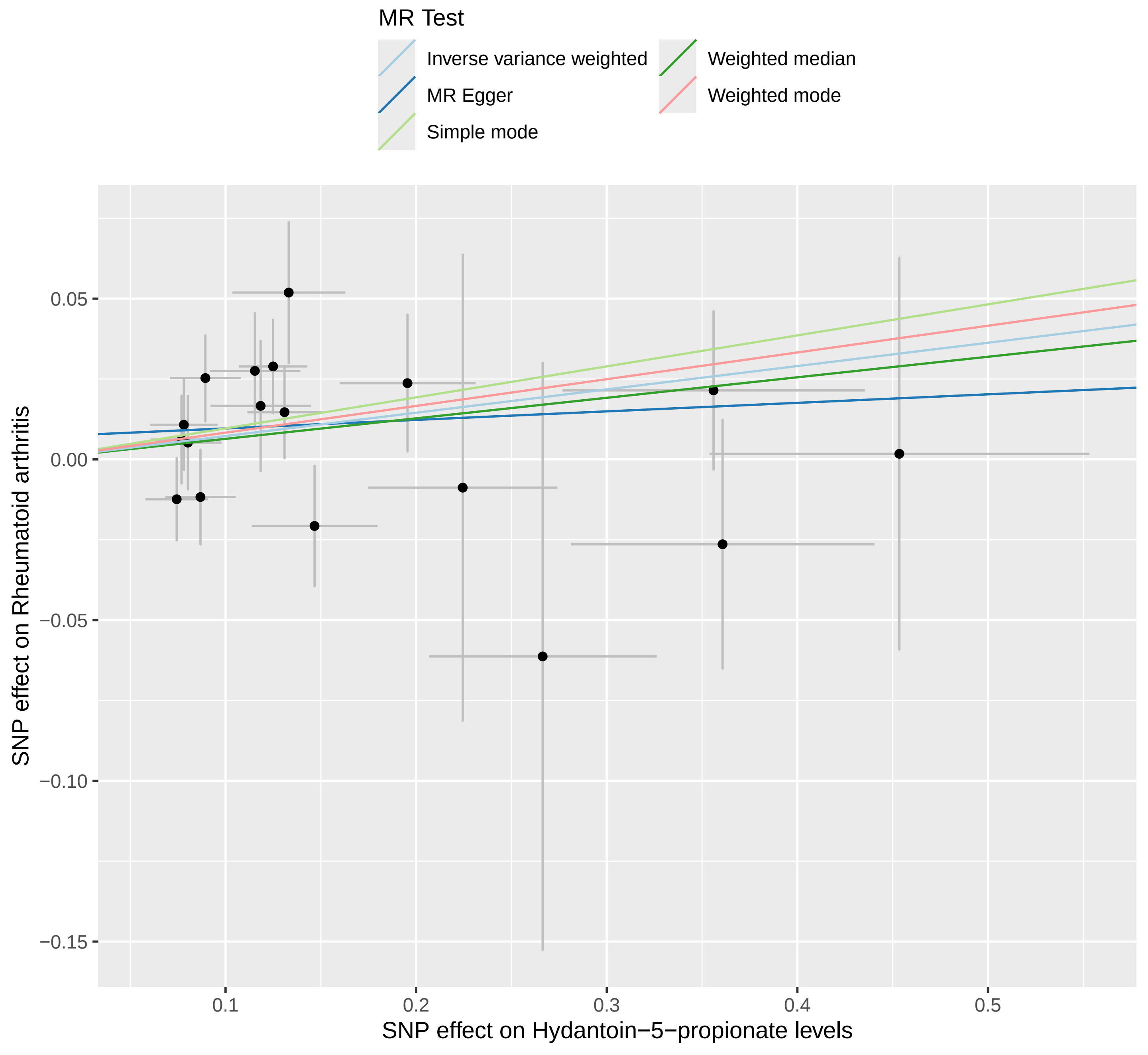
MR Test
Inverse variance weighted
MR Egger
Simple mode
Weighted median
Weighted mode
0.05
0.00
−0.05
−0.10
−0.15
0.1
0.2
0.3
0.4
0.5
SNP effect on Rheumatoid arthritis
SNP effect on Hydantoin−5−propionate levels

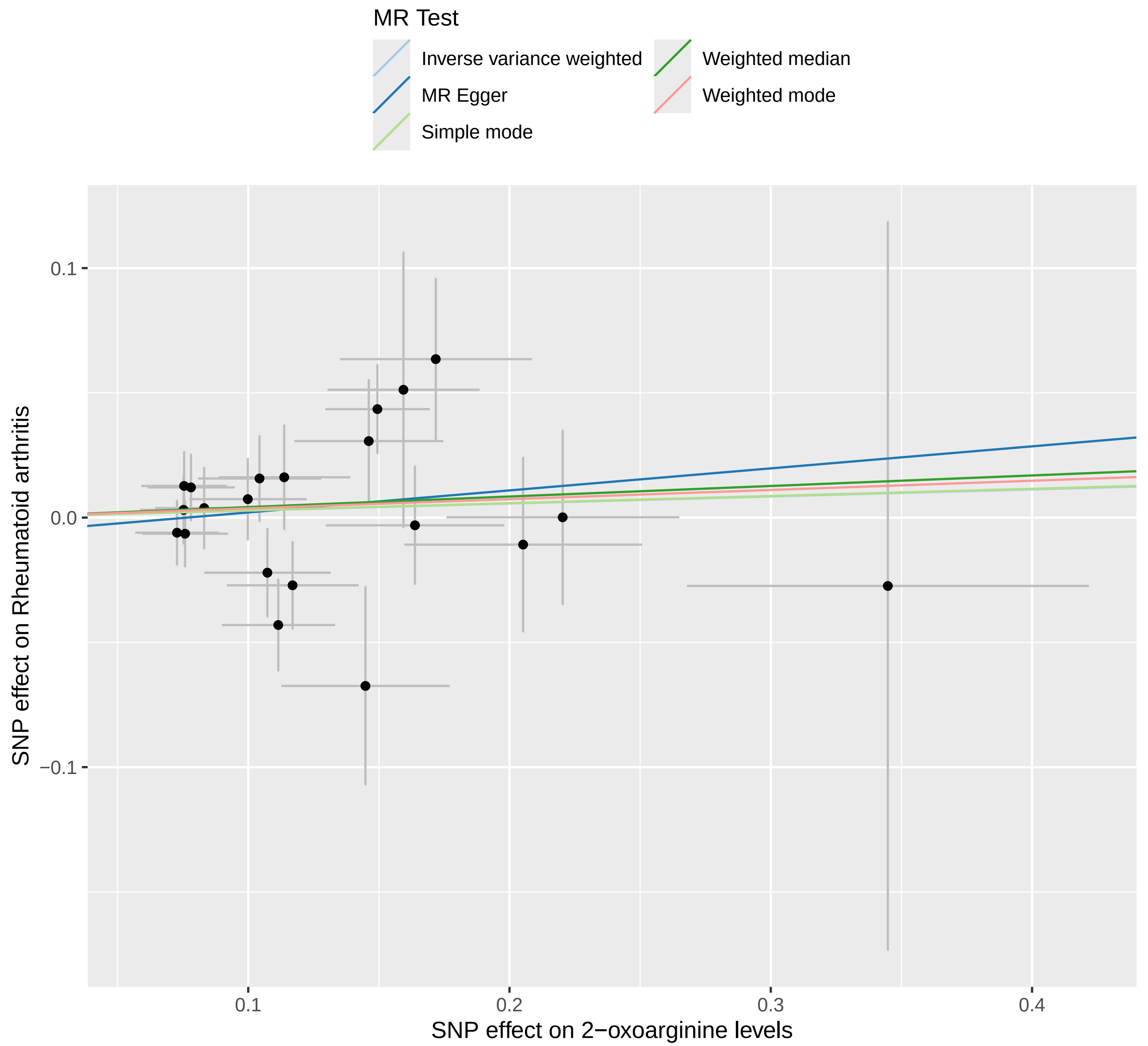

MR Test
Inverse variance weighted
MR Egger
Simple mode
Weighted median
Weighted mode
0.1
0.0
−0.1
SNP effect on Rheumatoid arthritis
0.1
0.2
0.3
0.4
SNP effect on 2−oxoarginine levels

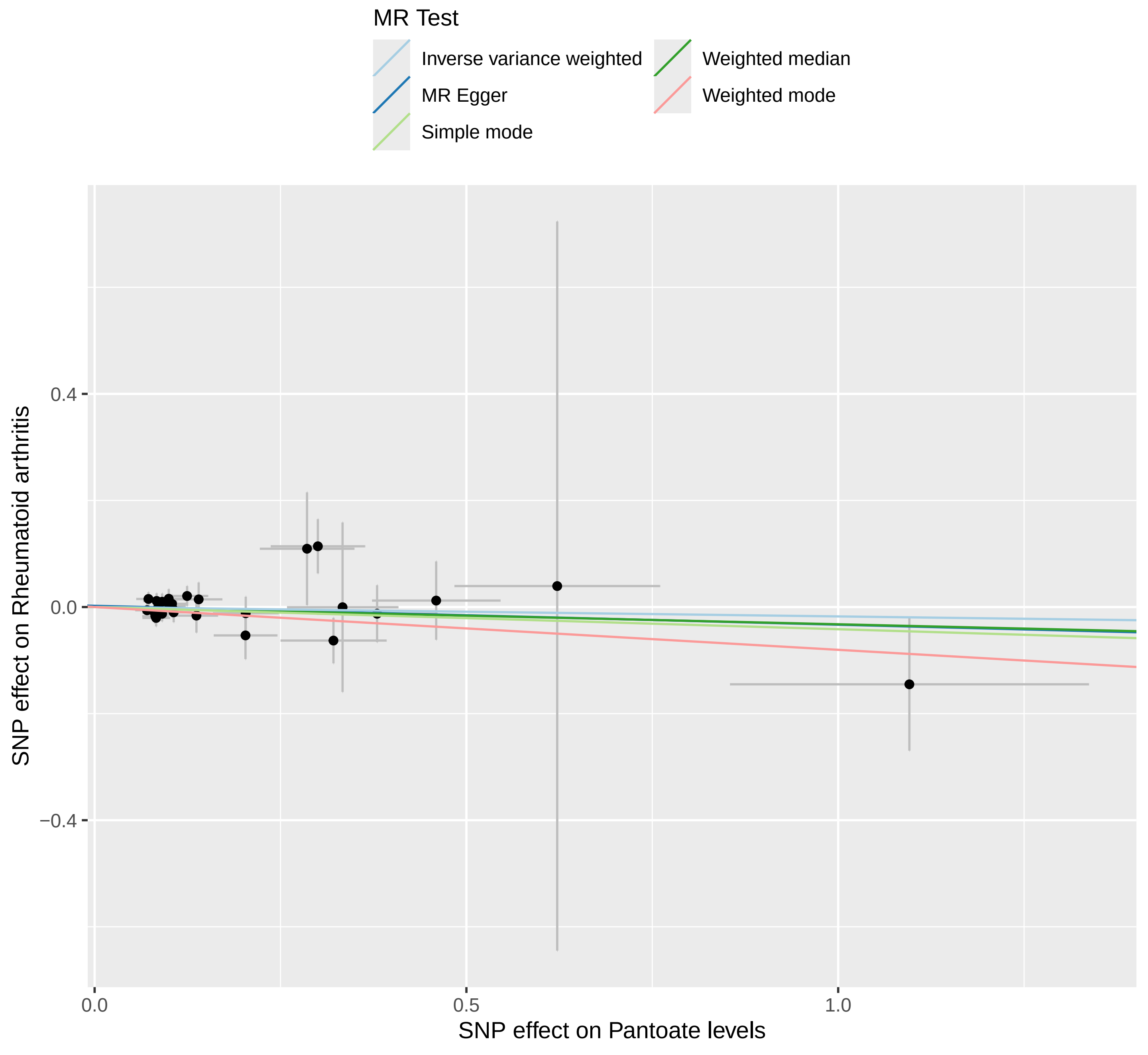
MR Test
Inverse variance weighted
MR Egger
Simple mode
Weighted median
Weighted mode
0.4
0.0
−0.4
SNP effect on Rheumatoid arthritis
0.0
0.5
1.0
SNP effect on Pantoate levels

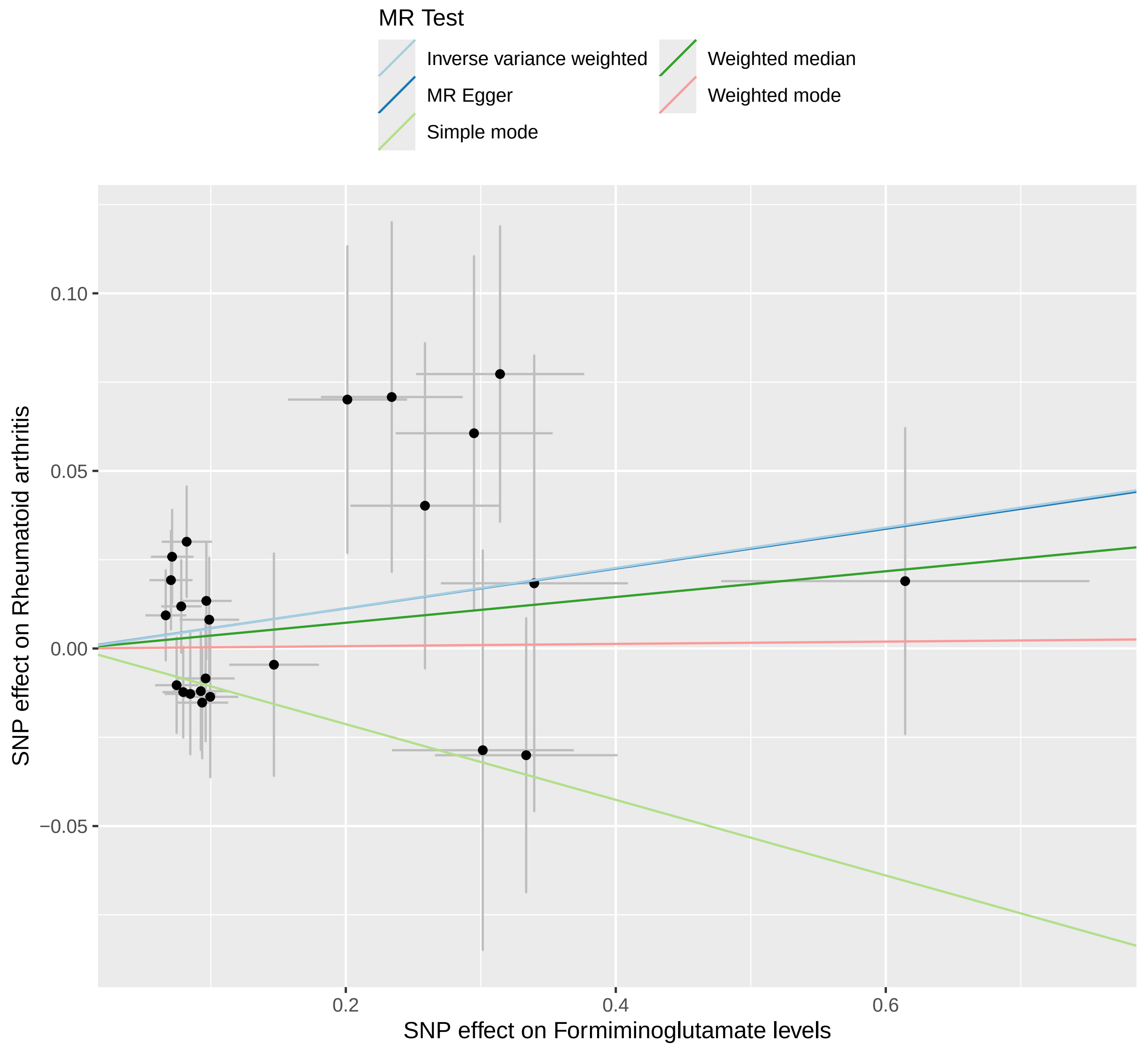
MR Test
Inverse variance weighted
MR Egger
Simple mode
Weighted median
Weighted mode
0.10
0.05
0.00
−0.05
0.2
0.4
0.6
SNP effect on Rheumatoid arthritis
SNP effect on Formiminoglutamate levels

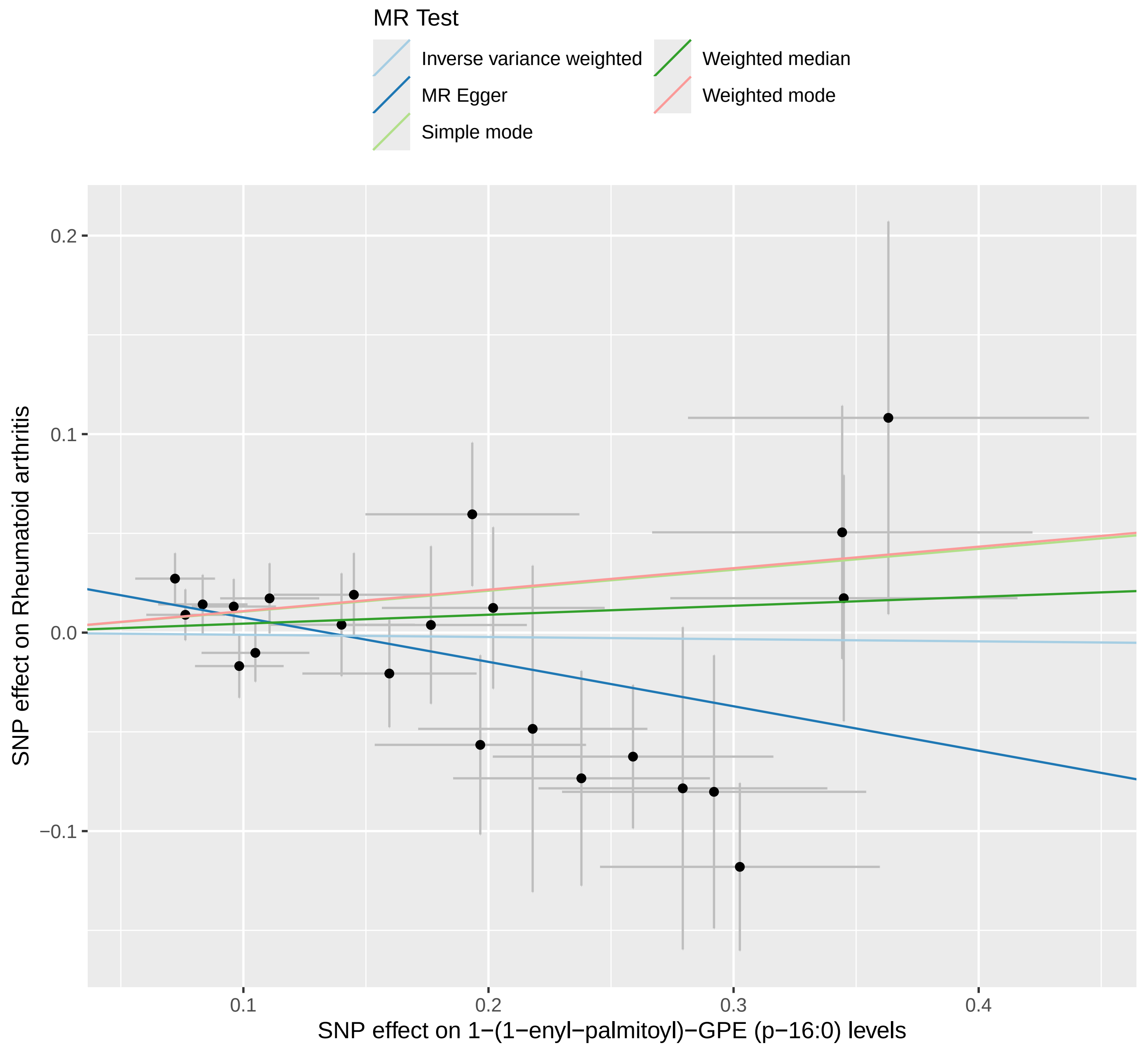
MR Test
Inverse variance weighted
MR Egger
Simple mode
Weighted median
Weighted mode
0.2
0.1
0.0
−0.1
SNP effect on Rheumatoid arthritis
0.1
0.2
0.3
0.4
SNP effect on 1−(1−enyl−palmitoyl)−GPE (p−16:0) levels

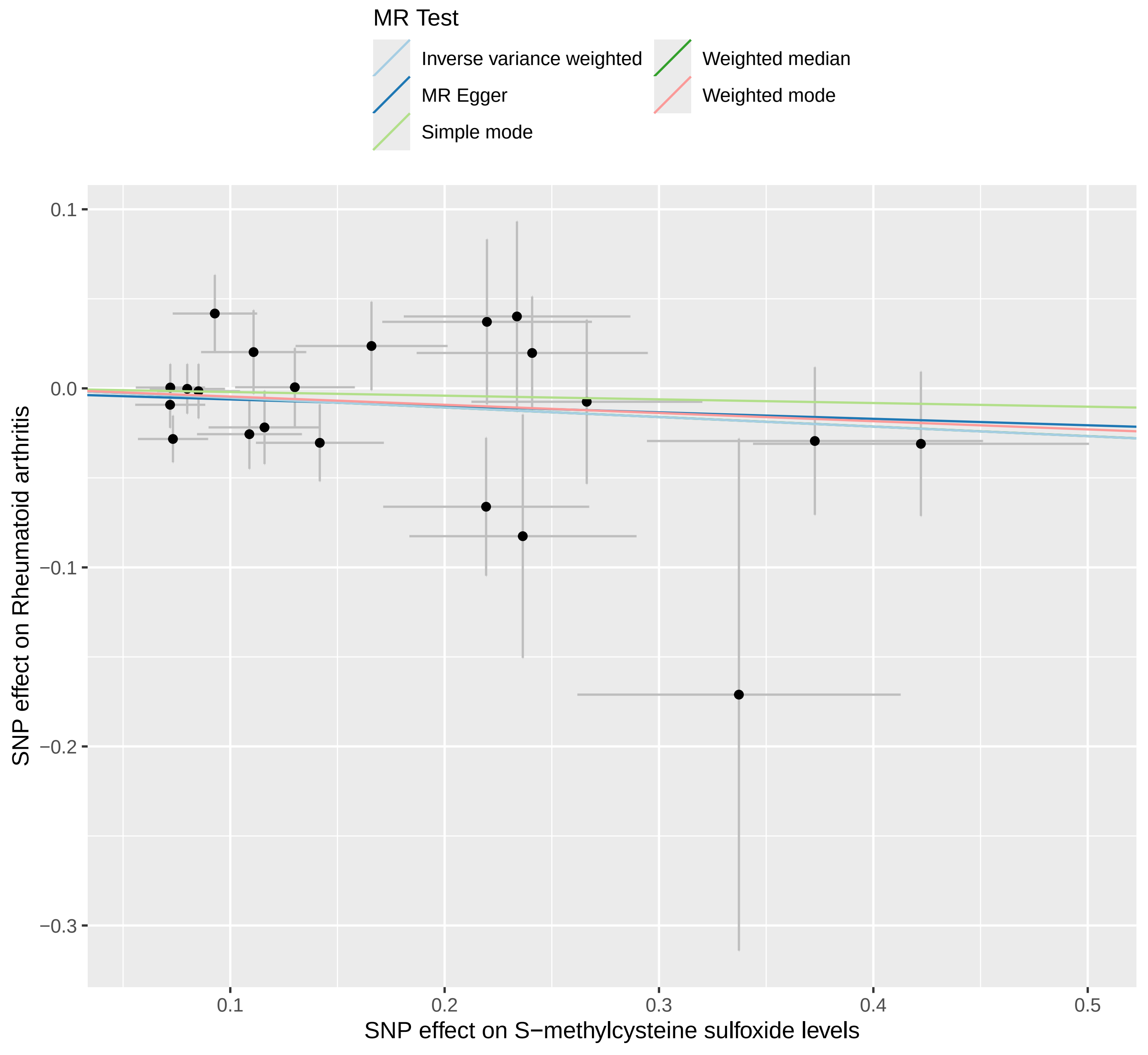

MR Test
Inverse variance weighted
MR Egger
Simple mode
Weighted median
Weighted mode
0.1
0.0
−0.1
−0.2
−0.3
0.1
0.2
0.3
0.4
0.5
SNP effect on Rheumatoid arthritis
SNP effect on S−methylcysteine sulfoxide levels

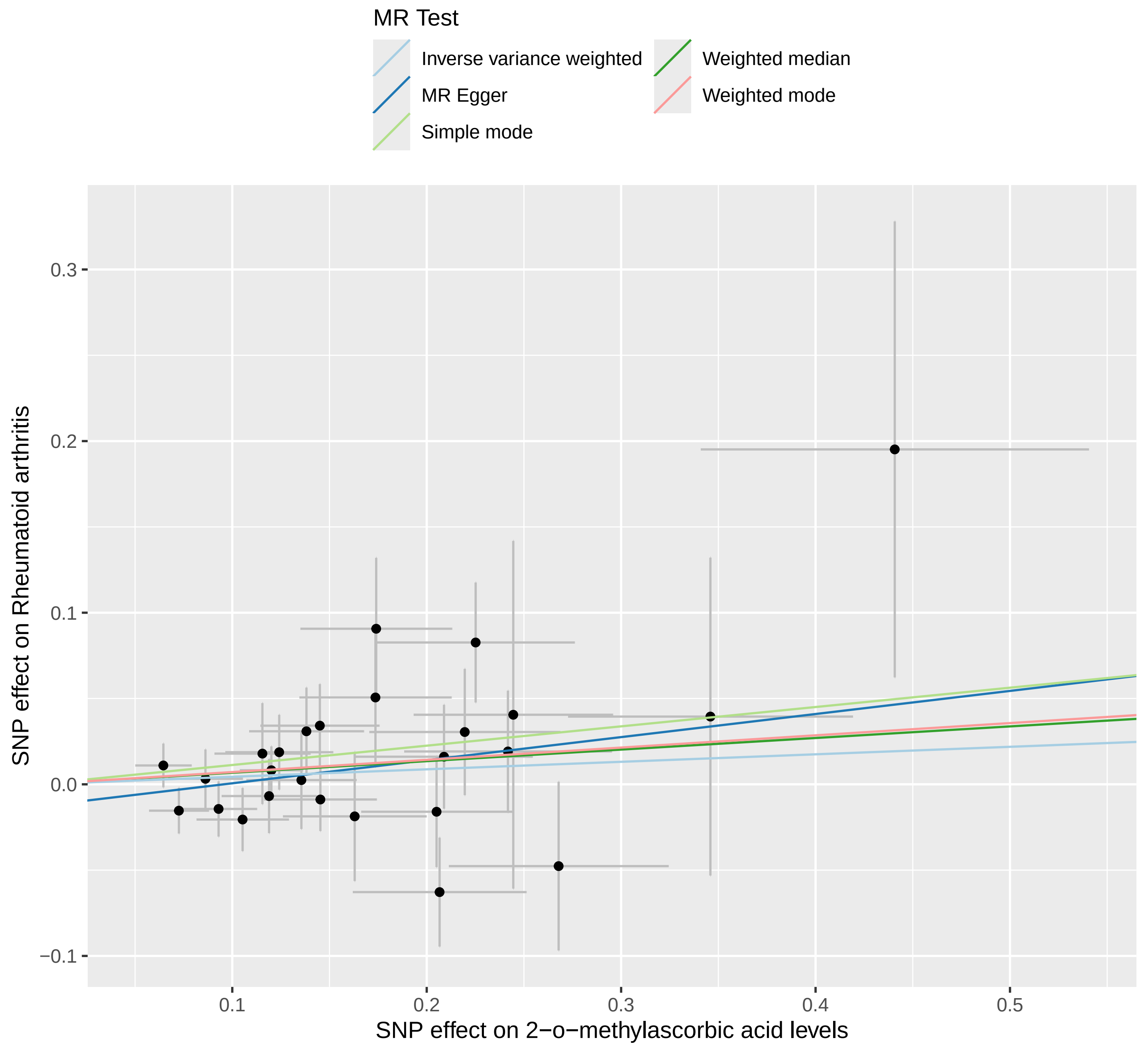
MR Test
Inverse variance weighted
MR Egger
Simple mode
Weighted median
Weighted mode
SNP effect on Rheumatoid arthritis
0.3
0.2
0.1
0.0
−0.1
0.1
0.2
0.3
0.4
0.5
SNP effect on 2−o−methylascorbic acid levels

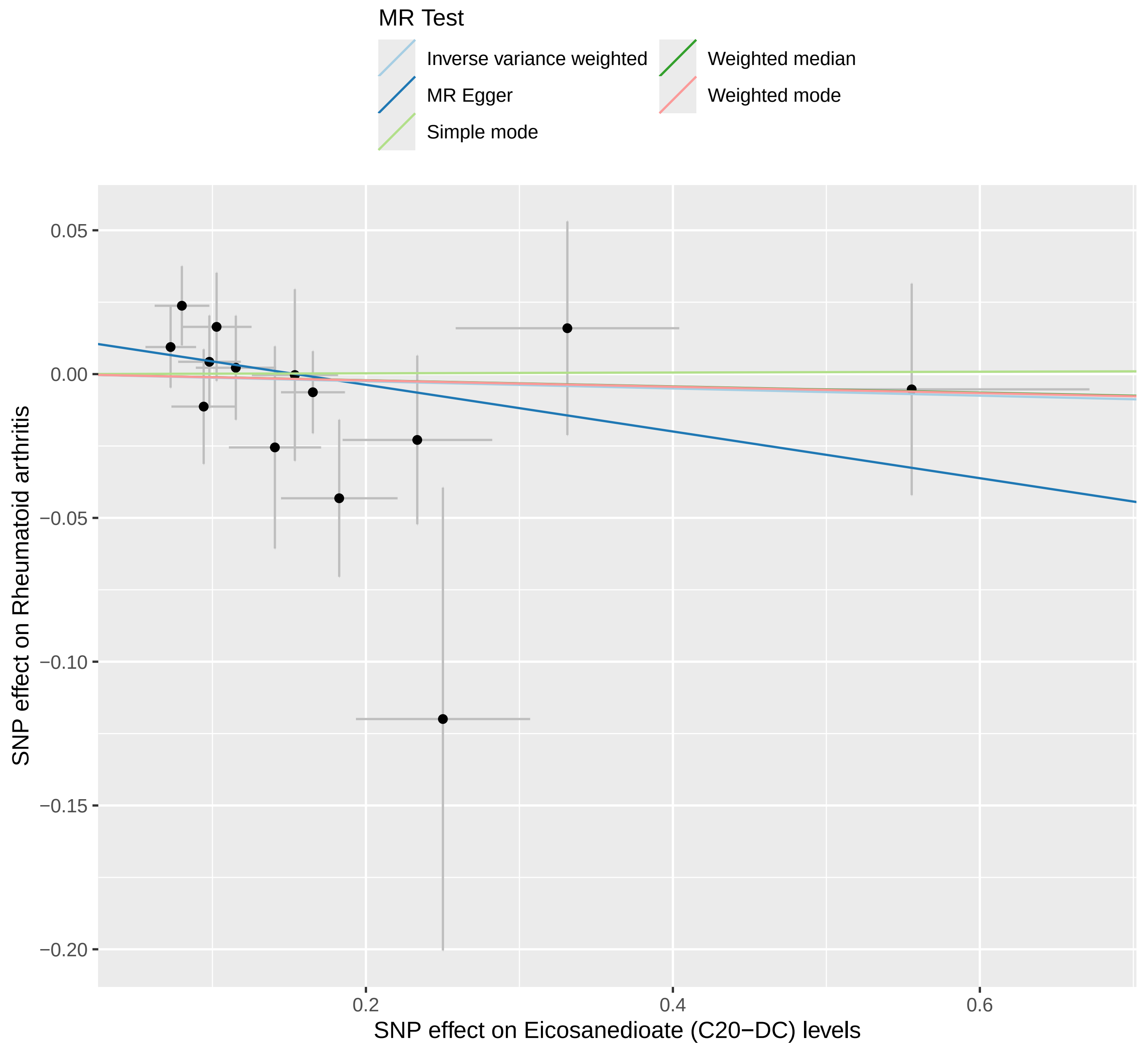

MR Test
Inverse variance weighted
MR Egger
Simple mode
Weighted median
Weighted mode
0.05
0.00
−0.05
−0.10
−0.15
−0.20
SNP effect on Rheumatoid arthritis
0.2
0.4
0.6
SNP effect on Eicosanedioate (C20−DC) levels

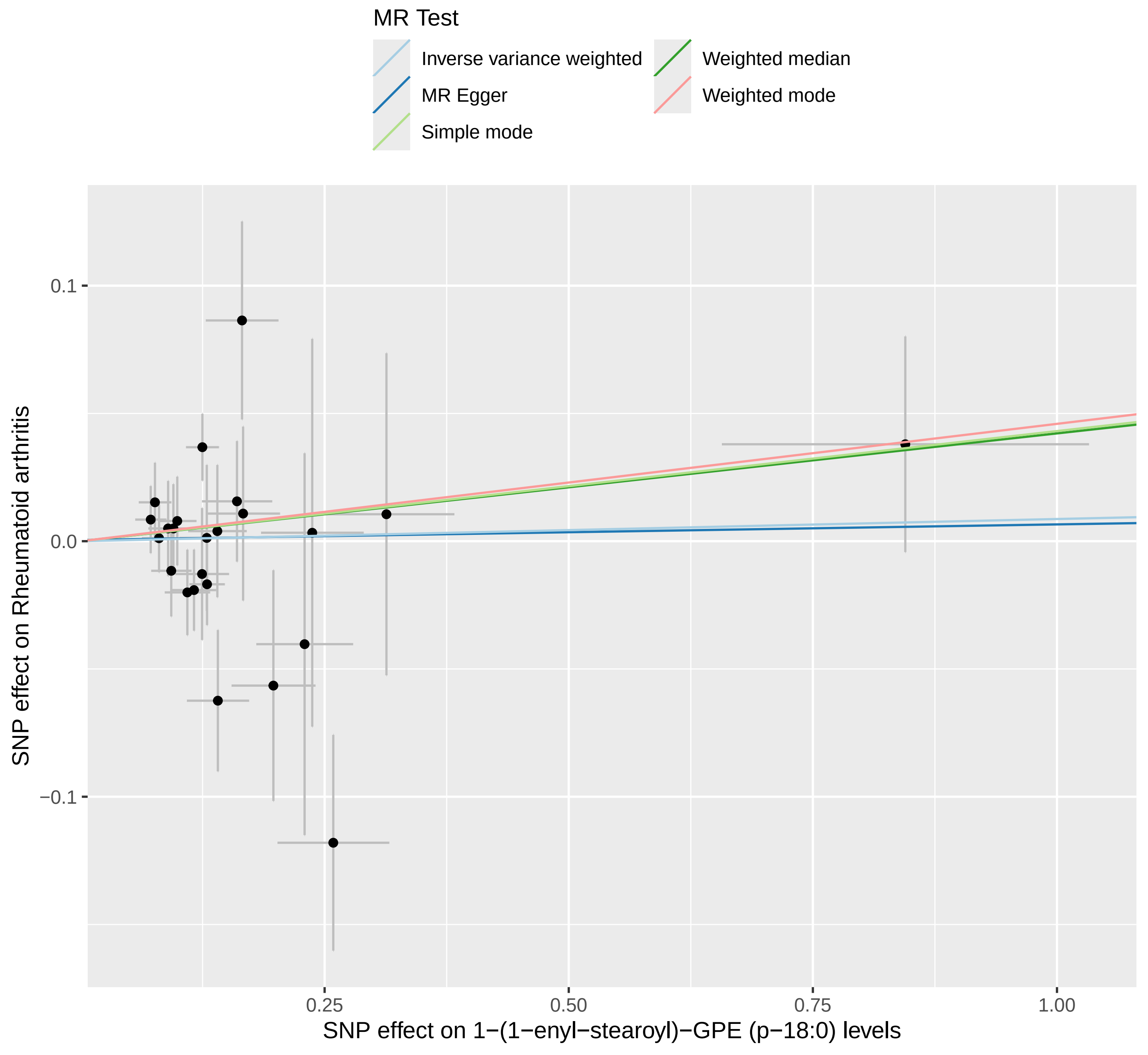
MR Test
Inverse variance weighted
MR Egger
Simple mode
Weighted median
Weighted mode
0.1
0.0
−0.1
SNP effect on Rheumatoid arthritis
0.25
0.50
0.75
1.00
SNP effect on 1−(1−enyl−stearoyl)−GPE (p−18:0) levels

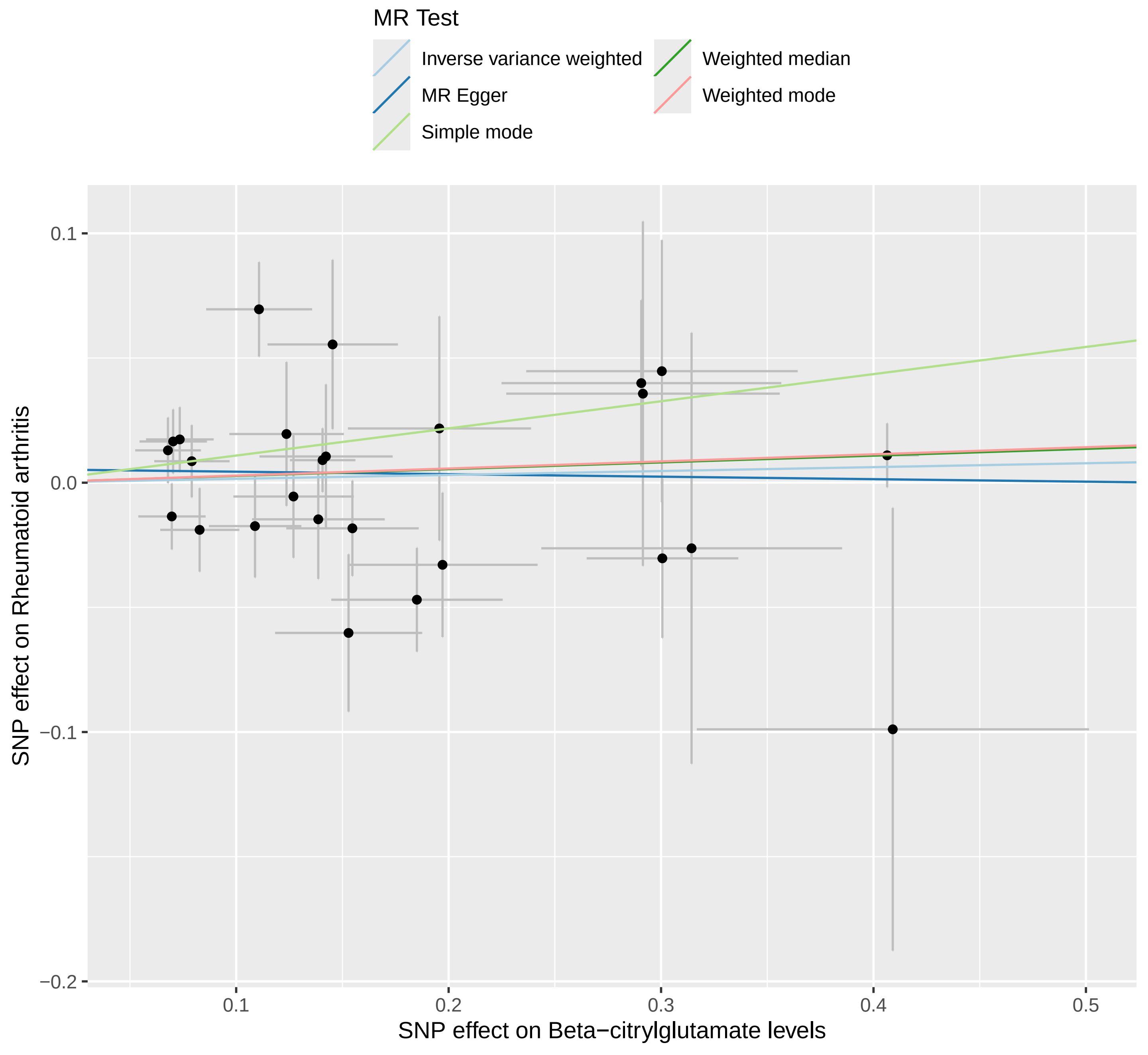

MR Test
Inverse variance weighted
MR Egger
Simple mode
Weighted median
Weighted mode
0.1
0.0
−0.1
−0.2
0.1
0.2
0.3
0.4
0.5
SNP effect on Rheumatoid arthritis
SNP effect on Beta−citrylglutamate levels

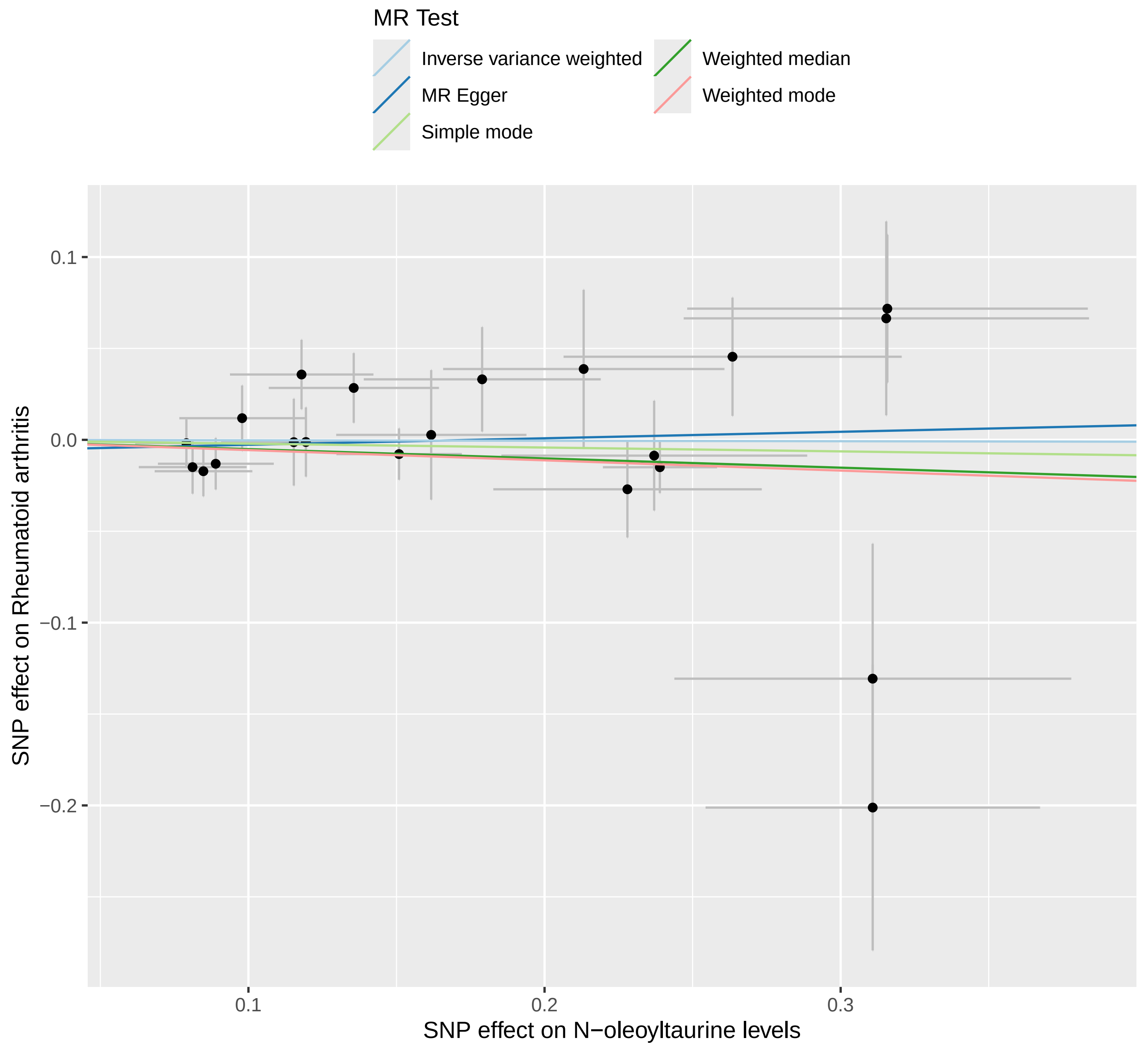
MR Test
Inverse variance weighted
MR Egger
Simple mode
Weighted median
Weighted mode
0.1
0.0
−0.1
−0.2
SNP effect on Rheumatoid arthritis
0.1
0.2
0.3
SNP effect on N−oleoyltaurine levels

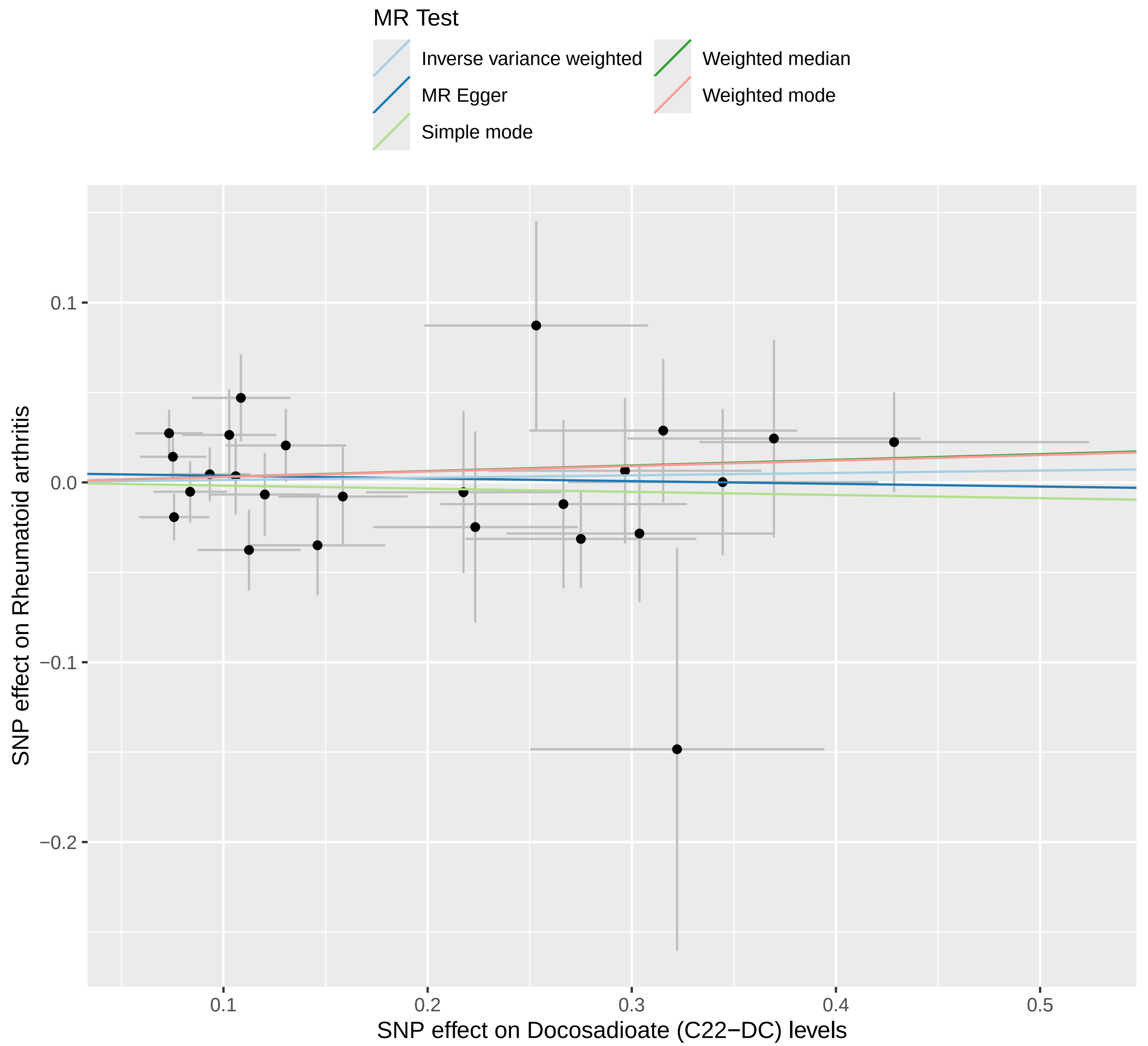
MR Test
Inverse variance weighted
MR Egger
Simple mode
Weighted median
Weighted mode
0.1
0.0
−0.1
−0.2
SNP effect on Rheumatoid arthritis
0.1
0.2
0.3
0.4
0.5
SNP effect on Docosadioate (C22−DC) levels

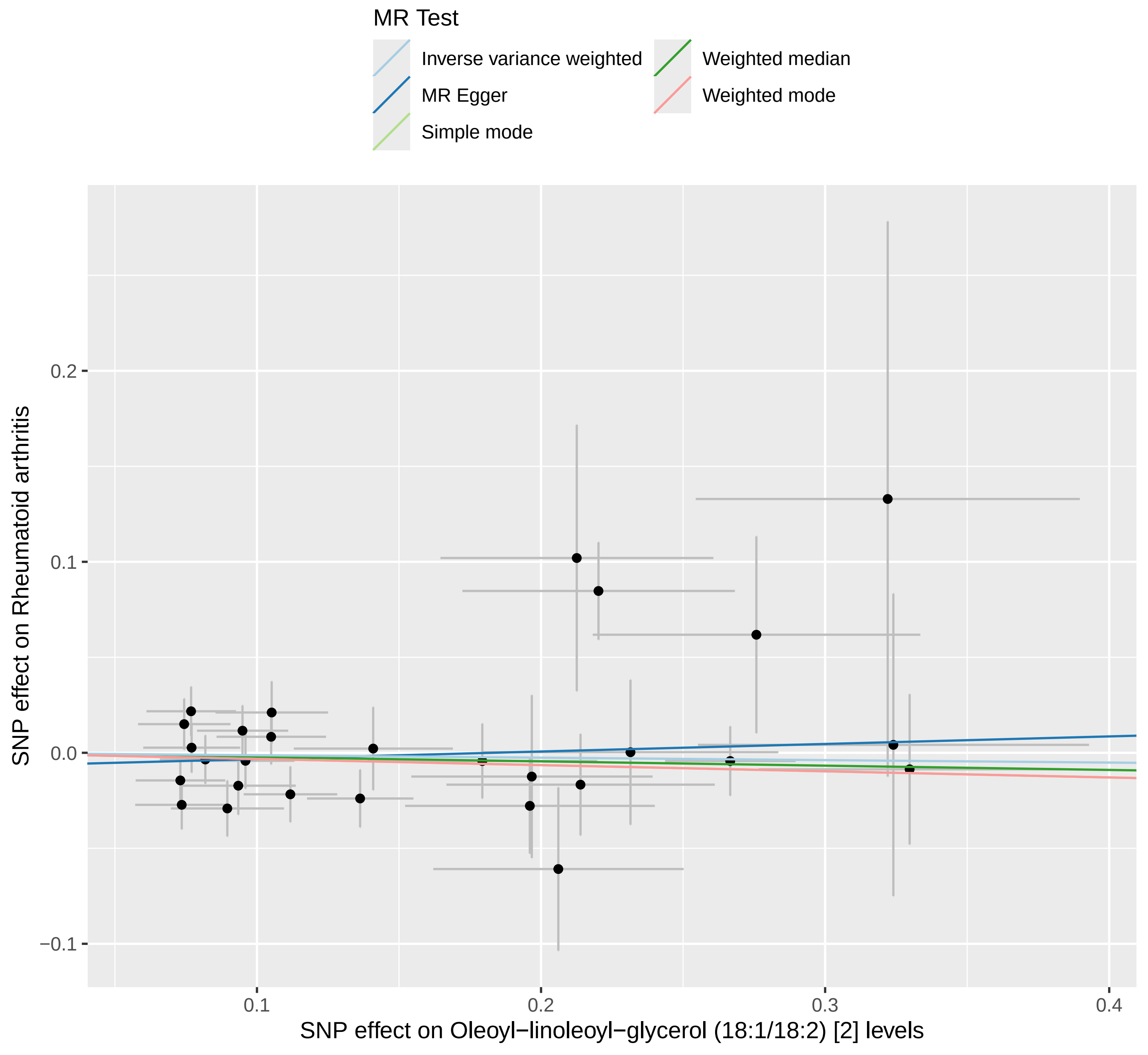
MR Test
Inverse variance weighted
MR Egger
Simple mode
Weighted median
Weighted mode
SNP effect on Rheumatoid arthritis
0.2
0.1
0.0
−0.1
0.1
0.2
0.3
0.4
SNP effect on Oleoyl−linoleoyl−glycerol (18:1/18:2) [2] levels

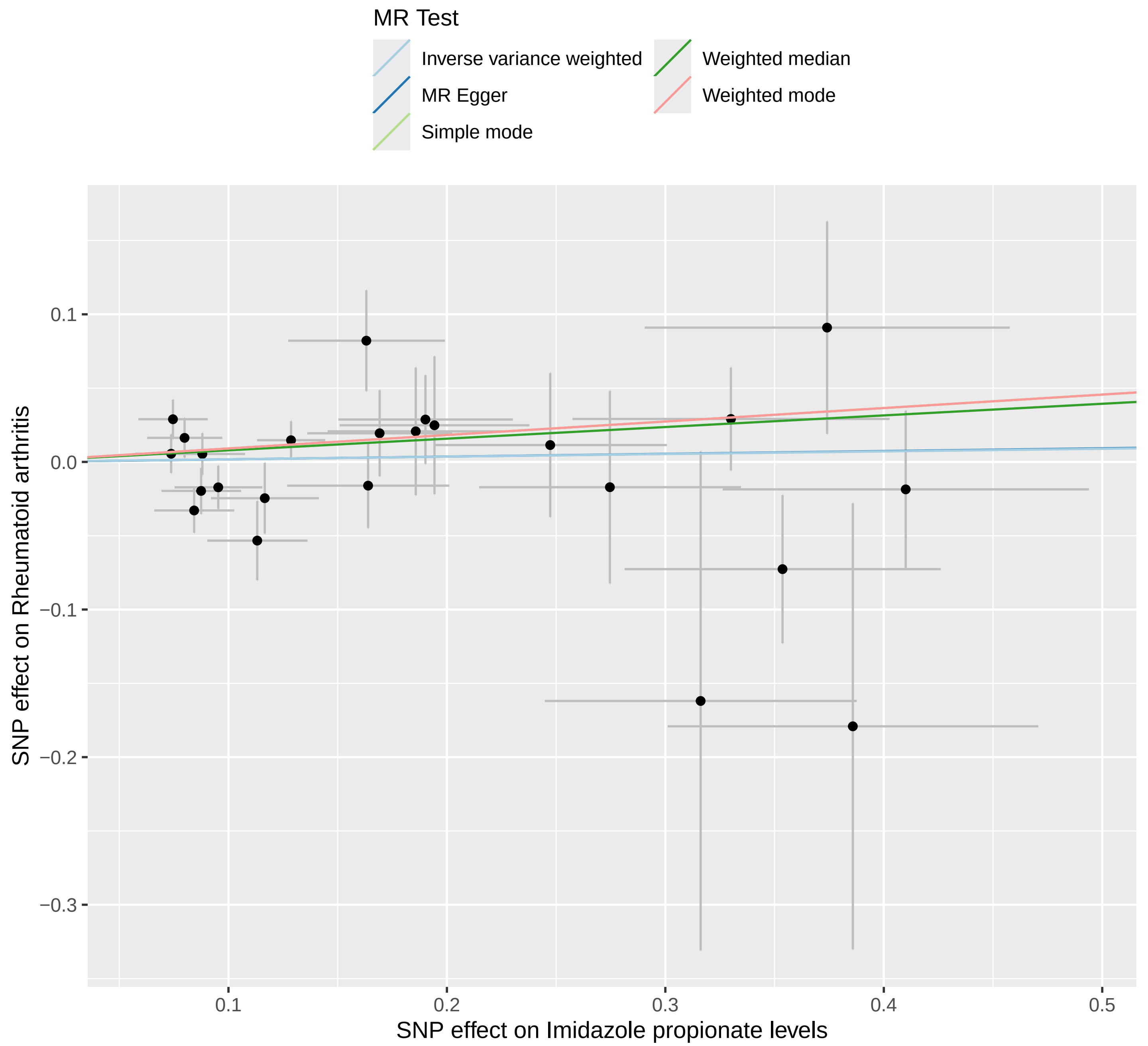

MR Test
Inverse variance weighted
MR Egger
Simple mode
Weighted median
Weighted mode
0.1
0.0
−0.1
−0.2
−0.3
0.1
0.2
0.3
0.4
0.5
SNP effect on Rheumatoid arthritis
SNP effect on Imidazole propionate levels

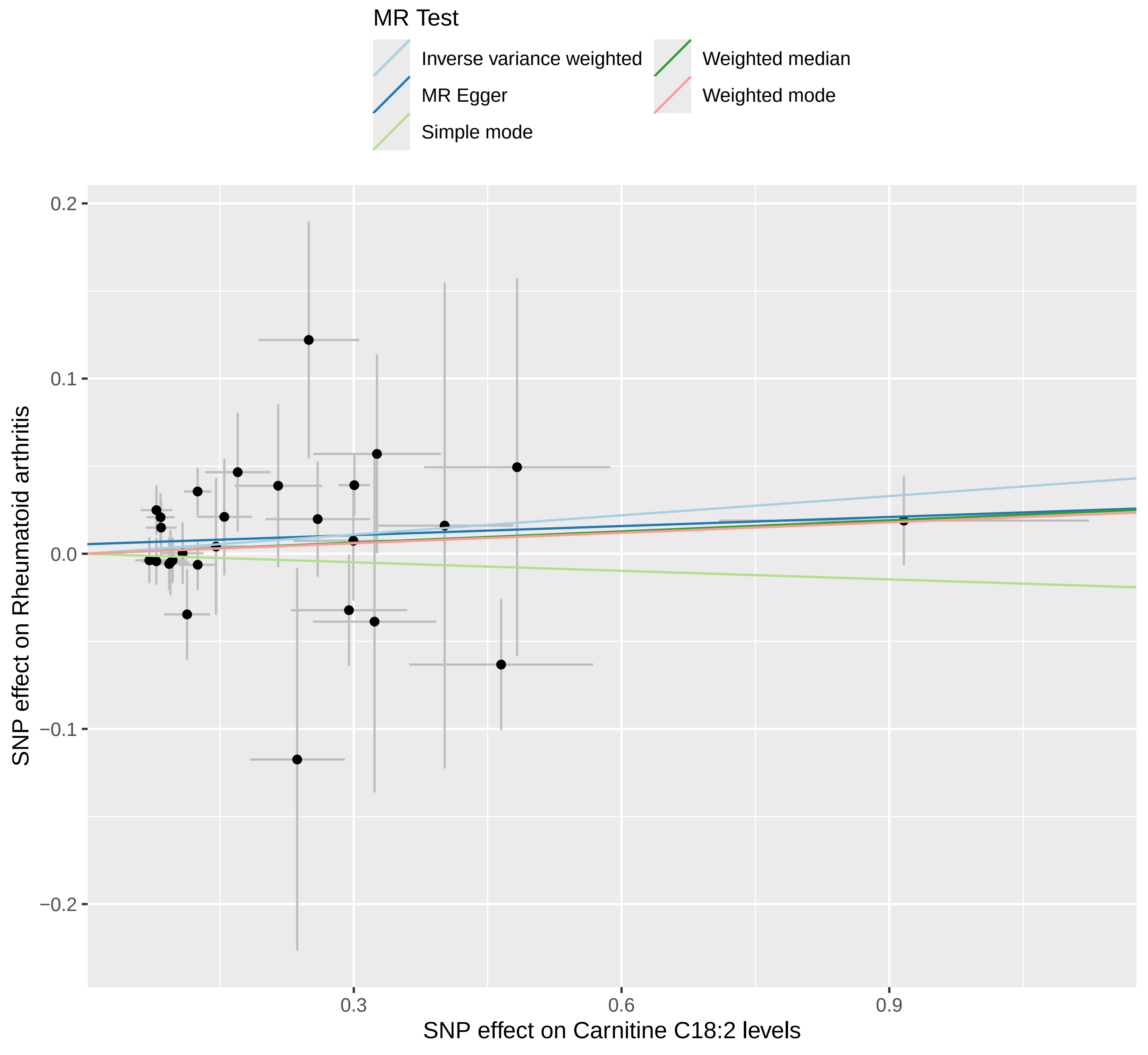
MR Test
Inverse variance weighted
MR Egger
Simple mode
Weighted median
Weighted mode
0.2
0.1
0.0
−0.1
−0.2
0.3
0.6
0.9
SNP effect on Rheumatoid arthritis
SNP effect on Carnitine C18:2 levels

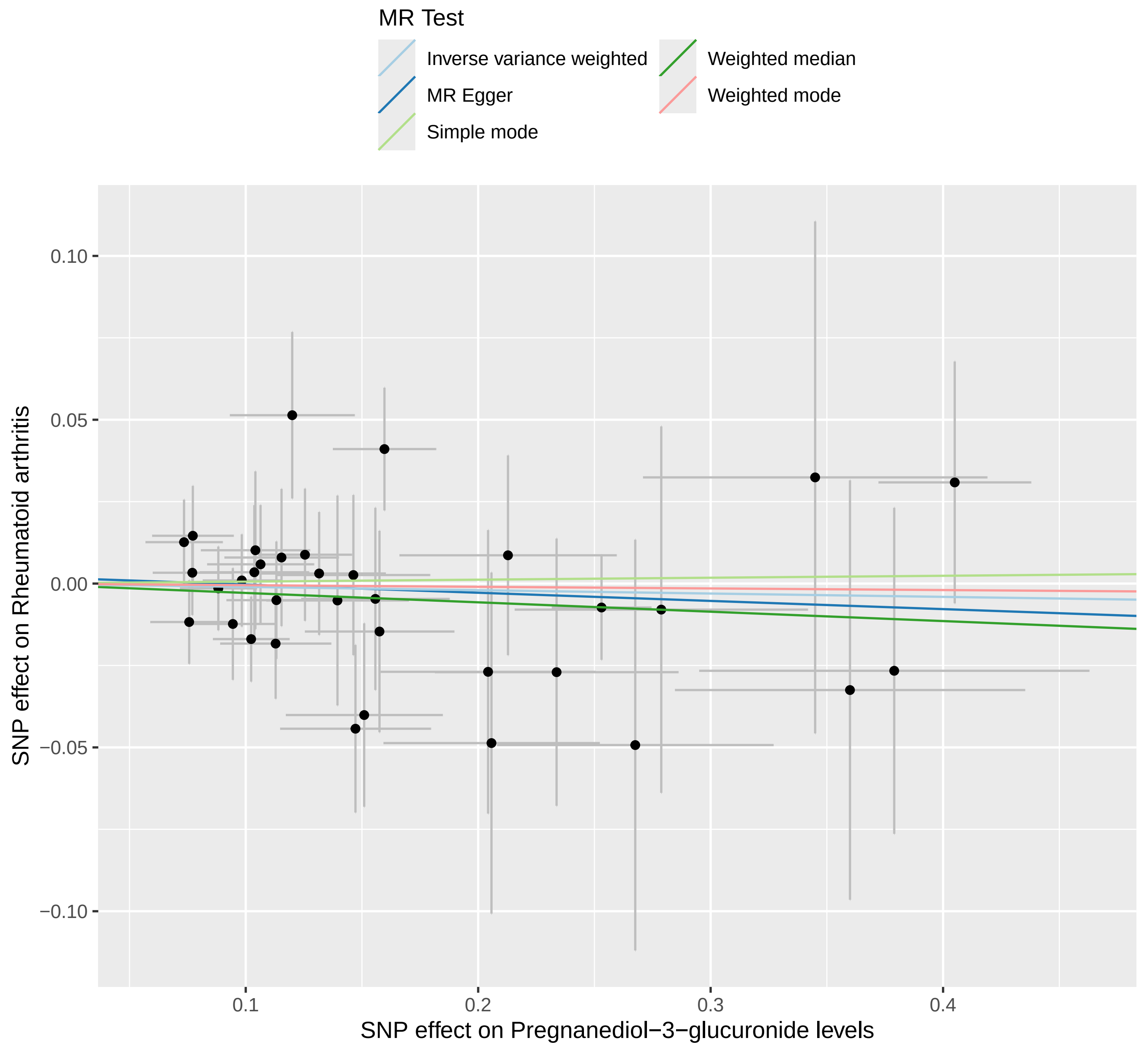

MR Test
Inverse variance weighted
MR Egger
Simple mode
Weighted median
Weighted mode
0.10
0.05
0.00
−0.05
−0.10
SNP effect on Rheumatoid arthritis
0.1
0.2
0.3
0.4
SNP effect on Pregnanediol−3−glucuronide levels

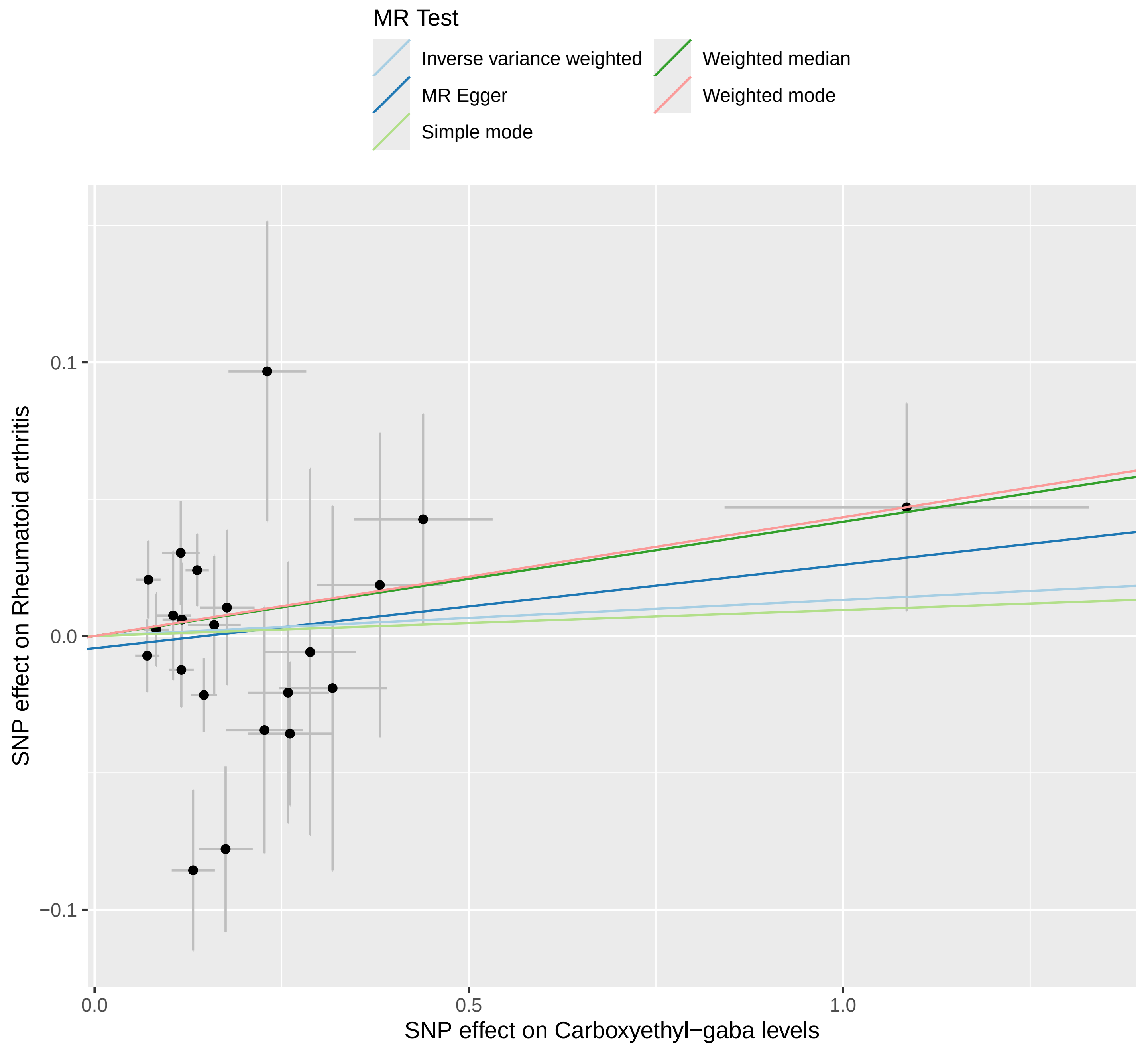

MR Test
Inverse variance weighted
MR Egger
Simple mode
Weighted median
Weighted mode
0.1
0.0
−0.1
0.0
0.5
1.0
SNP effect on Rheumatoid arthritis
SNP effect on Carboxyethyl−gaba levels

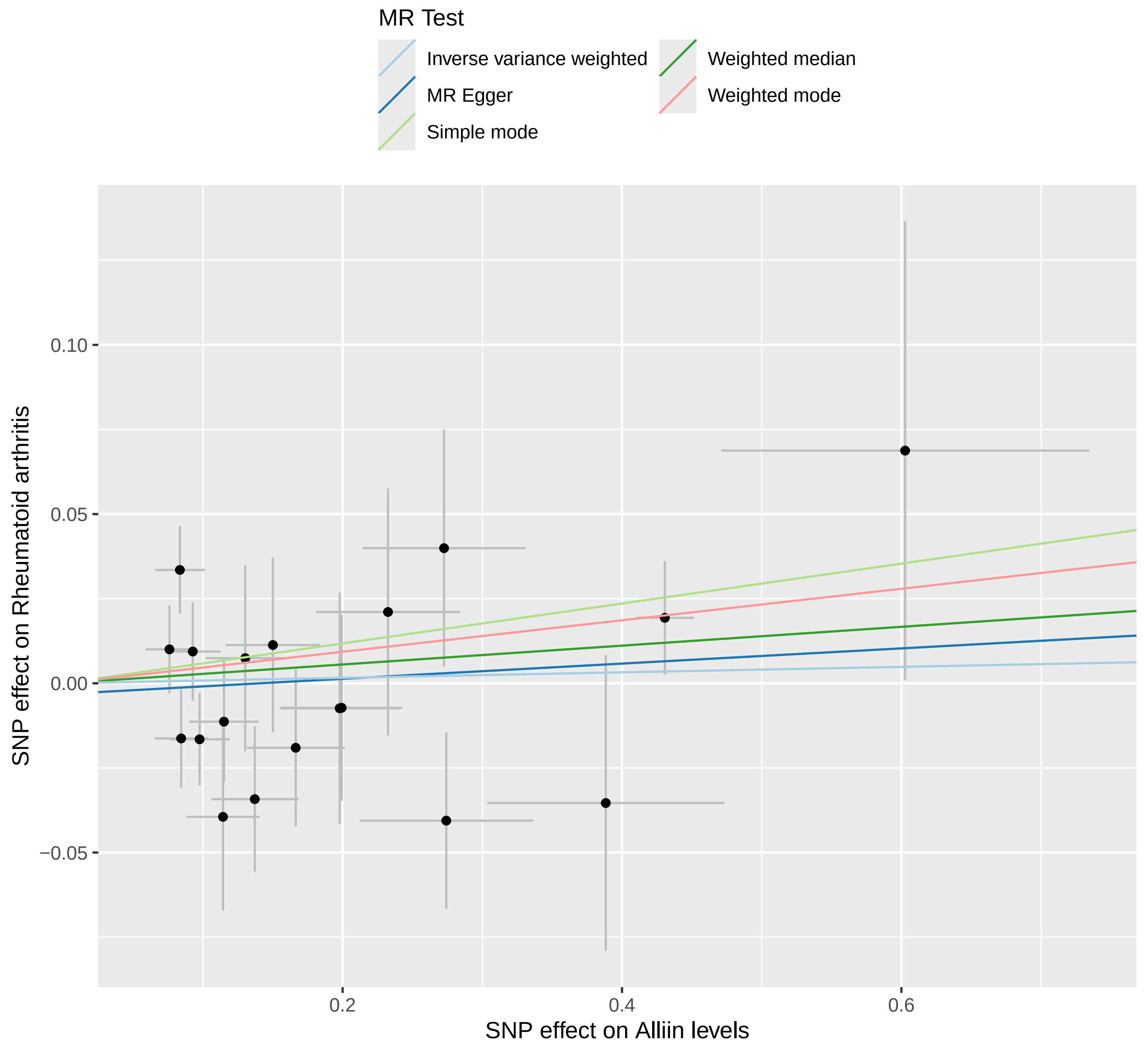
MR Test
Inverse variance weighted
MR Egger
Simple mode
Weighted median
Weighted mode
0.10
0.05
0.00
−0.05
SNP effect on Rheumatoid arthritis
0.2
0.4
0.6
SNP effect on Alliin levels

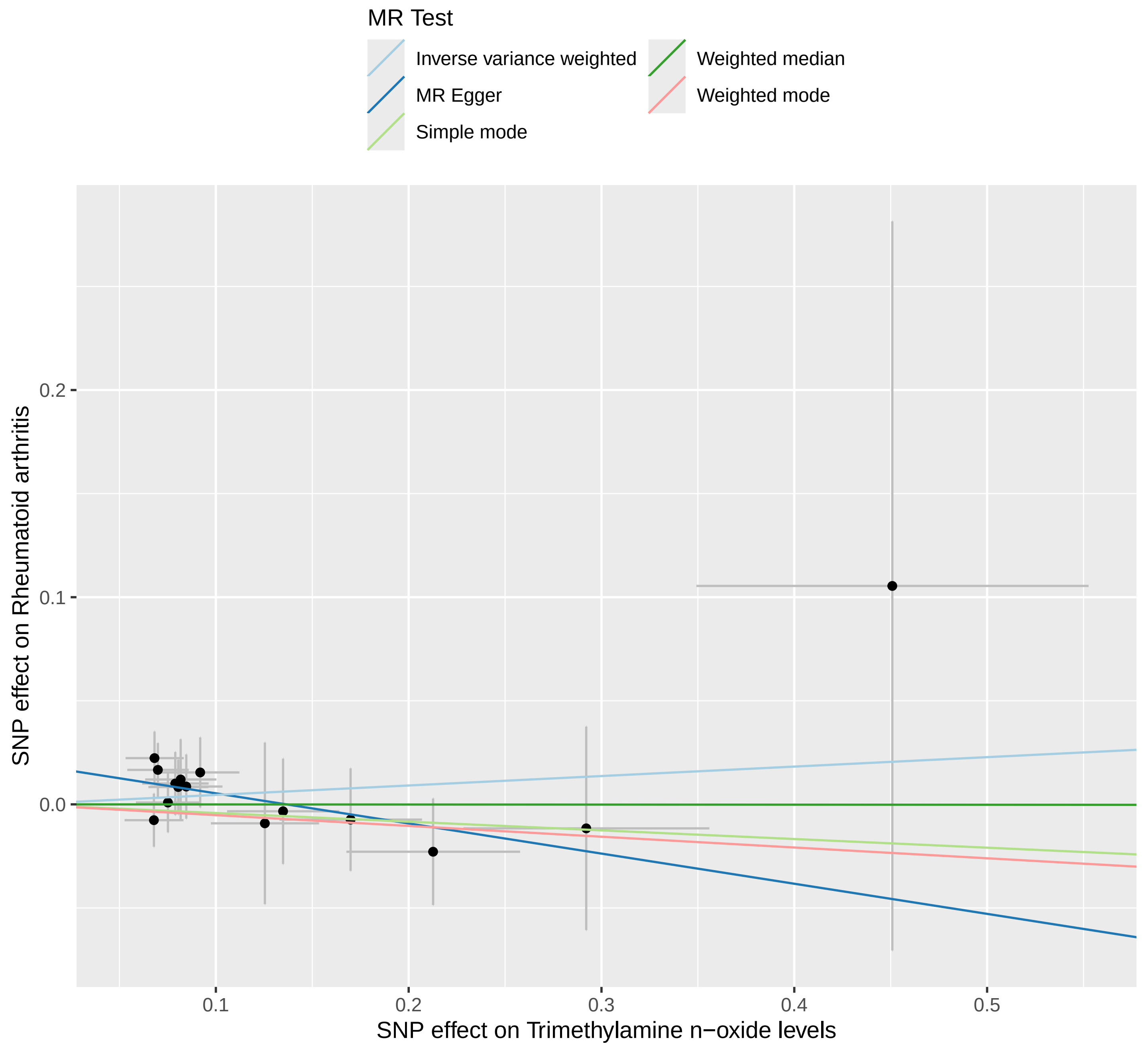
MR Test
Inverse variance weighted
MR Egger
Simple mode
Weighted median
Weighted mode
SNP effect on Rheumatoid arthritis
0.2
0.1
0.0
0.1
0.2
0.3
0.4
0.5
SNP effect on Trimethylamine n−oxide levels

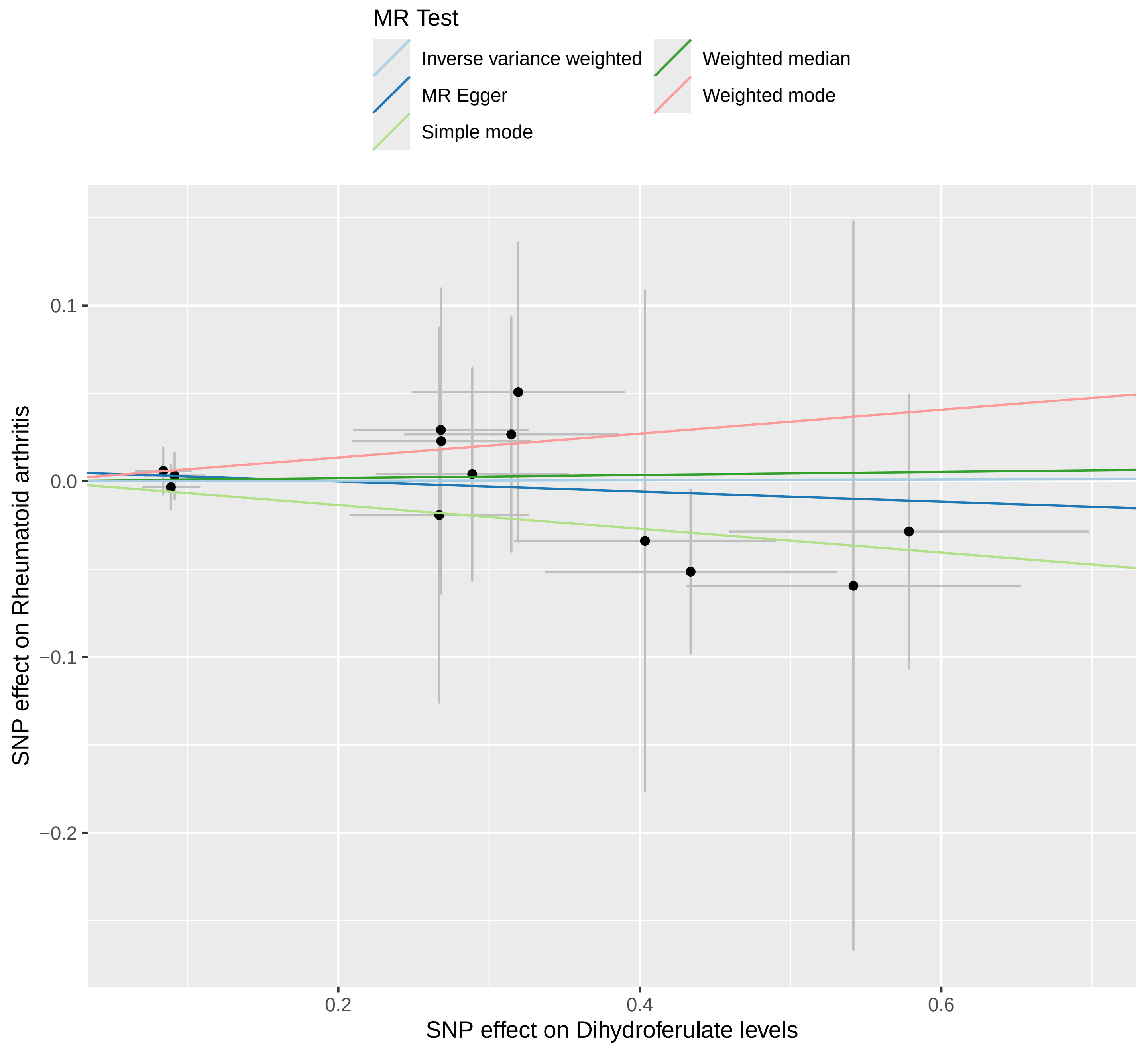
MR Test
Inverse variance weighted
MR Egger
Simple mode
Weighted median
Weighted mode
0.1
0.0
−0.1
−0.2
0.2
0.4
0.6
SNP effect on Dihydroferulate levels
SNP effect on Rheumatoid arthritis

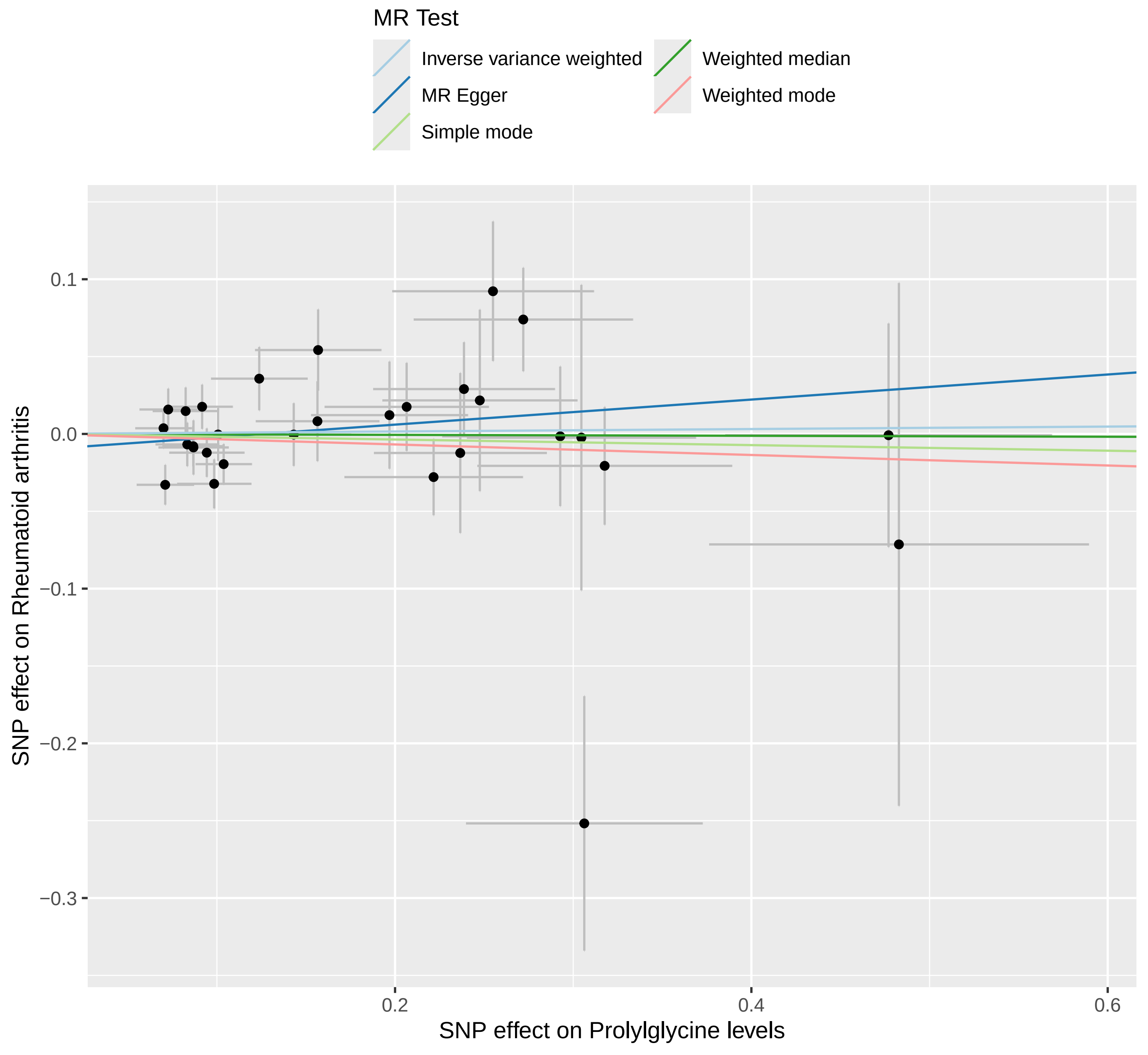

MR Test
Inverse variance weighted
MR Egger
Simple mode
Weighted median
Weighted mode
SNP effect on Rheumatoid arthritis
0.1
0.0
−0.1
−0.2
−0.3
0.2
0.4
0.6
SNP effect on Prolylglycine levels

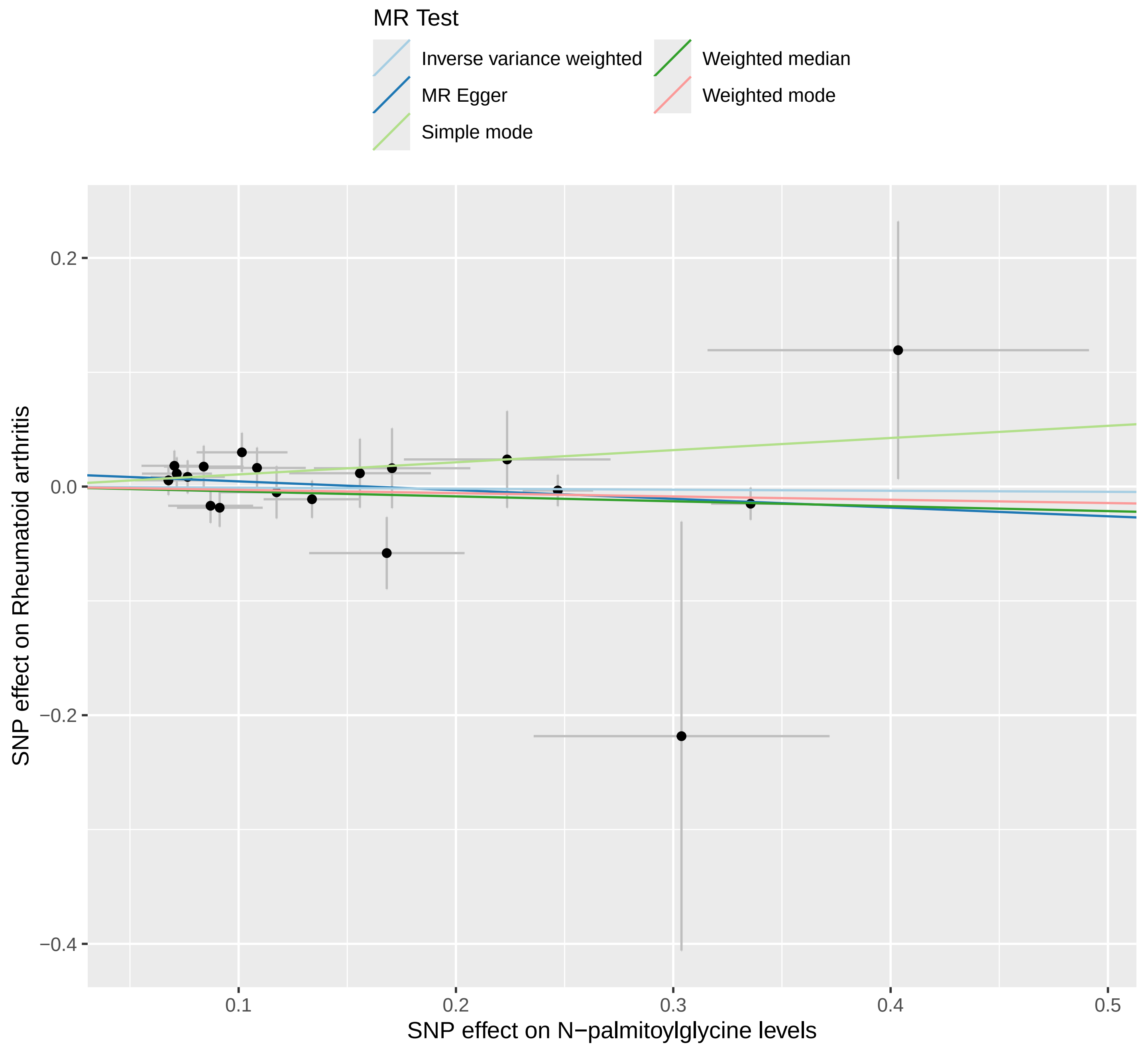
MR Test
Inverse variance weighted
MR Egger
Simple mode
Weighted median
Weighted mode
0.2
0.0
−0.2
−0.4
0.1
0.2
0.3
0.4
0.5
SNP effect on N−palmitoylglycine levels
SNP effect on Rheumatoid arthritis

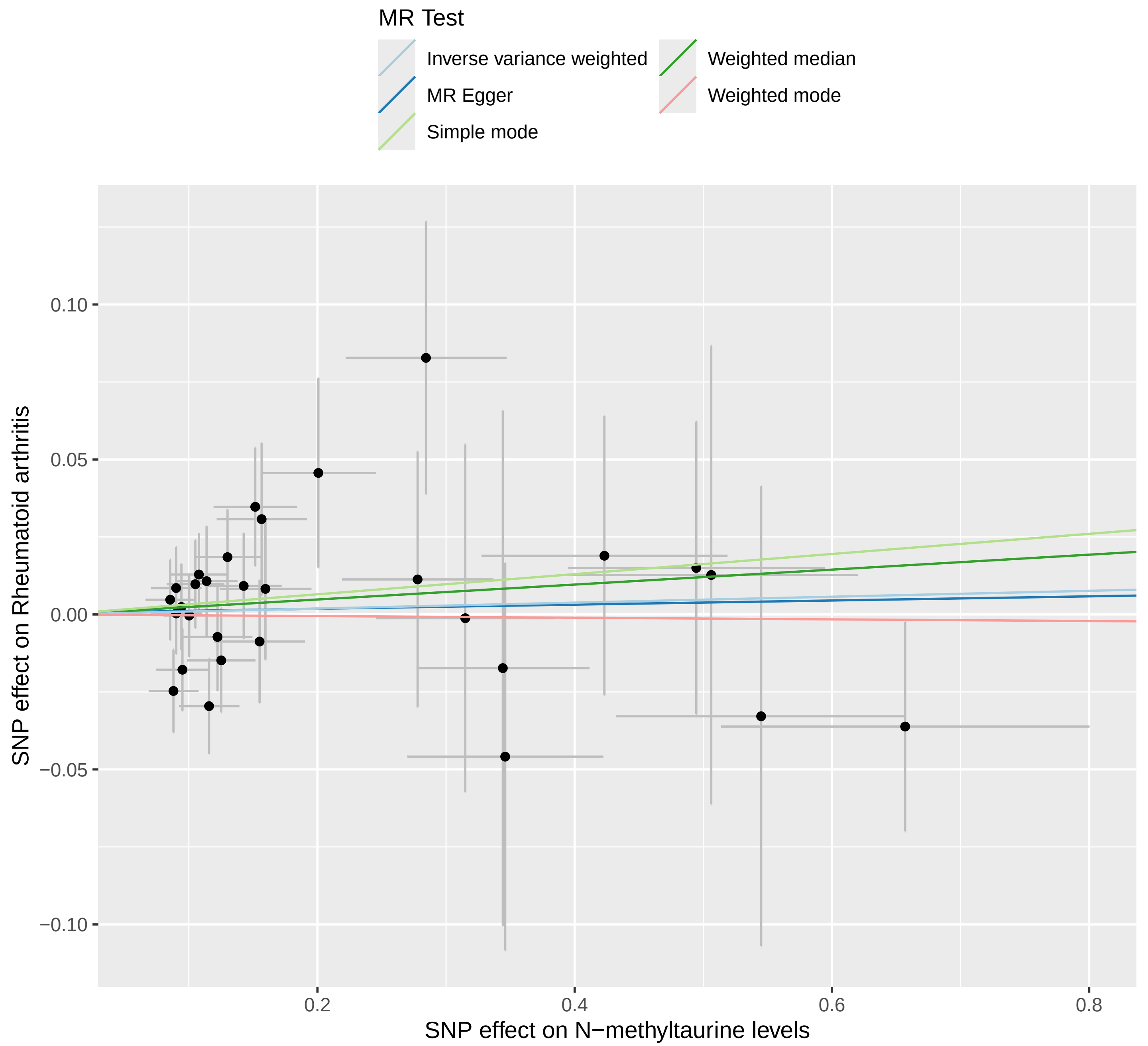

MR Test
Inverse variance weighted
MR Egger
Simple mode
Weighted median
Weighted mode
0.10
0.05
0.00
−0.05
−0.10
SNP effect on Rheumatoid arthritis
0.2
0.4
0.6
0.8
SNP effect on N−methyltaurine levels

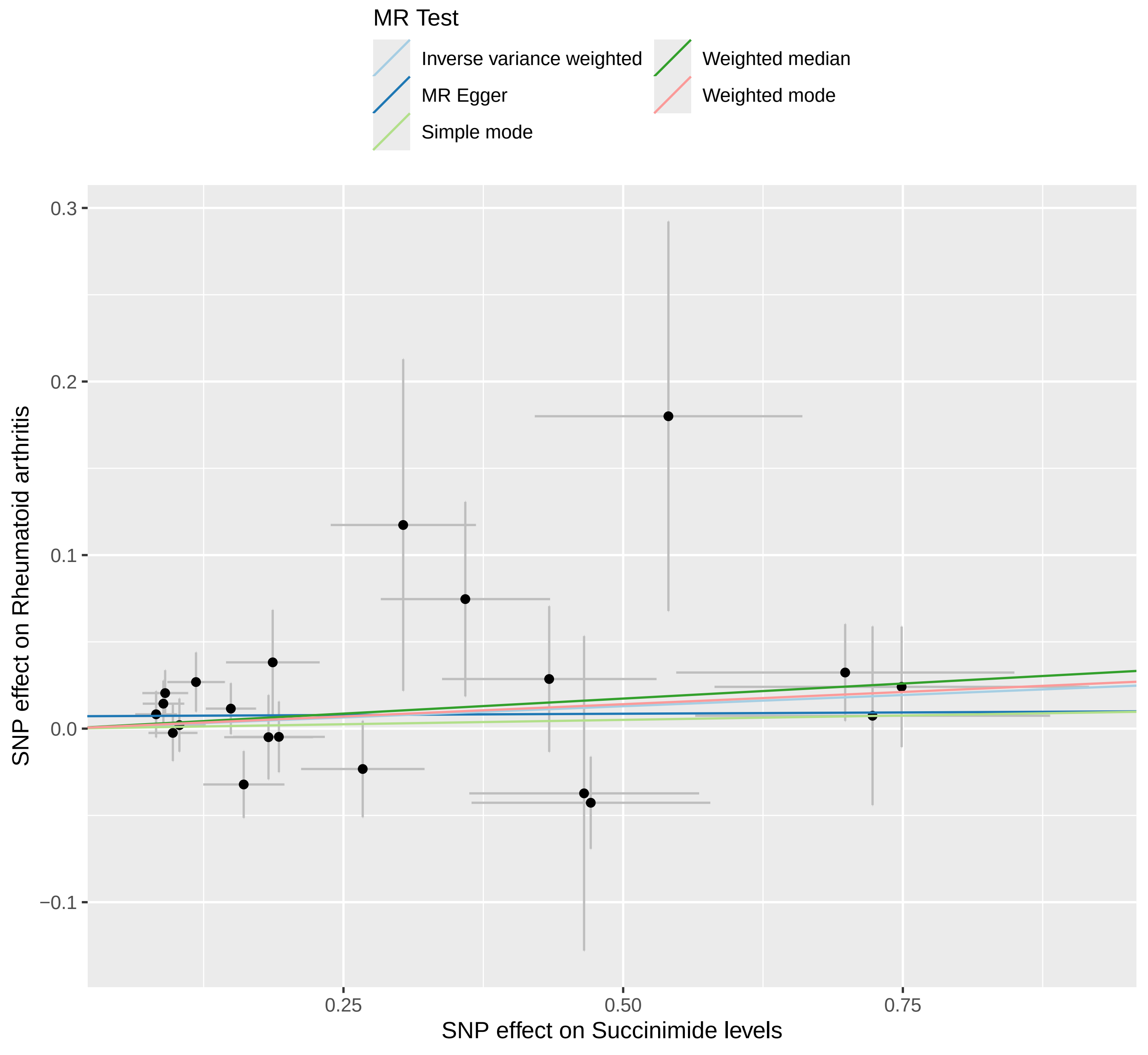

MR Test
Inverse variance weighted
MR Egger
Simple mode
Weighted median
Weighted mode
0.3
0.2
0.1
0.0
−0.1
0.25
0.50
0.75
SNP effect on Rheumatoid arthritis
SNP effect on Succinimide levels

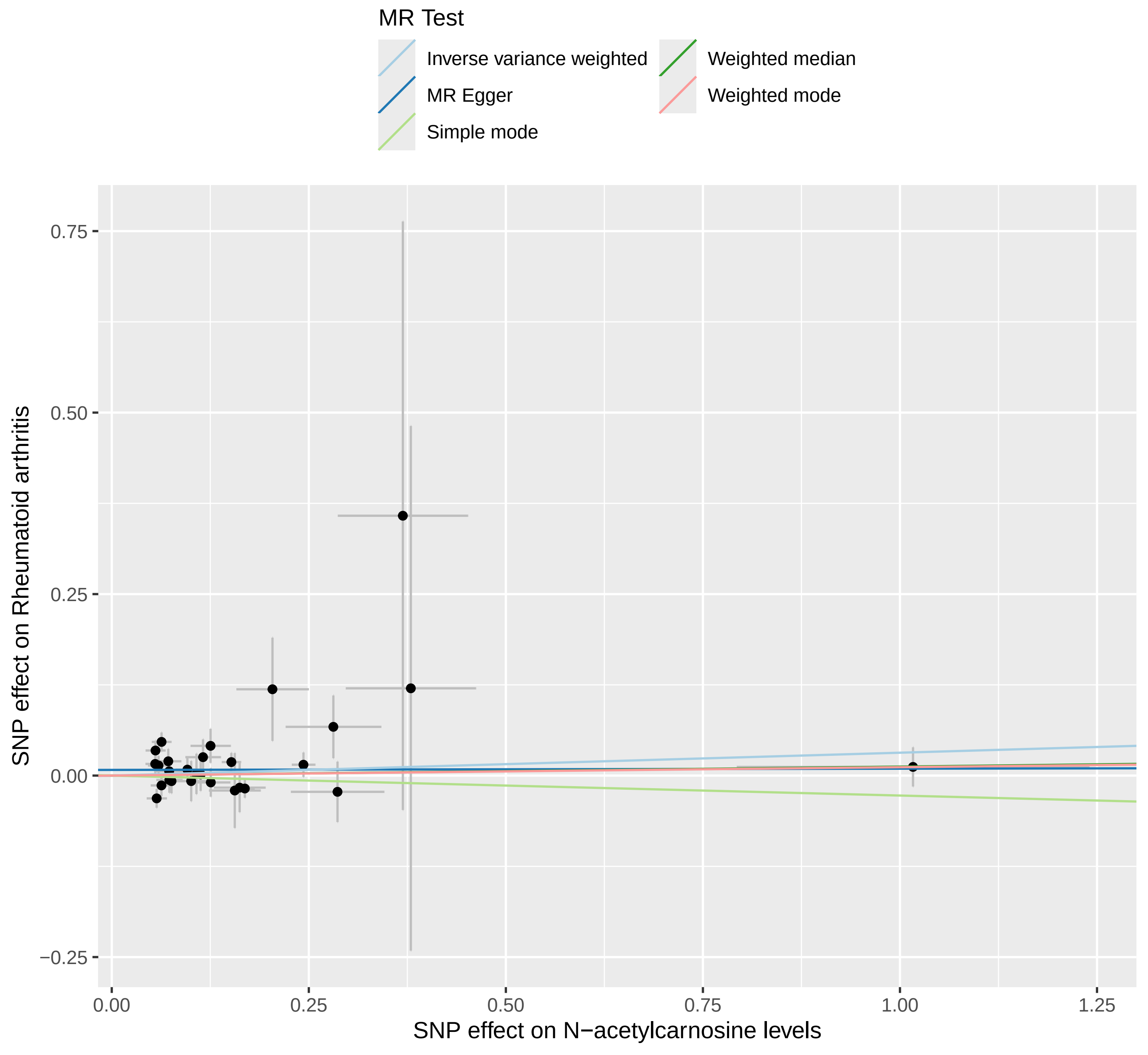
MR Test
Inverse variance weighted
MR Egger
Simple mode
Weighted median
Weighted mode
0.75
0.50
0.25
0.00
−0.25
0.00
0.25
0.50
0.75
1.00
1.25
SNP effect on Rheumatoid arthritis
SNP effect on N−acetylcarnosine levels

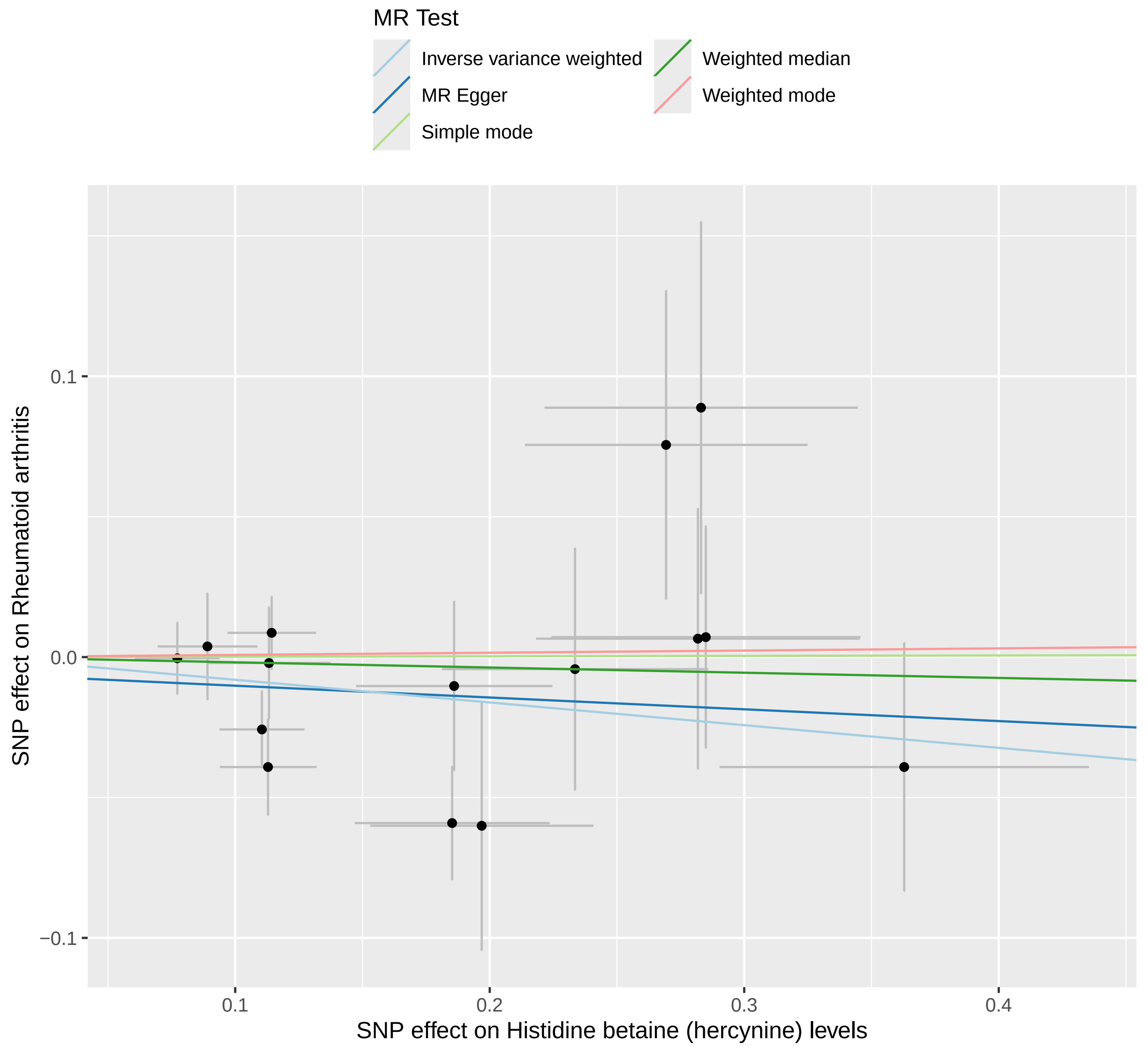

MR Test
Inverse variance weighted
MR Egger
Simple mode
Weighted median
Weighted mode
SNP effect on Rheumatoid arthritis
0.1
0.0
−0.1
0.1
0.2
0.3
0.4
SNP effect on Histidine betaine (hercynine) levels

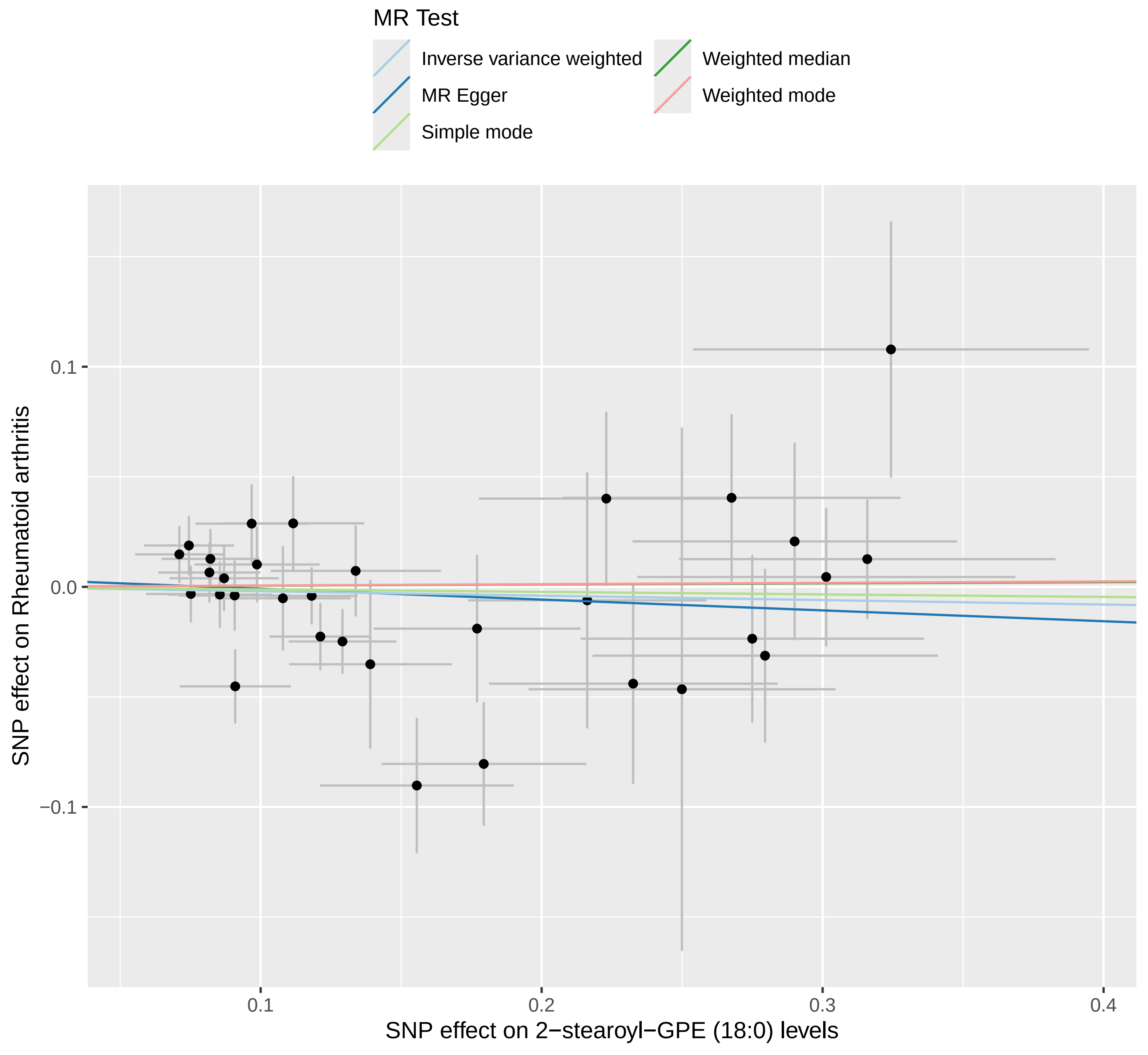

MR Test
Inverse variance weighted
MR Egger
Simple mode
Weighted median
Weighted mode
0.1
0.0
−0.1
SNP effect on Rheumatoid arthritis
0.1
0.2
0.3
0.4
SNP effect on 2−stearoyl−GPE (18:0) levels

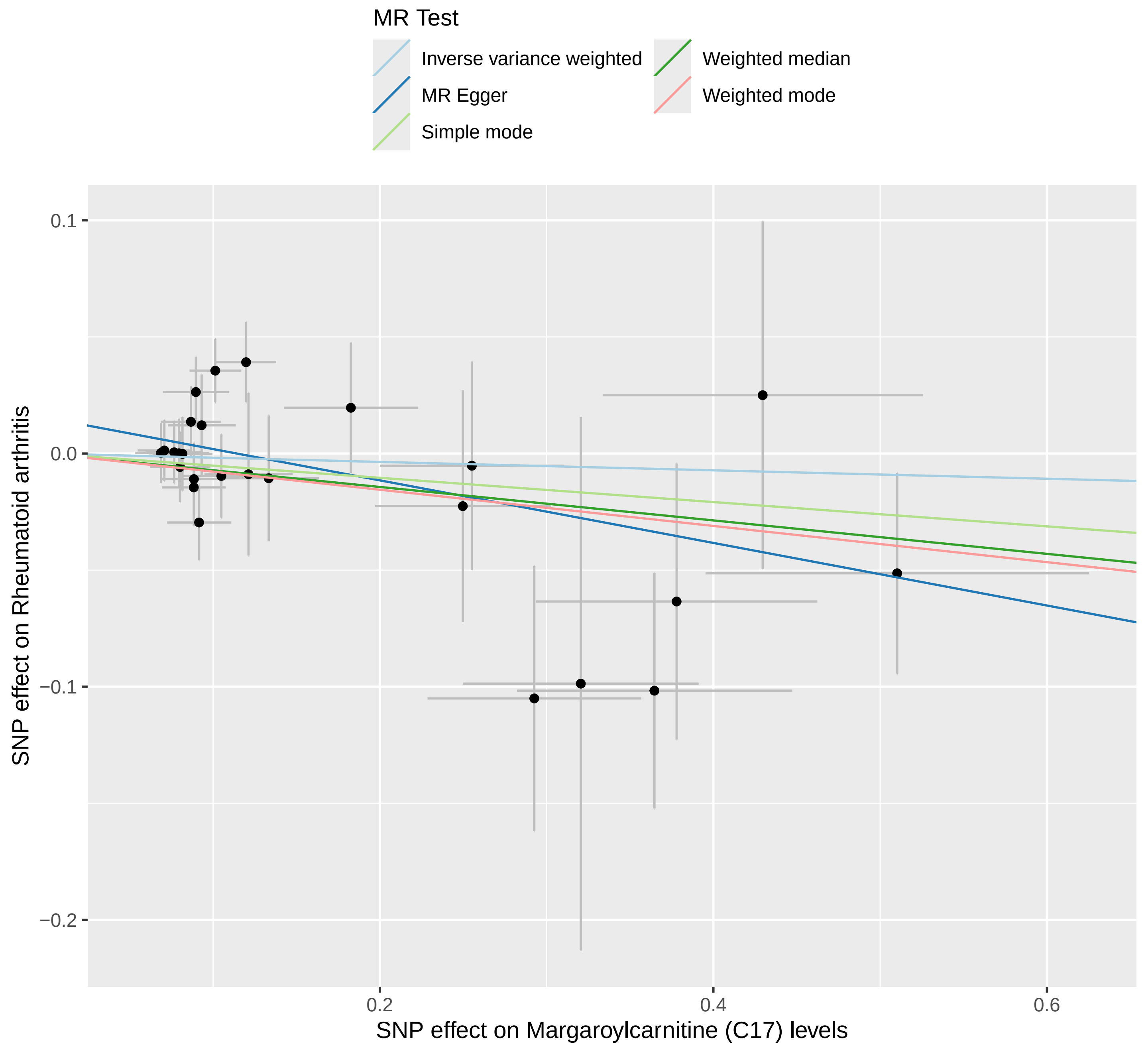
MR Test
Inverse variance weighted
MR Egger
Simple mode
Weighted median
Weighted mode
SNP effect on Rheumatoid arthritis
0.1
0.0
−0.1
−0.2
0.2
0.4
0.6
SNP effect on Margaroylcarnitine (C17) levels

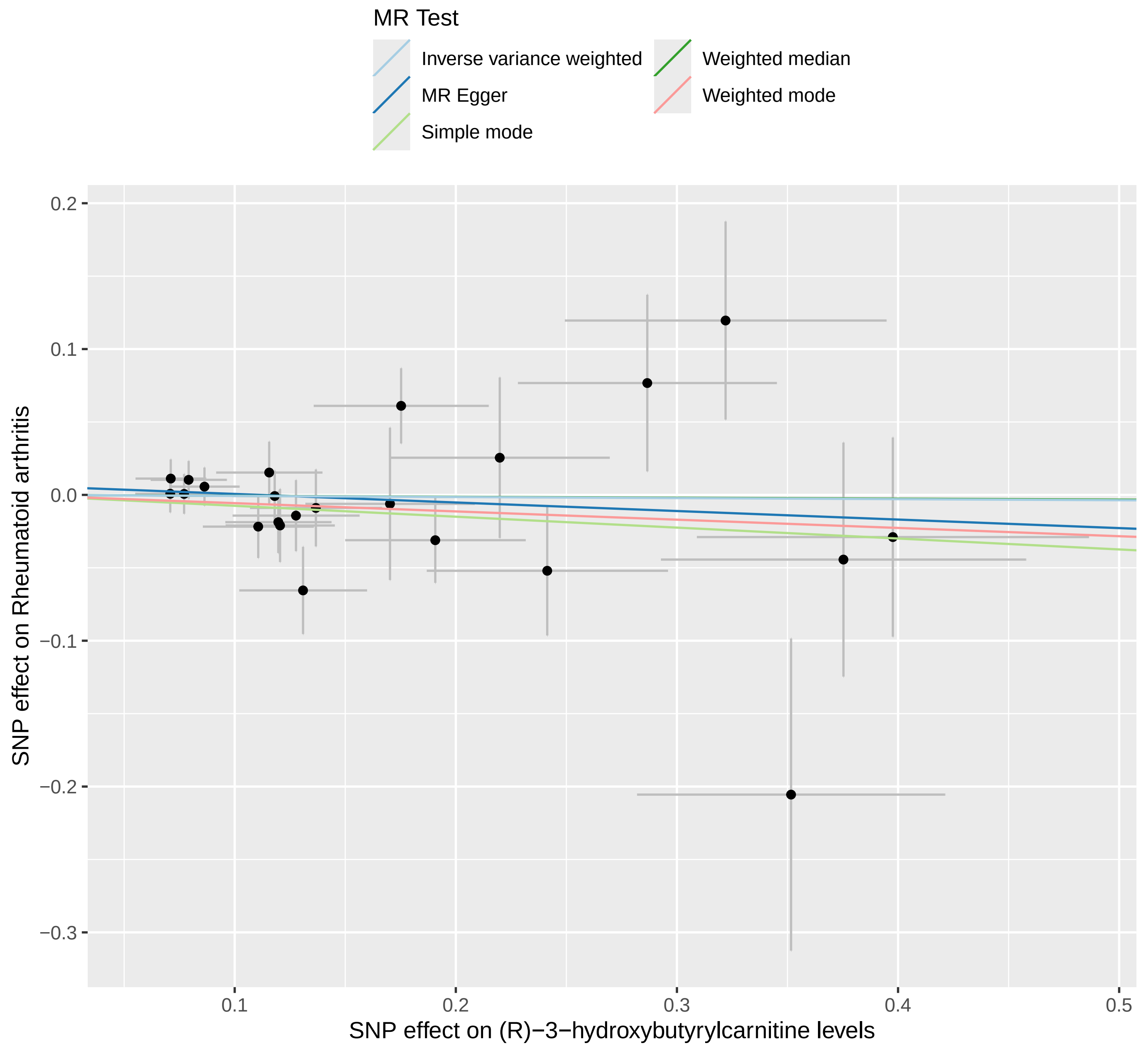

MR Test
Inverse variance weighted
MR Egger
Simple mode
Weighted median
Weighted mode
0.2
0.1
0.0
−0.1
−0.2
−0.3
SNP effect on Rheumatoid arthritis
0.1
0.2
0.3
0.4
0.5
SNP effect on (R)−3−hydroxybutyrylcarnitine levels

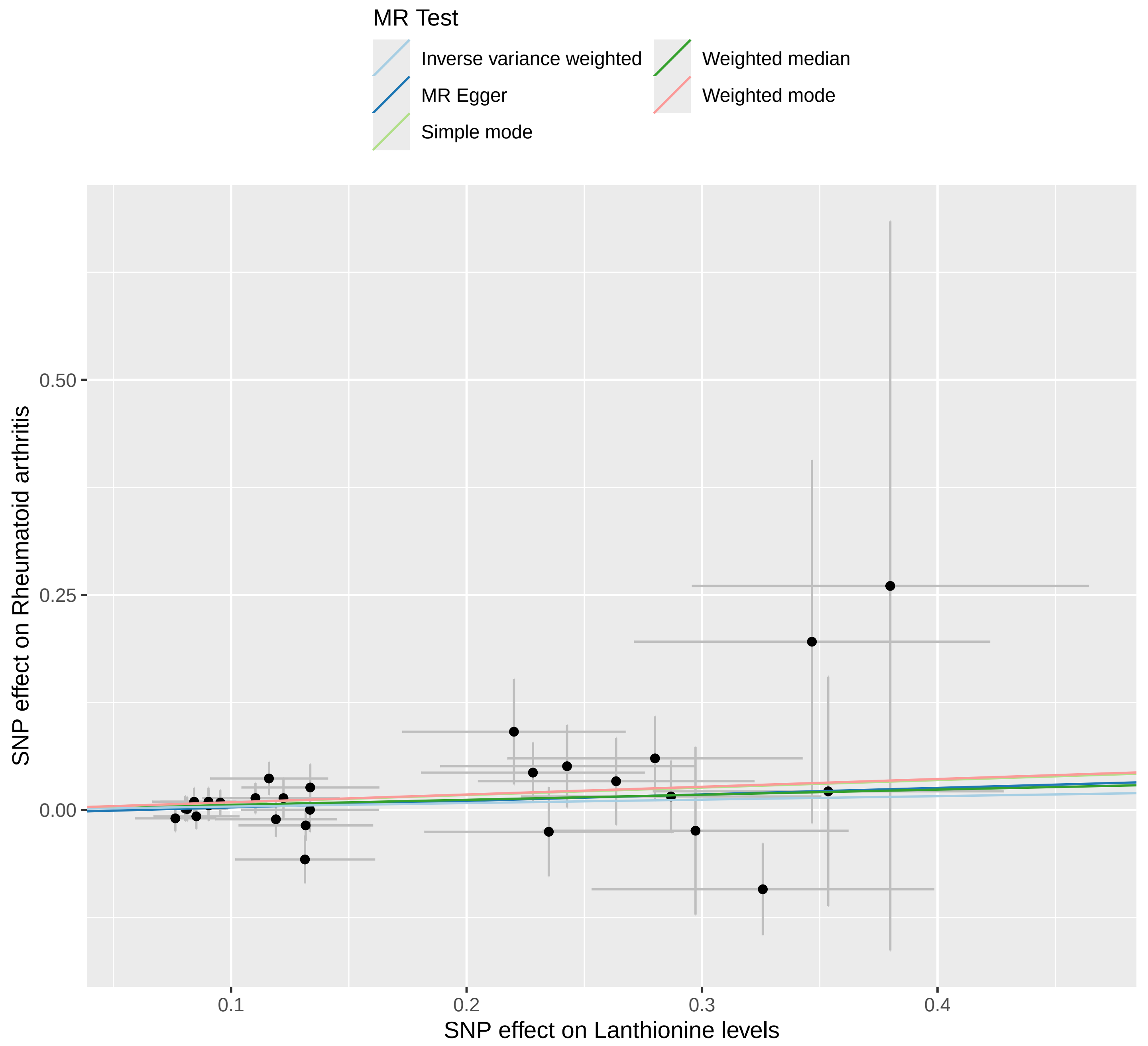

MR Test
Inverse variance weighted
MR Egger
Simple mode
Weighted median
Weighted mode
0.50
0.25
0.00
SNP effect on Rheumatoid arthritis
0.1
0.2
0.3
0.4
SNP effect on Lanthionine levels

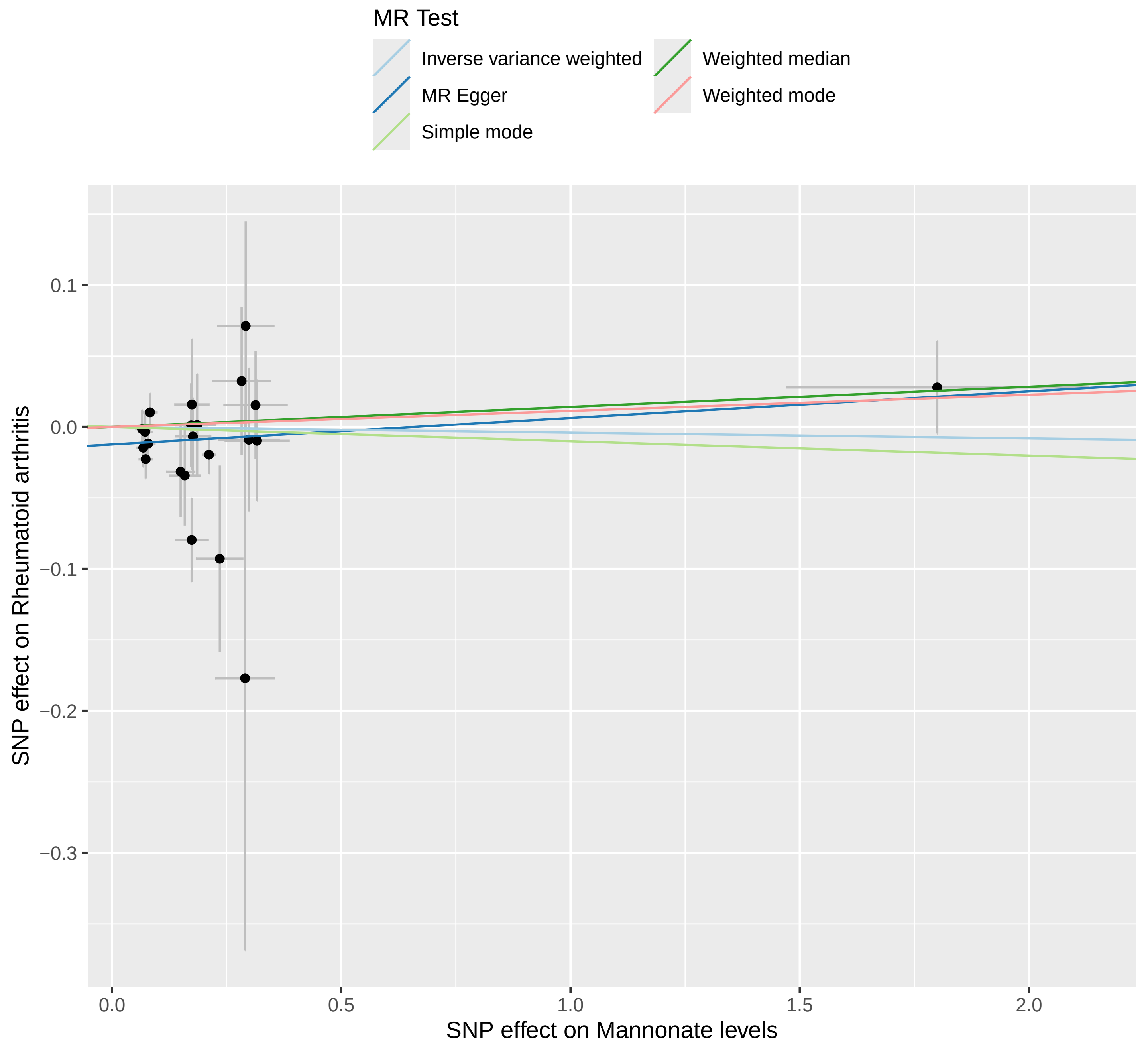
MR Test
Inverse variance weighted
MR Egger
Simple mode
Weighted median
Weighted mode
0.1
0.0
−0.1
−0.2
−0.3
0.0
0.5
1.0
1.5
2.0
SNP effect on Rheumatoid arthritis
SNP effect on Mannonate levels

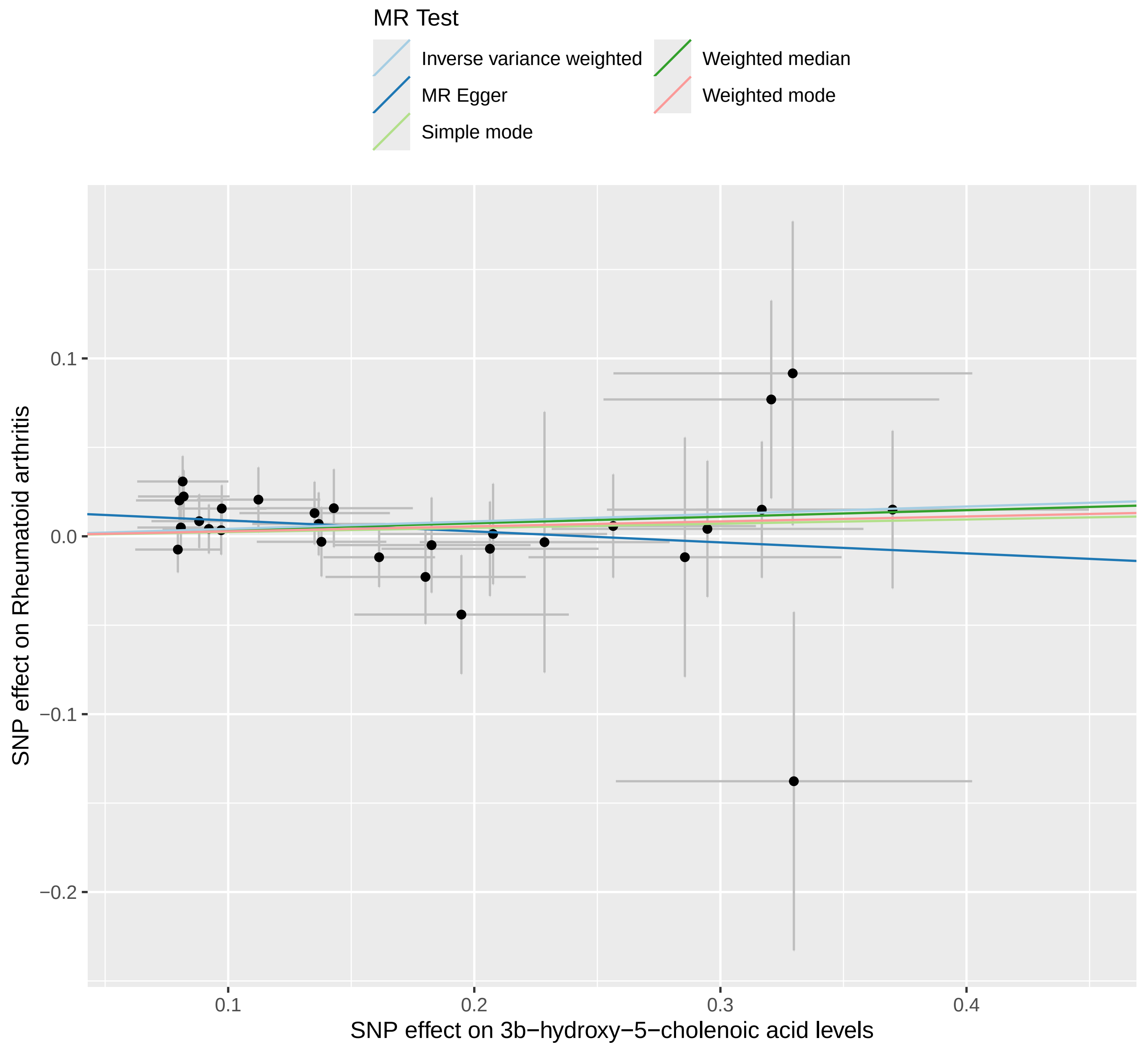

MR Test
Inverse variance weighted
MR Egger
Simple mode
Weighted median
Weighted mode
SNP effect on Rheumatoid arthritis
0.1
0.0
−0.1
−0.2
0.1
0.2
0.3
0.4
SNP effect on 3b−hydroxy−5−cholenoic acid levels

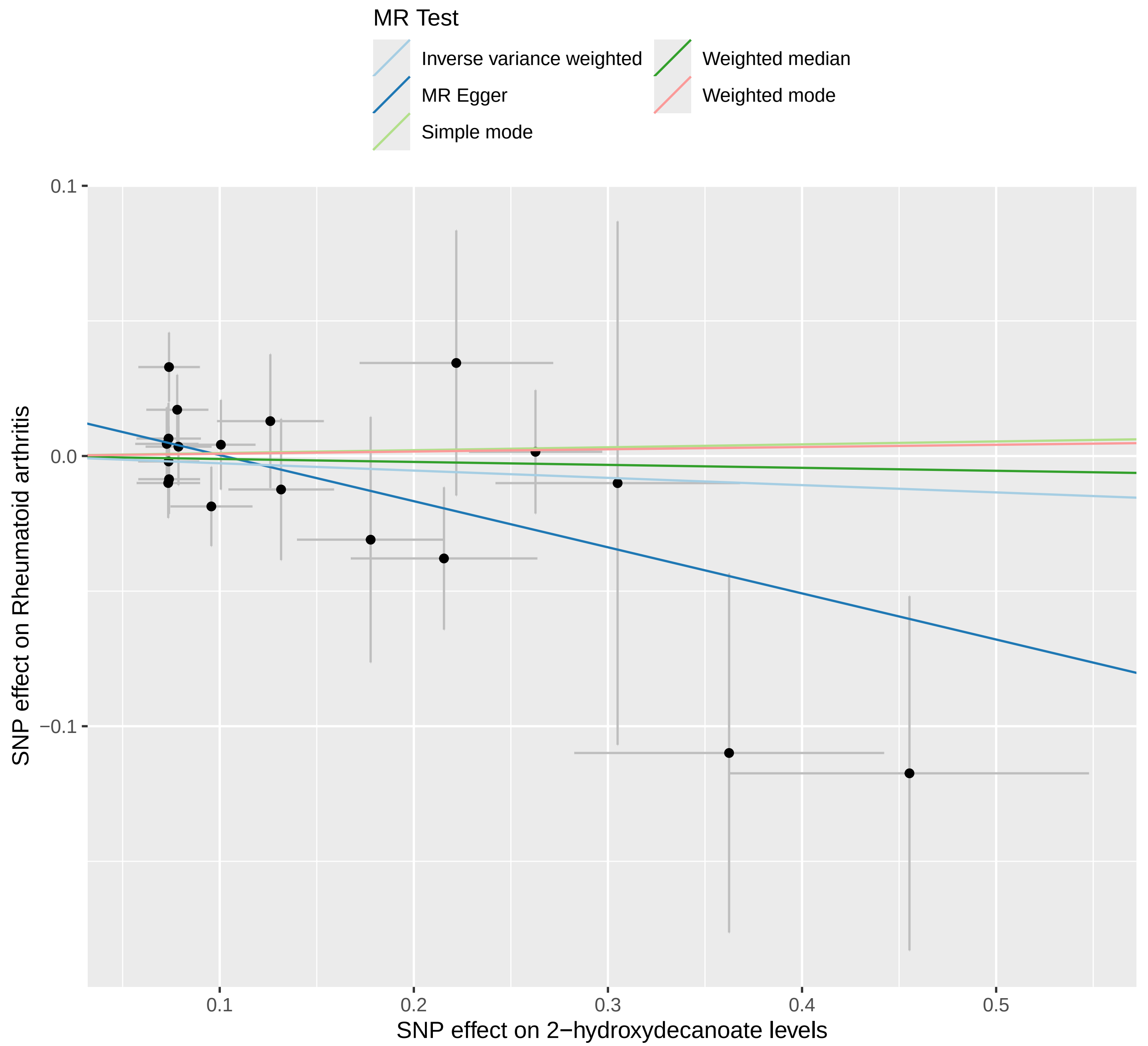
MR Test
Inverse variance weighted
MR Egger
Simple mode
Weighted median
Weighted mode
0.1
0.0
−0.1
0.1
0.2
0.3
0.4
0.5
SNP effect on Rheumatoid arthritis
SNP effect on 2−hydroxydecanoate levels

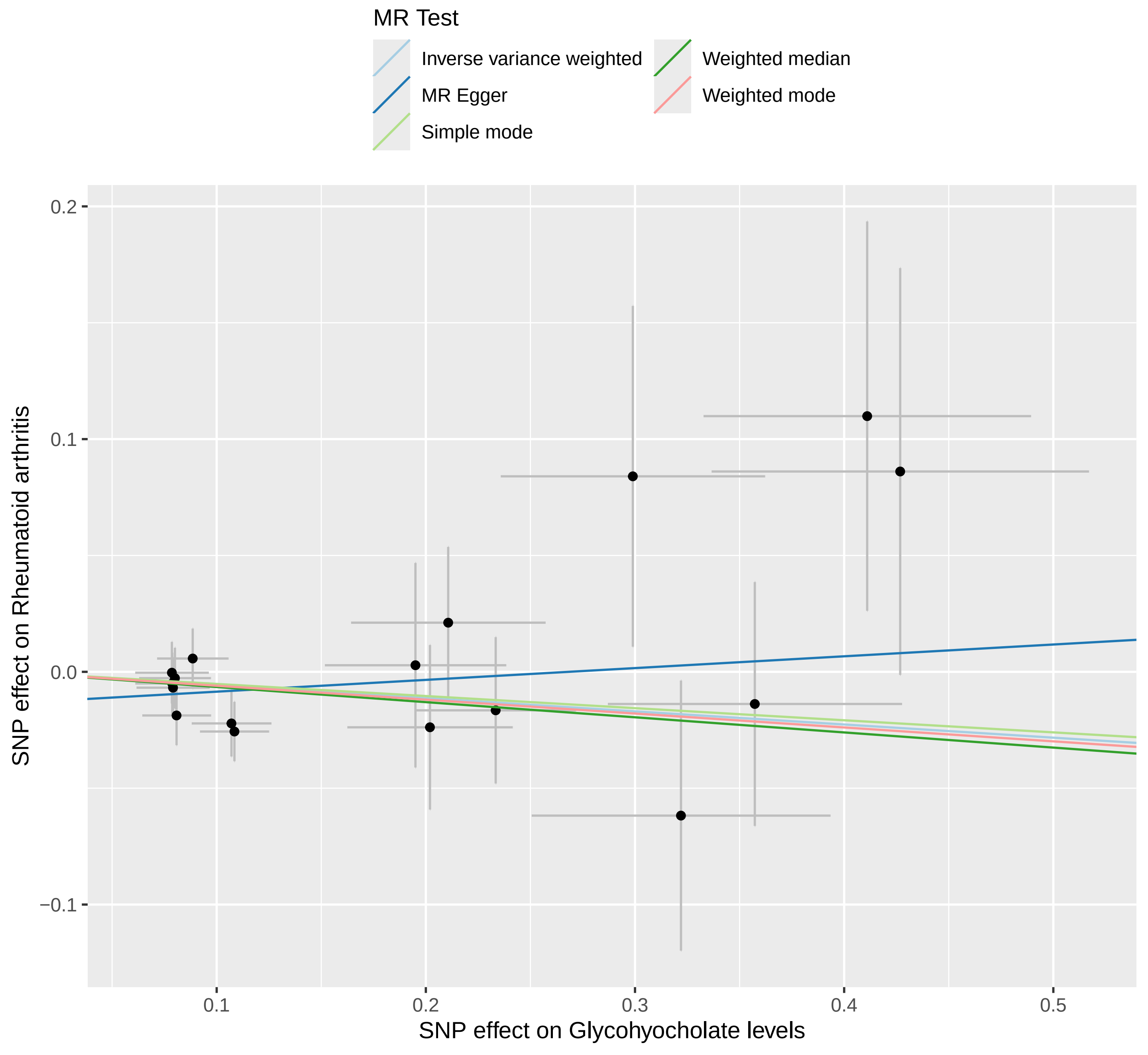
MR Test
Inverse variance weighted
MR Egger
Simple mode
Weighted median
Weighted mode
0.2
0.1
0.0
−0.1
0.1
0.2
0.3
0.4
0.5
SNP effect on Glycohyocholate levels
SNP effect on Rheumatoid arthritis

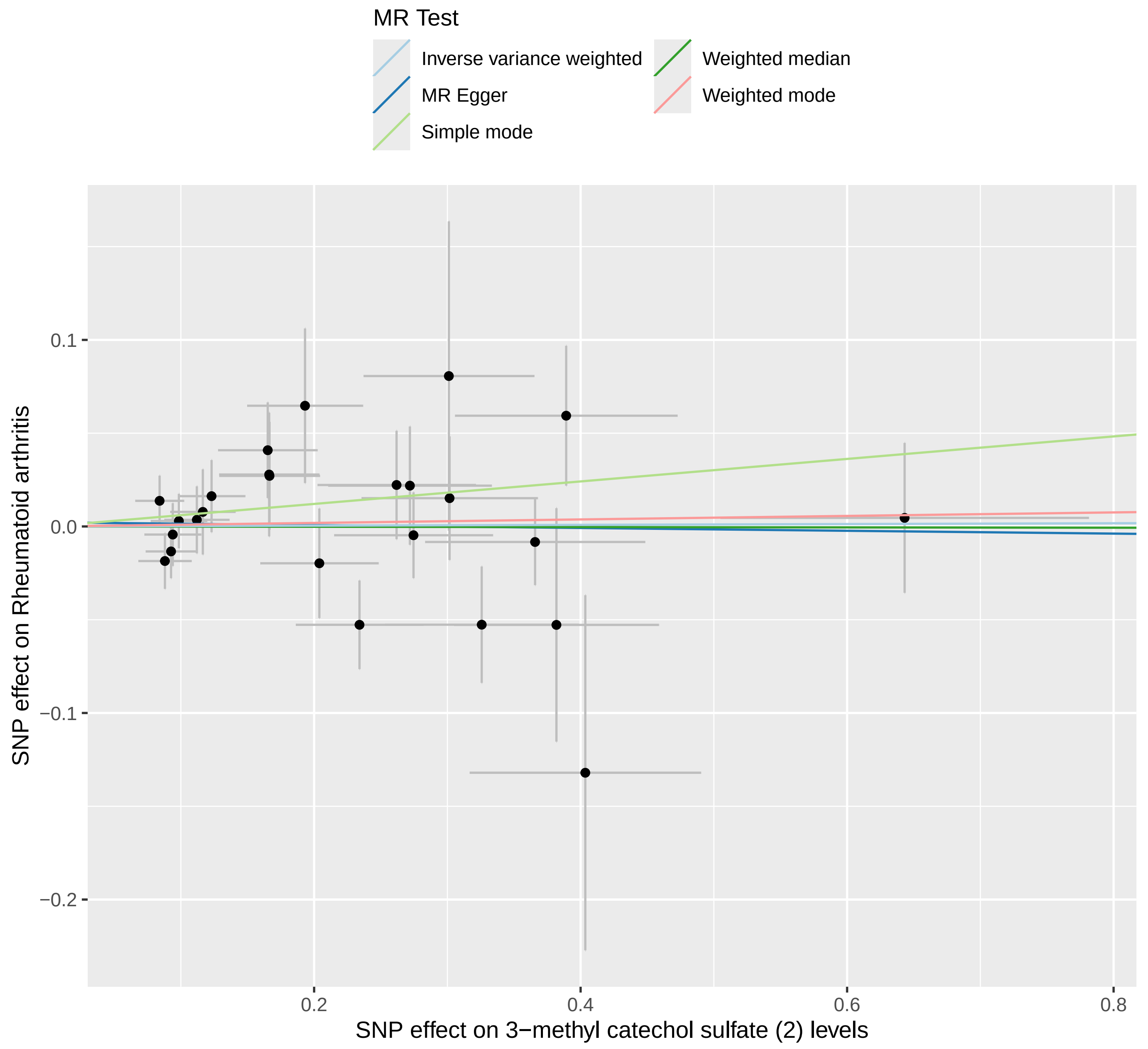
MR Test
Inverse variance weighted
Weighted median
MR Egger
Weighted mode
Simple mode
0.1
0.0
−0.1
−0.2
0.2
0.4
0.6
0.8
SNP effect on Rheumatoid arthritis
SNP effect on 3−methyl catechol sulfate (2) levels

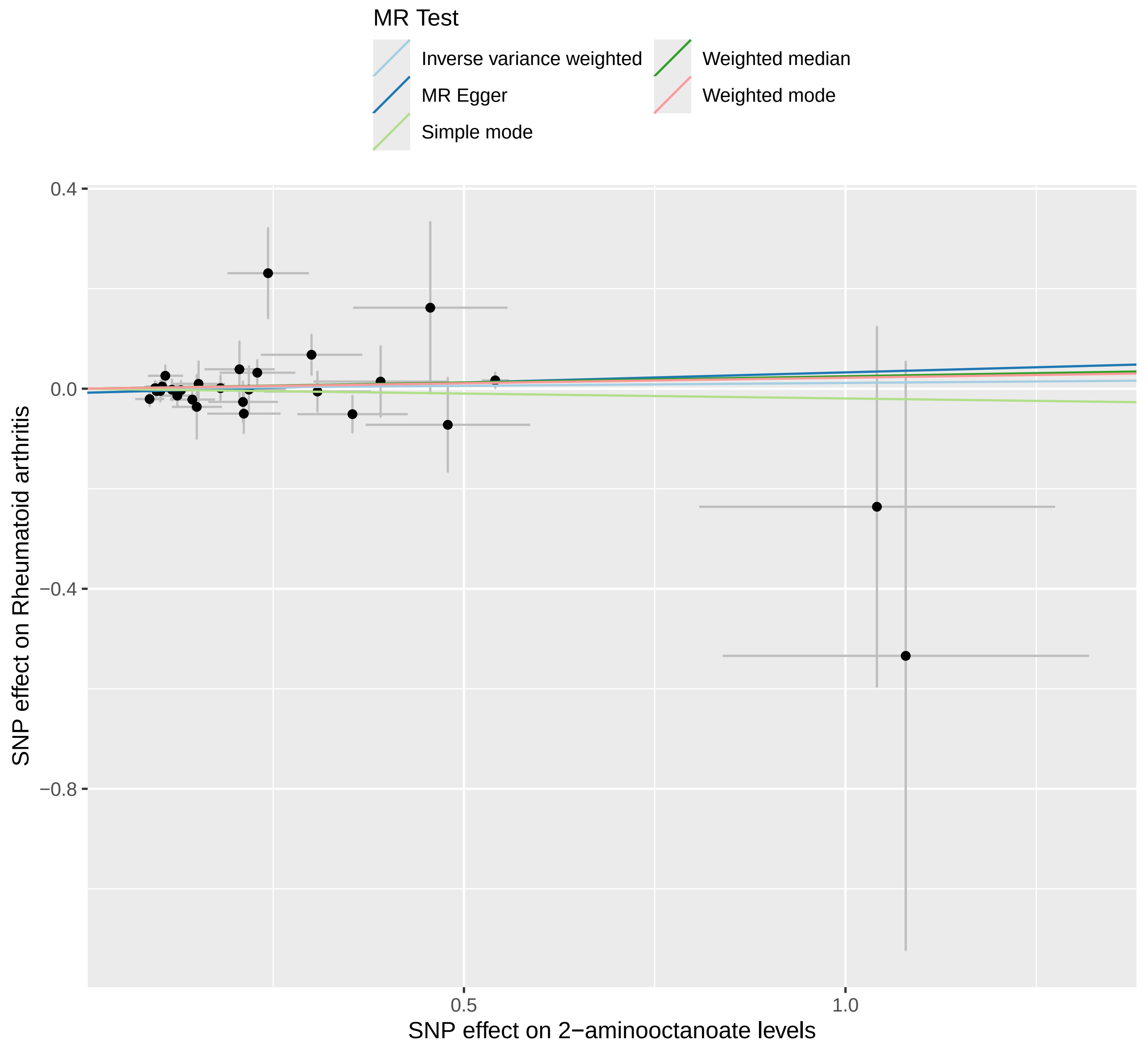

MR Test
Inverse variance weighted
MR Egger
Simple mode
Weighted median
Weighted mode
0.4
0.0
−0.4
−0.8
SNP effect on Rheumatoid arthritis
0.5
1.0
SNP effect on 2−aminooctanoate levels

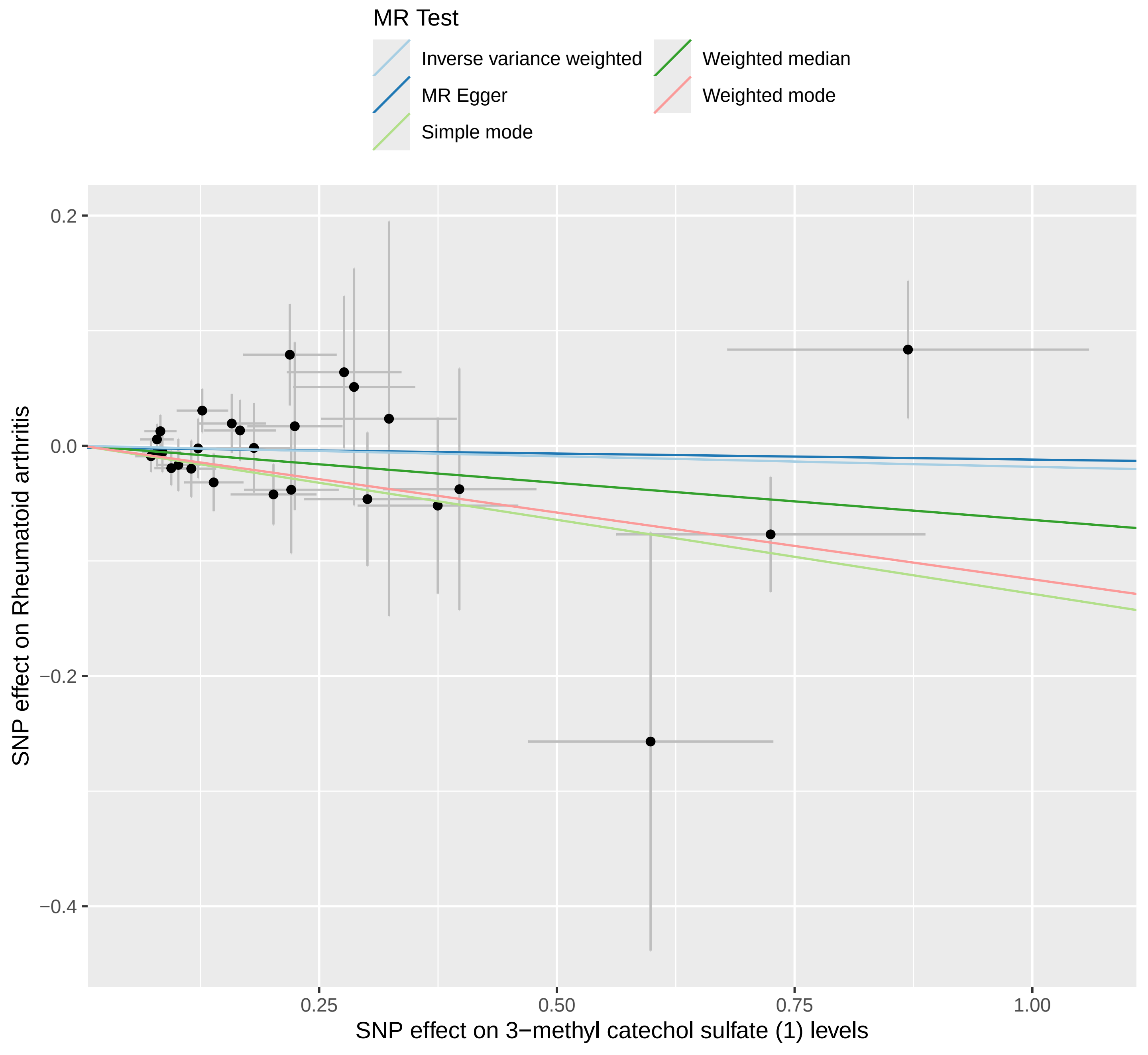
MR Test
Inverse variance weighted
MR Egger
Simple mode
Weighted median
Weighted mode
0.2
0.0
−0.2
−0.4
SNP effect on Rheumatoid arthritis
0.25
0.50
0.75
1.00
SNP effect on 3−methyl catechol sulfate (1) levels

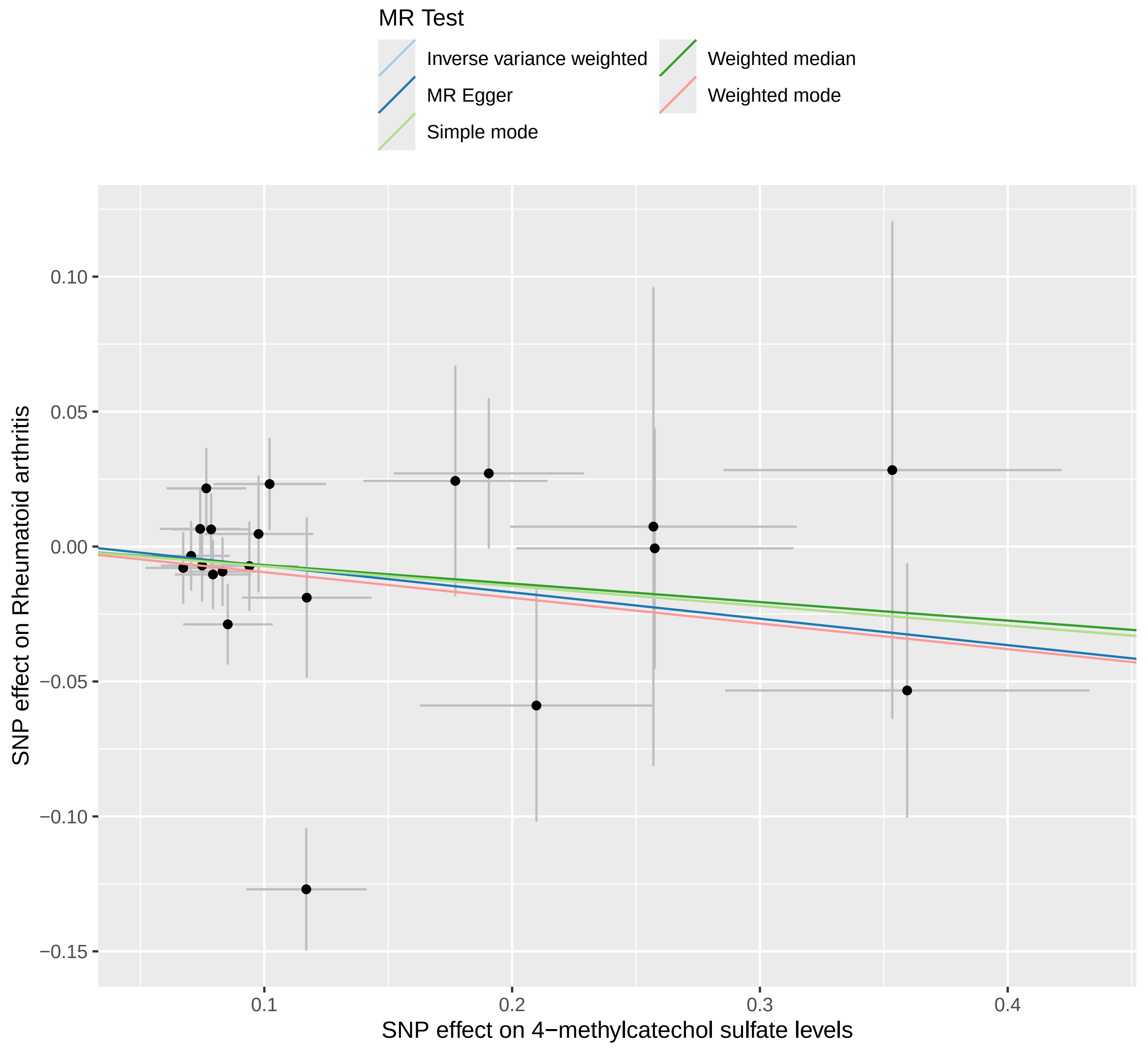
MR Test
Inverse variance weighted
MR Egger
Simple mode
Weighted median
Weighted mode
0.10
0.05
0.00
−0.05
−0.10
−0.15
0.1
0.2
0.3
0.4
SNP effect on Rheumatoid arthritis
SNP effect on 4−methylcatechol sulfate levels

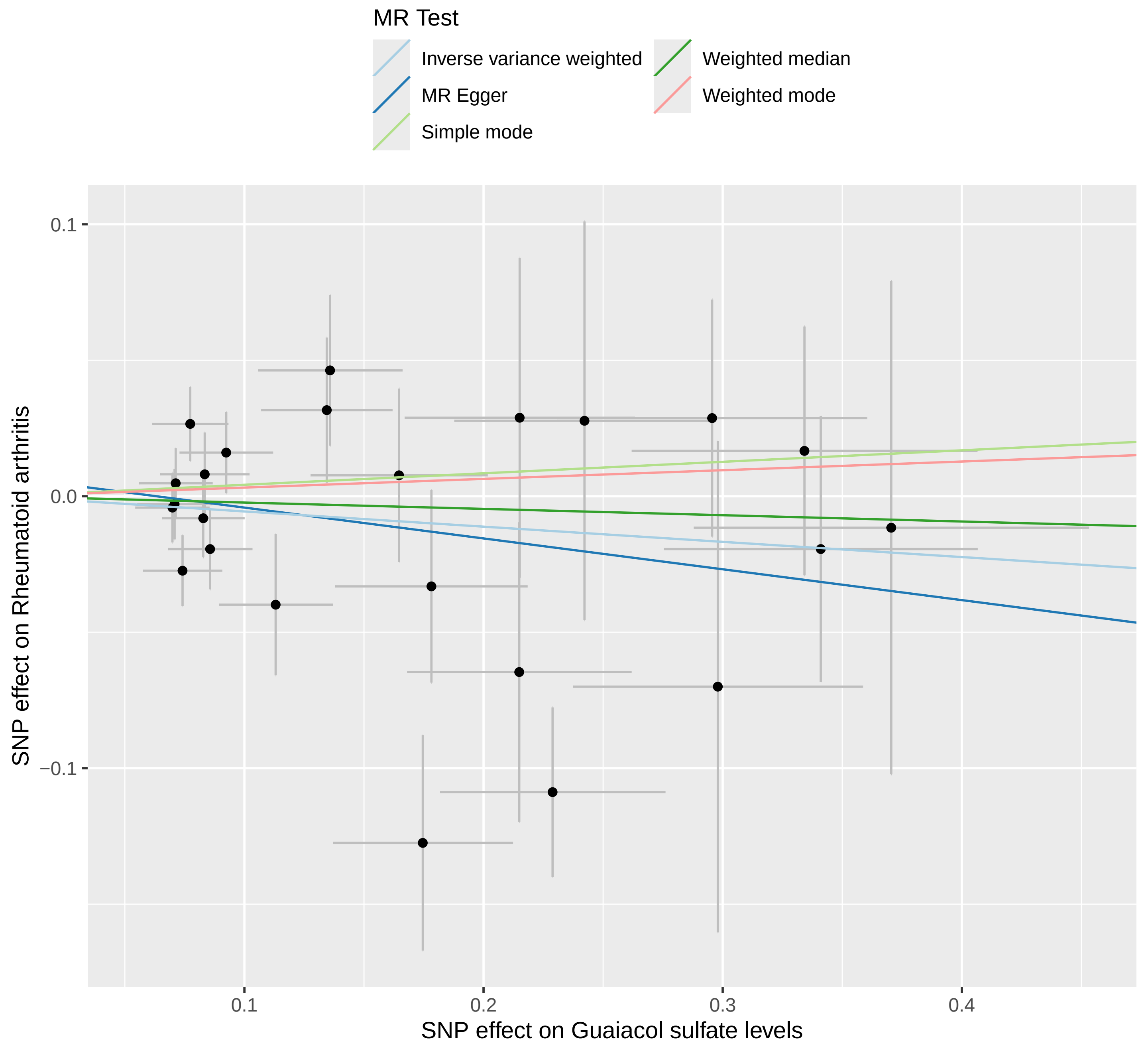
MR Test
Inverse variance weighted
MR Egger
Simple mode
Weighted median
Weighted mode
0.1
0.0
−0.1
0.1
0.2
0.3
0.4
SNP effect on Rheumatoid arthritis
SNP effect on Guaiacol sulfate levels

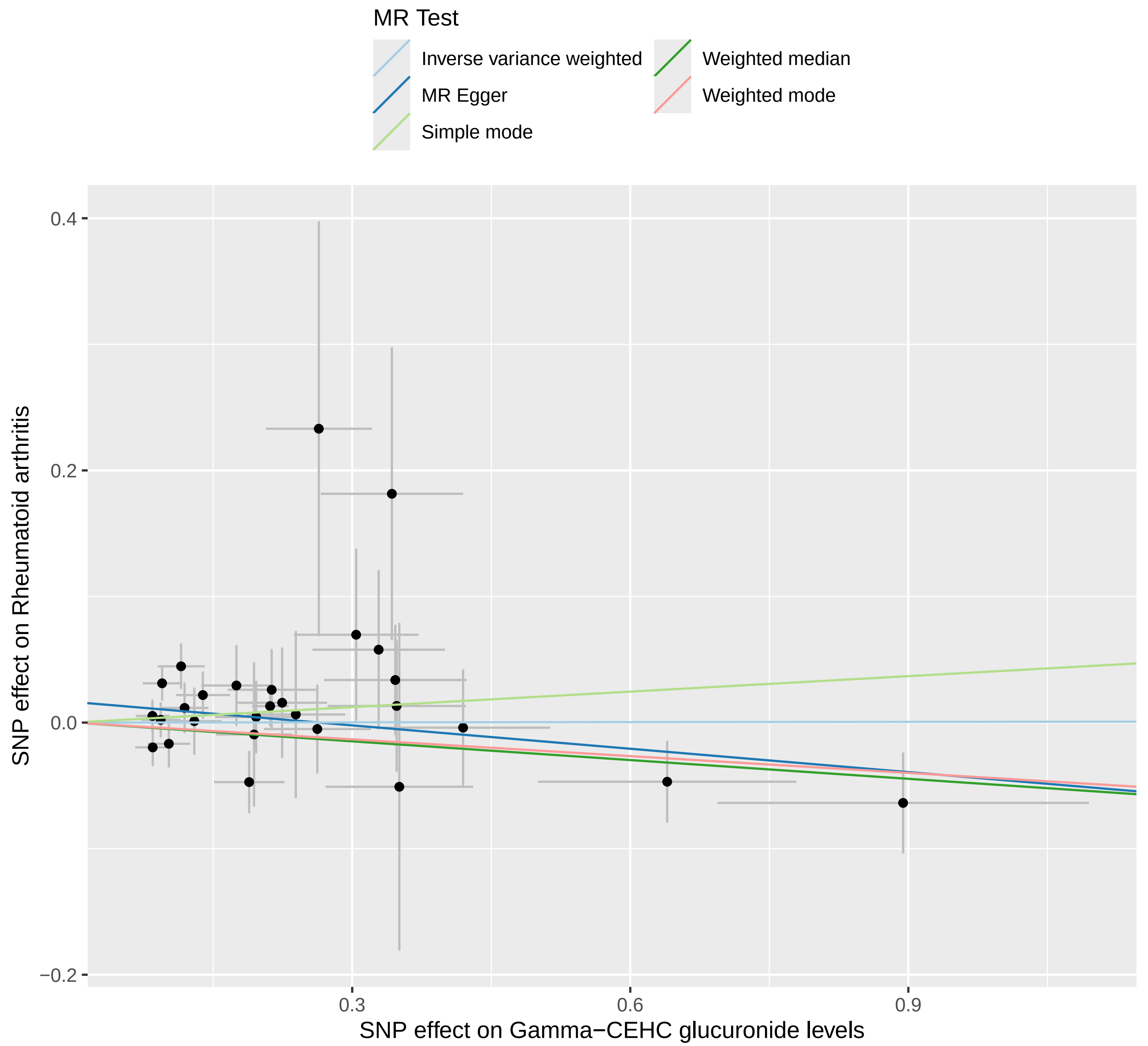

MR Test
Inverse variance weighted
MR Egger
Simple mode
Weighted median
Weighted mode
0.4
0.2
0.0
−0.2
0.3
0.6
0.9
SNP effect on Rheumatoid arthritis
SNP effect on Gamma−CEHC glucuronide levels

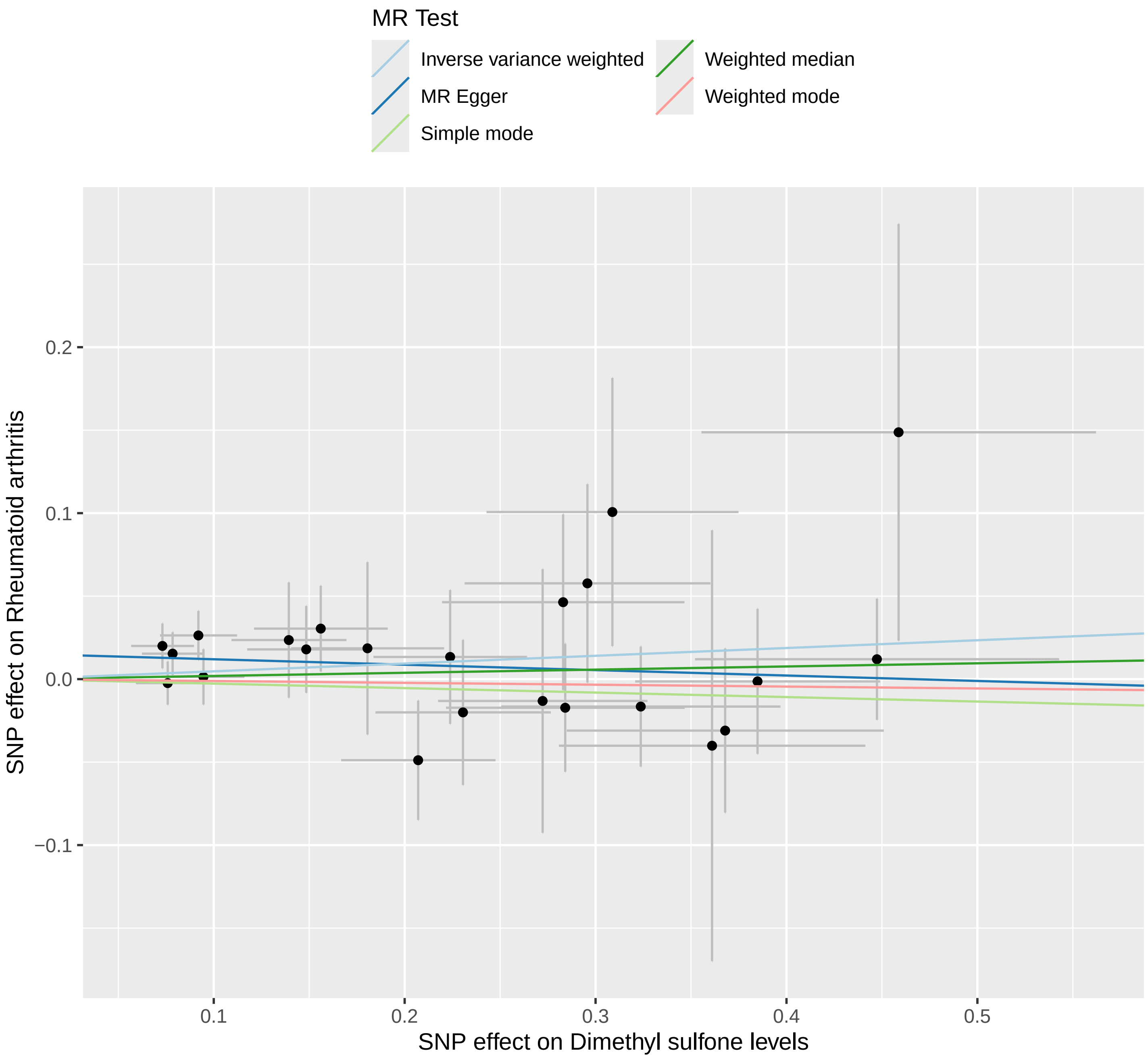

MR Test
Inverse variance weighted
MR Egger
Simple mode
Weighted median
Weighted mode
SNP effect on Rheumatoid arthritis
0.2
0.1
0.0
−0.1
0.1
0.2
0.3
0.4
0.5
SNP effect on Dimethyl sulfone levels

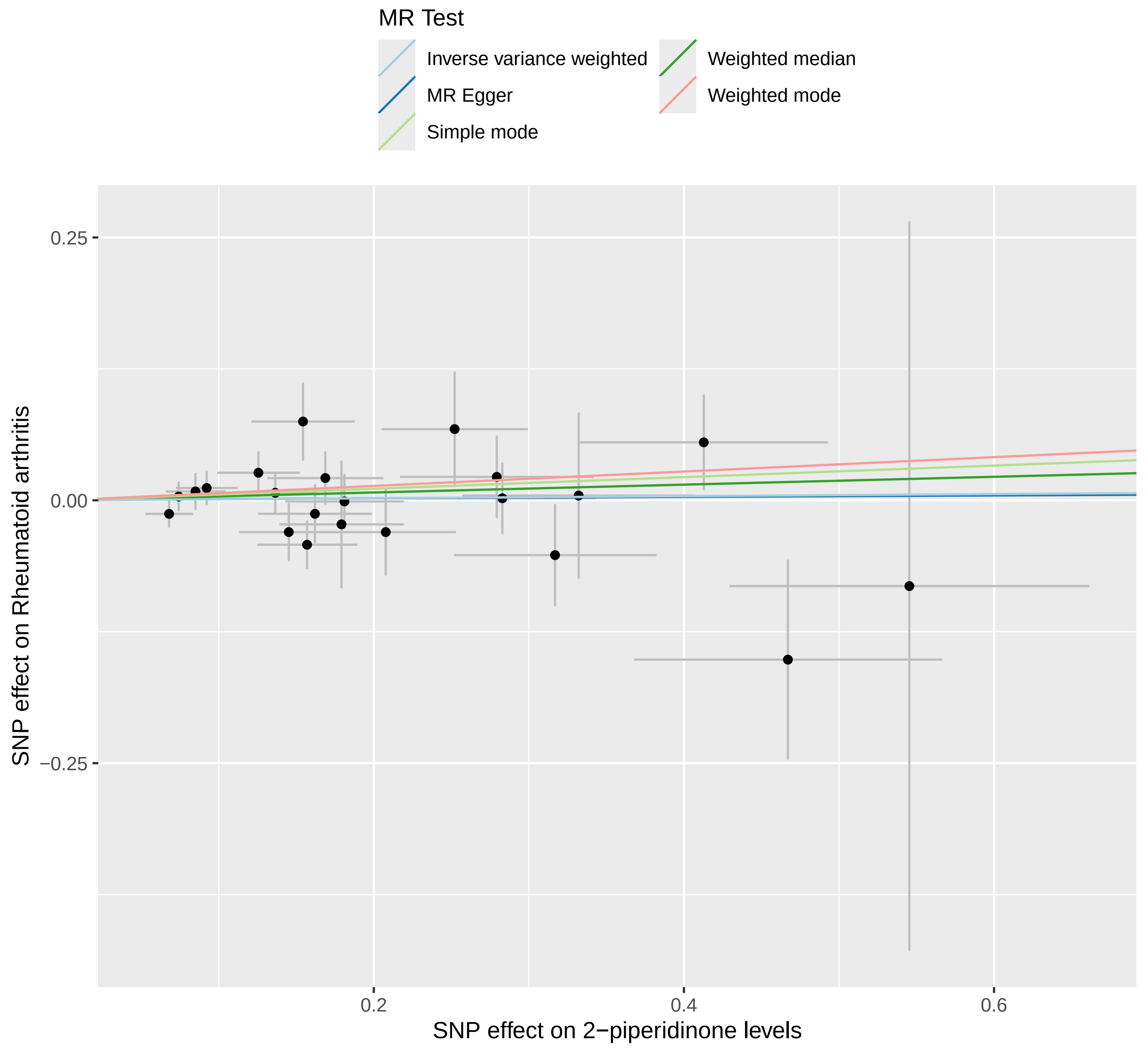
MR Test
Inverse variance weighted
MR Egger
Simple mode
Weighted median
Weighted mode
0.25
0.00
−0.25
0.2
0.4
0.6
SNP effect on Rheumatoid arthritis
SNP effect on 2−piperidinone levels

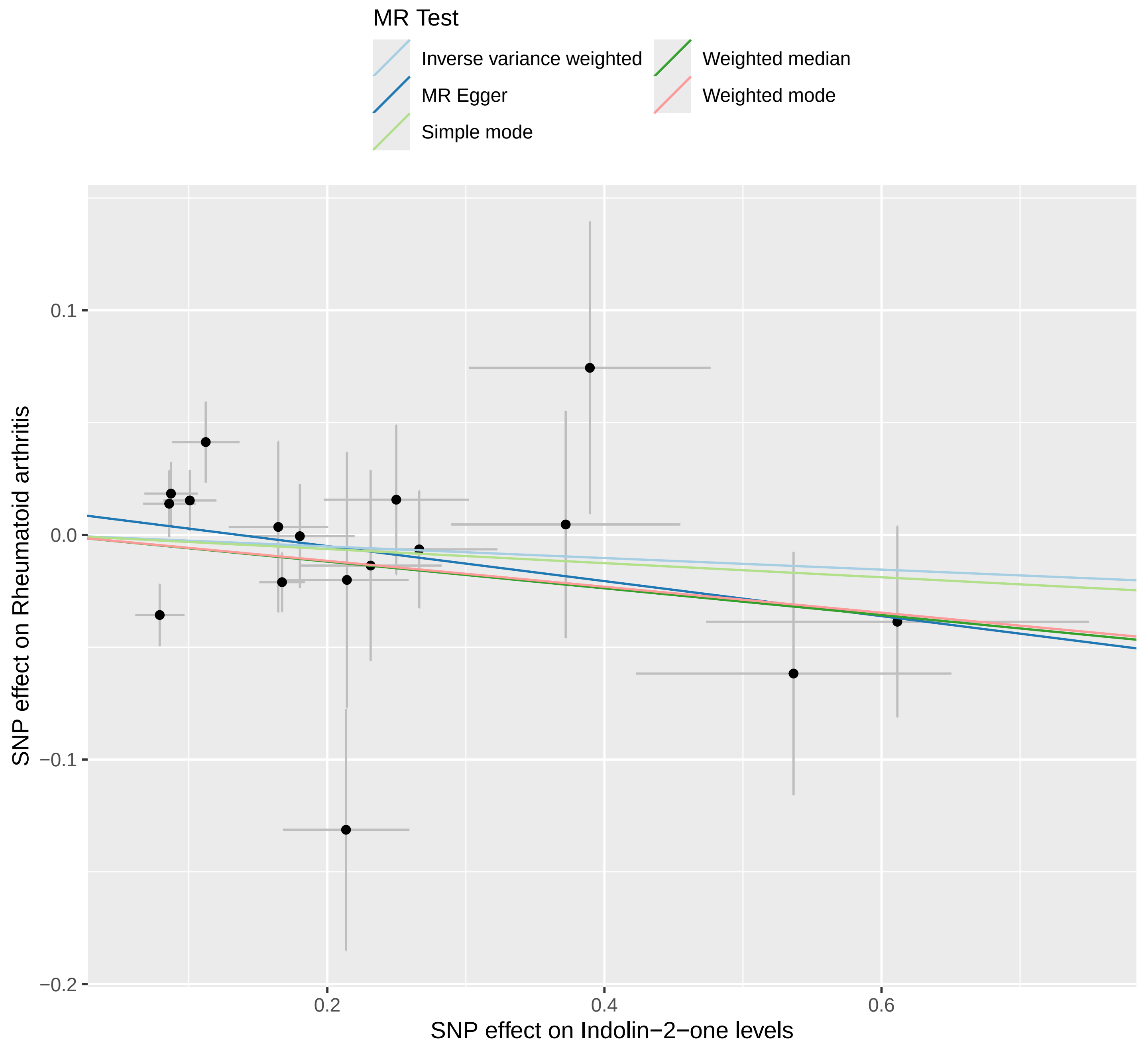
MR Test
Inverse variance weighted
MR Egger
Simple mode
Weighted median
Weighted mode
0.1
0.0
−0.1
−0.2
0.2
0.4
0.6
SNP effect on Rheumatoid arthritis
SNP effect on Indolin−2−one levels

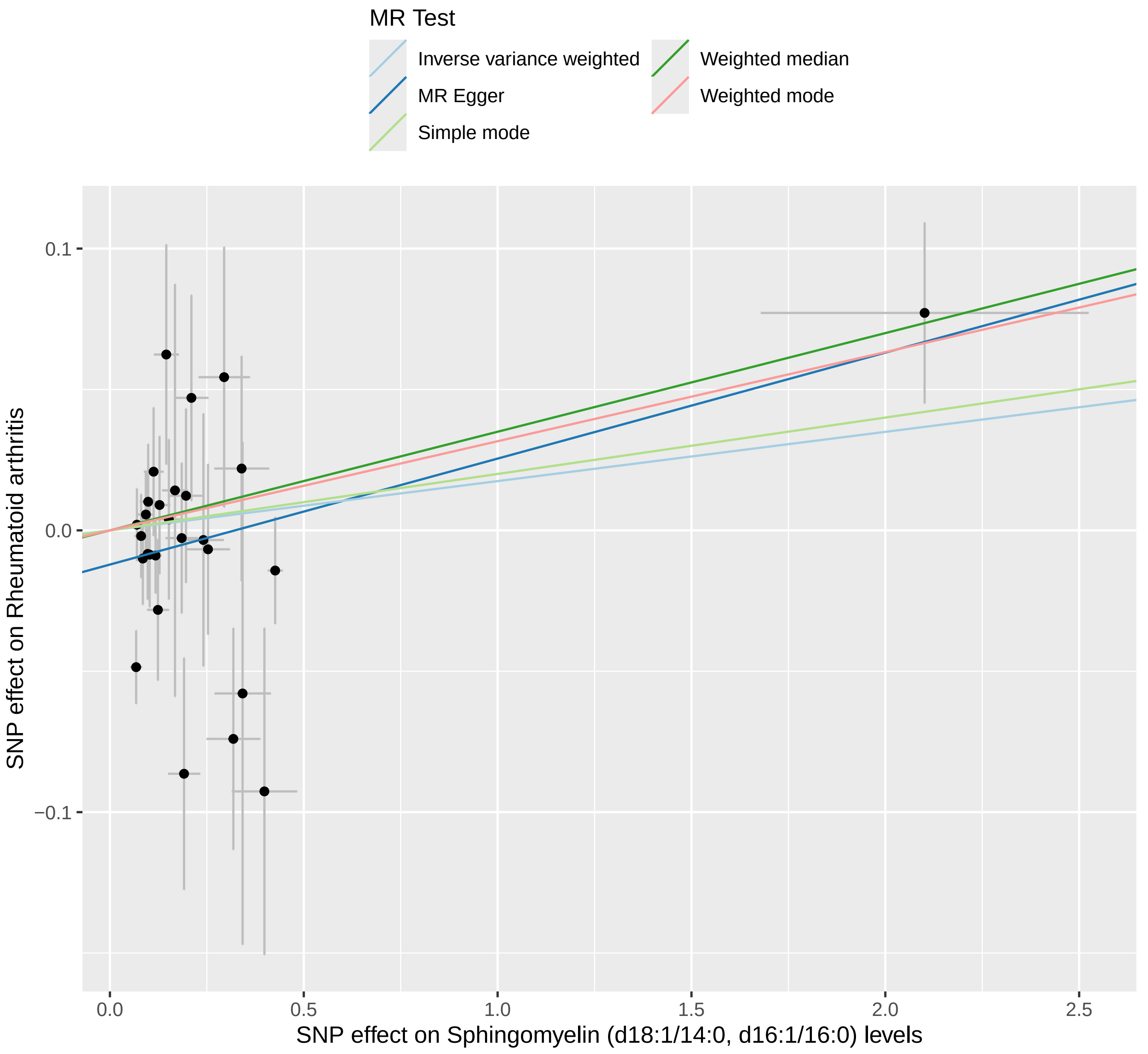
MR Test
Inverse variance weighted
MR Egger
Simple mode
Weighted median
Weighted mode
0.1
0.0
−0.1
0.0
0.5
1.0
1.5
2.0
2.5
SNP effect on Rheumatoid arthritis
SNP effect on Sphingomyelin (d18:1/14:0, d16:1/16:0) levels

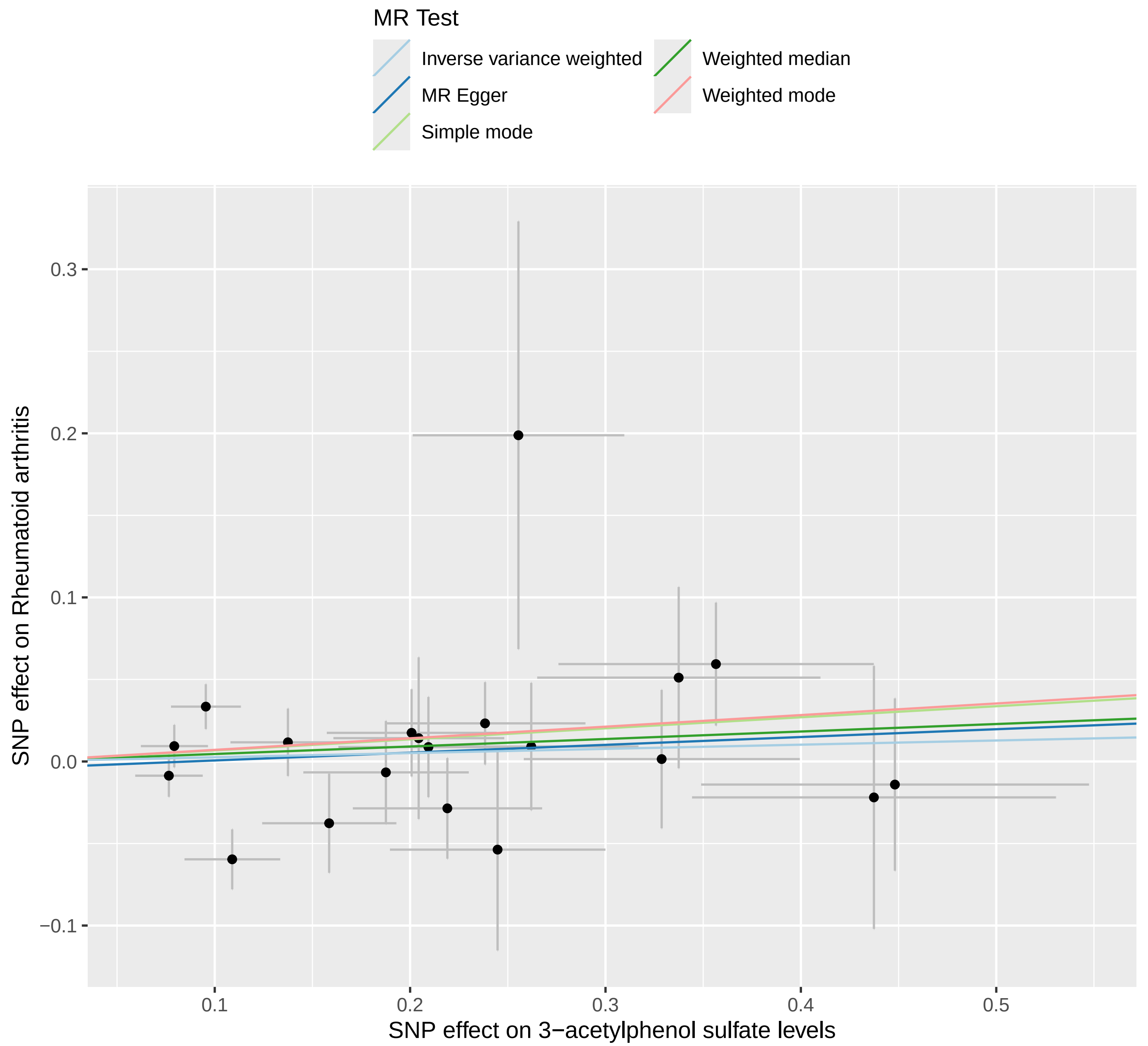

MR Test
Inverse variance weighted
MR Egger
Simple mode
Weighted median
Weighted mode
0.3
0.2
0.1
0.0
−0.1
0.1
0.2
0.3
0.4
0.5
SNP effect on Rheumatoid arthritis
SNP effect on 3−acetylphenol sulfate levels

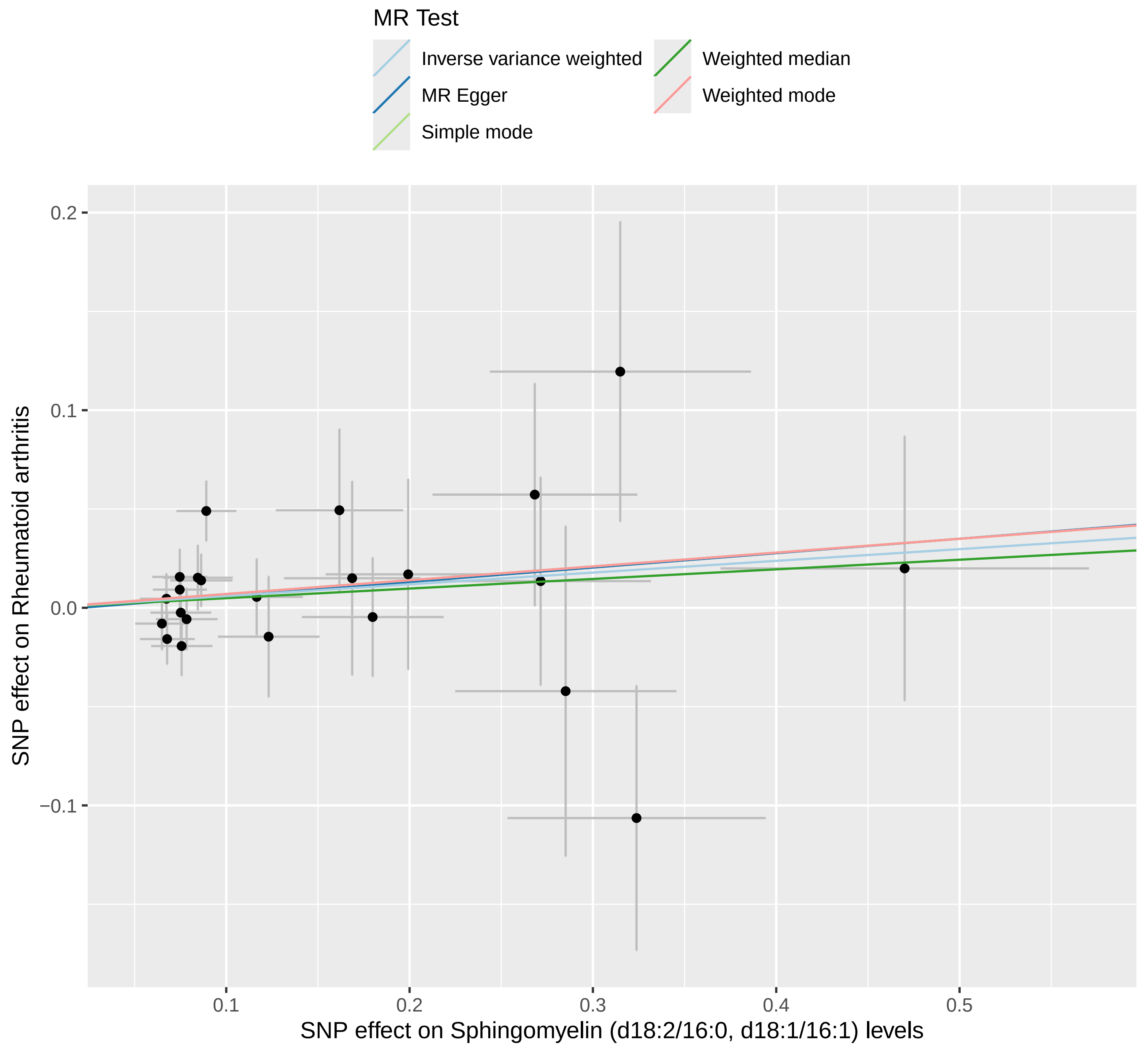

MR Test
Inverse variance weighted
MR Egger
Simple mode
Weighted median
Weighted mode
0.2
0.1
0.0
−0.1
0.1
0.2
0.3
0.4
0.5
SNP effect on Rheumatoid arthritis
SNP effect on Sphingomyelin (d18:2/16:0, d18:1/16:1) levels

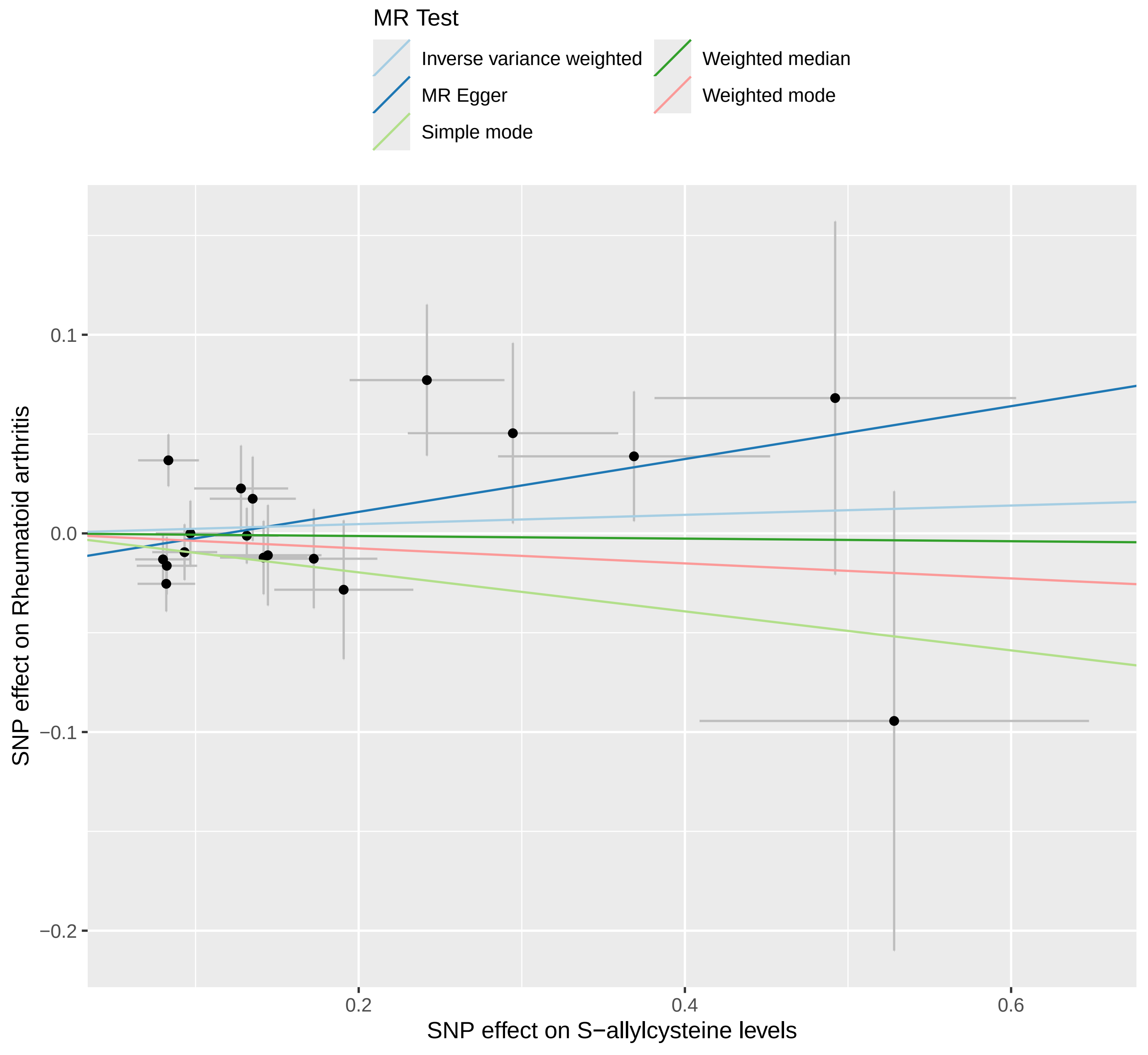

MR Test
Inverse variance weighted
MR Egger
Simple mode
Weighted median
Weighted mode
0.1
0.0
−0.1
−0.2
0.2
0.4
0.6
SNP effect on Rheumatoid arthritis
SNP effect on S−allylcysteine levels

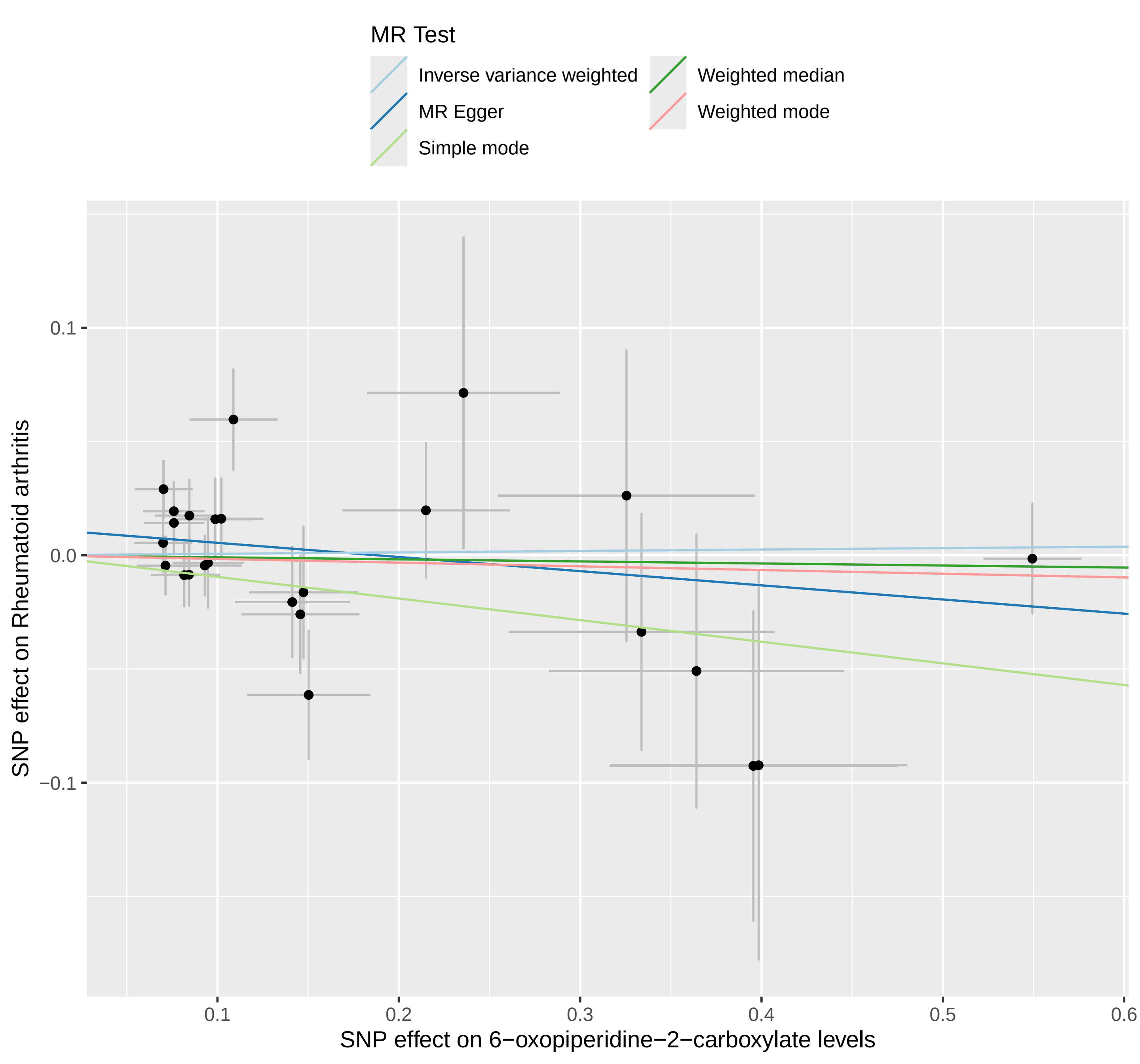
MR Test
Inverse variance weighted
Weighted median
MR Egger
Weighted mode
Simple mode
0.1
0.0
−0.1
0.1
0.2
0.3
0.4
0.5
0.6
SNP effect on Rheumatoid arthritis
SNP effect on 6−oxopiperidine−2−carboxylate levels

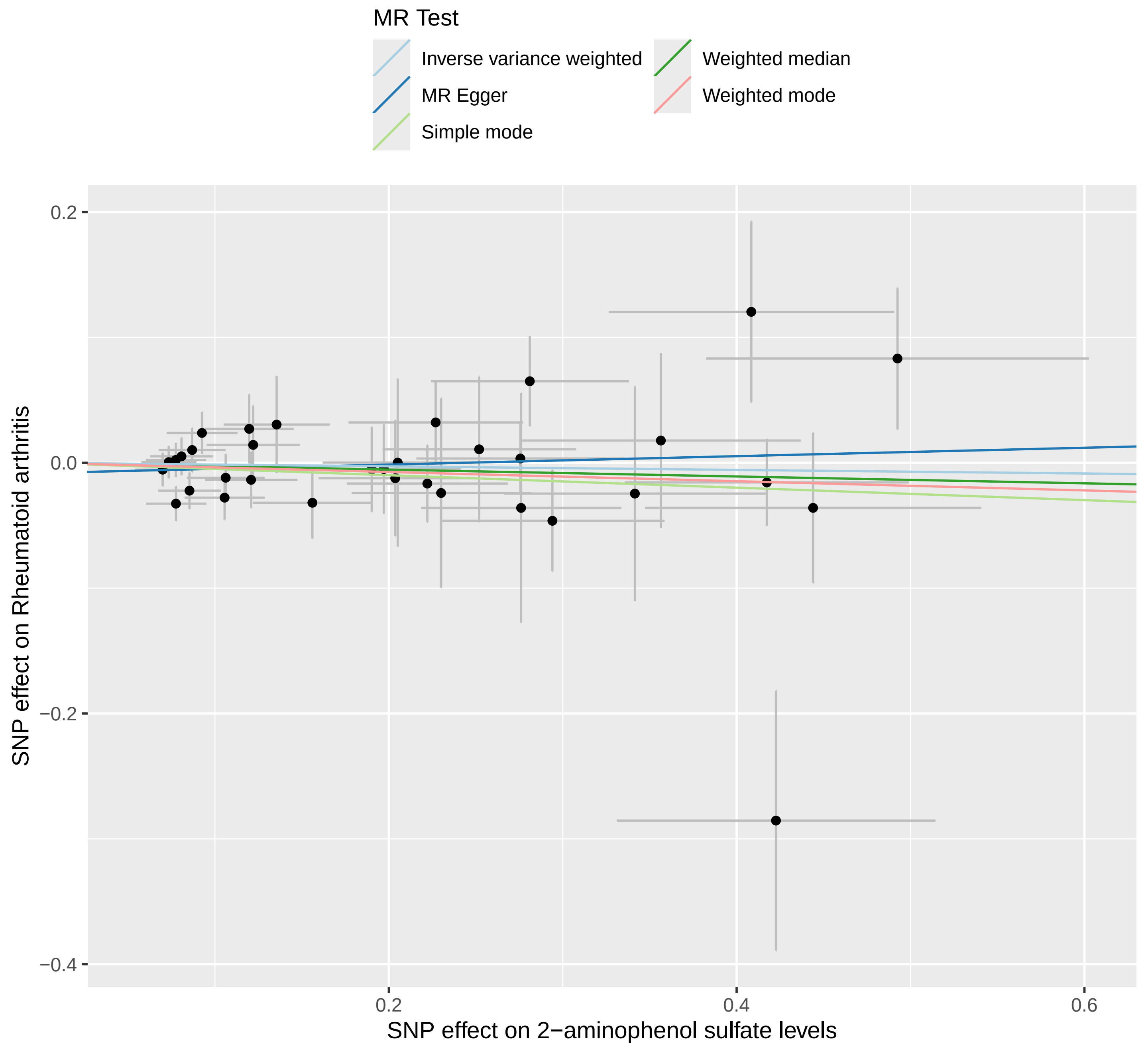

MR Test
Inverse variance weighted
MR Egger
Simple mode
Weighted median
Weighted mode
0.2
0.0
−0.2
−0.4
SNP effect on Rheumatoid arthritis
0.2
0.4
0.6
SNP effect on 2−aminophenol sulfate levels

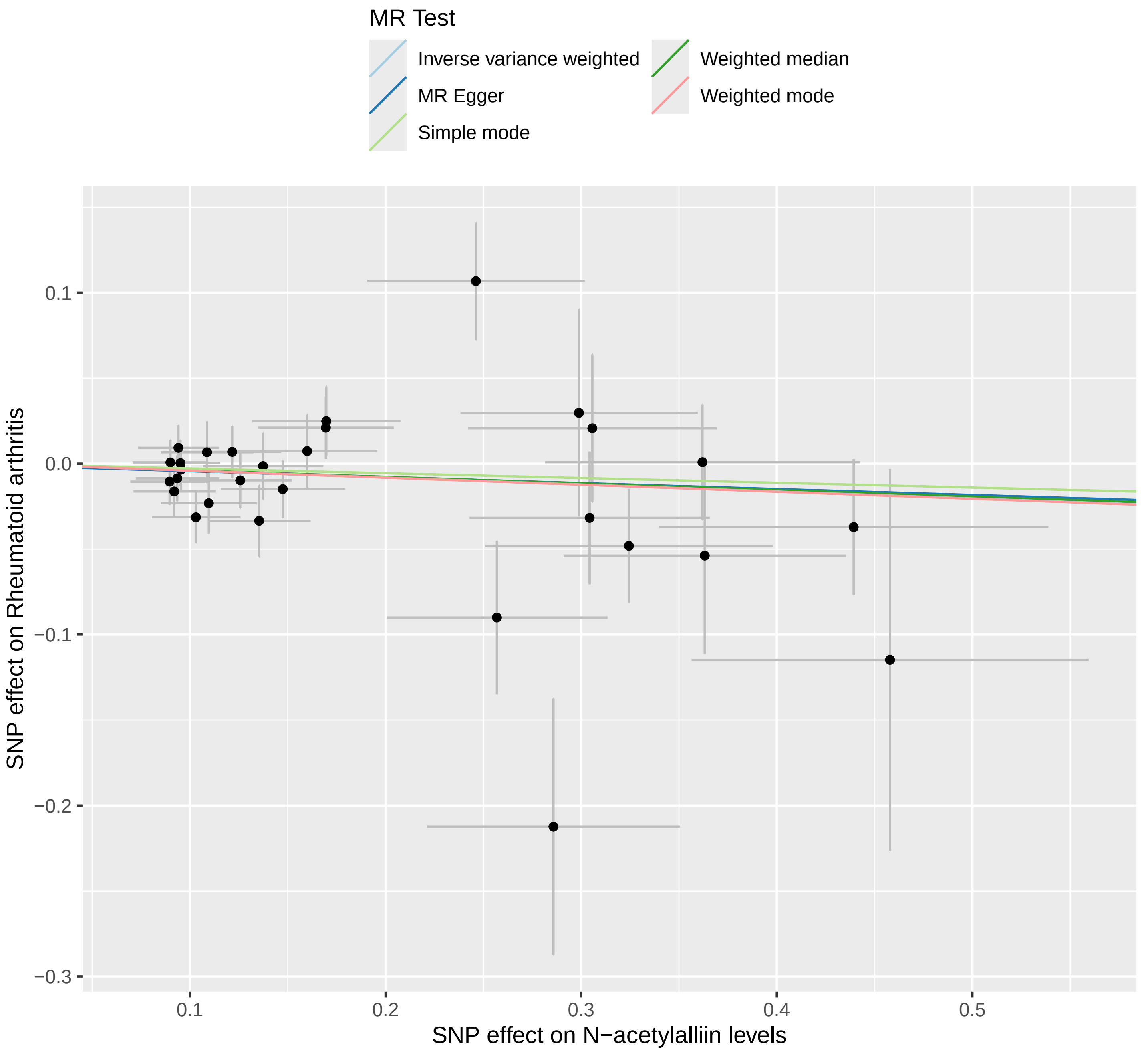
MR Test
Inverse variance weighted
MR Egger
Simple mode
Weighted median
Weighted mode
0.1
0.0
−0.1
−0.2
−0.3
SNP effect on Rheumatoid arthritis
0.1
0.2
0.3
0.4
0.5
SNP effect on N−acetylalliin levels

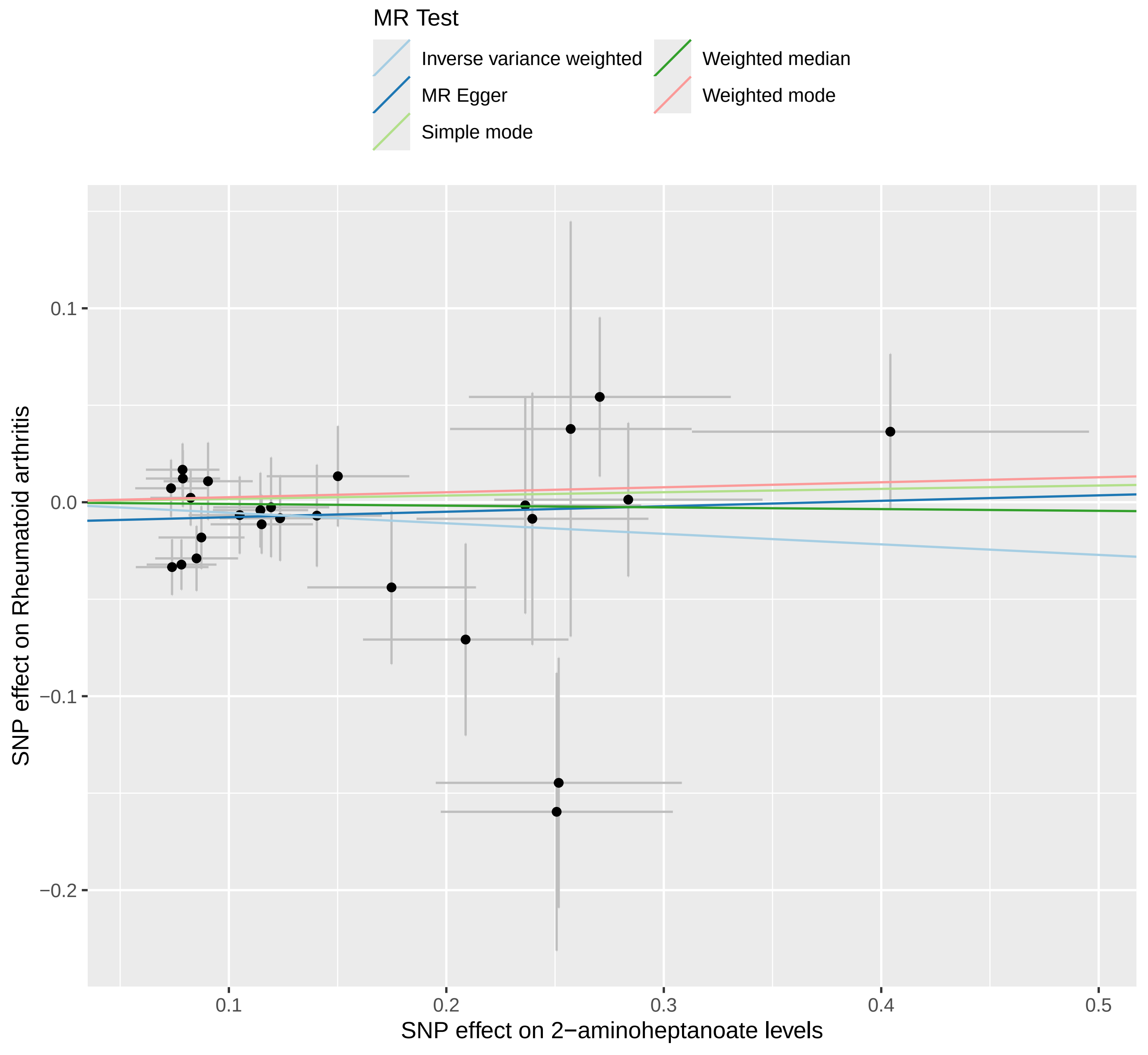
MR Test
Inverse variance weighted
MR Egger
Simple mode
Weighted median
Weighted mode
SNP effect on Rheumatoid arthritis
0.1
0.0
−0.1
−0.2
0.1
0.2
0.3
0.4
0.5
SNP effect on 2−aminoheptanoate levels

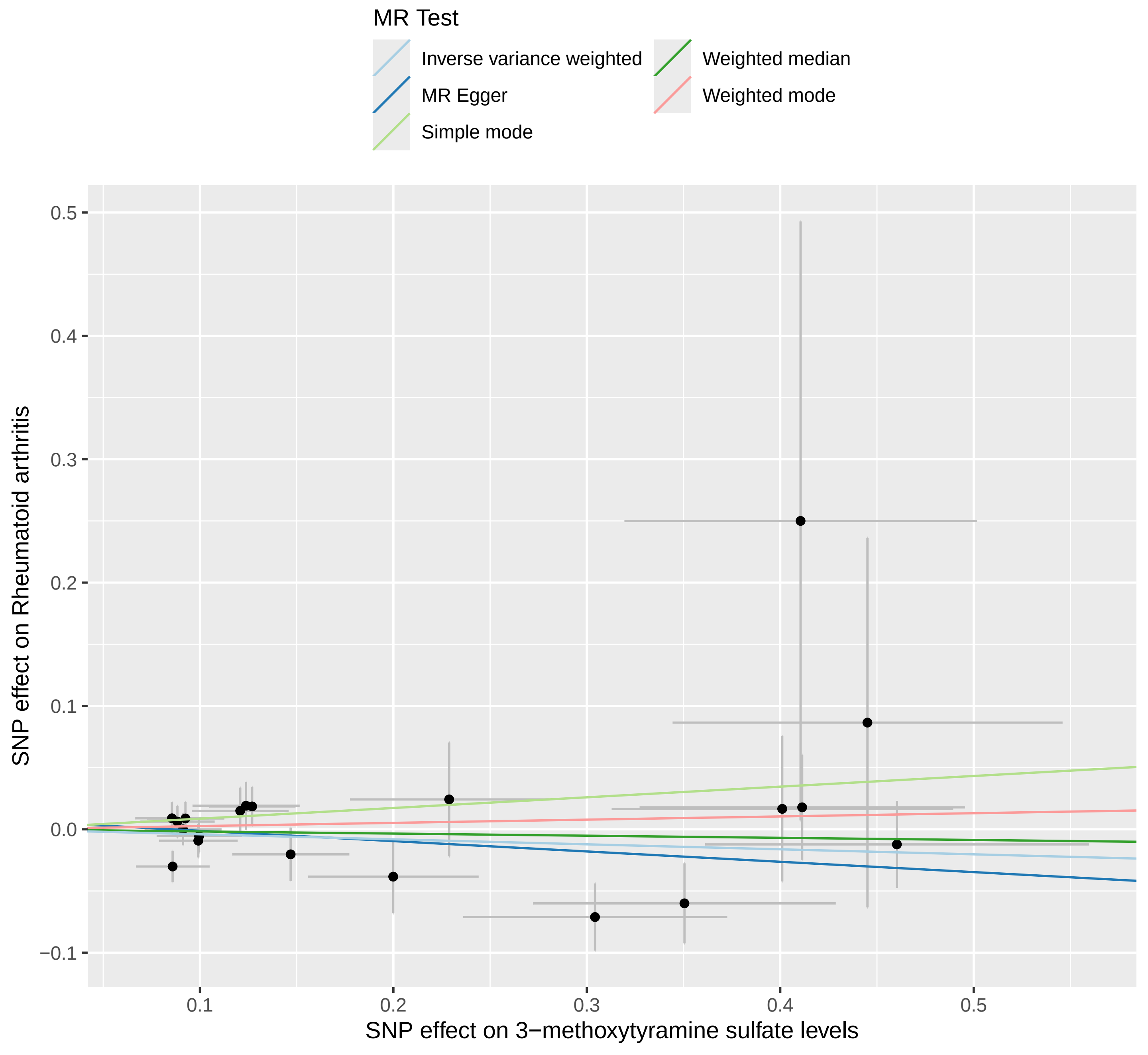

MR Test
Inverse variance weighted
MR Egger
Simple mode
Weighted median
Weighted mode
0.5
0.4
0.3
0.2
0.1
0.0
−0.1
SNP effect on Rheumatoid arthritis
0.1
0.2
0.3
0.4
0.5
SNP effect on 3−methoxytyramine sulfate levels

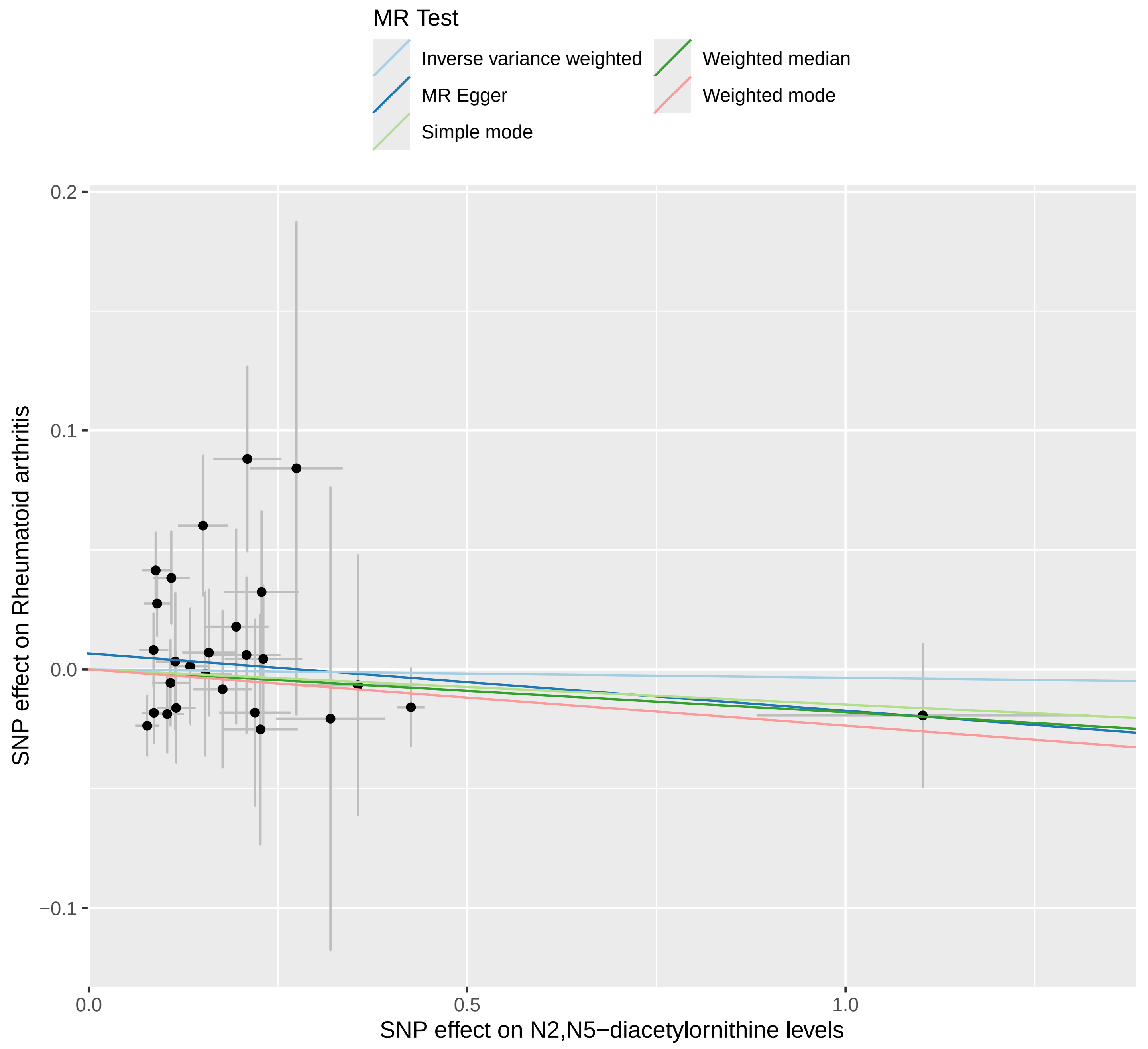
MR Test
Inverse variance weighted
MR Egger
Simple mode
Weighted median
Weighted mode
0.2
0.1
0.0
−0.1
0.0
0.5
1.0
SNP effect on Rheumatoid arthritis
SNP effect on N2,N5−diacetylornithine levels

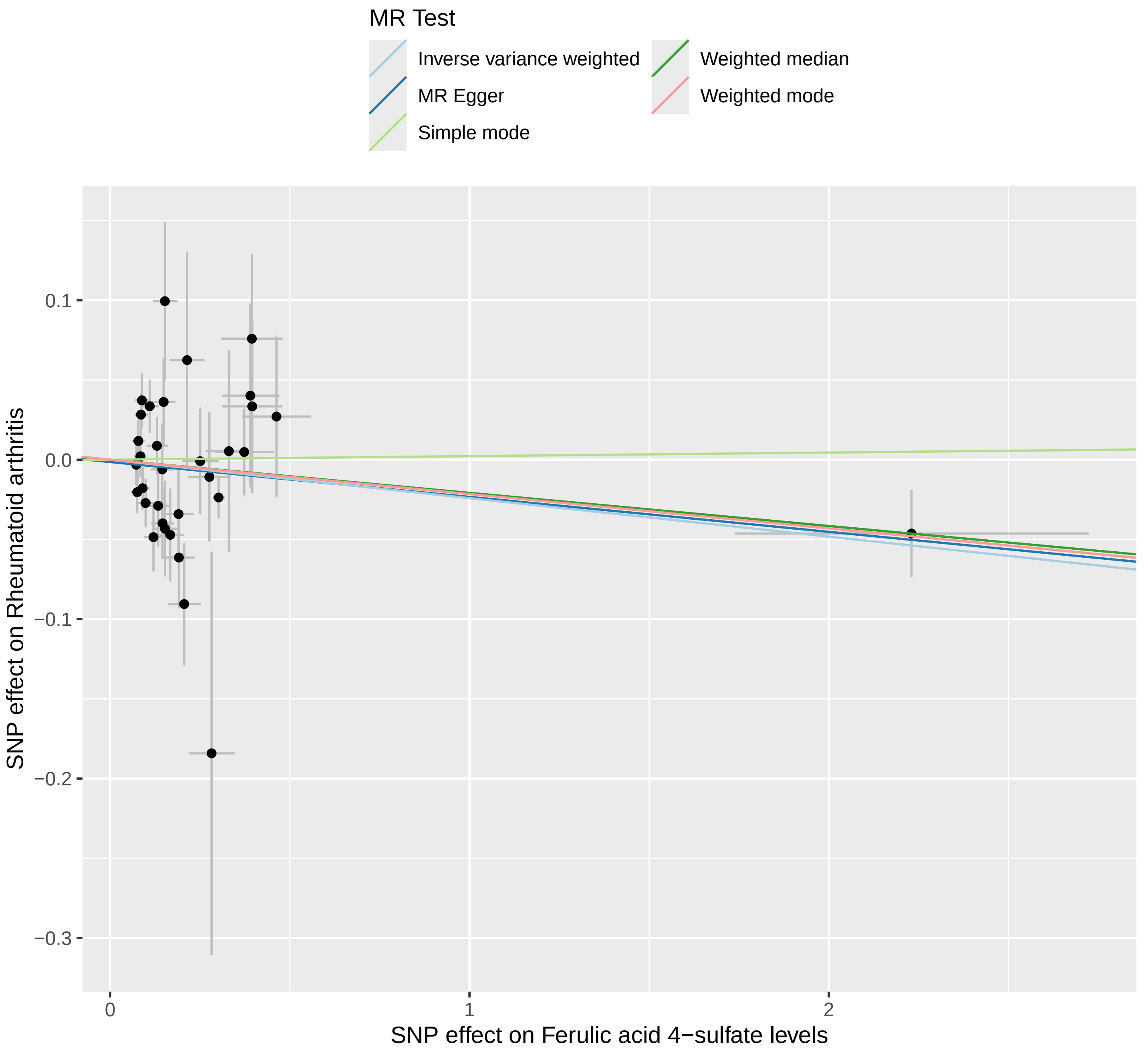

MR Test
Inverse variance weighted
MR Egger
Simple mode
Weighted median
Weighted mode
0.1
0.0
−0.1
−0.2
−0.3
0
1
2
SNP effect on Rheumatoid arthritis
SNP effect on Ferulic acid 4−sulfate levels

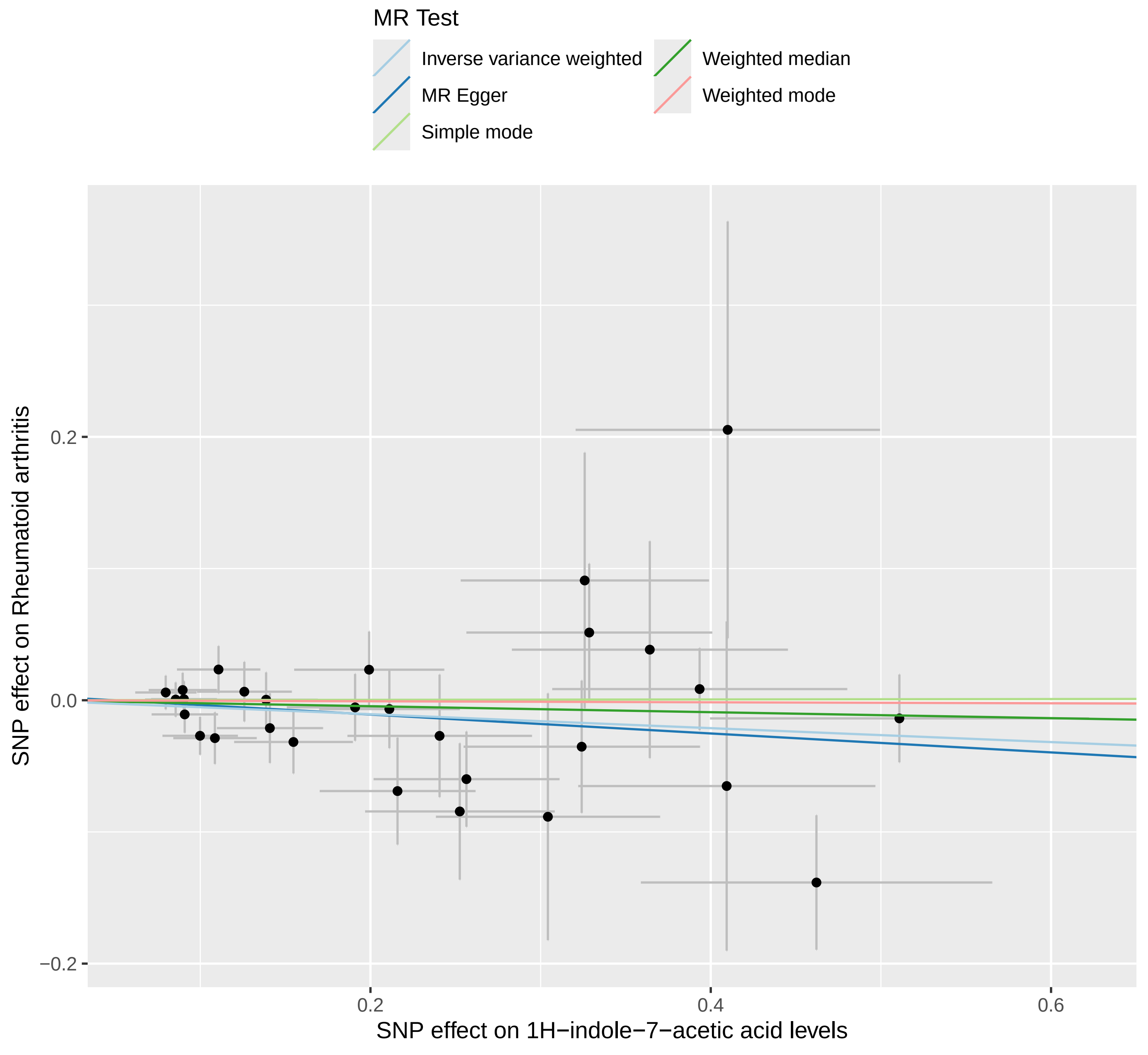
MR Test
Inverse variance weighted
MR Egger
Simple mode
Weighted median
Weighted mode
0.2
0.0
−0.2
SNP effect on Rheumatoid arthritis
0.2
0.4
0.6
SNP effect on 1H−indole−7−acetic acid levels

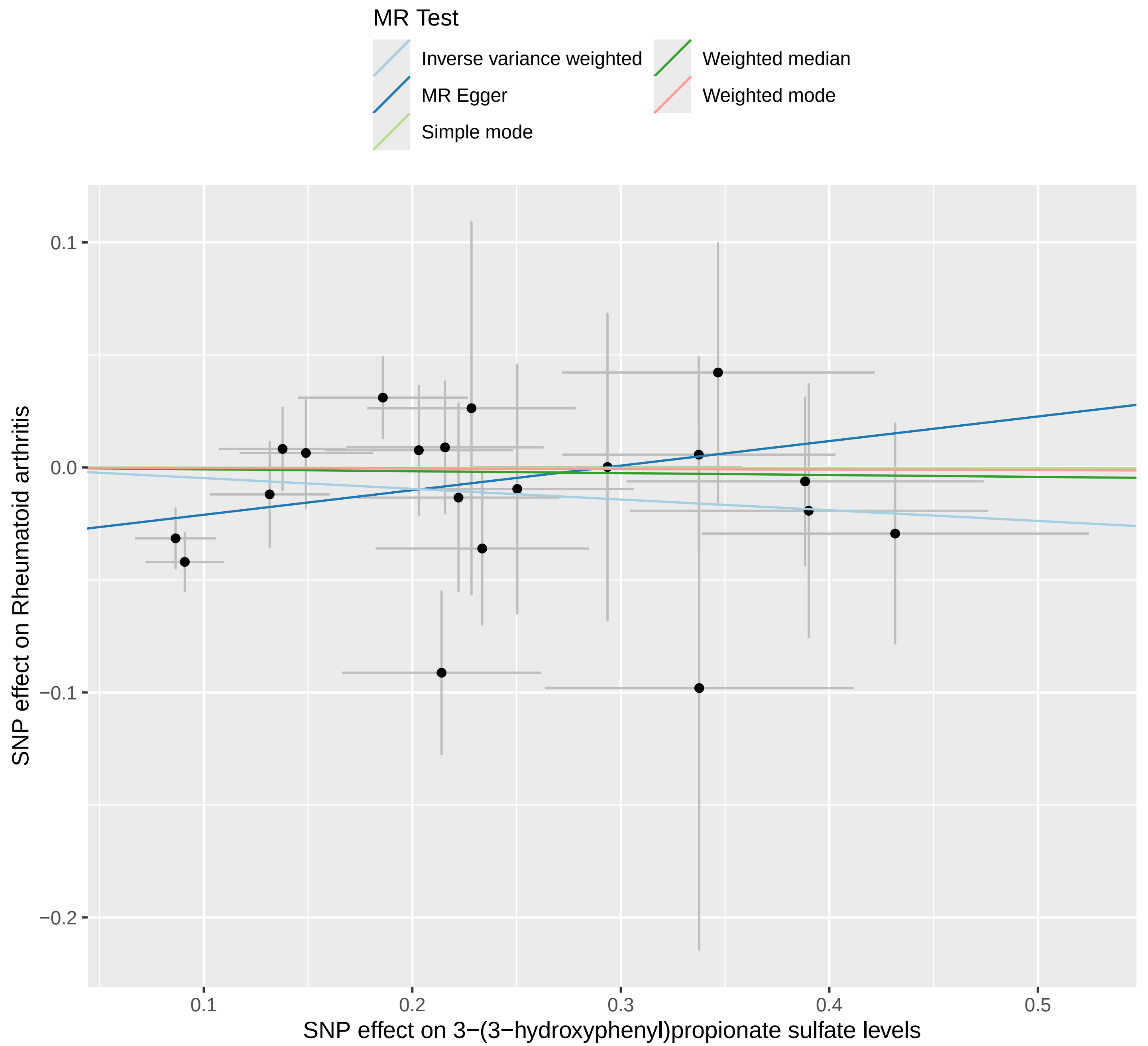

MR Test
Inverse variance weighted
MR Egger
Simple mode
Weighted median
Weighted mode
0.1
0.0
−0.1
−0.2
SNP effect on Rheumatoid arthritis
0.1
0.2
0.3
0.4
0.5
SNP effect on 3−(3−hydroxyphenyl)propionate sulfate levels

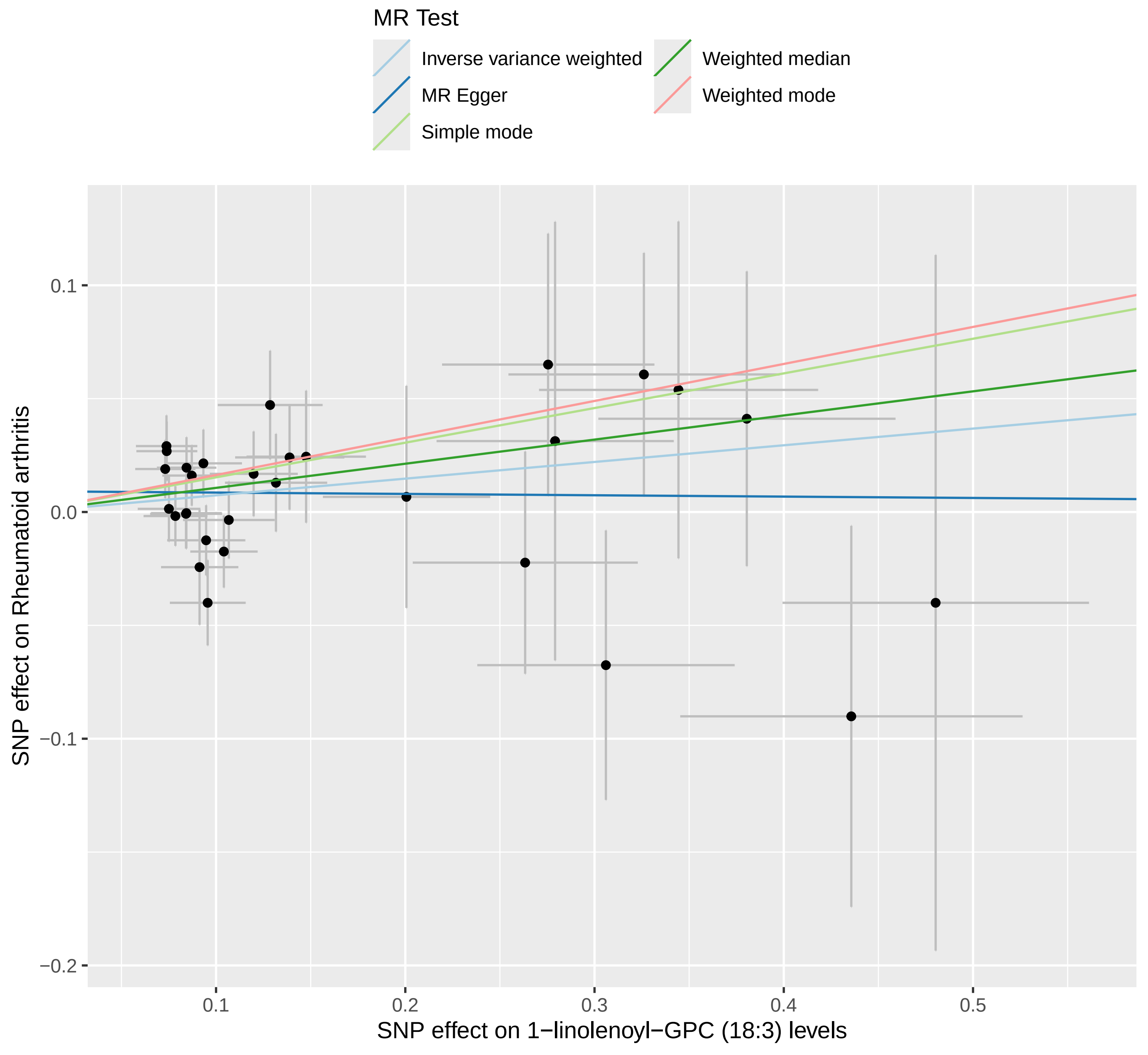

MR Test
Inverse variance weighted
MR Egger
Simple mode
Weighted median
Weighted mode
0.1
0.0
−0.1
−0.2
SNP effect on Rheumatoid arthritis
0.1
0.2
0.3
0.4
0.5
SNP effect on 1−linolenoyl−GPC (18:3) levels

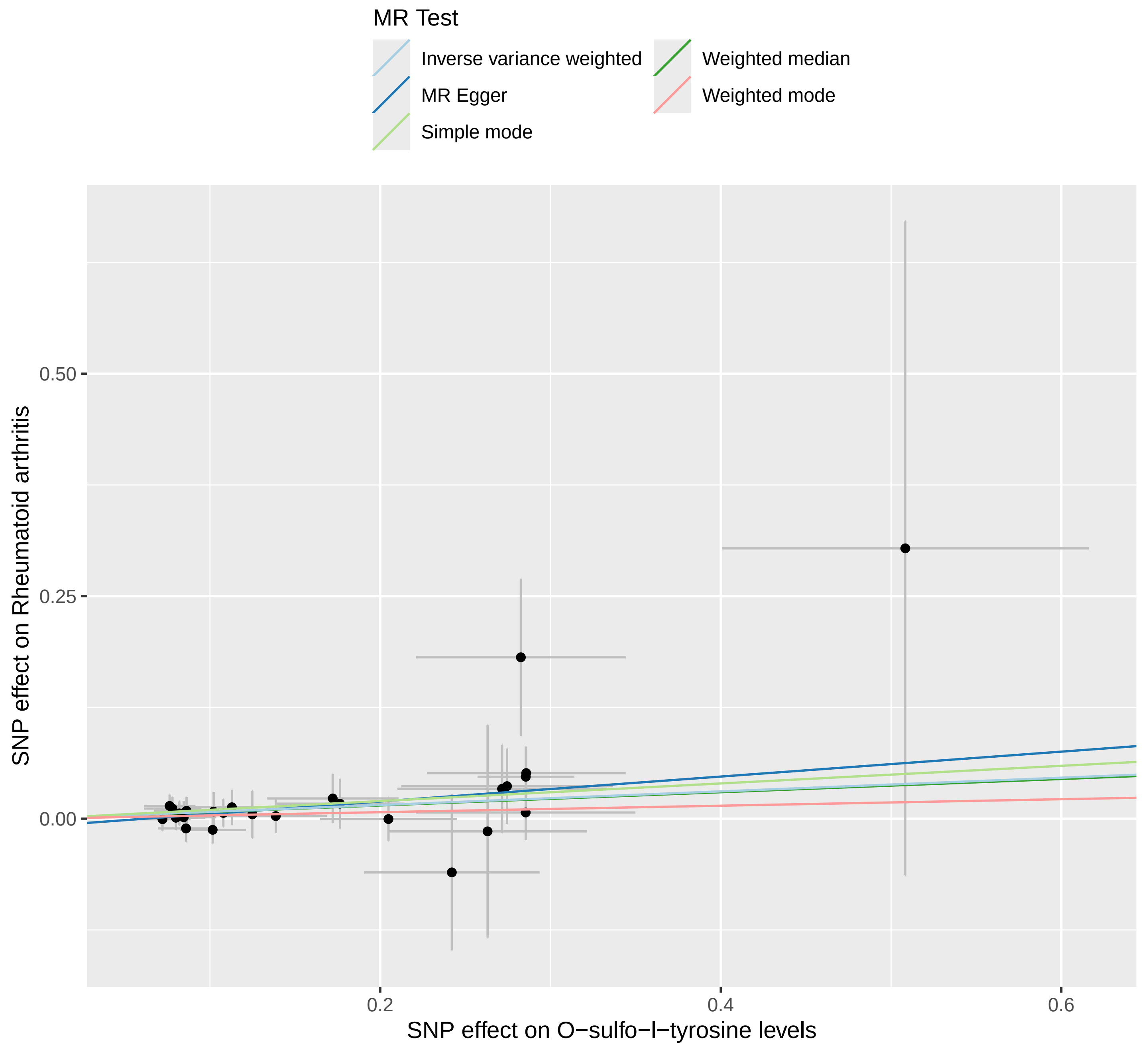

MR Test
Inverse variance weighted
MR Egger
Simple mode
Weighted median
Weighted mode
0.50
0.25
0.00
0.2
0.4
0.6
SNP effect on Rheumatoid arthritis
SNP effect on O−sulfo−l−tyrosine levels

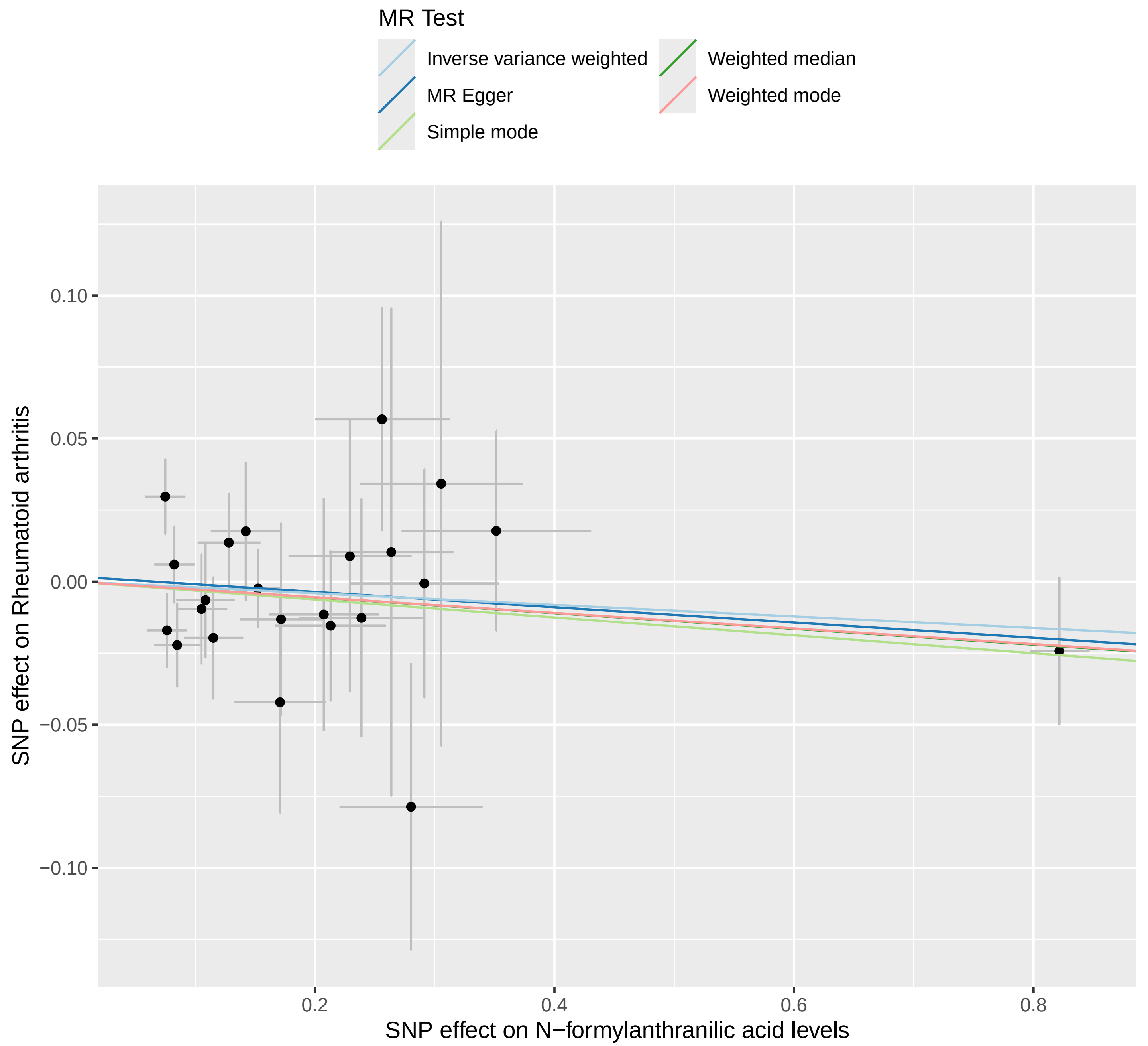
MR Test
Inverse variance weighted
MR Egger
Simple mode
Weighted median
Weighted mode
SNP effect on Rheumatoid arthritis
0.10
0.05
0.00
−0.05
−0.10
0.2
0.4
0.6
0.8
SNP effect on N−formylanthranilic acid levels

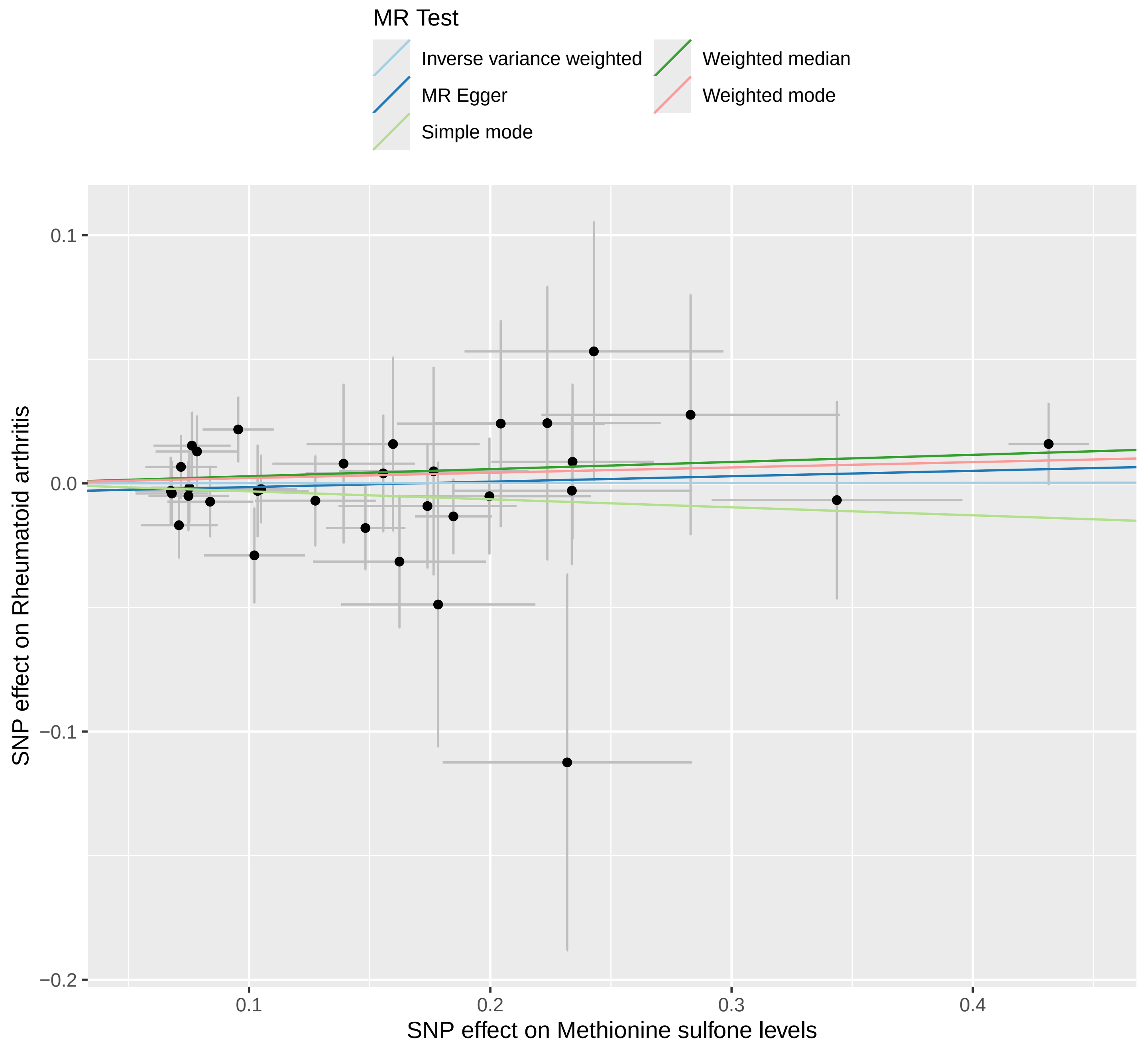

MR Test
Inverse variance weighted
MR Egger
Simple mode
Weighted median
Weighted mode
0.1
0.0
−0.1
−0.2
0.1
0.2
0.3
0.4
SNP effect on Rheumatoid arthritis
SNP effect on Methionine sulfone levels

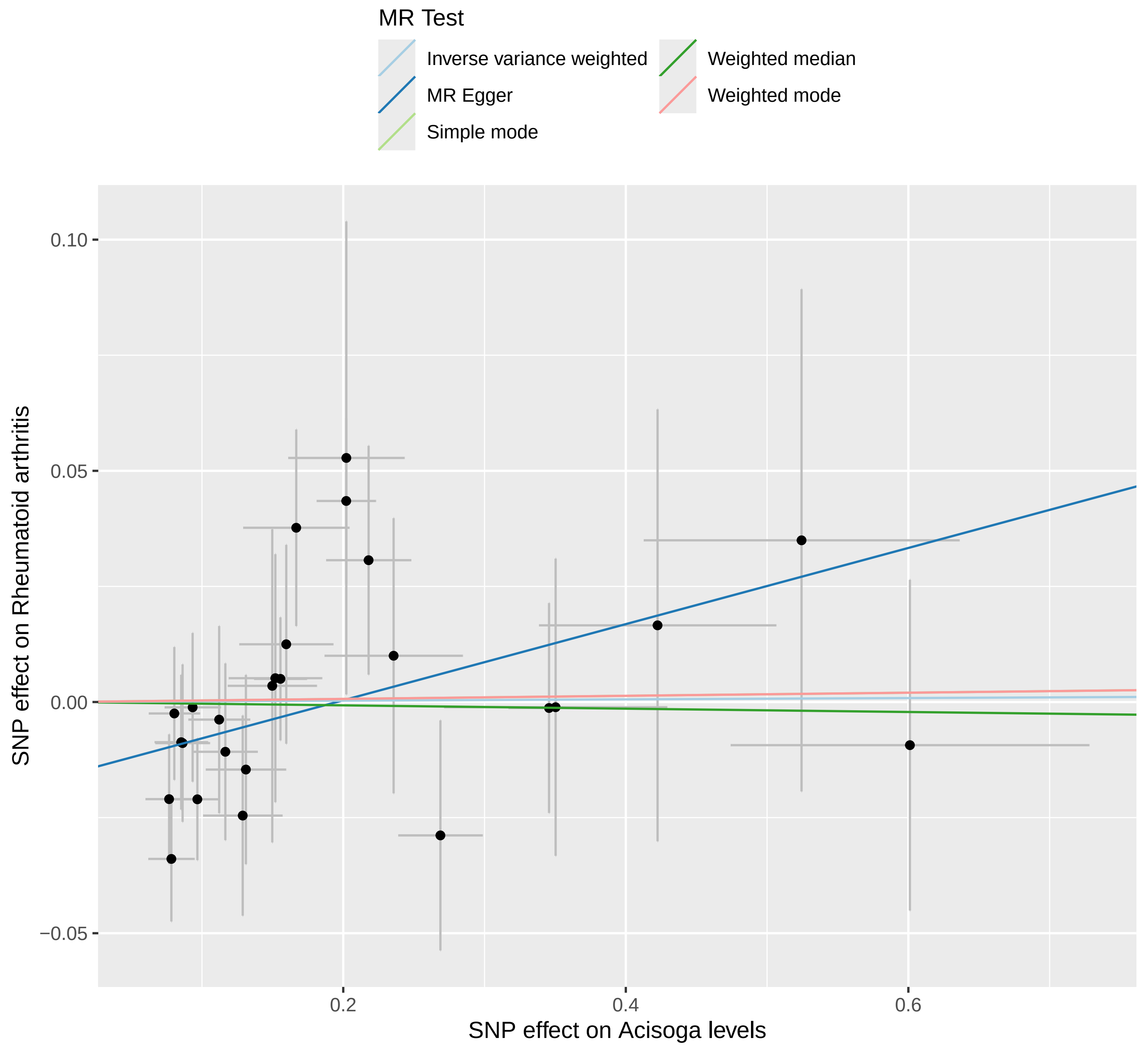
MR Test
Inverse variance weighted
MR Egger
Simple mode
Weighted median
Weighted mode
0.10
0.05
0.00
−0.05
0.2
0.4
0.6
SNP effect on Rheumatoid arthritis
SNP effect on Acisoga levels

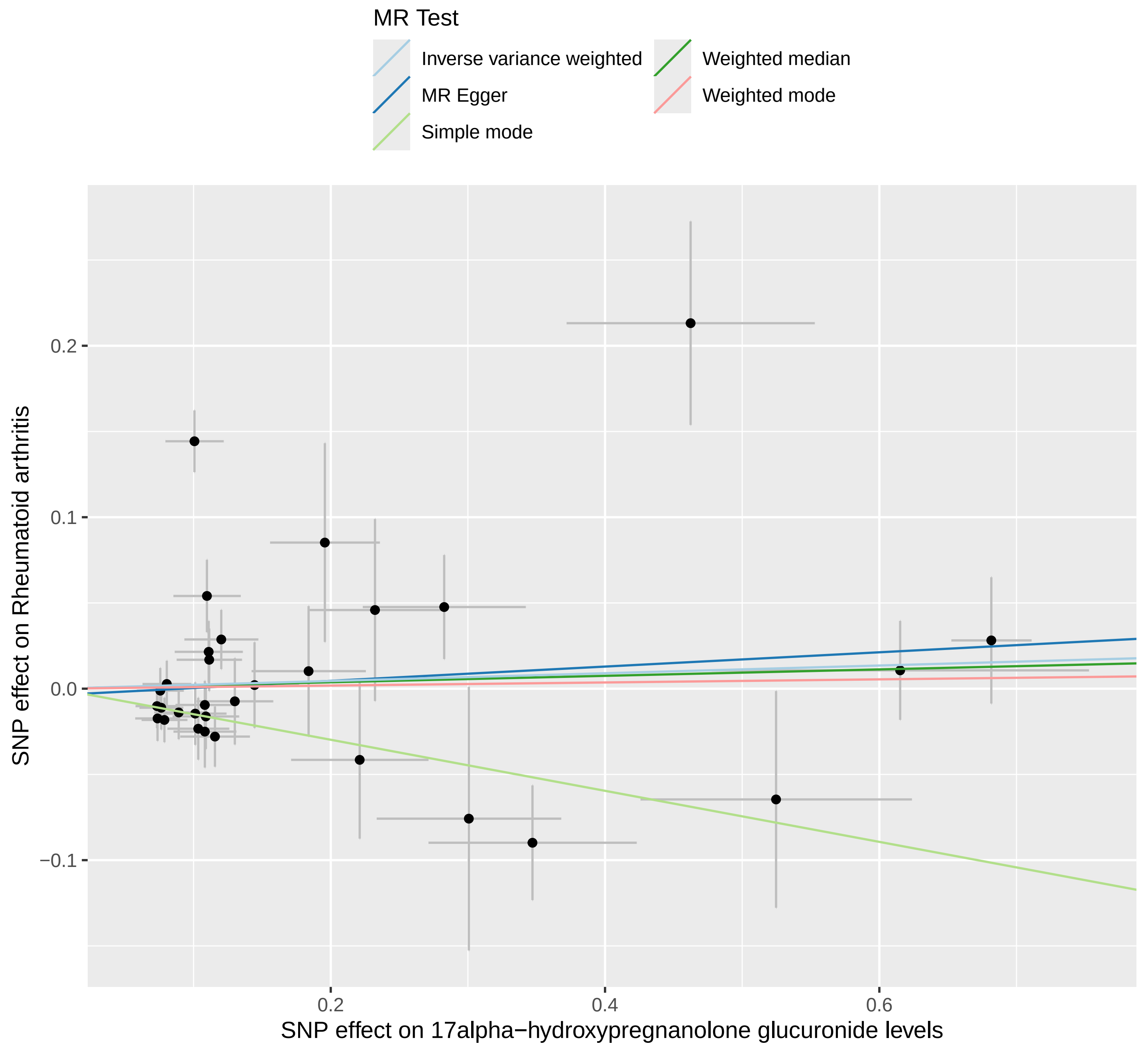

MR Test
Inverse variance weighted
MR Egger
Simple mode
Weighted median
Weighted mode
SNP effect on Rheumatoid arthritis
0.2
0.1
0.0
−0.1
0.2
0.4
0.6
SNP effect on 17alpha−hydroxypregnanolone glucuronide levels

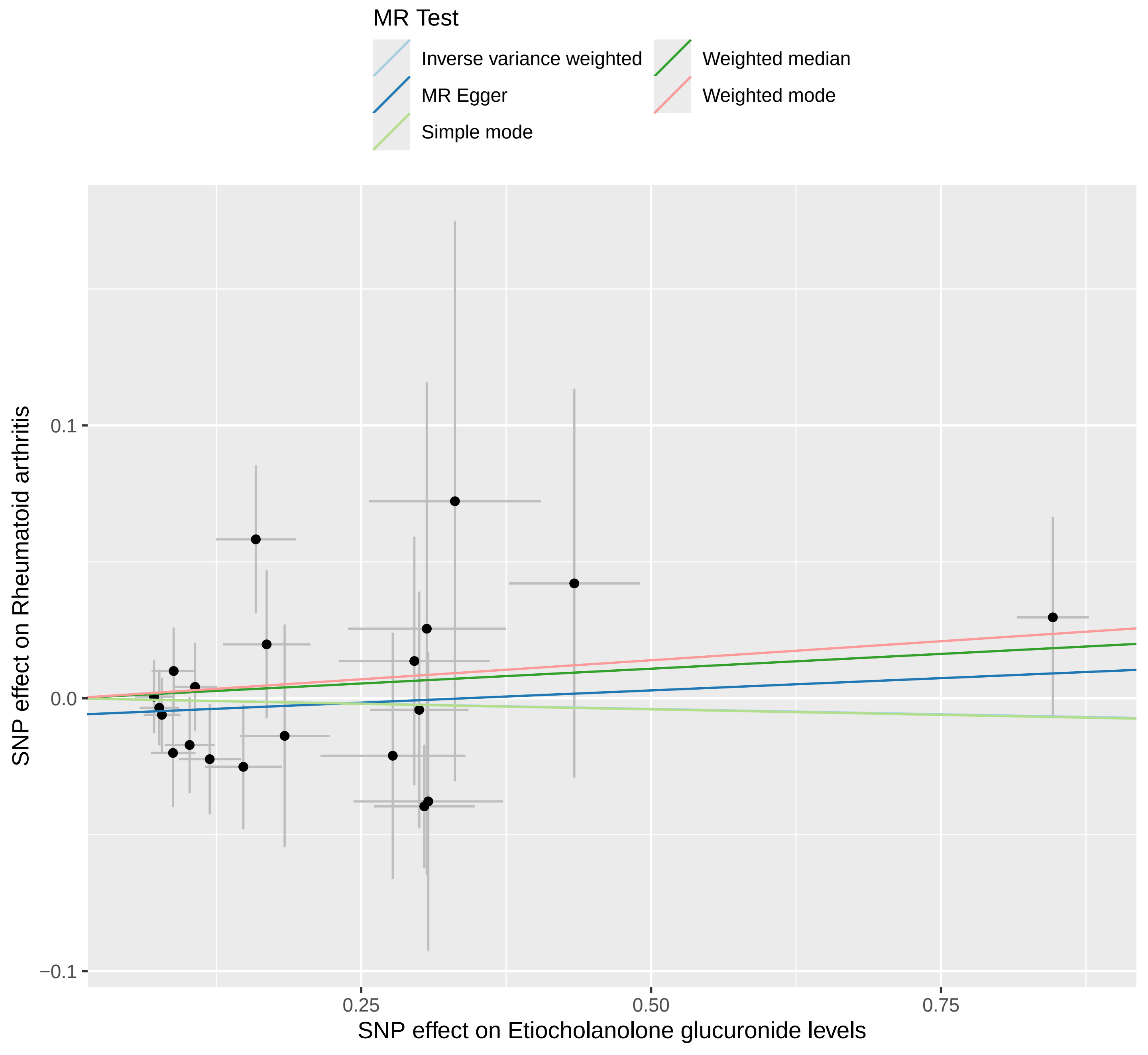
MR Test
Inverse variance weighted
MR Egger
Simple mode
Weighted median
Weighted mode
0.1
0.0
−0.1
0.25
0.50
0.75
SNP effect on Etiocholanolone glucuronide levels
SNP effect on Rheumatoid arthritis

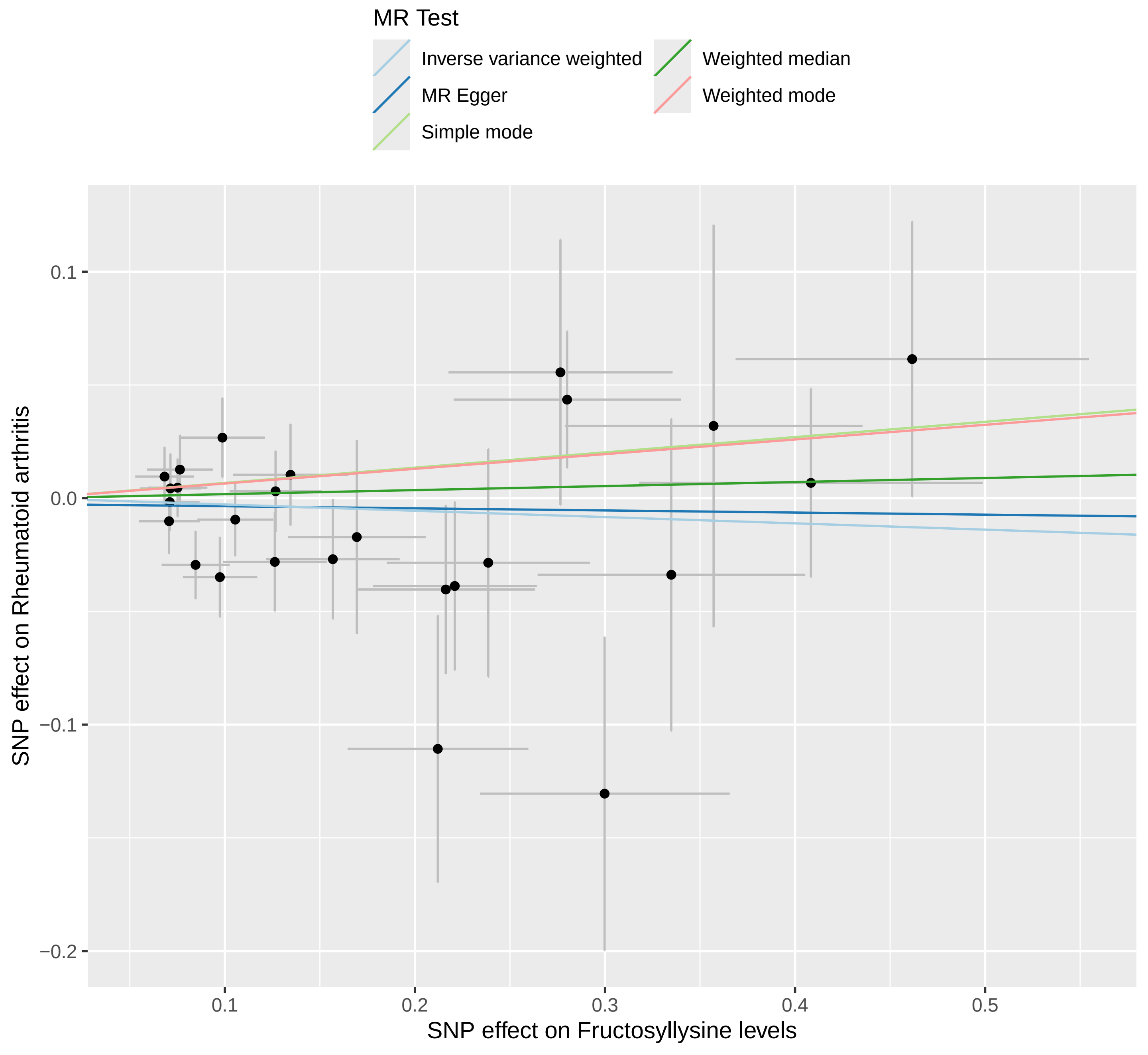
MR Test
Inverse variance weighted
MR Egger
Simple mode
Weighted median
Weighted mode
0.1
0.0
−0.1
−0.2
0.1
0.2
0.3
0.4
0.5
SNP effect on Rheumatoid arthritis
SNP effect on Fructosyllysine levels

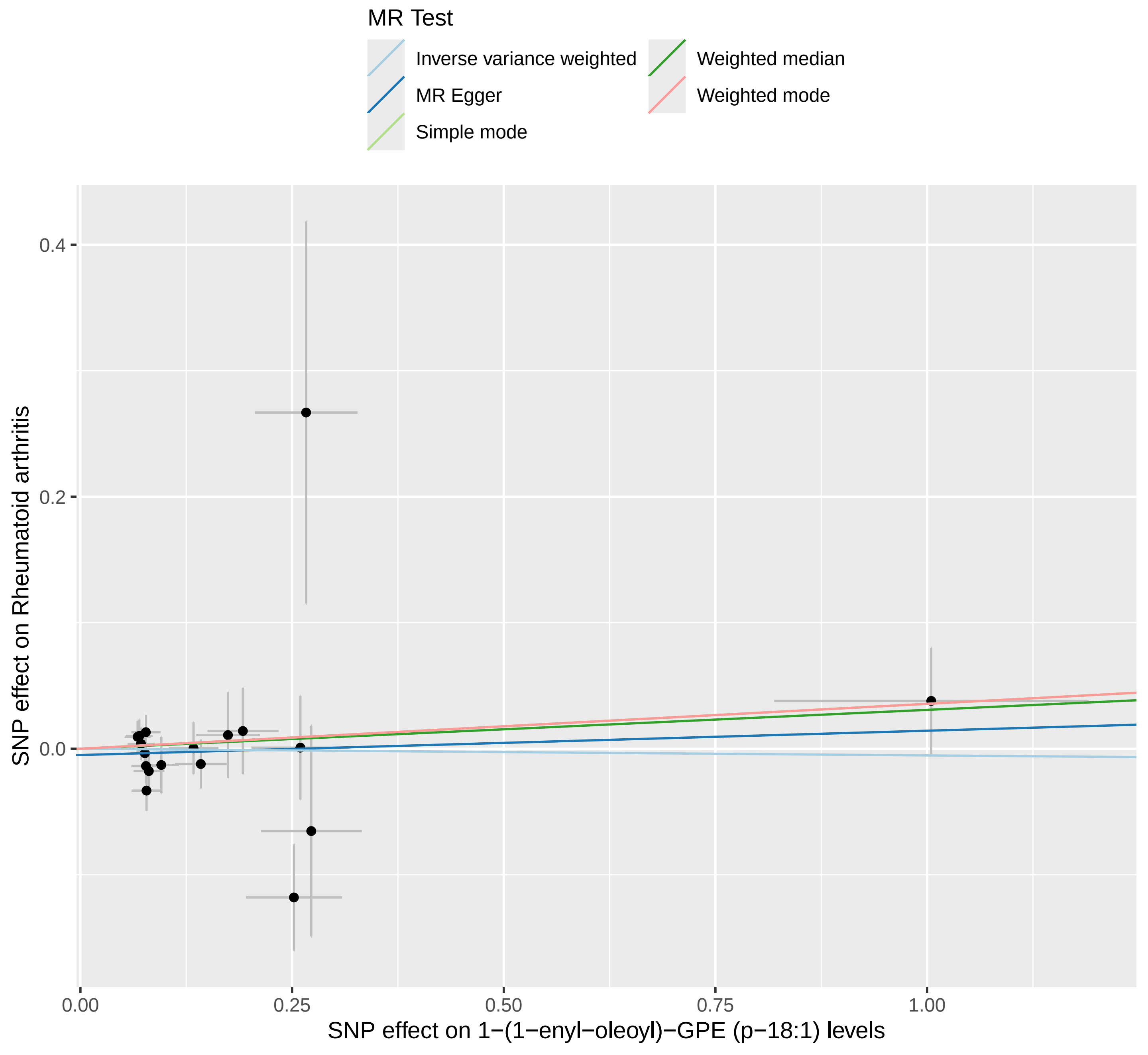
MR Test
Inverse variance weighted
MR Egger
Simple mode
Weighted median
Weighted mode
0.4
0.2
0.0
SNP effect on Rheumatoid arthritis
0.00
0.25
0.50
0.75
1.00
SNP effect on 1−(1−enyl−oleoyl)−GPE (p−18:1) levels

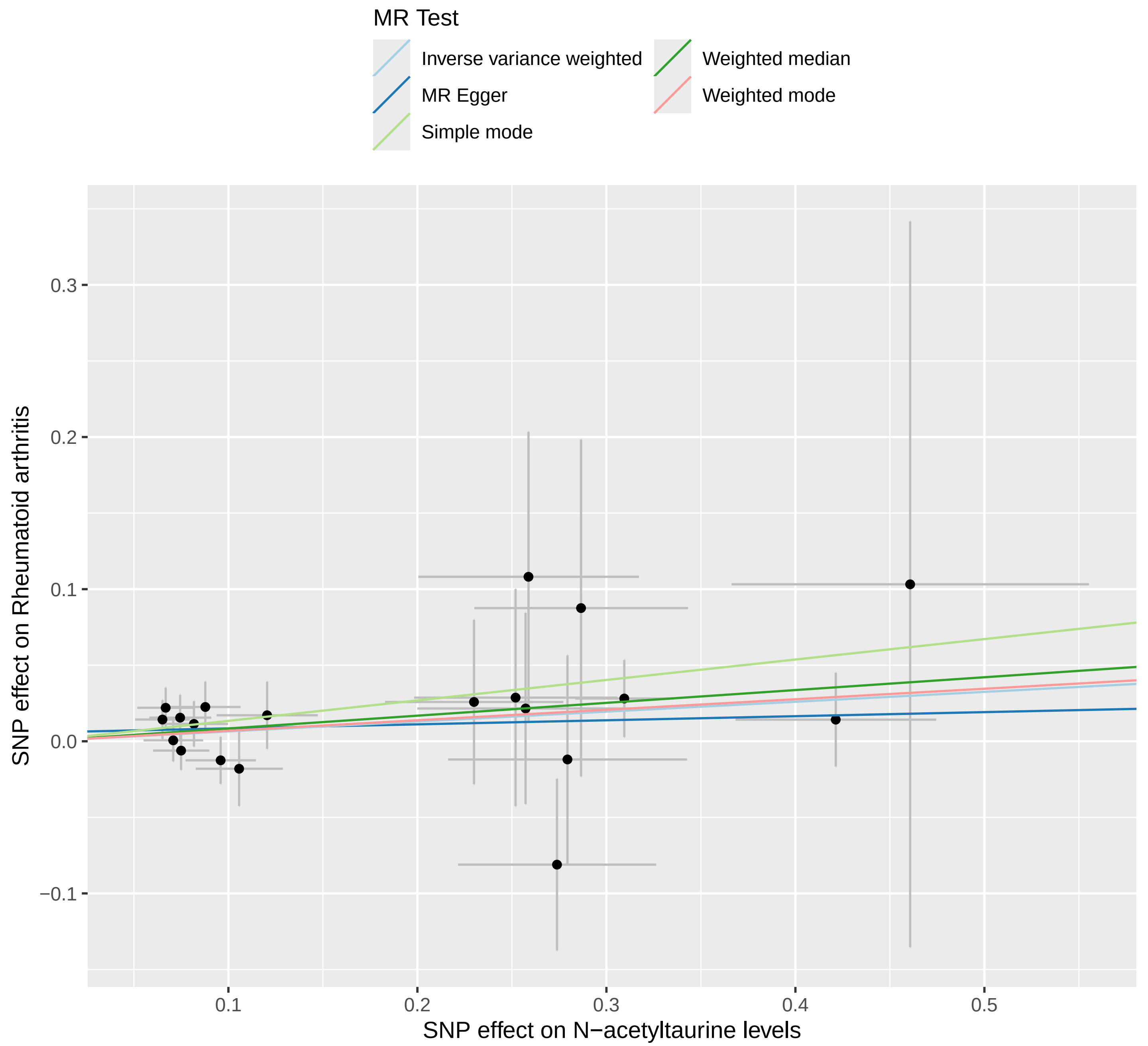

MR Test
Inverse variance weighted
MR Egger
Simple mode
Weighted median
Weighted mode
SNP effect on Rheumatoid arthritis
0.3
0.2
0.1
0.0
−0.1
0.1
0.2
0.3
0.4
0.5
SNP effect on N−acetyltaurine levels

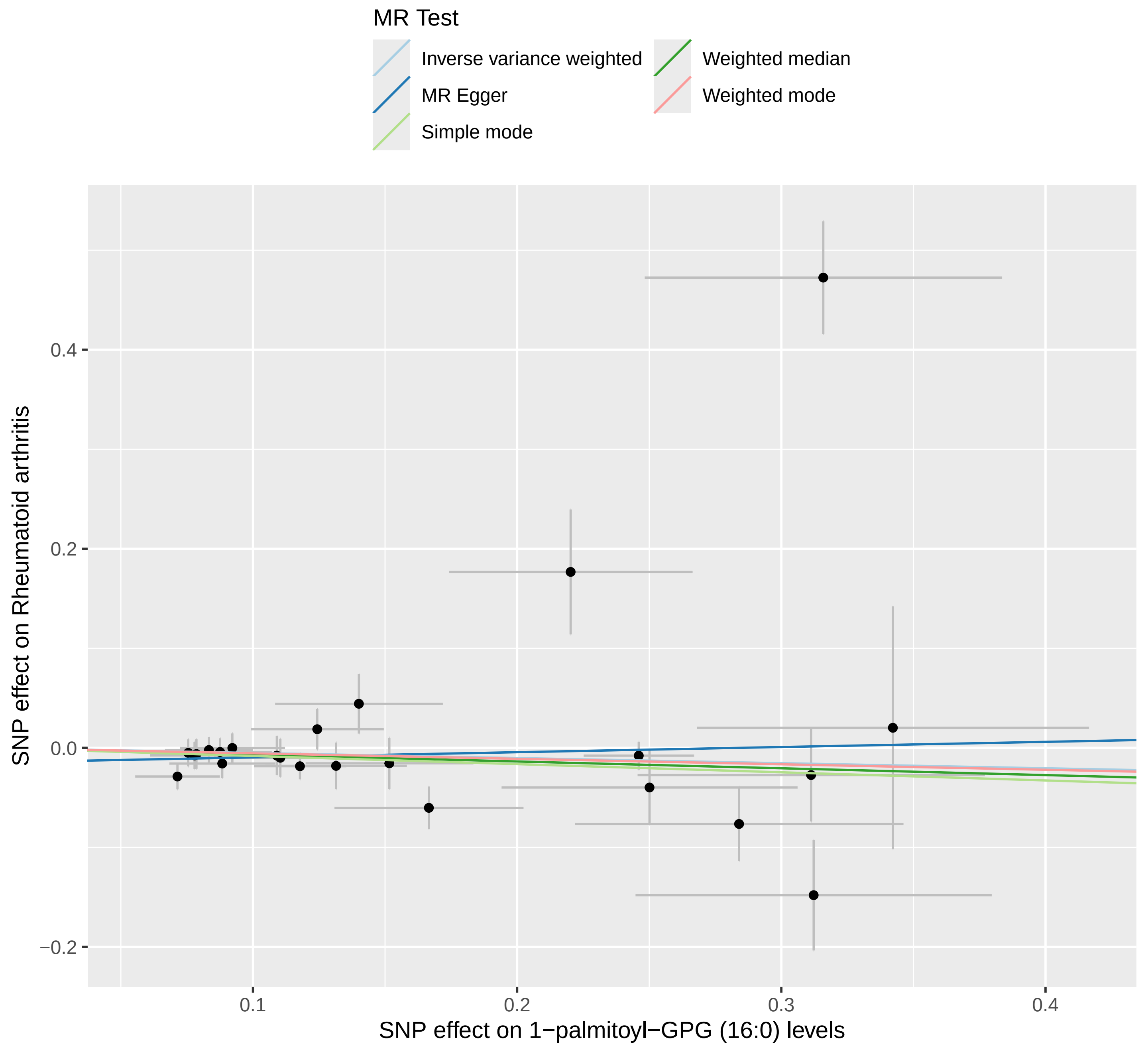
MR Test
Inverse variance weighted
MR Egger
Simple mode
Weighted median
Weighted mode
SNP effect on Rheumatoid arthritis
0.4
0.2
0.0
−0.2
0.1
0.2
0.3
0.4
SNP effect on 1−palmitoyl−GPG (16:0) levels

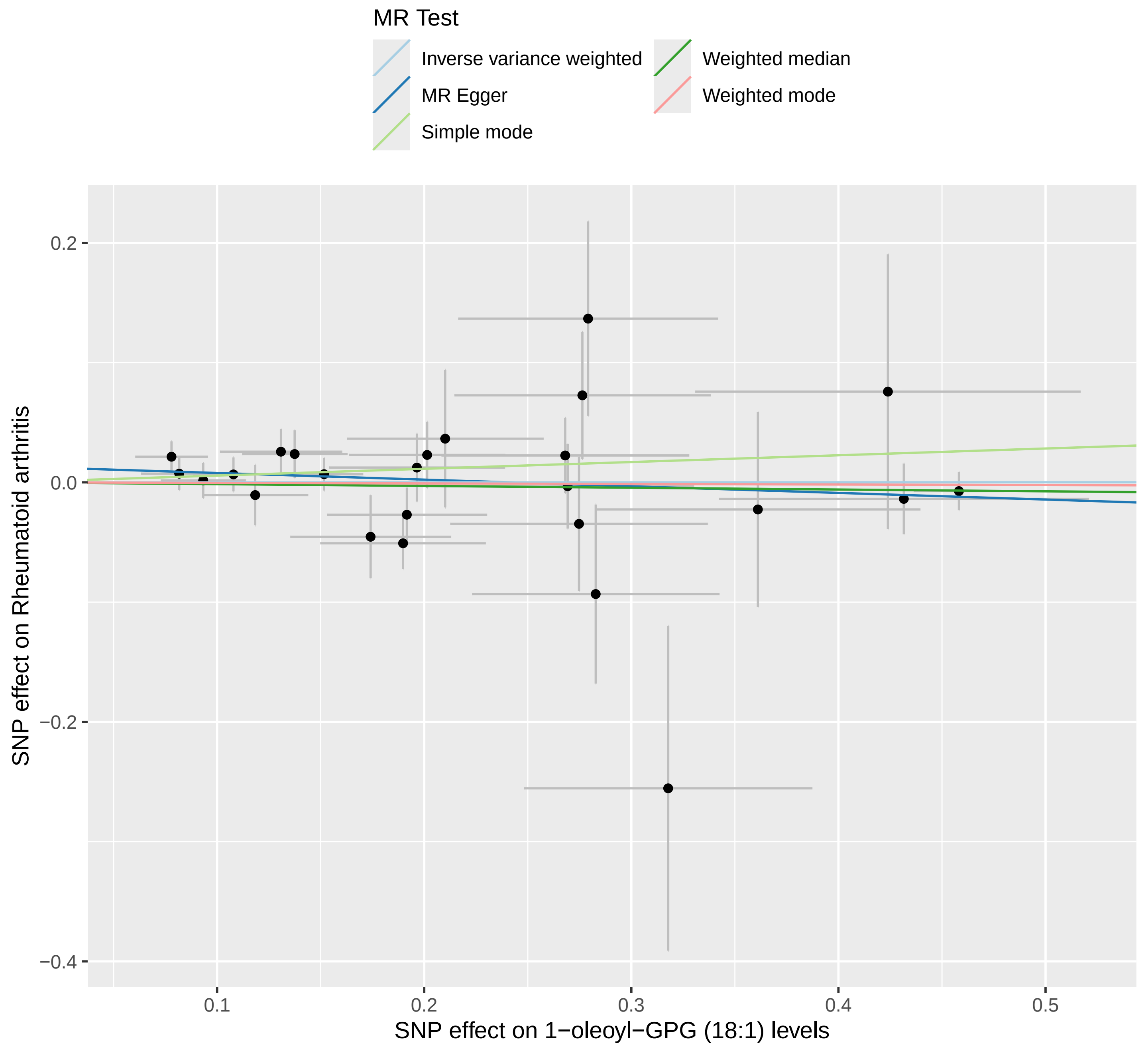

MR Test
Inverse variance weighted
MR Egger
Simple mode
Weighted median
Weighted mode
0.2
0.0
−0.2
−0.4
0.1
0.2
0.3
0.4
0.5
SNP effect on Rheumatoid arthritis
SNP effect on 1−oleoyl−GPG (18:1) levels

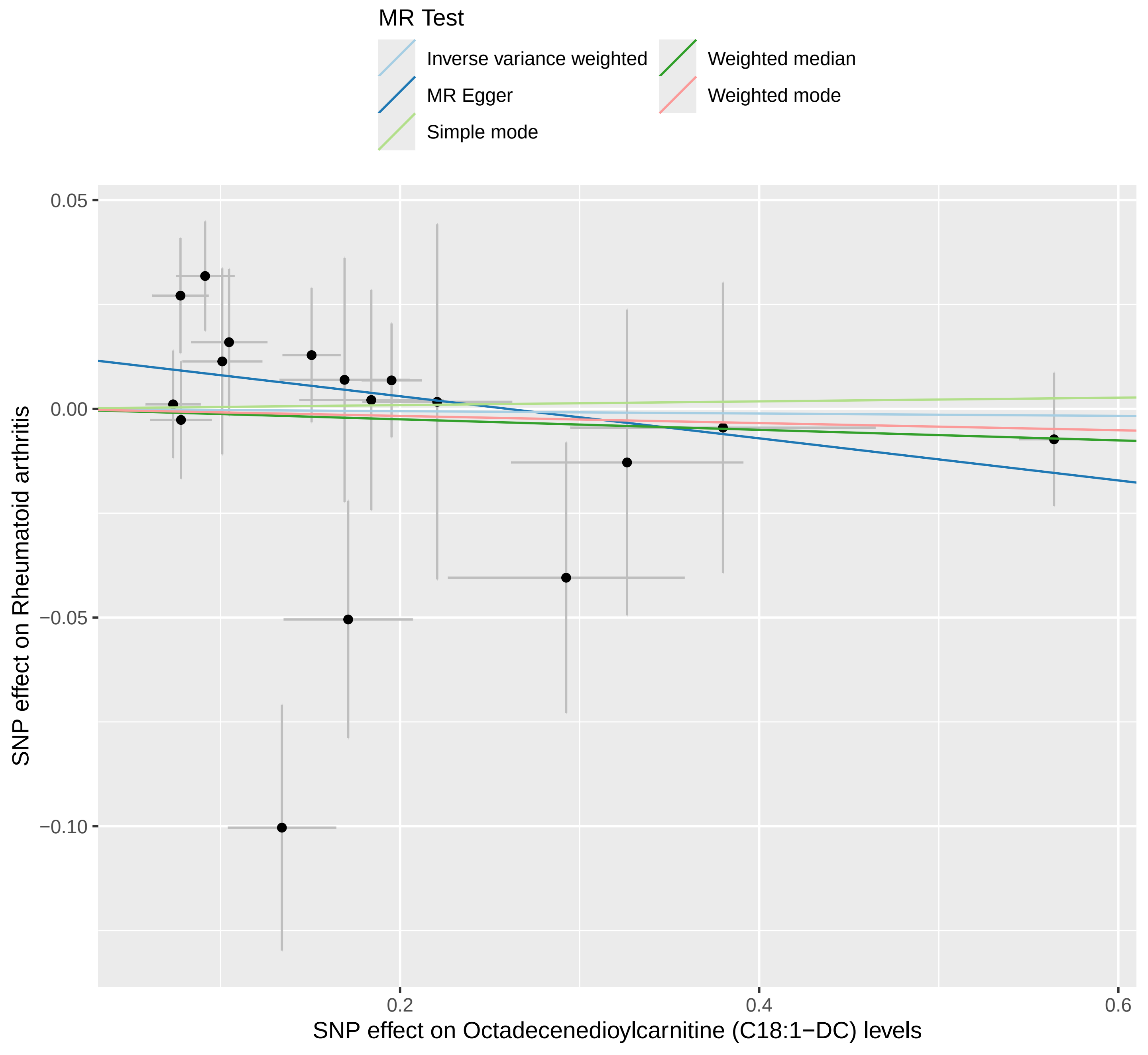

MR Test
Inverse variance weighted
MR Egger
Simple mode
Weighted median
Weighted mode
0.05
0.00
−0.05
−0.10
SNP effect on Rheumatoid arthritis
0.2
0.4
0.6
SNP effect on Octadecenedioylcarnitine (C18:1−DC) levels

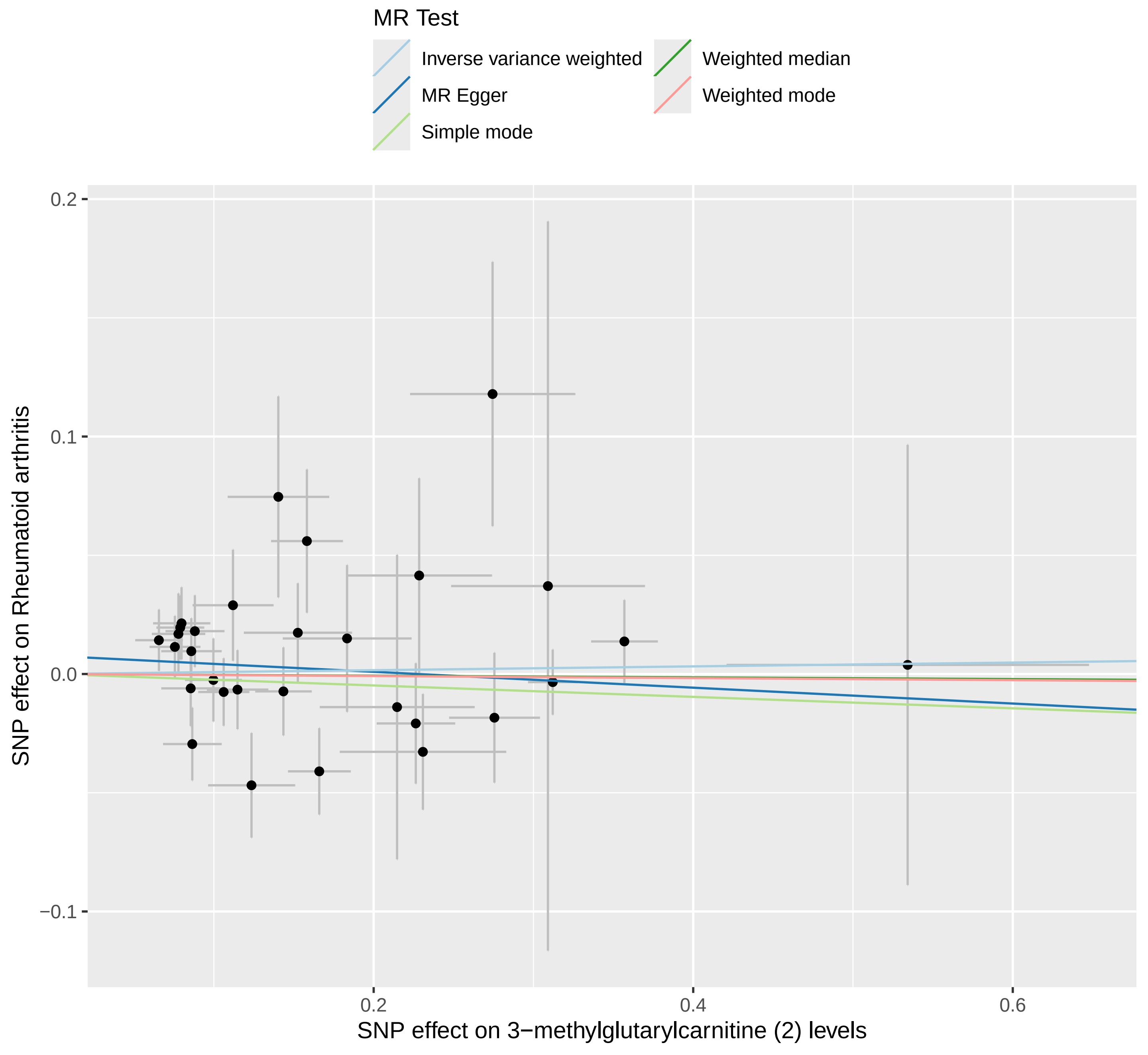
MR Test
Inverse variance weighted
MR Egger
Simple mode
Weighted median
Weighted mode
0.2
0.1
0.0
−0.1
SNP effect on Rheumatoid arthritis
0.2
0.4
0.6
SNP effect on 3−methylglutarylcarnitine (2) levels

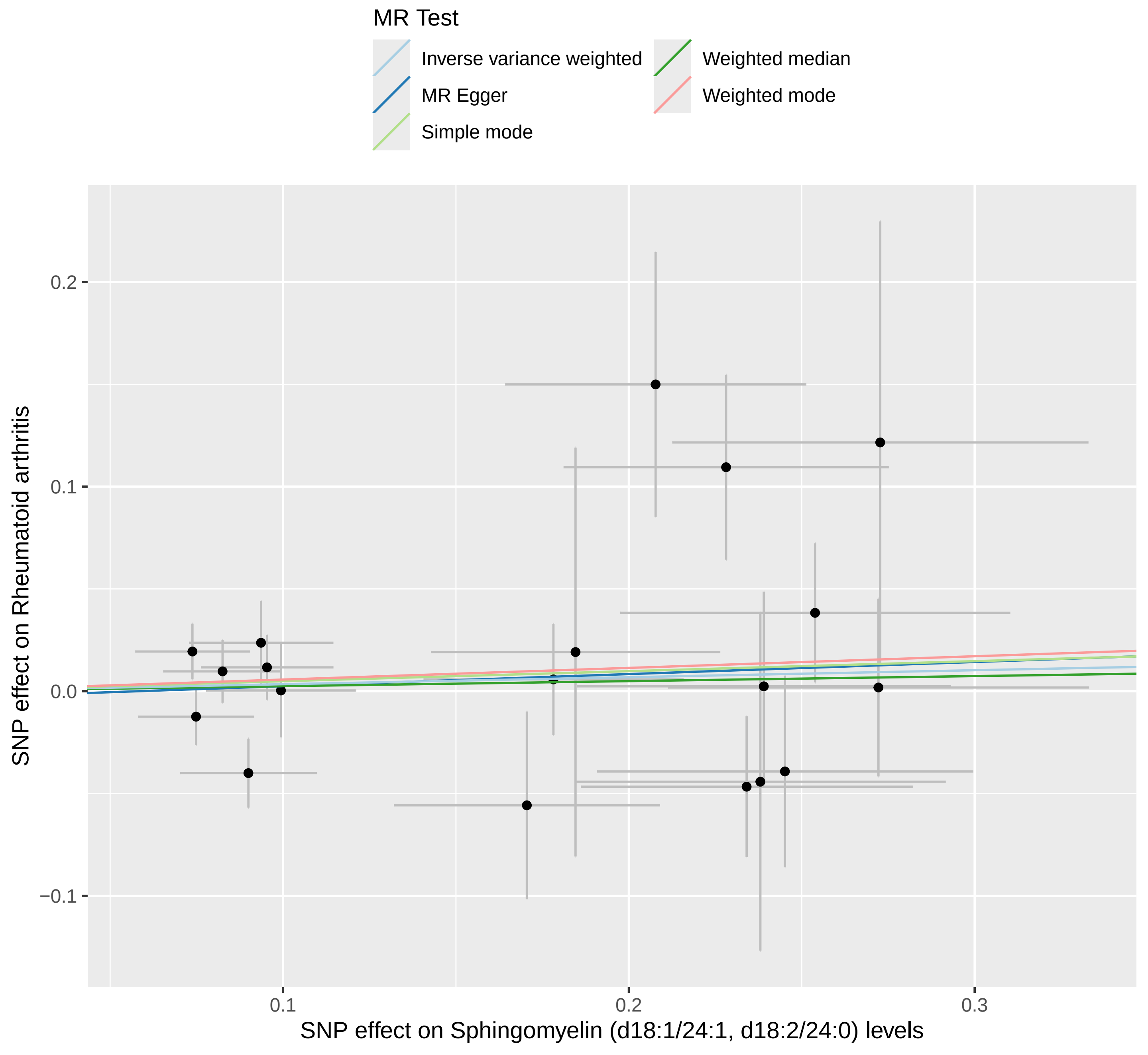
MR Test
Inverse variance weighted
MR Egger
Simple mode
Weighted median
Weighted mode
SNP effect on Rheumatoid arthritis
0.2
0.1
0.0
−0.1
0.1
0.2
0.3
SNP effect on Sphingomyelin (d18:1/24:1, d18:2/24:0) levels

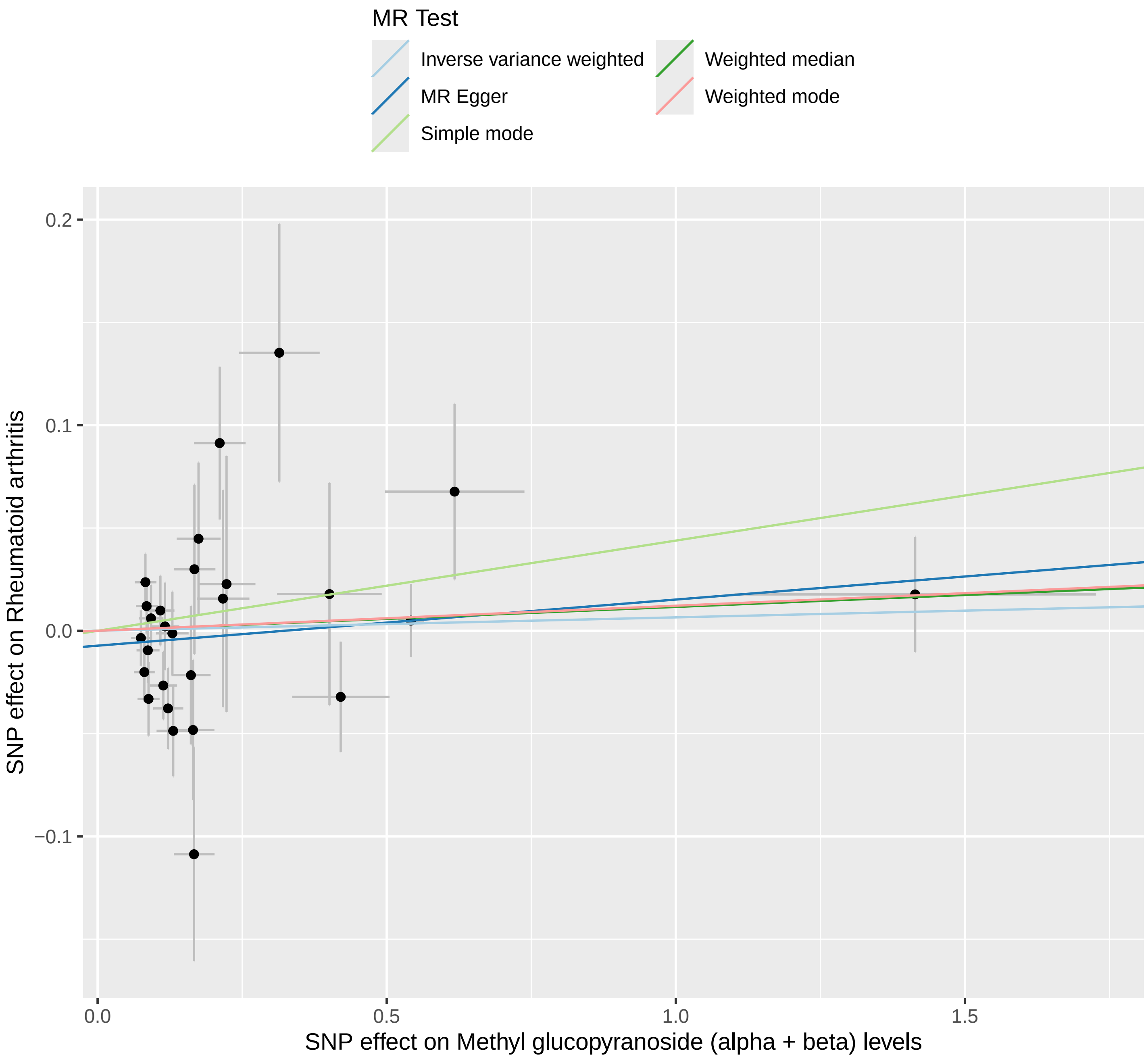
MR Test
Inverse variance weighted
MR Egger
Simple mode
Weighted median
Weighted mode
SNP effect on Rheumatoid arthritis
0.2
0.1
0.0
−0.1
0.0
0.5
1.0
1.5
SNP effect on Methyl glucopyranoside (alpha + beta) levels

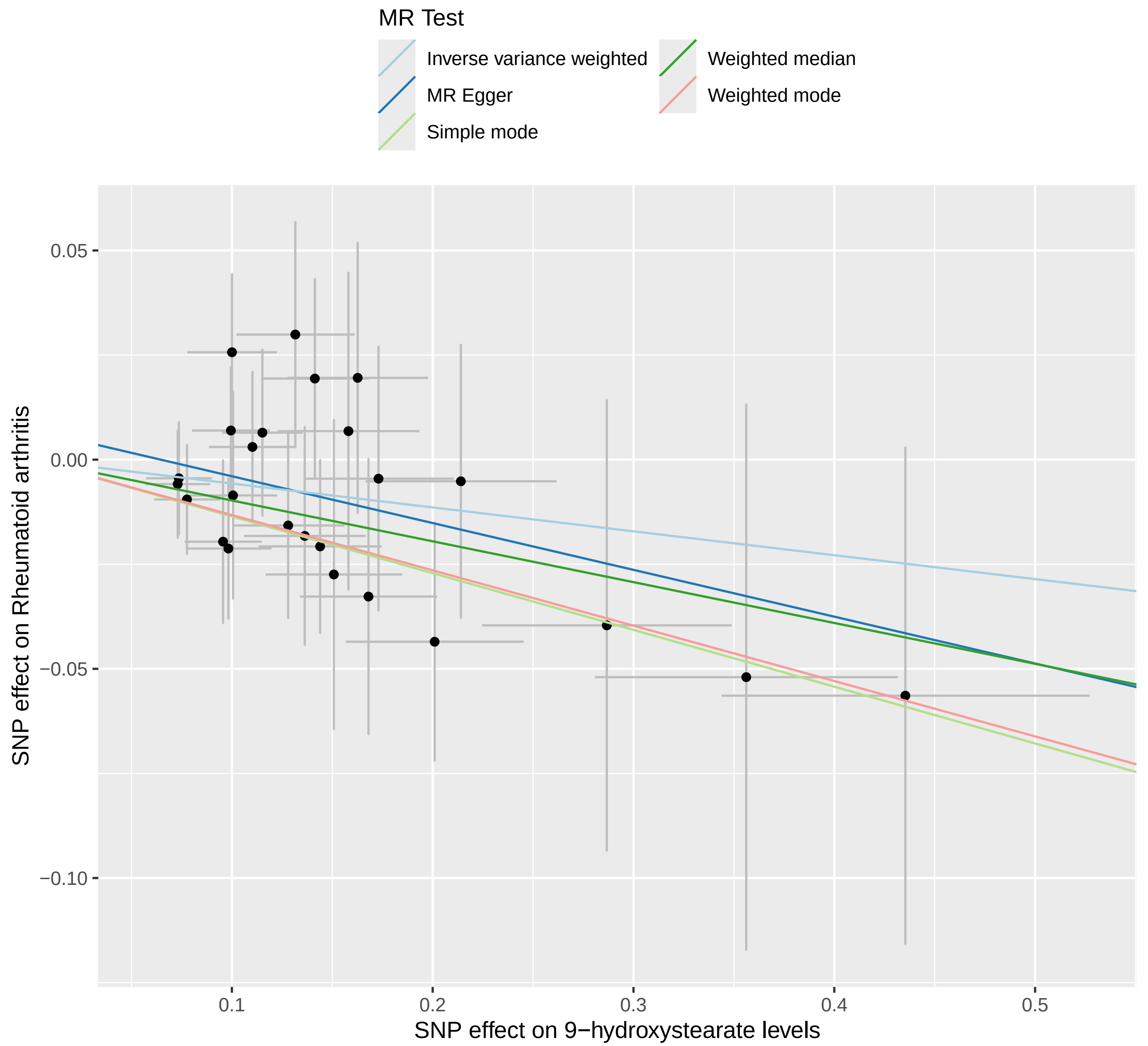
MR Test
Inverse variance weighted
MR Egger
Simple mode
Weighted median
Weighted mode
0.05
0.00
−0.05
−0.10
SNP effect on Rheumatoid arthritis
0.1
0.2
0.3
0.4
0.5
SNP effect on 9−hydroxystearate levels

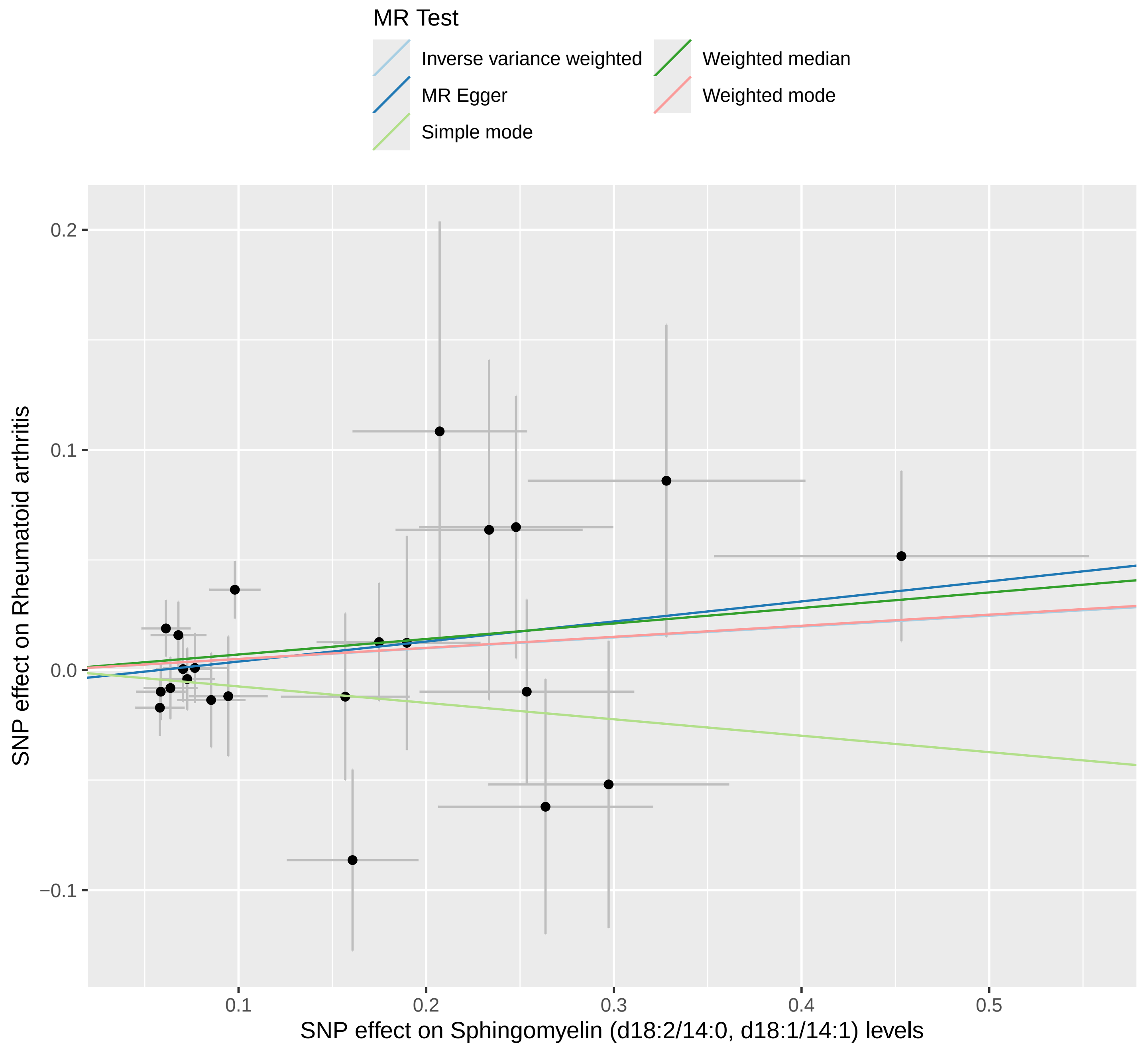
MR Test
Inverse variance weighted
MR Egger
Simple mode
Weighted median
Weighted mode
SNP effect on Rheumatoid arthritis
0.2
0.1
0.0
−0.1
0.1
0.2
0.3
0.4
0.5
SNP effect on Sphingomyelin (d18:2/14:0, d18:1/14:1) levels

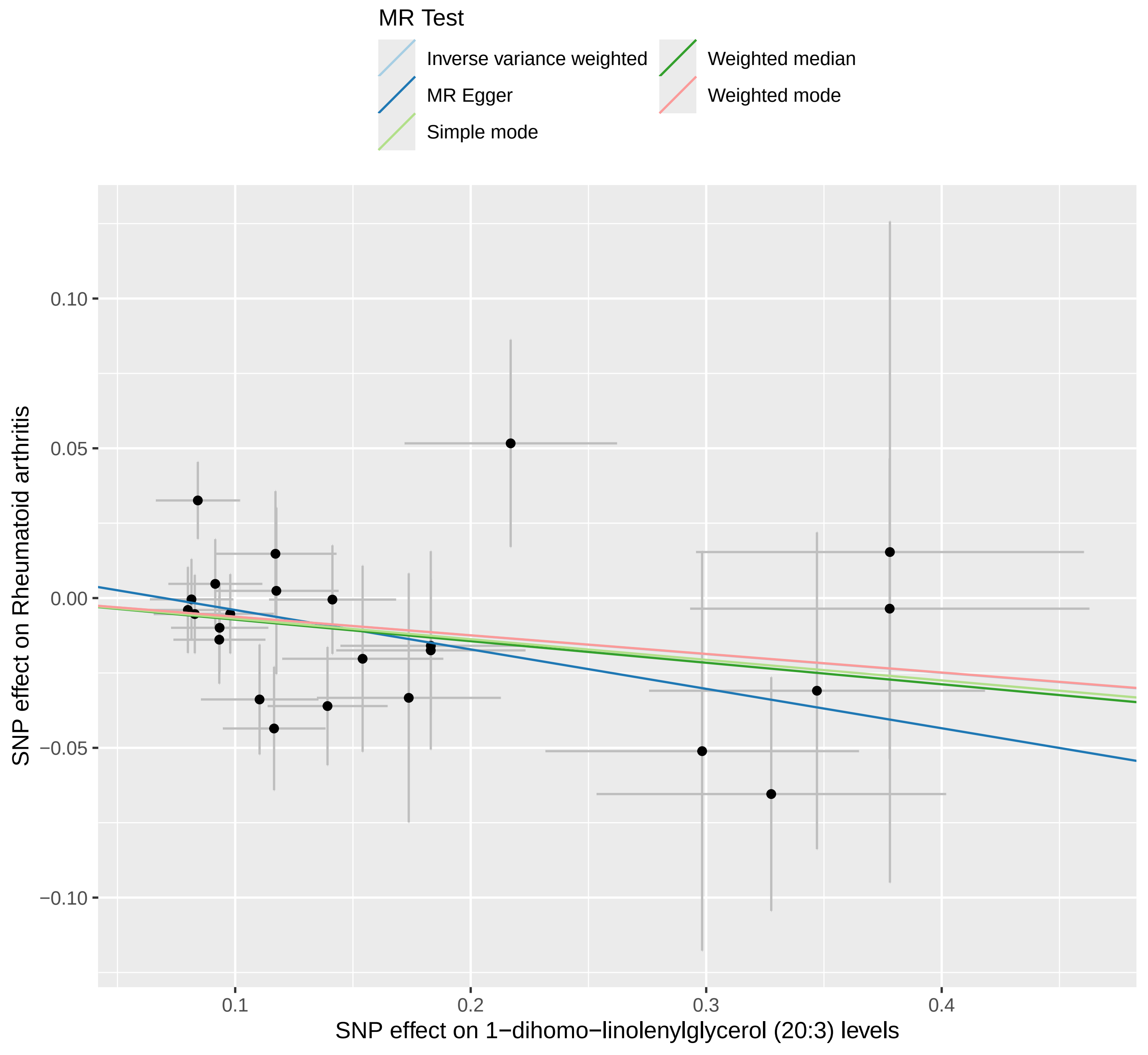

MR Test
Inverse variance weighted
MR Egger
Simple mode
Weighted median
Weighted mode
0.10
0.05
0.00
−0.05
−0.10
0.1
0.2
0.3
0.4
SNP effect on Rheumatoid arthritis
SNP effect on 1−dihomo−linolenylglycerol (20:3) levels

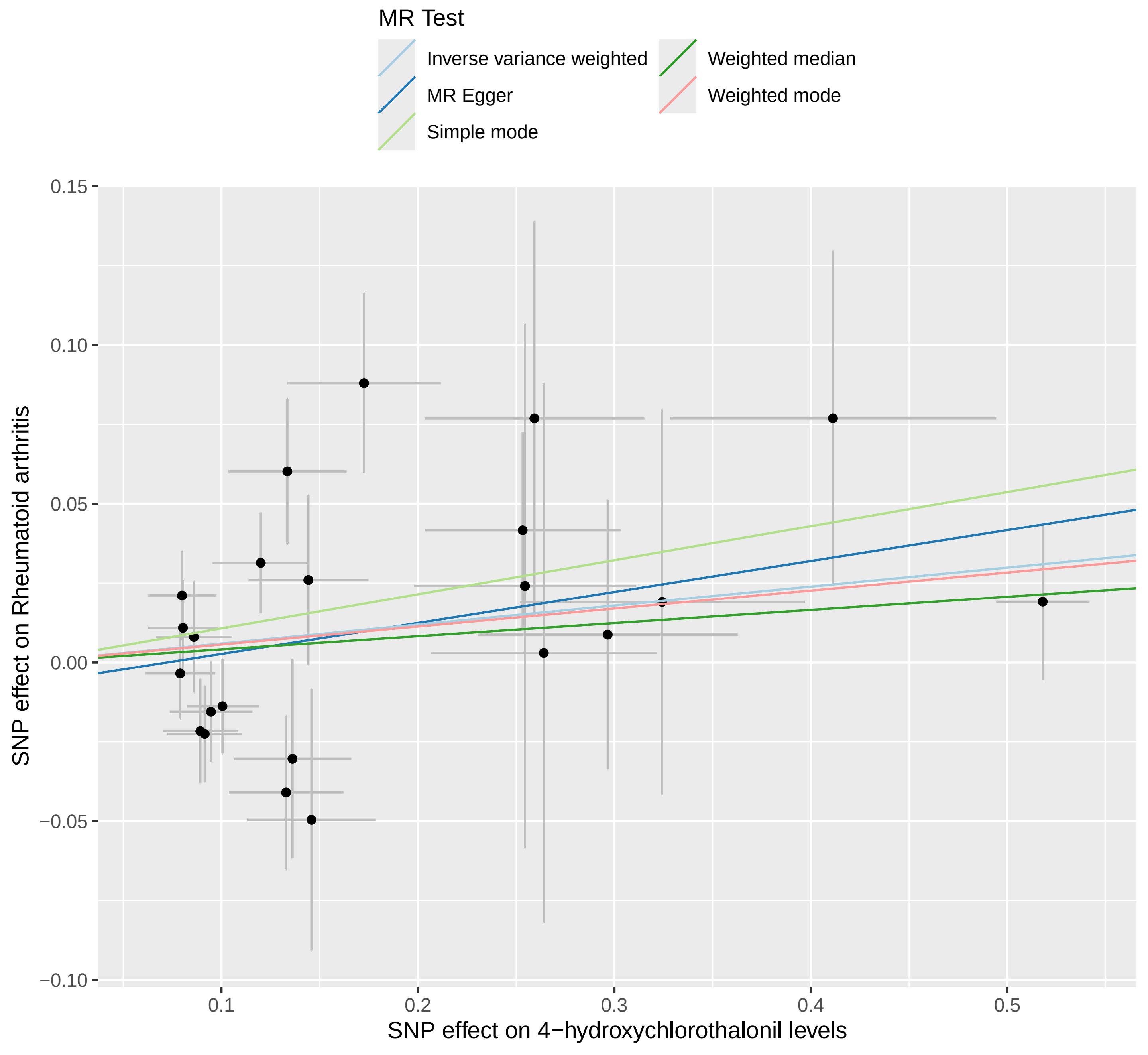

MR Test
Inverse variance weighted
MR Egger
Simple mode
Weighted median
Weighted mode
0.15
0.10
0.05
0.00
−0.05
−0.10
SNP effect on Rheumatoid arthritis
0.1
0.2
0.3
0.4
0.5
SNP effect on 4−hydroxychlorothalonil levels

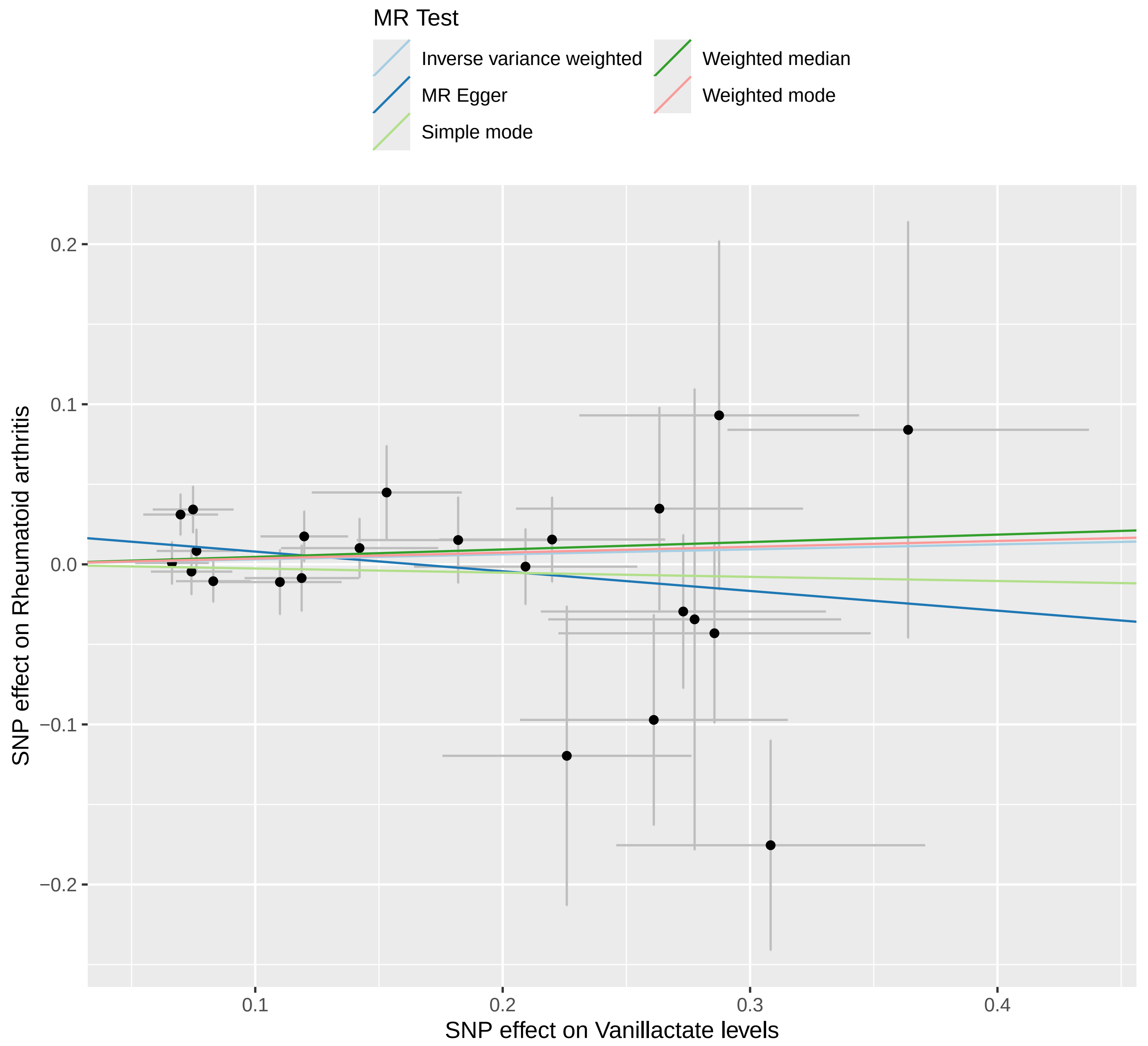

MR Test
Inverse variance weighted
MR Egger
Simple mode
Weighted median
Weighted mode
0.2
0.1
0.0
−0.1
−0.2
0.1
0.2
0.3
0.4
SNP effect on Rheumatoid arthritis
SNP effect on Vanillactate levels

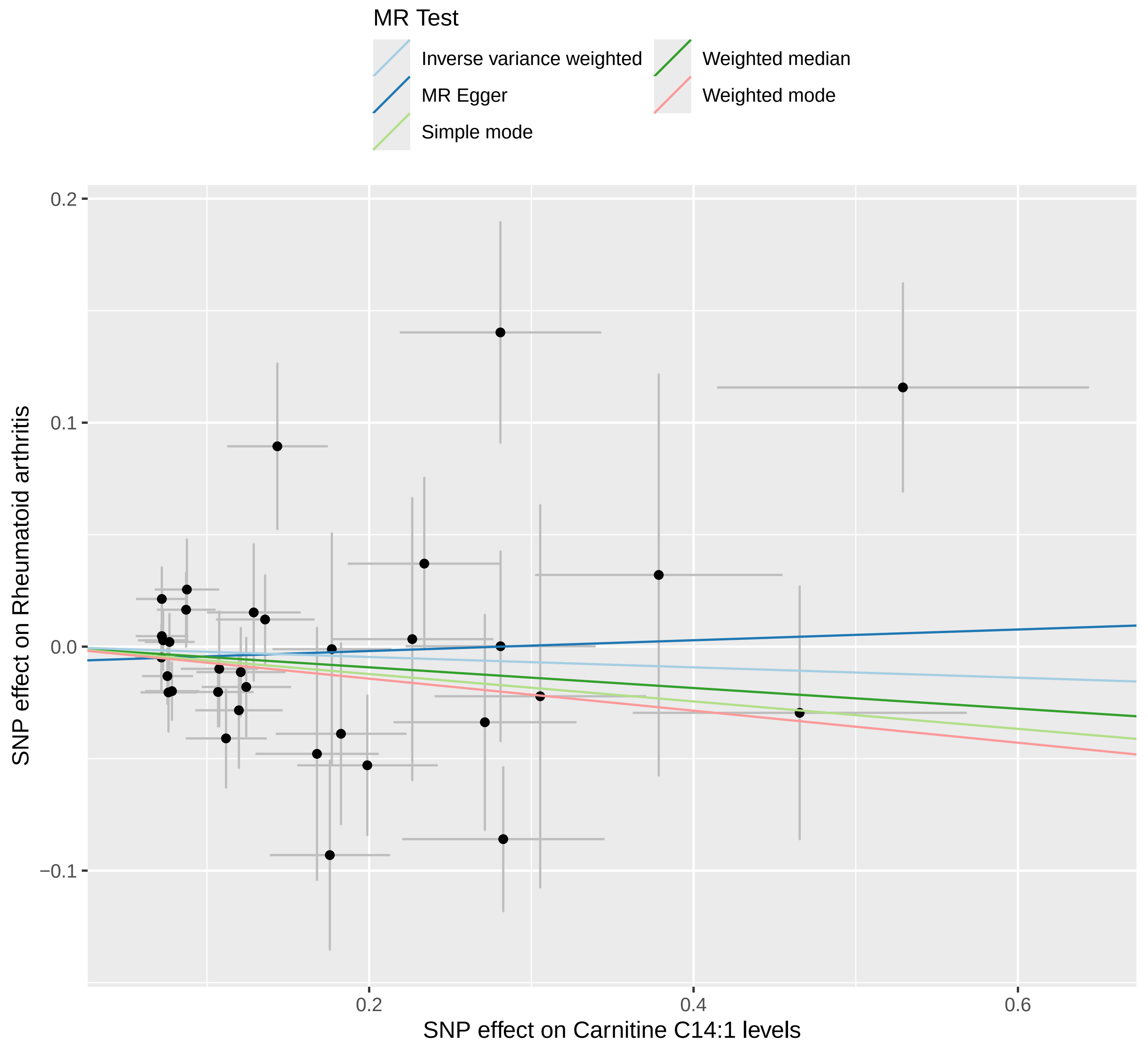

MR Test
Inverse variance weighted
MR Egger
Simple mode
Weighted median
Weighted mode
0.2
0.1
0.0
−0.1
SNP effect on Rheumatoid arthritis
0.2
0.4
0.6
SNP effect on Carnitine C14:1 levels

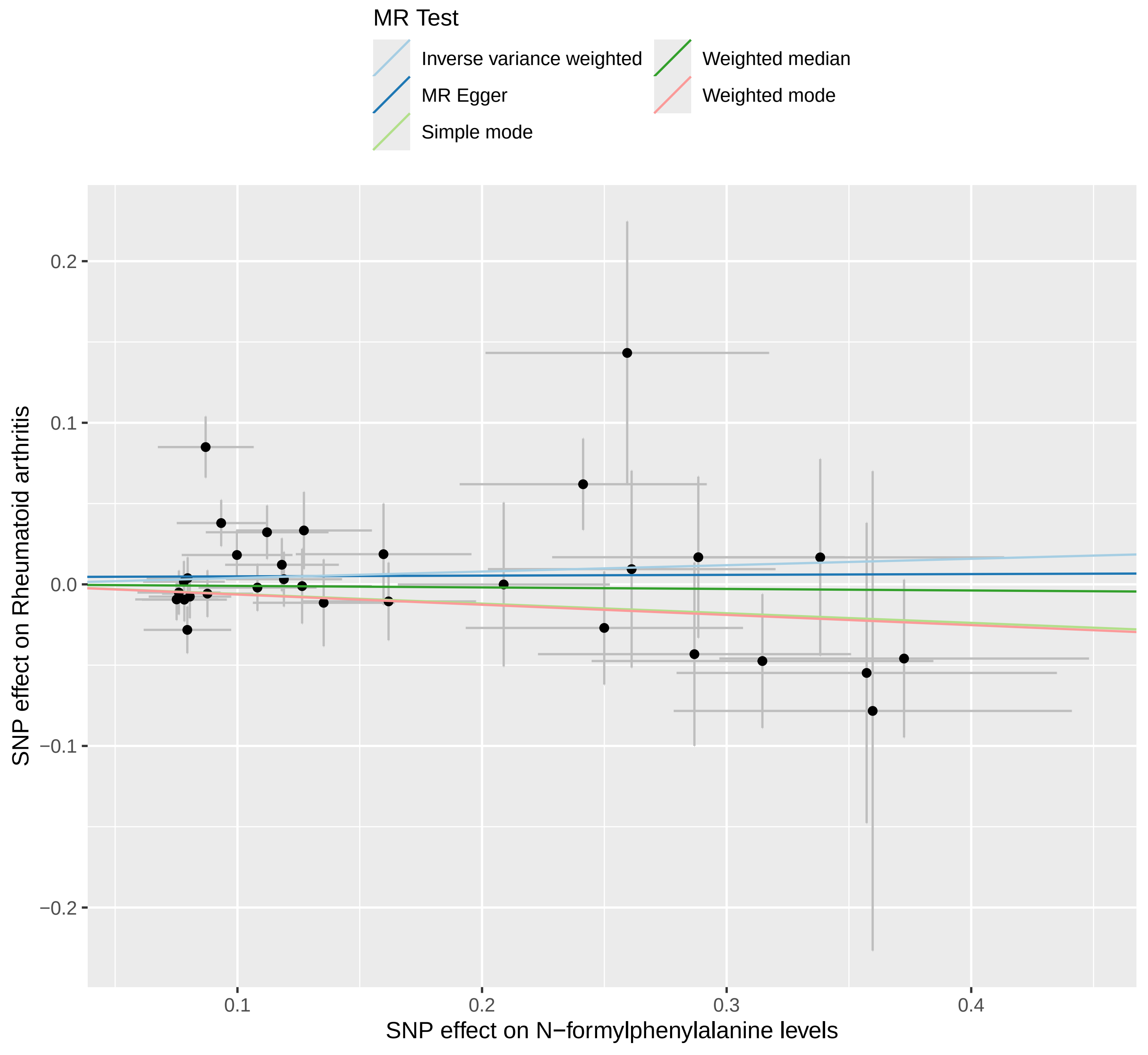

MR Test
Inverse variance weighted
MR Egger
Simple mode
Weighted median
Weighted mode
0.2
0.1
0.0
−0.1
−0.2
SNP effect on Rheumatoid arthritis
0.1
0.2
0.3
0.4
SNP effect on N−formylphenylalanine levels

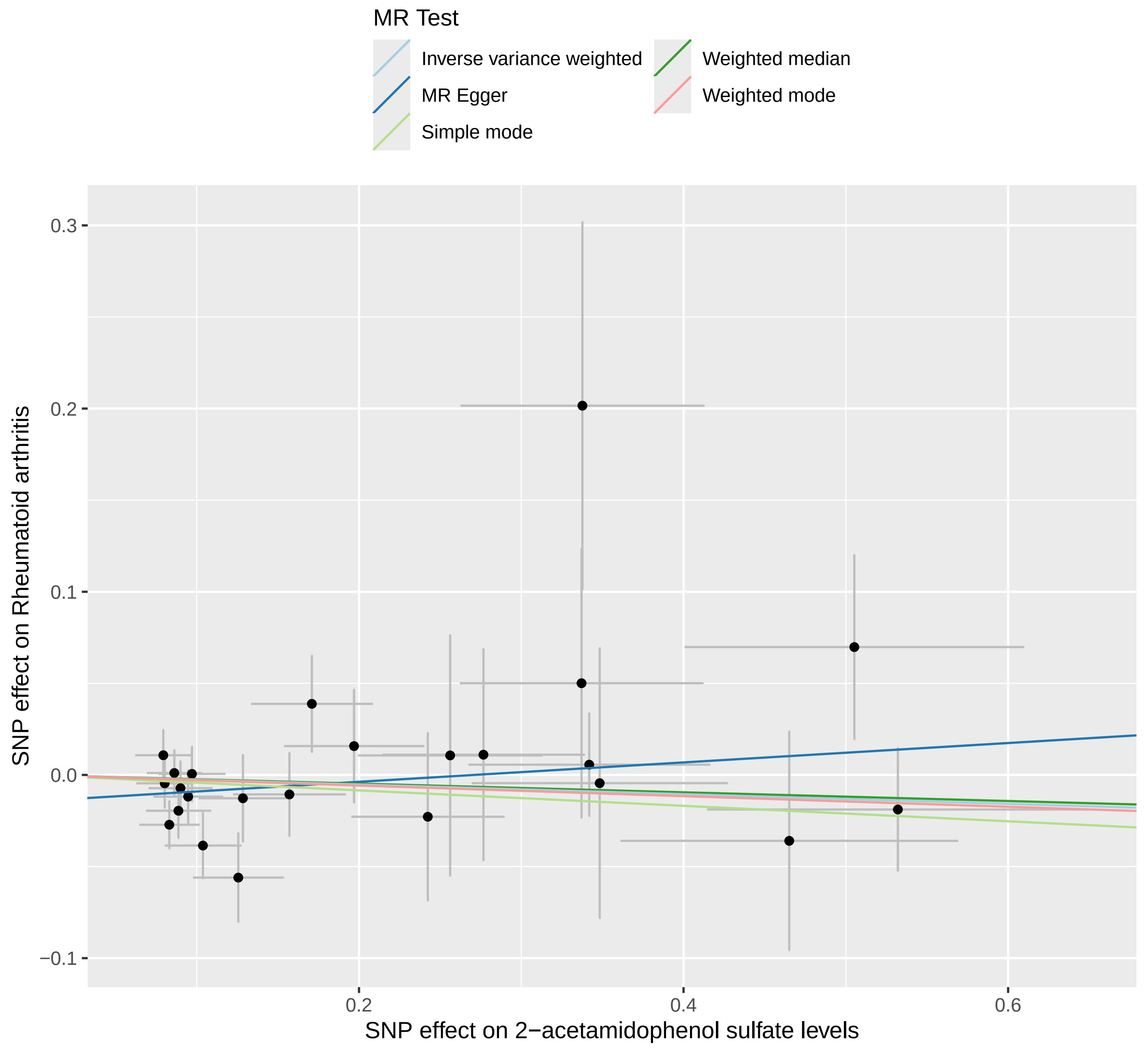

MR Test
Inverse variance weighted
MR Egger
Simple mode
Weighted median
Weighted mode
0.3
0.2
0.1
0.0
−0.1
0.2
0.4
0.6
SNP effect on Rheumatoid arthritis
SNP effect on 2−acetamidophenol sulfate levels

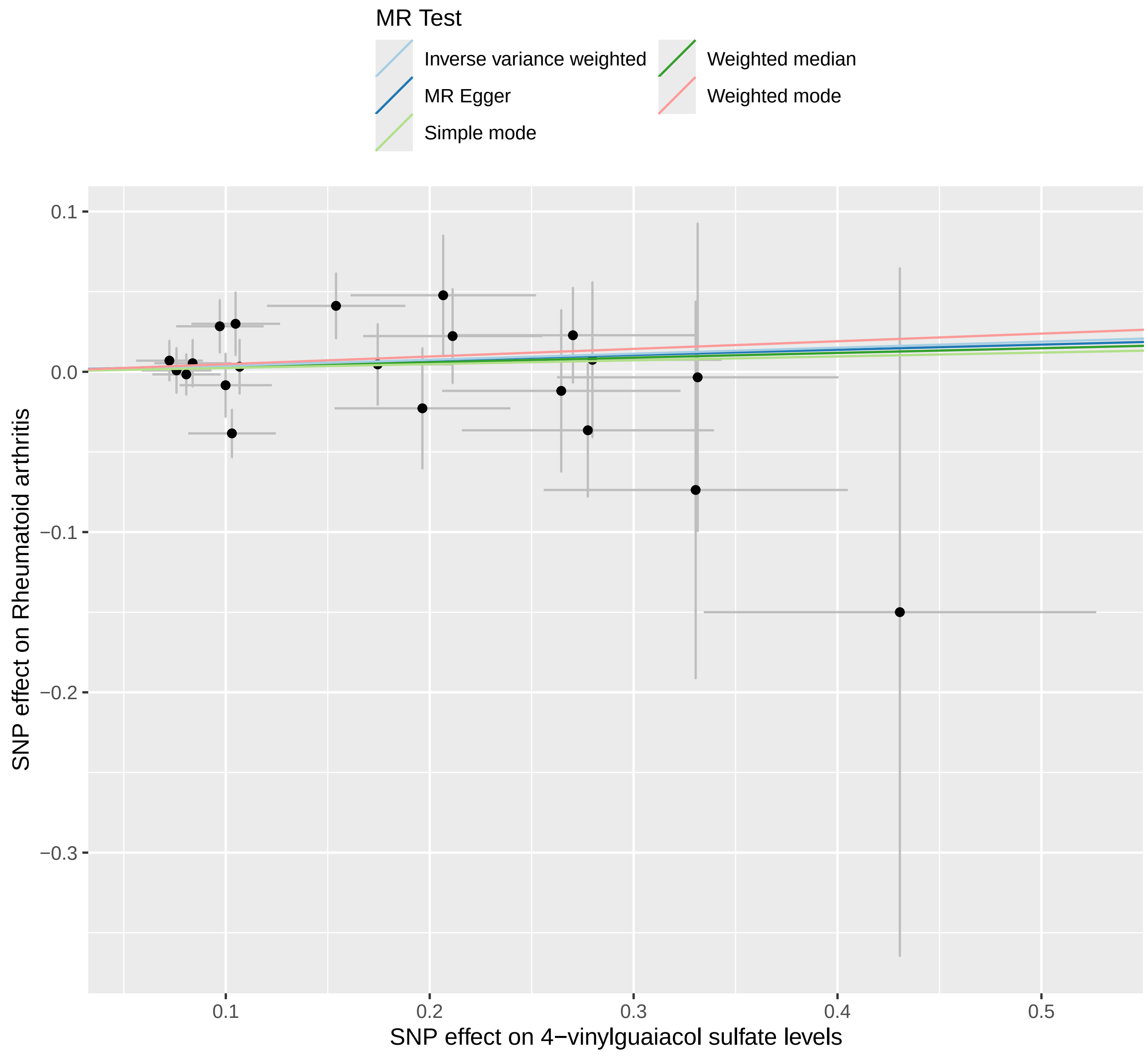
MR Test
Inverse variance weighted
MR Egger
Simple mode
Weighted median
Weighted mode
0.1
0.0
−0.1
−0.2
−0.3
0.1
0.2
0.3
0.4
0.5
SNP effect on Rheumatoid arthritis
SNP effect on 4−vinylguaiacol sulfate levels

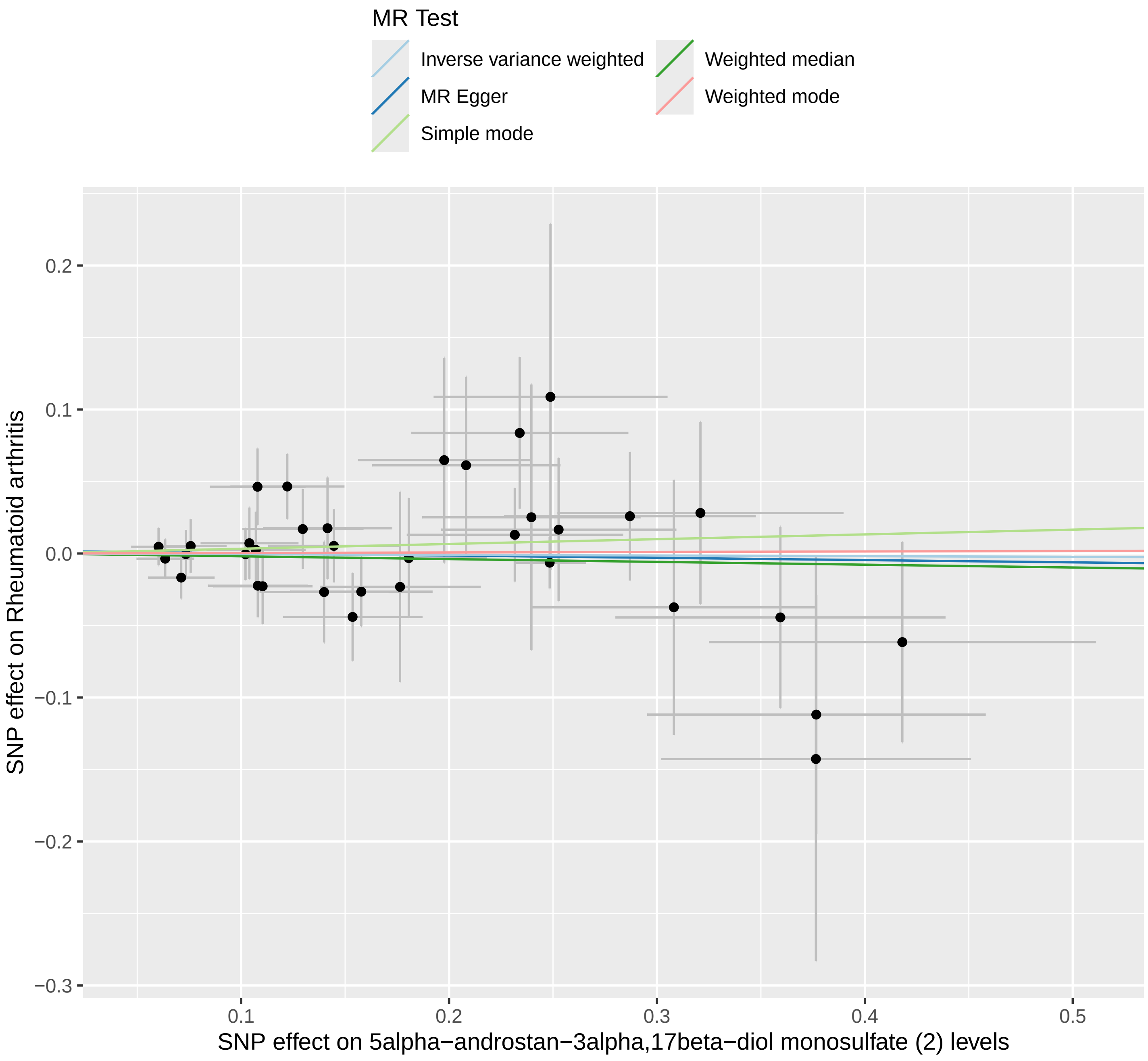
MR Test
Inverse variance weighted
MR Egger
Simple mode
Weighted median
Weighted mode
SNP effect on Rheumatoid arthritis
SNP effect on 5alpha−androstan−3alpha,17beta−diol monosulfate (2) levels
0.2
0.1
0.0
−0.1
−0.2
−0.3
0.1
0.2
0.3
0.4
0.5

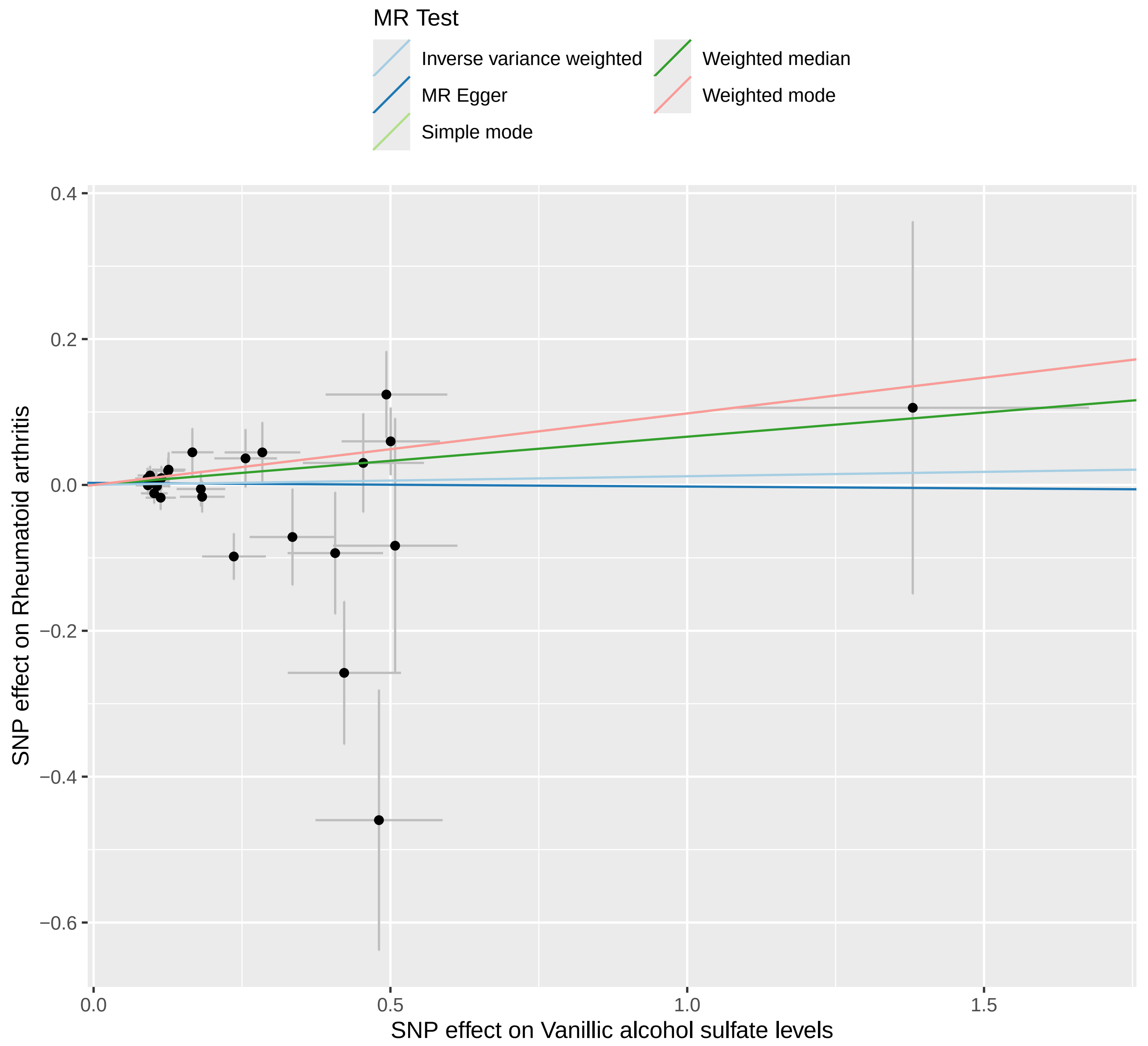
MR Test
Inverse variance weighted
MR Egger
Simple mode
Weighted median
Weighted mode
0.4
0.2
0.0
−0.2
−0.4
−0.6
SNP effect on Rheumatoid arthritis
0.0
0.5
1.0
1.5
SNP effect on Vanillic alcohol sulfate levels

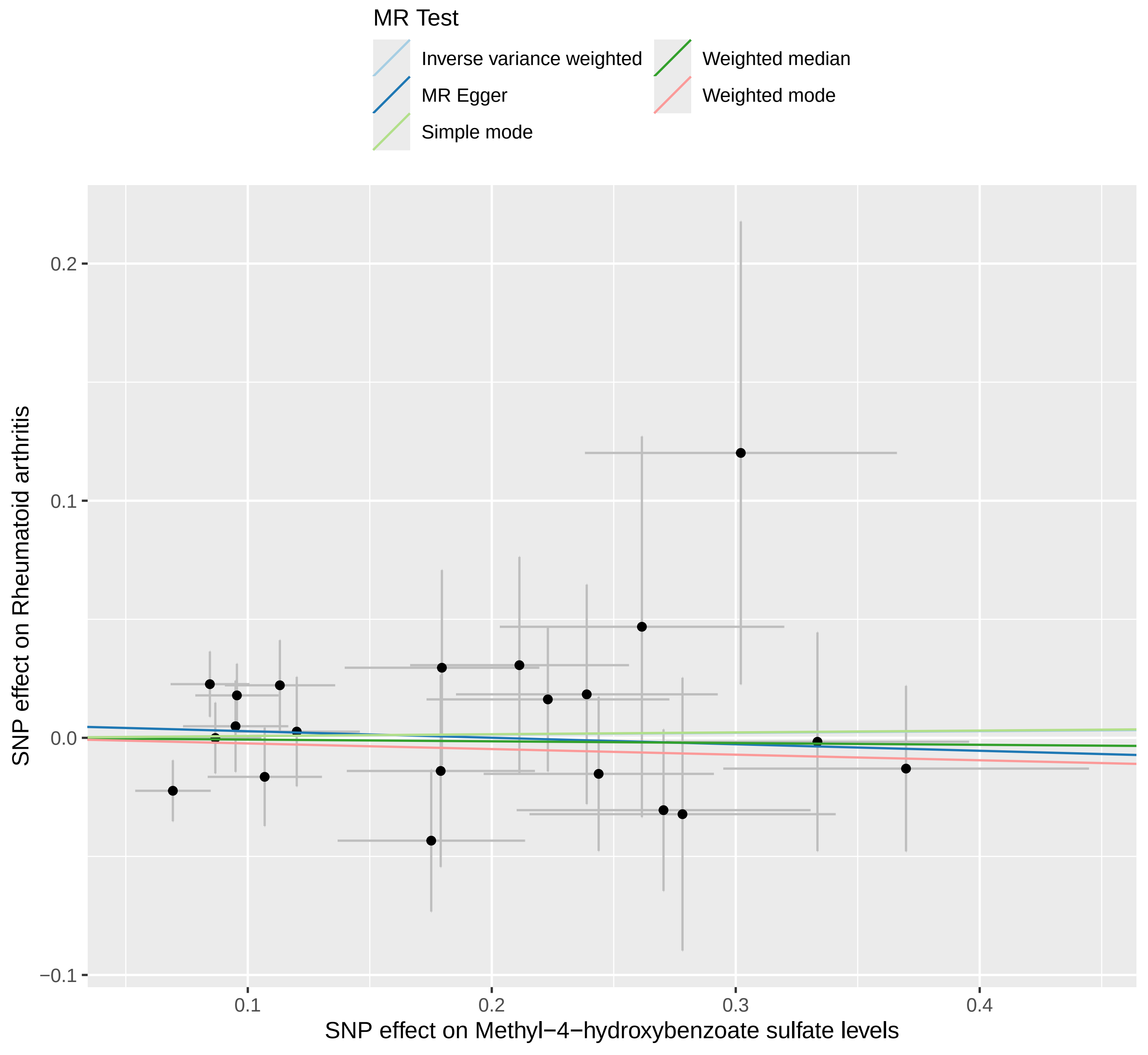
MR Test
Inverse variance weighted
MR Egger
Simple mode
Weighted median
Weighted mode
SNP effect on Rheumatoid arthritis
0.2
0.1
0.0
−0.1
0.1
0.2
0.3
0.4
SNP effect on Methyl−4−hydroxybenzoate sulfate levels

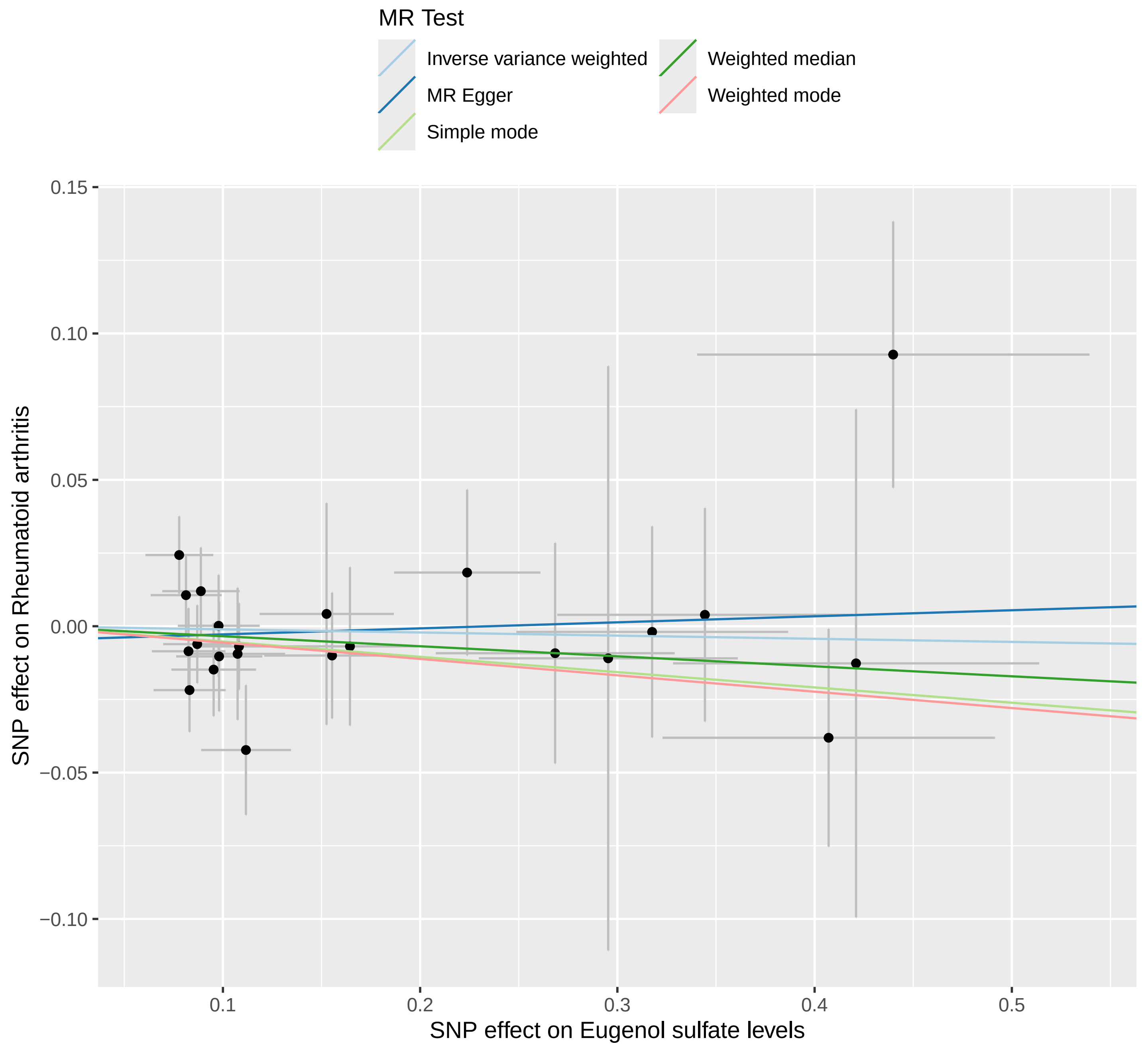

MR Test
Inverse variance weighted
MR Egger
Simple mode
Weighted median
Weighted mode
SNP effect on Rheumatoid arthritis
0.15
0.10
0.05
0.00
−0.05
−0.10
0.1
0.2
0.3
0.4
0.5
SNP effect on Eugenol sulfate levels

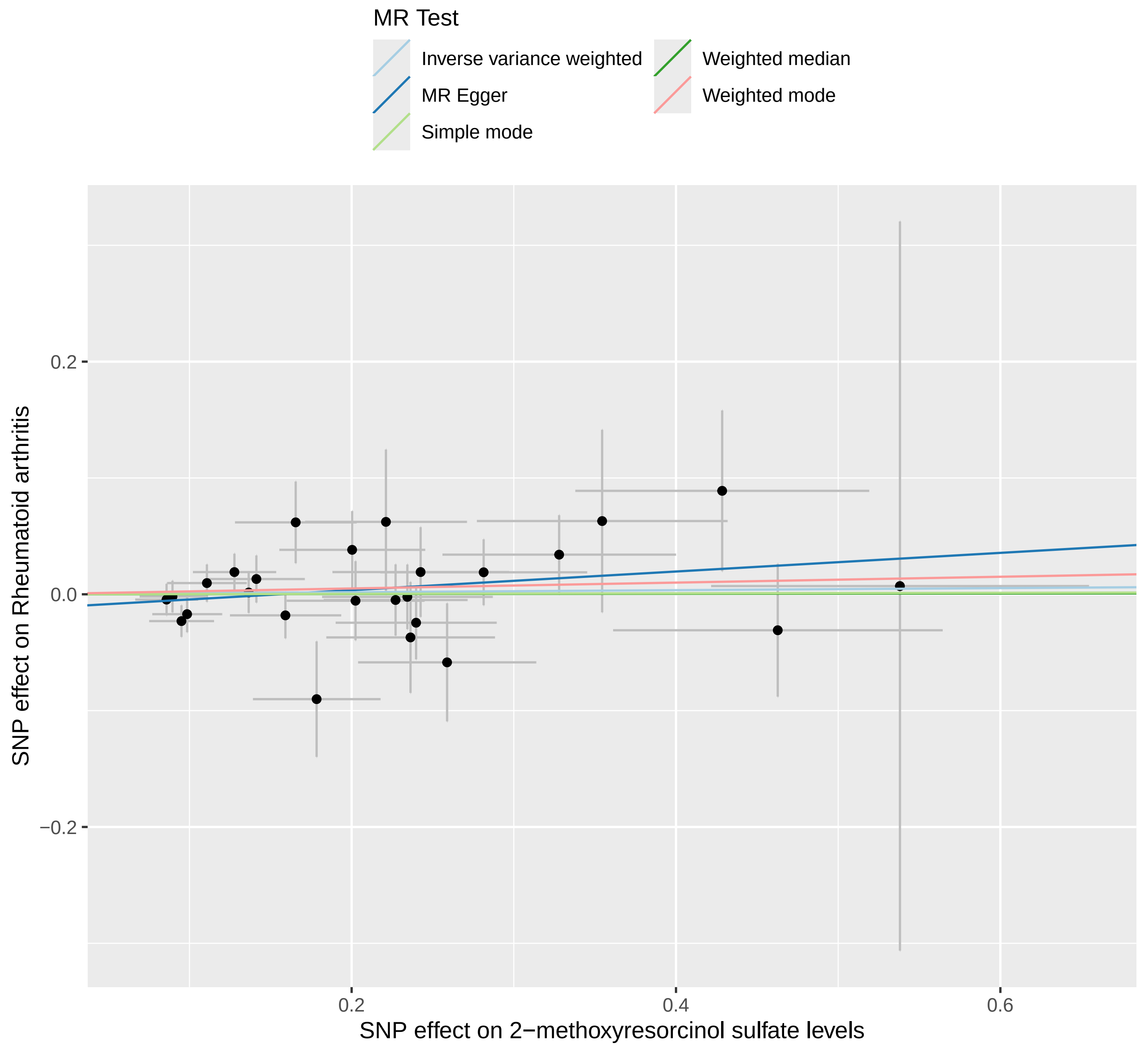

MR Test
Inverse variance weighted
MR Egger
Simple mode
Weighted median
Weighted mode
0.2
0.0
−0.2
SNP effect on Rheumatoid arthritis
0.2
0.4
0.6
SNP effect on 2−methoxyresorcinol sulfate levels

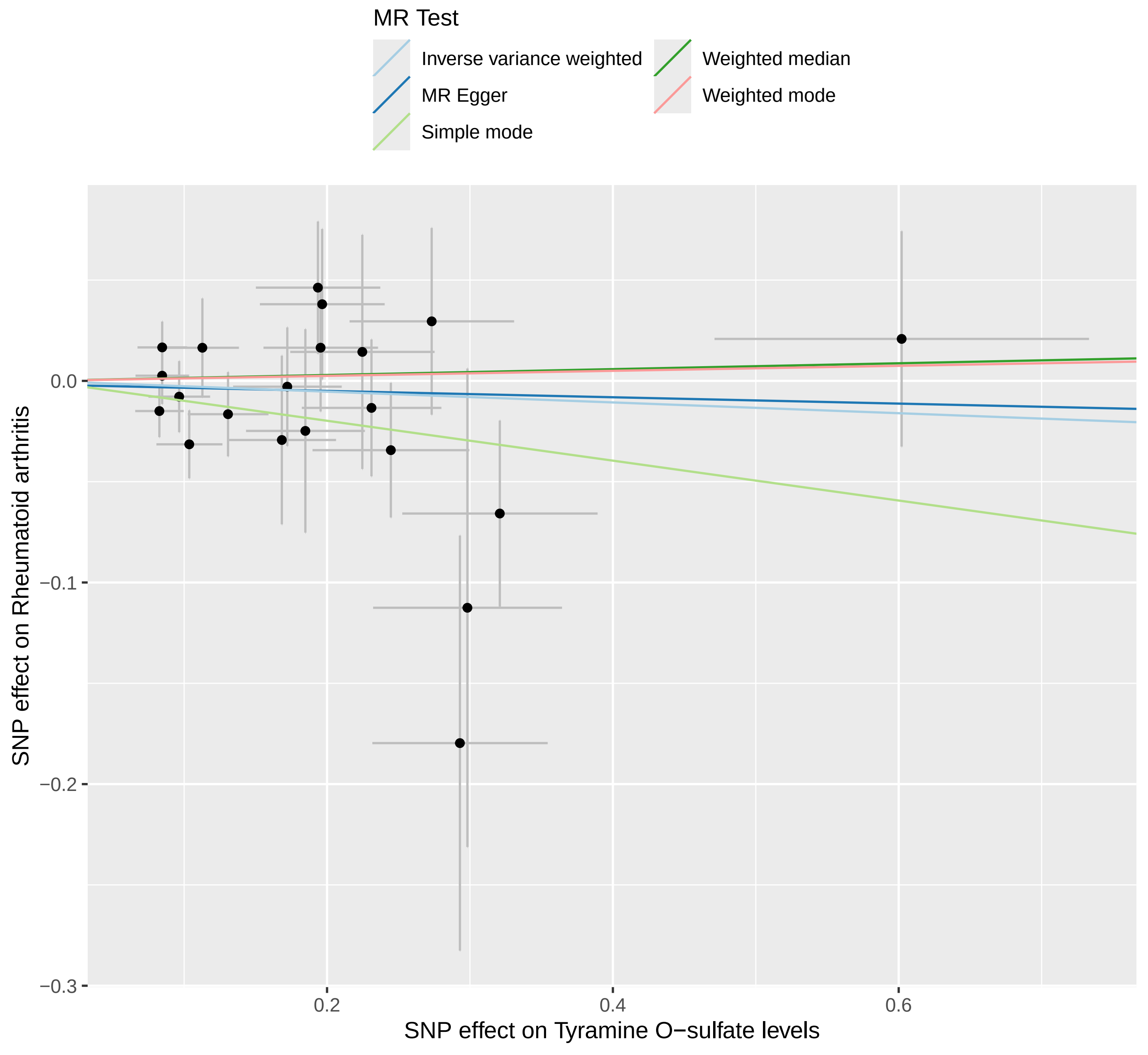
MR Test
Inverse variance weighted
MR Egger
Simple mode
Weighted median
Weighted mode
0.0
−0.1
−0.2
−0.3
0.2
0.4
0.6
SNP effect on Rheumatoid arthritis
SNP effect on Tyramine O−sulfate levels

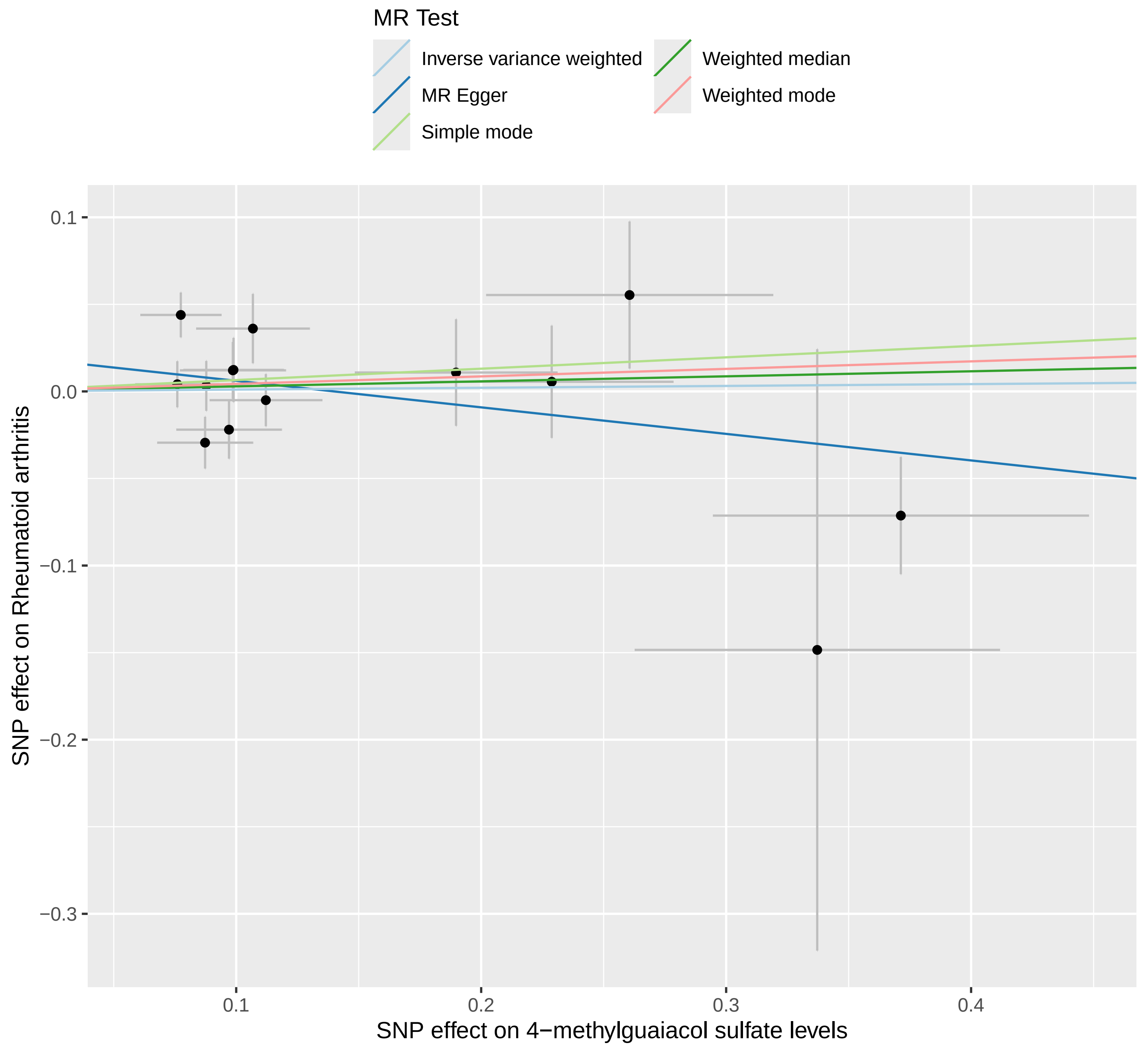
MR Test
Inverse variance weighted
MR Egger
Simple mode
Weighted median
Weighted mode
0.1
0.0
−0.1
−0.2
−0.3
SNP effect on Rheumatoid arthritis
0.1
0.2
0.3
0.4
SNP effect on 4−methylguaiacol sulfate levels

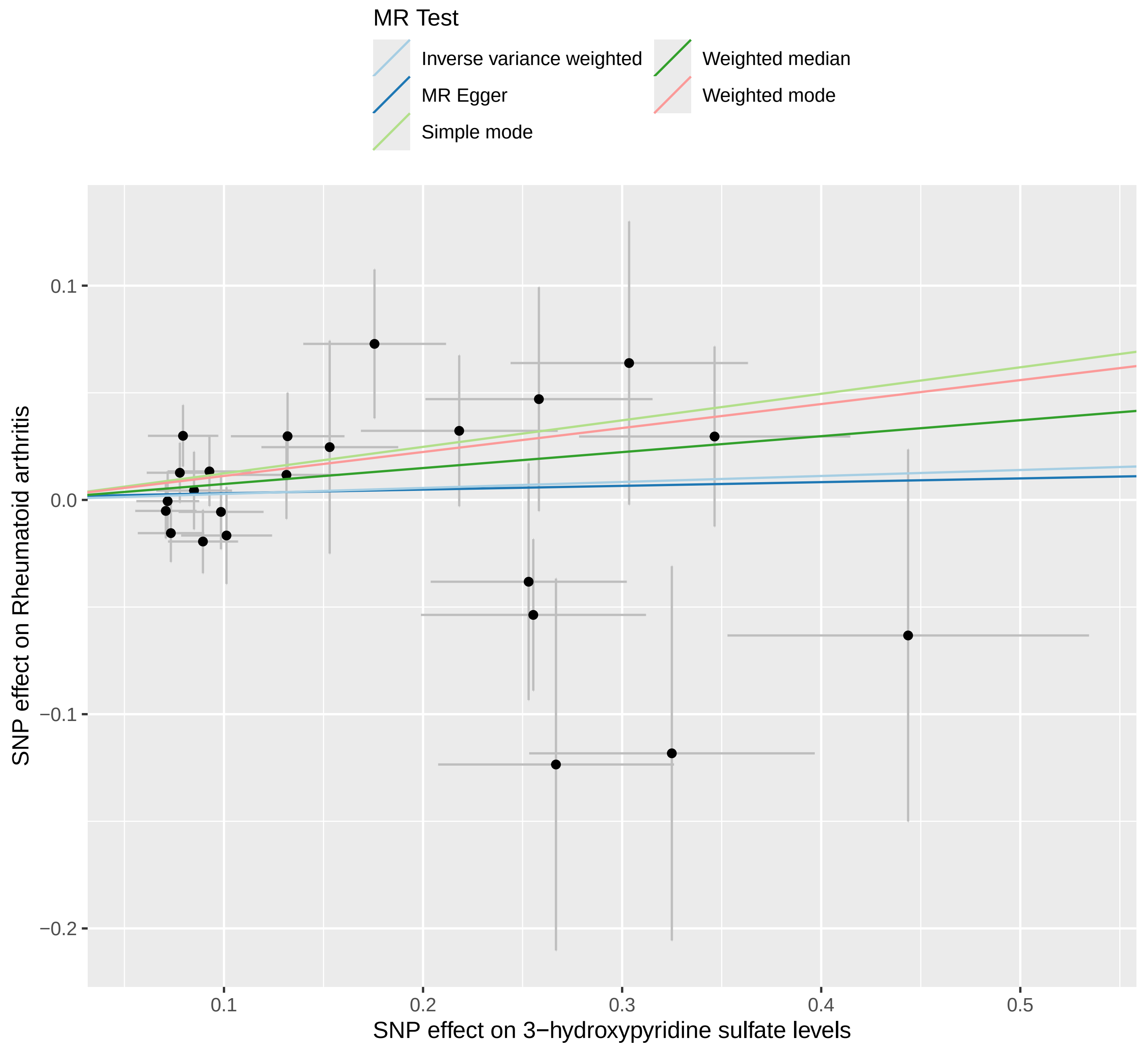

MR Test
Inverse variance weighted
MR Egger
Simple mode
Weighted median
Weighted mode
0.1
0.0
−0.1
−0.2
0.1
0.2
0.3
0.4
0.5
SNP effect on Rheumatoid arthritis
SNP effect on 3−hydroxypyridine sulfate levels

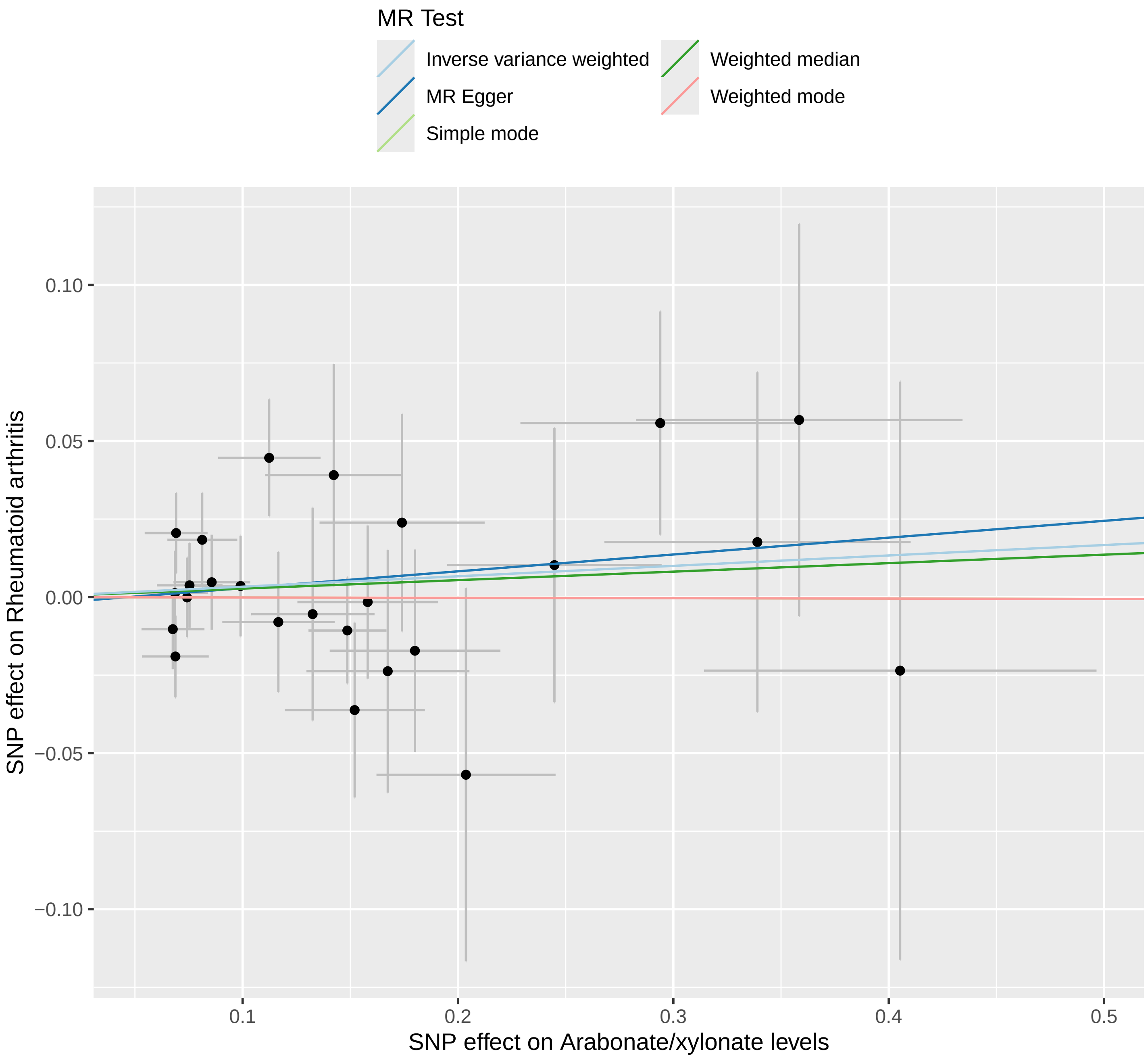
MR Test
Inverse variance weighted
MR Egger
Simple mode
Weighted median
Weighted mode
SNP effect on Rheumatoid arthritis
0.10
0.05
0.00
−0.05
−0.10
0.1
0.2
0.3
0.4
0.5
SNP effect on Arabonate/xylonate levels

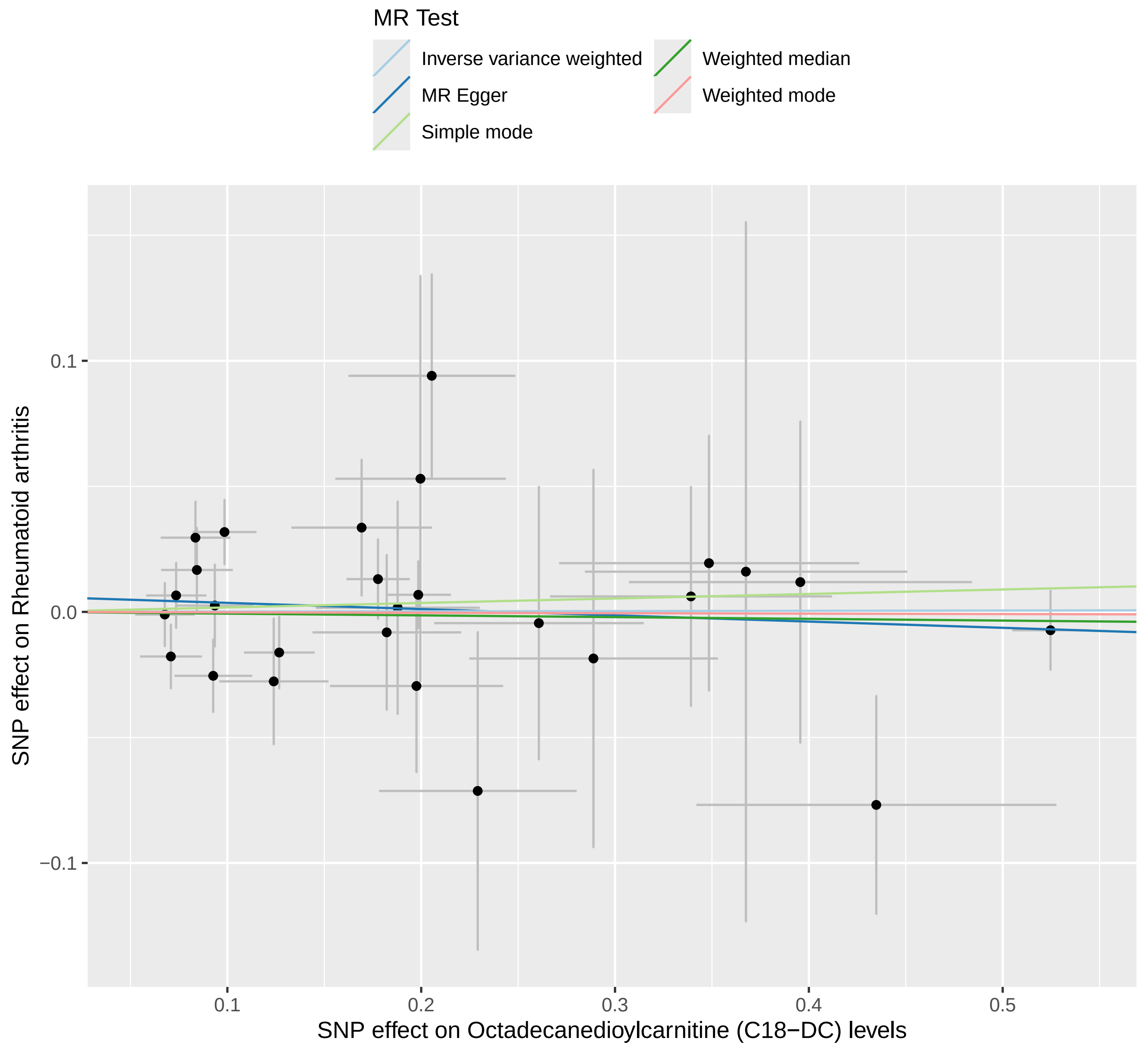
MR Test
Inverse variance weighted
MR Egger
Simple mode
Weighted median
Weighted mode
0.1
0.0
−0.1
SNP effect on Rheumatoid arthritis
0.1
0.2
0.3
0.4
0.5
SNP effect on Octadecanedioylcarnitine (C18−DC) levels

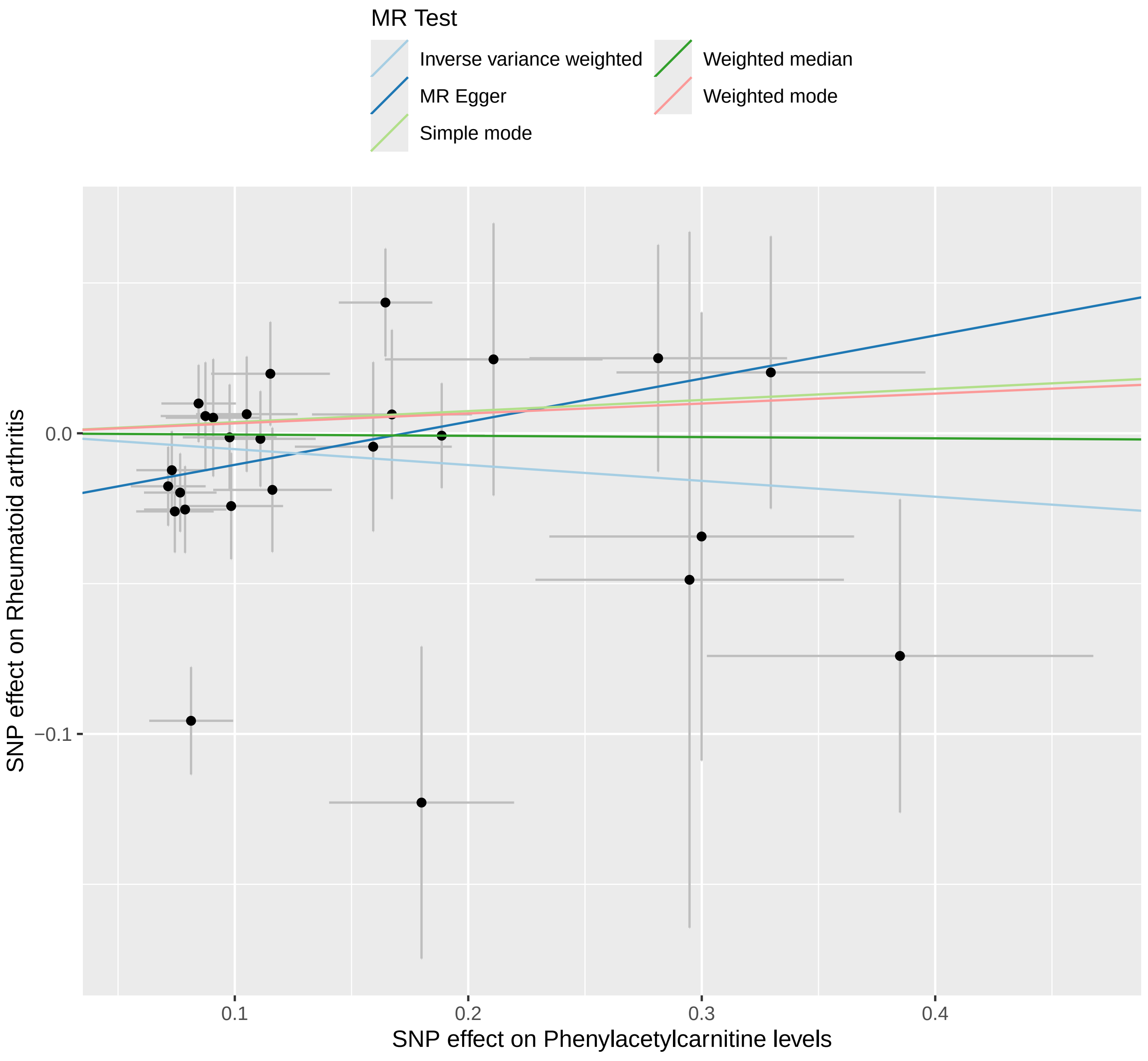

MR Test
Inverse variance weighted
MR Egger
Simple mode
Weighted median
Weighted mode
0.0
−0.1
0.1
0.2
0.3
0.4
SNP effect on Rheumatoid arthritis
SNP effect on Phenylacetylcarnitine levels

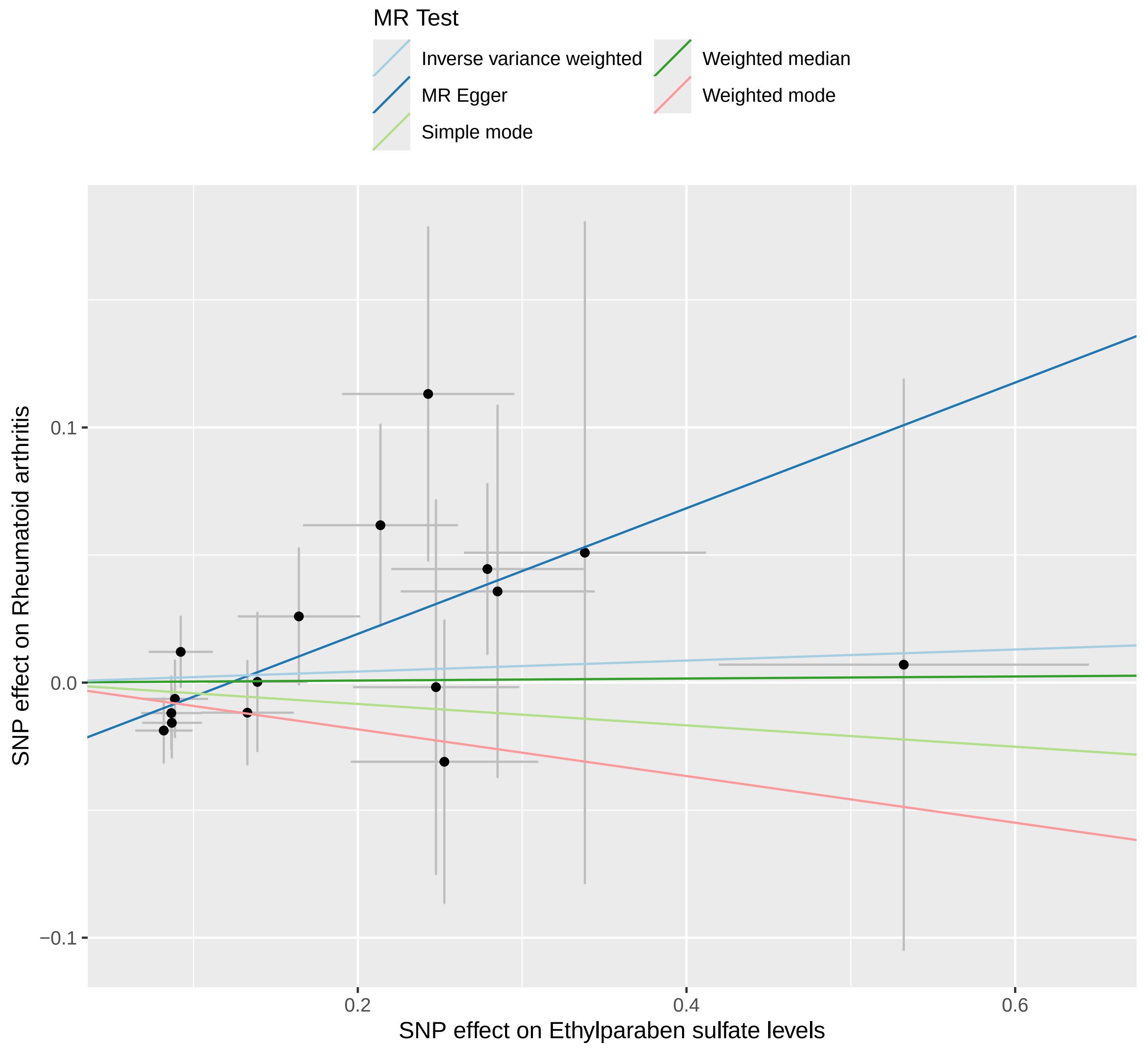
MR Test
Inverse variance weighted
MR Egger
Simple mode
Weighted median
Weighted mode
SNP effect on Rheumatoid arthritis
0.1
0.0
−0.1
0.2
0.4
0.6
SNP effect on Ethylparaben sulfate levels

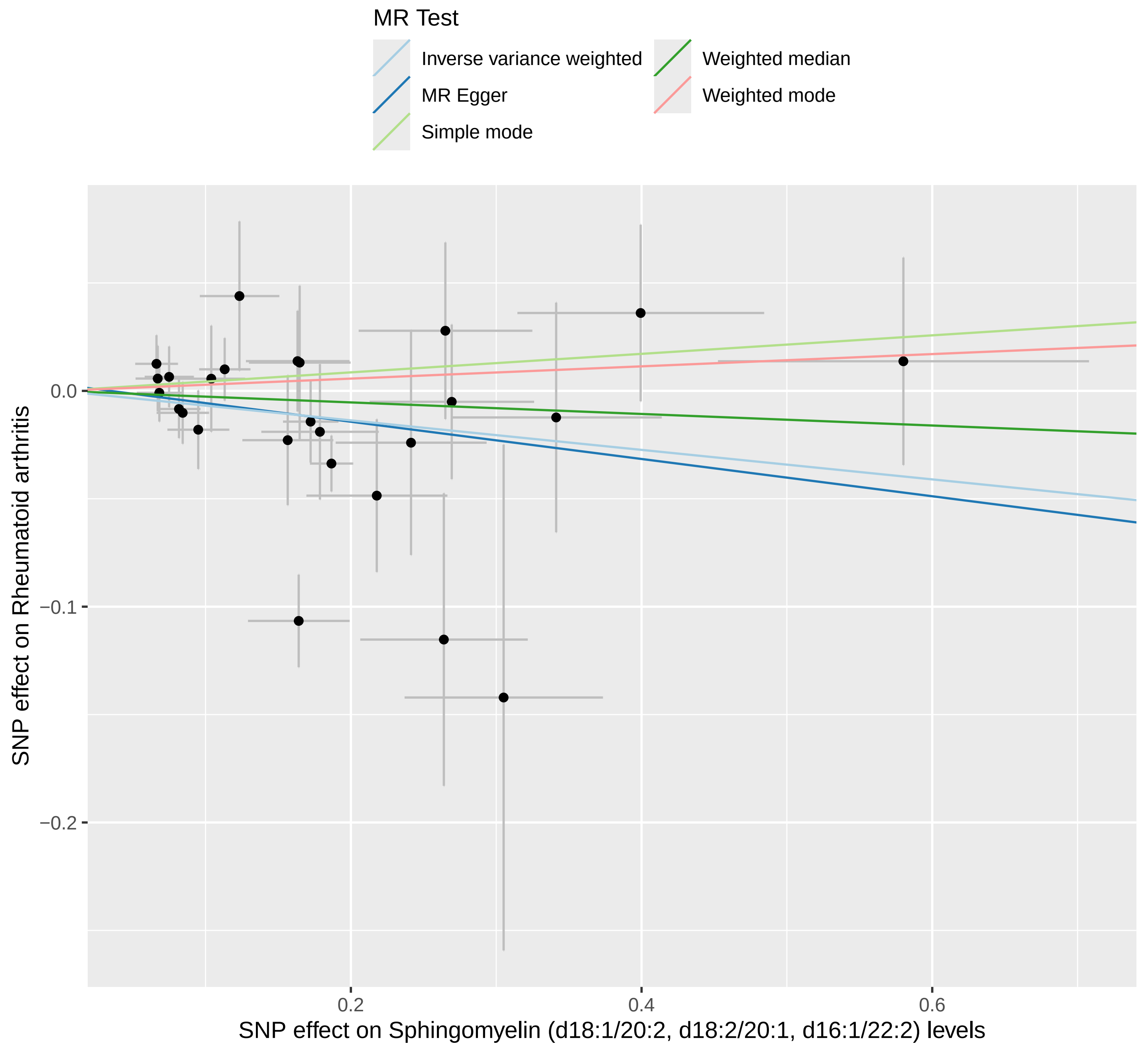

MR Test
Inverse variance weighted
MR Egger
Simple mode
Weighted median
Weighted mode
SNP effect on Rheumatoid arthritis
0.0
−0.1
−0.2
0.2
0.4
0.6
SNP effect on Sphingomyelin (d18:1/20:2, d18:2/20:1, d16:1/22:2) levels

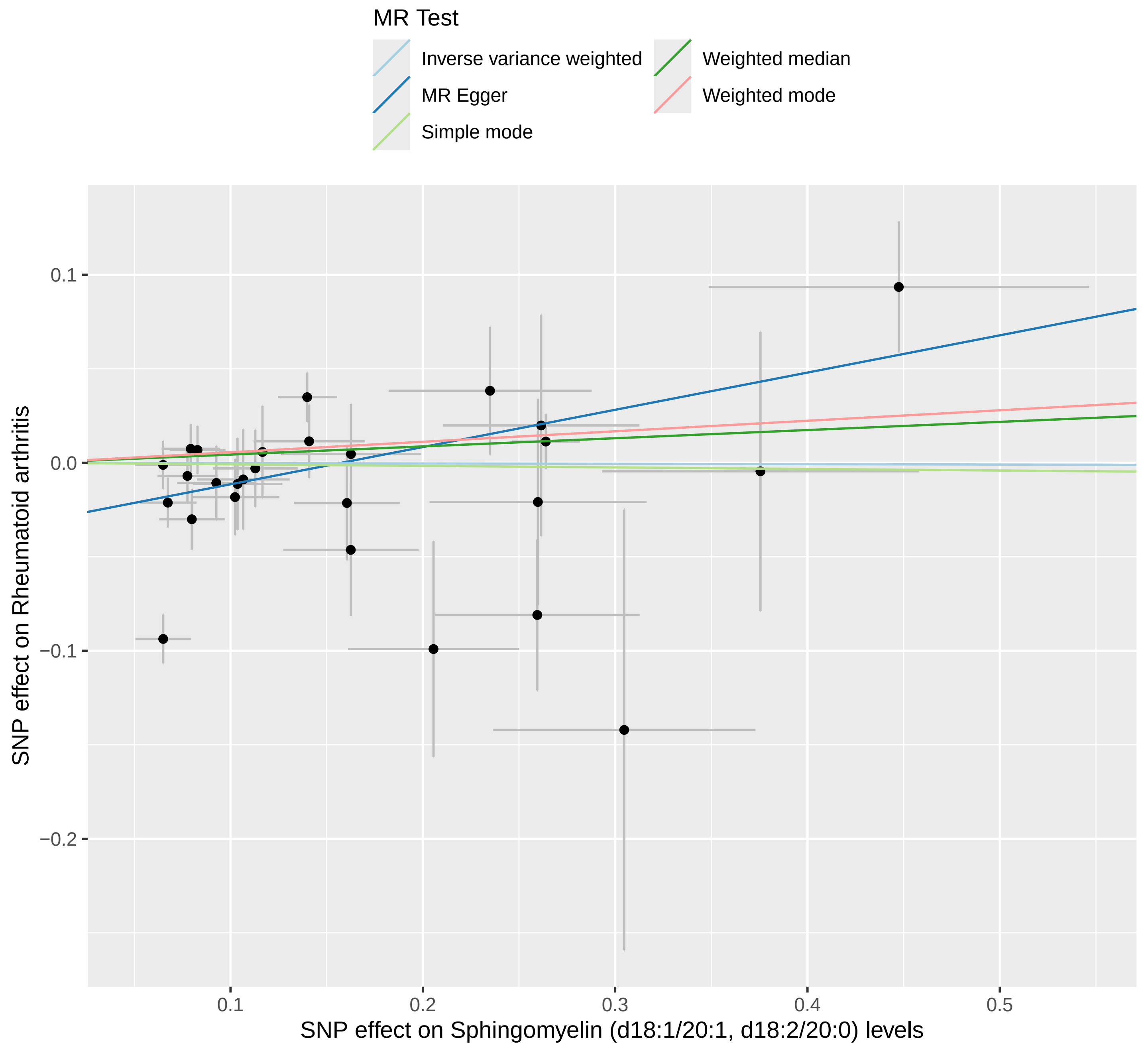

MR Test
Inverse variance weighted
MR Egger
Simple mode
Weighted median
Weighted mode
0.1
0.0
−0.1
−0.2
SNP effect on Rheumatoid arthritis
0.1
0.2
0.3
0.4
0.5
SNP effect on Sphingomyelin (d18:1/20:1, d18:2/20:0) levels

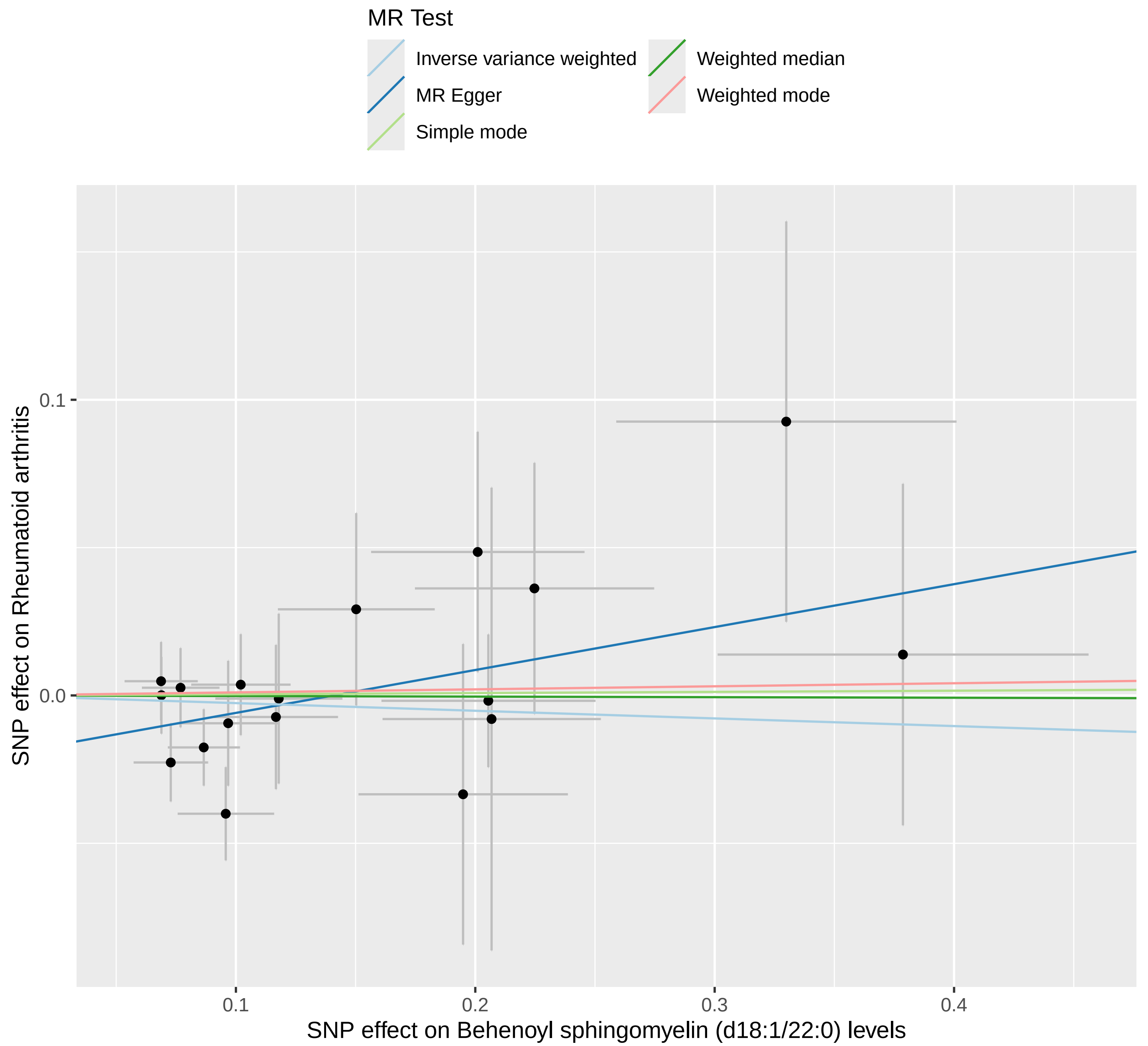

MR Test
Inverse variance weighted
MR Egger
Simple mode
Weighted median
Weighted mode
SNP effect on Rheumatoid arthritis
0.1
0.0
0.1
0.2
0.3
0.4
SNP effect on Behenoyl sphingomyelin (d18:1/22:0) levels

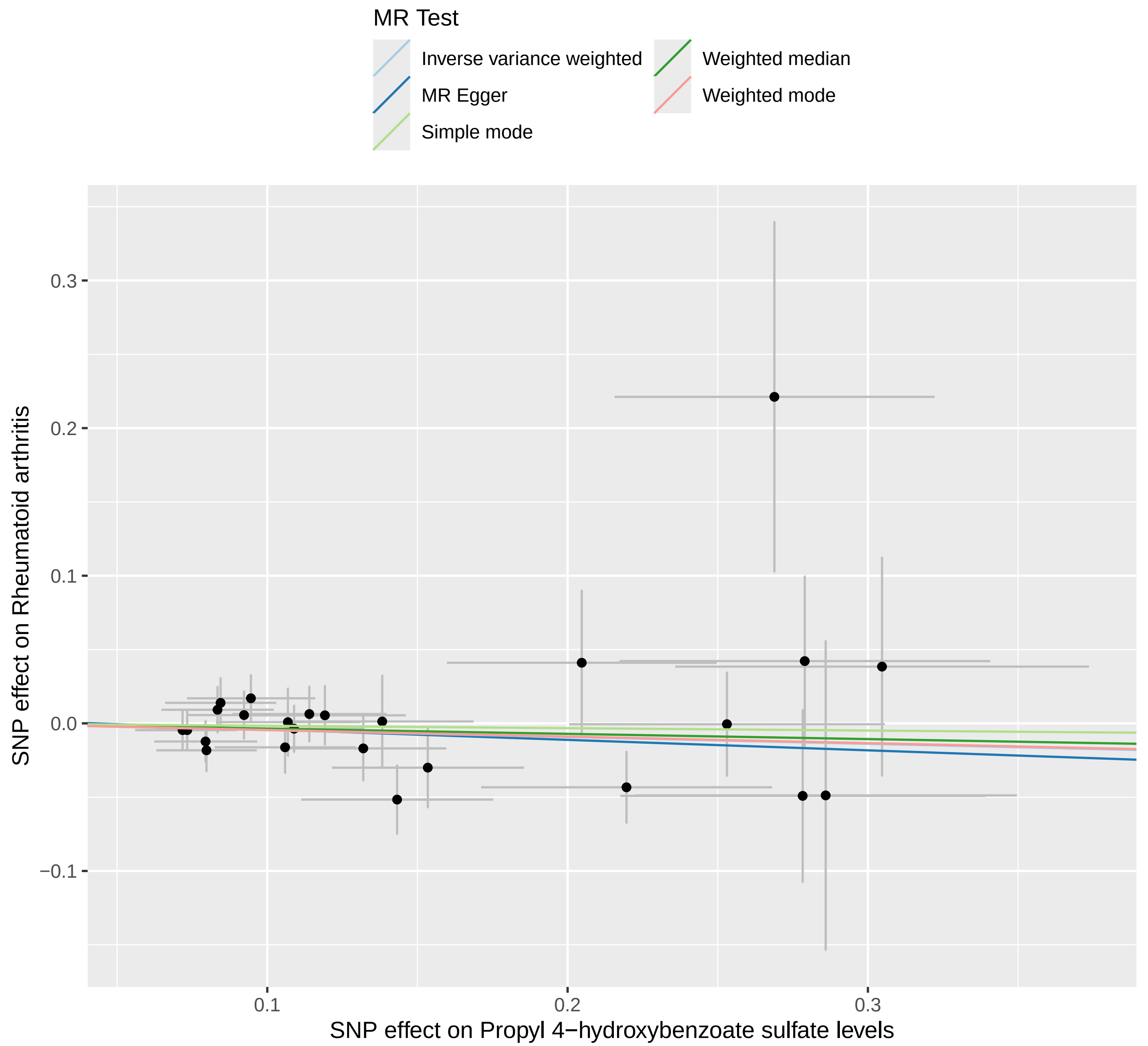
MR Test
Inverse variance weighted
MR Egger
Simple mode
Weighted median
Weighted mode
SNP effect on Rheumatoid arthritis
0.3
0.2
0.1
0.0
−0.1
0.1
0.2
0.3
SNP effect on Propyl 4−hydroxybenzoate sulfate levels

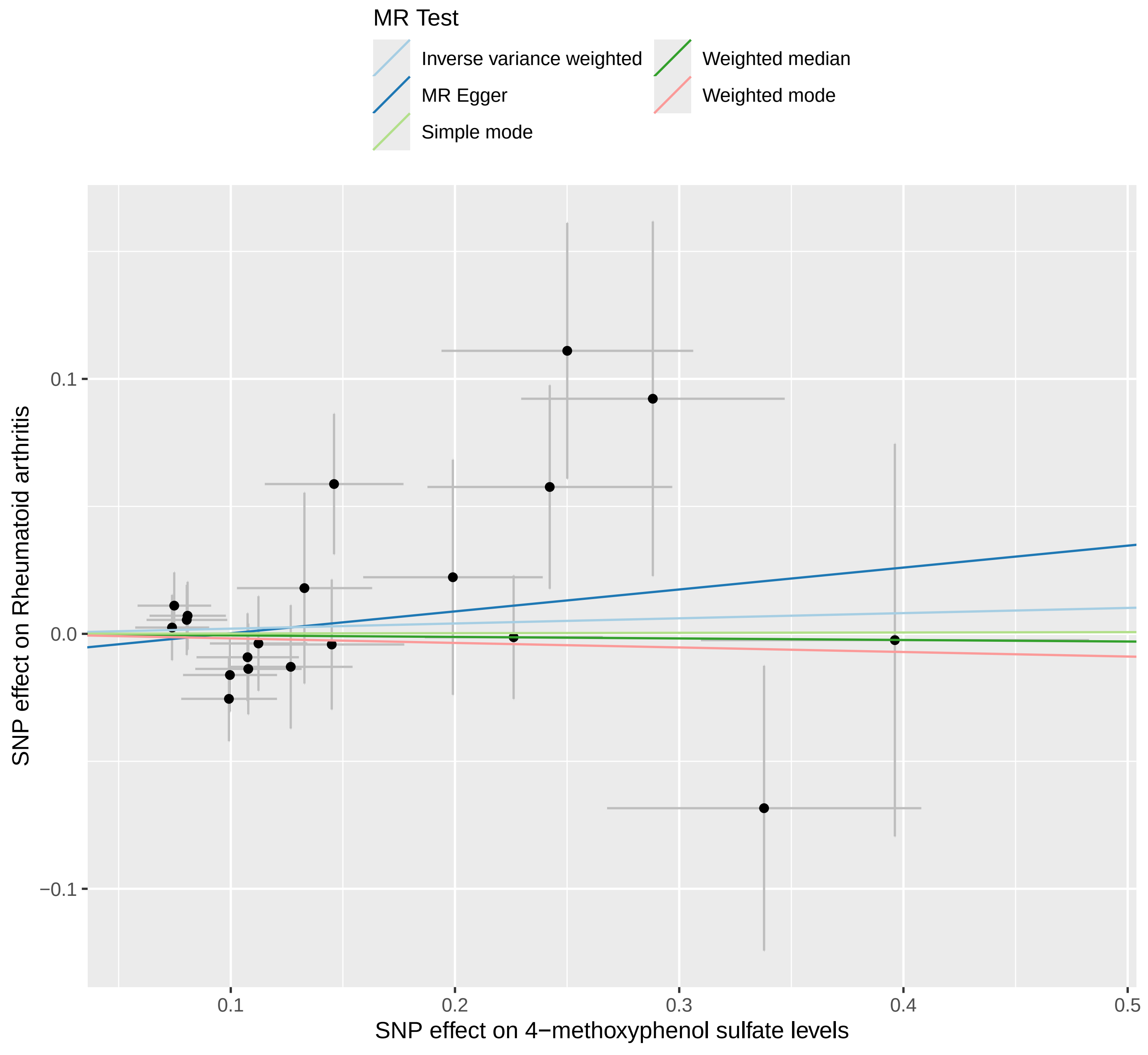
MR Test
Inverse variance weighted
MR Egger
Simple mode
Weighted median
Weighted mode
0.1
0.0
−0.1
0.1
0.2
0.3
0.4
0.5
SNP effect on Rheumatoid arthritis
SNP effect on 4−methoxyphenol sulfate levels

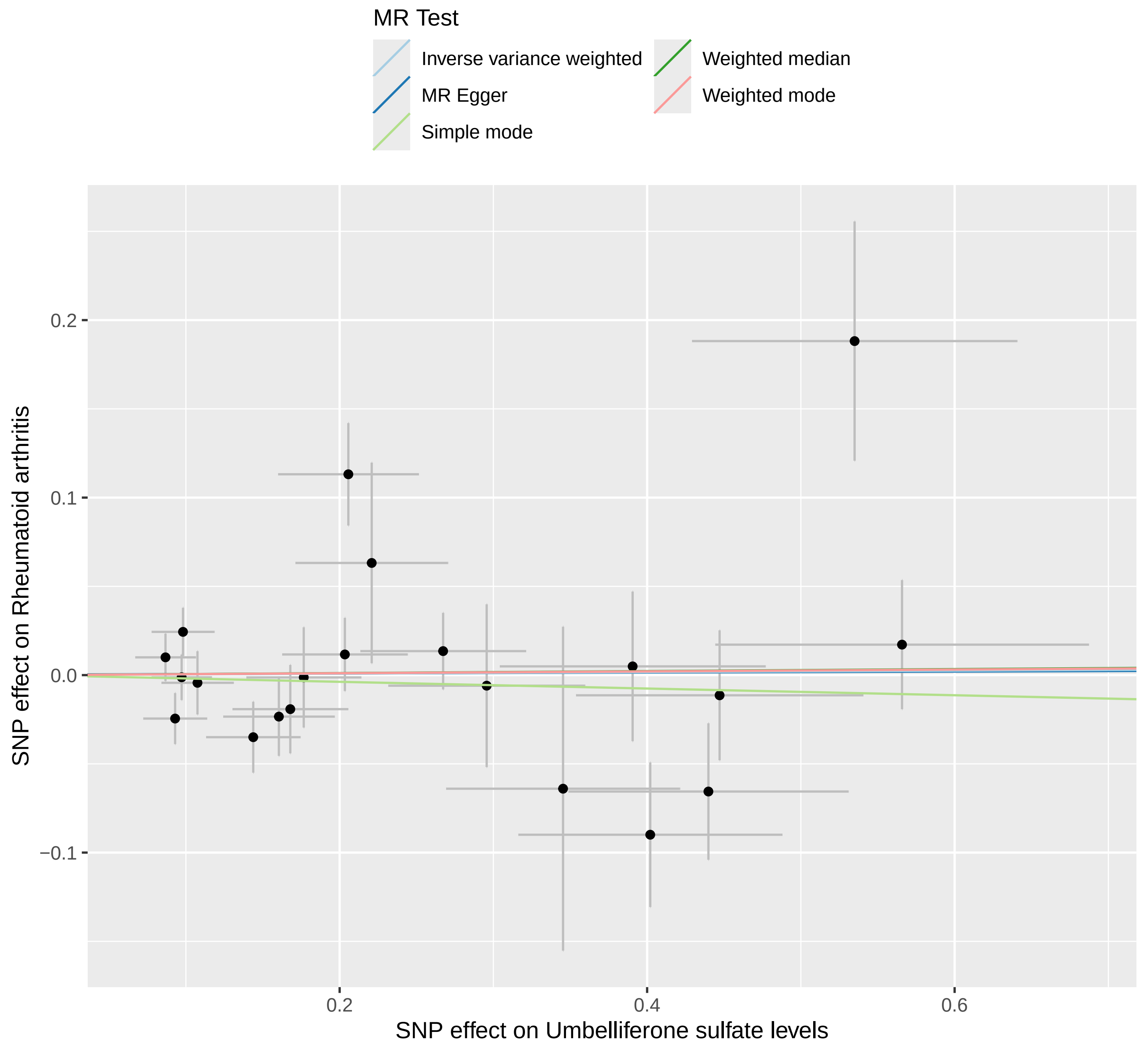
MR Test
Inverse variance weighted
MR Egger
Simple mode
Weighted median
Weighted mode
0.2
0.1
0.0
−0.1
SNP effect on Rheumatoid arthritis
0.2
0.4
0.6
SNP effect on Umbelliferone sulfate levels

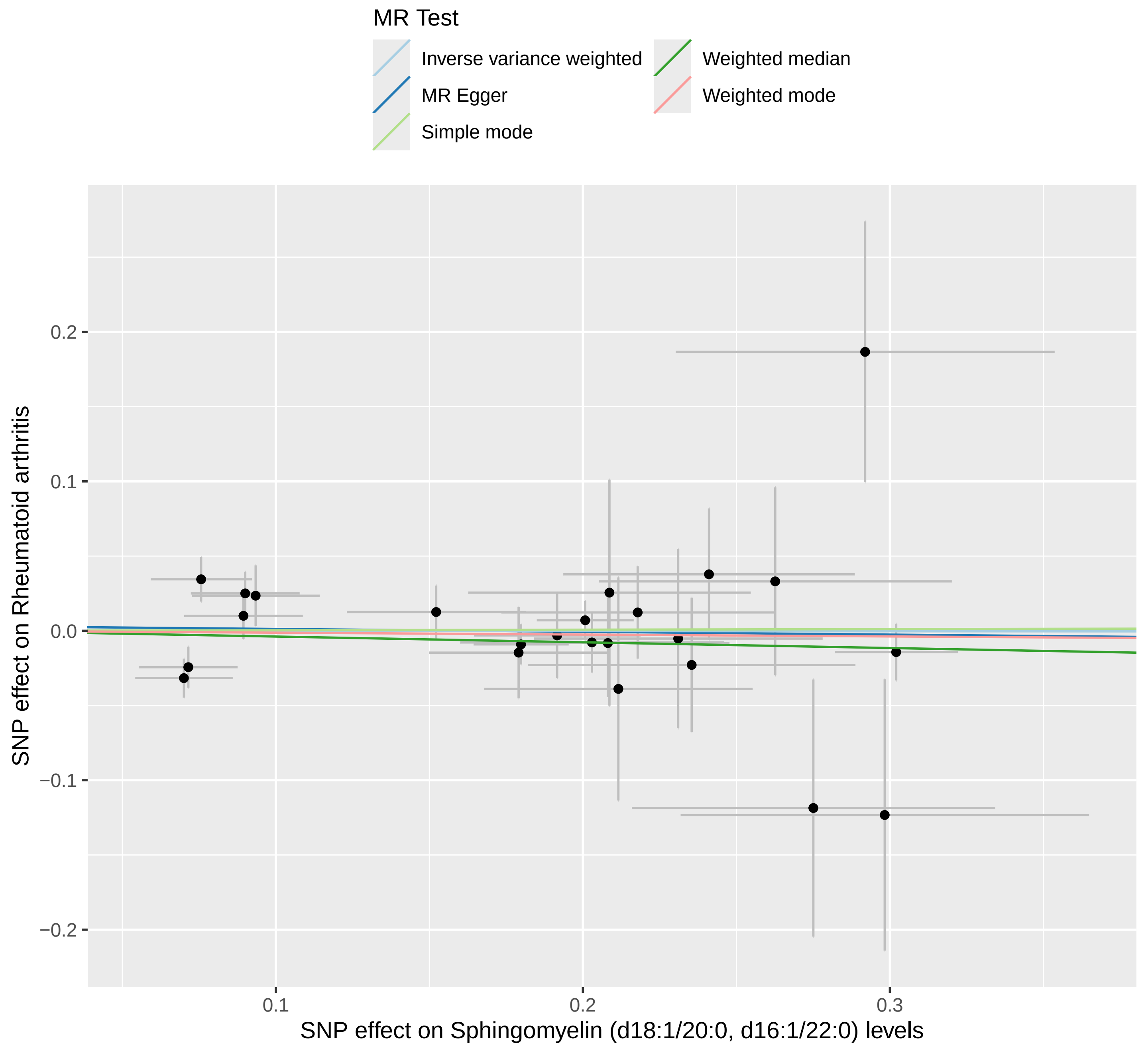
MR Test
Inverse variance weighted
MR Egger
Simple mode
Weighted median
Weighted mode
SNP effect on Rheumatoid arthritis
0.2
0.1
0.0
−0.1
−0.2
0.1
0.2
0.3
SNP effect on Sphingomyelin (d18:1/20:0, d16:1/22:0) levels

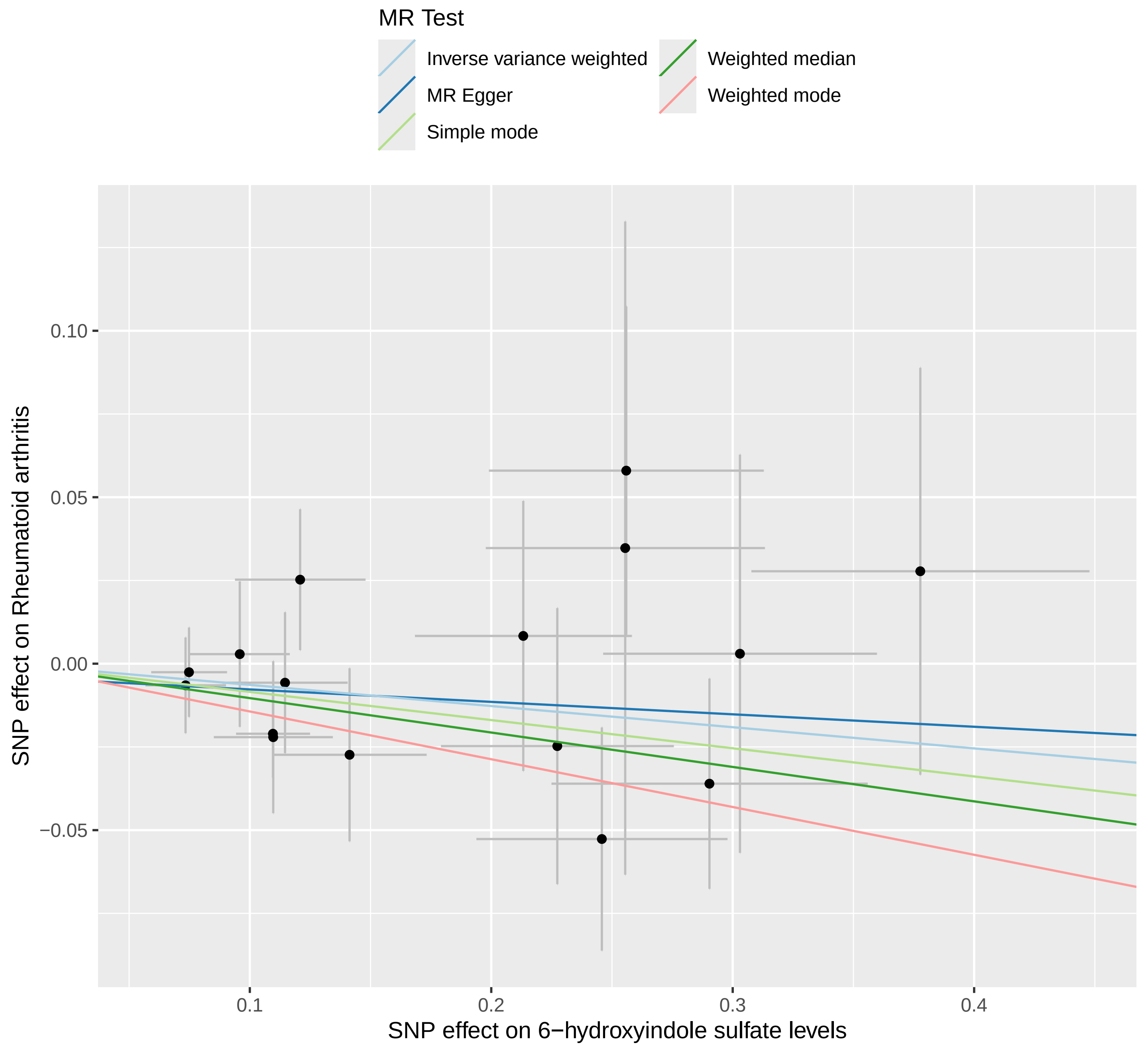

MR Test
Inverse variance weighted
MR Egger
Simple mode
Weighted median
Weighted mode
0.10
0.05
0.00
−0.05
0.1
0.2
0.3
0.4
SNP effect on Rheumatoid arthritis
SNP effect on 6−hydroxyindole sulfate levels

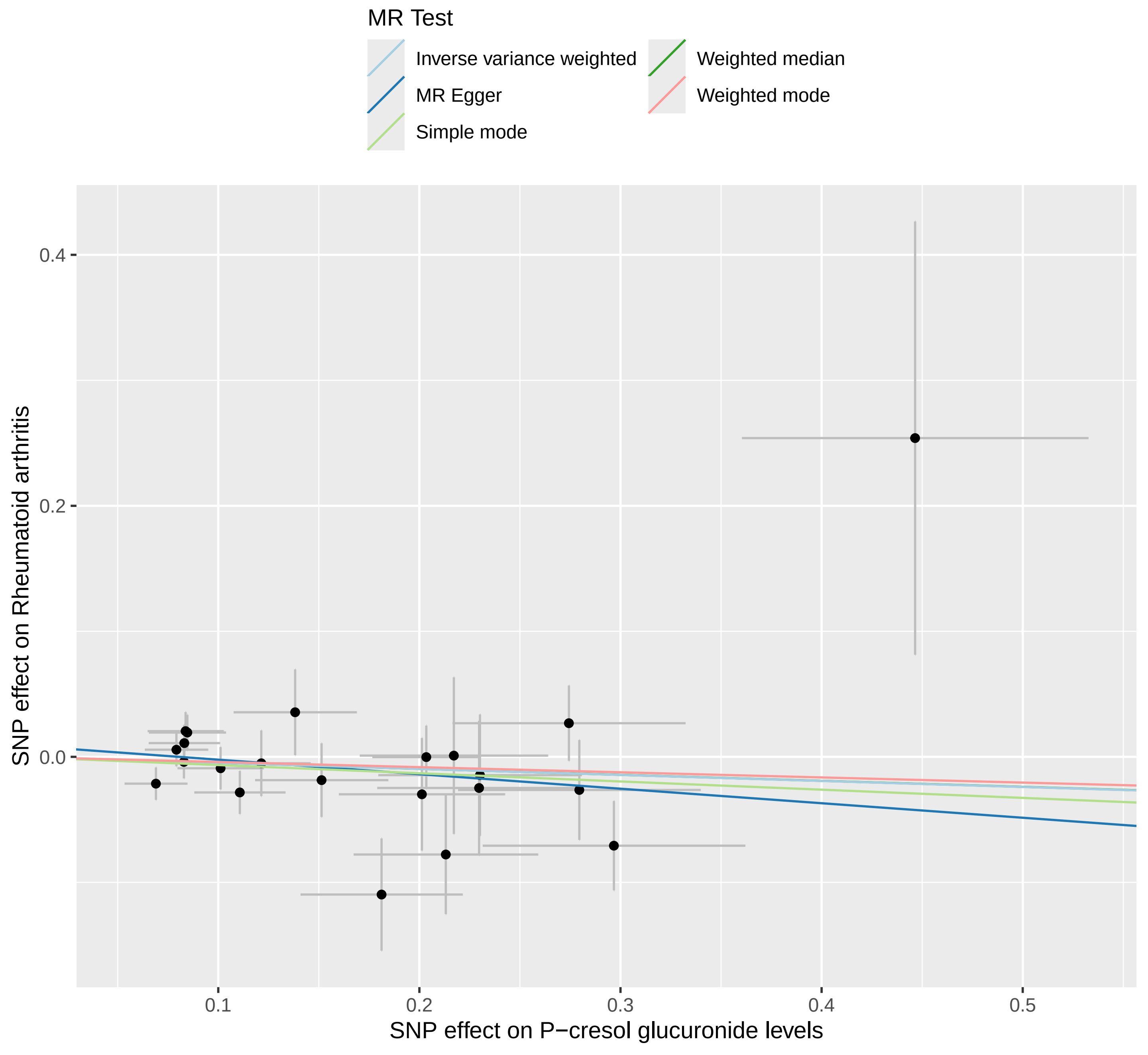

MR Test
Inverse variance weighted
MR Egger
Simple mode
Weighted median
Weighted mode
0.4
0.2
0.0
0.1
0.2
0.3
0.4
0.5
SNP effect on Rheumatoid arthritis
SNP effect on P−cresol glucuronide levels

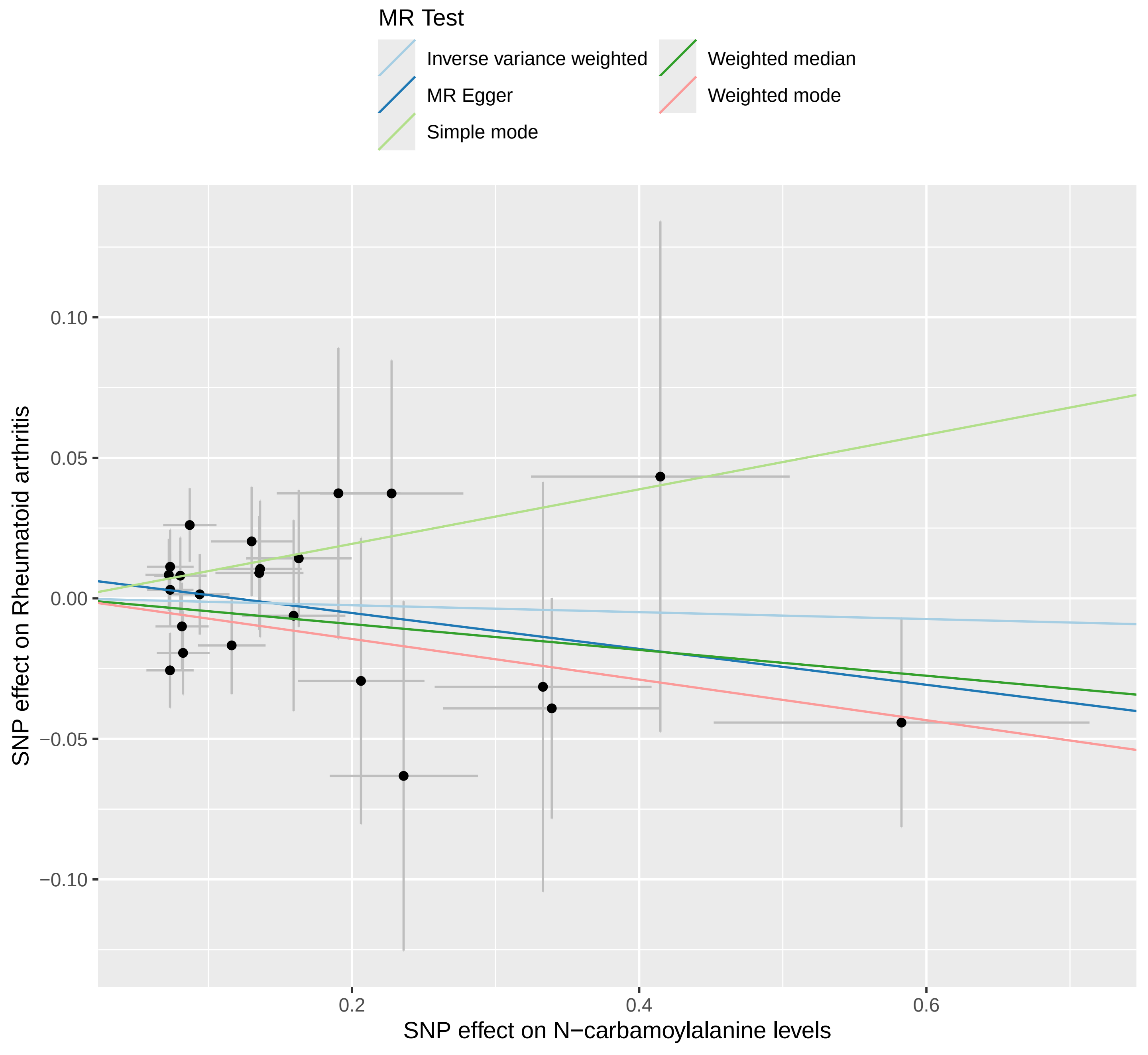

MR Test
Inverse variance weighted
MR Egger
Simple mode
Weighted median
Weighted mode
0.10
0.05
0.00
−0.05
−0.10
0.2
0.4
0.6
SNP effect on Rheumatoid arthritis
SNP effect on N−carbamoylalanine levels

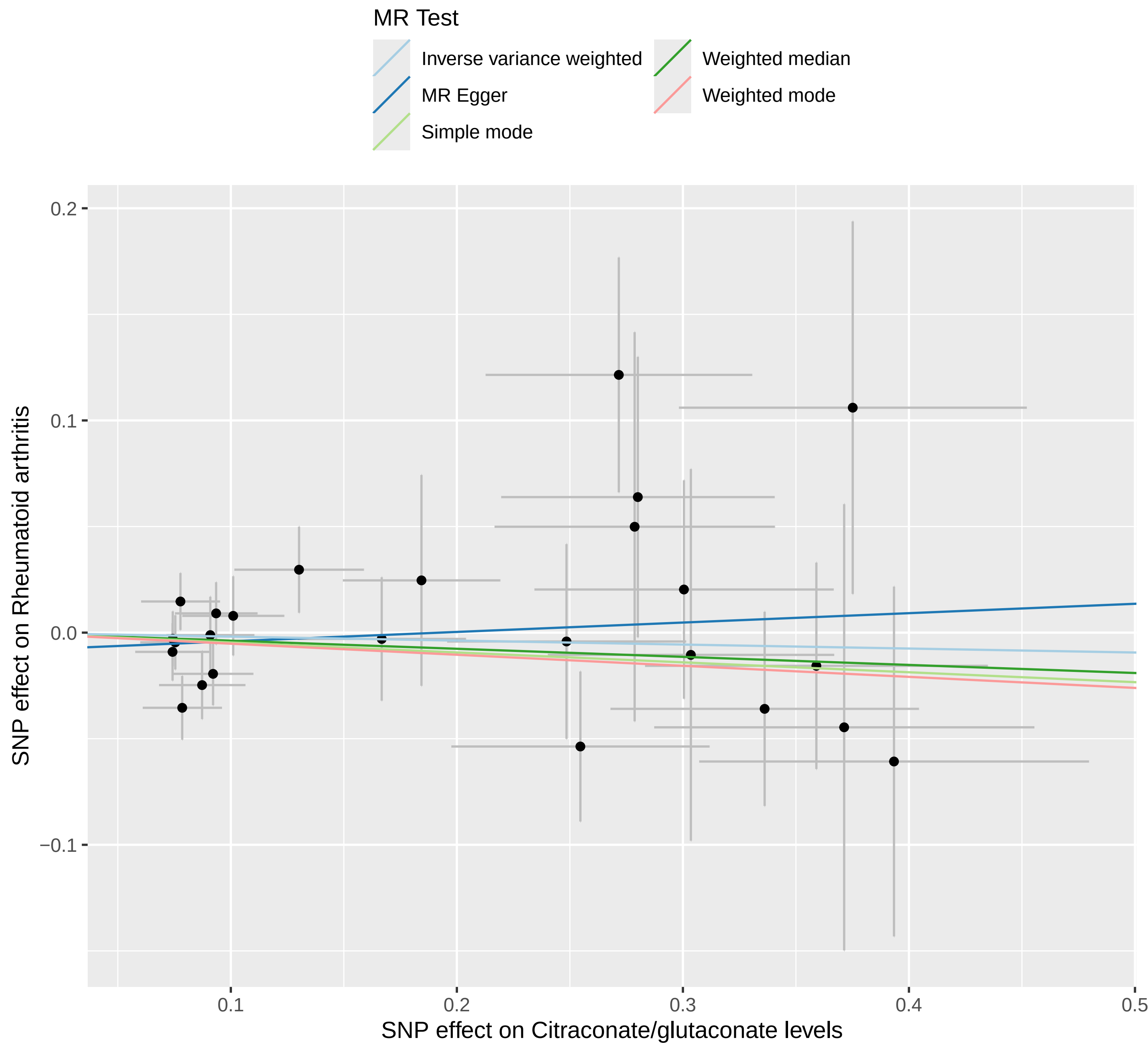

MR Test
Inverse variance weighted
MR Egger
Simple mode
Weighted median
Weighted mode
0.2
0.1
0.0
−0.1
SNP effect on Rheumatoid arthritis
0.1
0.2
0.3
0.4
0.5
SNP effect on Citraconate/glutaconate levels

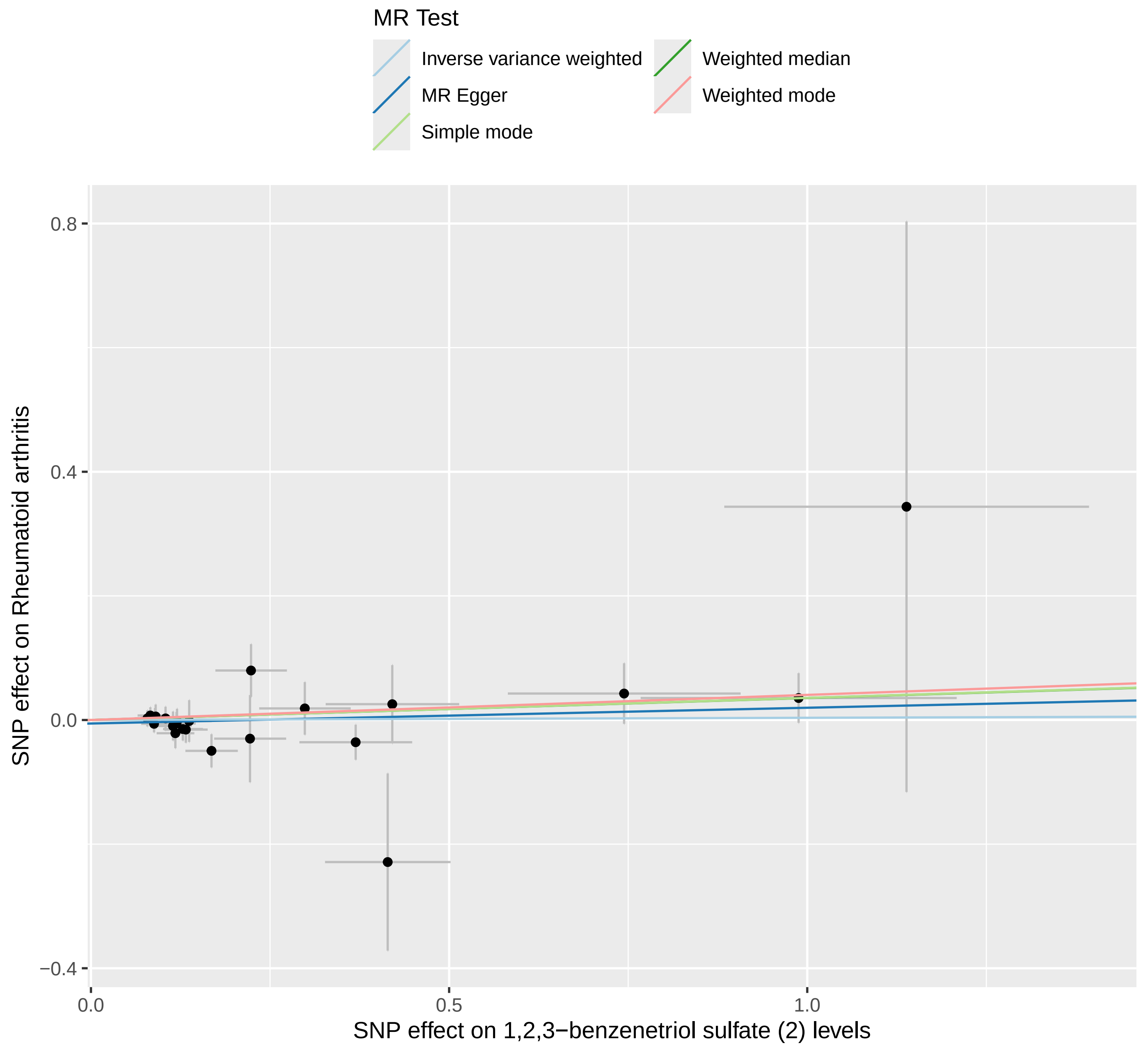

MR Test
Inverse variance weighted
MR Egger
Simple mode
Weighted median
Weighted mode
SNP effect on Rheumatoid arthritis
0.8
0.4
0.0
−0.4
0.0
0.5
1.0
SNP effect on 1,2,3−benzenetriol sulfate (2) levels

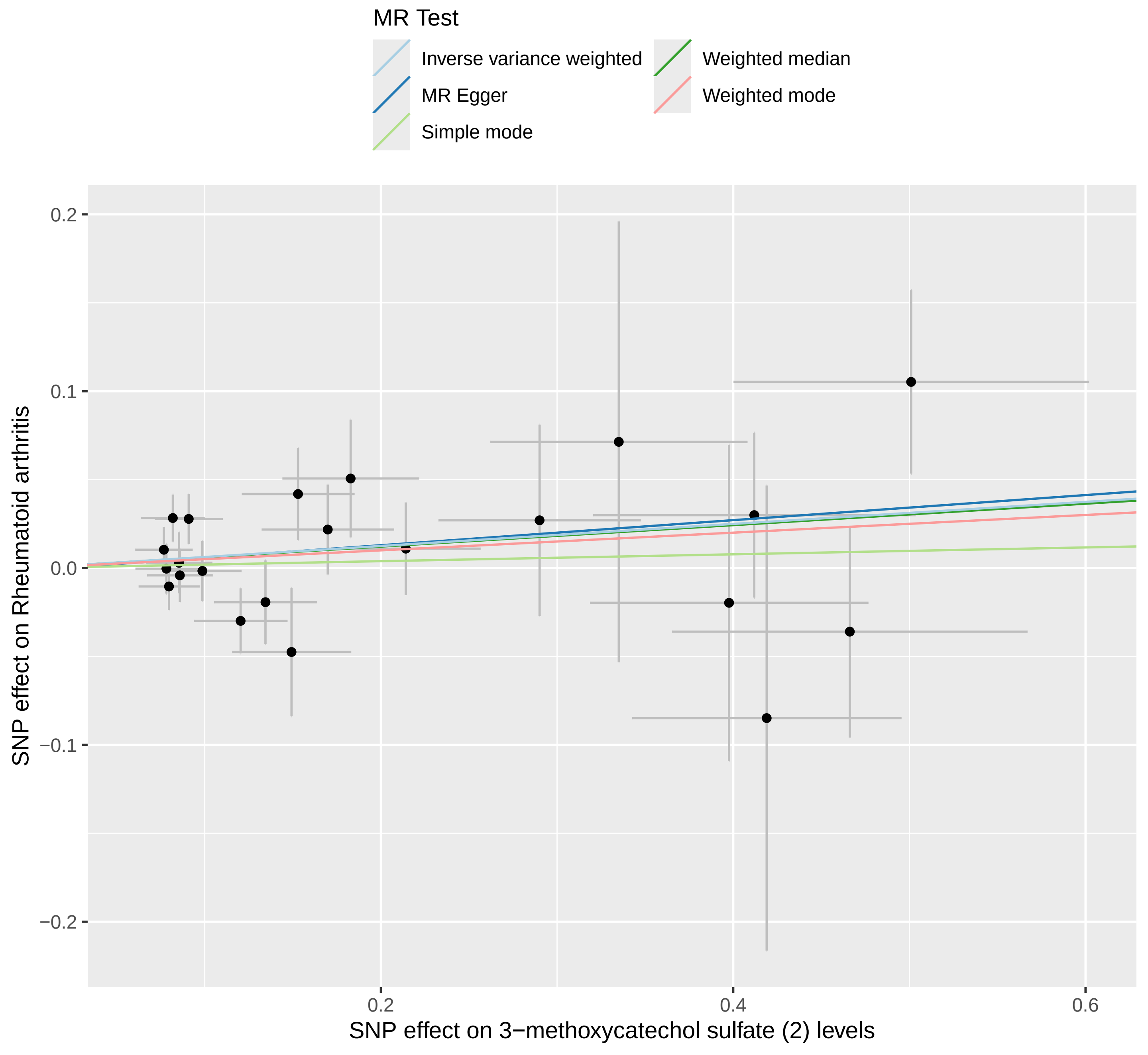

MR Test
Inverse variance weighted
MR Egger
Simple mode
Weighted median
Weighted mode
SNP effect on Rheumatoid arthritis
0.2
0.1
0.0
−0.1
−0.2
0.2
0.4
0.6
SNP effect on 3−methoxycatechol sulfate (2) levels

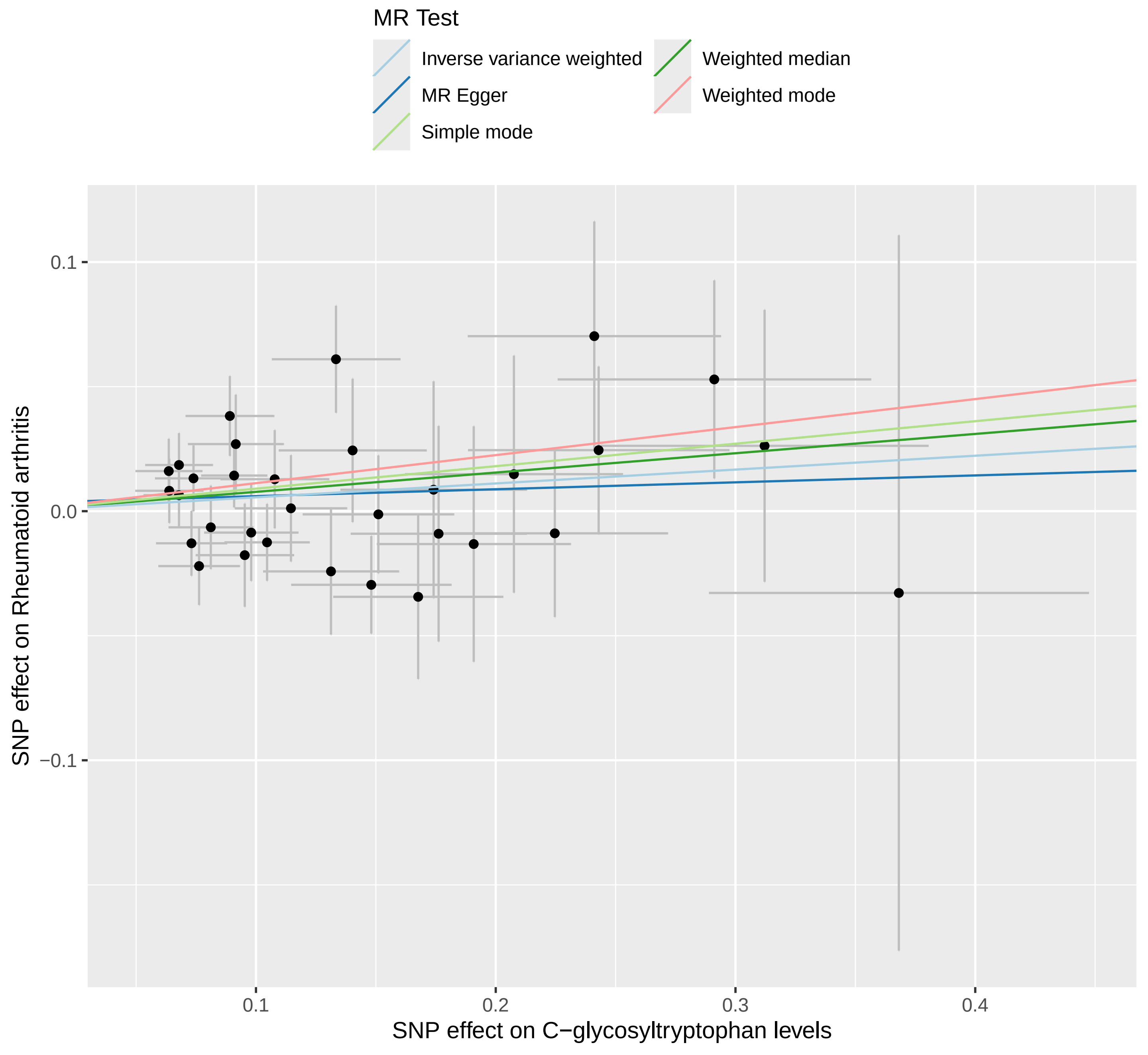

MR Test
Inverse variance weighted
MR Egger
Simple mode
Weighted median
Weighted mode
0.1
0.0
−0.1
0.1
0.2
0.3
0.4
SNP effect on Rheumatoid arthritis
SNP effect on C−glycosyltryptophan levels

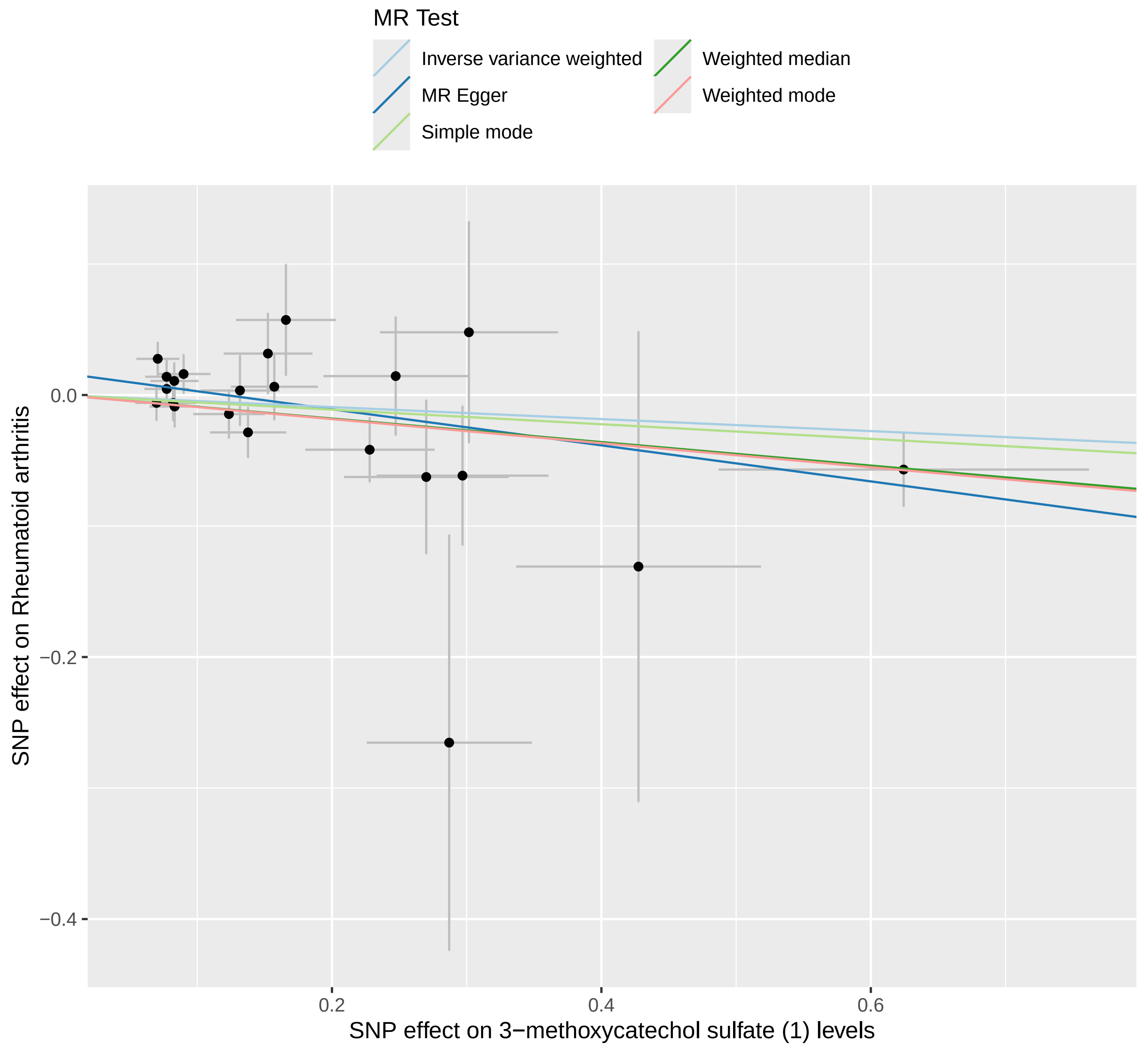
MR Test
Inverse variance weighted
MR Egger
Simple mode
Weighted median
Weighted mode
SNP effect on Rheumatoid arthritis
0.0
−0.2
−0.4
0.2
0.4
0.6
SNP effect on 3−methoxycatechol sulfate (1) levels

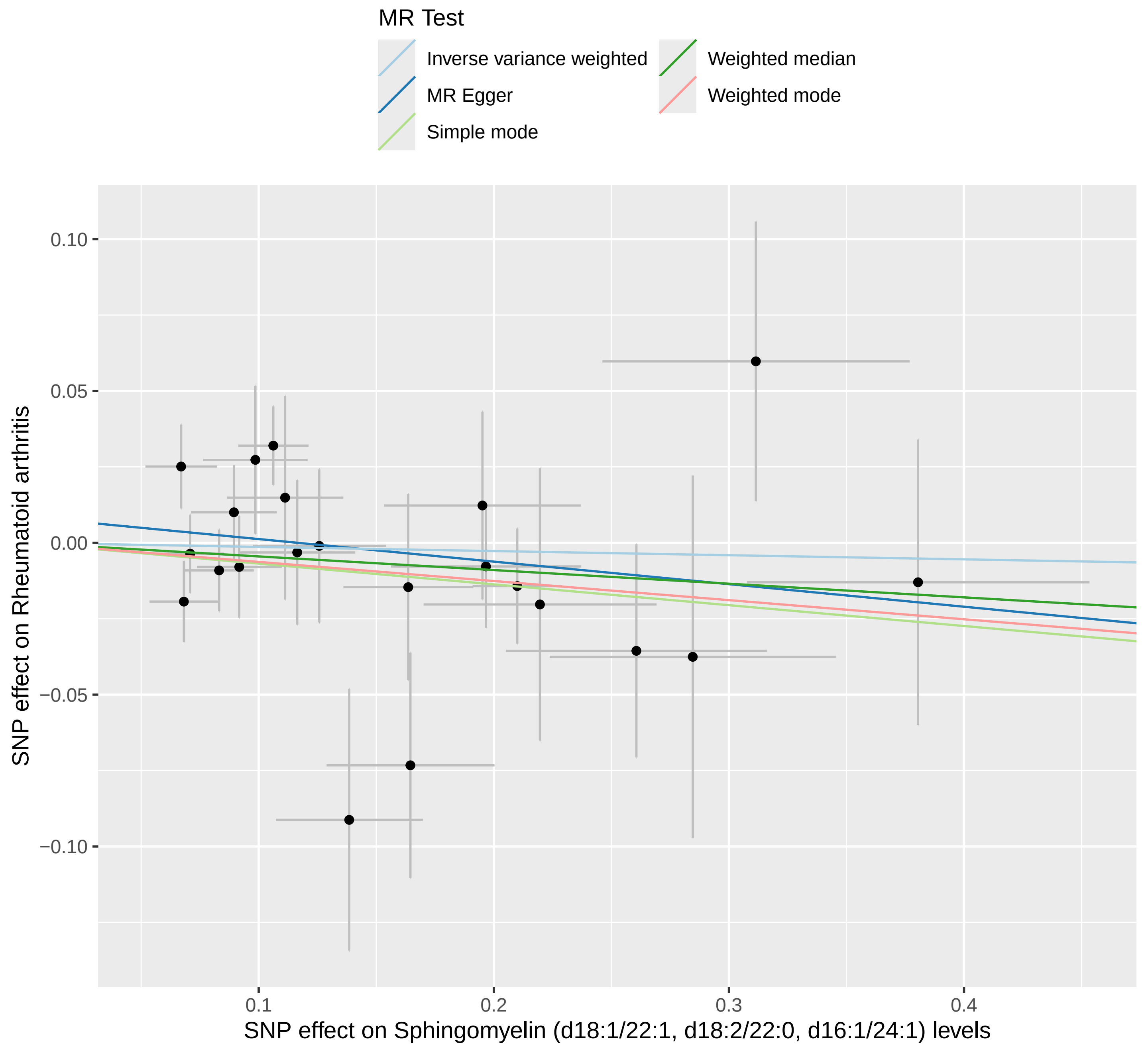

MR Test
Inverse variance weighted
MR Egger
Simple mode
Weighted median
Weighted mode
0.10
0.05
0.00
−0.05
−0.10
0.1
0.2
0.3
0.4
SNP effect on Rheumatoid arthritis
SNP effect on Sphingomyelin (d18:1/22:1, d18:2/22:0, d16:1/24:1) levels

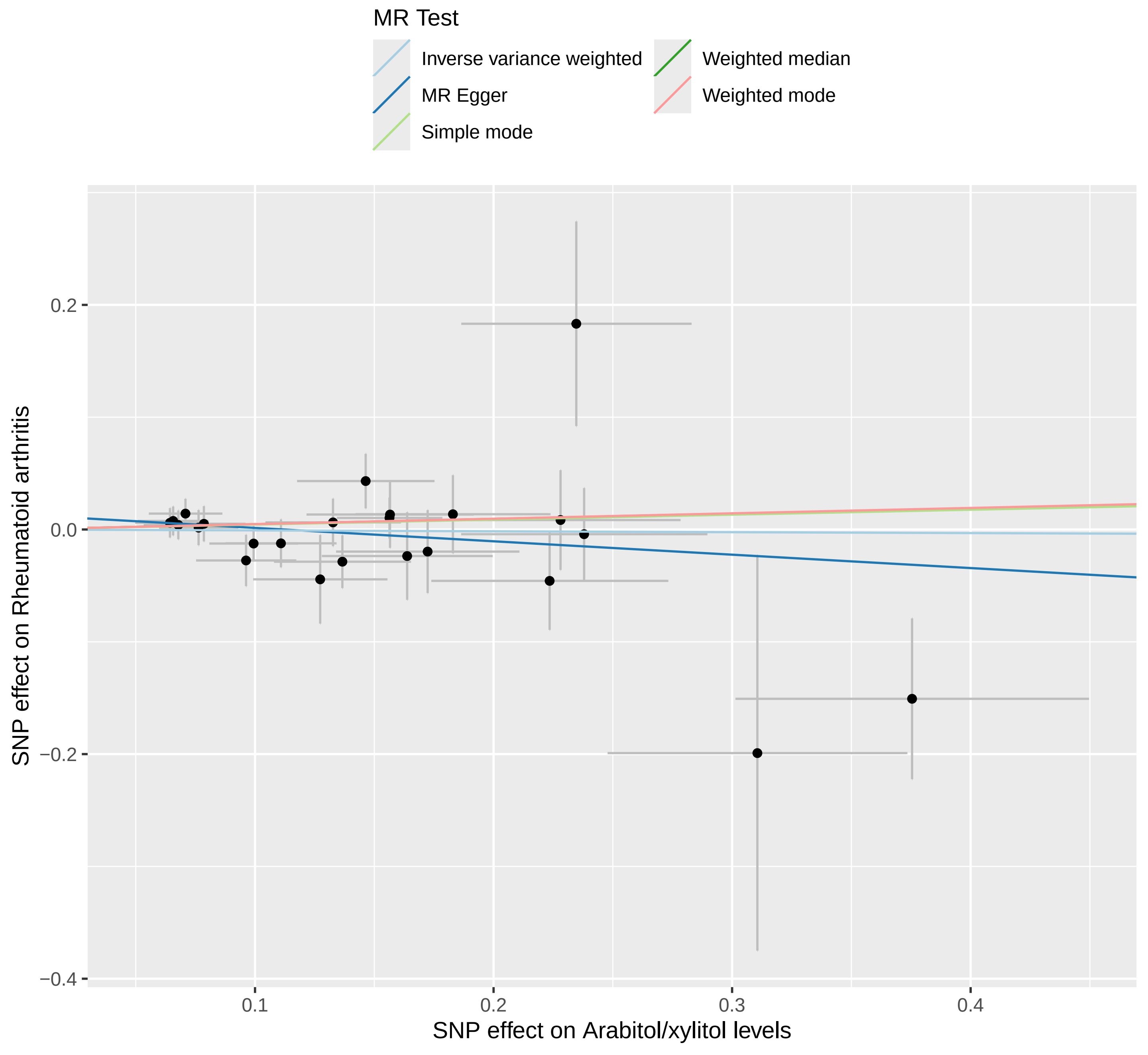
MR Test
Inverse variance weighted
MR Egger
Simple mode
Weighted median
Weighted mode
0.2
0.0
−0.2
−0.4
0.1
0.2
0.3
0.4
SNP effect on Rheumatoid arthritis
SNP effect on Arabitol/xylitol levels

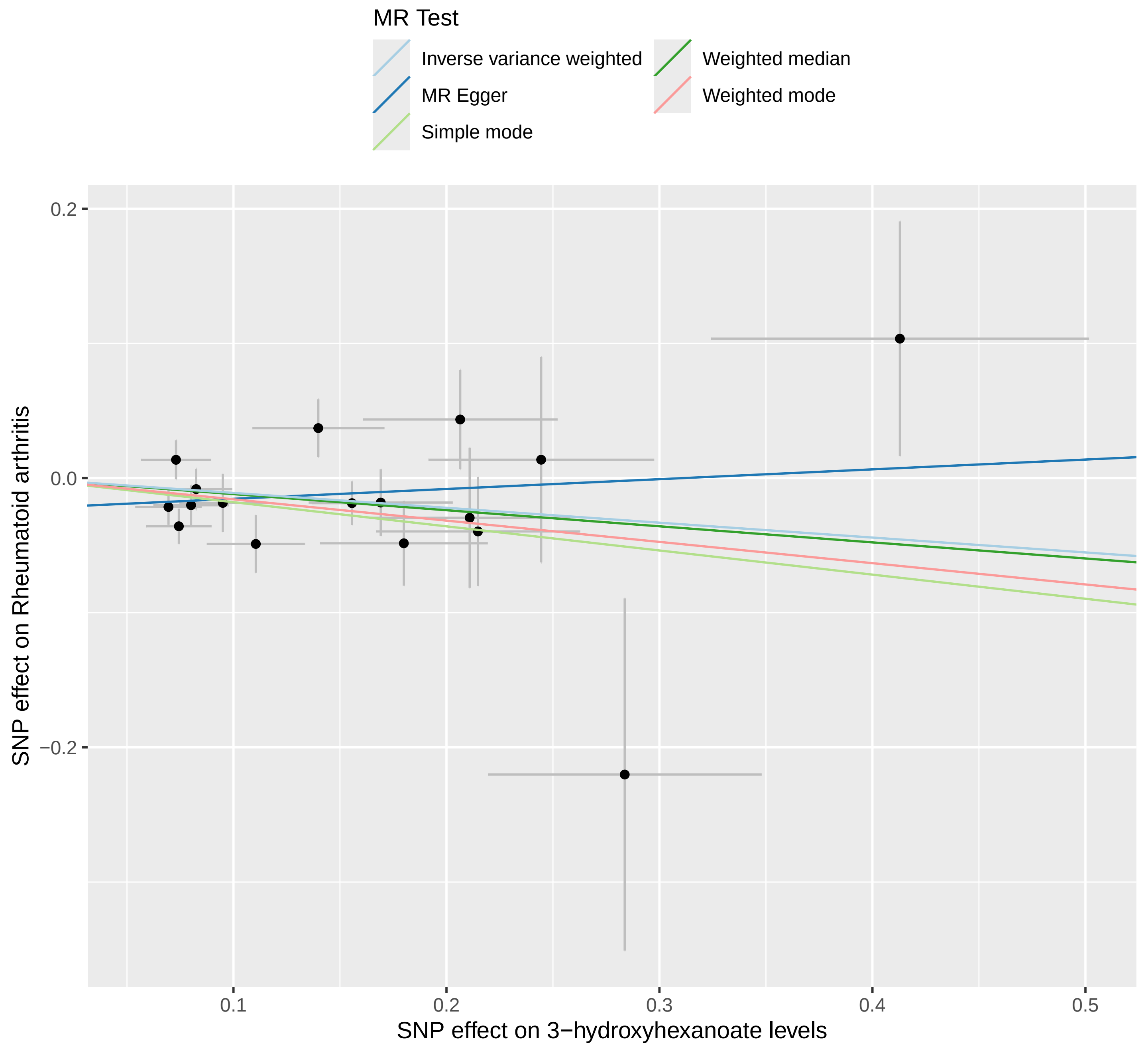

MR Test
Inverse variance weighted
MR Egger
Simple mode
Weighted median
Weighted mode
0.2
0.0
−0.2
0.1
0.2
0.3
0.4
0.5
SNP effect on Rheumatoid arthritis
SNP effect on 3−hydroxyhexanoate levels

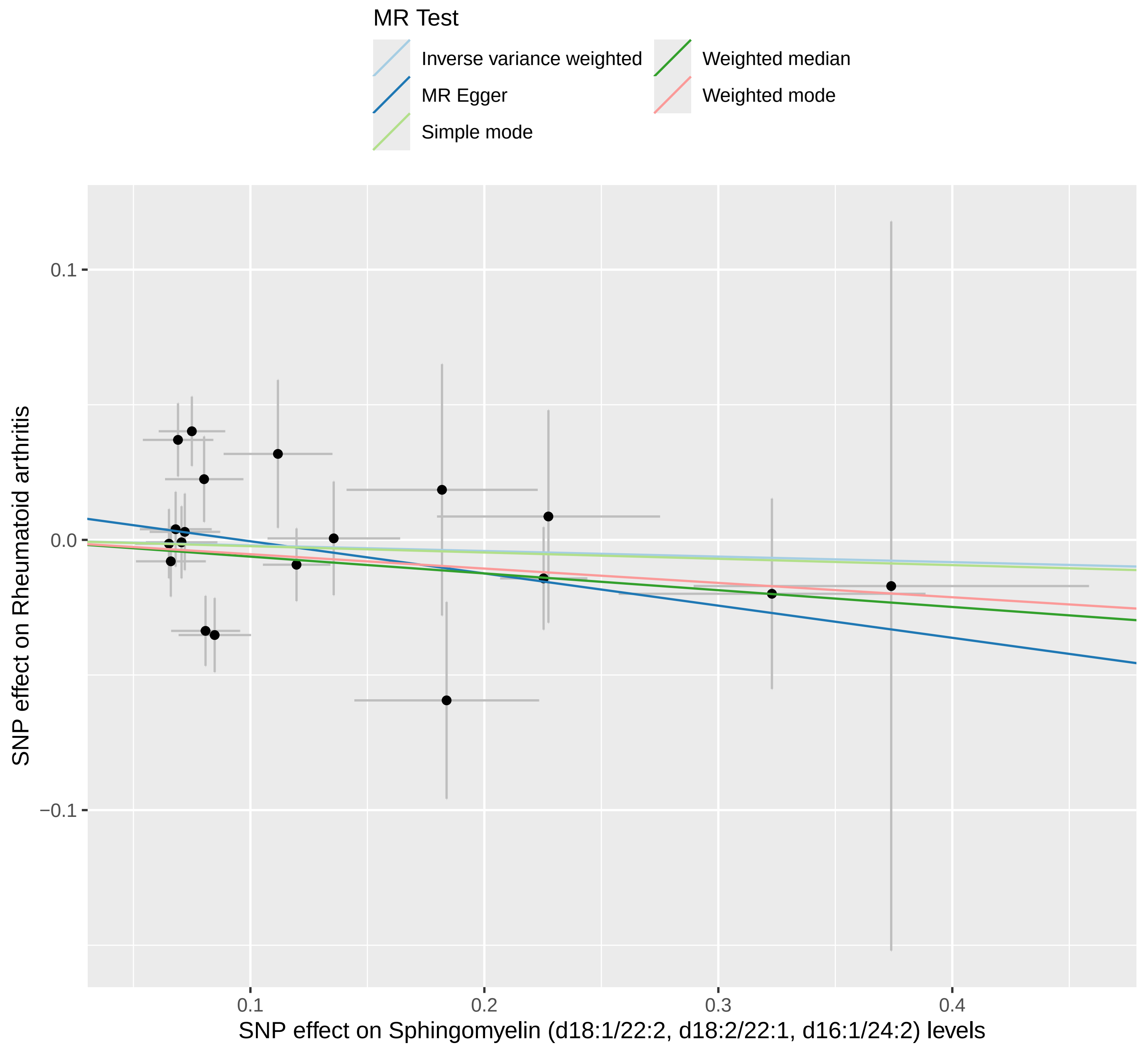

MR Test
Inverse variance weighted
MR Egger
Simple mode
Weighted median
Weighted mode
0.1
0.0
−0.1
SNP effect on Rheumatoid arthritis
0.1
0.2
0.3
0.4
SNP effect on Sphingomyelin (d18:1/22:2, d18:2/22:1, d16:1/24:2) levels

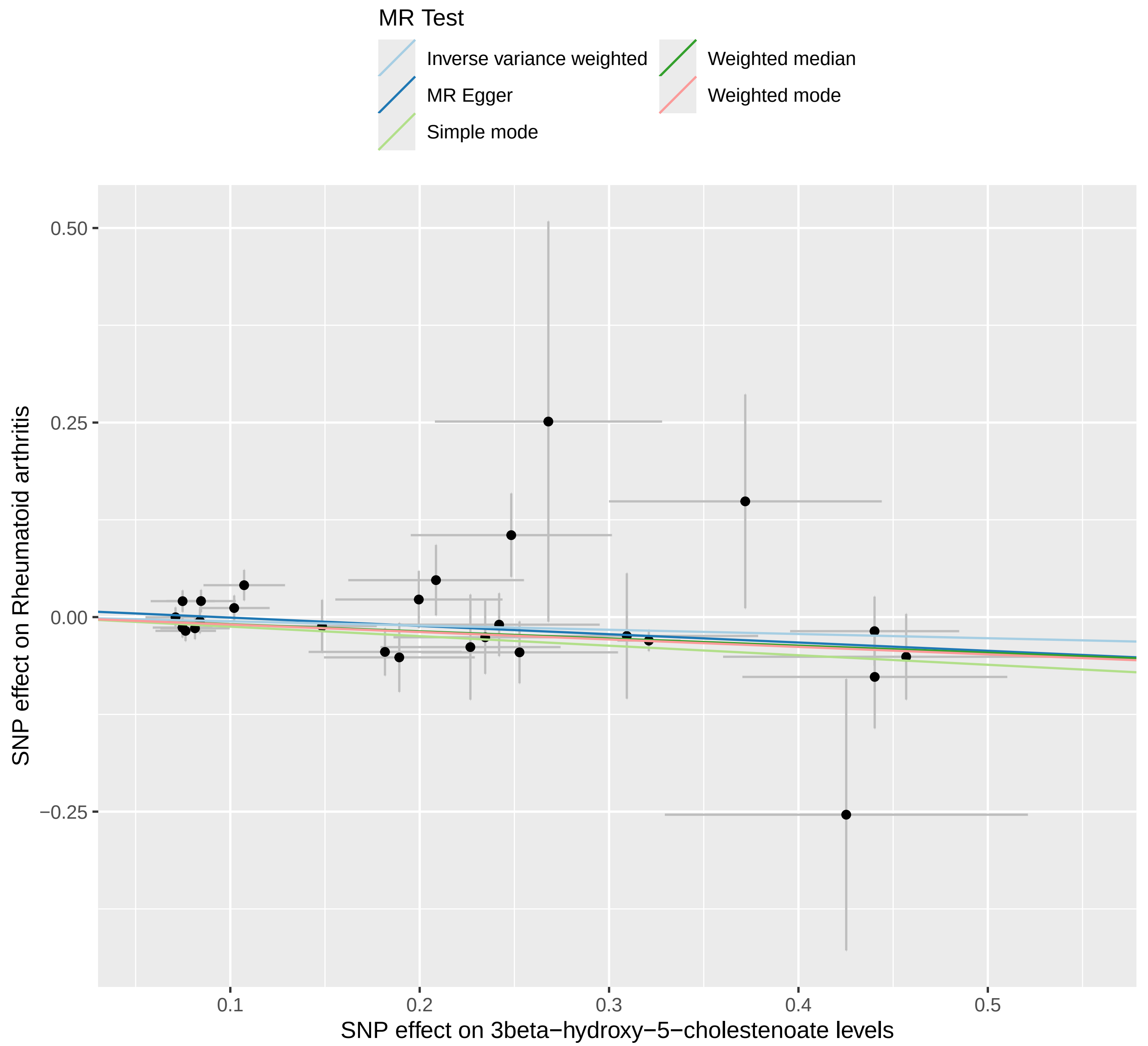
MR Test
Inverse variance weighted
MR Egger
Simple mode
Weighted median
Weighted mode
0.50
0.25
0.00
−0.25
SNP effect on Rheumatoid arthritis
0.1
0.2
0.3
0.4
0.5
SNP effect on 3beta−hydroxy−5−cholestenoate levels

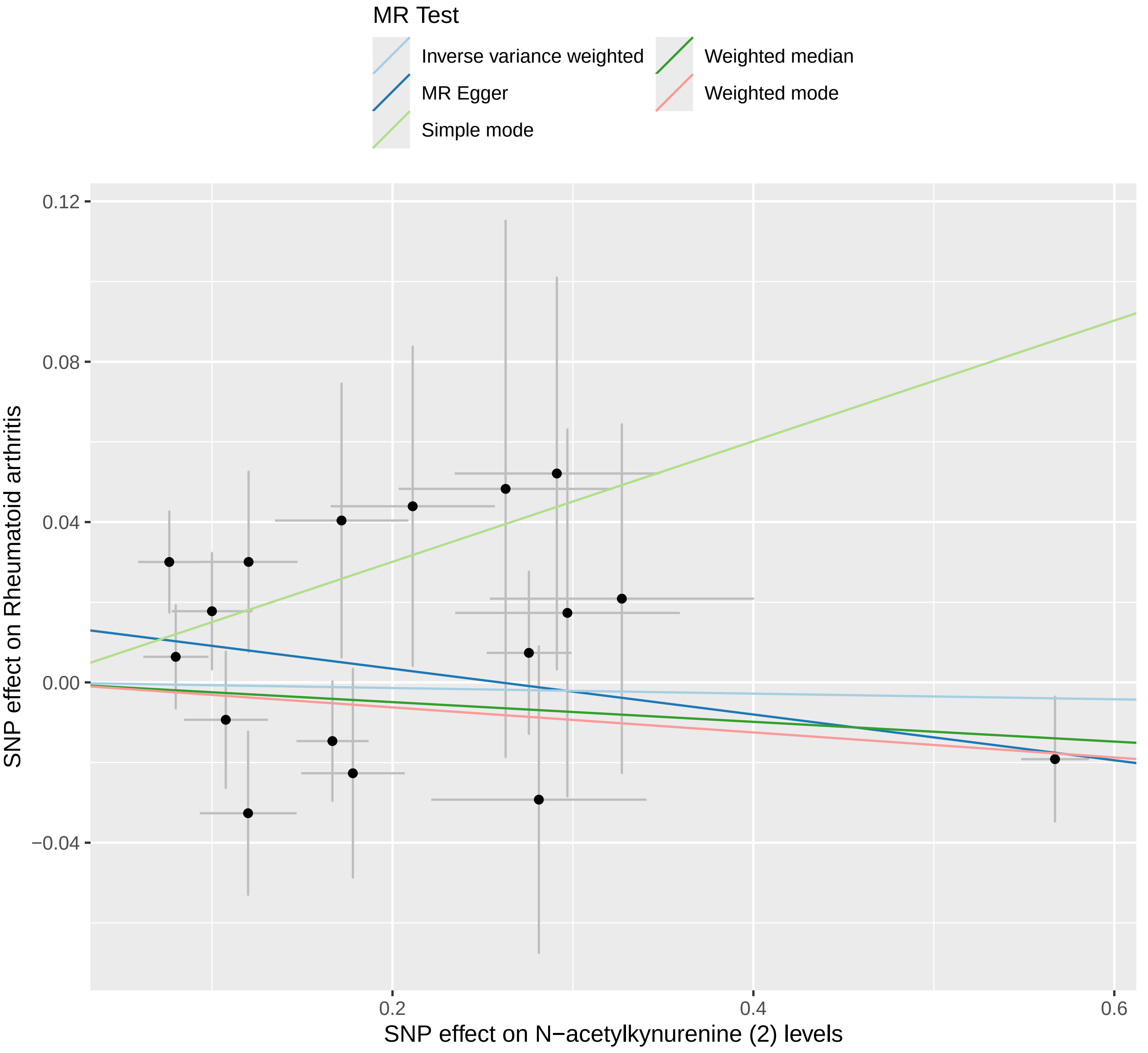
MR Test
Inverse variance weighted
MR Egger
Simple mode
Weighted median
Weighted mode
0.12
0.08
0.04
0.00
−0.04
0.2
0.4
0.6
SNP effect on Rheumatoid arthritis
SNP effect on N−acetylkynurenine (2) levels

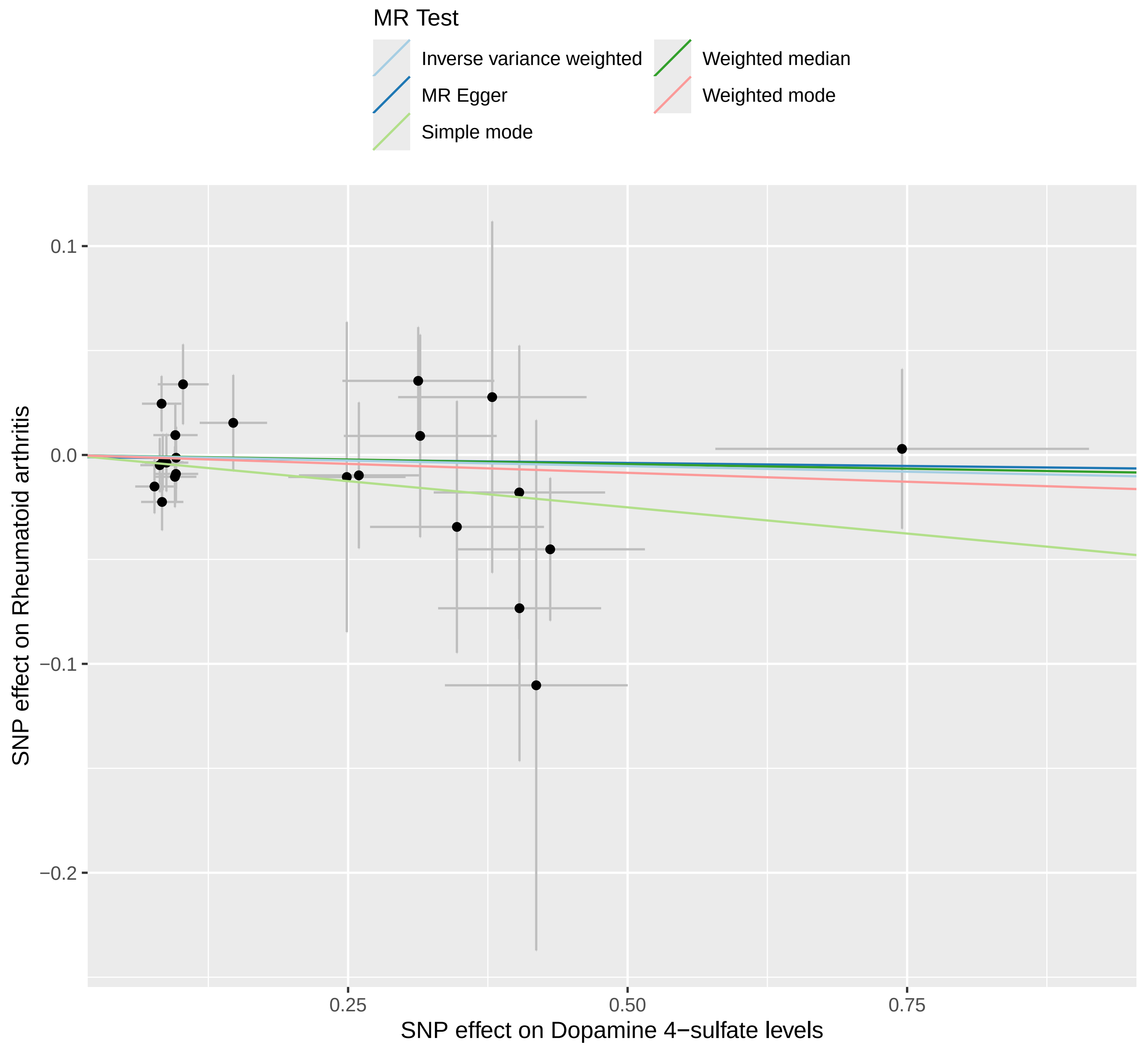
MR Test
Inverse variance weighted
MR Egger
Simple mode
Weighted median
Weighted mode
SNP effect on Rheumatoid arthritis
0.1
0.0
−0.1
−0.2
0.25
0.50
0.75
SNP effect on Dopamine 4−sulfate levels

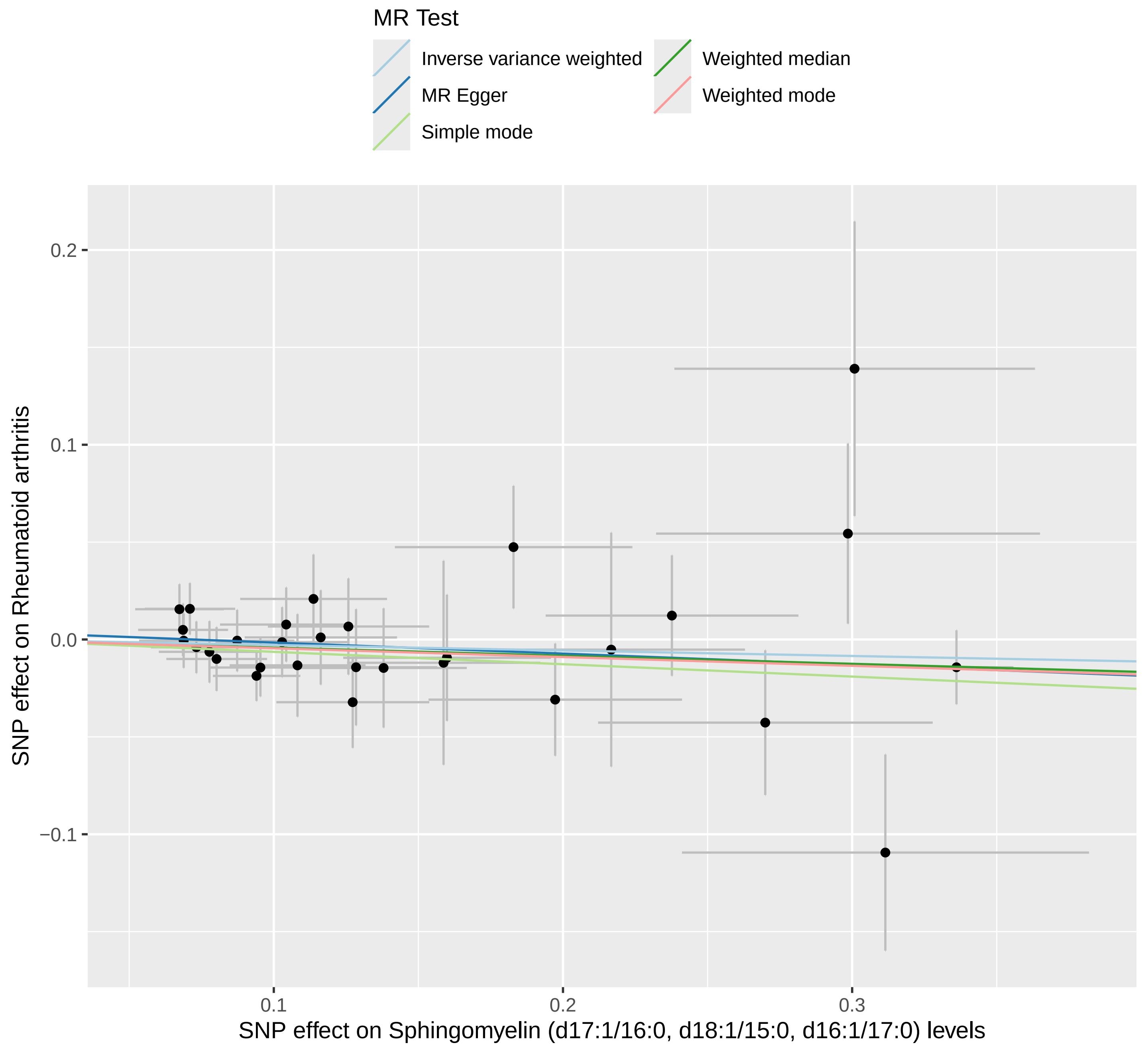
MR Test
Inverse variance weighted
MR Egger
Simple mode
Weighted median
Weighted mode
0.2
0.1
0.0
−0.1
0.1
0.2
0.3
SNP effect on Rheumatoid arthritis
SNP effect on Sphingomyelin (d17:1/16:0, d18:1/15:0, d16:1/17:0) levels

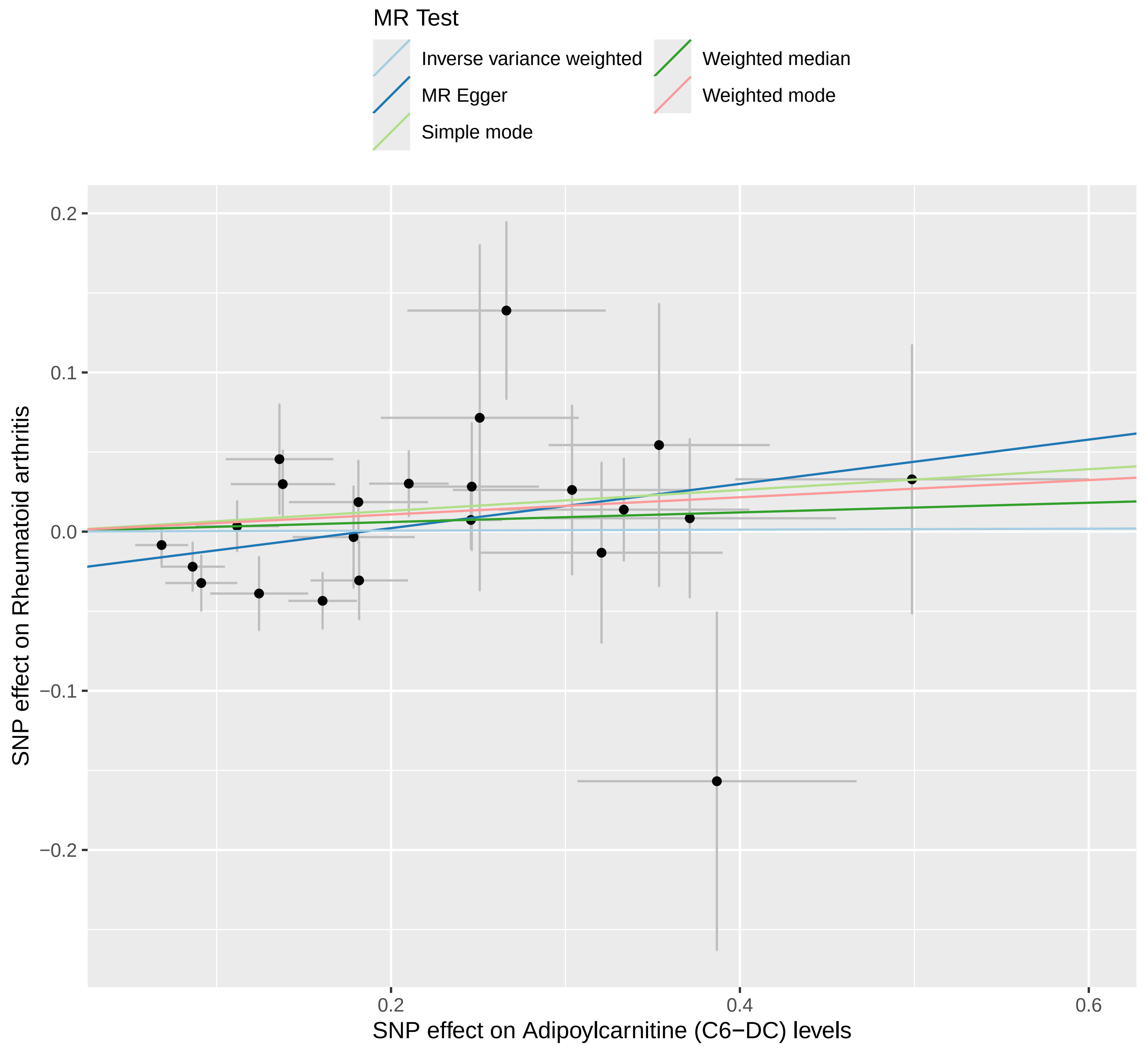
MR Test
Inverse variance weighted
MR Egger
Simple mode
Weighted median
Weighted mode
SNP effect on Rheumatoid arthritis
0.2
0.1
0.0
−0.1
−0.2
0.2
0.4
0.6
SNP effect on Adipoylcarnitine (C6−DC) levels

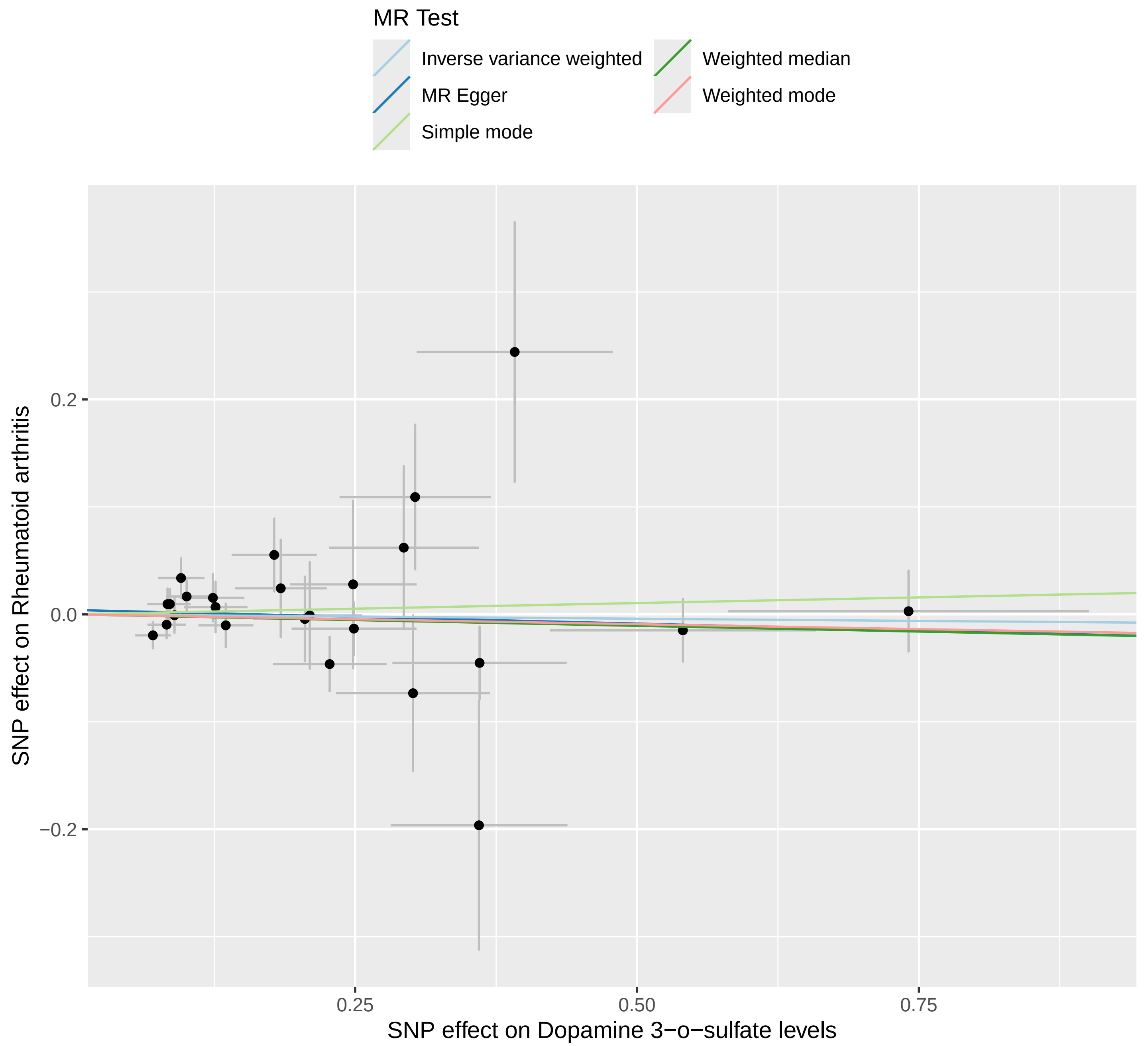
MR Test
Inverse variance weighted
MR Egger
Simple mode
Weighted median
Weighted mode
SNP effect on Rheumatoid arthritis
0.2
0.0
−0.2
0.25
0.50
0.75
SNP effect on Dopamine 3−o−sulfate levels

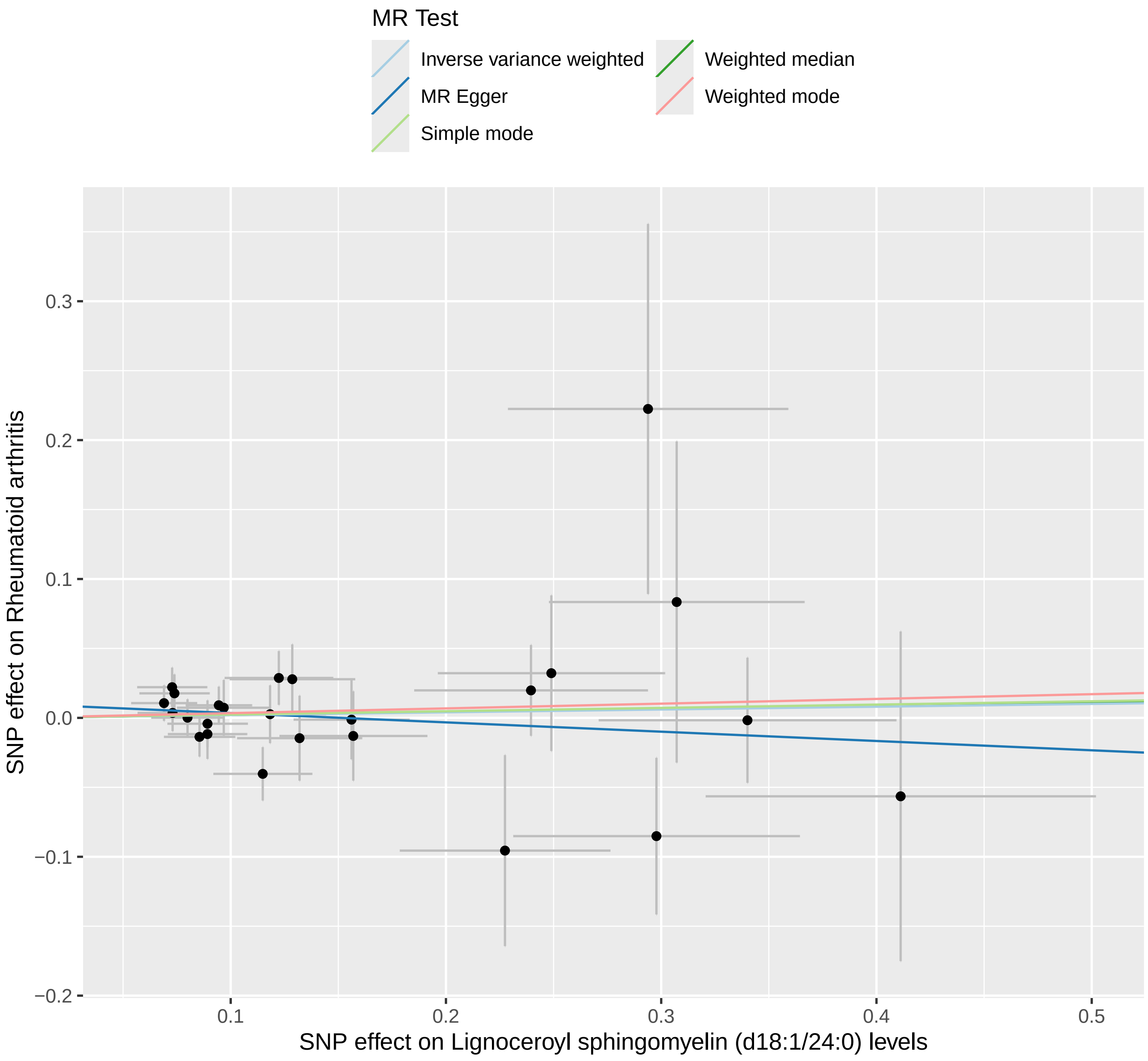
MR Test
Inverse variance weighted
MR Egger
Simple mode
Weighted median
Weighted mode
SNP effect on Rheumatoid arthritis
0.3
0.2
0.1
0.0
−0.1
−0.2
0.1
0.2
0.3
0.4
0.5
SNP effect on Lignoceroyl sphingomyelin (d18:1/24:0) levels

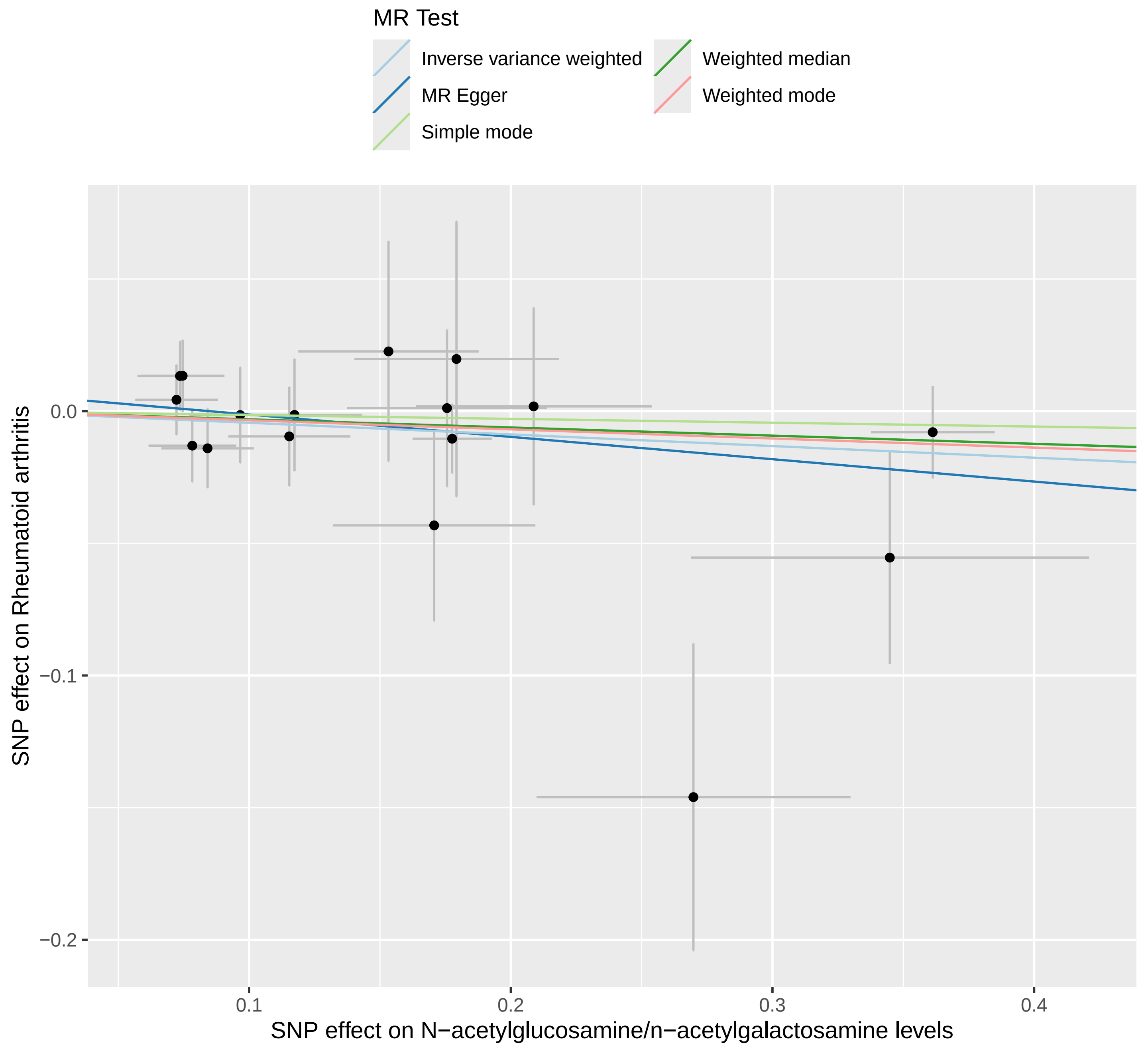
MR Test
Inverse variance weighted
MR Egger
Simple mode
Weighted median
Weighted mode
SNP effect on Rheumatoid arthritis
0.0
−0.1
−0.2
0.1
0.2
0.3
0.4
SNP effect on N−acetylglucosamine/n−acetylgalactosamine levels

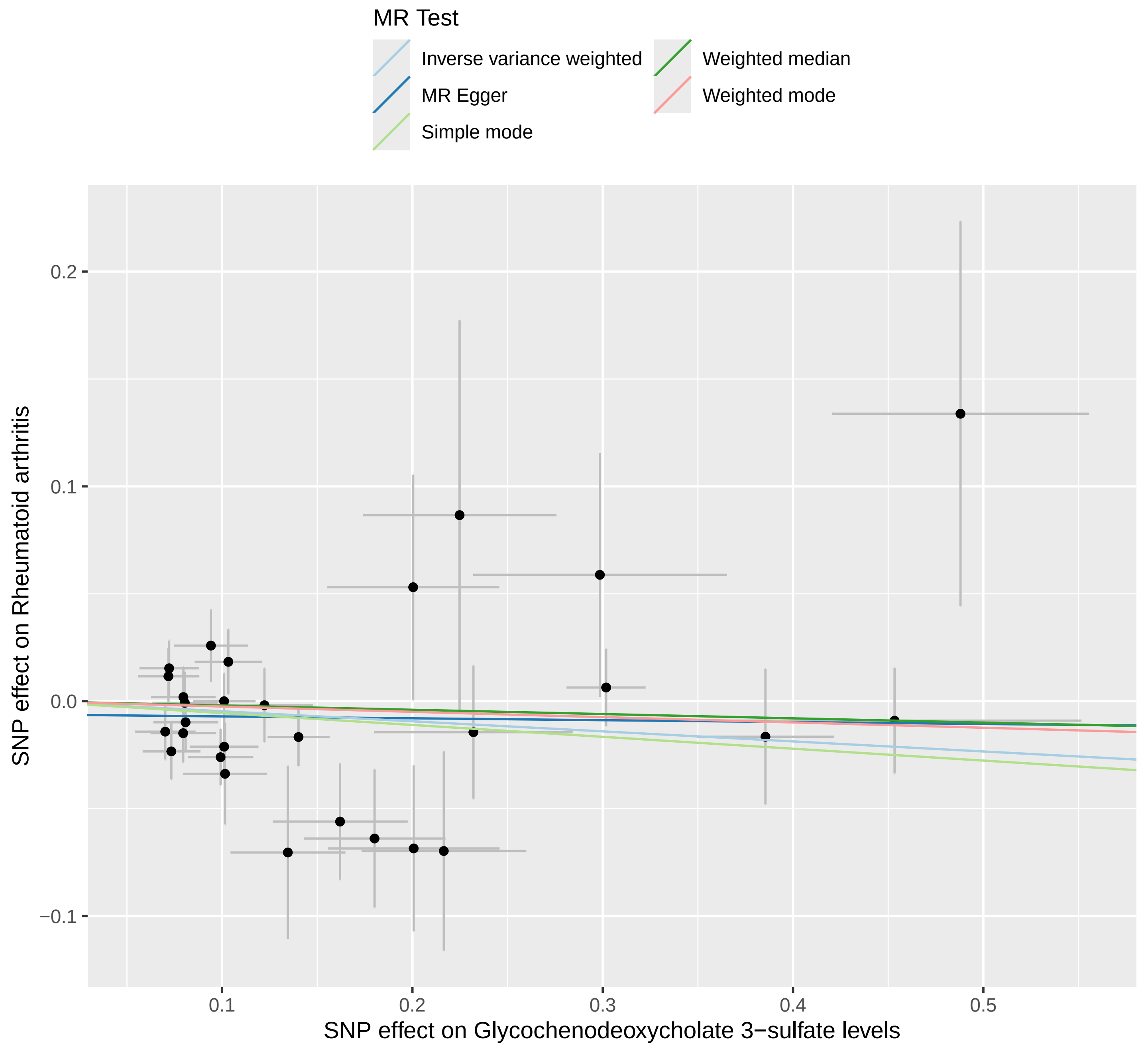

MR Test
Inverse variance weighted
MR Egger
Simple mode
Weighted median
Weighted mode
0.2
0.1
0.0
−0.1
0.1
0.2
0.3
0.4
0.5
SNP effect on Rheumatoid arthritis
SNP effect on Glycochenodeoxycholate 3−sulfate levels

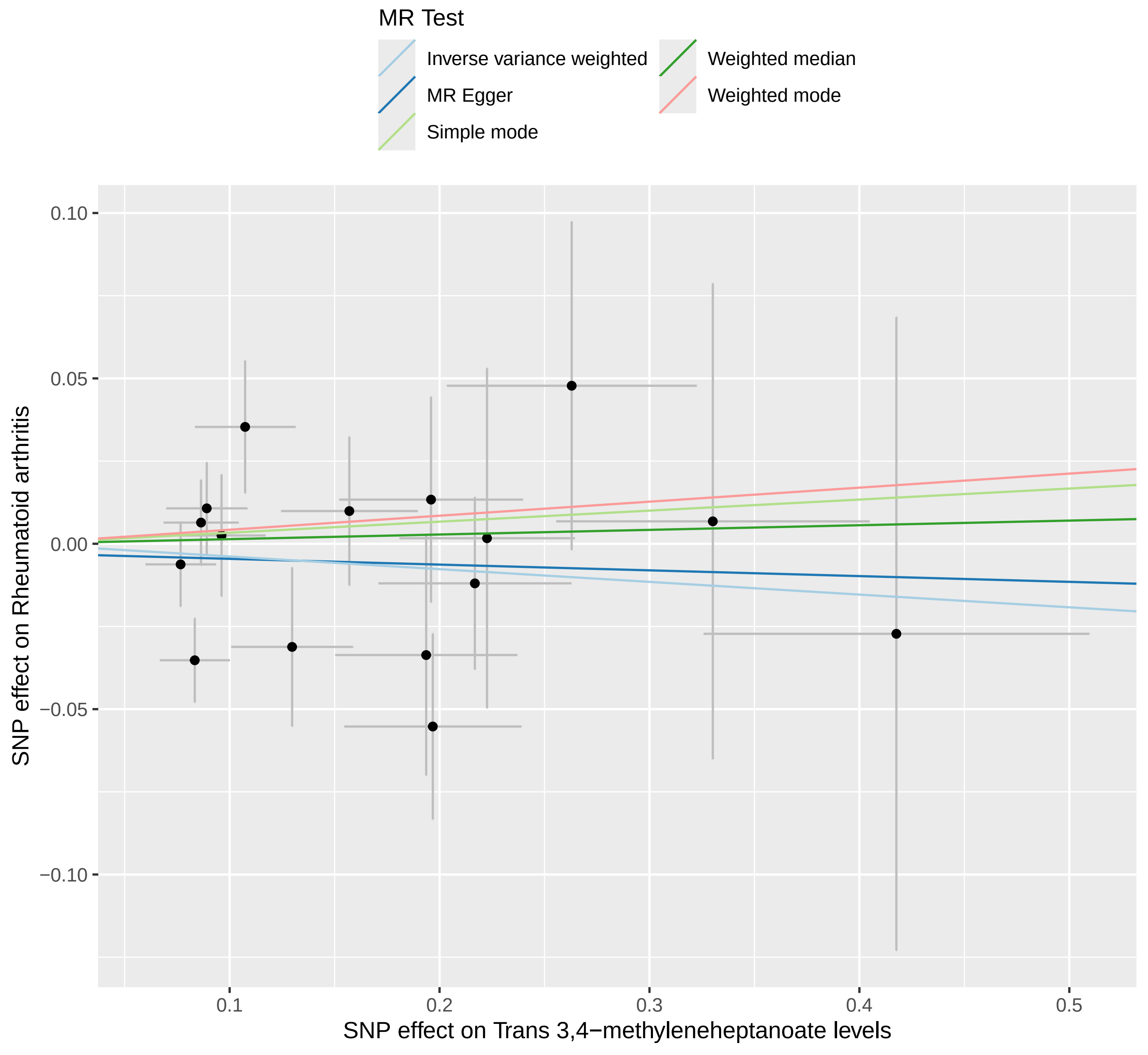
MR Test
Inverse variance weighted
MR Egger
Simple mode
Weighted median
Weighted mode
0.10
0.05
0.00
−0.05
−0.10
0.1
0.2
0.3
0.4
0.5
SNP effect on Rheumatoid arthritis
SNP effect on Trans 3,4−methyleneheptanoate levels

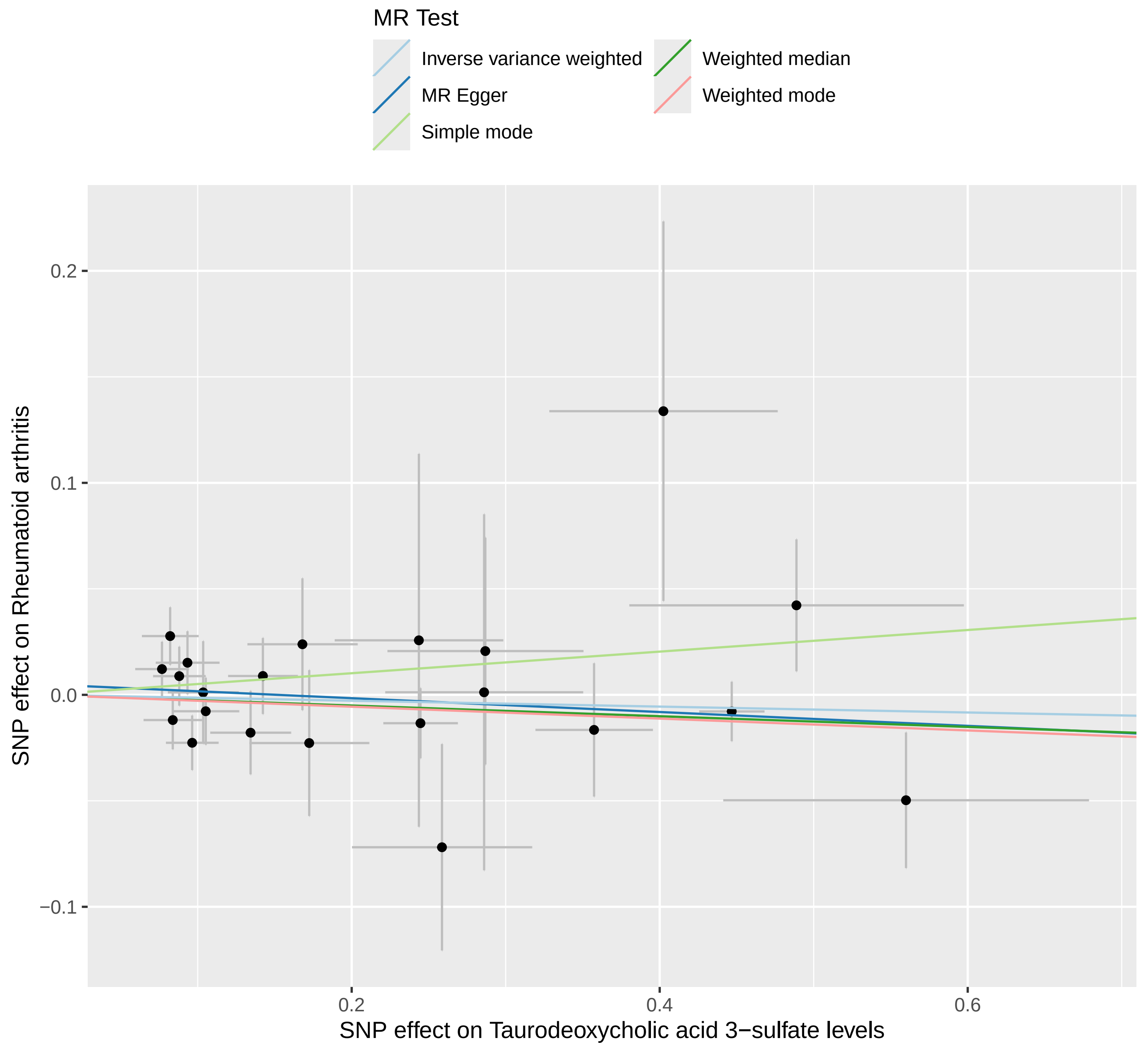
MR Test
Inverse variance weighted
MR Egger
Simple mode
Weighted median
Weighted mode
0.2
0.1
0.0
−0.1
0.2
0.4
0.6
SNP effect on Rheumatoid arthritis
SNP effect on Taurodeoxycholic acid 3−sulfate levels

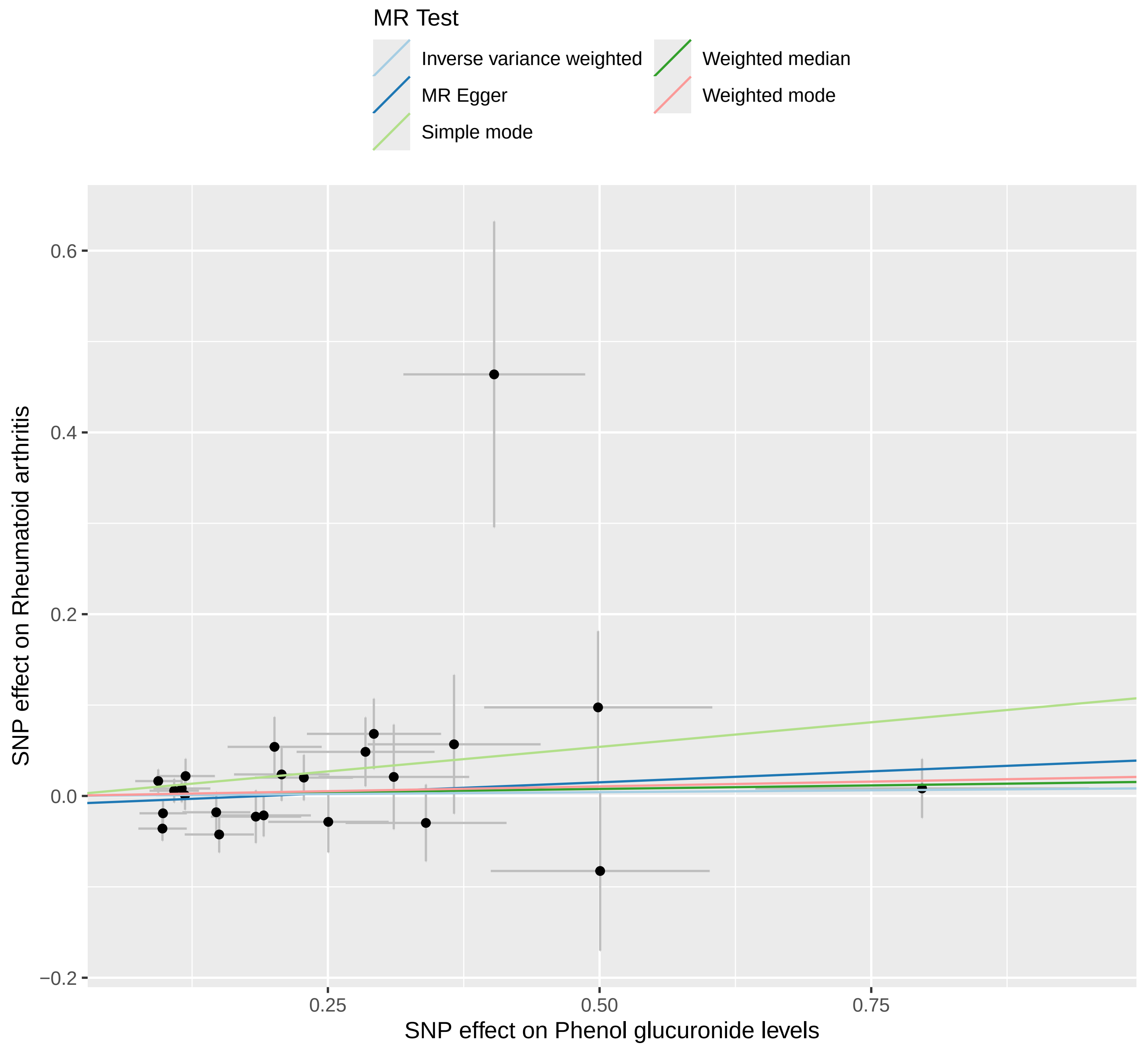

MR Test
Inverse variance weighted
MR Egger
Simple mode
Weighted median
Weighted mode
0.6
0.4
0.2
0.0
−0.2
0.25
0.50
0.75
SNP effect on Rheumatoid arthritis
SNP effect on Phenol glucuronide levels

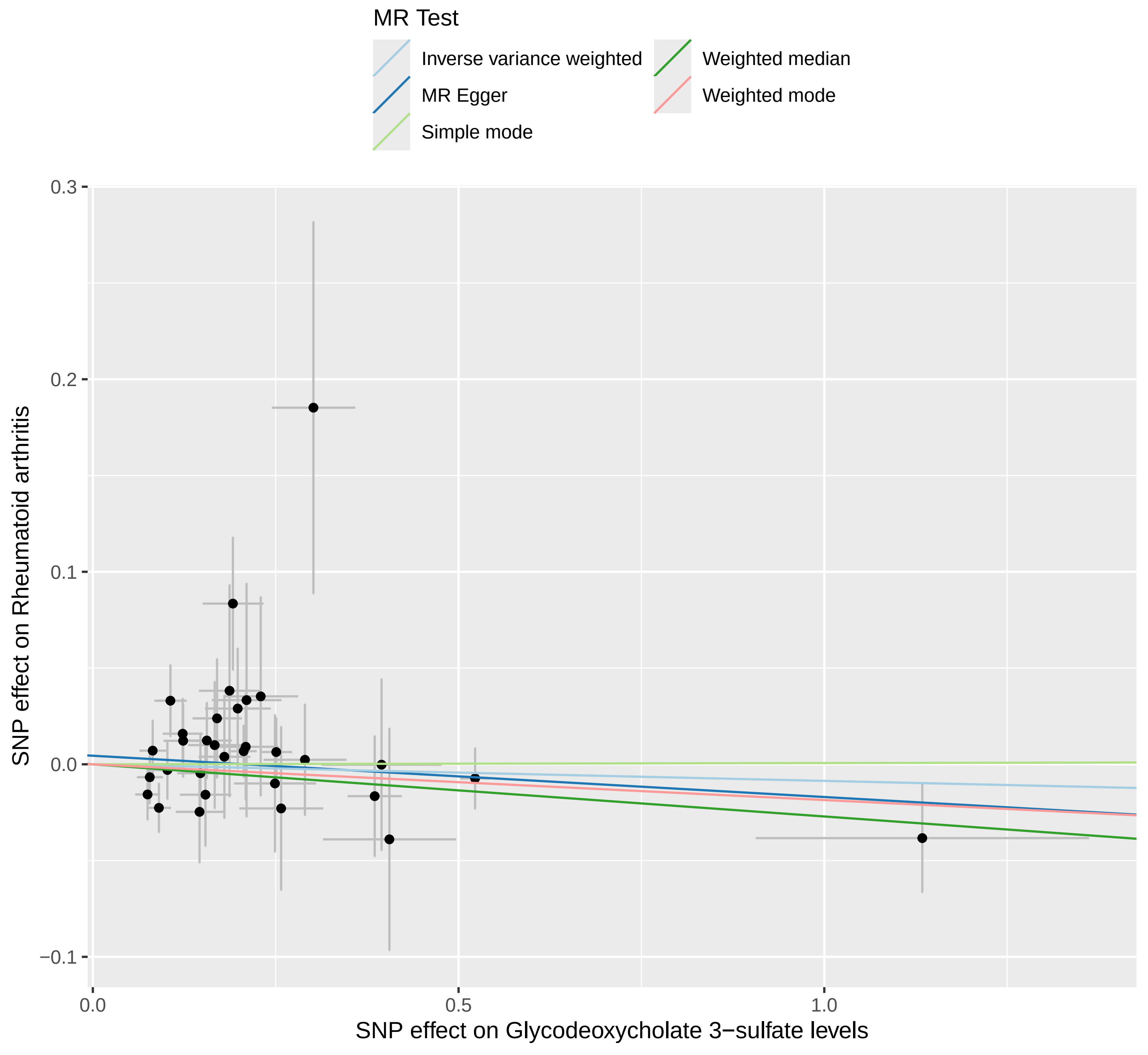
MR Test
Inverse variance weighted
MR Egger
Simple mode
Weighted median
Weighted mode
0.3
0.2
0.1
0.0
−0.1
SNP effect on Rheumatoid arthritis
0.0
0.5
1.0
SNP effect on Glycodeoxycholate 3−sulfate levels

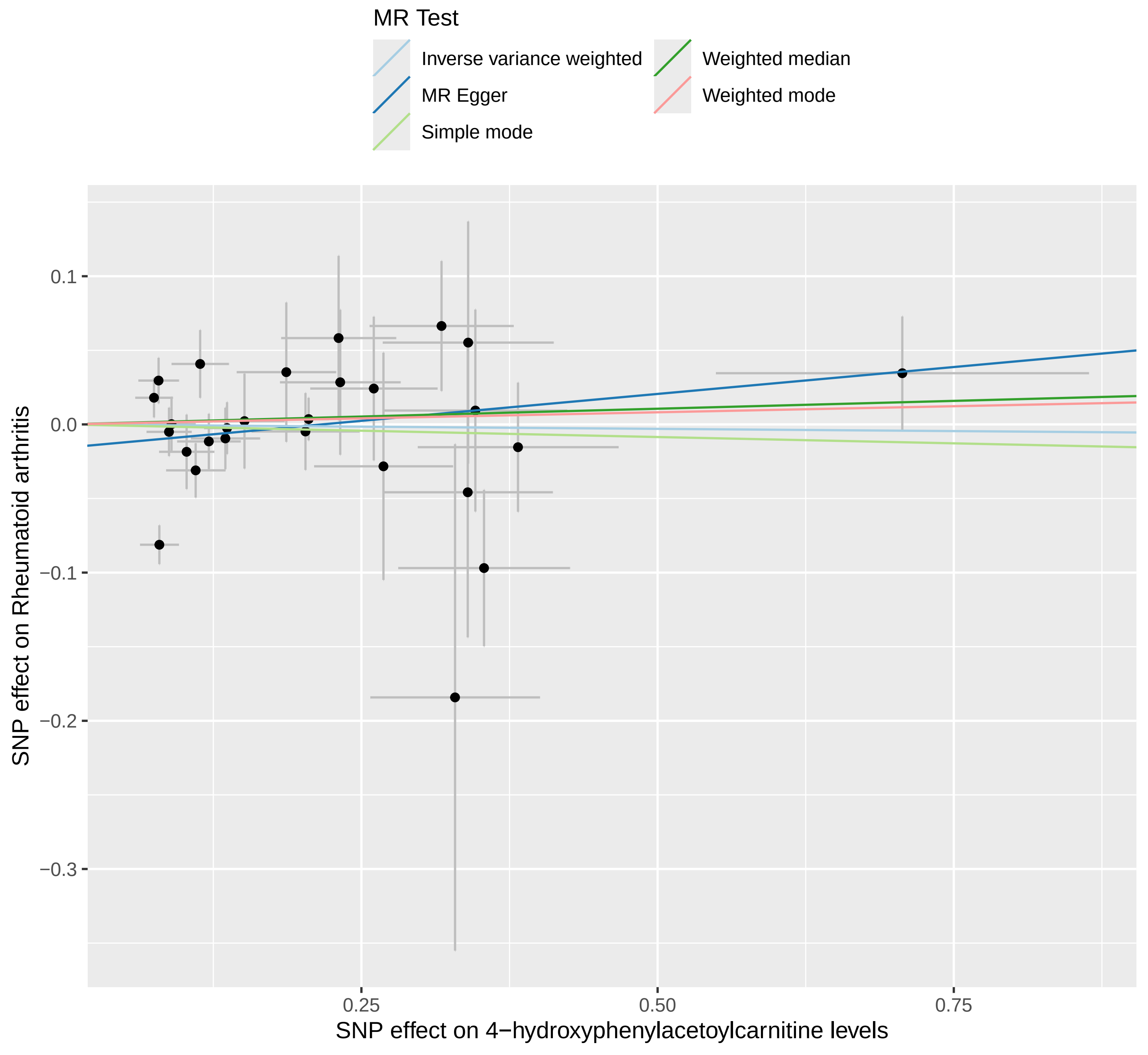

MR Test
Inverse variance weighted
MR Egger
Simple mode
Weighted median
Weighted mode
0.1
0.0
−0.1
−0.2
−0.3
SNP effect on Rheumatoid arthritis
0.25
0.50
0.75
SNP effect on 4−hydroxyphenylacetoylcarnitine levels

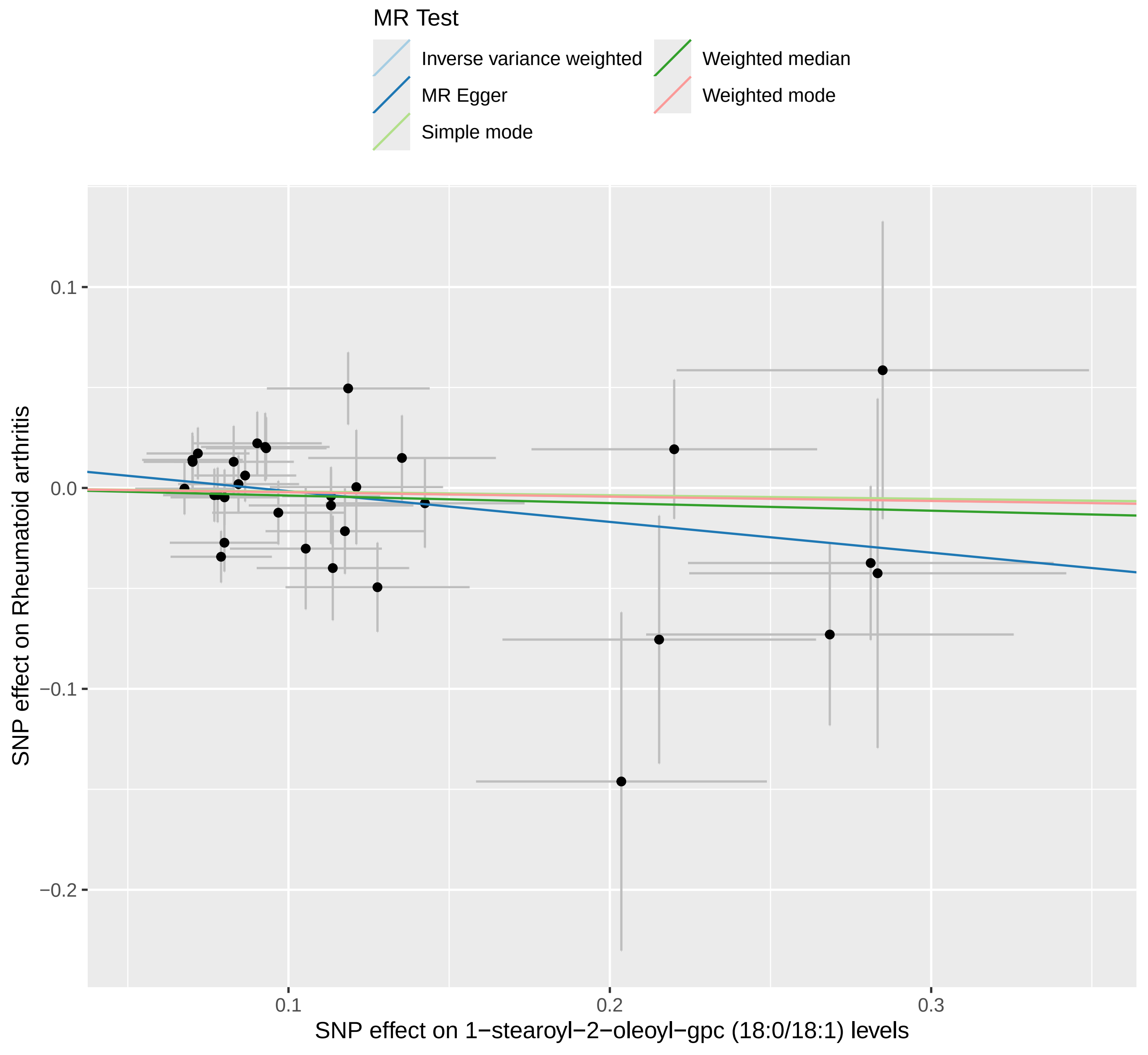
MR Test
Inverse variance weighted
MR Egger
Simple mode
Weighted median
Weighted mode
0.1
0.0
−0.1
−0.2
SNP effect on Rheumatoid arthritis
0.1
0.2
0.3
SNP effect on 1−stearoyl−2−oleoyl−gpc (18:0/18:1) levels

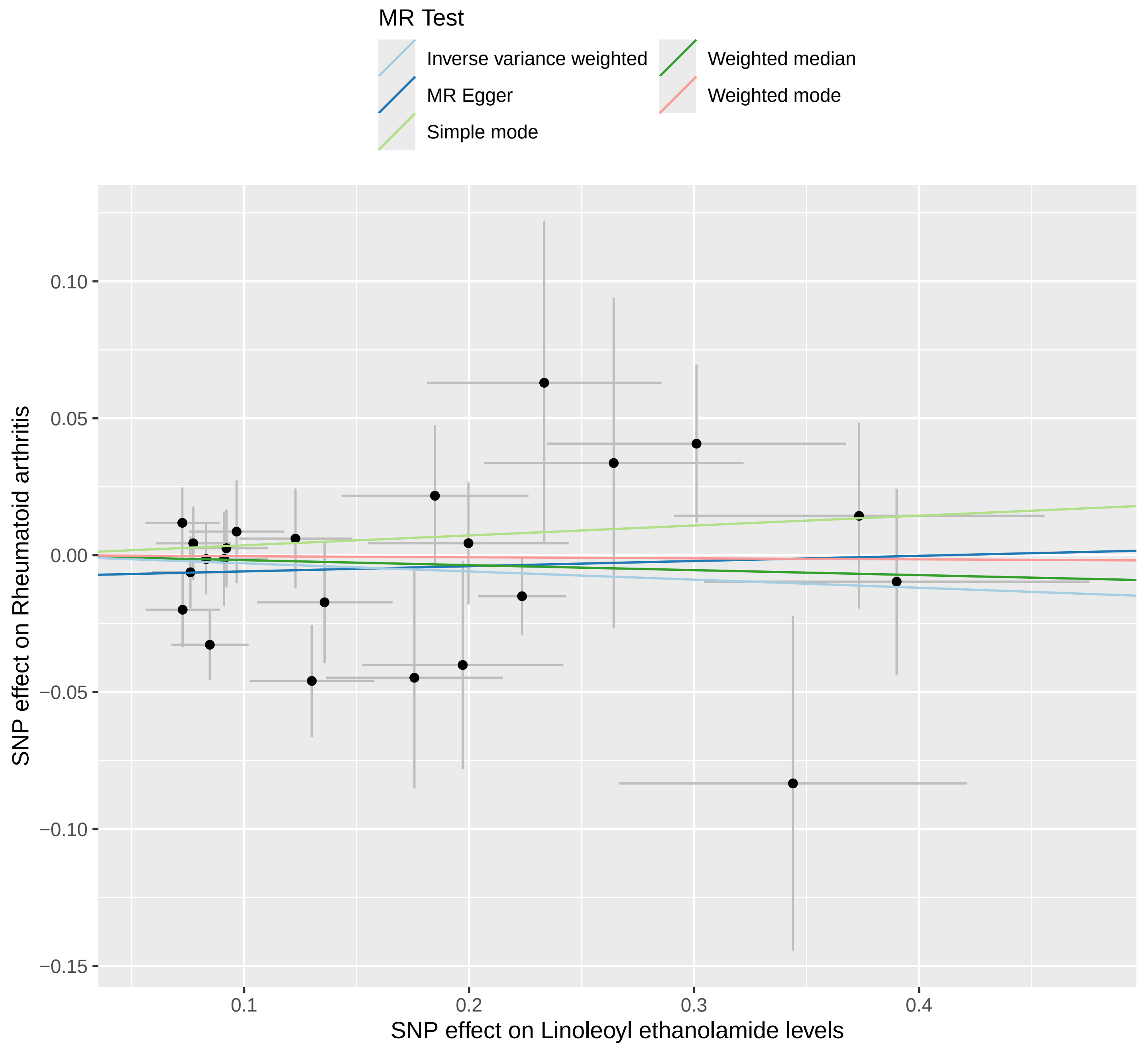
MR Test
Inverse variance weighted
MR Egger
Simple mode
Weighted median
Weighted mode
0.10
0.05
0.00
−0.05
−0.10
−0.15
SNP effect on Rheumatoid arthritis
0.1
0.2
0.3
0.4
SNP effect on Linoleoyl ethanolamide levels

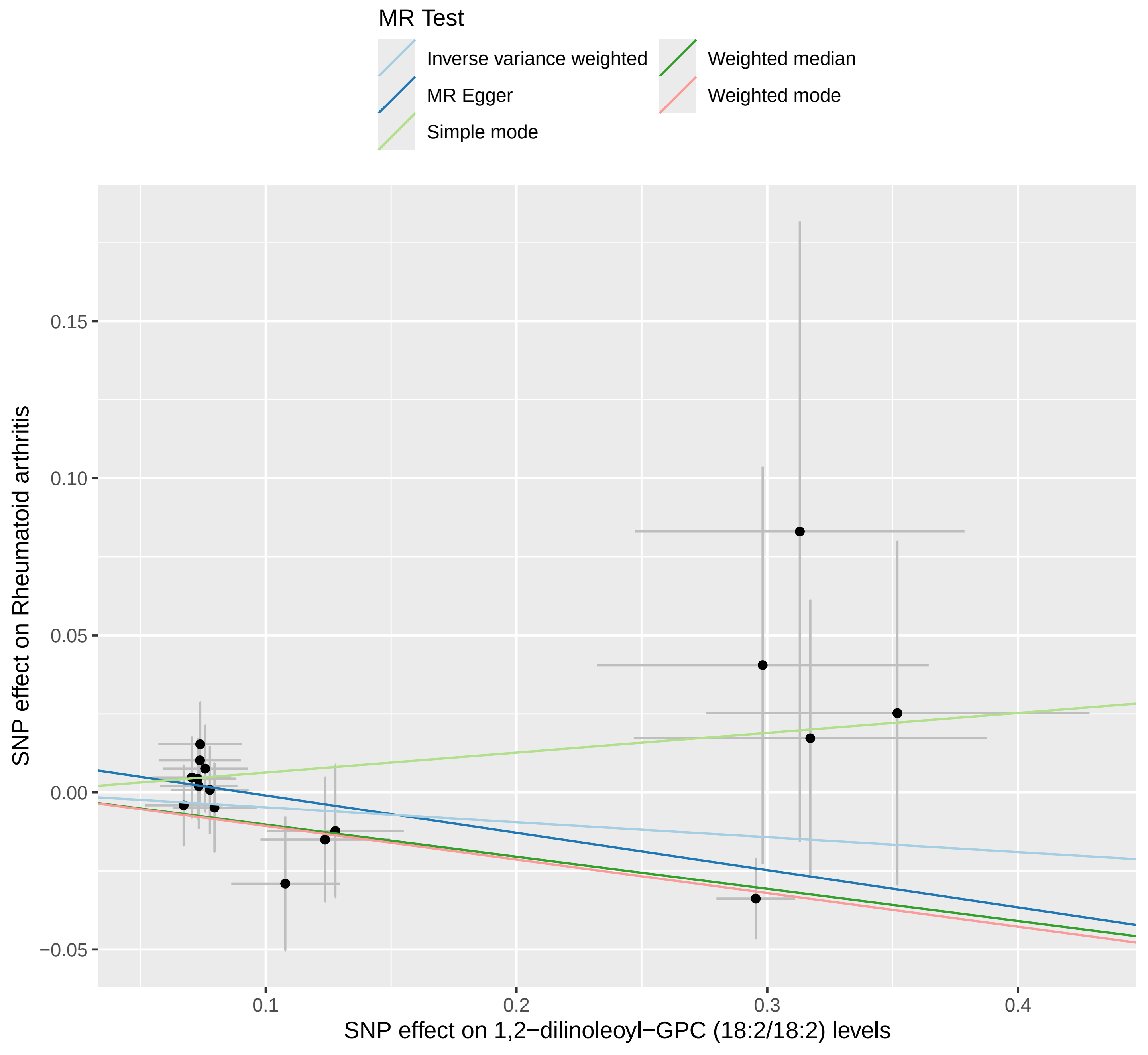

MR Test
Inverse variance weighted
MR Egger
Simple mode
Weighted median
Weighted mode
SNP effect on Rheumatoid arthritis
0.15
0.10
0.05
0.00
−0.05
0.1
0.2
0.3
0.4
SNP effect on 1,2−dilinoleoyl−GPC (18:2/18:2) levels

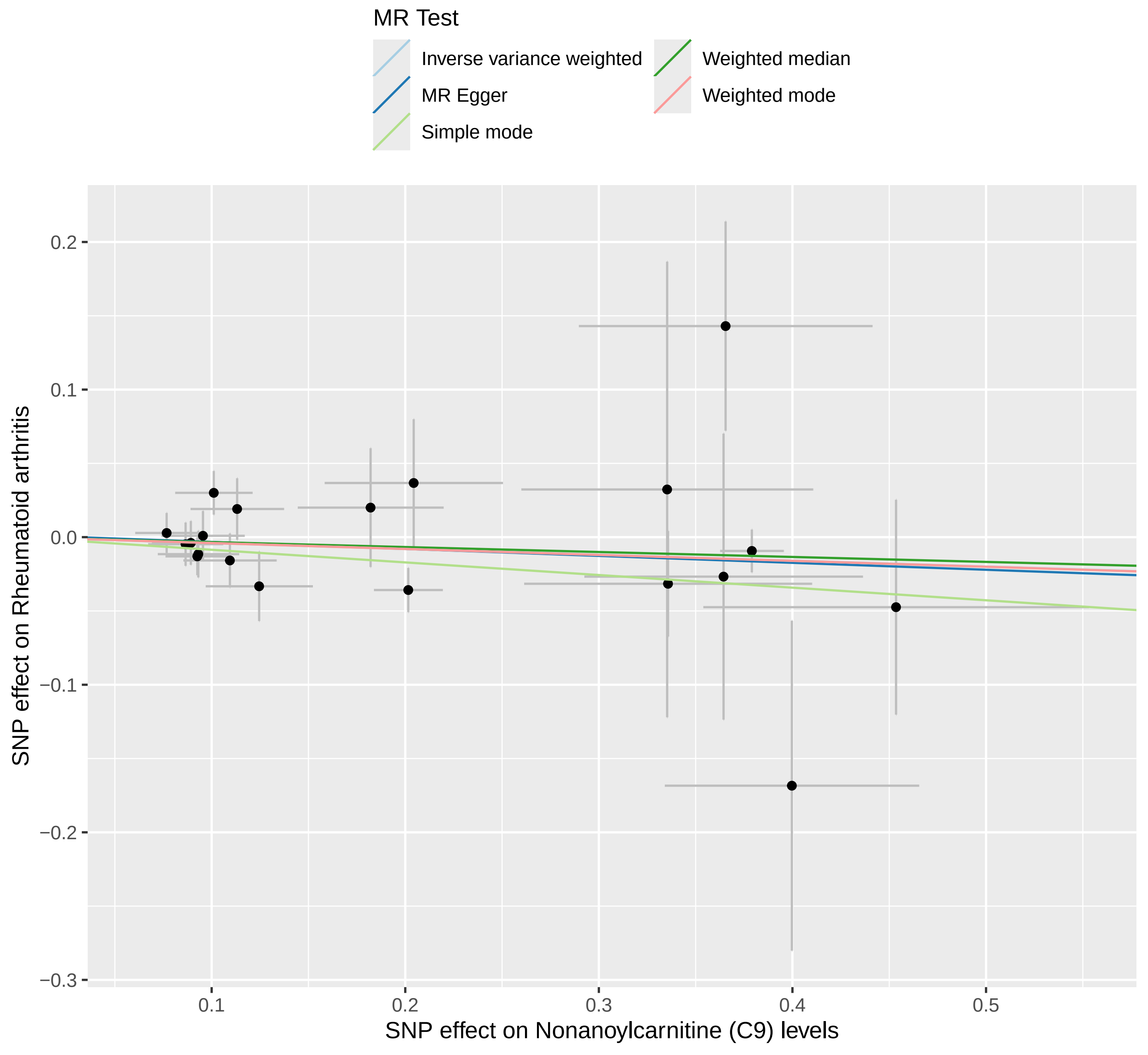
MR Test
Inverse variance weighted
MR Egger
Simple mode
Weighted median
Weighted mode
0.2
0.1
0.0
−0.1
−0.2
−0.3
0.1
0.2
0.3
0.4
0.5
SNP effect on Rheumatoid arthritis
SNP effect on Nonanoylcarnitine (C9) levels

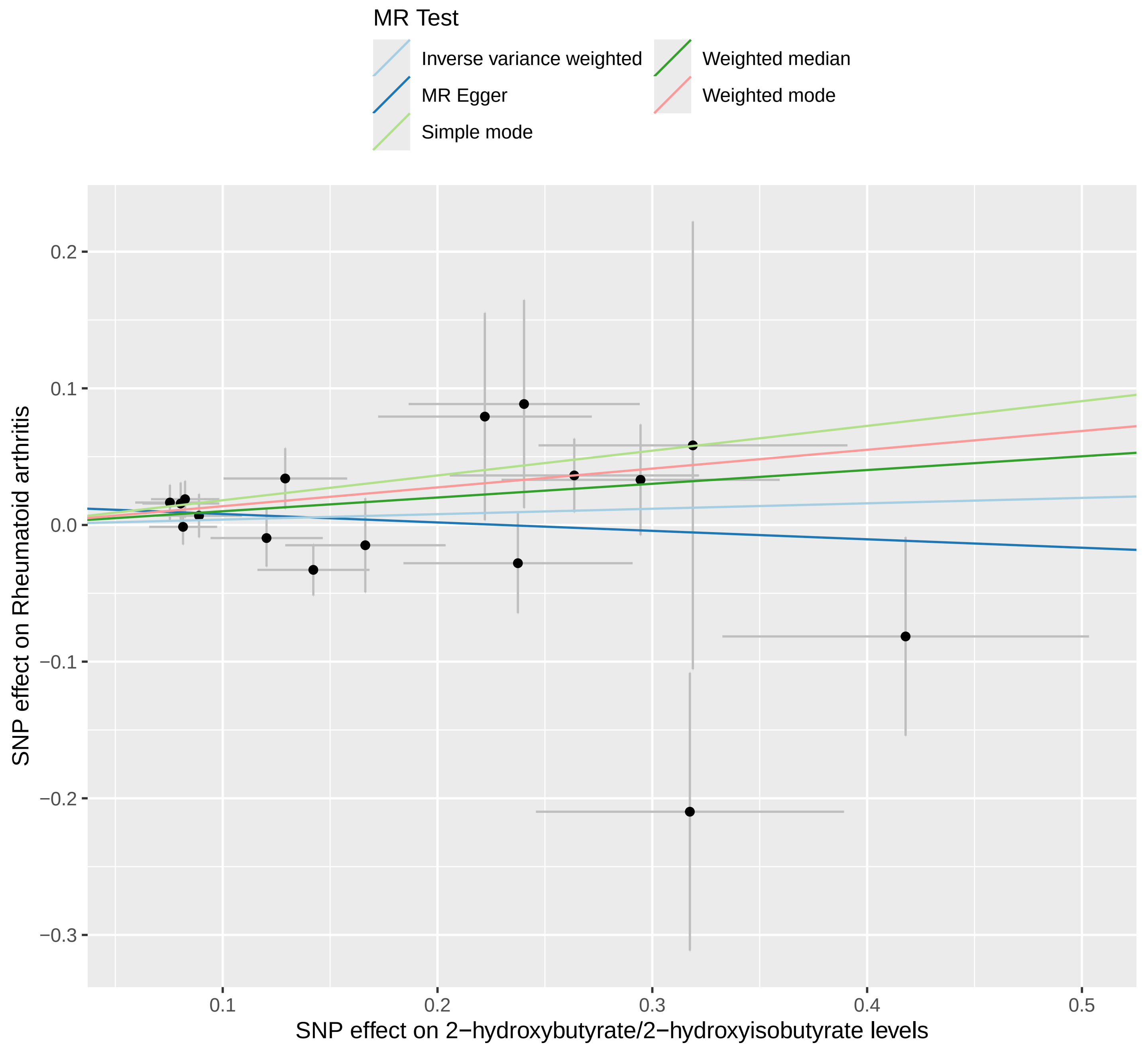
MR Test
Inverse variance weighted
MR Egger
Simple mode
Weighted median
Weighted mode
0.2
0.1
0.0
−0.1
−0.2
−0.3
SNP effect on Rheumatoid arthritis
0.1
0.2
0.3
0.4
0.5
SNP effect on 2−hydroxybutyrate/2−hydroxyisobutyrate levels

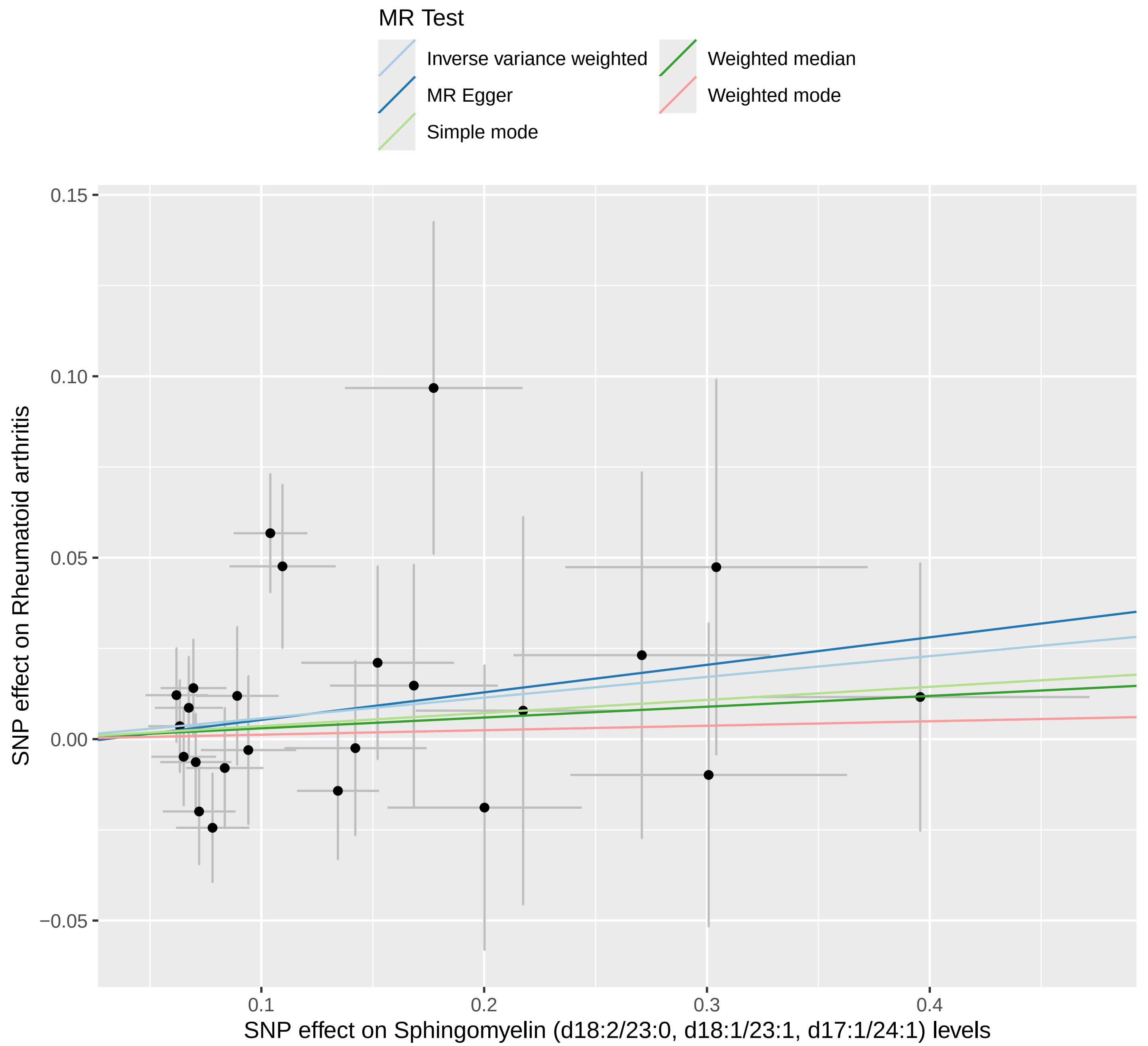

MR Test
Inverse variance weighted
MR Egger
Simple mode
Weighted median
Weighted mode
0.15
0.10
0.05
0.00
−0.05
0.1
0.2
0.3
0.4
SNP effect on Rheumatoid arthritis
SNP effect on Sphingomyelin (d18:2/23:0, d18:1/23:1, d17:1/24:1) levels

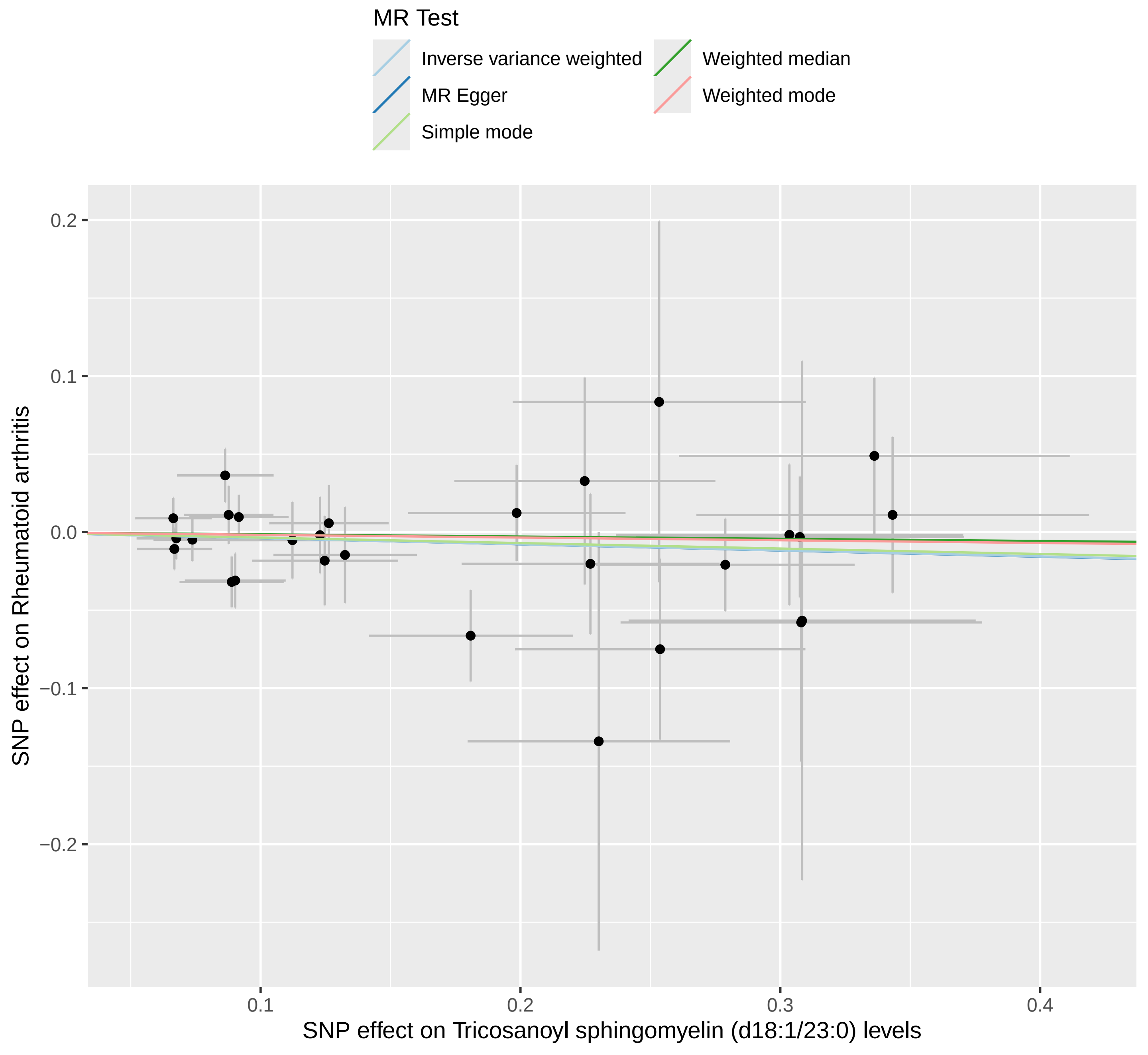

MR Test
Inverse variance weighted
MR Egger
Simple mode
Weighted median
Weighted mode
0.2
0.1
0.0
−0.1
−0.2
0.1
0.2
0.3
0.4
SNP effect on Rheumatoid arthritis
SNP effect on Tricosanoyl sphingomyelin (d18:1/23:0) levels

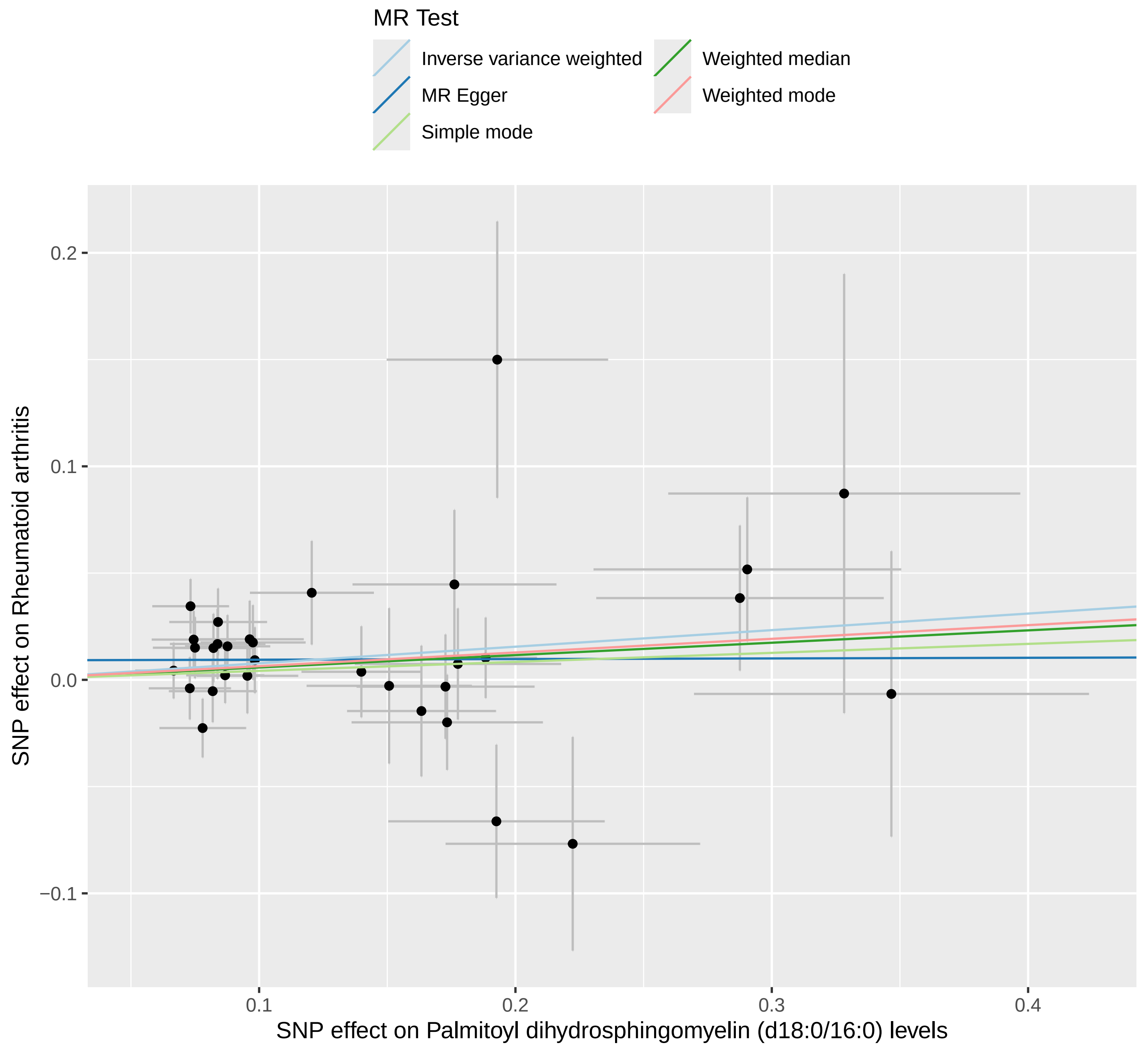

MR Test
Inverse variance weighted
MR Egger
Simple mode
Weighted median
Weighted mode
0.2
0.1
0.0
−0.1
SNP effect on Rheumatoid arthritis
0.1
0.2
0.3
0.4
SNP effect on Palmitoyl dihydrosphingomyelin (d18:0/16:0) levels

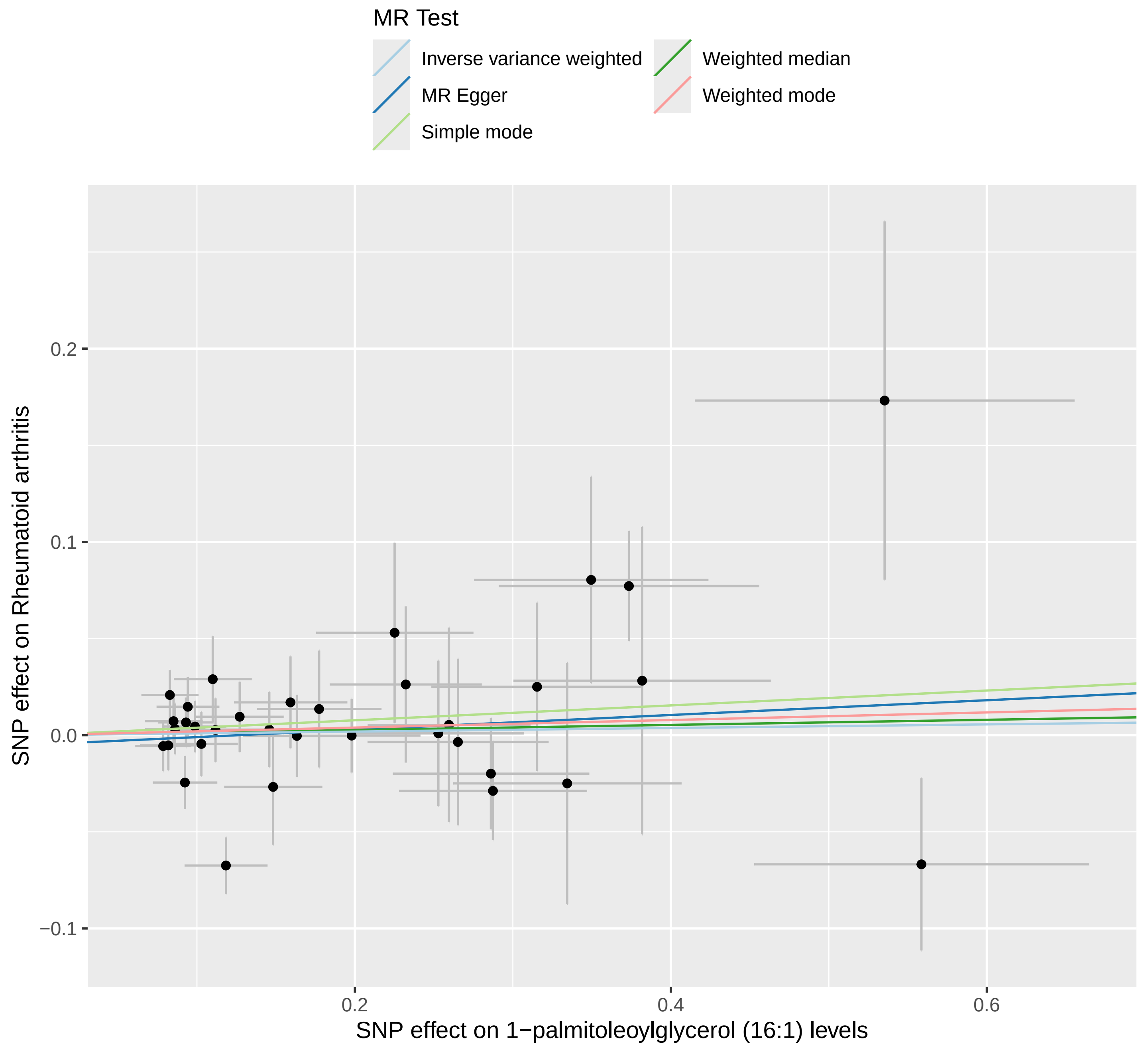
MR Test
Inverse variance weighted
MR Egger
Simple mode
Weighted median
Weighted mode
SNP effect on Rheumatoid arthritis
0.2
0.1
0.0
−0.1
0.2
0.4
0.6
SNP effect on 1−palmitoleoylglycerol (16:1) levels

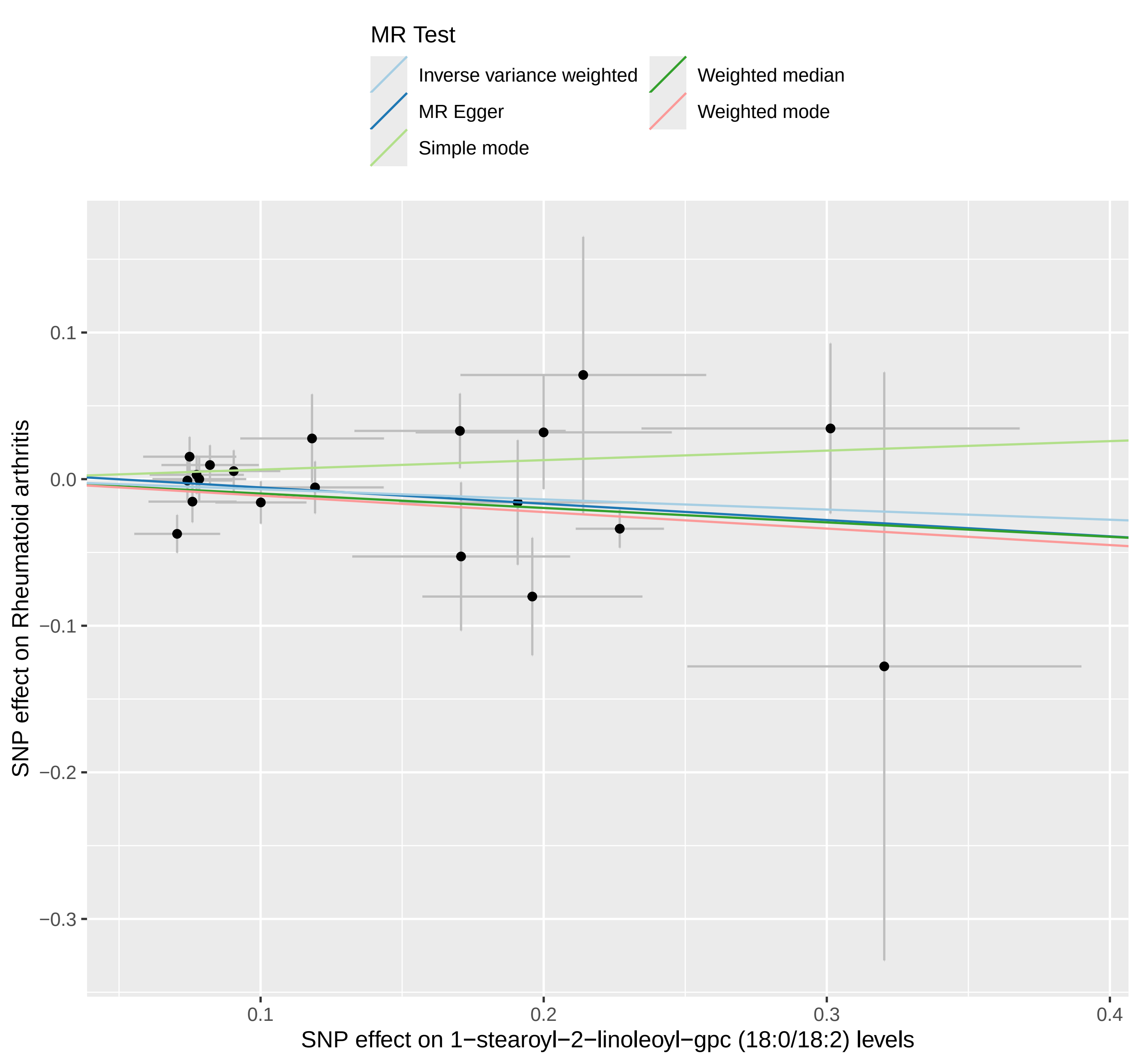

MR Test
Inverse variance weighted
MR Egger
Simple mode
Weighted median
Weighted mode
SNP effect on Rheumatoid arthritis
0.1
0.0
−0.1
−0.2
−0.3
0.1
0.2
0.3
0.4
SNP effect on 1−stearoyl−2−linoleoyl−gpc (18:0/18:2) levels

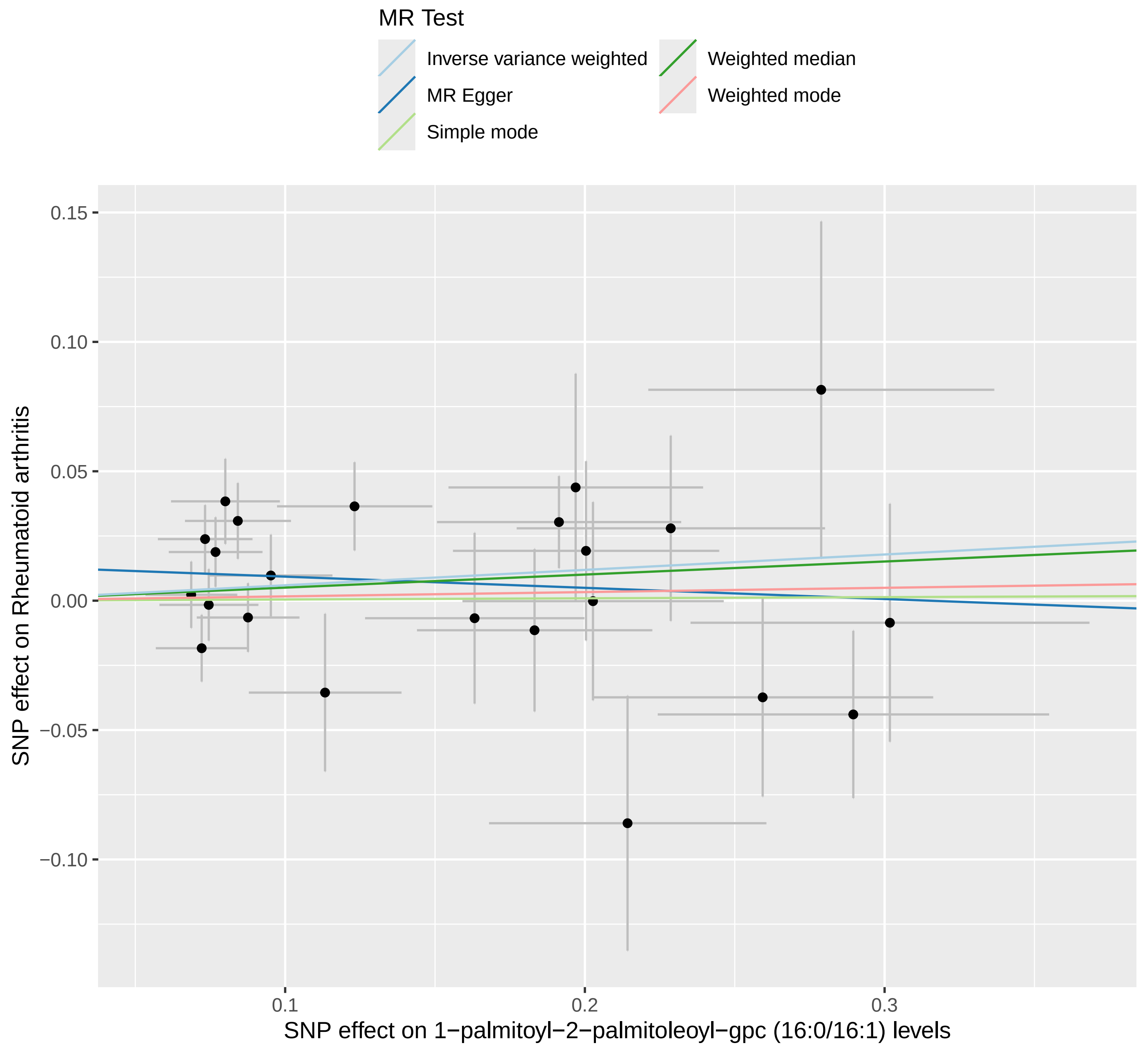

MR Test
Inverse variance weighted
MR Egger
Simple mode
Weighted median
Weighted mode
0.15
0.10
0.05
0.00
−0.05
−0.10
SNP effect on Rheumatoid arthritis
0.1
0.2
0.3
SNP effect on 1−palmitoyl−2−palmitoleoyl−gpc (16:0/16:1) levels

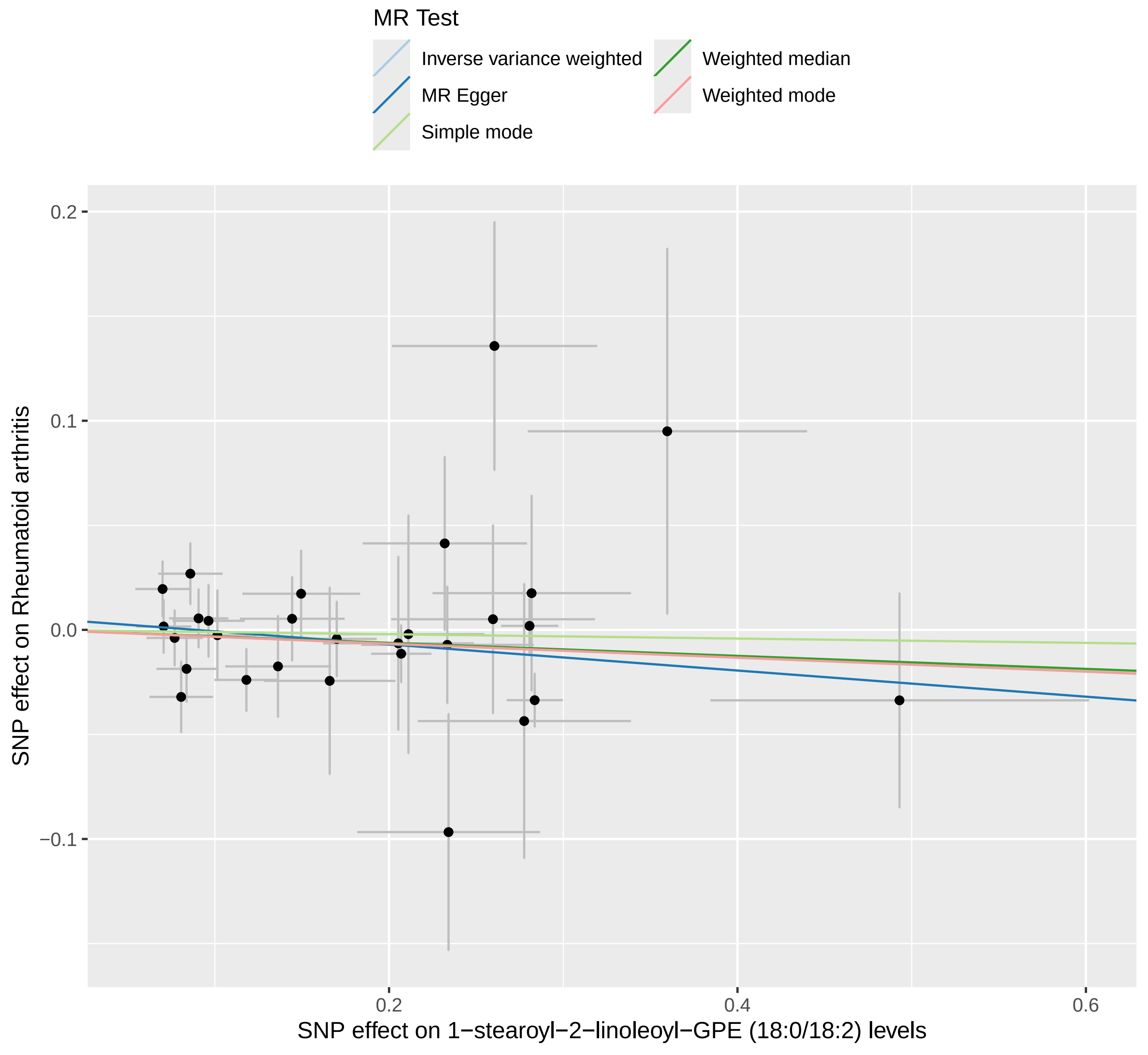
MR Test
Inverse variance weighted
MR Egger
Simple mode
Weighted median
Weighted mode
0.2
0.1
0.0
−0.1
SNP effect on Rheumatoid arthritis
0.2
0.4
0.6
SNP effect on 1−stearoyl−2−linoleoyl−GPE (18:0/18:2) levels

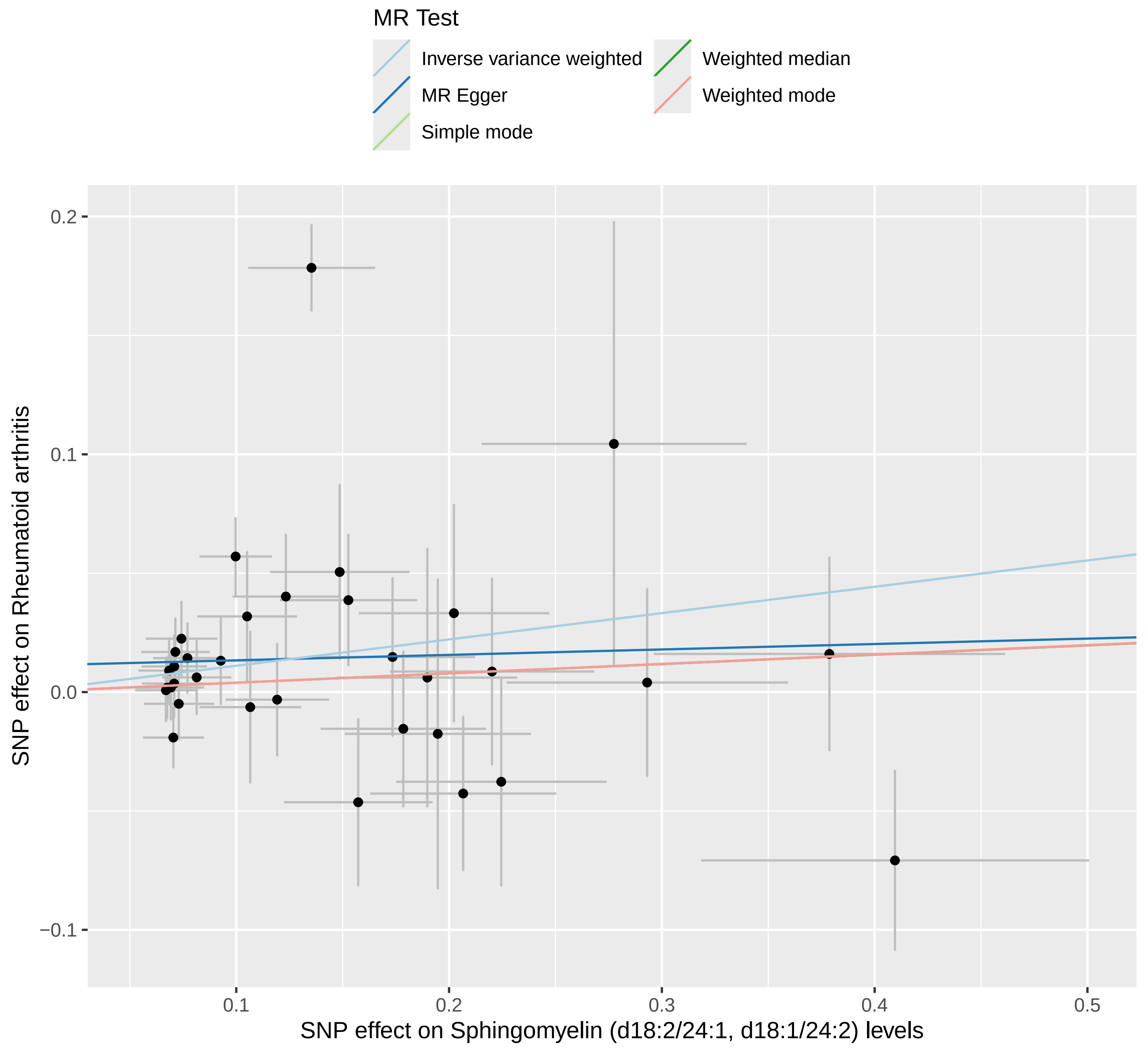

MR Test
Inverse variance weighted
MR Egger
Simple mode
Weighted median
Weighted mode
0.2
0.1
0.0
−0.1
0.1
0.2
0.3
0.4
0.5
SNP effect on Rheumatoid arthritis
SNP effect on Sphingomyelin (d18:2/24:1, d18:1/24:2) levels

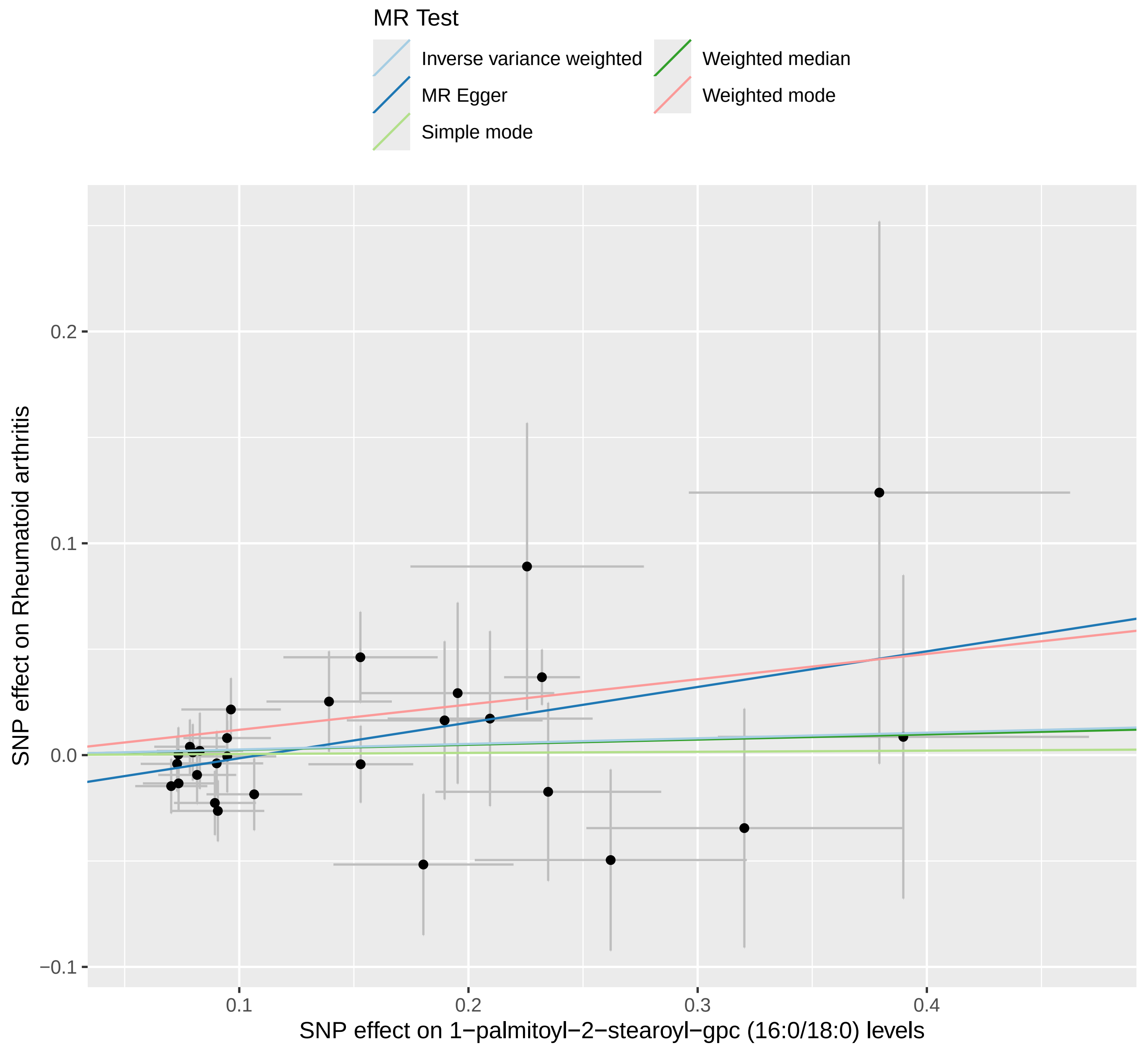
MR Test
Inverse variance weighted
MR Egger
Simple mode
Weighted median
Weighted mode
0.2
0.1
0.0
−0.1
0.1
0.2
0.3
0.4
SNP effect on Rheumatoid arthritis
SNP effect on 1−palmitoyl−2−stearoyl−gpc (16:0/18:0) levels

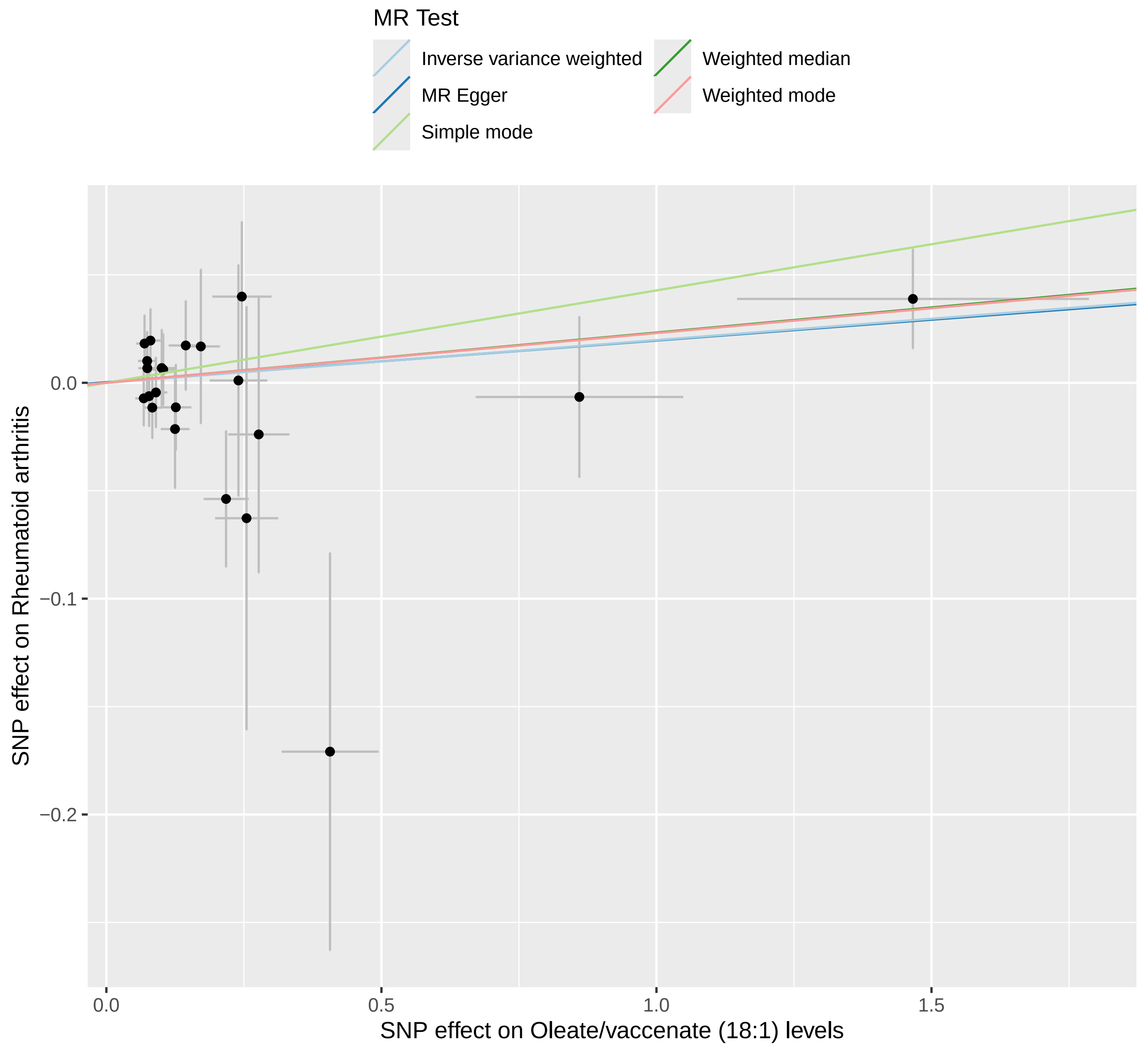
MR Test
Inverse variance weighted
MR Egger
Simple mode
Weighted median
Weighted mode
0.0
−0.1
−0.2
0.0
0.5
1.0
1.5
SNP effect on Rheumatoid arthritis
SNP effect on Oleate/vaccenate (18:1) levels

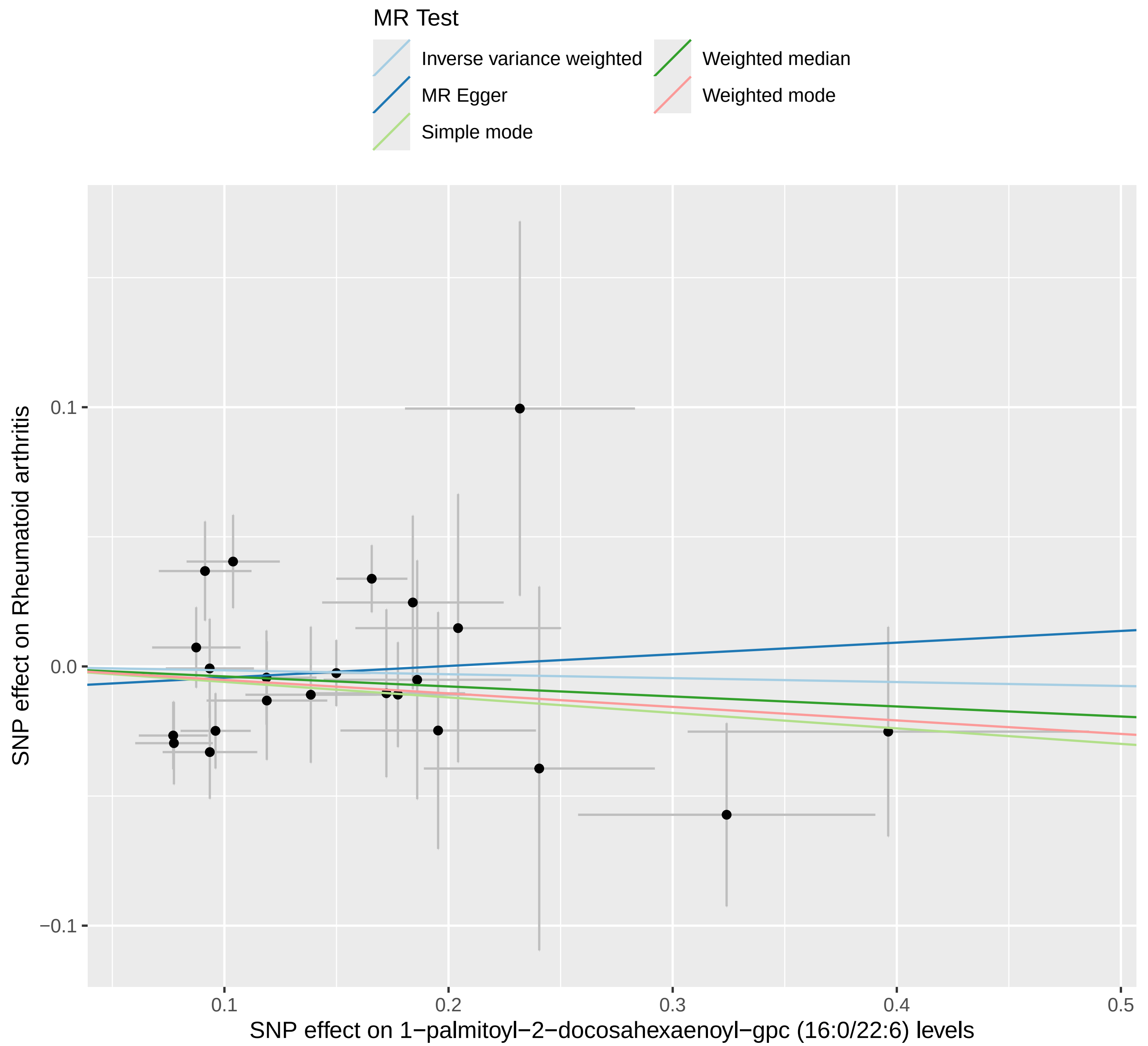

MR Test
Inverse variance weighted
MR Egger
Simple mode
Weighted median
Weighted mode
0.1
0.0
−0.1
SNP effect on Rheumatoid arthritis
0.1
0.2
0.3
0.4
0.5
SNP effect on 1−palmitoyl−2−docosahexaenoyl−gpc (16:0/22:6) levels

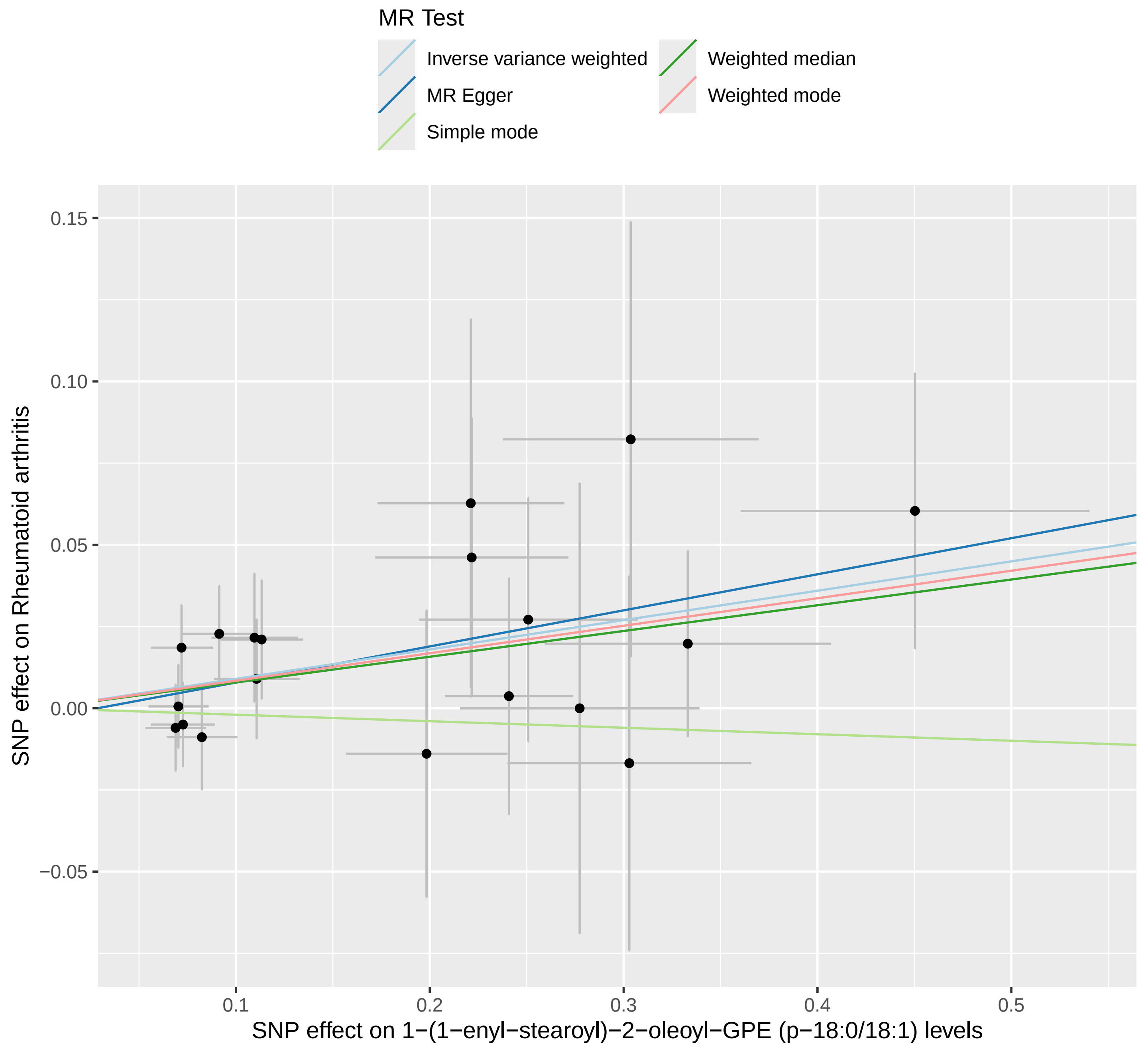
MR Test
Inverse variance weighted
MR Egger
Simple mode
Weighted median
Weighted mode
0.15
0.10
0.05
0.00
−0.05
SNP effect on Rheumatoid arthritis
0.1
0.2
0.3
0.4
0.5
SNP effect on 1−(1−enyl−stearoyl)−2−oleoyl−GPE (p−18:0/18:1) levels

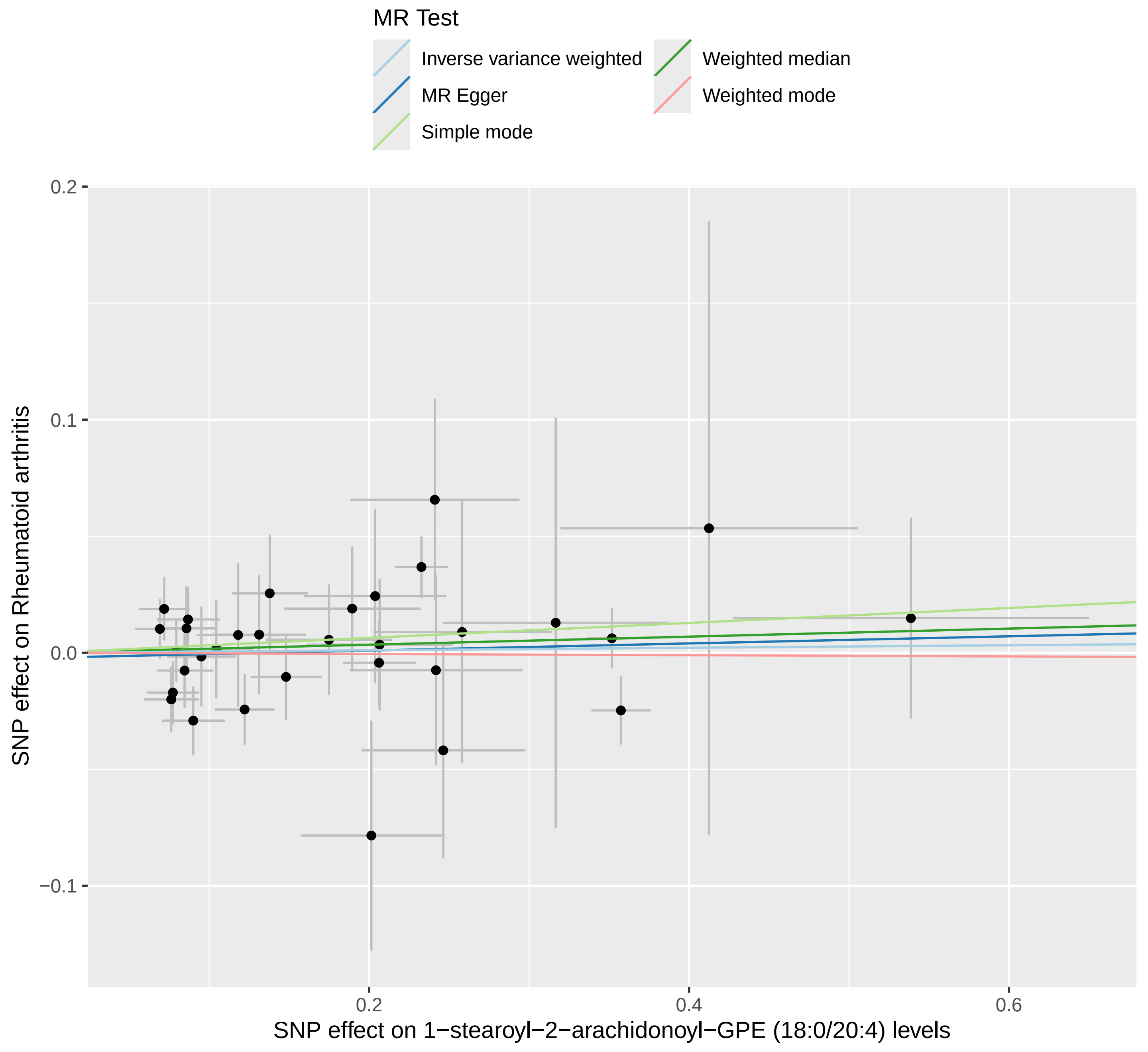

MR Test
Inverse variance weighted
MR Egger
Simple mode
Weighted median
Weighted mode
0.2
0.1
0.0
−0.1
SNP effect on Rheumatoid arthritis
0.2
0.4
0.6
SNP effect on 1−stearoyl−2−arachidonoyl−GPE (18:0/20:4) levels

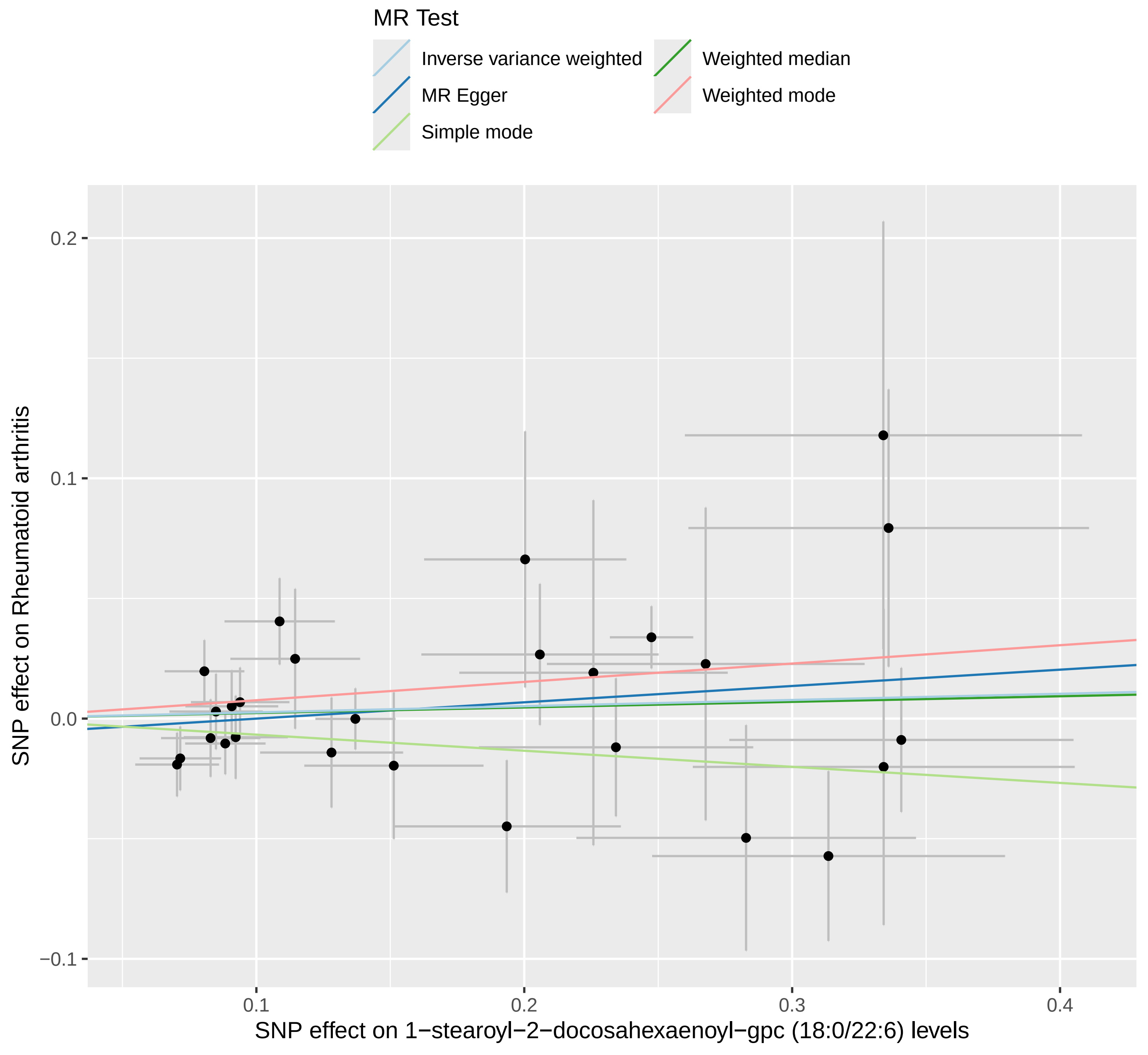
MR Test
Inverse variance weighted
MR Egger
Simple mode
Weighted median
Weighted mode
0.2
0.1
0.0
−0.1
SNP effect on Rheumatoid arthritis
0.1
0.2
0.3
0.4
SNP effect on 1−stearoyl−2−docosahexaenoyl−gpc (18:0/22:6) levels

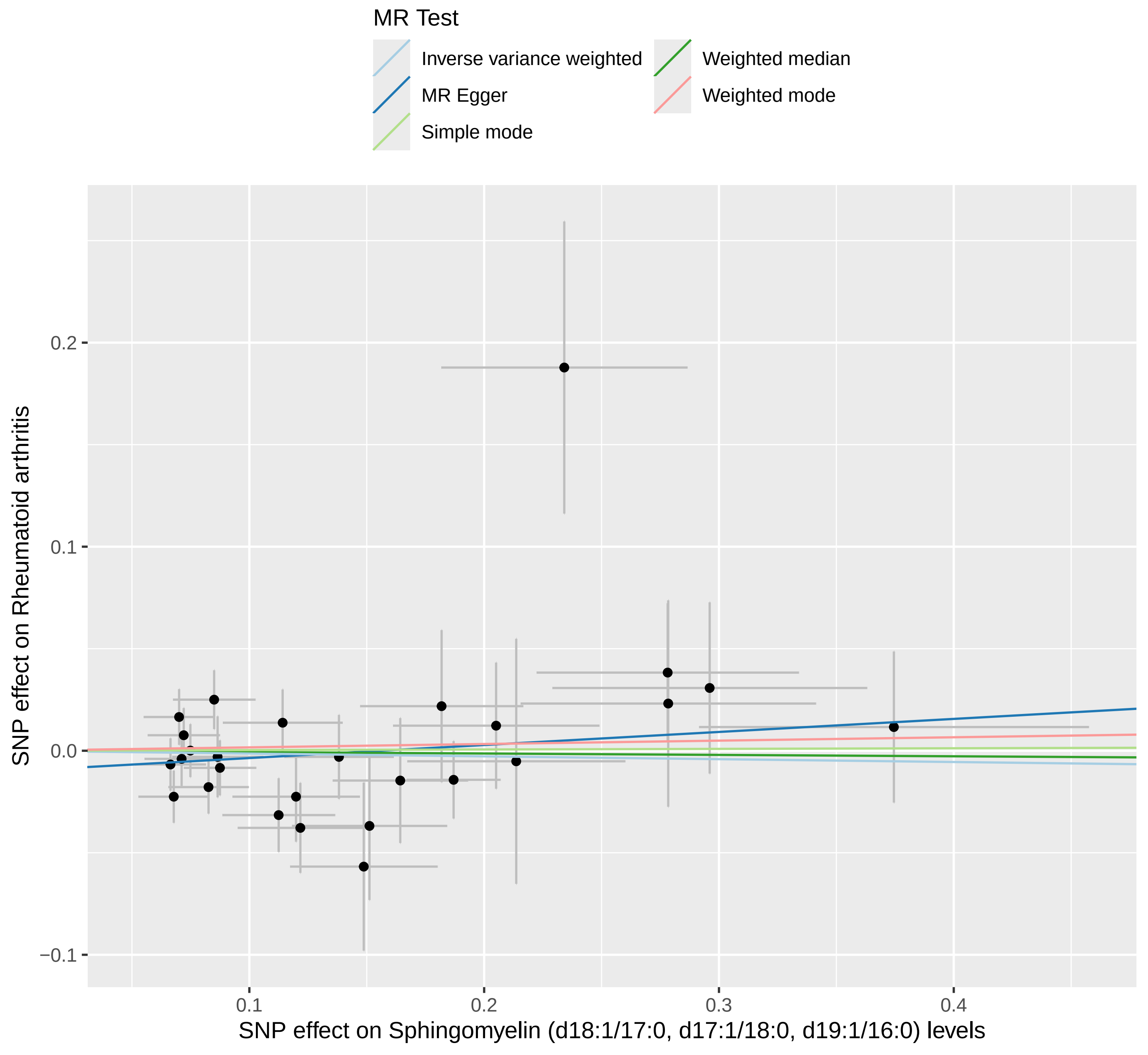
MR Test
Inverse variance weighted
MR Egger
Simple mode
Weighted median
Weighted mode
0.2
0.1
0.0
−0.1
0.1
0.2
0.3
0.4
SNP effect on Rheumatoid arthritis
SNP effect on Sphingomyelin (d18:1/17:0, d17:1/18:0, d19:1/16:0) levels

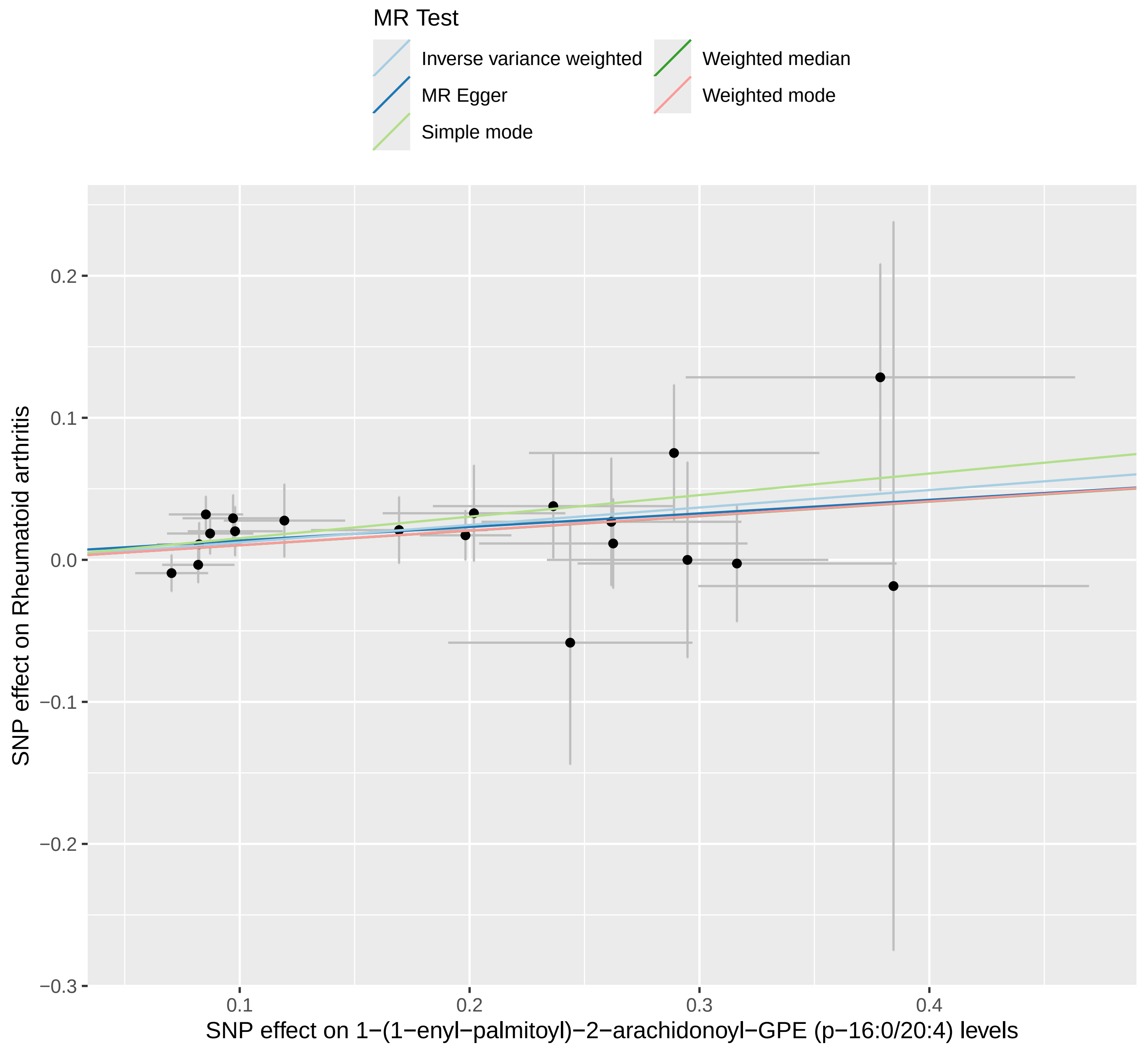

MR Test
Inverse variance weighted
MR Egger
Simple mode
Weighted median
Weighted mode
SNP effect on Rheumatoid arthritis
0.2
0.1
0.0
−0.1
−0.2
−0.3
0.1
0.2
0.3
0.4
SNP effect on 1−(1−enyl−palmitoyl)−2−arachidonoyl−GPE (p−16:0/20:4) levels

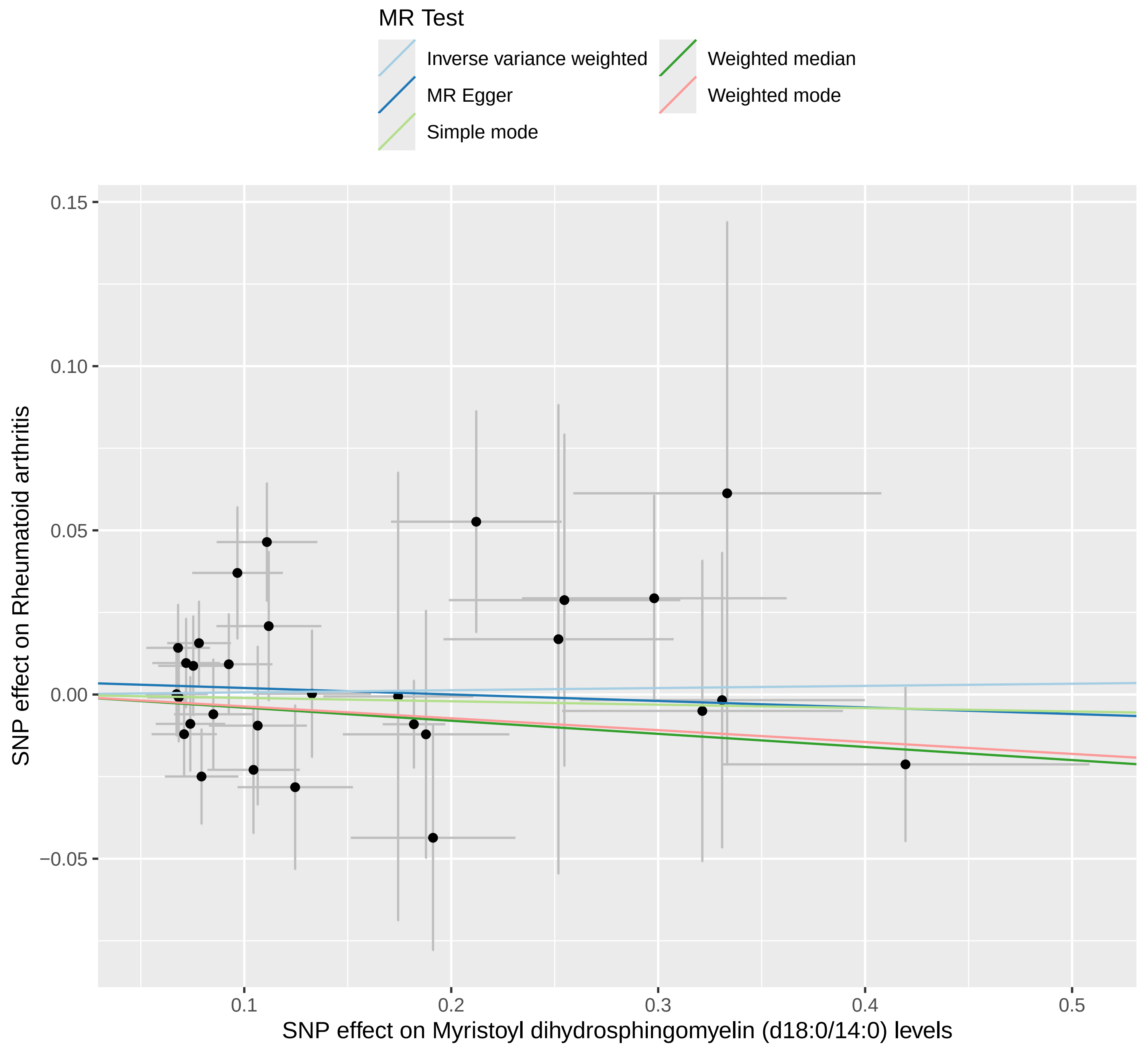

MR Test
Inverse variance weighted
MR Egger
Simple mode
Weighted median
Weighted mode
0.15
0.10
0.05
0.00
−0.05
0.1
0.2
0.3
0.4
0.5
SNP effect on Rheumatoid arthritis
SNP effect on Myristoyl dihydrosphingomyelin (d18:0/14:0) levels

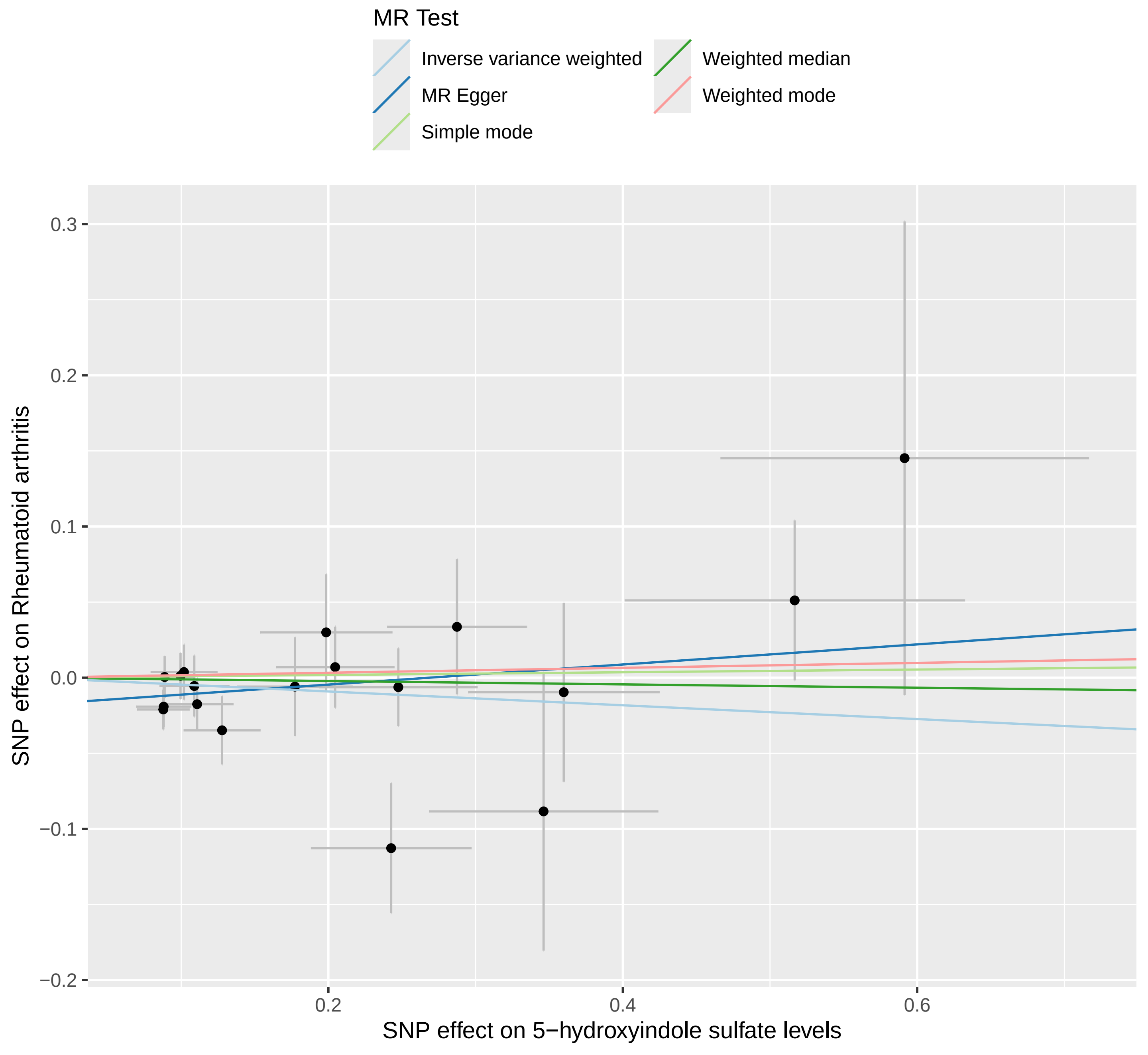
MR Test
Inverse variance weighted
MR Egger
Simple mode
Weighted median
Weighted mode
SNP effect on Rheumatoid arthritis
0.3
0.2
0.1
0.0
−0.1
−0.2
0.2
0.4
0.6
SNP effect on 5−hydroxyindole sulfate levels

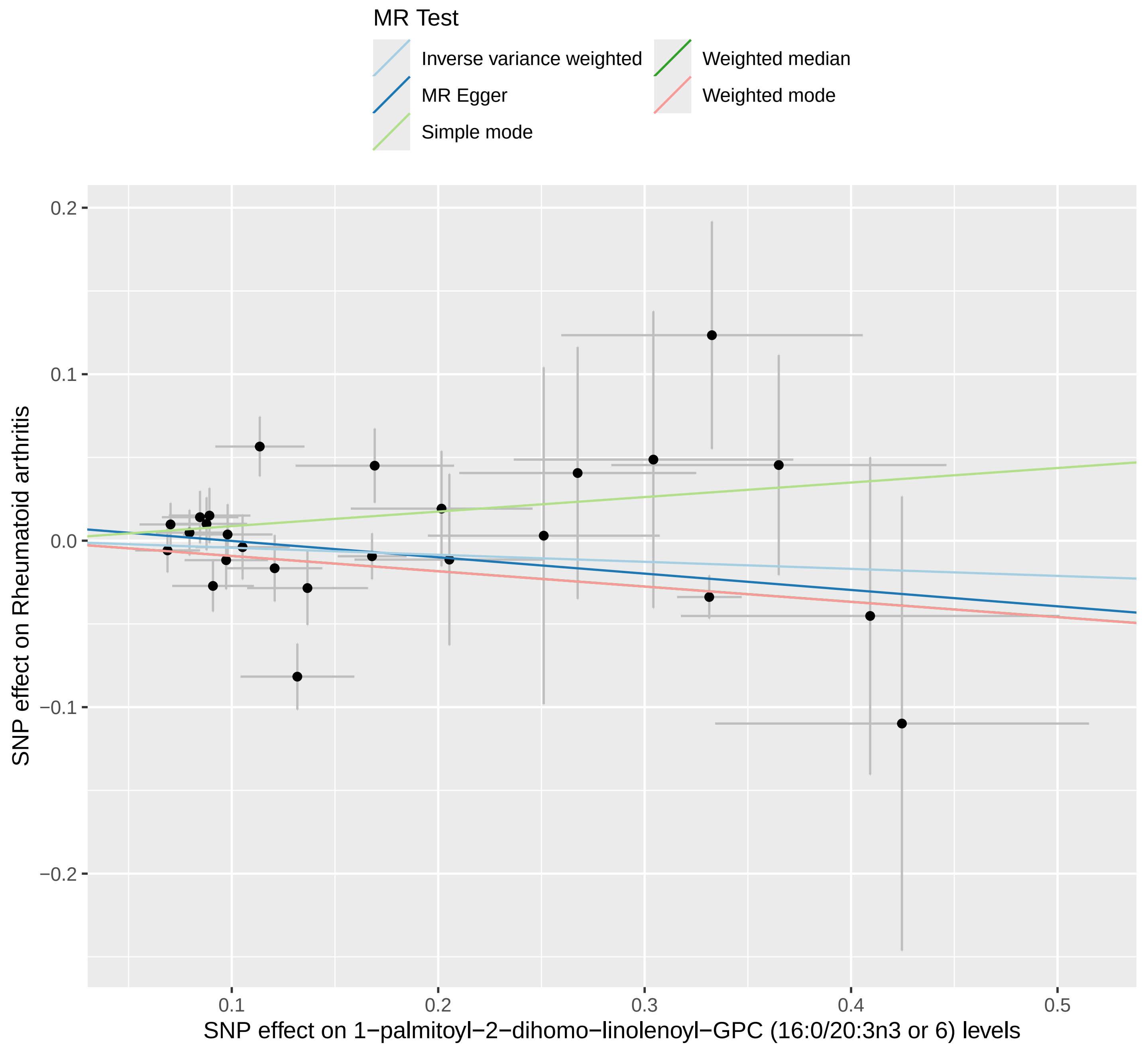

MR Test
Inverse variance weighted
MR Egger
Simple mode
Weighted median
Weighted mode
0.2
0.1
0.0
−0.1
−0.2
SNP effect on Rheumatoid arthritis
0.1
0.2
0.3
0.4
0.5
SNP effect on 1−palmitoyl−2−dihomo−linolenoyl−GPC (16:0/20:3n3 or 6) levels

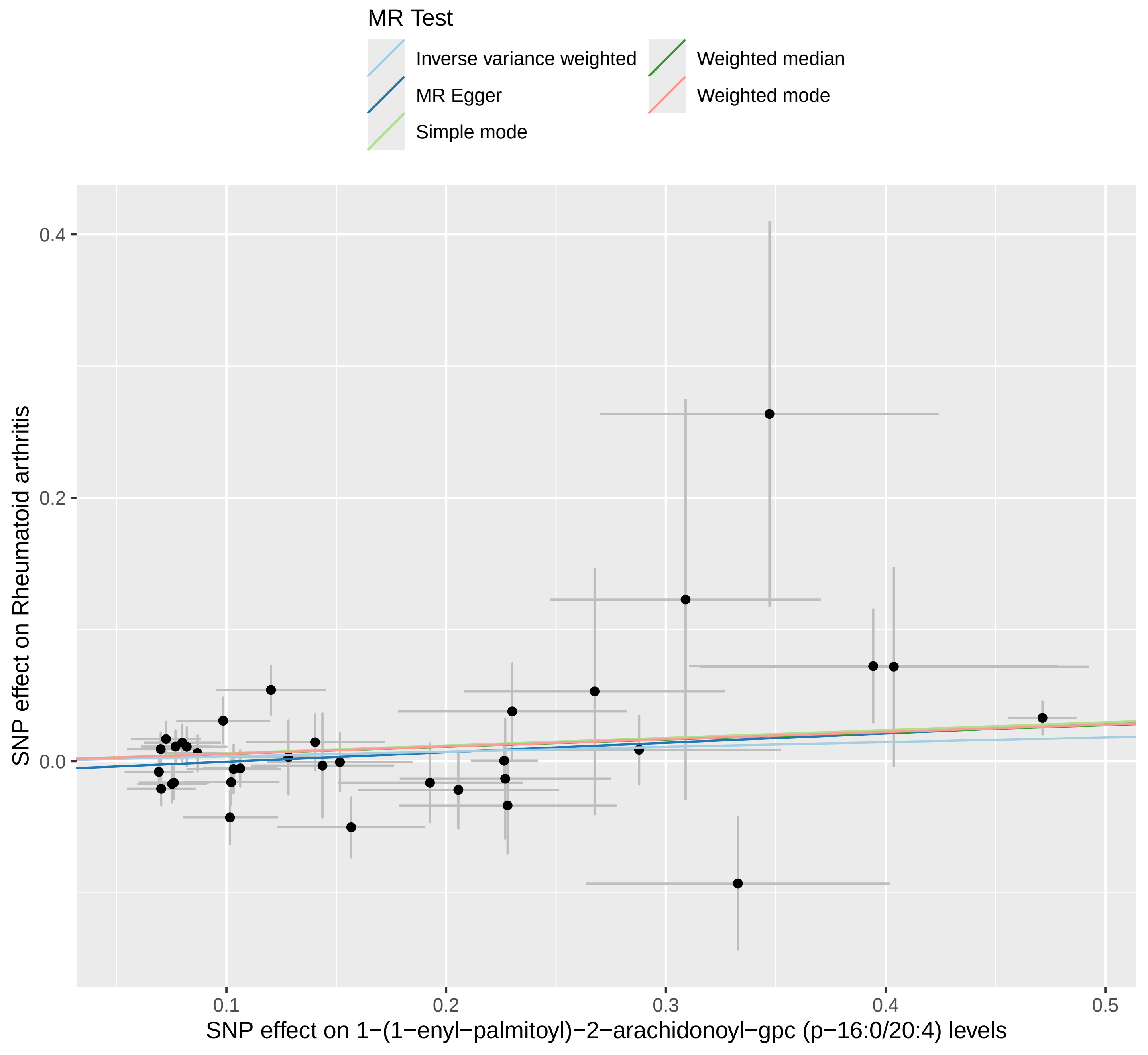

MR Test
Inverse variance weighted
MR Egger
Simple mode
Weighted median
Weighted mode
SNP effect on Rheumatoid arthritis
0.4
0.2
0.0
0.1
0.2
0.3
0.4
0.5
SNP effect on 1−(1−enyl−palmitoyl)−2−arachidonoyl−gpc (p−16:0/20:4) levels

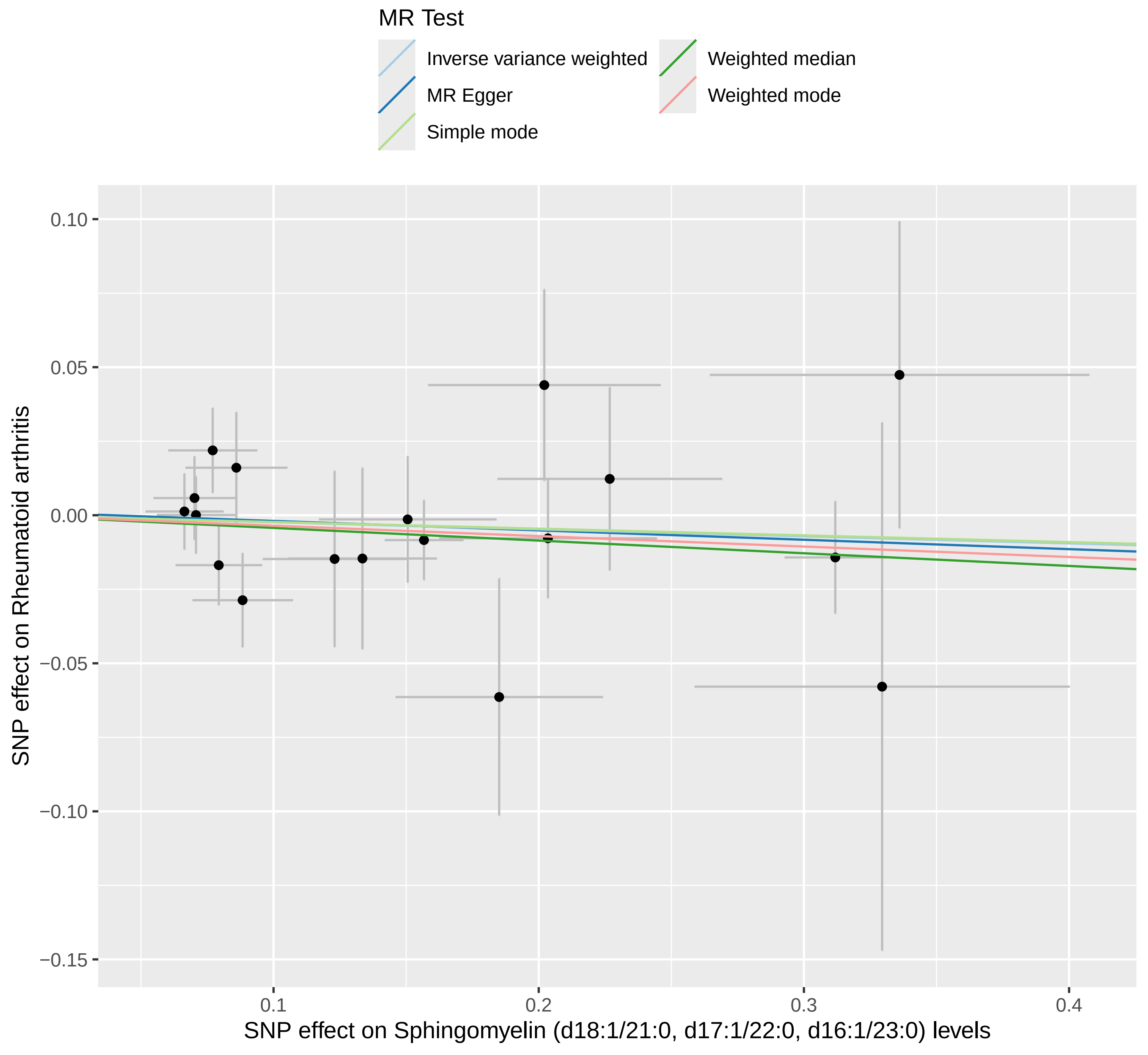

MR Test
Inverse variance weighted
MR Egger
Simple mode
Weighted median
Weighted mode
0.10
0.05
0.00
−0.05
−0.10
−0.15
SNP effect on Rheumatoid arthritis
0.1
0.2
0.3
0.4
SNP effect on Sphingomyelin (d18:1/21:0, d17:1/22:0, d16:1/23:0) levels

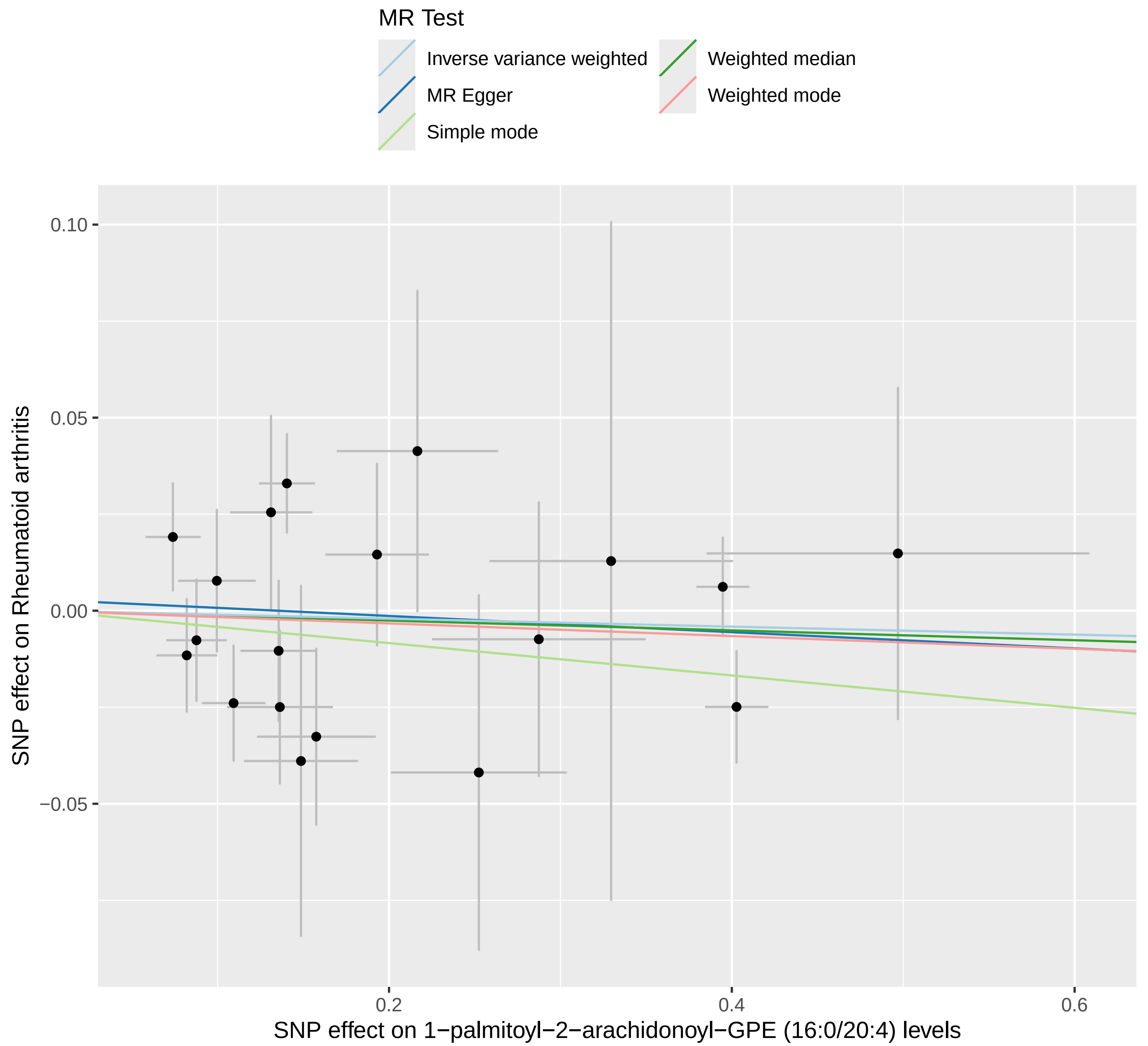
MR Test
Inverse variance weighted
MR Egger
Simple mode
Weighted median
Weighted mode
0.10
0.05
0.00
−0.05
SNP effect on Rheumatoid arthritis
0.2
0.4
0.6
SNP effect on 1−palmitoyl−2−arachidonoyl−GPE (16:0/20:4) levels

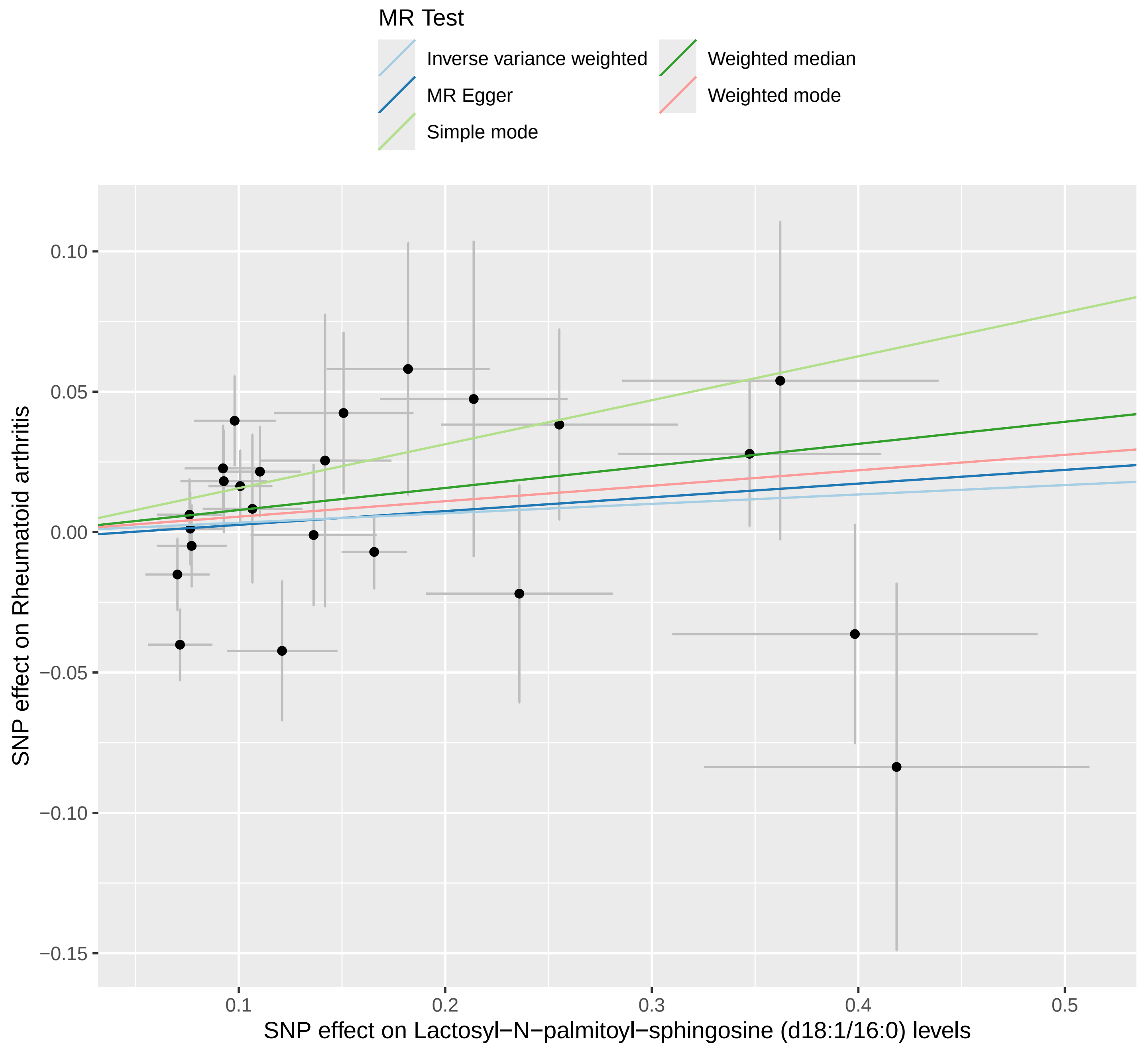
MR Test
Inverse variance weighted
MR Egger
Simple mode
Weighted median
Weighted mode
0.10
0.05
0.00
−0.05
−0.10
−0.15
SNP effect on Rheumatoid arthritis
0.1
0.2
0.3
0.4
0.5
SNP effect on Lactosyl−N−palmitoyl−sphingosine (d18:1/16:0) levels

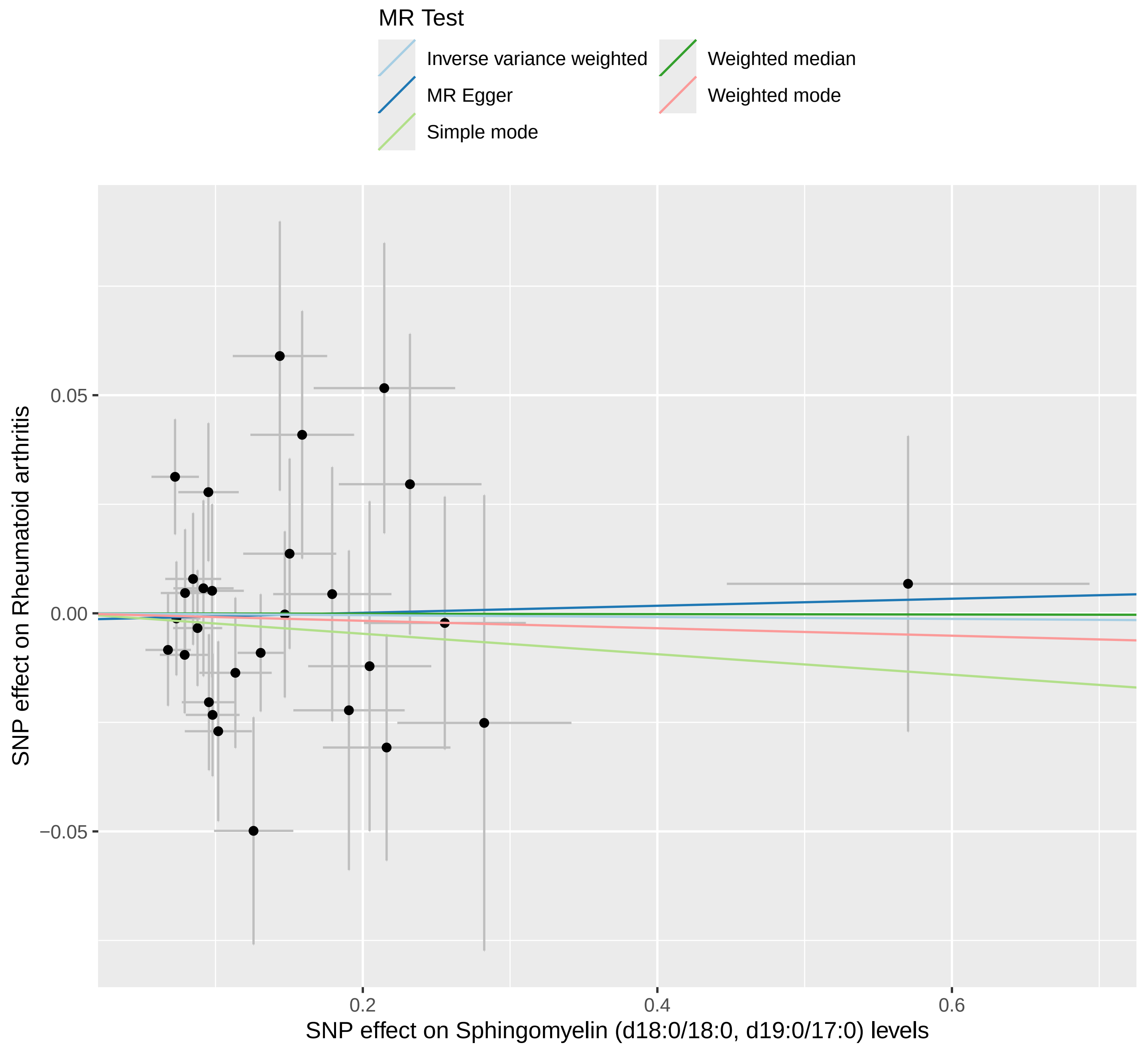
MR Test
Inverse variance weighted
MR Egger
Simple mode
Weighted median
Weighted mode
0.05
0.00
−0.05
0.2
0.4
0.6
SNP effect on Rheumatoid arthritis
SNP effect on Sphingomyelin (d18:0/18:0, d19:0/17:0) levels

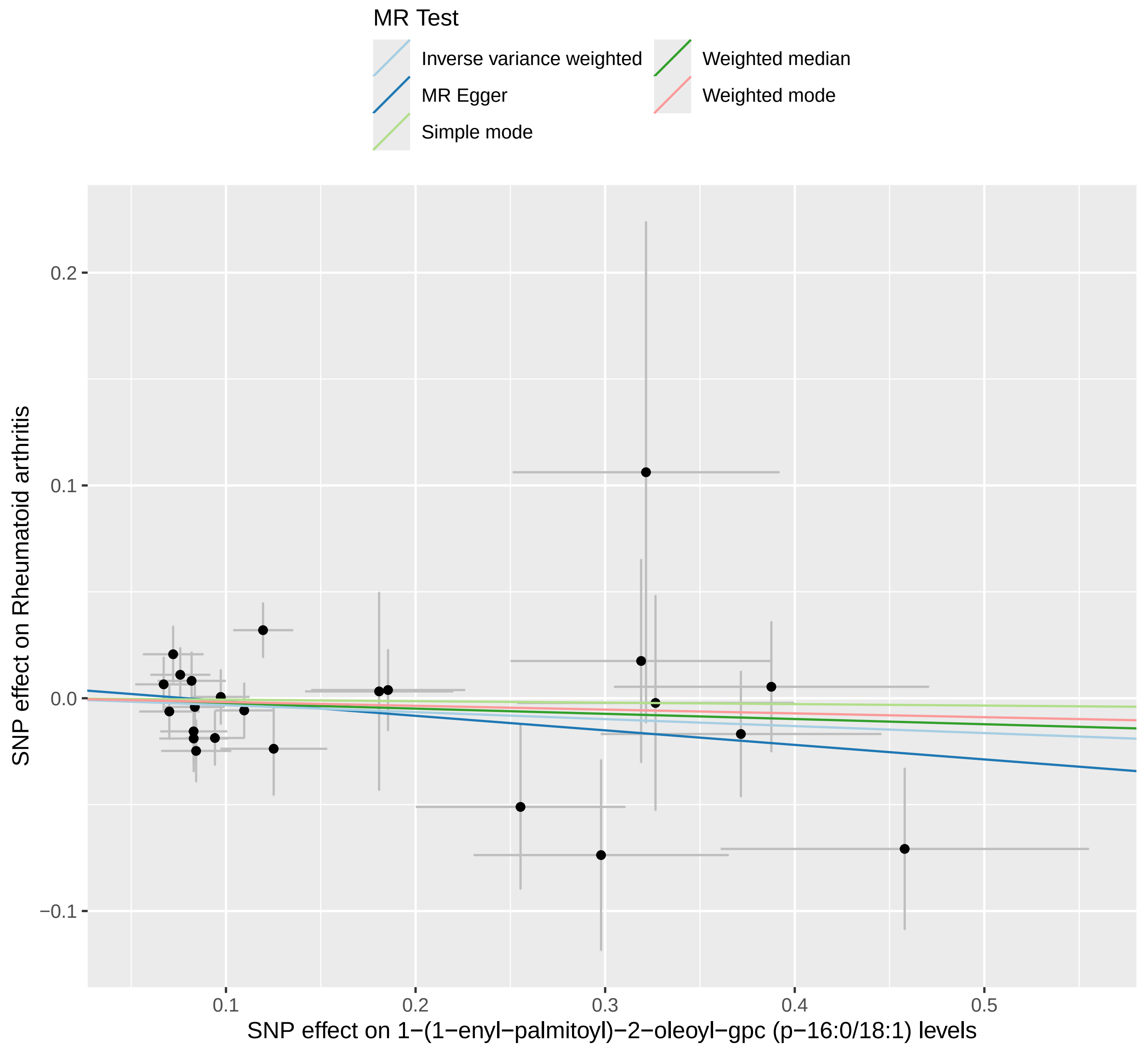
MR Test
Inverse variance weighted
MR Egger
Simple mode
Weighted median
Weighted mode
0.2
0.1
0.0
−0.1
0.1
0.2
0.3
0.4
0.5
SNP effect on Rheumatoid arthritis
SNP effect on 1−(1−enyl−palmitoyl)−2−oleoyl−gpc (p−16:0/18:1) levels

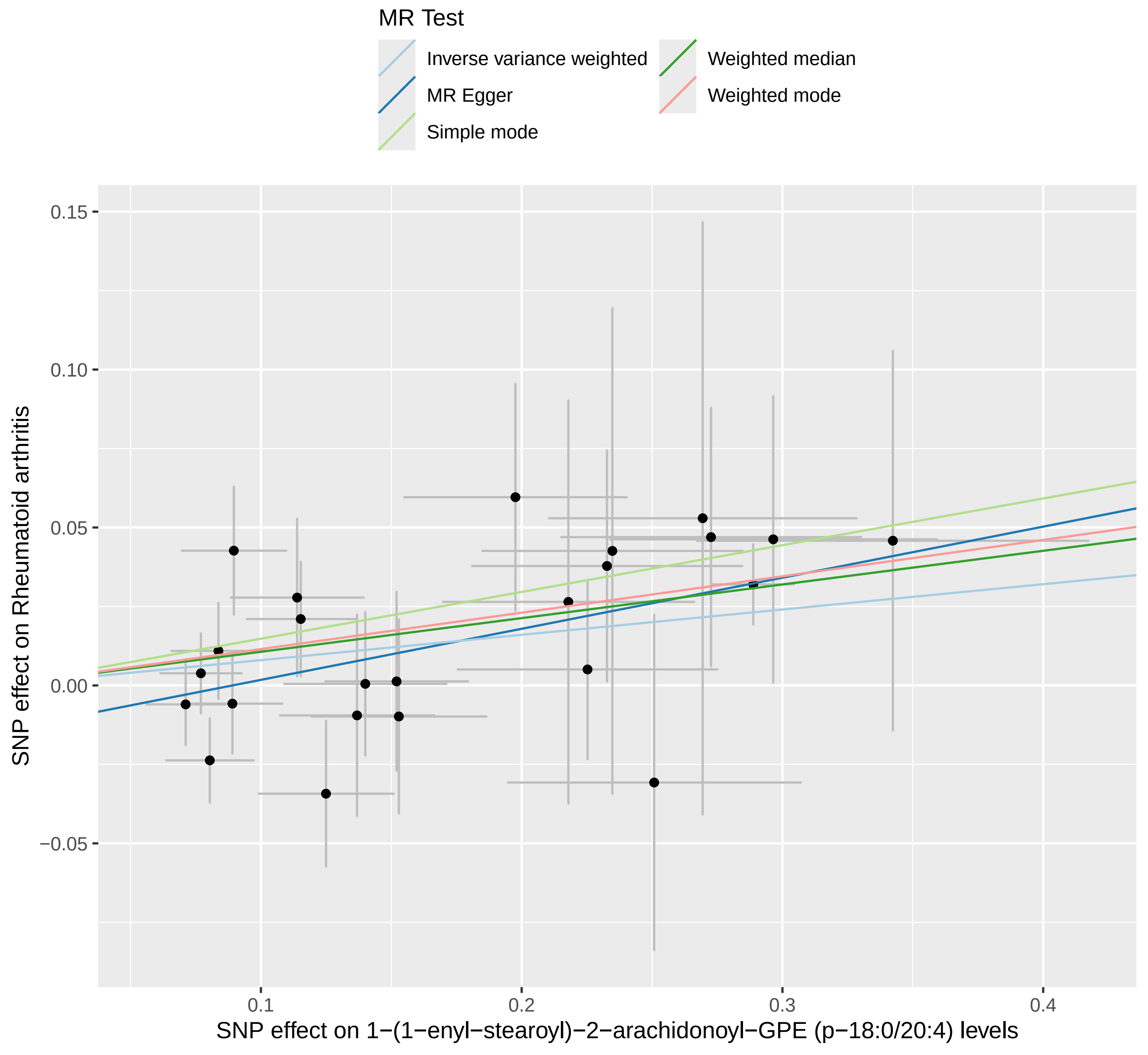

MR Test
Inverse variance weighted
MR Egger
Simple mode
Weighted median
Weighted mode
0.15
0.10
0.05
0.00
−0.05
0.1
0.2
0.3
0.4
SNP effect on Rheumatoid arthritis
SNP effect on 1−(1−enyl−stearoyl)−2−arachidonoyl−GPE (p−18:0/20:4) levels

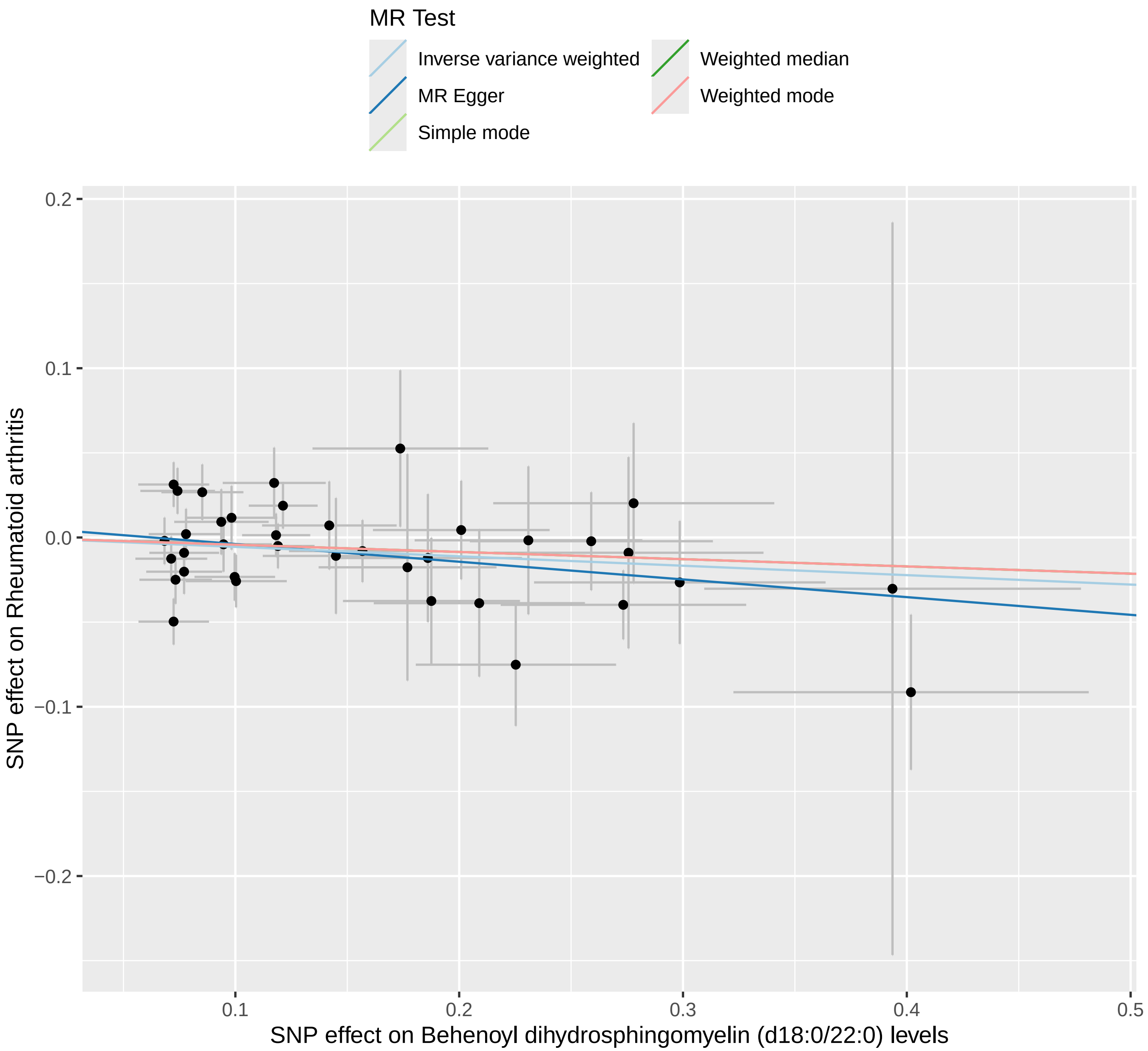

MR Test
Inverse variance weighted
MR Egger
Simple mode
Weighted median
Weighted mode
0.2
0.1
0.0
−0.1
−0.2
SNP effect on Rheumatoid arthritis
0.1
0.2
0.3
0.4
0.5
SNP effect on Behenoyl dihydrosphingomyelin (d18:0/22:0) levels

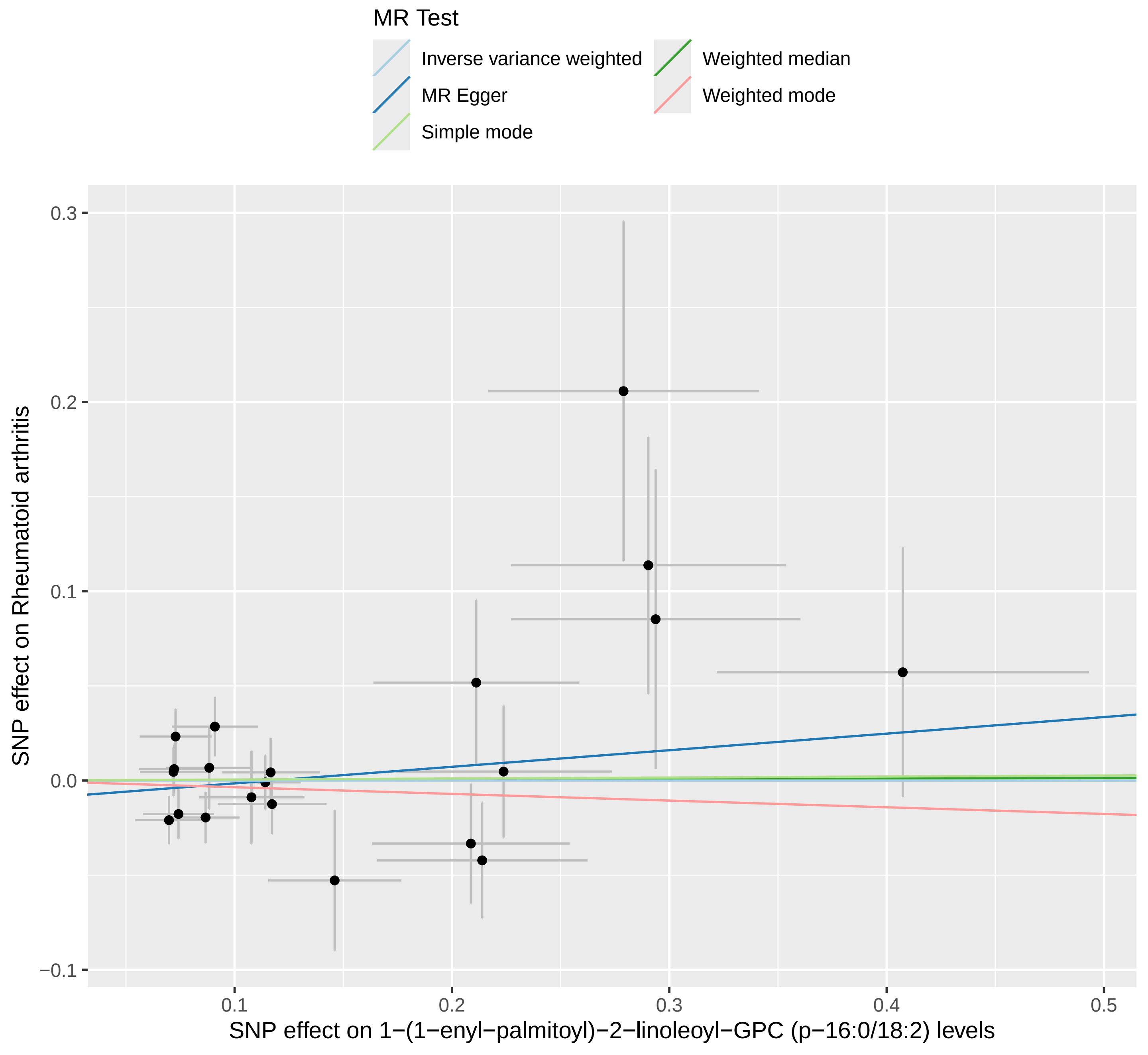
MR Test
Inverse variance weighted
MR Egger
Simple mode
Weighted median
Weighted mode
0.3
0.2
0.1
0.0
−0.1
0.1
0.2
0.3
0.4
0.5
SNP effect on Rheumatoid arthritis
SNP effect on 1−(1−enyl−palmitoyl)−2−linoleoyl−GPC (p−16:0/18:2) levels

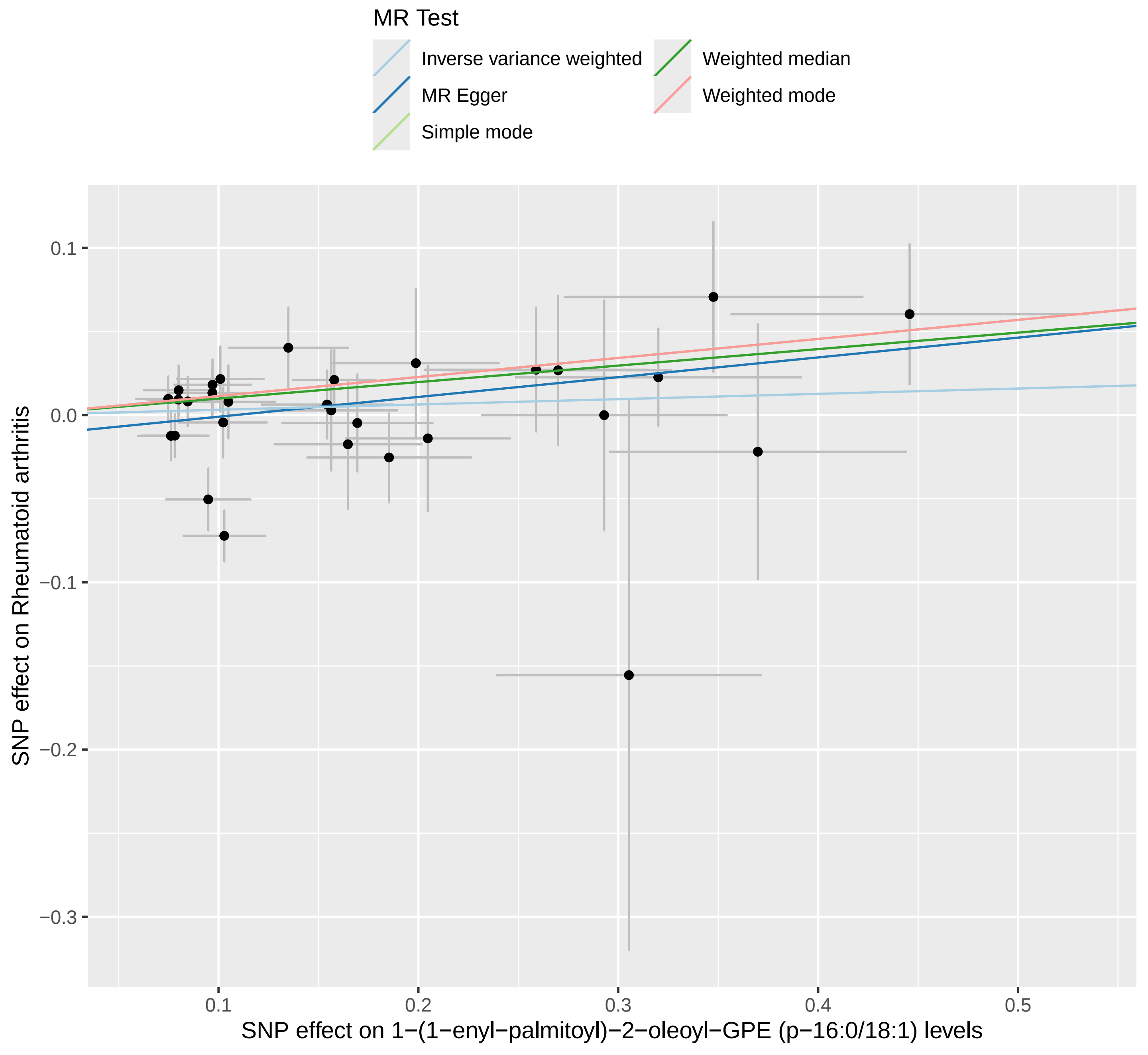
MR Test
Inverse variance weighted
MR Egger
Simple mode
Weighted median
Weighted mode
0.1
0.0
−0.1
−0.2
−0.3
SNP effect on Rheumatoid arthritis
0.1
0.2
0.3
0.4
0.5
SNP effect on 1−(1−enyl−palmitoyl)−2−oleoyl−GPE (p−16:0/18:1) levels

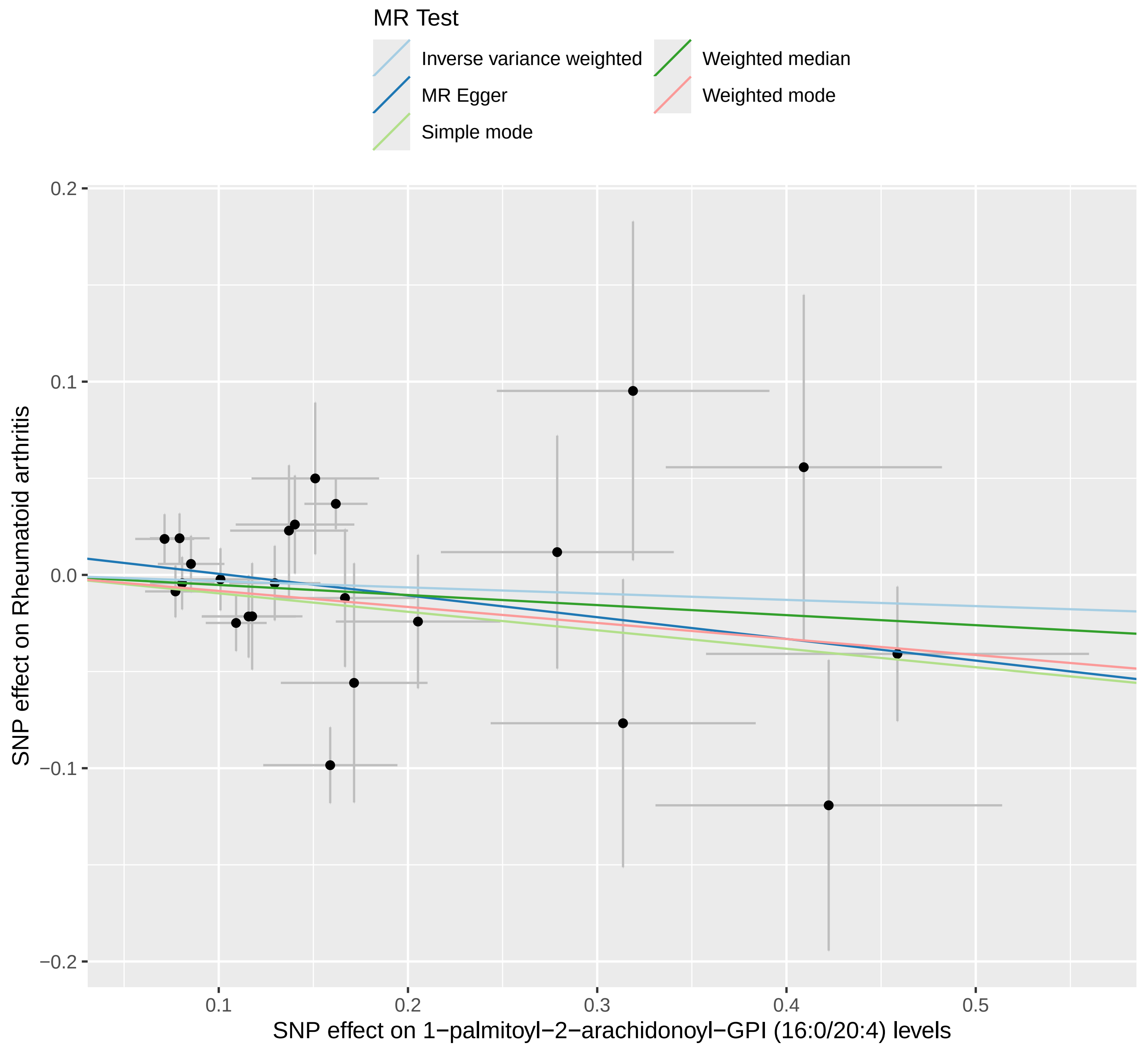
MR Test
Inverse variance weighted
MR Egger
Simple mode
Weighted median
Weighted mode
0.2
0.1
0.0
−0.1
−0.2
SNP effect on Rheumatoid arthritis
0.1
0.2
0.3
0.4
0.5
SNP effect on 1−palmitoyl−2−arachidonoyl−GPI (16:0/20:4) levels

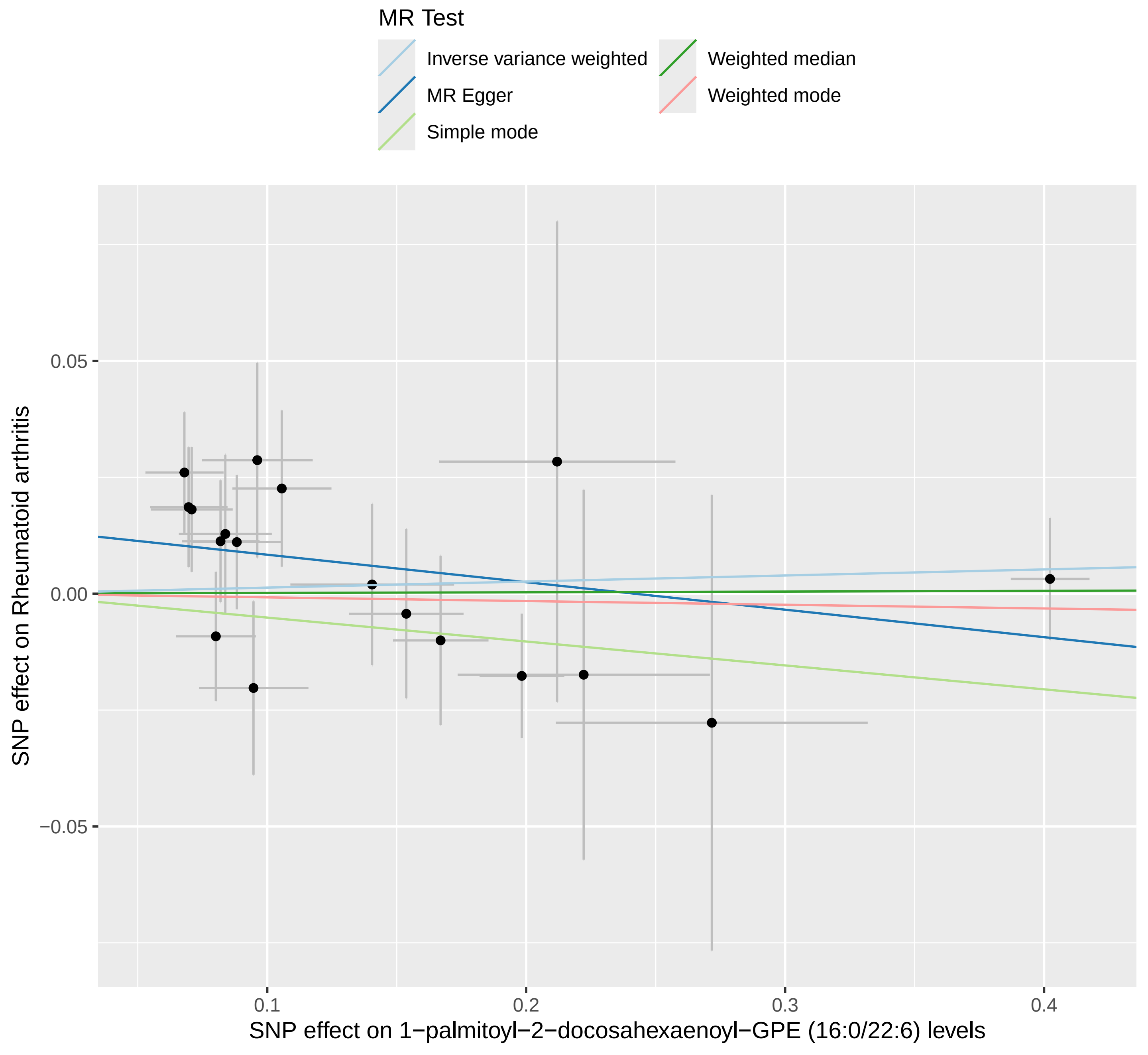
MR Test
Inverse variance weighted
MR Egger
Simple mode
Weighted median
Weighted mode
0.05
0.00
−0.05
0.1
0.2
0.3
0.4
SNP effect on Rheumatoid arthritis
SNP effect on 1−palmitoyl−2−docosahexaenoyl−GPE (16:0/22:6) levels

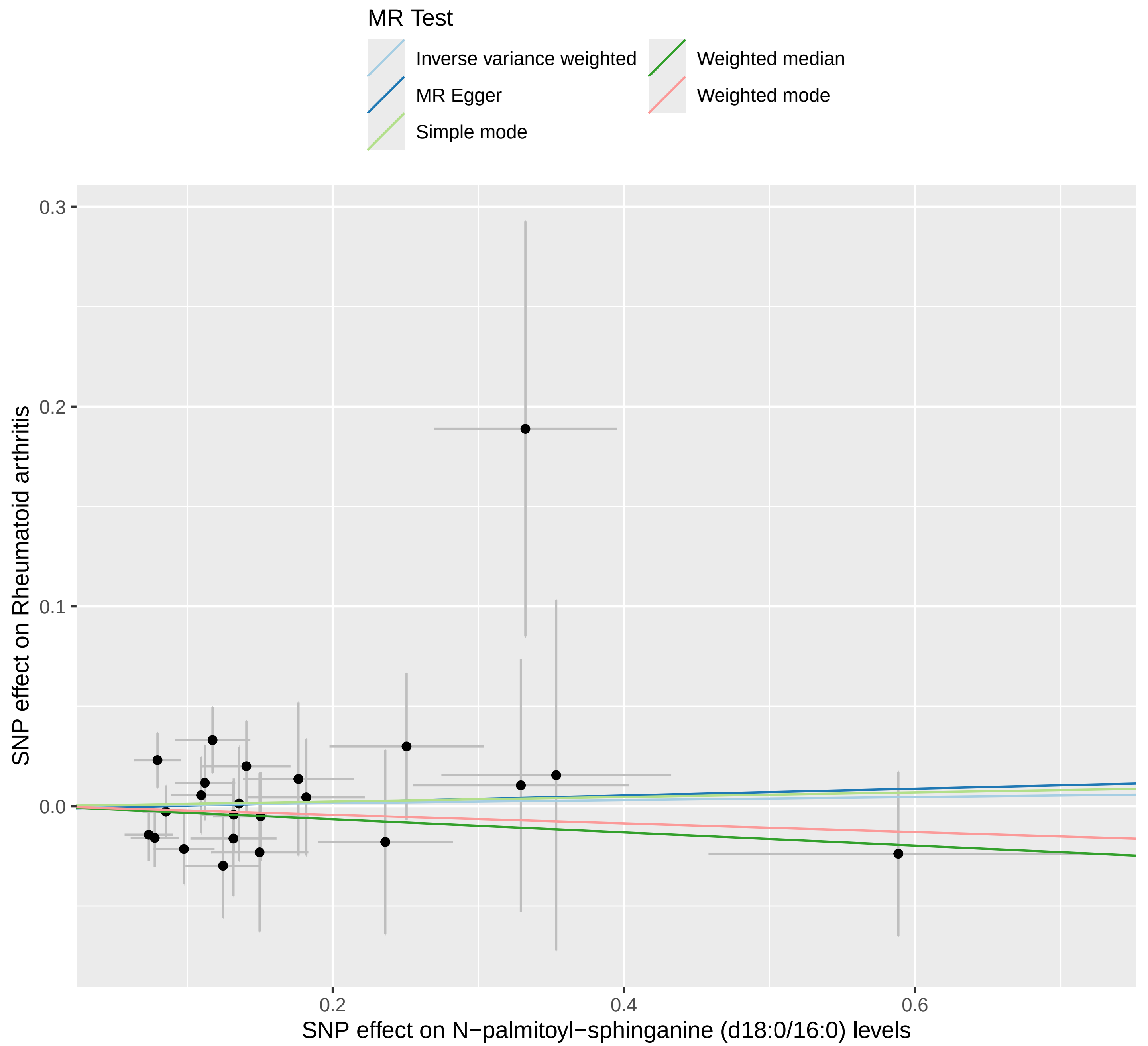
MR Test
Inverse variance weighted
MR Egger
Simple mode
Weighted median
Weighted mode
0.3
0.2
0.1
0.0
0.2
0.4
0.6
SNP effect on Rheumatoid arthritis
SNP effect on N−palmitoyl−sphinganine (d18:0/16:0) levels

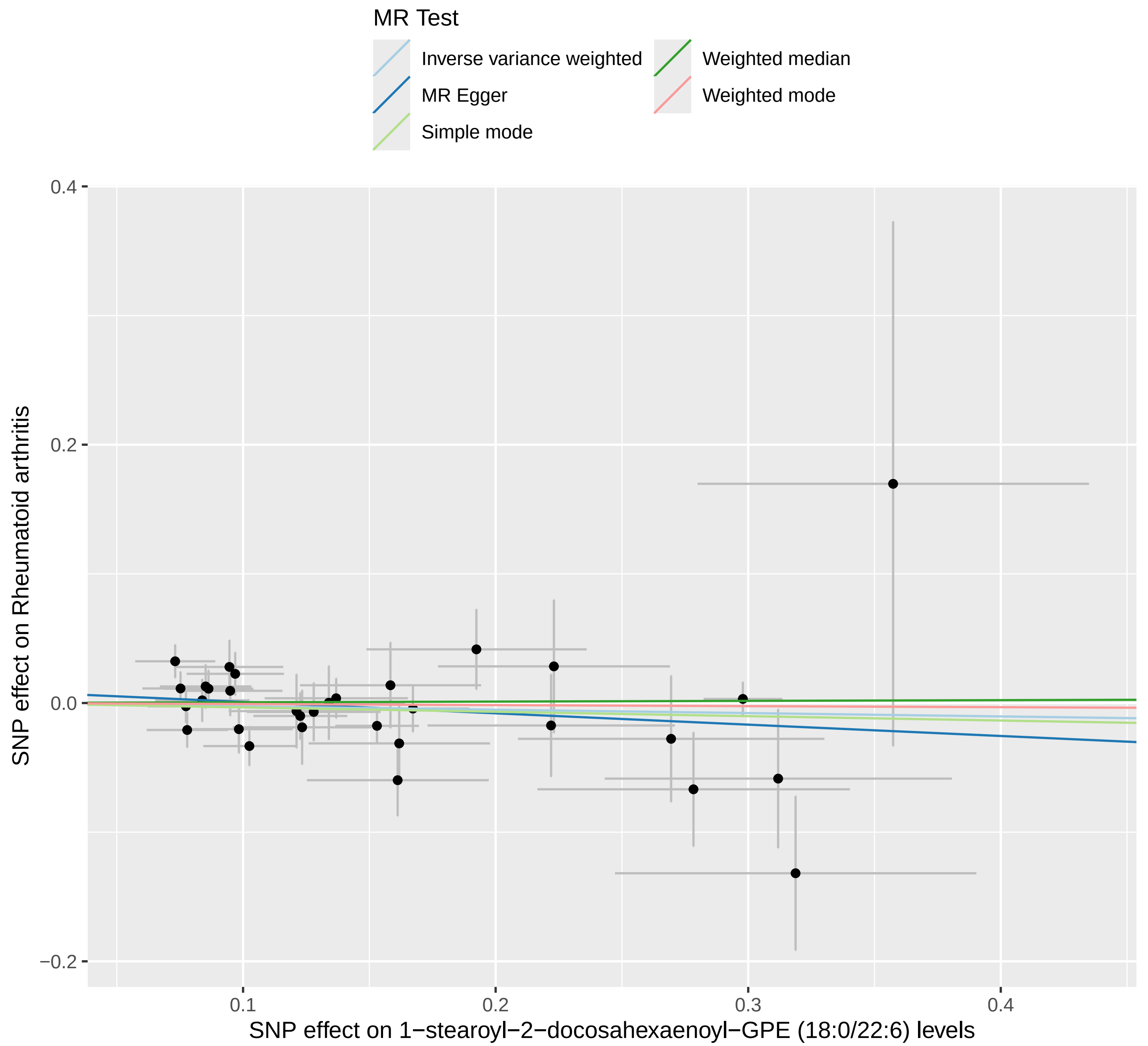

MR Test
Inverse variance weighted
MR Egger
Simple mode
Weighted median
Weighted mode
0.4
0.2
0.0
−0.2
0.1
0.2
0.3
0.4
SNP effect on Rheumatoid arthritis
SNP effect on 1−stearoyl−2−docosahexaenoyl−GPE (18:0/22:6) levels

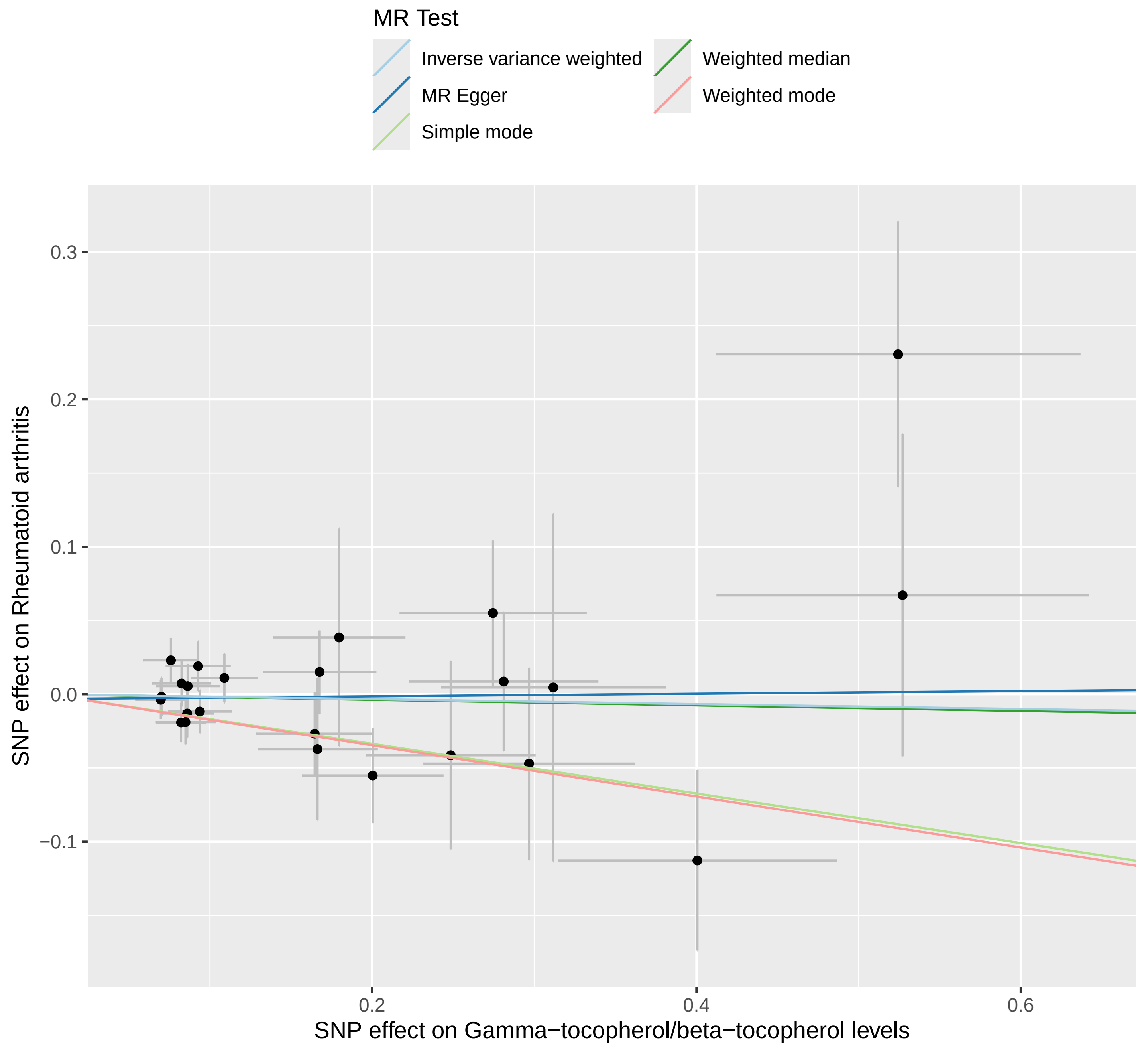
MR Test
Inverse variance weighted
MR Egger
Simple mode
Weighted median
Weighted mode
0.3
0.2
0.1
0.0
−0.1
0.2
0.4
0.6
SNP effect on Rheumatoid arthritis
SNP effect on Gamma−tocopherol/beta−tocopherol levels

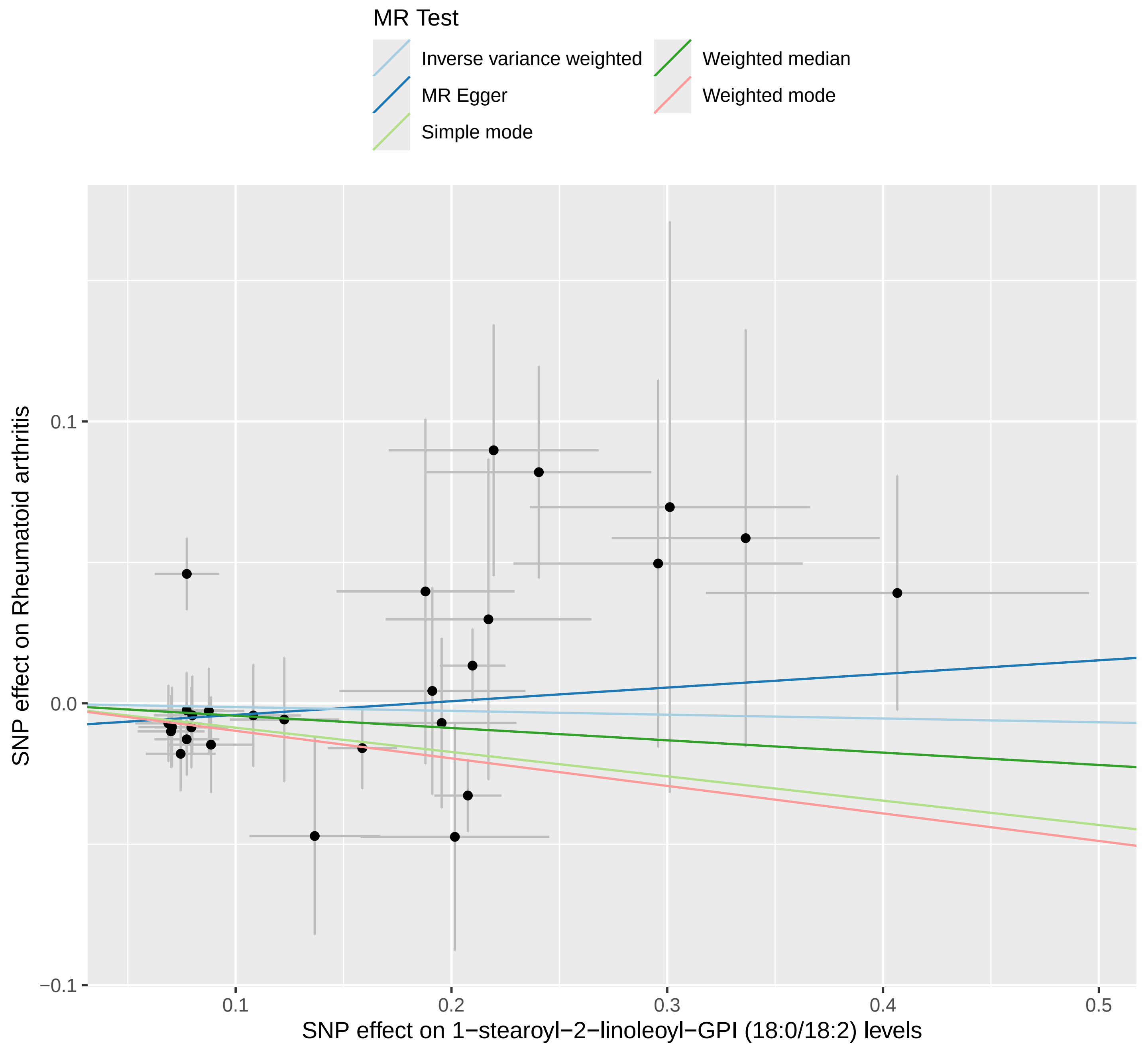
MR Test
Inverse variance weighted
MR Egger
Simple mode
Weighted median
Weighted mode
SNP effect on Rheumatoid arthritis
0.1
0.0
−0.1
0.1
0.2
0.3
0.4
0.5
SNP effect on 1−stearoyl−2−linoleoyl−GPI (18:0/18:2) levels

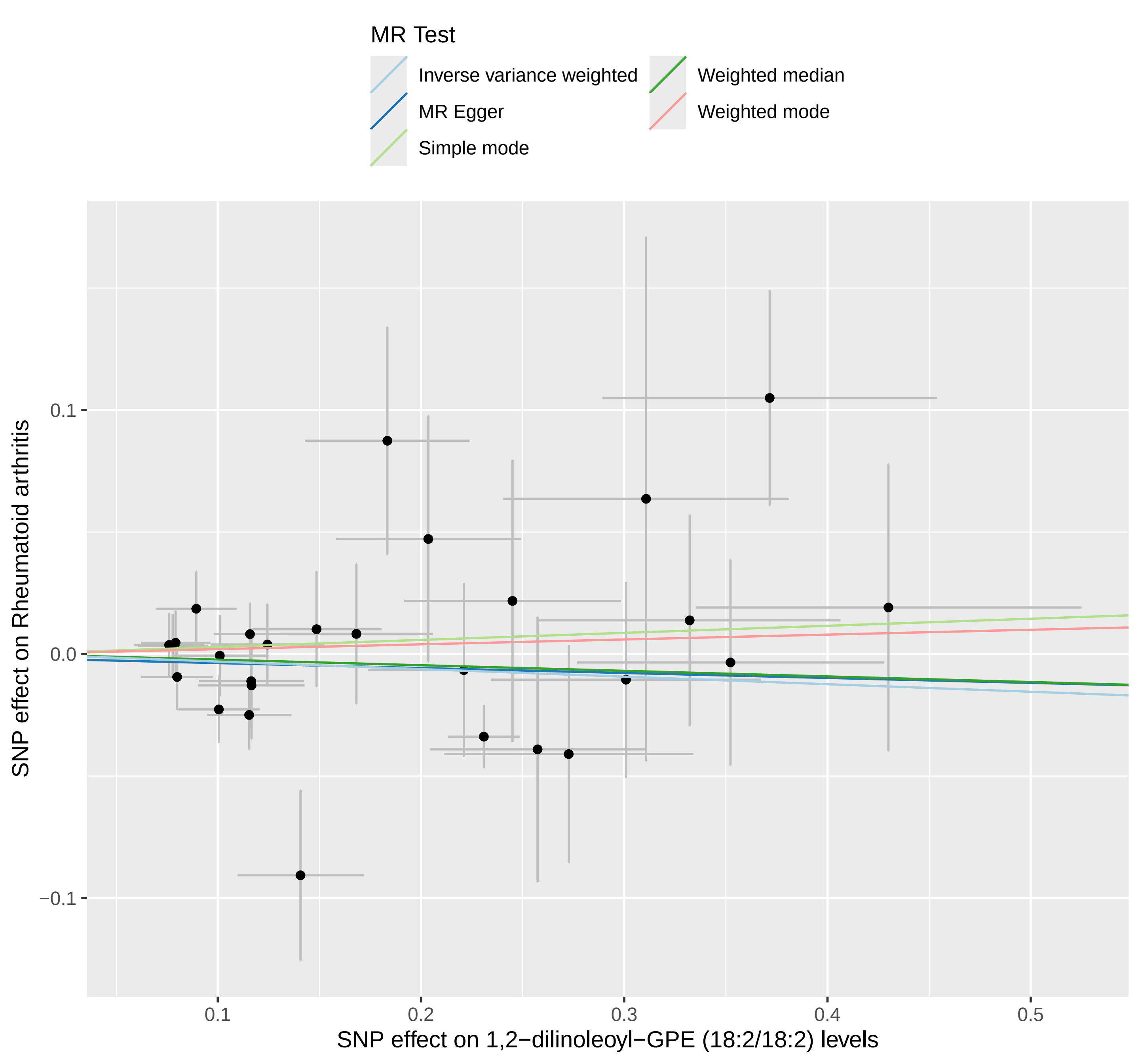
MR Test
Inverse variance weighted
MR Egger
Simple mode
Weighted median
Weighted mode
SNP effect on Rheumatoid arthritis
0.1
0.0
−0.1
0.1
0.2
0.3
0.4
0.5
SNP effect on 1,2−dilinoleoyl−GPE (18:2/18:2) levels

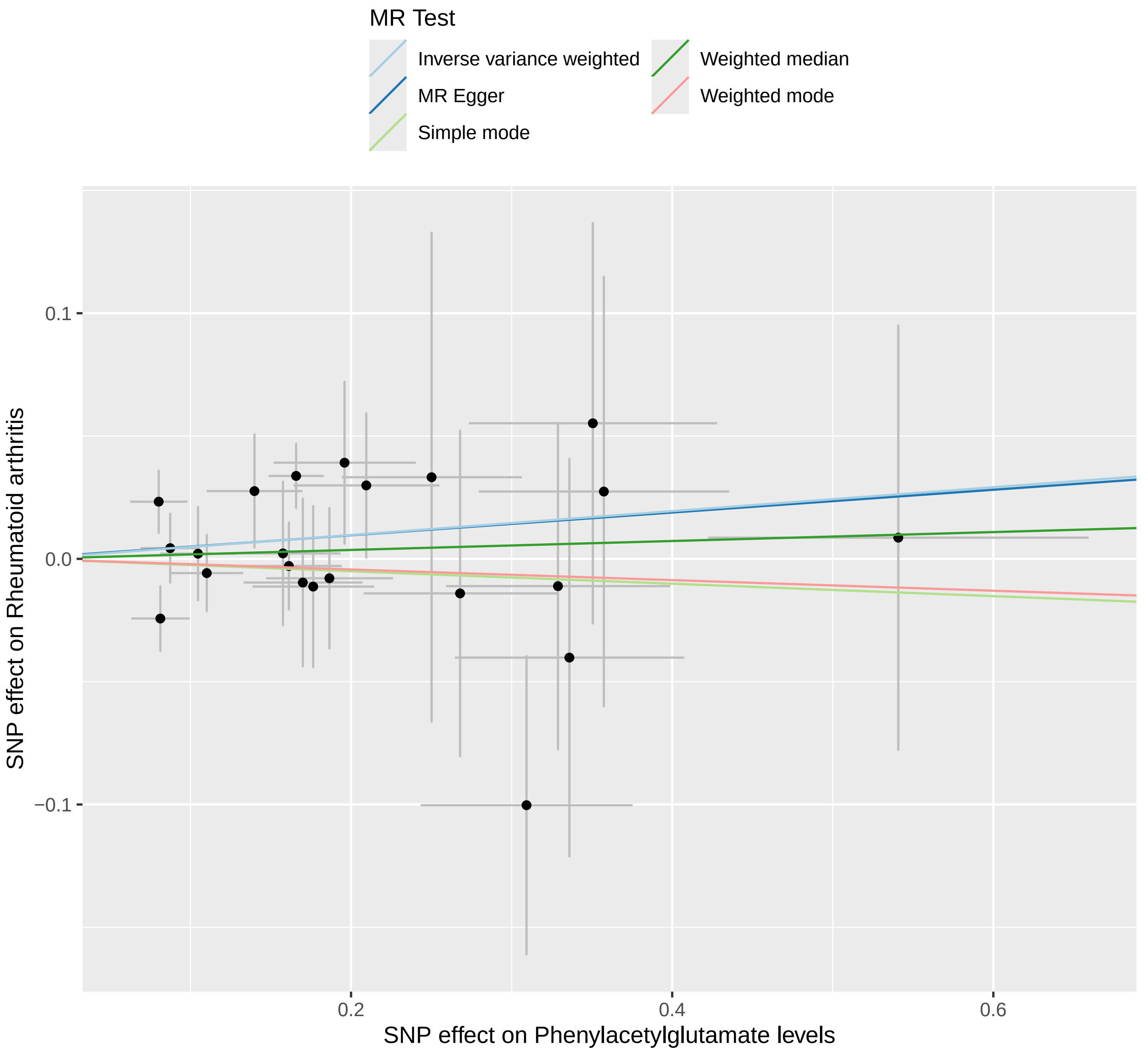
MR Test
Inverse variance weighted
MR Egger
Simple mode
Weighted median
Weighted mode
SNP effect on Rheumatoid arthritis
0.1
0.0
−0.1
0.2
0.4
0.6
SNP effect on Phenylacetylglutamate levels

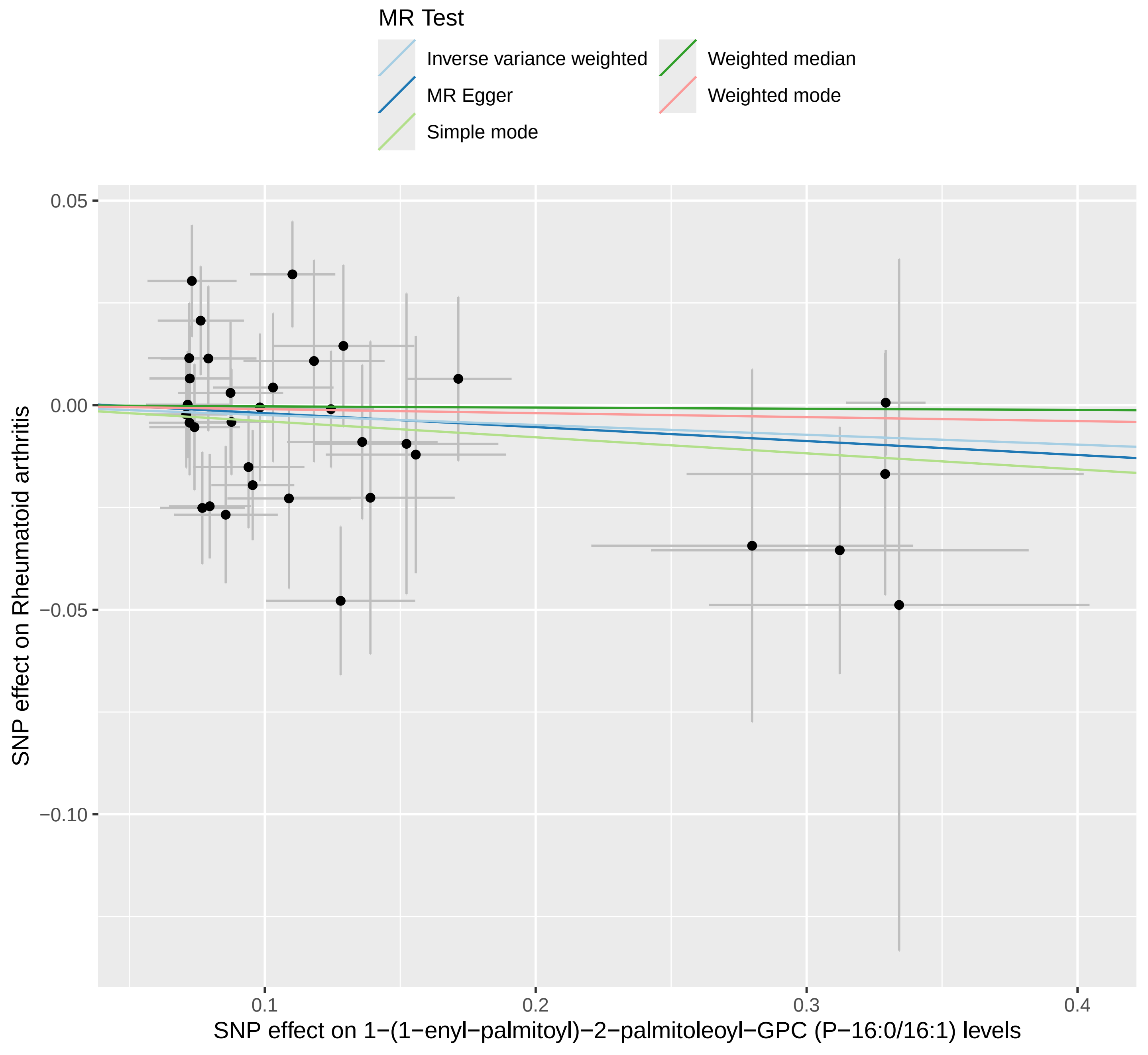
MR Test
Inverse variance weighted
MR Egger
Simple mode
Weighted median
Weighted mode
0.05
0.00
−0.05
−0.10
0.1
0.2
0.3
0.4
SNP effect on Rheumatoid arthritis
SNP effect on 1−(1−enyl−palmitoyl)−2−palmitoleoyl−GPC (P−16:0/16:1) levels

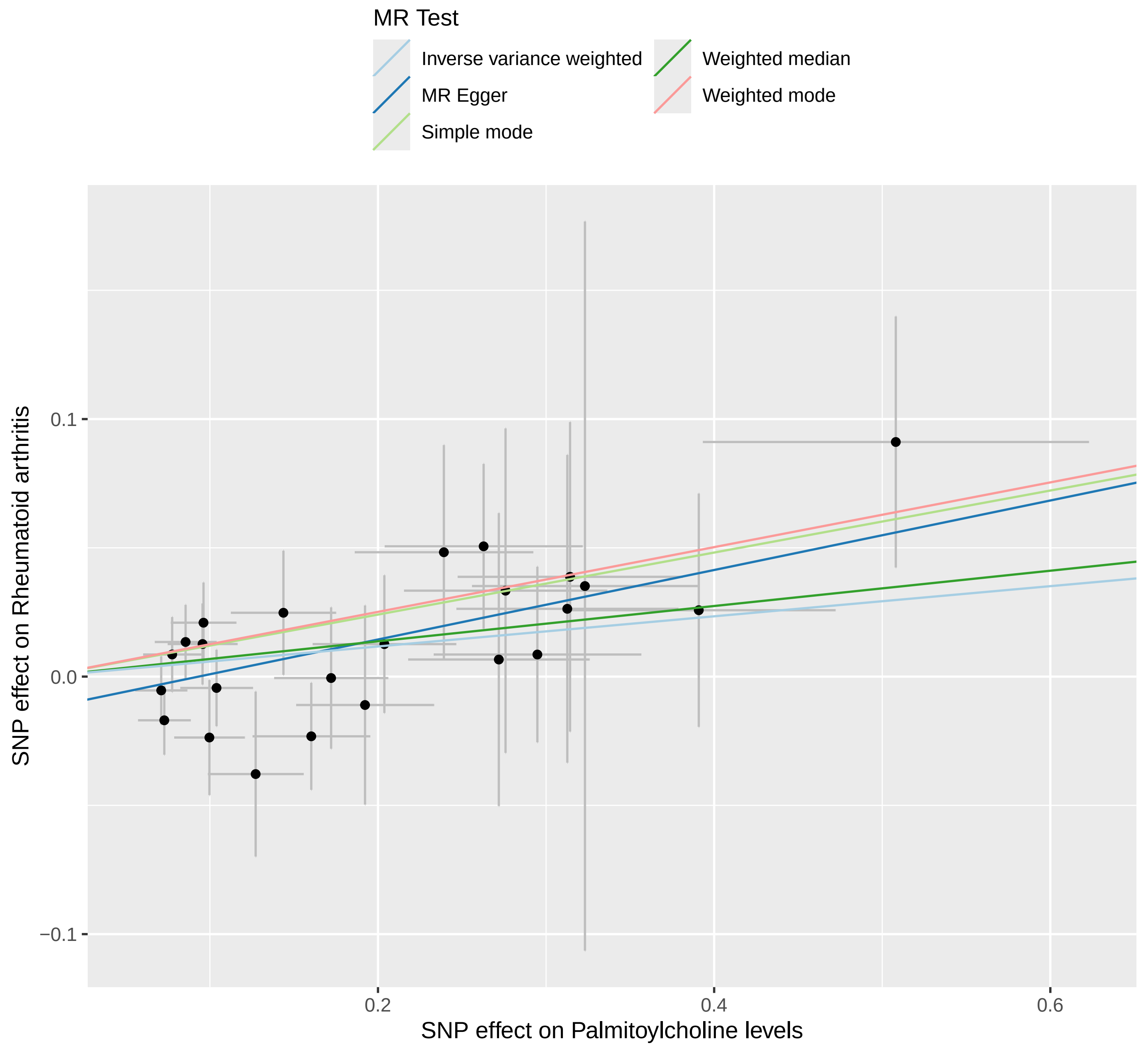
MR Test
Inverse variance weighted
MR Egger
Simple mode
Weighted median
Weighted mode
0.1
0.0
−0.1
0.2
0.4
0.6
SNP effect on Rheumatoid arthritis
SNP effect on Palmitoylcholine levels

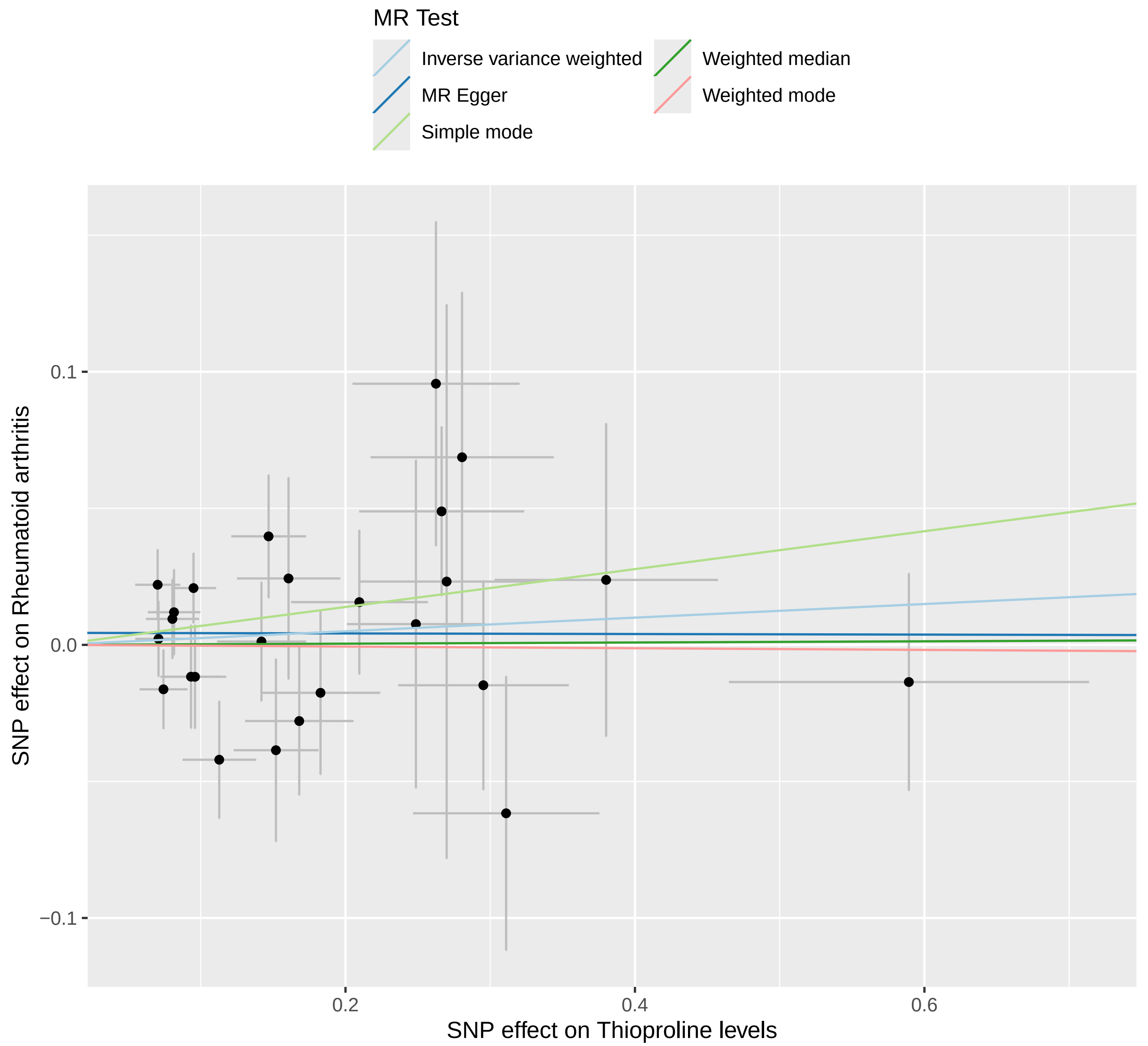
MR Test
Inverse variance weighted
MR Egger
Simple mode
Weighted median
Weighted mode
SNP effect on Rheumatoid arthritis
0.1
0.0
−0.1
0.2
0.4
0.6
SNP effect on Thioproline levels

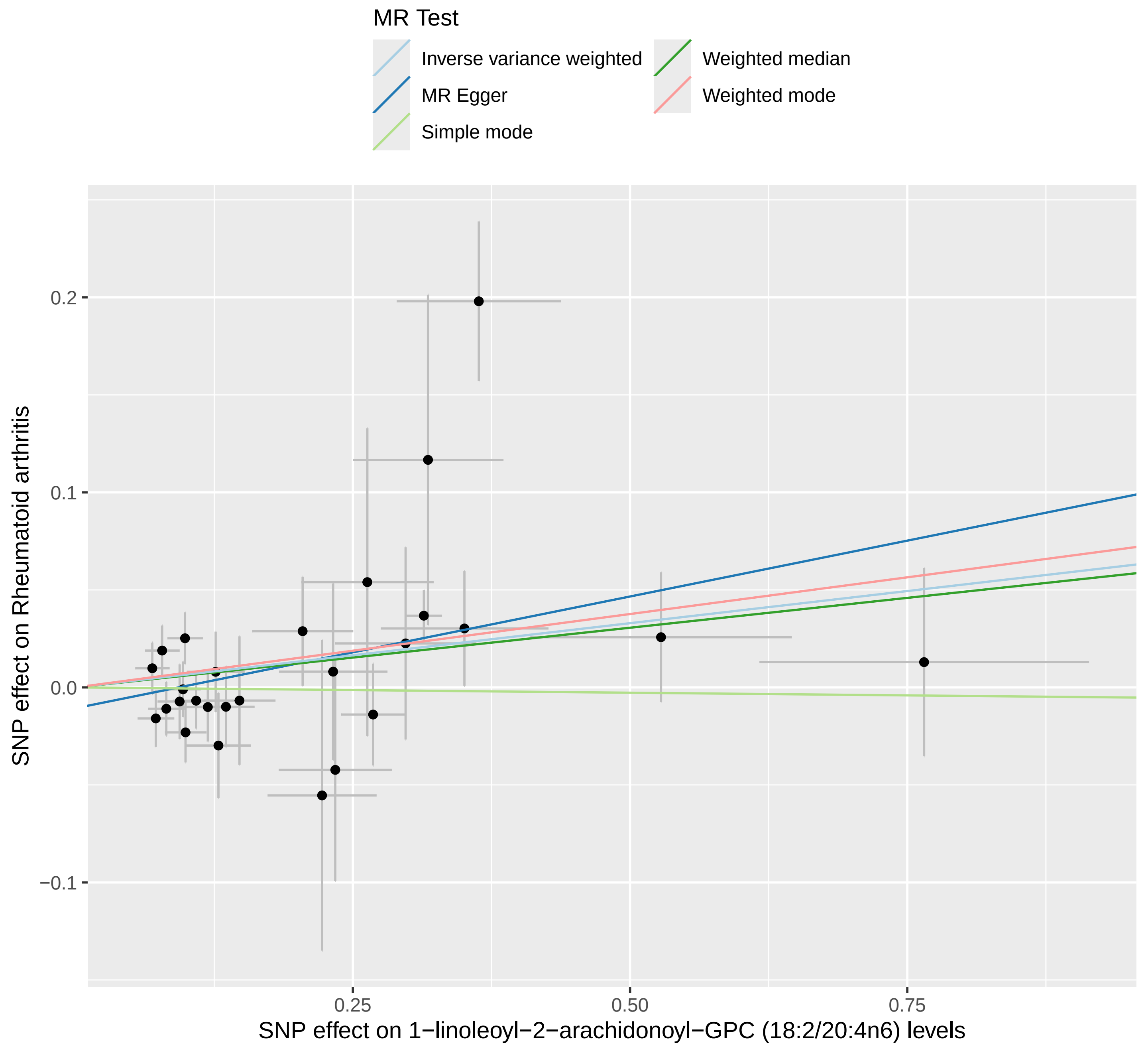

MR Test
Inverse variance weighted
MR Egger
Simple mode
Weighted median
Weighted mode
0.2
0.1
0.0
−0.1
0.25
0.50
0.75
SNP effect on Rheumatoid arthritis
SNP effect on 1−linoleoyl−2−arachidonoyl−GPC (18:2/20:4n6) levels

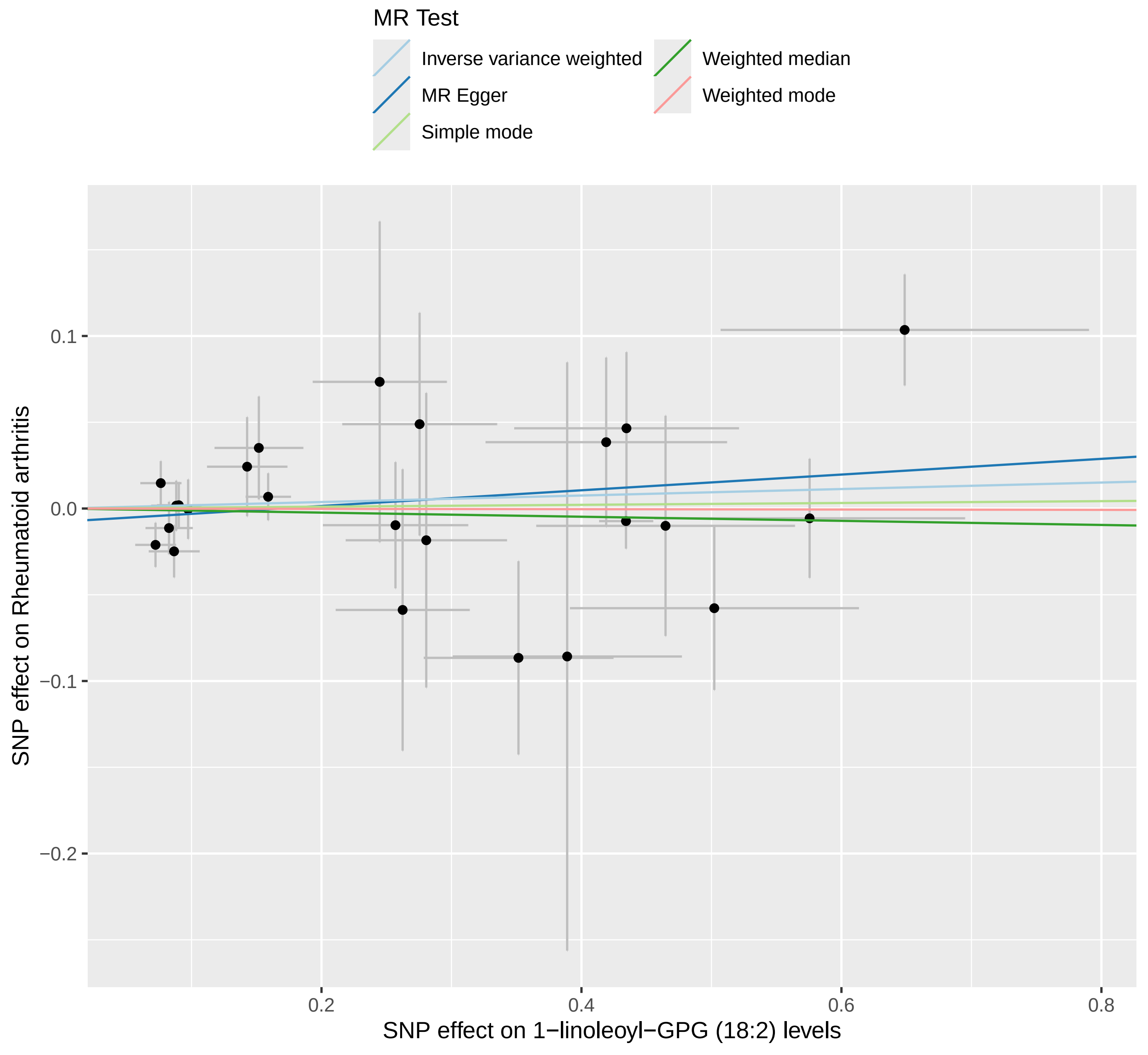

MR Test
Inverse variance weighted
MR Egger
Simple mode
Weighted median
Weighted mode
0.1
0.0
−0.1
−0.2
0.2
0.4
0.6
0.8
SNP effect on Rheumatoid arthritis
SNP effect on 1−linoleoyl−GPG (18:2) levels

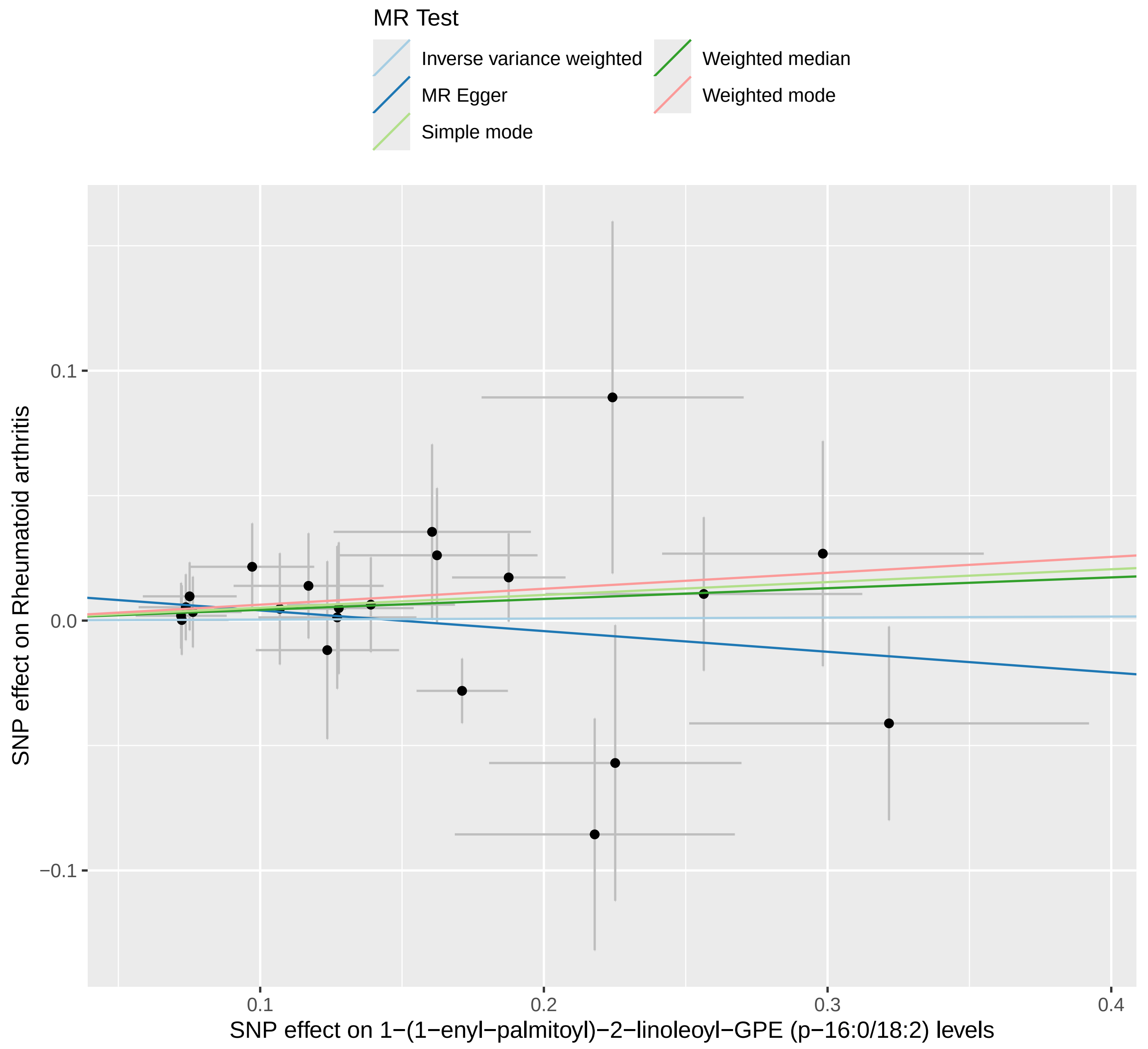

MR Test
Inverse variance weighted
MR Egger
Simple mode
Weighted median
Weighted mode
0.1
0.0
−0.1
SNP effect on Rheumatoid arthritis
0.1
0.2
0.3
0.4
SNP effect on 1−(1−enyl−palmitoyl)−2−linoleoyl−GPE (p−16:0/18:2) levels

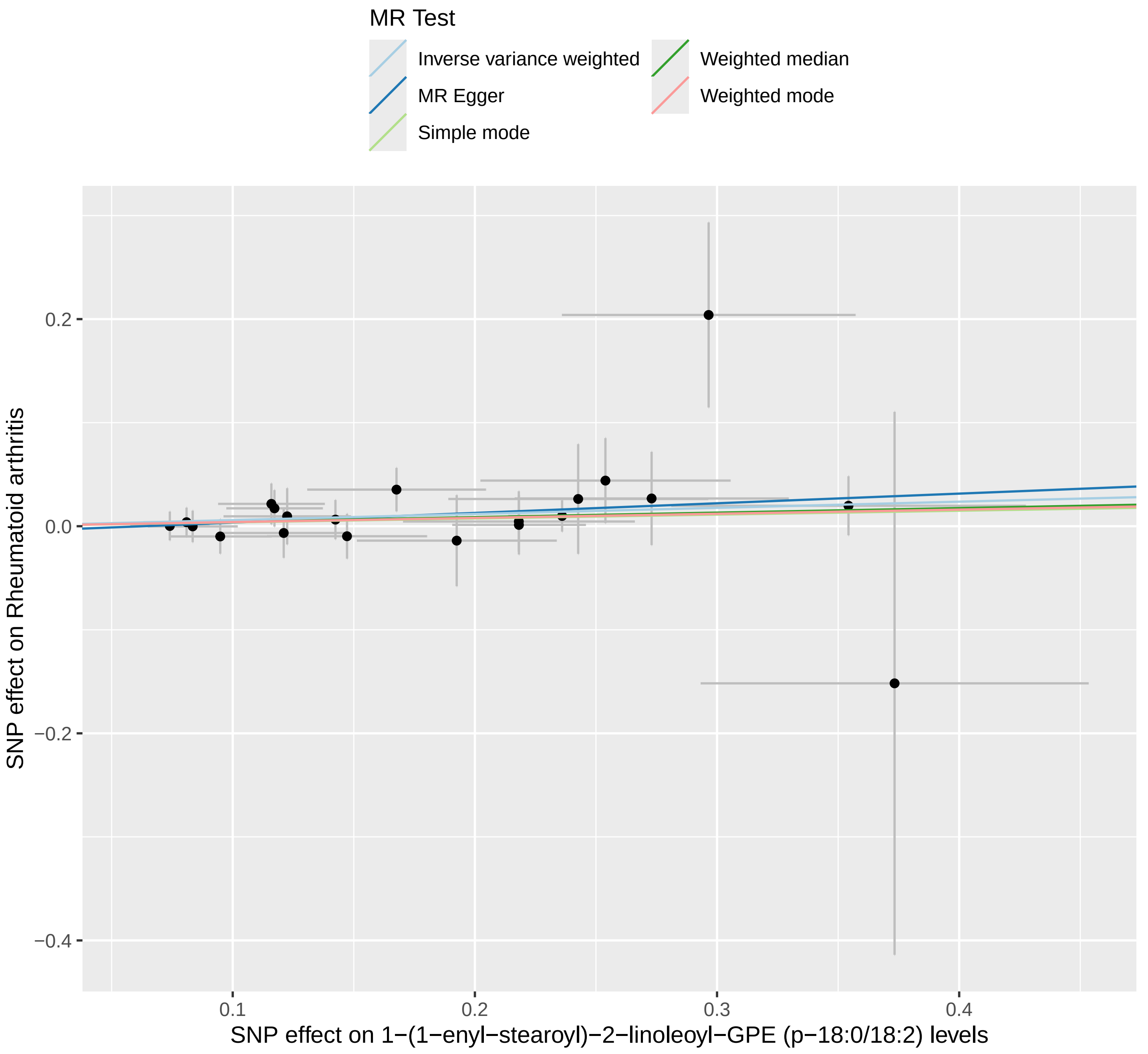
MR Test
Inverse variance weighted
MR Egger
Simple mode
Weighted median
Weighted mode
0.2
0.0
−0.2
−0.4
0.1
0.2
0.3
0.4
SNP effect on Rheumatoid arthritis
SNP effect on 1−(1−enyl−stearoyl)−2−linoleoyl−GPE (p−18:0/18:2) levels

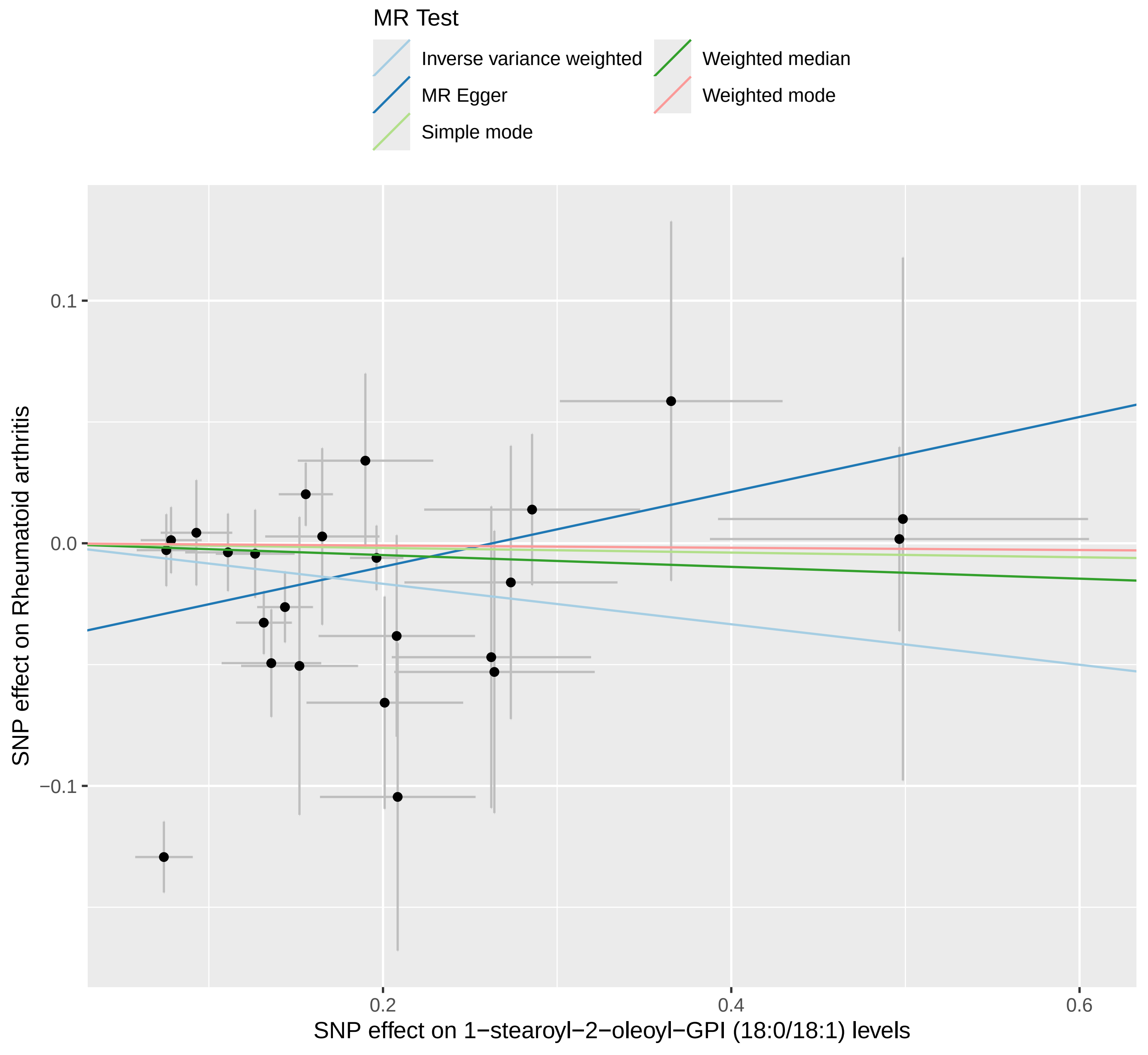

MR Test
Inverse variance weighted
MR Egger
Simple mode
Weighted median
Weighted mode
0.1
0.0
−0.1
0.2
0.4
0.6
SNP effect on Rheumatoid arthritis
SNP effect on 1−stearoyl−2−oleoyl−GPI (18:0/18:1) levels

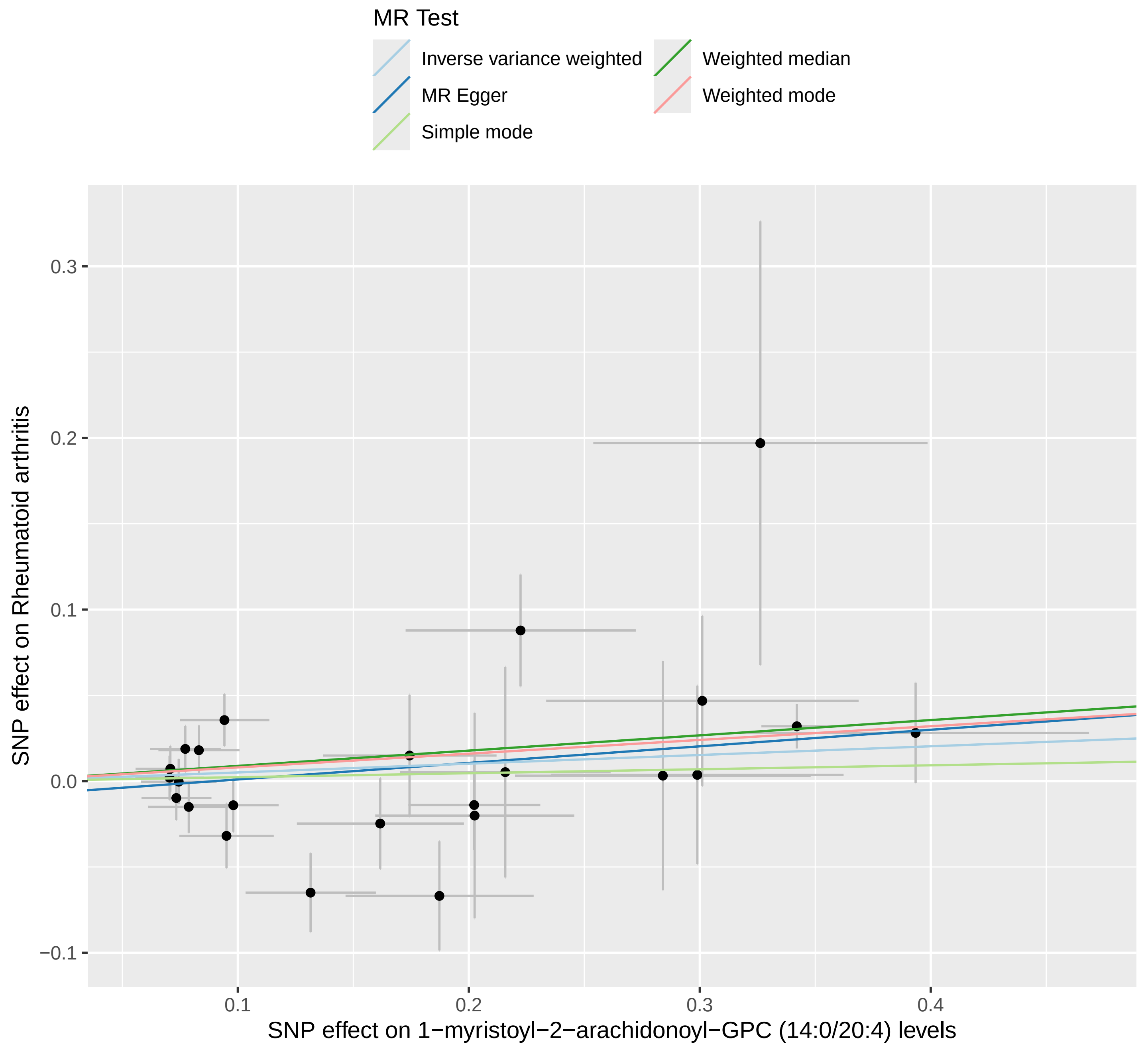

MR Test
Inverse variance weighted
MR Egger
Simple mode
Weighted median
Weighted mode
SNP effect on Rheumatoid arthritis
0.3
0.2
0.1
0.0
−0.1
0.1
0.2
0.3
0.4
SNP effect on 1−myristoyl−2−arachidonoyl−GPC (14:0/20:4) levels

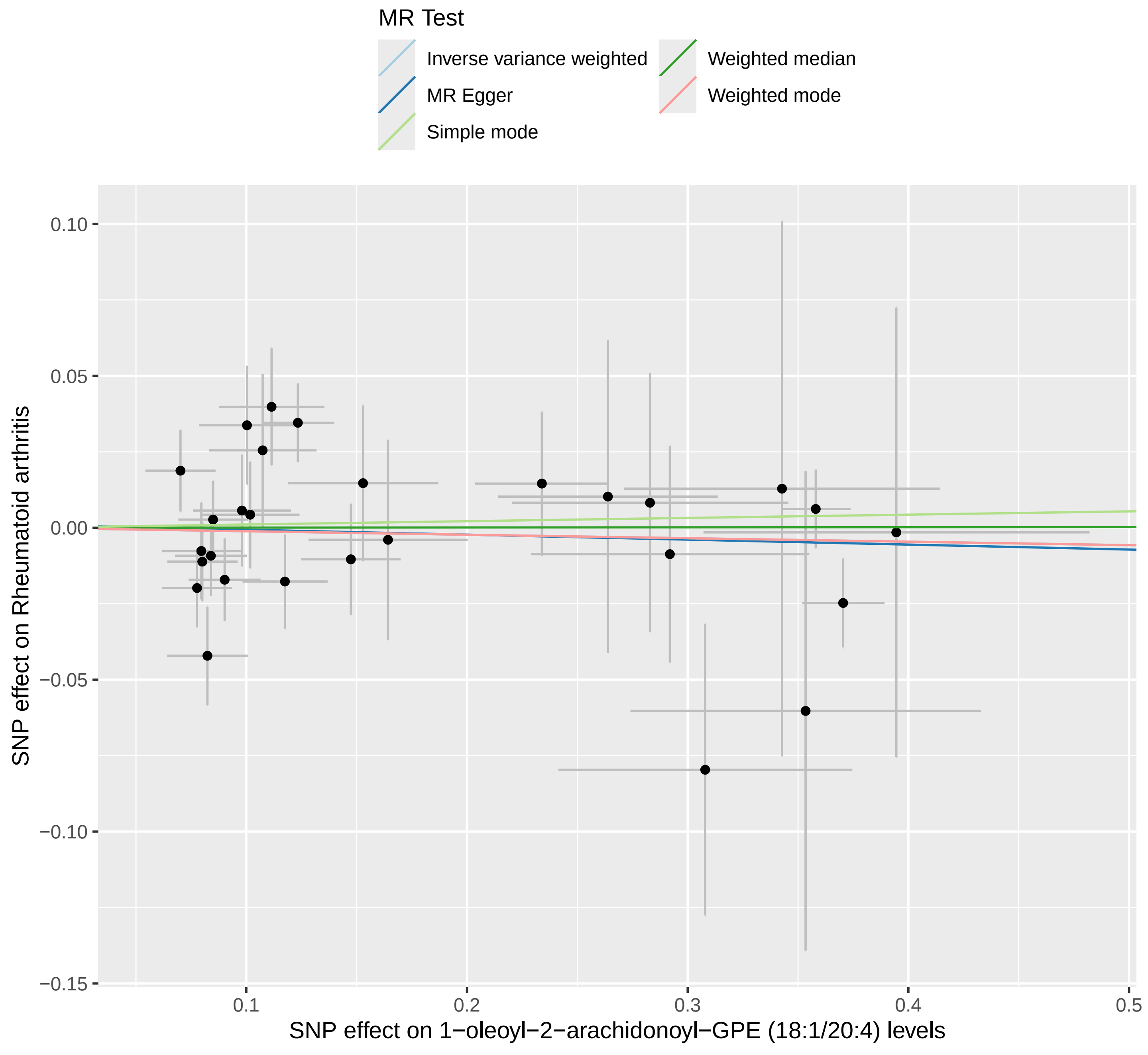
MR Test
Inverse variance weighted
MR Egger
Simple mode
Weighted median
Weighted mode
0.10
0.05
0.00
−0.05
−0.10
−0.15
0.1
0.2
0.3
0.4
0.5
SNP effect on Rheumatoid arthritis
SNP effect on 1−oleoyl−2−arachidonoyl−GPE (18:1/20:4) levels

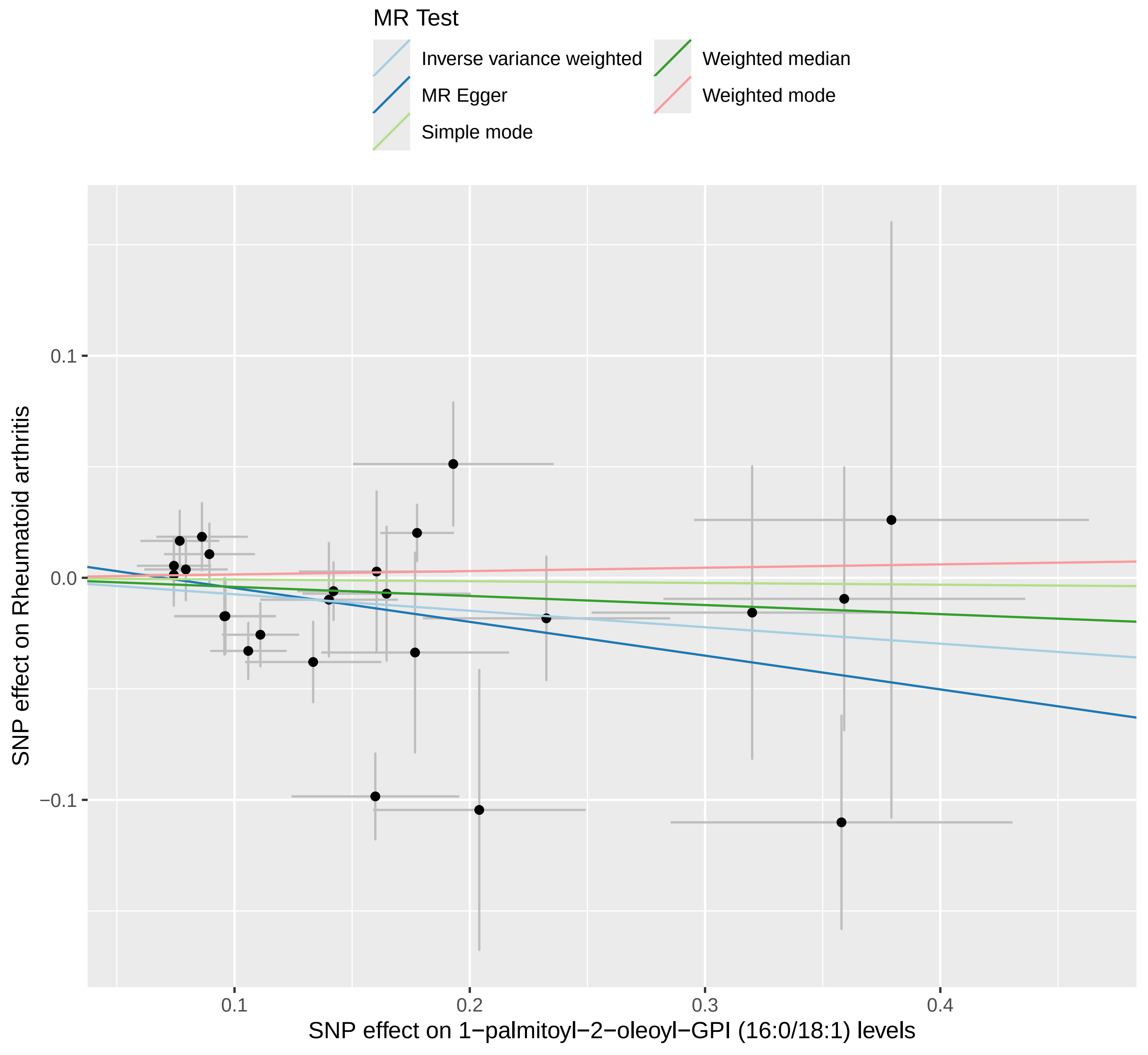

MR Test
Inverse variance weighted
MR Egger
Simple mode
Weighted median
Weighted mode
0.1
0.0
−0.1
SNP effect on Rheumatoid arthritis
0.1
0.2
0.3
0.4
SNP effect on 1−palmitoyl−2−oleoyl−GPI (16:0/18:1) levels

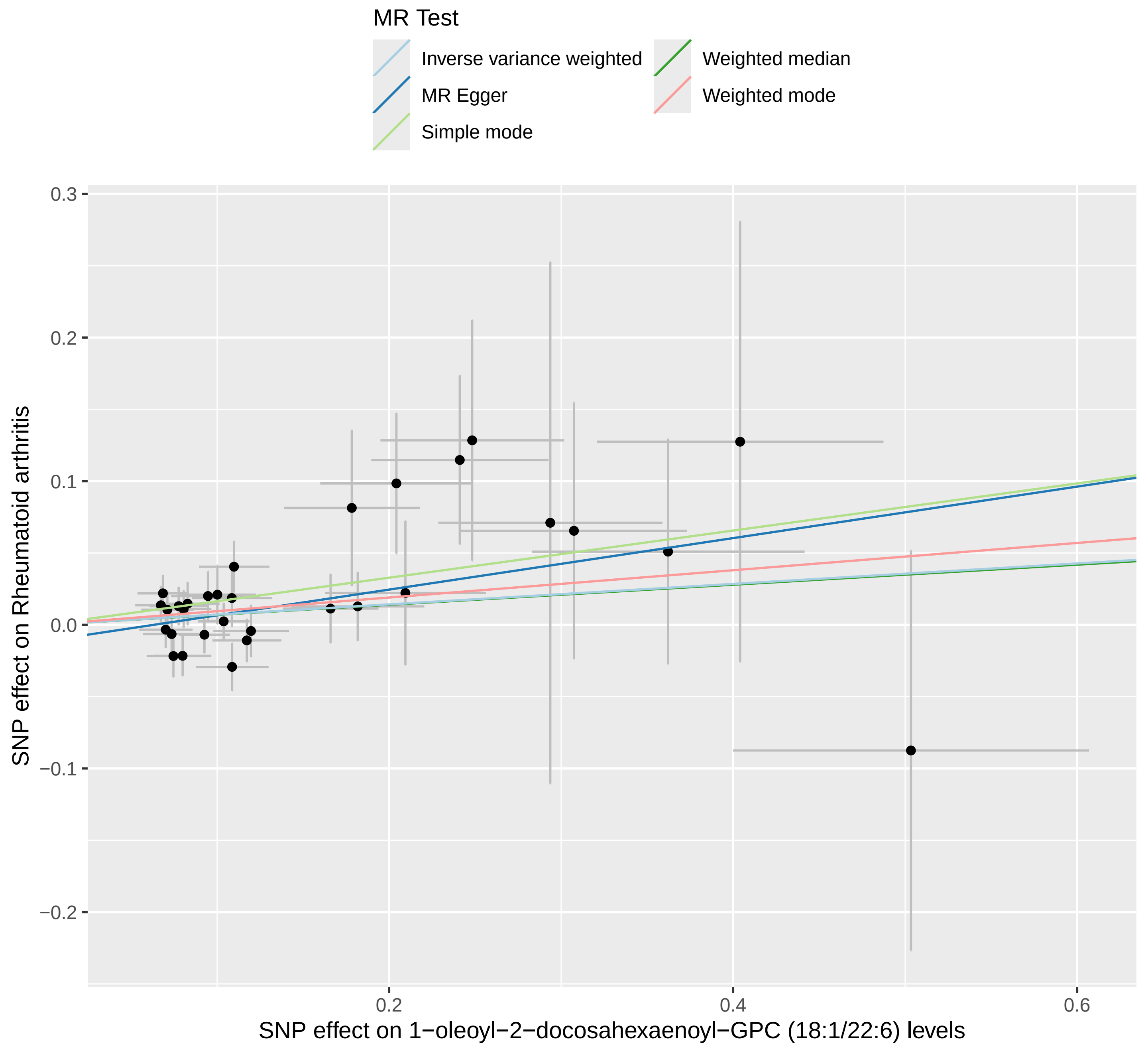
MR Test
Inverse variance weighted
MR Egger
Simple mode
Weighted median
Weighted mode
0.3
0.2
0.1
0.0
−0.1
−0.2
SNP effect on Rheumatoid arthritis
0.2
0.4
0.6
SNP effect on 1−oleoyl−2−docosahexaenoyl−GPC (18:1/22:6) levels

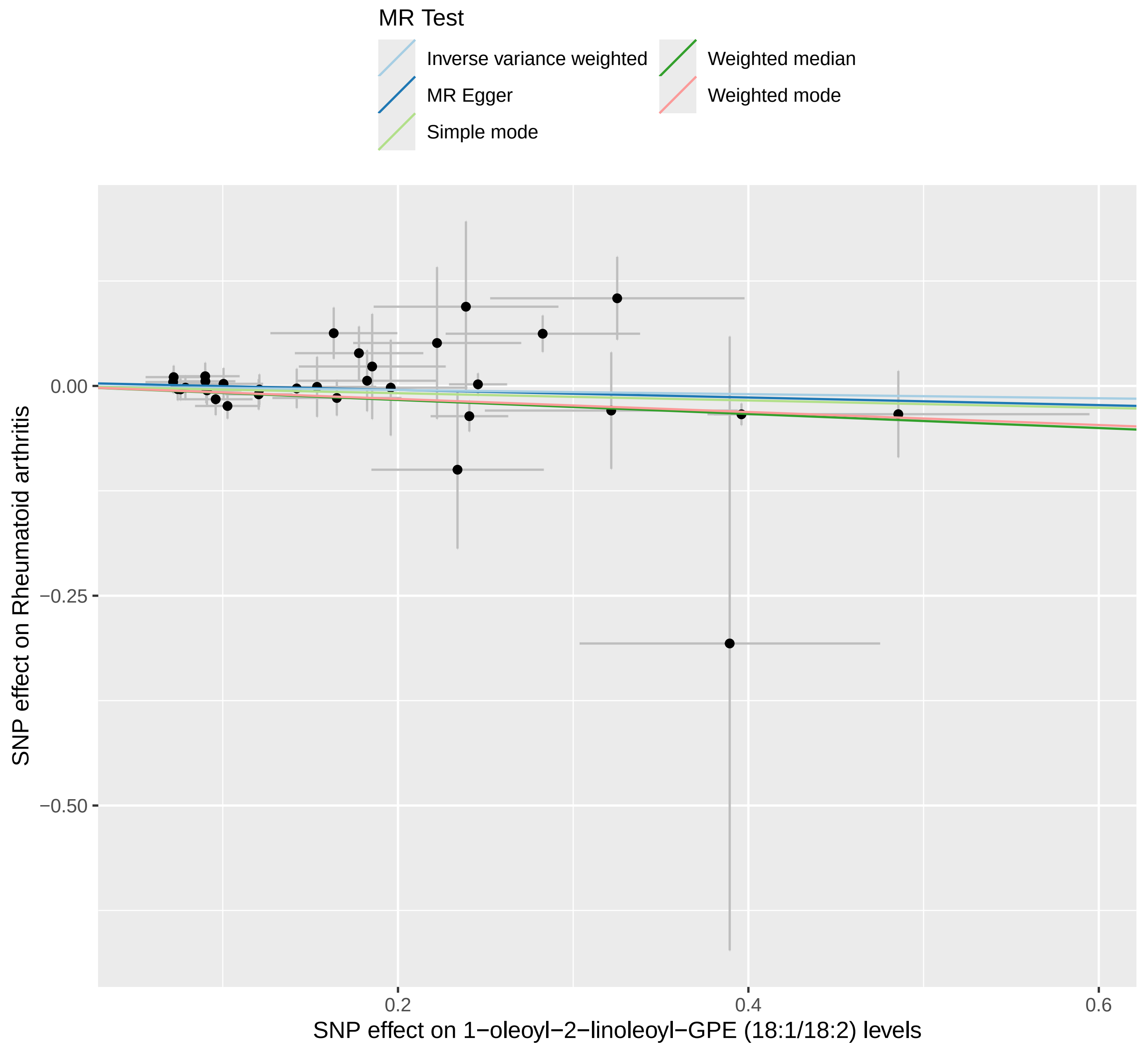

MR Test
Inverse variance weighted
MR Egger
Simple mode
Weighted median
Weighted mode
SNP effect on Rheumatoid arthritis
0.00
−0.25
−0.50
0.2
0.4
0.6
SNP effect on 1−oleoyl−2−linoleoyl−GPE (18:1/18:2) levels

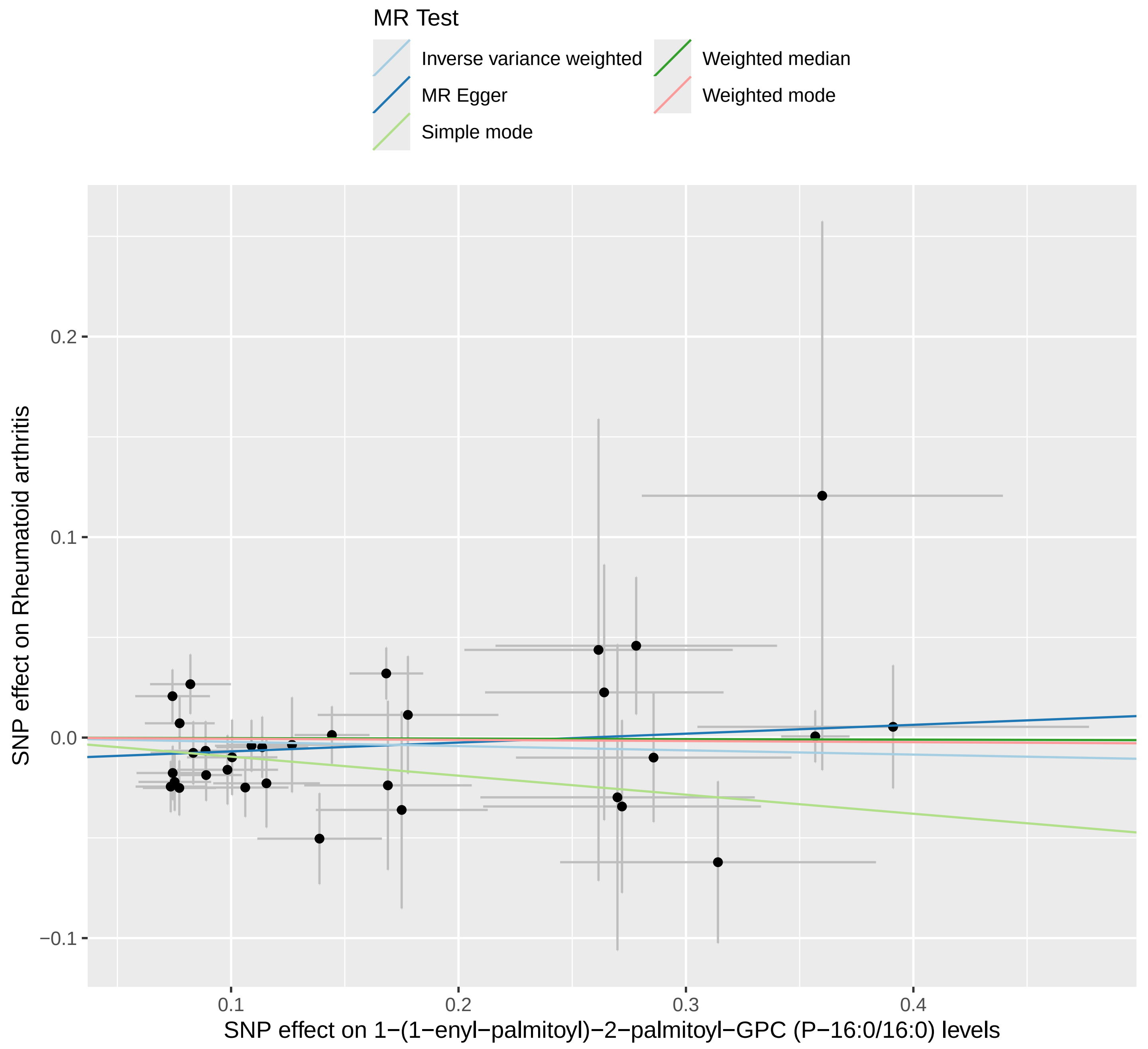

MR Test
Inverse variance weighted
MR Egger
Simple mode
Weighted median
Weighted mode
0.2
0.1
0.0
−0.1
0.1
0.2
0.3
0.4
SNP effect on Rheumatoid arthritis
SNP effect on 1−(1−enyl−palmitoyl)−2−palmitoyl−GPC (P−16:0/16:0) levels

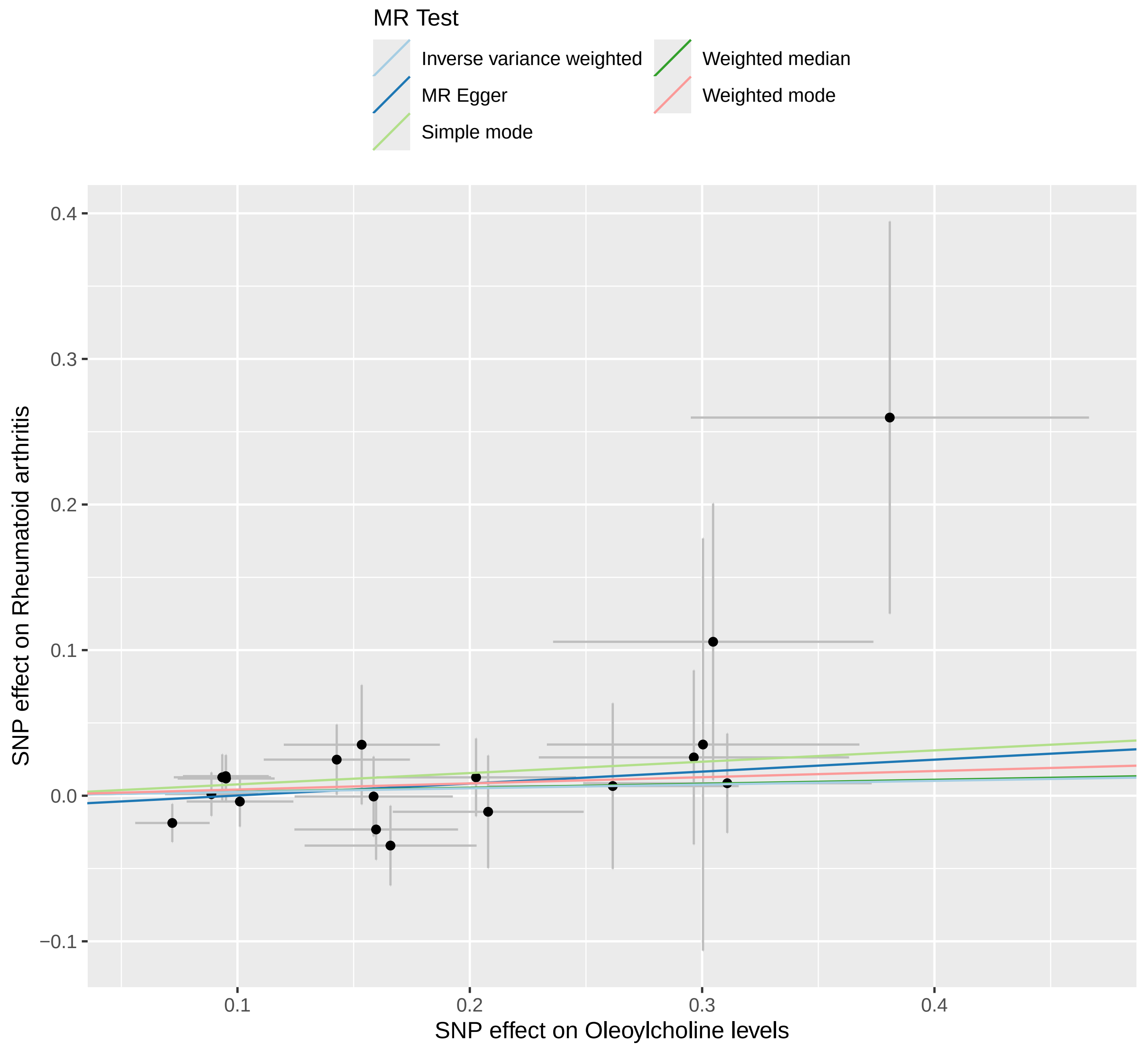
MR Test
Inverse variance weighted
MR Egger
Simple mode
Weighted median
Weighted mode
0.4
0.3
0.2
0.1
0.0
−0.1
0.1
0.2
0.3
0.4
SNP effect on Rheumatoid arthritis
SNP effect on Oleoylcholine levels

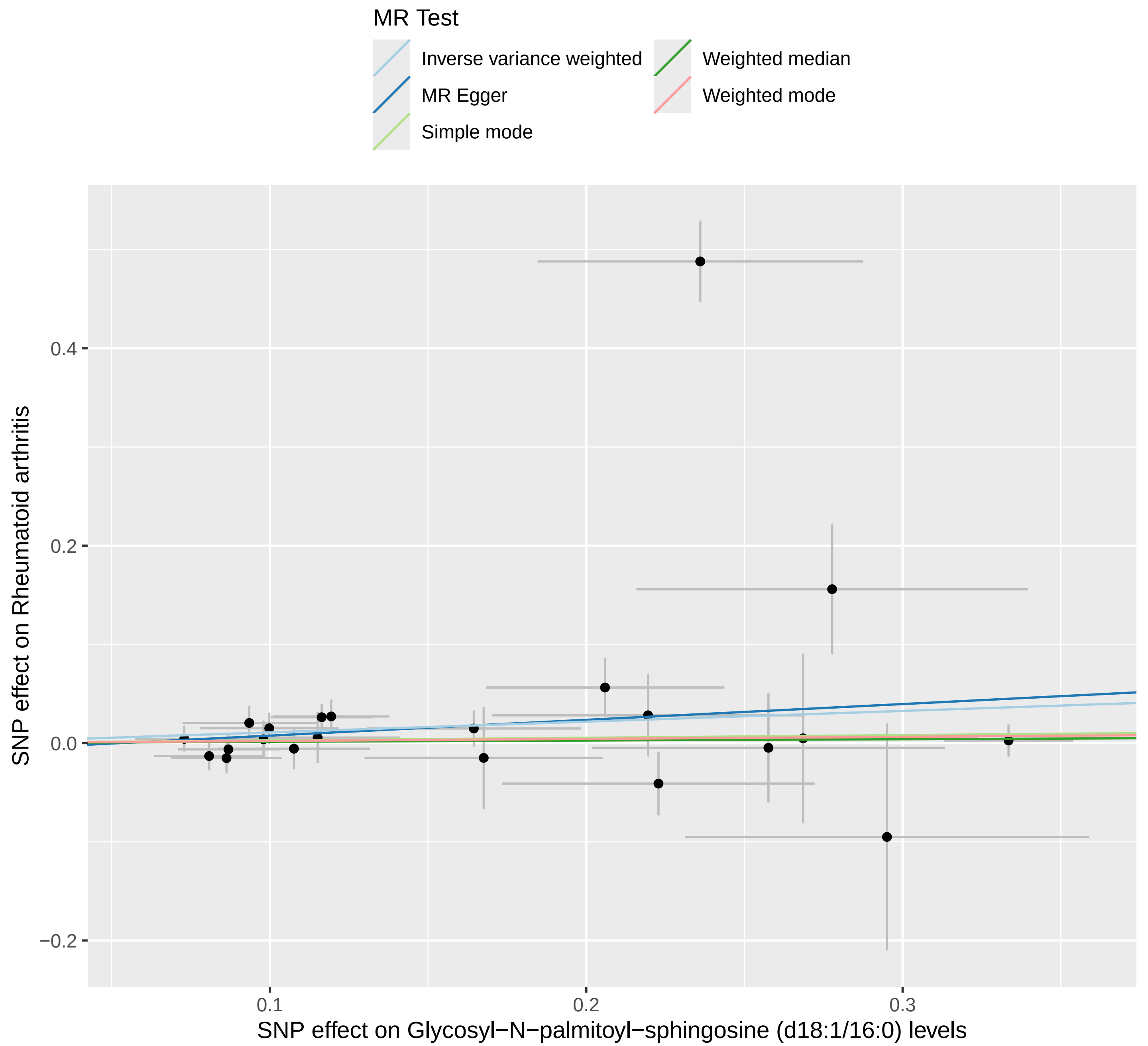

MR Test
Inverse variance weighted
MR Egger
Simple mode
Weighted median
Weighted mode
SNP effect on Rheumatoid arthritis
0.4
0.2
0.0
−0.2
0.1
0.2
0.3
SNP effect on Glycosyl−N−palmitoyl−sphingosine (d18:1/16:0) levels

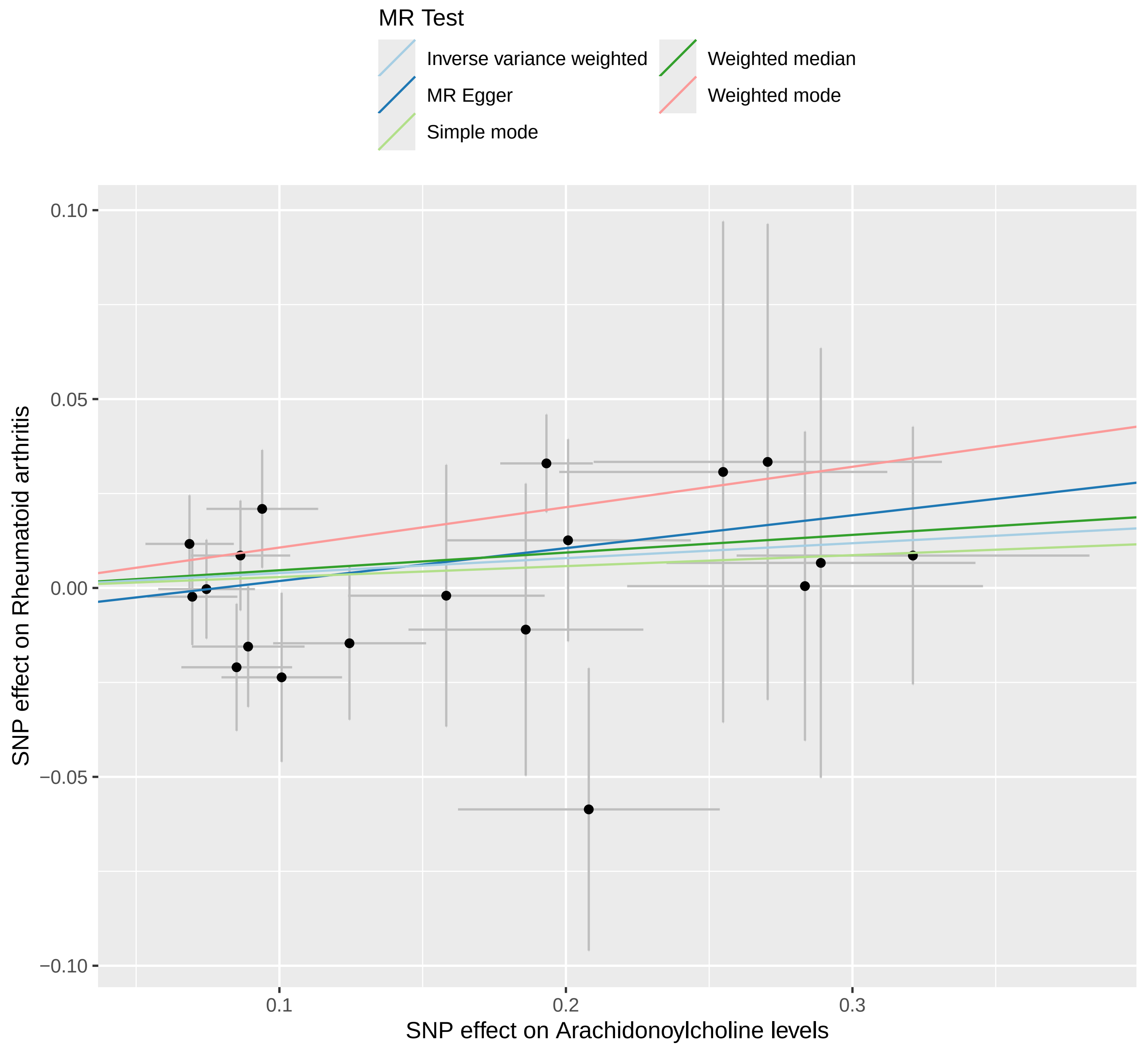

MR Test
Inverse variance weighted
MR Egger
Simple mode
Weighted median
Weighted mode
0.10
0.05
0.00
−0.05
−0.10
SNP effect on Rheumatoid arthritis
0.1
0.2
0.3
SNP effect on Arachidonoylcholine levels

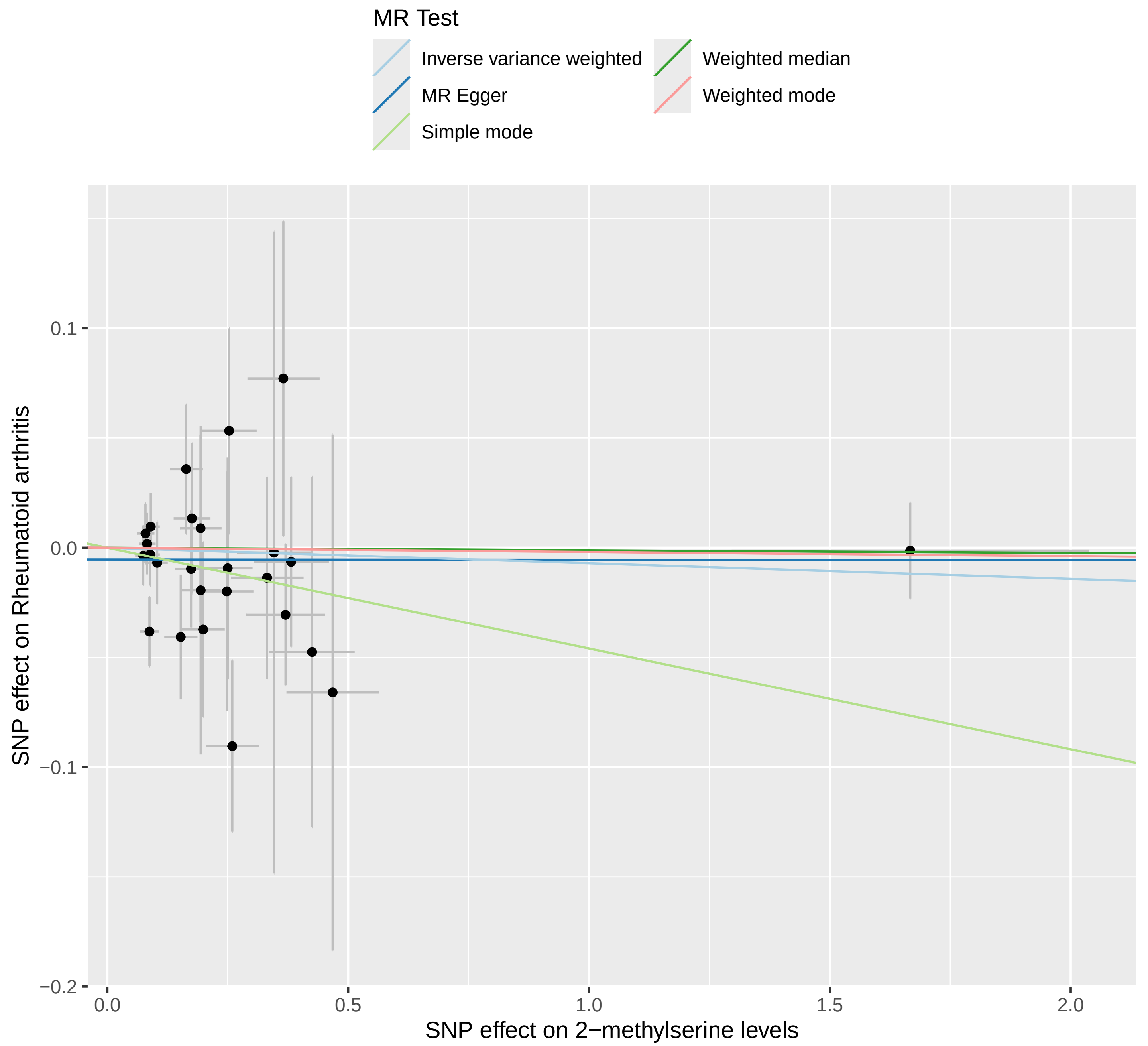
MR Test
Inverse variance weighted
MR Egger
Simple mode
Weighted median
Weighted mode
SNP effect on Rheumatoid arthritis
0.1
0.0
−0.1
−0.2
0.0
0.5
1.0
1.5
2.0
SNP effect on 2−methylserine levels

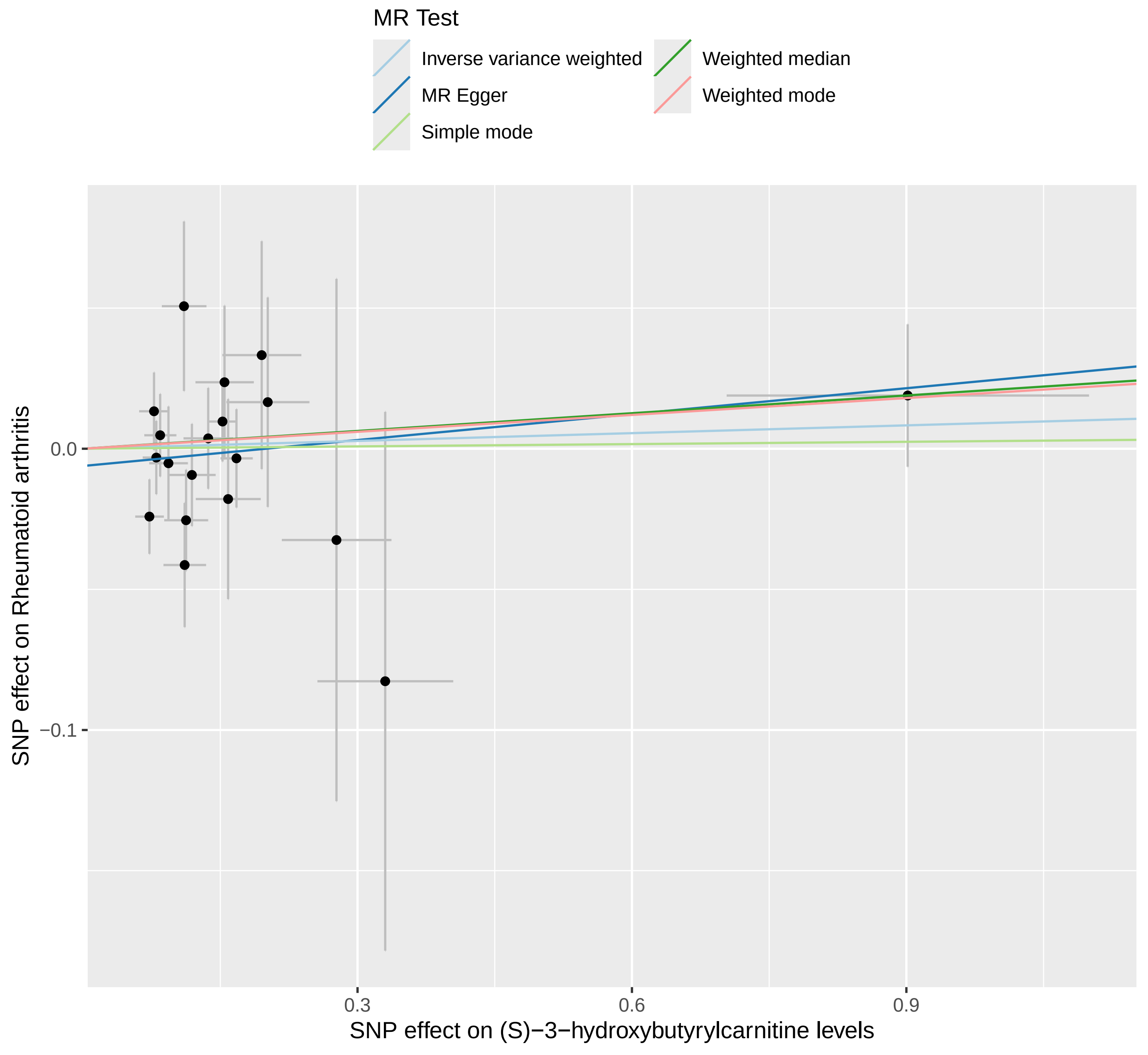
MR Test
Inverse variance weighted
MR Egger
Simple mode
Weighted median
Weighted mode
0.0
−0.1
0.3
0.6
0.9
SNP effect on (S)−3−hydroxybutyrylcarnitine levels
SNP effect on Rheumatoid arthritis

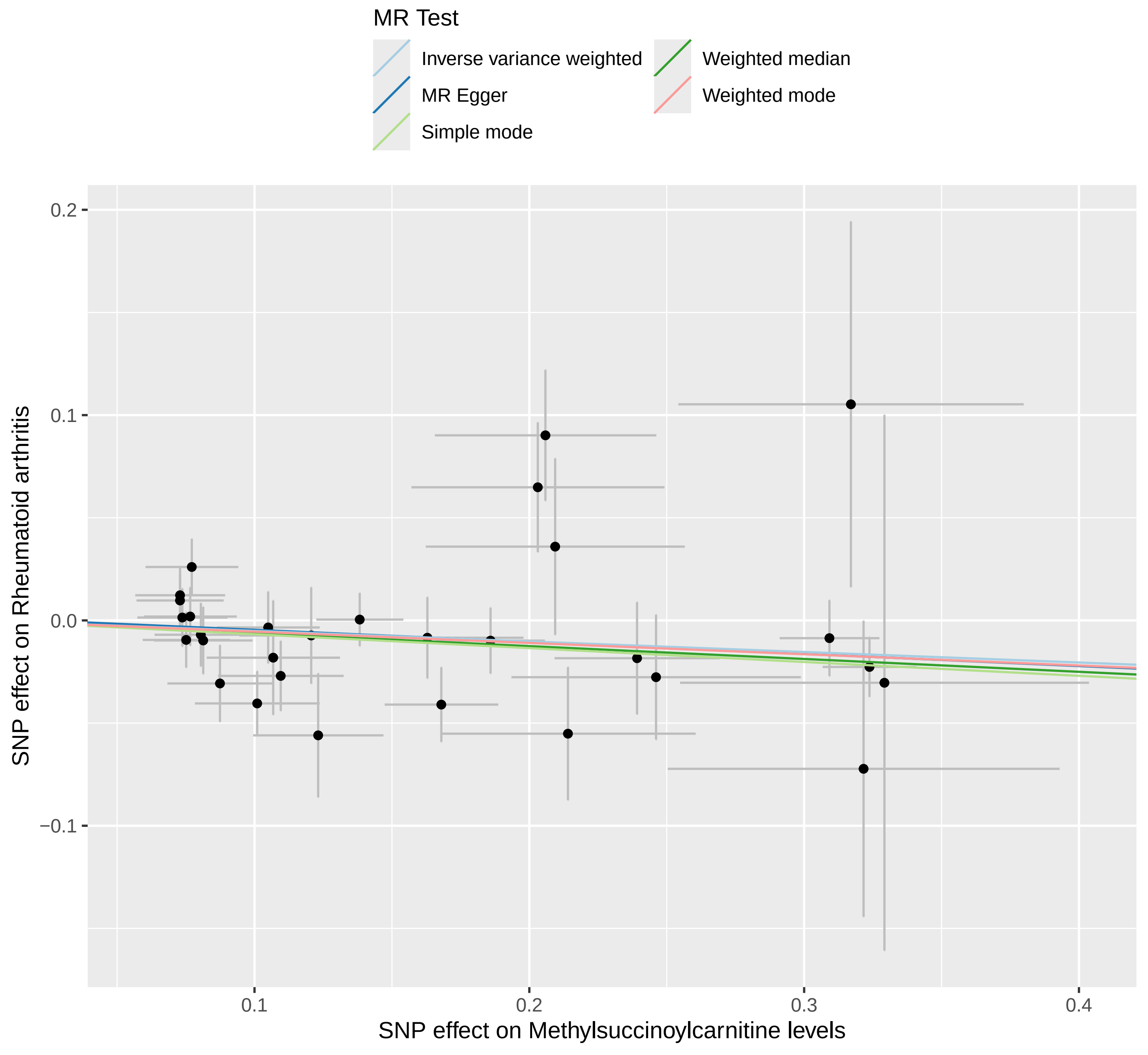

MR Test
Inverse variance weighted
MR Egger
Simple mode
Weighted median
Weighted mode
0.2
0.1
0.0
−0.1
0.1
0.2
0.3
0.4
SNP effect on Rheumatoid arthritis
SNP effect on Methylsuccinoylcarnitine levels

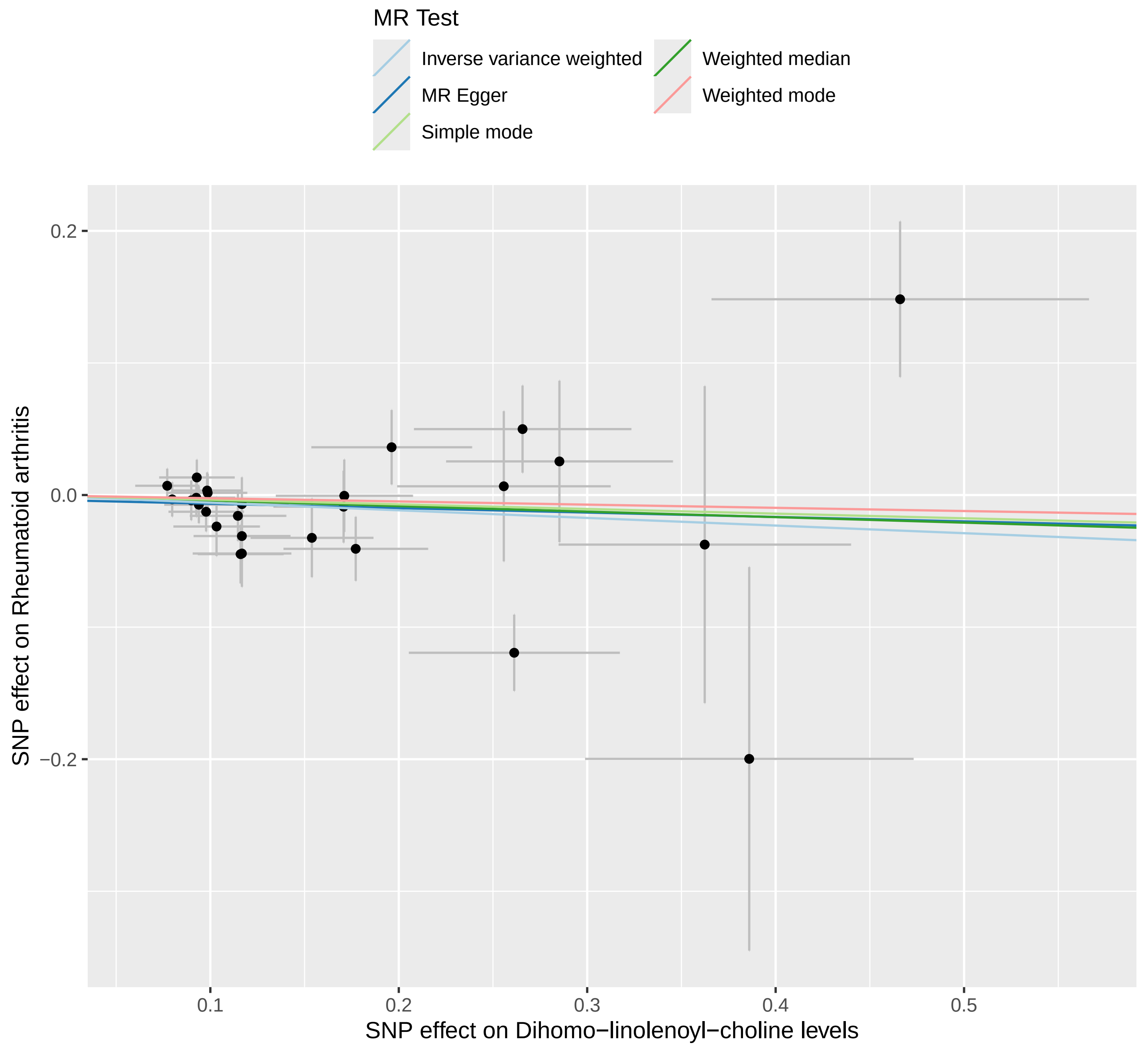
MR Test
Inverse variance weighted
MR Egger
Simple mode
Weighted median
Weighted mode
0.2
0.0
−0.2
0.1
0.2
0.3
0.4
0.5
SNP effect on Rheumatoid arthritis
SNP effect on Dihomo−linolenoyl−choline levels

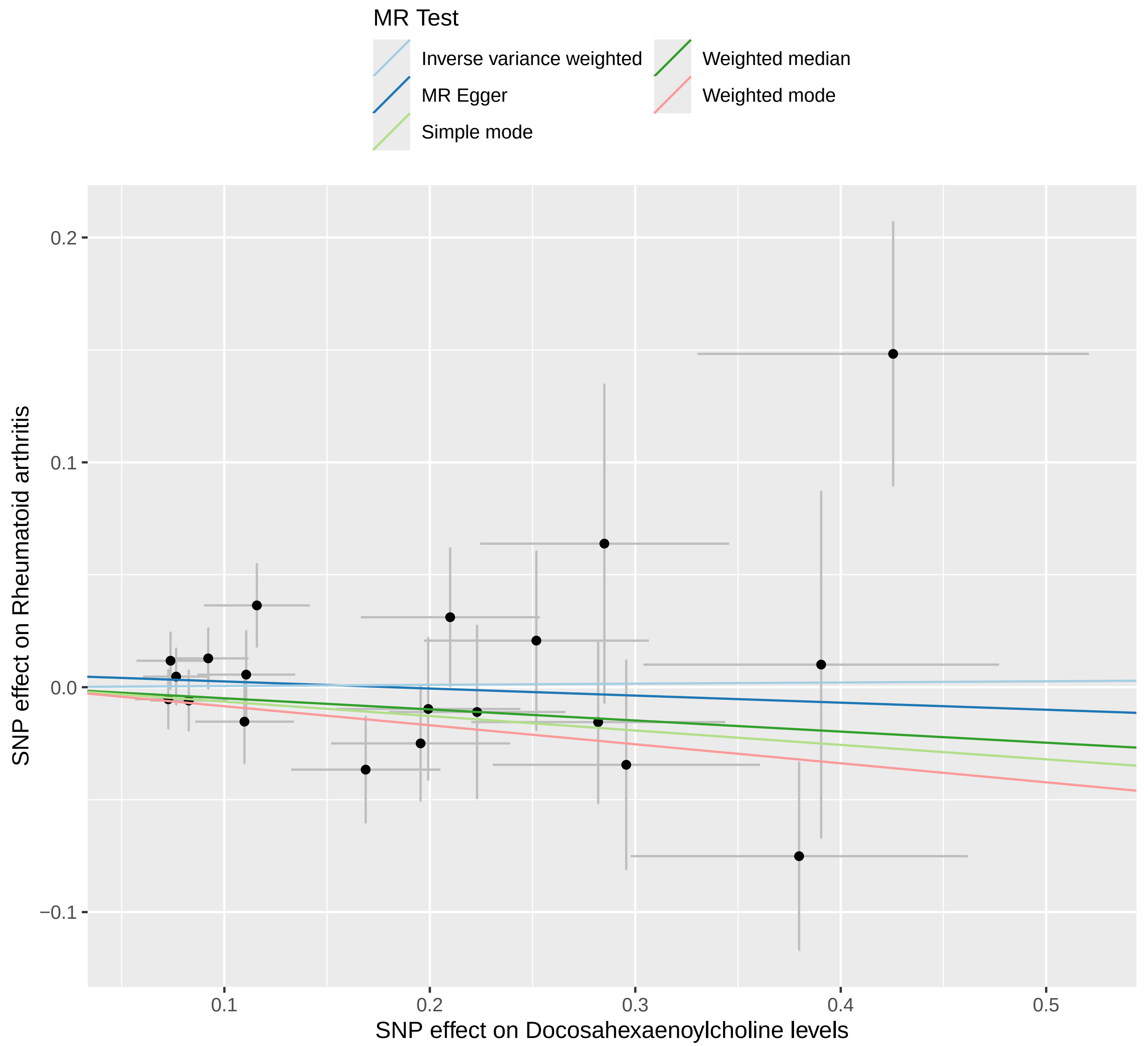

MR Test
Inverse variance weighted
MR Egger
Simple mode
Weighted median
Weighted mode
0.2
0.1
0.0
−0.1
SNP effect on Rheumatoid arthritis
0.1
0.2
0.3
0.4
0.5
SNP effect on Docosahexaenoylcholine levels

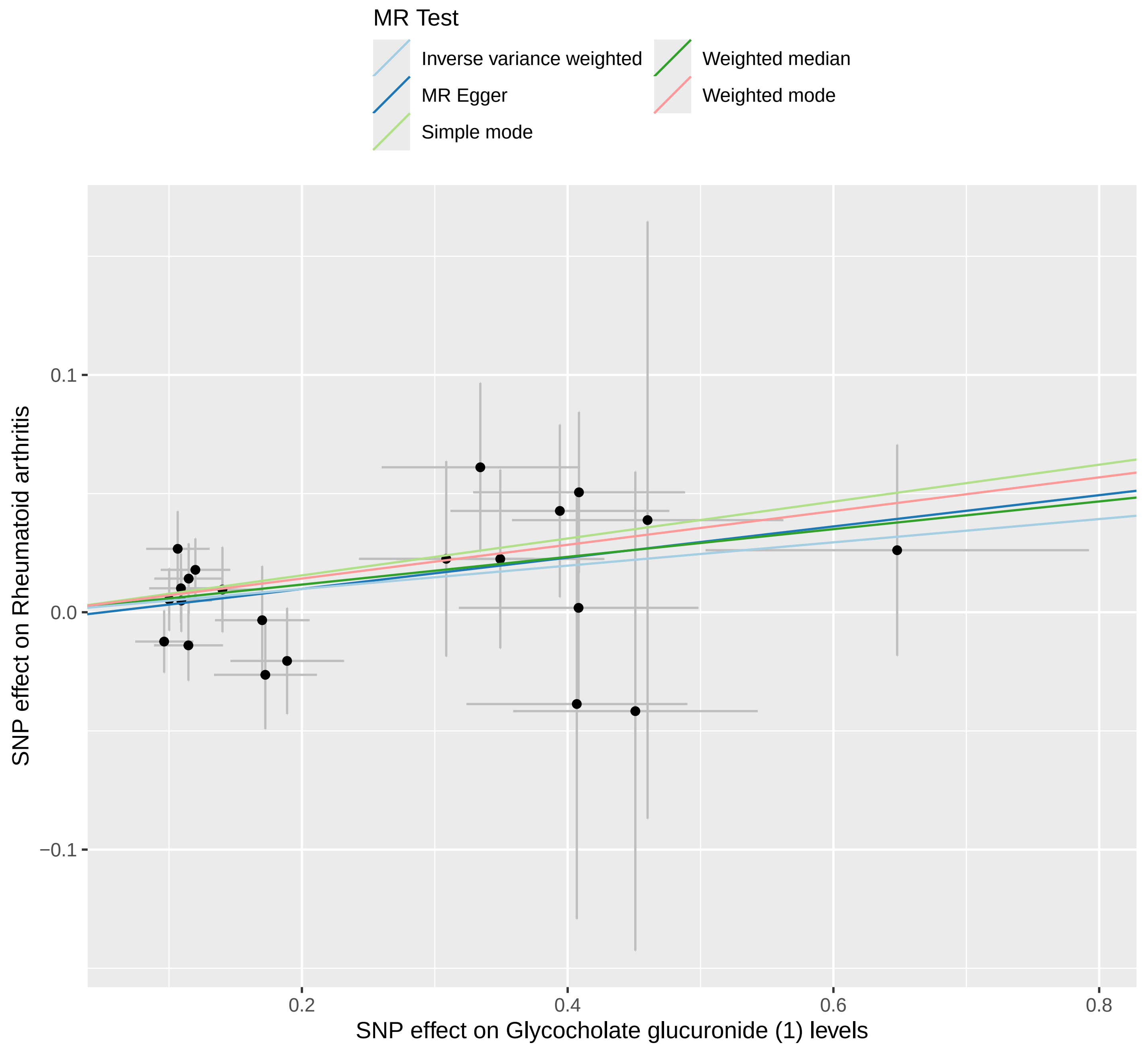

MR Test
Inverse variance weighted
MR Egger
Simple mode
Weighted median
Weighted mode
0.1
0.0
−0.1
0.2
0.4
0.6
0.8
SNP effect on Rheumatoid arthritis
SNP effect on Glycocholate glucuronide (1) levels

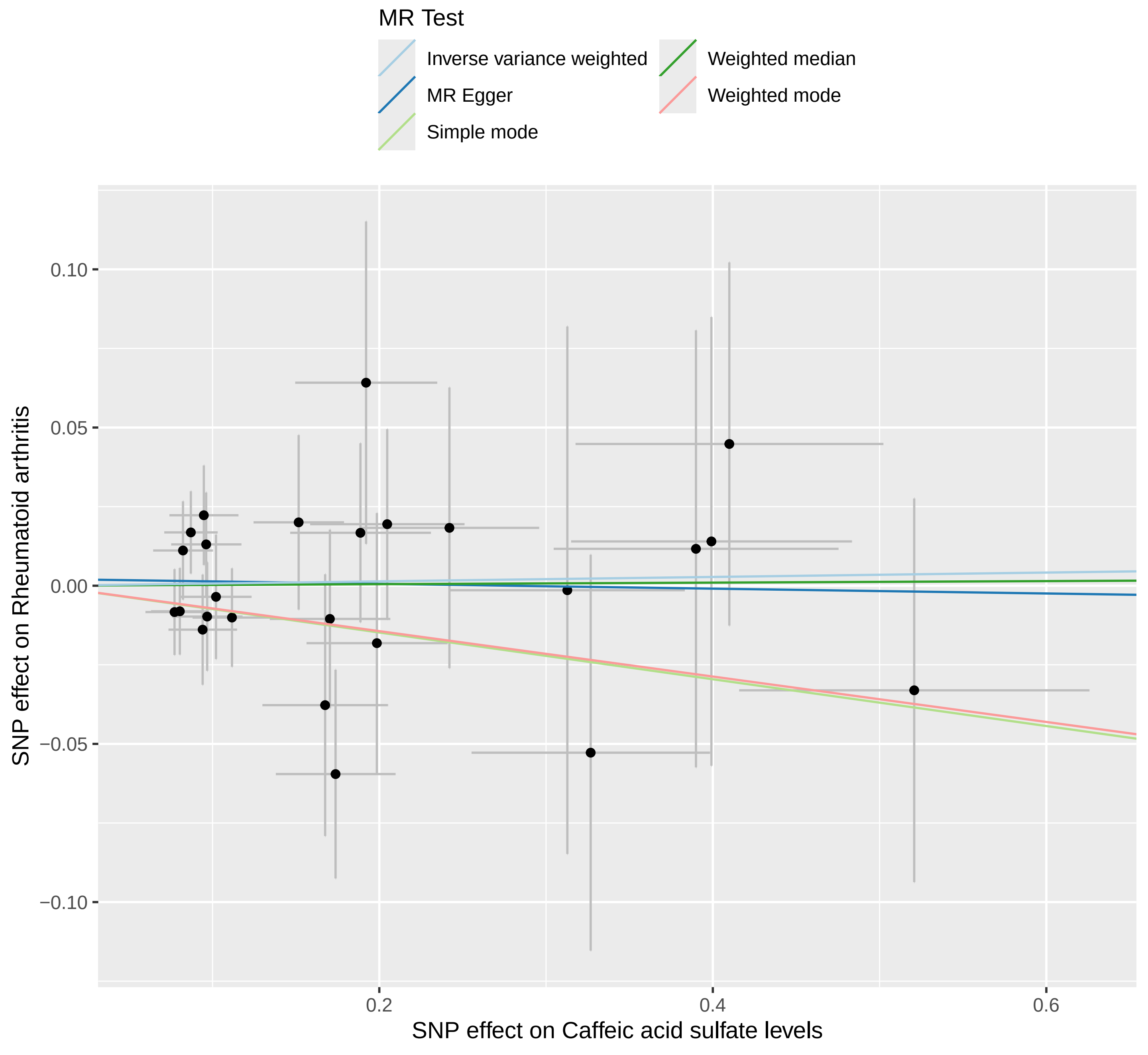

MR Test
Inverse variance weighted
MR Egger
Simple mode
Weighted median
Weighted mode
0.10
0.05
0.00
−0.05
−0.10
0.2
0.4
0.6
SNP effect on Rheumatoid arthritis
SNP effect on Caffeic acid sulfate levels

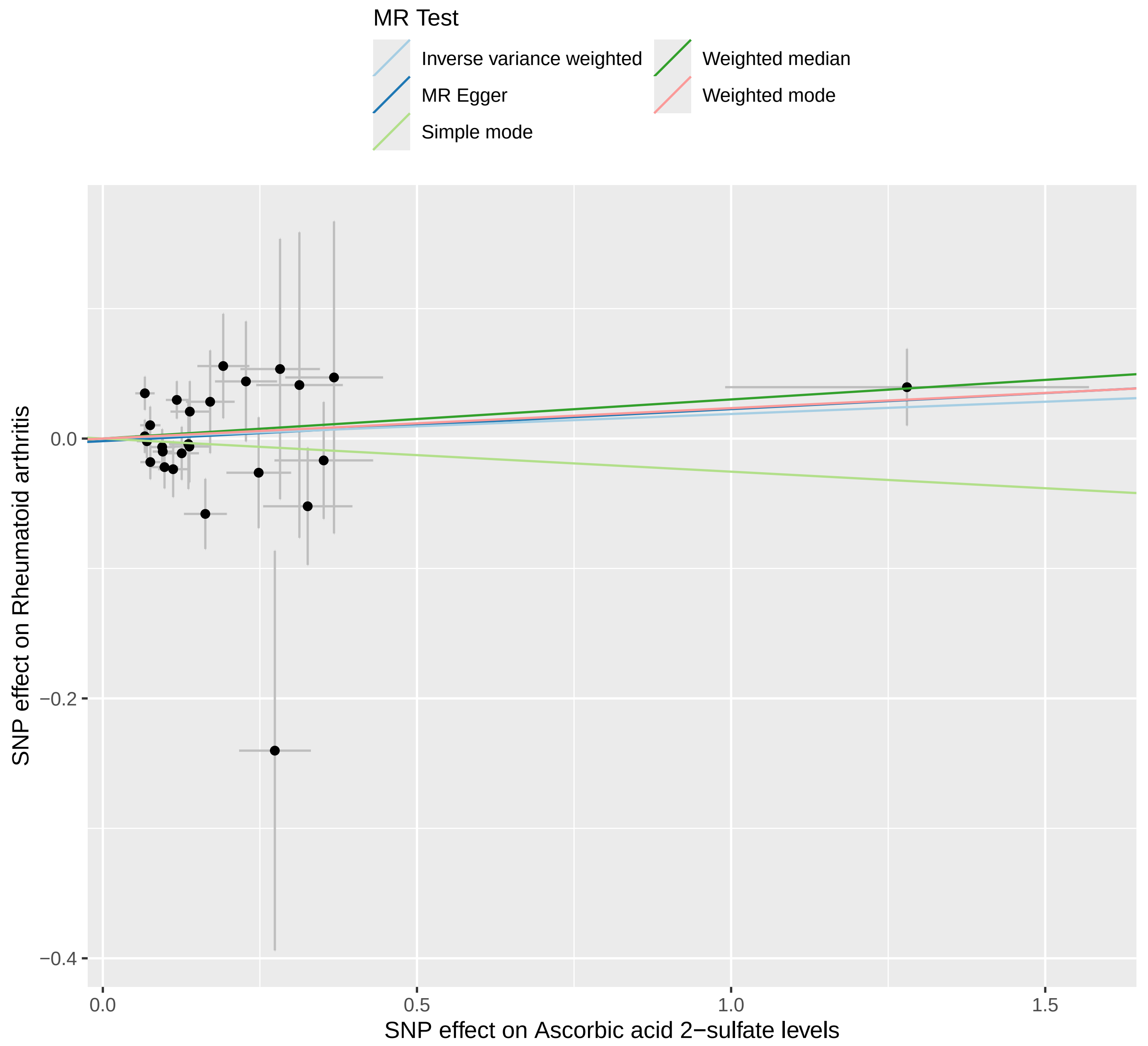
MR Test
Inverse variance weighted
MR Egger
Simple mode
Weighted median
Weighted mode
SNP effect on Rheumatoid arthritis
0.0
−0.2
−0.4
0.0
0.5
1.0
1.5
SNP effect on Ascorbic acid 2−sulfate levels

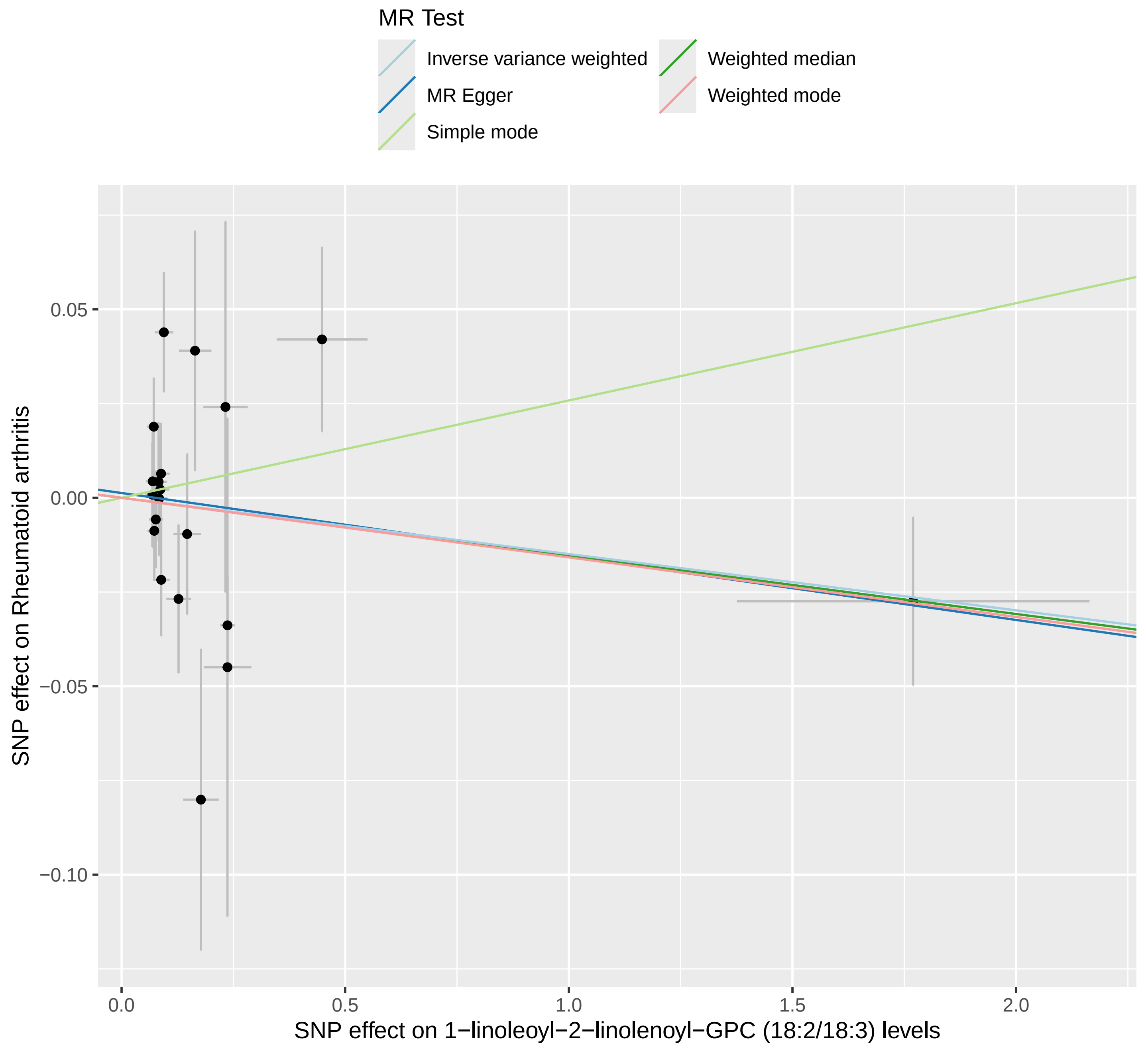
MR Test
Inverse variance weighted
MR Egger
Simple mode
Weighted median
Weighted mode
0.05
0.00
−0.05
−0.10
0.0
0.5
1.0
1.5
2.0
SNP effect on Rheumatoid arthritis
SNP effect on 1−linoleoyl−2−linolenoyl−GPC (18:2/18:3) levels

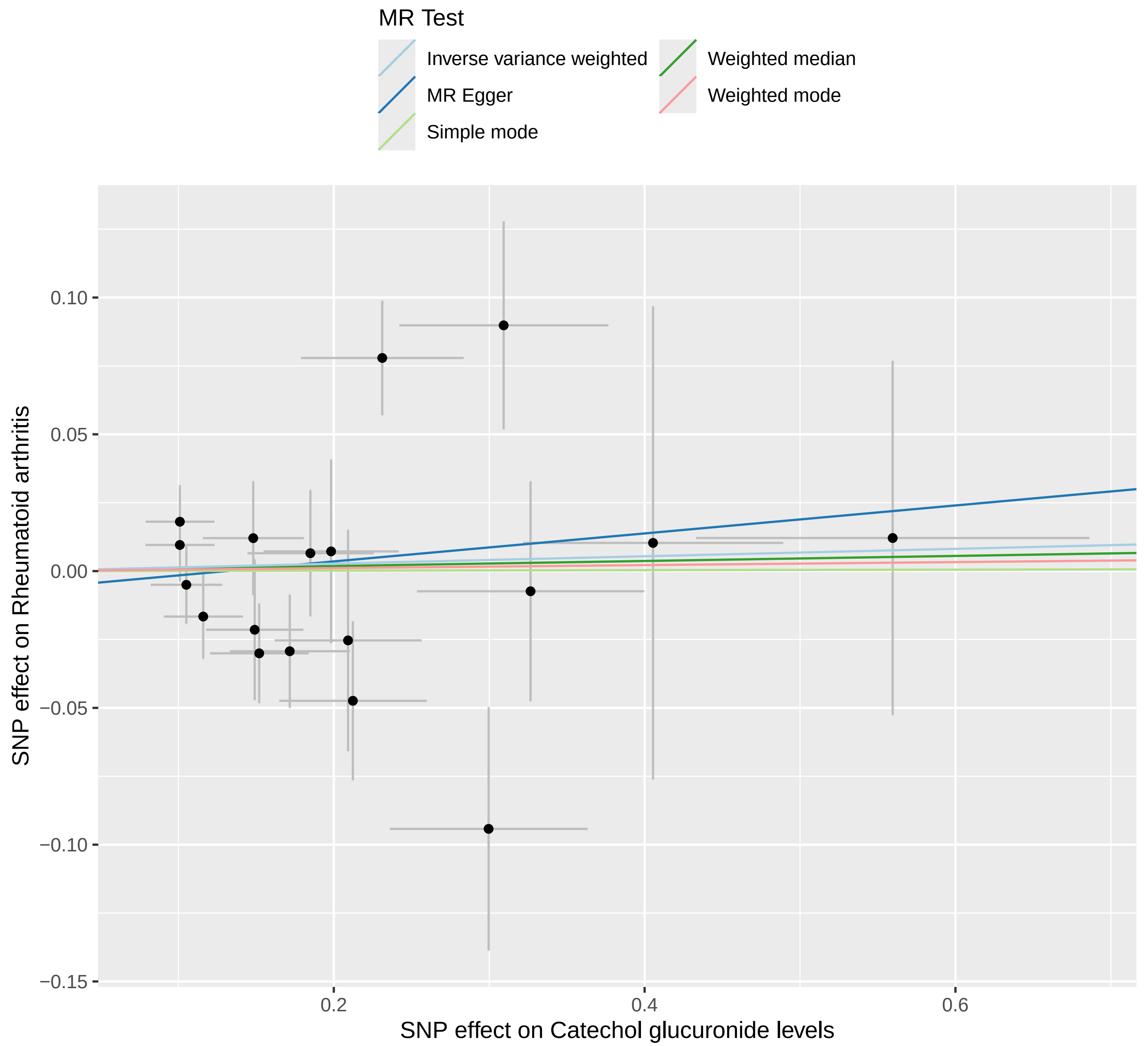
MR Test
Inverse variance weighted
MR Egger
Simple mode
Weighted median
Weighted mode
0.10
0.05
0.00
−0.05
−0.10
−0.15
SNP effect on Rheumatoid arthritis
0.2
0.4
0.6
SNP effect on Catechol glucuronide levels

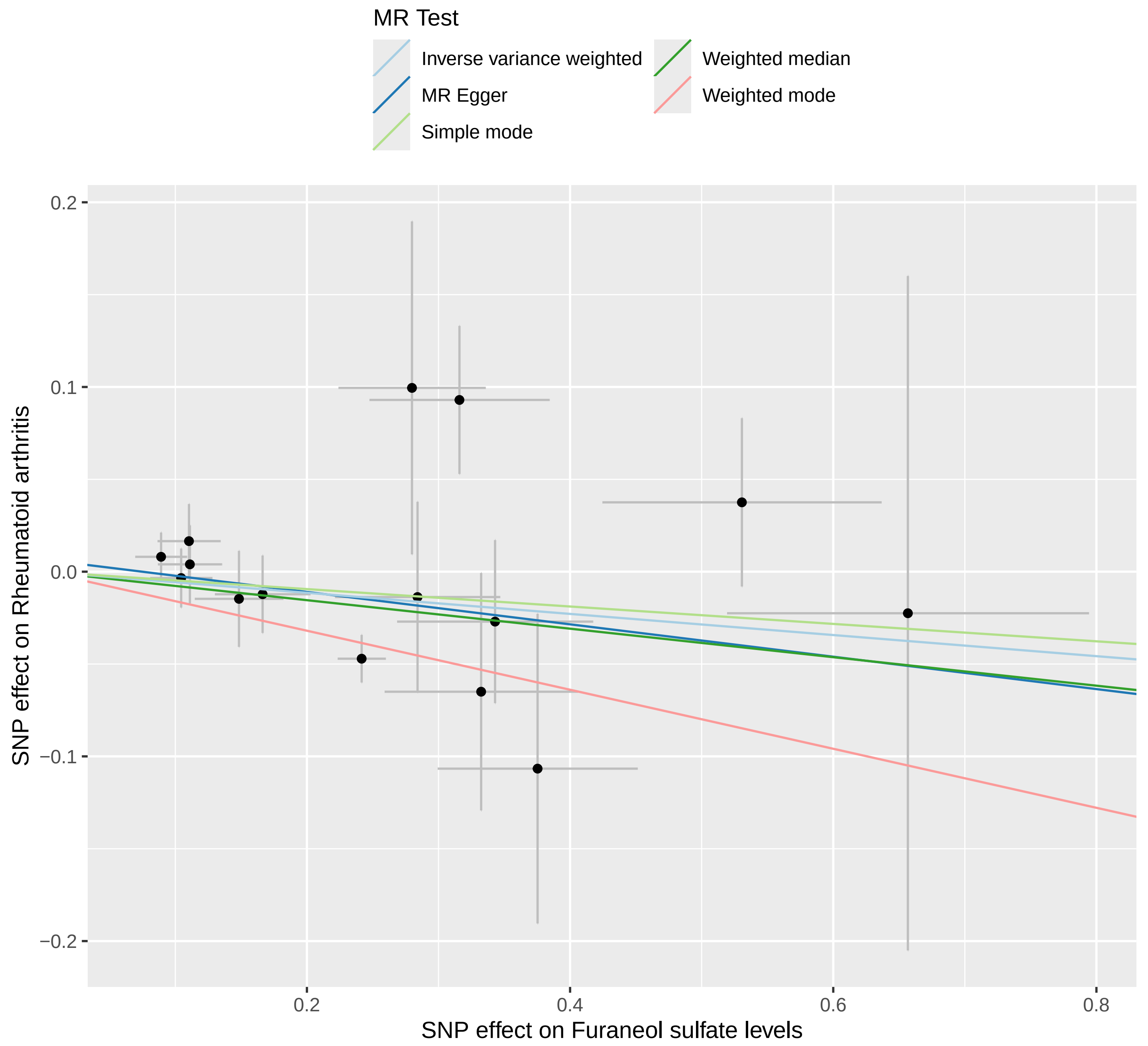
MR Test
Inverse variance weighted
MR Egger
Simple mode
Weighted median
Weighted mode
0.2
0.1
0.0
−0.1
−0.2
0.2
0.4
0.6
0.8
SNP effect on Rheumatoid arthritis
SNP effect on Furaneol sulfate levels

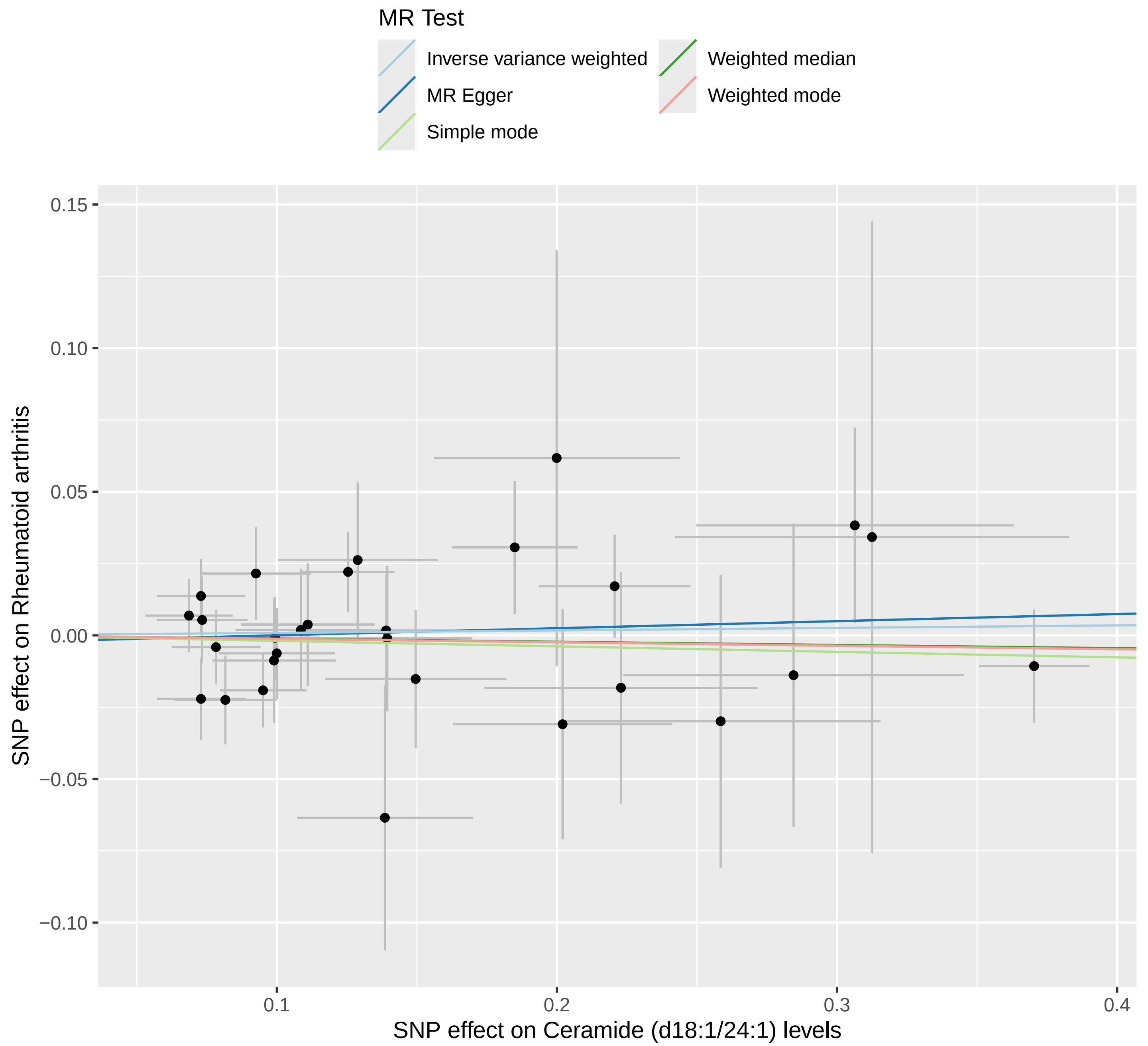

MR Test
Inverse variance weighted
MR Egger
Simple mode
Weighted median
Weighted mode
0.15
0.10
0.05
0.00
−0.05
−0.10
SNP effect on Rheumatoid arthritis
0.1
0.2
0.3
0.4
SNP effect on Ceramide (d18:1/24:1) levels

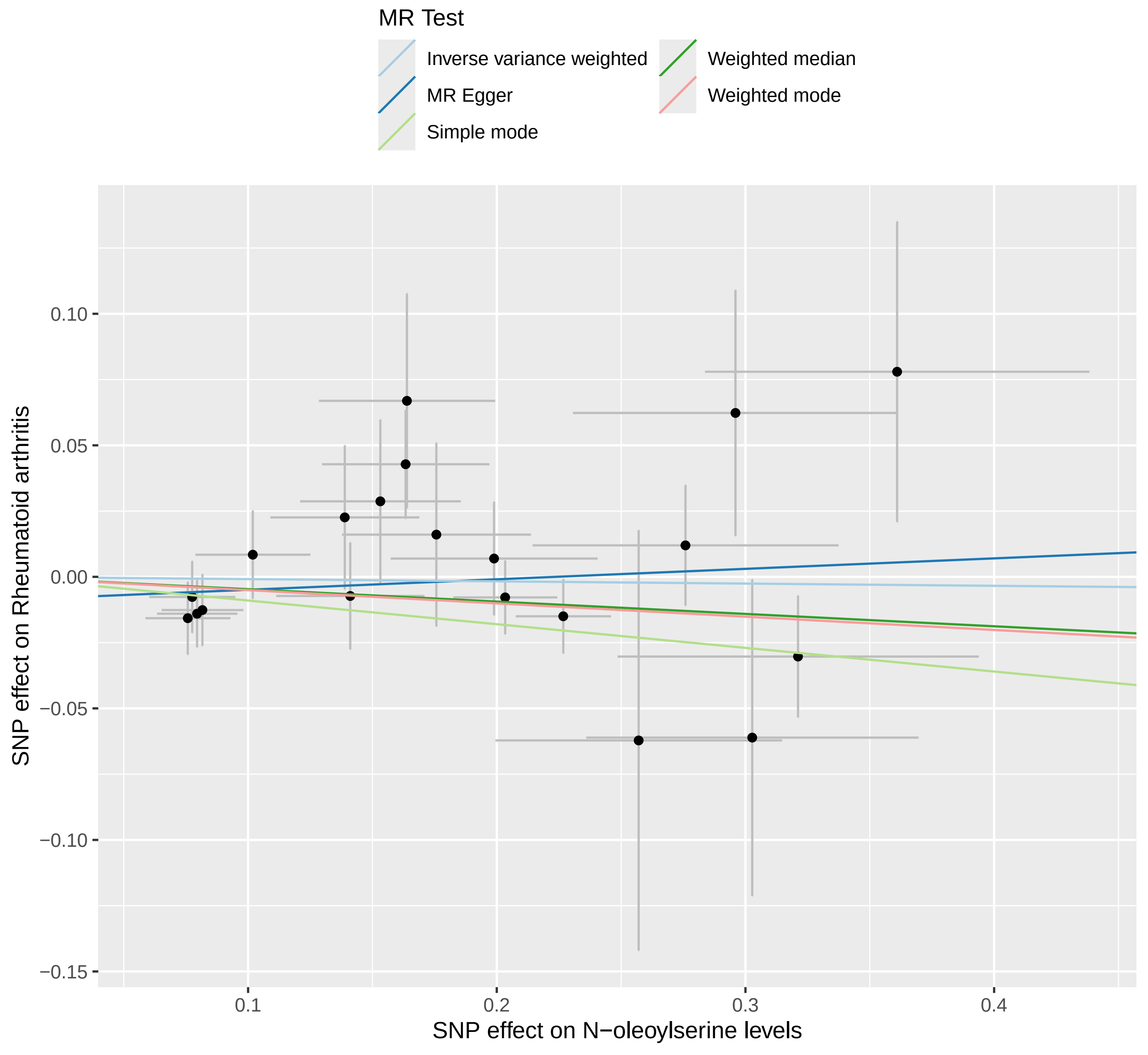
MR Test
Inverse variance weighted
MR Egger
Simple mode
Weighted median
Weighted mode
SNP effect on Rheumatoid arthritis
0.10
0.05
0.00
−0.05
−0.10
−0.15
0.1
0.2
0.3
0.4
SNP effect on N−oleoylserine levels

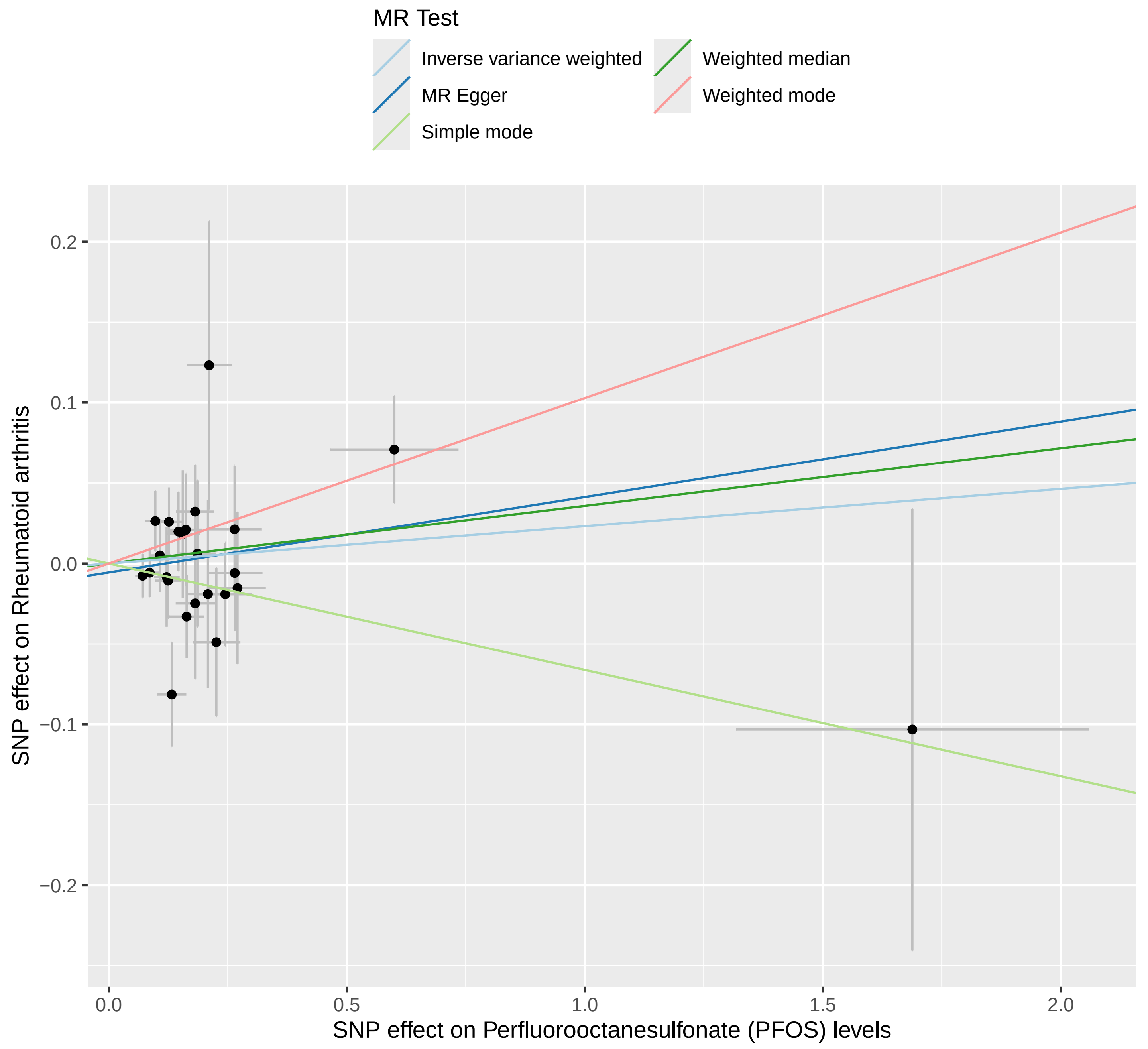
MR Test
Inverse variance weighted
MR Egger
Simple mode
Weighted median
Weighted mode
0.2
0.1
0.0
−0.1
−0.2
0.0
0.5
1.0
1.5
2.0
SNP effect on Rheumatoid arthritis
SNP effect on Perfluorooctanesulfonate (PFOS) levels

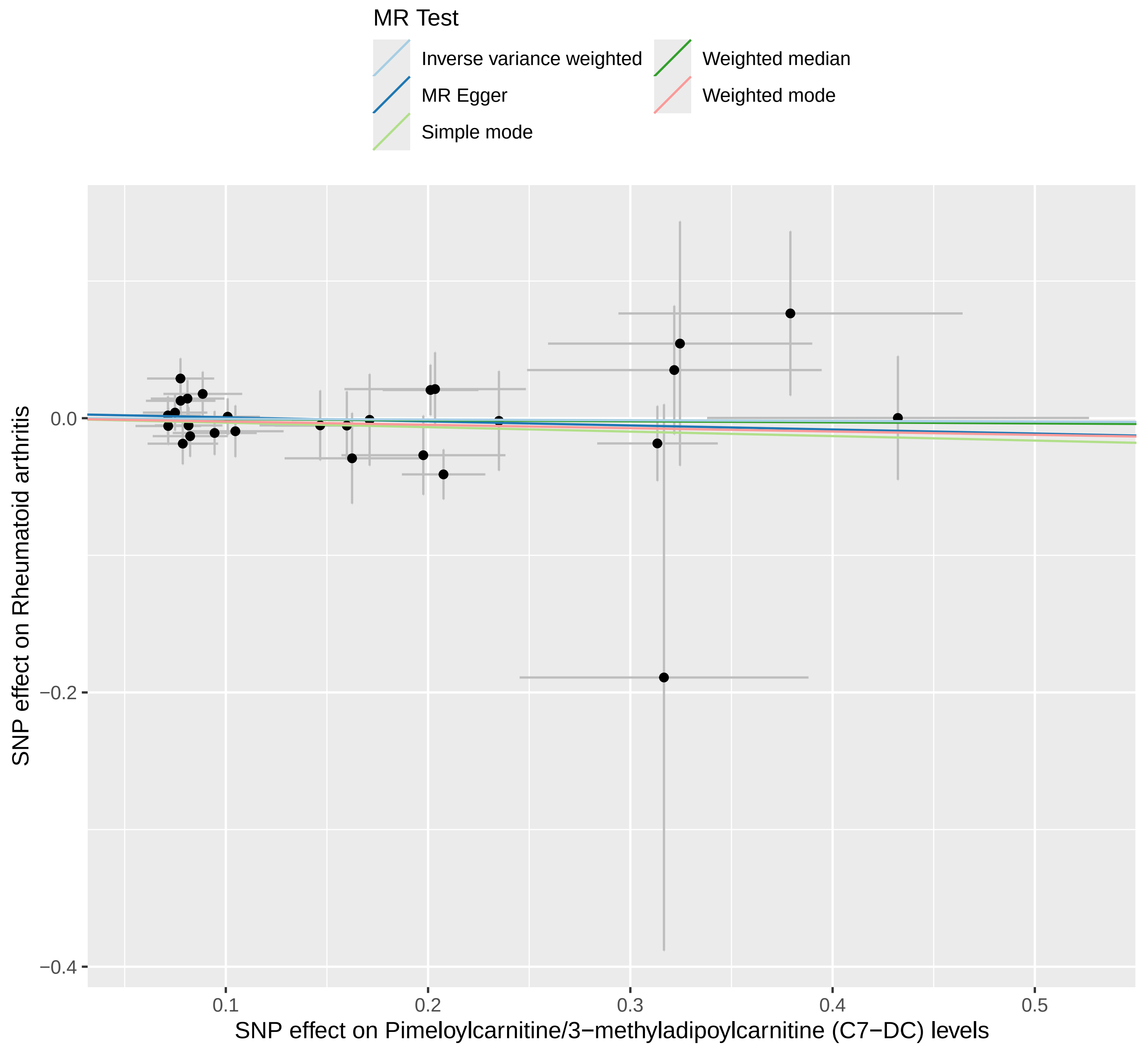

MR Test
Inverse variance weighted
MR Egger
Simple mode
Weighted median
Weighted mode
SNP effect on Rheumatoid arthritis
0.0
−0.2
−0.4
0.1
0.2
0.3
0.4
0.5
SNP effect on Pimeloylcarnitine/3−methyladipoylcarnitine (C7−DC) levels

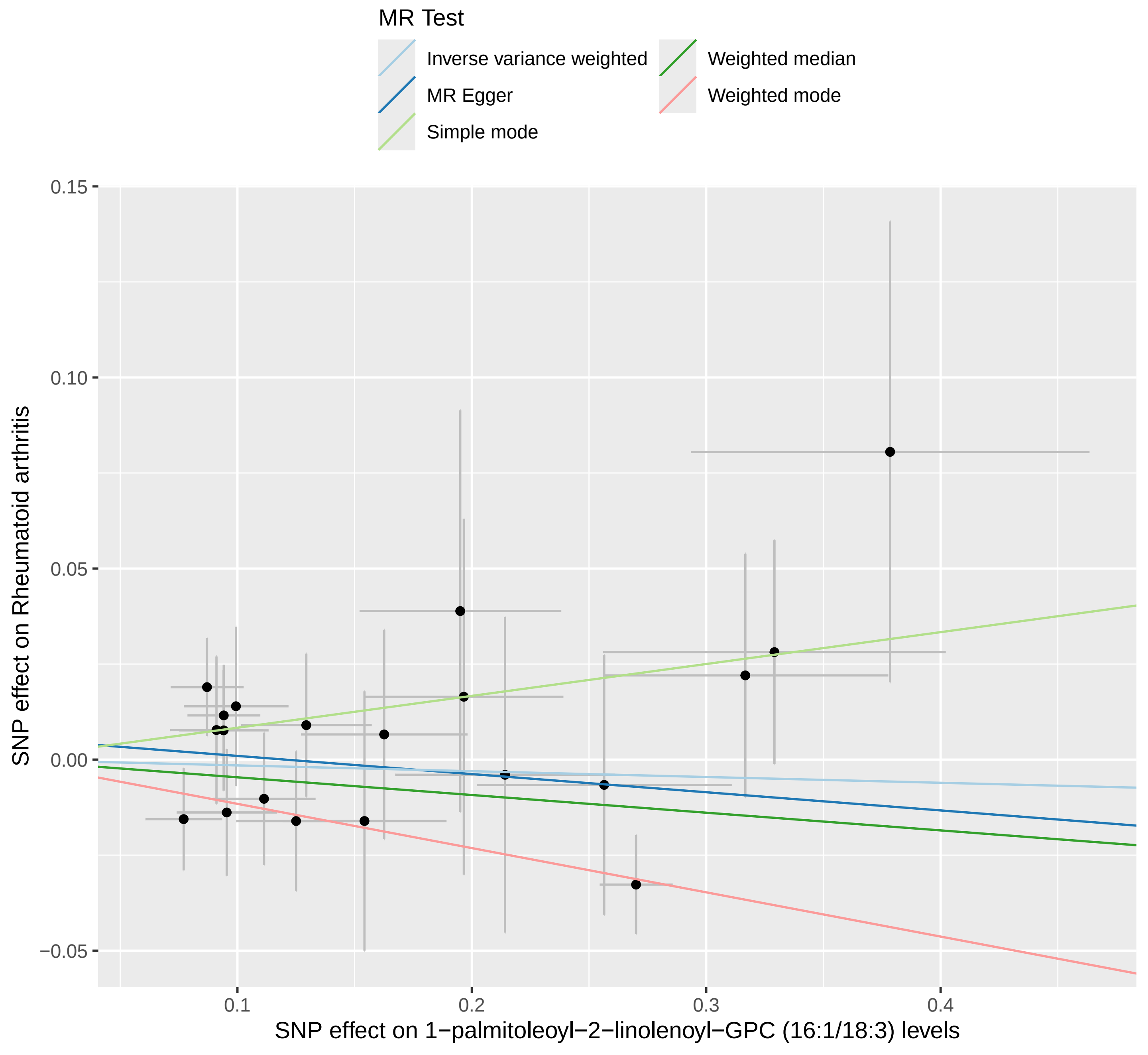

MR Test
Inverse variance weighted
MR Egger
Simple mode
Weighted median
Weighted mode
0.15
0.10
0.05
0.00
−0.05
0.1
0.2
0.3
0.4
SNP effect on Rheumatoid arthritis
SNP effect on 1−palmitoleoyl−2−linolenoyl−GPC (16:1/18:3) levels

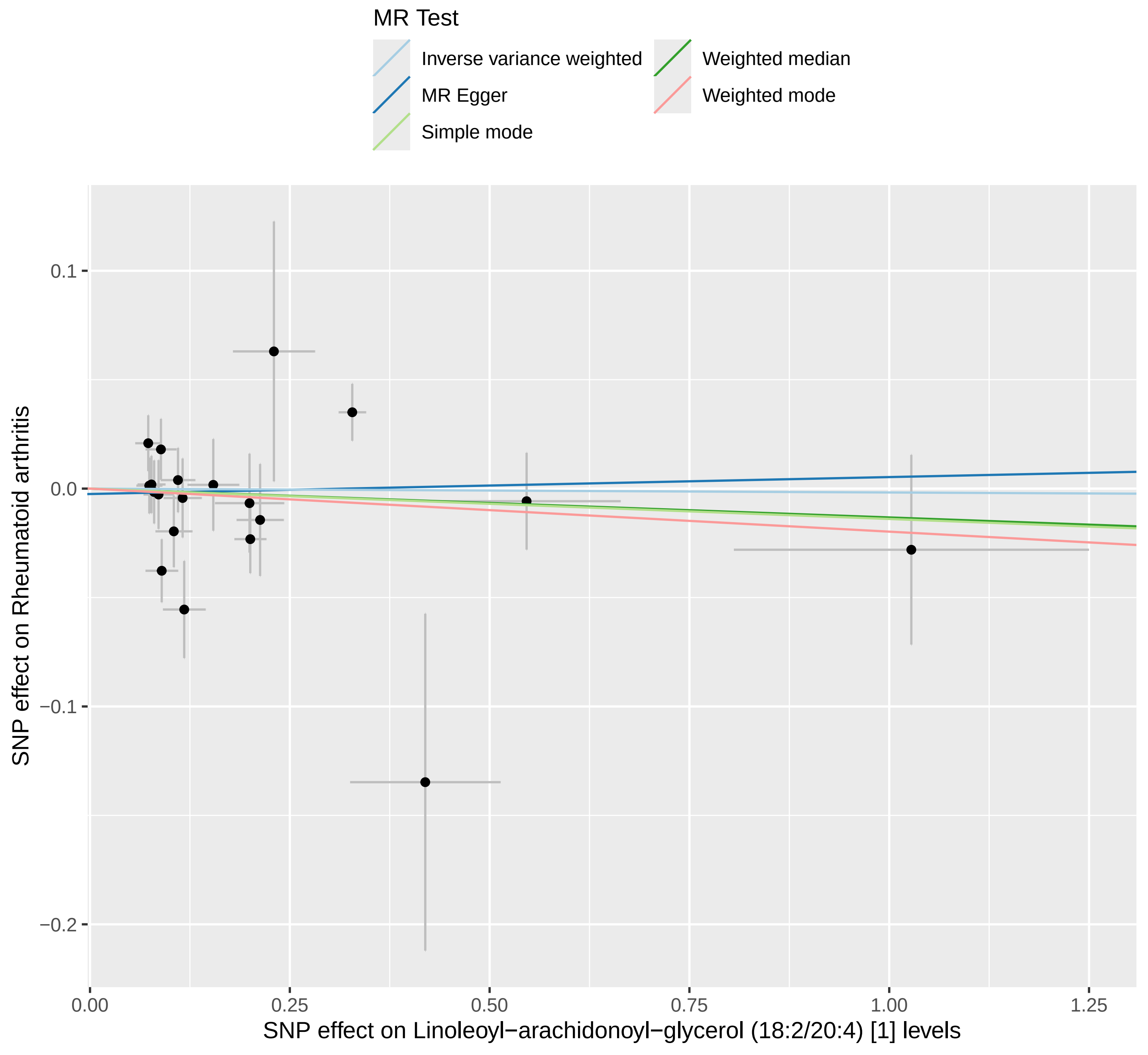

MR Test
Inverse variance weighted
MR Egger
Simple mode
Weighted median
Weighted mode
0.1
0.0
−0.1
−0.2
0.00
0.25
0.50
0.75
1.00
1.25
SNP effect on Rheumatoid arthritis
SNP effect on Linoleoyl−arachidonoyl−glycerol (18:2/20:4) [1] levels

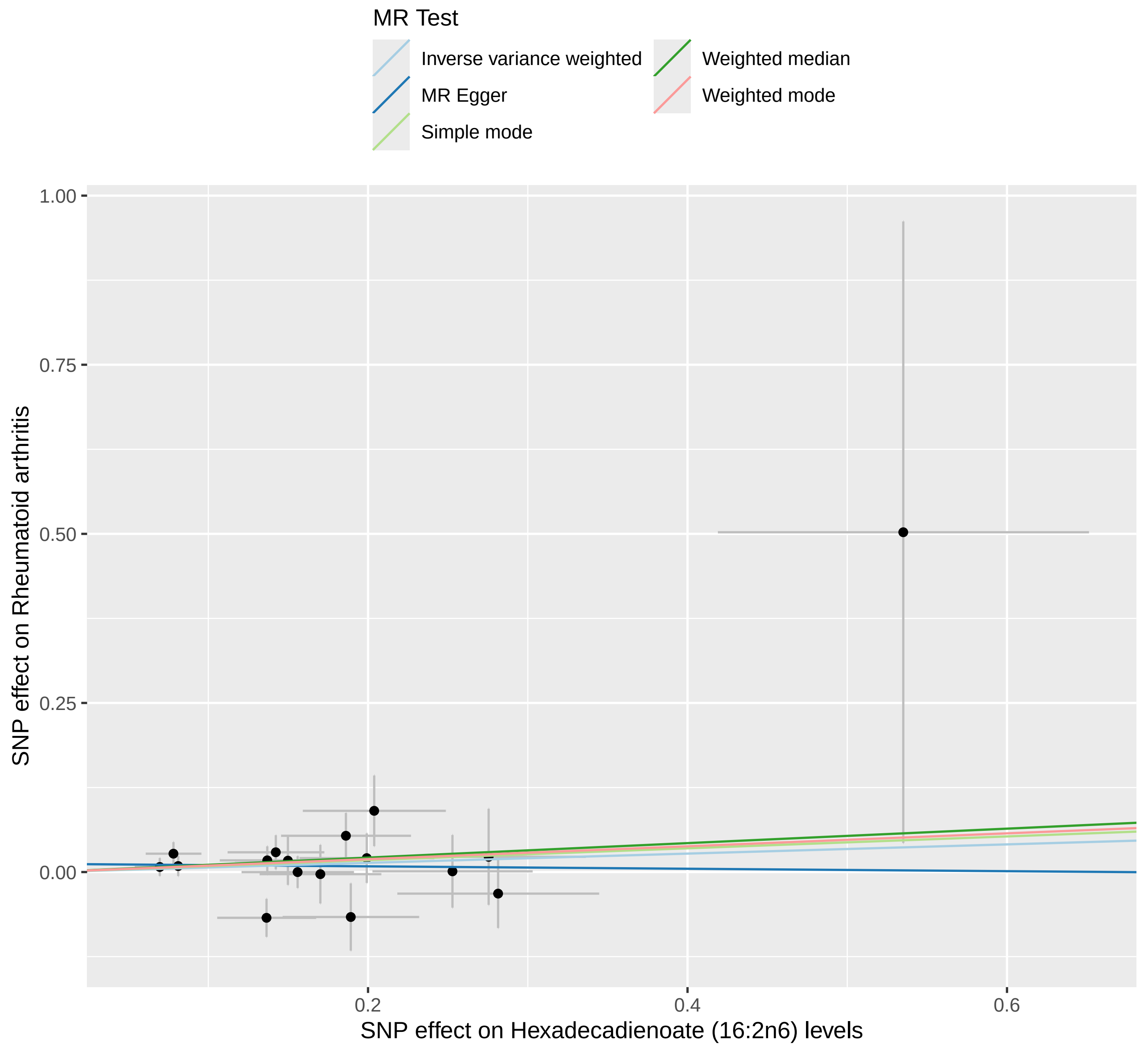

MR Test
Inverse variance weighted
MR Egger
Simple mode
Weighted median
Weighted mode
1.00
0.75
0.50
0.25
0.00
SNP effect on Rheumatoid arthritis
0.2
0.4
0.6
SNP effect on Hexadecadienoate (16:2n6) levels

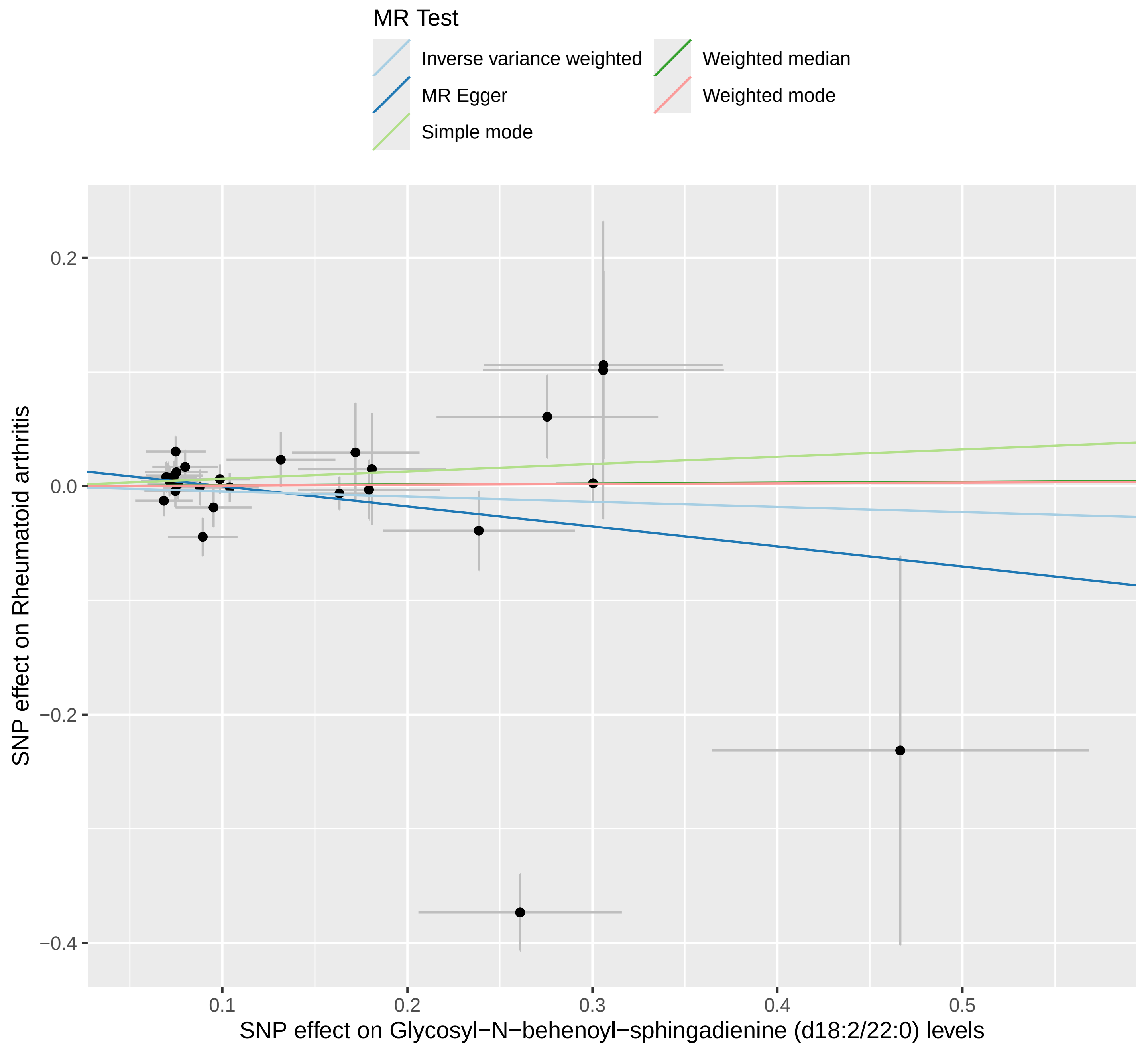

MR Test
Inverse variance weighted
MR Egger
Simple mode
Weighted median
Weighted mode
0.2
0.0
−0.2
−0.4
0.1
0.2
0.3
0.4
0.5
SNP effect on Rheumatoid arthritis
SNP effect on Glycosyl−N−behenoyl−sphingadienine (d18:2/22:0) levels

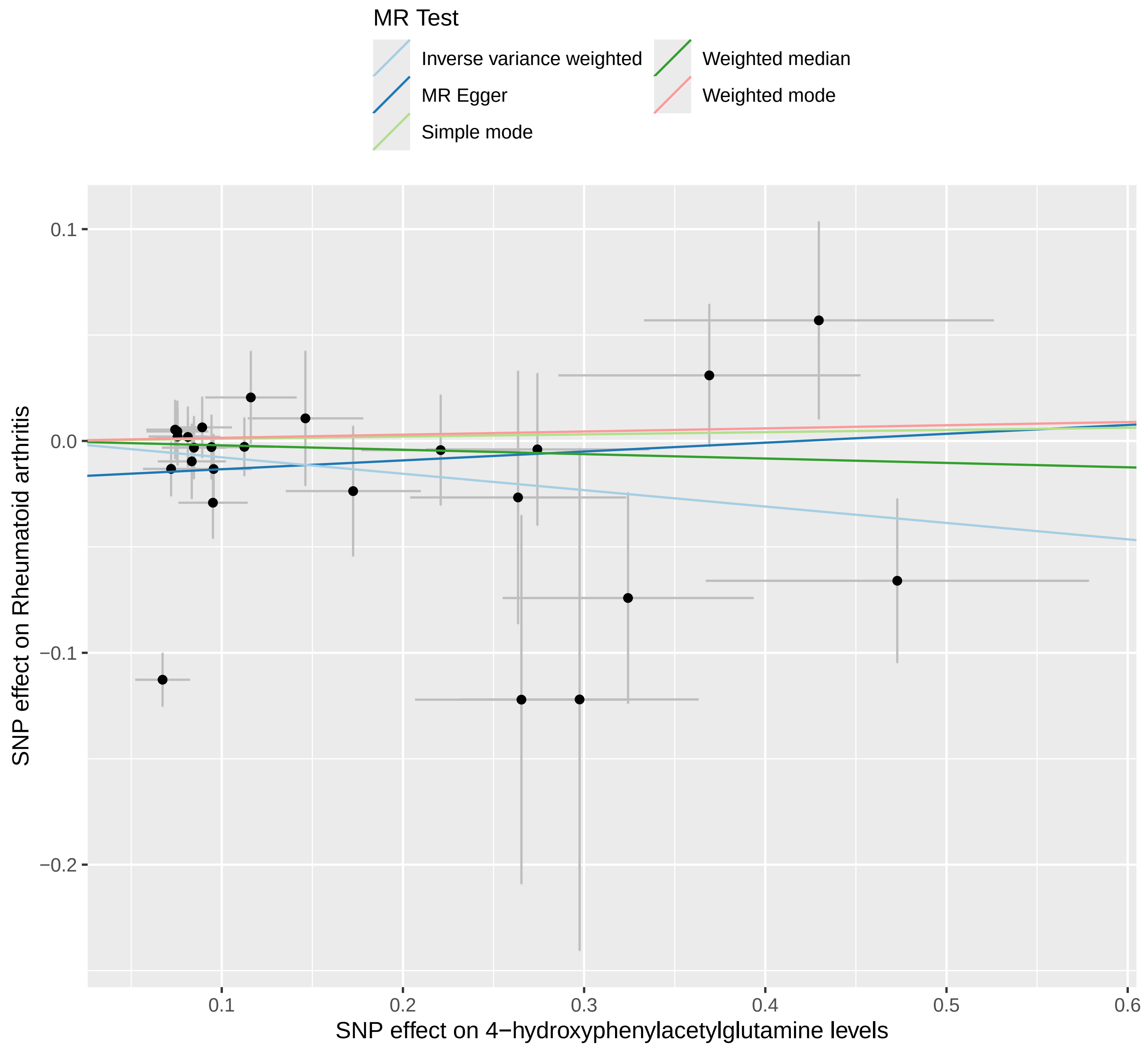
MR Test
Inverse variance weighted
MR Egger
Simple mode
Weighted median
Weighted mode
0.1
0.0
−0.1
−0.2
0.1
0.2
0.3
0.4
0.5
0.6
SNP effect on Rheumatoid arthritis
SNP effect on 4−hydroxyphenylacetylglutamine levels

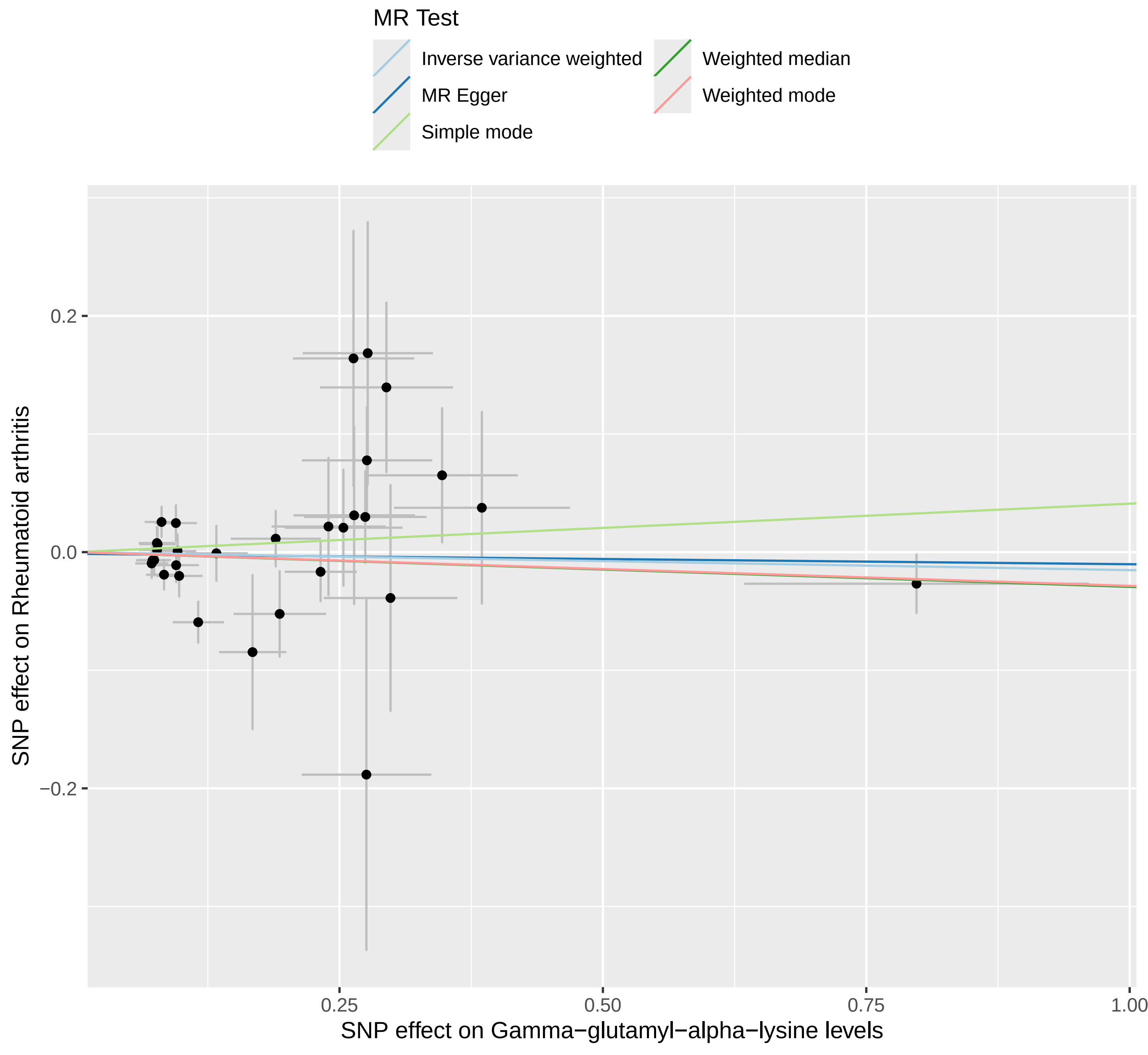
MR Test
Inverse variance weighted
MR Egger
Simple mode
Weighted median
Weighted mode
0.2
0.0
−0.2
0.25
0.50
0.75
1.00
SNP effect on Rheumatoid arthritis
SNP effect on Gamma−glutamyl−alpha−lysine levels

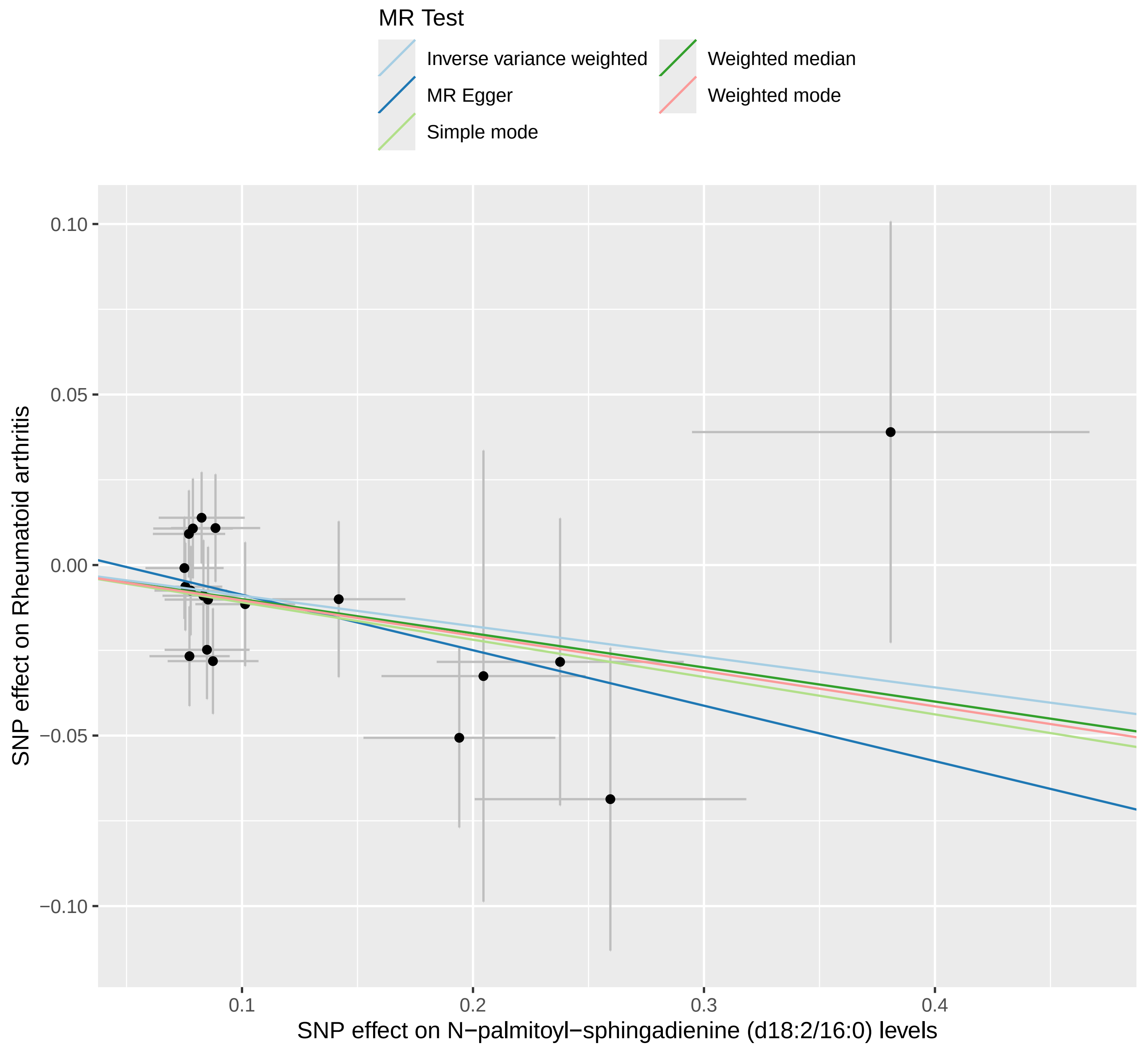
MR Test
Inverse variance weighted
MR Egger
Simple mode
Weighted median
Weighted mode
0.10
0.05
0.00
−0.05
−0.10
SNP effect on Rheumatoid arthritis
0.1
0.2
0.3
0.4
SNP effect on N−palmitoyl−sphingadienine (d18:2/16:0) levels

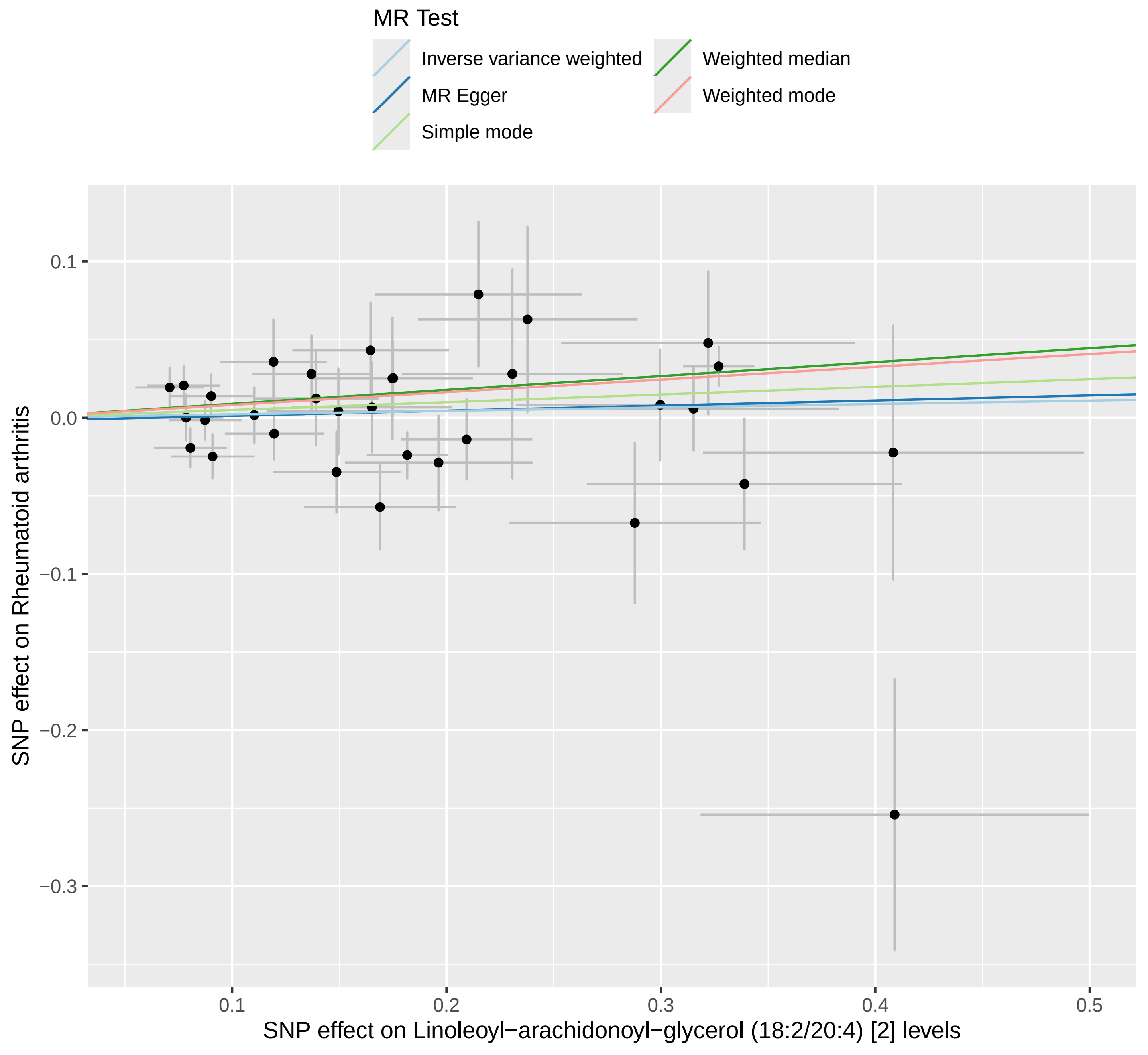
MR Test
Inverse variance weighted
MR Egger
Simple mode
Weighted median
Weighted mode
0.1
0.0
−0.1
−0.2
−0.3
SNP effect on Rheumatoid arthritis
0.1
0.2
0.3
0.4
0.5
SNP effect on Linoleoyl−arachidonoyl−glycerol (18:2/20:4) [2] levels

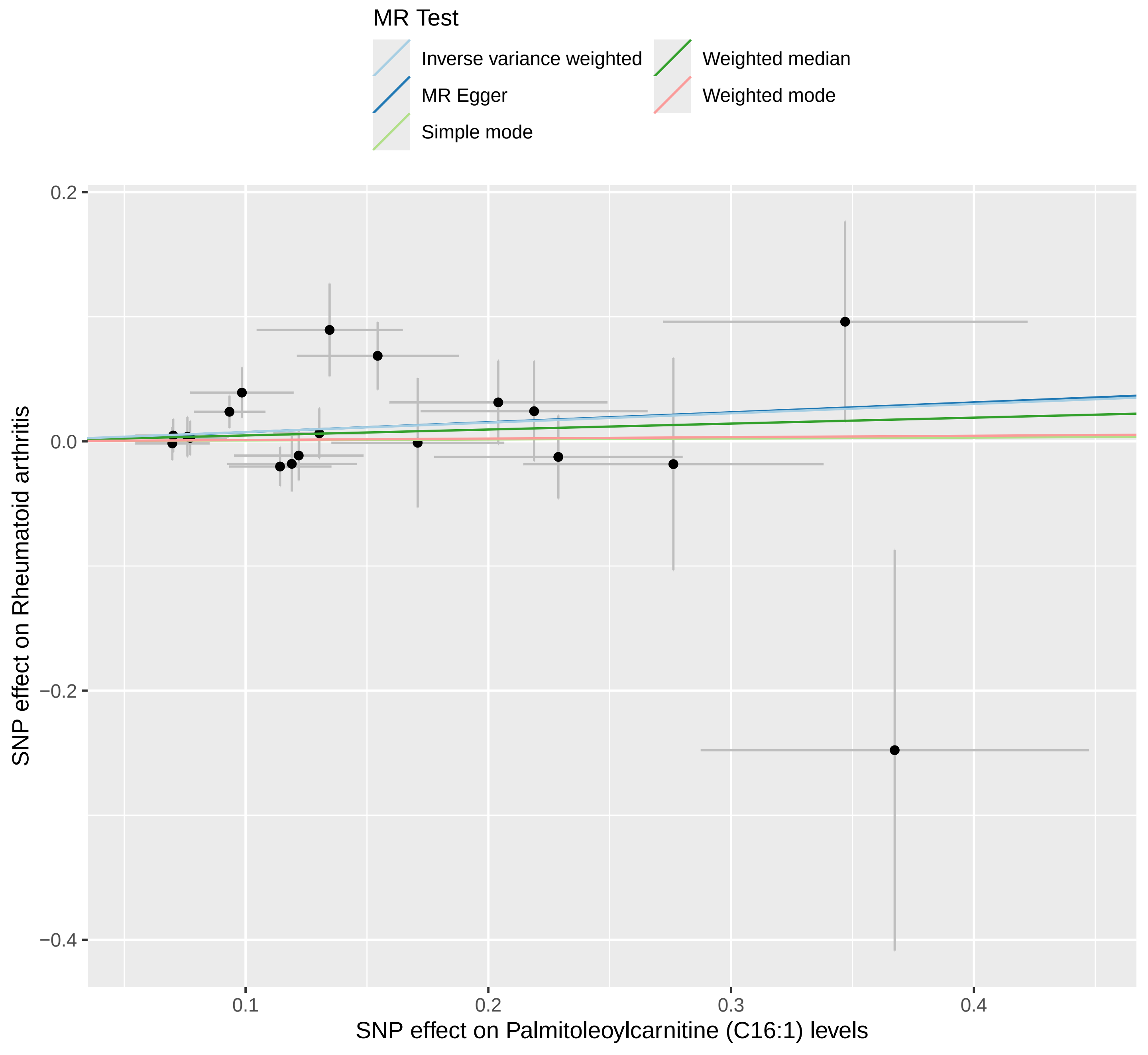
MR Test
Inverse variance weighted
MR Egger
Simple mode
Weighted median
Weighted mode
0.2
0.0
−0.2
−0.4
0.1
0.2
0.3
0.4
SNP effect on Rheumatoid arthritis
SNP effect on Palmitoleoylcarnitine (C16:1) levels

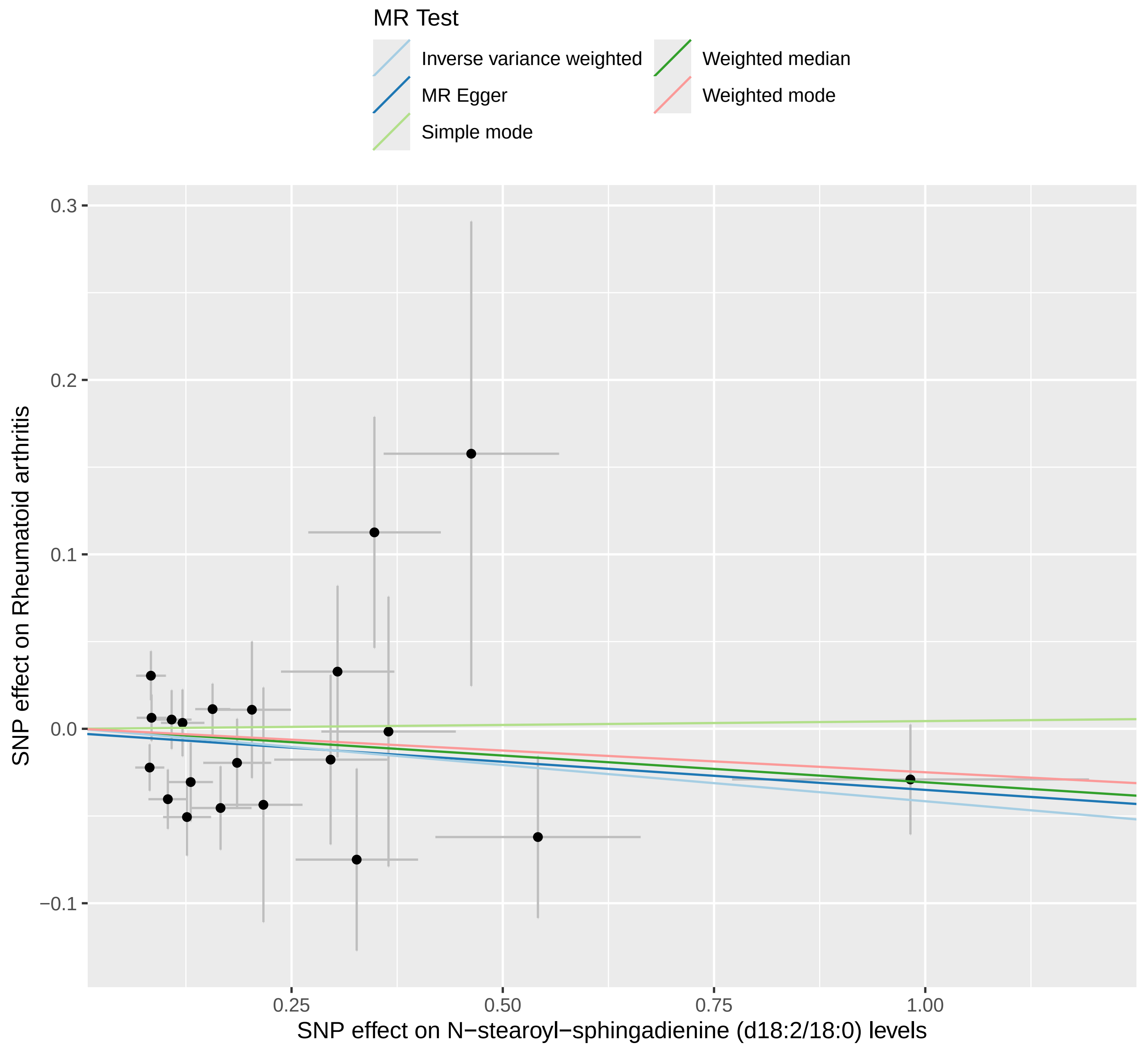
MR Test
Inverse variance weighted
MR Egger
Simple mode
Weighted median
Weighted mode
0.3
0.2
0.1
0.0
−0.1
SNP effect on Rheumatoid arthritis
0.25
0.50
0.75
1.00
SNP effect on N−stearoyl−sphingadienine (d18:2/18:0) levels

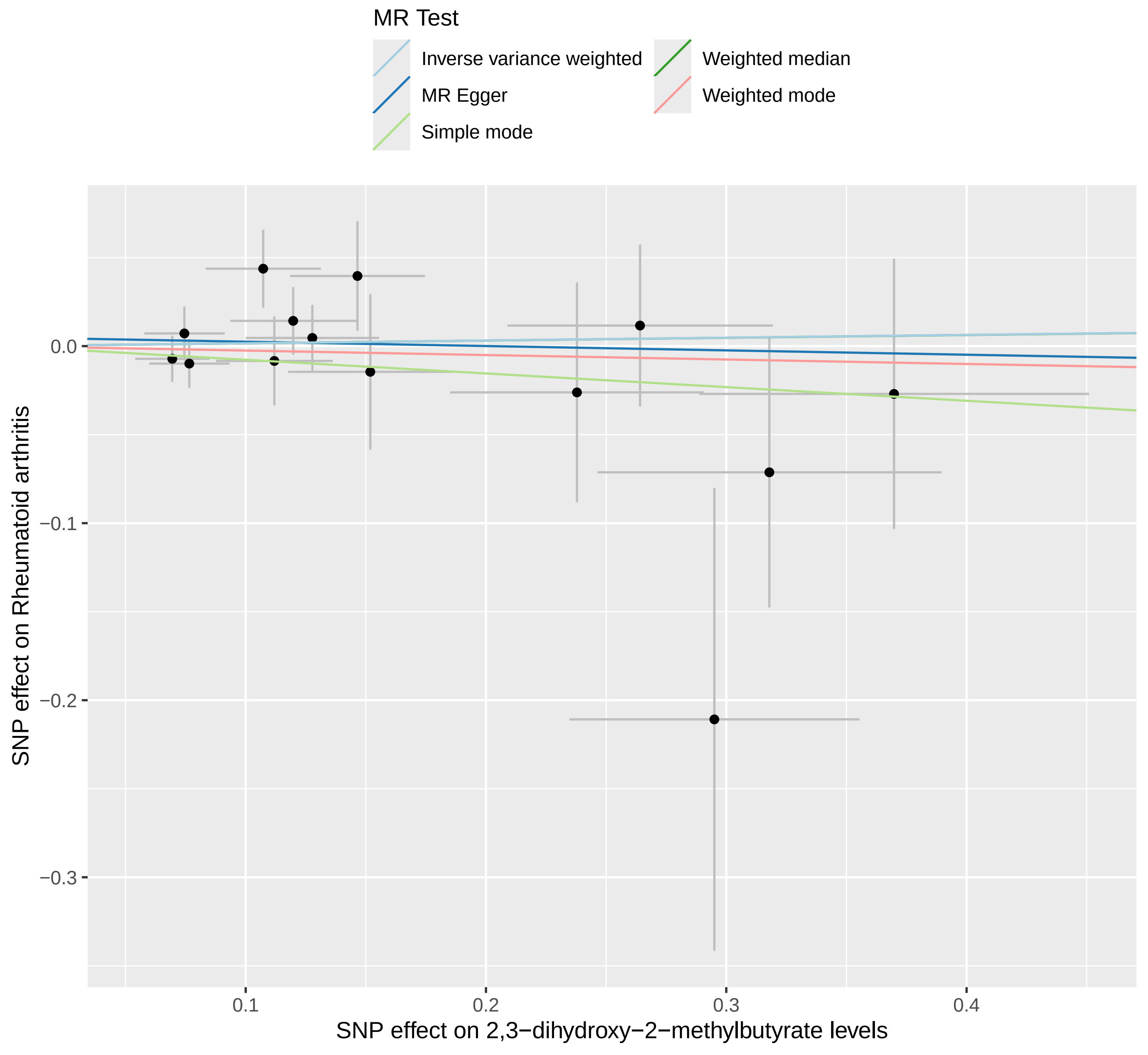
MR Test
Inverse variance weighted
MR Egger
Simple mode
Weighted median
Weighted mode
SNP effect on Rheumatoid arthritis
0.0
−0.1
−0.2
−0.3
0.1
0.2
0.3
0.4
SNP effect on 2,3−dihydroxy−2−methylbutyrate levels

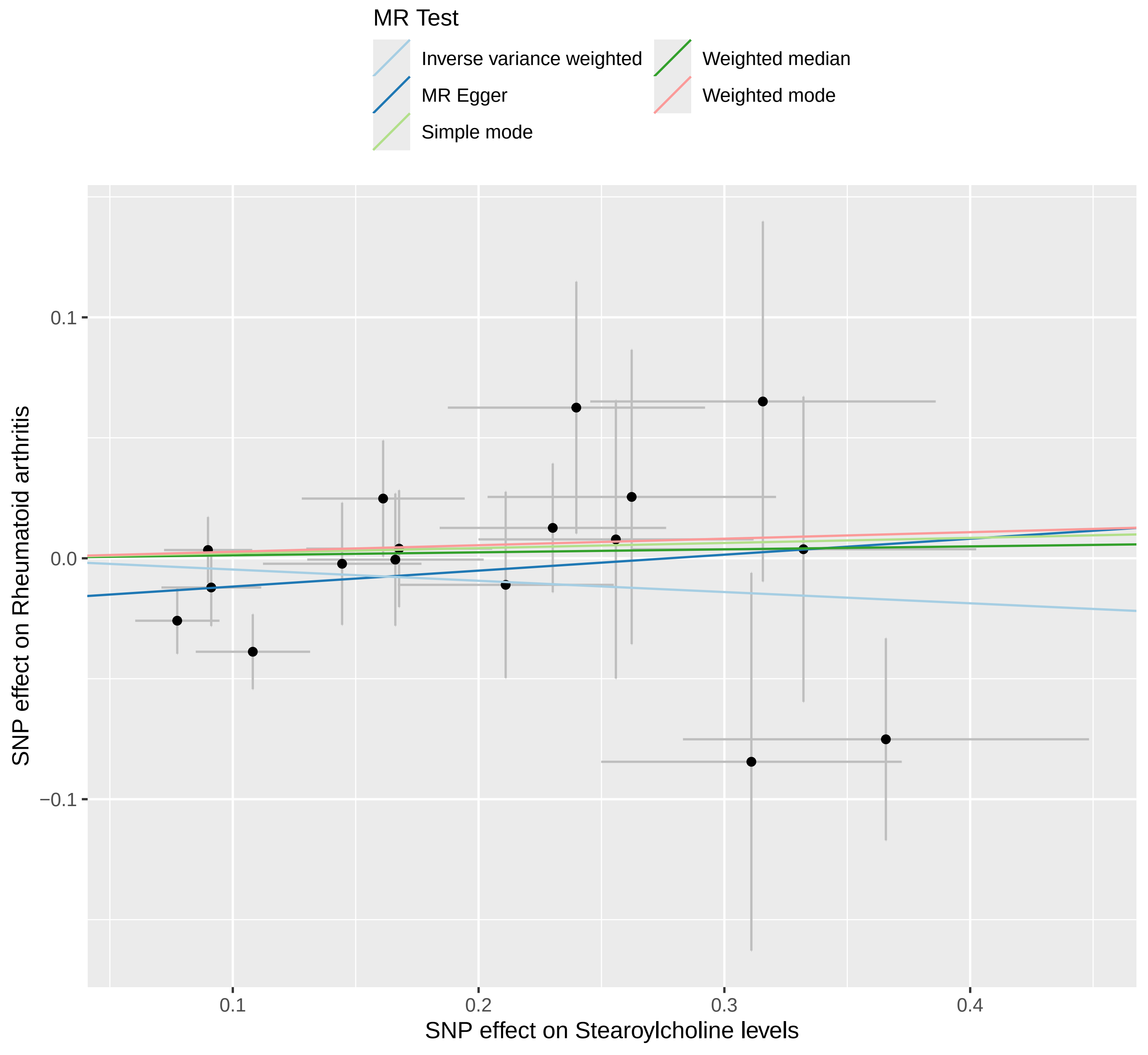
MR Test
Inverse variance weighted
MR Egger
Simple mode
Weighted median
Weighted mode
SNP effect on Rheumatoid arthritis
SNP effect on Stearoylcholine levels
0.1
0.0
−0.1
0.1
0.2
0.3
0.4

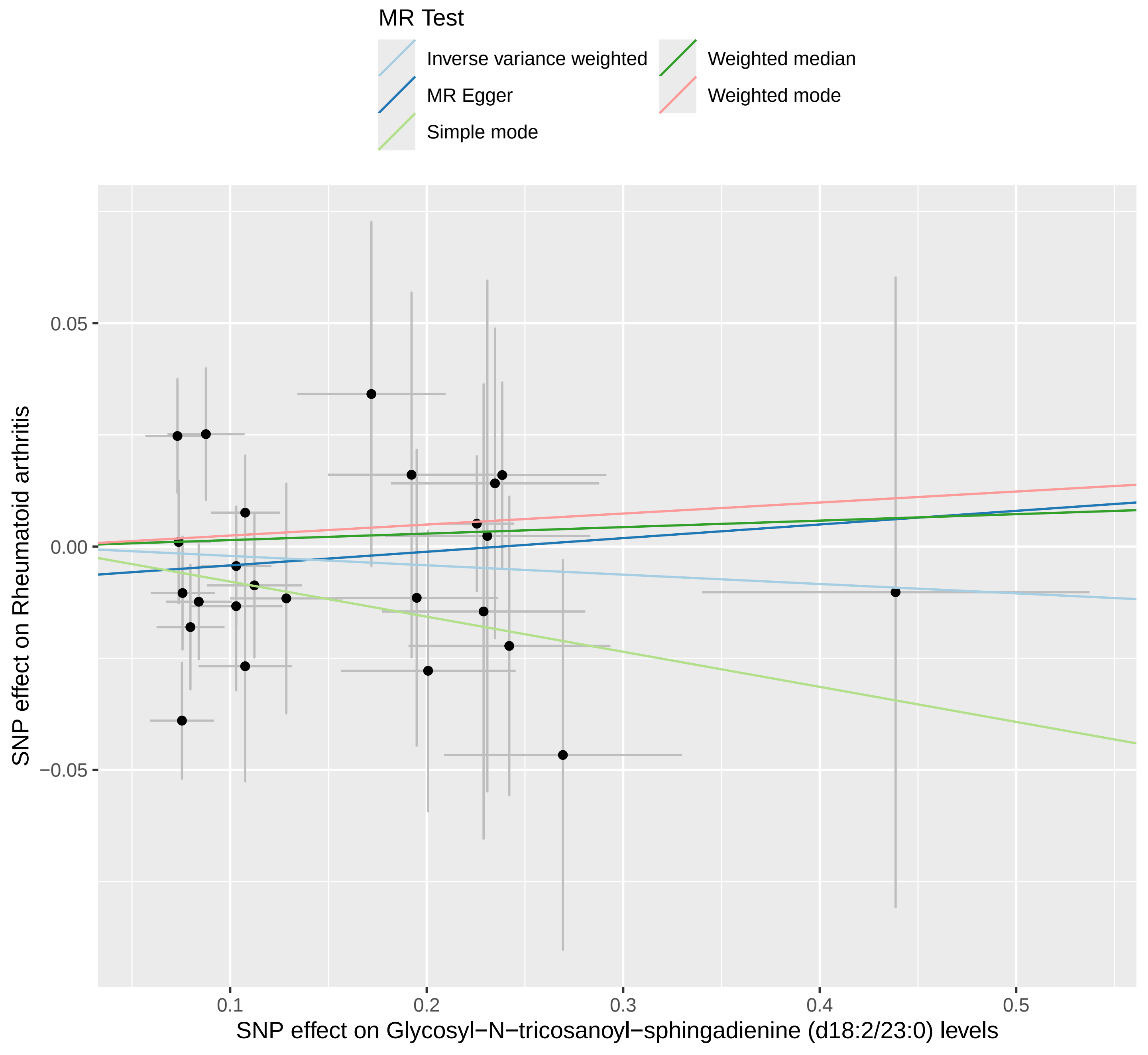
MR Test
Inverse variance weighted
MR Egger
Simple mode
Weighted median
Weighted mode
0.05
0.00
−0.05
0.1
0.2
0.3
0.4
0.5
SNP effect on Rheumatoid arthritis
SNP effect on Glycosyl−N−tricosanoyl−sphingadienine (d18:2/23:0) levels

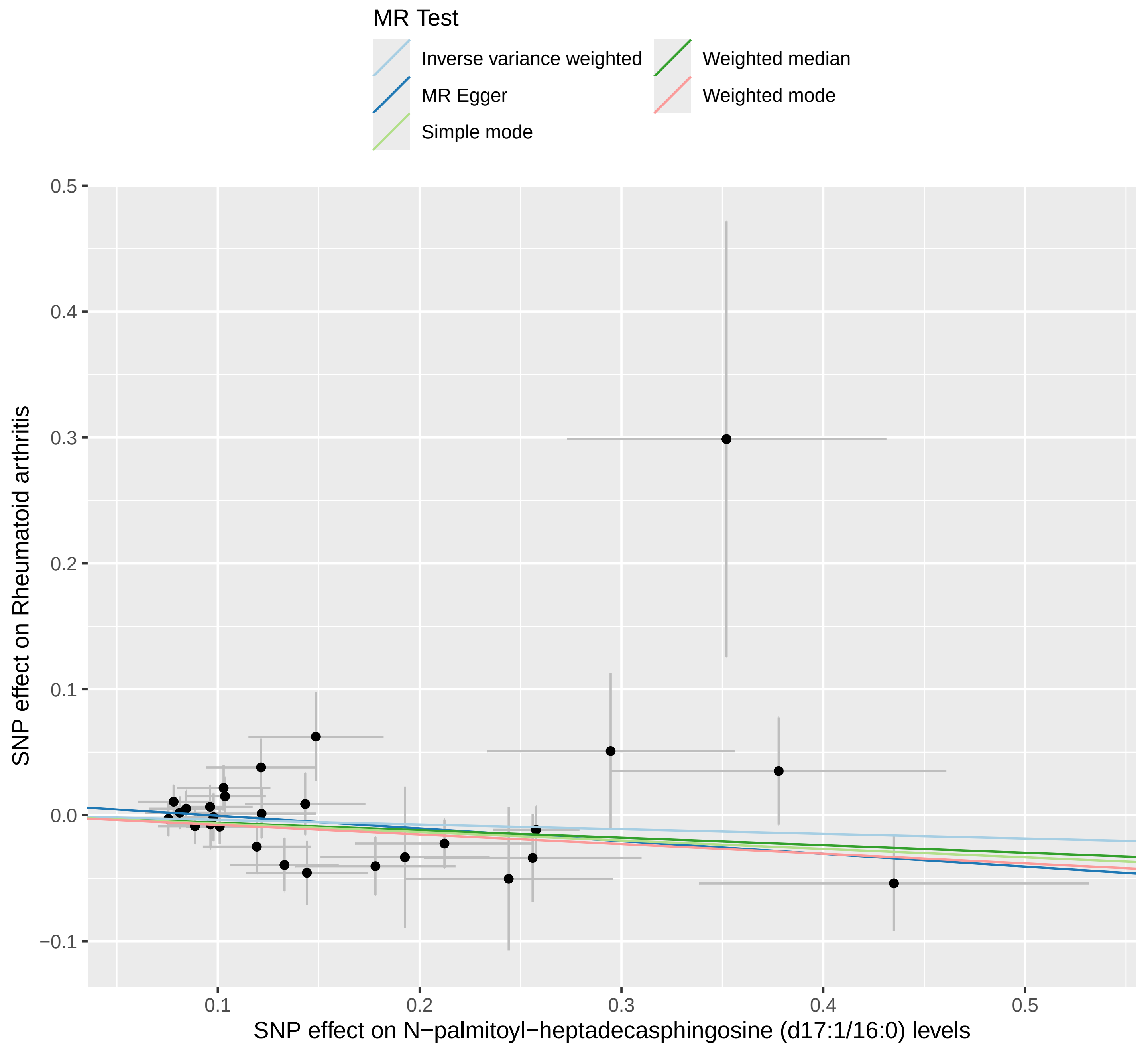
MR Test
Inverse variance weighted
MR Egger
Simple mode
Weighted median
Weighted mode
0.5
0.4
0.3
0.2
0.1
0.0
−0.1
0.1
0.2
0.3
0.4
0.5
SNP effect on Rheumatoid arthritis
SNP effect on N−palmitoyl−heptadecasphingosine (d17:1/16:0) levels

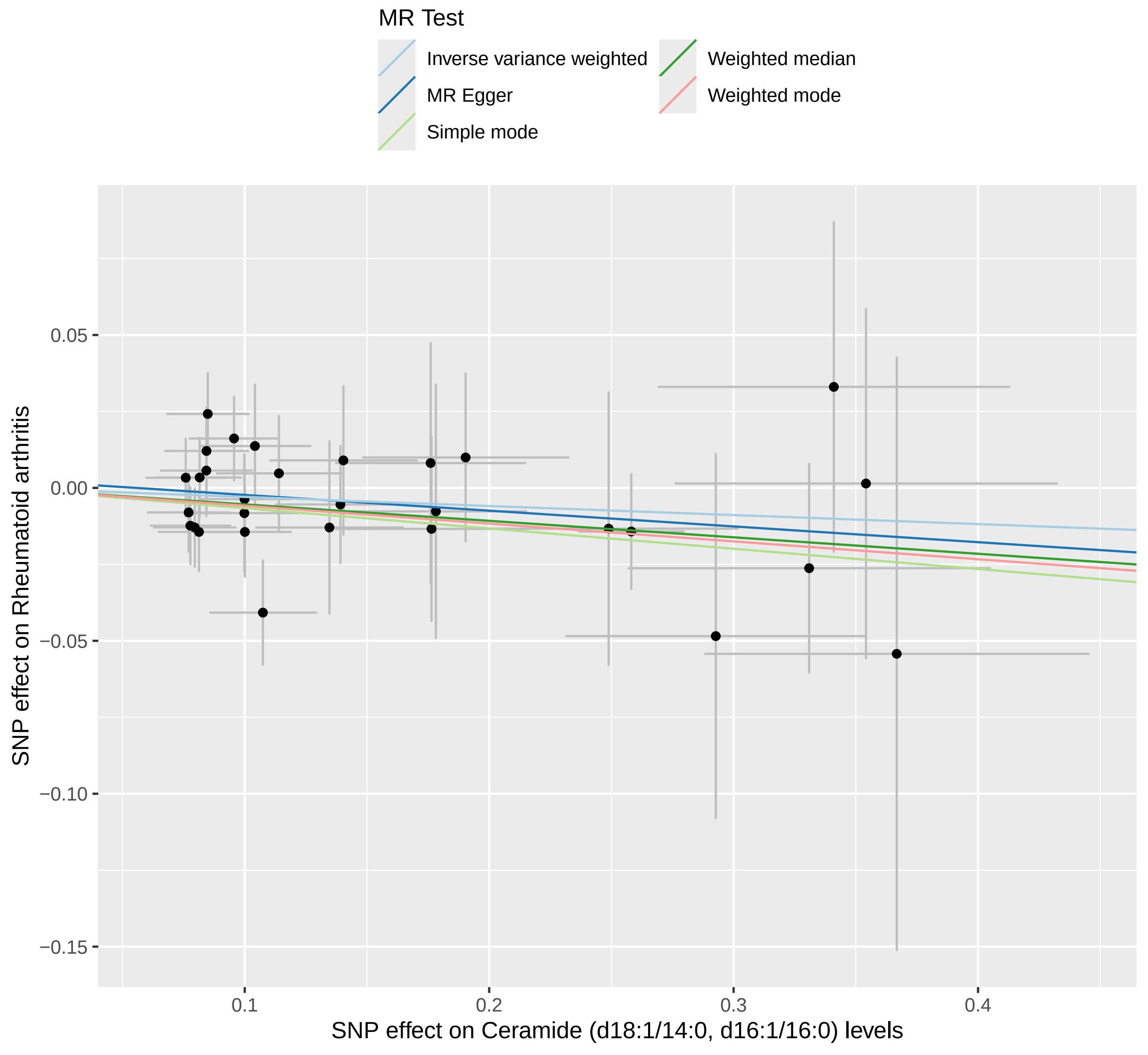

MR Test
Inverse variance weighted
MR Egger
Simple mode
Weighted median
Weighted mode
0.05
0.00
−0.05
−0.10
−0.15
0.1
0.2
0.3
0.4
SNP effect on Rheumatoid arthritis
SNP effect on Ceramide (d18:1/14:0, d16:1/16:0) levels

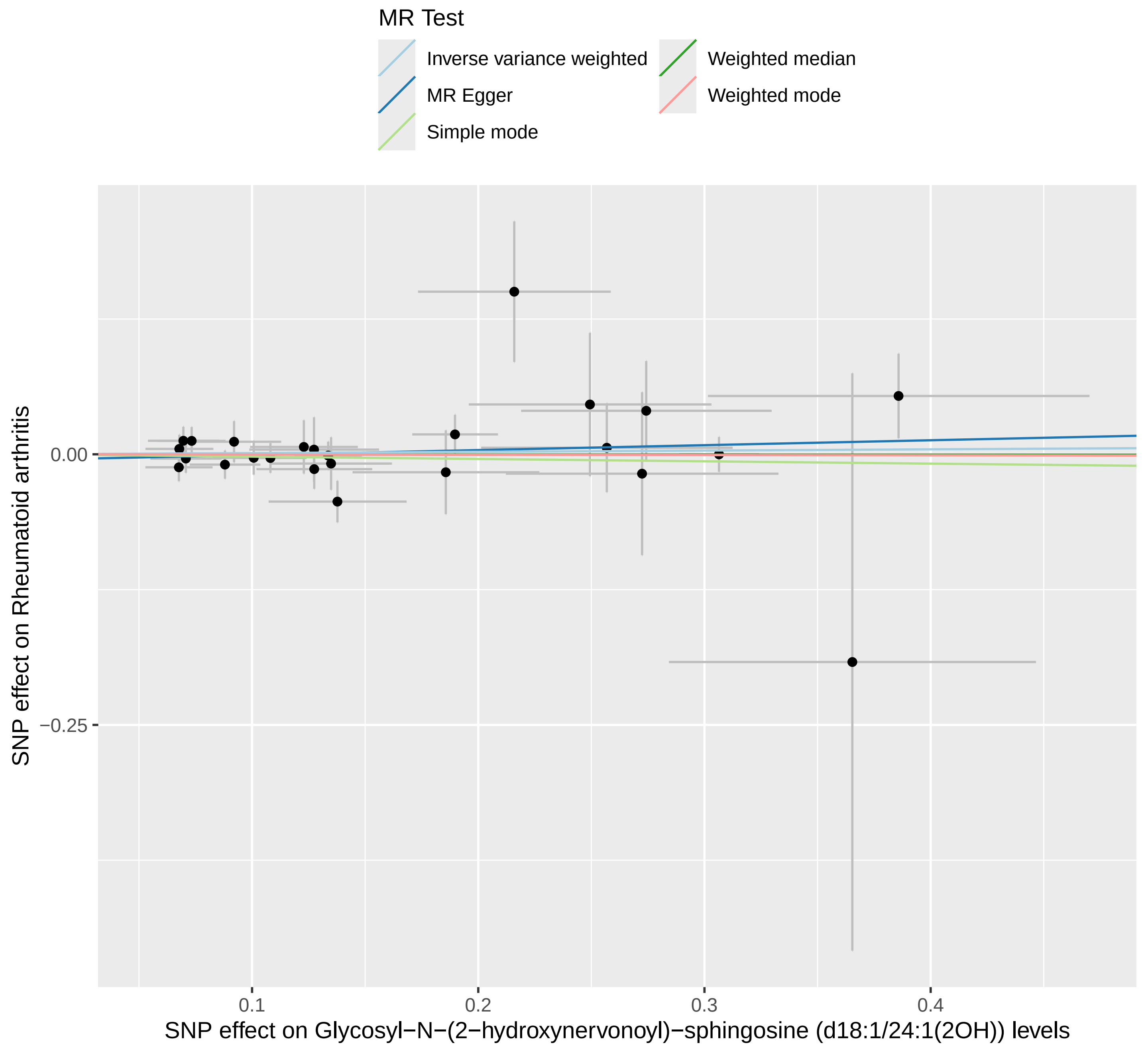
MR Test
Inverse variance weighted
MR Egger
Simple mode
Weighted median
Weighted mode
0.00
−0.25
0.1
0.2
0.3
0.4
SNP effect on Rheumatoid arthritis
SNP effect on Glycosyl−N−(2−hydroxynervonoyl)−sphingosine (d18:1/24:1(2OH)) levels

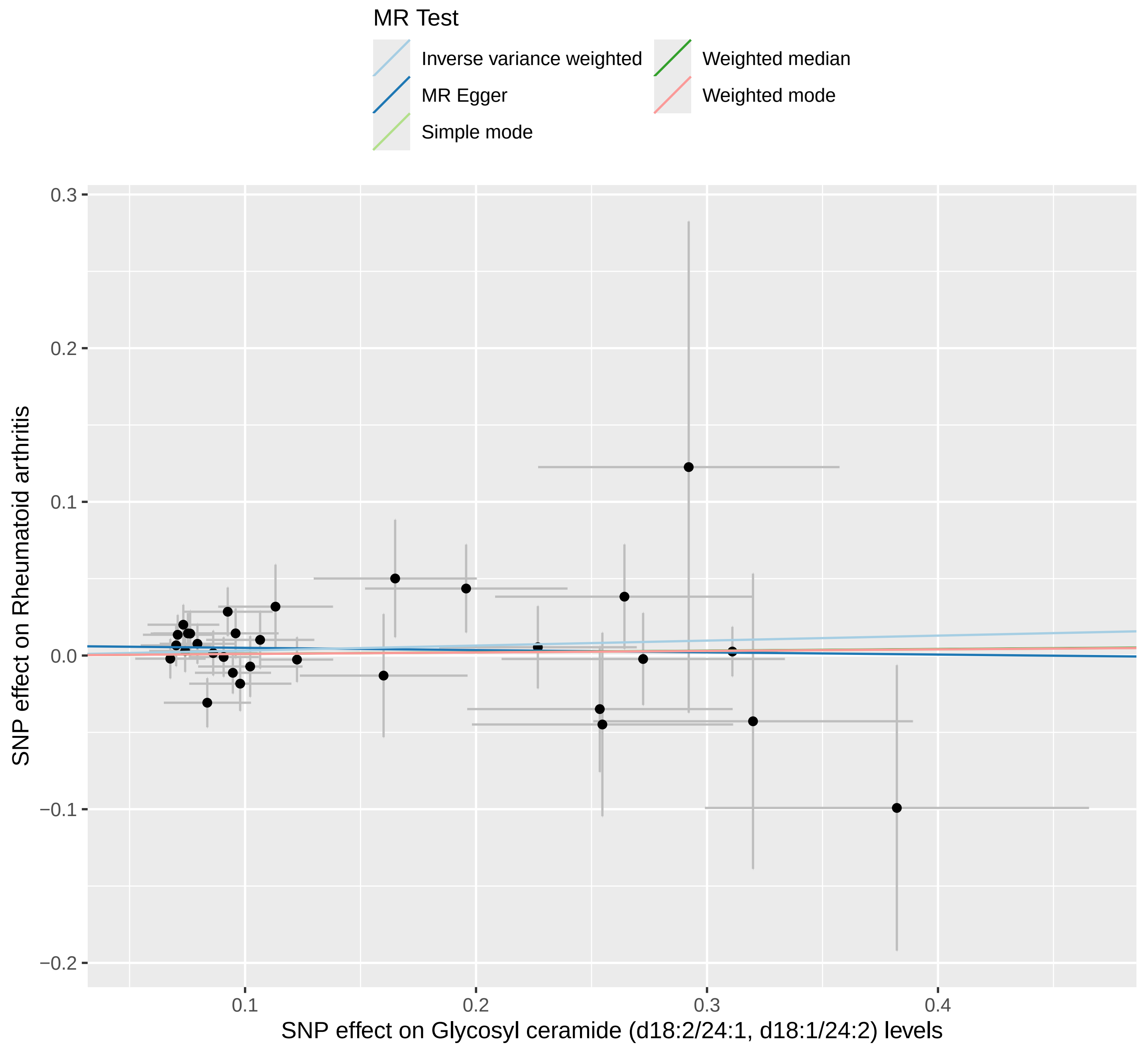

MR Test
Inverse variance weighted
MR Egger
Simple mode
Weighted median
Weighted mode
0.3
0.2
0.1
0.0
−0.1
−0.2
SNP effect on Rheumatoid arthritis
0.1
0.2
0.3
0.4
SNP effect on Glycosyl ceramide (d18:2/24:1, d18:1/24:2) levels

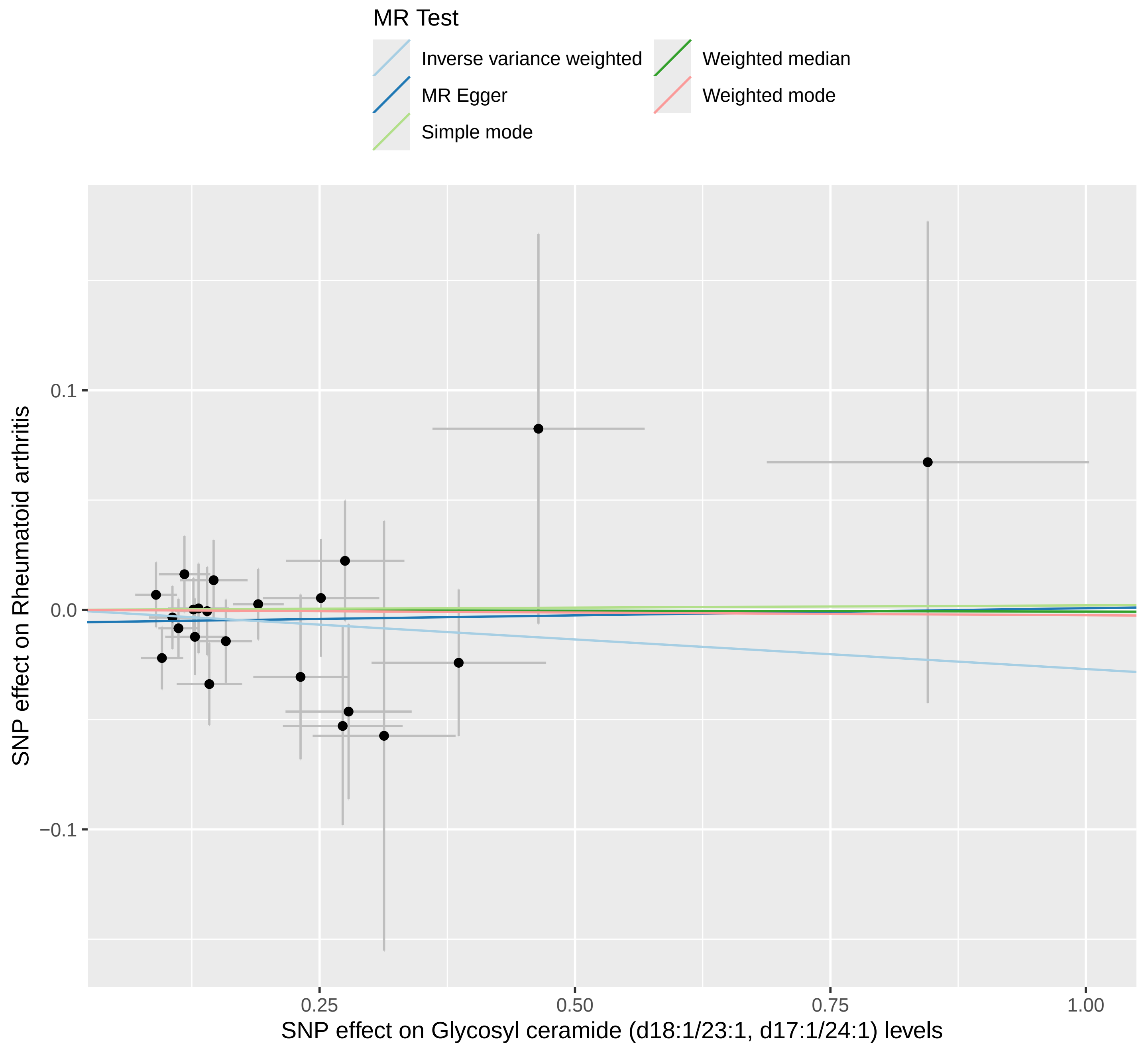
MR Test
Inverse variance weighted
MR Egger
Simple mode
Weighted median
Weighted mode
SNP effect on Rheumatoid arthritis
0.1
0.0
−0.1
0.25
0.50
0.75
1.00
SNP effect on Glycosyl ceramide (d18:1/23:1, d17:1/24:1) levels

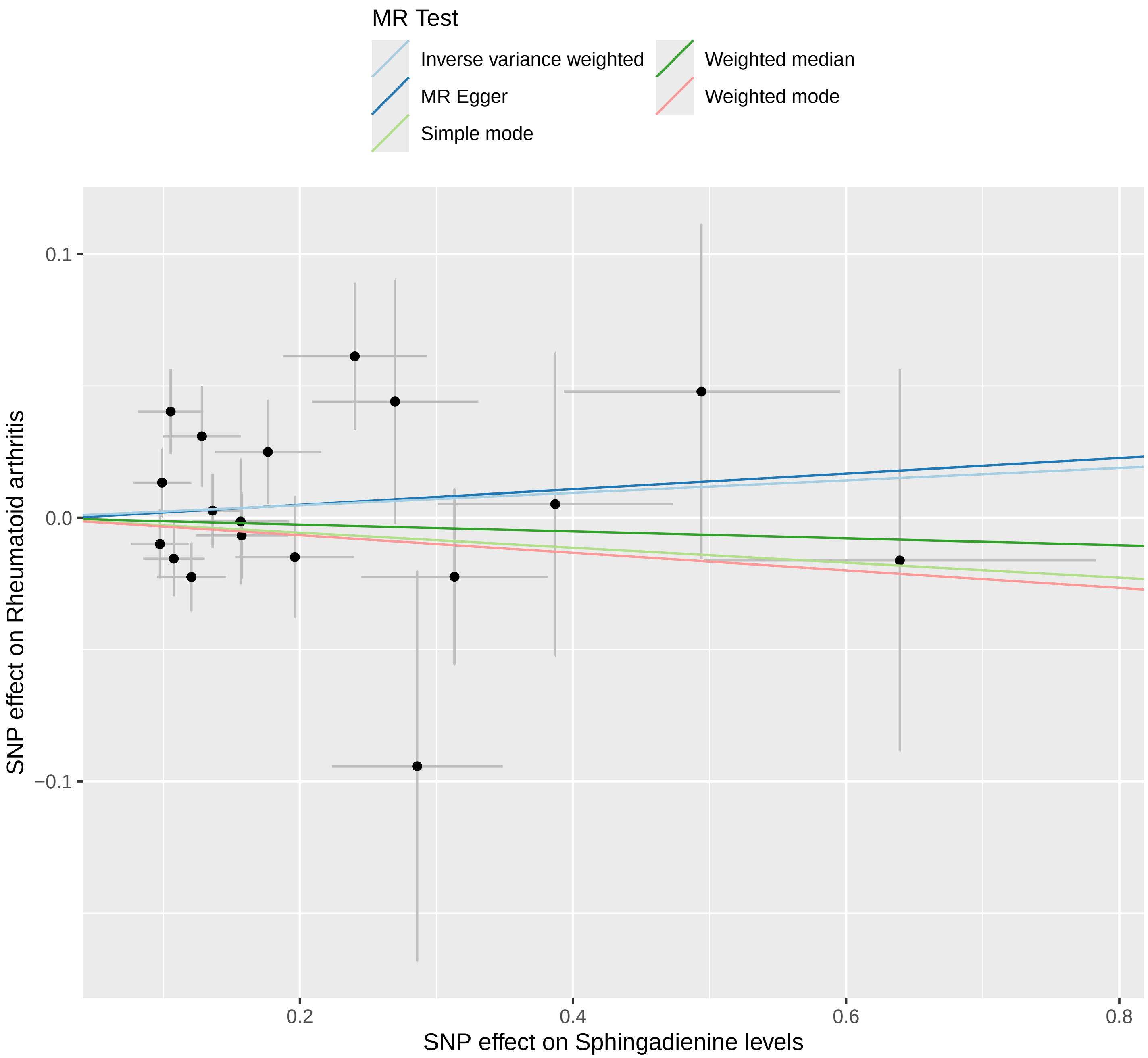
MR Test
Inverse variance weighted
MR Egger
Simple mode
Weighted median
Weighted mode
0.1
0.0
−0.1
0.2
0.4
0.6
0.8
SNP effect on Rheumatoid arthritis
SNP effect on Sphingadienine levels

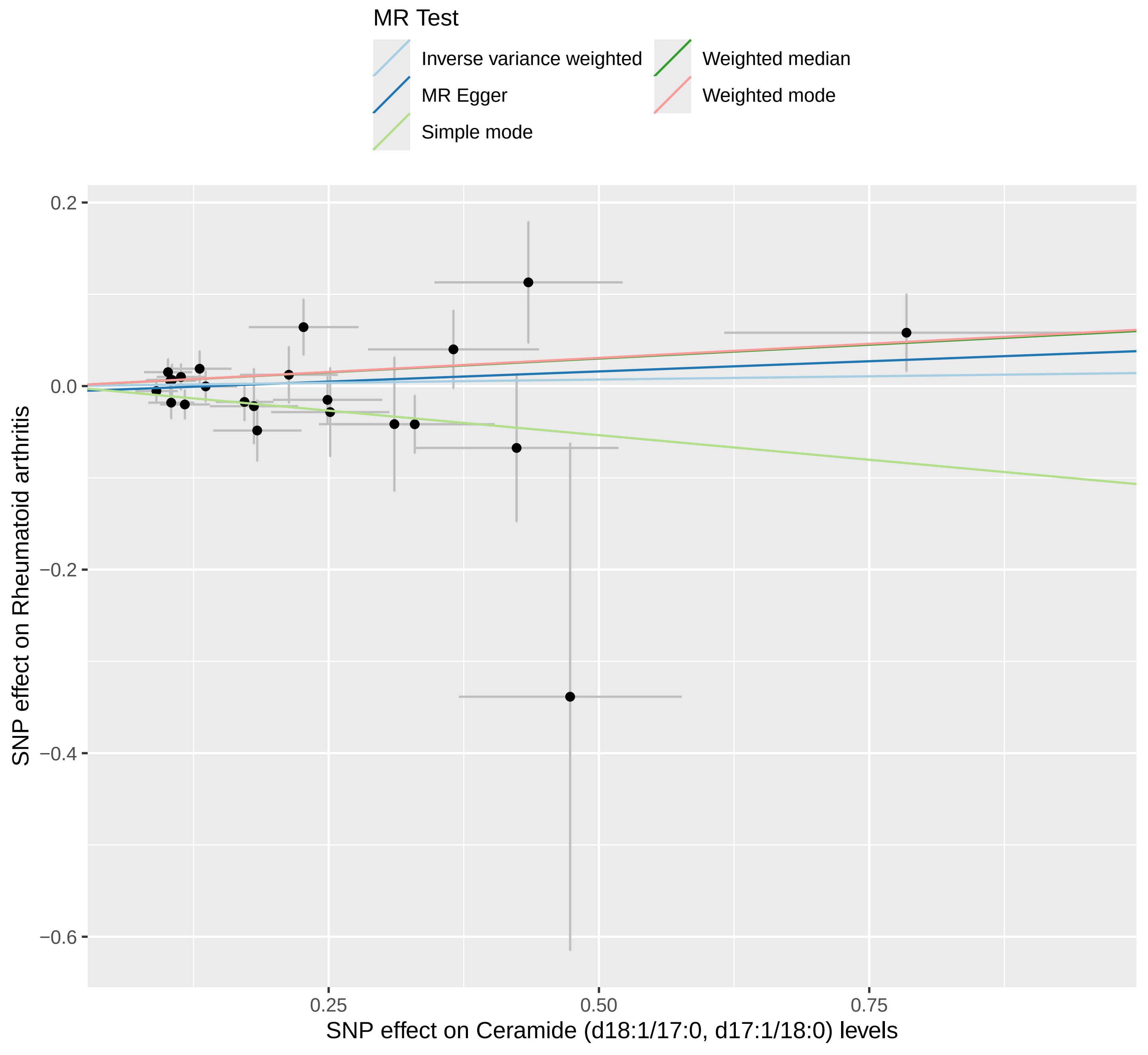

MR Test
Inverse variance weighted
MR Egger
Simple mode
Weighted median
Weighted mode
0.2
0.0
−0.2
−0.4
−0.6
SNP effect on Rheumatoid arthritis
0.25
0.50
0.75
SNP effect on Ceramide (d18:1/17:0, d17:1/18:0) levels

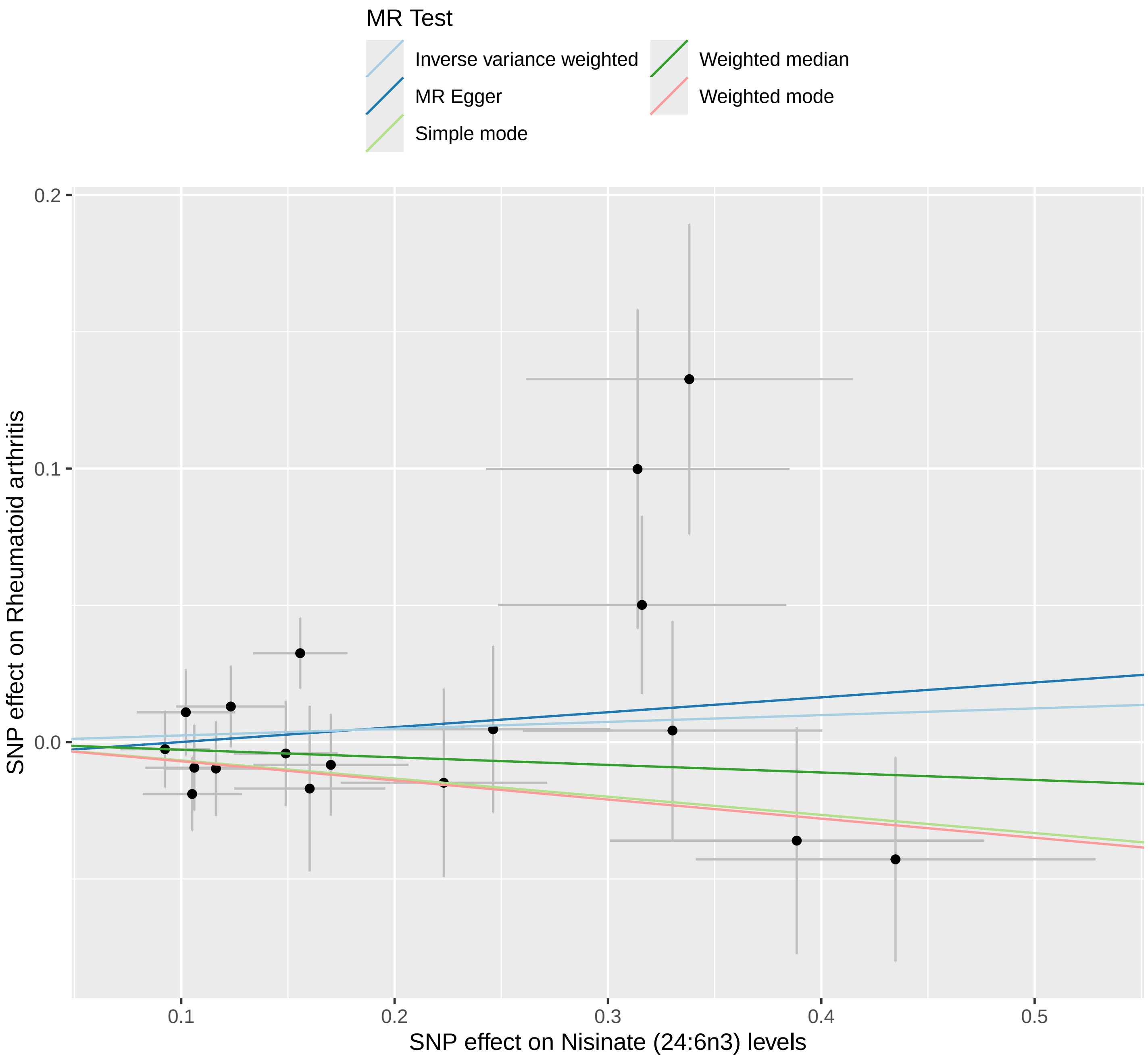
MR Test
Inverse variance weighted
MR Egger
Simple mode
Weighted median
Weighted mode
0.2
0.1
0.0
0.1
0.2
0.3
0.4
0.5
SNP effect on Rheumatoid arthritis
SNP effect on Nisinate (24:6n3) levels

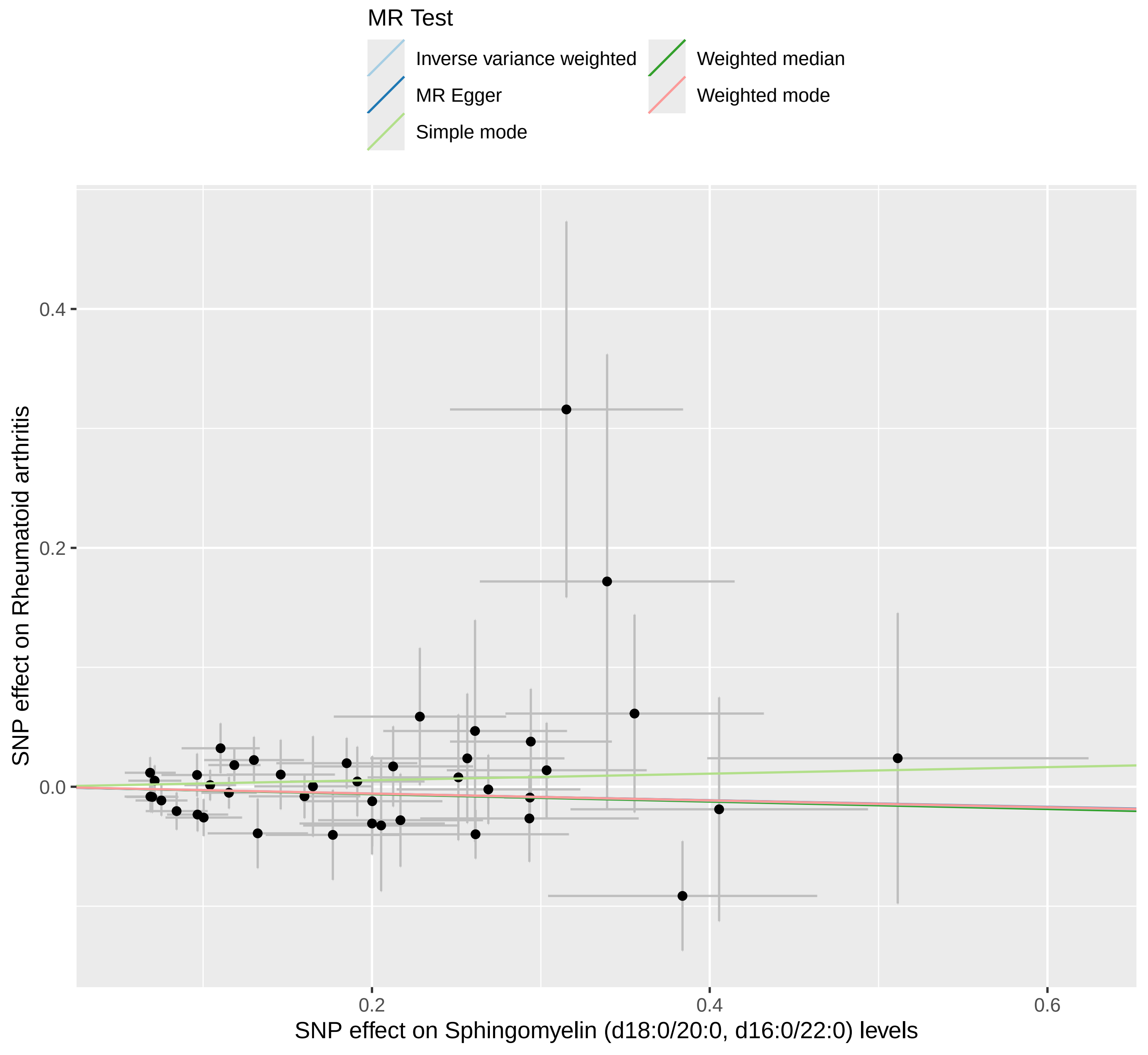
MR Test
Inverse variance weighted
MR Egger
Simple mode
Weighted median
Weighted mode
0.4
0.2
0.0
SNP effect on Rheumatoid arthritis
0.2
0.4
0.6
SNP effect on Sphingomyelin (d18:0/20:0, d16:0/22:0) levels

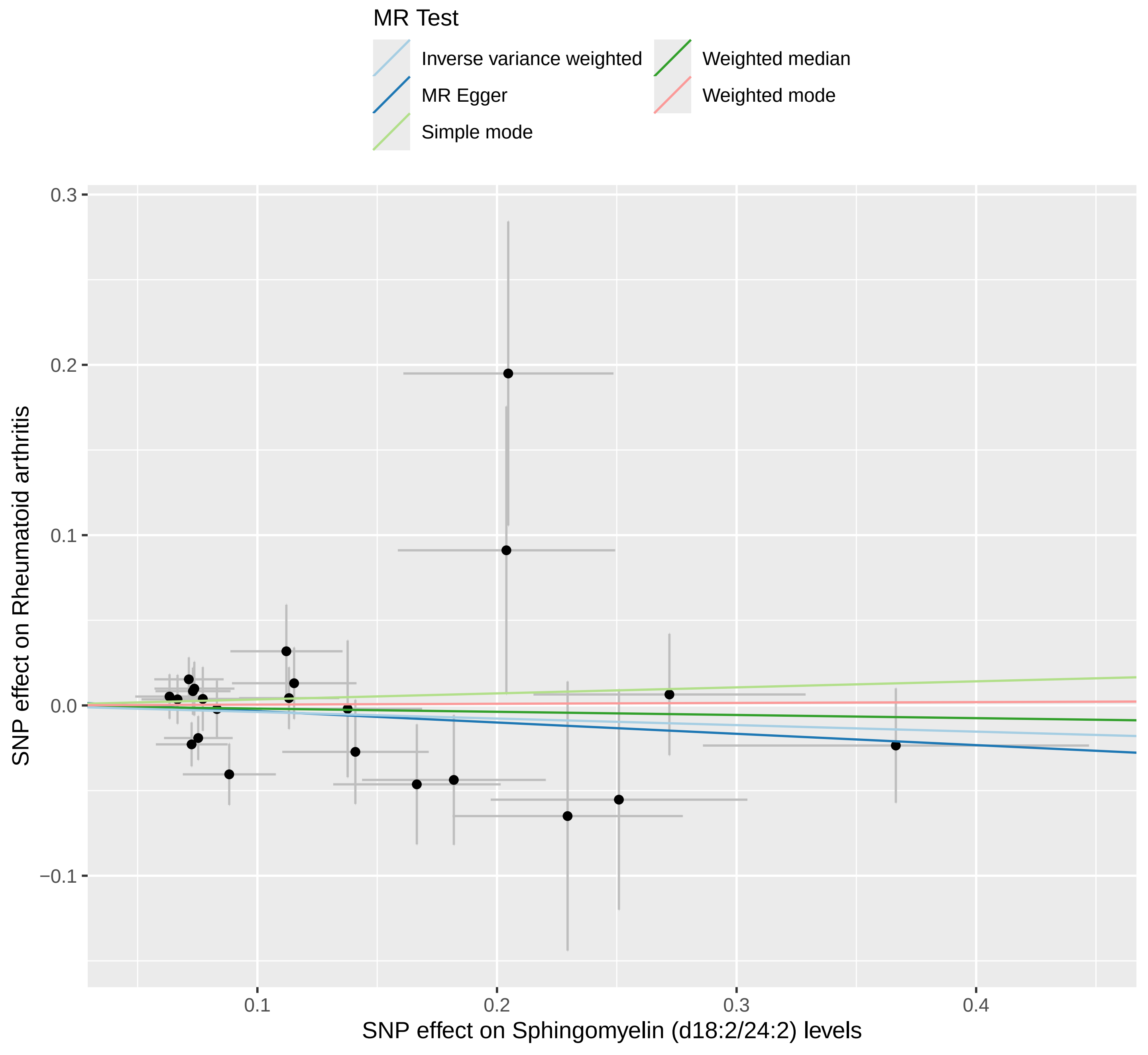

MR Test
Inverse variance weighted
MR Egger
Simple mode
Weighted median
Weighted mode
SNP effect on Rheumatoid arthritis
SNP effect on Sphingomyelin (d18:2/24:2) levels
0.3
0.2
0.1
0.0
−0.1
0.1
0.2
0.3
0.4

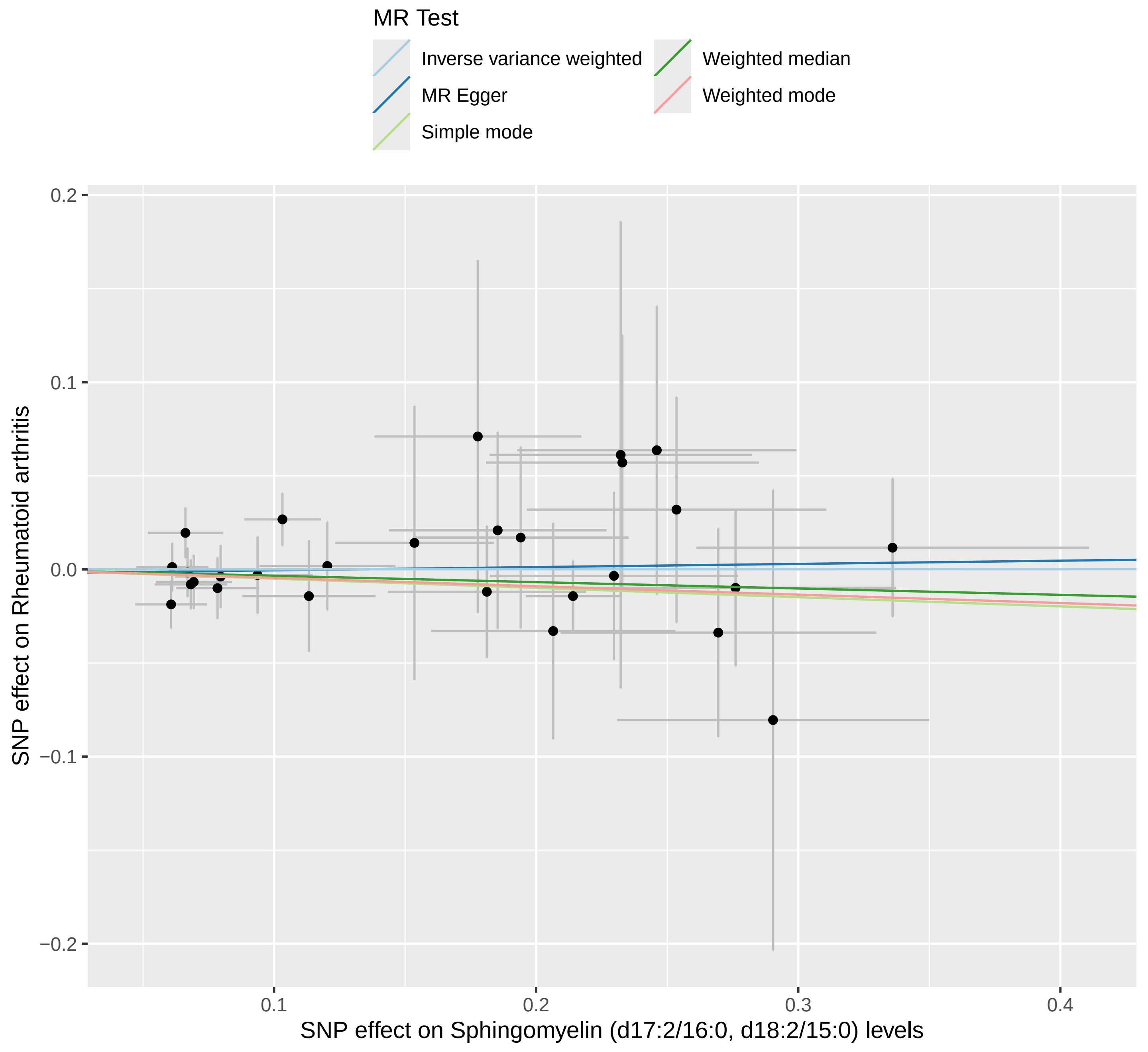
MR Test
Inverse variance weighted
MR Egger
Simple mode
Weighted median
Weighted mode
0.2
0.1
0.0
−0.1
−0.2
SNP effect on Rheumatoid arthritis
0.1
0.2
0.3
0.4
SNP effect on Sphingomyelin (d17:2/16:0, d18:2/15:0) levels

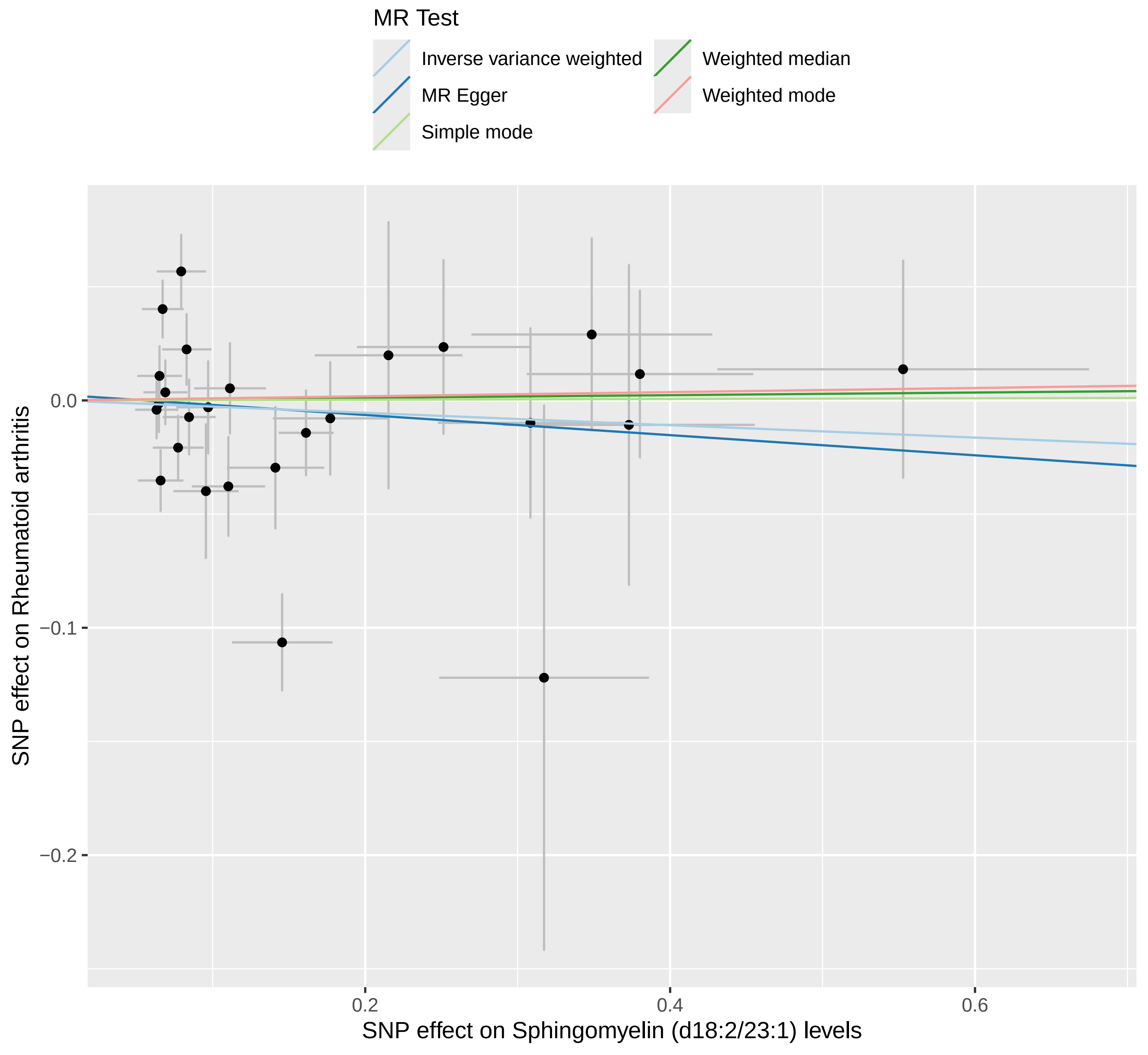
MR Test
Inverse variance weighted
MR Egger
Simple mode
Weighted median
Weighted mode
0.0
−0.1
−0.2
0.2
0.4
0.6
SNP effect on Rheumatoid arthritis
SNP effect on Sphingomyelin (d18:2/23:1) levels

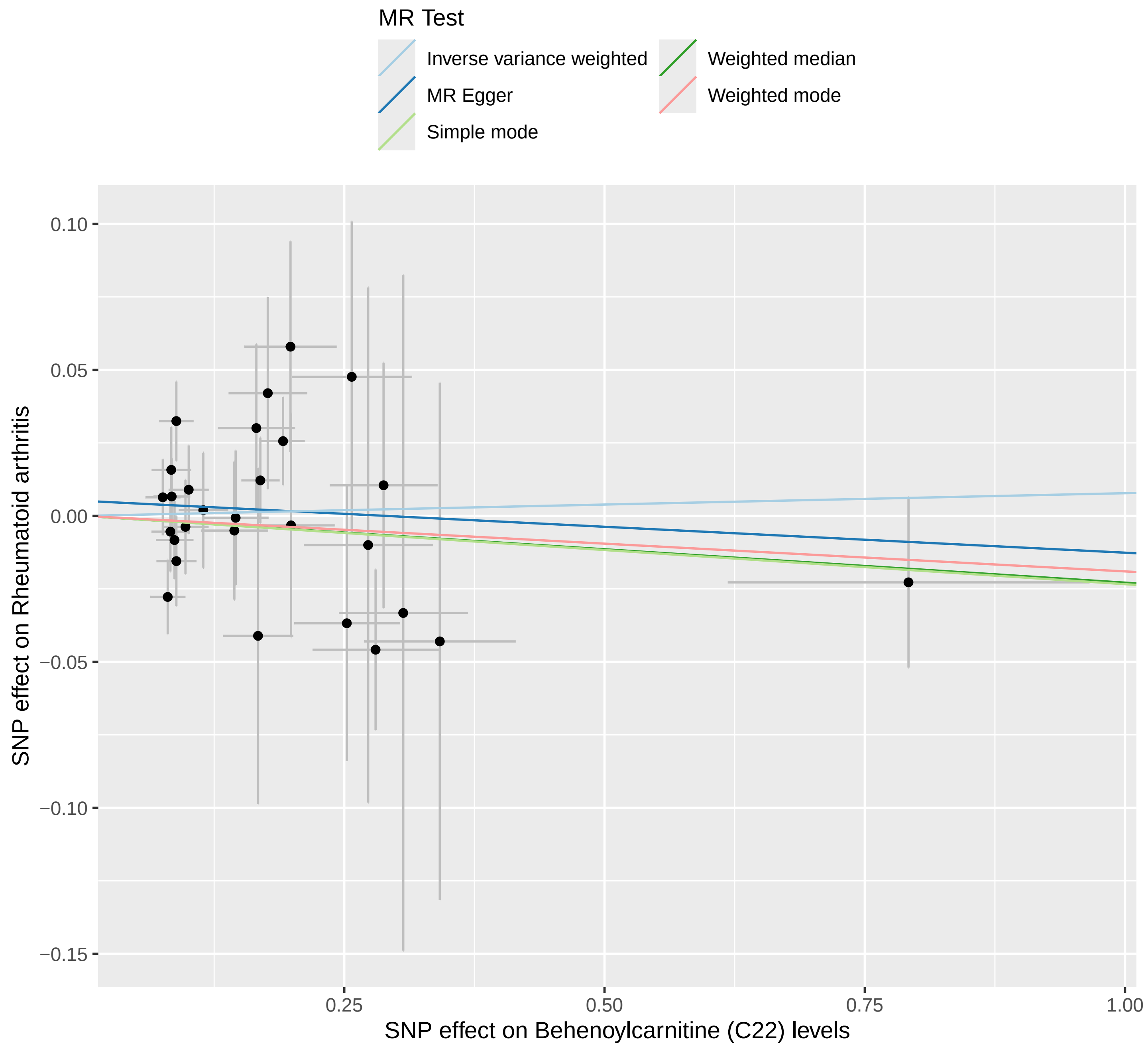
MR Test
Inverse variance weighted
MR Egger
Simple mode
Weighted median
Weighted mode
SNP effect on Rheumatoid arthritis
SNP effect on Behenoylcarnitine (C22) levels
0.10
0.05
0.00
−0.05
−0.10
−0.15
0.25
0.50
0.75
1.00

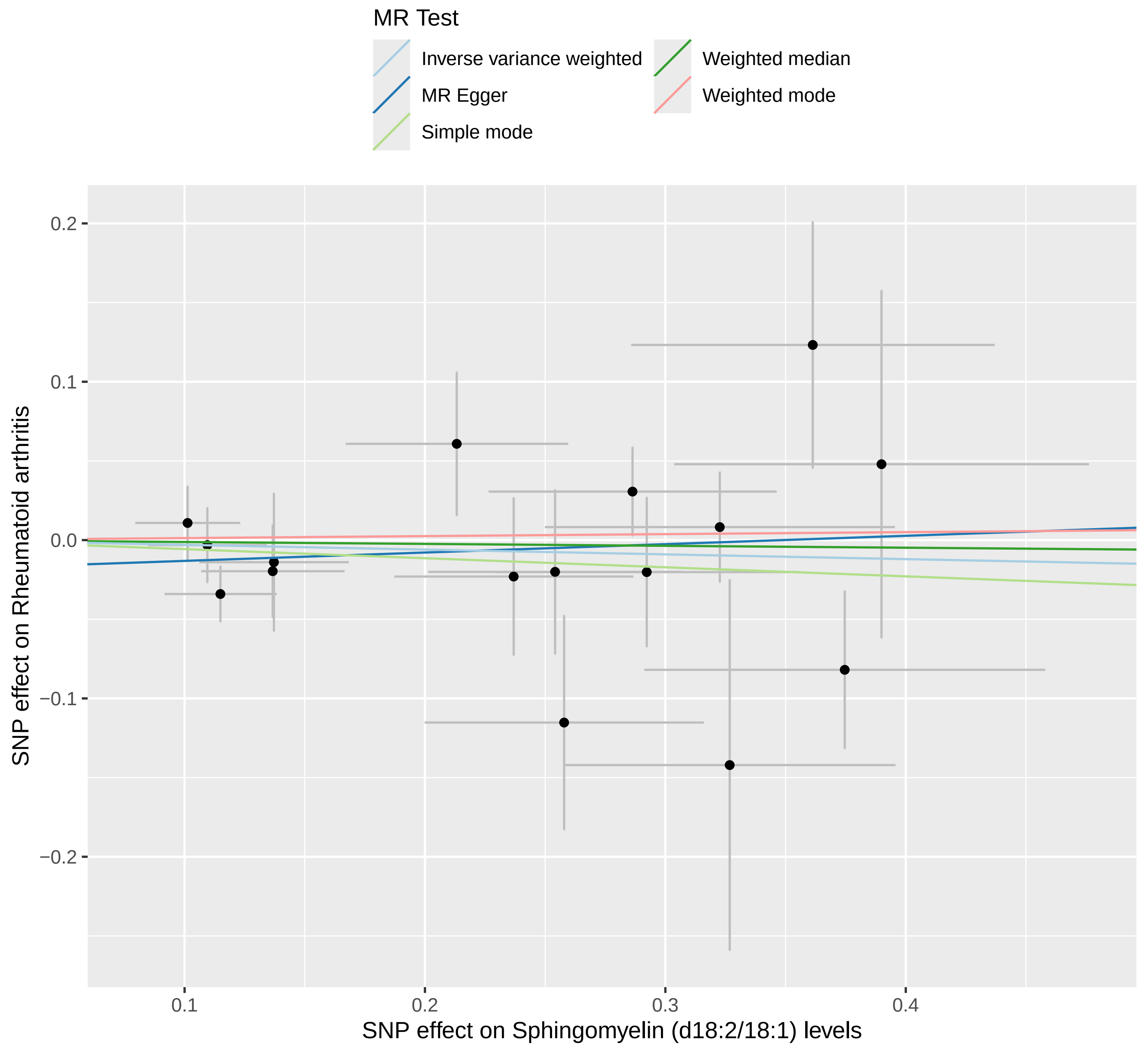

MR Test
Inverse variance weighted
MR Egger
Simple mode
Weighted median
Weighted mode
0.2
0.1
0.0
−0.1
−0.2
SNP effect on Rheumatoid arthritis
0.1
0.2
0.3
0.4
SNP effect on Sphingomyelin (d18:2/18:1) levels

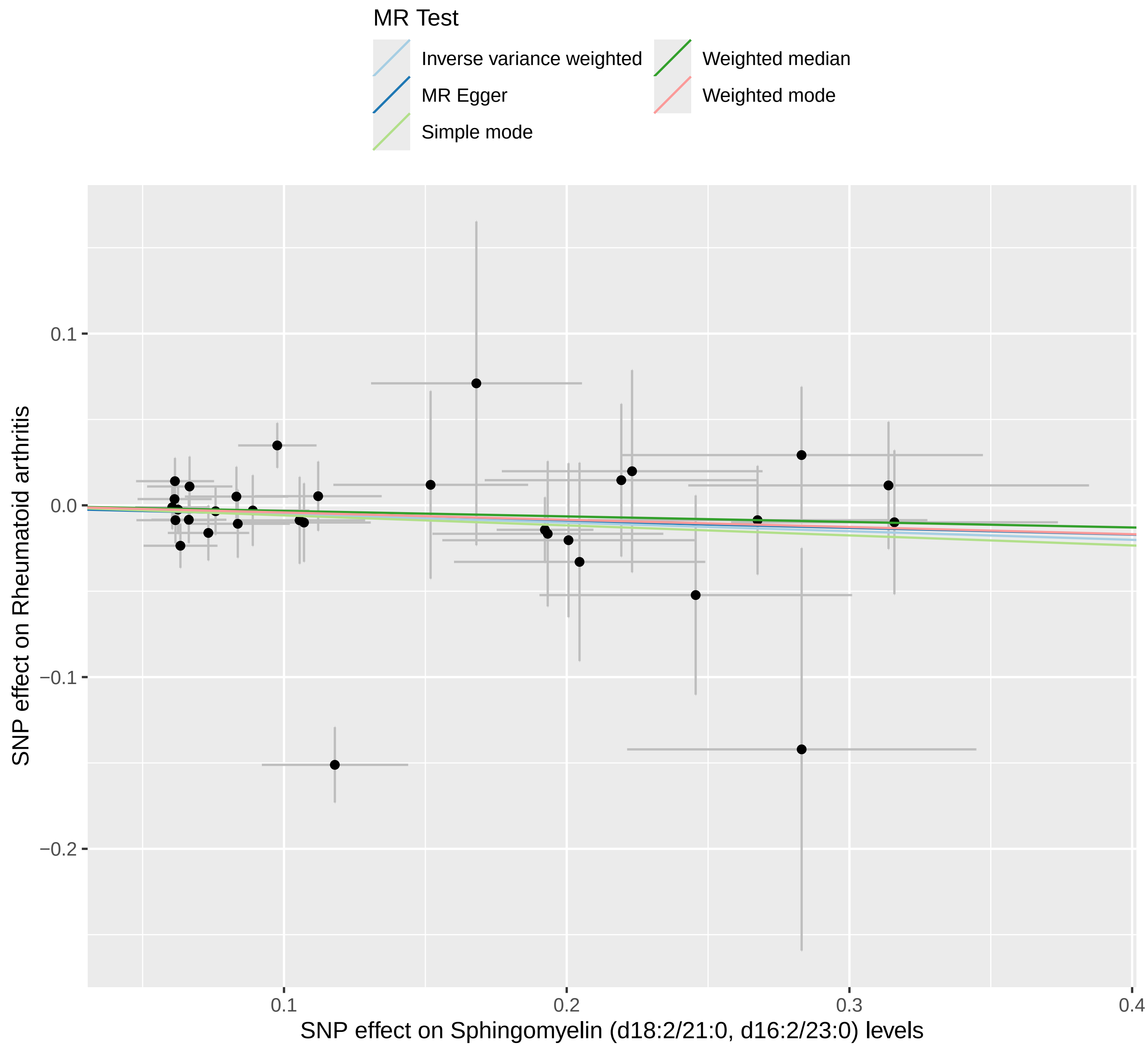

MR Test
Inverse variance weighted
MR Egger
Simple mode
Weighted median
Weighted mode
SNP effect on Rheumatoid arthritis
0.1
0.0
−0.1
−0.2
0.1
0.2
0.3
0.4
SNP effect on Sphingomyelin (d18:2/21:0, d16:2/23:0) levels

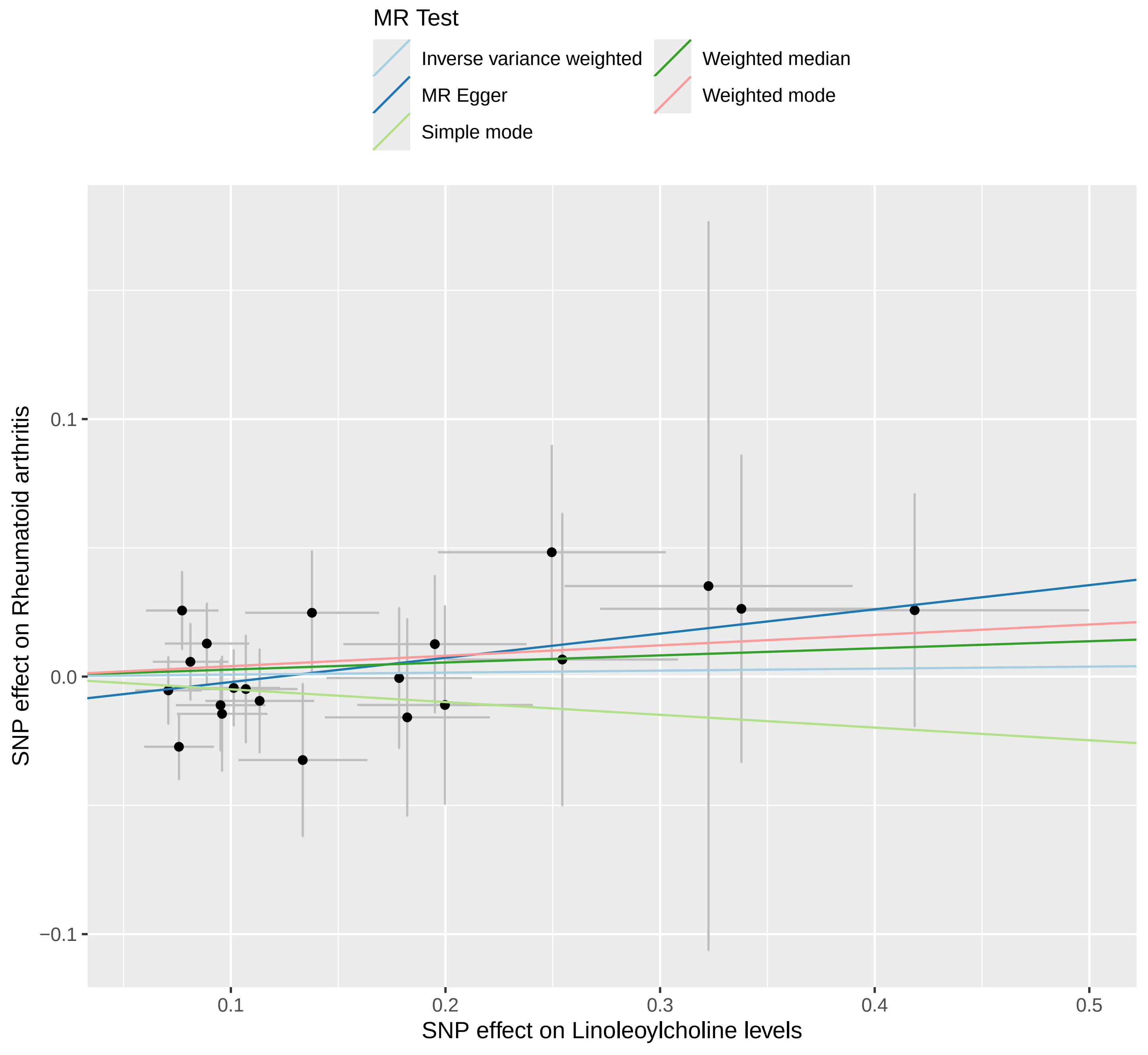
MR Test
Inverse variance weighted
MR Egger
Simple mode
Weighted median
Weighted mode
0.1
0.0
−0.1
0.1
0.2
0.3
0.4
0.5
SNP effect on Rheumatoid arthritis
SNP effect on Linoleoylcholine levels

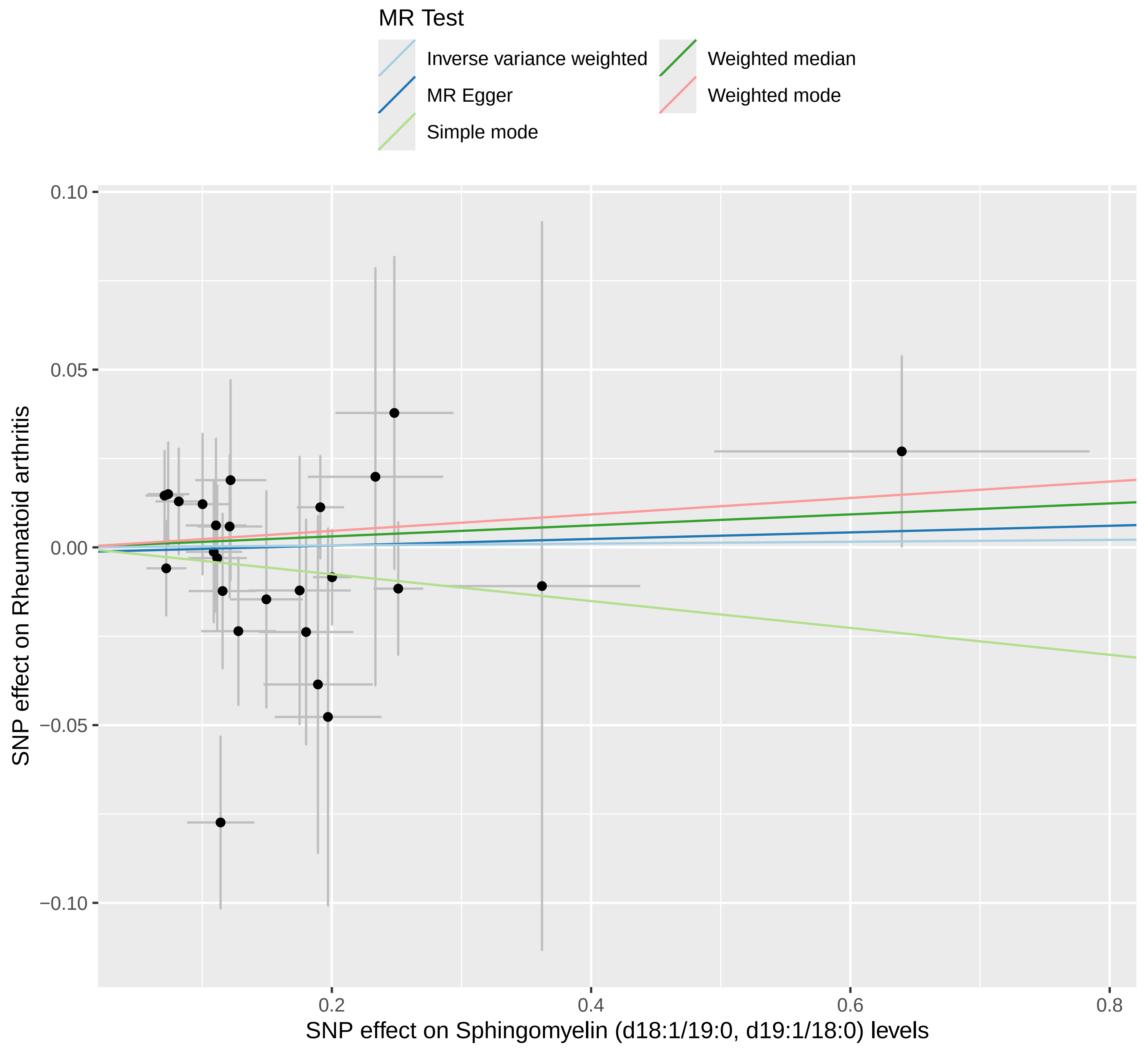
MR Test
Inverse variance weighted
MR Egger
Simple mode
Weighted median
Weighted mode
0.10
0.05
0.00
−0.05
−0.10
SNP effect on Rheumatoid arthritis
0.2
0.4
0.6
0.8
SNP effect on Sphingomyelin (d18:1/19:0, d19:1/18:0) levels

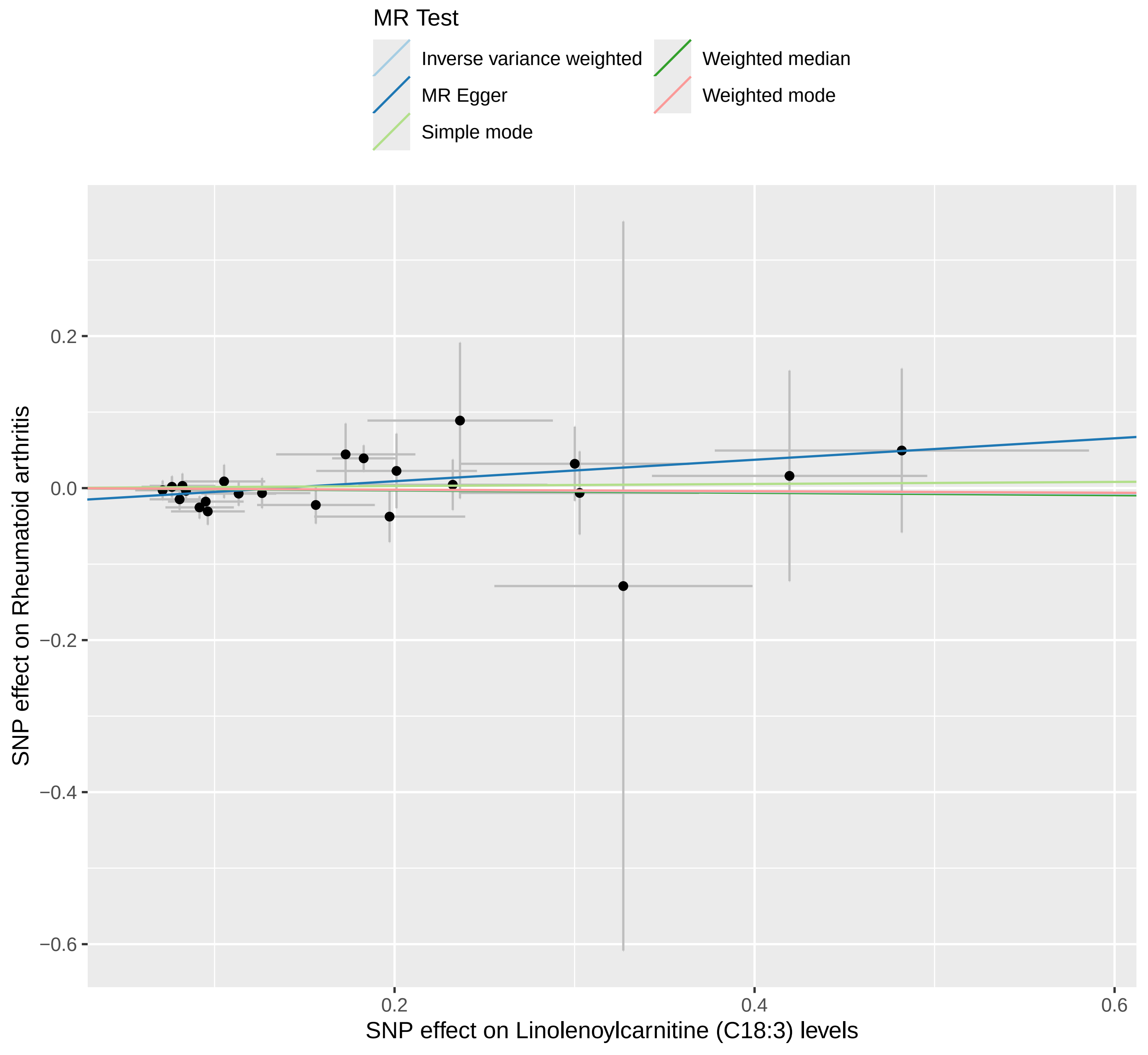
MR Test
Inverse variance weighted
MR Egger
Simple mode
Weighted median
Weighted mode
0.2
0.0
−0.2
−0.4
−0.6
SNP effect on Rheumatoid arthritis
0.2
0.4
0.6
SNP effect on Linolenoylcarnitine (C18:3) levels

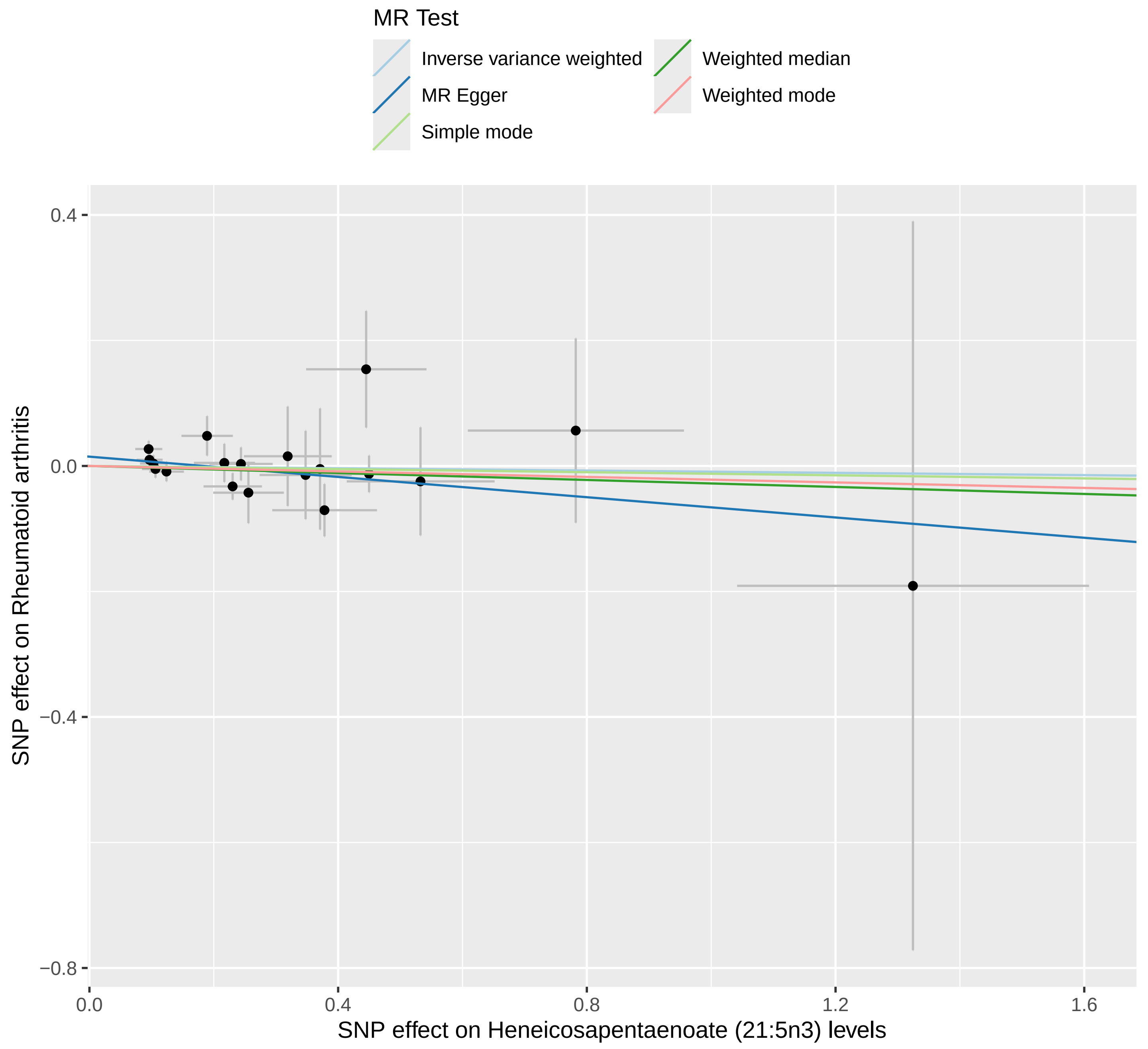

MR Test
Inverse variance weighted
MR Egger
Simple mode
Weighted median
Weighted mode
0.4
0.0
−0.4
−0.8
SNP effect on Rheumatoid arthritis
0.0
0.4
0.8
1.2
1.6
SNP effect on Heneicosapentaenoate (21:5n3) levels

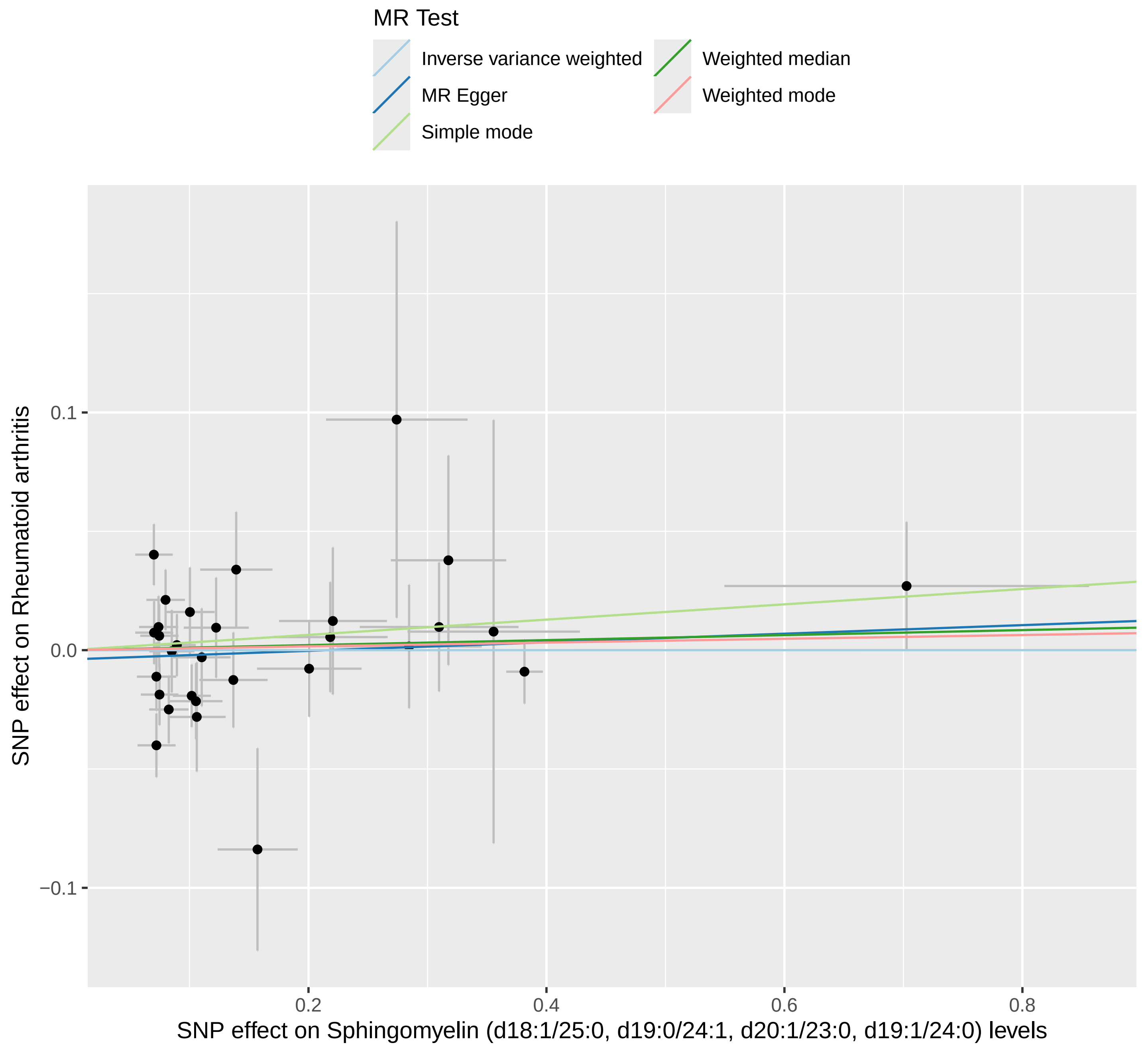

MR Test
Inverse variance weighted
MR Egger
Simple mode
Weighted median
Weighted mode
SNP effect on Rheumatoid arthritis
0.1
0.0
−0.1
0.2
0.4
0.6
0.8
SNP effect on Sphingomyelin (d18:1/25:0, d19:0/24:1, d20:1/23:0, d19:1/24:0) levels

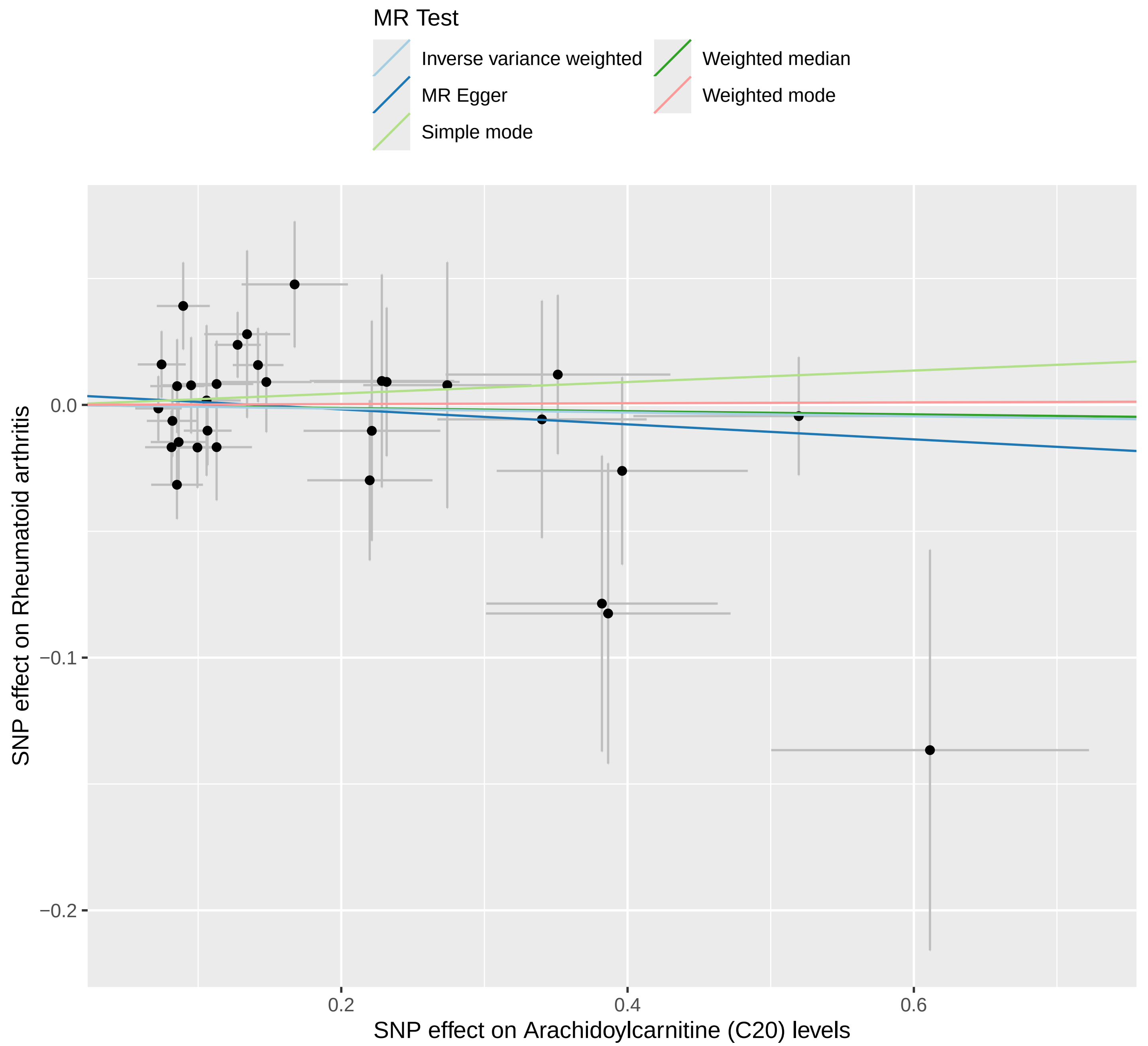

MR Test
Inverse variance weighted
MR Egger
Simple mode
Weighted median
Weighted mode
SNP effect on Rheumatoid arthritis
0.0
−0.1
−0.2
0.2
0.4
0.6
SNP effect on Arachidoylcarnitine (C20) levels

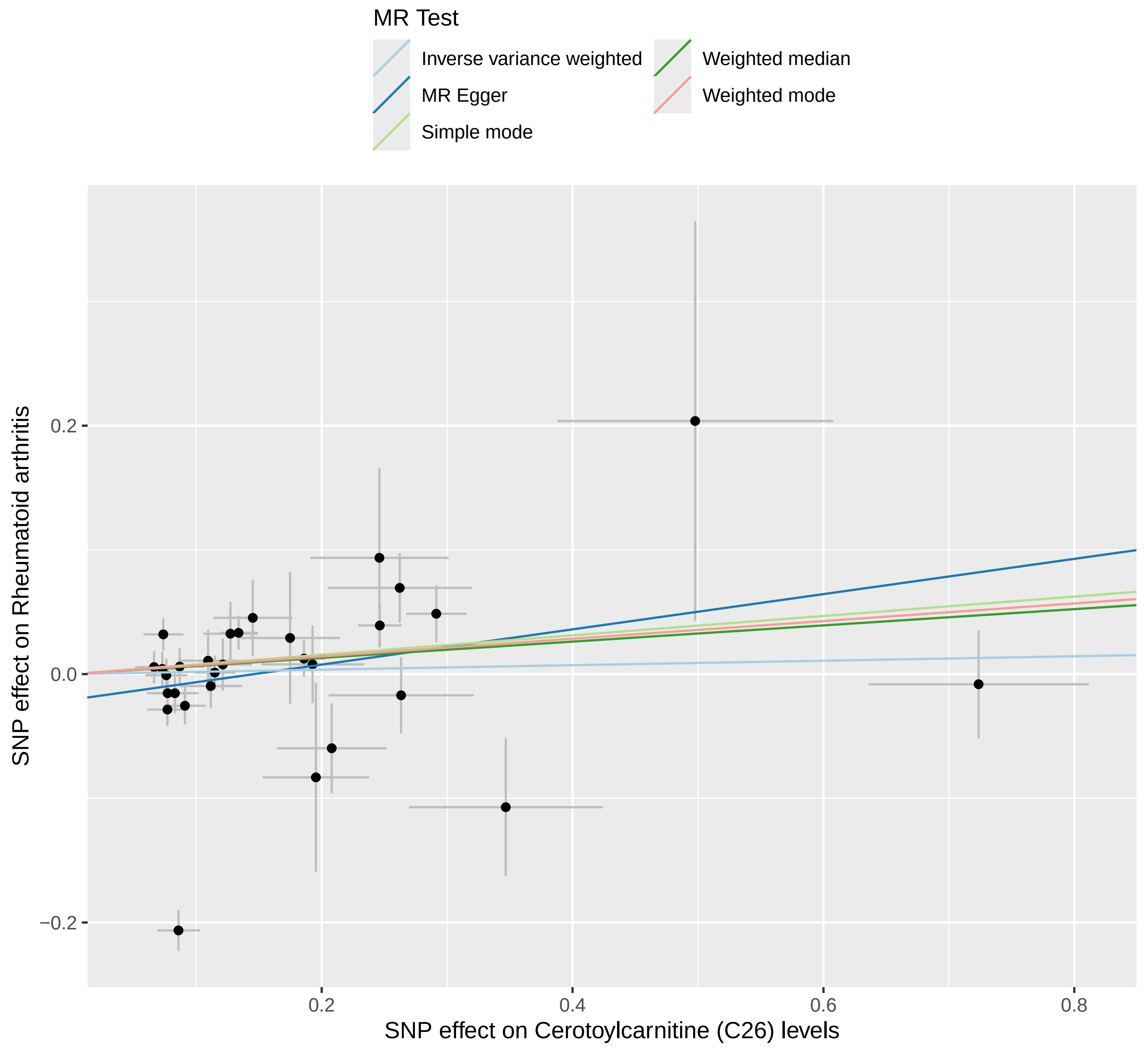
MR Test
Inverse variance weighted
MR Egger
Simple mode
Weighted median
Weighted mode
0.2
0.0
−0.2
0.2
0.4
0.6
0.8
SNP effect on Rheumatoid arthritis
SNP effect on Cerotoylcarnitine (C26) levels

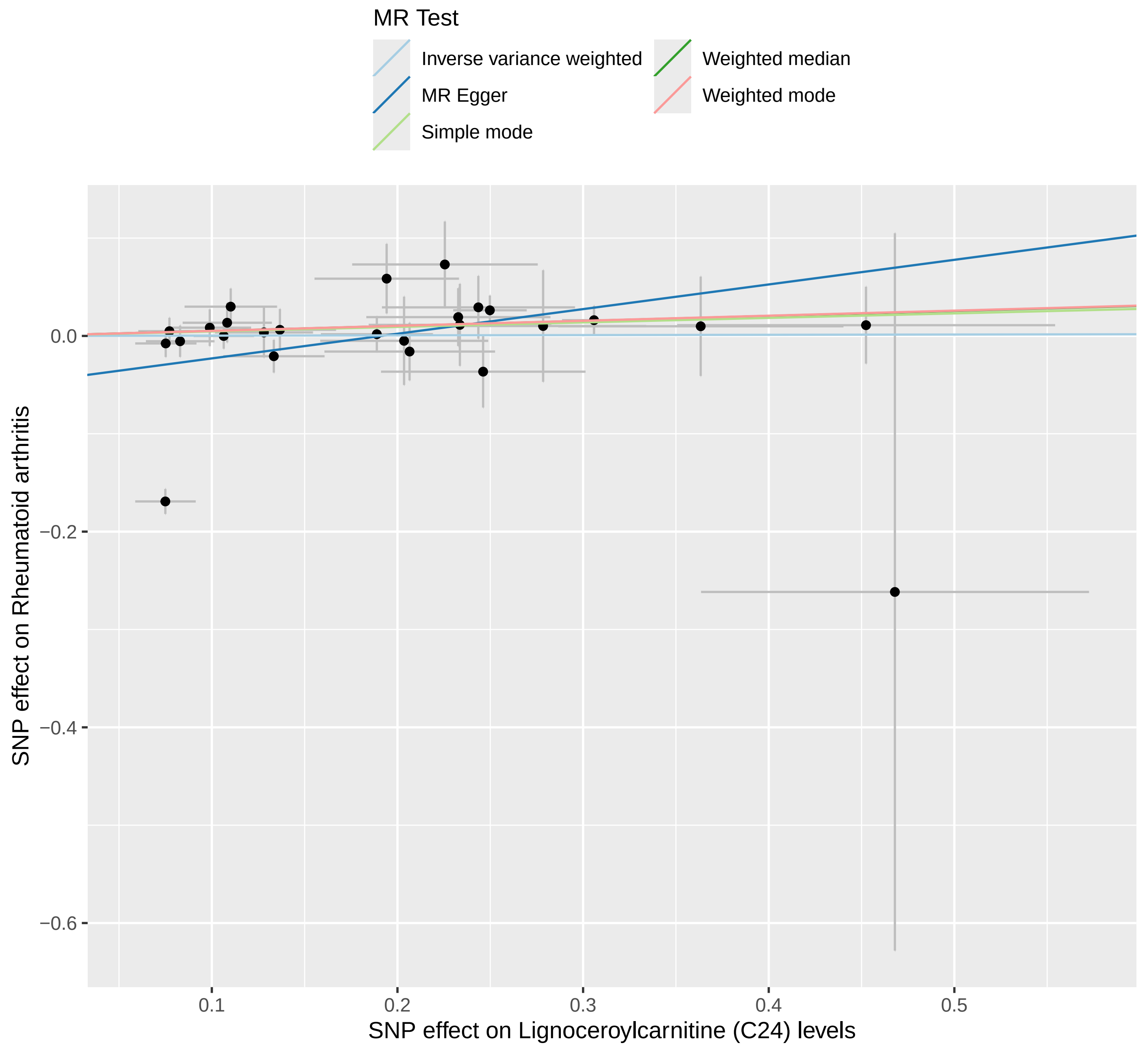
MR Test
Inverse variance weighted
MR Egger
Simple mode
Weighted median
Weighted mode
0.0
−0.2
−0.4
−0.6
0.1
0.2
0.3
0.4
0.5
SNP effect on Rheumatoid arthritis
SNP effect on Lignoceroylcarnitine (C24) levels

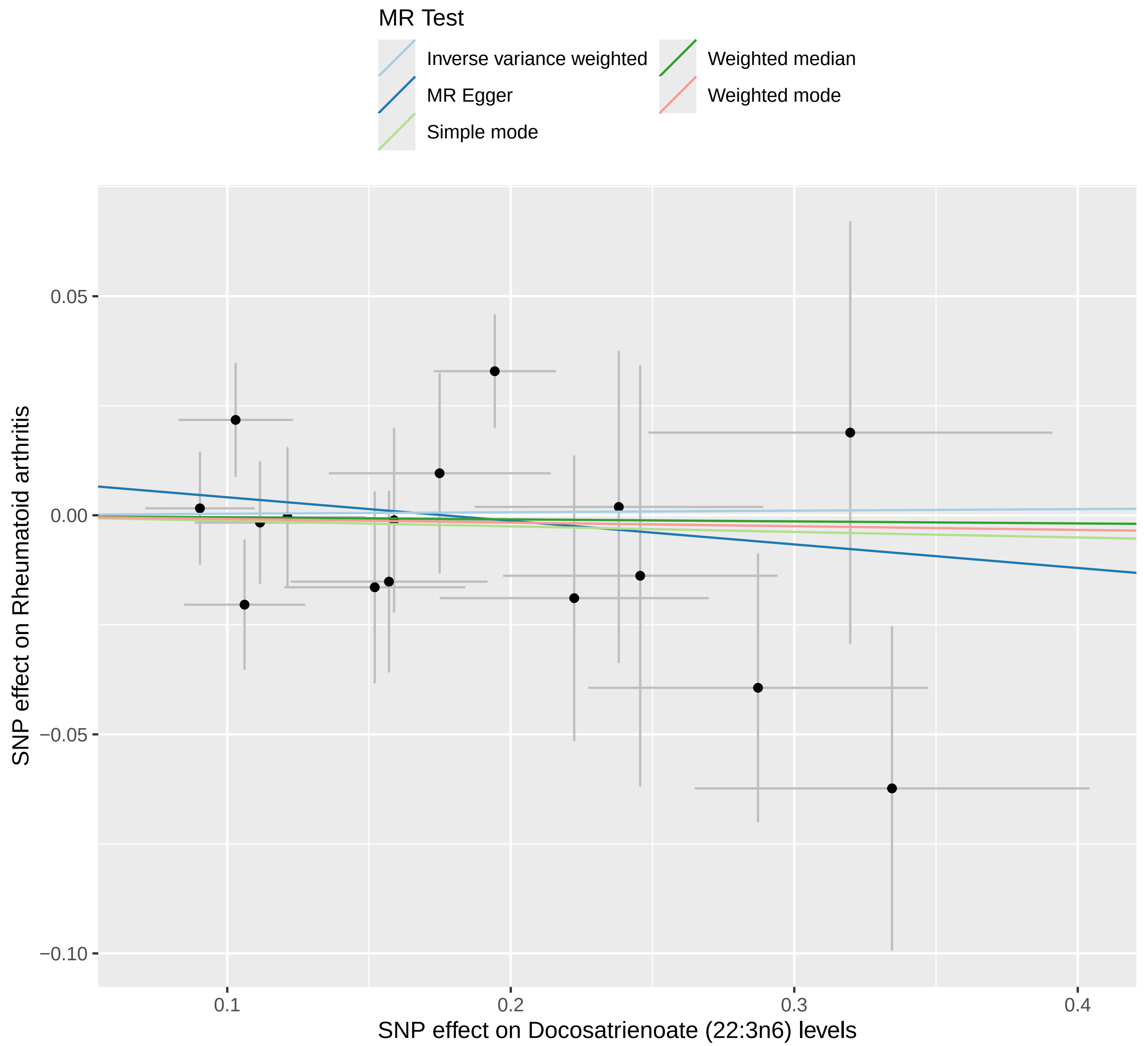
MR Test
Inverse variance weighted
MR Egger
Simple mode
Weighted median
Weighted mode
0.05
0.00
−0.05
−0.10
0.1
0.2
0.3
0.4
SNP effect on Rheumatoid arthritis
SNP effect on Docosatrienoate (22:3n6) levels

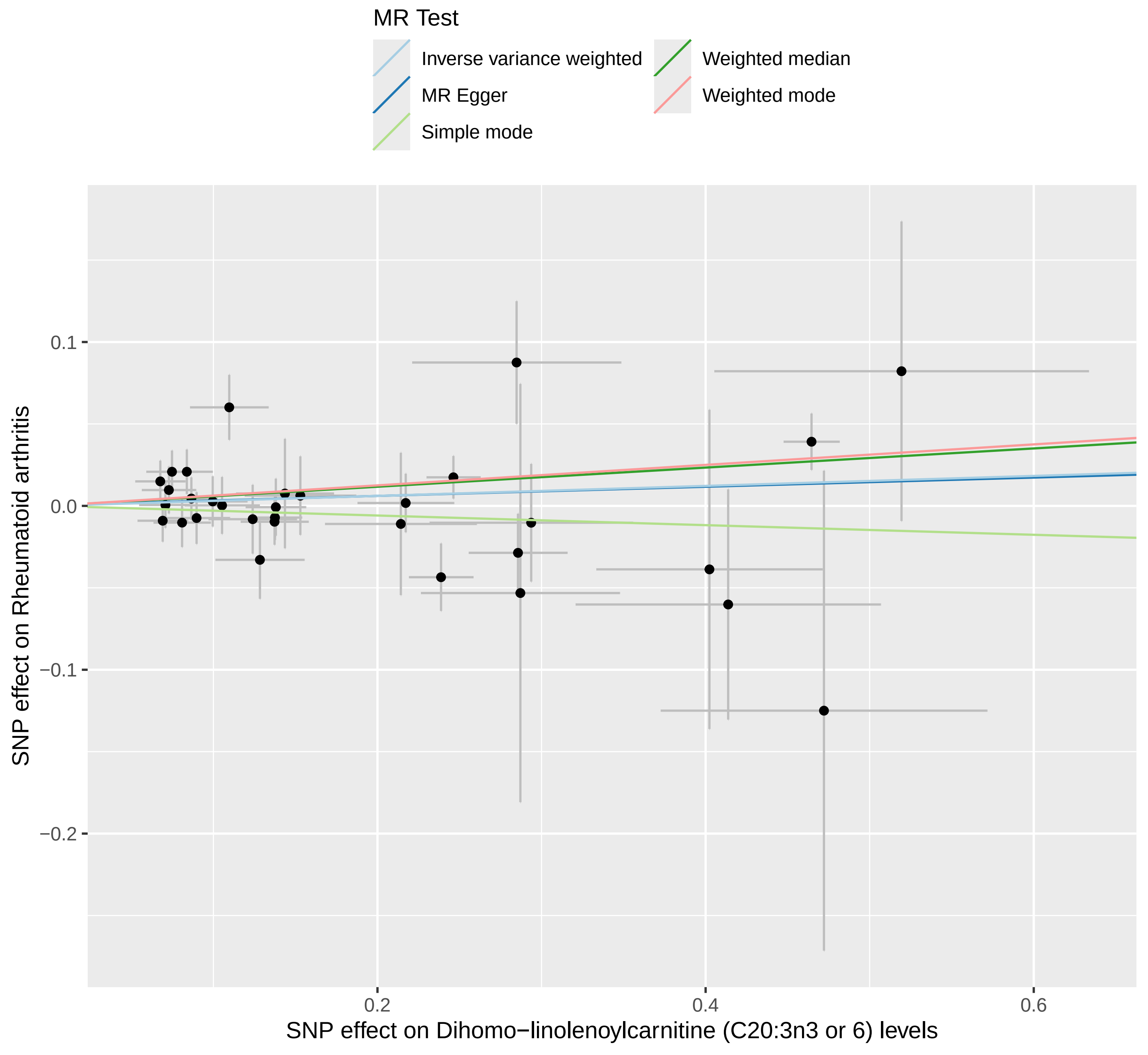
MR Test
Inverse variance weighted
MR Egger
Simple mode
Weighted median
Weighted mode
0.1
0.0
−0.1
−0.2
0.2
0.4
0.6
SNP effect on Rheumatoid arthritis
SNP effect on Dihomo−linolenoylcarnitine (C20:3n3 or 6) levels

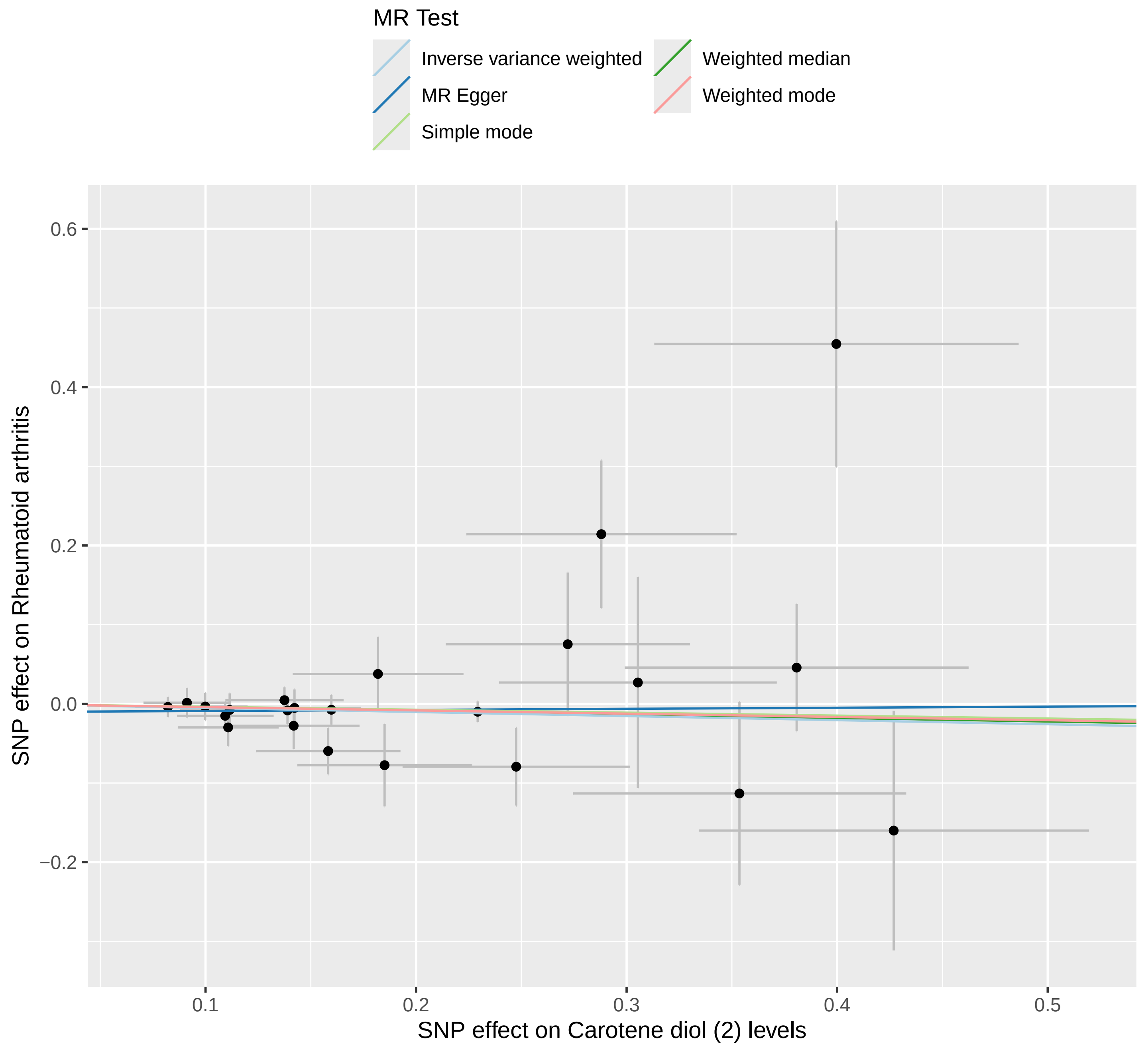
MR Test
Inverse variance weighted
MR Egger
Simple mode
Weighted median
Weighted mode
0.6
0.4
0.2
0.0
−0.2
SNP effect on Rheumatoid arthritis
0.1
0.2
0.3
0.4
0.5
SNP effect on Carotene diol (2) levels

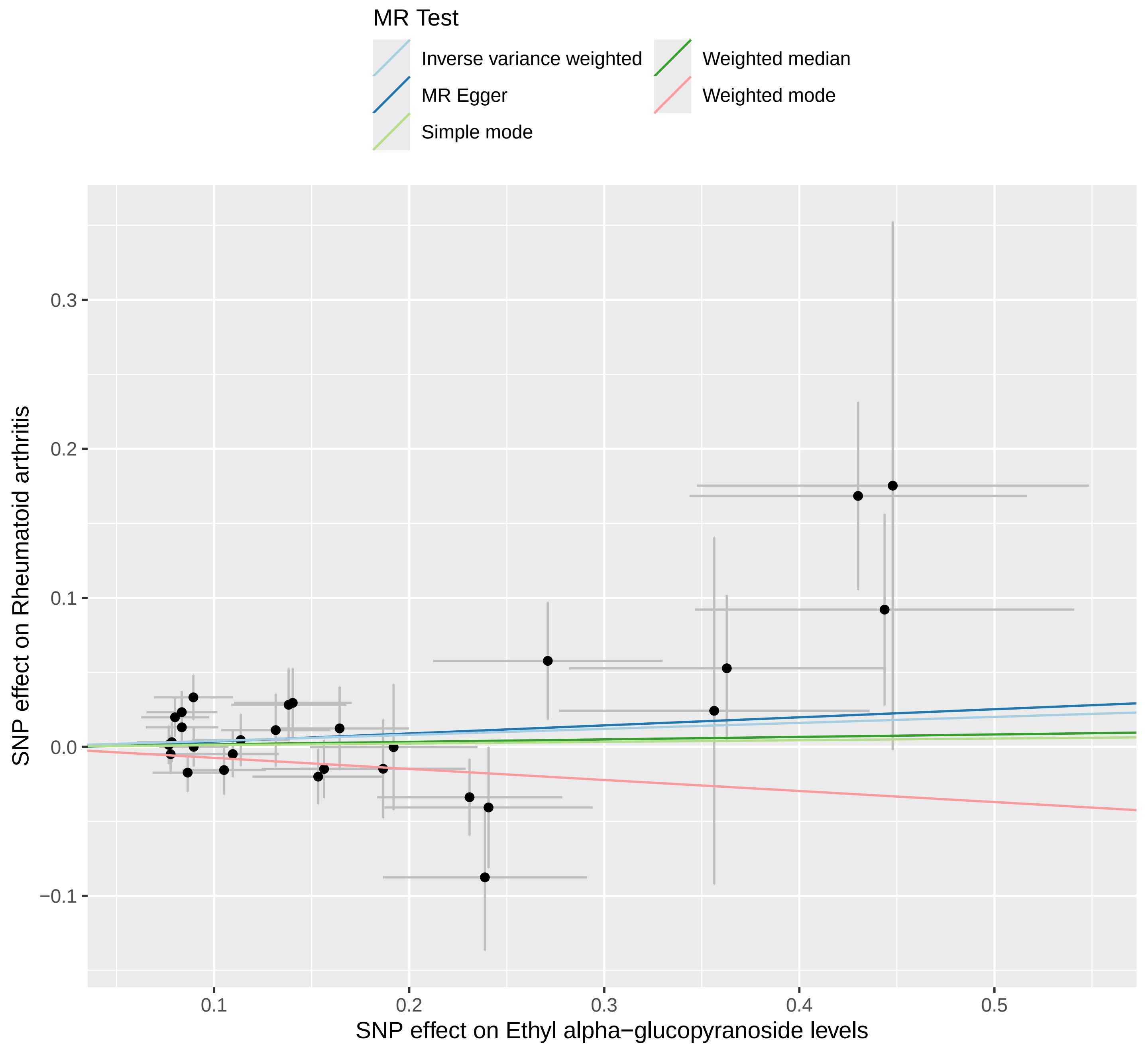
MR Test
Inverse variance weighted
MR Egger
Simple mode
Weighted median
Weighted mode
SNP effect on Rheumatoid arthritis
0.3
0.2
0.1
0.0
−0.1
0.1
0.2
0.3
0.4
0.5
SNP effect on Ethyl alpha−glucopyranoside levels

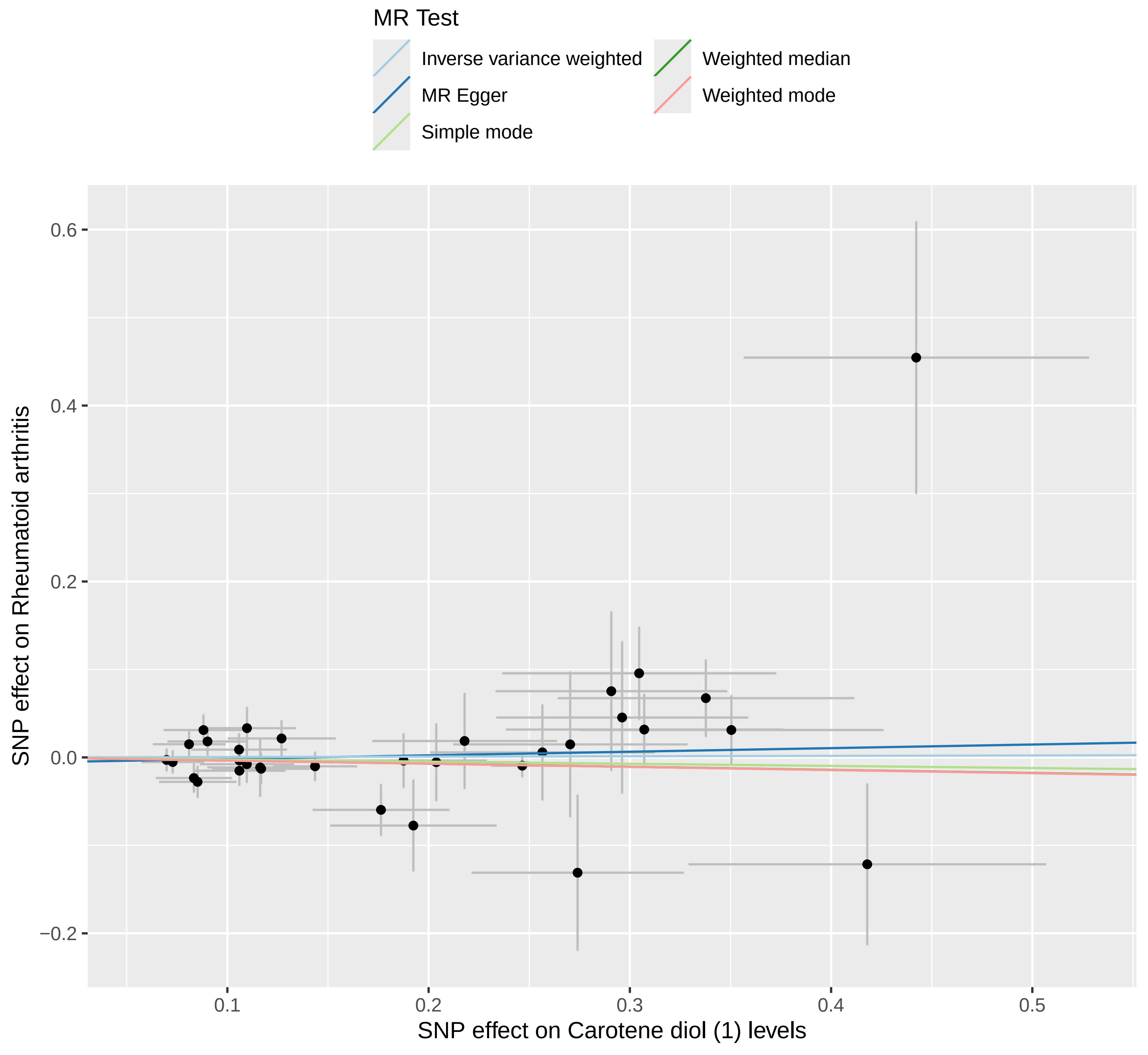
MR Test
Inverse variance weighted
MR Egger
Simple mode
Weighted median
Weighted mode
0.6
0.4
0.2
0.0
−0.2
0.1
0.2
0.3
0.4
0.5
SNP effect on Rheumatoid arthritis
SNP effect on Carotene diol (1) levels

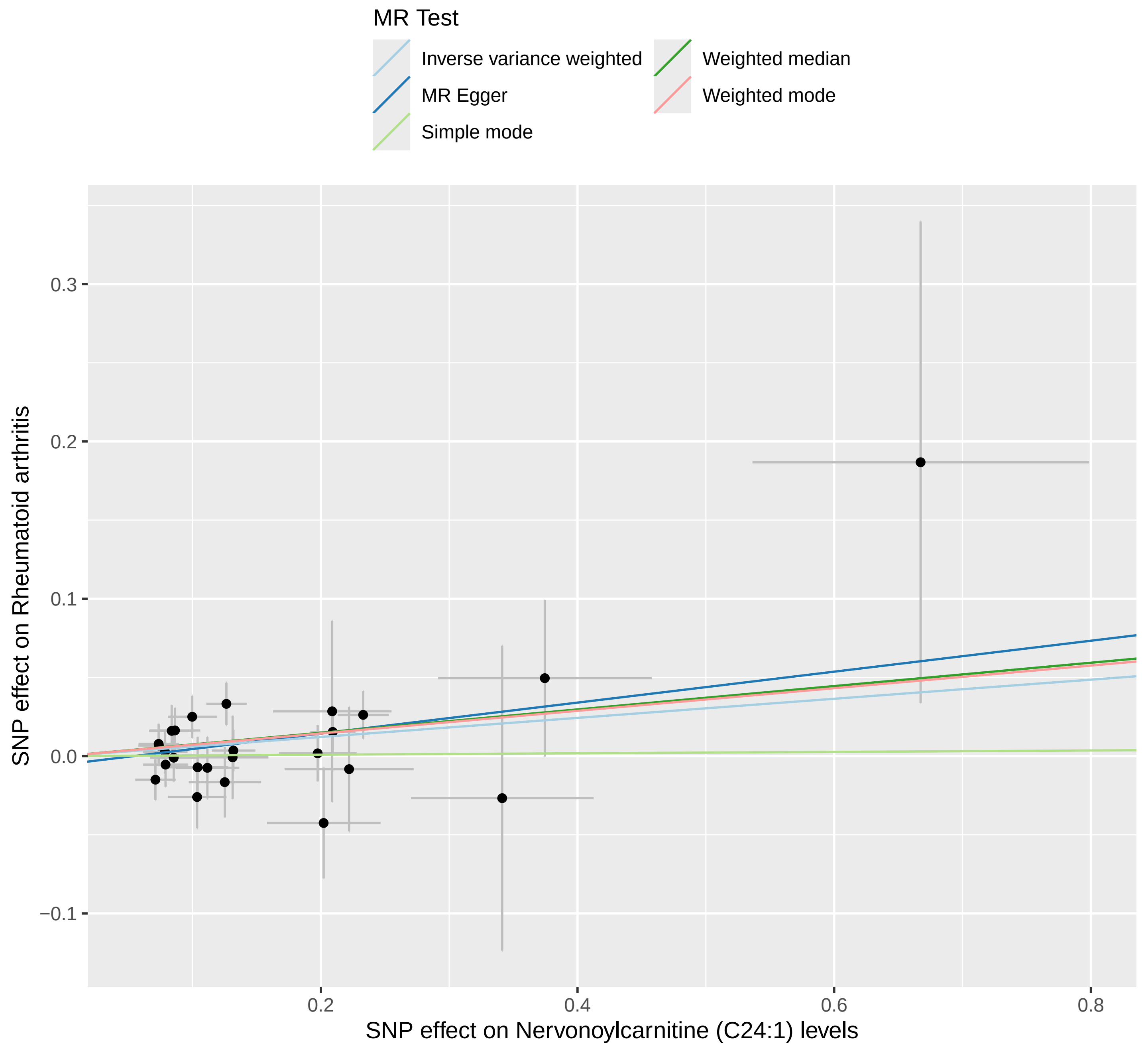
MR Test
Inverse variance weighted
MR Egger
Simple mode
Weighted median
Weighted mode
SNP effect on Rheumatoid arthritis
0.3
0.2
0.1
0.0
−0.1
0.2
0.4
0.6
0.8
SNP effect on Nervonoylcarnitine (C24:1) levels

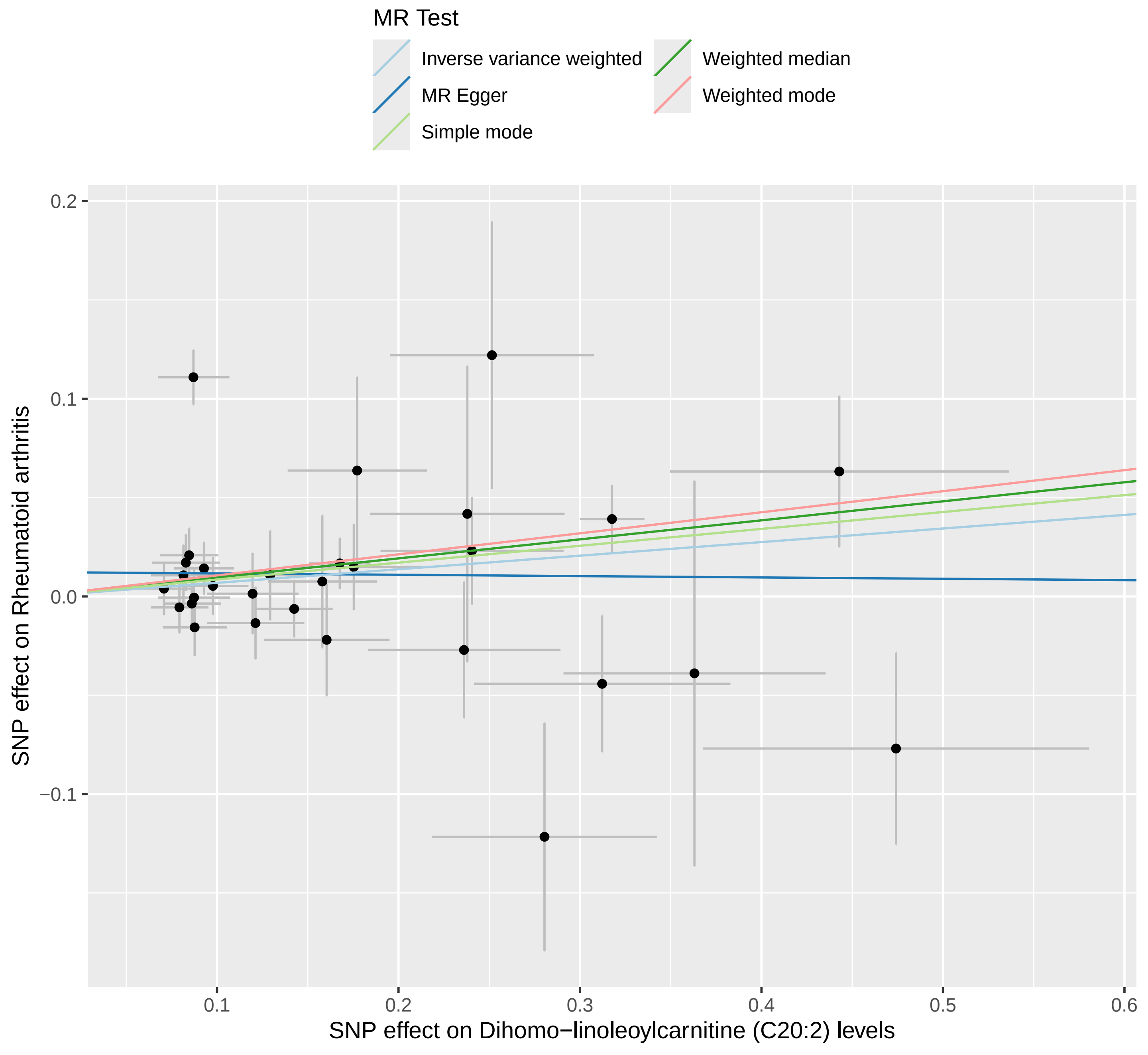
MR Test
Inverse variance weighted
MR Egger
Simple mode
Weighted median
Weighted mode
0.2
0.1
0.0
−0.1
0.1
0.2
0.3
0.4
0.5
0.6
SNP effect on Rheumatoid arthritis
SNP effect on Dihomo−linoleoylcarnitine (C20:2) levels

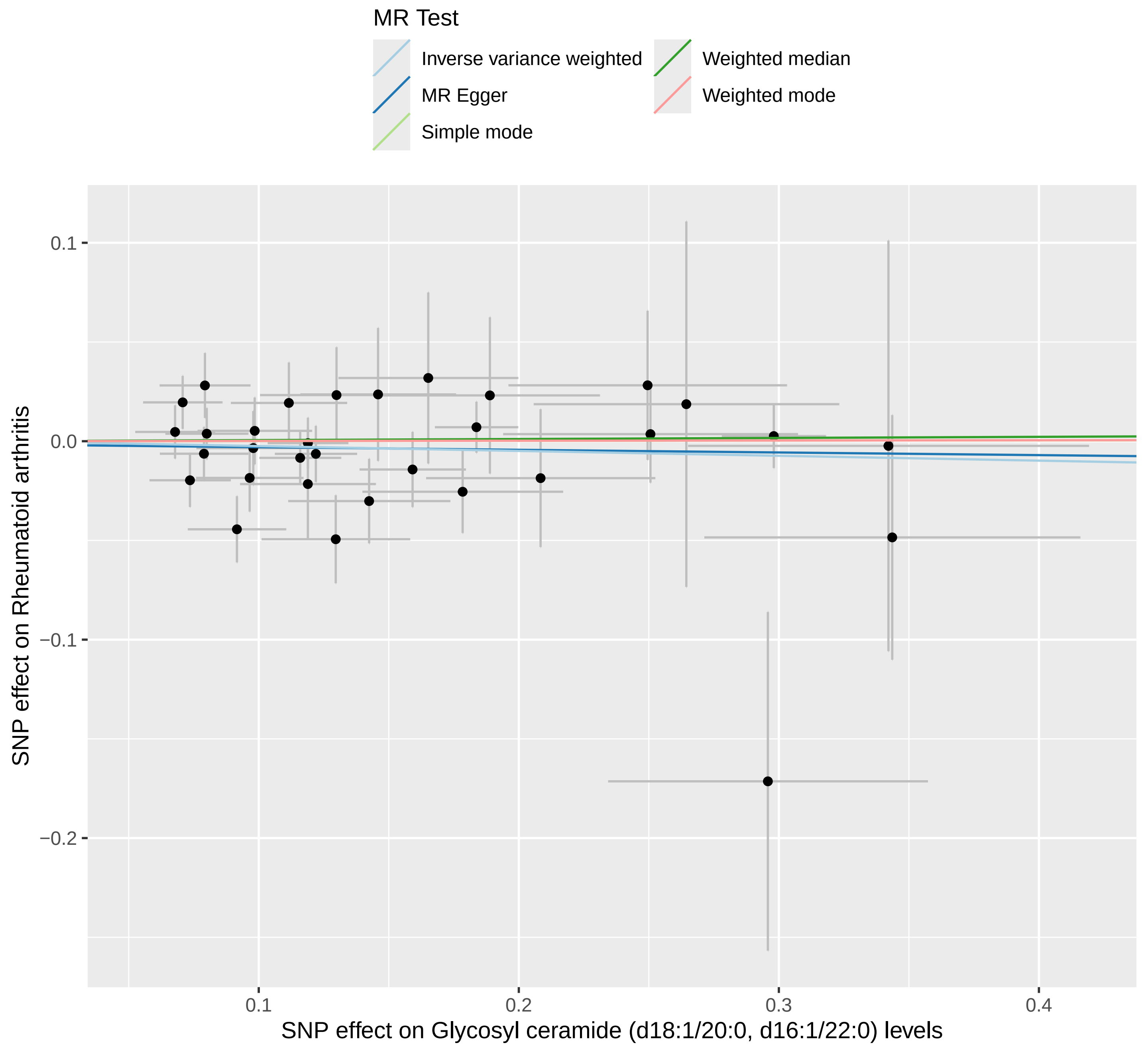
MR Test
Inverse variance weighted
MR Egger
Simple mode
Weighted median
Weighted mode
0.1
0.0
−0.1
−0.2
0.1
0.2
0.3
0.4
SNP effect on Rheumatoid arthritis
SNP effect on Glycosyl ceramide (d18:1/20:0, d16:1/22:0) levels

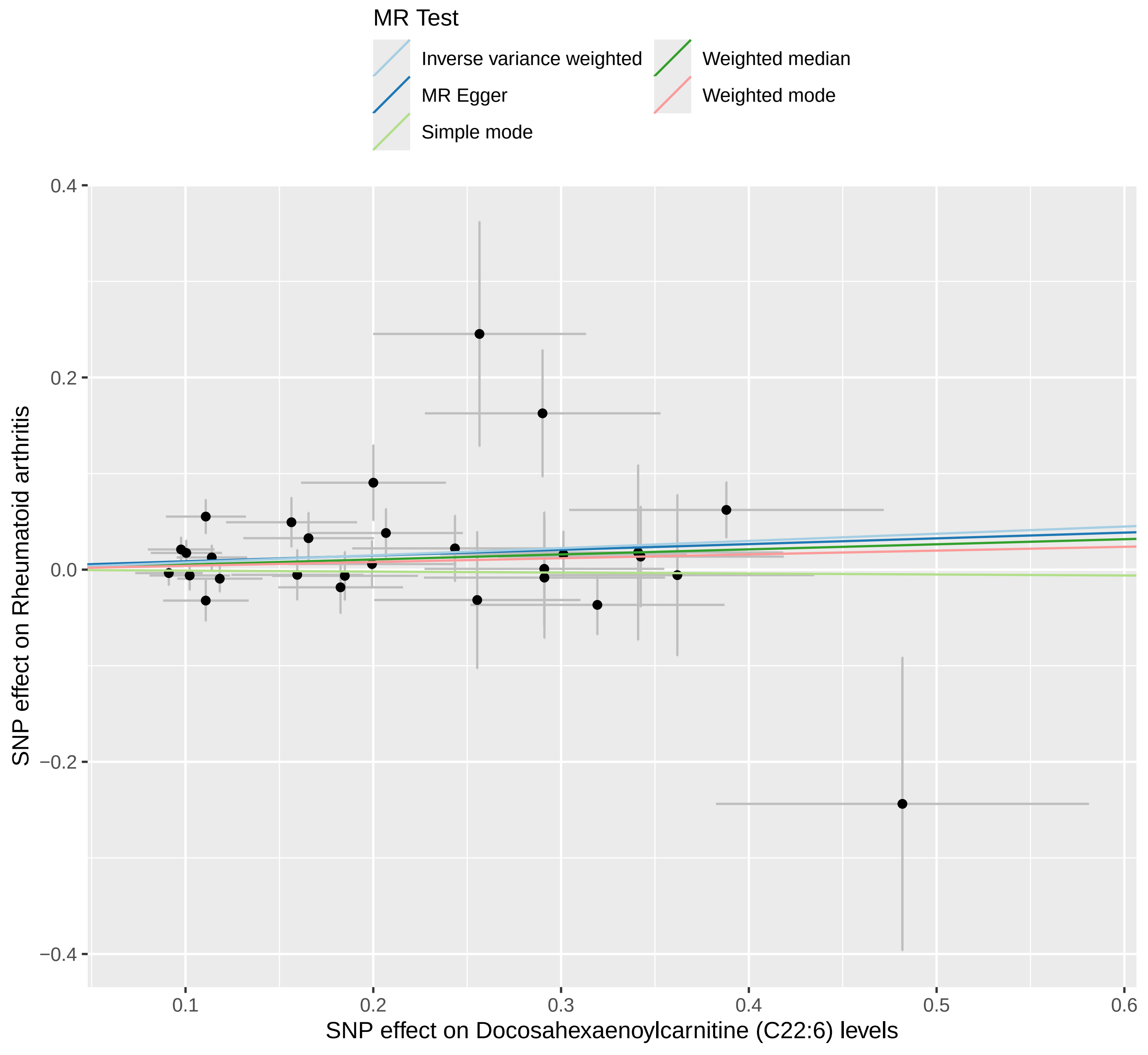
MR Test
Inverse variance weighted
MR Egger
Simple mode
Weighted median
Weighted mode
0.4
0.2
0.0
−0.2
−0.4
SNP effect on Rheumatoid arthritis
0.1
0.2
0.3
0.4
0.5
0.6
SNP effect on Docosahexaenoylcarnitine (C22:6) levels

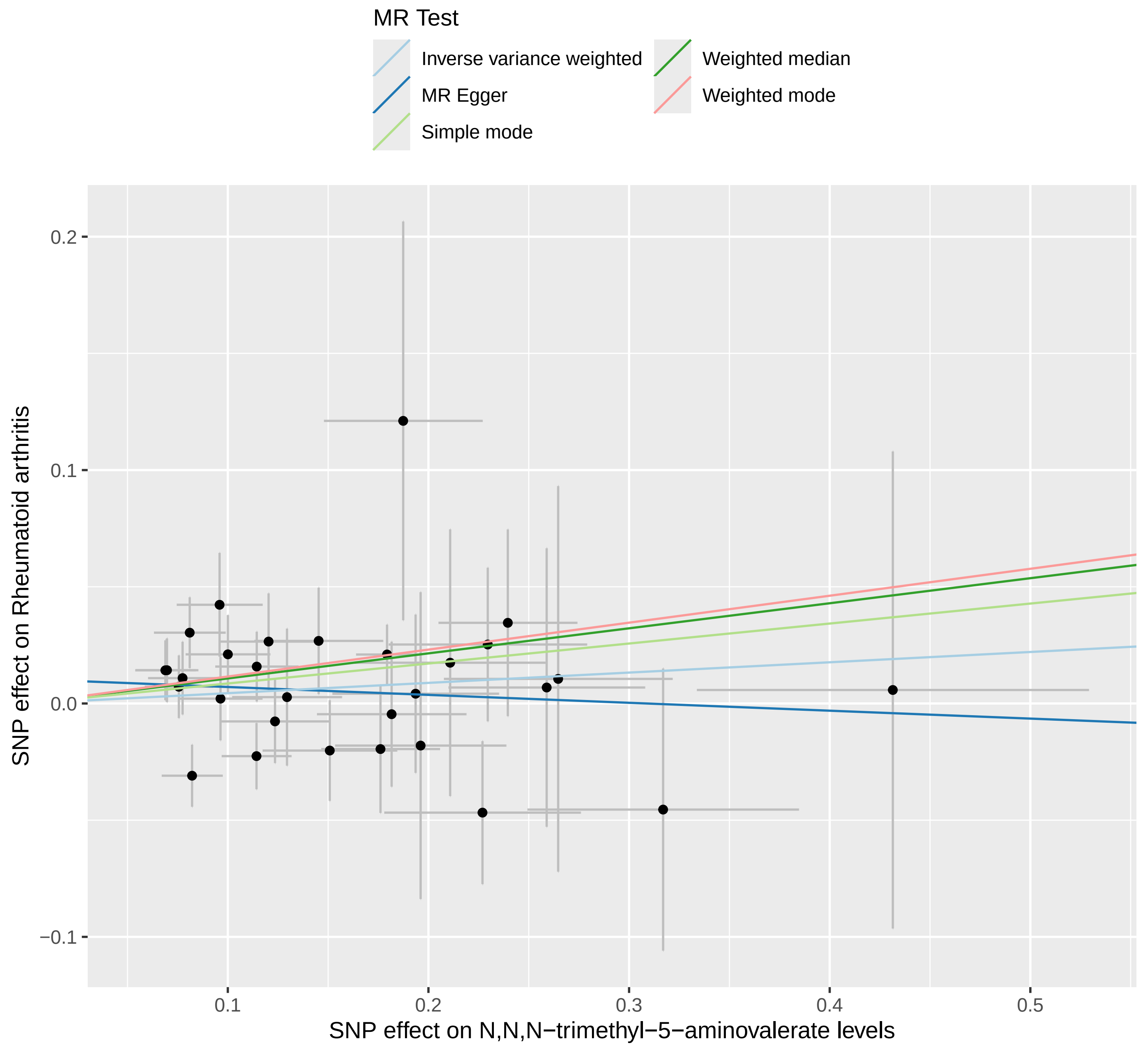
MR Test
Inverse variance weighted
MR Egger
Simple mode
Weighted median
Weighted mode
0.2
0.1
0.0
−0.1
0.1
0.2
0.3
0.4
0.5
SNP effect on Rheumatoid arthritis
SNP effect on N,N,N−trimethyl−5−aminovalerate levels

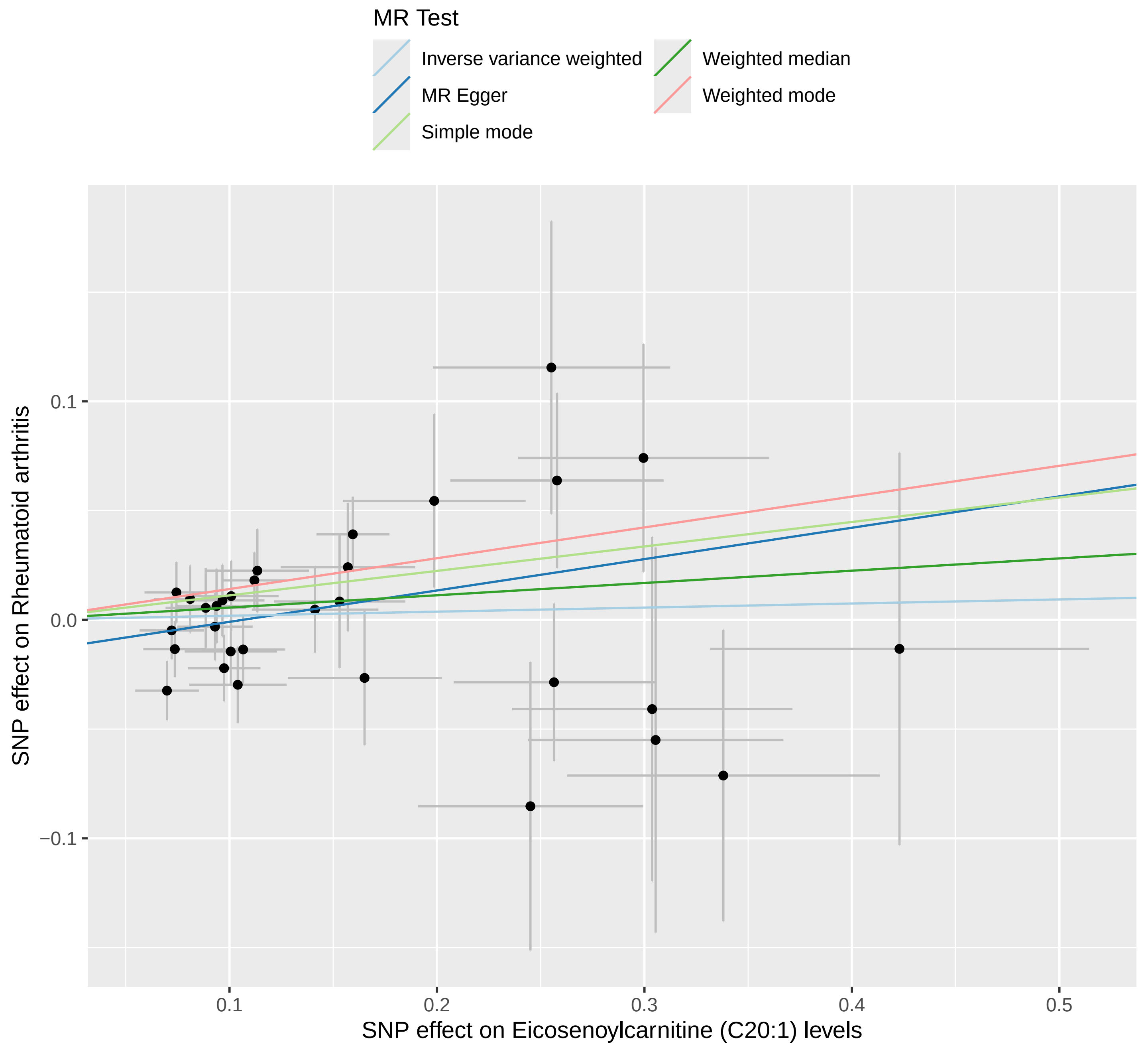
MR Test
Inverse variance weighted
MR Egger
Simple mode
Weighted median
Weighted mode
0.1
0.0
−0.1
SNP effect on Rheumatoid arthritis
0.1
0.2
0.3
0.4
0.5
SNP effect on Eicosenoylcarnitine (C20:1) levels

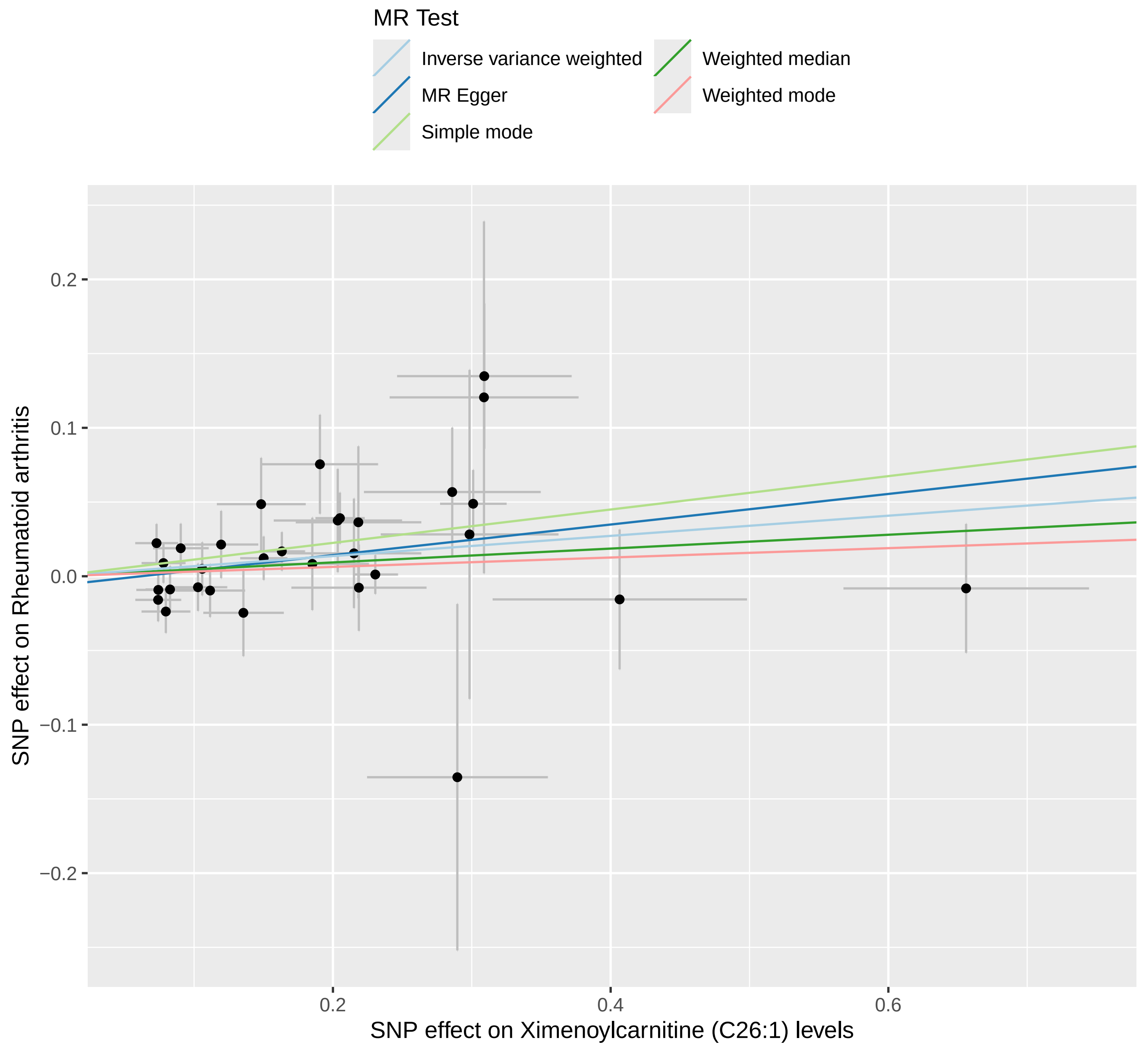

MR Test
Inverse variance weighted
MR Egger
Simple mode
Weighted median
Weighted mode
0.2
0.1
0.0
−0.1
−0.2
SNP effect on Rheumatoid arthritis
0.2
0.4
0.6
SNP effect on Ximenoylcarnitine (C26:1) levels

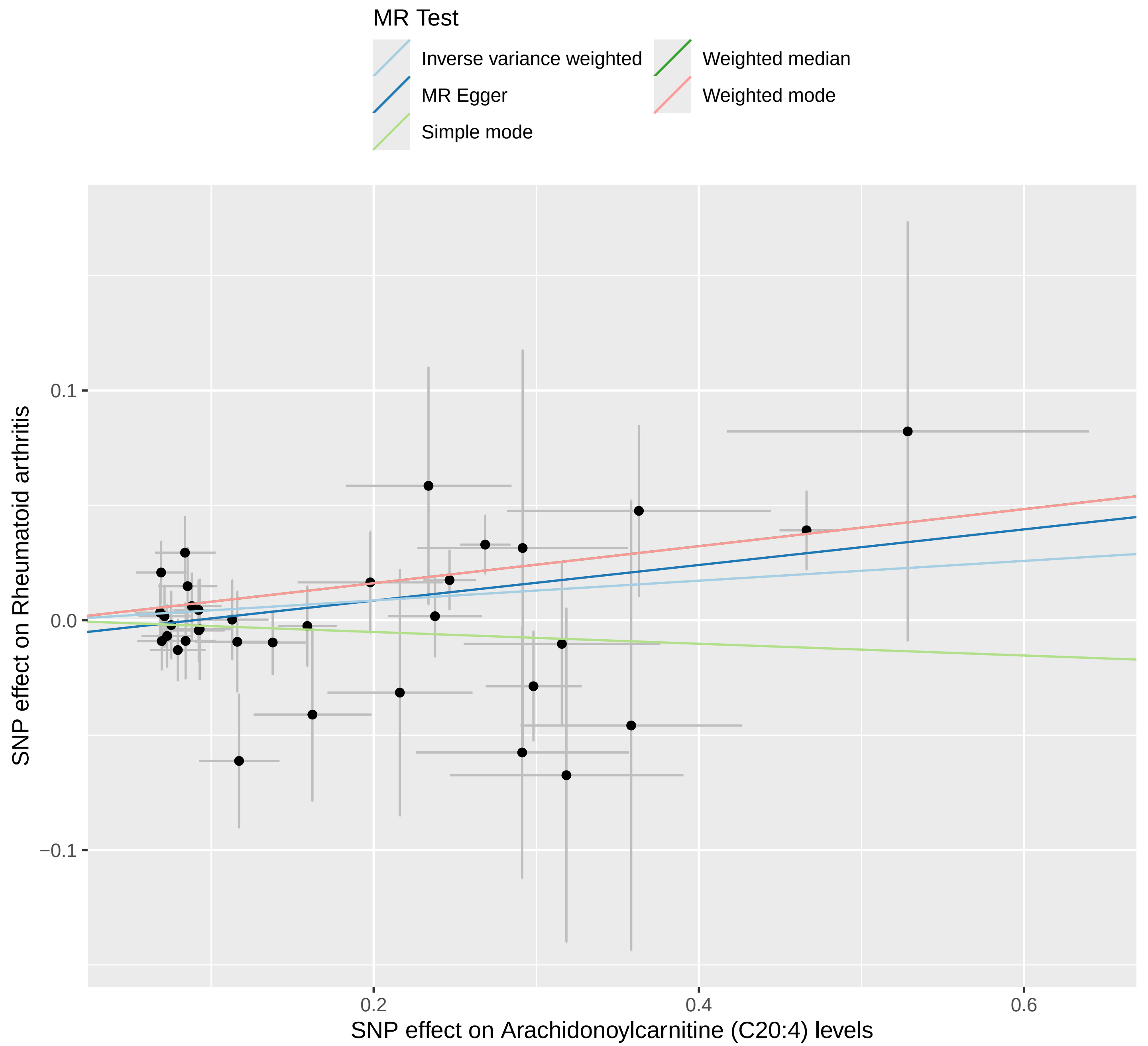
MR Test
Inverse variance weighted
MR Egger
Simple mode
Weighted median
Weighted mode
SNP effect on Rheumatoid arthritis
0.1
0.0
−0.1
0.2
0.4
0.6
SNP effect on Arachidonoylcarnitine (C20:4) levels

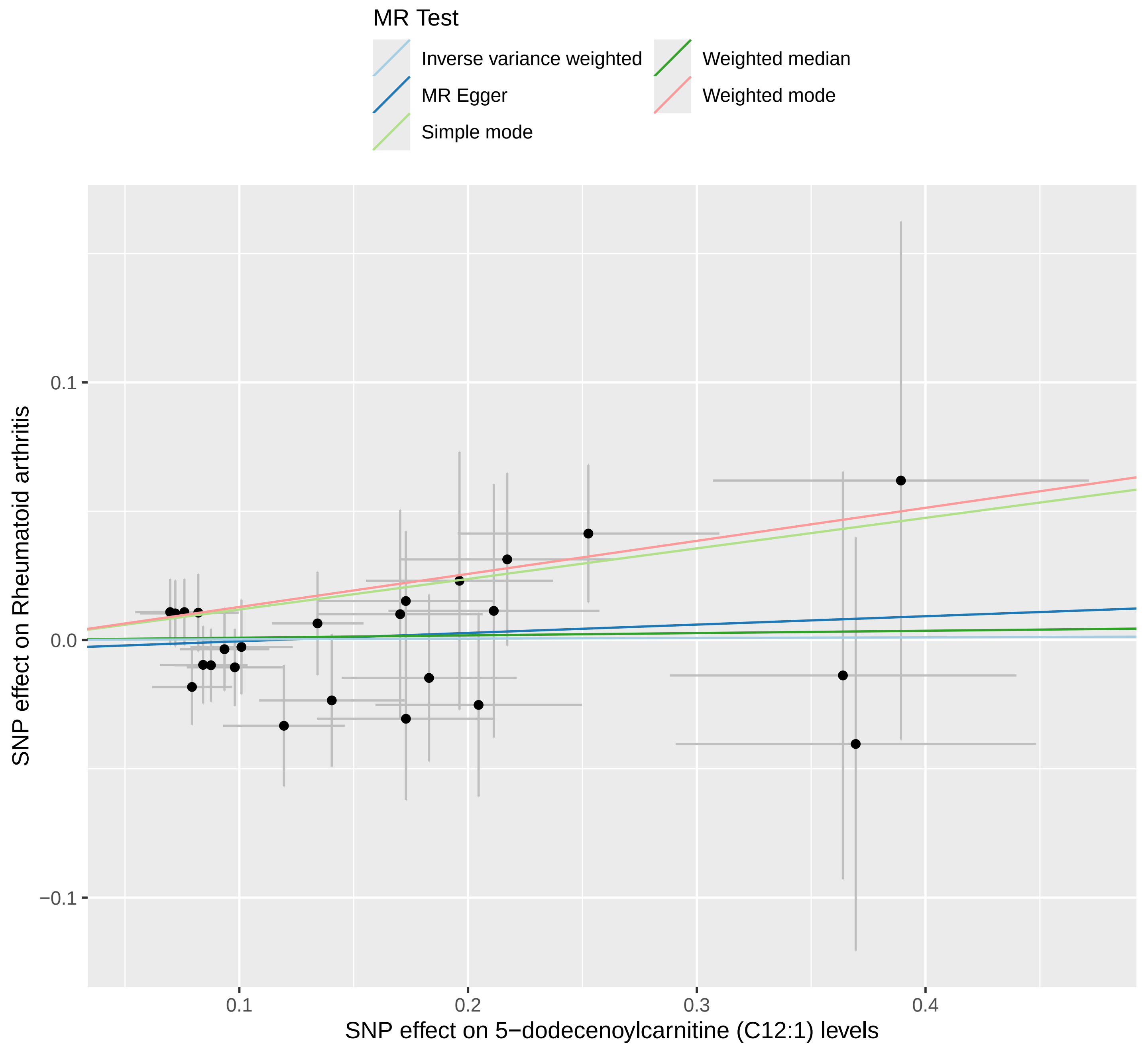
MR Test
Inverse variance weighted
MR Egger
Simple mode
Weighted median
Weighted mode
SNP effect on Rheumatoid arthritis
0.1
0.0
−0.1
0.1
0.2
0.3
0.4
SNP effect on 5−dodecenoylcarnitine (C12:1) levels

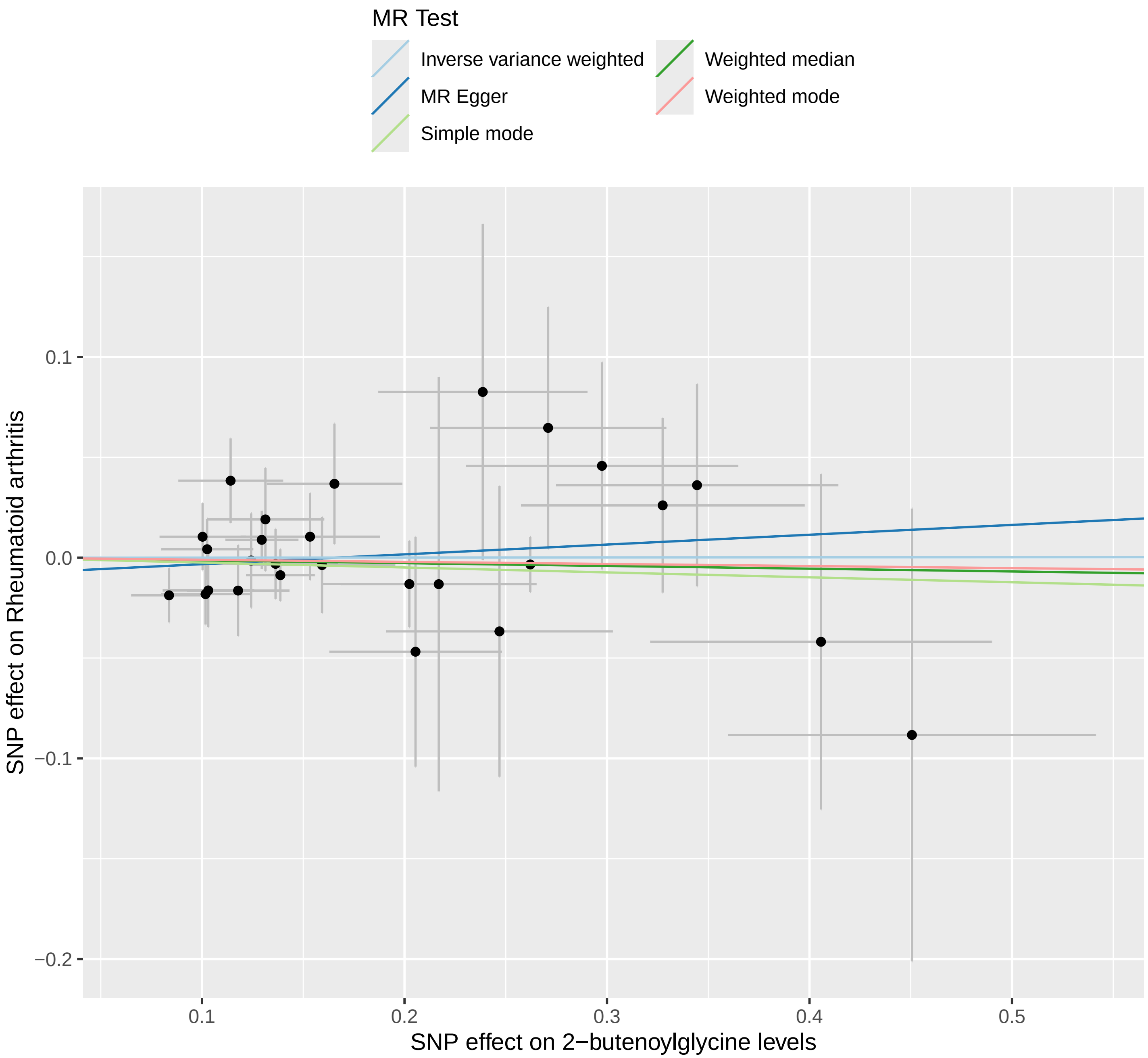
MR Test
Inverse variance weighted
MR Egger
Simple mode
Weighted median
Weighted mode
0.1
0.0
−0.1
−0.2
SNP effect on Rheumatoid arthritis
0.1
0.2
0.3
0.4
0.5
SNP effect on 2−butenoylglycine levels

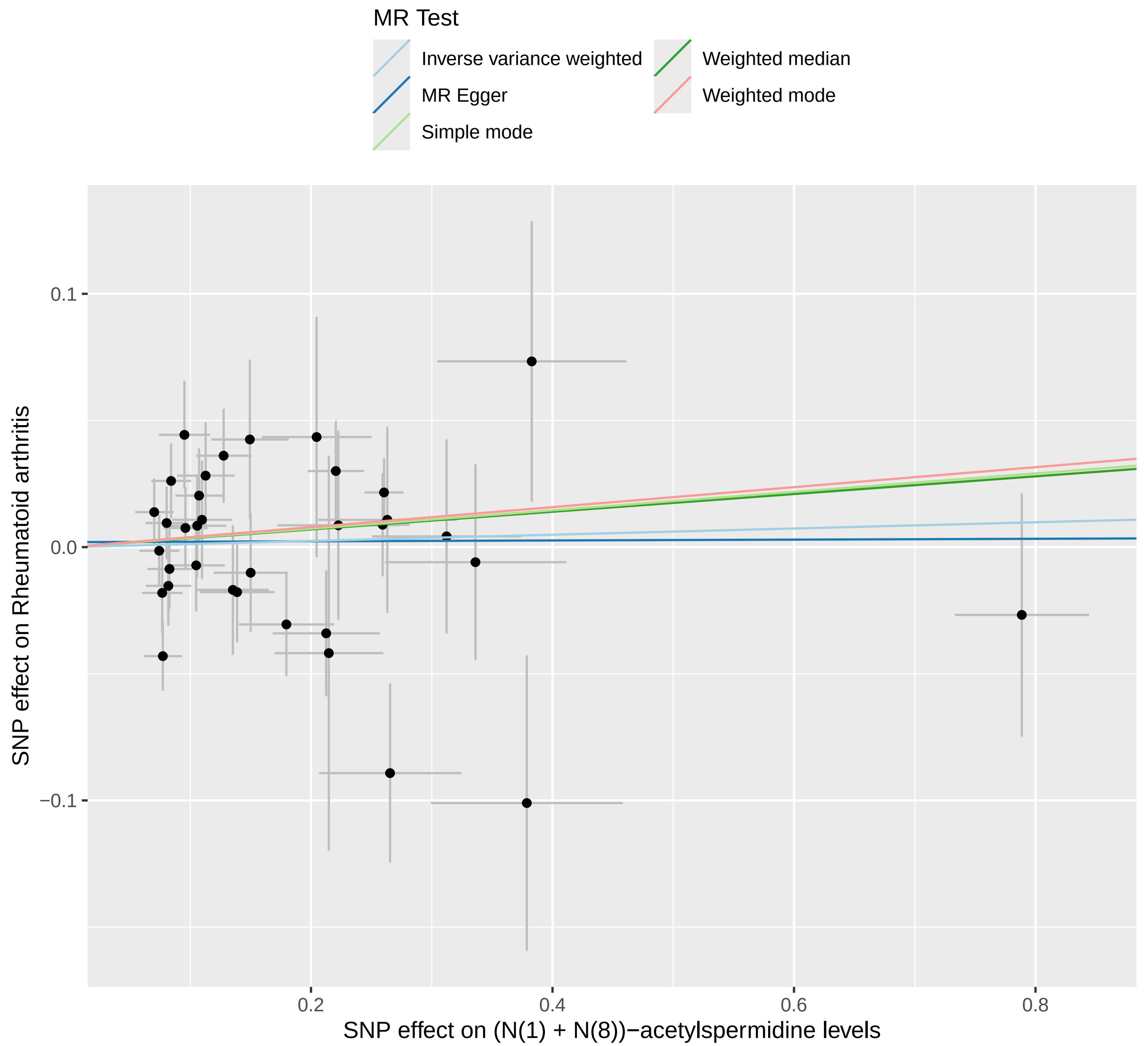

MR Test
Inverse variance weighted
MR Egger
Simple mode
Weighted median
Weighted mode
0.1
0.0
−0.1
SNP effect on Rheumatoid arthritis
0.2
0.4
0.6
0.8
SNP effect on (N(1) + N(8))−acetylspermidine levels

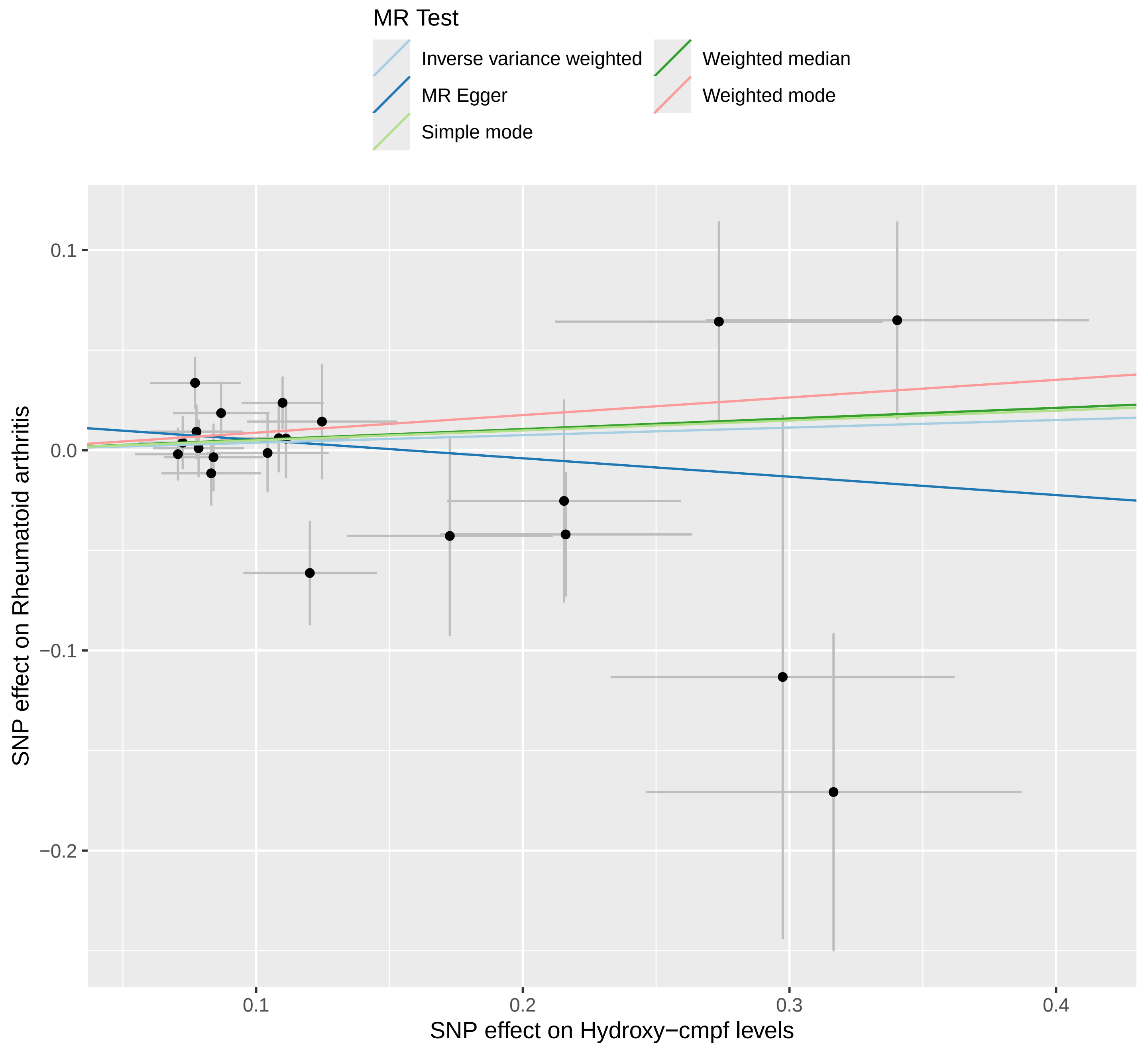

MR Test
Inverse variance weighted
MR Egger
Simple mode
Weighted median
Weighted mode
0.1
0.0
−0.1
−0.2
SNP effect on Rheumatoid arthritis
0.1
0.2
0.3
0.4
SNP effect on Hydroxy−cmpf levels

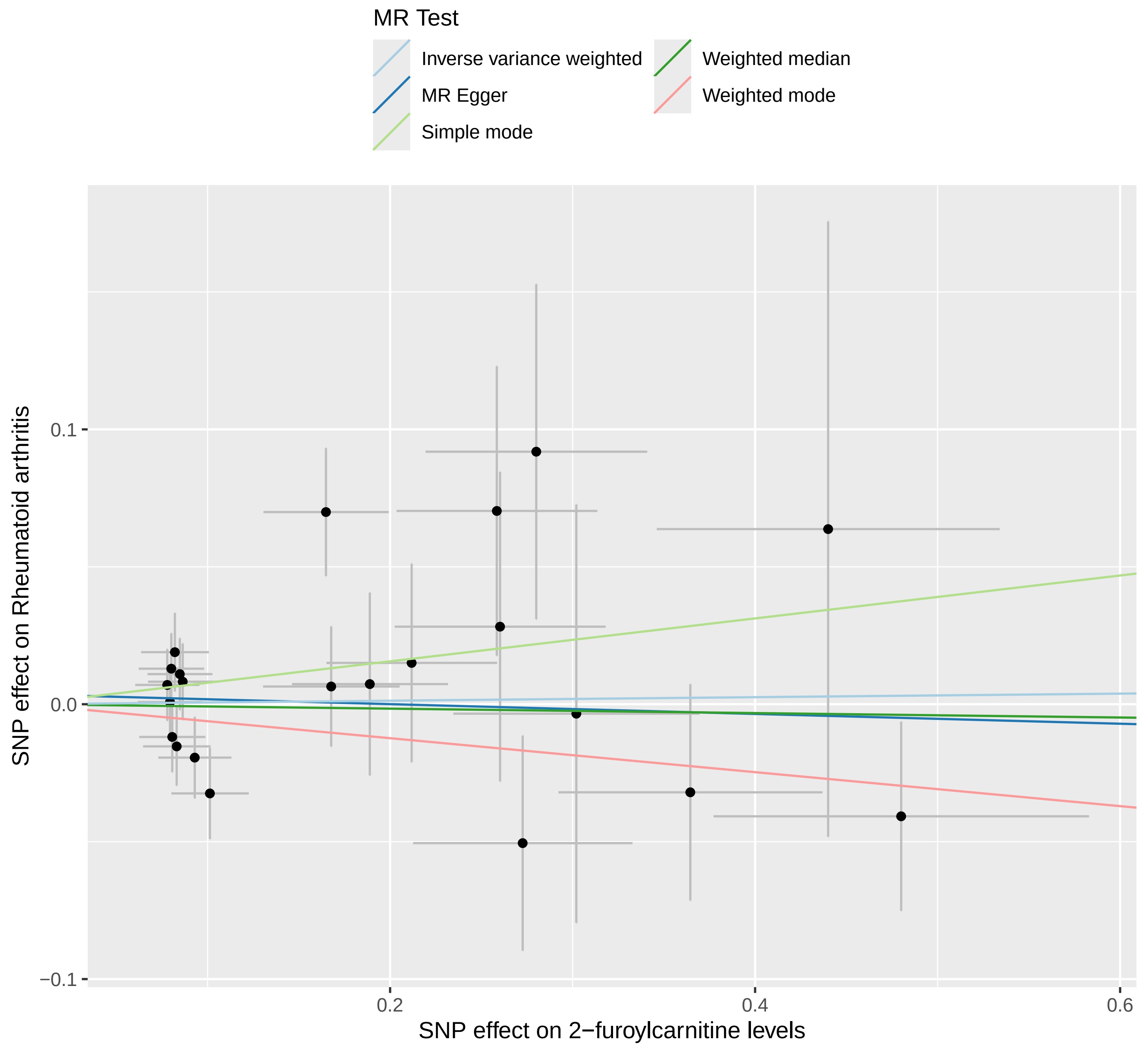

MR Test
Inverse variance weighted
MR Egger
Simple mode
Weighted median
Weighted mode
SNP effect on Rheumatoid arthritis
0.1
0.0
−0.1
0.2
0.4
0.6
SNP effect on 2−furoylcarnitine levels

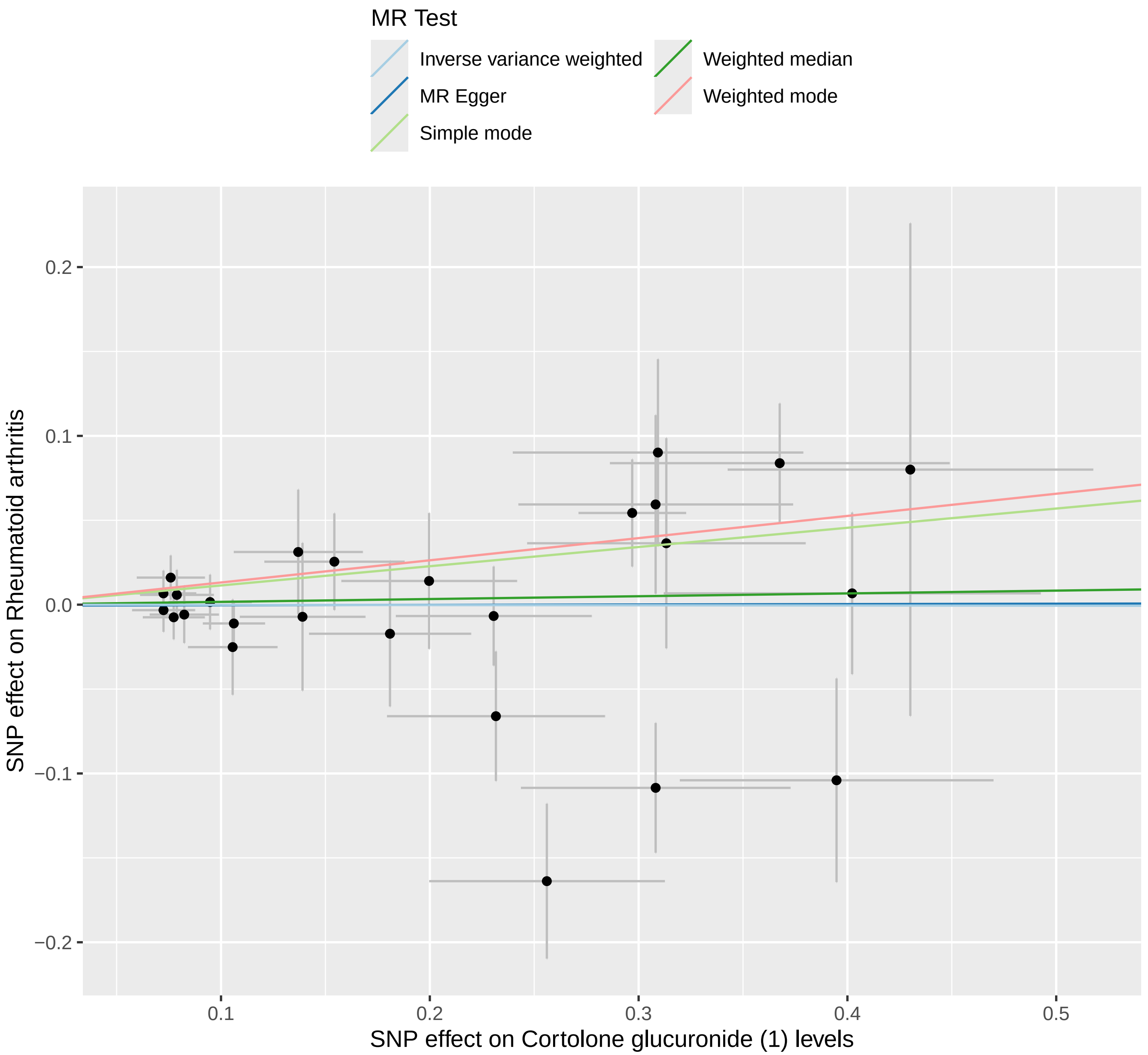
MR Test
Inverse variance weighted
MR Egger
Simple mode
Weighted median
Weighted mode
0.2
0.1
0.0
−0.1
−0.2
SNP effect on Rheumatoid arthritis
0.1
0.2
0.3
0.4
0.5
SNP effect on Cortolone glucuronide (1) levels

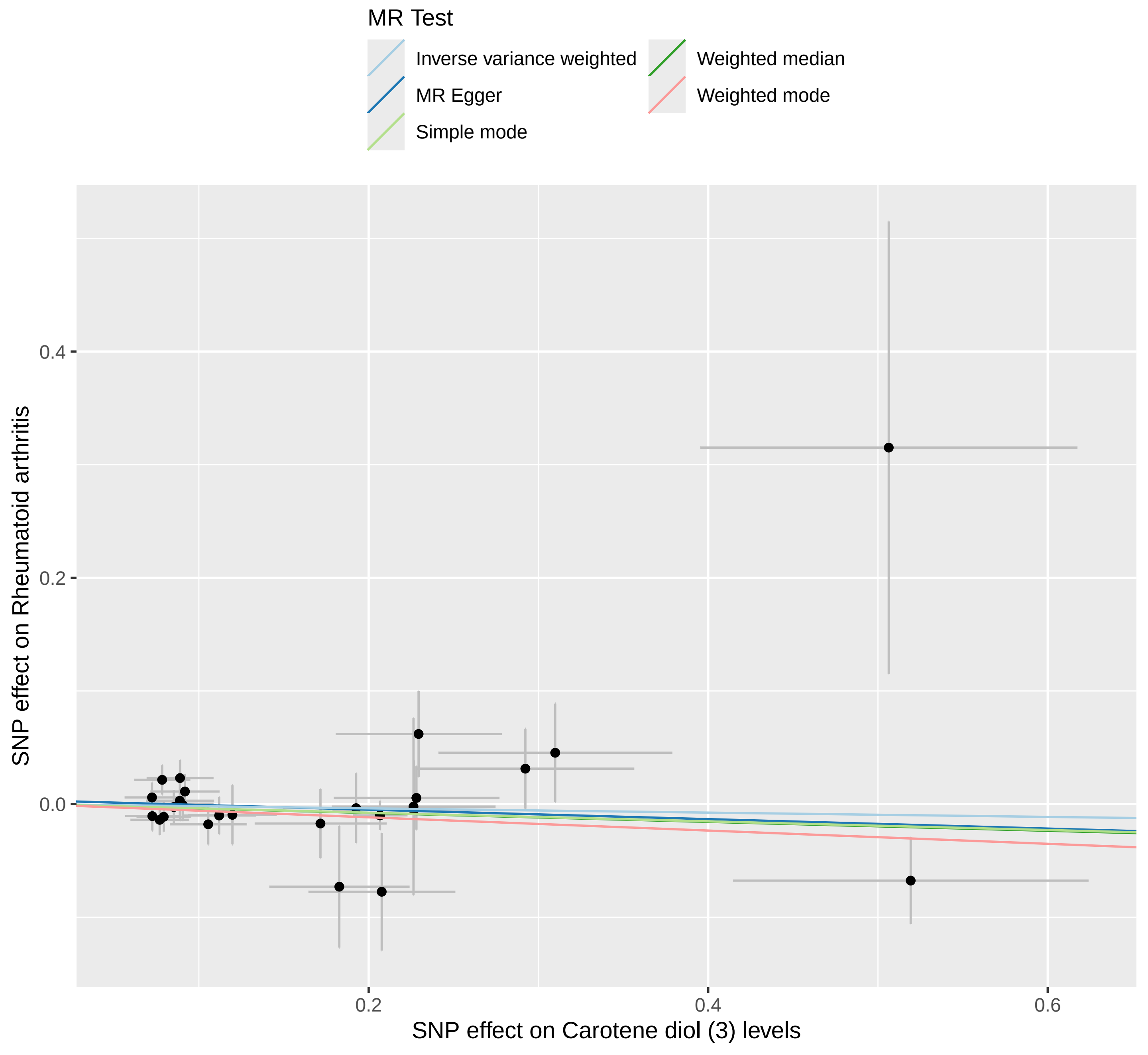

MR Test
Inverse variance weighted
MR Egger
Simple mode
Weighted median
Weighted mode
SNP effect on Rheumatoid arthritis
0.4
0.2
0.0
0.2
0.4
0.6
SNP effect on Carotene diol (3) levels

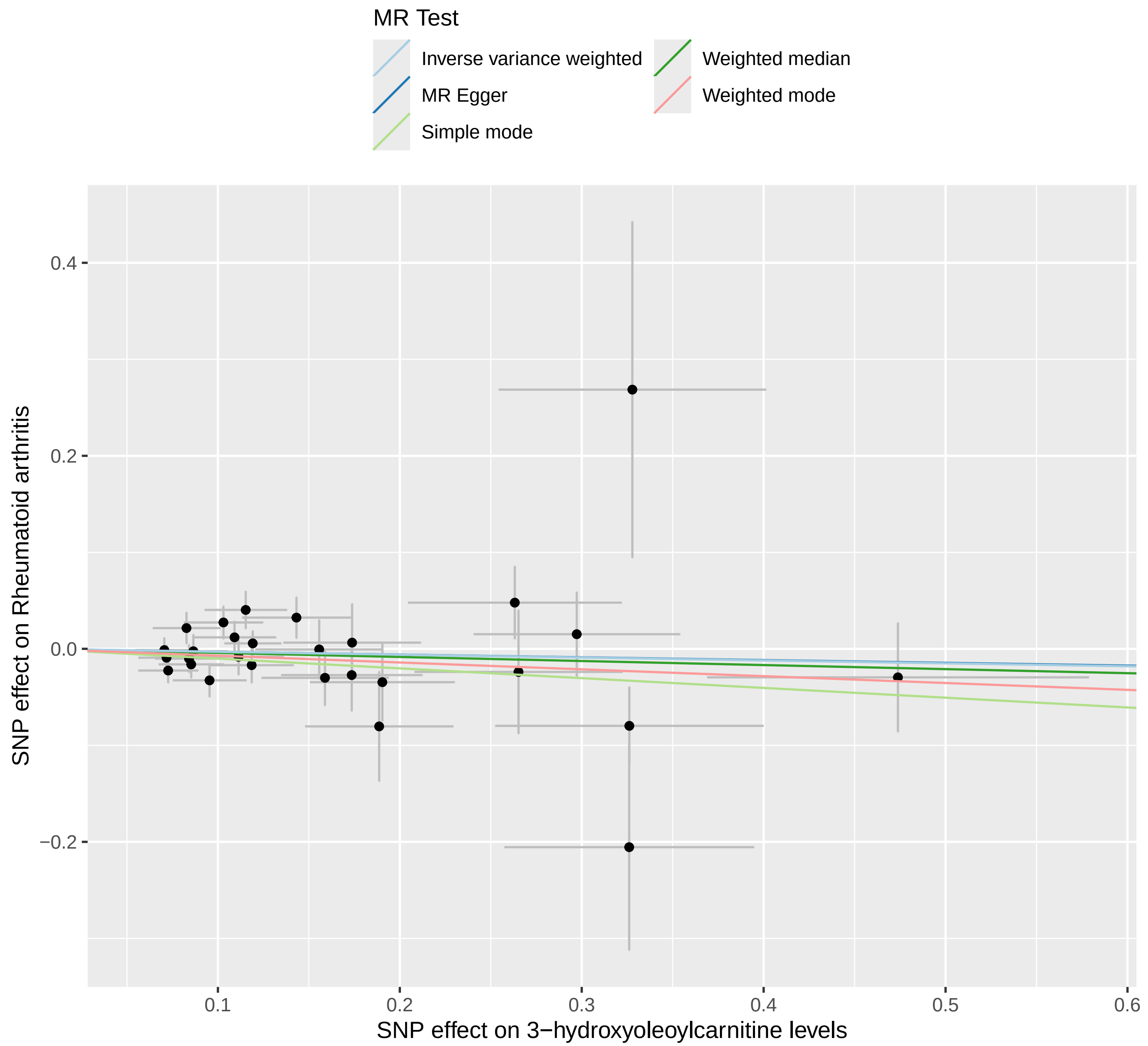
MR Test
Inverse variance weighted
MR Egger
Simple mode
Weighted median
Weighted mode
0.4
0.2
0.0
−0.2
0.1
0.2
0.3
0.4
0.5
0.6
SNP effect on Rheumatoid arthritis
SNP effect on 3−hydroxyoleoylcarnitine levels

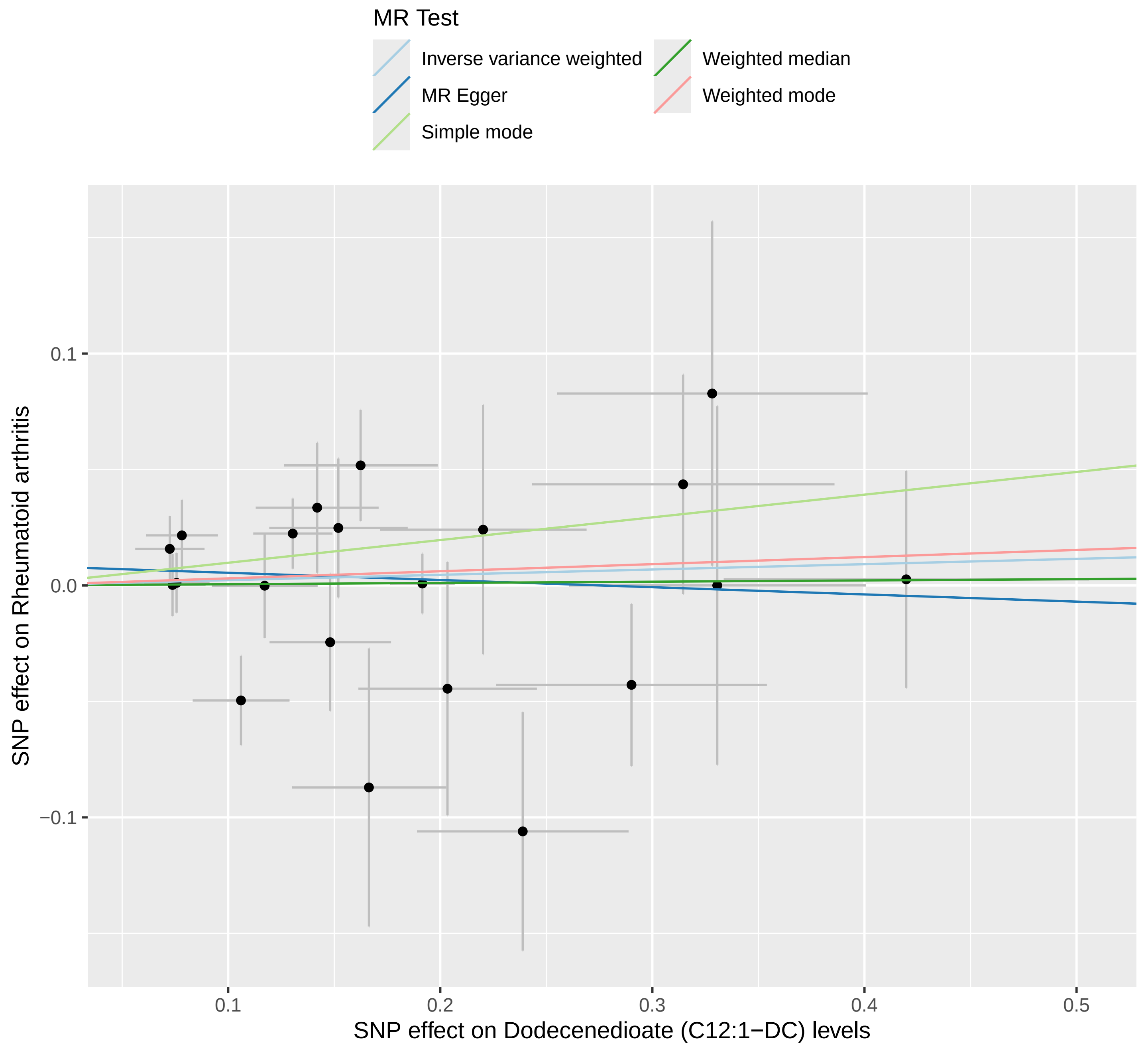
MR Test
Inverse variance weighted
MR Egger
Simple mode
Weighted median
Weighted mode
SNP effect on Rheumatoid arthritis
0.1
0.0
−0.1
0.1
0.2
0.3
0.4
0.5
SNP effect on Dodecenedioate (C12:1−DC) levels

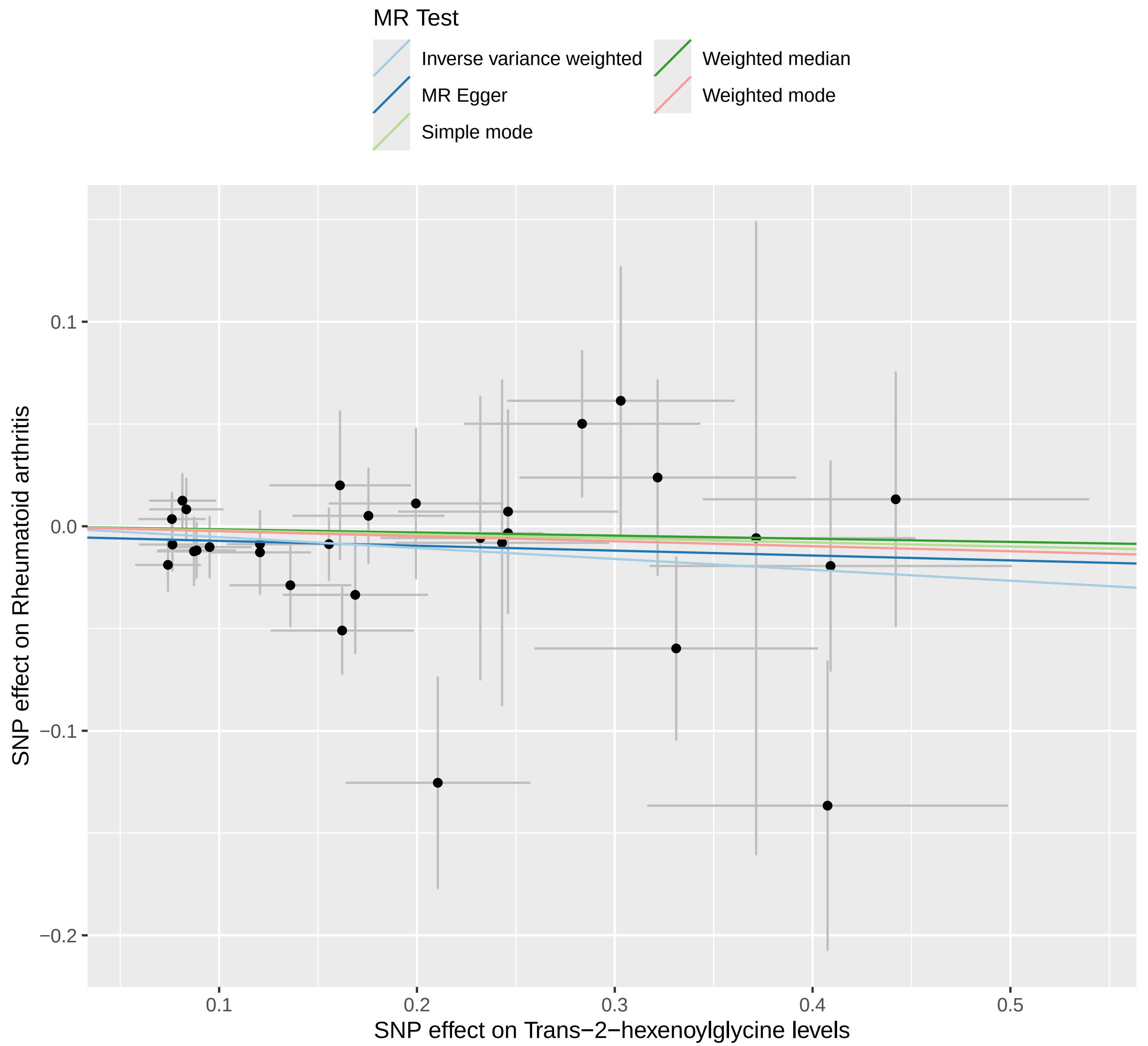
MR Test
Inverse variance weighted
MR Egger
Simple mode
Weighted median
Weighted mode
0.1
0.0
−0.1
−0.2
0.1
0.2
0.3
0.4
0.5
SNP effect on Rheumatoid arthritis
SNP effect on Trans−2−hexenoylglycine levels

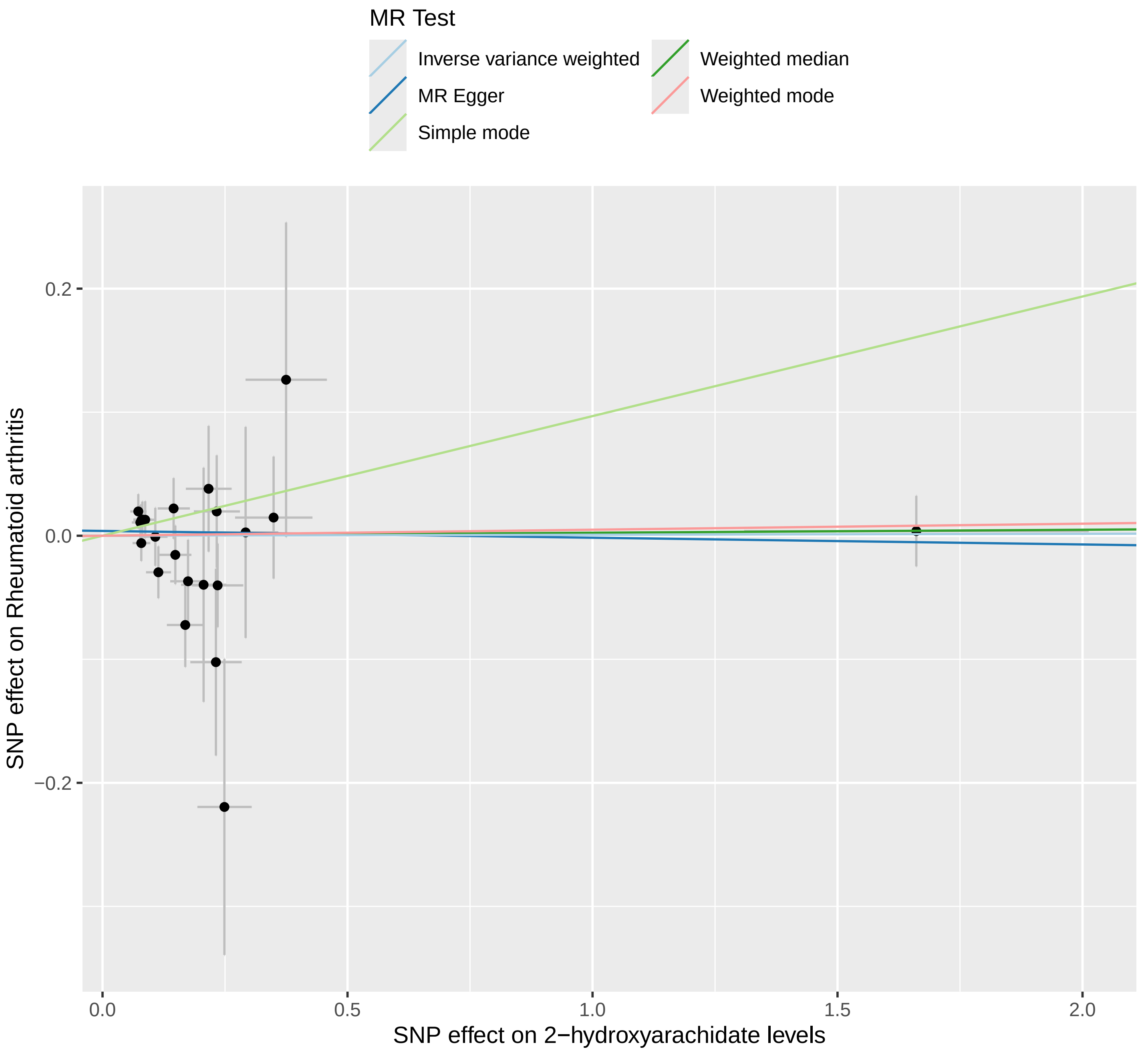
MR Test
Inverse variance weighted
MR Egger
Simple mode
Weighted median
Weighted mode
0.2
0.0
−0.2
0.0
0.5
1.0
1.5
2.0
SNP effect on Rheumatoid arthritis
SNP effect on 2−hydroxyarachidate levels

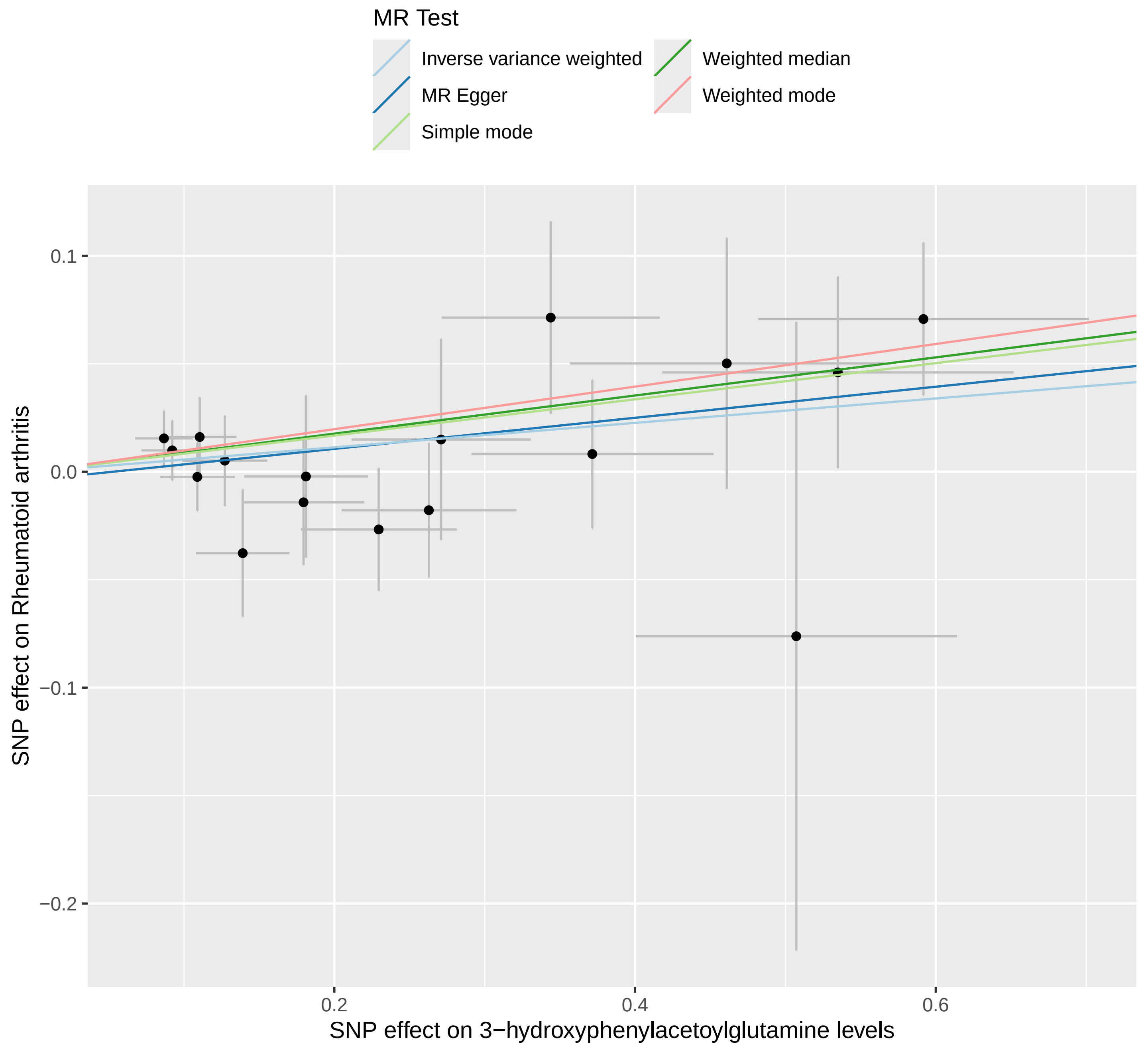

MR Test
Inverse variance weighted
MR Egger
Simple mode
Weighted median
Weighted mode
0.1
0.0
−0.1
−0.2
0.2
0.4
0.6
SNP effect on Rheumatoid arthritis
SNP effect on 3−hydroxyphenylacetoylglutamine levels

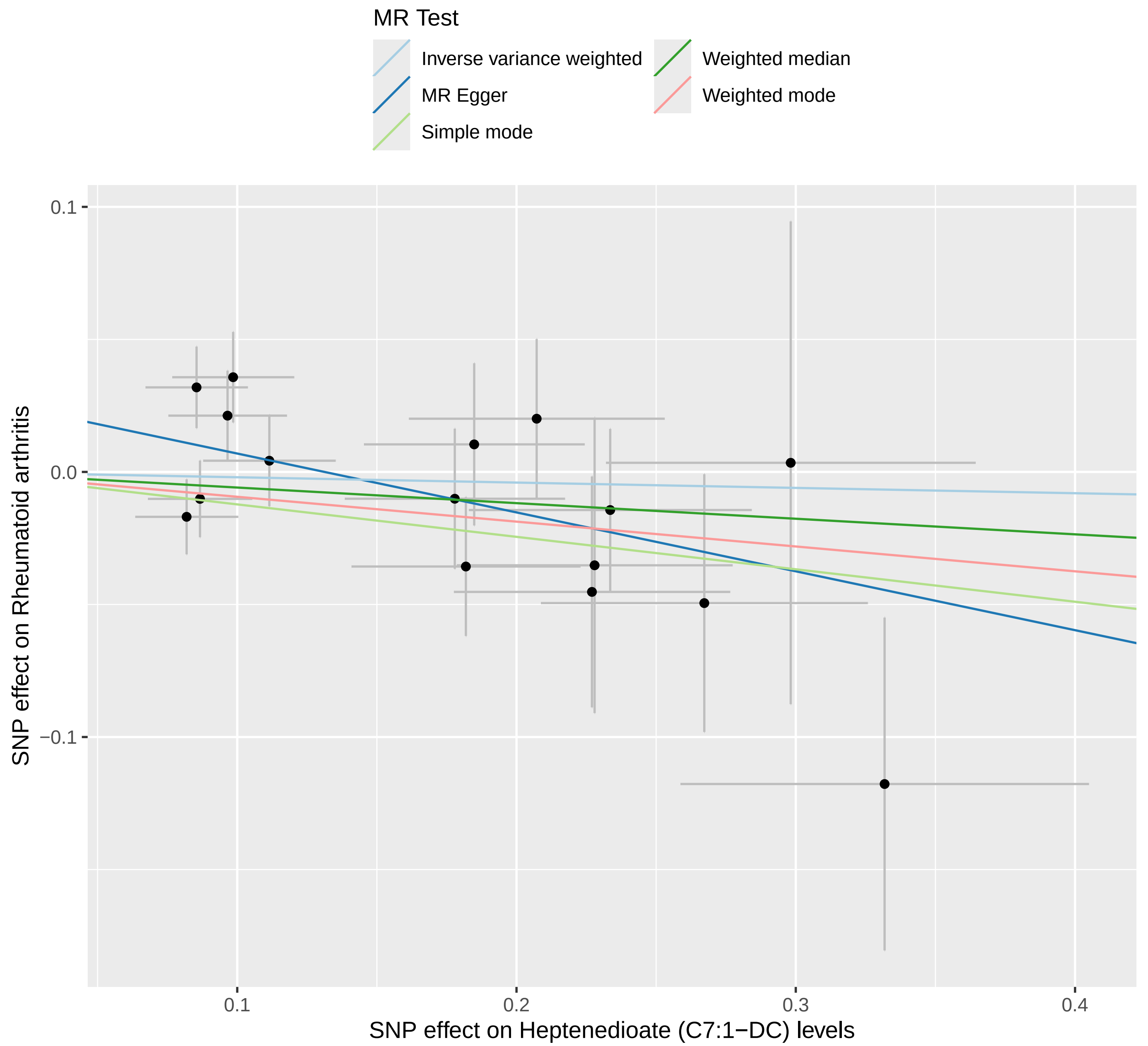
MR Test
Inverse variance weighted
MR Egger
Simple mode
Weighted median
Weighted mode
0.1
0.0
−0.1
0.1
0.2
0.3
0.4
SNP effect on Rheumatoid arthritis
SNP effect on Heptenedioate (C7:1−DC) levels

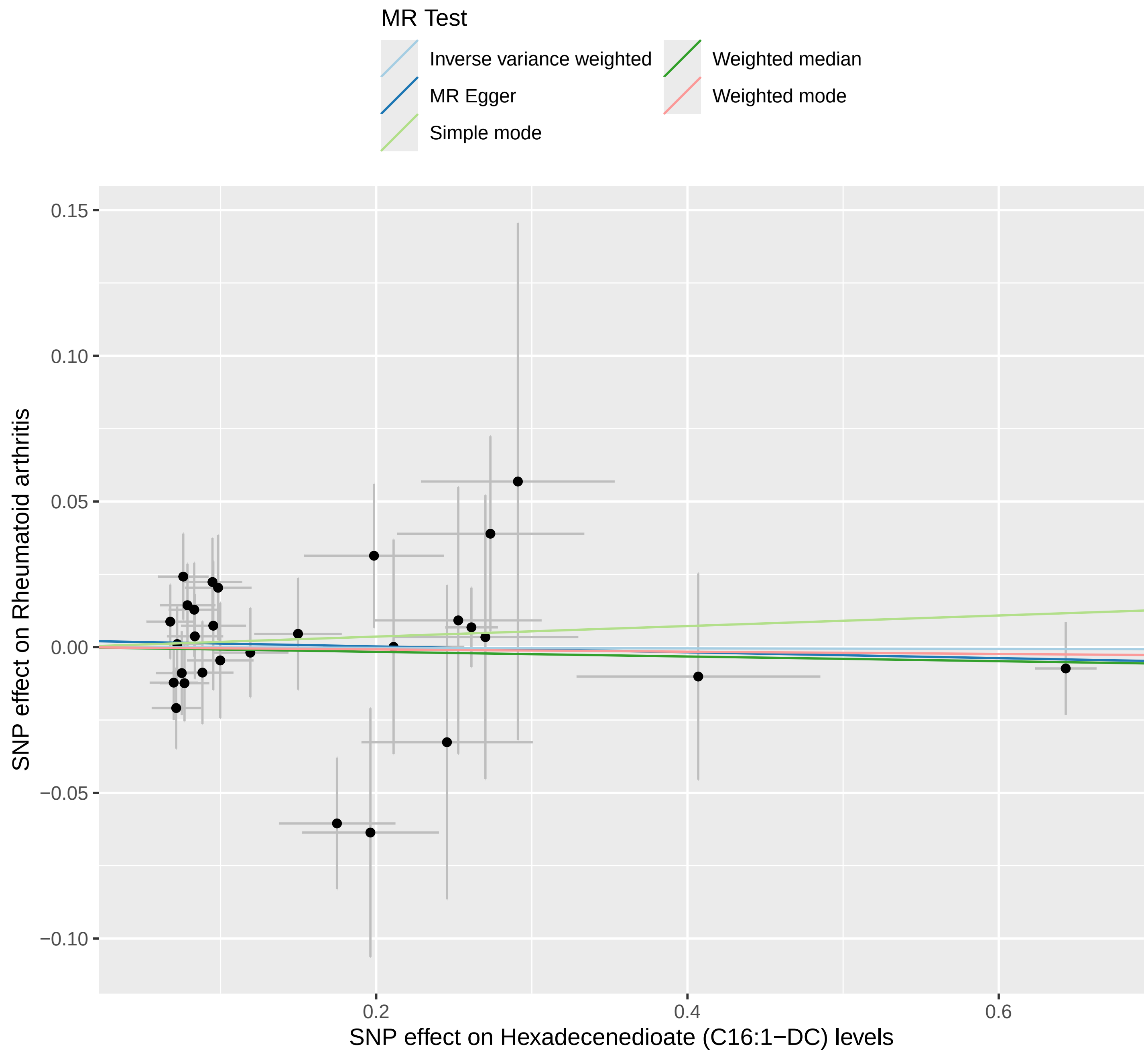
MR Test
Inverse variance weighted
MR Egger
Simple mode
Weighted median
Weighted mode
0.15
0.10
0.05
0.00
−0.05
−0.10
SNP effect on Rheumatoid arthritis
0.2
0.4
0.6
SNP effect on Hexadecenedioate (C16:1−DC) levels

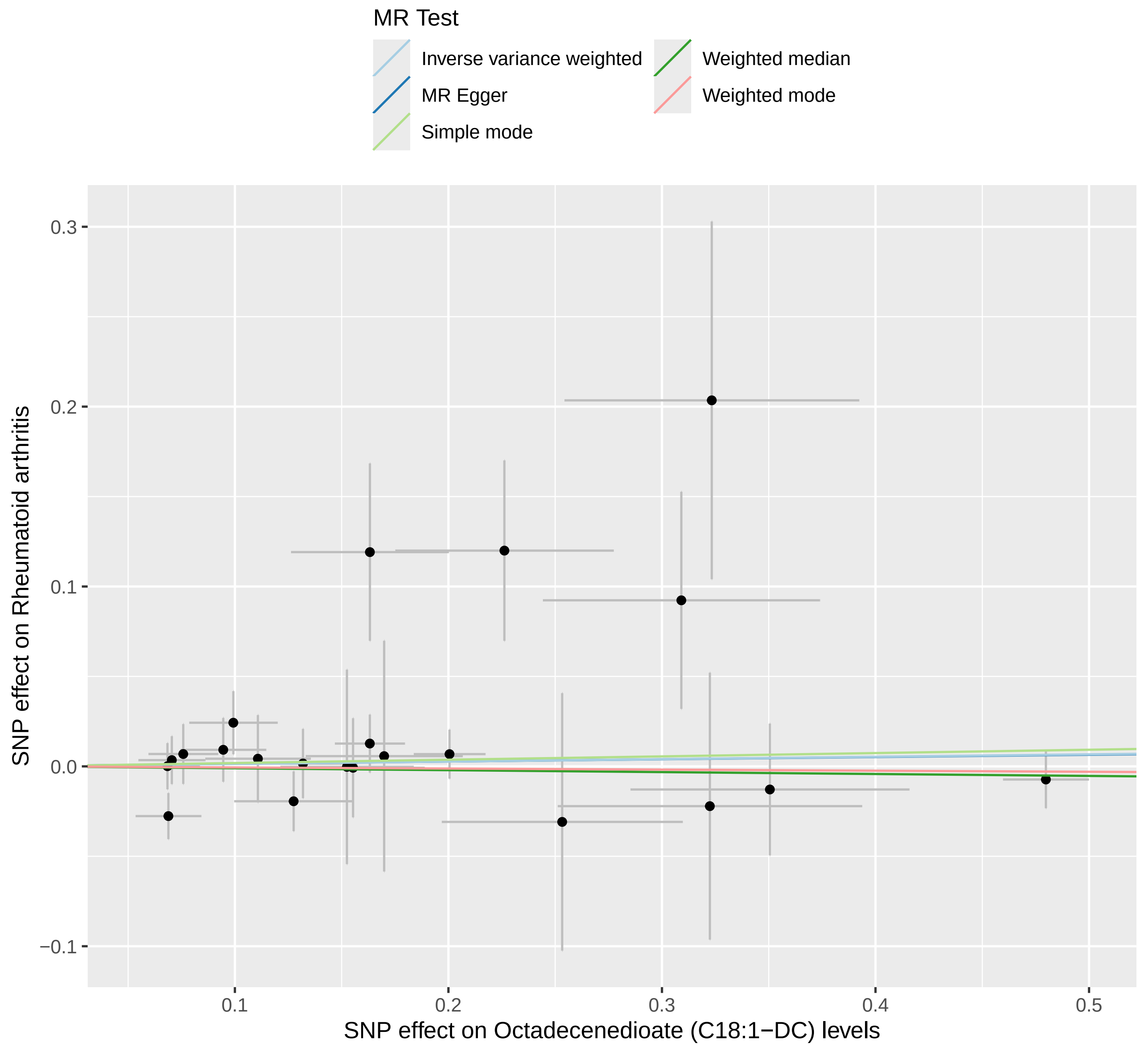
MR Test
Inverse variance weighted
MR Egger
Simple mode
Weighted median
Weighted mode
SNP effect on Rheumatoid arthritis
0.3
0.2
0.1
0.0
−0.1
0.1
0.2
0.3
0.4
0.5
SNP effect on Octadecenedioate (C18:1−DC) levels

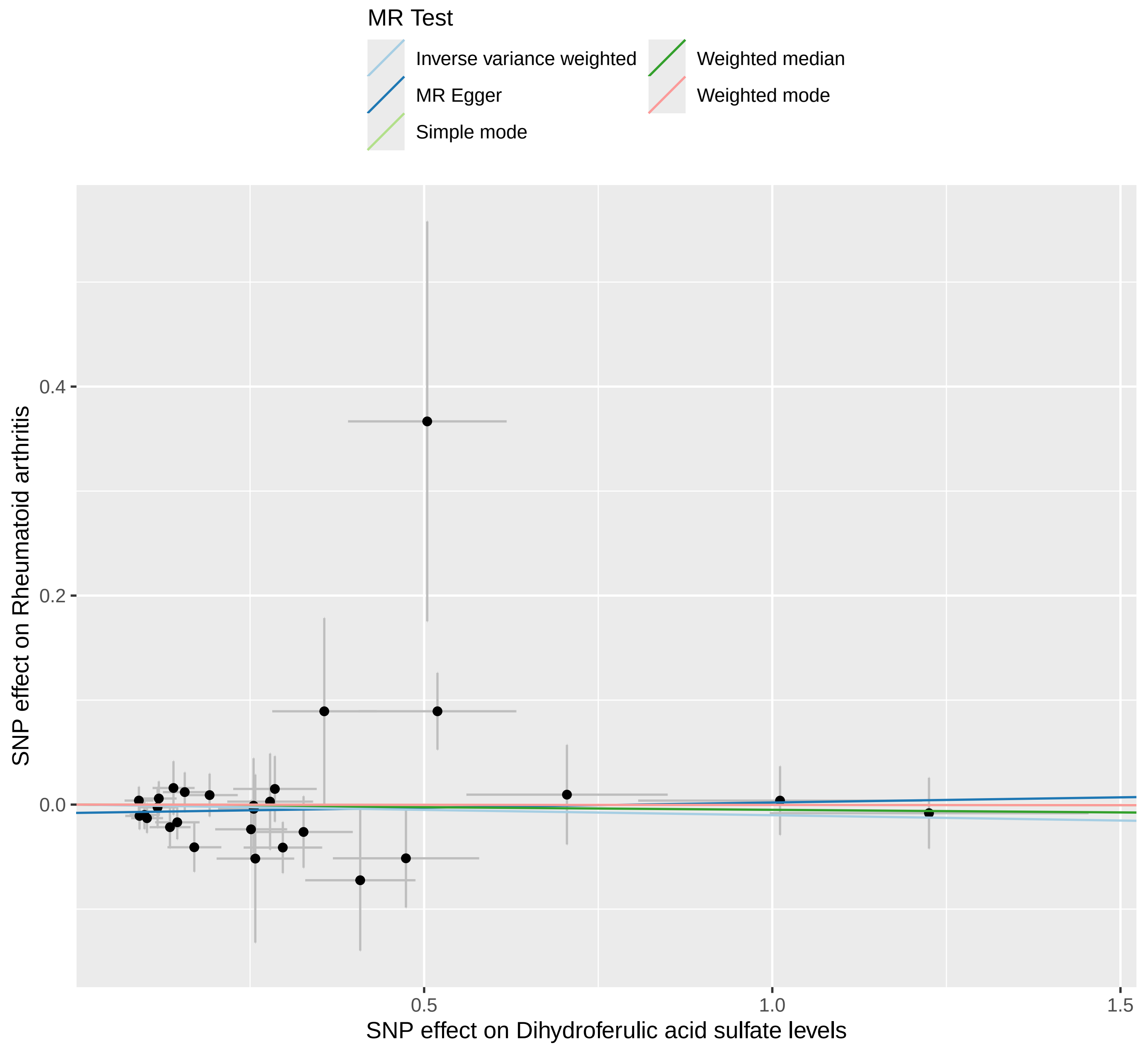

MR Test
Inverse variance weighted
MR Egger
Simple mode
Weighted median
Weighted mode
SNP effect on Rheumatoid arthritis
0.4
0.2
0.0
0.5
1.0
1.5
SNP effect on Dihydroferulic acid sulfate levels

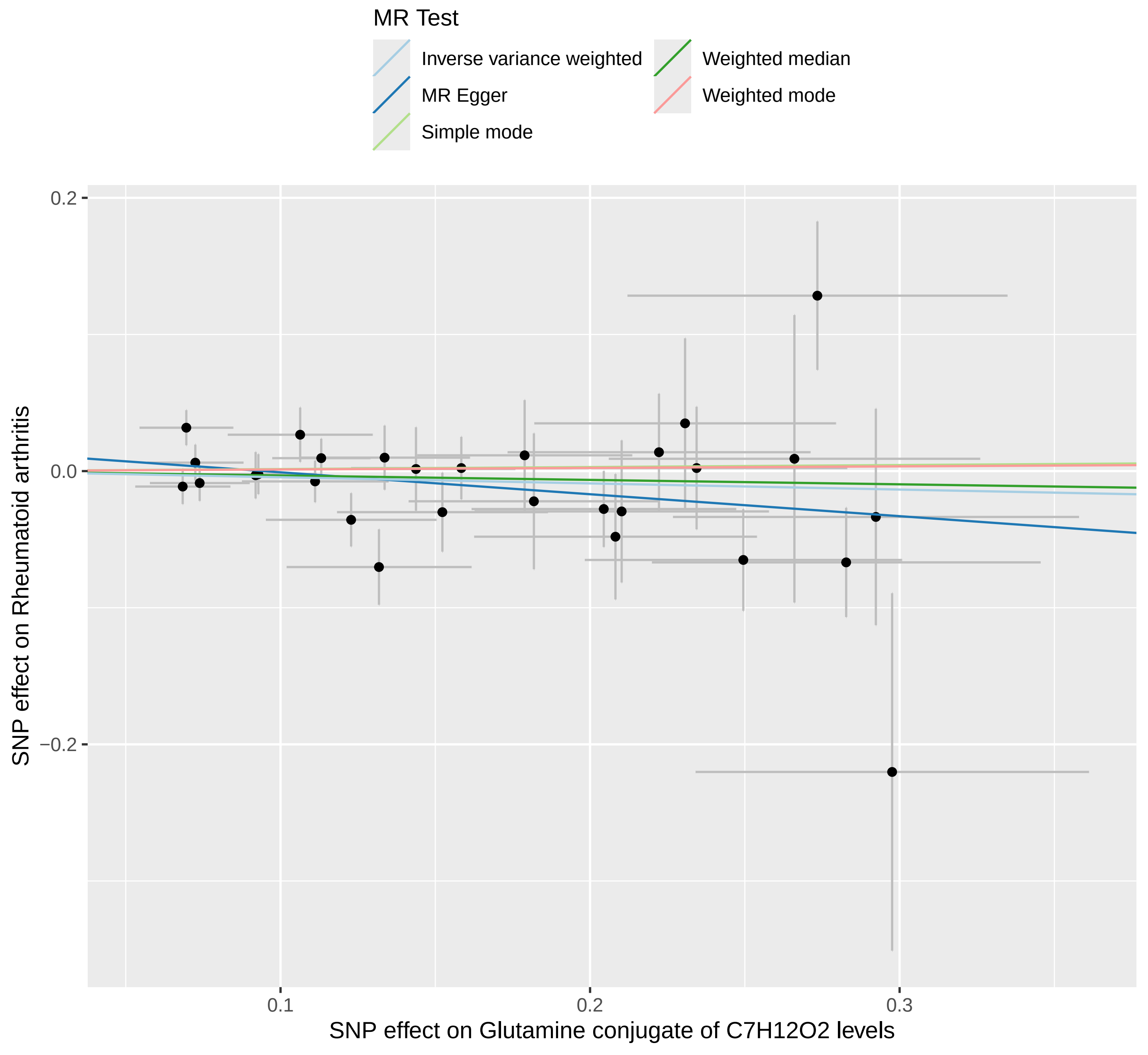

MR Test
Inverse variance weighted
MR Egger
Simple mode
Weighted median
Weighted mode
0.2
0.0
−0.2
0.1
0.2
0.3
SNP effect on Rheumatoid arthritis
SNP effect on Glutamine conjugate of C7H12O2 levels

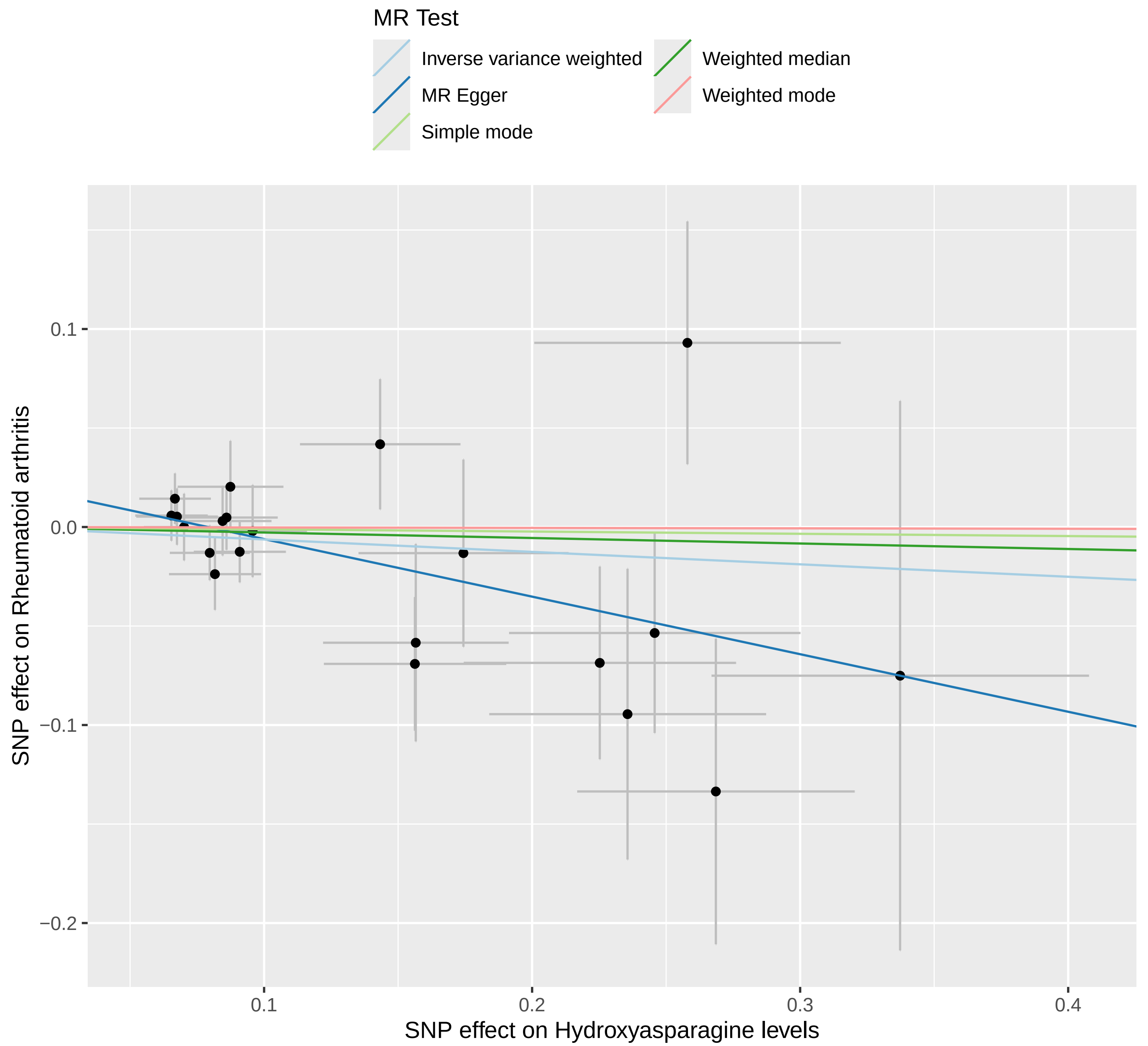

MR Test
Inverse variance weighted
MR Egger
Simple mode
Weighted median
Weighted mode
0.1
0.0
−0.1
−0.2
SNP effect on Rheumatoid arthritis
0.1
0.2
0.3
0.4
SNP effect on Hydroxyasparagine levels

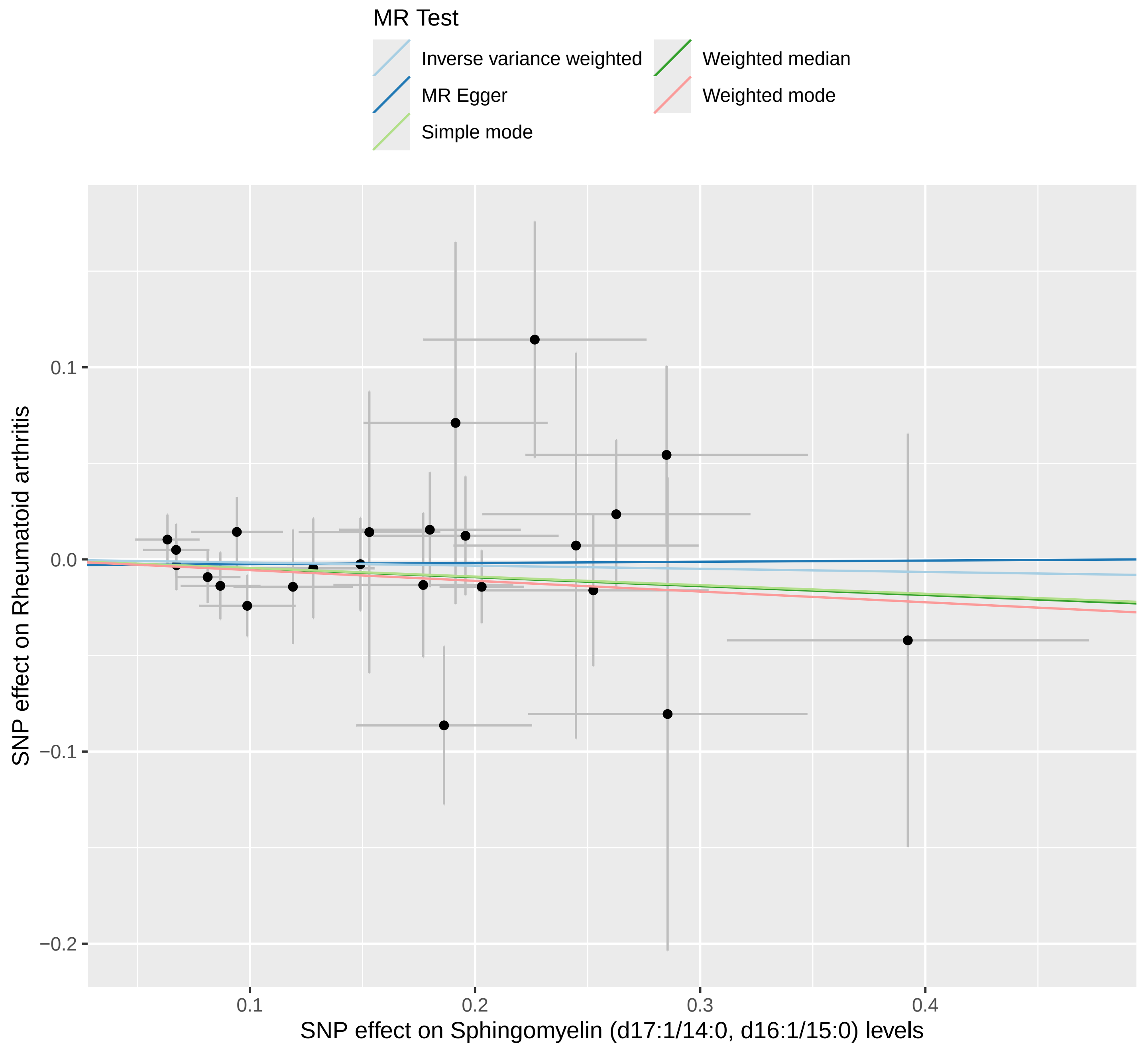
MR Test
Inverse variance weighted
MR Egger
Simple mode
Weighted median
Weighted mode
SNP effect on Rheumatoid arthritis
0.1
0.0
−0.1
−0.2
0.1
0.2
0.3
0.4
SNP effect on Sphingomyelin (d17:1/14:0, d16:1/15:0) levels

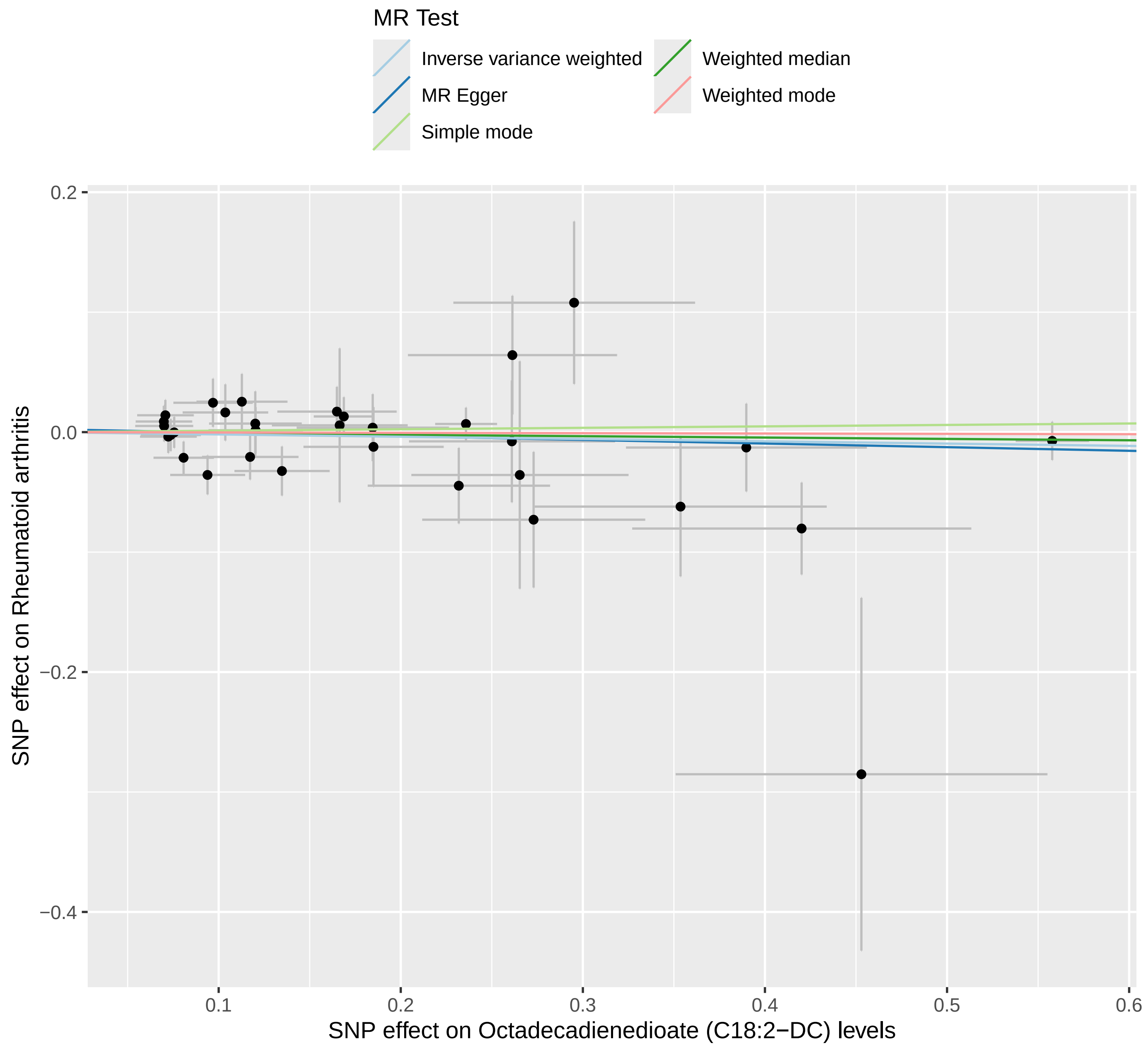

MR Test
Inverse variance weighted
MR Egger
Simple mode
Weighted median
Weighted mode
0.2
0.0
−0.2
−0.4
SNP effect on Rheumatoid arthritis
0.1
0.2
0.3
0.4
0.5
0.6
SNP effect on Octadecadienedioate (C18:2−DC) levels

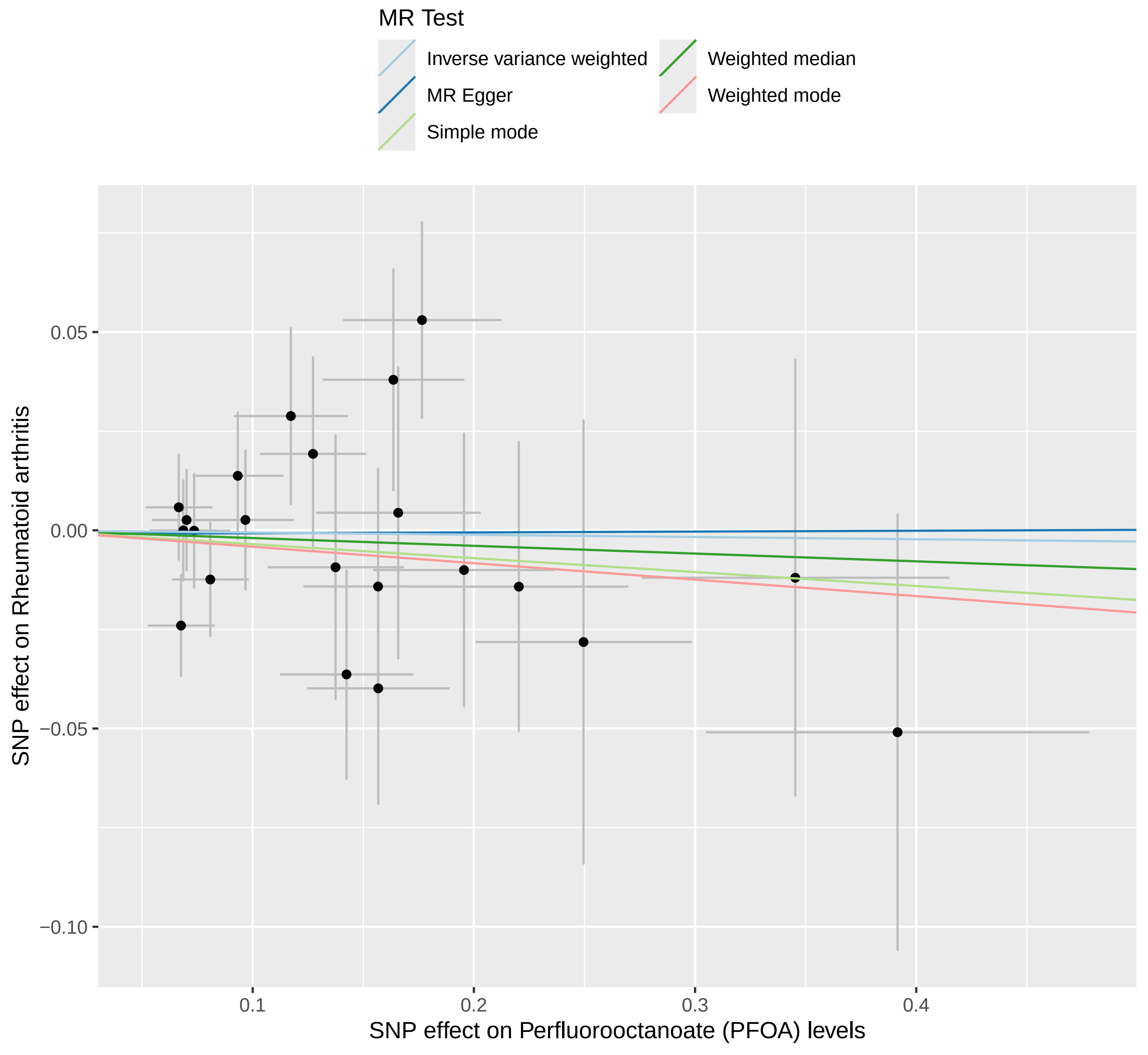
MR Test
Inverse variance weighted
MR Egger
Simple mode
Weighted median
Weighted mode
0.05
0.00
−0.05
−0.10
SNP effect on Rheumatoid arthritis
0.1
0.2
0.3
0.4
SNP effect on Perfluorooctanoate (PFOA) levels

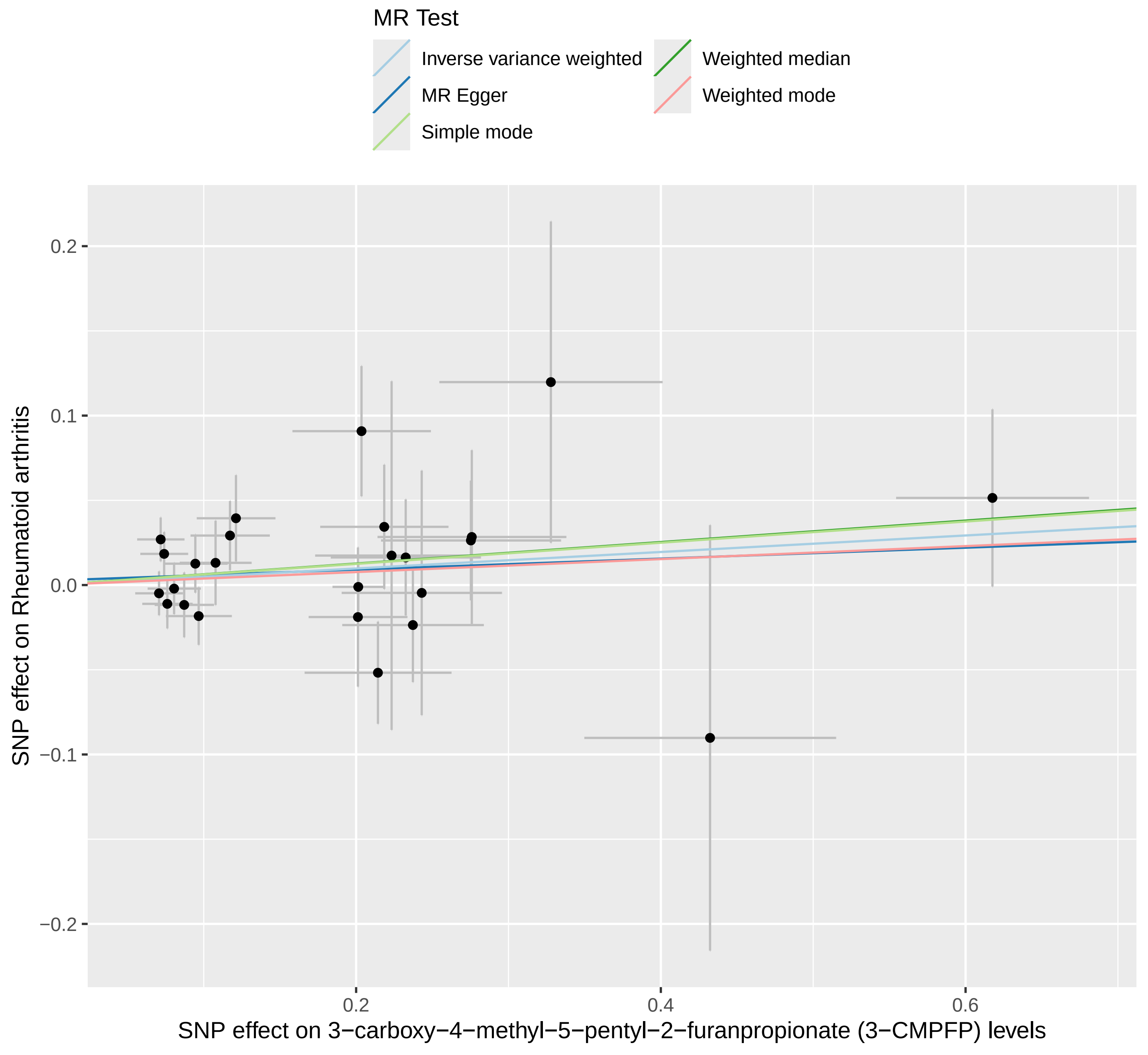
MR Test
Inverse variance weighted
MR Egger
Simple mode
Weighted median
Weighted mode
0.2
0.1
0.0
−0.1
−0.2
SNP effect on Rheumatoid arthritis
0.2
0.4
0.6
SNP effect on 3−carboxy−4−methyl−5−pentyl−2−furanpropionate (3−CMPFP) levels

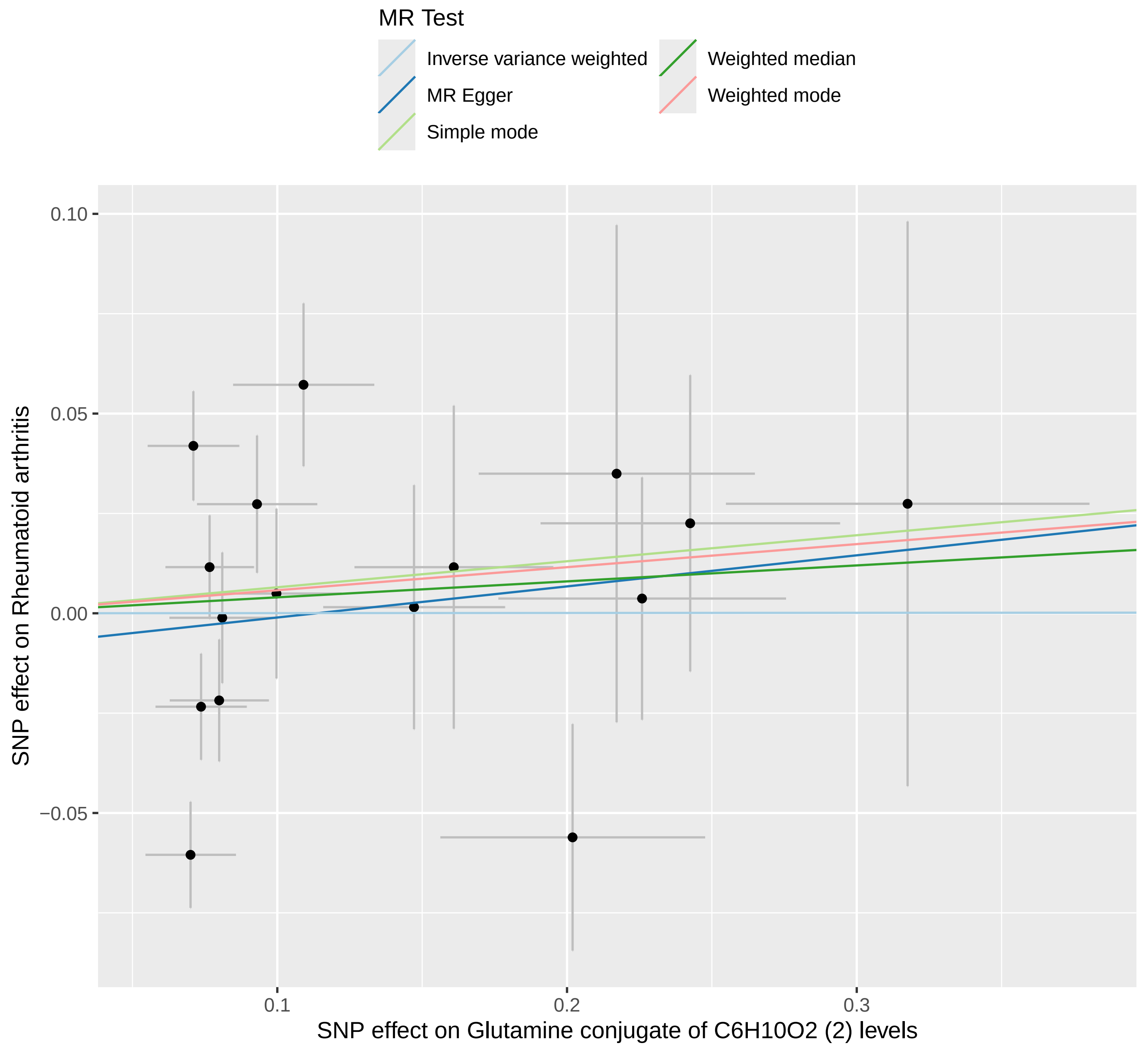
MR Test
Inverse variance weighted
MR Egger
Simple mode
Weighted median
Weighted mode
0.10
0.05
0.00
−0.05
0.1
0.2
0.3
SNP effect on Rheumatoid arthritis
SNP effect on Glutamine conjugate of C6H10O2 (2) levels

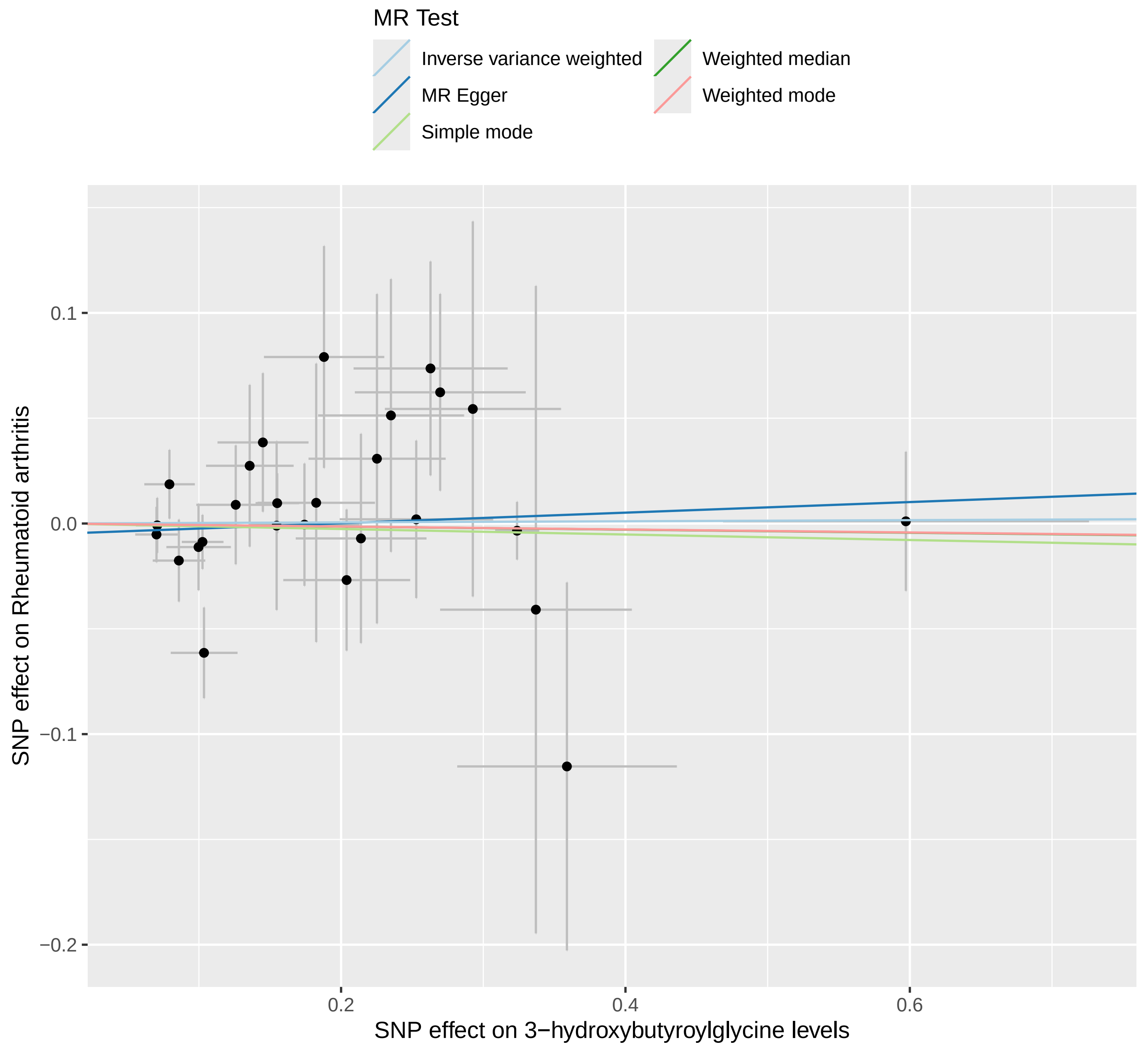
MR Test
Inverse variance weighted
MR Egger
Simple mode
Weighted median
Weighted mode
0.1
0.0
−0.1
−0.2
0.2
0.4
0.6
SNP effect on Rheumatoid arthritis
SNP effect on 3−hydroxybutyroylglycine levels

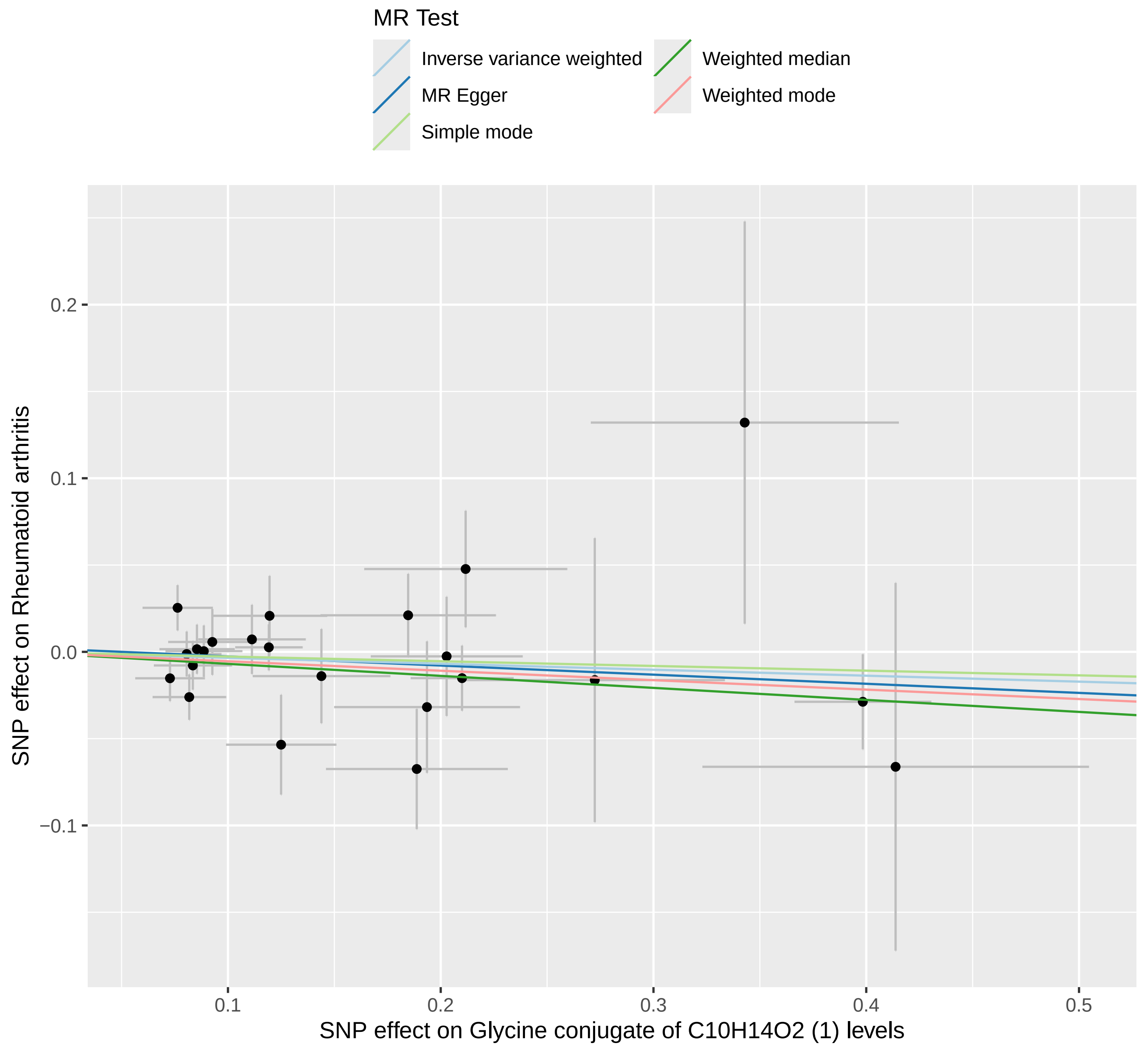

MR Test
Inverse variance weighted
MR Egger
Simple mode
Weighted median
Weighted mode
0.2
0.1
0.0
−0.1
SNP effect on Rheumatoid arthritis
0.1
0.2
0.3
0.4
0.5
SNP effect on Glycine conjugate of C10H14O2 (1) levels

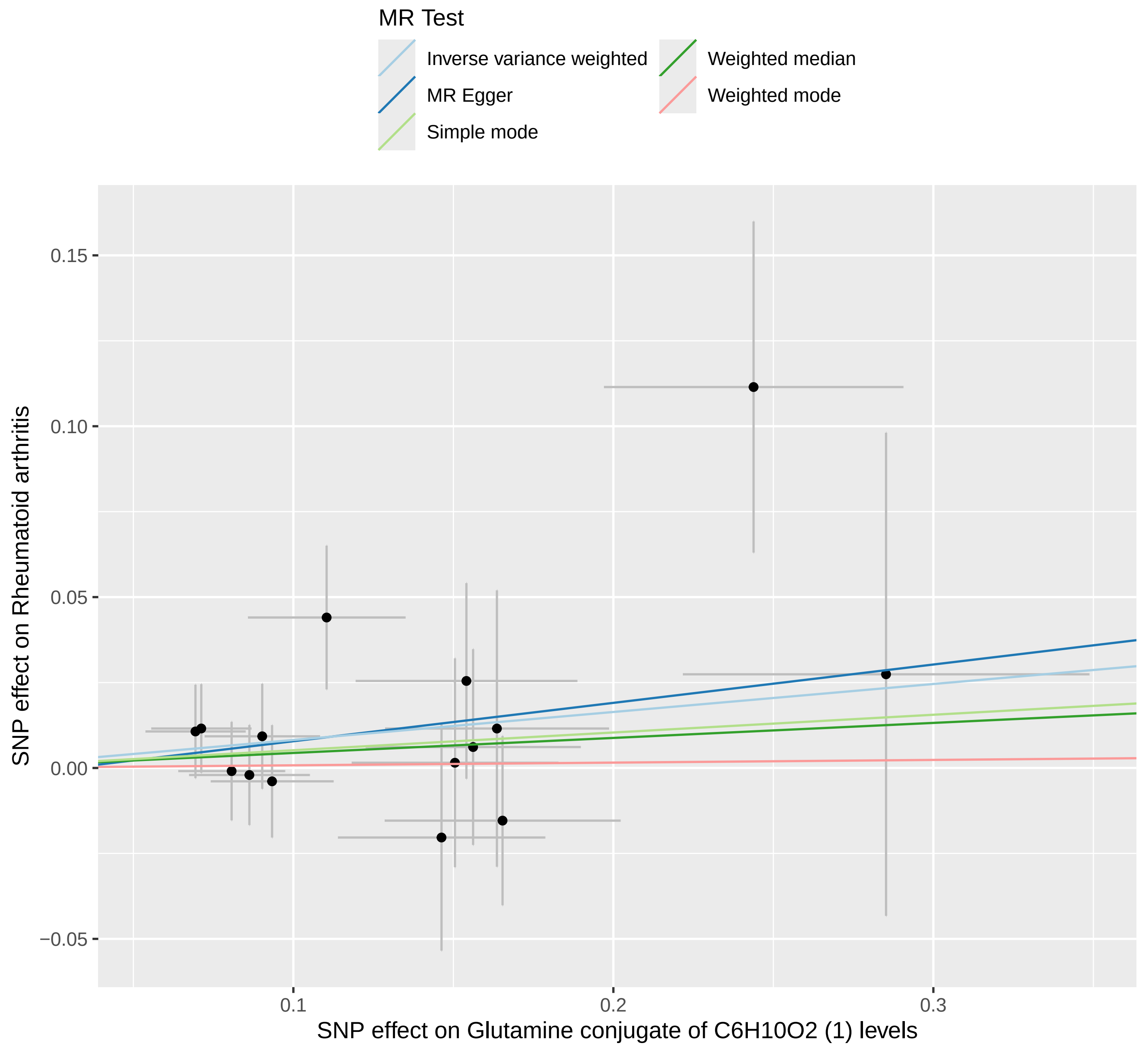
MR Test
Inverse variance weighted
MR Egger
Simple mode
Weighted median
Weighted mode
SNP effect on Rheumatoid arthritis
0.15
0.10
0.05
0.00
−0.05
0.1
0.2
0.3
SNP effect on Glutamine conjugate of C6H10O2 (1) levels

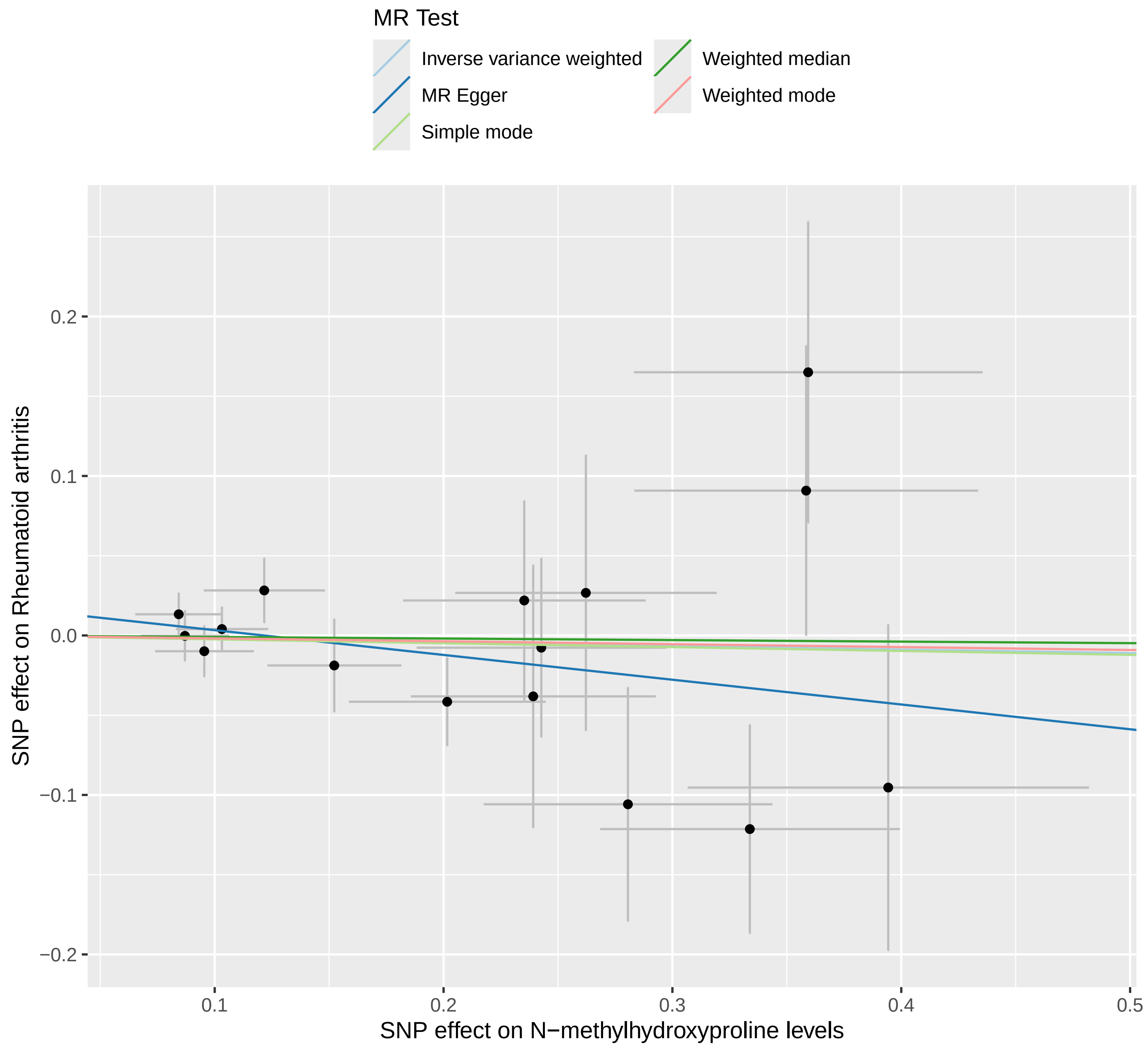
MR Test
Inverse variance weighted
MR Egger
Simple mode
Weighted median
Weighted mode
SNP effect on Rheumatoid arthritis
0.2
0.1
0.0
−0.1
−0.2
0.1
0.2
0.3
0.4
0.5
SNP effect on N−methylhydroxyproline levels

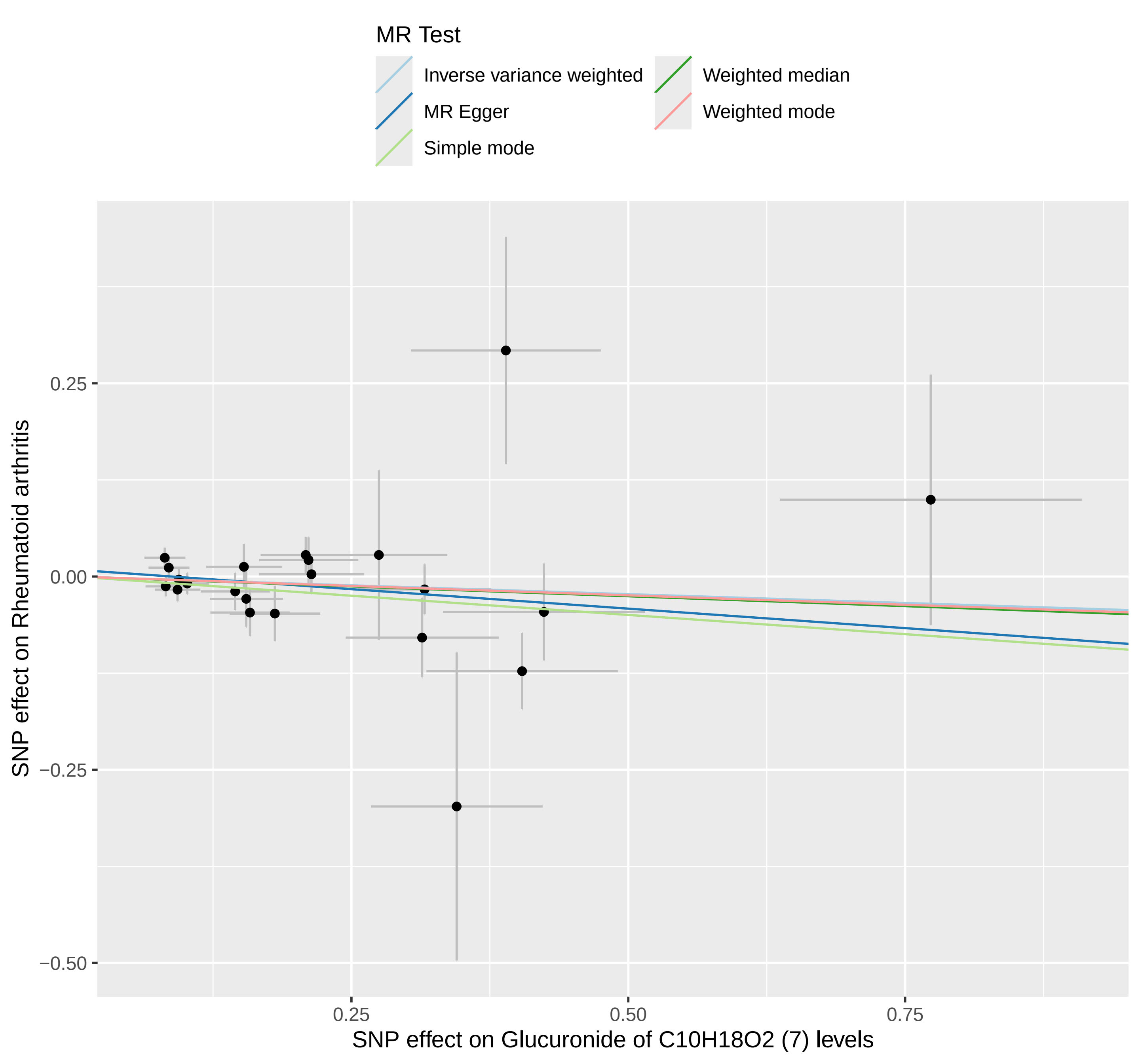
MR Test
Inverse variance weighted
MR Egger
Simple mode
Weighted median
Weighted mode
0.25
0.00
−0.25
−0.50
0.25
0.50
0.75
SNP effect on Rheumatoid arthritis
SNP effect on Glucuronide of C10H18O2 (7) levels

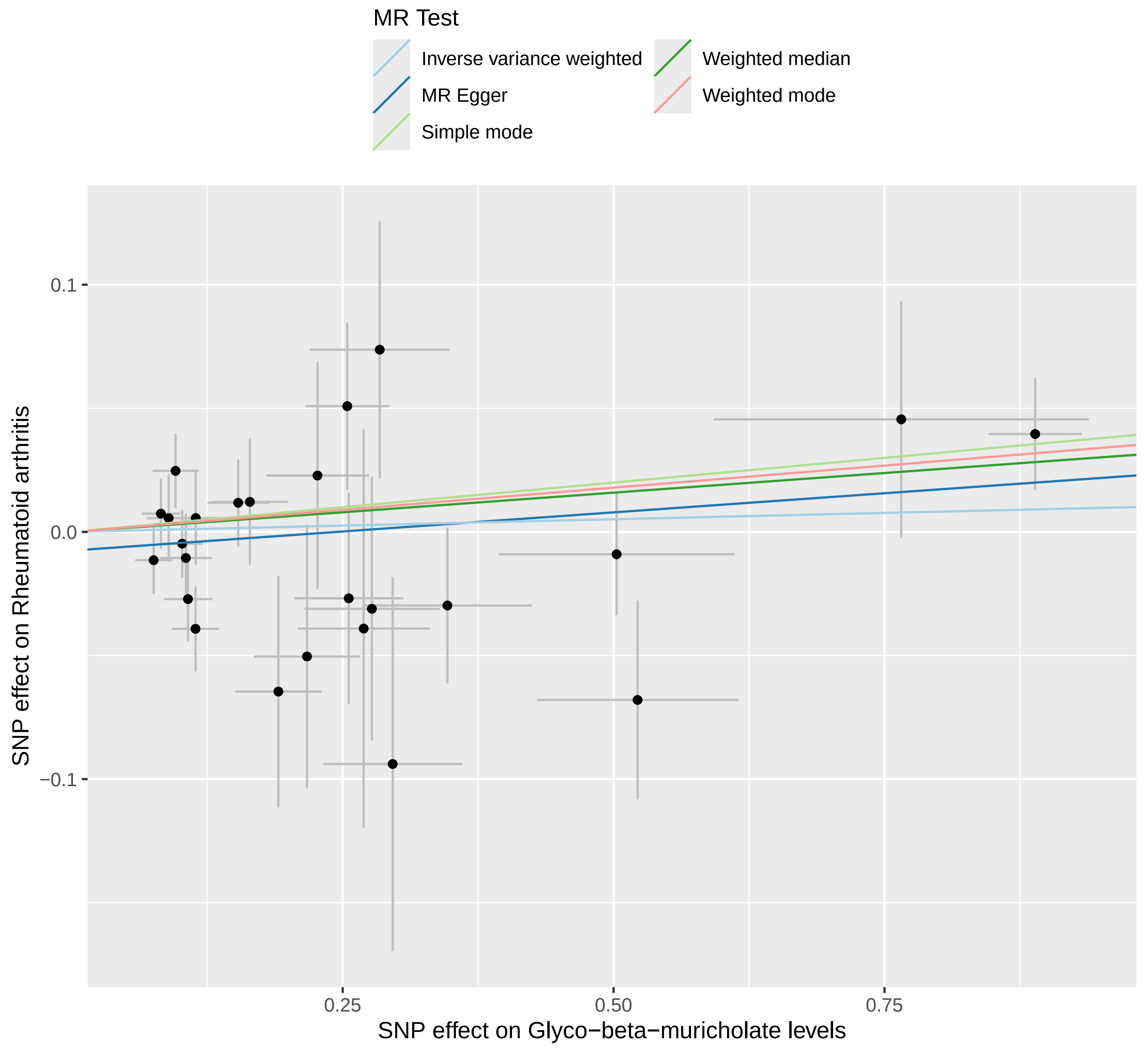
MR Test
Inverse variance weighted
MR Egger
Simple mode
Weighted median
Weighted mode
0.1
0.0
−0.1
SNP effect on Rheumatoid arthritis
0.25
0.50
0.75
SNP effect on Glyco−beta−muricholate levels

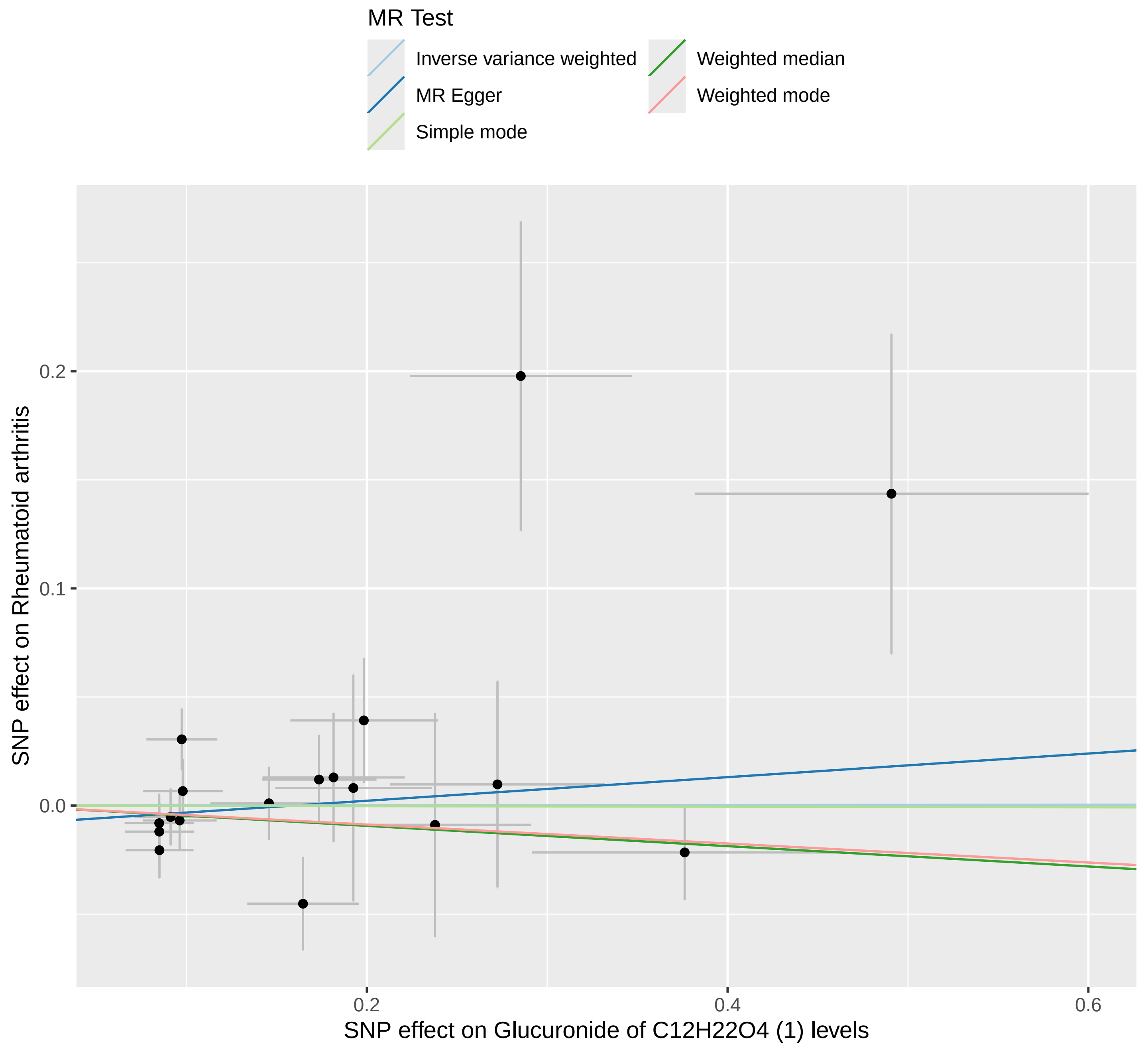

MR Test
Inverse variance weighted
MR Egger
Simple mode
Weighted median
Weighted mode
0.2
0.1
0.0
SNP effect on Rheumatoid arthritis
0.2
0.4
0.6
SNP effect on Glucuronide of C12H22O4 (1) levels

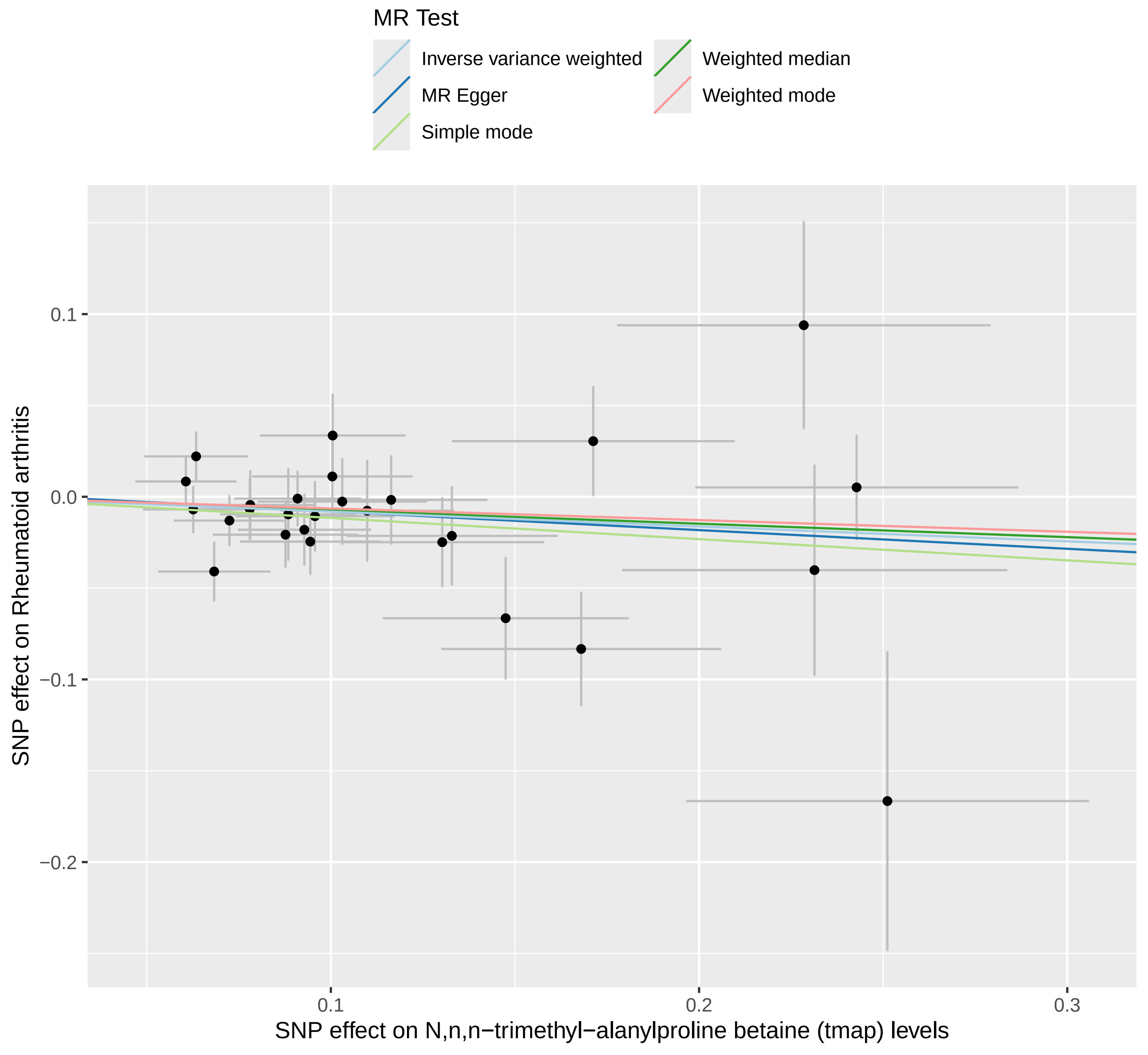

MR Test
Inverse variance weighted
MR Egger
Simple mode
Weighted median
Weighted mode
SNP effect on Rheumatoid arthritis
0.1
0.0
−0.1
−0.2
0.1
0.2
0.3
SNP effect on N,n,n−trimethyl−alanylproline betaine (tmap) levels

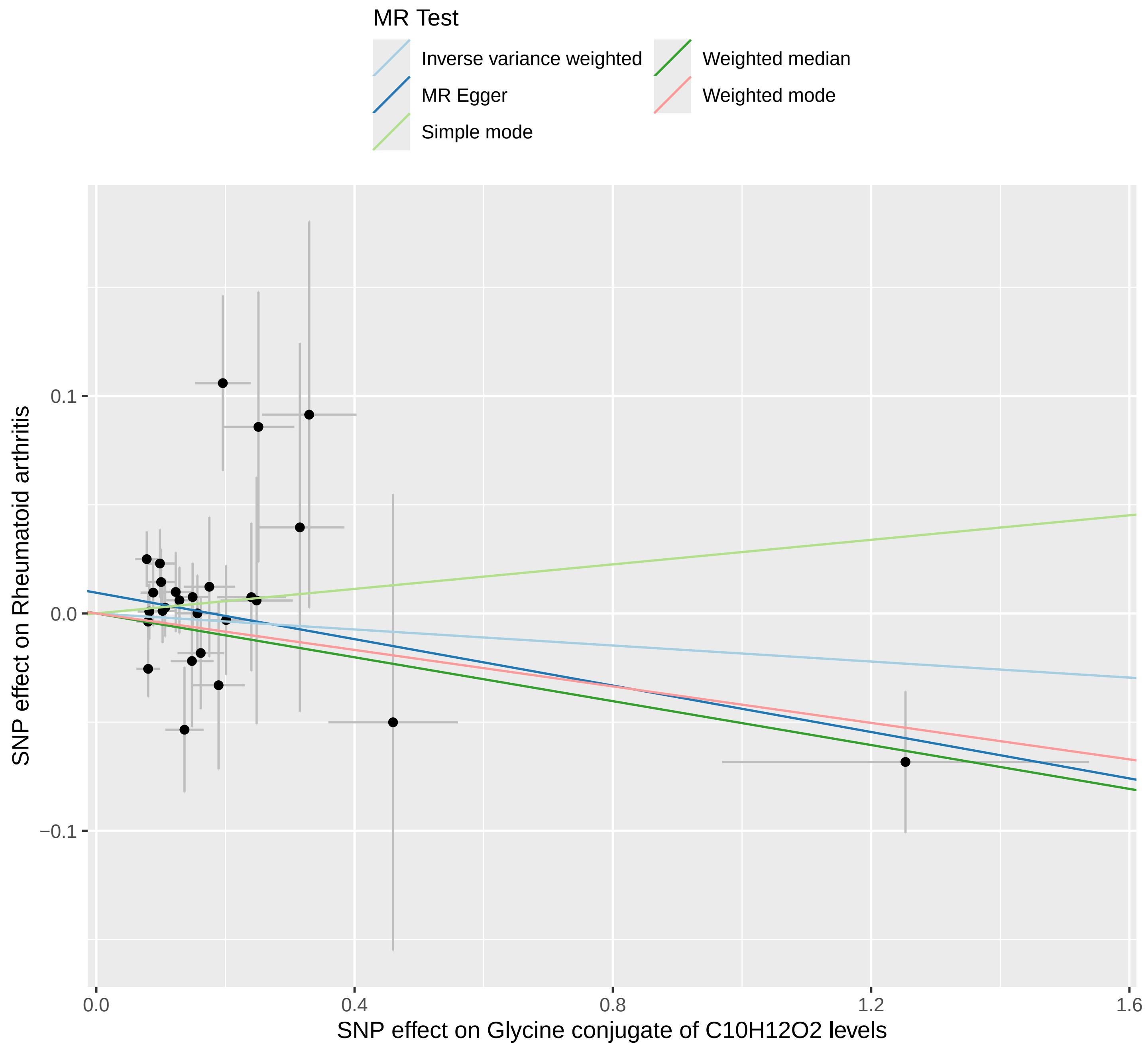
MR Test
Inverse variance weighted
MR Egger
Simple mode
Weighted median
Weighted mode
SNP effect on Rheumatoid arthritis
0.1
0.0
−0.1
0.0
0.4
0.8
1.2
1.6
SNP effect on Glycine conjugate of C10H12O2 levels

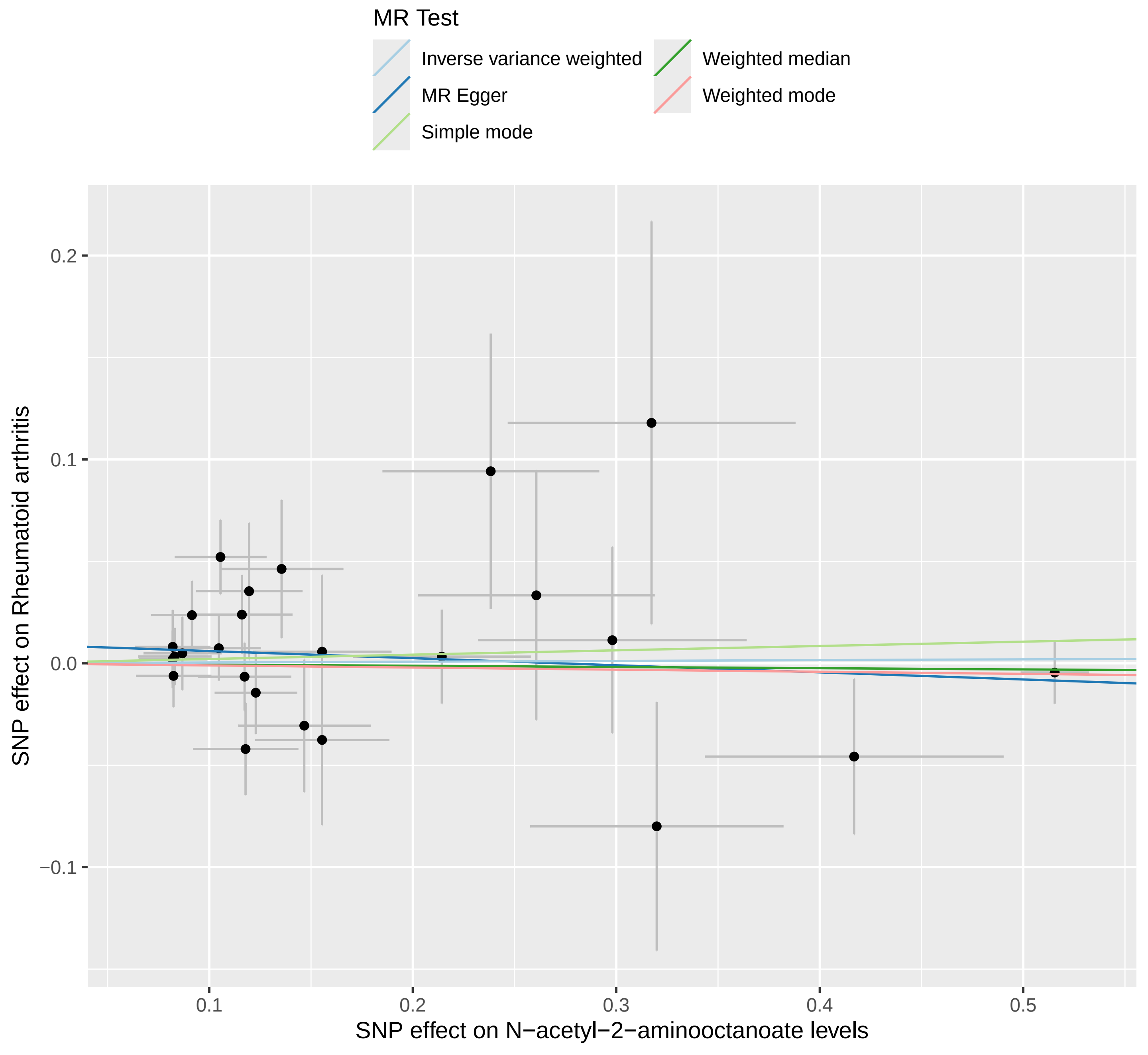

MR Test
Inverse variance weighted
MR Egger
Simple mode
Weighted median
Weighted mode
0.2
0.1
0.0
−0.1
0.1
0.2
0.3
0.4
0.5
SNP effect on Rheumatoid arthritis
SNP effect on N−acetyl−2−aminooctanoate levels

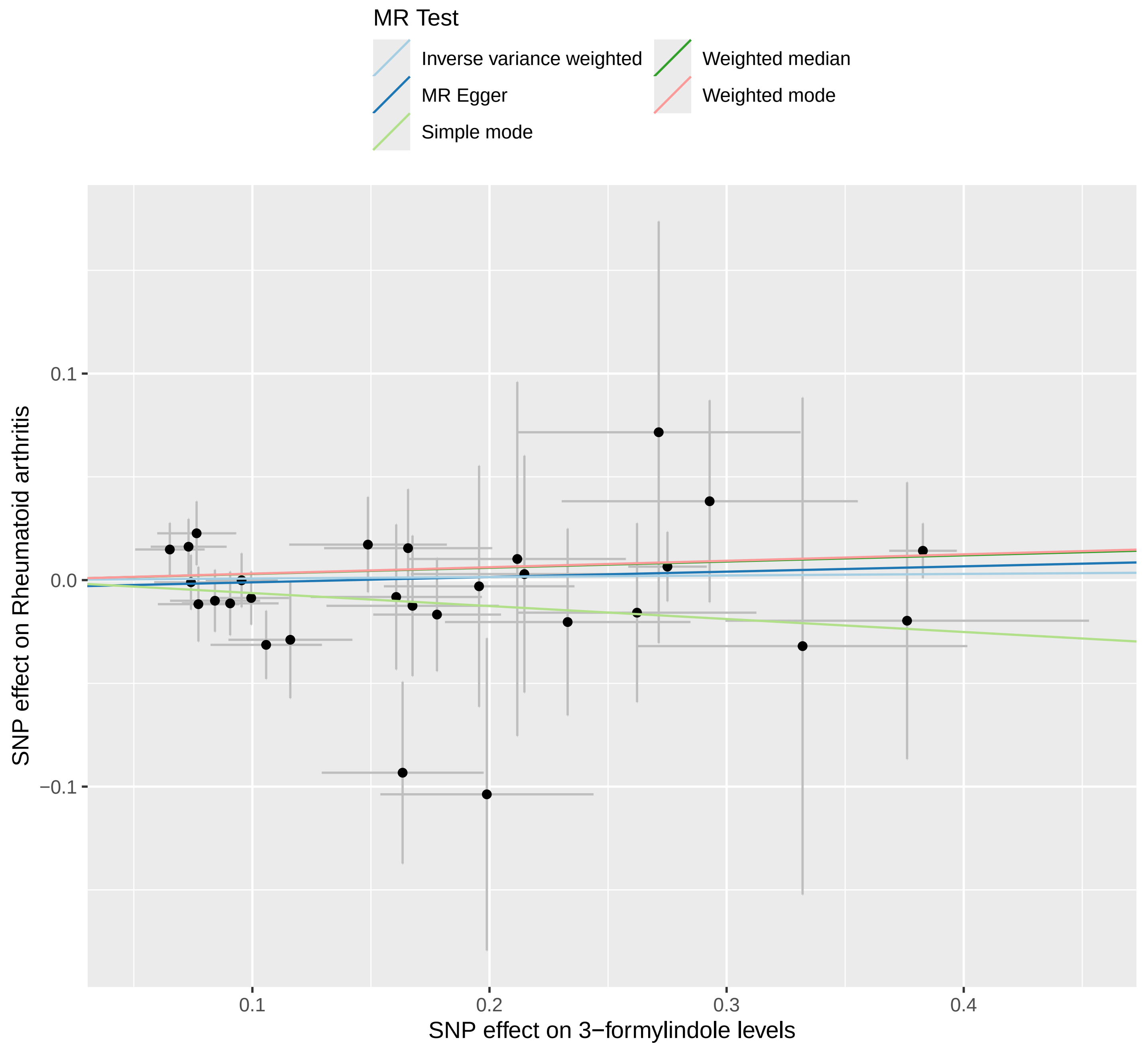
MR Test
Inverse variance weighted
MR Egger
Simple mode
Weighted median
Weighted mode
0.1
0.0
−0.1
0.1
0.2
0.3
0.4
SNP effect on Rheumatoid arthritis
SNP effect on 3−formylindole levels

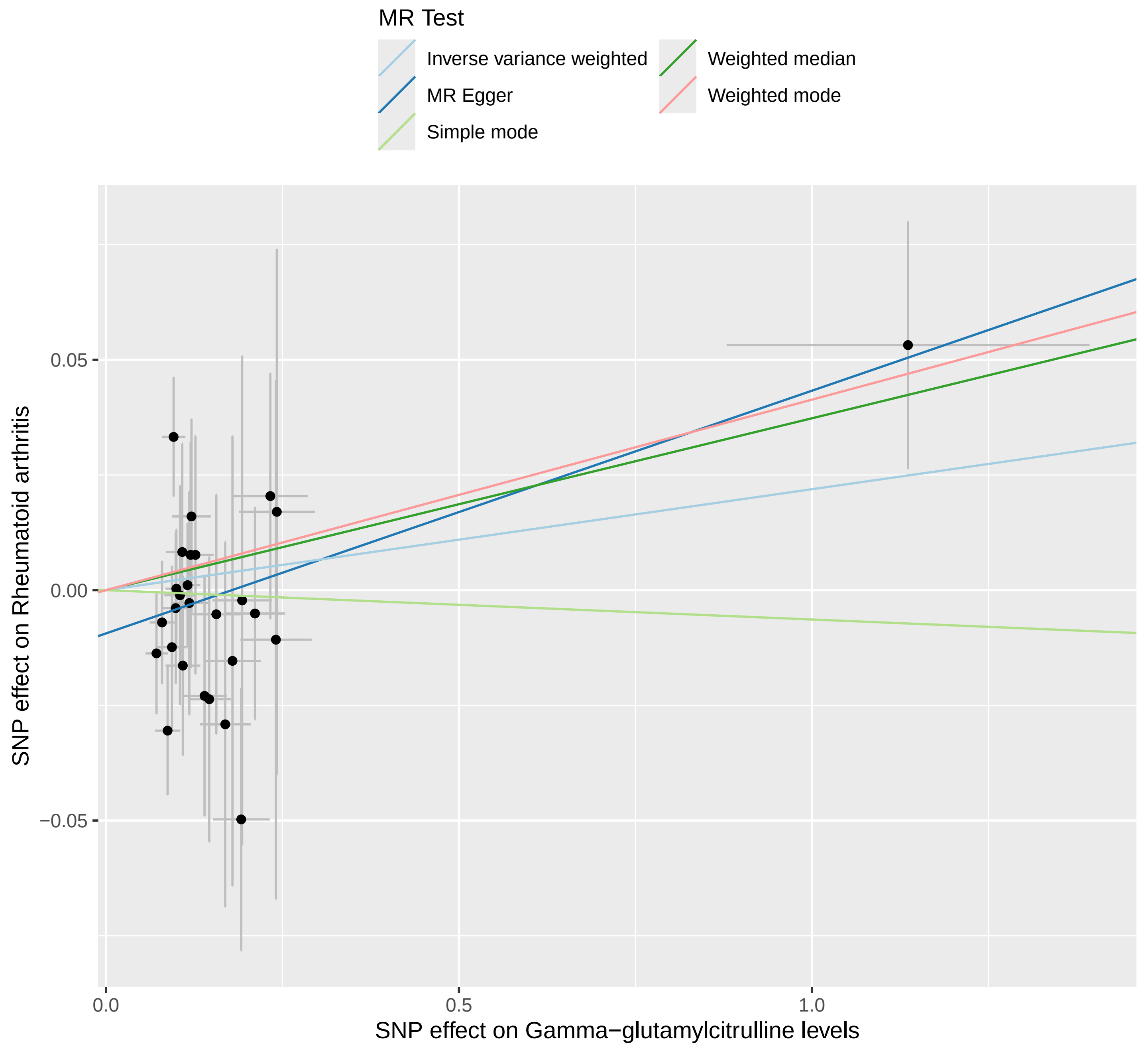
MR Test
Inverse variance weighted
MR Egger
Simple mode
Weighted median
Weighted mode
SNP effect on Rheumatoid arthritis
0.05
0.00
−0.05
0.0
0.5
1.0
SNP effect on Gamma−glutamylcitrulline levels

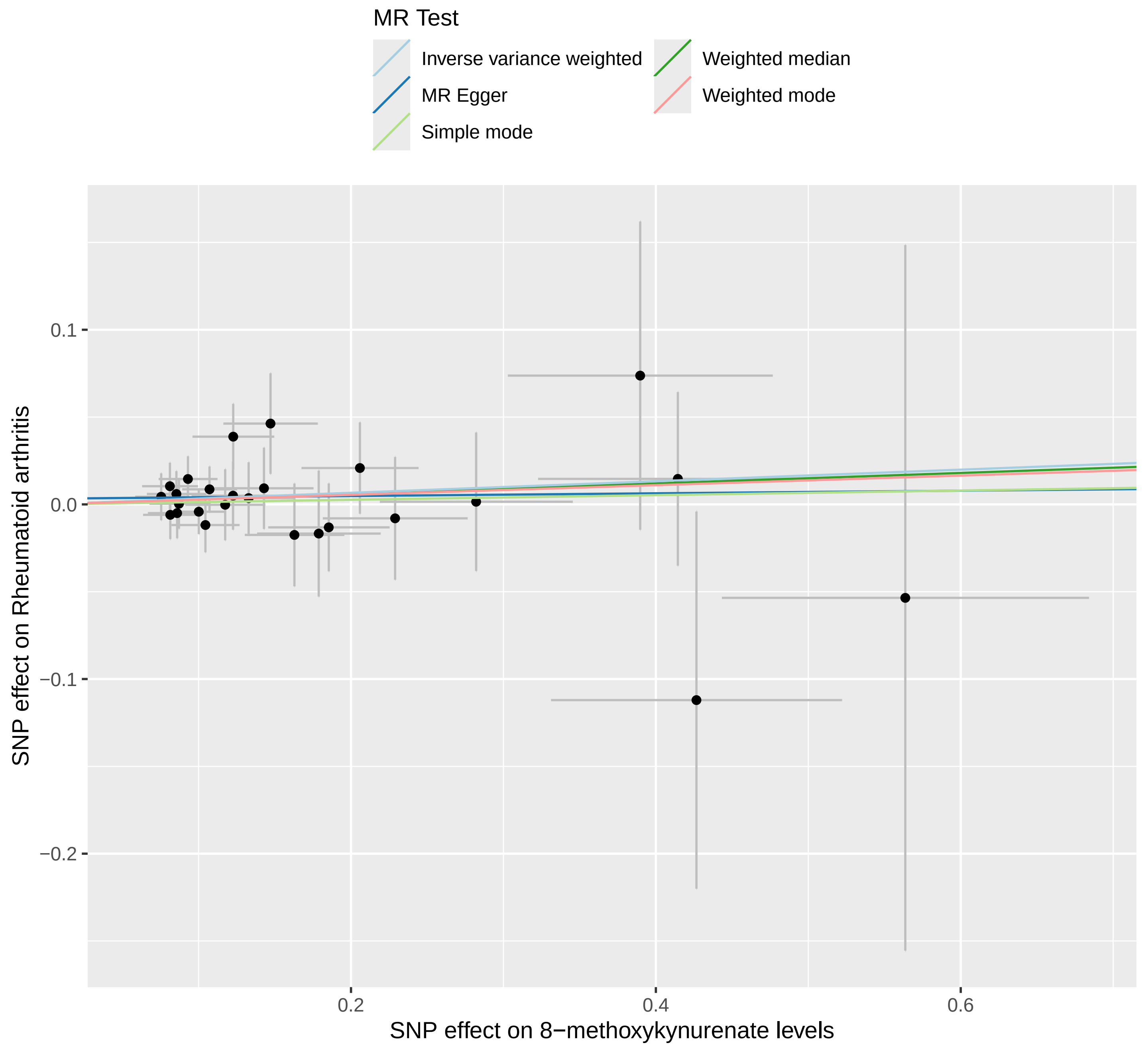
MR Test
Inverse variance weighted
MR Egger
Simple mode
Weighted median
Weighted mode
0.1
0.0
−0.1
−0.2
SNP effect on Rheumatoid arthritis
0.2
0.4
0.6
SNP effect on 8−methoxykynurenate levels

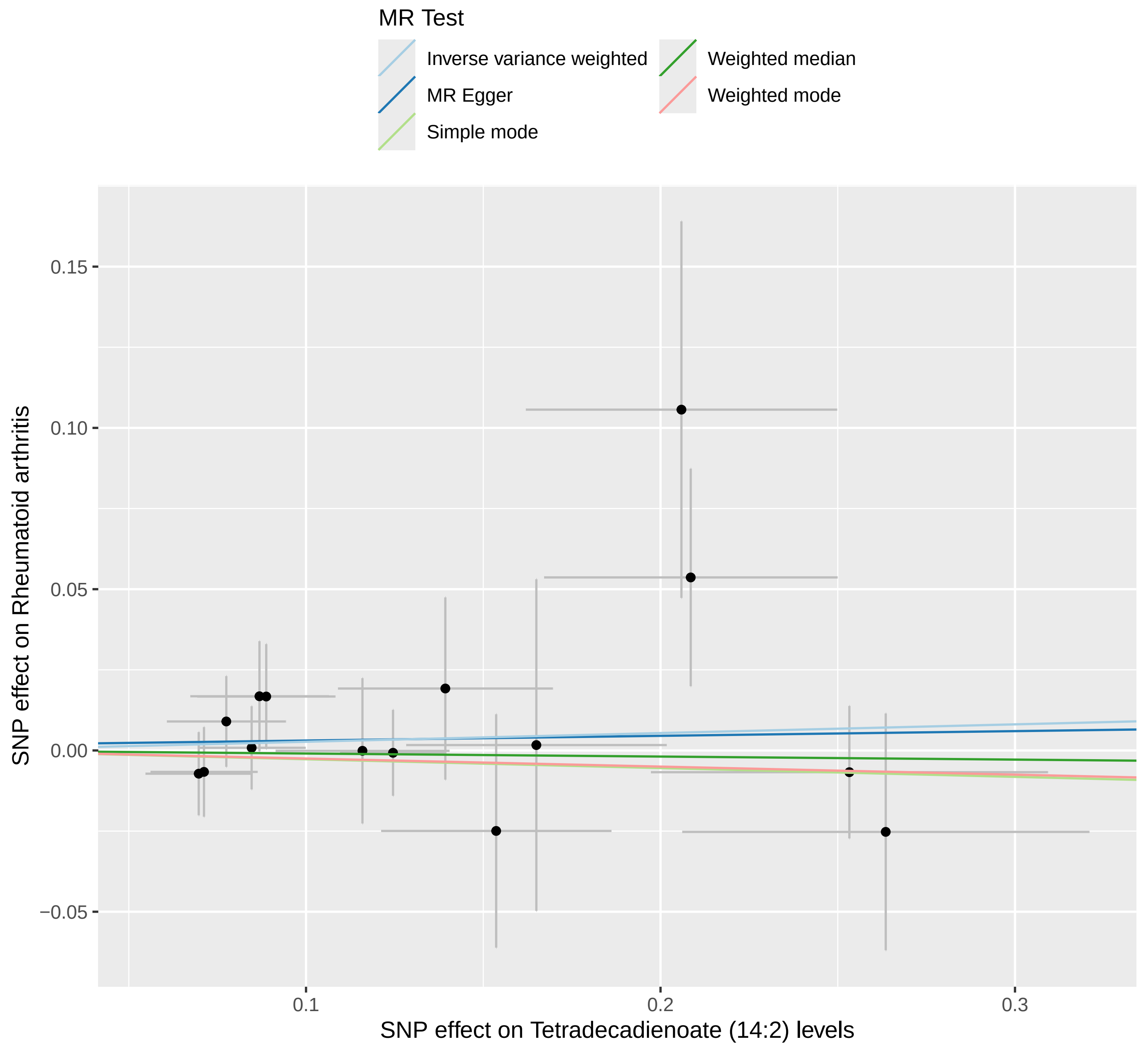

MR Test
Inverse variance weighted
MR Egger
Simple mode
Weighted median
Weighted mode
0.15
0.10
0.05
0.00
−0.05
0.1
0.2
0.3
SNP effect on Rheumatoid arthritis
SNP effect on Tetradecadienoate (14:2) levels

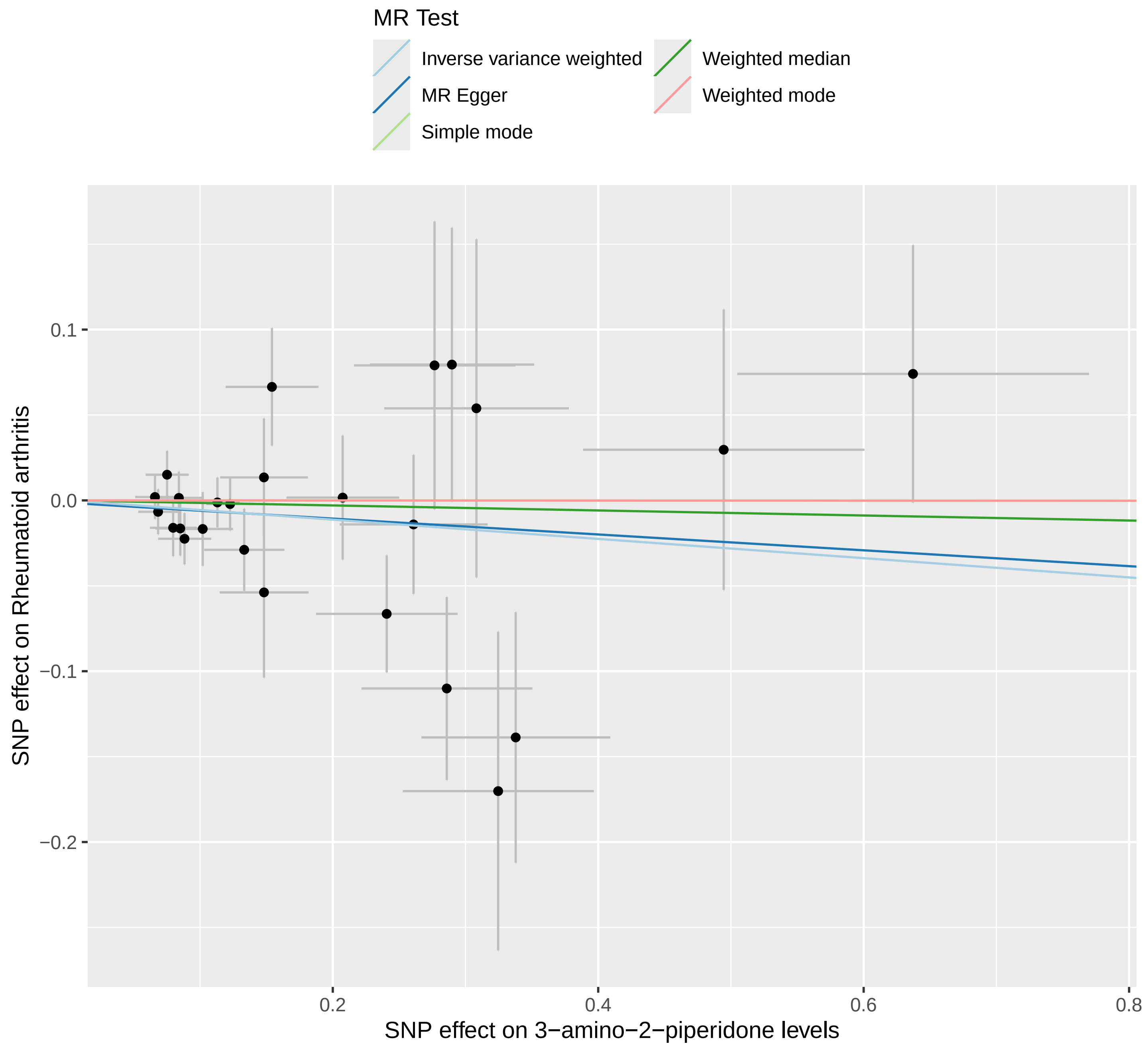
MR Test
Inverse variance weighted
MR Egger
Simple mode
Weighted median
Weighted mode
0.1
0.0
−0.1
−0.2
0.2
0.4
0.6
0.8
SNP effect on Rheumatoid arthritis
SNP effect on 3−amino−2−piperidone levels

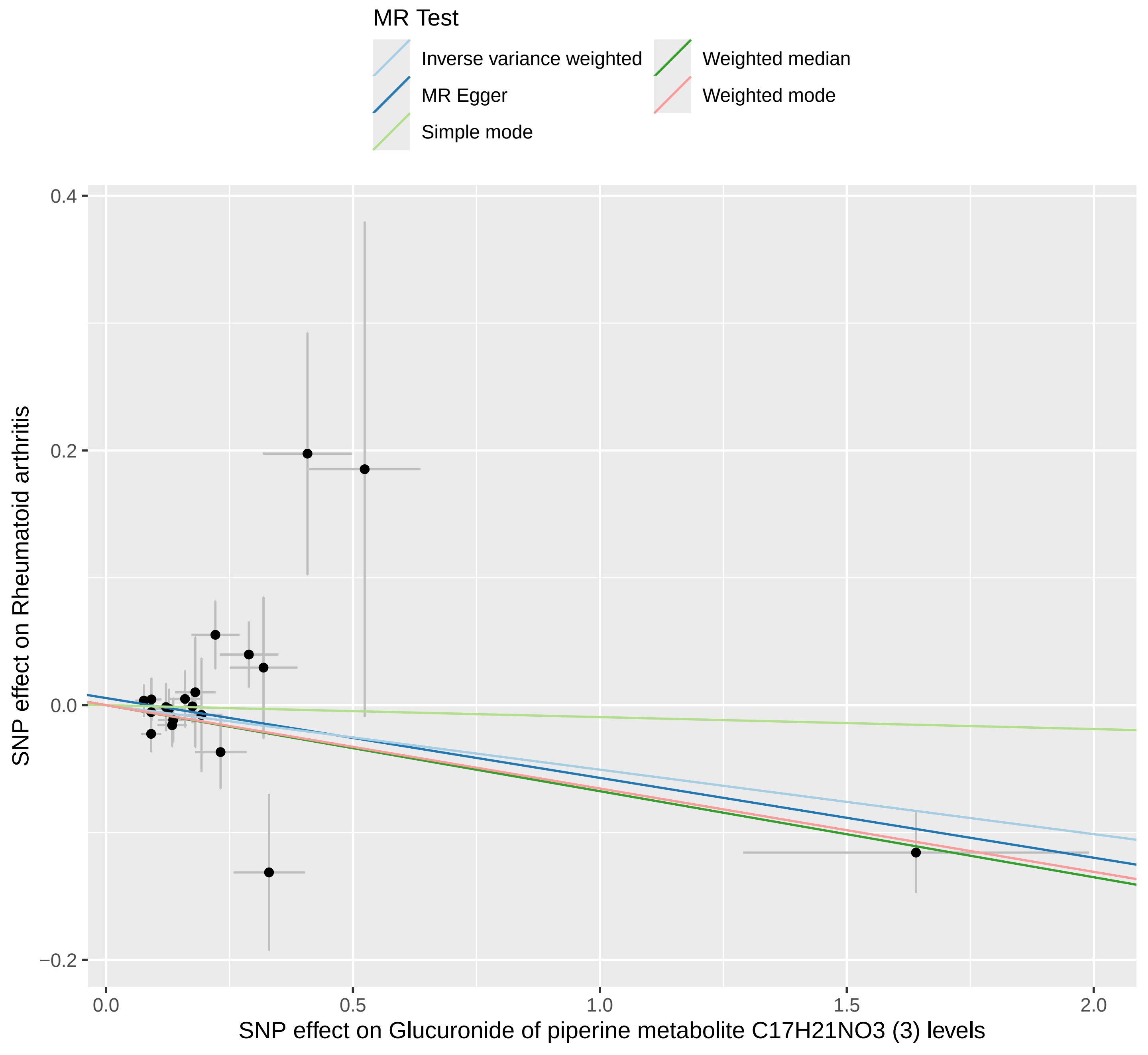

MR Test
Inverse variance weighted
MR Egger
Simple mode
Weighted median
Weighted mode
0.4
0.2
0.0
−0.2
SNP effect on Rheumatoid arthritis
0.0
0.5
1.0
1.5
2.0
SNP effect on Glucuronide of piperine metabolite C17H21NO3 (3) levels

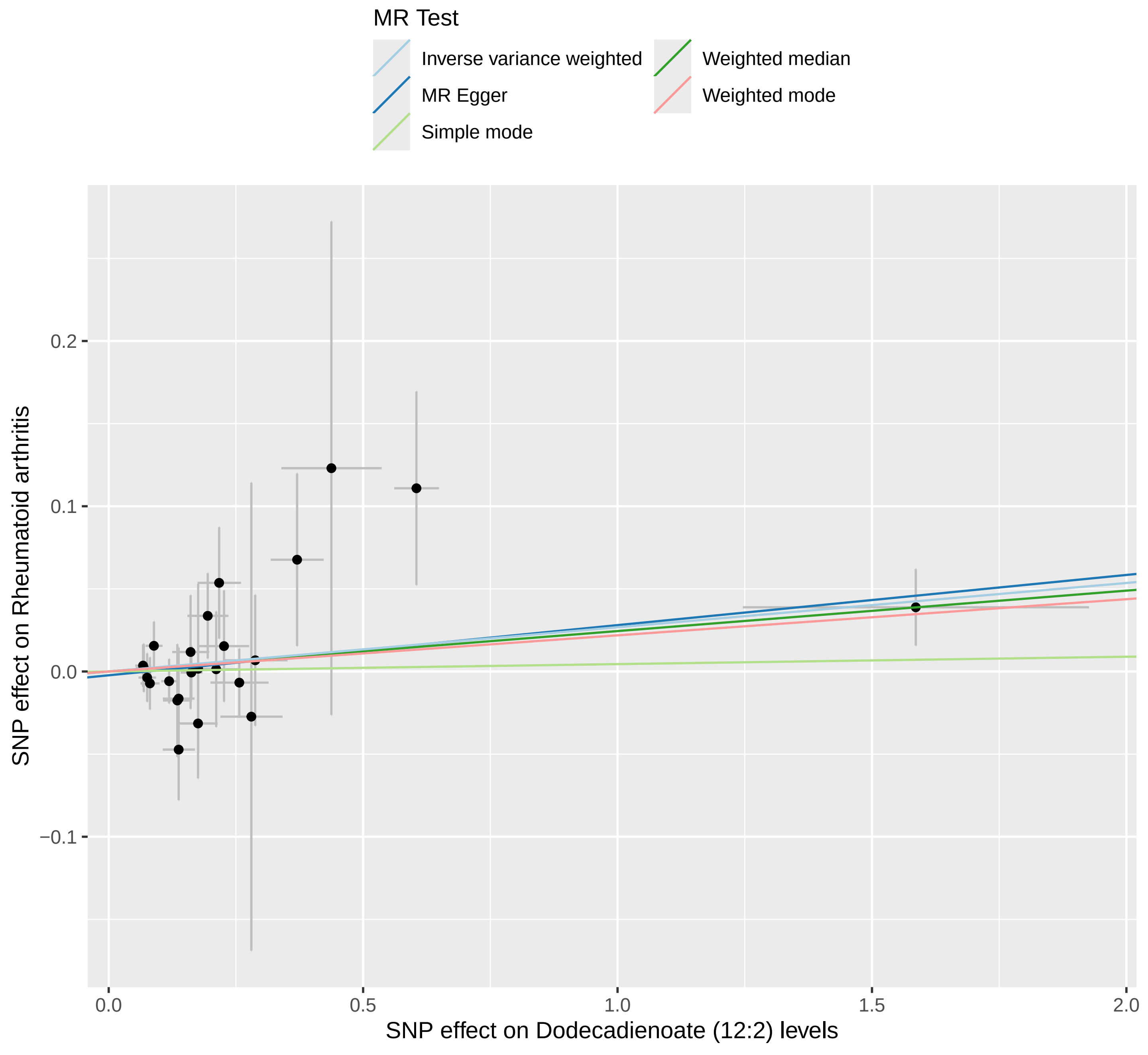

MR Test
Inverse variance weighted
MR Egger
Simple mode
Weighted median
Weighted mode
SNP effect on Rheumatoid arthritis
0.2
0.1
0.0
−0.1
0.0
0.5
1.0
1.5
2.0
SNP effect on Dodecadienoate (12:2) levels

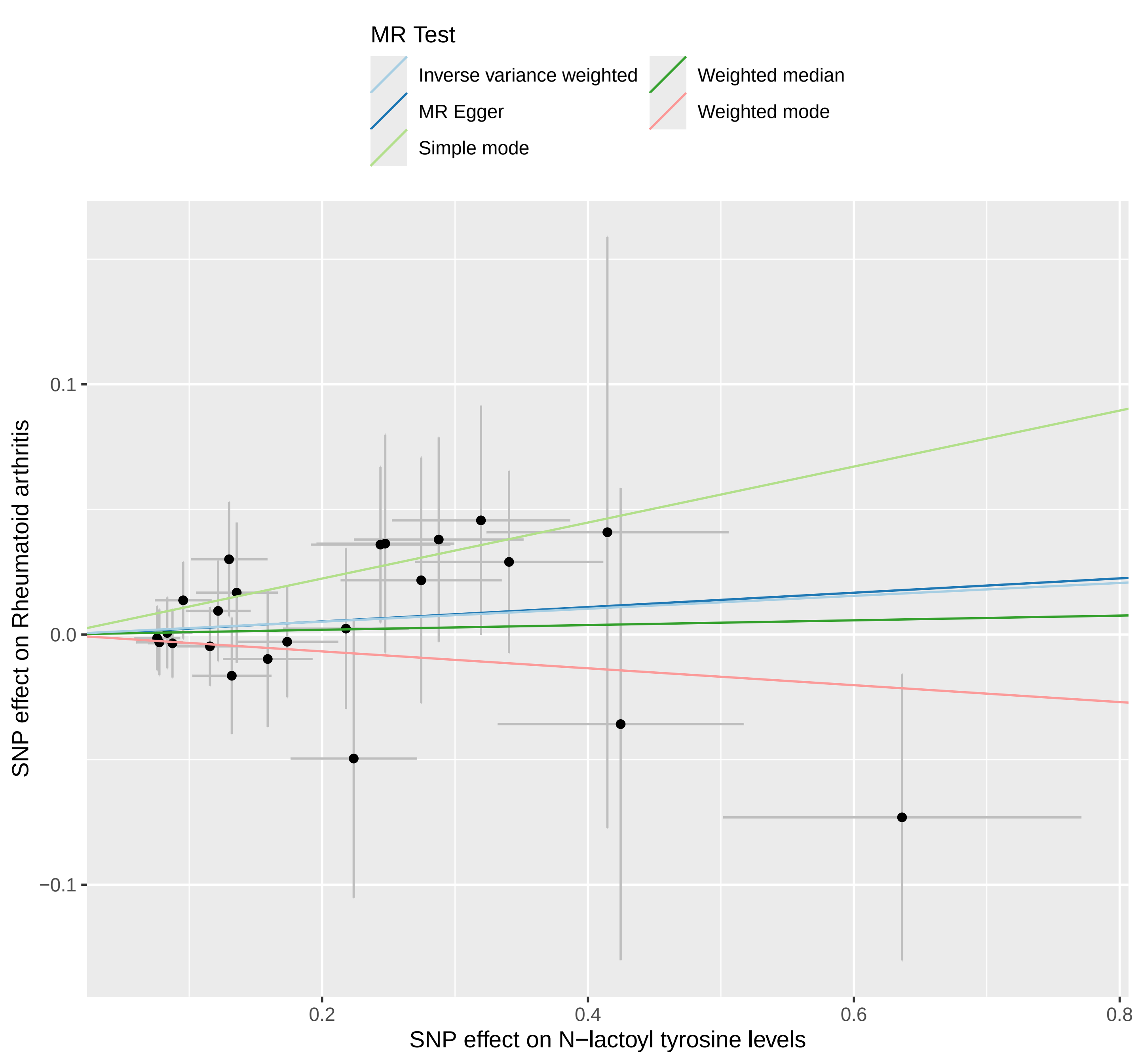

MR Test
Inverse variance weighted
MR Egger
Simple mode
Weighted median
Weighted mode
0.1
0.0
−0.1
SNP effect on Rheumatoid arthritis
0.2
0.4
0.6
0.8
SNP effect on N−lactoyl tyrosine levels

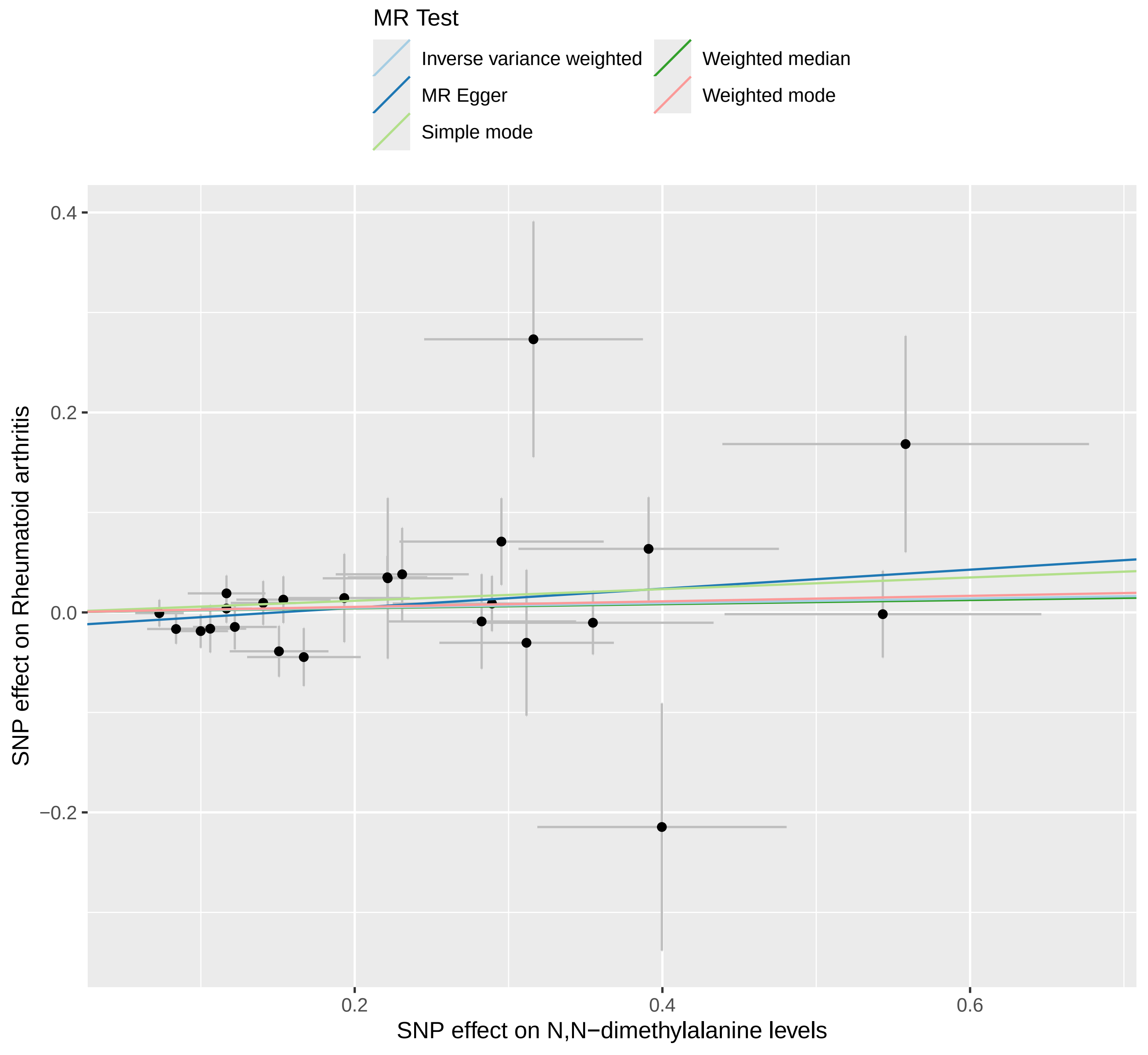
MR Test
Inverse variance weighted
MR Egger
Simple mode
Weighted median
Weighted mode
0.4
0.2
0.0
−0.2
SNP effect on Rheumatoid arthritis
0.2
0.4
0.6
SNP effect on N,N−dimethylalanine levels

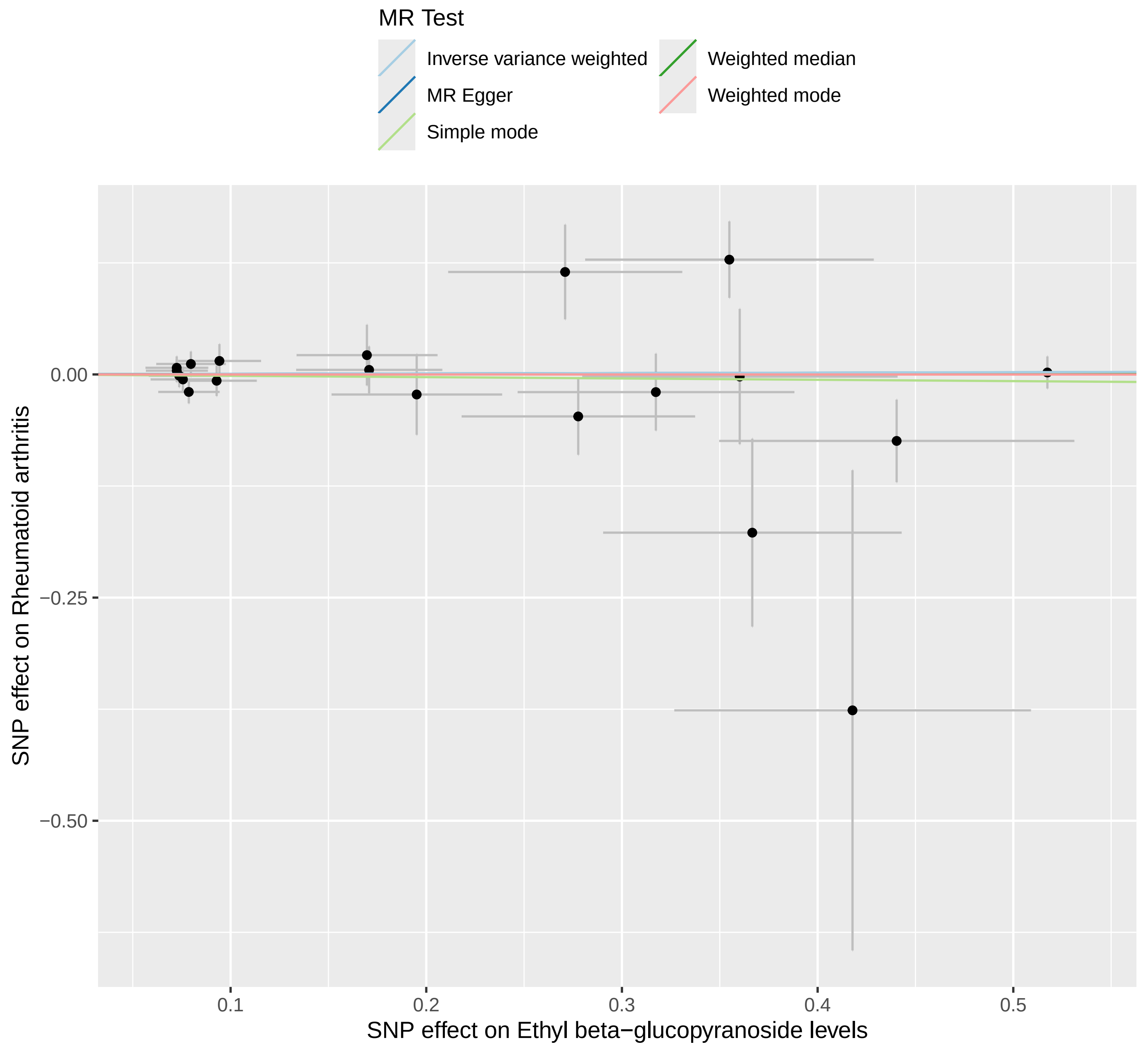
MR Test
Inverse variance weighted
MR Egger
Simple mode
Weighted median
Weighted mode
0.00
−0.25
−0.50
0.1
0.2
0.3
0.4
0.5
SNP effect on Rheumatoid arthritis
SNP effect on Ethyl beta−glucopyranoside levels

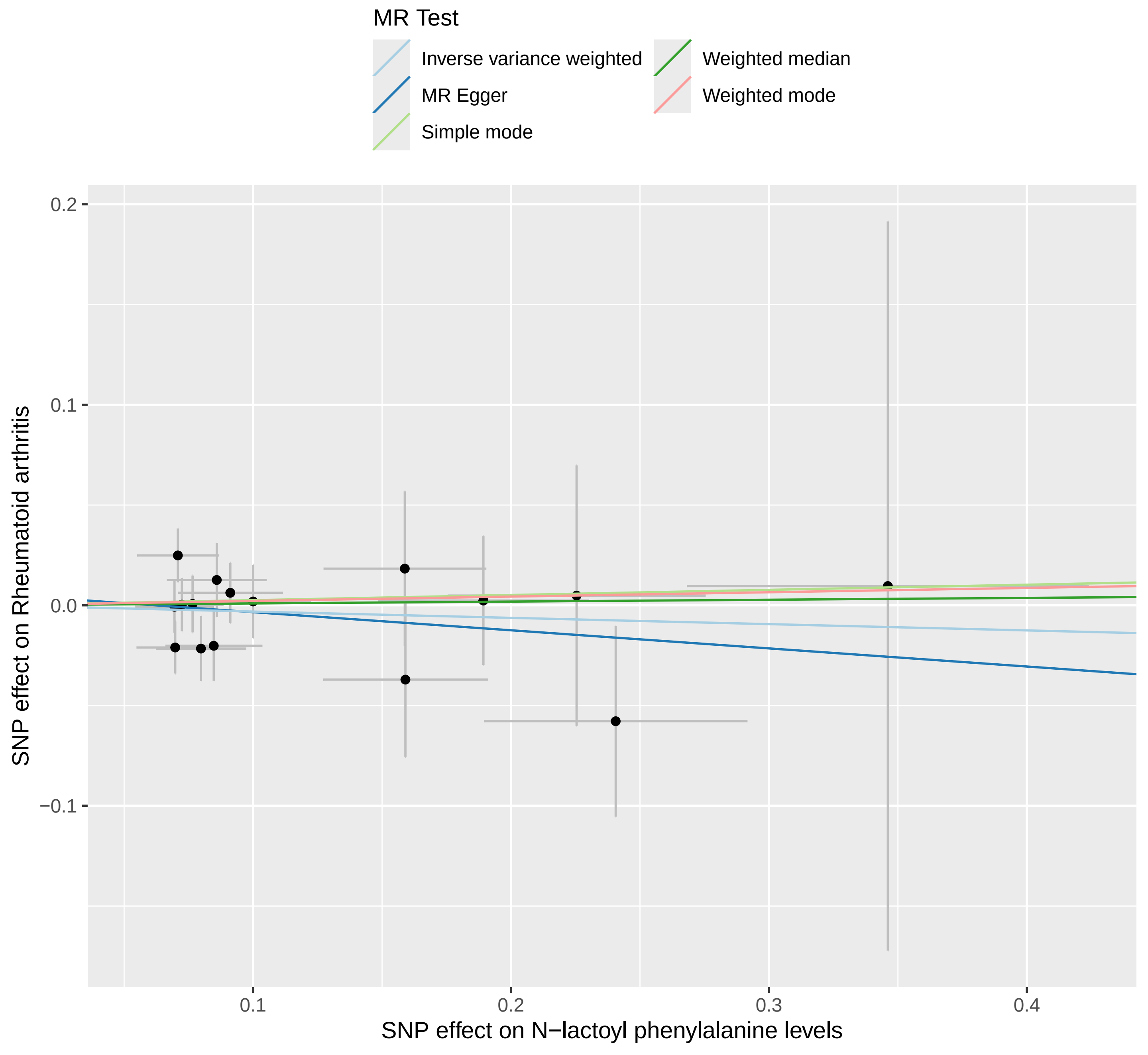

MR Test
Inverse variance weighted
MR Egger
Simple mode
Weighted median
Weighted mode
0.2
0.1
0.0
−0.1
0.1
0.2
0.3
0.4
SNP effect on Rheumatoid arthritis
SNP effect on N−lactoyl phenylalanine levels

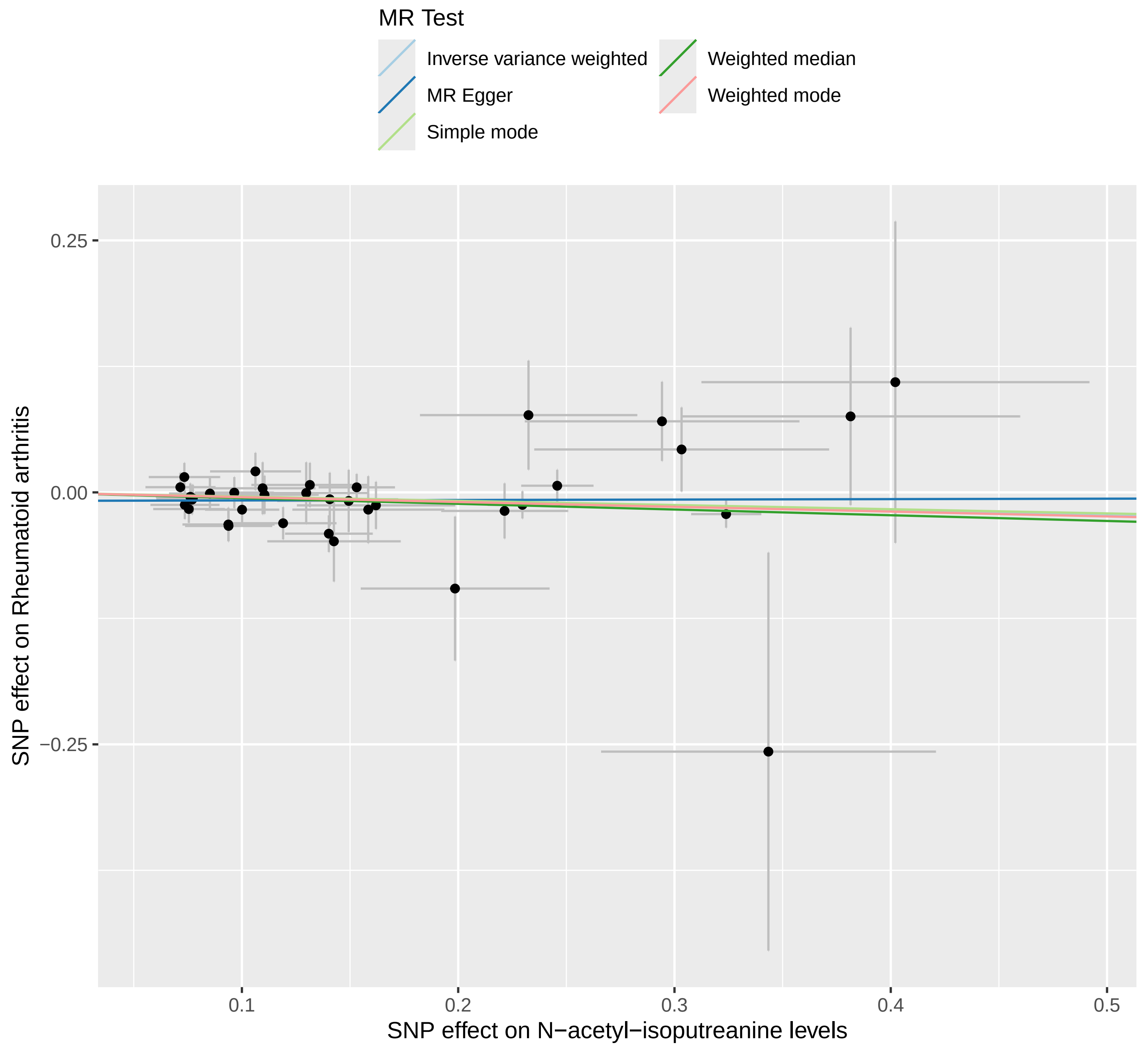
MR Test
Inverse variance weighted
MR Egger
Simple mode
Weighted median
Weighted mode
0.25
0.00
−0.25
0.1
0.2
0.3
0.4
0.5
SNP effect on Rheumatoid arthritis
SNP effect on N−acetyl−isoputreanine levels

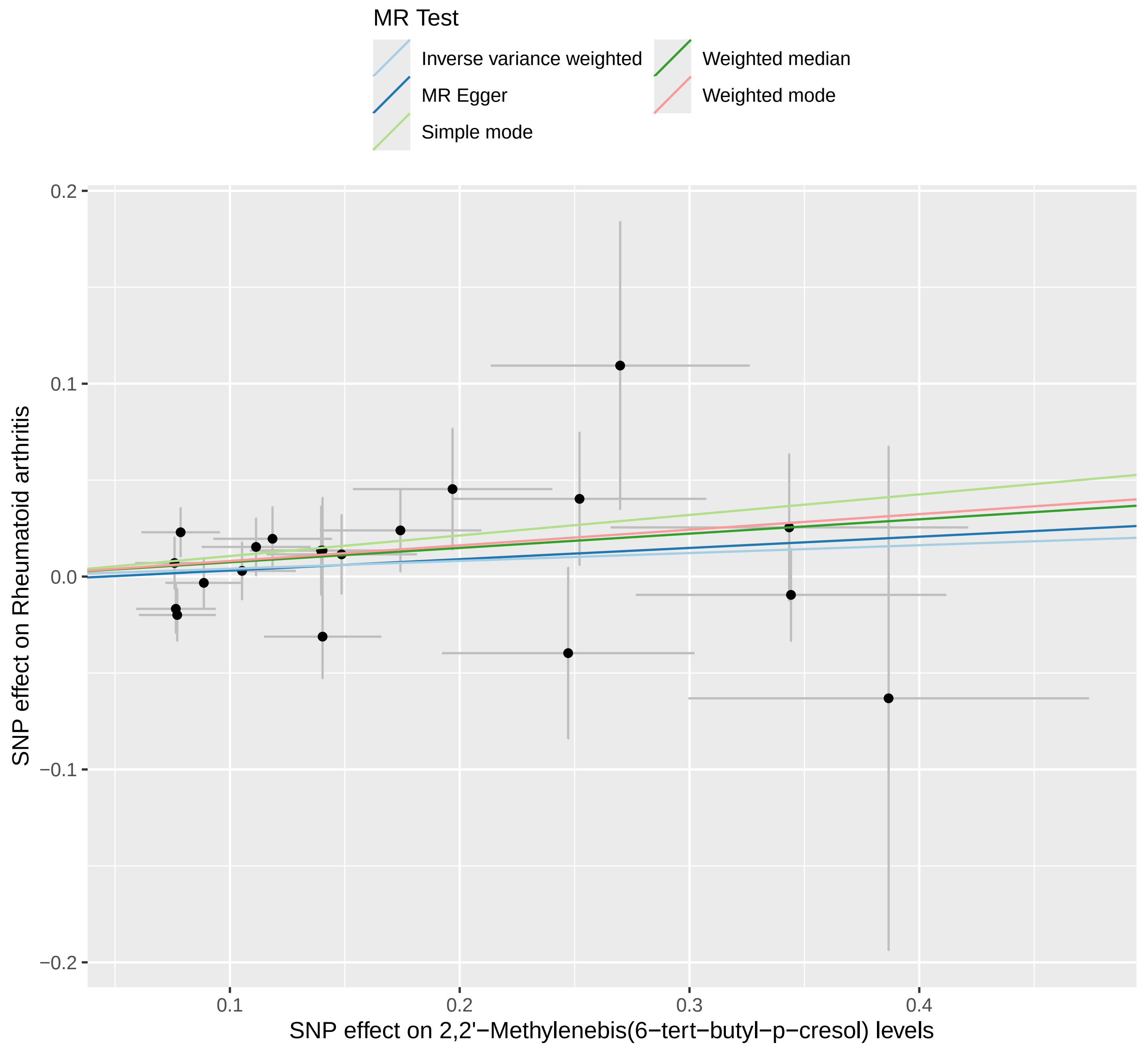

MR Test
Inverse variance weighted
MR Egger
Simple mode
Weighted median
Weighted mode
0.2
0.1
0.0
−0.1
−0.2
SNP effect on Rheumatoid arthritis
0.1
0.2
0.3
0.4
SNP effect on 2,2'−Methylenebis(6−tert−butyl−p−cresol) levels

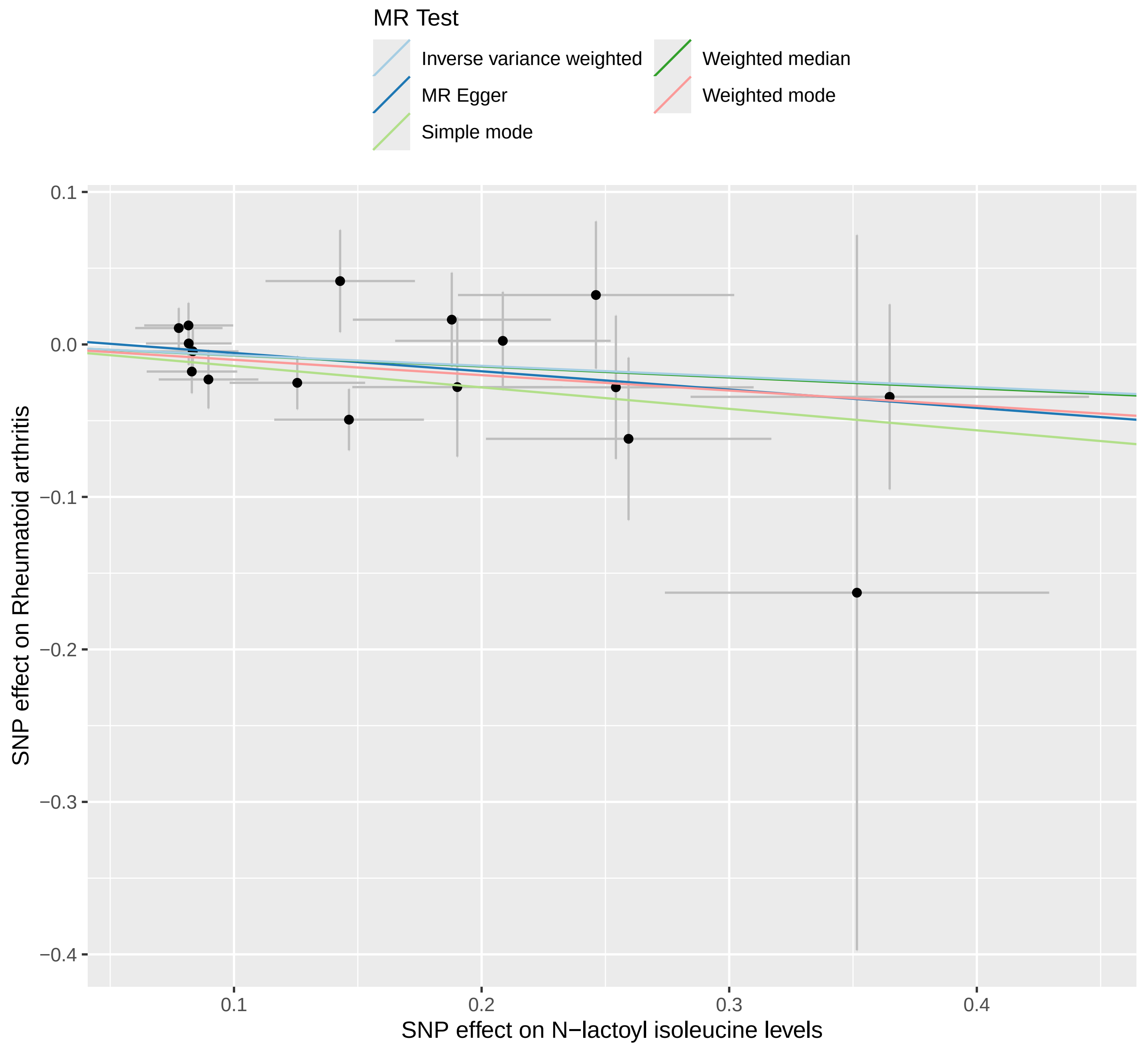
MR Test
Inverse variance weighted
MR Egger
Simple mode
Weighted median
Weighted mode
0.1
0.0
−0.1
−0.2
−0.3
−0.4
SNP effect on Rheumatoid arthritis
0.1
0.2
0.3
0.4
SNP effect on N−lactoyl isoleucine levels

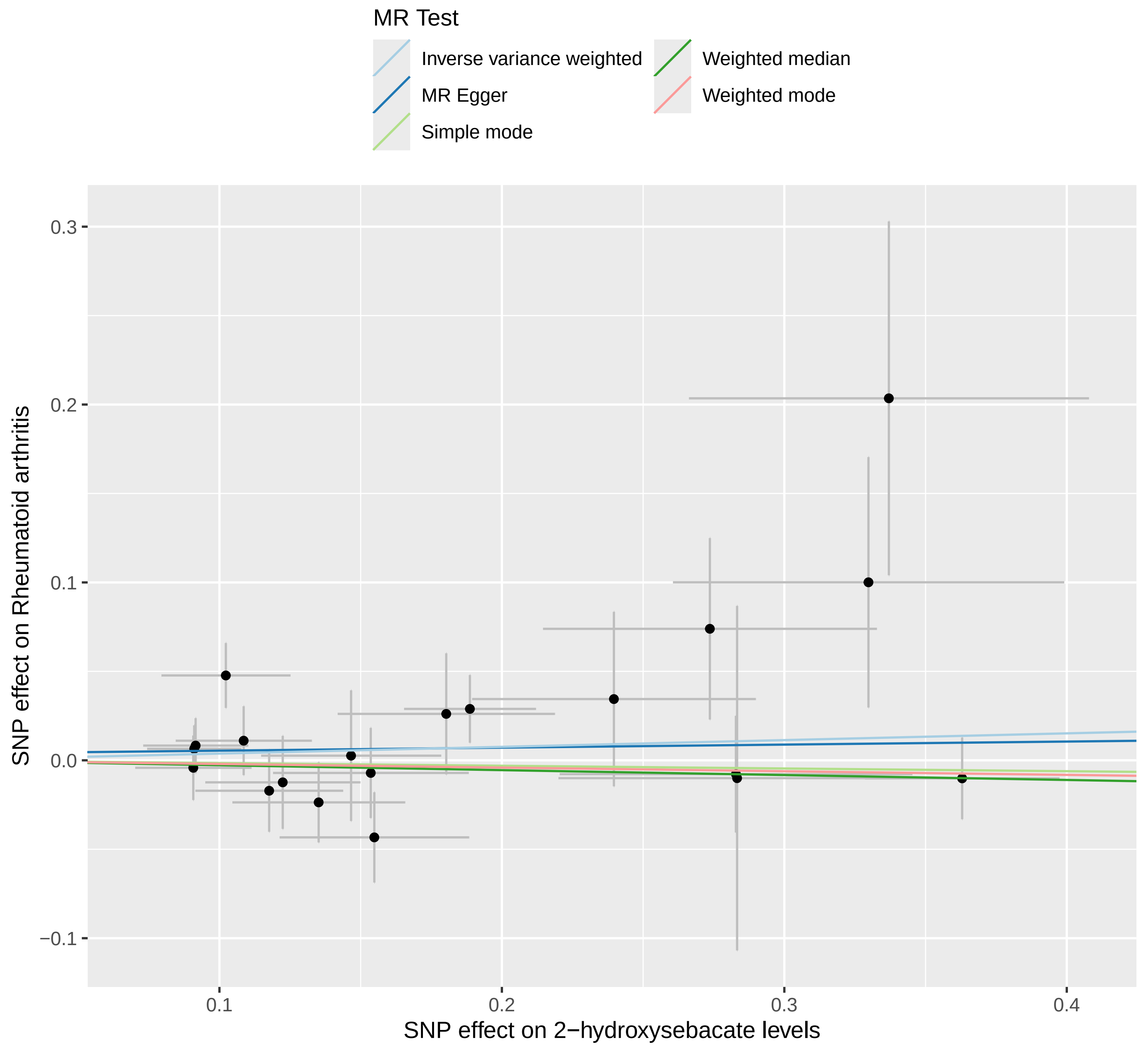
MR Test
Inverse variance weighted
MR Egger
Simple mode
Weighted median
Weighted mode
SNP effect on Rheumatoid arthritis
0.3
0.2
0.1
0.0
−0.1
0.1
0.2
0.3
0.4
SNP effect on 2−hydroxysebacate levels

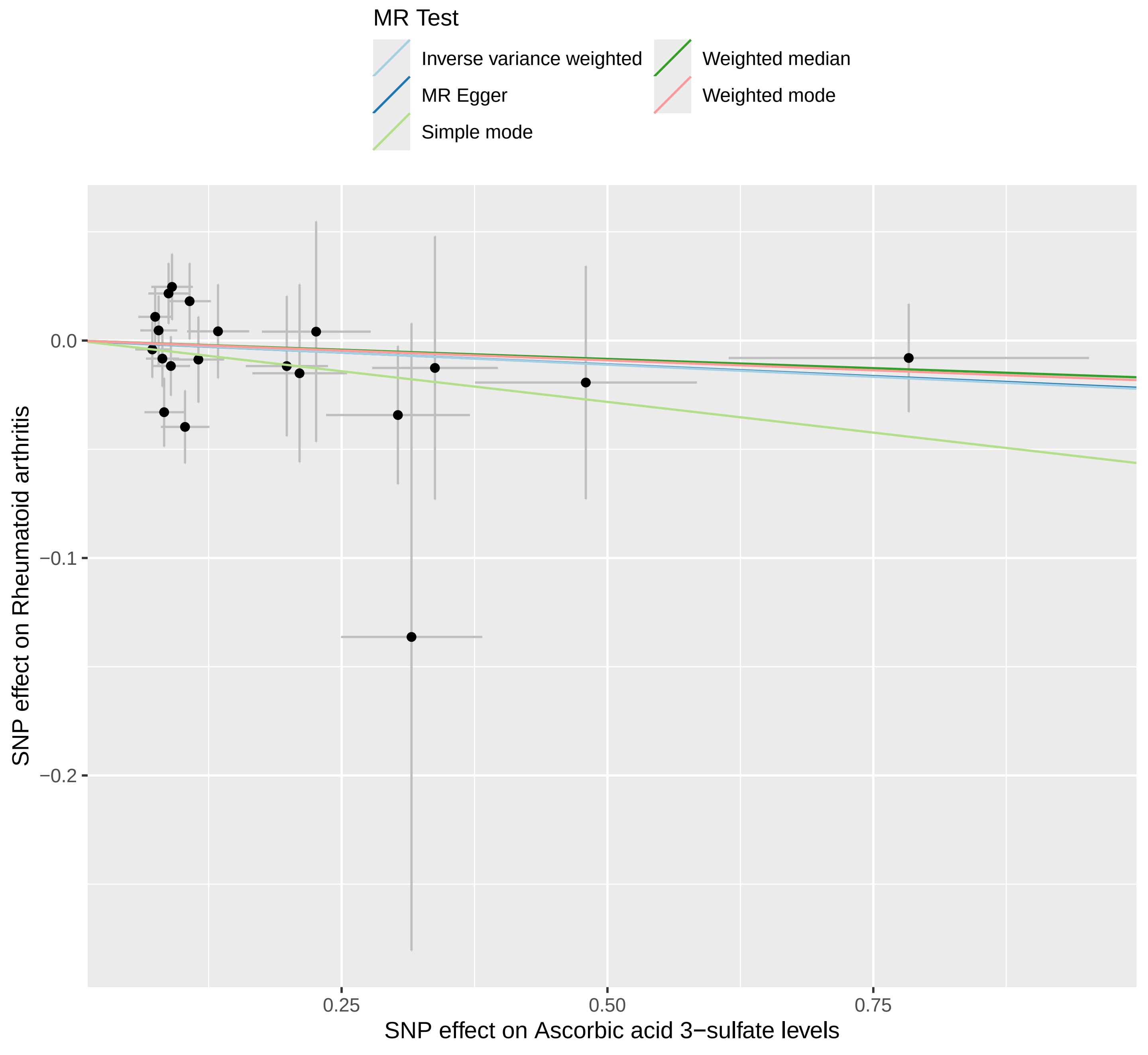
MR Test
Inverse variance weighted
MR Egger
Simple mode
Weighted median
Weighted mode
0.0
−0.1
−0.2
0.25
0.50
0.75
SNP effect on Rheumatoid arthritis
SNP effect on Ascorbic acid 3−sulfate levels

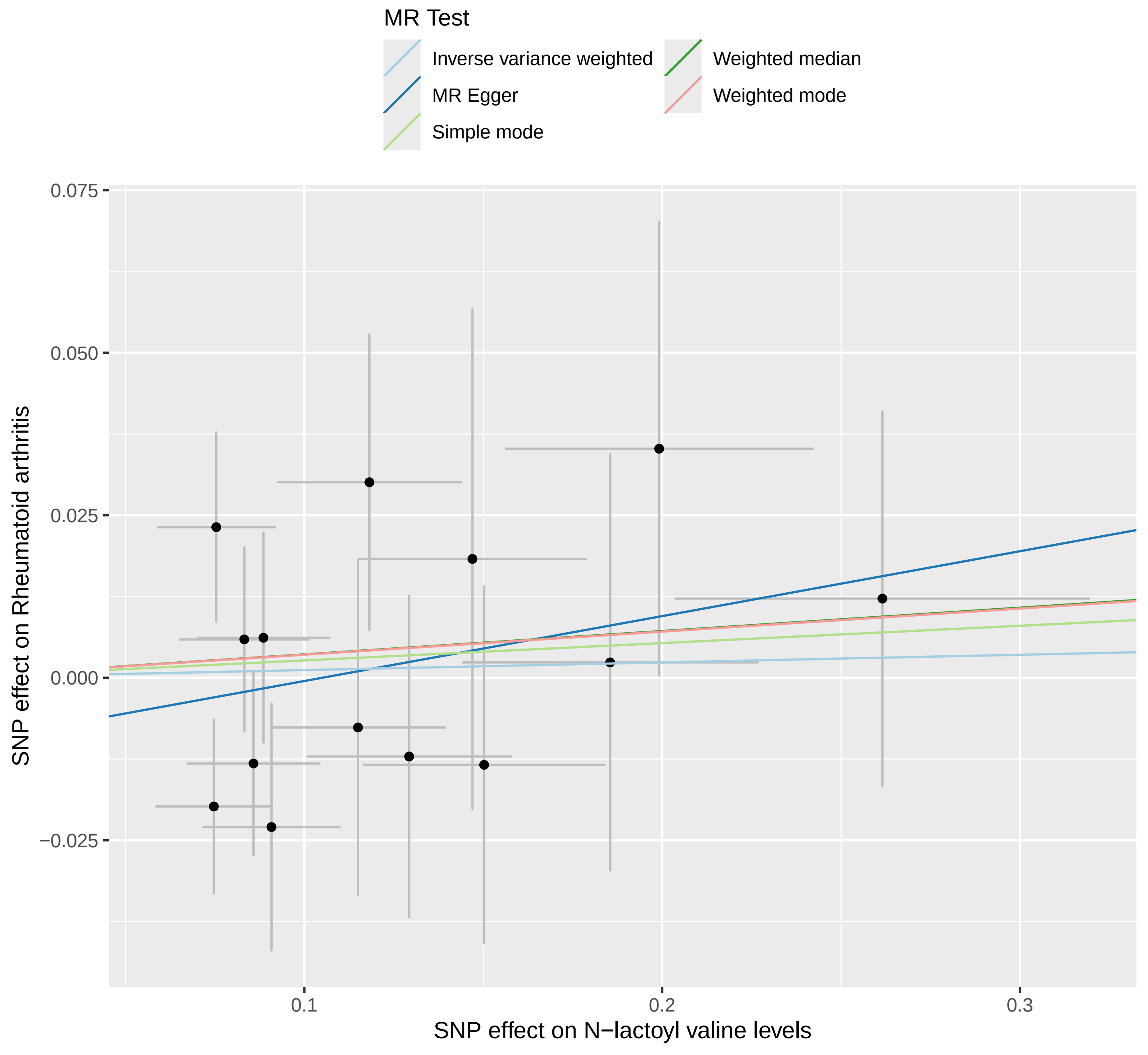
MR Test
Inverse variance weighted
MR Egger
Simple mode
Weighted median
Weighted mode
0.075
0.050
0.025
0.000
−0.025
0.1
0.2
0.3
SNP effect on Rheumatoid arthritis
SNP effect on N−lactoyl valine levels

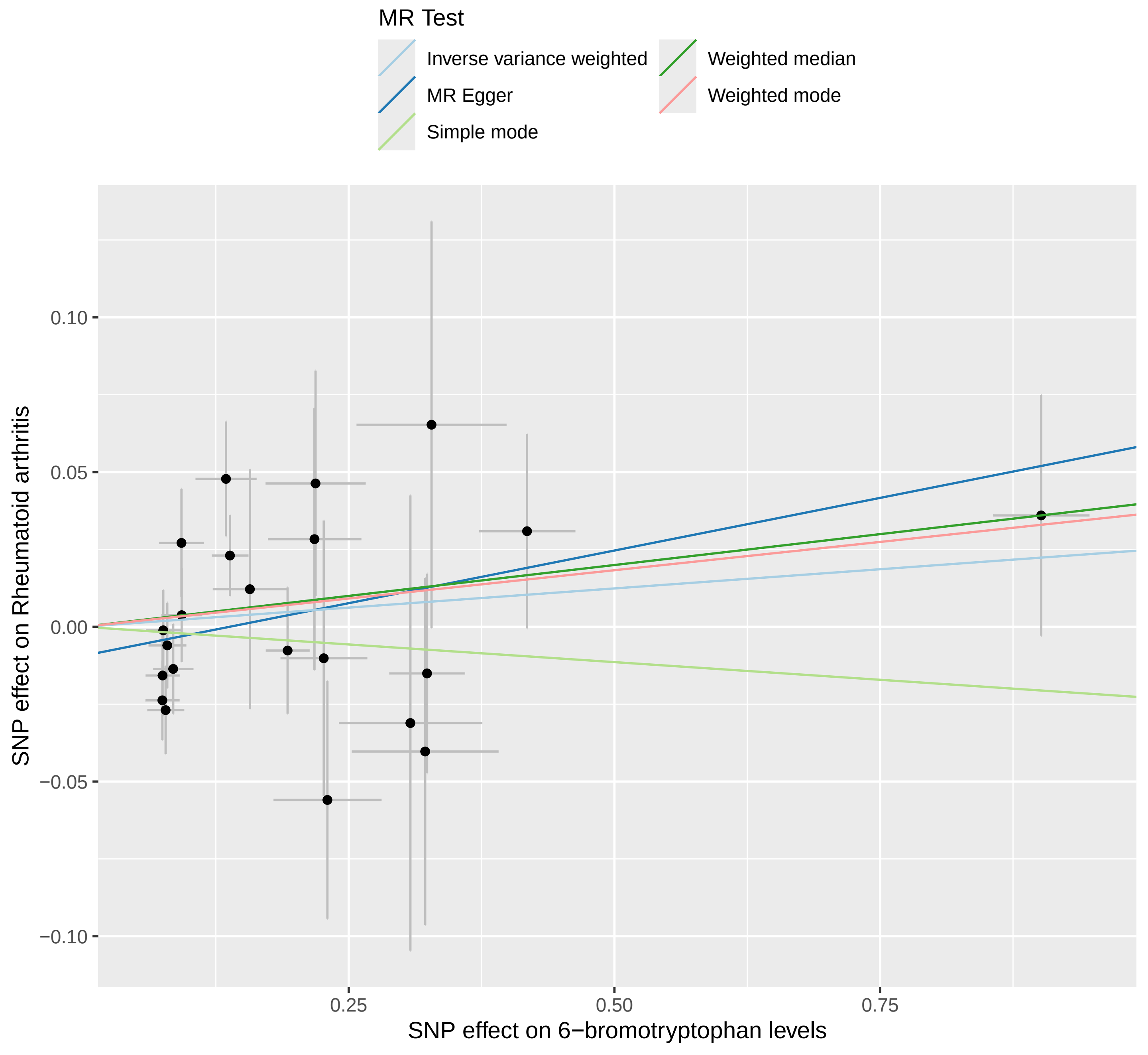
MR Test
Inverse variance weighted
MR Egger
Simple mode
Weighted median
Weighted mode
0.10
0.05
0.00
−0.05
−0.10
SNP effect on Rheumatoid arthritis
0.25
0.50
0.75
SNP effect on 6−bromotryptophan levels

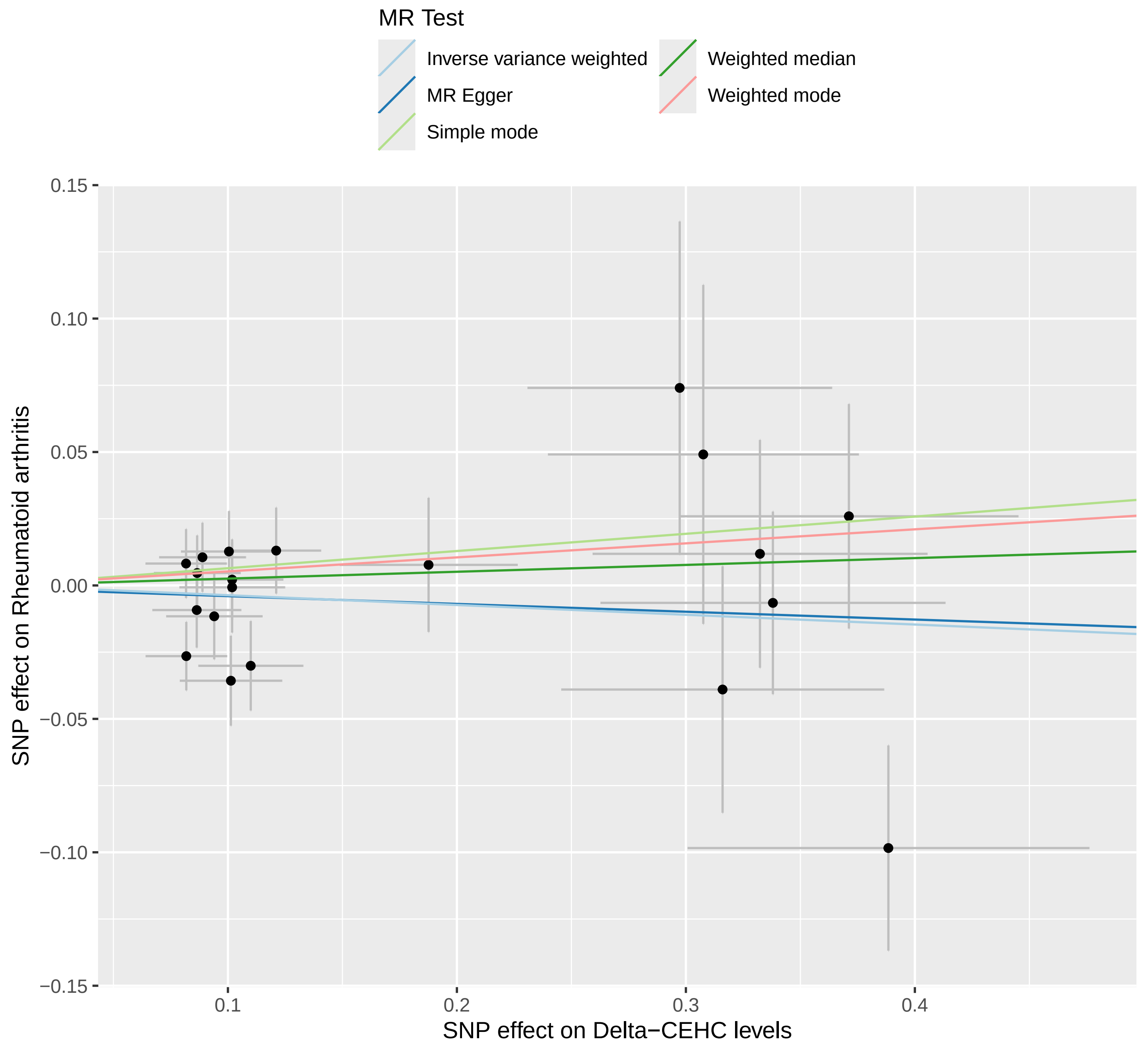
MR Test
Inverse variance weighted
MR Egger
Simple mode
Weighted median
Weighted mode
0.15
0.10
0.05
0.00
−0.05
−0.10
−0.15
0.1
0.2
0.3
0.4
SNP effect on Rheumatoid arthritis
SNP effect on Delta−CEHC levels

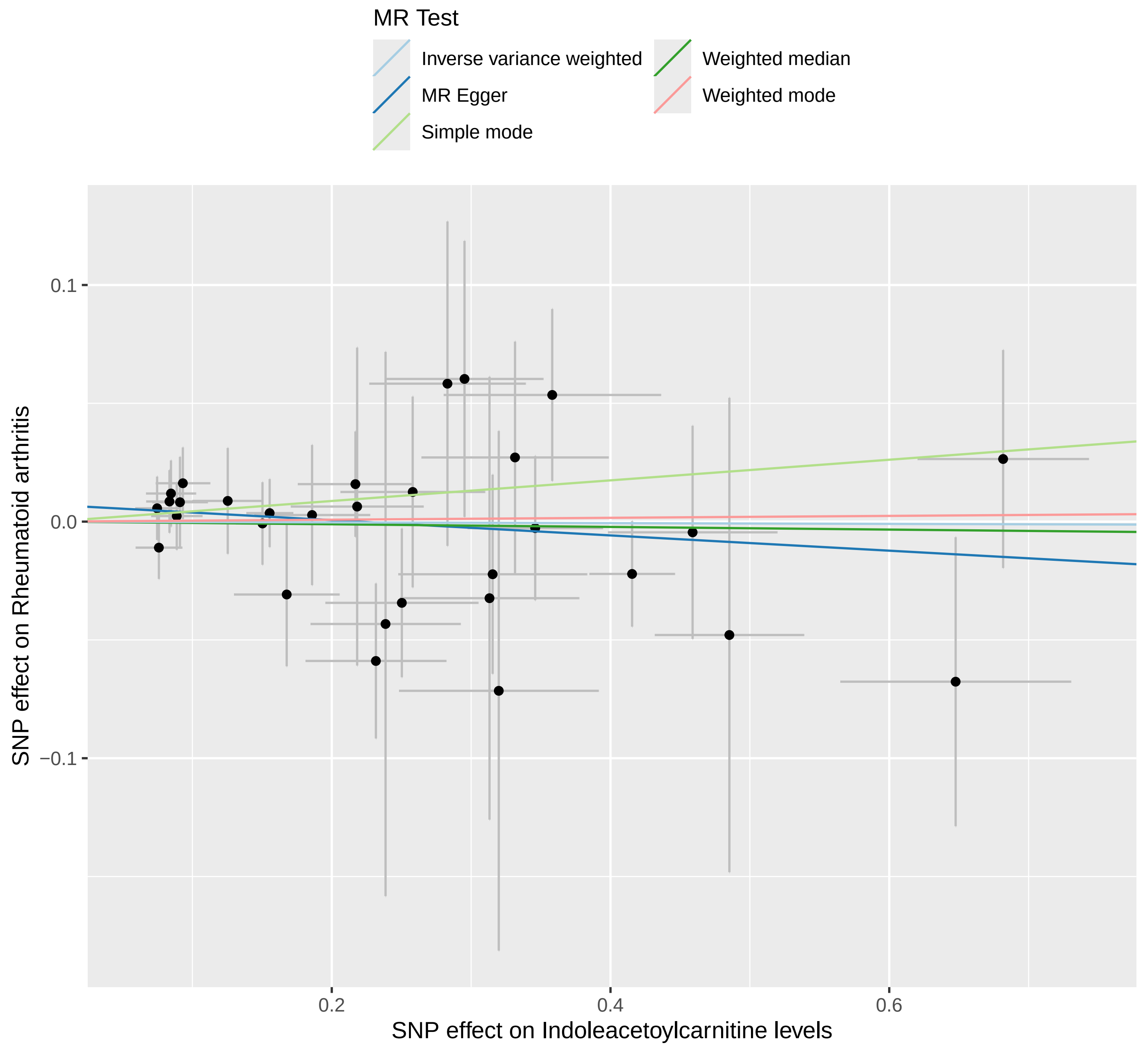

MR Test
Inverse variance weighted
MR Egger
Simple mode
Weighted median
Weighted mode
0.1
0.0
−0.1
0.2
0.4
0.6
SNP effect on Rheumatoid arthritis
SNP effect on Indoleacetoylcarnitine levels

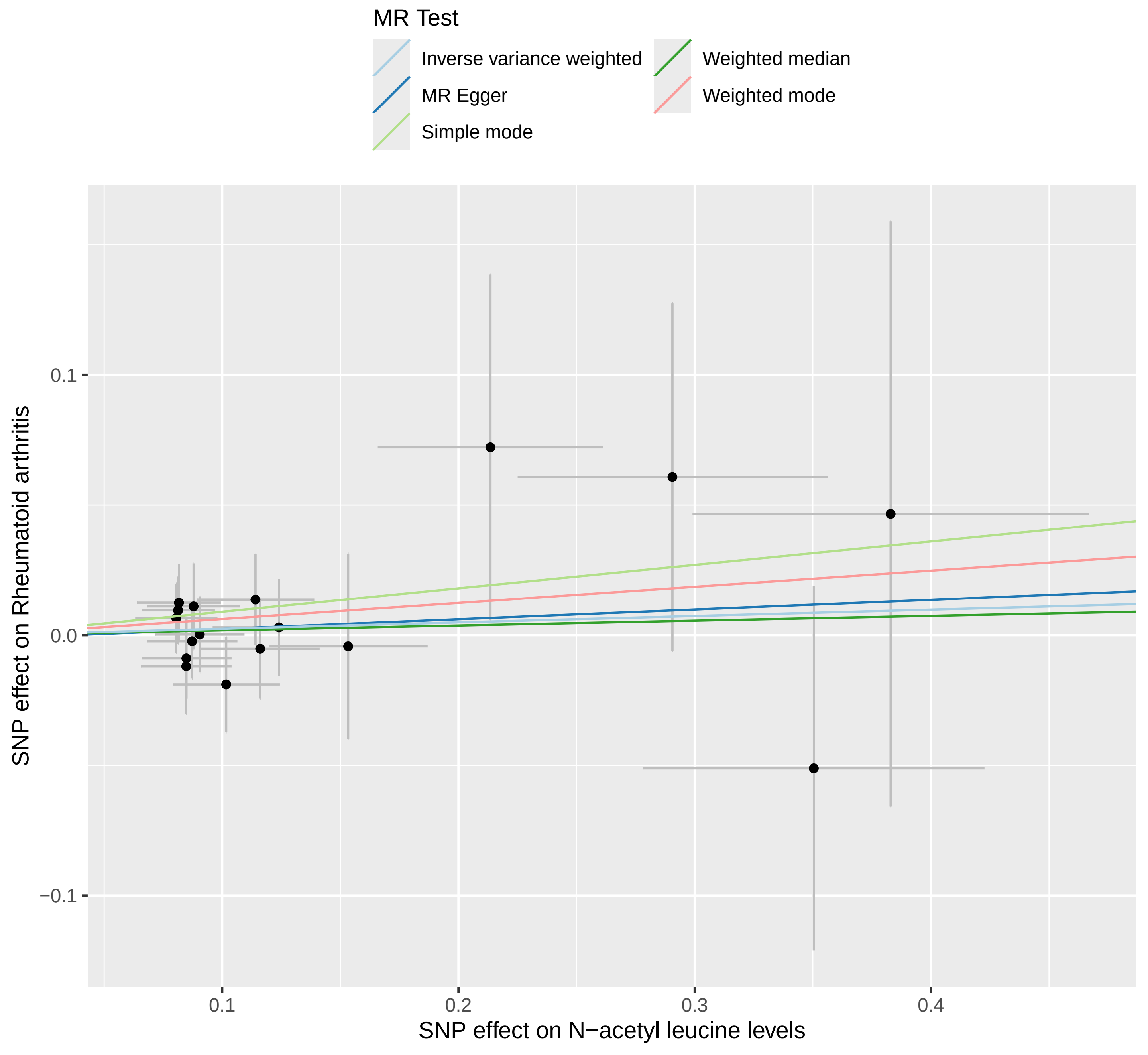
MR Test
Inverse variance weighted
MR Egger
Simple mode
Weighted median
Weighted mode
0.1
0.0
−0.1
0.1
0.2
0.3
0.4
SNP effect on Rheumatoid arthritis
SNP effect on N−acetyl leucine levels

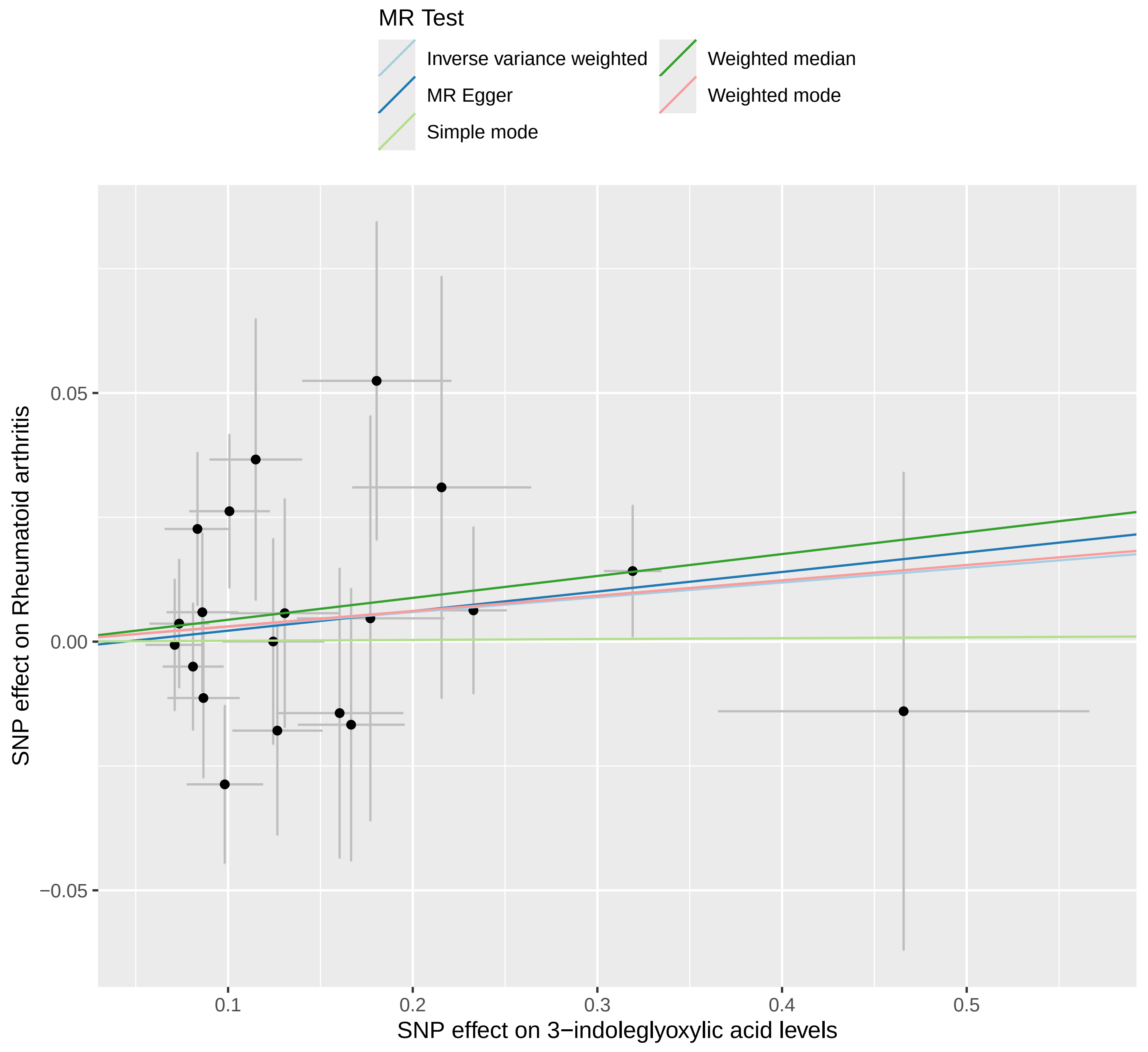

MR Test
Inverse variance weighted
MR Egger
Simple mode
Weighted median
Weighted mode
0.05
0.00
−0.05
0.1
0.2
0.3
0.4
0.5
SNP effect on Rheumatoid arthritis
SNP effect on 3−indoleglyoxylic acid levels

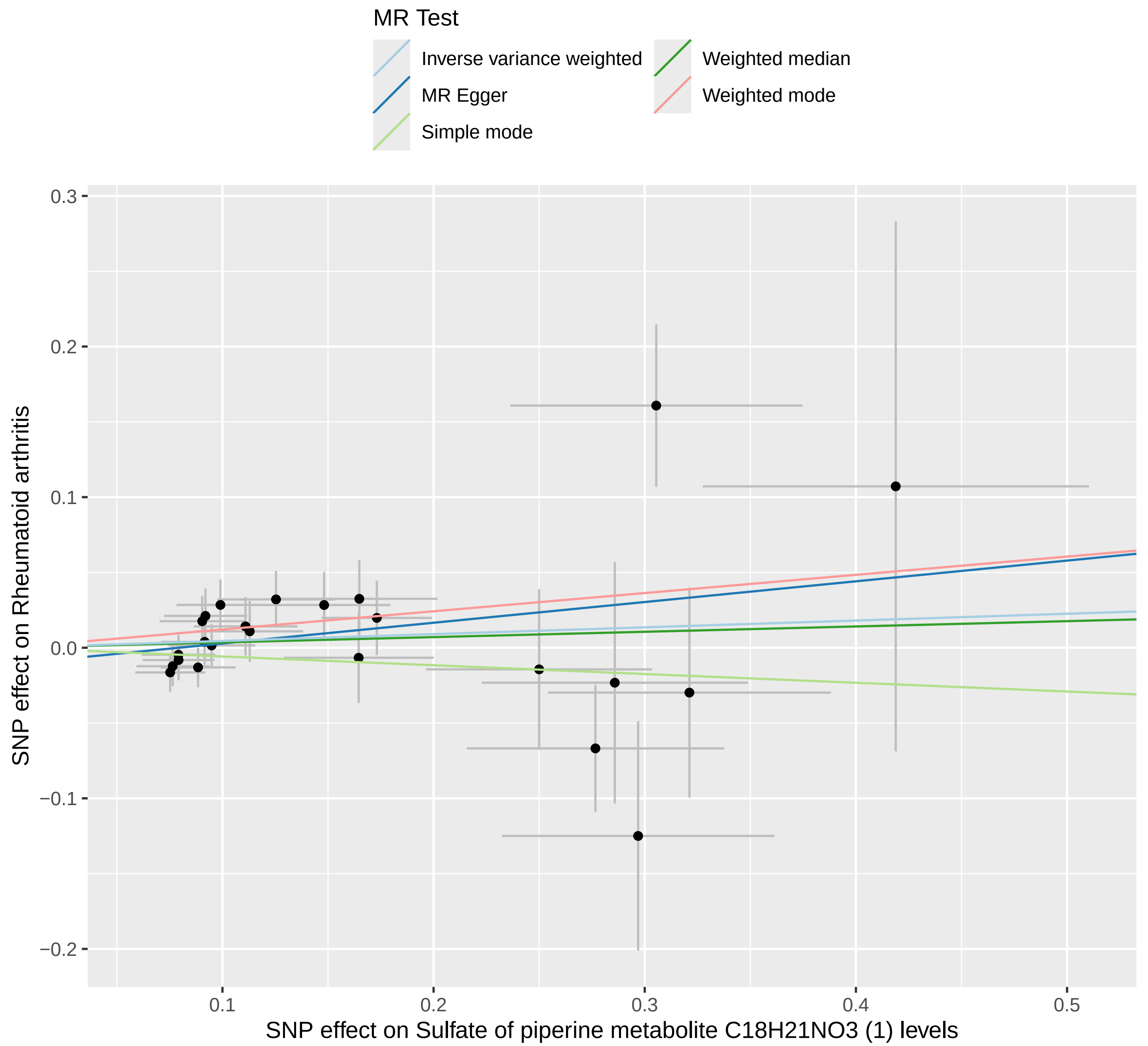
MR Test
Inverse variance weighted
MR Egger
Simple mode
Weighted median
Weighted mode
SNP effect on Rheumatoid arthritis
SNP effect on Sulfate of piperine metabolite C18H21NO3 (1) levels
0.3
0.2
0.1
0.0
−0.1
−0.2
0.1
0.2
0.3
0.4
0.5

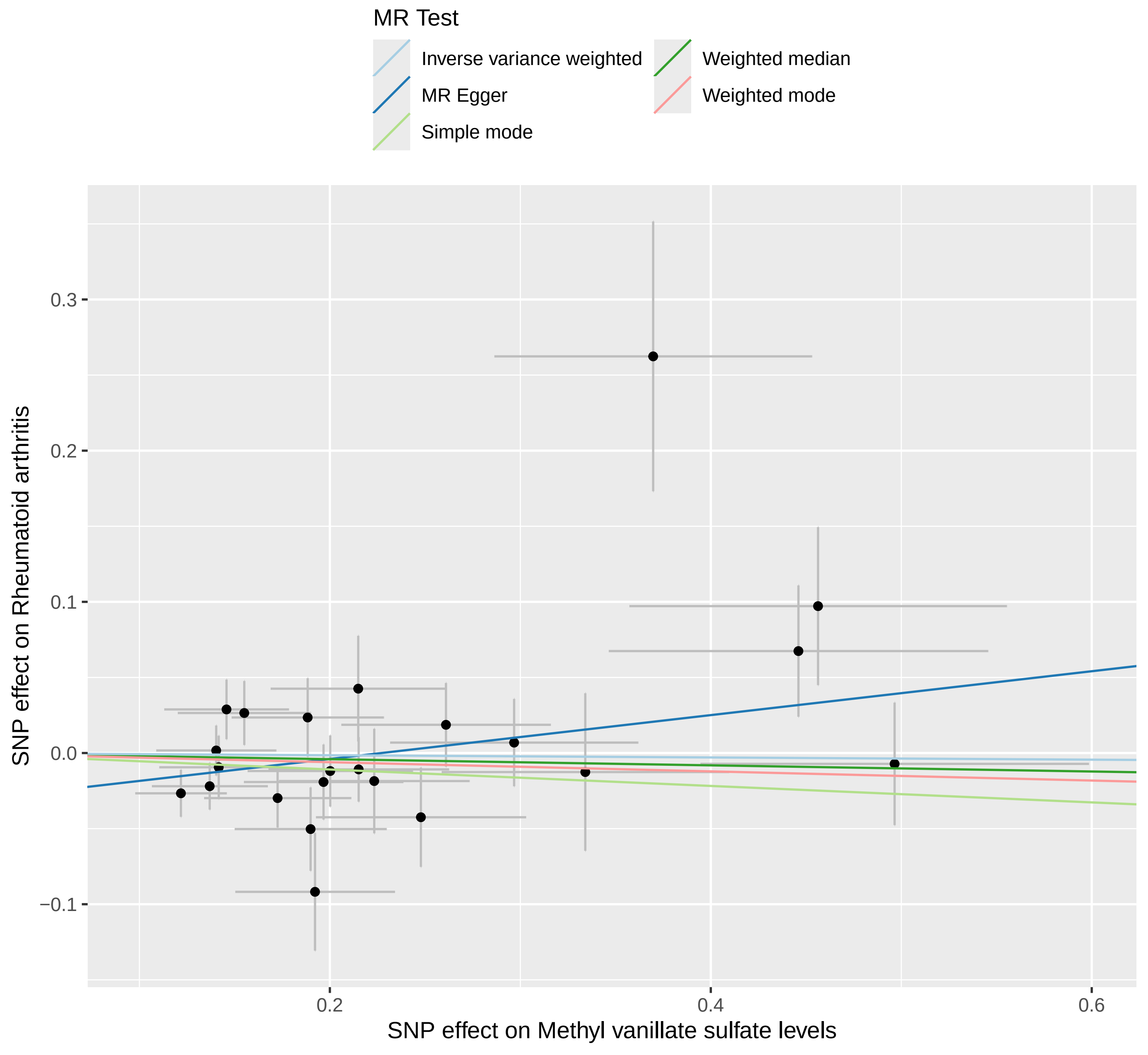
MR Test
Inverse variance weighted
MR Egger
Simple mode
Weighted median
Weighted mode
SNP effect on Rheumatoid arthritis
0.3
0.2
0.1
0.0
−0.1
0.2
0.4
0.6
SNP effect on Methyl vanillate sulfate levels

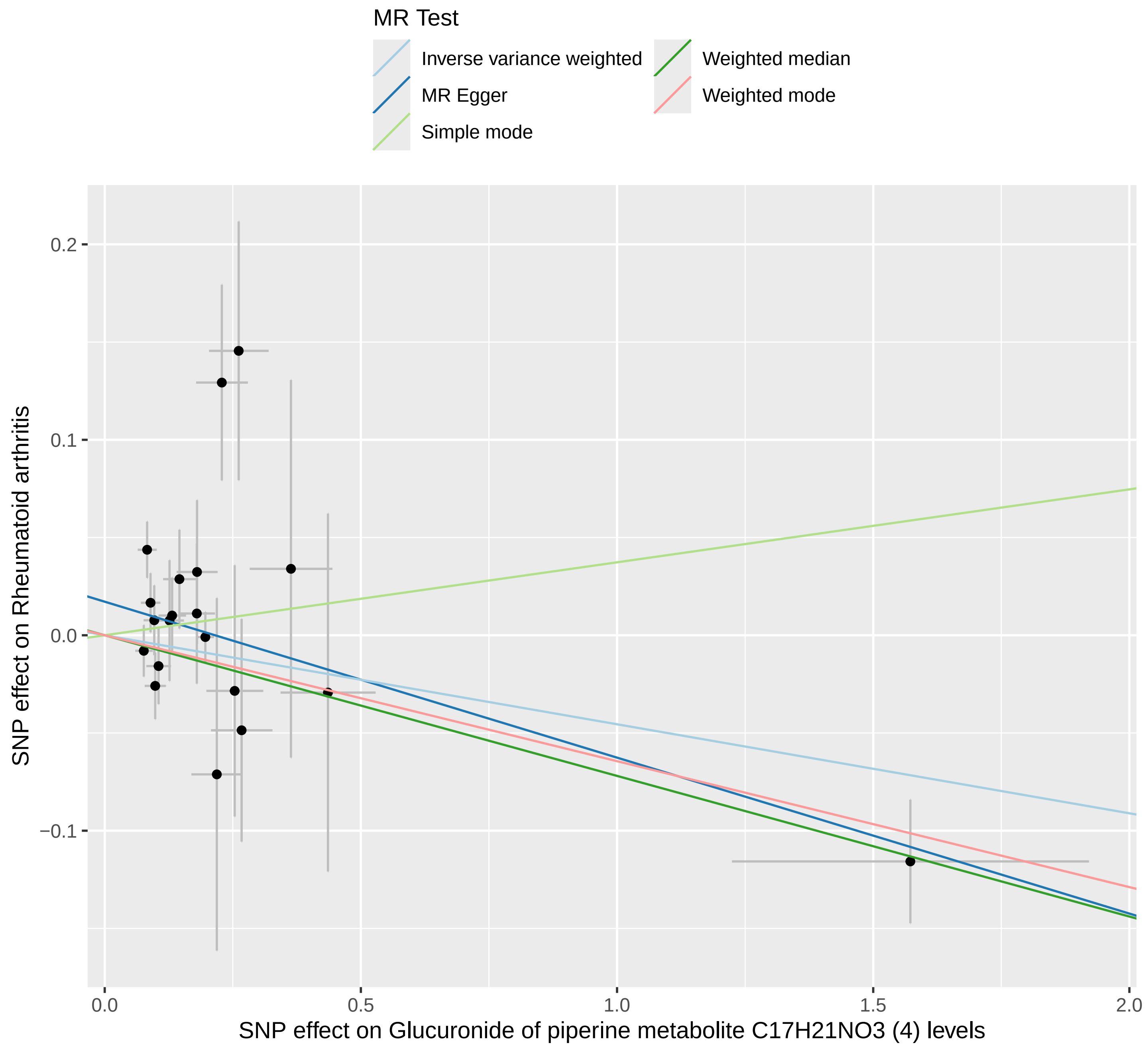

MR Test
Inverse variance weighted
MR Egger
Simple mode
Weighted median
Weighted mode
0.2
0.1
0.0
−0.1
0.0
0.5
1.0
1.5
2.0
SNP effect on Rheumatoid arthritis
SNP effect on Glucuronide of piperine metabolite C17H21NO3 (4) levels

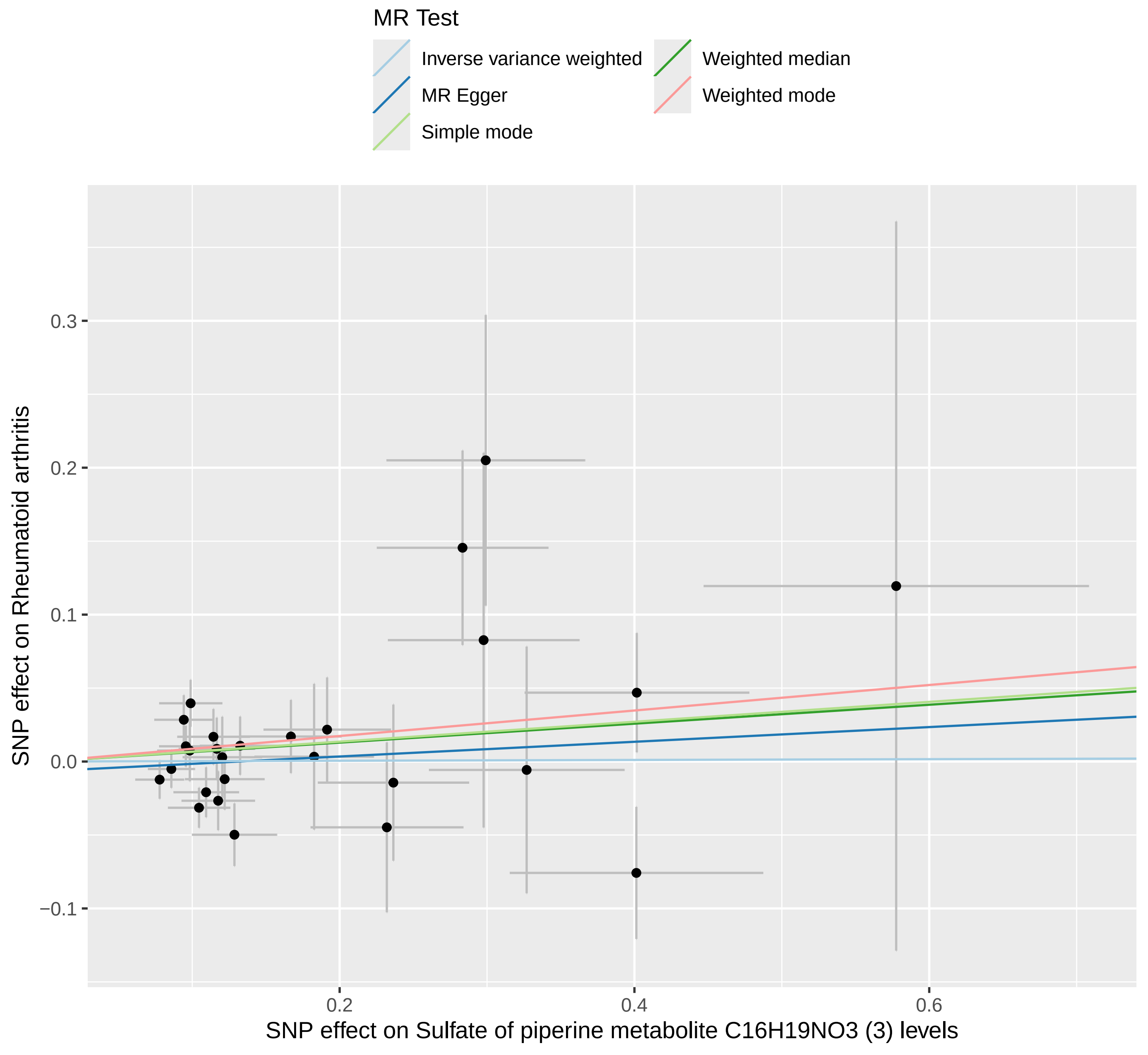

MR Test
Inverse variance weighted
MR Egger
Simple mode
Weighted median
Weighted mode
0.3
0.2
0.1
0.0
−0.1
0.2
0.4
0.6
SNP effect on Rheumatoid arthritis
SNP effect on Sulfate of piperine metabolite C16H19NO3 (3) levels

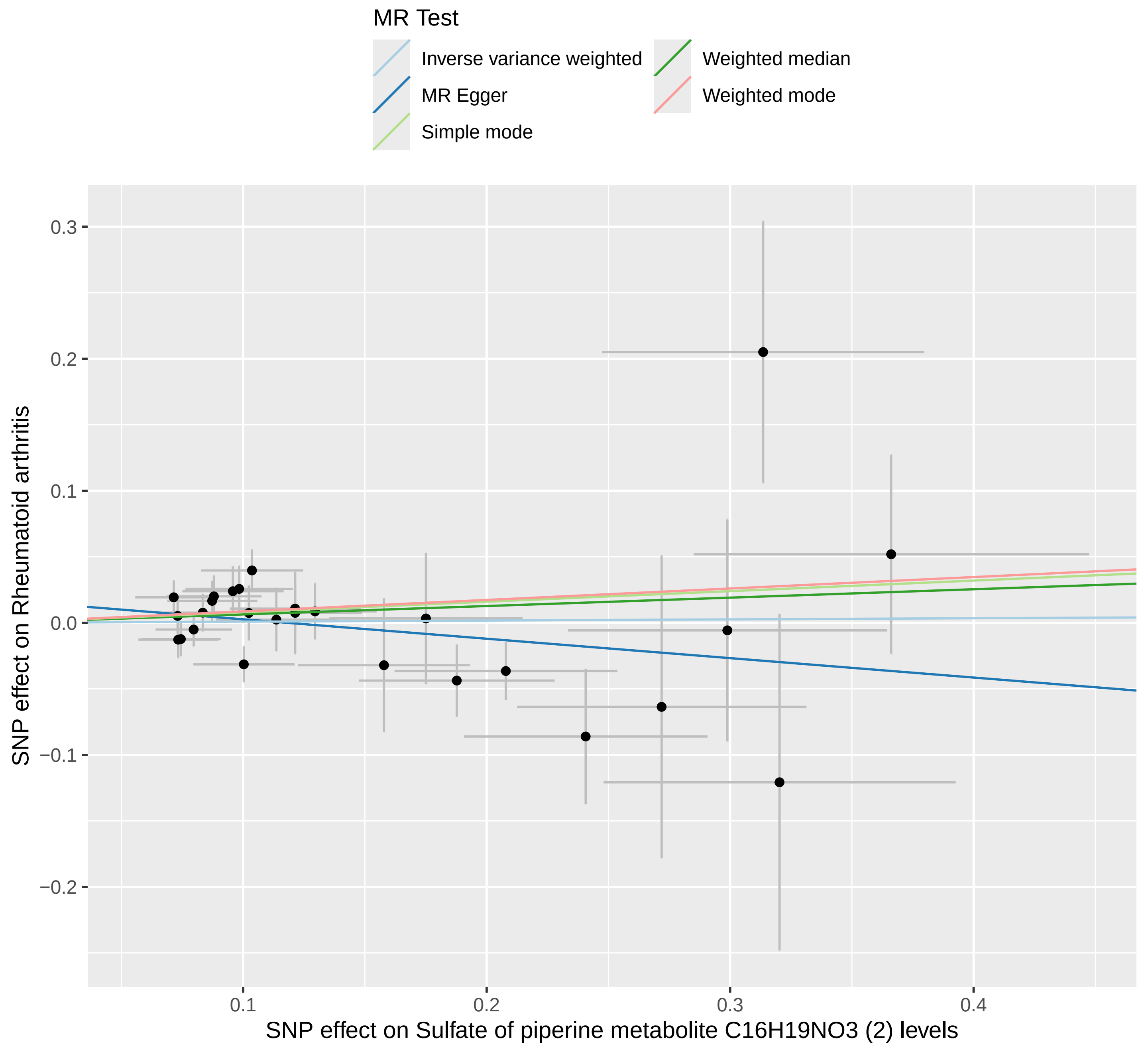
MR Test
Inverse variance weighted
MR Egger
Simple mode
Weighted median
Weighted mode
SNP effect on Rheumatoid arthritis
0.3
0.2
0.1
0.0
−0.1
−0.2
0.1
0.2
0.3
0.4
SNP effect on Sulfate of piperine metabolite C16H19NO3 (2) levels

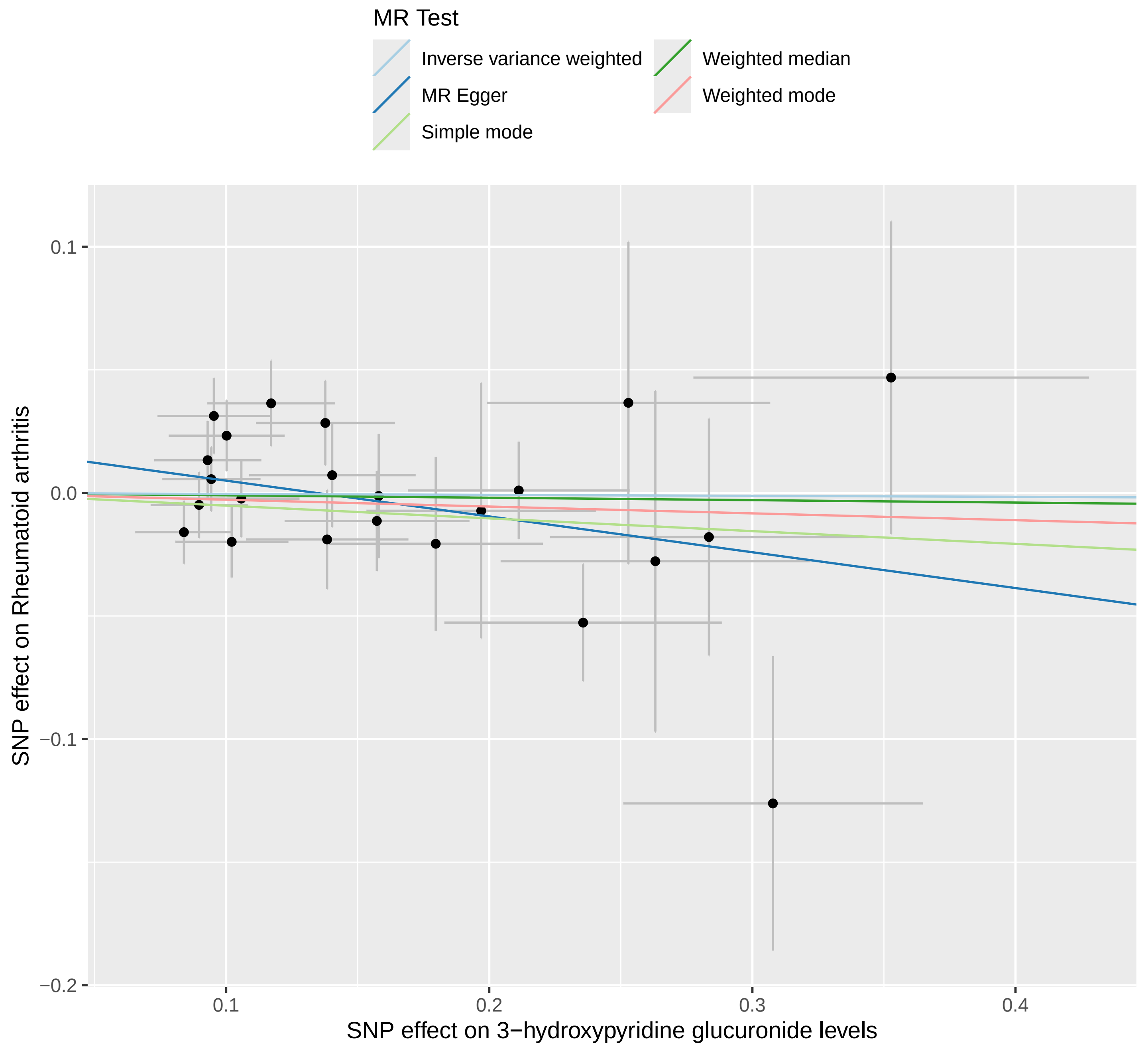
MR Test
Inverse variance weighted
MR Egger
Simple mode
Weighted median
Weighted mode
0.1
0.0
−0.1
−0.2
SNP effect on Rheumatoid arthritis
0.1
0.2
0.3
0.4
SNP effect on 3−hydroxypyridine glucuronide levels

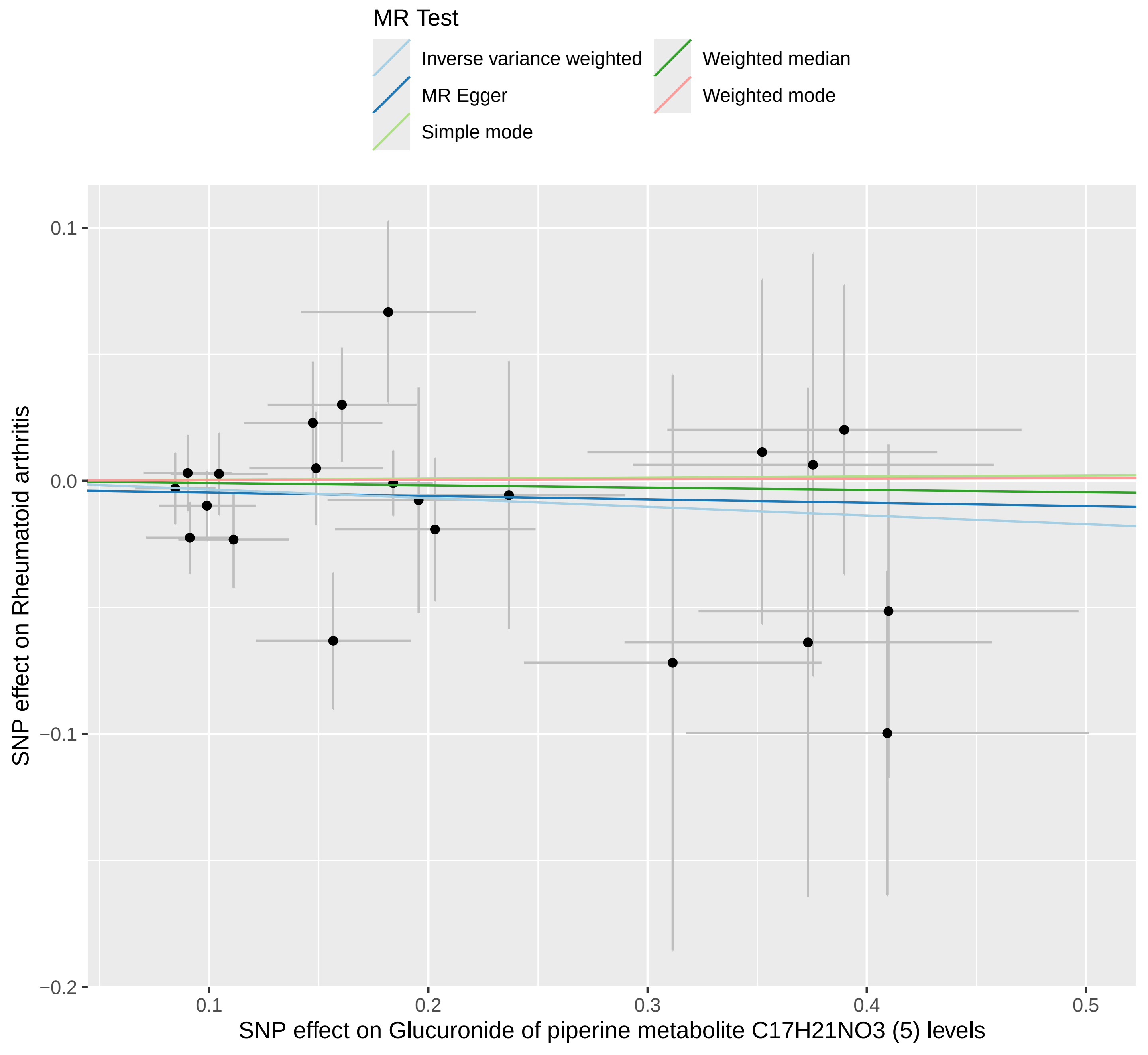

MR Test
Inverse variance weighted
MR Egger
Simple mode
Weighted median
Weighted mode
0.1
0.0
−0.1
−0.2
0.1
0.2
0.3
0.4
0.5
SNP effect on Rheumatoid arthritis
SNP effect on Glucuronide of piperine metabolite C17H21NO3 (5) levels

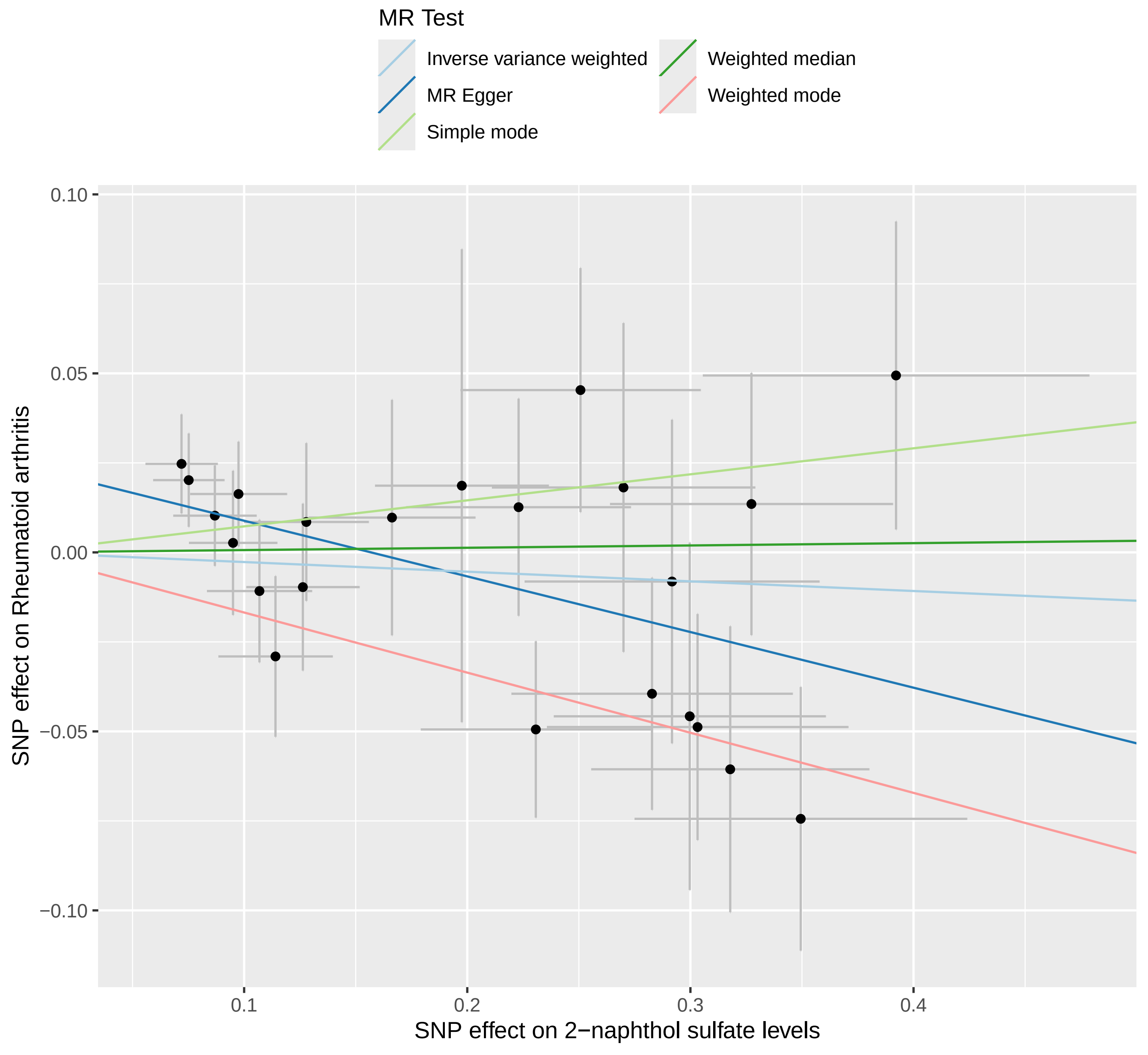

MR Test
Inverse variance weighted
MR Egger
Simple mode
Weighted median
Weighted mode
0.10
0.05
0.00
−0.05
−0.10
0.1
0.2
0.3
0.4
SNP effect on Rheumatoid arthritis
SNP effect on 2−naphthol sulfate levels

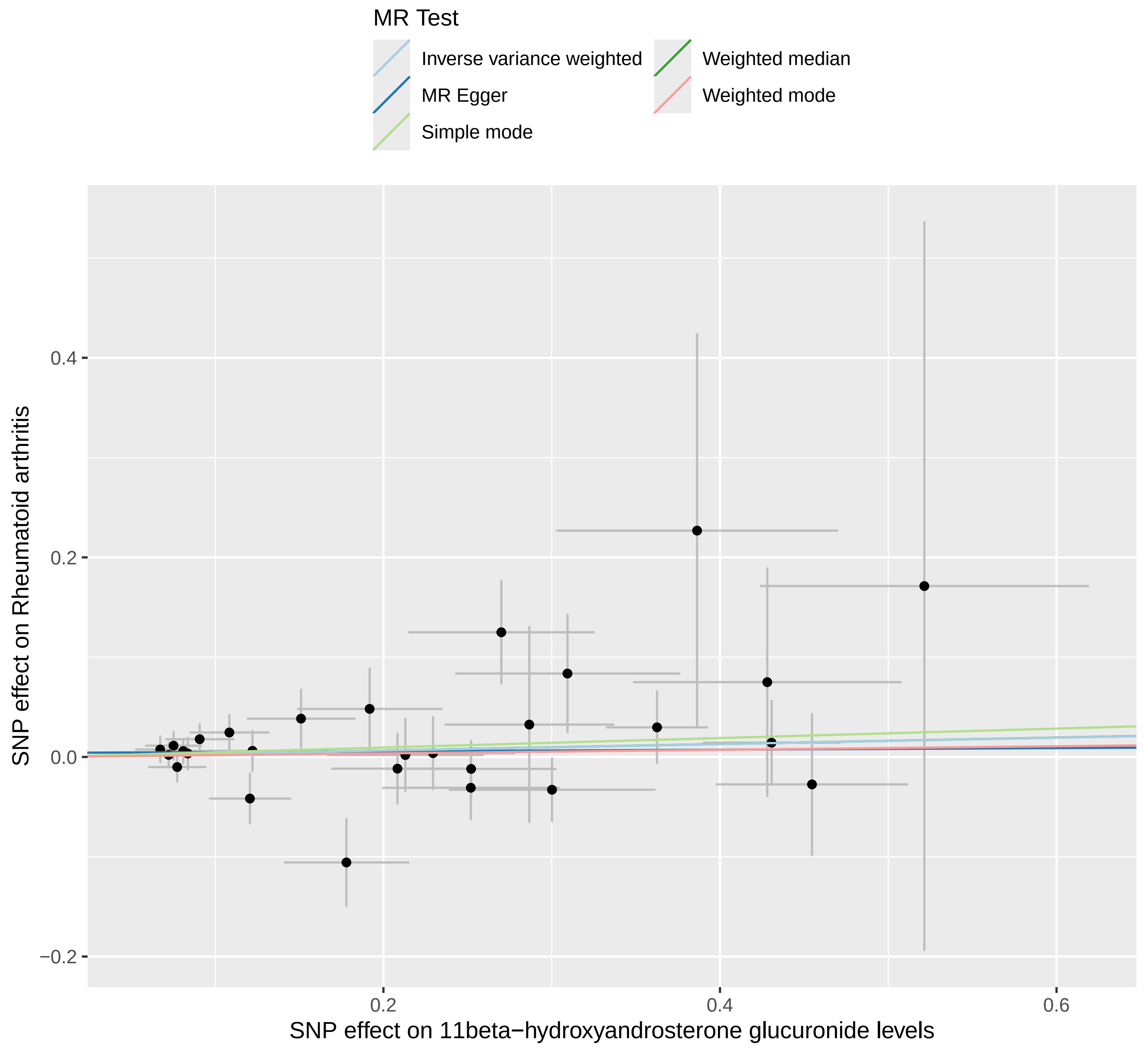
MR Test
Inverse variance weighted
MR Egger
Simple mode
Weighted median
Weighted mode
0.4
0.2
0.0
−0.2
SNP effect on Rheumatoid arthritis
0.2
0.4
0.6
SNP effect on 11beta−hydroxyandrosterone glucuronide levels

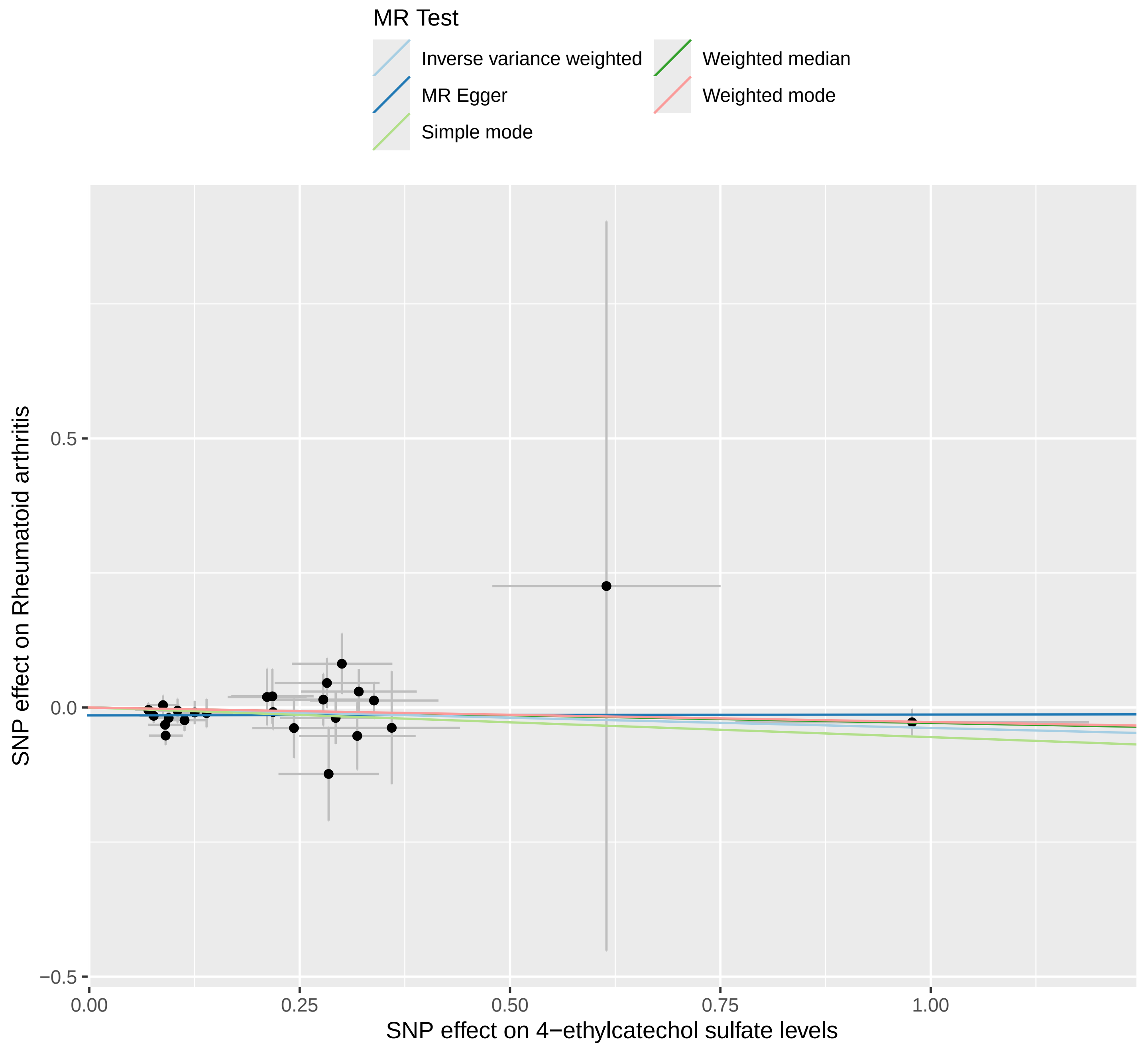
MR Test
Inverse variance weighted
MR Egger
Simple mode
Weighted median
Weighted mode
SNP effect on Rheumatoid arthritis
0.5
0.0
−0.5
0.00
0.25
0.50
0.75
1.00
SNP effect on 4−ethylcatechol sulfate levels

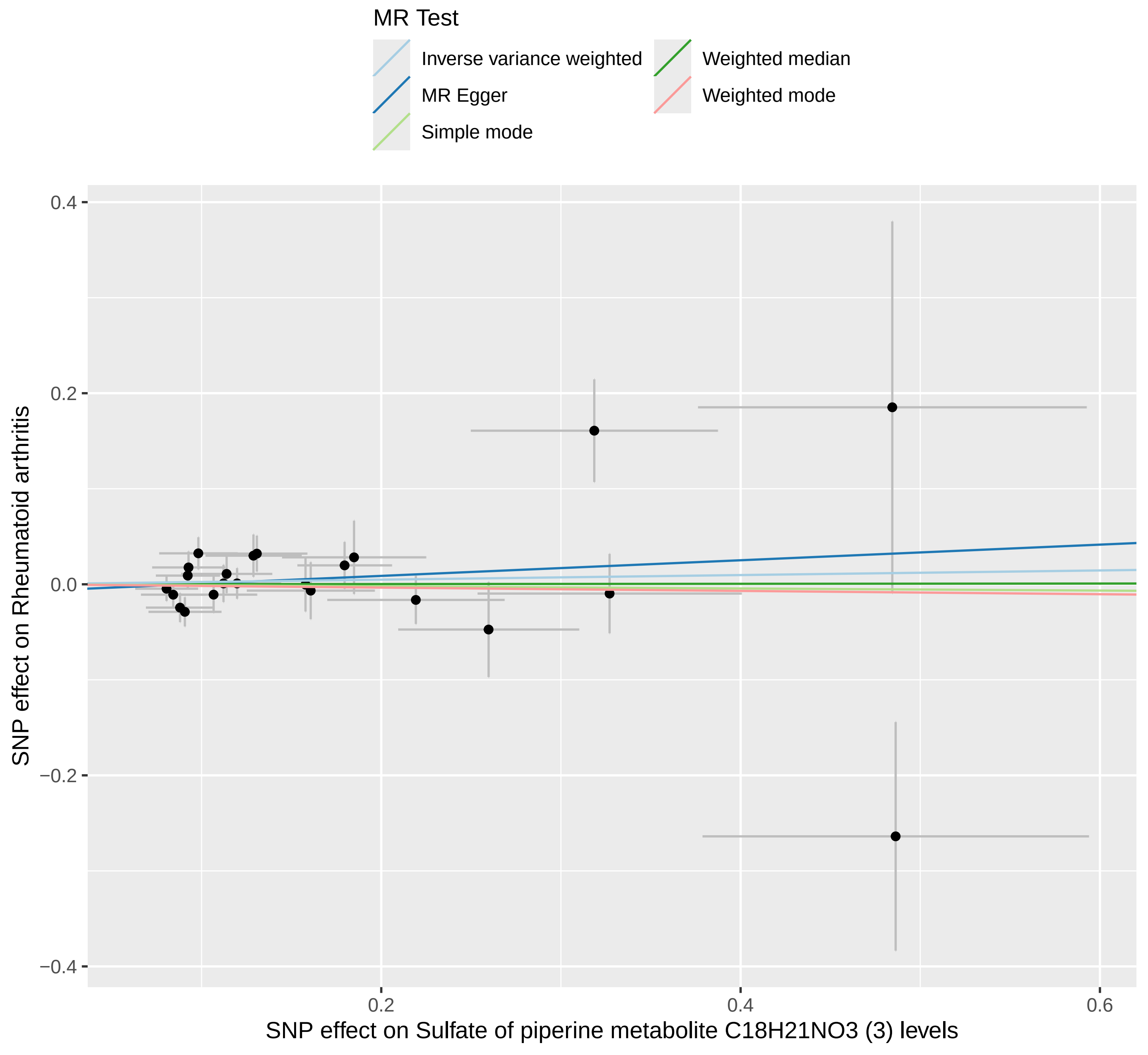
MR Test
Inverse variance weighted
MR Egger
Simple mode
Weighted median
Weighted mode
0.4
0.2
0.0
−0.2
−0.4
SNP effect on Rheumatoid arthritis
0.2
0.4
0.6
SNP effect on Sulfate of piperine metabolite C18H21NO3 (3) levels

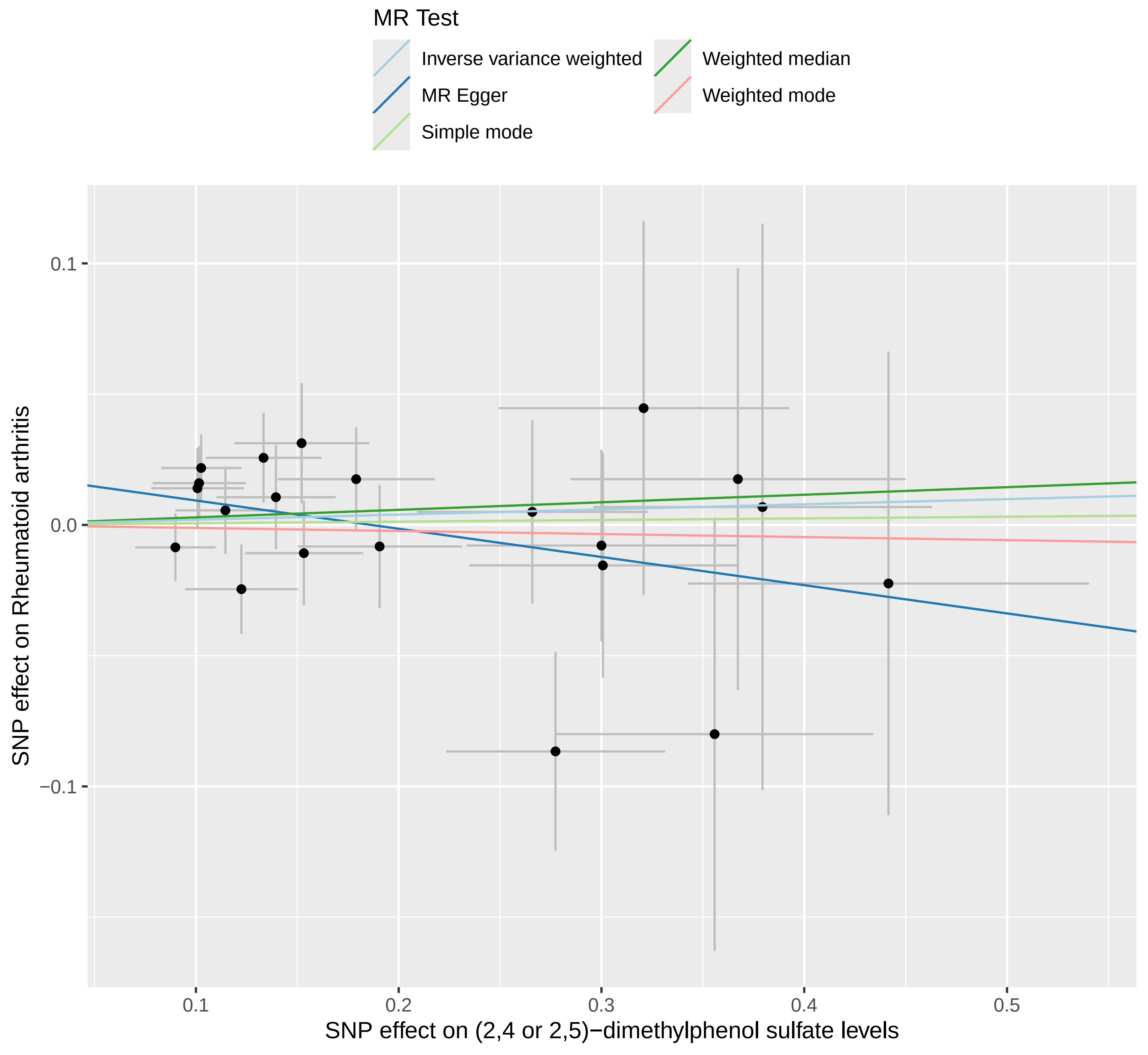
MR Test
Inverse variance weighted
MR Egger
Simple mode
Weighted median
Weighted mode
SNP effect on Rheumatoid arthritis
0.1
0.0
−0.1
0.1
0.2
0.3
0.4
0.5
SNP effect on (2,4 or 2,5)−dimethylphenol sulfate levels

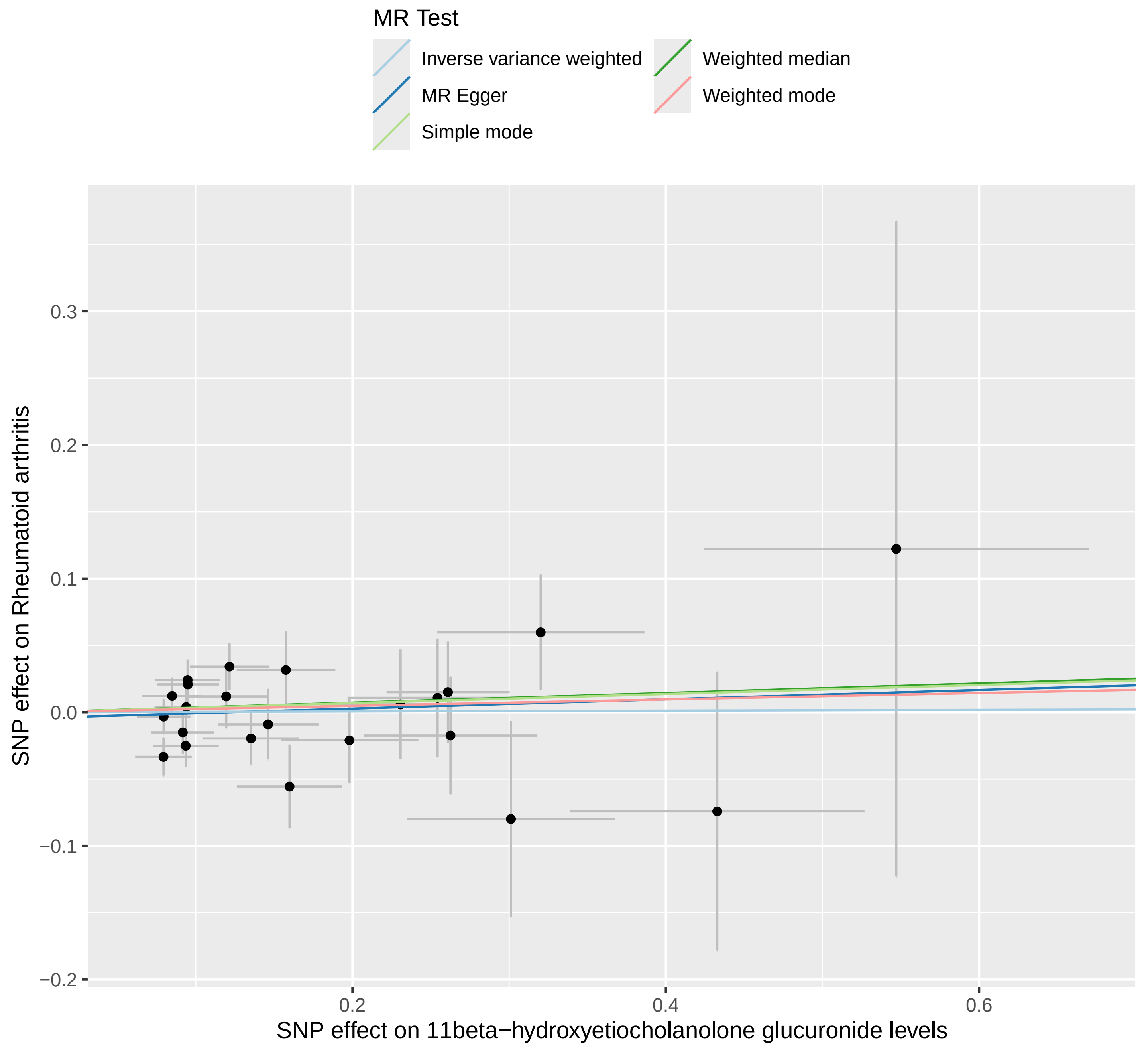

MR Test
Inverse variance weighted
MR Egger
Simple mode
Weighted median
Weighted mode
0.3
0.2
0.1
0.0
−0.1
−0.2
0.2
0.4
0.6
SNP effect on Rheumatoid arthritis
SNP effect on 11beta−hydroxyetiocholanolone glucuronide levels

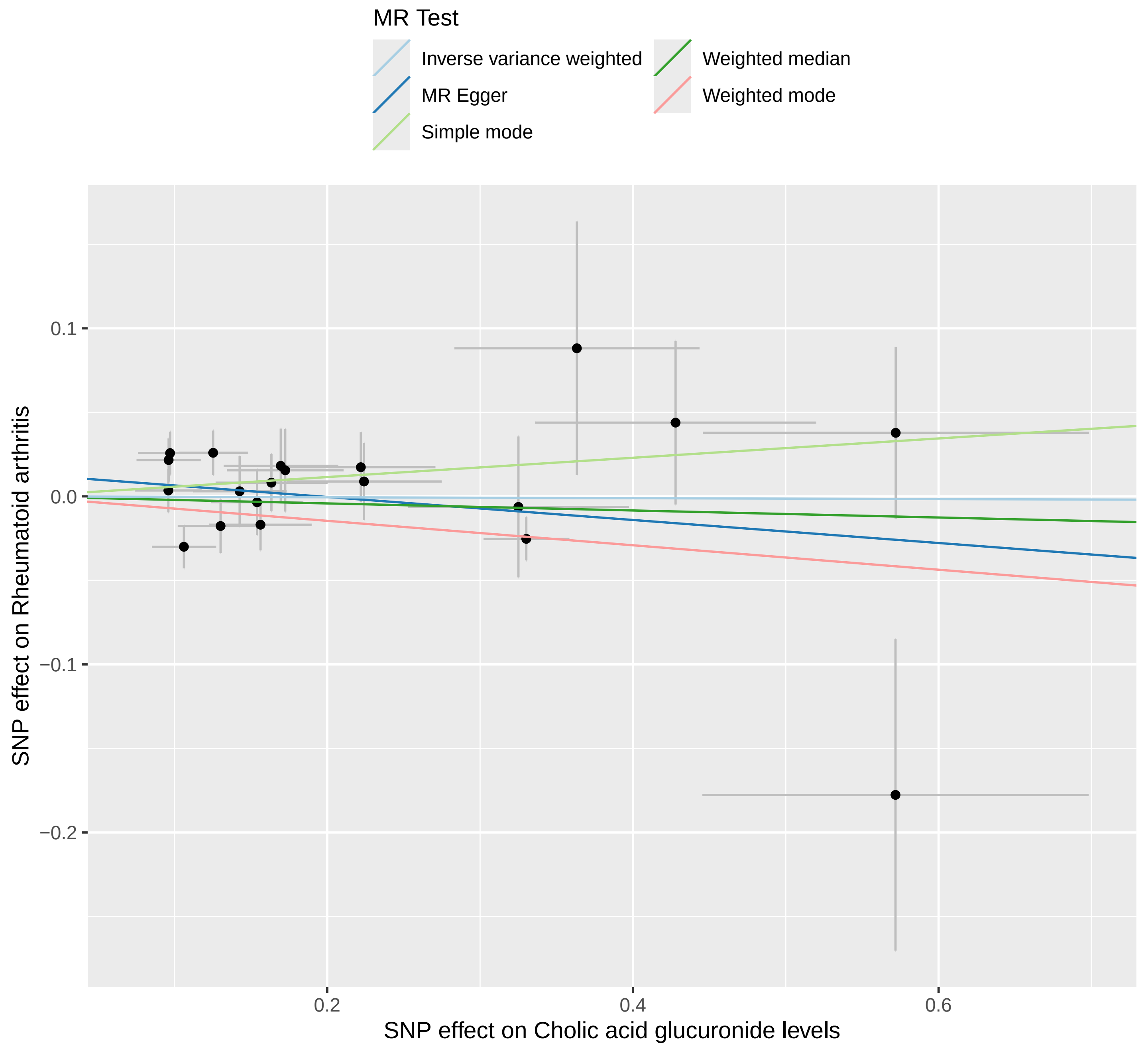

MR Test
Inverse variance weighted
MR Egger
Simple mode
Weighted median
Weighted mode
SNP effect on Rheumatoid arthritis
0.1
0.0
−0.1
−0.2
0.2
0.4
0.6
SNP effect on Cholic acid glucuronide levels

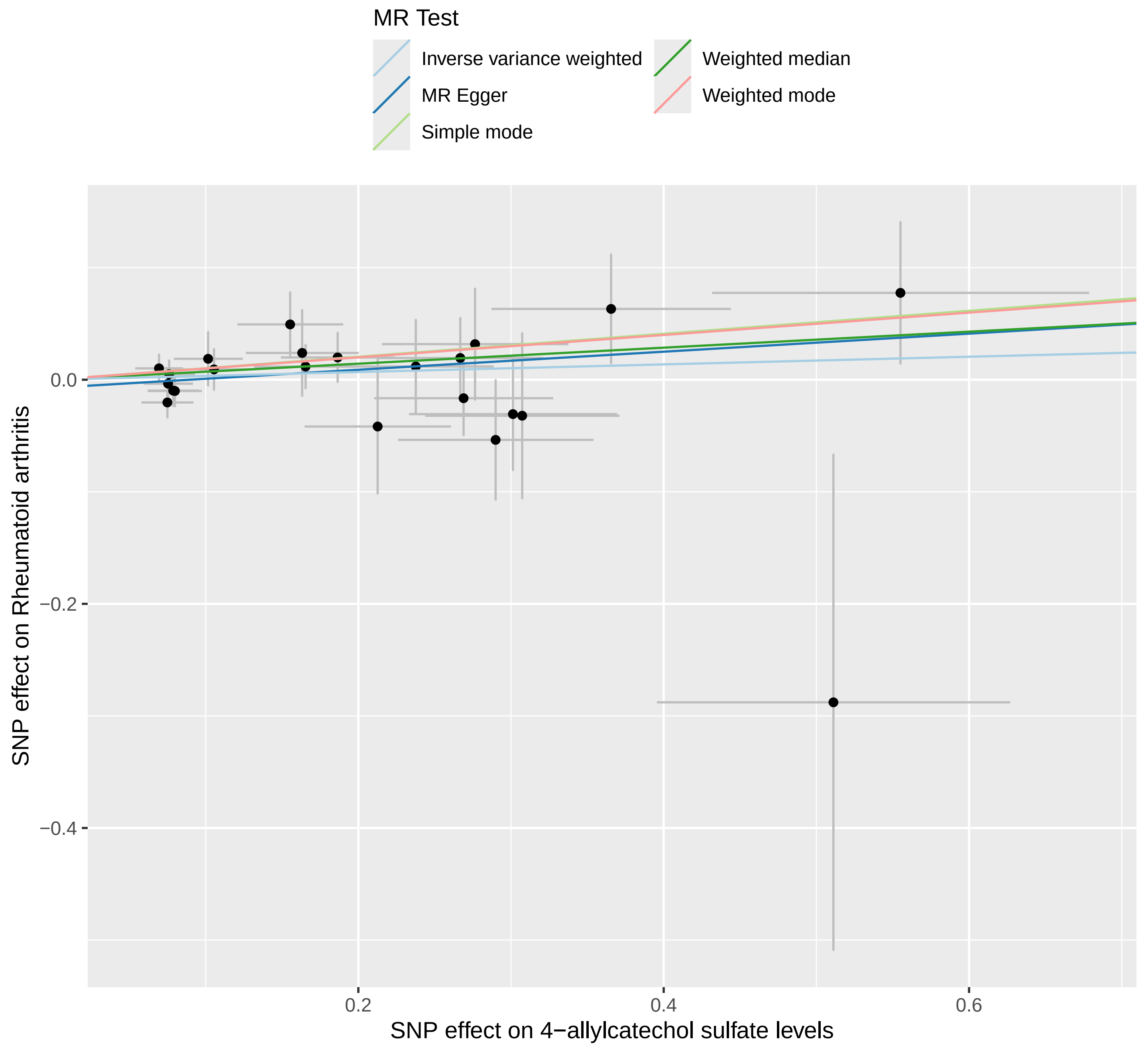
MR Test
Inverse variance weighted
MR Egger
Simple mode
Weighted median
Weighted mode
0.0
−0.2
−0.4
0.2
0.4
0.6
SNP effect on Rheumatoid arthritis
SNP effect on 4−allylcatechol sulfate levels

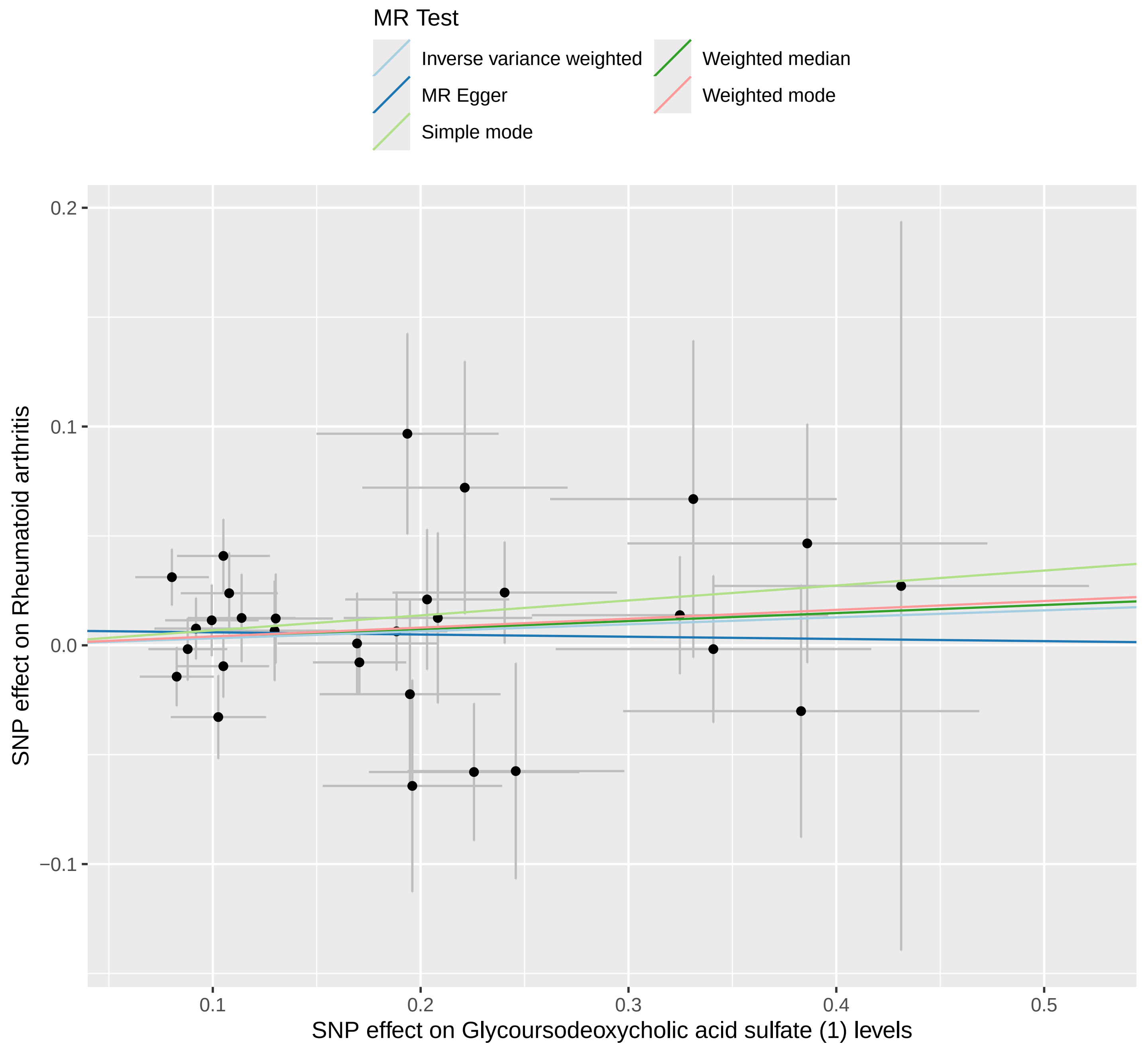
MR Test
Inverse variance weighted
MR Egger
Simple mode
Weighted median
Weighted mode
0.2
0.1
0.0
−0.1
0.1
0.2
0.3
0.4
0.5
SNP effect on Rheumatoid arthritis
SNP effect on Glycoursodeoxycholic acid sulfate (1) levels

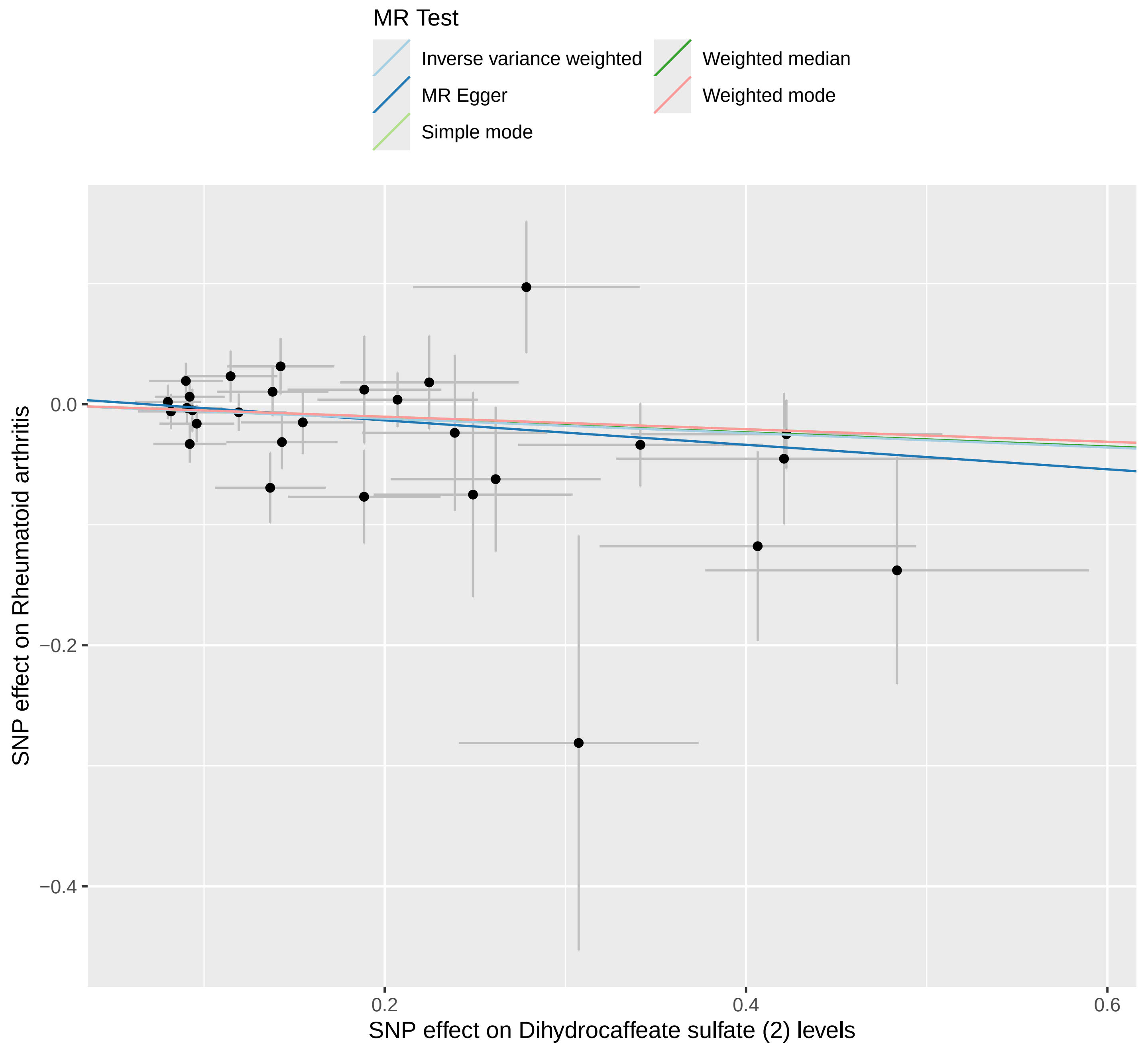

MR Test
Inverse variance weighted
MR Egger
Simple mode
Weighted median
Weighted mode
SNP effect on Rheumatoid arthritis
0.0
−0.2
−0.4
0.2
0.4
0.6
SNP effect on Dihydrocaffeate sulfate (2) levels

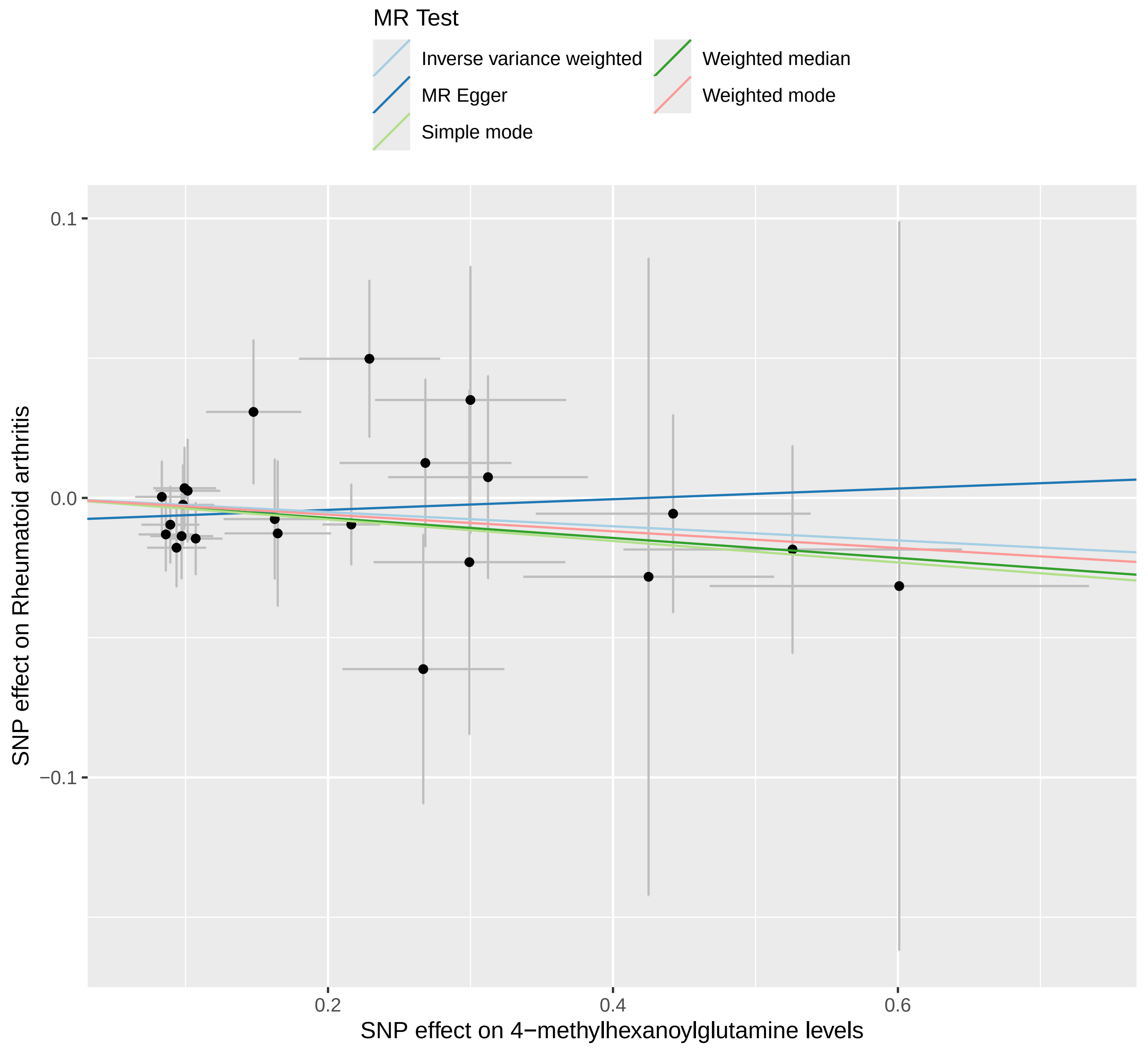
MR Test
Inverse variance weighted
MR Egger
Simple mode
Weighted median
Weighted mode
0.1
0.0
−0.1
0.2
0.4
0.6
SNP effect on Rheumatoid arthritis
SNP effect on 4−methylhexanoylglutamine levels

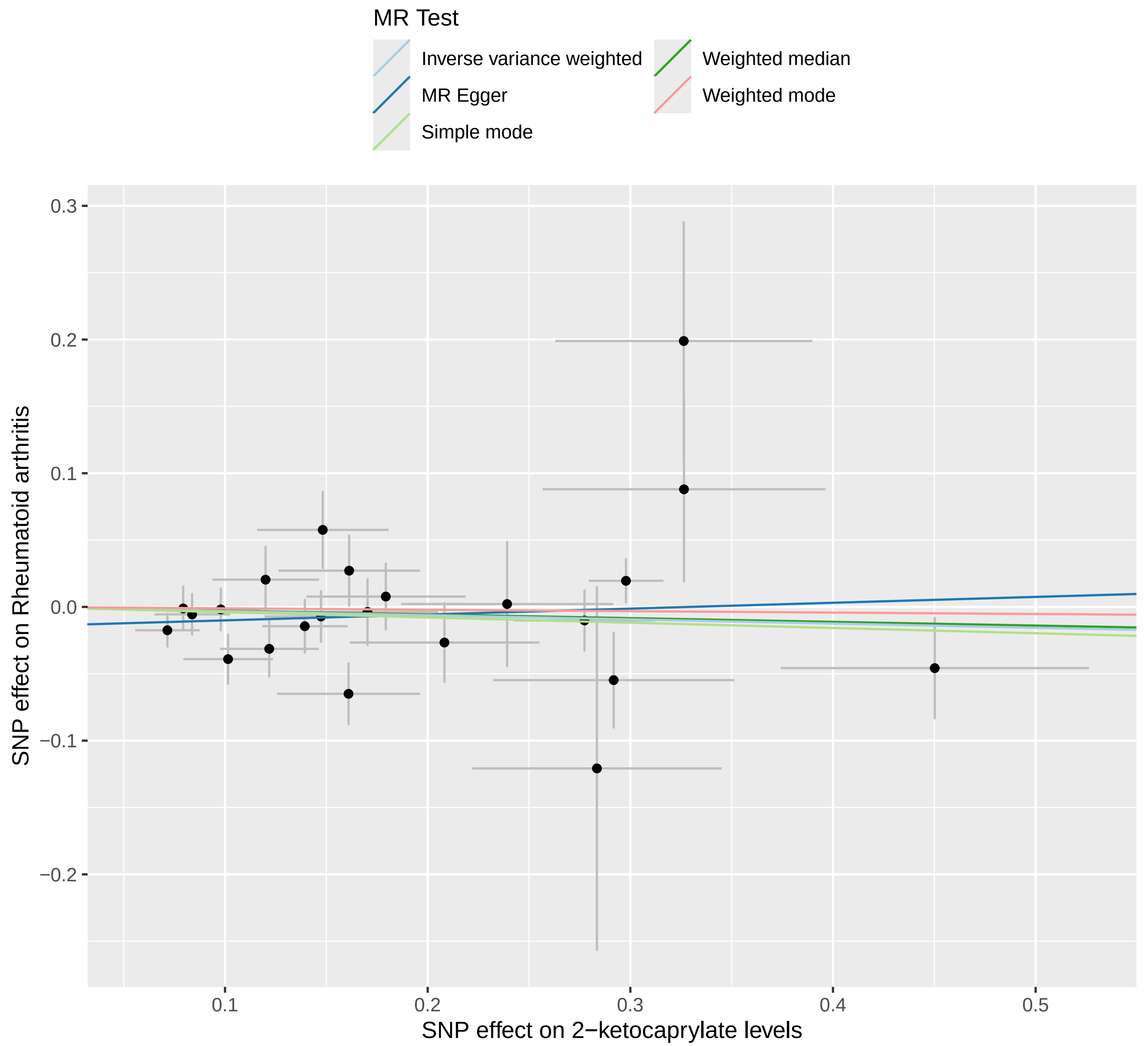

MR Test
Inverse variance weighted
MR Egger
Simple mode
Weighted median
Weighted mode
0.3
0.2
0.1
0.0
−0.1
−0.2
SNP effect on Rheumatoid arthritis
0.1
0.2
0.3
0.4
0.5
SNP effect on 2−ketocaprylate levels

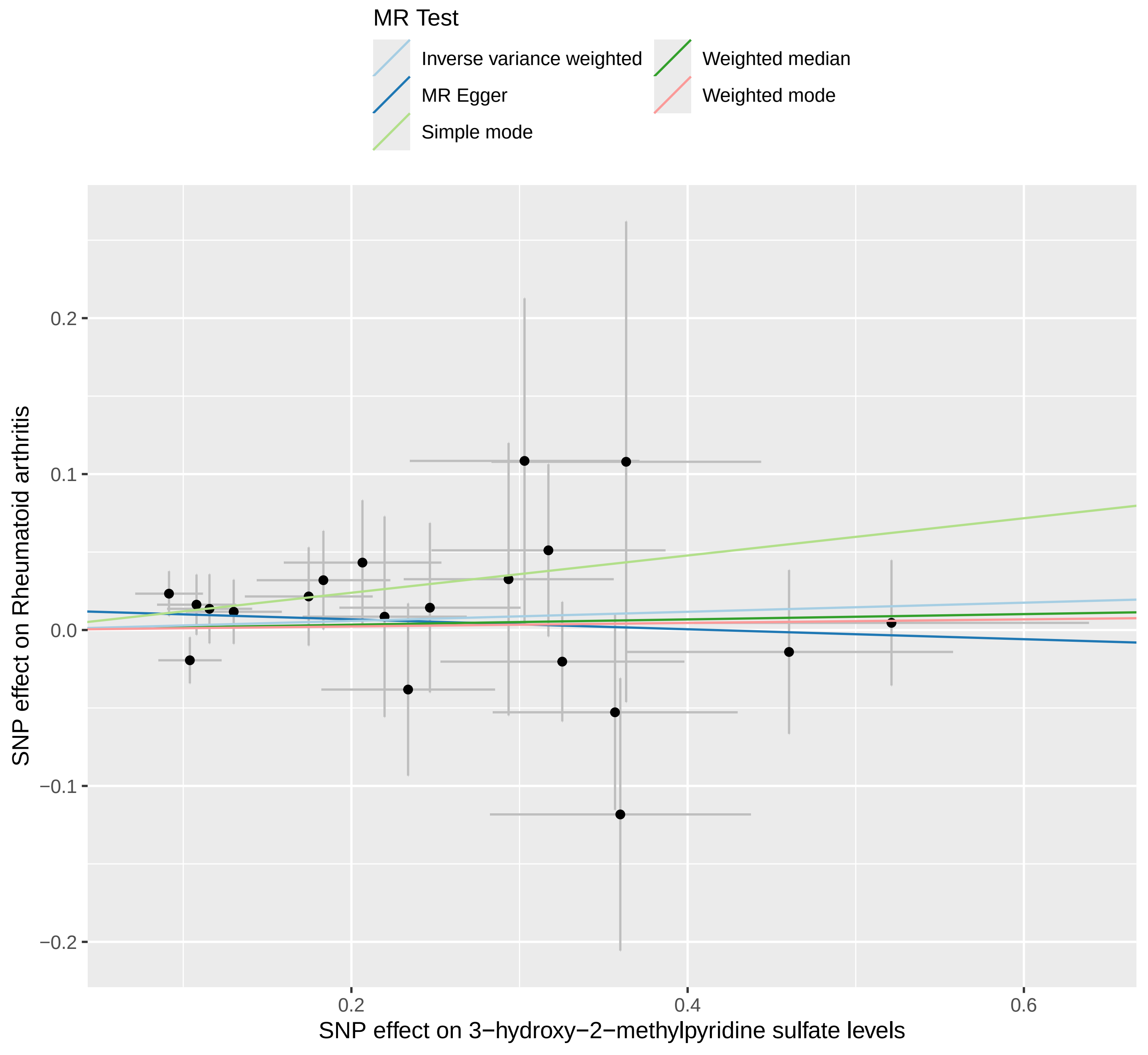

MR Test
Inverse variance weighted
MR Egger
Simple mode
Weighted median
Weighted mode
0.2
0.1
0.0
−0.1
−0.2
SNP effect on Rheumatoid arthritis
0.2
0.4
0.6
SNP effect on 3−hydroxy−2−methylpyridine sulfate levels

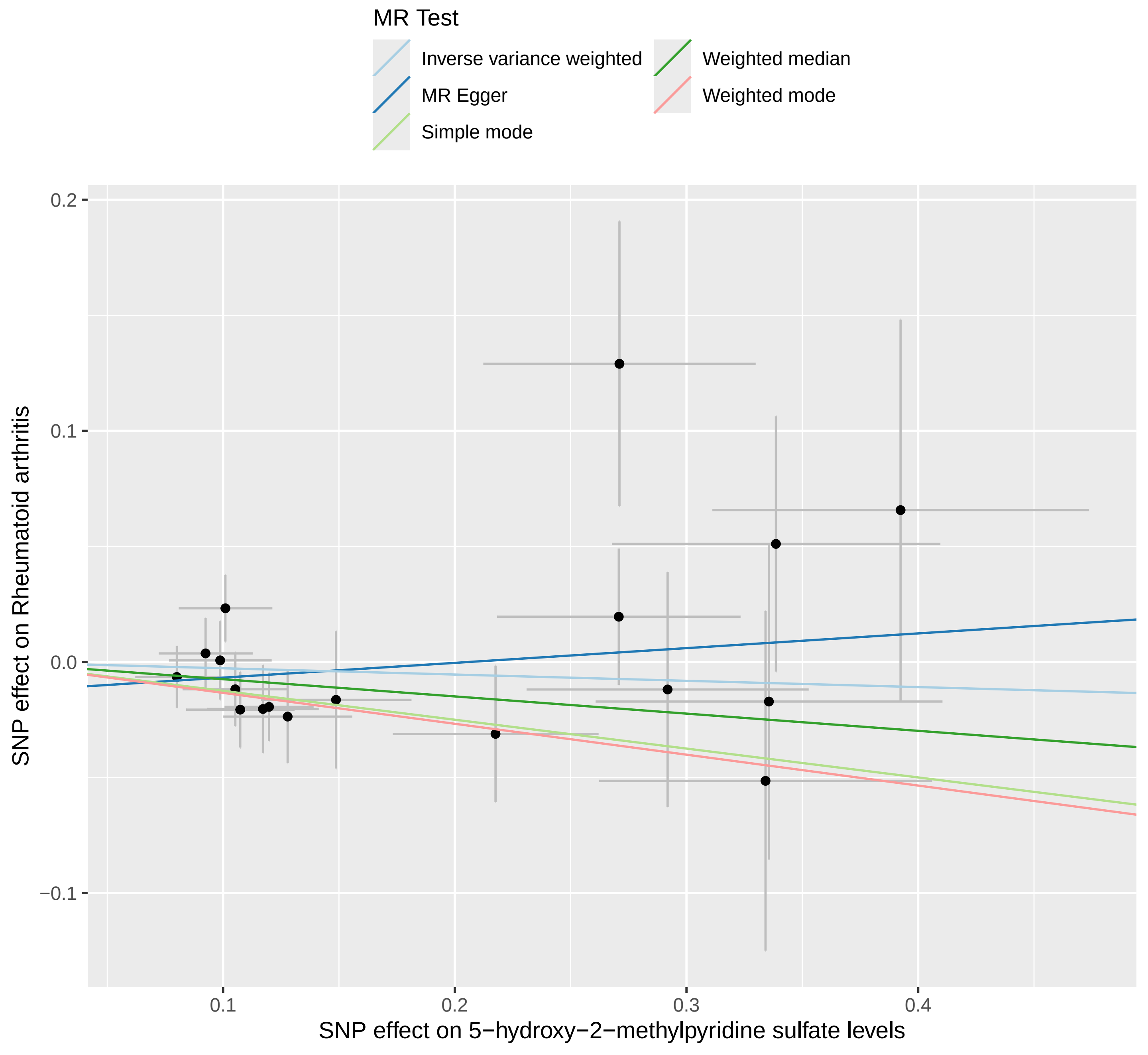

MR Test
Inverse variance weighted
MR Egger
Simple mode
Weighted median
Weighted mode
0.2
0.1
0.0
−0.1
0.1
0.2
0.3
0.4
SNP effect on Rheumatoid arthritis
SNP effect on 5−hydroxy−2−methylpyridine sulfate levels

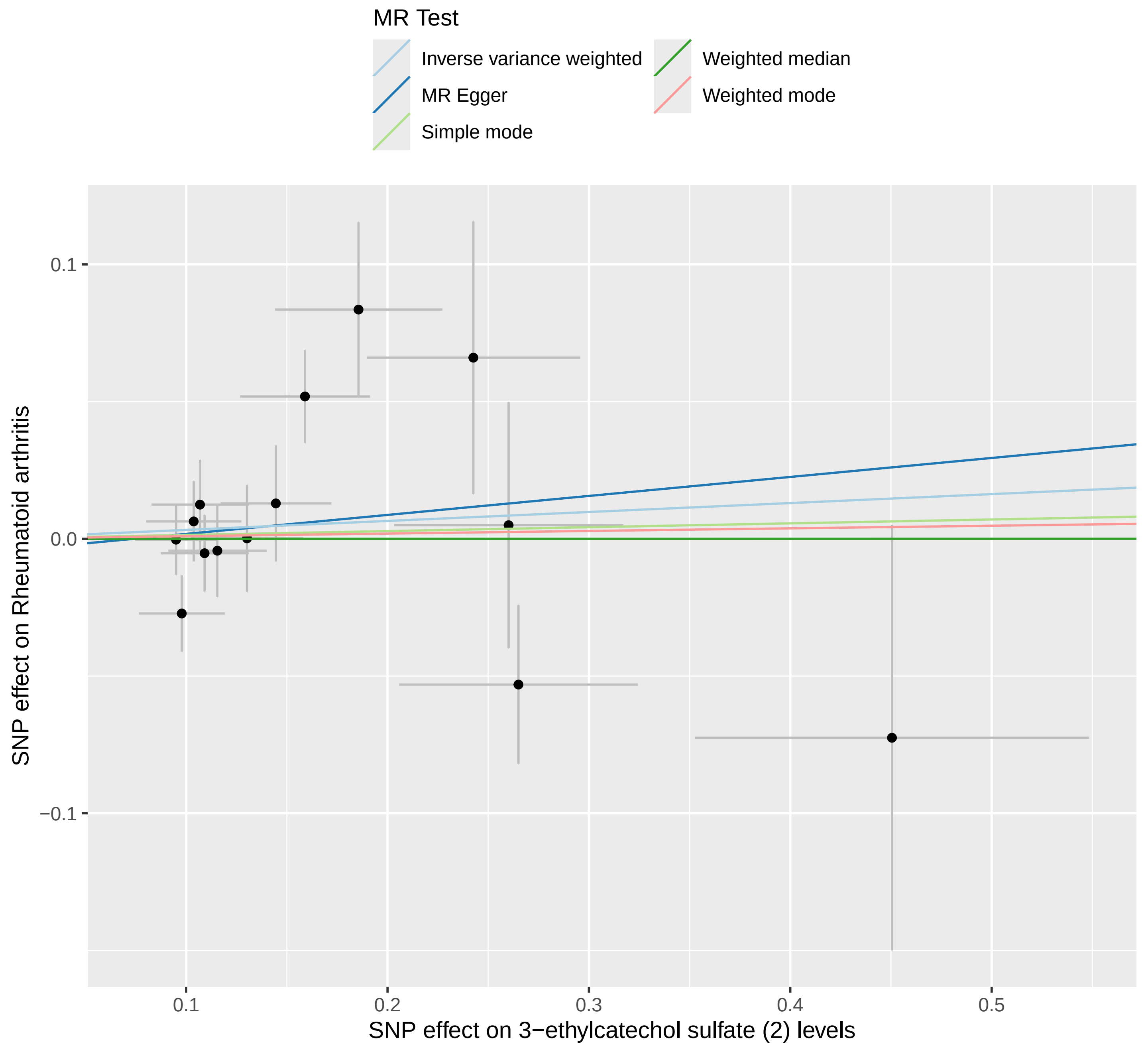

MR Test
Inverse variance weighted
MR Egger
Simple mode
Weighted median
Weighted mode
SNP effect on Rheumatoid arthritis
0.1
0.0
−0.1
0.1
0.2
0.3
0.4
0.5
SNP effect on 3−ethylcatechol sulfate (2) levels

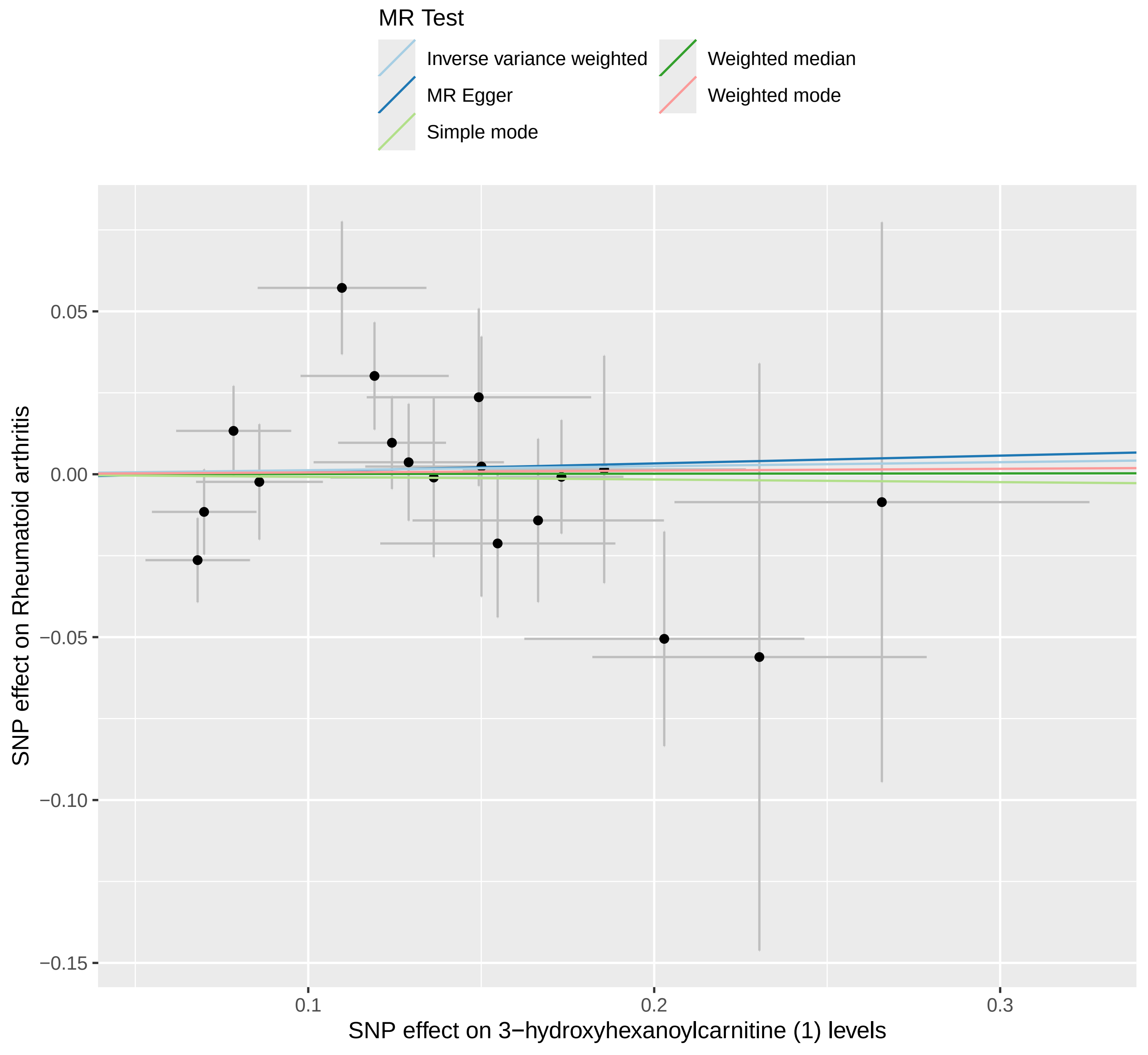
MR Test
Inverse variance weighted
MR Egger
Simple mode
Weighted median
Weighted mode
0.05
0.00
−0.05
−0.10
−0.15
SNP effect on Rheumatoid arthritis
0.1
0.2
0.3
SNP effect on 3−hydroxyhexanoylcarnitine (1) levels

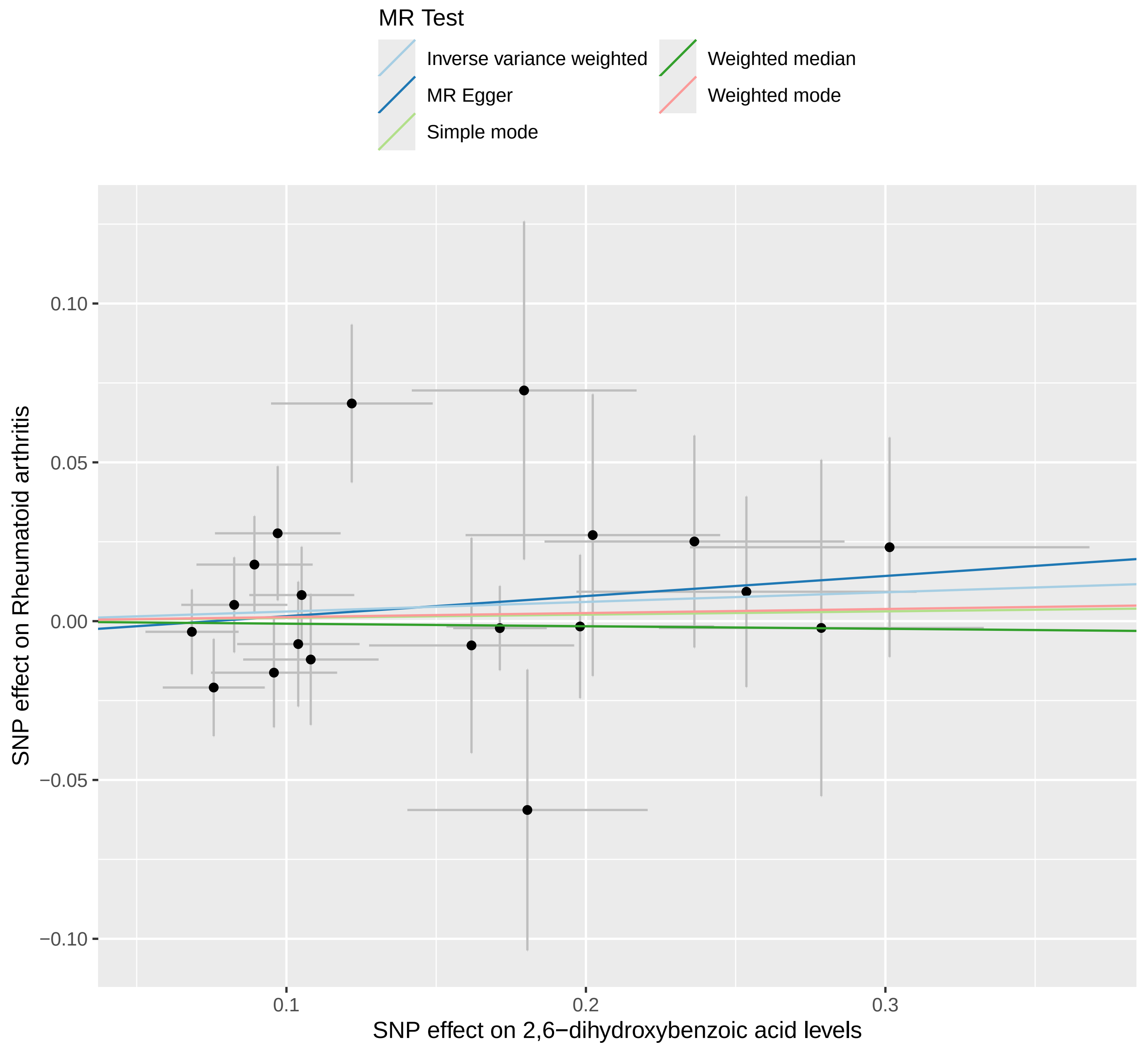

MR Test
Inverse variance weighted
MR Egger
Simple mode
Weighted median
Weighted mode
0.10
0.05
0.00
−0.05
−0.10
SNP effect on Rheumatoid arthritis
0.1
0.2
0.3
SNP effect on 2,6−dihydroxybenzoic acid levels

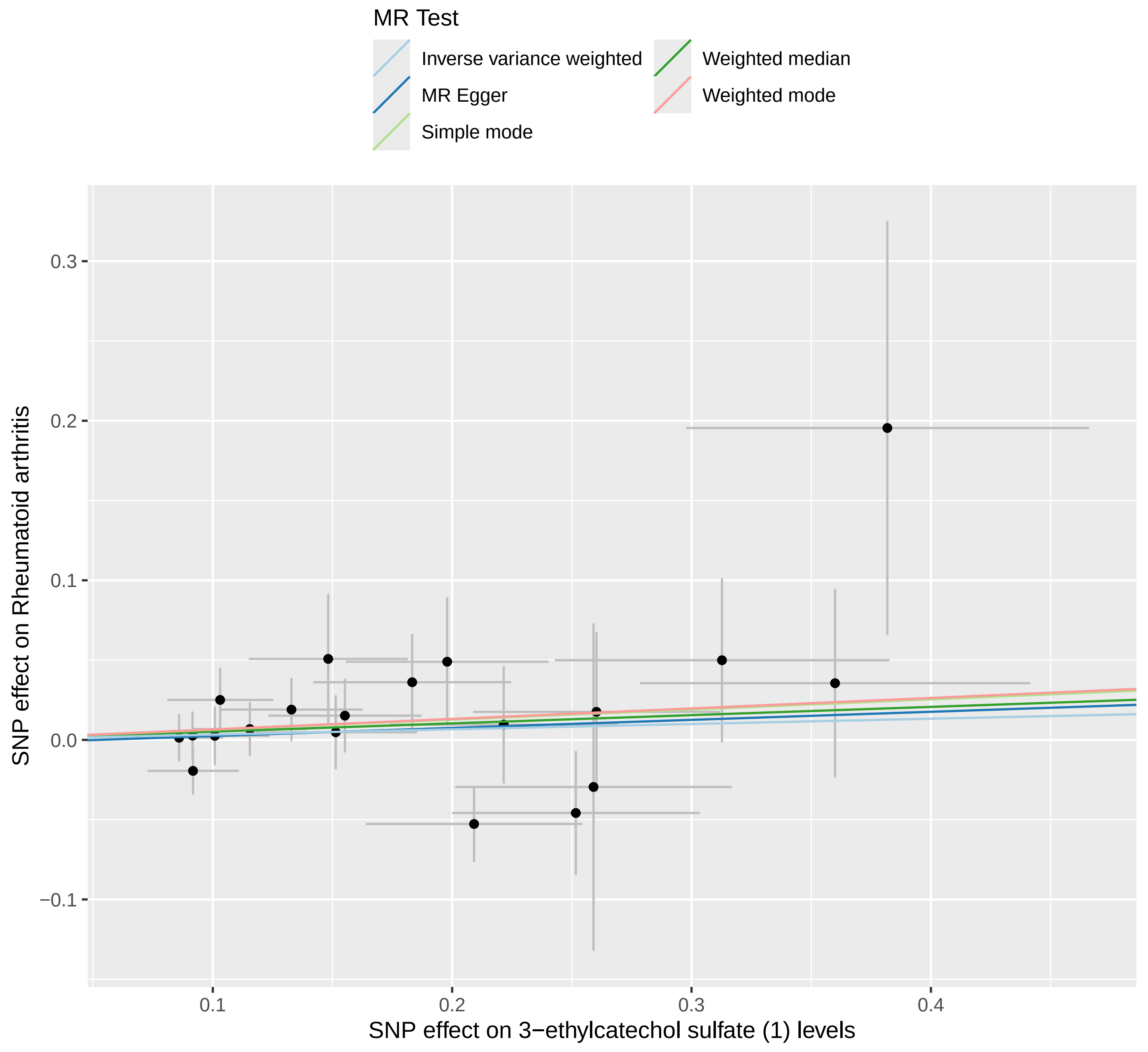
MR Test
Inverse variance weighted
MR Egger
Simple mode
Weighted median
Weighted mode
SNP effect on Rheumatoid arthritis
0.3
0.2
0.1
0.0
−0.1
0.1
0.2
0.3
0.4
SNP effect on 3−ethylcatechol sulfate (1) levels

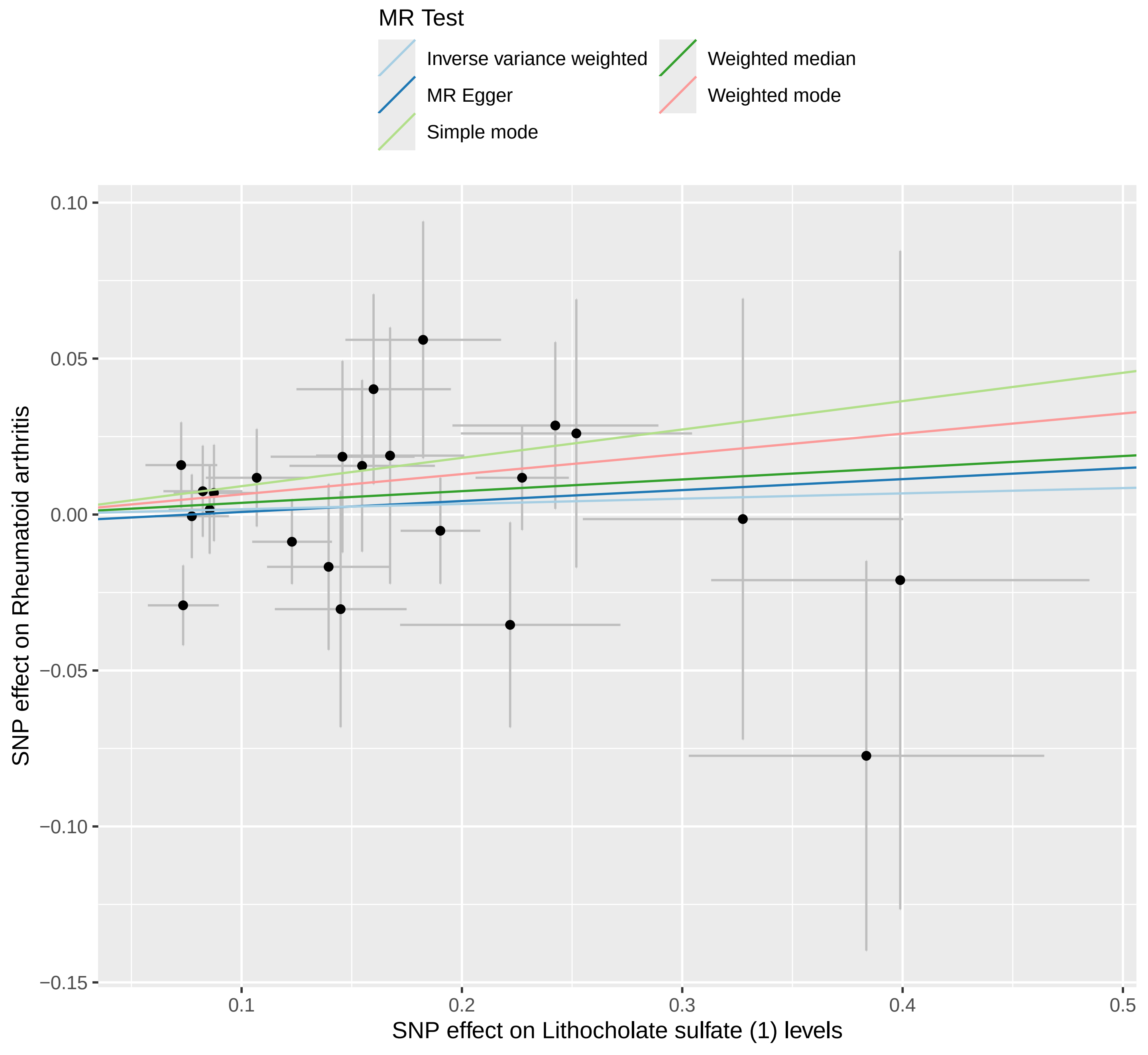
MR Test
Inverse variance weighted
MR Egger
Simple mode
Weighted median
Weighted mode
0.10
0.05
0.00
−0.05
−0.10
−0.15
0.1
0.2
0.3
0.4
0.5
SNP effect on Rheumatoid arthritis
SNP effect on Lithocholate sulfate (1) levels

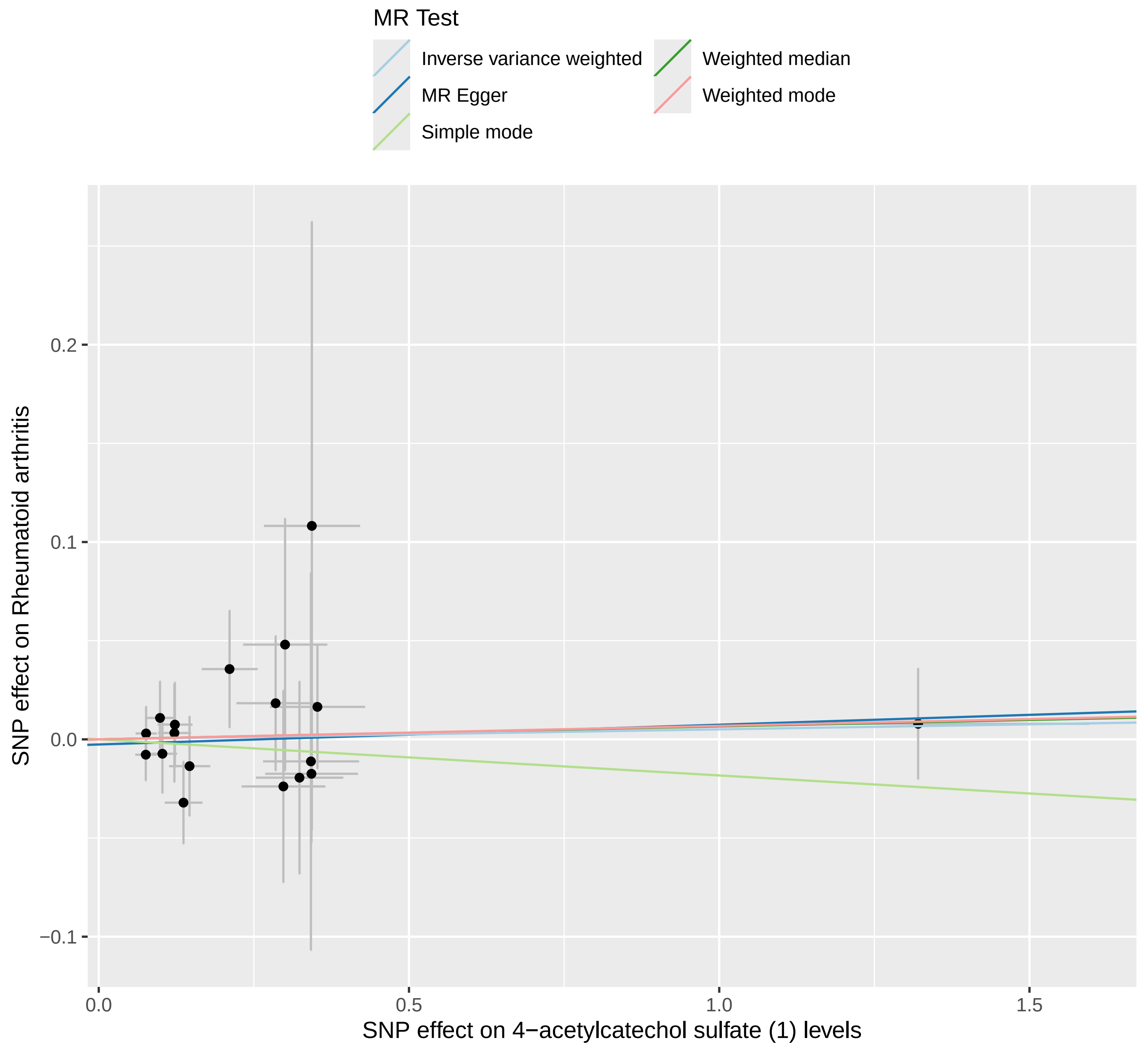
MR Test
Inverse variance weighted
MR Egger
Simple mode
Weighted median
Weighted mode
SNP effect on Rheumatoid arthritis
0.2
0.1
0.0
−0.1
0.0
0.5
1.0
1.5
SNP effect on 4−acetylcatechol sulfate (1) levels

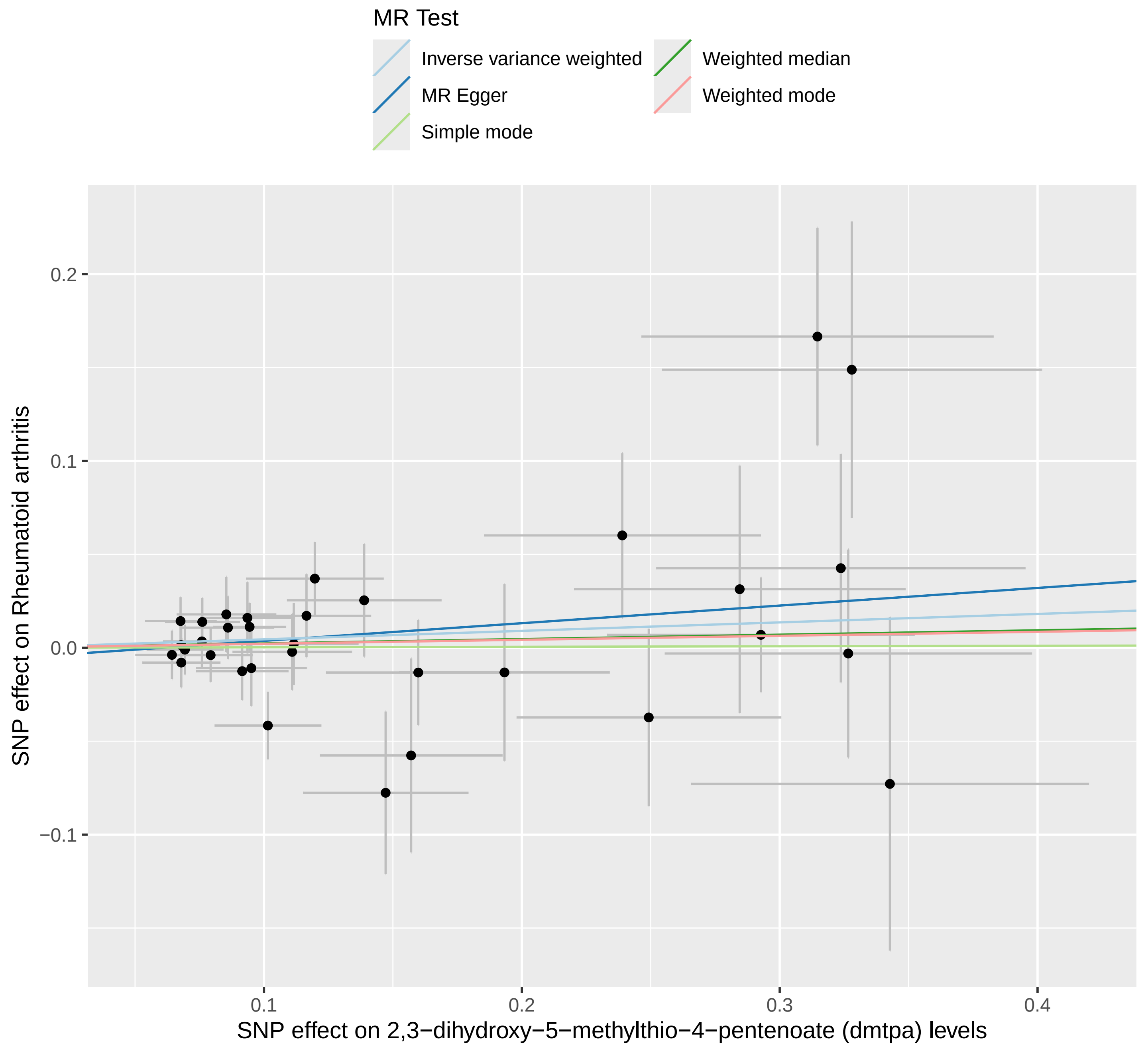

MR Test
Inverse variance weighted
MR Egger
Simple mode
Weighted median
Weighted mode
SNP effect on Rheumatoid arthritis
0.2
0.1
0.0
−0.1
0.1
0.2
0.3
0.4
SNP effect on 2,3−dihydroxy−5−methylthio−4−pentenoate (dmtpa) levels

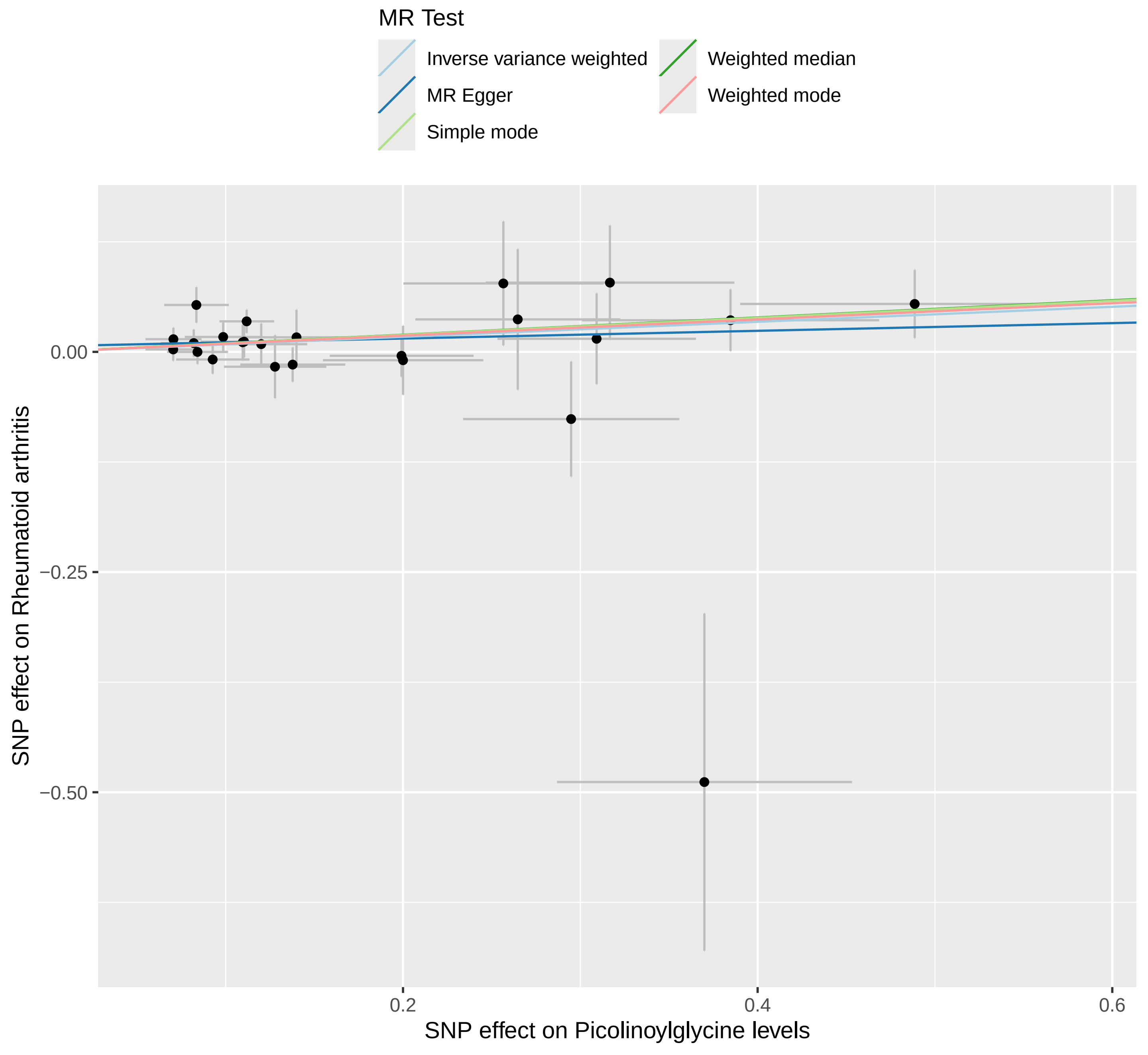
MR Test
Inverse variance weighted
MR Egger
Simple mode
Weighted median
Weighted mode
0.00
−0.25
−0.50
0.2
0.4
0.6
SNP effect on Rheumatoid arthritis
SNP effect on Picolinoylglycine levels

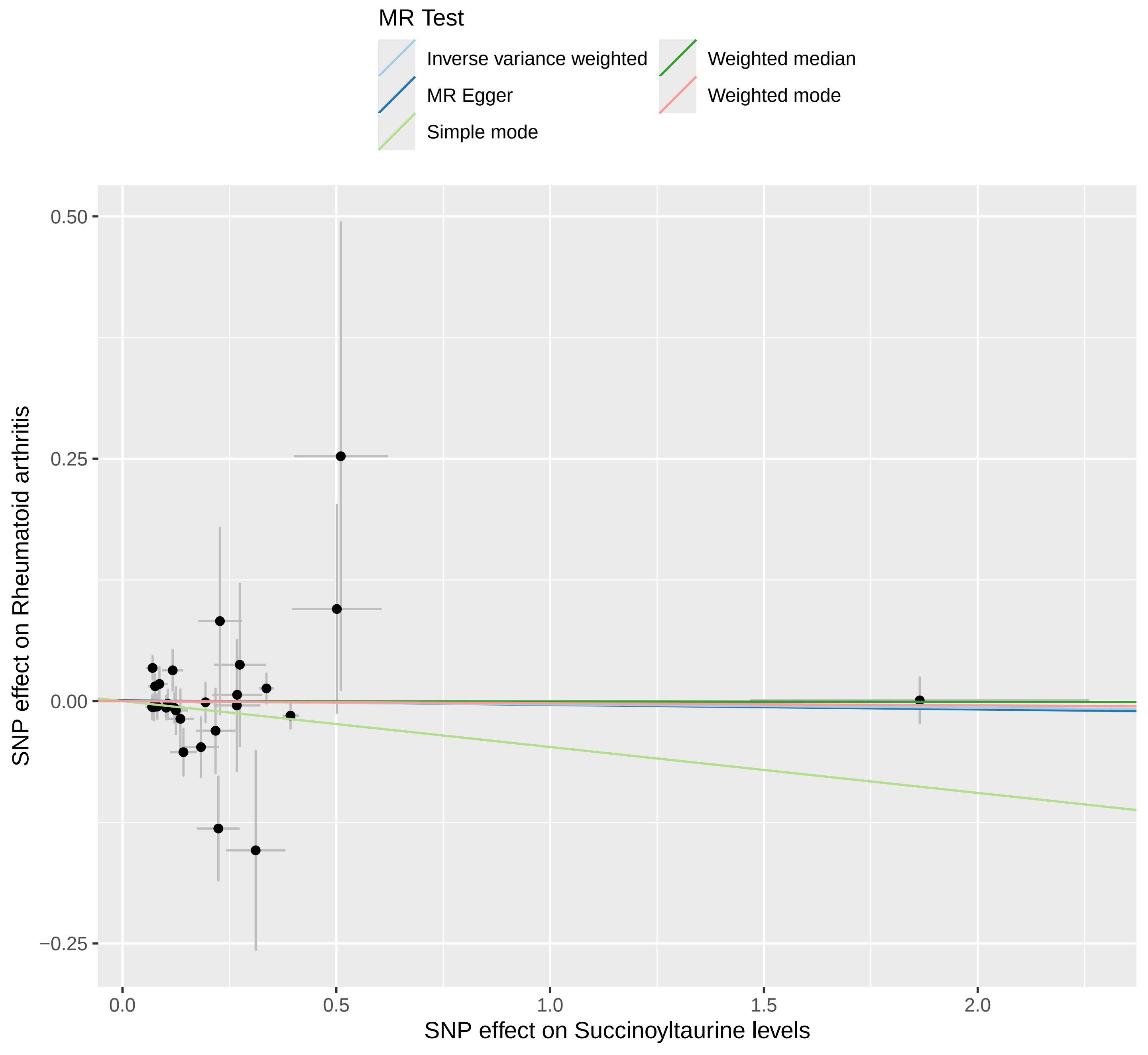
MR Test
Inverse variance weighted
MR Egger
Simple mode
Weighted median
Weighted mode
0.50
0.25
0.00
−0.25
0.0
0.5
1.0
1.5
2.0
SNP effect on Rheumatoid arthritis
SNP effect on Succinoyltaurine levels

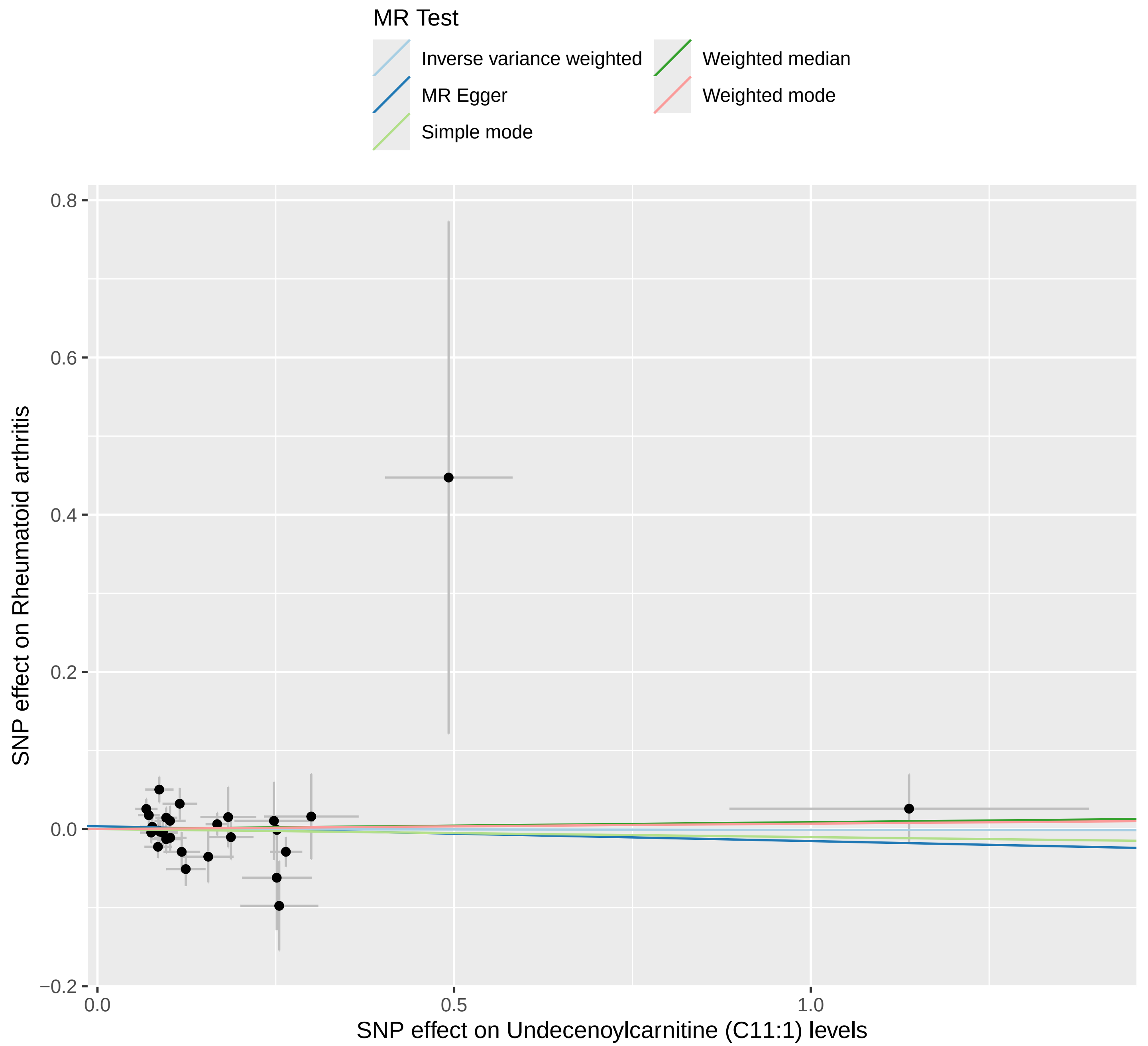

MR Test
Inverse variance weighted
MR Egger
Simple mode
Weighted median
Weighted mode
SNP effect on Rheumatoid arthritis
SNP effect on Undecenoylcarnitine (C11:1) levels
0.8
0.6
0.4
0.2
0.0
−0.2
0.0
0.5
1.0

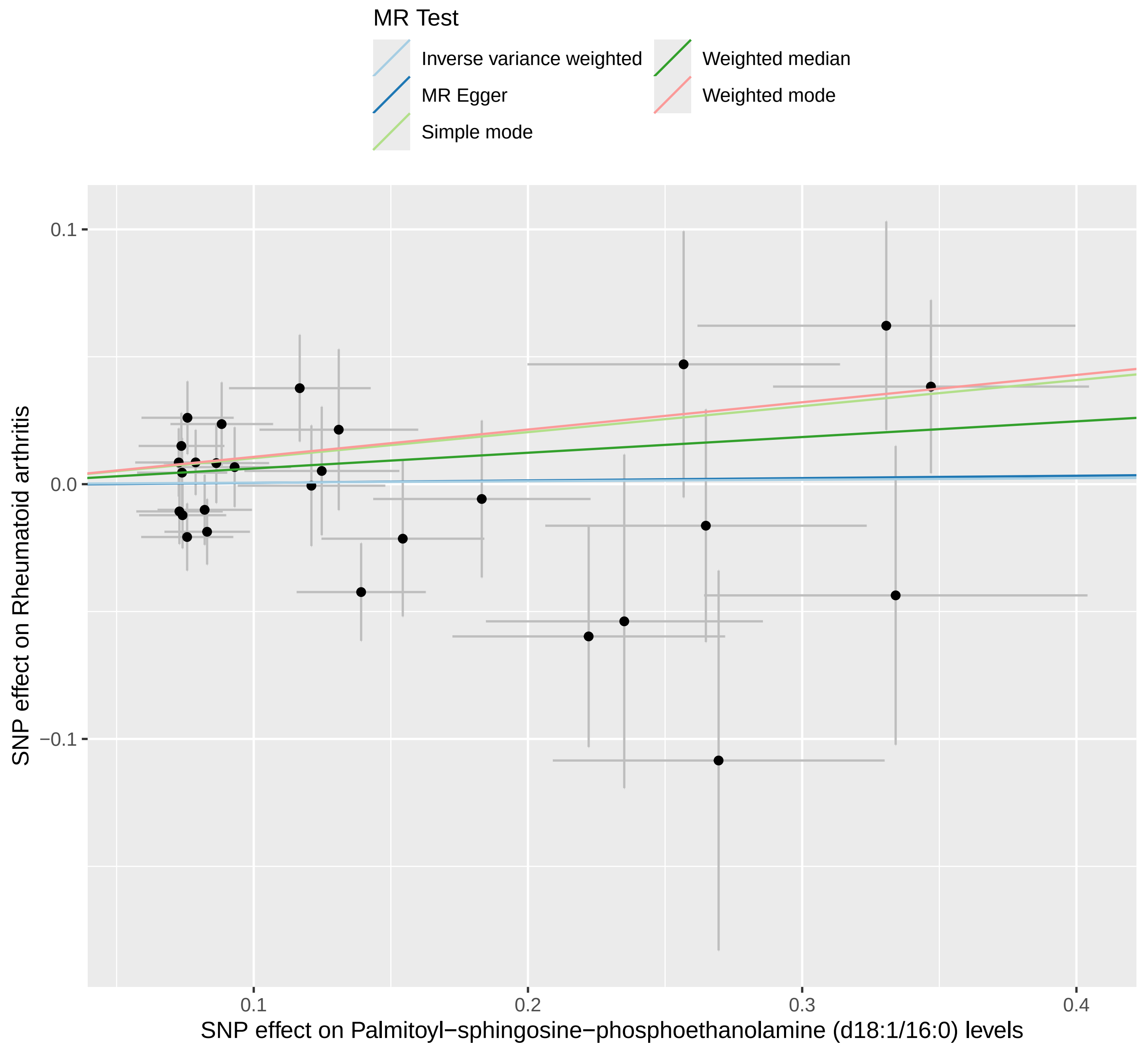

MR Test
Inverse variance weighted
MR Egger
Simple mode
Weighted median
Weighted mode
0.1
0.0
−0.1
0.1
0.2
0.3
0.4
SNP effect on Rheumatoid arthritis
SNP effect on Palmitoyl−sphingosine−phosphoethanolamine (d18:1/16:0) levels

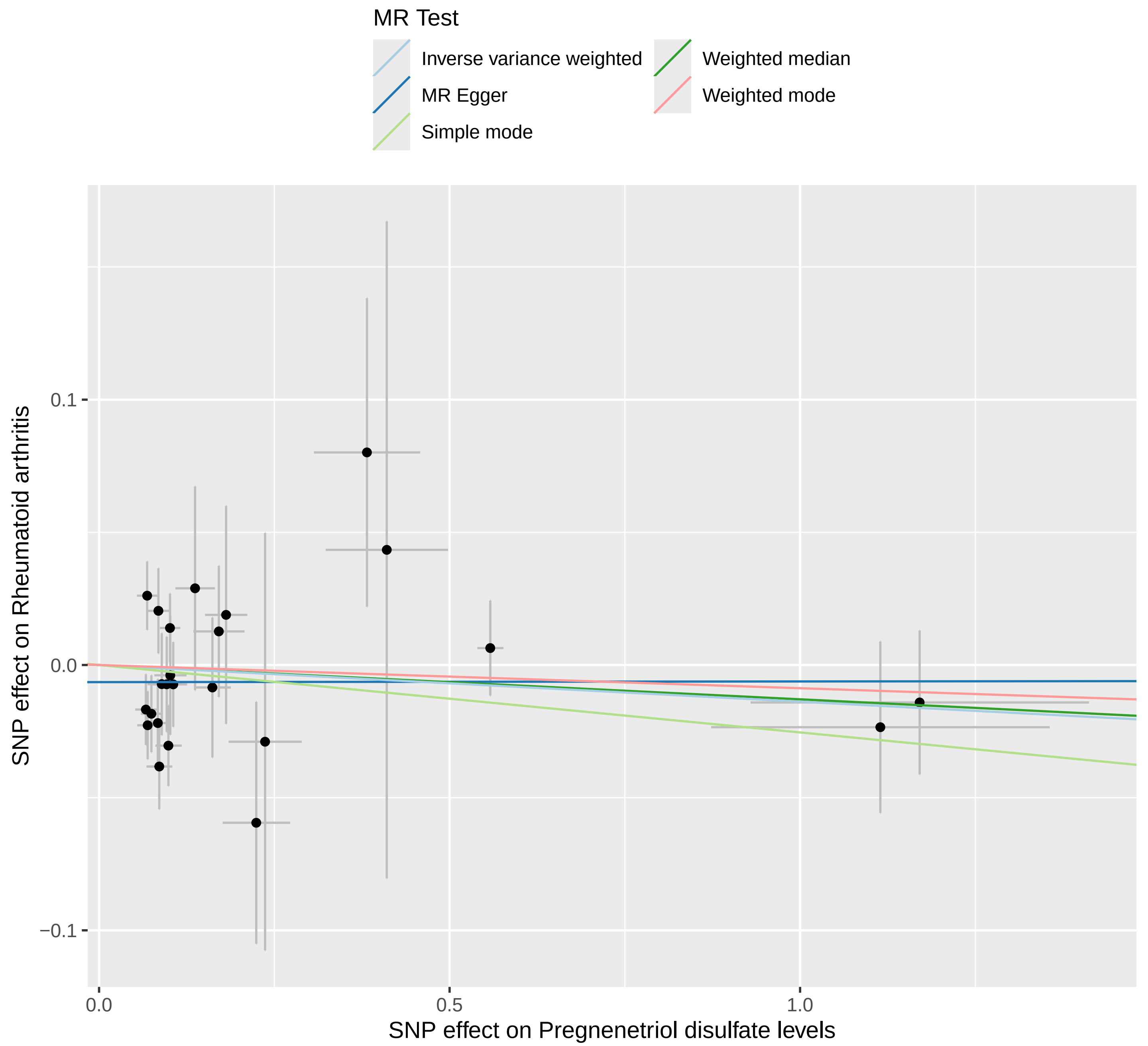
MR Test
Inverse variance weighted
MR Egger
Simple mode
Weighted median
Weighted mode
SNP effect on Rheumatoid arthritis
0.1
0.0
−0.1
0.0
0.5
1.0
SNP effect on Pregnenetriol disulfate levels

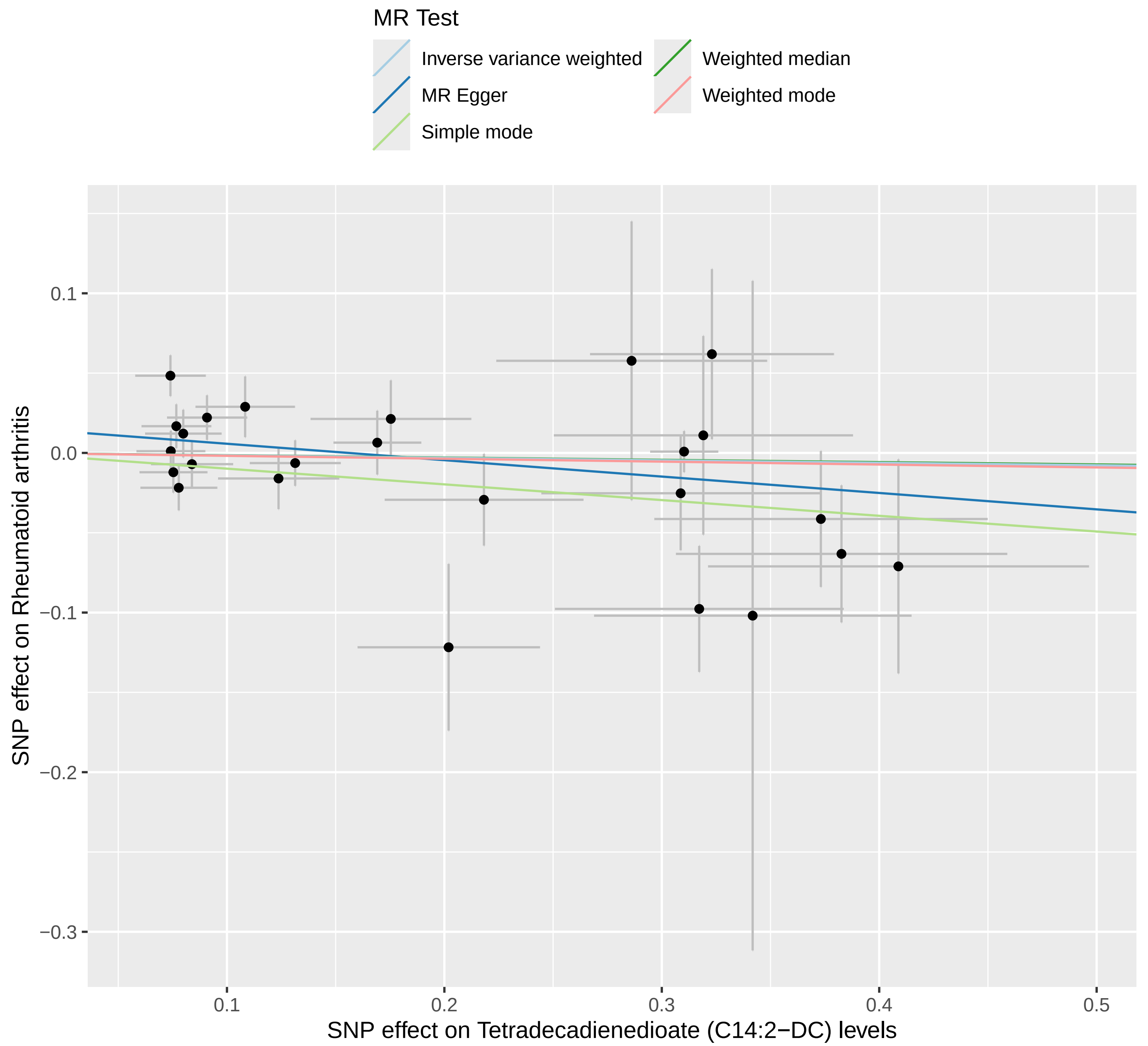

MR Test
Inverse variance weighted
MR Egger
Simple mode
Weighted median
Weighted mode
0.1
0.0
−0.1
−0.2
−0.3
0.1
0.2
0.3
0.4
0.5
SNP effect on Rheumatoid arthritis
SNP effect on Tetradecadienedioate (C14:2−DC) levels

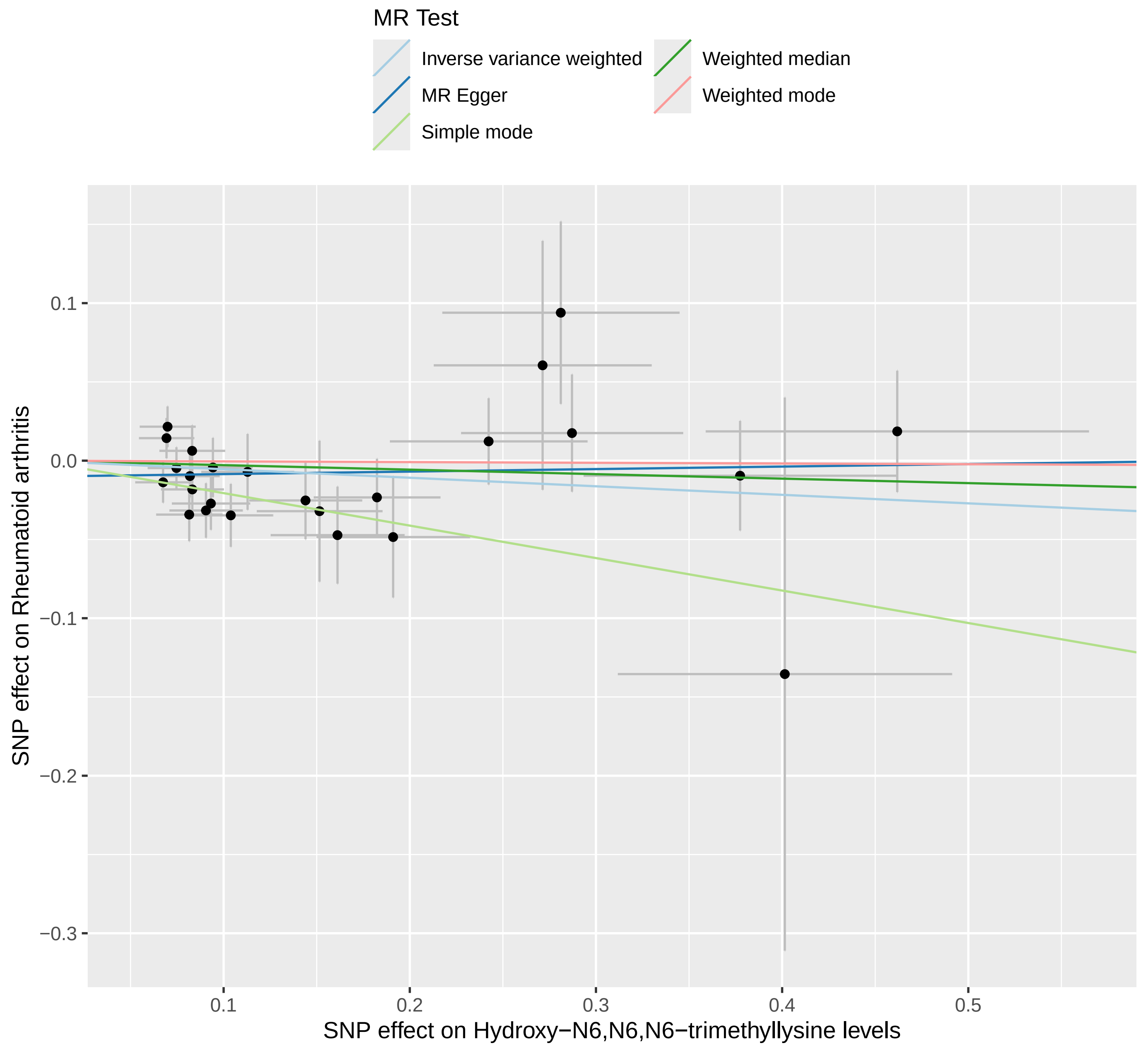
MR Test
Inverse variance weighted
MR Egger
Simple mode
Weighted median
Weighted mode
SNP effect on Rheumatoid arthritis
0.1
0.0
−0.1
−0.2
−0.3
0.1
0.2
0.3
0.4
0.5
SNP effect on Hydroxy−N6,N6,N6−trimethyllysine levels

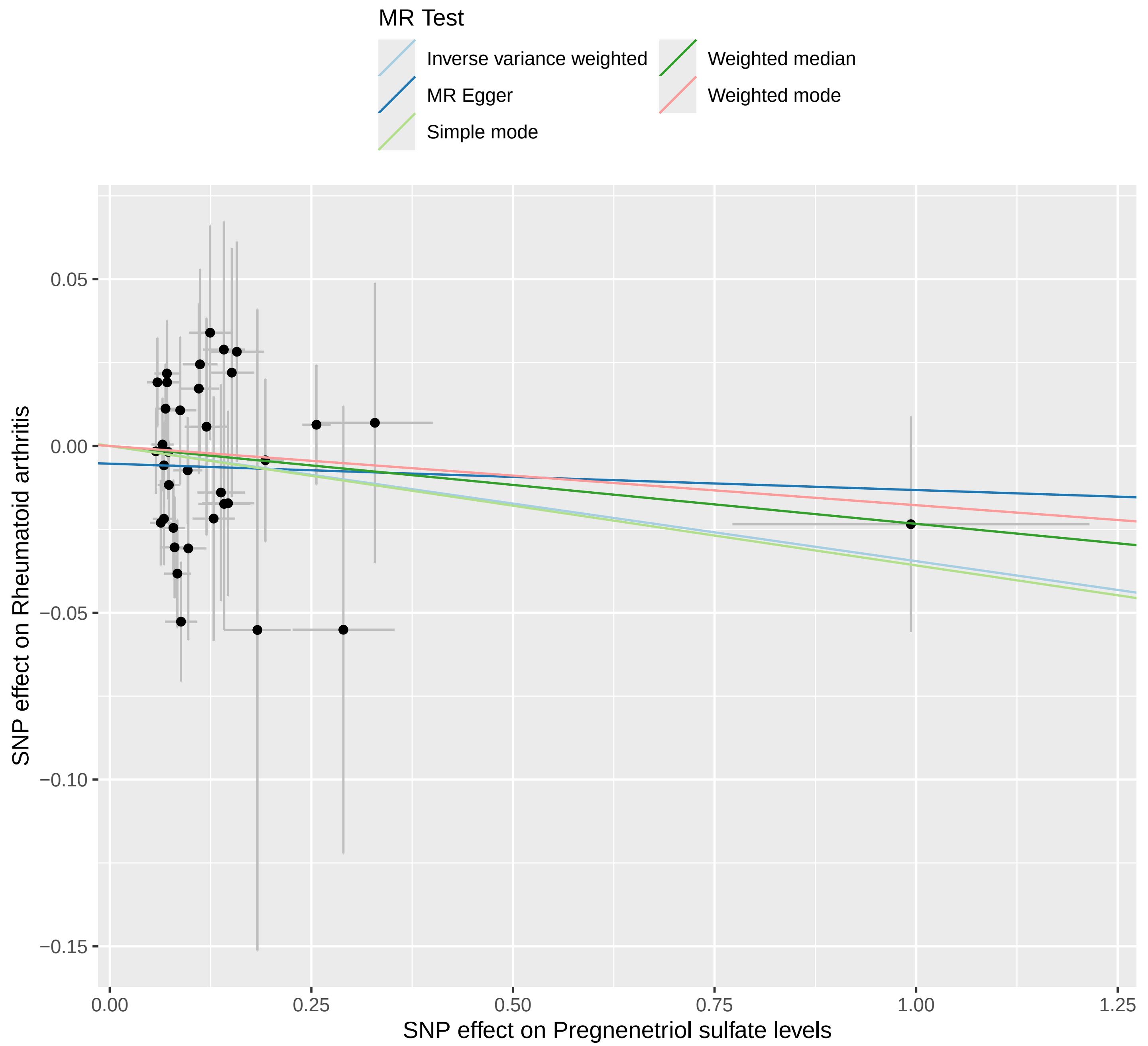
MR Test
Inverse variance weighted
MR Egger
Simple mode
Weighted median
Weighted mode
0.05
0.00
−0.05
−0.10
−0.15
SNP effect on Rheumatoid arthritis
0.00
0.25
0.50
0.75
1.00
1.25
SNP effect on Pregnenetriol sulfate levels

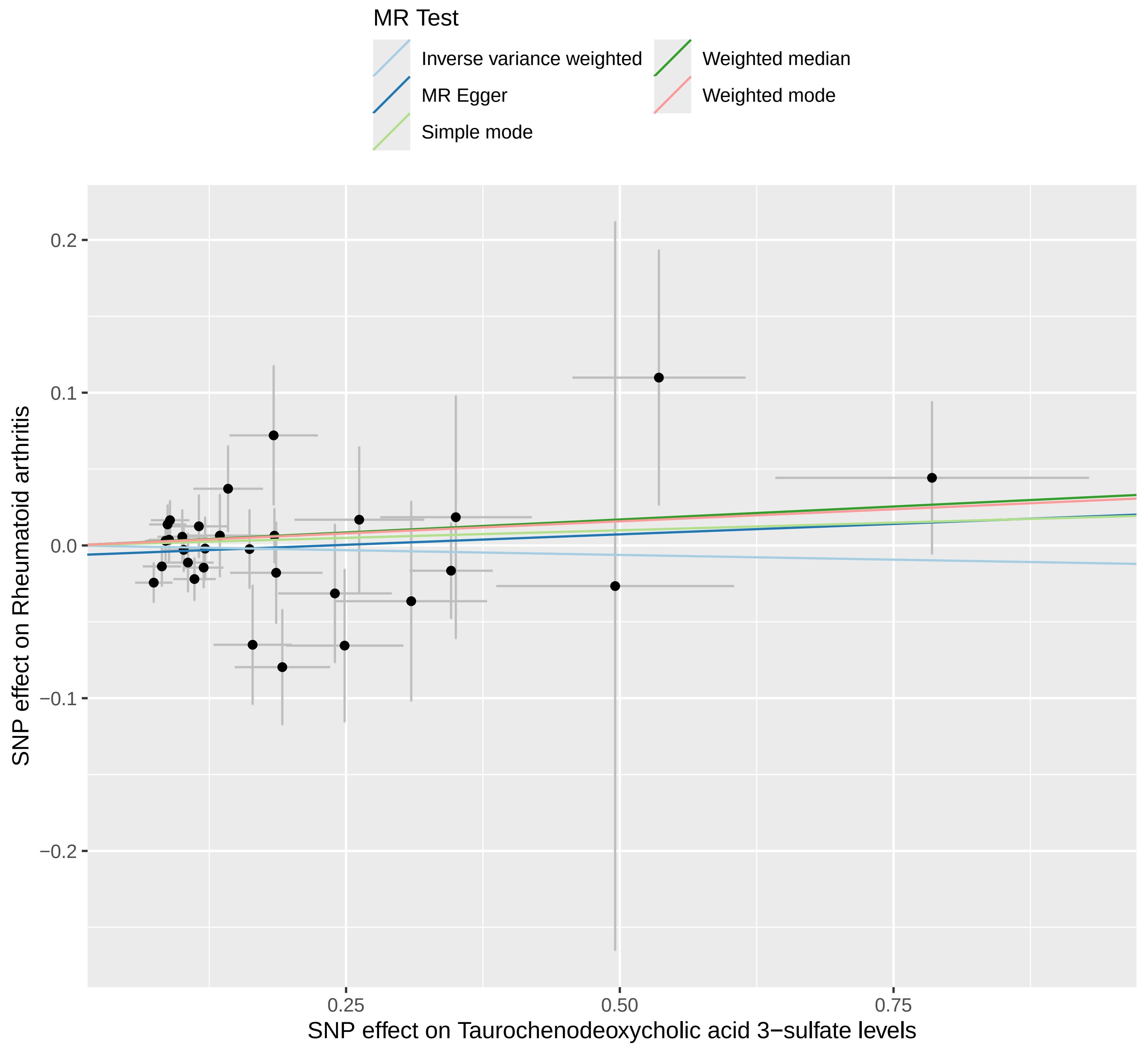
MR Test
Inverse variance weighted
MR Egger
Simple mode
Weighted median
Weighted mode
SNP effect on Rheumatoid arthritis
0.2
0.1
0.0
−0.1
−0.2
0.25
0.50
0.75
SNP effect on Taurochenodeoxycholic acid 3−sulfate levels

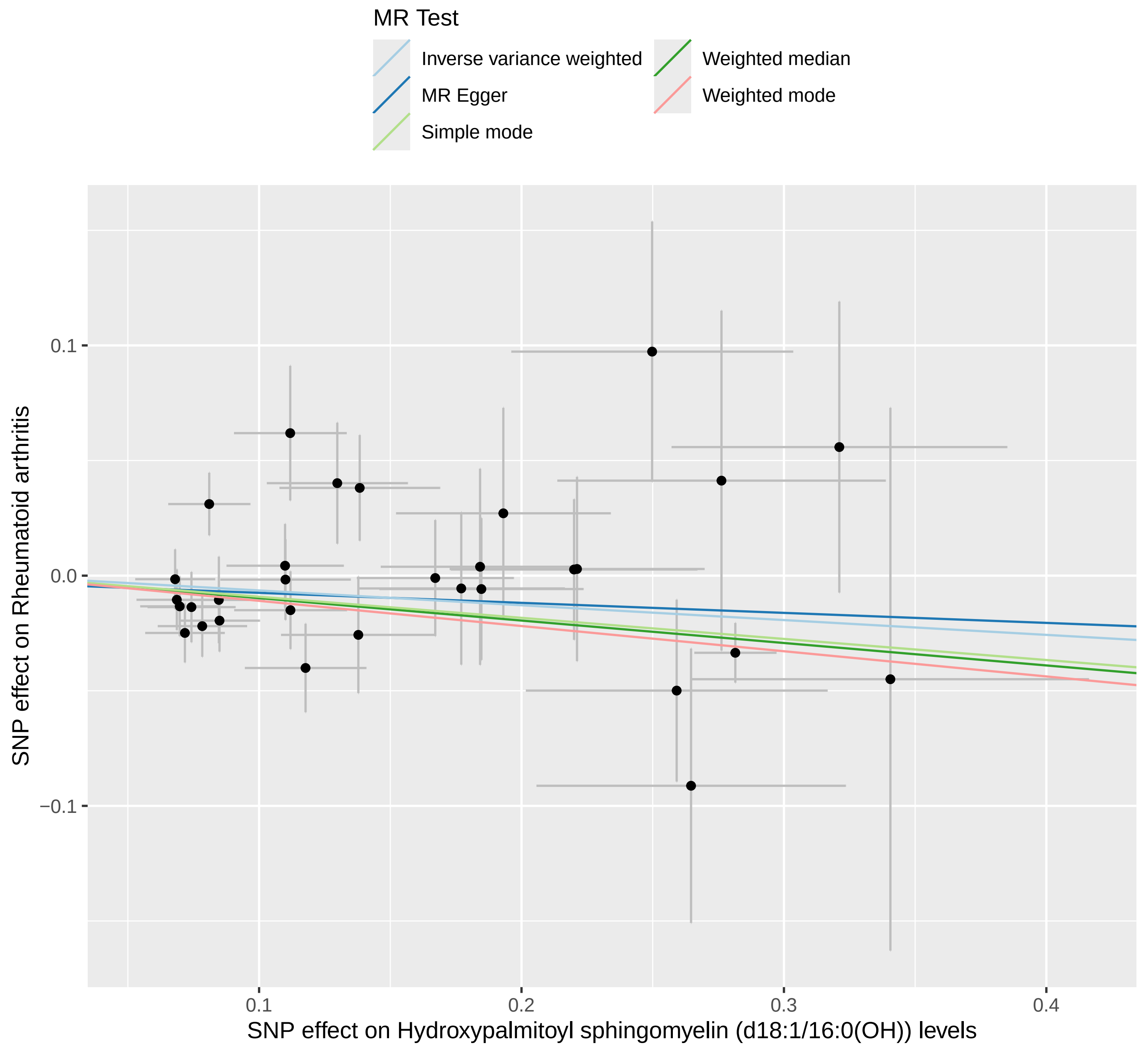

MR Test
Inverse variance weighted
MR Egger
Simple mode
Weighted median
Weighted mode
0.1
0.0
−0.1
SNP effect on Rheumatoid arthritis
0.1
0.2
0.3
0.4
SNP effect on Hydroxypalmitoyl sphingomyelin (d18:1/16:0(OH)) levels

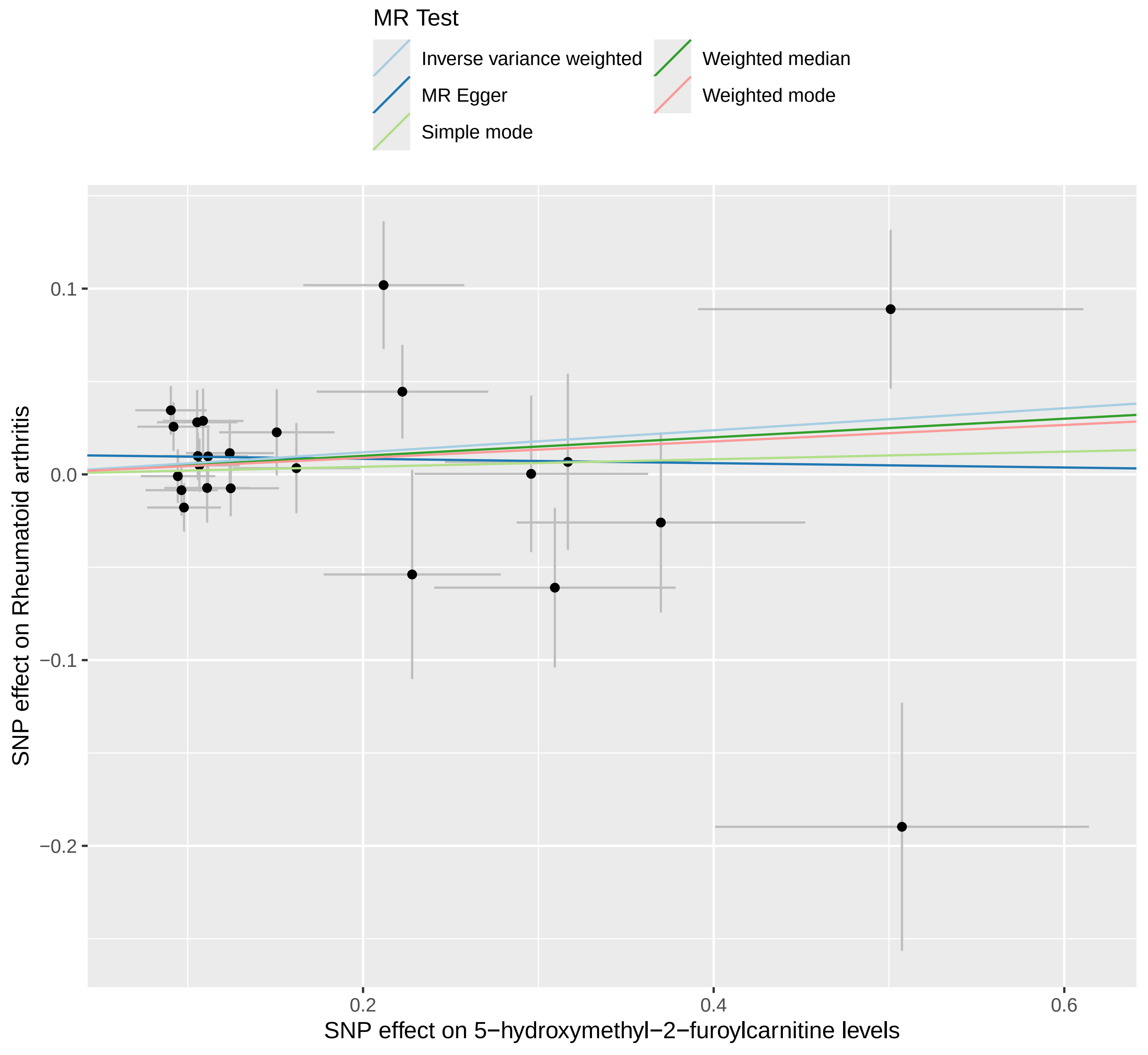

MR Test
Inverse variance weighted
MR Egger
Simple mode
Weighted median
Weighted mode
0.1
0.0
−0.1
−0.2
0.2
0.4
0.6
SNP effect on Rheumatoid arthritis
SNP effect on 5−hydroxymethyl−2−furoylcarnitine levels

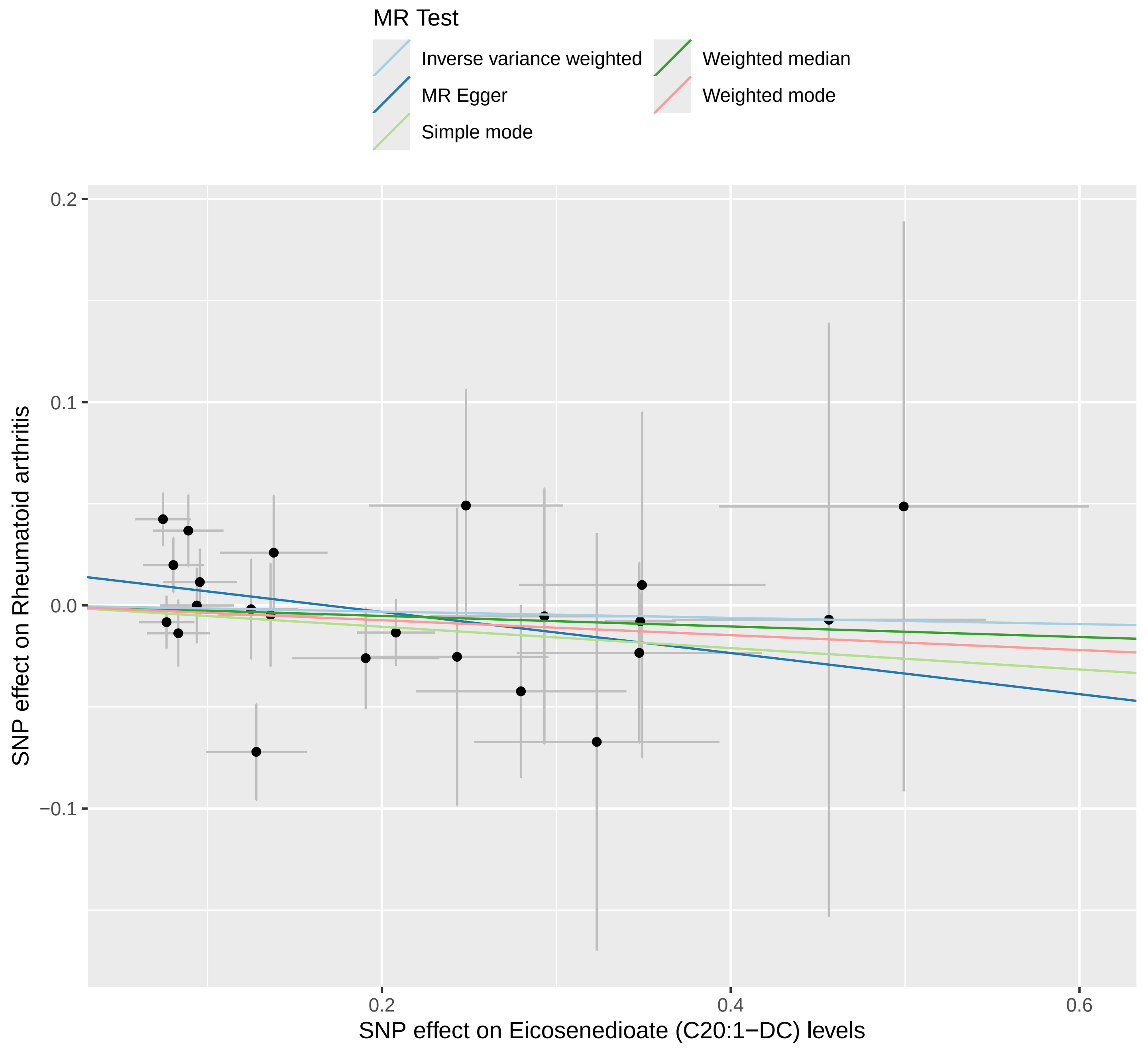

MR Test
Inverse variance weighted
MR Egger
Simple mode
Weighted median
Weighted mode
0.2
0.1
0.0
−0.1
0.2
0.4
0.6
SNP effect on Rheumatoid arthritis
SNP effect on Eicosenedioate (C20:1−DC) levels

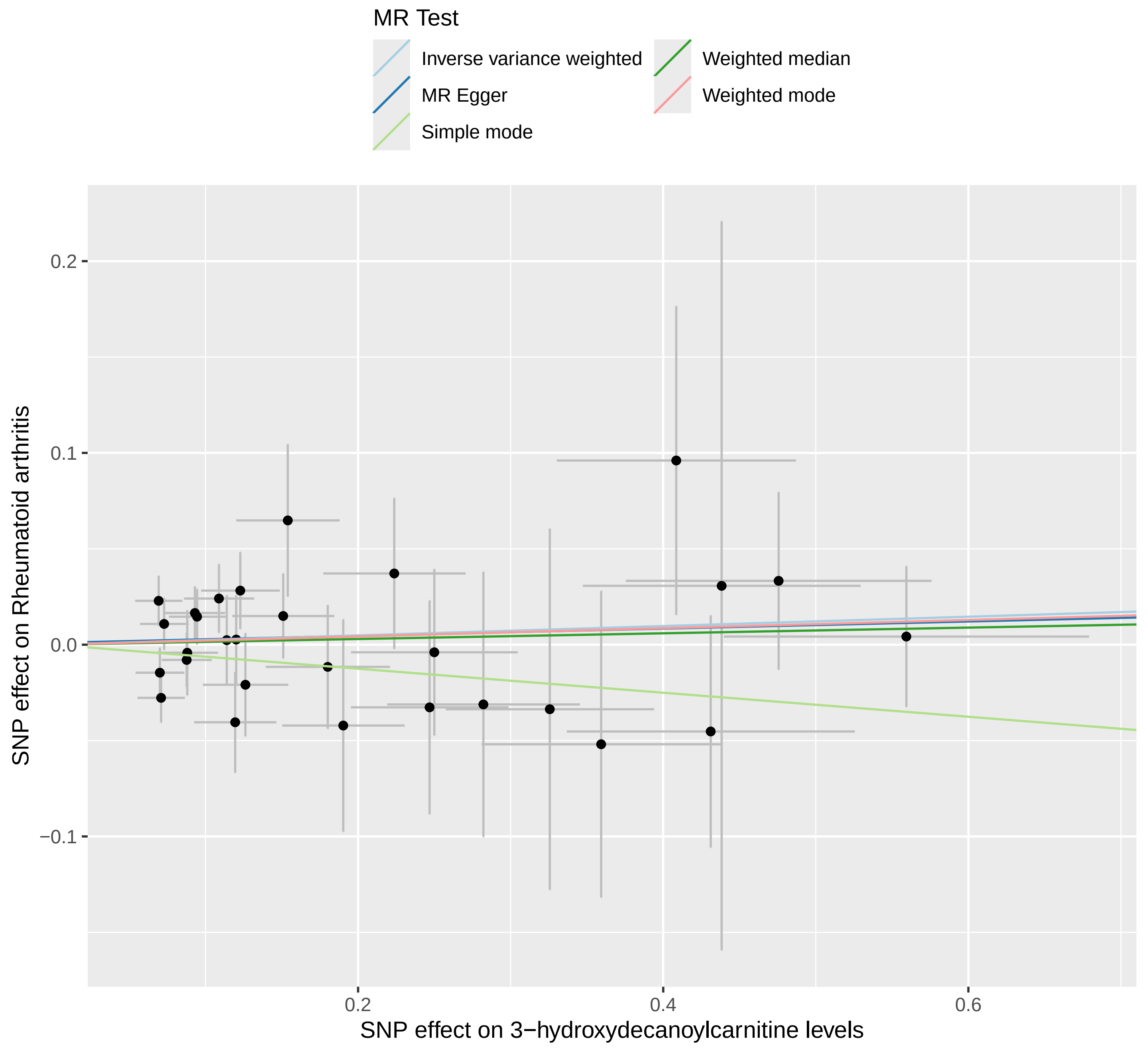
MR Test
Inverse variance weighted
MR Egger
Simple mode
Weighted median
Weighted mode
0.2
0.1
0.0
−0.1
SNP effect on Rheumatoid arthritis
0.2
0.4
0.6
SNP effect on 3−hydroxydecanoylcarnitine levels

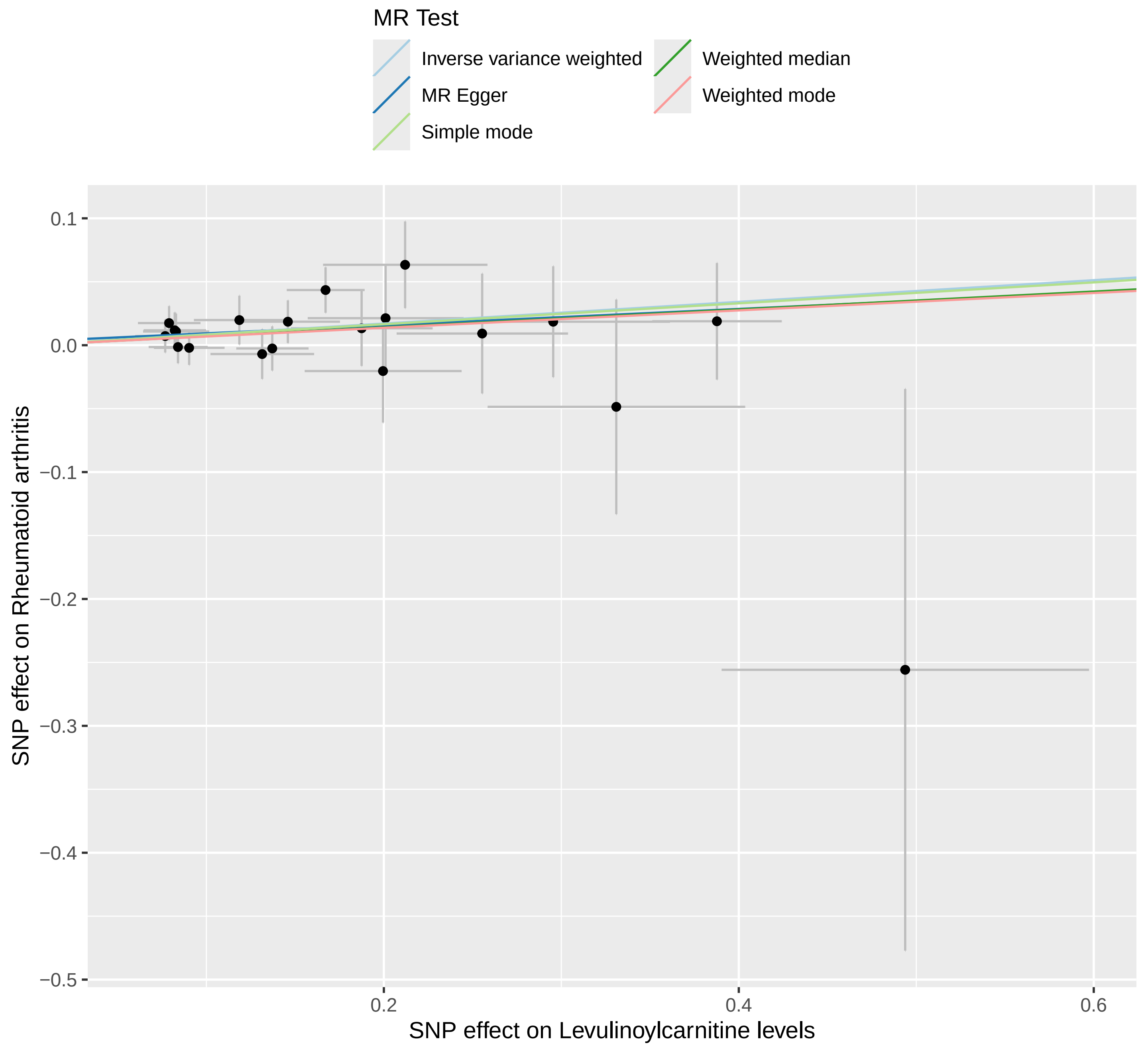

MR Test
Inverse variance weighted
MR Egger
Simple mode
Weighted median
Weighted mode
0.1
0.0
−0.1
−0.2
−0.3
−0.4
−0.5
0.2
0.4
0.6
SNP effect on Rheumatoid arthritis
SNP effect on Levulinoylcarnitine levels

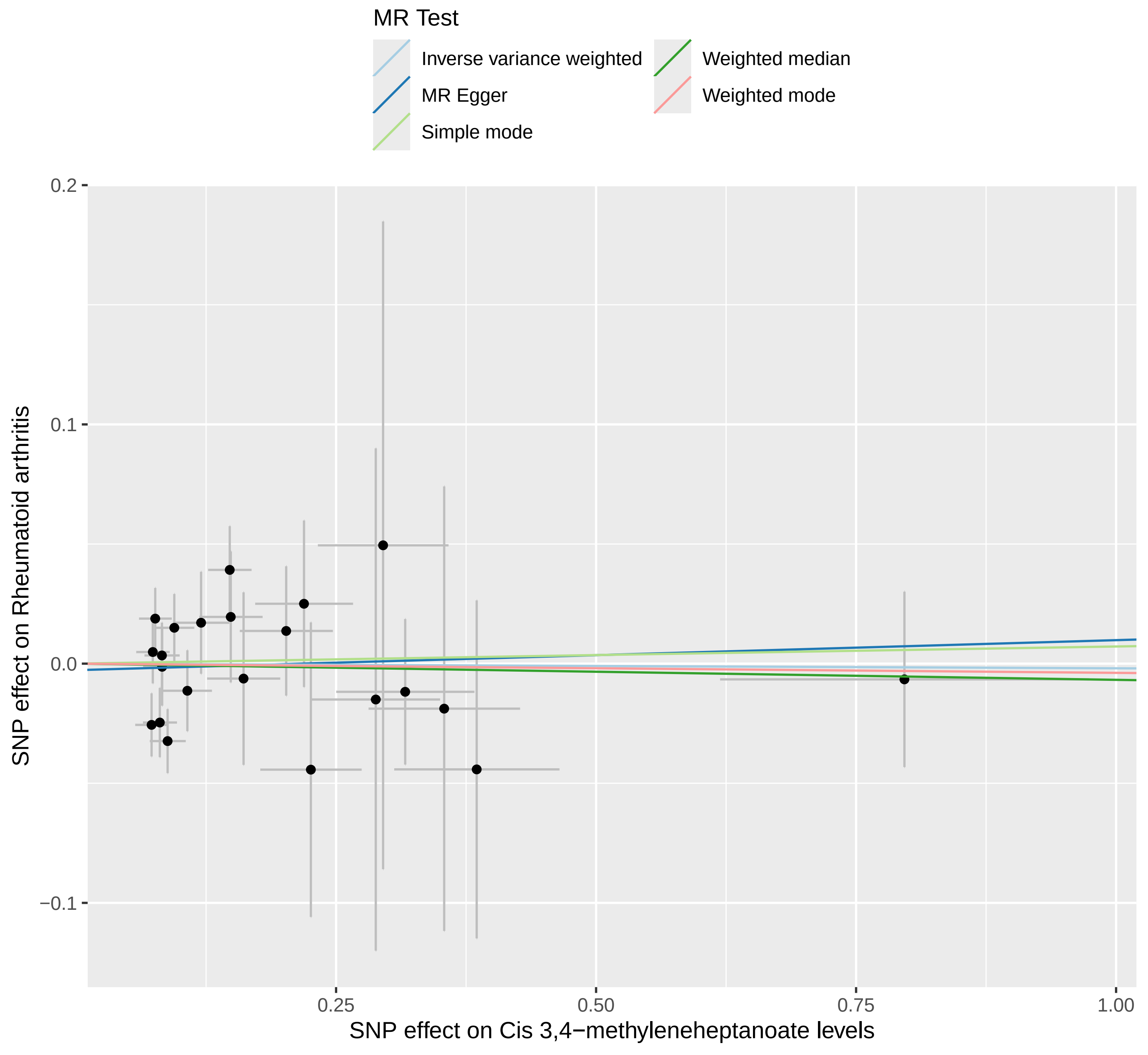
MR Test
Inverse variance weighted
MR Egger
Simple mode
Weighted median
Weighted mode
0.2
0.1
0.0
−0.1
0.25
0.50
0.75
1.00
SNP effect on Rheumatoid arthritis
SNP effect on Cis 3,4−methyleneheptanoate levels

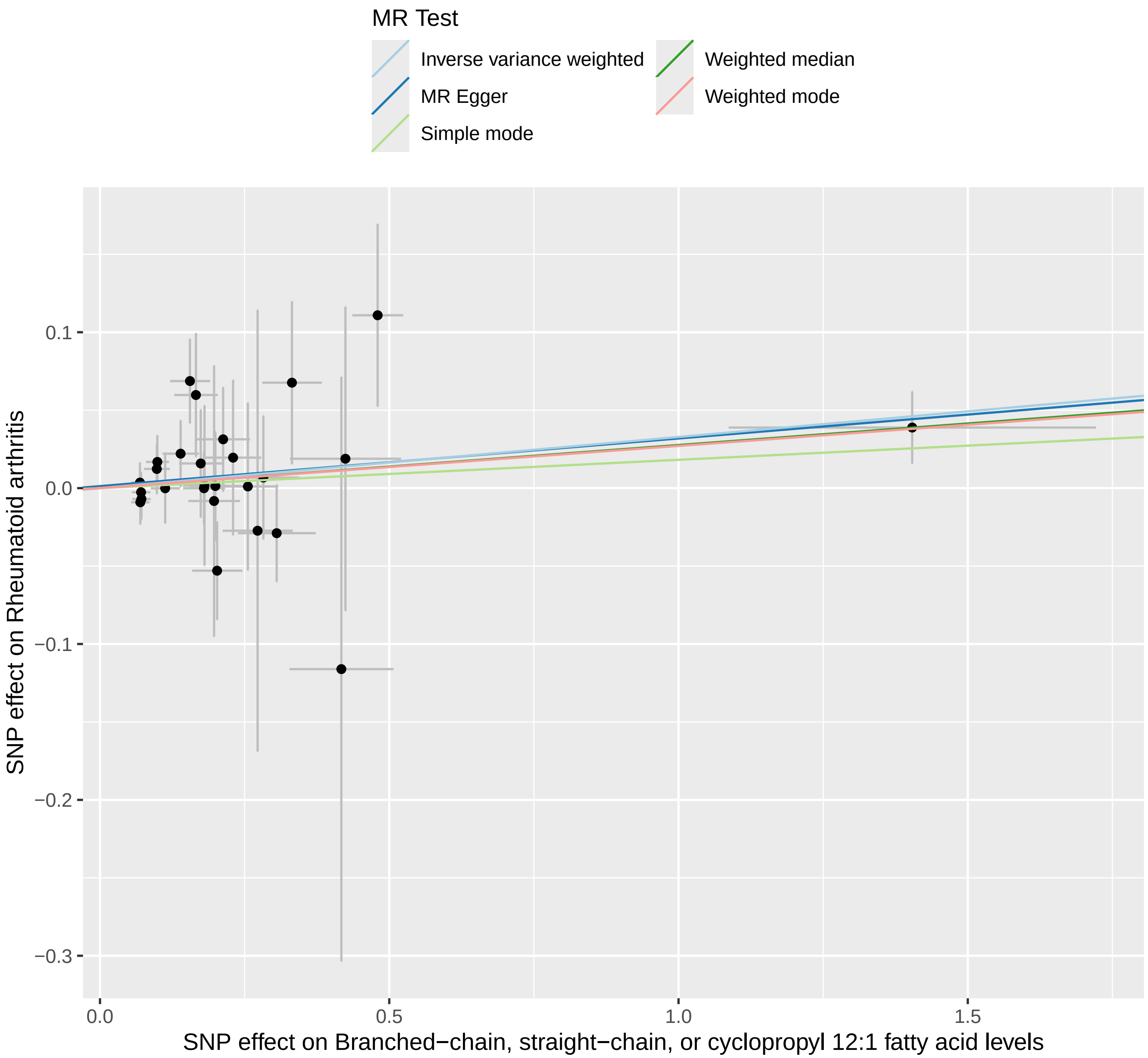

MR Test
Inverse variance weighted
MR Egger
Simple mode
Weighted median
Weighted mode
SNP effect on Rheumatoid arthritis
0.1
0.0
−0.1
−0.2
−0.3
0.0
0.5
1.0
1.5
SNP effect on Branched−chain, straight−chain, or cyclopropyl 12:1 fatty acid levels

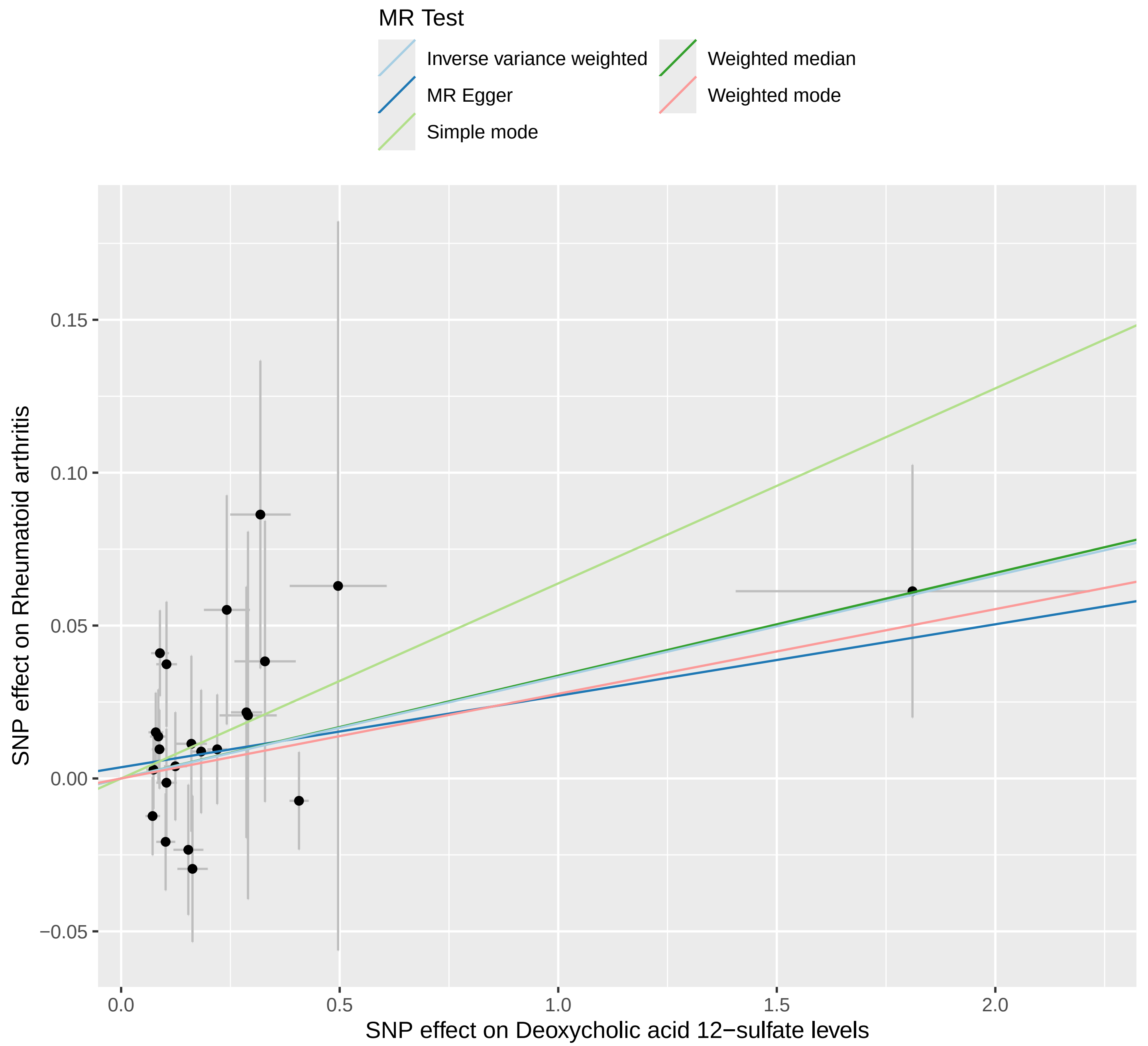
MR Test
Inverse variance weighted
MR Egger
Simple mode
Weighted median
Weighted mode
SNP effect on Rheumatoid arthritis
0.15
0.10
0.05
0.00
−0.05
0.0
0.5
1.0
1.5
2.0
SNP effect on Deoxycholic acid 12−sulfate levels

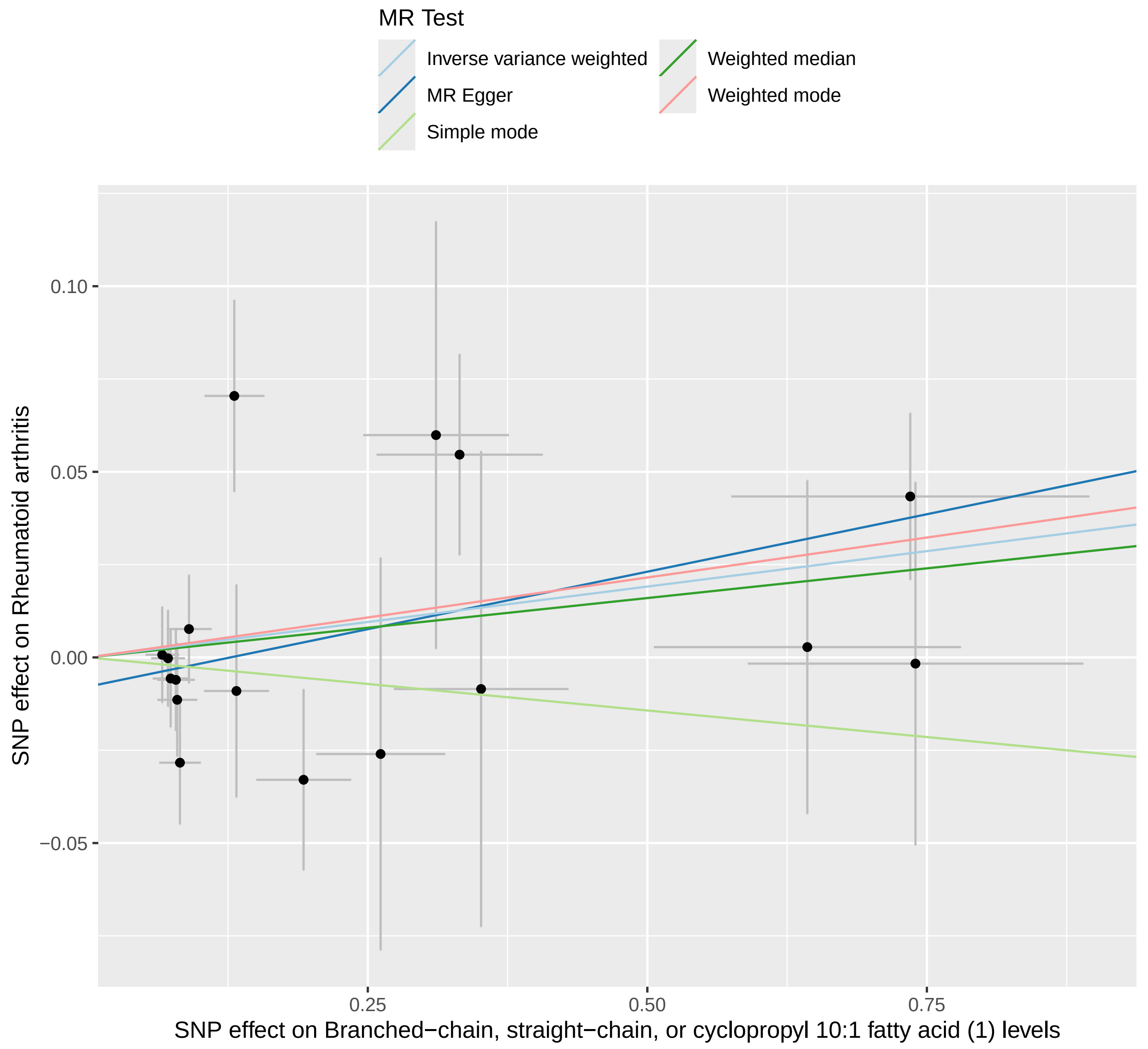
MR Test
Inverse variance weighted
MR Egger
Simple mode
Weighted median
Weighted mode
0.10
0.05
0.00
−0.05
0.25
0.50
0.75
SNP effect on Rheumatoid arthritis
SNP effect on Branched−chain, straight−chain, or cyclopropyl 10:1 fatty acid (1) levels

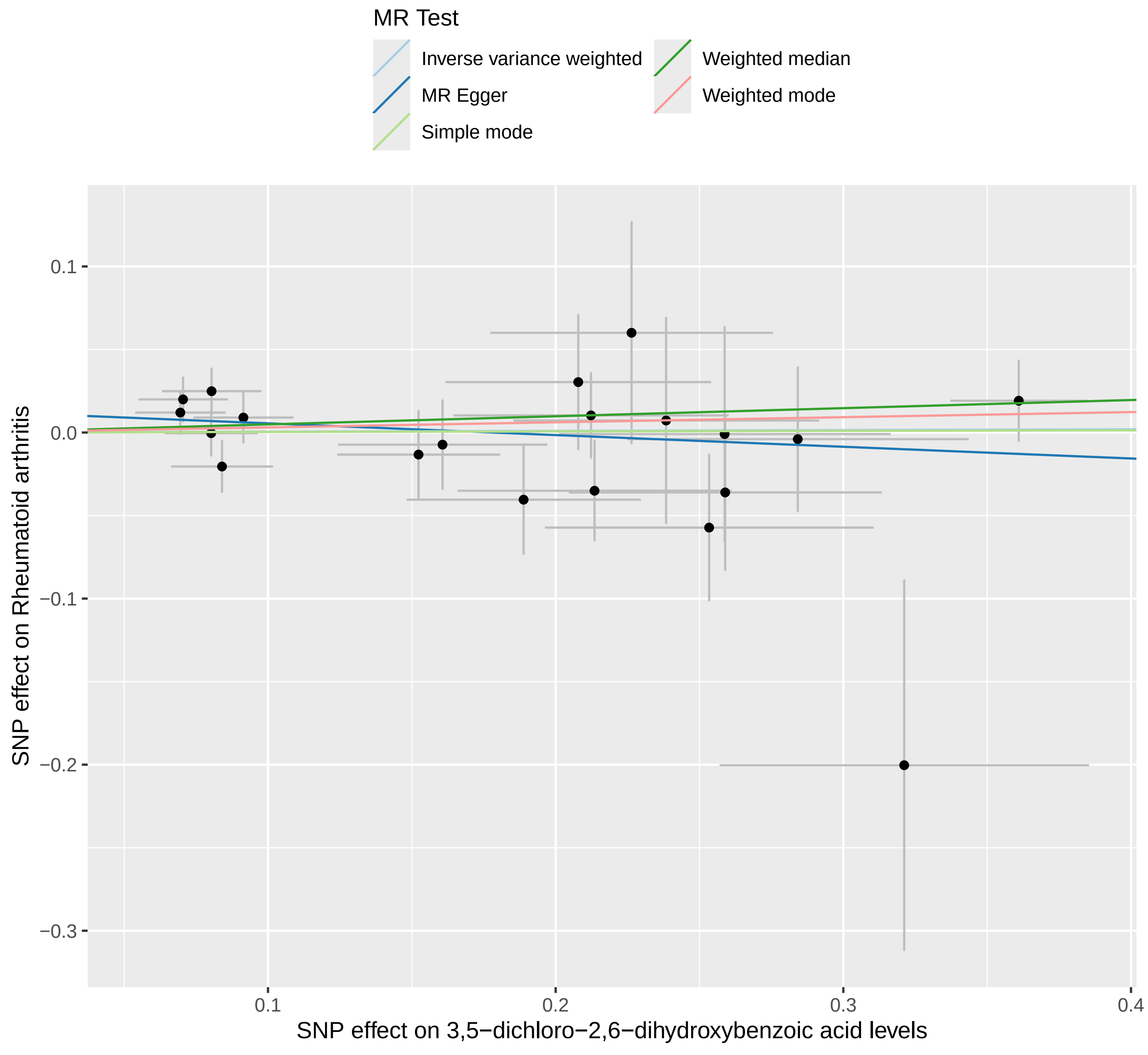
MR Test
Inverse variance weighted
MR Egger
Simple mode
Weighted median
Weighted mode
SNP effect on Rheumatoid arthritis
0.1
0.0
−0.1
−0.2
−0.3
0.1
0.2
0.3
0.4
SNP effect on 3,5−dichloro−2,6−dihydroxybenzoic acid levels

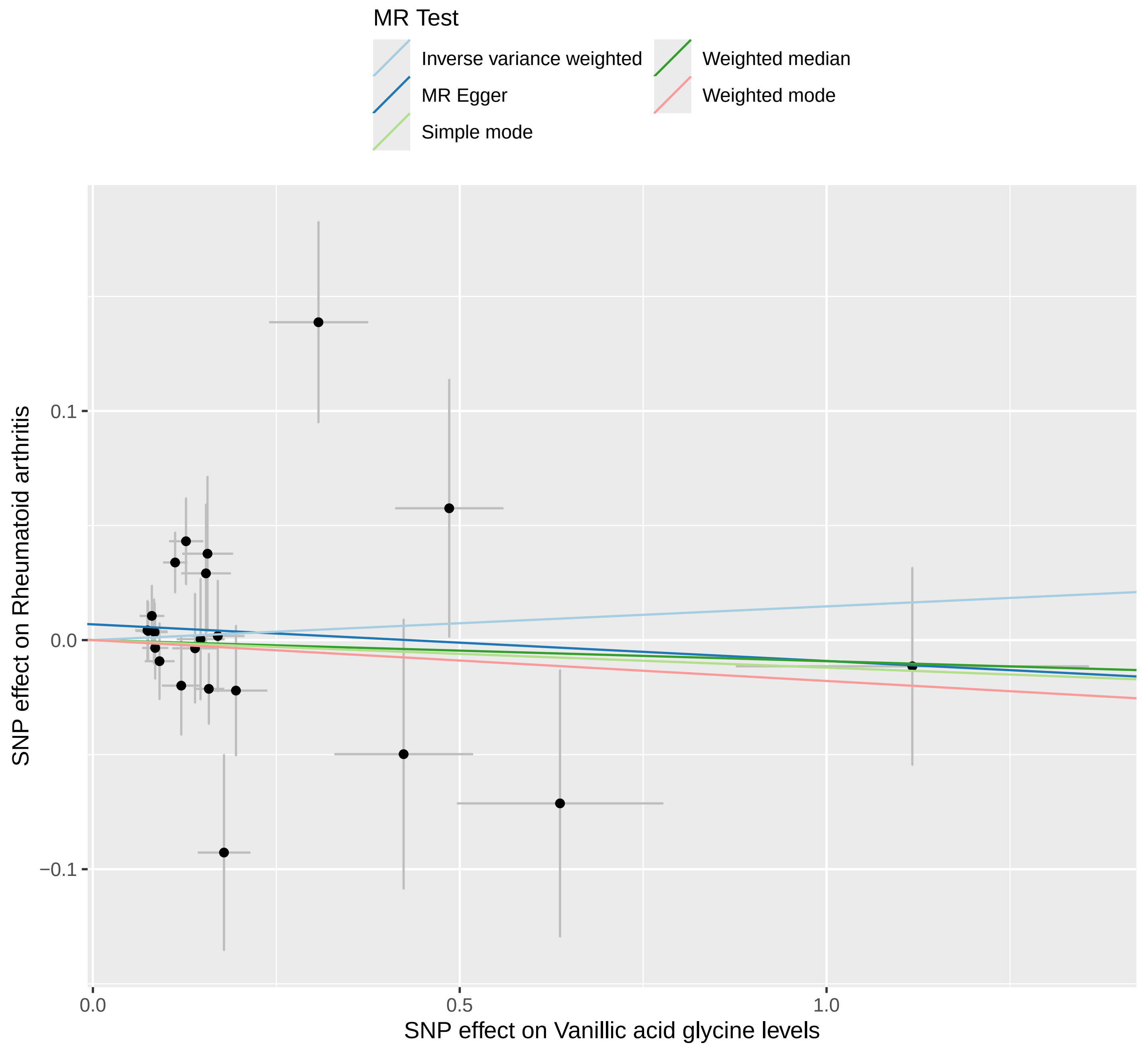

MR Test
Inverse variance weighted
MR Egger
Simple mode
Weighted median
Weighted mode
0.1
0.0
−0.1
SNP effect on Rheumatoid arthritis
0.0
0.5
1.0
SNP effect on Vanillic acid glycine levels

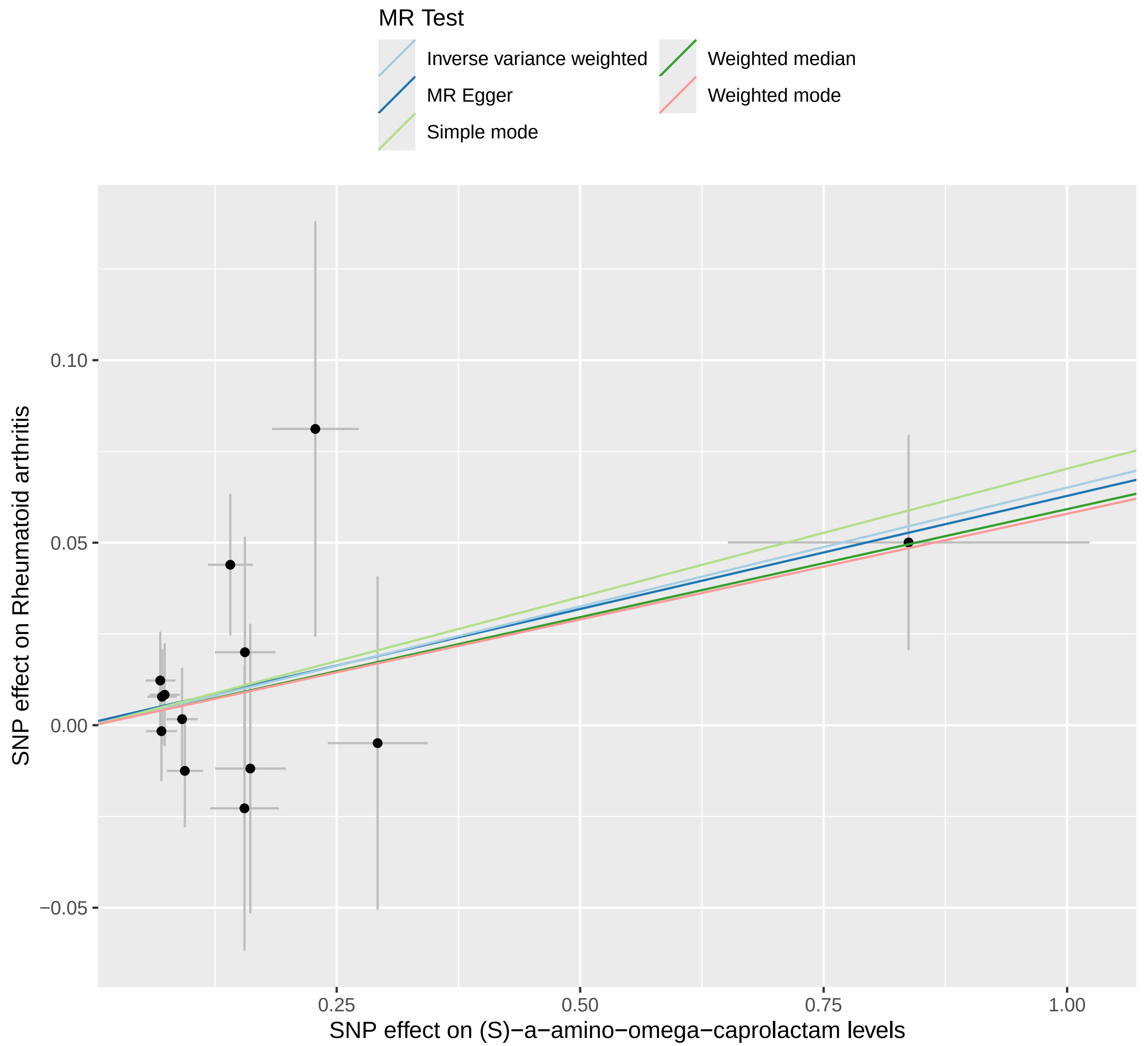

MR Test
Inverse variance weighted
MR Egger
Simple mode
Weighted median
Weighted mode
SNP effect on Rheumatoid arthritis
0.10
0.05
0.00
−0.05
0.25
0.50
0.75
1.00
SNP effect on (S)−a−amino−omega−caprolactam levels

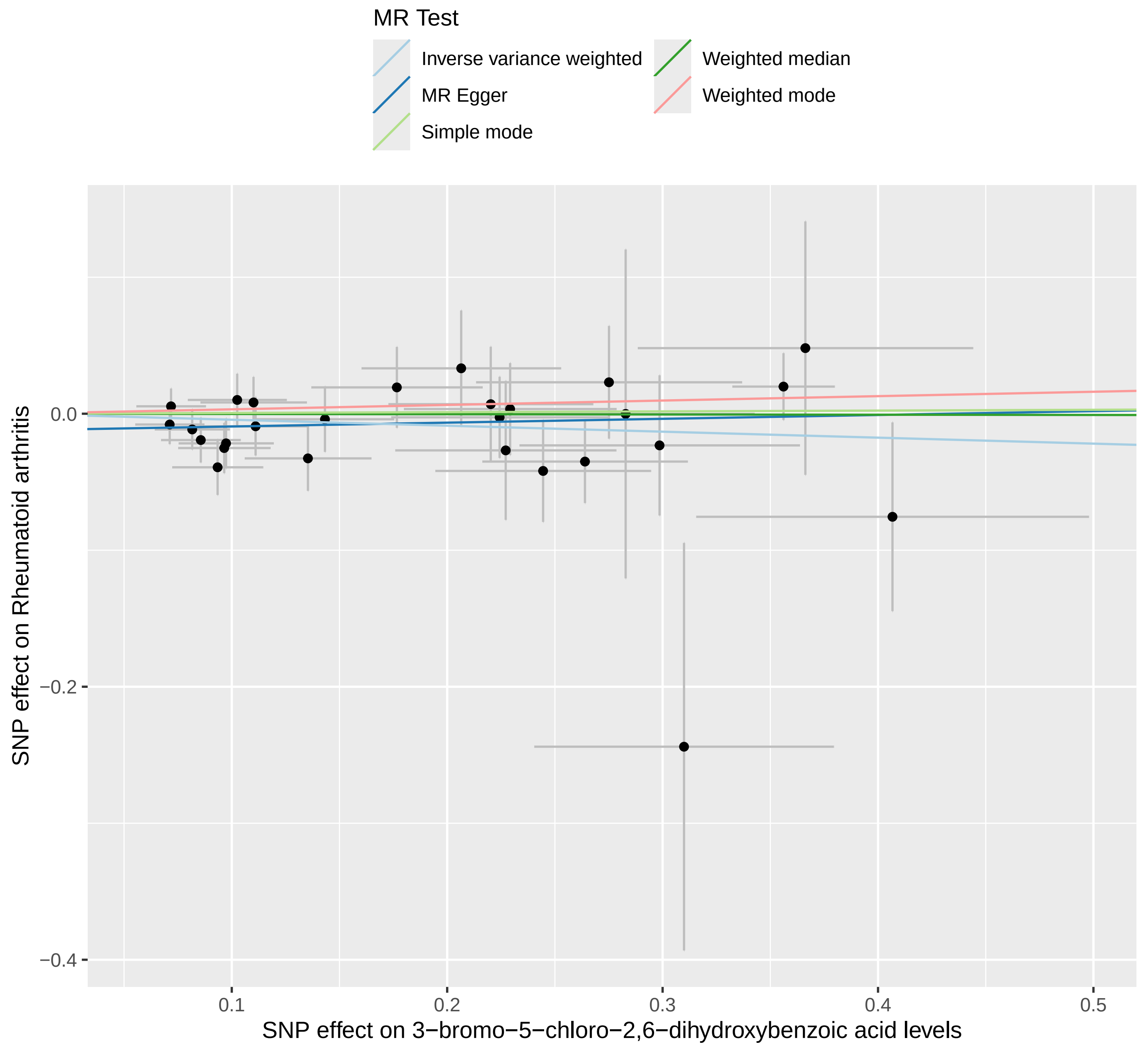
MR Test
Inverse variance weighted
MR Egger
Simple mode
Weighted median
Weighted mode
SNP effect on Rheumatoid arthritis
0.0
−0.2
−0.4
0.1
0.2
0.3
0.4
0.5
SNP effect on 3−bromo−5−chloro−2,6−dihydroxybenzoic acid levels

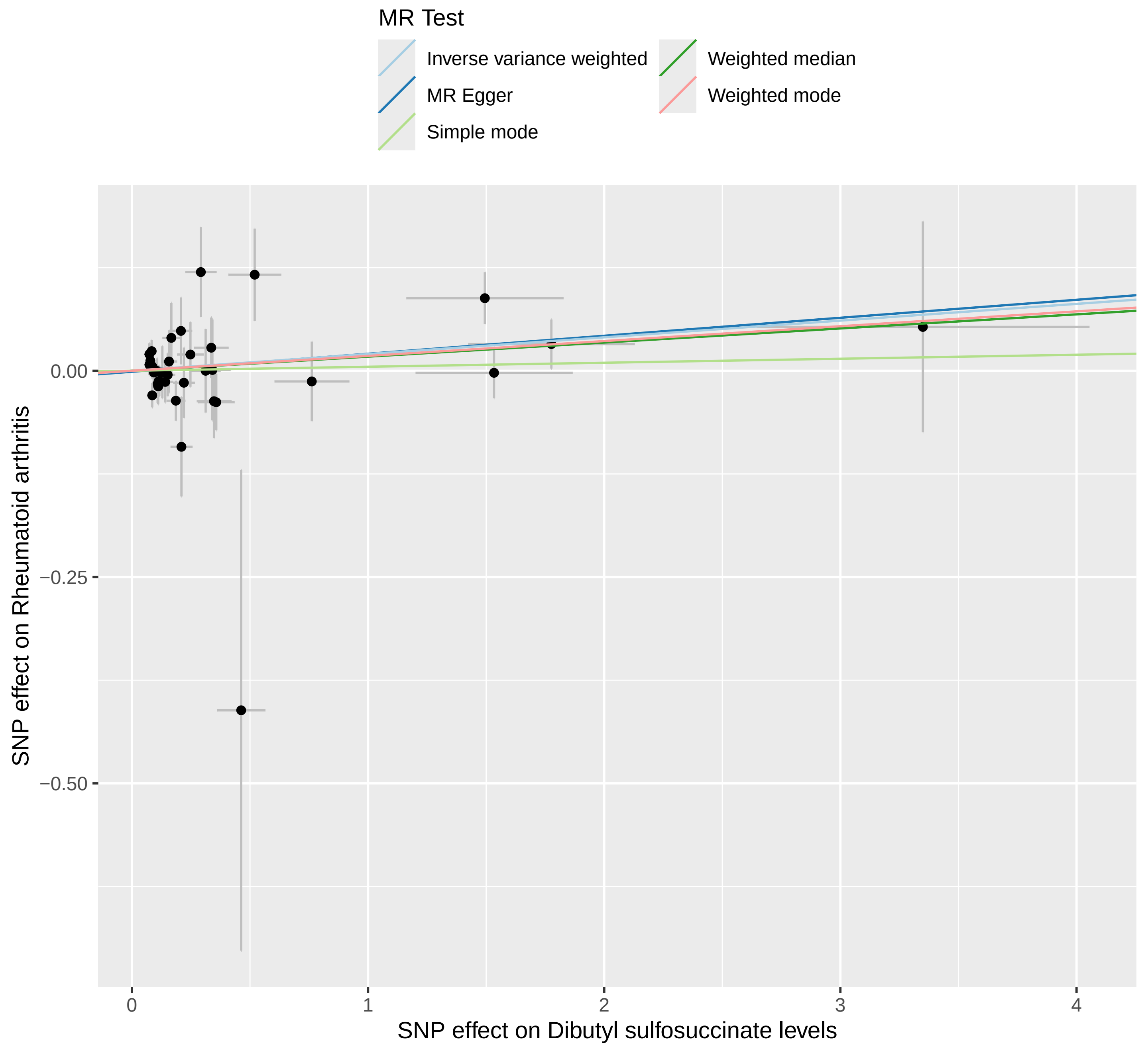
MR Test
Inverse variance weighted
MR Egger
Simple mode
Weighted median
Weighted mode
SNP effect on Rheumatoid arthritis
0.00
−0.25
−0.50
0
1
2
3
4
SNP effect on Dibutyl sulfosuccinate levels

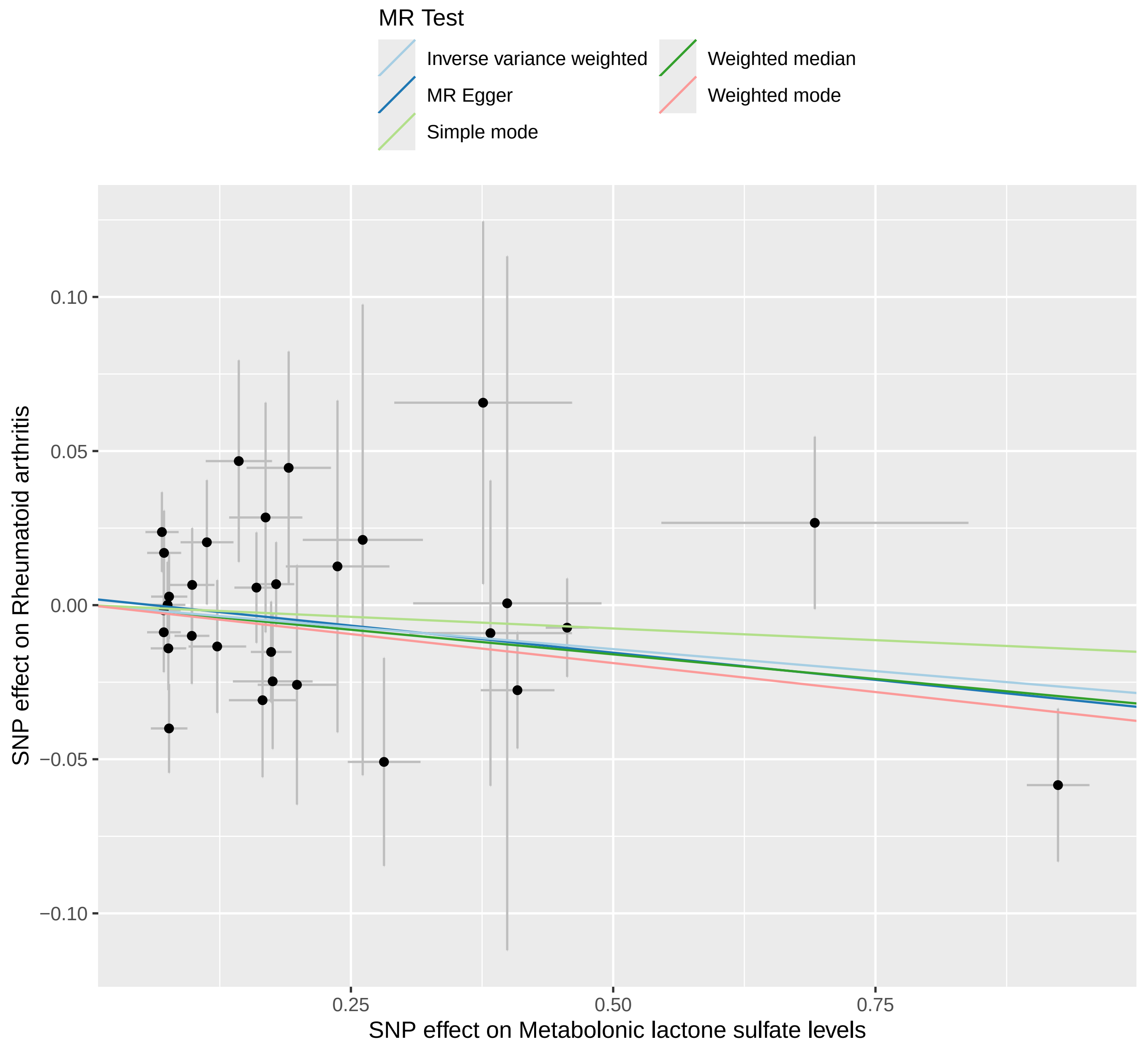

MR Test
Inverse variance weighted
MR Egger
Simple mode
Weighted median
Weighted mode
0.10
0.05
0.00
−0.05
−0.10
0.25
0.50
0.75
SNP effect on Rheumatoid arthritis
SNP effect on Metabolonic lactone sulfate levels

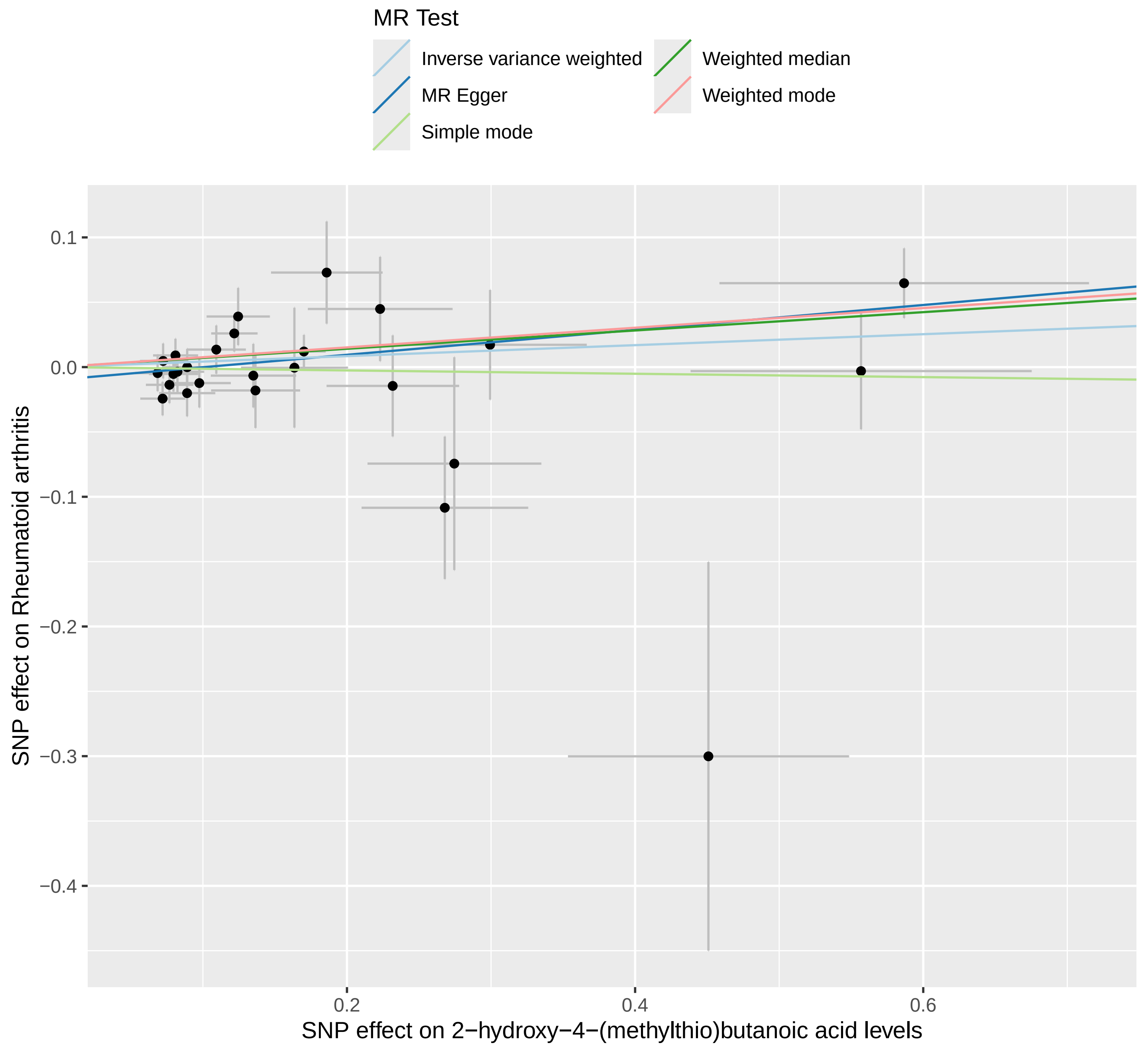

MR Test
Inverse variance weighted
MR Egger
Simple mode
Weighted median
Weighted mode
0.1
0.0
−0.1
−0.2
−0.3
−0.4
0.2
0.4
0.6
SNP effect on Rheumatoid arthritis
SNP effect on 2−hydroxy−4−(methylthio)butanoic acid levels

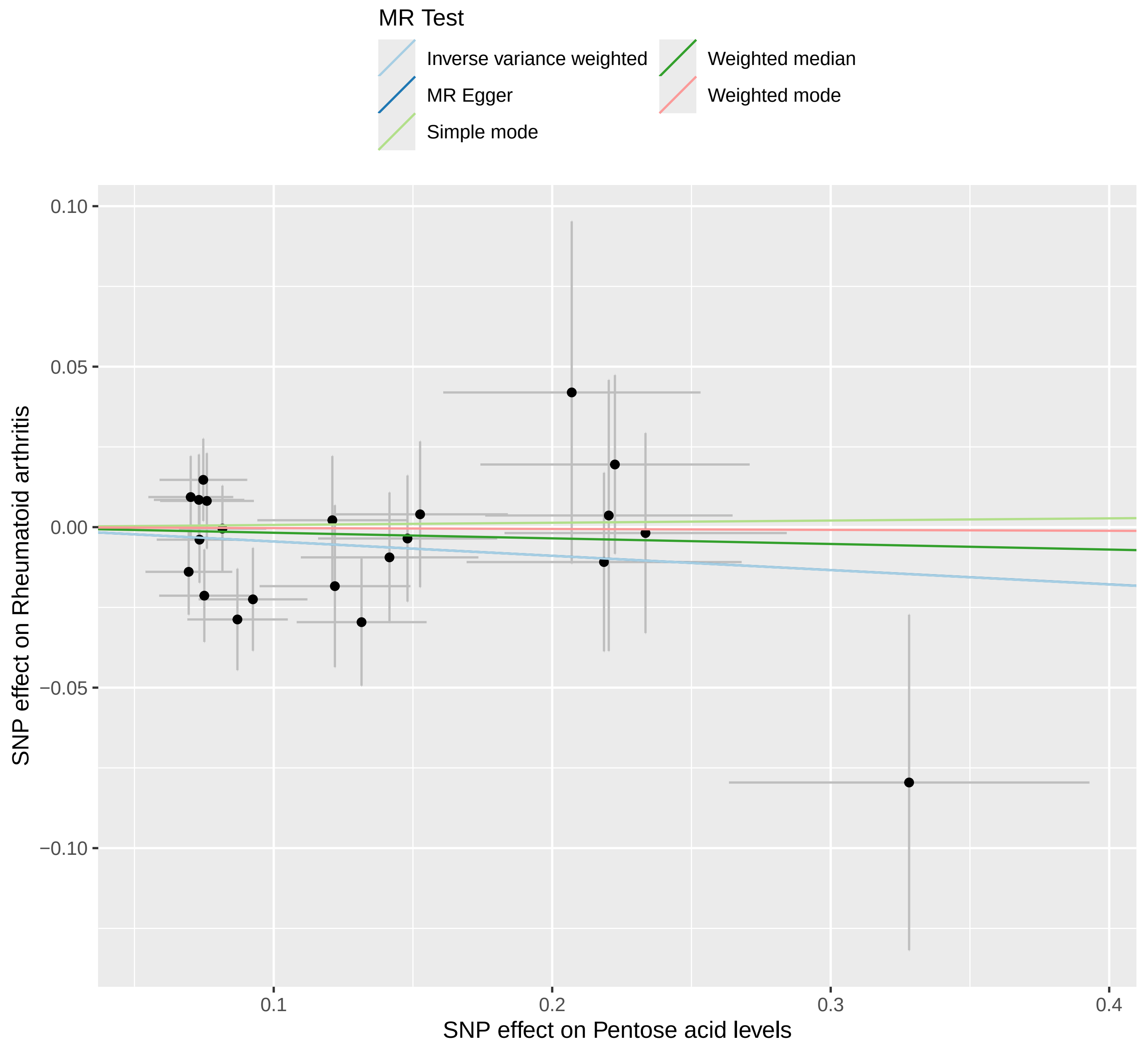

MR Test
Inverse variance weighted
MR Egger
Simple mode
Weighted median
Weighted mode
0.10
0.05
0.00
−0.05
−0.10
0.1
0.2
0.3
0.4
SNP effect on Rheumatoid arthritis
SNP effect on Pentose acid levels

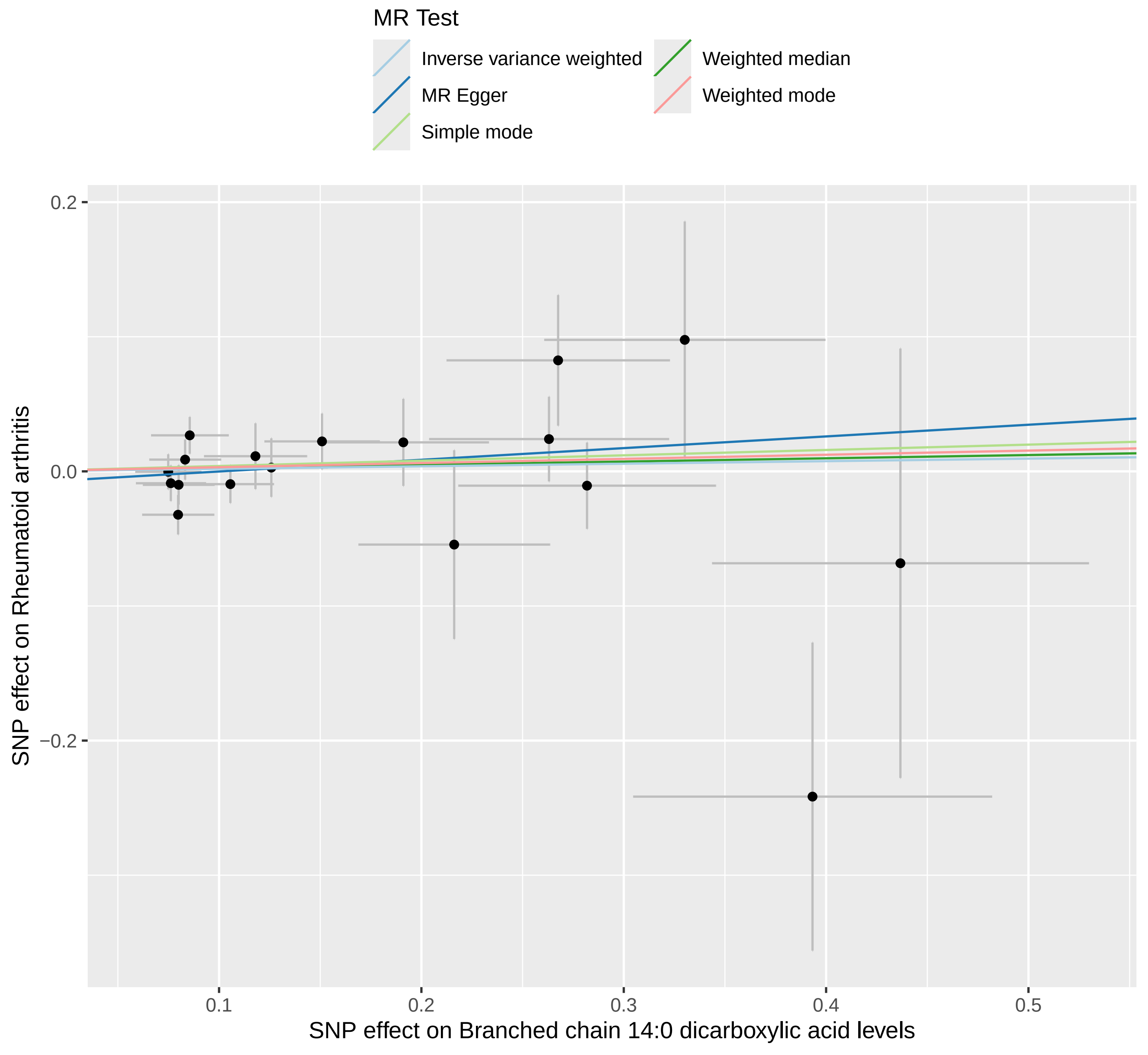
MR Test
Inverse variance weighted
MR Egger
Simple mode
Weighted median
Weighted mode
0.2
0.0
−0.2
0.1
0.2
0.3
0.4
0.5
SNP effect on Rheumatoid arthritis
SNP effect on Branched chain 14:0 dicarboxylic acid levels

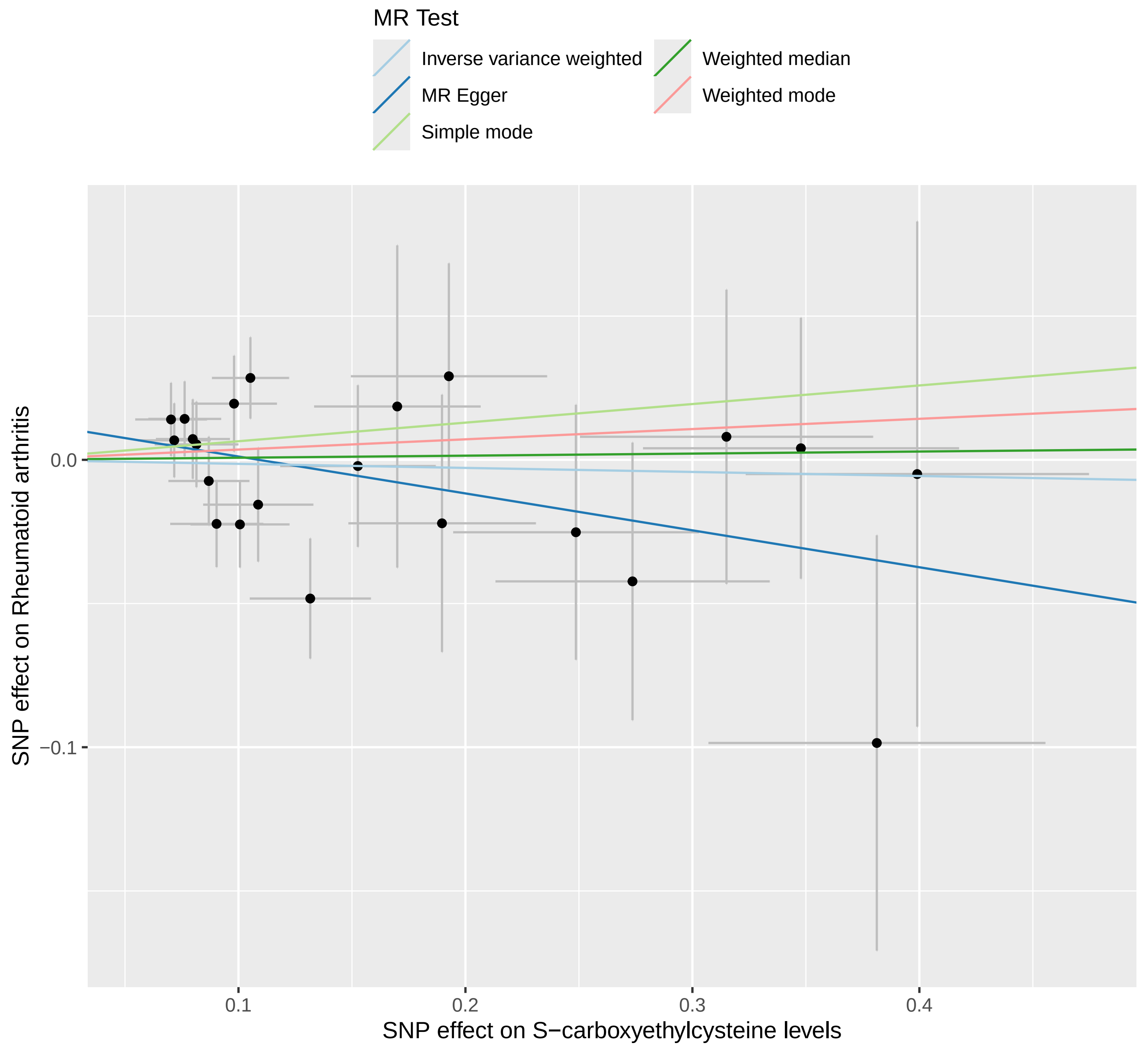
MR Test
Inverse variance weighted
MR Egger
Simple mode
Weighted median
Weighted mode
0.0
−0.1
0.1
0.2
0.3
0.4
SNP effect on Rheumatoid arthritis
SNP effect on S−carboxyethylcysteine levels

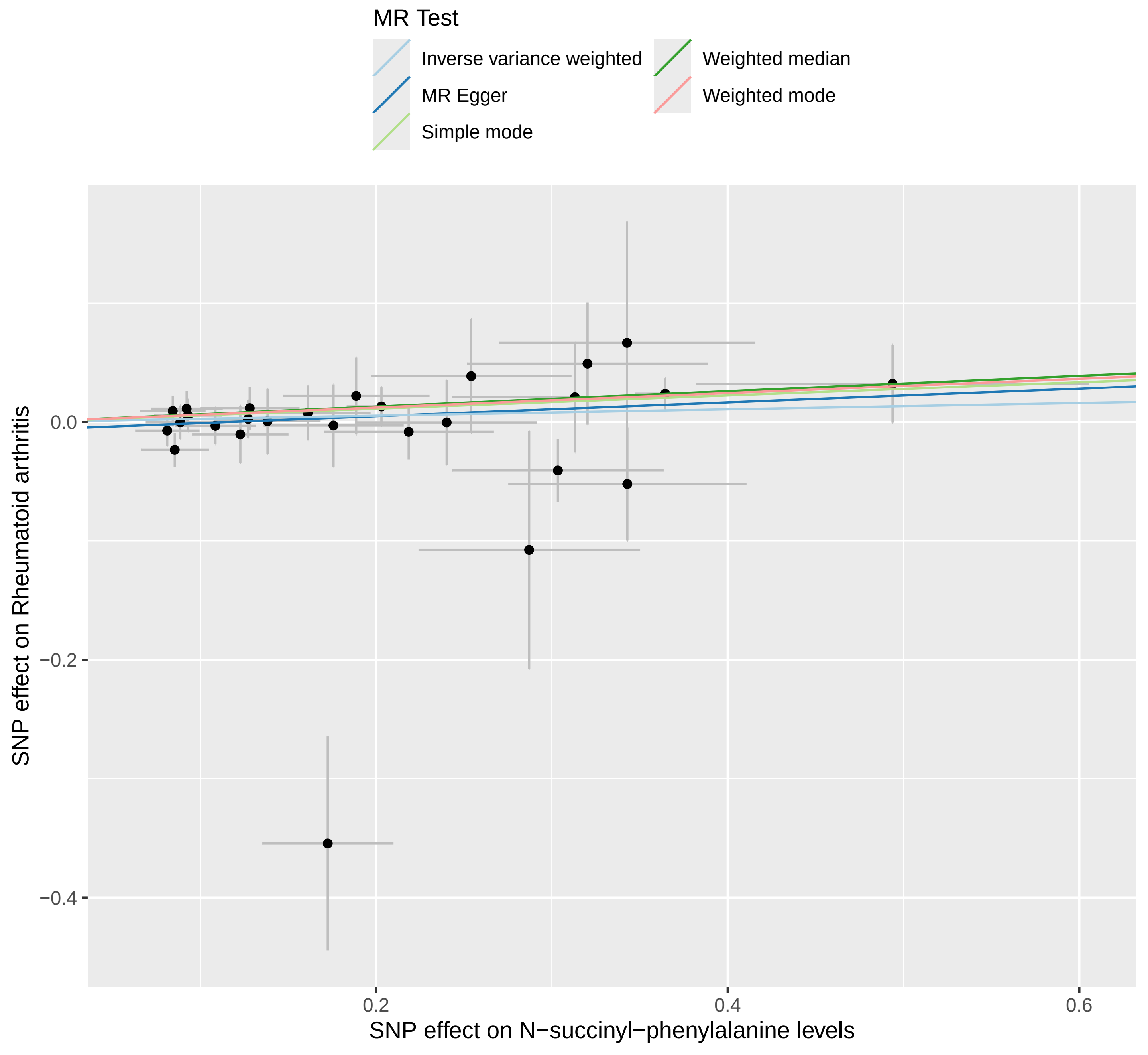
MR Test
Inverse variance weighted
MR Egger
Simple mode
Weighted median
Weighted mode
SNP effect on Rheumatoid arthritis
0.0
−0.2
−0.4
0.2
0.4
0.6
SNP effect on N−succinyl−phenylalanine levels

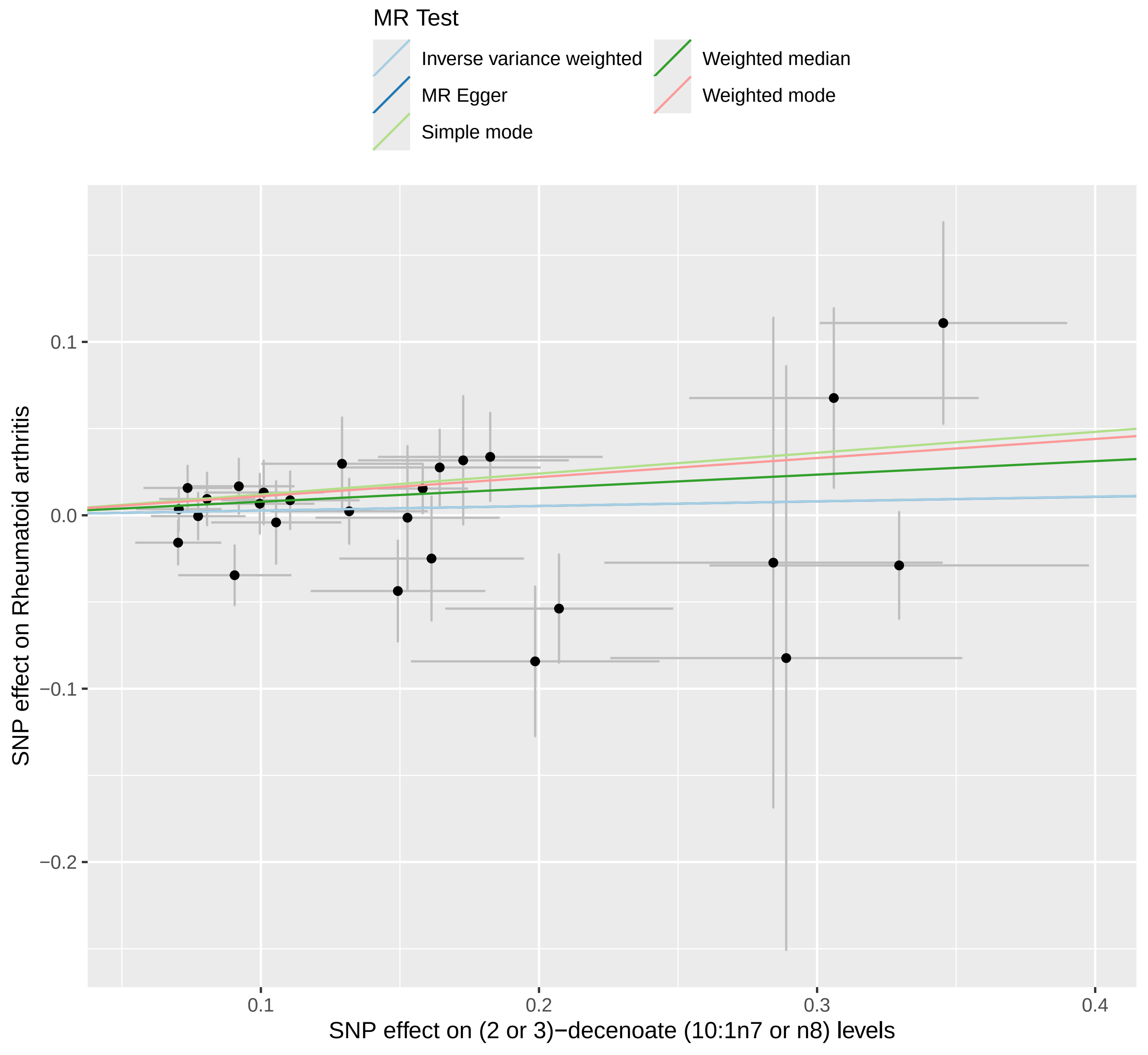

MR Test
Inverse variance weighted
MR Egger
Simple mode
Weighted median
Weighted mode
0.1
0.0
−0.1
−0.2
0.1
0.2
0.3
0.4
SNP effect on Rheumatoid arthritis
SNP effect on (2 or 3)−decenoate (10:1n7 or n8) levels

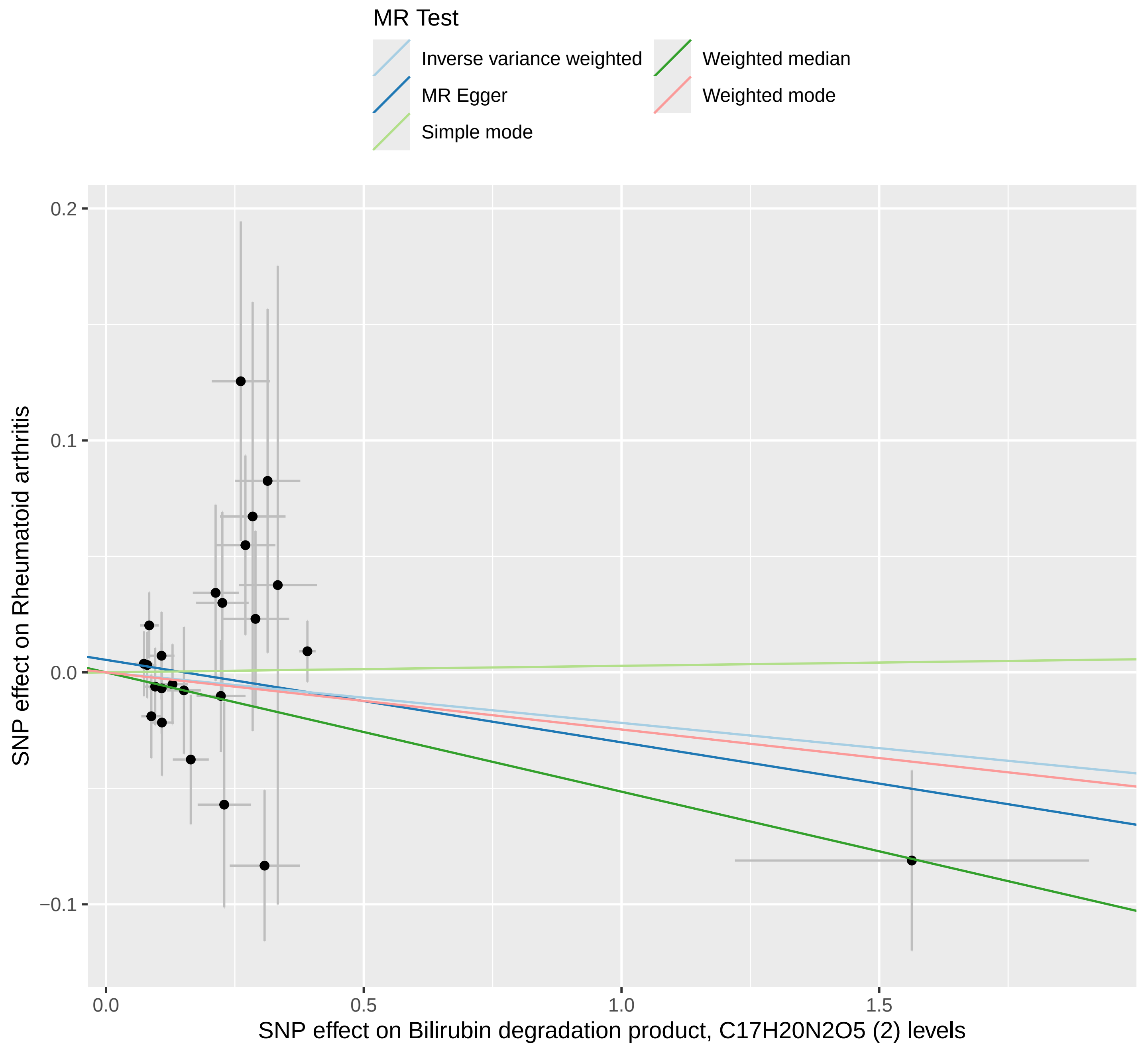
MR Test
Inverse variance weighted
MR Egger
Simple mode
Weighted median
Weighted mode
0.2
0.1
0.0
−0.1
0.0
0.5
1.0
1.5
SNP effect on Rheumatoid arthritis
SNP effect on Bilirubin degradation product, C17H20N2O5 (2) levels

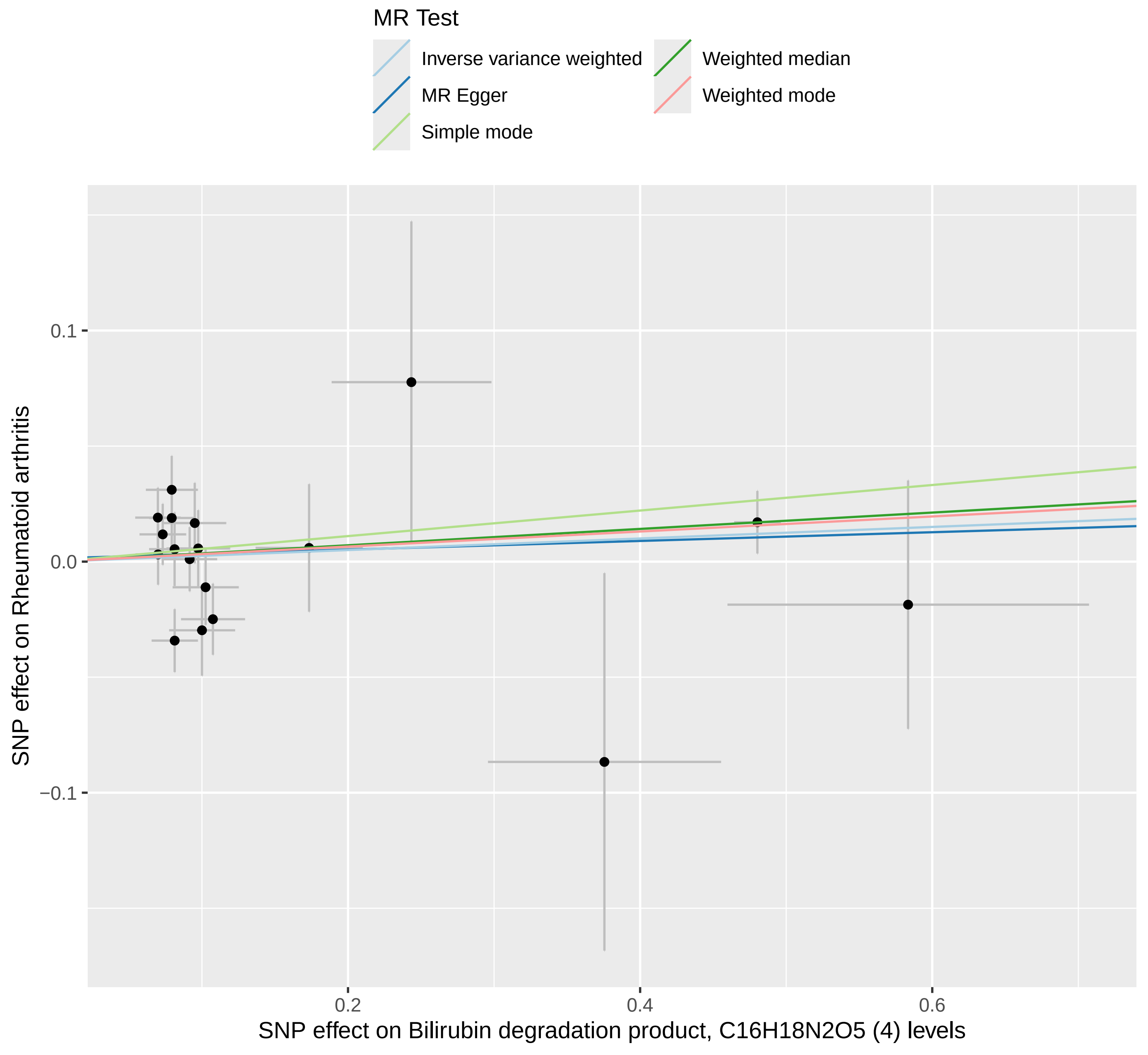

MR Test
Inverse variance weighted
MR Egger
Simple mode
Weighted median
Weighted mode
SNP effect on Rheumatoid arthritis
0.1
0.0
−0.1
0.2
0.4
0.6
SNP effect on Bilirubin degradation product, C16H18N2O5 (4) levels

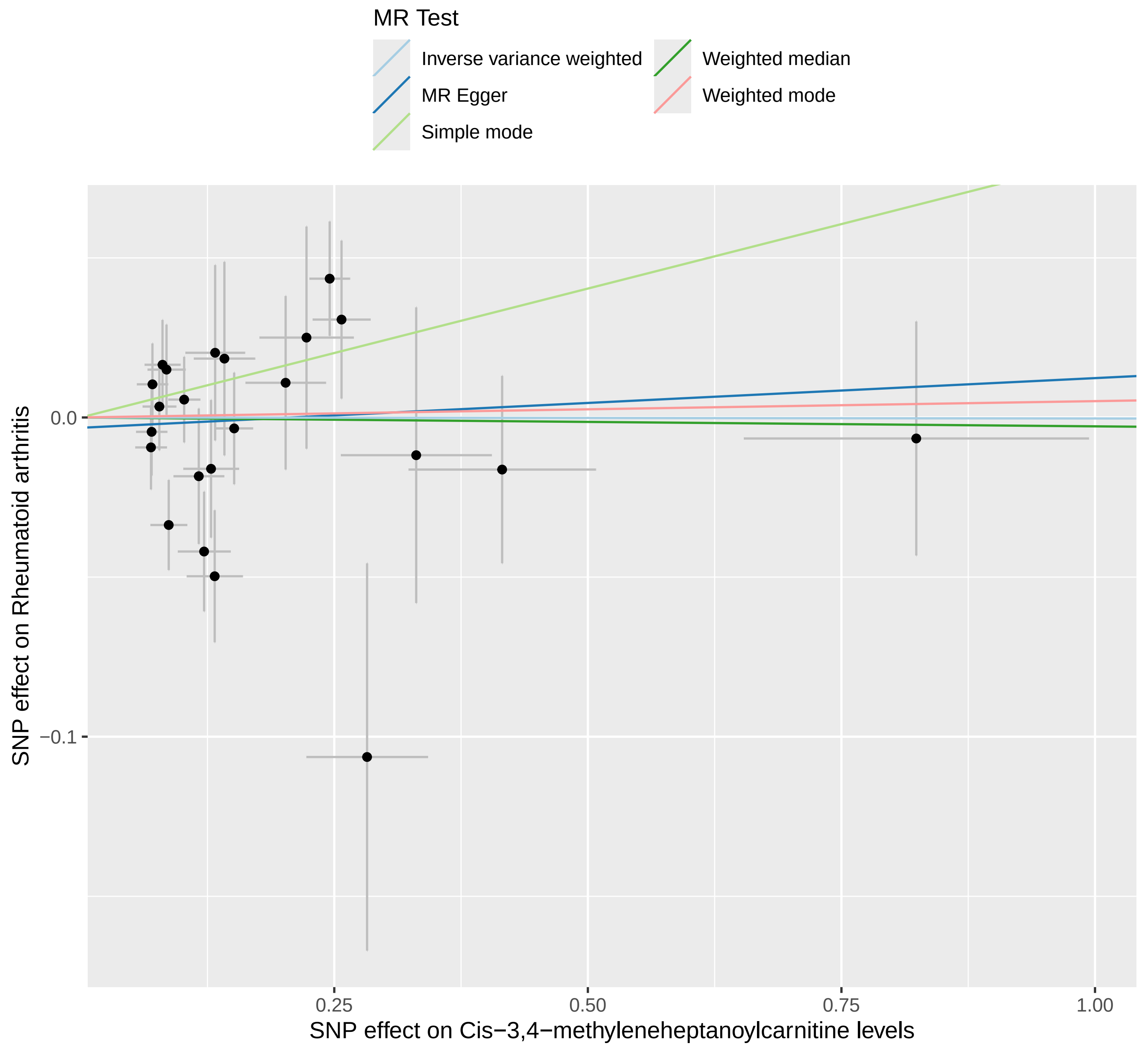
MR Test
Inverse variance weighted
MR Egger
Simple mode
Weighted median
Weighted mode
0.0
−0.1
0.25
0.50
0.75
1.00
SNP effect on Rheumatoid arthritis
SNP effect on Cis−3,4−methyleneheptanoylcarnitine levels

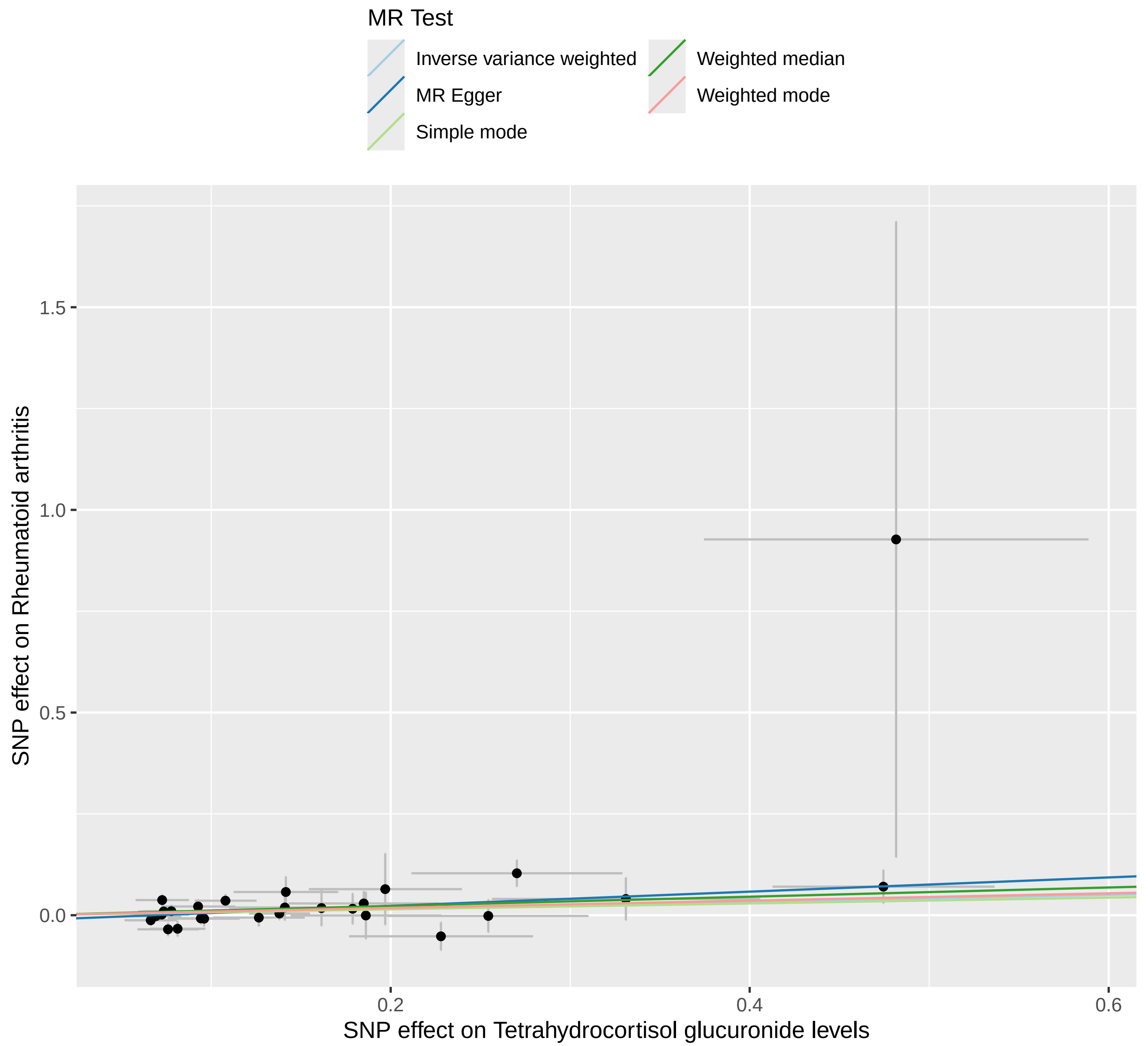
MR Test
Inverse variance weighted
MR Egger
Simple mode
Weighted median
Weighted mode
1.5
1.0
0.5
0.0
0.2
0.4
0.6
SNP effect on Rheumatoid arthritis
SNP effect on Tetrahydrocortisol glucuronide levels

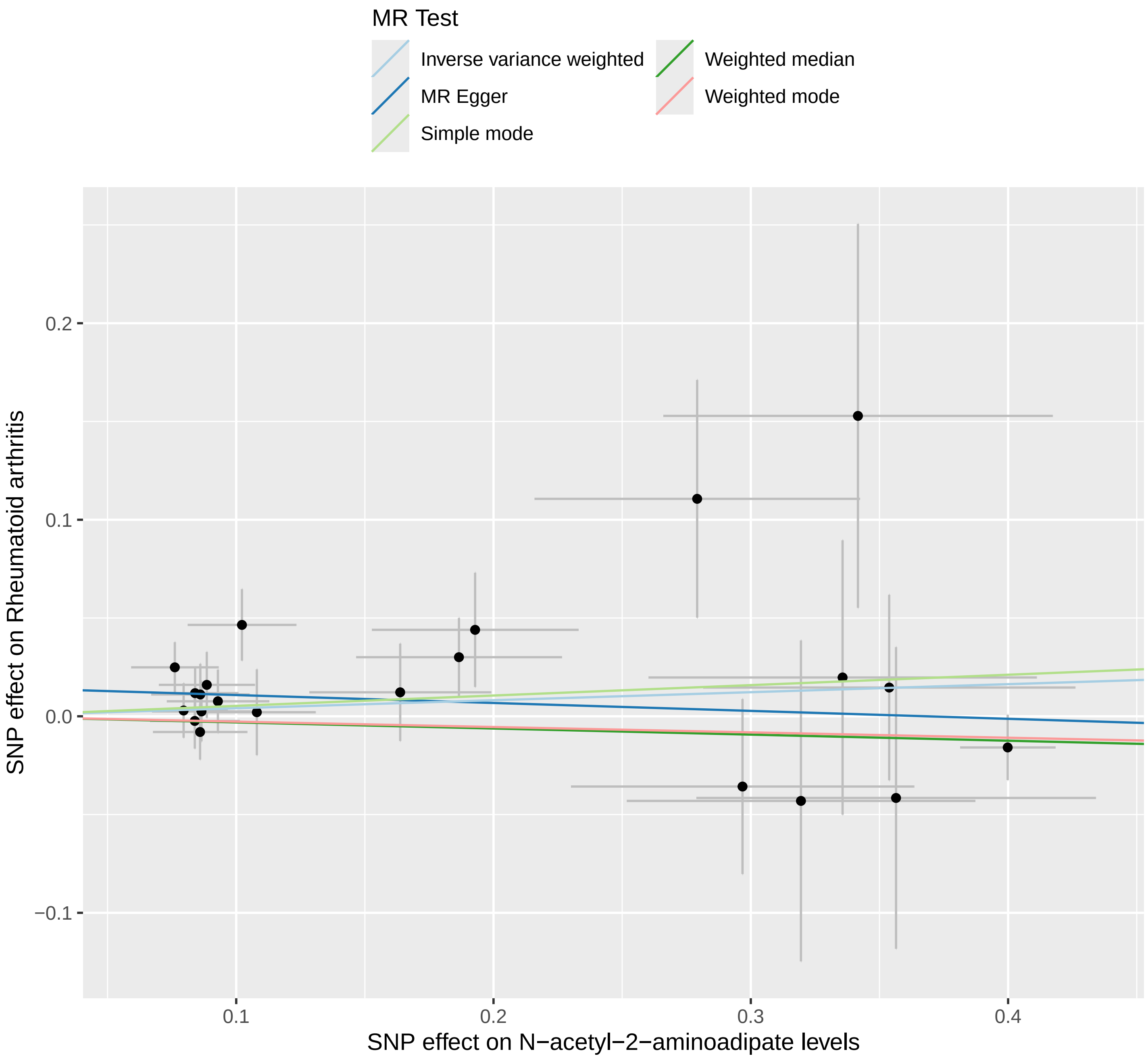
MR Test
Inverse variance weighted
MR Egger
Simple mode
Weighted median
Weighted mode
0.2
0.1
0.0
−0.1
0.1
0.2
0.3
0.4
SNP effect on Rheumatoid arthritis
SNP effect on N−acetyl−2−aminoadipate levels

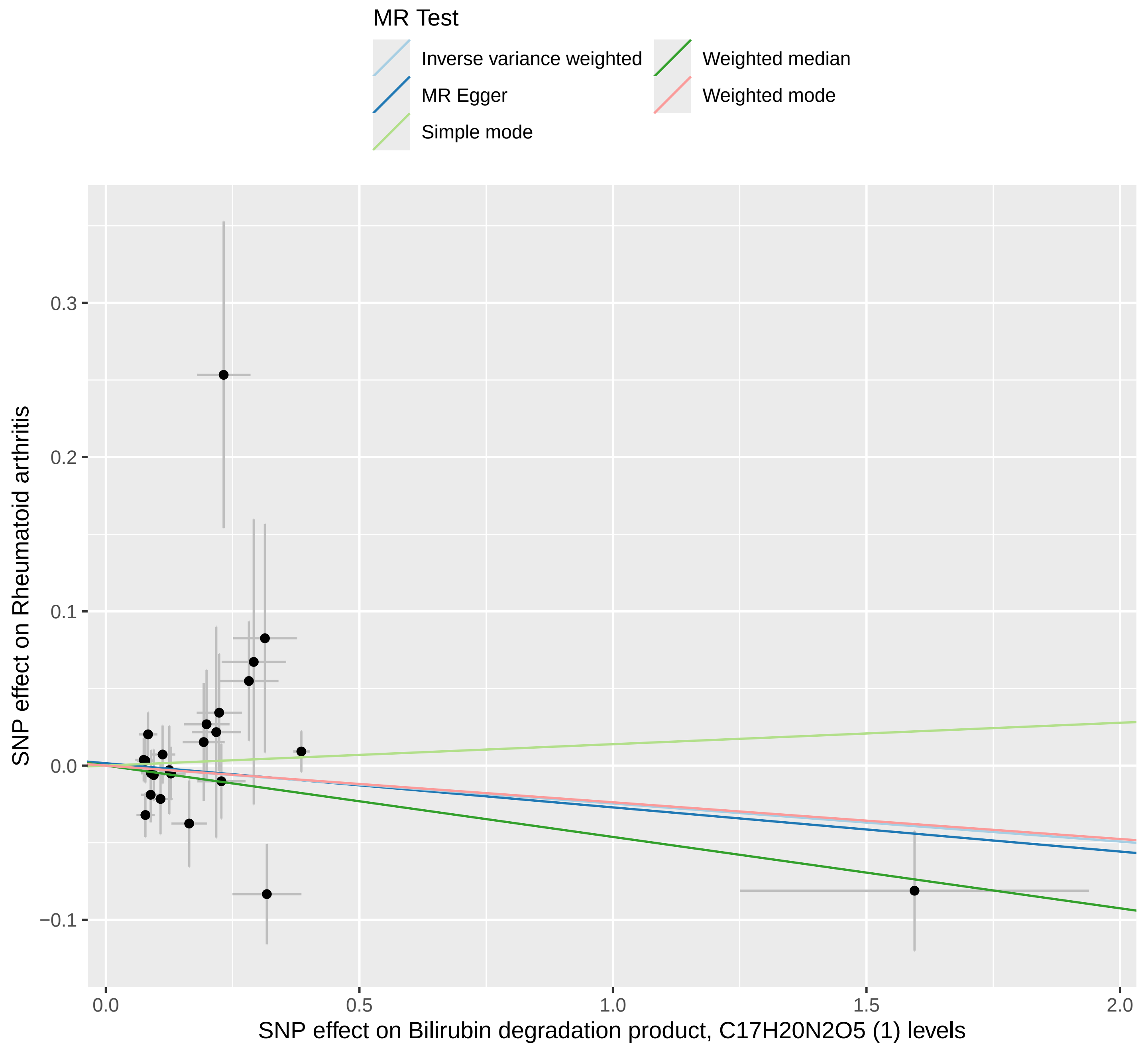

MR Test
Inverse variance weighted
MR Egger
Simple mode
Weighted median
Weighted mode
SNP effect on Rheumatoid arthritis
0.3
0.2
0.1
0.0
−0.1
0.0
0.5
1.0
1.5
2.0
SNP effect on Bilirubin degradation product, C17H20N2O5 (1) levels

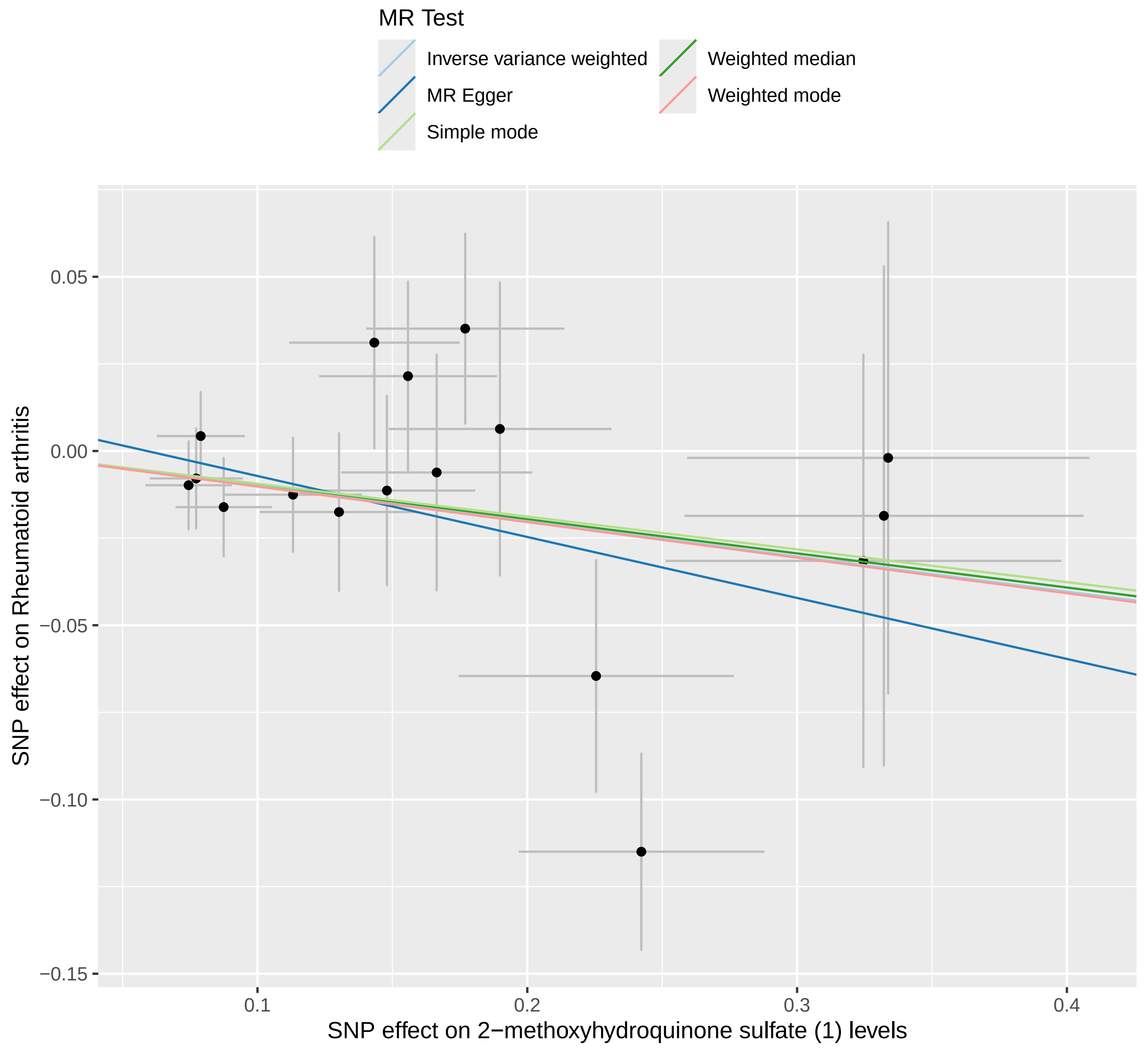

MR Test
Inverse variance weighted
MR Egger
Simple mode
Weighted median
Weighted mode
SNP effect on Rheumatoid arthritis
0.05
0.00
−0.05
−0.10
−0.15
0.1
0.2
0.3
0.4
SNP effect on 2−methoxyhydroquinone sulfate (1) levels

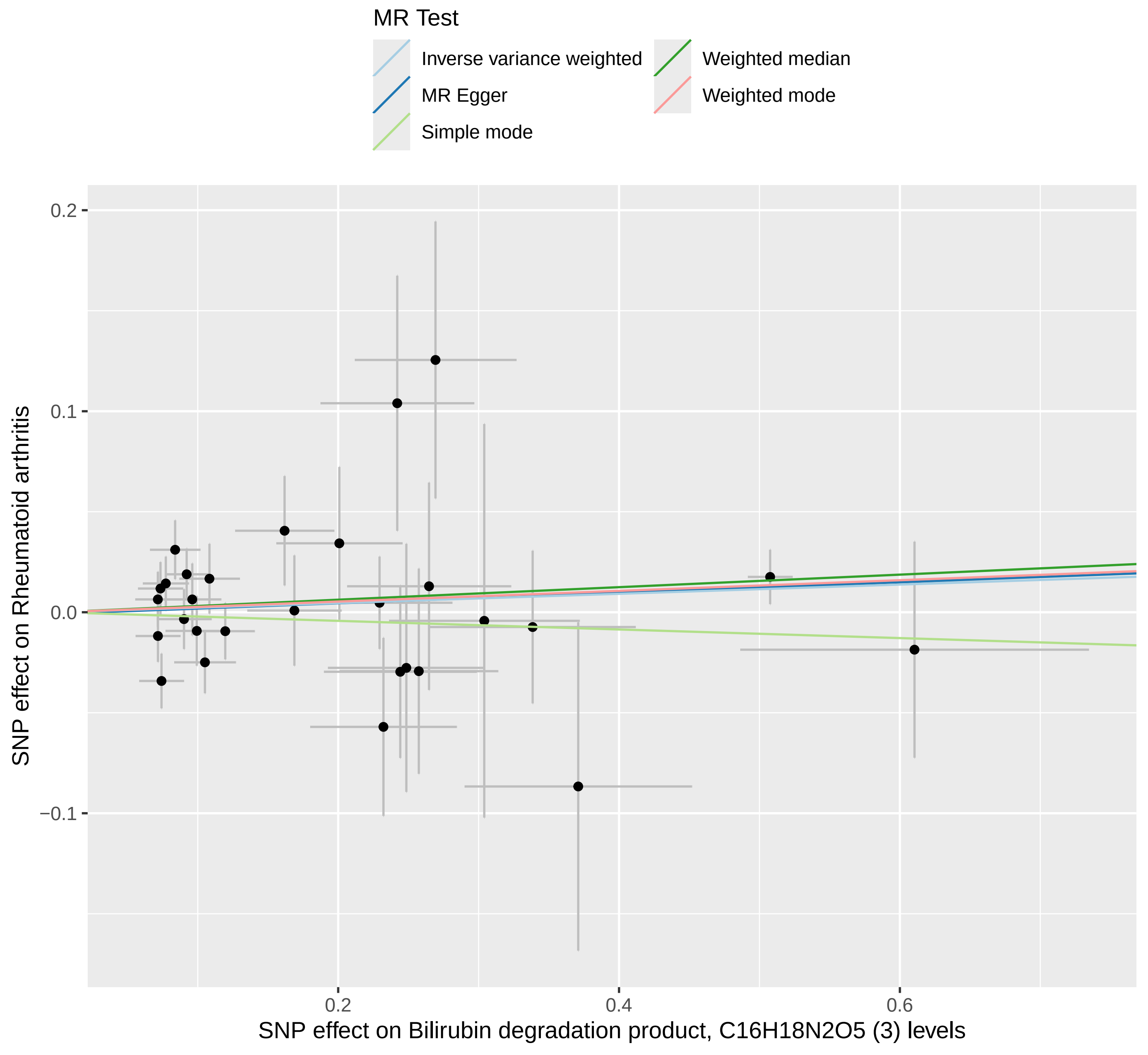
MR Test
Inverse variance weighted
MR Egger
Simple mode
Weighted median
Weighted mode
0.2
0.1
0.0
−0.1
0.2
0.4
0.6
SNP effect on Rheumatoid arthritis
SNP effect on Bilirubin degradation product, C16H18N2O5 (3) levels

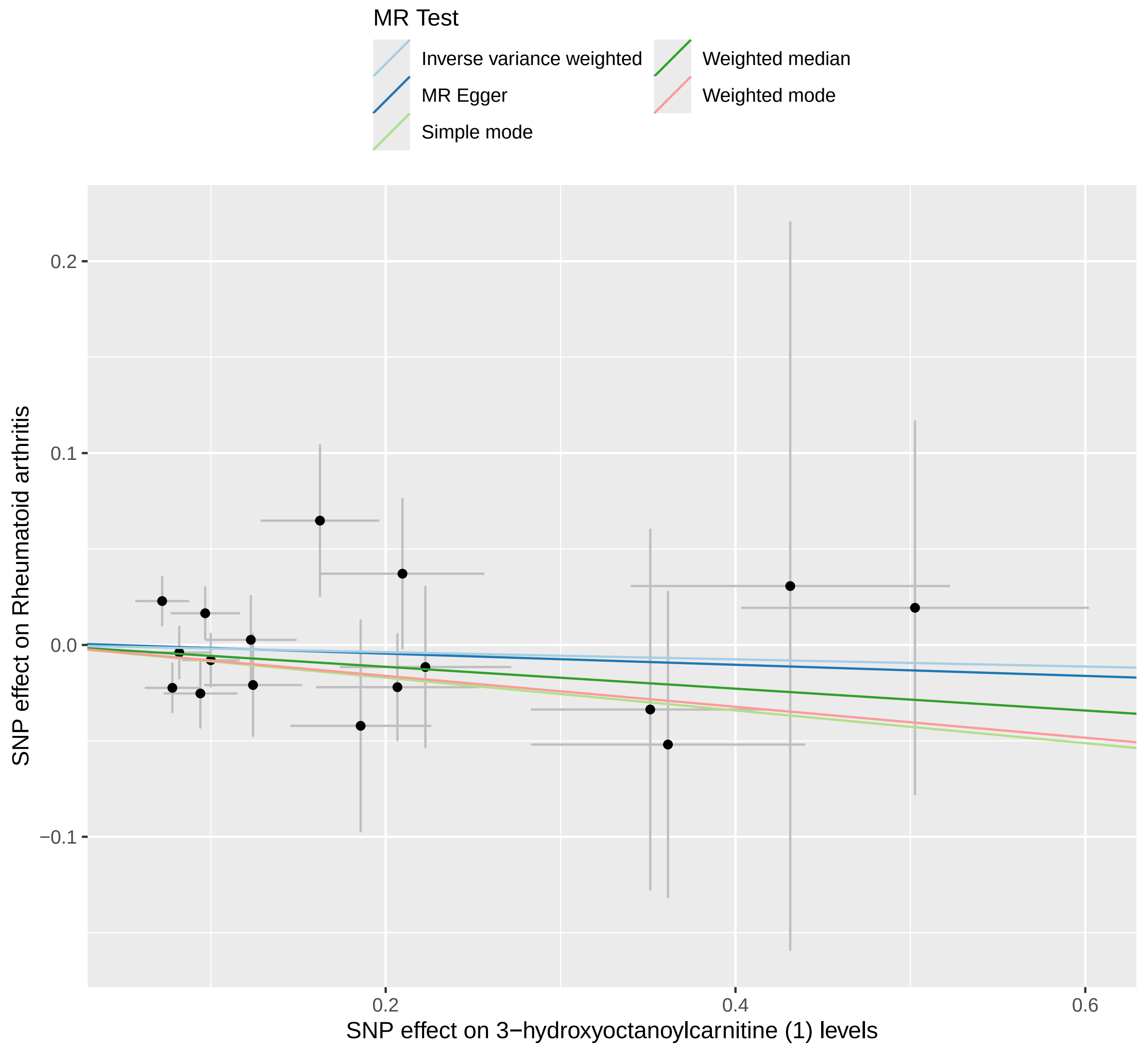
MR Test
Inverse variance weighted
MR Egger
Simple mode
Weighted median
Weighted mode
0.2
0.1
0.0
−0.1
SNP effect on Rheumatoid arthritis
0.2
0.4
0.6
SNP effect on 3−hydroxyoctanoylcarnitine (1) levels

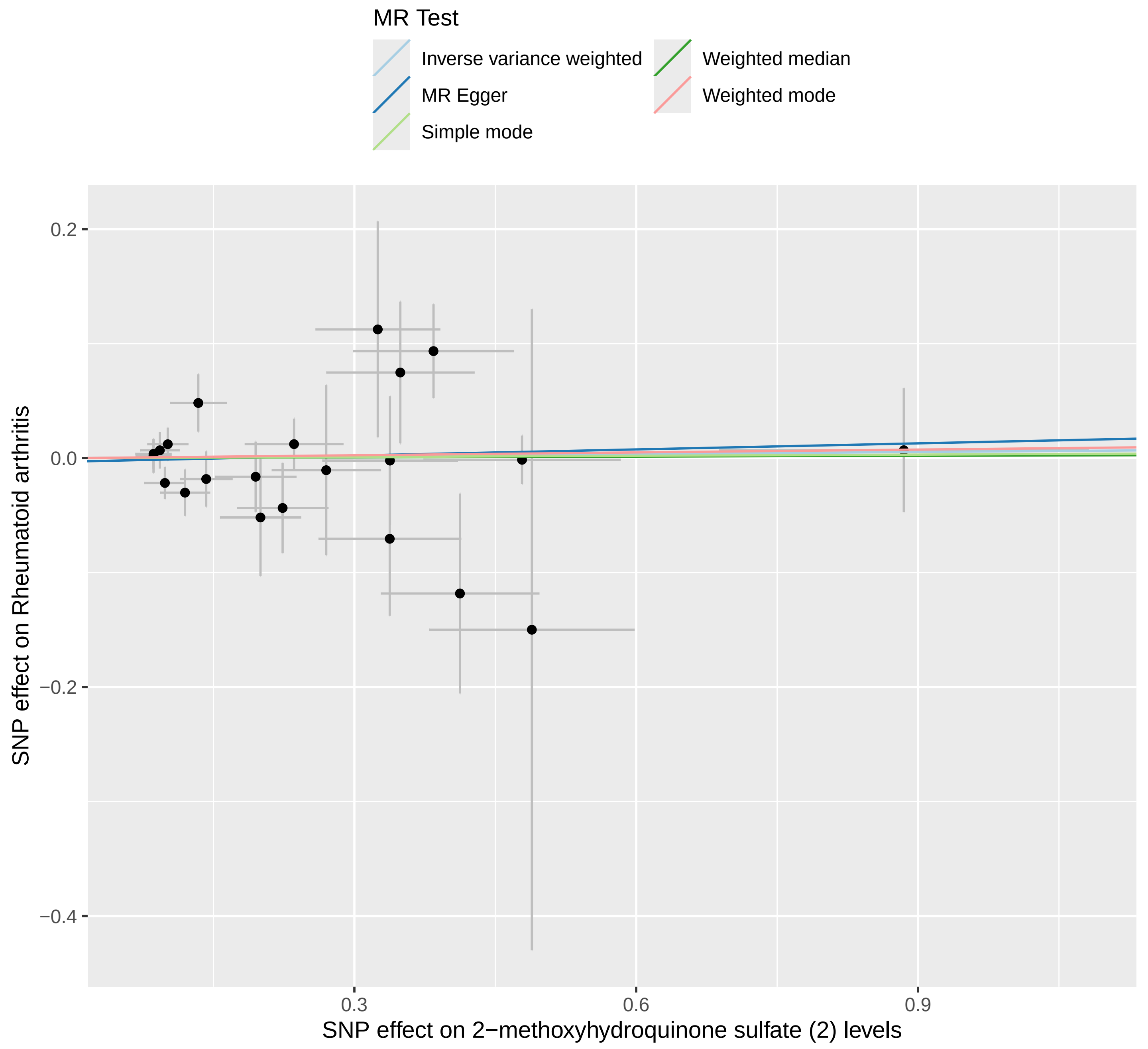
MR Test
Inverse variance weighted
MR Egger
Simple mode
Weighted median
Weighted mode
0.2
0.0
−0.2
−0.4
SNP effect on Rheumatoid arthritis
0.3
0.6
0.9
SNP effect on 2−methoxyhydroquinone sulfate (2) levels

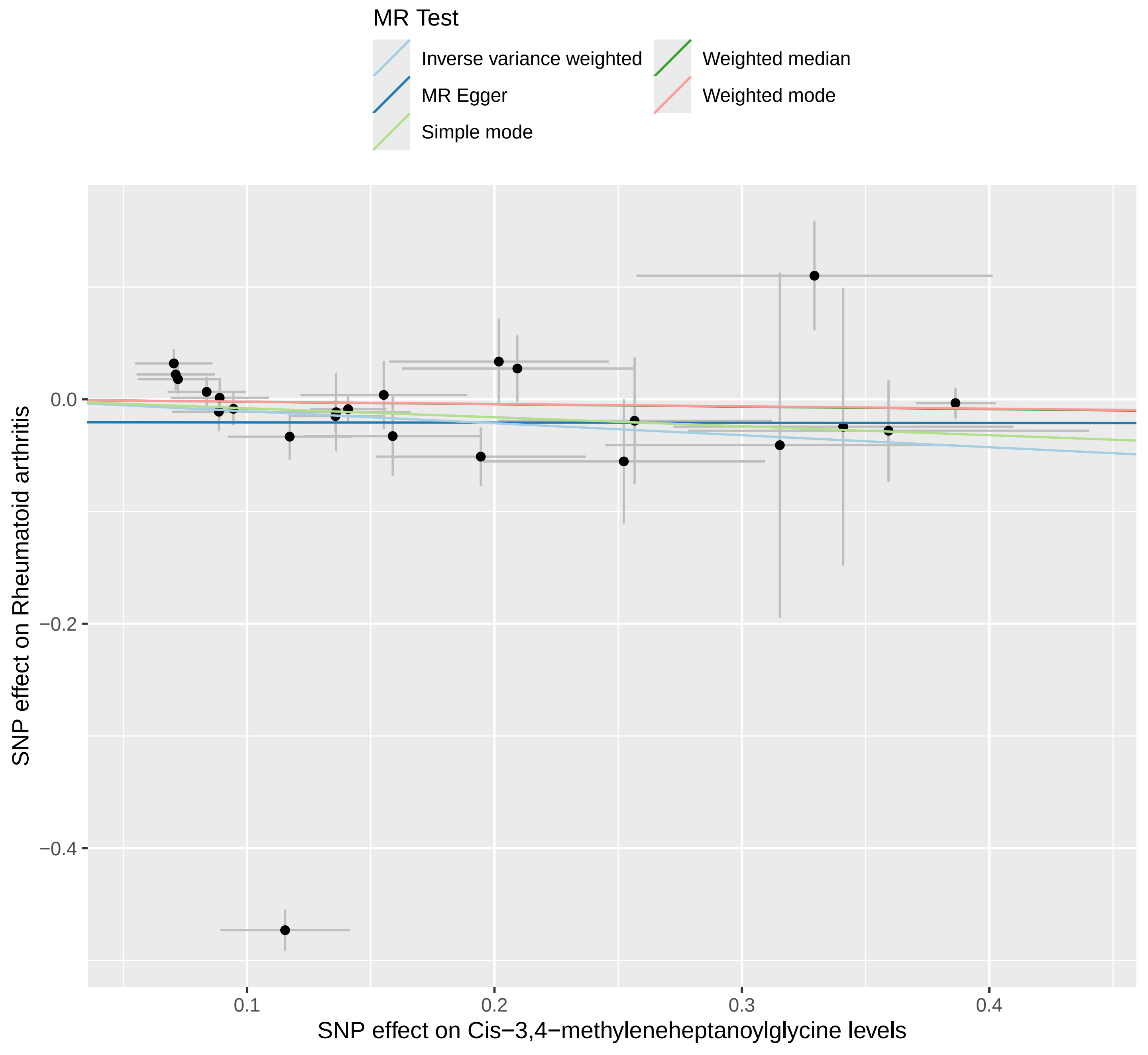

MR Test
Inverse variance weighted
MR Egger
Simple mode
Weighted median
Weighted mode
0.0
−0.2
−0.4
SNP effect on Rheumatoid arthritis
0.1
0.2
0.3
0.4
SNP effect on Cis−3,4−methyleneheptanoylglycine levels

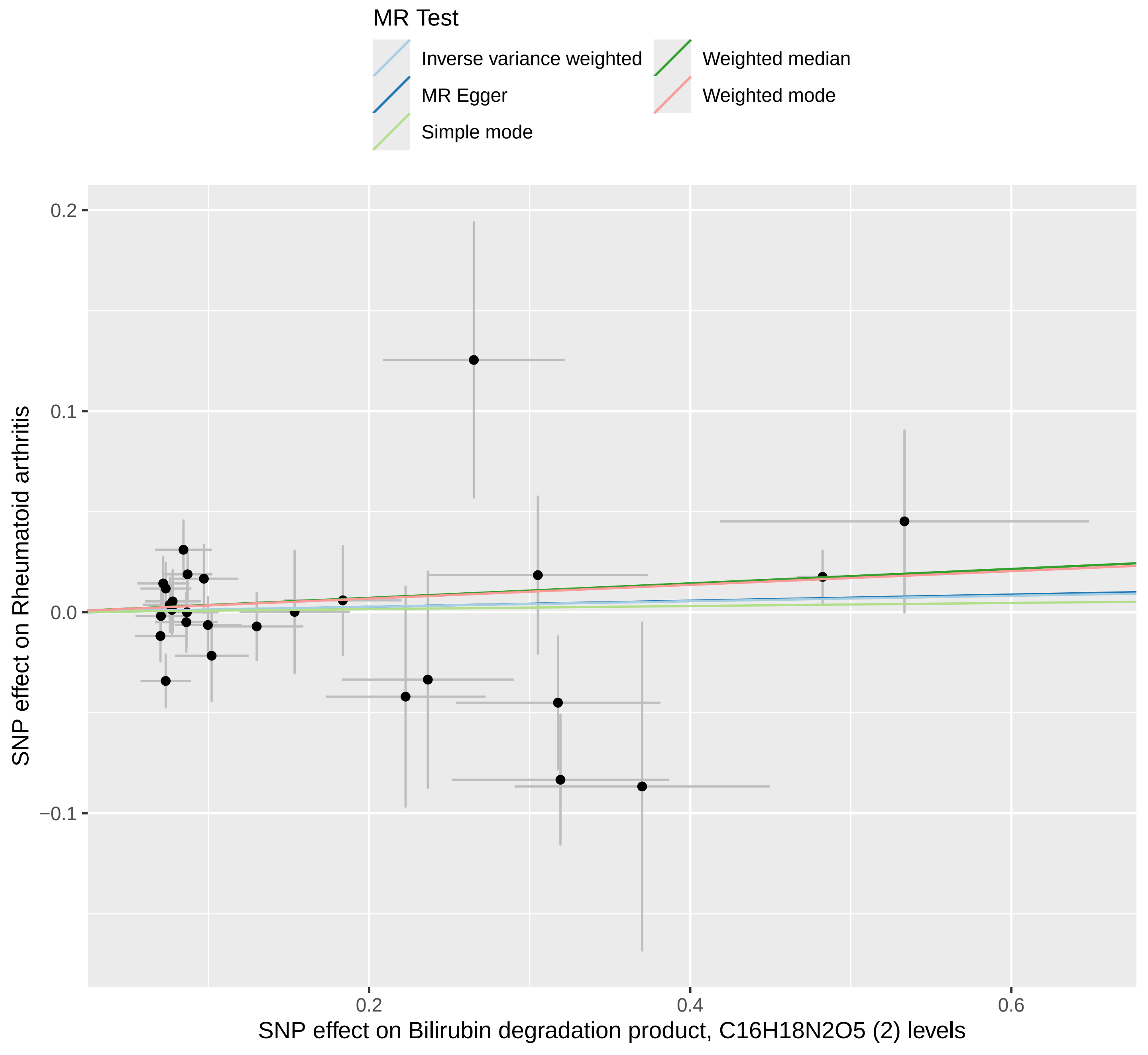

MR Test
Inverse variance weighted
MR Egger
Simple mode
Weighted median
Weighted mode
0.2
0.1
0.0
−0.1
0.2
0.4
0.6
SNP effect on Rheumatoid arthritis
SNP effect on Bilirubin degradation product, C16H18N2O5 (2) levels

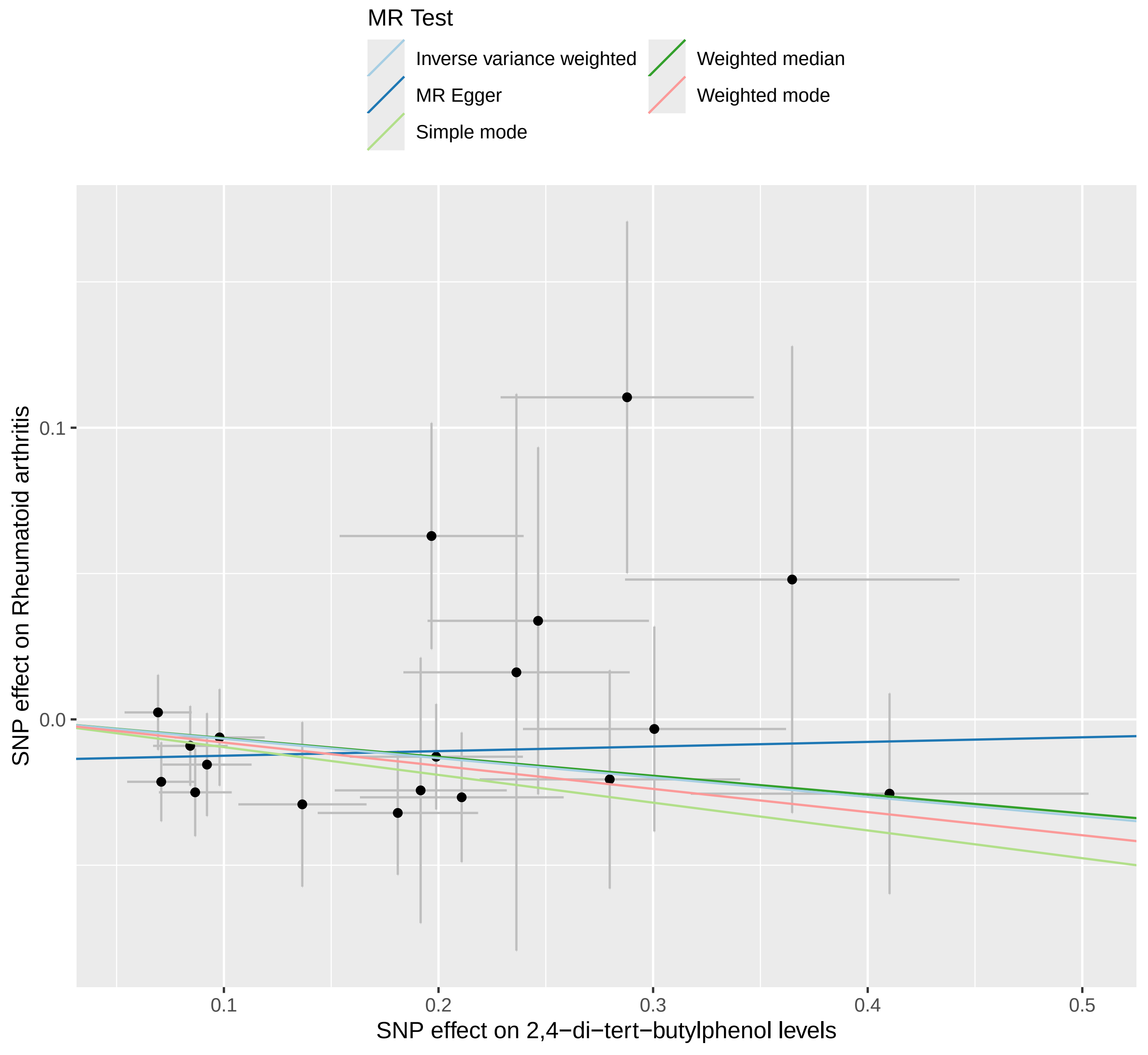

MR Test
Inverse variance weighted
MR Egger
Simple mode
Weighted median
Weighted mode
SNP effect on Rheumatoid arthritis
0.1
0.0
0.1
0.2
0.3
0.4
0.5
SNP effect on 2,4−di−tert−butylphenol levels

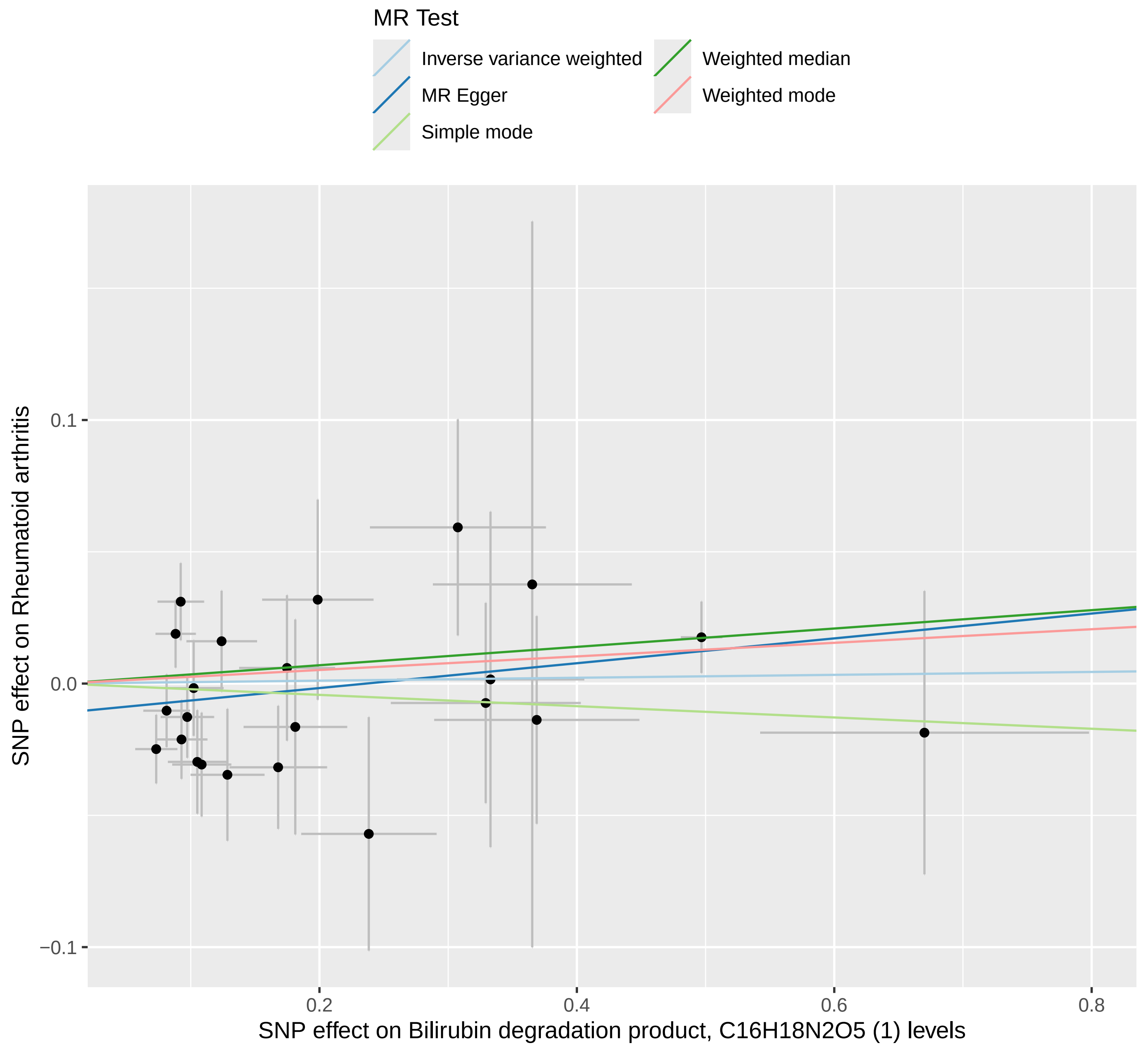

MR Test
Inverse variance weighted
MR Egger
Simple mode
Weighted median
Weighted mode
0.1
0.0
−0.1
0.2
0.4
0.6
0.8
SNP effect on Rheumatoid arthritis
SNP effect on Bilirubin degradation product, C16H18N2O5 (1) levels

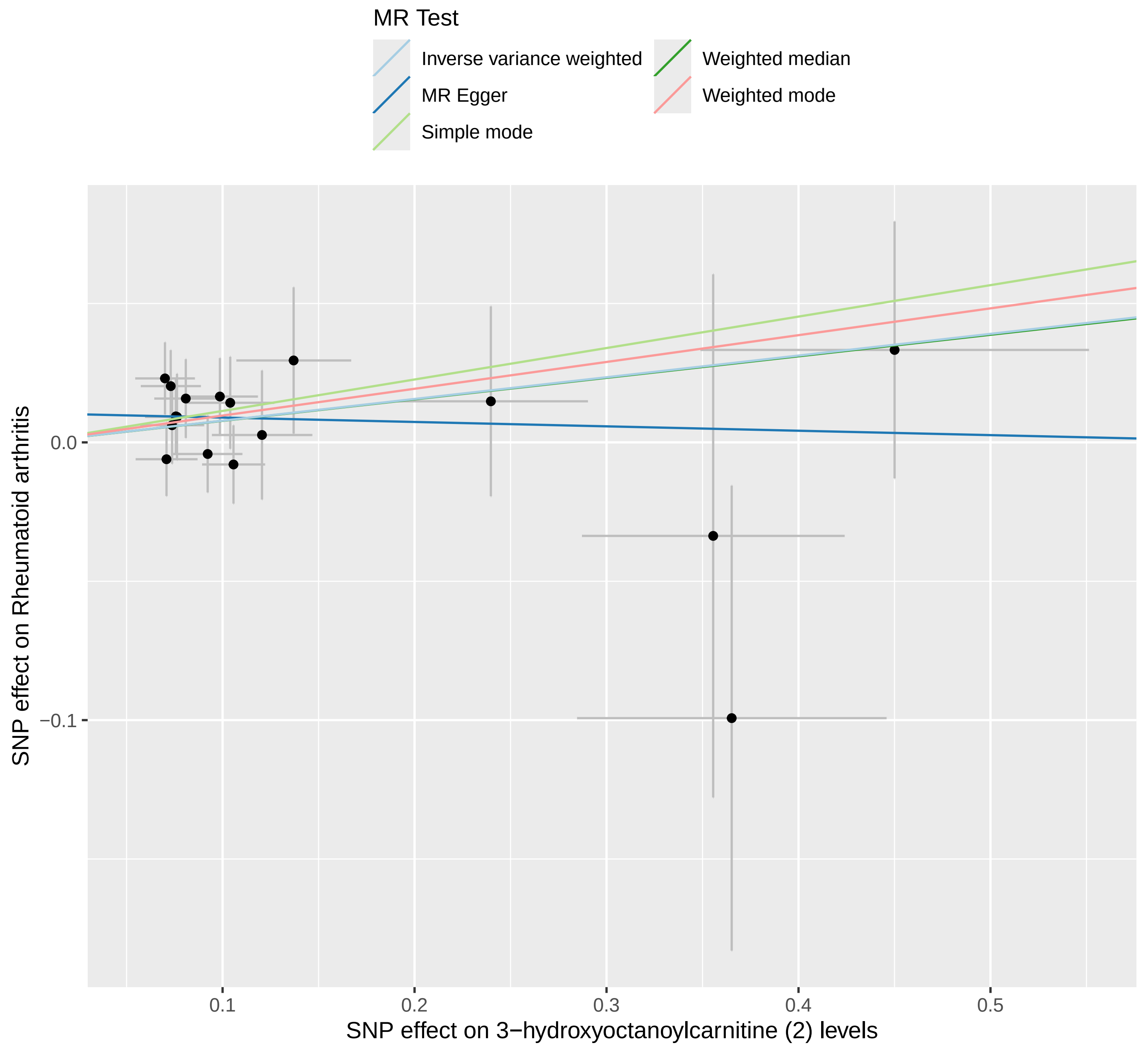
MR Test
Inverse variance weighted
MR Egger
Simple mode
Weighted median
Weighted mode
SNP effect on Rheumatoid arthritis
0.0
−0.1
0.1
0.2
0.3
0.4
0.5
SNP effect on 3−hydroxyoctanoylcarnitine (2) levels

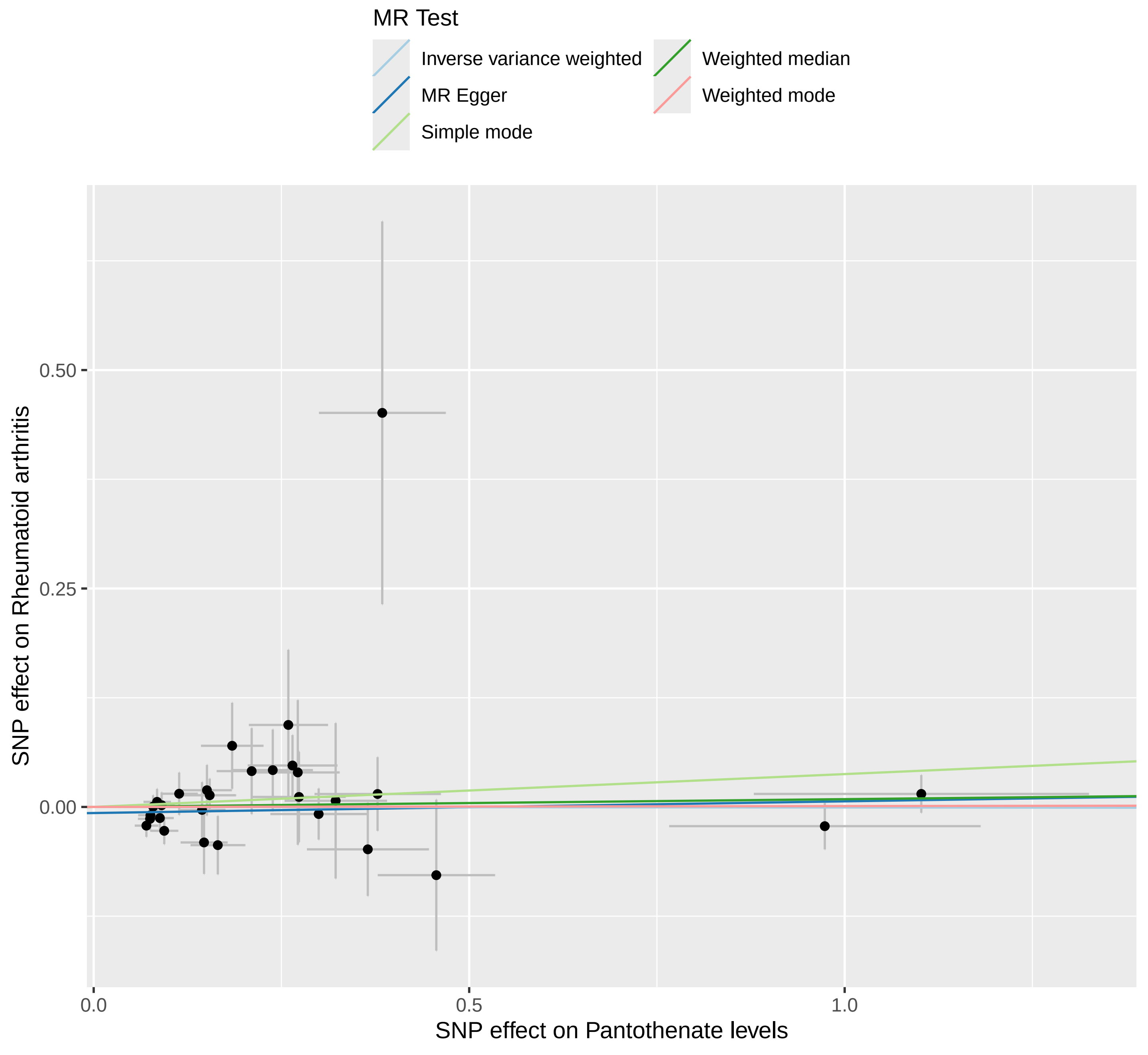

MR Test
Inverse variance weighted
MR Egger
Simple mode
Weighted median
Weighted mode
0.50
0.25
0.00
SNP effect on Rheumatoid arthritis
0.0
0.5
1.0
SNP effect on Pantothenate levels

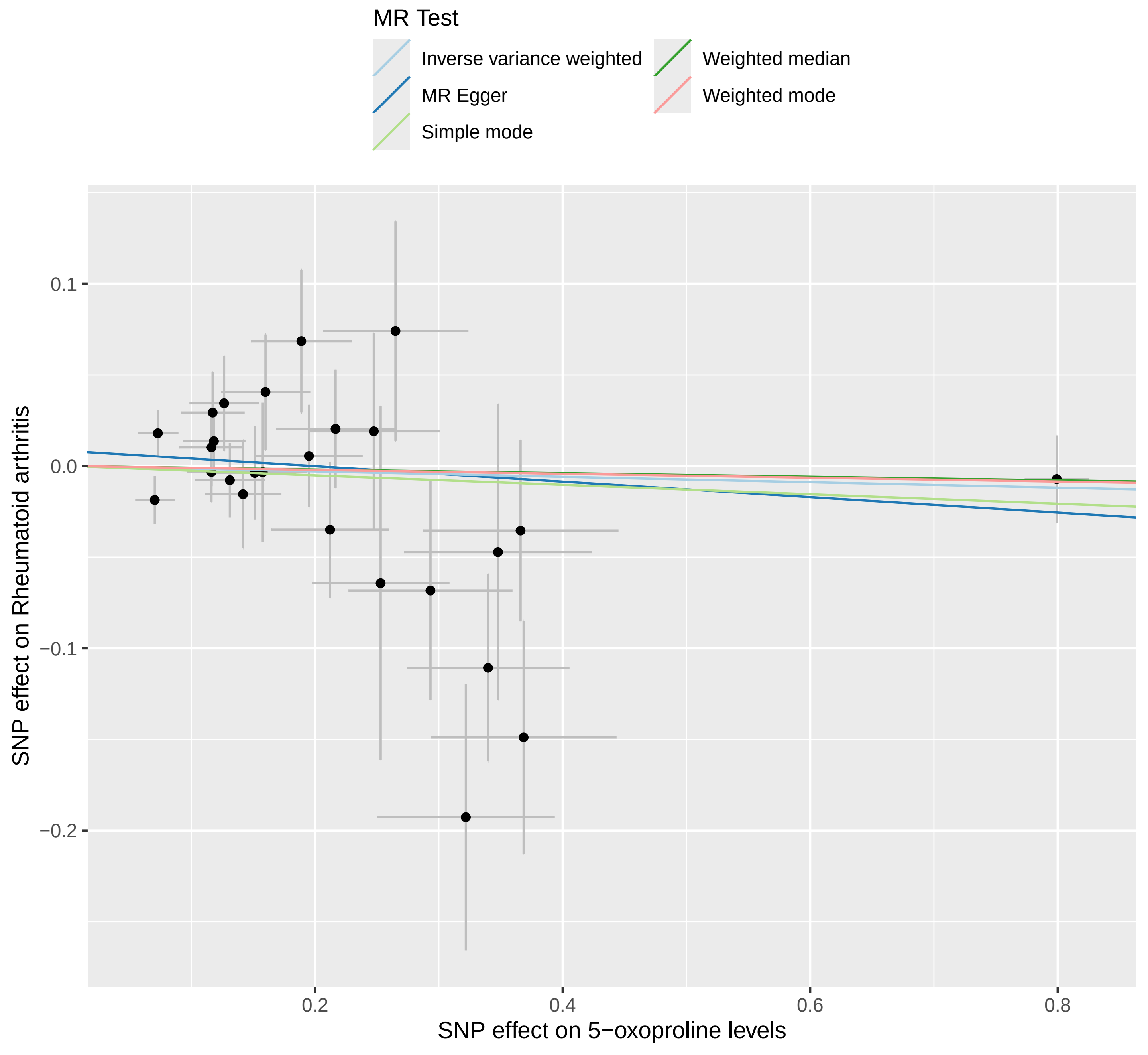
MR Test
Inverse variance weighted
MR Egger
Simple mode
Weighted median
Weighted mode
0.1
0.0
−0.1
−0.2
SNP effect on Rheumatoid arthritis
0.2
0.4
0.6
0.8
SNP effect on 5−oxoproline levels

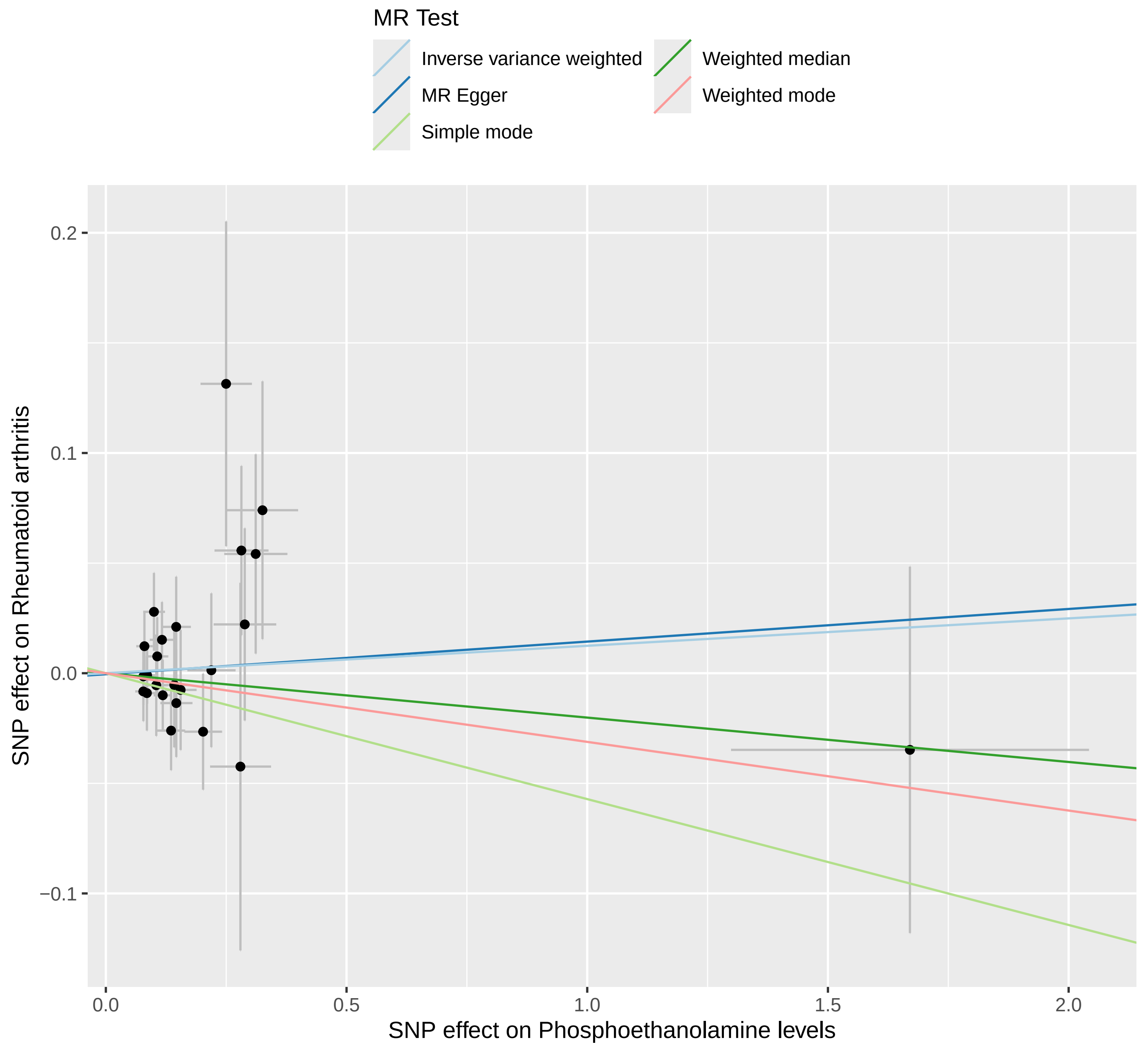

MR Test
Inverse variance weighted
MR Egger
Simple mode
Weighted median
Weighted mode
0.2
0.1
0.0
−0.1
0.0
0.5
1.0
1.5
2.0
SNP effect on Rheumatoid arthritis
SNP effect on Phosphoethanolamine levels

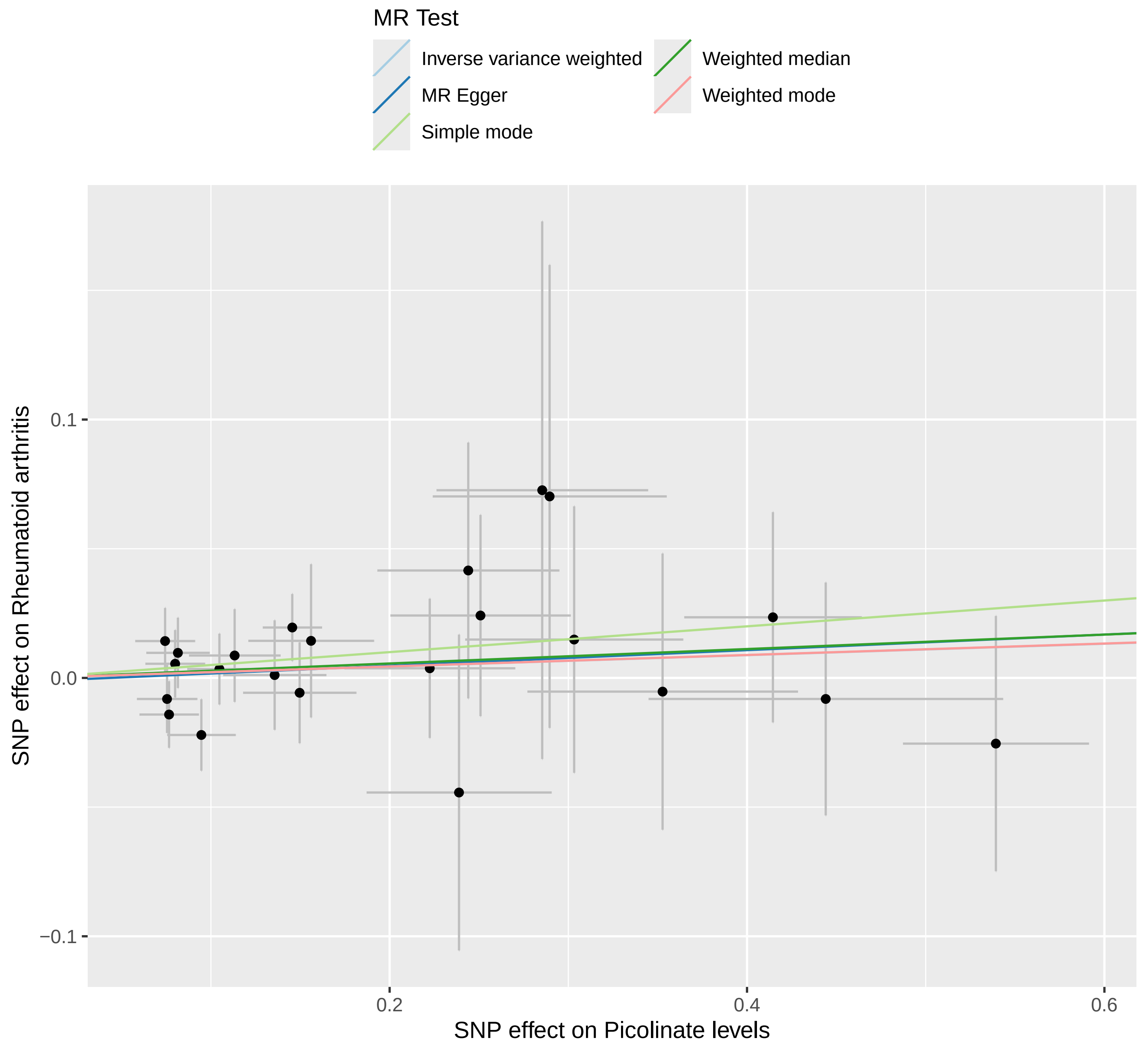

MR Test
Inverse variance weighted
MR Egger
Simple mode
Weighted median
Weighted mode
SNP effect on Rheumatoid arthritis
0.1
0.0
−0.1
0.2
0.4
0.6
SNP effect on Picolinate levels

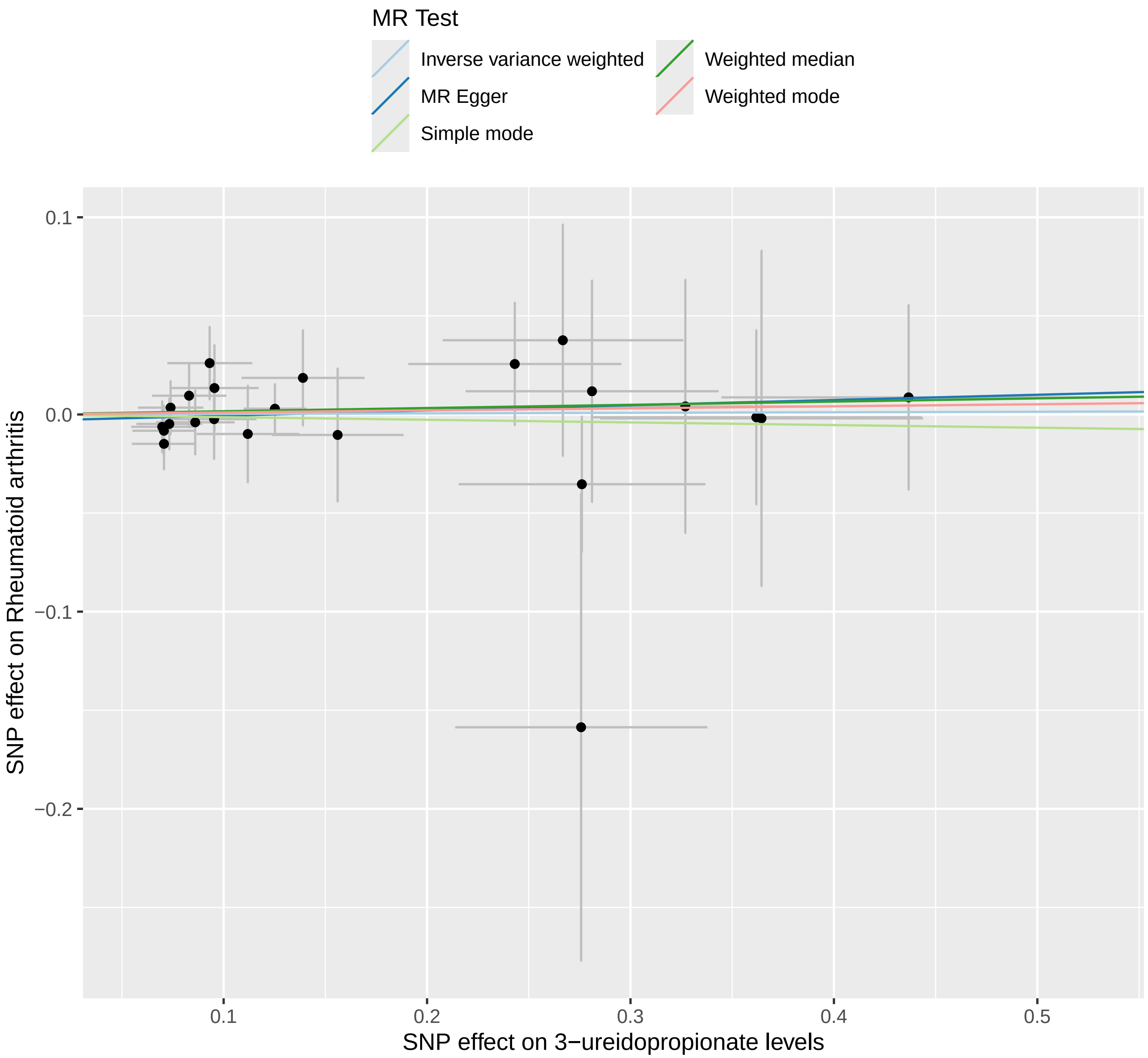
MR Test
Inverse variance weighted
MR Egger
Simple mode
Weighted median
Weighted mode
0.1
0.0
−0.1
−0.2
0.1
0.2
0.3
0.4
0.5
SNP effect on Rheumatoid arthritis
SNP effect on 3−ureidopropionate levels

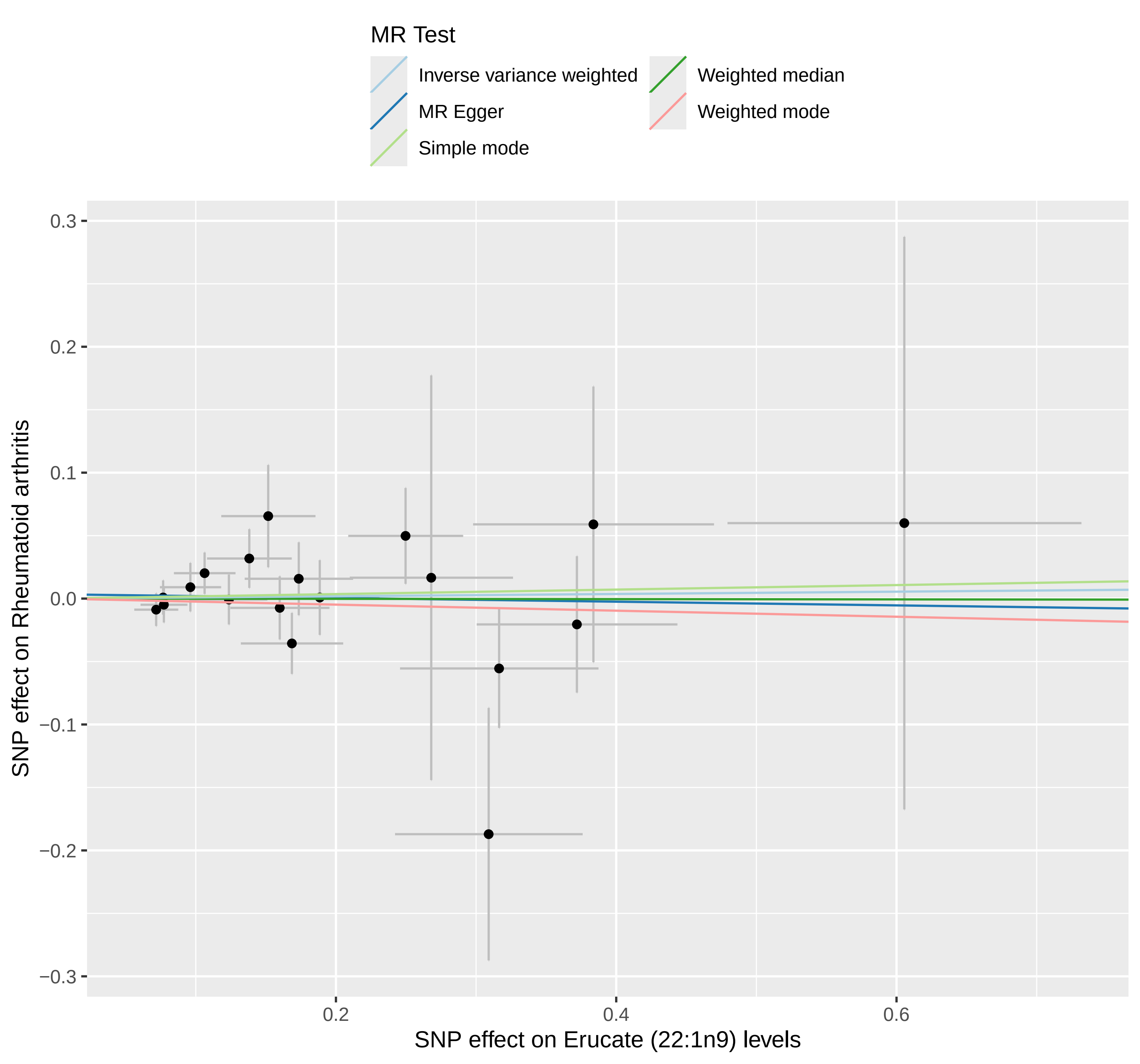

MR Test
Inverse variance weighted
MR Egger
Simple mode
Weighted median
Weighted mode
0.3
0.2
0.1
0.0
−0.1
−0.2
−0.3
0.2
0.4
0.6
SNP effect on Rheumatoid arthritis
SNP effect on Erucate (22:1n9) levels

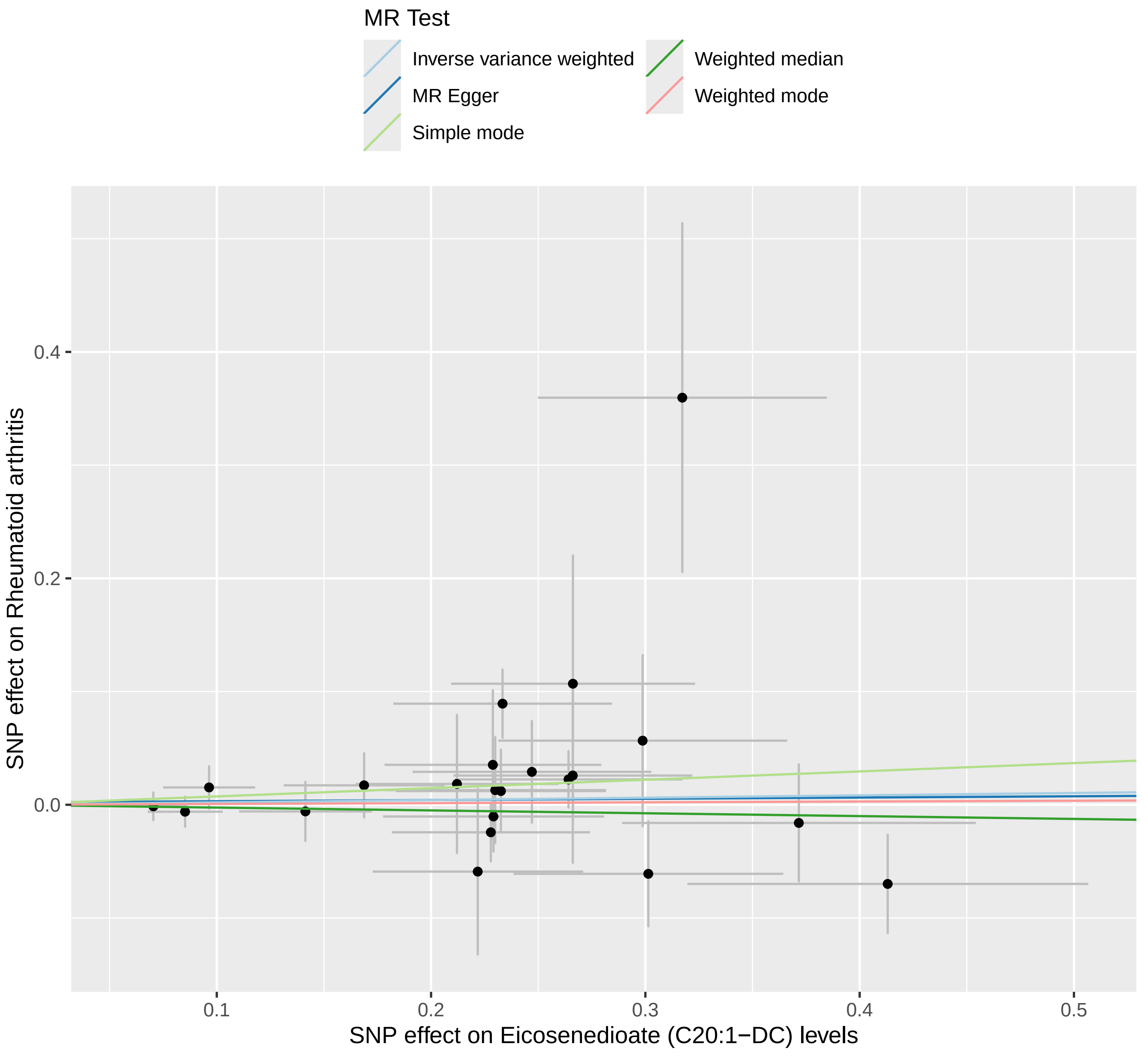
MR Test
Inverse variance weighted
MR Egger
Simple mode
Weighted median
Weighted mode
SNP effect on Rheumatoid arthritis
0.4
0.2
0.0
0.1
0.2
0.3
0.4
0.5
SNP effect on Eicosenedioate (C20:1−DC) levels

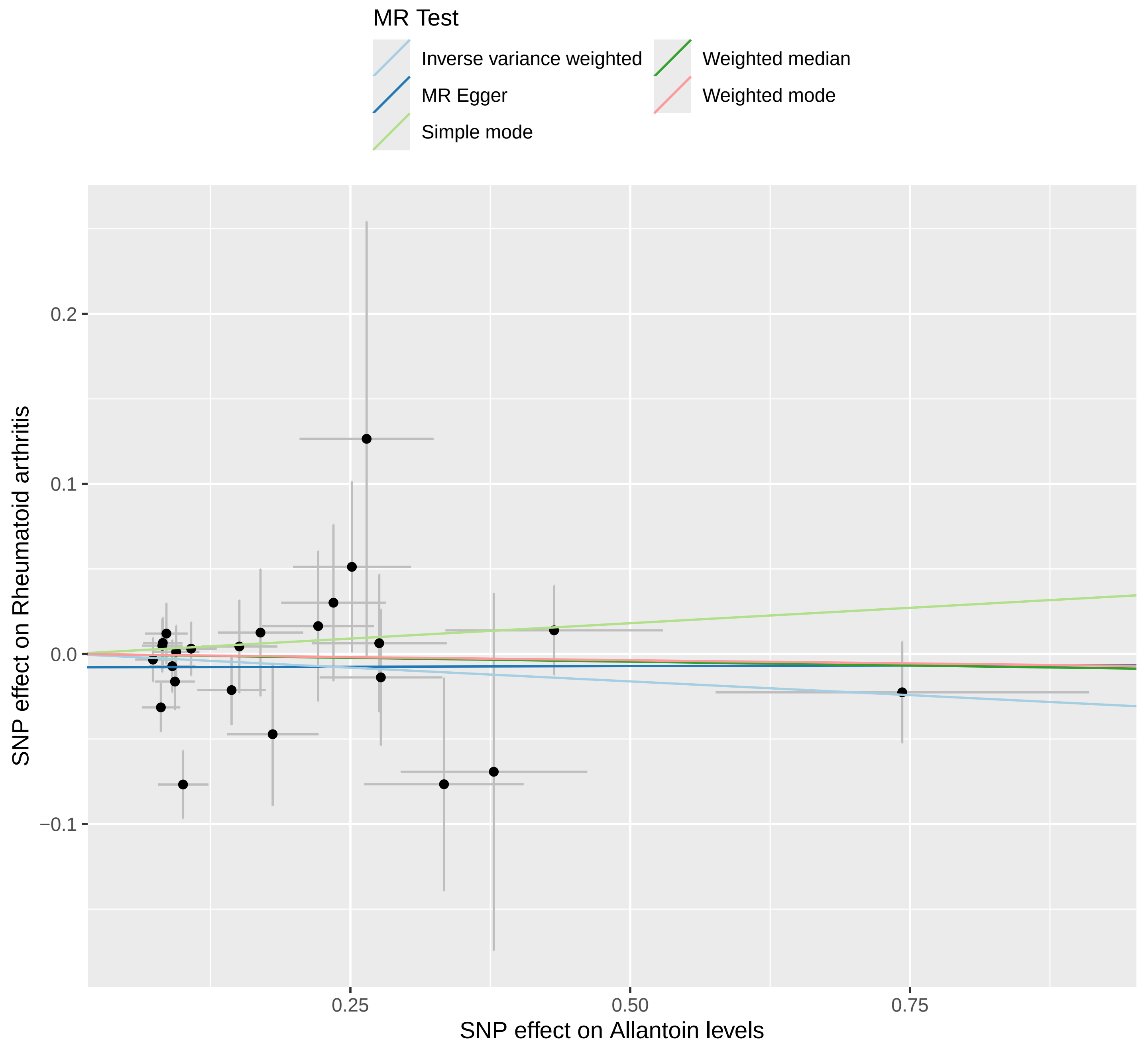
MR Test
Inverse variance weighted
Weighted median
MR Egger
Weighted mode
Simple mode
SNP effect on Rheumatoid arthritis
0.2
0.1
0.0
−0.1
0.25
0.50
0.75
SNP effect on Allantoin levels

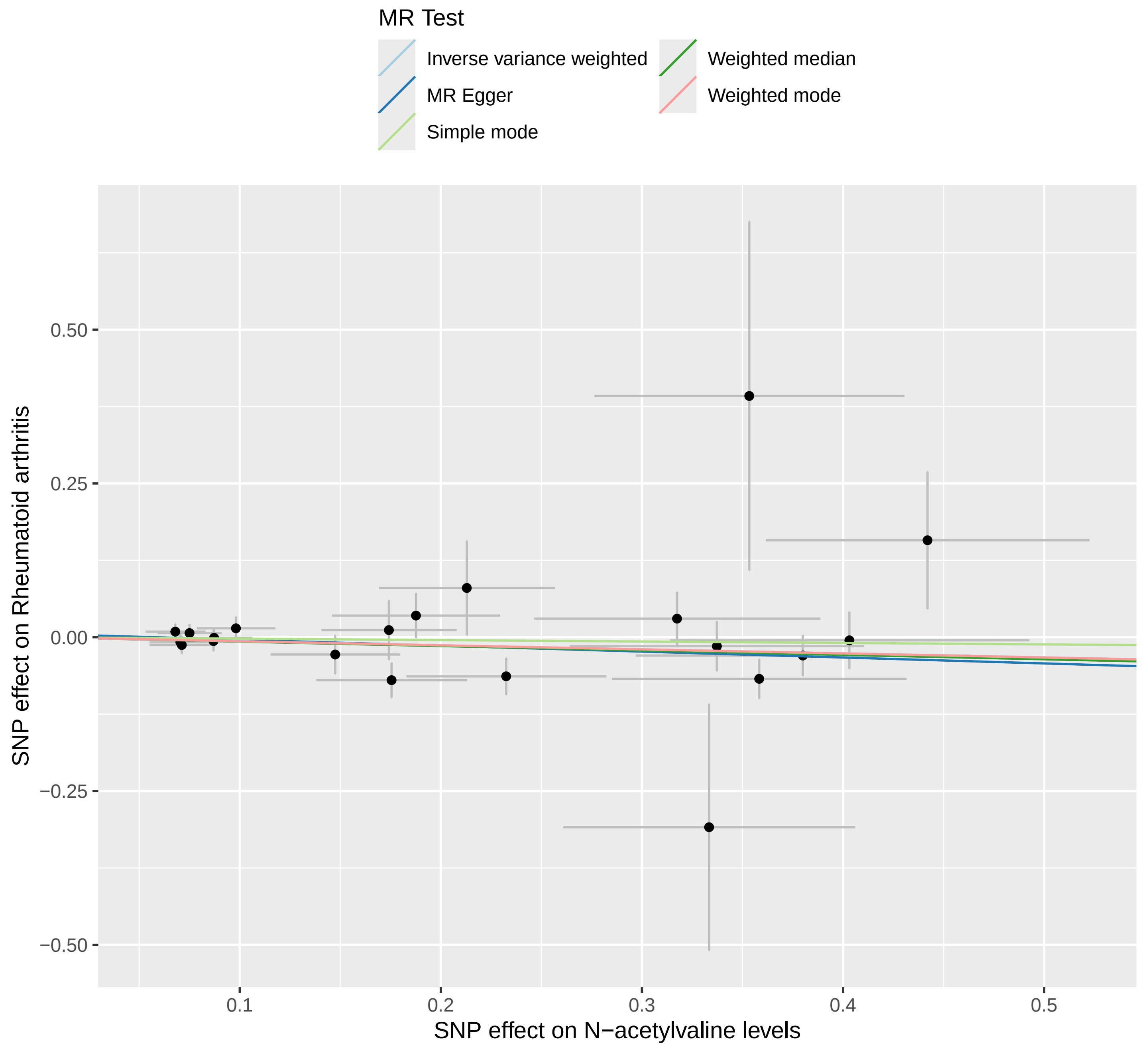

MR Test
Inverse variance weighted
MR Egger
Simple mode
Weighted median
Weighted mode
SNP effect on Rheumatoid arthritis
0.50
0.25
0.00
−0.25
−0.50
0.1
0.2
0.3
0.4
0.5
SNP effect on N−acetylvaline levels

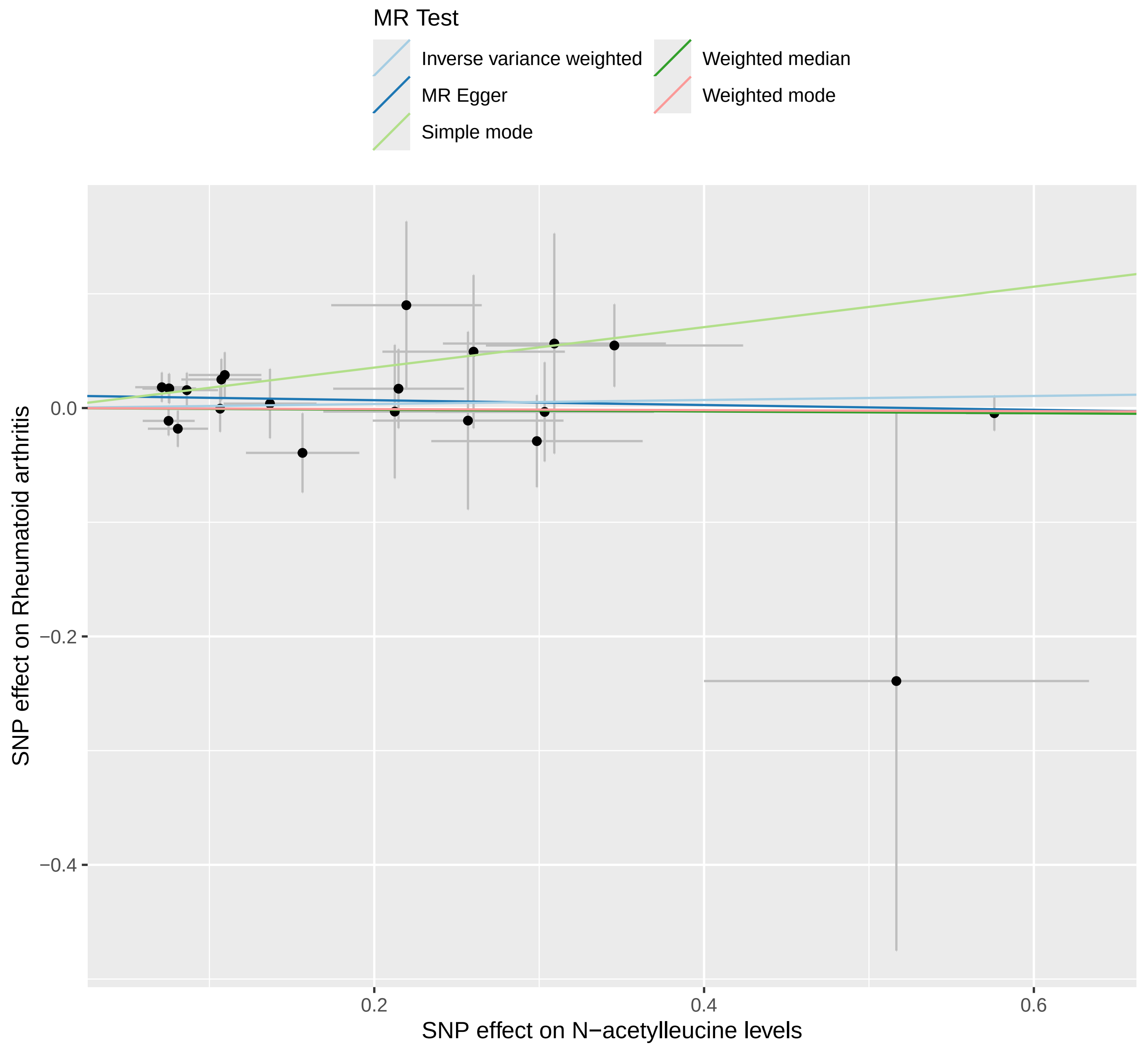
MR Test
Inverse variance weighted
MR Egger
Simple mode
Weighted median
Weighted mode
SNP effect on Rheumatoid arthritis
0.0
−0.2
−0.4
0.2
0.4
0.6
SNP effect on N−acetylleucine levels

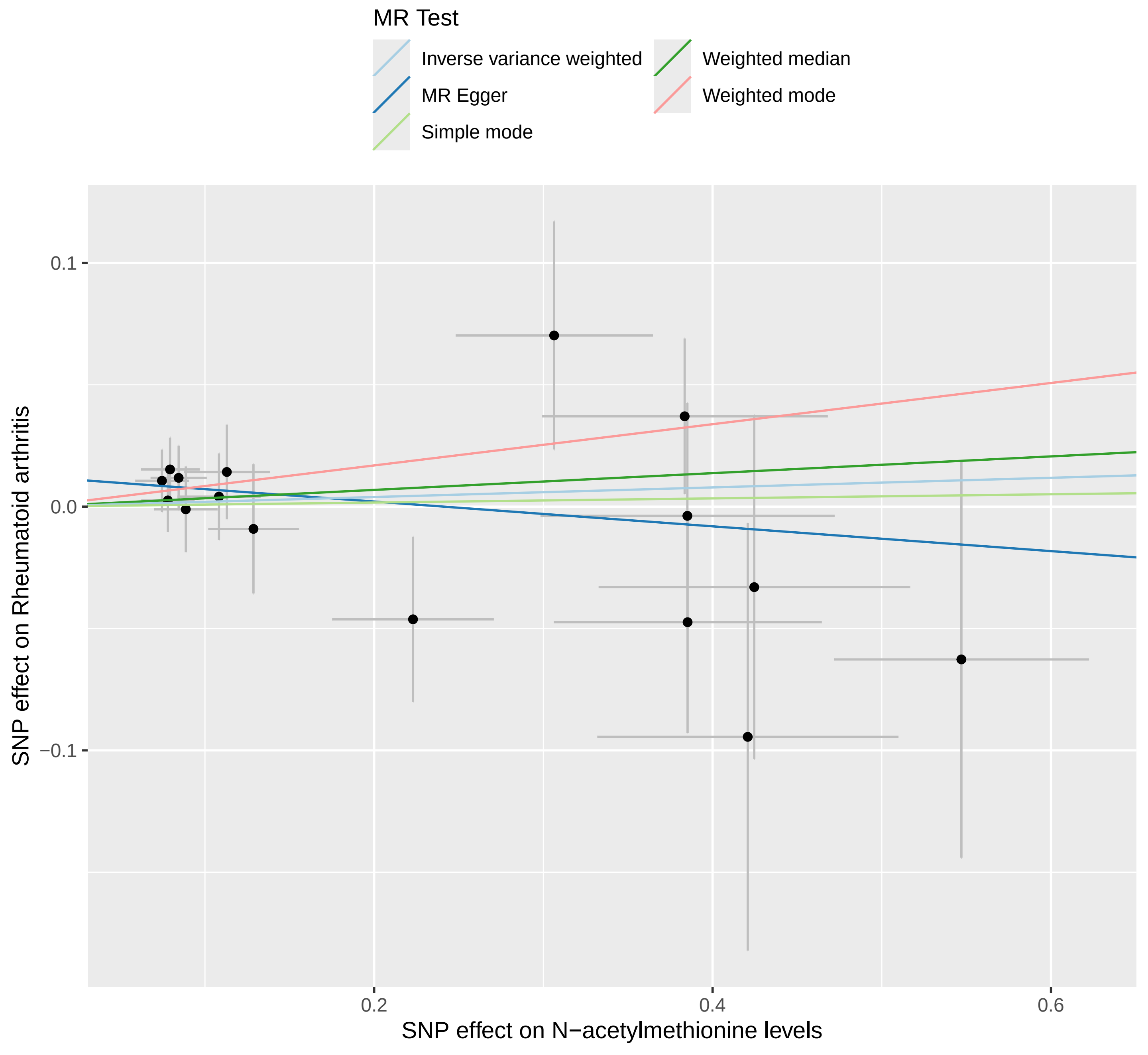

MR Test
Inverse variance weighted
MR Egger
Simple mode
Weighted median
Weighted mode
0.1
0.0
−0.1
0.2
0.4
0.6
SNP effect on Rheumatoid arthritis
SNP effect on N−acetylmethionine levels

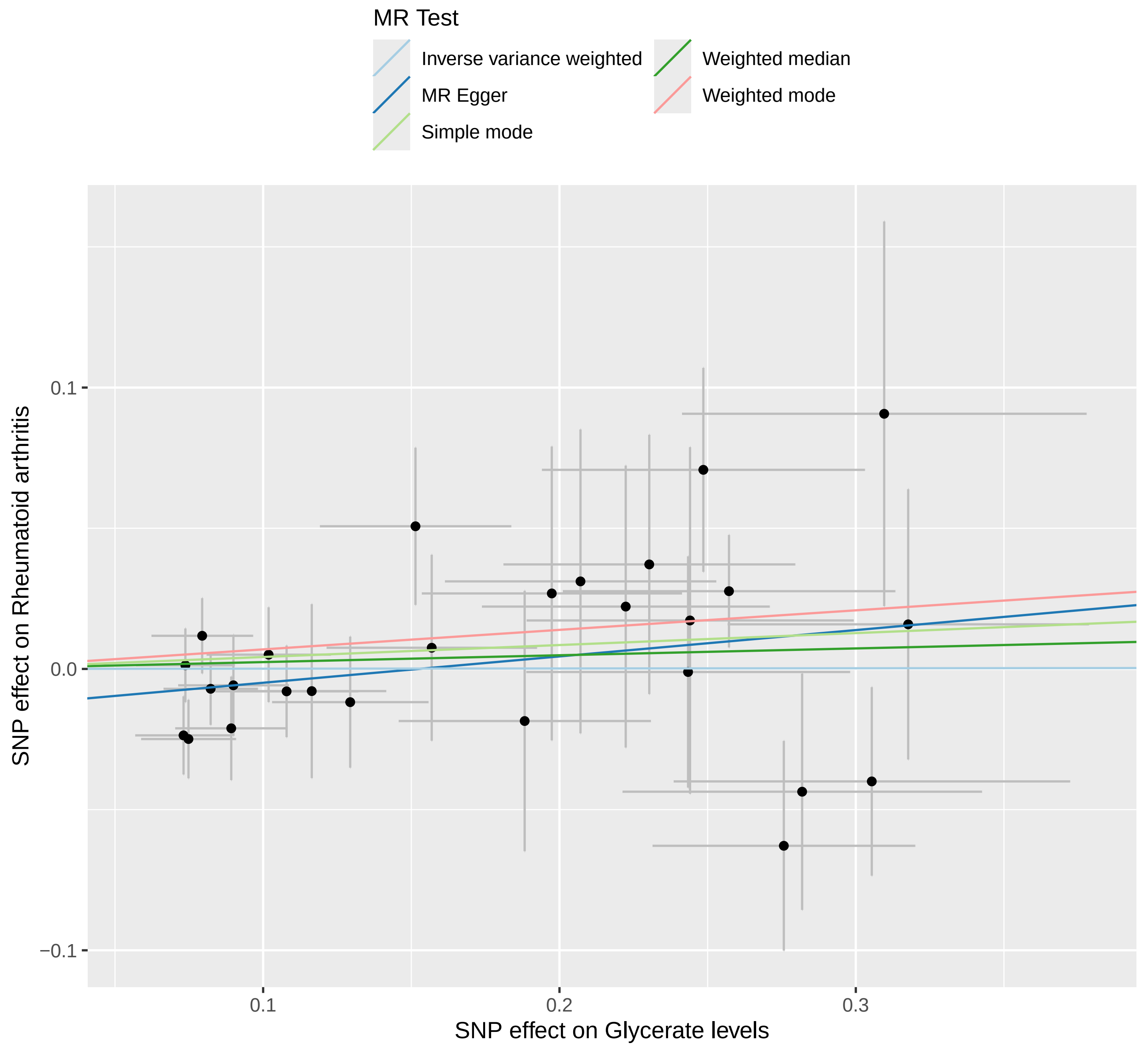

MR Test
Inverse variance weighted
MR Egger
Simple mode
Weighted median
Weighted mode
0.1
0.0
−0.1
SNP effect on Rheumatoid arthritis
0.1
0.2
0.3
SNP effect on Glycerate levels

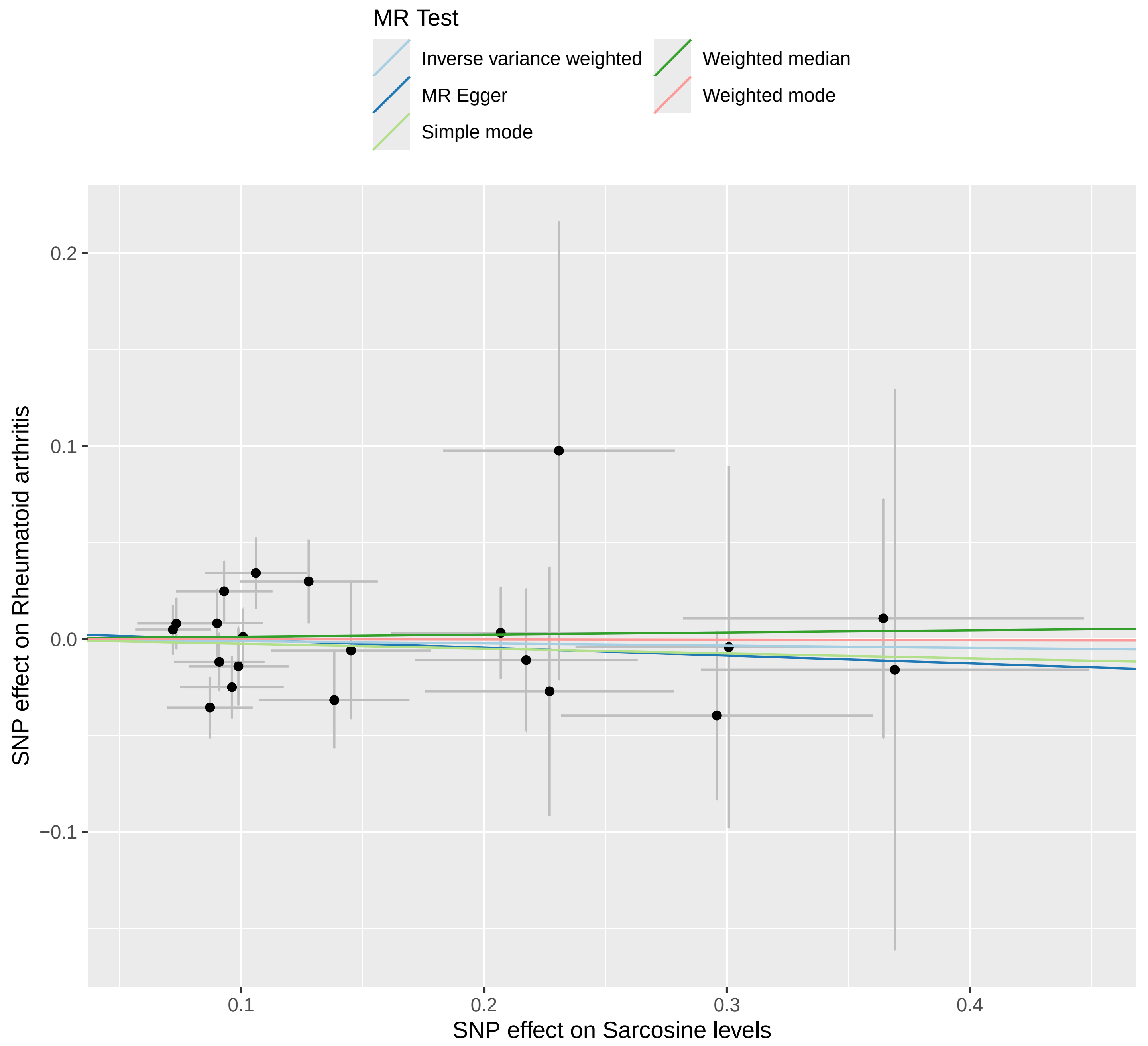

MR Test
Inverse variance weighted
MR Egger
Simple mode
Weighted median
Weighted mode
0.2
0.1
0.0
−0.1
SNP effect on Rheumatoid arthritis
0.1
0.2
0.3
0.4
SNP effect on Sarcosine levels

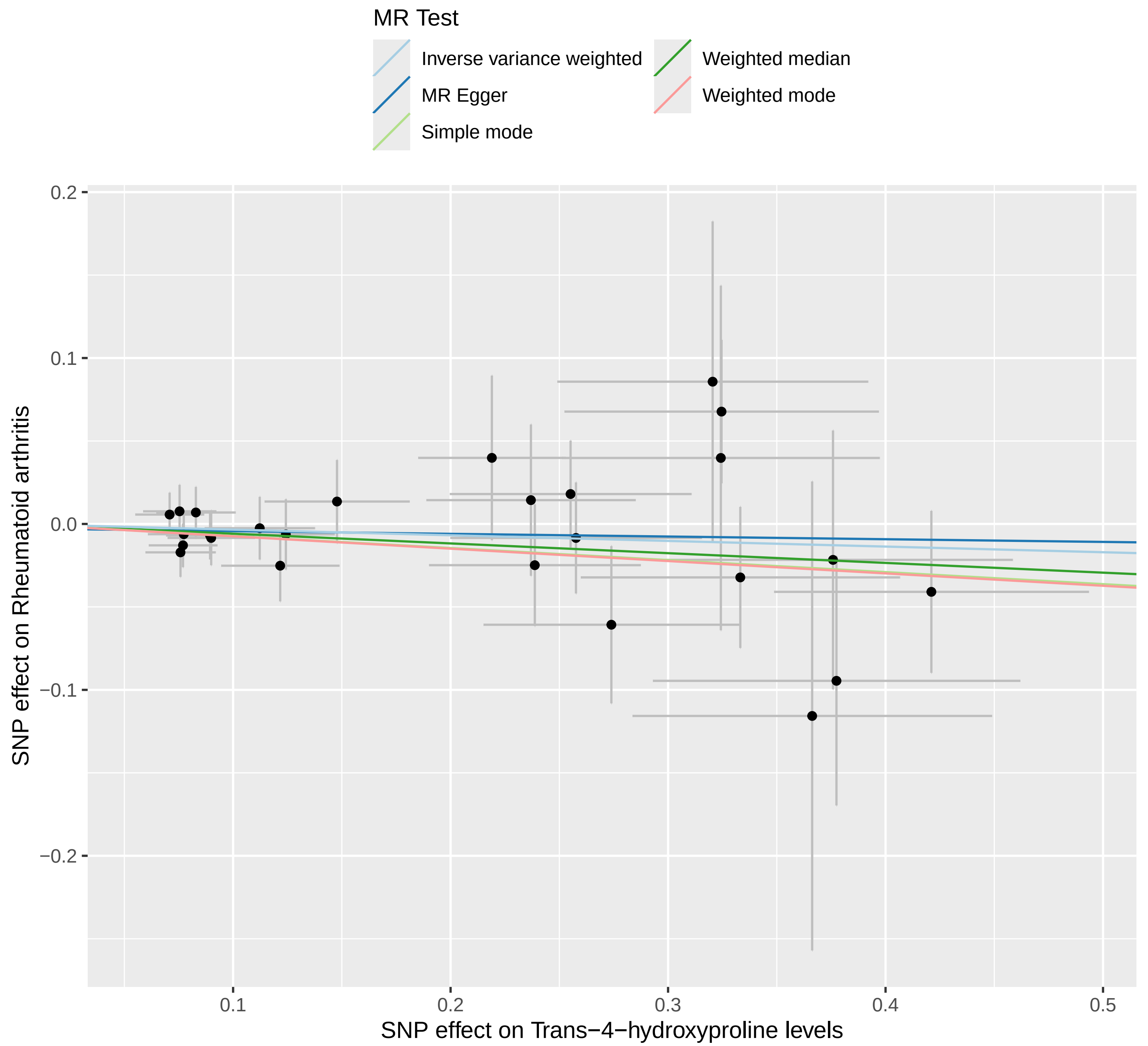
MR Test
Inverse variance weighted
MR Egger
Simple mode
Weighted median
Weighted mode
0.2
0.1
0.0
−0.1
−0.2
0.1
0.2
0.3
0.4
0.5
SNP effect on Rheumatoid arthritis
SNP effect on Trans−4−hydroxyproline levels

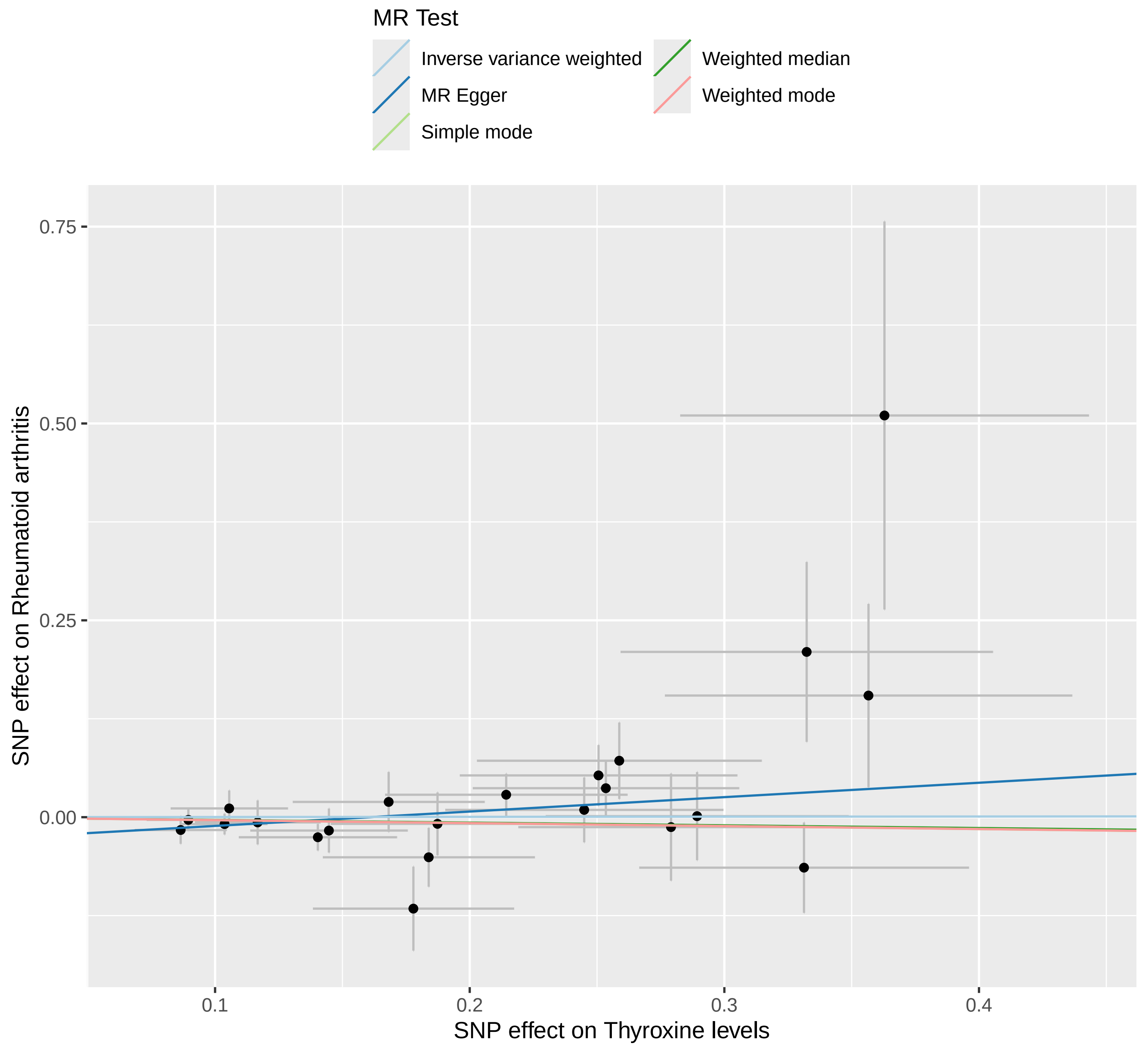

MR Test
Inverse variance weighted
MR Egger
Simple mode
Weighted median
Weighted mode
0.75
0.50
0.25
0.00
0.1
0.2
0.3
0.4
SNP effect on Rheumatoid arthritis
SNP effect on Thyroxine levels

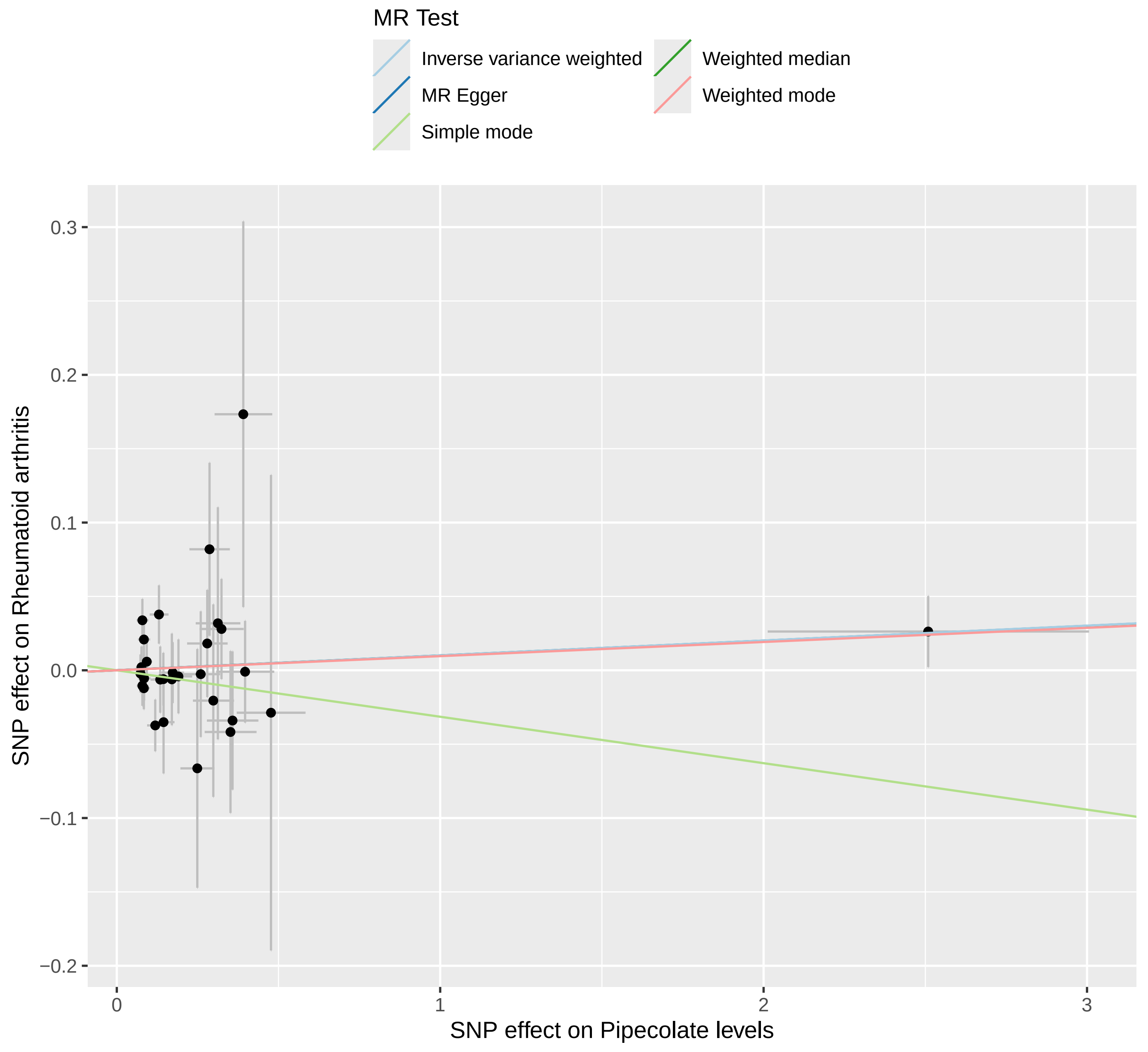
MR Test
Inverse variance weighted
MR Egger
Simple mode
Weighted median
Weighted mode
0.3
0.2
0.1
0.0
−0.1
−0.2
SNP effect on Rheumatoid arthritis
0
1
2
3
SNP effect on Pipecolate levels

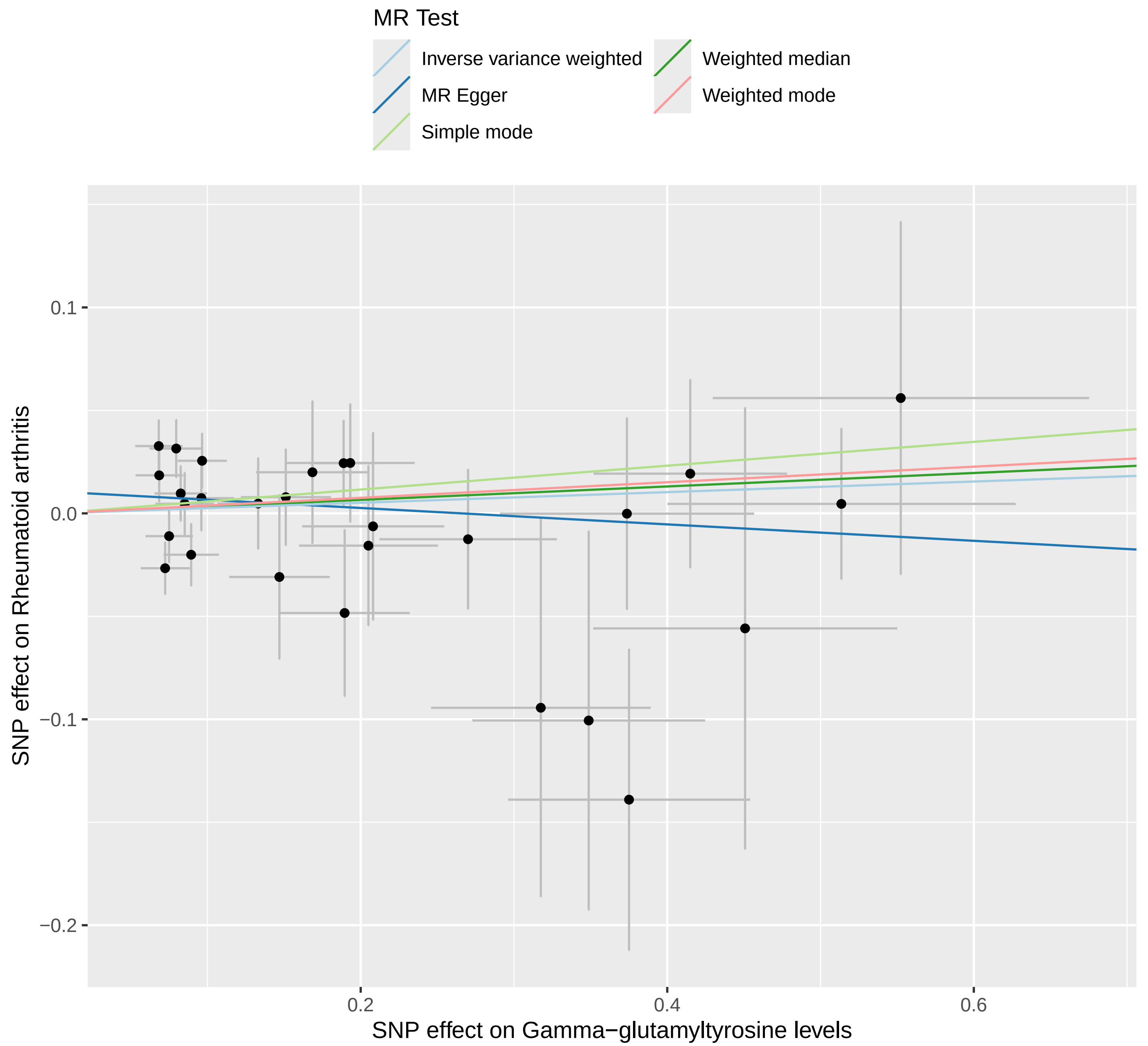
MR Test
Inverse variance weighted
MR Egger
Simple mode
Weighted median
Weighted mode
SNP effect on Rheumatoid arthritis
0.1
0.0
−0.1
−0.2
0.2
0.4
0.6
SNP effect on Gamma−glutamyltyrosine levels

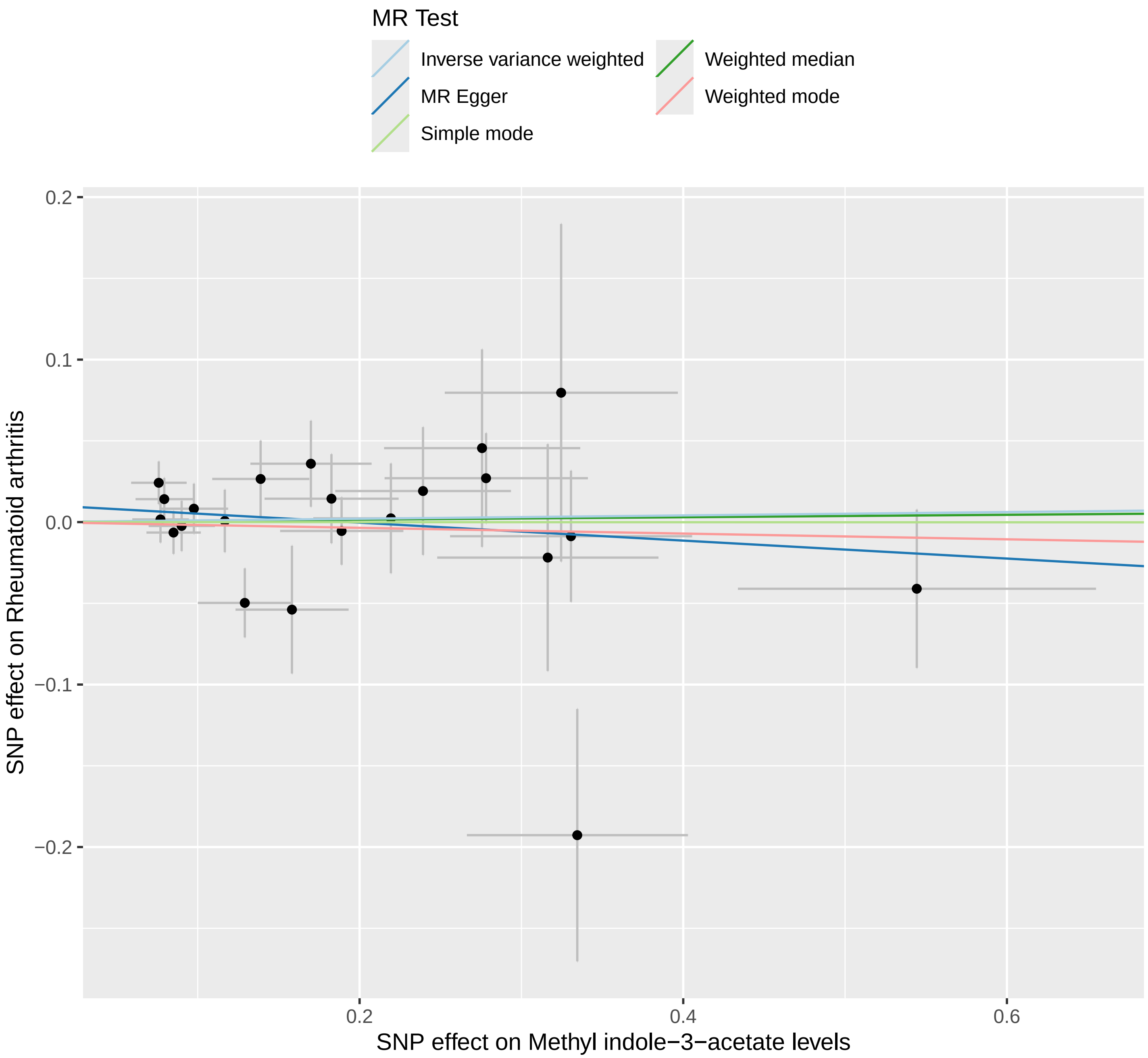
MR Test
Inverse variance weighted
MR Egger
Simple mode
Weighted median
Weighted mode
0.2
0.1
0.0
−0.1
−0.2
SNP effect on Rheumatoid arthritis
0.2
0.4
0.6
SNP effect on Methyl indole−3−acetate levels

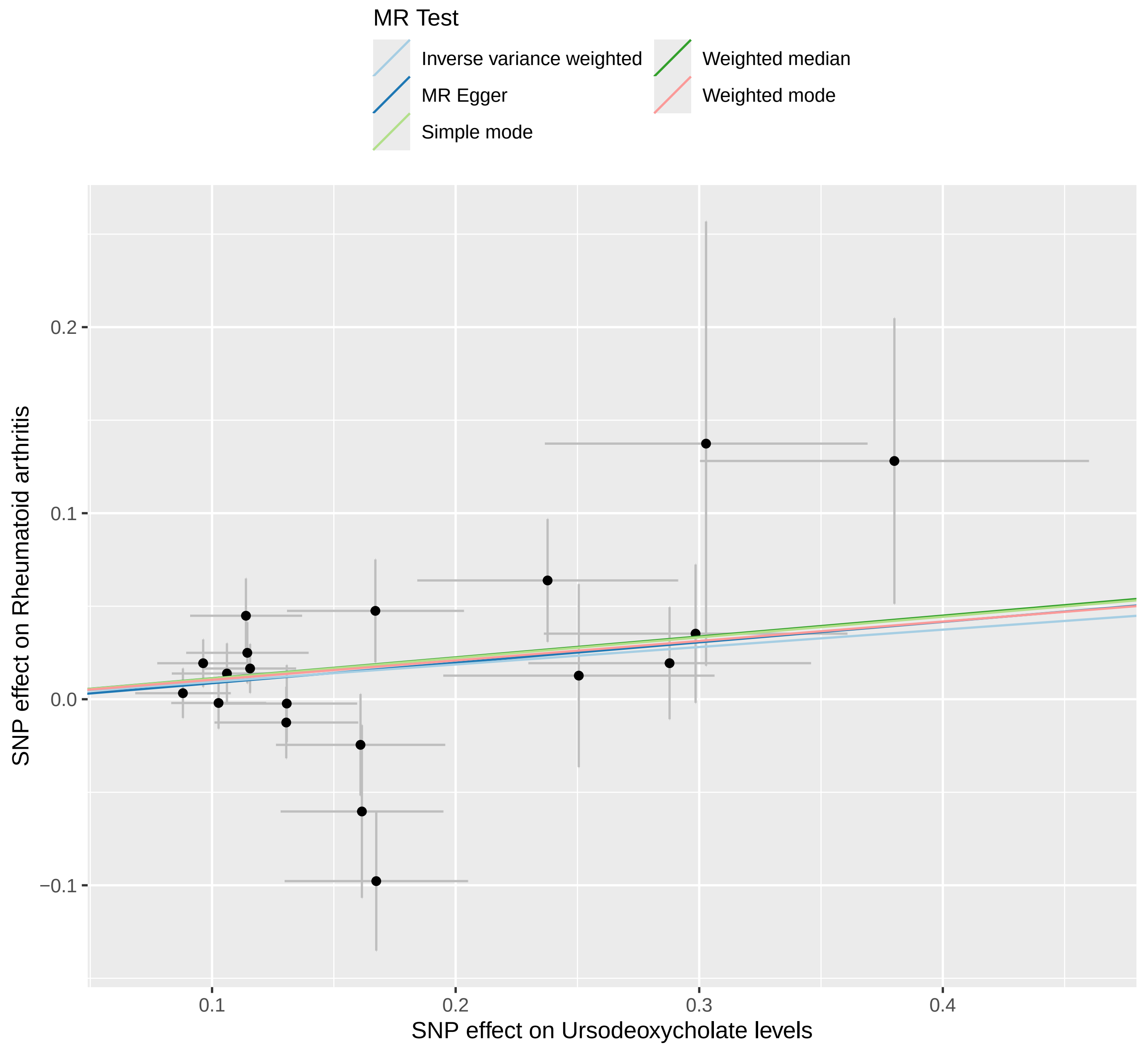

MR Test
Inverse variance weighted
MR Egger
Simple mode
Weighted median
Weighted mode
0.2
0.1
0.0
−0.1
SNP effect on Rheumatoid arthritis
0.1
0.2
0.3
0.4
SNP effect on Ursodeoxycholate levels

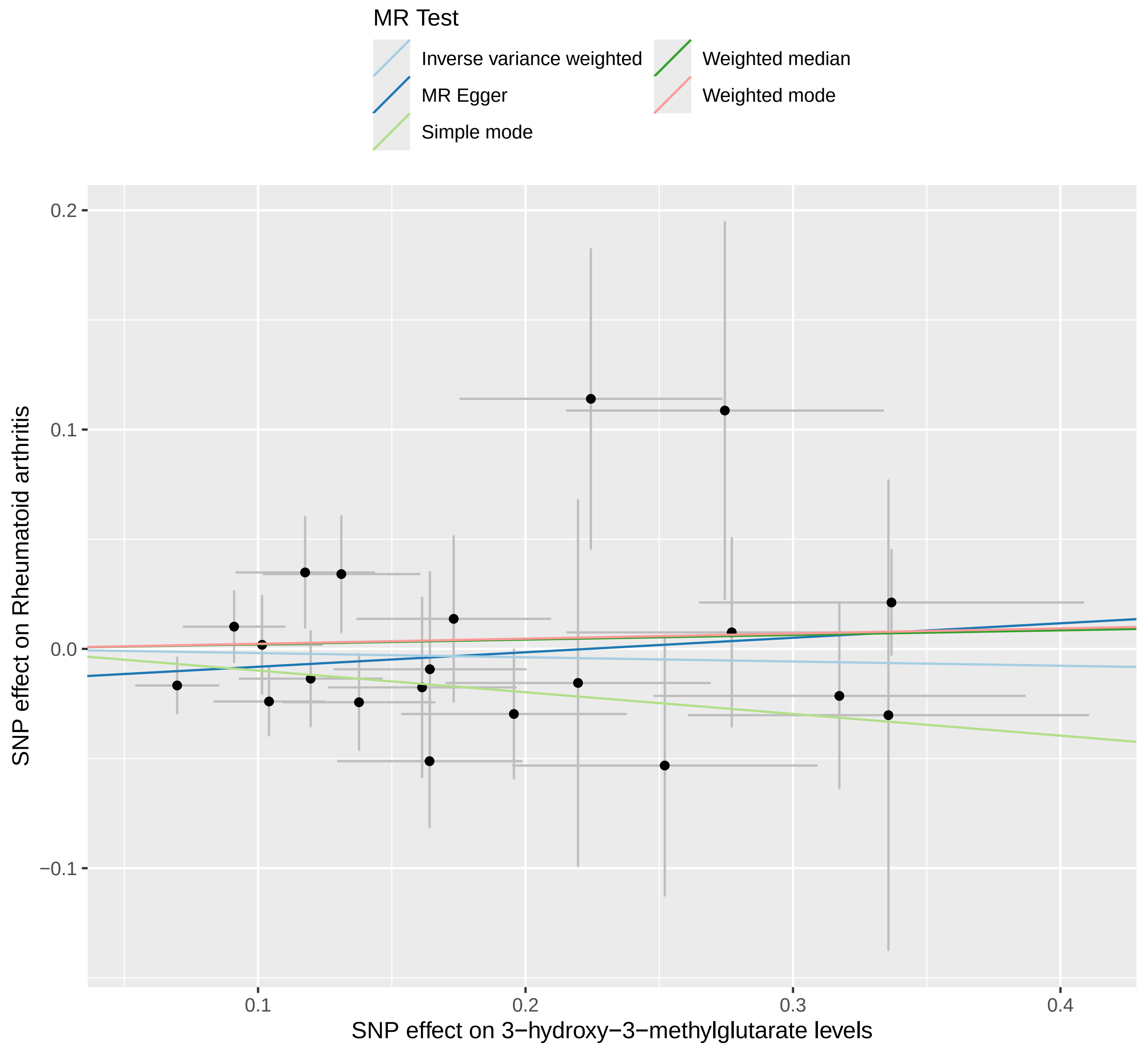
MR Test
Inverse variance weighted
MR Egger
Simple mode
Weighted median
Weighted mode
0.2
0.1
0.0
−0.1
SNP effect on Rheumatoid arthritis
0.1
0.2
0.3
0.4
SNP effect on 3−hydroxy−3−methylglutarate levels

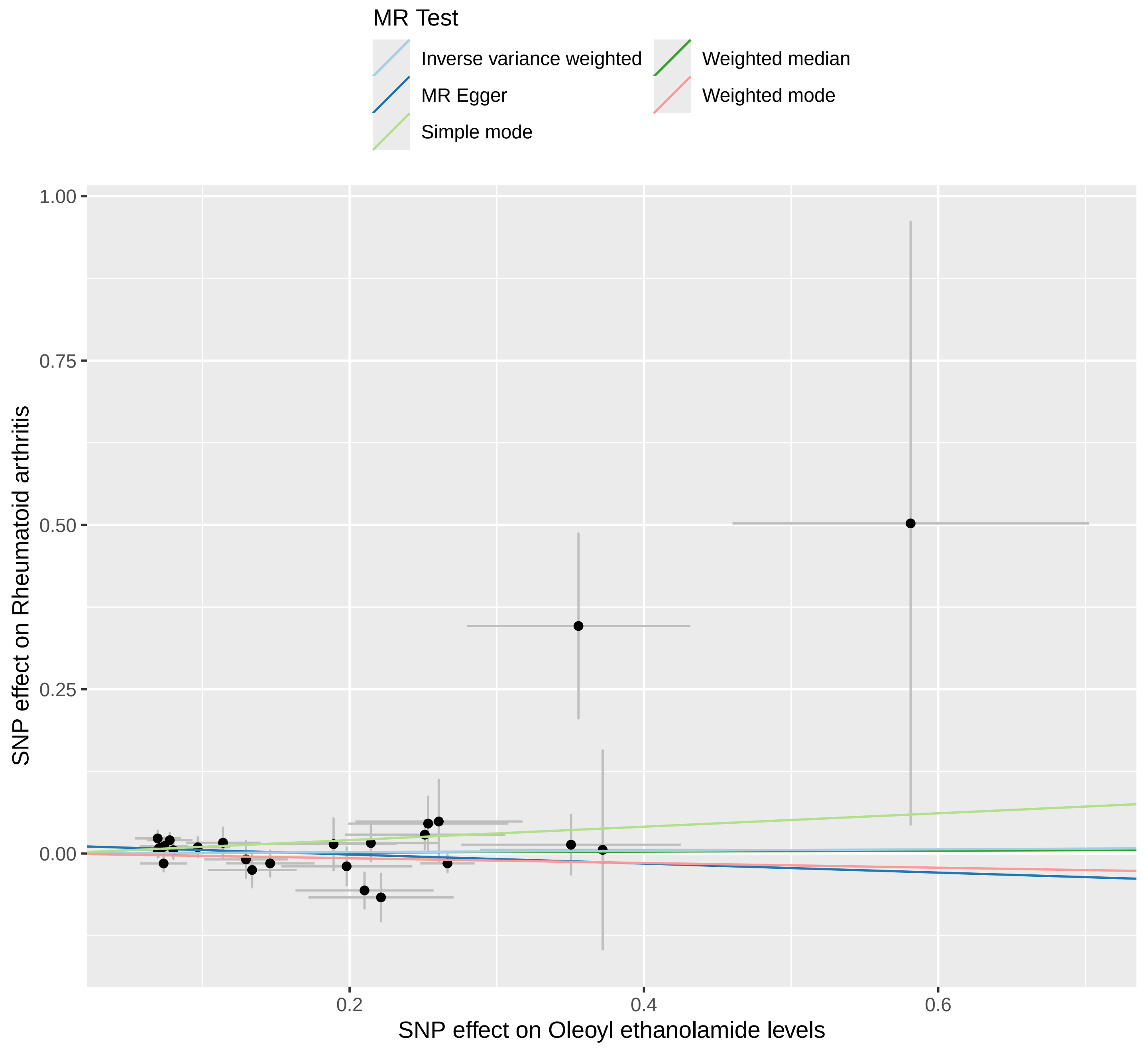
MR Test
Inverse variance weighted
MR Egger
Simple mode
Weighted median
Weighted mode
1.00
0.75
0.50
0.25
0.00
SNP effect on Rheumatoid arthritis
0.2
0.4
0.6
SNP effect on Oleoyl ethanolamide levels

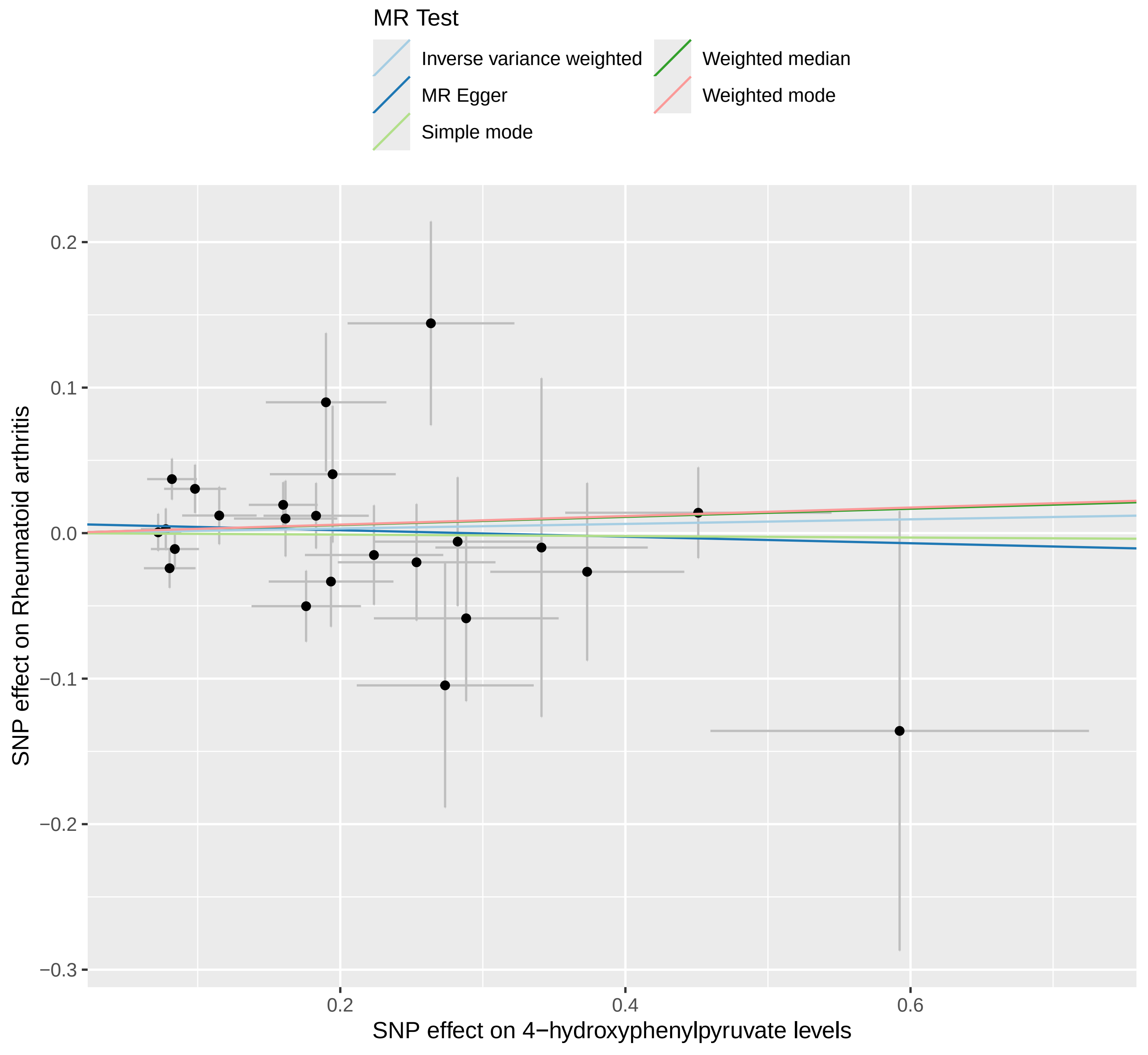
MR Test
Inverse variance weighted
MR Egger
Simple mode
Weighted median
Weighted mode
0.2
0.1
0.0
−0.1
−0.2
−0.3
SNP effect on Rheumatoid arthritis
0.2
0.4
0.6
SNP effect on 4−hydroxyphenylpyruvate levels

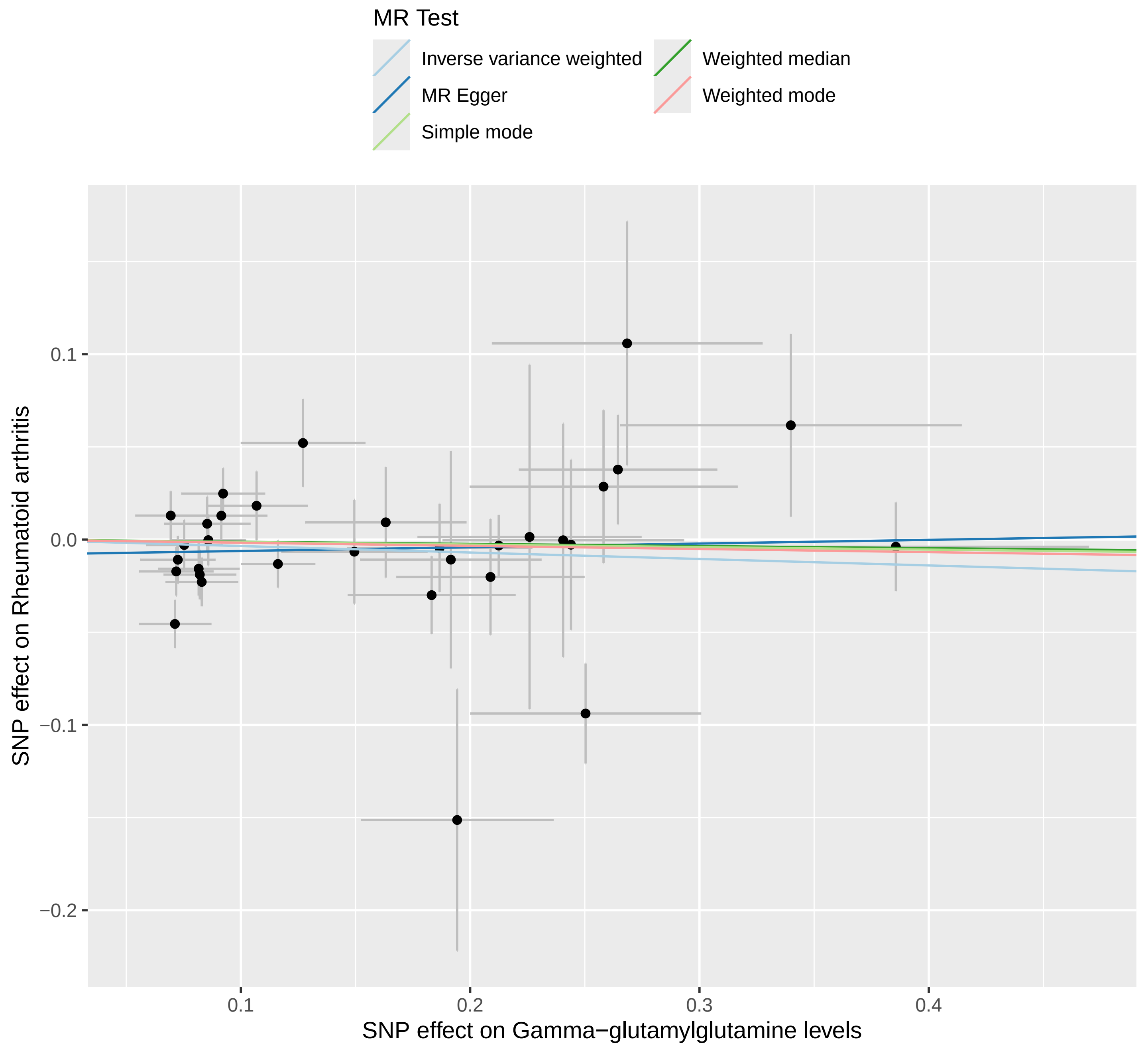
MR Test
Inverse variance weighted
MR Egger
Simple mode
Weighted median
Weighted mode
0.1
0.0
−0.1
−0.2
SNP effect on Rheumatoid arthritis
0.1
0.2
0.3
0.4
SNP effect on Gamma−glutamylglutamine levels

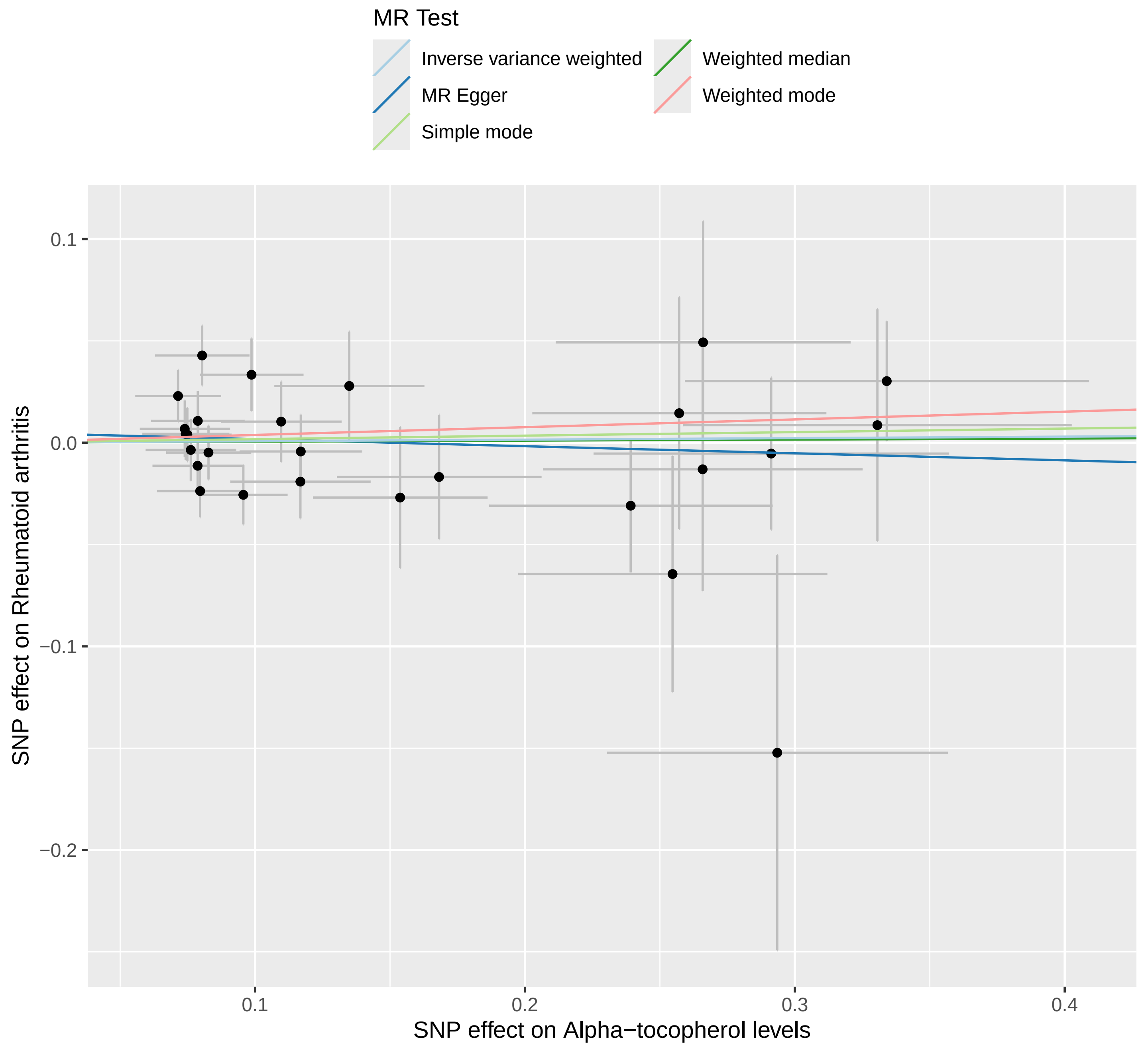
MR Test
Inverse variance weighted
MR Egger
Simple mode
Weighted median
Weighted mode
0.1
0.0
−0.1
−0.2
0.1
0.2
0.3
0.4
SNP effect on Rheumatoid arthritis
SNP effect on Alpha−tocopherol levels

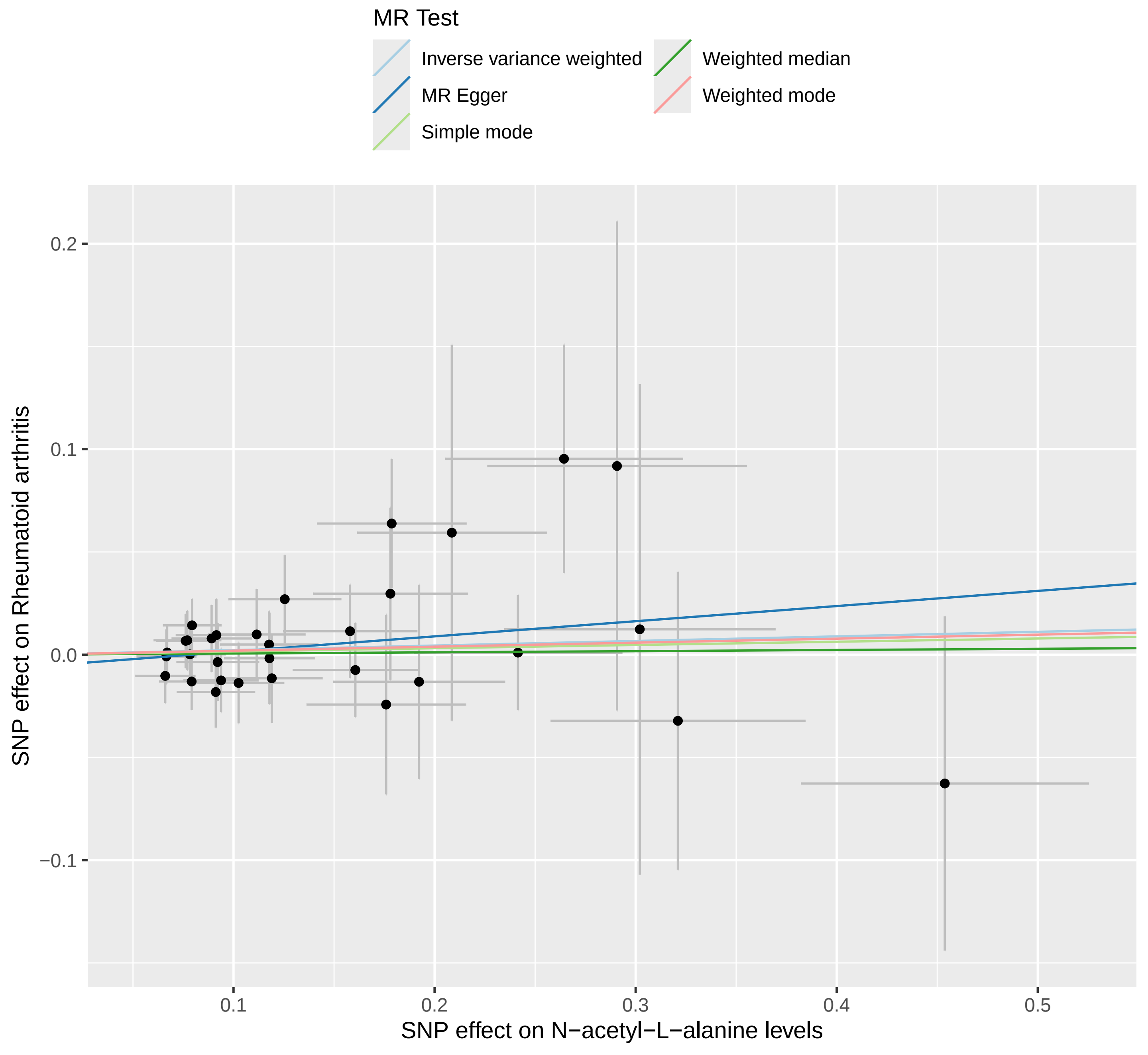

MR Test
Inverse variance weighted
MR Egger
Simple mode
Weighted median
Weighted mode
0.2
0.1
0.0
−0.1
SNP effect on Rheumatoid arthritis
0.1
0.2
0.3
0.4
0.5
SNP effect on N−acetyl−L−alanine levels

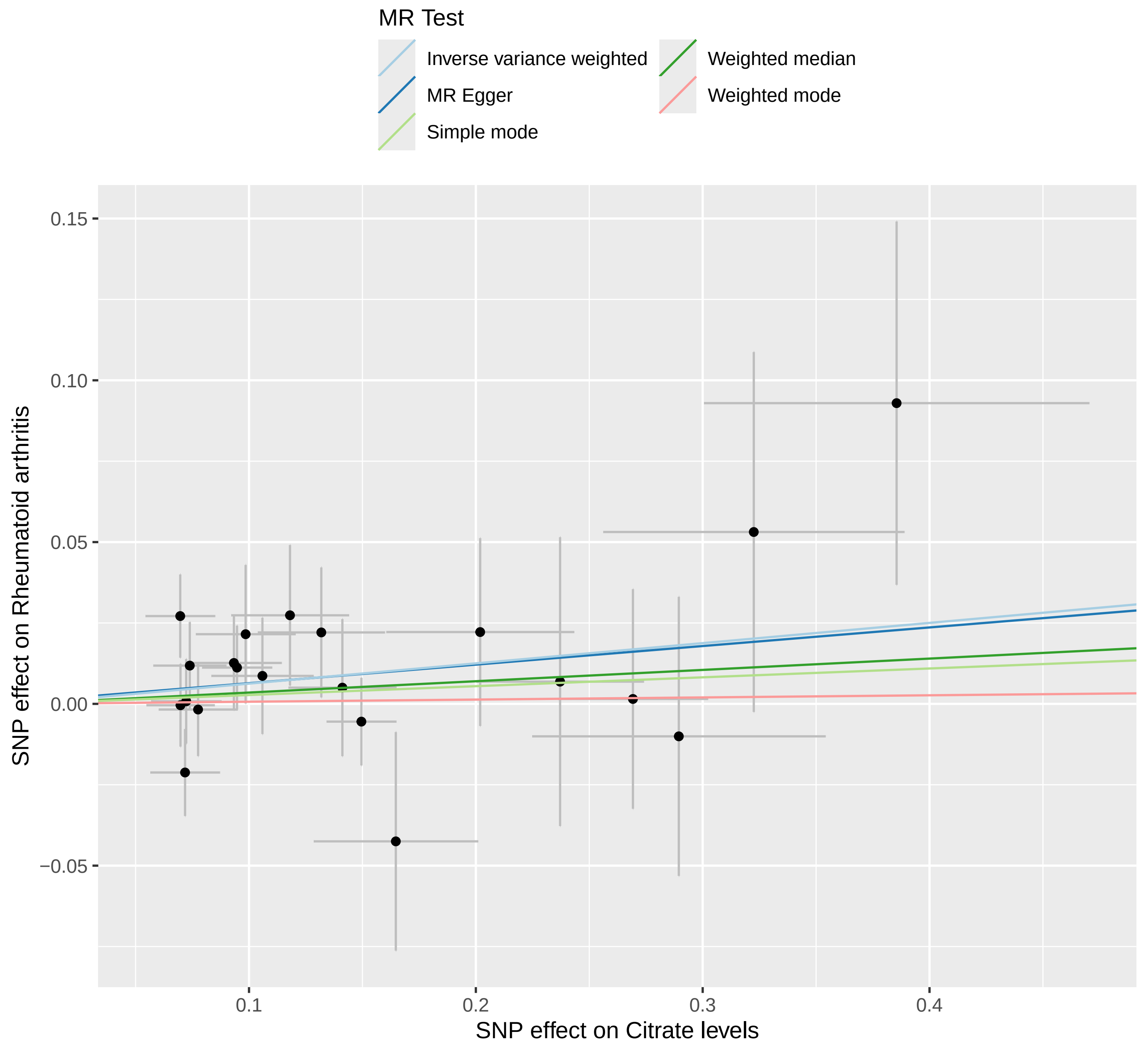

MR Test
Inverse variance weighted
MR Egger
Simple mode
Weighted median
Weighted mode
0.15
0.10
0.05
0.00
−0.05
SNP effect on Rheumatoid arthritis
0.1
0.2
0.3
0.4
SNP effect on Citrate levels

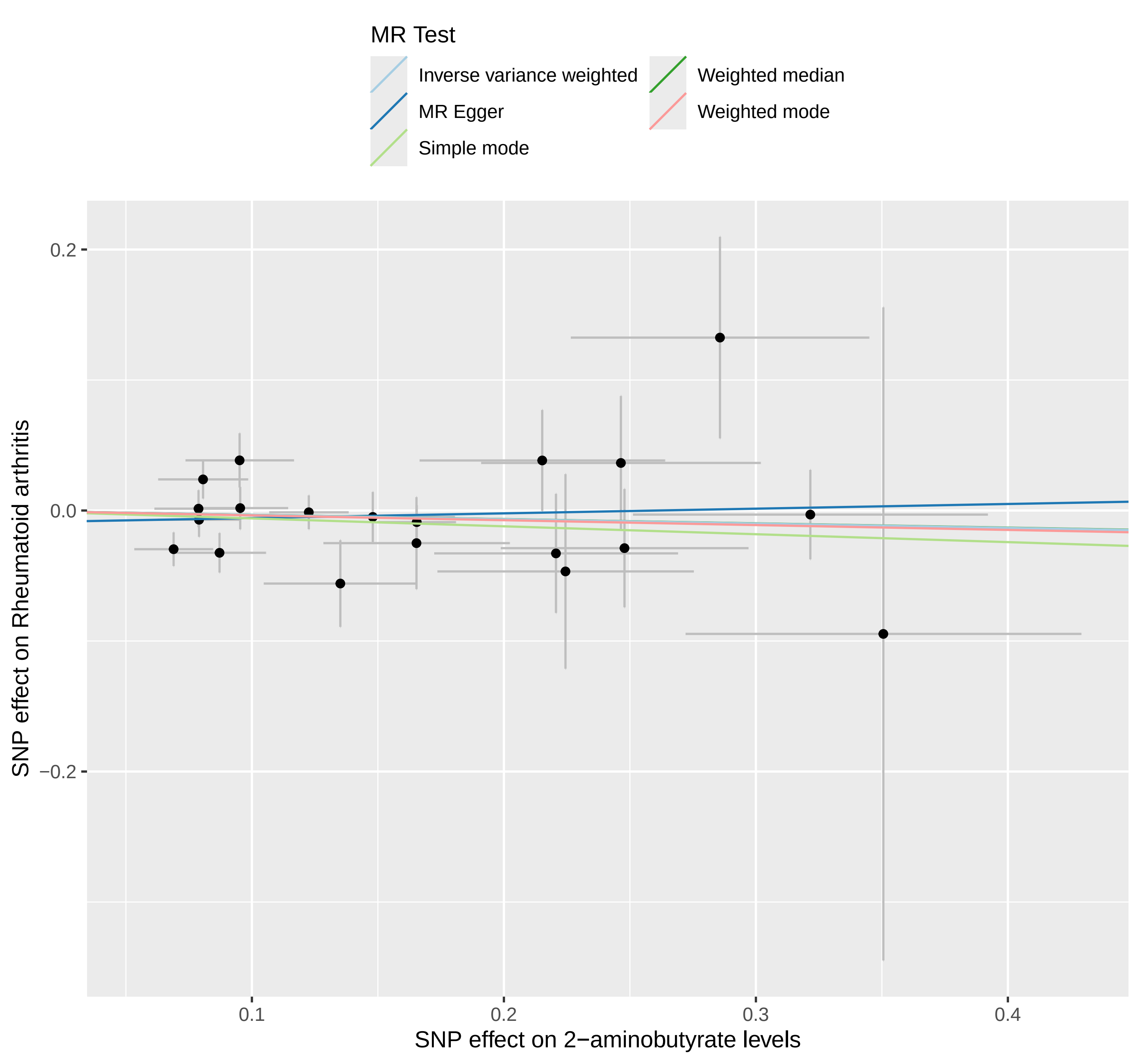
MR Test
Inverse variance weighted
MR Egger
Simple mode
Weighted median
Weighted mode
0.2
0.0
−0.2
0.1
0.2
0.3
0.4
SNP effect on Rheumatoid arthritis
SNP effect on 2−aminobutyrate levels

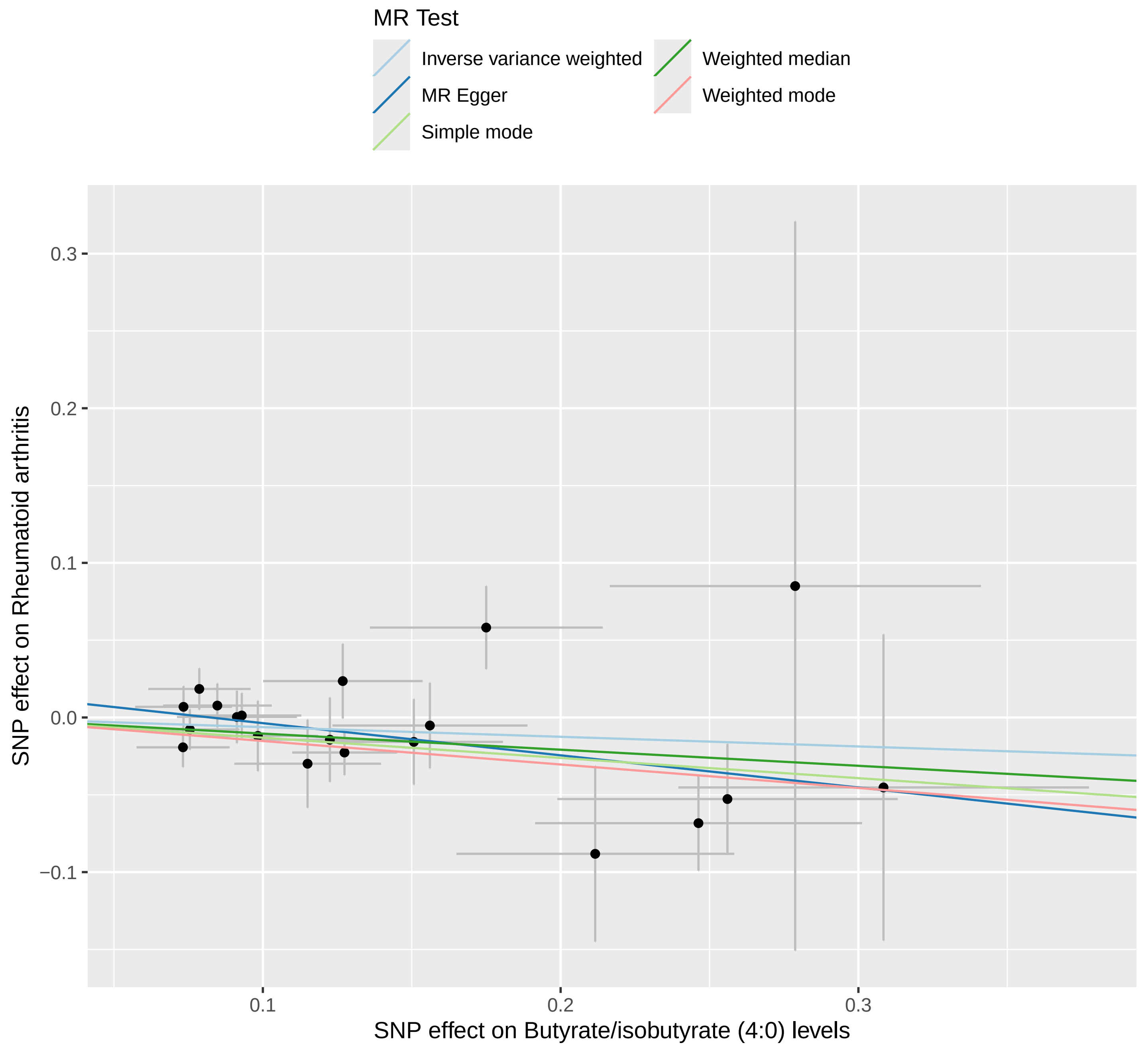

MR Test
Inverse variance weighted
MR Egger
Simple mode
Weighted median
Weighted mode
SNP effect on Rheumatoid arthritis
0.3
0.2
0.1
0.0
−0.1
0.1
0.2
0.3
SNP effect on Butyrate/isobutyrate (4:0) levels

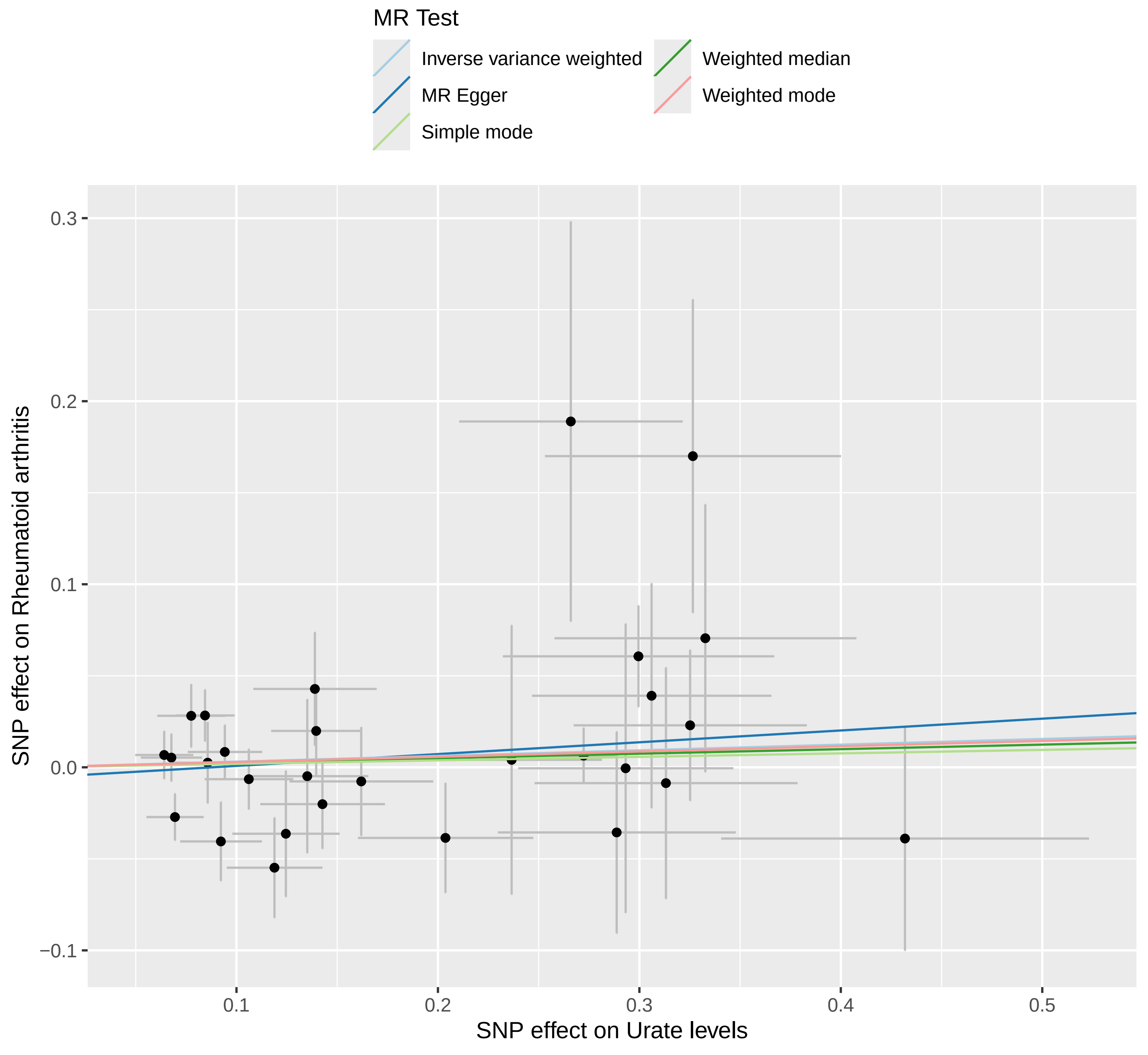

MR Test
Inverse variance weighted
MR Egger
Simple mode
Weighted median
Weighted mode
0.3
0.2
0.1
0.0
−0.1
0.1
0.2
0.3
0.4
0.5
SNP effect on Rheumatoid arthritis
SNP effect on Urate levels

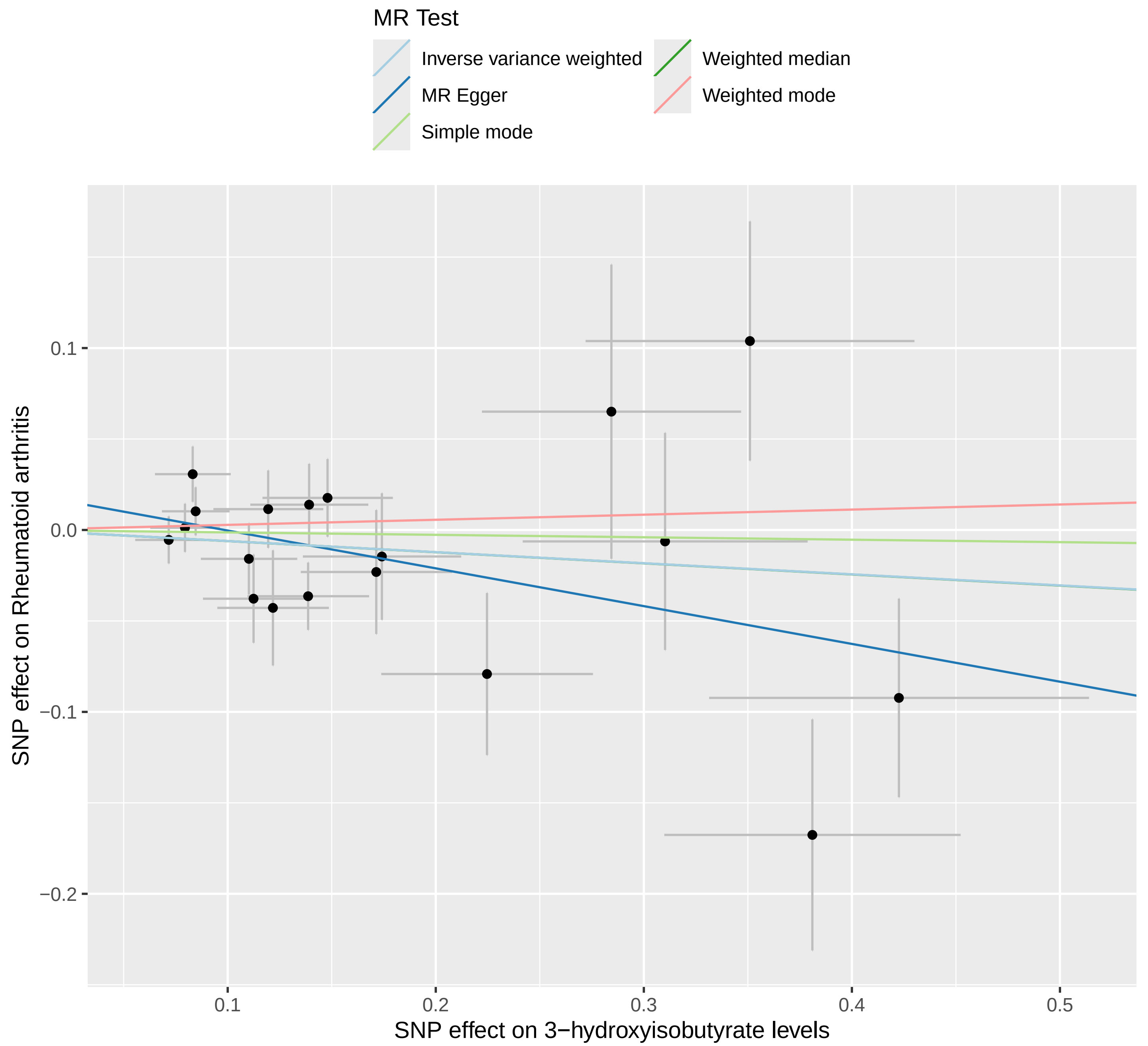

MR Test
Inverse variance weighted
MR Egger
Simple mode
Weighted median
Weighted mode
0.1
0.0
−0.1
−0.2
0.1
0.2
0.3
0.4
0.5
SNP effect on Rheumatoid arthritis
SNP effect on 3−hydroxyisobutyrate levels

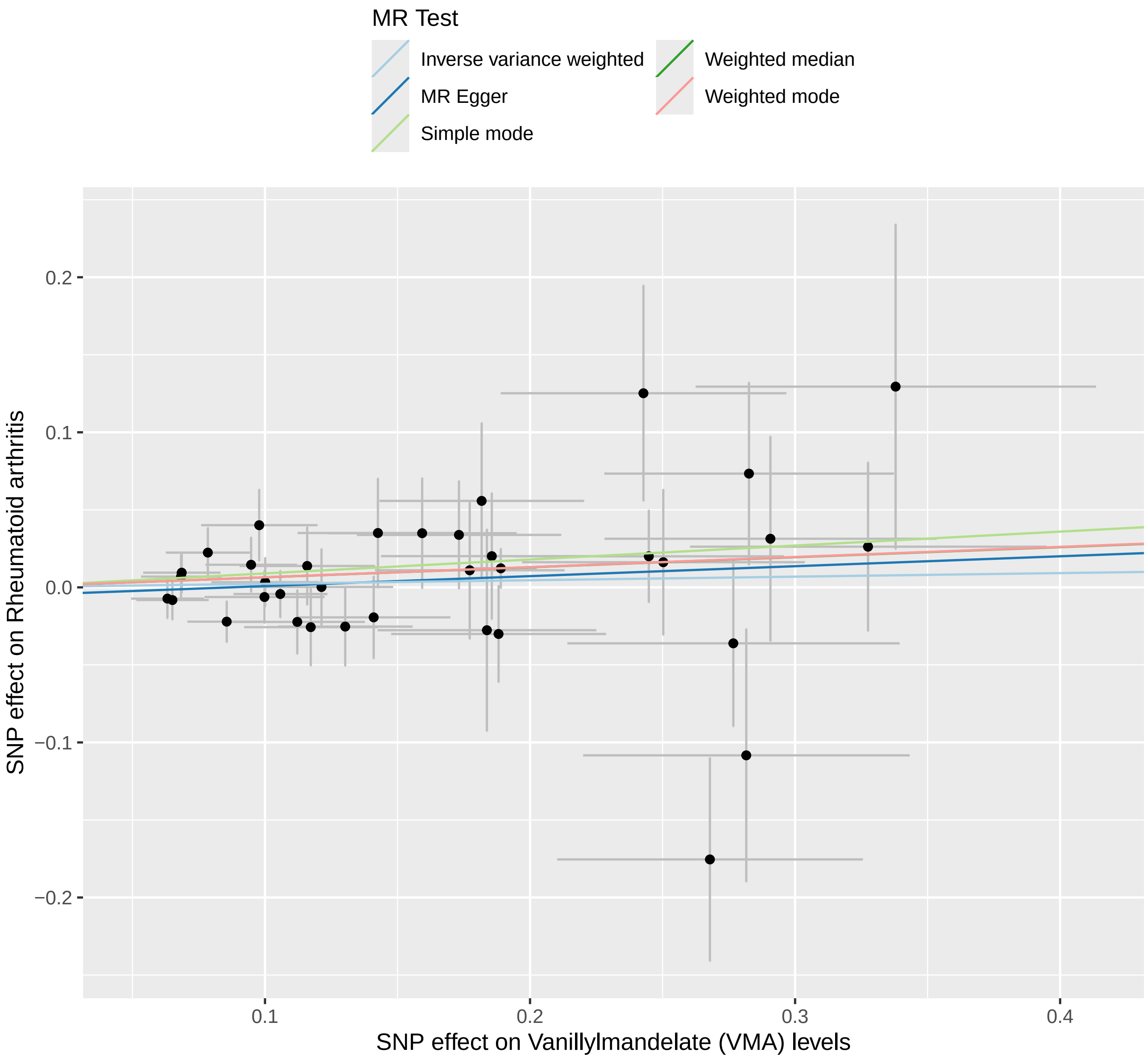
MR Test
Inverse variance weighted
MR Egger
Simple mode
Weighted median
Weighted mode
0.2
0.1
0.0
−0.1
−0.2
SNP effect on Rheumatoid arthritis
0.1
0.2
0.3
0.4
SNP effect on Vanillylmandelate (VMA) levels

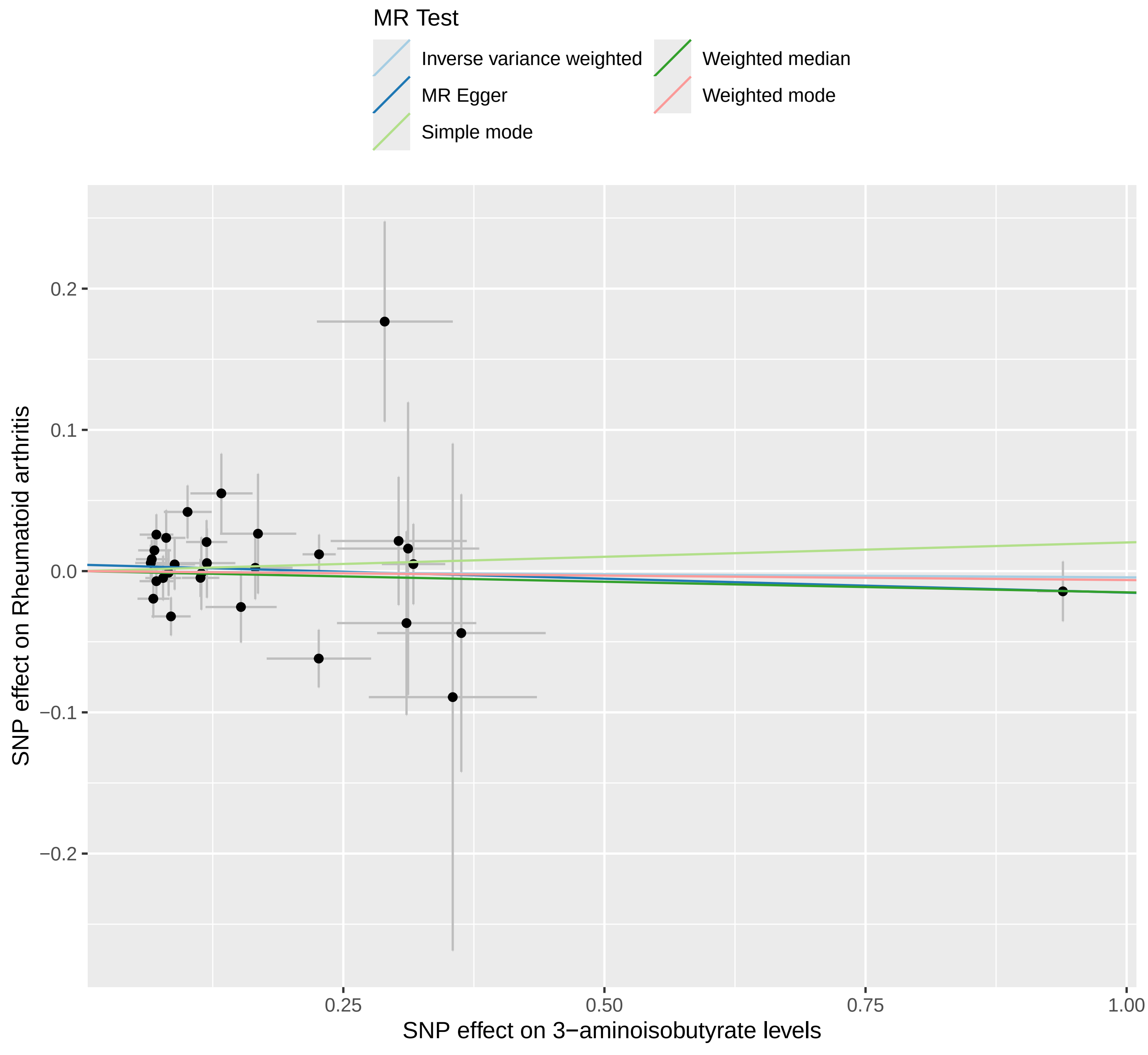
MR Test
Inverse variance weighted
MR Egger
Simple mode
Weighted median
Weighted mode
SNP effect on Rheumatoid arthritis
0.2
0.1
0.0
−0.1
−0.2
0.25
0.50
0.75
1.00
SNP effect on 3−aminoisobutyrate levels

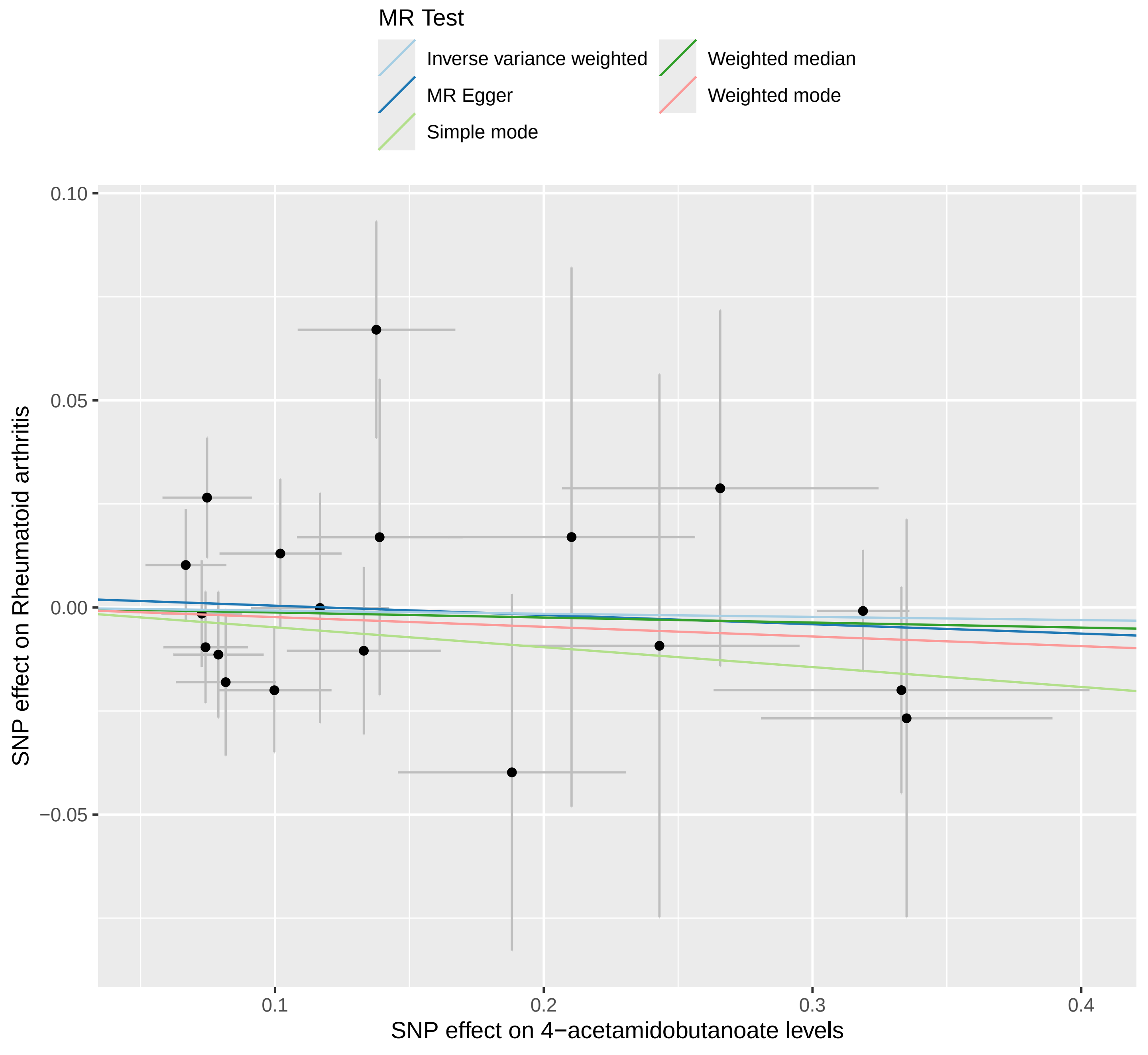
MR Test
Inverse variance weighted
MR Egger
Simple mode
Weighted median
Weighted mode
0.10
0.05
0.00
−0.05
0.1
0.2
0.3
0.4
SNP effect on Rheumatoid arthritis
SNP effect on 4−acetamidobutanoate levels

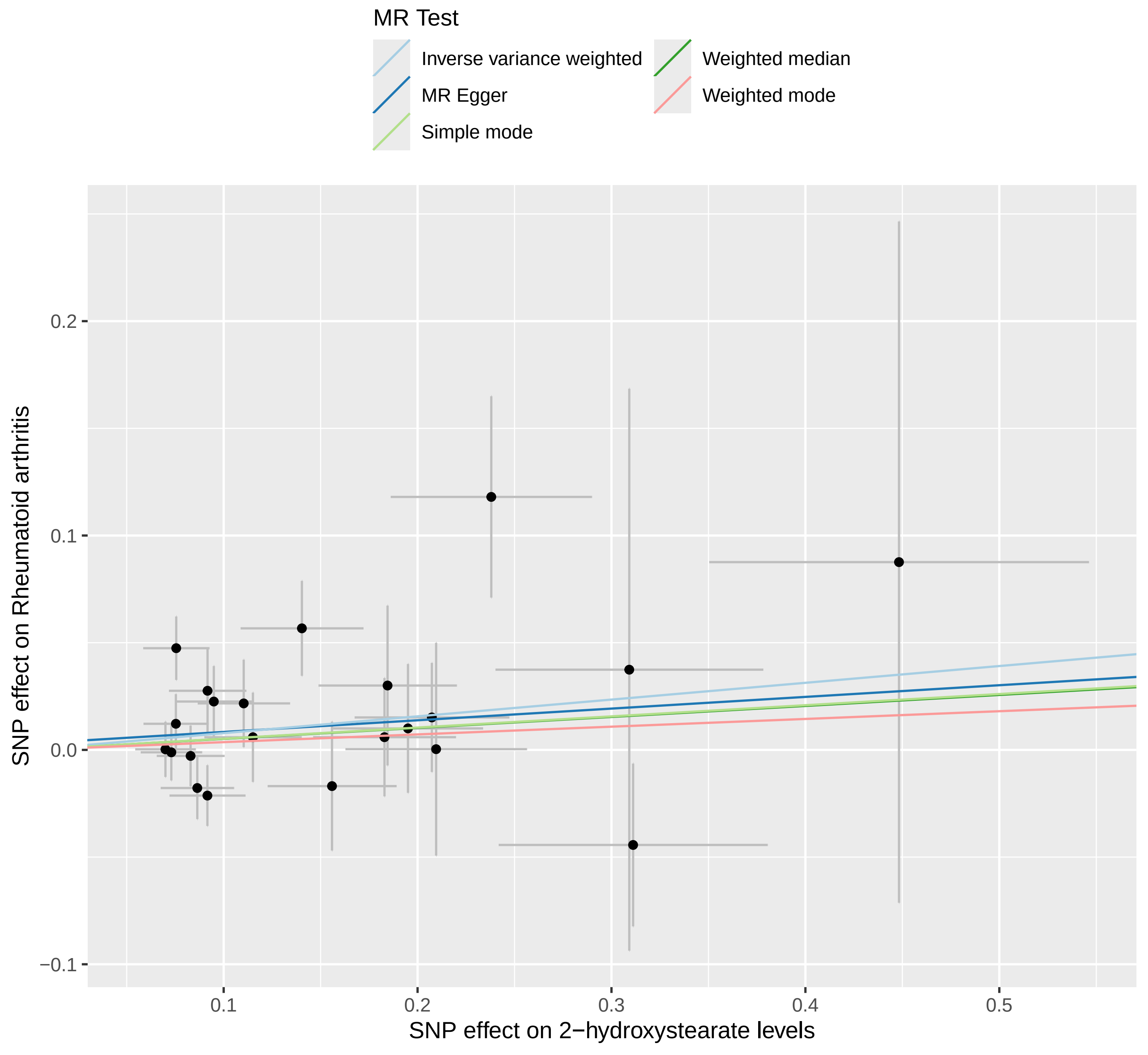

MR Test
Inverse variance weighted
MR Egger
Simple mode
Weighted median
Weighted mode
SNP effect on Rheumatoid arthritis
0.2
0.1
0.0
−0.1
0.1
0.2
0.3
0.4
0.5
SNP effect on 2−hydroxystearate levels

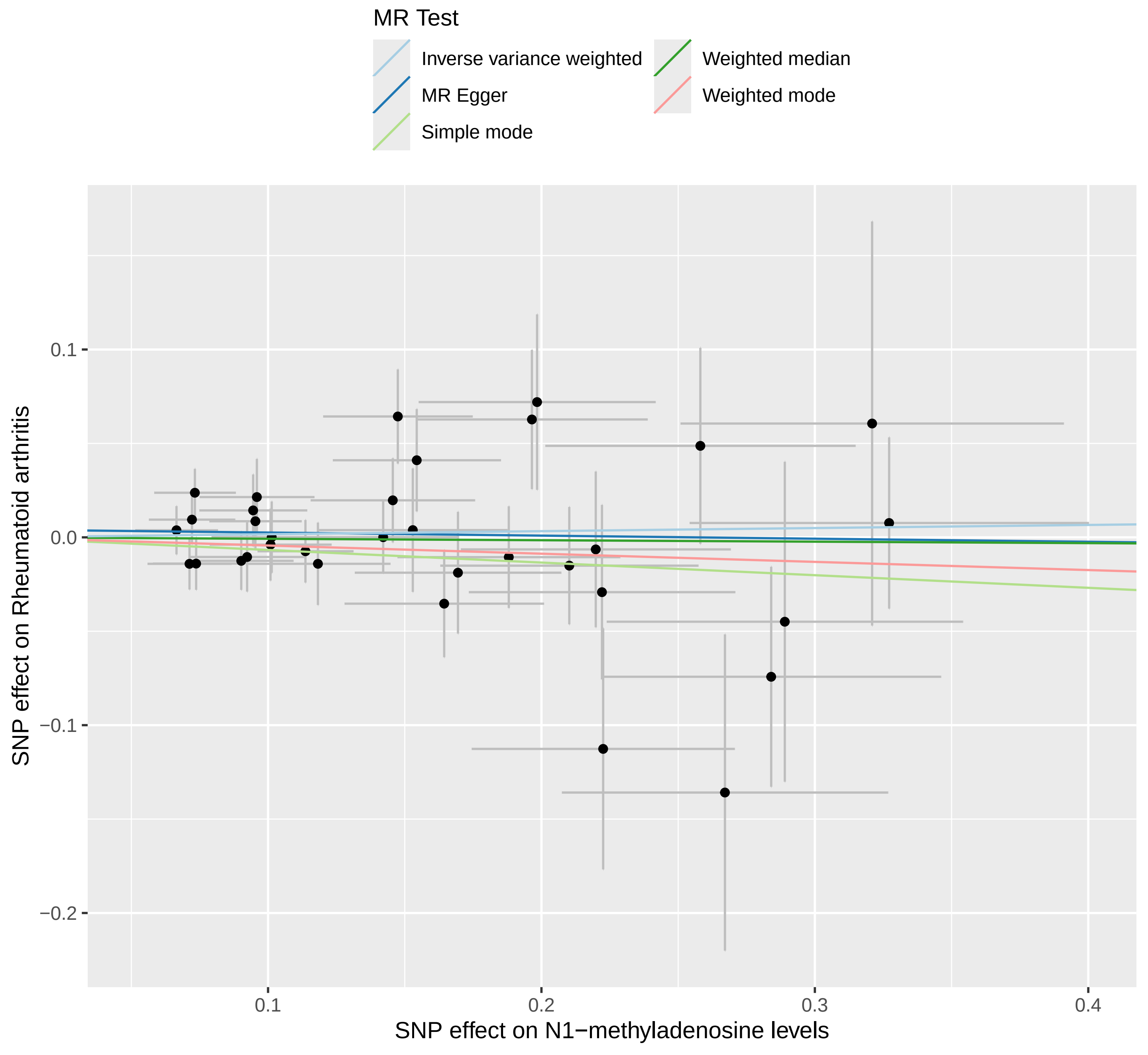
MR Test
Inverse variance weighted
MR Egger
Simple mode
Weighted median
Weighted mode
0.1
0.0
−0.1
−0.2
0.1
0.2
0.3
0.4
SNP effect on Rheumatoid arthritis
SNP effect on N1−methyladenosine levels

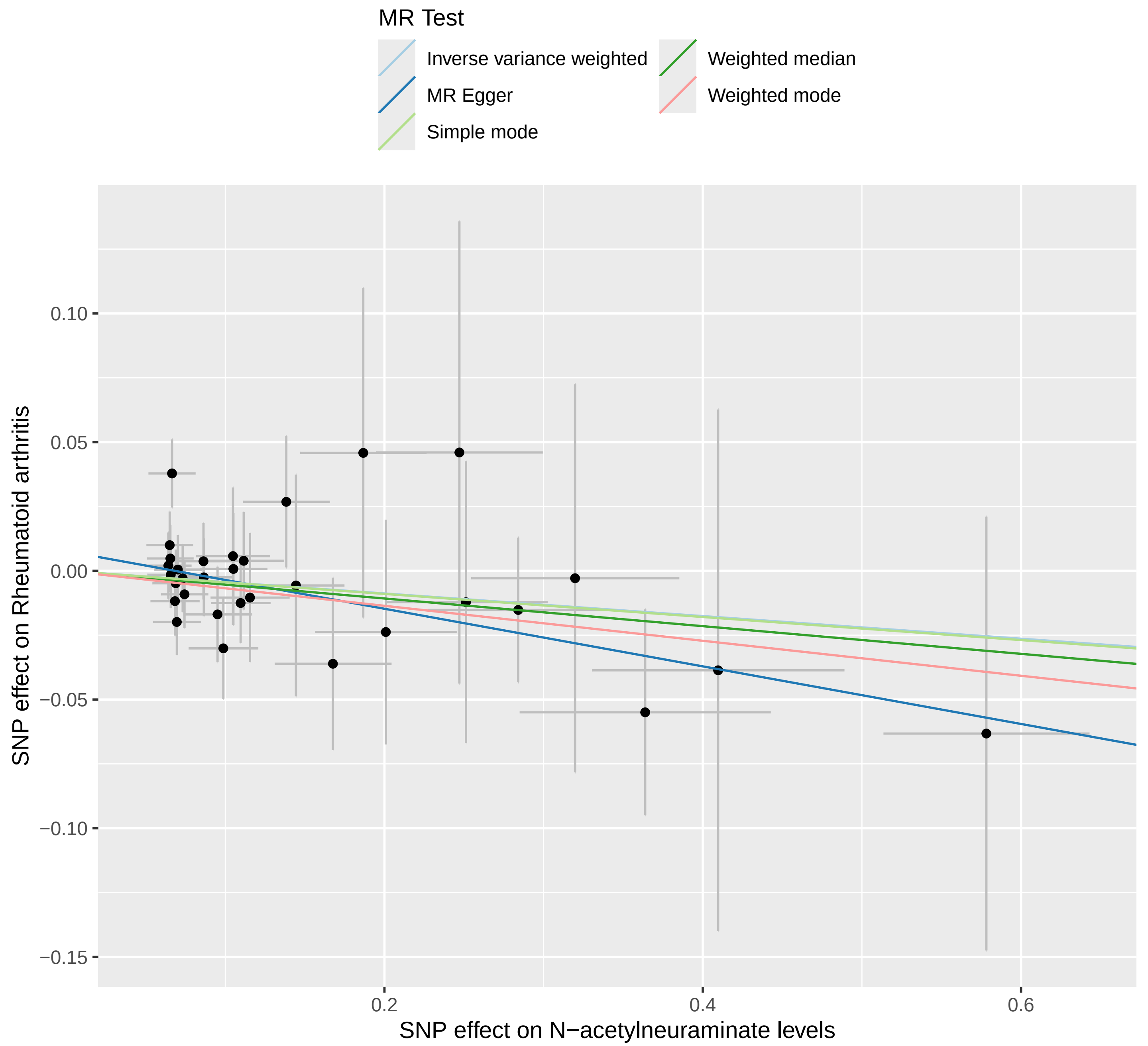

MR Test
Inverse variance weighted
MR Egger
Simple mode
Weighted median
Weighted mode
0.10
0.05
0.00
−0.05
−0.10
−0.15
SNP effect on Rheumatoid arthritis
0.2
0.4
0.6
SNP effect on N−acetylneuraminate levels

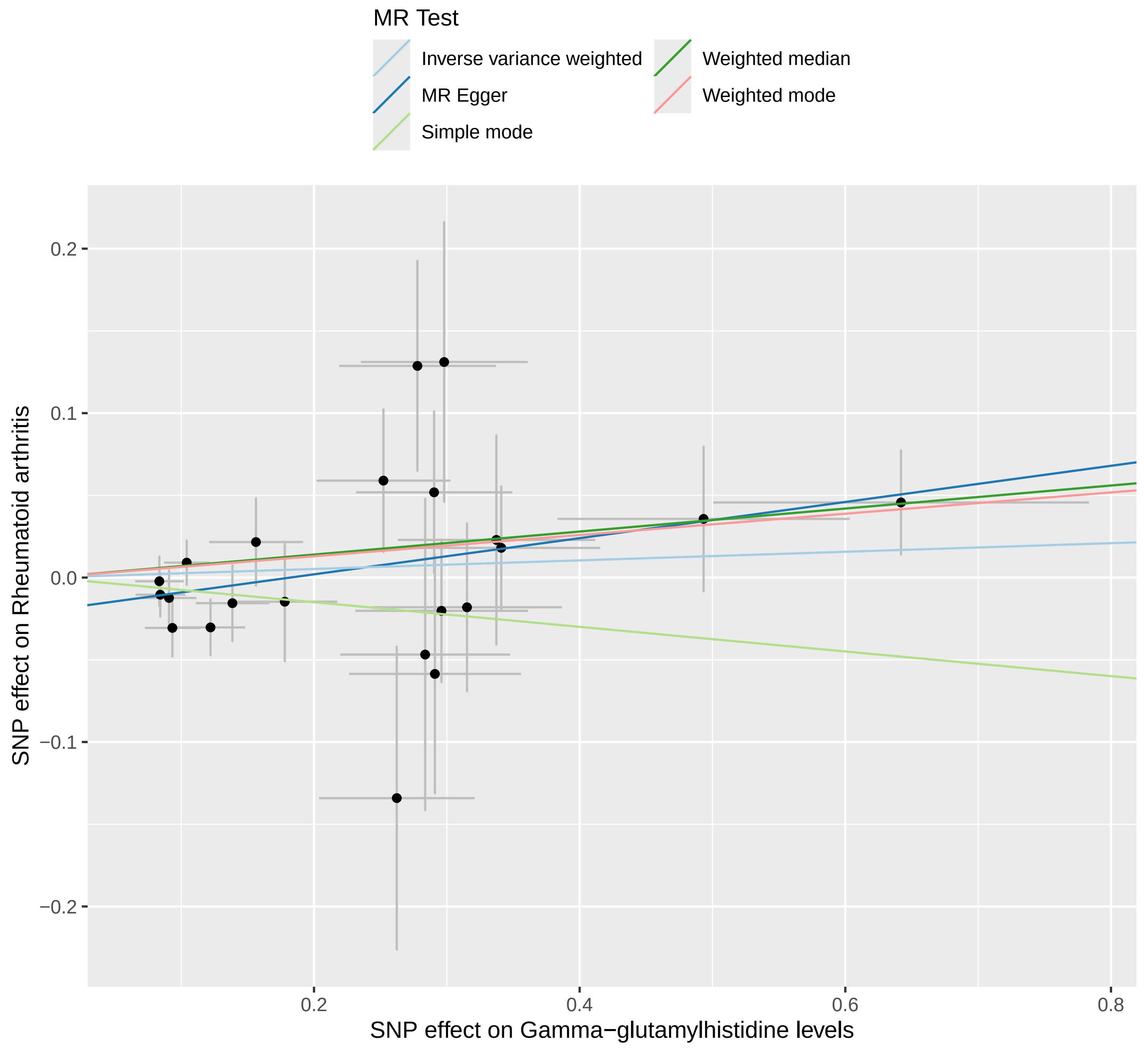

MR Test
Inverse variance weighted
MR Egger
Simple mode
Weighted median
Weighted mode
0.2
0.1
0.0
−0.1
−0.2
SNP effect on Rheumatoid arthritis
0.2
0.4
0.6
0.8
SNP effect on Gamma−glutamylhistidine levels

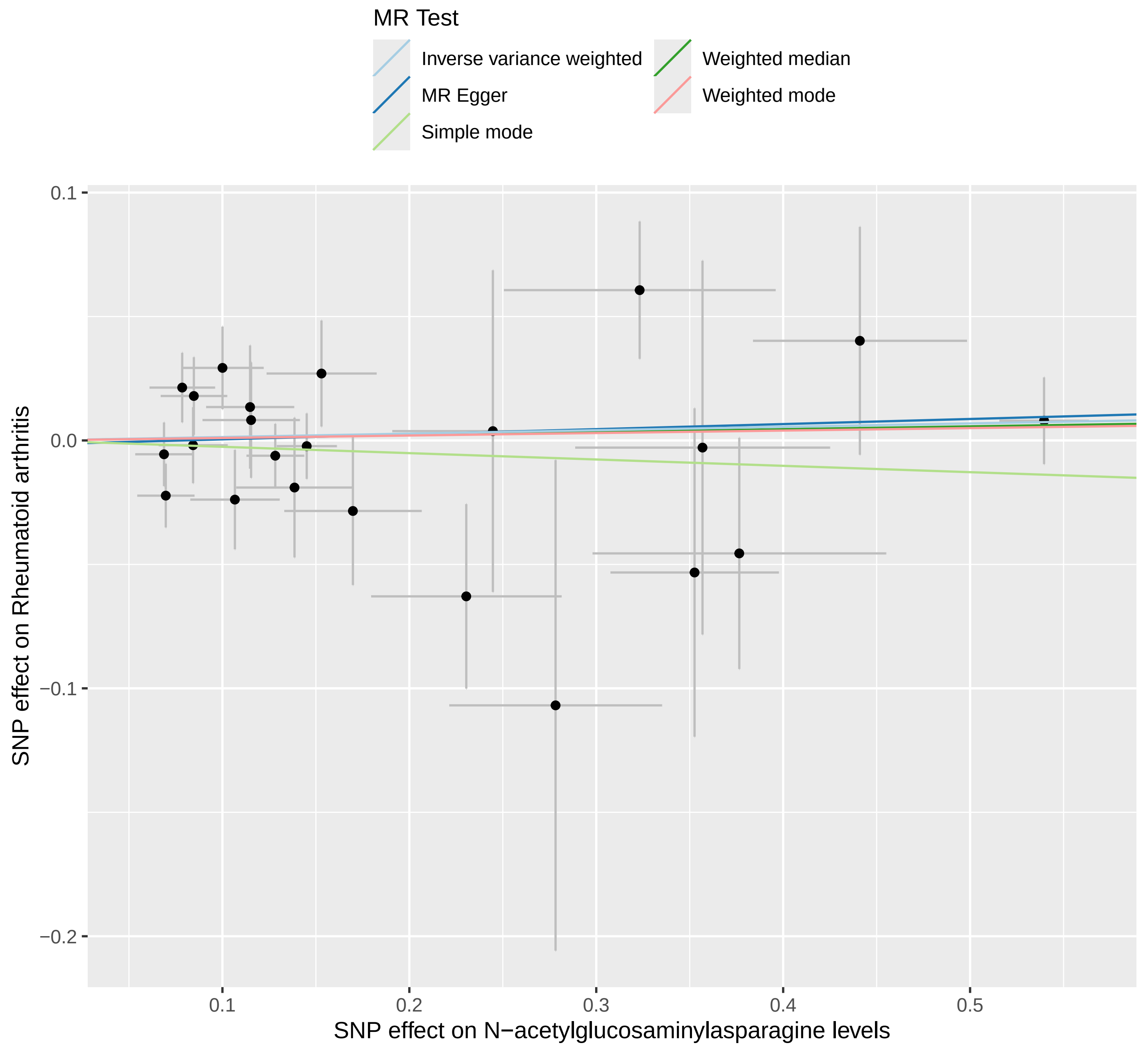
MR Test
Inverse variance weighted
MR Egger
Simple mode
Weighted median
Weighted mode
0.1
0.0
−0.1
−0.2
SNP effect on Rheumatoid arthritis
0.1
0.2
0.3
0.4
0.5
SNP effect on N−acetylglucosaminylasparagine levels

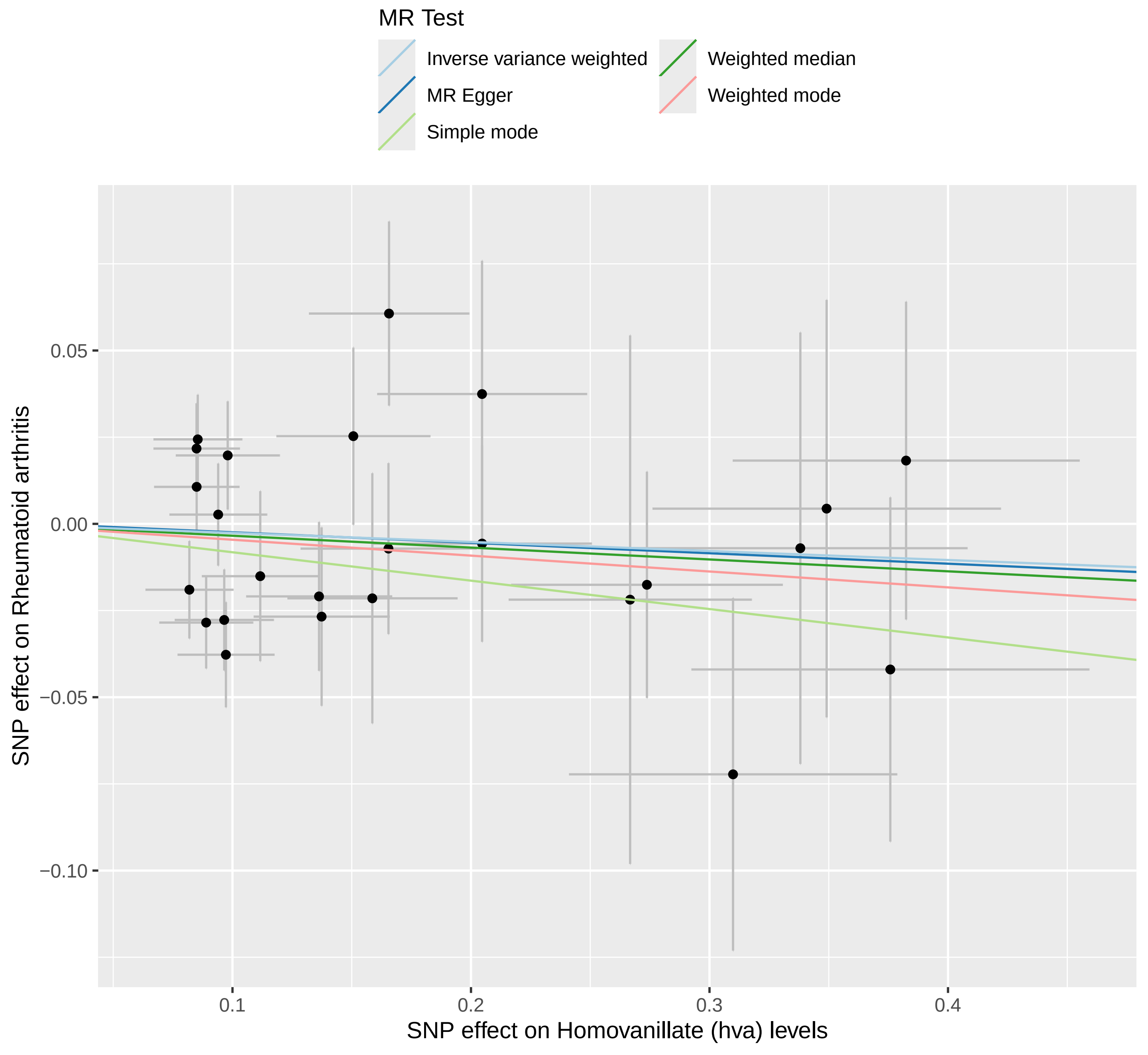

MR Test
Inverse variance weighted
MR Egger
Simple mode
Weighted median
Weighted mode
0.05
0.00
−0.05
−0.10
0.1
0.2
0.3
0.4
SNP effect on Rheumatoid arthritis
SNP effect on Homovanillate (hva) levels

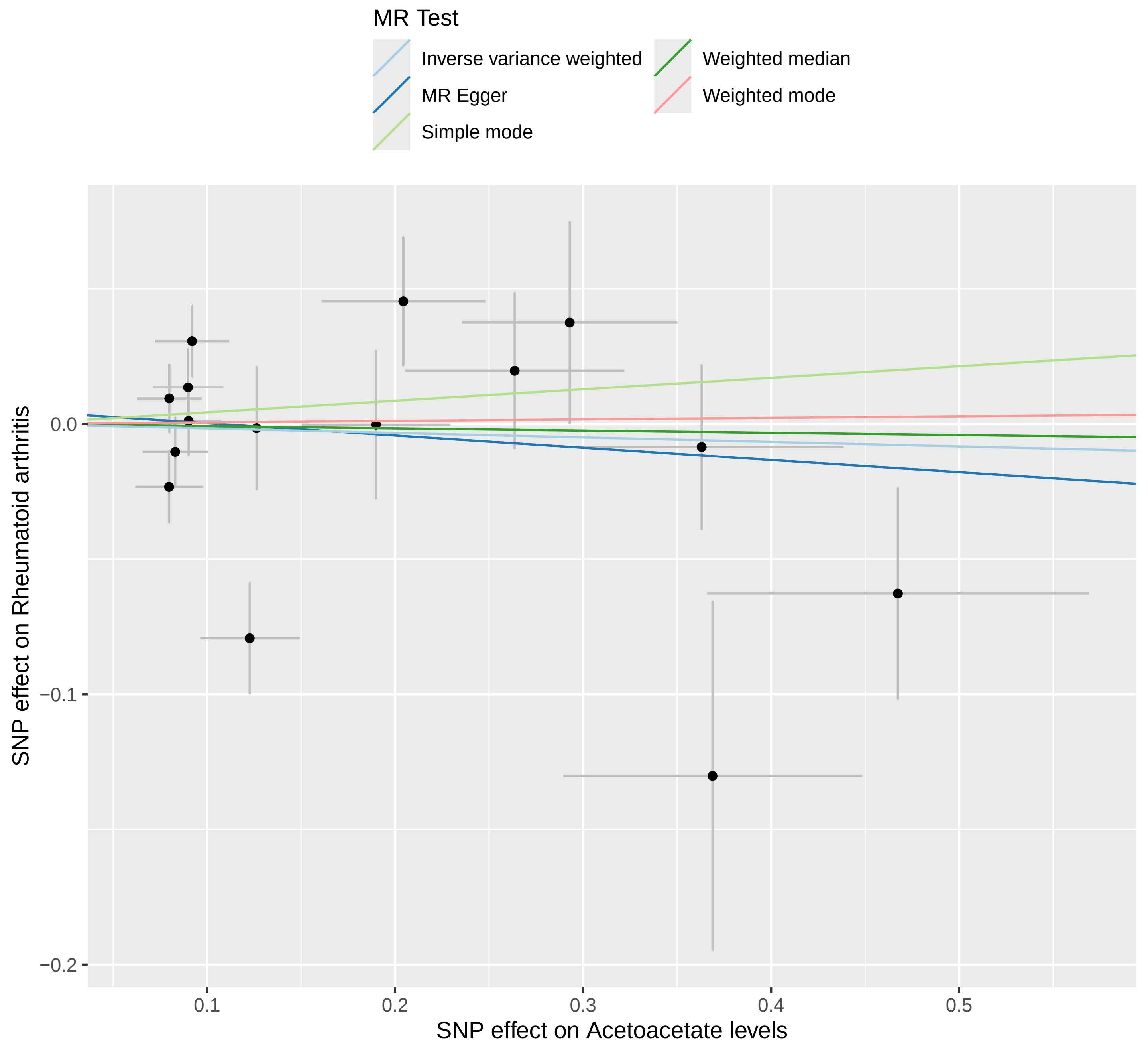

MR Test
Inverse variance weighted
MR Egger
Simple mode
Weighted median
Weighted mode
SNP effect on Rheumatoid arthritis
0.0
−0.1
−0.2
0.1
0.2
0.3
0.4
0.5
SNP effect on Acetoacetate levels

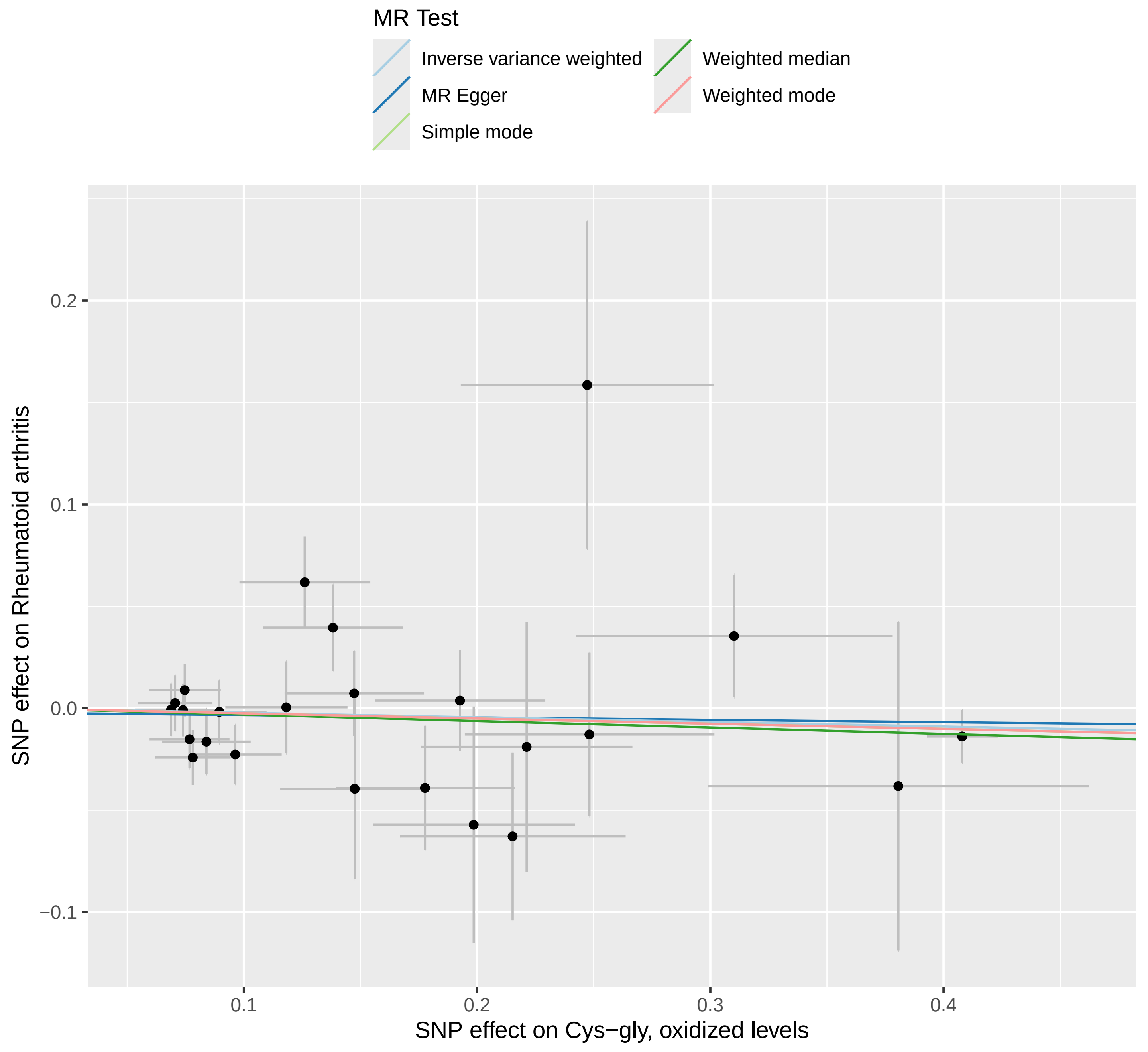

MR Test
Inverse variance weighted
MR Egger
Simple mode
Weighted median
Weighted mode
0.2
0.1
0.0
−0.1
0.1
0.2
0.3
0.4
SNP effect on Rheumatoid arthritis
SNP effect on Cys−gly, oxidized levels

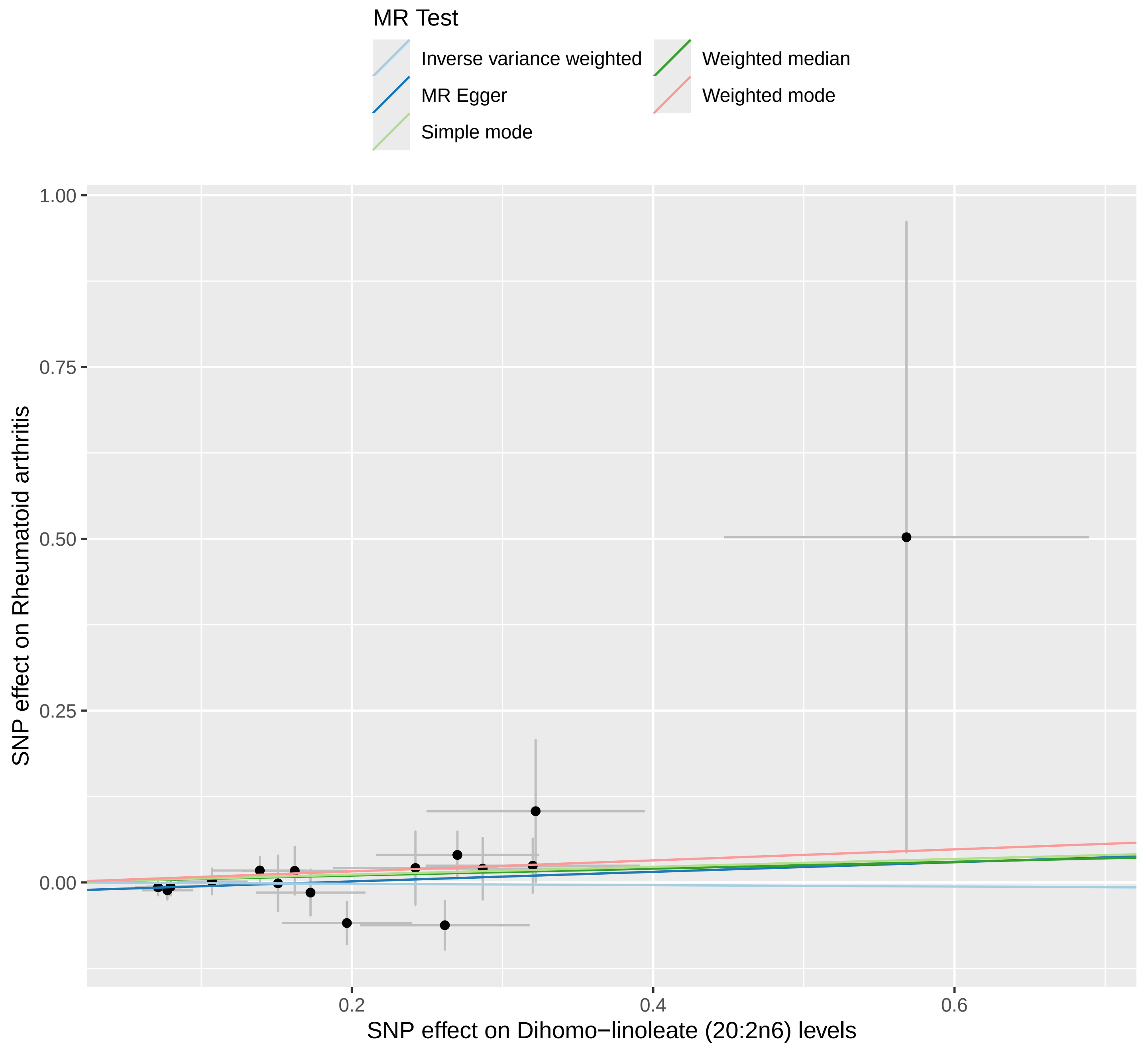
MR Test
Inverse variance weighted
MR Egger
Simple mode
Weighted median
Weighted mode
1.00
0.75
0.50
0.25
0.00
0.2
0.4
0.6
SNP effect on Rheumatoid arthritis
SNP effect on Dihomo−linoleate (20:2n6) levels

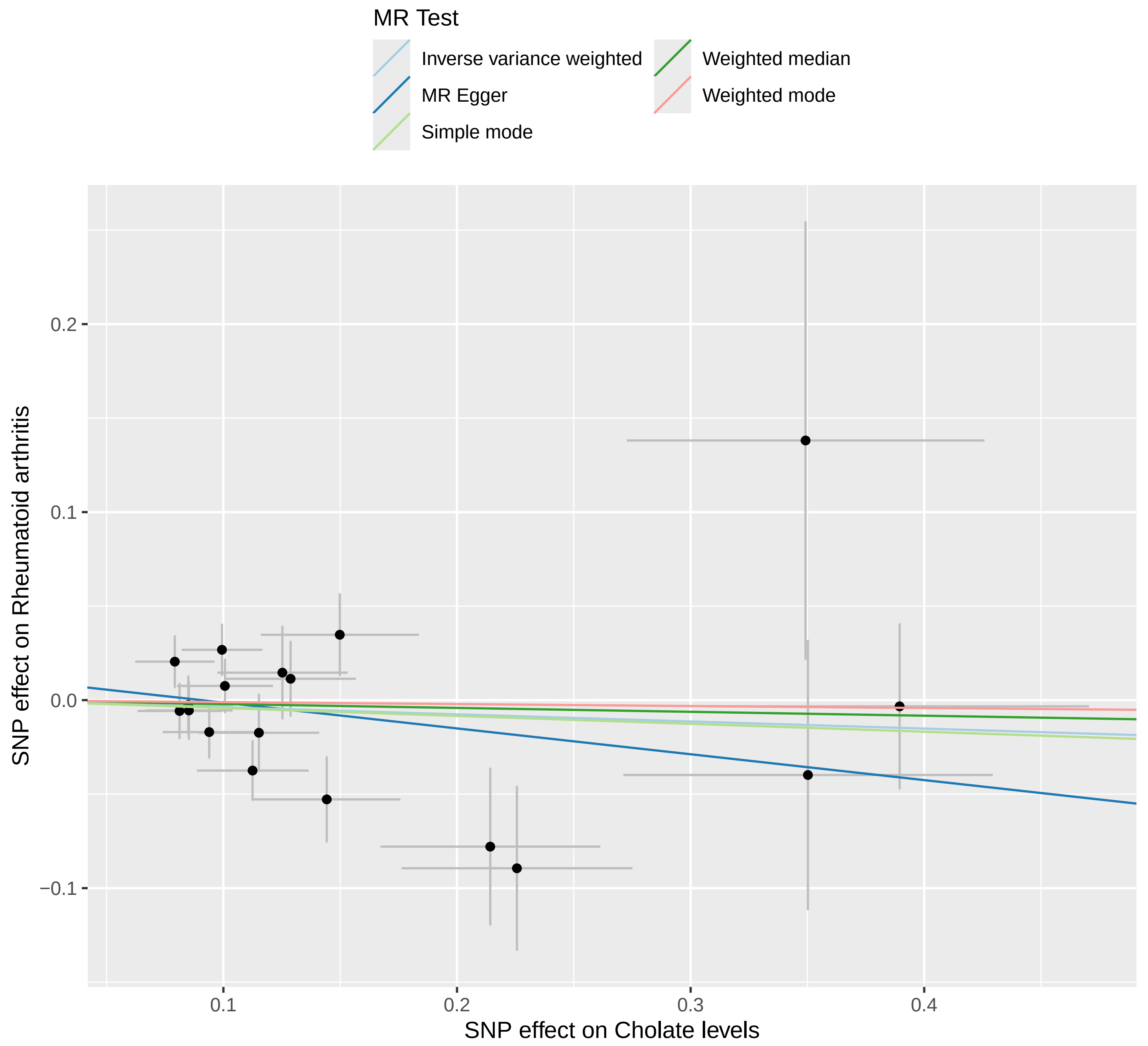
MR Test
Inverse variance weighted
MR Egger
Simple mode
Weighted median
Weighted mode
0.2
0.1
0.0
−0.1
0.1
0.2
0.3
0.4
SNP effect on Rheumatoid arthritis
SNP effect on Cholate levels

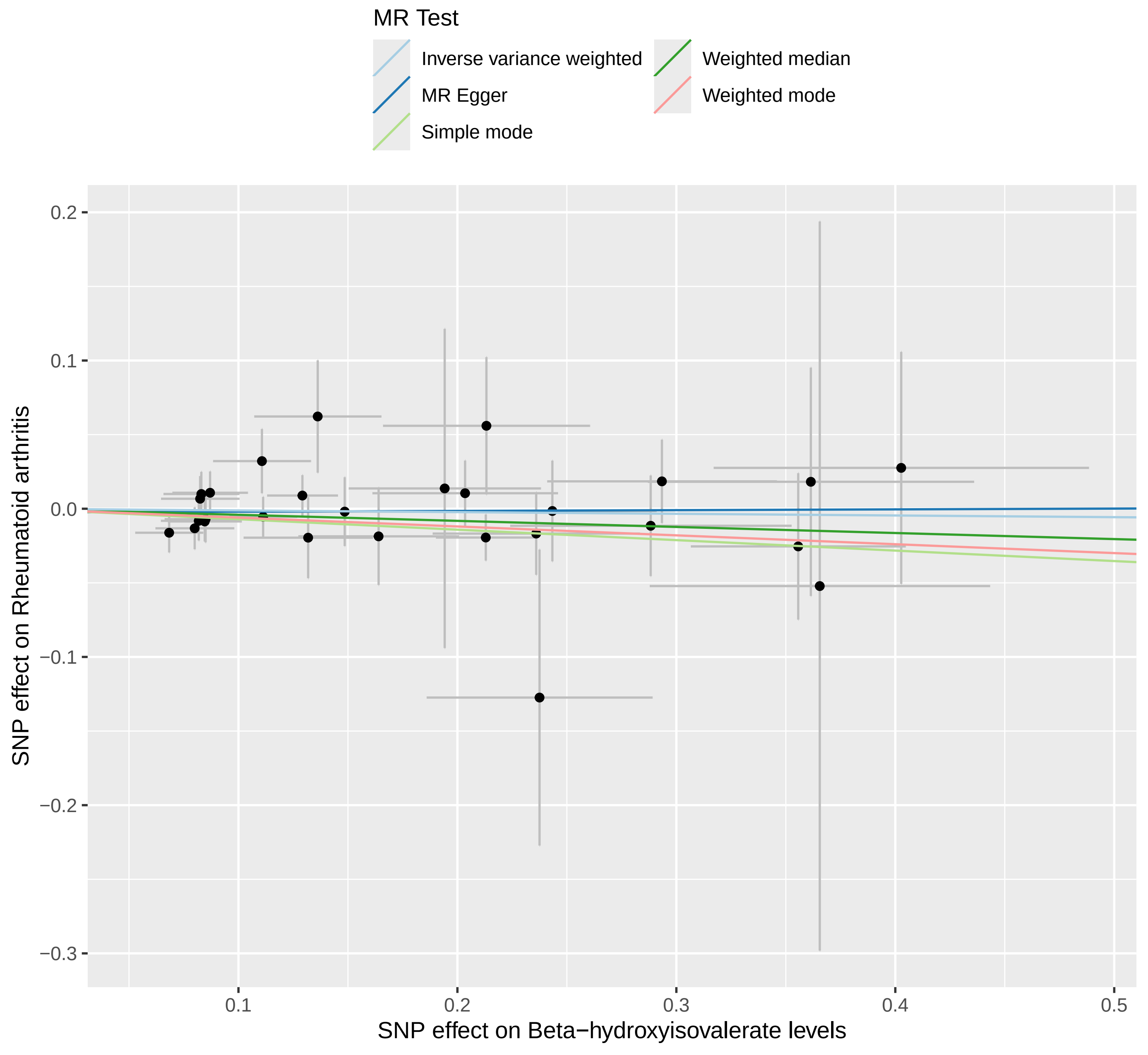
MR Test
Inverse variance weighted
MR Egger
Simple mode
Weighted median
Weighted mode
0.2
0.1
0.0
−0.1
−0.2
−0.3
SNP effect on Rheumatoid arthritis
0.1
0.2
0.3
0.4
0.5
SNP effect on Beta−hydroxyisovalerate levels

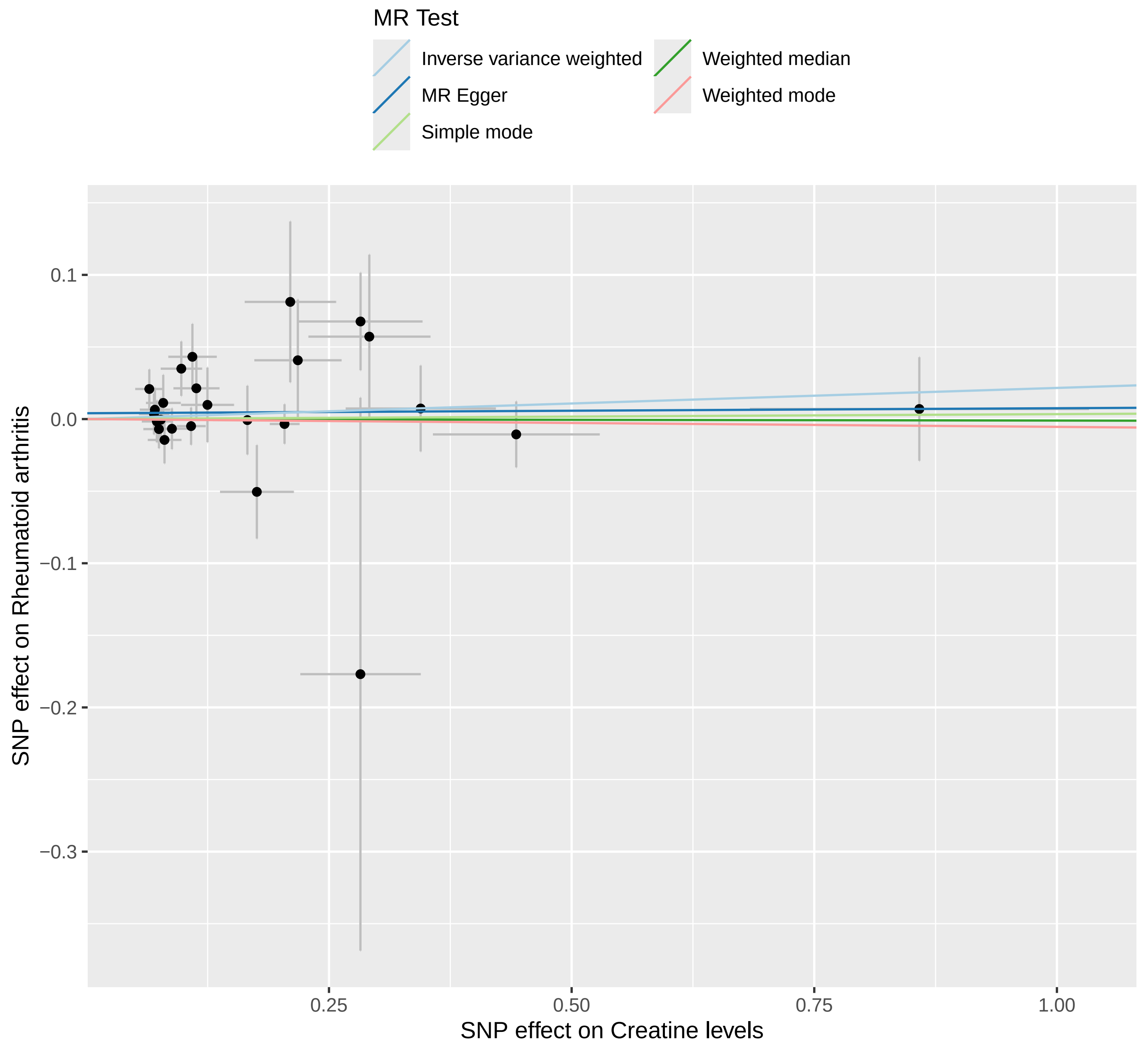
MR Test
Inverse variance weighted
MR Egger
Simple mode
Weighted median
Weighted mode
0.1
0.0
−0.1
−0.2
−0.3
0.25
0.50
0.75
1.00
SNP effect on Rheumatoid arthritis
SNP effect on Creatine levels

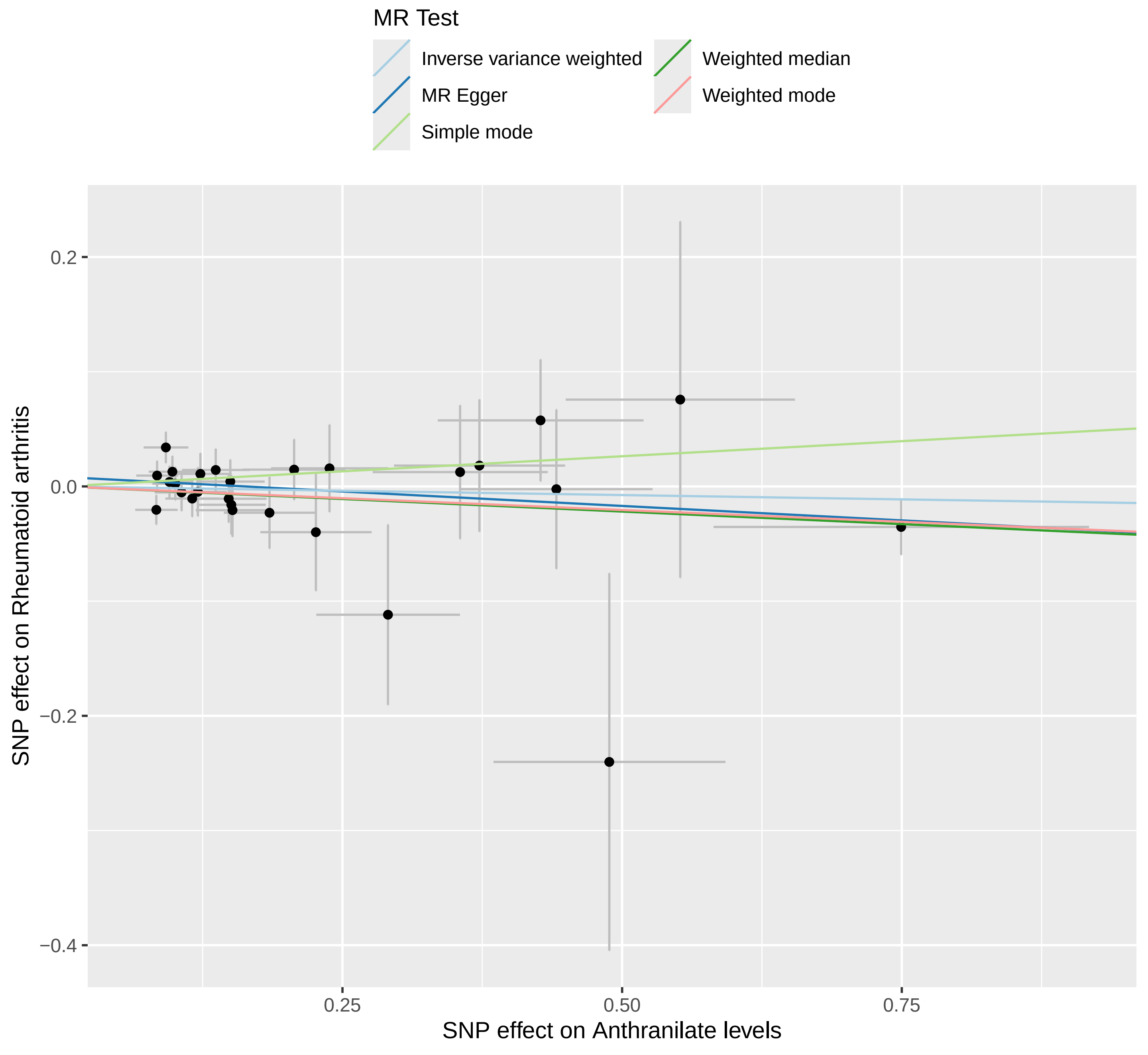
MR Test
Inverse variance weighted
MR Egger
Simple mode
Weighted median
Weighted mode
0.2
0.0
−0.2
−0.4
0.25
0.50
0.75
SNP effect on Rheumatoid arthritis
SNP effect on Anthranilate levels

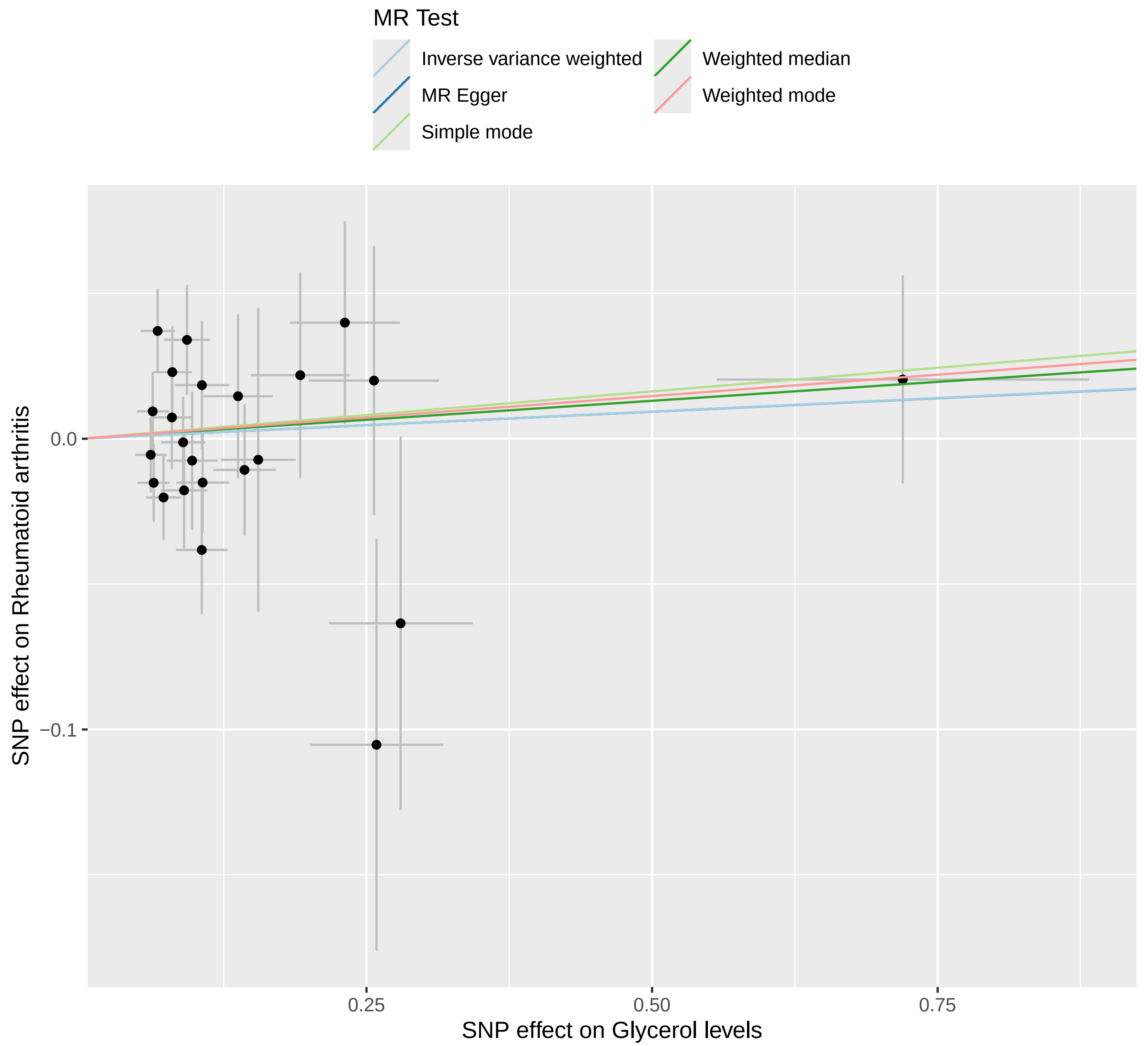
MR Test
Inverse variance weighted
MR Egger
Simple mode
Weighted median
Weighted mode
SNP effect on Rheumatoid arthritis
0.0
−0.1
0.25
0.50
0.75
SNP effect on Glycerol levels

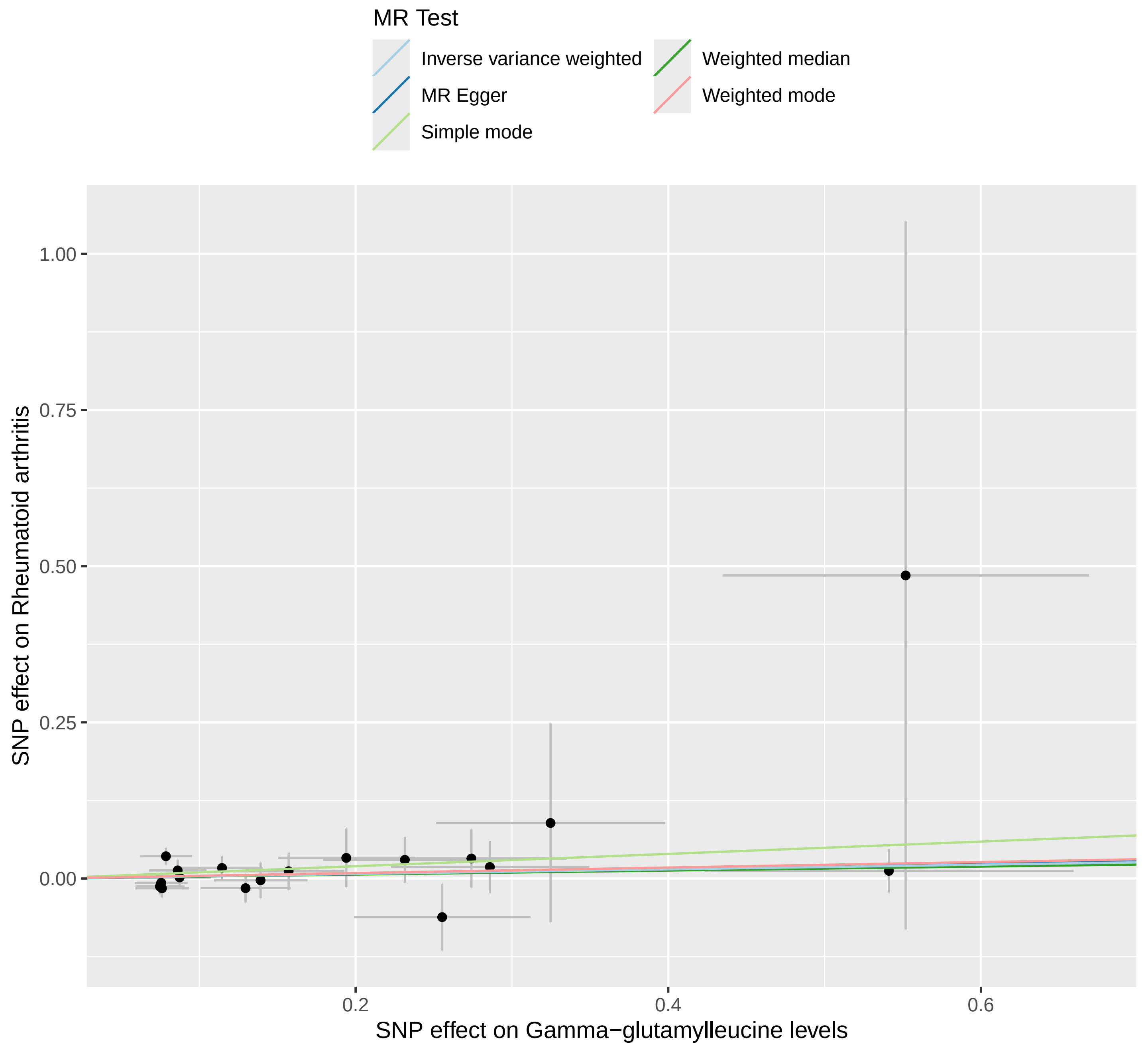

MR Test
Inverse variance weighted
MR Egger
Simple mode
Weighted median
Weighted mode
1.00
0.75
0.50
0.25
0.00
0.2
0.4
0.6
SNP effect on Rheumatoid arthritis
SNP effect on Gamma−glutamylleucine levels

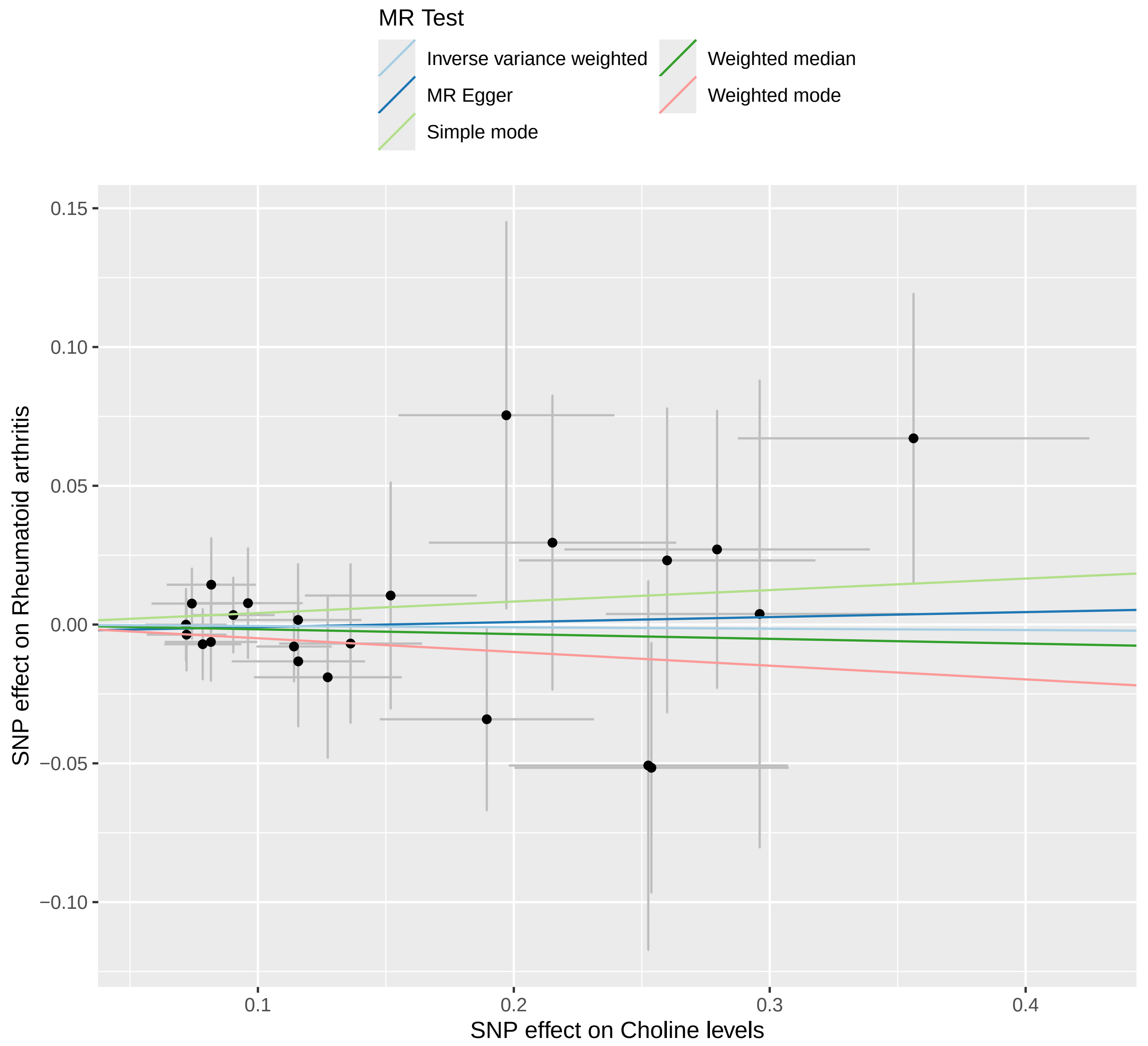

MR Test
Inverse variance weighted
MR Egger
Simple mode
Weighted median
Weighted mode
0.15
0.10
0.05
0.00
−0.05
−0.10
SNP effect on Rheumatoid arthritis
0.1
0.2
0.3
0.4
SNP effect on Choline levels

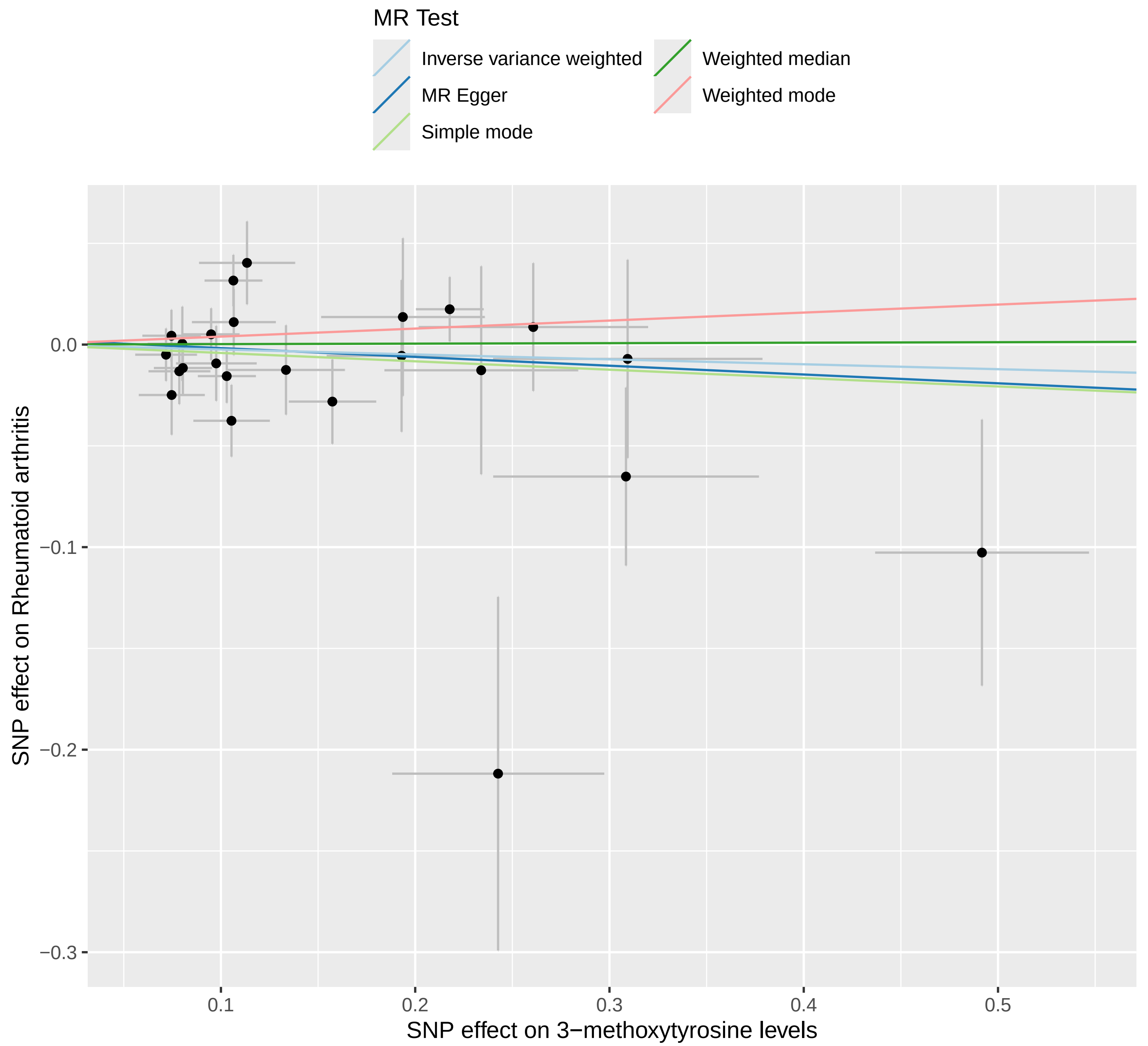
MR Test
Inverse variance weighted
MR Egger
Simple mode
Weighted median
Weighted mode
0.0
−0.1
−0.2
−0.3
0.1
0.2
0.3
0.4
0.5
SNP effect on Rheumatoid arthritis
SNP effect on 3−methoxytyrosine levels

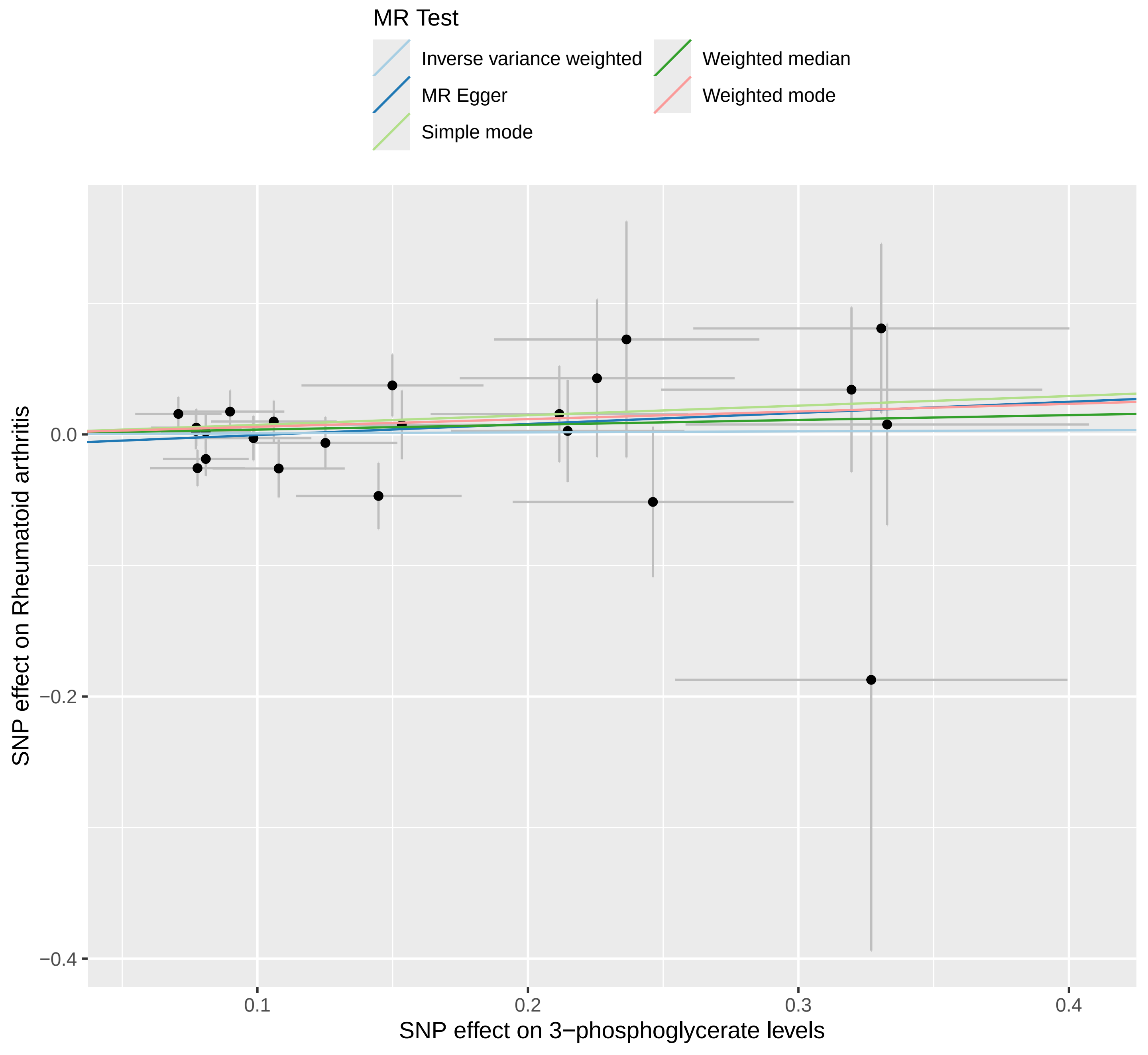
MR Test
Inverse variance weighted
MR Egger
Simple mode
Weighted median
Weighted mode
SNP effect on Rheumatoid arthritis
0.0
−0.2
−0.4
0.1
0.2
0.3
0.4
SNP effect on 3−phosphoglycerate levels

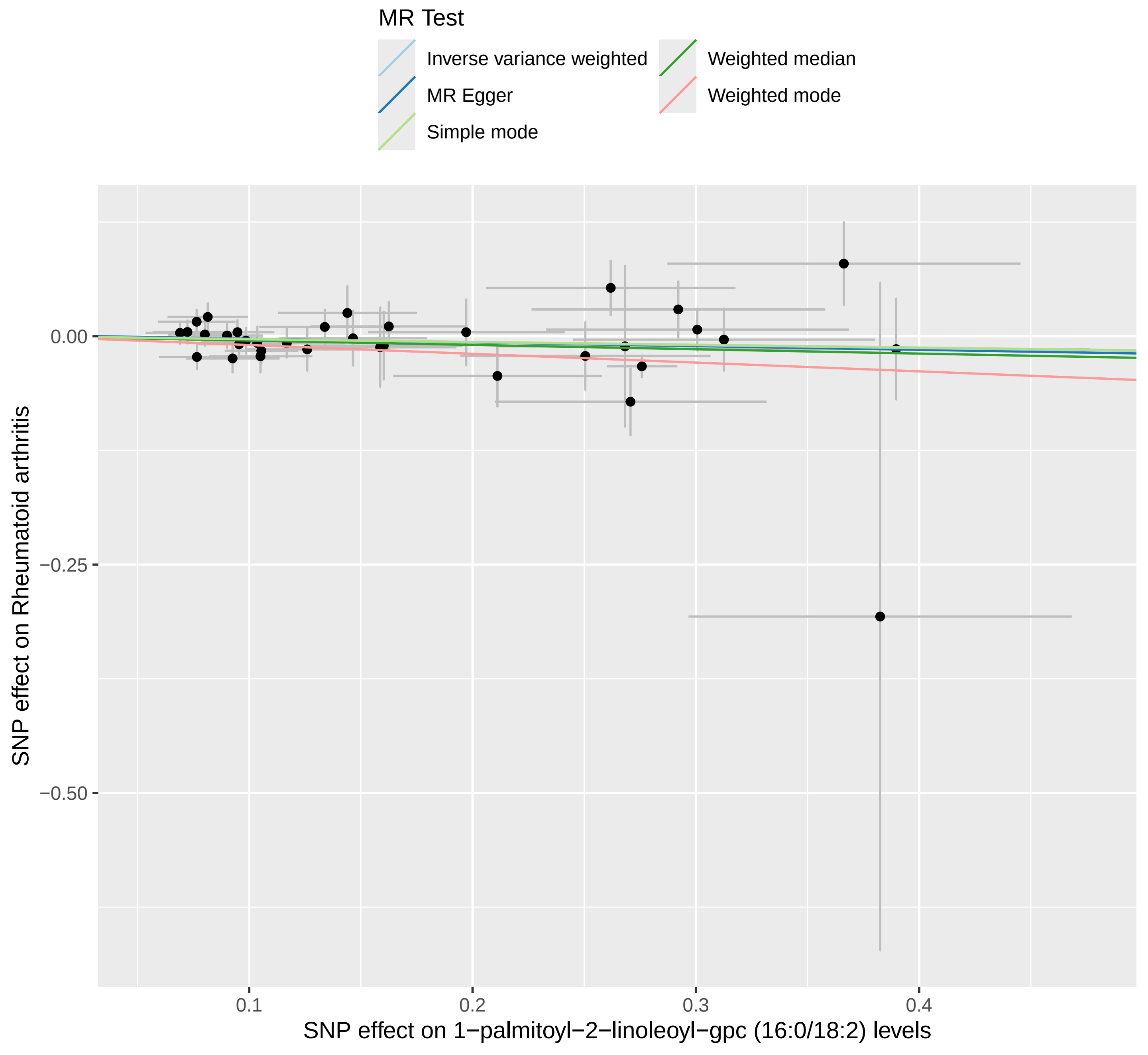

MR Test
Inverse variance weighted
MR Egger
Simple mode
Weighted median
Weighted mode
0.00
−0.25
−0.50
0.1
0.2
0.3
0.4
SNP effect on Rheumatoid arthritis
SNP effect on 1−palmitoyl−2−linoleoyl−gpc (16:0/18:2) levels

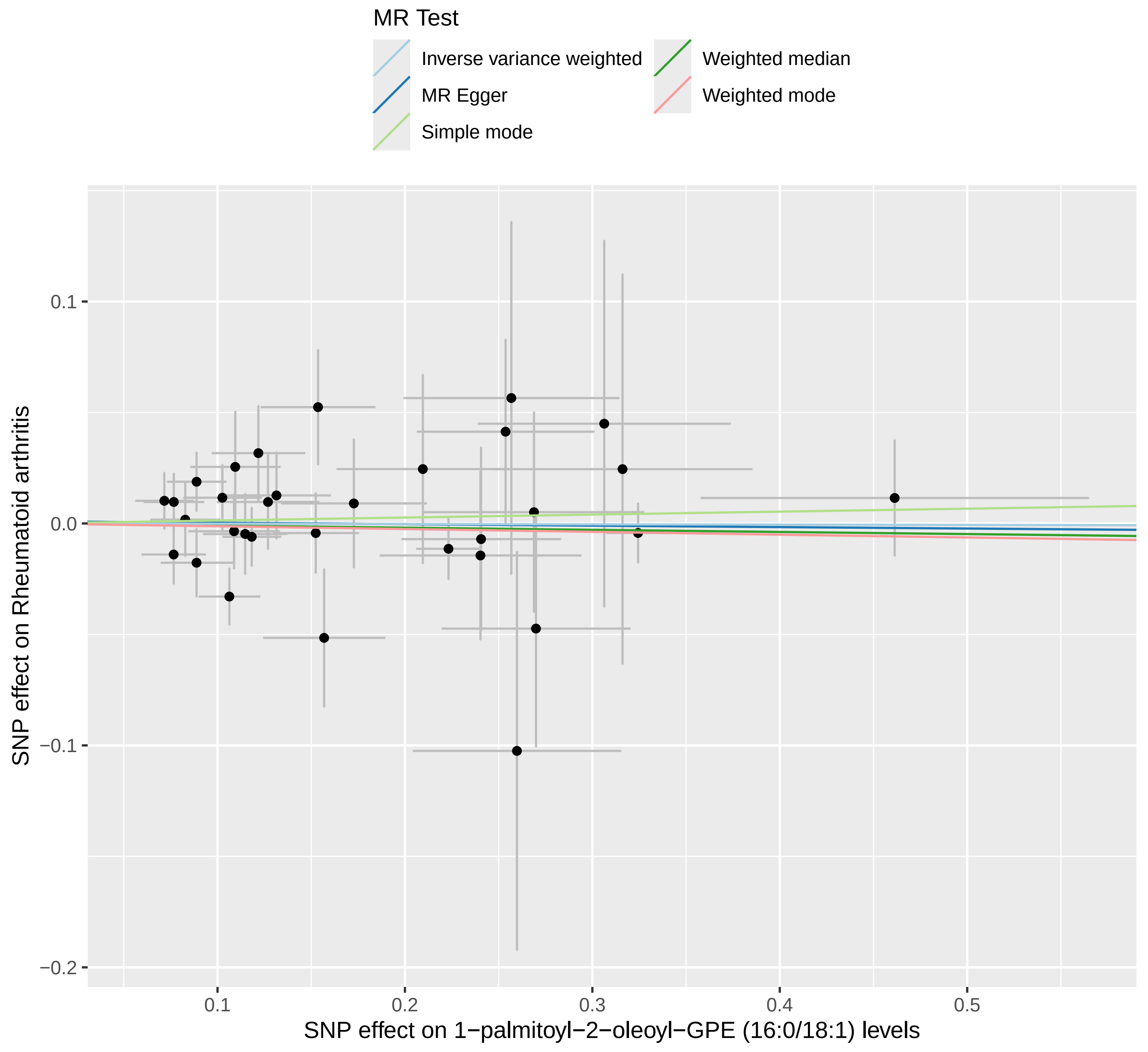
MR Test
Inverse variance weighted
MR Egger
Simple mode
Weighted median
Weighted mode
0.1
0.0
−0.1
−0.2
0.1
0.2
0.3
0.4
0.5
SNP effect on Rheumatoid arthritis
SNP effect on 1−palmitoyl−2−oleoyl−GPE (16:0/18:1) levels

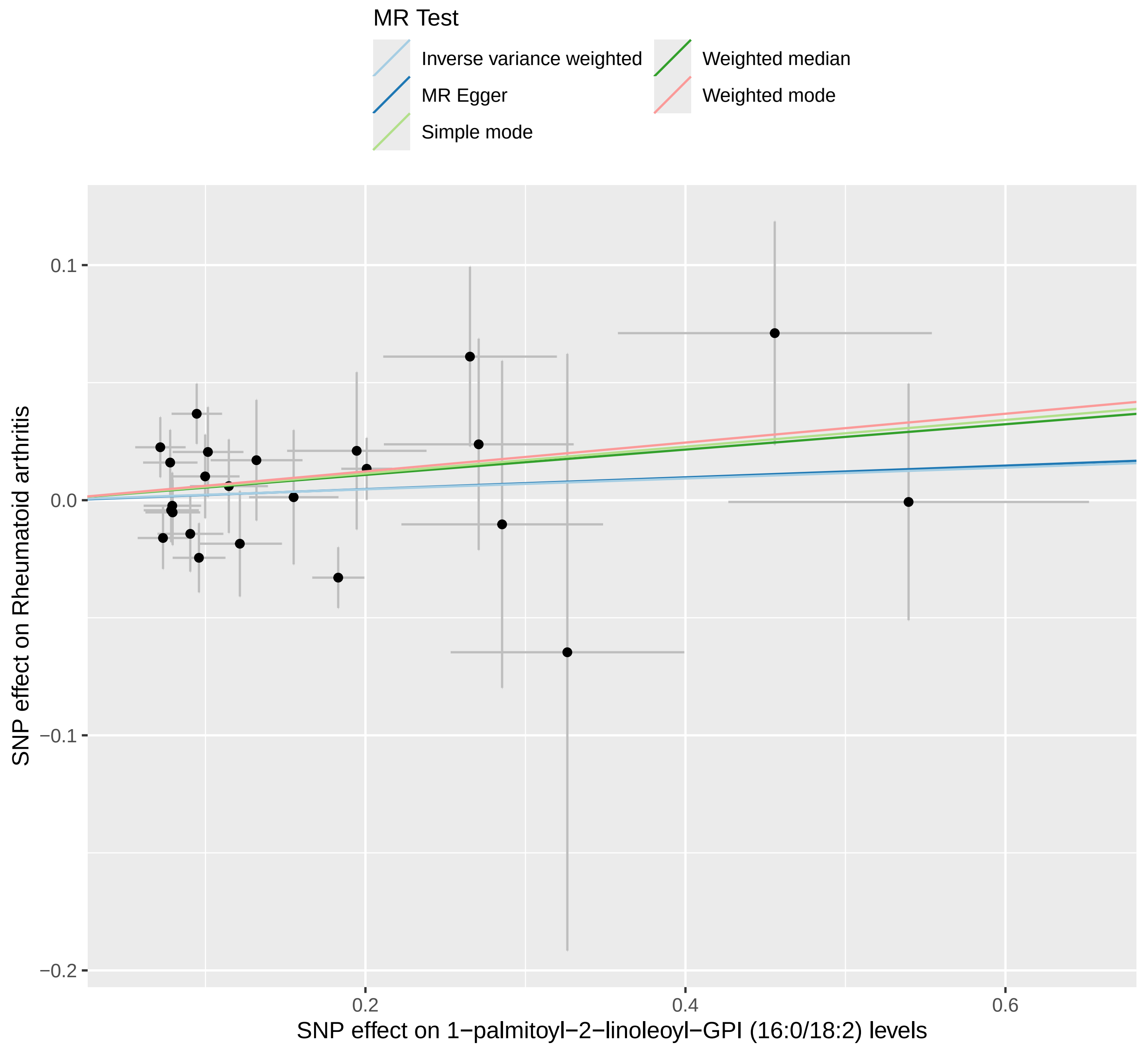

MR Test
Inverse variance weighted
MR Egger
Simple mode
Weighted median
Weighted mode
0.1
0.0
−0.1
−0.2
0.2
0.4
0.6
SNP effect on Rheumatoid arthritis
SNP effect on 1−palmitoyl−2−linoleoyl−GPI (16:0/18:2) levels

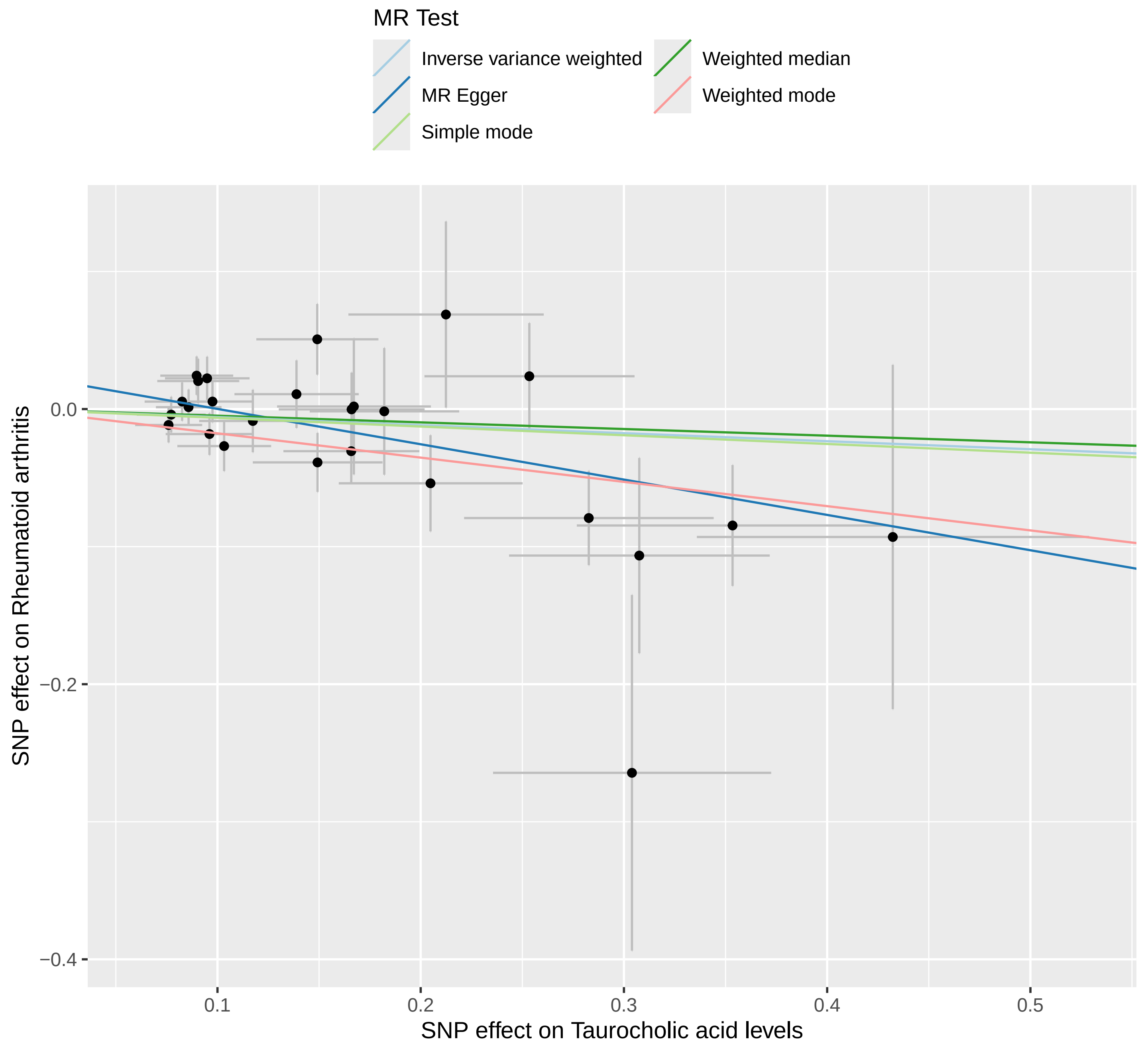
MR Test
Inverse variance weighted
MR Egger
Simple mode
Weighted median
Weighted mode
SNP effect on Rheumatoid arthritis
0.0
−0.2
−0.4
0.1
0.2
0.3
0.4
0.5
SNP effect on Taurocholic acid levels

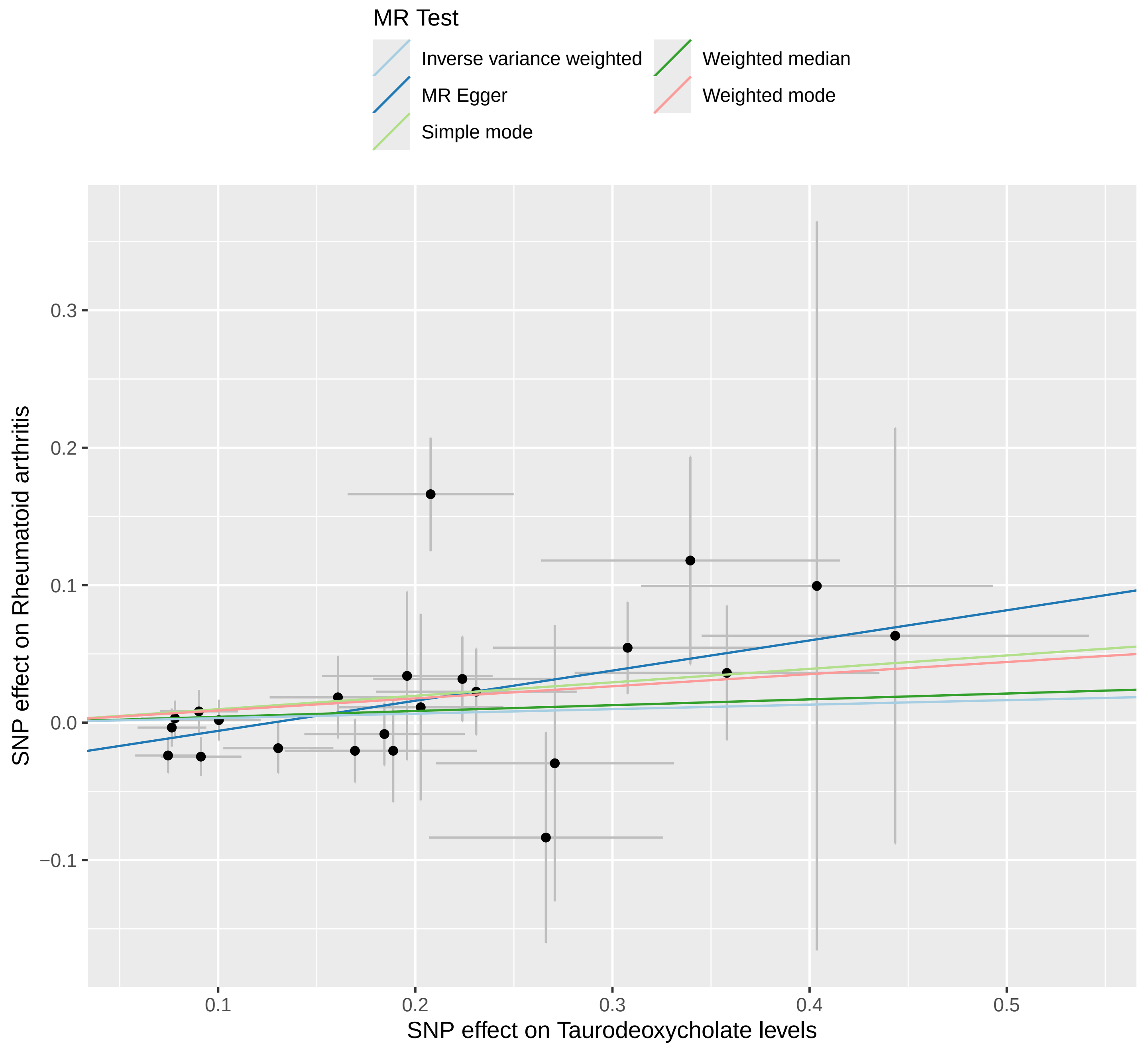
MR Test
Inverse variance weighted
MR Egger
Simple mode
Weighted median
Weighted mode
0.3
0.2
0.1
0.0
−0.1
0.1
0.2
0.3
0.4
0.5
SNP effect on Rheumatoid arthritis
SNP effect on Taurodeoxycholate levels

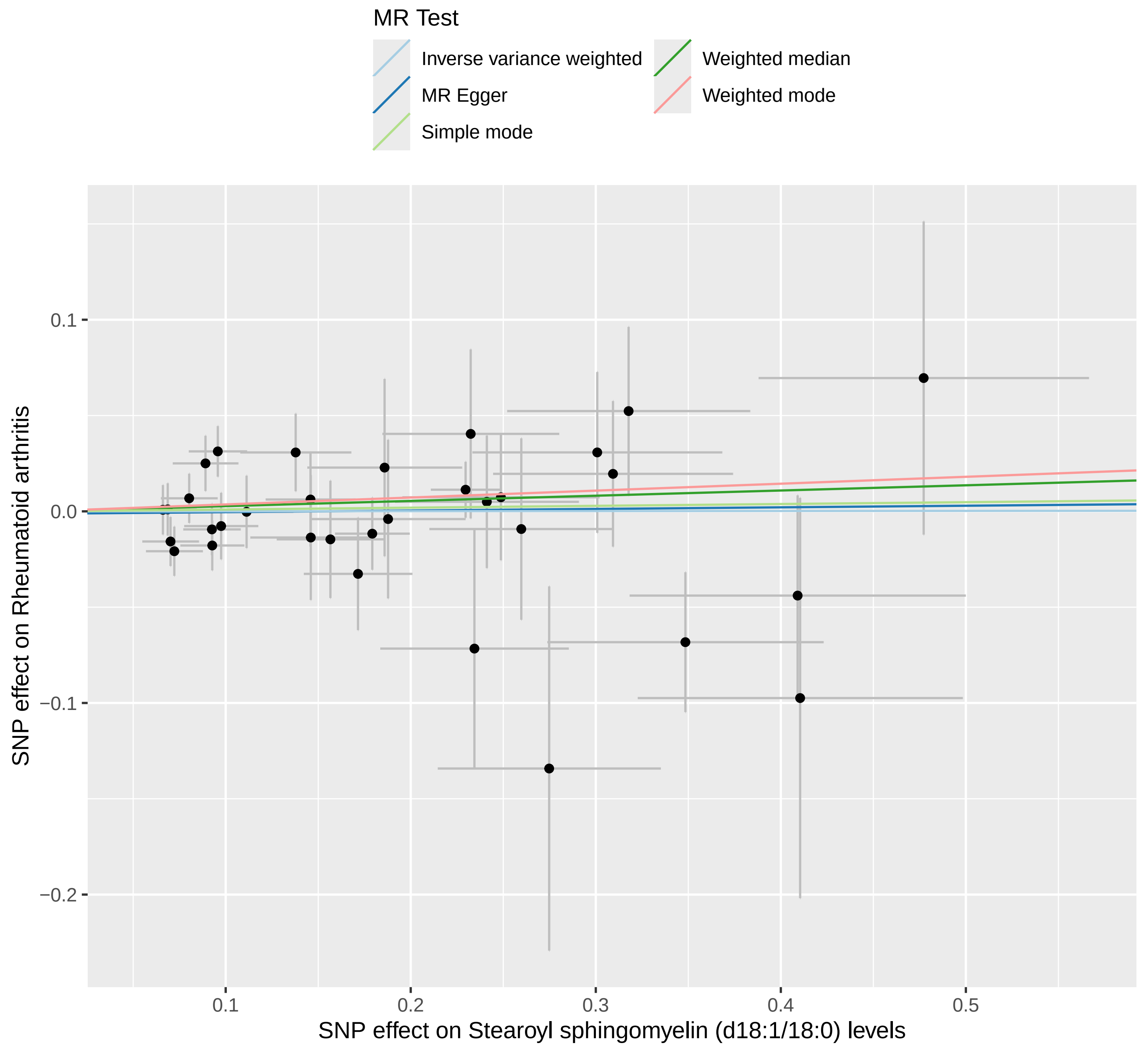
MR Test
Inverse variance weighted
MR Egger
Simple mode
Weighted median
Weighted mode
SNP effect on Rheumatoid arthritis
0.1
0.0
−0.1
−0.2
0.1
0.2
0.3
0.4
0.5
SNP effect on Stearoyl sphingomyelin (d18:1/18:0) levels

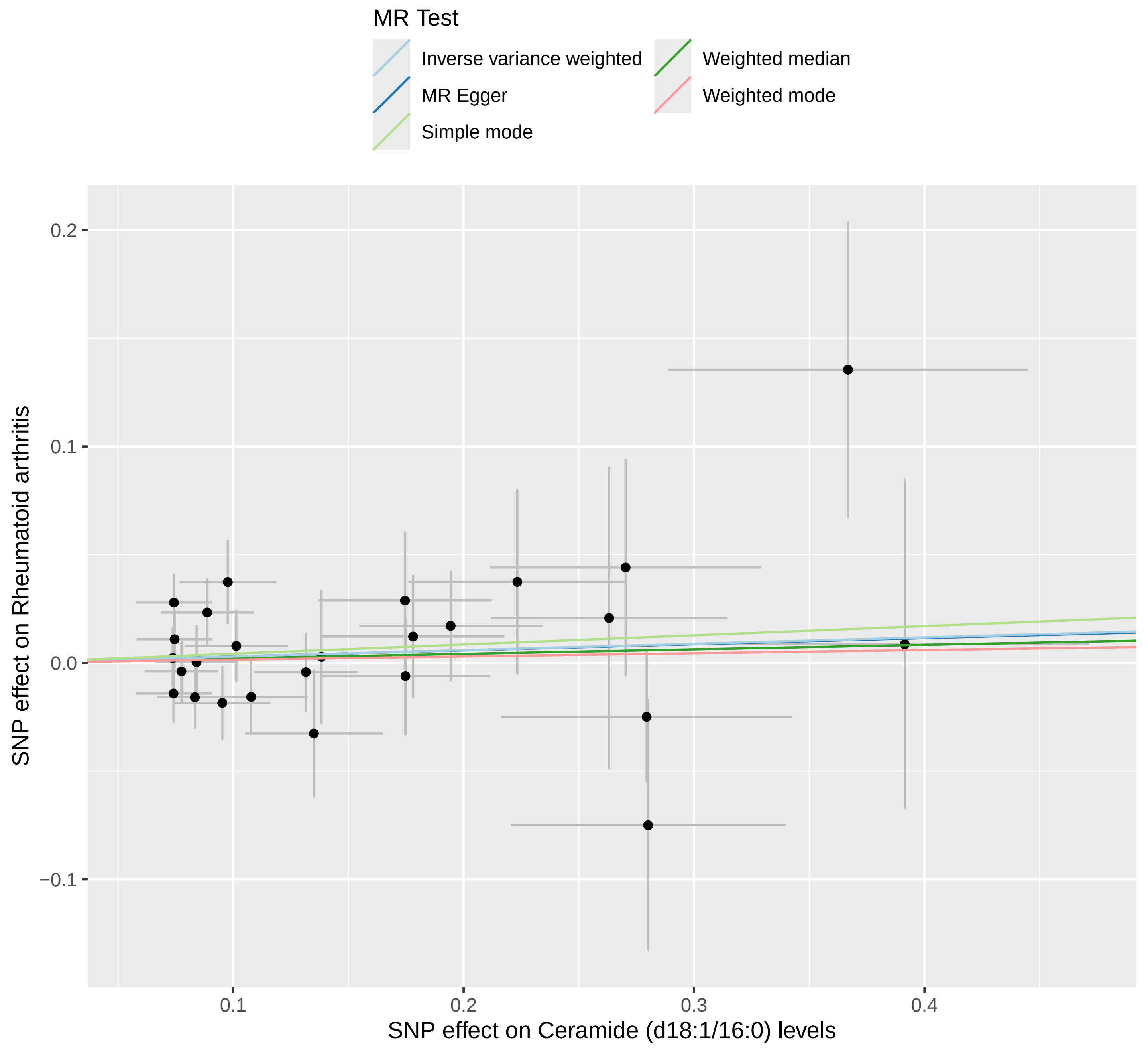
MR Test
Inverse variance weighted
MR Egger
Simple mode
Weighted median
Weighted mode
0.2
0.1
0.0
−0.1
0.1
0.2
0.3
0.4
SNP effect on Rheumatoid arthritis
SNP effect on Ceramide (d18:1/16:0) levels

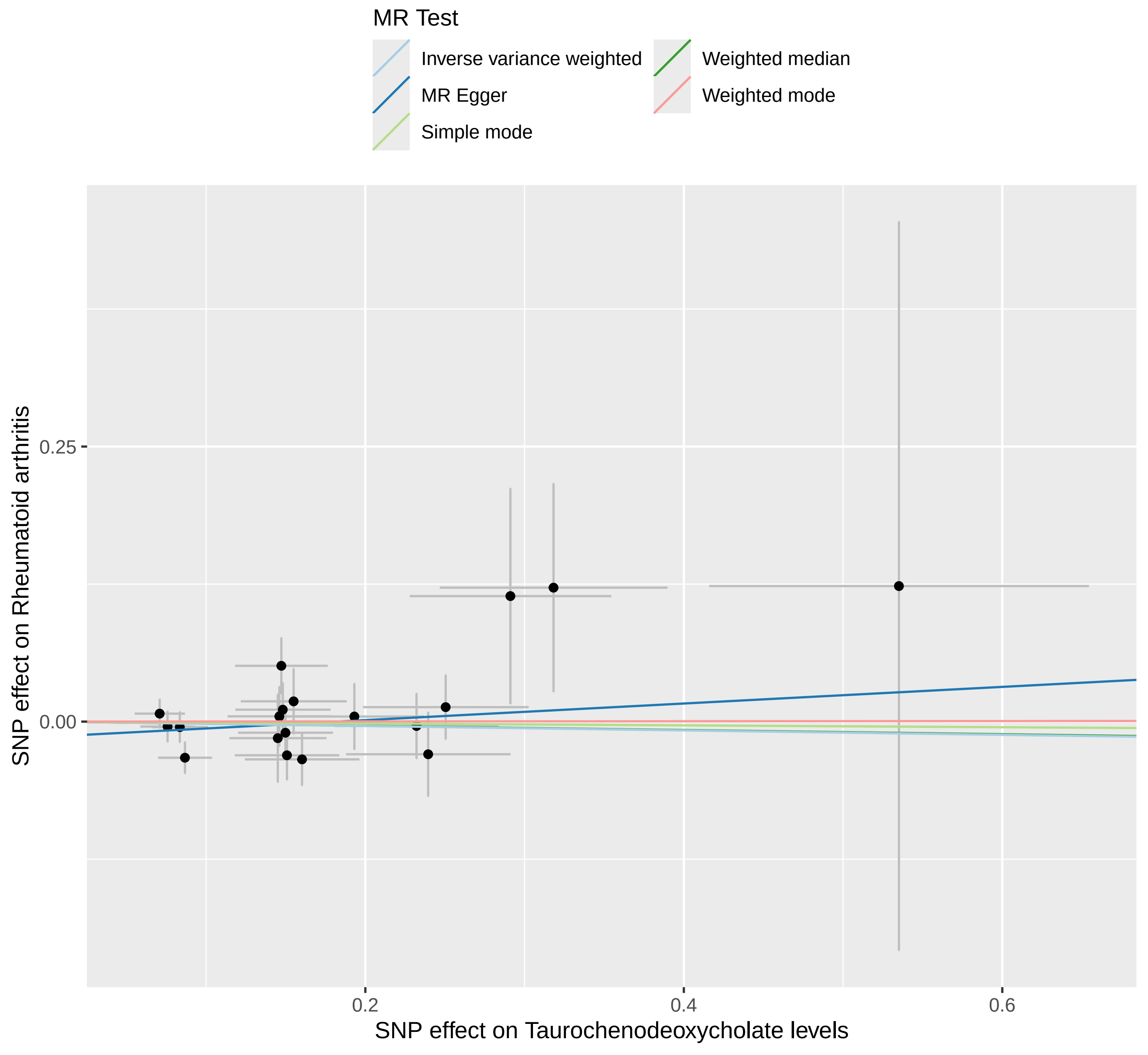
MR Test
Inverse variance weighted
MR Egger
Simple mode
Weighted median
Weighted mode
0.25
0.00
0.2
0.4
0.6
SNP effect on Rheumatoid arthritis
SNP effect on Taurochenodeoxycholate levels

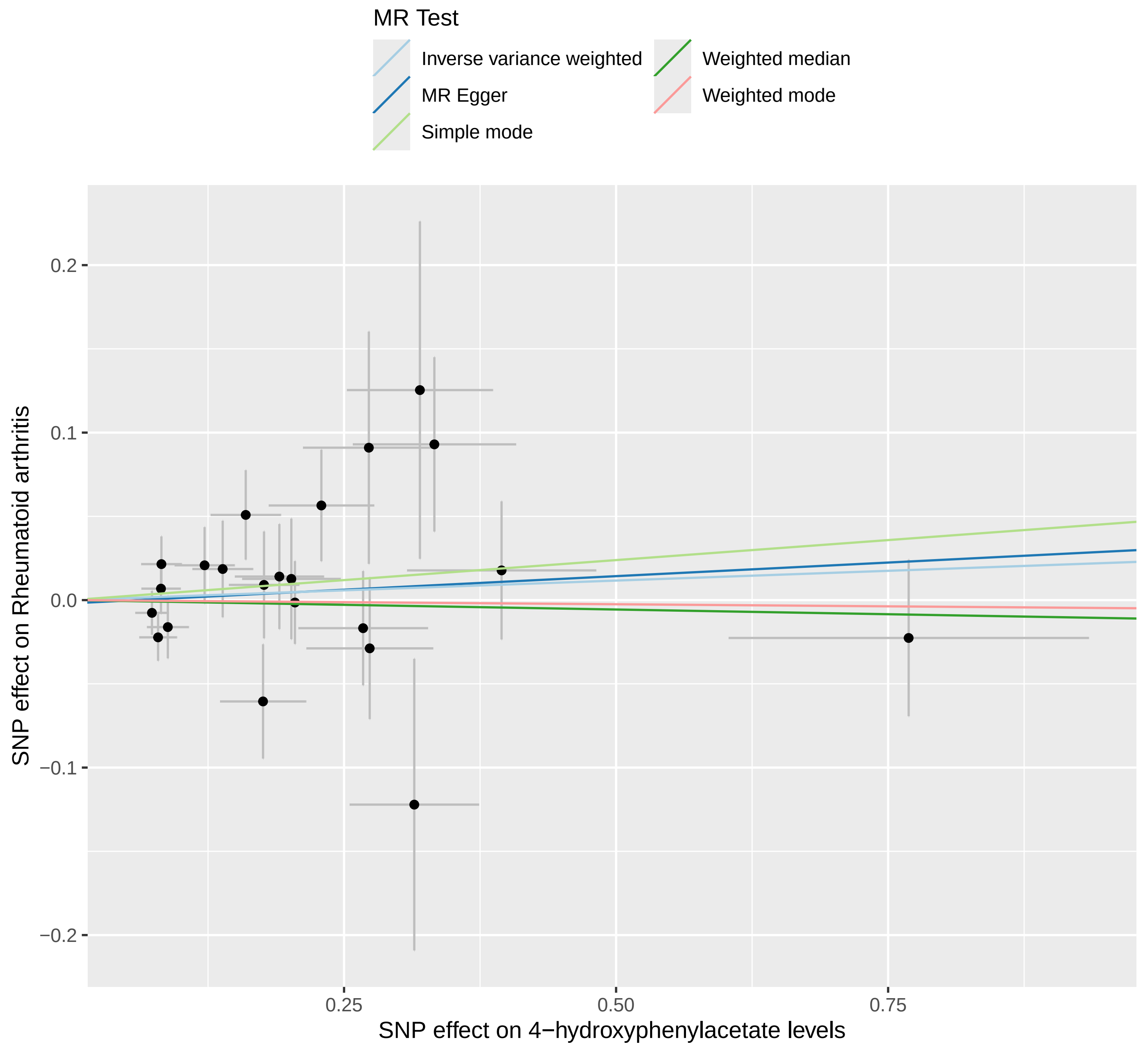

MR Test
Inverse variance weighted
MR Egger
Simple mode
Weighted median
Weighted mode
0.2
0.1
0.0
−0.1
−0.2
SNP effect on Rheumatoid arthritis
0.25
0.50
0.75
SNP effect on 4−hydroxyphenylacetate levels

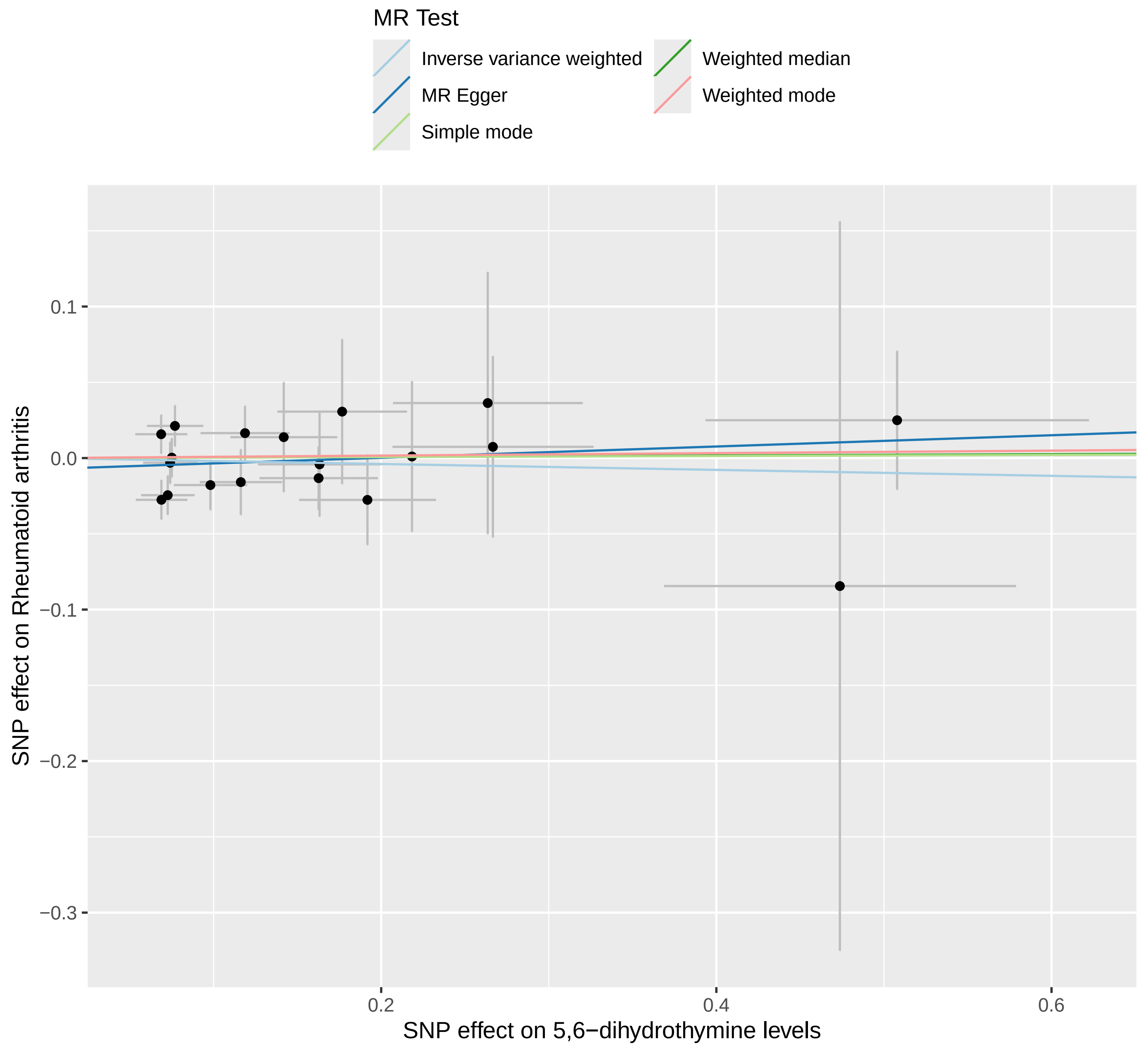
MR Test
Inverse variance weighted
MR Egger
Simple mode
Weighted median
Weighted mode
0.1
0.0
−0.1
−0.2
−0.3
0.2
0.4
0.6
SNP effect on Rheumatoid arthritis
SNP effect on 5,6−dihydrothymine levels

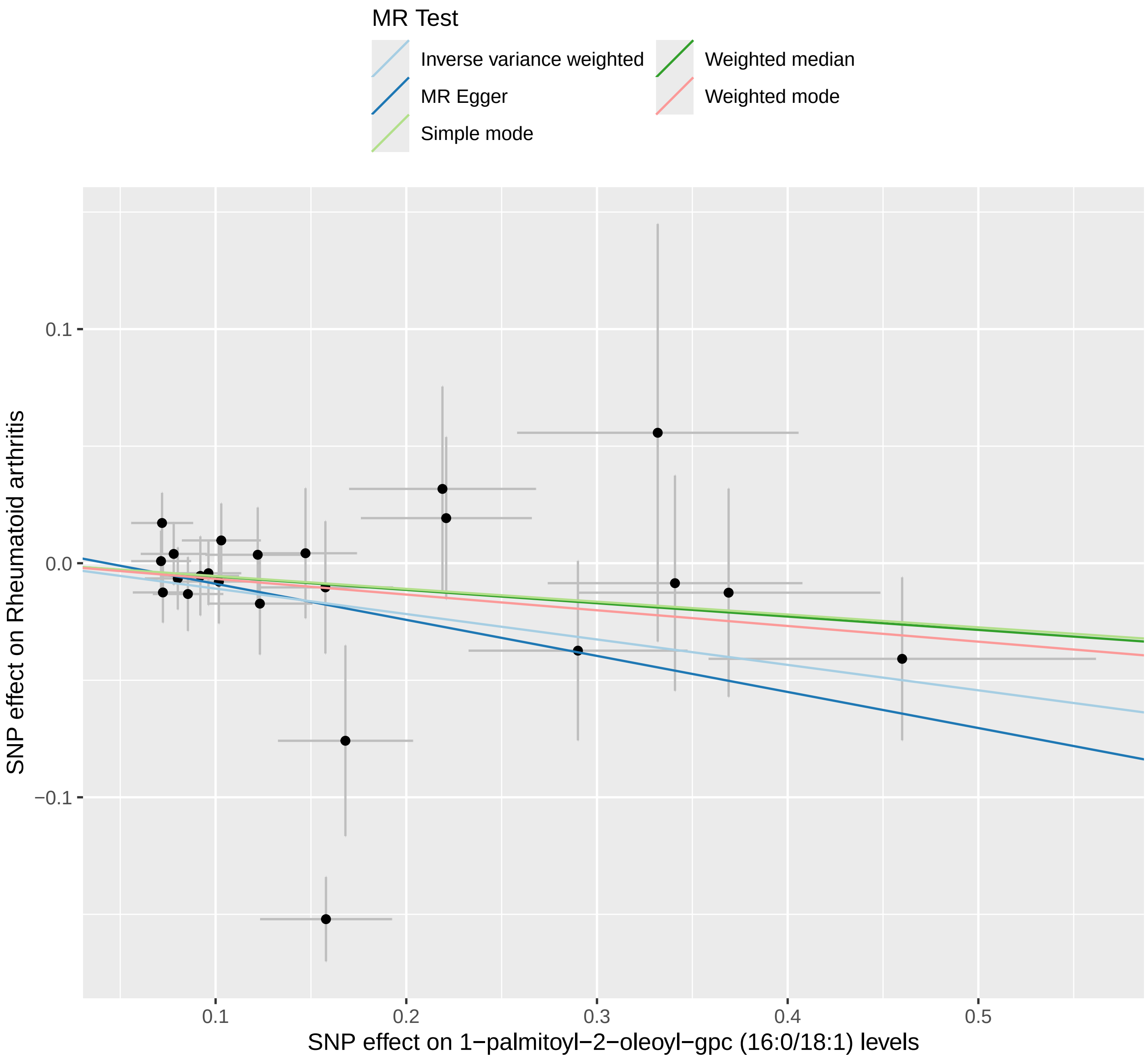
MR Test
Inverse variance weighted
MR Egger
Simple mode
Weighted median
Weighted mode
SNP effect on Rheumatoid arthritis
0.1
0.0
−0.1
0.1
0.2
0.3
0.4
0.5
SNP effect on 1−palmitoyl−2−oleoyl−gpc (16:0/18:1) levels

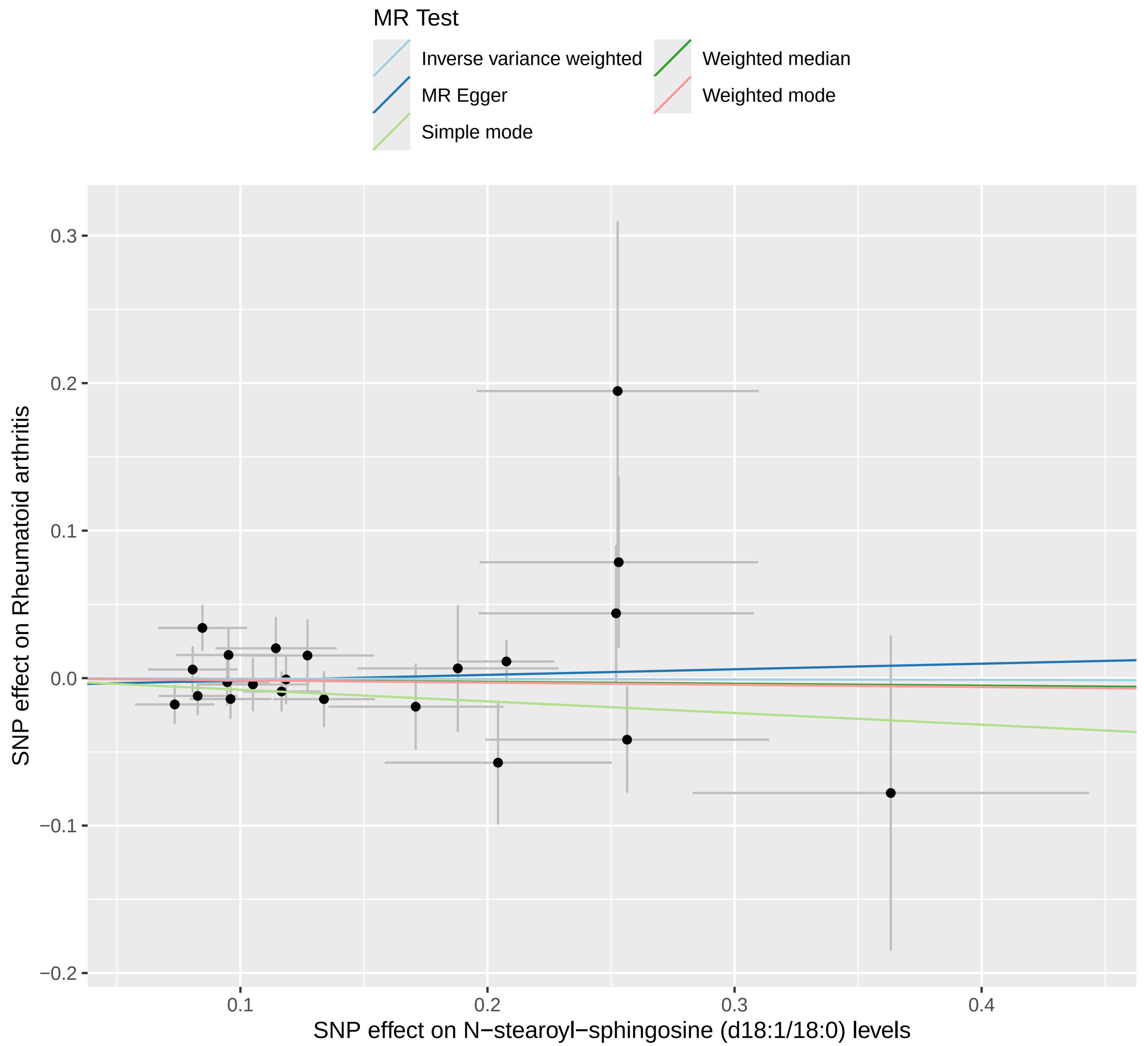
MR Test
Inverse variance weighted
MR Egger
Simple mode
Weighted median
Weighted mode
SNP effect on Rheumatoid arthritis
0.3
0.2
0.1
0.0
−0.1
−0.2
0.1
0.2
0.3
0.4
SNP effect on N−stearoyl−sphingosine (d18:1/18:0) levels

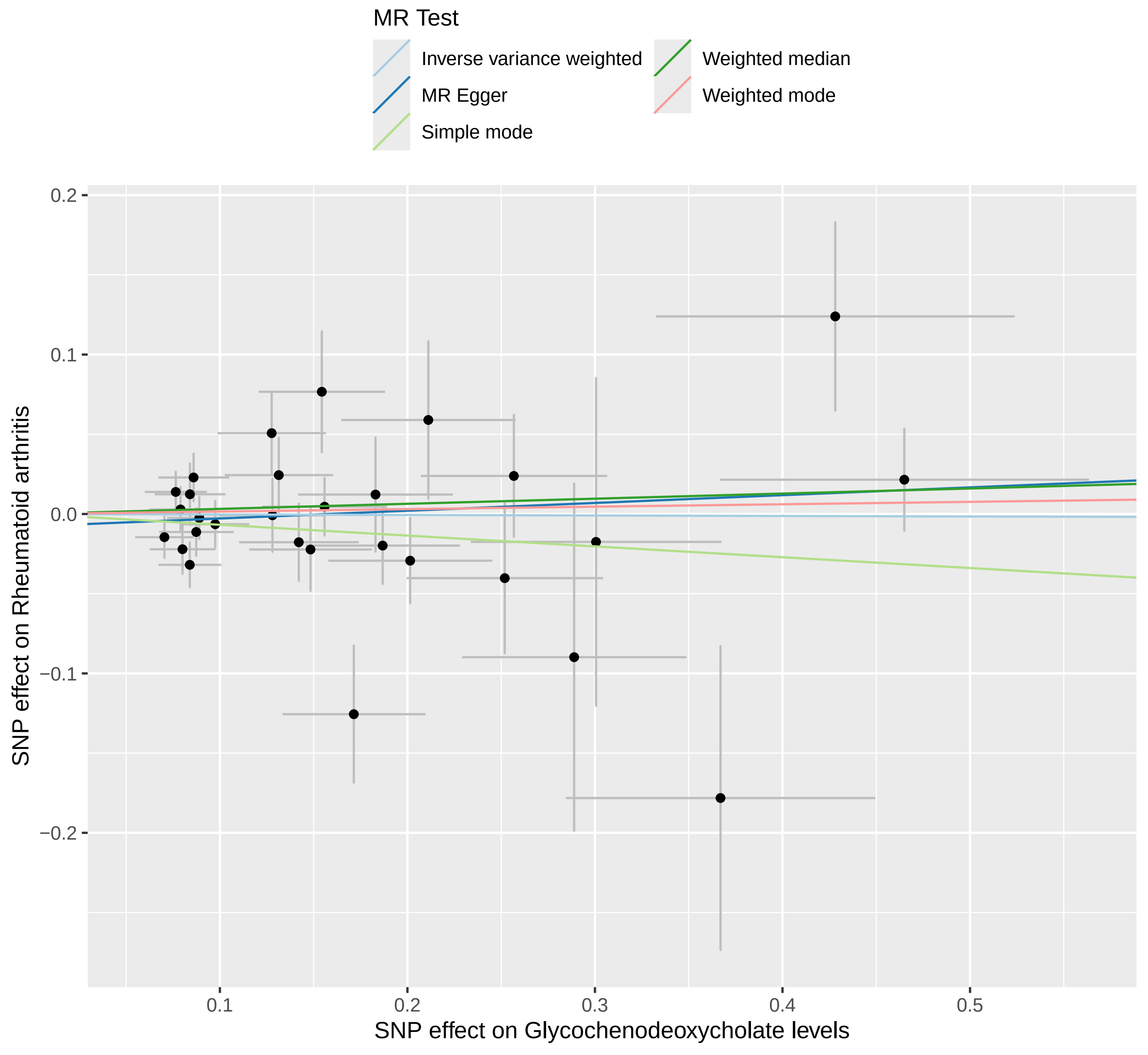
MR Test
Inverse variance weighted
MR Egger
Simple mode
Weighted median
Weighted mode
0.2
0.1
0.0
−0.1
−0.2
SNP effect on Rheumatoid arthritis
0.1
0.2
0.3
0.4
0.5
SNP effect on Glycochenodeoxycholate levels

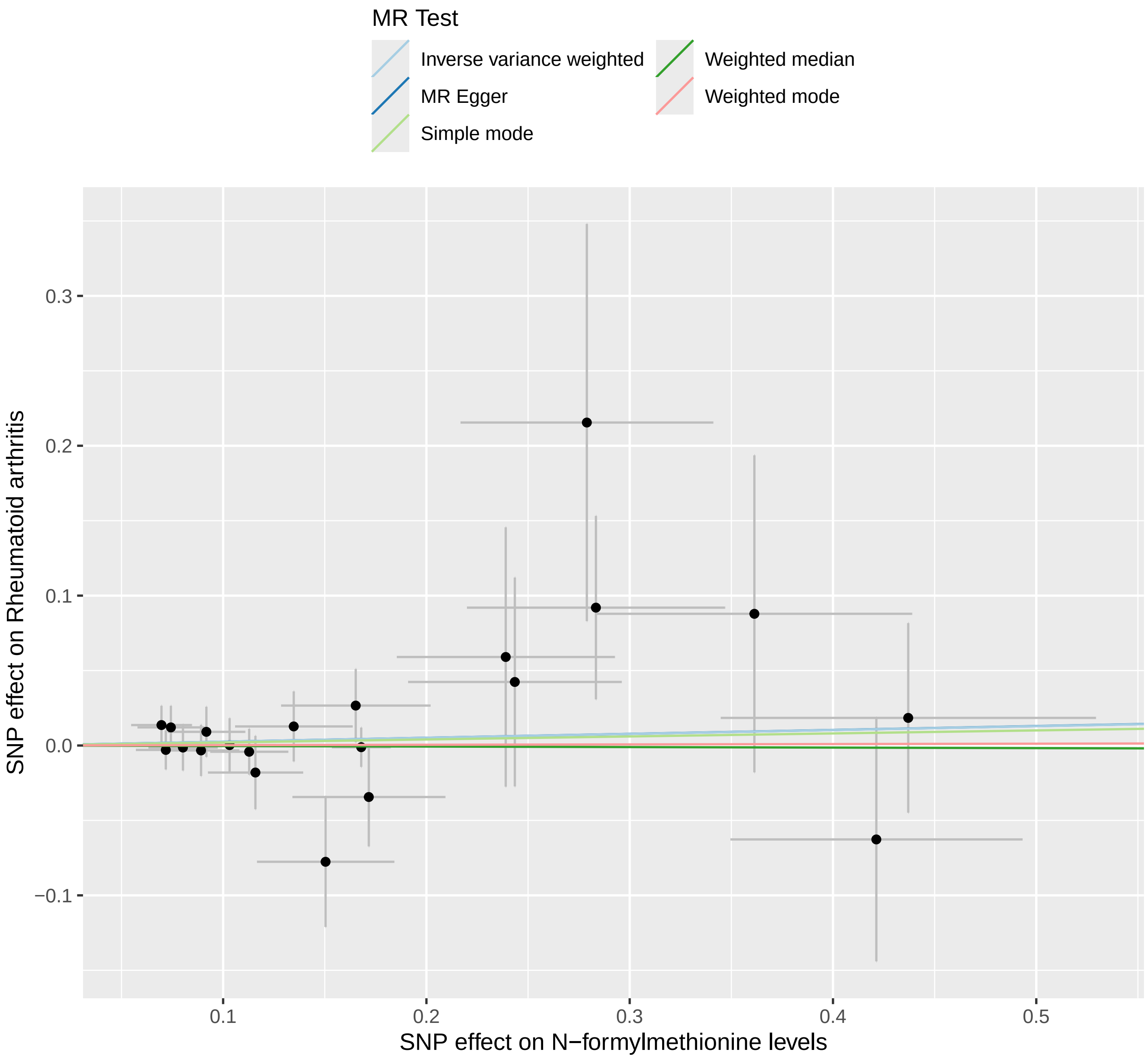
MR Test
Inverse variance weighted
MR Egger
Simple mode
Weighted median
Weighted mode
0.3
0.2
0.1
0.0
−0.1
SNP effect on Rheumatoid arthritis
0.1
0.2
0.3
0.4
0.5
SNP effect on N−formylmethionine levels

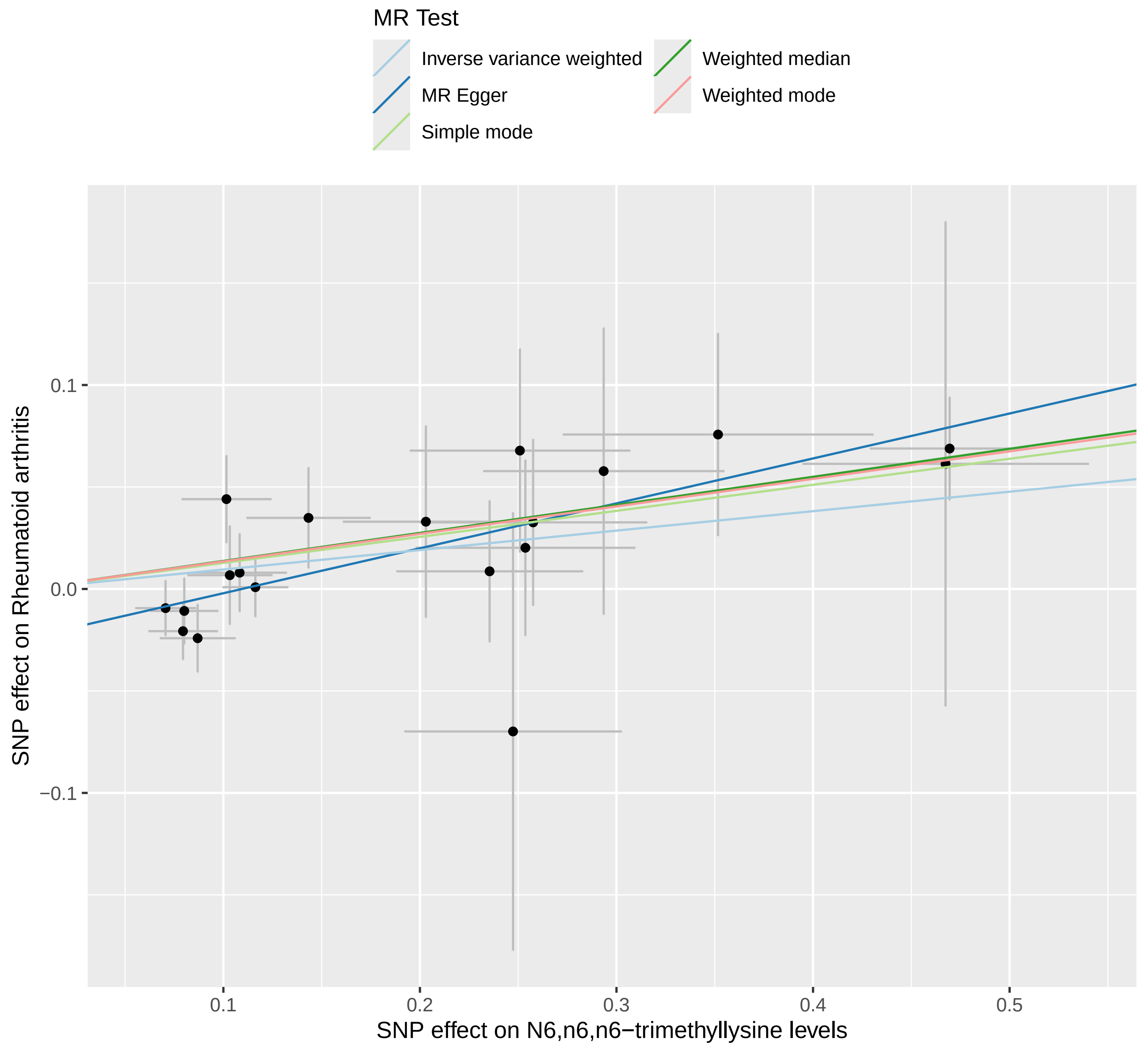
MR Test
Inverse variance weighted
MR Egger
Simple mode
Weighted median
Weighted mode
SNP effect on Rheumatoid arthritis
0.1
0.0
−0.1
0.1
0.2
0.3
0.4
0.5
SNP effect on N6,n6,n6−trimethyllysine levels

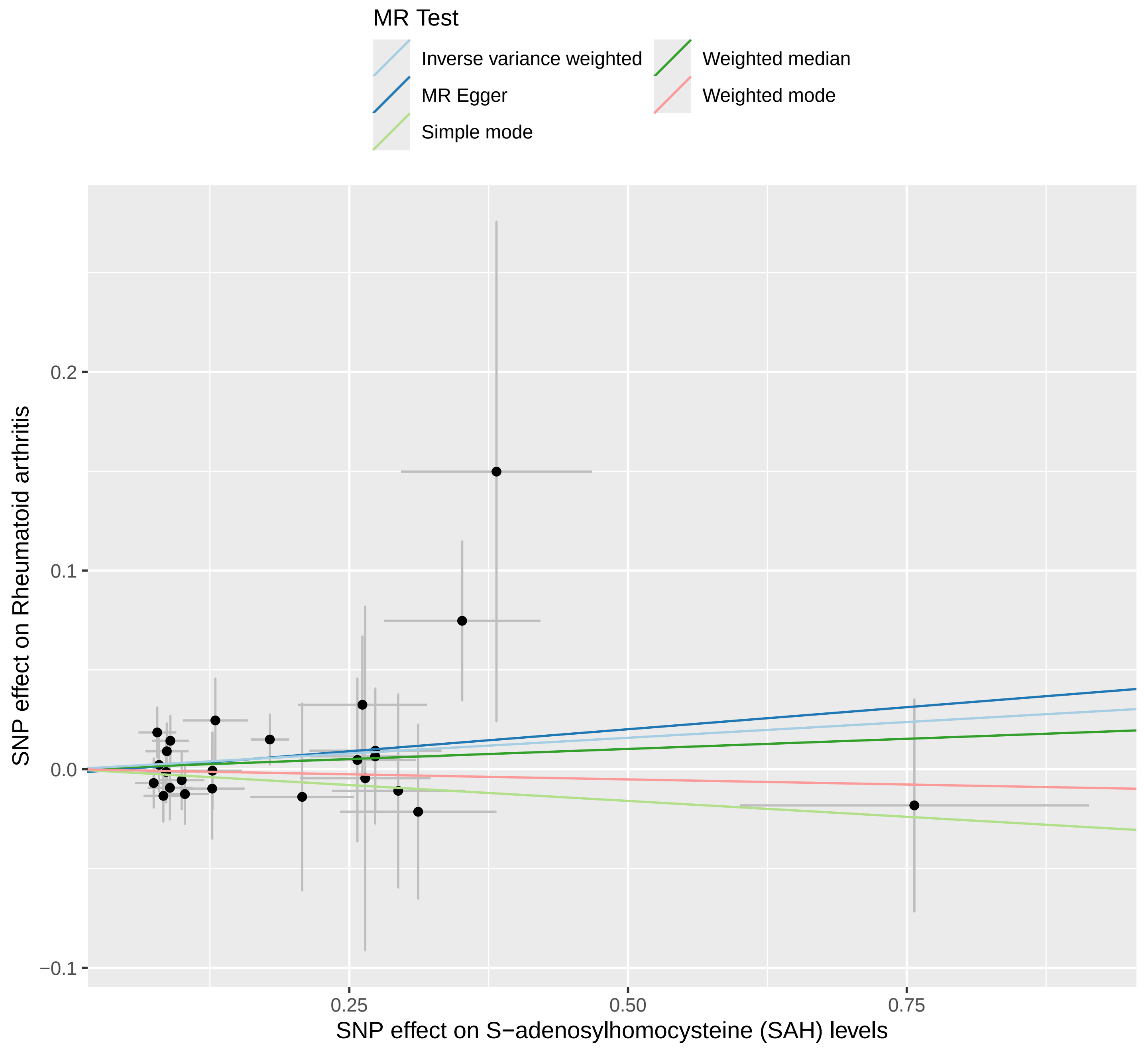
MR Test
Inverse variance weighted
MR Egger
Simple mode
Weighted median
Weighted mode
0.2
0.1
0.0
−0.1
SNP effect on Rheumatoid arthritis
0.25
0.50
0.75
SNP effect on S−adenosylhomocysteine (SAH) levels

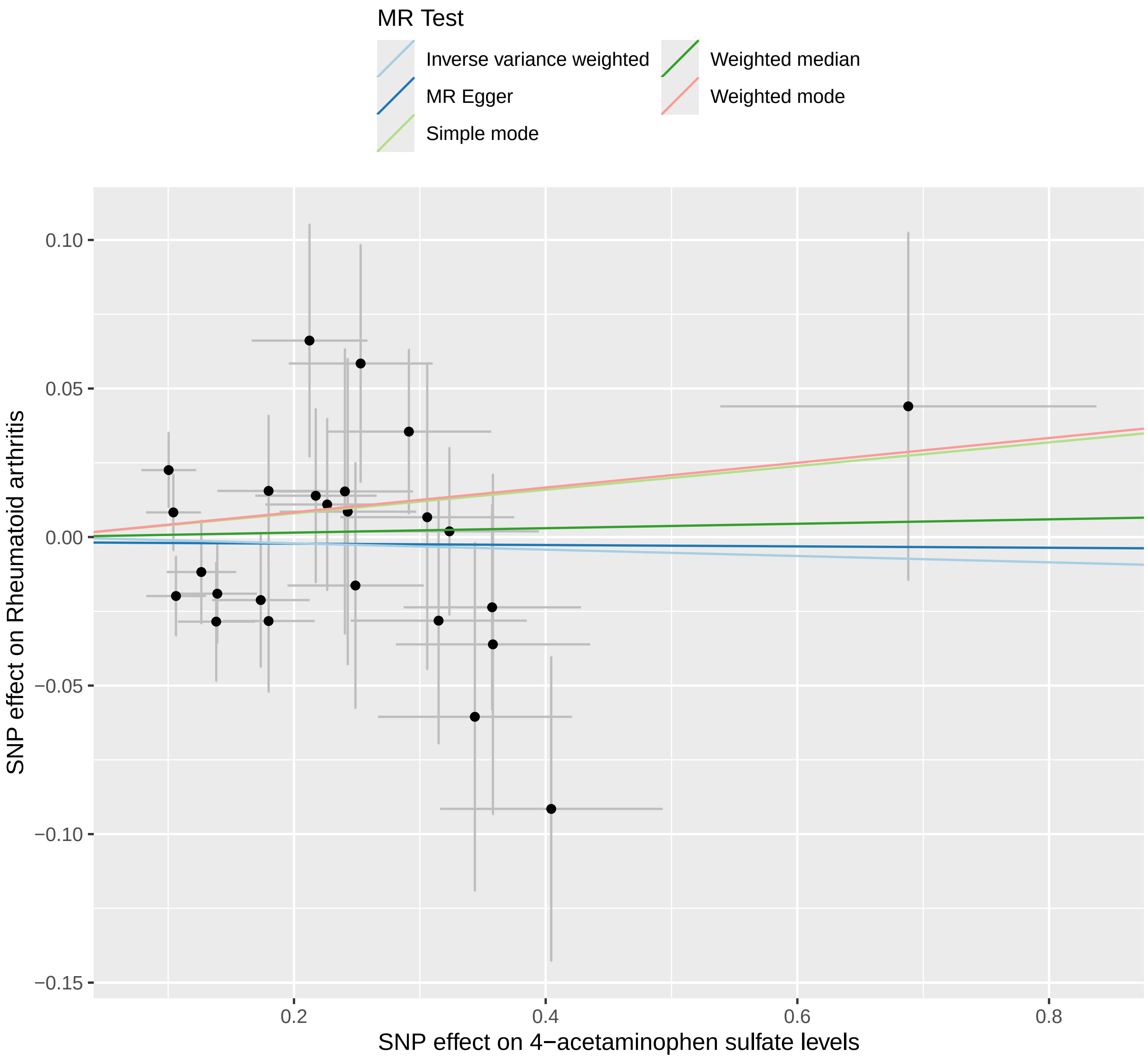

MR Test
Inverse variance weighted
MR Egger
Simple mode
Weighted median
Weighted mode
0.10
0.05
0.00
−0.05
−0.10
−0.15
SNP effect on Rheumatoid arthritis
0.2
0.4
0.6
0.8
SNP effect on 4−acetaminophen sulfate levels

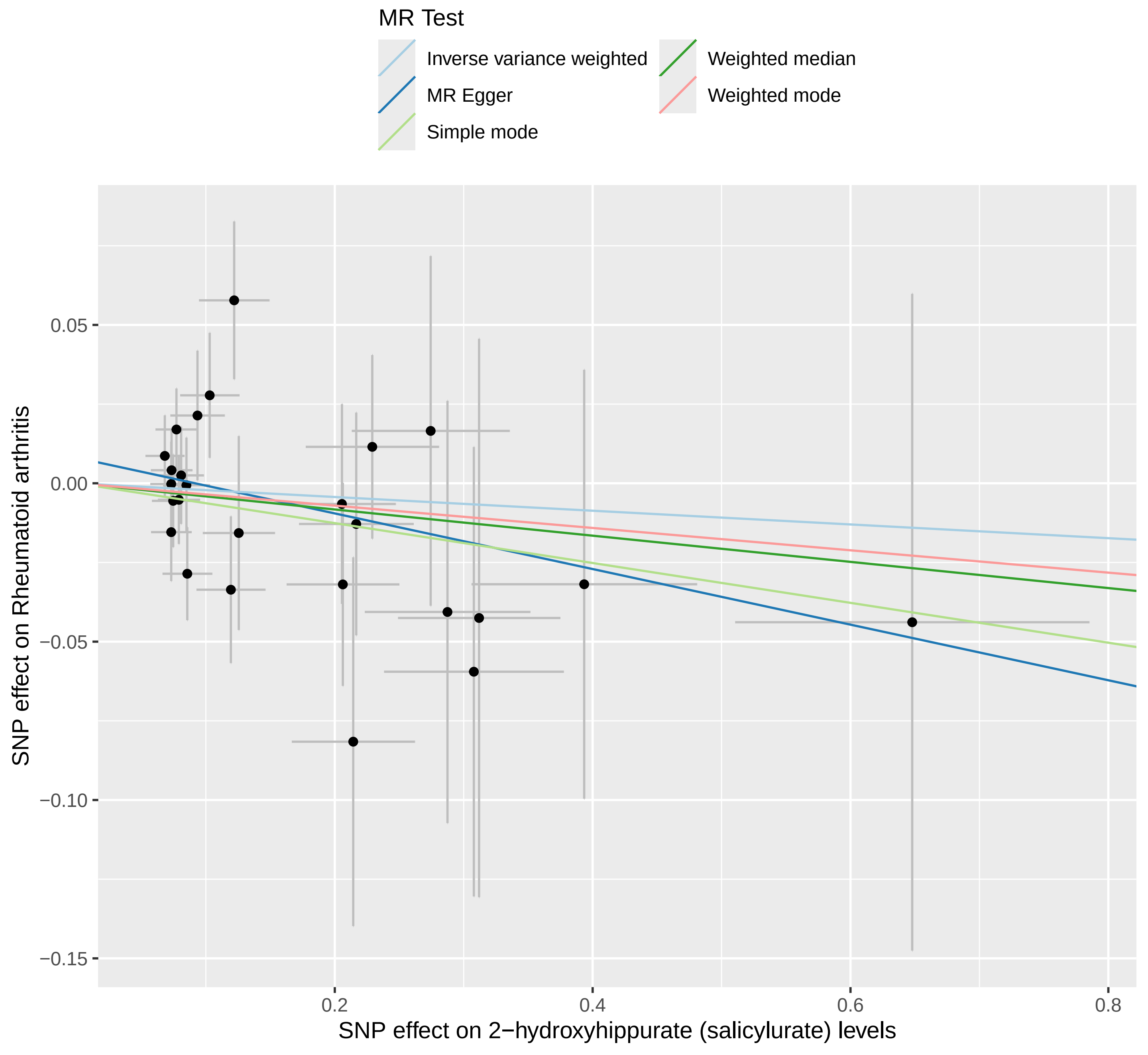

MR Test
Inverse variance weighted
MR Egger
Simple mode
Weighted median
Weighted mode
SNP effect on Rheumatoid arthritis
0.05
0.00
−0.05
−0.10
−0.15
0.2
0.4
0.6
0.8
SNP effect on 2−hydroxyhippurate (salicylurate) levels

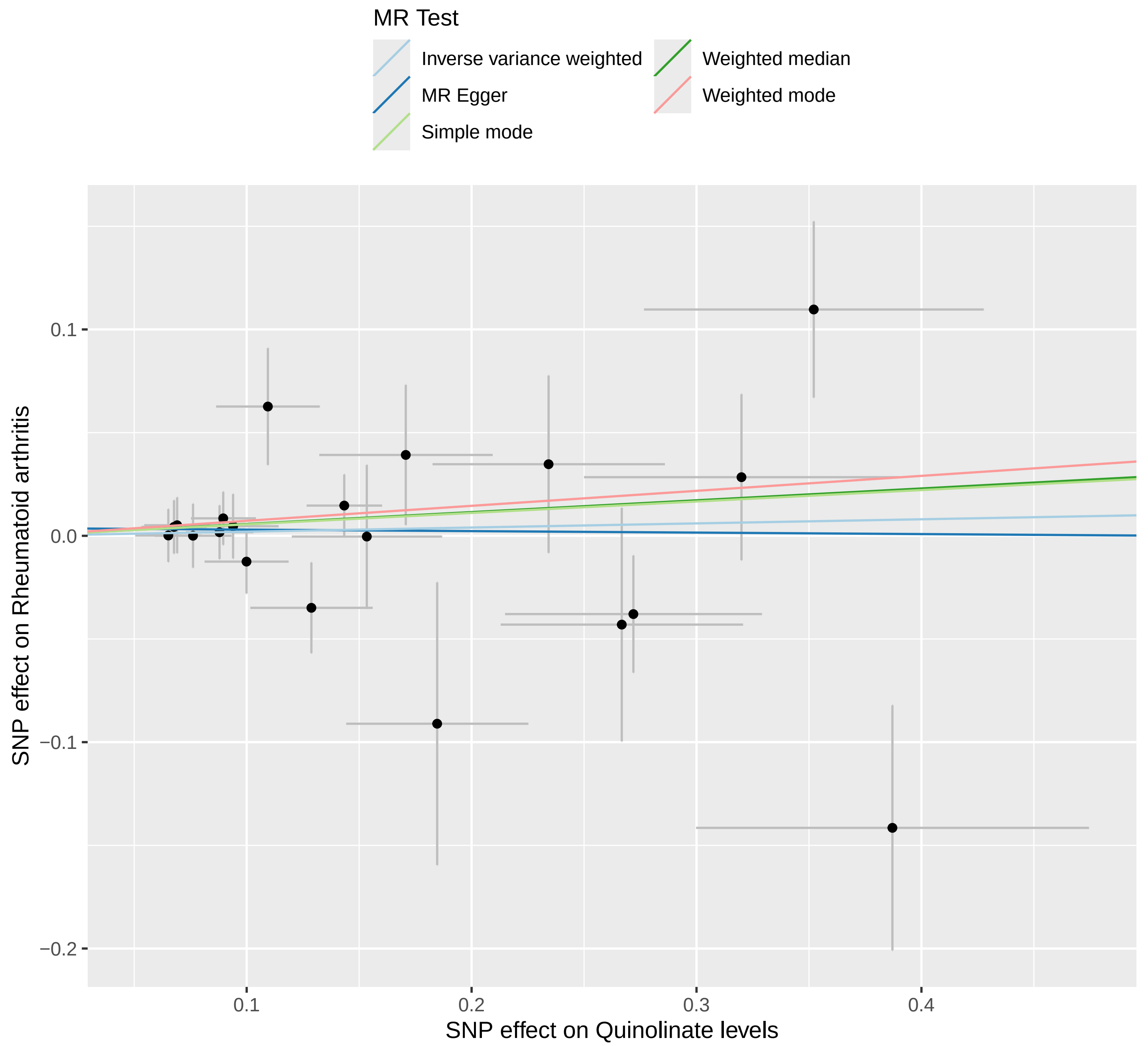
MR Test
Inverse variance weighted
MR Egger
Simple mode
Weighted median
Weighted mode
0.1
0.0
−0.1
−0.2
0.1
0.2
0.3
0.4
SNP effect on Rheumatoid arthritis
SNP effect on Quinolinate levels

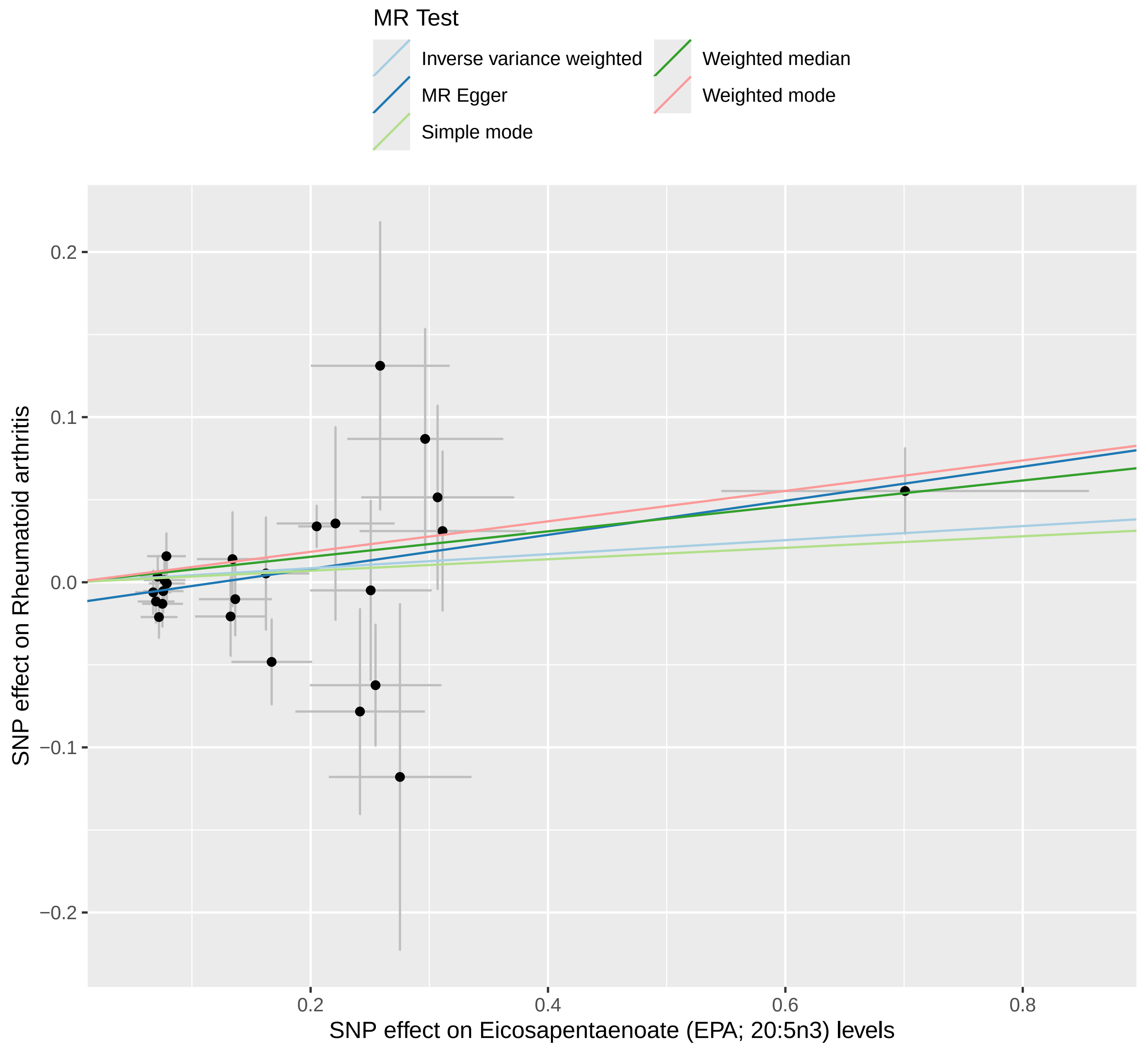
MR Test
Inverse variance weighted
MR Egger
Simple mode
Weighted median
Weighted mode
0.2
0.1
0.0
−0.1
−0.2
0.2
0.4
0.6
0.8
SNP effect on Rheumatoid arthritis
SNP effect on Eicosapentaenoate (EPA; 20:5n3) levels

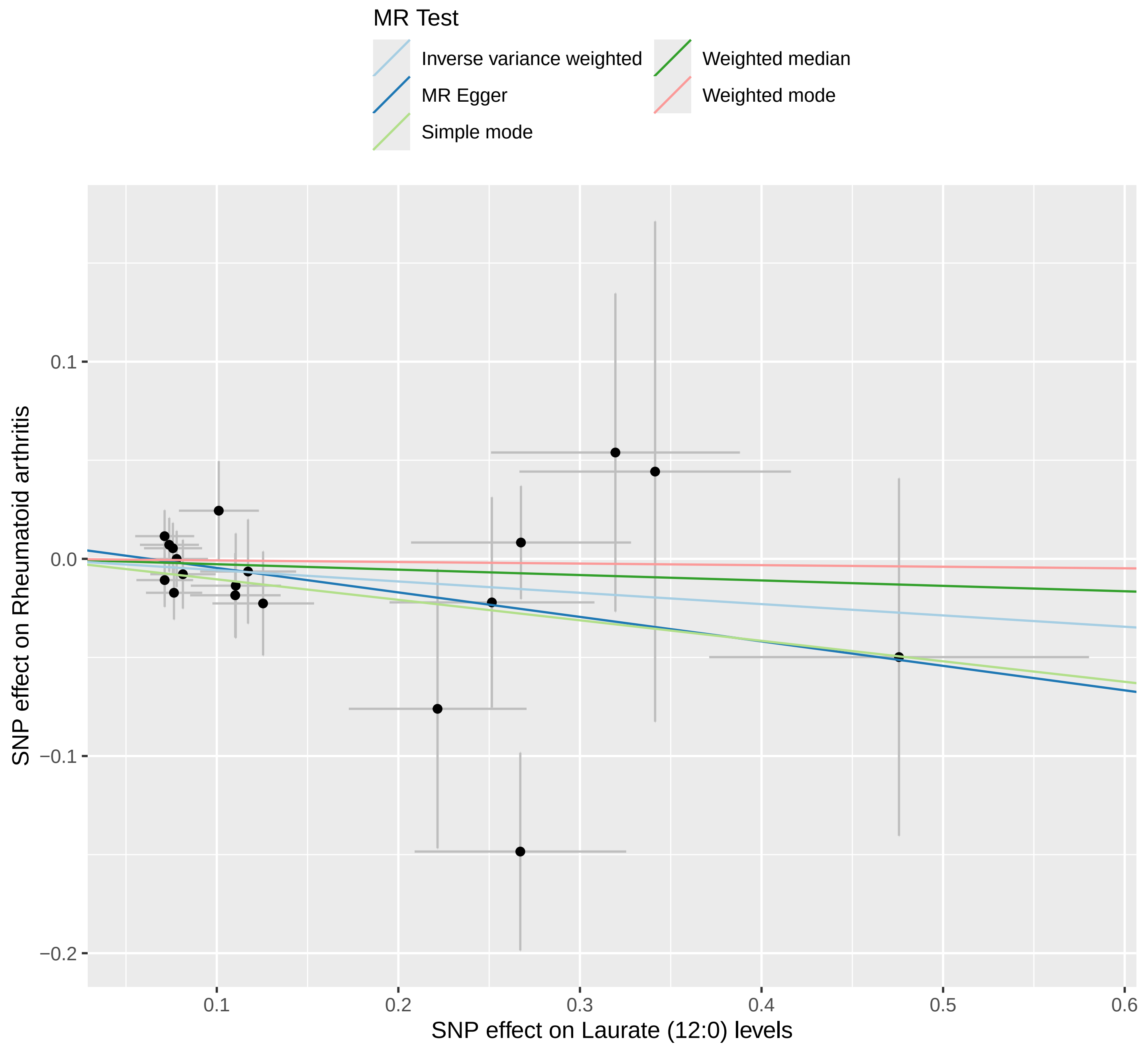

MR Test
Inverse variance weighted
MR Egger
Simple mode
Weighted median
Weighted mode
0.1
0.0
−0.1
−0.2
SNP effect on Rheumatoid arthritis
0.1
0.2
0.3
0.4
0.5
0.6
SNP effect on Laurate (12:0) levels

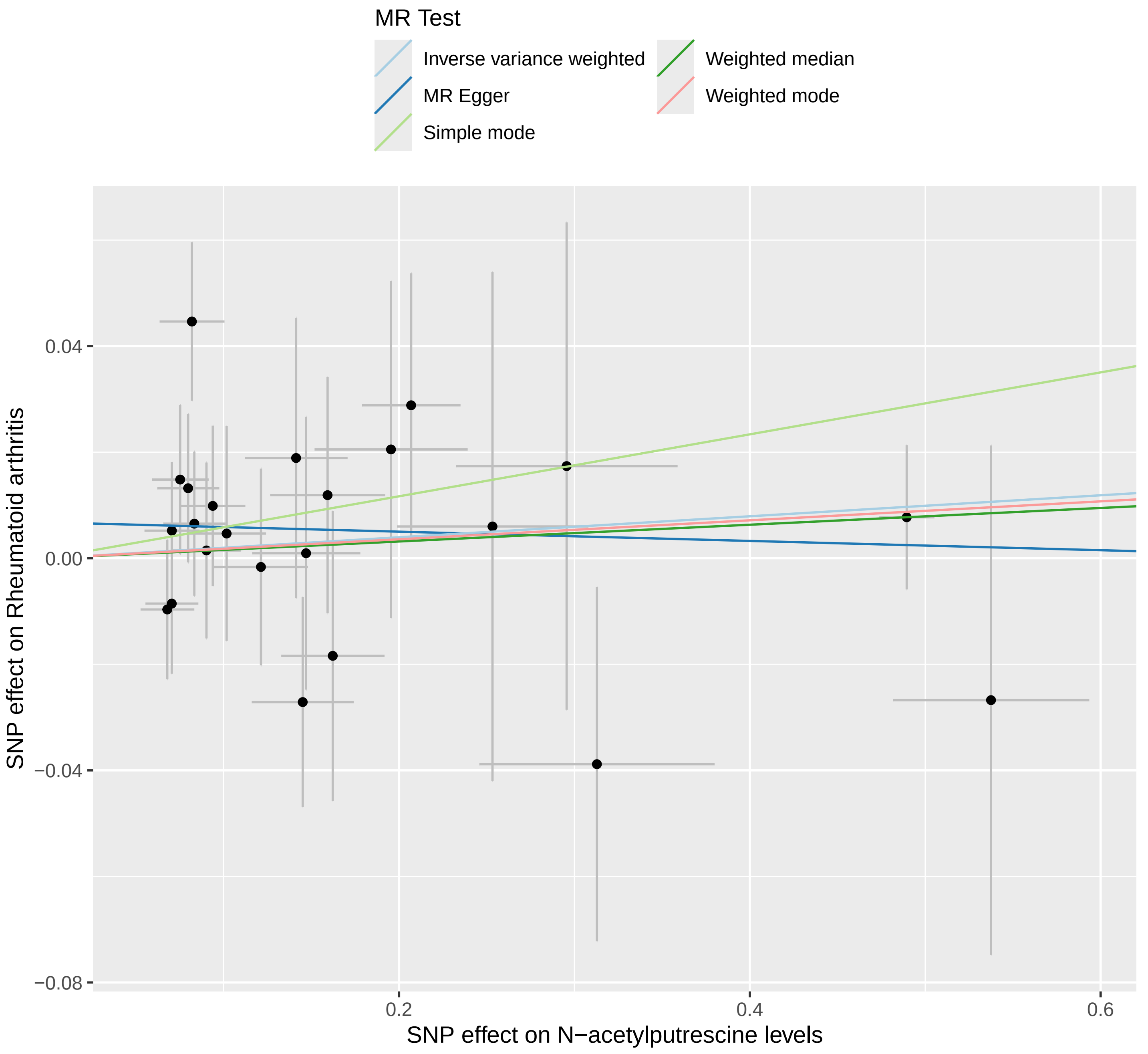

MR Test
Inverse variance weighted
MR Egger
Simple mode
Weighted median
Weighted mode
SNP effect on Rheumatoid arthritis
0.04
0.00
−0.04
−0.08
0.2
0.4
0.6
SNP effect on N−acetylputrescine levels

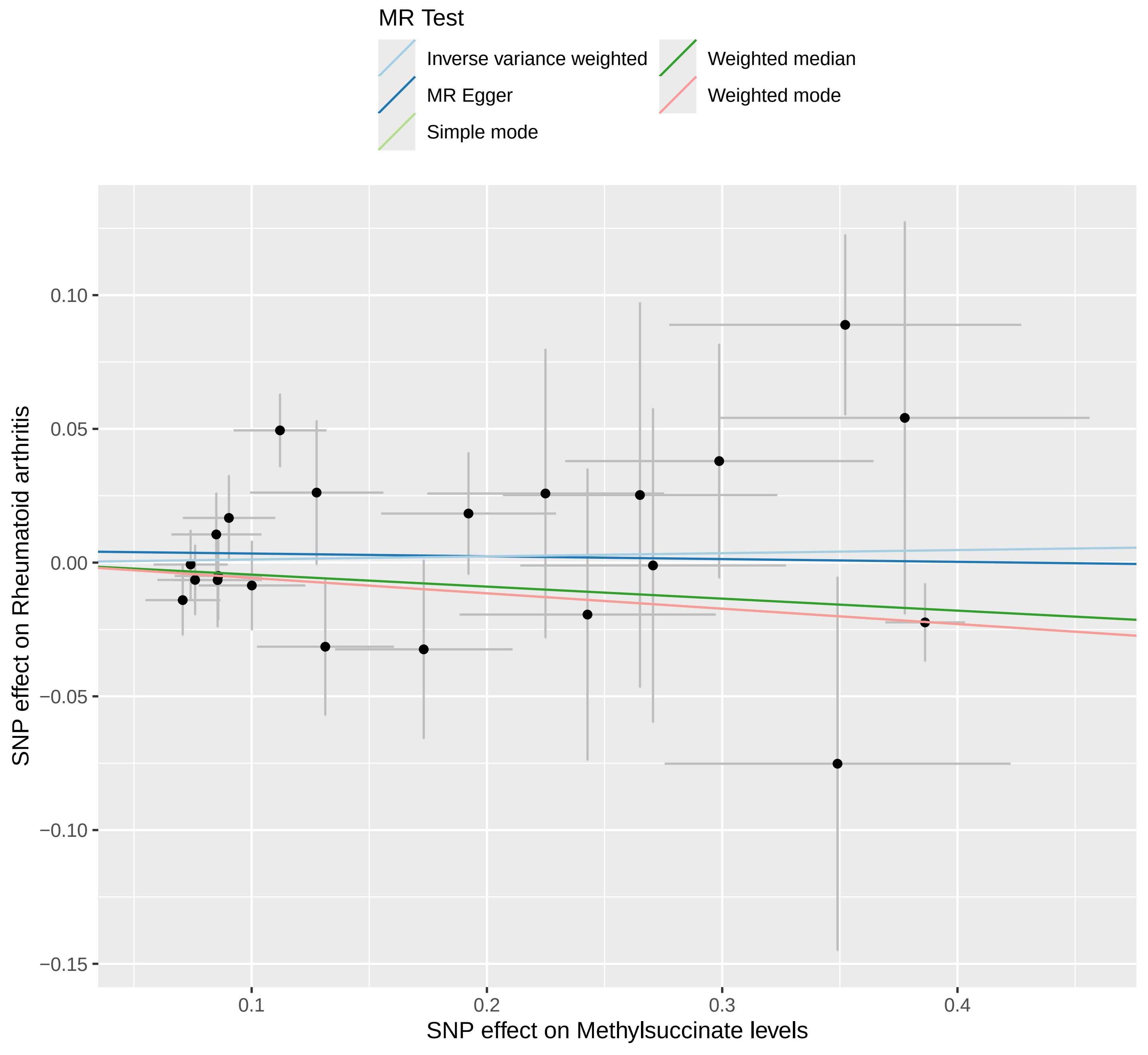
MR Test
Inverse variance weighted
MR Egger
Simple mode
Weighted median
Weighted mode
SNP effect on Rheumatoid arthritis
SNP effect on Methylsuccinate levels
0.10
0.05
0.00
−0.05
−0.10
−0.15
0.1
0.2
0.3
0.4

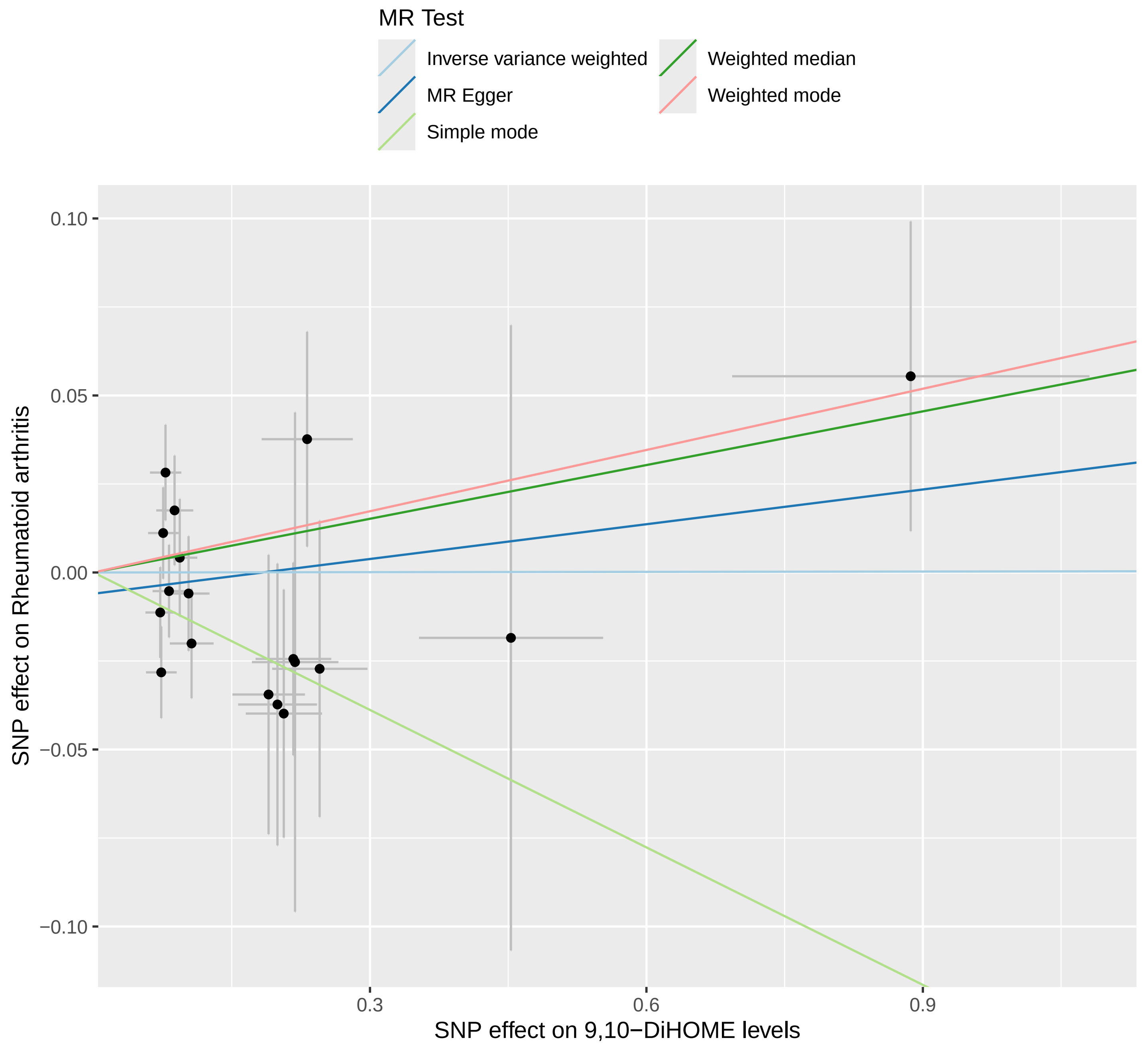

MR Test
Inverse variance weighted
MR Egger
Simple mode
Weighted median
Weighted mode
0.10
0.05
0.00
−0.05
−0.10
SNP effect on Rheumatoid arthritis
0.3
0.6
0.9
SNP effect on 9,10−DiHOME levels

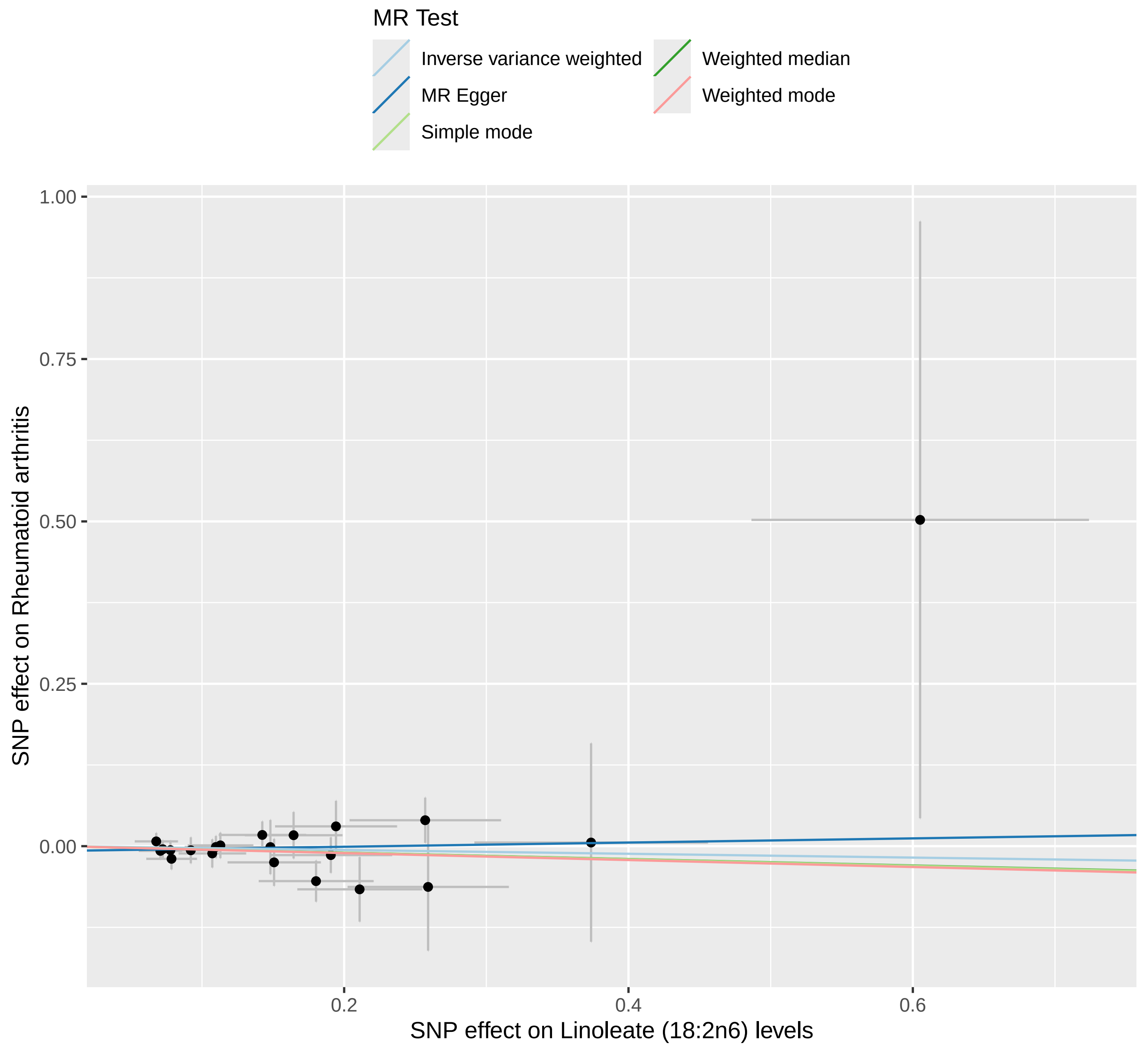
MR Test
Inverse variance weighted
MR Egger
Simple mode
Weighted median
Weighted mode
1.00
0.75
0.50
0.25
0.00
0.2
0.4
0.6
SNP effect on Rheumatoid arthritis
SNP effect on Linoleate (18:2n6) levels

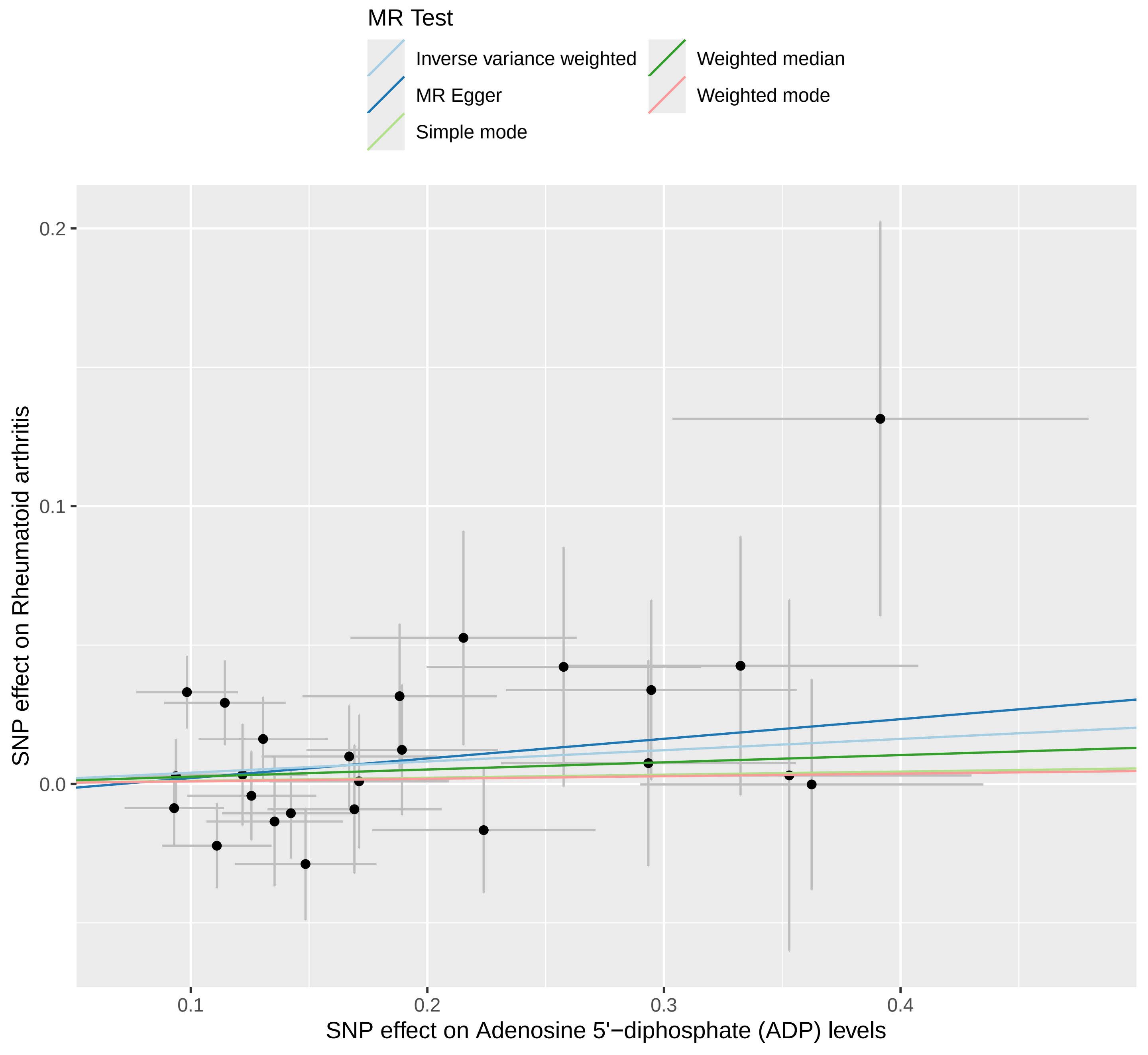
MR Test
Inverse variance weighted
MR Egger
Simple mode
Weighted median
Weighted mode
0.2
0.1
0.0
0.1
0.2
0.3
0.4
SNP effect on Rheumatoid arthritis
SNP effect on Adenosine 5'−diphosphate (ADP) levels

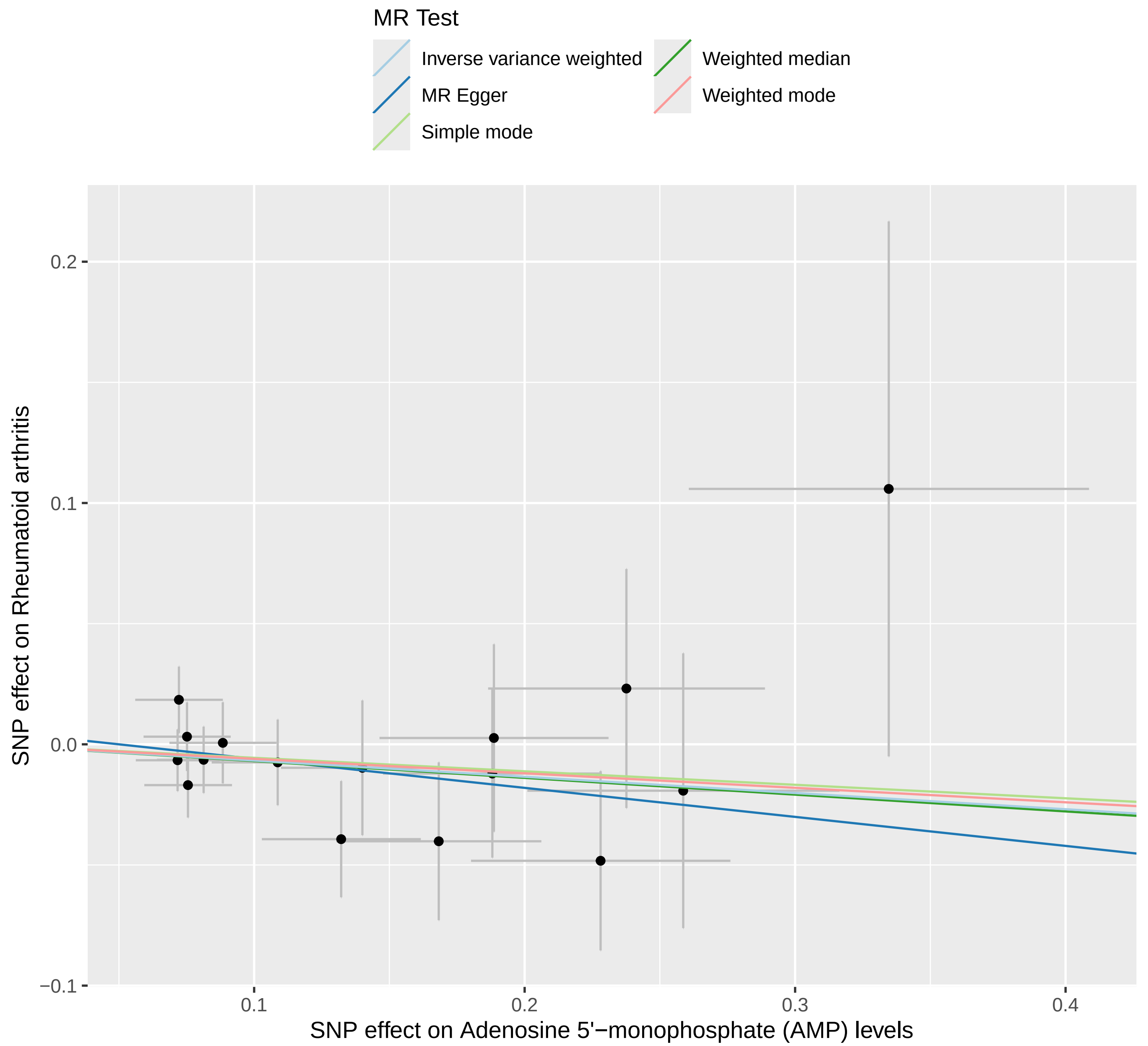

MR Test
Inverse variance weighted
MR Egger
Simple mode
Weighted median
Weighted mode
0.2
0.1
0.0
−0.1
0.1
0.2
0.3
0.4
SNP effect on Rheumatoid arthritis
SNP effect on Adenosine 5'−monophosphate (AMP) levels

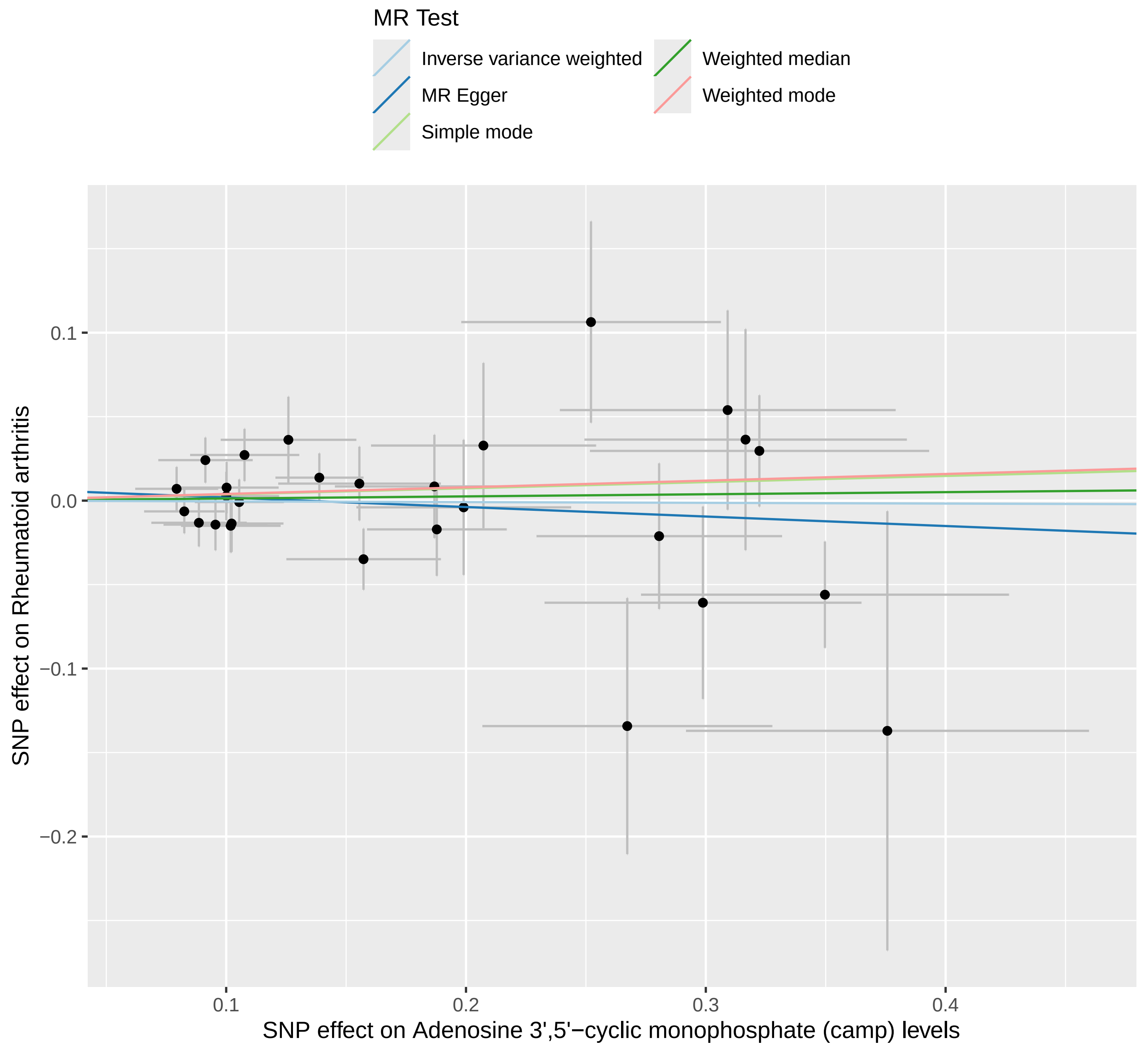
MR Test
Inverse variance weighted
MR Egger
Simple mode
Weighted median
Weighted mode
SNP effect on Rheumatoid arthritis
0.1
0.0
−0.1
−0.2
0.1
0.2
0.3
0.4
SNP effect on Adenosine 3',5'−cyclic monophosphate (camp) levels

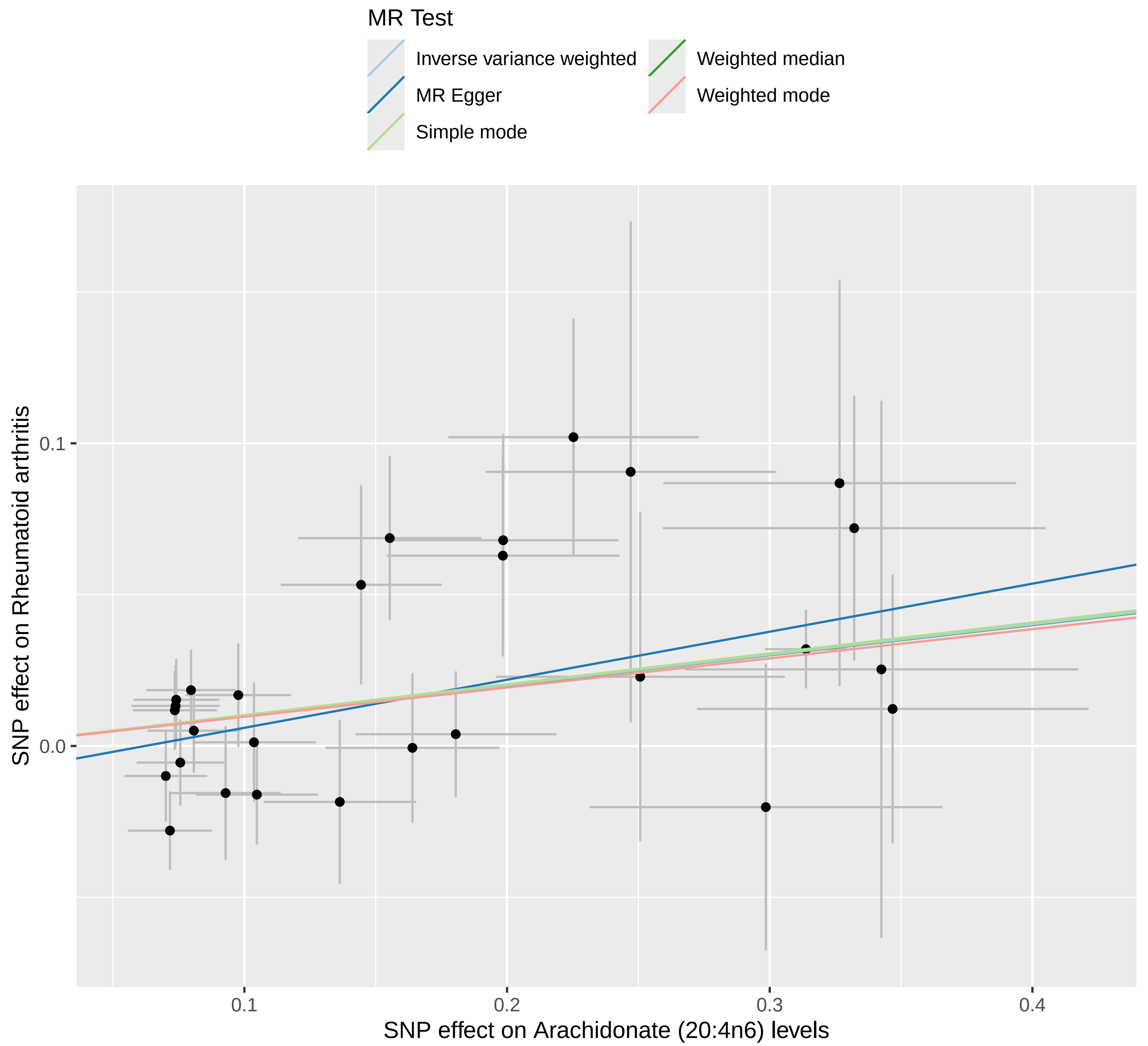
MR Test
Inverse variance weighted
MR Egger
Simple mode
Weighted median
Weighted mode
SNP effect on Rheumatoid arthritis
0.1
0.0
0.1
0.2
0.3
0.4
SNP effect on Arachidonate (20:4n6) levels

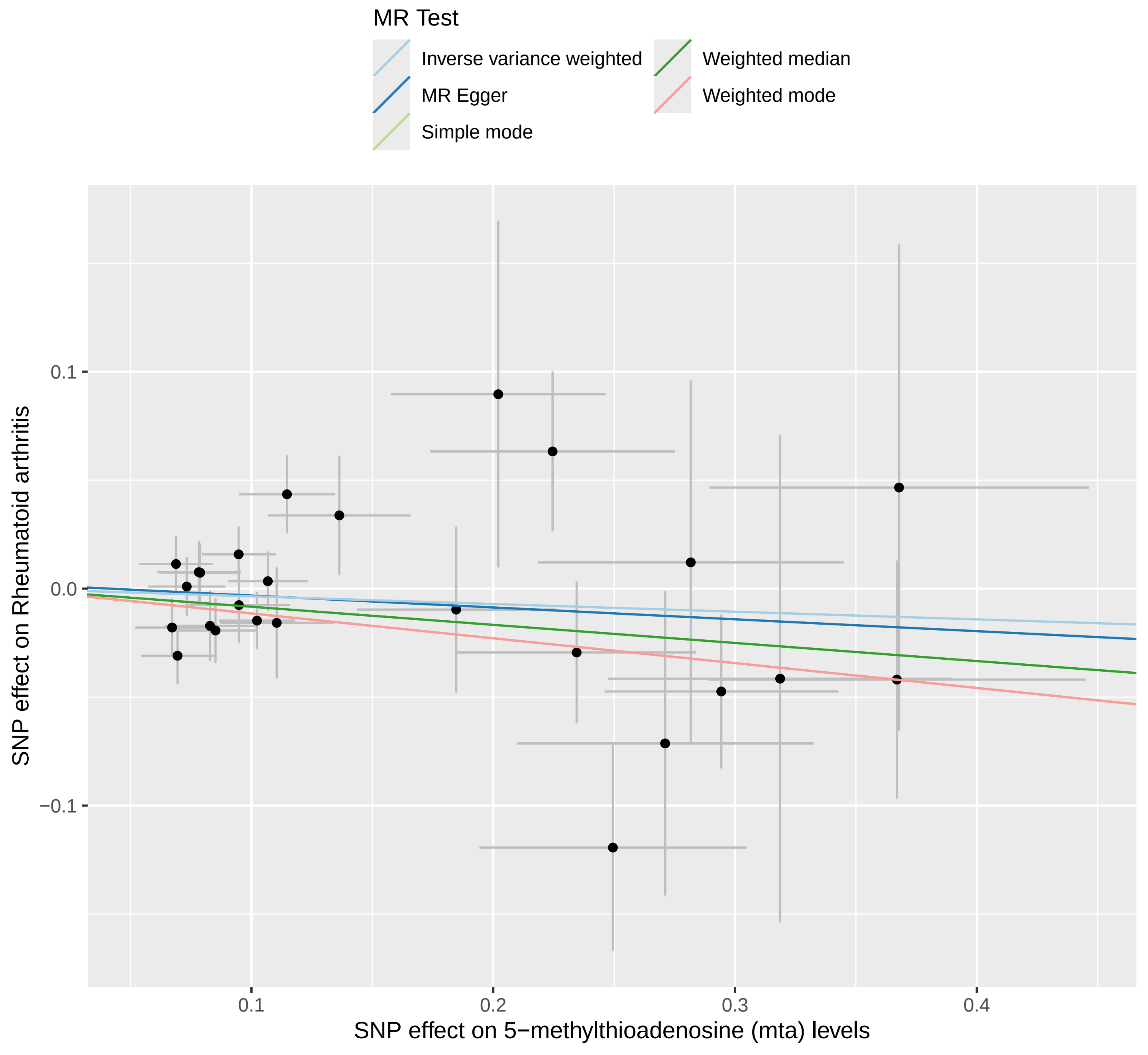

MR Test
Inverse variance weighted
MR Egger
Simple mode
Weighted median
Weighted mode
0.1
0.0
−0.1
SNP effect on Rheumatoid arthritis
0.1
0.2
0.3
0.4
SNP effect on 5−methylthioadenosine (mta) levels

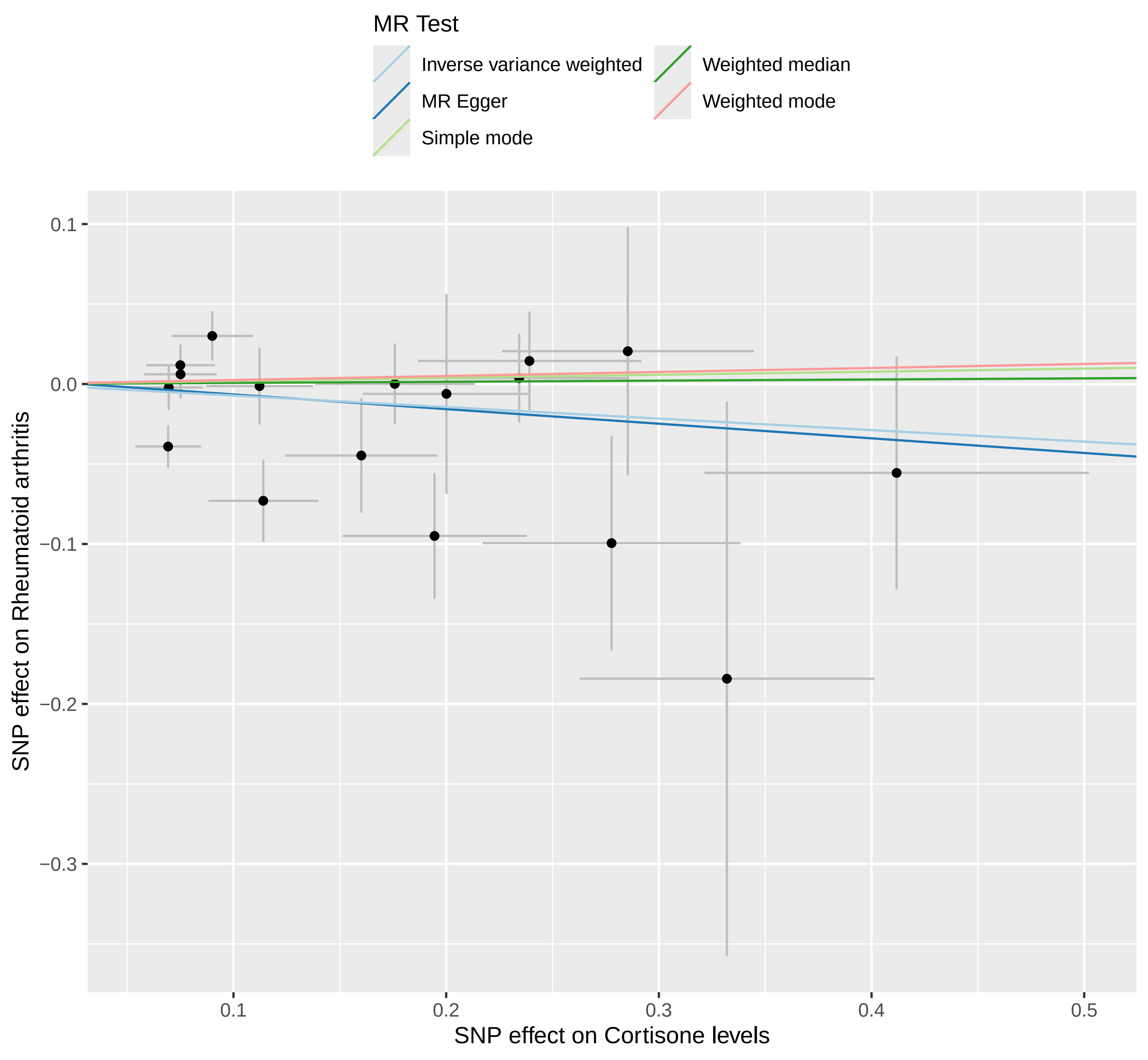

MR Test
Inverse variance weighted
MR Egger
Simple mode
Weighted median
Weighted mode
SNP effect on Rheumatoid arthritis
SNP effect on Cortisone levels
0.1
0.0
−0.1
−0.2
−0.3
0.1
0.2
0.3
0.4
0.5

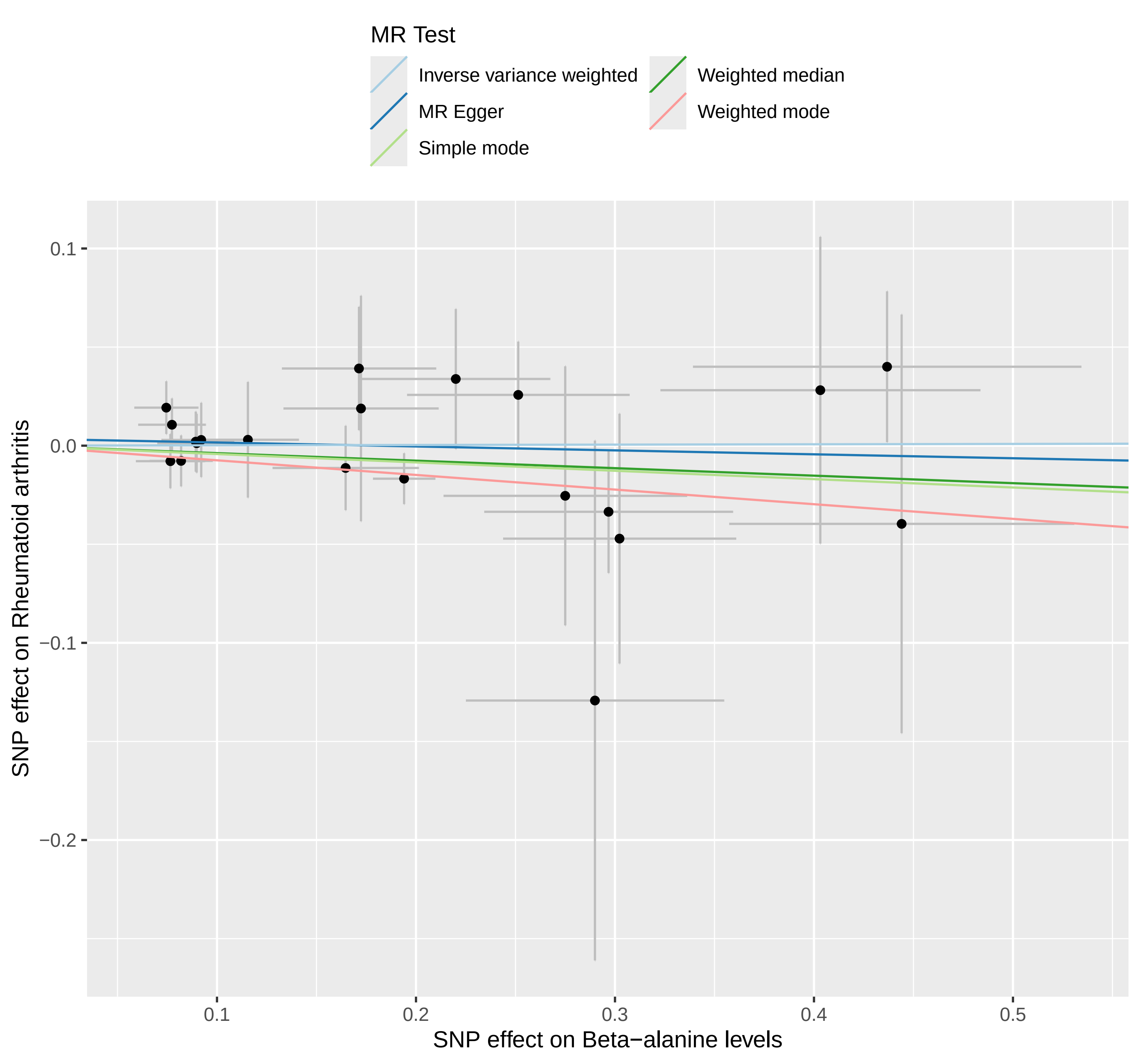
MR Test
Inverse variance weighted
MR Egger
Simple mode
Weighted median
Weighted mode
0.1
0.0
−0.1
−0.2
0.1
0.2
0.3
0.4
0.5
SNP effect on Rheumatoid arthritis
SNP effect on Beta−alanine levels

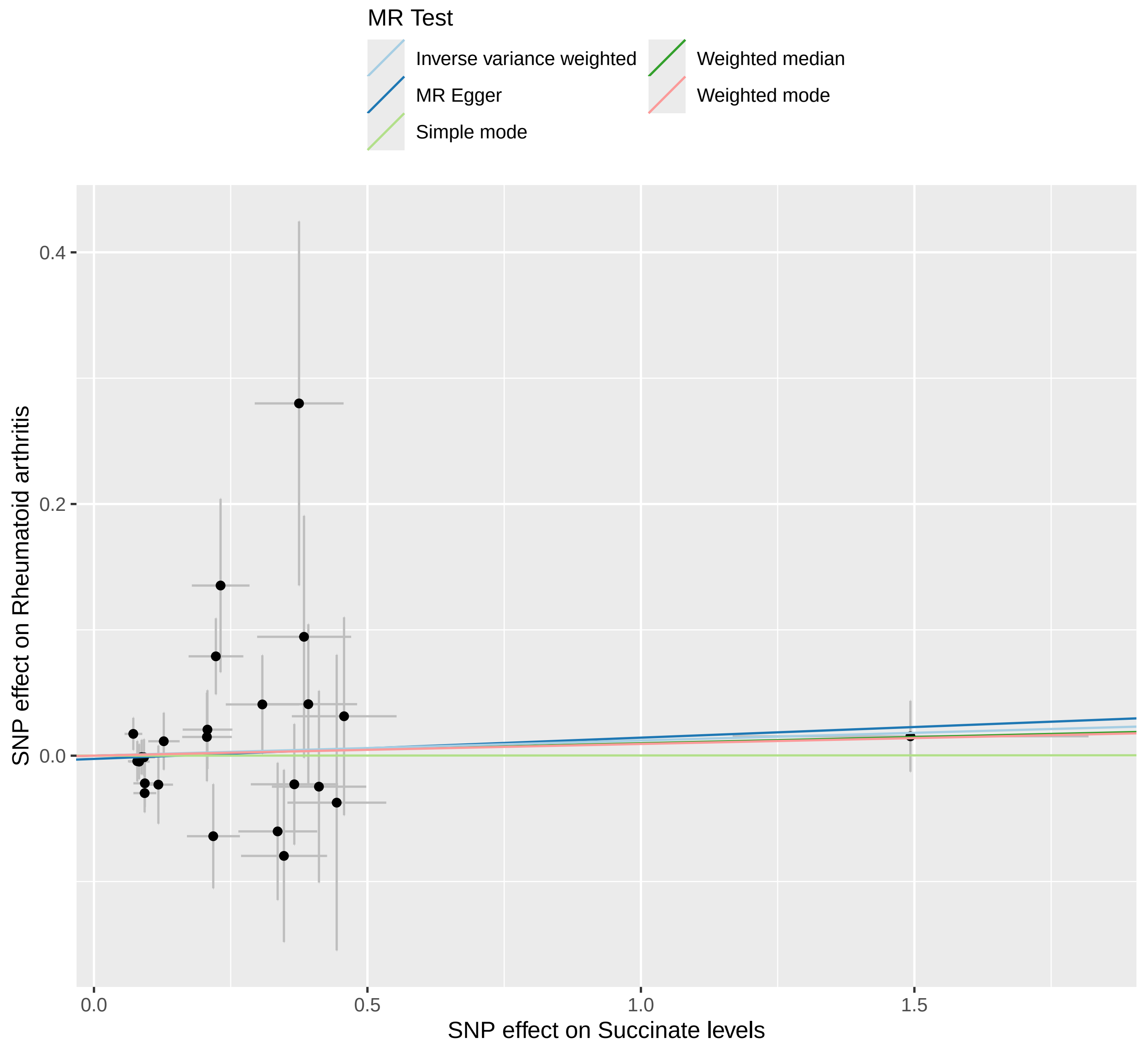
MR Test
Inverse variance weighted
MR Egger
Simple mode
Weighted median
Weighted mode
SNP effect on Rheumatoid arthritis
0.4
0.2
0.0
0.0
0.5
1.0
1.5
SNP effect on Succinate levels

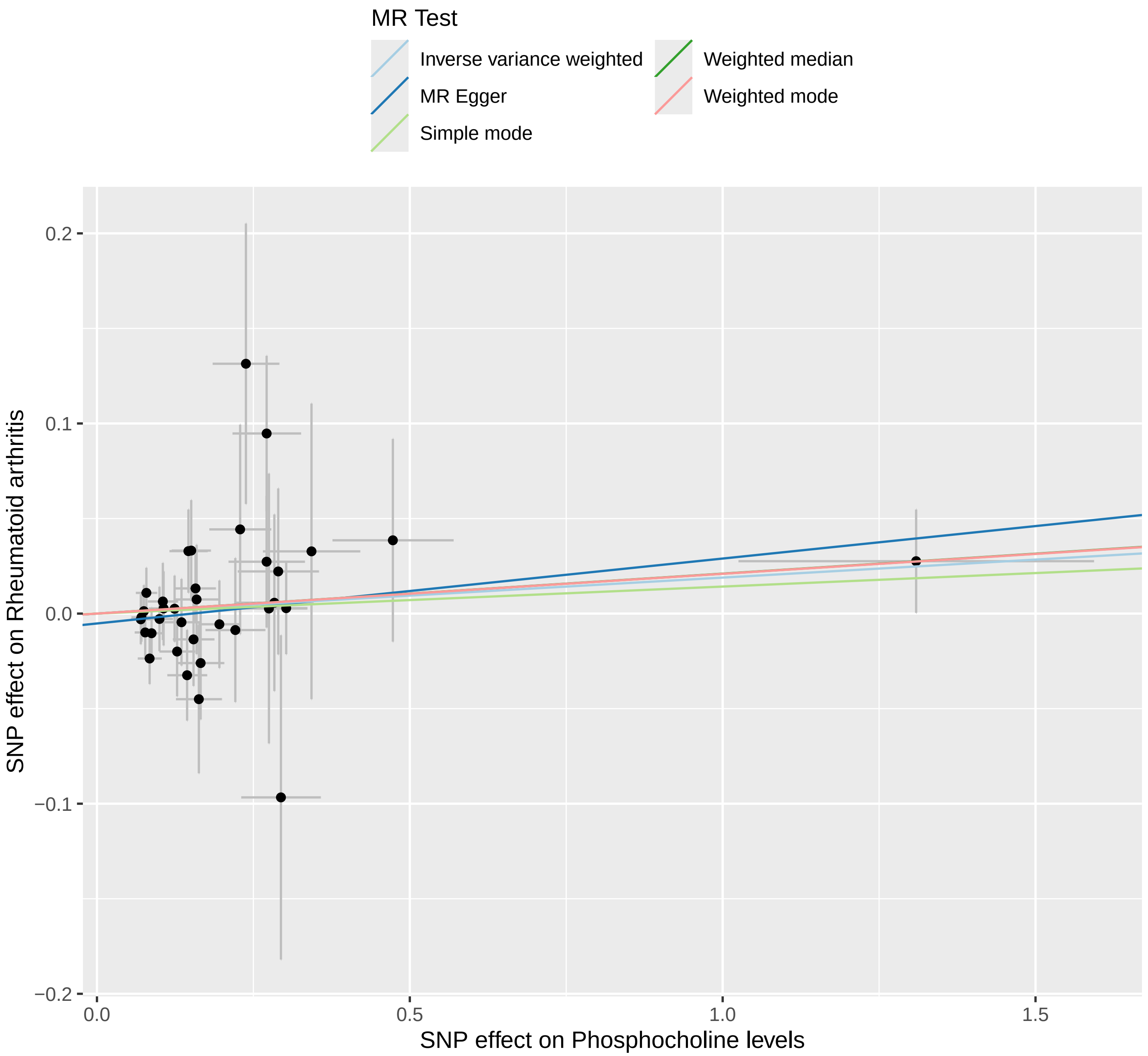

MR Test
Inverse variance weighted
MR Egger
Simple mode
Weighted median
Weighted mode
SNP effect on Rheumatoid arthritis
0.2
0.1
0.0
−0.1
−0.2
0.0
0.5
1.0
1.5
SNP effect on Phosphocholine levels

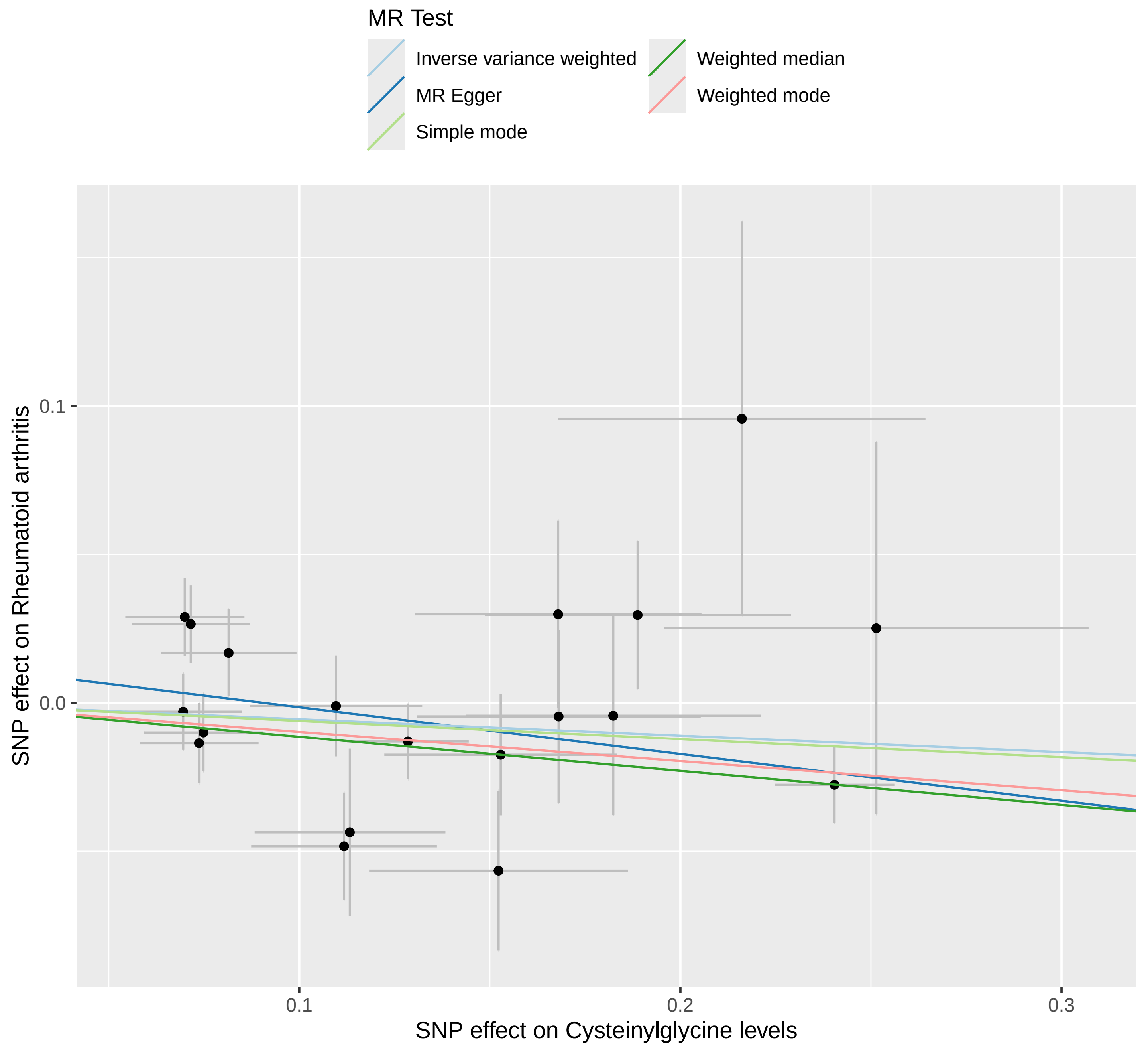

MR Test
Inverse variance weighted
MR Egger
Simple mode
Weighted median
Weighted mode
SNP effect on Rheumatoid arthritis
0.1
0.0
0.1
0.2
0.3
SNP effect on Cysteinylglycine levels

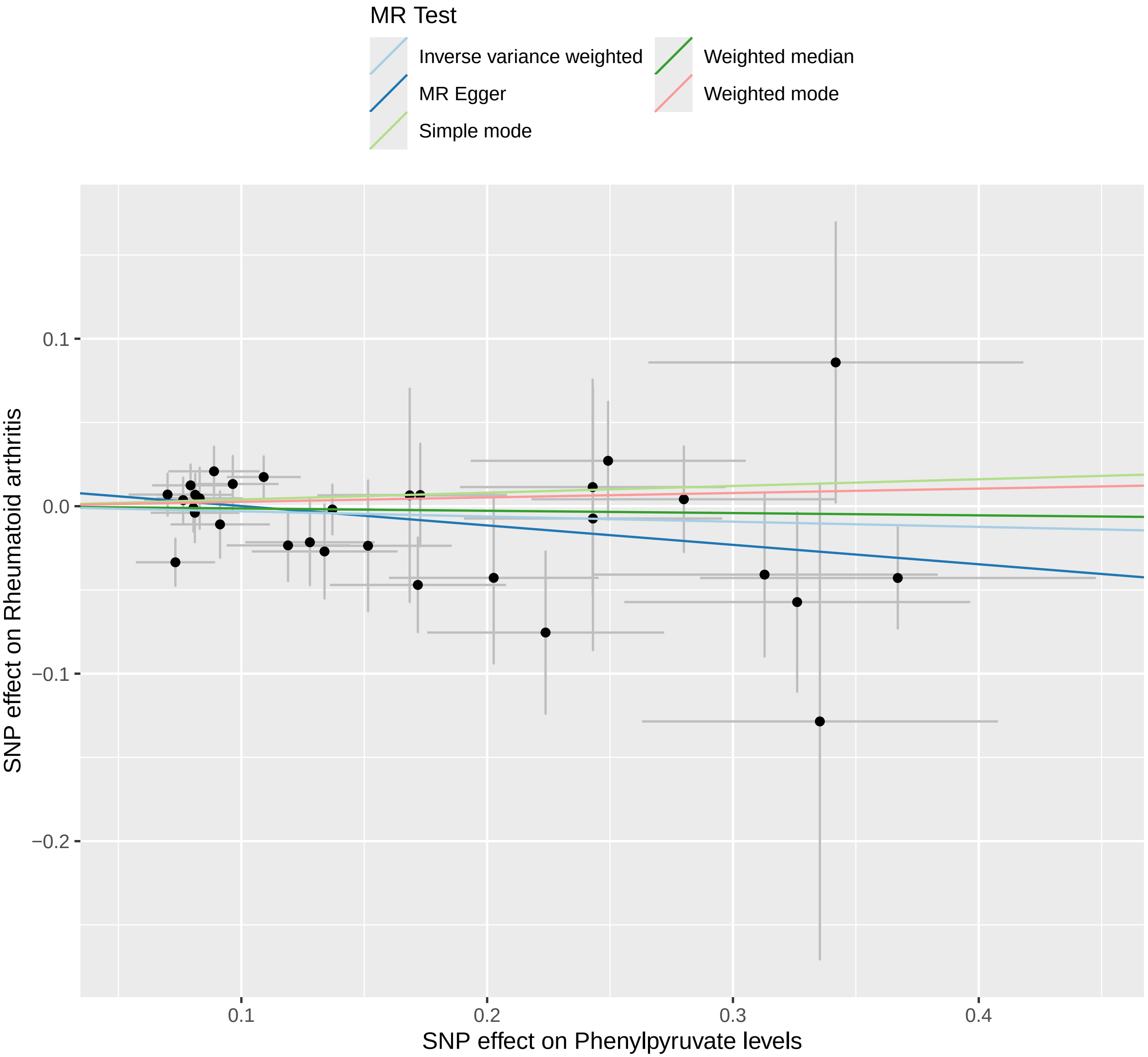
MR Test
Inverse variance weighted
MR Egger
Simple mode
Weighted median
Weighted mode
0.1
0.0
−0.1
−0.2
0.1
0.2
0.3
0.4
SNP effect on Rheumatoid arthritis
SNP effect on Phenylpyruvate levels

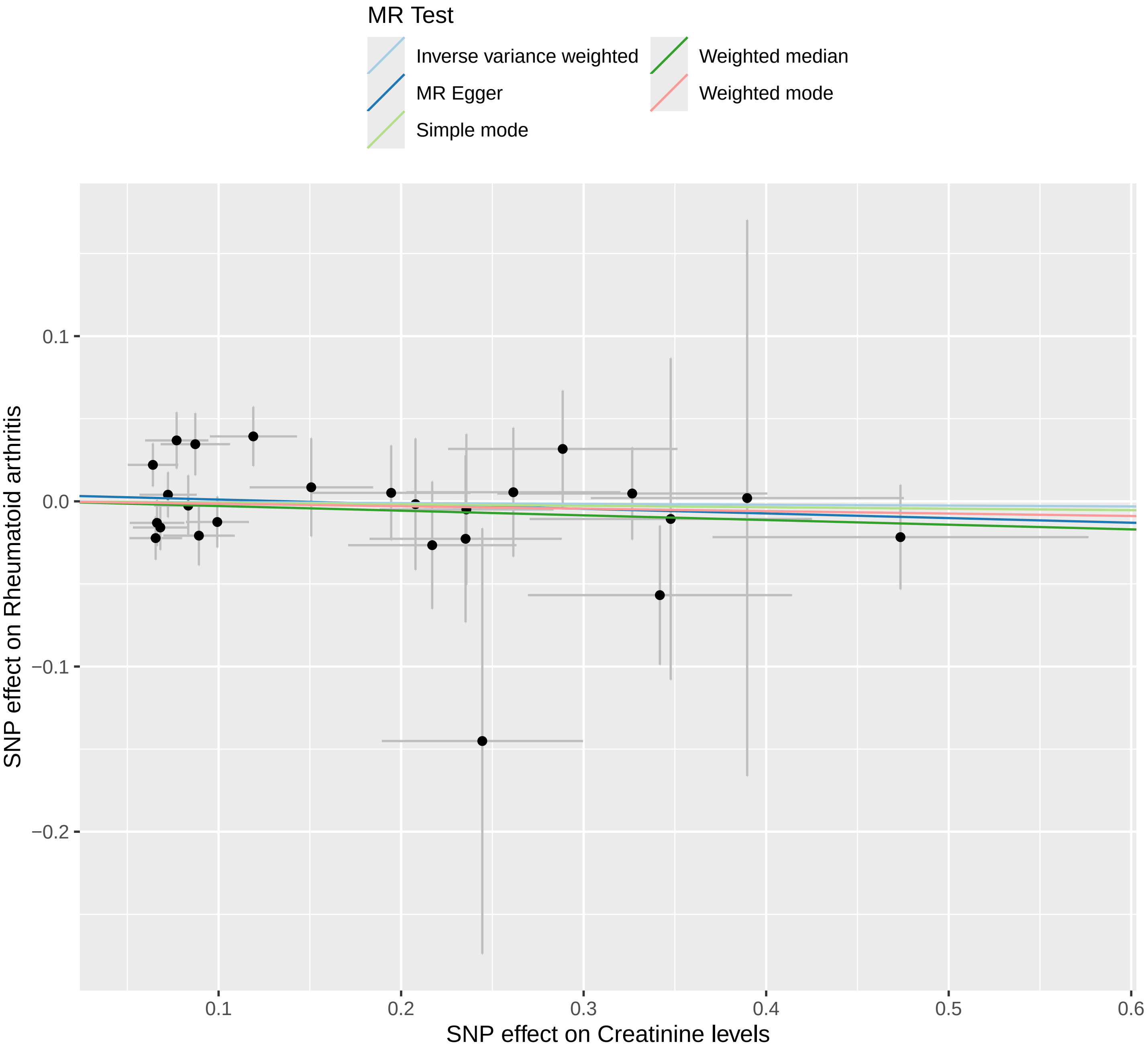
MR Test
Inverse variance weighted
MR Egger
Simple mode
Weighted median
Weighted mode
0.1
0.0
−0.1
−0.2
0.1
0.2
0.3
0.4
0.5
0.6
SNP effect on Rheumatoid arthritis
SNP effect on Creatinine levels

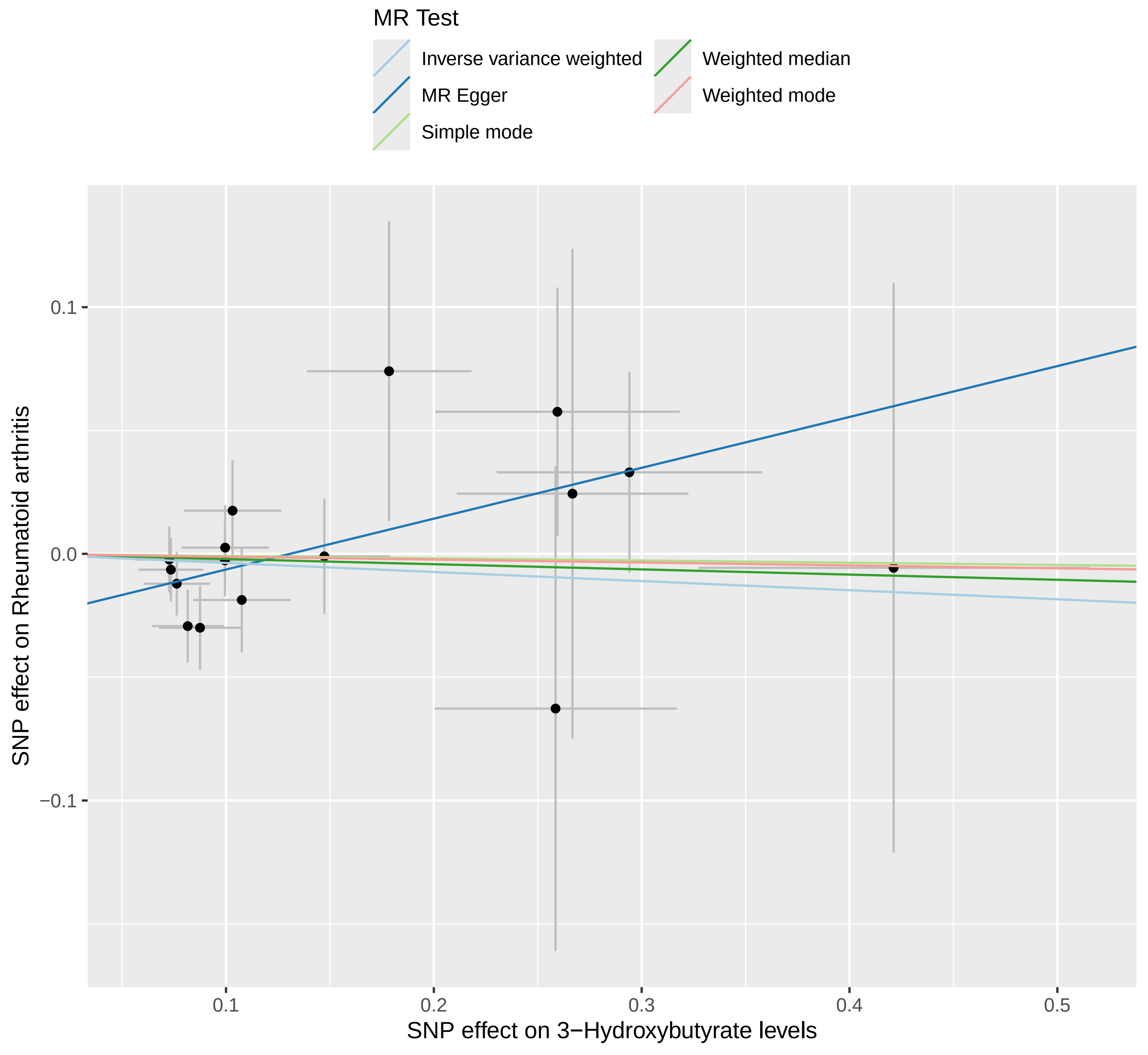
MR Test
Inverse variance weighted
MR Egger
Simple mode
Weighted median
Weighted mode
0.1
0.0
−0.1
SNP effect on Rheumatoid arthritis
0.1
0.2
0.3
0.4
0.5
SNP effect on 3−Hydroxybutyrate levels

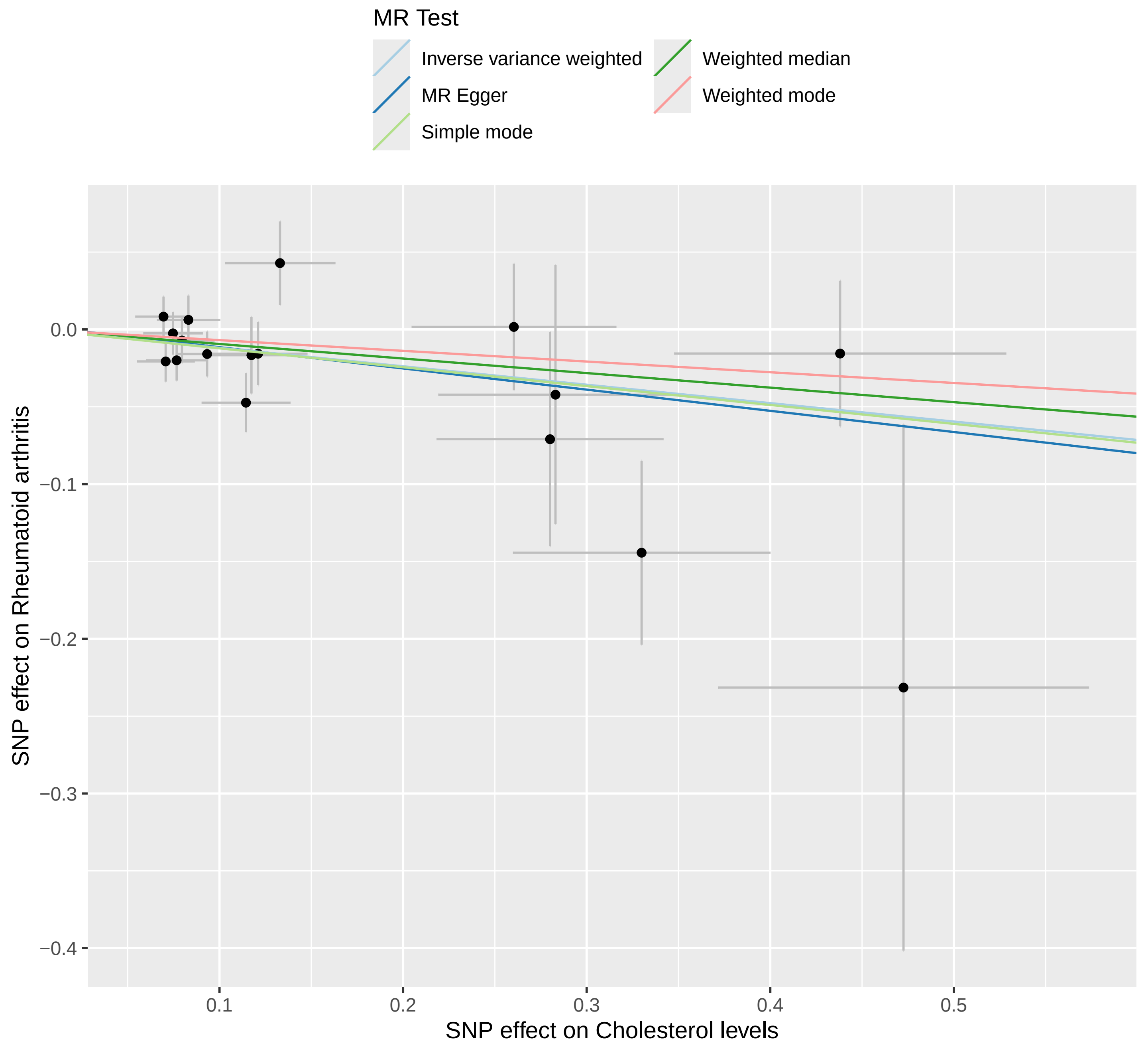

MR Test
Inverse variance weighted
MR Egger
Simple mode
Weighted median
Weighted mode
SNP effect on Rheumatoid arthritis
0.0
−0.1
−0.2
−0.3
−0.4
0.1
0.2
0.3
0.4
0.5
SNP effect on Cholesterol levels

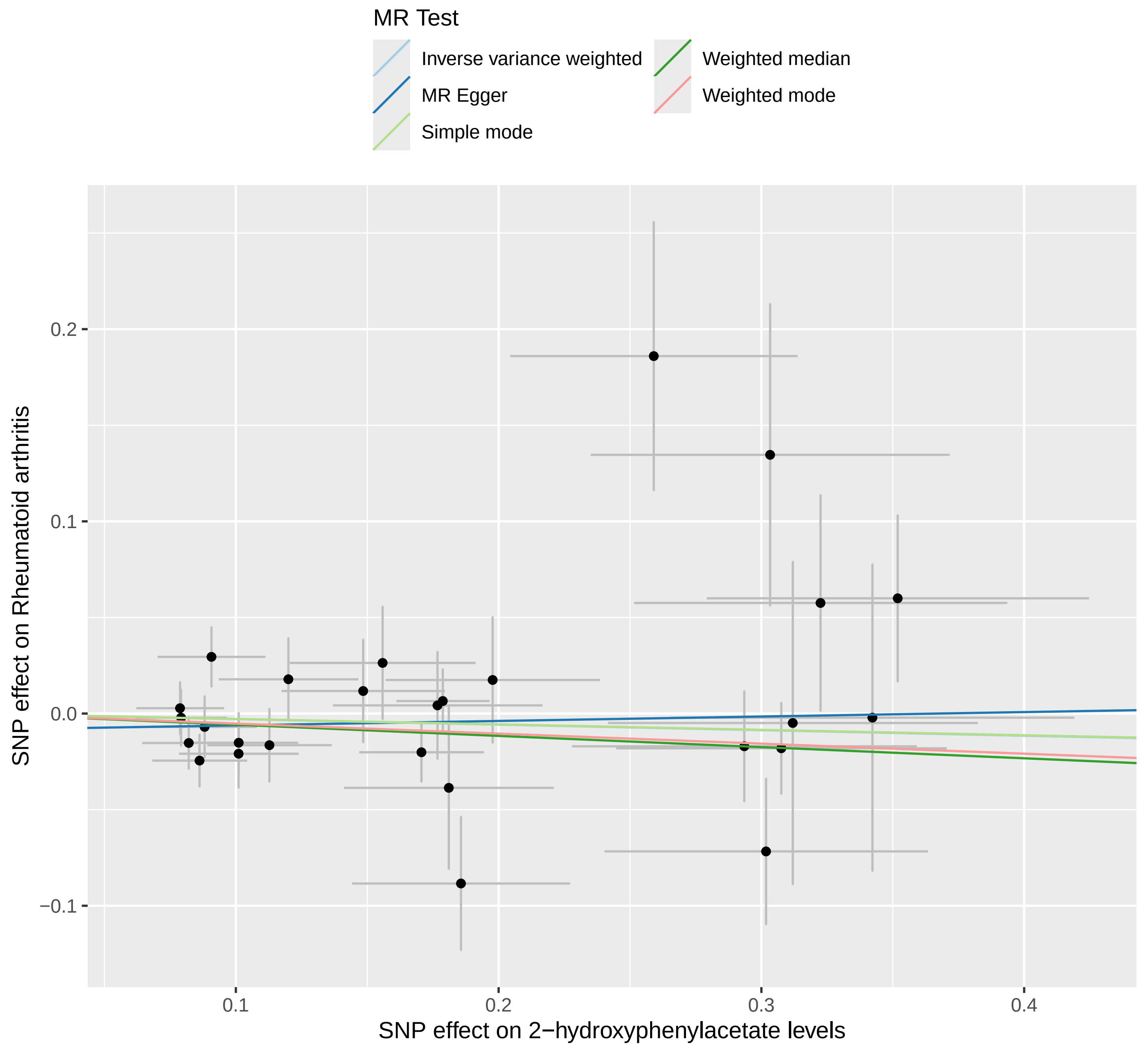
MR Test
Inverse variance weighted
MR Egger
Simple mode
Weighted median
Weighted mode
SNP effect on Rheumatoid arthritis
0.2
0.1
0.0
−0.1
0.1
0.2
0.3
0.4
SNP effect on 2−hydroxyphenylacetate levels

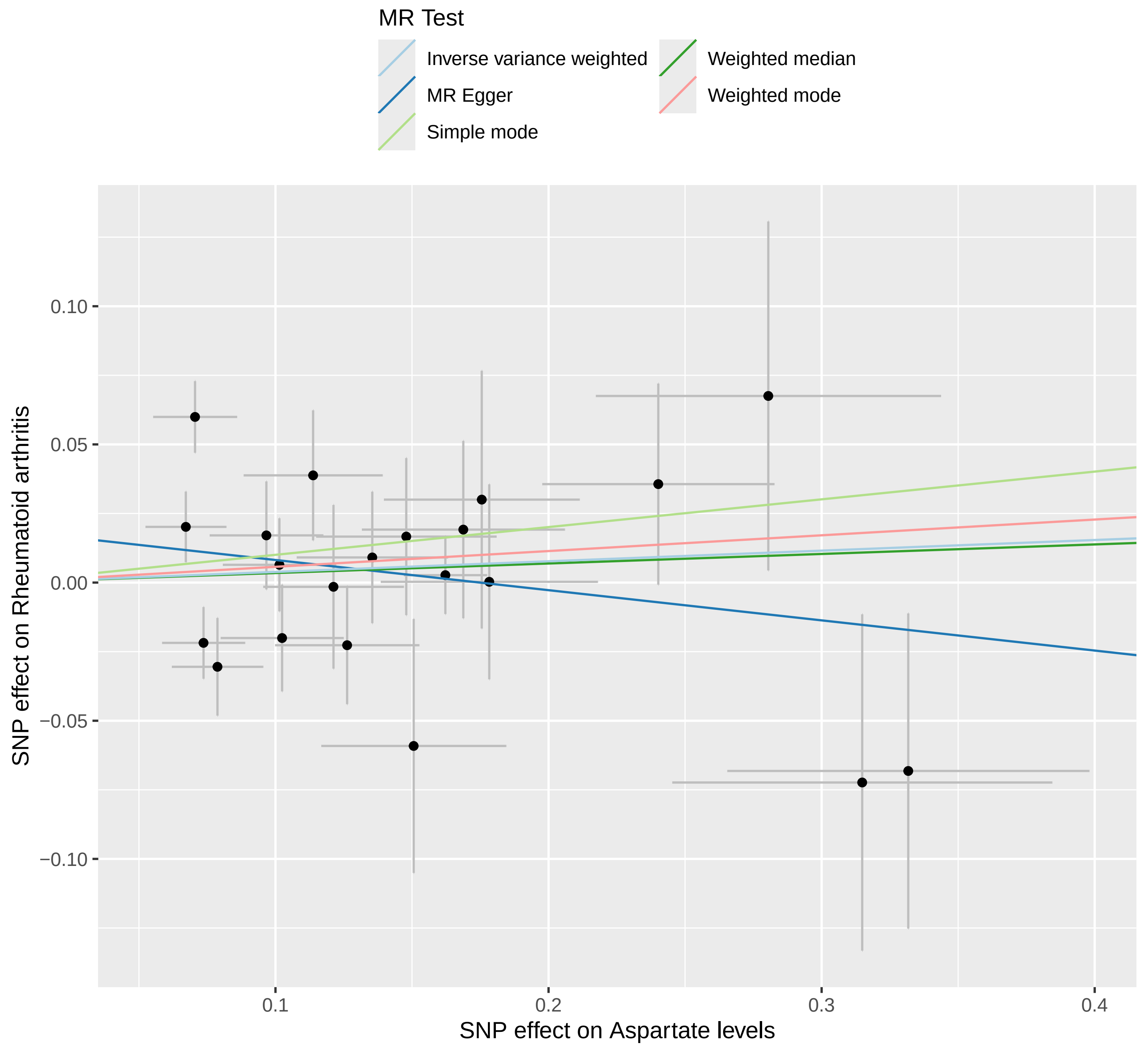

MR Test
Inverse variance weighted
MR Egger
Simple mode
Weighted median
Weighted mode
0.10
0.05
0.00
−0.05
−0.10
0.1
0.2
0.3
0.4
SNP effect on Rheumatoid arthritis
SNP effect on Aspartate levels

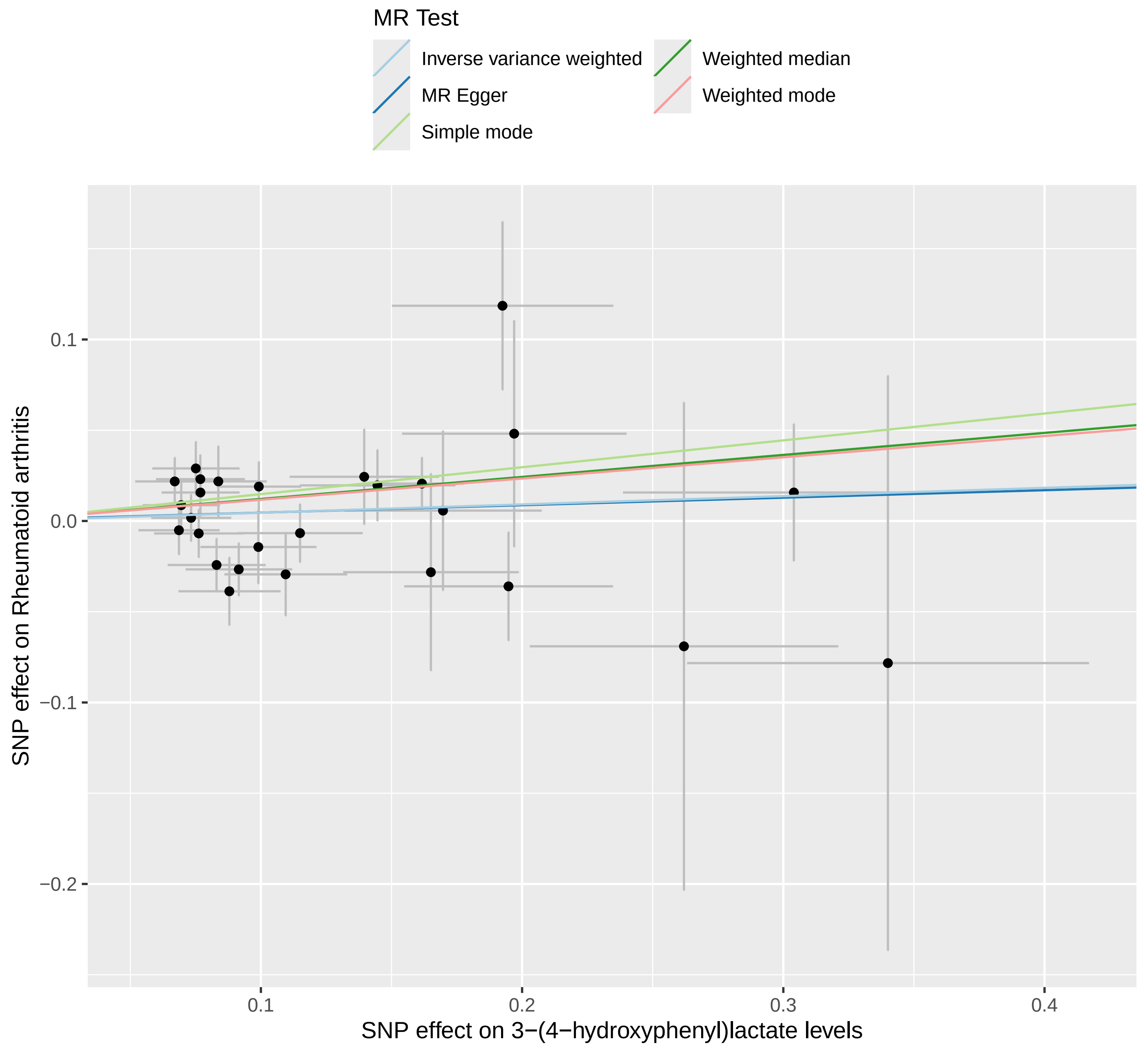
MR Test
Inverse variance weighted
MR Egger
Simple mode
Weighted median
Weighted mode
0.1
0.0
−0.1
−0.2
0.1
0.2
0.3
0.4
SNP effect on Rheumatoid arthritis
SNP effect on 3−(4−hydroxyphenyl)lactate levels

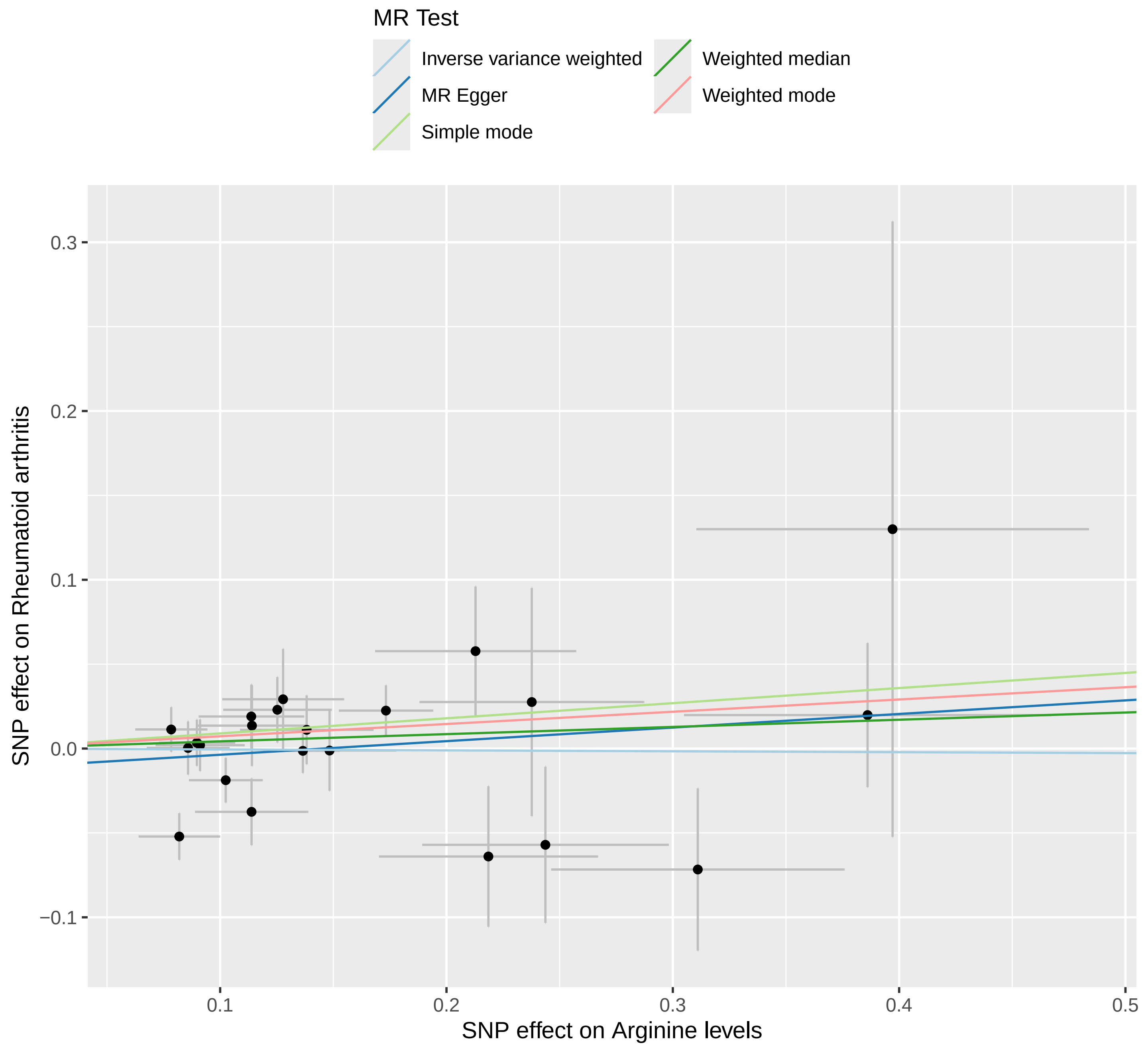
MR Test
Inverse variance weighted
MR Egger
Simple mode
Weighted median
Weighted mode
SNP effect on Rheumatoid arthritis
0.3
0.2
0.1
0.0
−0.1
0.1
0.2
0.3
0.4
0.5
SNP effect on Arginine levels

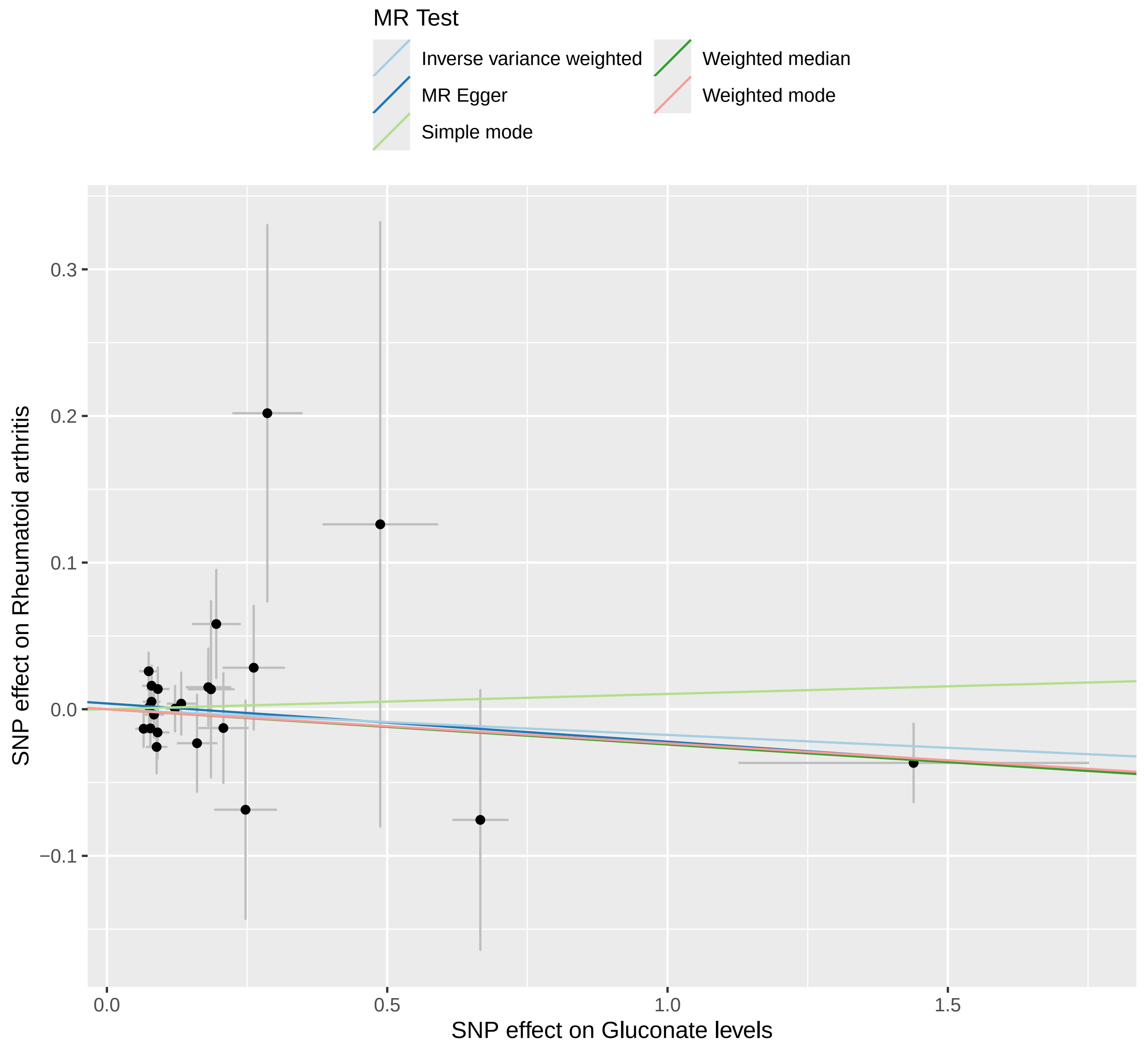

MR Test
Inverse variance weighted
MR Egger
Simple mode
Weighted median
Weighted mode
0.3
0.2
0.1
0.0
−0.1
0.0
0.5
1.0
1.5
SNP effect on Rheumatoid arthritis
SNP effect on Gluconate levels

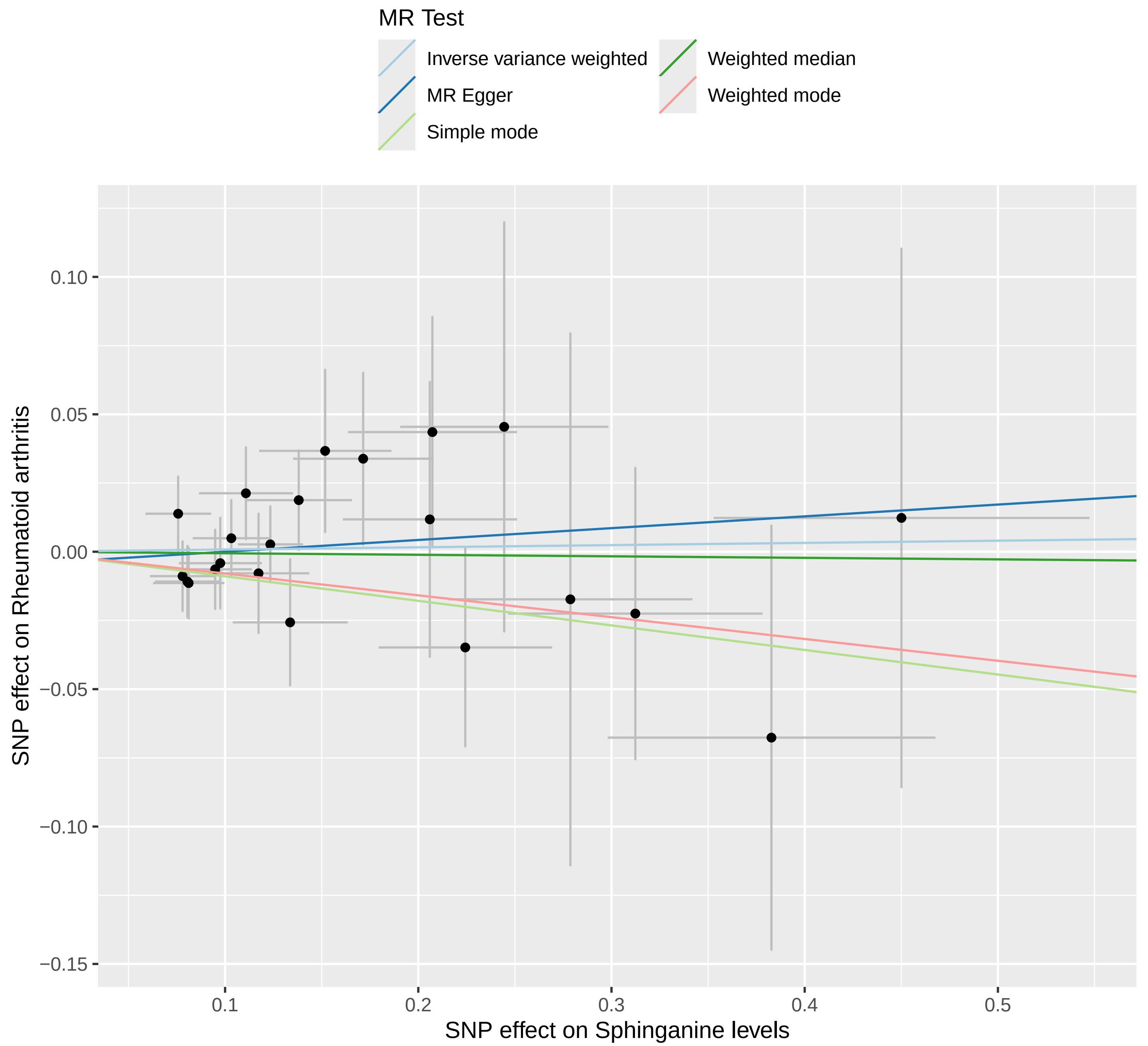

MR Test
Inverse variance weighted
MR Egger
Simple mode
Weighted median
Weighted mode
0.10
0.05
0.00
−0.05
−0.10
−0.15
SNP effect on Rheumatoid arthritis
0.1
0.2
0.3
0.4
0.5
SNP effect on Sphinganine levels

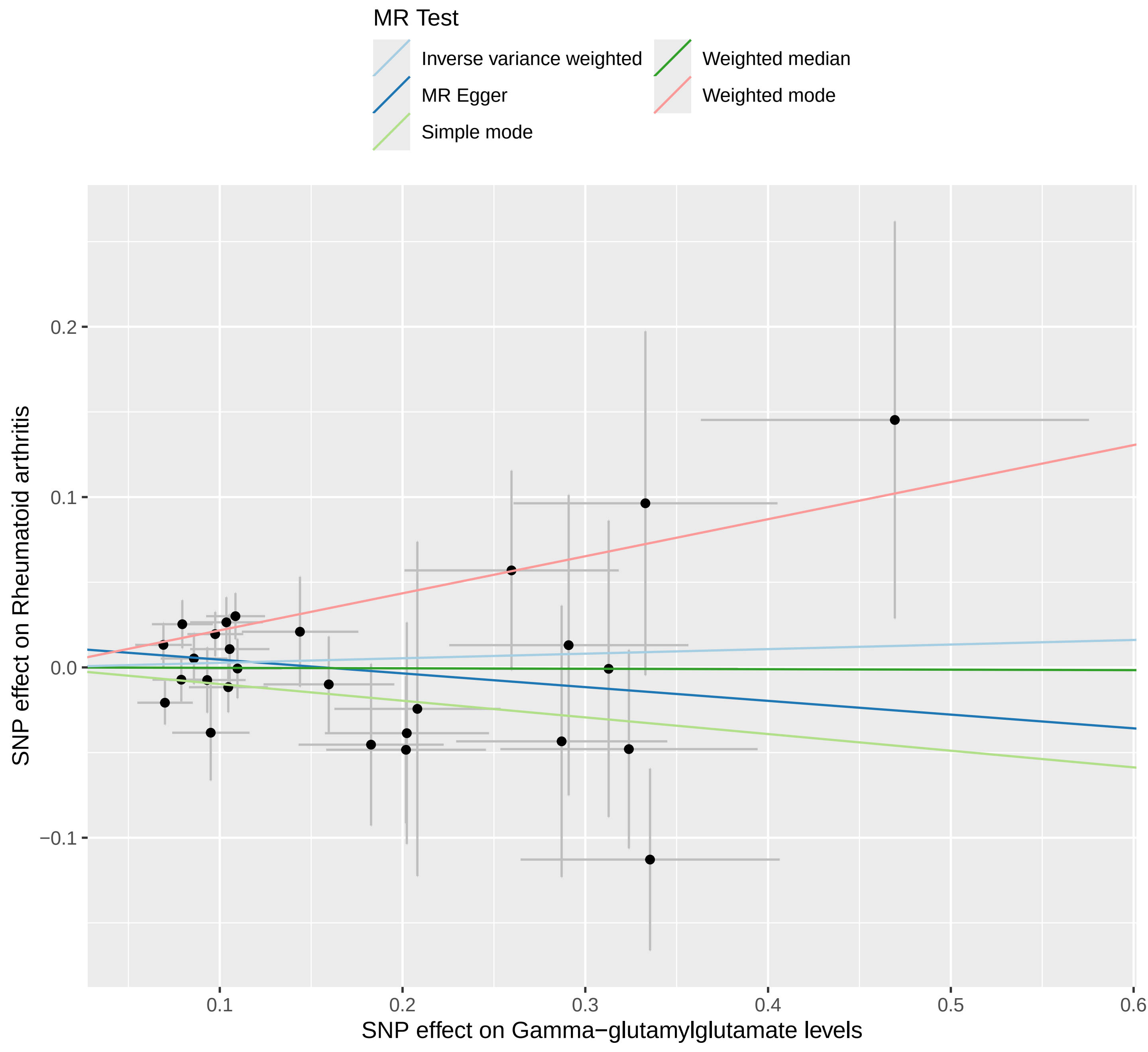
MR Test
Inverse variance weighted
MR Egger
Simple mode
Weighted median
Weighted mode
SNP effect on Rheumatoid arthritis
0.2
0.1
0.0
−0.1
0.1
0.2
0.3
0.4
0.5
0.6
SNP effect on Gamma−glutamylglutamate levels

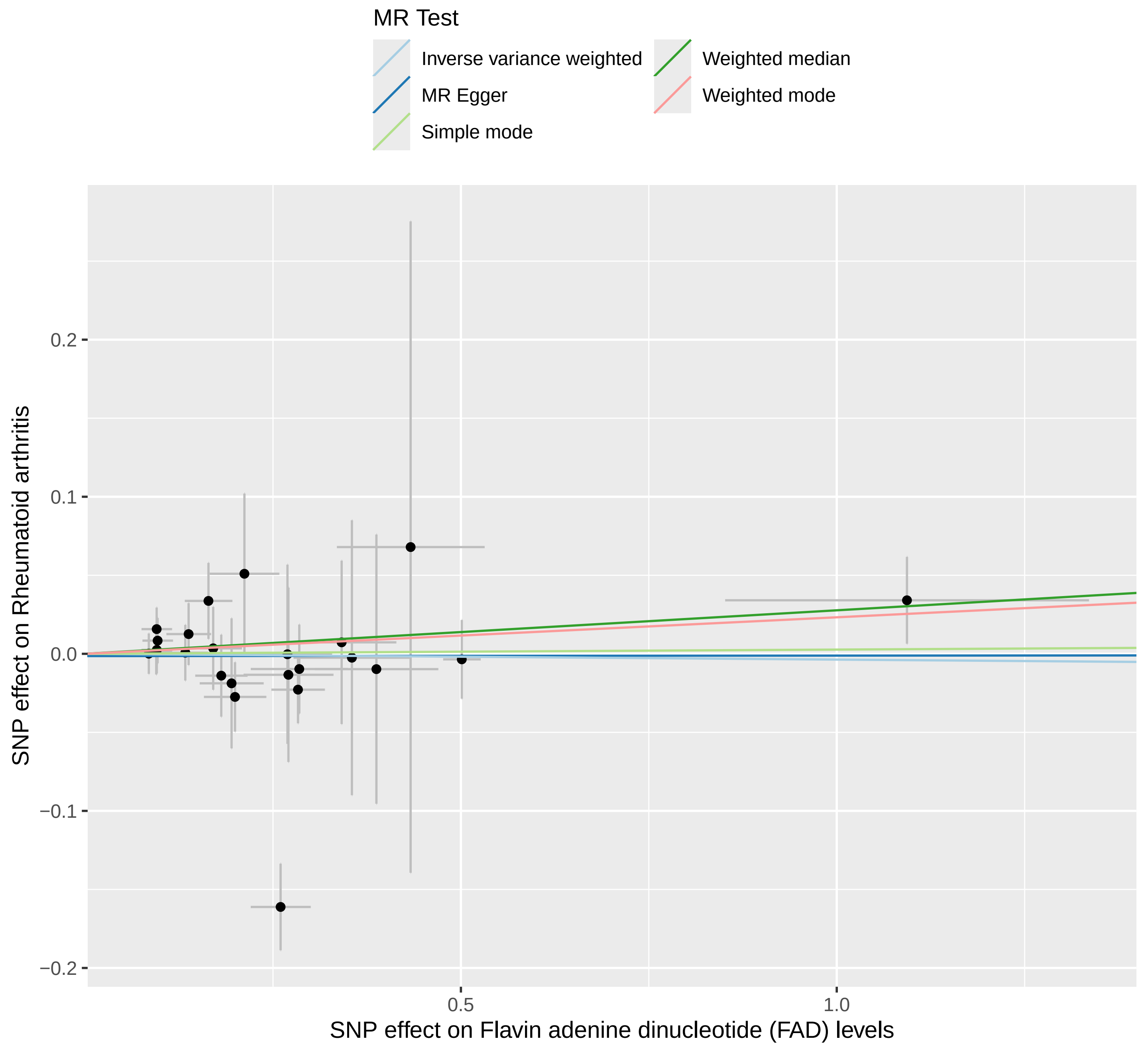
MR Test
Inverse variance weighted
MR Egger
Simple mode
Weighted median
Weighted mode
SNP effect on Rheumatoid arthritis
0.2
0.1
0.0
−0.1
−0.2
0.5
1.0
SNP effect on Flavin adenine dinucleotide (FAD) levels

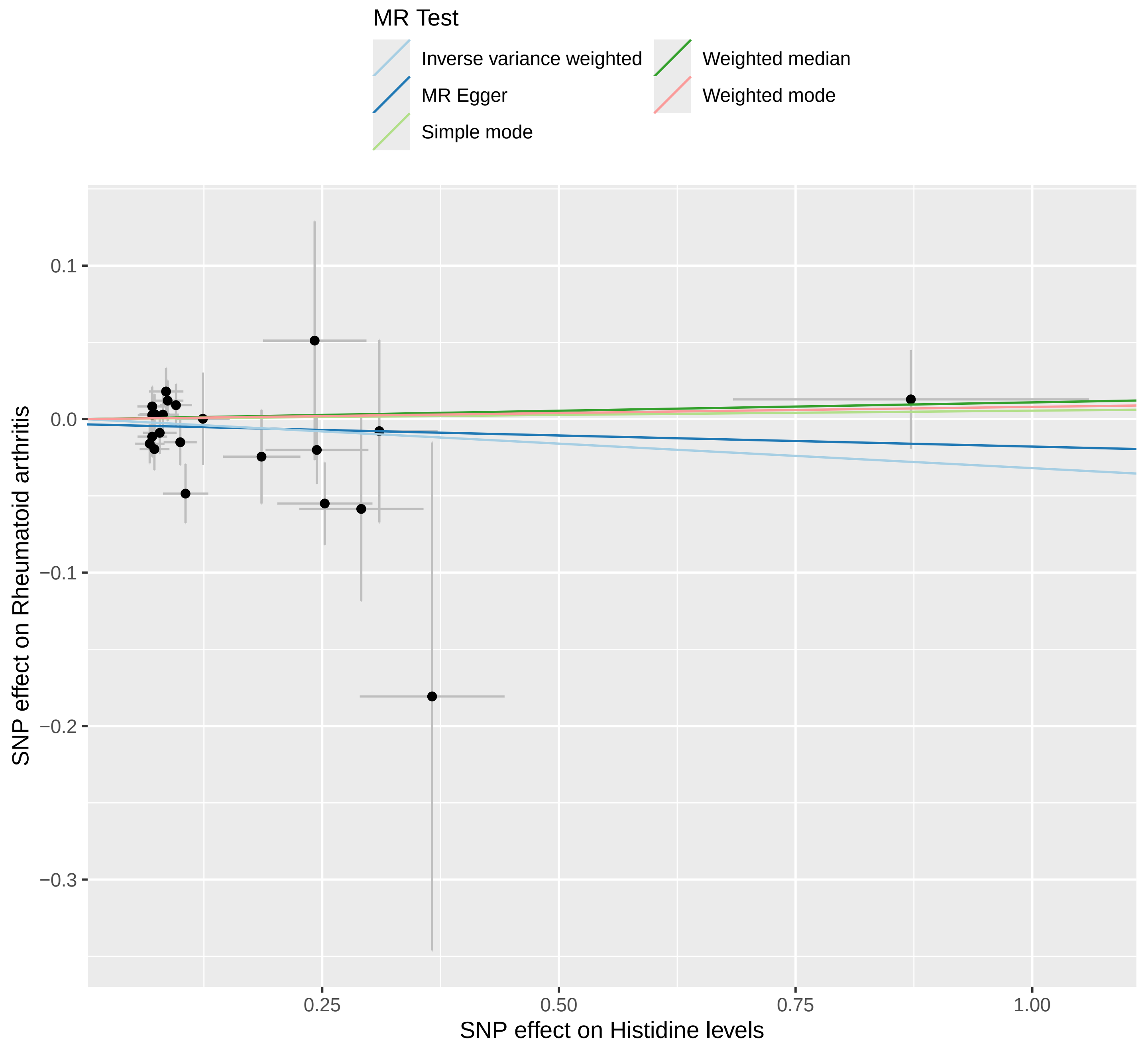

MR Test
Inverse variance weighted
MR Egger
Simple mode
Weighted median
Weighted mode
0.1
0.0
−0.1
−0.2
−0.3
SNP effect on Rheumatoid arthritis
0.25
0.50
0.75
1.00
SNP effect on Histidine levels

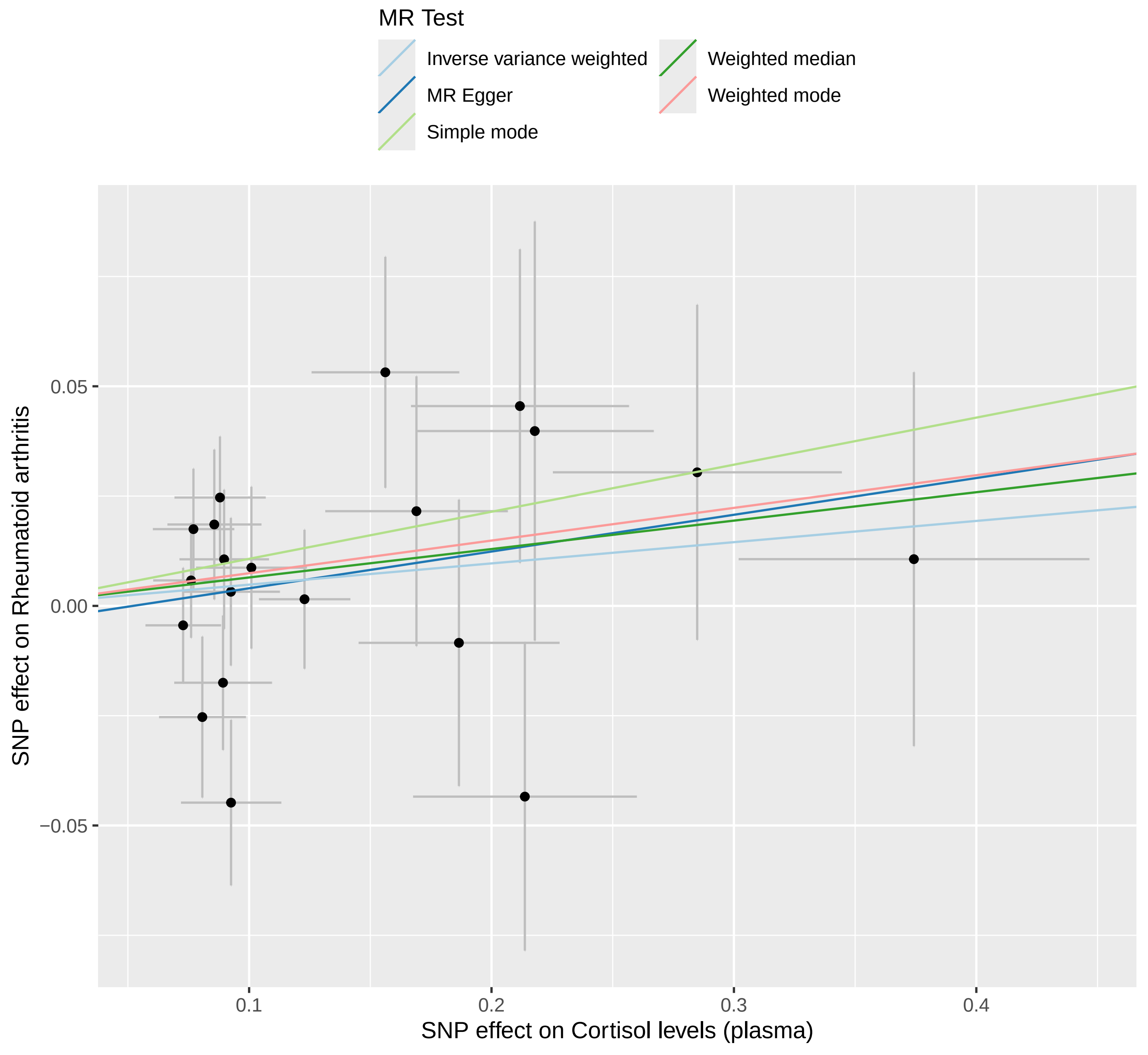

MR Test
Inverse variance weighted
MR Egger
Simple mode
Weighted median
Weighted mode
SNP effect on Rheumatoid arthritis
0.05
0.00
−0.05
0.1
0.2
0.3
0.4
SNP effect on Cortisol levels (plasma)

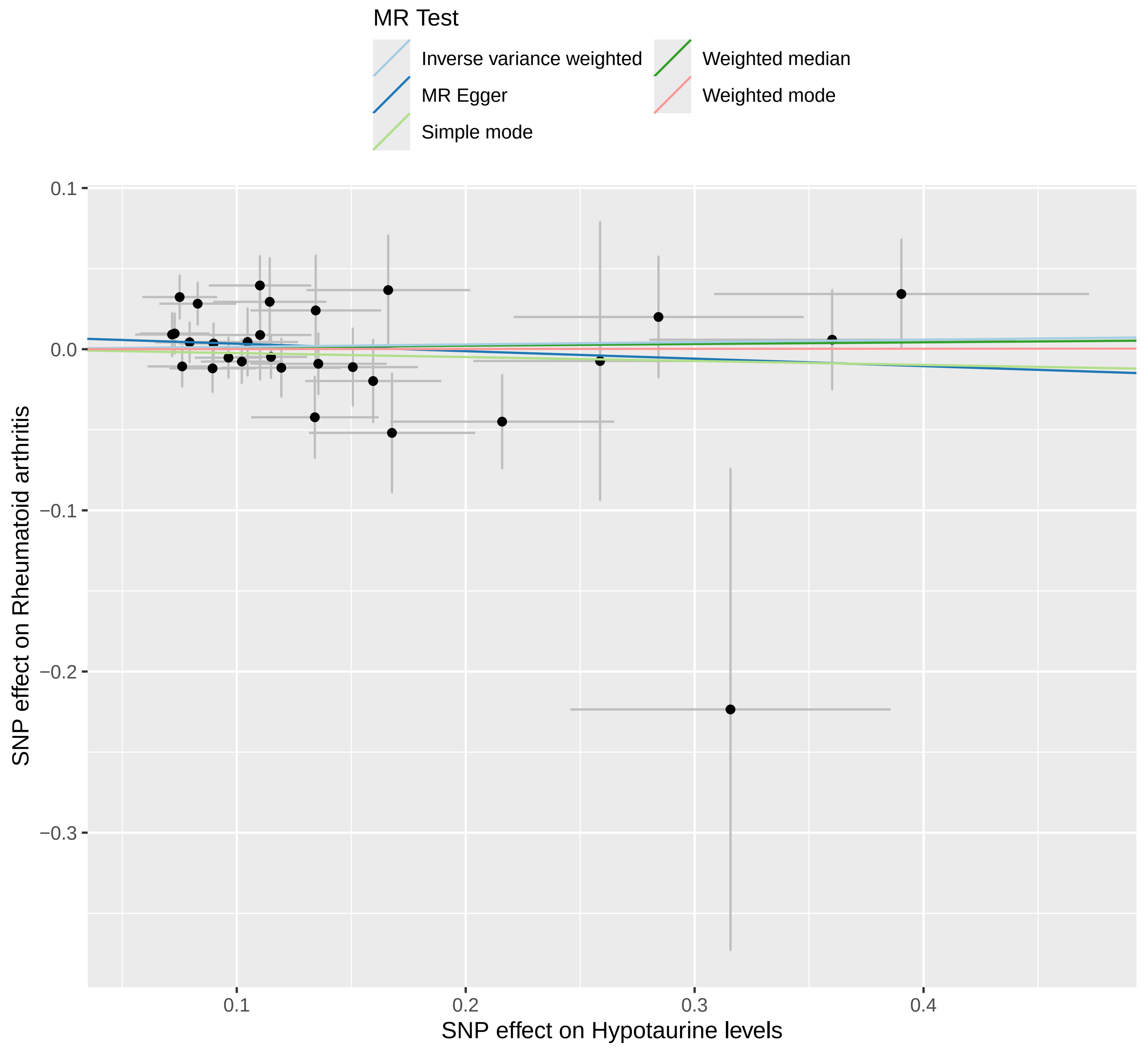
MR Test
Inverse variance weighted
MR Egger
Simple mode
Weighted median
Weighted mode
0.1
0.0
−0.1
−0.2
−0.3
0.1
0.2
0.3
0.4
SNP effect on Rheumatoid arthritis
SNP effect on Hypotaurine levels

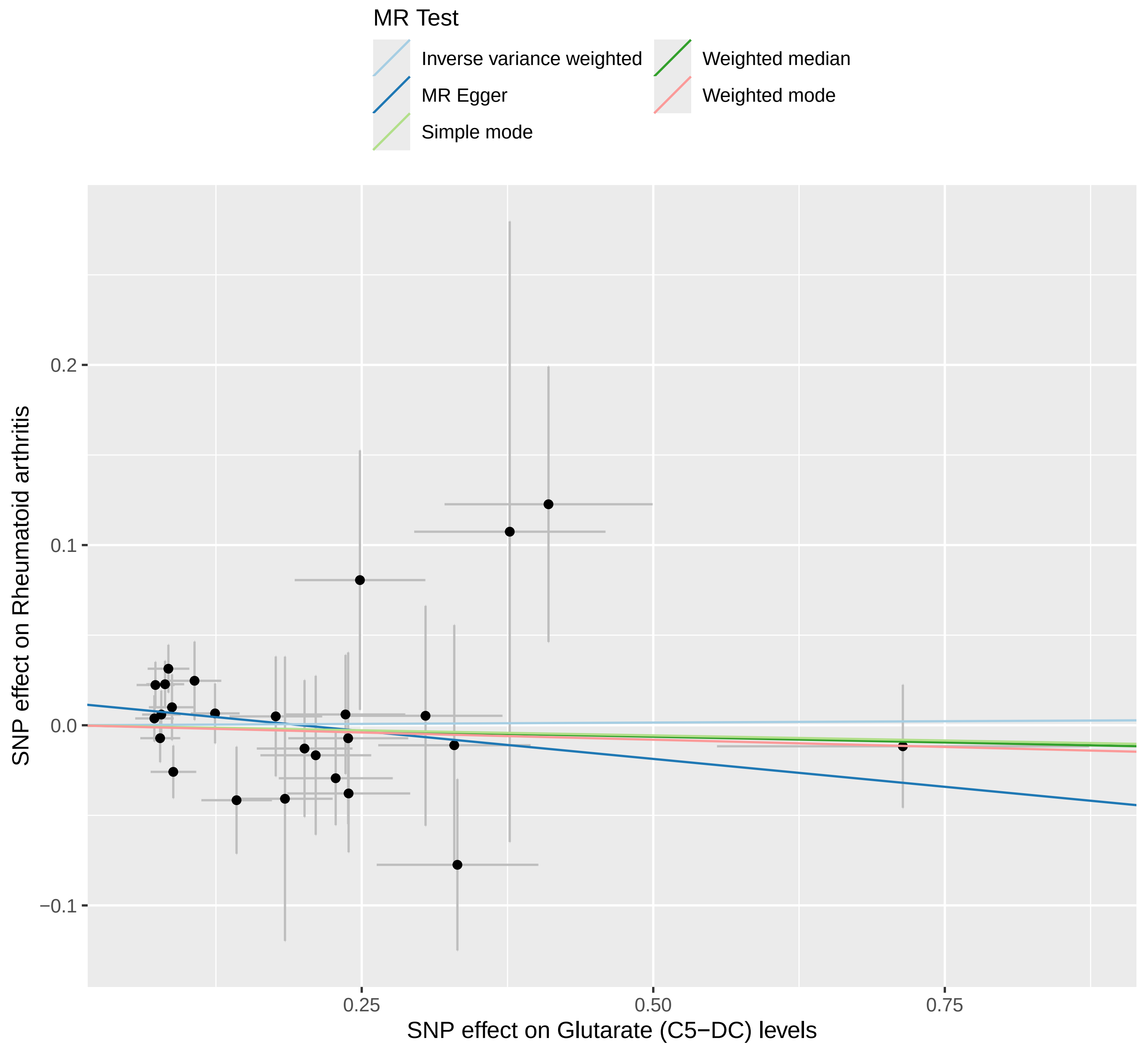
MR Test
Inverse variance weighted
MR Egger
Simple mode
Weighted median
Weighted mode
0.2
0.1
0.0
−0.1
SNP effect on Rheumatoid arthritis
0.25
0.50
0.75
SNP effect on Glutarate (C5−DC) levels

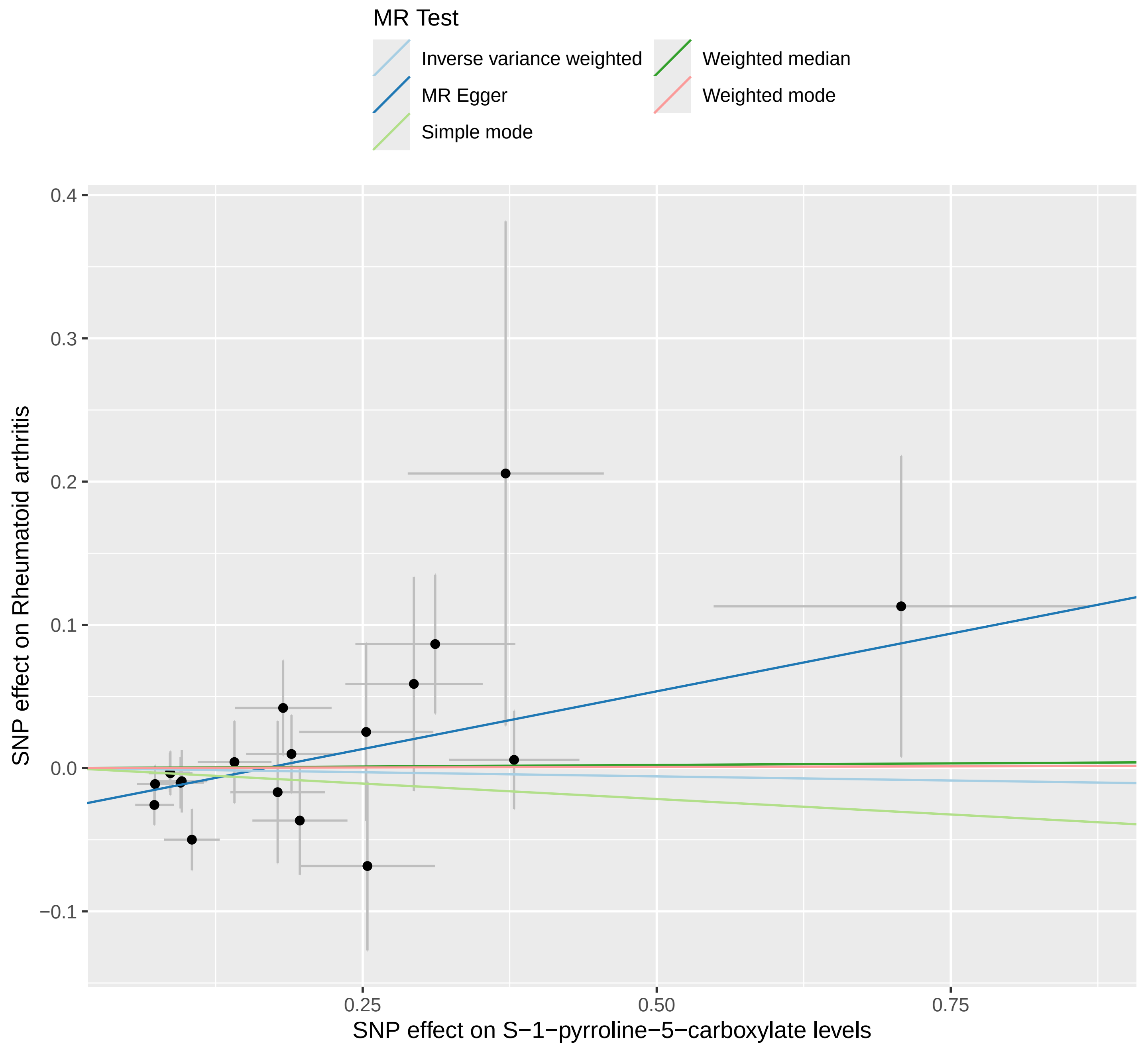

MR Test
Inverse variance weighted
MR Egger
Simple mode
Weighted median
Weighted mode
0.4
0.3
0.2
0.1
0.0
−0.1
0.25
0.50
0.75
SNP effect on Rheumatoid arthritis
SNP effect on S−1−pyrroline−5−carboxylate levels

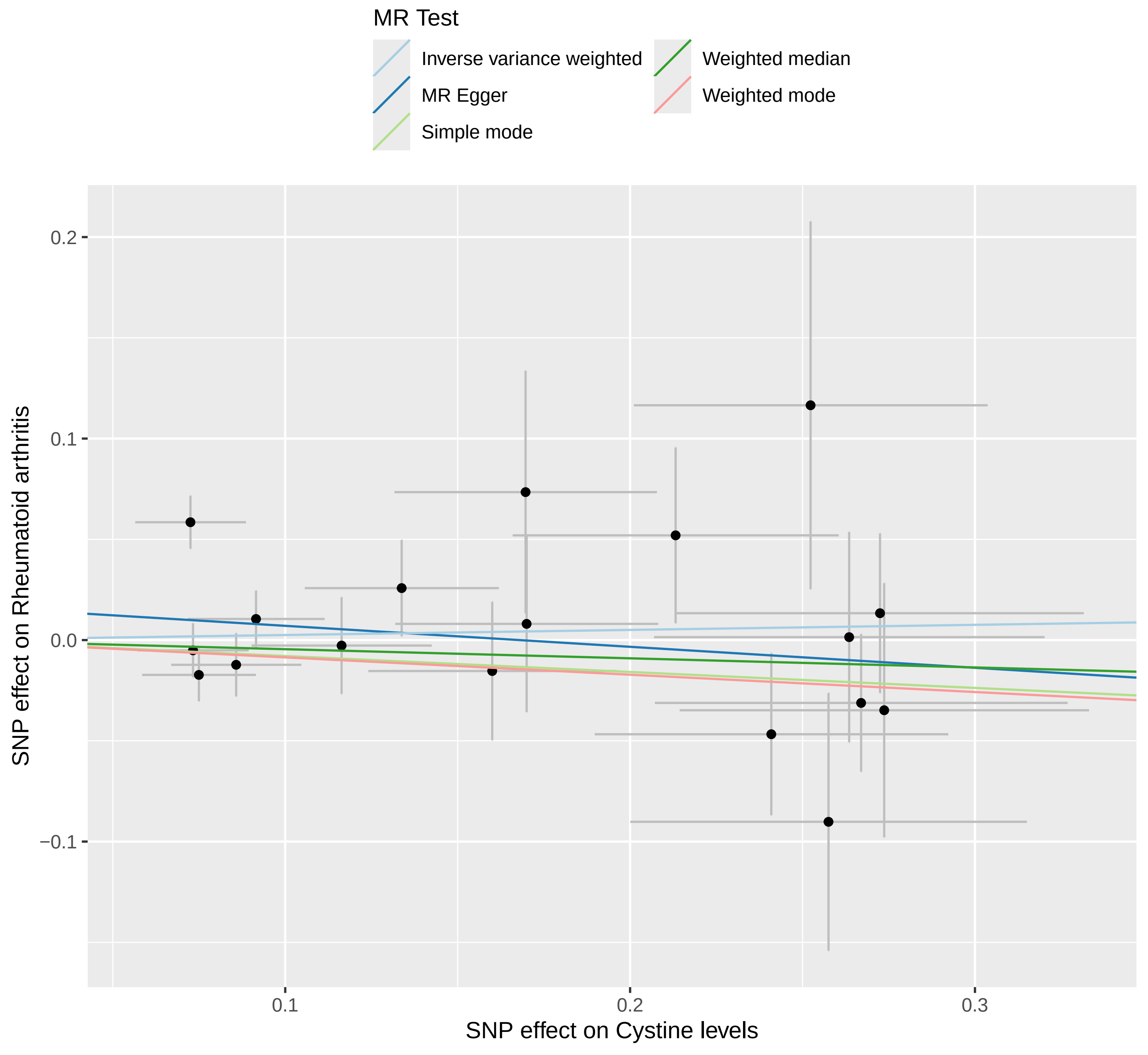
MR Test
Inverse variance weighted
MR Egger
Simple mode
Weighted median
Weighted mode
0.2
0.1
0.0
−0.1
0.1
0.2
0.3
SNP effect on Rheumatoid arthritis
SNP effect on Cystine levels

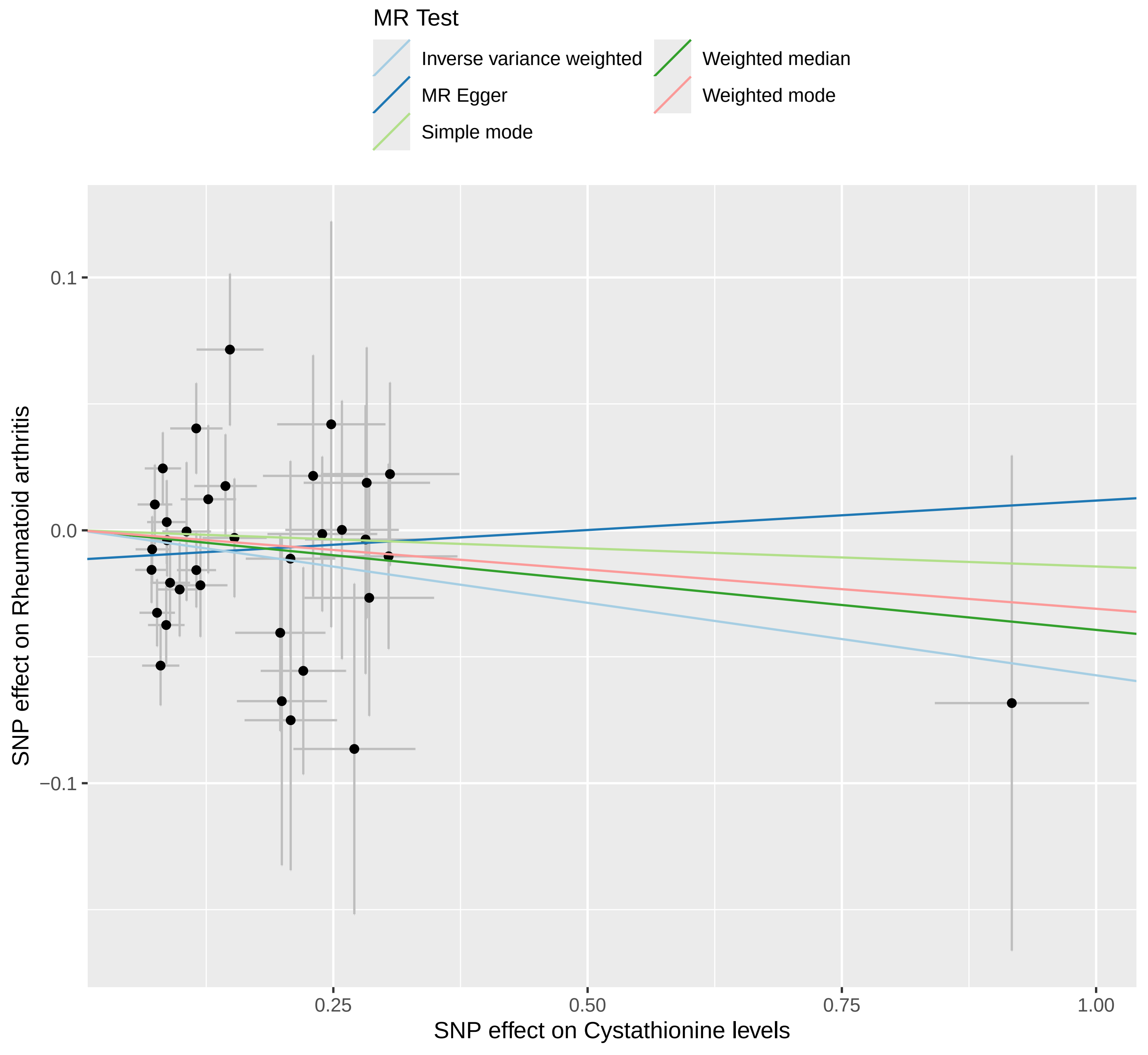

MR Test
Inverse variance weighted
MR Egger
Simple mode
Weighted median
Weighted mode
0.1
0.0
−0.1
0.25
0.50
0.75
1.00
SNP effect on Rheumatoid arthritis
SNP effect on Cystathionine levels

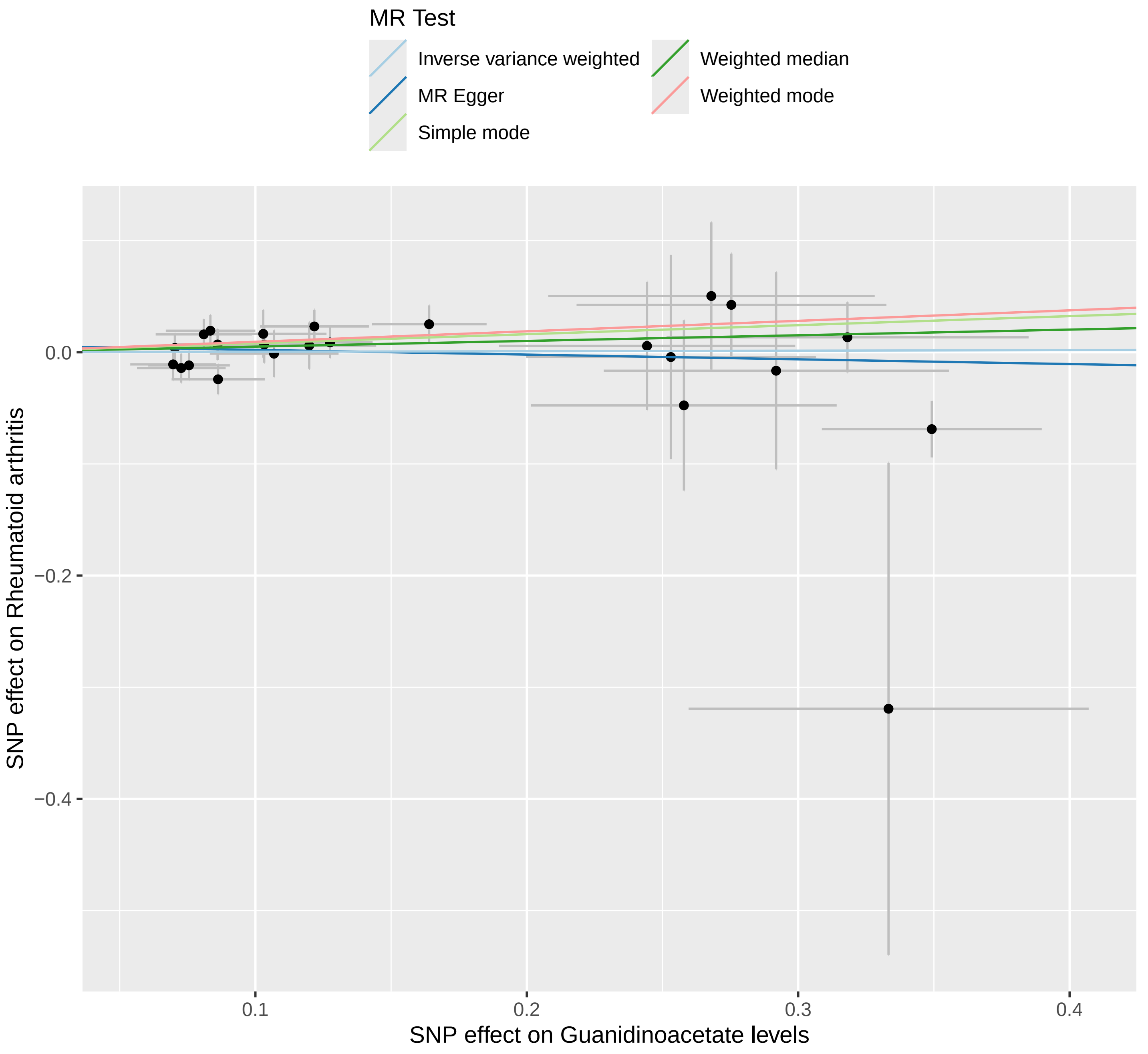
MR Test
Inverse variance weighted
MR Egger
Simple mode
Weighted median
Weighted mode
0.0
−0.2
−0.4
0.1
0.2
0.3
0.4
SNP effect on Rheumatoid arthritis
SNP effect on Guanidinoacetate levels

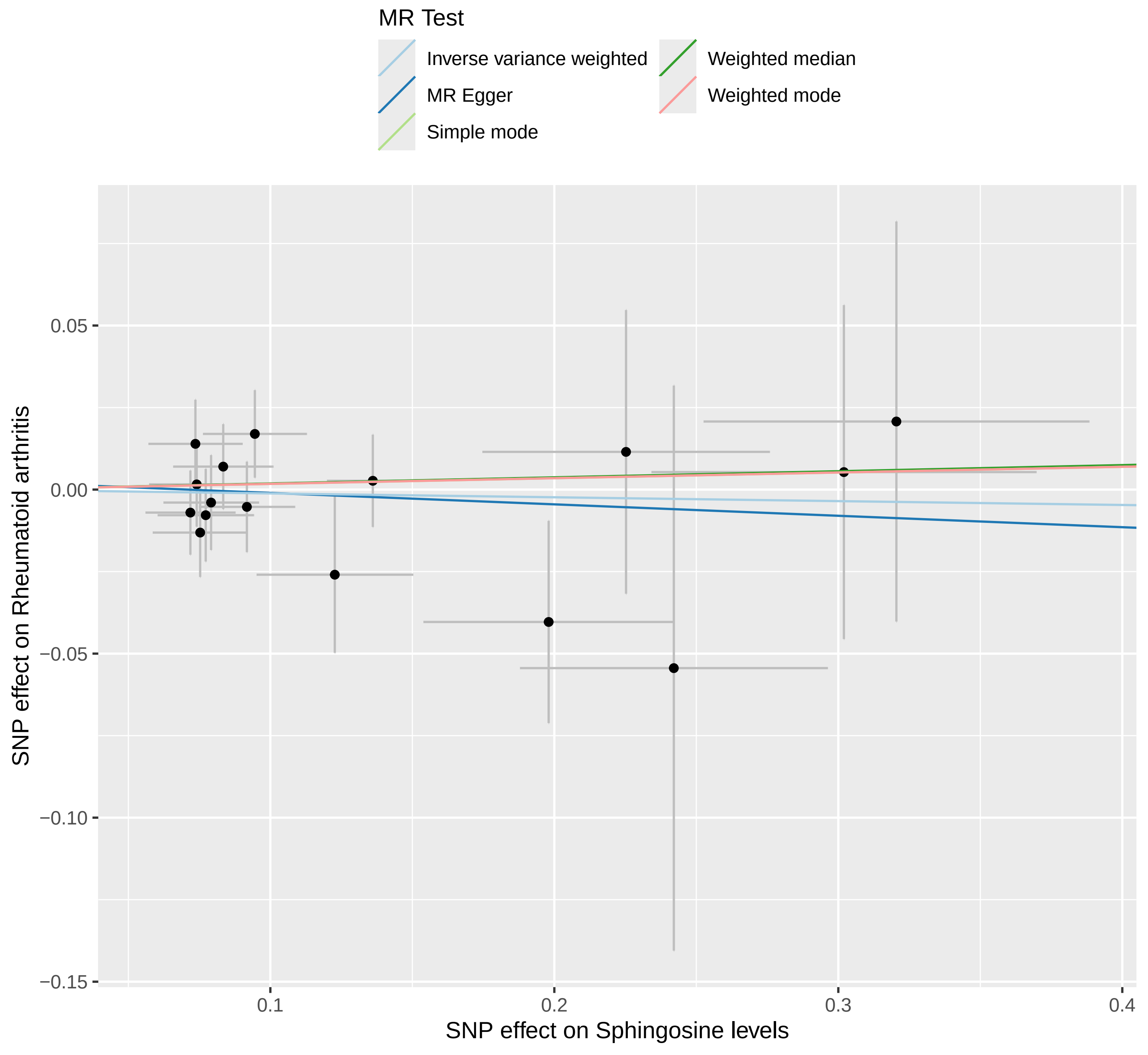
MR Test
Inverse variance weighted
MR Egger
Simple mode
Weighted median
Weighted mode
0.05
0.00
−0.05
−0.10
−0.15
0.1
0.2
0.3
0.4
SNP effect on Rheumatoid arthritis
SNP effect on Sphingosine levels

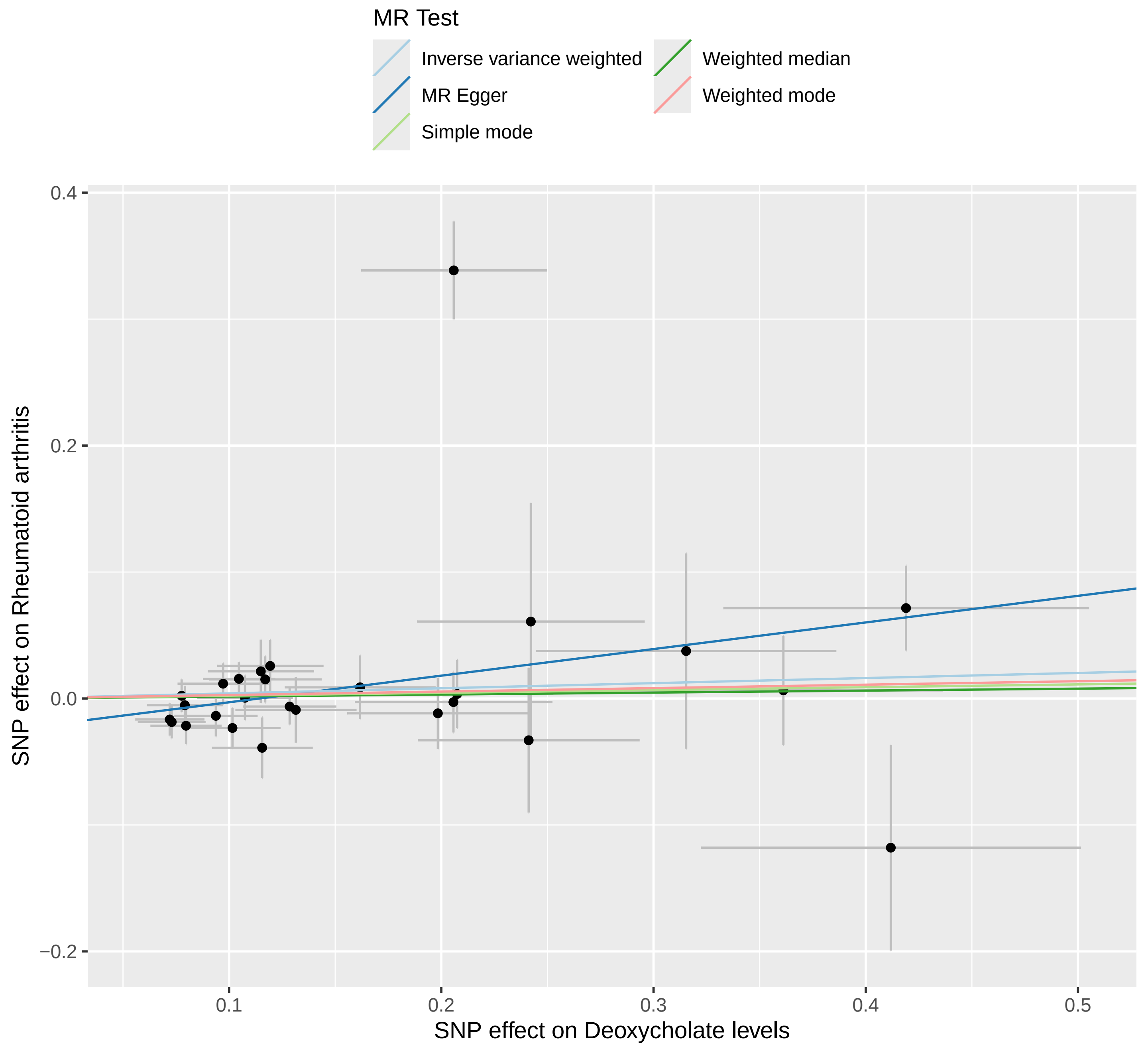

MR Test
Inverse variance weighted
MR Egger
Simple mode
Weighted median
Weighted mode
0.4
0.2
0.0
−0.2
0.1
0.2
0.3
0.4
0.5
SNP effect on Deoxycholate levels
SNP effect on Rheumatoid arthritis

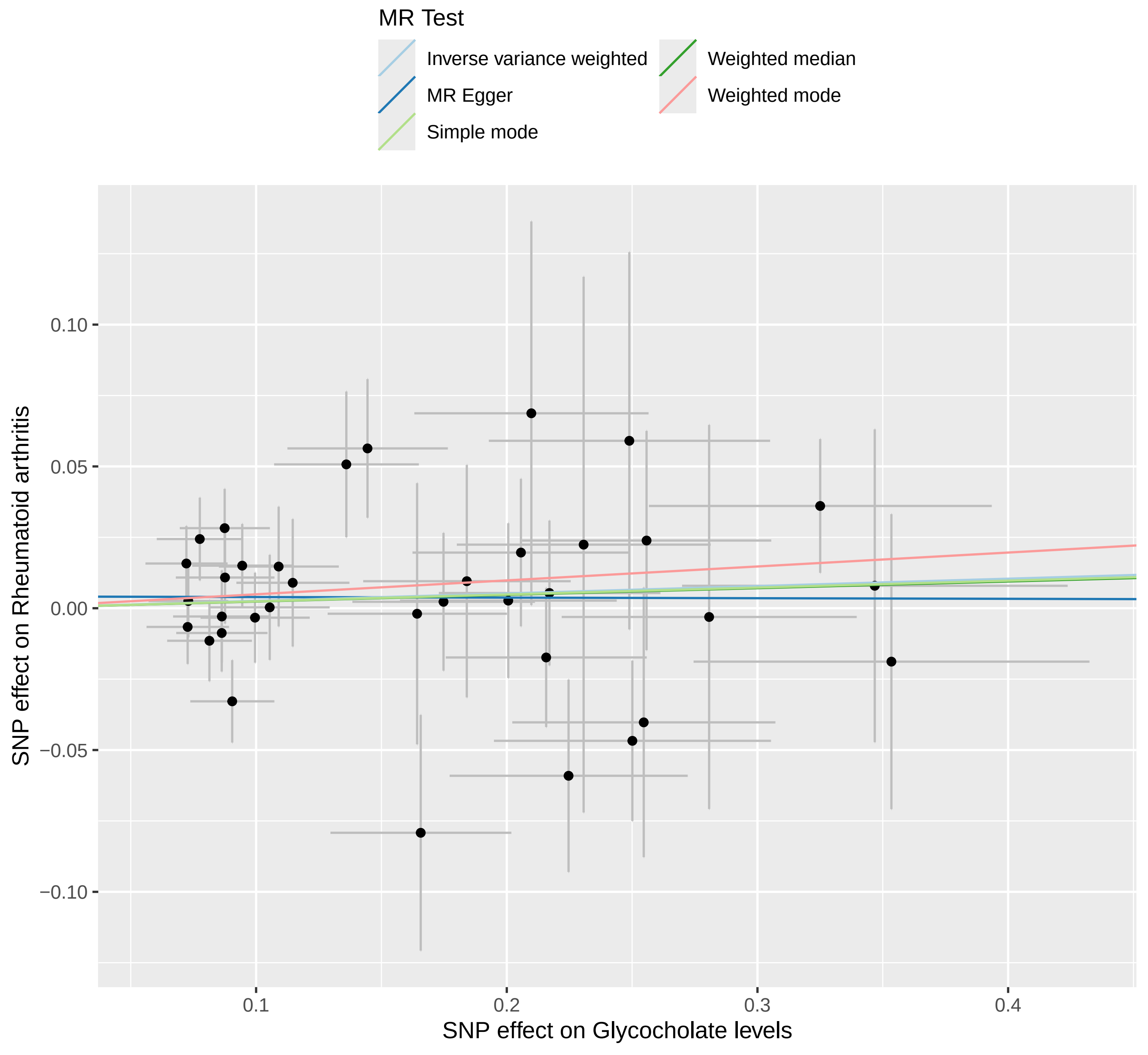
MR Test
Inverse variance weighted
MR Egger
Simple mode
Weighted median
Weighted mode
0.10
0.05
0.00
−0.05
−0.10
0.1
0.2
0.3
0.4
SNP effect on Rheumatoid arthritis
SNP effect on Glycocholate levels

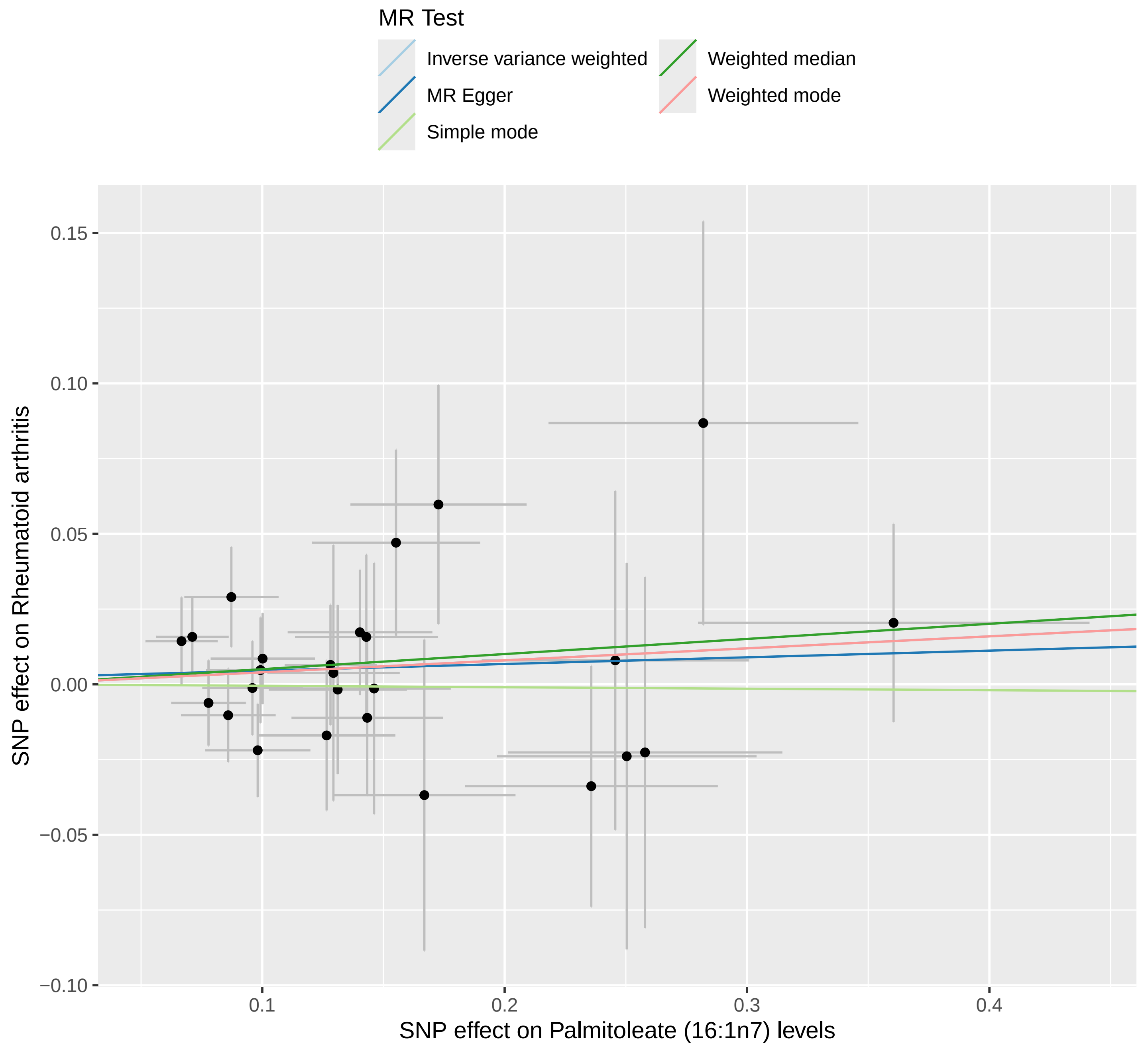

MR Test
Inverse variance weighted
MR Egger
Simple mode
Weighted median
Weighted mode
0.15
0.10
0.05
0.00
−0.05
−0.10
0.1
0.2
0.3
0.4
SNP effect on Rheumatoid arthritis
SNP effect on Palmitoleate (16:1n7) levels

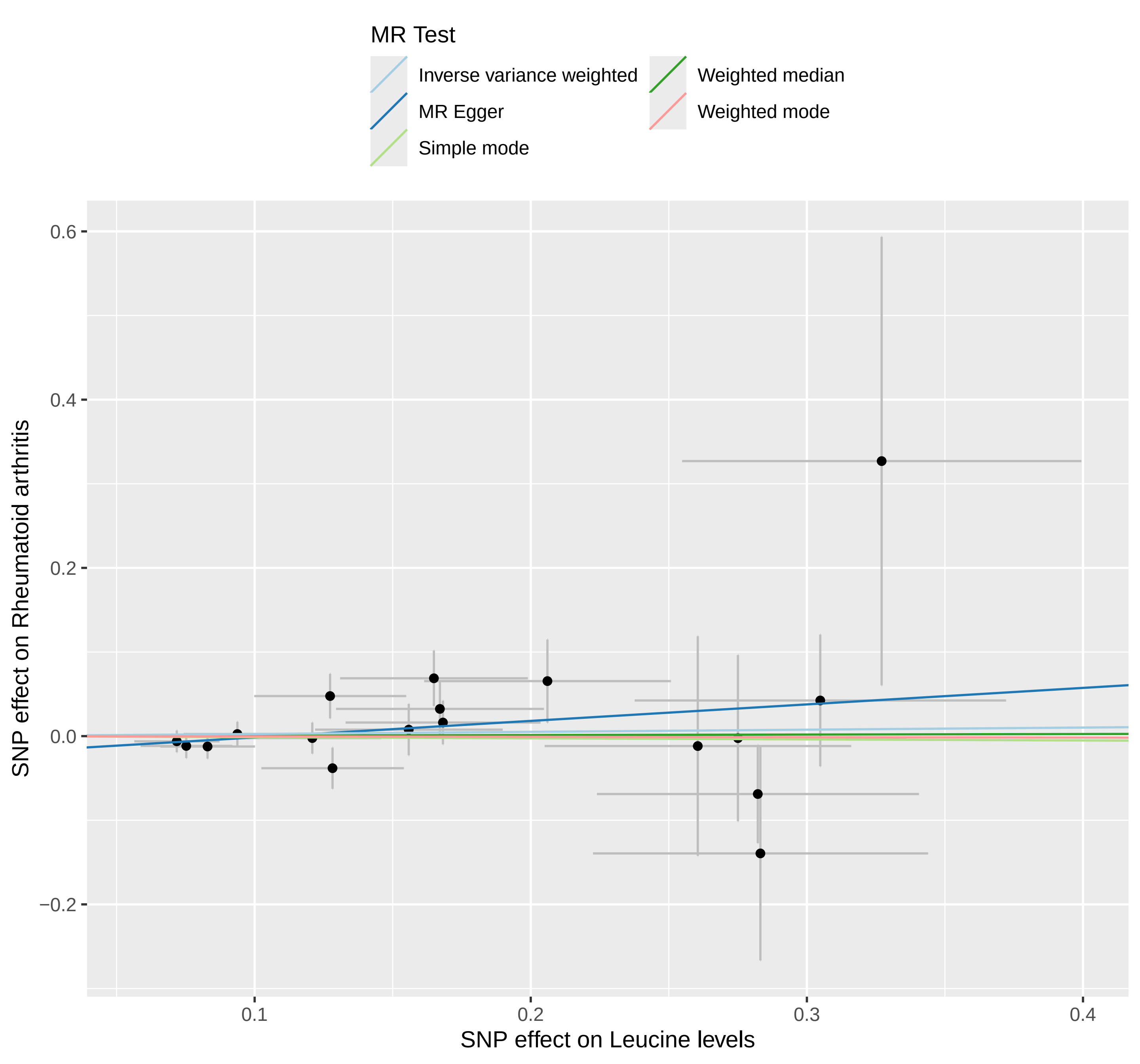

MR Test
Inverse variance weighted
MR Egger
Simple mode
Weighted median
Weighted mode
0.6
0.4
0.2
0.0
−0.2
0.1
0.2
0.3
0.4
SNP effect on Rheumatoid arthritis
SNP effect on Leucine levels

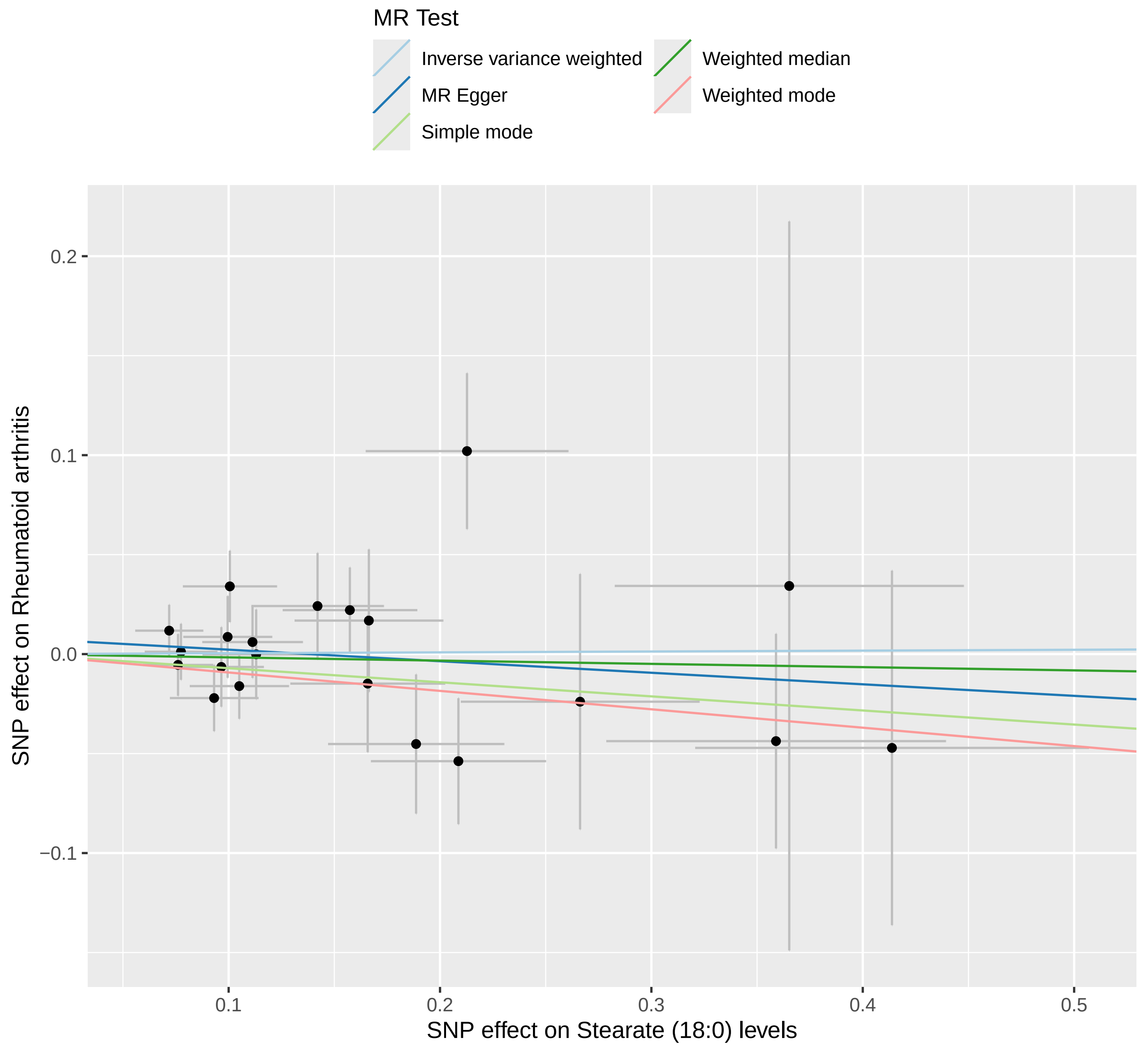
MR Test
Inverse variance weighted
MR Egger
Simple mode
Weighted median
Weighted mode
0.2
0.1
0.0
−0.1
0.1
0.2
0.3
0.4
0.5
SNP effect on Rheumatoid arthritis
SNP effect on Stearate (18:0) levels

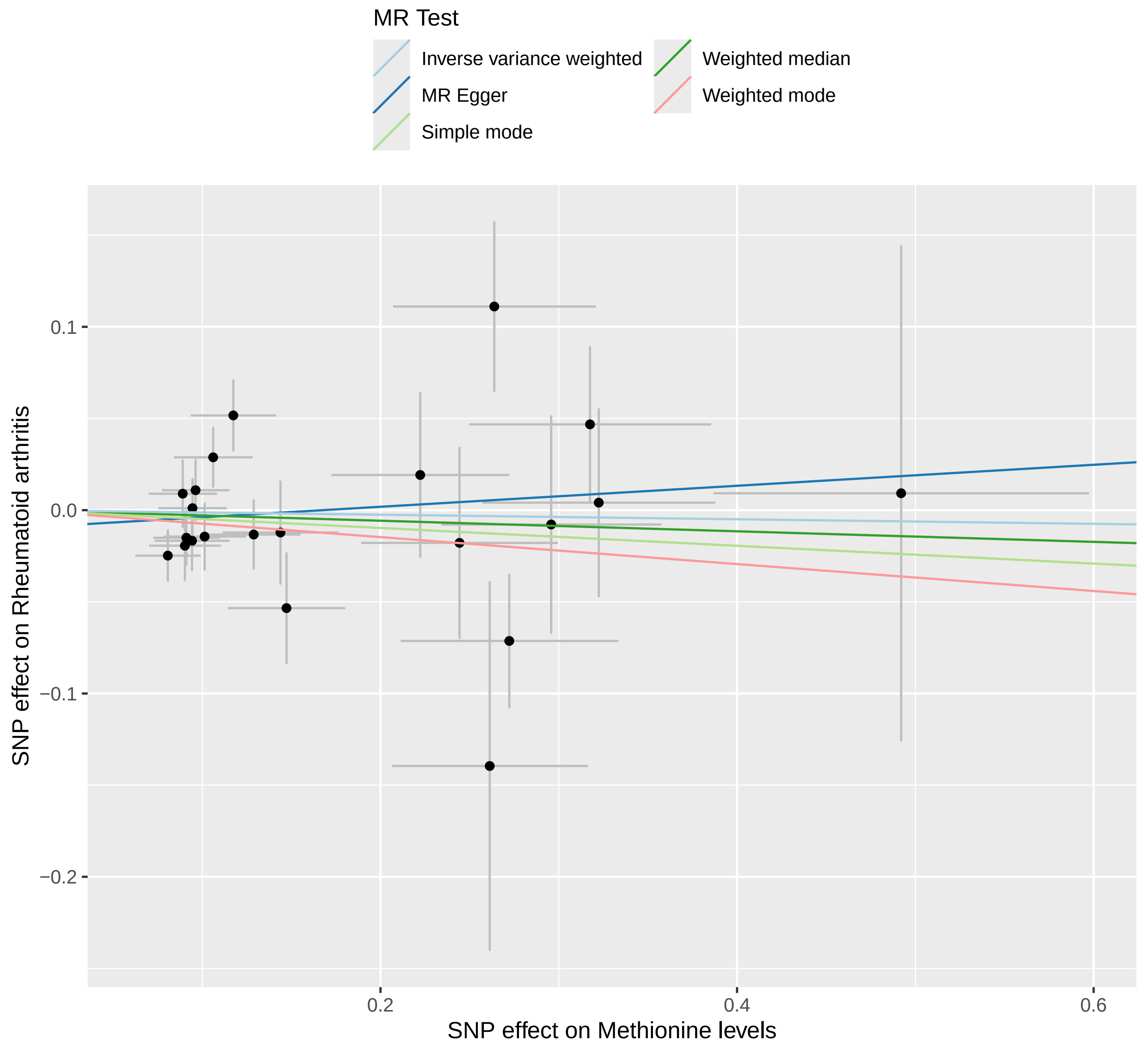
MR Test
Inverse variance weighted
MR Egger
Simple mode
Weighted median
Weighted mode
SNP effect on Rheumatoid arthritis
0.1
0.0
−0.1
−0.2
0.2
0.4
0.6
SNP effect on Methionine levels

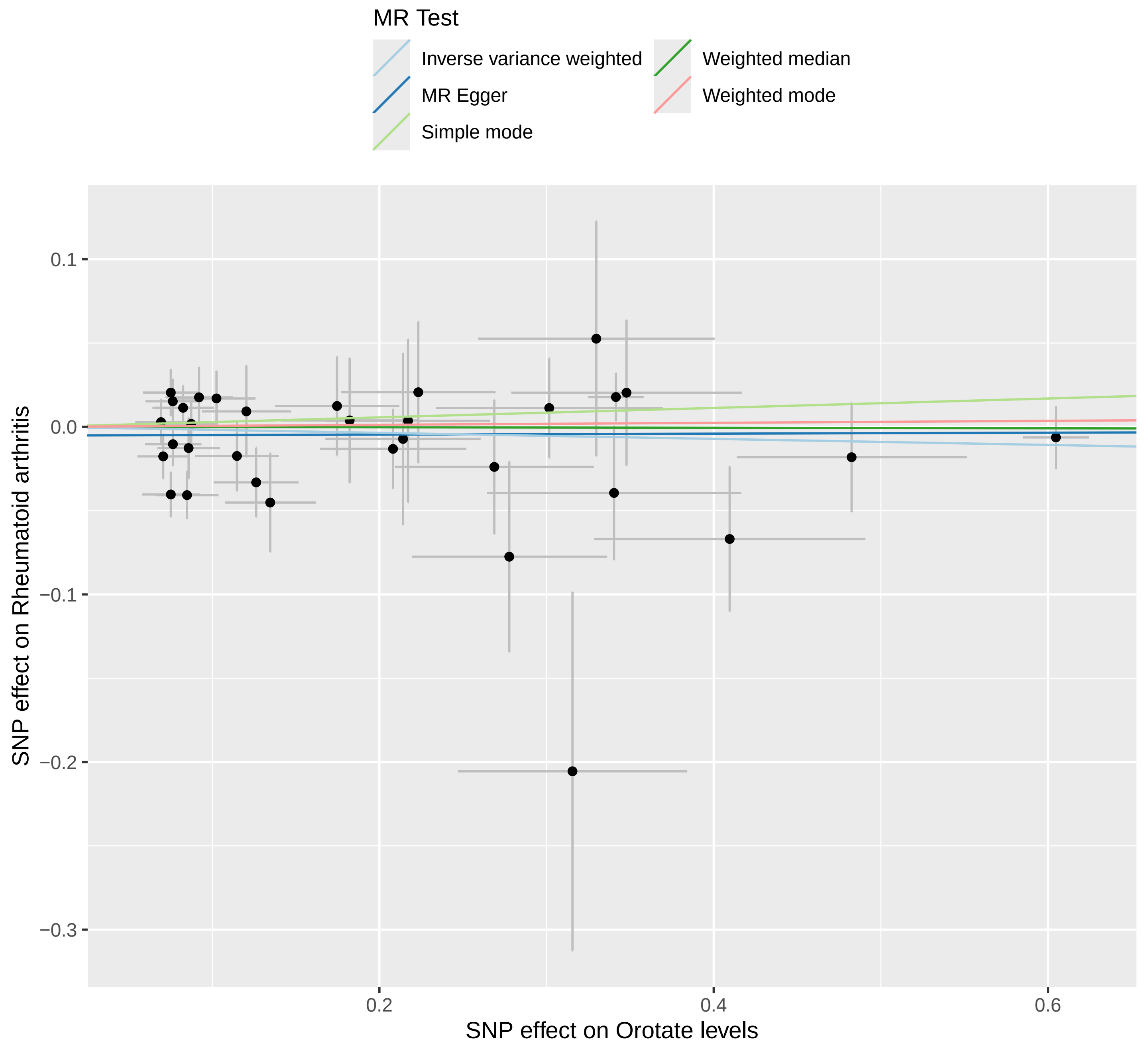
MR Test
Inverse variance weighted
MR Egger
Simple mode
Weighted median
Weighted mode
0.1
0.0
−0.1
−0.2
−0.3
SNP effect on Rheumatoid arthritis
0.2
0.4
0.6
SNP effect on Orotate levels

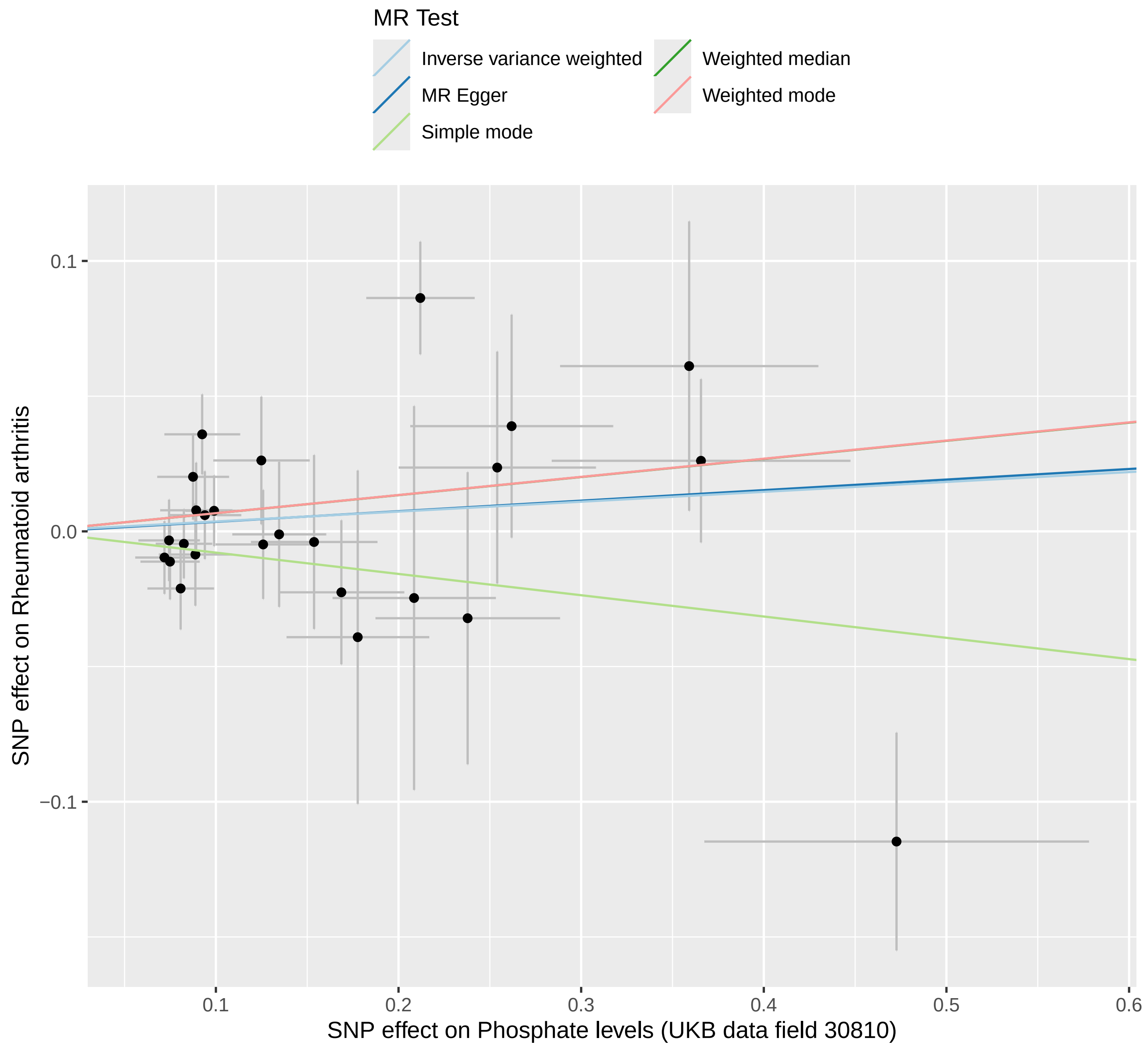

MR Test
Inverse variance weighted
MR Egger
Simple mode
Weighted median
Weighted mode
0.1
0.0
−0.1
0.1
0.2
0.3
0.4
0.5
0.6
SNP effect on Rheumatoid arthritis
SNP effect on Phosphate levels (UKB data field 30810)

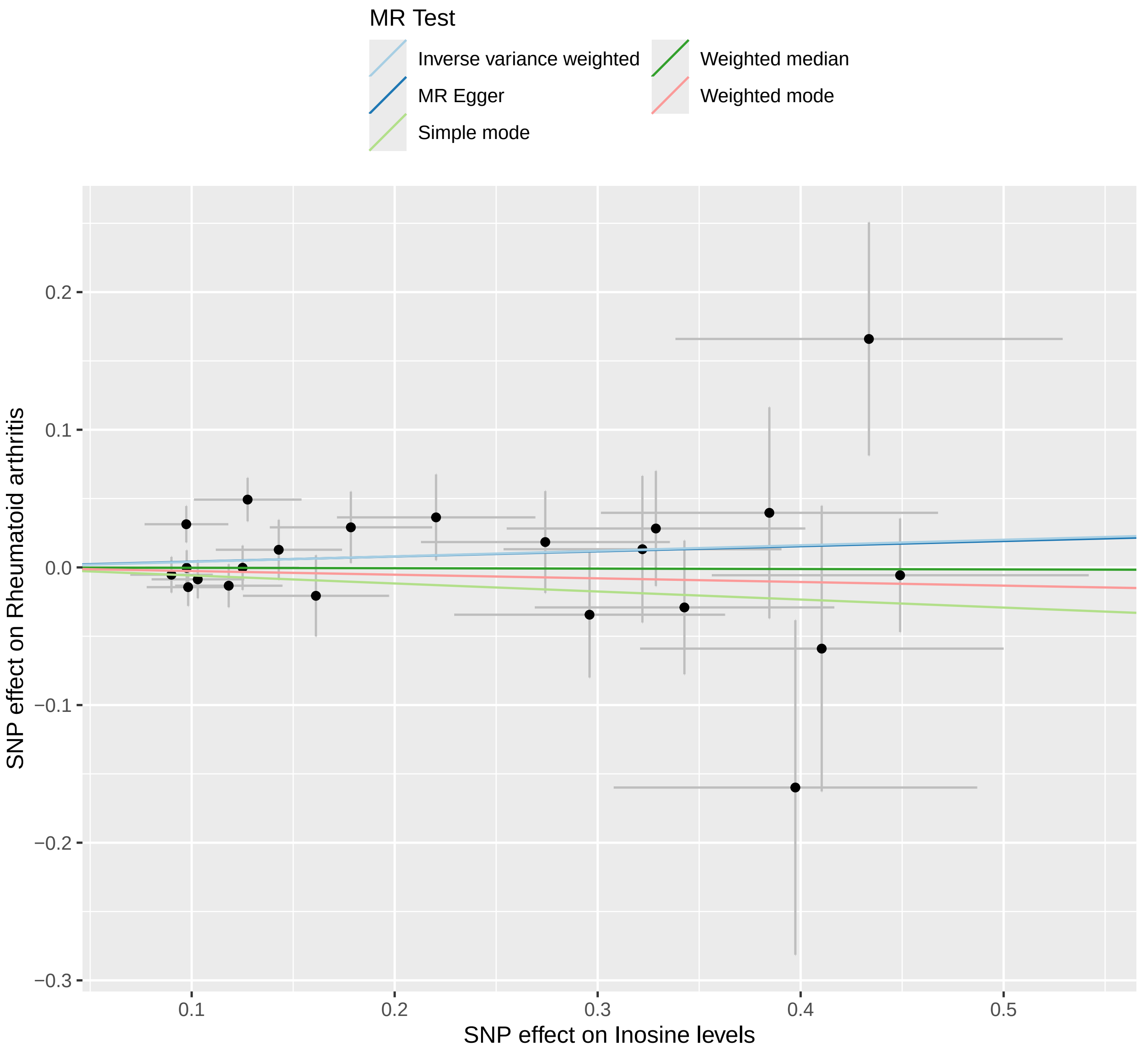

MR Test
Inverse variance weighted
MR Egger
Simple mode
Weighted median
Weighted mode
0.2
0.1
0.0
−0.1
−0.2
−0.3
SNP effect on Rheumatoid arthritis
0.1
0.2
0.3
0.4
0.5
SNP effect on Inosine levels

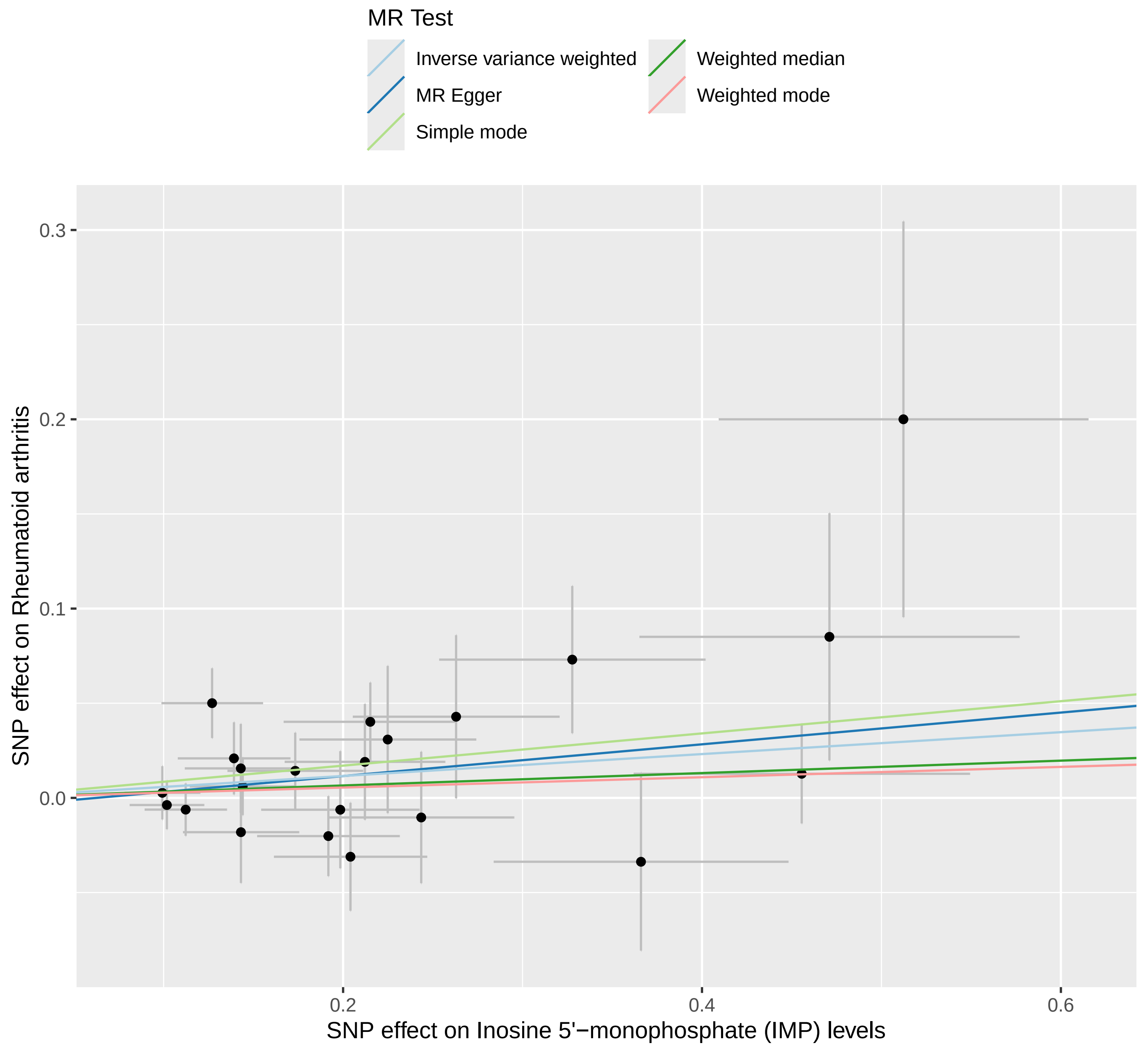
MR Test
Inverse variance weighted
MR Egger
Simple mode
Weighted median
Weighted mode
0.3
0.2
0.1
0.0
0.2
0.4
0.6
SNP effect on Rheumatoid arthritis
SNP effect on Inosine 5'−monophosphate (IMP) levels

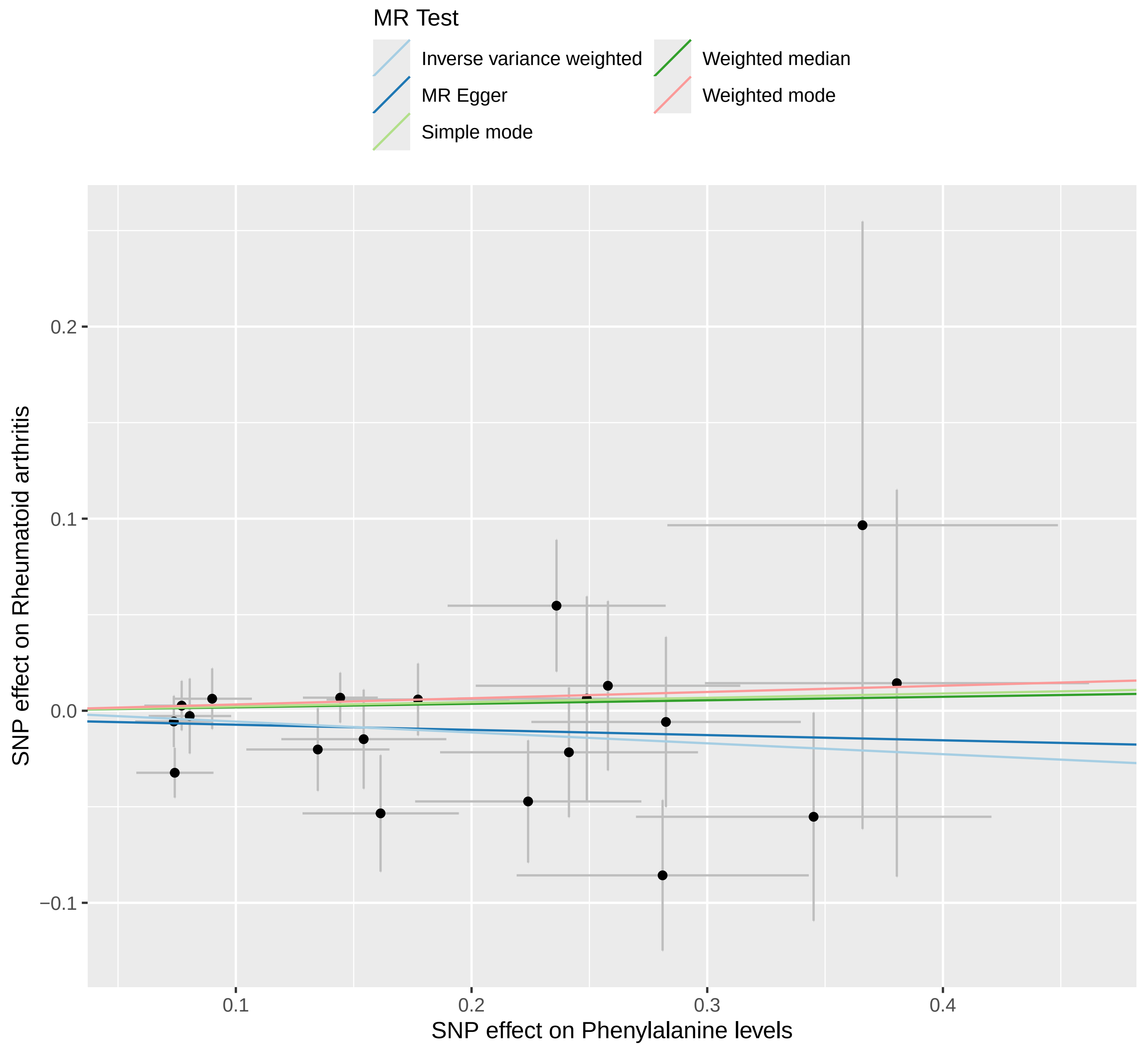
MR Test
Inverse variance weighted
MR Egger
Simple mode
Weighted median
Weighted mode
SNP effect on Rheumatoid arthritis
0.2
0.1
0.0
−0.1
0.1
0.2
0.3
0.4
SNP effect on Phenylalanine levels

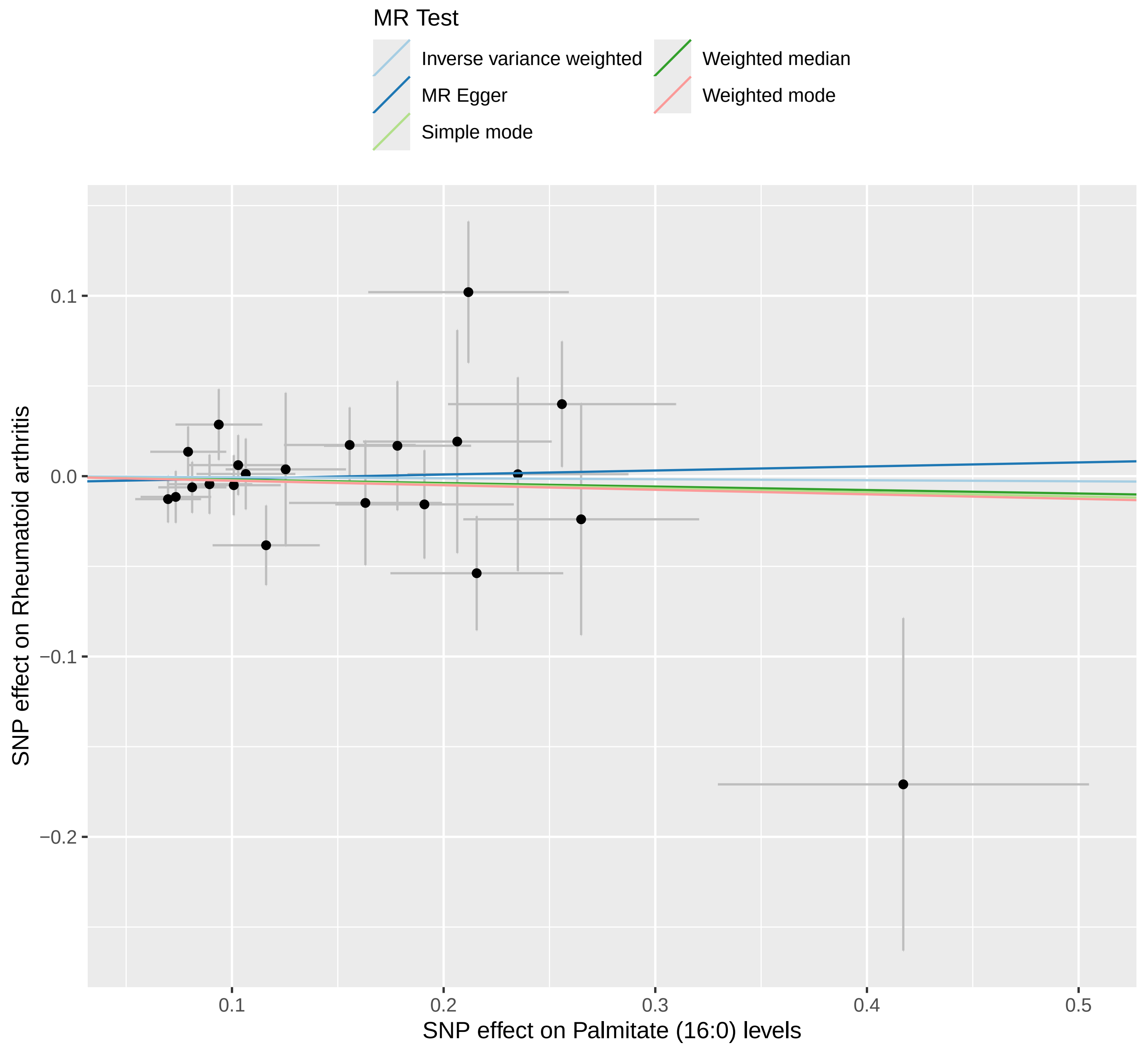
MR Test
Inverse variance weighted
MR Egger
Simple mode
Weighted median
Weighted mode
0.1
0.0
−0.1
−0.2
SNP effect on Rheumatoid arthritis
0.1
0.2
0.3
0.4
0.5
SNP effect on Palmitate (16:0) levels

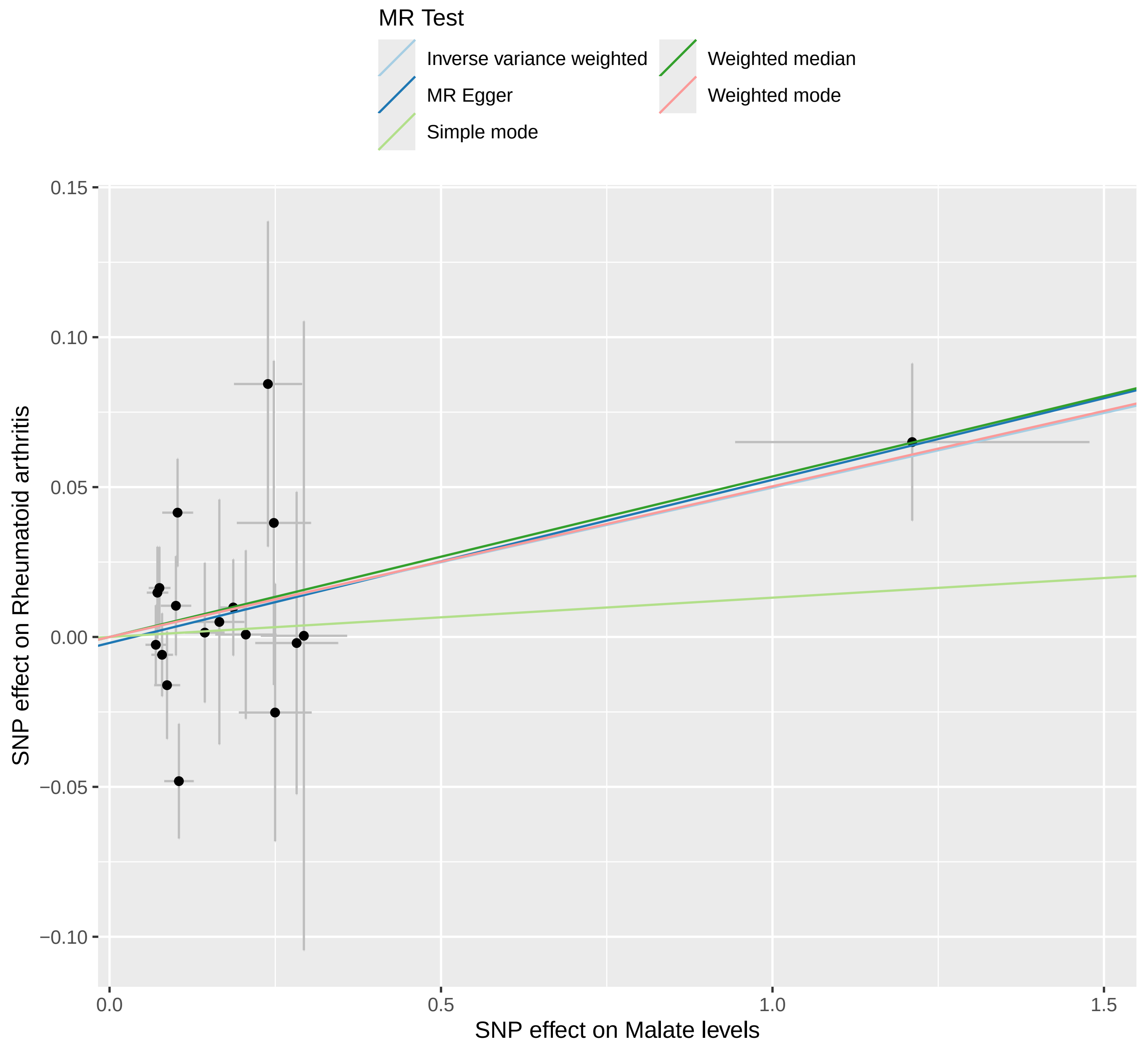

MR Test
Inverse variance weighted
MR Egger
Simple mode
Weighted median
Weighted mode
0.15
0.10
0.05
0.00
−0.05
−0.10
0.0
0.5
1.0
1.5
SNP effect on Rheumatoid arthritis
SNP effect on Malate levels

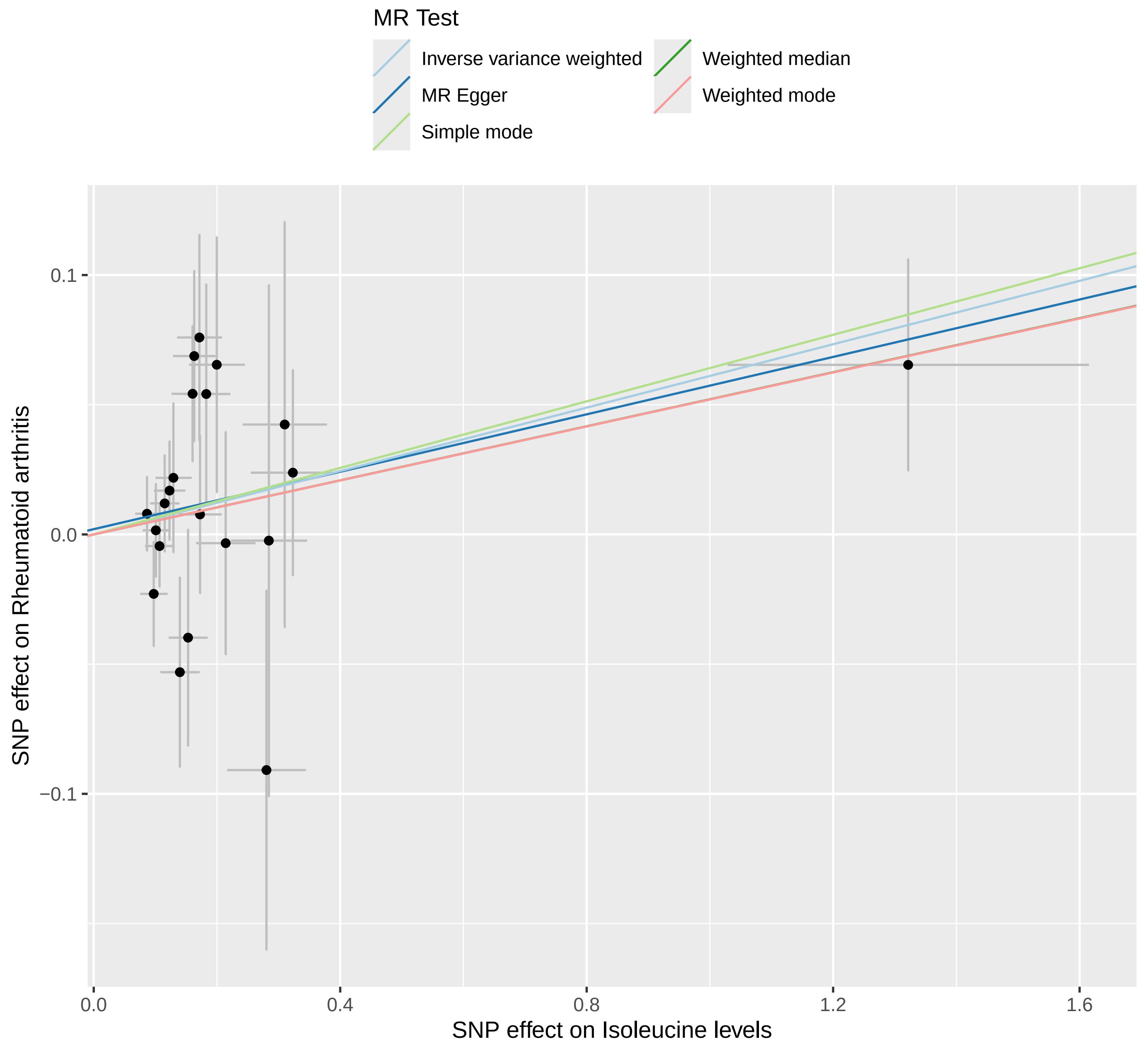
MR Test
Inverse variance weighted
MR Egger
Simple mode
Weighted median
Weighted mode
0.1
0.0
−0.1
0.0
0.4
0.8
1.2
1.6
SNP effect on Rheumatoid arthritis
SNP effect on Isoleucine levels

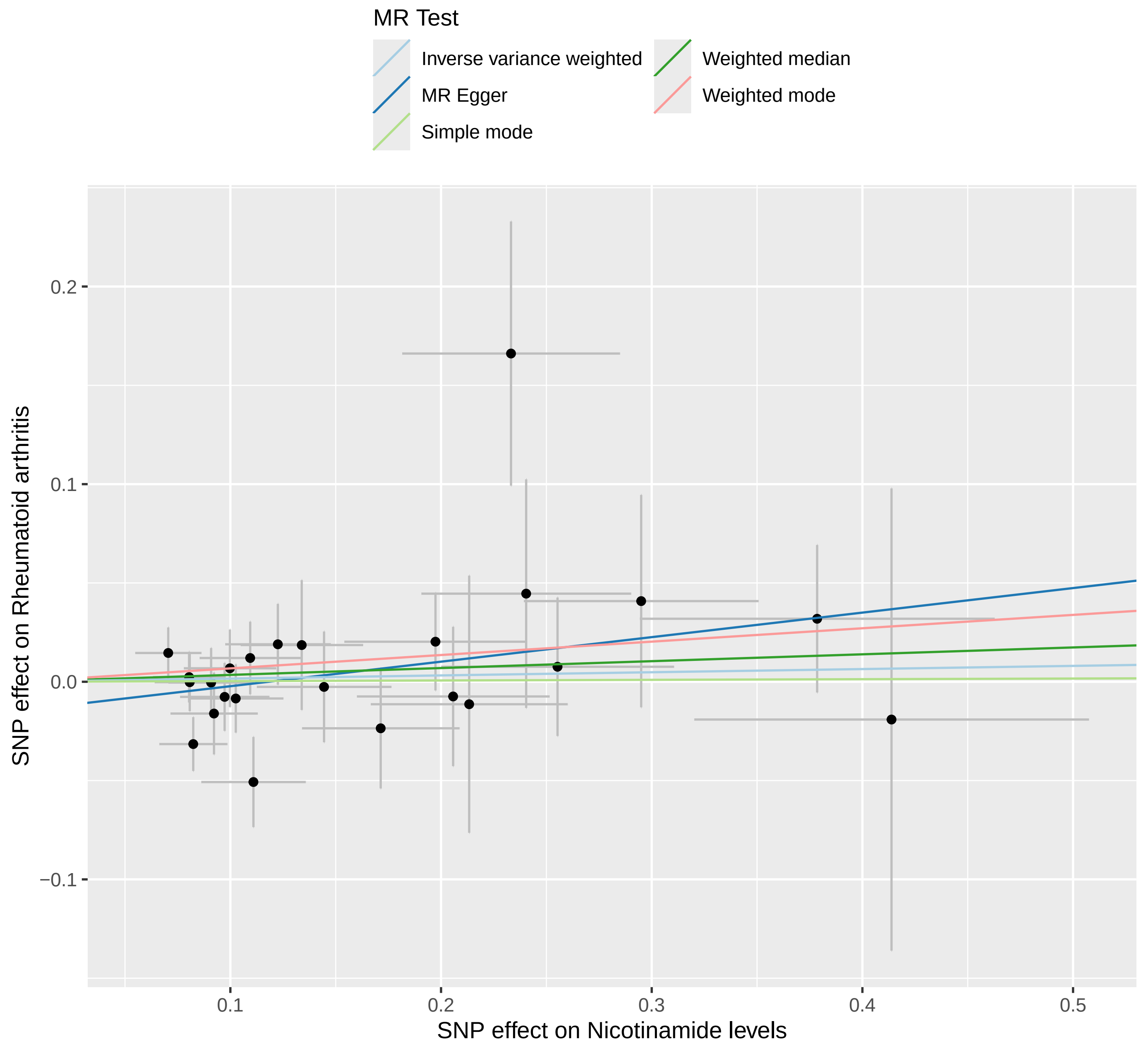

MR Test
Inverse variance weighted
MR Egger
Simple mode
Weighted median
Weighted mode
SNP effect on Rheumatoid arthritis
0.2
0.1
0.0
−0.1
0.1
0.2
0.3
0.4
0.5
SNP effect on Nicotinamide levels

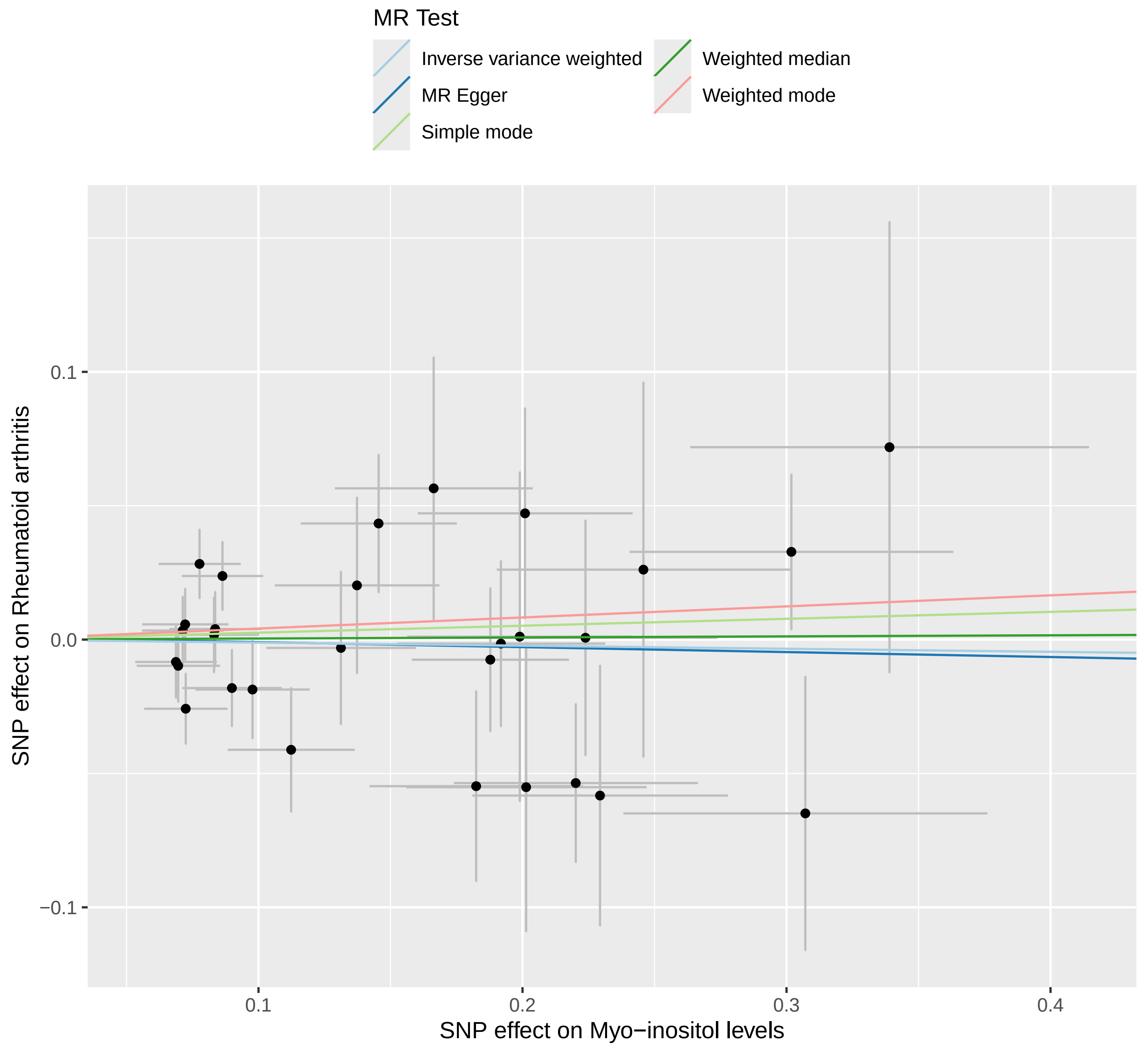
MR Test
Inverse variance weighted
MR Egger
Simple mode
Weighted median
Weighted mode
0.1
0.0
−0.1
0.1
0.2
0.3
0.4
SNP effect on Rheumatoid arthritis
SNP effect on Myo−inositol levels

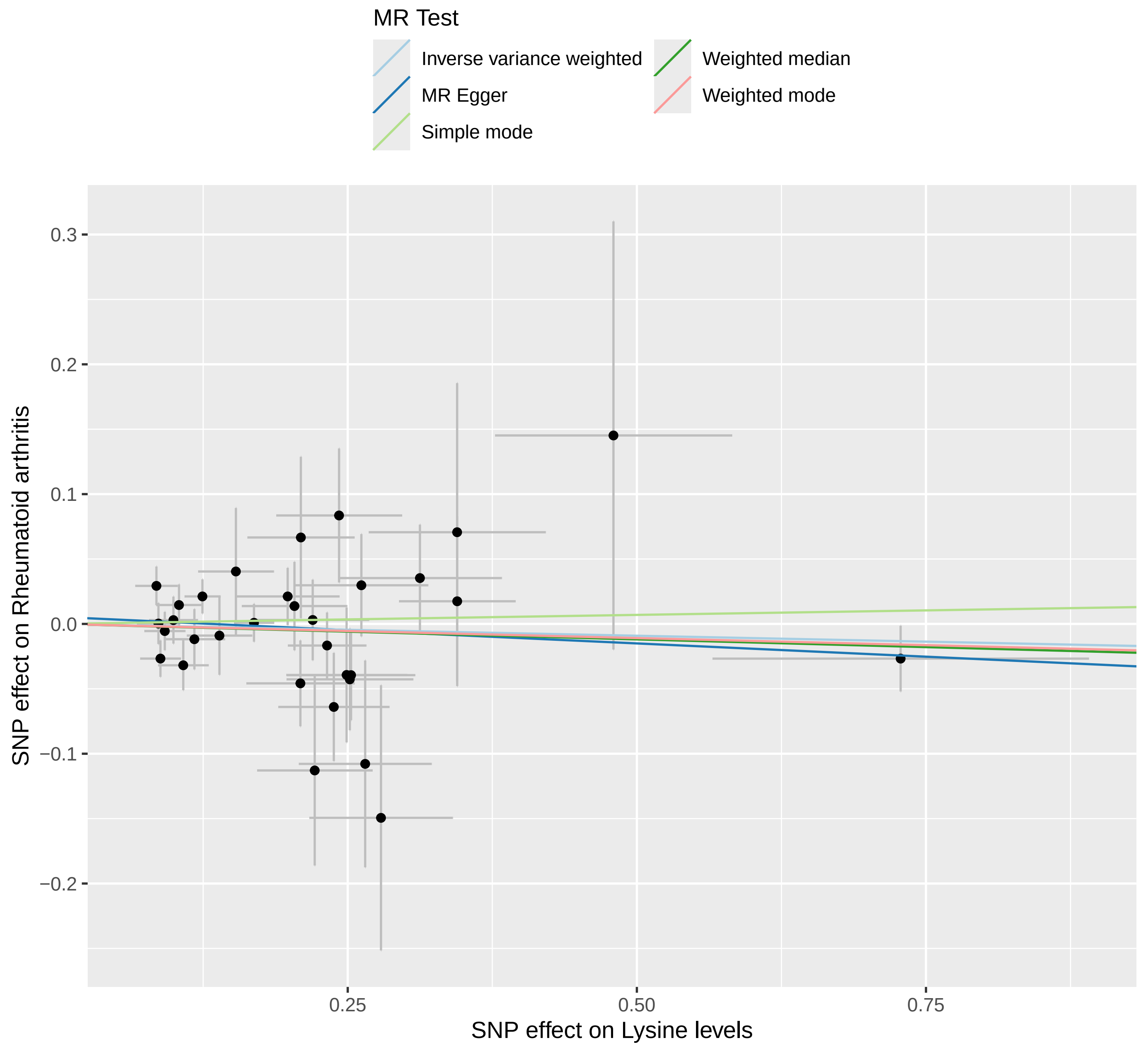
MR Test
Inverse variance weighted
MR Egger
Simple mode
Weighted median
Weighted mode
0.3
0.2
0.1
0.0
−0.1
−0.2
SNP effect on Rheumatoid arthritis
0.25
0.50
0.75
SNP effect on Lysine levels

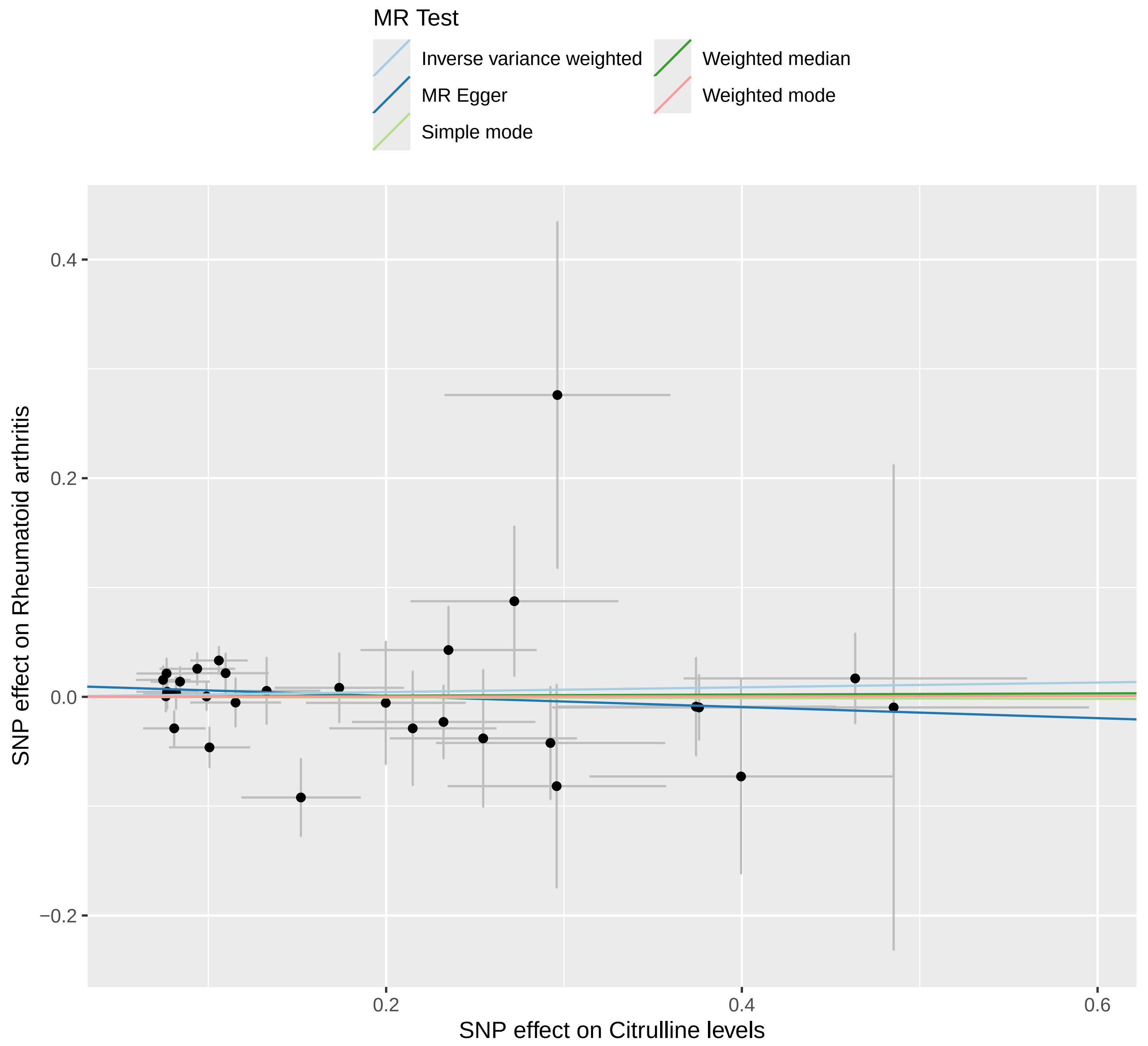
MR Test
Inverse variance weighted
MR Egger
Simple mode
Weighted median
Weighted mode
0.4
0.2
0.0
−0.2
SNP effect on Rheumatoid arthritis
0.2
0.4
0.6
SNP effect on Citrulline levels

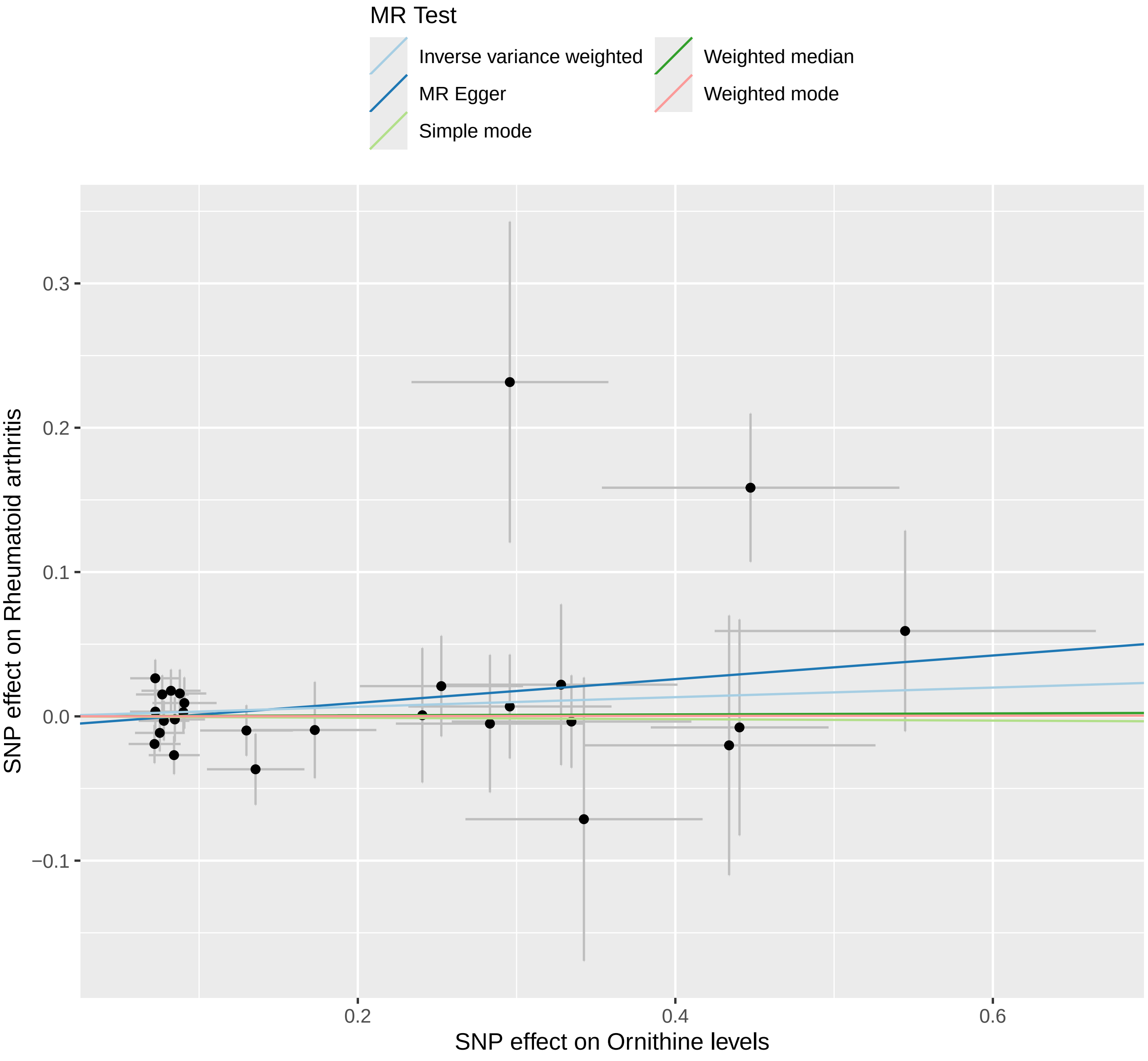
MR Test
Inverse variance weighted
MR Egger
Simple mode
Weighted median
Weighted mode
0.3
0.2
0.1
0.0
−0.1
0.2
0.4
0.6
SNP effect on Rheumatoid arthritis
SNP effect on Ornithine levels

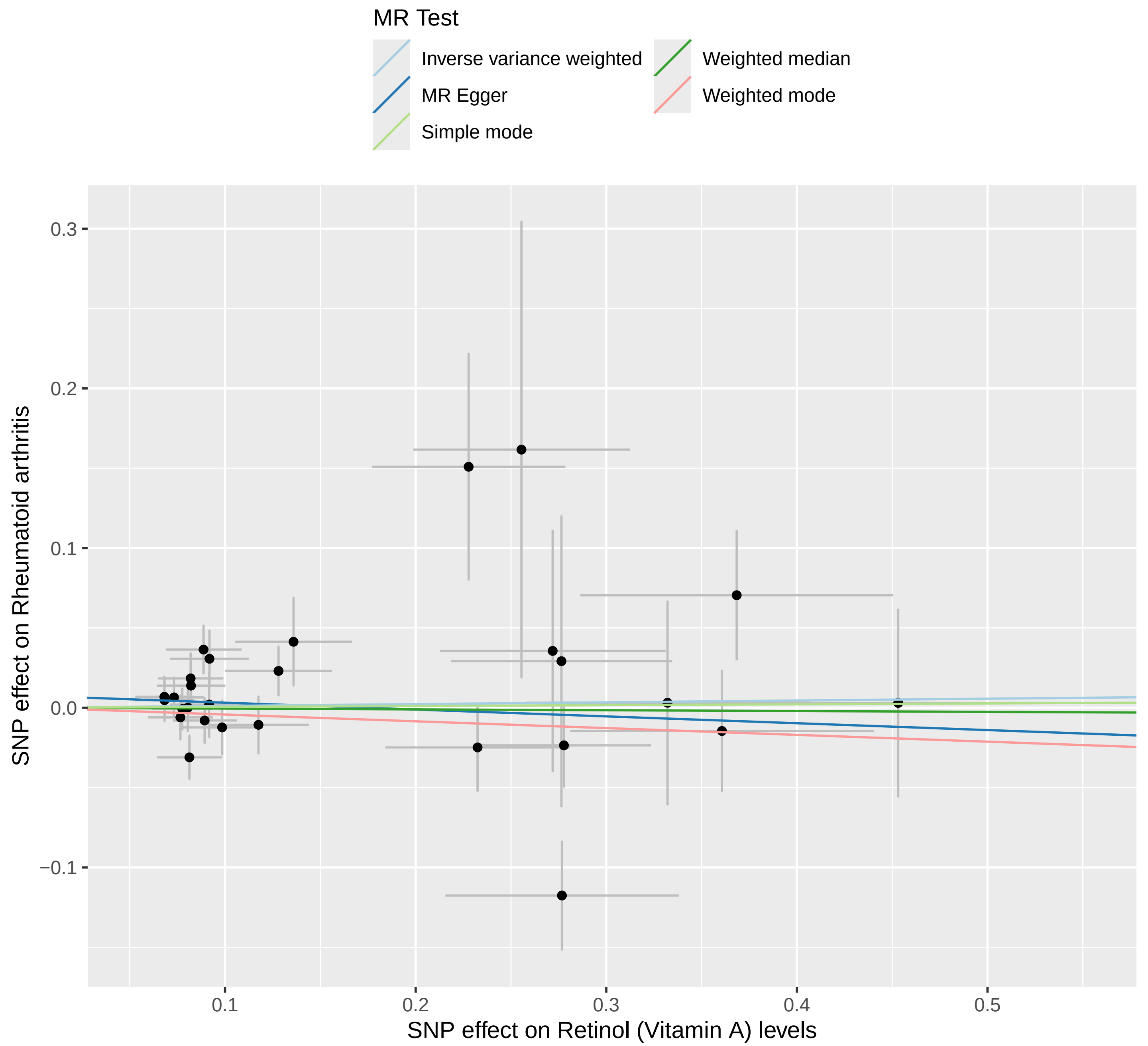

MR Test
Inverse variance weighted
MR Egger
Simple mode
Weighted median
Weighted mode
0.3
0.2
0.1
0.0
−0.1
SNP effect on Rheumatoid arthritis
0.1
0.2
0.3
0.4
0.5
SNP effect on Retinol (Vitamin A) levels

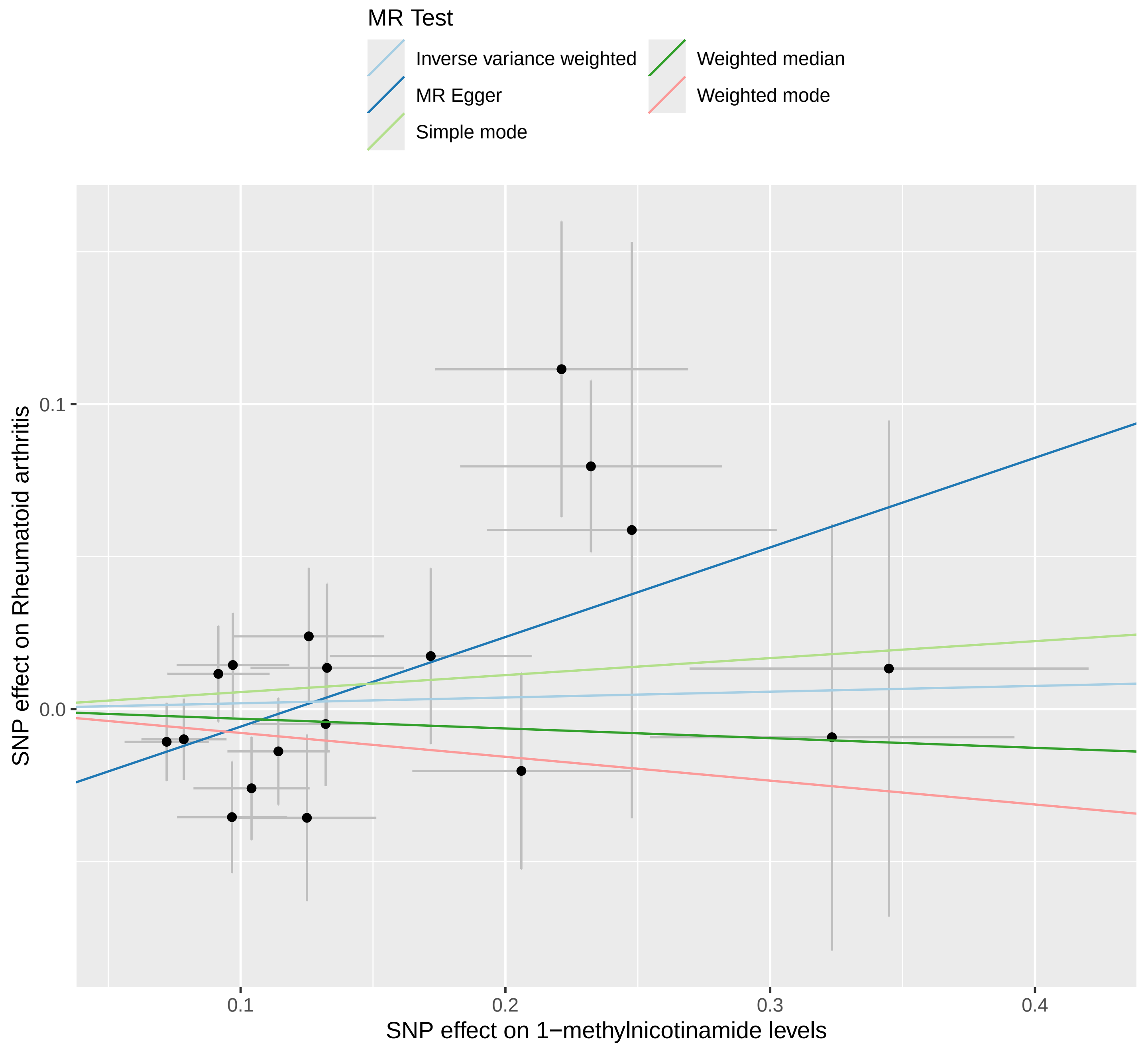

MR Test
Inverse variance weighted
MR Egger
Simple mode
Weighted median
Weighted mode
0.1
0.0
SNP effect on Rheumatoid arthritis
0.1
0.2
0.3
0.4
SNP effect on 1−methylnicotinamide levels

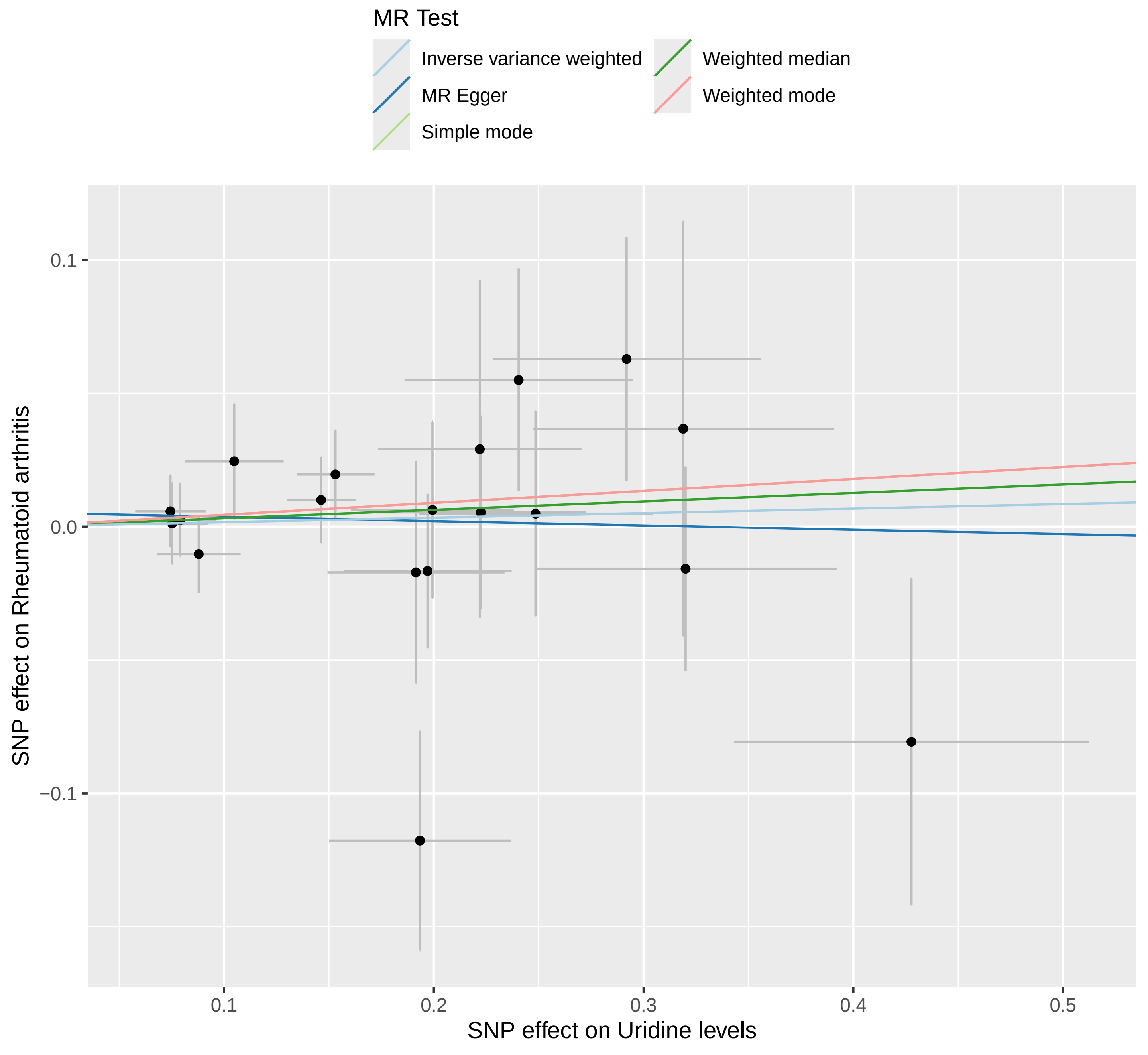
MR Test
Inverse variance weighted
MR Egger
Simple mode
Weighted median
Weighted mode
0.1
0.0
−0.1
0.1
0.2
0.3
0.4
0.5
SNP effect on Rheumatoid arthritis
SNP effect on Uridine levels

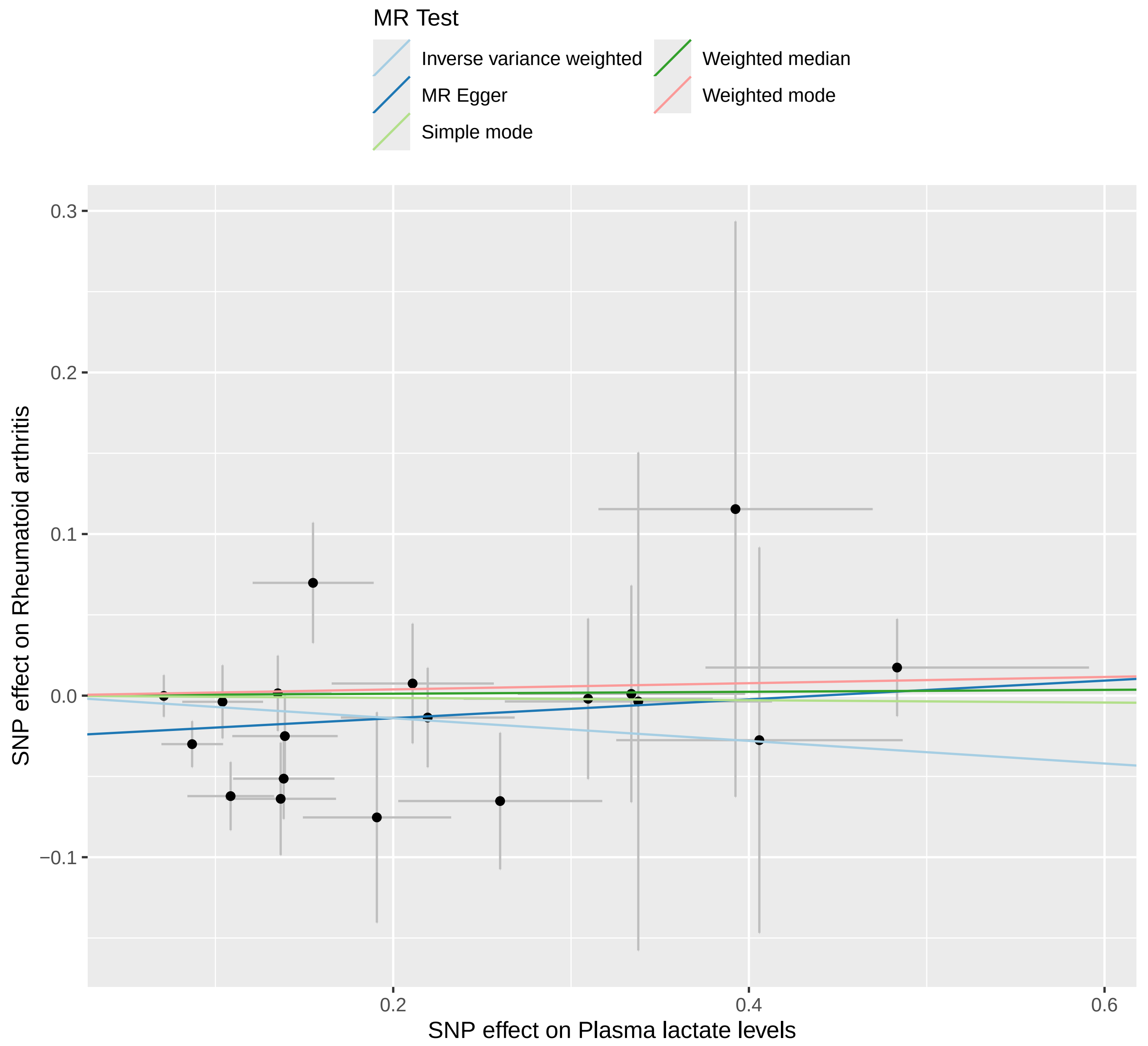

MR Test
Inverse variance weighted
MR Egger
Simple mode
Weighted median
Weighted mode
0.3
0.2
0.1
0.0
−0.1
0.2
0.4
0.6
SNP effect on Rheumatoid arthritis
SNP effect on Plasma lactate levels

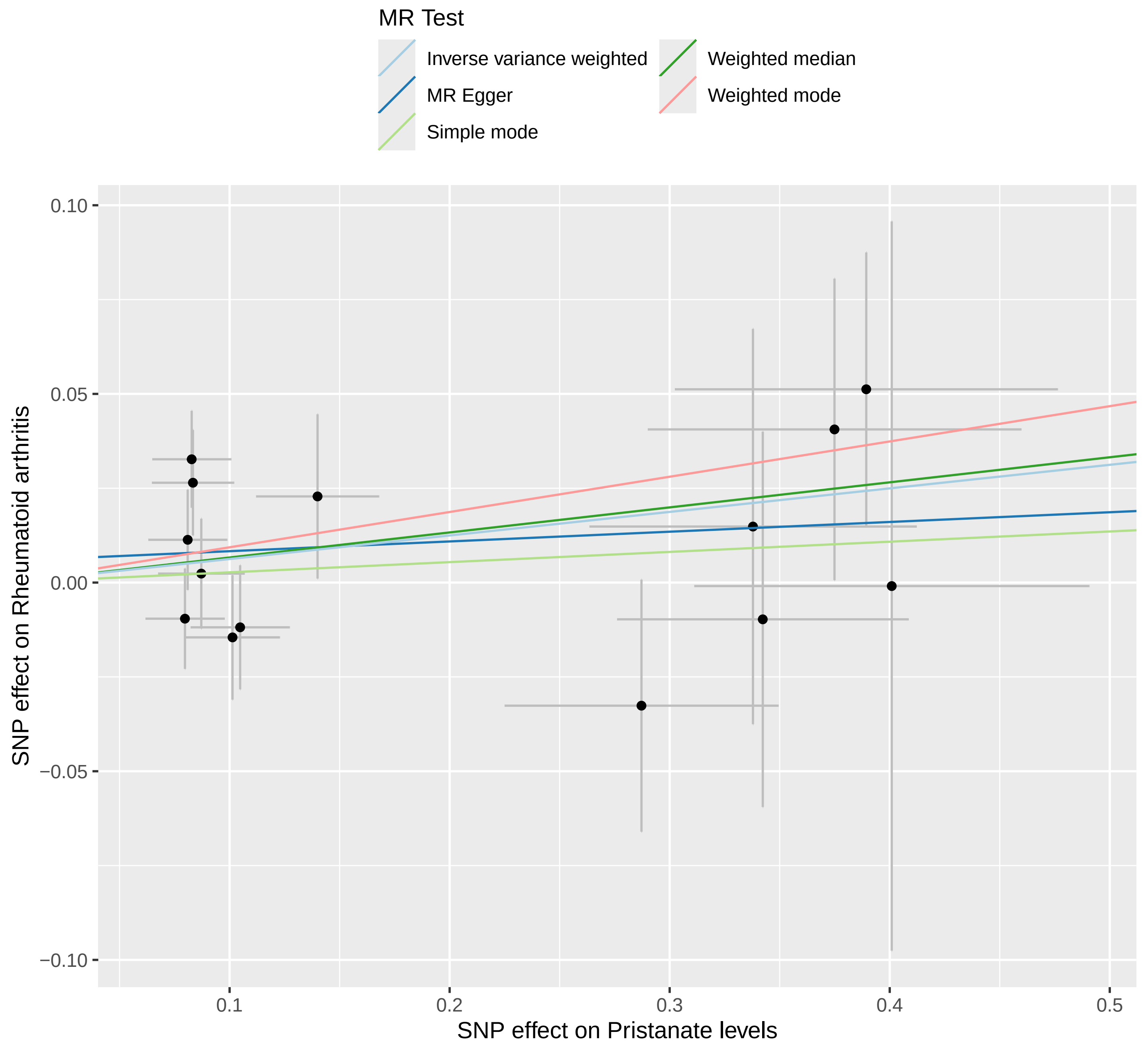

MR Test
Inverse variance weighted
MR Egger
Simple mode
Weighted median
Weighted mode
0.10
0.05
0.00
−0.05
−0.10
SNP effect on Rheumatoid arthritis
0.1
0.2
0.3
0.4
0.5
SNP effect on Pristanate levels

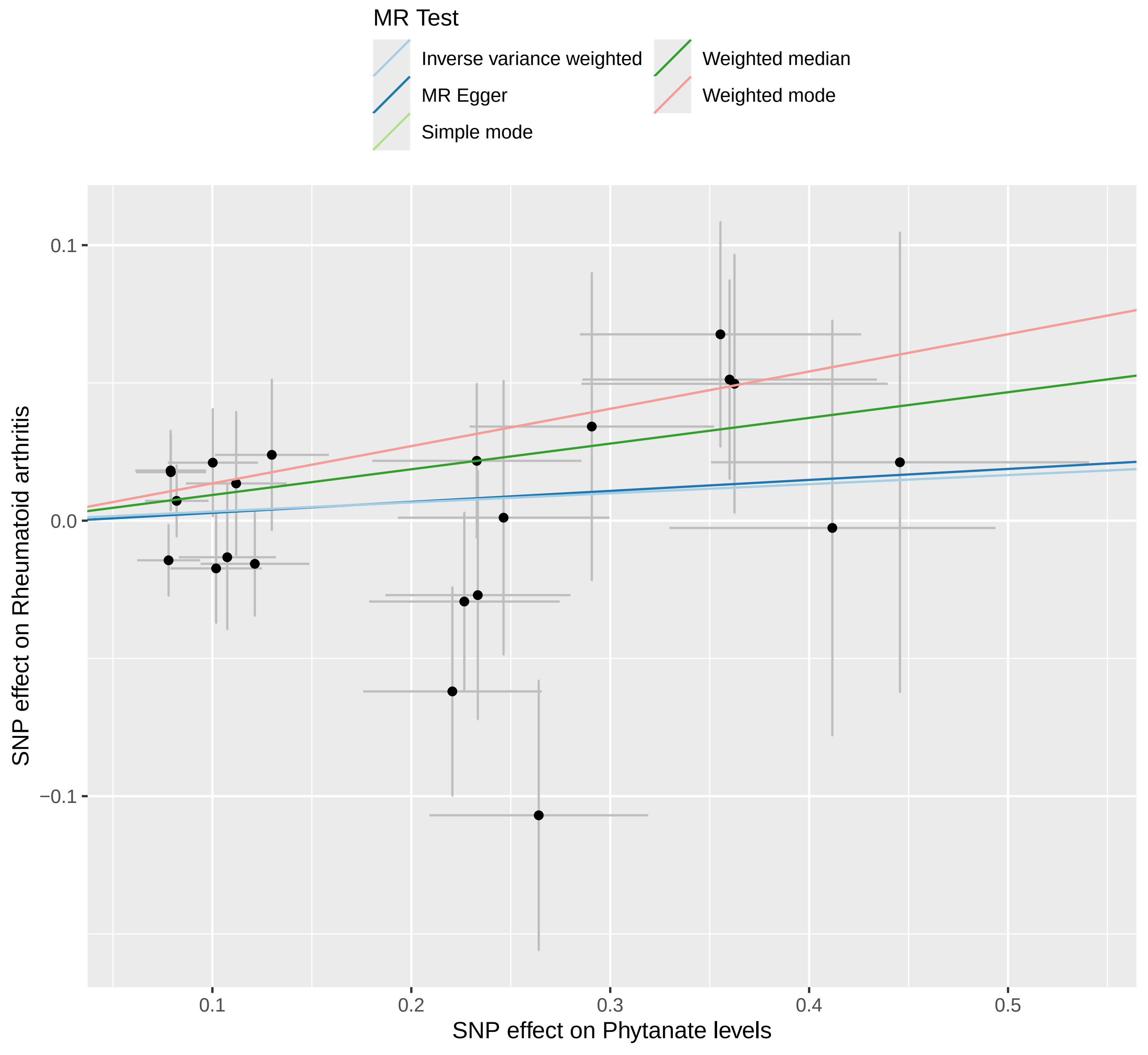

MR Test
Inverse variance weighted
MR Egger
Simple mode
Weighted median
Weighted mode
0.1
0.0
−0.1
0.1
0.2
0.3
0.4
0.5
SNP effect on Rheumatoid arthritis
SNP effect on Phytanate levels

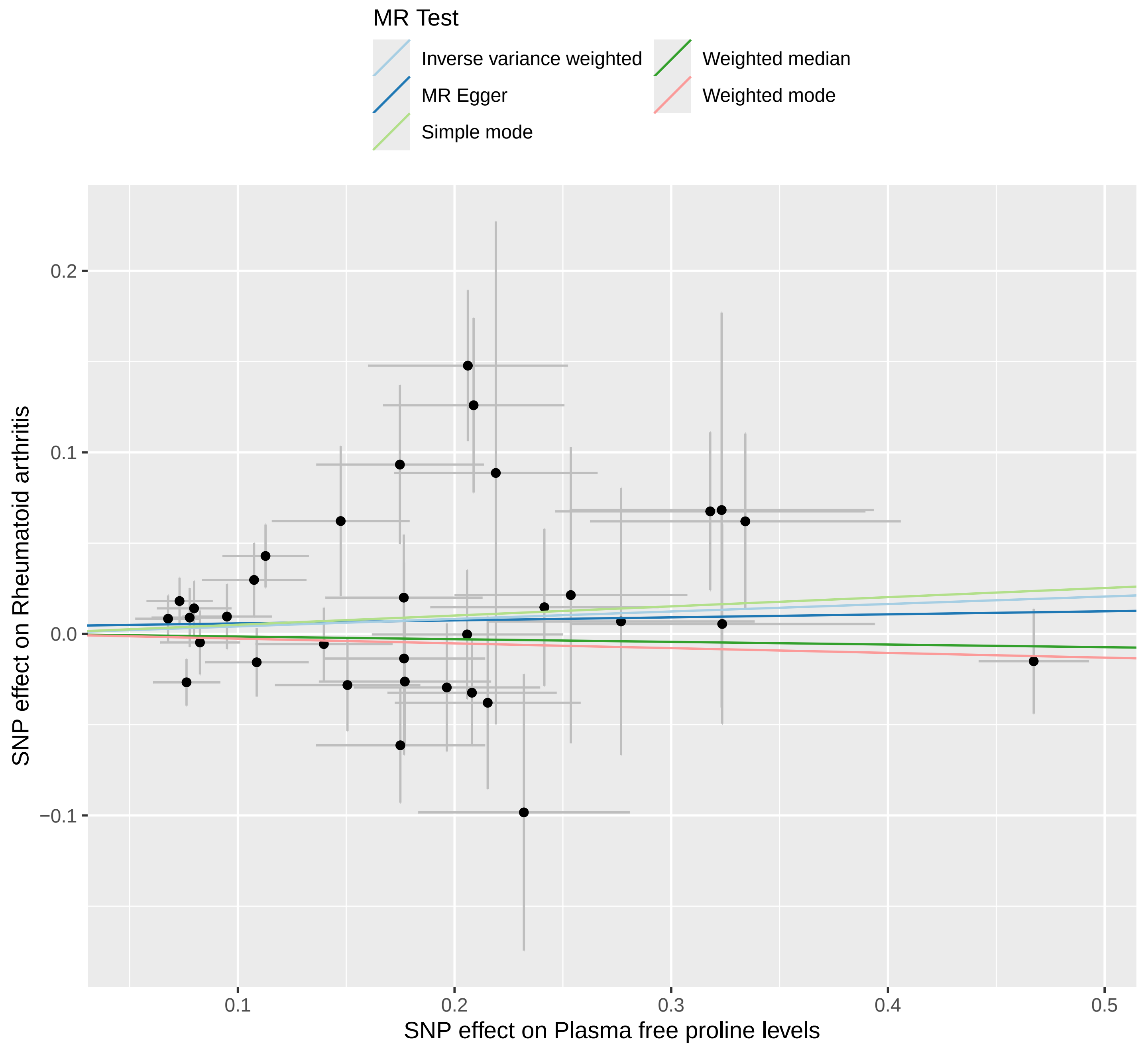

MR Test
Inverse variance weighted
MR Egger
Simple mode
Weighted median
Weighted mode
0.2
0.1
0.0
−0.1
SNP effect on Rheumatoid arthritis
0.1
0.2
0.3
0.4
0.5
SNP effect on Plasma free proline levels

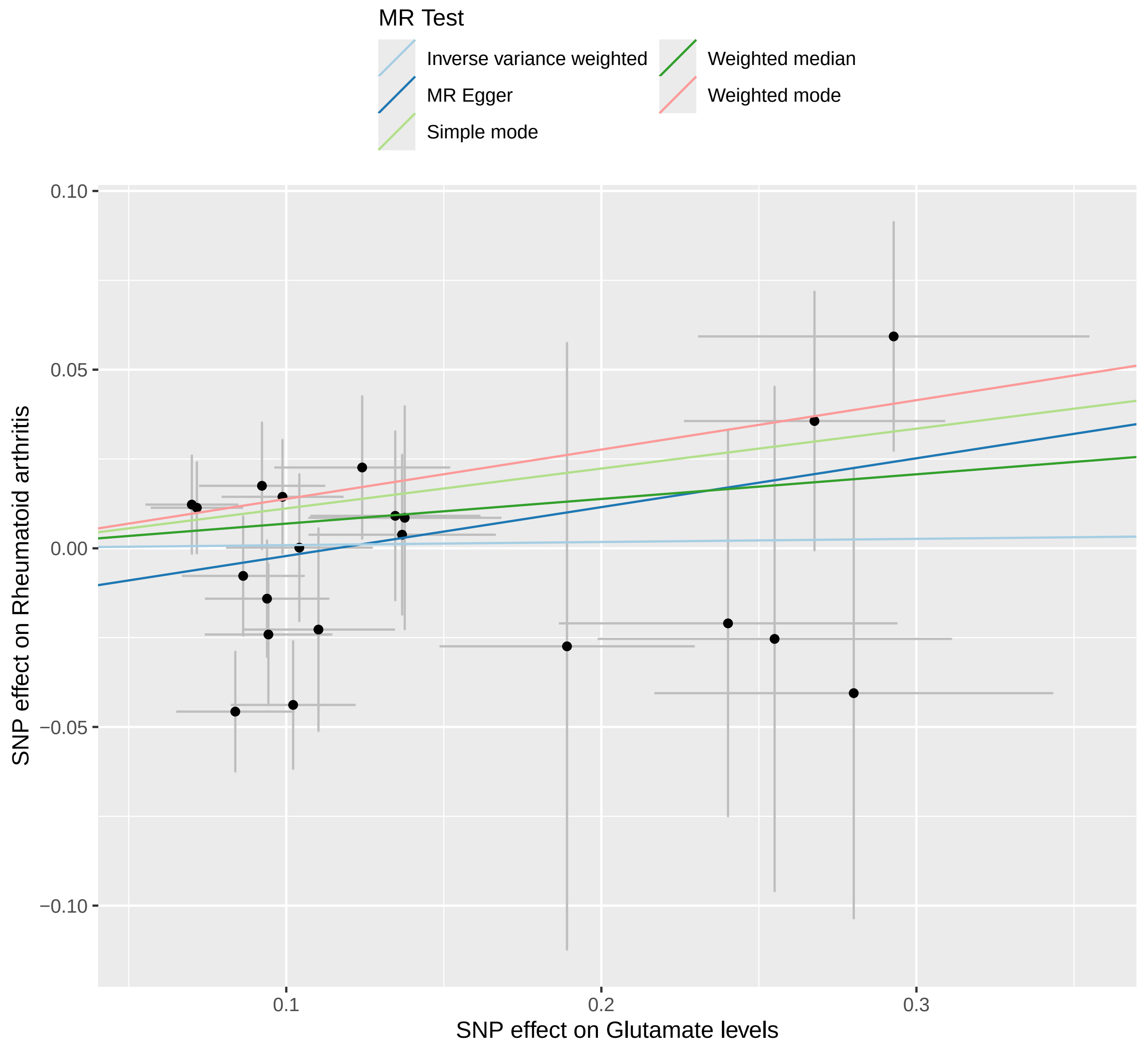
MR Test
Inverse variance weighted
MR Egger
Simple mode
Weighted median
Weighted mode
0.10
0.05
0.00
−0.05
−0.10
SNP effect on Rheumatoid arthritis
0.1
0.2
0.3
SNP effect on Glutamate levels

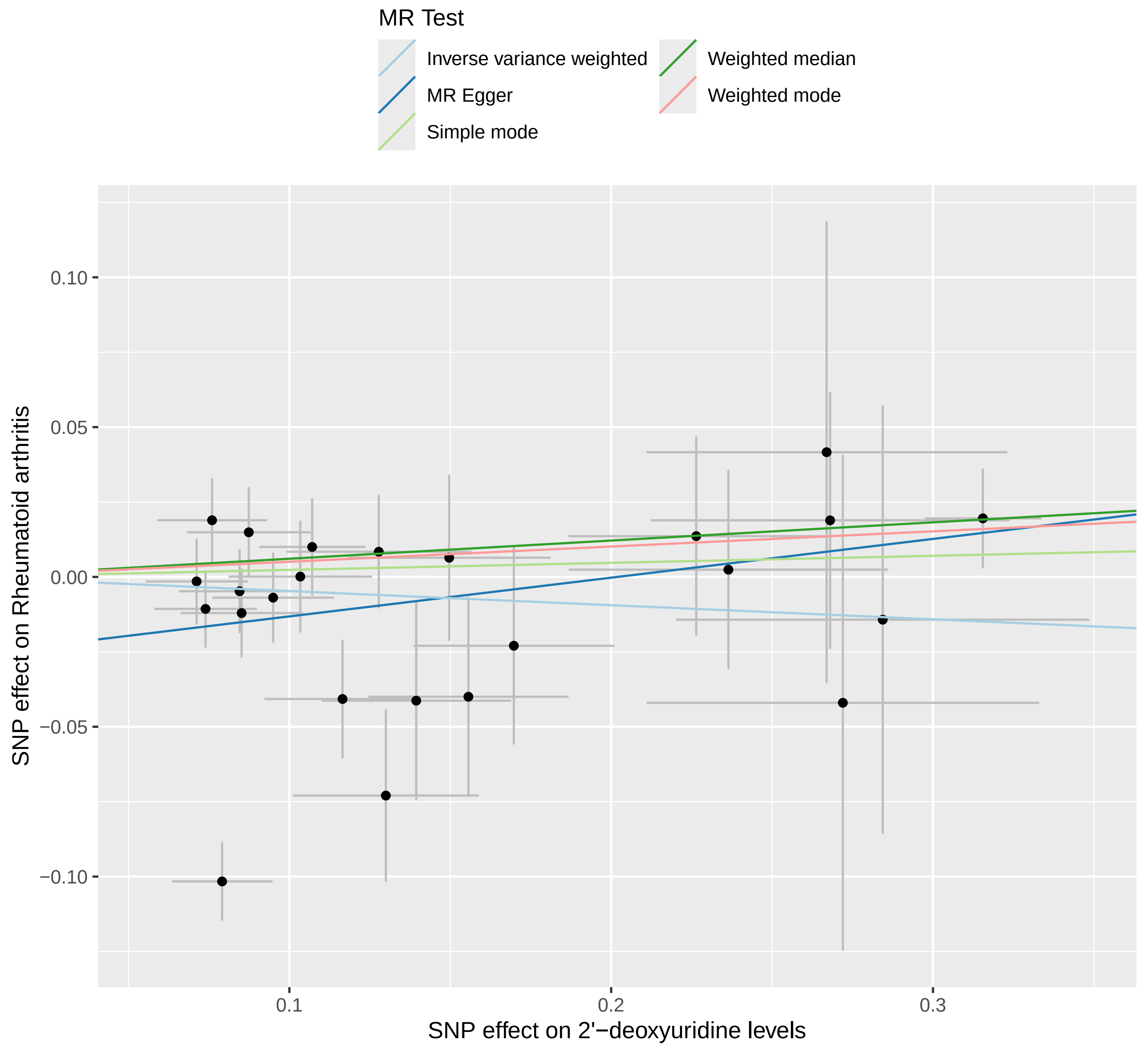

MR Test
Inverse variance weighted
MR Egger
Simple mode
Weighted median
Weighted mode
0.10
0.05
0.00
−0.05
−0.10
SNP effect on Rheumatoid arthritis
0.1
0.2
0.3
SNP effect on 2'−deoxyuridine levels

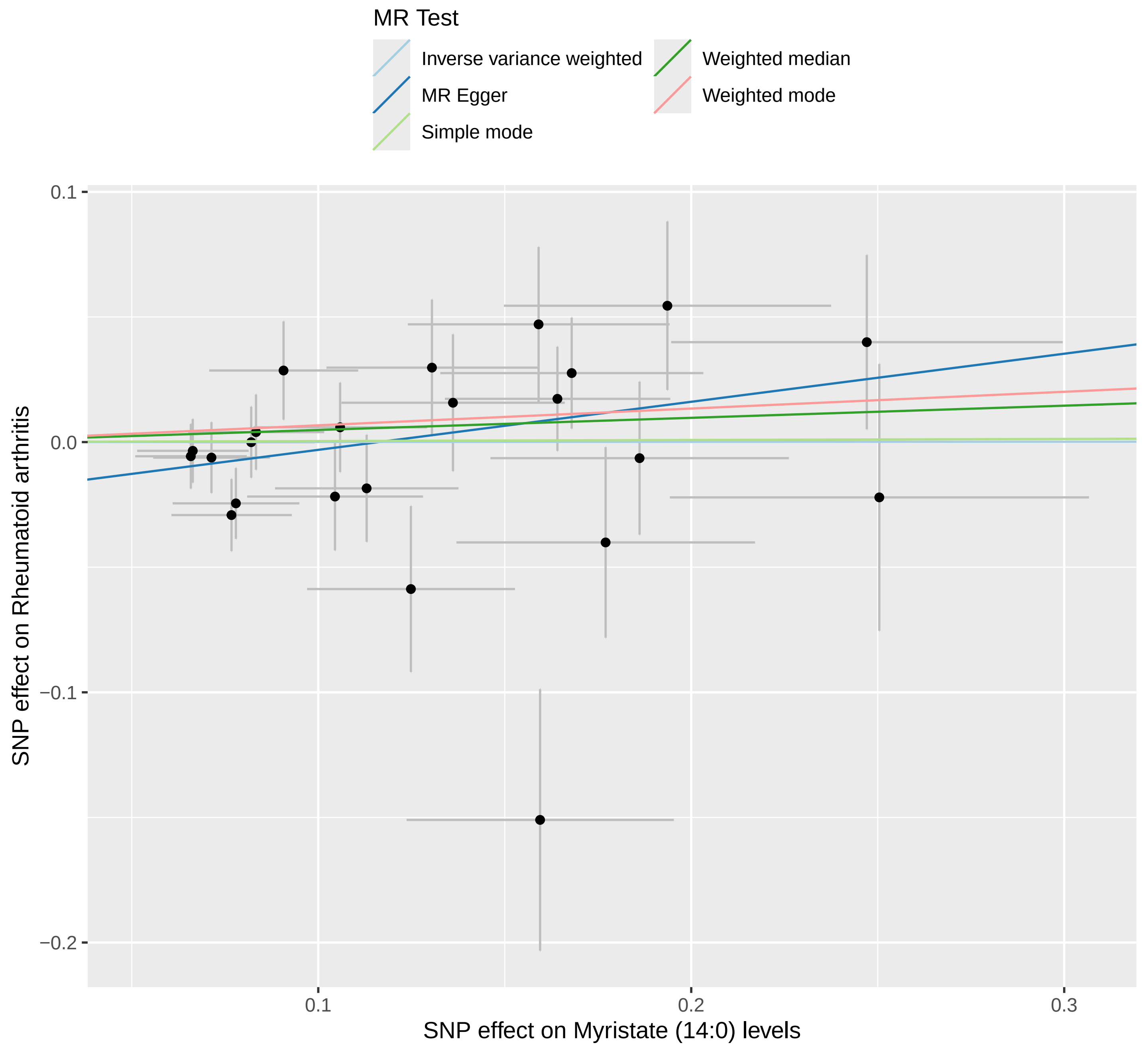
MR Test
Inverse variance weighted
MR Egger
Simple mode
Weighted median
Weighted mode
0.1
0.0
−0.1
−0.2
SNP effect on Rheumatoid arthritis
0.1
0.2
0.3
SNP effect on Myristate (14:0) levels

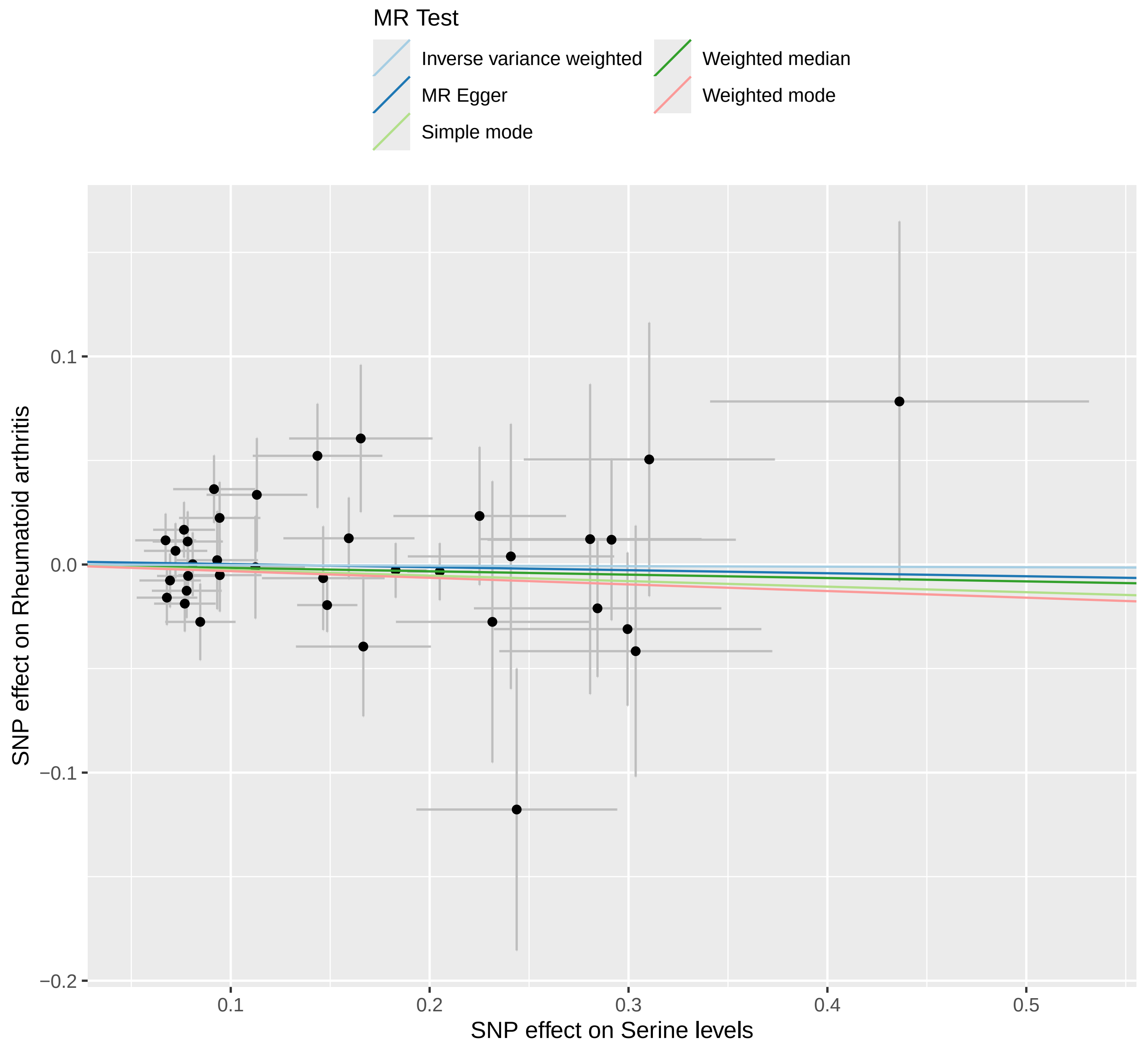
MR Test
Inverse variance weighted
MR Egger
Simple mode
Weighted median
Weighted mode
SNP effect on Rheumatoid arthritis
0.1
0.0
−0.1
−0.2
0.1
0.2
0.3
0.4
0.5
SNP effect on Serine levels

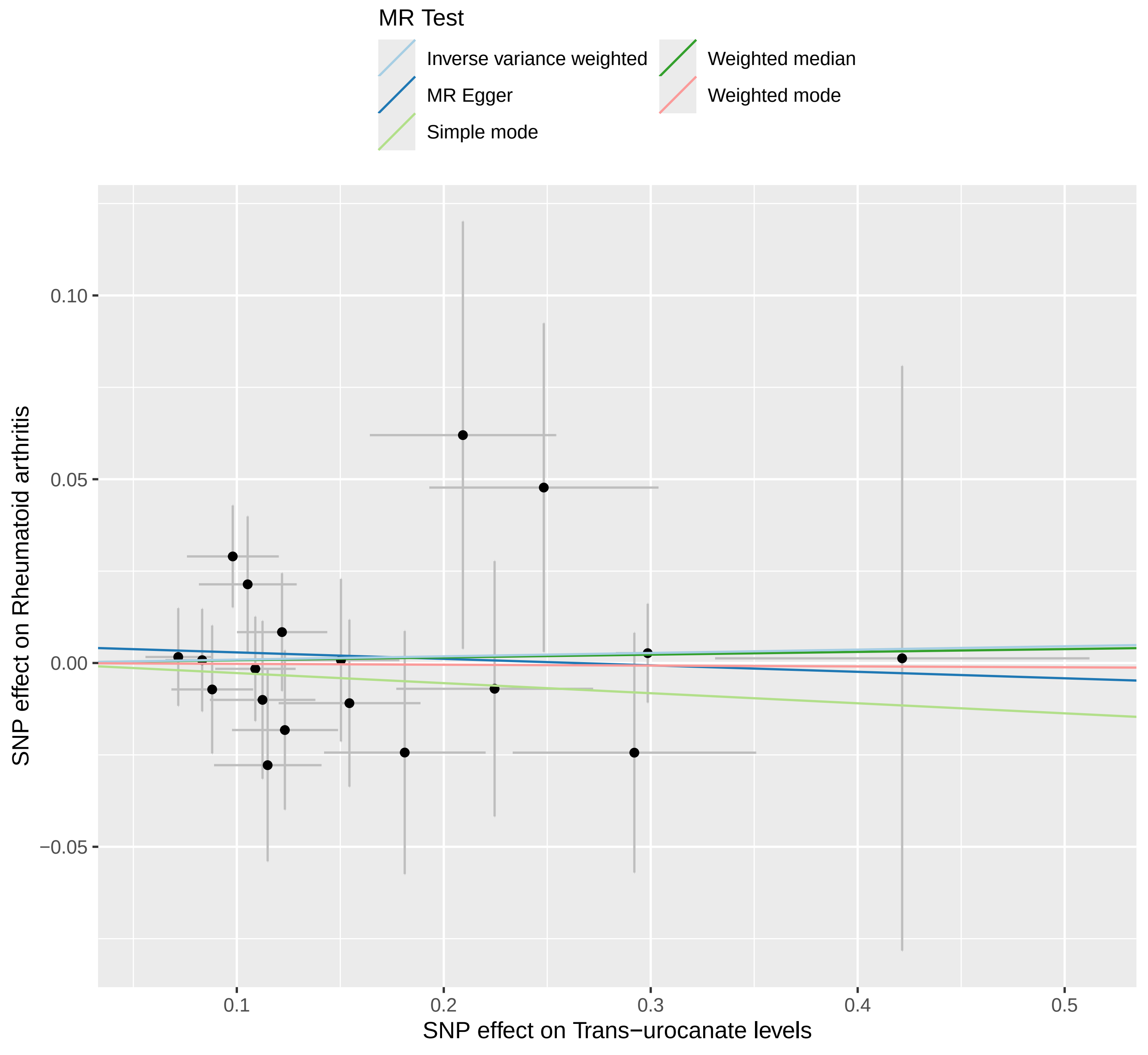
MR Test
Inverse variance weighted
MR Egger
Simple mode
Weighted median
Weighted mode
0.10
0.05
0.00
−0.05
SNP effect on Rheumatoid arthritis
0.1
0.2
0.3
0.4
0.5
SNP effect on Trans−urocanate levels

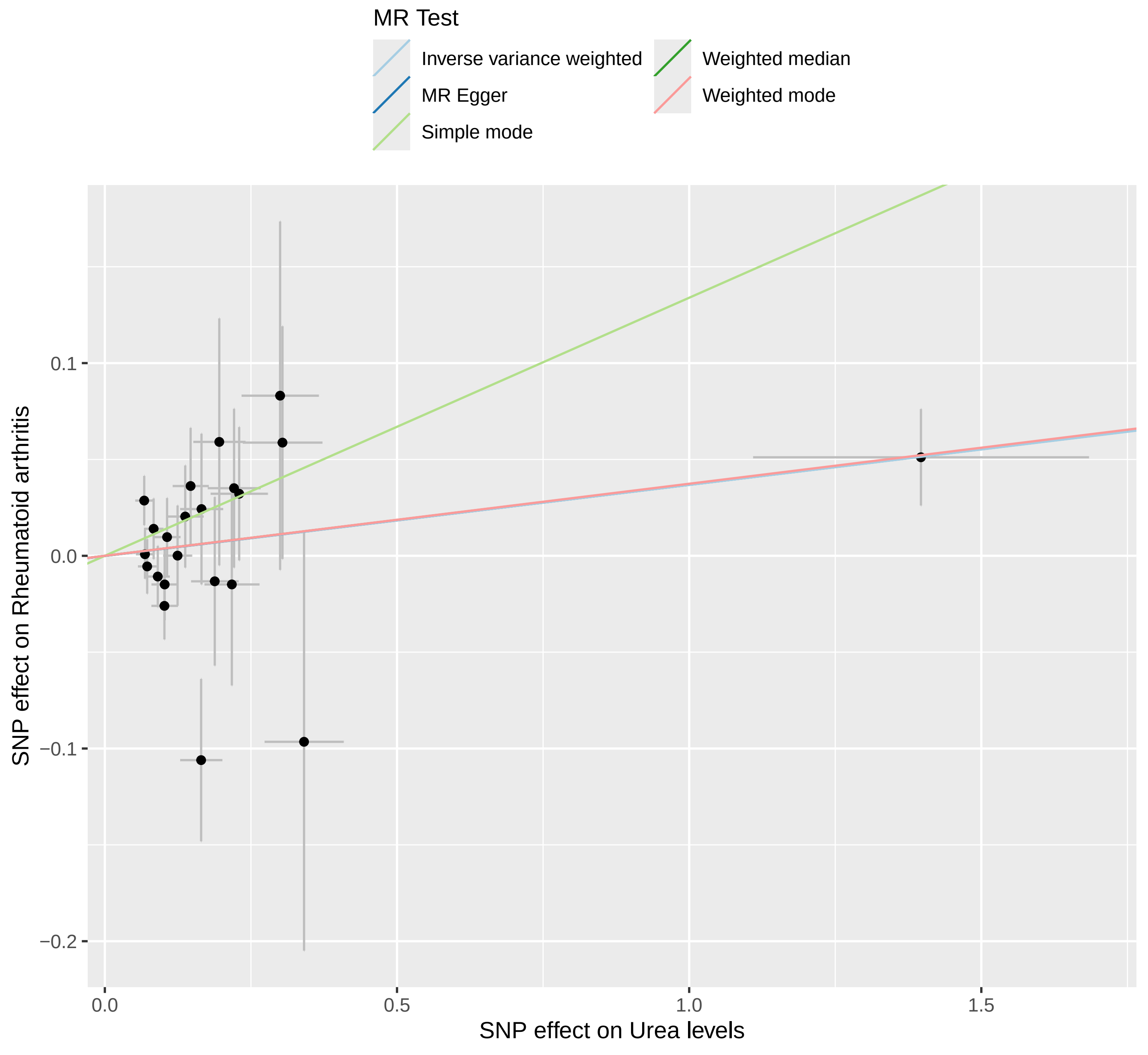

MR Test
Inverse variance weighted
MR Egger
Simple mode
Weighted median
Weighted mode
0.1
0.0
−0.1
−0.2
SNP effect on Rheumatoid arthritis
0.0
0.5
1.0
1.5
SNP effect on Urea levels

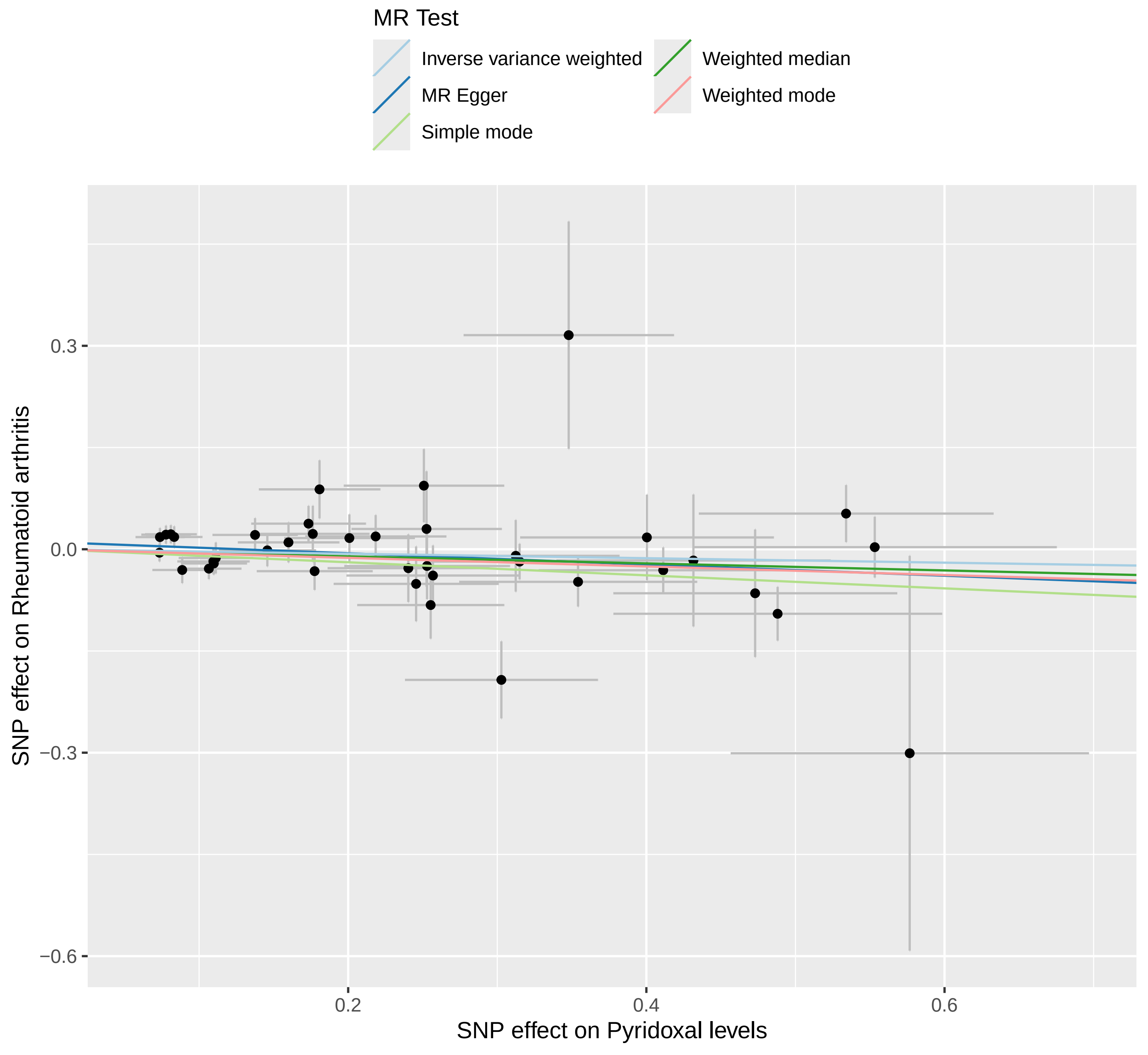
MR Test
Inverse variance weighted
MR Egger
Simple mode
Weighted median
Weighted mode
0.3
0.0
−0.3
−0.6
0.2
0.4
0.6
SNP effect on Rheumatoid arthritis
SNP effect on Pyridoxal levels

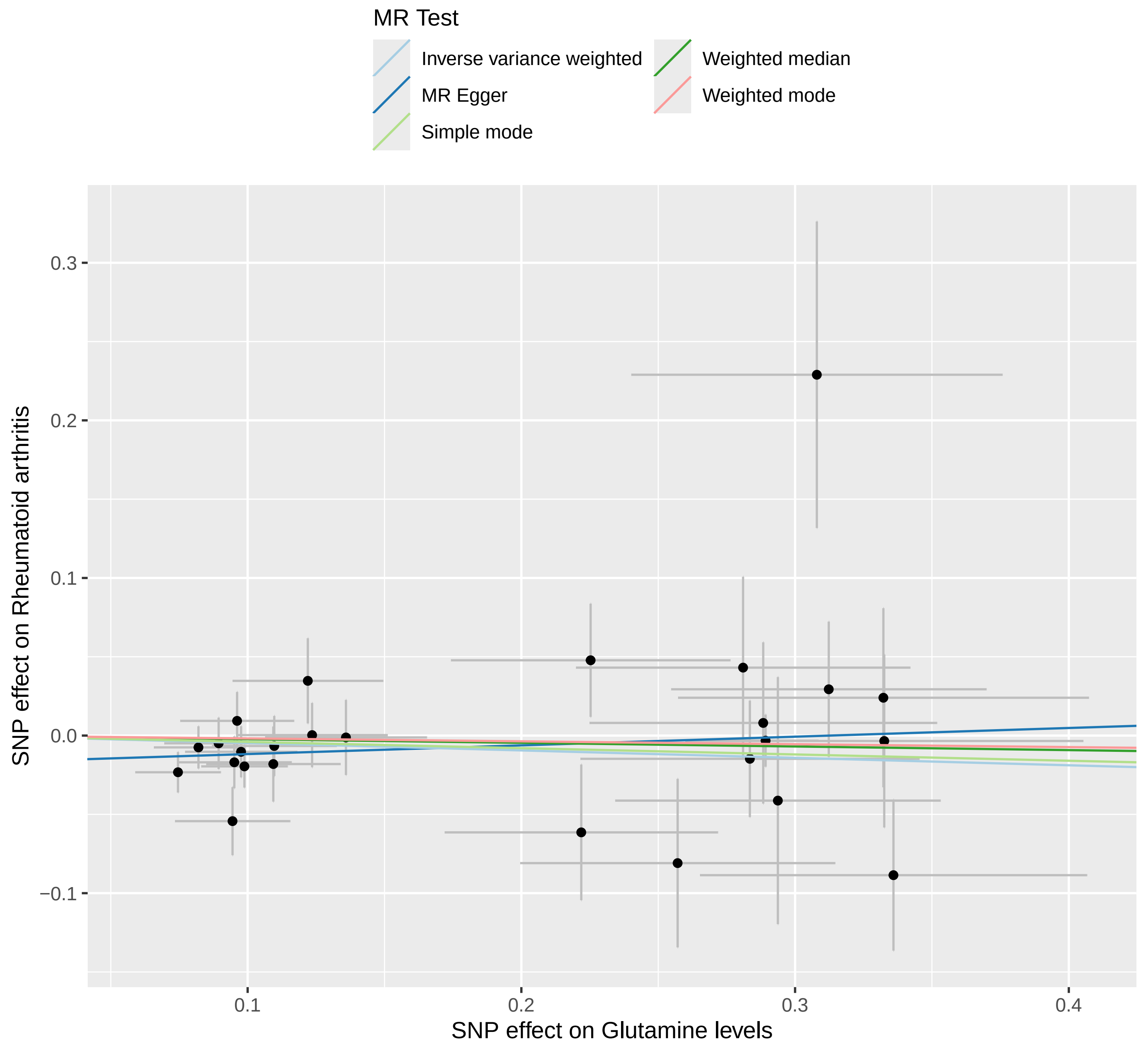

MR Test
Inverse variance weighted
MR Egger
Simple mode
Weighted median
Weighted mode
0.3
0.2
0.1
0.0
−0.1
0.1
0.2
0.3
0.4
SNP effect on Rheumatoid arthritis
SNP effect on Glutamine levels

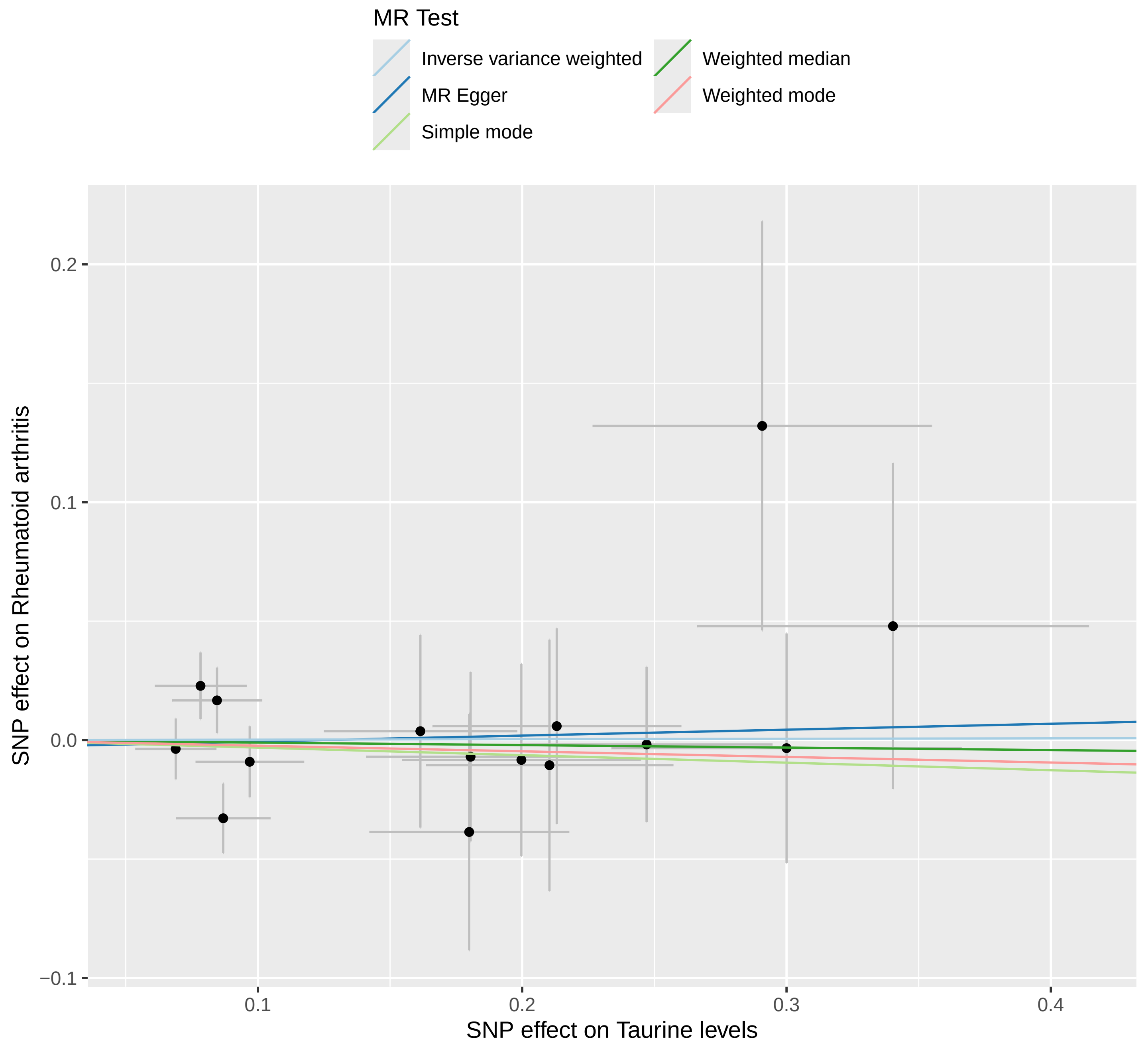
MR Test
Inverse variance weighted
MR Egger
Simple mode
Weighted median
Weighted mode
SNP effect on Rheumatoid arthritis
SNP effect on Taurine levels
0.2
0.1
0.0
−0.1
0.1
0.2
0.3
0.4

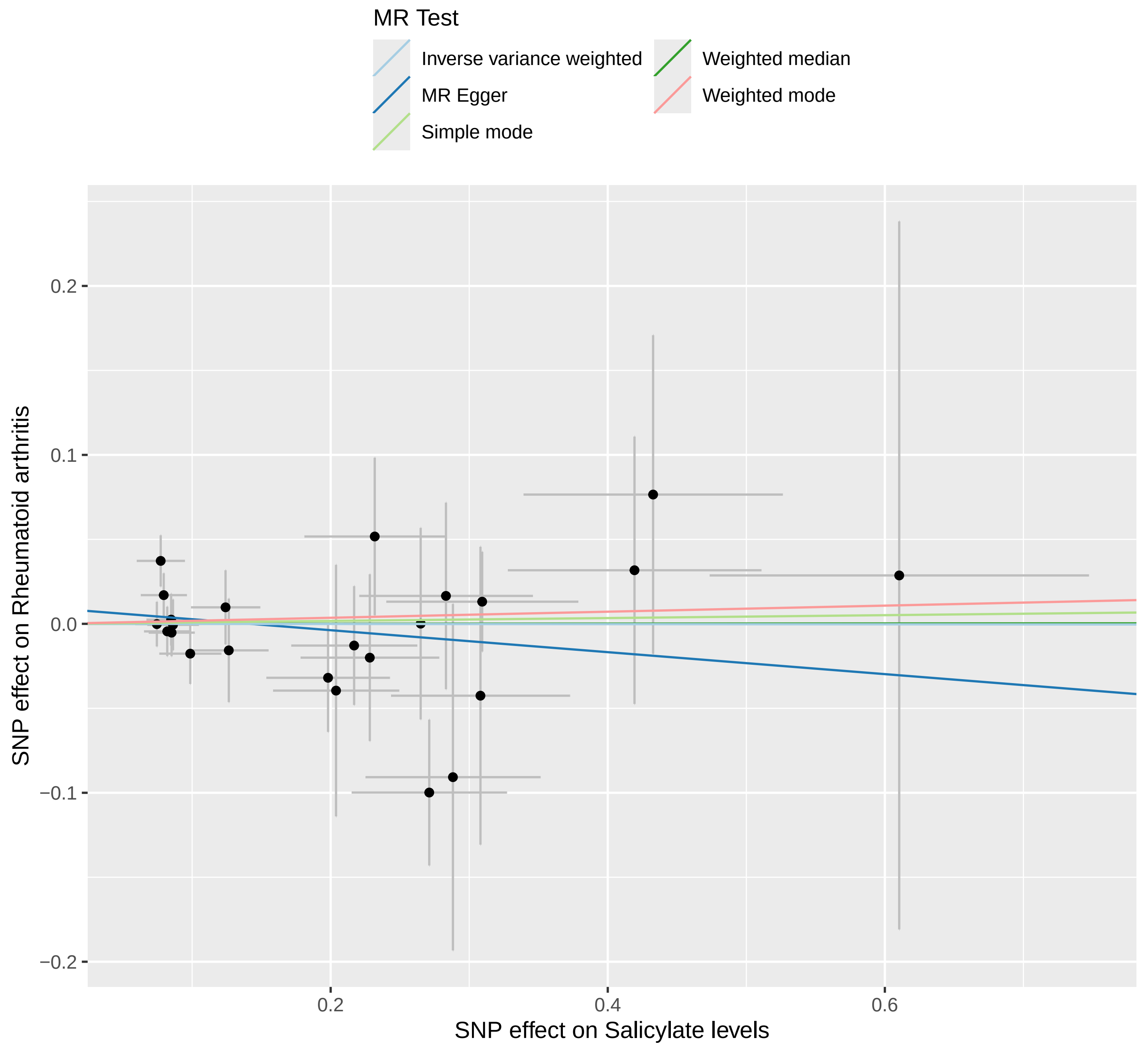
MR Test
Inverse variance weighted
MR Egger
Simple mode
Weighted median
Weighted mode
0.2
0.1
0.0
−0.1
−0.2
SNP effect on Rheumatoid arthritis
0.2
0.4
0.6
SNP effect on Salicylate levels

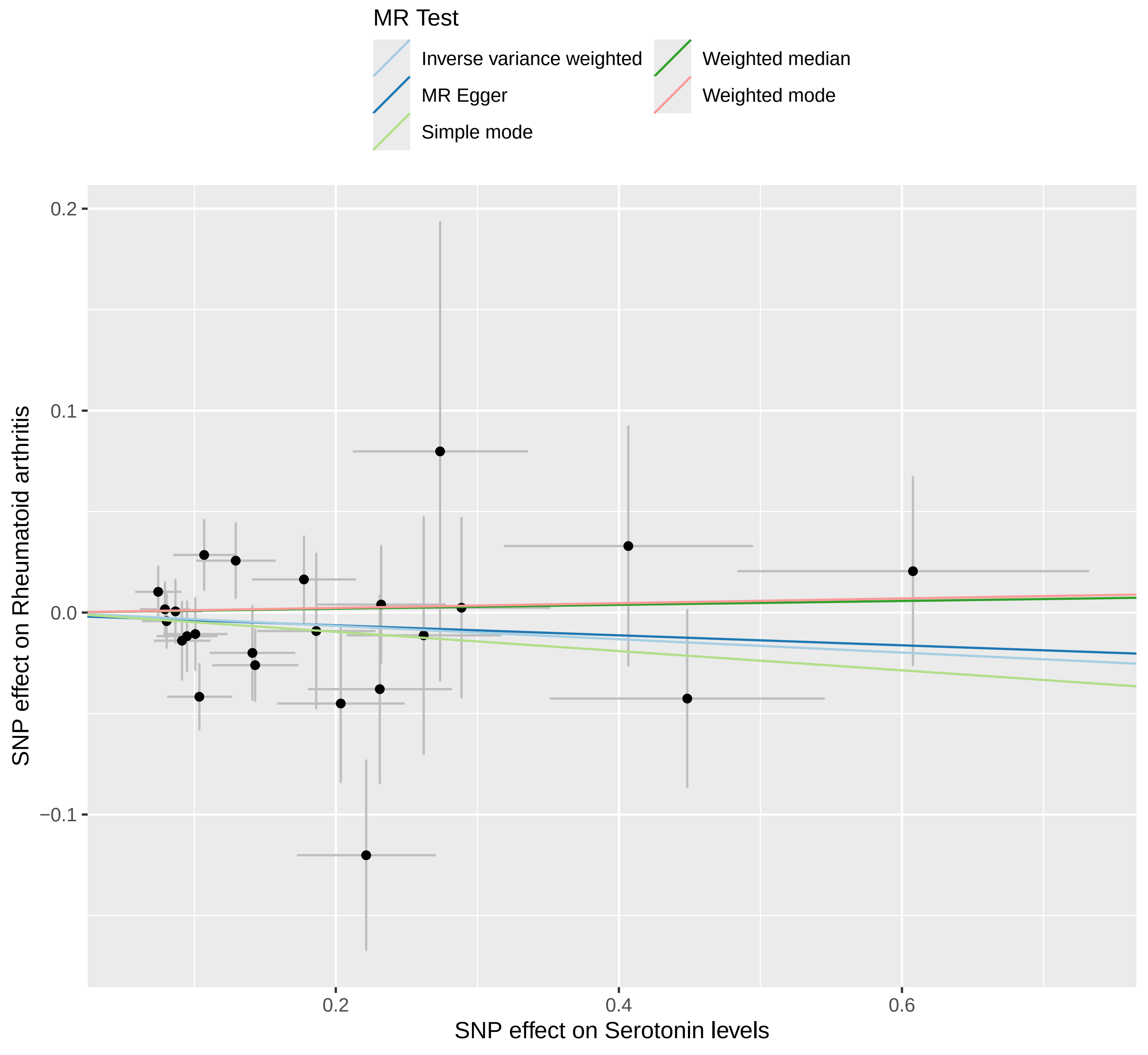

MR Test
Inverse variance weighted
MR Egger
Simple mode
Weighted median
Weighted mode
0.2
0.1
0.0
−0.1
SNP effect on Rheumatoid arthritis
0.2
0.4
0.6
SNP effect on Serotonin levels

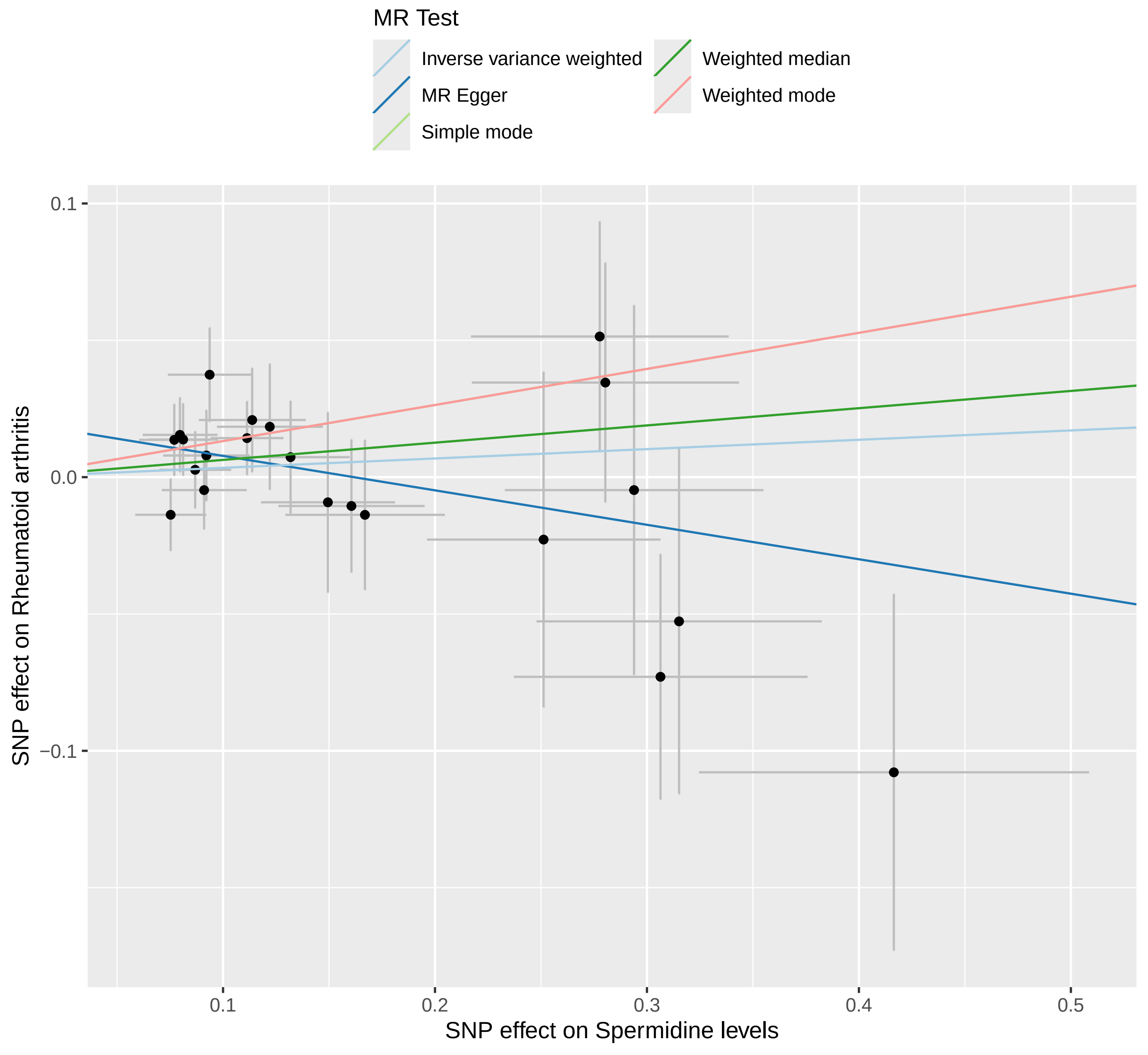

MR Test
Inverse variance weighted
MR Egger
Simple mode
Weighted median
Weighted mode
0.1
0.0
−0.1
SNP effect on Rheumatoid arthritis
0.1
0.2
0.3
0.4
0.5
SNP effect on Spermidine levels

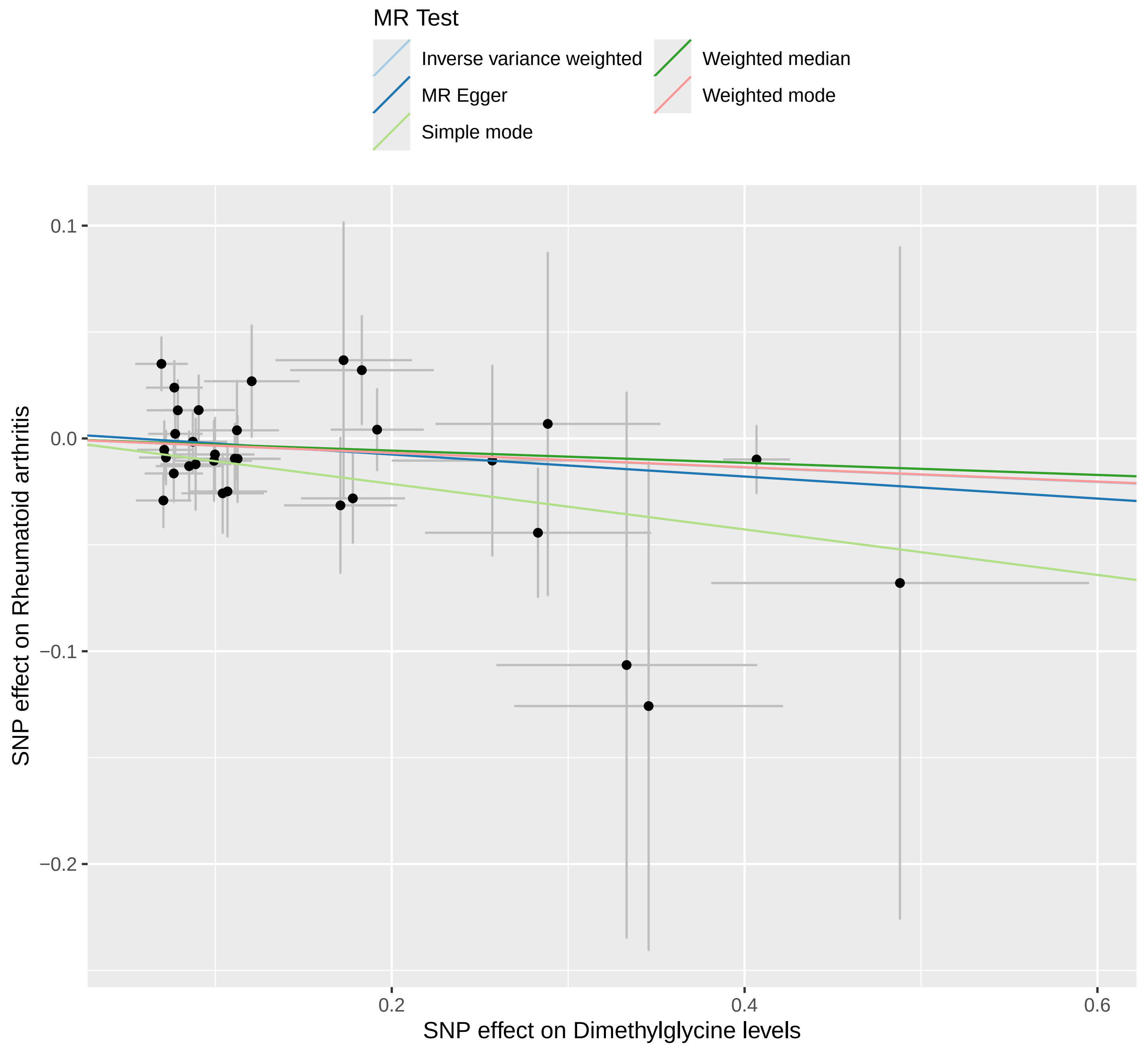
MR Test
Inverse variance weighted
MR Egger
Simple mode
Weighted median
Weighted mode
SNP effect on Rheumatoid arthritis
0.1
0.0
−0.1
−0.2
0.2
0.4
0.6
SNP effect on Dimethylglycine levels

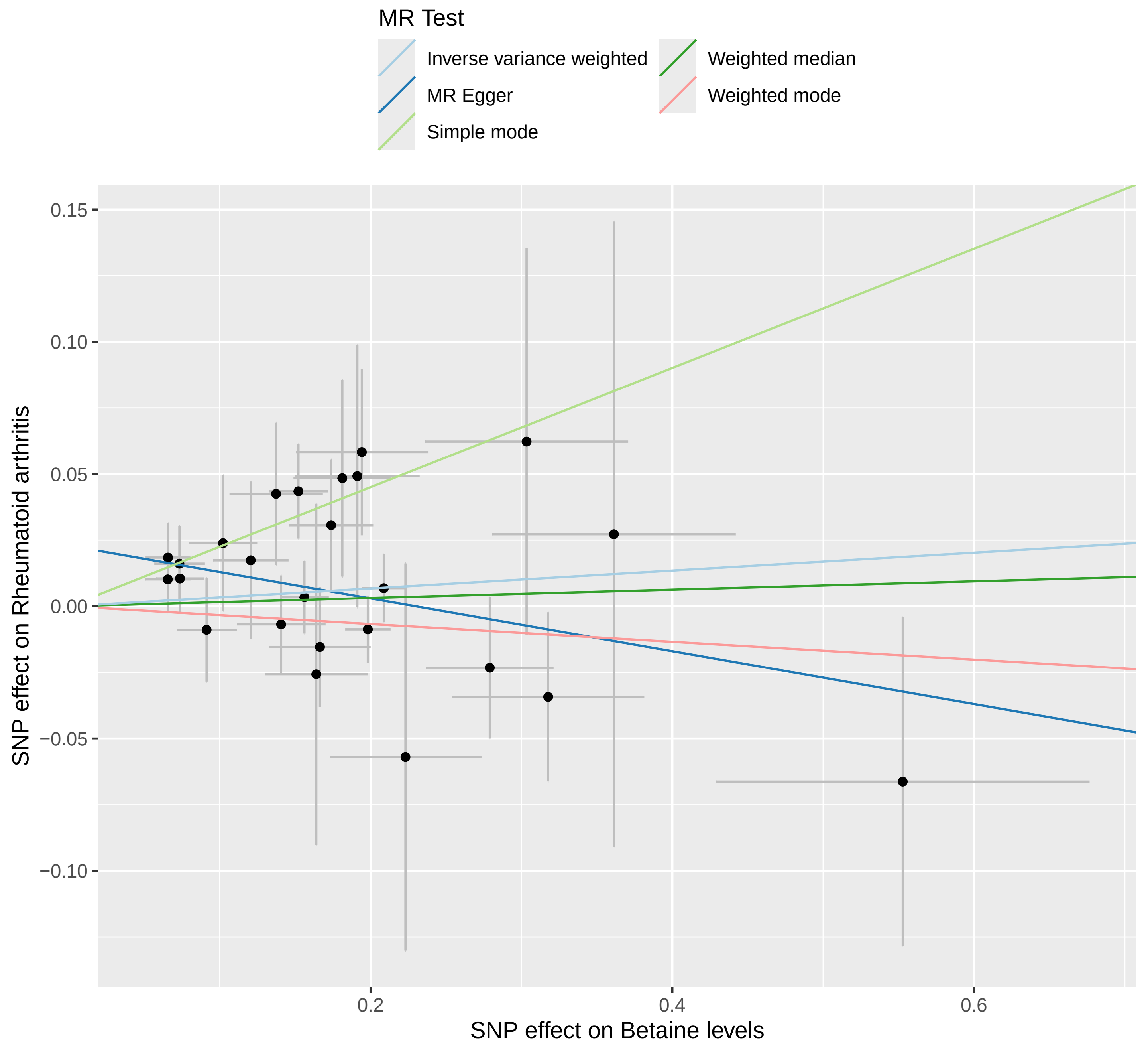
MR Test
Inverse variance weighted
MR Egger
Simple mode
Weighted median
Weighted mode
0.15
0.10
0.05
0.00
−0.05
−0.10
SNP effect on Rheumatoid arthritis
0.2
0.4
0.6
SNP effect on Betaine levels

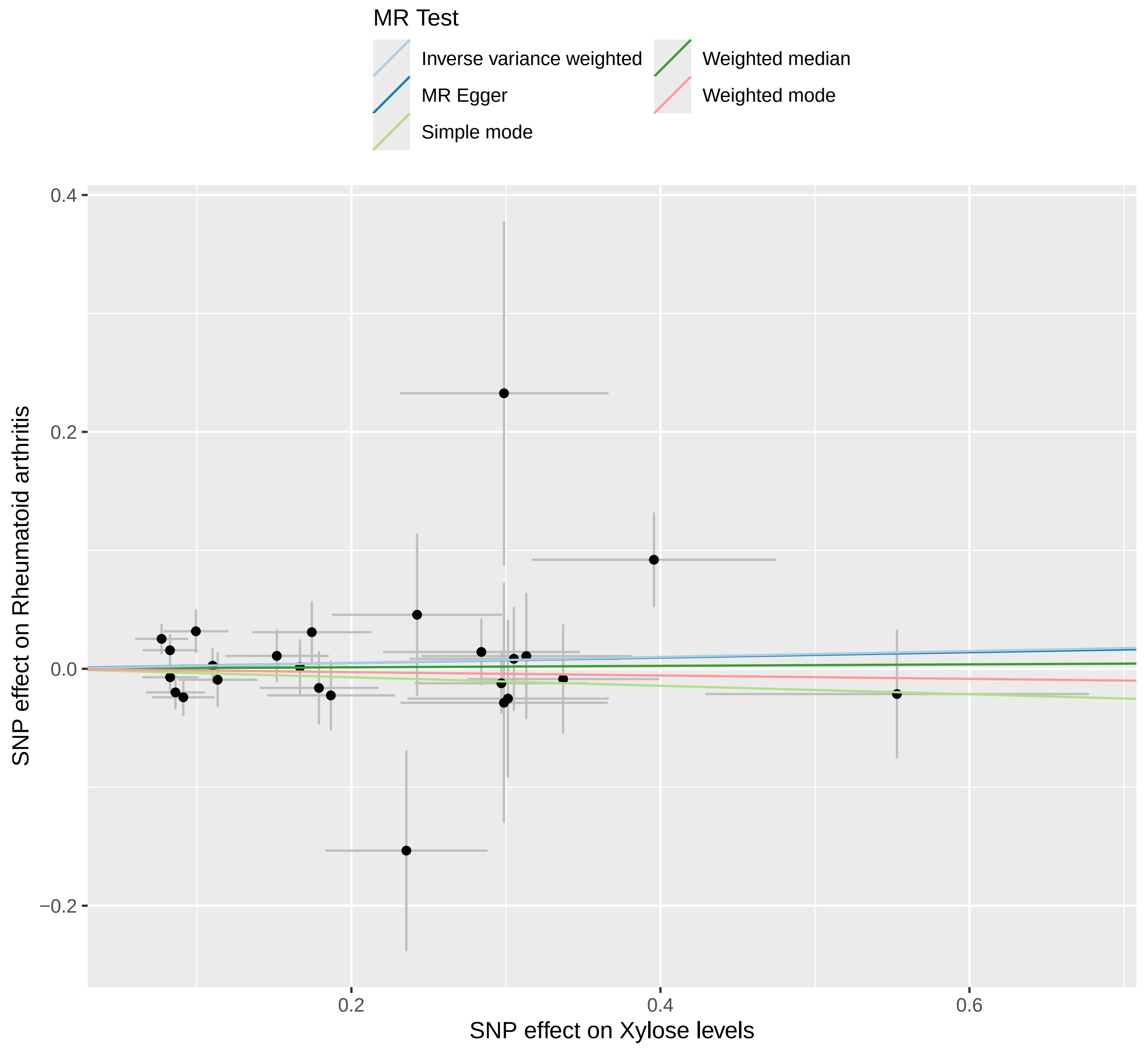
MR Test
Inverse variance weighted
MR Egger
Simple mode
Weighted median
Weighted mode
0.4
0.2
0.0
−0.2
0.2
0.4
0.6
SNP effect on Rheumatoid arthritis
SNP effect on Xylose levels

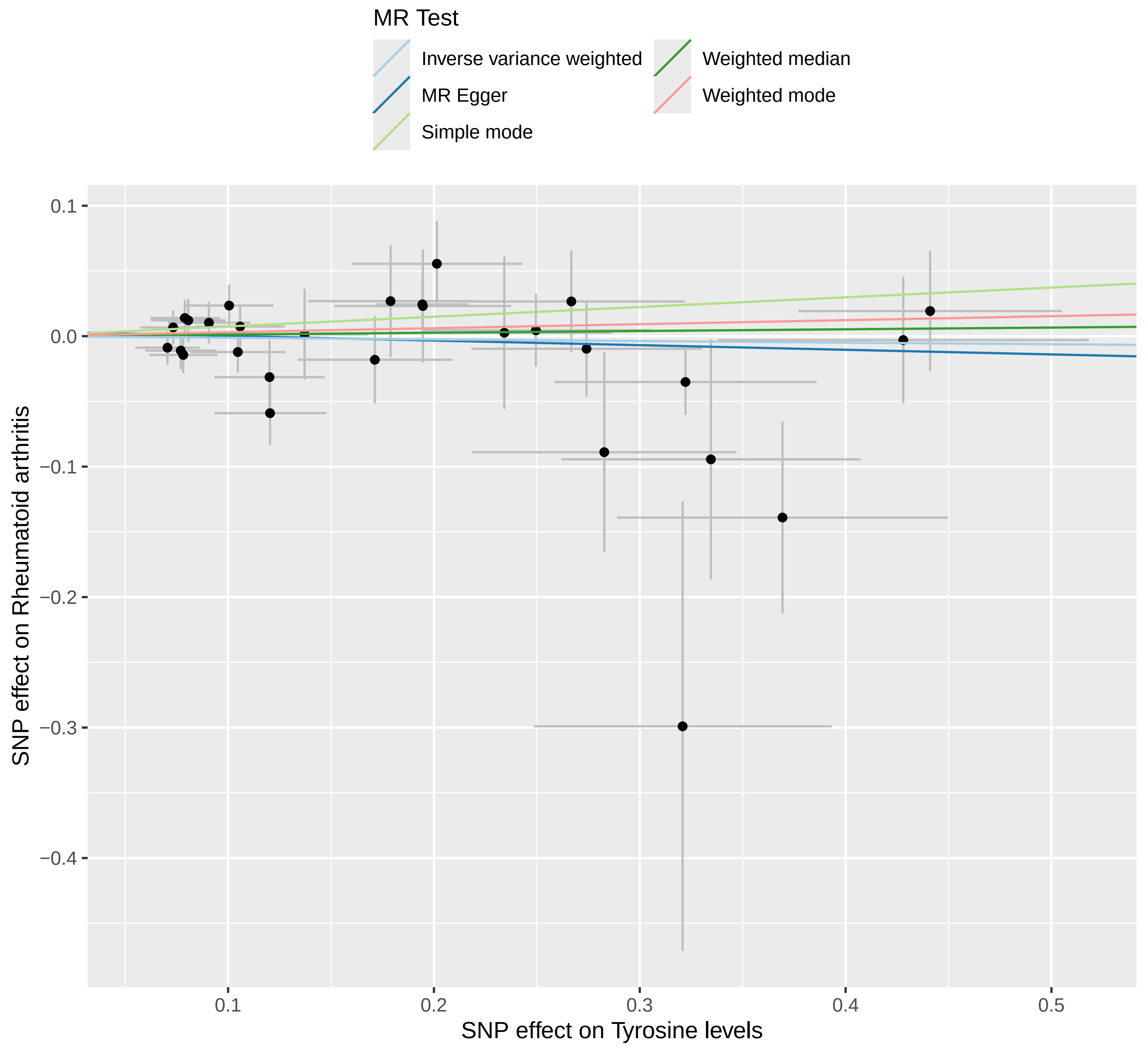

MR Test
Inverse variance weighted
MR Egger
Simple mode
Weighted median
Weighted mode
SNP effect on Rheumatoid arthritis
0.1
0.0
−0.1
−0.2
−0.3
−0.4
0.1
0.2
0.3
0.4
0.5
SNP effect on Tyrosine levels

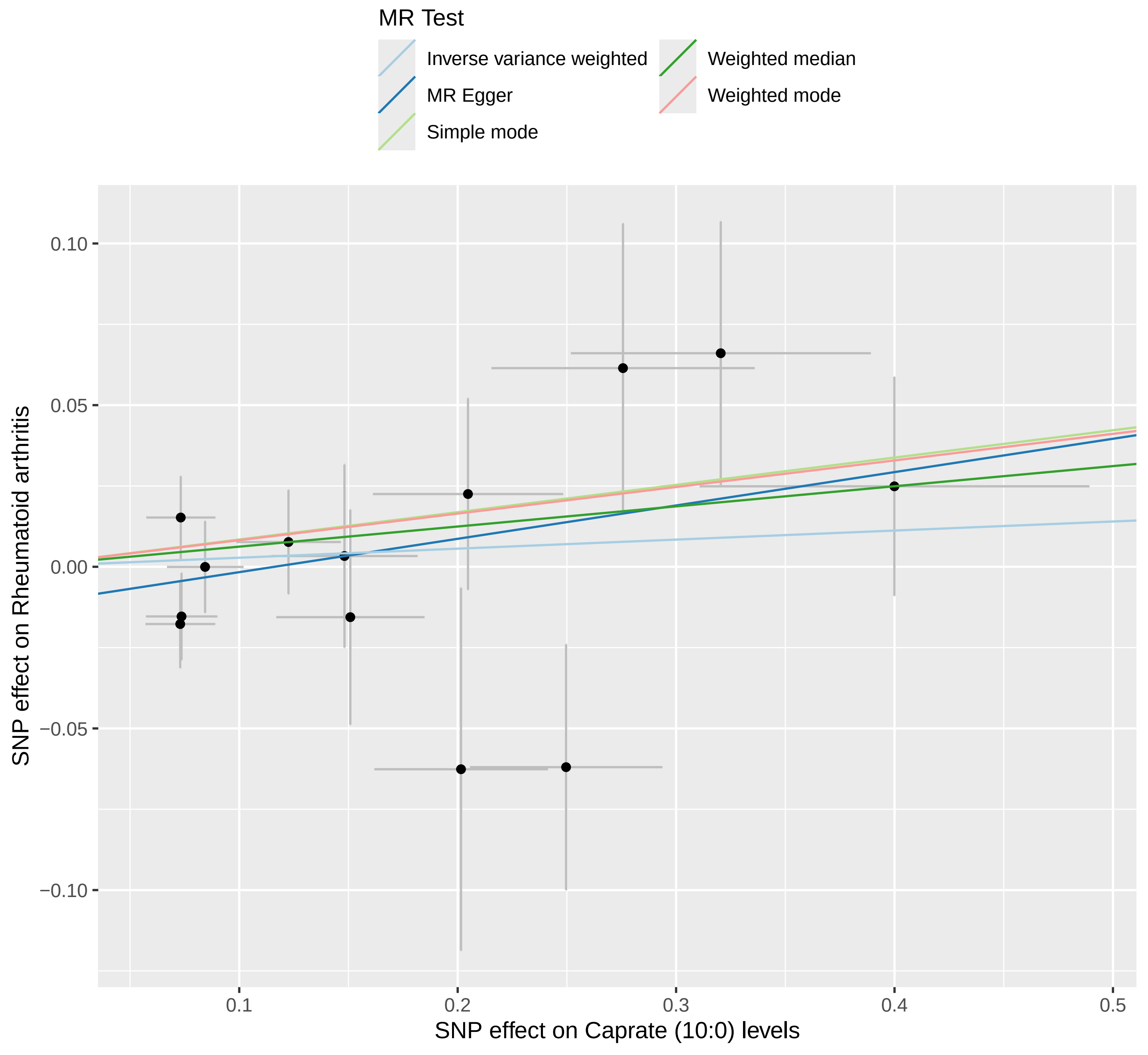

MR Test
Inverse variance weighted
MR Egger
Simple mode
Weighted median
Weighted mode
0.10
0.05
0.00
−0.05
−0.10
SNP effect on Rheumatoid arthritis
0.1
0.2
0.3
0.4
0.5
SNP effect on Caprate (10:0) levels

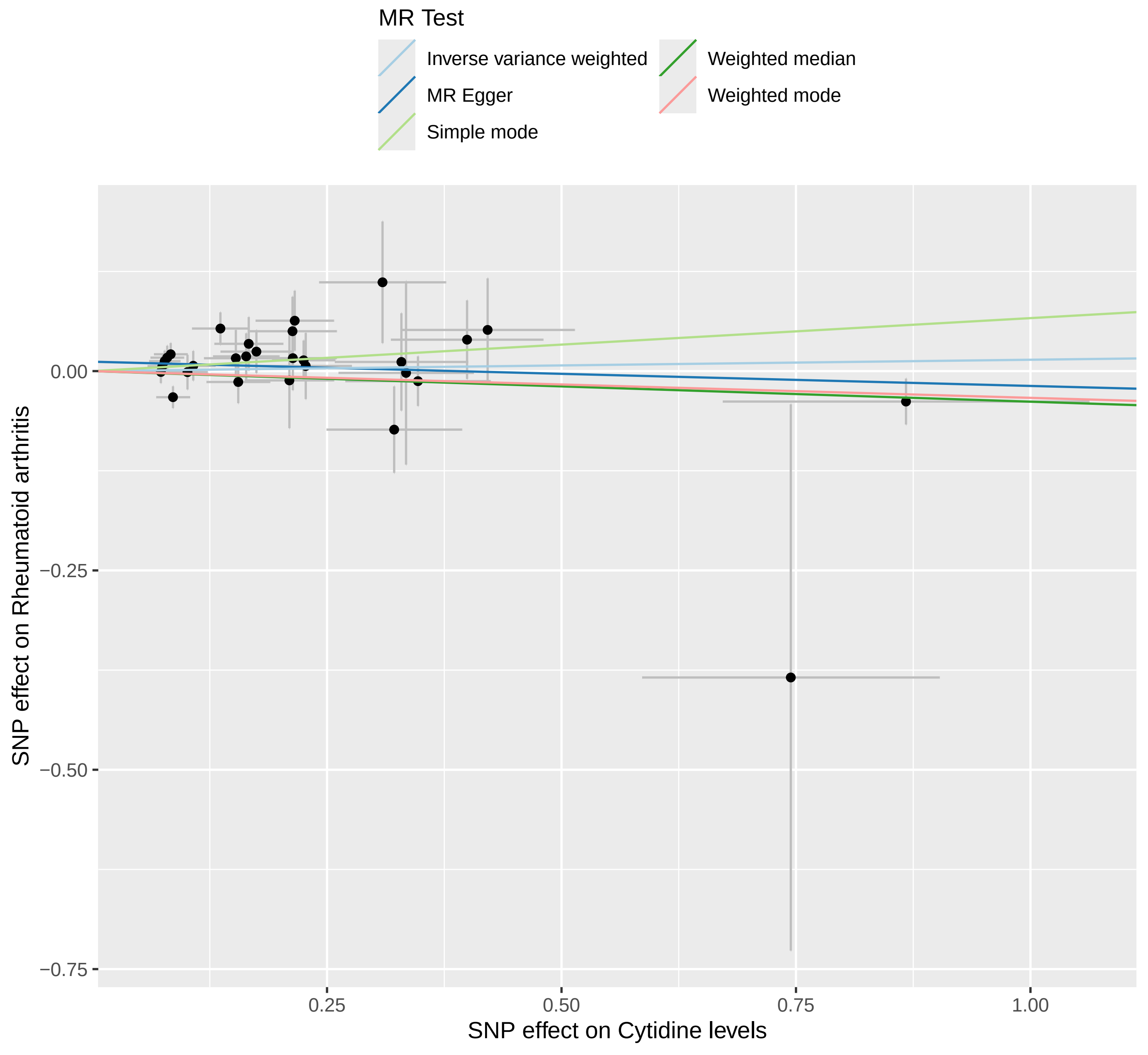

MR Test
Inverse variance weighted
MR Egger
Simple mode
Weighted median
Weighted mode
SNP effect on Rheumatoid arthritis
0.00
−0.25
−0.50
−0.75
0.25
0.50
0.75
1.00
SNP effect on Cytidine levels

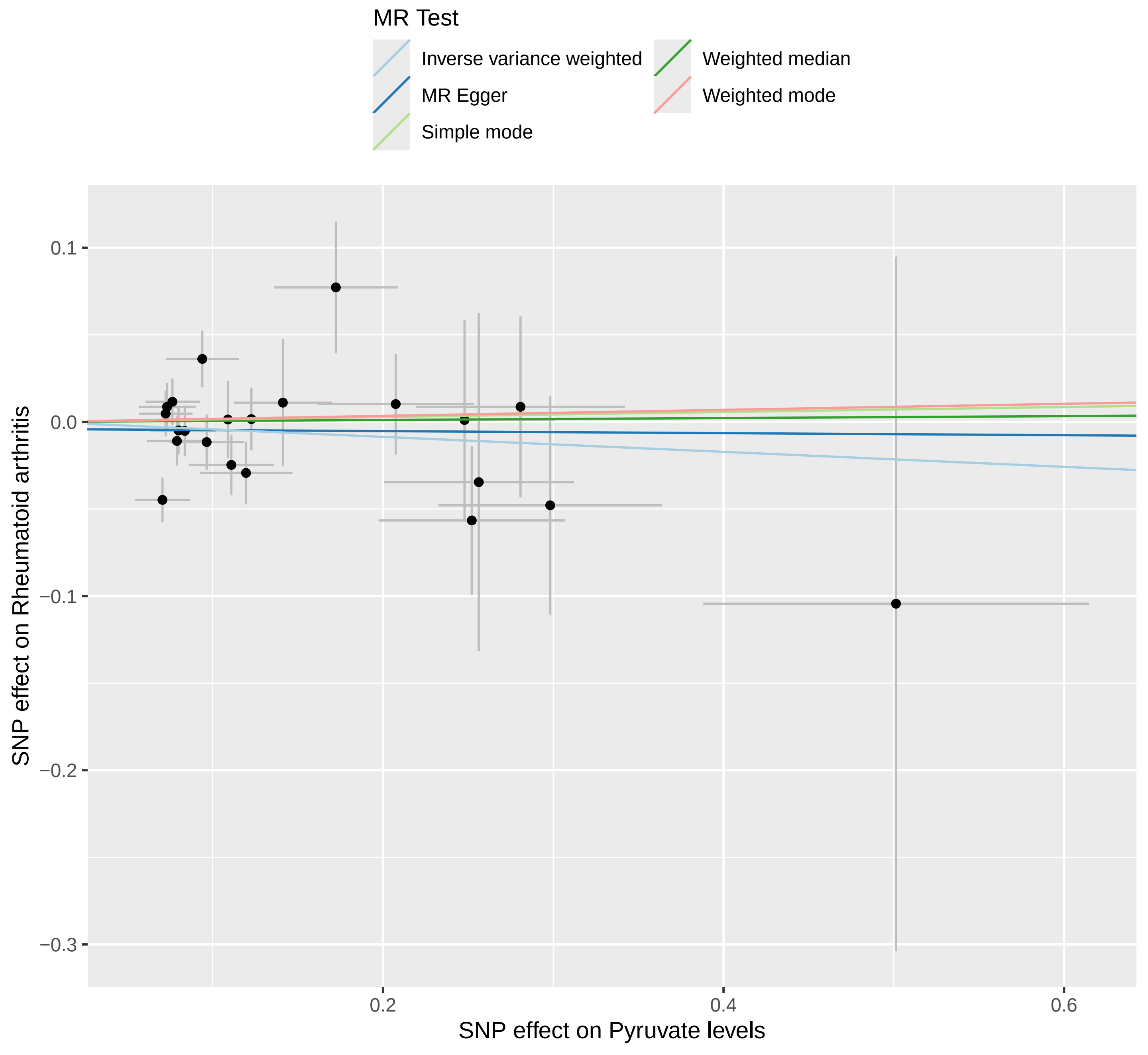

MR Test
Inverse variance weighted
MR Egger
Simple mode
Weighted median
Weighted mode
0.1
0.0
−0.1
−0.2
−0.3
0.2
0.4
0.6
SNP effect on Rheumatoid arthritis
SNP effect on Pyruvate levels

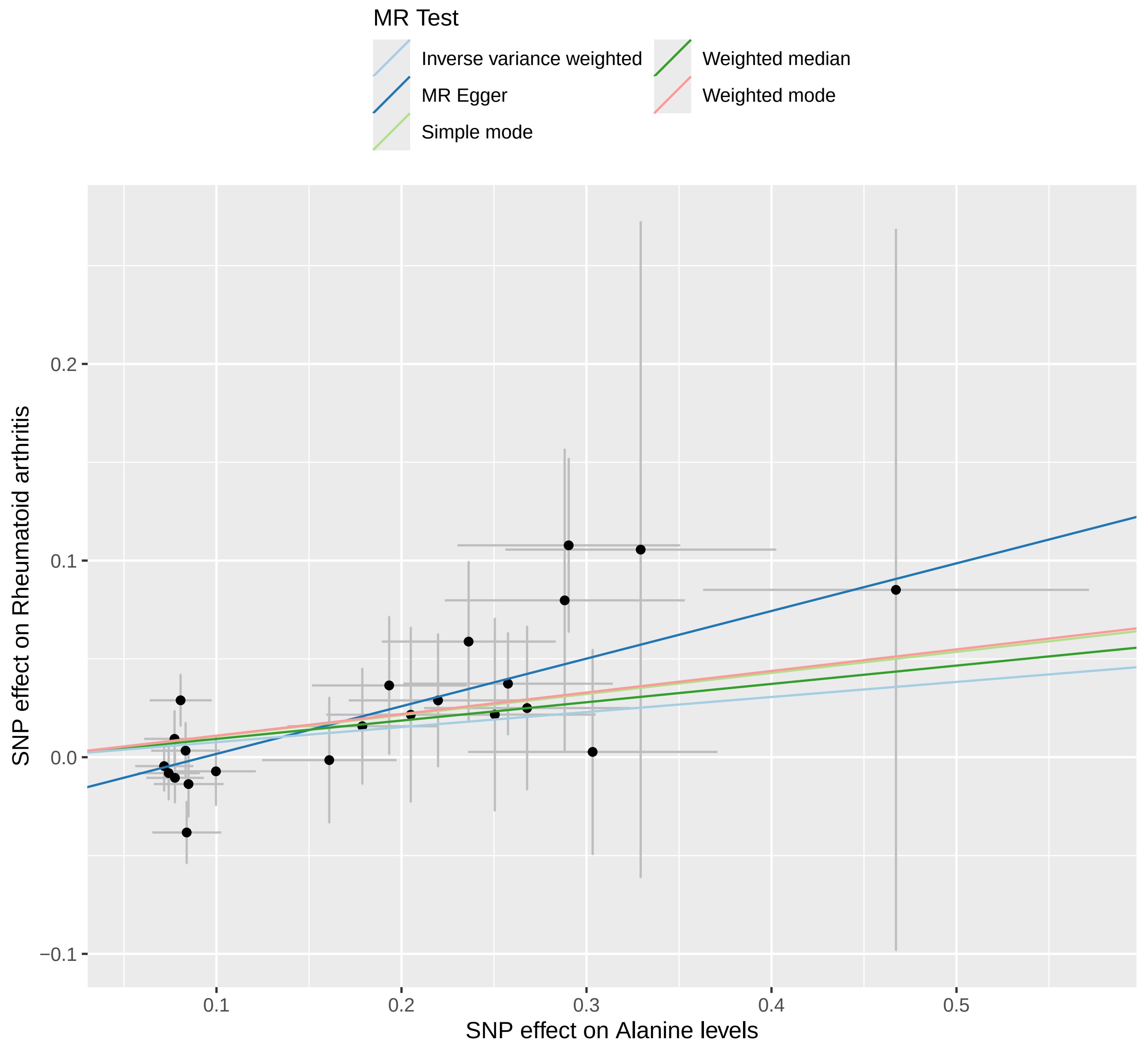
MR Test
Inverse variance weighted
MR Egger
Simple mode
Weighted median
Weighted mode
SNP effect on Rheumatoid arthritis
0.2
0.1
0.0
−0.1
0.1
0.2
0.3
0.4
0.5
SNP effect on Alanine levels

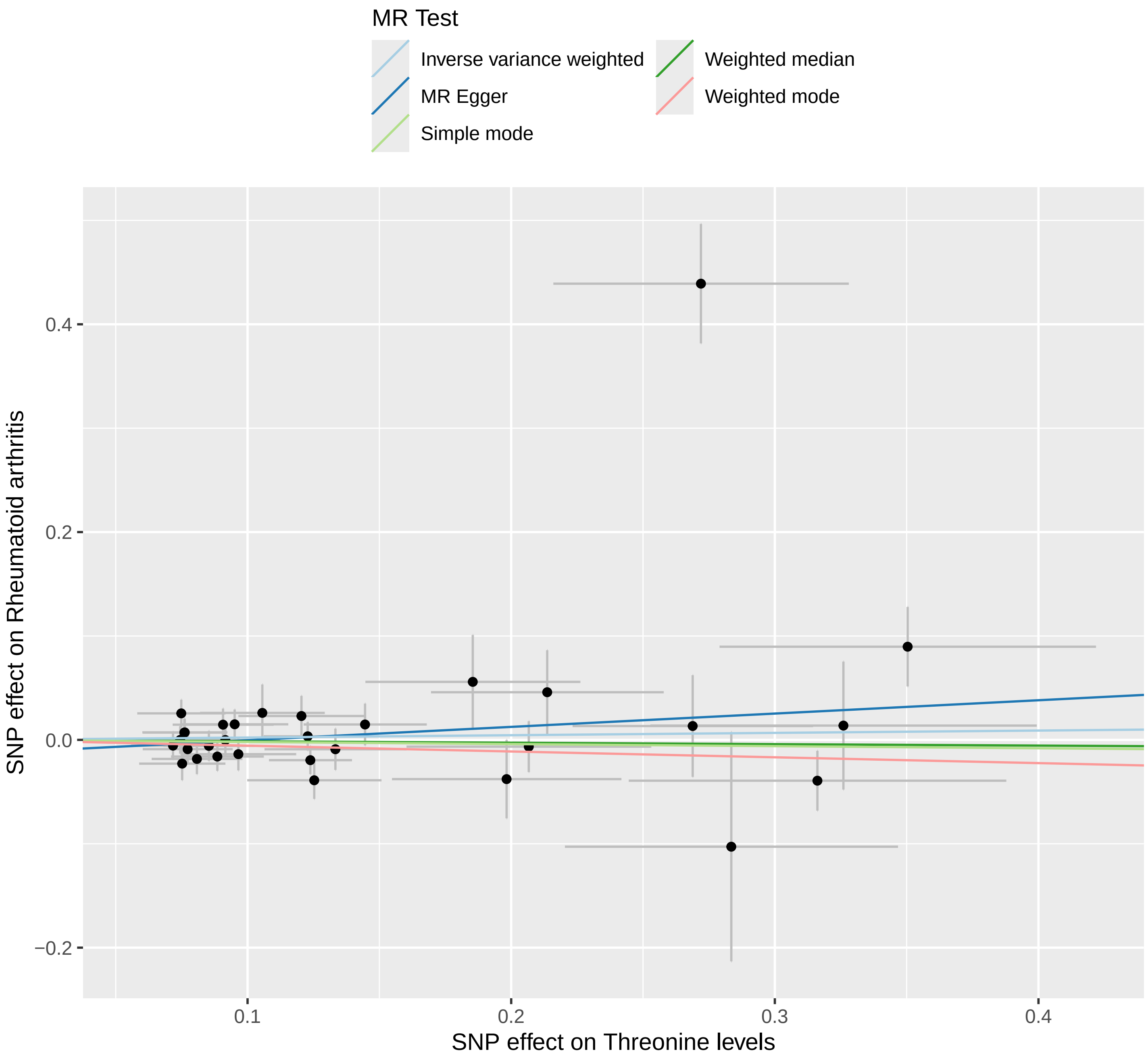
MR Test
Inverse variance weighted
MR Egger
Simple mode
Weighted median
Weighted mode
SNP effect on Rheumatoid arthritis
SNP effect on Threonine levels
0.4
0.2
0.0
−0.2
0.1
0.2
0.3
0.4

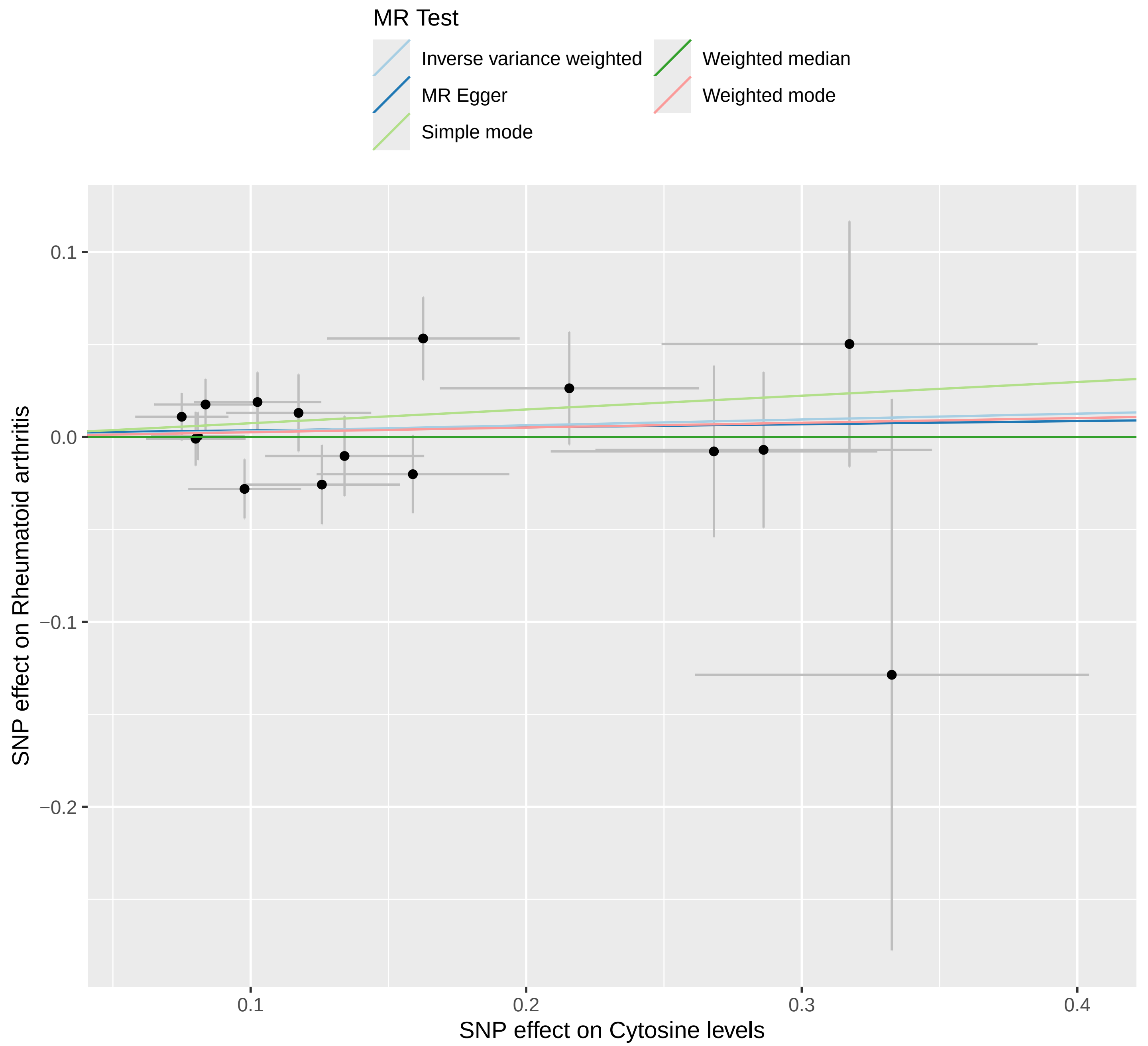
MR Test
Inverse variance weighted
MR Egger
Simple mode
Weighted median
Weighted mode
0.1
0.0
−0.1
−0.2
SNP effect on Rheumatoid arthritis
0.1
0.2
0.3
0.4
SNP effect on Cytosine levels

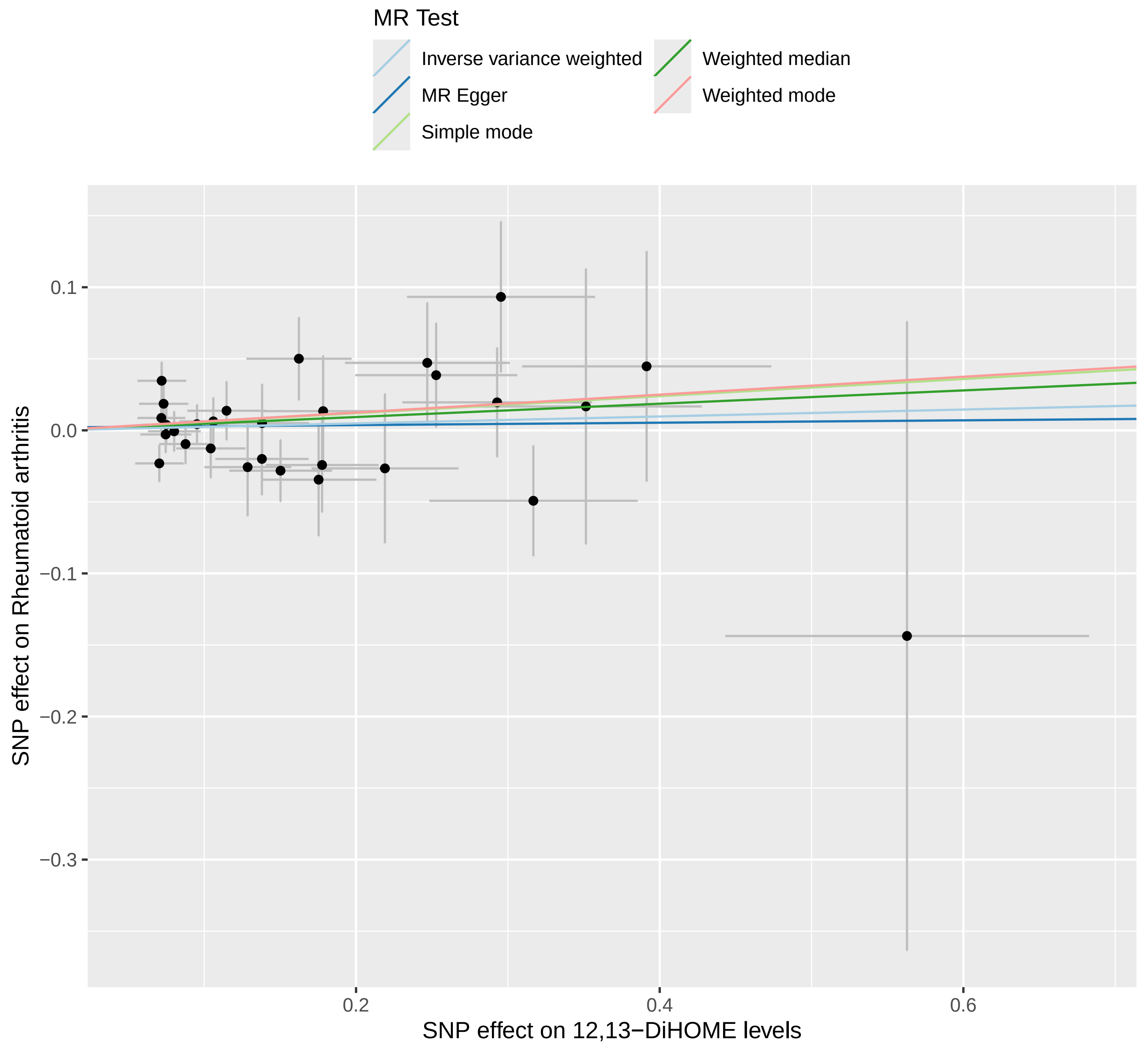
MR Test
Inverse variance weighted
MR Egger
Simple mode
Weighted median
Weighted mode
0.1
0.0
−0.1
−0.2
−0.3
0.2
0.4
0.6
SNP effect on Rheumatoid arthritis
SNP effect on 12,13−DiHOME levels

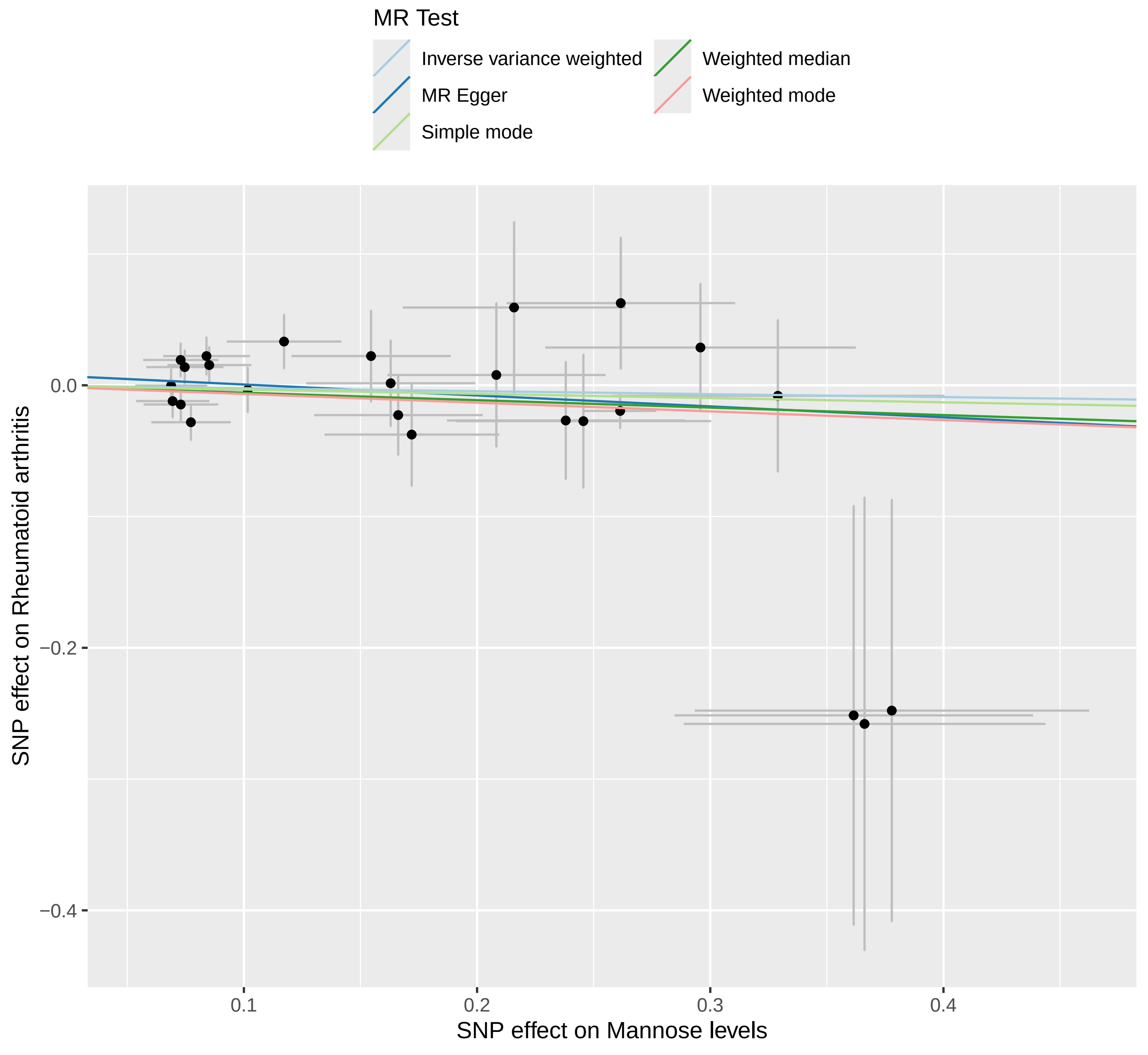
MR Test
Inverse variance weighted
MR Egger
Simple mode
Weighted median
Weighted mode
0.0
−0.2
−0.4
SNP effect on Rheumatoid arthritis
0.1
0.2
0.3
0.4
SNP effect on Mannose levels

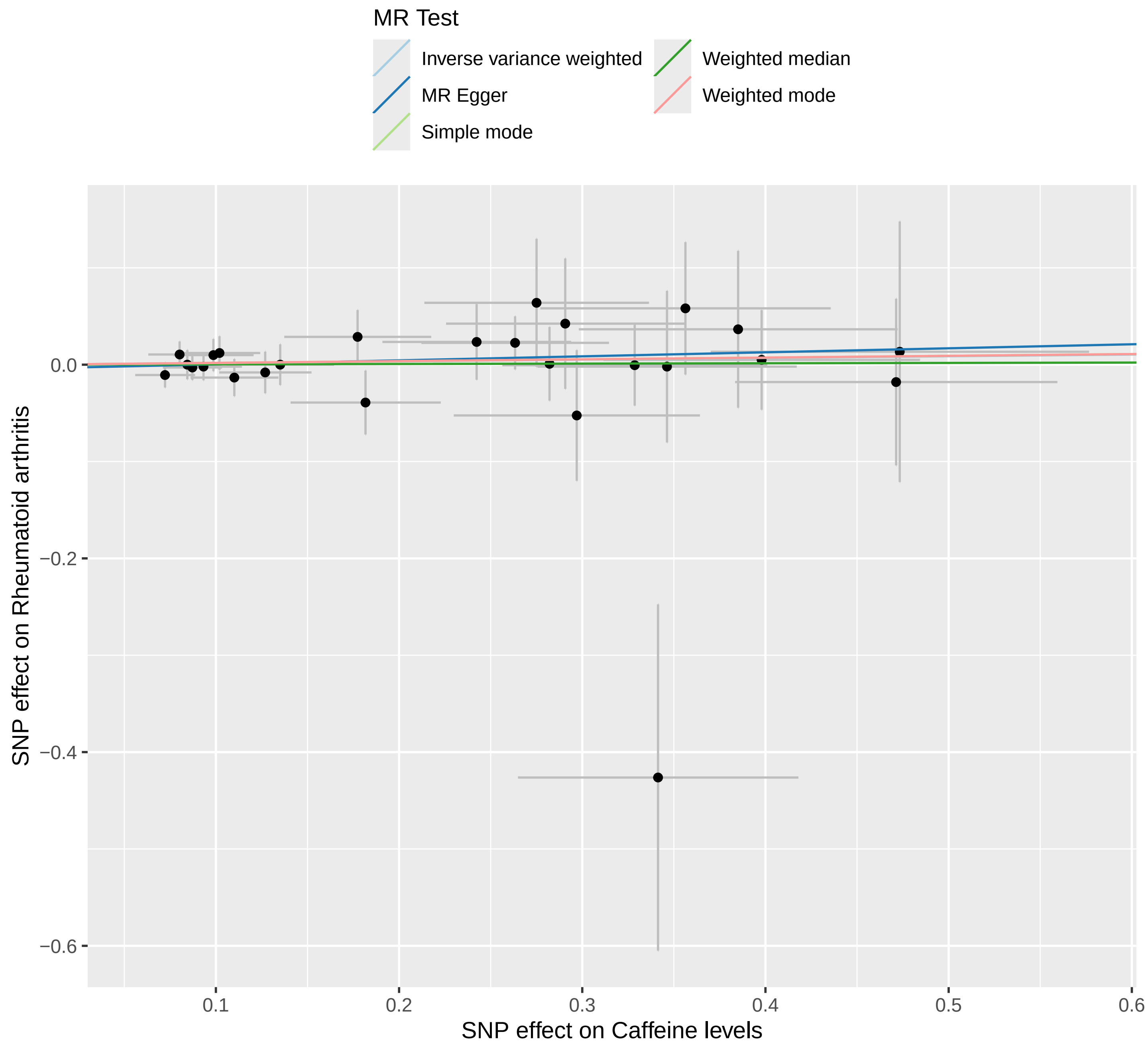

MR Test
Inverse variance weighted
MR Egger
Simple mode
Weighted median
Weighted mode
0.0
−0.2
−0.4
−0.6
0.1
0.2
0.3
0.4
0.5
0.6
SNP effect on Rheumatoid arthritis
SNP effect on Caffeine levels

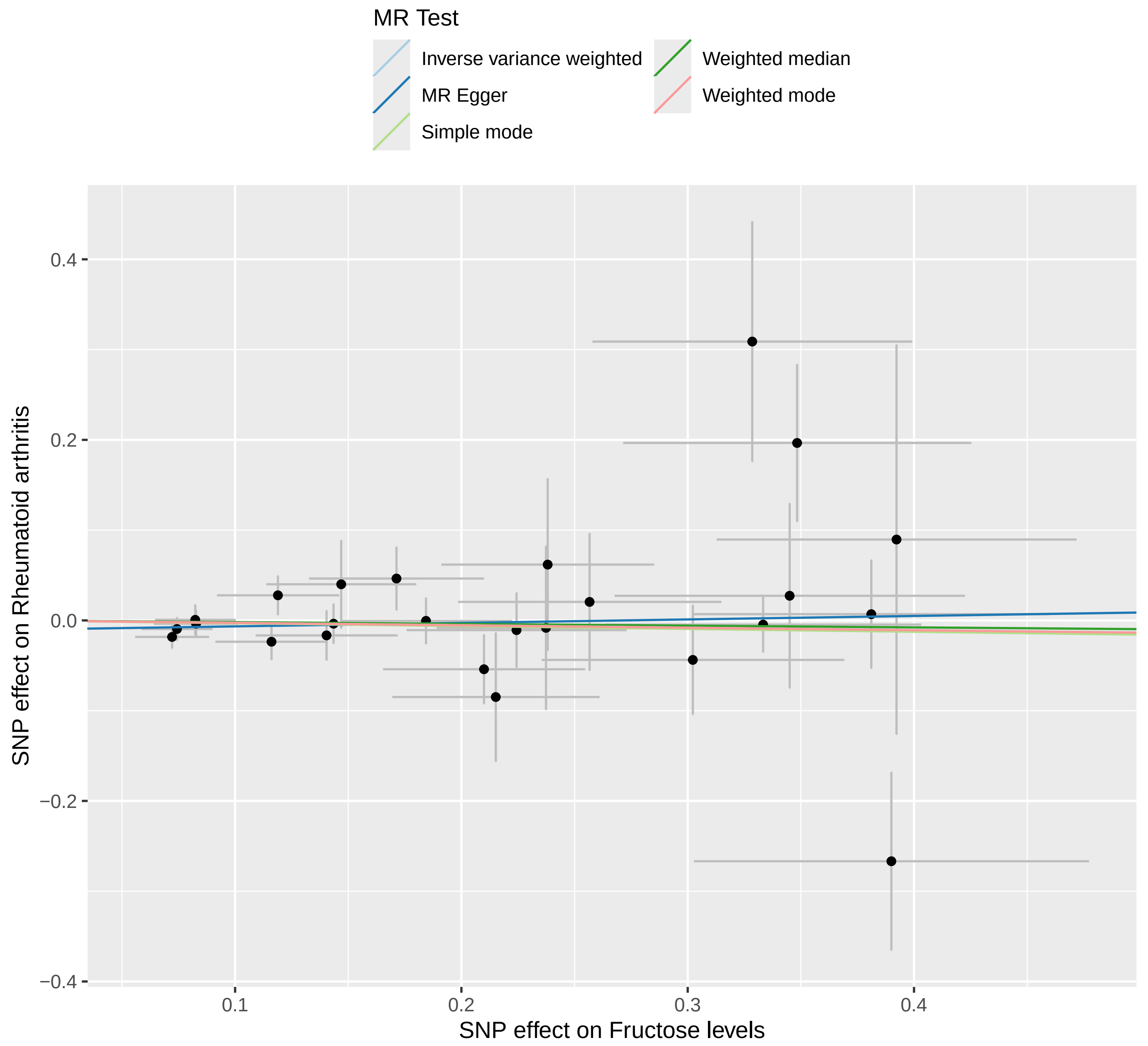

MR Test
Inverse variance weighted
MR Egger
Simple mode
Weighted median
Weighted mode
SNP effect on Rheumatoid arthritis
0.4
0.2
0.0
−0.2
−0.4
0.1
0.2
0.3
0.4
SNP effect on Fructose levels

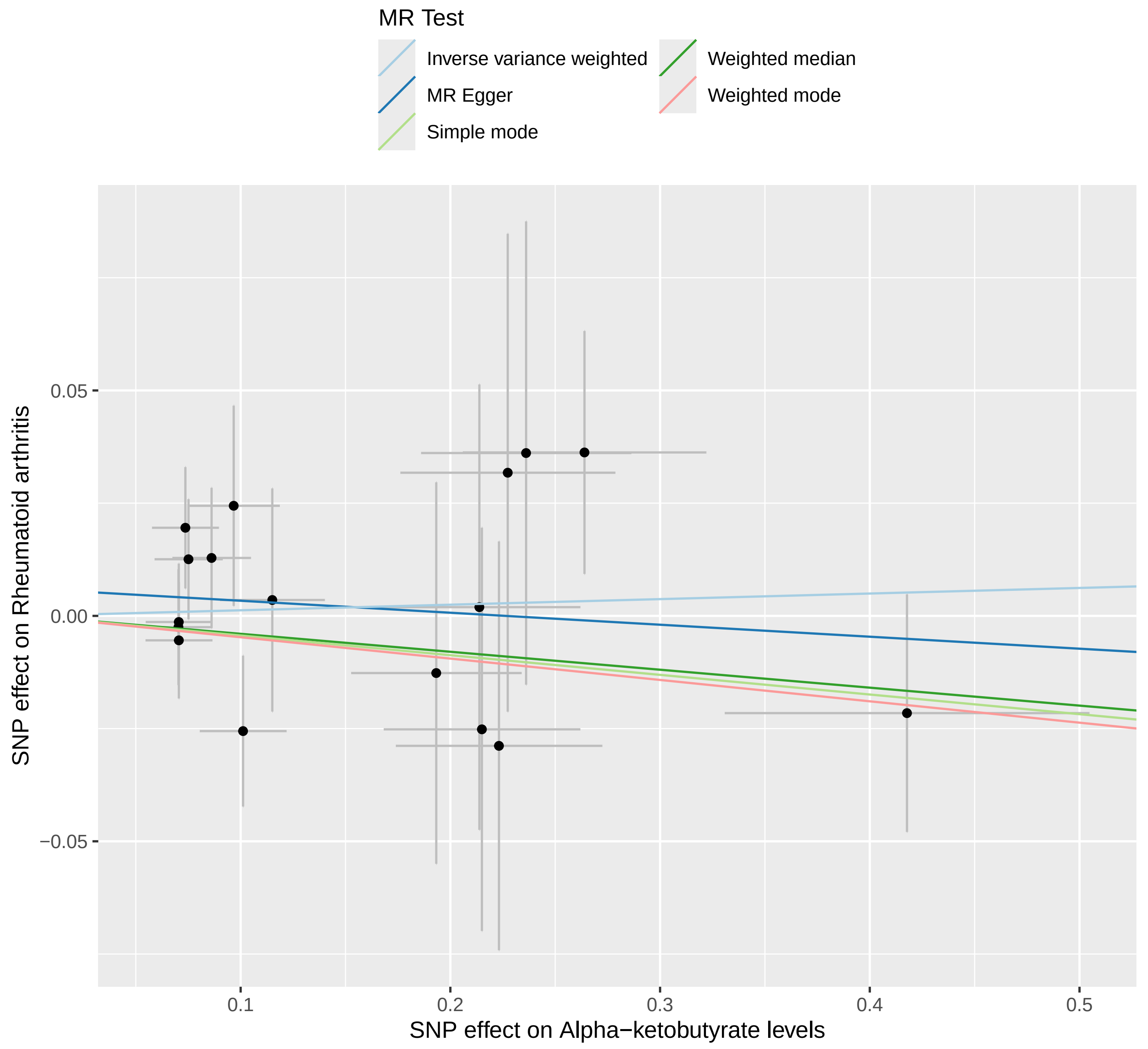
MR Test
Inverse variance weighted
MR Egger
Simple mode
Weighted median
Weighted mode
0.05
0.00
−0.05
0.1
0.2
0.3
0.4
0.5
SNP effect on Rheumatoid arthritis
SNP effect on Alpha−ketobutyrate levels

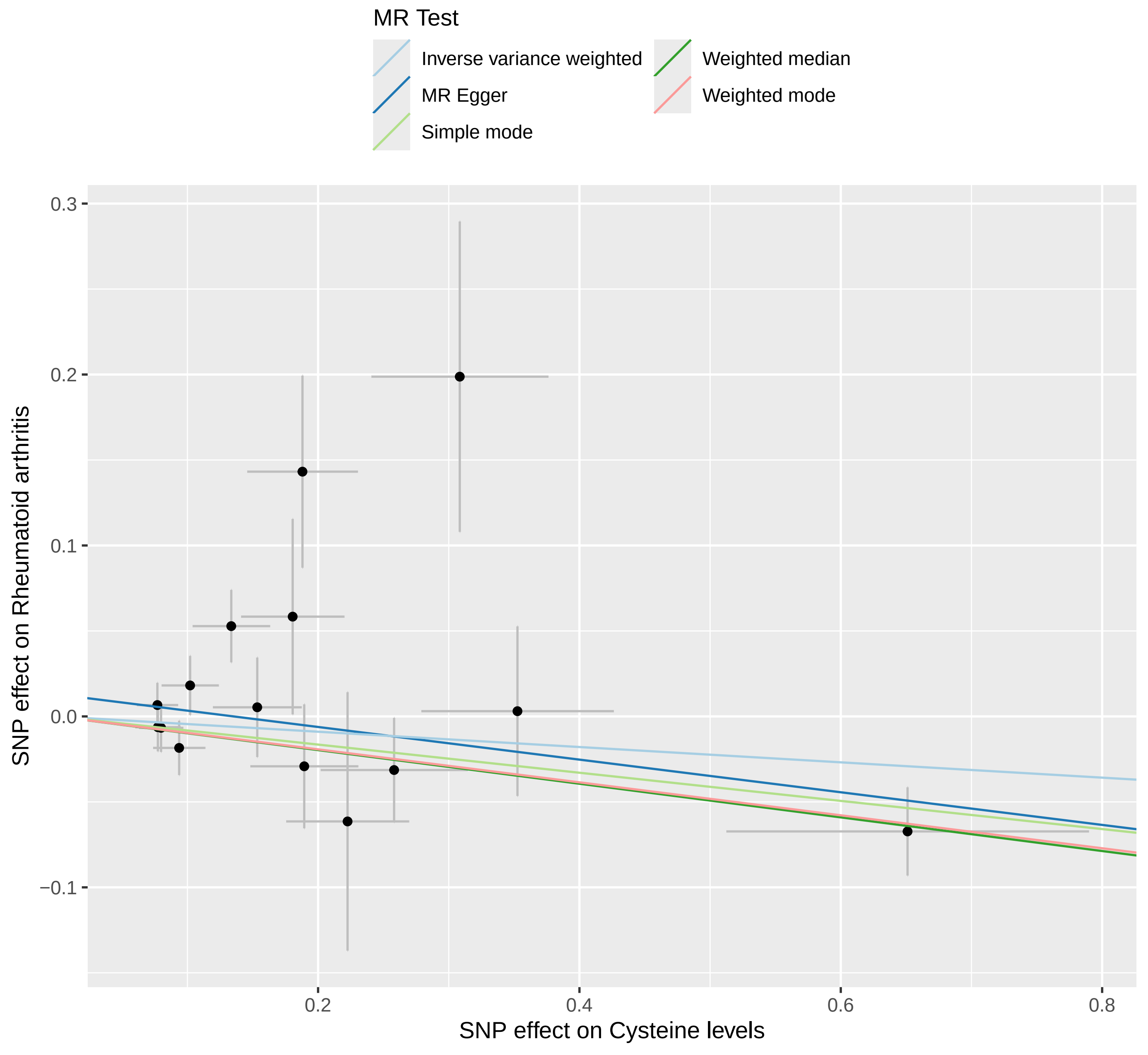
MR Test
Inverse variance weighted
MR Egger
Simple mode
Weighted median
Weighted mode
SNP effect on Rheumatoid arthritis
0.3
0.2
0.1
0.0
−0.1
0.2
0.4
0.6
0.8
SNP effect on Cysteine levels

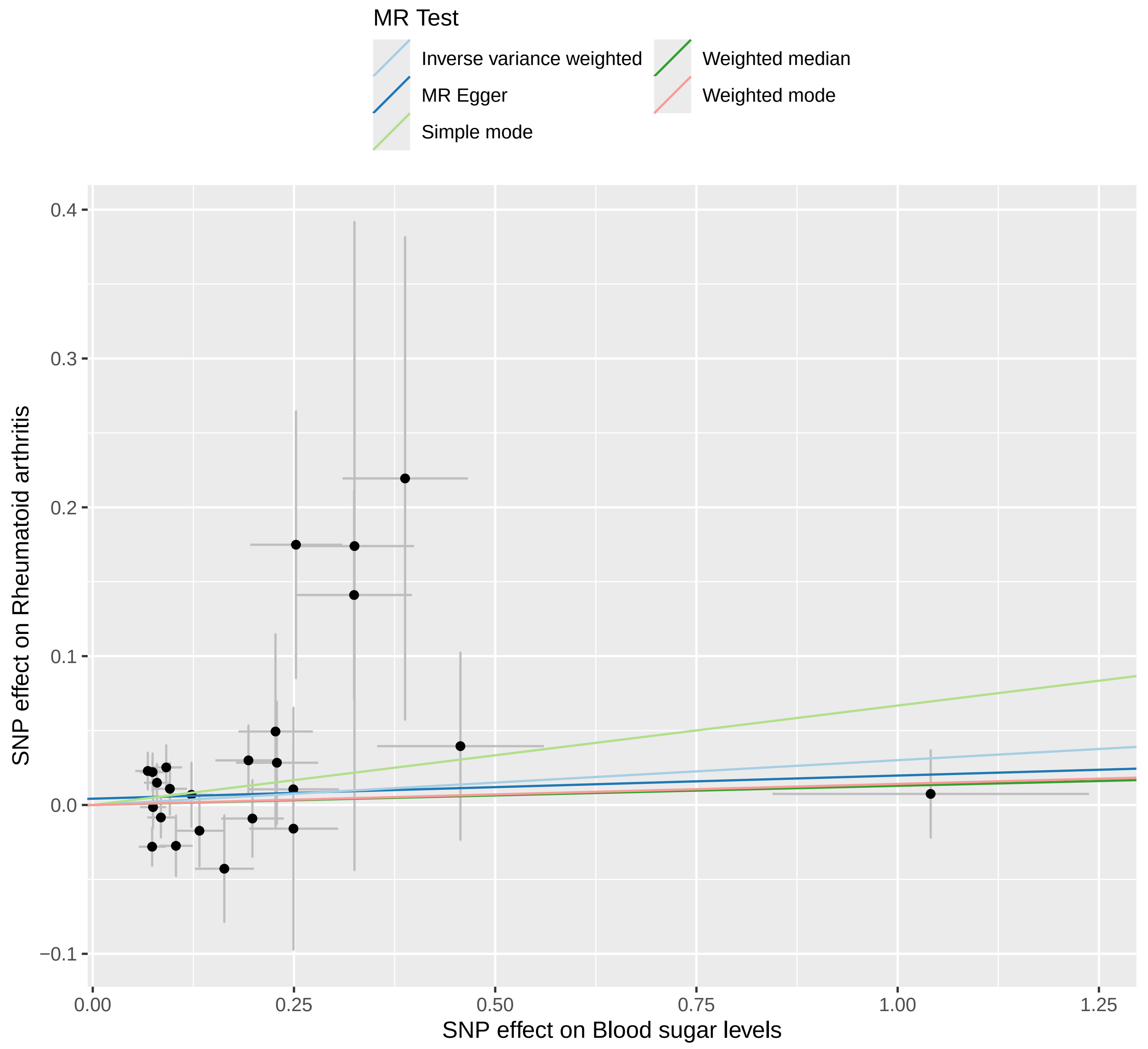

MR Test
Inverse variance weighted
MR Egger
Simple mode
Weighted median
Weighted mode
0.4
0.3
0.2
0.1
0.0
−0.1
0.00
0.25
0.50
0.75
1.00
1.25
SNP effect on Rheumatoid arthritis
SNP effect on Blood sugar levels

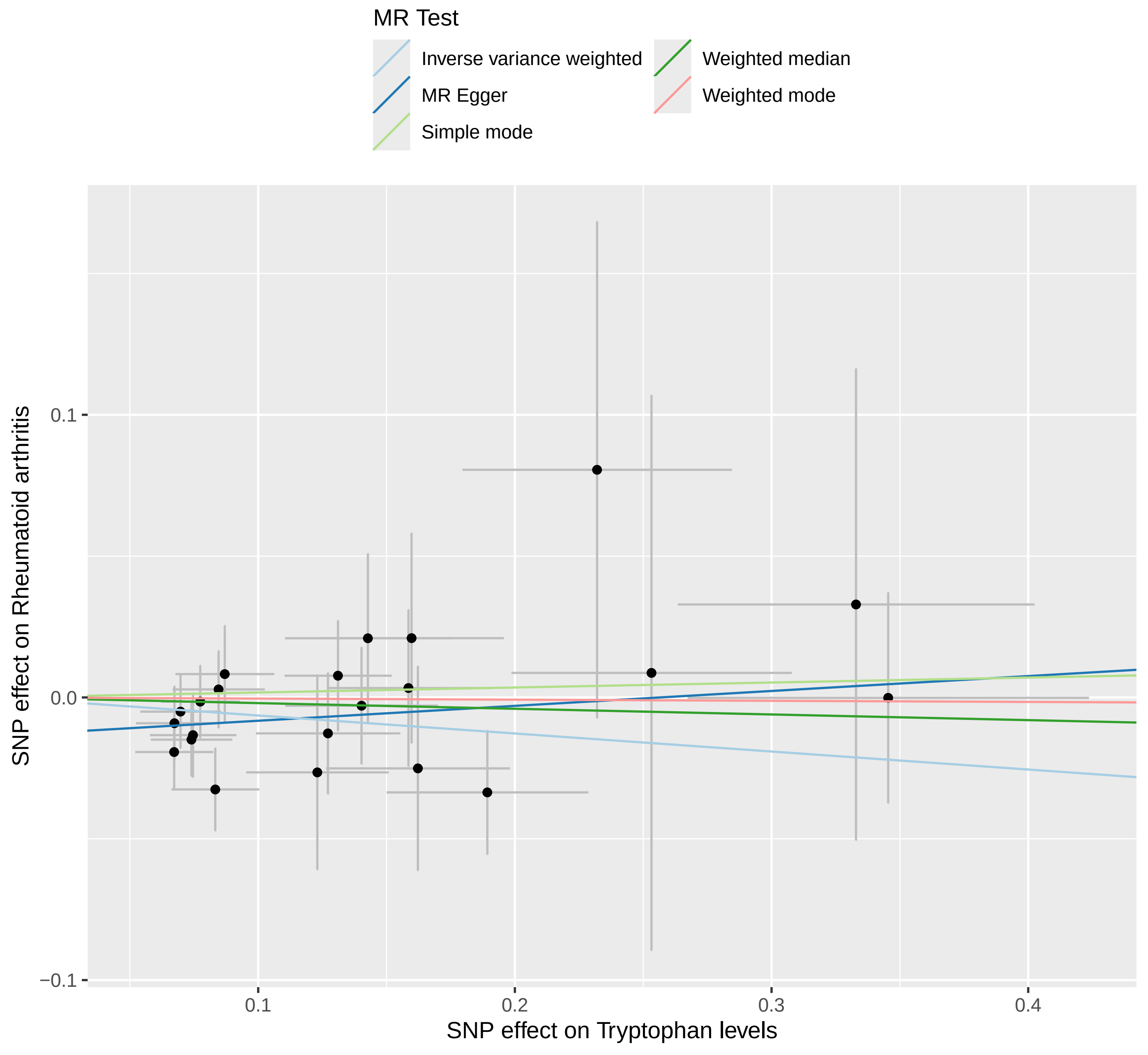
MR Test
Inverse variance weighted
MR Egger
Simple mode
Weighted median
Weighted mode
0.1
0.0
−0.1
0.1
0.2
0.3
0.4
SNP effect on Rheumatoid arthritis
SNP effect on Tryptophan levels

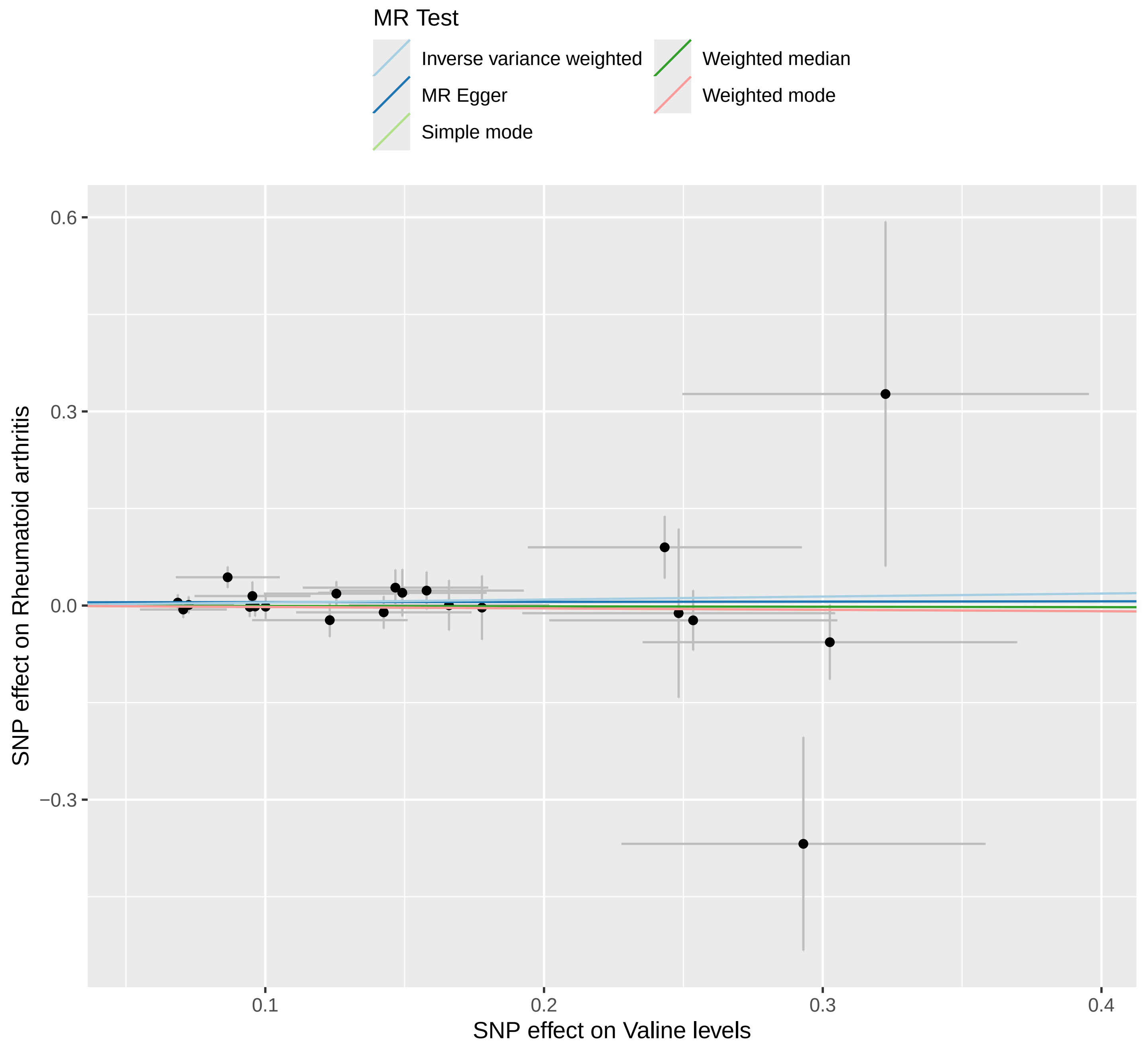
MR Test
Inverse variance weighted
MR Egger
Simple mode
Weighted median
Weighted mode
0.6
0.3
0.0
−0.3
0.1
0.2
0.3
0.4
SNP effect on Rheumatoid arthritis
SNP effect on Valine levels

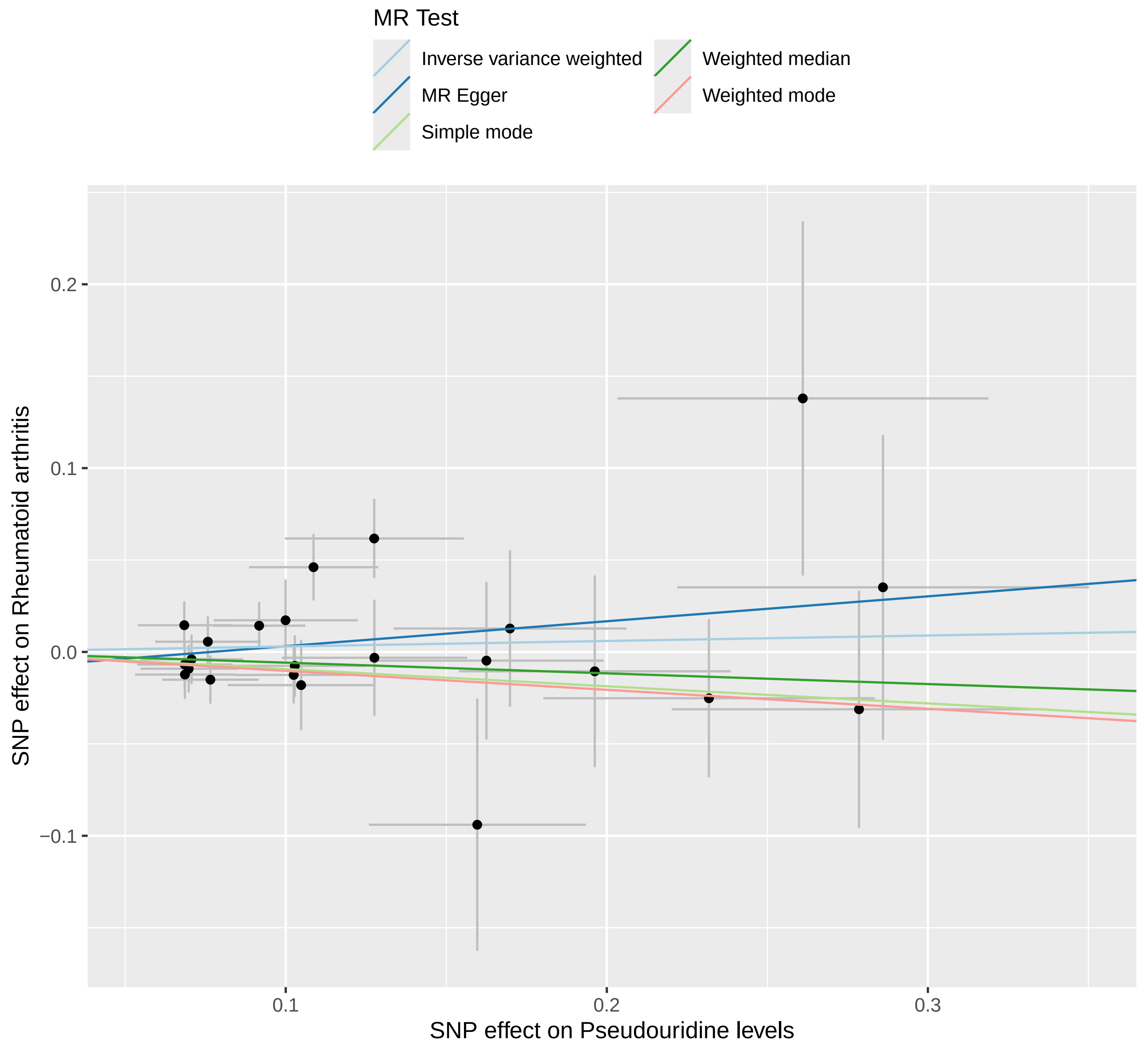
MR Test
Inverse variance weighted
MR Egger
Simple mode
Weighted median
Weighted mode
0.2
0.1
0.0
−0.1
0.1
0.2
0.3
SNP effect on Rheumatoid arthritis
SNP effect on Pseudouridine levels

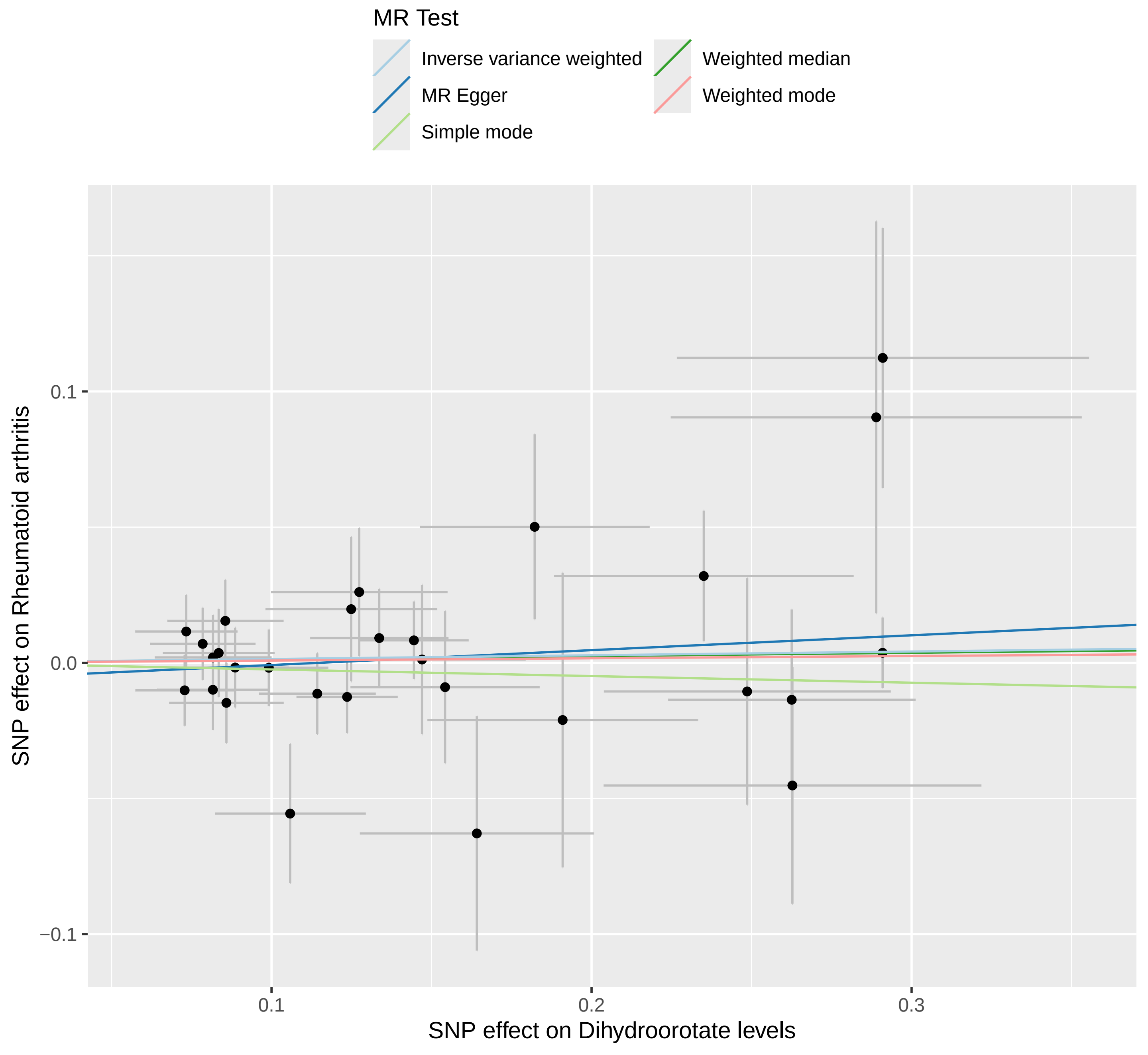
MR Test
Inverse variance weighted
MR Egger
Simple mode
Weighted median
Weighted mode
0.1
0.0
−0.1
SNP effect on Rheumatoid arthritis
0.1
0.2
0.3
SNP effect on Dihydroorotate levels

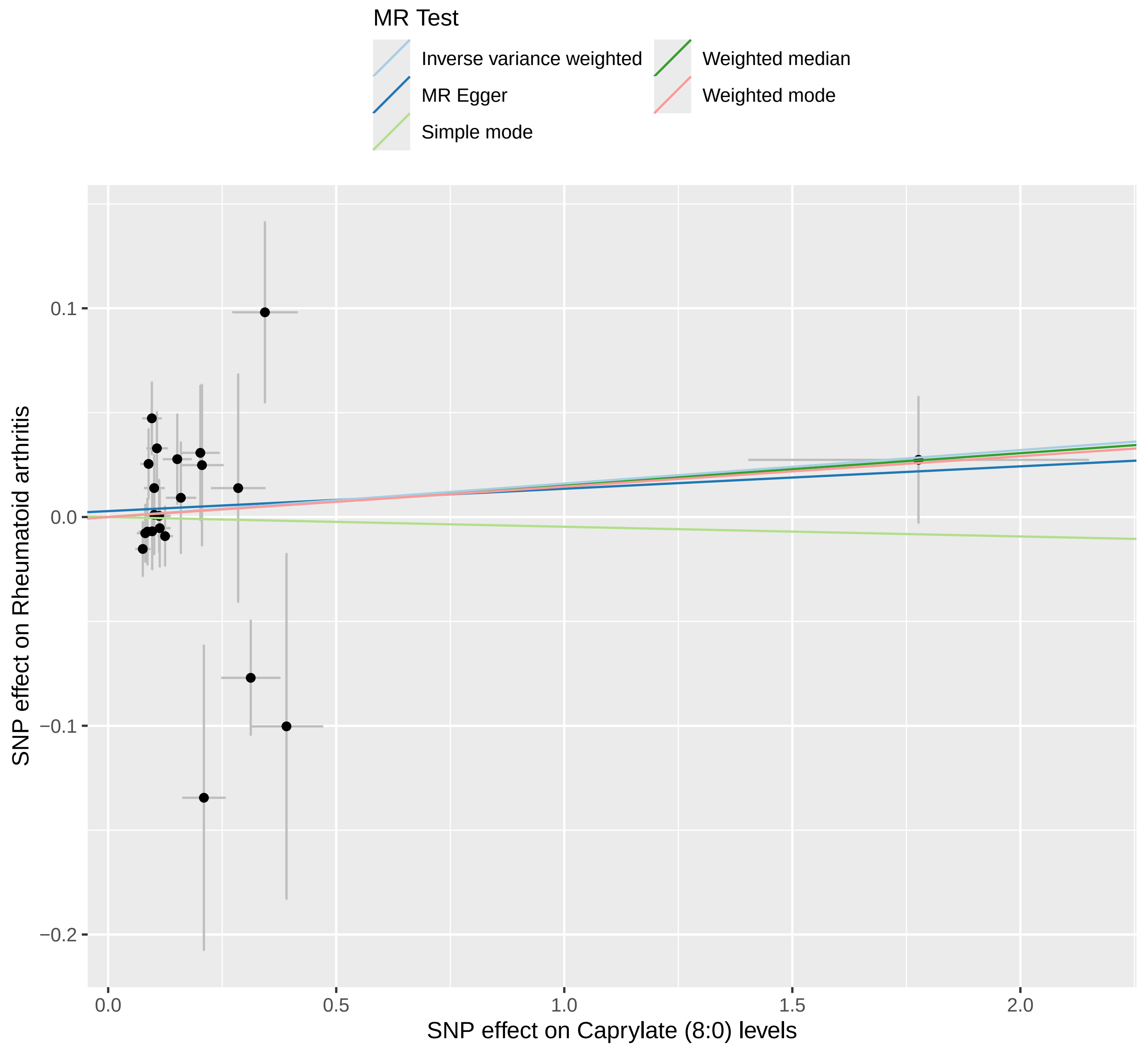
MR Test
Inverse variance weighted
MR Egger
Simple mode
Weighted median
Weighted mode
0.1
0.0
−0.1
−0.2
SNP effect on Rheumatoid arthritis
0.0
0.5
1.0
1.5
2.0
SNP effect on Caprylate (8:0) levels

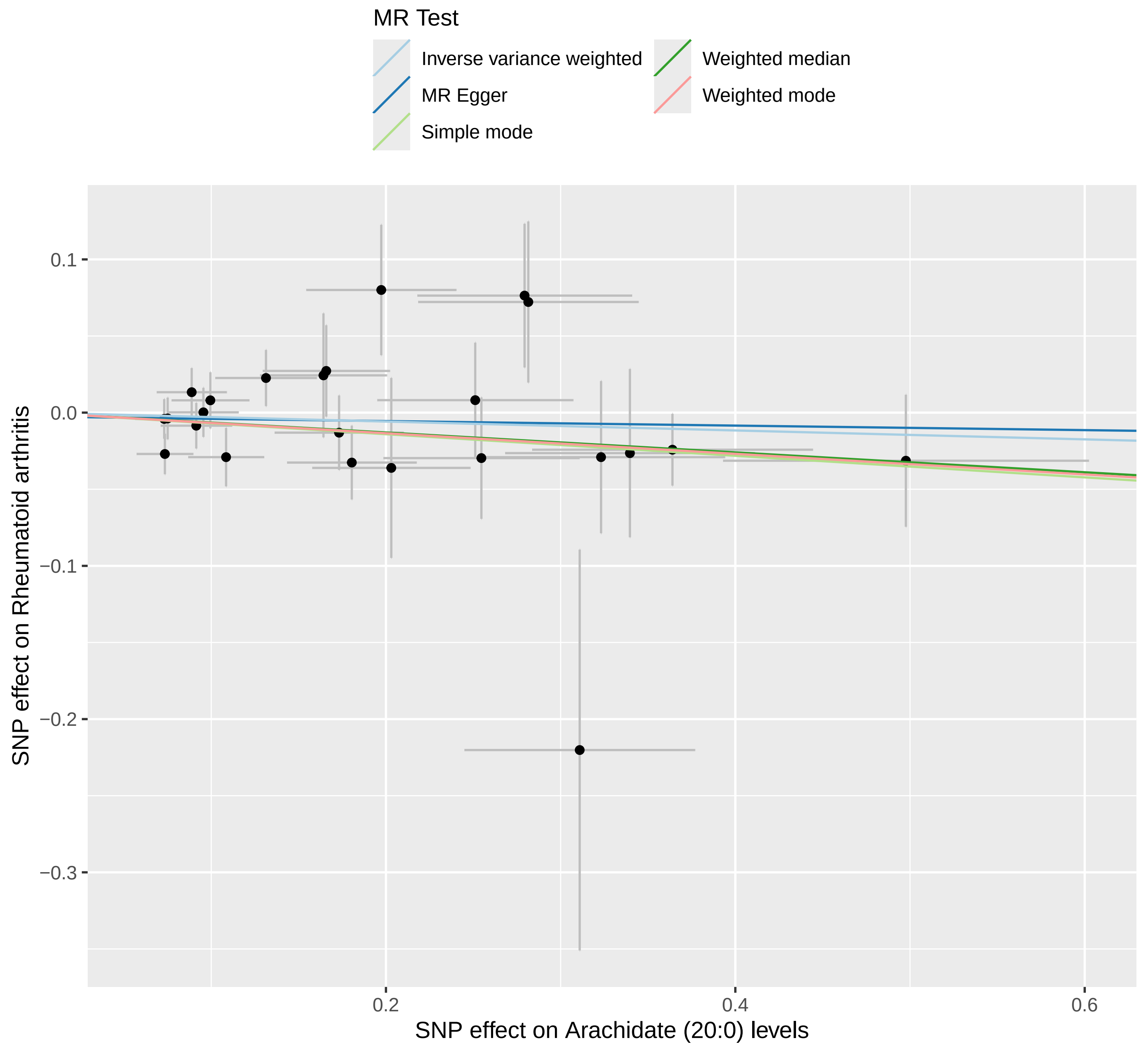
MR Test
Inverse variance weighted
MR Egger
Simple mode
Weighted median
Weighted mode
0.1
0.0
−0.1
−0.2
−0.3
0.2
0.4
0.6
SNP effect on Rheumatoid arthritis
SNP effect on Arachidate (20:0) levels

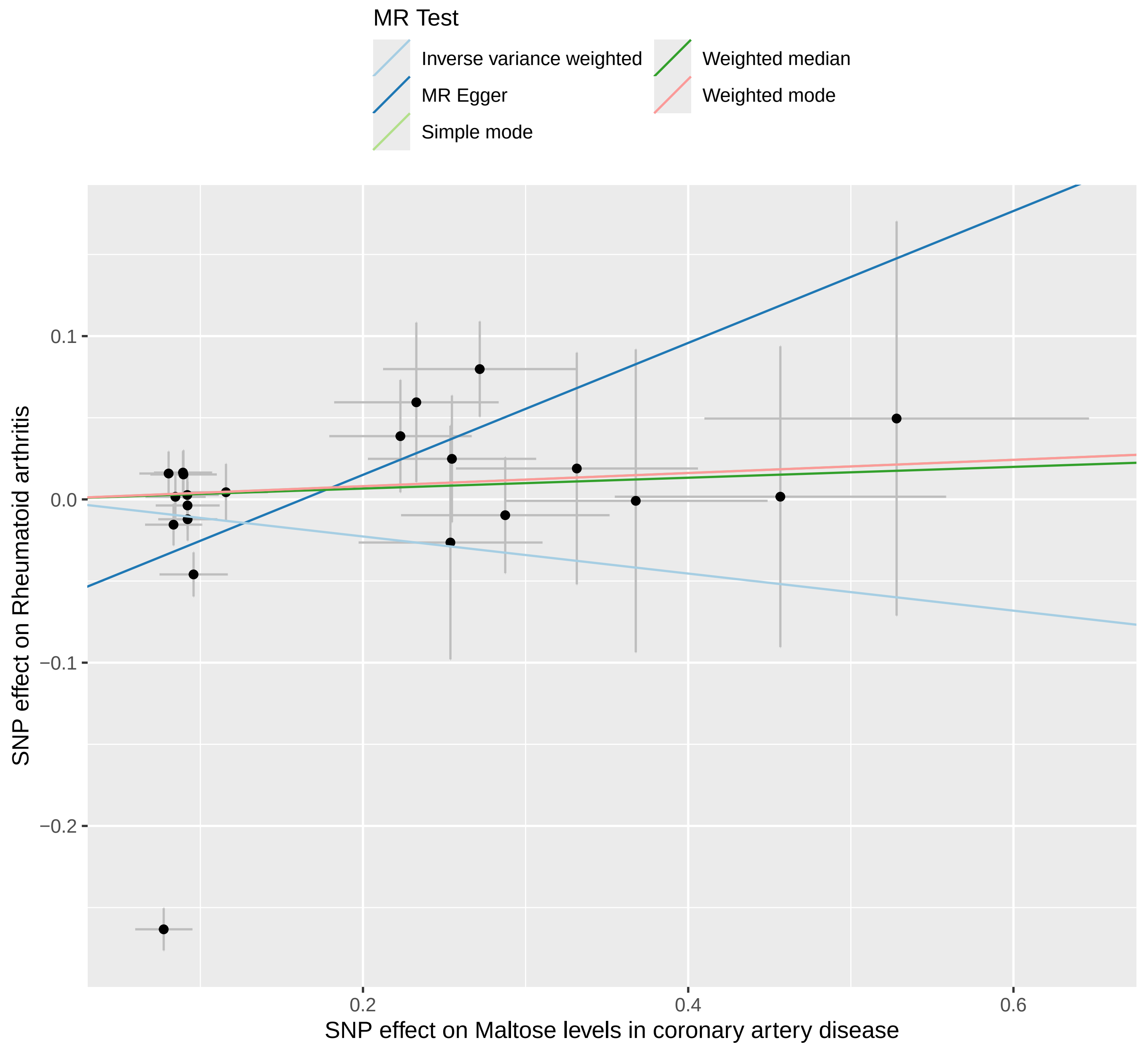

MR Test
Inverse variance weighted
MR Egger
Simple mode
Weighted median
Weighted mode
0.1
0.0
−0.1
−0.2
0.2
0.4
0.6
SNP effect on Rheumatoid arthritis
SNP effect on Maltose levels in coronary artery disease

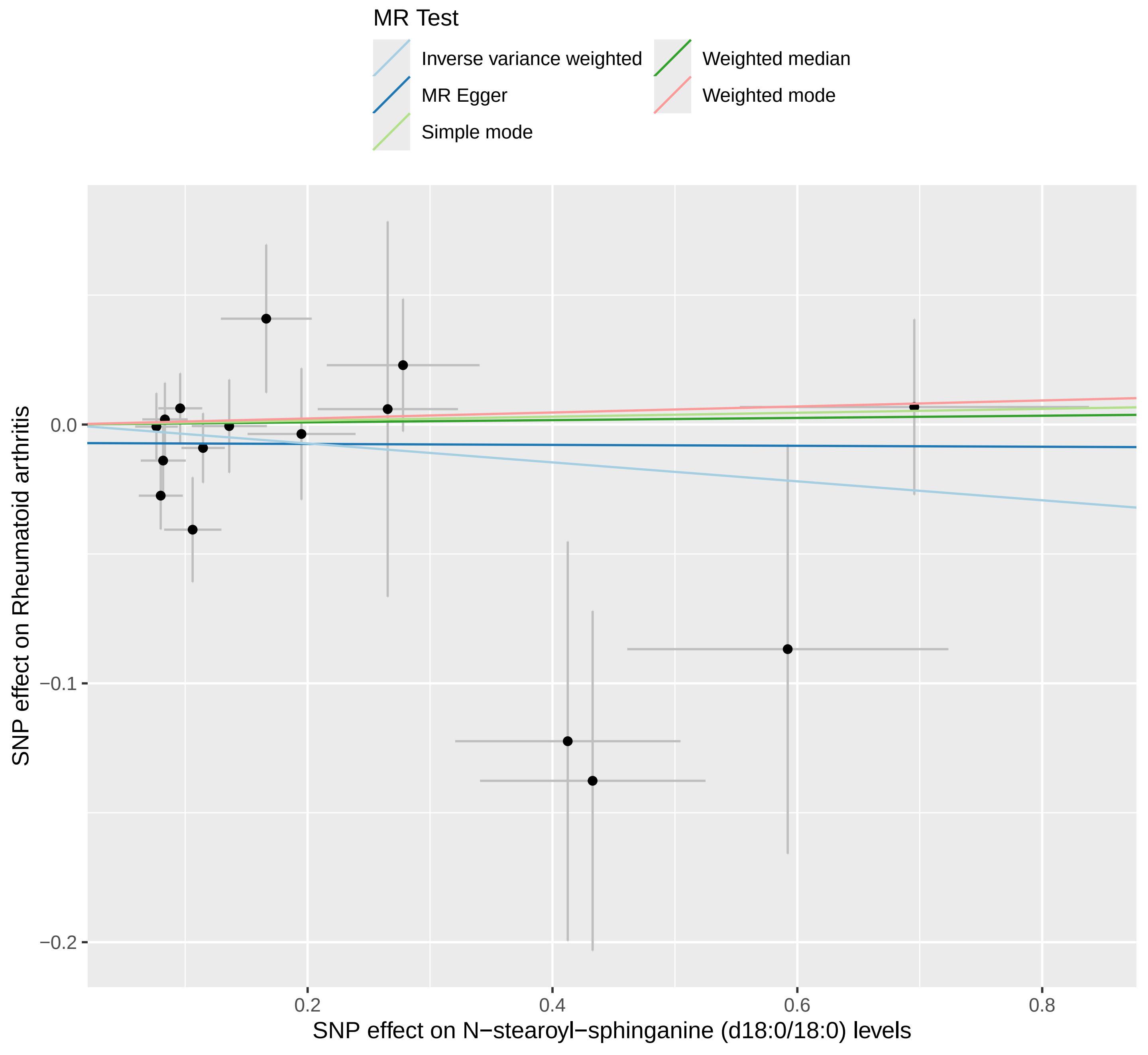

MR Test
Inverse variance weighted
MR Egger
Simple mode
Weighted median
Weighted mode
SNP effect on Rheumatoid arthritis
0.0
−0.1
−0.2
0.2
0.4
0.6
0.8
SNP effect on N−stearoyl−sphinganine (d18:0/18:0) levels

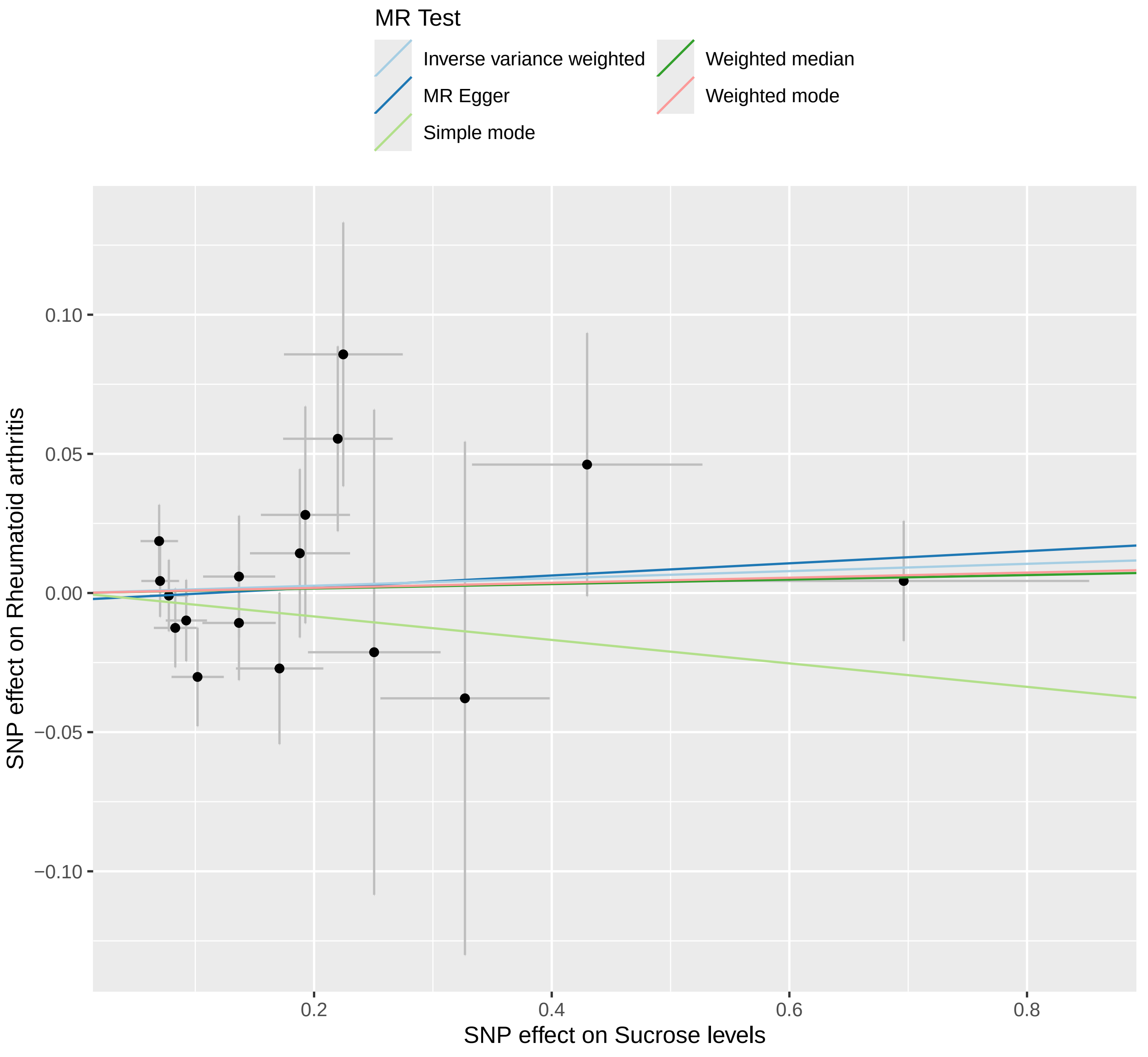
MR Test
Inverse variance weighted
MR Egger
Simple mode
Weighted median
Weighted mode
0.10
0.05
0.00
−0.05
−0.10
SNP effect on Rheumatoid arthritis
0.2
0.4
0.6
0.8
SNP effect on Sucrose levels

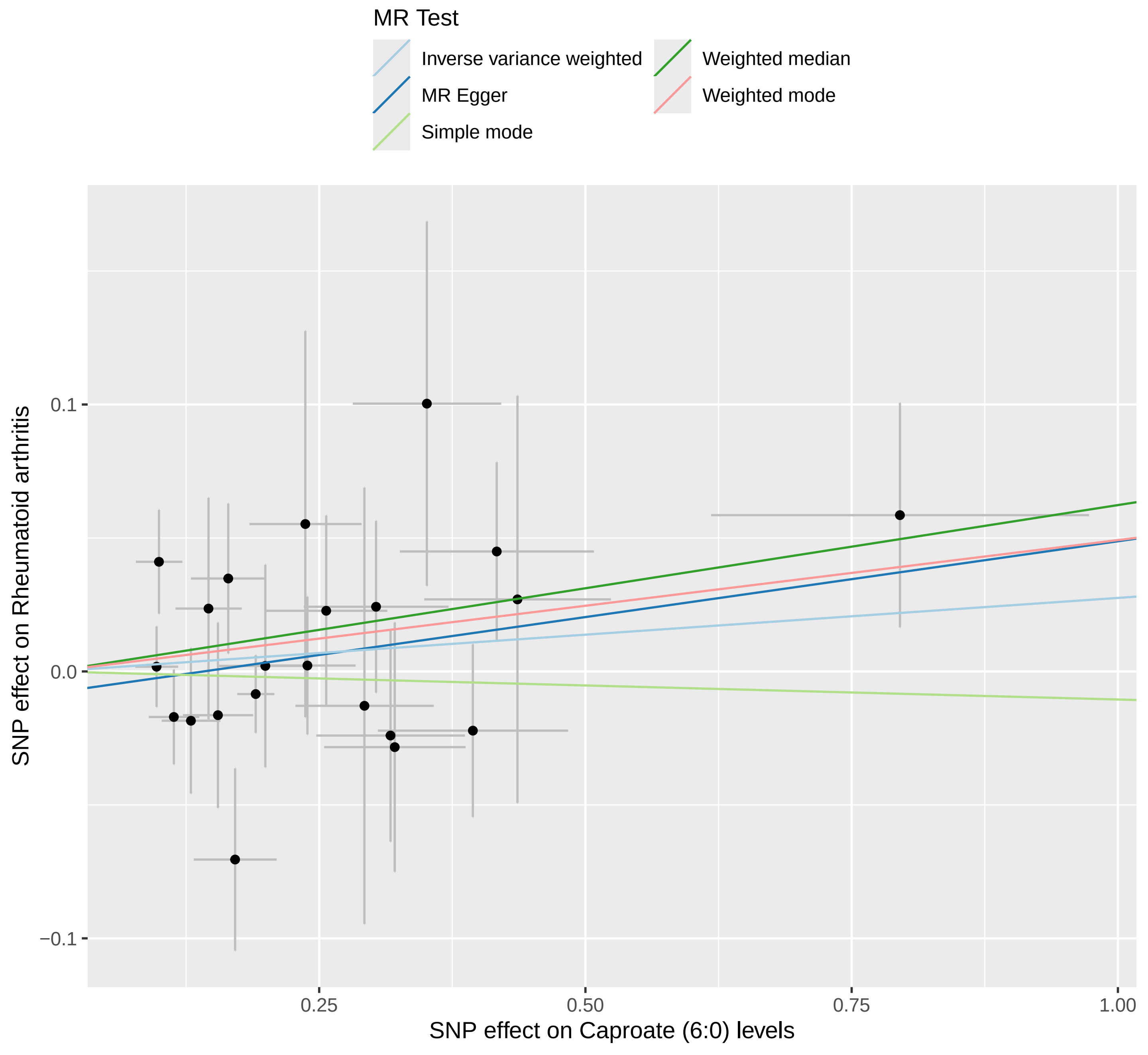

MR Test
Inverse variance weighted
MR Egger
Simple mode
Weighted median
Weighted mode
0.1
0.0
−0.1
0.25
0.50
0.75
1.00
SNP effect on Rheumatoid arthritis
SNP effect on Caproate (6:0) levels

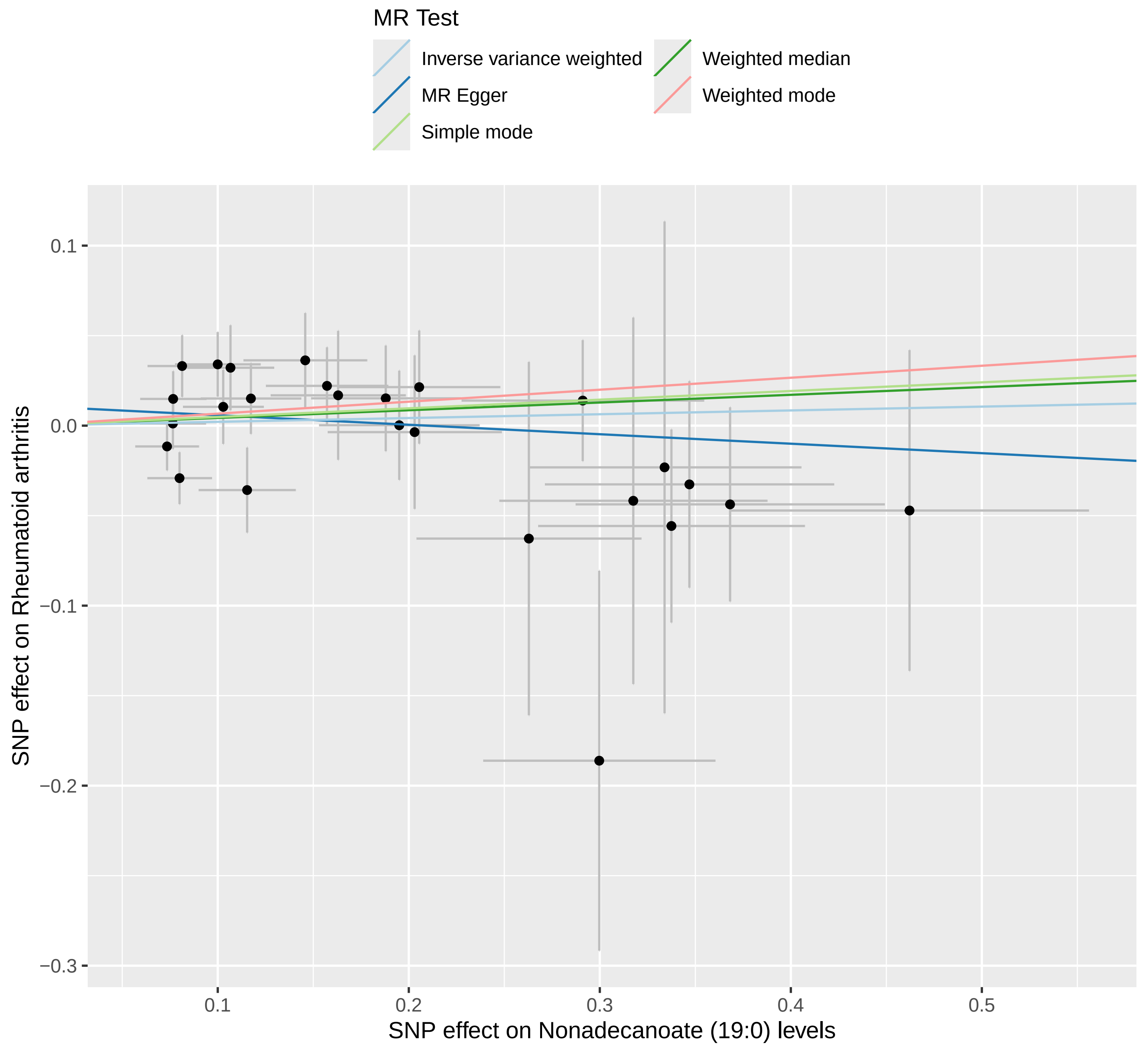

MR Test
Inverse variance weighted
MR Egger
Simple mode
Weighted median
Weighted mode
SNP effect on Rheumatoid arthritis
0.1
0.0
−0.1
−0.2
−0.3
0.1
0.2
0.3
0.4
0.5
SNP effect on Nonadecanoate (19:0) levels

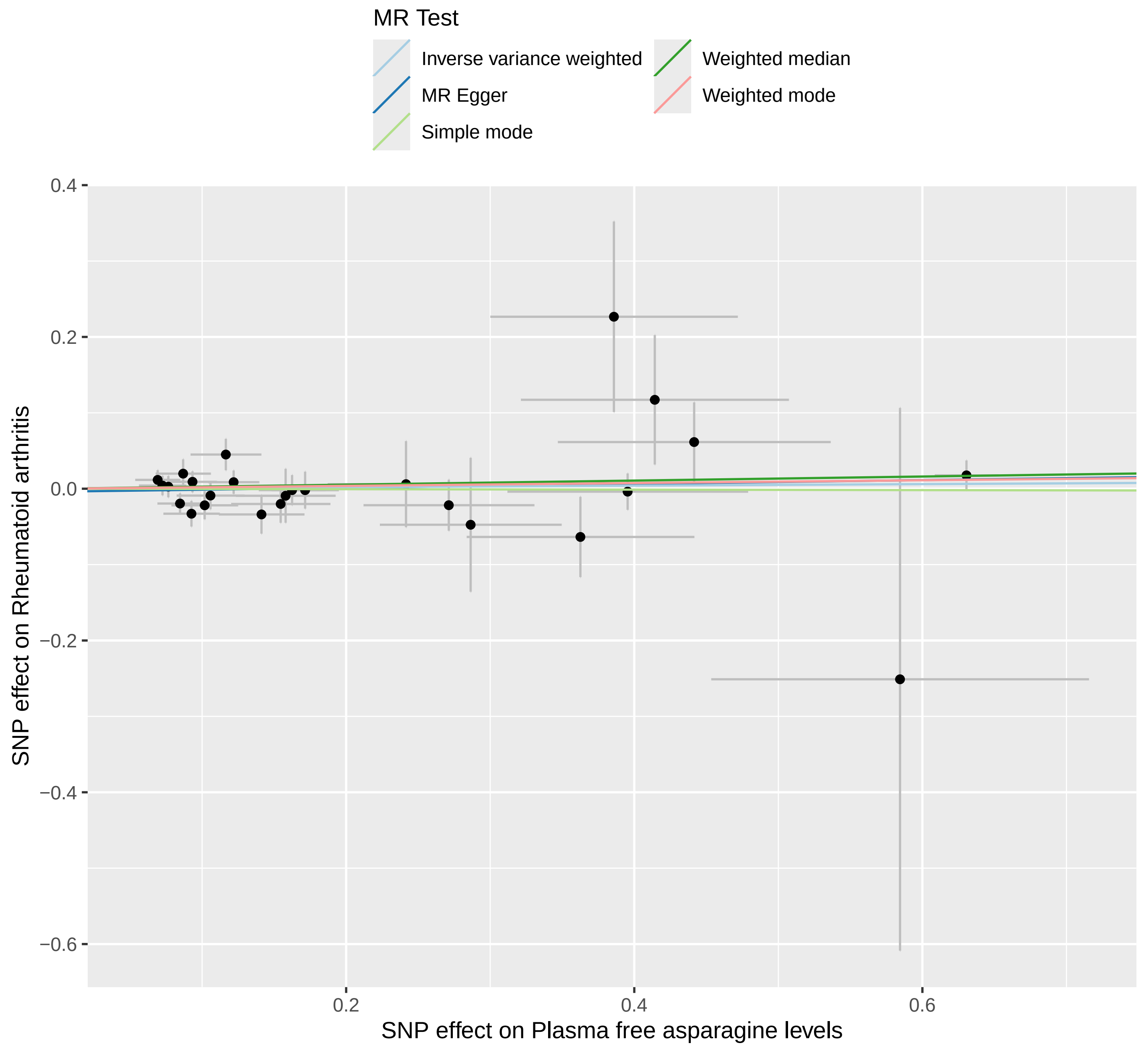

MR Test
Inverse variance weighted
MR Egger
Simple mode
Weighted median
Weighted mode
0.4
0.2
0.0
−0.2
−0.4
−0.6
0.2
0.4
0.6
SNP effect on Rheumatoid arthritis
SNP effect on Plasma free asparagine levels

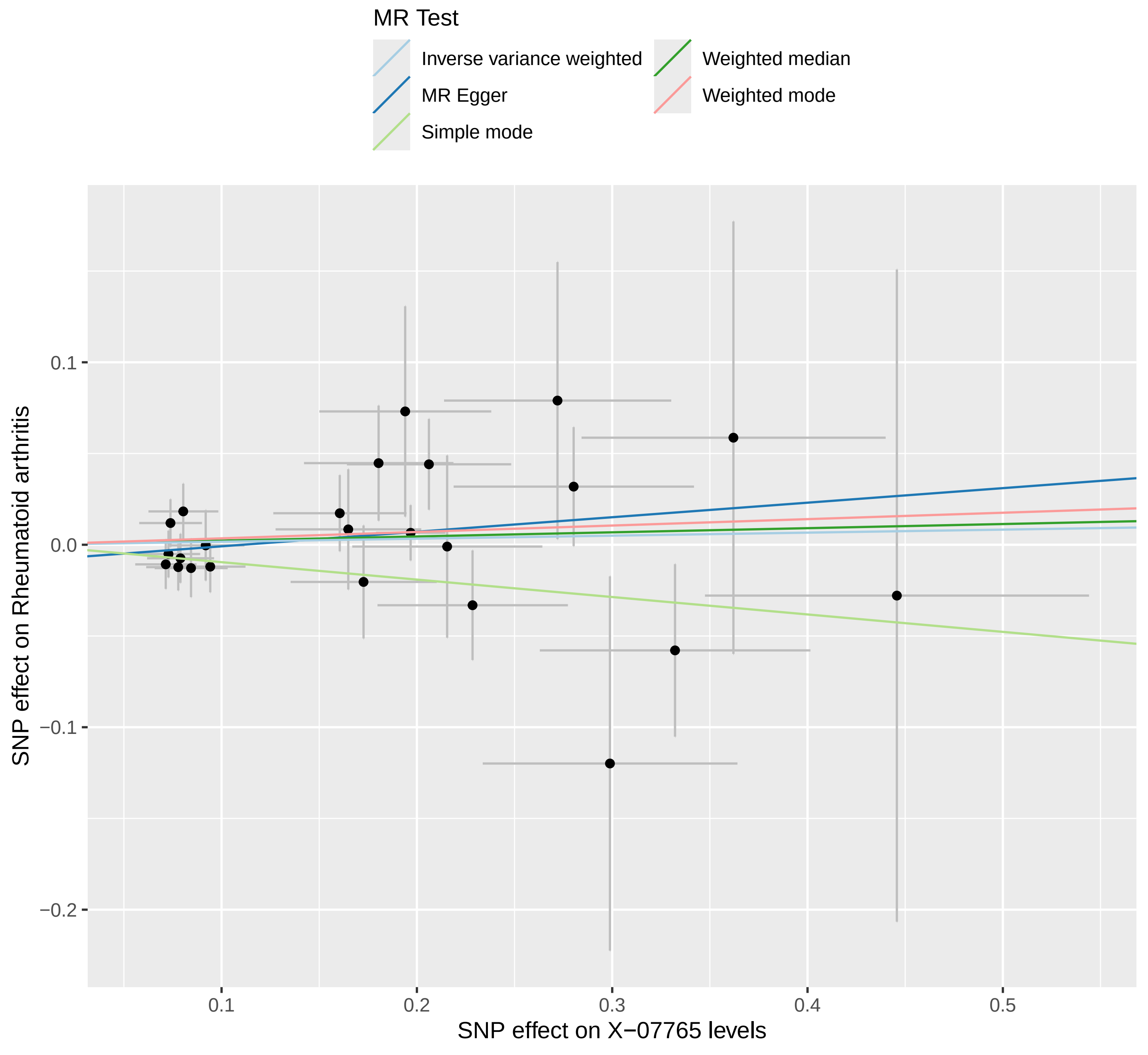

MR Test
Inverse variance weighted
MR Egger
Simple mode
Weighted median
Weighted mode
0.1
0.0
−0.1
−0.2
SNP effect on Rheumatoid arthritis
0.1
0.2
0.3
0.4
0.5
SNP effect on X−07765 levels

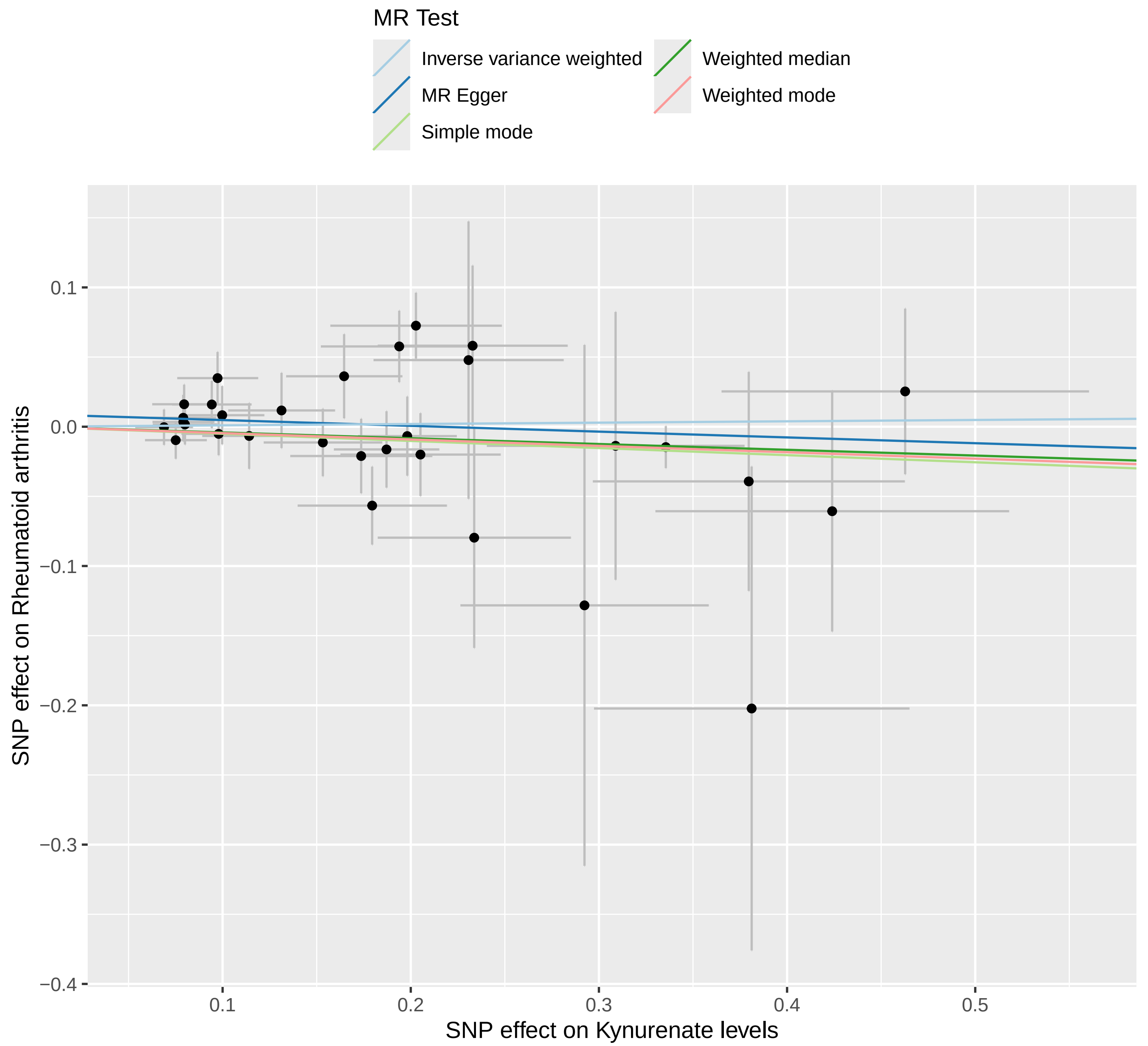

MR Test
Inverse variance weighted
MR Egger
Simple mode
Weighted median
Weighted mode
0.1
0.0
−0.1
−0.2
−0.3
−0.4
SNP effect on Rheumatoid arthritis
0.1
0.2
0.3
0.4
0.5
SNP effect on Kynurenate levels

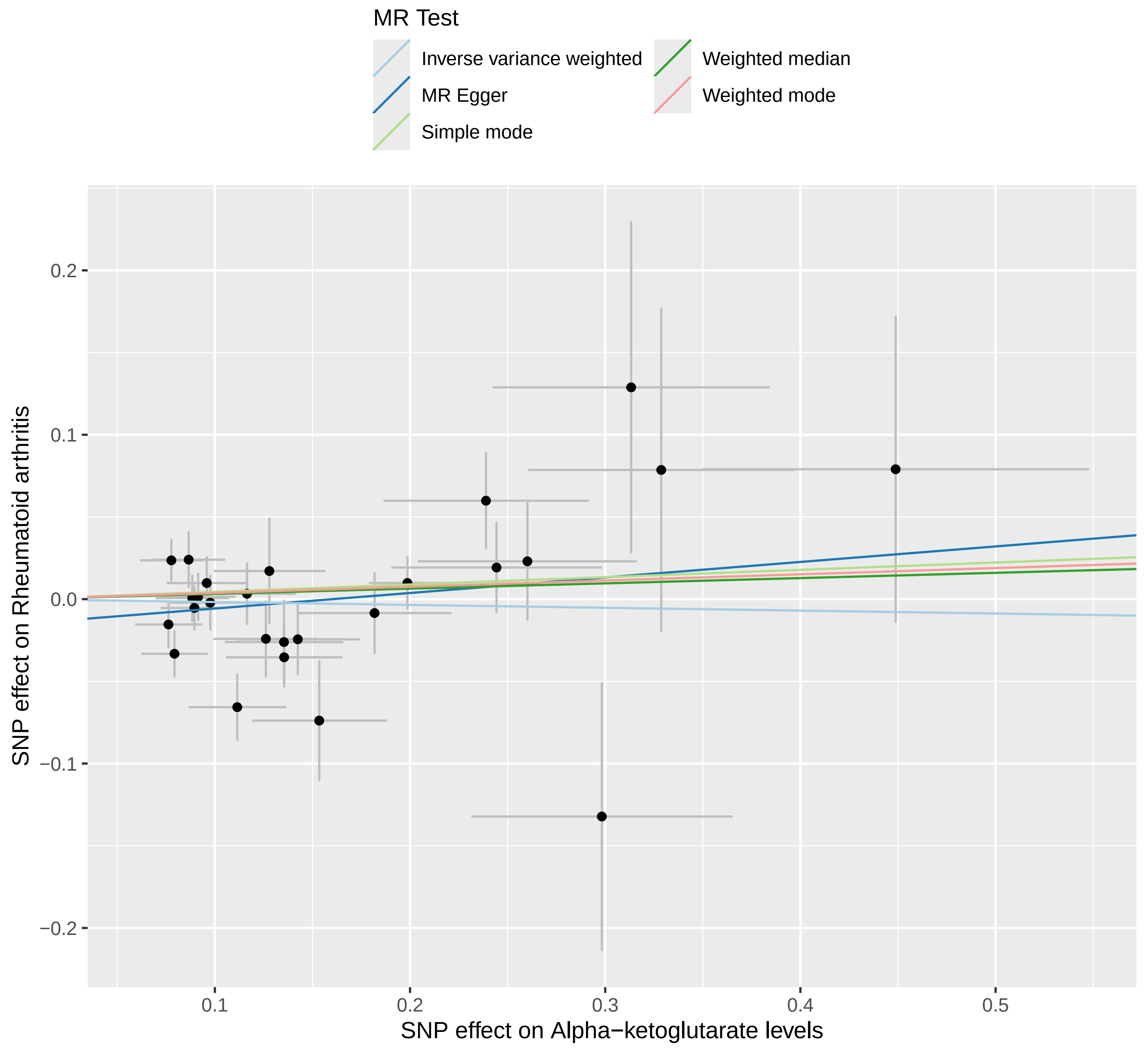
MR Test
Inverse variance weighted
MR Egger
Simple mode
Weighted median
Weighted mode
SNP effect on Rheumatoid arthritis
0.2
0.1
0.0
−0.1
−0.2
0.1
0.2
0.3
0.4
0.5
SNP effect on Alpha−ketoglutarate levels

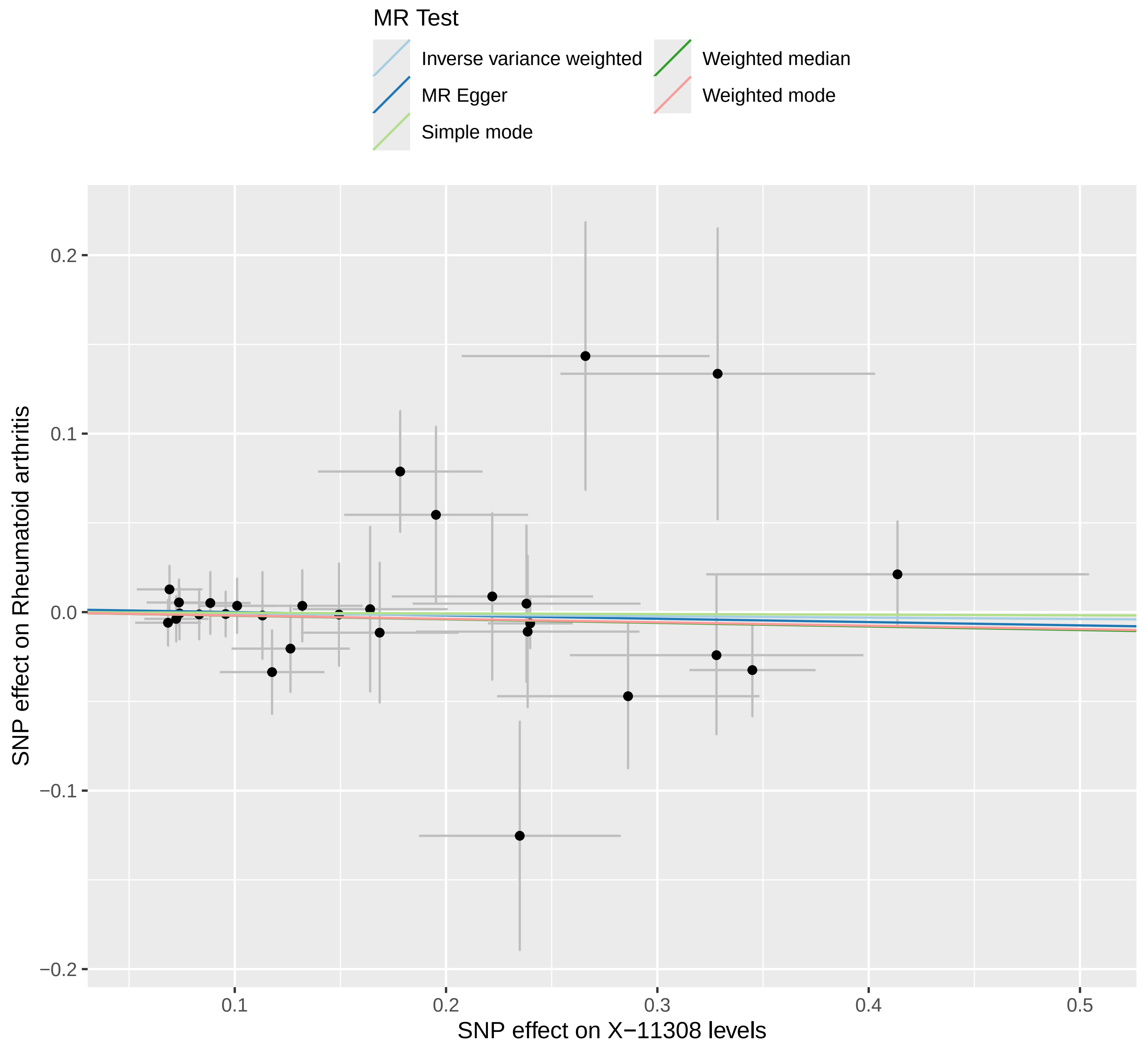

MR Test
Inverse variance weighted
MR Egger
Simple mode
Weighted median
Weighted mode
0.2
0.1
0.0
−0.1
−0.2
SNP effect on Rheumatoid arthritis
0.1
0.2
0.3
0.4
0.5
SNP effect on X−11308 levels

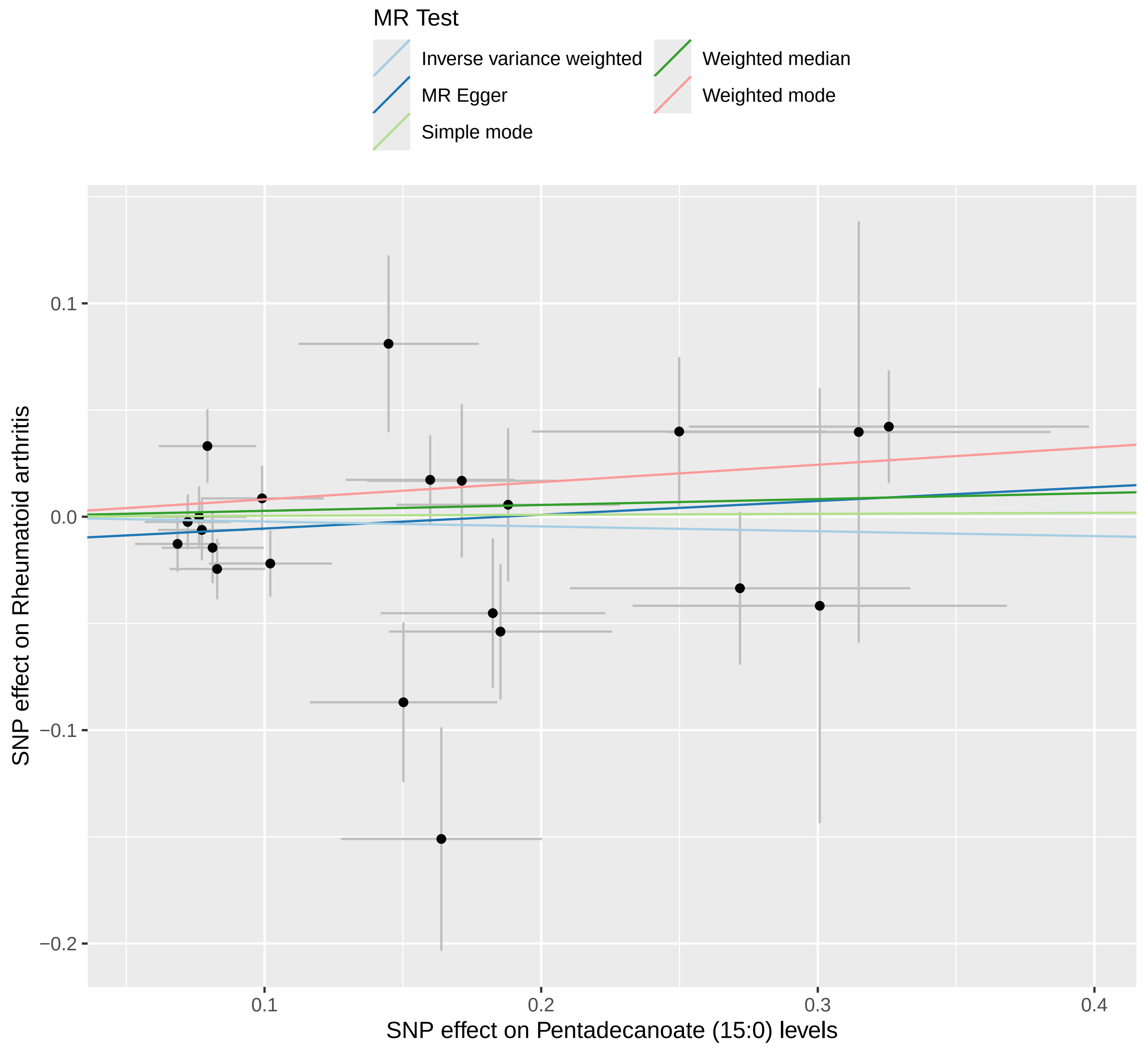
MR Test
Inverse variance weighted
MR Egger
Simple mode
Weighted median
Weighted mode
0.1
0.0
−0.1
−0.2
0.1
0.2
0.3
0.4
SNP effect on Rheumatoid arthritis
SNP effect on Pentadecanoate (15:0) levels

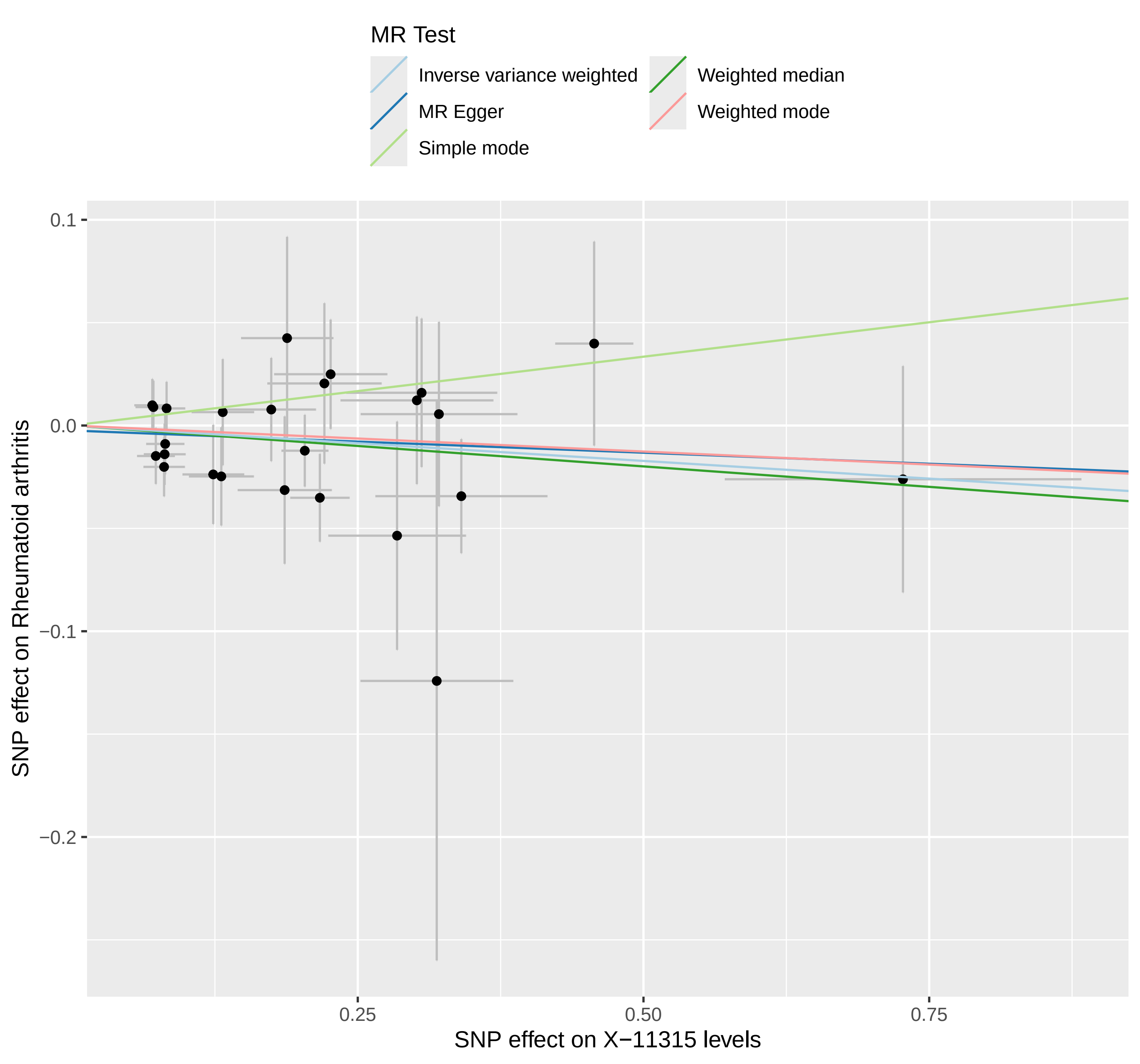
MR Test
Inverse variance weighted
MR Egger
Simple mode
Weighted median
Weighted mode
0.1
0.0
−0.1
−0.2
0.25
0.50
0.75
SNP effect on Rheumatoid arthritis
SNP effect on X−11315 levels

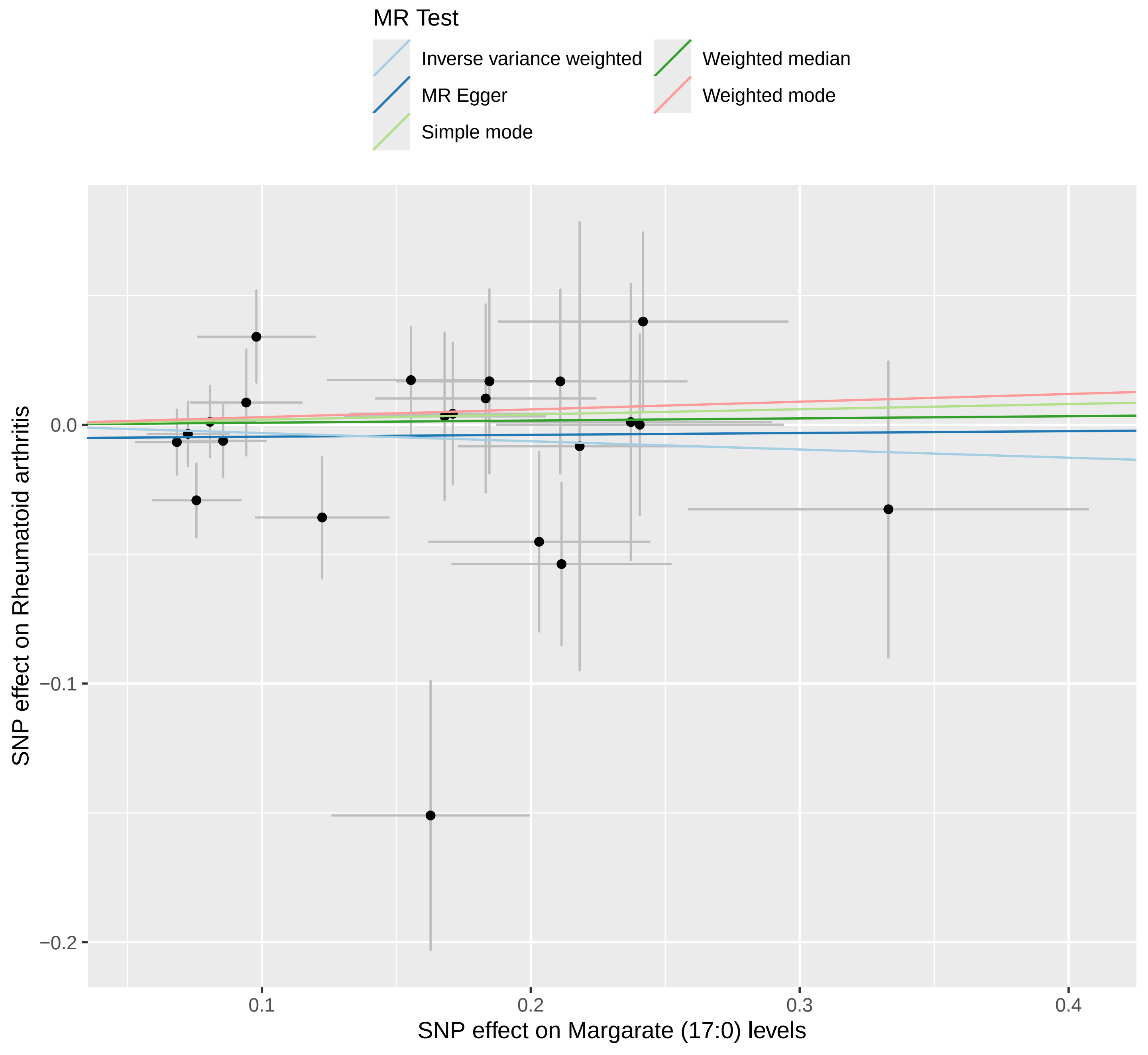
MR Test
Inverse variance weighted
MR Egger
Simple mode
Weighted median
Weighted mode
SNP effect on Rheumatoid arthritis
0.0
−0.1
−0.2
0.1
0.2
0.3
0.4
SNP effect on Margarate (17:0) levels

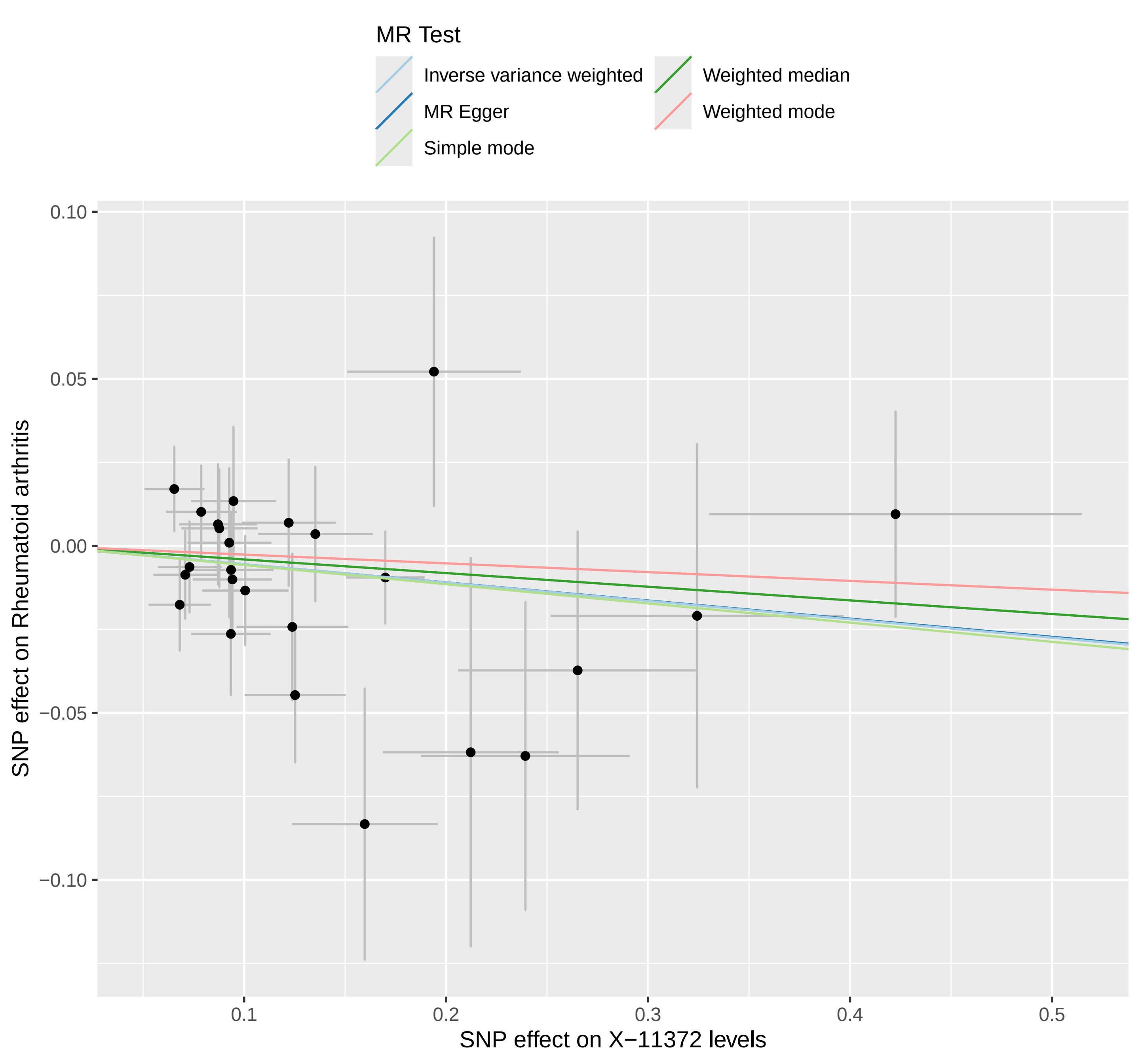
MR Test
Inverse variance weighted
MR Egger
Simple mode
Weighted median
Weighted mode
0.10
0.05
0.00
−0.05
−0.10
SNP effect on Rheumatoid arthritis
0.1
0.2
0.3
0.4
0.5
SNP effect on X−11372 levels

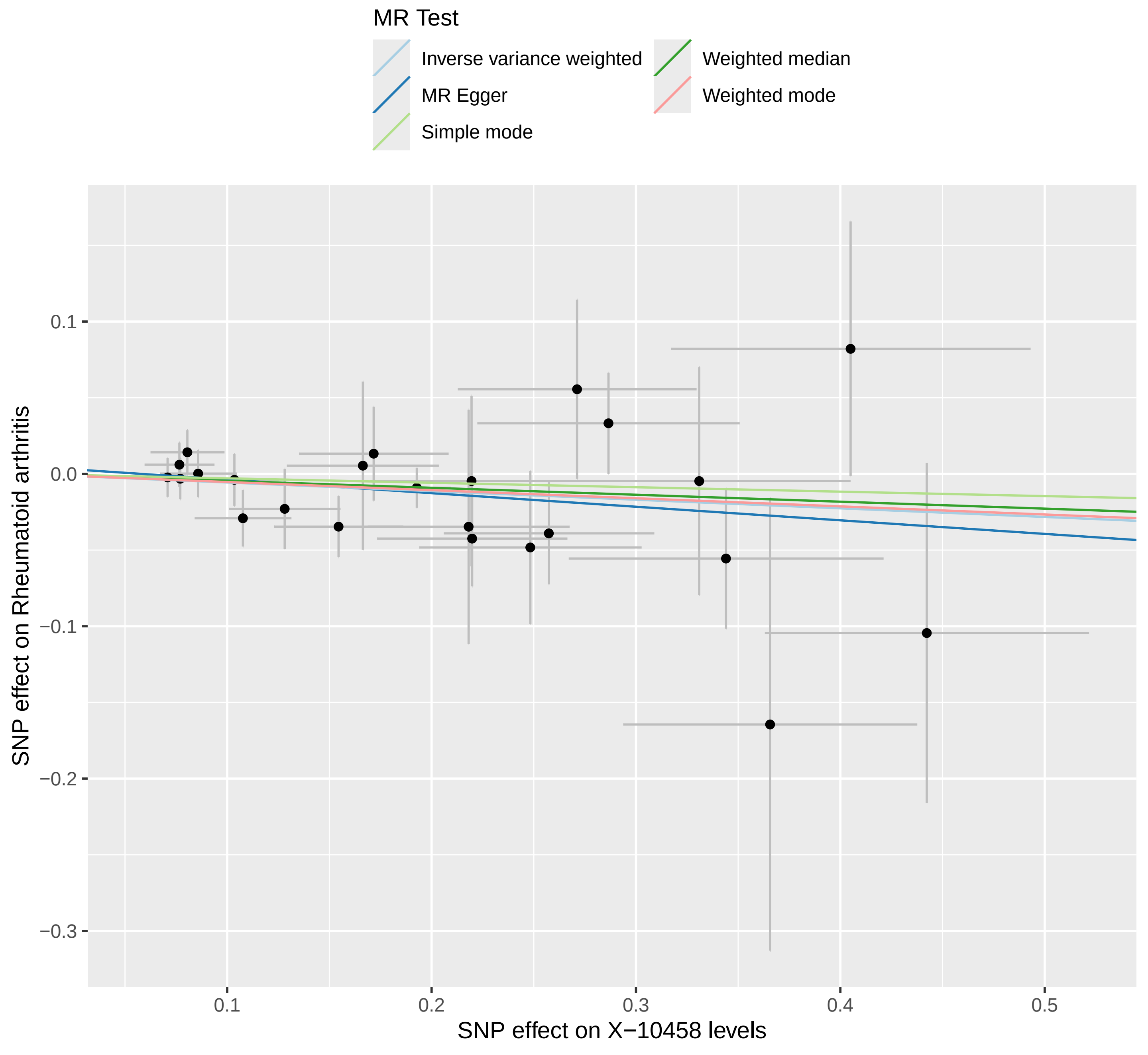

MR Test
Inverse variance weighted
MR Egger
Simple mode
Weighted median
Weighted mode
SNP effect on Rheumatoid arthritis
0.1
0.0
−0.1
−0.2
−0.3
0.1
0.2
0.3
0.4
0.5
SNP effect on X−10458 levels

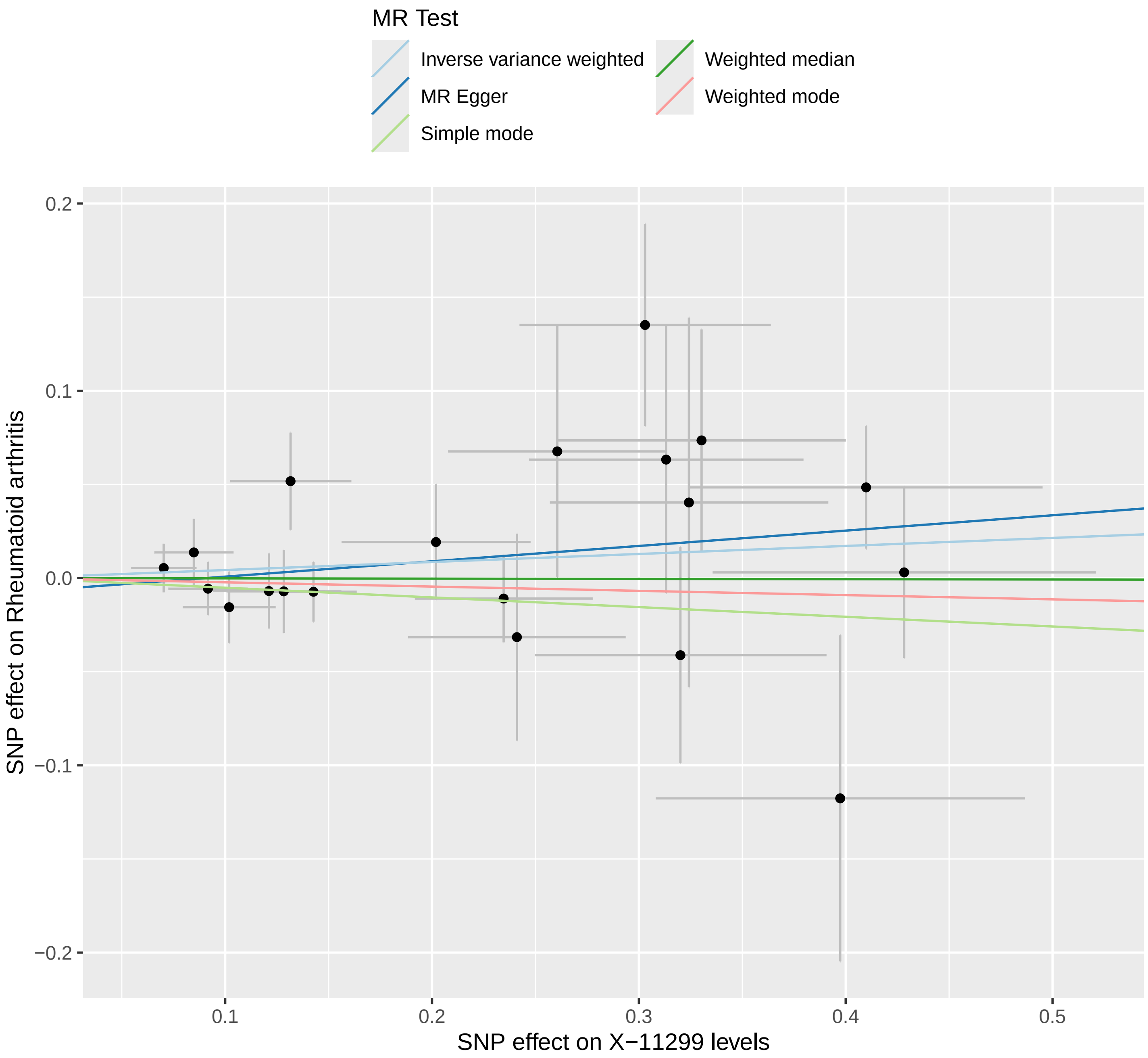
MR Test
Inverse variance weighted
MR Egger
Simple mode
Weighted median
Weighted mode
0.2
0.1
0.0
−0.1
−0.2
SNP effect on Rheumatoid arthritis
0.1
0.2
0.3
0.4
0.5
SNP effect on X−11299 levels

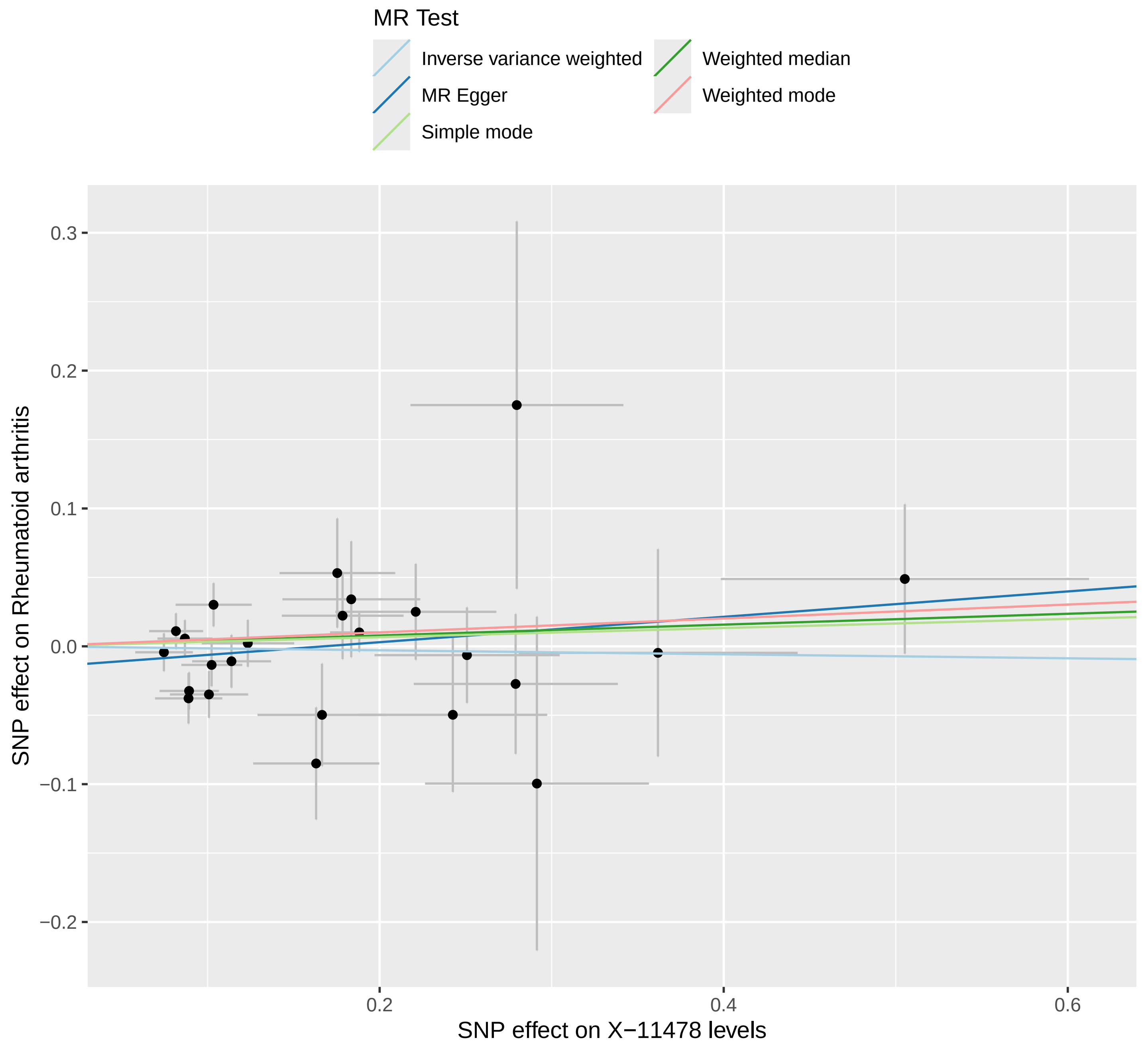

MR Test
Inverse variance weighted
MR Egger
Simple mode
Weighted median
Weighted mode
SNP effect on Rheumatoid arthritis
0.3
0.2
0.1
0.0
−0.1
−0.2
0.2
0.4
0.6
SNP effect on X−11478 levels

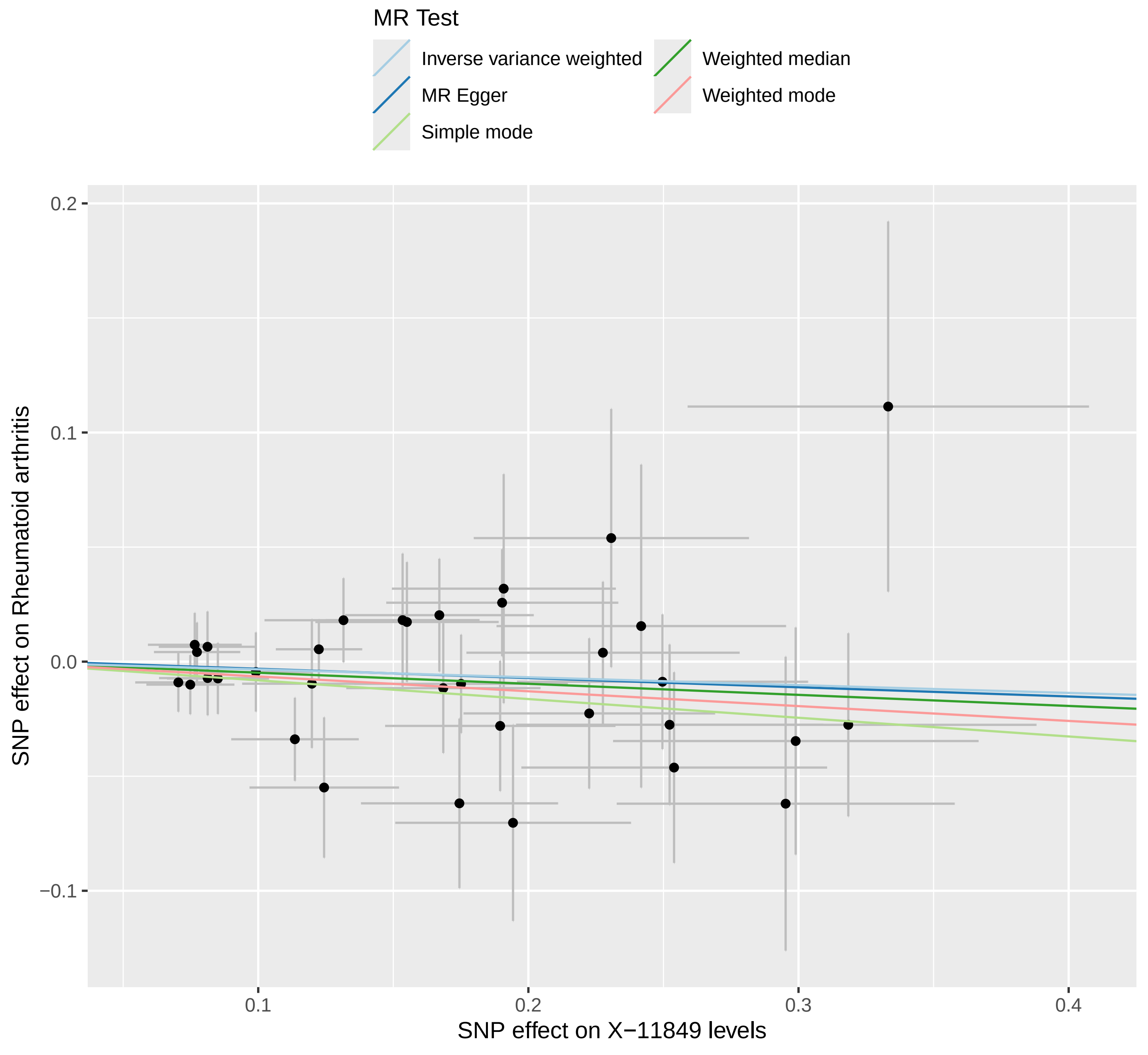
MR Test
Inverse variance weighted
MR Egger
Simple mode
Weighted median
Weighted mode
0.2
0.1
0.0
−0.1
0.1
0.2
0.3
0.4
SNP effect on X−11849 levels
SNP effect on Rheumatoid arthritis

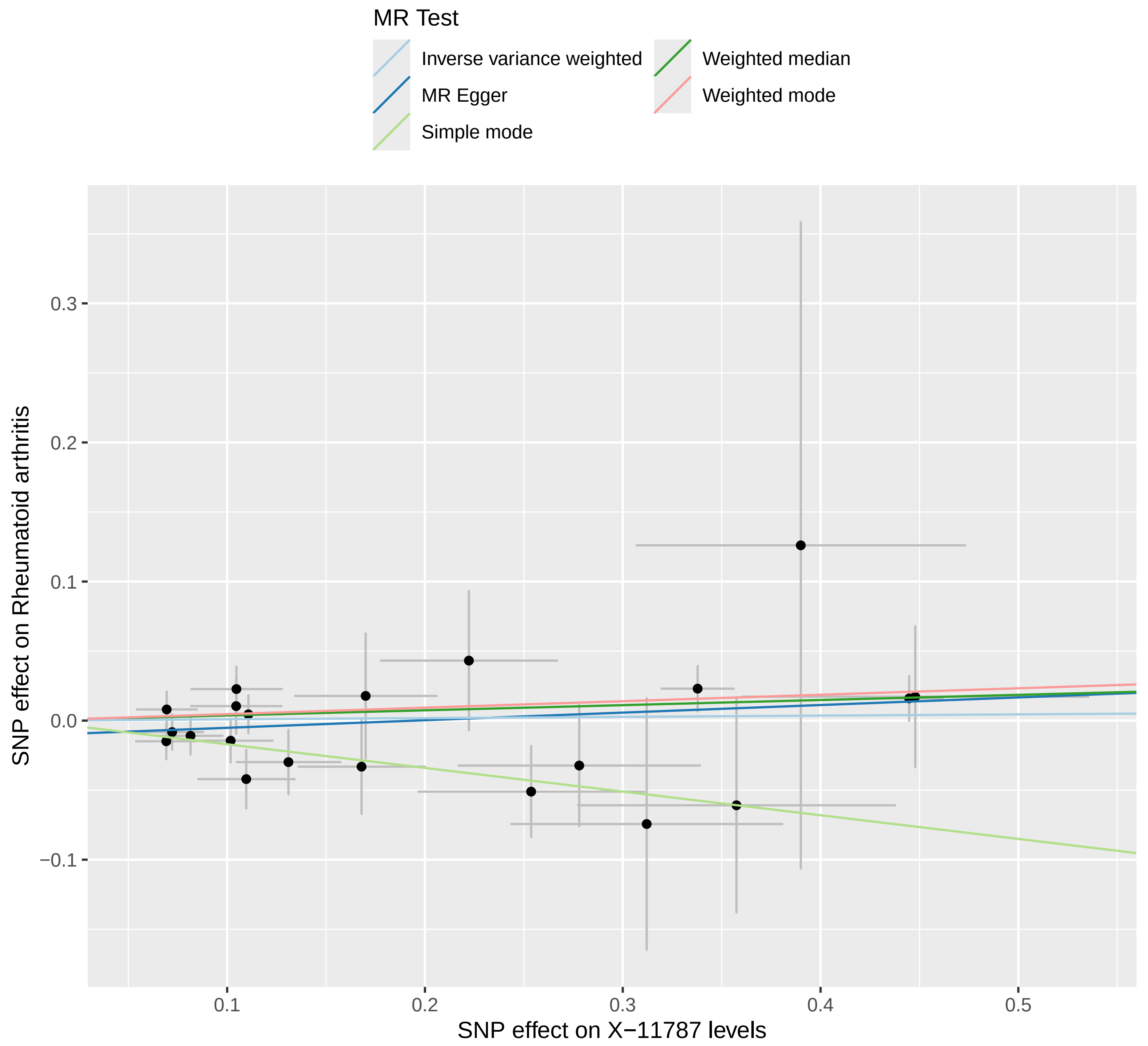
MR Test
Inverse variance weighted
MR Egger
Simple mode
Weighted median
Weighted mode
SNP effect on Rheumatoid arthritis
0.3
0.2
0.1
0.0
−0.1
0.1
0.2
0.3
0.4
0.5
SNP effect on X−11787 levels

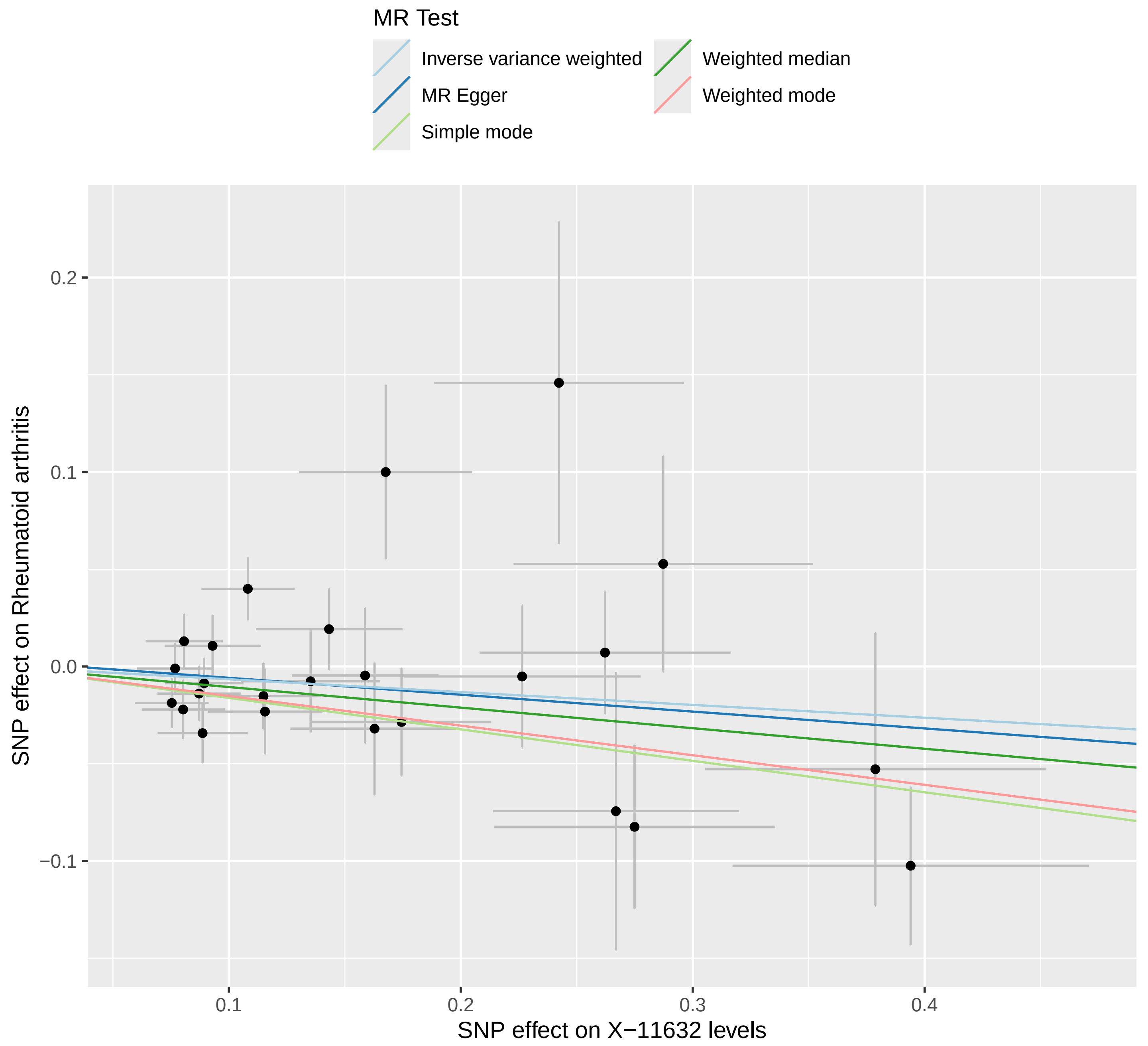
MR Test
Inverse variance weighted
MR Egger
Simple mode
Weighted median
Weighted mode
0.2
0.1
0.0
−0.1
0.1
0.2
0.3
0.4
SNP effect on Rheumatoid arthritis
SNP effect on X−11632 levels

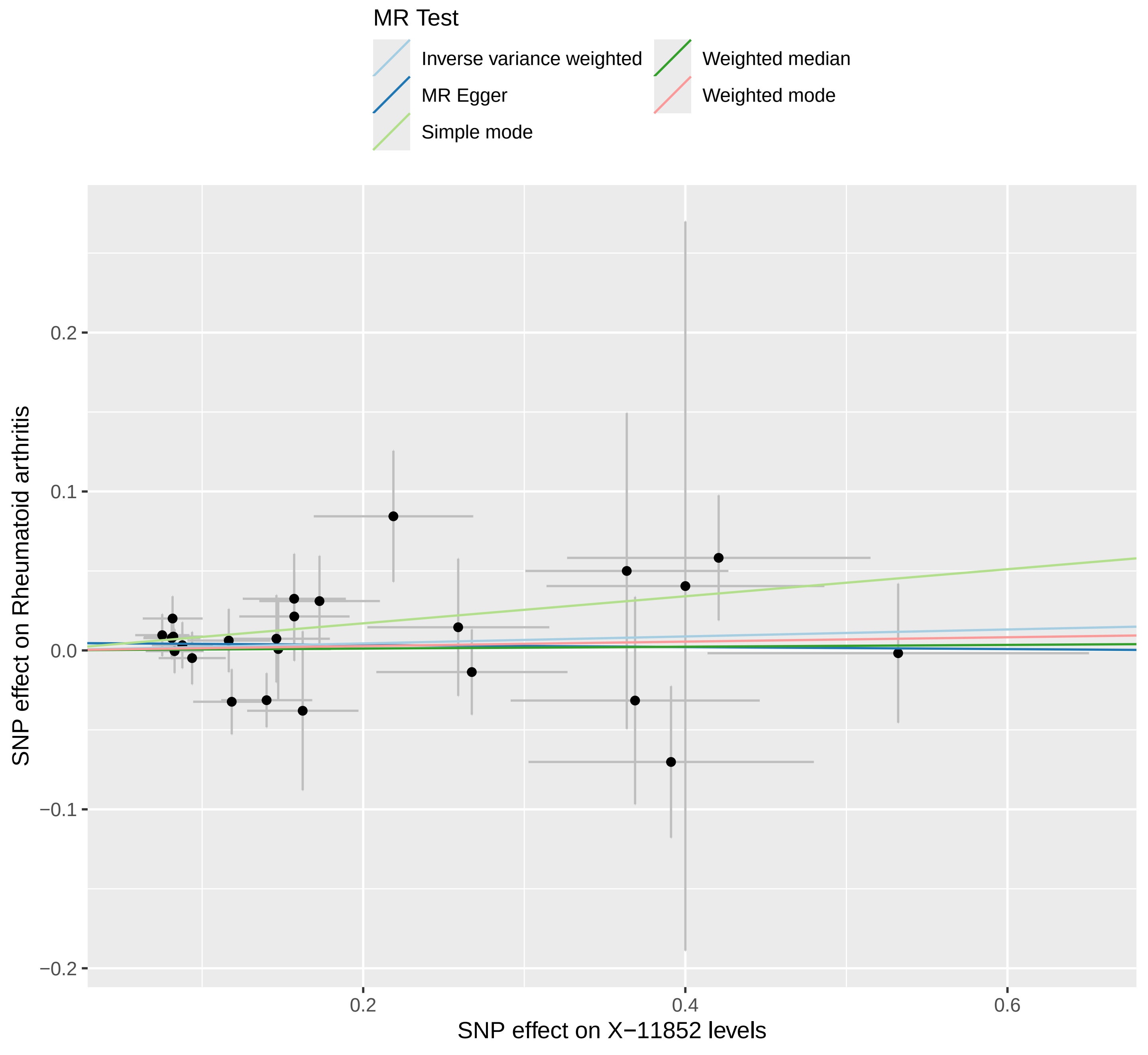
MR Test
Inverse variance weighted
MR Egger
Simple mode
Weighted median
Weighted mode
SNP effect on Rheumatoid arthritis
0.2
0.1
0.0
−0.1
−0.2
0.2
0.4
0.6
SNP effect on X−11852 levels

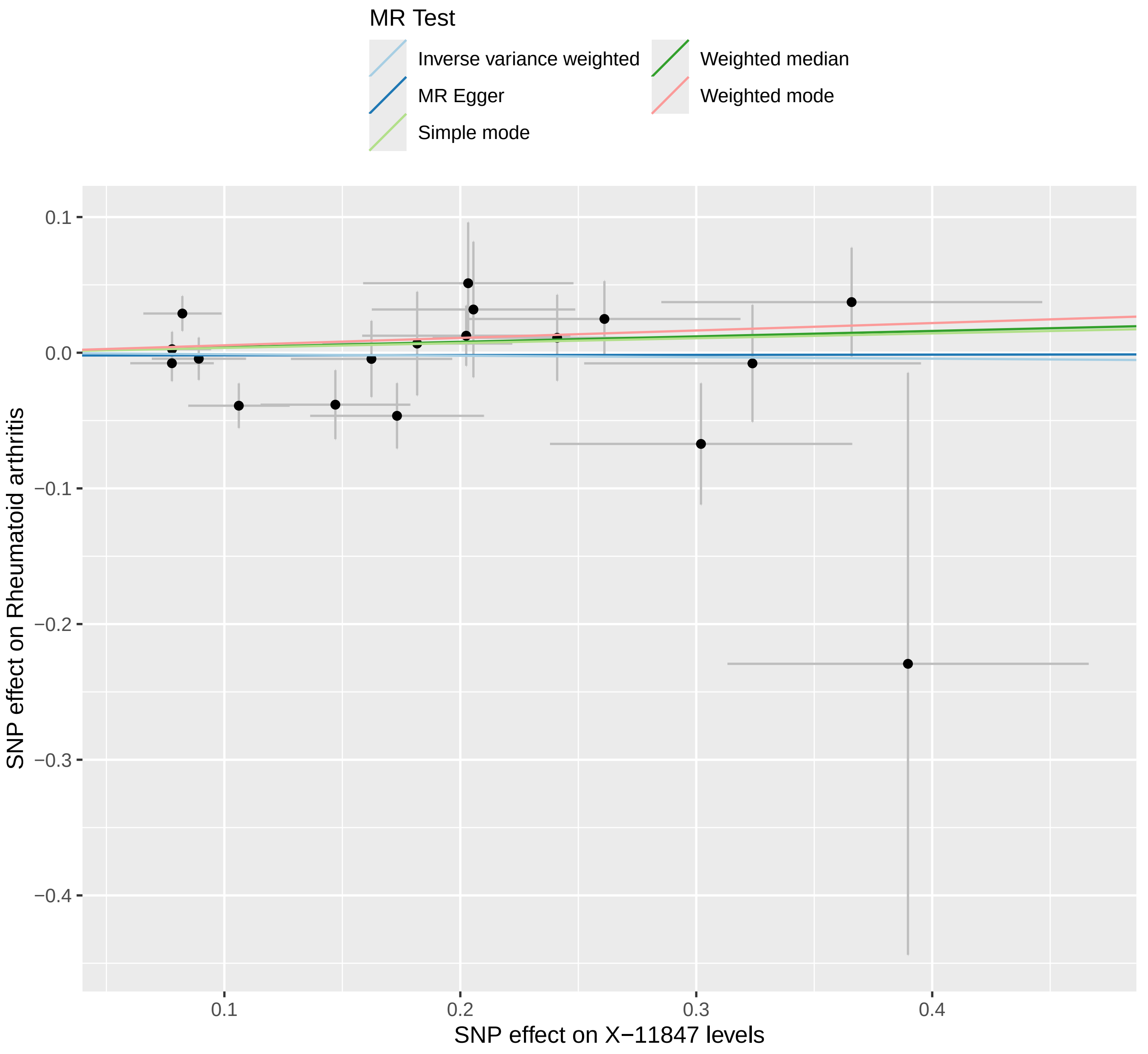
MR Test
Inverse variance weighted
MR Egger
Simple mode
Weighted median
Weighted mode
0.1
0.0
−0.1
−0.2
−0.3
−0.4
0.1
0.2
0.3
0.4
SNP effect on Rheumatoid arthritis
SNP effect on X−11847 levels

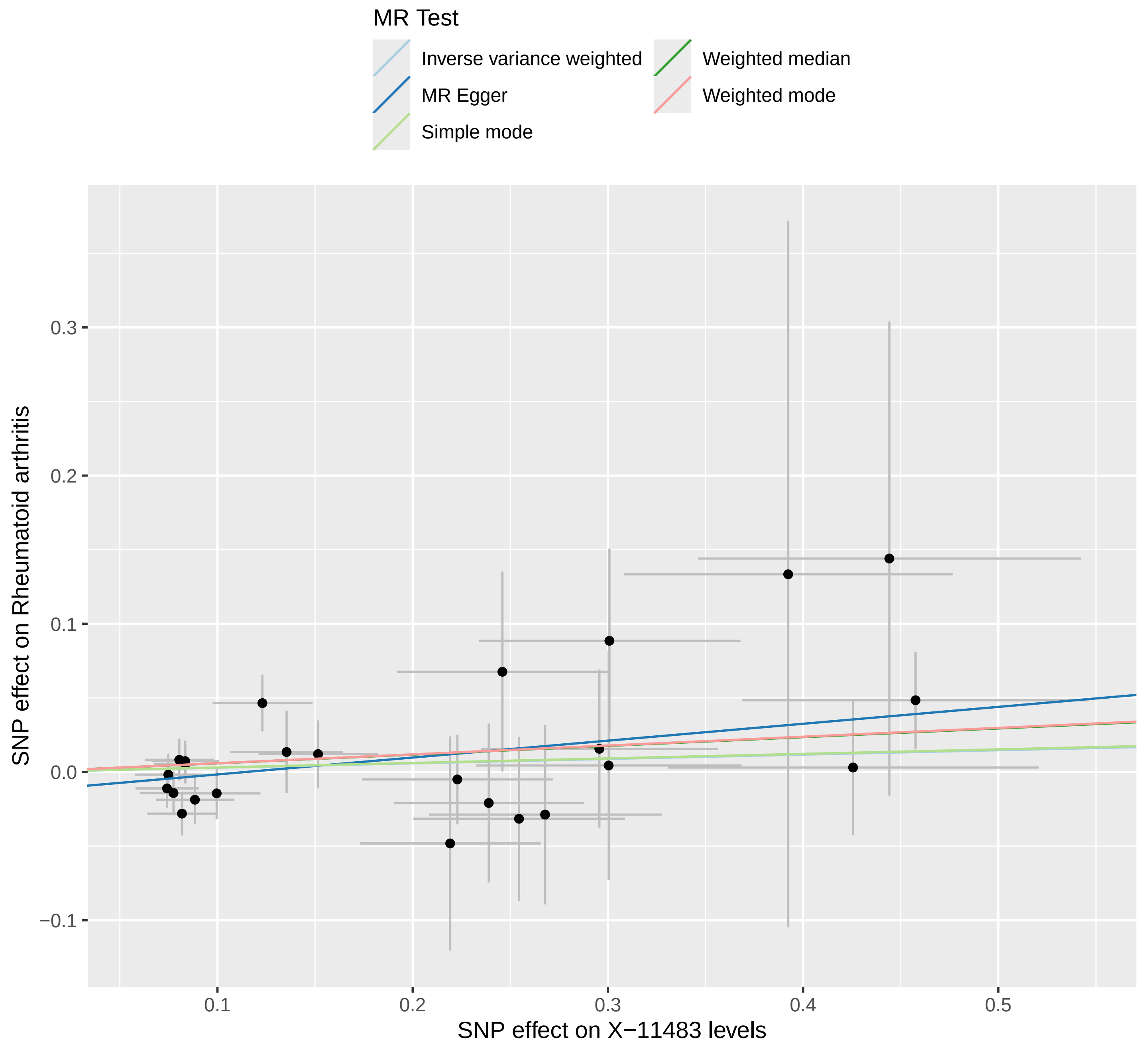
MR Test
Inverse variance weighted
MR Egger
Simple mode
Weighted median
Weighted mode
SNP effect on Rheumatoid arthritis
0.3
0.2
0.1
0.0
−0.1
0.1
0.2
0.3
0.4
0.5
SNP effect on X−11483 levels

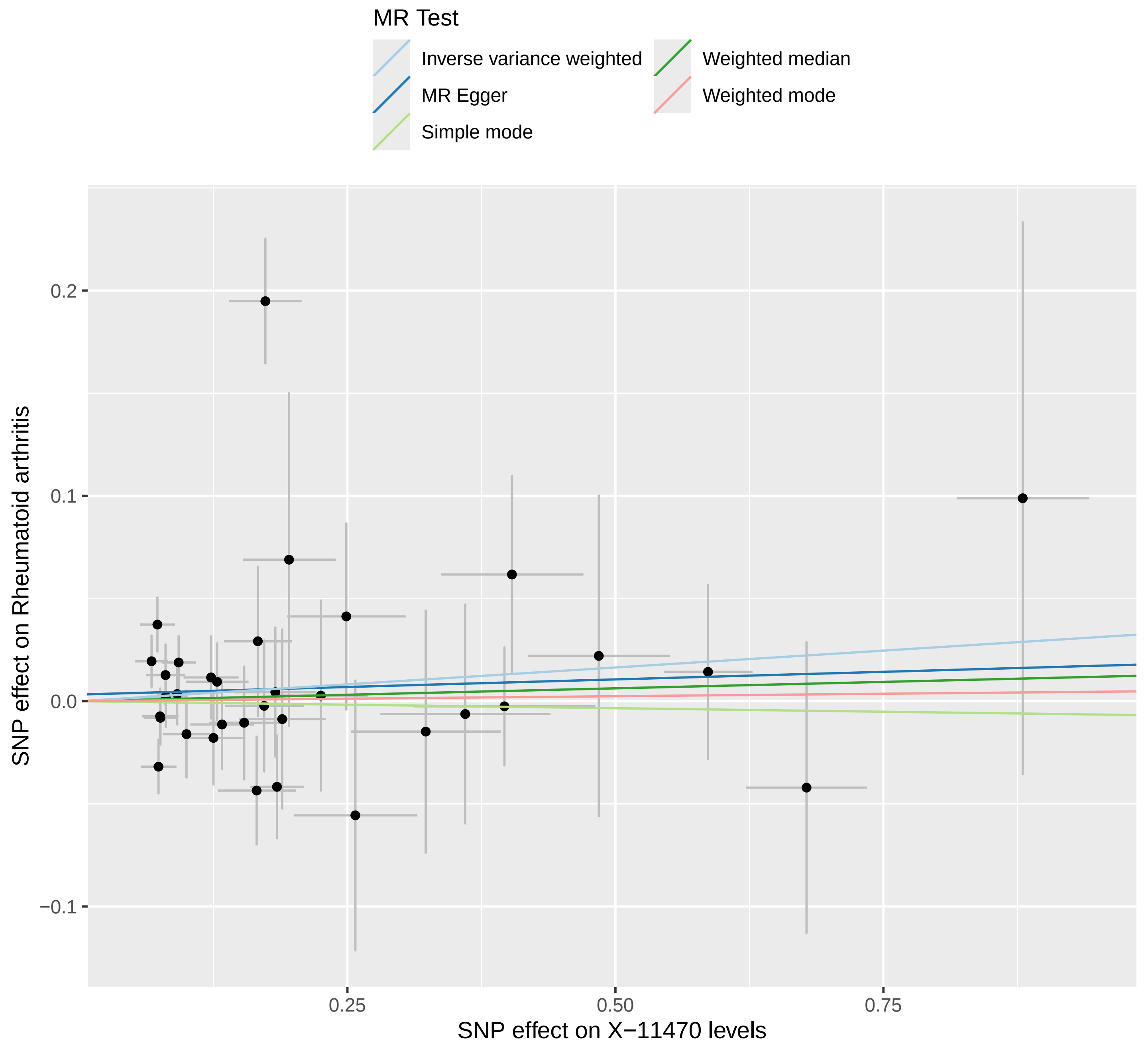
MR Test
Inverse variance weighted
MR Egger
Simple mode
Weighted median
Weighted mode
SNP effect on Rheumatoid arthritis
0.2
0.1
0.0
−0.1
0.25
0.50
0.75
SNP effect on X−11470 levels

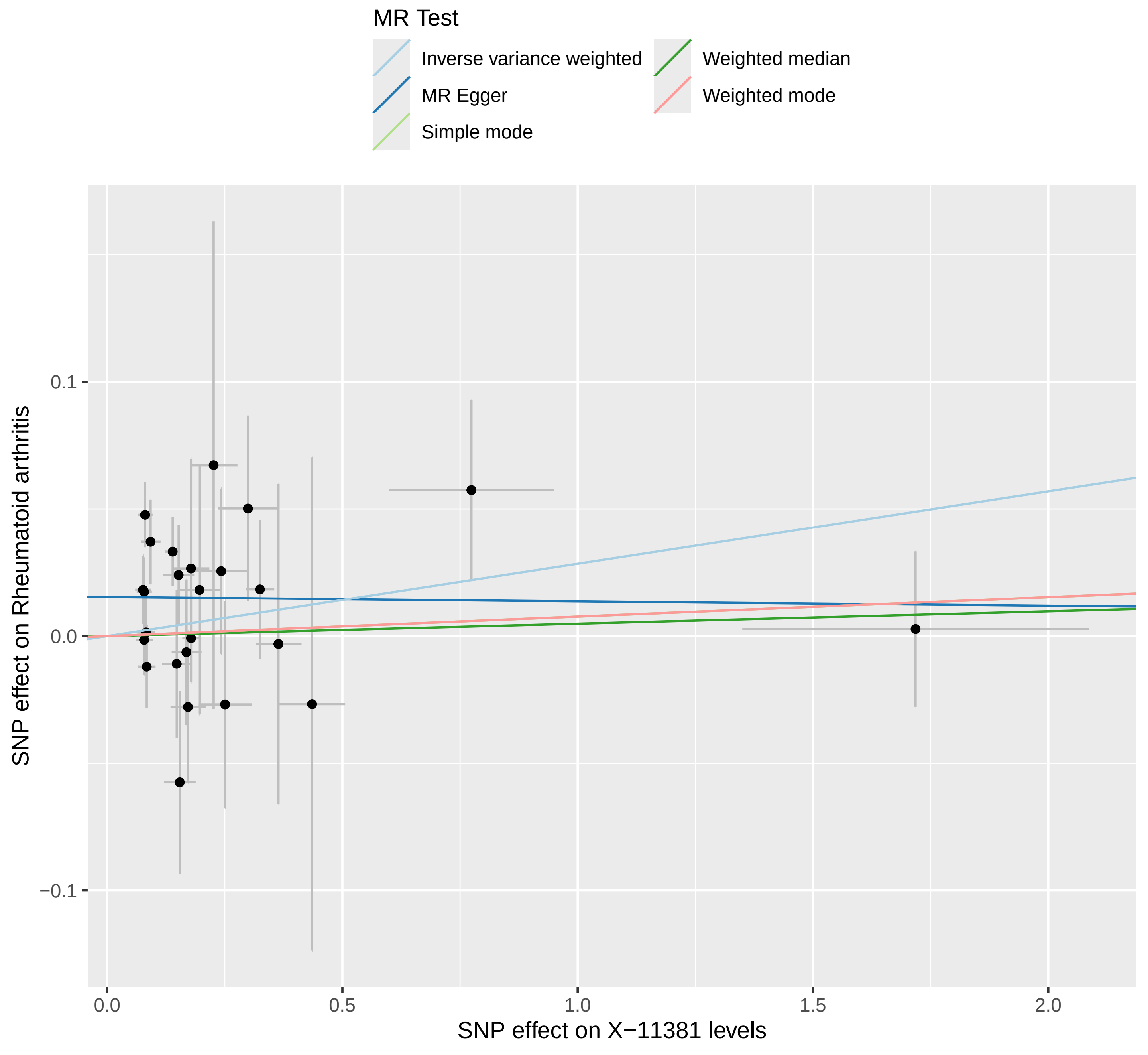
MR Test
Inverse variance weighted
MR Egger
Simple mode
Weighted median
Weighted mode
SNP effect on Rheumatoid arthritis
SNP effect on X−11381 levels
0.1
0.0
−0.1
0.0
0.5
1.0
1.5
2.0

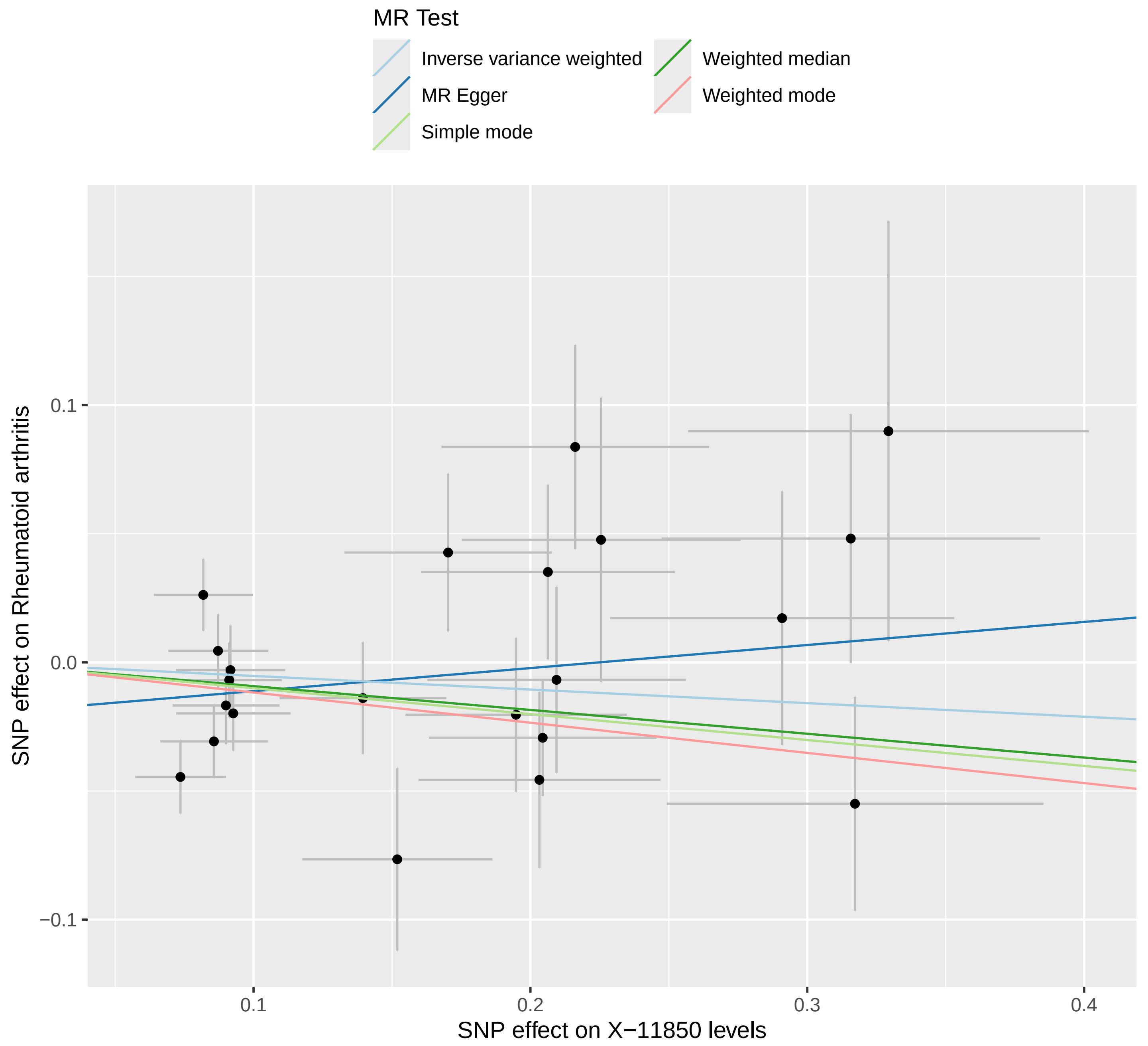
MR Test
Inverse variance weighted
MR Egger
Simple mode
Weighted median
Weighted mode
0.1
0.0
−0.1
0.1
0.2
0.3
0.4
SNP effect on Rheumatoid arthritis
SNP effect on X−11850 levels

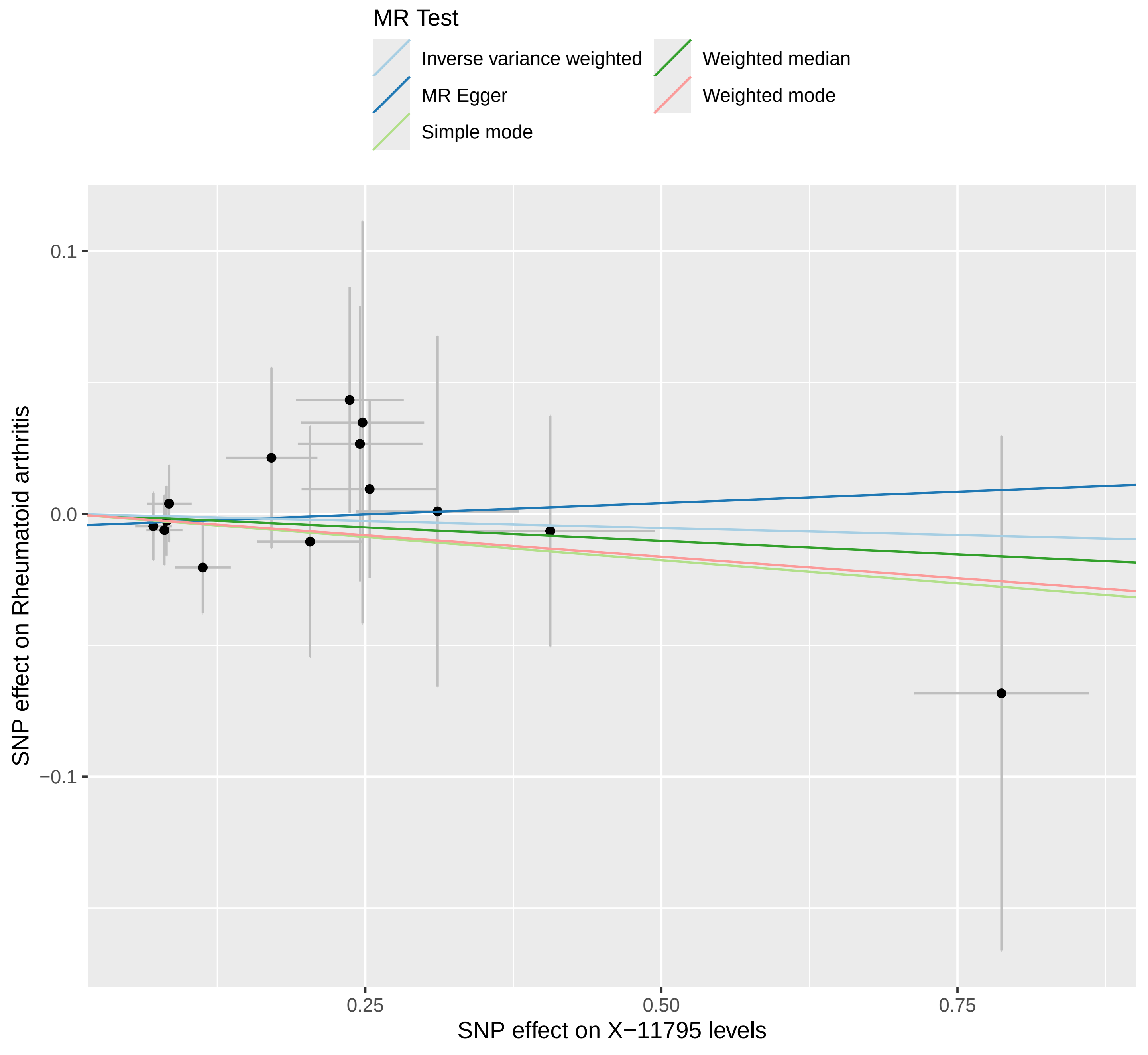
MR Test
Inverse variance weighted
MR Egger
Simple mode
Weighted median
Weighted mode
0.1
0.0
−0.1
0.25
0.50
0.75
SNP effect on Rheumatoid arthritis
SNP effect on X−11795 levels

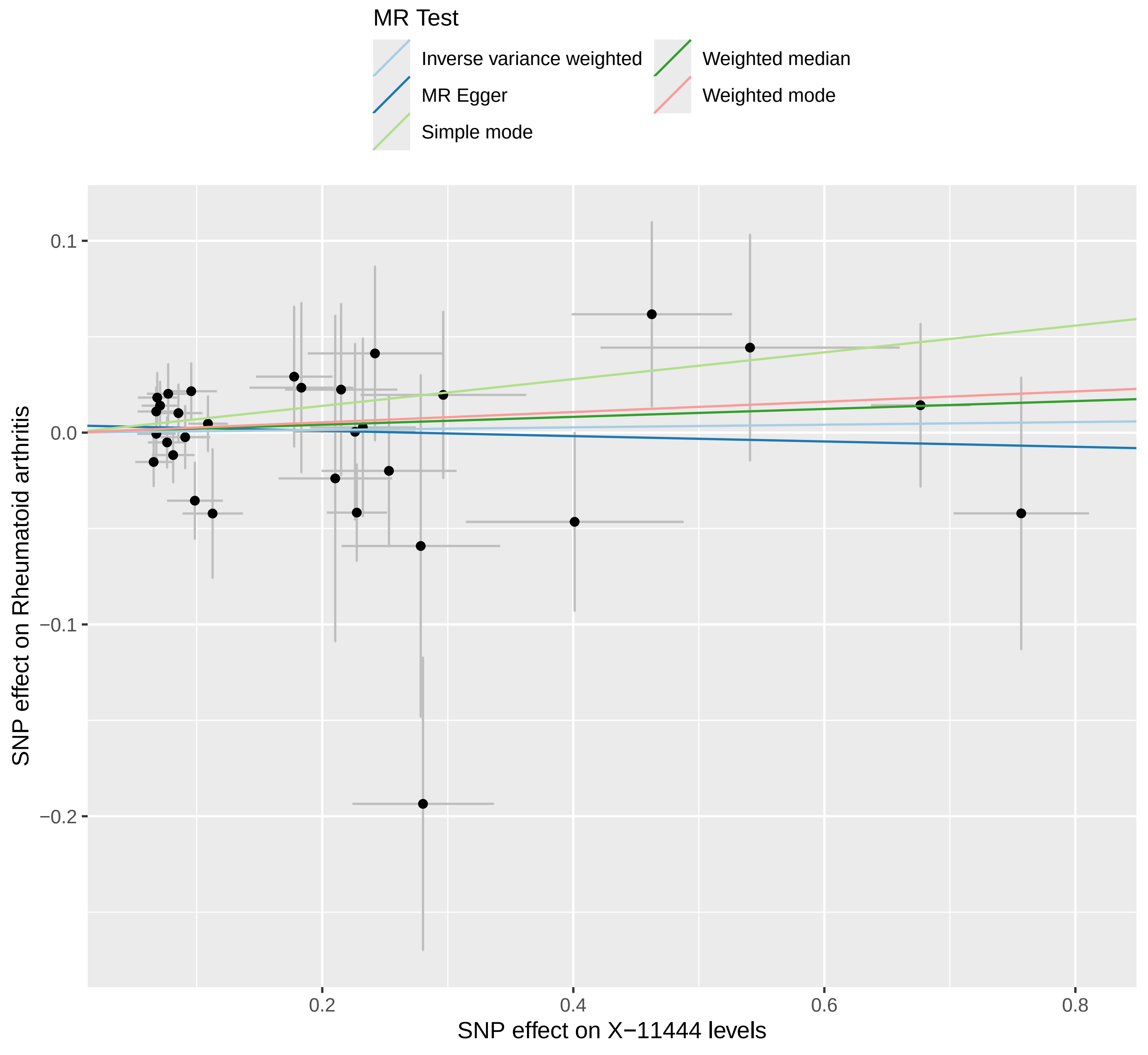

MR Test
Inverse variance weighted
MR Egger
Simple mode
Weighted median
Weighted mode
0.1
0.0
−0.1
−0.2
0.2
0.4
0.6
0.8
SNP effect on Rheumatoid arthritis
SNP effect on X−11444 levels

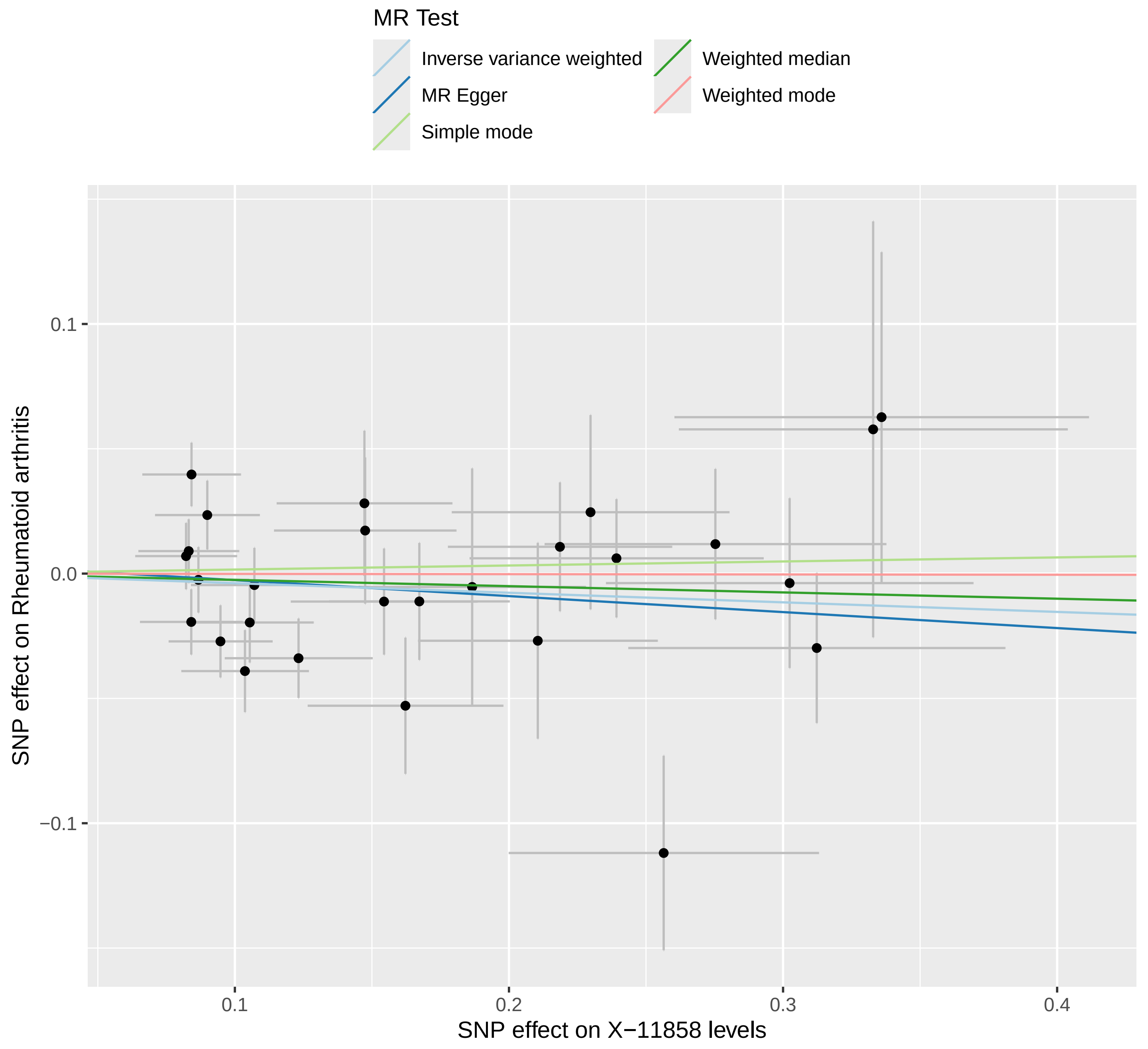
MR Test
Inverse variance weighted
MR Egger
Simple mode
Weighted median
Weighted mode
SNP effect on Rheumatoid arthritis
0.1
0.0
−0.1
0.1
0.2
0.3
0.4
SNP effect on X−11858 levels

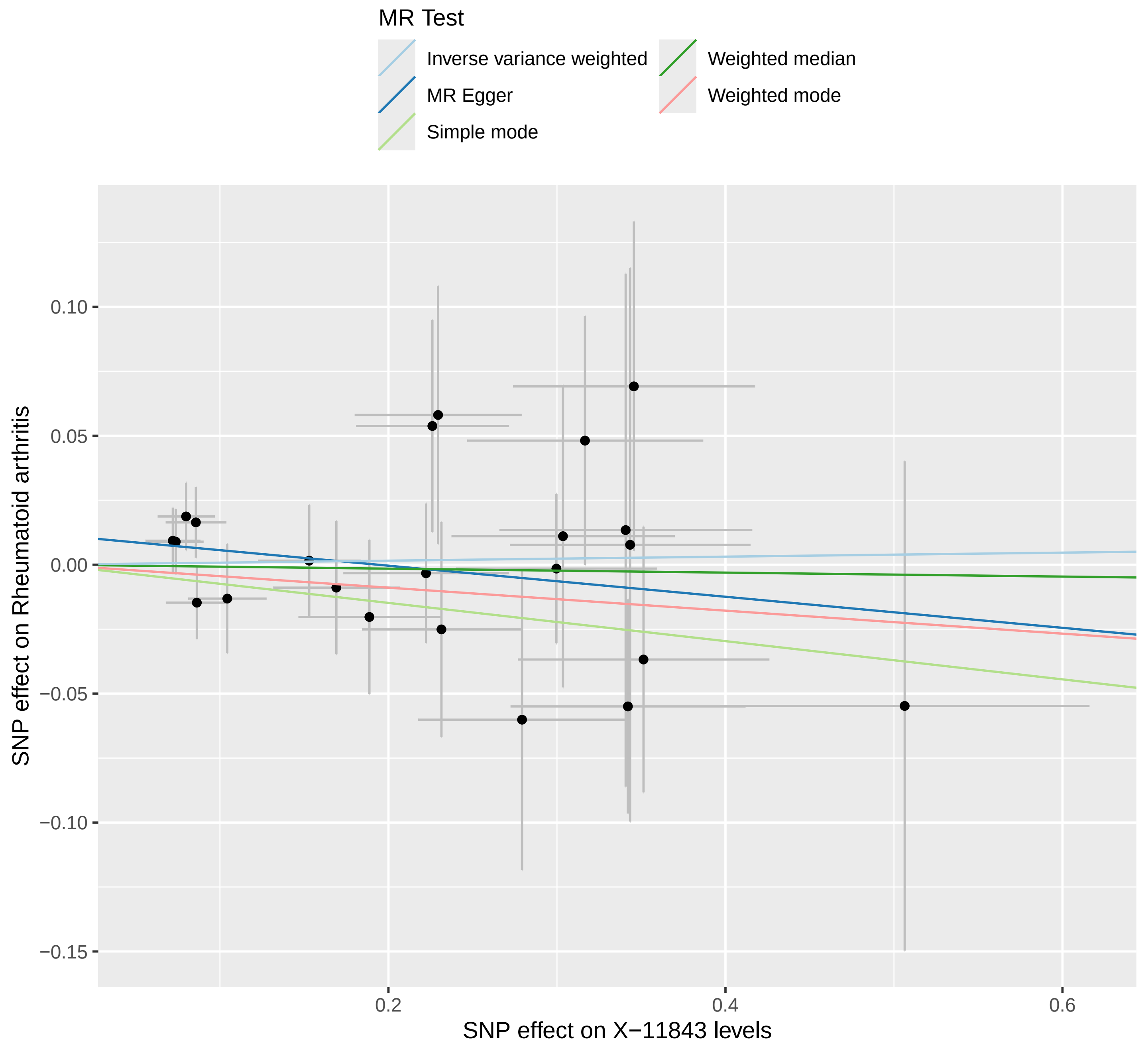
MR Test
Inverse variance weighted
MR Egger
Simple mode
Weighted median
Weighted mode
SNP effect on Rheumatoid arthritis
0.10
0.05
0.00
−0.05
−0.10
−0.15
0.2
0.4
0.6
SNP effect on X−11843 levels

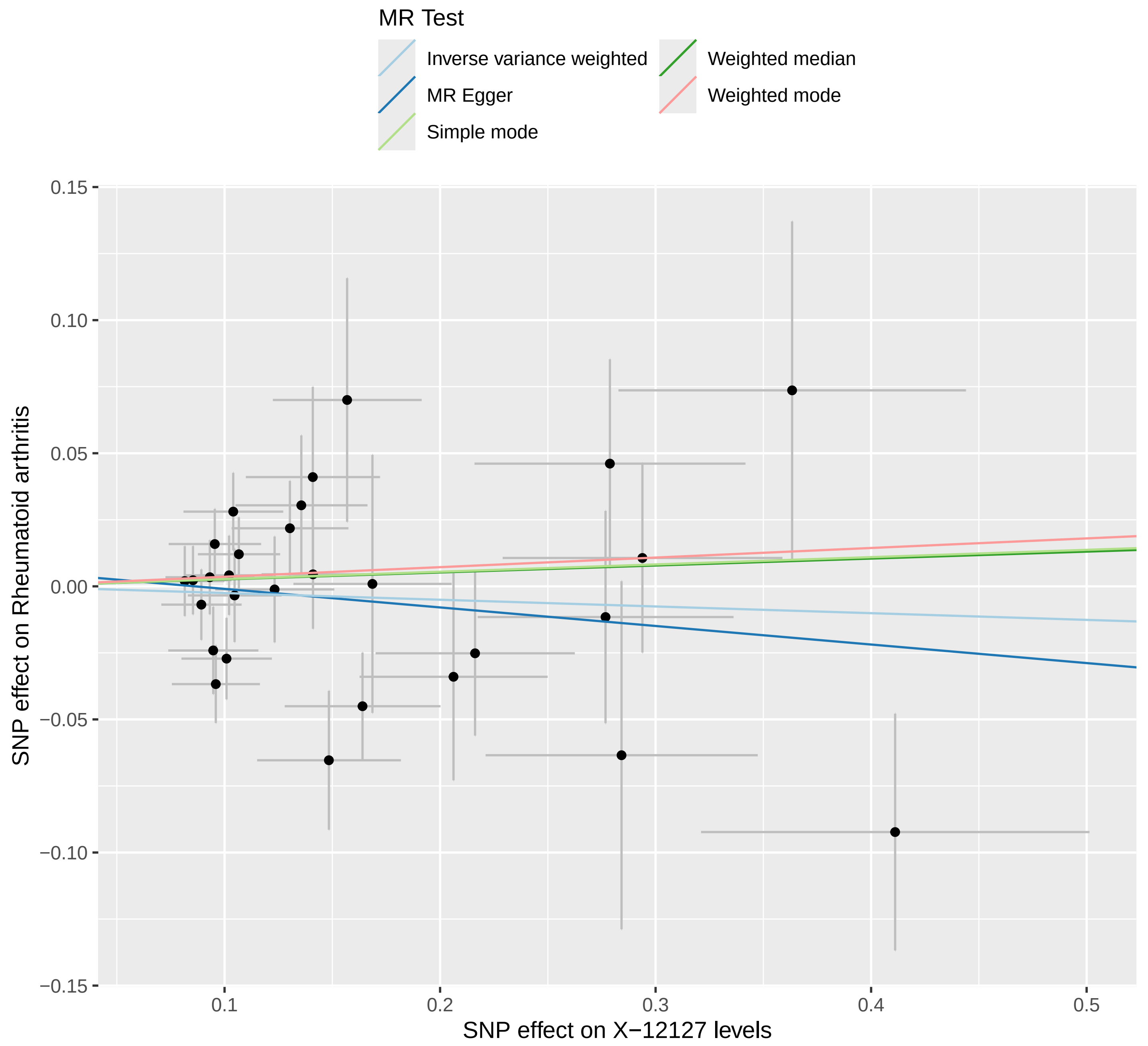
MR Test
Inverse variance weighted
MR Egger
Simple mode
Weighted median
Weighted mode
0.15
0.10
0.05
0.00
−0.05
−0.10
−0.15
SNP effect on Rheumatoid arthritis
0.1
0.2
0.3
0.4
0.5
SNP effect on X−12127 levels

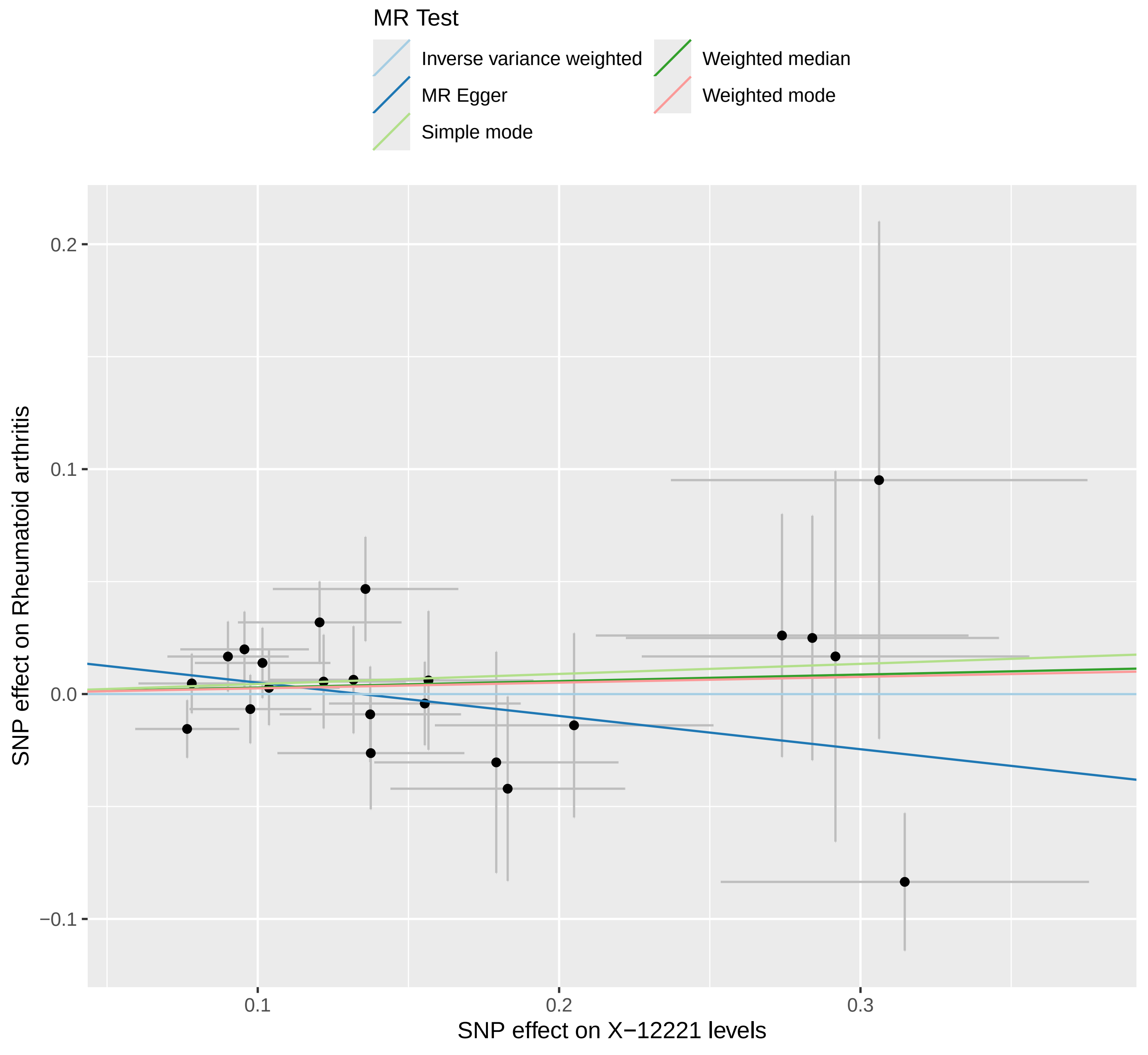
MR Test
Inverse variance weighted
MR Egger
Simple mode
Weighted median
Weighted mode
0.2
0.1
0.0
−0.1
SNP effect on Rheumatoid arthritis
0.1
0.2
0.3
SNP effect on X−12221 levels

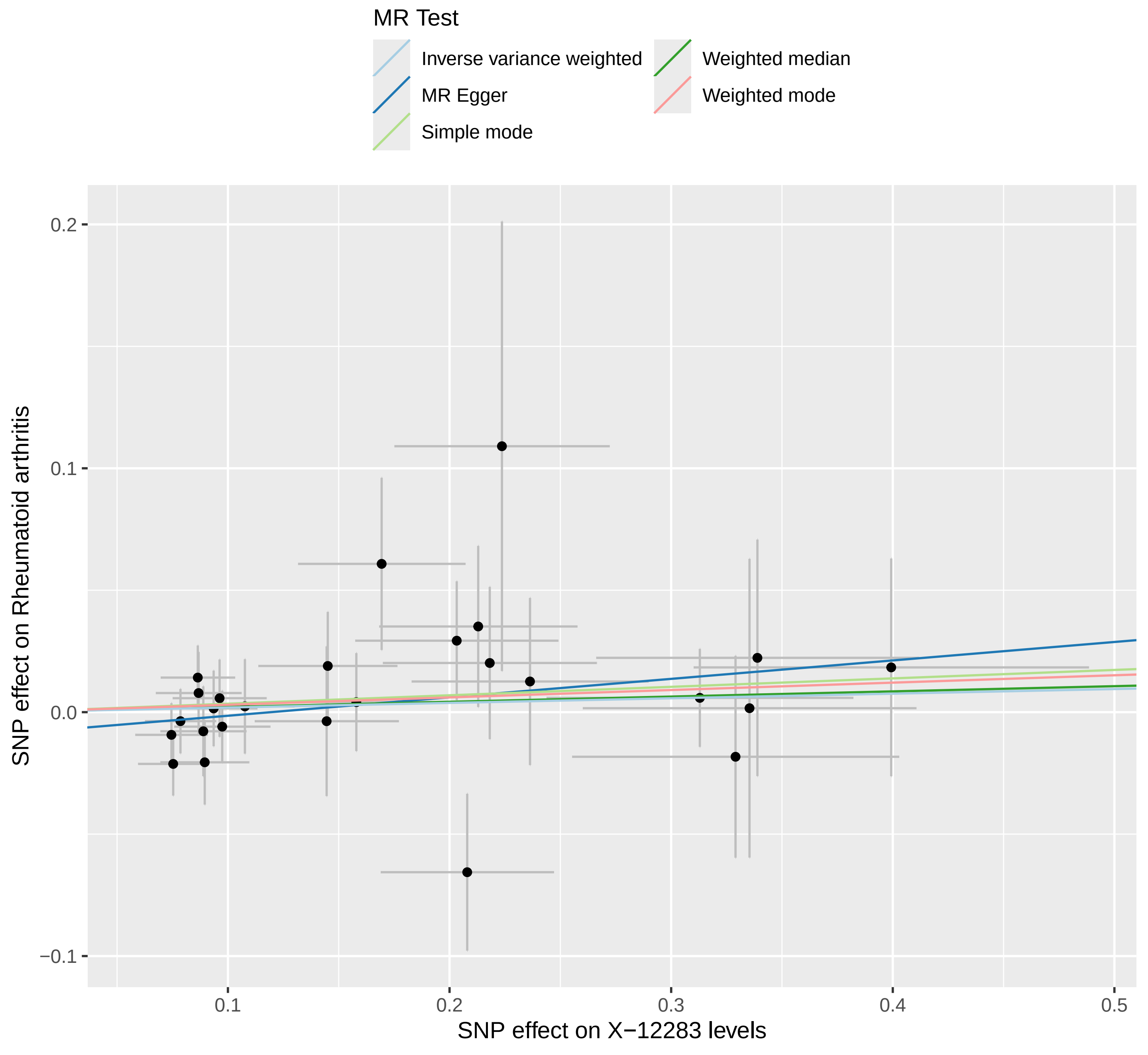
MR Test
Inverse variance weighted
Weighted median
MR Egger
Weighted mode
Simple mode
0.2
0.1
0.0
−0.1
SNP effect on Rheumatoid arthritis
0.1
0.2
0.3
0.4
0.5
SNP effect on X−12283 levels

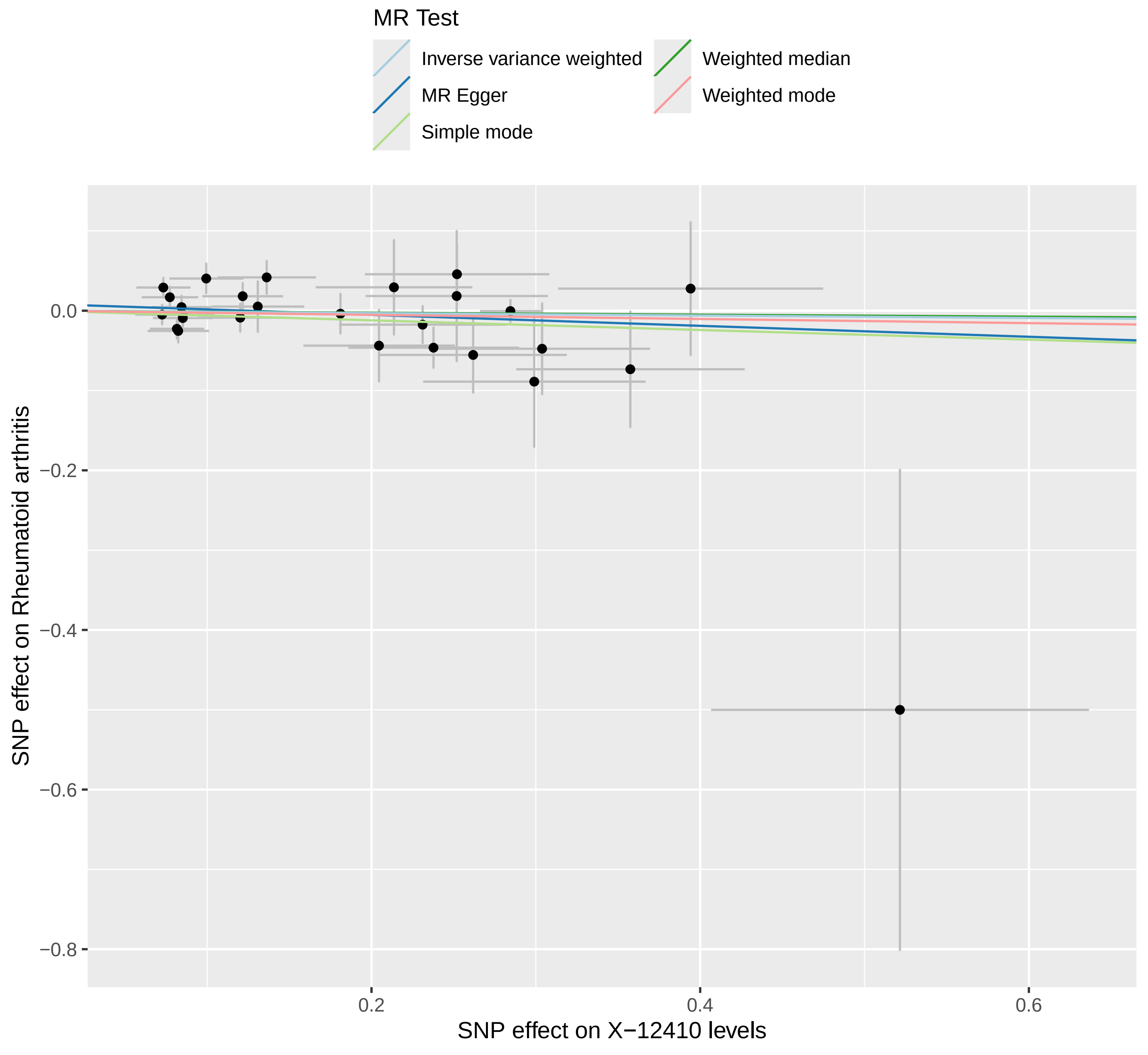

MR Test
Inverse variance weighted
MR Egger
Simple mode
Weighted median
Weighted mode
0.0
−0.2
−0.4
−0.6
−0.8
0.2
0.4
0.6
SNP effect on Rheumatoid arthritis
SNP effect on X−12410 levels

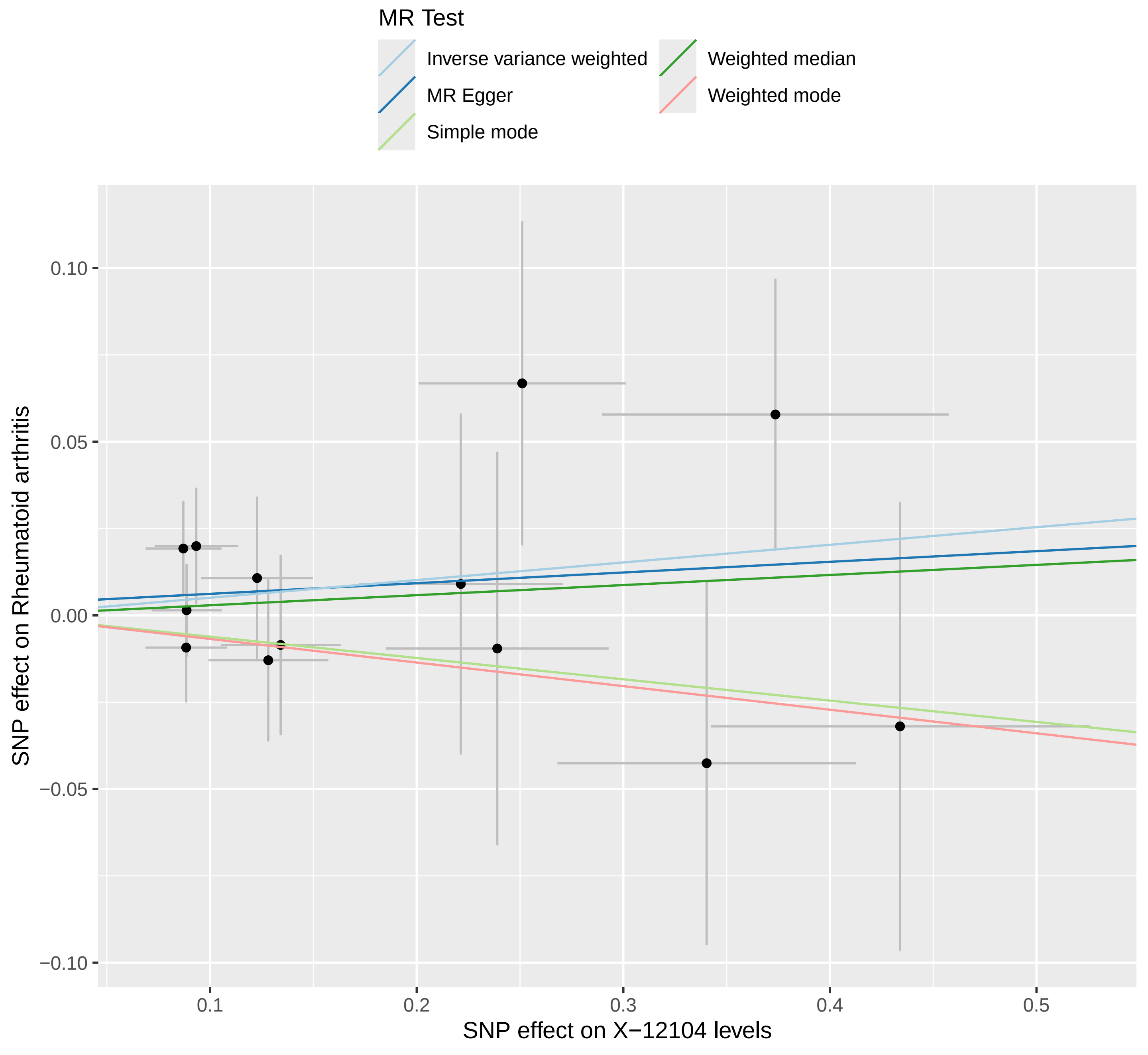

MR Test
Inverse variance weighted
MR Egger
Simple mode
Weighted median
Weighted mode
0.10
0.05
0.00
−0.05
−0.10
SNP effect on Rheumatoid arthritis
0.1
0.2
0.3
0.4
0.5
SNP effect on X−12104 levels

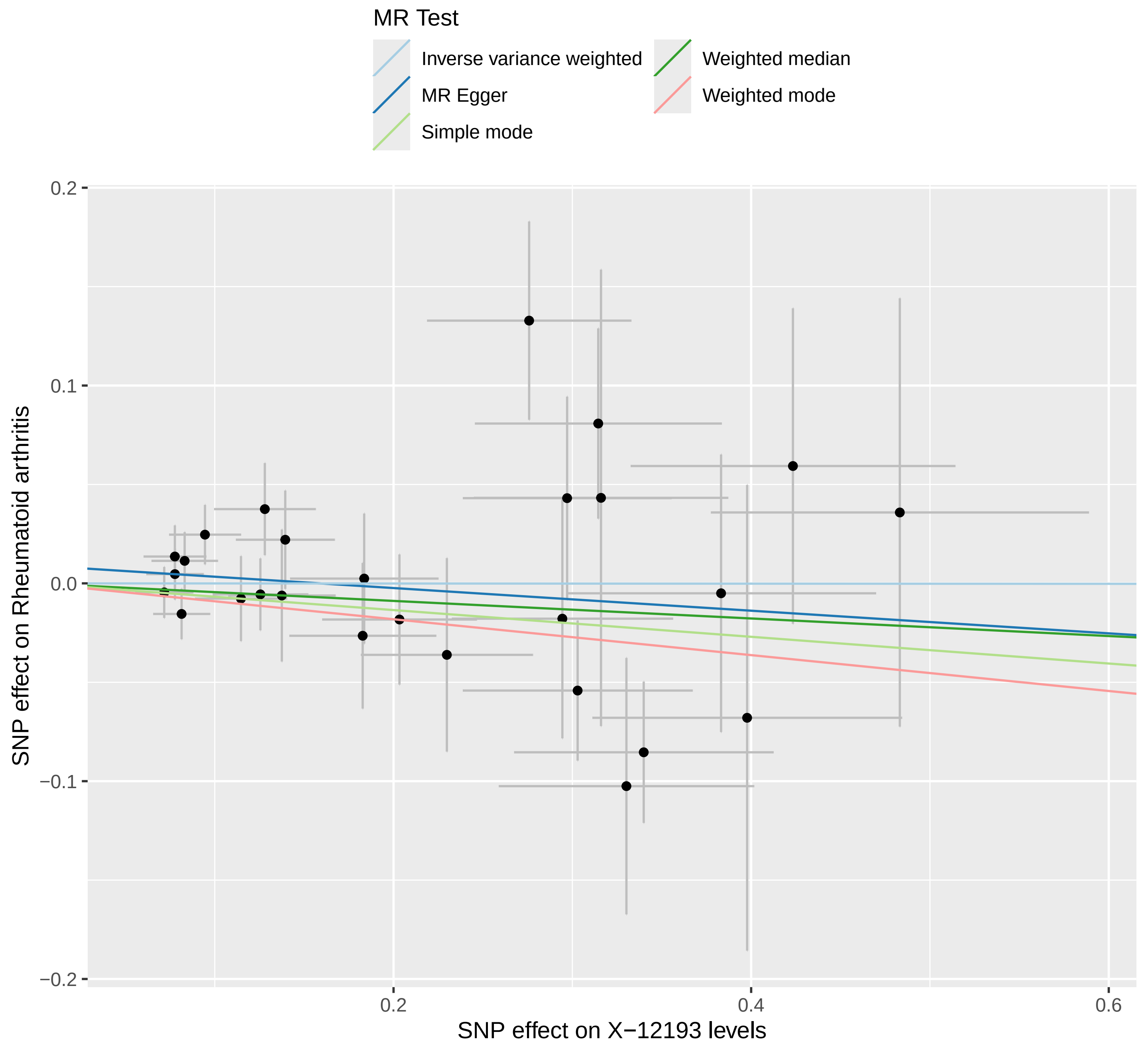
MR Test
Inverse variance weighted
MR Egger
Simple mode
Weighted median
Weighted mode
0.2
0.1
0.0
−0.1
−0.2
SNP effect on Rheumatoid arthritis
0.2
0.4
0.6
SNP effect on X−12193 levels

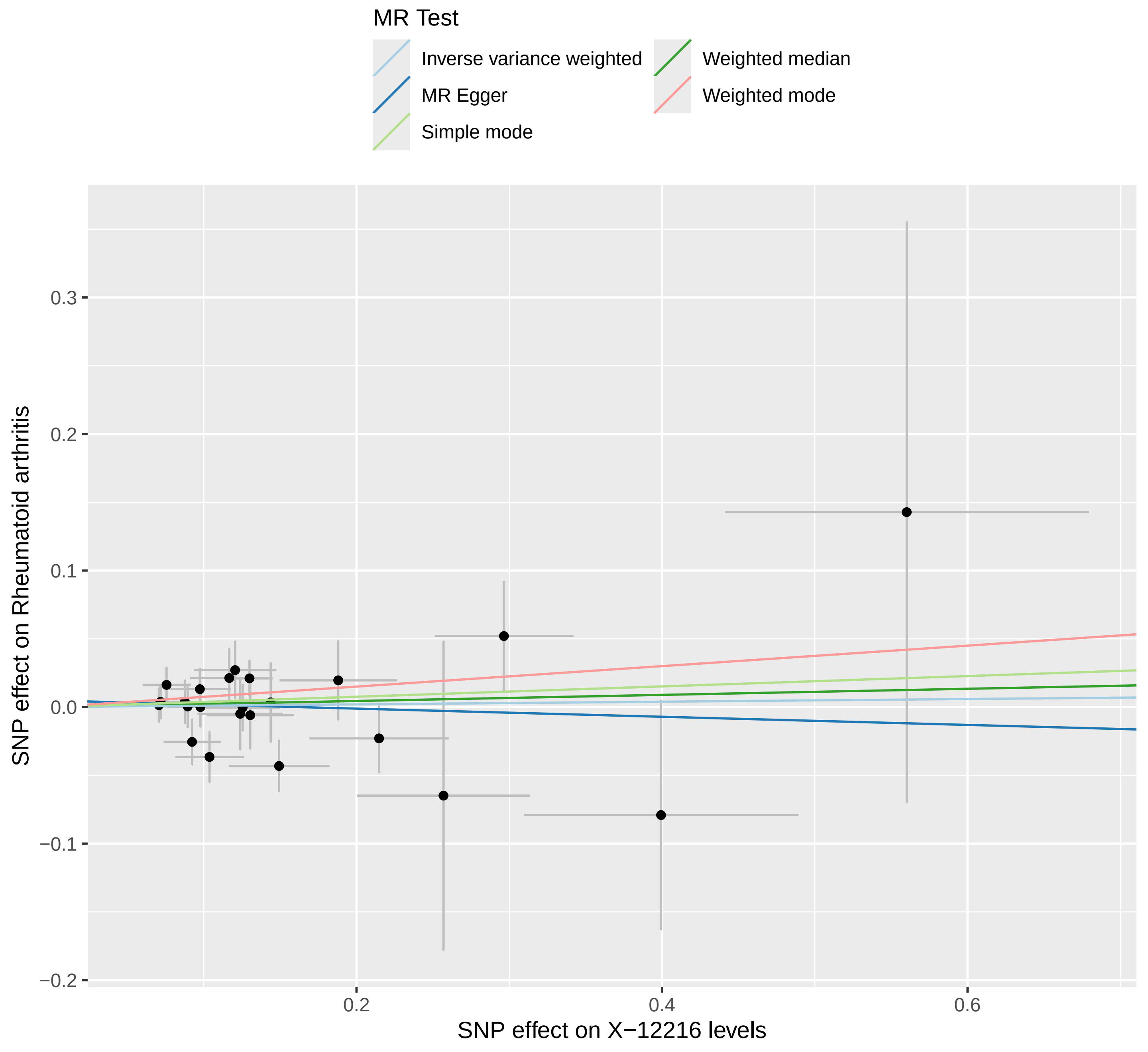

MR Test
Inverse variance weighted
MR Egger
Simple mode
Weighted median
Weighted mode
0.3
0.2
0.1
0.0
−0.1
−0.2
0.2
0.4
0.6
SNP effect on Rheumatoid arthritis
SNP effect on X−12216 levels

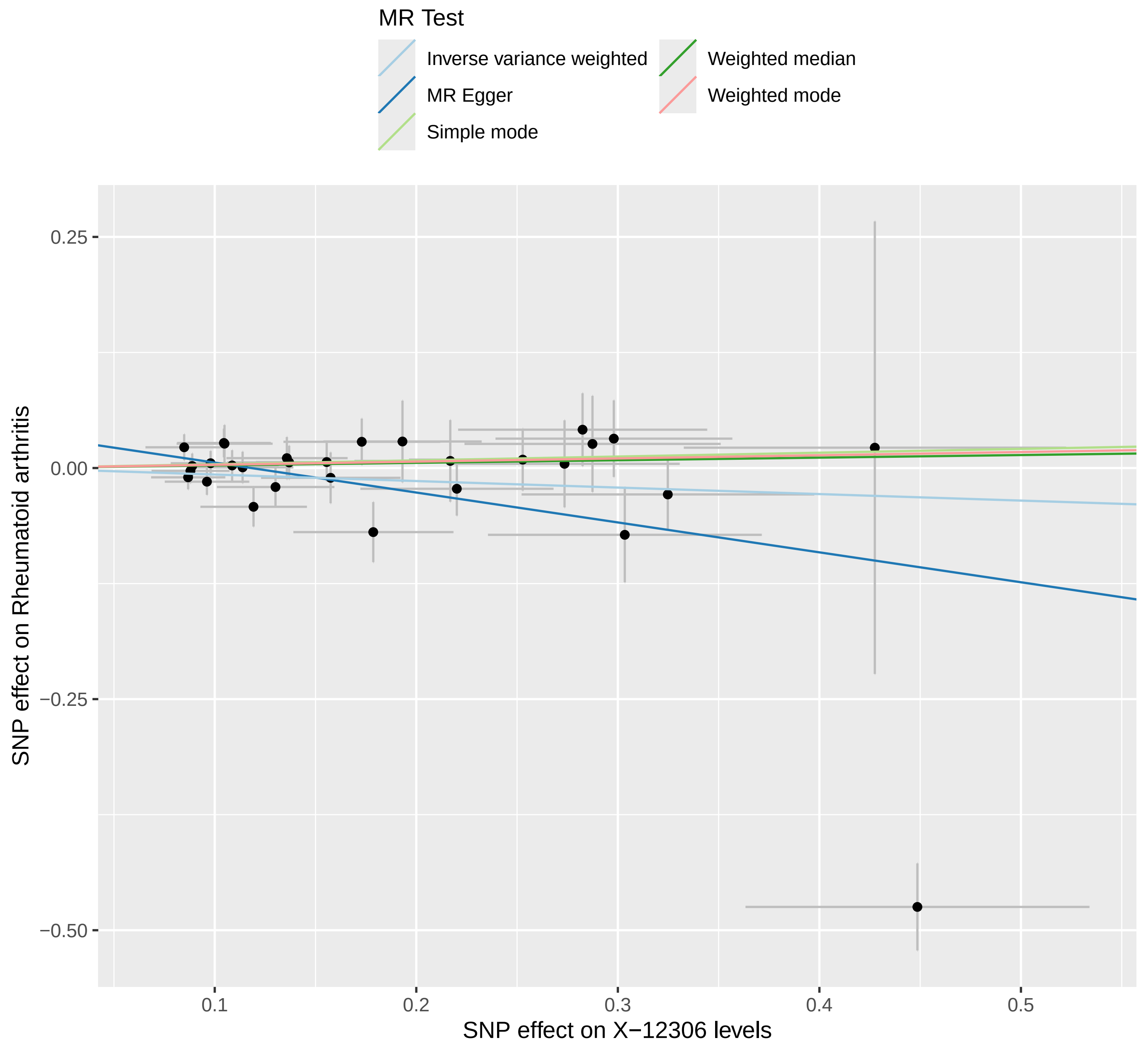

MR Test
Inverse variance weighted
MR Egger
Simple mode
Weighted median
Weighted mode
0.25
0.00
−0.25
−0.50
0.1
0.2
0.3
0.4
0.5
SNP effect on X−12306 levels
SNP effect on Rheumatoid arthritis

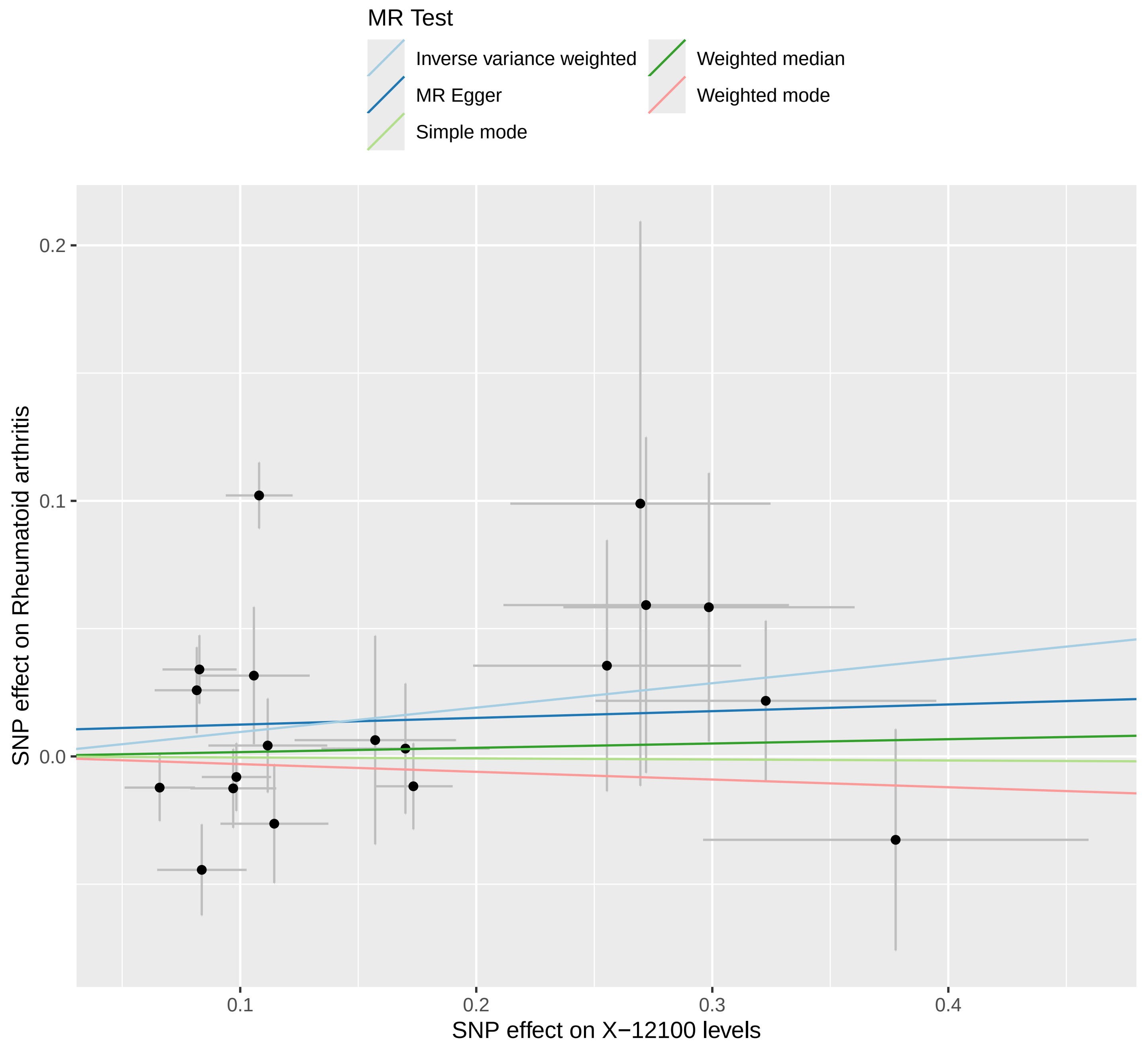
MR Test
Inverse variance weighted
MR Egger
Simple mode
Weighted median
Weighted mode
SNP effect on Rheumatoid arthritis
0.2
0.1
0.0
0.1
0.2
0.3
0.4
SNP effect on X−12100 levels

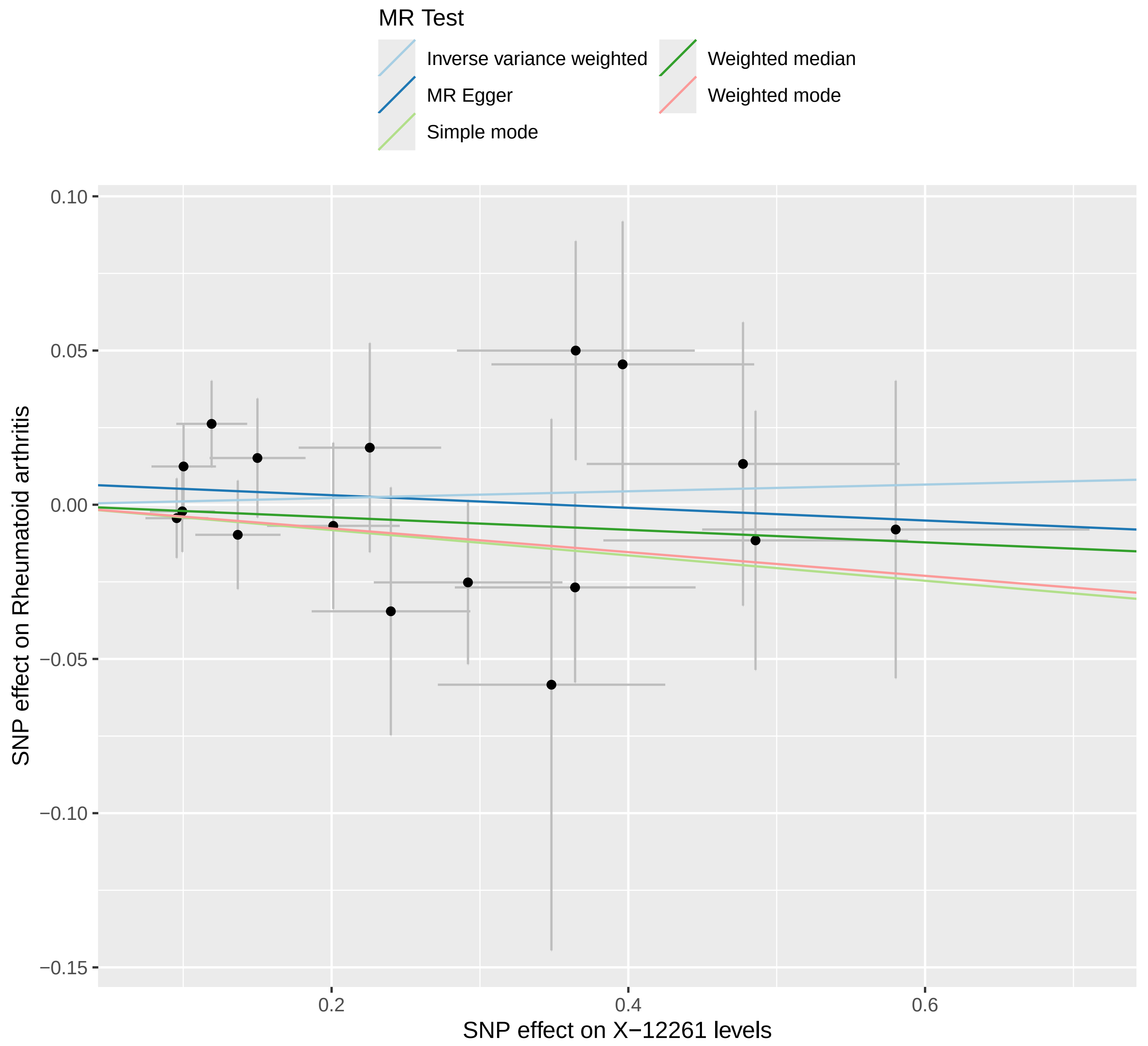

MR Test
Inverse variance weighted
MR Egger
Simple mode
Weighted median
Weighted mode
0.10
0.05
0.00
−0.05
−0.10
−0.15
0.2
0.4
0.6
SNP effect on Rheumatoid arthritis
SNP effect on X−12261 levels

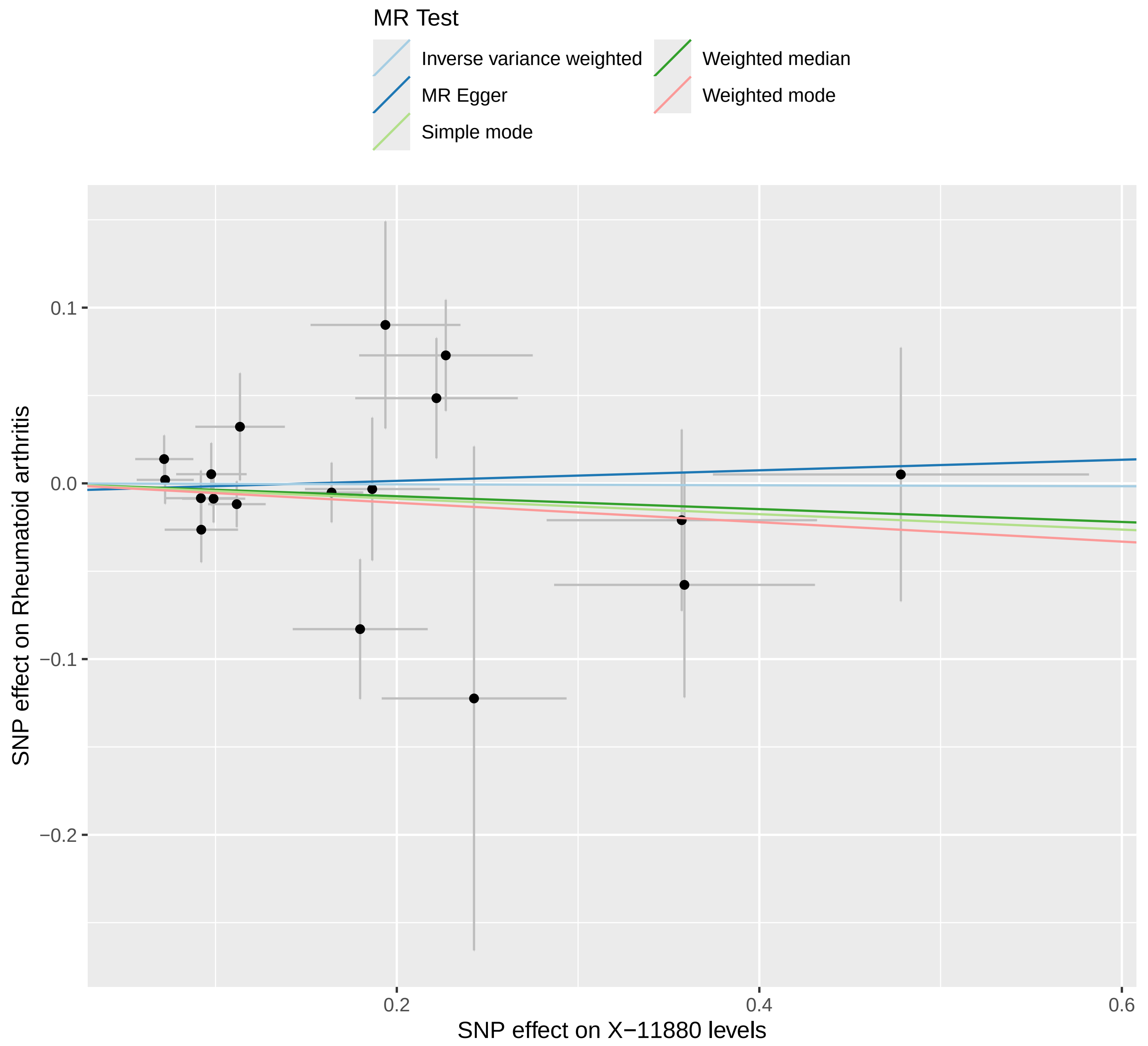
MR Test
Inverse variance weighted
MR Egger
Simple mode
Weighted median
Weighted mode
0.1
0.0
−0.1
−0.2
0.2
0.4
0.6
SNP effect on Rheumatoid arthritis
SNP effect on X−11880 levels

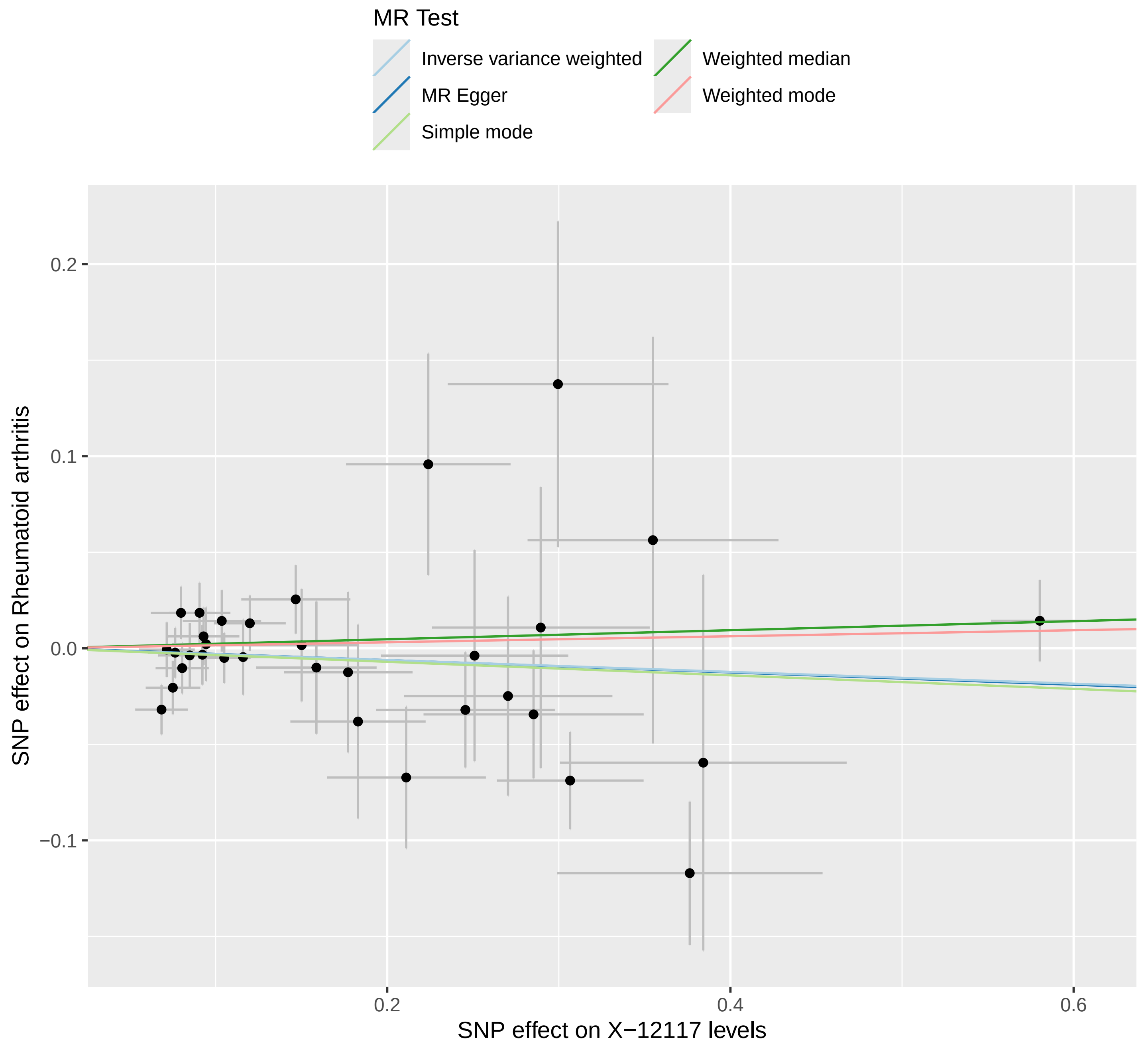
MR Test
Inverse variance weighted
MR Egger
Simple mode
Weighted median
Weighted mode
SNP effect on Rheumatoid arthritis
0.2
0.1
0.0
−0.1
0.2
0.4
0.6
SNP effect on X−12117 levels

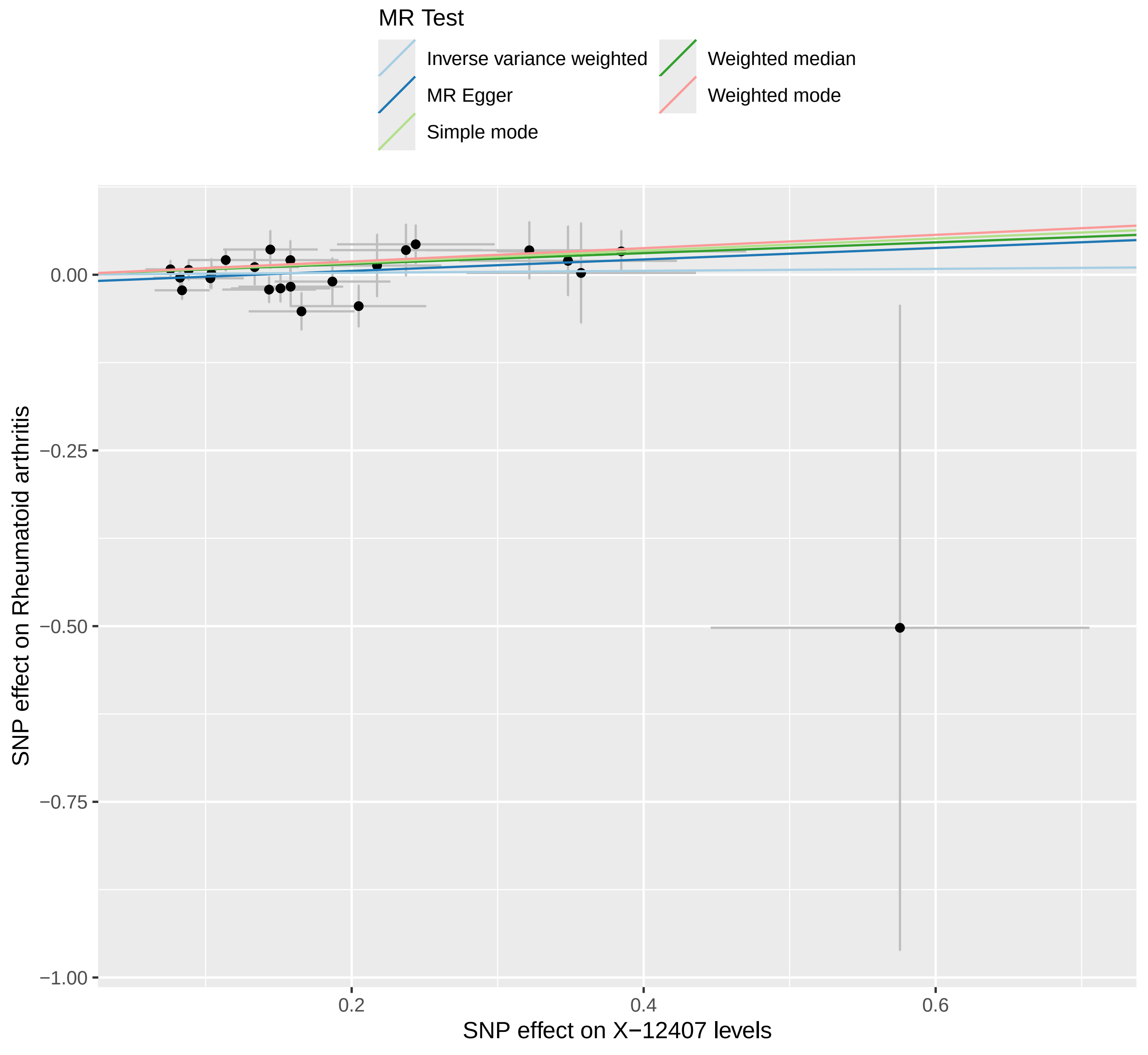
MR Test
Inverse variance weighted
MR Egger
Simple mode
Weighted median
Weighted mode
0.00
−0.25
−0.50
−0.75
−1.00
0.2
0.4
0.6
SNP effect on Rheumatoid arthritis
SNP effect on X−12407 levels

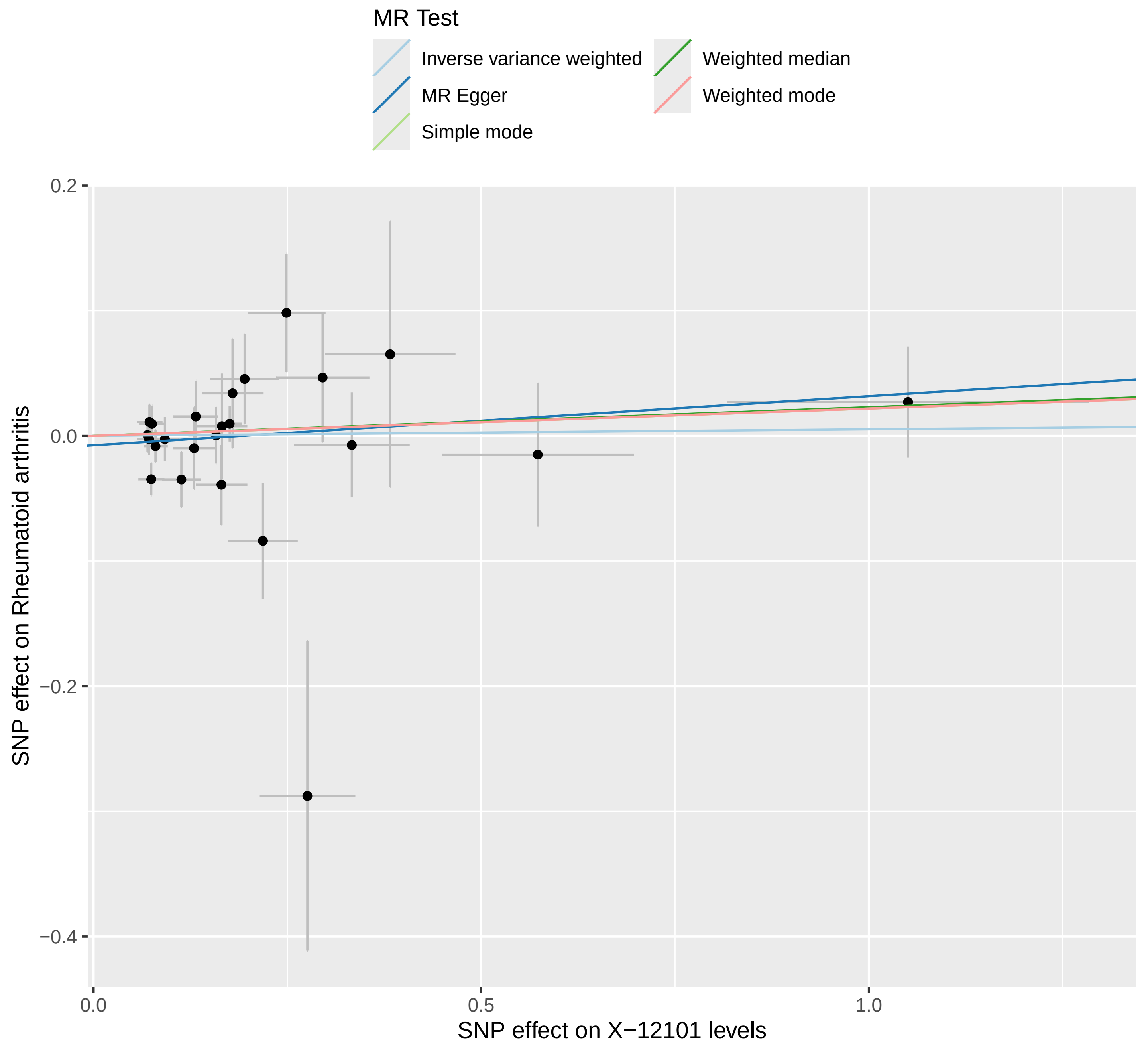
MR Test
Inverse variance weighted
MR Egger
Simple mode
Weighted median
Weighted mode
0.2
0.0
−0.2
−0.4
0.0
0.5
1.0
SNP effect on Rheumatoid arthritis
SNP effect on X−12101 levels

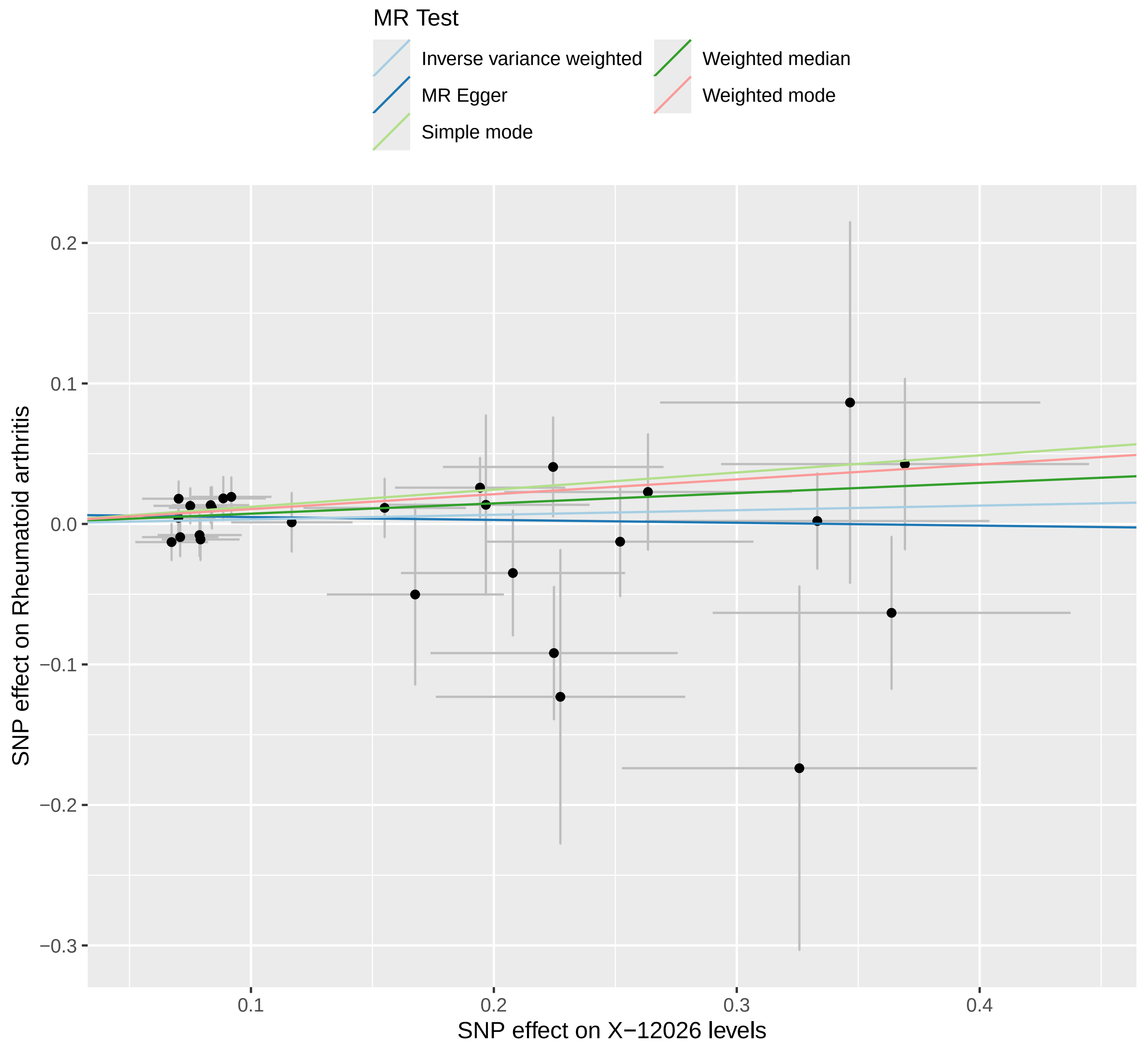
MR Test
Inverse variance weighted
MR Egger
Simple mode
Weighted median
Weighted mode
0.2
0.1
0.0
−0.1
−0.2
−0.3
SNP effect on Rheumatoid arthritis
0.1
0.2
0.3
0.4
SNP effect on X−12026 levels

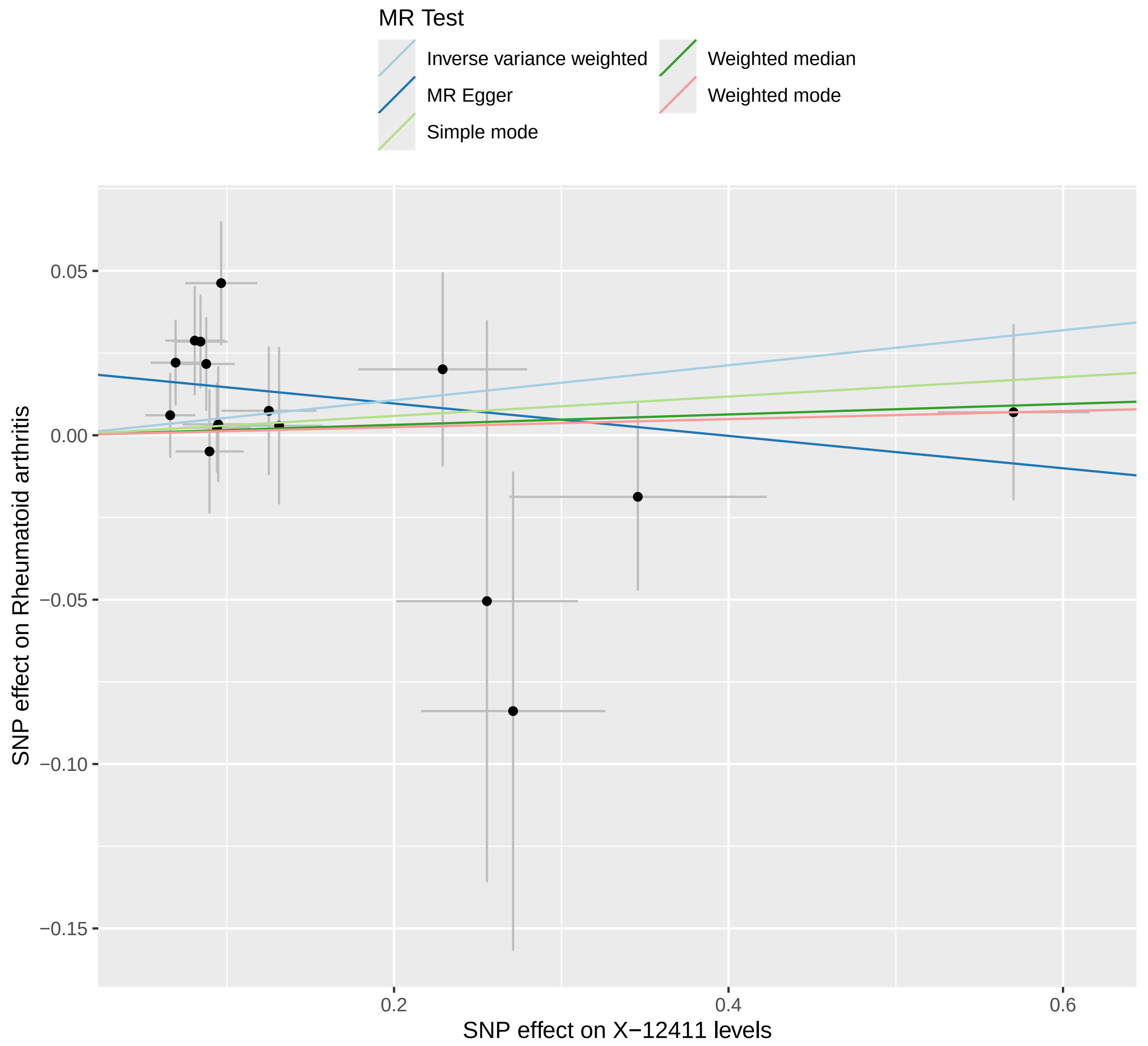

MR Test
Inverse variance weighted
MR Egger
Simple mode
Weighted median
Weighted mode
0.05
0.00
−0.05
−0.10
−0.15
0.2
0.4
0.6
SNP effect on Rheumatoid arthritis
SNP effect on X−12411 levels

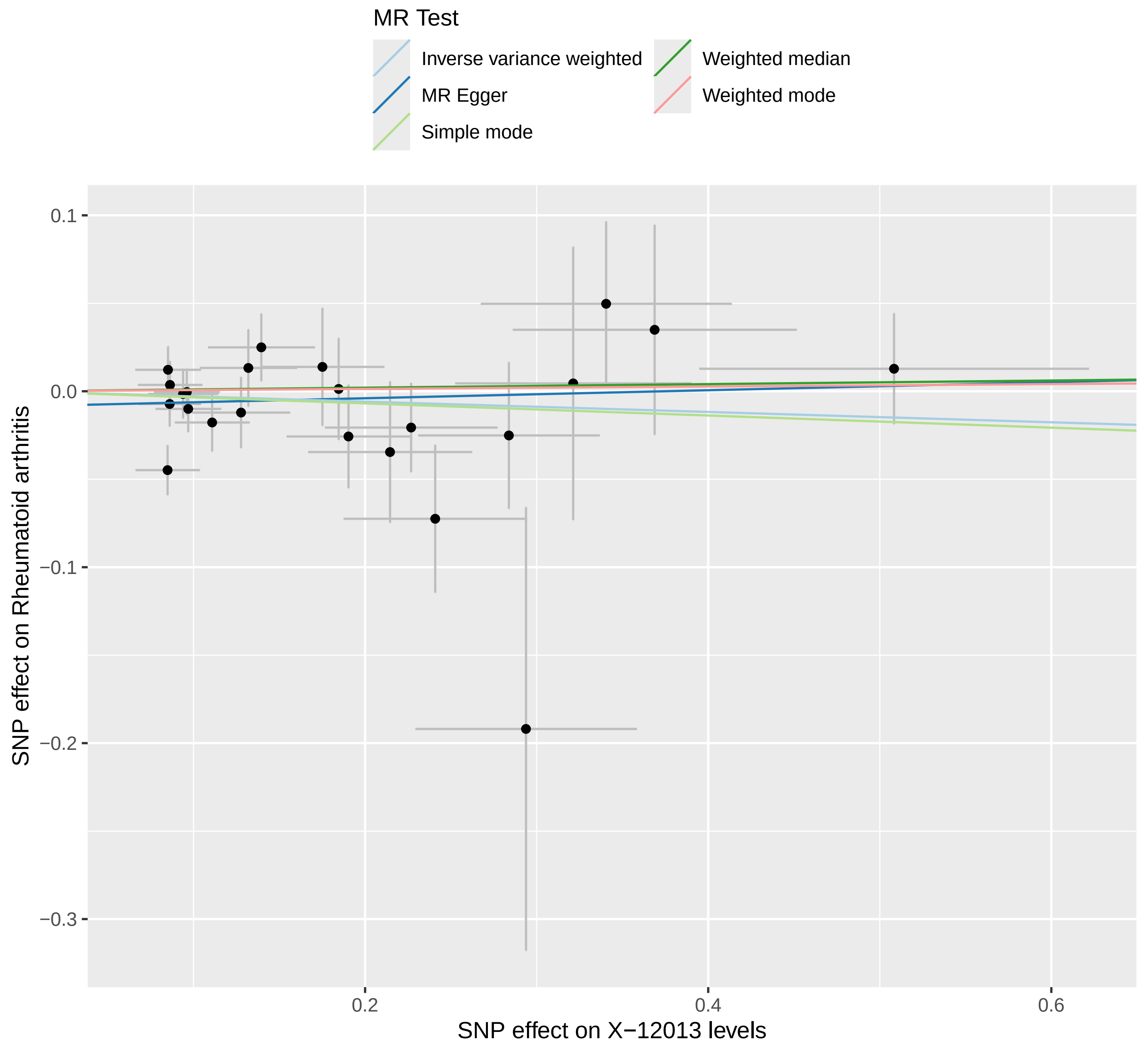

MR Test
Inverse variance weighted
MR Egger
Simple mode
Weighted median
Weighted mode
0.1
0.0
−0.1
−0.2
−0.3
0.2
0.4
0.6
SNP effect on Rheumatoid arthritis
SNP effect on X−12013 levels

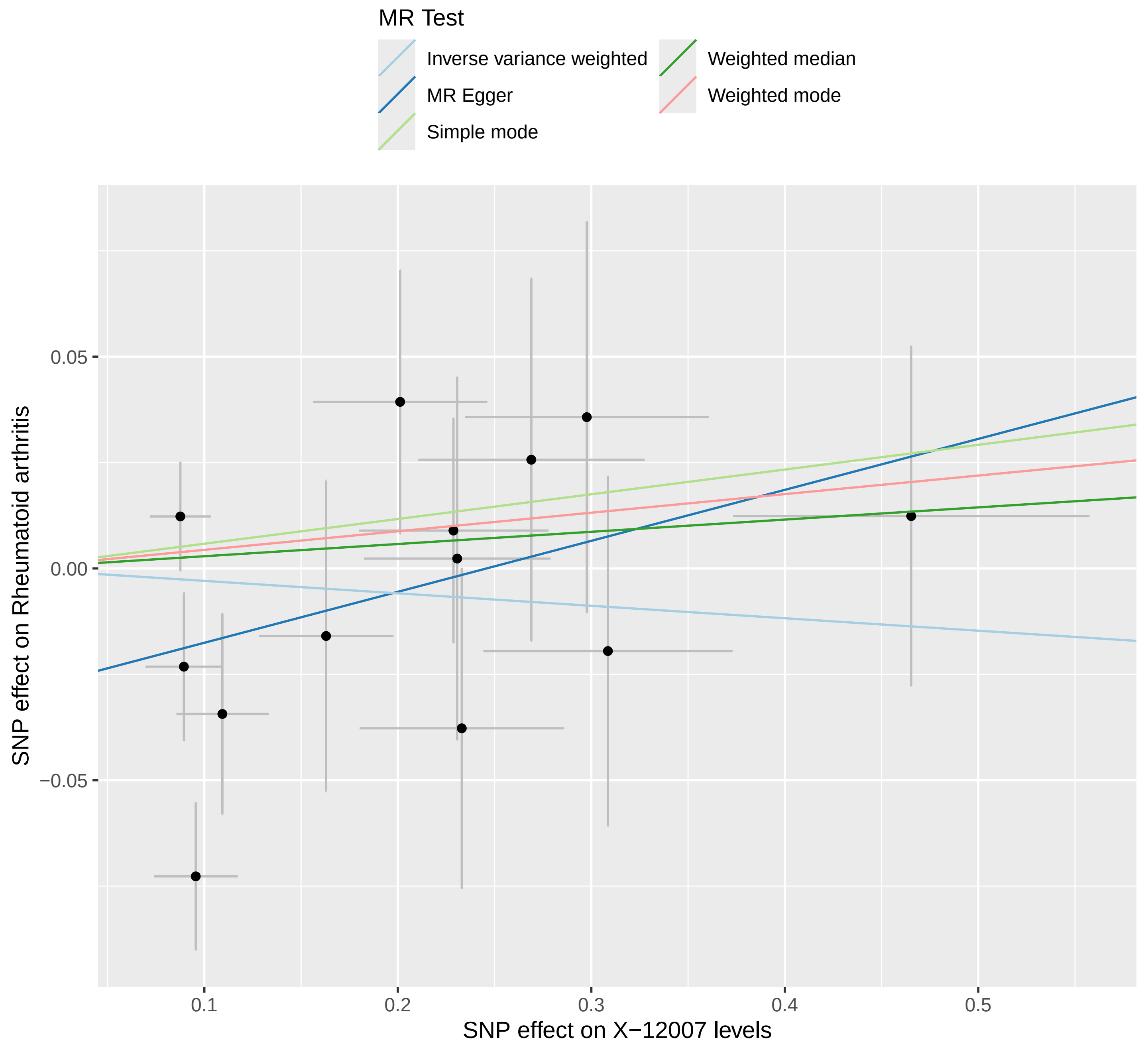
MR Test
Inverse variance weighted
MR Egger
Simple mode
Weighted median
Weighted mode
SNP effect on Rheumatoid arthritis
0.05
0.00
−0.05
0.1
0.2
0.3
0.4
0.5
SNP effect on X−12007 levels

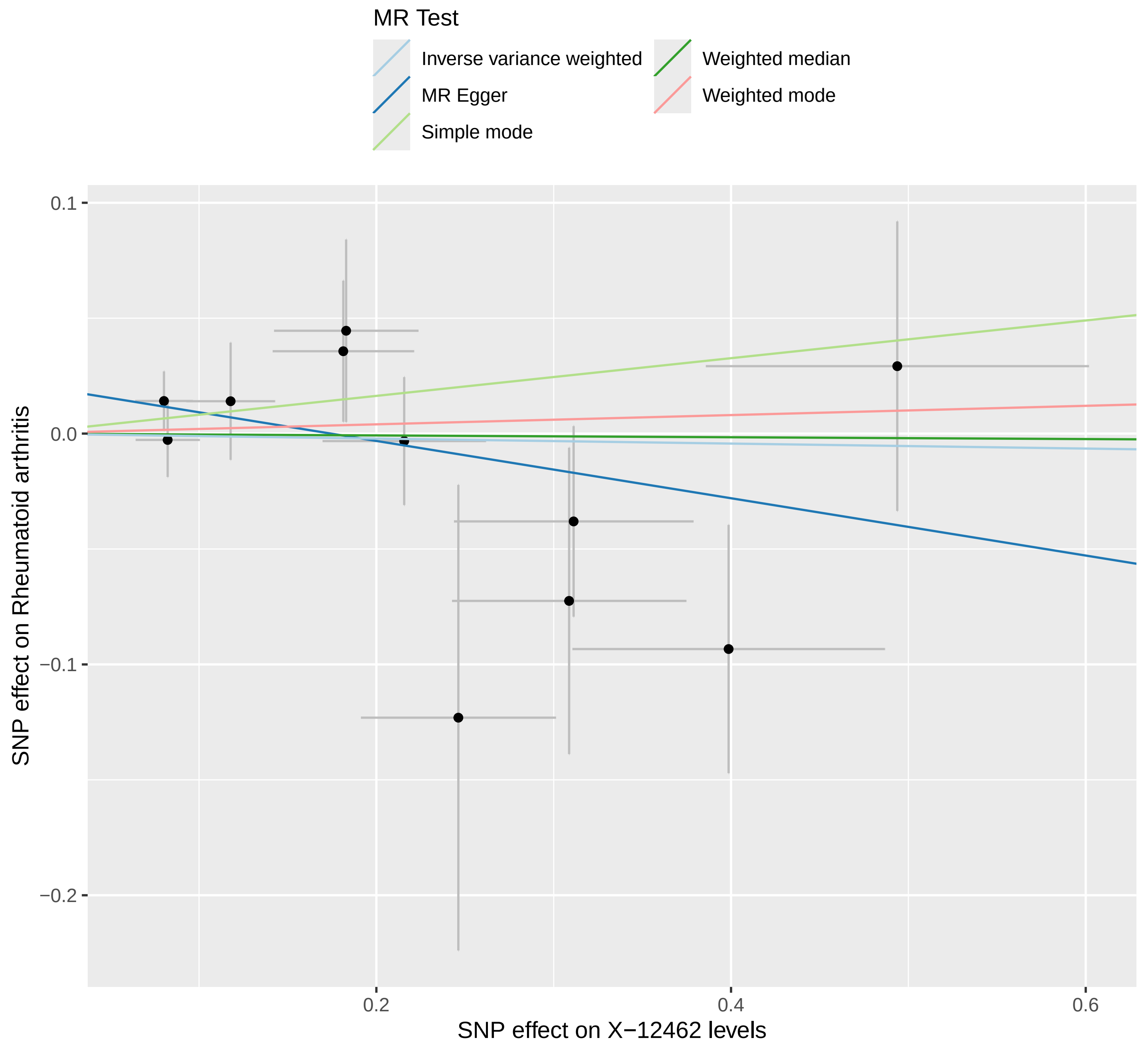
MR Test
Inverse variance weighted
MR Egger
Simple mode
Weighted median
Weighted mode
0.1
0.0
−0.1
−0.2
0.2
0.4
0.6
SNP effect on Rheumatoid arthritis
SNP effect on X−12462 levels

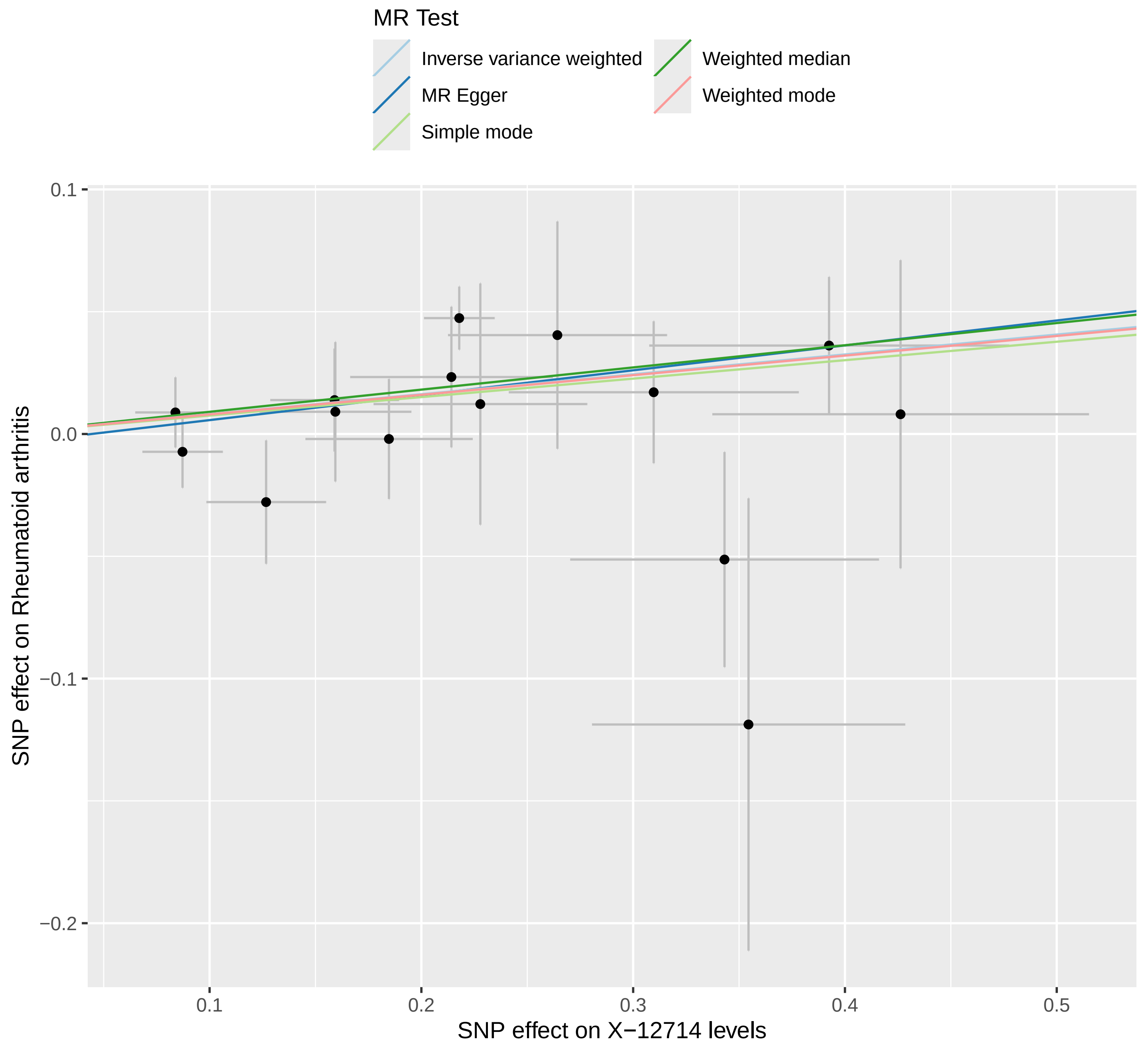

MR Test
Inverse variance weighted
MR Egger
Simple mode
Weighted median
Weighted mode
0.1
0.0
−0.1
−0.2
SNP effect on Rheumatoid arthritis
0.1
0.2
0.3
0.4
0.5
SNP effect on X−12714 levels

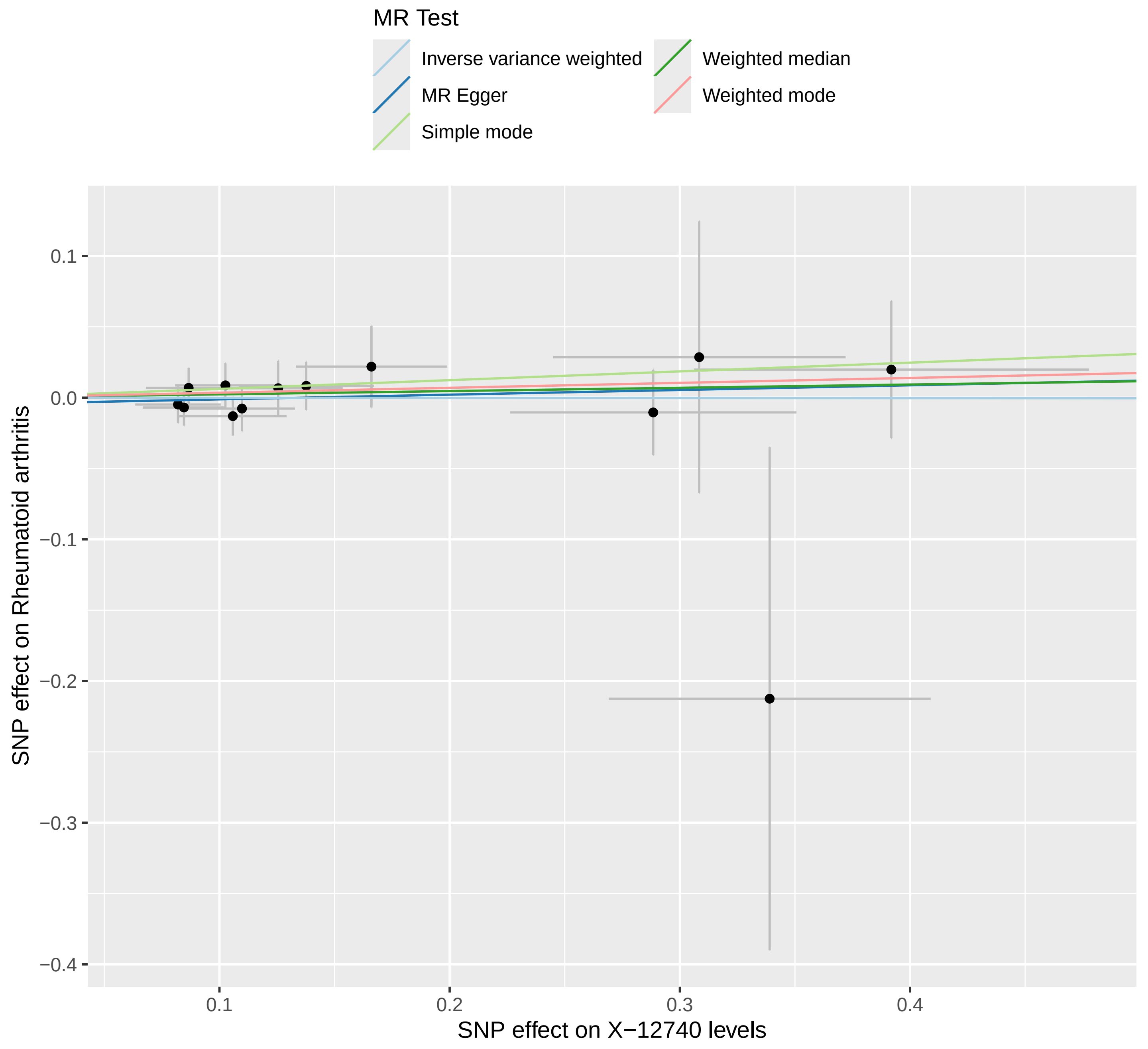
MR Test
Inverse variance weighted
MR Egger
Simple mode
Weighted median
Weighted mode
0.1
0.0
−0.1
−0.2
−0.3
−0.4
SNP effect on Rheumatoid arthritis
0.1
0.2
0.3
0.4
SNP effect on X−12740 levels

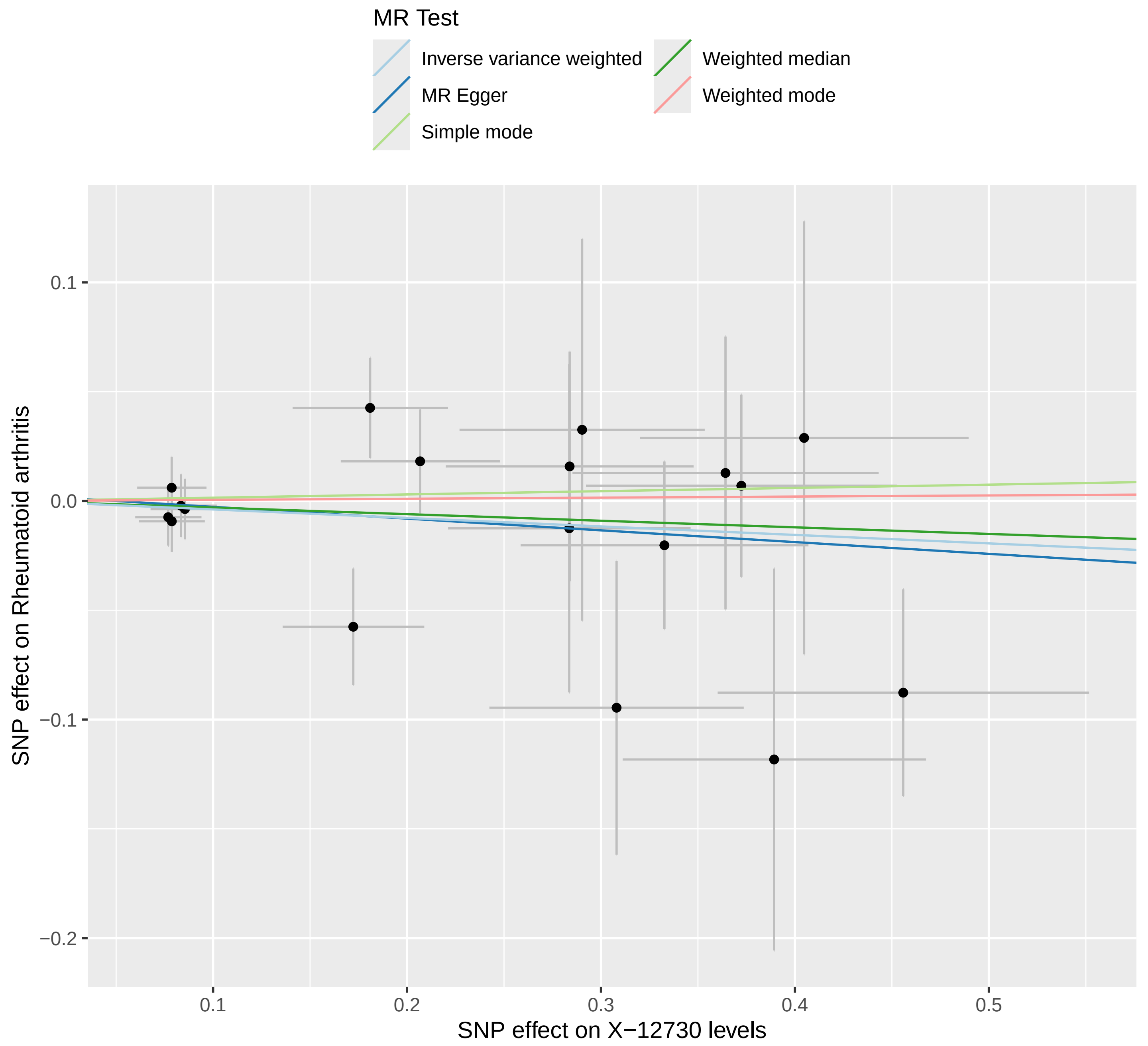
MR Test
Inverse variance weighted
MR Egger
Simple mode
Weighted median
Weighted mode
0.1
0.0
−0.1
−0.2
SNP effect on Rheumatoid arthritis
0.1
0.2
0.3
0.4
0.5
SNP effect on X−12730 levels

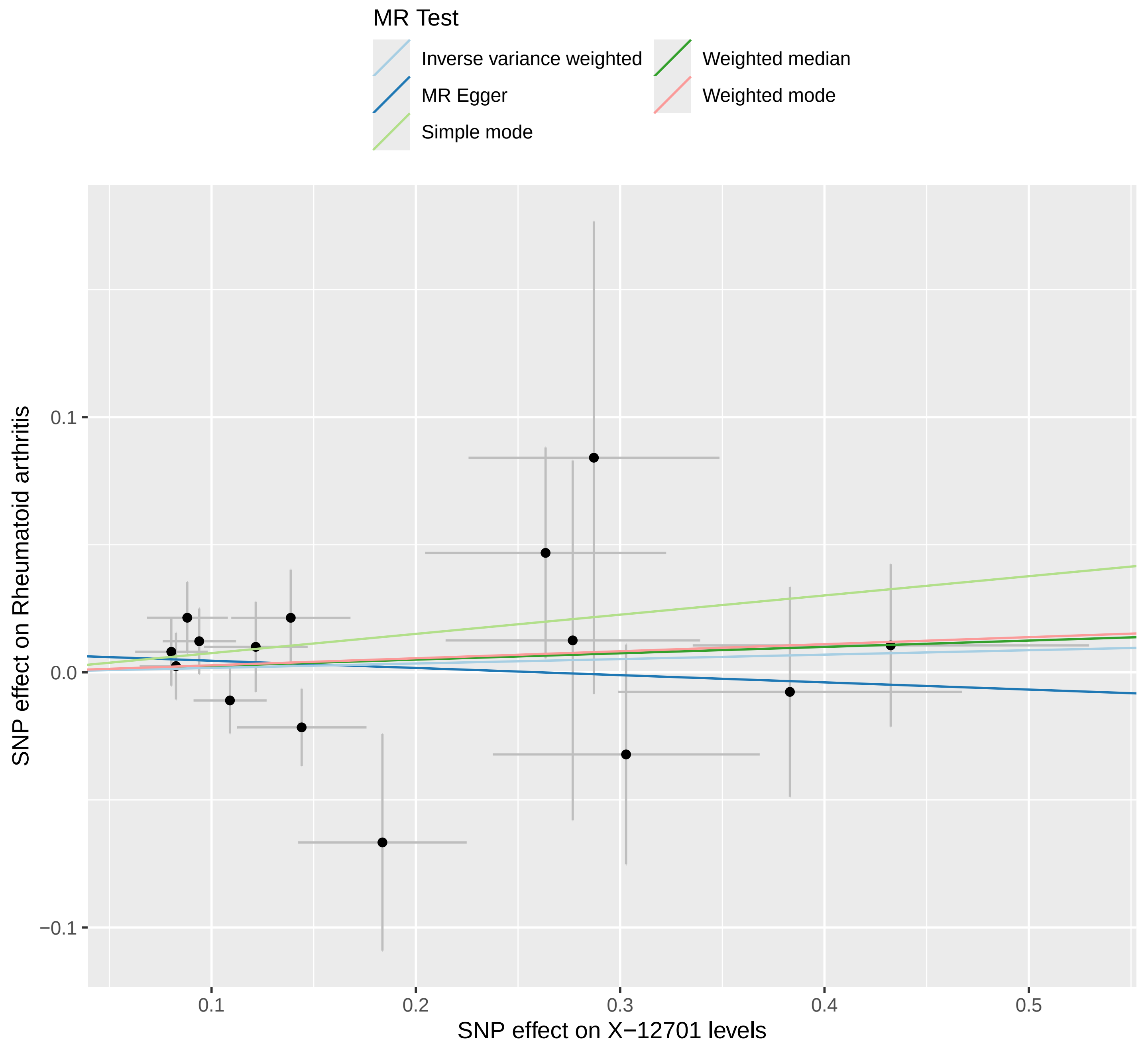
MR Test
Inverse variance weighted
MR Egger
Simple mode
Weighted median
Weighted mode
SNP effect on Rheumatoid arthritis
0.1
0.0
−0.1
0.1
0.2
0.3
0.4
0.5
SNP effect on X−12701 levels

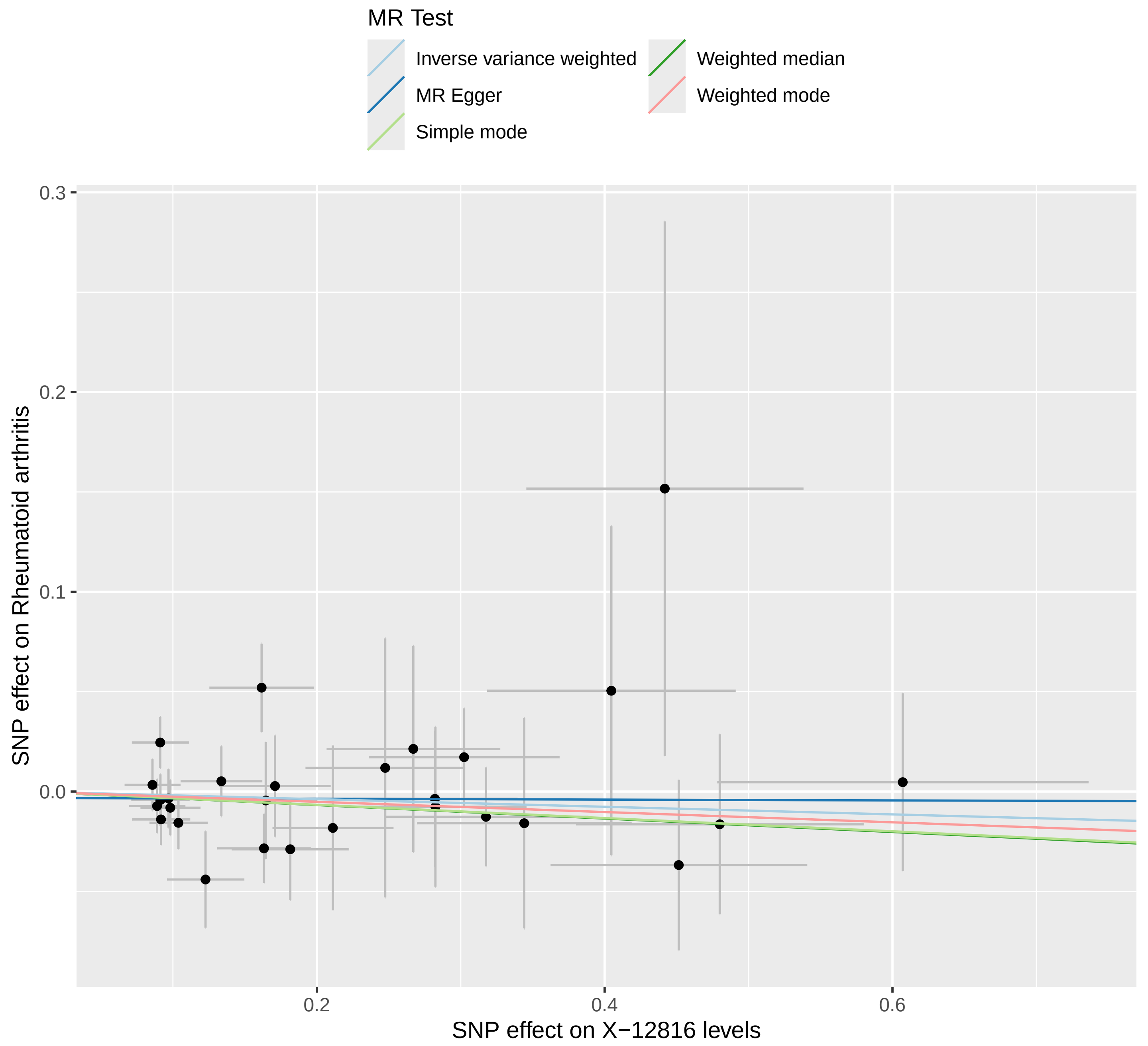
MR Test
Inverse variance weighted
MR Egger
Simple mode
Weighted median
Weighted mode
0.3
0.2
0.1
0.0
0.2
0.4
0.6
SNP effect on Rheumatoid arthritis
SNP effect on X−12816 levels

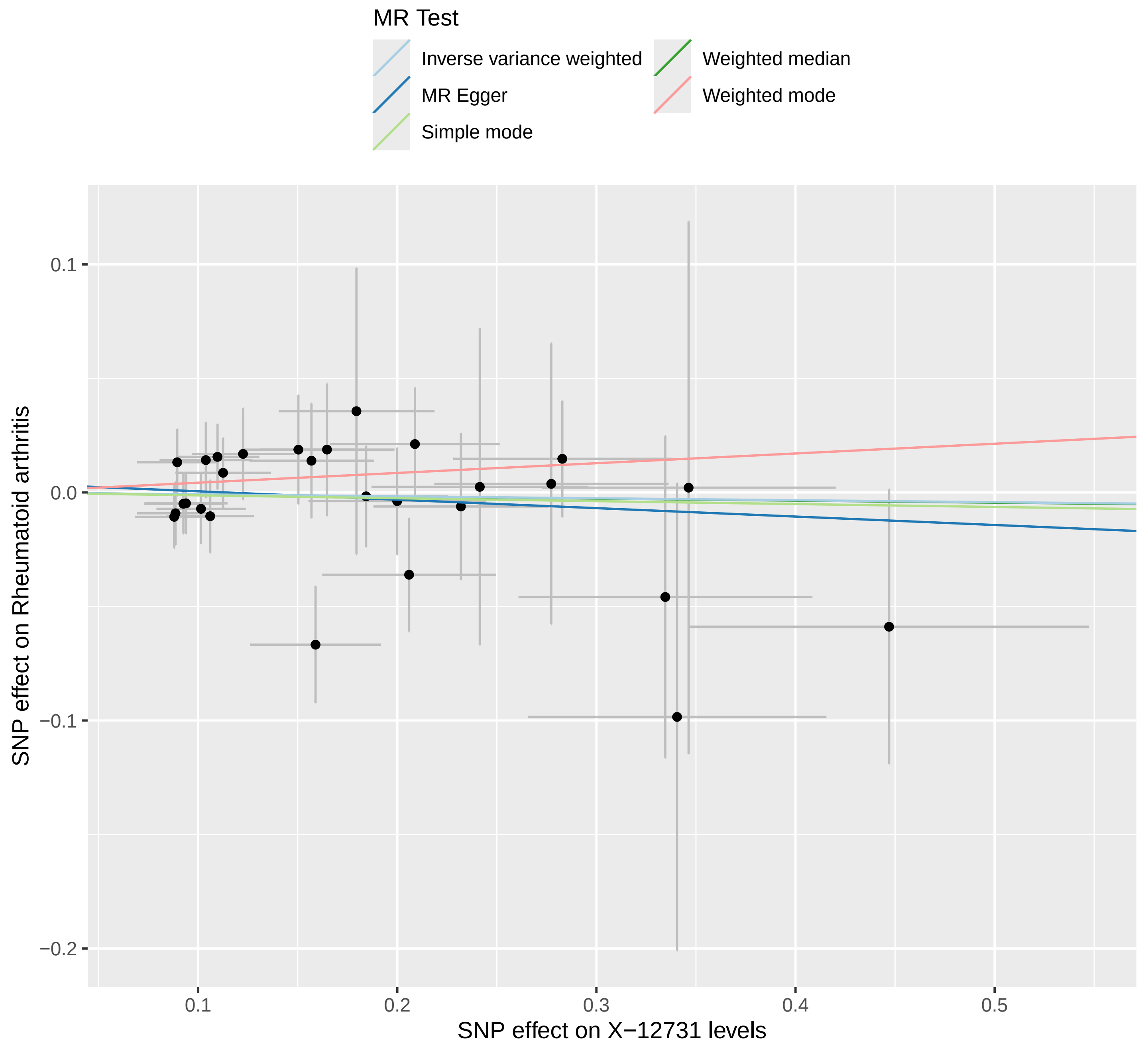

MR Test
Inverse variance weighted
MR Egger
Simple mode
Weighted median
Weighted mode
0.1
0.0
−0.1
−0.2
SNP effect on Rheumatoid arthritis
0.1
0.2
0.3
0.4
0.5
SNP effect on X−12731 levels

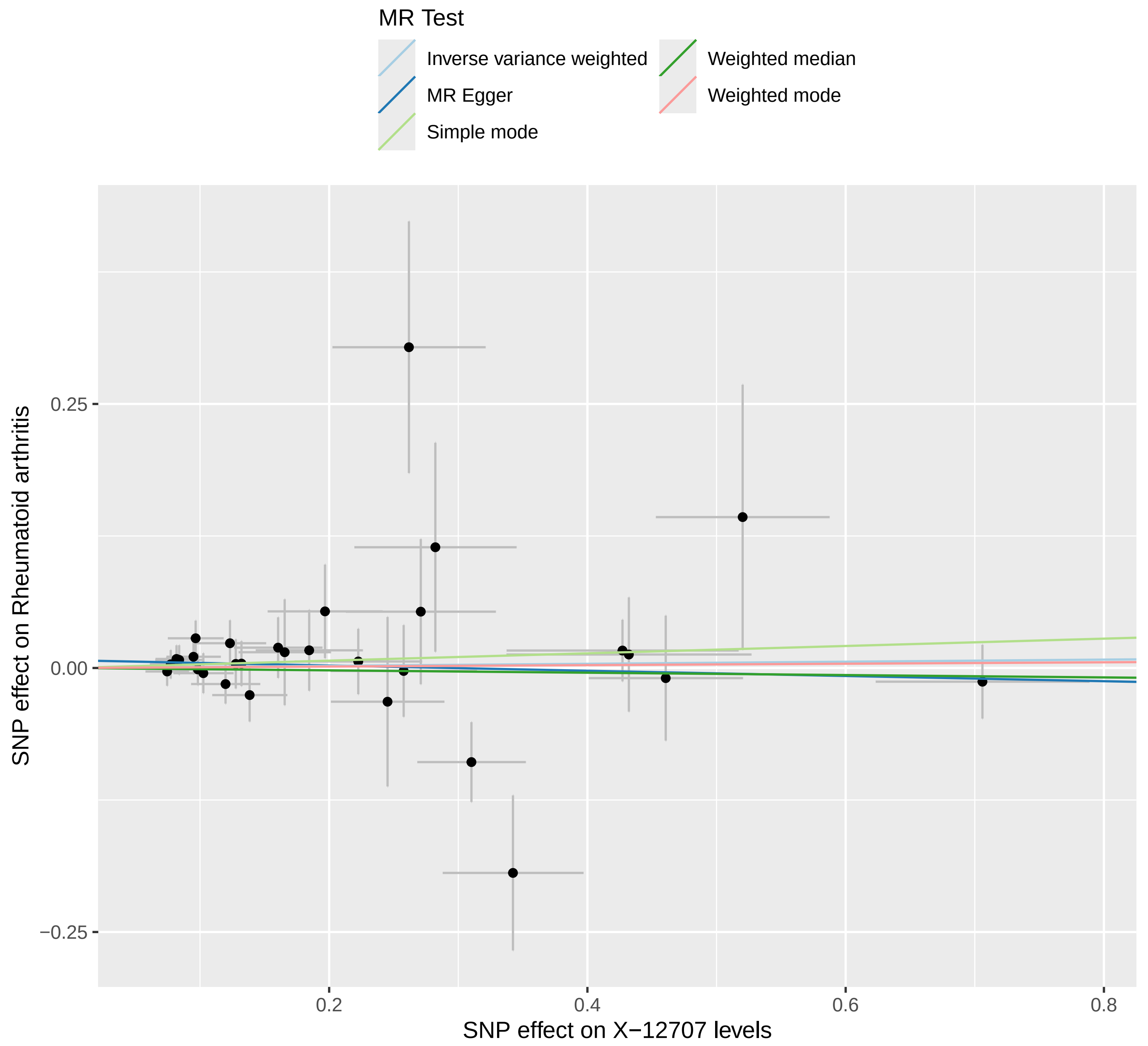
MR Test
Inverse variance weighted
MR Egger
Simple mode
Weighted median
Weighted mode
0.25
0.00
−0.25
0.2
0.4
0.6
0.8
SNP effect on Rheumatoid arthritis
SNP effect on X−12707 levels

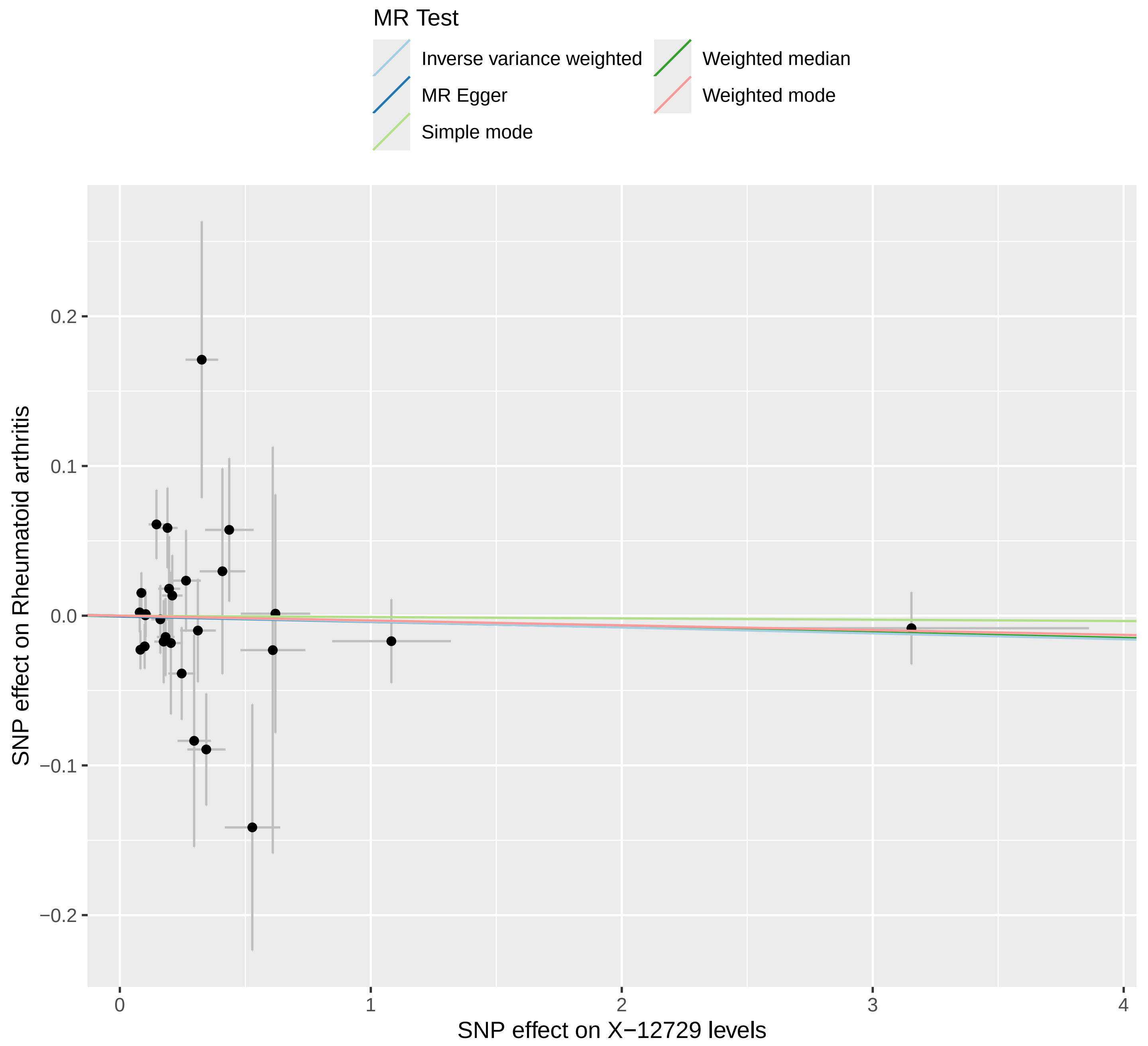

MR Test
Inverse variance weighted
MR Egger
Simple mode
Weighted median
Weighted mode
0.2
0.1
0.0
−0.1
−0.2
SNP effect on Rheumatoid arthritis
0
1
2
3
4
SNP effect on X−12729 levels

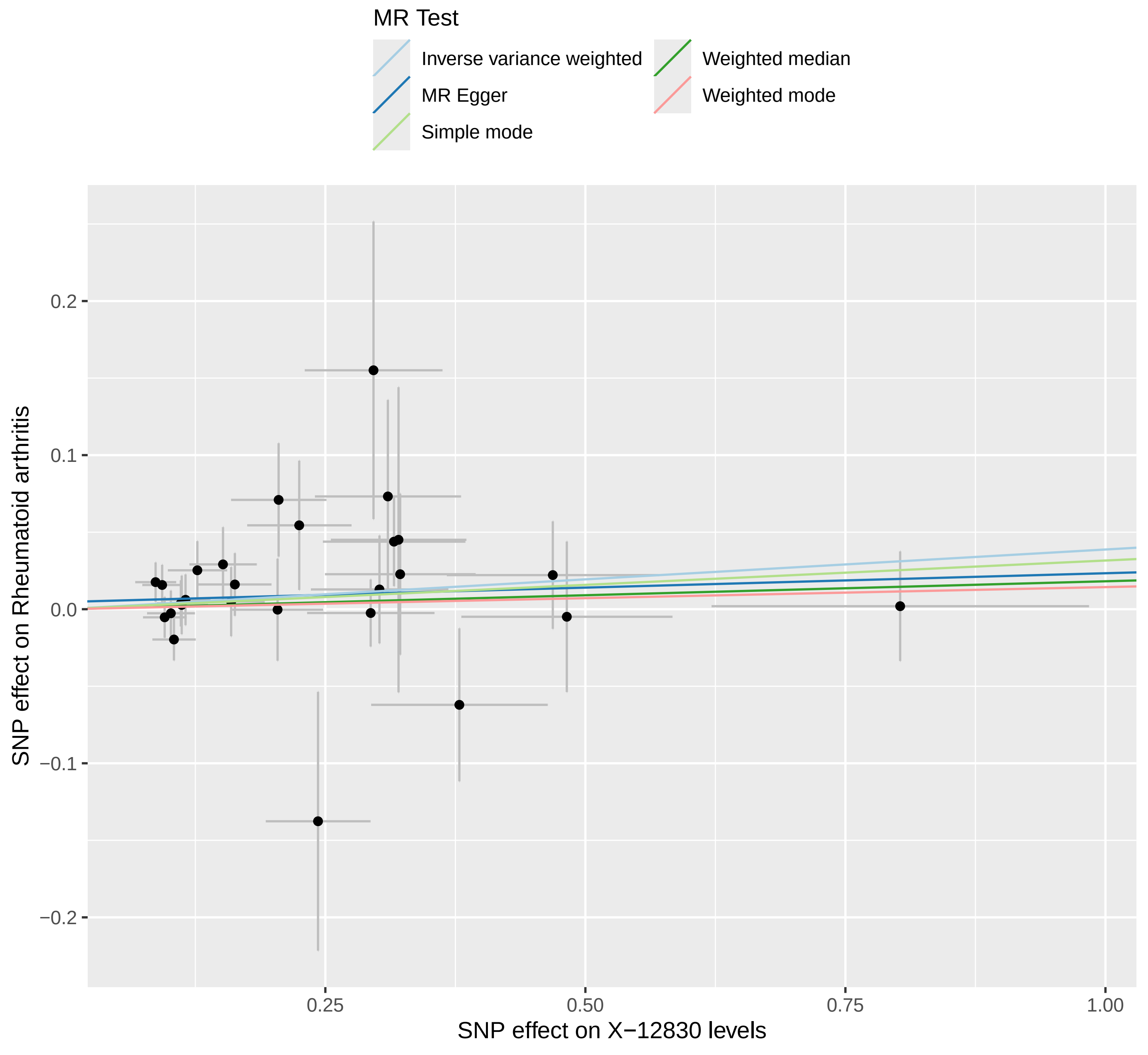

MR Test
Inverse variance weighted
MR Egger
Simple mode
Weighted median
Weighted mode
0.2
0.1
0.0
−0.1
−0.2
SNP effect on Rheumatoid arthritis
0.25
0.50
0.75
1.00
SNP effect on X−12830 levels

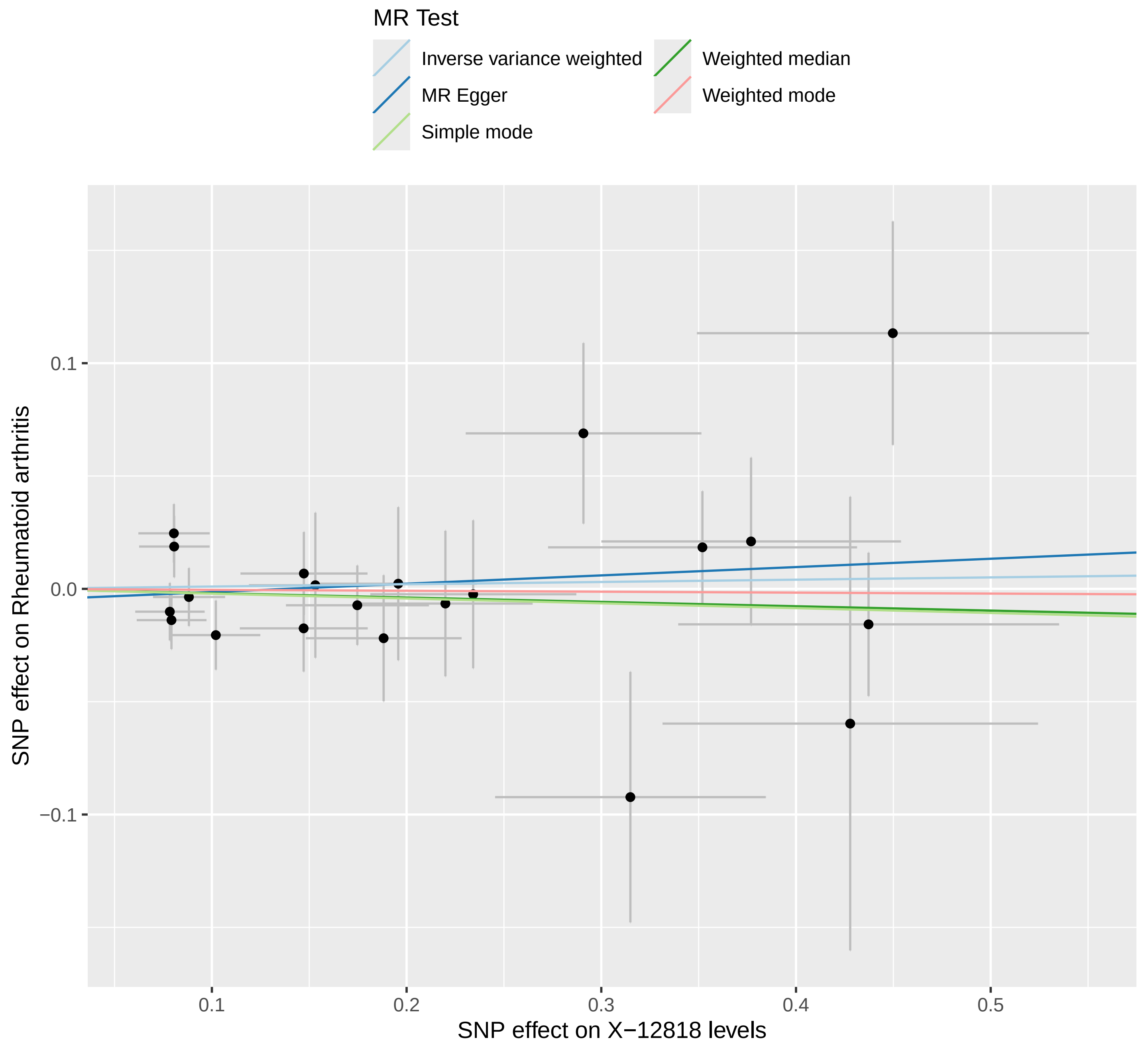
MR Test
Inverse variance weighted
MR Egger
Simple mode
Weighted median
Weighted mode
0.1
0.0
−0.1
SNP effect on Rheumatoid arthritis
0.1
0.2
0.3
0.4
0.5
SNP effect on X−12818 levels

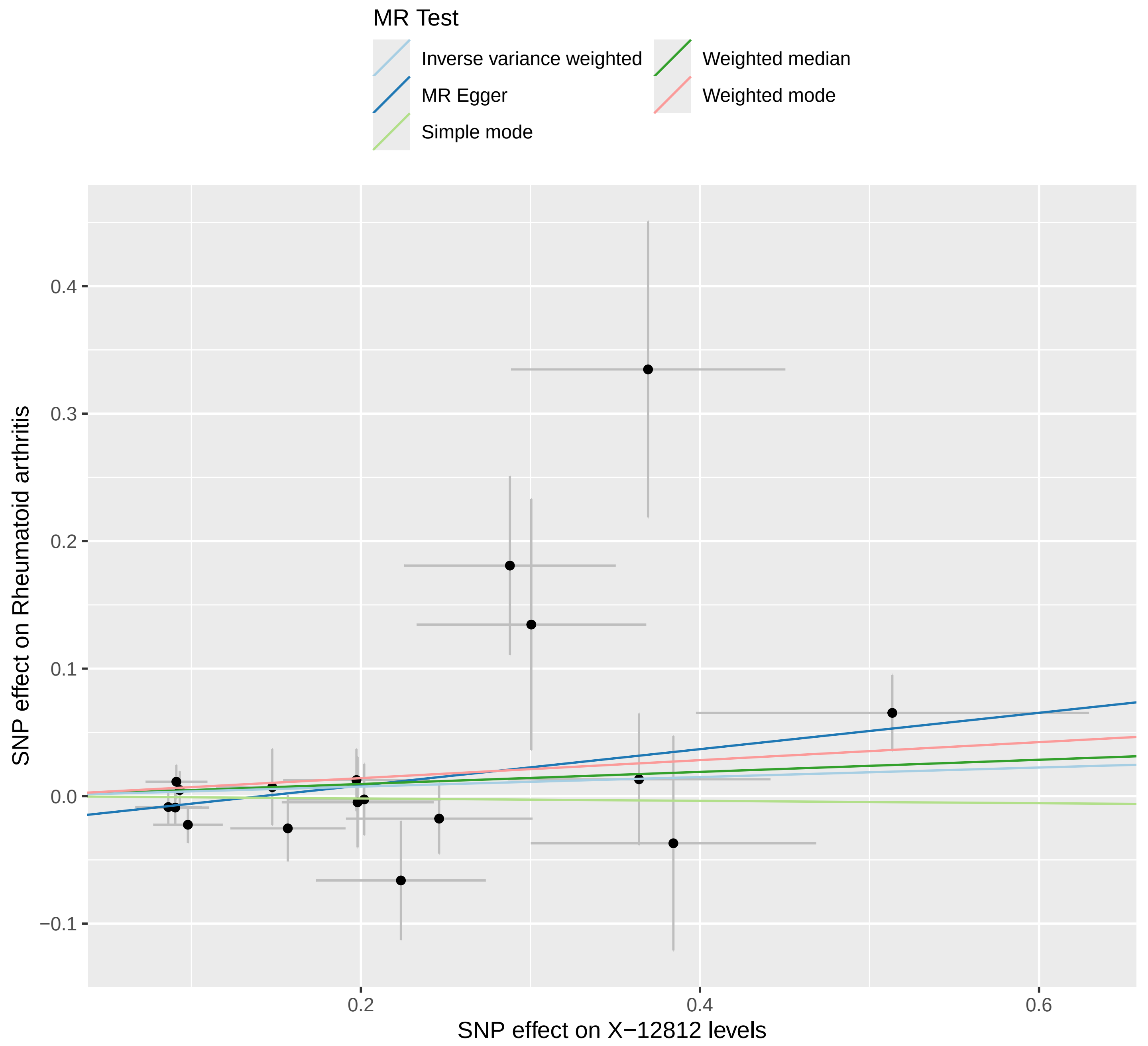
MR Test
Inverse variance weighted
MR Egger
Simple mode
Weighted median
Weighted mode
SNP effect on Rheumatoid arthritis
0.4
0.3
0.2
0.1
0.0
−0.1
0.2
0.4
0.6
SNP effect on X−12812 levels

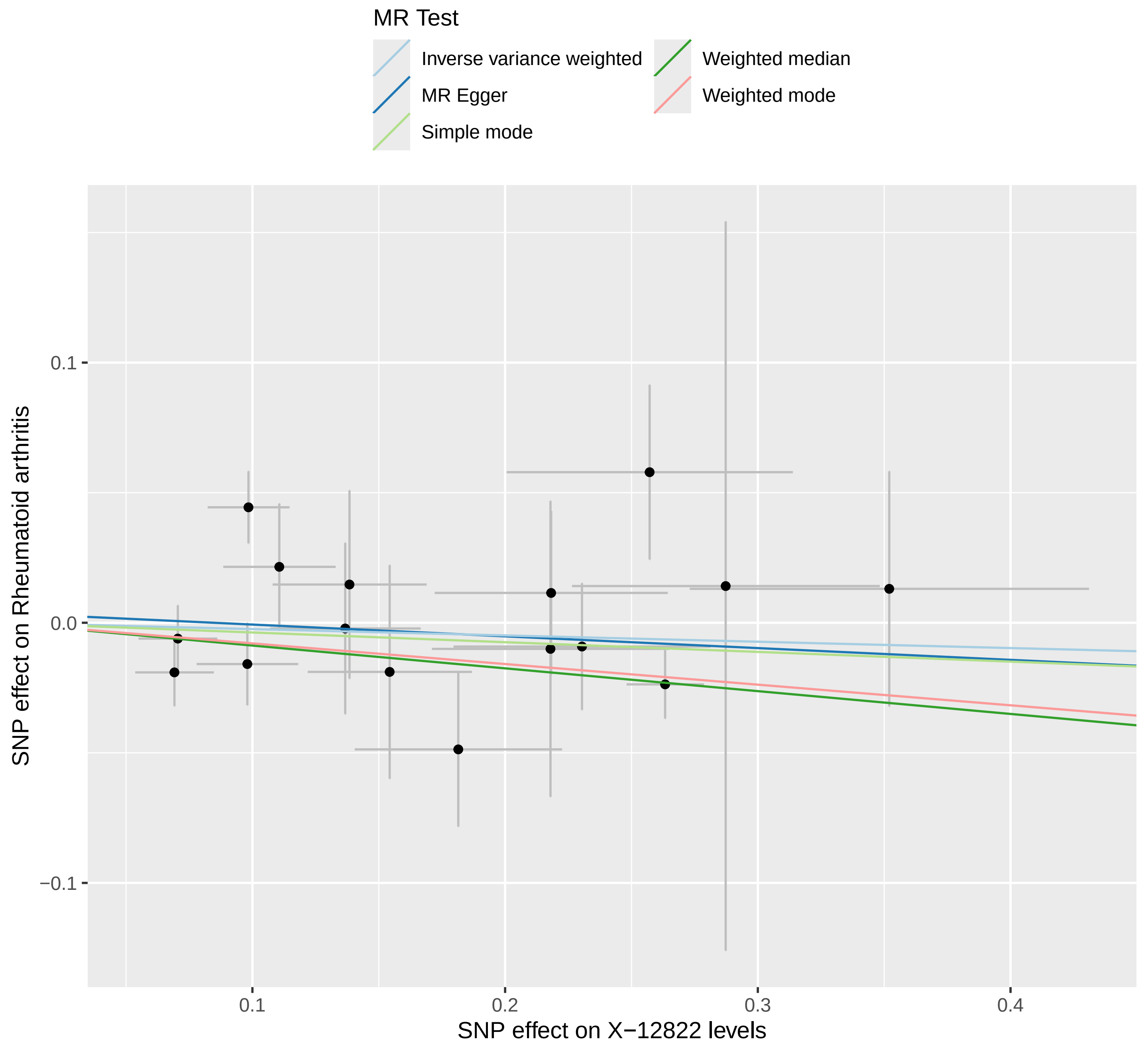

MR Test
Inverse variance weighted
MR Egger
Simple mode
Weighted median
Weighted mode
0.1
0.0
−0.1
0.1
0.2
0.3
0.4
SNP effect on Rheumatoid arthritis
SNP effect on X−12822 levels

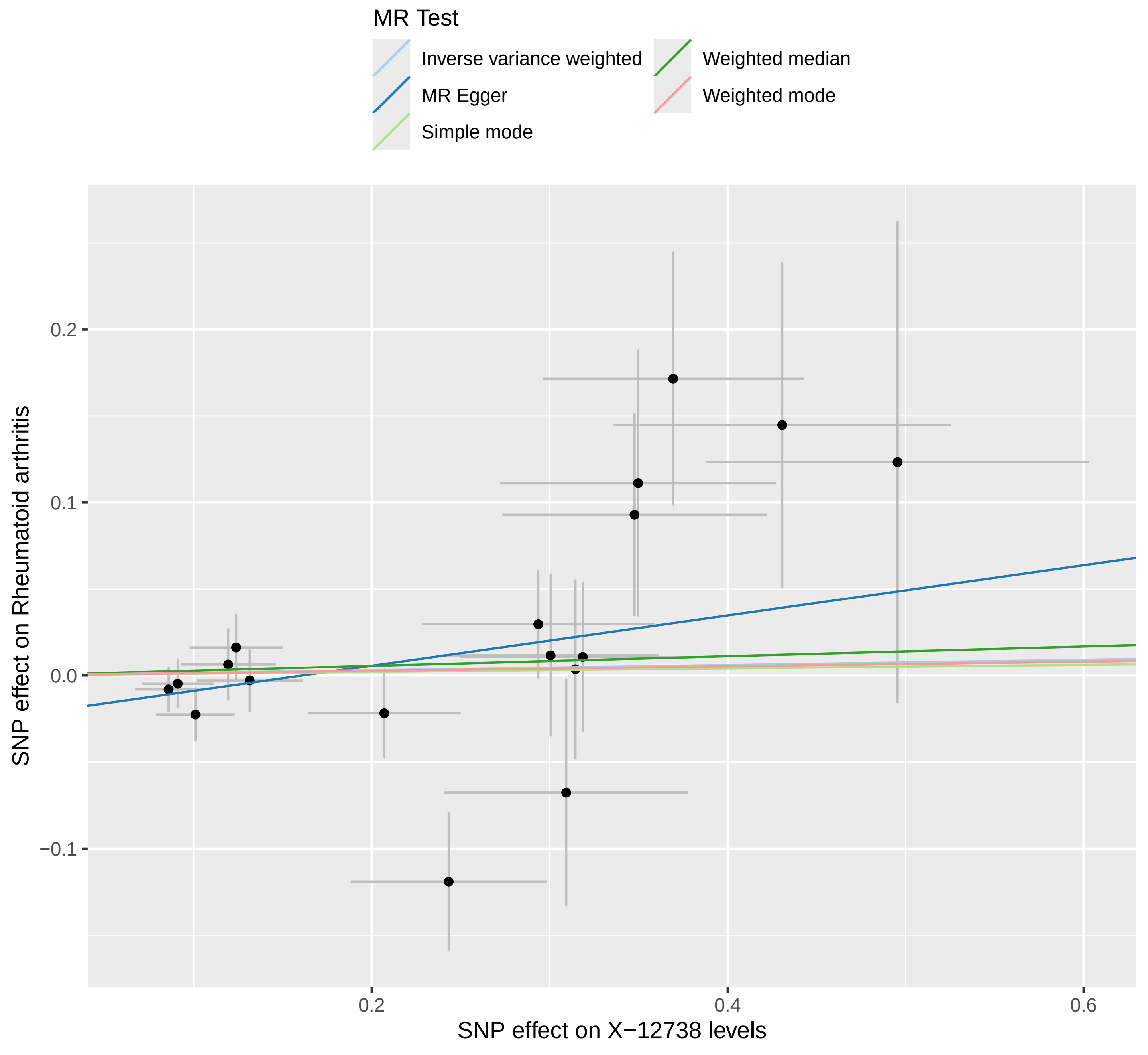

MR Test
Inverse variance weighted
MR Egger
Simple mode
Weighted median
Weighted mode
0.2
0.1
0.0
−0.1
0.2
0.4
0.6
SNP effect on Rheumatoid arthritis
SNP effect on X−12738 levels

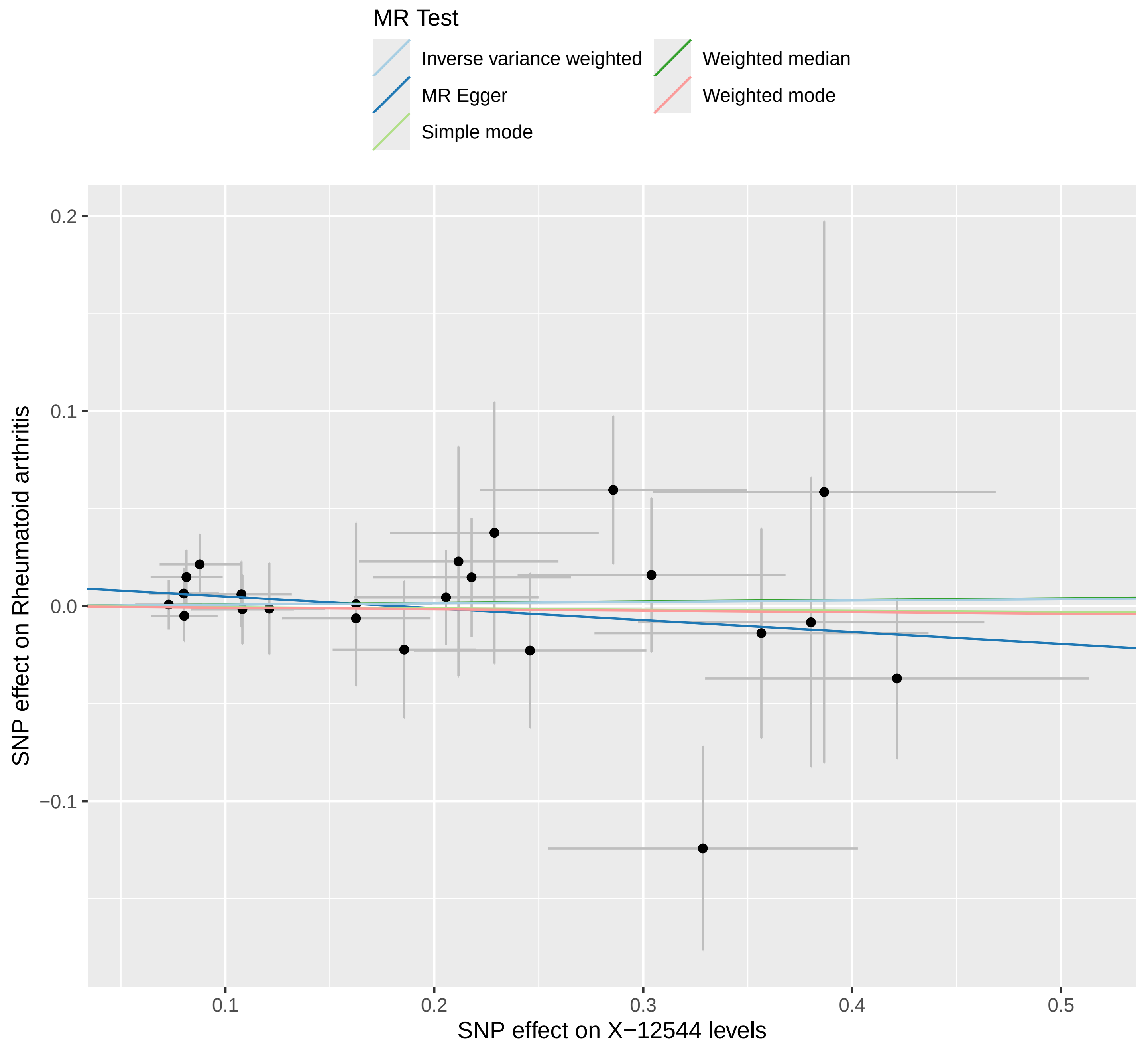
MR Test
Inverse variance weighted
MR Egger
Simple mode
Weighted median
Weighted mode
0.2
0.1
0.0
−0.1
0.1
0.2
0.3
0.4
0.5
SNP effect on Rheumatoid arthritis
SNP effect on X−12544 levels

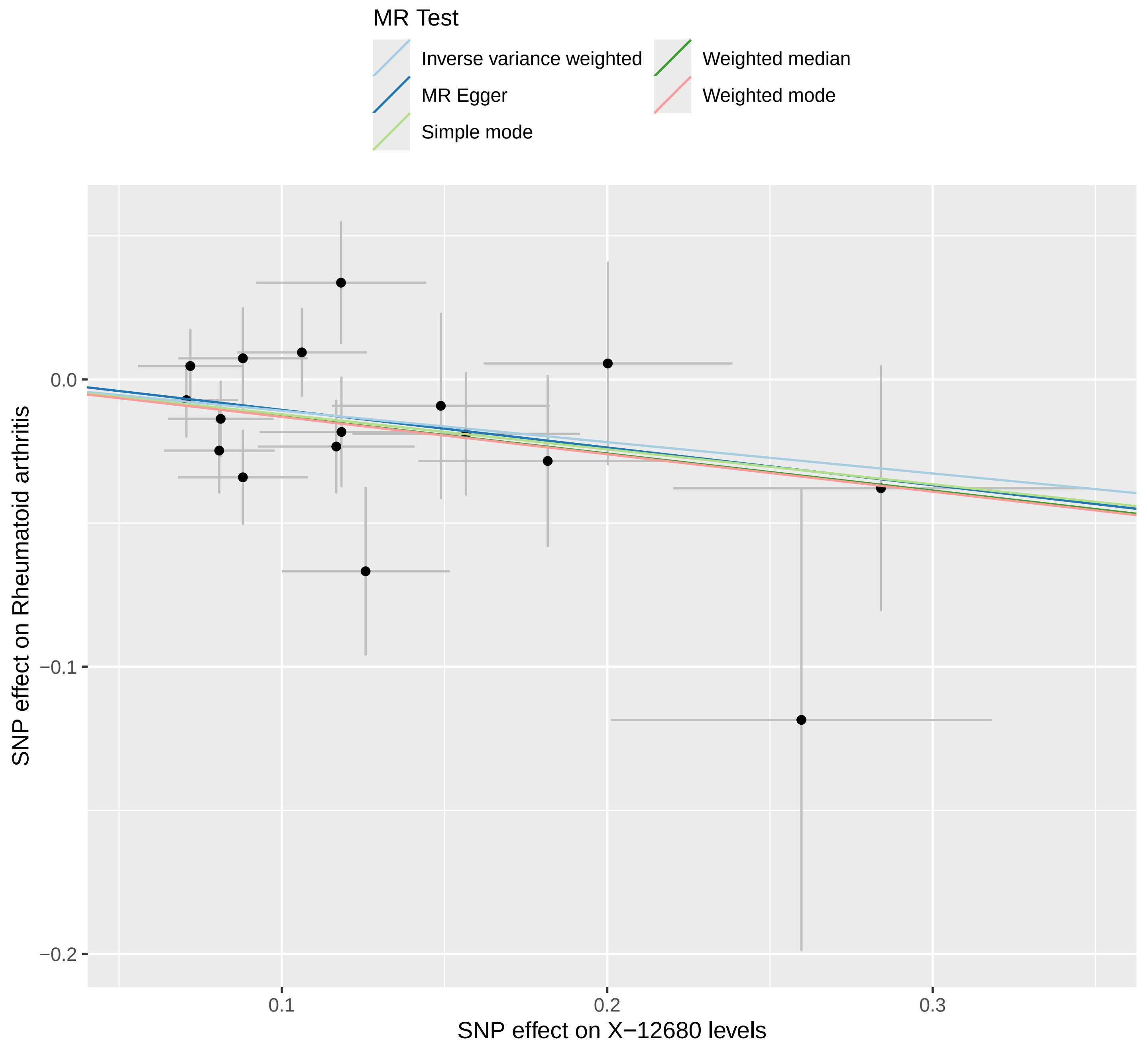

MR Test
Inverse variance weighted
MR Egger
Simple mode
Weighted median
Weighted mode
0.0
−0.1
−0.2
0.1
0.2
0.3
SNP effect on Rheumatoid arthritis
SNP effect on X−12680 levels

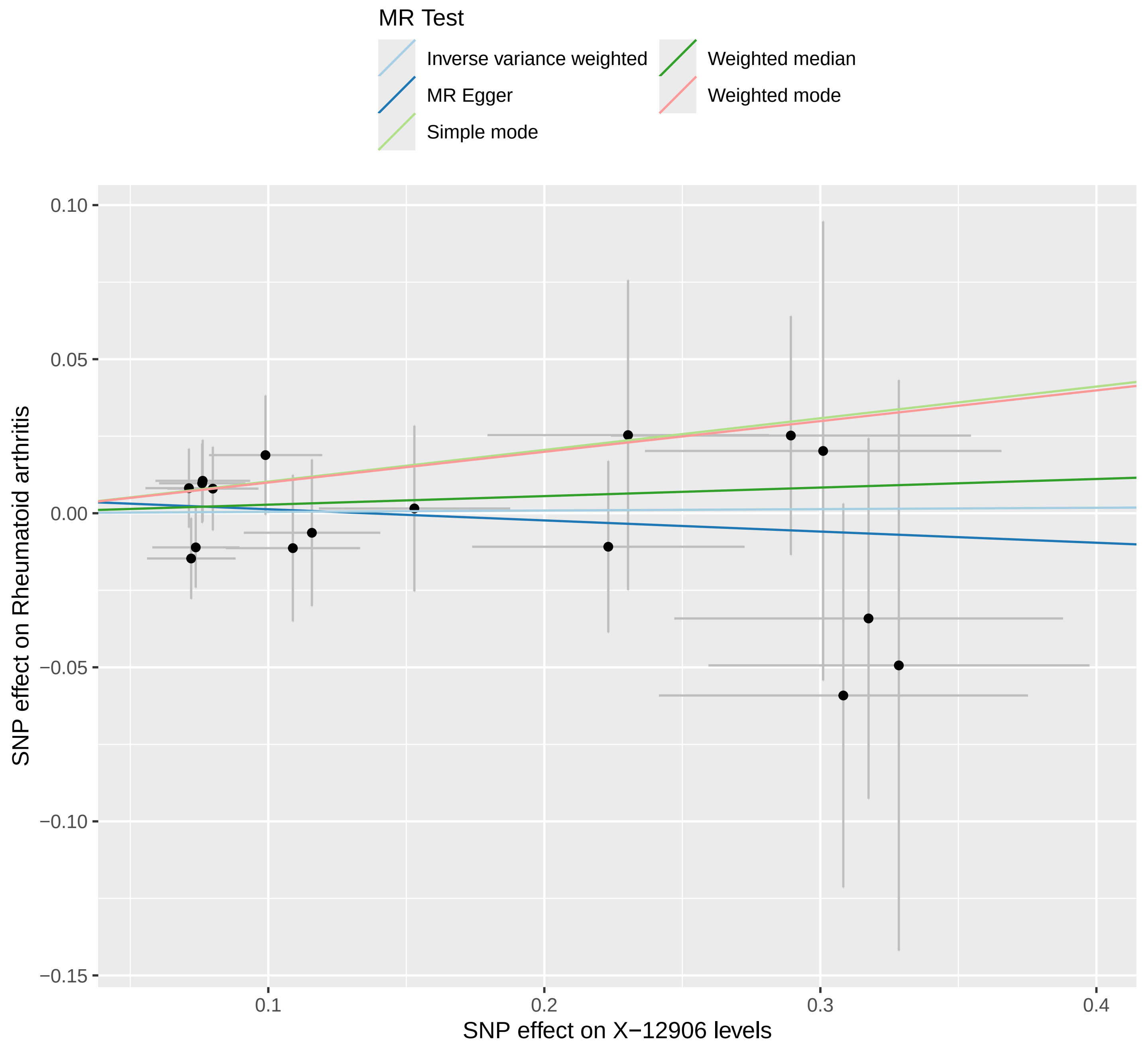
MR Test
Inverse variance weighted
MR Egger
Simple mode
Weighted median
Weighted mode
0.10
0.05
0.00
−0.05
−0.10
−0.15
0.1
0.2
0.3
0.4
SNP effect on Rheumatoid arthritis
SNP effect on X−12906 levels

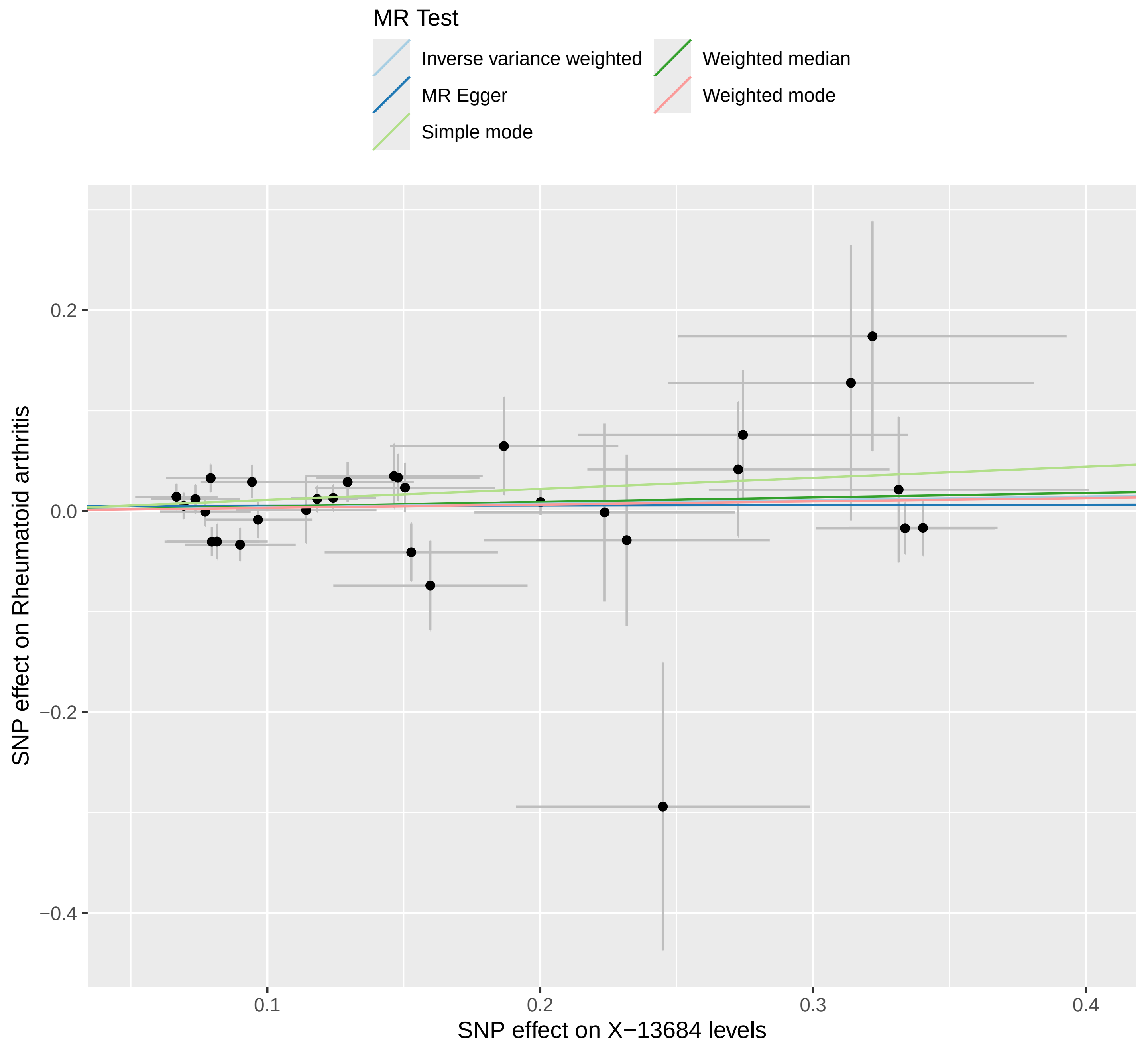
MR Test
Inverse variance weighted
MR Egger
Simple mode
Weighted median
Weighted mode
SNP effect on Rheumatoid arthritis
0.2
0.0
−0.2
−0.4
0.1
0.2
0.3
0.4
SNP effect on X−13684 levels

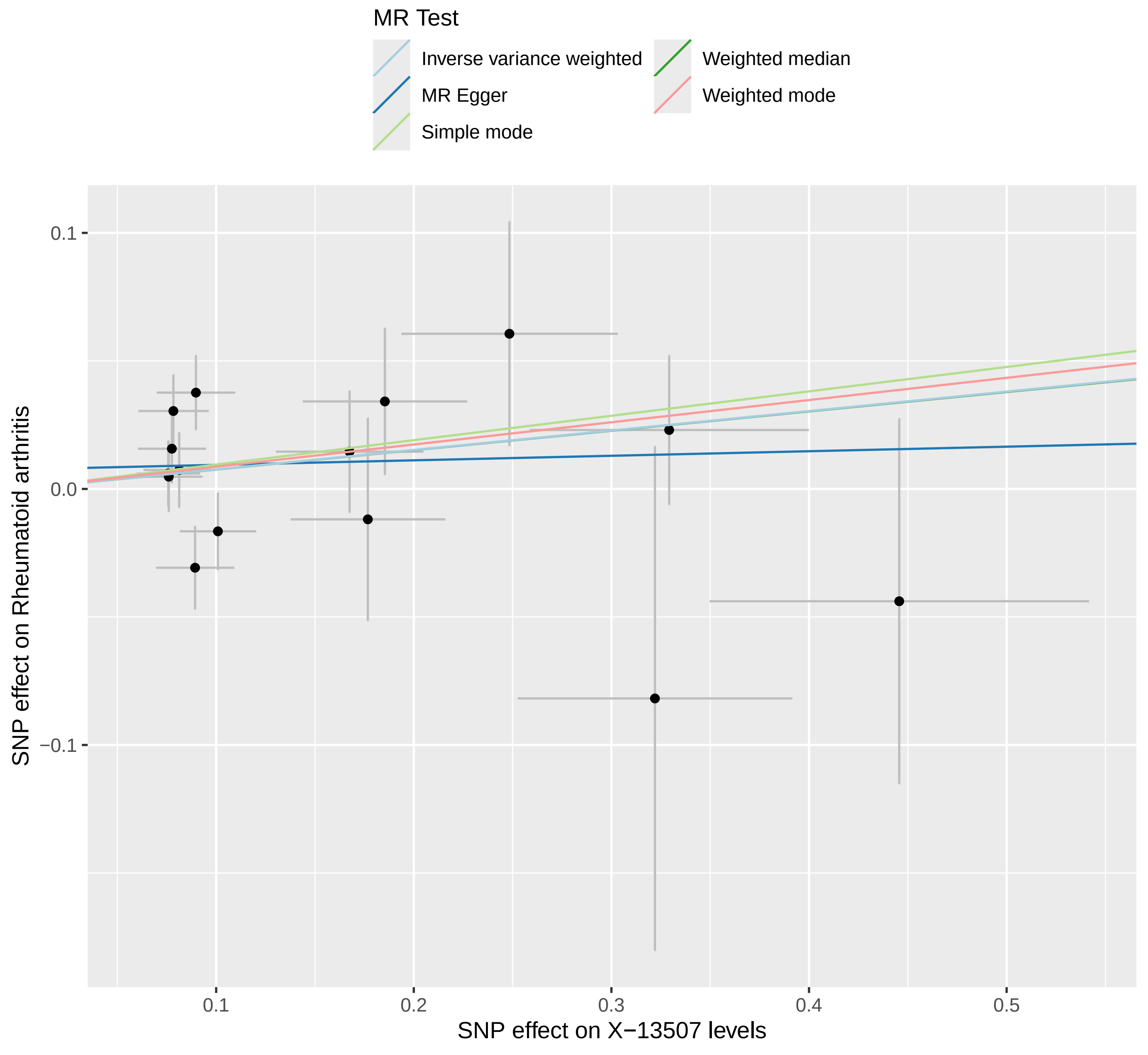
MR Test
Inverse variance weighted
MR Egger
Simple mode
Weighted median
Weighted mode
0.1
0.0
−0.1
0.1
0.2
0.3
0.4
0.5
SNP effect on Rheumatoid arthritis
SNP effect on X−13507 levels

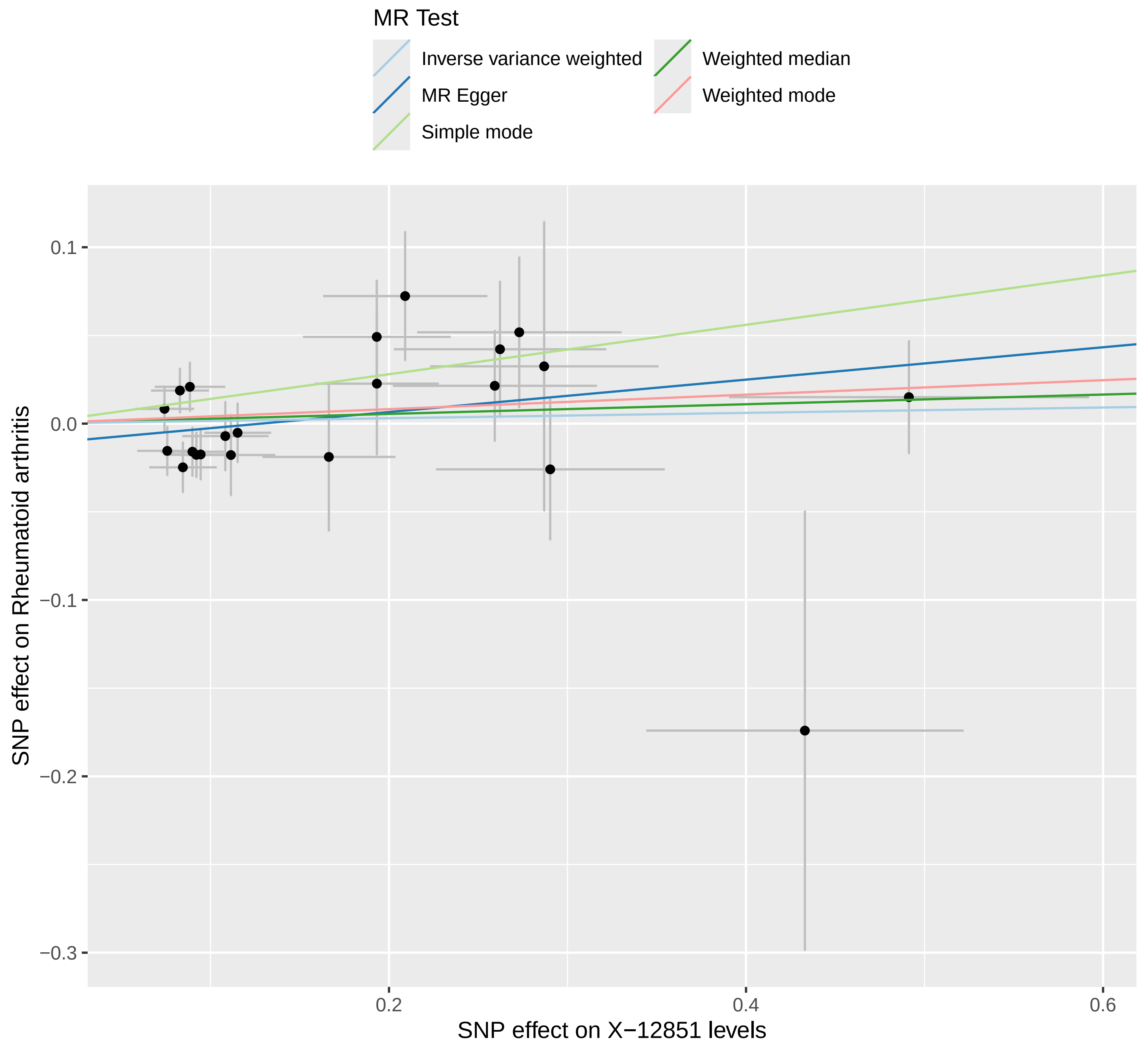

MR Test
Inverse variance weighted
MR Egger
Simple mode
Weighted median
Weighted mode
0.1
0.0
−0.1
−0.2
−0.3
0.2
0.4
0.6
SNP effect on Rheumatoid arthritis
SNP effect on X−12851 levels

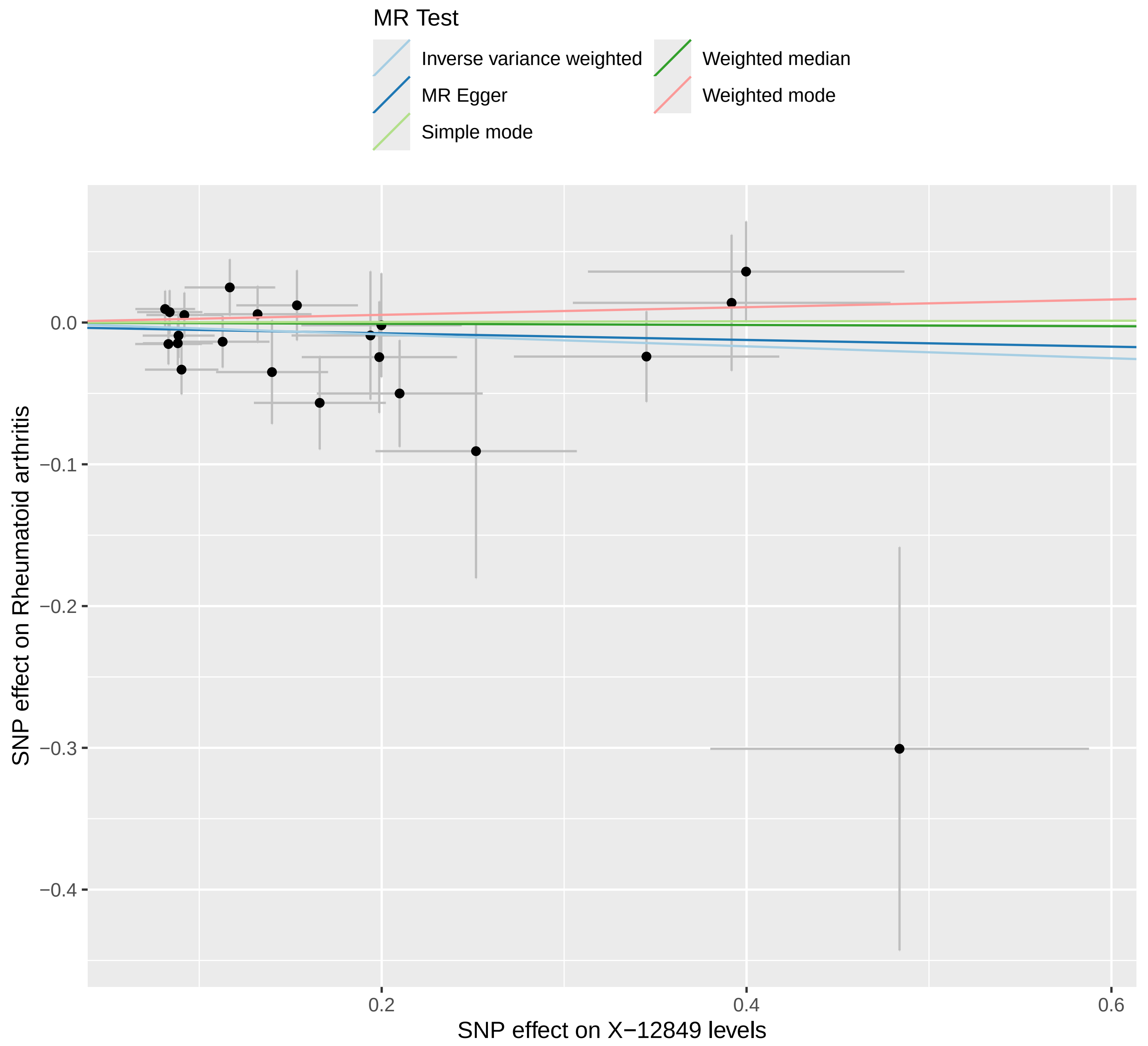

MR Test
Inverse variance weighted
MR Egger
Simple mode
Weighted median
Weighted mode
0.0
−0.1
−0.2
−0.3
−0.4
0.2
0.4
0.6
SNP effect on Rheumatoid arthritis
SNP effect on X−12849 levels

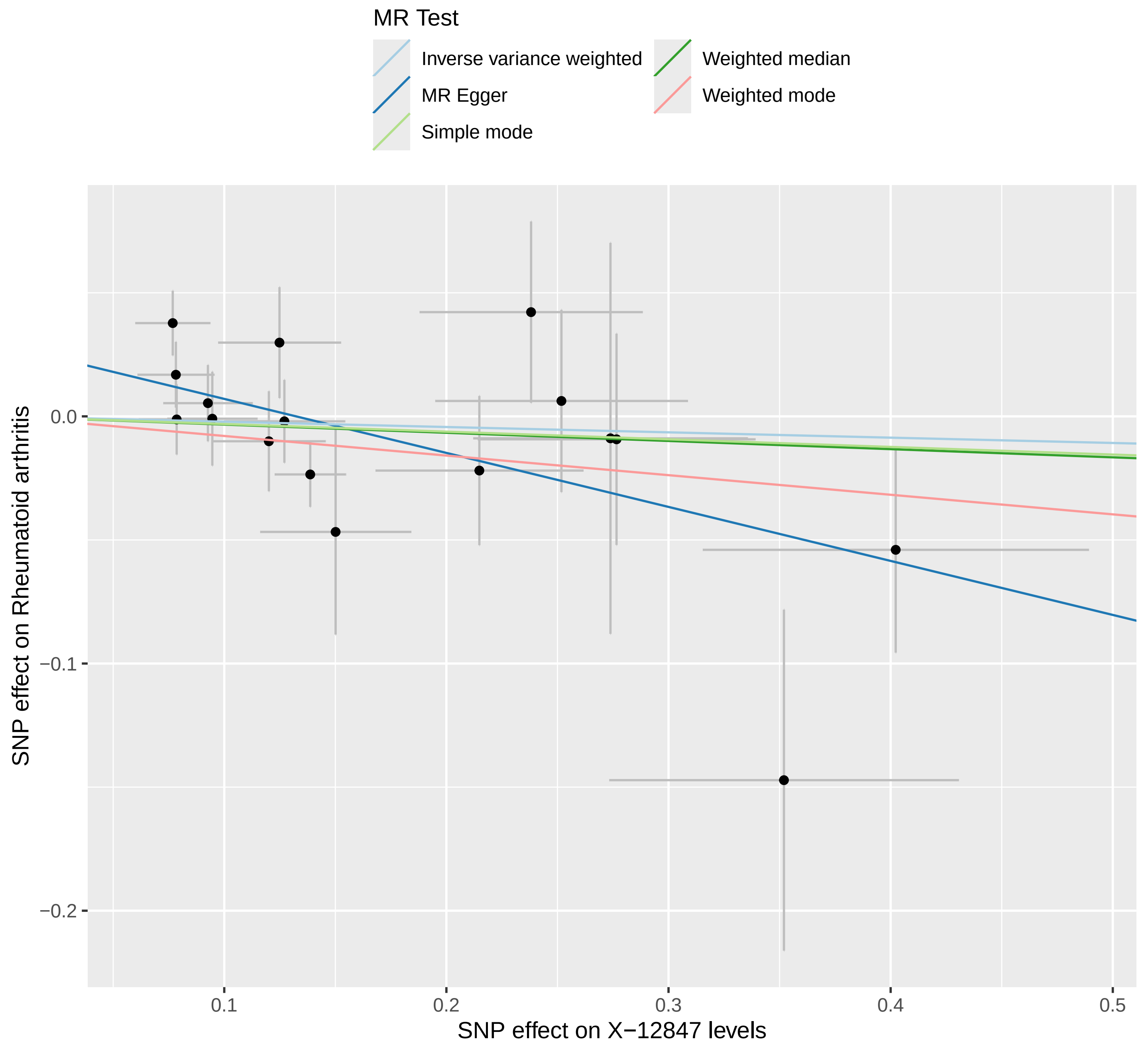

MR Test
Inverse variance weighted
MR Egger
Simple mode
Weighted median
Weighted mode
SNP effect on Rheumatoid arthritis
0.0
−0.1
−0.2
0.1
0.2
0.3
0.4
0.5
SNP effect on X−12847 levels

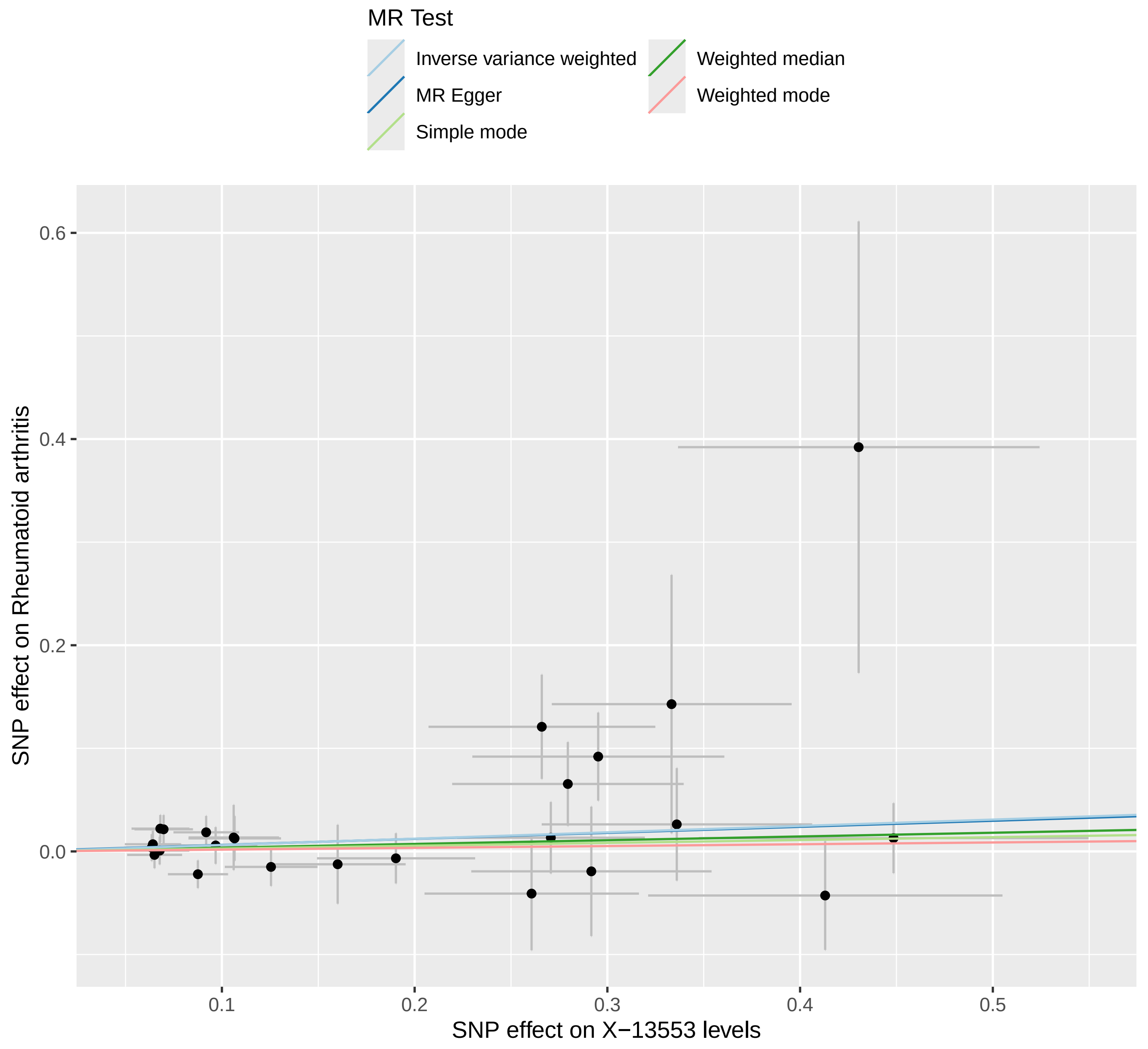
MR Test
Inverse variance weighted
MR Egger
Simple mode
Weighted median
Weighted mode
SNP effect on Rheumatoid arthritis
0.6
0.4
0.2
0.0
0.1
0.2
0.3
0.4
0.5
SNP effect on X−13553 levels

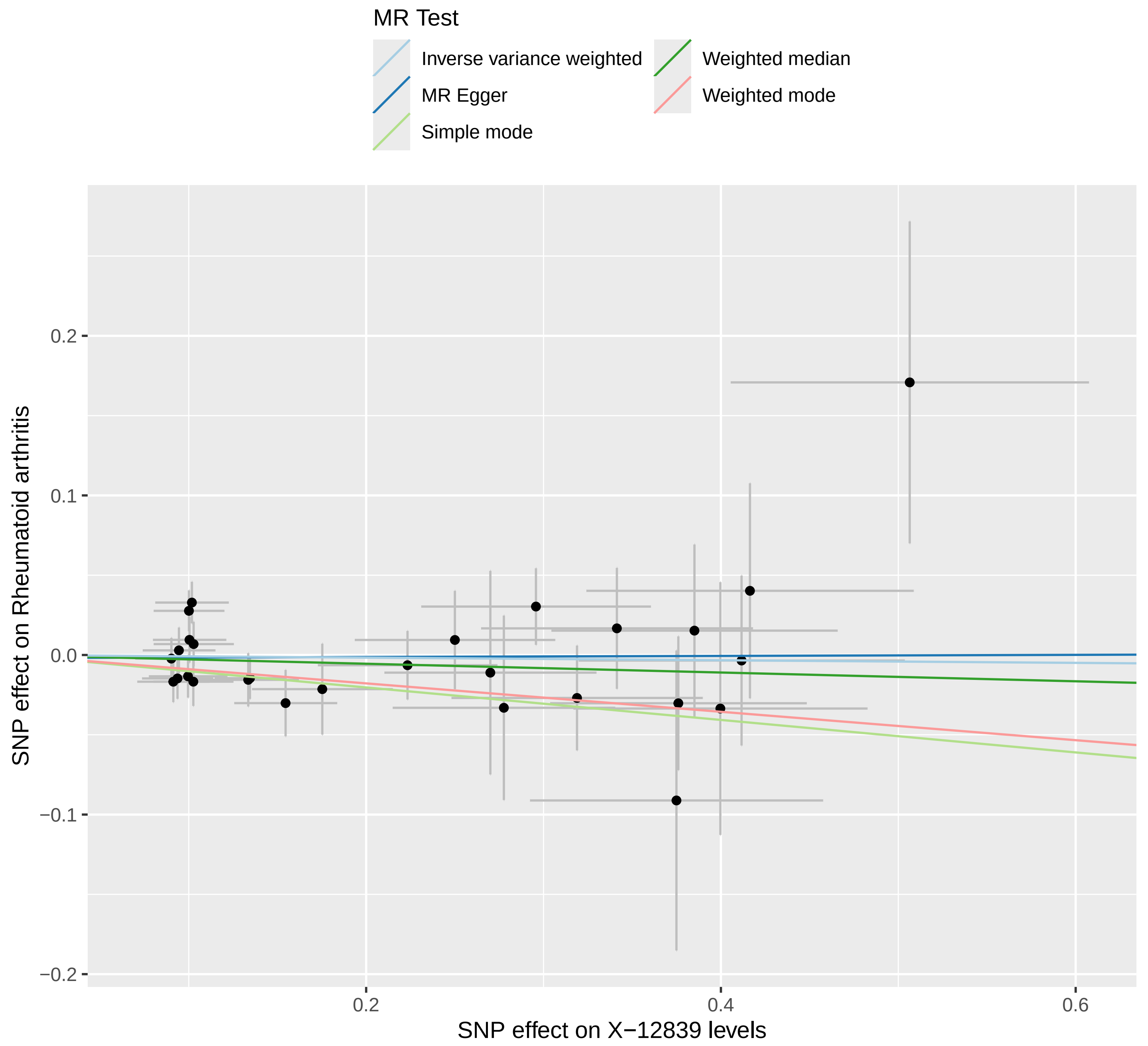

MR Test
Inverse variance weighted
MR Egger
Simple mode
Weighted median
Weighted mode
0.2
0.1
0.0
−0.1
−0.2
SNP effect on Rheumatoid arthritis
0.2
0.4
0.6
SNP effect on X−12839 levels

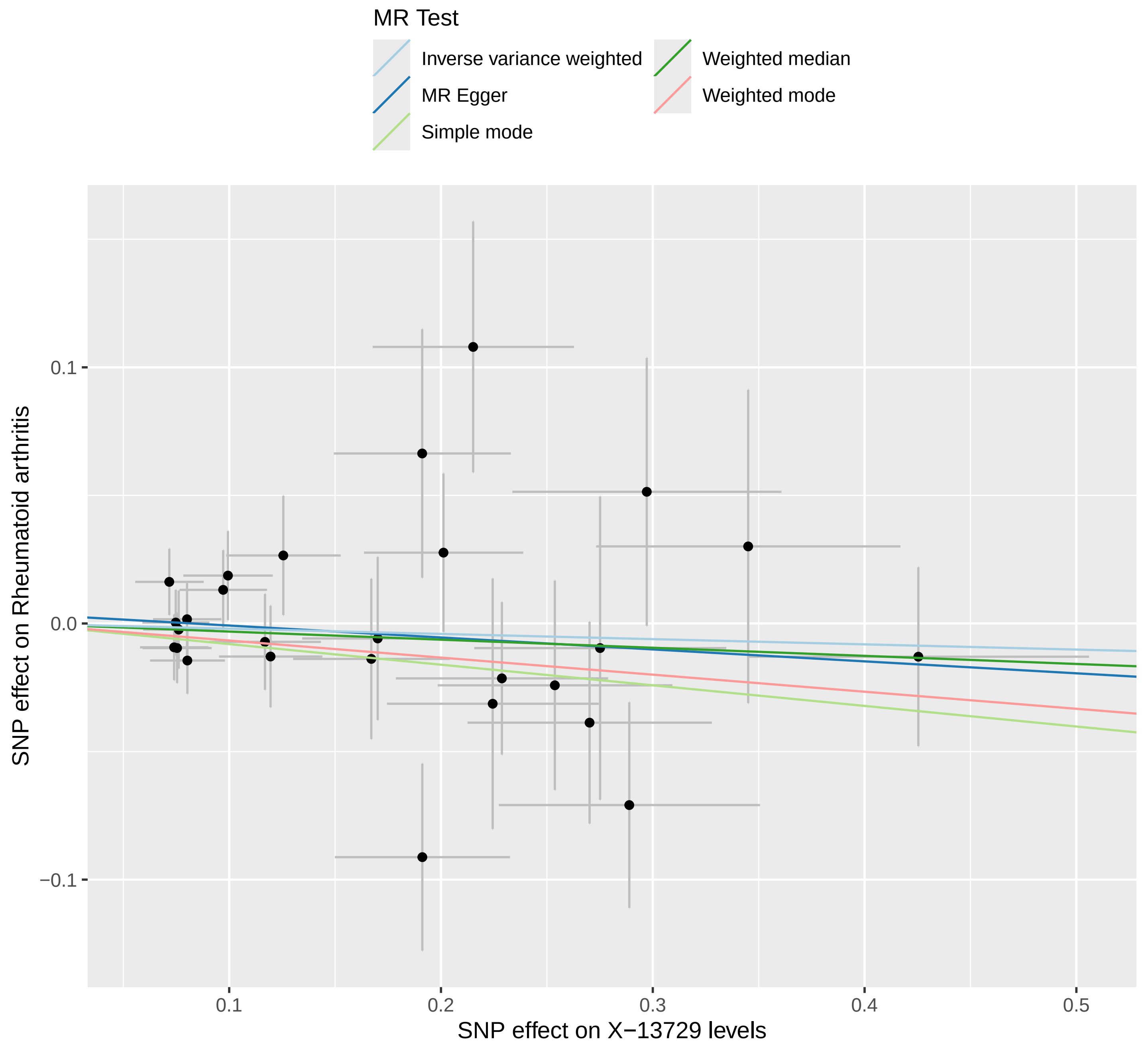

MR Test
Inverse variance weighted
MR Egger
Simple mode
Weighted median
Weighted mode
SNP effect on Rheumatoid arthritis
0.1
0.0
−0.1
0.1
0.2
0.3
0.4
0.5
SNP effect on X−13729 levels

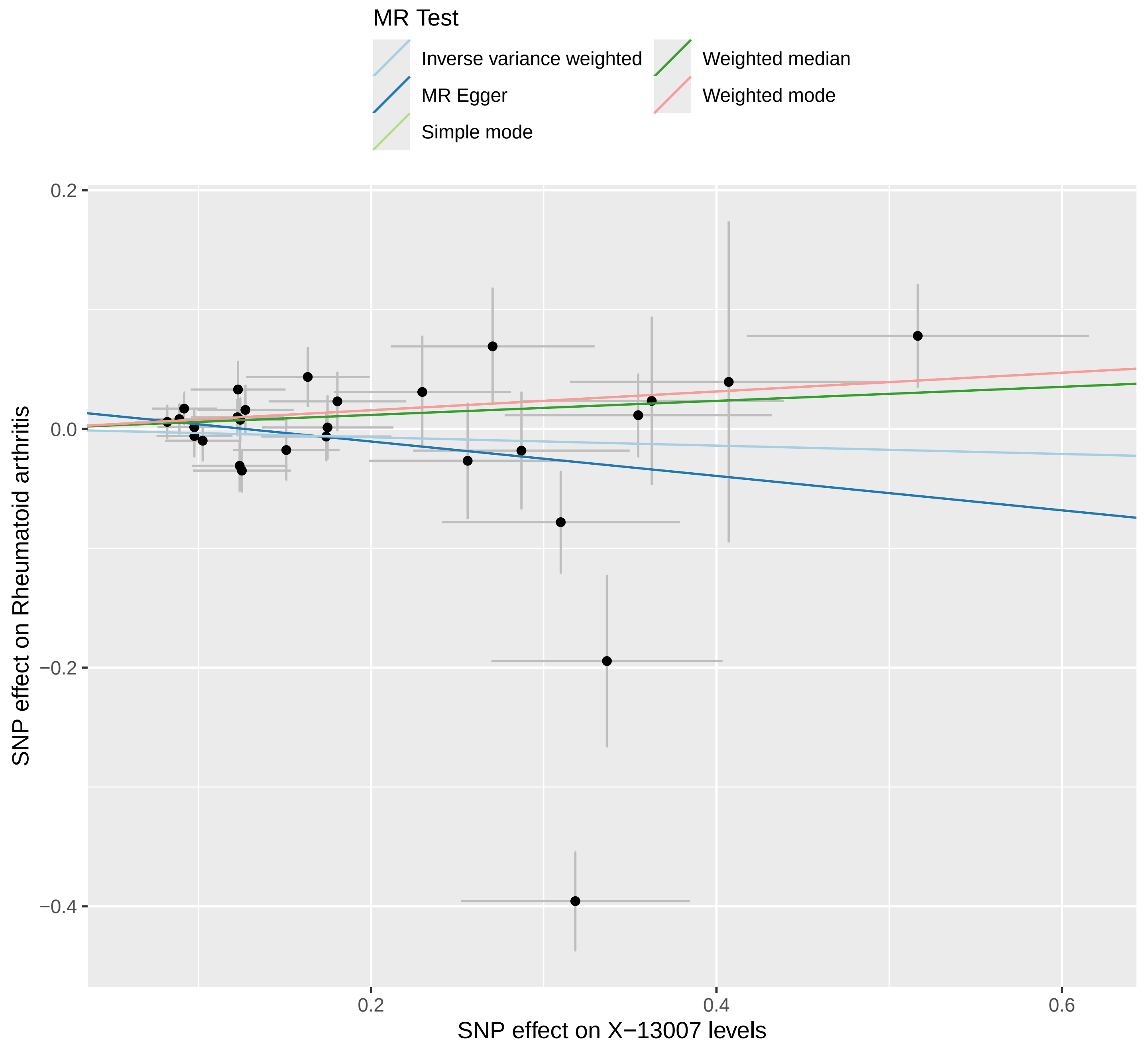

MR Test
Inverse variance weighted
MR Egger
Simple mode
Weighted median
Weighted mode
0.2
0.0
−0.2
−0.4
0.2
0.4
0.6
SNP effect on Rheumatoid arthritis
SNP effect on X−13007 levels

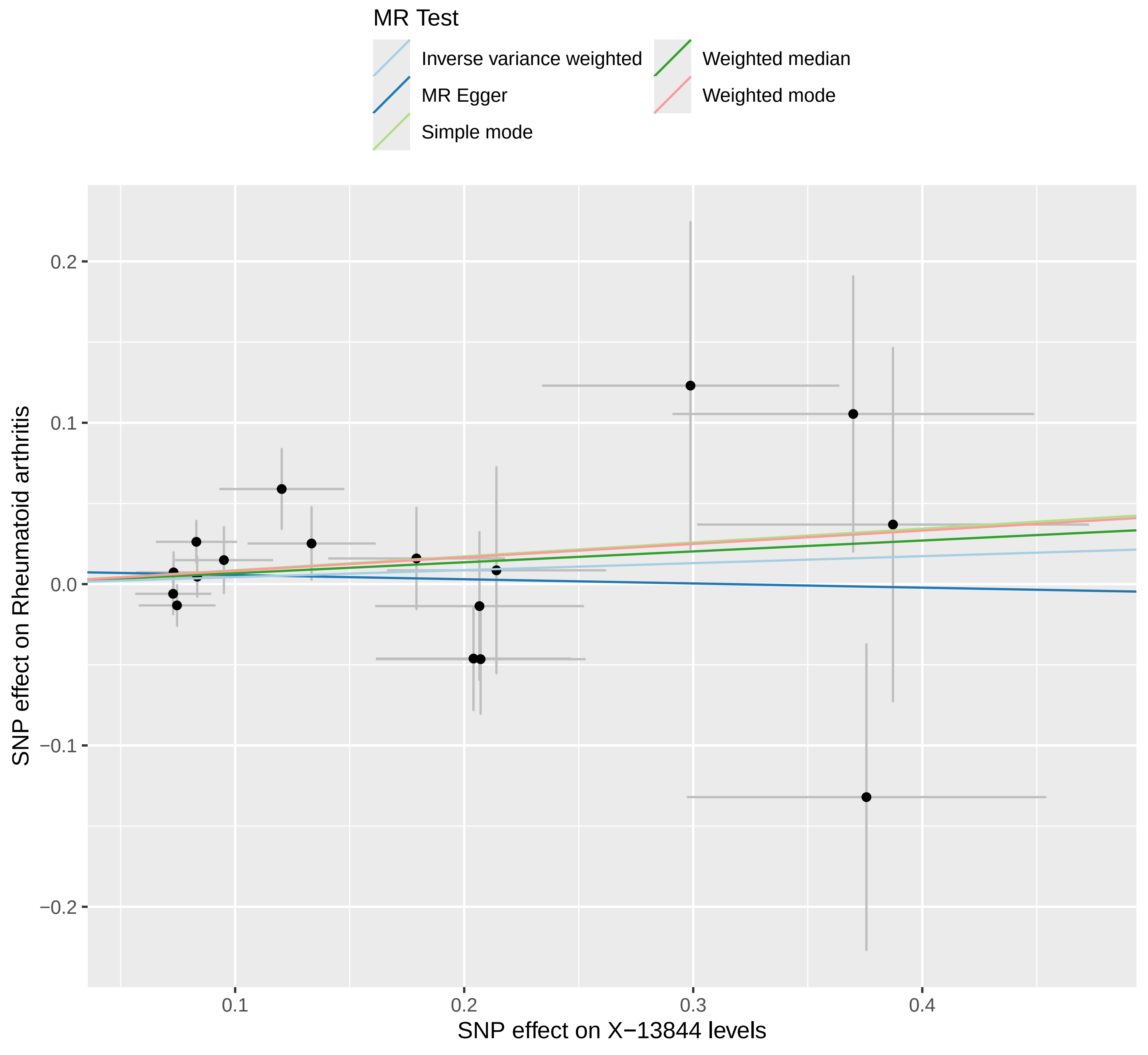
MR Test
Inverse variance weighted
MR Egger
Simple mode
Weighted median
Weighted mode
0.2
0.1
0.0
−0.1
−0.2
0.1
0.2
0.3
0.4
SNP effect on Rheumatoid arthritis
SNP effect on X−13844 levels

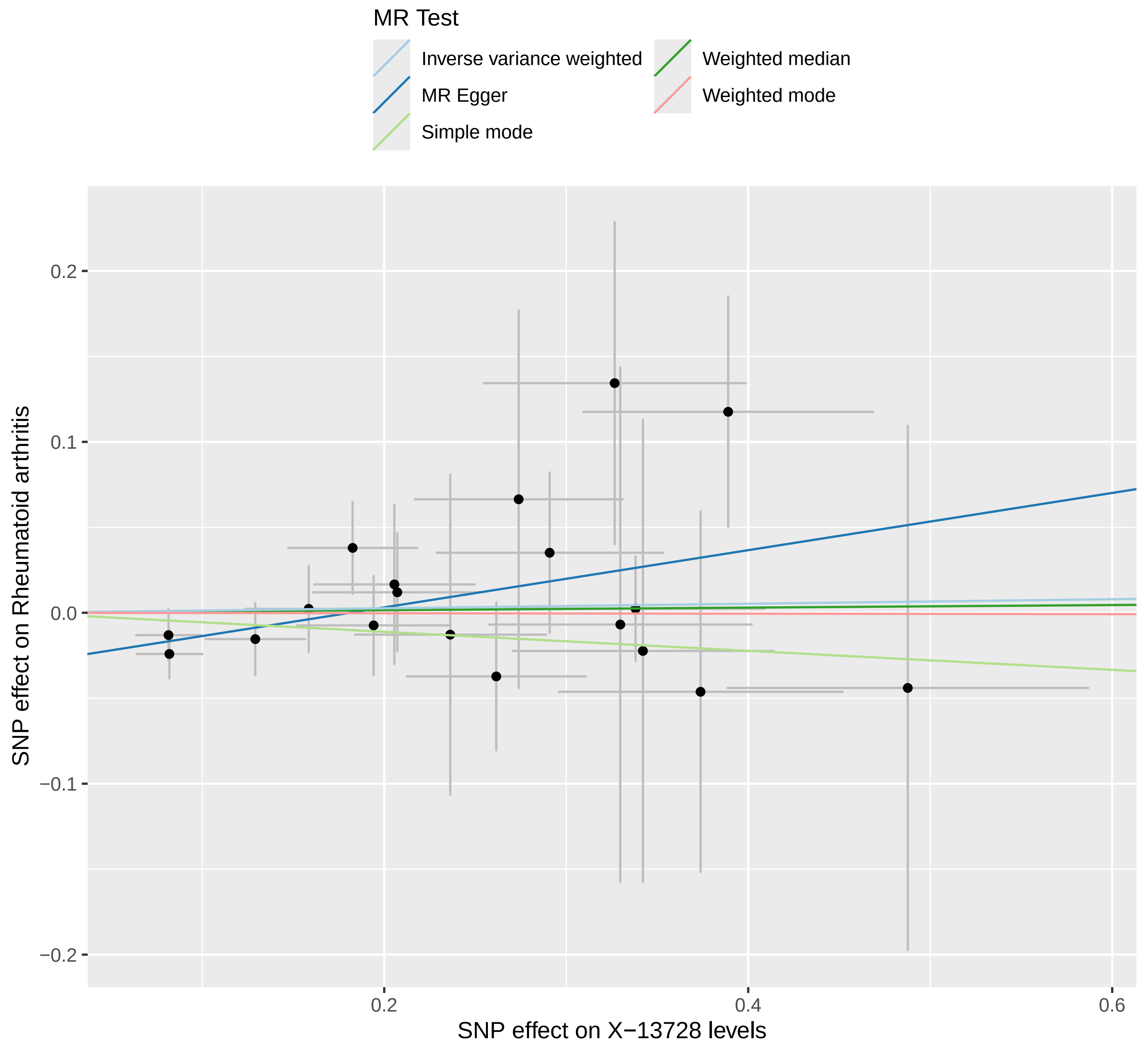

MR Test
Inverse variance weighted
MR Egger
Simple mode
Weighted median
Weighted mode
SNP effect on Rheumatoid arthritis
0.2
0.1
0.0
−0.1
−0.2
0.2
0.4
0.6
SNP effect on X−13728 levels

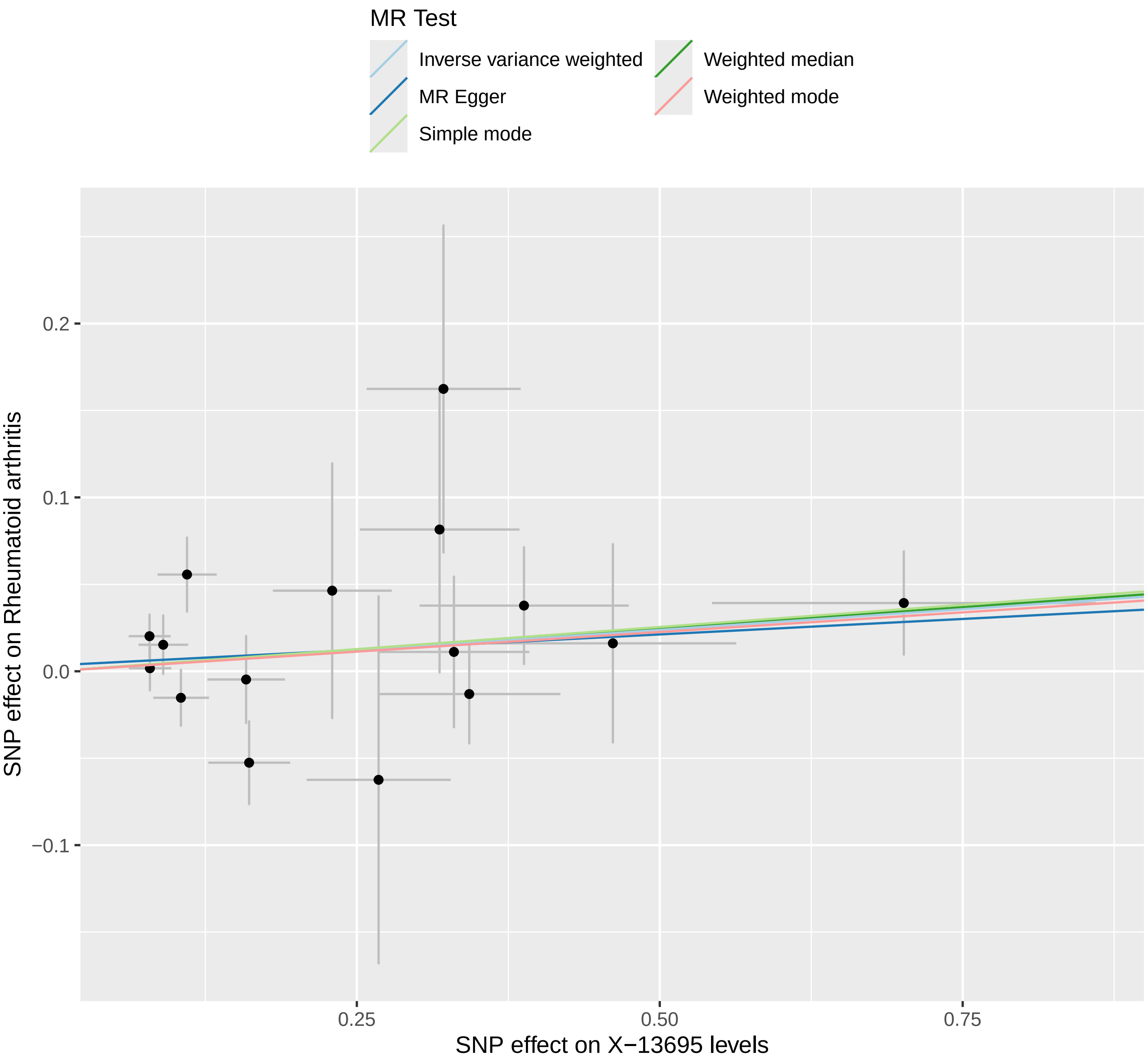
MR Test
Inverse variance weighted
MR Egger
Simple mode
Weighted median
Weighted mode
0.2
0.1
0.0
−0.1
0.25
0.50
0.75
SNP effect on Rheumatoid arthritis
SNP effect on X−13695 levels

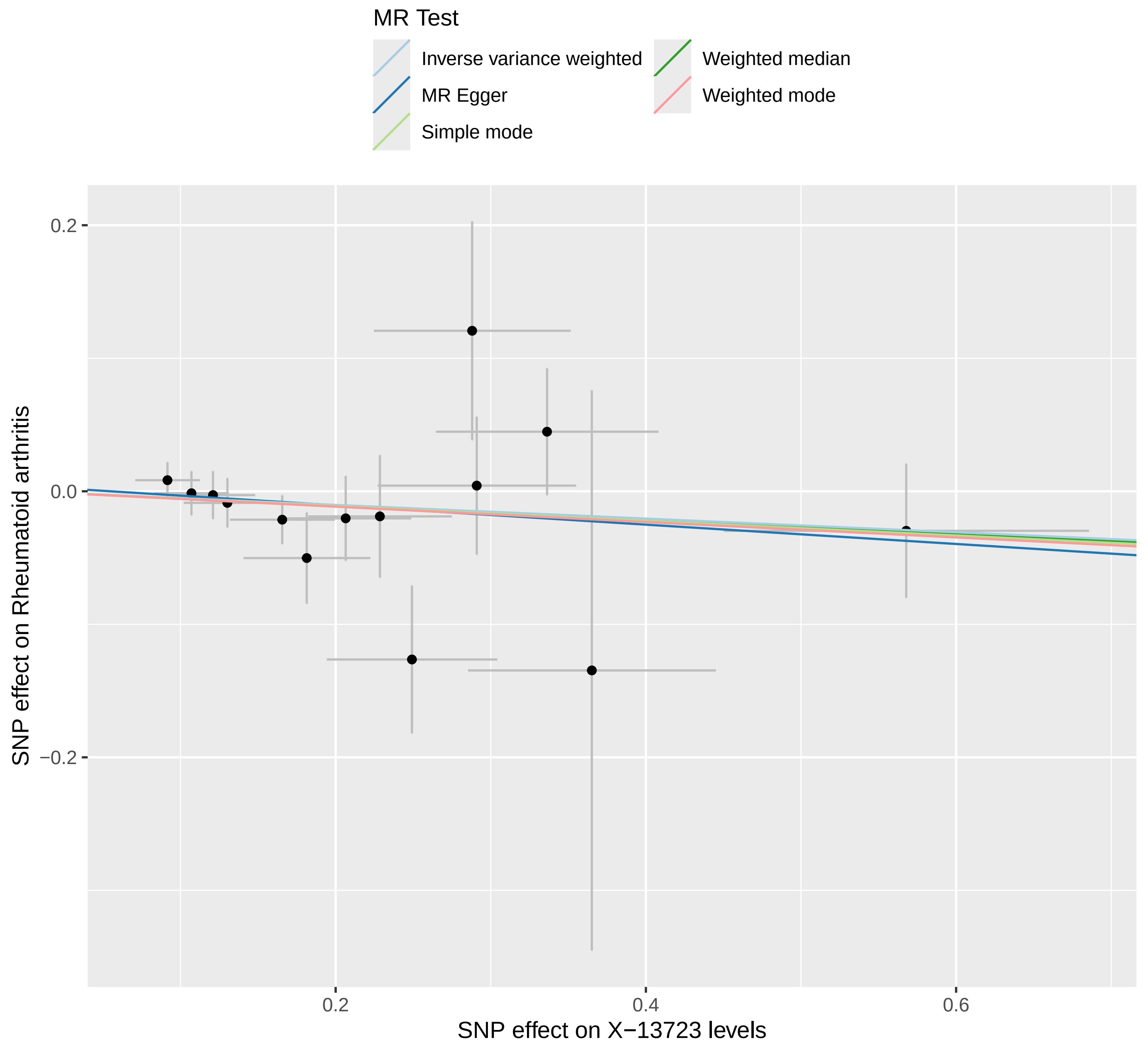
MR Test
Inverse variance weighted
MR Egger
Simple mode
Weighted median
Weighted mode
0.2
0.0
−0.2
0.2
0.4
0.6
SNP effect on Rheumatoid arthritis
SNP effect on X−13723 levels

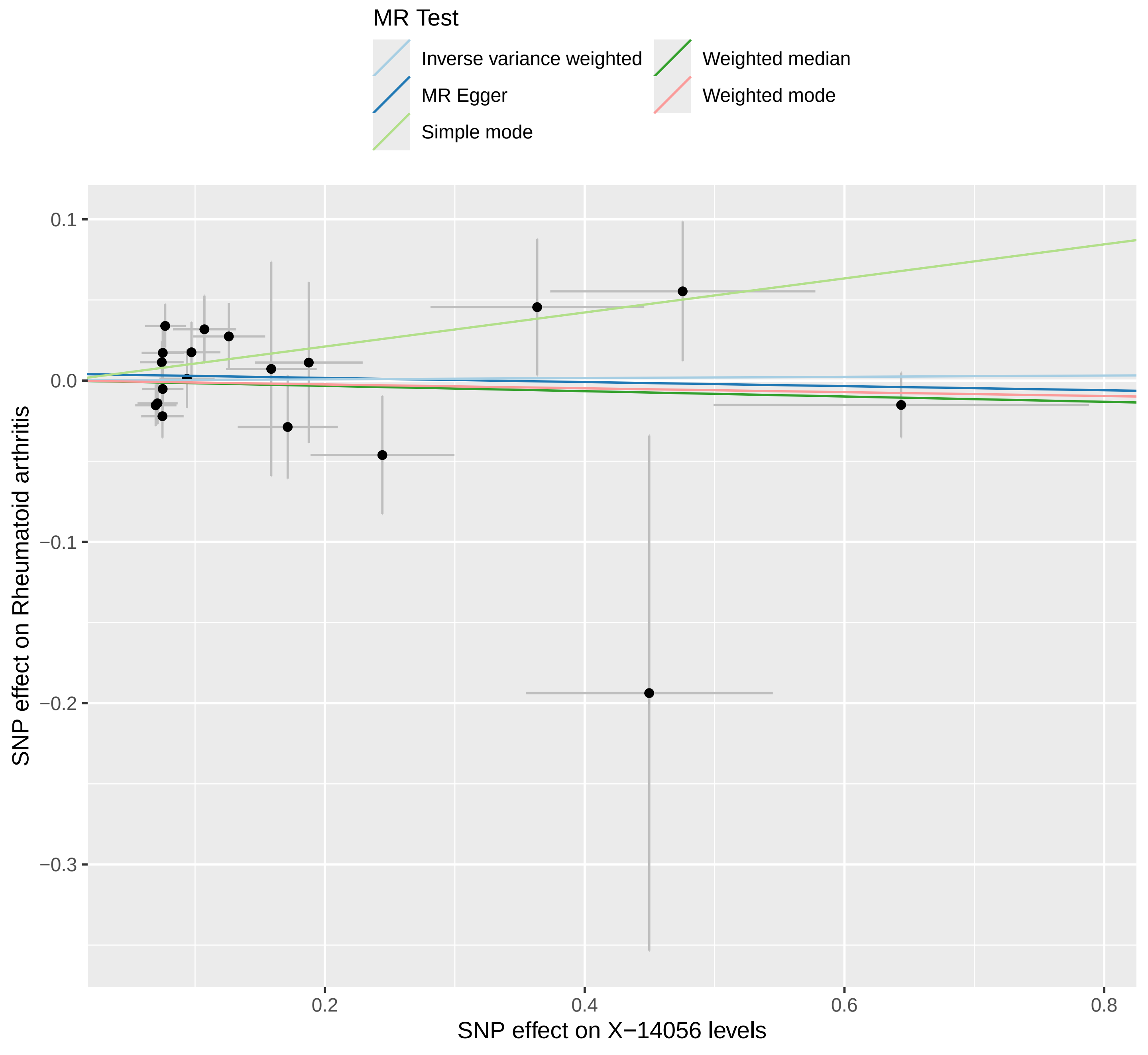

MR Test
Inverse variance weighted
MR Egger
Simple mode
Weighted median
Weighted mode
0.1
0.0
−0.1
−0.2
−0.3
0.2
0.4
0.6
0.8
SNP effect on Rheumatoid arthritis
SNP effect on X−14056 levels

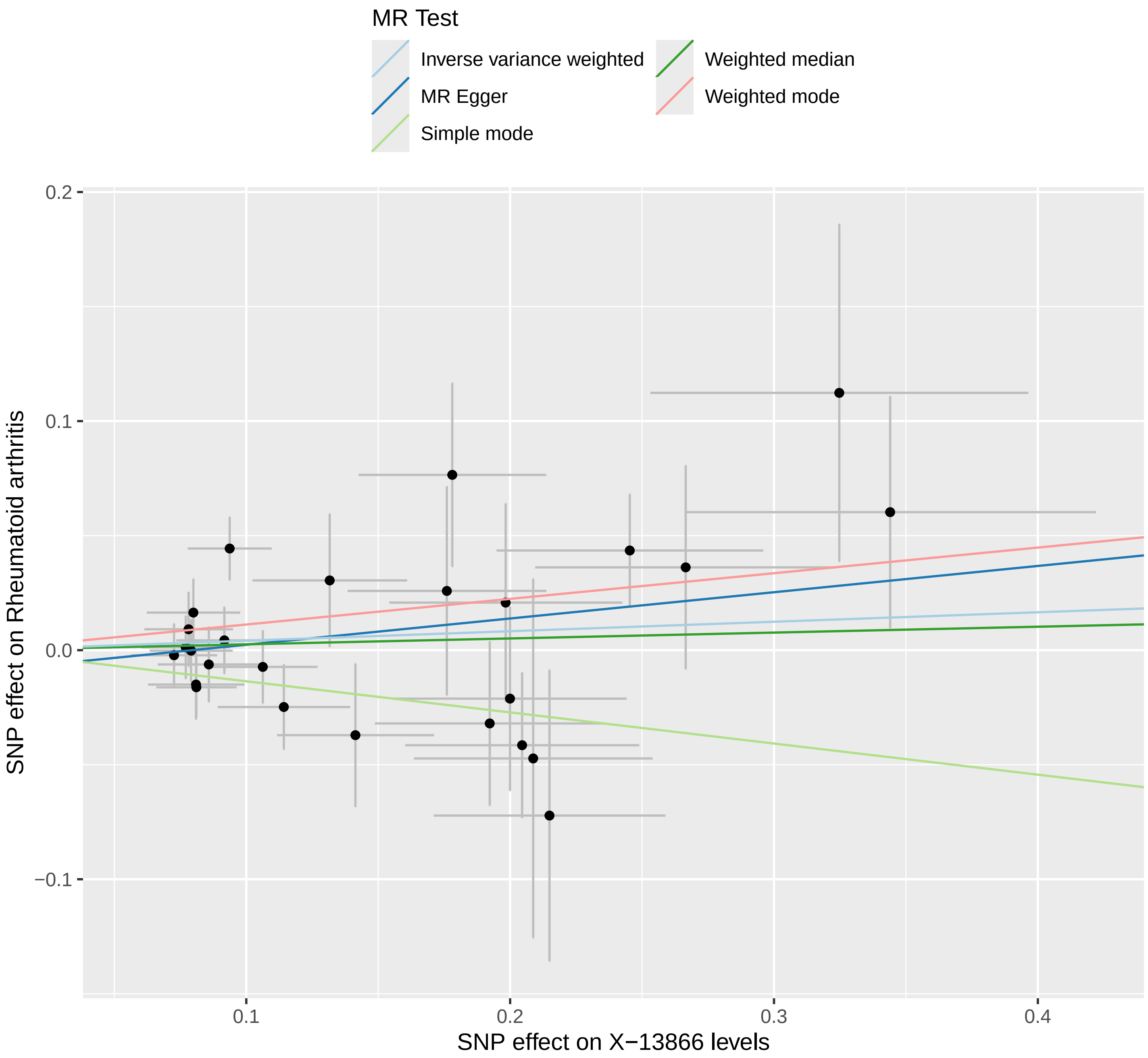
MR Test
Inverse variance weighted
MR Egger
Simple mode
Weighted median
Weighted mode
0.2
0.1
0.0
−0.1
SNP effect on Rheumatoid arthritis
0.1
0.2
0.3
0.4
SNP effect on X−13866 levels

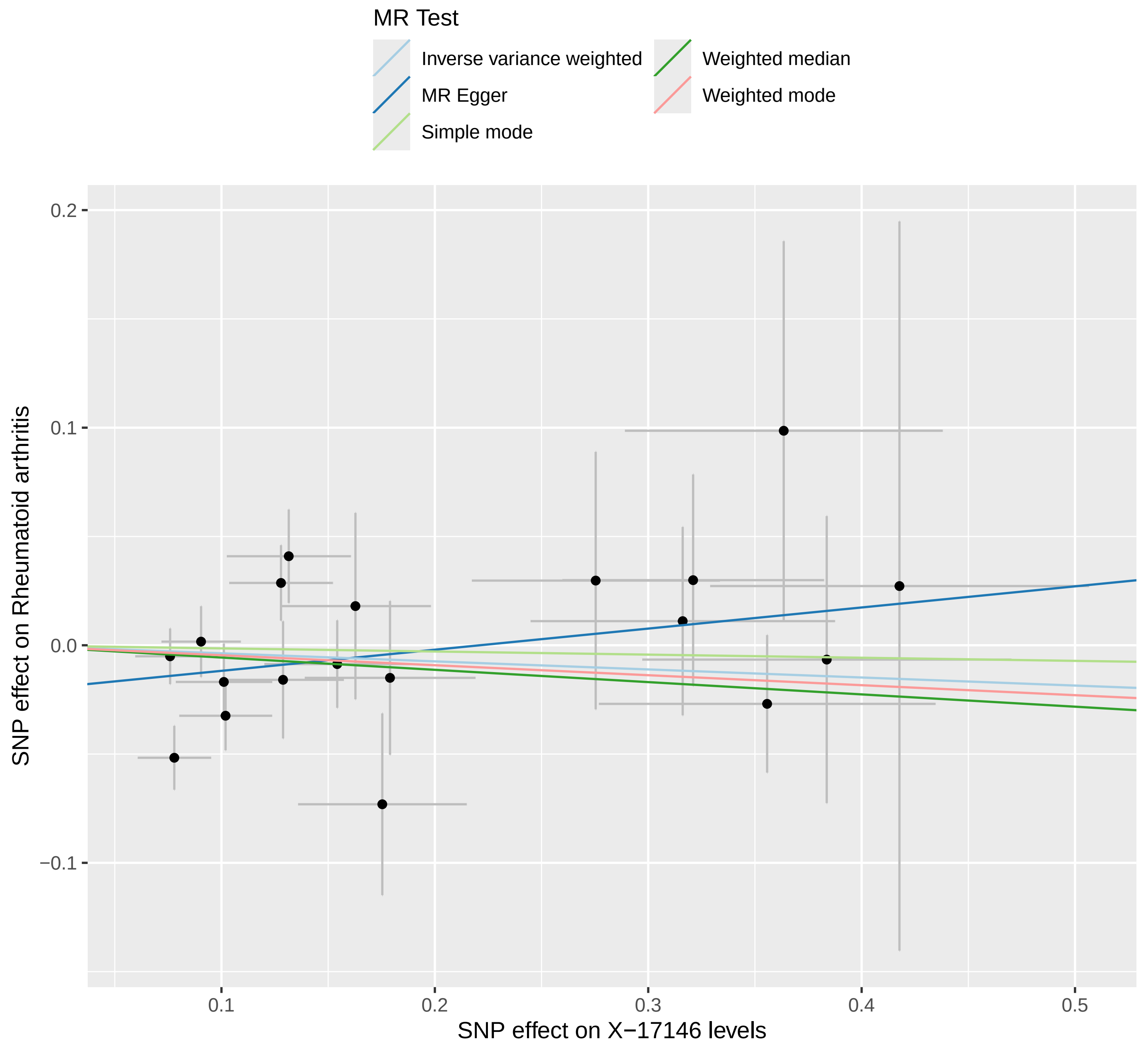
MR Test
Inverse variance weighted
MR Egger
Simple mode
Weighted median
Weighted mode
0.2
0.1
0.0
−0.1
0.1
0.2
0.3
0.4
0.5
SNP effect on Rheumatoid arthritis
SNP effect on X−17146 levels

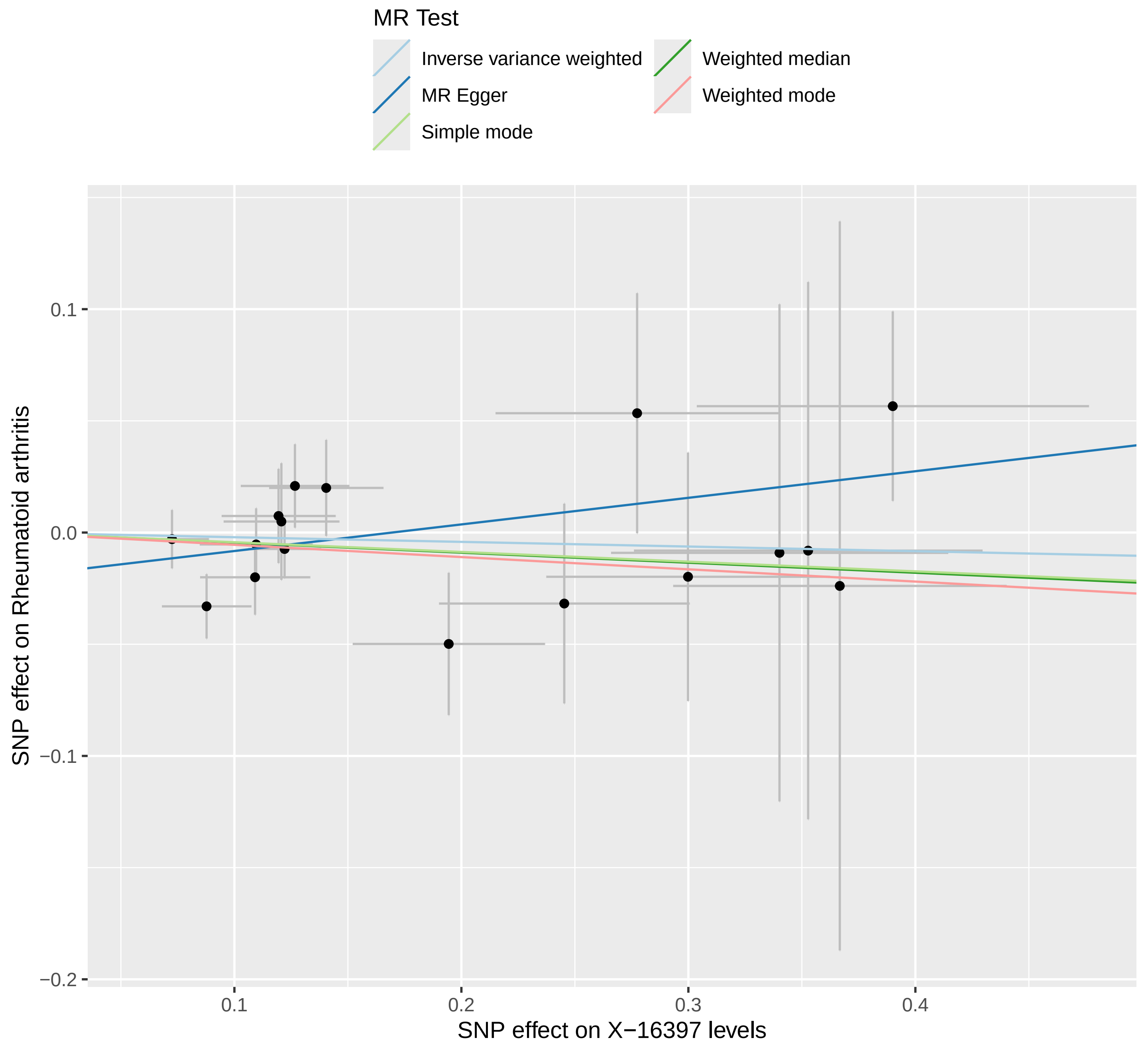

MR Test
Inverse variance weighted
MR Egger
Simple mode
Weighted median
Weighted mode
0.1
0.0
−0.1
−0.2
0.1
0.2
0.3
0.4
SNP effect on Rheumatoid arthritis
SNP effect on X−16397 levels

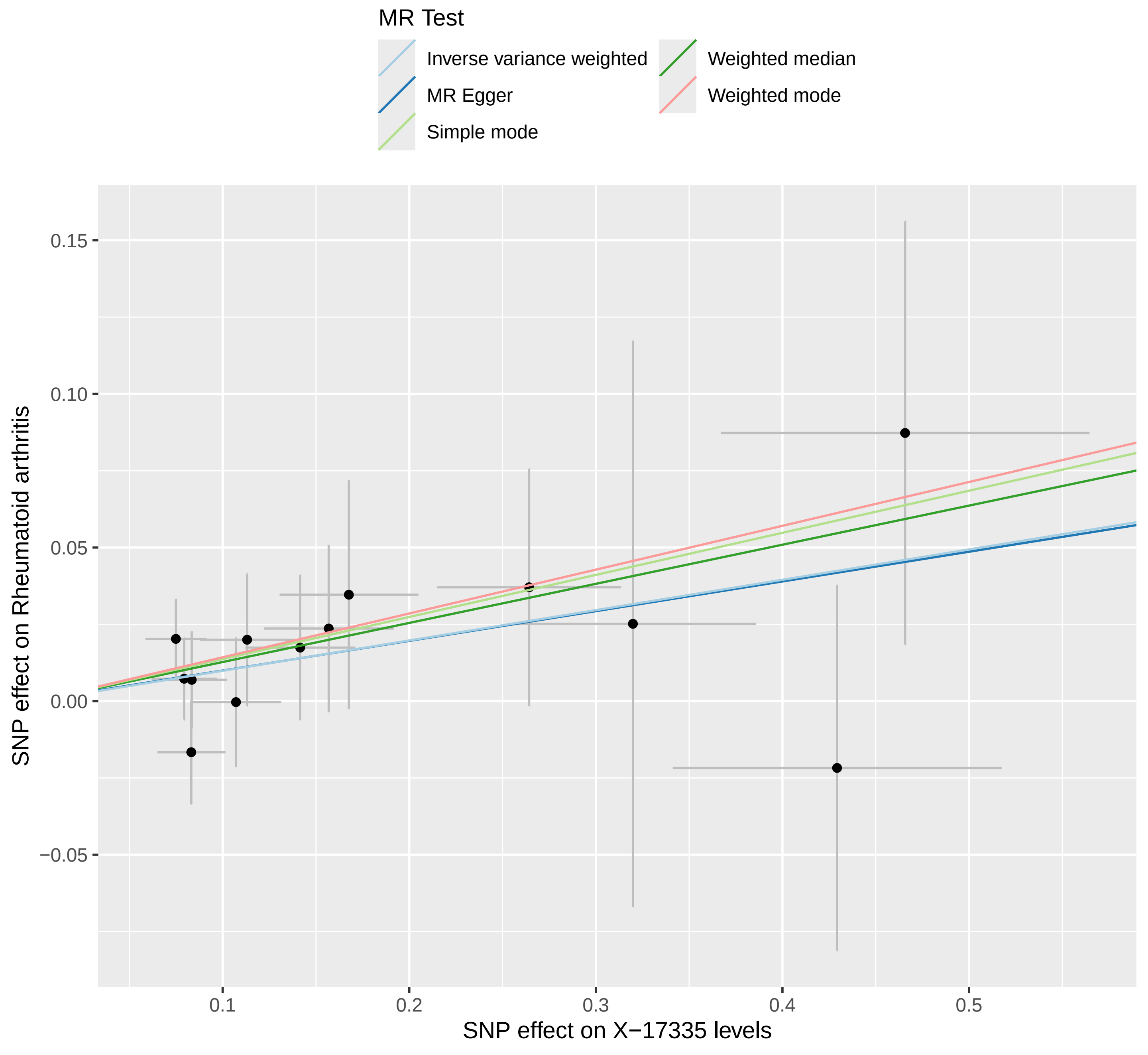
MR Test
Inverse variance weighted
MR Egger
Simple mode
Weighted median
Weighted mode
0.15
0.10
0.05
0.00
−0.05
0.1
0.2
0.3
0.4
0.5
SNP effect on Rheumatoid arthritis
SNP effect on X−17335 levels

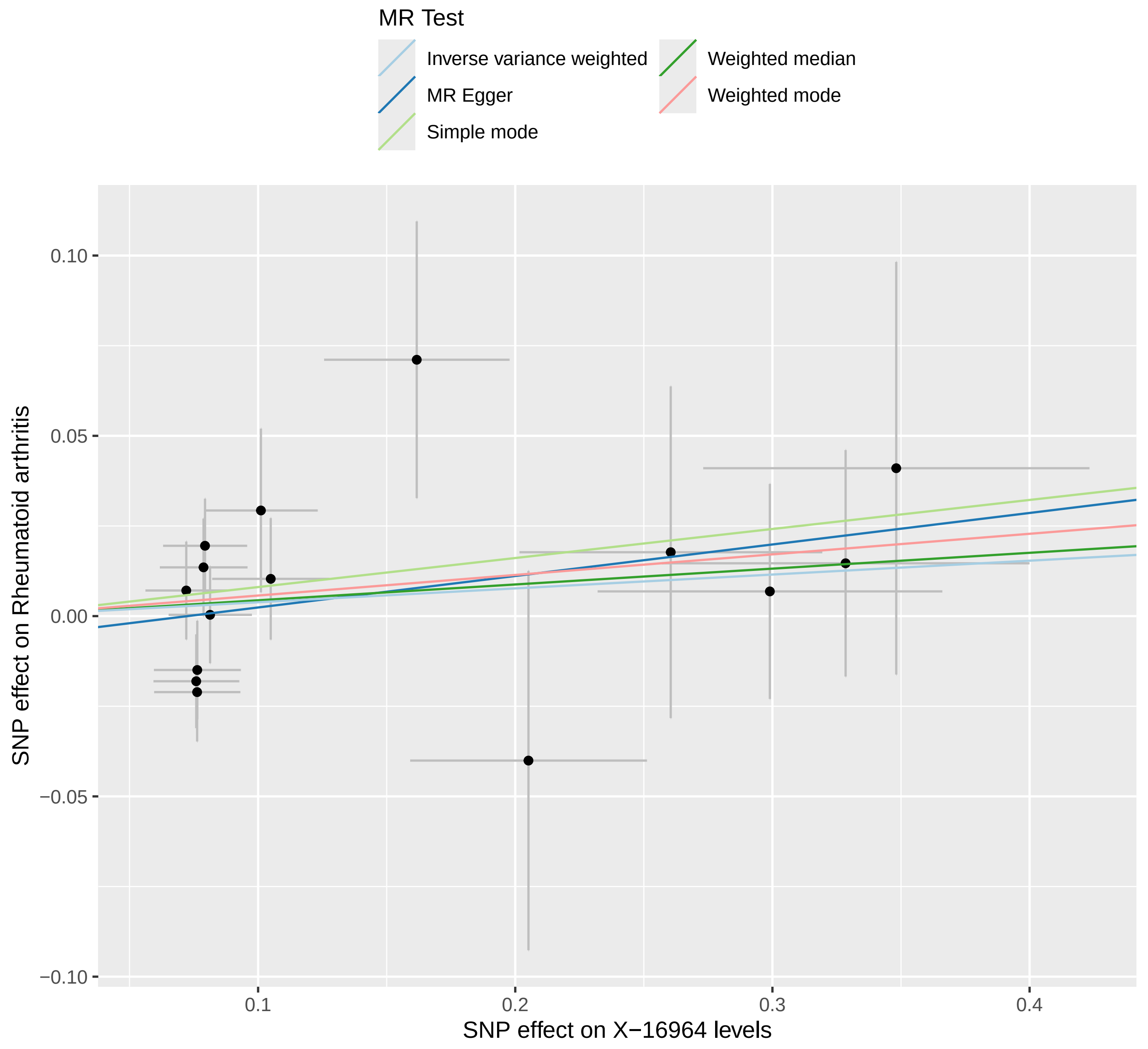
MR Test
Inverse variance weighted
MR Egger
Simple mode
Weighted median
Weighted mode
0.10
0.05
0.00
−0.05
−0.10
SNP effect on Rheumatoid arthritis
0.1
0.2
0.3
0.4
SNP effect on X−16964 levels

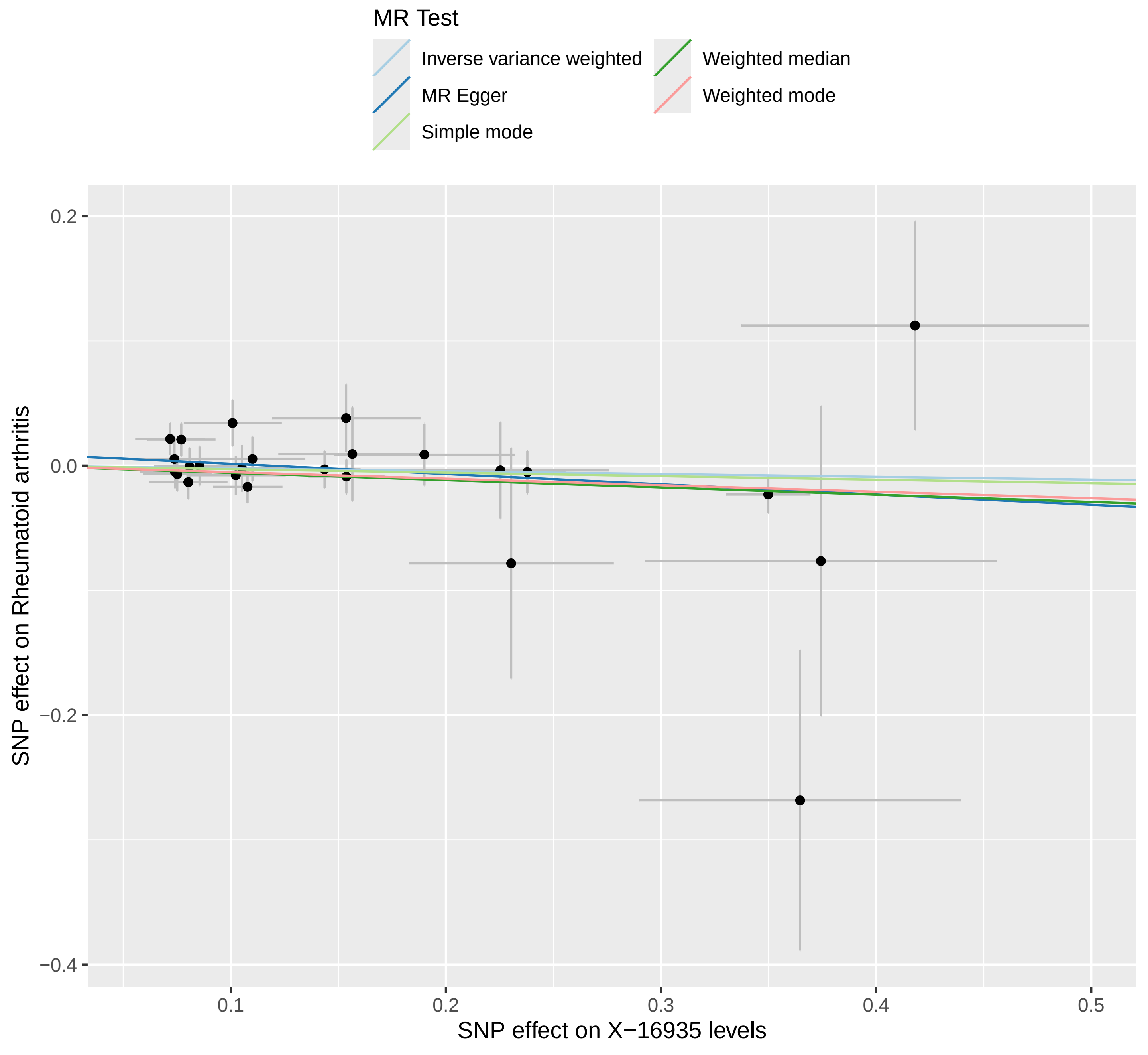
MR Test
Inverse variance weighted
MR Egger
Simple mode
Weighted median
Weighted mode
0.2
0.0
−0.2
−0.4
SNP effect on Rheumatoid arthritis
0.1
0.2
0.3
0.4
0.5
SNP effect on X−16935 levels

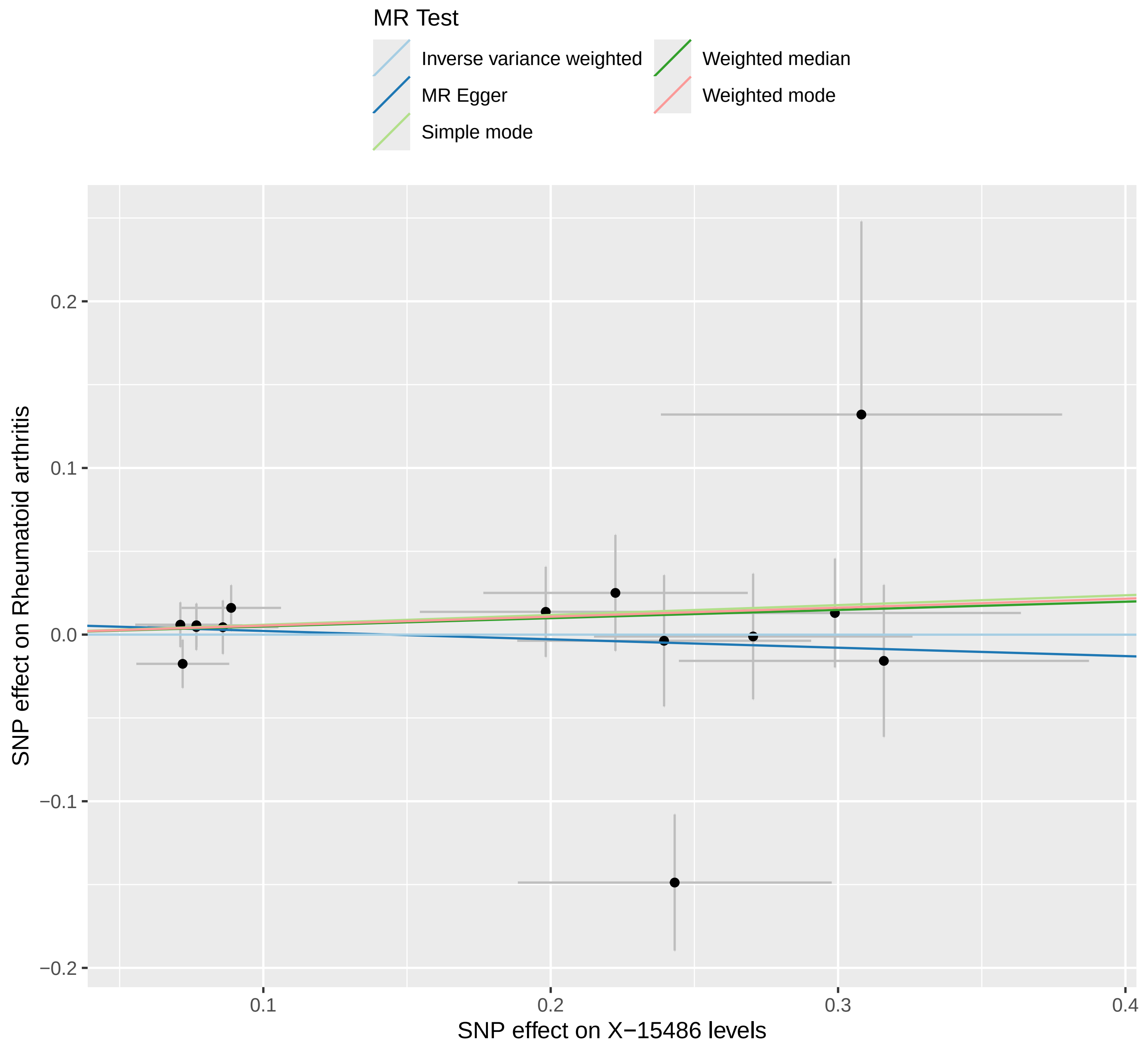
MR Test
Inverse variance weighted
MR Egger
Simple mode
Weighted median
Weighted mode
0.2
0.1
0.0
−0.1
−0.2
SNP effect on Rheumatoid arthritis
0.1
0.2
0.3
0.4
SNP effect on X−15486 levels

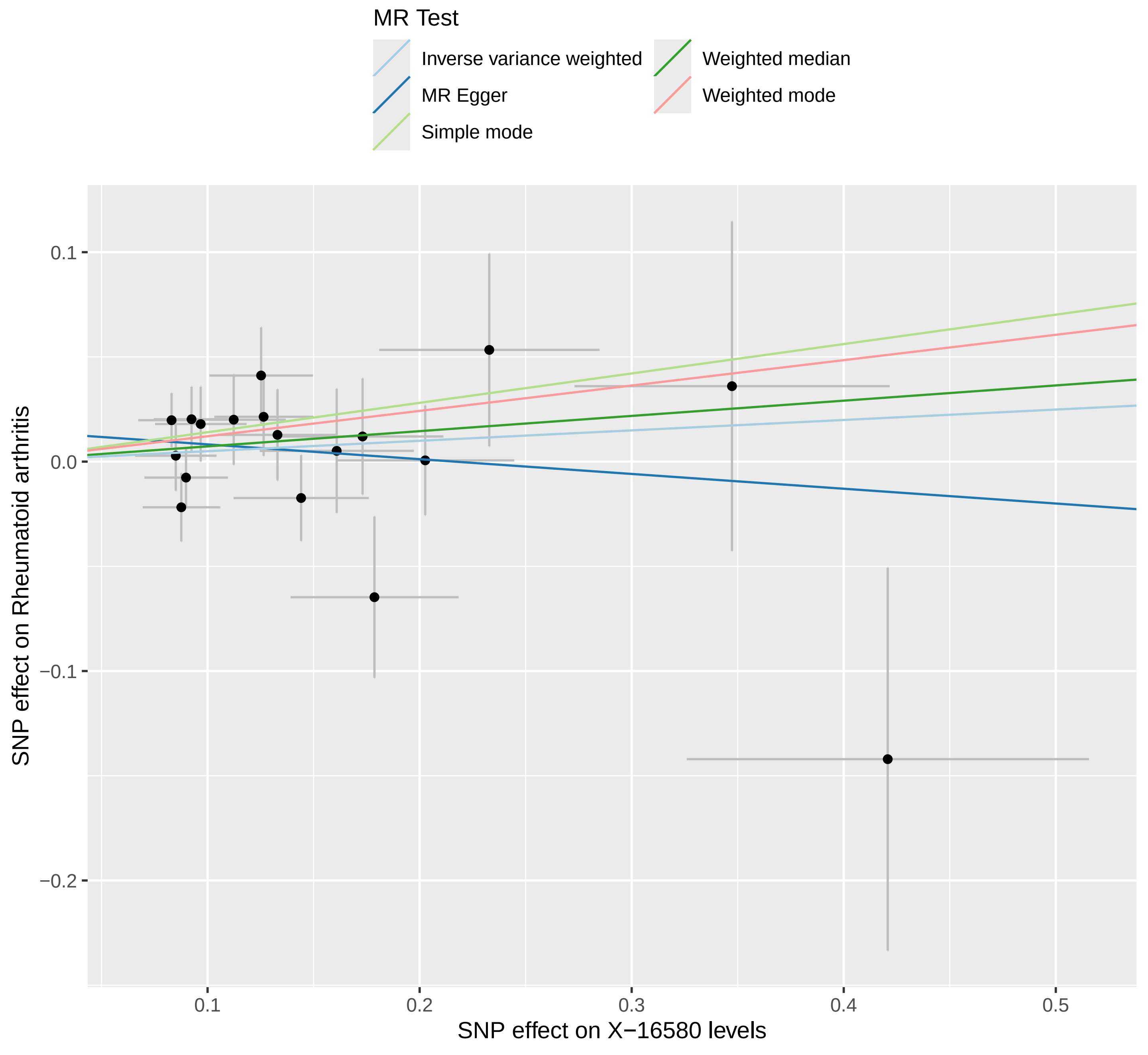
MR Test
Inverse variance weighted
MR Egger
Simple mode
Weighted median
Weighted mode
0.1
0.0
−0.1
−0.2
SNP effect on Rheumatoid arthritis
0.1
0.2
0.3
0.4
0.5
SNP effect on X−16580 levels

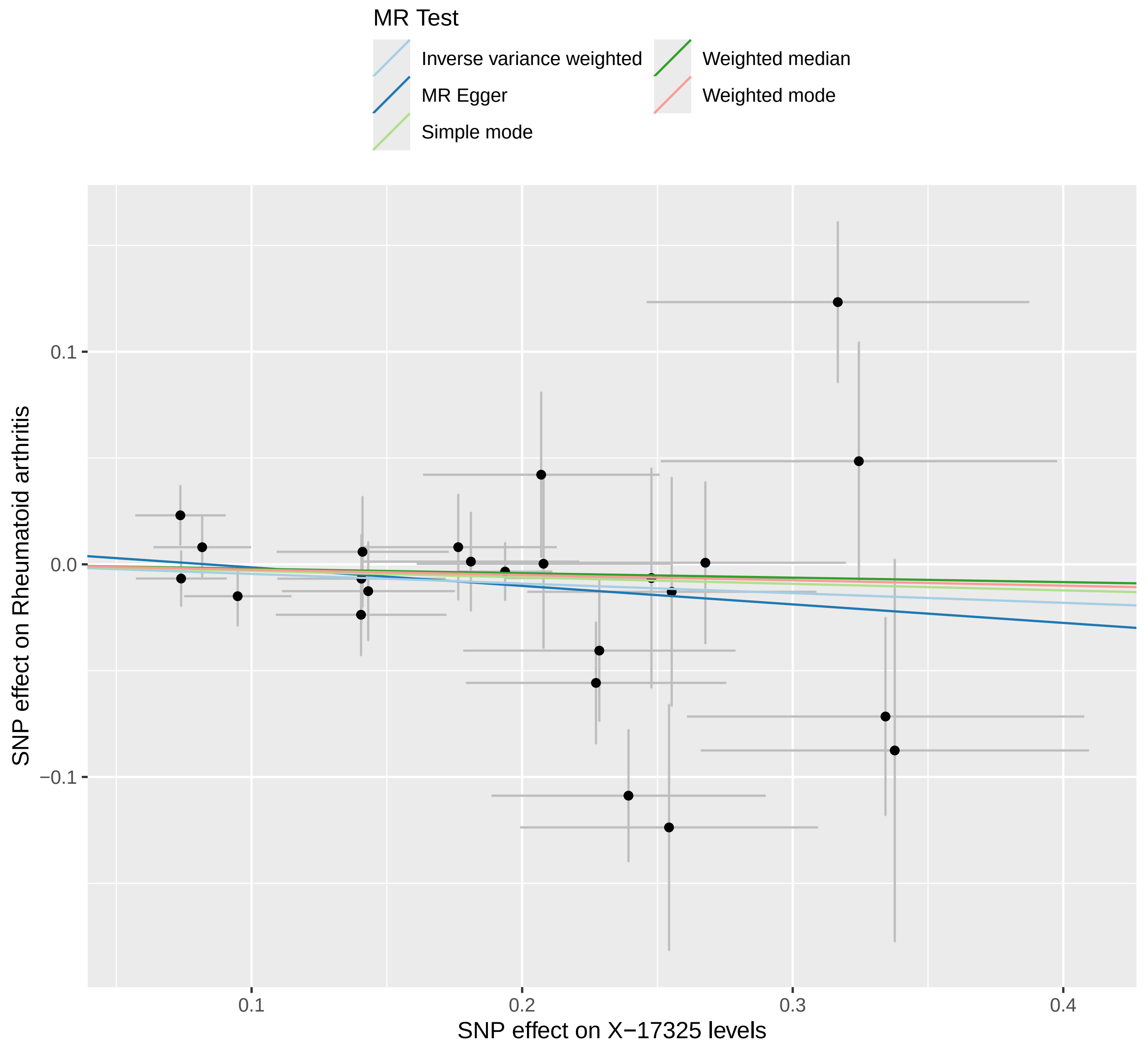
MR Test
Inverse variance weighted
MR Egger
Simple mode
Weighted median
Weighted mode
SNP effect on Rheumatoid arthritis
0.1
0.0
−0.1
0.1
0.2
0.3
0.4
SNP effect on X−17325 levels

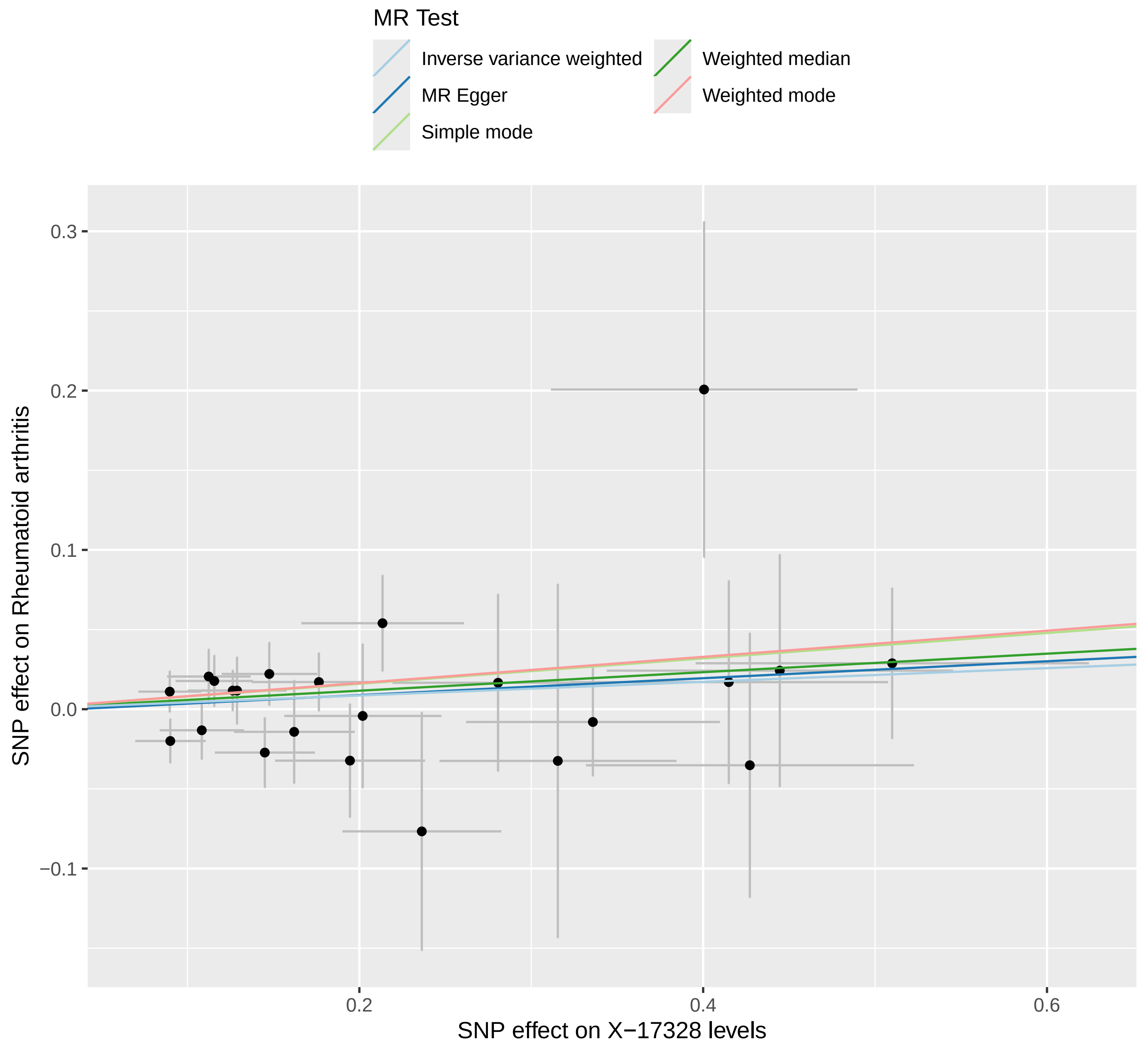
MR Test
Inverse variance weighted
MR Egger
Simple mode
Weighted median
Weighted mode
SNP effect on Rheumatoid arthritis
0.3
0.2
0.1
0.0
−0.1
0.2
0.4
0.6
SNP effect on X−17328 levels

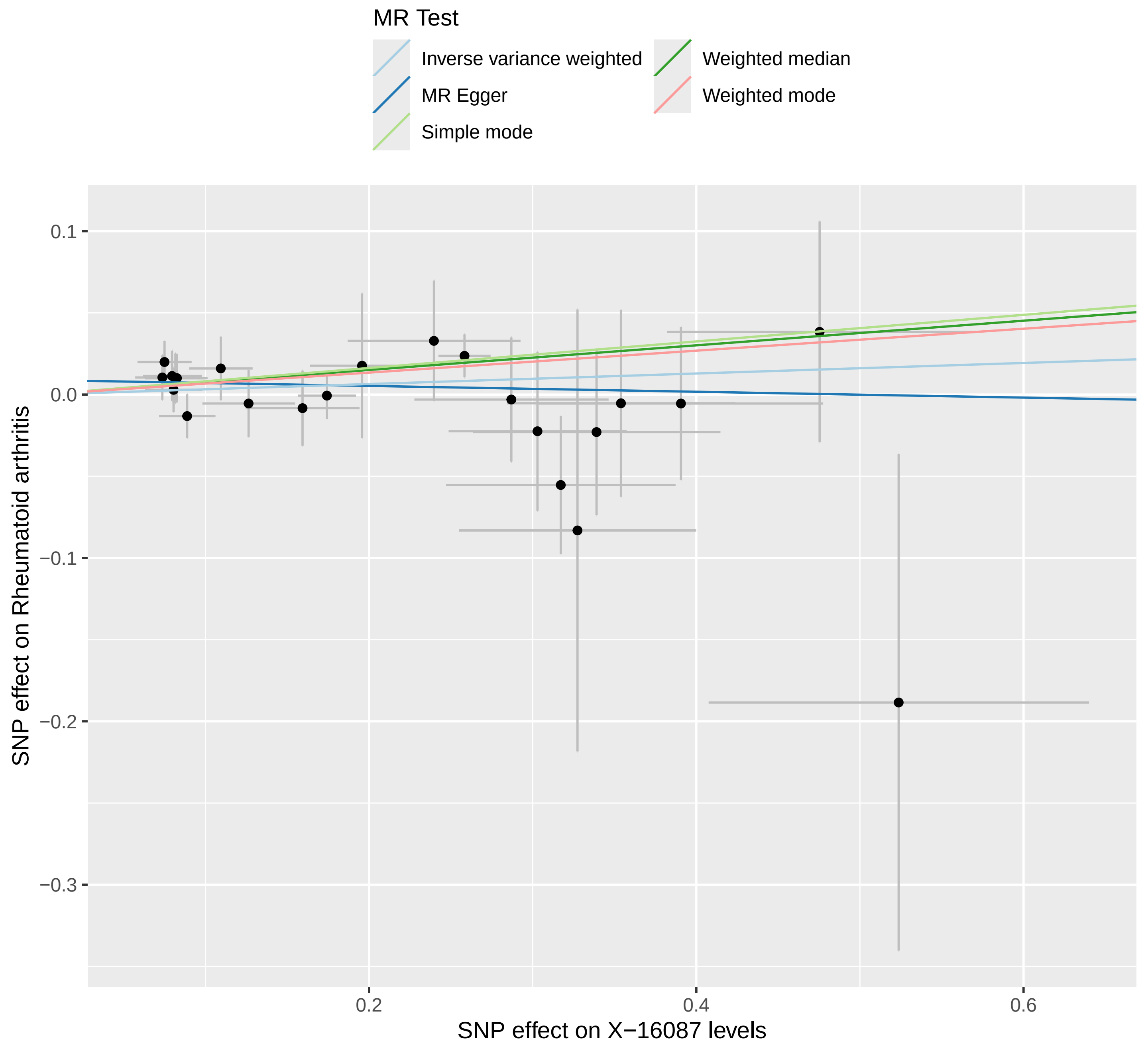
MR Test
Inverse variance weighted
MR Egger
Simple mode
Weighted median
Weighted mode
0.1
0.0
−0.1
−0.2
−0.3
0.2
0.4
0.6
SNP effect on Rheumatoid arthritis
SNP effect on X−16087 levels

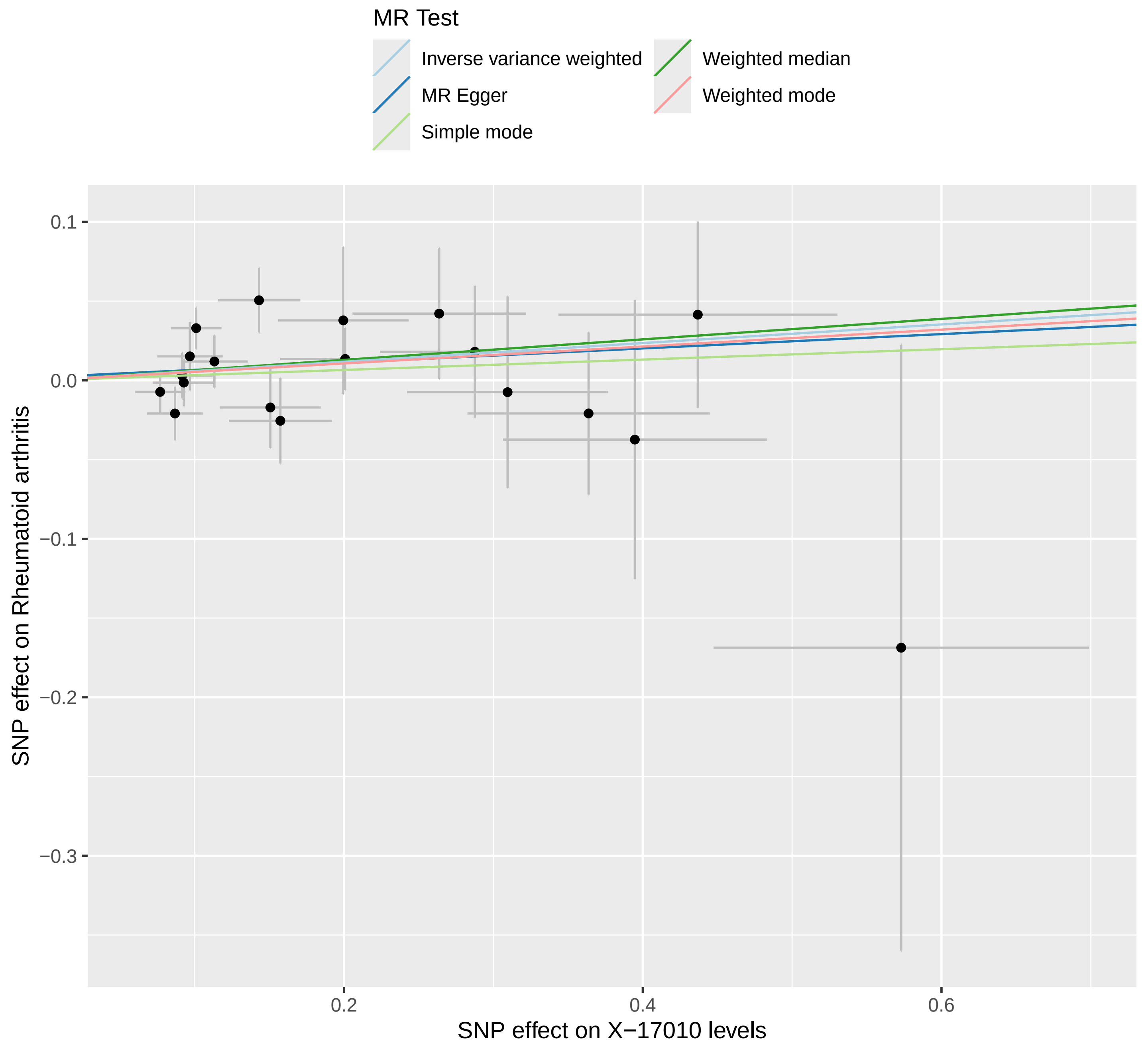
MR Test
Inverse variance weighted
MR Egger
Simple mode
Weighted median
Weighted mode
0.1
0.0
−0.1
−0.2
−0.3
0.2
0.4
0.6
SNP effect on Rheumatoid arthritis
SNP effect on X−17010 levels

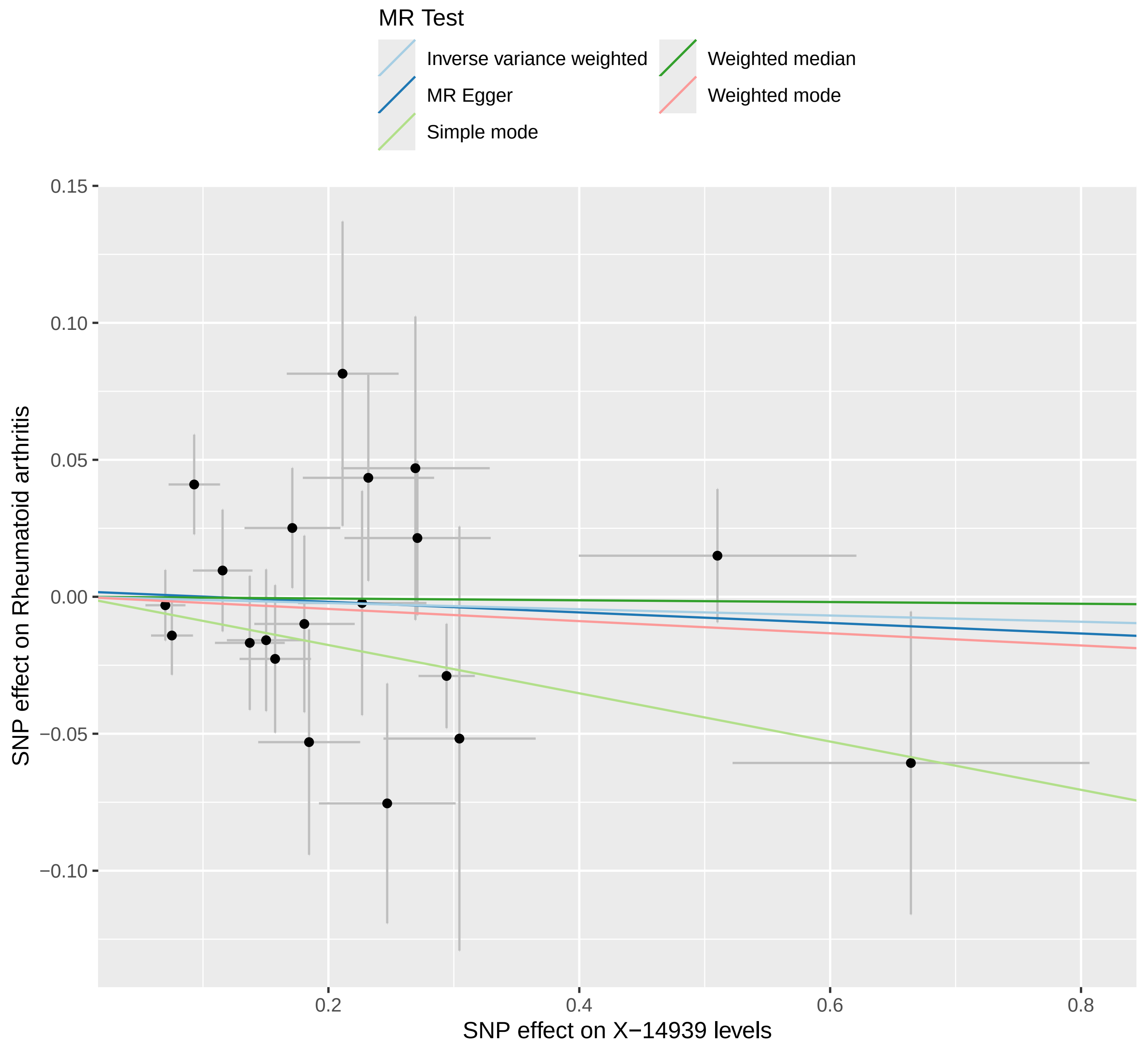
MR Test
Inverse variance weighted
MR Egger
Simple mode
Weighted median
Weighted mode
0.15
0.10
0.05
0.00
−0.05
−0.10
SNP effect on Rheumatoid arthritis
0.2
0.4
0.6
0.8
SNP effect on X−14939 levels

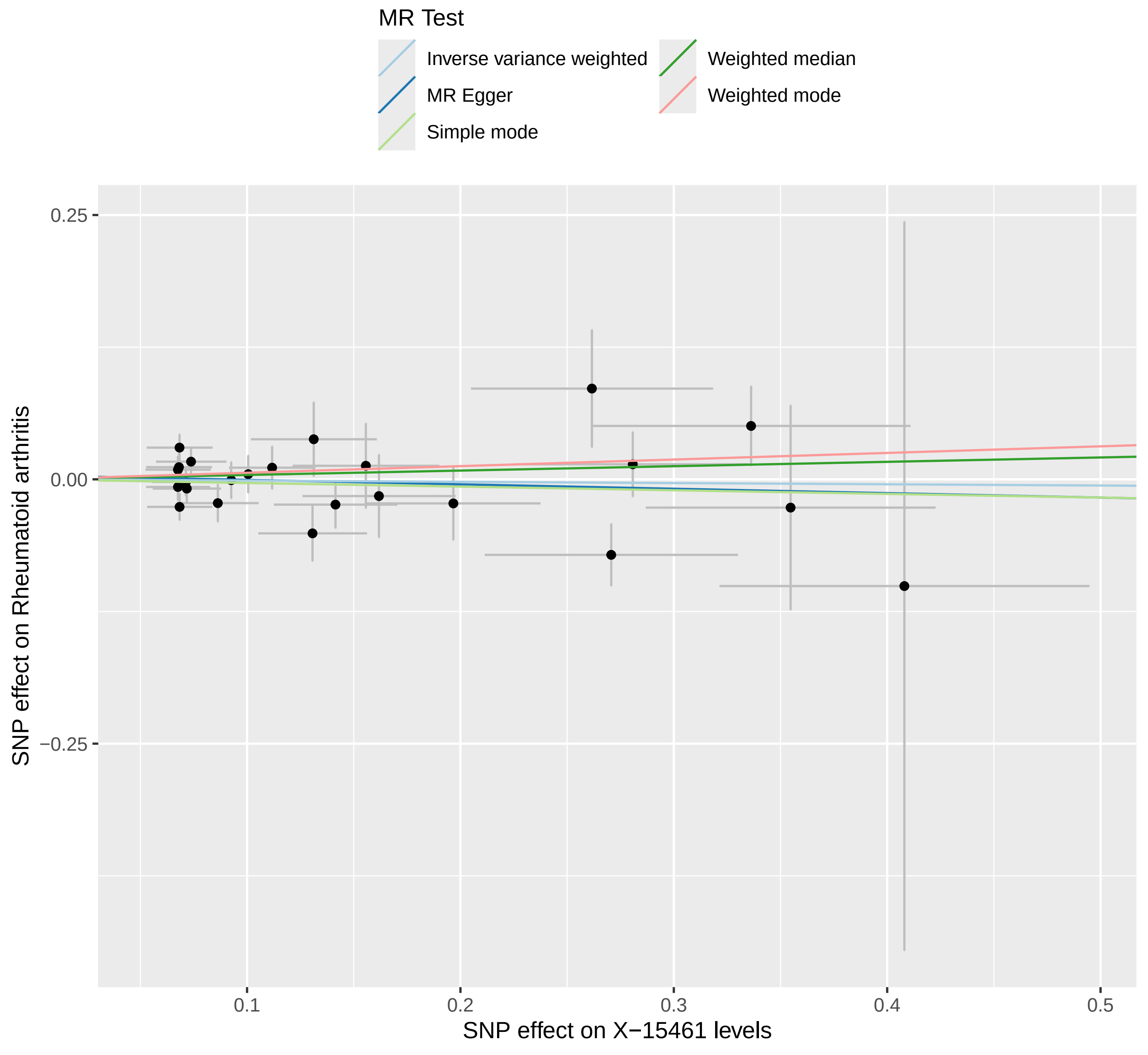

MR Test
Inverse variance weighted
MR Egger
Simple mode
Weighted median
Weighted mode
0.25
0.00
−0.25
0.1
0.2
0.3
0.4
0.5
SNP effect on Rheumatoid arthritis
SNP effect on X−15461 levels

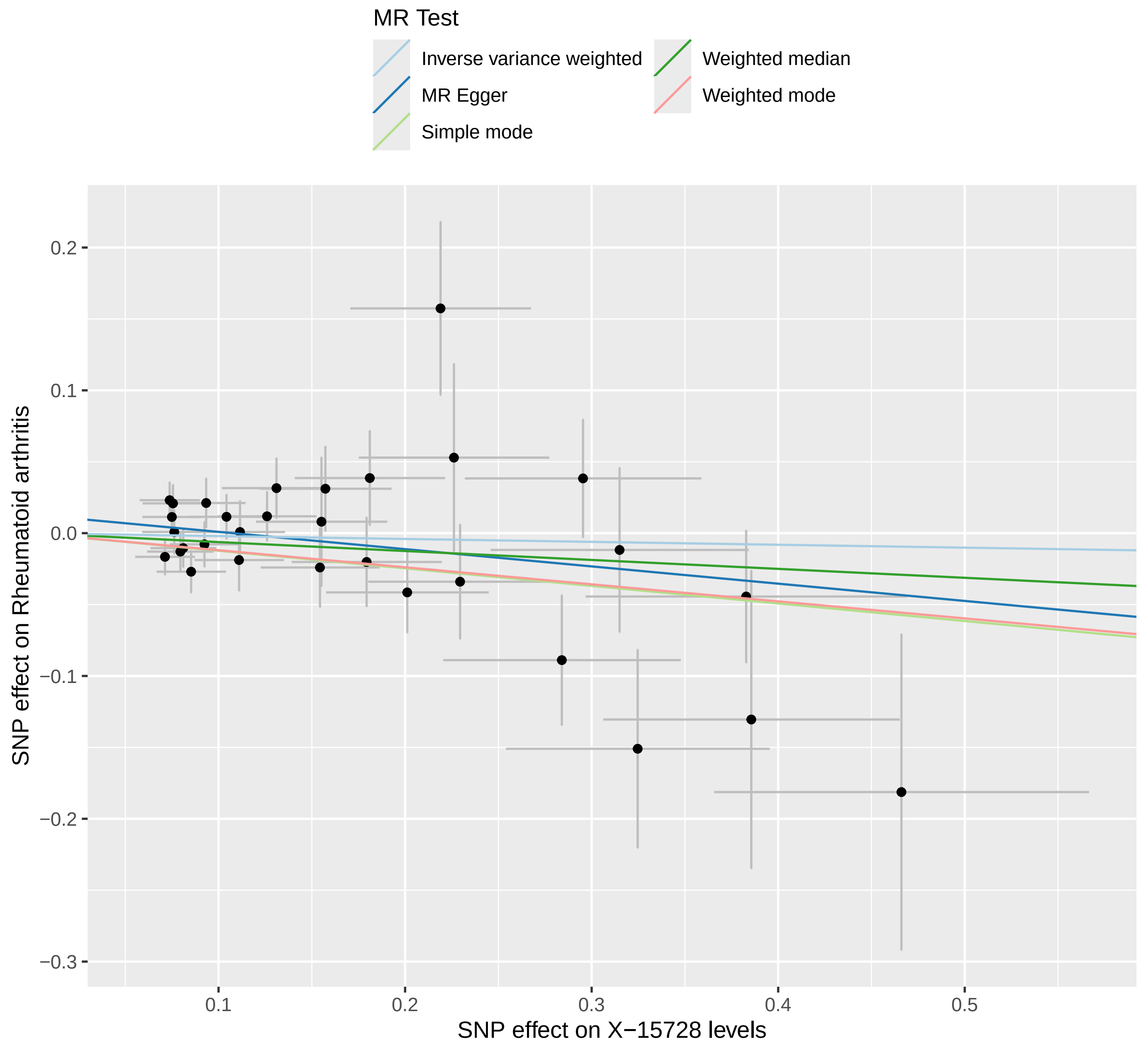
MR Test
Inverse variance weighted
MR Egger
Simple mode
Weighted median
Weighted mode
0.2
0.1
0.0
−0.1
−0.2
−0.3
SNP effect on Rheumatoid arthritis
0.1
0.2
0.3
0.4
0.5
SNP effect on X−15728 levels

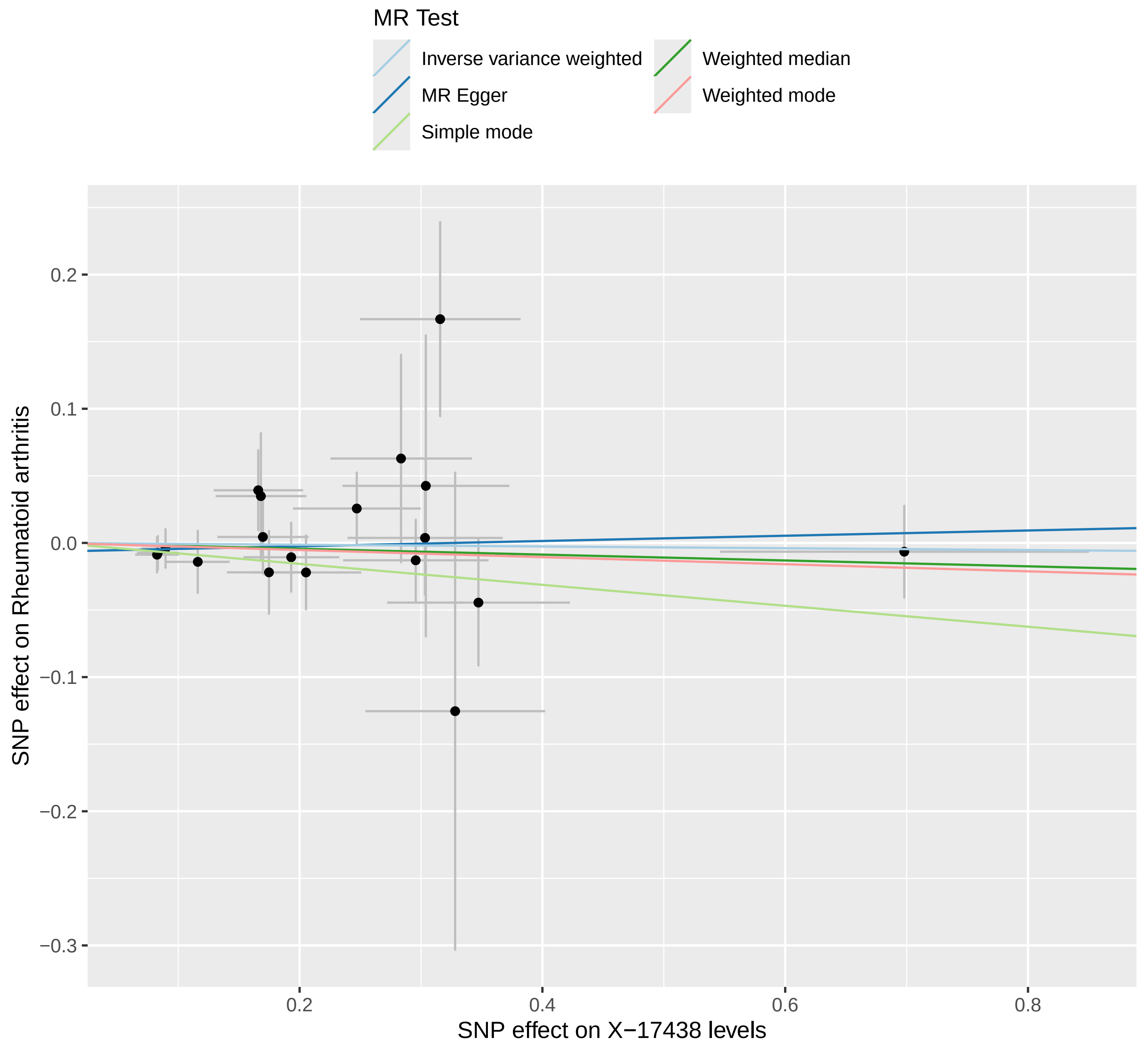
MR Test
Inverse variance weighted
MR Egger
Simple mode
Weighted median
Weighted mode
0.2
0.1
0.0
−0.1
−0.2
−0.3
0.2
0.4
0.6
0.8
SNP effect on Rheumatoid arthritis
SNP effect on X−17438 levels

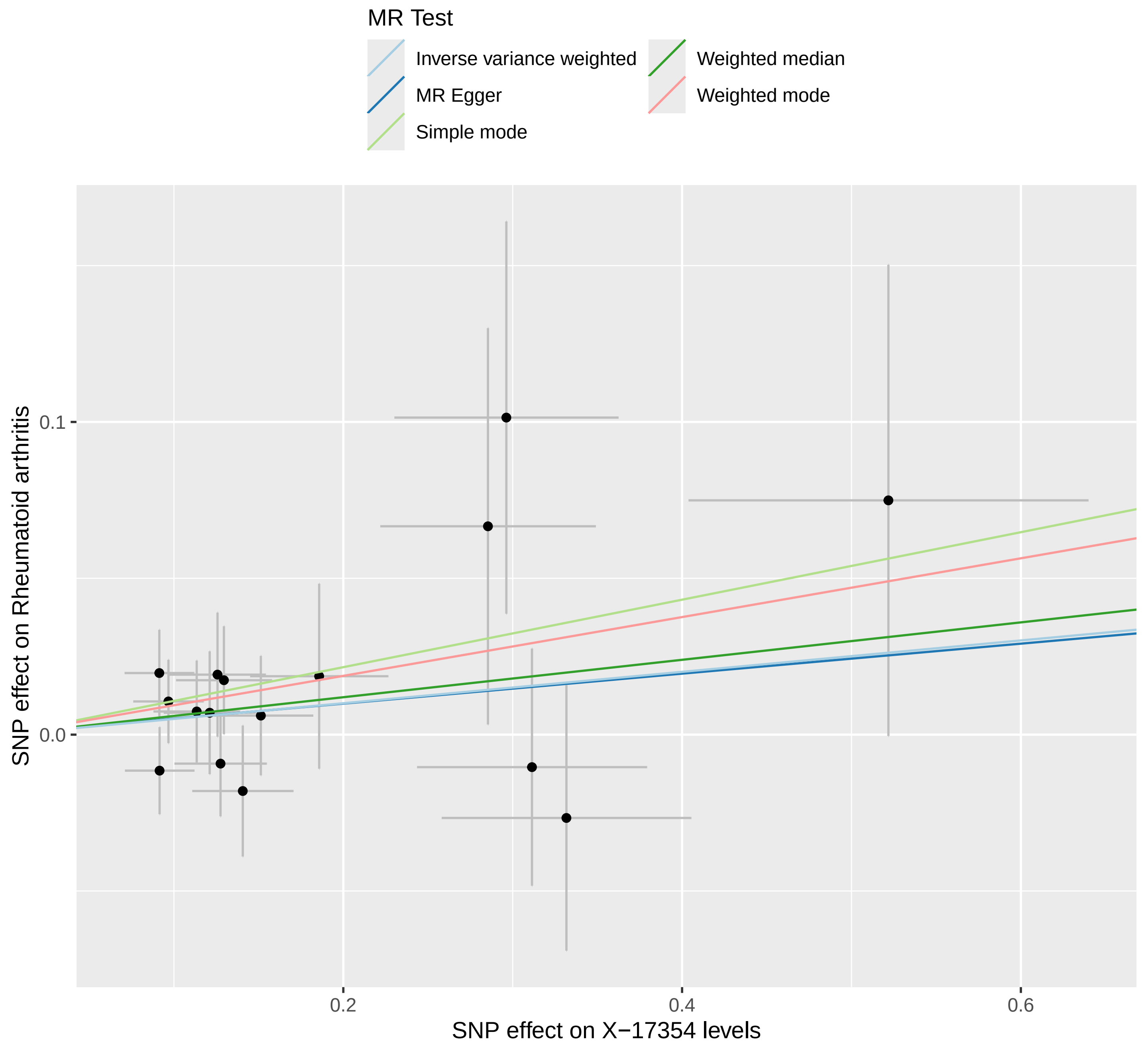

MR Test
Inverse variance weighted
MR Egger
Simple mode
Weighted median
Weighted mode
0.1
0.0
0.2
0.4
0.6
SNP effect on Rheumatoid arthritis
SNP effect on X−17354 levels

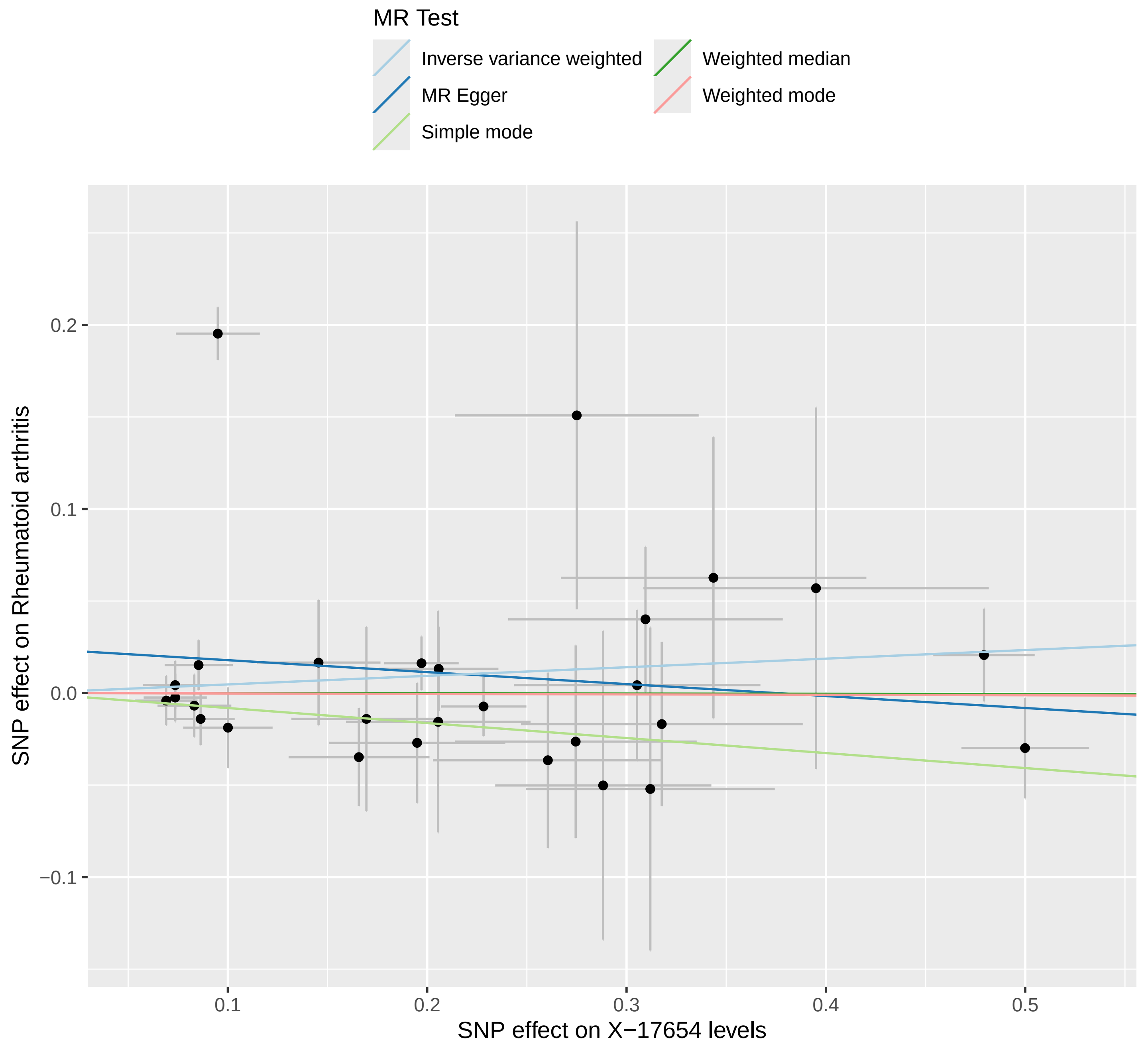

MR Test
Inverse variance weighted
MR Egger
Simple mode
Weighted median
Weighted mode
0.2
0.1
0.0
−0.1
0.1
0.2
0.3
0.4
0.5
SNP effect on Rheumatoid arthritis
SNP effect on X−17654 levels

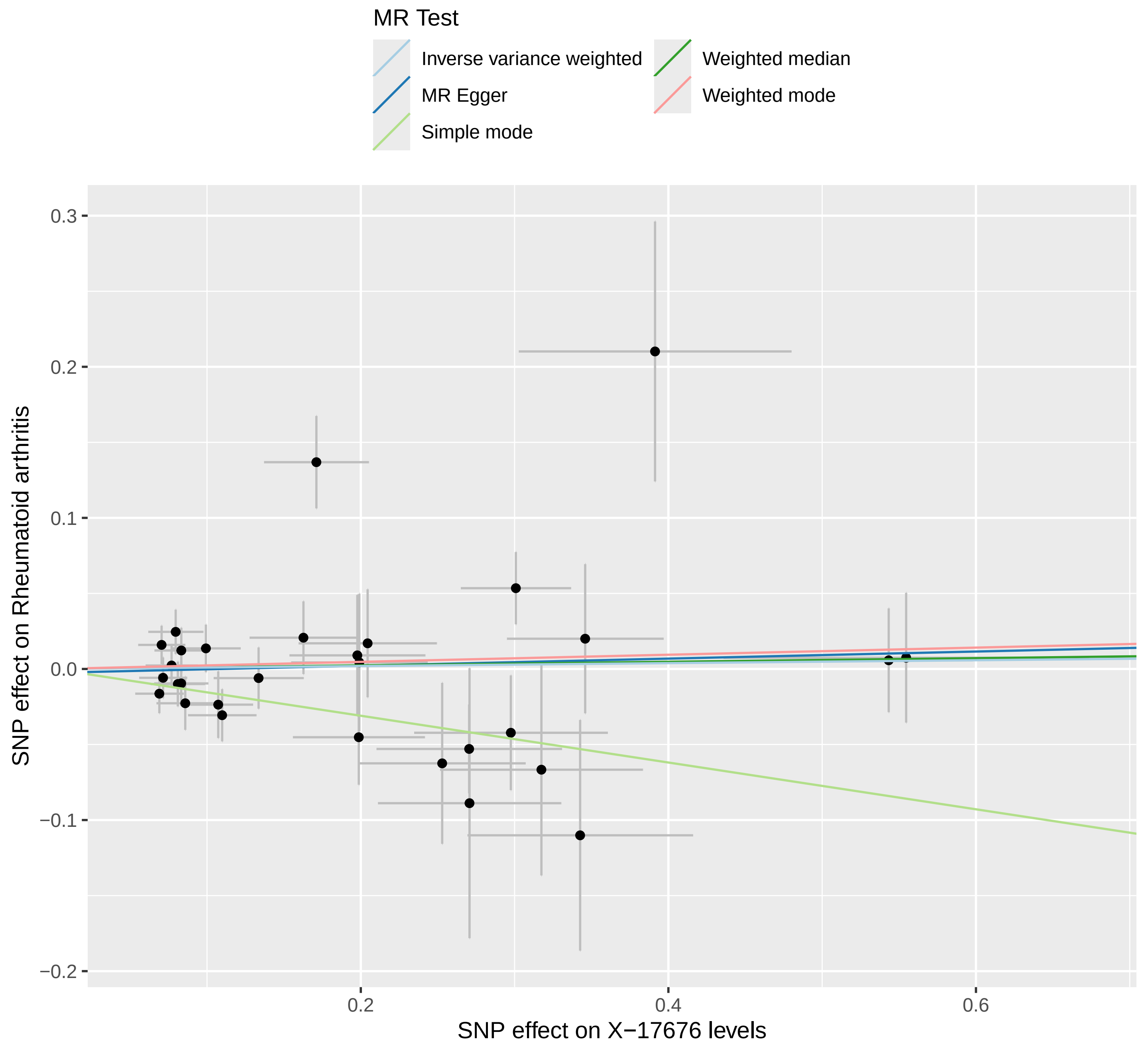
MR Test
Inverse variance weighted
MR Egger
Simple mode
Weighted median
Weighted mode
0.3
0.2
0.1
0.0
−0.1
−0.2
SNP effect on Rheumatoid arthritis
0.2
0.4
0.6
SNP effect on X−17676 levels

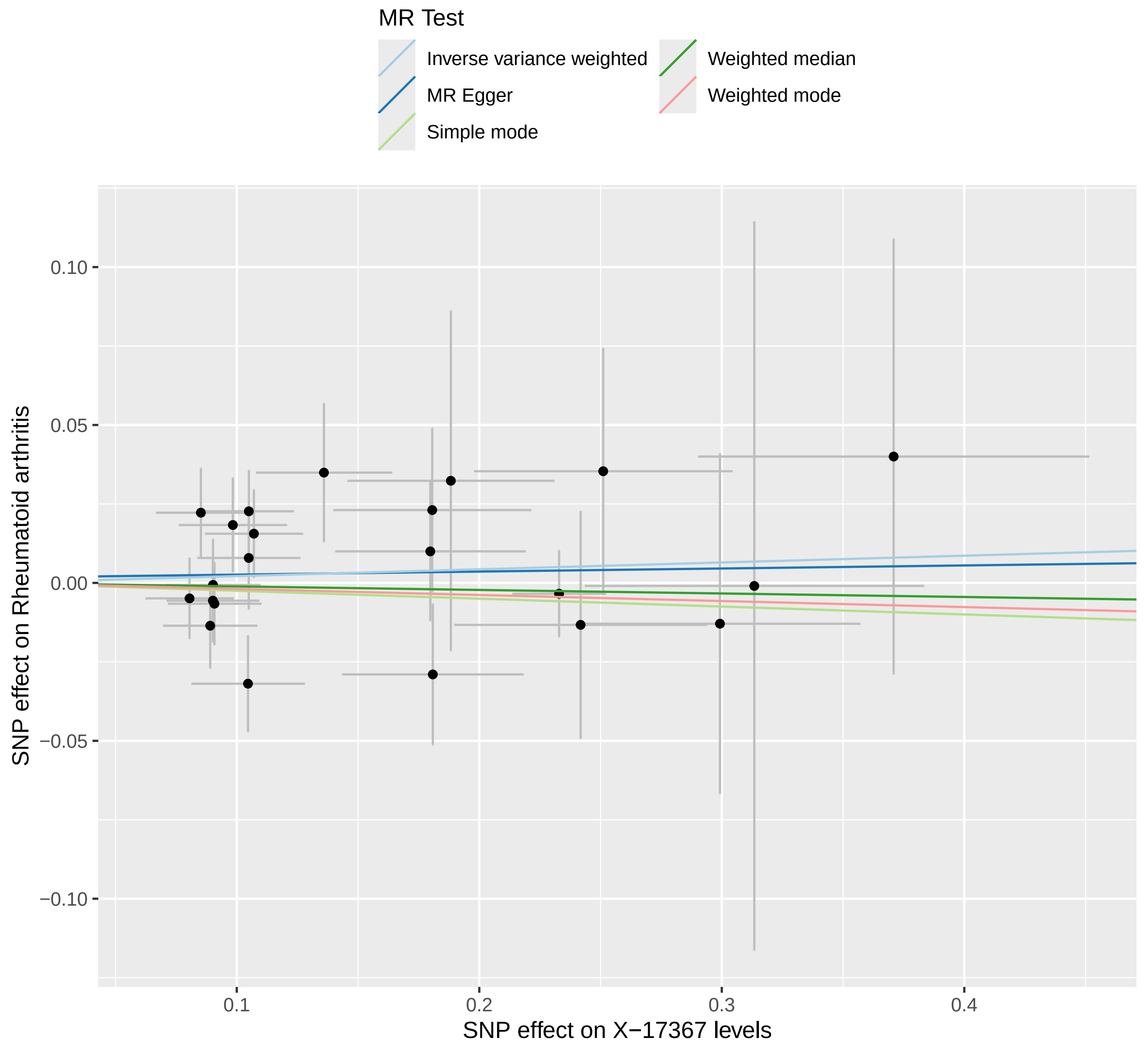
MR Test
Inverse variance weighted
MR Egger
Simple mode
Weighted median
Weighted mode
0.10
0.05
0.00
−0.05
−0.10
SNP effect on Rheumatoid arthritis
0.1
0.2
0.3
0.4
SNP effect on X−17367 levels

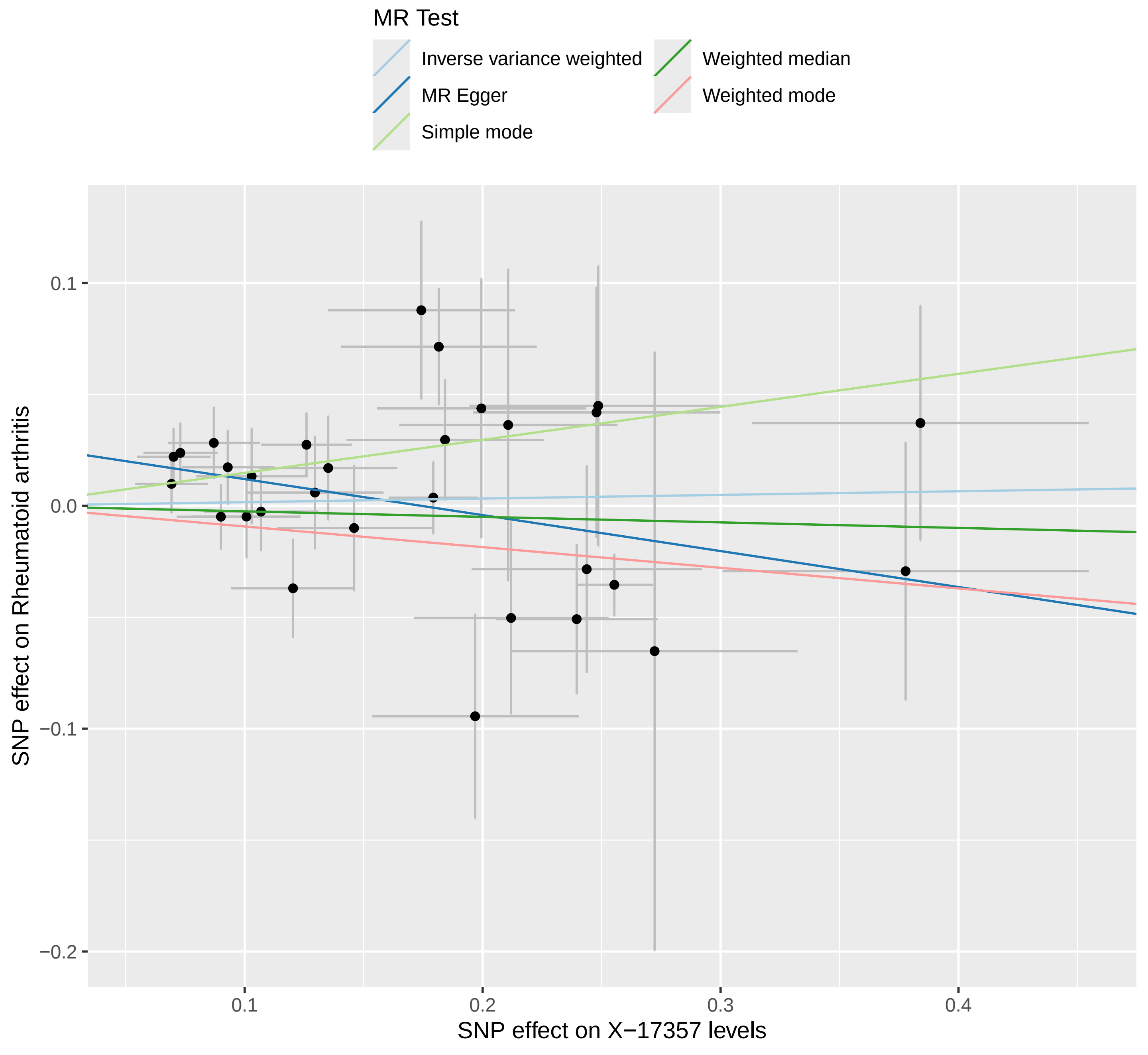

MR Test
Inverse variance weighted
MR Egger
Simple mode
Weighted median
Weighted mode
SNP effect on Rheumatoid arthritis
0.1
0.0
−0.1
−0.2
0.1
0.2
0.3
0.4
SNP effect on X−17357 levels

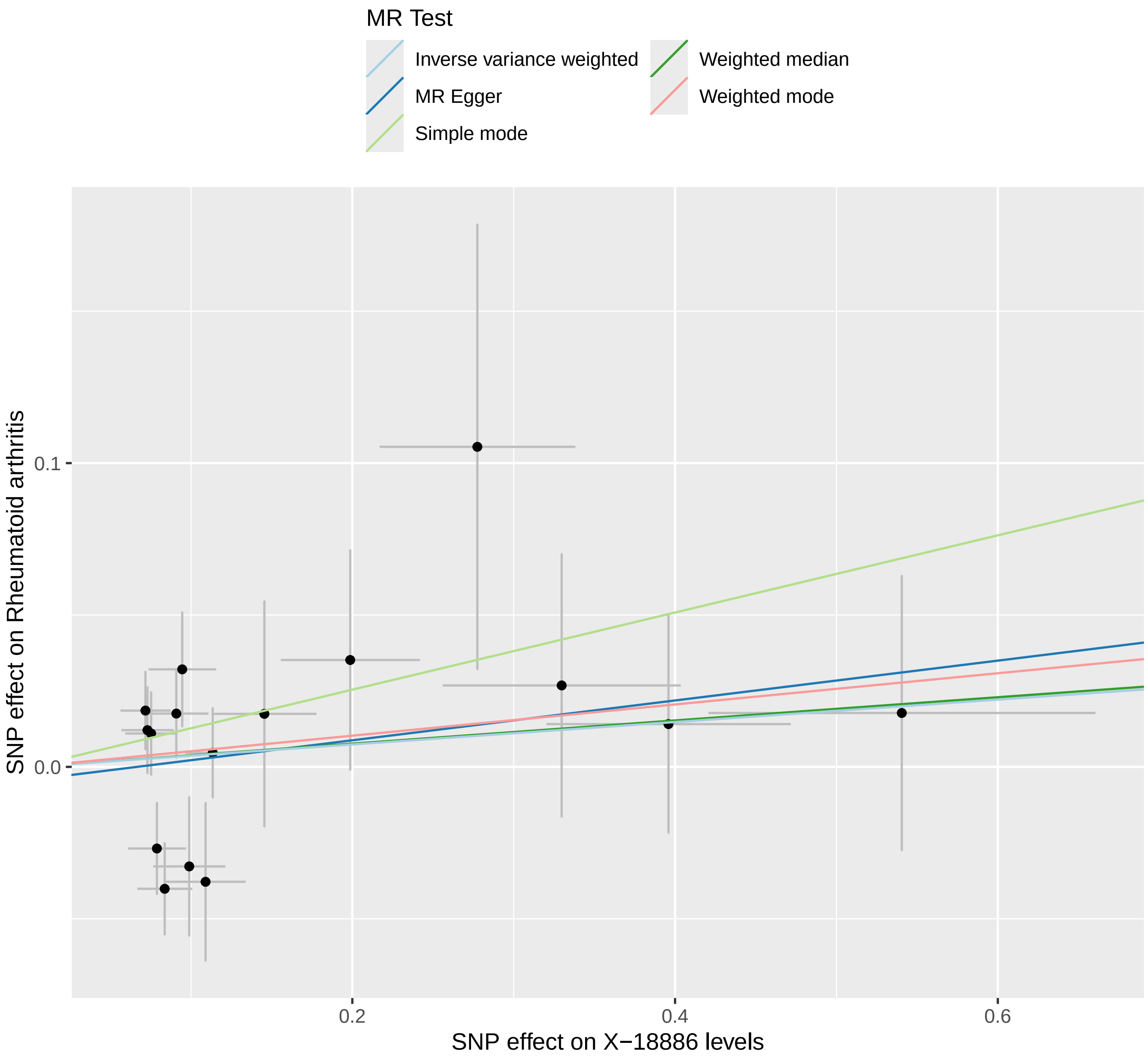

MR Test
Inverse variance weighted
MR Egger
Simple mode
Weighted median
Weighted mode
0.1
0.0
0.2
0.4
0.6
SNP effect on Rheumatoid arthritis
SNP effect on X−18886 levels

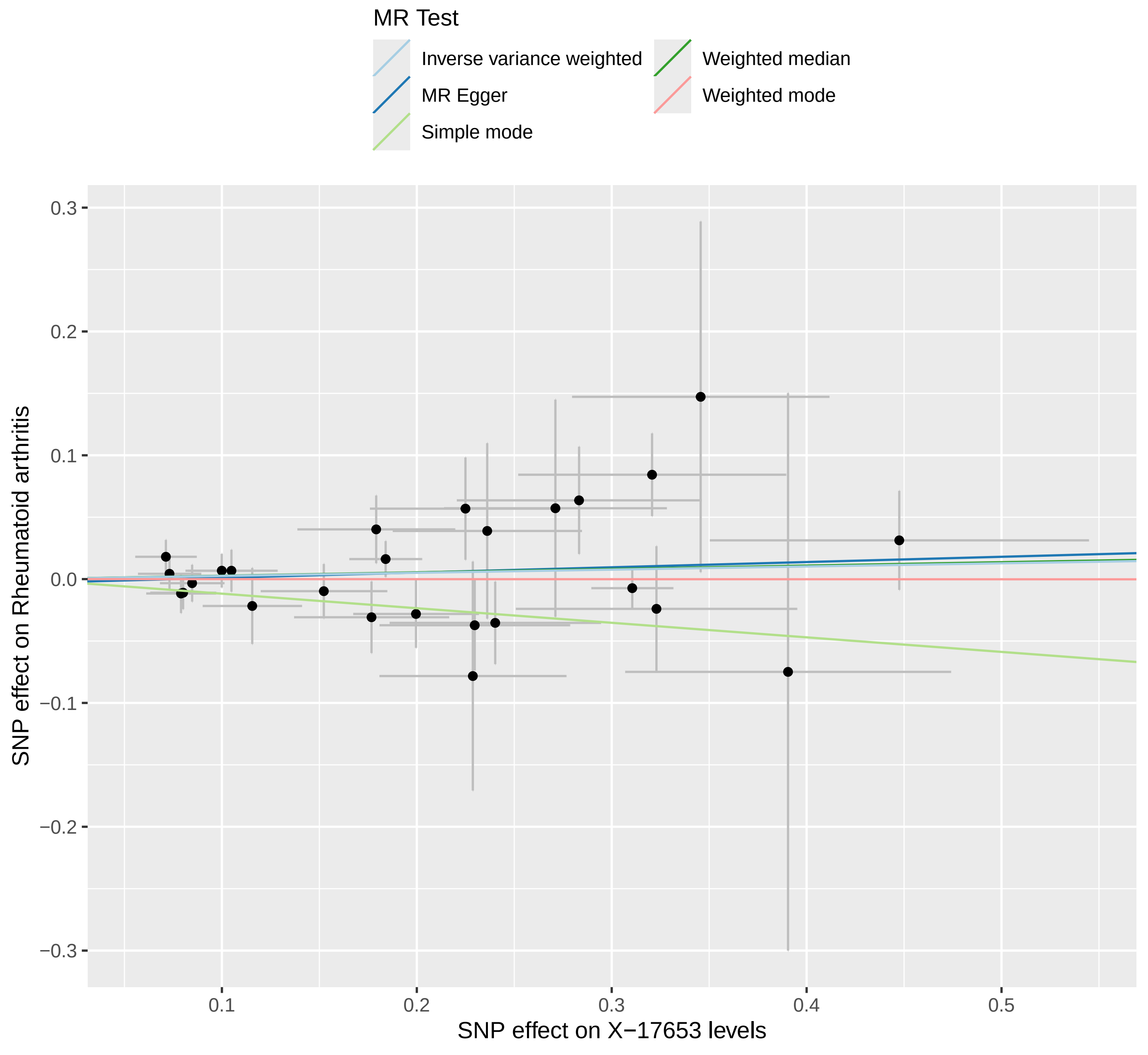
MR Test
Inverse variance weighted
MR Egger
Simple mode
Weighted median
Weighted mode
0.3
0.2
0.1
0.0
−0.1
−0.2
−0.3
SNP effect on Rheumatoid arthritis
0.1
0.2
0.3
0.4
0.5
SNP effect on X−17653 levels

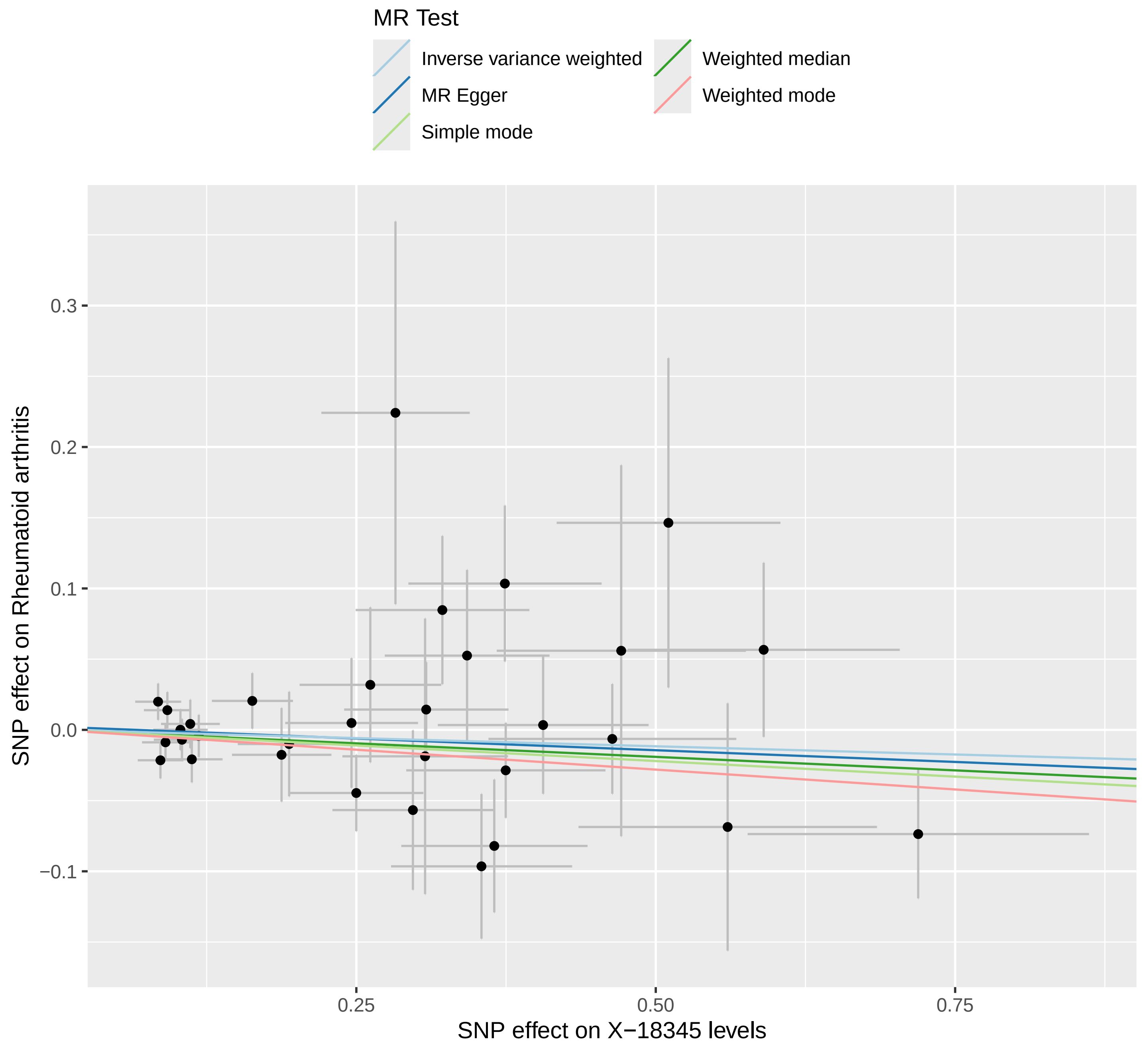
MR Test
Inverse variance weighted
MR Egger
Simple mode
Weighted median
Weighted mode
0.3
0.2
0.1
0.0
−0.1
0.25
0.50
0.75
SNP effect on Rheumatoid arthritis
SNP effect on X−18345 levels

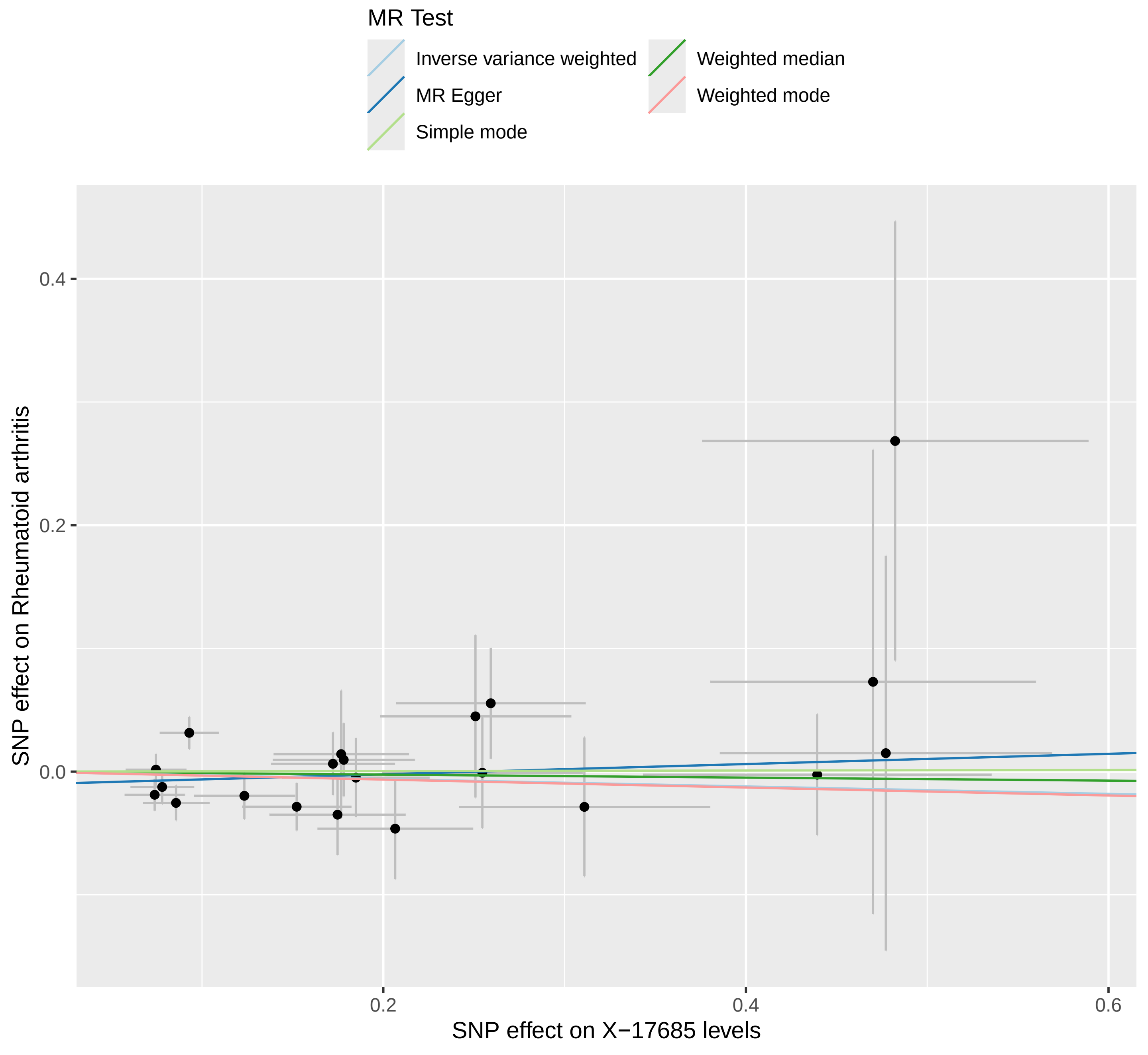
MR Test
Inverse variance weighted
MR Egger
Simple mode
Weighted median
Weighted mode
0.4
0.2
0.0
SNP effect on Rheumatoid arthritis
0.2
0.4
0.6
SNP effect on X−17685 levels

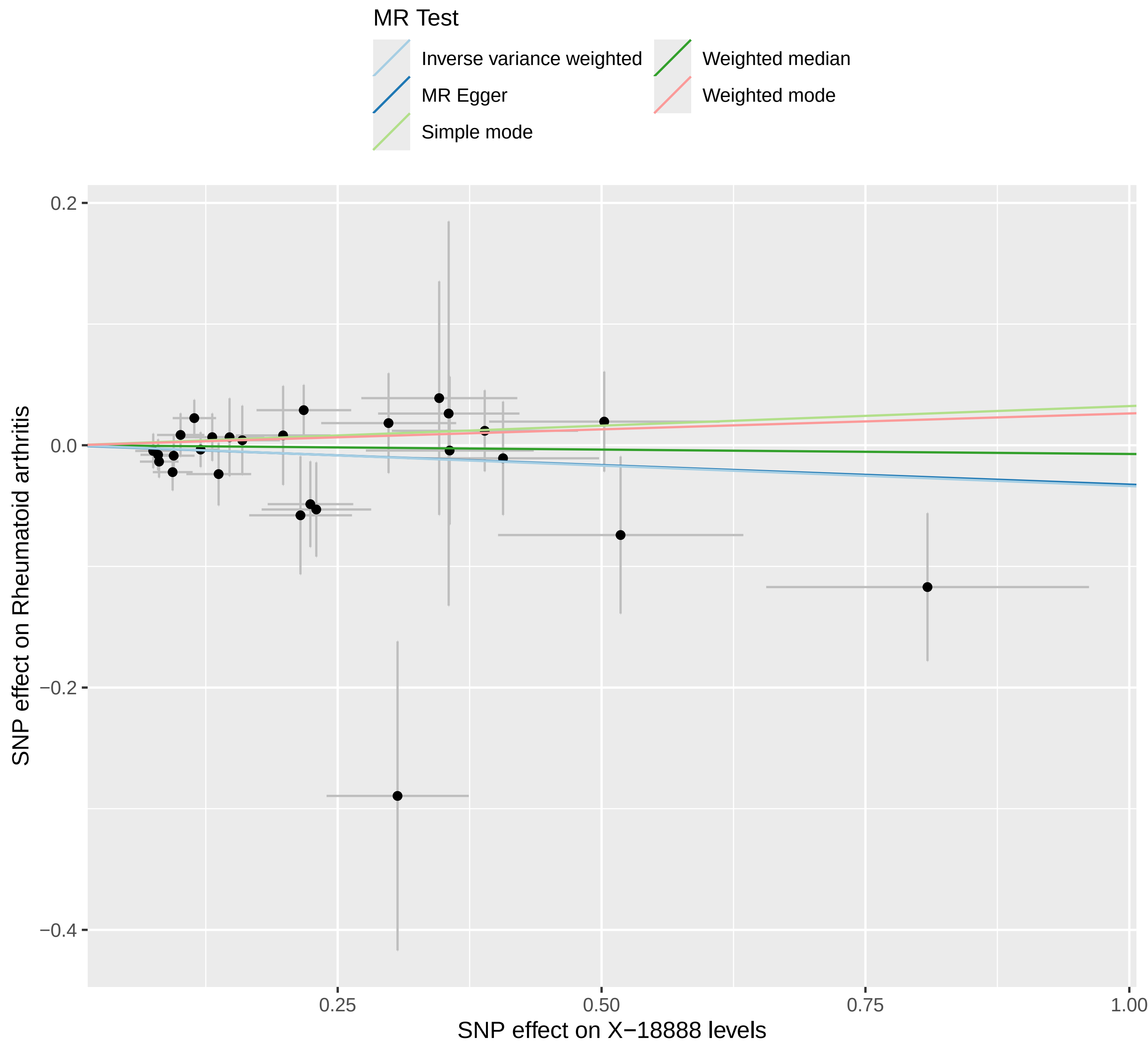

MR Test
Inverse variance weighted
MR Egger
Simple mode
Weighted median
Weighted mode
0.2
0.0
−0.2
−0.4
0.25
0.50
0.75
1.00
SNP effect on Rheumatoid arthritis
SNP effect on X−18888 levels

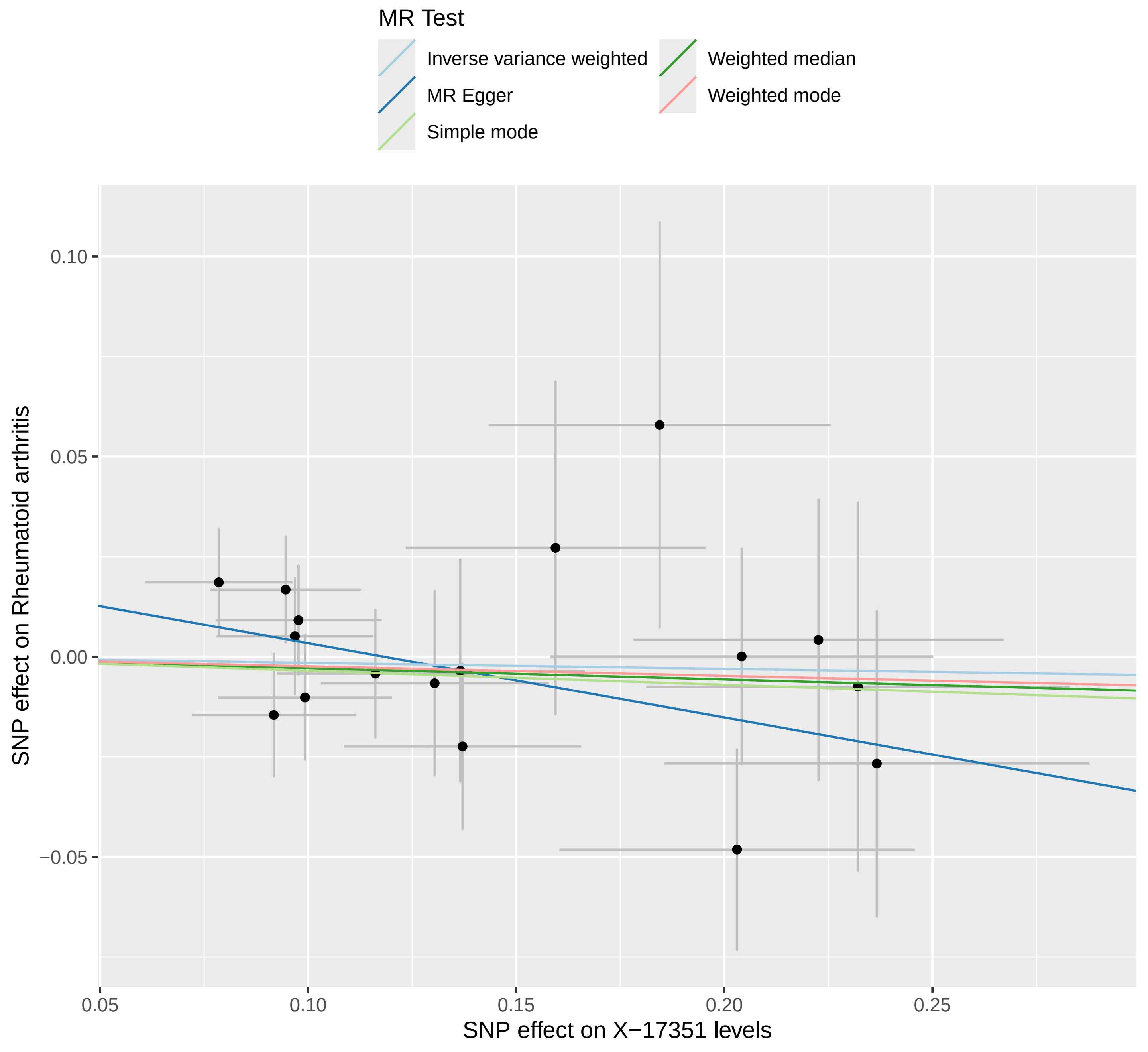
MR Test
Inverse variance weighted
MR Egger
Simple mode
Weighted median
Weighted mode
0.10
0.05
0.00
−0.05
SNP effect on Rheumatoid arthritis
0.05
0.10
0.15
0.20
0.25
SNP effect on X−17351 levels

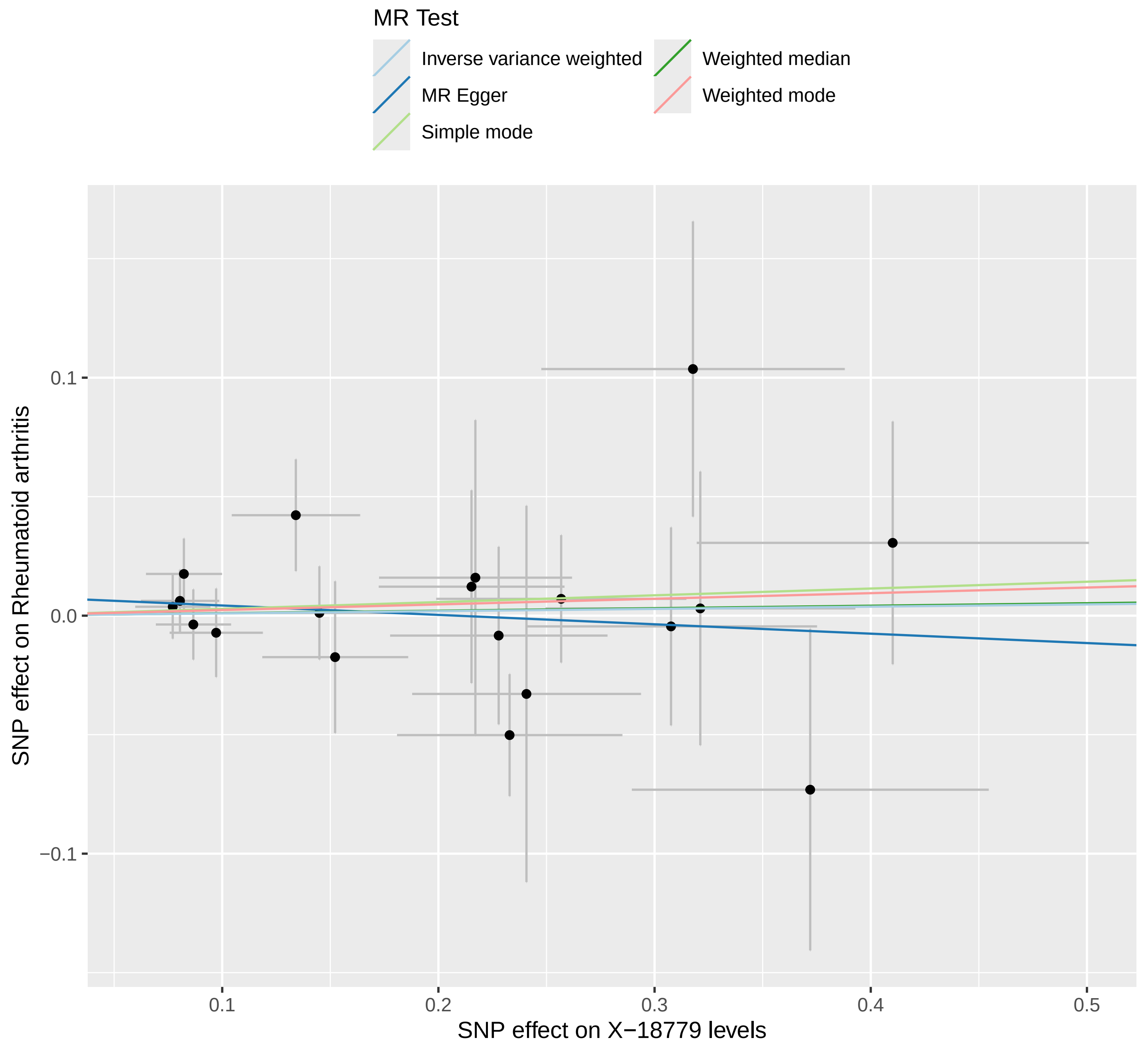

MR Test
Inverse variance weighted
MR Egger
Simple mode
Weighted median
Weighted mode
0.1
0.0
−0.1
0.1
0.2
0.3
0.4
0.5
SNP effect on Rheumatoid arthritis
SNP effect on X−18779 levels

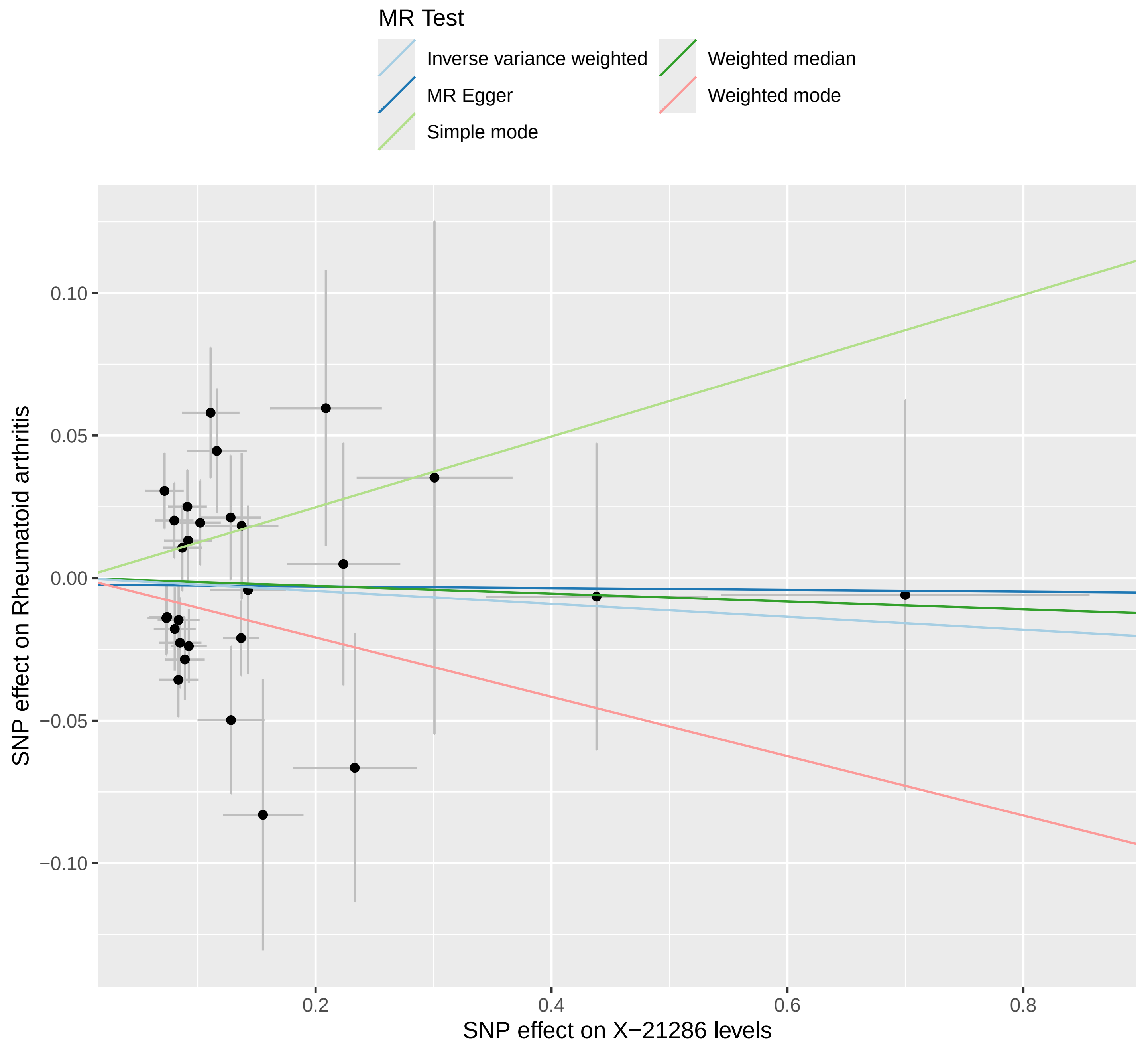
MR Test
Inverse variance weighted
MR Egger
Simple mode
Weighted median
Weighted mode
0.10
0.05
0.00
−0.05
−0.10
SNP effect on Rheumatoid arthritis
0.2
0.4
0.6
0.8
SNP effect on X−21286 levels

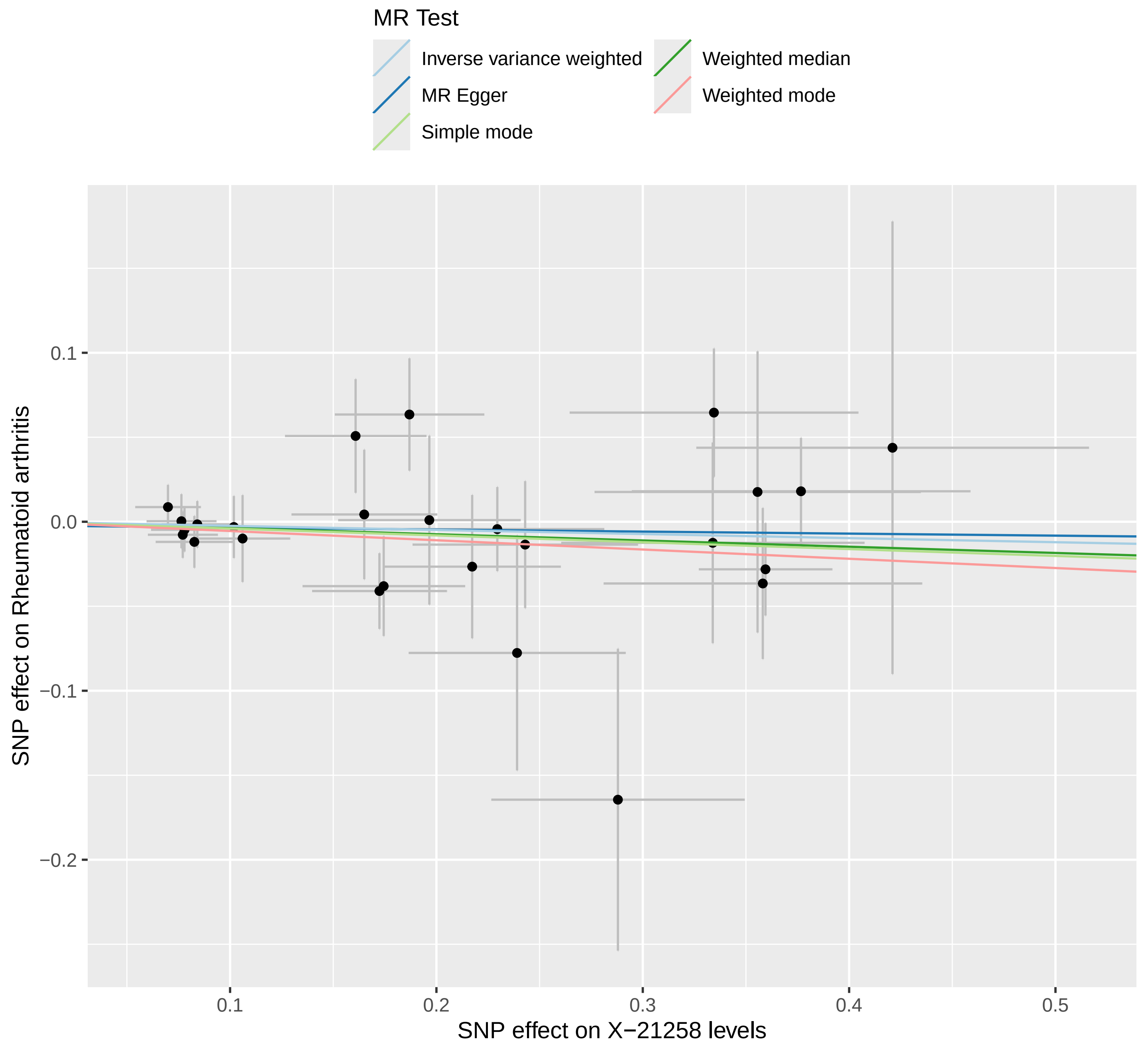
MR Test
Inverse variance weighted
MR Egger
Simple mode
Weighted median
Weighted mode
SNP effect on Rheumatoid arthritis
0.1
0.0
−0.1
−0.2
0.1
0.2
0.3
0.4
0.5
SNP effect on X−21258 levels

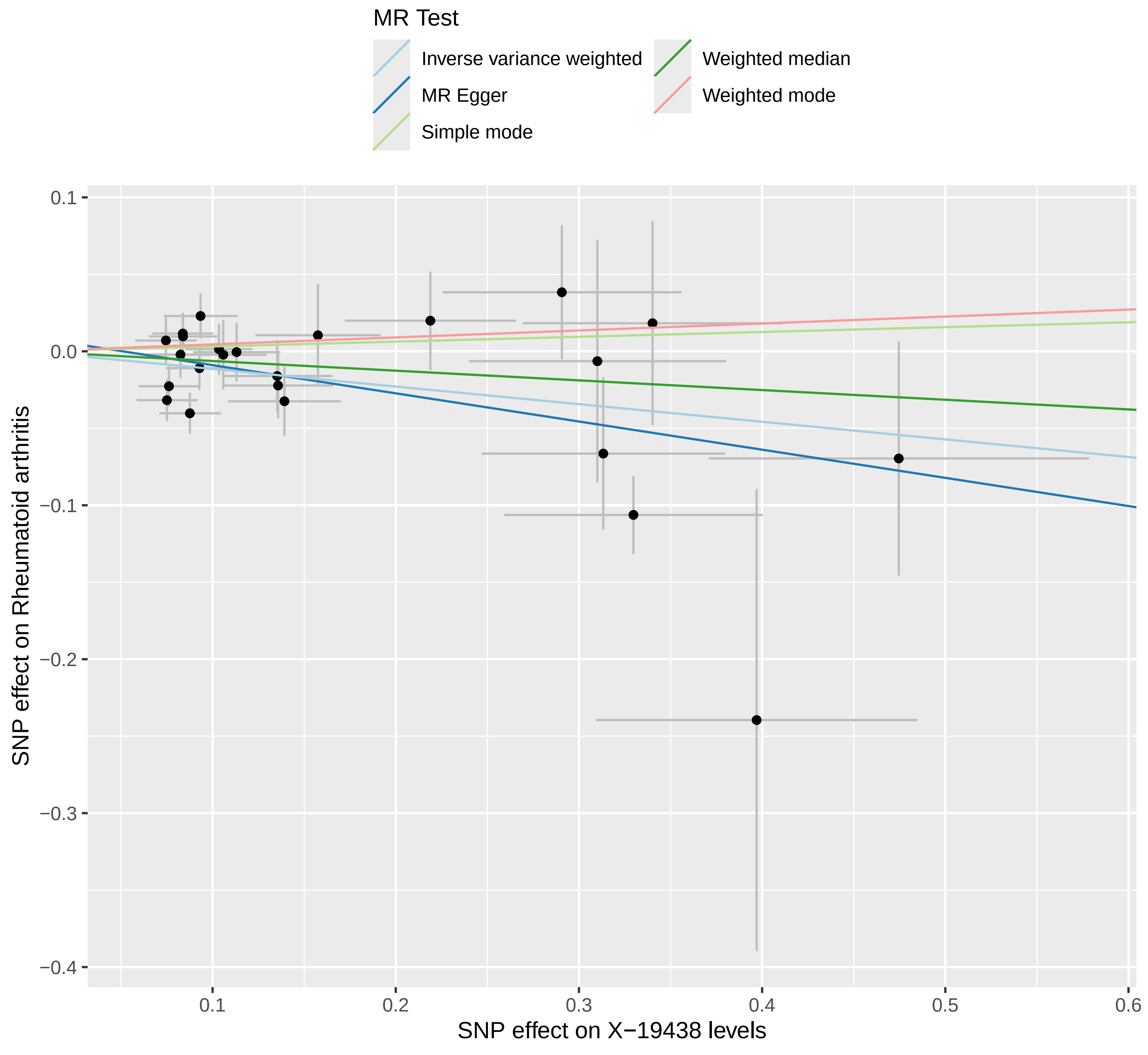
MR Test
Inverse variance weighted
MR Egger
Simple mode
Weighted median
Weighted mode
SNP effect on Rheumatoid arthritis
0.1
0.0
−0.1
−0.2
−0.3
−0.4
0.1
0.2
0.3
0.4
0.5
0.6
SNP effect on X−19438 levels

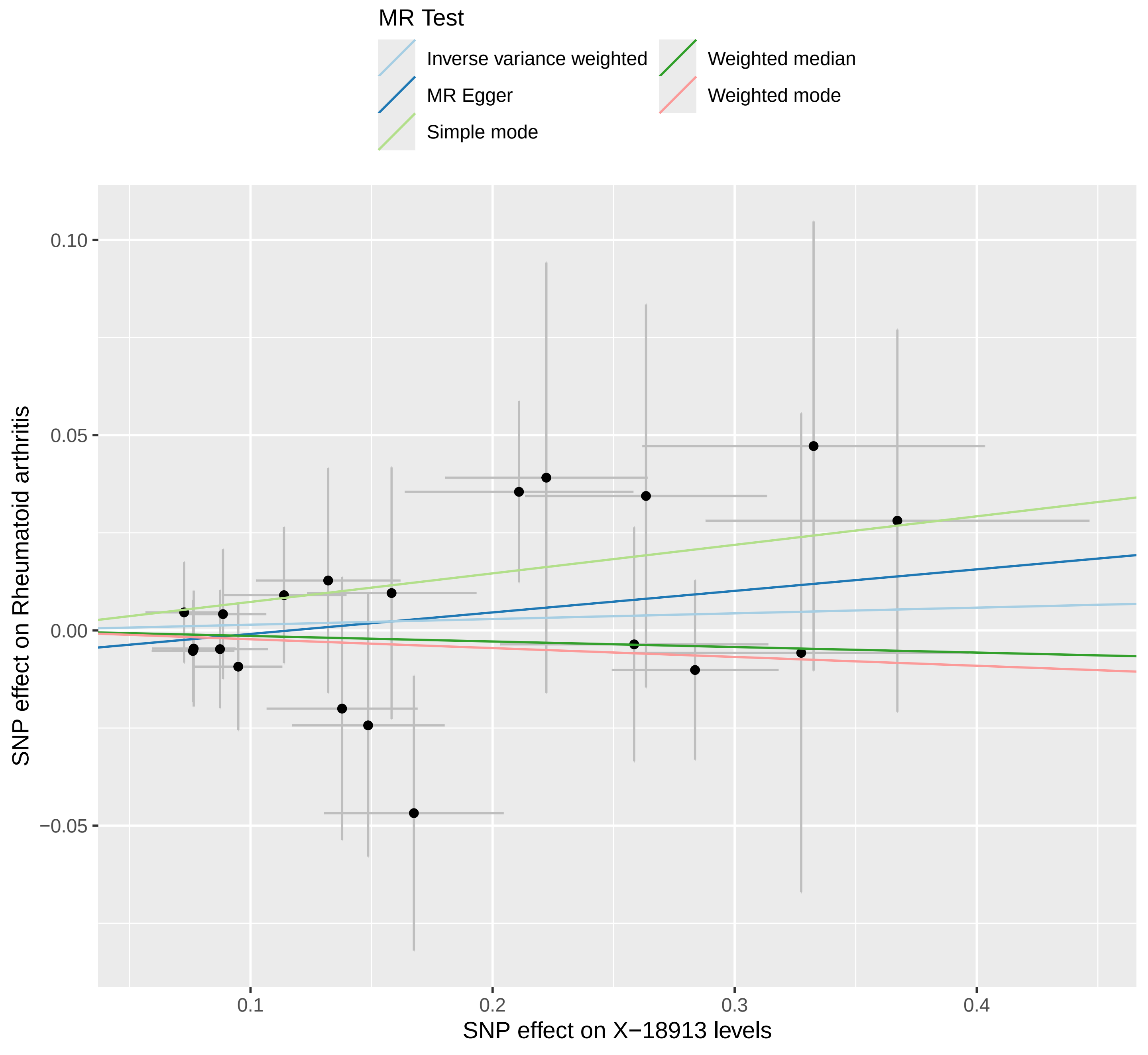
MR Test
Inverse variance weighted
MR Egger
Simple mode
Weighted median
Weighted mode
0.10
0.05
0.00
−0.05
SNP effect on Rheumatoid arthritis
0.1
0.2
0.3
0.4
SNP effect on X−18913 levels

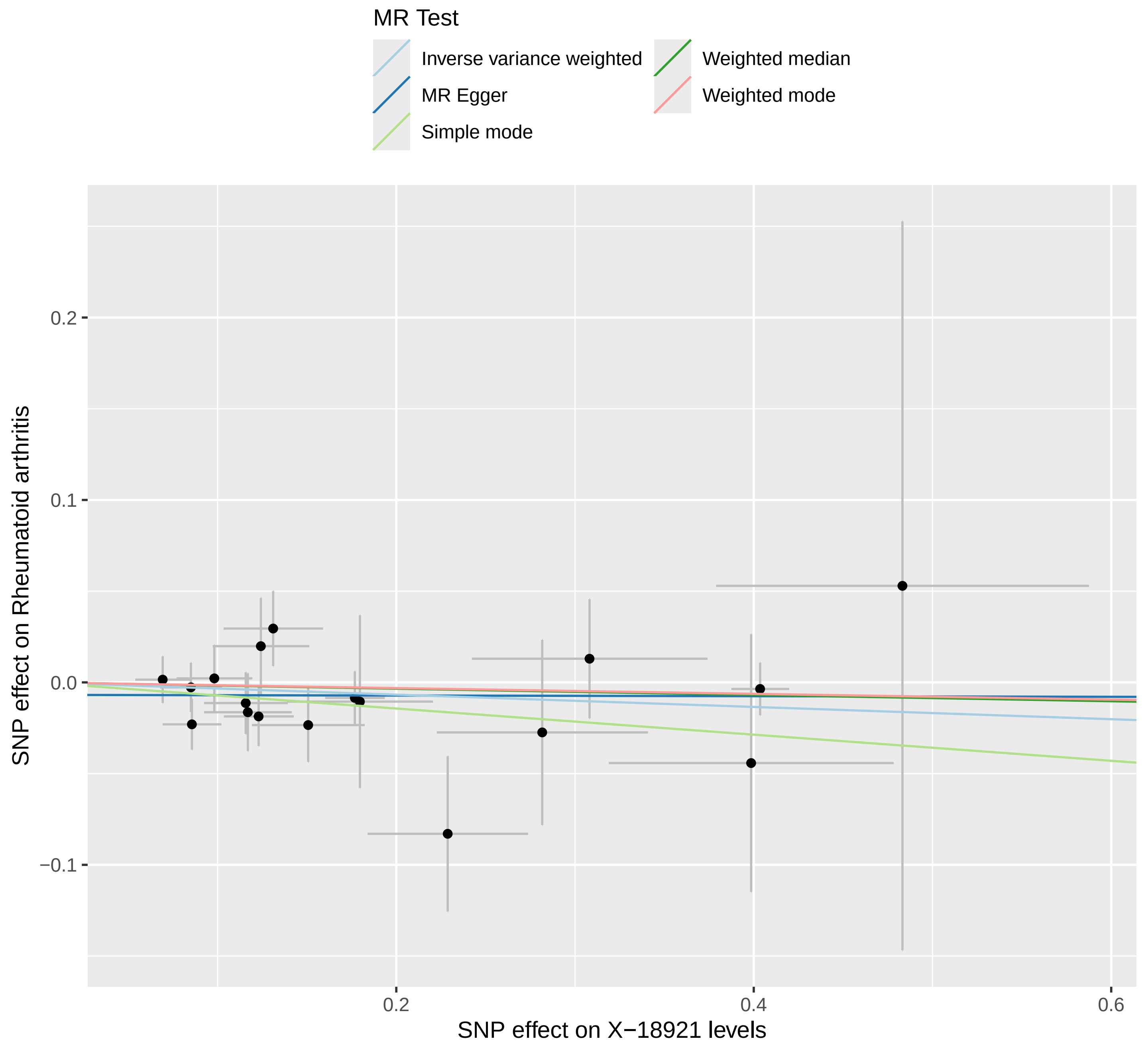

MR Test
Inverse variance weighted
MR Egger
Simple mode
Weighted median
Weighted mode
0.2
0.1
0.0
−0.1
0.2
0.4
0.6
SNP effect on Rheumatoid arthritis
SNP effect on X−18921 levels

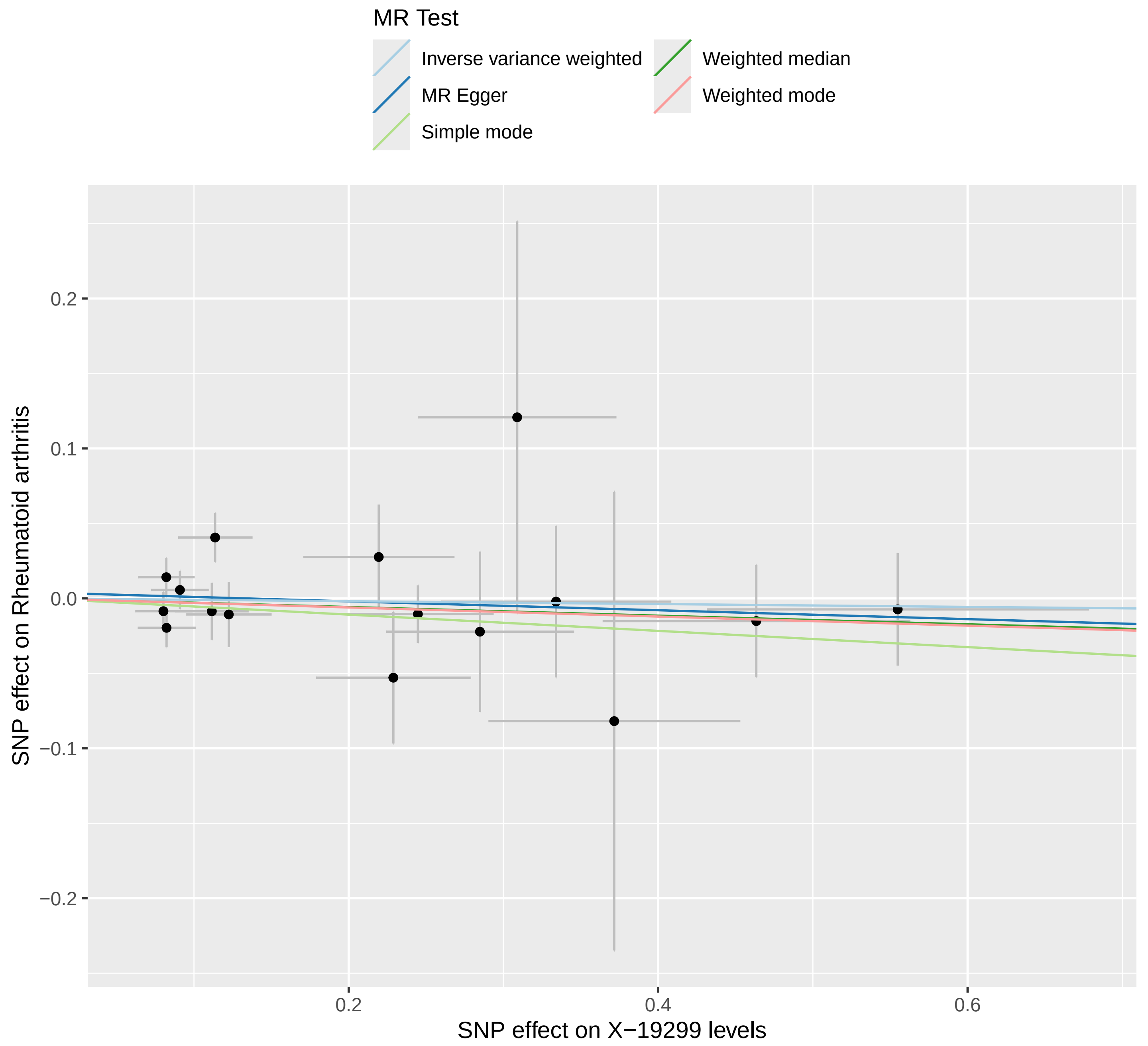
MR Test
Inverse variance weighted
MR Egger
Simple mode
Weighted median
Weighted mode
0.2
0.1
0.0
−0.1
−0.2
SNP effect on Rheumatoid arthritis
0.2
0.4
0.6
SNP effect on X−19299 levels

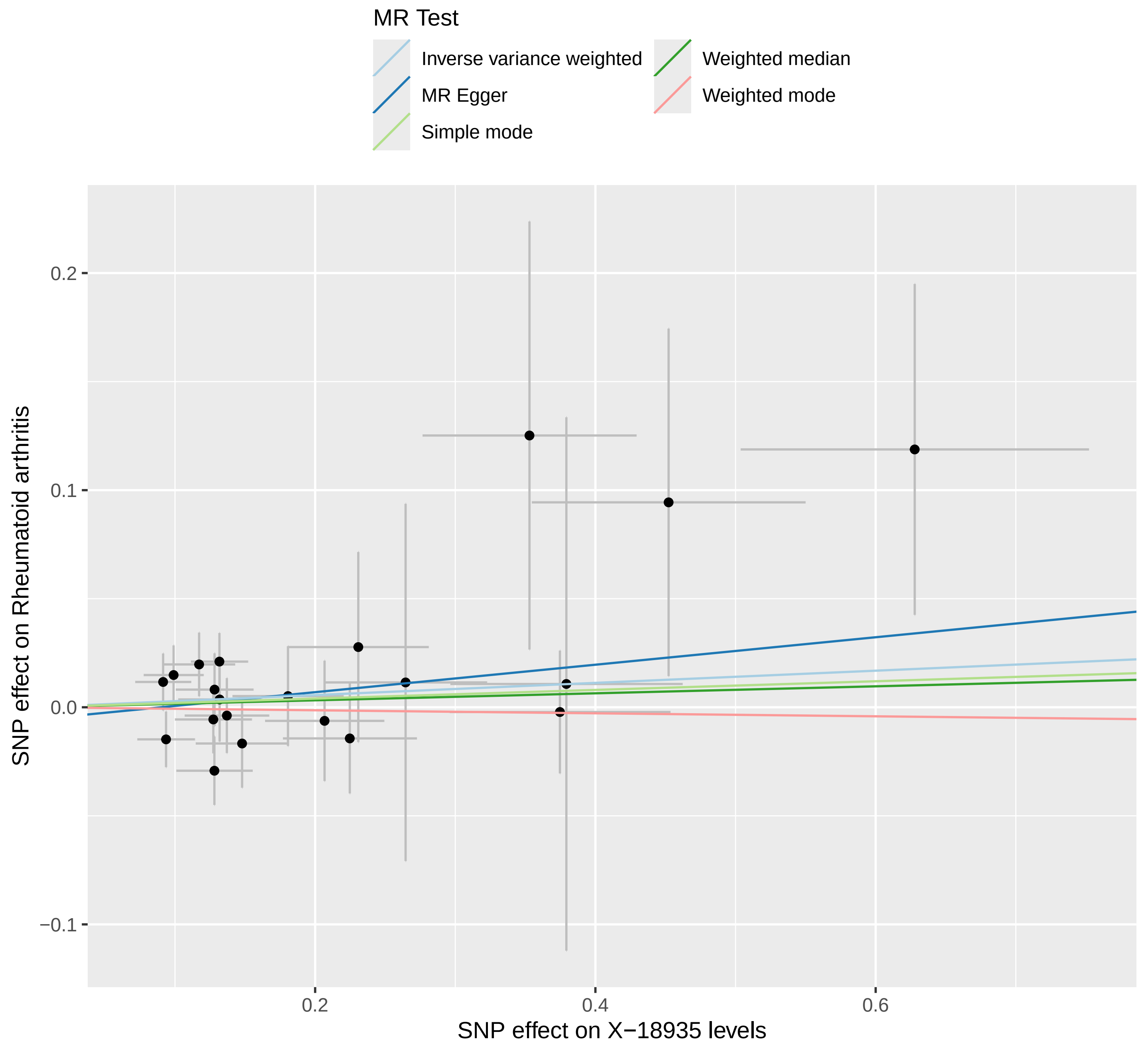
MR Test
Inverse variance weighted
MR Egger
Simple mode
Weighted median
Weighted mode
0.2
0.1
0.0
−0.1
0.2
0.4
0.6
SNP effect on Rheumatoid arthritis
SNP effect on X−18935 levels

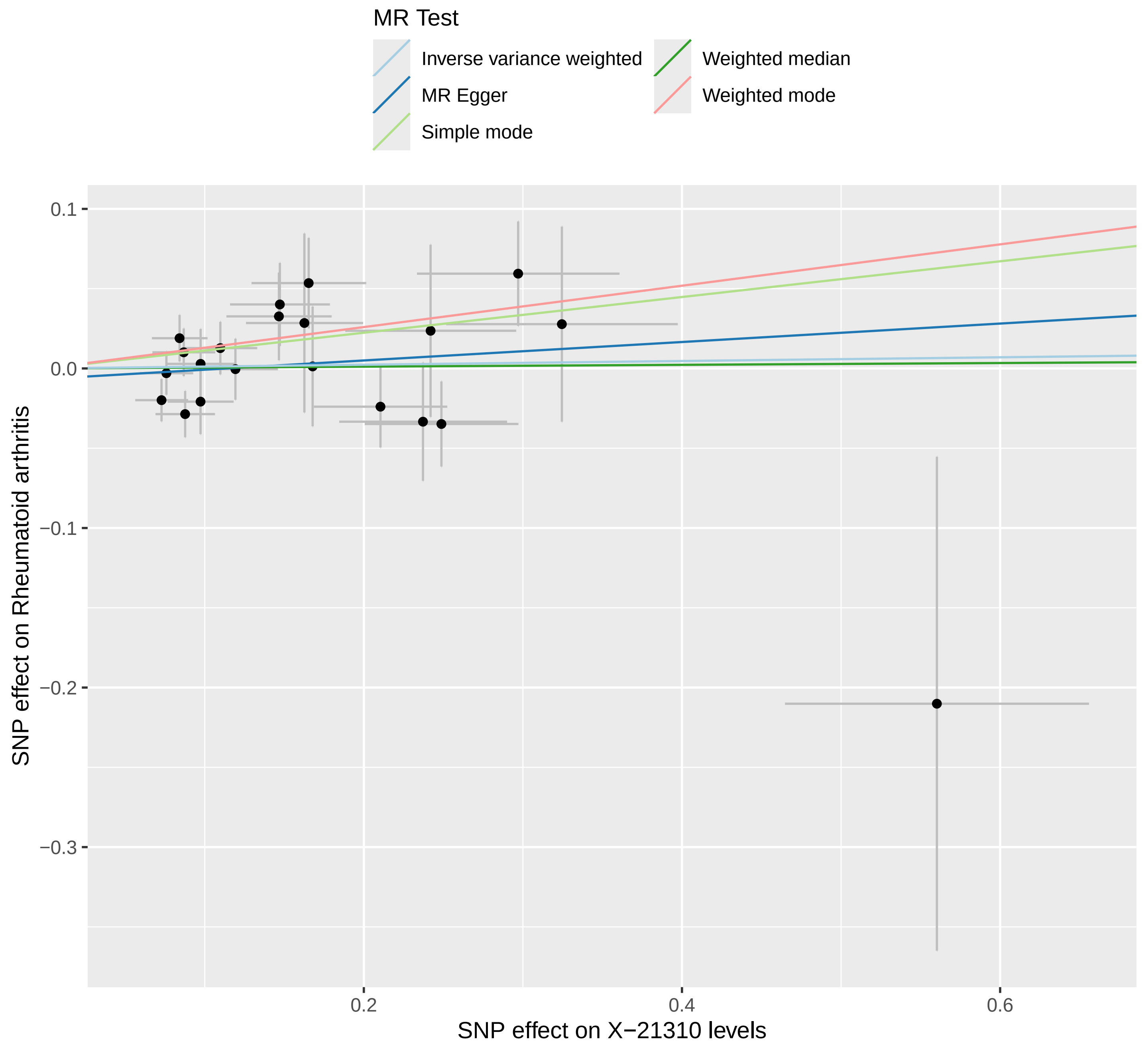

MR Test
Inverse variance weighted
MR Egger
Simple mode
Weighted median
Weighted mode
0.1
0.0
−0.1
−0.2
−0.3
0.2
0.4
0.6
SNP effect on Rheumatoid arthritis
SNP effect on X−21310 levels

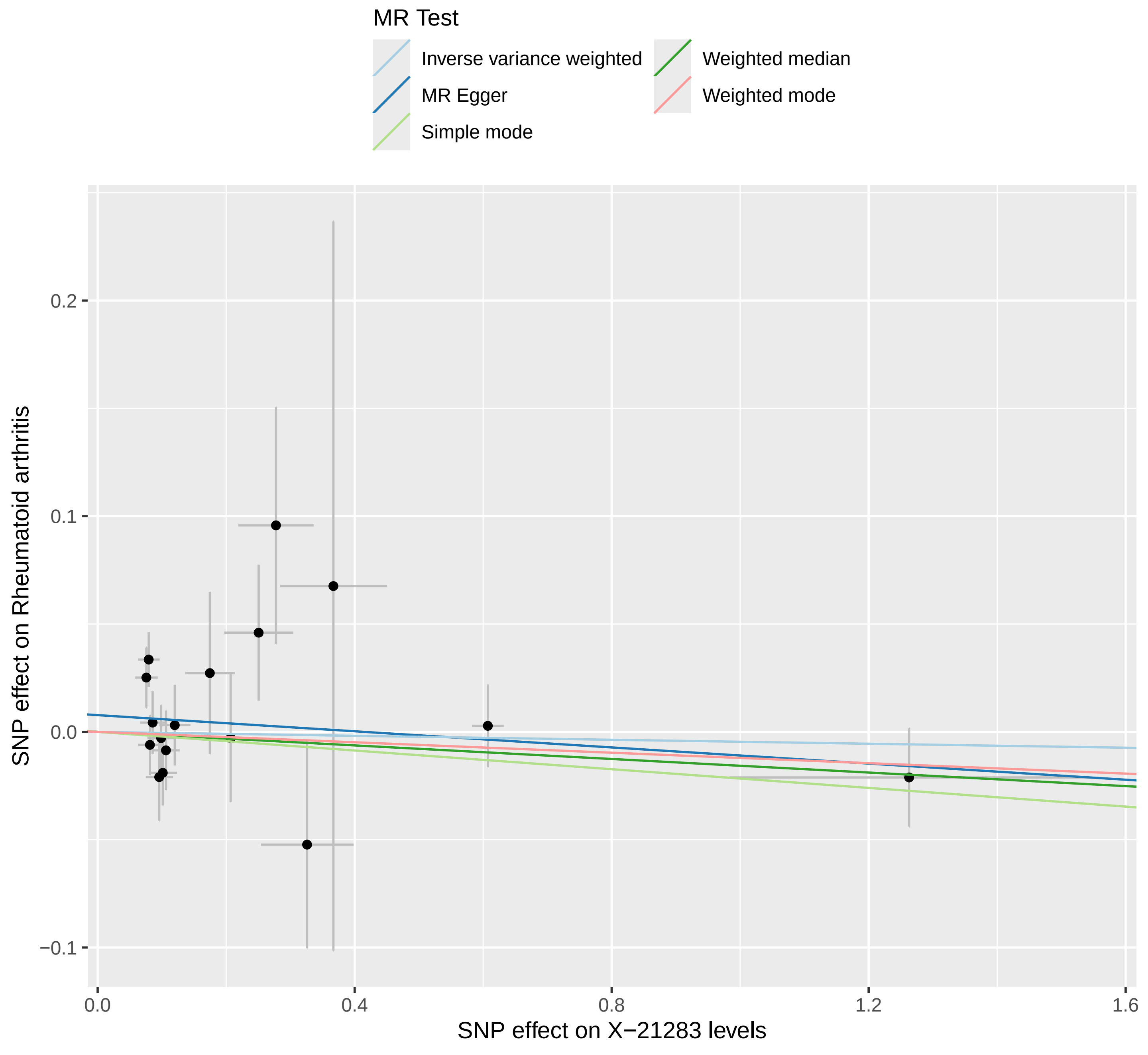
MR Test
Inverse variance weighted
Weighted median
MR Egger
Weighted mode
Simple mode
0.2
0.1
0.0
−0.1
0.0
0.4
0.8
1.2
1.6
SNP effect on Rheumatoid arthritis
SNP effect on X−21283 levels

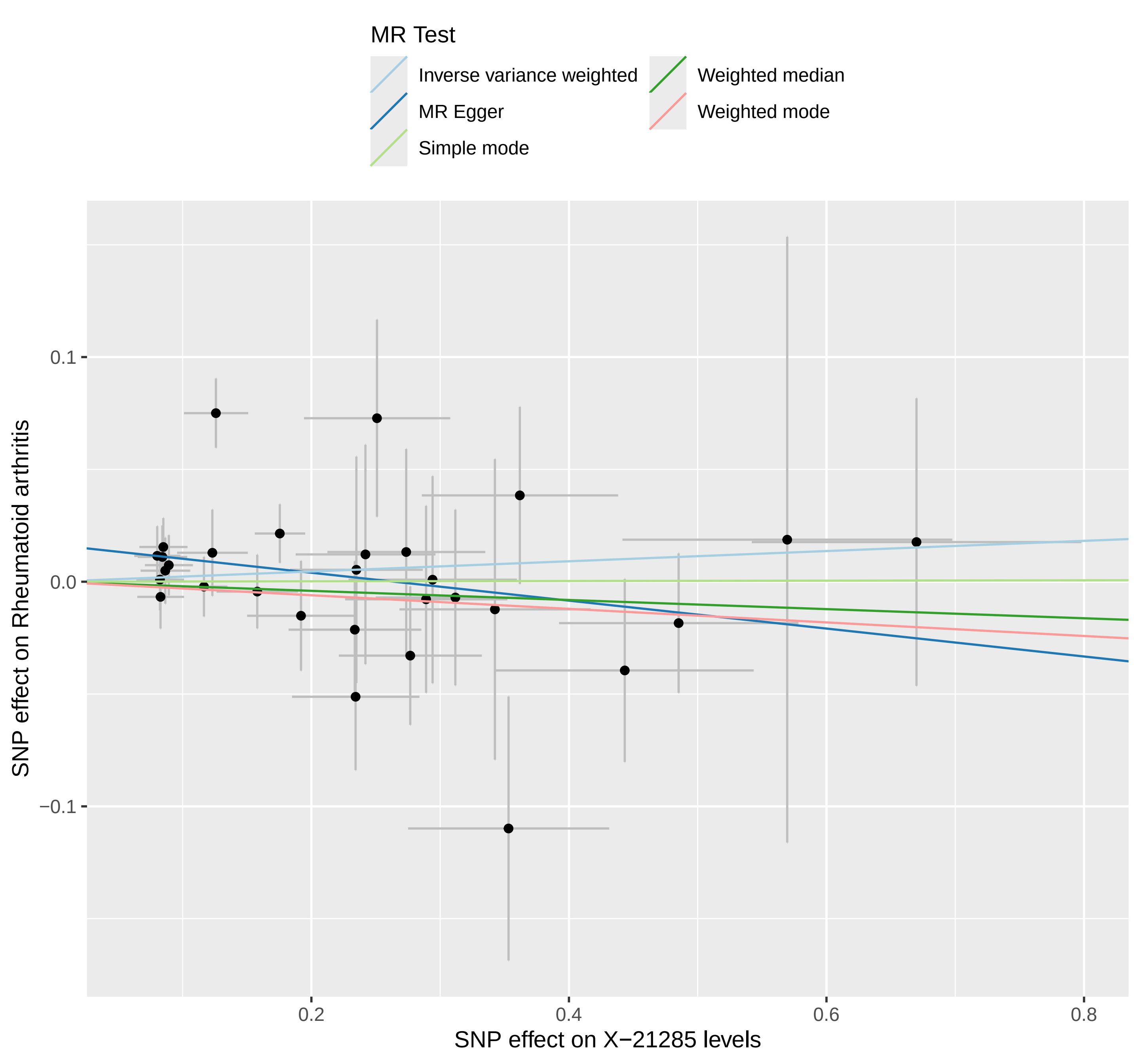
MR Test
Inverse variance weighted
MR Egger
Simple mode
Weighted median
Weighted mode
0.1
0.0
−0.1
0.2
0.4
0.6
0.8
SNP effect on Rheumatoid arthritis
SNP effect on X−21285 levels

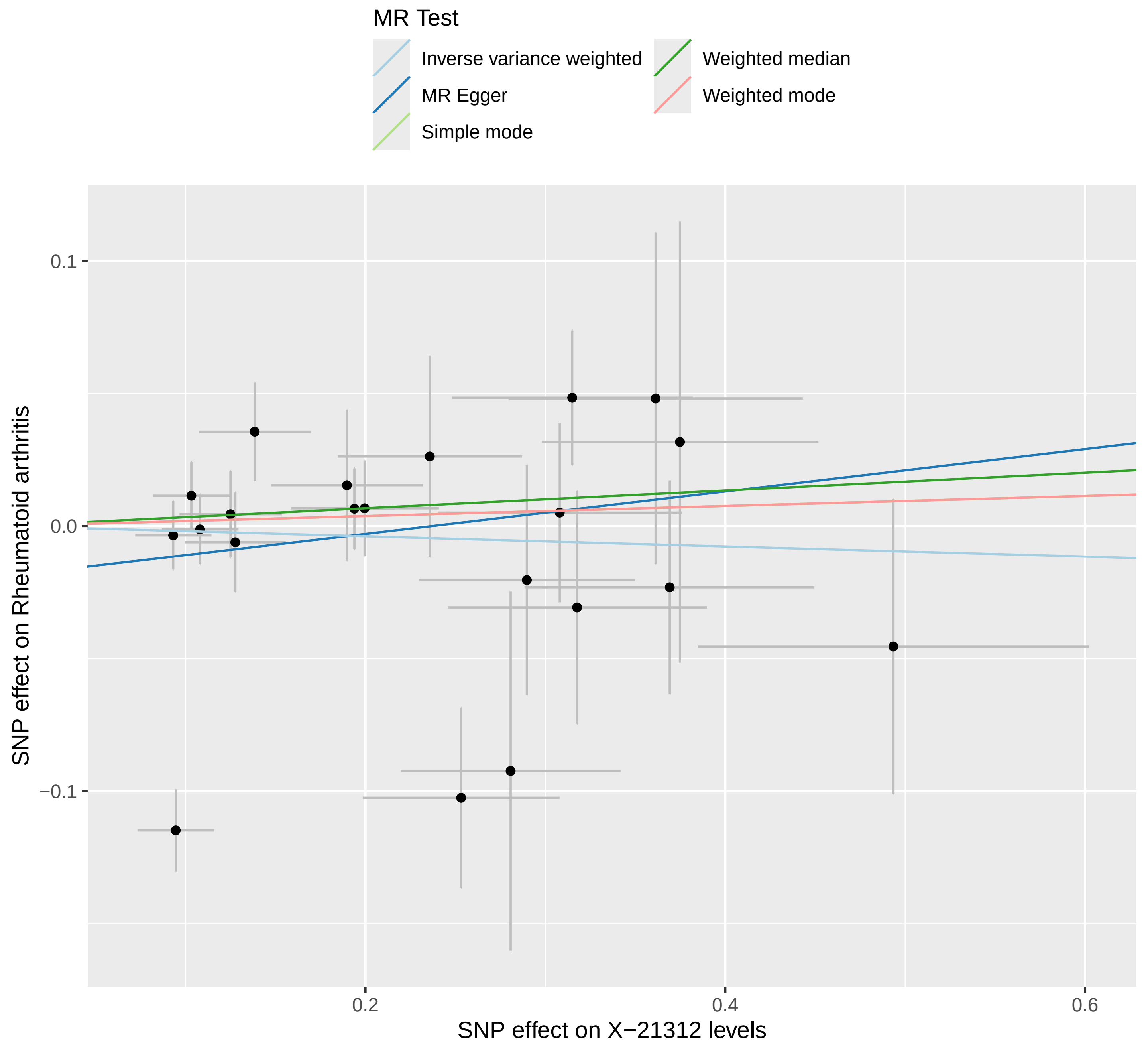

MR Test
Inverse variance weighted
MR Egger
Simple mode
Weighted median
Weighted mode
0.1
0.0
−0.1
0.2
0.4
0.6
SNP effect on Rheumatoid arthritis
SNP effect on X−21312 levels

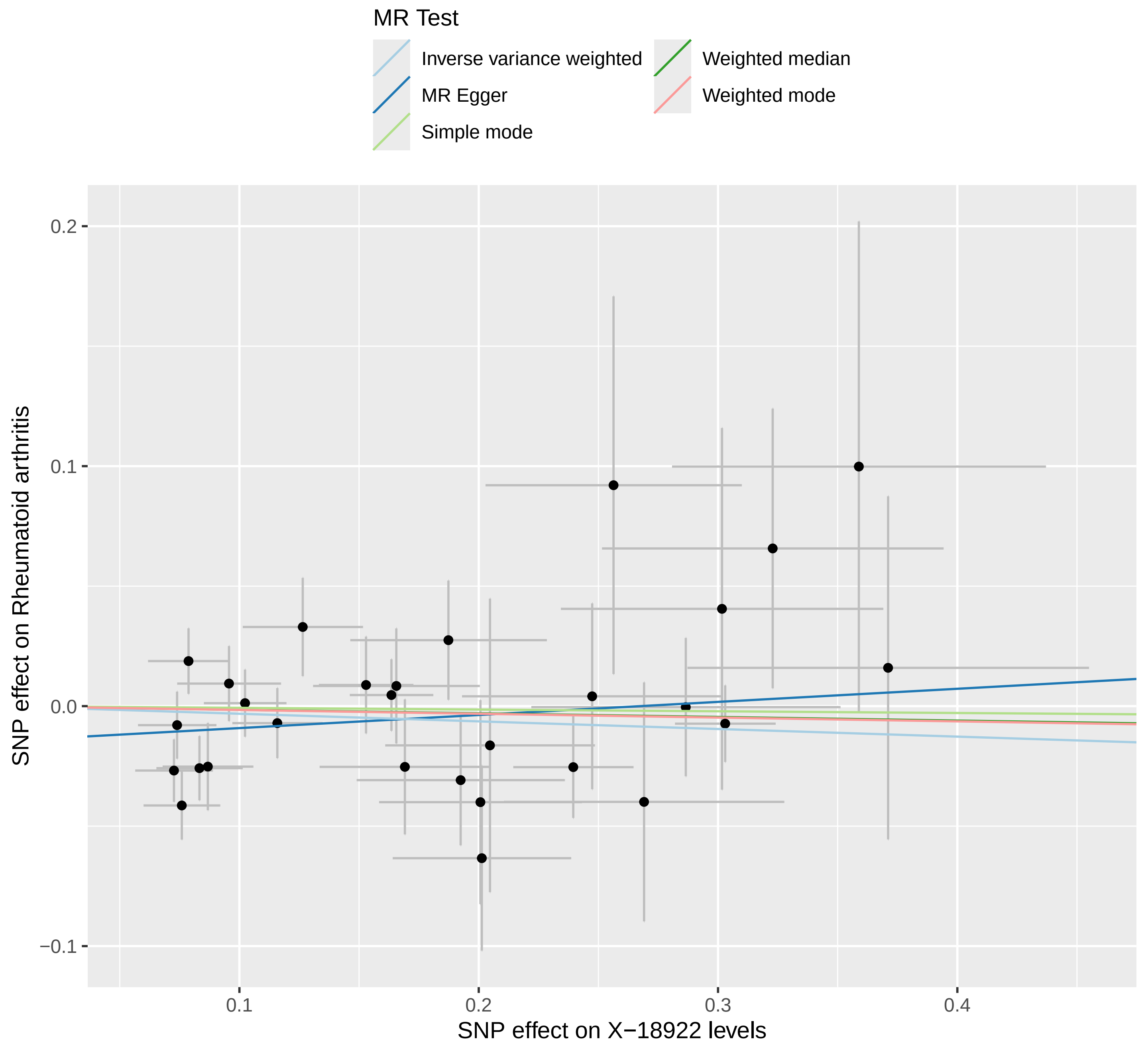
MR Test
Inverse variance weighted
MR Egger
Simple mode
Weighted median
Weighted mode
SNP effect on Rheumatoid arthritis
SNP effect on X−18922 levels
0.2
0.1
0.0
−0.1
0.1
0.2
0.3
0.4

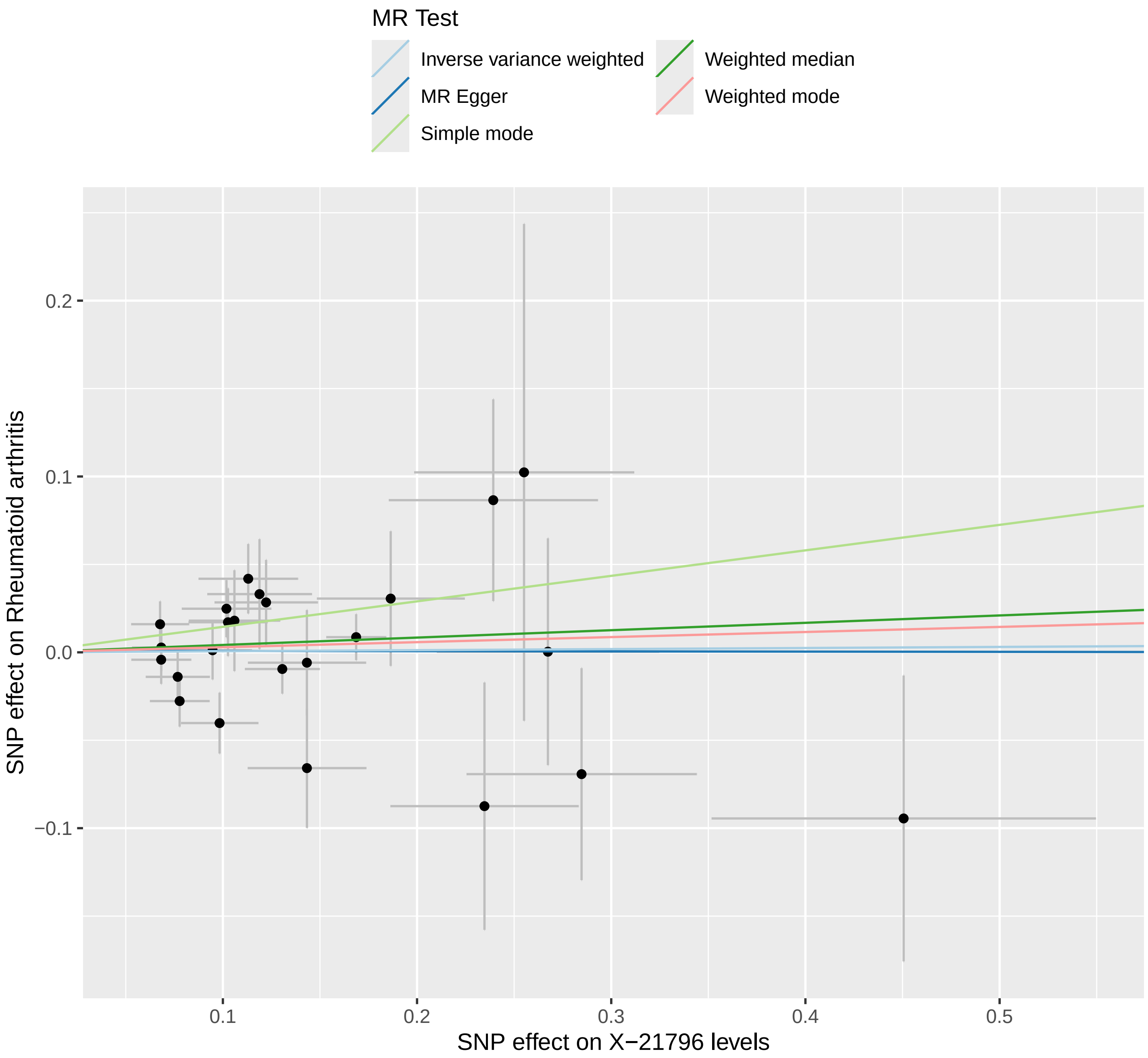
MR Test
Inverse variance weighted
MR Egger
Simple mode
Weighted median
Weighted mode
0.2
0.1
0.0
−0.1
SNP effect on Rheumatoid arthritis
0.1
0.2
0.3
0.4
0.5
SNP effect on X−21796 levels

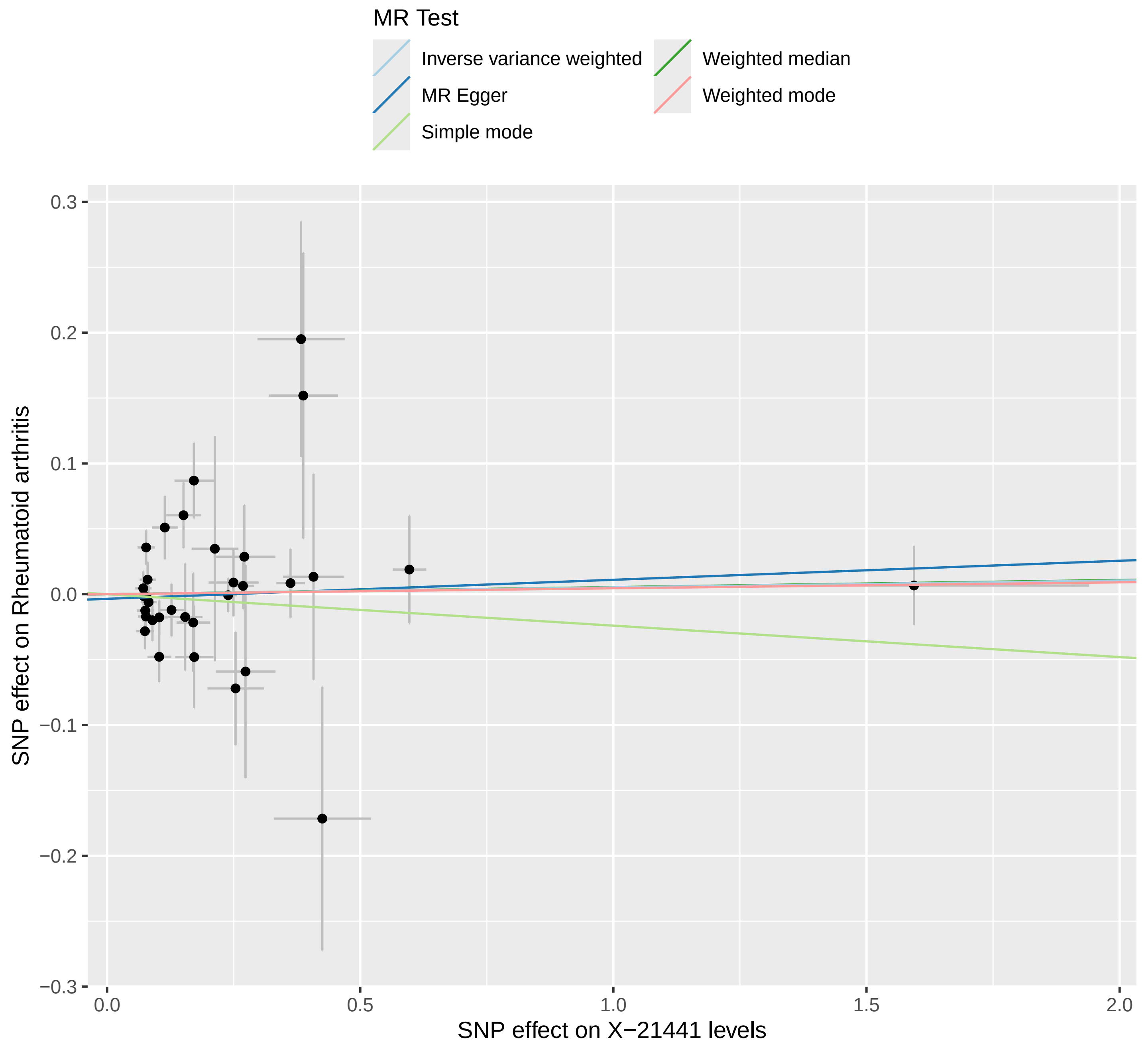
MR Test
Inverse variance weighted
MR Egger
Simple mode
Weighted median
Weighted mode
0.3
0.2
0.1
0.0
−0.1
−0.2
−0.3
SNP effect on Rheumatoid arthritis
0.0
0.5
1.0
1.5
2.0
SNP effect on X−21441 levels

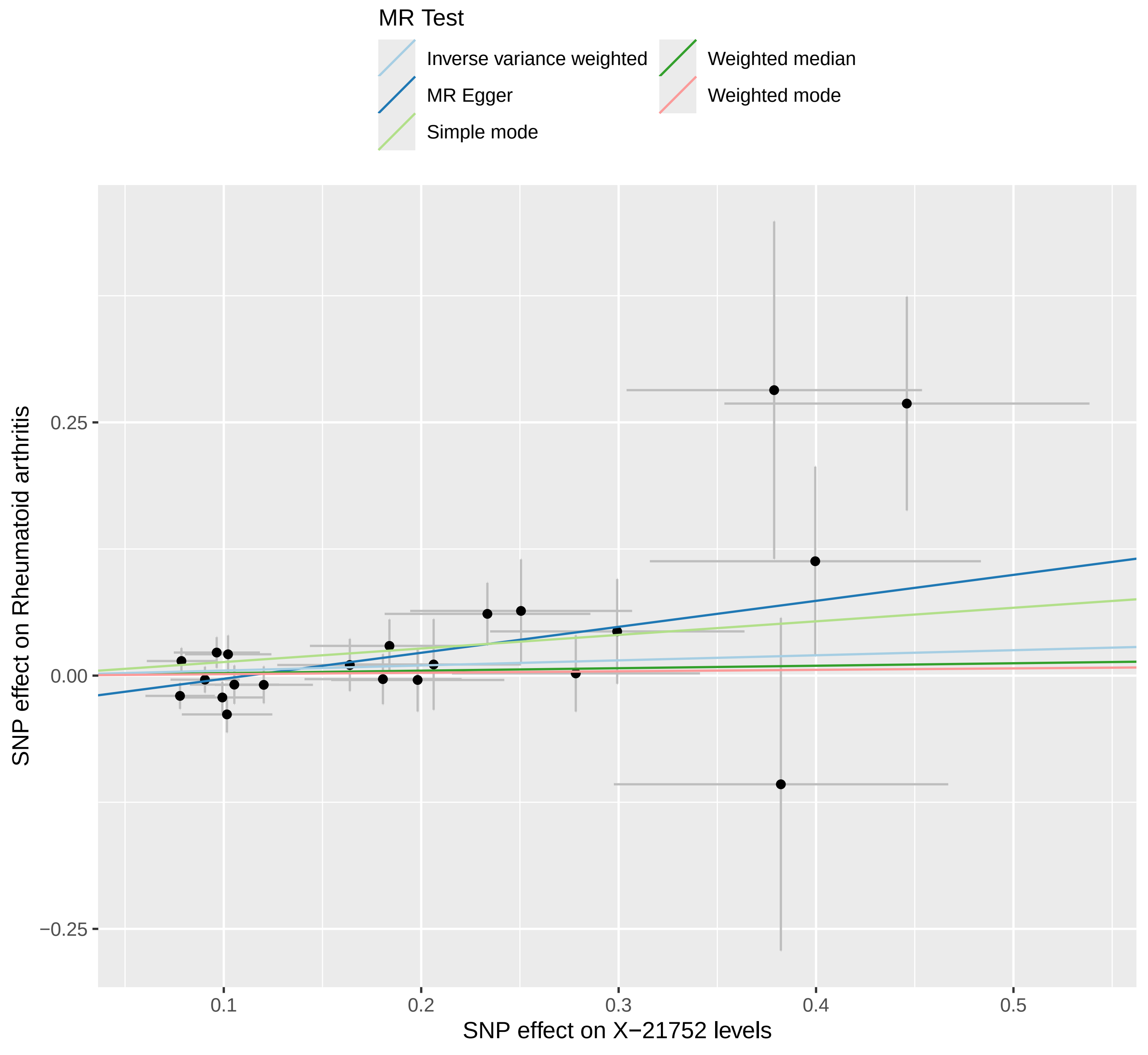

MR Test
Inverse variance weighted
MR Egger
Simple mode
Weighted median
Weighted mode
SNP effect on Rheumatoid arthritis
0.25
0.00
−0.25
0.1
0.2
0.3
0.4
0.5
SNP effect on X−21752 levels

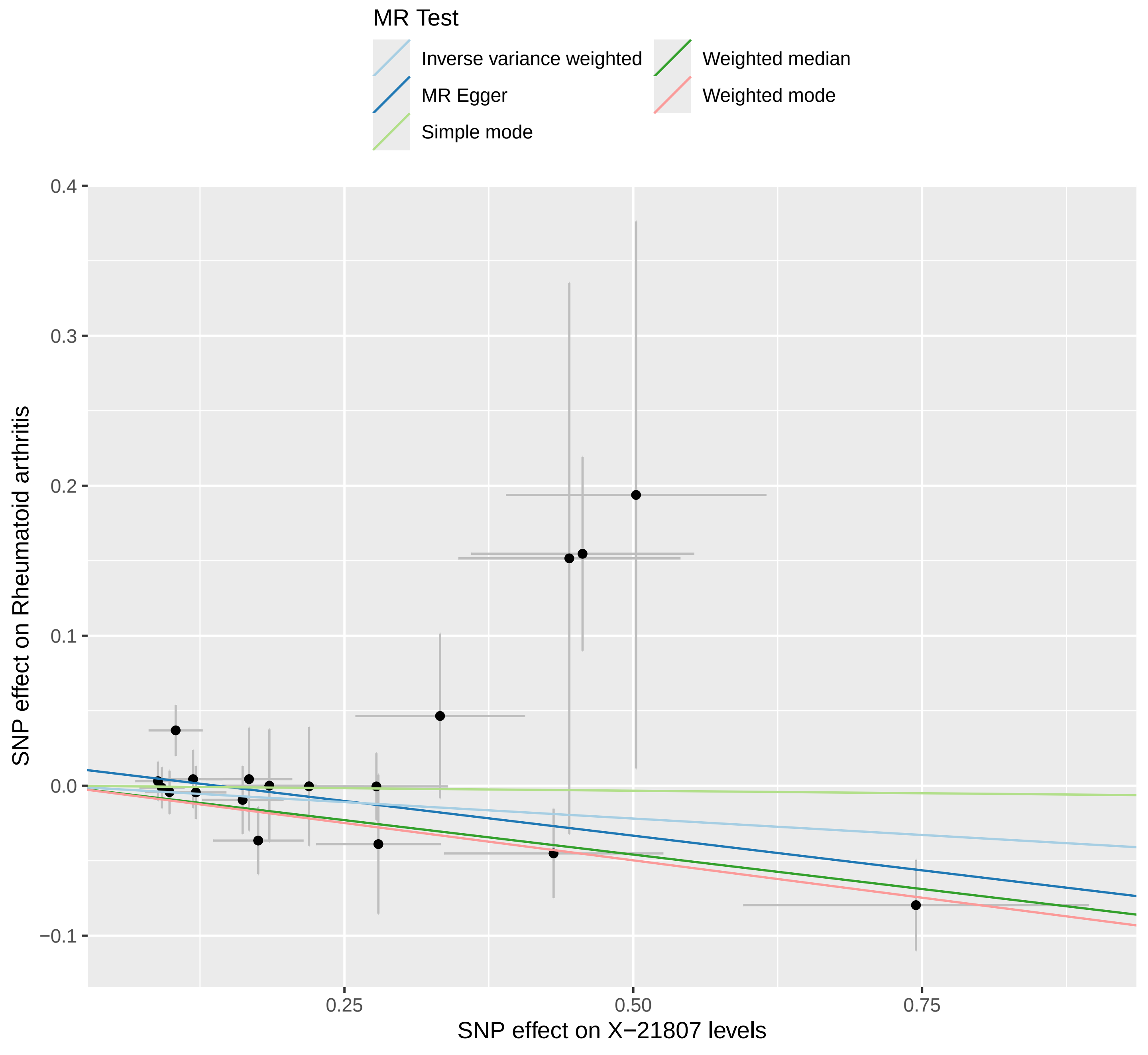

MR Test
Inverse variance weighted
MR Egger
Simple mode
Weighted median
Weighted mode
0.4
0.3
0.2
0.1
0.0
−0.1
SNP effect on Rheumatoid arthritis
0.25
0.50
0.75
SNP effect on X−21807 levels

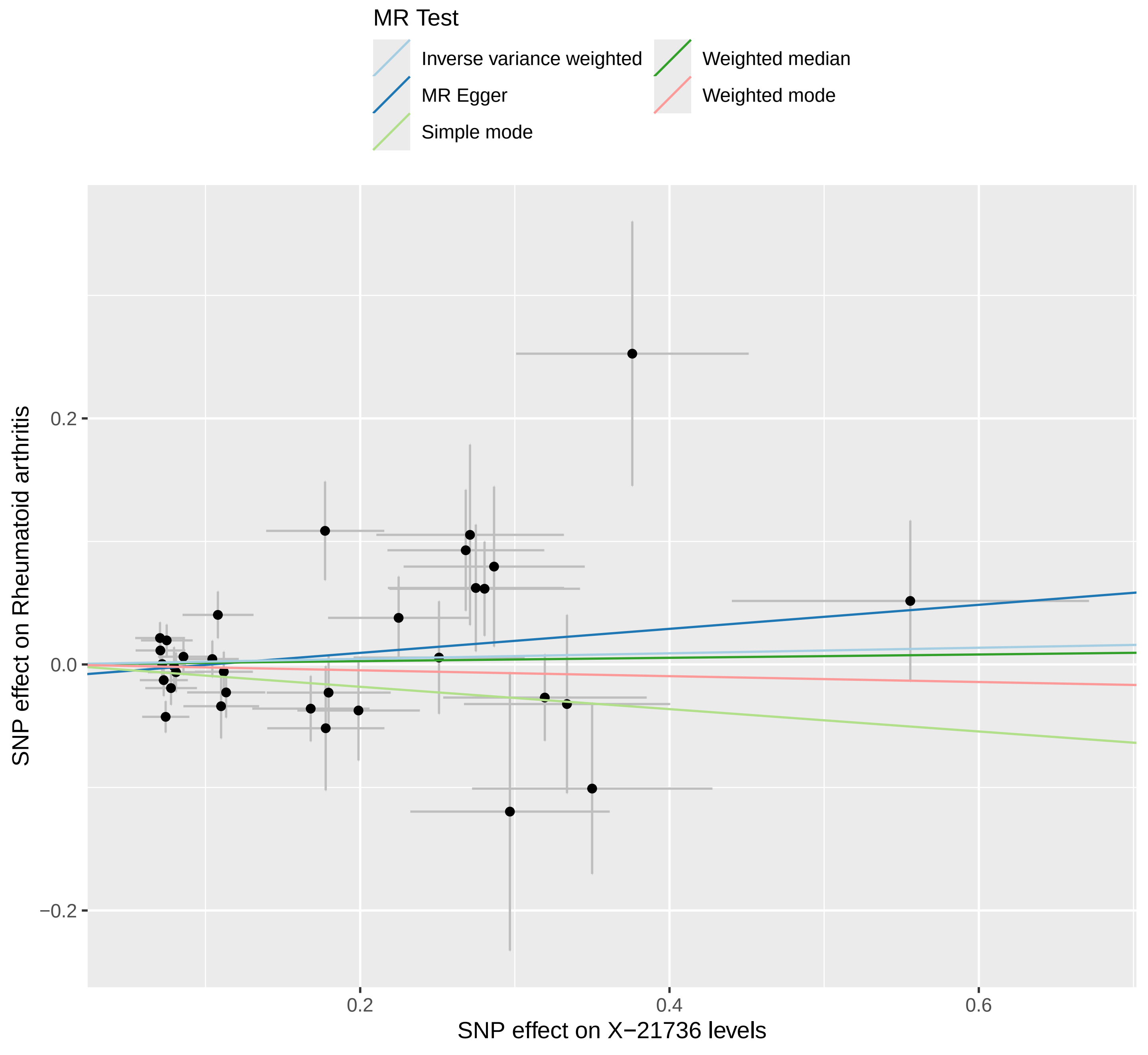

MR Test
Inverse variance weighted
MR Egger
Simple mode
Weighted median
Weighted mode
SNP effect on Rheumatoid arthritis
0.2
0.0
−0.2
0.2
0.4
0.6
SNP effect on X−21736 levels

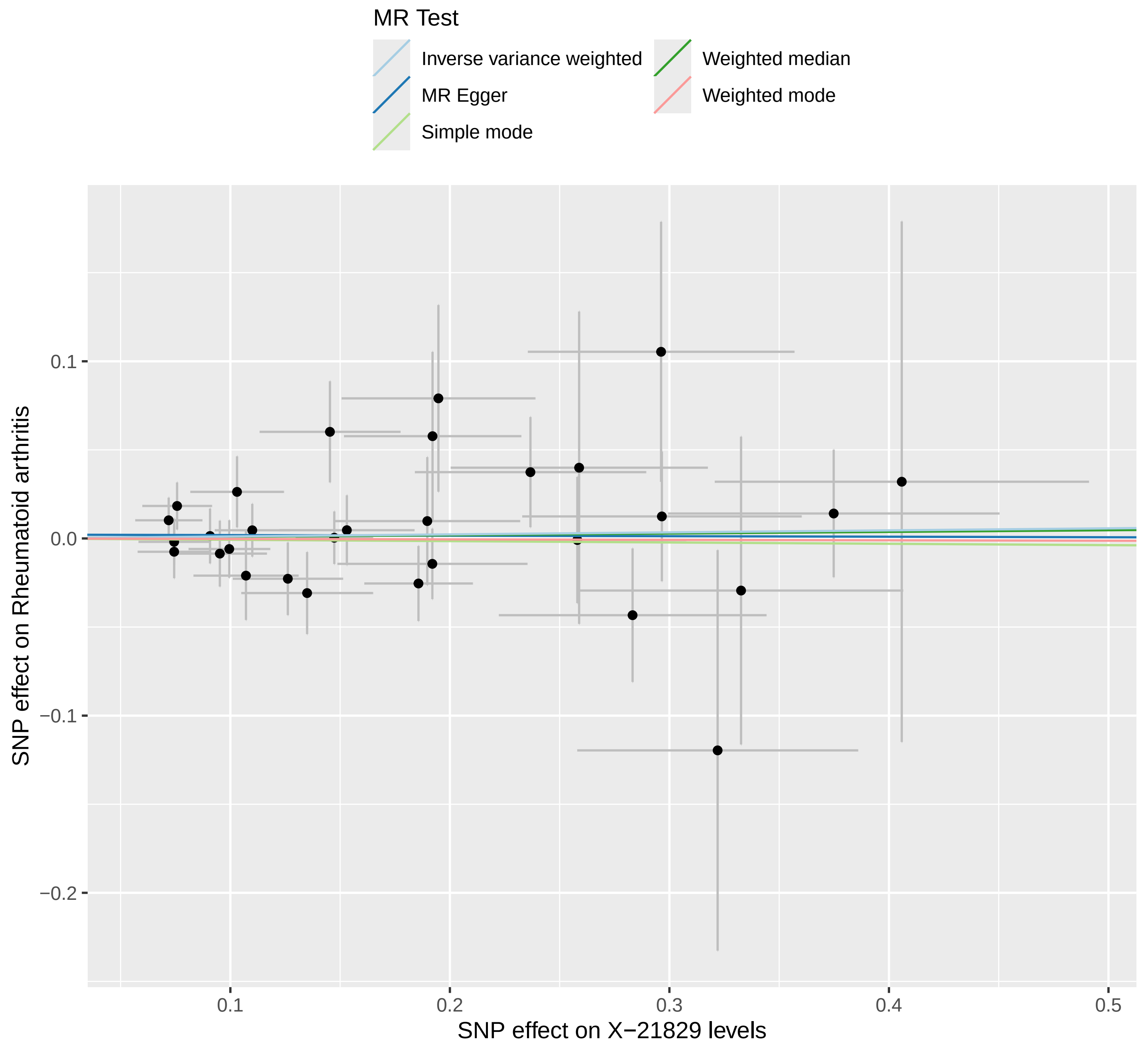
MR Test
Inverse variance weighted
MR Egger
Simple mode
Weighted median
Weighted mode
0.1
0.0
−0.1
−0.2
0.1
0.2
0.3
0.4
0.5
SNP effect on Rheumatoid arthritis
SNP effect on X−21829 levels

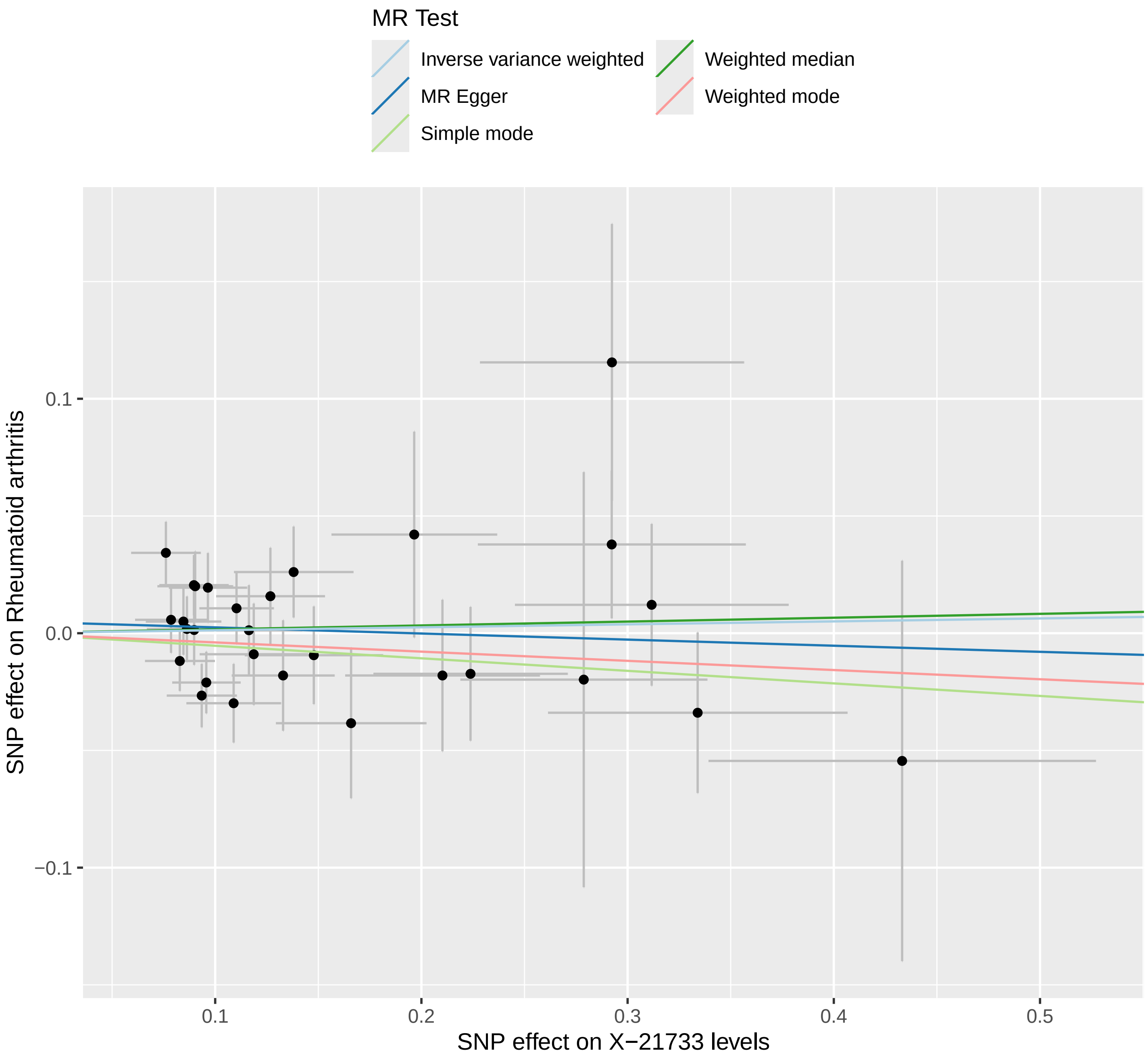
MR Test
Inverse variance weighted
MR Egger
Simple mode
Weighted median
Weighted mode
SNP effect on Rheumatoid arthritis
0.1
0.0
−0.1
0.1
0.2
0.3
0.4
0.5
SNP effect on X−21733 levels

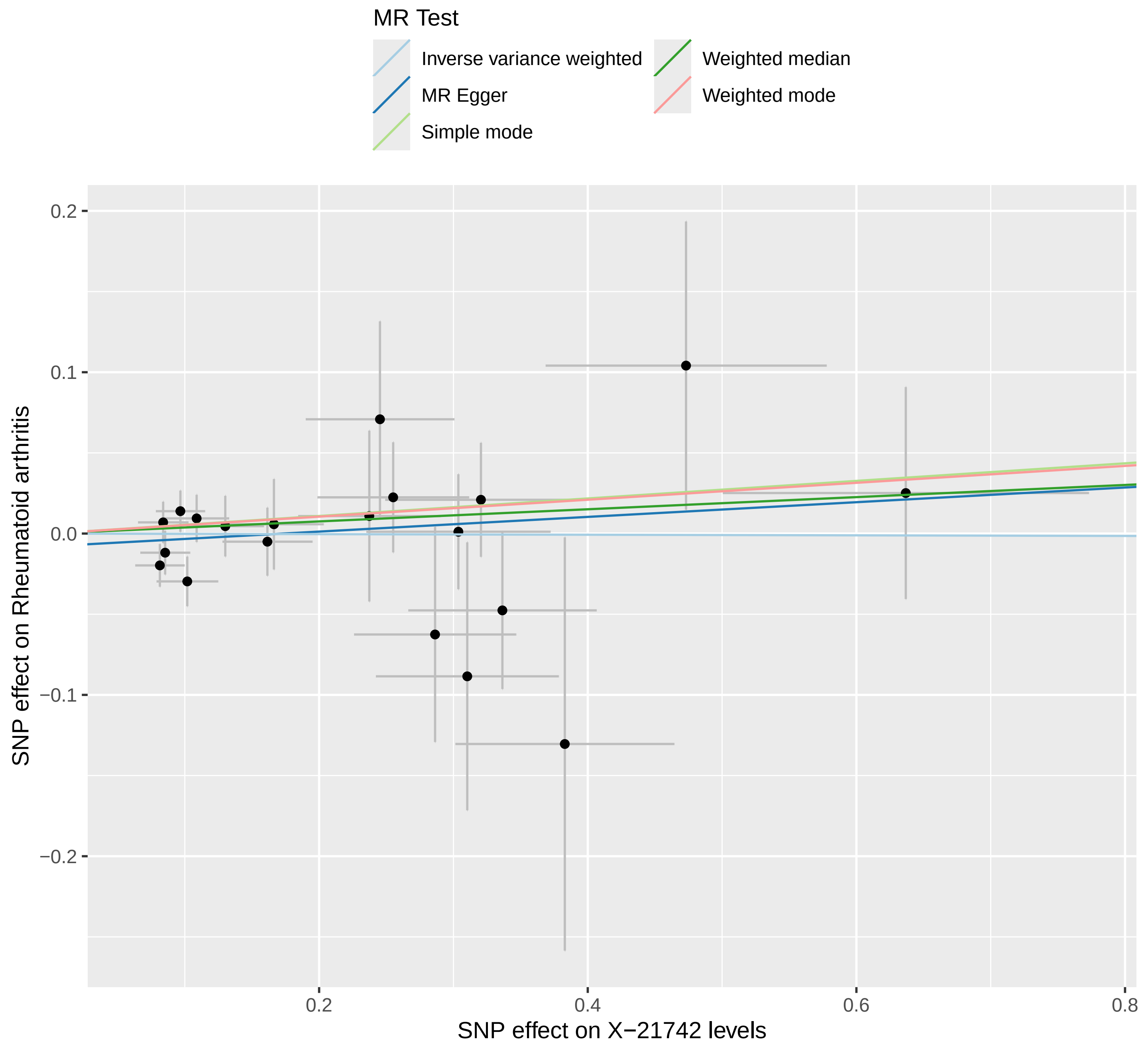
MR Test
Inverse variance weighted
MR Egger
Simple mode
Weighted median
Weighted mode
0.2
0.1
0.0
−0.1
−0.2
SNP effect on Rheumatoid arthritis
0.2
0.4
0.6
0.8
SNP effect on X−21742 levels

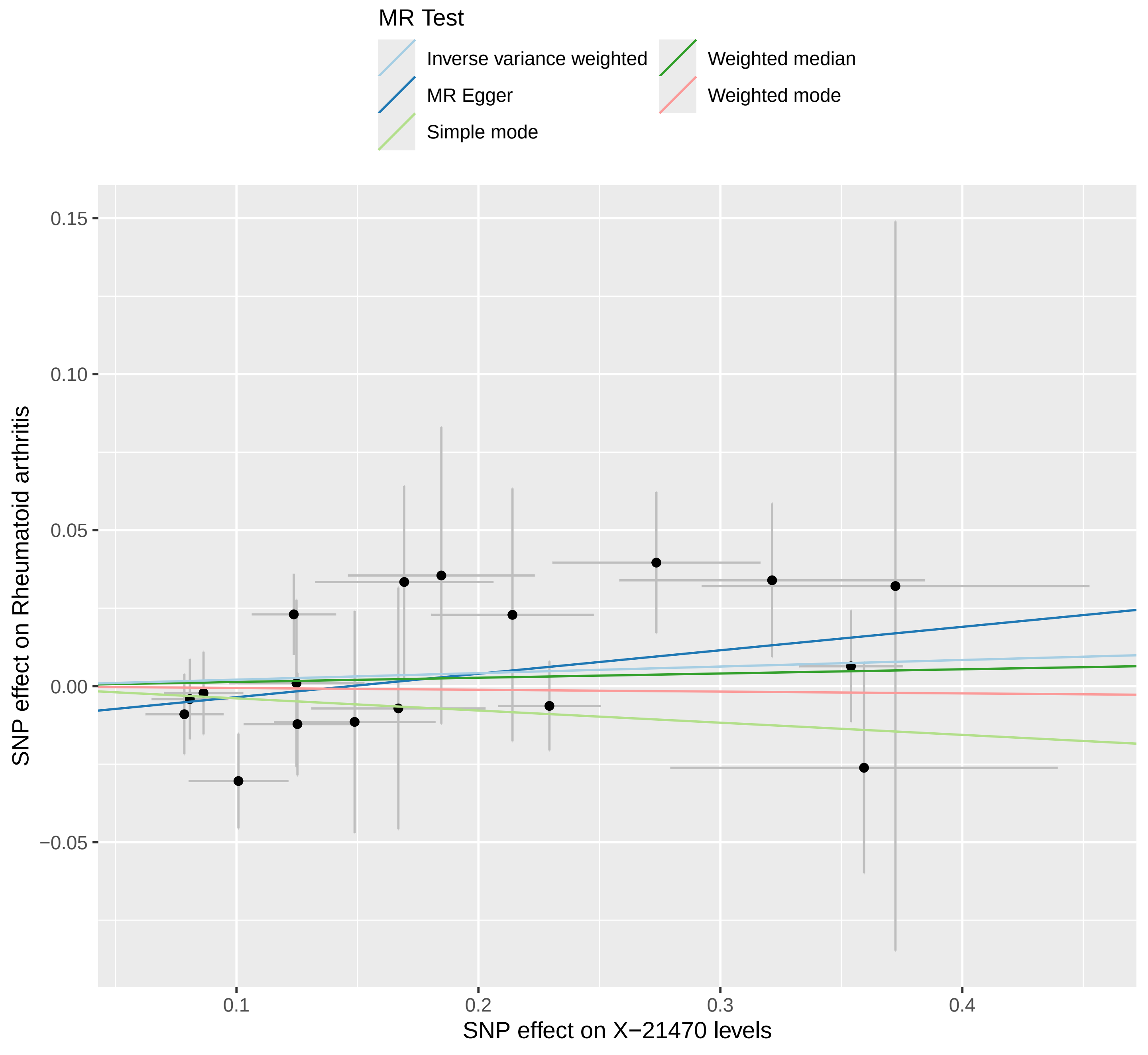

MR Test
Inverse variance weighted
MR Egger
Simple mode
Weighted median
Weighted mode
0.15
0.10
0.05
0.00
−0.05
SNP effect on Rheumatoid arthritis
0.1
0.2
0.3
0.4
SNP effect on X−21470 levels

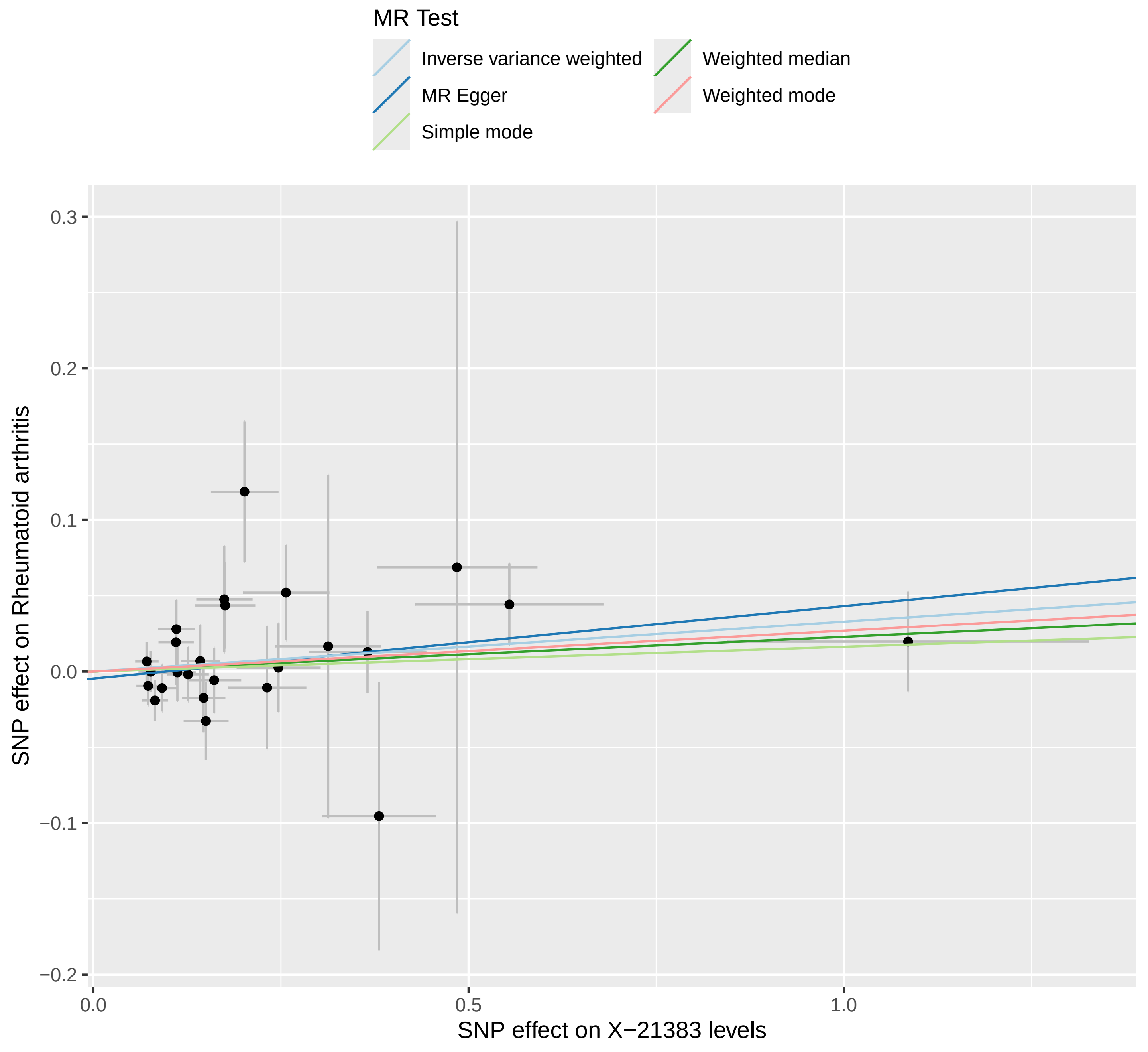
MR Test
Inverse variance weighted
MR Egger
Simple mode
Weighted median
Weighted mode
SNP effect on Rheumatoid arthritis
0.3
0.2
0.1
0.0
−0.1
−0.2
0.0
0.5
1.0
SNP effect on X−21383 levels

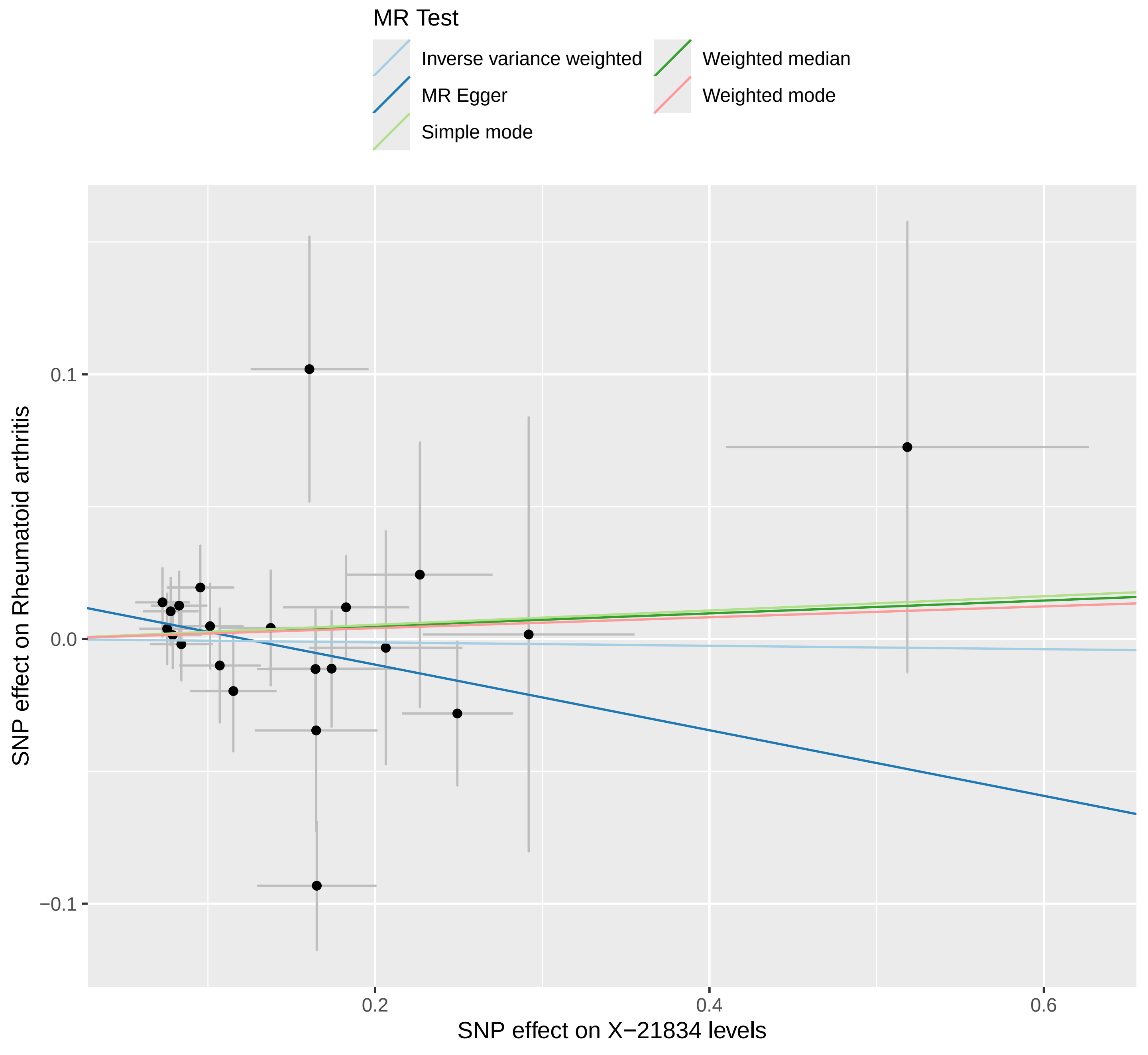

MR Test
Inverse variance weighted
MR Egger
Simple mode
Weighted median
Weighted mode
0.1
0.0
−0.1
0.2
0.4
0.6
SNP effect on Rheumatoid arthritis
SNP effect on X−21834 levels

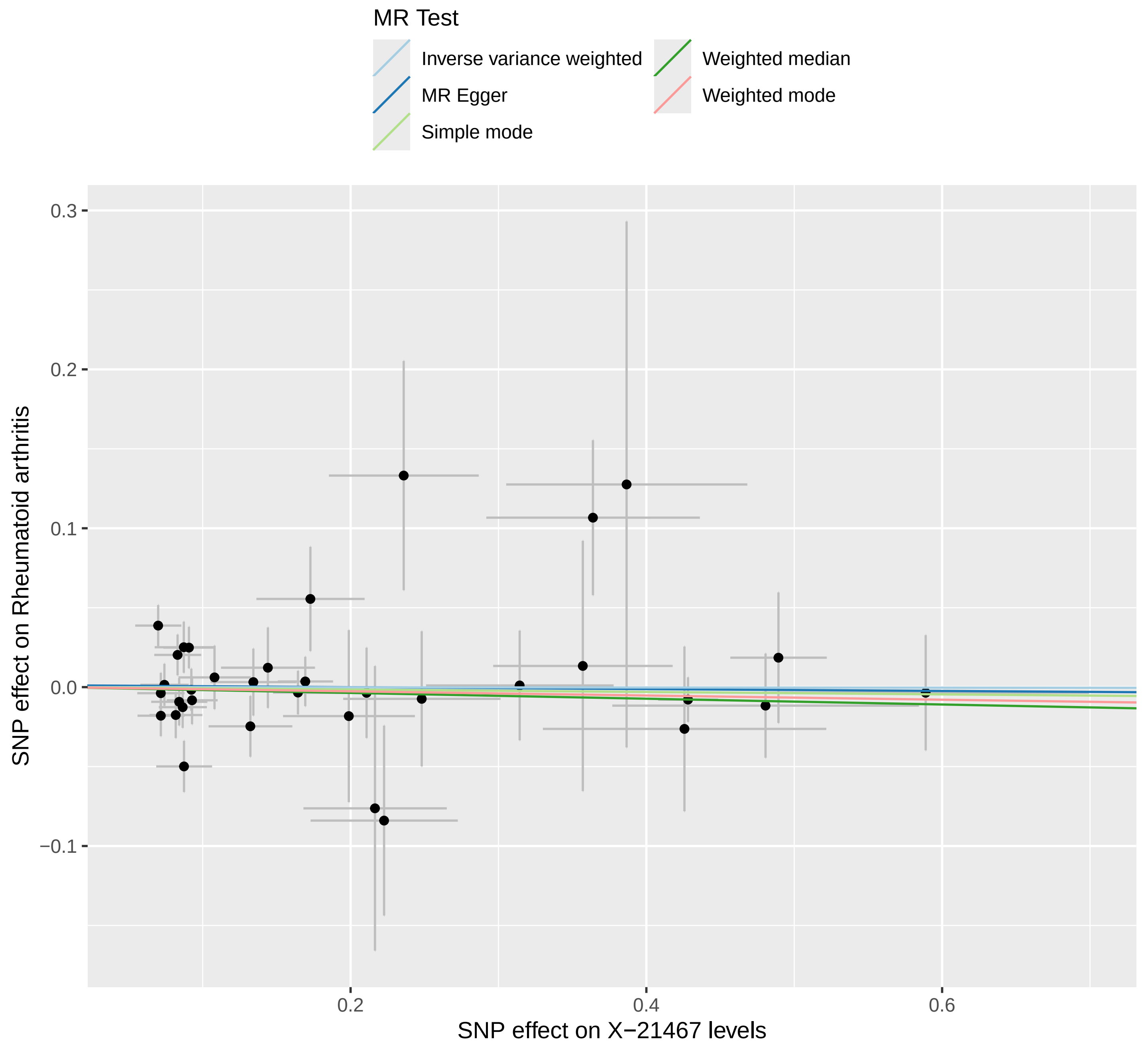
MR Test
Inverse variance weighted
MR Egger
Simple mode
Weighted median
Weighted mode
0.3
0.2
0.1
0.0
−0.1
0.2
0.4
0.6
SNP effect on Rheumatoid arthritis
SNP effect on X−21467 levels

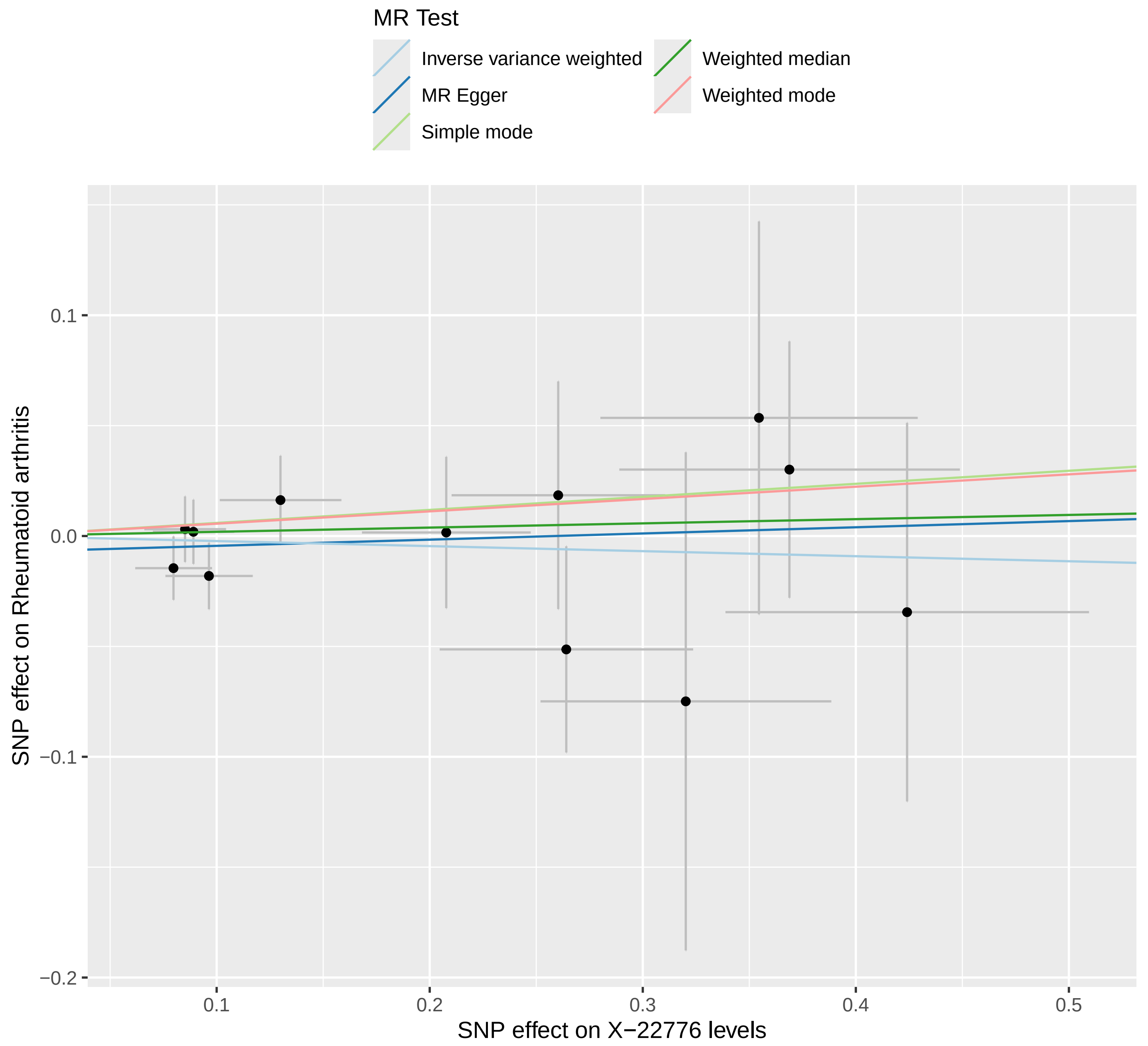
MR Test
Inverse variance weighted
MR Egger
Simple mode
Weighted median
Weighted mode
0.1
0.0
−0.1
−0.2
SNP effect on Rheumatoid arthritis
0.1
0.2
0.3
0.4
0.5
SNP effect on X−22776 levels

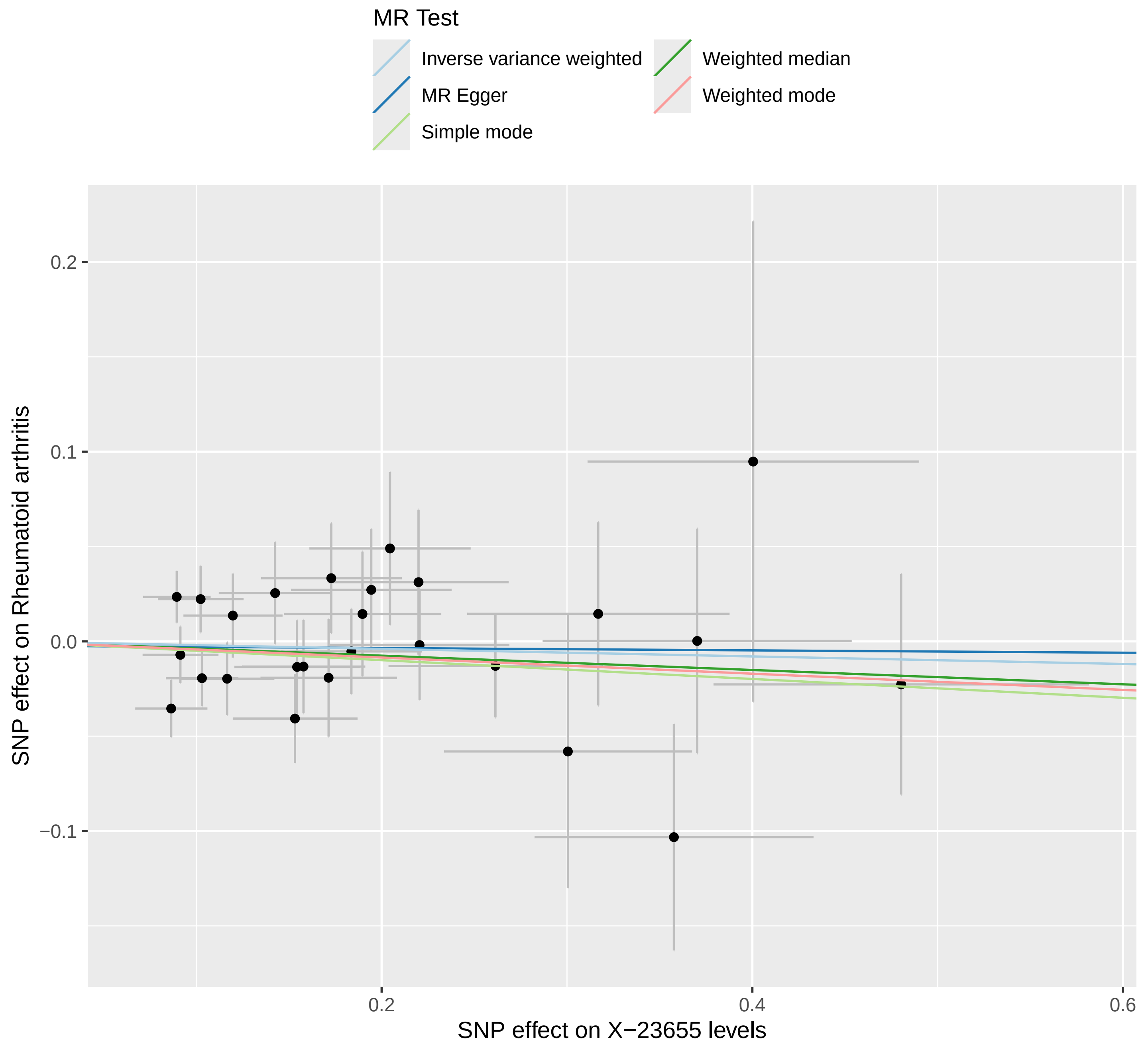
MR Test
Inverse variance weighted
MR Egger
Simple mode
Weighted median
Weighted mode
0.2
0.1
0.0
−0.1
SNP effect on Rheumatoid arthritis
0.2
0.4
0.6
SNP effect on X−23655 levels

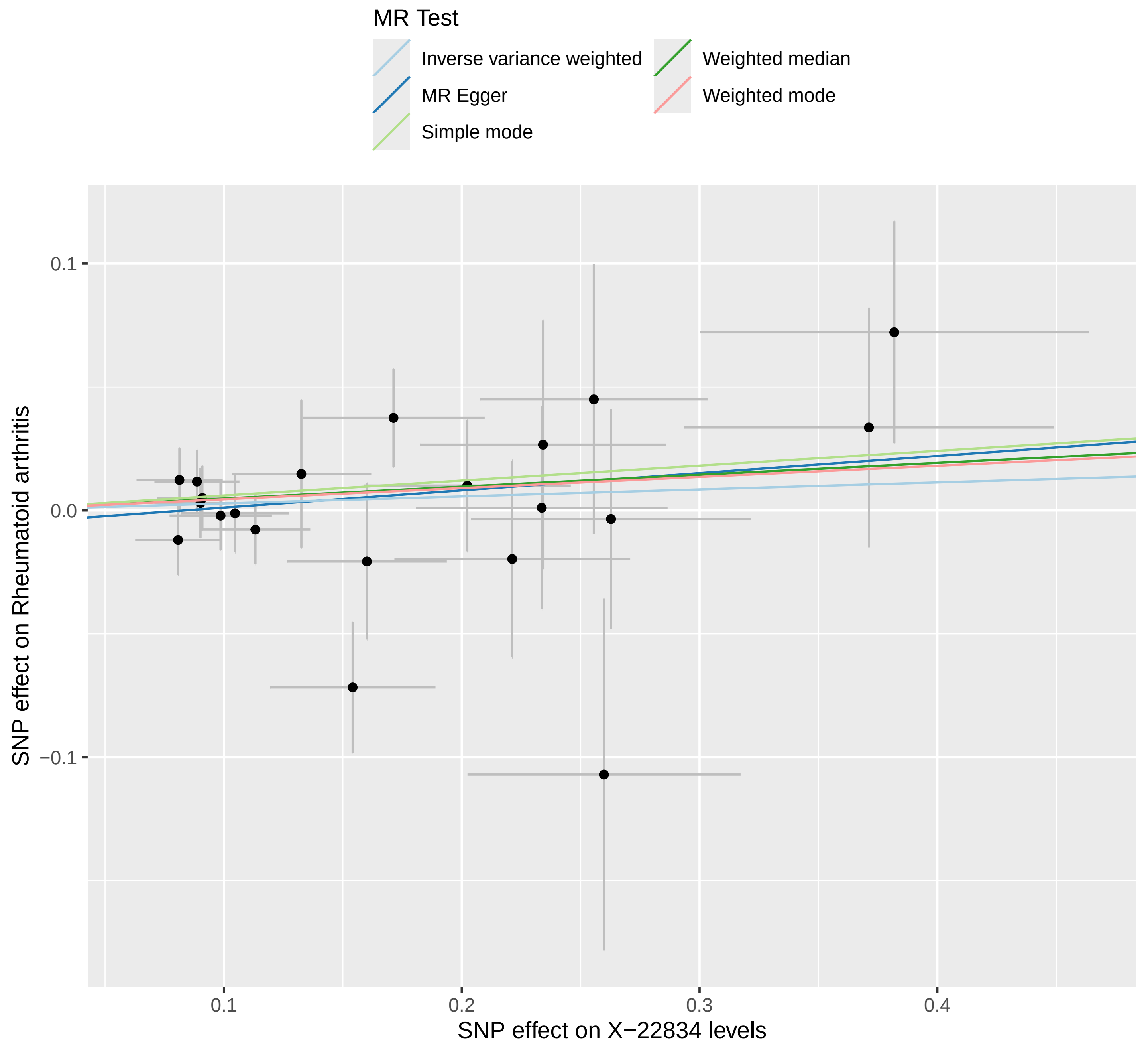

MR Test
Inverse variance weighted
MR Egger
Simple mode
Weighted median
Weighted mode
0.1
0.0
−0.1
0.1
0.2
0.3
0.4
SNP effect on Rheumatoid arthritis
SNP effect on X−22834 levels

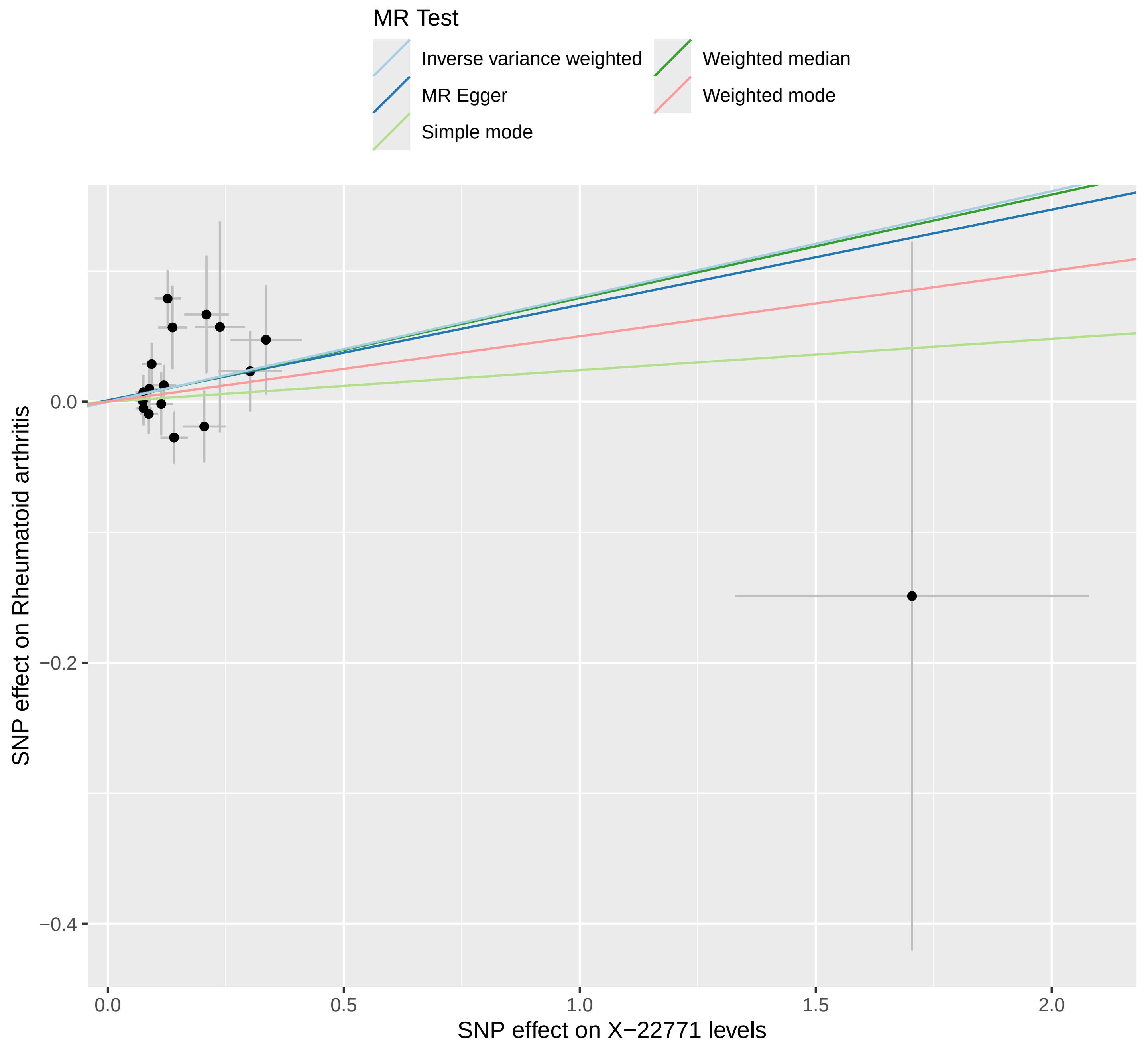

MR Test
Inverse variance weighted
MR Egger
Simple mode
Weighted median
Weighted mode
SNP effect on Rheumatoid arthritis
0.0
−0.2
−0.4
0.0
0.5
1.0
1.5
2.0
SNP effect on X−22771 levels

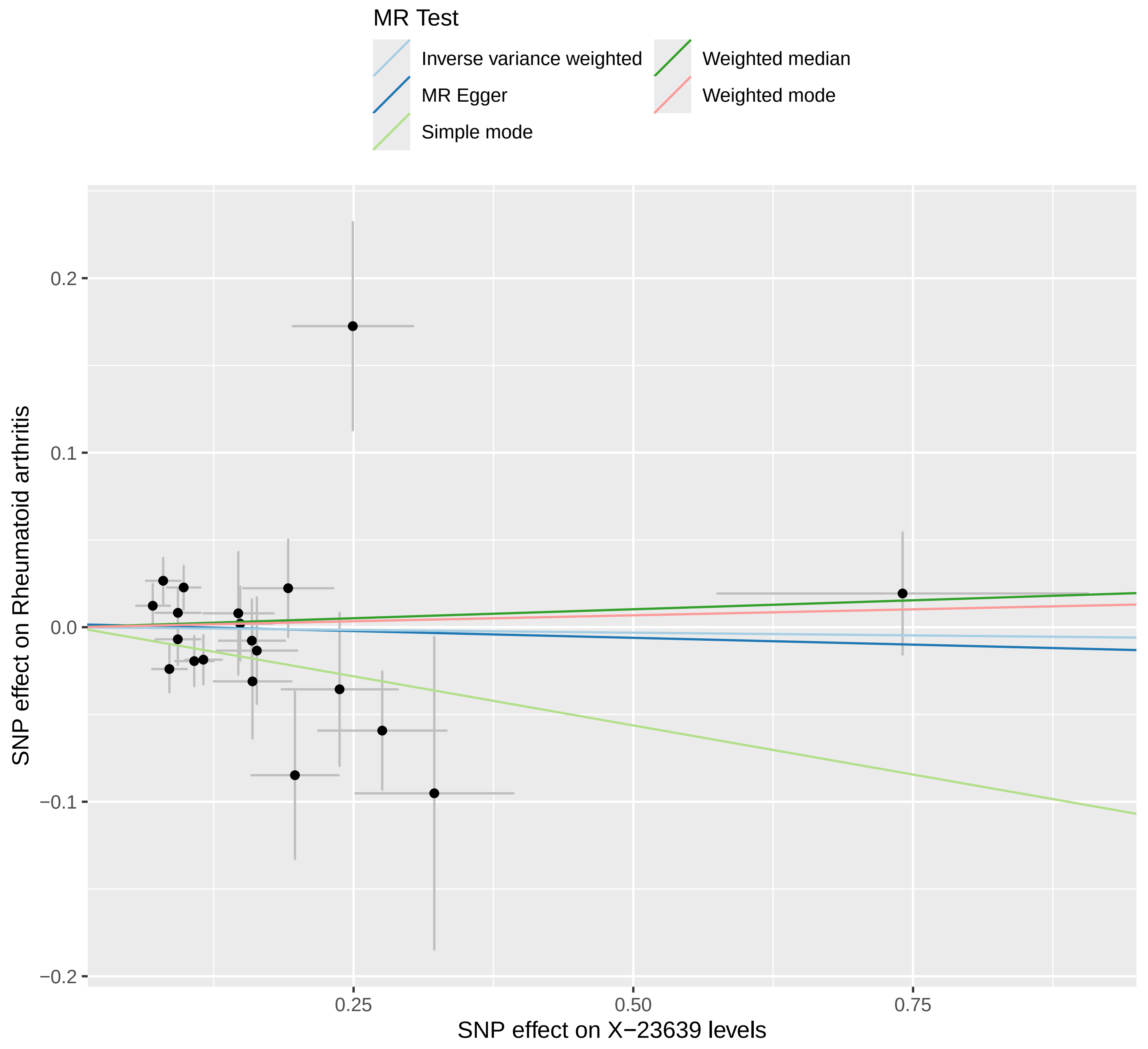
MR Test
Inverse variance weighted
MR Egger
Simple mode
Weighted median
Weighted mode
SNP effect on Rheumatoid arthritis
0.2
0.1
0.0
−0.1
−0.2
0.25
0.50
0.75
SNP effect on X−23639 levels

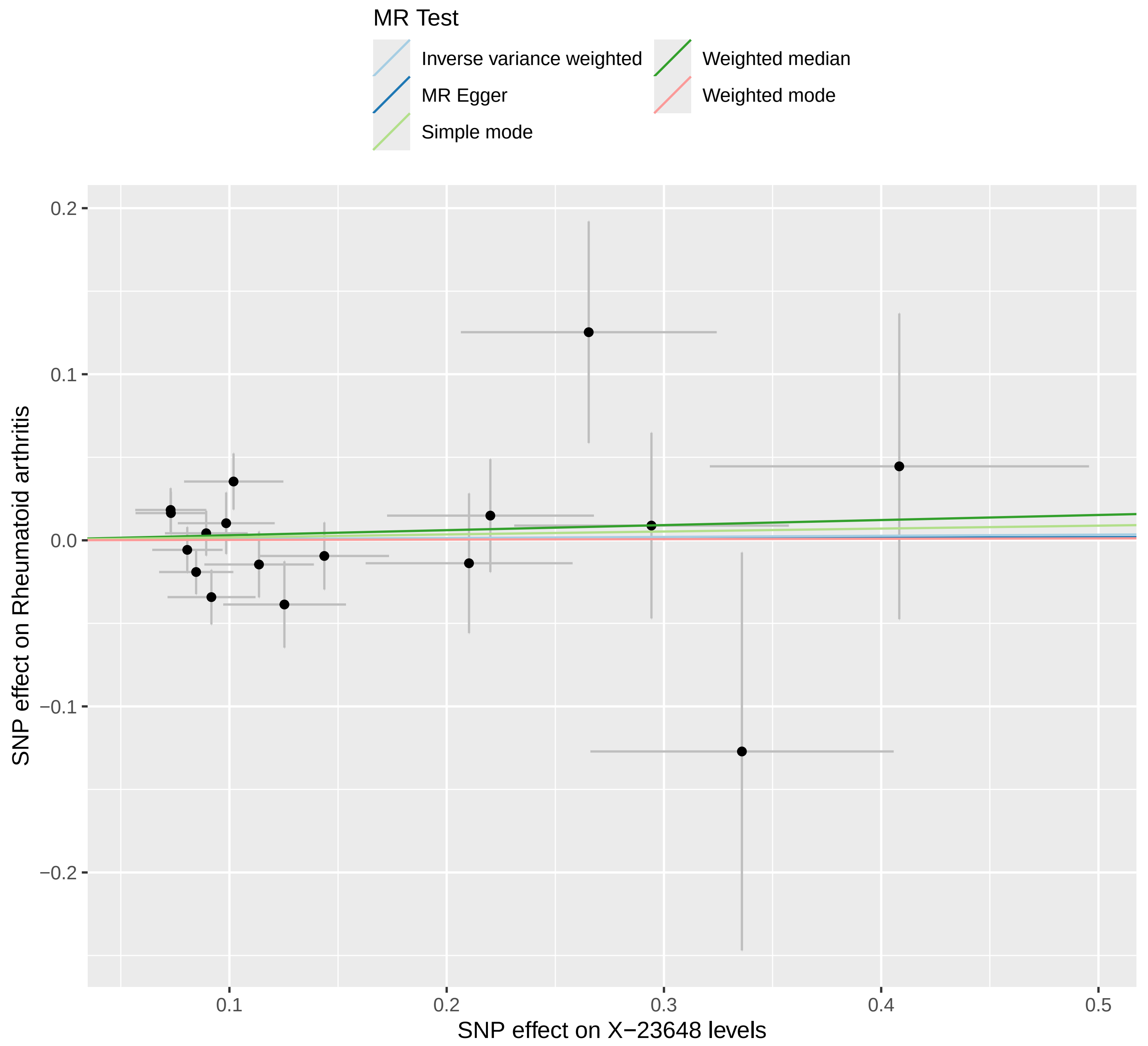
MR Test
Inverse variance weighted
MR Egger
Simple mode
Weighted median
Weighted mode
0.2
0.1
0.0
−0.1
−0.2
SNP effect on Rheumatoid arthritis
0.1
0.2
0.3
0.4
0.5
SNP effect on X−23648 levels

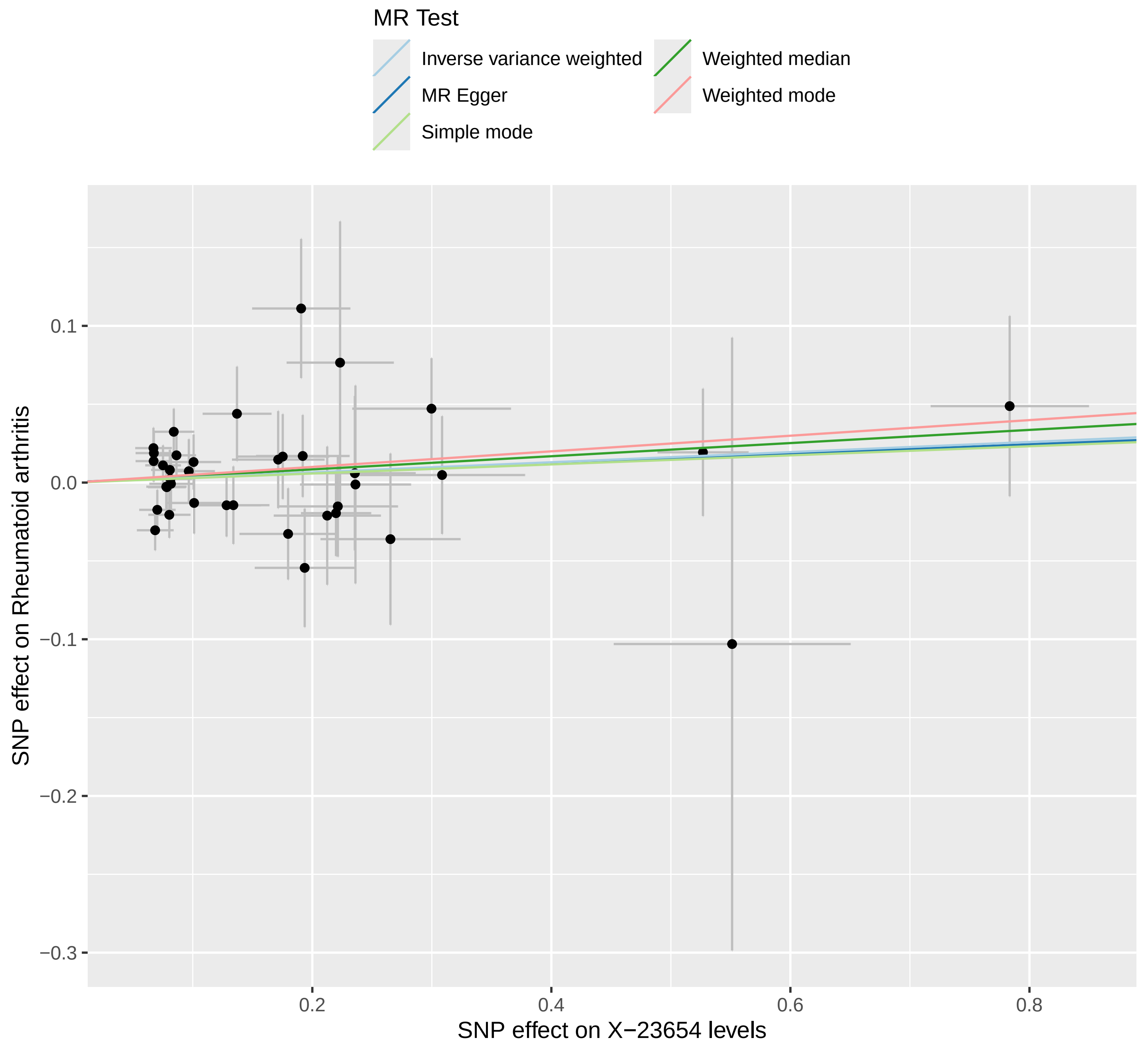

MR Test
Inverse variance weighted
MR Egger
Simple mode
Weighted median
Weighted mode
0.1
0.0
−0.1
−0.2
−0.3
SNP effect on Rheumatoid arthritis
0.2
0.4
0.6
0.8
SNP effect on X−23654 levels

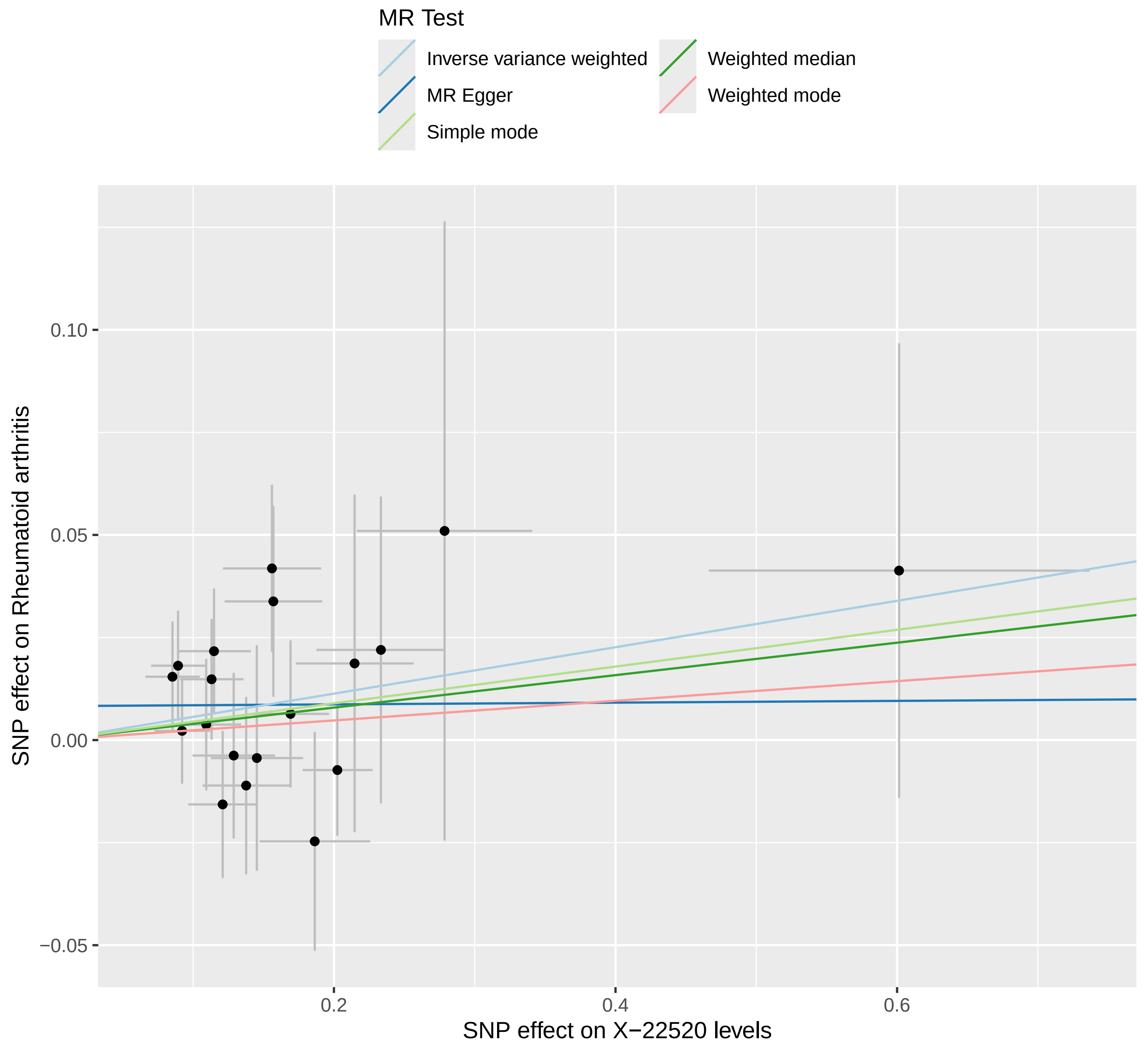

MR Test
Inverse variance weighted
MR Egger
Simple mode
Weighted median
Weighted mode
0.10
0.05
0.00
−0.05
0.2
0.4
0.6
SNP effect on Rheumatoid arthritis
SNP effect on X−22520 levels

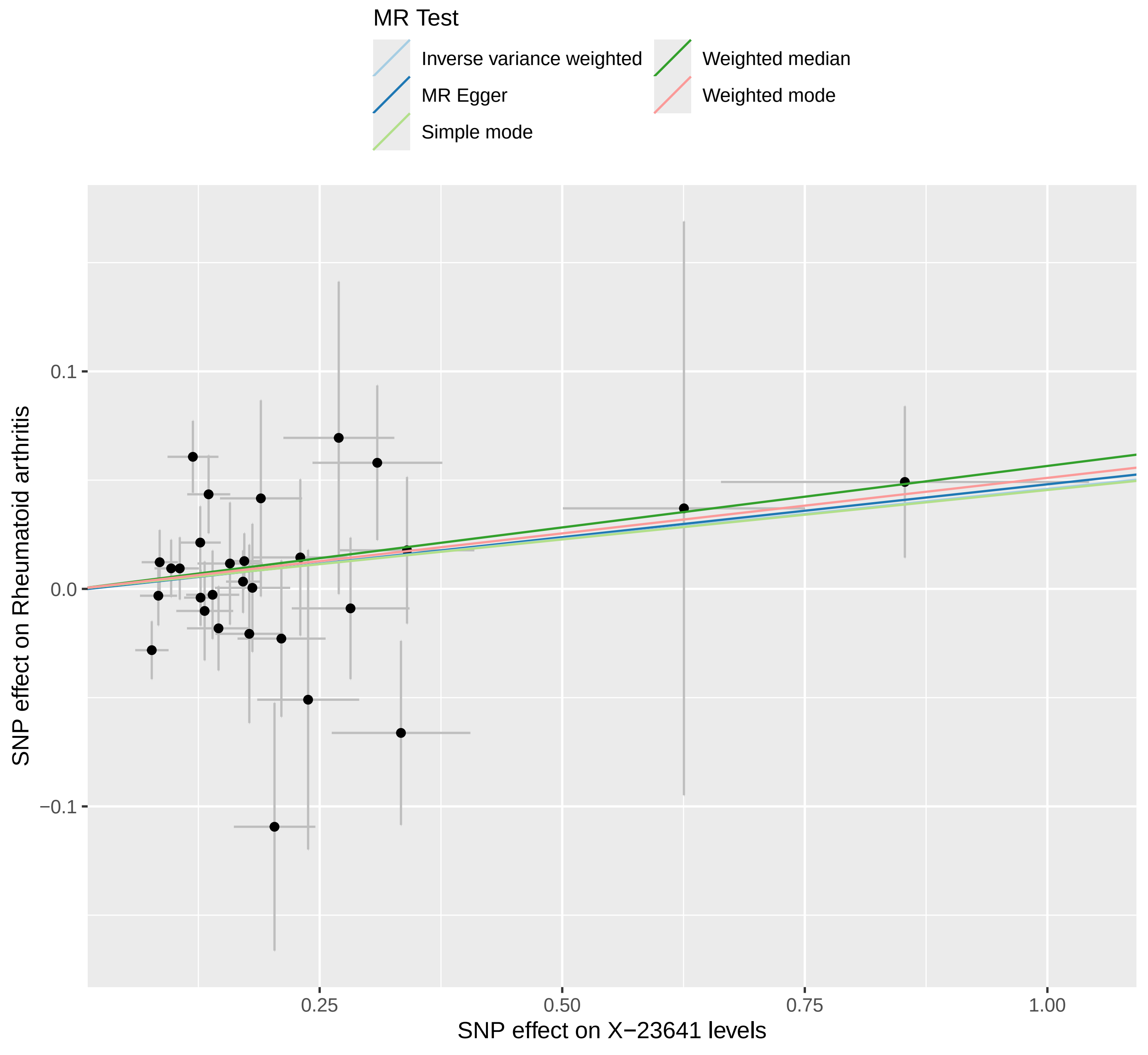
MR Test
Inverse variance weighted
MR Egger
Simple mode
Weighted median
Weighted mode
SNP effect on Rheumatoid arthritis
0.1
0.0
−0.1
0.25
0.50
0.75
1.00
SNP effect on X−23641 levels

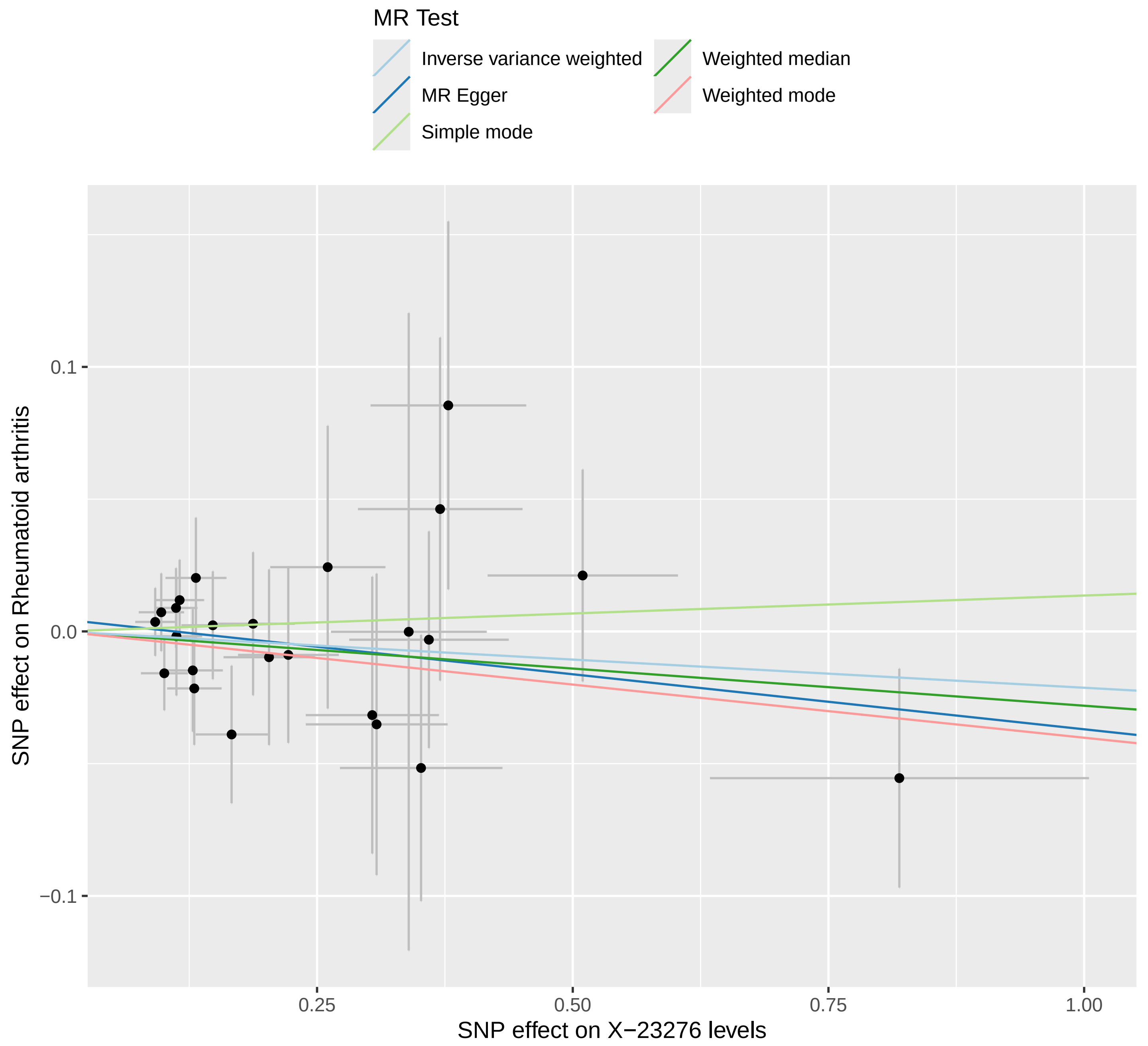

MR Test
Inverse variance weighted
MR Egger
Simple mode
Weighted median
Weighted mode
SNP effect on Rheumatoid arthritis
0.1
0.0
−0.1
0.25
0.50
0.75
1.00
SNP effect on X−23276 levels

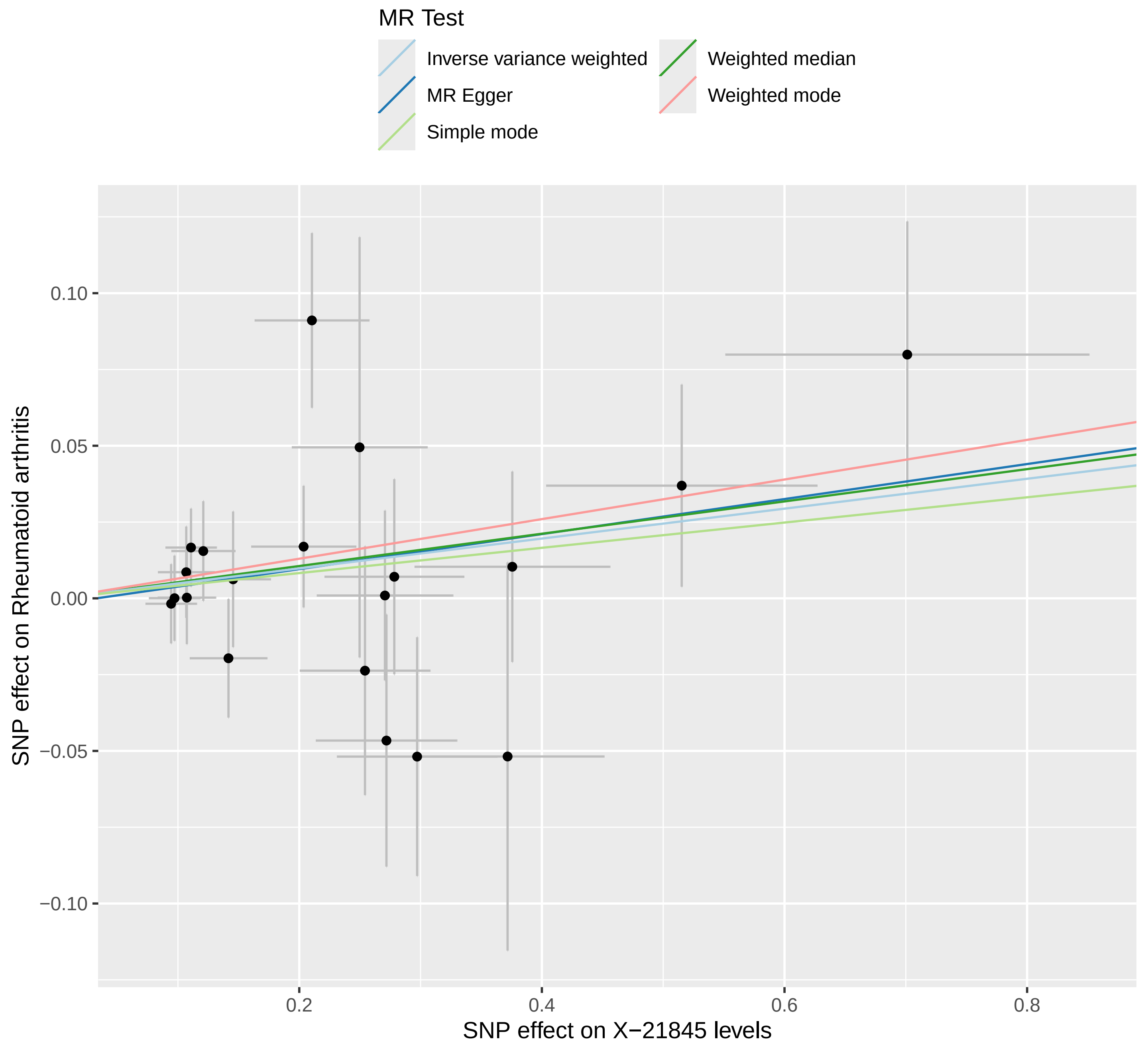
MR Test
Inverse variance weighted
MR Egger
Simple mode
Weighted median
Weighted mode
SNP effect on Rheumatoid arthritis
0.10
0.05
0.00
−0.05
−0.10
0.2
0.4
0.6
0.8
SNP effect on X−21845 levels

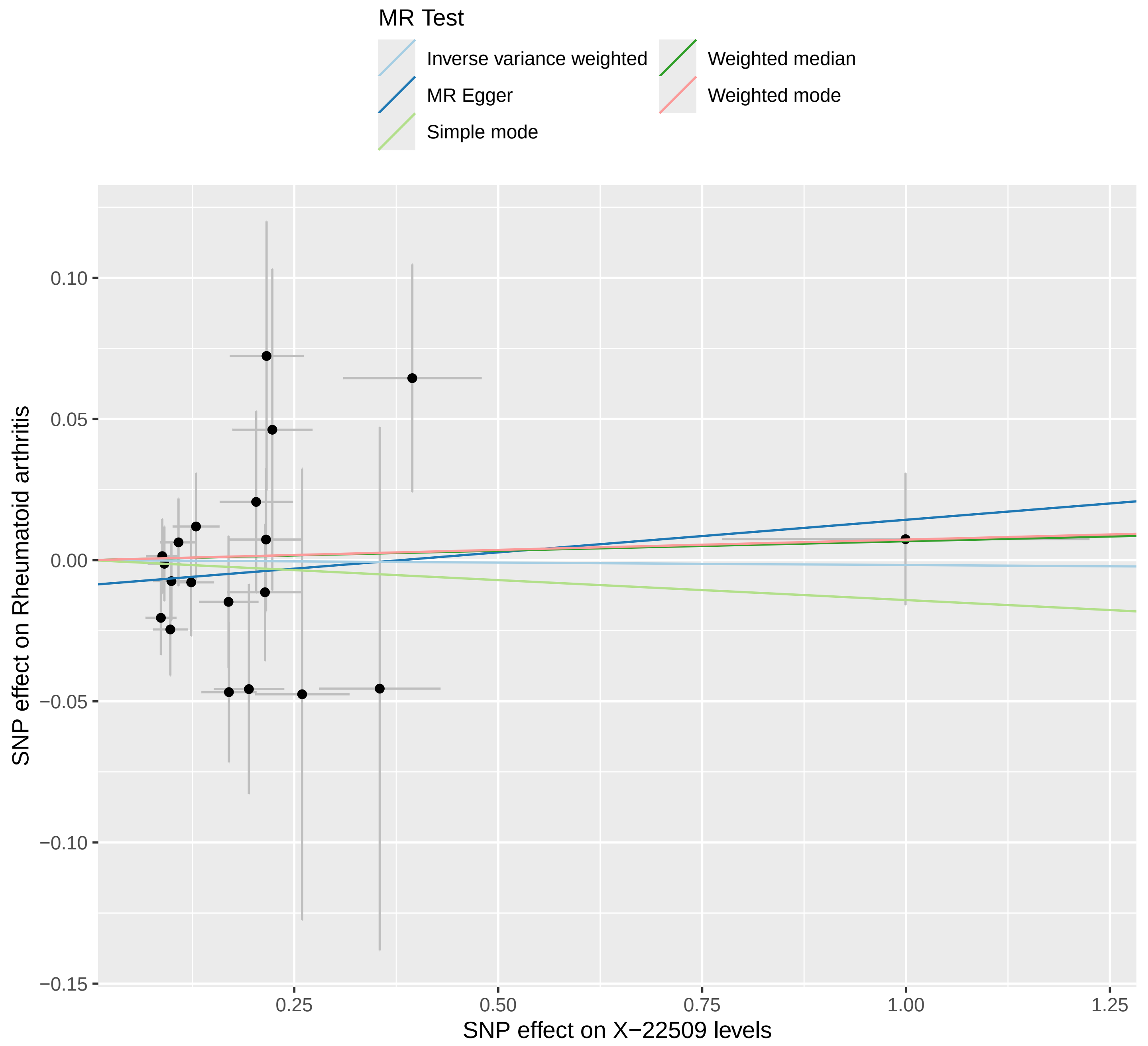
MR Test
Inverse variance weighted
MR Egger
Simple mode
Weighted median
Weighted mode
0.10
0.05
0.00
−0.05
−0.10
−0.15
SNP effect on Rheumatoid arthritis
0.25
0.50
0.75
1.00
1.25
SNP effect on X−22509 levels

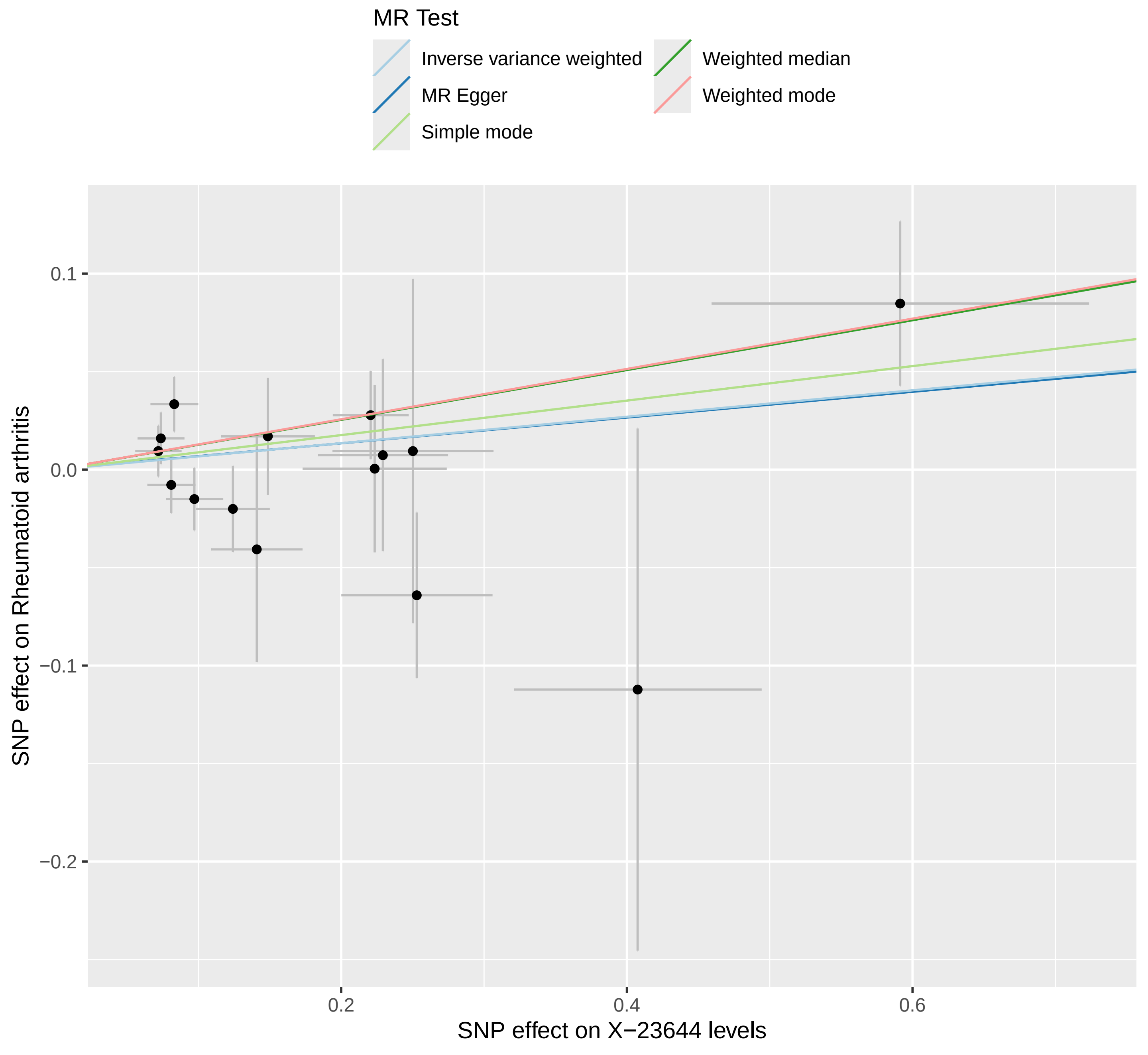

MR Test
Inverse variance weighted
MR Egger
Simple mode
Weighted median
Weighted mode
0.1
0.0
−0.1
−0.2
0.2
0.4
0.6
SNP effect on Rheumatoid arthritis
SNP effect on X−23644 levels

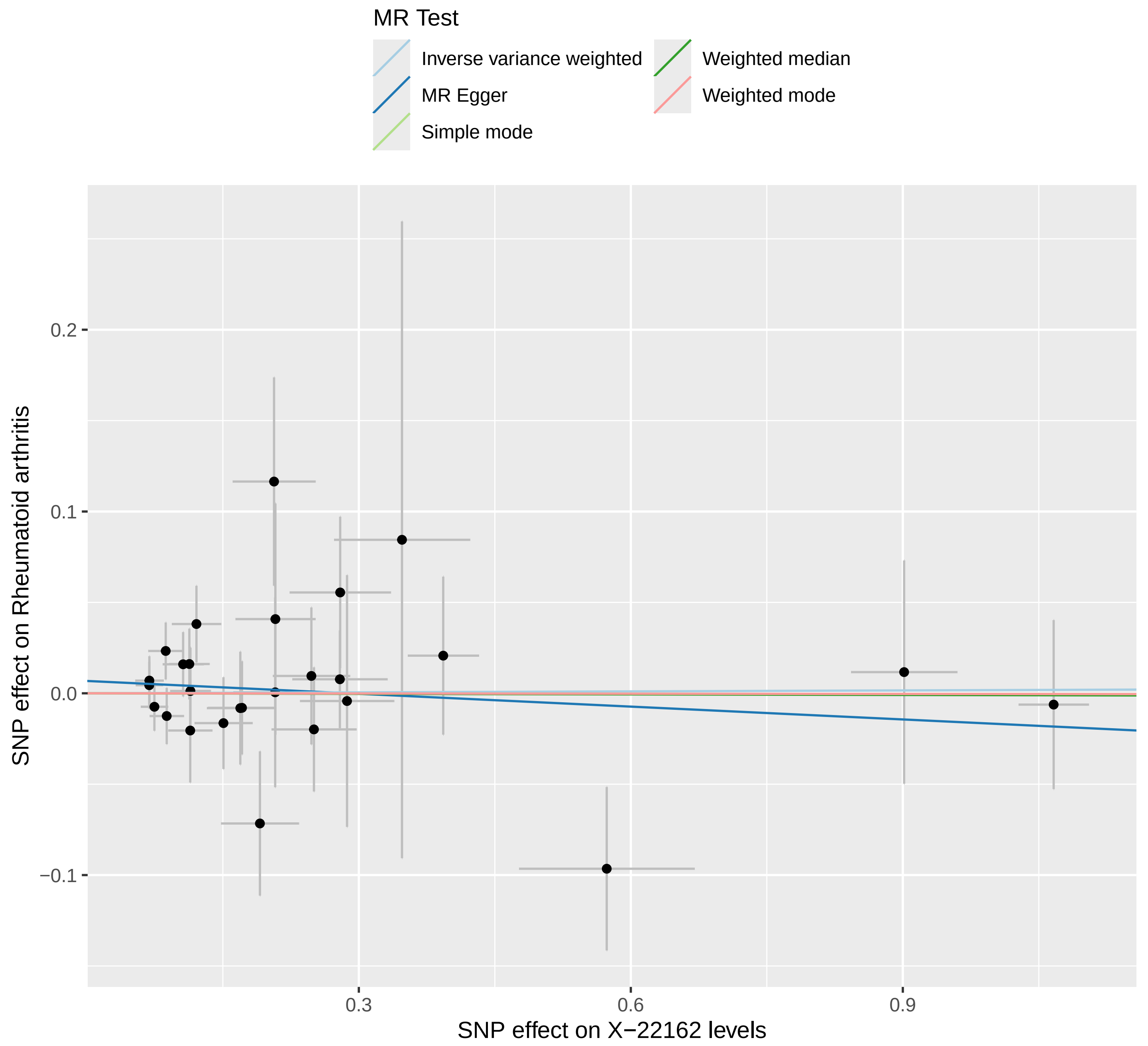

MR Test
Inverse variance weighted
MR Egger
Simple mode
Weighted median
Weighted mode
0.2
0.1
0.0
−0.1
0.3
0.6
0.9
SNP effect on Rheumatoid arthritis
SNP effect on X−22162 levels

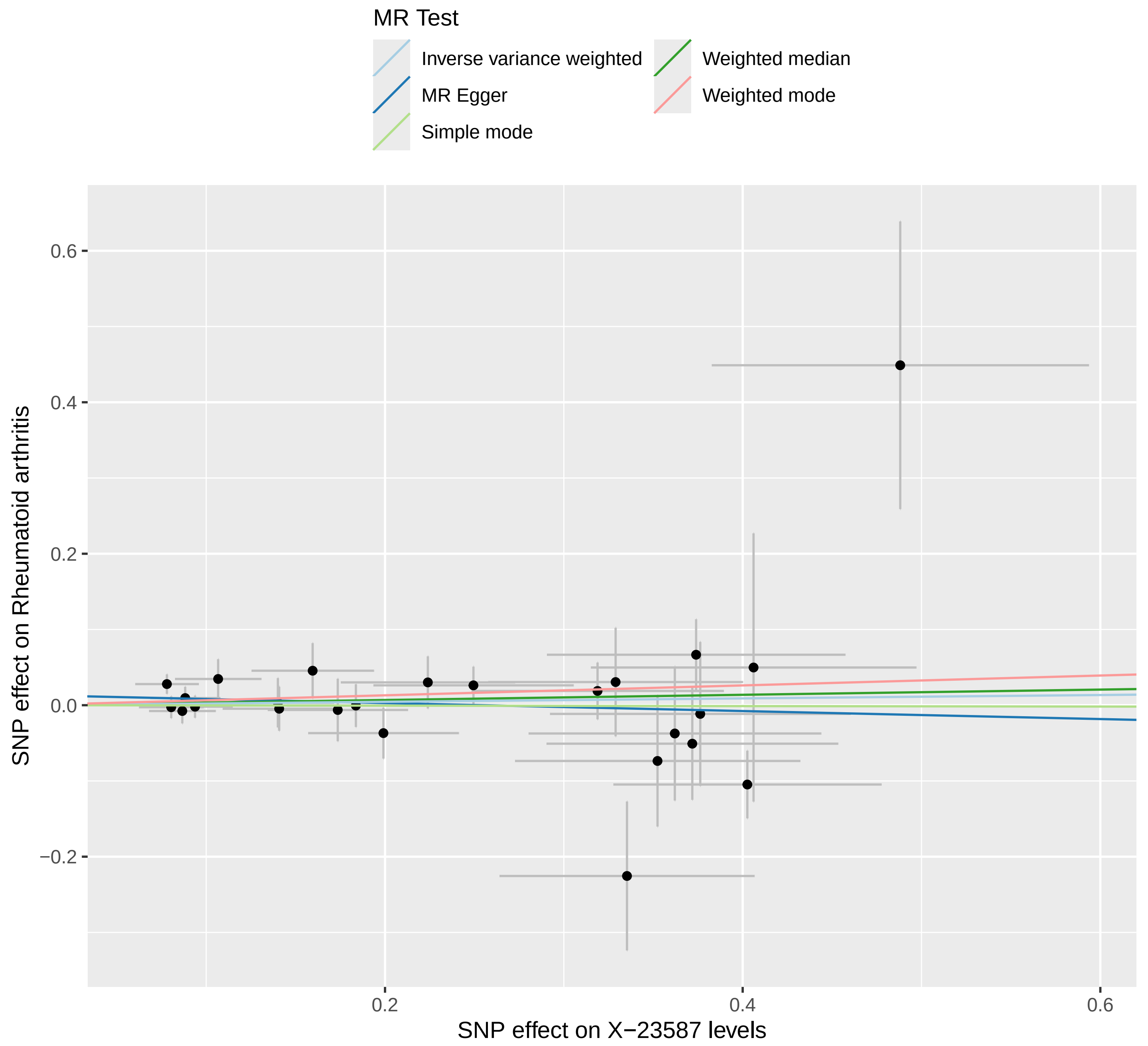
MR Test
Inverse variance weighted
MR Egger
Simple mode
Weighted median
Weighted mode
0.6
0.4
0.2
0.0
−0.2
SNP effect on Rheumatoid arthritis
0.2
0.4
0.6
SNP effect on X−23587 levels

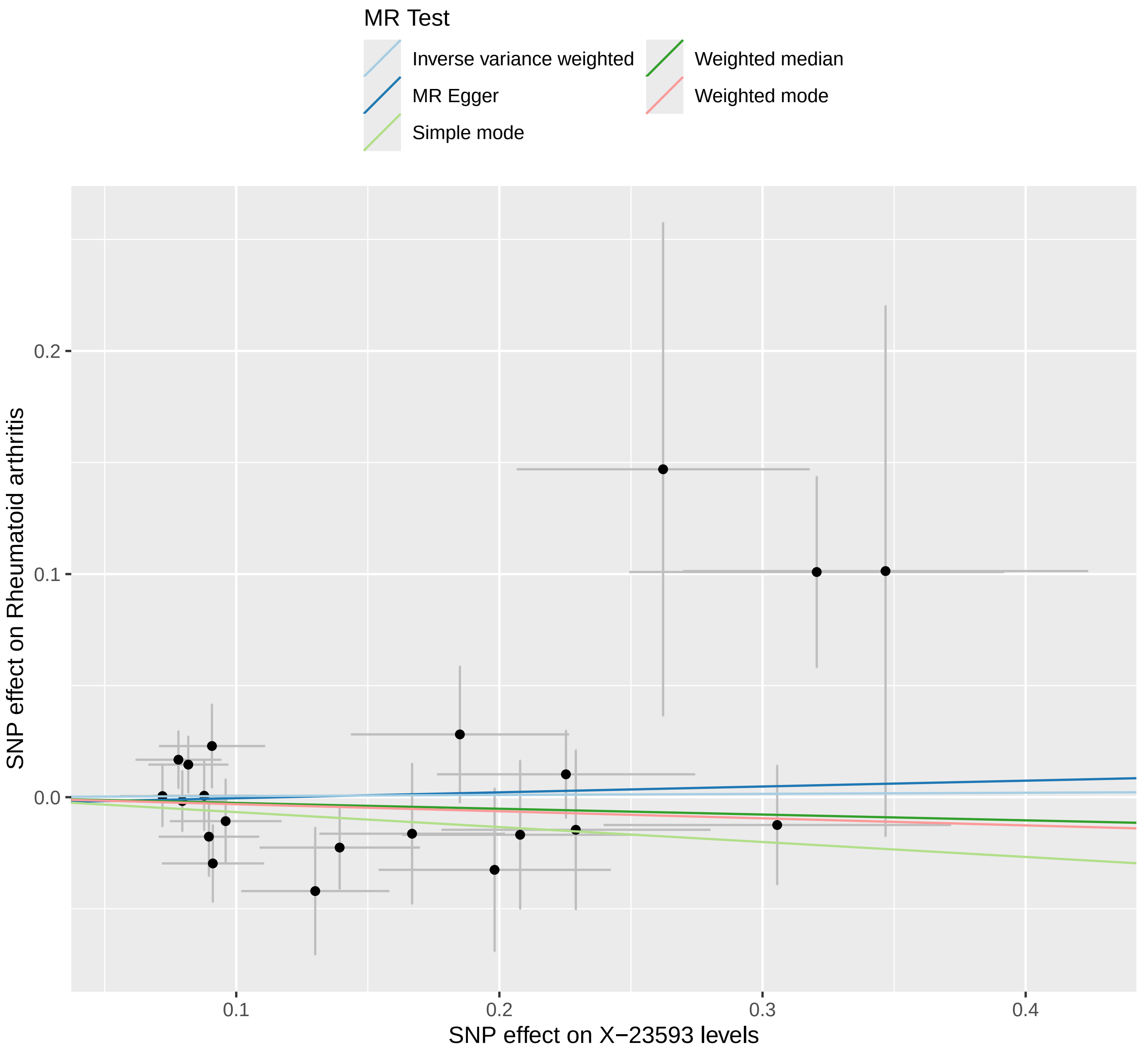
MR Test
Inverse variance weighted
MR Egger
Simple mode
Weighted median
Weighted mode
SNP effect on Rheumatoid arthritis
0.2
0.1
0.0
0.1
0.2
0.3
0.4
SNP effect on X−23593 levels

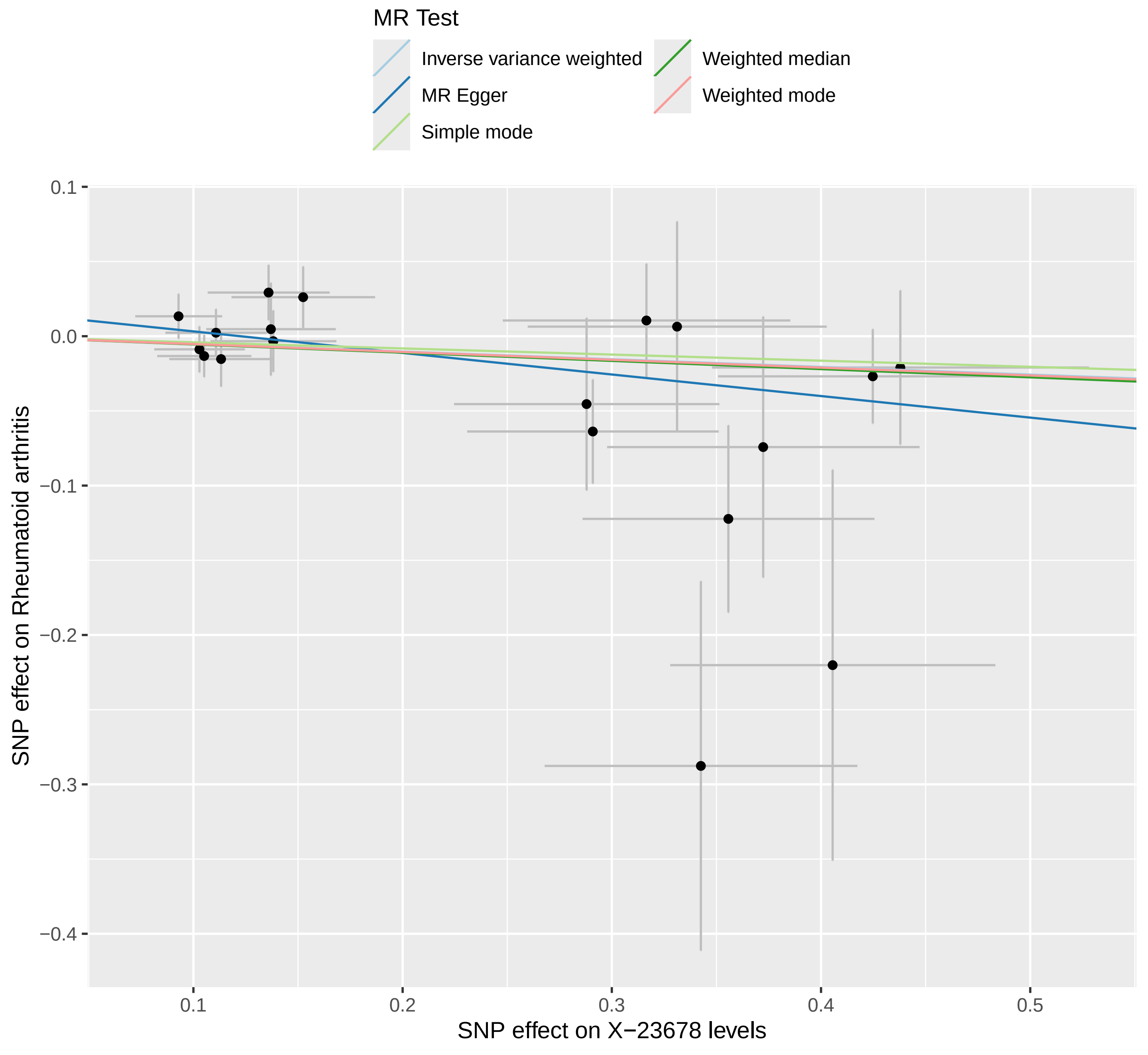
MR Test
Inverse variance weighted
MR Egger
Simple mode
Weighted median
Weighted mode
SNP effect on Rheumatoid arthritis
0.1
0.0
−0.1
−0.2
−0.3
−0.4
0.1
0.2
0.3
0.4
0.5
SNP effect on X−23678 levels

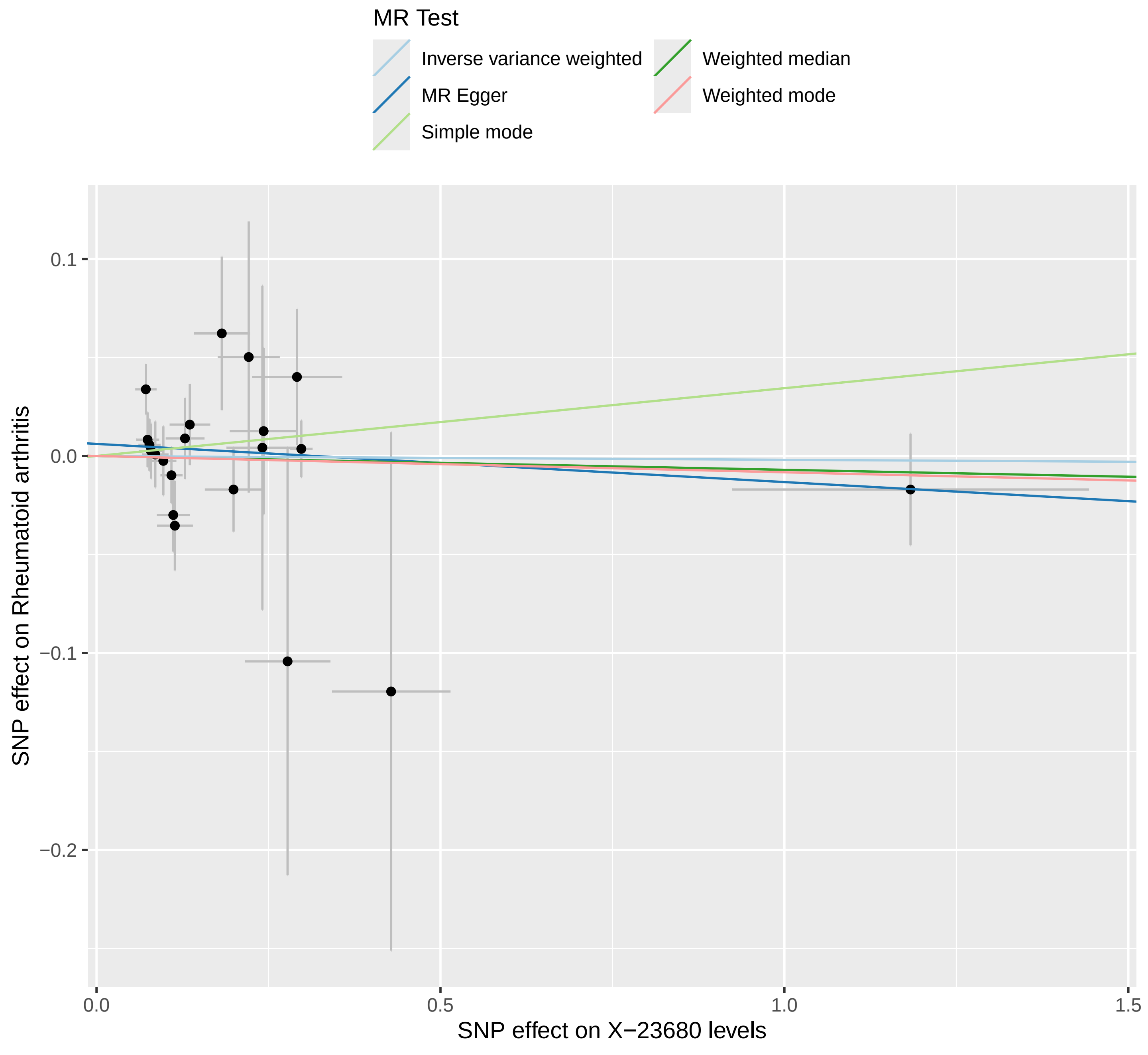

MR Test
Inverse variance weighted
MR Egger
Simple mode
Weighted median
Weighted mode
0.1
0.0
−0.1
−0.2
SNP effect on Rheumatoid arthritis
0.0
0.5
1.0
1.5
SNP effect on X−23680 levels

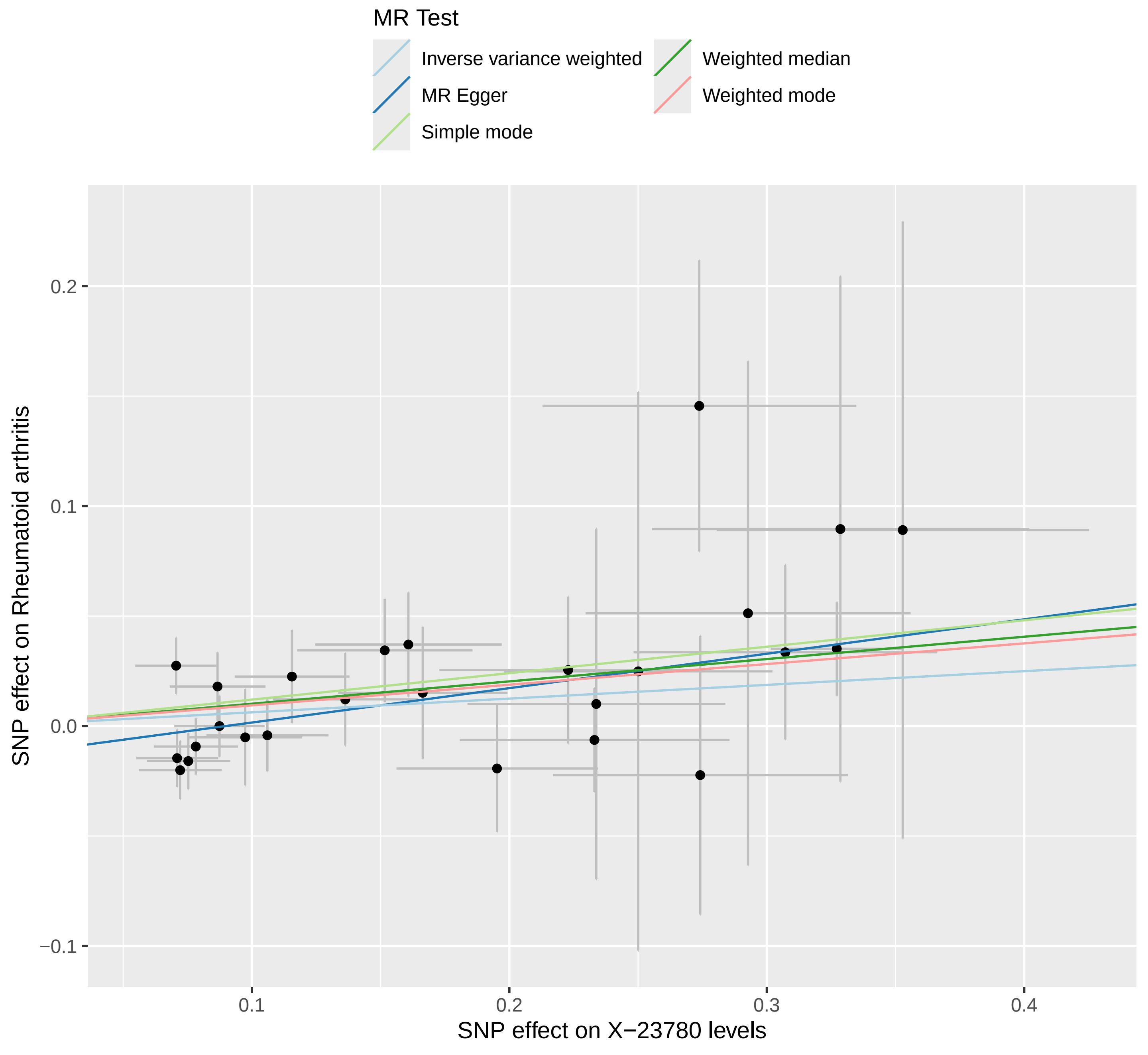
MR Test
Inverse variance weighted
MR Egger
Simple mode
Weighted median
Weighted mode
SNP effect on Rheumatoid arthritis
0.2
0.1
0.0
−0.1
0.1
0.2
0.3
0.4
SNP effect on X−23780 levels

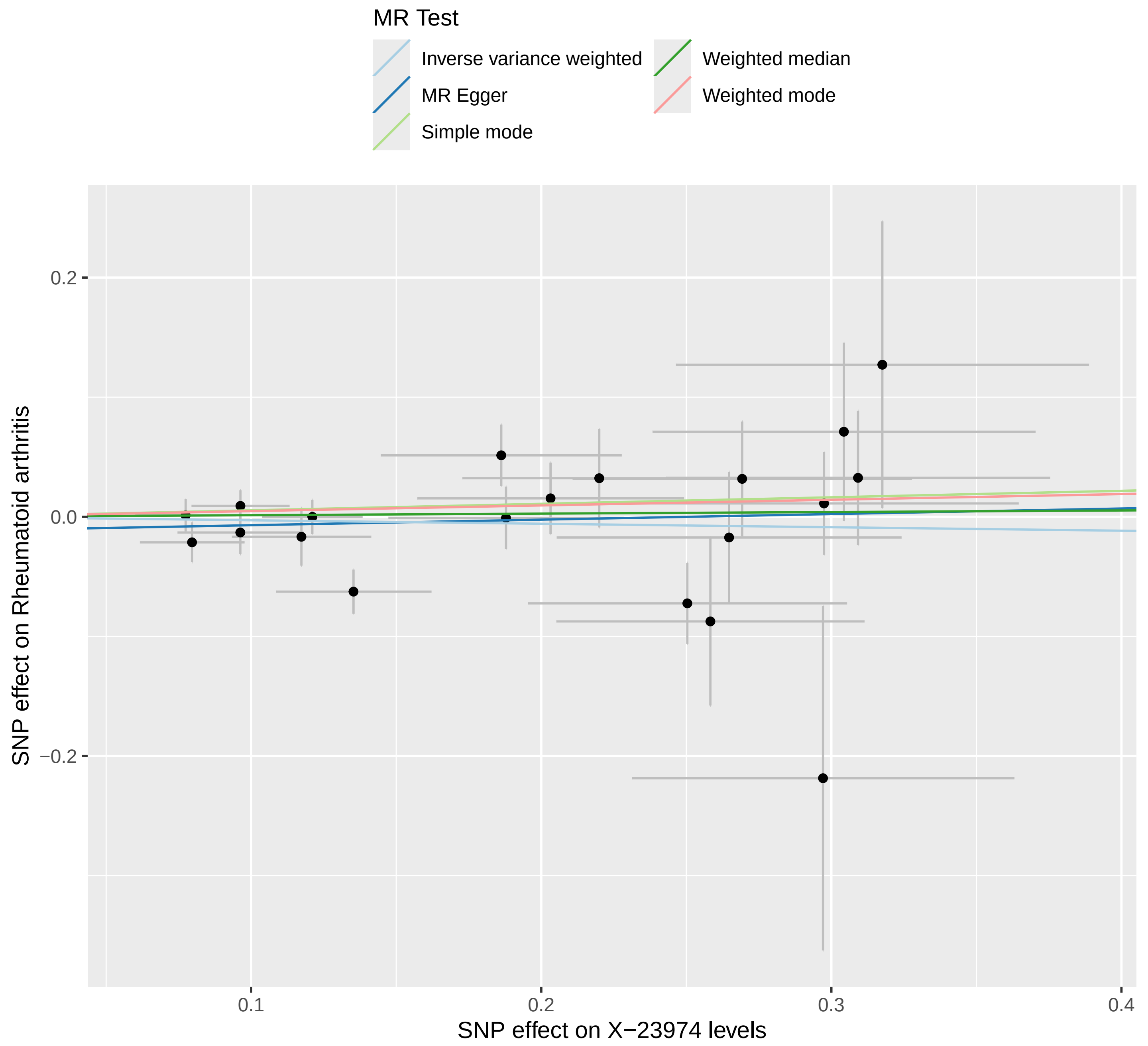
MR Test
Inverse variance weighted
MR Egger
Simple mode
Weighted median
Weighted mode
SNP effect on Rheumatoid arthritis
0.2
0.0
−0.2
0.1
0.2
0.3
0.4
SNP effect on X−23974 levels

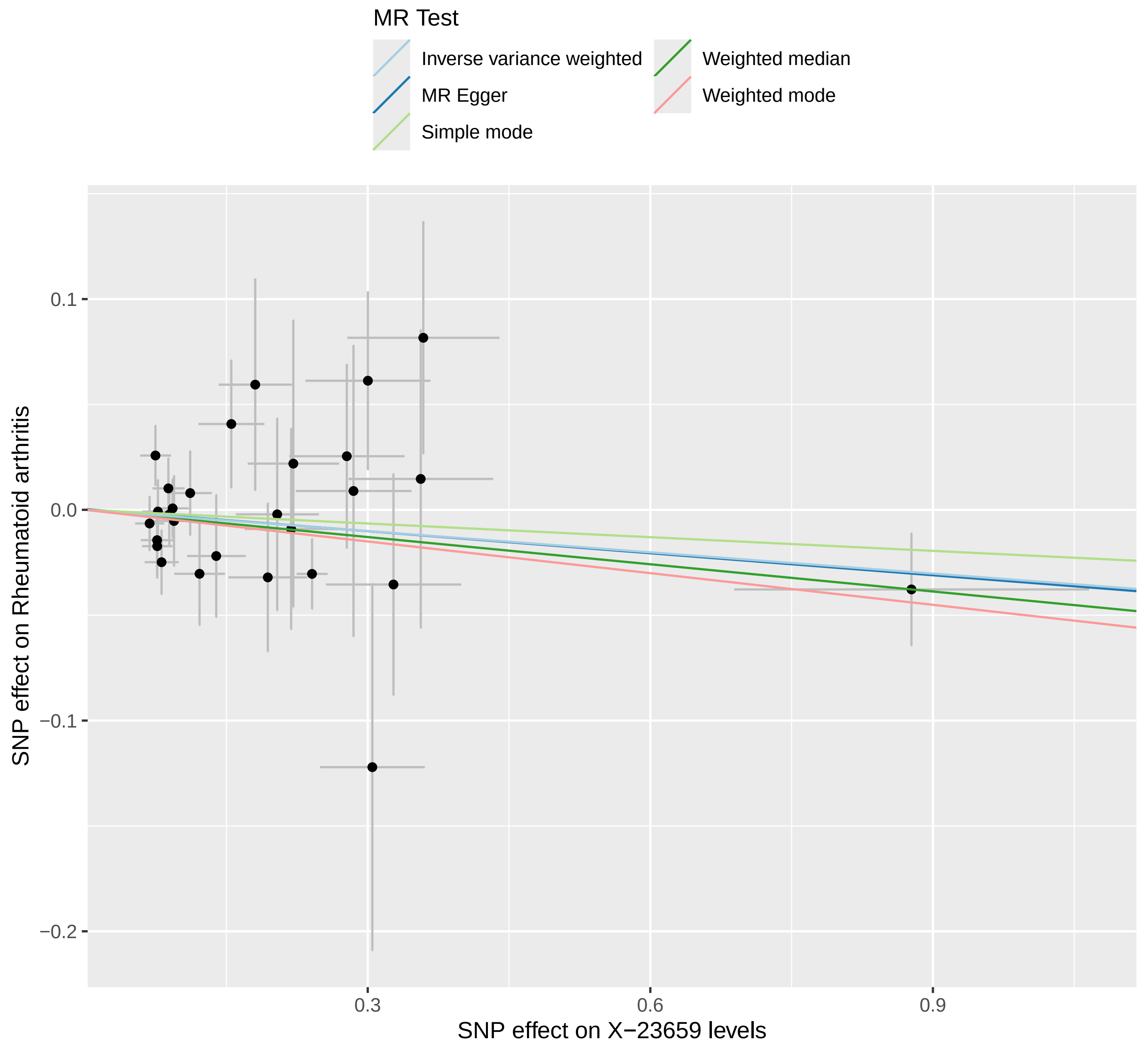

MR Test
Inverse variance weighted
MR Egger
Simple mode
Weighted median
Weighted mode
0.1
0.0
−0.1
−0.2
SNP effect on Rheumatoid arthritis
0.3
0.6
0.9
SNP effect on X−23659 levels

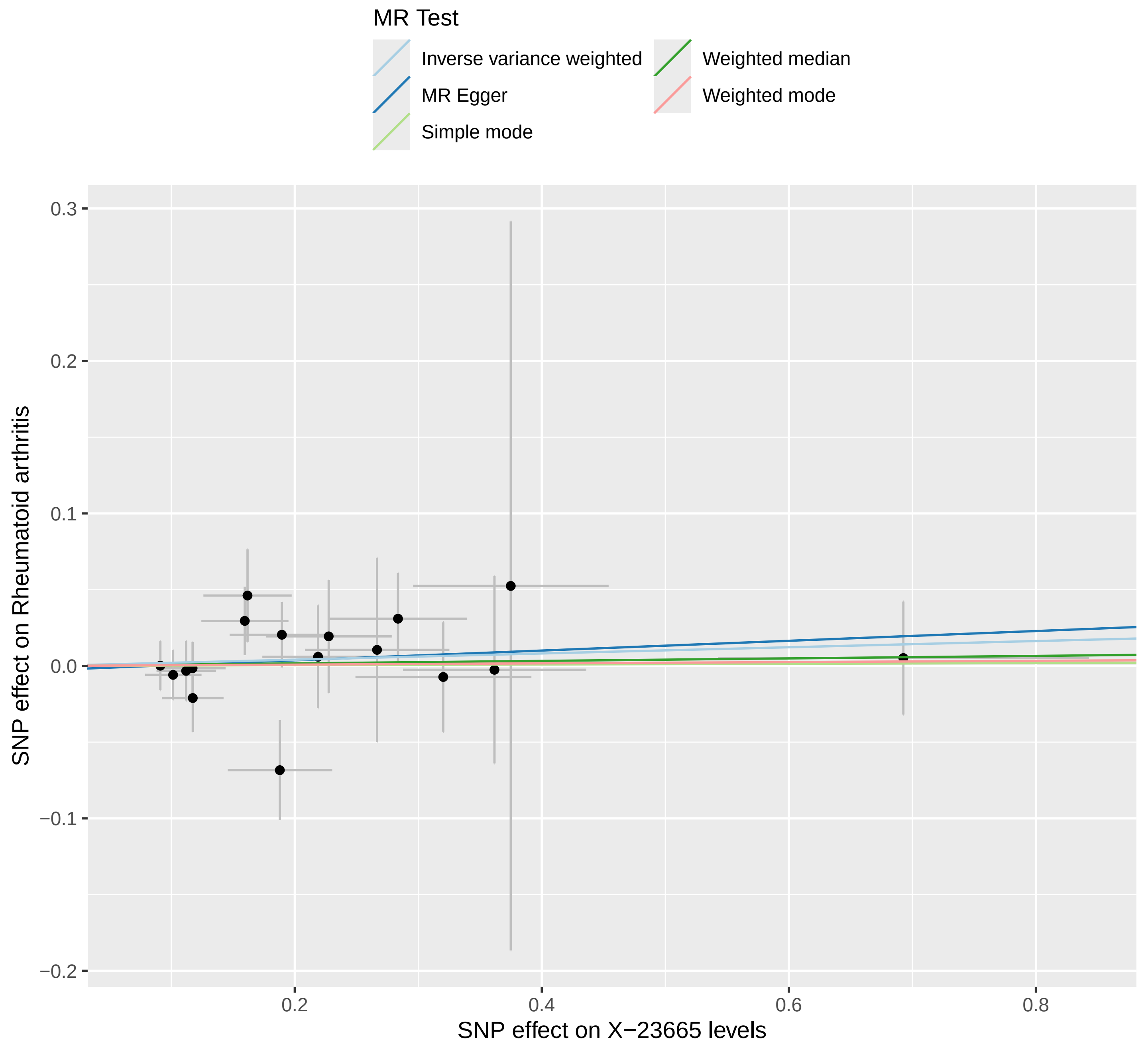
MR Test
Inverse variance weighted
MR Egger
Simple mode
Weighted median
Weighted mode
SNP effect on Rheumatoid arthritis
SNP effect on X−23665 levels
0.3
0.2
0.1
0.0
−0.1
−0.2
0.2
0.4
0.6
0.8

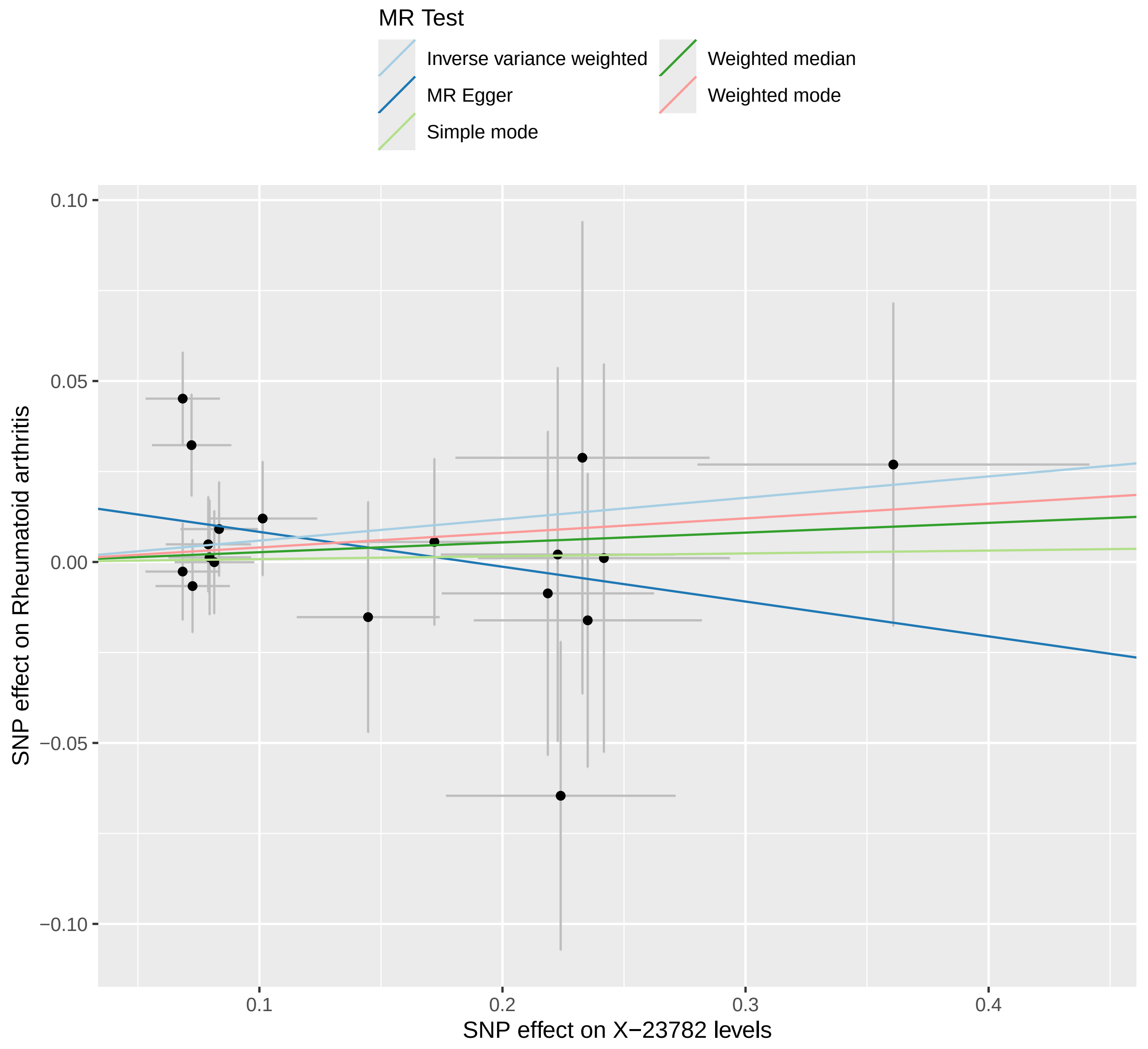
MR Test
Inverse variance weighted
MR Egger
Simple mode
Weighted median
Weighted mode
0.10
0.05
0.00
−0.05
−0.10
SNP effect on Rheumatoid arthritis
0.1
0.2
0.3
0.4
SNP effect on X−23782 levels

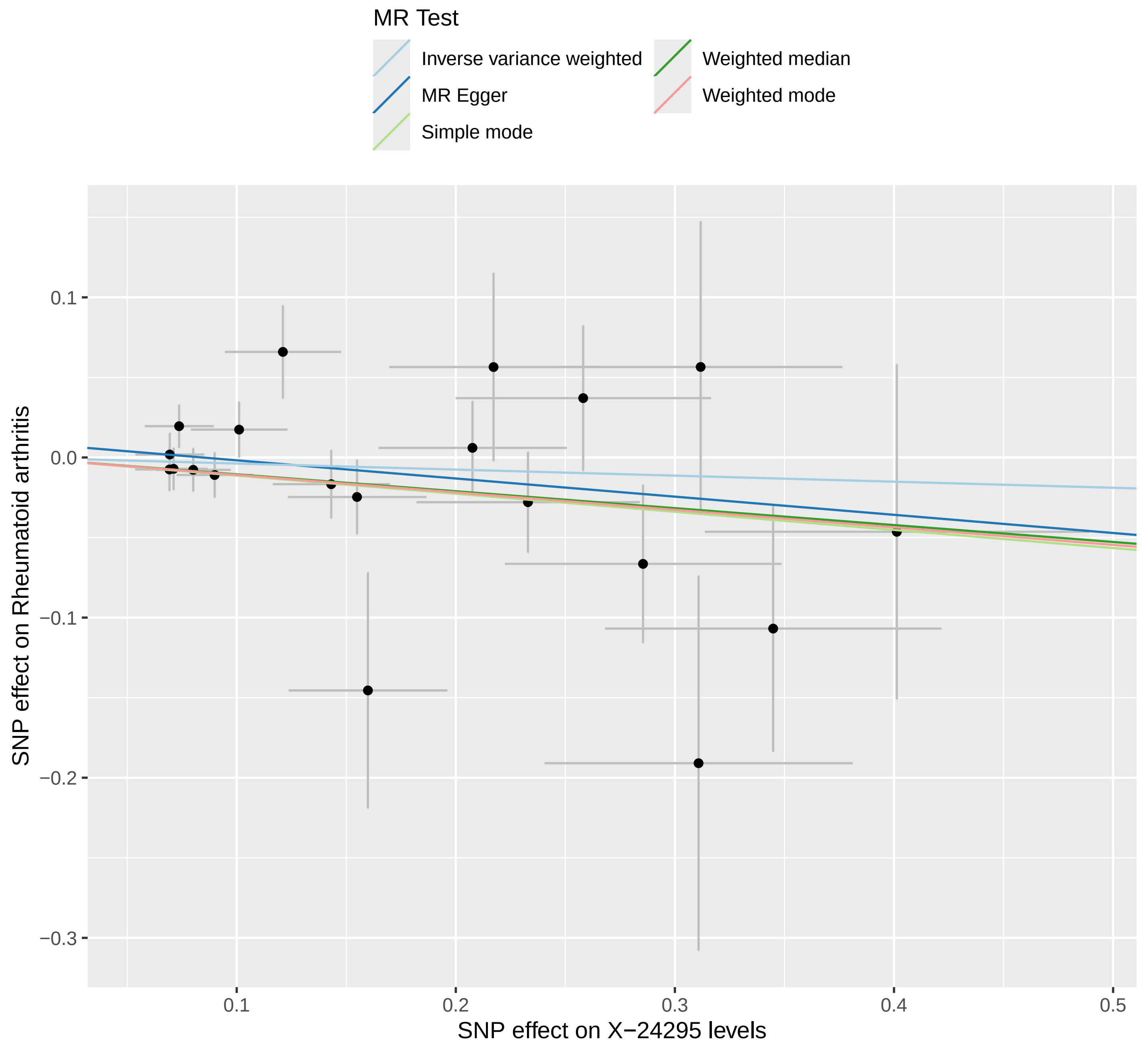

MR Test
Inverse variance weighted
MR Egger
Simple mode
Weighted median
Weighted mode
SNP effect on Rheumatoid arthritis
0.1
0.0
−0.1
−0.2
−0.3
0.1
0.2
0.3
0.4
0.5
SNP effect on X−24295 levels

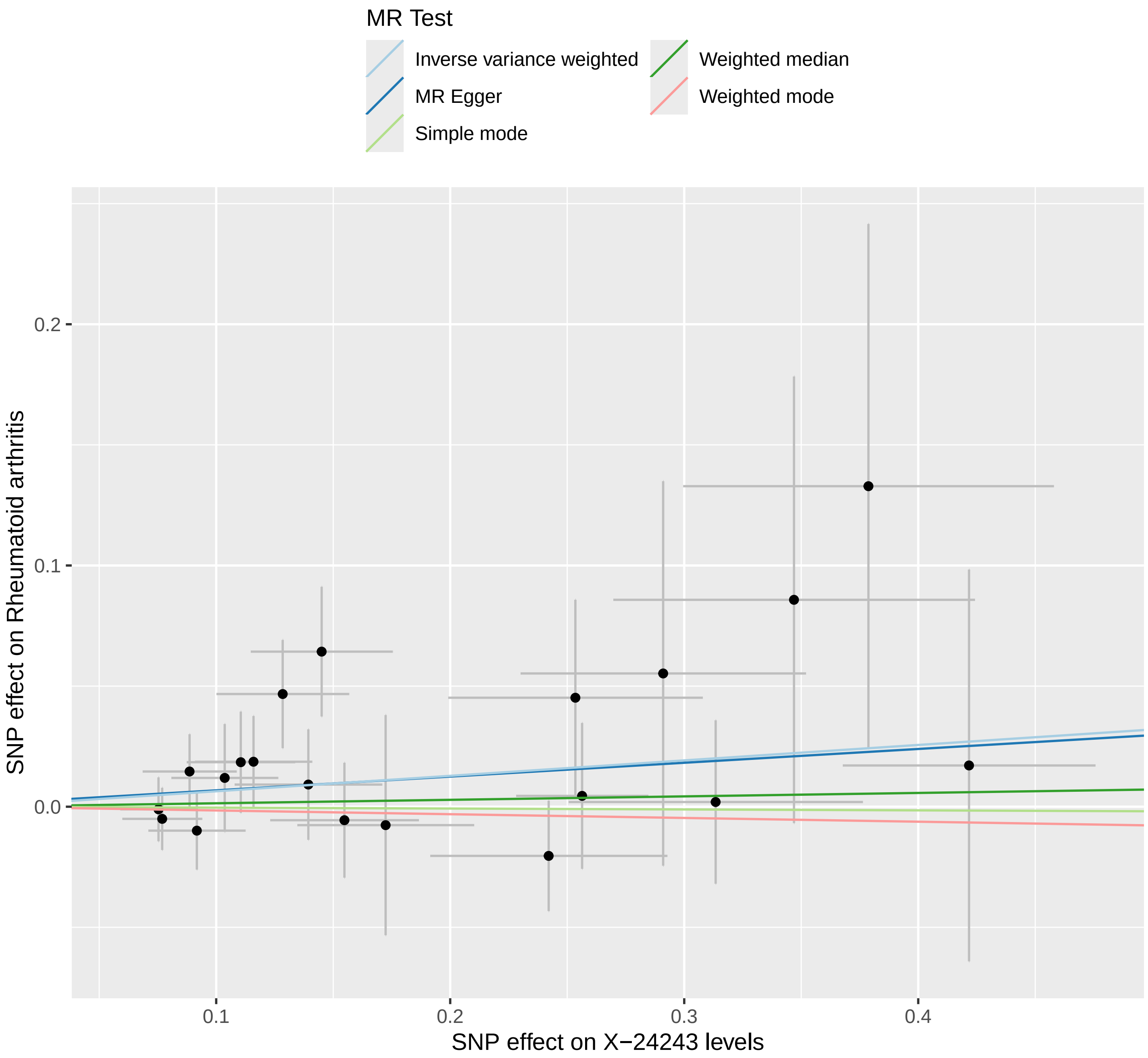
MR Test
Inverse variance weighted
MR Egger
Simple mode
Weighted median
Weighted mode
0.2
0.1
0.0
0.1
0.2
0.3
0.4
SNP effect on Rheumatoid arthritis
SNP effect on X−24243 levels

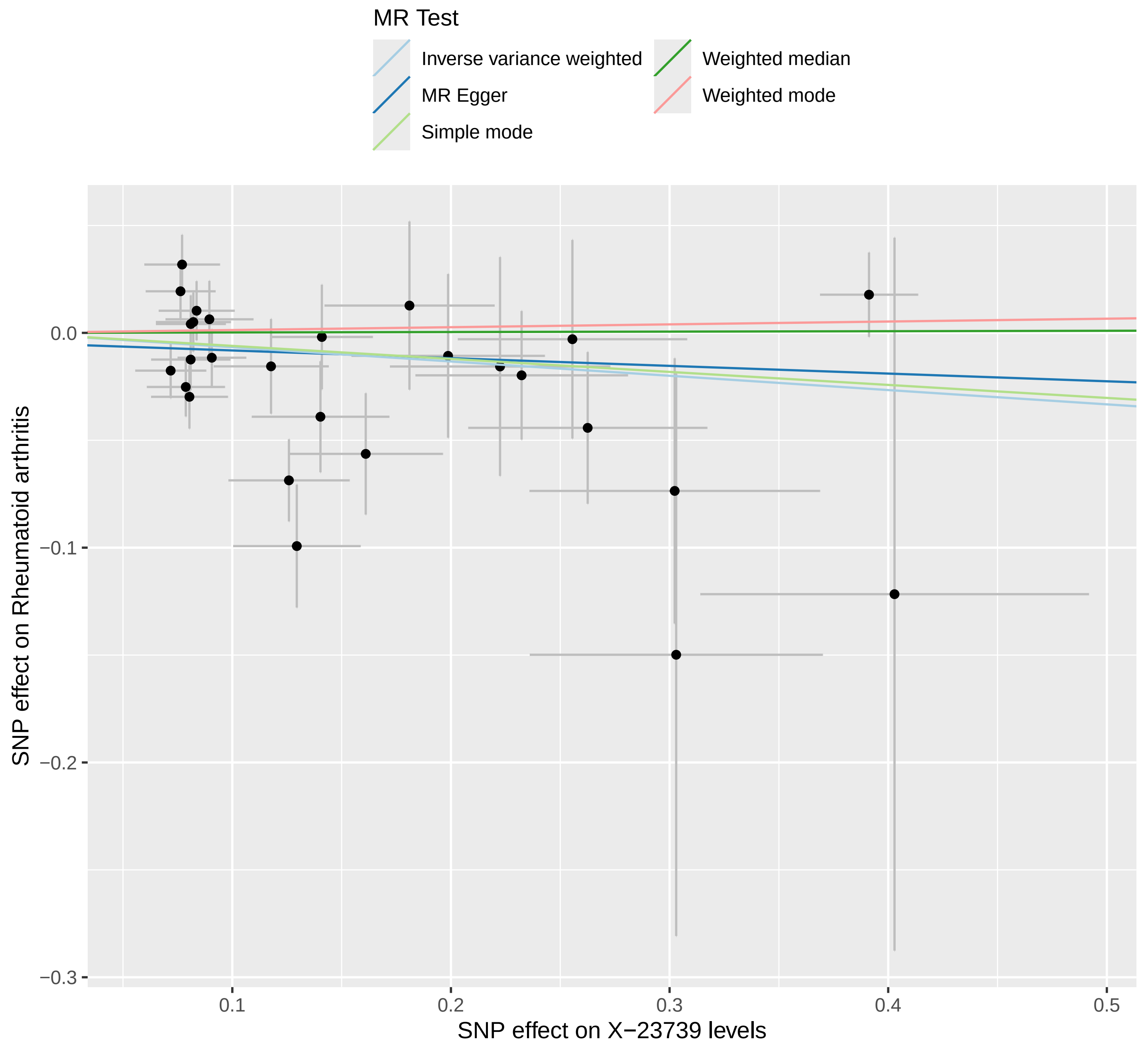
MR Test
Inverse variance weighted
MR Egger
Simple mode
Weighted median
Weighted mode
0.0
−0.1
−0.2
−0.3
0.1
0.2
0.3
0.4
0.5
SNP effect on Rheumatoid arthritis
SNP effect on X−23739 levels

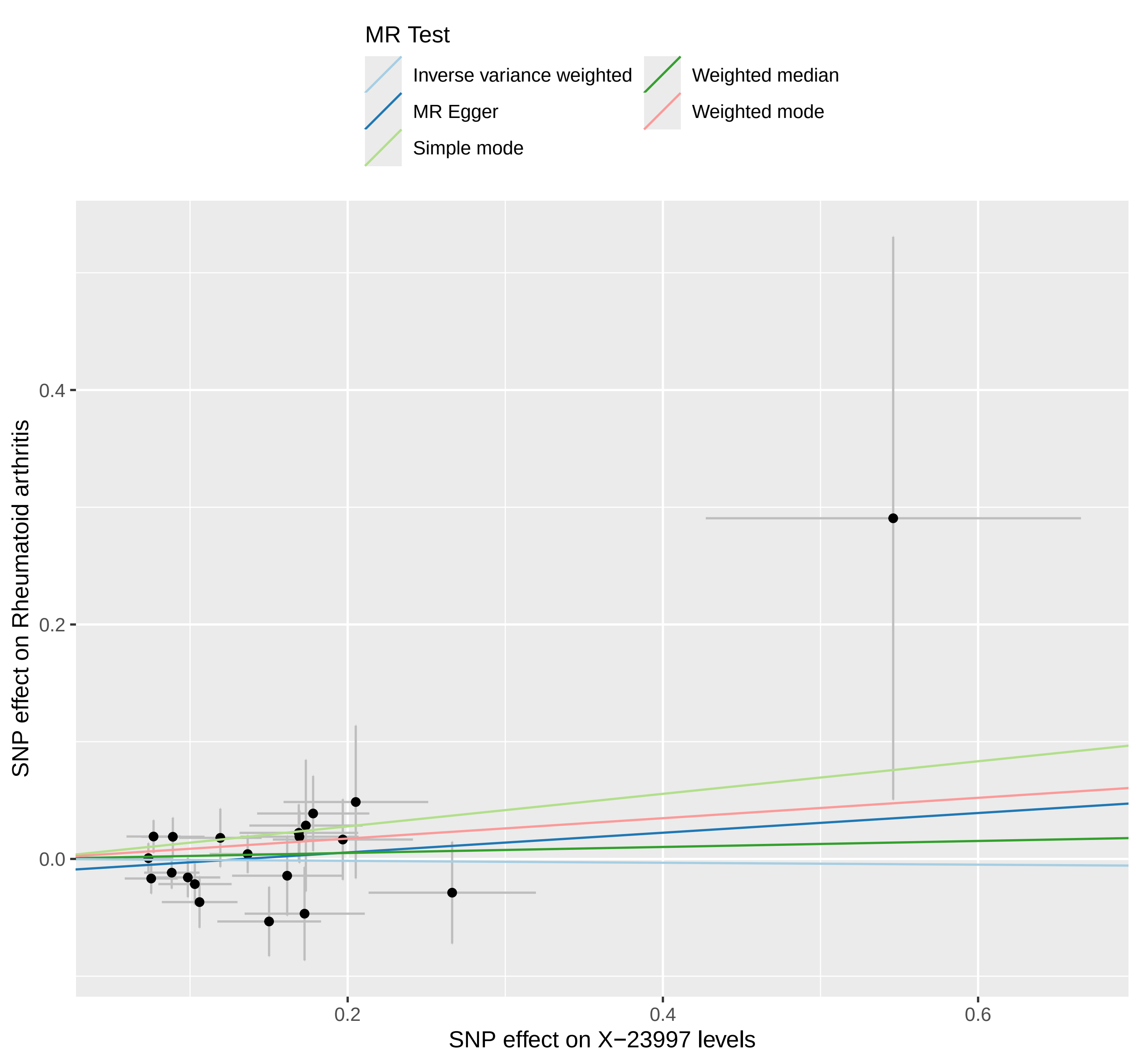
MR Test
Inverse variance weighted
MR Egger
Simple mode
Weighted median
Weighted mode
0.4
0.2
0.0
SNP effect on Rheumatoid arthritis
0.2
0.4
0.6
SNP effect on X−23997 levels

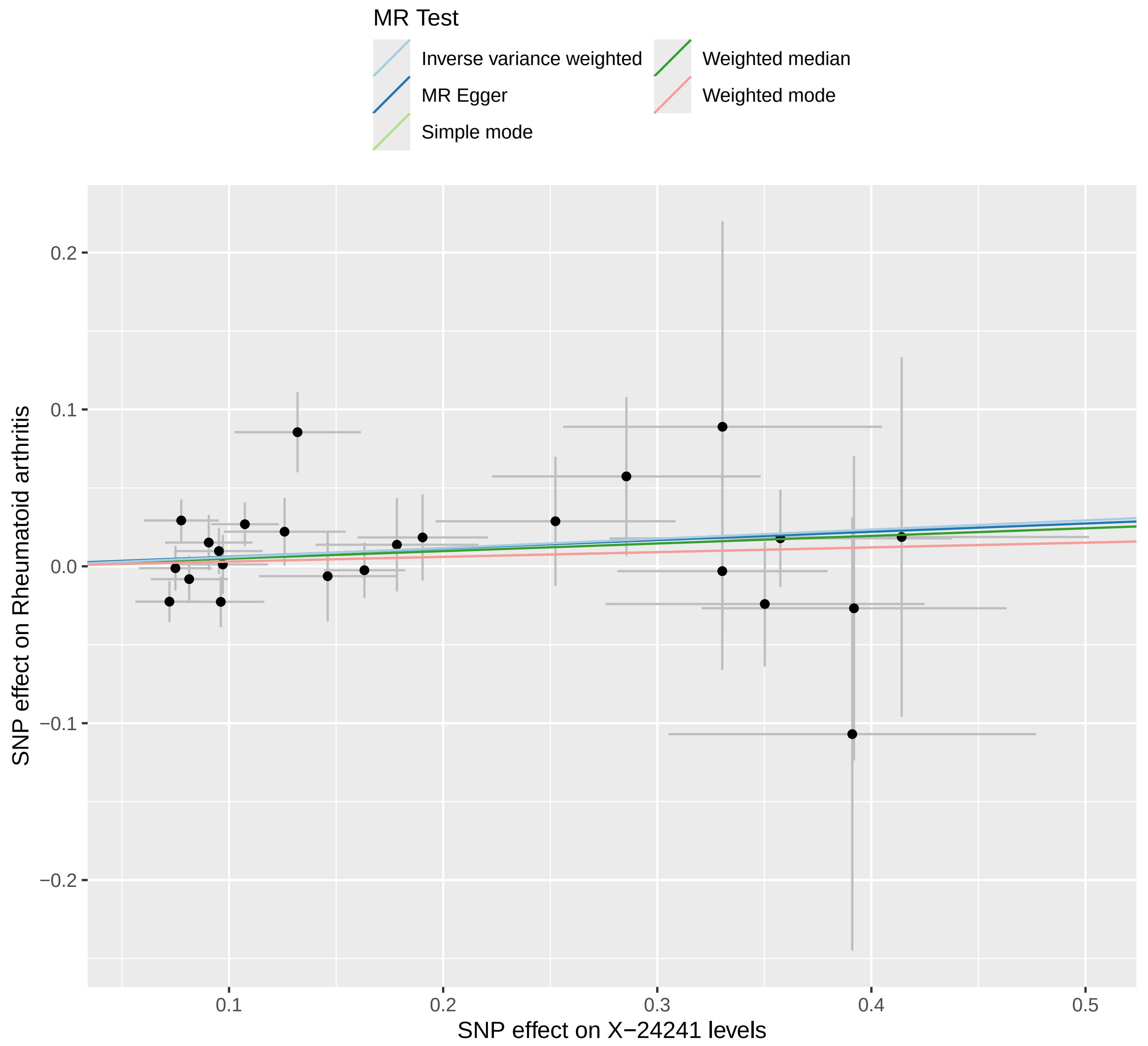
MR Test
Inverse variance weighted
MR Egger
Simple mode
Weighted median
Weighted mode
SNP effect on Rheumatoid arthritis
SNP effect on X−24241 levels
0.2
0.1
0.0
−0.1
−0.2
0.1
0.2
0.3
0.4
0.5

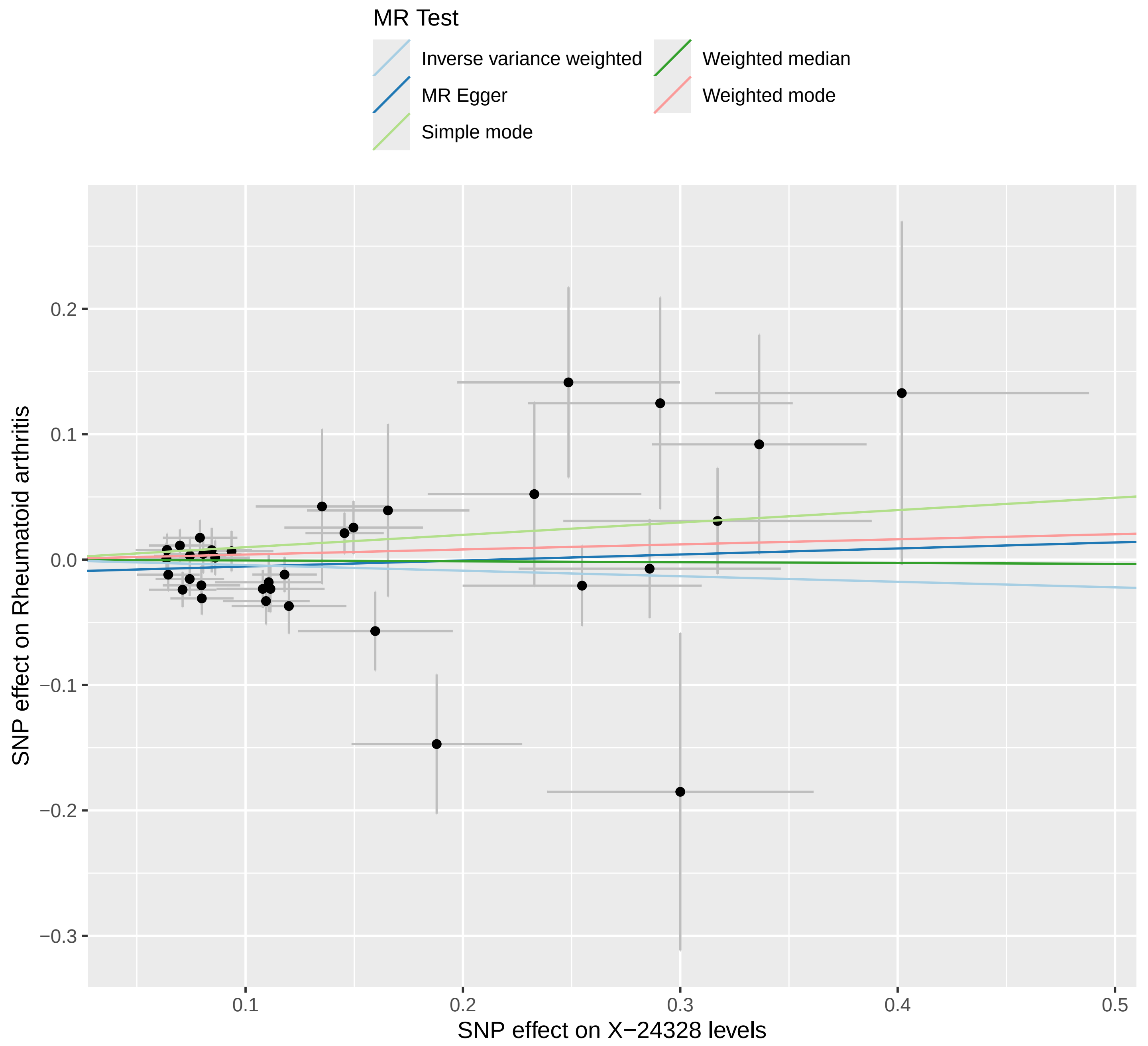
MR Test
Inverse variance weighted
MR Egger
Simple mode
Weighted median
Weighted mode
SNP effect on Rheumatoid arthritis
0.2
0.1
0.0
−0.1
−0.2
−0.3
0.1
0.2
0.3
0.4
0.5
SNP effect on X−24328 levels

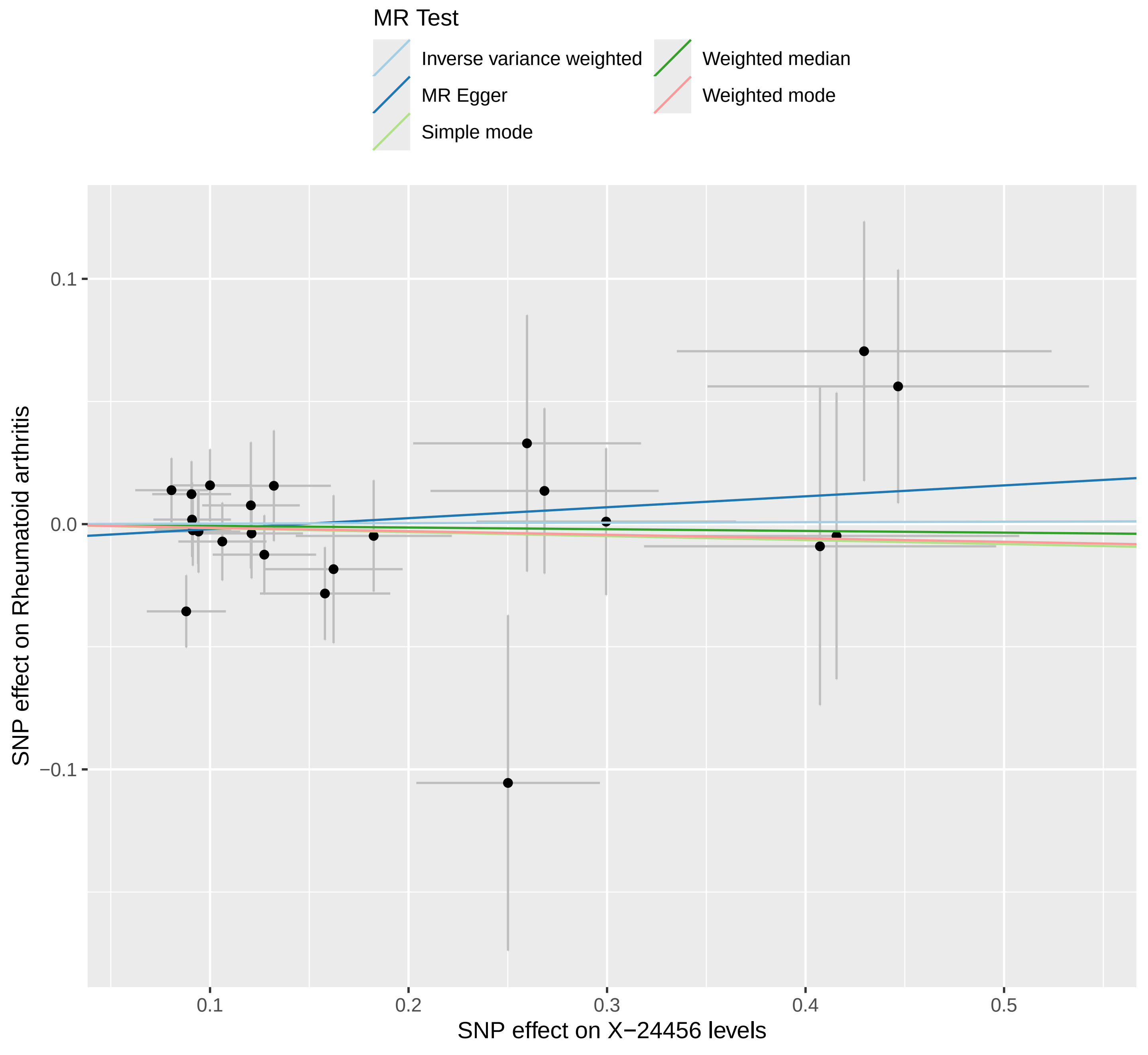

MR Test
Inverse variance weighted
MR Egger
Simple mode
Weighted median
Weighted mode
0.1
0.0
−0.1
SNP effect on Rheumatoid arthritis
0.1
0.2
0.3
0.4
0.5
SNP effect on X−24456 levels

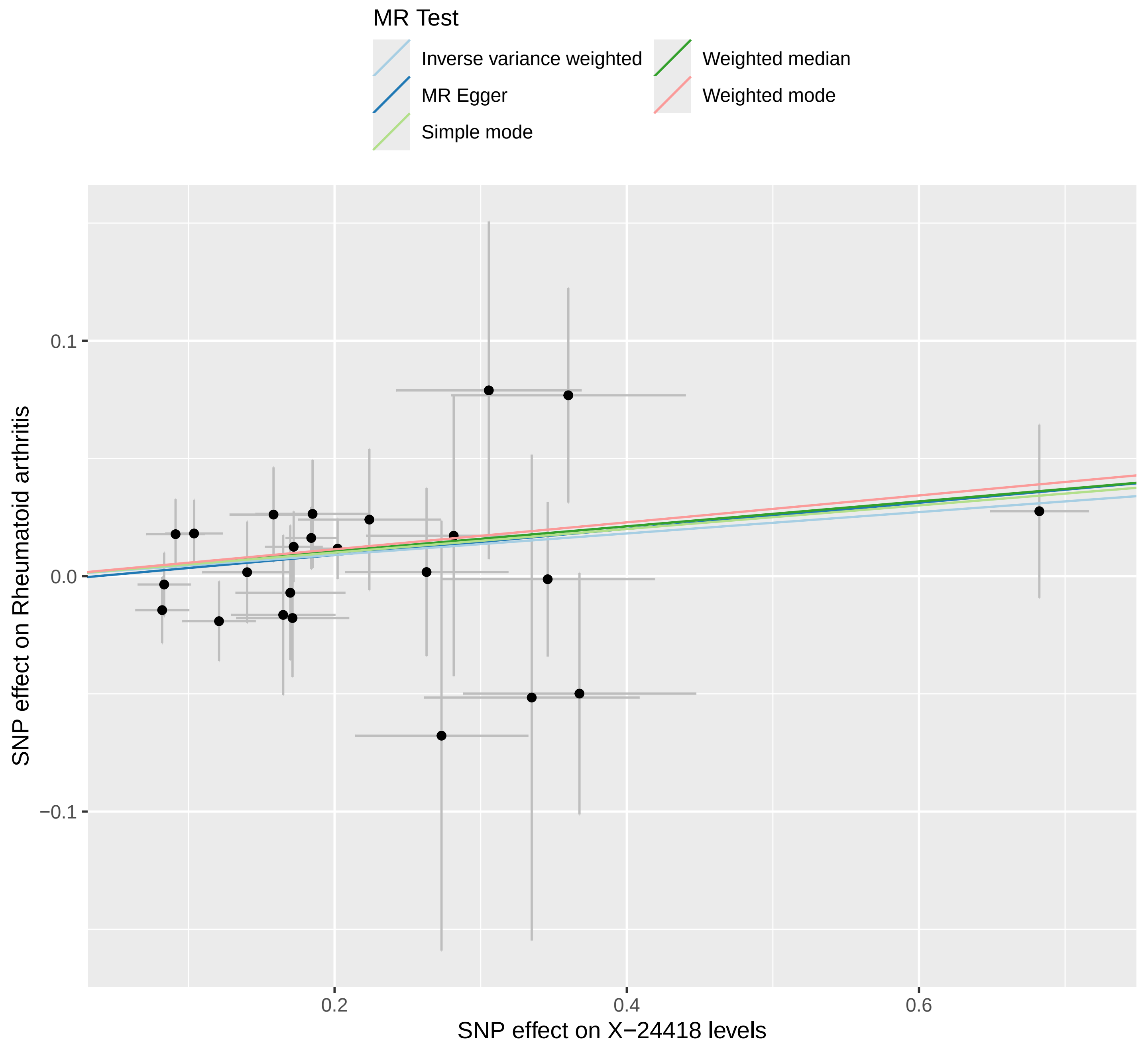
MR Test
Inverse variance weighted
MR Egger
Simple mode
Weighted median
Weighted mode
0.1
0.0
−0.1
0.2
0.4
0.6
SNP effect on Rheumatoid arthritis
SNP effect on X−24418 levels

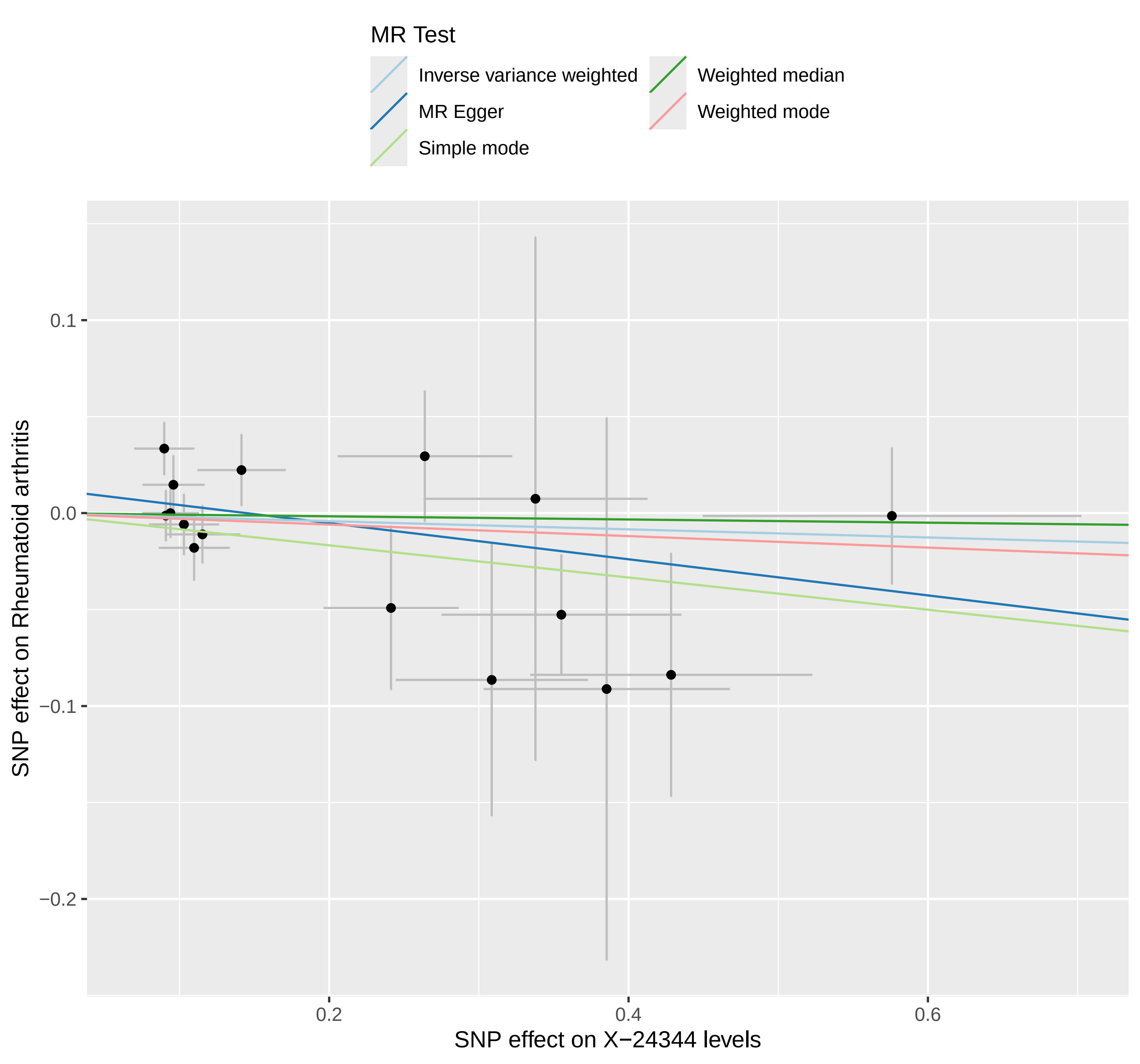
MR Test
Inverse variance weighted
MR Egger
Simple mode
Weighted median
Weighted mode
0.1
0.0
−0.1
−0.2
0.2
0.4
0.6
SNP effect on Rheumatoid arthritis
SNP effect on X−24344 levels

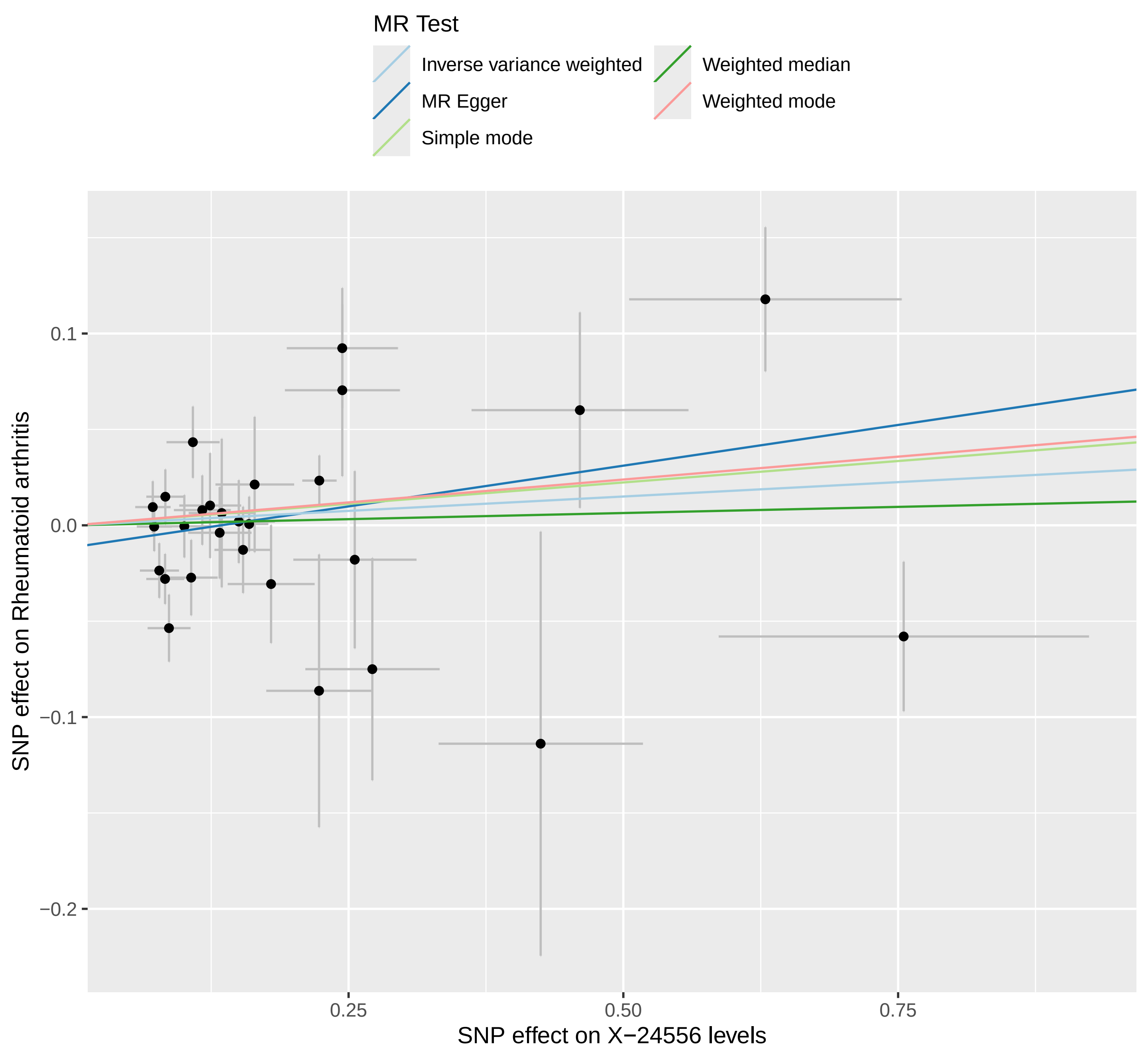
MR Test
Inverse variance weighted
MR Egger
Simple mode
Weighted median
Weighted mode
0.1
0.0
−0.1
−0.2
SNP effect on Rheumatoid arthritis
0.25
0.50
0.75
SNP effect on X−24556 levels

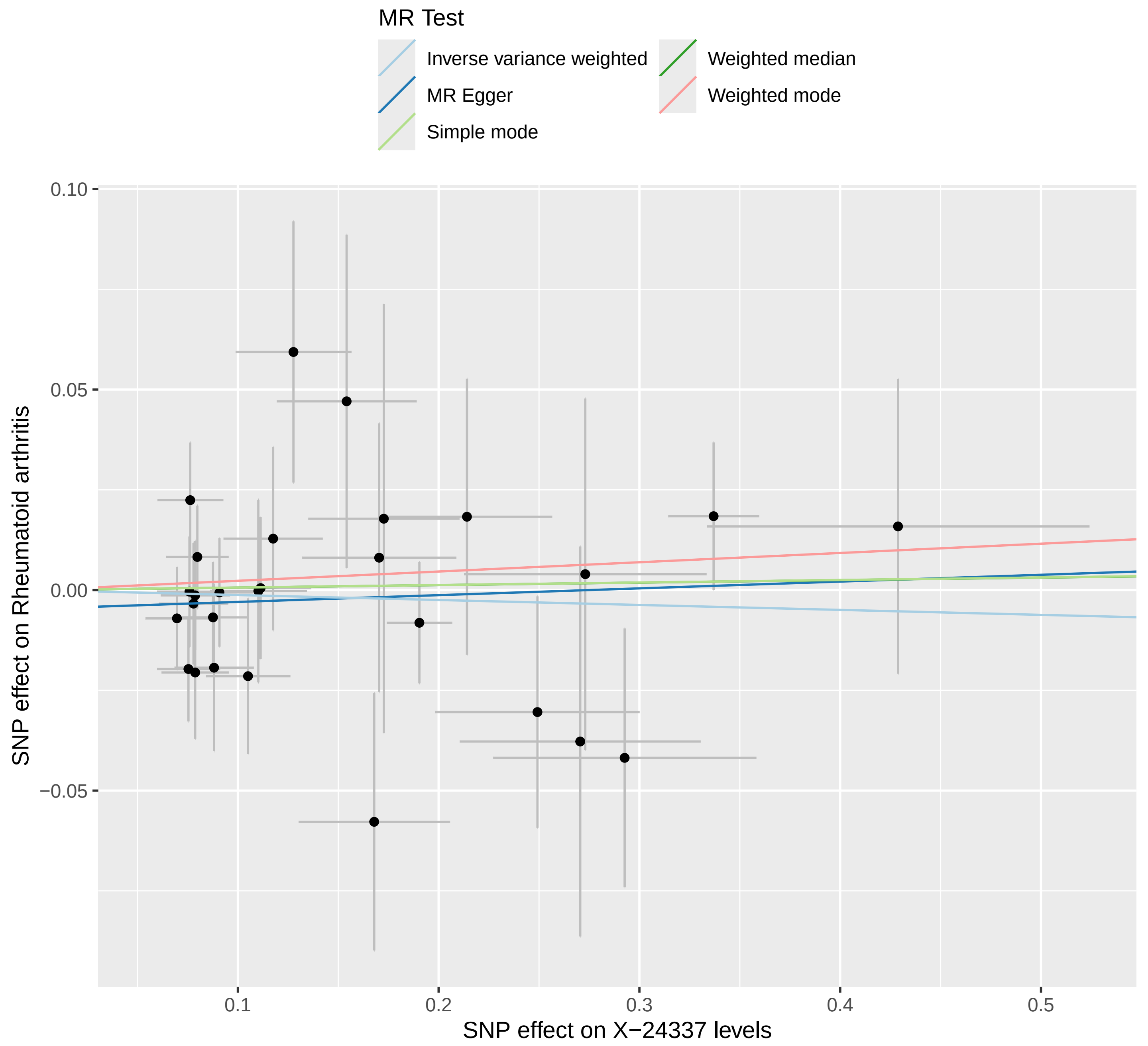
MR Test
Inverse variance weighted
MR Egger
Simple mode
Weighted median
Weighted mode
SNP effect on Rheumatoid arthritis
0.10
0.05
0.00
−0.05
0.1
0.2
0.3
0.4
0.5
SNP effect on X−24337 levels

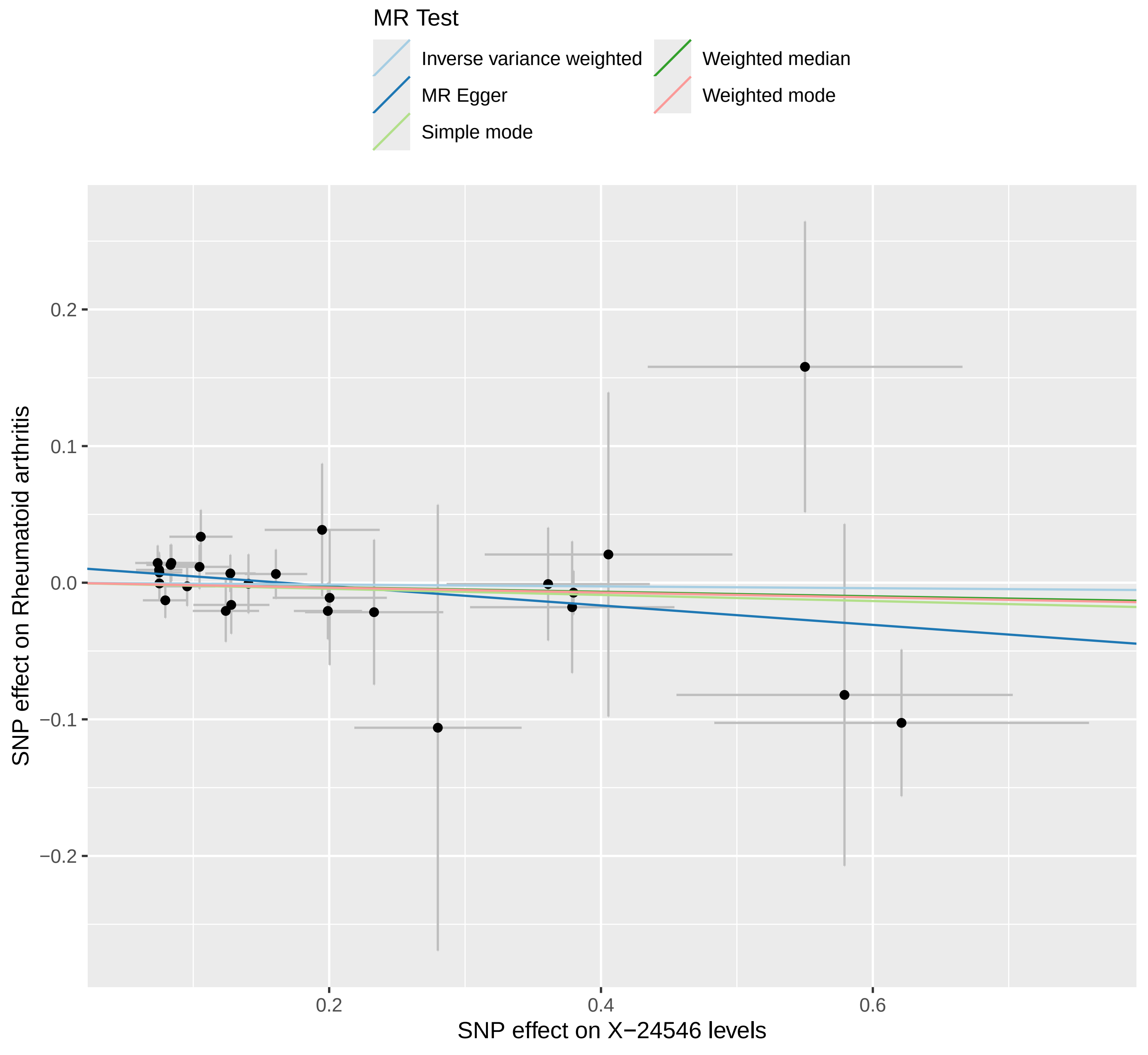
MR Test
Inverse variance weighted
MR Egger
Simple mode
Weighted median
Weighted mode
SNP effect on Rheumatoid arthritis
0.2
0.1
0.0
−0.1
−0.2
0.2
0.4
0.6
SNP effect on X−24546 levels

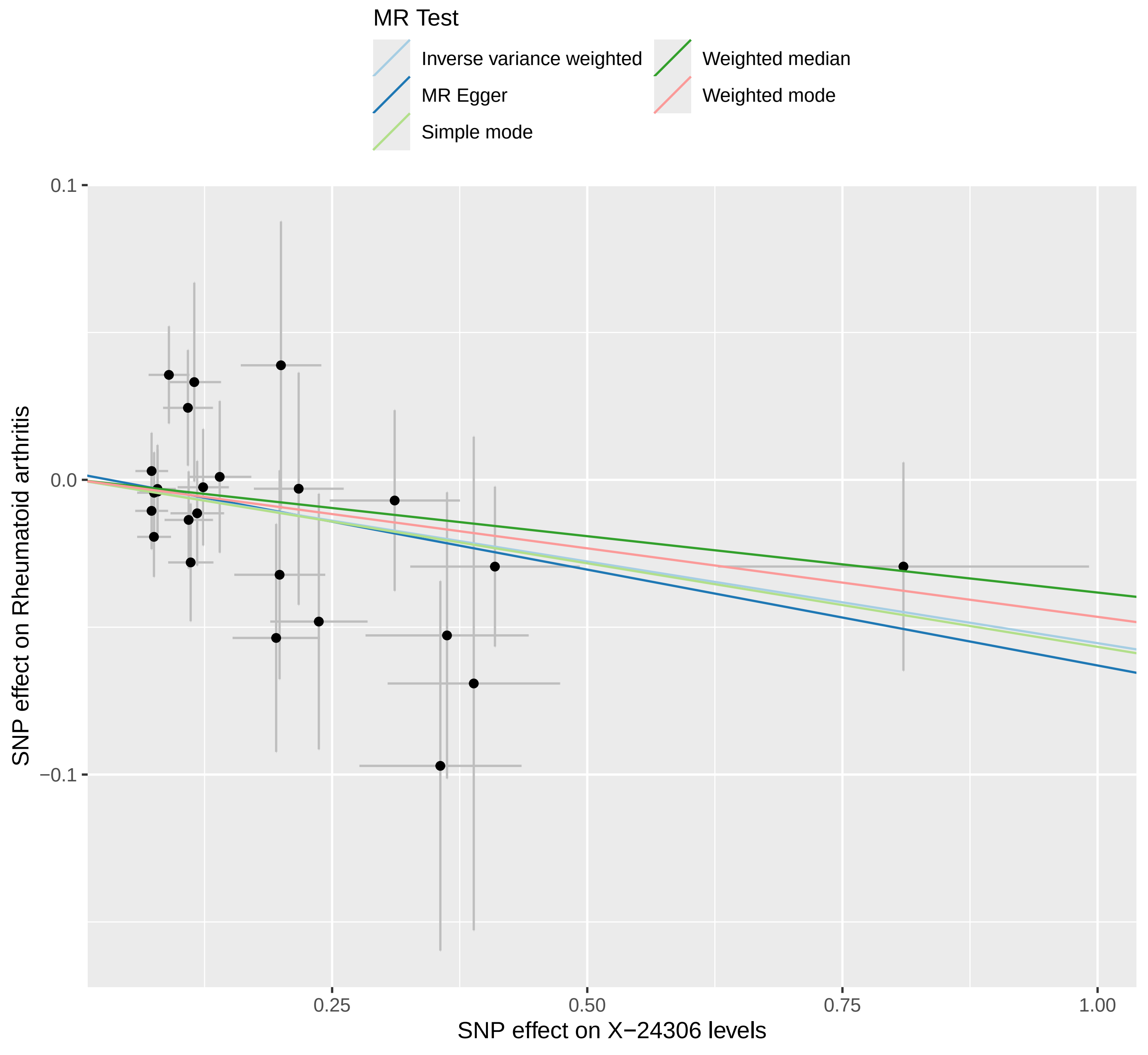
MR Test
Inverse variance weighted
MR Egger
Simple mode
Weighted median
Weighted mode
0.1
0.0
−0.1
0.25
0.50
0.75
1.00
SNP effect on Rheumatoid arthritis
SNP effect on X−24306 levels

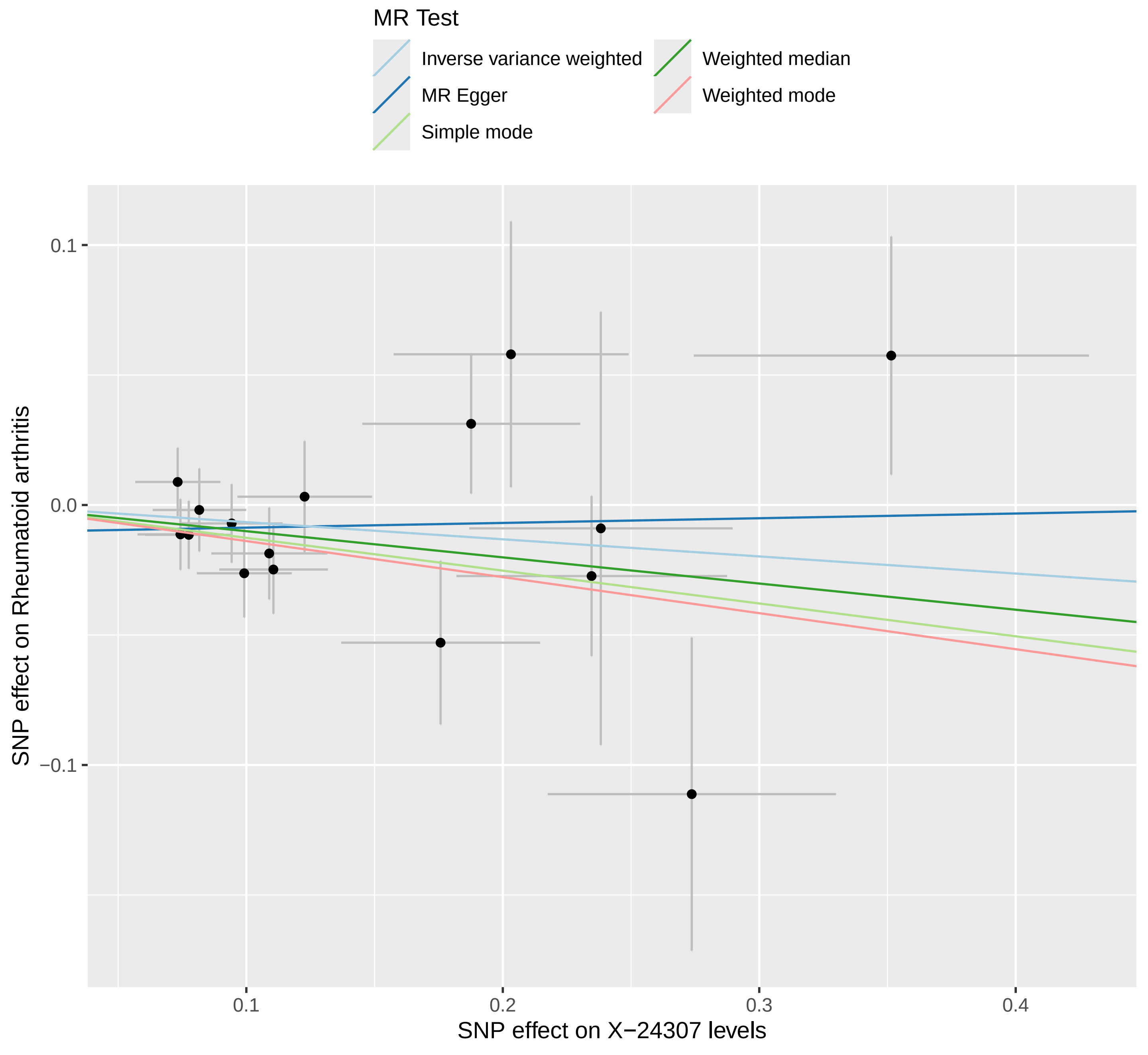
MR Test
Inverse variance weighted
MR Egger
Simple mode
Weighted median
Weighted mode
0.1
0.0
−0.1
0.1
0.2
0.3
0.4
SNP effect on Rheumatoid arthritis
SNP effect on X−24307 levels

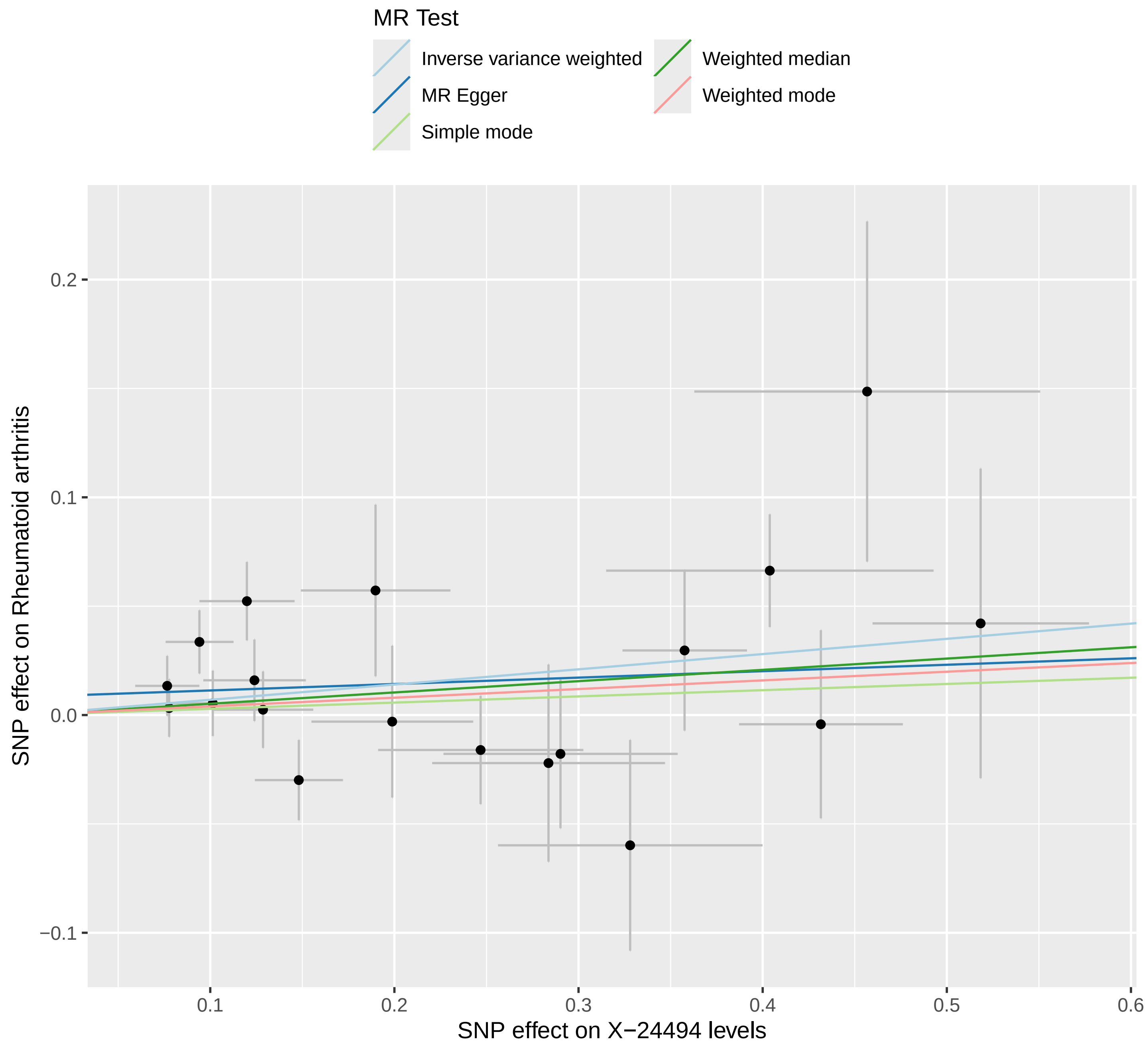
MR Test
Inverse variance weighted
MR Egger
Simple mode
Weighted median
Weighted mode
0.2
0.1
0.0
−0.1
0.1
0.2
0.3
0.4
0.5
0.6
SNP effect on Rheumatoid arthritis
SNP effect on X−24494 levels

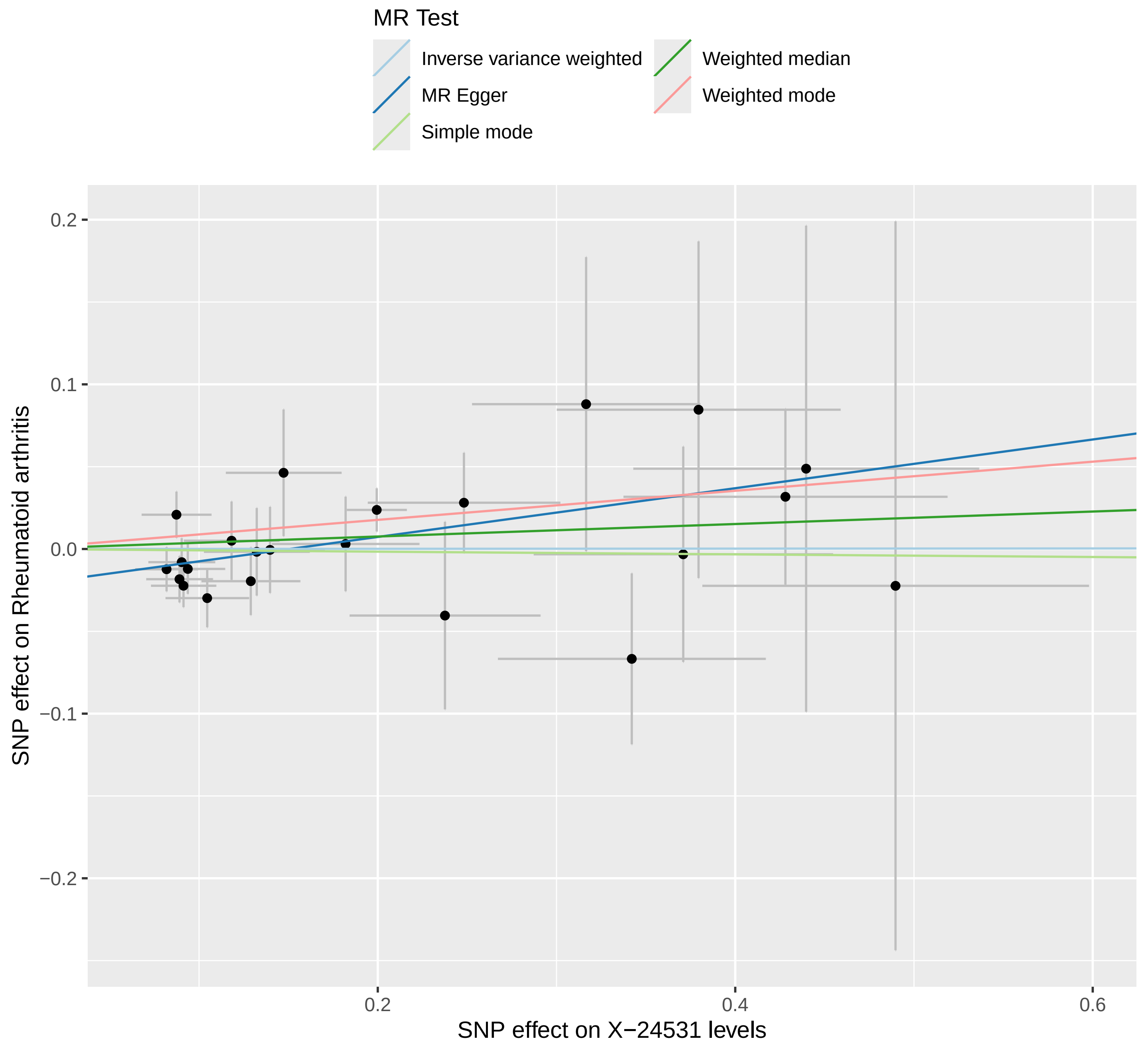

MR Test
Inverse variance weighted
MR Egger
Simple mode
Weighted median
Weighted mode
0.2
0.1
0.0
−0.1
−0.2
SNP effect on Rheumatoid arthritis
0.2
0.4
0.6
SNP effect on X−24531 levels

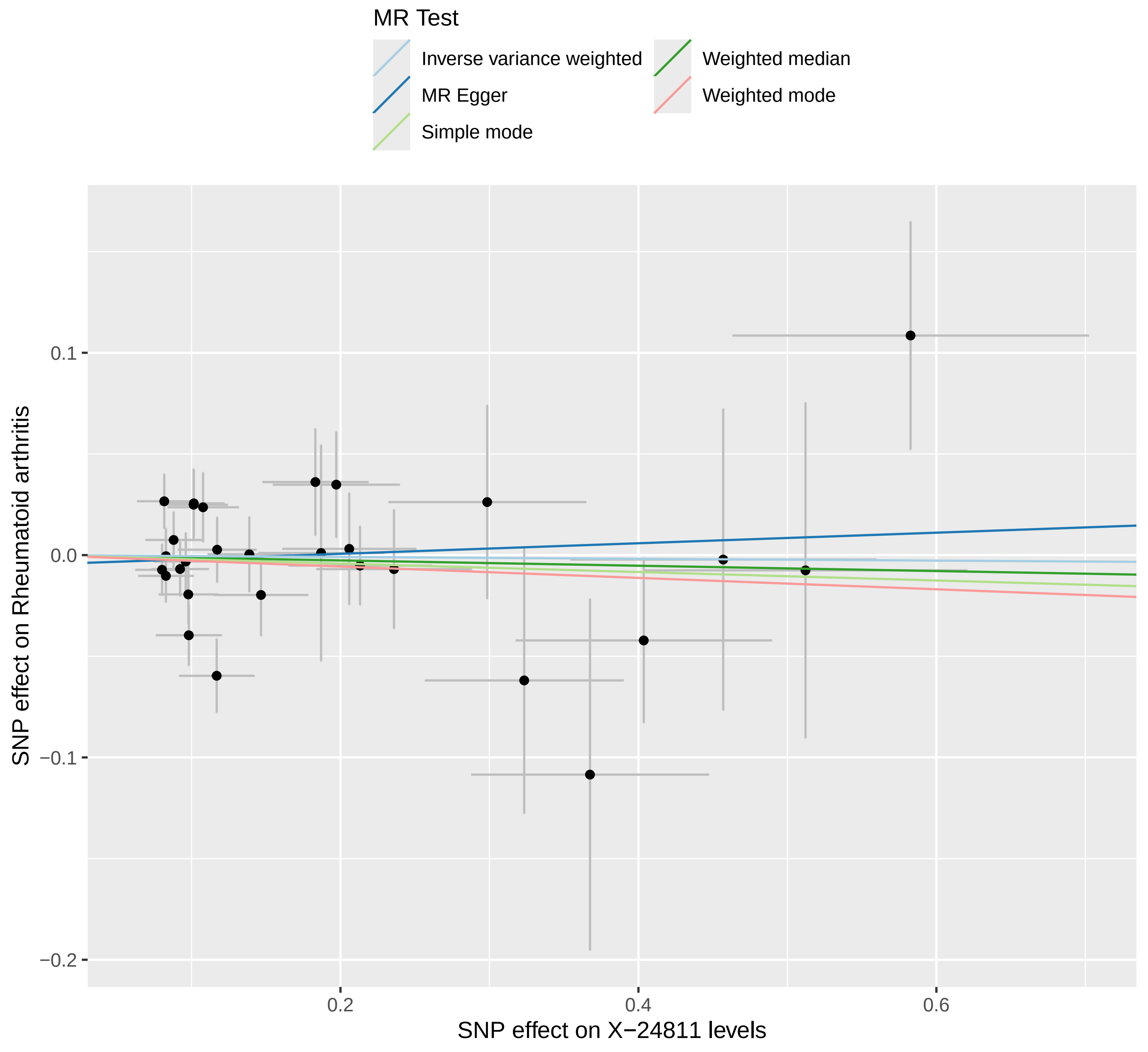

MR Test
Inverse variance weighted
MR Egger
Simple mode
Weighted median
Weighted mode
0.1
0.0
−0.1
−0.2
0.2
0.4
0.6
SNP effect on Rheumatoid arthritis
SNP effect on X−24811 levels

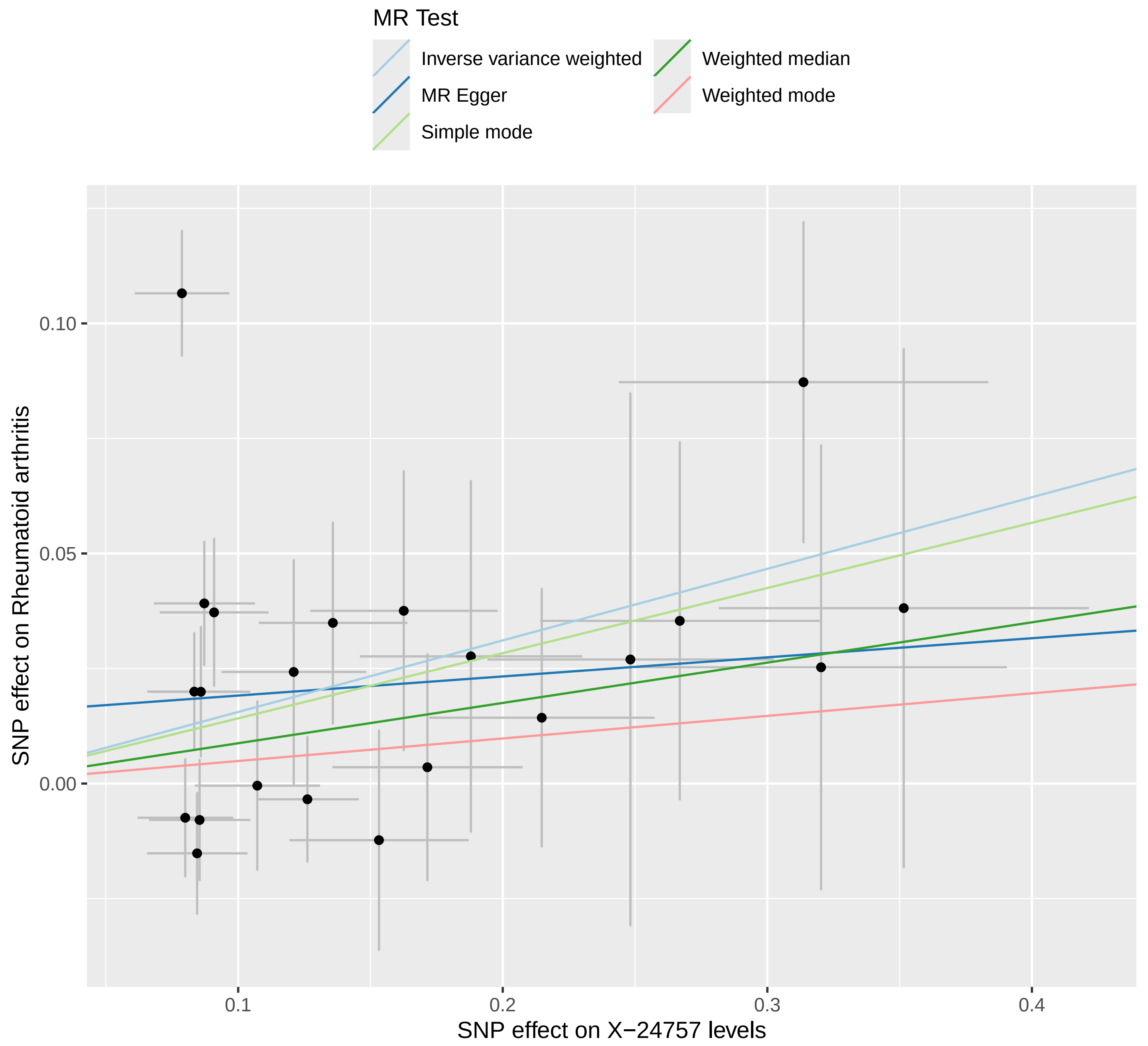
MR Test
Inverse variance weighted
MR Egger
Simple mode
Weighted median
Weighted mode
0.10
0.05
0.00
0.1
0.2
0.3
0.4
SNP effect on Rheumatoid arthritis
SNP effect on X−24757 levels

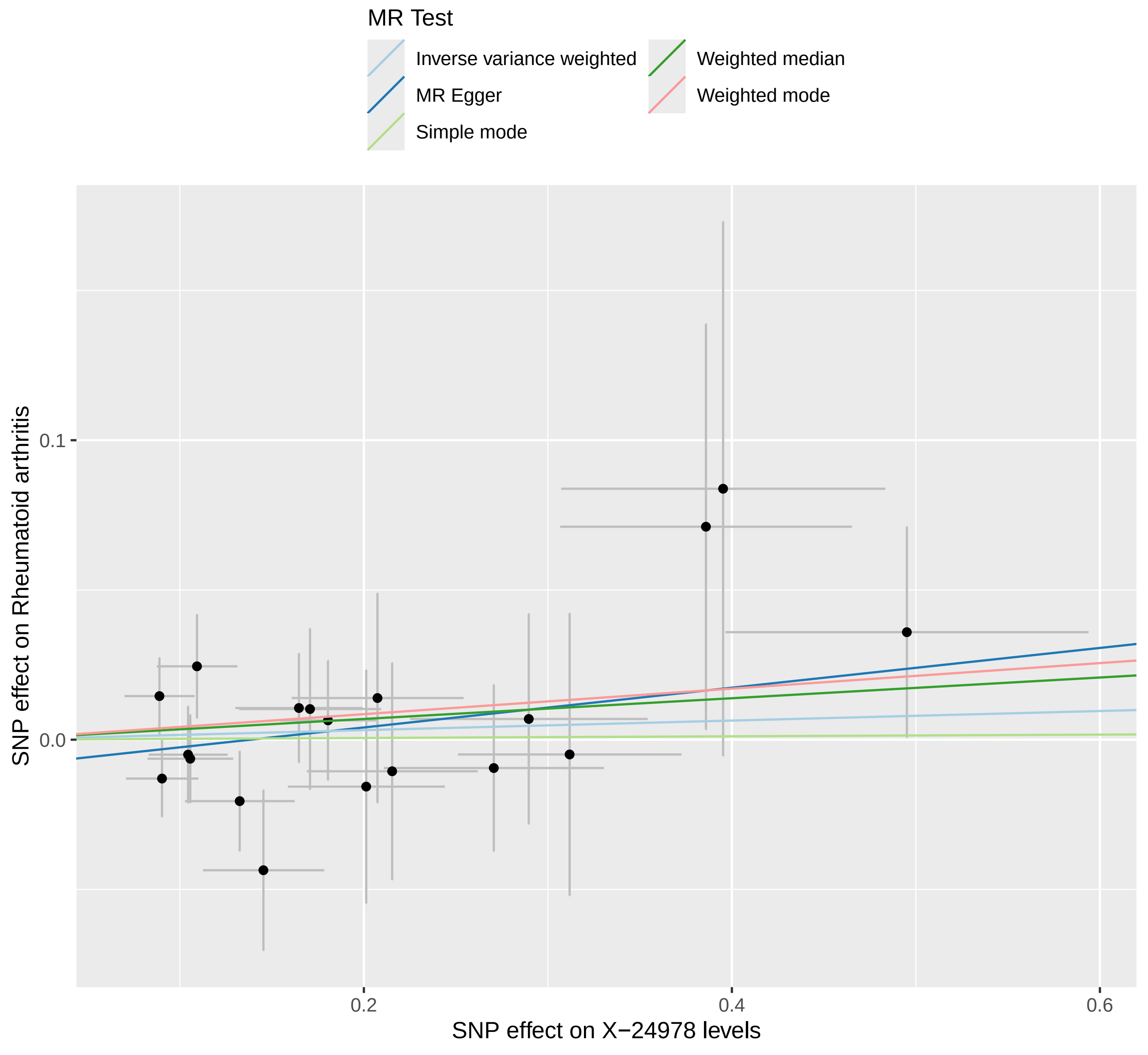
MR Test
Inverse variance weighted
MR Egger
Simple mode
Weighted median
Weighted mode
SNP effect on Rheumatoid arthritis
0.1
0.0
0.2
0.4
0.6
SNP effect on X−24978 levels

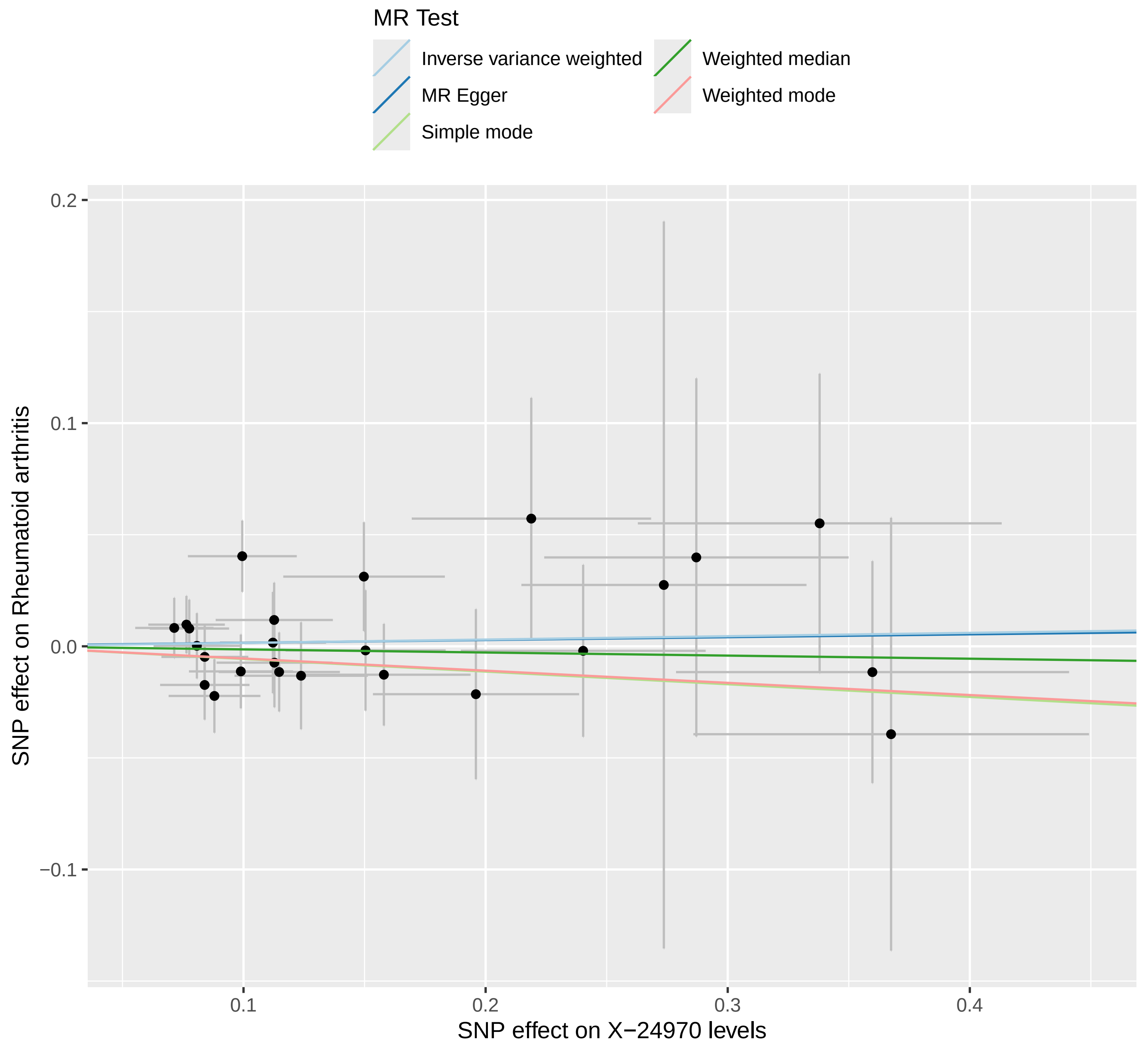
MR Test
Inverse variance weighted
MR Egger
Simple mode
Weighted median
Weighted mode
SNP effect on Rheumatoid arthritis
0.2
0.1
0.0
−0.1
0.1
0.2
0.3
0.4
SNP effect on X−24970 levels

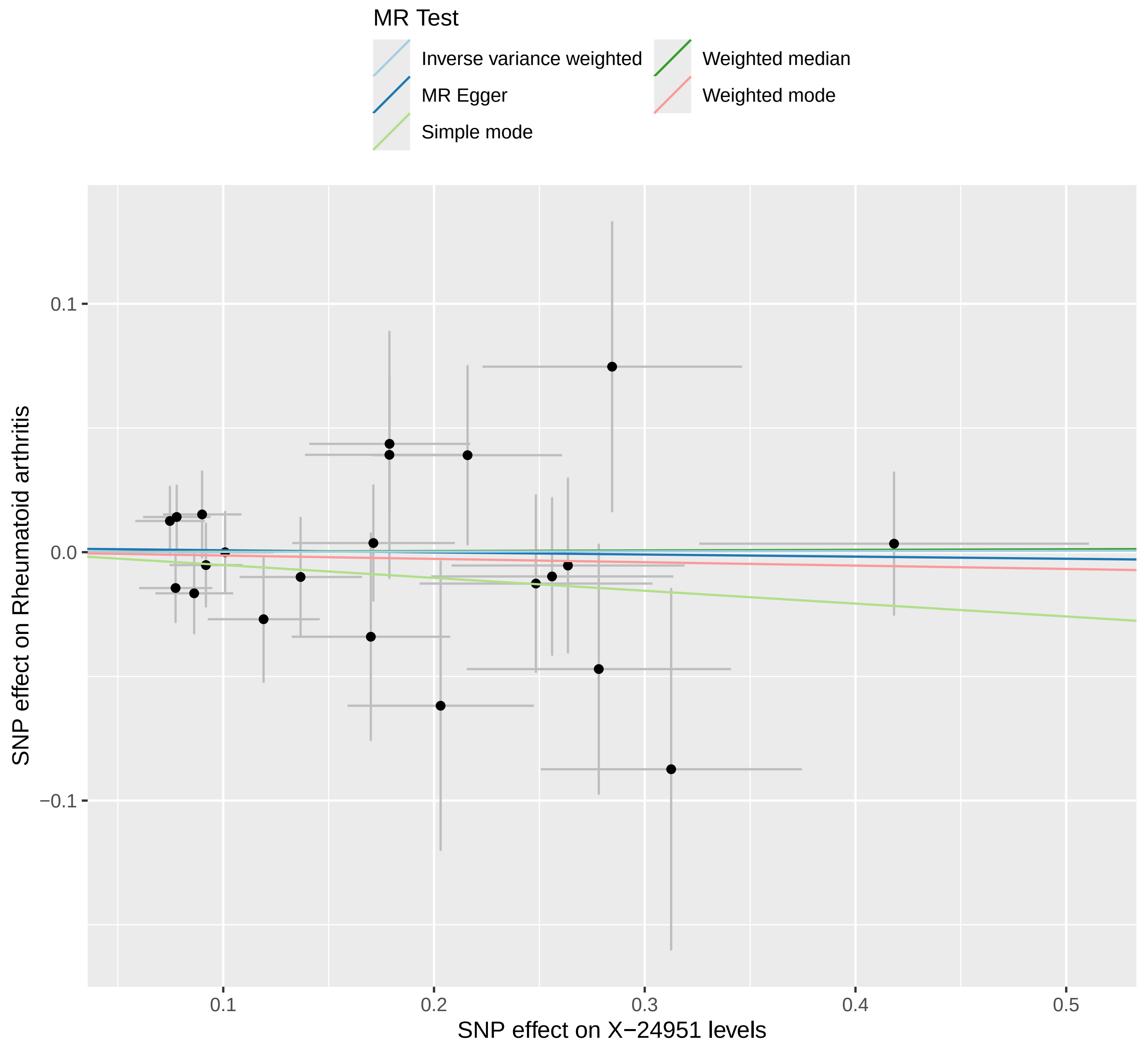
MR Test
Inverse variance weighted
MR Egger
Simple mode
Weighted median
Weighted mode
0.1
0.0
−0.1
0.1
0.2
0.3
0.4
0.5
SNP effect on Rheumatoid arthritis
SNP effect on X−24951 levels

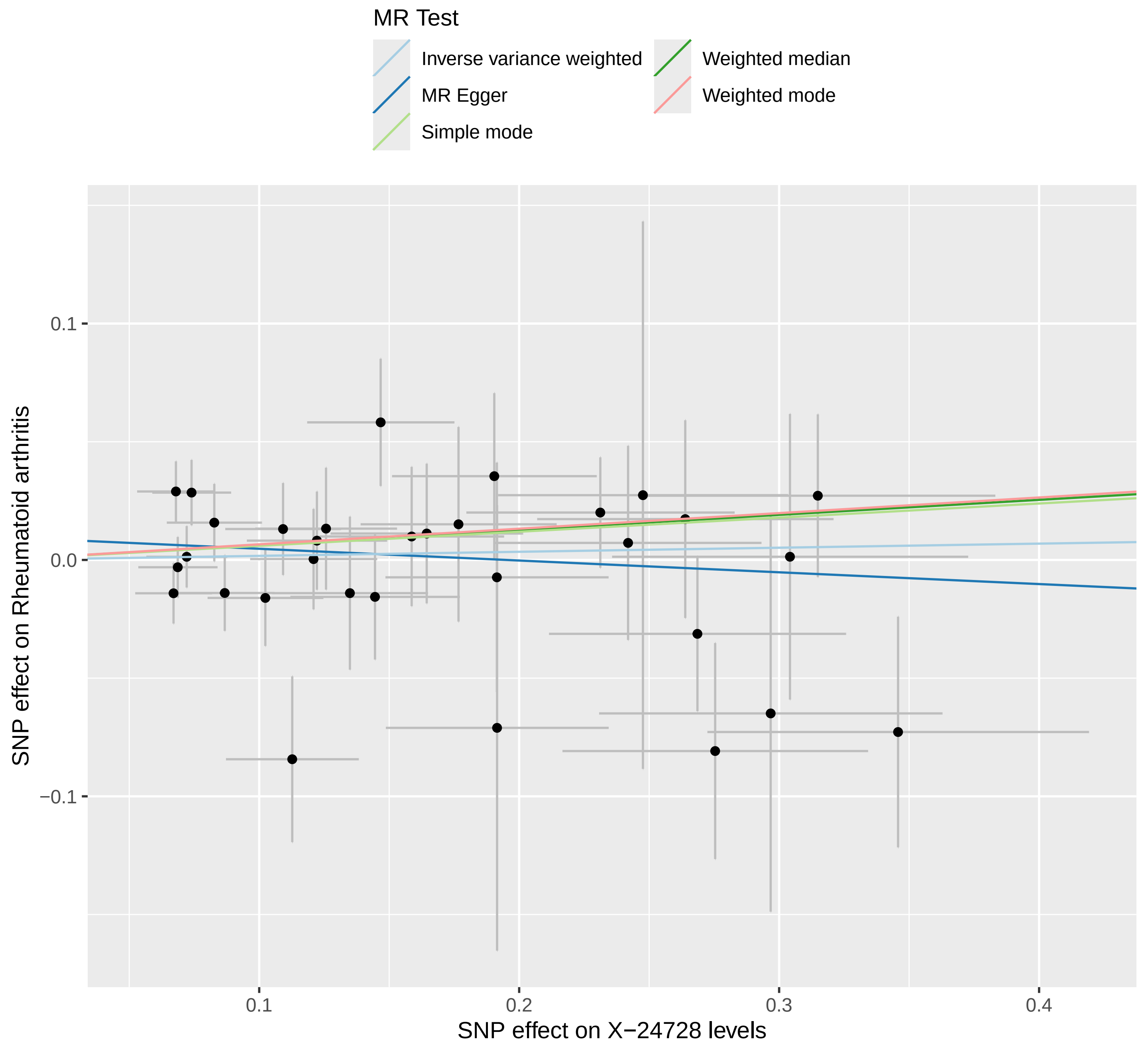

MR Test
Inverse variance weighted
MR Egger
Simple mode
Weighted median
Weighted mode
0.1
0.0
−0.1
SNP effect on Rheumatoid arthritis
0.1
0.2
0.3
0.4
SNP effect on X−24728 levels

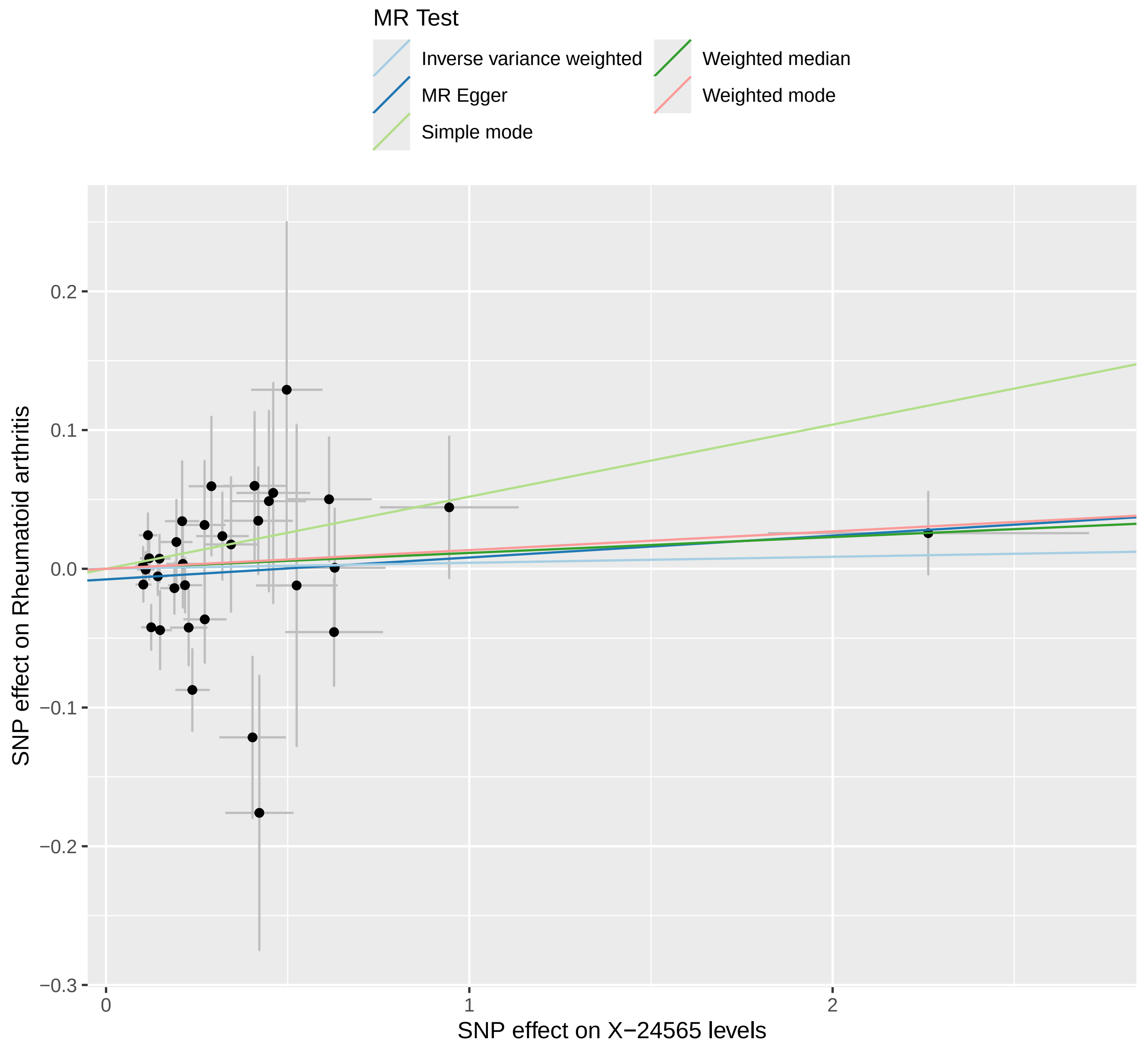
MR Test
Inverse variance weighted
MR Egger
Simple mode
Weighted median
Weighted mode
0.2
0.1
0.0
−0.1
−0.2
−0.3
0
1
2
SNP effect on Rheumatoid arthritis
SNP effect on X−24565 levels

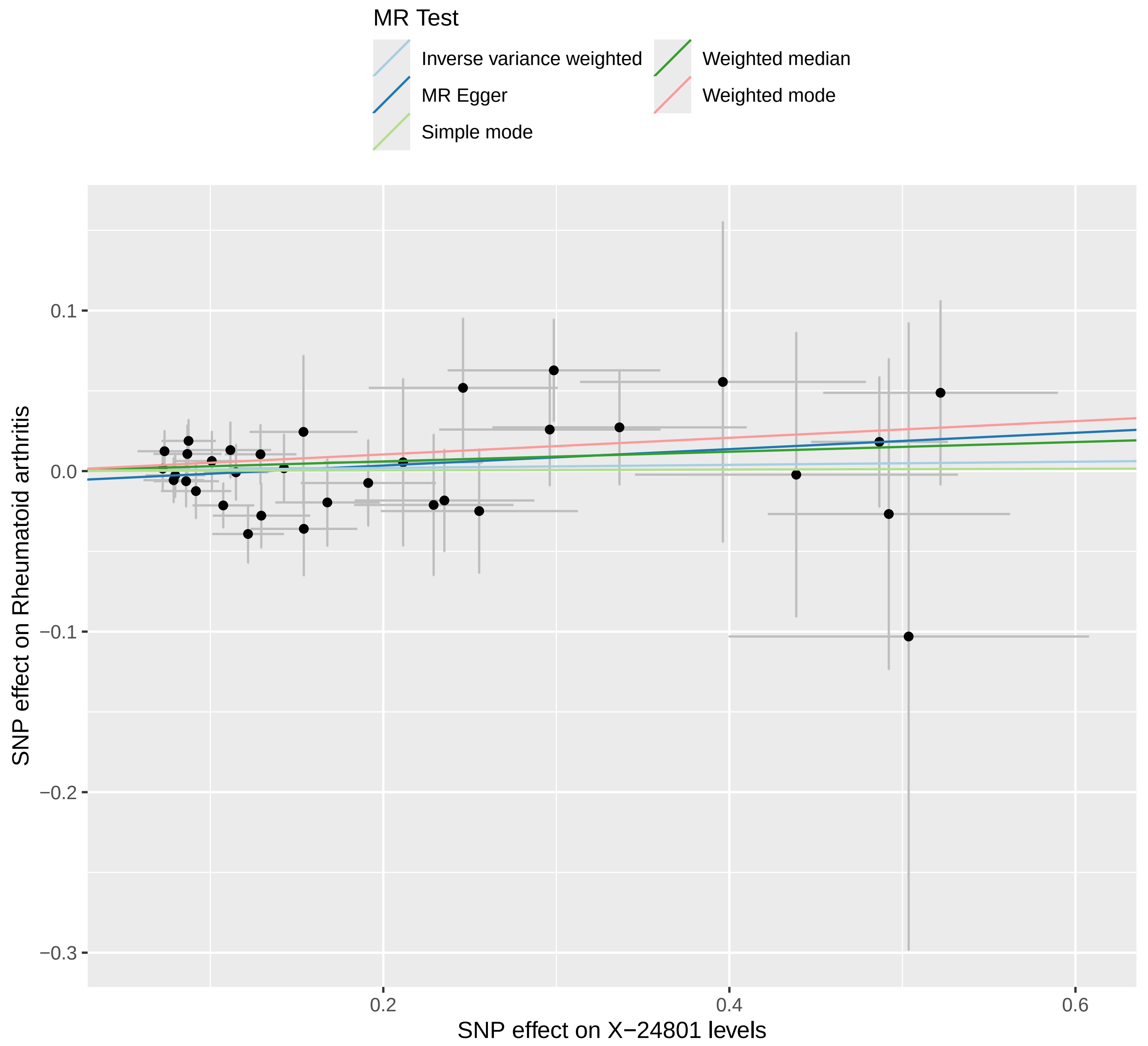

MR Test
Inverse variance weighted
MR Egger
Simple mode
Weighted median
Weighted mode
0.1
0.0
−0.1
−0.2
−0.3
SNP effect on Rheumatoid arthritis
0.2
0.4
0.6
SNP effect on X−24801 levels

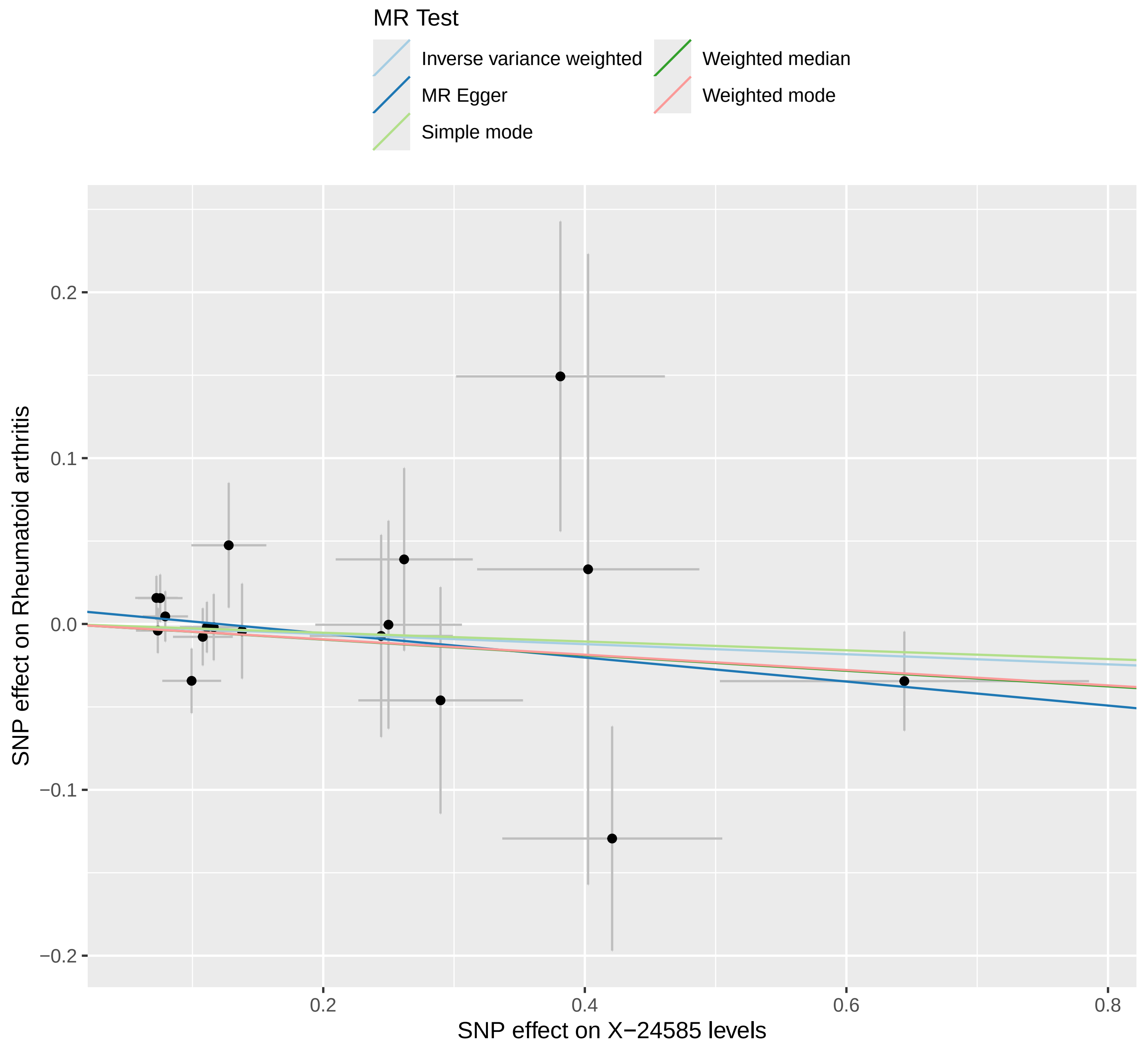
MR Test
Inverse variance weighted
MR Egger
Simple mode
Weighted median
Weighted mode
SNP effect on Rheumatoid arthritis
0.2
0.1
0.0
−0.1
−0.2
0.2
0.4
0.6
0.8
SNP effect on X−24585 levels

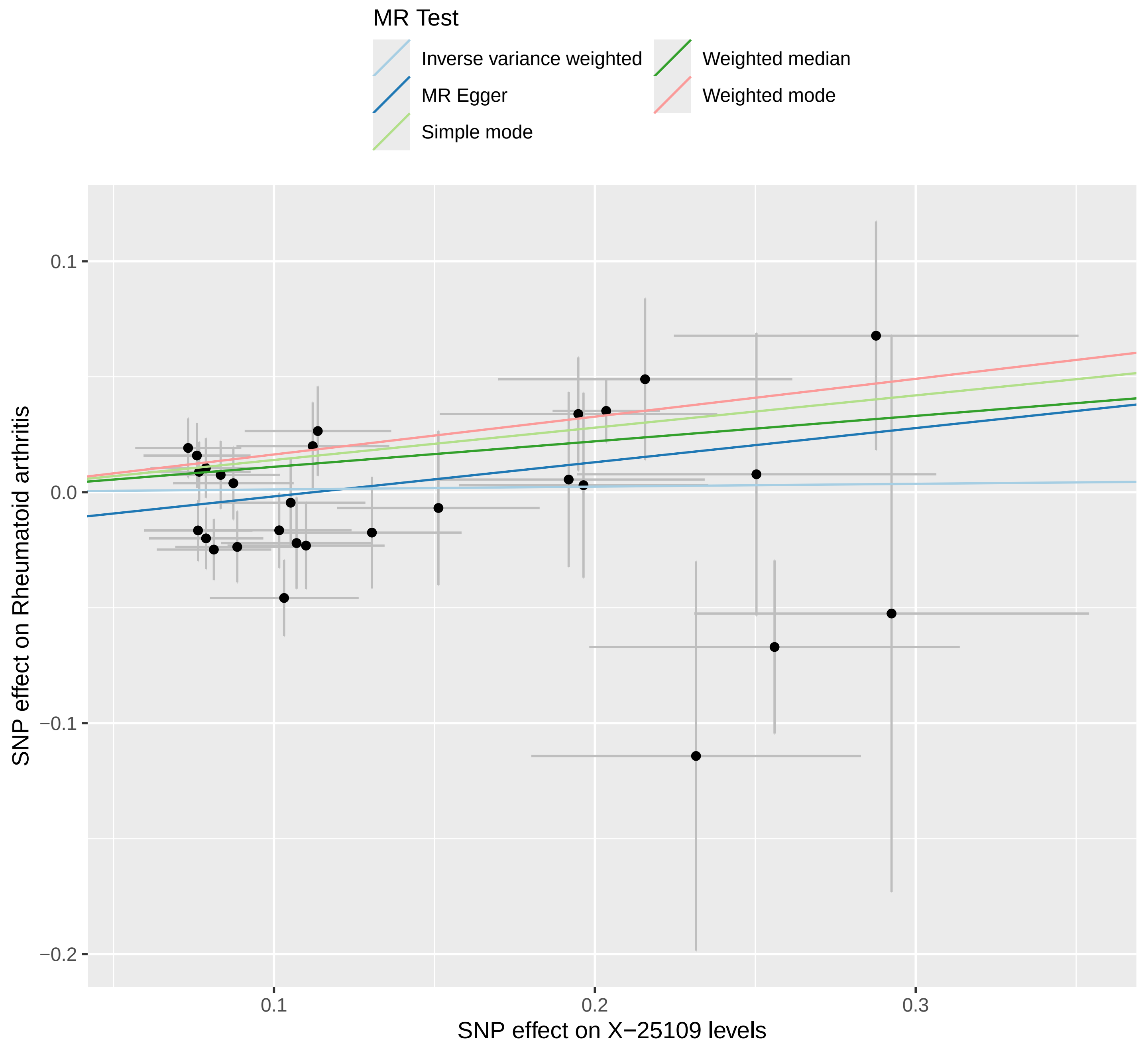
MR Test
Inverse variance weighted
MR Egger
Simple mode
Weighted median
Weighted mode
0.1
0.0
−0.1
−0.2
SNP effect on Rheumatoid arthritis
0.1
0.2
0.3
SNP effect on X−25109 levels

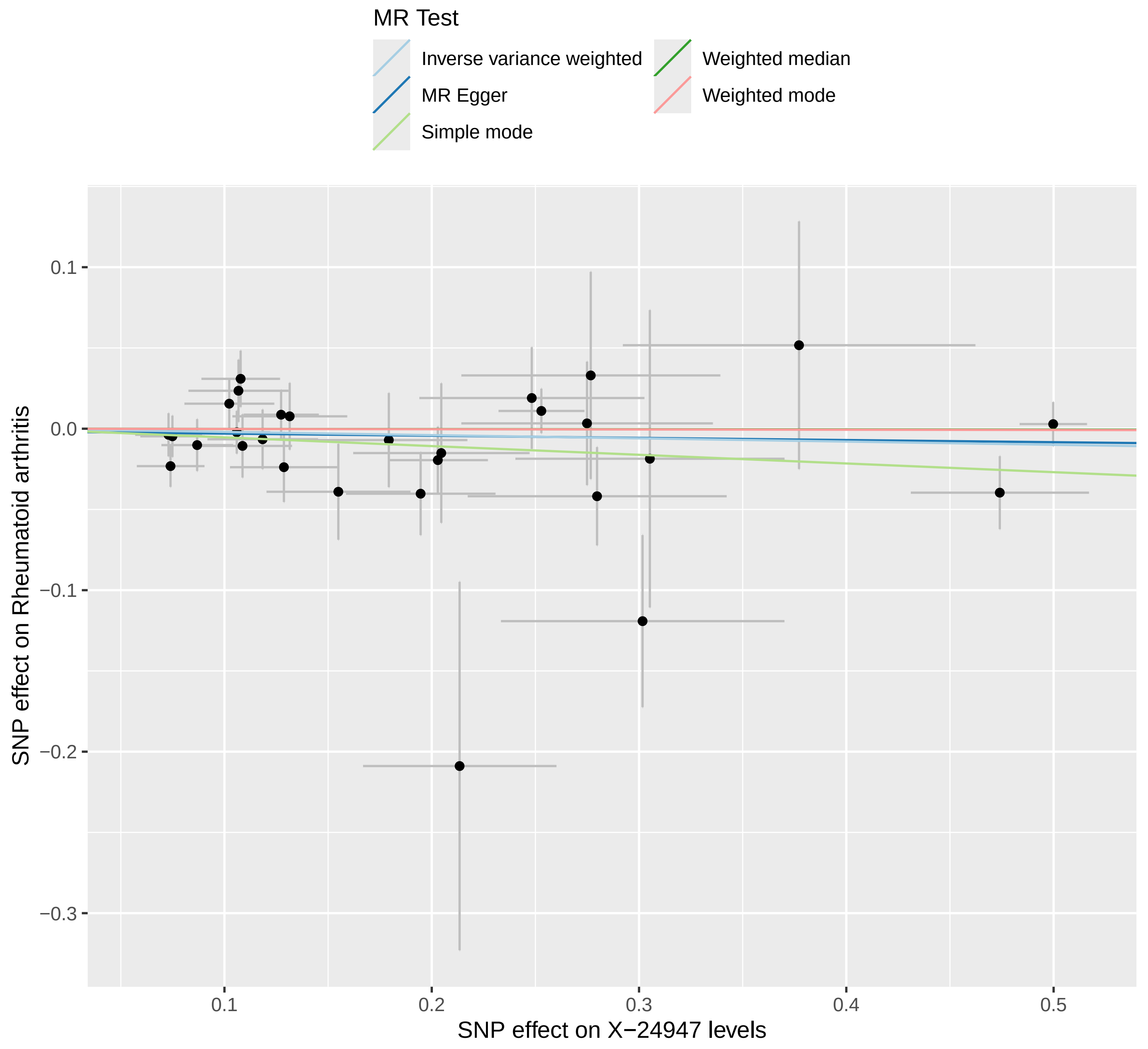

MR Test
Inverse variance weighted
MR Egger
Simple mode
Weighted median
Weighted mode
0.1
0.0
−0.1
−0.2
−0.3
0.1
0.2
0.3
0.4
0.5
SNP effect on Rheumatoid arthritis
SNP effect on X−24947 levels

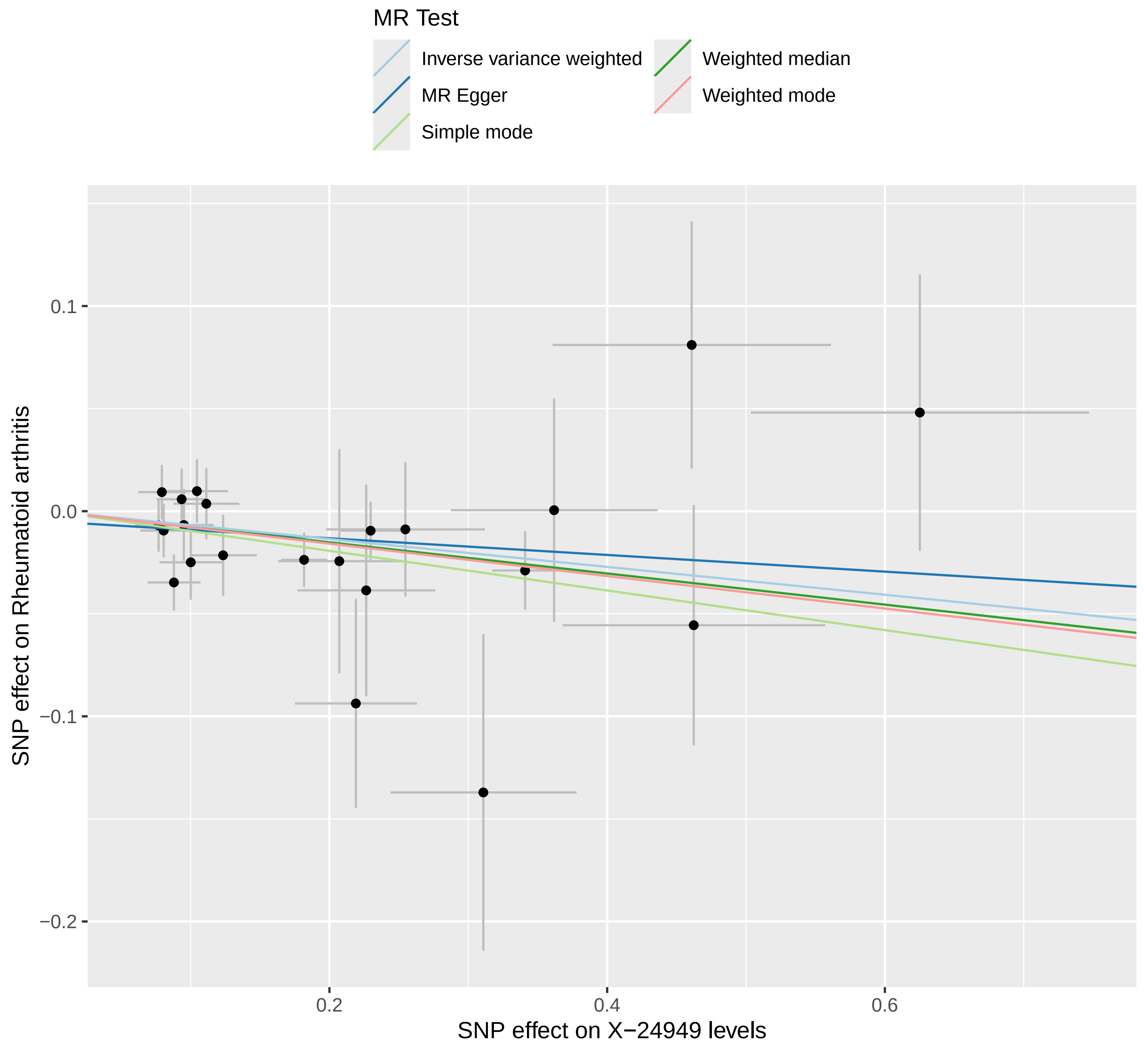
MR Test
Inverse variance weighted
MR Egger
Simple mode
Weighted median
Weighted mode
0.1
0.0
−0.1
−0.2
SNP effect on Rheumatoid arthritis
0.2
0.4
0.6
SNP effect on X−24949 levels

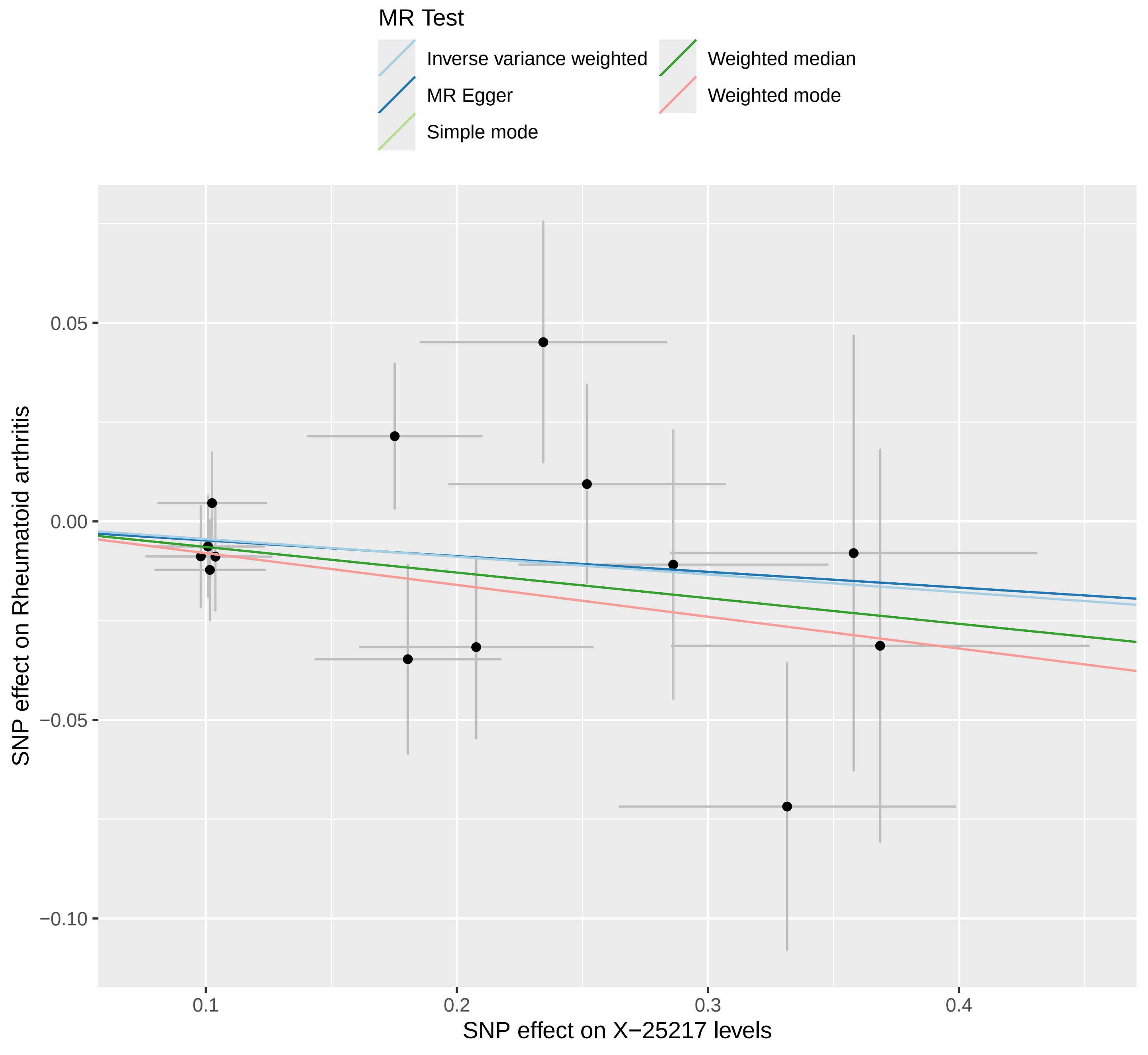

MR Test
Inverse variance weighted
MR Egger
Simple mode
Weighted median
Weighted mode
0.05
0.00
−0.05
−0.10
0.1
0.2
0.3
0.4
SNP effect on Rheumatoid arthritis
SNP effect on X−25217 levels

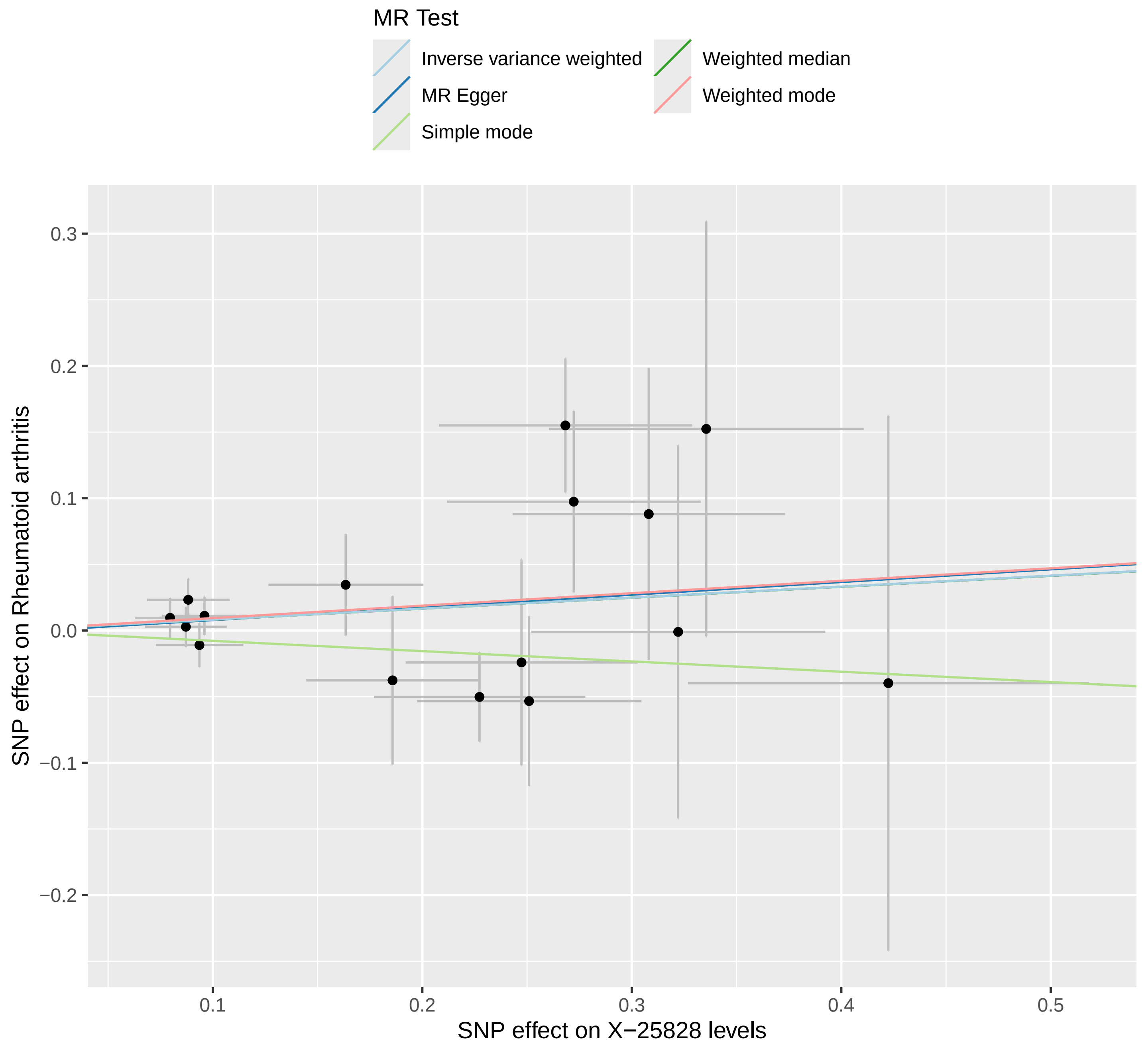

MR Test
Inverse variance weighted
MR Egger
Simple mode
Weighted median
Weighted mode
0.3
0.2
0.1
0.0
−0.1
−0.2
0.1
0.2
0.3
0.4
0.5
SNP effect on Rheumatoid arthritis
SNP effect on X−25828 levels

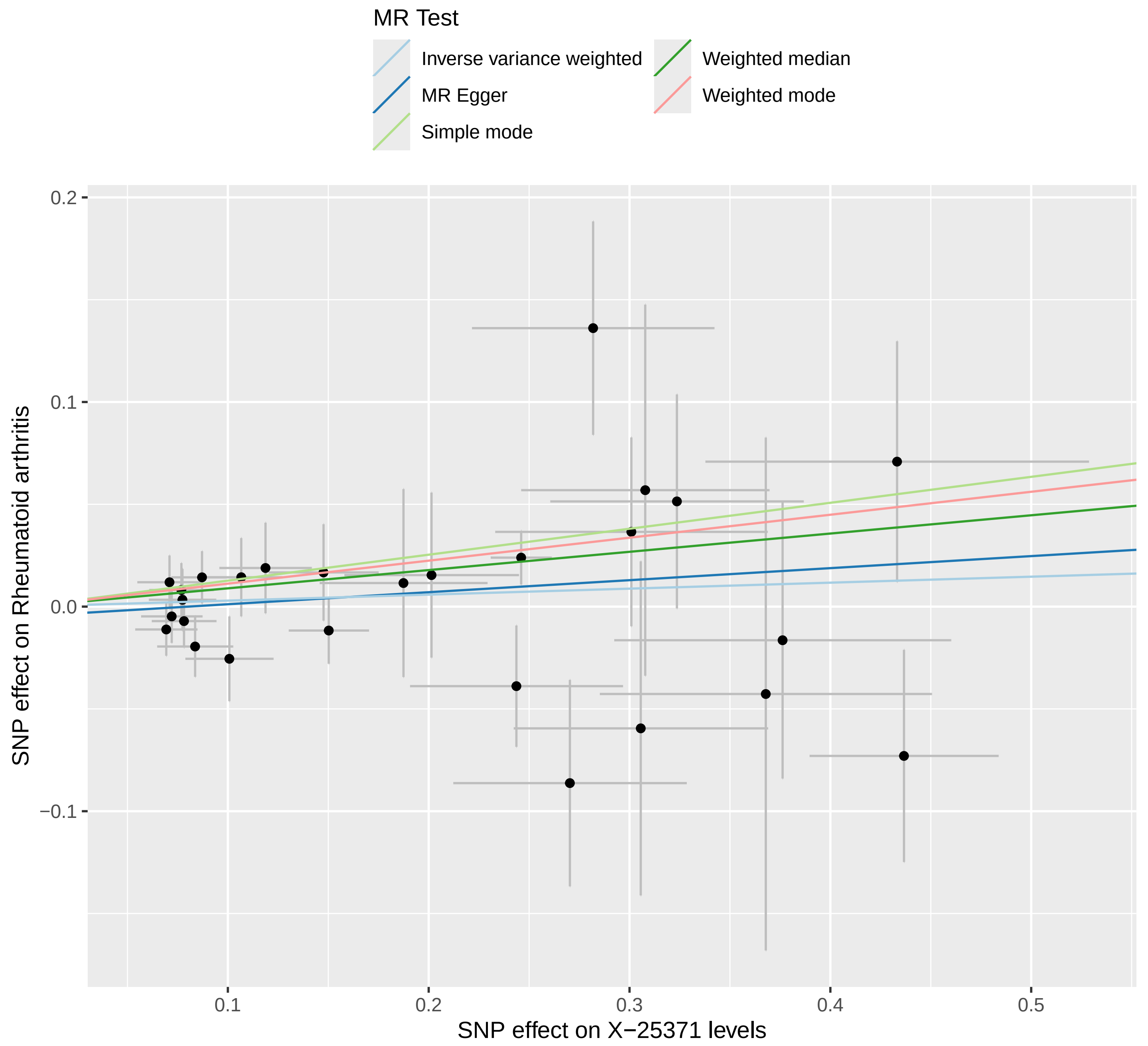

MR Test
Inverse variance weighted
MR Egger
Simple mode
Weighted median
Weighted mode
0.2
0.1
0.0
−0.1
SNP effect on Rheumatoid arthritis
0.1
0.2
0.3
0.4
0.5
SNP effect on X−25371 levels

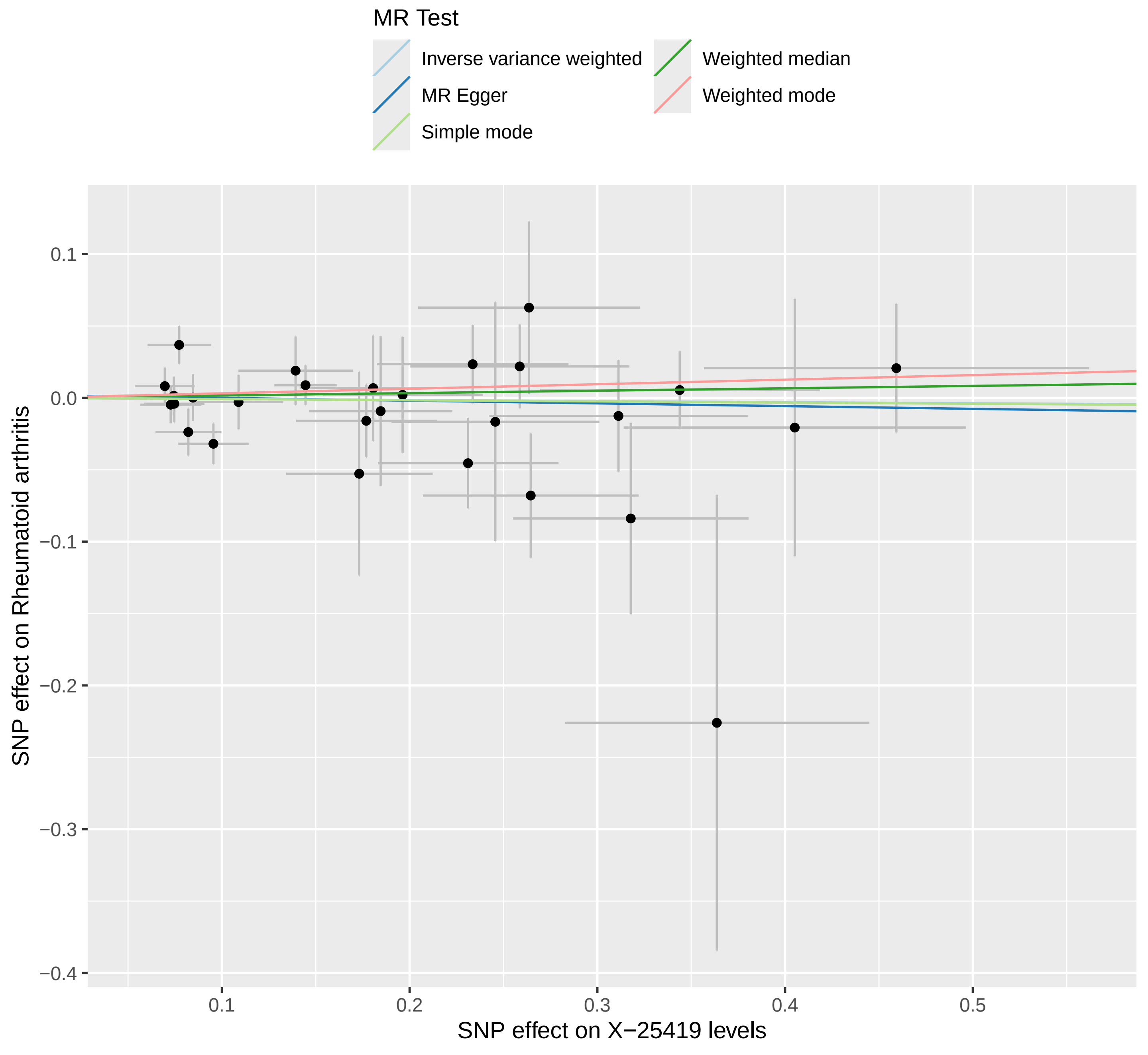

MR Test
Inverse variance weighted
MR Egger
Simple mode
Weighted median
Weighted mode
0.1
0.0
−0.1
−0.2
−0.3
−0.4
0.1
0.2
0.3
0.4
0.5
SNP effect on Rheumatoid arthritis
SNP effect on X−25419 levels

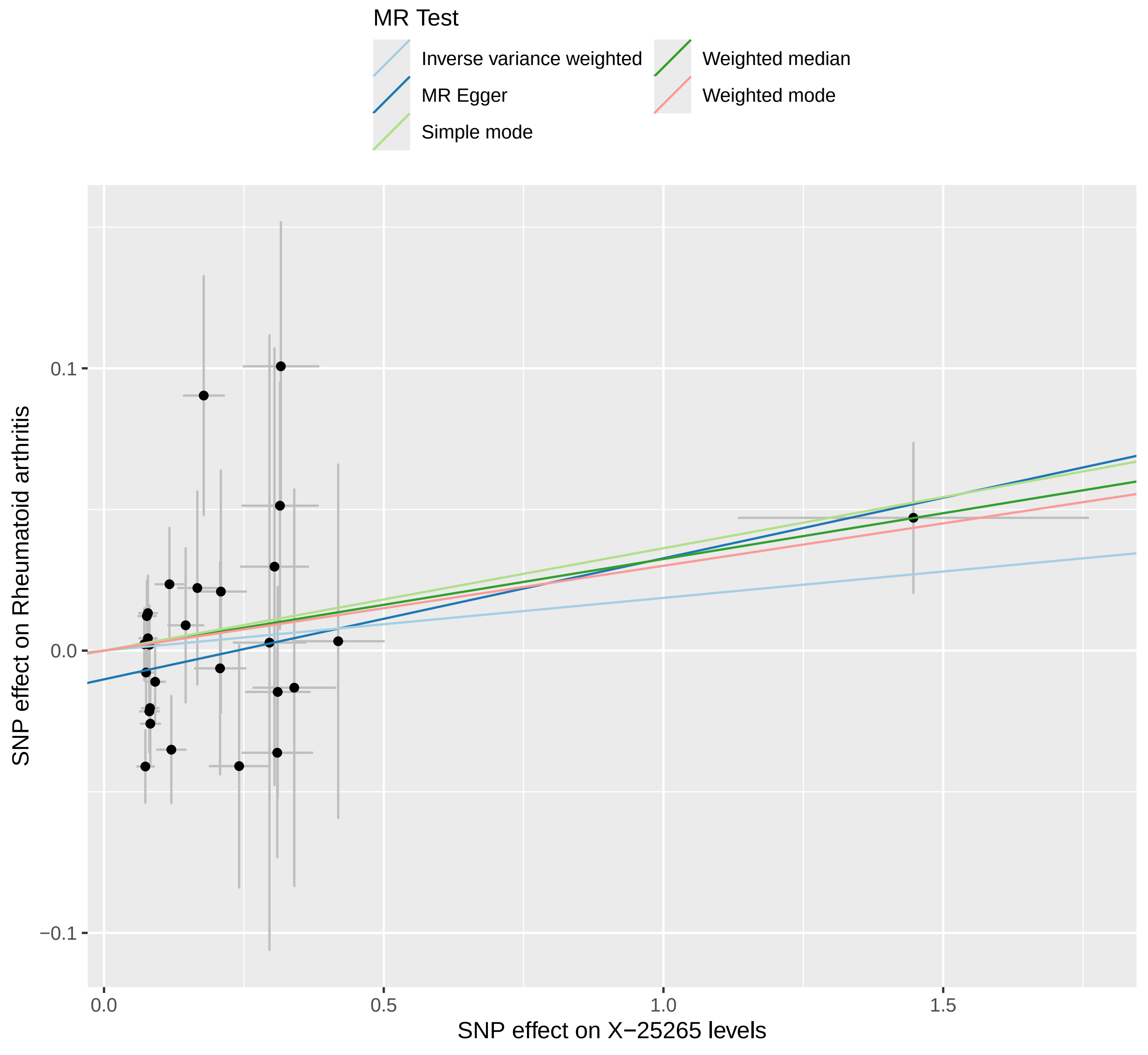
MR Test
Inverse variance weighted
MR Egger
Simple mode
Weighted median
Weighted mode
SNP effect on Rheumatoid arthritis
0.1
0.0
−0.1
0.0
0.5
1.0
1.5
SNP effect on X−25265 levels

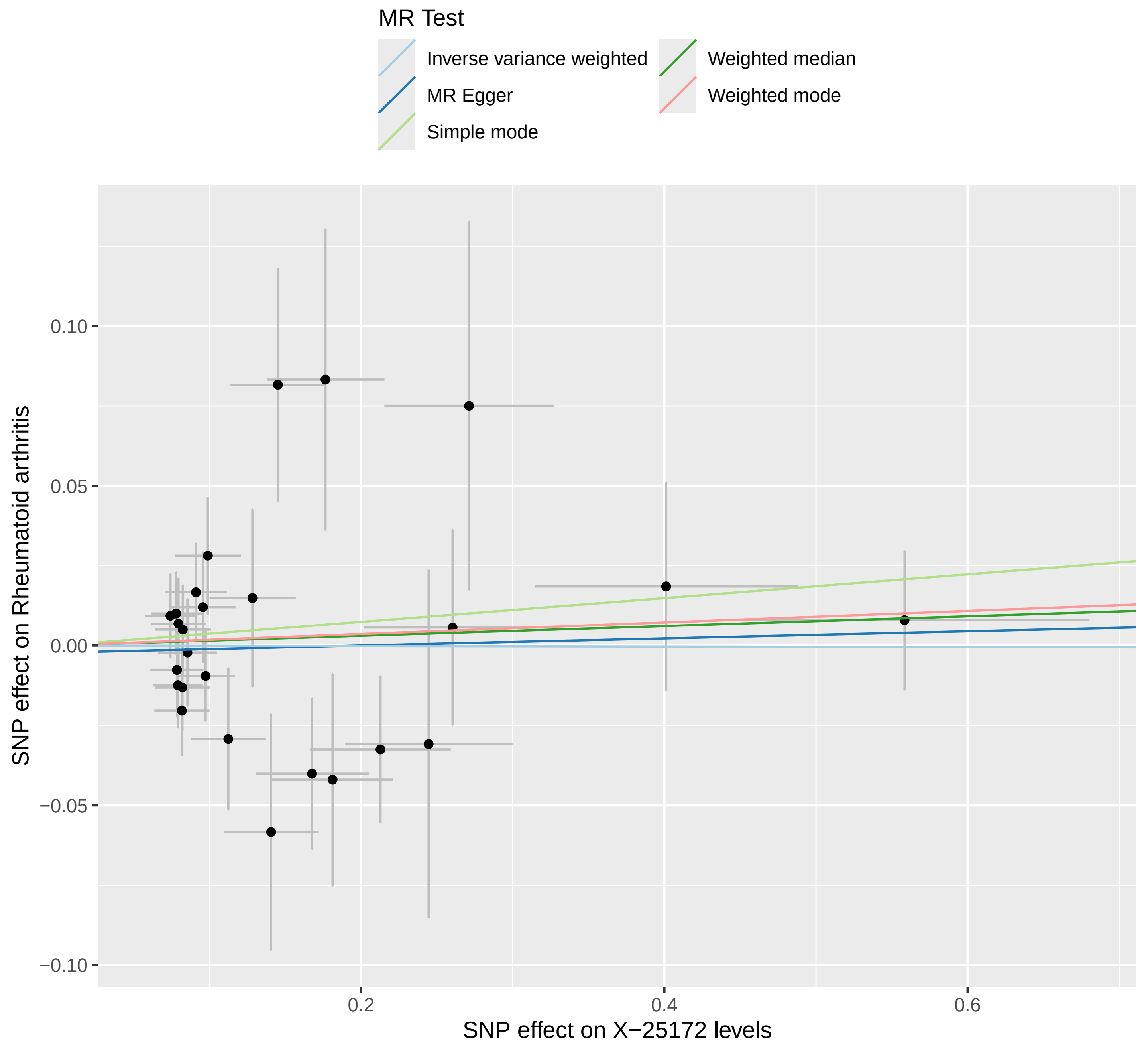

MR Test
Inverse variance weighted
MR Egger
Simple mode
Weighted median
Weighted mode
0.10
0.05
0.00
−0.05
−0.10
0.2
0.4
0.6
SNP effect on Rheumatoid arthritis
SNP effect on X−25172 levels

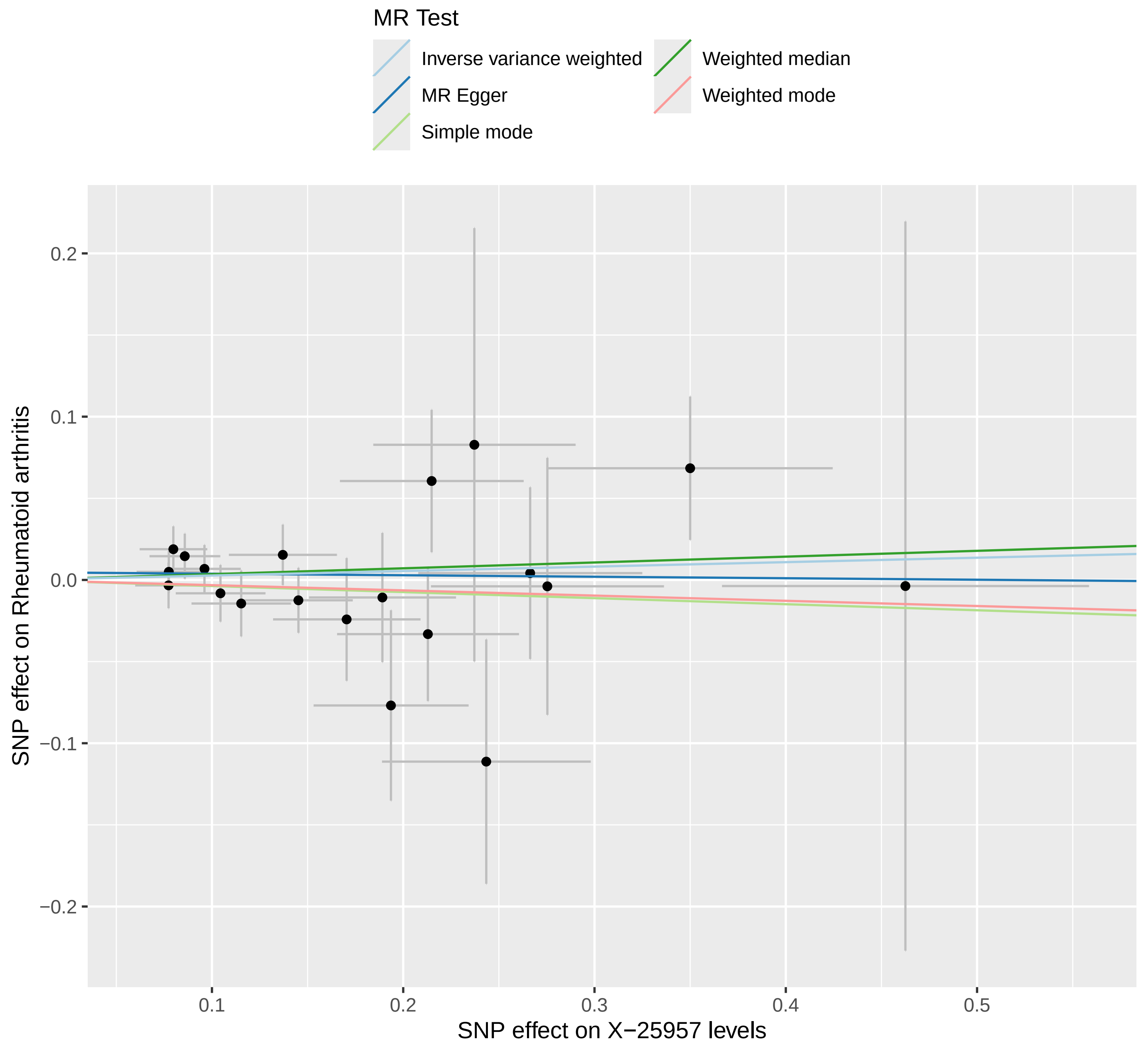

MR Test
Inverse variance weighted
MR Egger
Simple mode
Weighted median
Weighted mode
SNP effect on Rheumatoid arthritis
0.2
0.1
0.0
−0.1
−0.2
0.1
0.2
0.3
0.4
0.5
SNP effect on X−25957 levels

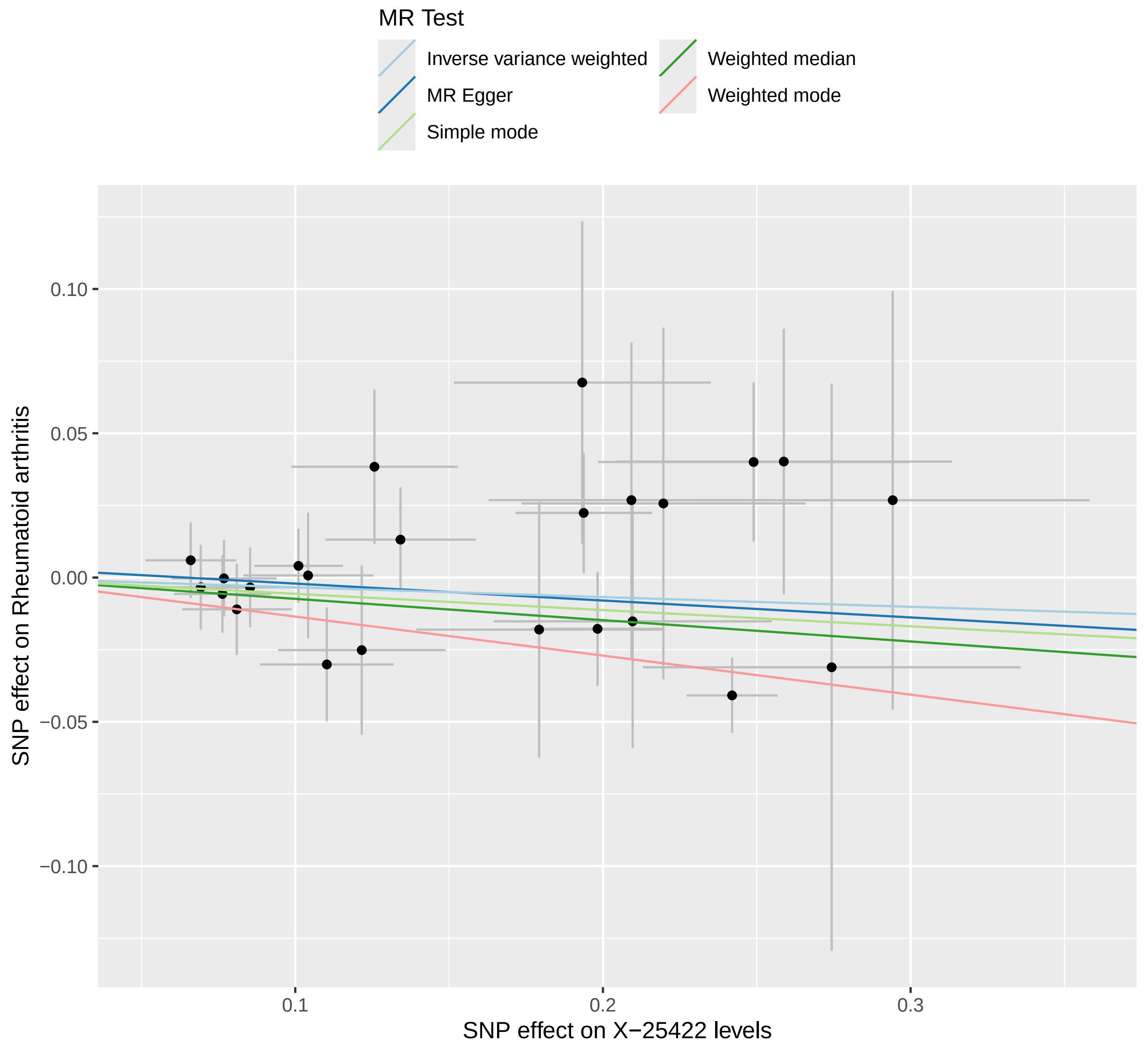

MR Test
Inverse variance weighted
MR Egger
Simple mode
Weighted median
Weighted mode
0.10
0.05
0.00
−0.05
−0.10
0.1
0.2
0.3
SNP effect on Rheumatoid arthritis
SNP effect on X−25422 levels

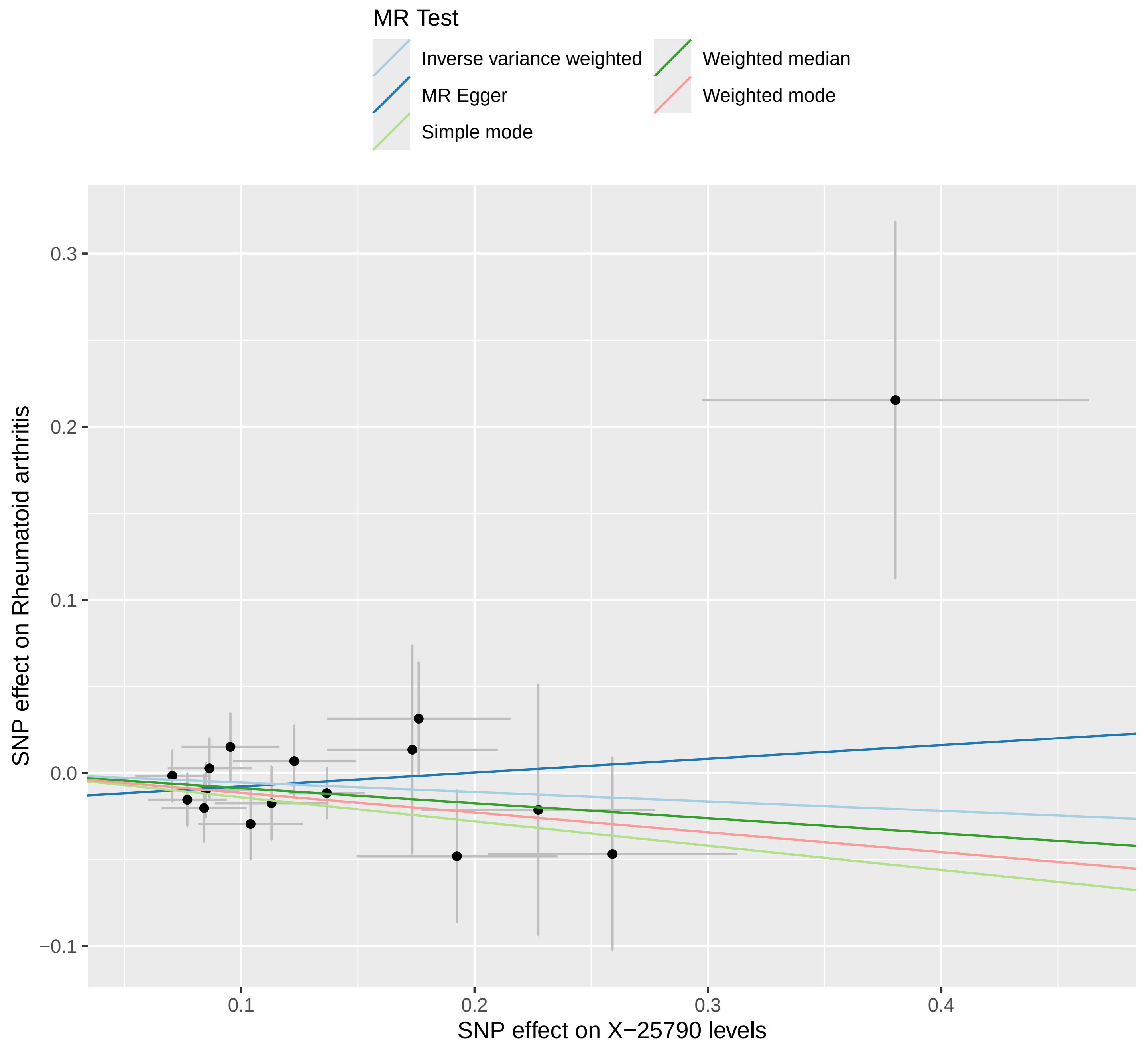

MR Test
Inverse variance weighted
MR Egger
Simple mode
Weighted median
Weighted mode
SNP effect on Rheumatoid arthritis
0.3
0.2
0.1
0.0
−0.1
0.1
0.2
0.3
0.4
SNP effect on X−25790 levels

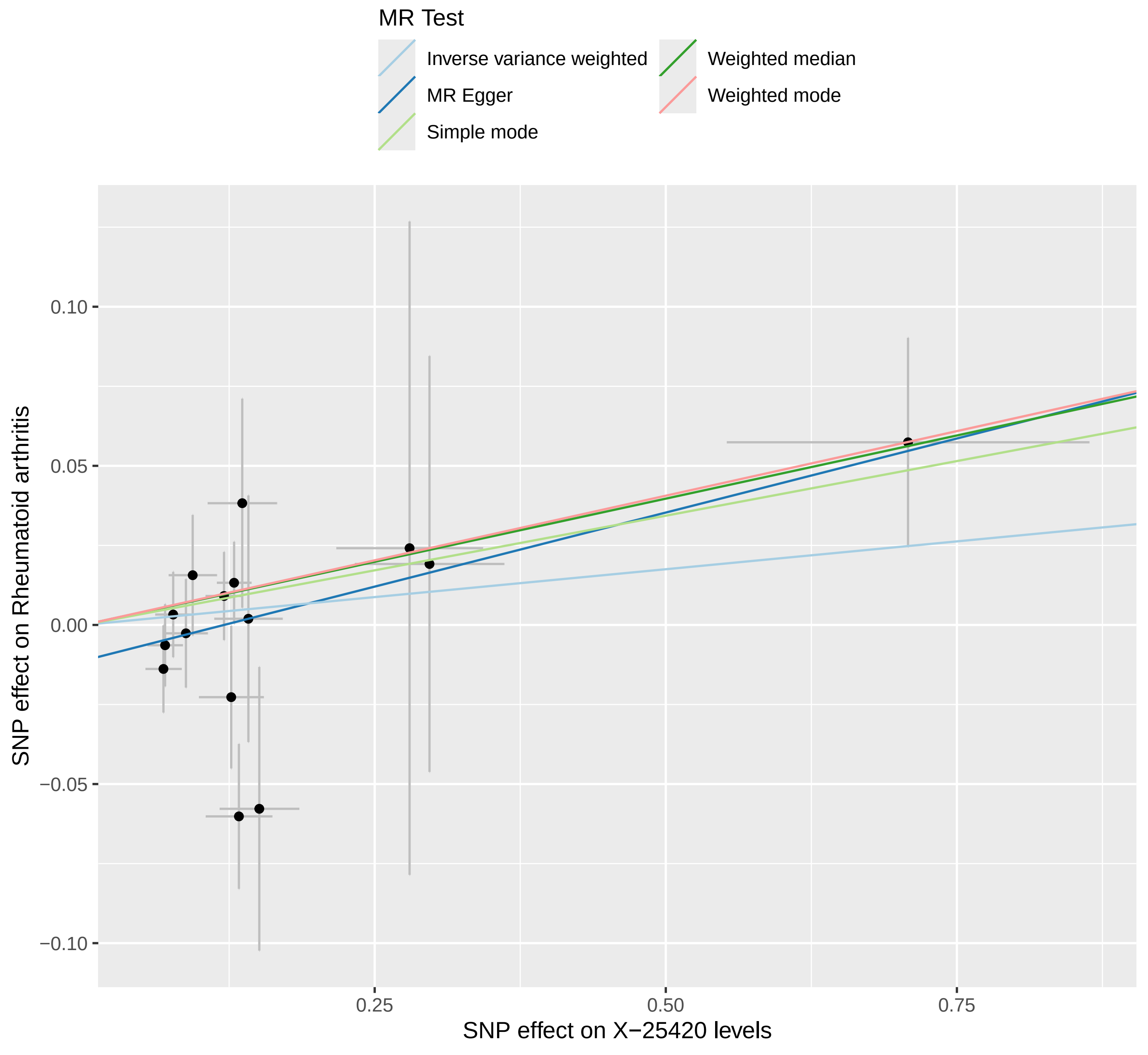
MR Test
Inverse variance weighted
MR Egger
Simple mode
Weighted median
Weighted mode
0.10
0.05
0.00
−0.05
−0.10
SNP effect on Rheumatoid arthritis
0.25
0.50
0.75
SNP effect on X−25420 levels

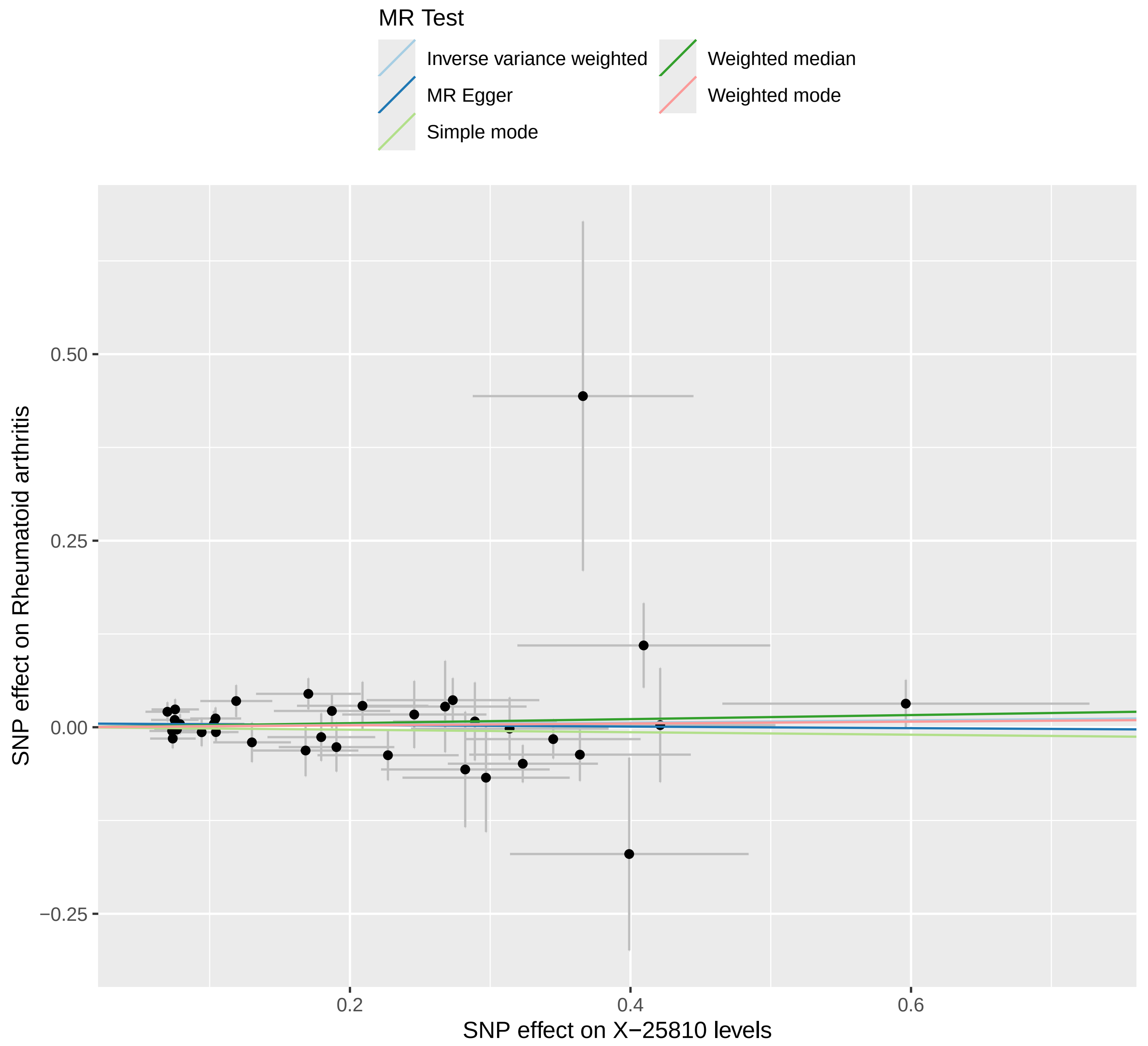
MR Test
Inverse variance weighted
MR Egger
Simple mode
Weighted median
Weighted mode
0.50
0.25
0.00
−0.25
0.2
0.4
0.6
SNP effect on Rheumatoid arthritis
SNP effect on X−25810 levels

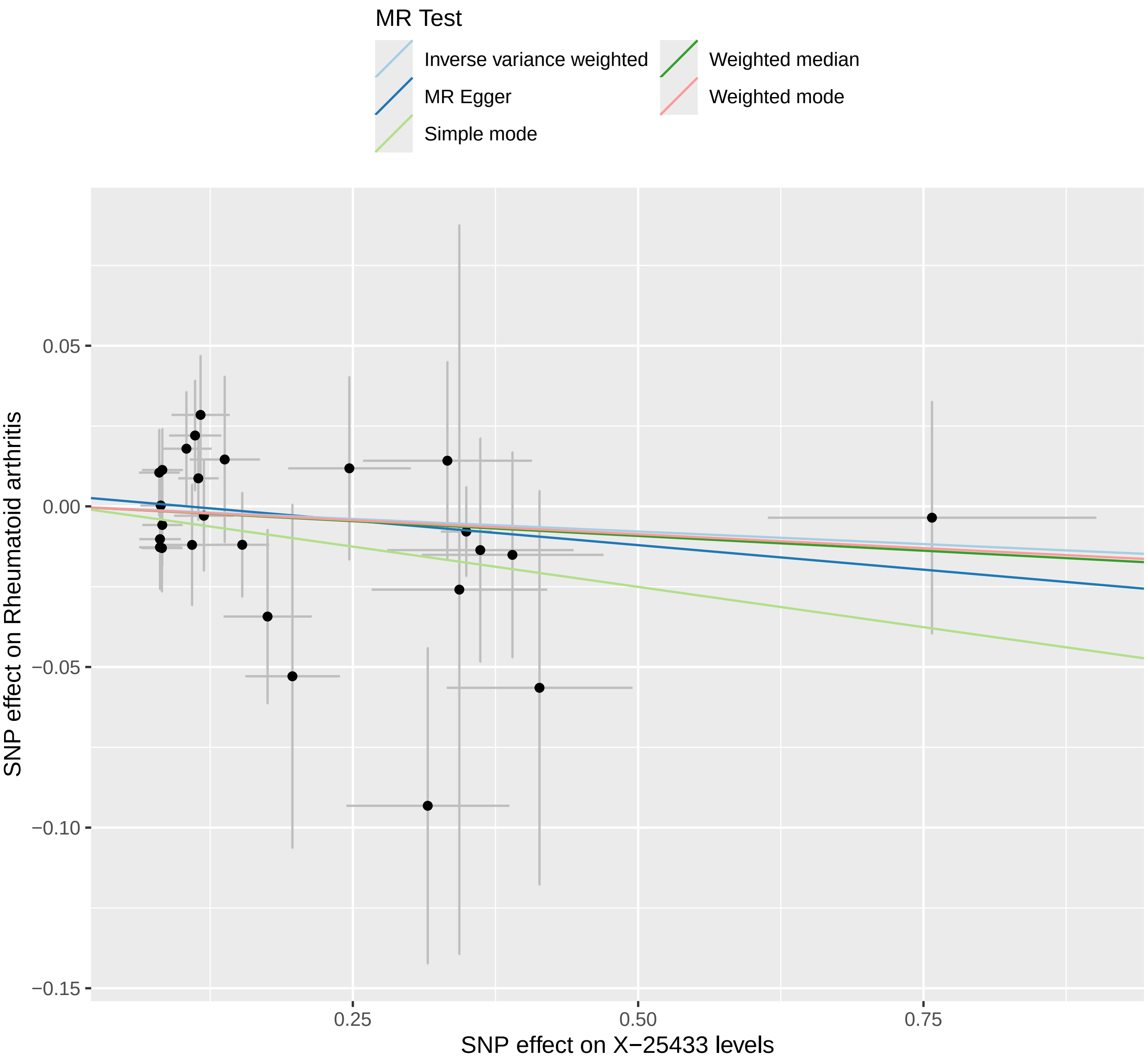

MR Test
Inverse variance weighted
MR Egger
Simple mode
Weighted median
Weighted mode
0.05
0.00
−0.05
−0.10
−0.15
SNP effect on Rheumatoid arthritis
0.25
0.50
0.75
SNP effect on X−25433 levels

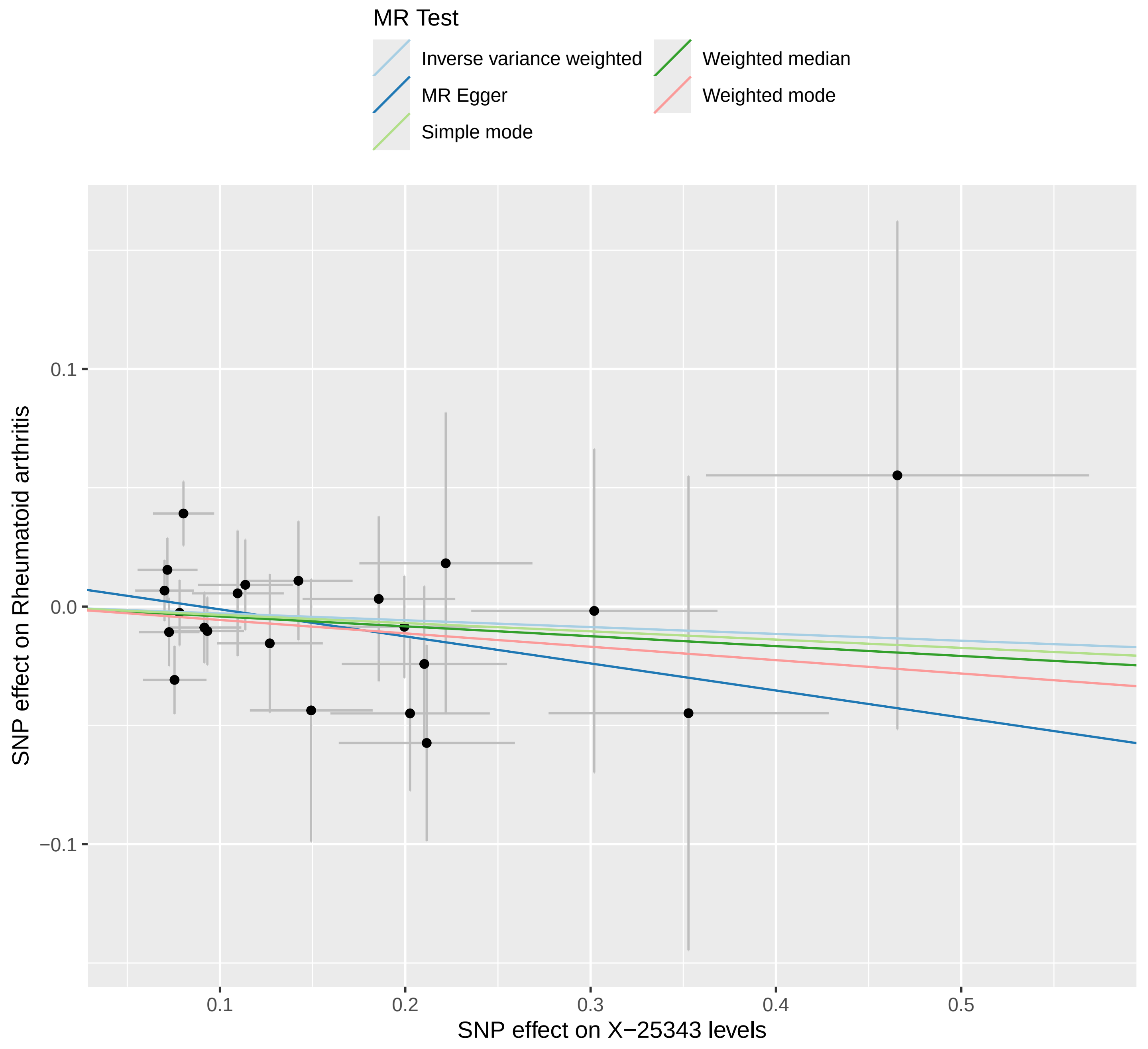
MR Test
Inverse variance weighted
MR Egger
Simple mode
Weighted median
Weighted mode
0.1
0.0
−0.1
SNP effect on Rheumatoid arthritis
0.1
0.2
0.3
0.4
0.5
SNP effect on X−25343 levels

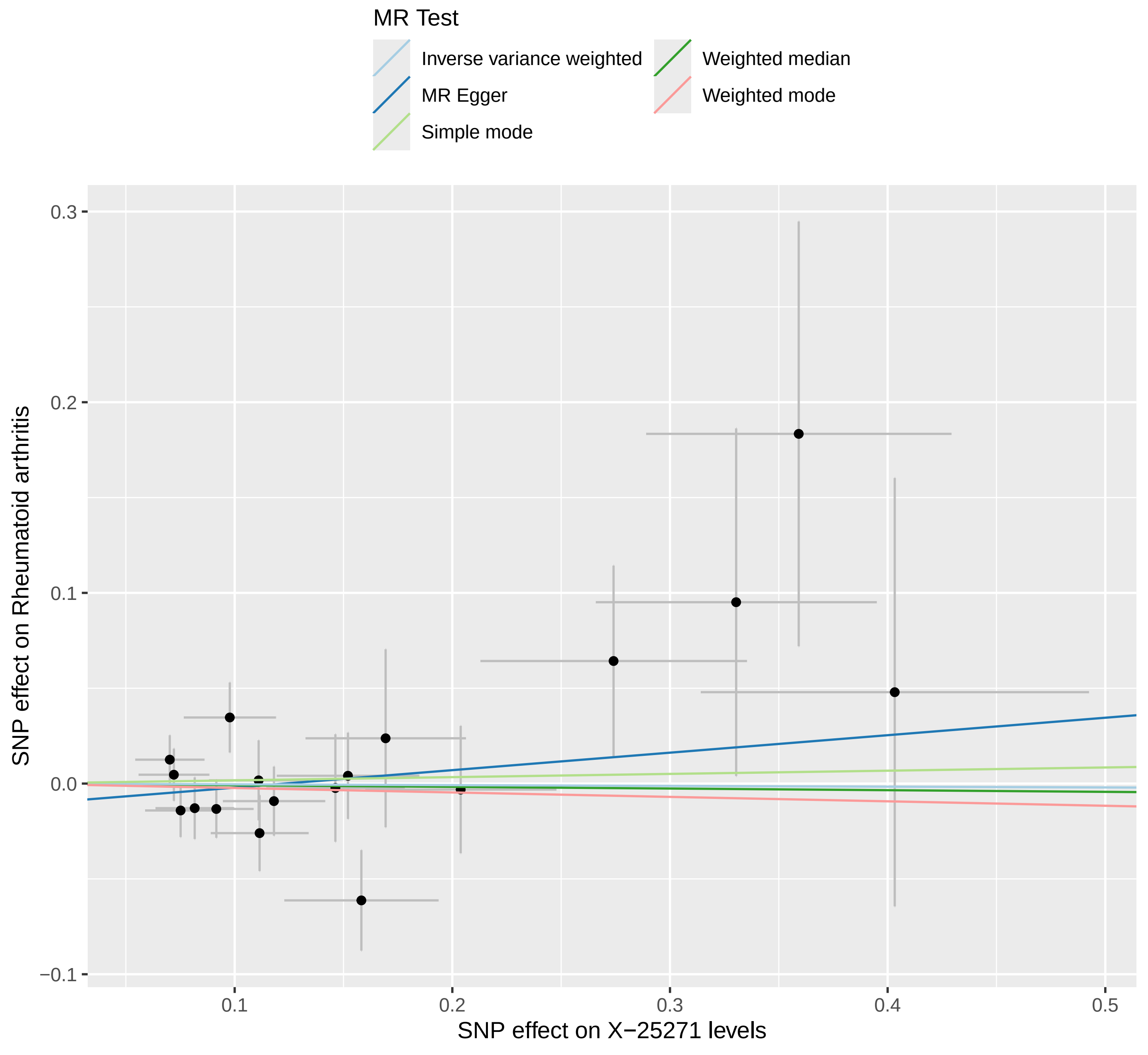

MR Test
Inverse variance weighted
MR Egger
Simple mode
Weighted median
Weighted mode
SNP effect on Rheumatoid arthritis
0.3
0.2
0.1
0.0
−0.1
0.1
0.2
0.3
0.4
0.5
SNP effect on X−25271 levels

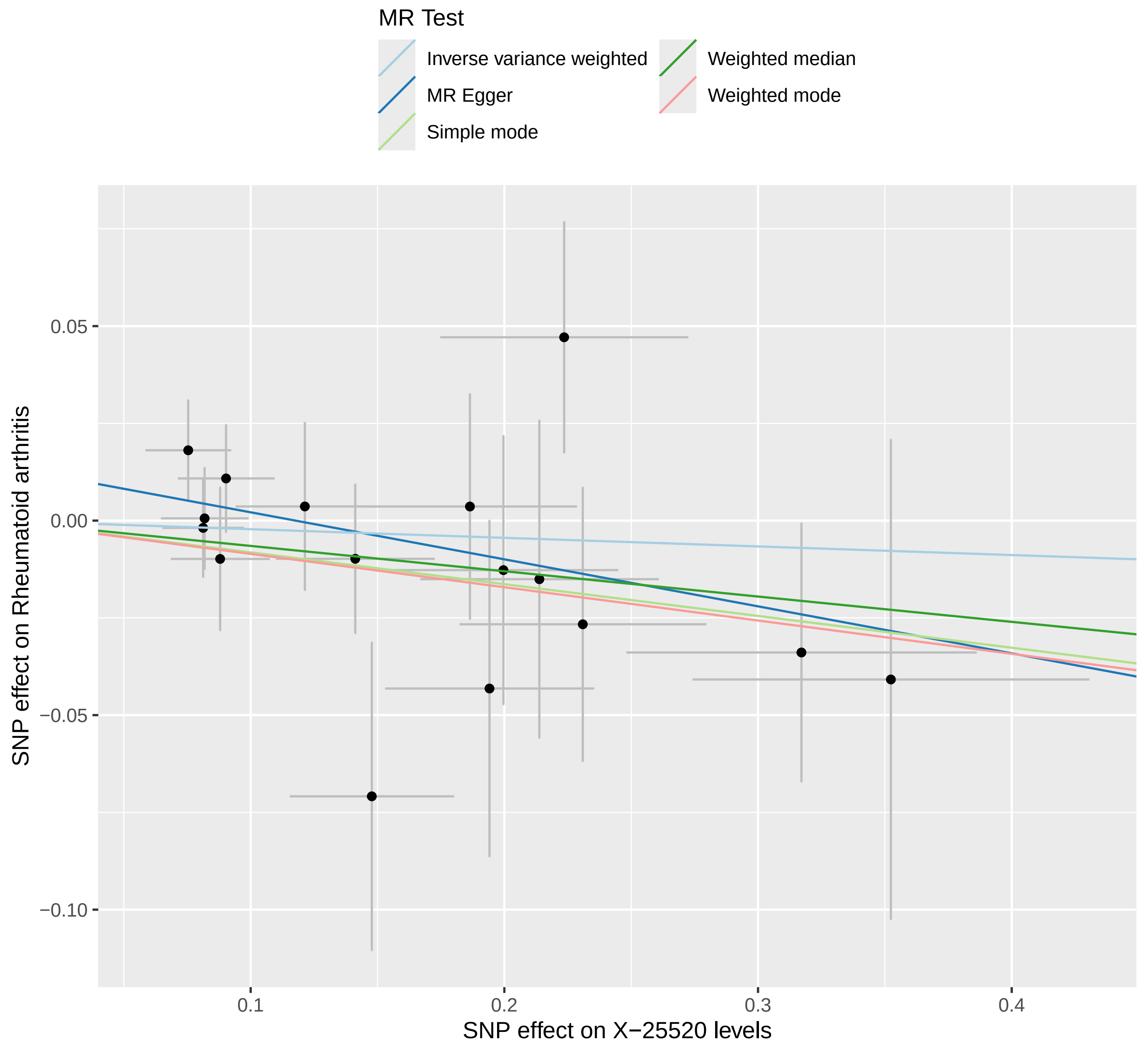
MR Test
Inverse variance weighted
MR Egger
Simple mode
Weighted median
Weighted mode
0.05
0.00
−0.05
−0.10
0.1
0.2
0.3
0.4
SNP effect on Rheumatoid arthritis
SNP effect on X−25520 levels

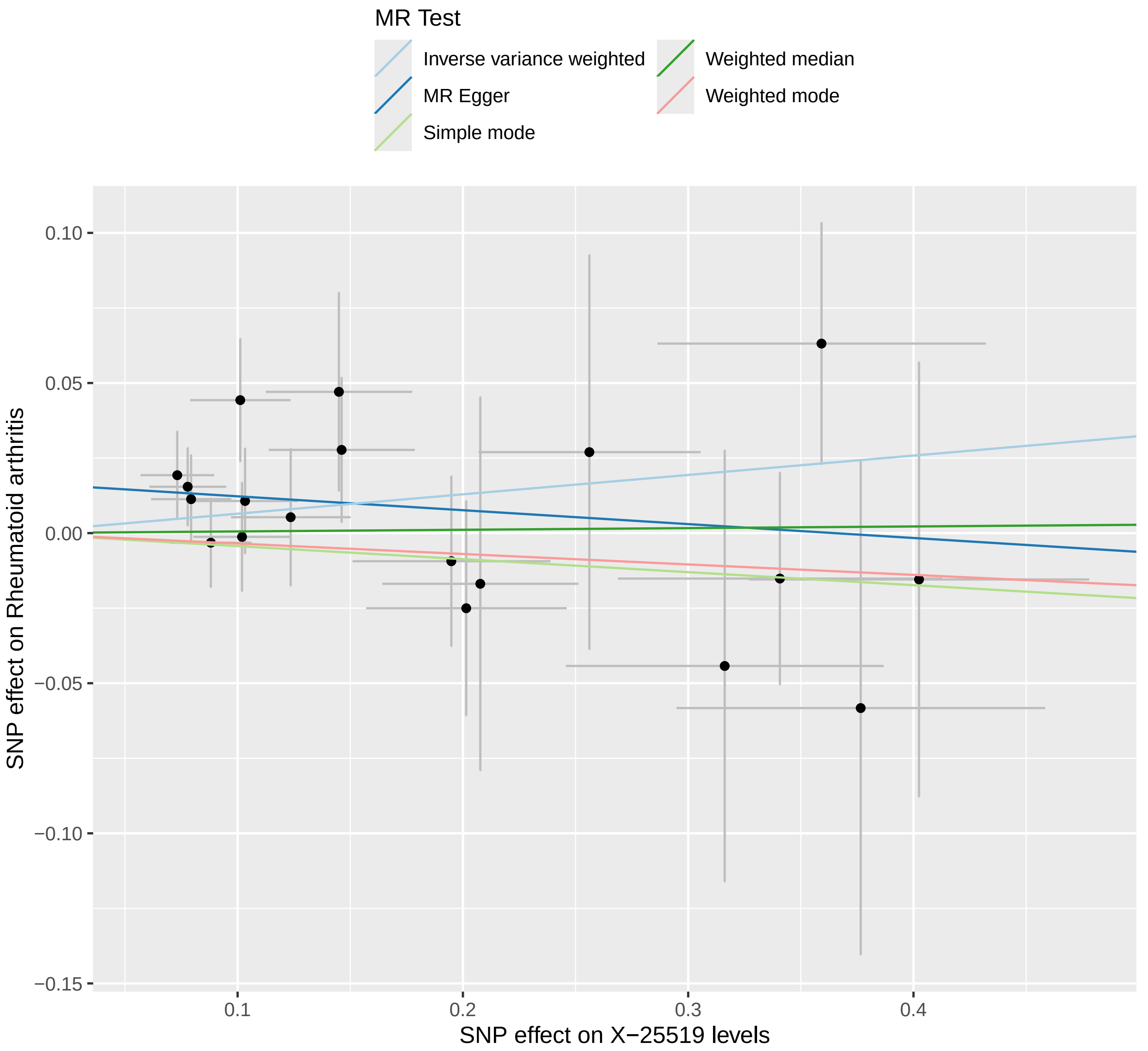
MR Test
Inverse variance weighted
MR Egger
Simple mode
Weighted median
Weighted mode
0.10
0.05
0.00
−0.05
−0.10
−0.15
SNP effect on Rheumatoid arthritis
0.1
0.2
0.3
0.4
SNP effect on X−25519 levels

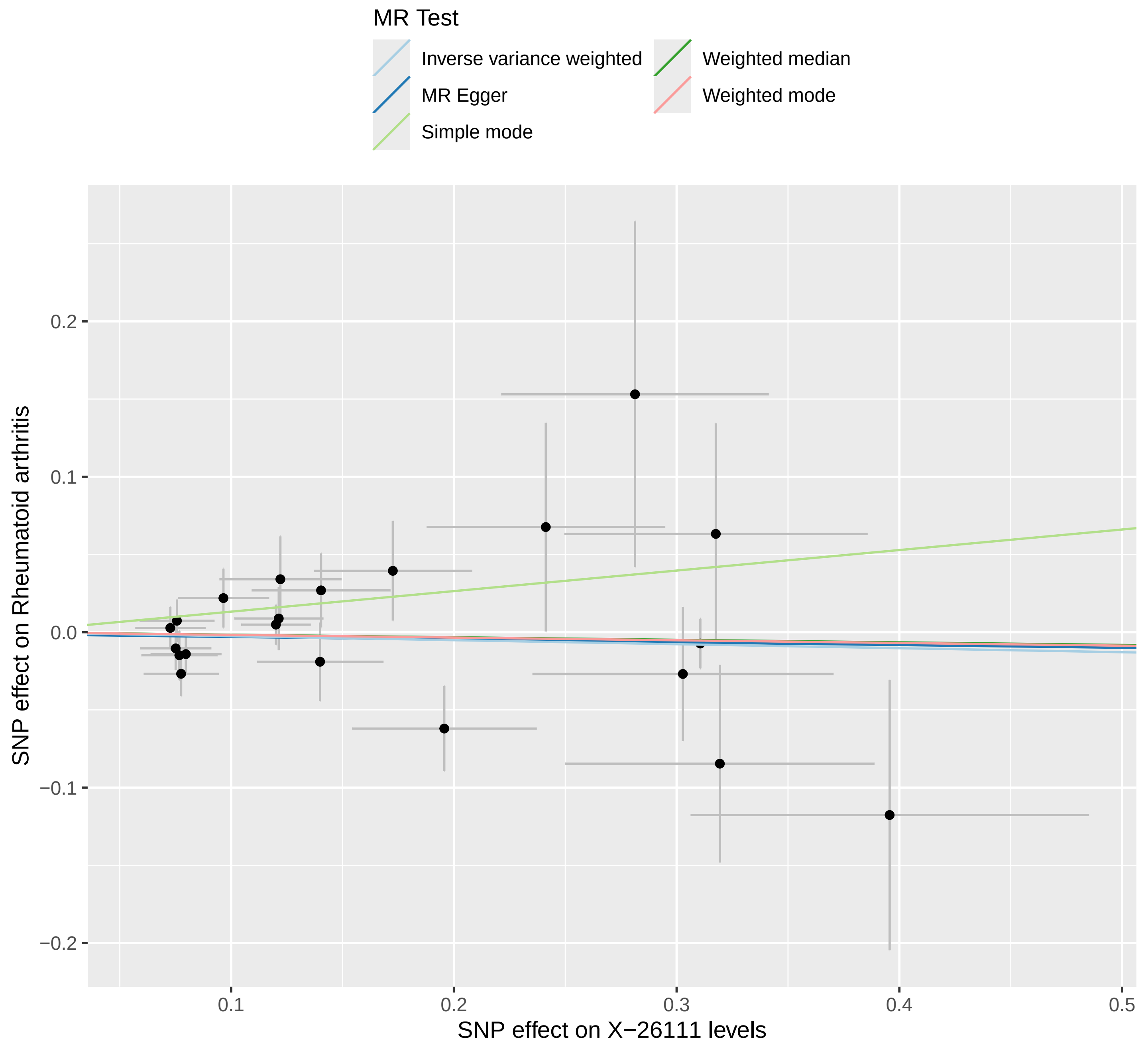
MR Test
Inverse variance weighted
MR Egger
Simple mode
Weighted median
Weighted mode
SNP effect on Rheumatoid arthritis
SNP effect on X−26111 levels
0.2
0.1
0.0
−0.1
−0.2
0.1
0.2
0.3
0.4
0.5

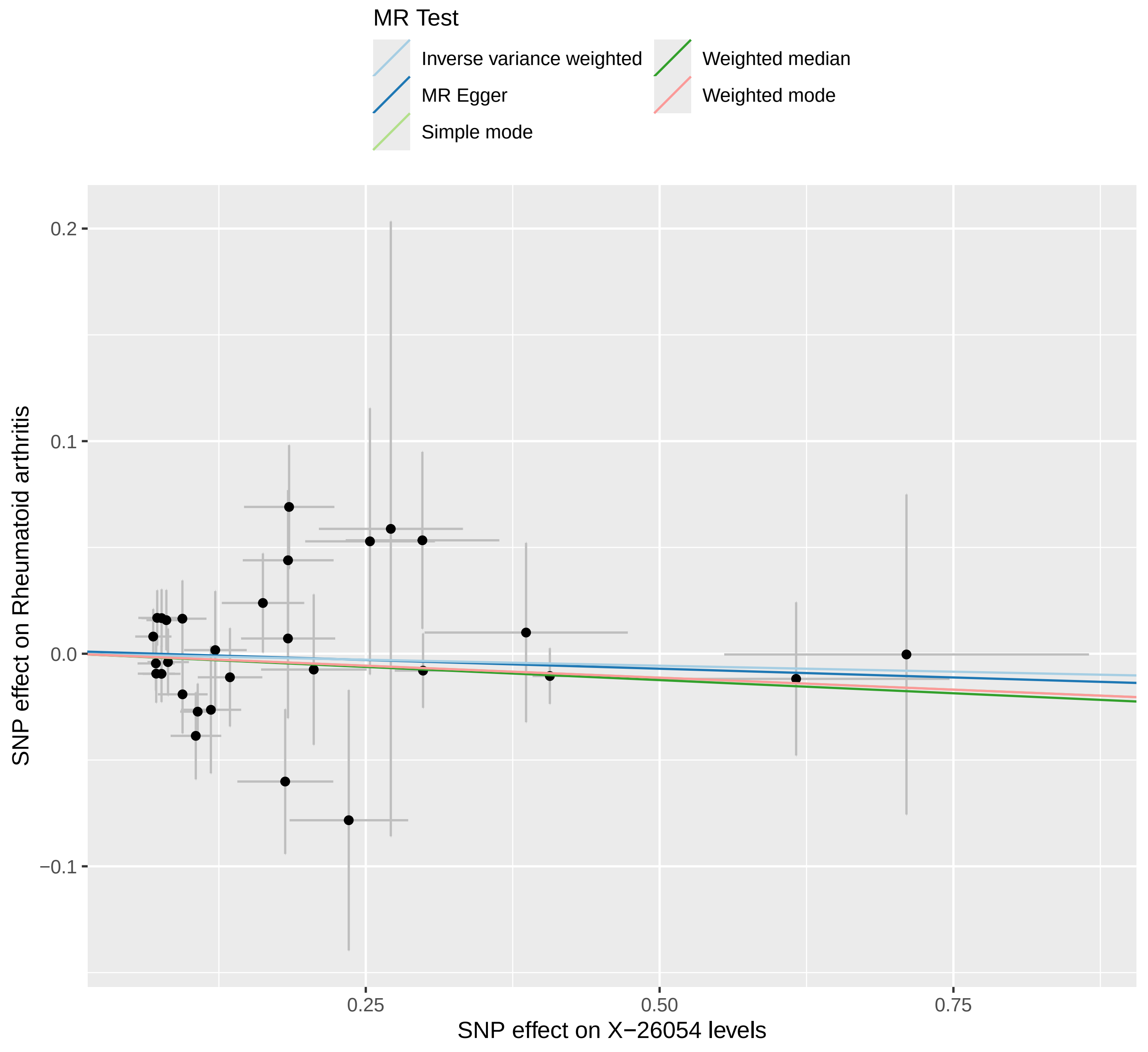

MR Test
Inverse variance weighted
MR Egger
Simple mode
Weighted median
Weighted mode
0.2
0.1
0.0
−0.1
0.25
0.50
0.75
SNP effect on Rheumatoid arthritis
SNP effect on X−26054 levels

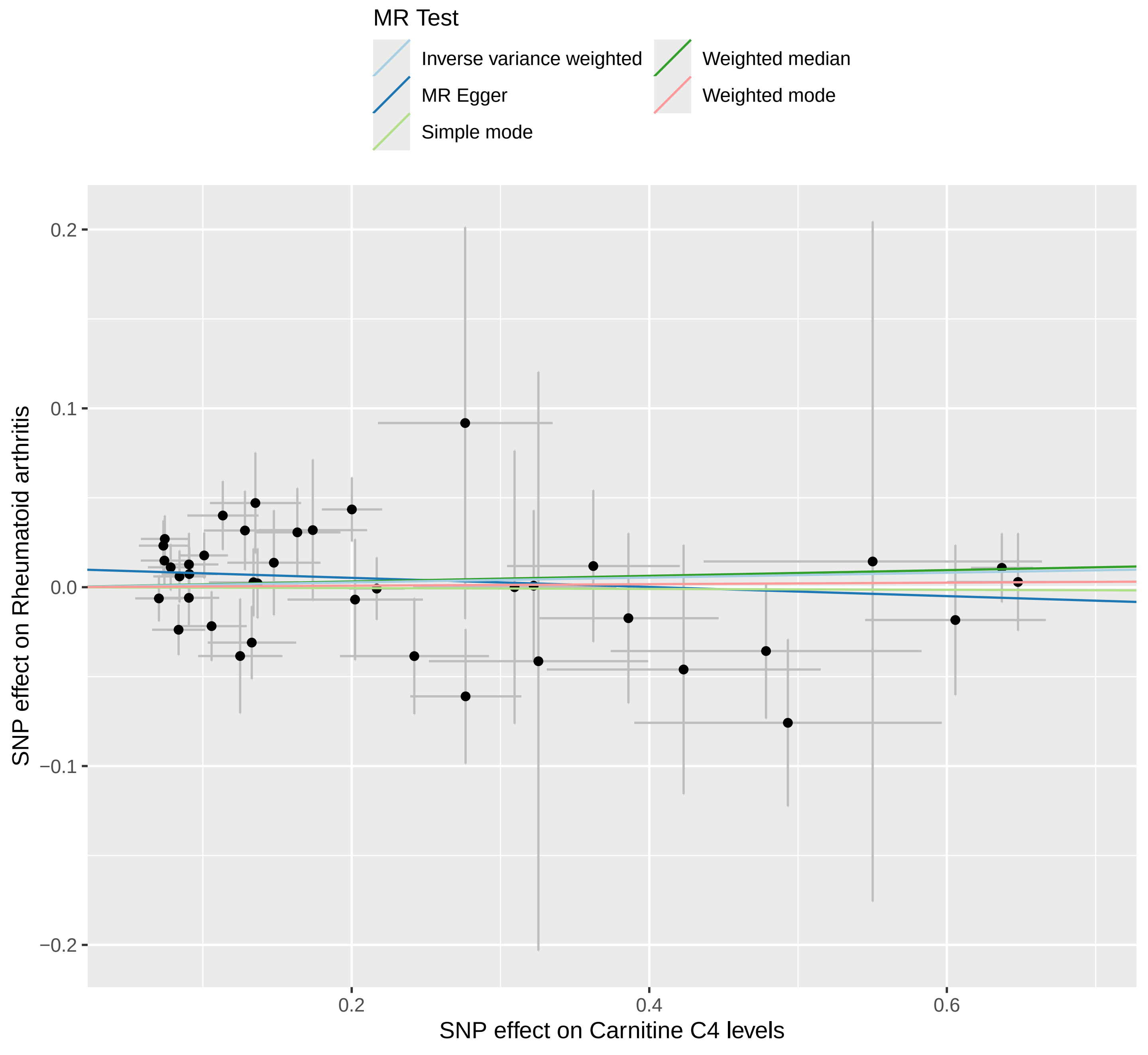
MR Test
Inverse variance weighted
MR Egger
Simple mode
Weighted median
Weighted mode
SNP effect on Rheumatoid arthritis
0.2
0.1
0.0
−0.1
−0.2
0.2
0.4
0.6
SNP effect on Carnitine C4 levels

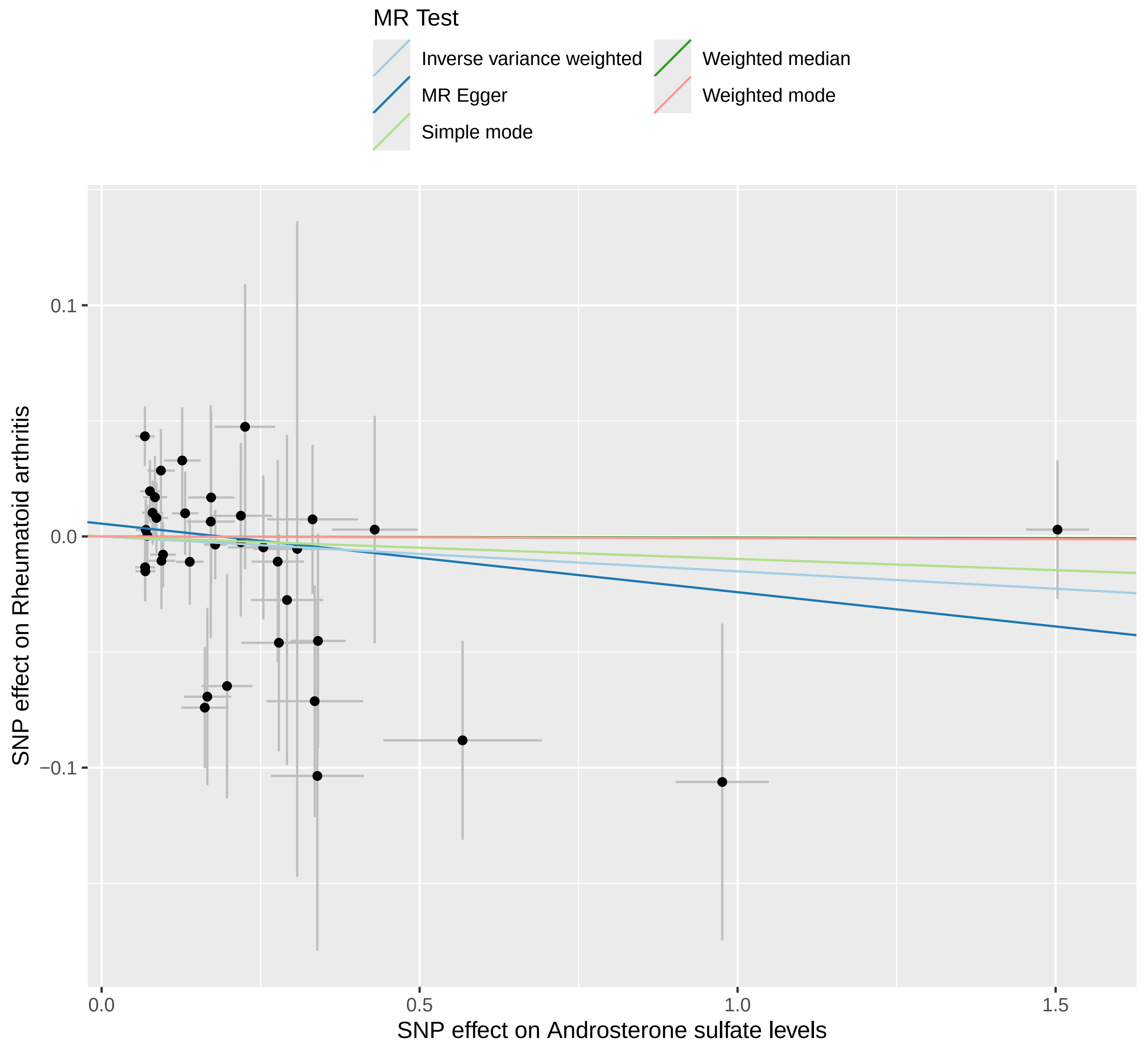

MR Test
Inverse variance weighted
MR Egger
Simple mode
Weighted median
Weighted mode
0.1
0.0
−0.1
SNP effect on Rheumatoid arthritis
0.0
0.5
1.0
1.5
SNP effect on Androsterone sulfate levels

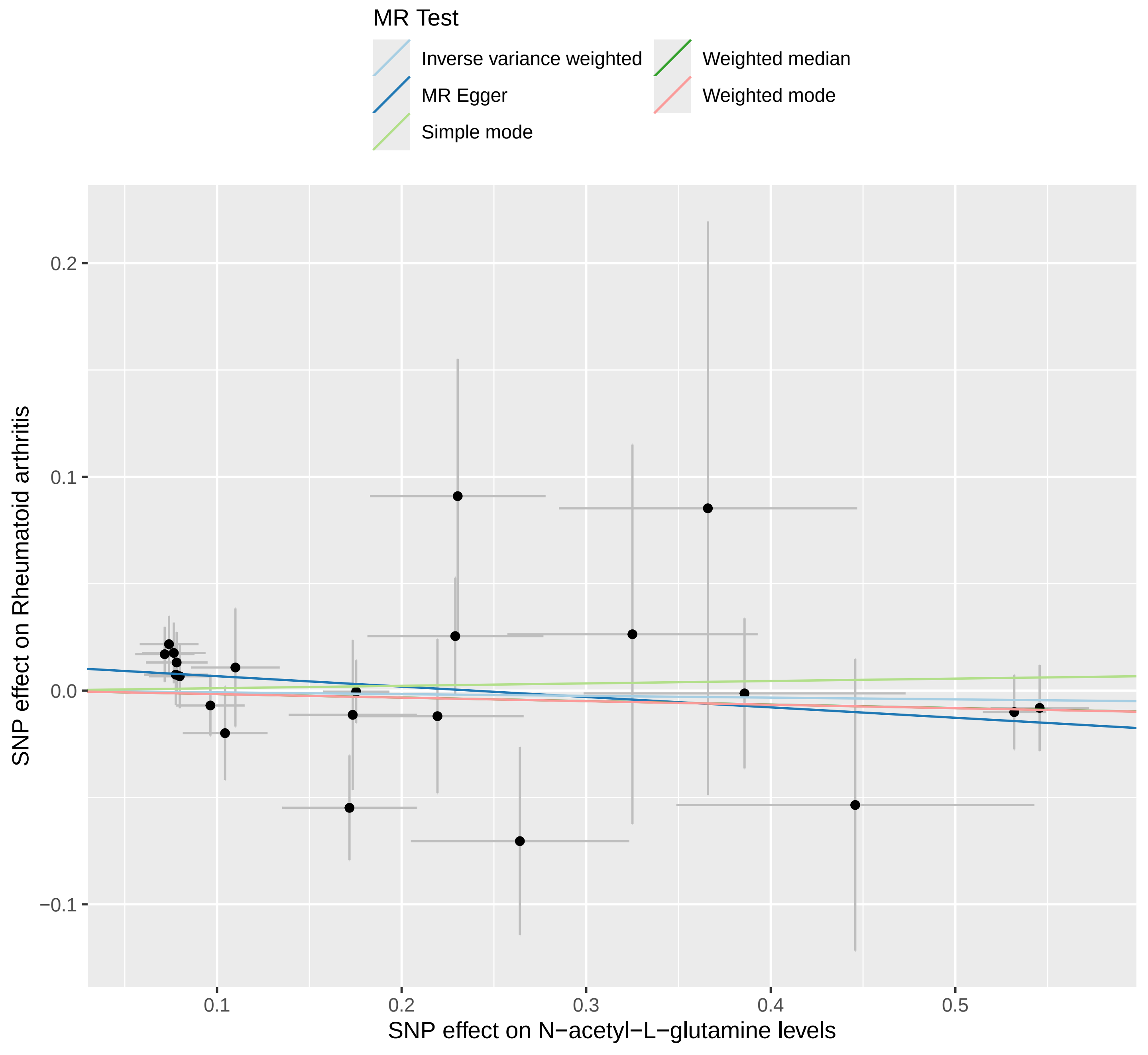
MR Test
Inverse variance weighted
MR Egger
Simple mode
Weighted median
Weighted mode
0.2
0.1
0.0
−0.1
0.1
0.2
0.3
0.4
0.5
SNP effect on Rheumatoid arthritis
SNP effect on N−acetyl−L−glutamine levels

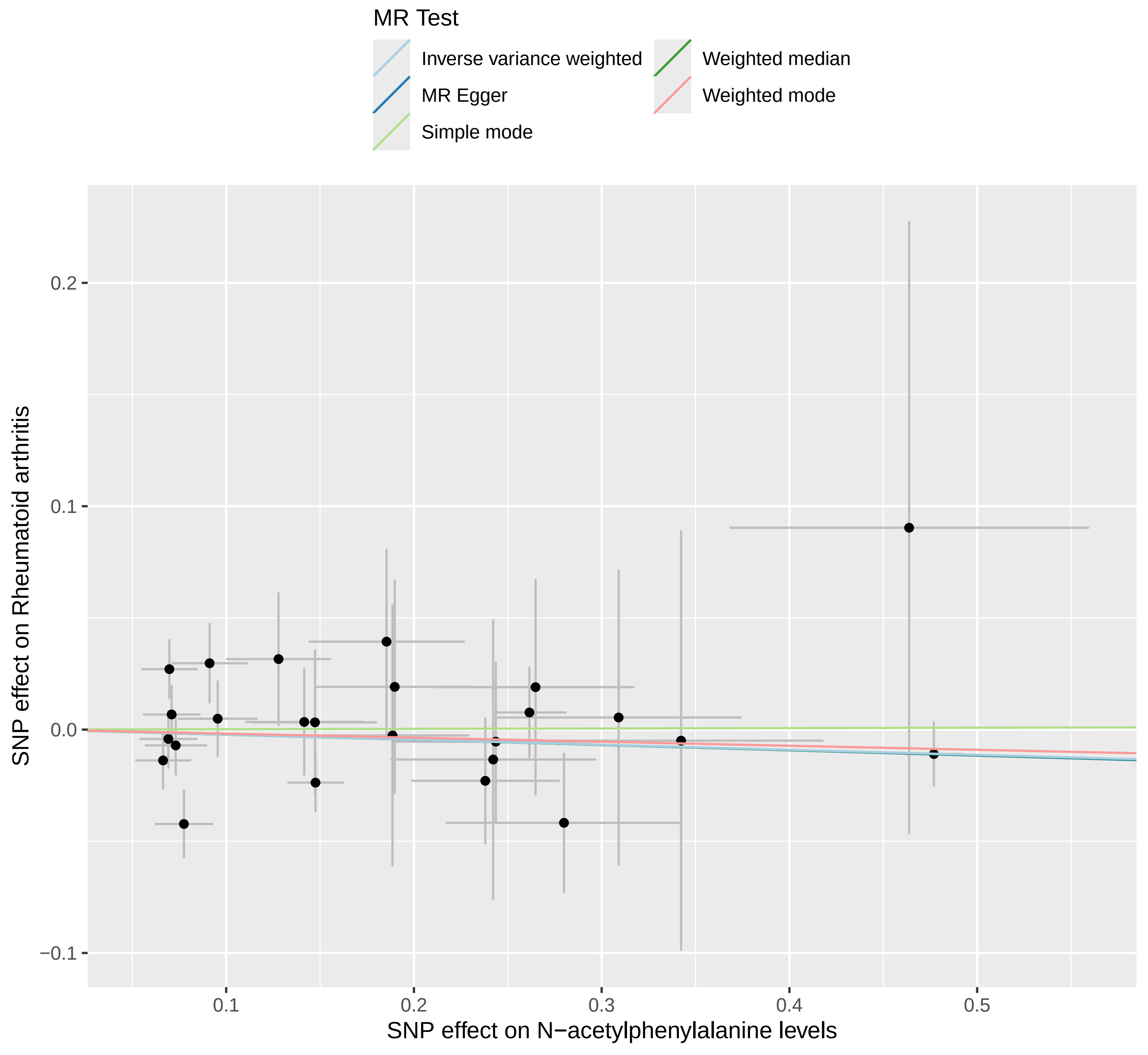
MR Test
Inverse variance weighted
MR Egger
Simple mode
Weighted median
Weighted mode
0.2
0.1
0.0
−0.1
0.1
0.2
0.3
0.4
0.5
SNP effect on Rheumatoid arthritis
SNP effect on N−acetylphenylalanine levels

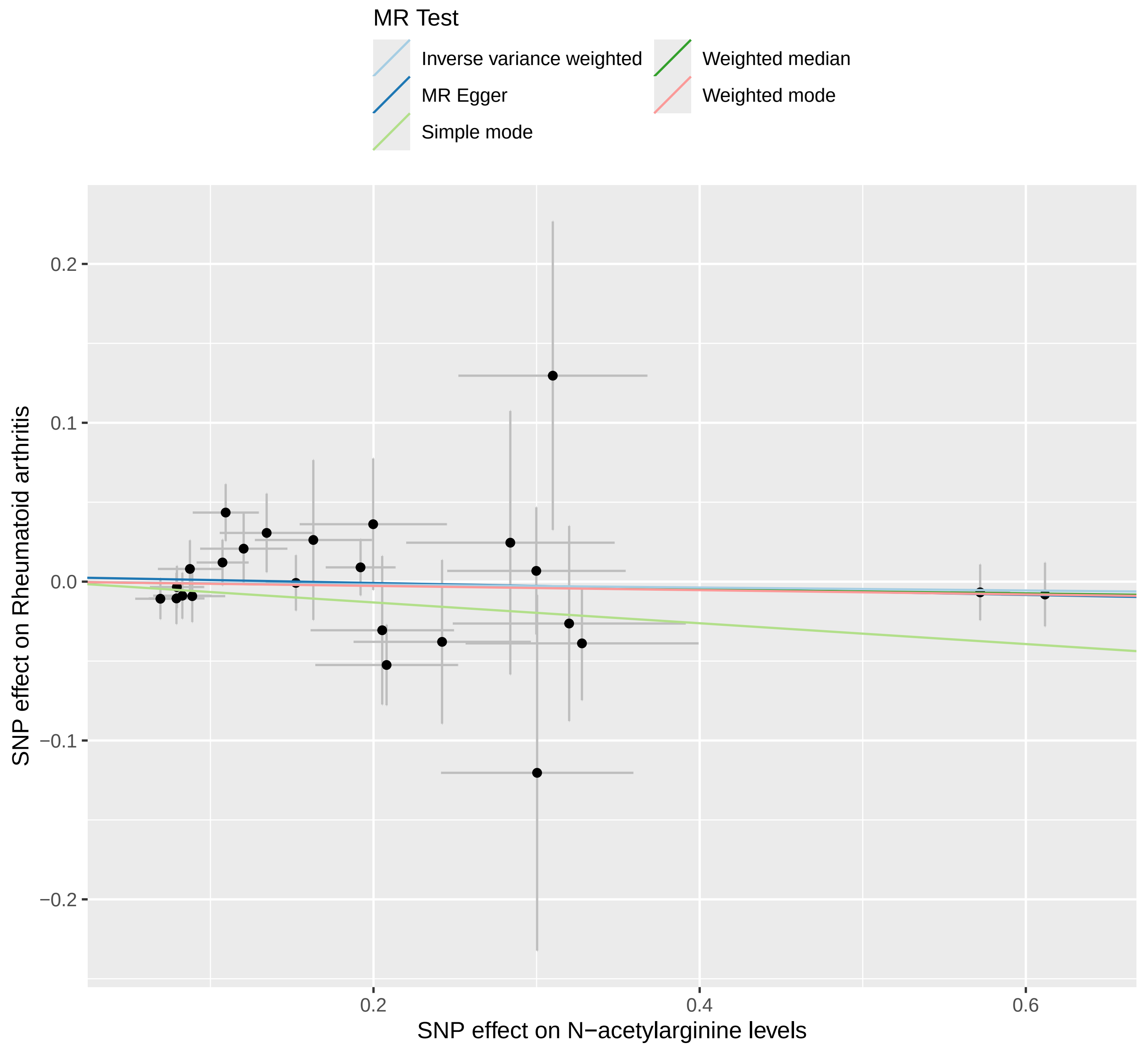
MR Test
Inverse variance weighted
MR Egger
Simple mode
Weighted median
Weighted mode
0.2
0.1
0.0
−0.1
−0.2
0.2
0.4
0.6
SNP effect on Rheumatoid arthritis
SNP effect on N−acetylarginine levels

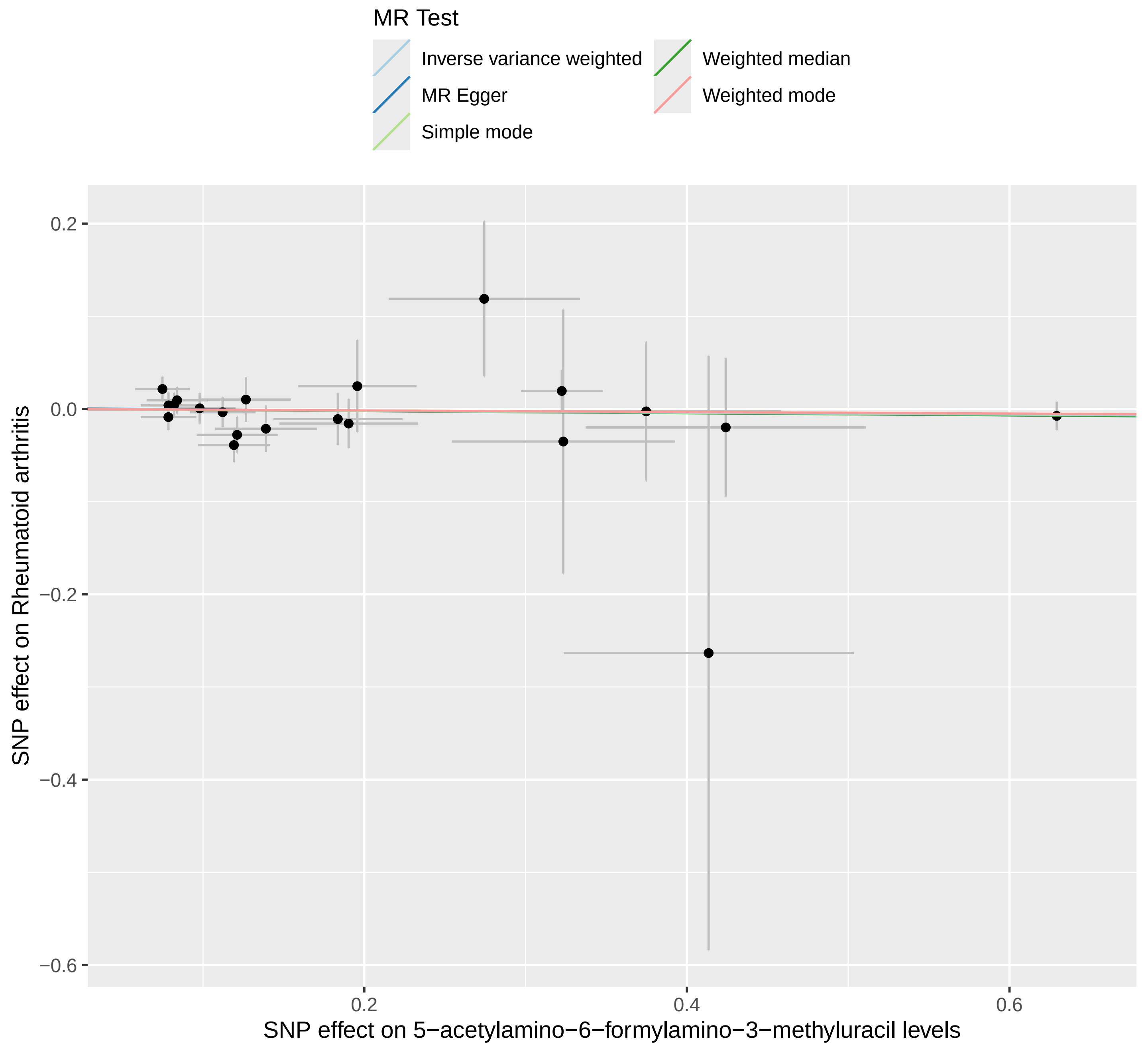
MR Test
Inverse variance weighted
MR Egger
Simple mode
Weighted median
Weighted mode
0.2
0.0
−0.2
−0.4
−0.6
SNP effect on Rheumatoid arthritis
0.2
0.4
0.6
SNP effect on 5−acetylamino−6−formylamino−3−methyluracil levels

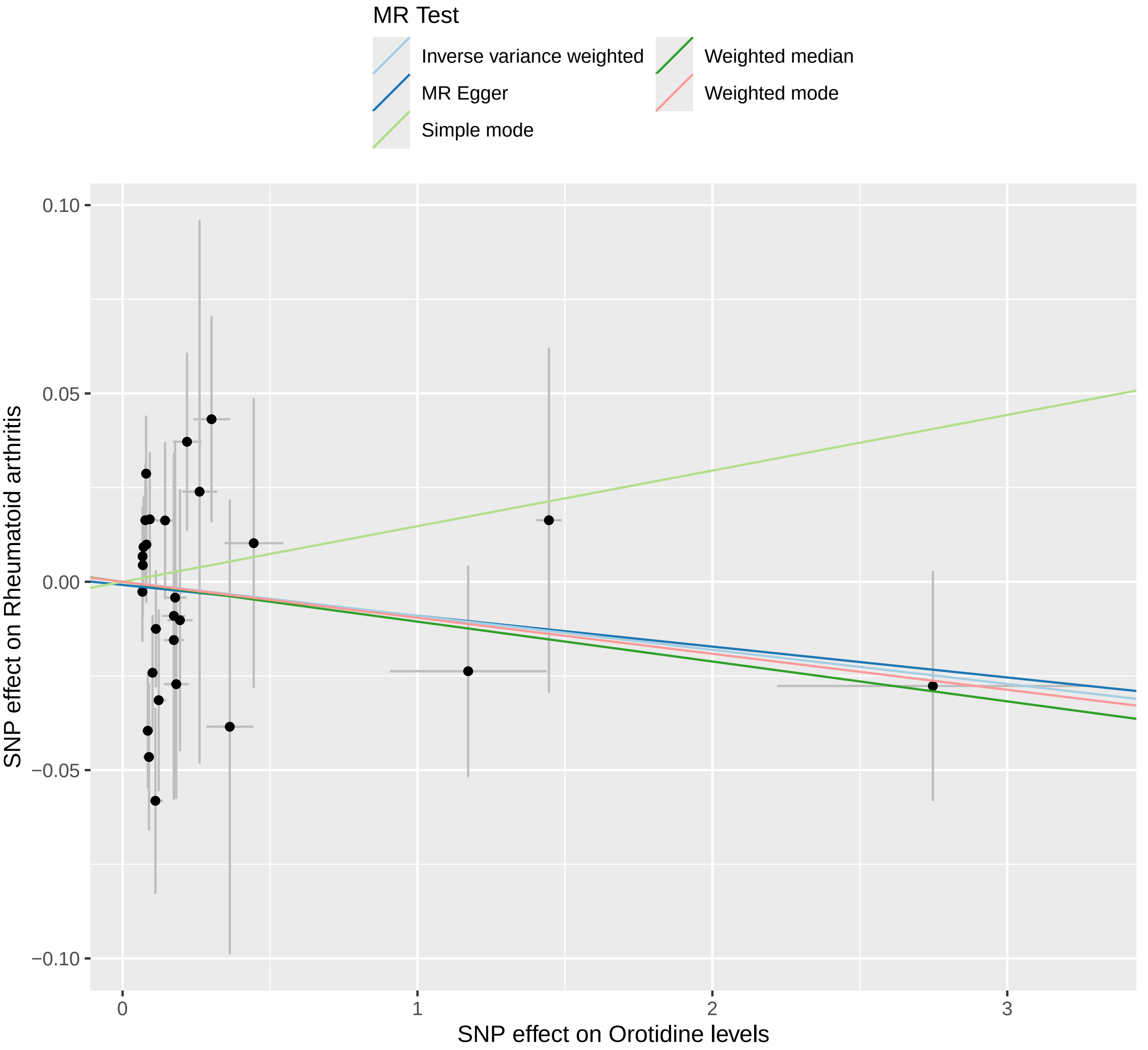
MR Test
Inverse variance weighted
MR Egger
Simple mode
Weighted median
Weighted mode
0.10
0.05
0.00
−0.05
−0.10
0
1
2
3
SNP effect on Rheumatoid arthritis
SNP effect on Orotidine levels

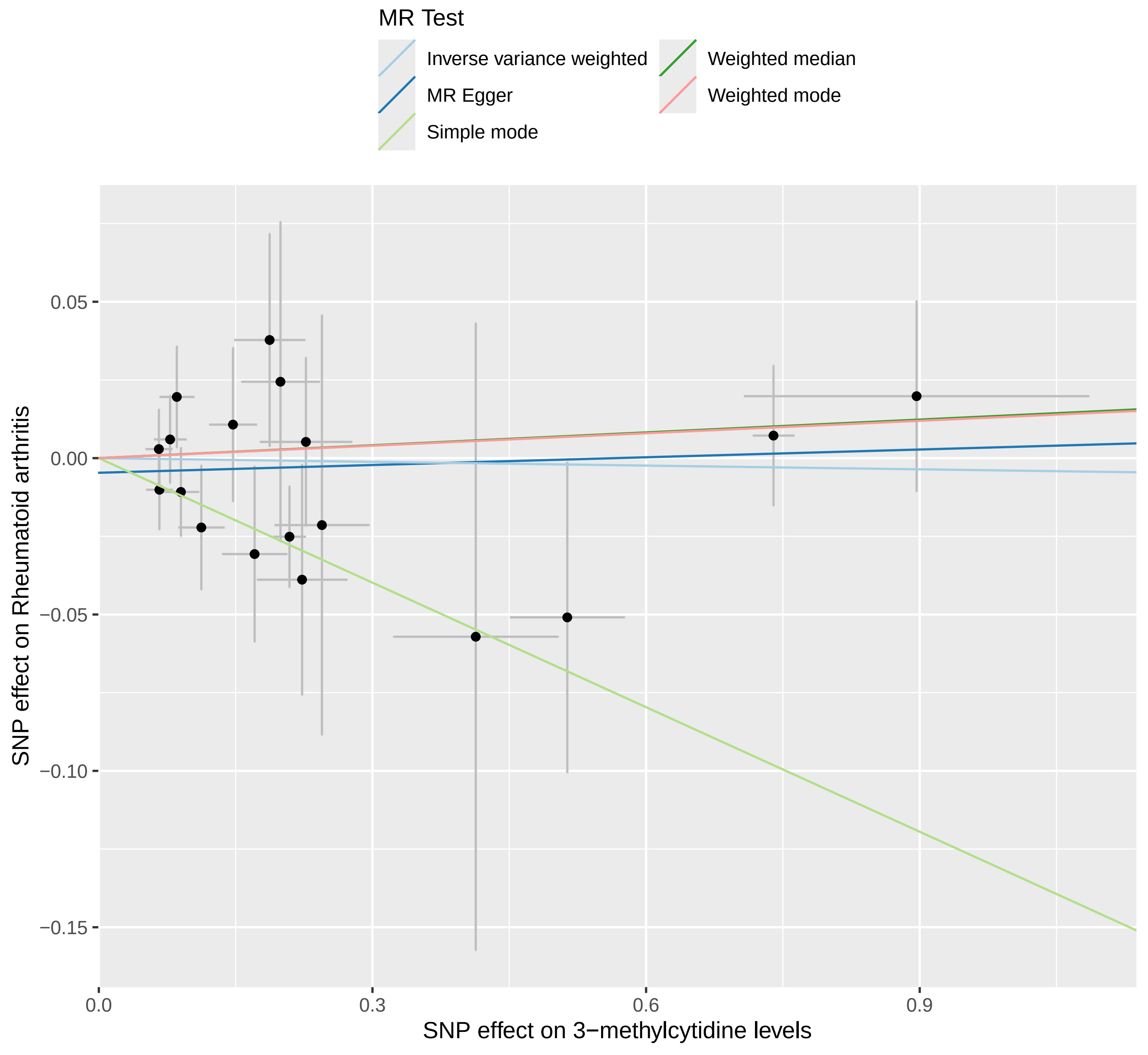
MR Test
Inverse variance weighted
MR Egger
Simple mode
Weighted median
Weighted mode
0.05
0.00
−0.05
−0.10
−0.15
0.0
0.3
0.6
0.9
SNP effect on 3−methylcytidine levels
SNP effect on Rheumatoid arthritis

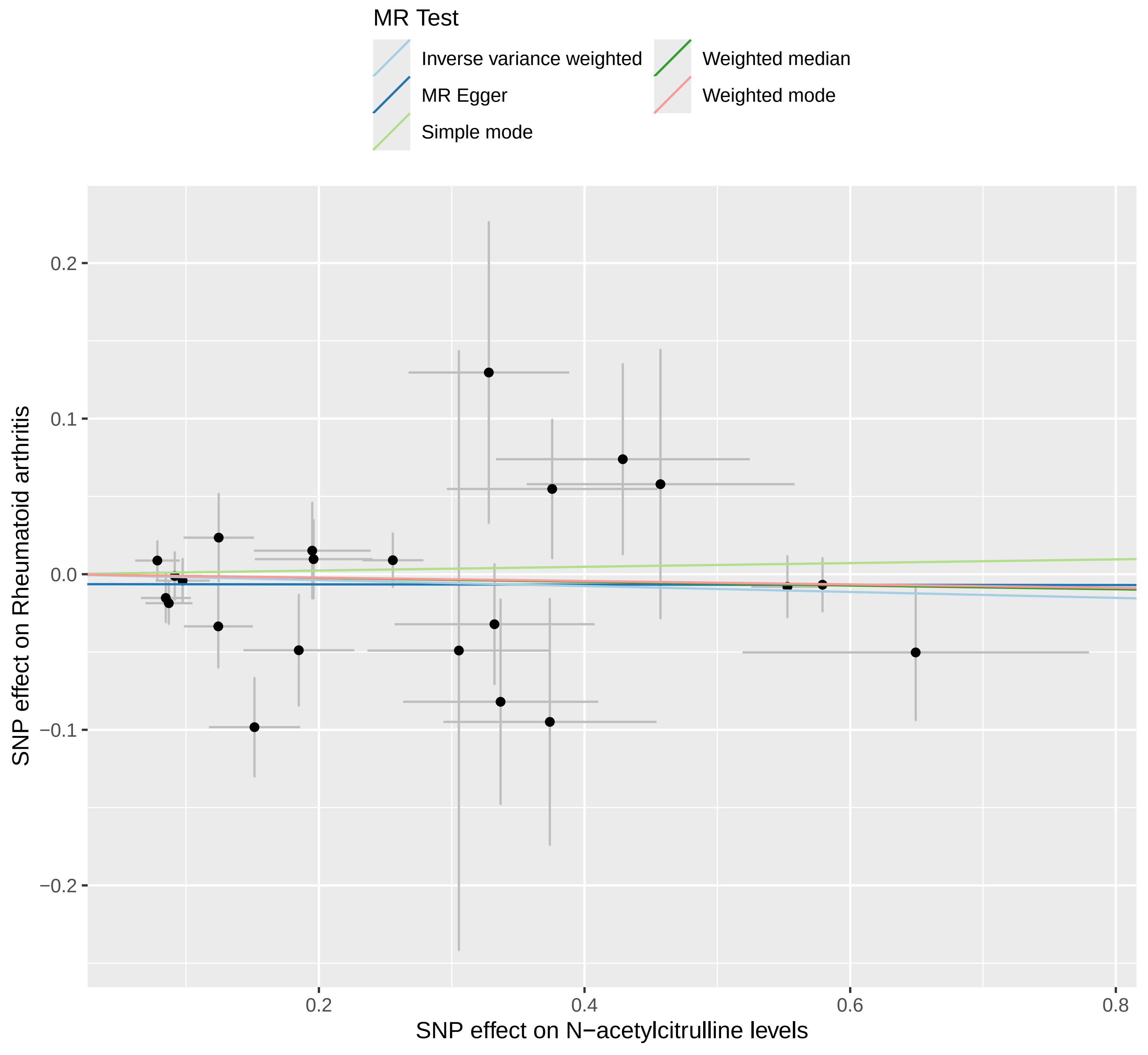

MR Test
Inverse variance weighted
MR Egger
Simple mode
Weighted median
Weighted mode
SNP effect on Rheumatoid arthritis
0.2
0.1
0.0
−0.1
−0.2
0.2
0.4
0.6
0.8
SNP effect on N−acetylcitrulline levels

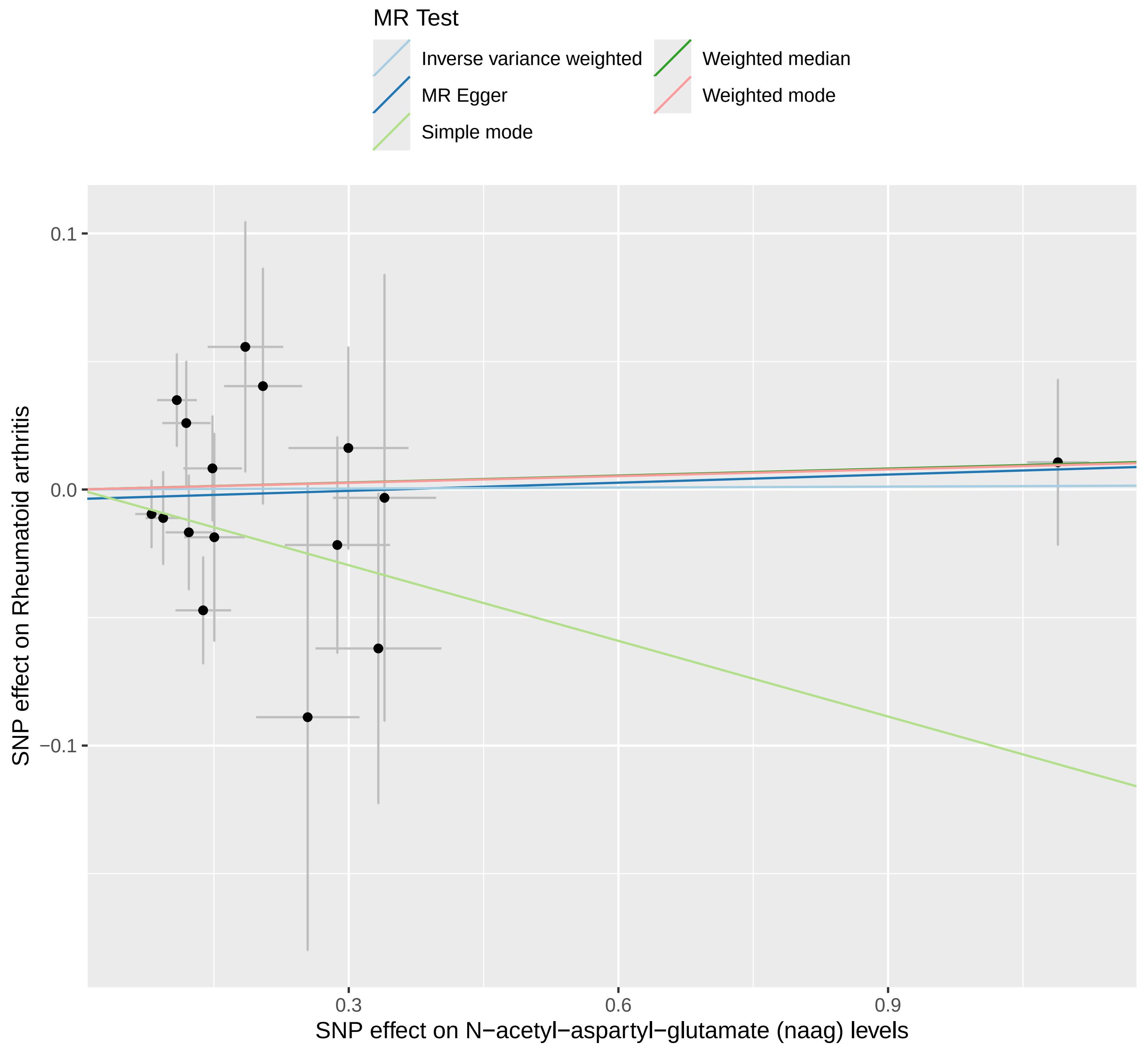

MR Test
Inverse variance weighted
MR Egger
Simple mode
Weighted median
Weighted mode
0.1
0.0
−0.1
0.3
0.6
0.9
SNP effect on Rheumatoid arthritis
SNP effect on N−acetyl−aspartyl−glutamate (naag) levels

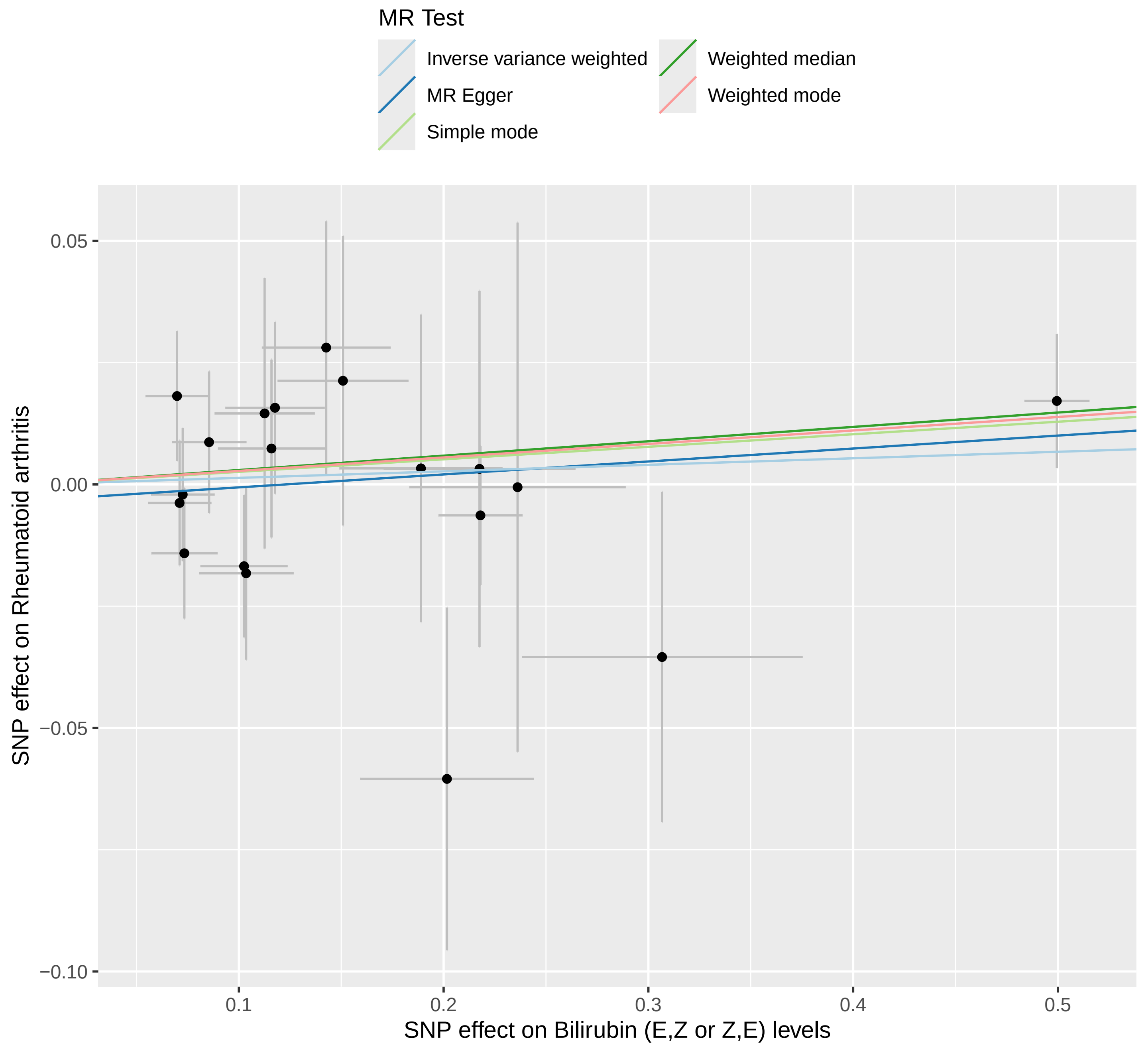
MR Test
Inverse variance weighted
MR Egger
Simple mode
Weighted median
Weighted mode
0.05
0.00
−0.05
−0.10
SNP effect on Rheumatoid arthritis
0.1
0.2
0.3
0.4
0.5
SNP effect on Bilirubin (E,Z or Z,E) levels

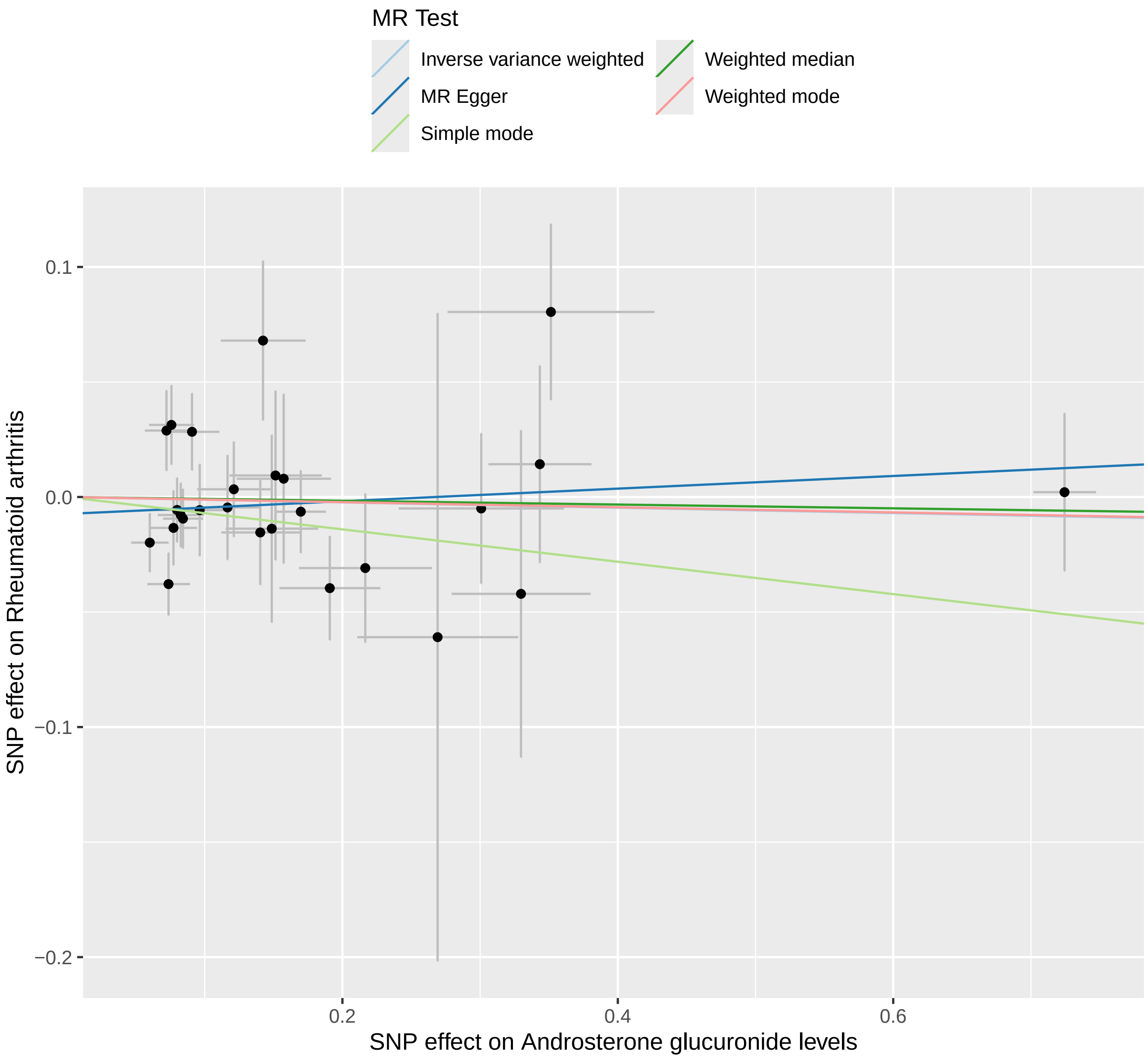
MR Test
Inverse variance weighted
MR Egger
Simple mode
Weighted median
Weighted mode
0.1
0.0
−0.1
−0.2
0.2
0.4
0.6
SNP effect on Rheumatoid arthritis
SNP effect on Androsterone glucuronide levels

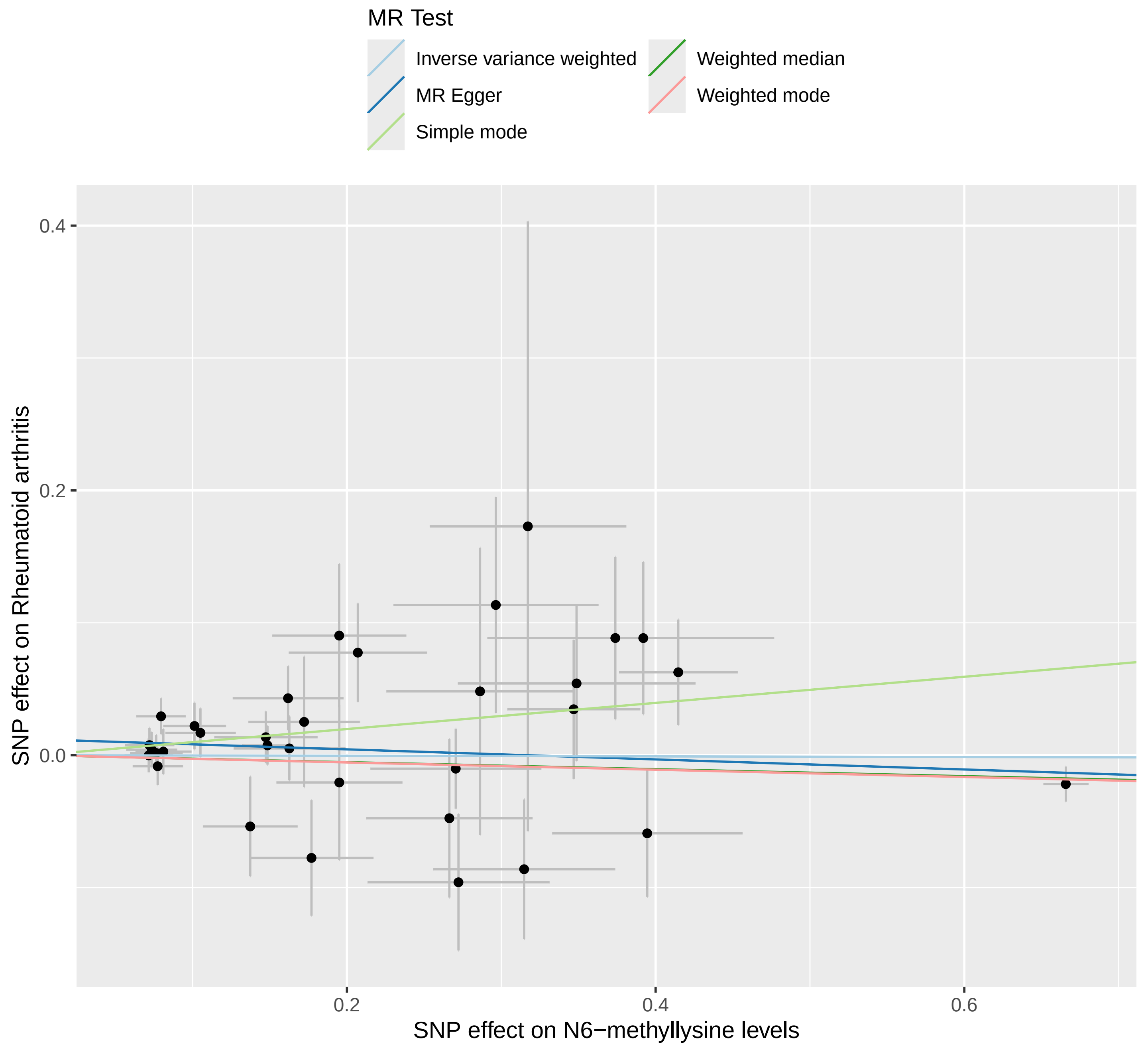

MR Test
Inverse variance weighted
MR Egger
Simple mode
Weighted median
Weighted mode
SNP effect on Rheumatoid arthritis
0.4
0.2
0.0
0.2
0.4
0.6
SNP effect on N6−methyllysine levels

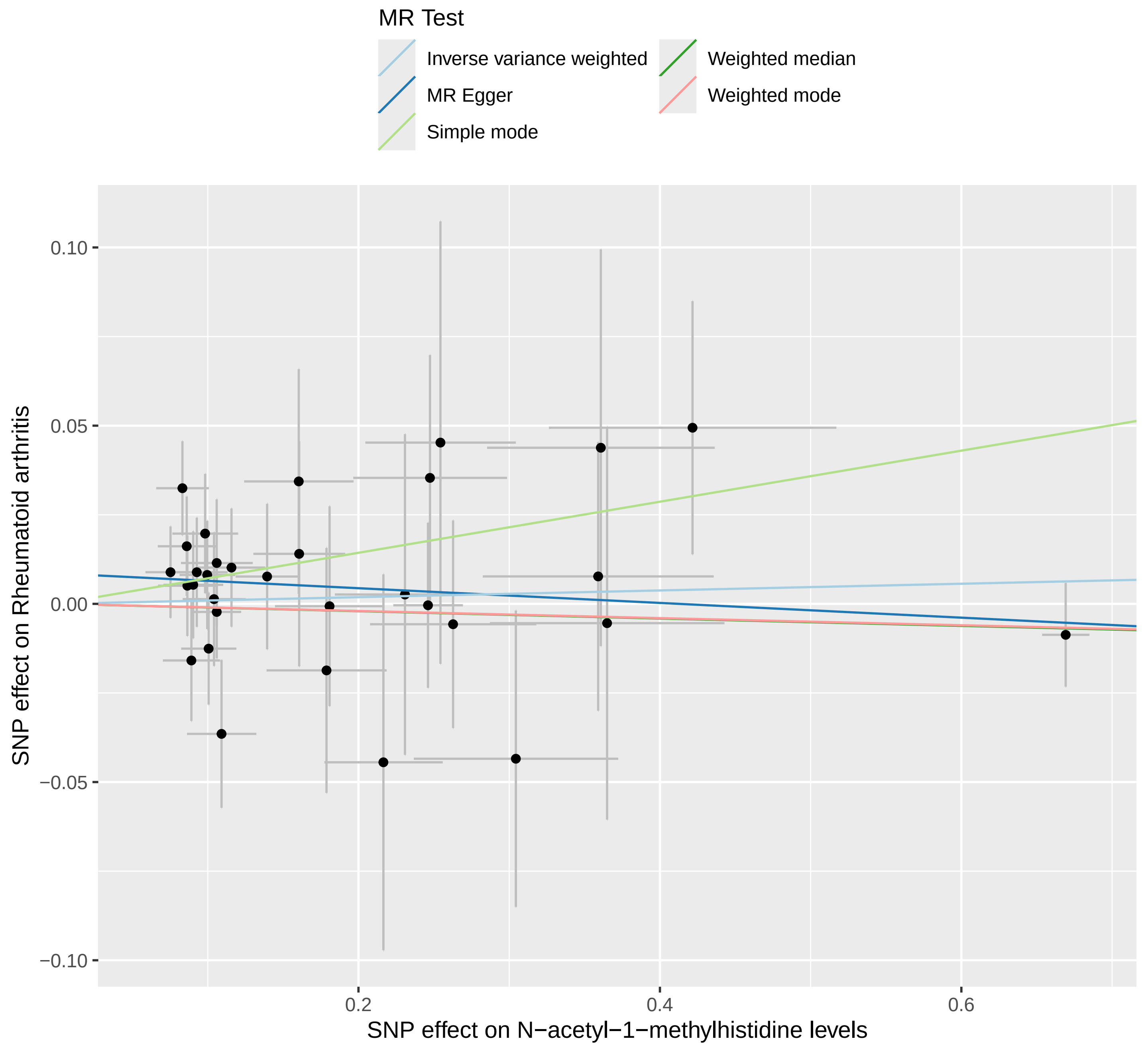
MR Test
Inverse variance weighted
MR Egger
Simple mode
Weighted median
Weighted mode
0.10
0.05
0.00
−0.05
−0.10
0.2
0.4
0.6
SNP effect on Rheumatoid arthritis
SNP effect on N−acetyl−1−methylhistidine levels

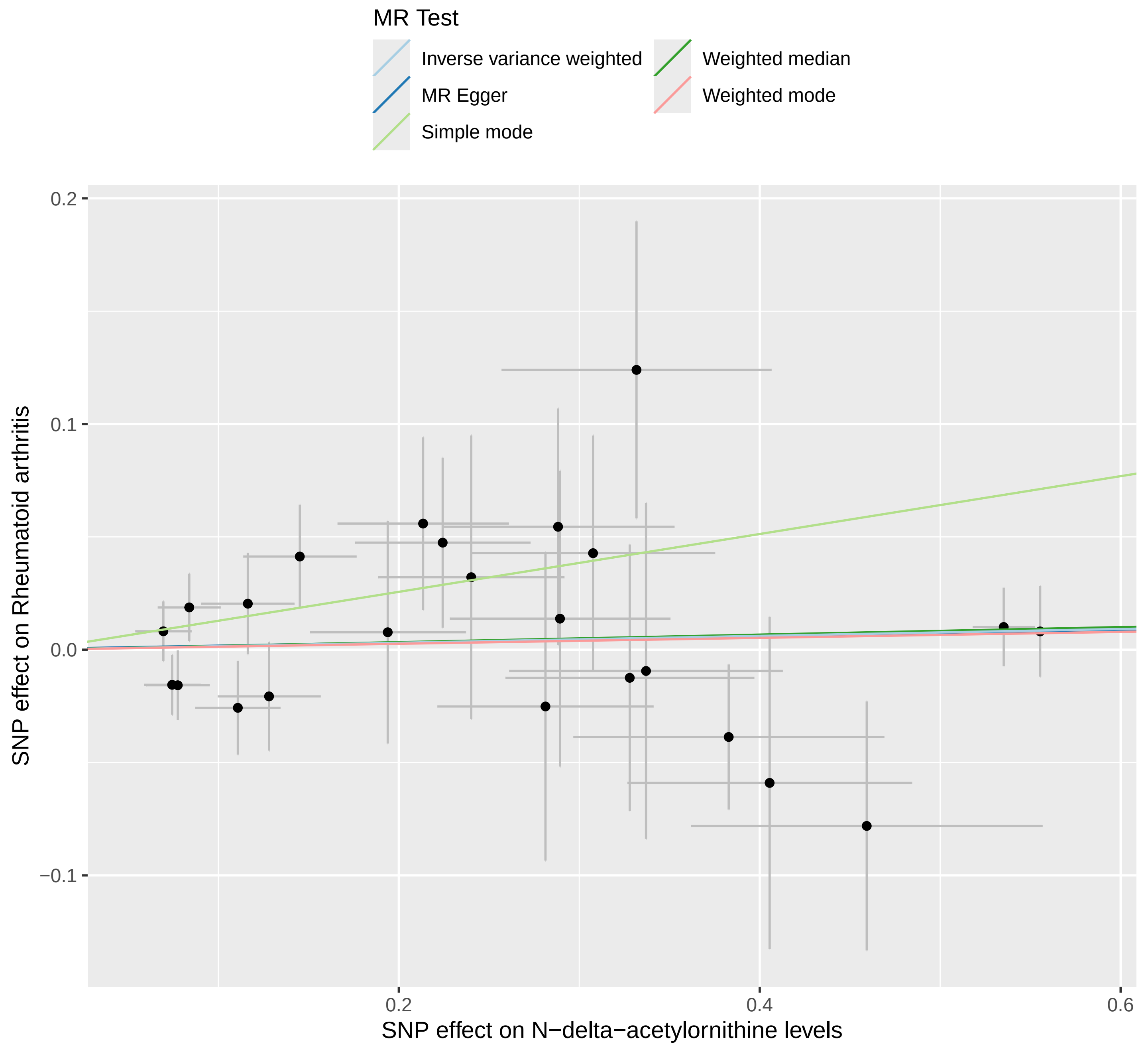
MR Test
Inverse variance weighted
MR Egger
Simple mode
Weighted median
Weighted mode
SNP effect on Rheumatoid arthritis
0.2
0.1
0.0
−0.1
0.2
0.4
0.6
SNP effect on N−delta−acetylornithine levels

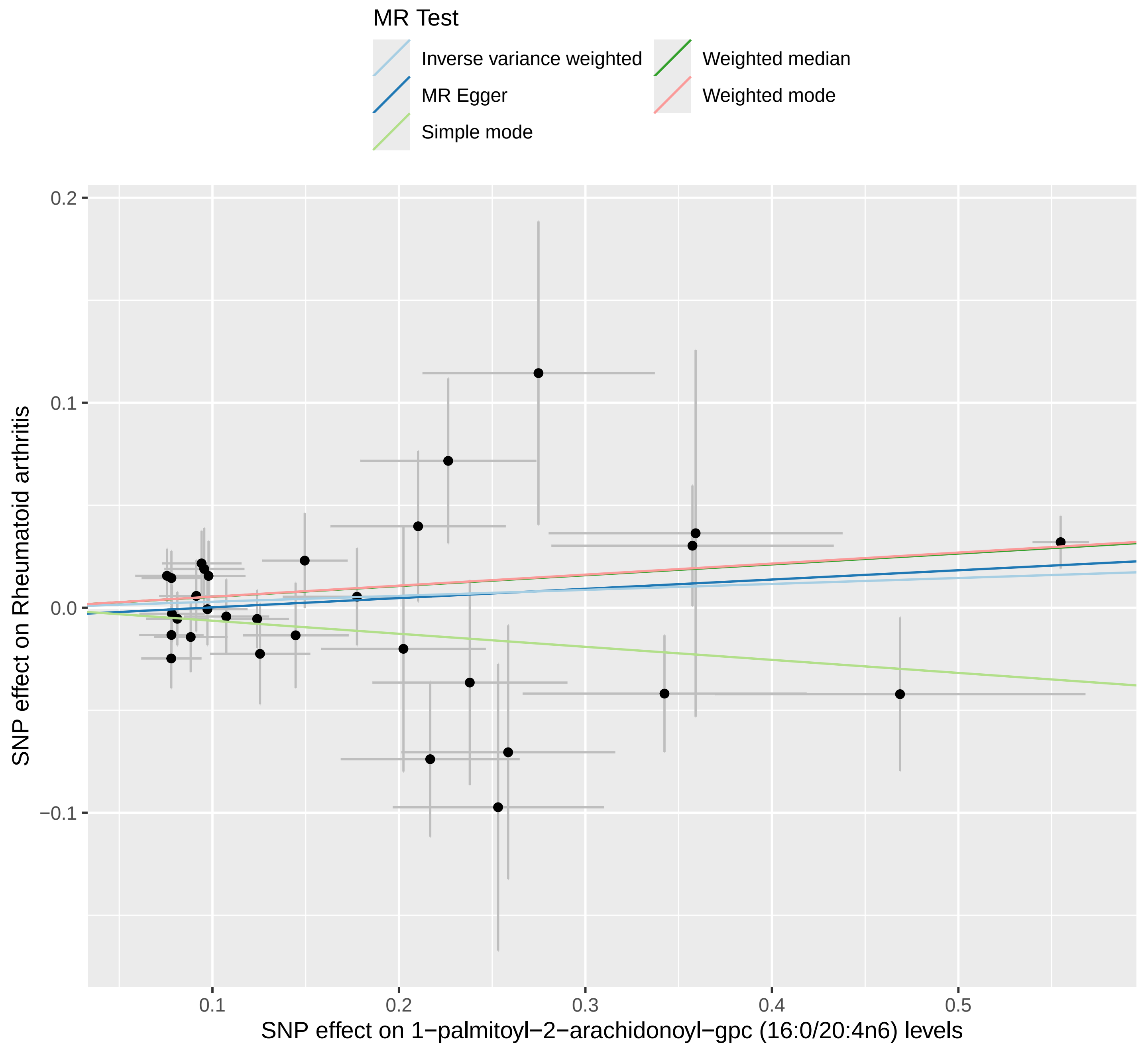

MR Test
Inverse variance weighted
MR Egger
Simple mode
Weighted median
Weighted mode
0.2
0.1
0.0
−0.1
SNP effect on Rheumatoid arthritis
0.1
0.2
0.3
0.4
0.5
SNP effect on 1−palmitoyl−2−arachidonoyl−gpc (16:0/20:4n6) levels

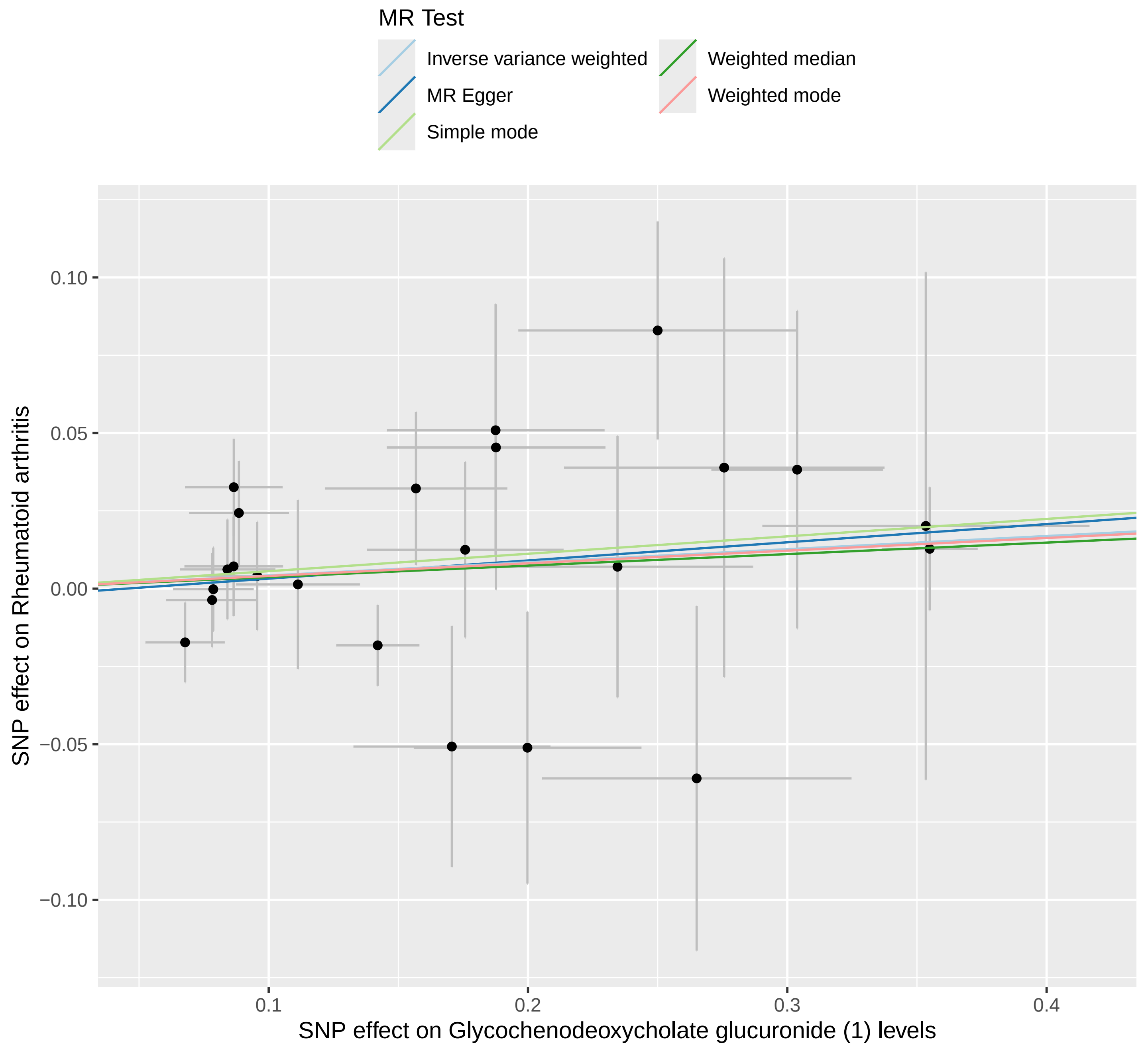
MR Test
Inverse variance weighted
MR Egger
Simple mode
Weighted median
Weighted mode
0.10
0.05
0.00
−0.05
−0.10
0.1
0.2
0.3
0.4
SNP effect on Rheumatoid arthritis
SNP effect on Glycochenodeoxycholate glucuronide (1) levels

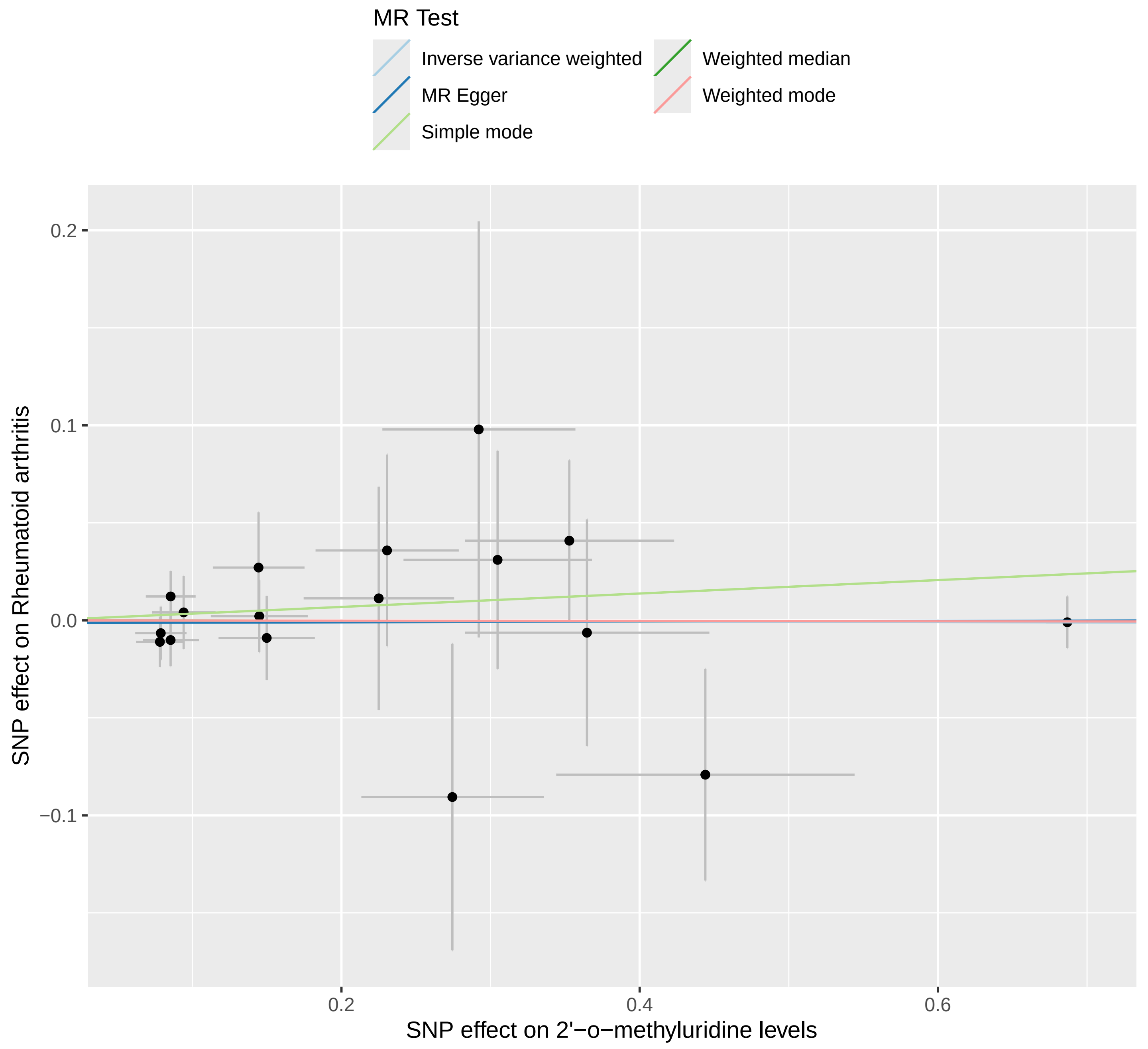

MR Test
Inverse variance weighted
MR Egger
Simple mode
Weighted median
Weighted mode
0.2
0.1
0.0
−0.1
SNP effect on Rheumatoid arthritis
0.2
0.4
0.6
SNP effect on 2'−o−methyluridine levels

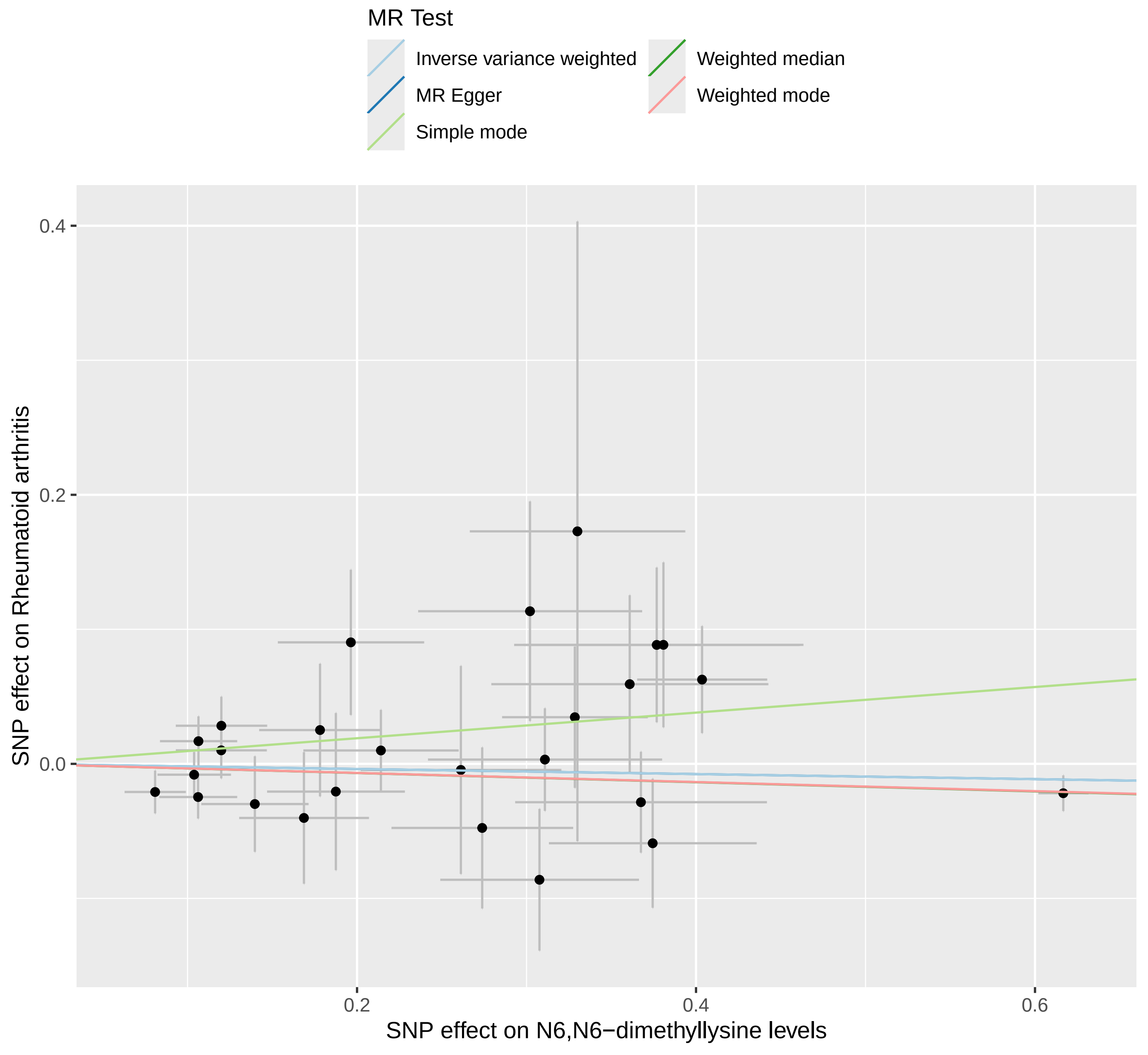
MR Test
Inverse variance weighted
MR Egger
Simple mode
Weighted median
Weighted mode
0.4
0.2
0.0
0.2
0.4
0.6
SNP effect on Rheumatoid arthritis
SNP effect on N6,N6−dimethyllysine levels

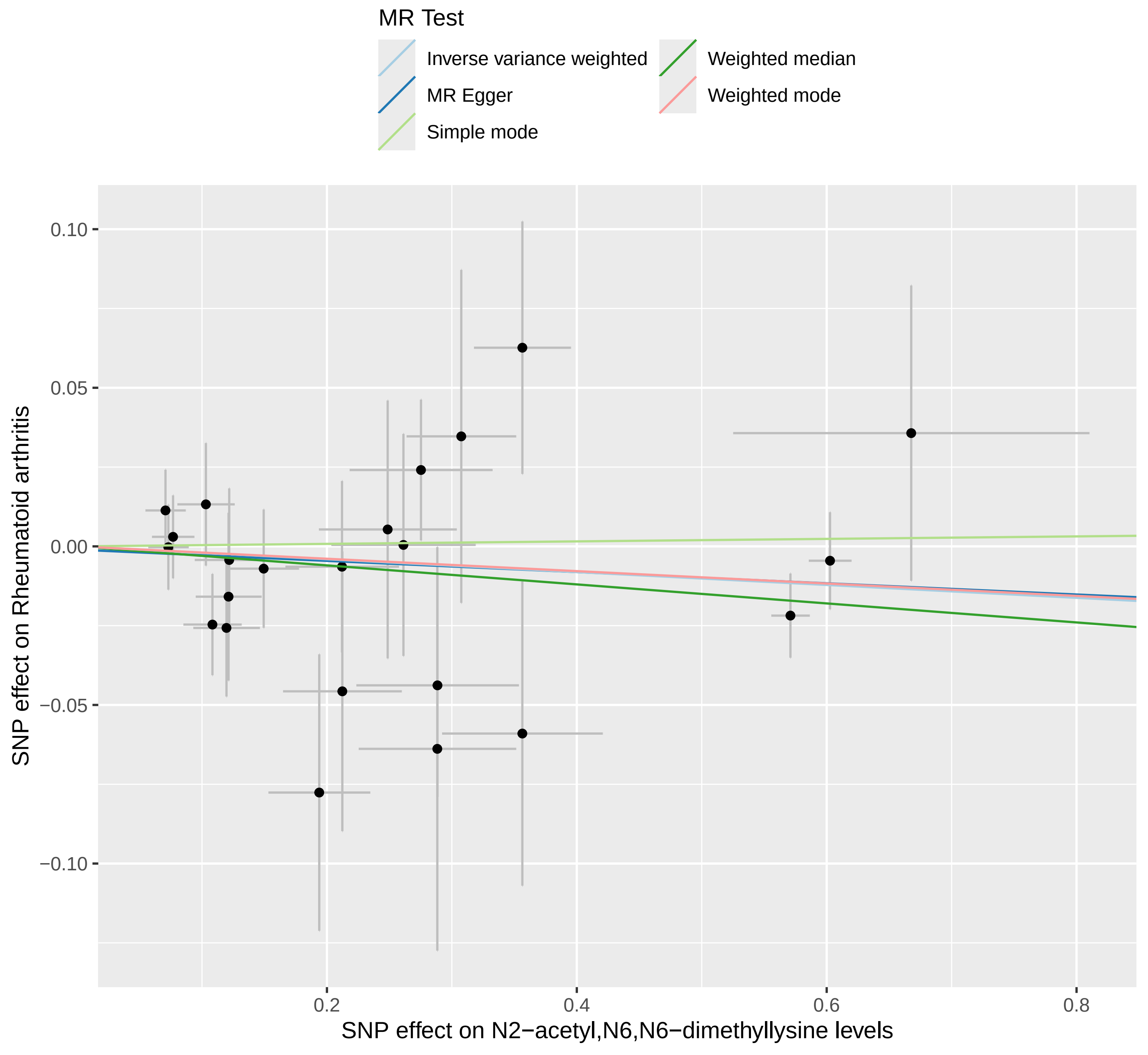

MR Test
Inverse variance weighted
MR Egger
Simple mode
Weighted median
Weighted mode
0.10
0.05
0.00
−0.05
−0.10
SNP effect on Rheumatoid arthritis
0.2
0.4
0.6
0.8
SNP effect on N2−acetyl,N6,N6−dimethyllysine levels

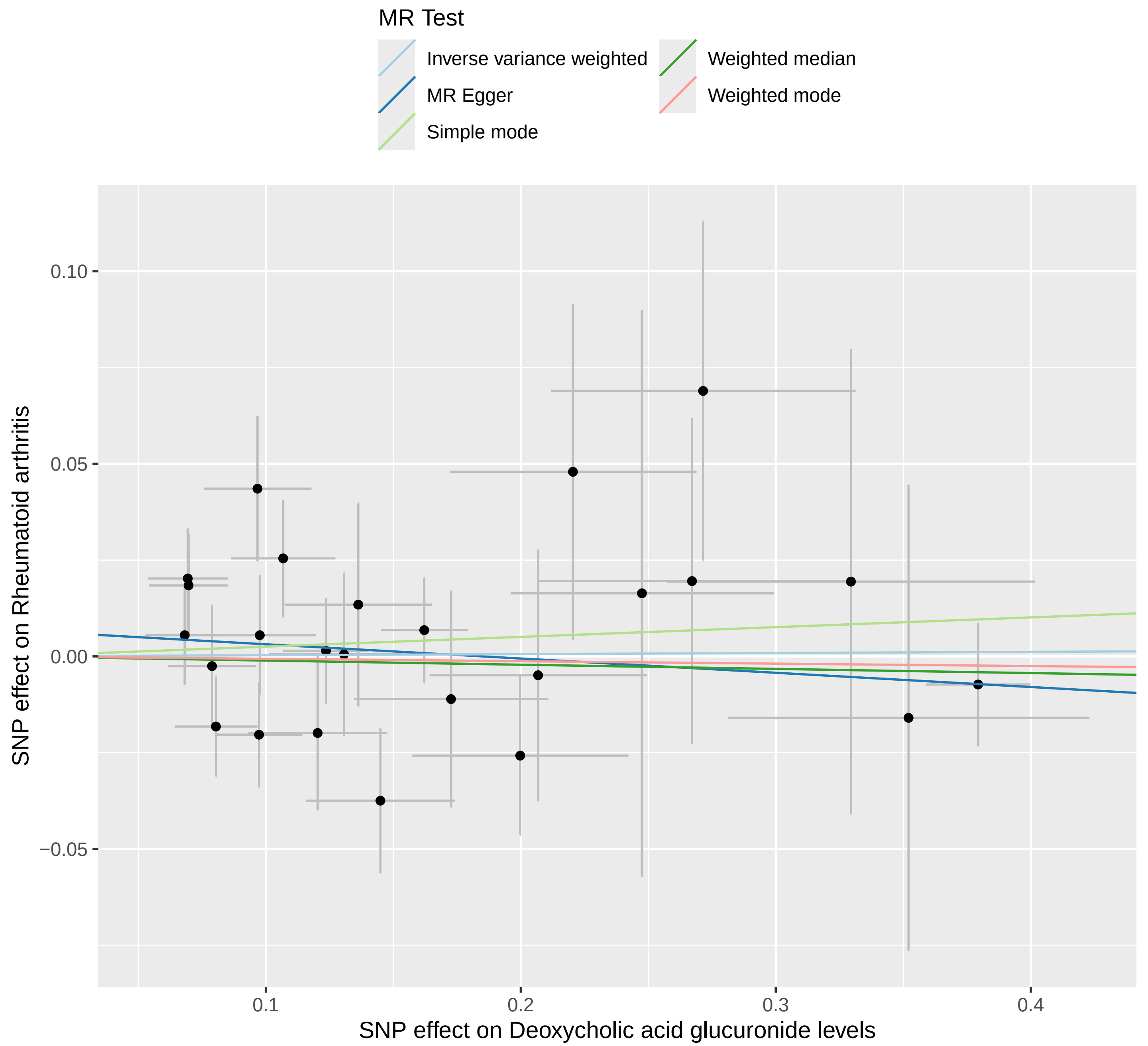
MR Test
Inverse variance weighted
MR Egger
Simple mode
Weighted median
Weighted mode
0.10
0.05
0.00
−0.05
0.1
0.2
0.3
0.4
SNP effect on Rheumatoid arthritis
SNP effect on Deoxycholic acid glucuronide levels

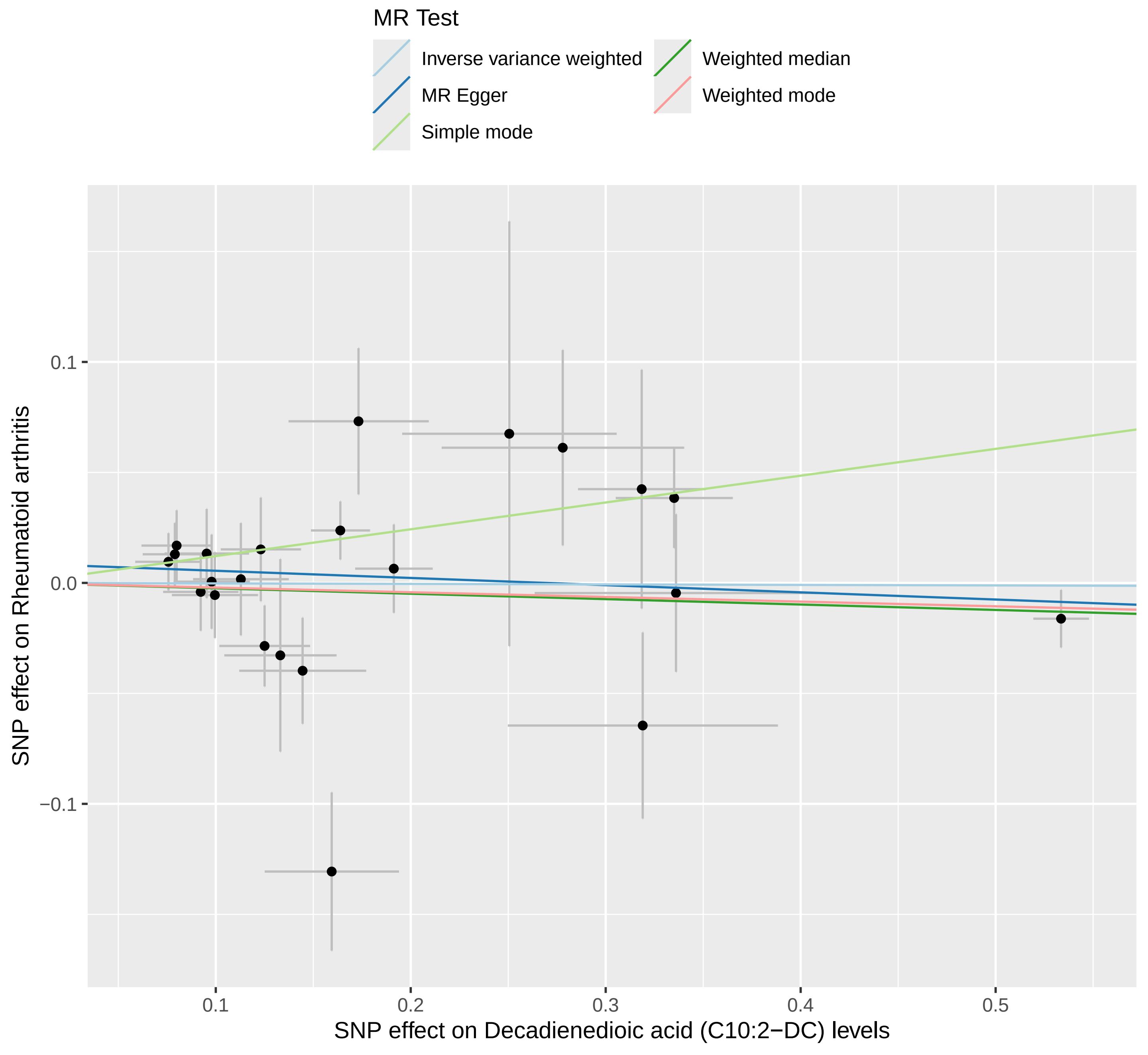

MR Test
Inverse variance weighted
MR Egger
Simple mode
Weighted median
Weighted mode
0.1
0.0
−0.1
SNP effect on Rheumatoid arthritis
0.1
0.2
0.3
0.4
0.5
SNP effect on Decadienedioic acid (C10:2−DC) levels

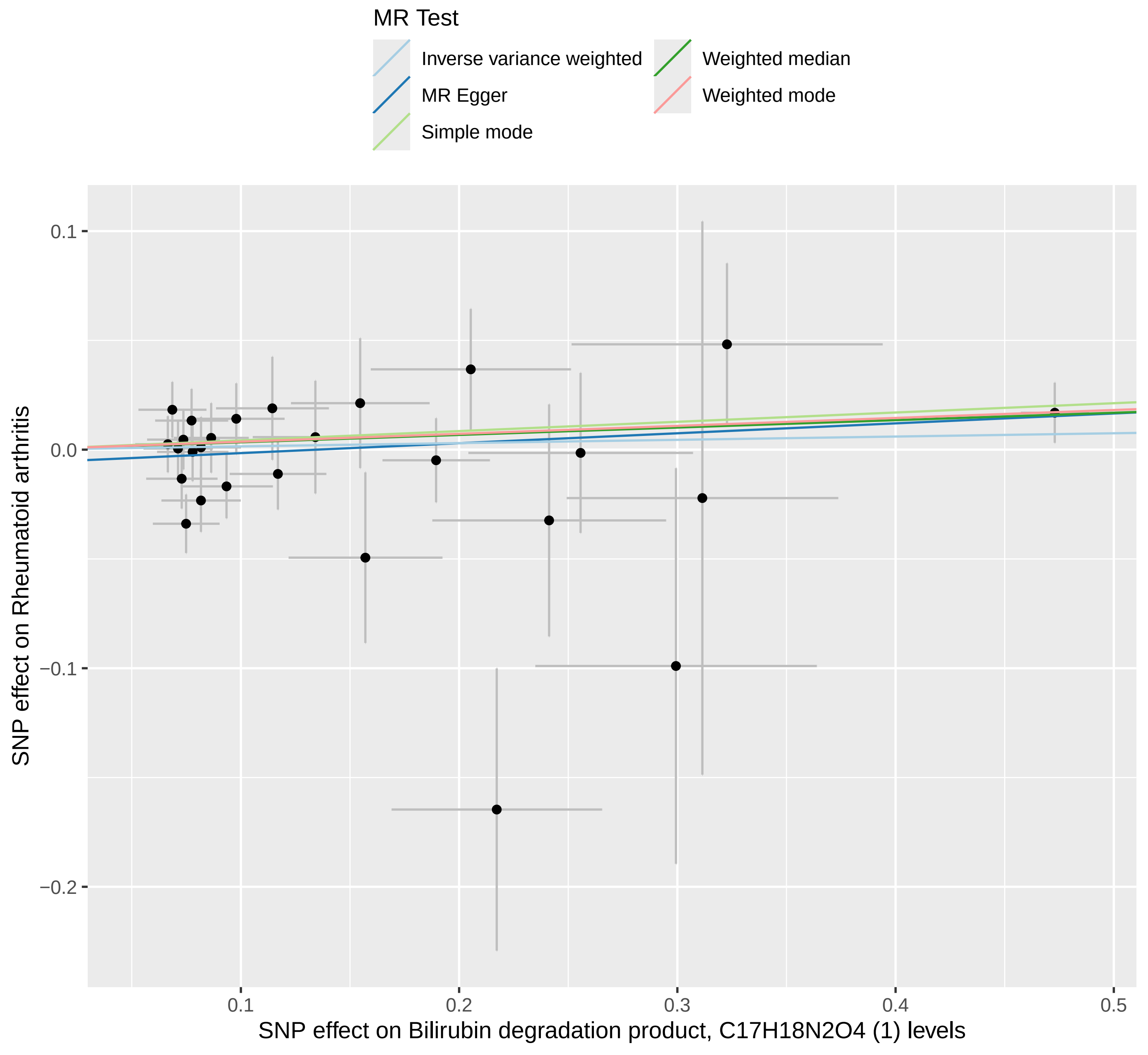

MR Test
Inverse variance weighted
MR Egger
Simple mode
Weighted median
Weighted mode
0.1
0.0
−0.1
−0.2
0.1
0.2
0.3
0.4
0.5
SNP effect on Rheumatoid arthritis
SNP effect on Bilirubin degradation product, C17H18N2O4 (1) levels

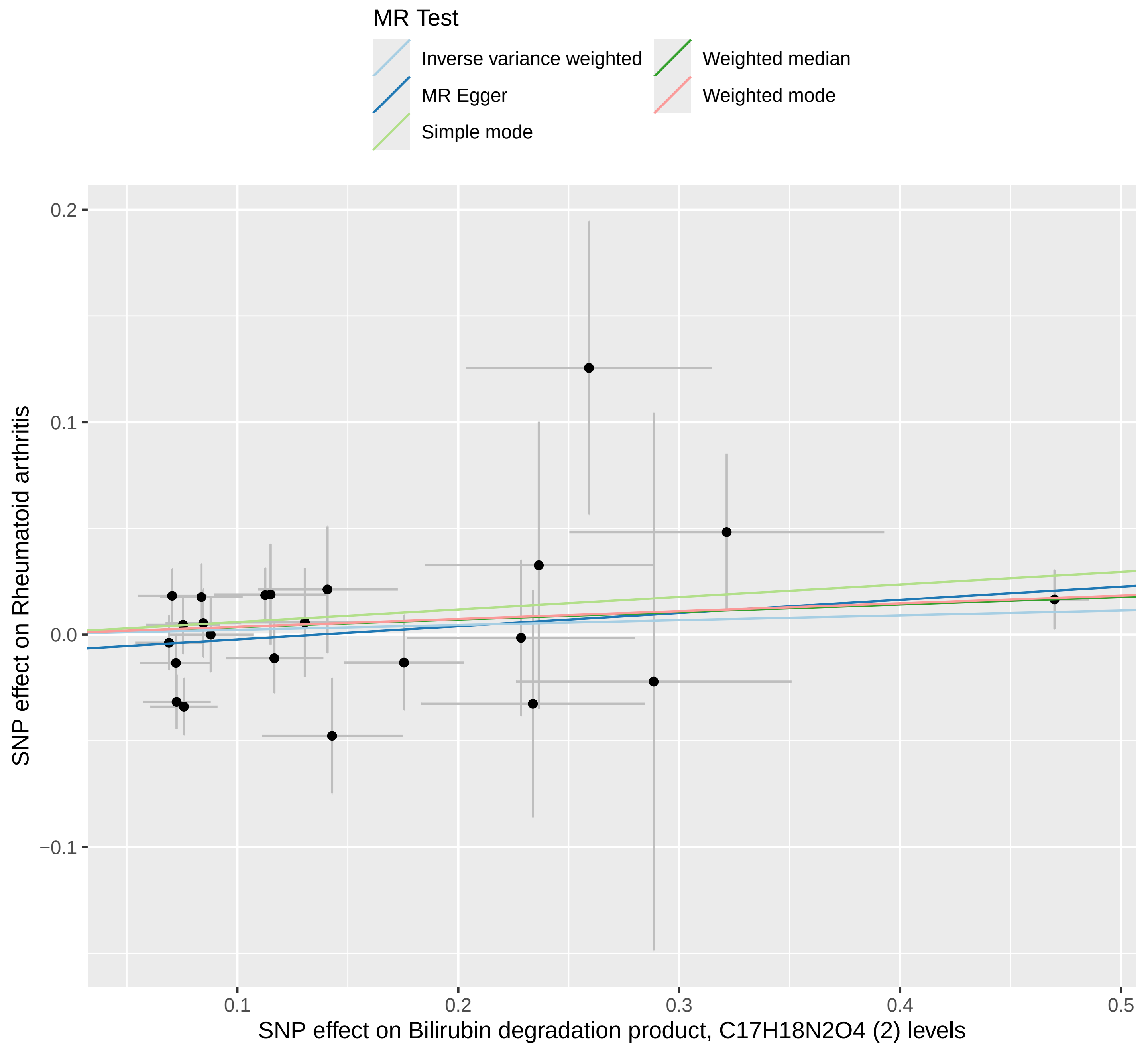

MR Test
Inverse variance weighted
MR Egger
Simple mode
Weighted median
Weighted mode
0.2
0.1
0.0
−0.1
0.1
0.2
0.3
0.4
0.5
SNP effect on Rheumatoid arthritis
SNP effect on Bilirubin degradation product, C17H18N2O4 (2) levels

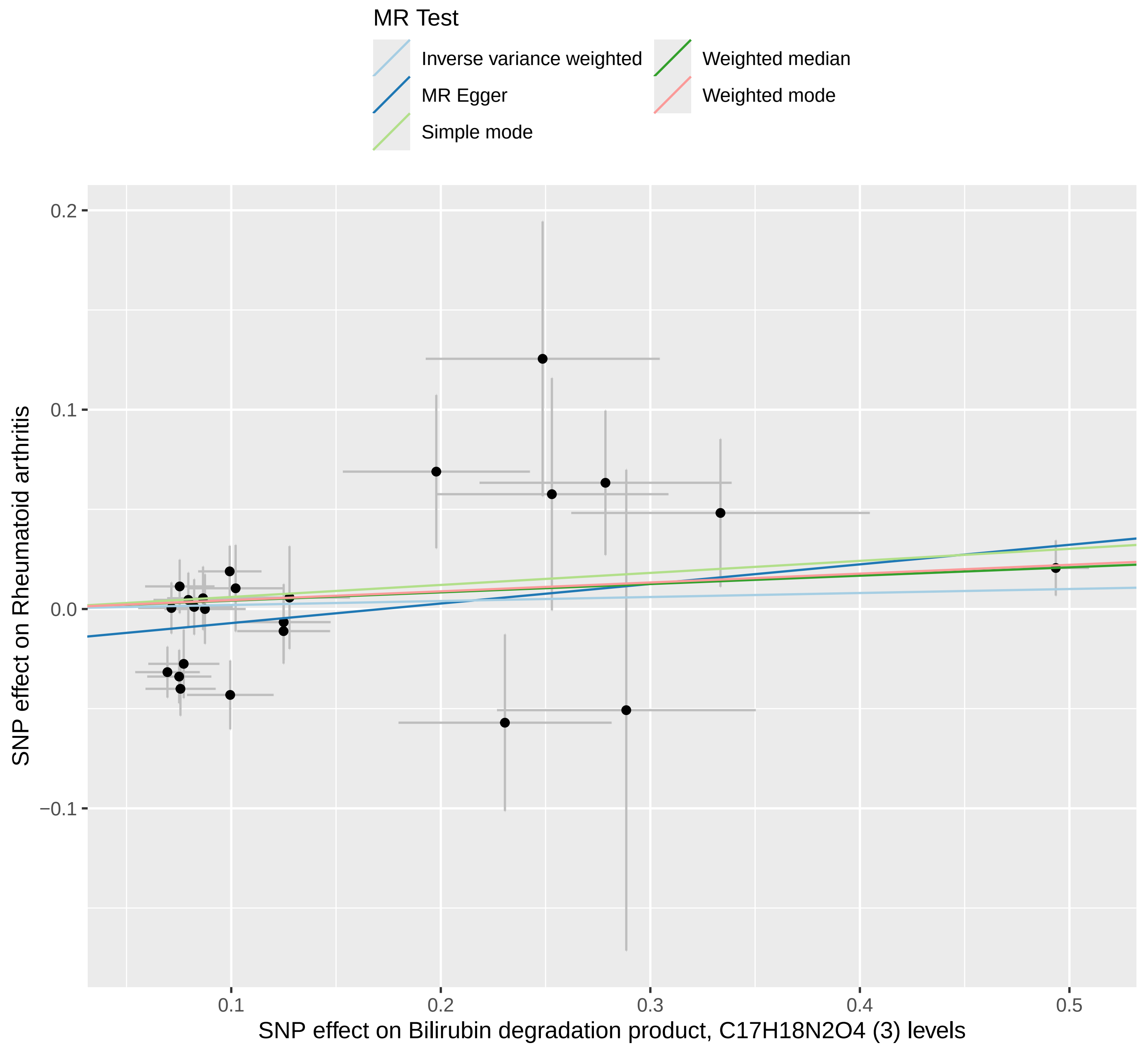

MR Test
Inverse variance weighted
MR Egger
Simple mode
Weighted median
Weighted mode
0.2
0.1
0.0
−0.1
0.1
0.2
0.3
0.4
0.5
SNP effect on Rheumatoid arthritis
SNP effect on Bilirubin degradation product, C17H18N2O4 (3) levels

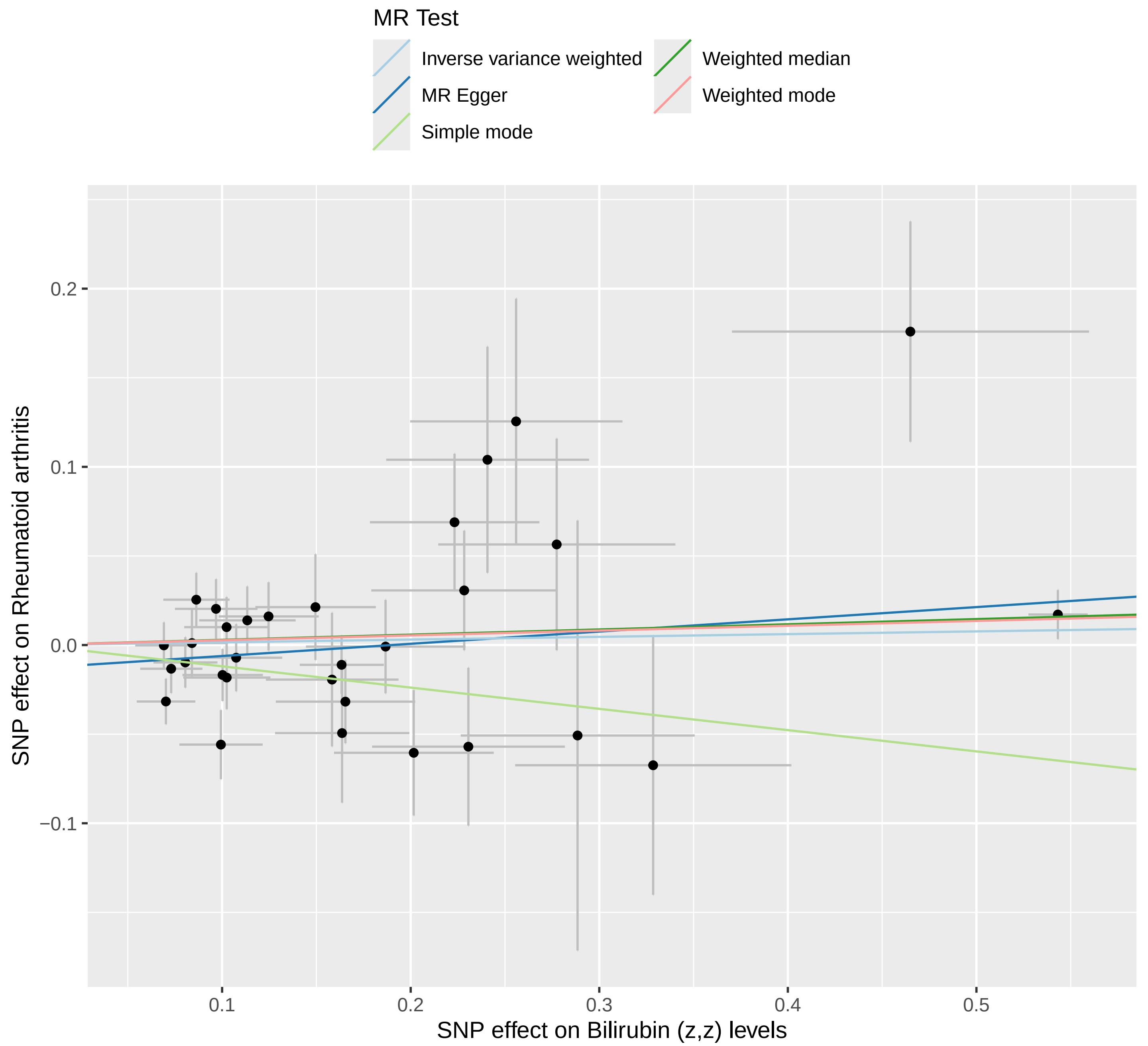
MR Test
Inverse variance weighted
MR Egger
Simple mode
Weighted median
Weighted mode
SNP effect on Rheumatoid arthritis
0.2
0.1
0.0
−0.1
0.1
0.2
0.3
0.4
0.5
SNP effect on Bilirubin (z,z) levels

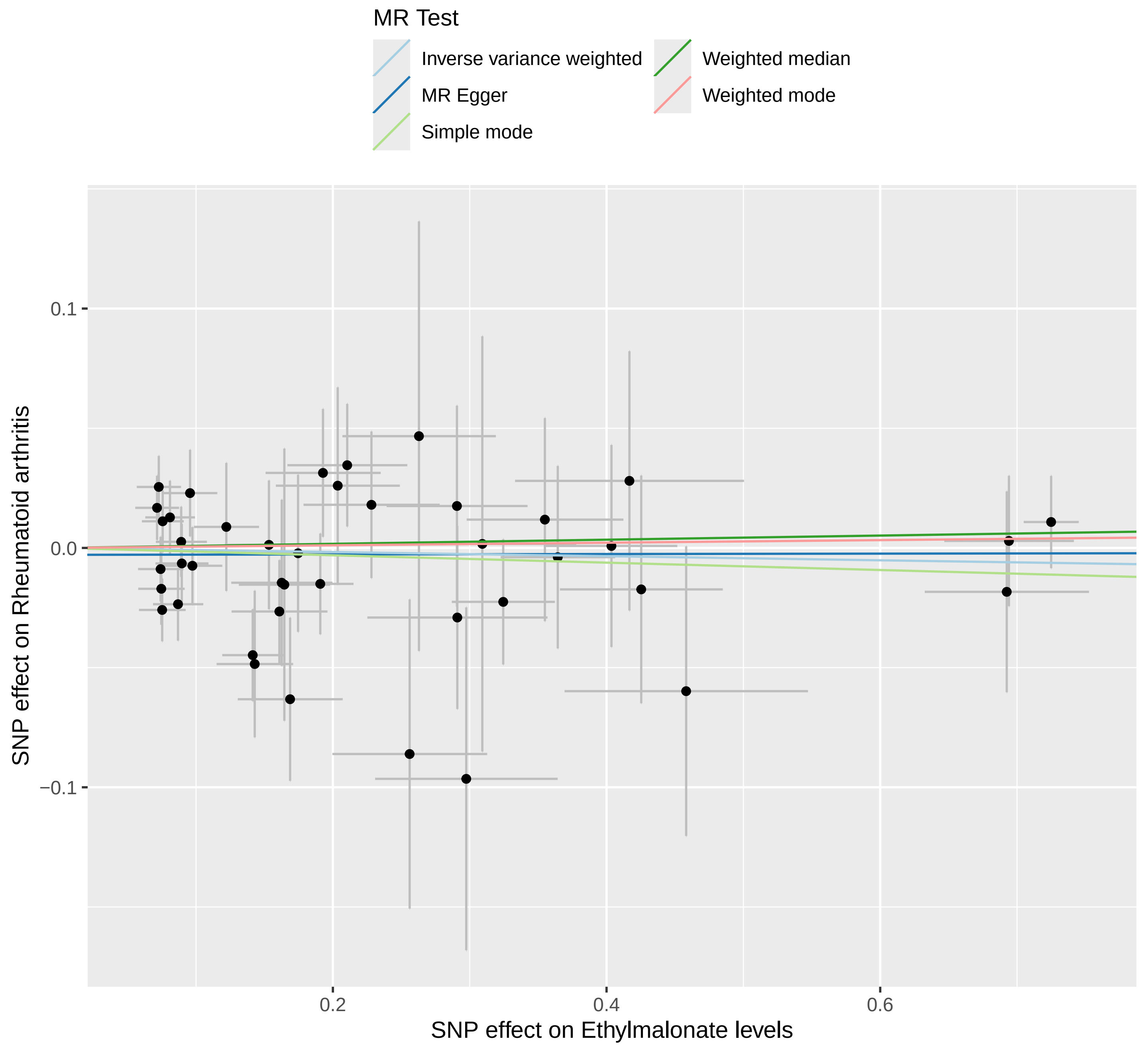
MR Test
Inverse variance weighted
MR Egger
Simple mode
Weighted median
Weighted mode
0.1
0.0
−0.1
0.2
0.4
0.6
SNP effect on Rheumatoid arthritis
SNP effect on Ethylmalonate levels

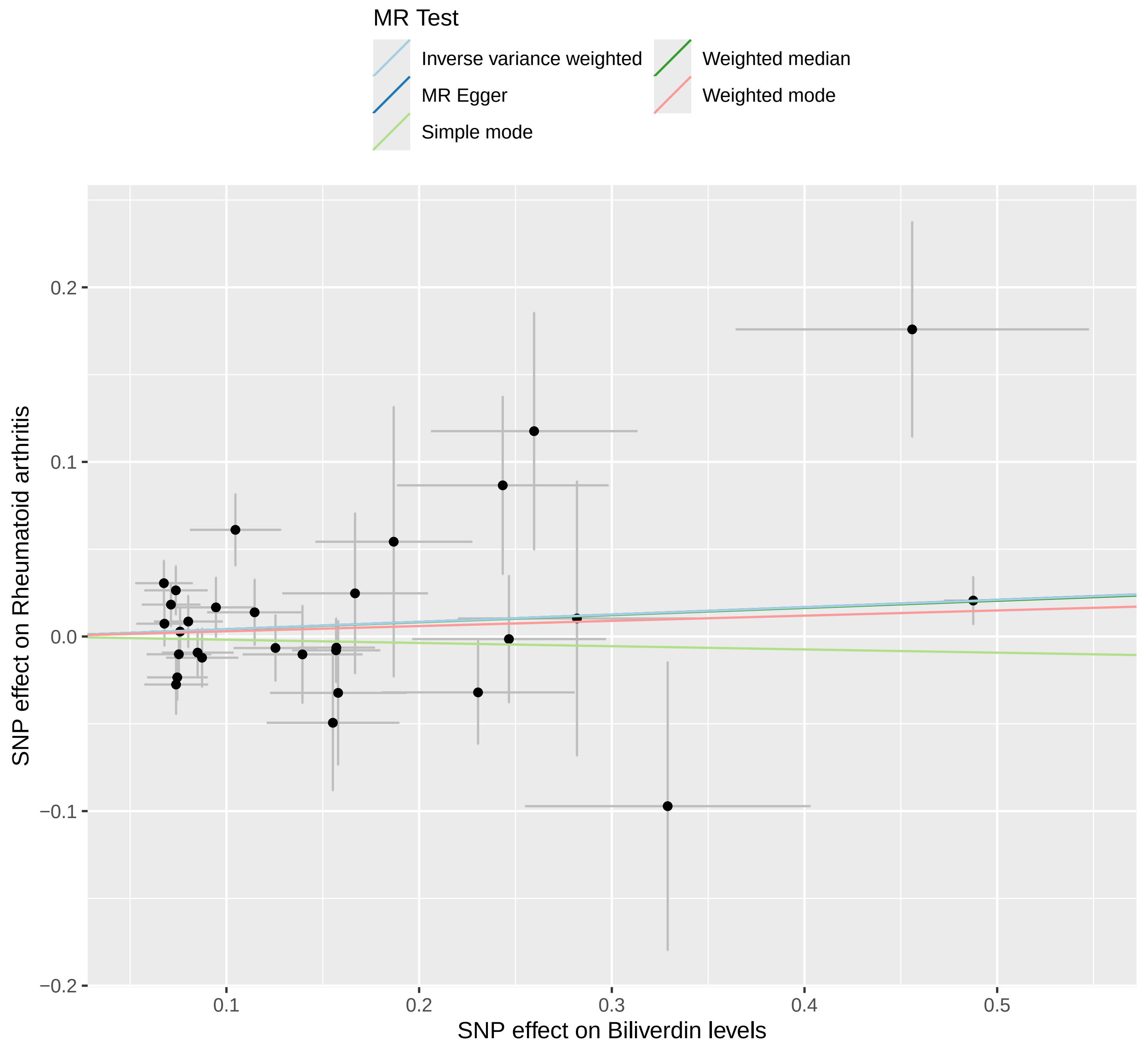
MR Test
Inverse variance weighted
MR Egger
Simple mode
Weighted median
Weighted mode
SNP effect on Rheumatoid arthritis
0.2
0.1
0.0
−0.1
−0.2
0.1
0.2
0.3
0.4
0.5
SNP effect on Biliverdin levels

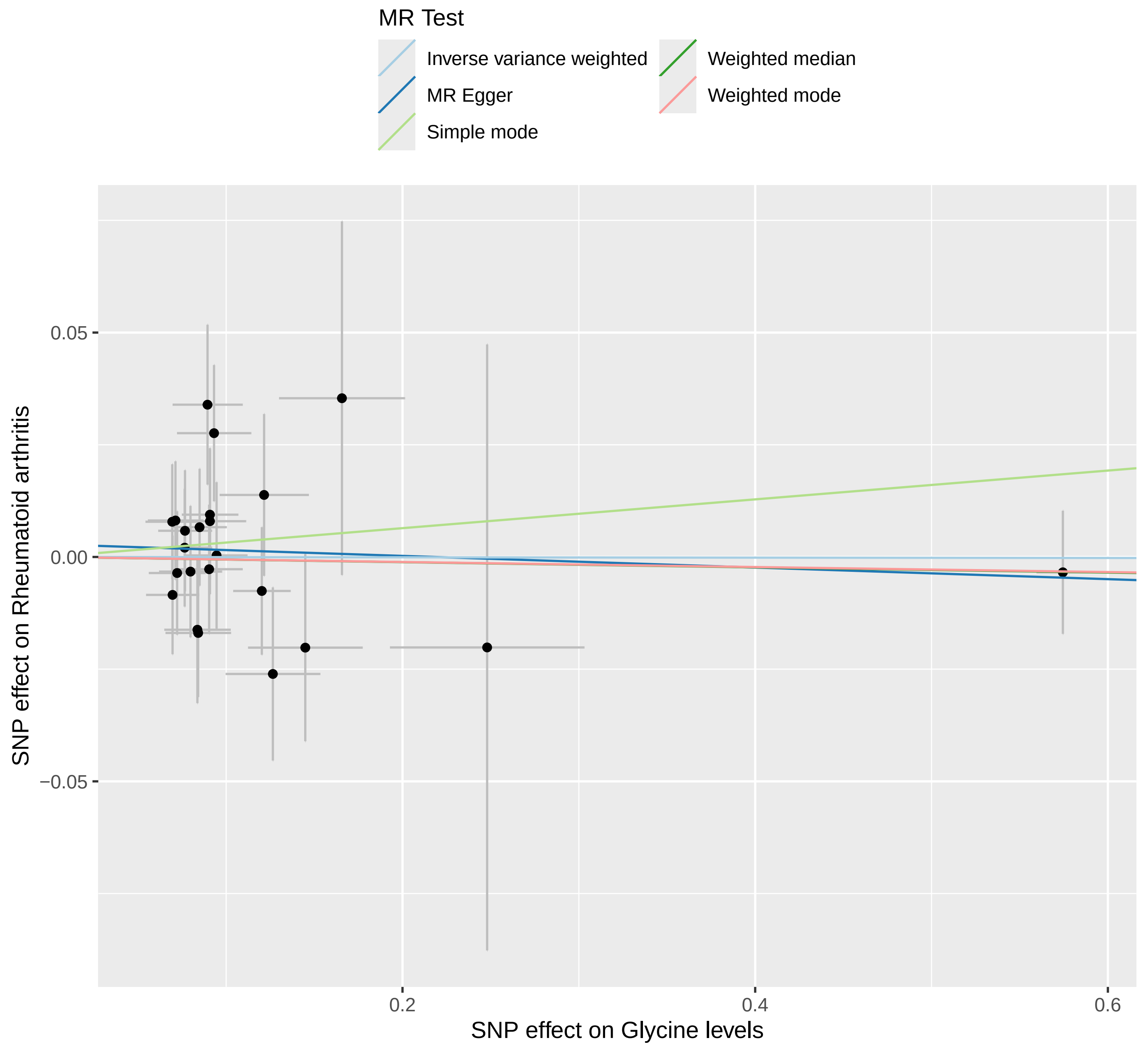

MR Test
Inverse variance weighted
MR Egger
Simple mode
Weighted median
Weighted mode
0.05
0.00
−0.05
0.2
0.4
0.6
SNP effect on Rheumatoid arthritis
SNP effect on Glycine levels

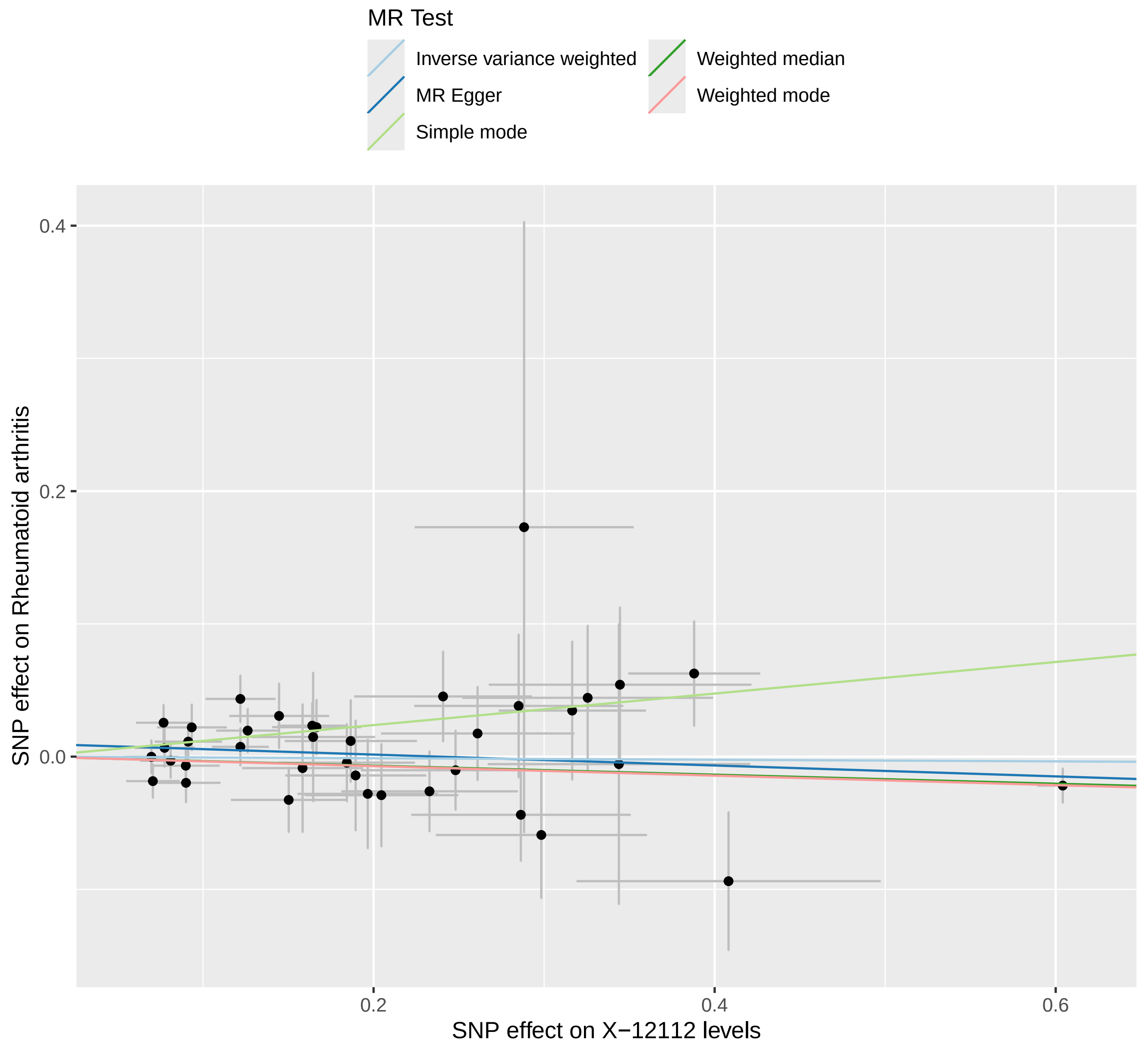

MR Test
Inverse variance weighted
MR Egger
Simple mode
Weighted median
Weighted mode
0.4
0.2
0.0
SNP effect on Rheumatoid arthritis
0.2
0.4
0.6
SNP effect on X−12112 levels

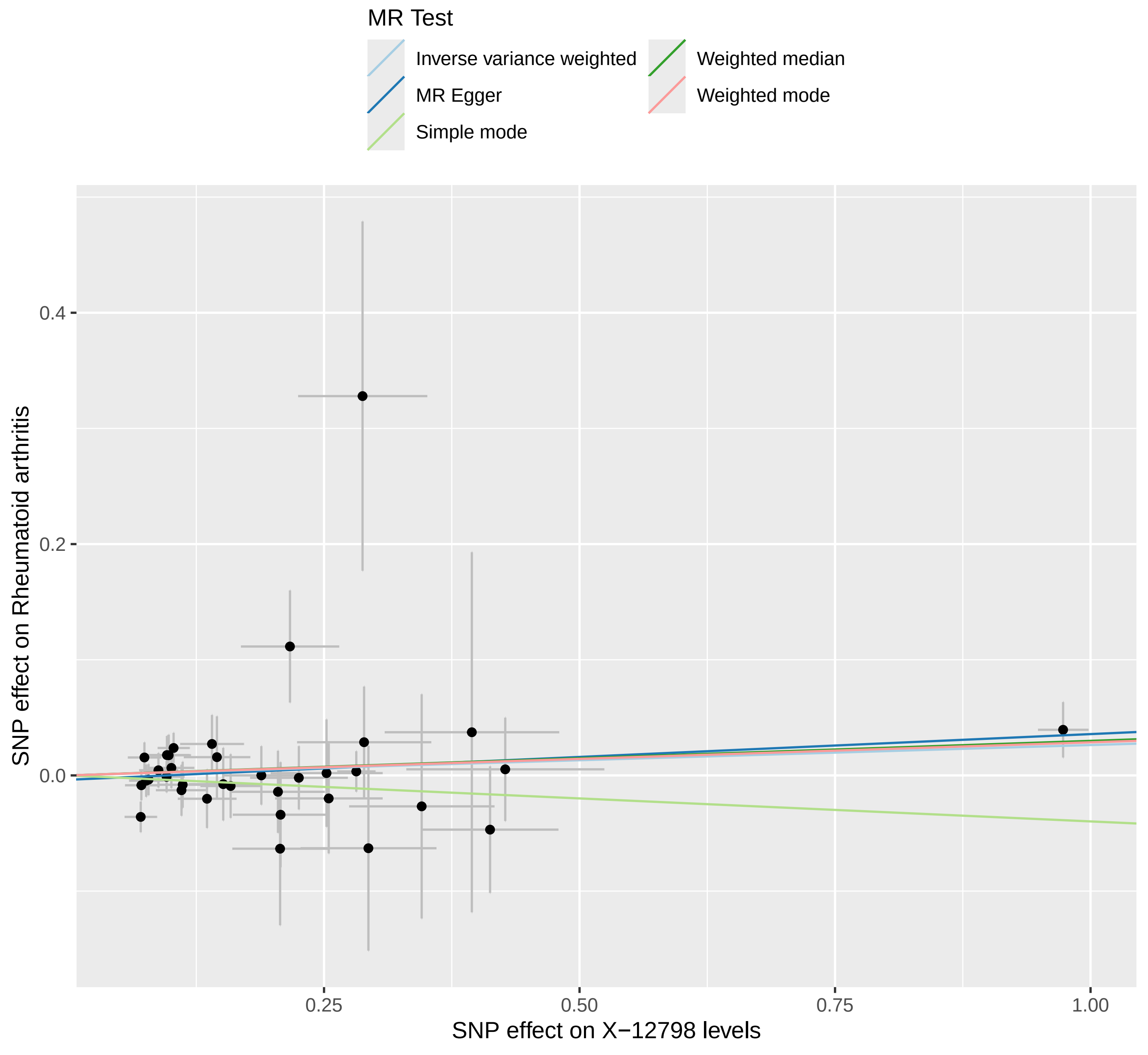
MR Test
Inverse variance weighted
MR Egger
Simple mode
Weighted median
Weighted mode
0.4
0.2
0.0
SNP effect on Rheumatoid arthritis
0.25
0.50
0.75
1.00
SNP effect on X−12798 levels

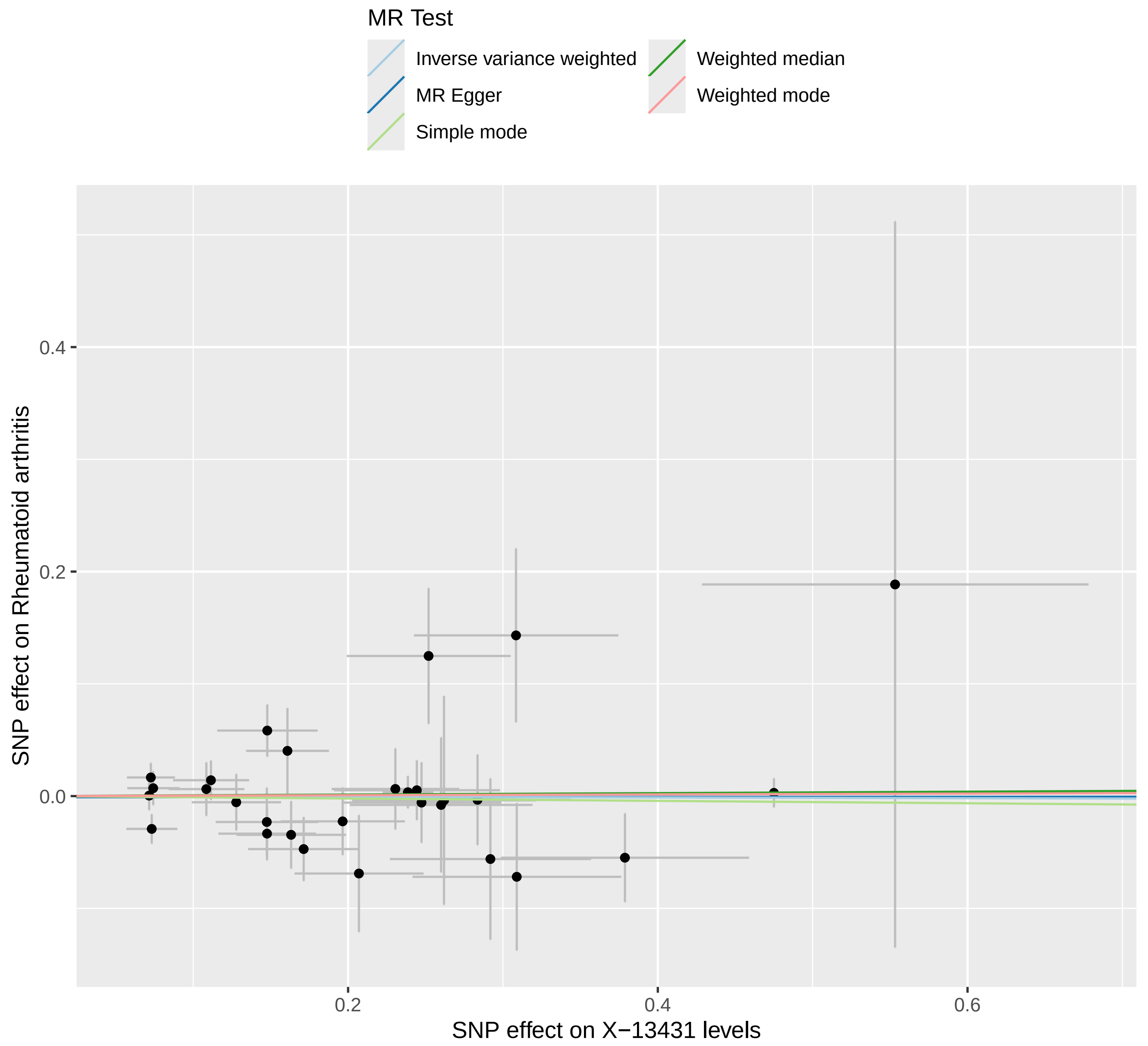

MR Test
Inverse variance weighted
MR Egger
Simple mode
Weighted median
Weighted mode
0.4
0.2
0.0
0.2
0.4
0.6
SNP effect on Rheumatoid arthritis
SNP effect on X−13431 levels

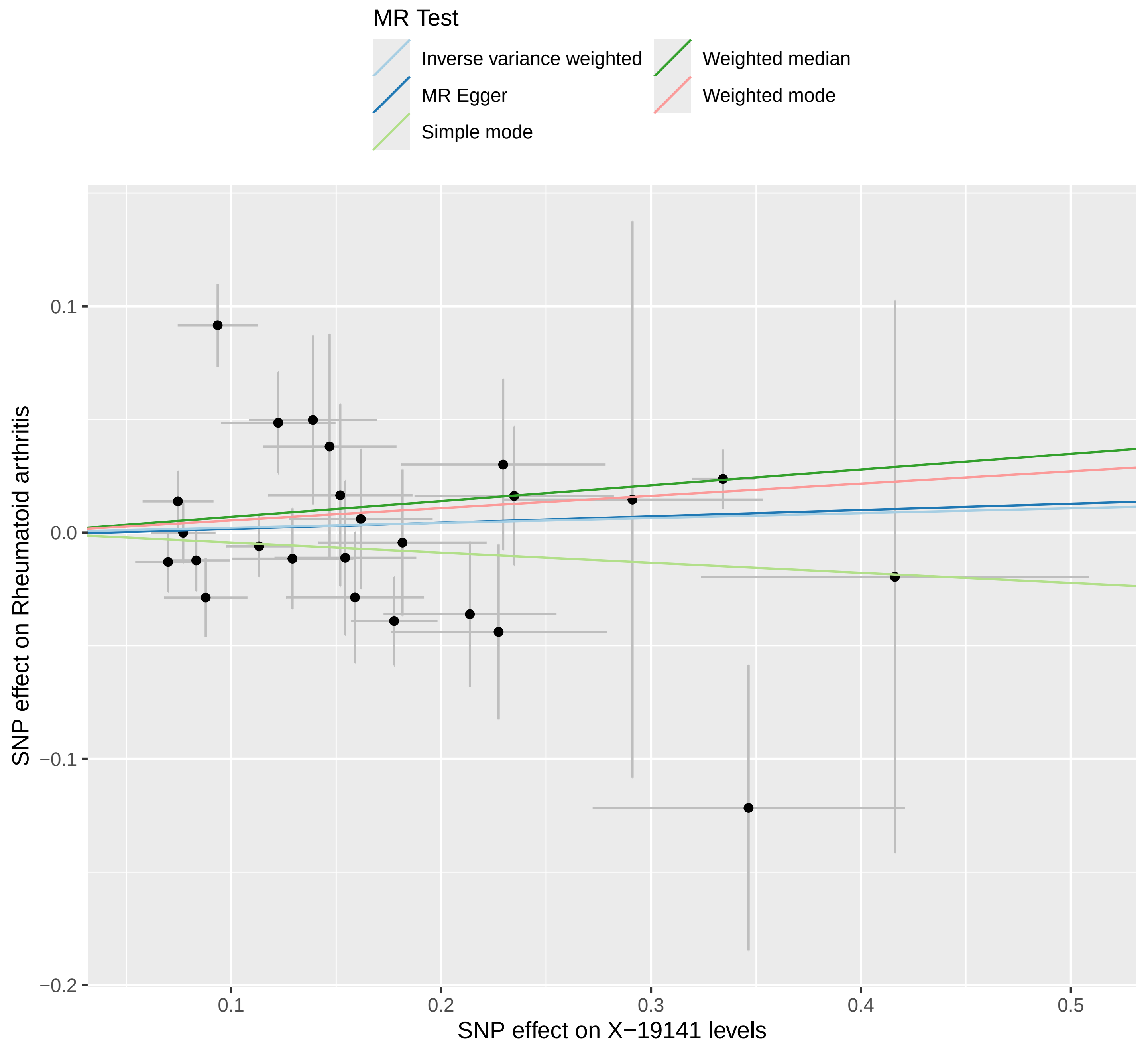
MR Test
Inverse variance weighted
MR Egger
Simple mode
Weighted median
Weighted mode
0.1
0.0
−0.1
−0.2
0.1
0.2
0.3
0.4
0.5
SNP effect on Rheumatoid arthritis
SNP effect on X−19141 levels

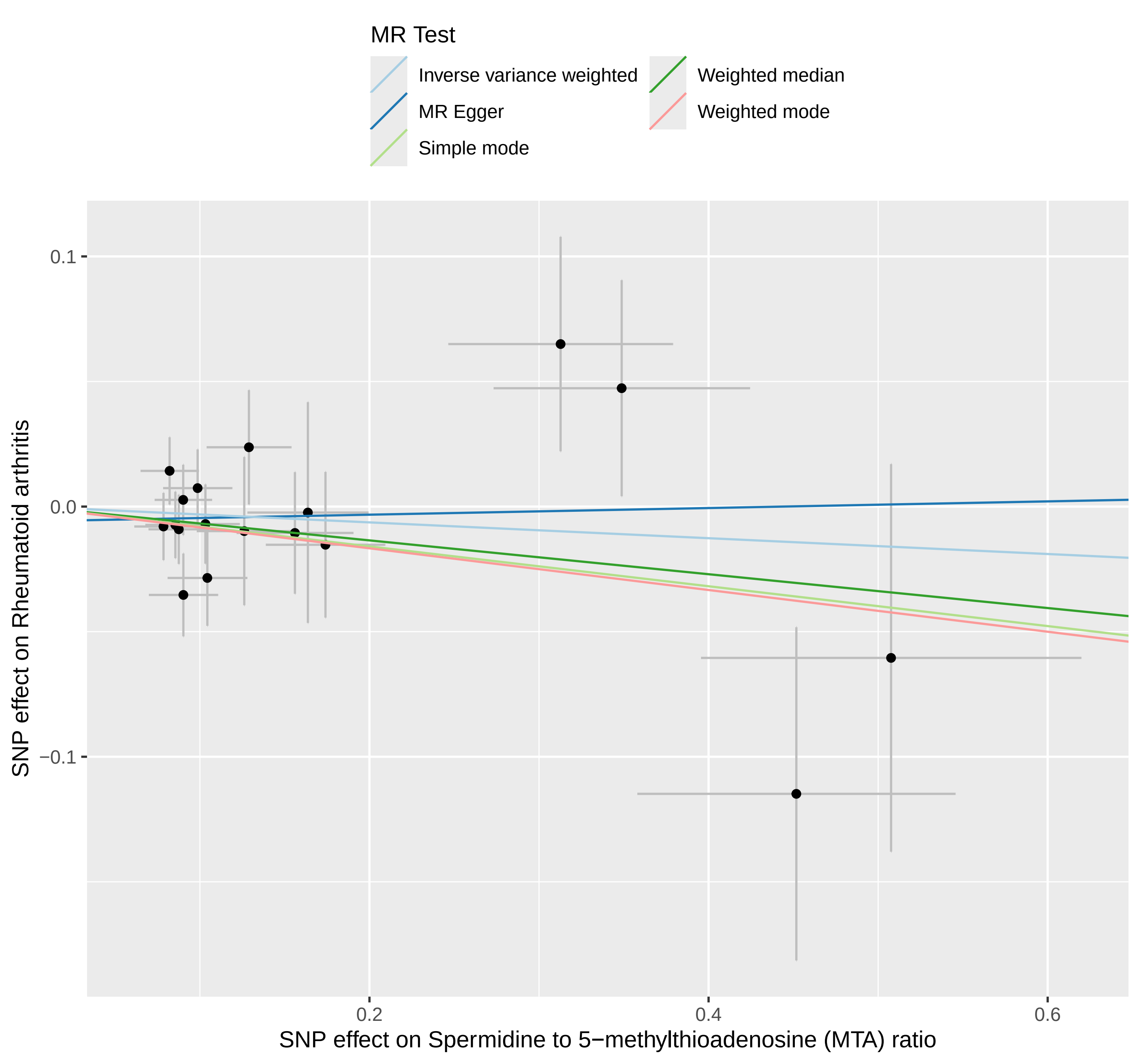
MR Test
Inverse variance weighted
MR Egger
Simple mode
Weighted median
Weighted mode
0.1
0.0
−0.1
0.2
0.4
0.6
SNP effect on Rheumatoid arthritis
SNP effect on Spermidine to 5−methylthioadenosine (MTA) ratio

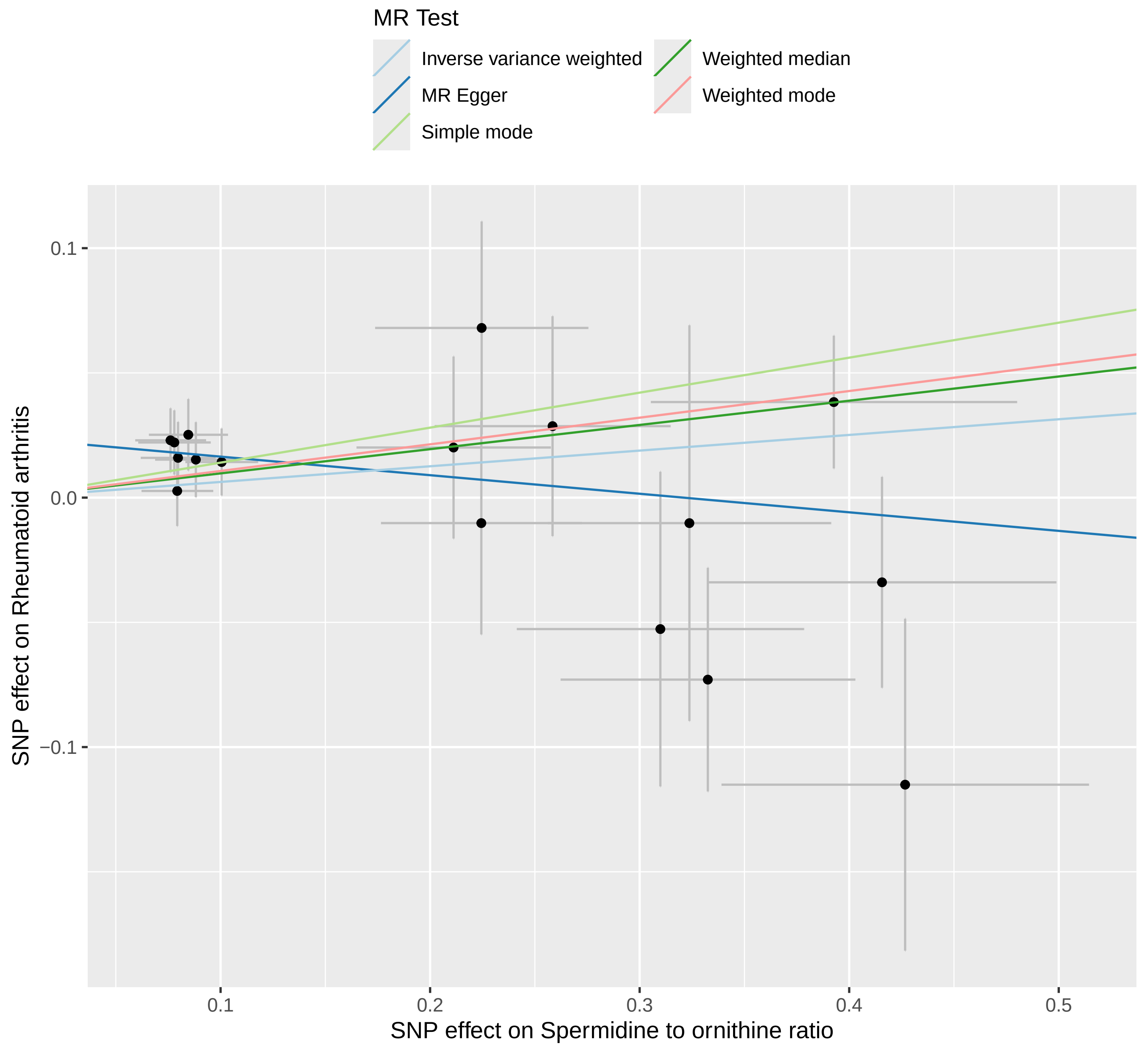
MR Test
Inverse variance weighted
MR Egger
Simple mode
Weighted median
Weighted mode
0.1
0.0
−0.1
SNP effect on Rheumatoid arthritis
0.1
0.2
0.3
0.4
0.5
SNP effect on Spermidine to ornithine ratio

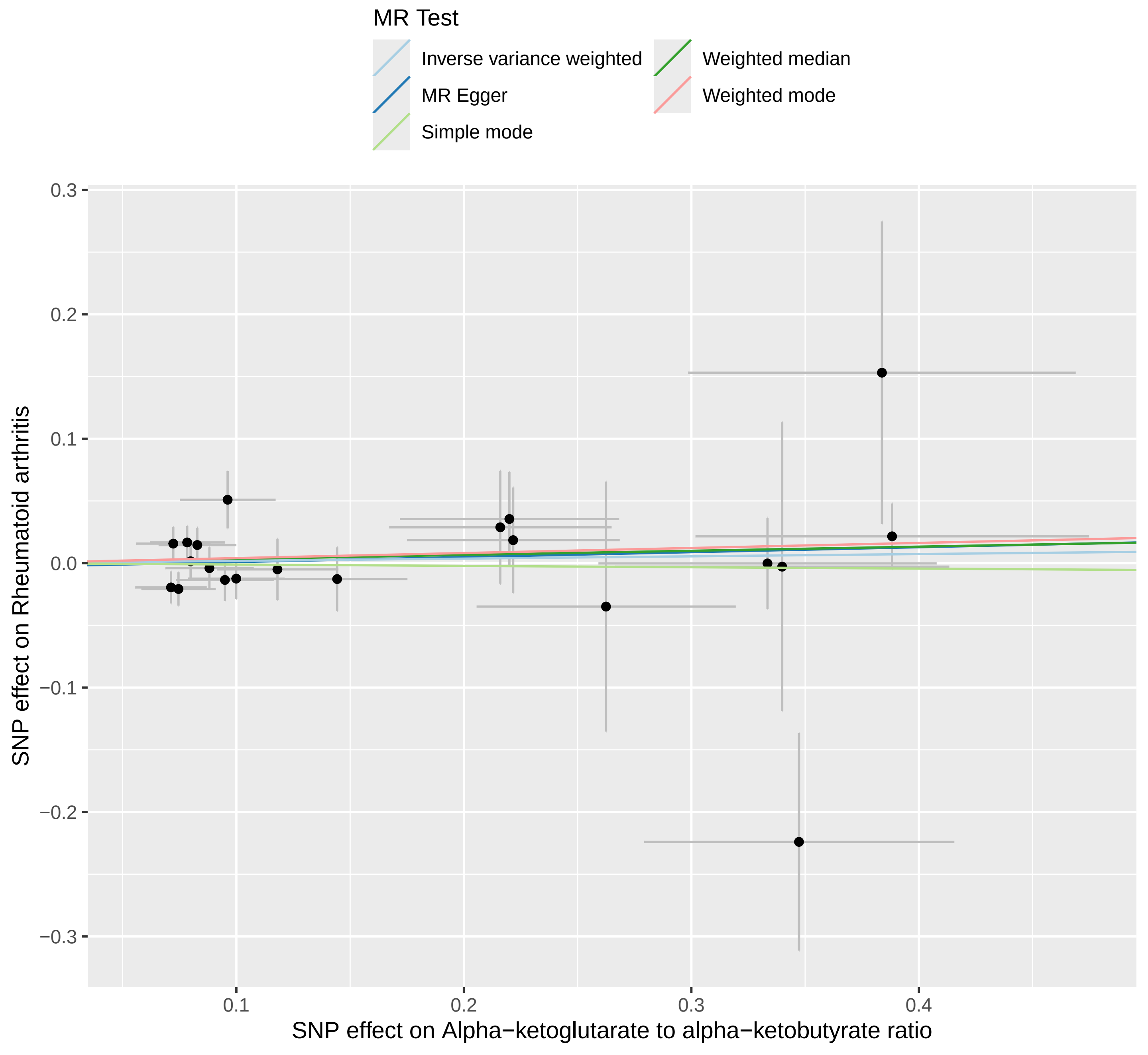

MR Test
Inverse variance weighted
MR Egger
Simple mode
Weighted median
Weighted mode
0.3
0.2
0.1
0.0
−0.1
−0.2
−0.3
0.1
0.2
0.3
0.4
SNP effect on Rheumatoid arthritis
SNP effect on Alpha−ketoglutarate to alpha−ketobutyrate ratio

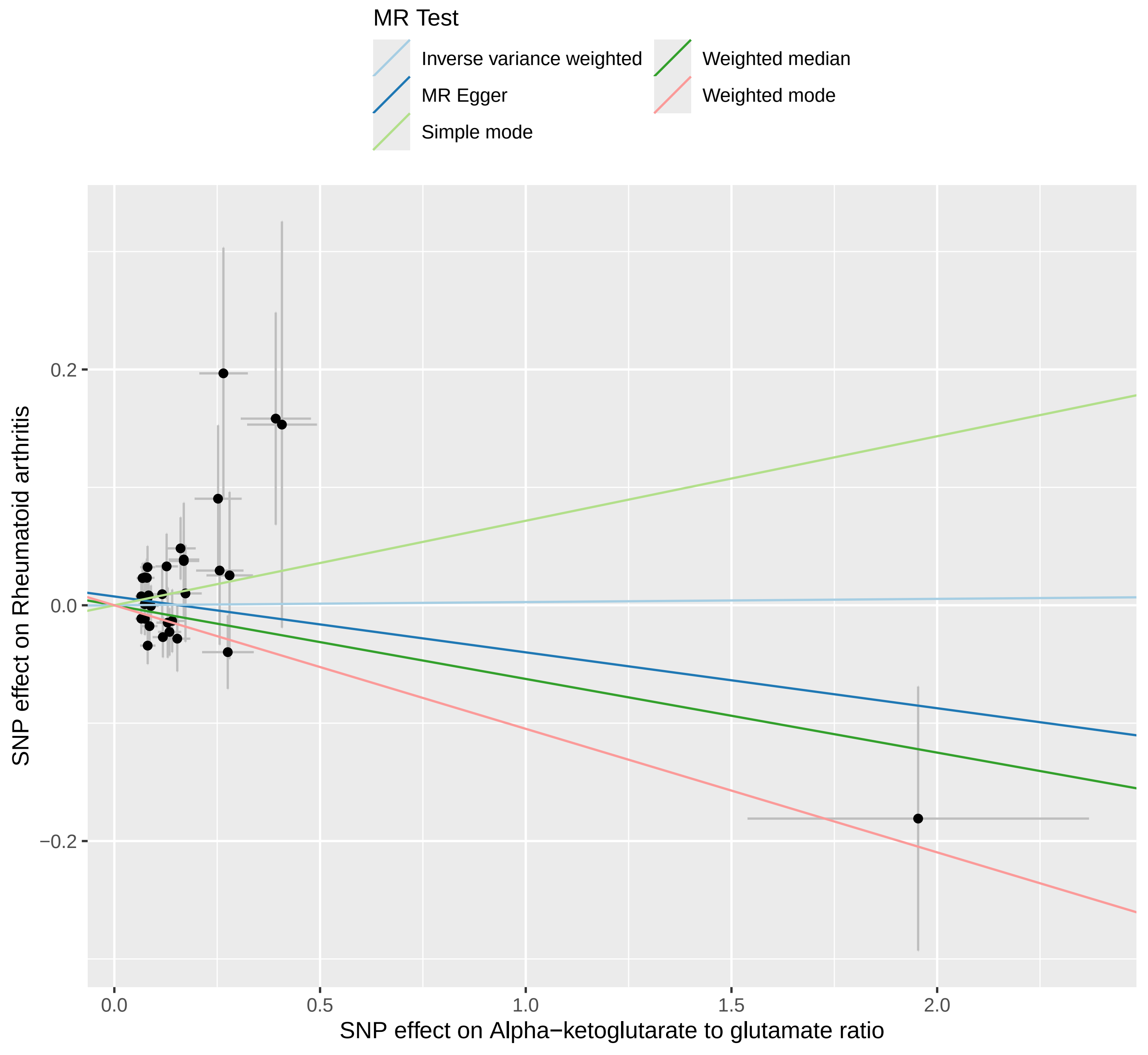

MR Test
Inverse variance weighted
MR Egger
Simple mode
Weighted median
Weighted mode
0.2
0.0
−0.2
SNP effect on Rheumatoid arthritis
0.0
0.5
1.0
1.5
2.0
SNP effect on Alpha−ketoglutarate to glutamate ratio

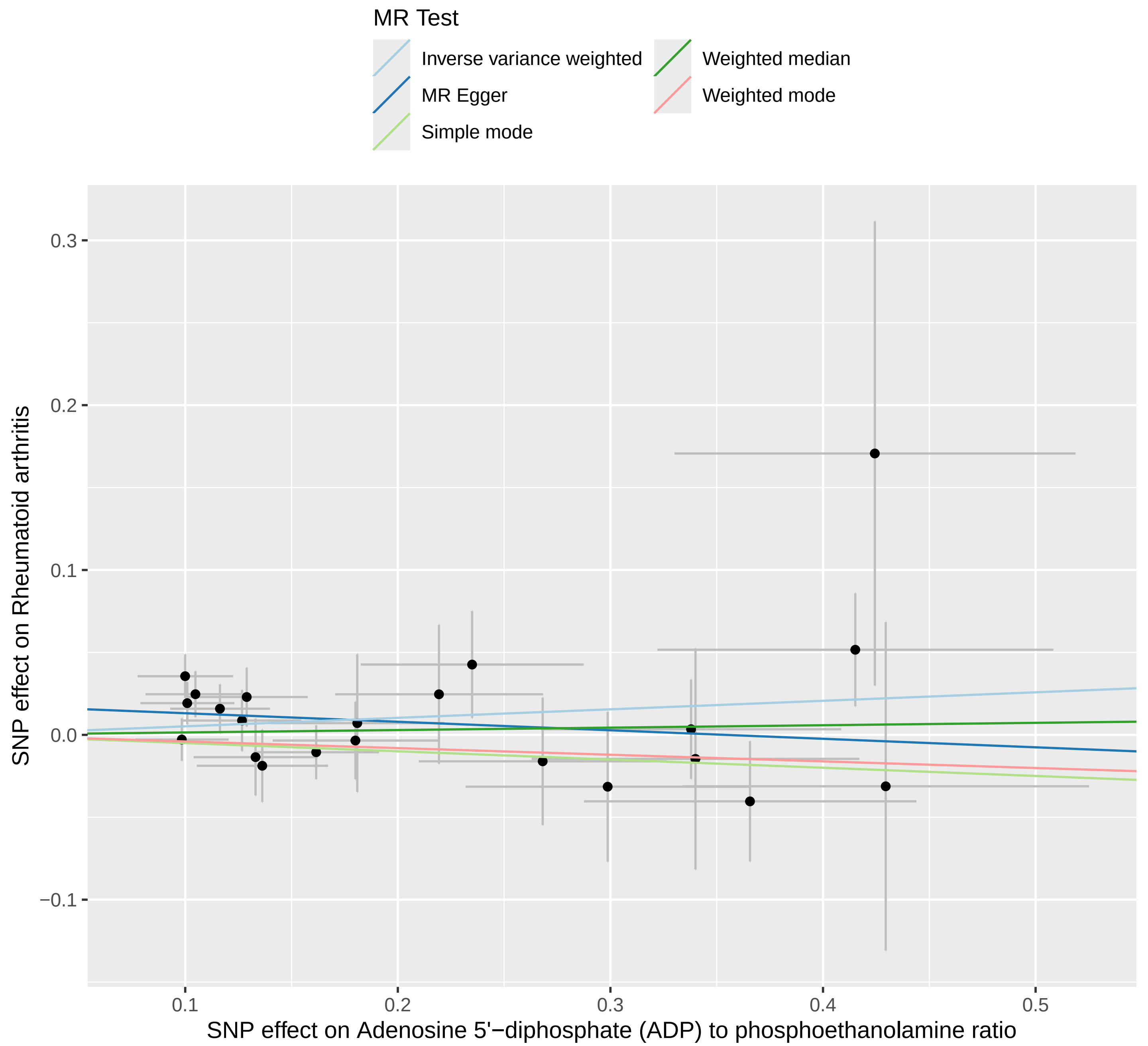

MR Test
Inverse variance weighted
MR Egger
Simple mode
Weighted median
Weighted mode
0.3
0.2
0.1
0.0
−0.1
0.1
0.2
0.3
0.4
0.5
SNP effect on Rheumatoid arthritis
SNP effect on Adenosine 5'−diphosphate (ADP) to phosphoethanolamine ratio

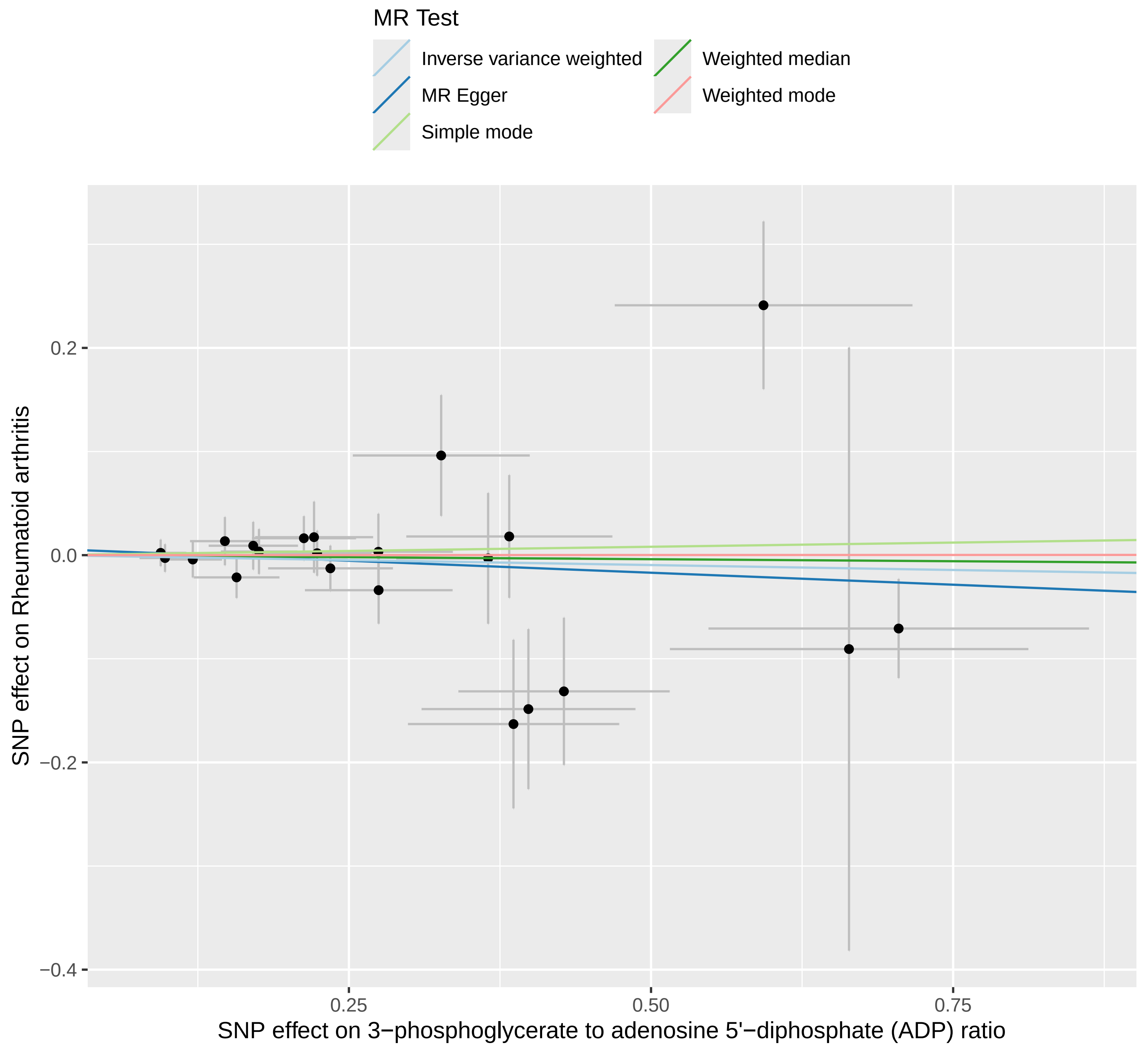

MR Test
Inverse variance weighted
MR Egger
Simple mode
Weighted median
Weighted mode
0.2
0.0
−0.2
−0.4
SNP effect on Rheumatoid arthritis
0.25
0.50
0.75
SNP effect on 3−phosphoglycerate to adenosine 5'−diphosphate (ADP) ratio

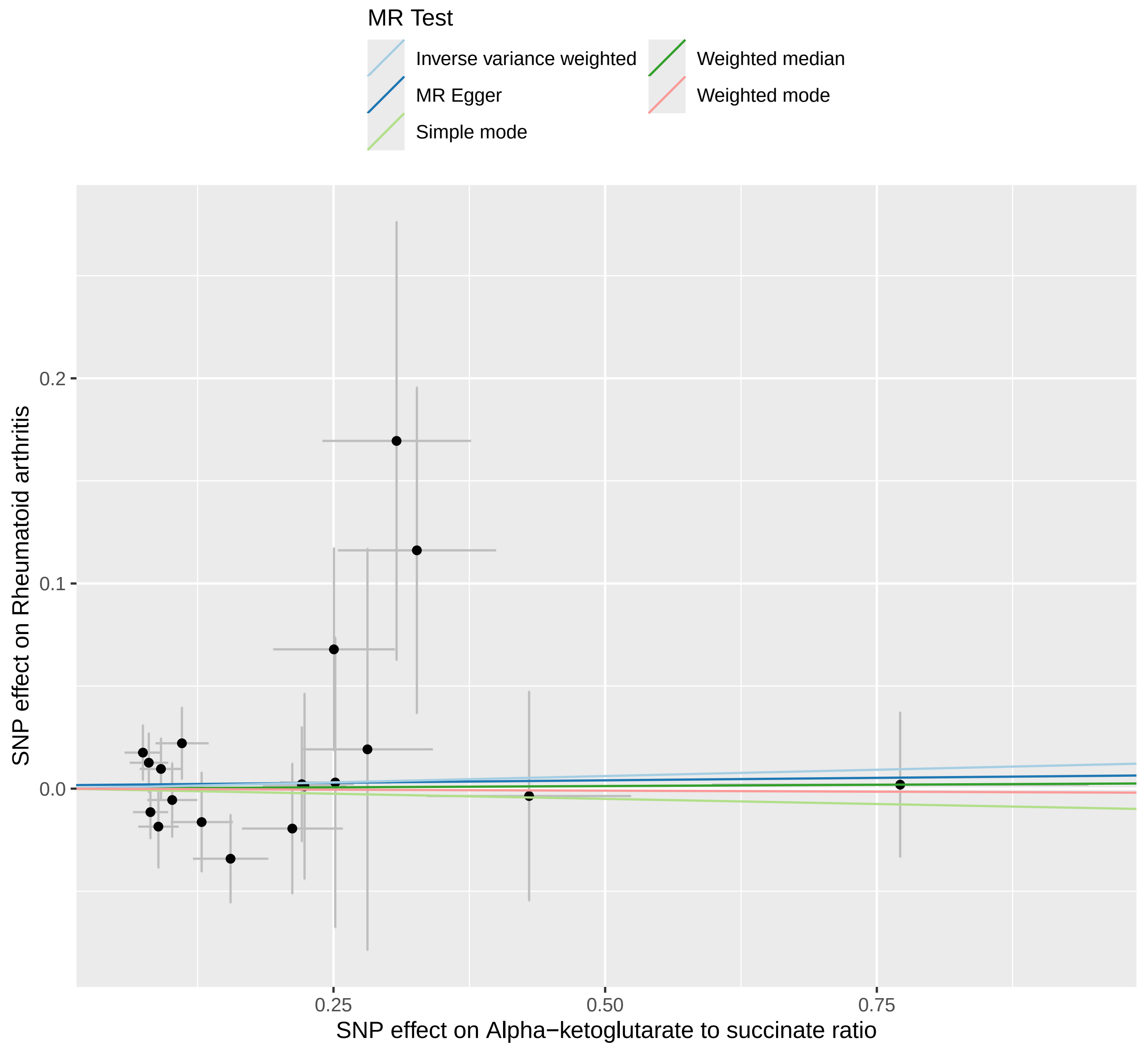
MR Test
Inverse variance weighted
MR Egger
Simple mode
Weighted median
Weighted mode
0.2
0.1
0.0
0.25
0.50
0.75
SNP effect on Rheumatoid arthritis
SNP effect on Alpha−ketoglutarate to succinate ratio

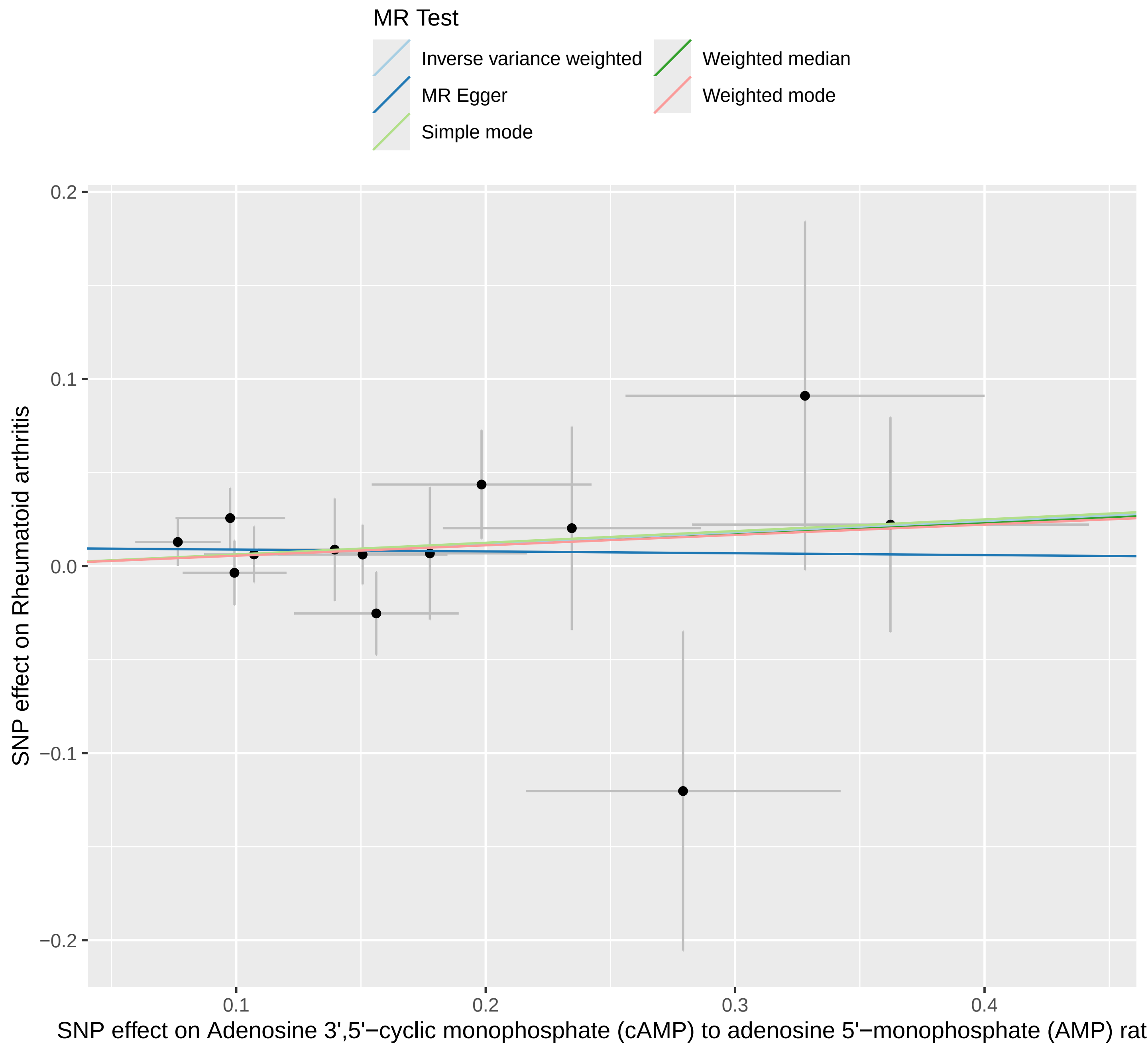

MR Test
Inverse variance weighted
MR Egger
Simple mode
Weighted median
Weighted mode
0.2
0.1
0.0
−0.1
−0.2
SNP effect on Rheumatoid arthritis
0.1
0.2
0.3
0.4
SNP effect on Adenosine 3',5'−cyclic monophosphate (cAMP) to adenosine 5'−monophosphate (AMP) rat

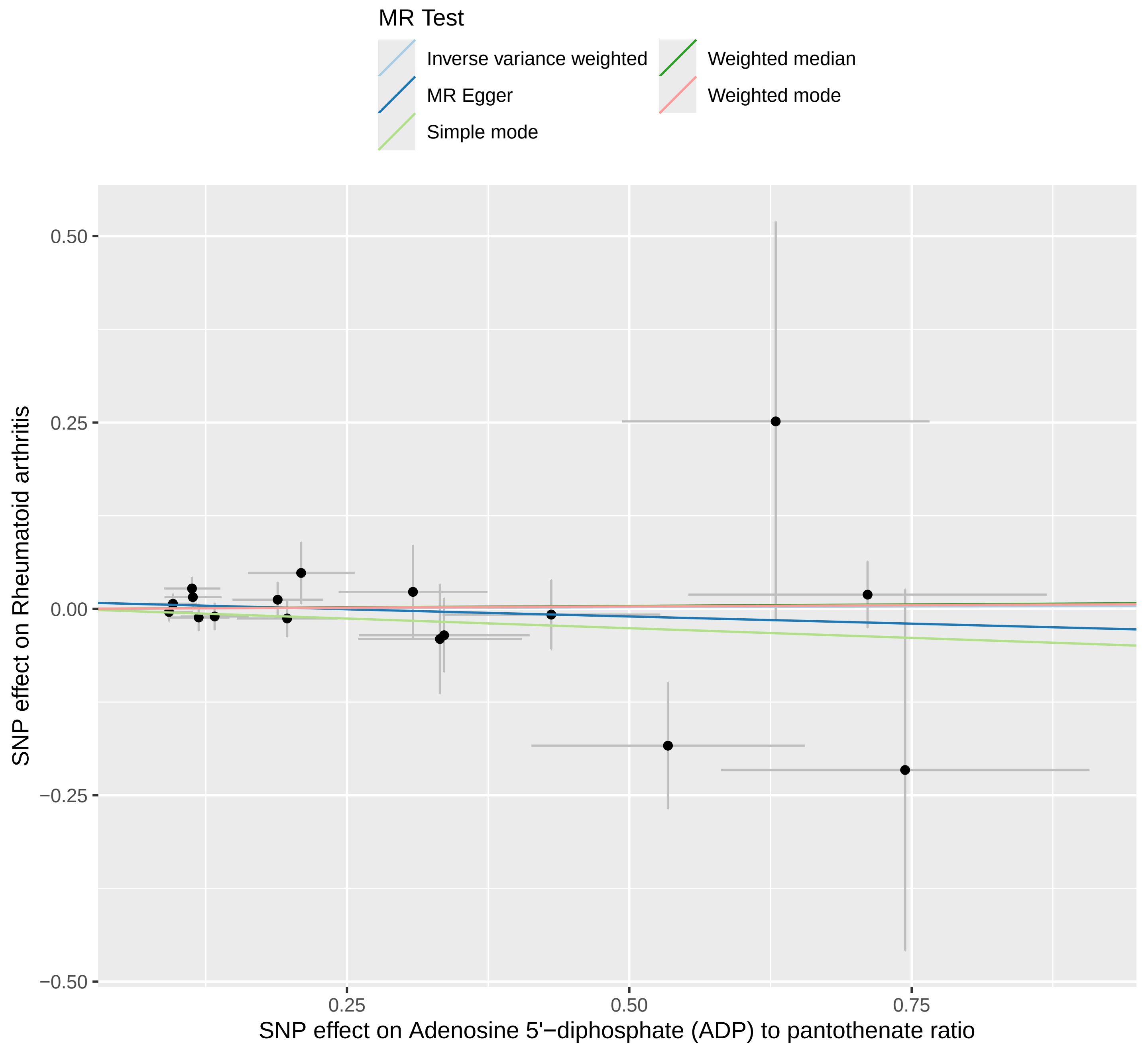

MR Test
Inverse variance weighted
MR Egger
Simple mode
Weighted median
Weighted mode
0.50
0.25
0.00
−0.25
−0.50
SNP effect on Rheumatoid arthritis
0.25
0.50
0.75
SNP effect on Adenosine 5'−diphosphate (ADP) to pantothenate ratio

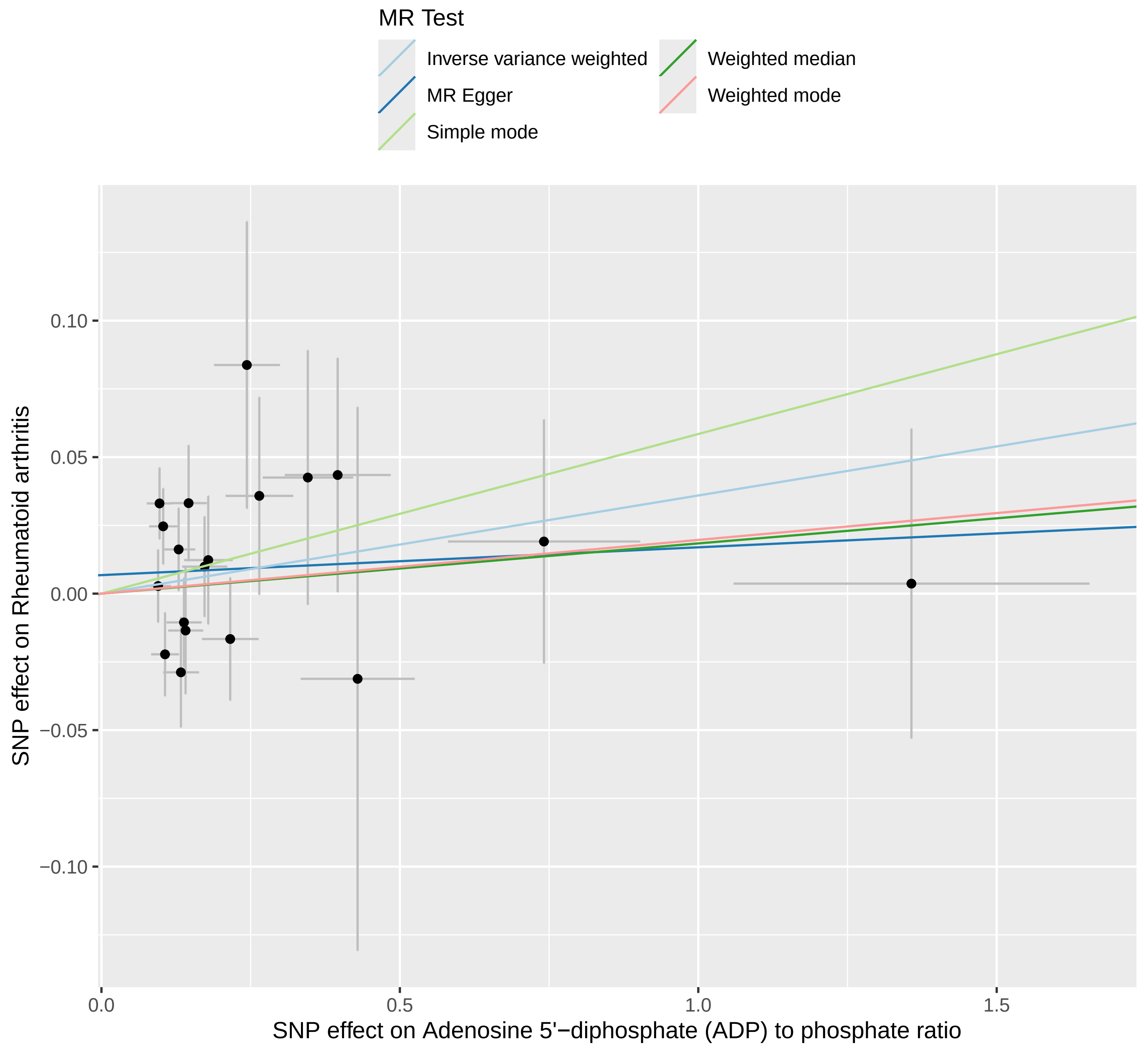

MR Test
Inverse variance weighted
MR Egger
Simple mode
Weighted median
Weighted mode
0.10
0.05
0.00
−0.05
−0.10
SNP effect on Rheumatoid arthritis
0.0
0.5
1.0
1.5
SNP effect on Adenosine 5'−diphosphate (ADP) to phosphate ratio

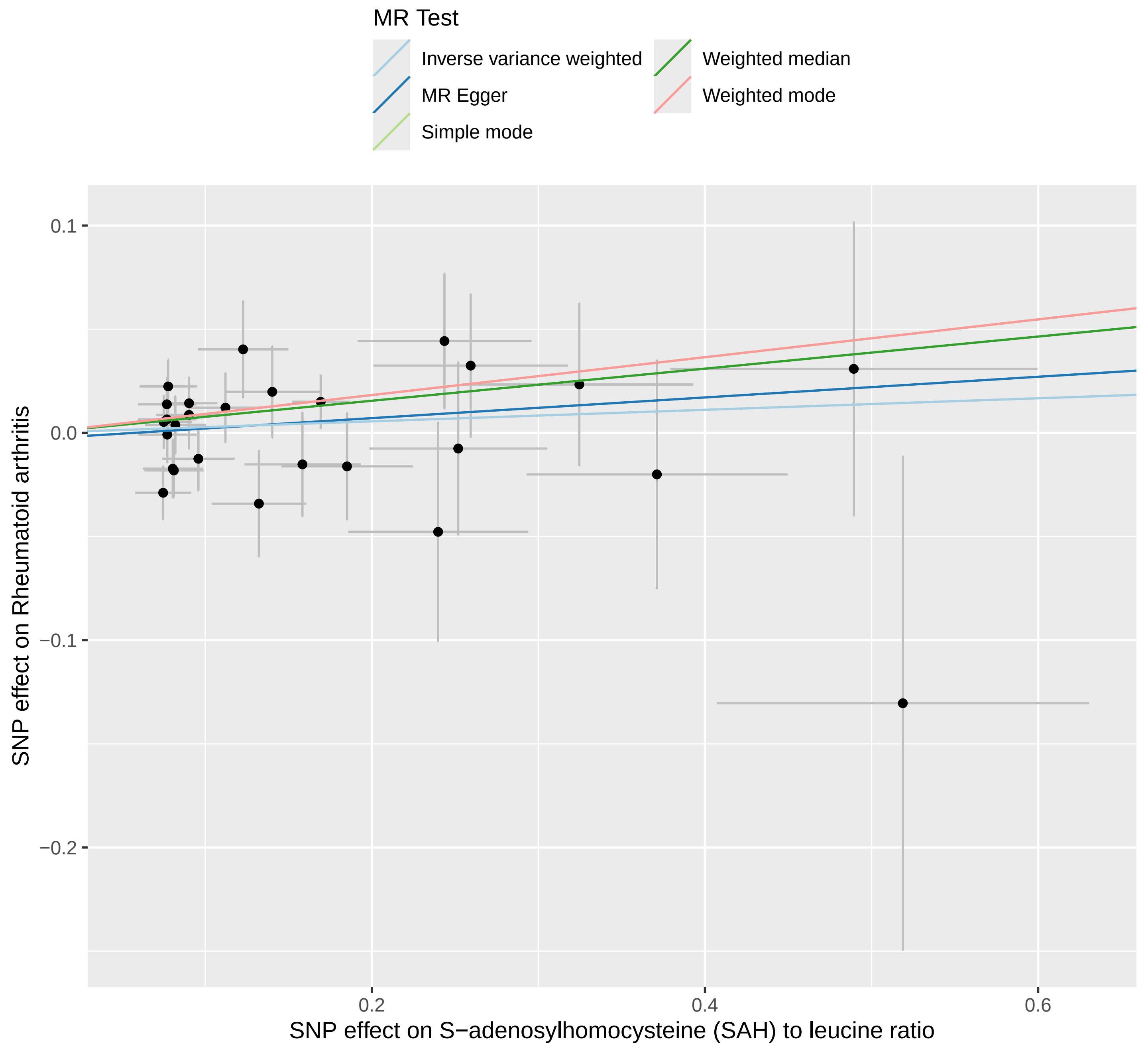

MR Test
Inverse variance weighted
MR Egger
Simple mode
Weighted median
Weighted mode
0.1
0.0
−0.1
−0.2
0.2
0.4
0.6
SNP effect on Rheumatoid arthritis
SNP effect on S−adenosylhomocysteine (SAH) to leucine ratio

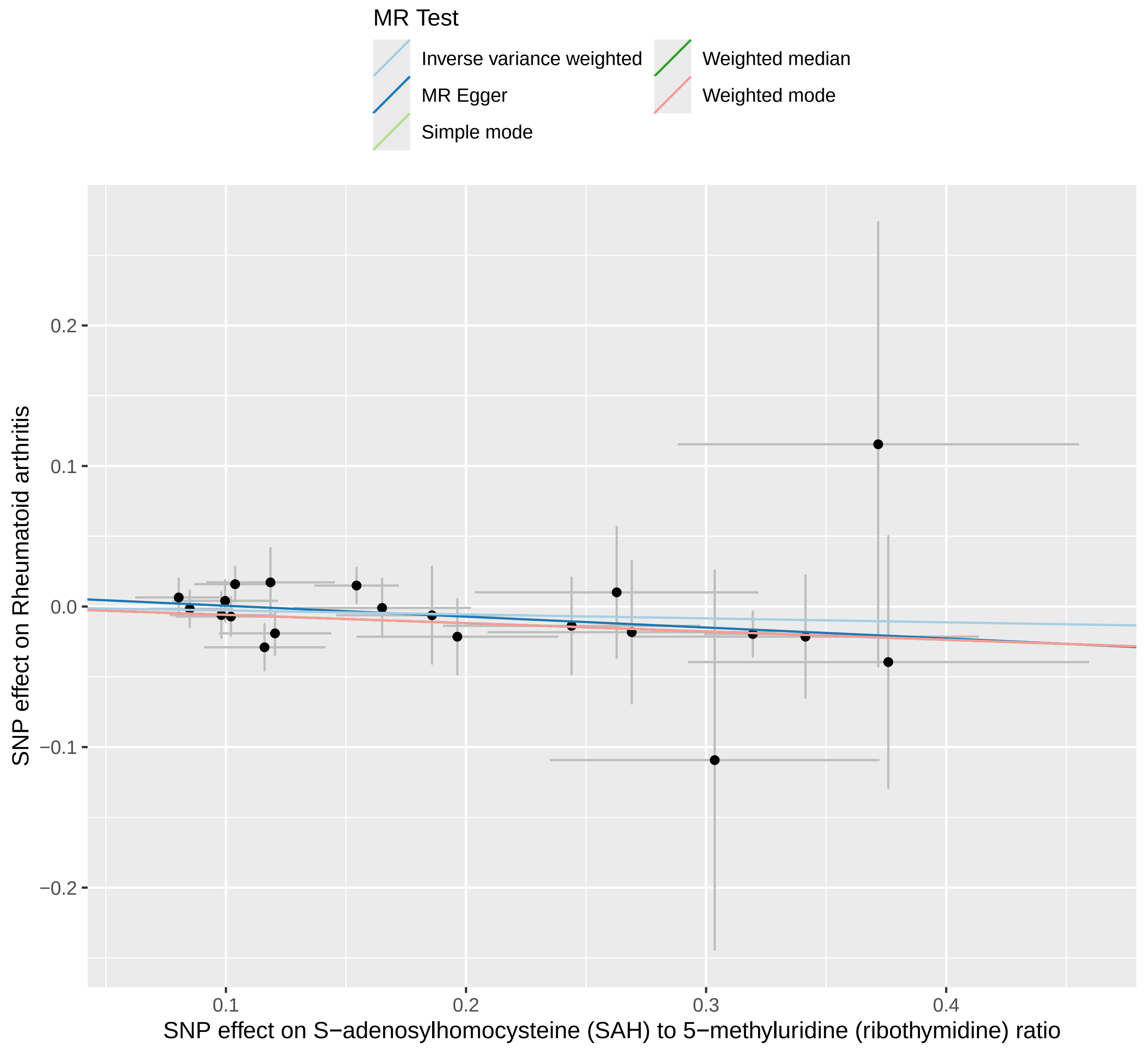
MR Test
Inverse variance weighted
MR Egger
Simple mode
Weighted median
Weighted mode
0.2
0.1
0.0
−0.1
−0.2
0.1
0.2
0.3
0.4
SNP effect on Rheumatoid arthritis
SNP effect on S−adenosylhomocysteine (SAH) to 5−methyluridine (ribothymidine) ratio

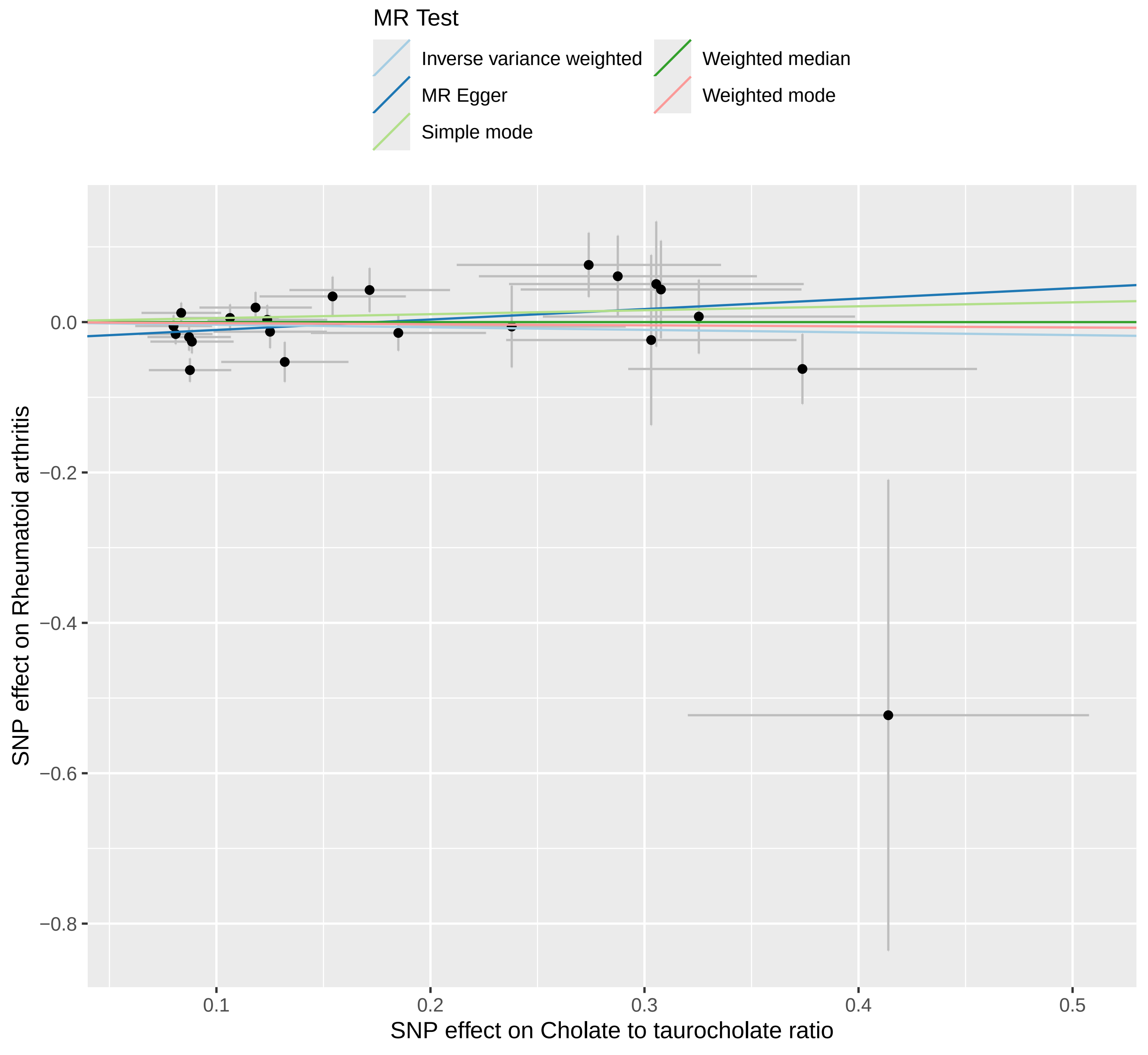

MR Test
Inverse variance weighted
MR Egger
Simple mode
Weighted median
Weighted mode
0.0
−0.2
−0.4
−0.6
−0.8
0.1
0.2
0.3
0.4
0.5
SNP effect on Rheumatoid arthritis
SNP effect on Cholate to taurocholate ratio

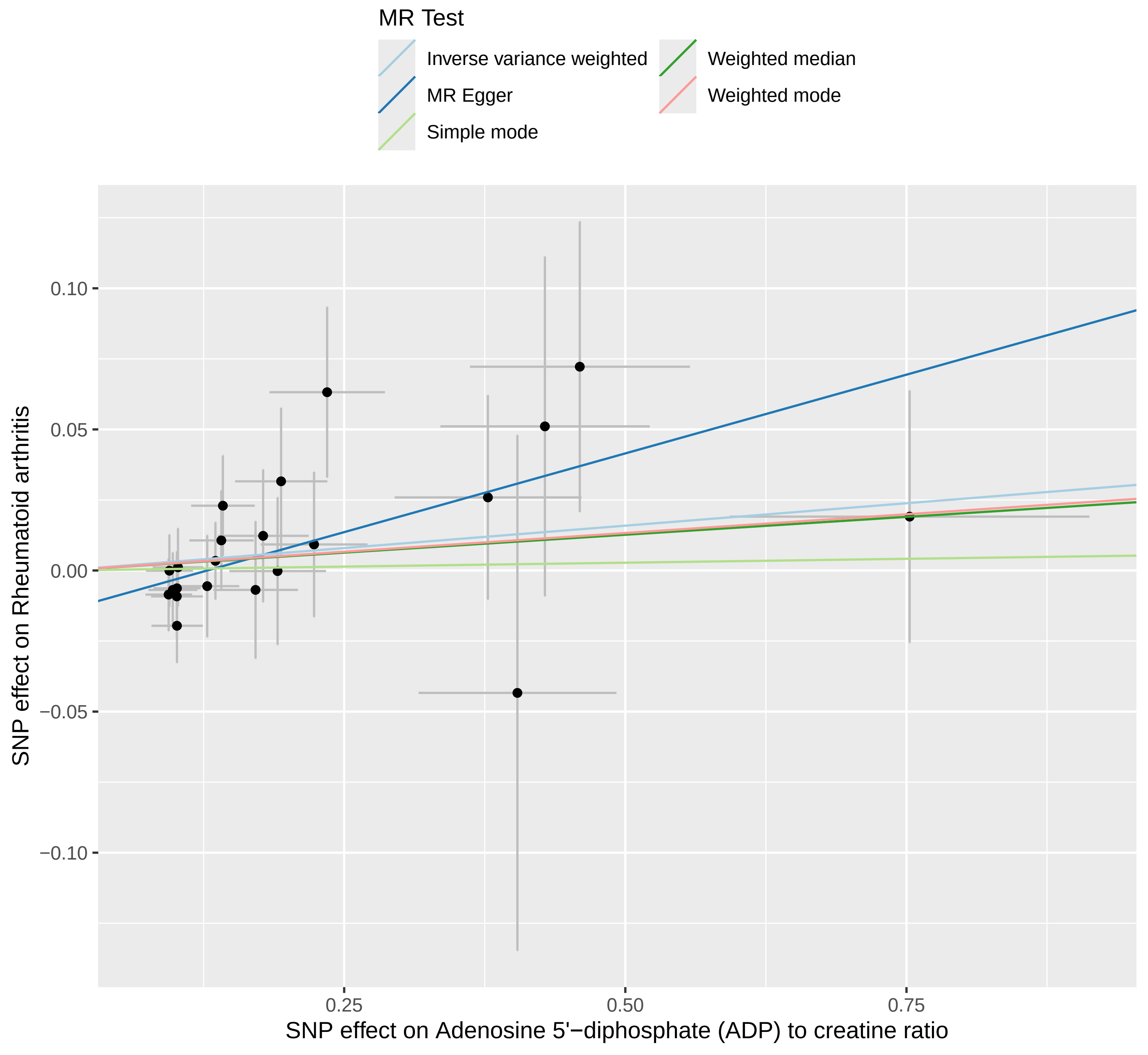
MR Test
Inverse variance weighted
MR Egger
Simple mode
Weighted median
Weighted mode
0.10
0.05
0.00
−0.05
−0.10
0.25
0.50
0.75
SNP effect on Rheumatoid arthritis
SNP effect on Adenosine 5'−diphosphate (ADP) to creatine ratio

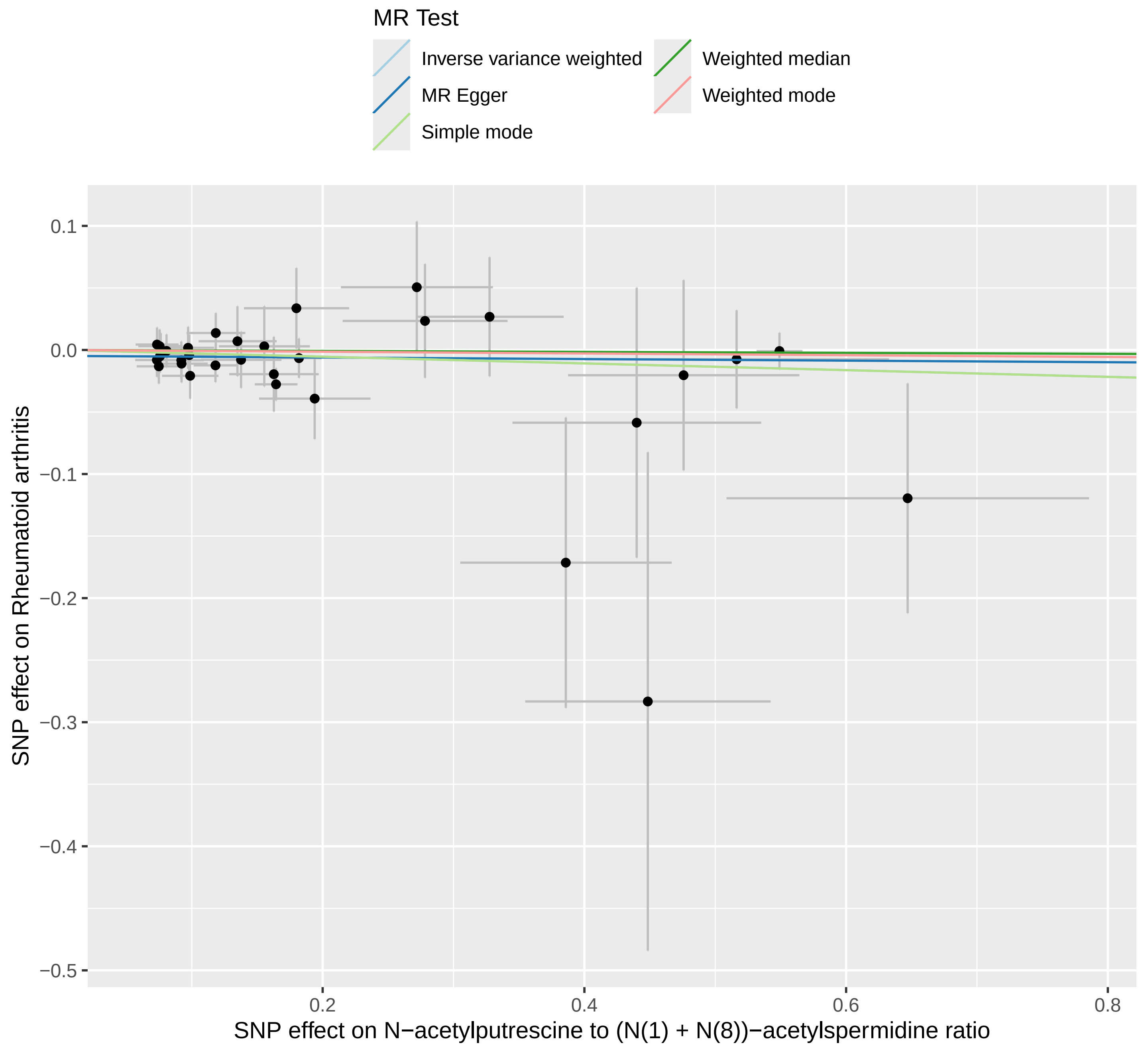
MR Test
Inverse variance weighted
MR Egger
Simple mode
Weighted median
Weighted mode
0.1
0.0
−0.1
−0.2
−0.3
−0.4
−0.5
0.2
0.4
0.6
0.8
SNP effect on Rheumatoid arthritis
SNP effect on N−acetylputrescine to (N(1) + N(8))−acetylspermidine ratio

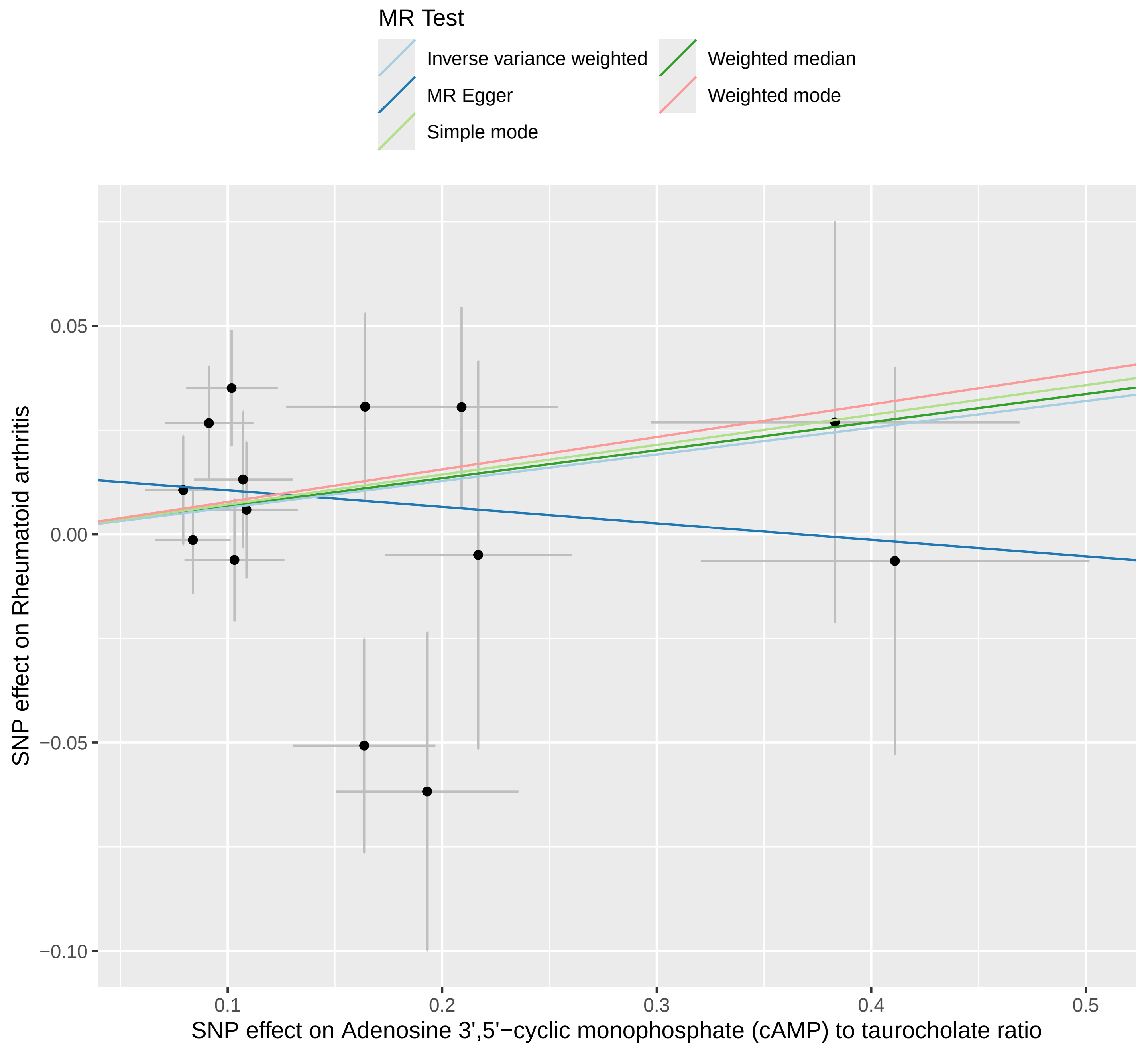
MR Test
Inverse variance weighted
MR Egger
Simple mode
Weighted median
Weighted mode
0.05
0.00
−0.05
−0.10
0.1
0.2
0.3
0.4
0.5
SNP effect on Rheumatoid arthritis
SNP effect on Adenosine 3',5'−cyclic monophosphate (cAMP) to taurocholate ratio

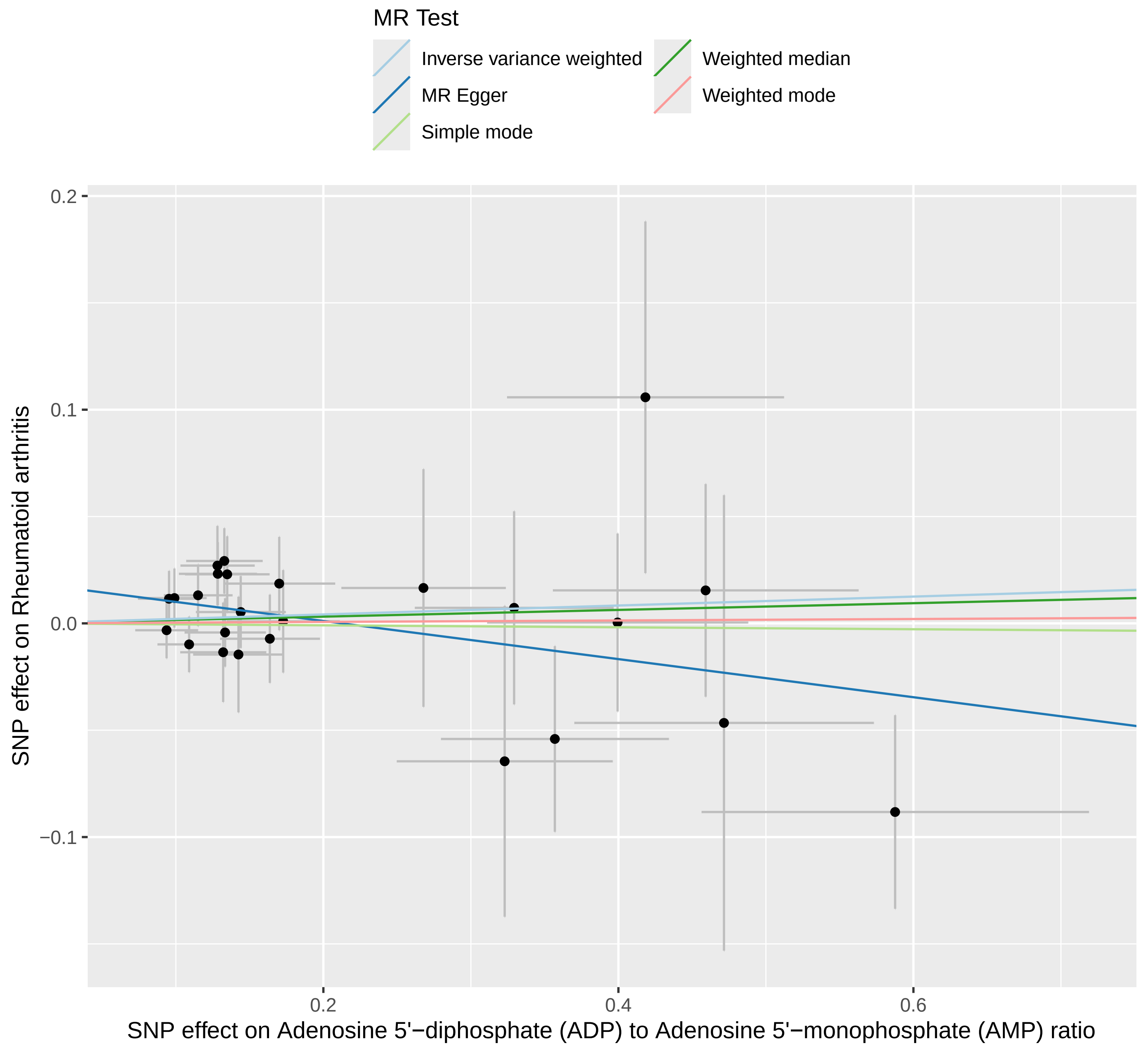

MR Test
Inverse variance weighted
MR Egger
Simple mode
Weighted median
Weighted mode
SNP effect on Rheumatoid arthritis
0.2
0.1
0.0
−0.1
0.2
0.4
0.6
SNP effect on Adenosine 5'−diphosphate (ADP) to Adenosine 5'−monophosphate (AMP) ratio

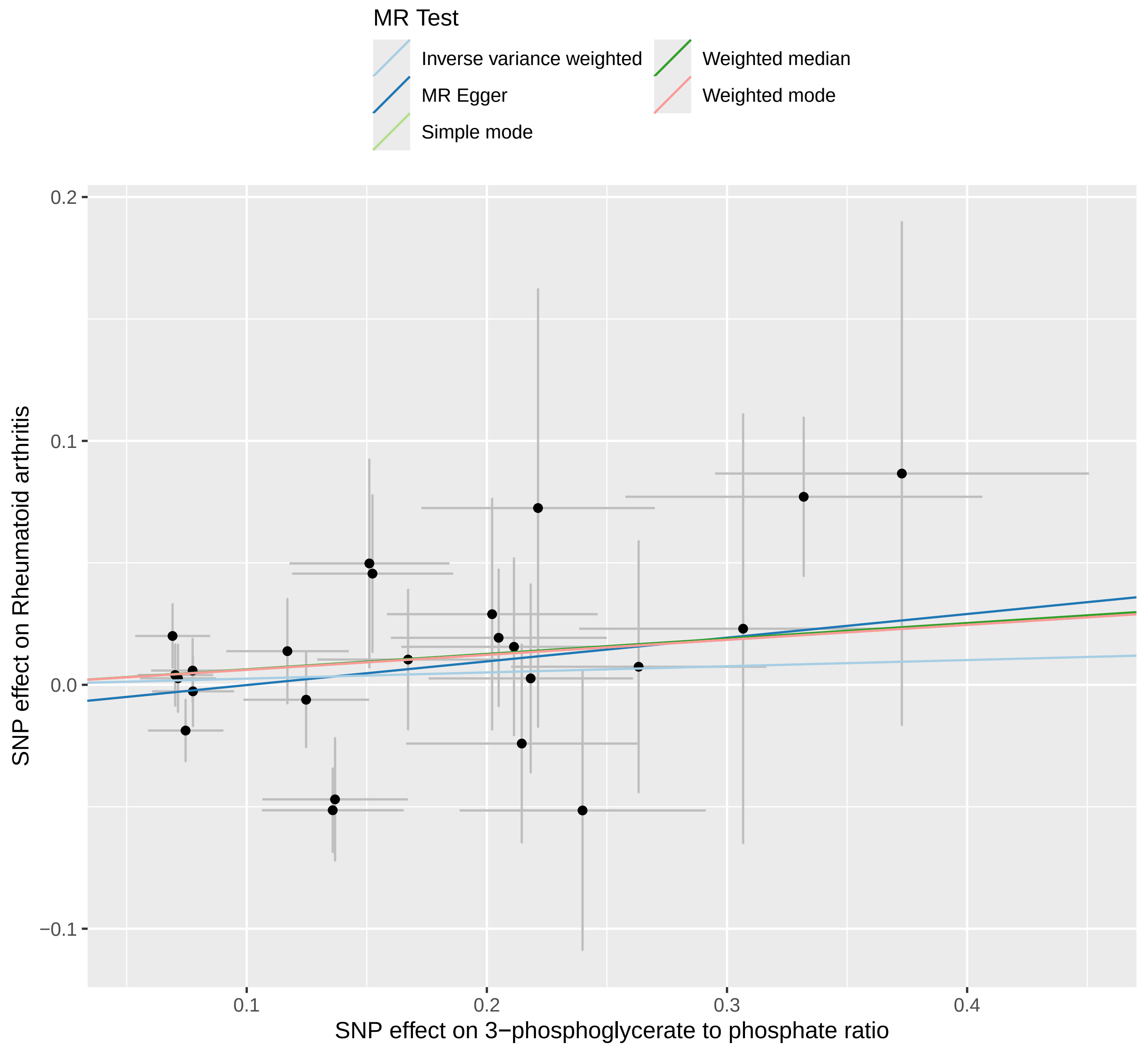
MR Test
Inverse variance weighted
MR Egger
Simple mode
Weighted median
Weighted mode
SNP effect on Rheumatoid arthritis
0.2
0.1
0.0
−0.1
0.1
0.2
0.3
0.4
SNP effect on 3−phosphoglycerate to phosphate ratio

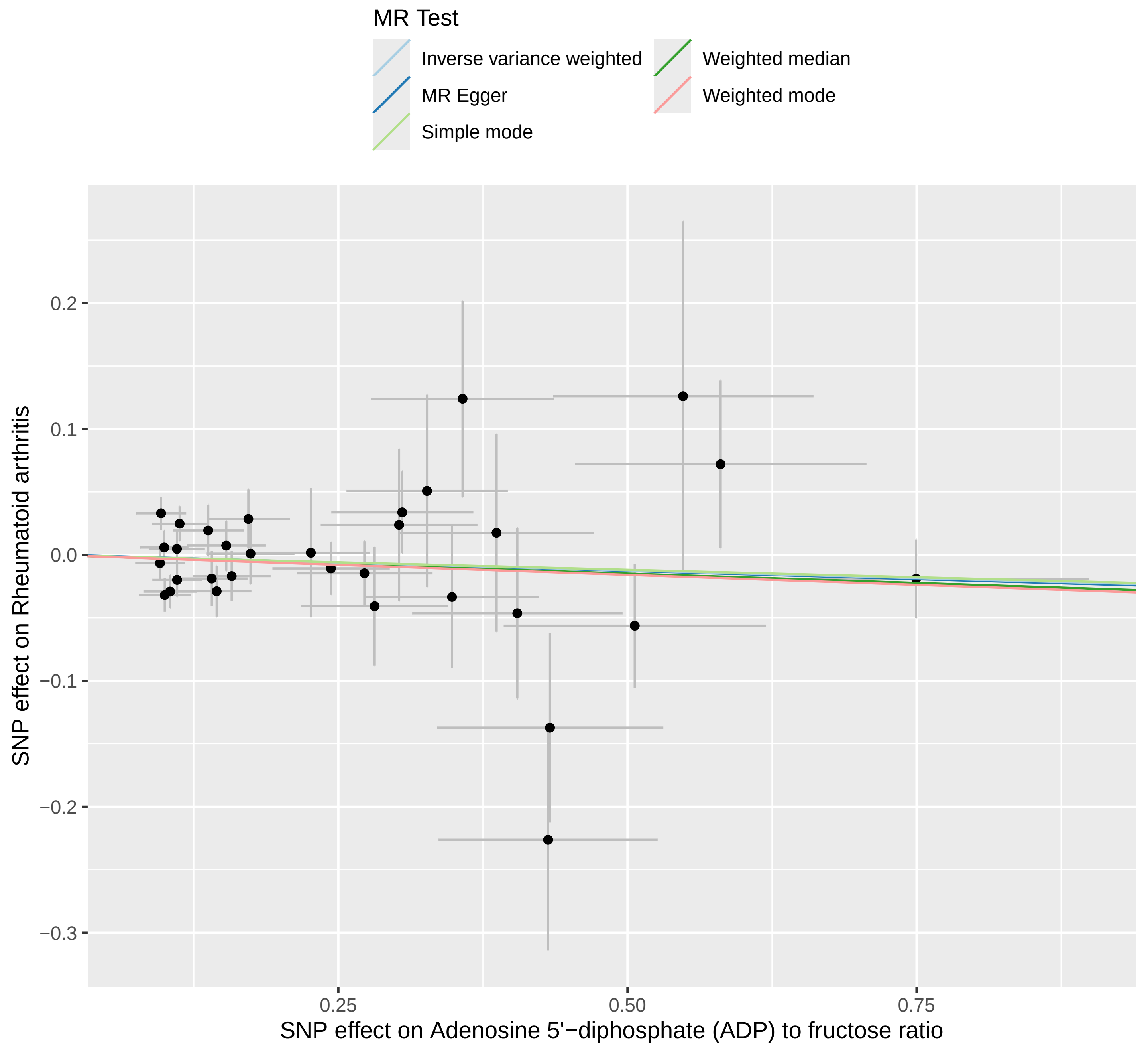

MR Test
Inverse variance weighted
MR Egger
Simple mode
Weighted median
Weighted mode
0.2
0.1
0.0
−0.1
−0.2
−0.3
SNP effect on Rheumatoid arthritis
0.25
0.50
0.75
SNP effect on Adenosine 5'−diphosphate (ADP) to fructose ratio

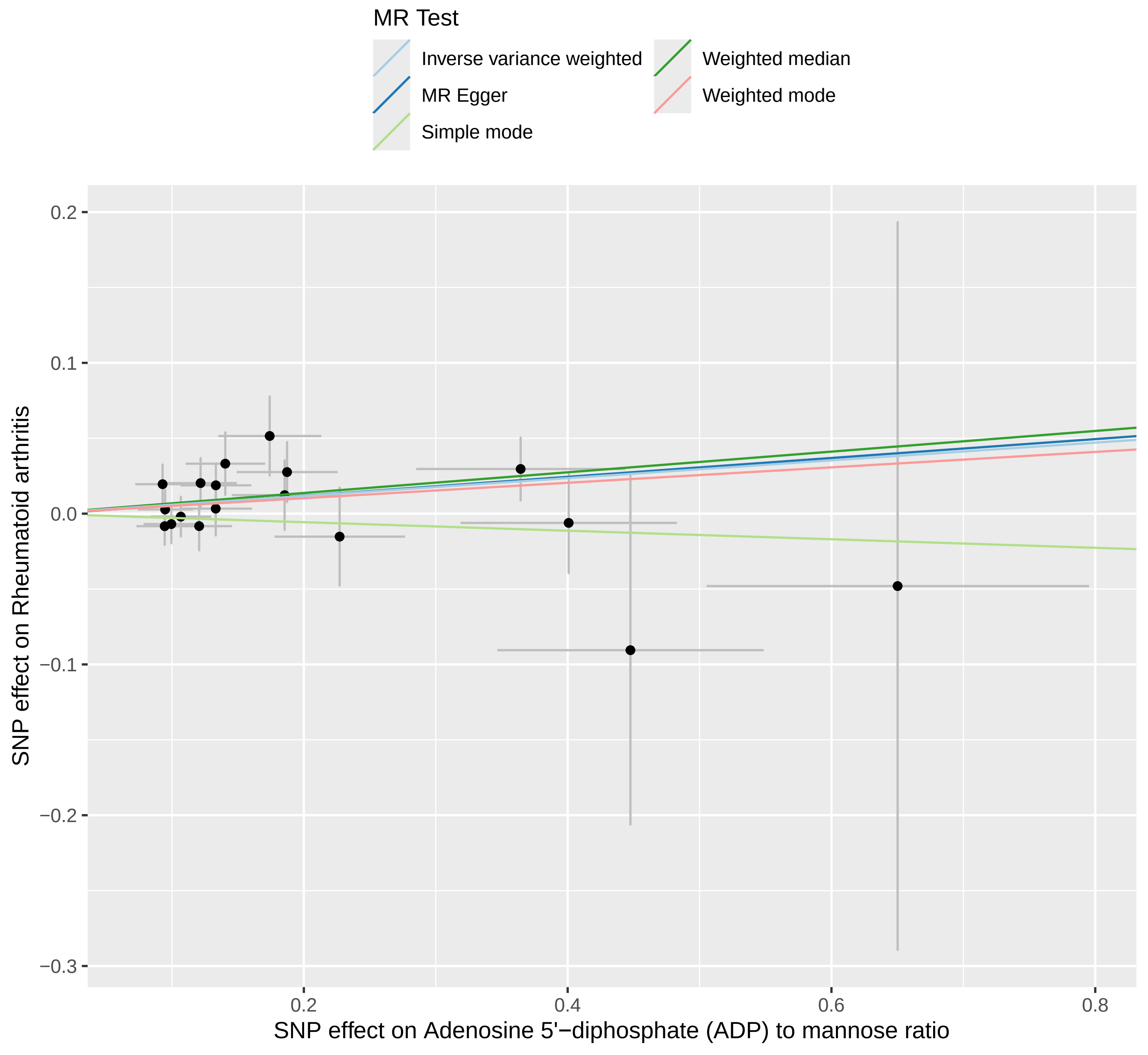

MR Test
Inverse variance weighted
MR Egger
Simple mode
Weighted median
Weighted mode
0.2
0.1
0.0
−0.1
−0.2
−0.3
SNP effect on Rheumatoid arthritis
0.2
0.4
0.6
0.8
SNP effect on Adenosine 5'−diphosphate (ADP) to mannose ratio

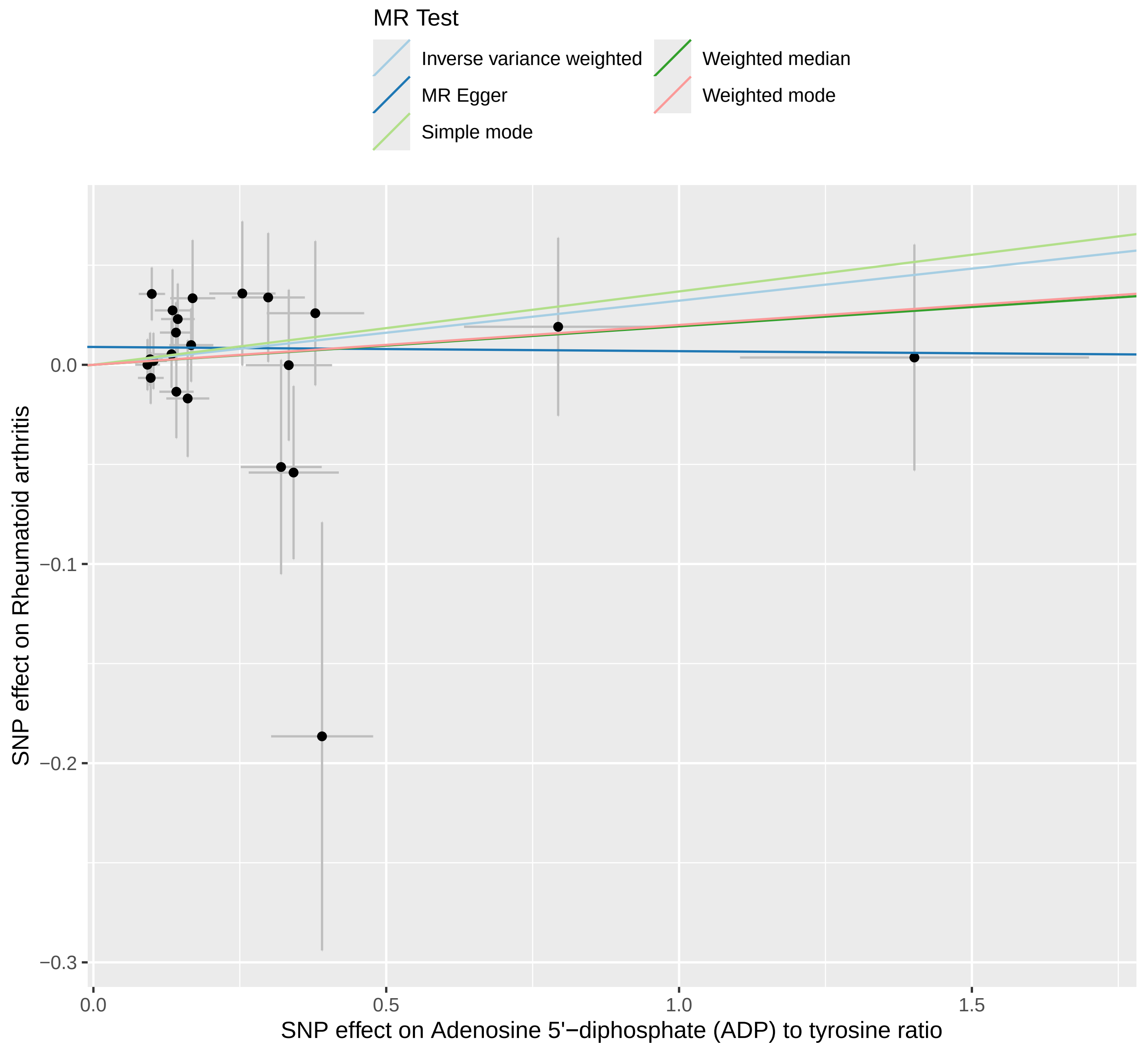
MR Test
Inverse variance weighted
MR Egger
Simple mode
Weighted median
Weighted mode
0.0
−0.1
−0.2
−0.3
0.0
0.5
1.0
1.5
SNP effect on Rheumatoid arthritis
SNP effect on Adenosine 5'−diphosphate (ADP) to tyrosine ratio

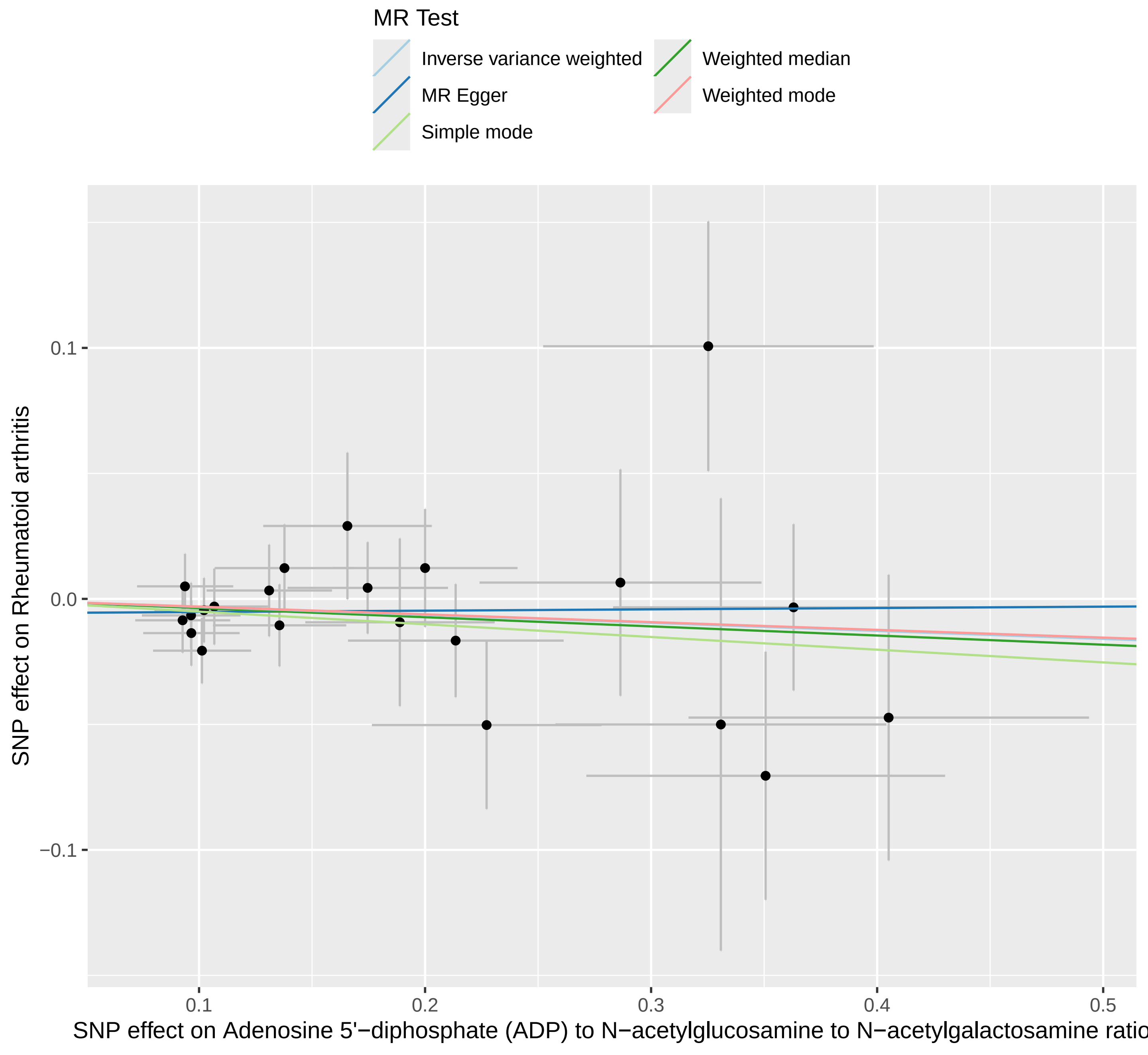

MR Test
Inverse variance weighted
MR Egger
Simple mode
Weighted median
Weighted mode
0.1
0.0
−0.1
0.1
0.2
0.3
0.4
0.5
SNP effect on Rheumatoid arthritis
SNP effect on Adenosine 5'−diphosphate (ADP) to N−acetylglucosamine to N−acetylgalactosamine ratio

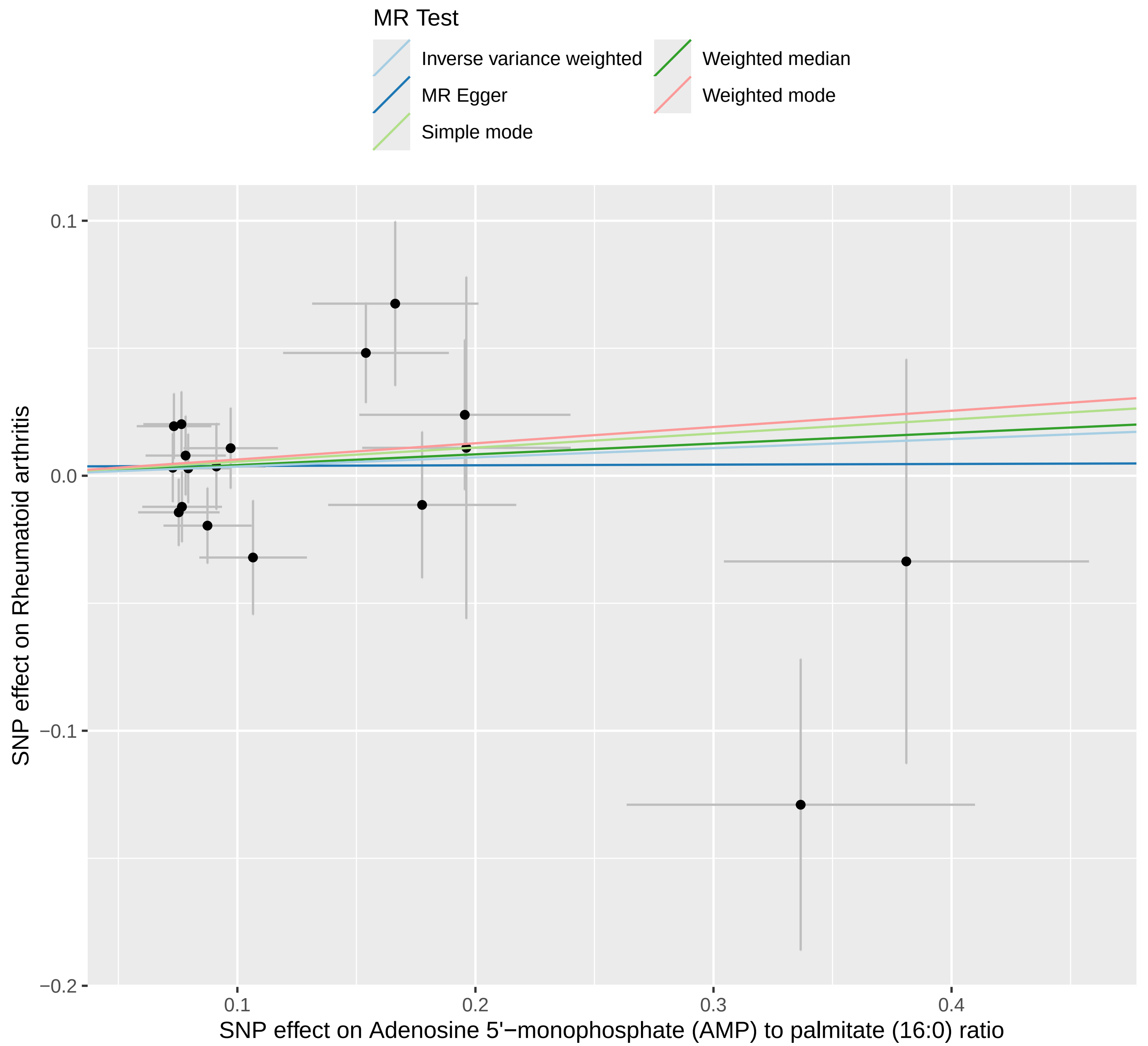

MR Test
Inverse variance weighted
MR Egger
Simple mode
Weighted median
Weighted mode
0.1
0.0
−0.1
−0.2
0.1
0.2
0.3
0.4
SNP effect on Rheumatoid arthritis
SNP effect on Adenosine 5'−monophosphate (AMP) to palmitate (16:0) ratio

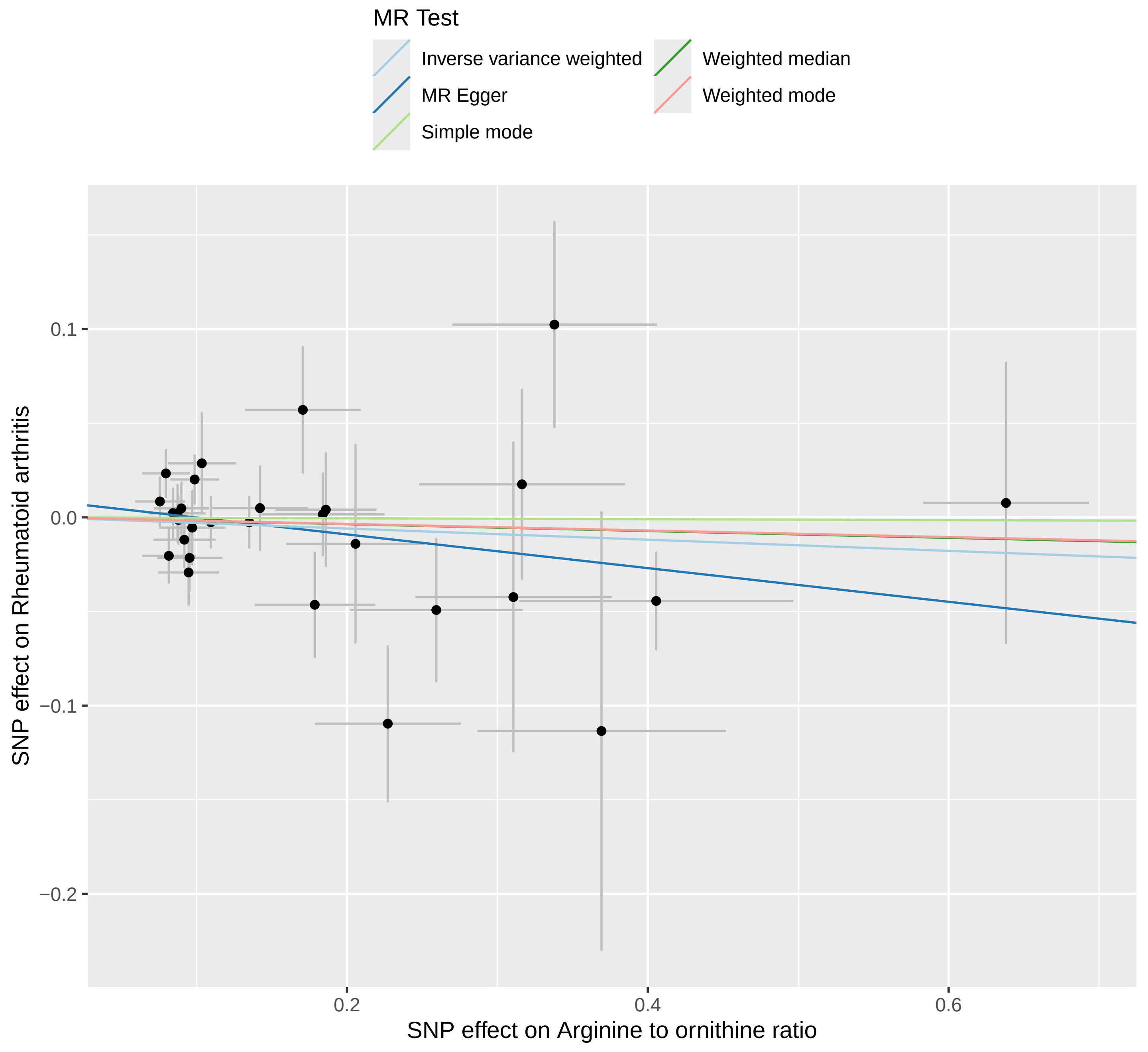
MR Test
Inverse variance weighted
MR Egger
Simple mode
Weighted median
Weighted mode
0.1
0.0
−0.1
−0.2
0.2
0.4
0.6
SNP effect on Rheumatoid arthritis
SNP effect on Arginine to ornithine ratio

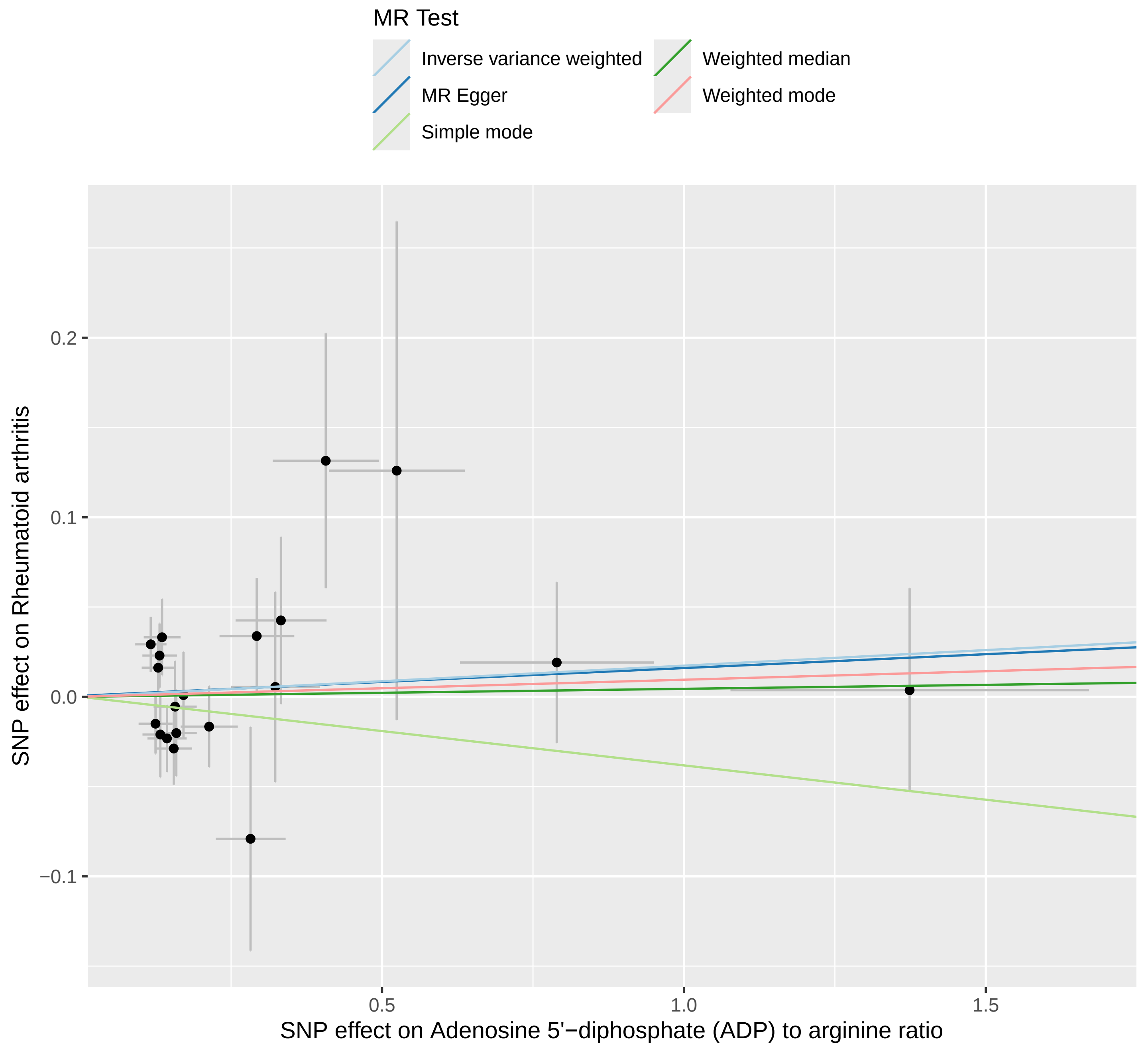
MR Test
Inverse variance weighted
MR Egger
Simple mode
Weighted median
Weighted mode
0.2
0.1
0.0
−0.1
SNP effect on Rheumatoid arthritis
0.5
1.0
1.5
SNP effect on Adenosine 5'−diphosphate (ADP) to arginine ratio

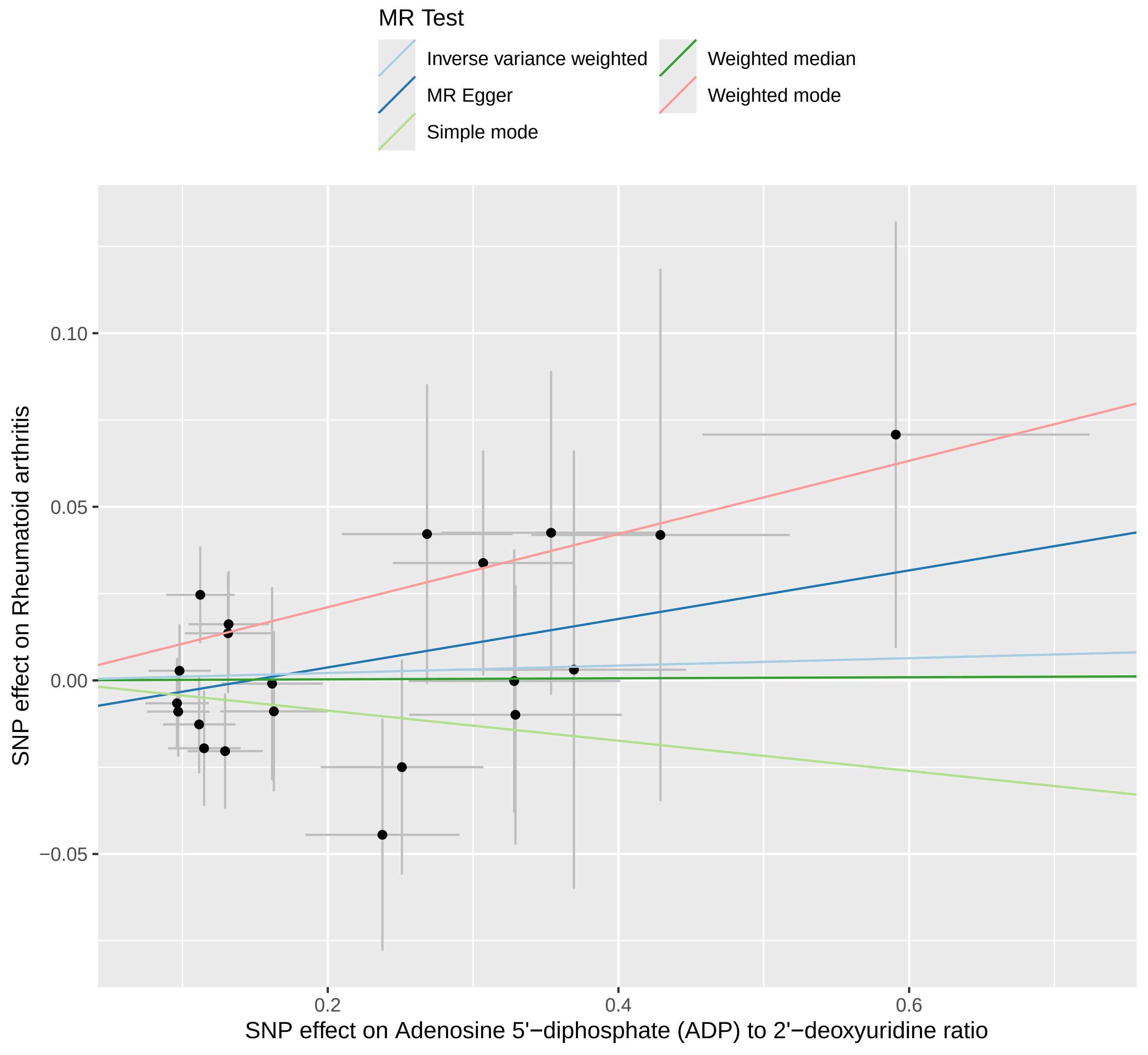

MR Test
Inverse variance weighted
MR Egger
Simple mode
Weighted median
Weighted mode
0.10
0.05
0.00
−0.05
0.2
0.4
0.6
SNP effect on Rheumatoid arthritis
SNP effect on Adenosine 5'−diphosphate (ADP) to 2'−deoxyuridine ratio

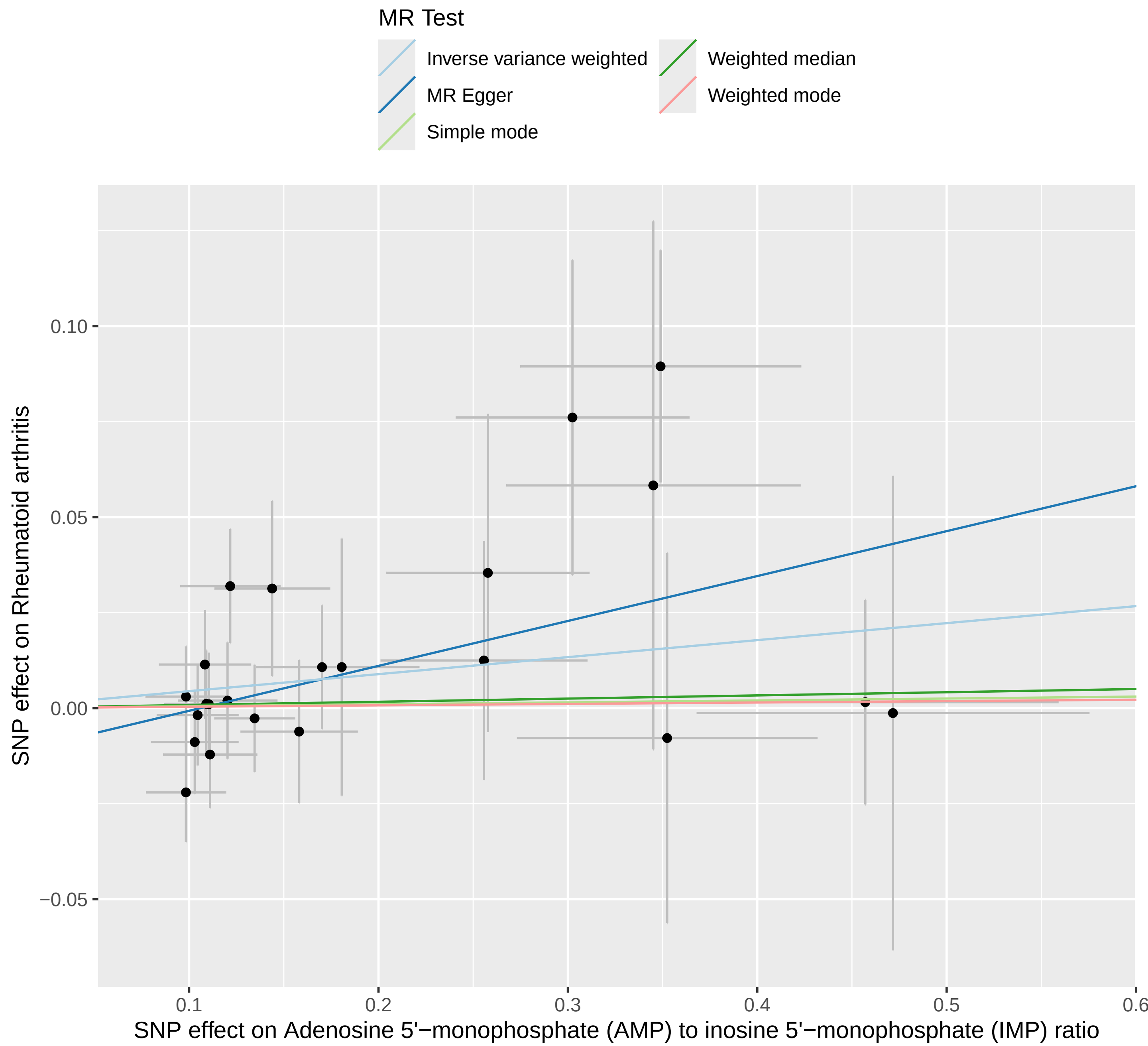
MR Test
Inverse variance weighted
MR Egger
Simple mode
Weighted median
Weighted mode
SNP effect on Rheumatoid arthritis
0.10
0.05
0.00
−0.05
0.1
0.2
0.3
0.4
0.5
0.6
SNP effect on Adenosine 5'−monophosphate (AMP) to inosine 5'−monophosphate (IMP) ratio

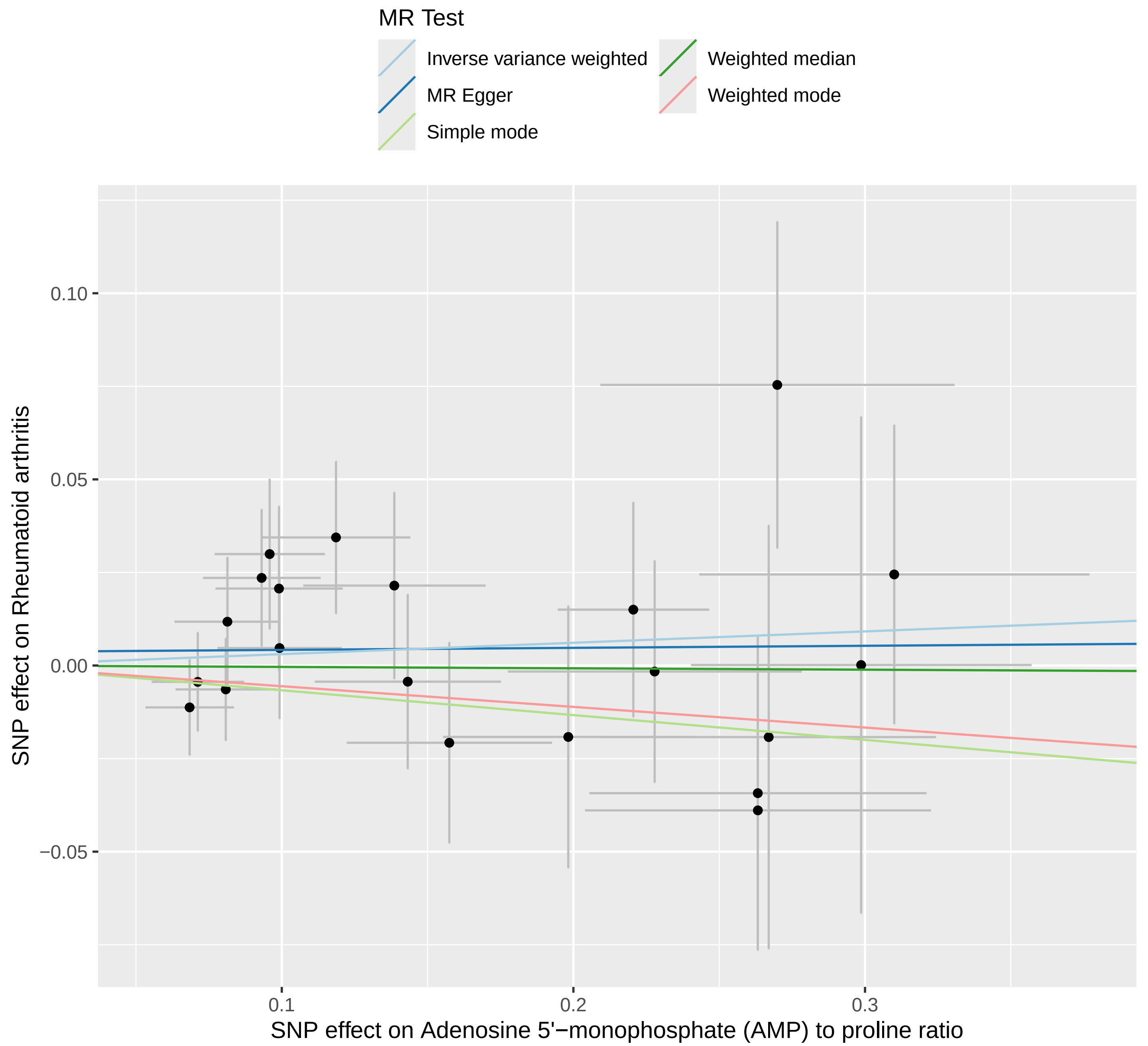
MR Test
Inverse variance weighted
MR Egger
Simple mode
Weighted median
Weighted mode
SNP effect on Rheumatoid arthritis
0.10
0.05
0.00
−0.05
0.1
0.2
0.3
SNP effect on Adenosine 5'−monophosphate (AMP) to proline ratio

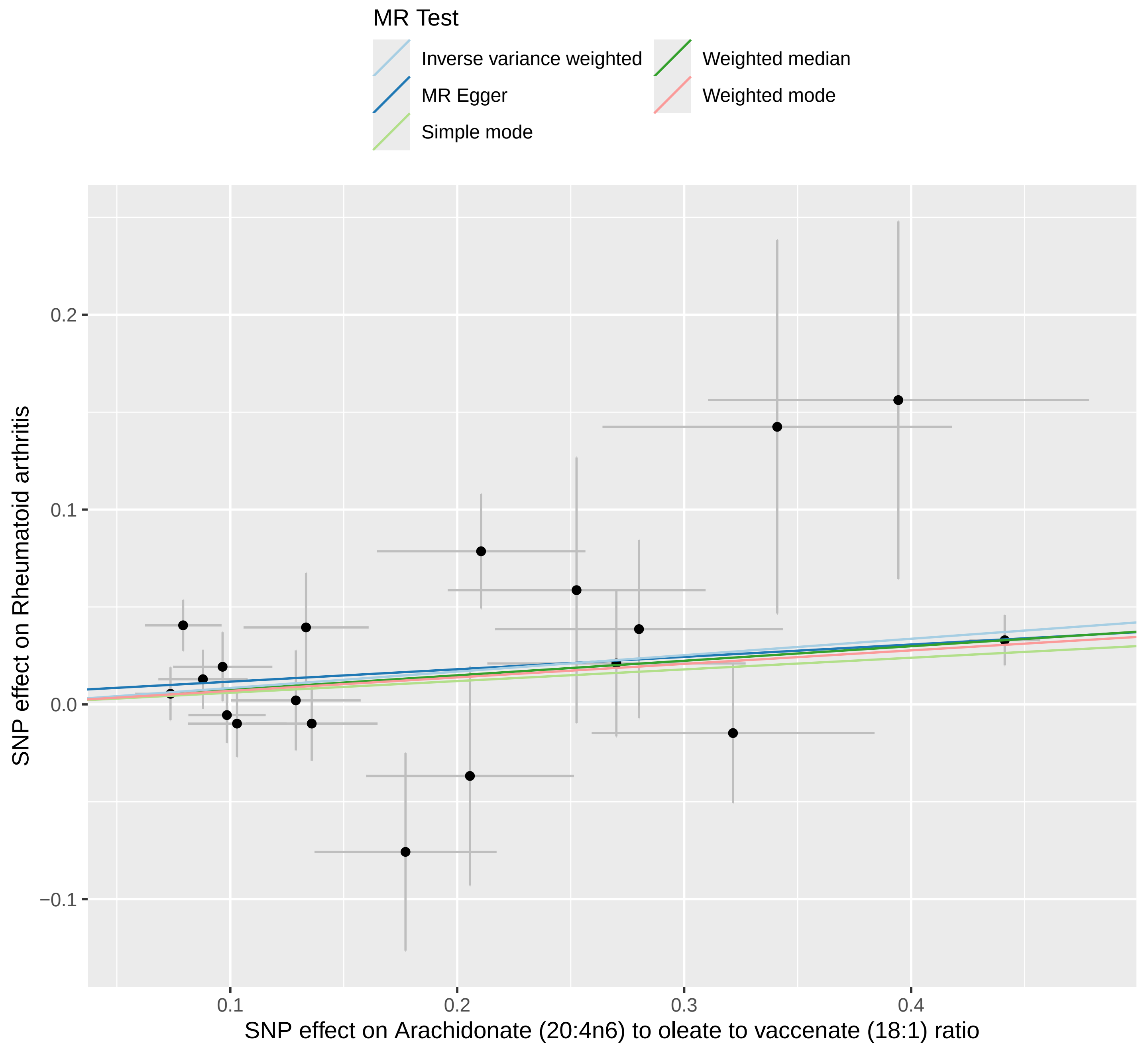

MR Test
Inverse variance weighted
MR Egger
Simple mode
Weighted median
Weighted mode
0.2
0.1
0.0
−0.1
0.1
0.2
0.3
0.4
SNP effect on Rheumatoid arthritis
SNP effect on Arachidonate (20:4n6) to oleate to vaccenate (18:1) ratio

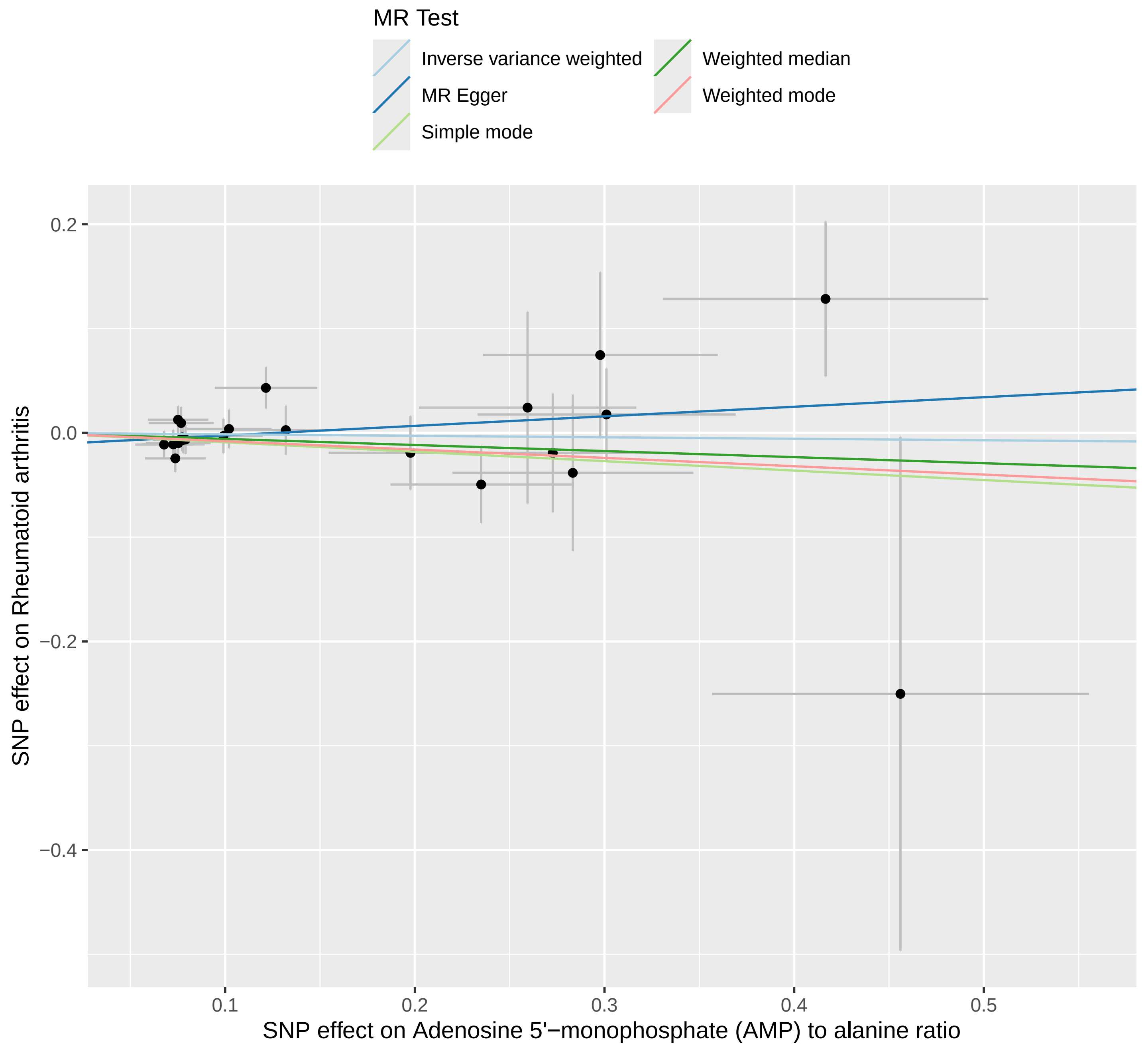
MR Test
Inverse variance weighted
MR Egger
Simple mode
Weighted median
Weighted mode
0.2
0.0
−0.2
−0.4
0.1
0.2
0.3
0.4
0.5
SNP effect on Rheumatoid arthritis
SNP effect on Adenosine 5'−monophosphate (AMP) to alanine ratio

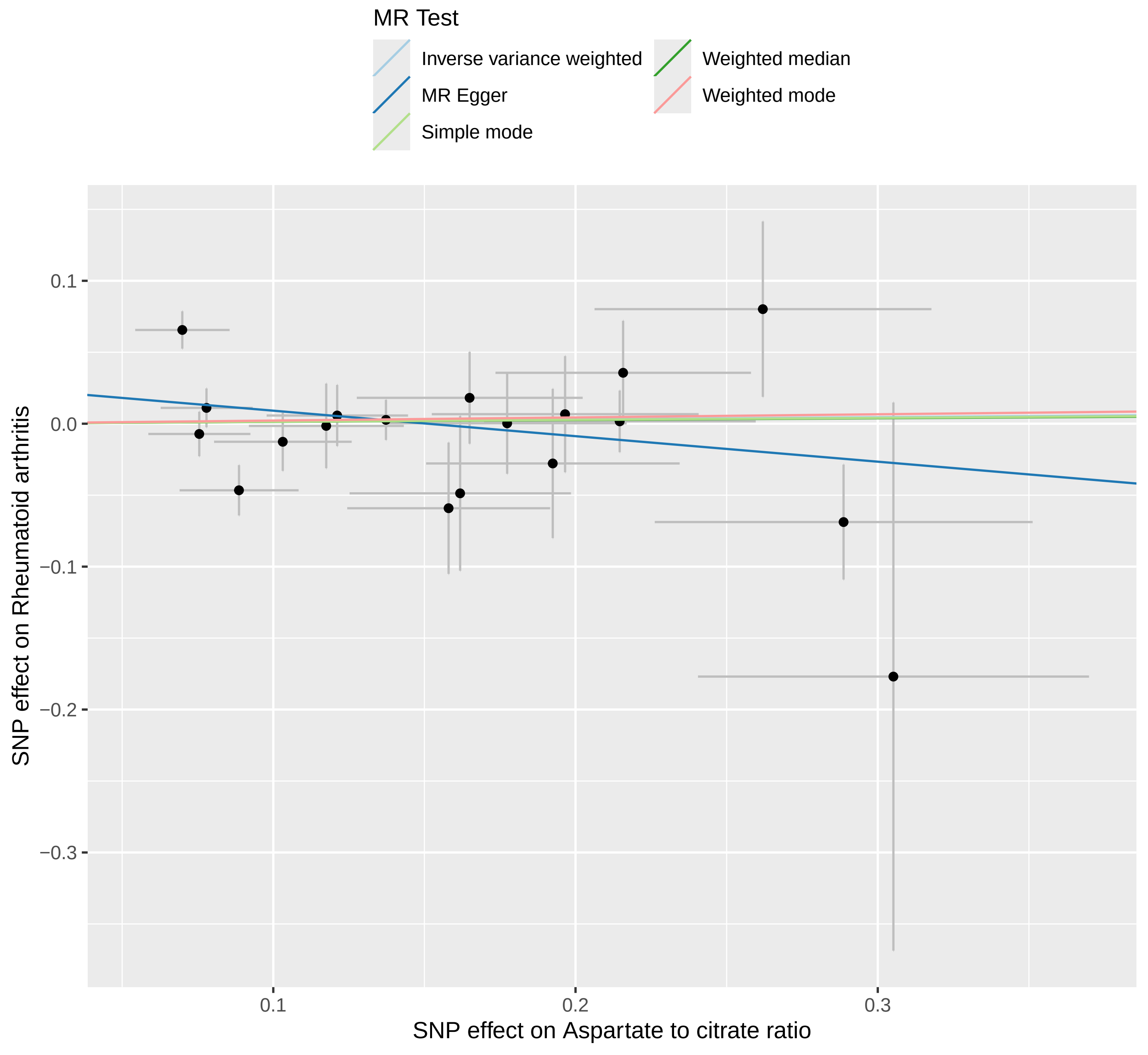

MR Test
Inverse variance weighted
MR Egger
Simple mode
Weighted median
Weighted mode
0.1
0.0
−0.1
−0.2
−0.3
0.1
0.2
0.3
SNP effect on Rheumatoid arthritis
SNP effect on Aspartate to citrate ratio

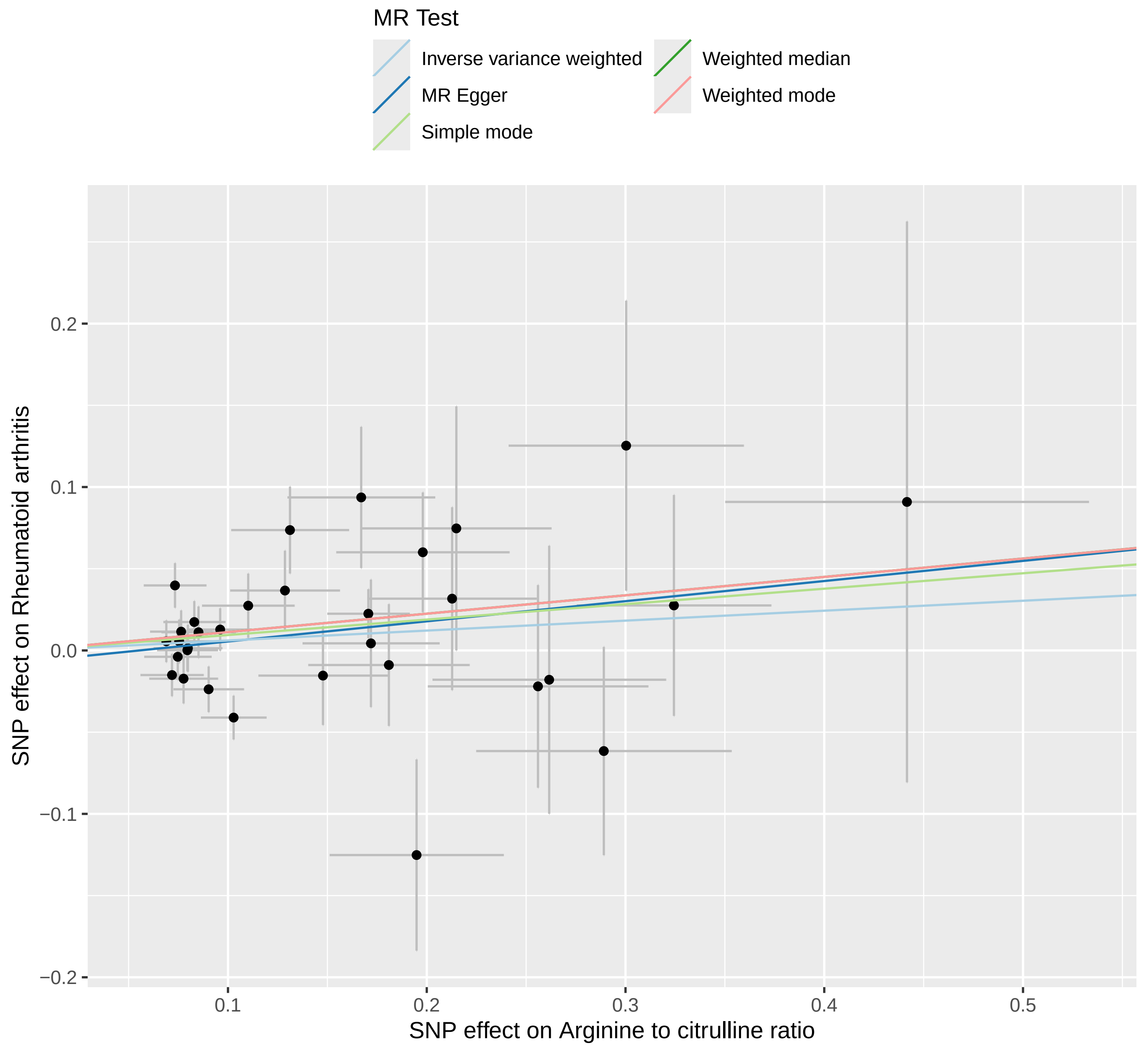

MR Test
Inverse variance weighted
MR Egger
Simple mode
Weighted median
Weighted mode
SNP effect on Rheumatoid arthritis
0.2
0.1
0.0
−0.1
−0.2
0.1
0.2
0.3
0.4
0.5
SNP effect on Arginine to citrulline ratio

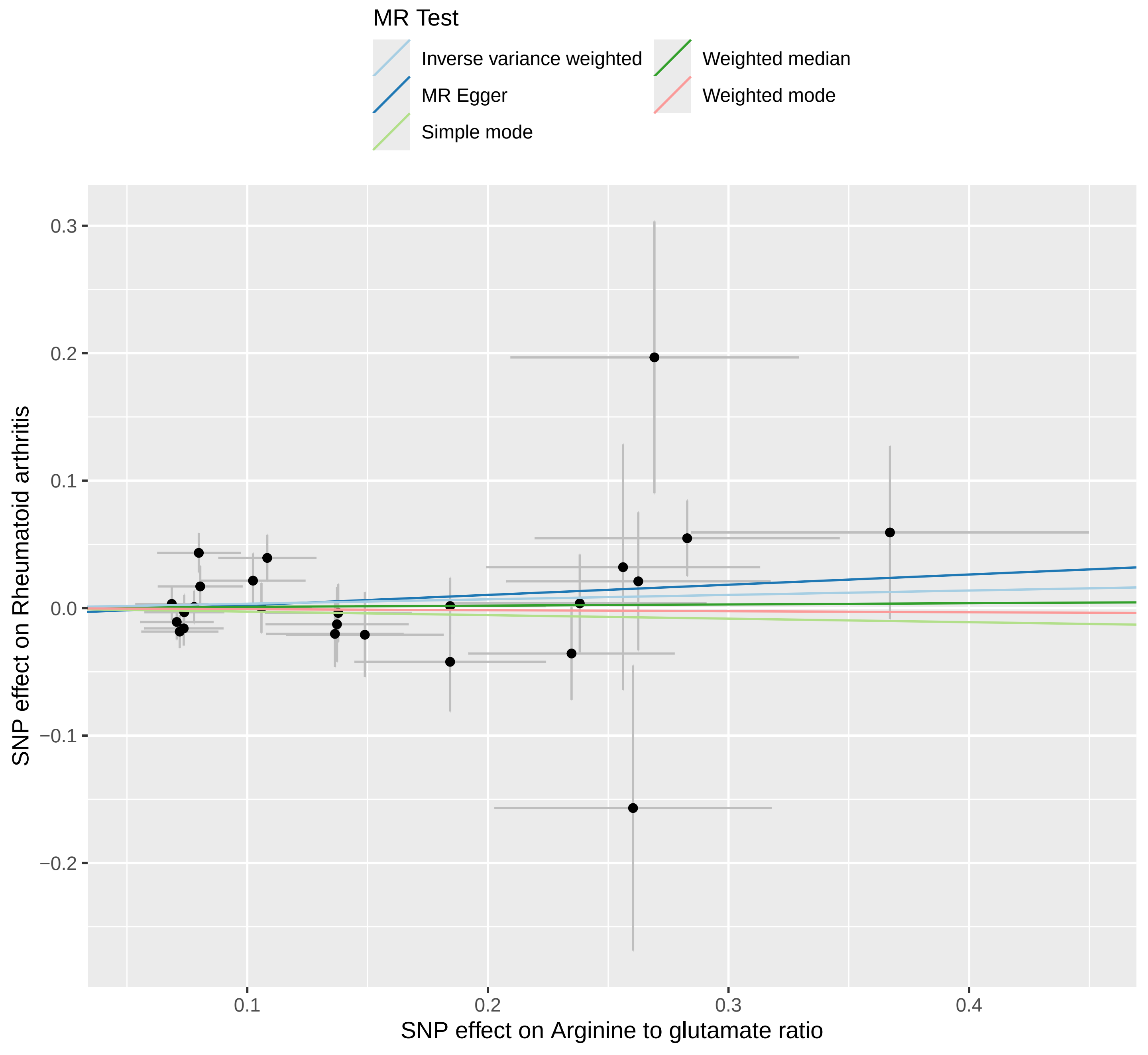

MR Test
Inverse variance weighted
MR Egger
Simple mode
Weighted median
Weighted mode
0.3
0.2
0.1
0.0
−0.1
−0.2
SNP effect on Rheumatoid arthritis
0.1
0.2
0.3
0.4
SNP effect on Arginine to glutamate ratio

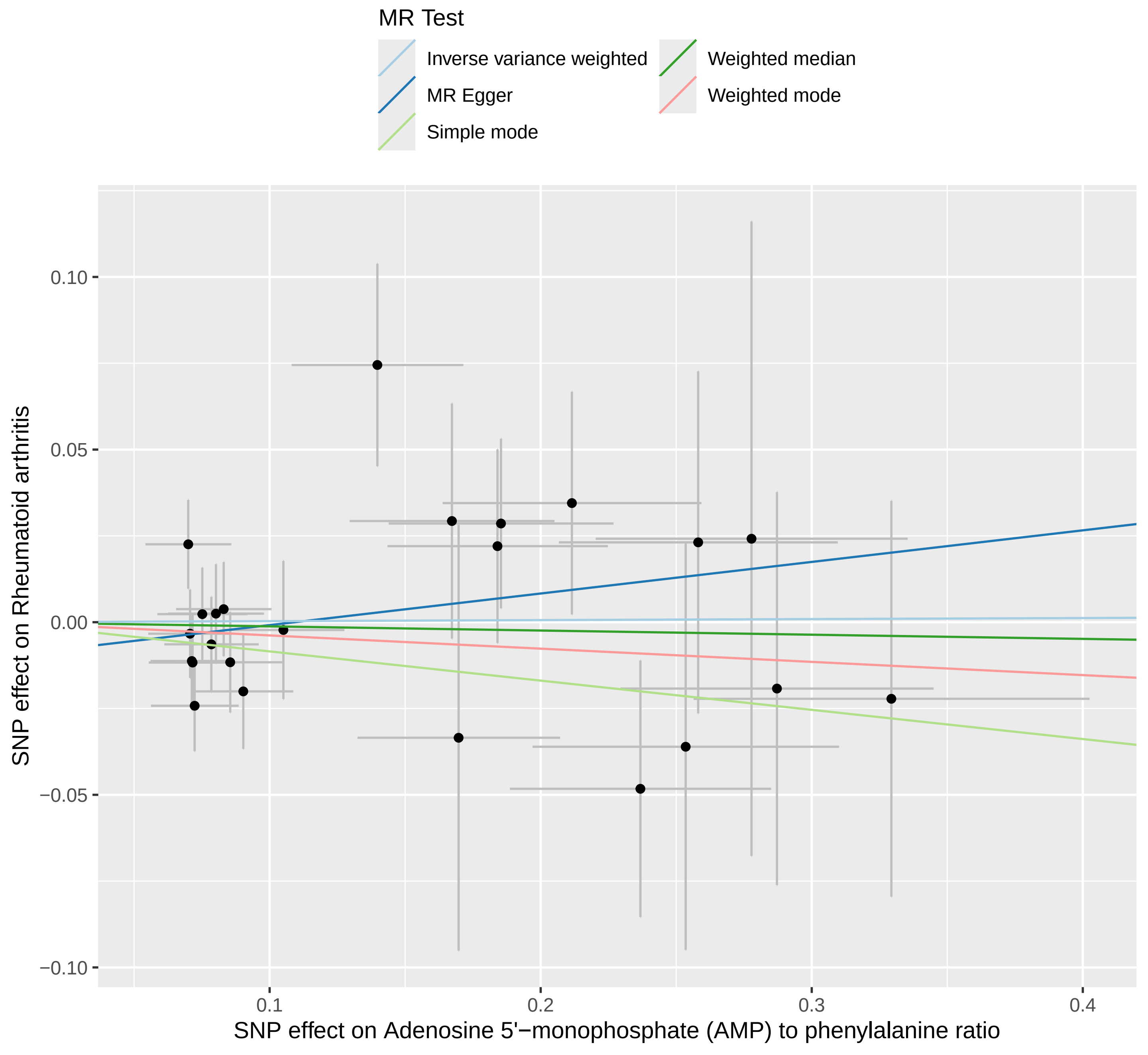
MR Test
Inverse variance weighted
MR Egger
Simple mode
Weighted median
Weighted mode
0.10
0.05
0.00
−0.05
−0.10
0.1
0.2
0.3
0.4
SNP effect on Rheumatoid arthritis
SNP effect on Adenosine 5'−monophosphate (AMP) to phenylalanine ratio

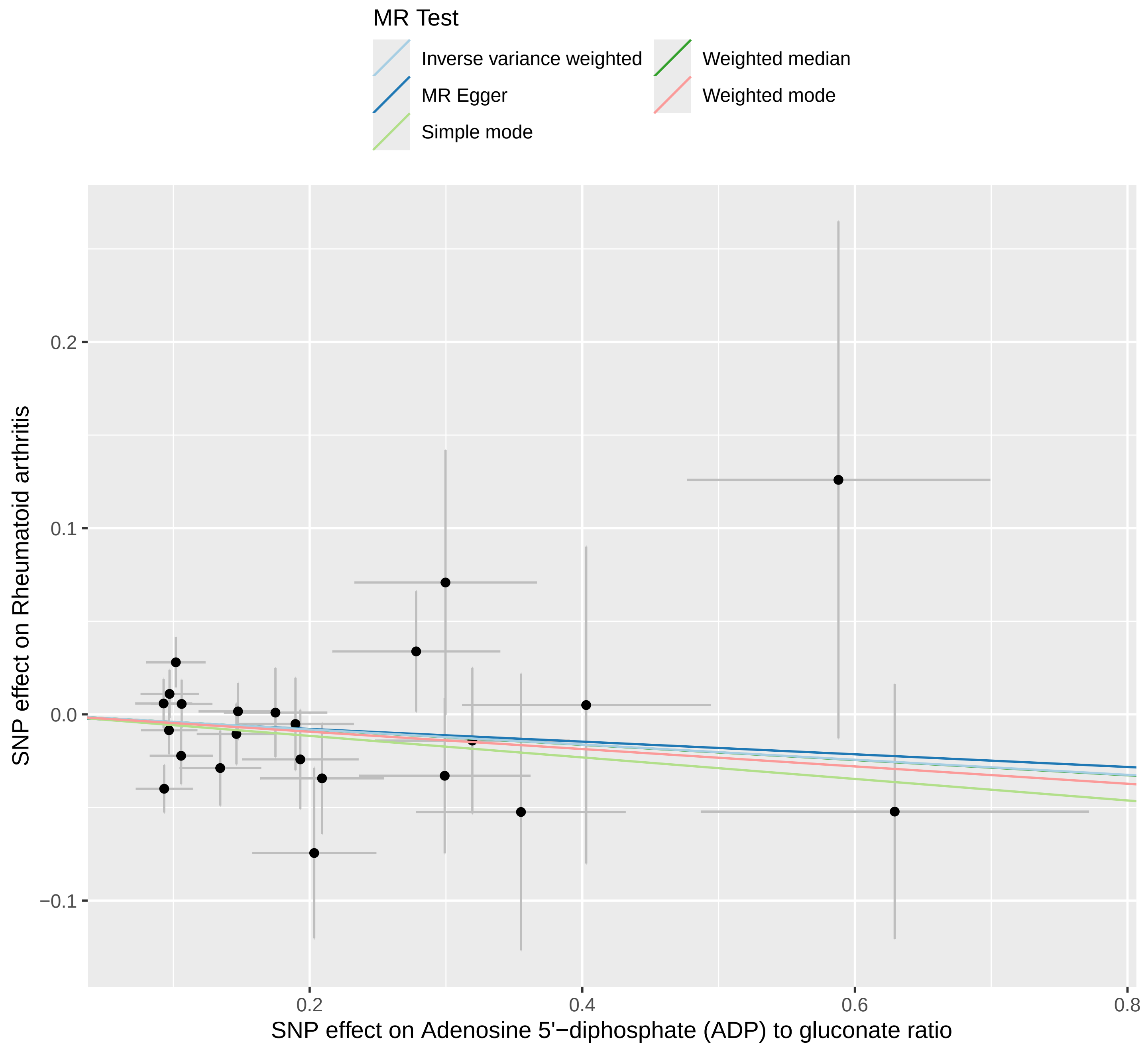

MR Test
Inverse variance weighted
MR Egger
Simple mode
Weighted median
Weighted mode
0.2
0.1
0.0
−0.1
SNP effect on Rheumatoid arthritis
0.2
0.4
0.6
0.8
SNP effect on Adenosine 5'−diphosphate (ADP) to gluconate ratio

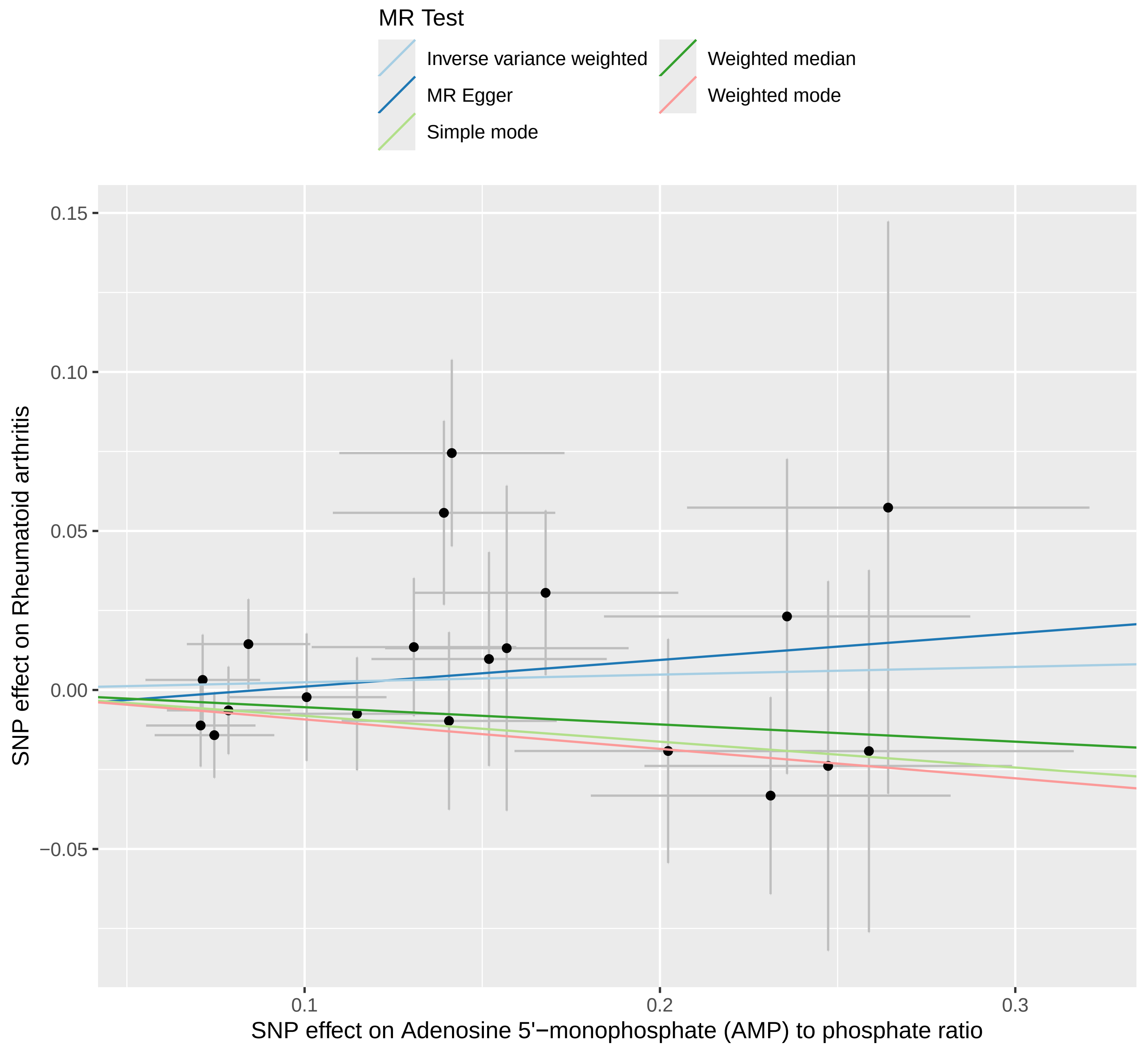
MR Test
Inverse variance weighted
MR Egger
Simple mode
Weighted median
Weighted mode
0.15
0.10
0.05
0.00
−0.05
SNP effect on Rheumatoid arthritis
0.1
0.2
0.3
SNP effect on Adenosine 5'−monophosphate (AMP) to phosphate ratio

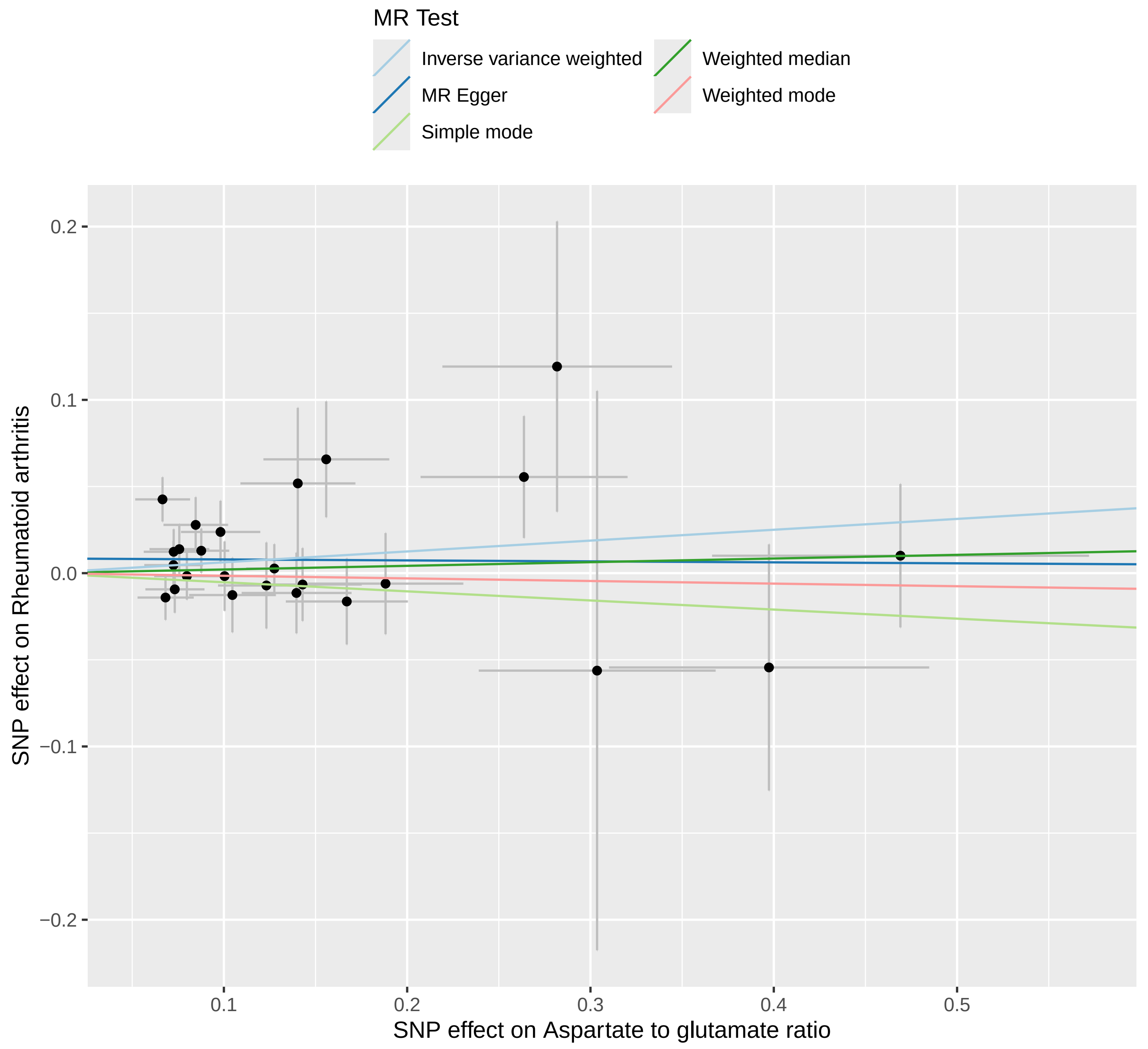

MR Test
Inverse variance weighted
MR Egger
Simple mode
Weighted median
Weighted mode
0.2
0.1
0.0
−0.1
−0.2
SNP effect on Rheumatoid arthritis
0.1
0.2
0.3
0.4
0.5
SNP effect on Aspartate to glutamate ratio

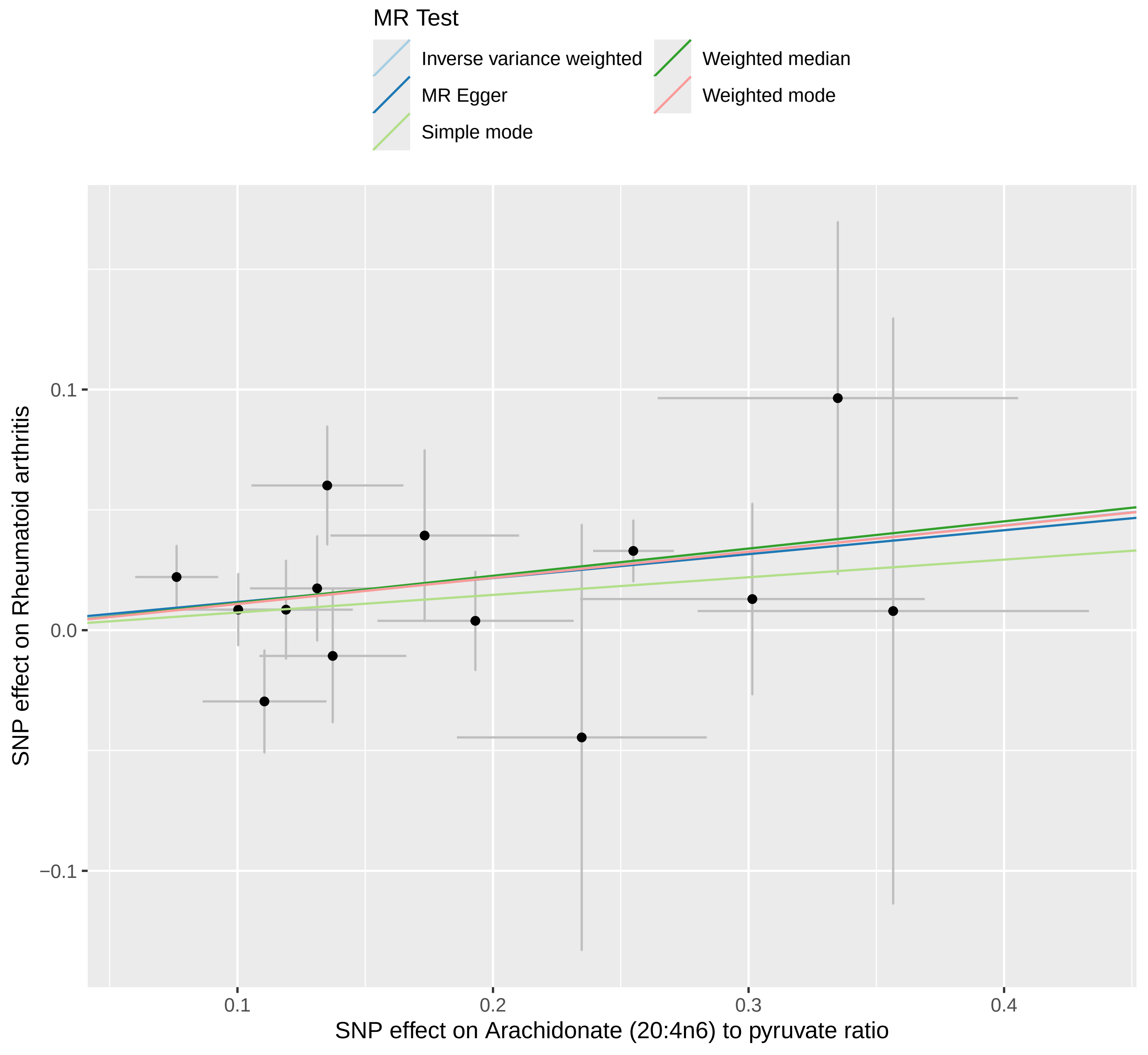

MR Test
Inverse variance weighted
MR Egger
Simple mode
Weighted median
Weighted mode
0.1
0.0
−0.1
SNP effect on Rheumatoid arthritis
0.1
0.2
0.3
0.4
SNP effect on Arachidonate (20:4n6) to pyruvate ratio

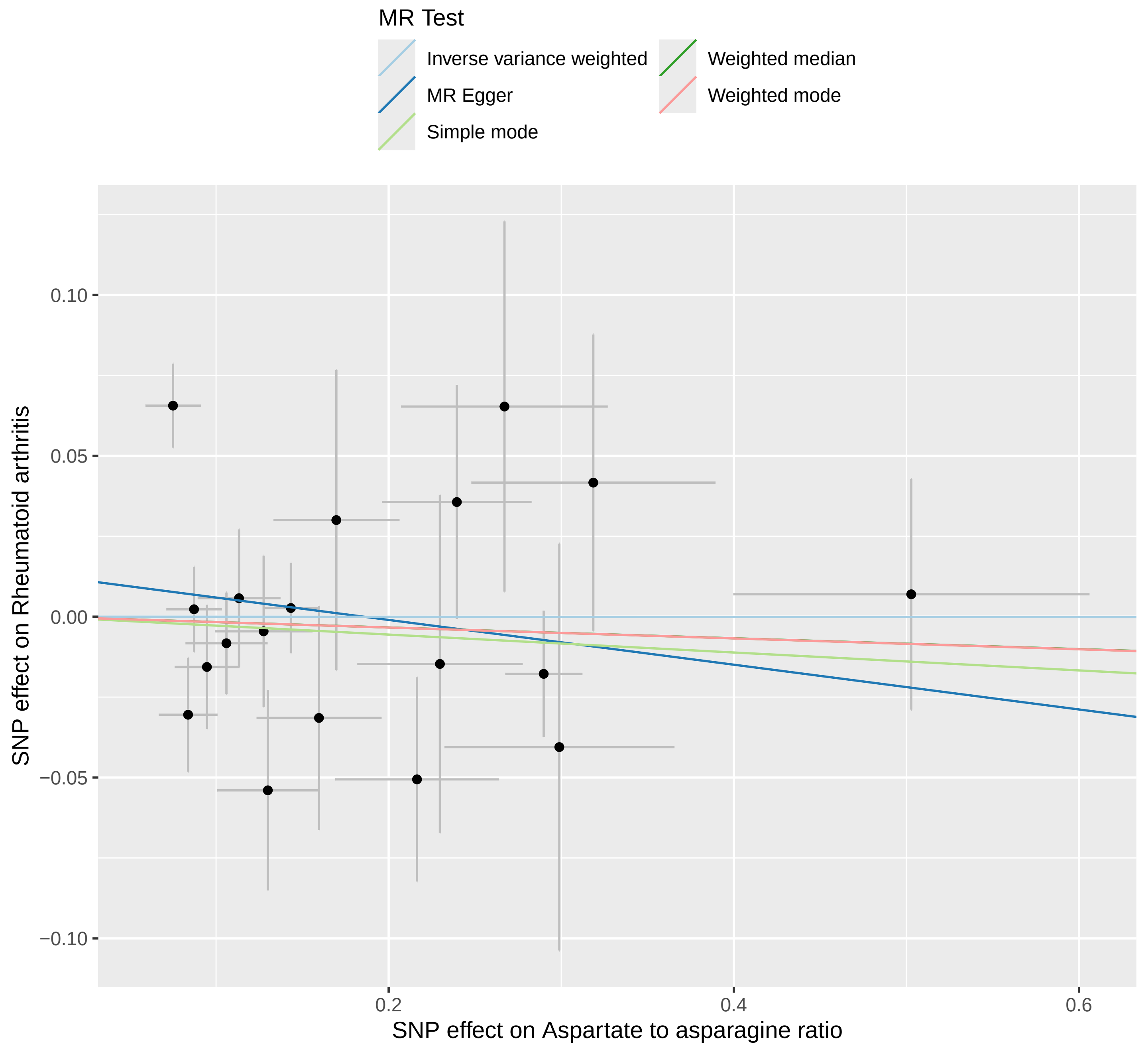
MR Test
Inverse variance weighted
MR Egger
Simple mode
Weighted median
Weighted mode
SNP effect on Rheumatoid arthritis
0.10
0.05
0.00
−0.05
−0.10
0.2
0.4
0.6
SNP effect on Aspartate to asparagine ratio

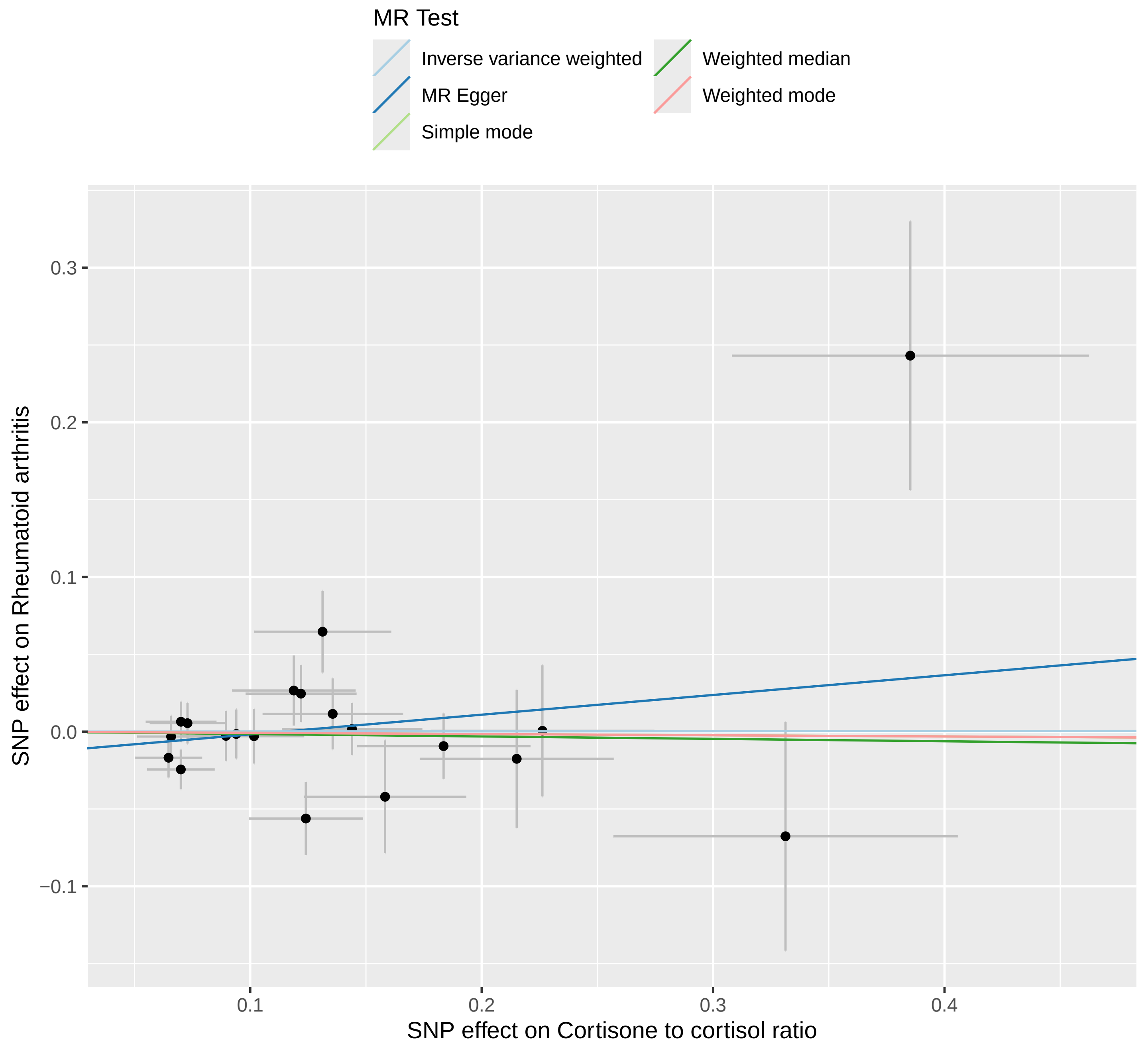
MR Test
Inverse variance weighted
MR Egger
Simple mode
Weighted median
Weighted mode
0.3
0.2
0.1
0.0
−0.1
0.1
0.2
0.3
0.4
SNP effect on Rheumatoid arthritis
SNP effect on Cortisone to cortisol ratio

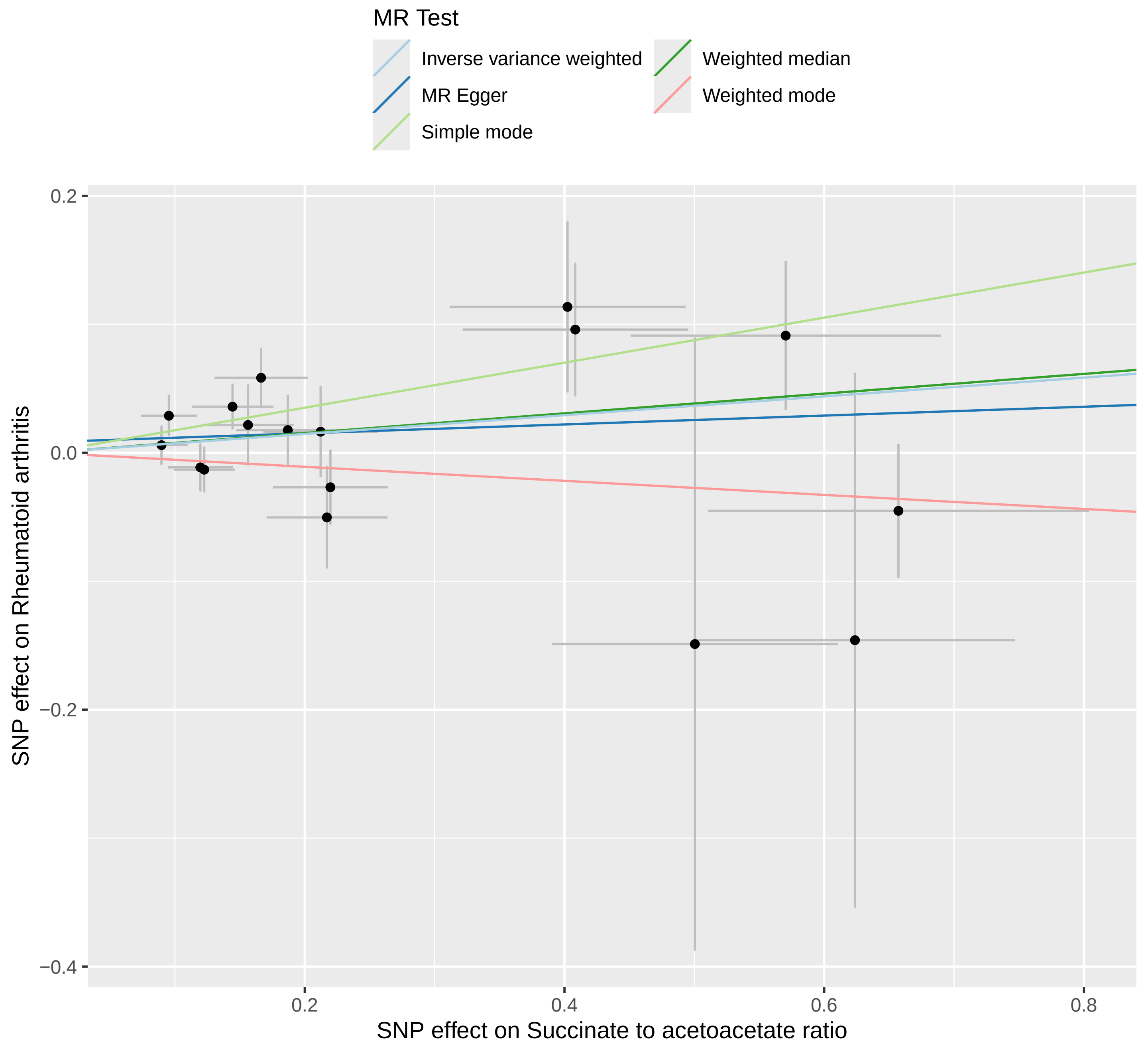
MR Test
Inverse variance weighted
MR Egger
Simple mode
Weighted median
Weighted mode
0.2
0.0
−0.2
−0.4
SNP effect on Rheumatoid arthritis
0.2
0.4
0.6
0.8
SNP effect on Succinate to acetoacetate ratio

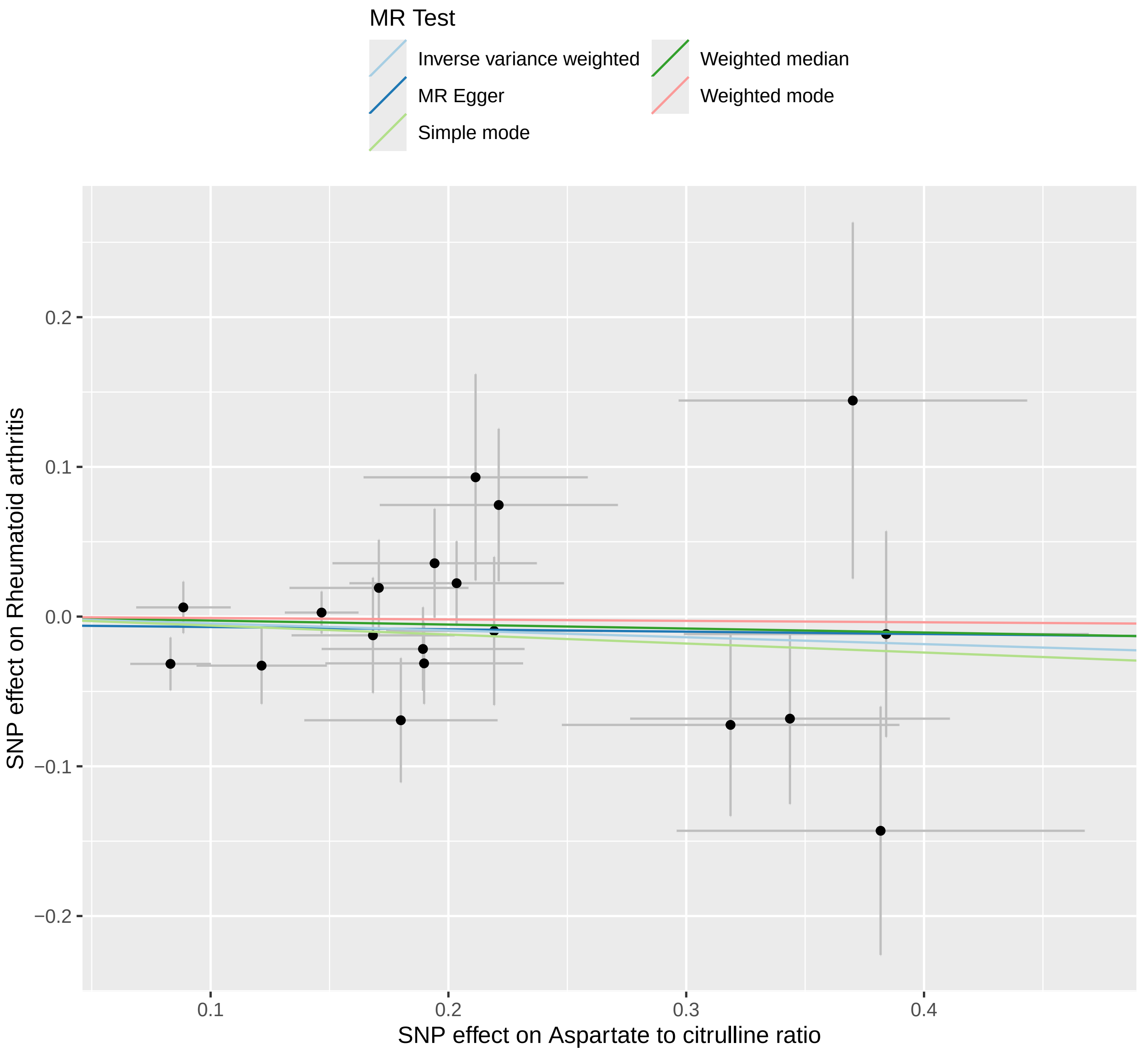
MR Test
Inverse variance weighted
MR Egger
Simple mode
Weighted median
Weighted mode
0.2
0.1
0.0
−0.1
−0.2
0.1
0.2
0.3
0.4
SNP effect on Rheumatoid arthritis
SNP effect on Aspartate to citrulline ratio

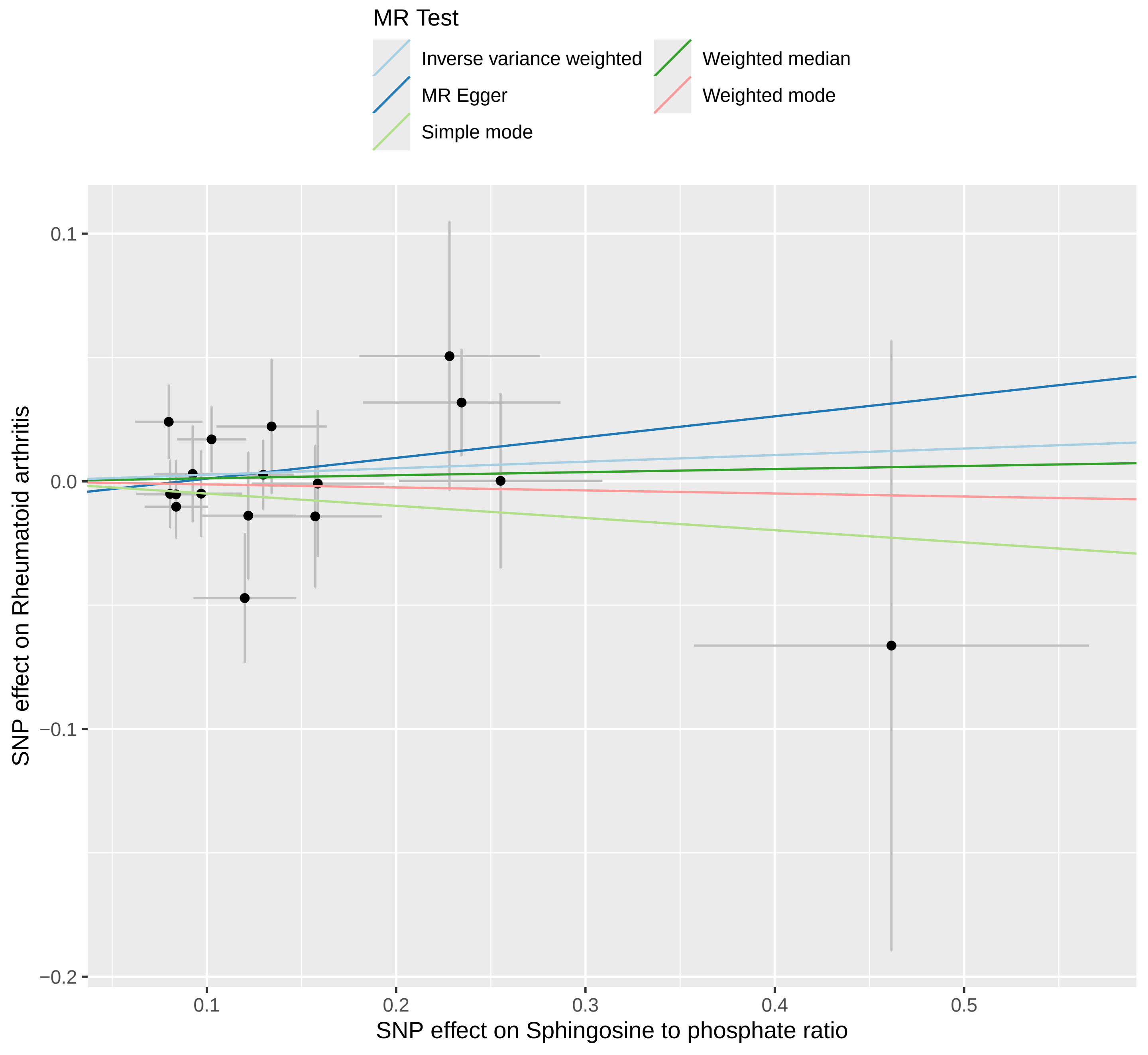

MR Test
Inverse variance weighted
MR Egger
Simple mode
Weighted median
Weighted mode
0.1
0.0
−0.1
−0.2
0.1
0.2
0.3
0.4
0.5
SNP effect on Rheumatoid arthritis
SNP effect on Sphingosine to phosphate ratio

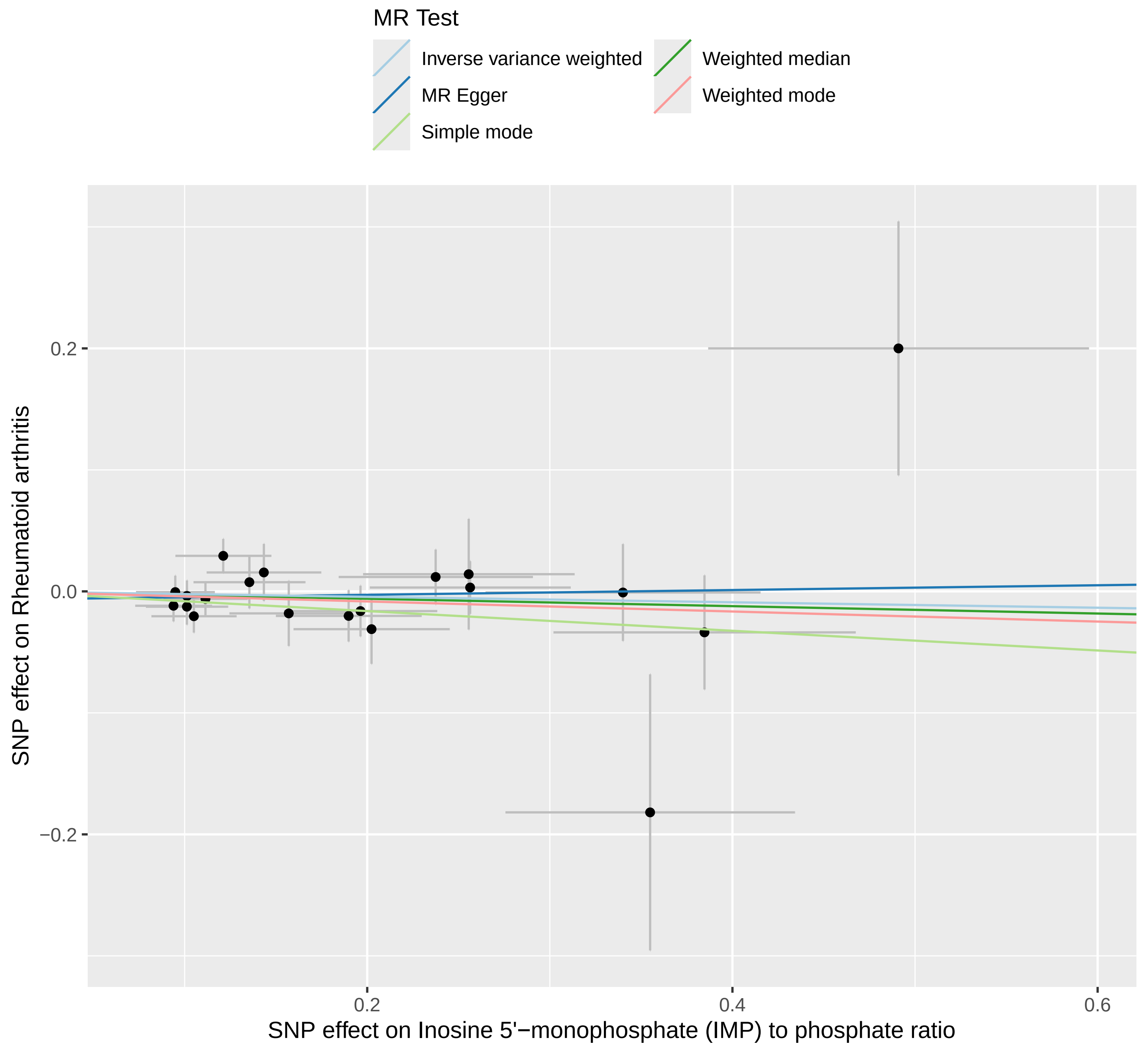
MR Test
Inverse variance weighted
MR Egger
Simple mode
Weighted median
Weighted mode
0.2
0.0
−0.2
SNP effect on Rheumatoid arthritis
0.2
0.4
0.6
SNP effect on Inosine 5'−monophosphate (IMP) to phosphate ratio

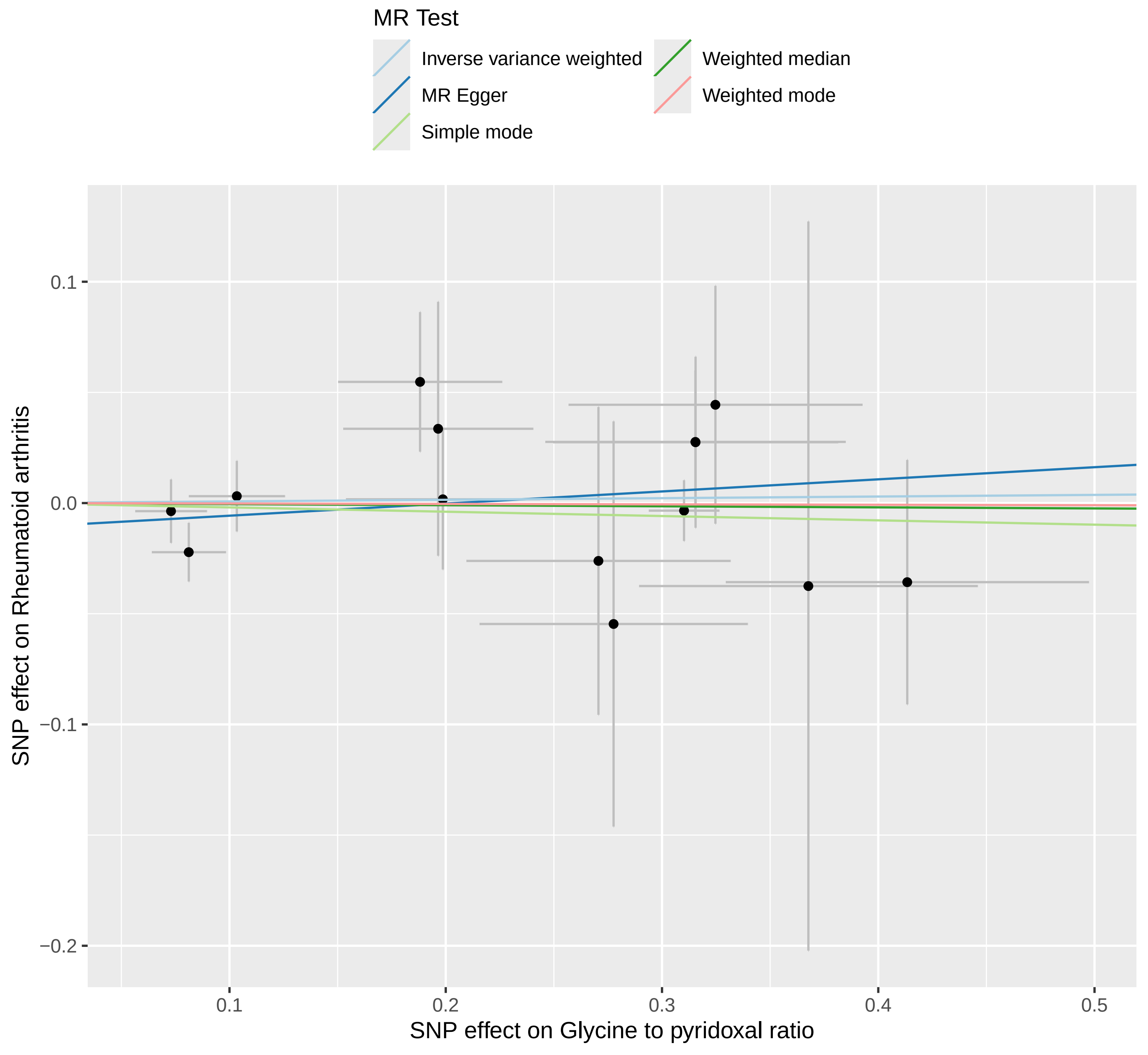
MR Test
Inverse variance weighted
MR Egger
Simple mode
Weighted median
Weighted mode
SNP effect on Rheumatoid arthritis
0.1
0.0
−0.1
−0.2
0.1
0.2
0.3
0.4
0.5
SNP effect on Glycine to pyridoxal ratio

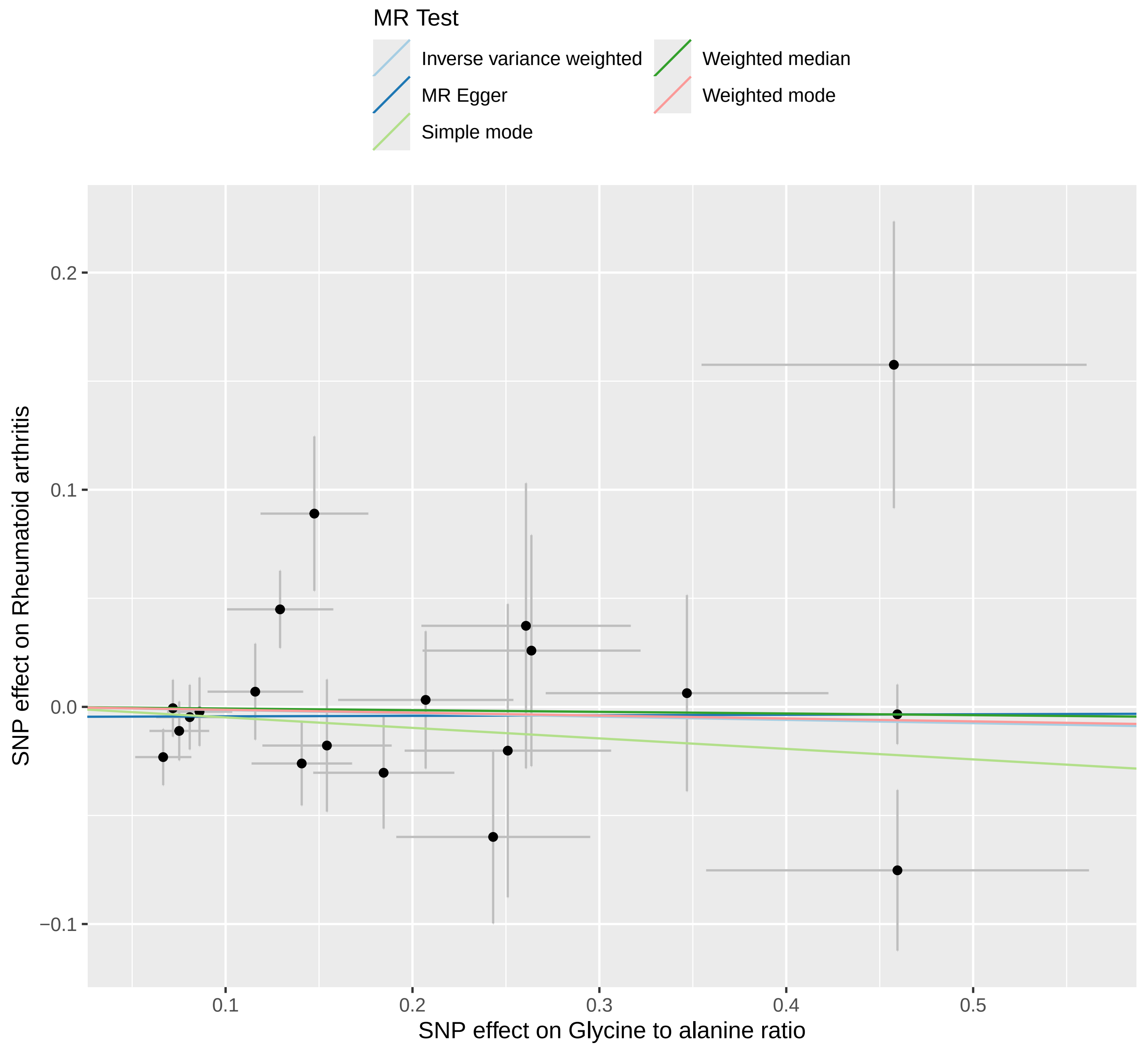

MR Test
Inverse variance weighted
MR Egger
Simple mode
Weighted median
Weighted mode
0.2
0.1
0.0
−0.1
0.1
0.2
0.3
0.4
0.5
SNP effect on Rheumatoid arthritis
SNP effect on Glycine to alanine ratio

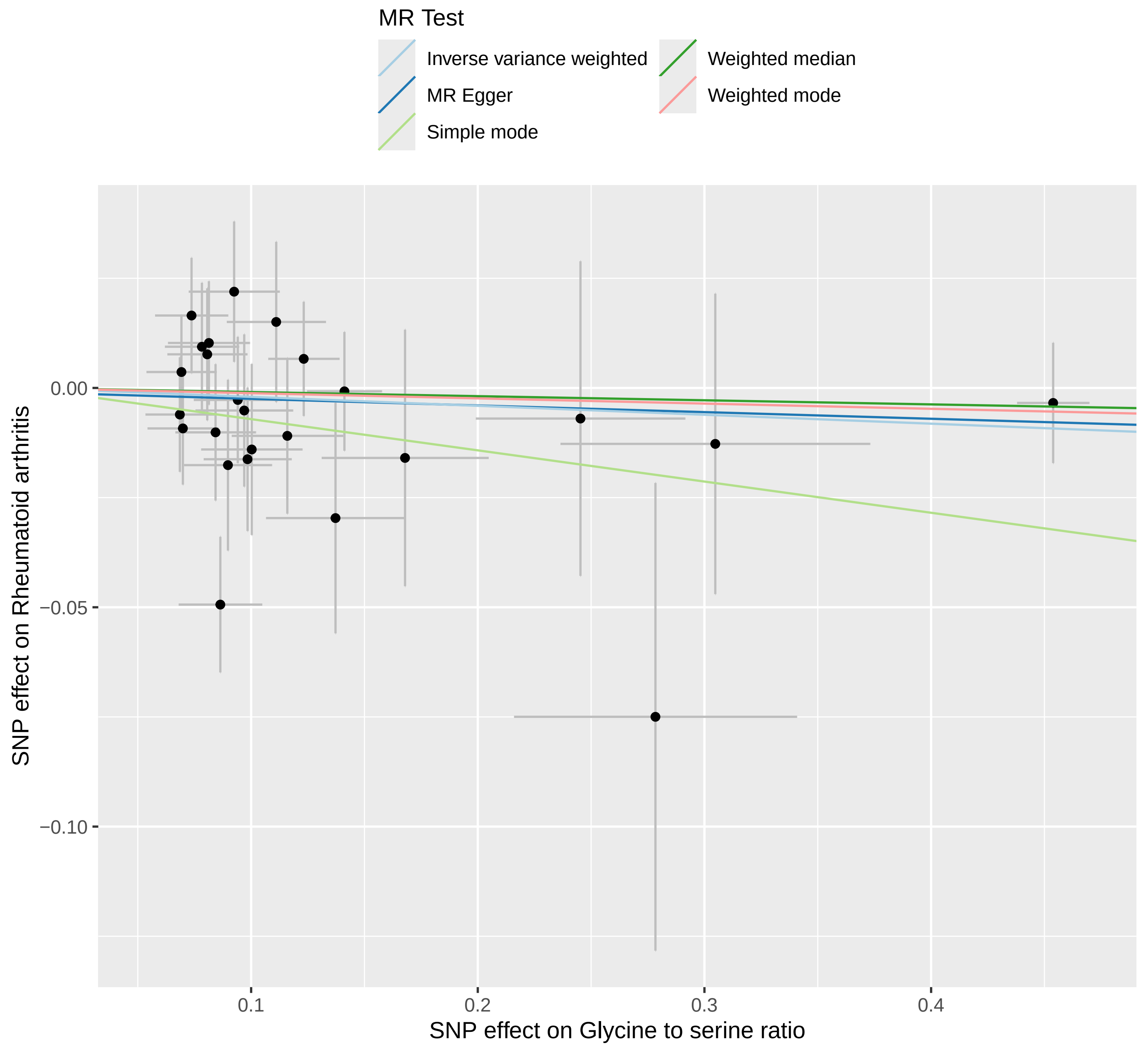
MR Test
Inverse variance weighted
MR Egger
Simple mode
Weighted median
Weighted mode
SNP effect on Rheumatoid arthritis
0.00
−0.05
−0.10
0.1
0.2
0.3
0.4
SNP effect on Glycine to serine ratio

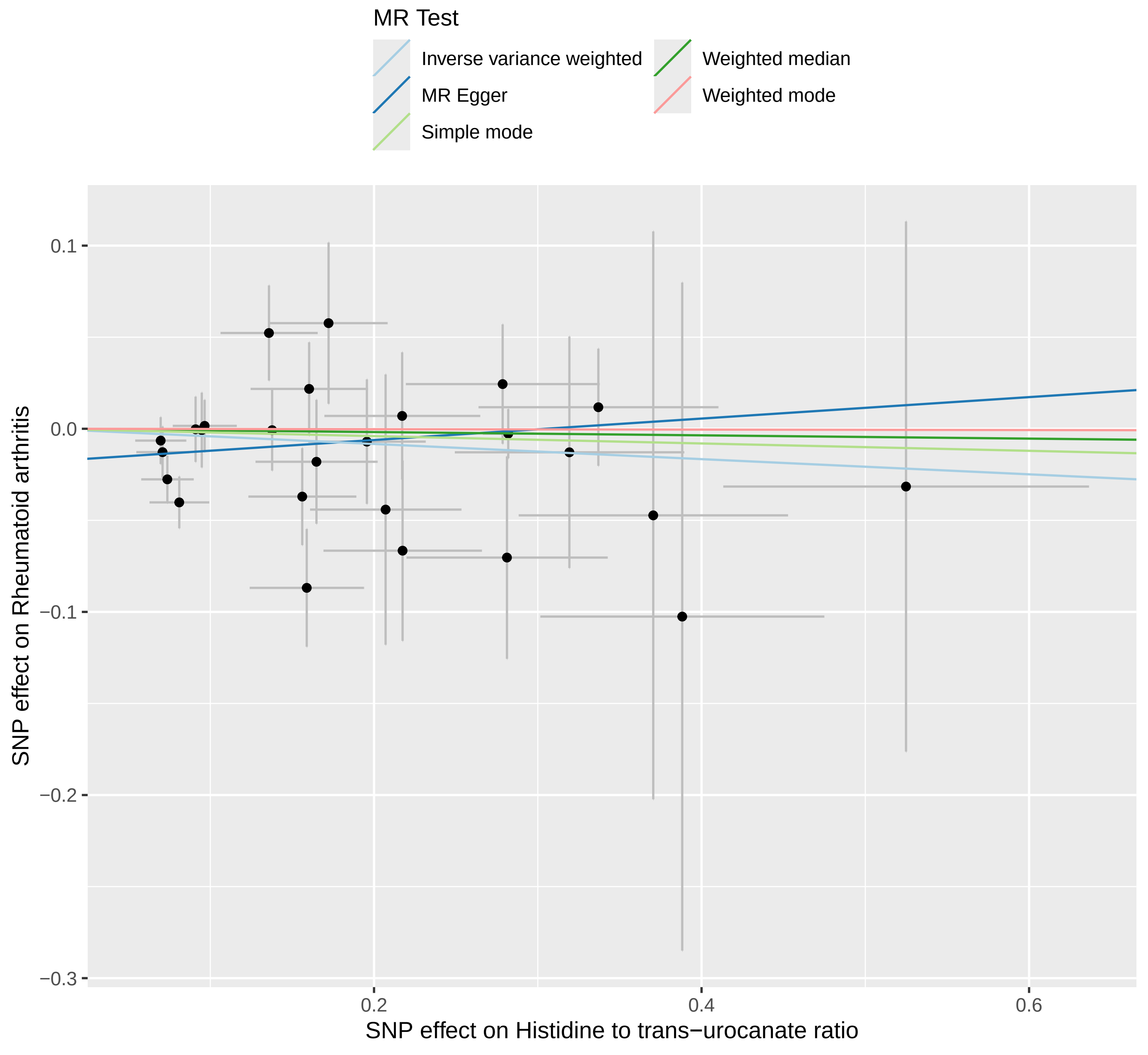
MR Test
Inverse variance weighted
MR Egger
Simple mode
Weighted median
Weighted mode
0.1
0.0
−0.1
−0.2
−0.3
0.2
0.4
0.6
SNP effect on Rheumatoid arthritis
SNP effect on Histidine to trans−urocanate ratio

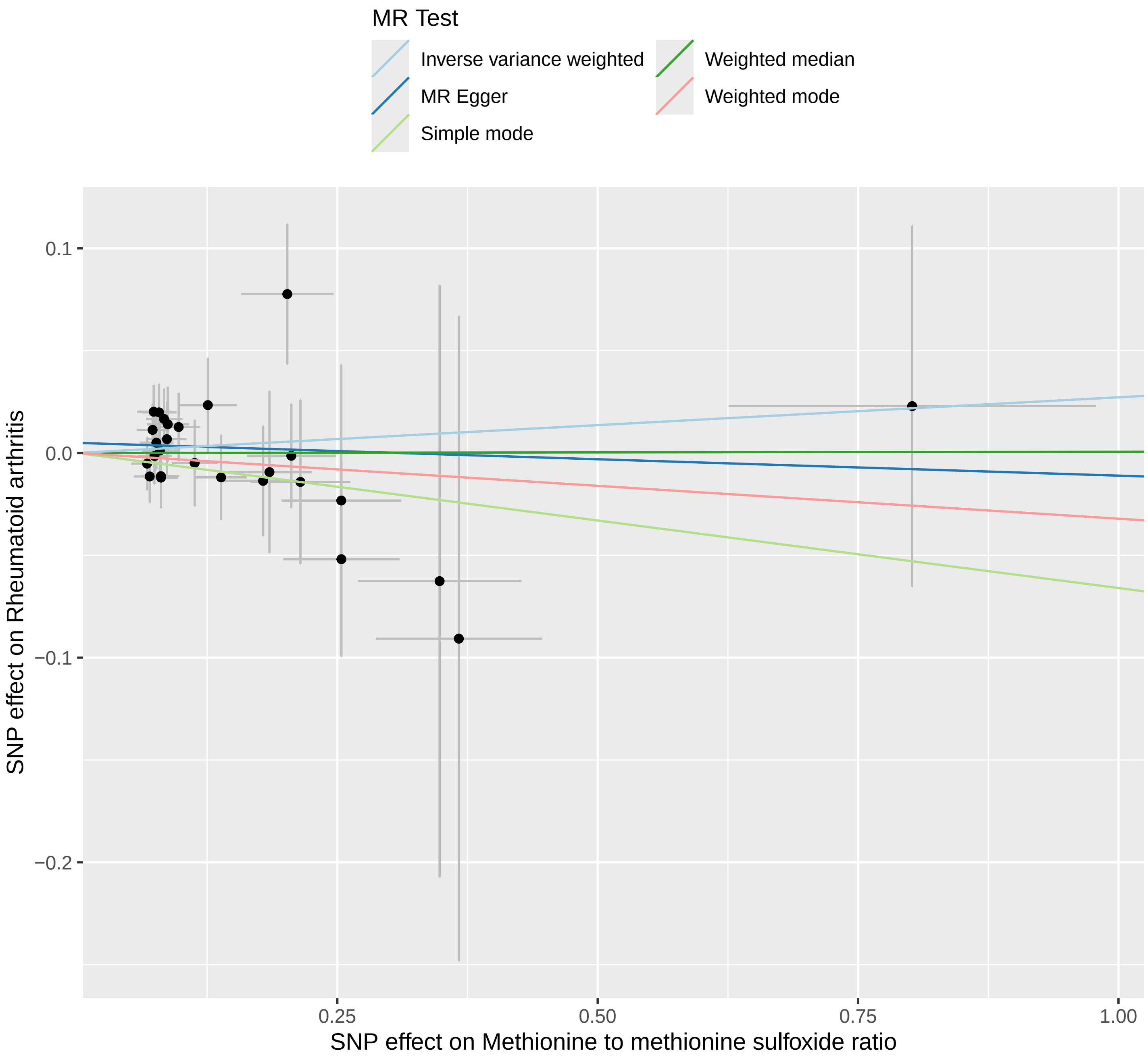
MR Test
Inverse variance weighted
MR Egger
Simple mode
Weighted median
Weighted mode
0.1
0.0
−0.1
−0.2
SNP effect on Rheumatoid arthritis
0.25
0.50
0.75
1.00
SNP effect on Methionine to methionine sulfoxide ratio

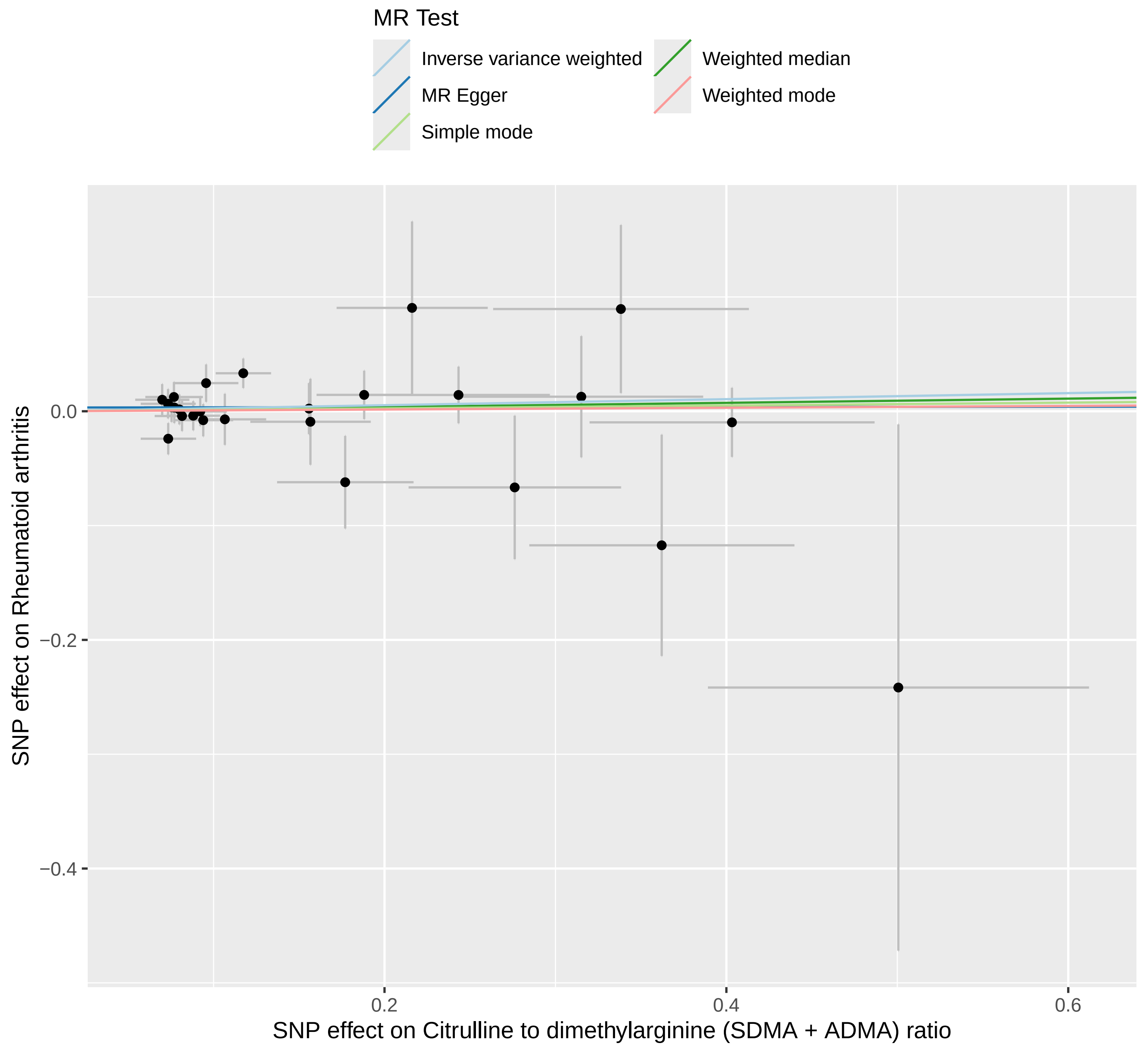
MR Test
Inverse variance weighted
MR Egger
Simple mode
Weighted median
Weighted mode
0.0
−0.2
−0.4
0.2
0.4
0.6
SNP effect on Rheumatoid arthritis
SNP effect on Citrulline to dimethylarginine (SDMA + ADMA) ratio

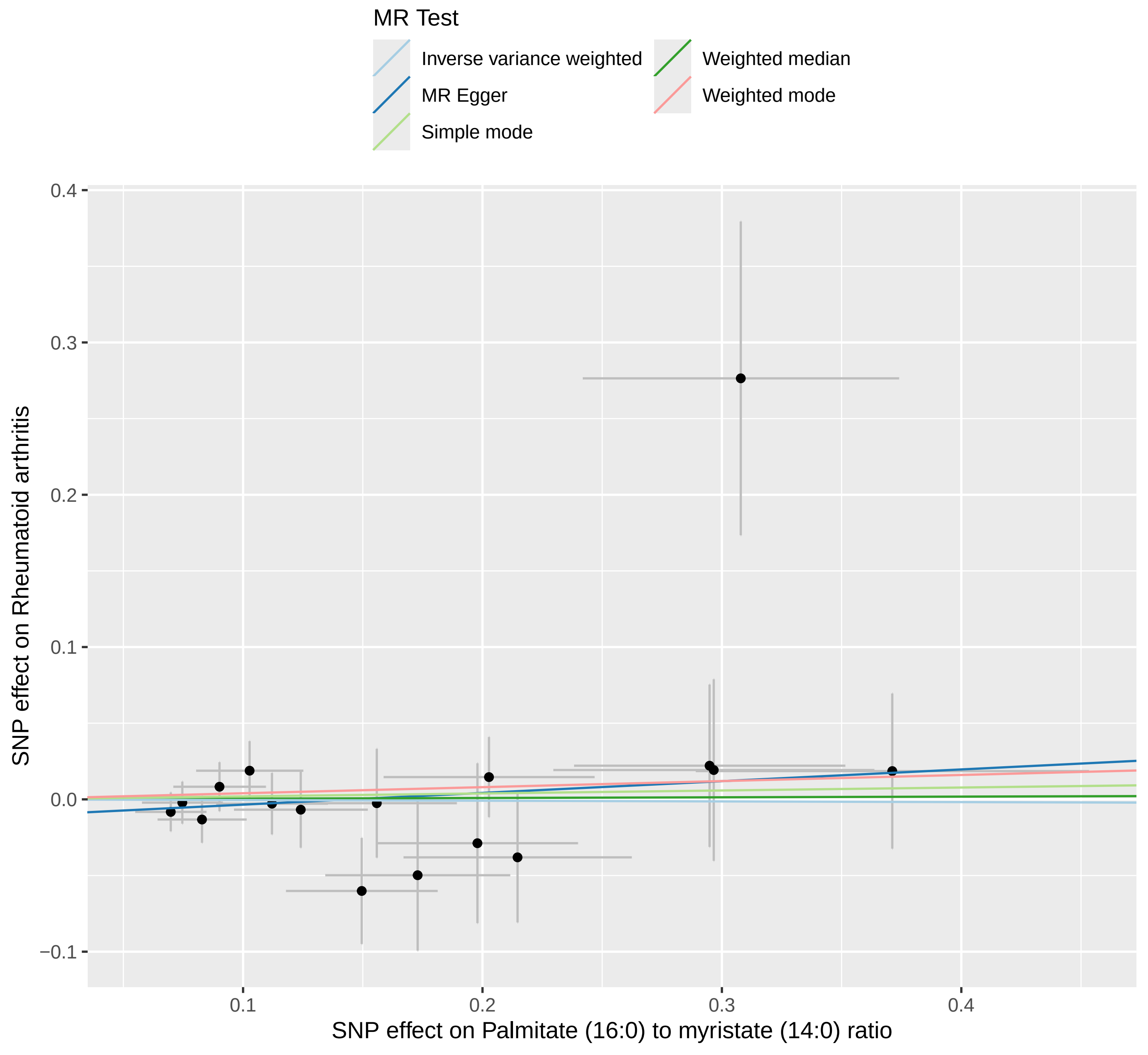
MR Test
Inverse variance weighted
MR Egger
Simple mode
Weighted median
Weighted mode
0.4
0.3
0.2
0.1
0.0
−0.1
0.1
0.2
0.3
0.4
SNP effect on Rheumatoid arthritis
SNP effect on Palmitate (16:0) to myristate (14:0) ratio

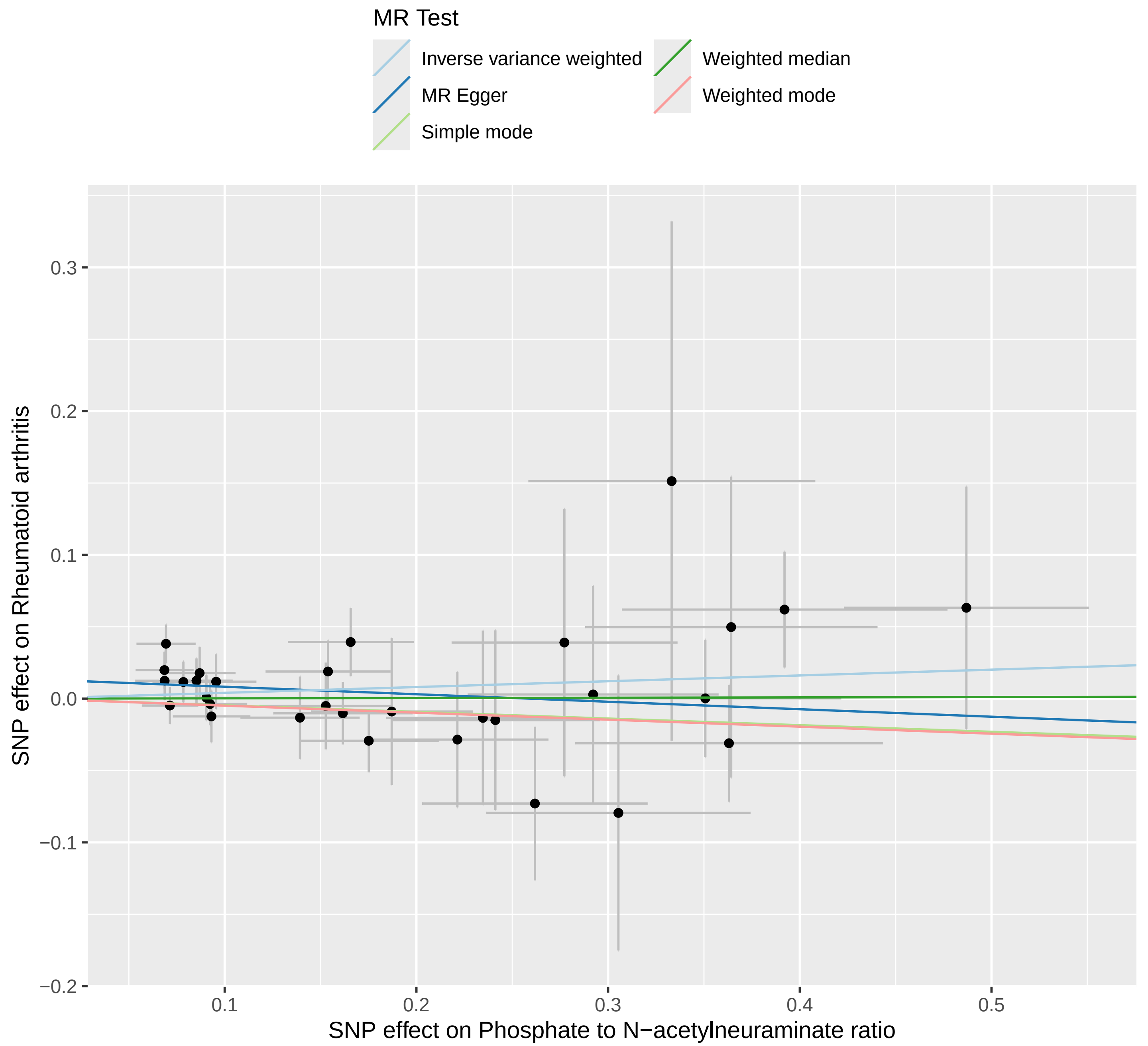
MR Test
Inverse variance weighted
MR Egger
Simple mode
Weighted median
Weighted mode
0.3
0.2
0.1
0.0
−0.1
−0.2
0.1
0.2
0.3
0.4
0.5
SNP effect on Rheumatoid arthritis
SNP effect on Phosphate to N−acetylneuraminate ratio

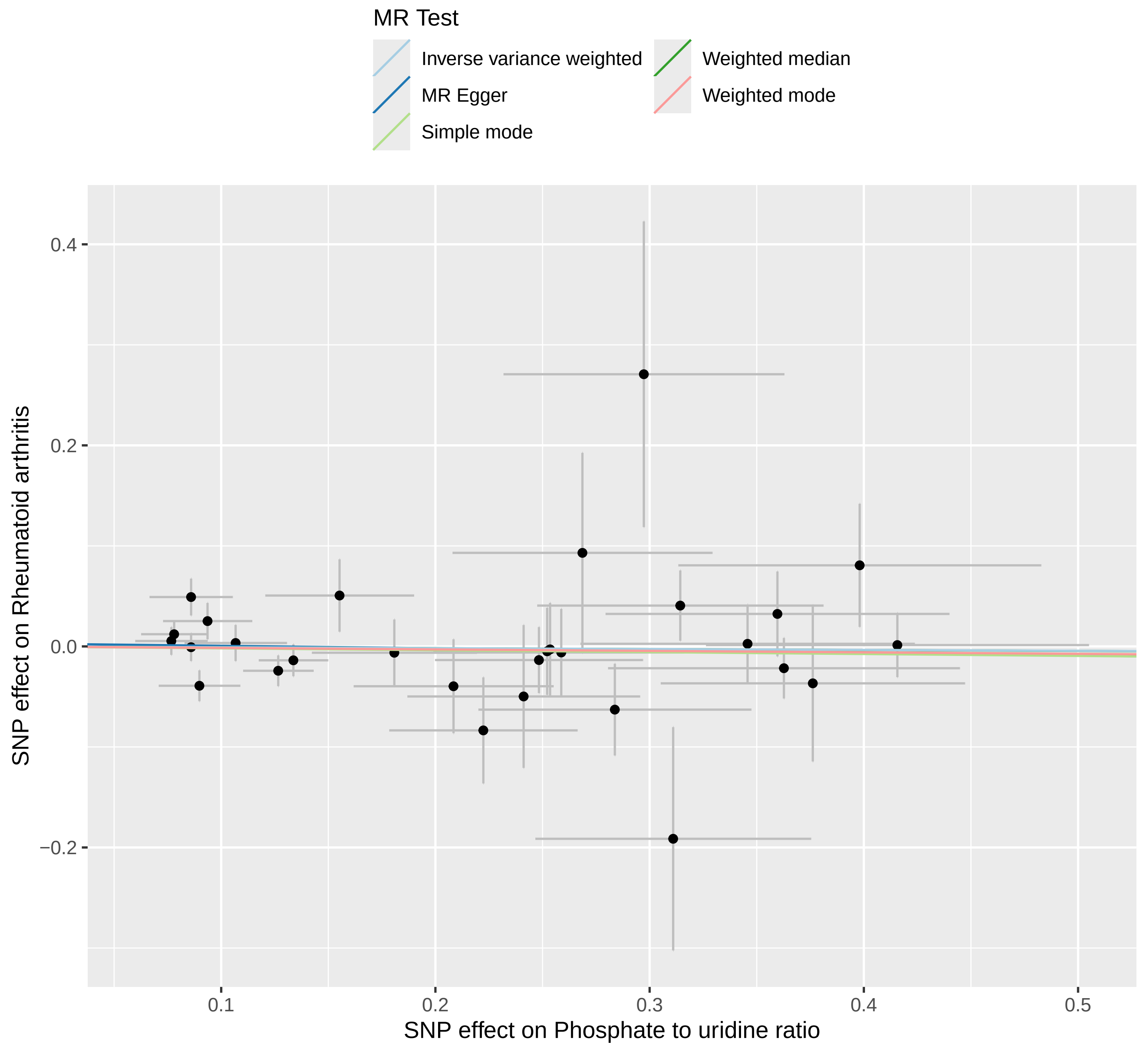
MR Test
Inverse variance weighted
MR Egger
Simple mode
Weighted median
Weighted mode
SNP effect on Rheumatoid arthritis
0.4
0.2
0.0
−0.2
0.1
0.2
0.3
0.4
0.5
SNP effect on Phosphate to uridine ratio

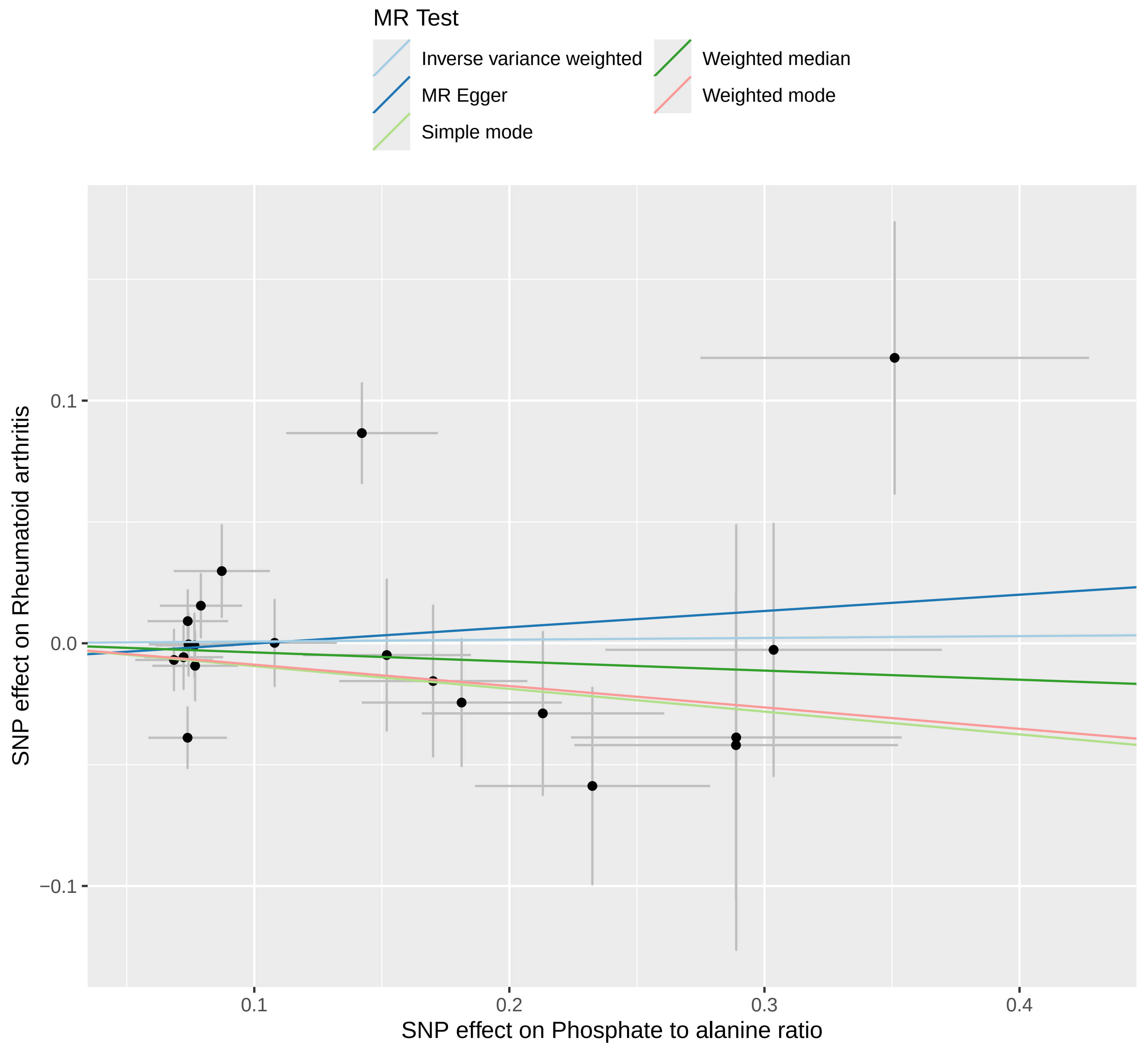
MR Test
Inverse variance weighted
MR Egger
Simple mode
Weighted median
Weighted mode
0.1
0.0
−0.1
SNP effect on Rheumatoid arthritis
0.1
0.2
0.3
0.4
SNP effect on Phosphate to alanine ratio

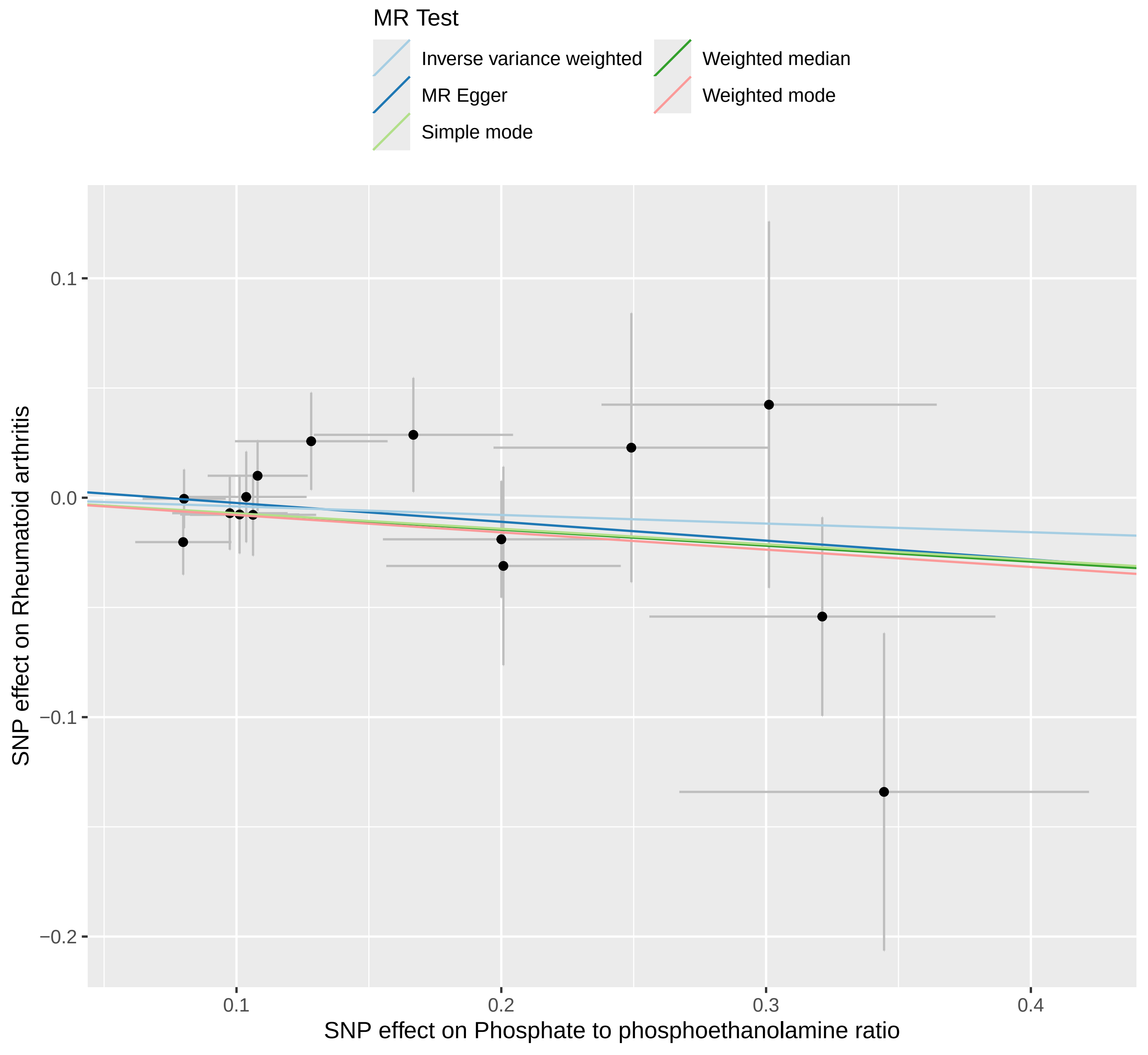
MR Test
Inverse variance weighted
MR Egger
Simple mode
Weighted median
Weighted mode
0.1
0.0
−0.1
−0.2
SNP effect on Rheumatoid arthritis
0.1
0.2
0.3
0.4
SNP effect on Phosphate to phosphoethanolamine ratio

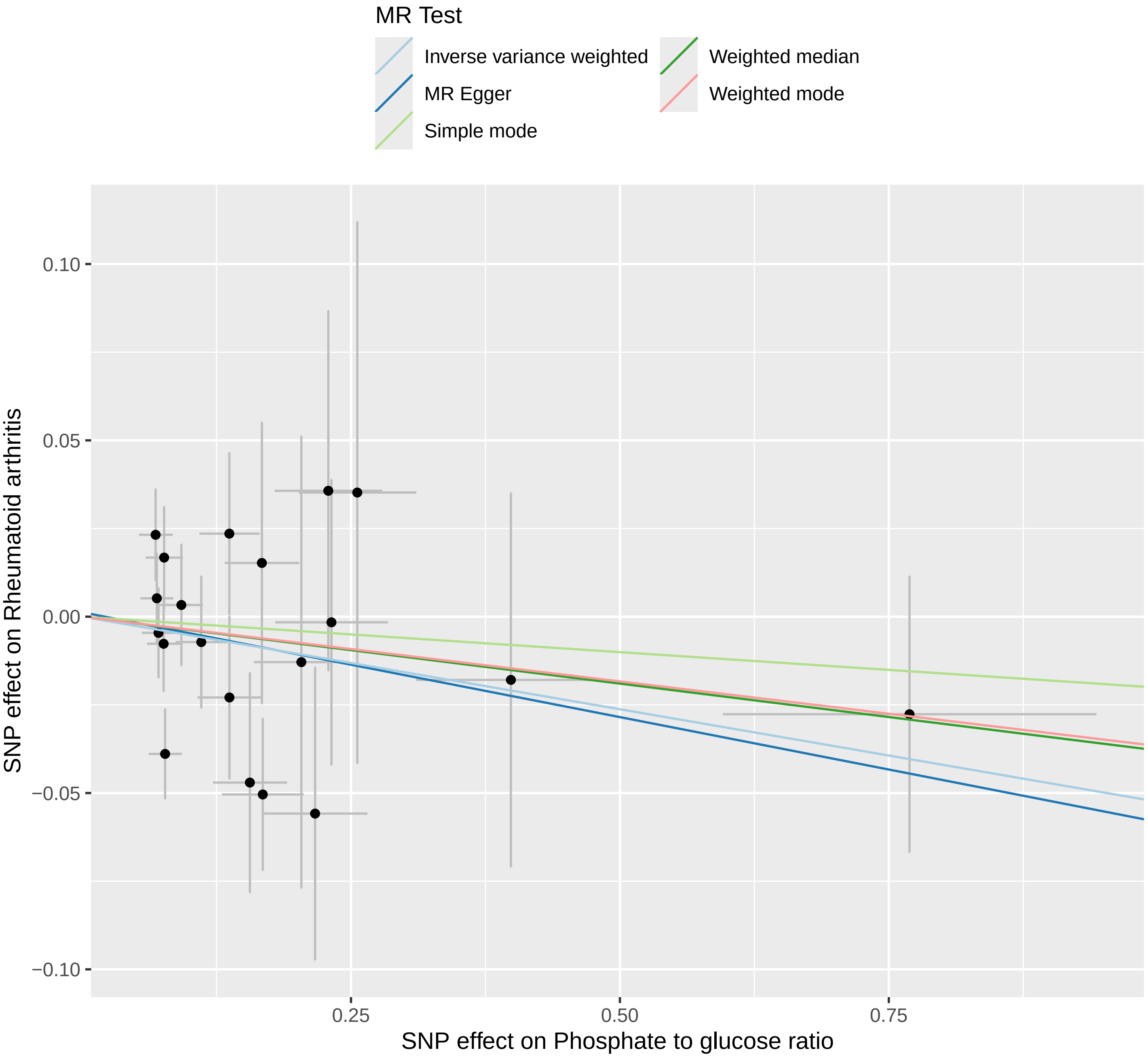

MR Test
Inverse variance weighted
MR Egger
Simple mode
Weighted median
Weighted mode
0.10
0.05
0.00
−0.05
−0.10
SNP effect on Rheumatoid arthritis
0.25
0.50
0.75
SNP effect on Phosphate to glucose ratio

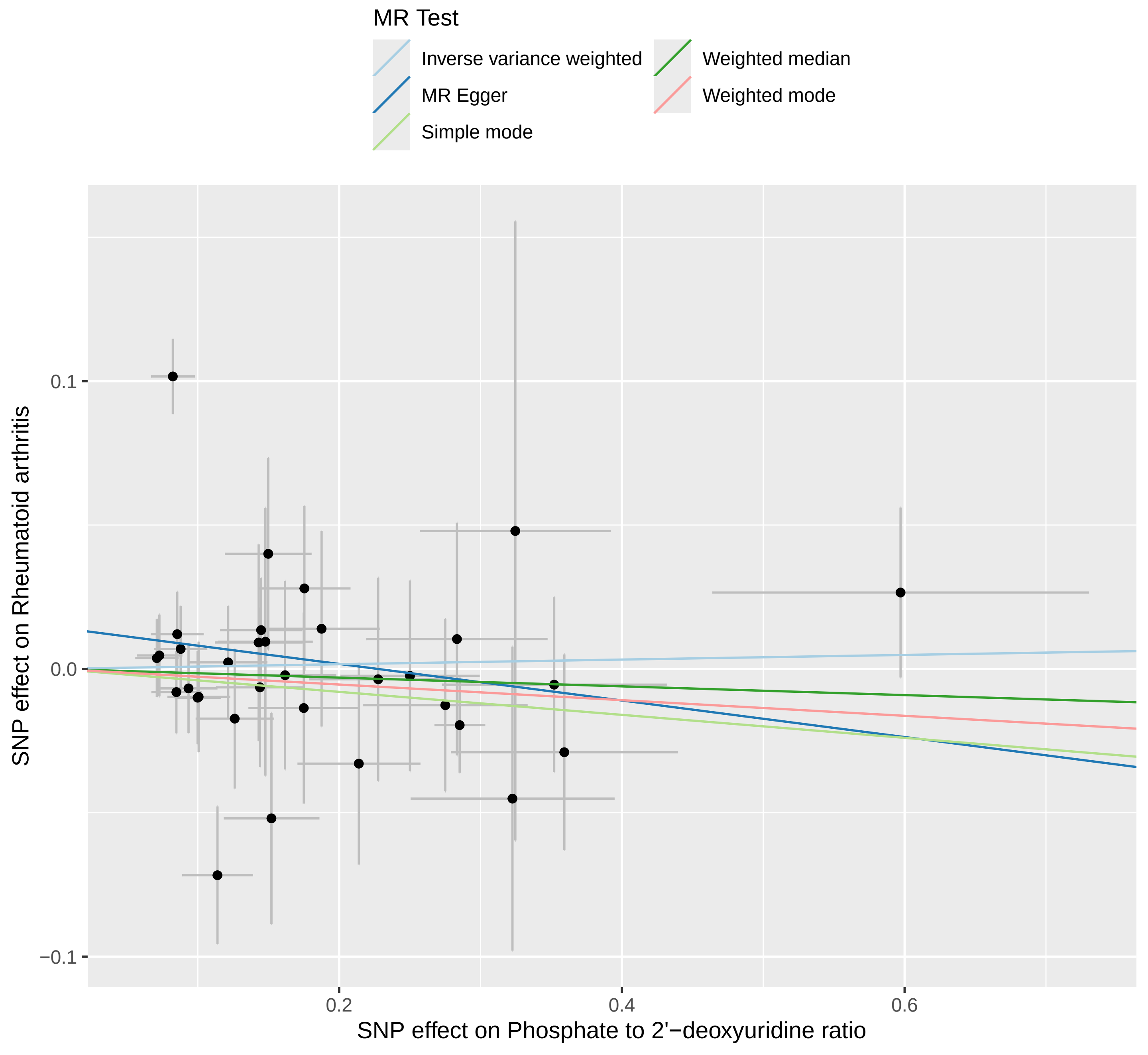
MR Test
Inverse variance weighted
MR Egger
Simple mode
Weighted median
Weighted mode
SNP effect on Rheumatoid arthritis
0.1
0.0
−0.1
0.2
0.4
0.6
SNP effect on Phosphate to 2'−deoxyuridine ratio

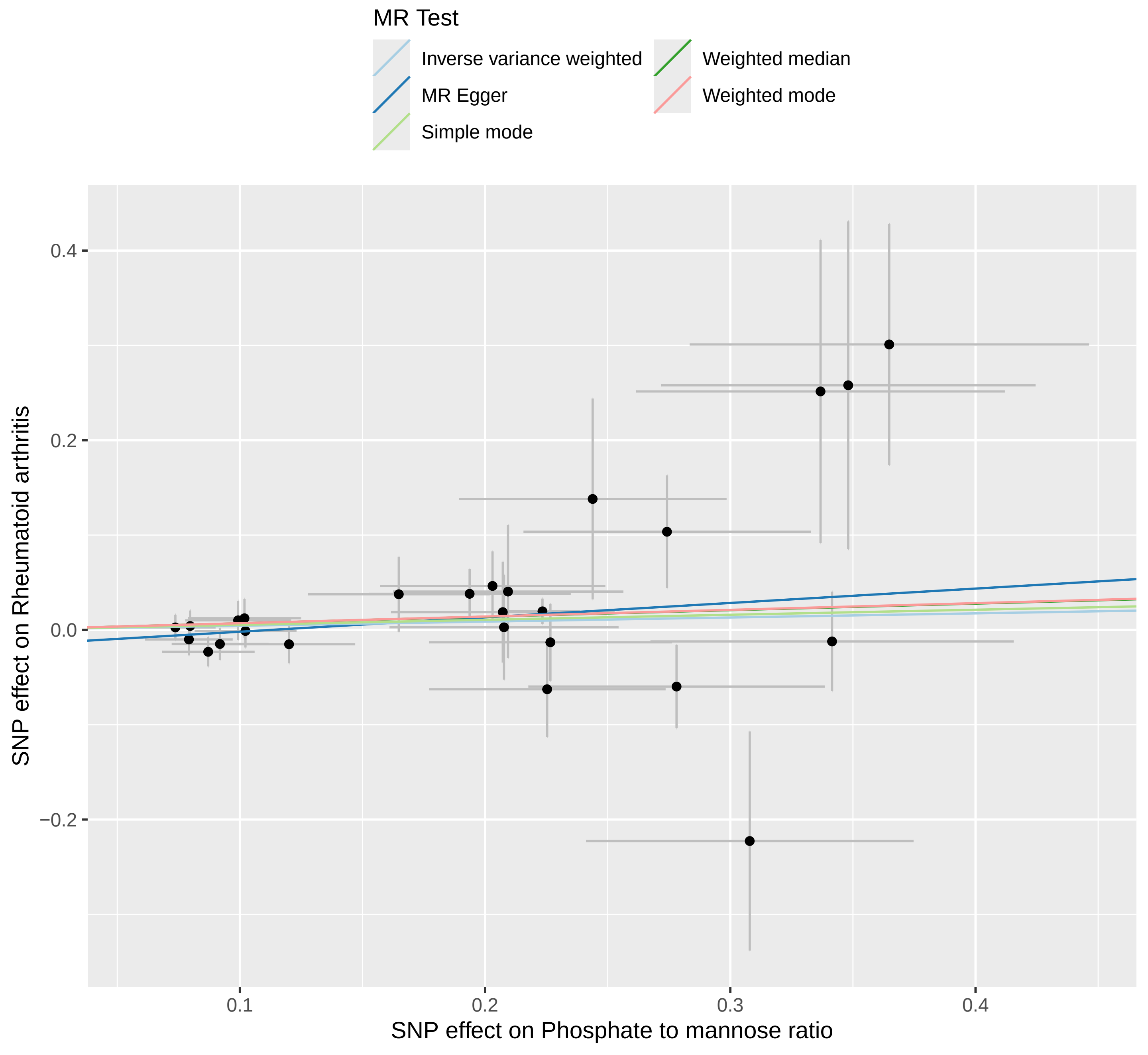
MR Test
Inverse variance weighted
MR Egger
Simple mode
Weighted median
Weighted mode
0.4
0.2
0.0
−0.2
SNP effect on Rheumatoid arthritis
0.1
0.2
0.3
0.4
SNP effect on Phosphate to mannose ratio

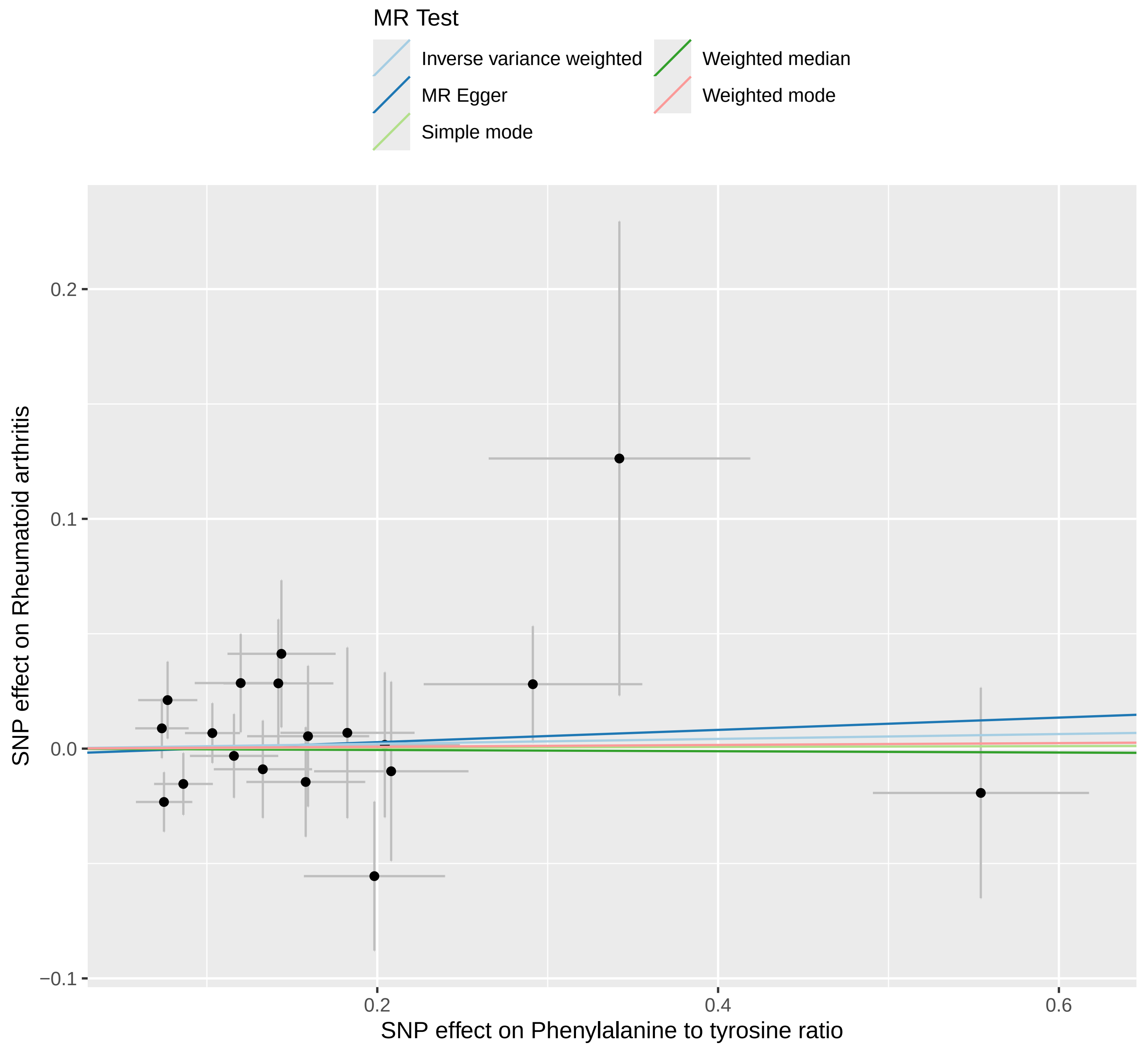
MR Test
Inverse variance weighted
MR Egger
Simple mode
Weighted median
Weighted mode
SNP effect on Rheumatoid arthritis
SNP effect on Phenylalanine to tyrosine ratio
0.2
0.1
0.0
−0.1
0.2
0.4
0.6

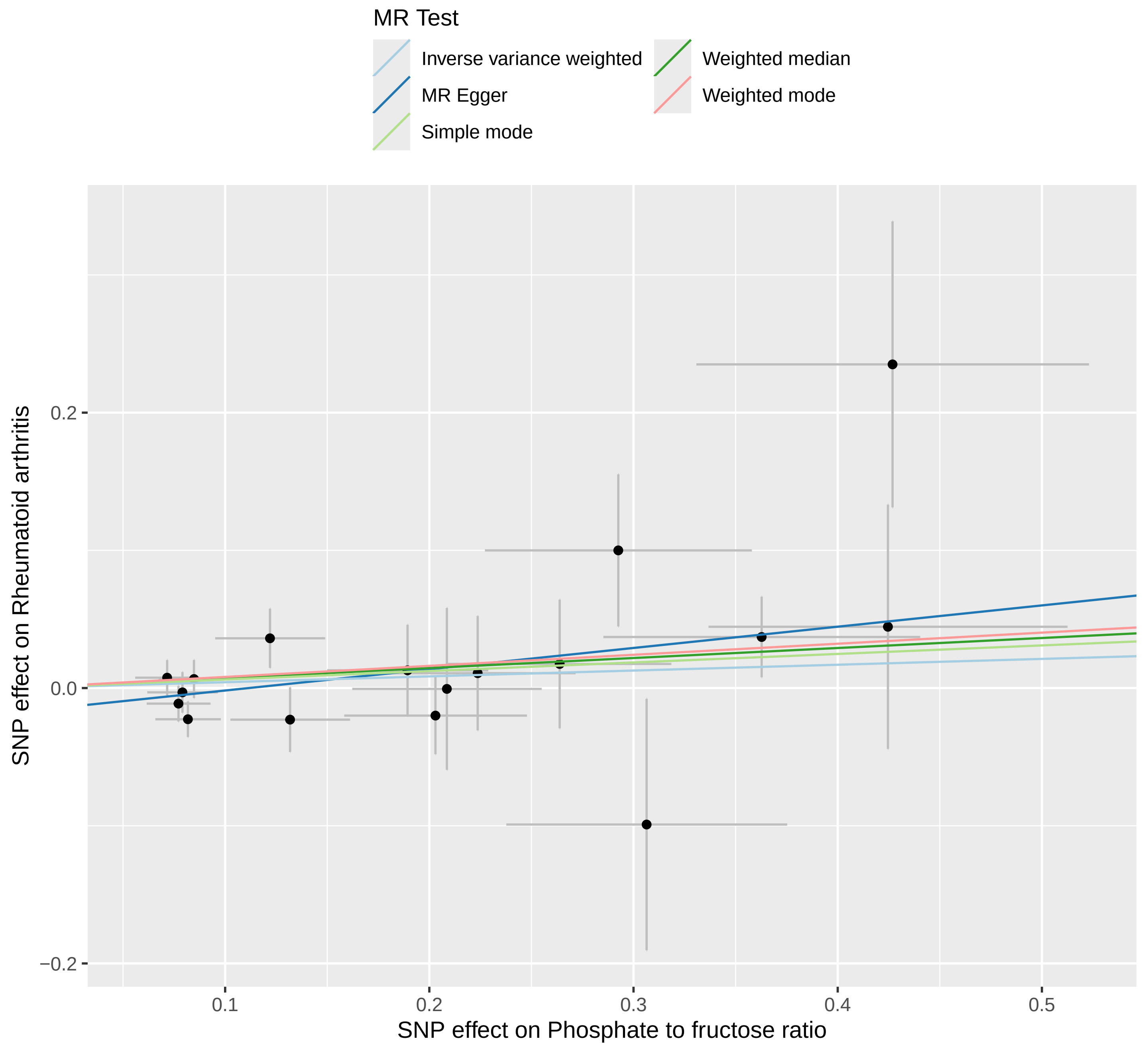

MR Test
Inverse variance weighted
MR Egger
Simple mode
Weighted median
Weighted mode
0.2
0.0
−0.2
0.1
0.2
0.3
0.4
0.5
SNP effect on Rheumatoid arthritis
SNP effect on Phosphate to fructose ratio

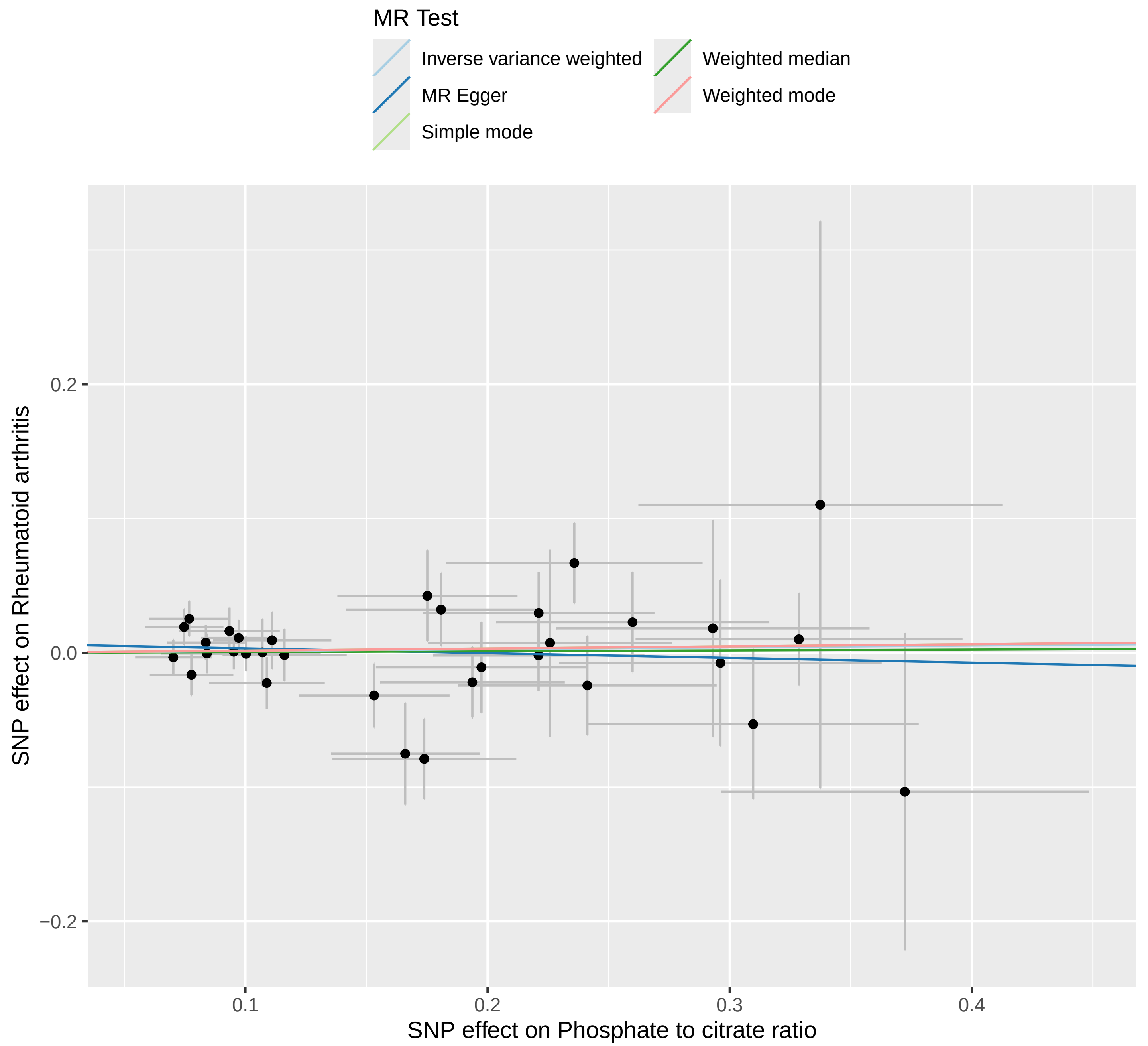
MR Test
Inverse variance weighted
MR Egger
Simple mode
Weighted median
Weighted mode
0.2
0.0
−0.2
0.1
0.2
0.3
0.4
SNP effect on Rheumatoid arthritis
SNP effect on Phosphate to citrate ratio

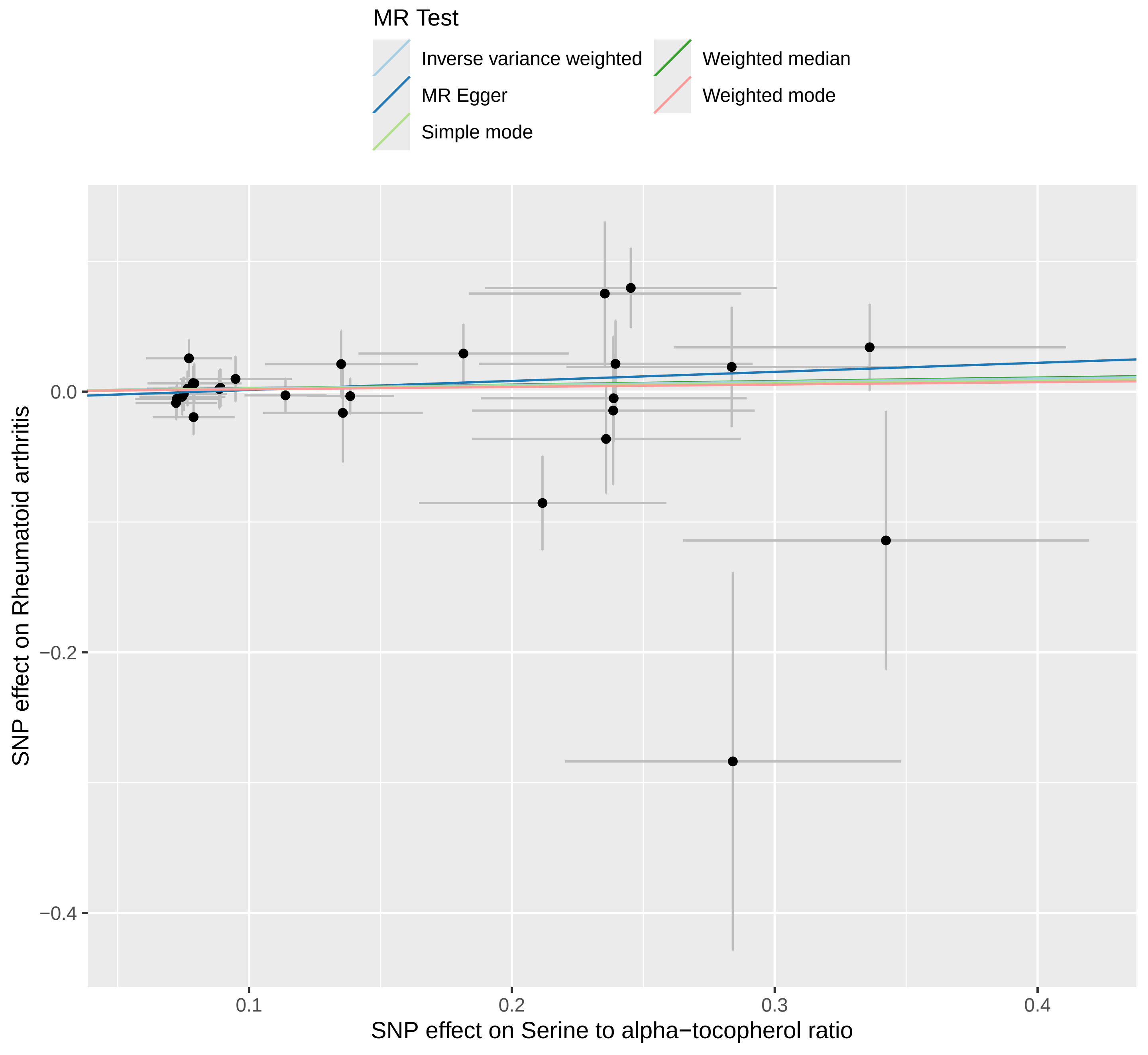

MR Test
Inverse variance weighted
MR Egger
Simple mode
Weighted median
Weighted mode
SNP effect on Rheumatoid arthritis
0.0
−0.2
−0.4
0.1
0.2
0.3
0.4
SNP effect on Serine to alpha−tocopherol ratio

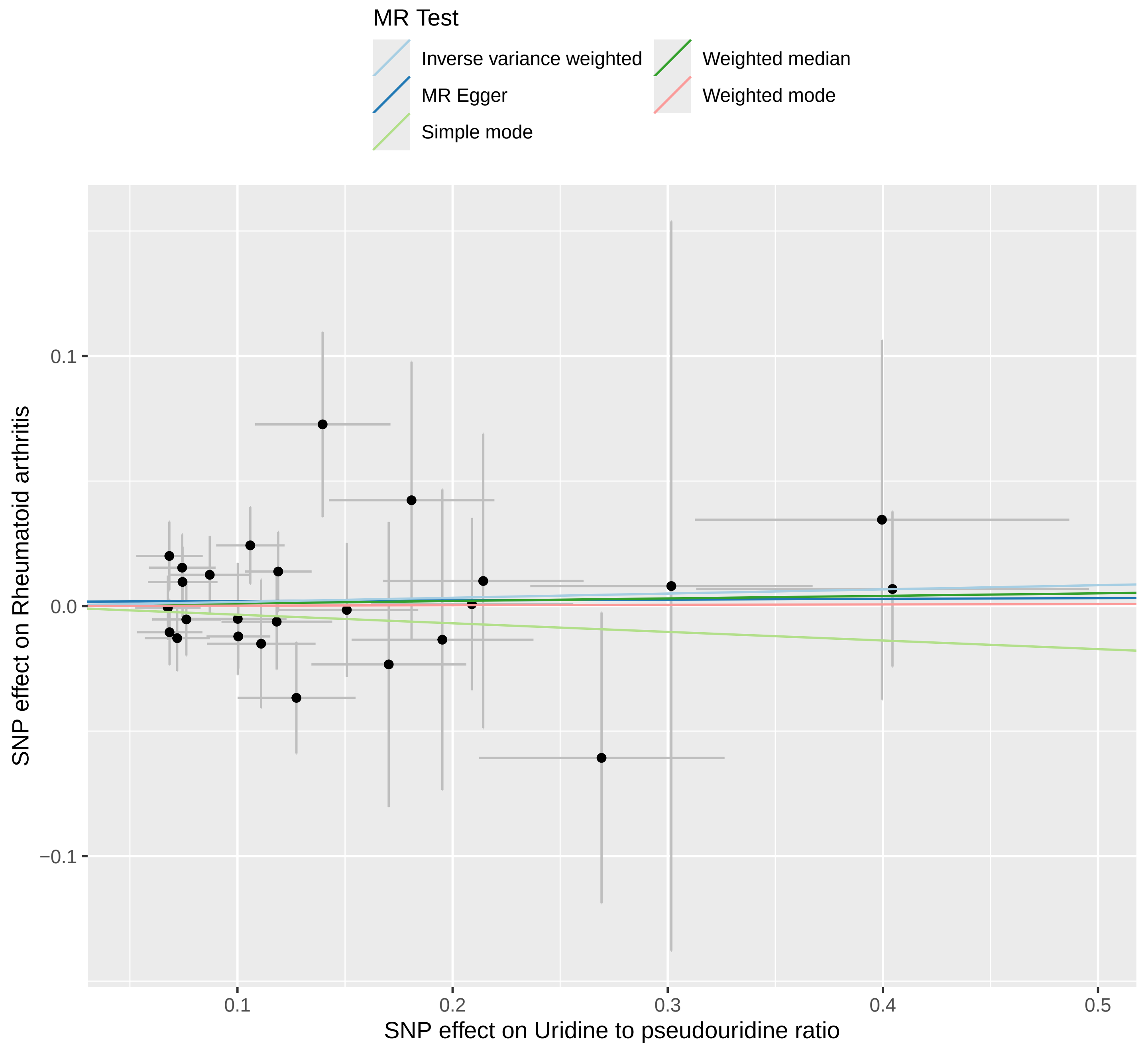
MR Test
Inverse variance weighted
MR Egger
Simple mode
Weighted median
Weighted mode
0.1
0.0
−0.1
SNP effect on Rheumatoid arthritis
0.1
0.2
0.3
0.4
0.5
SNP effect on Uridine to pseudouridine ratio

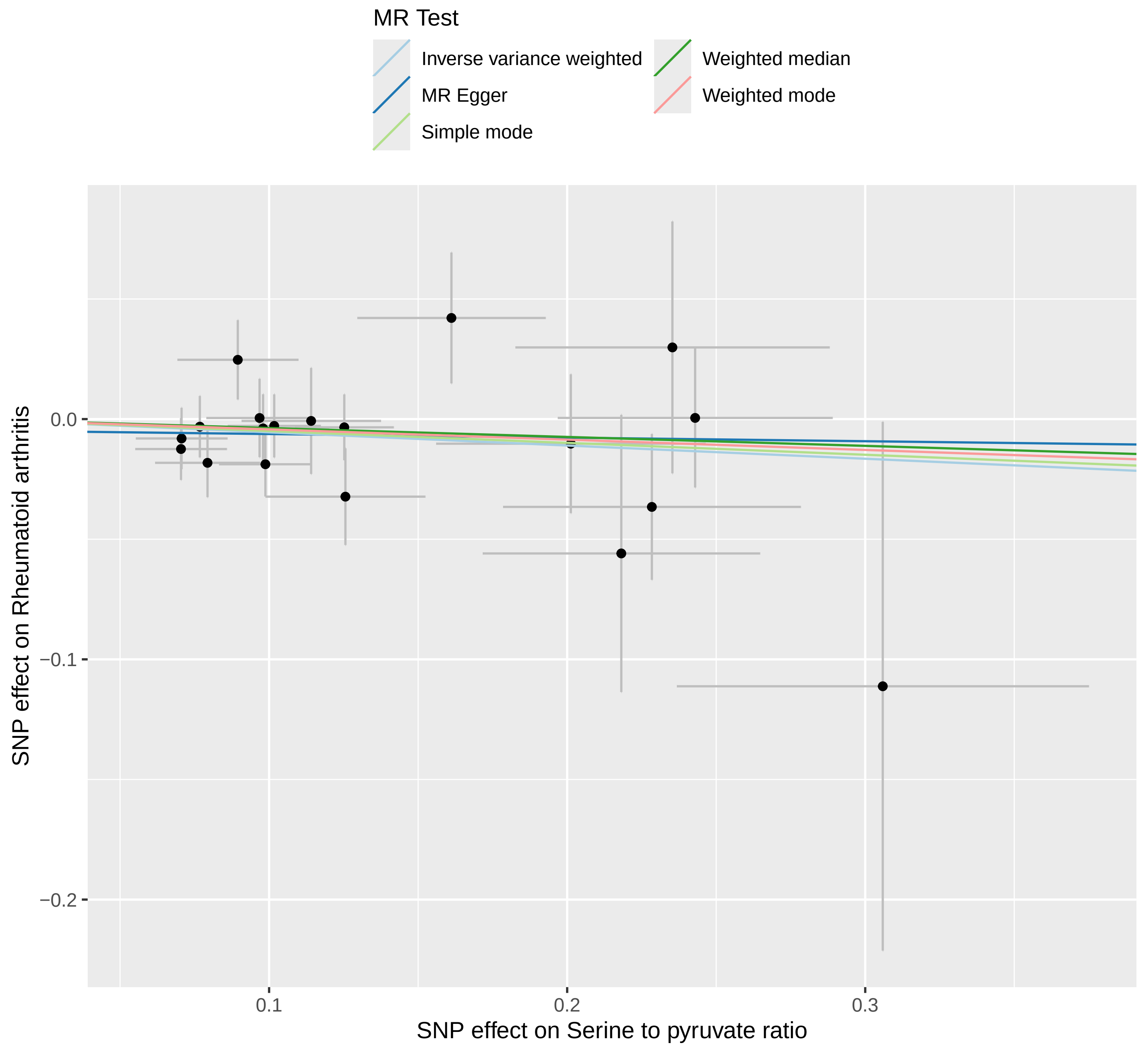
MR Test
Inverse variance weighted
MR Egger
Simple mode
Weighted median
Weighted mode
SNP effect on Rheumatoid arthritis
0.0
−0.1
−0.2
0.1
0.2
0.3
SNP effect on Serine to pyruvate ratio

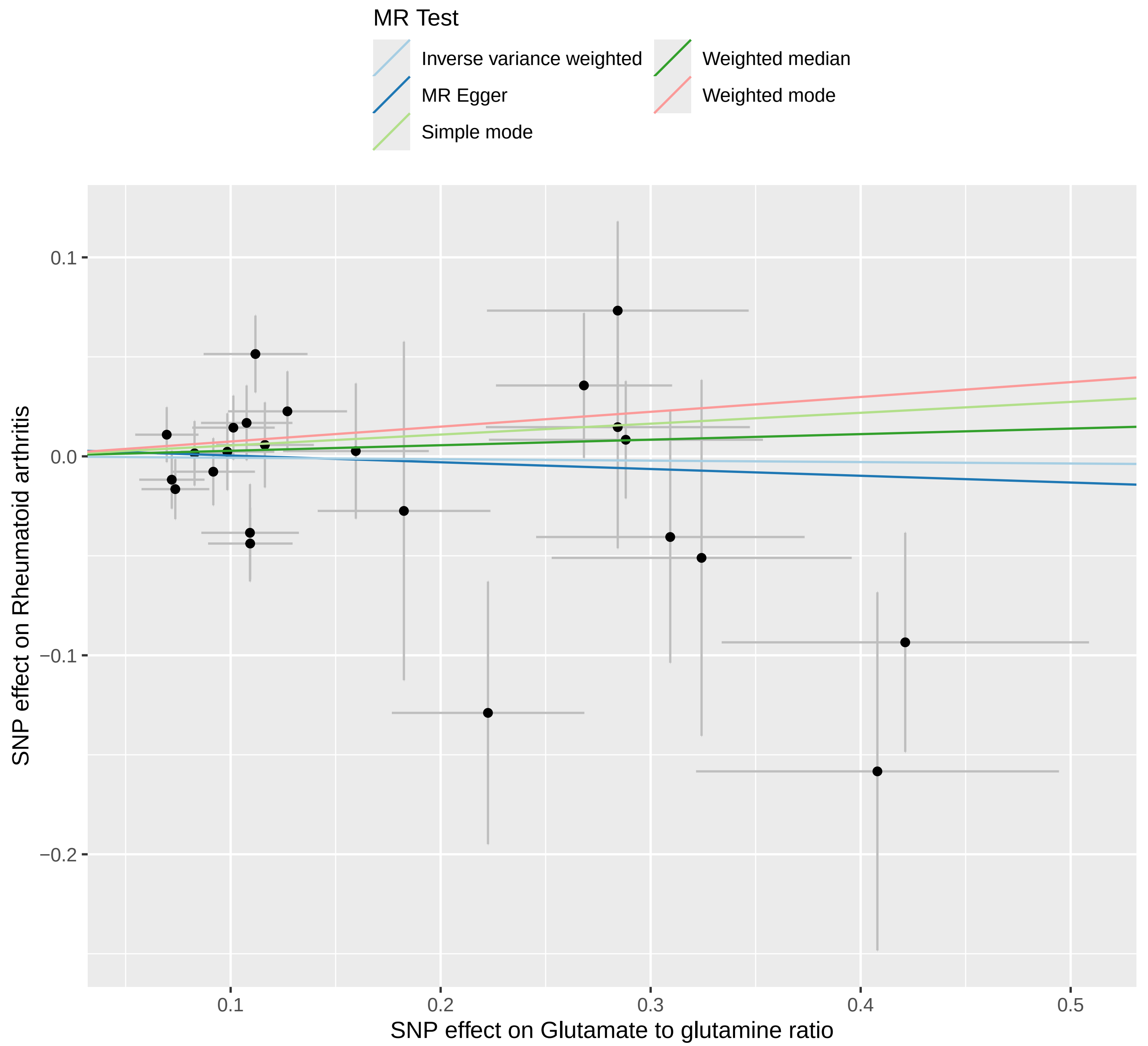
MR Test
Inverse variance weighted
MR Egger
Simple mode
Weighted median
Weighted mode
0.1
0.0
−0.1
−0.2
0.1
0.2
0.3
0.4
0.5
SNP effect on Rheumatoid arthritis
SNP effect on Glutamate to glutamine ratio

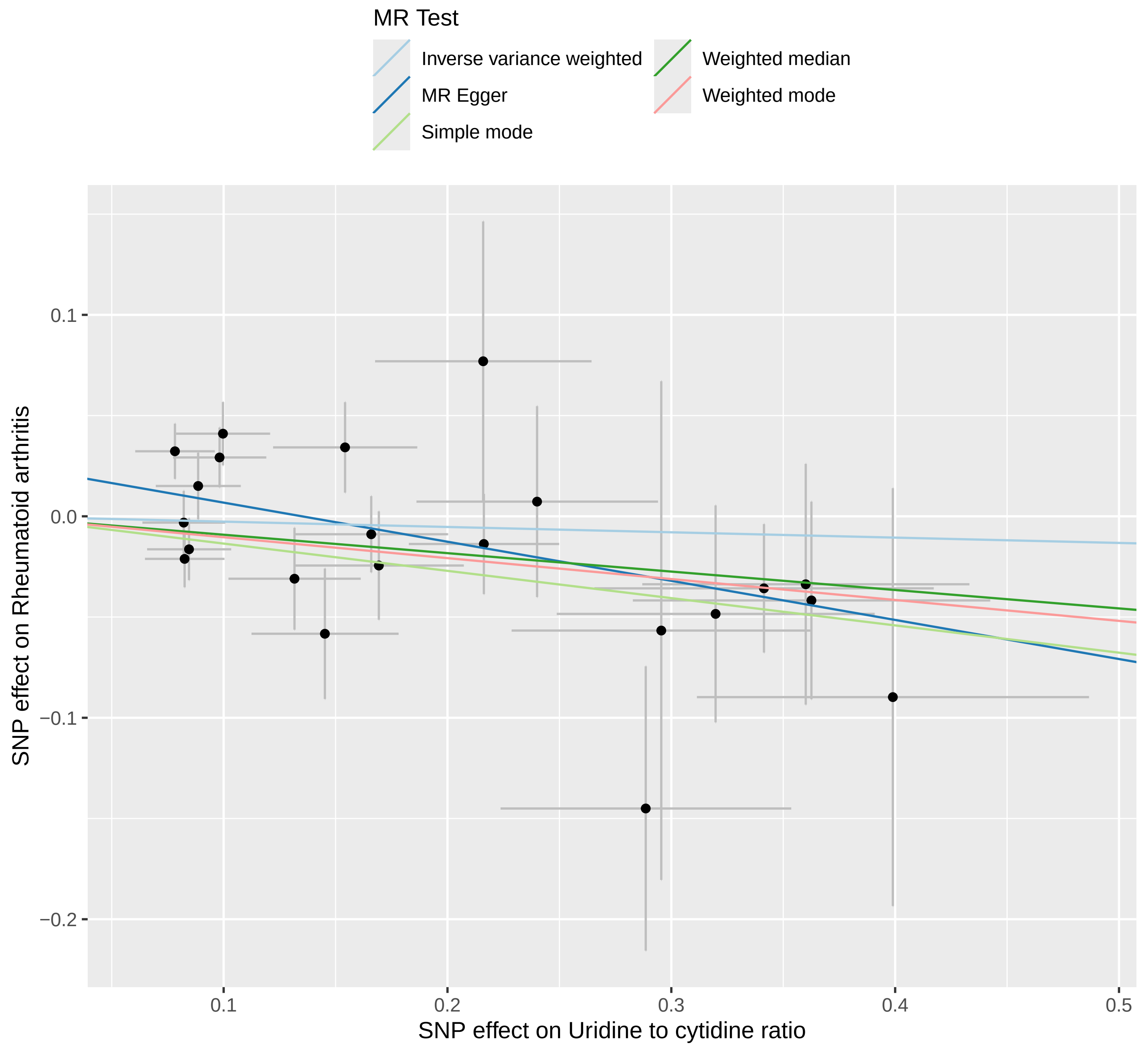
MR Test
Inverse variance weighted
MR Egger
Simple mode
Weighted median
Weighted mode
0.1
0.0
−0.1
−0.2
0.1
0.2
0.3
0.4
0.5
SNP effect on Rheumatoid arthritis
SNP effect on Uridine to cytidine ratio

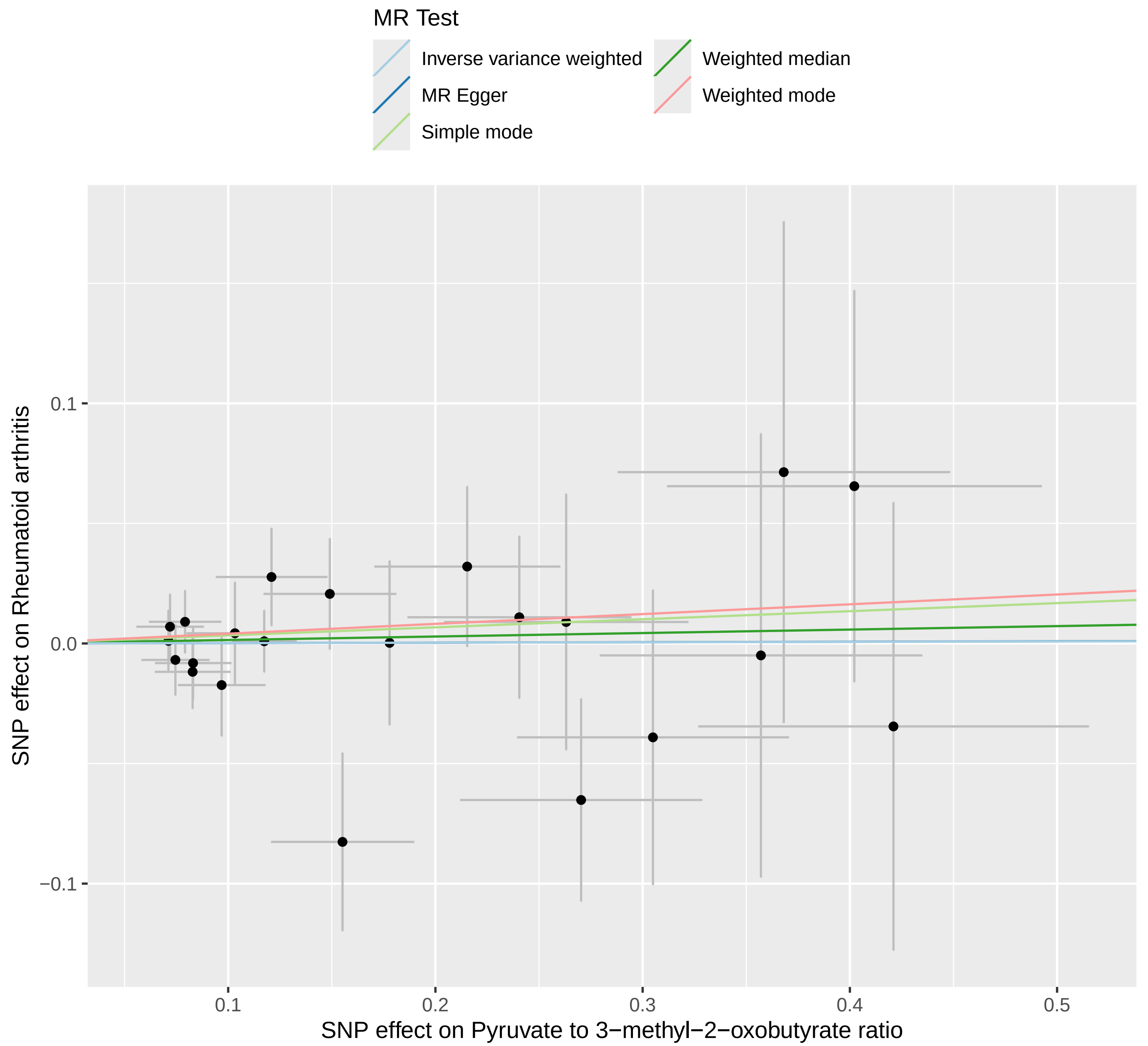
MR Test
Inverse variance weighted
MR Egger
Simple mode
Weighted median
Weighted mode
0.1
0.0
−0.1
0.1
0.2
0.3
0.4
0.5
SNP effect on Rheumatoid arthritis
SNP effect on Pyruvate to 3−methyl−2−oxobutyrate ratio

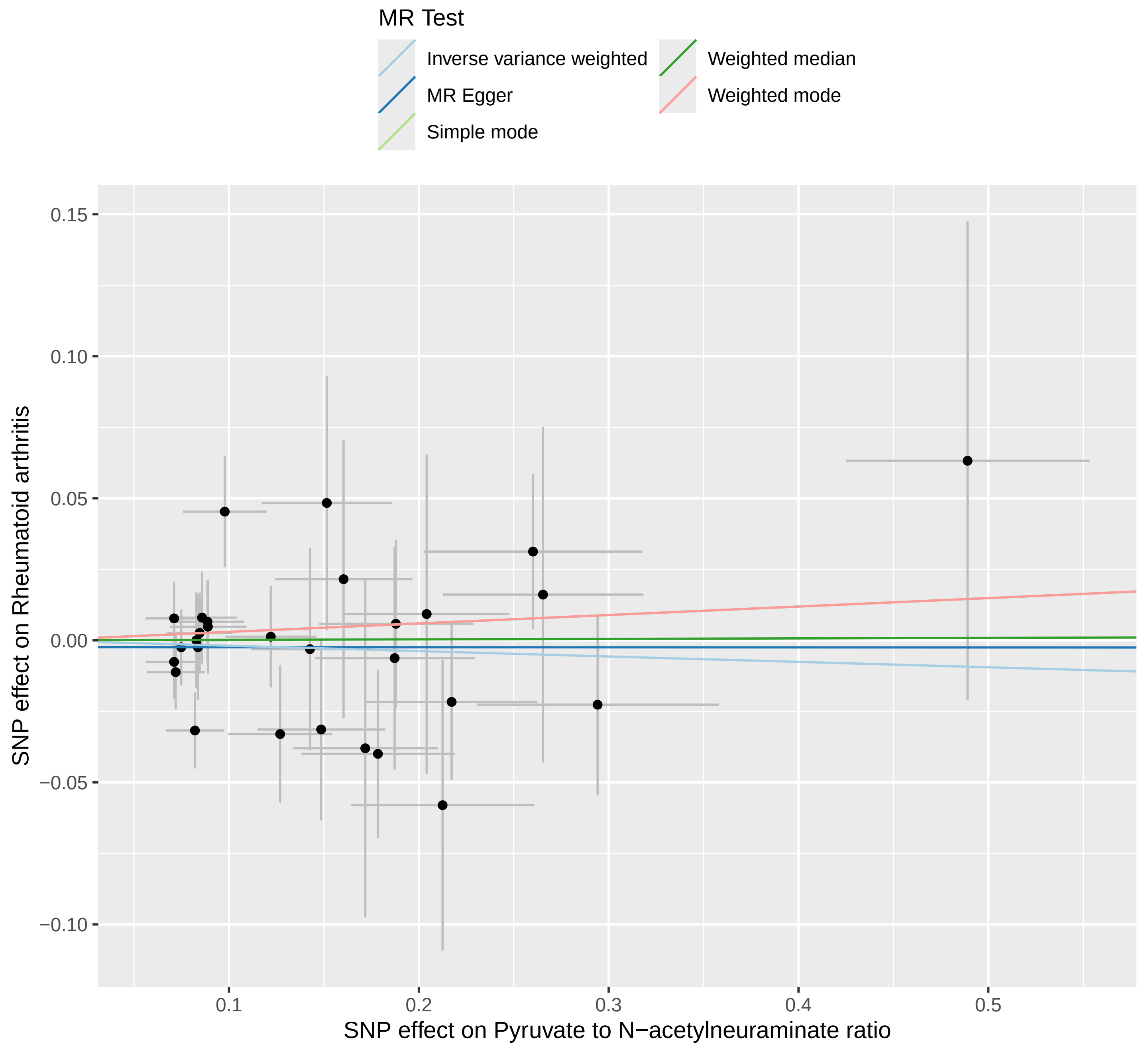
MR Test
Inverse variance weighted
MR Egger
Simple mode
Weighted median
Weighted mode
0.15
0.10
0.05
0.00
−0.05
−0.10
0.1
0.2
0.3
0.4
0.5
SNP effect on Rheumatoid arthritis
SNP effect on Pyruvate to N−acetylneuraminate ratio

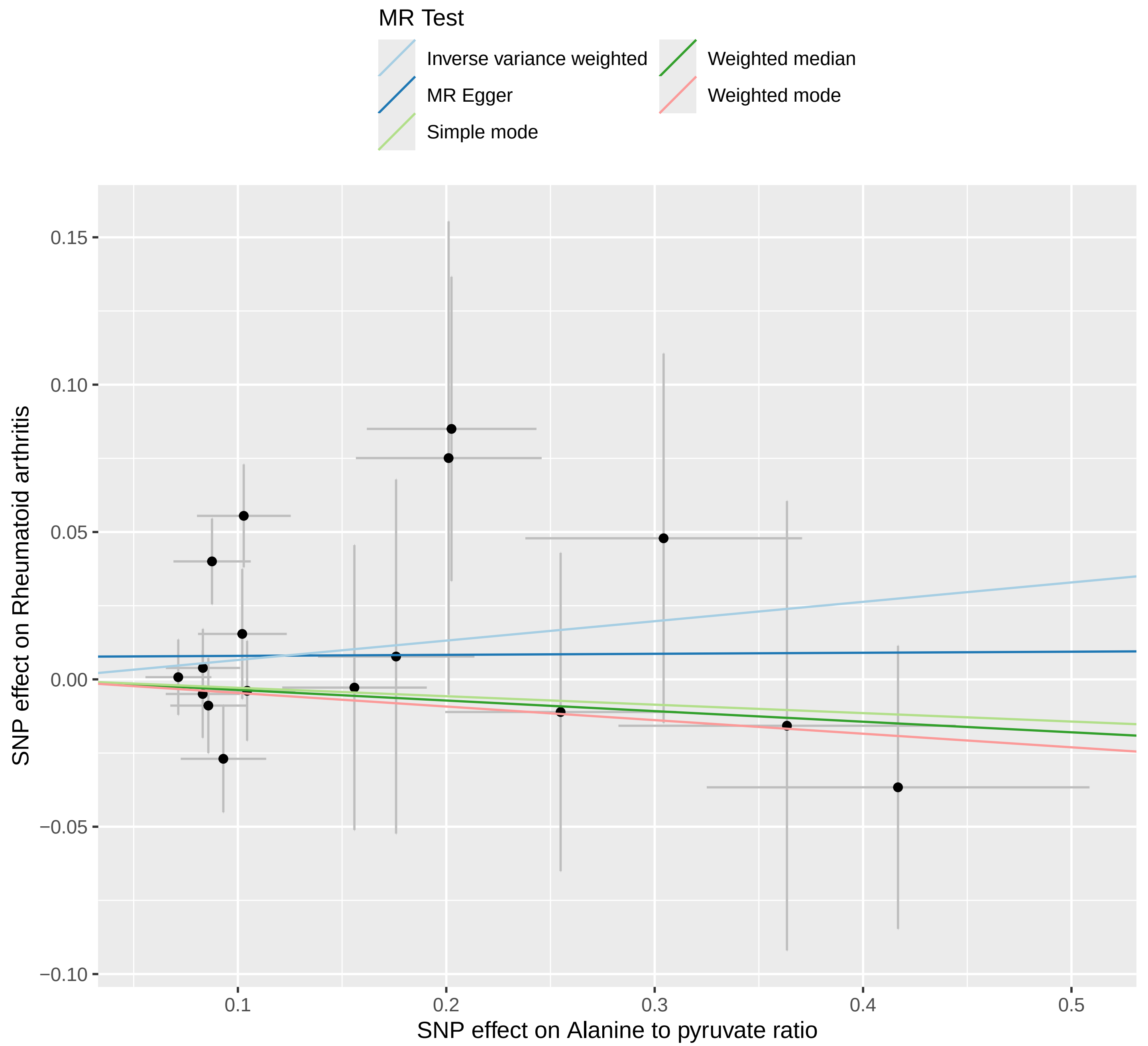

MR Test
Inverse variance weighted
MR Egger
Simple mode
Weighted median
Weighted mode
0.15
0.10
0.05
0.00
−0.05
−0.10
SNP effect on Rheumatoid arthritis
0.1
0.2
0.3
0.4
0.5
SNP effect on Alanine to pyruvate ratio

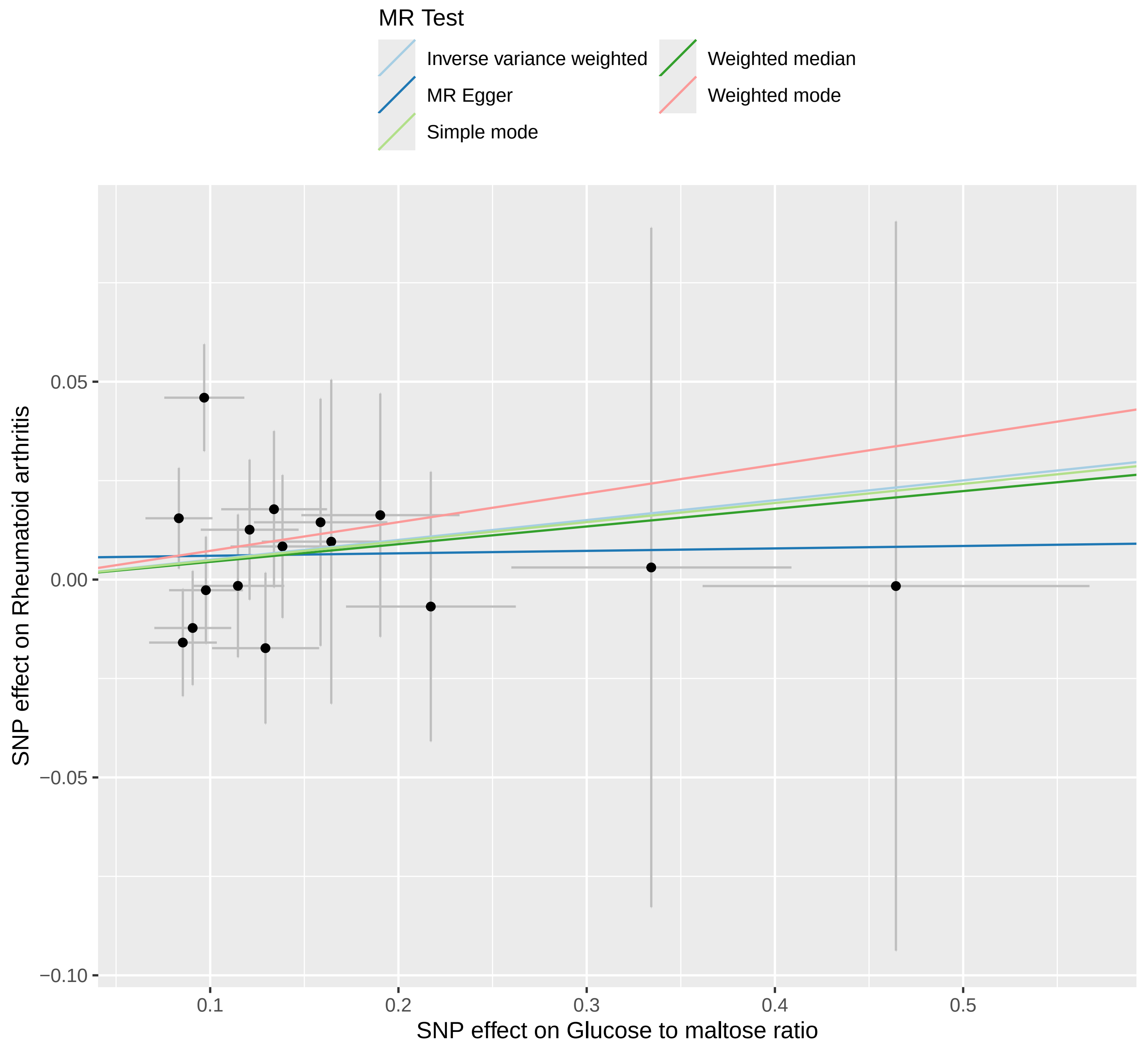
MR Test
Inverse variance weighted
MR Egger
Simple mode
Weighted median
Weighted mode
0.05
0.00
−0.05
−0.10
SNP effect on Rheumatoid arthritis
0.1
0.2
0.3
0.4
0.5
SNP effect on Glucose to maltose ratio

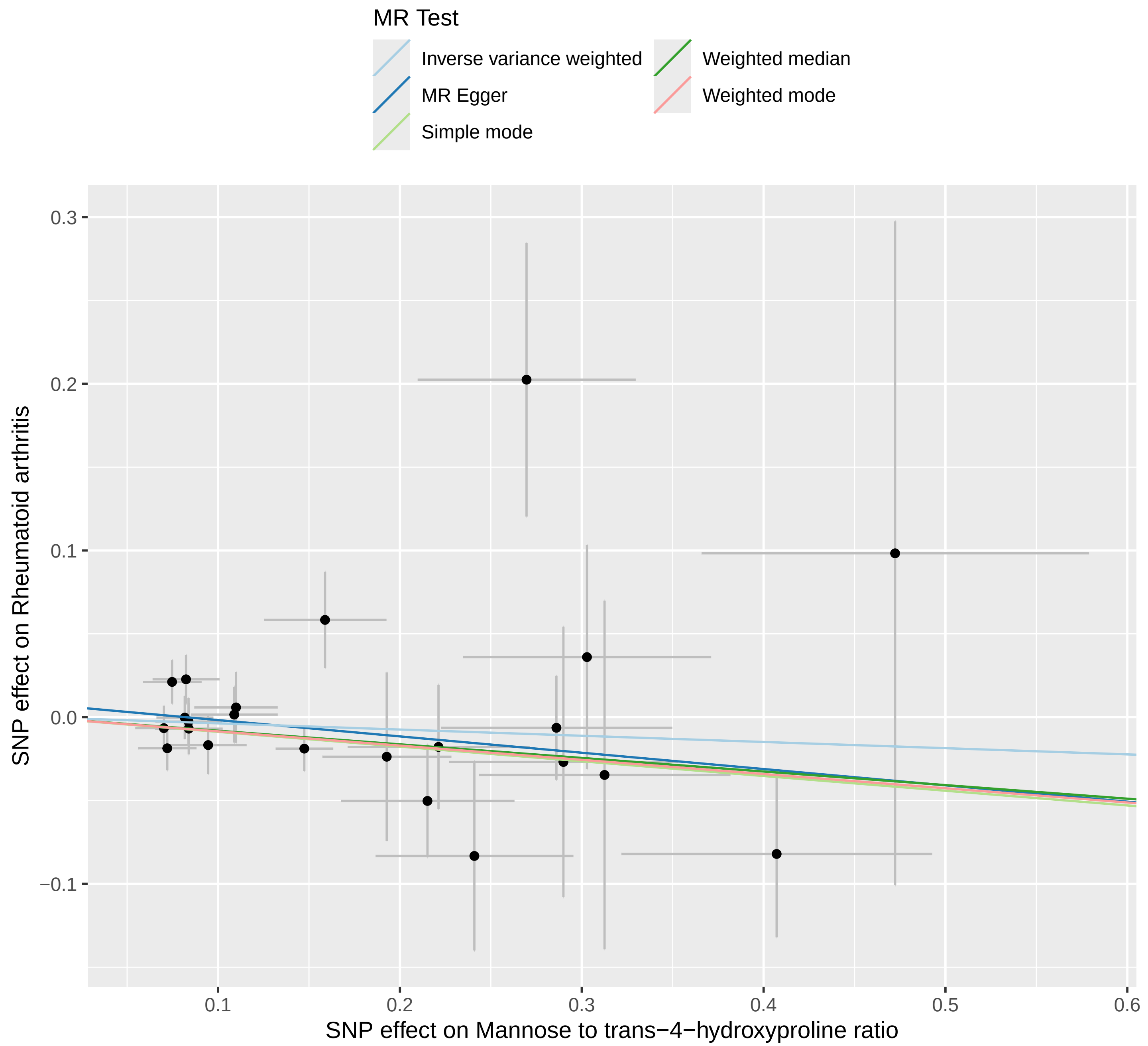
MR Test
Inverse variance weighted
MR Egger
Simple mode
Weighted median
Weighted mode
0.3
0.2
0.1
0.0
−0.1
0.1
0.2
0.3
0.4
0.5
0.6
SNP effect on Rheumatoid arthritis
SNP effect on Mannose to trans−4−hydroxyproline ratio

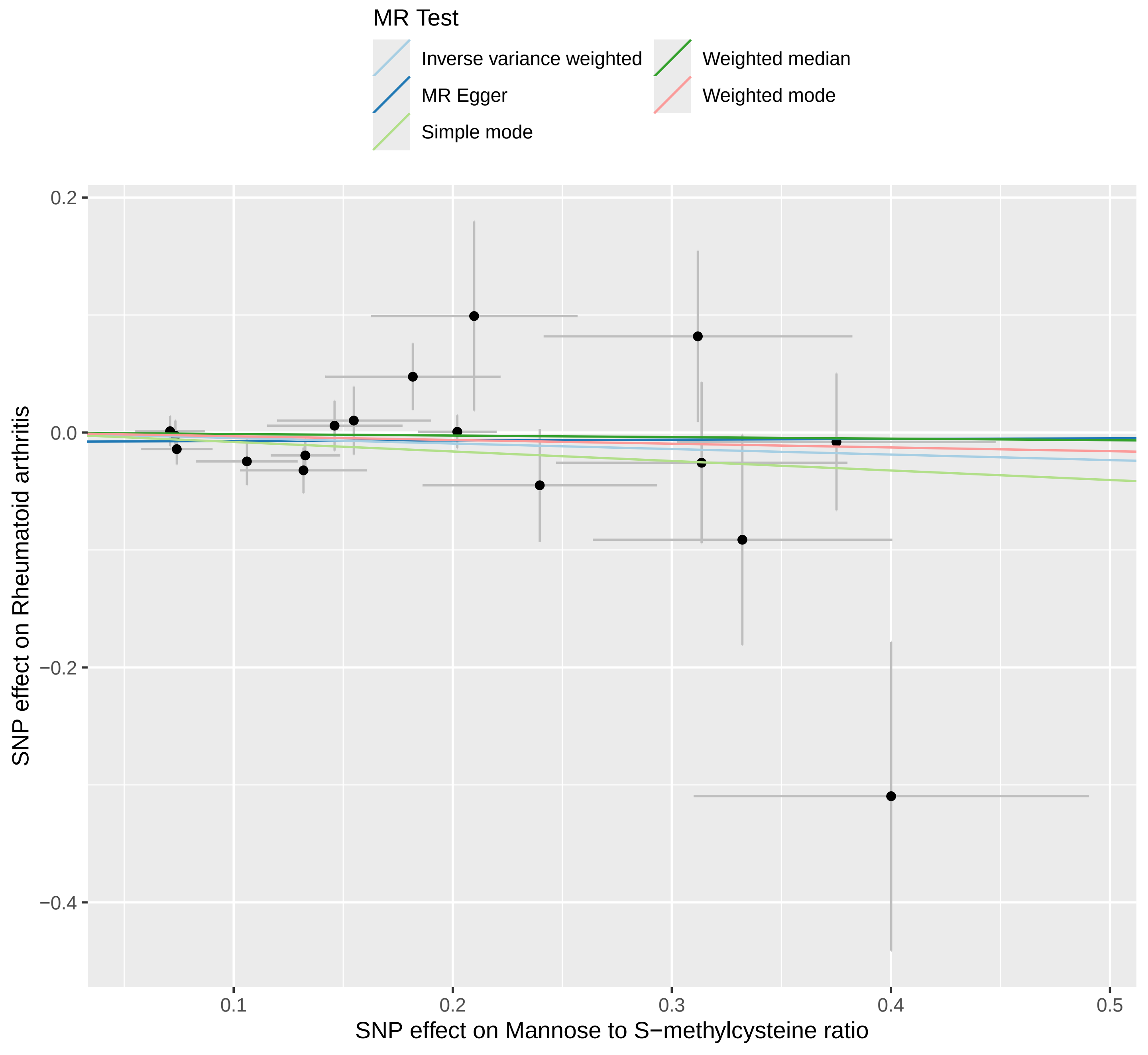

MR Test
Inverse variance weighted
MR Egger
Simple mode
Weighted median
Weighted mode
0.2
0.0
−0.2
−0.4
0.1
0.2
0.3
0.4
0.5
SNP effect on Rheumatoid arthritis
SNP effect on Mannose to S−methylcysteine ratio

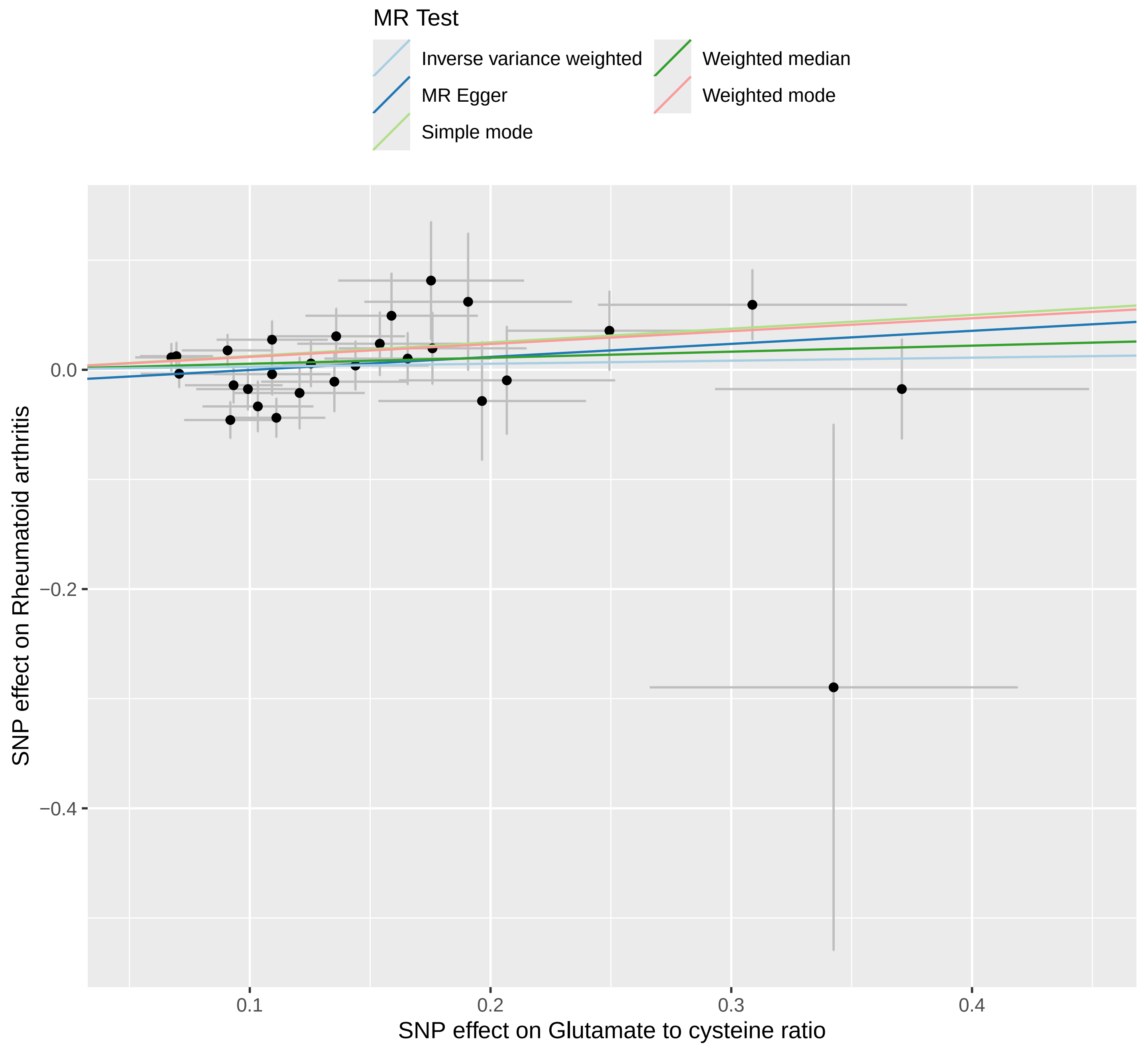

MR Test
Inverse variance weighted
MR Egger
Simple mode
Weighted median
Weighted mode
SNP effect on Rheumatoid arthritis
0.0
−0.2
−0.4
0.1
0.2
0.3
0.4
SNP effect on Glutamate to cysteine ratio

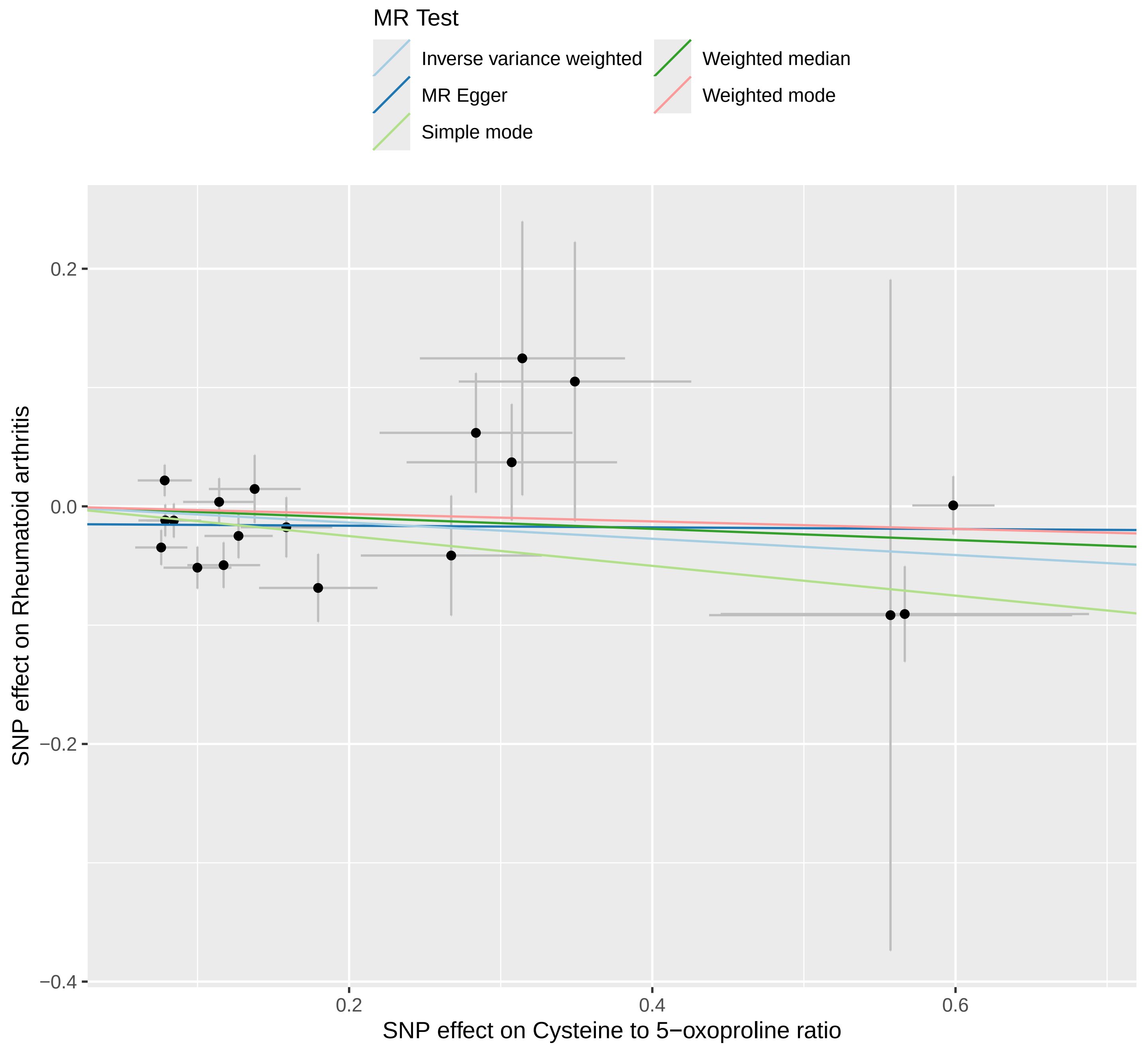
MR Test
Inverse variance weighted
MR Egger
Simple mode
Weighted median
Weighted mode
0.2
0.0
−0.2
−0.4
0.2
0.4
0.6
SNP effect on Rheumatoid arthritis
SNP effect on Cysteine to 5−oxoproline ratio

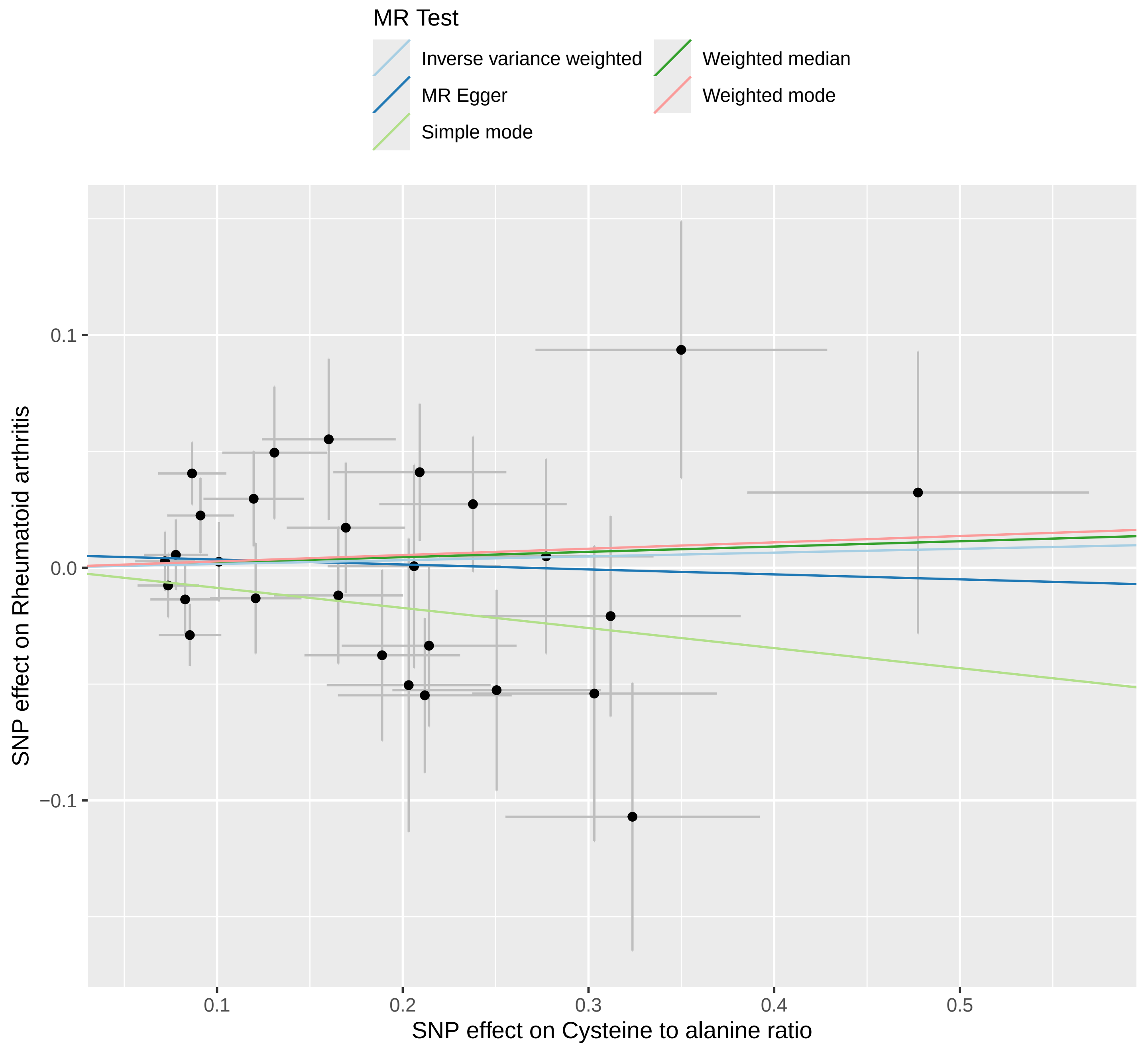

MR Test
Inverse variance weighted
MR Egger
Simple mode
Weighted median
Weighted mode
0.1
0.0
−0.1
0.1
0.2
0.3
0.4
0.5
SNP effect on Rheumatoid arthritis
SNP effect on Cysteine to alanine ratio

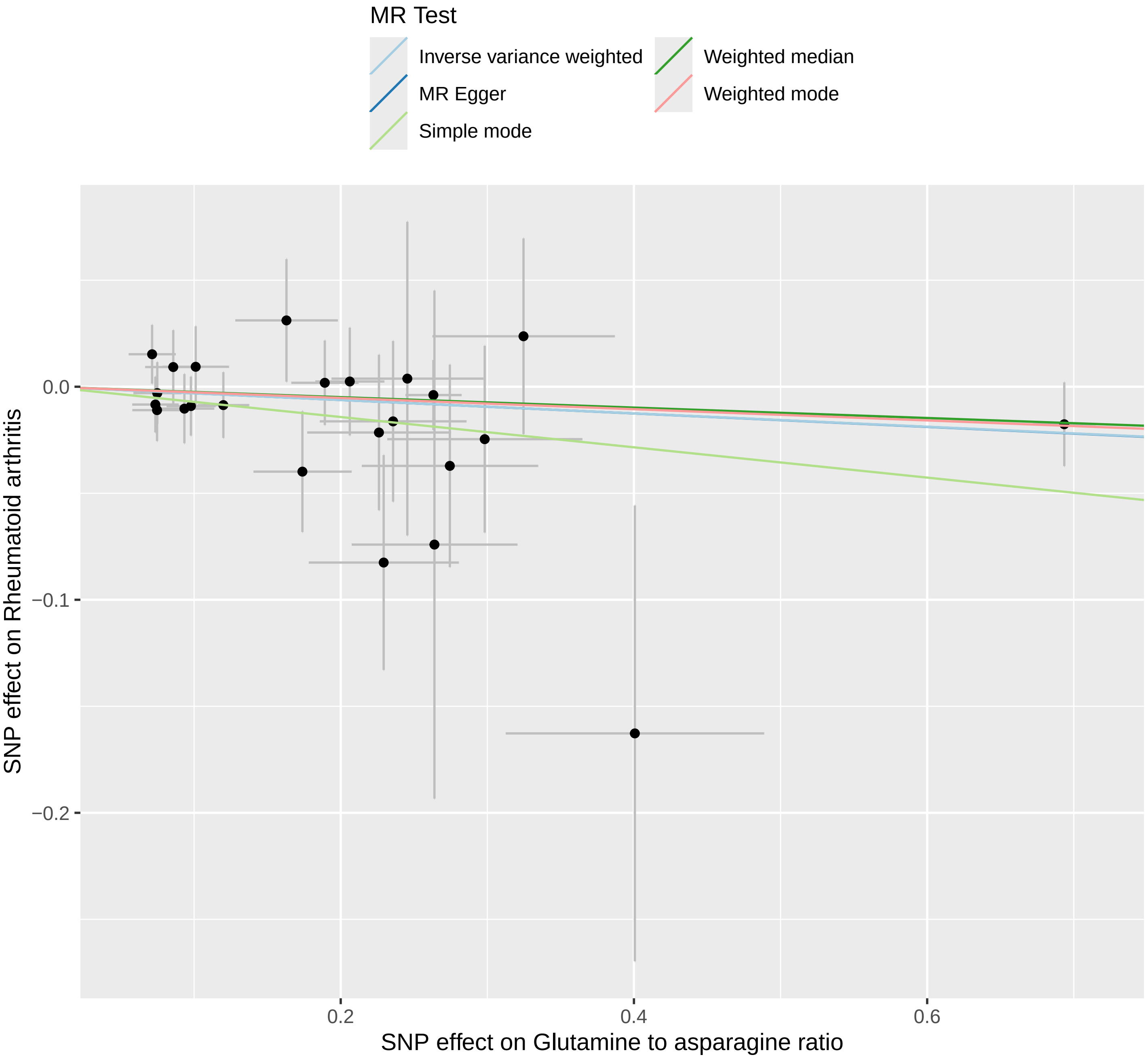

MR Test
Inverse variance weighted
MR Egger
Simple mode
Weighted median
Weighted mode
0.0
−0.1
−0.2
SNP effect on Rheumatoid arthritis
0.2
0.4
0.6
SNP effect on Glutamine to asparagine ratio

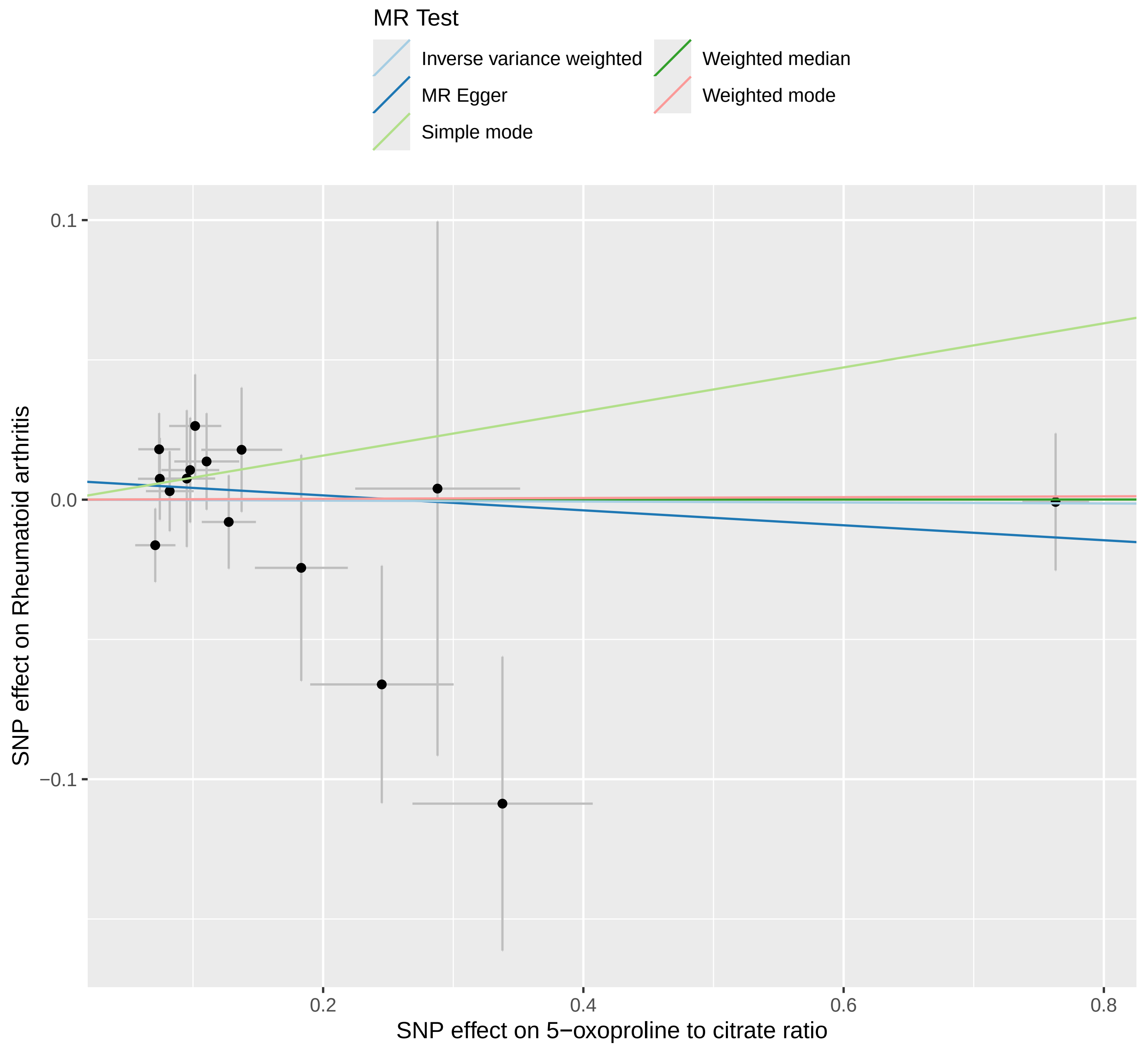
MR Test
Inverse variance weighted
MR Egger
Simple mode
Weighted median
Weighted mode
0.1
0.0
−0.1
0.2
0.4
0.6
0.8
SNP effect on Rheumatoid arthritis
SNP effect on 5−oxoproline to citrate ratio

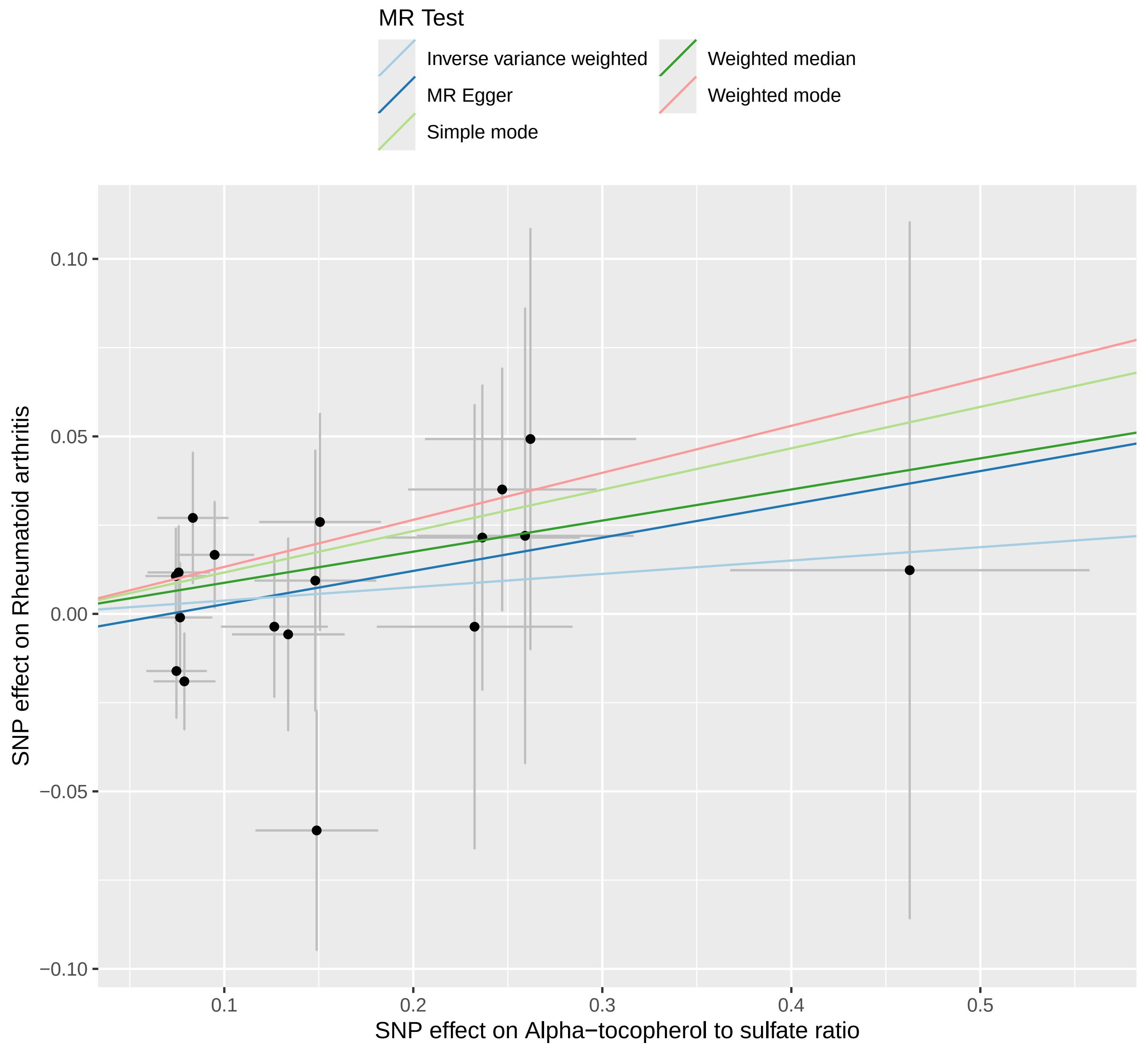
MR Test
Inverse variance weighted
MR Egger
Simple mode
Weighted median
Weighted mode
0.10
0.05
0.00
−0.05
−0.10
SNP effect on Rheumatoid arthritis
0.1
0.2
0.3
0.4
0.5
SNP effect on Alpha−tocopherol to sulfate ratio

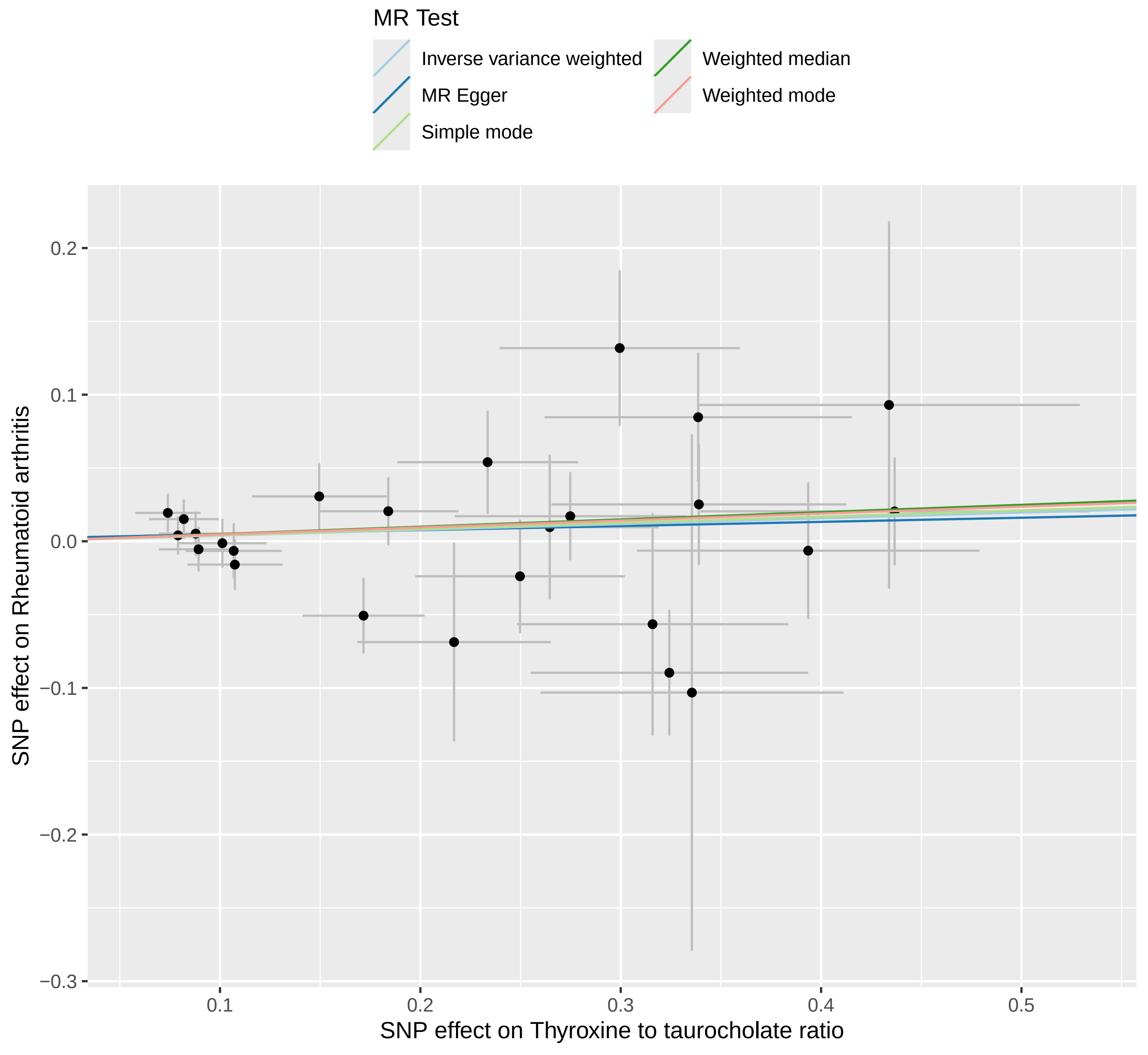

MR Test
Inverse variance weighted
MR Egger
Simple mode
Weighted median
Weighted mode
0.2
0.1
0.0
−0.1
−0.2
−0.3
0.1
0.2
0.3
0.4
0.5
SNP effect on Rheumatoid arthritis
SNP effect on Thyroxine to taurocholate ratio

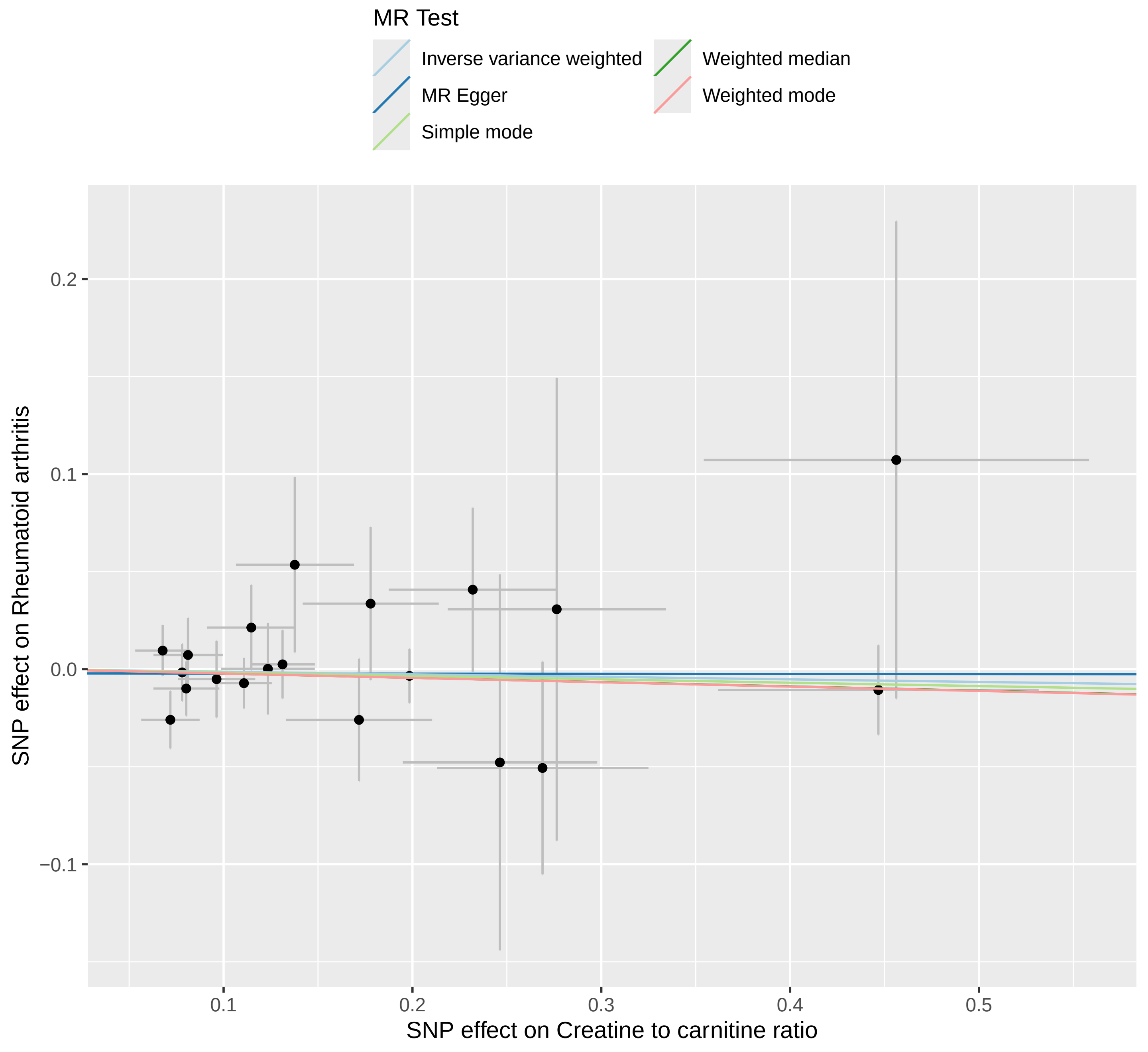
MR Test
Inverse variance weighted
MR Egger
Simple mode
Weighted median
Weighted mode
0.2
0.1
0.0
−0.1
SNP effect on Rheumatoid arthritis
0.1
0.2
0.3
0.4
0.5
SNP effect on Creatine to carnitine ratio

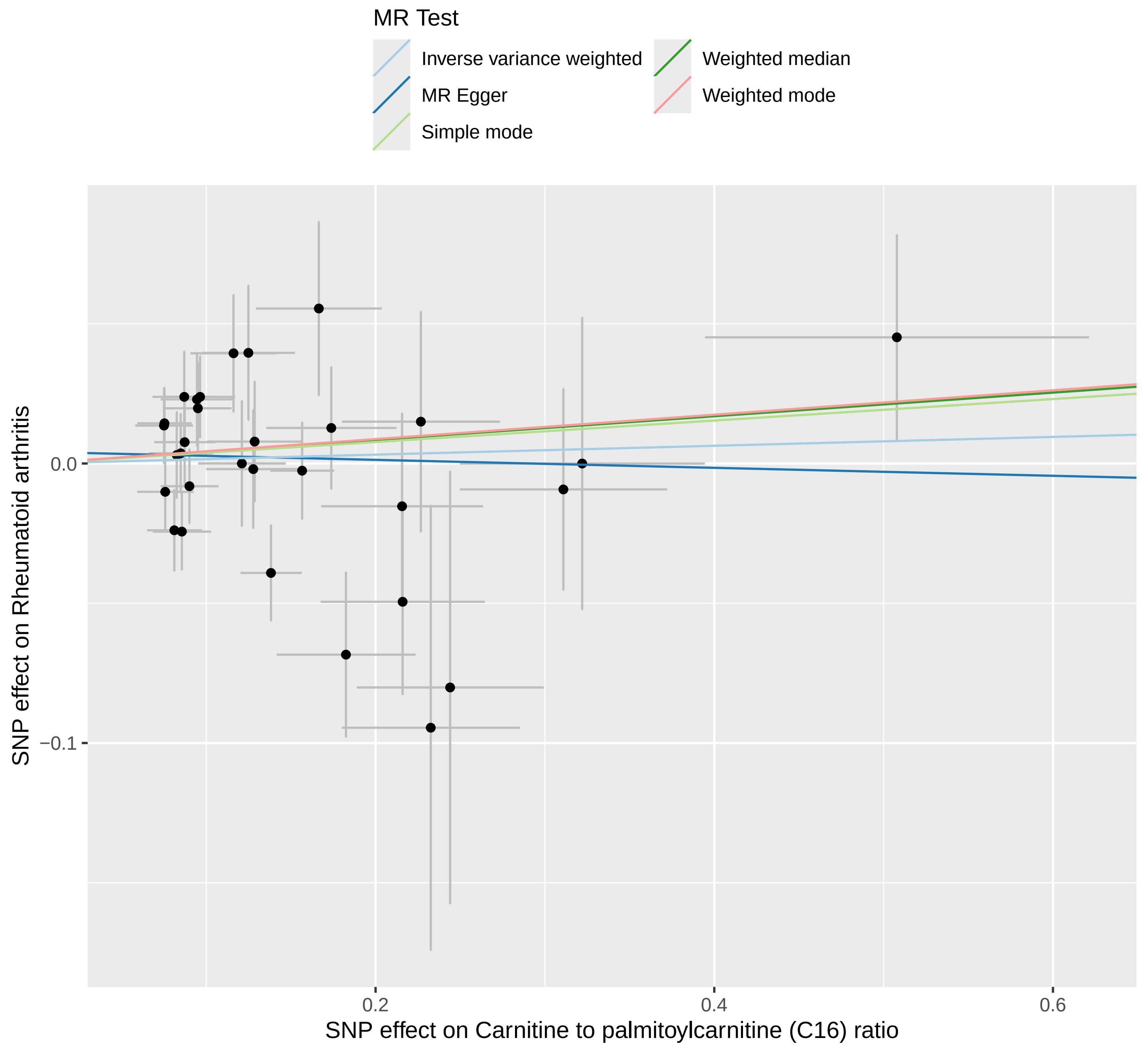

MR Test
Inverse variance weighted
MR Egger
Simple mode
Weighted median
Weighted mode
SNP effect on Rheumatoid arthritis
0.0
−0.1
0.2
0.4
0.6
SNP effect on Carnitine to palmitoylcarnitine (C16) ratio

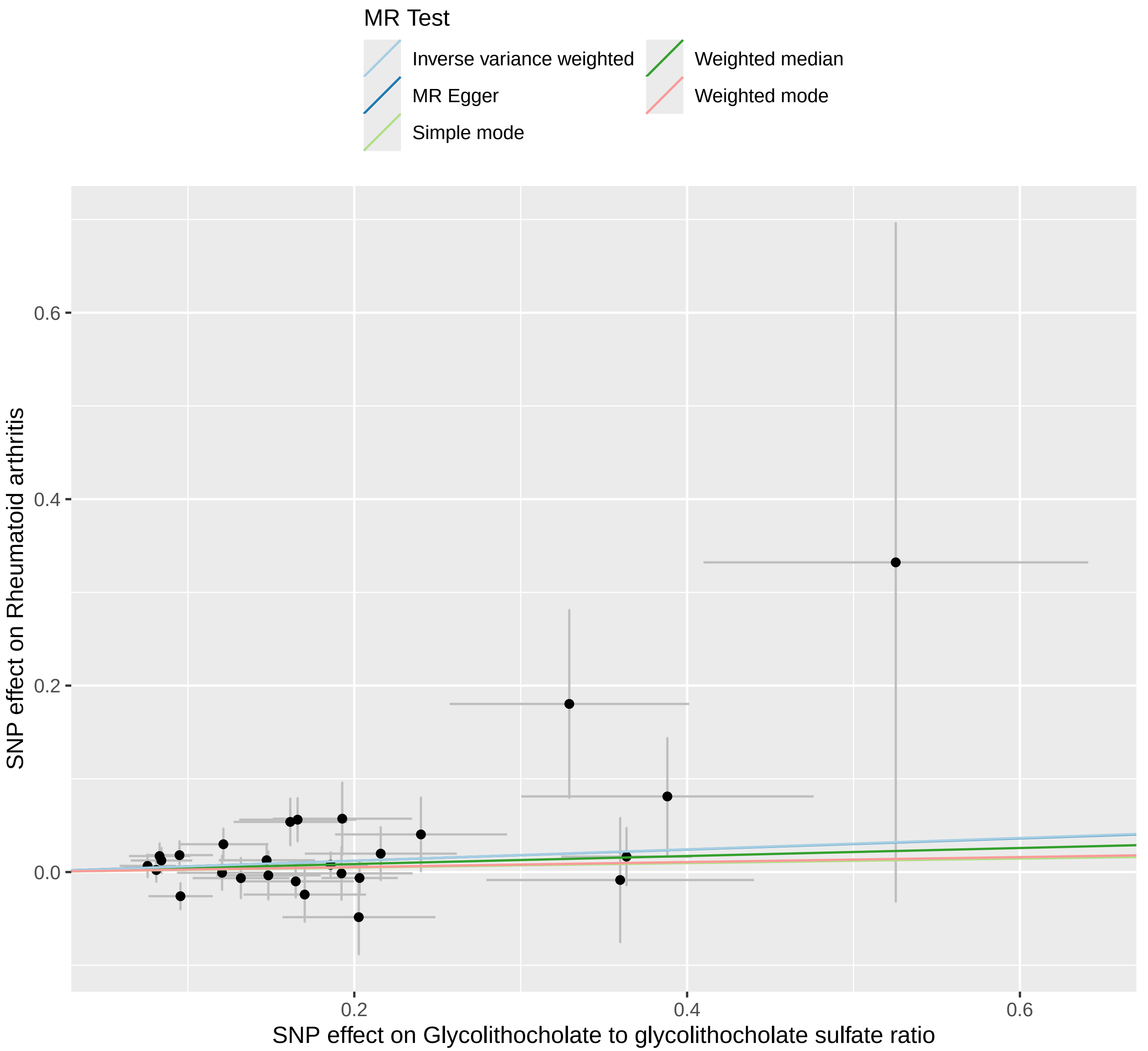
MR Test
Inverse variance weighted
MR Egger
Simple mode
Weighted median
Weighted mode
0.6
0.4
0.2
0.0
SNP effect on Rheumatoid arthritis
0.2
0.4
0.6
SNP effect on Glycolithocholate to glycolithocholate sulfate ratio

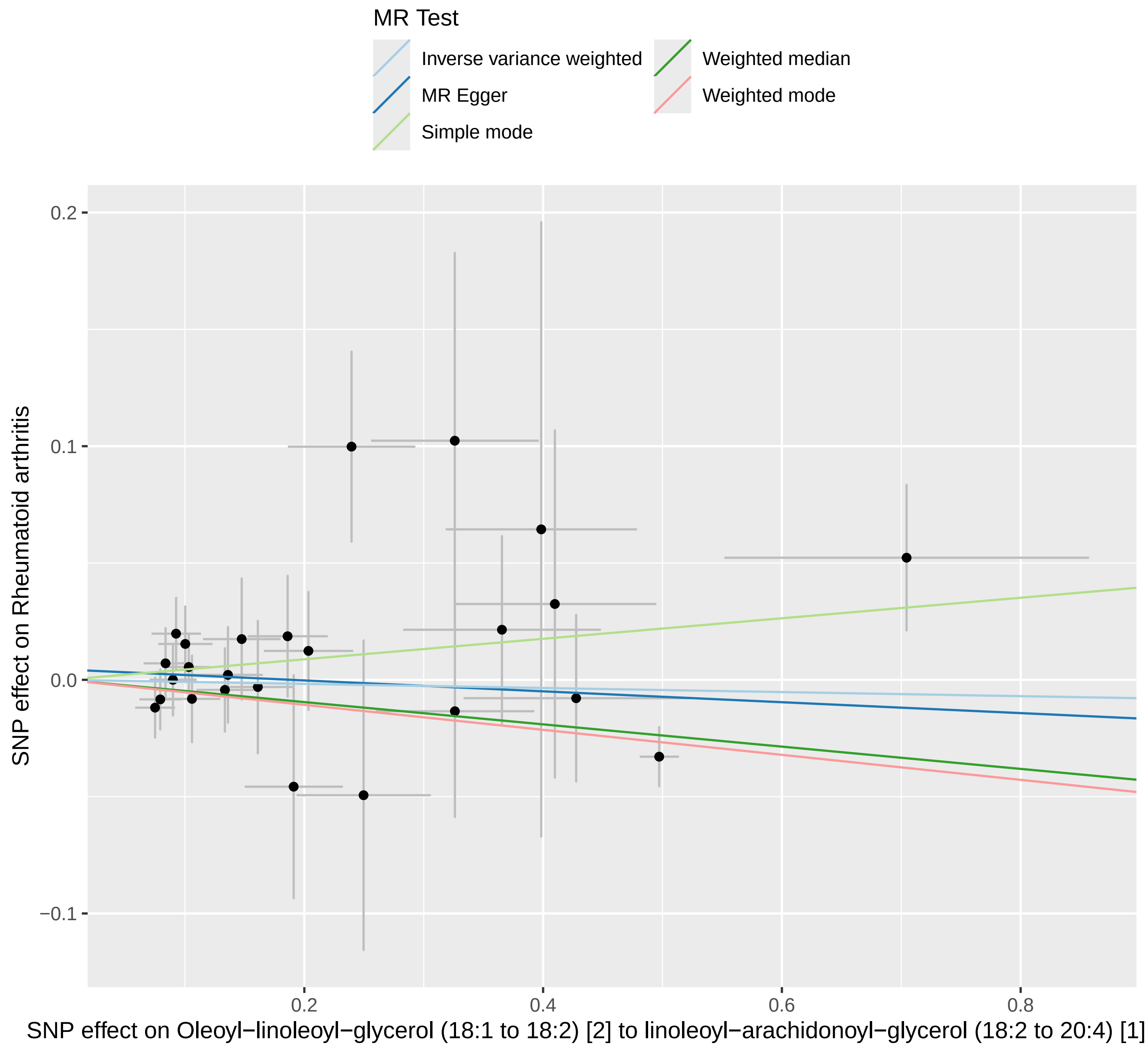
MR Test
Inverse variance weighted
MR Egger
Simple mode
Weighted median
Weighted mode
0.2
0.1
0.0
−0.1
SNP effect on Rheumatoid arthritis
0.2
0.4
0.6
0.8
SNP effect on Oleoyl−linoleoyl−glycerol (18:1 to 18:2) [2] to linoleoyl−arachidonoyl−glycerol (18:2 to 20:4) [1]

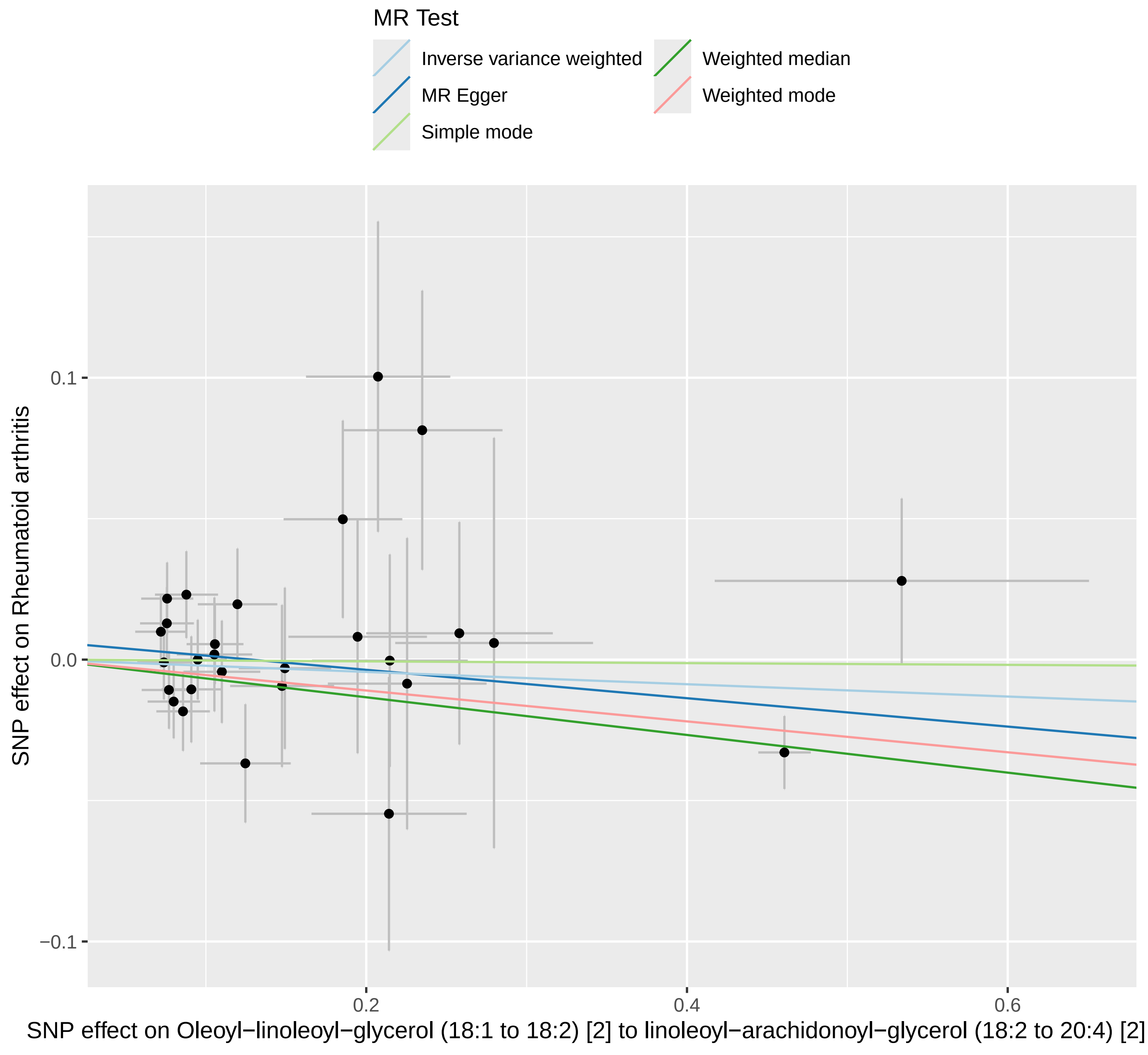

MR Test
Inverse variance weighted
MR Egger
Simple mode
Weighted median
Weighted mode
SNP effect on Rheumatoid arthritis
0.1
0.0
−0.1
0.2
0.4
0.6
SNP effect on Oleoyl−linoleoyl−glycerol (18:1 to 18:2) [2] to linoleoyl−arachidonoyl−glycerol (18:2 to 20:4) [2]

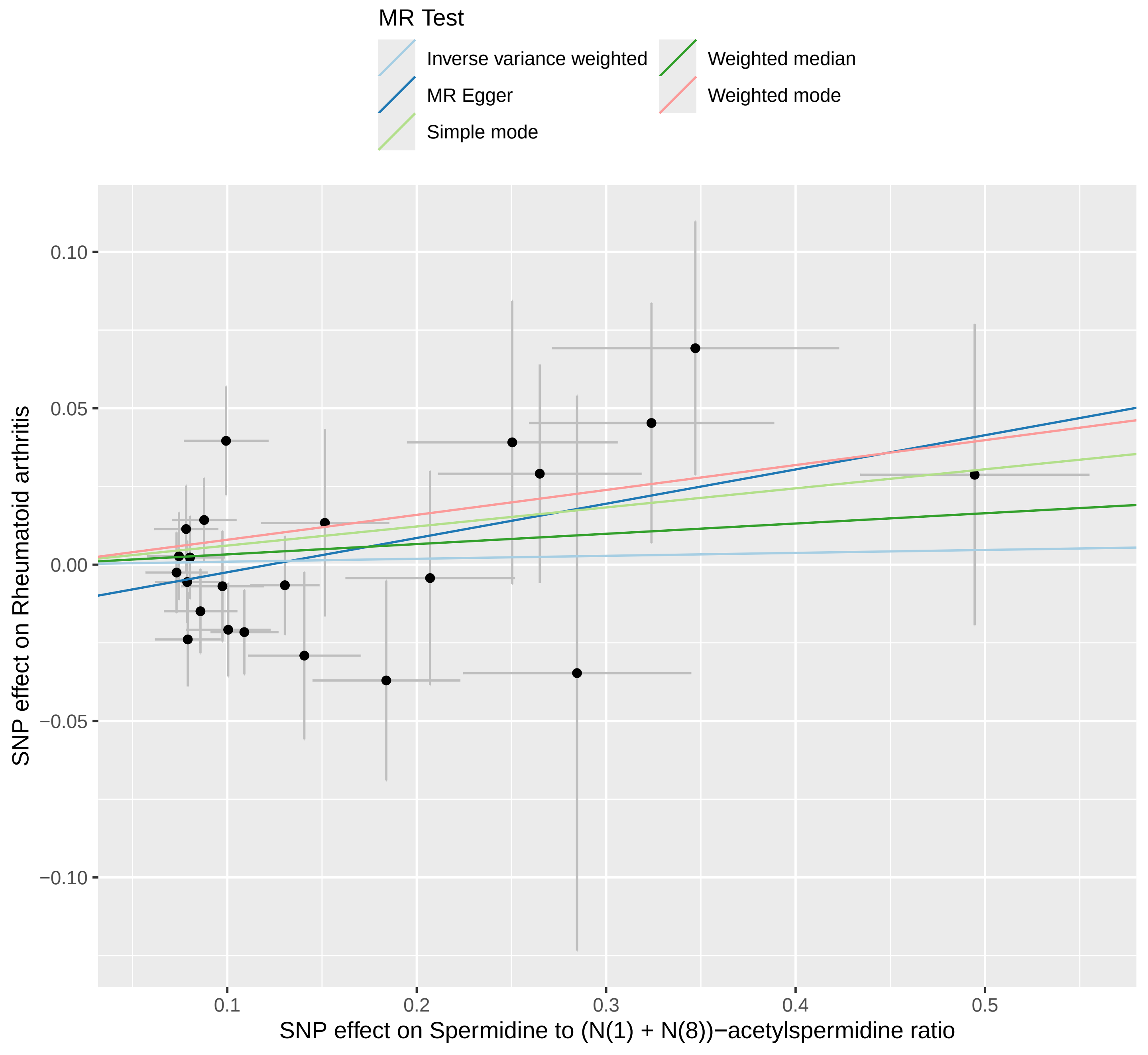

MR Test
Inverse variance weighted
MR Egger
Simple mode
Weighted median
Weighted mode
0.10
0.05
0.00
−0.05
−0.10
SNP effect on Rheumatoid arthritis
0.1
0.2
0.3
0.4
0.5
SNP effect on Spermidine to (N(1) + N(8))−acetylspermidine ratio

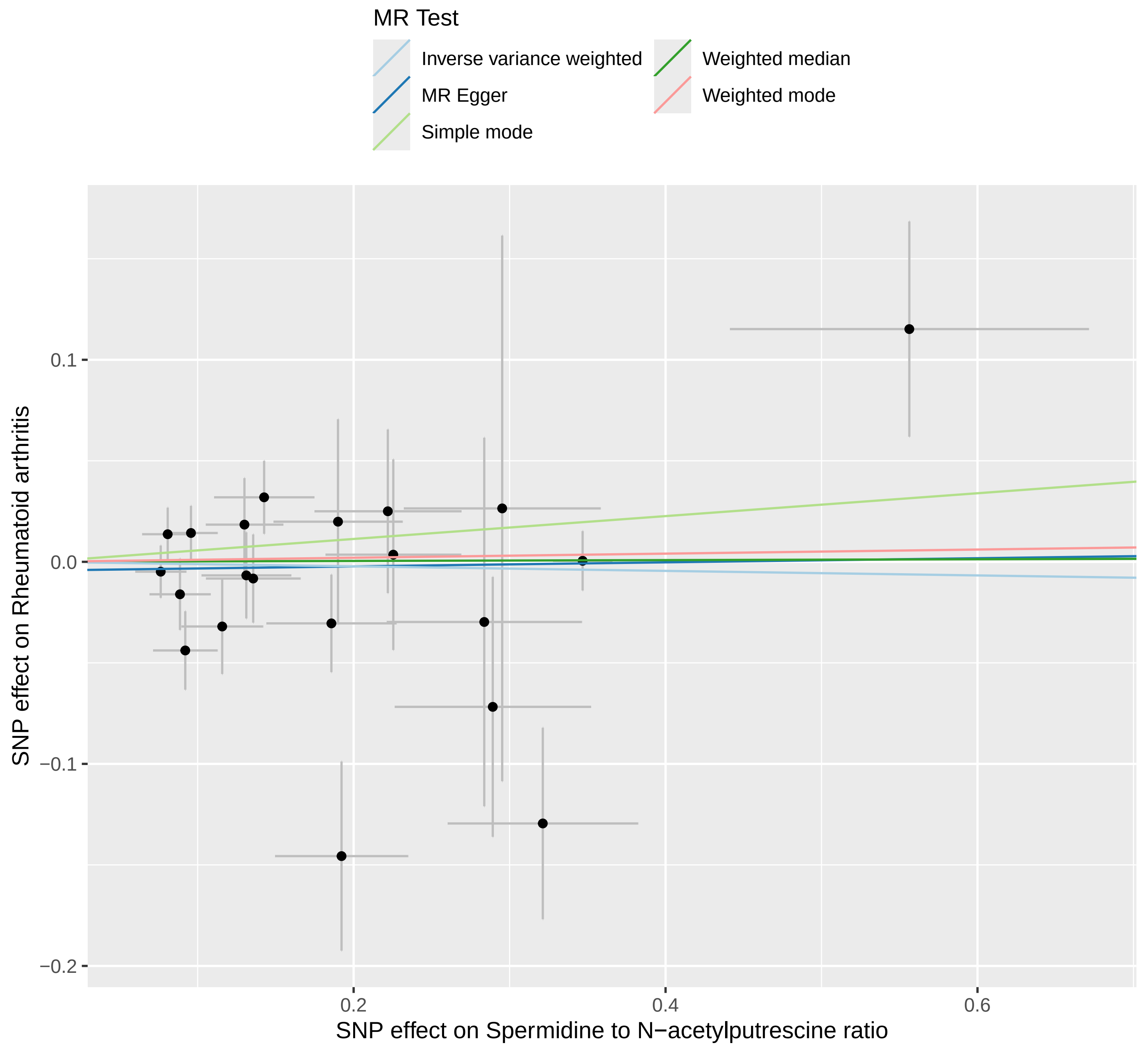

MR Test
Inverse variance weighted
MR Egger
Simple mode
Weighted median
Weighted mode
0.1
0.0
−0.1
−0.2
0.2
0.4
0.6
SNP effect on Rheumatoid arthritis
SNP effect on Spermidine to N−acetylputrescine ratio

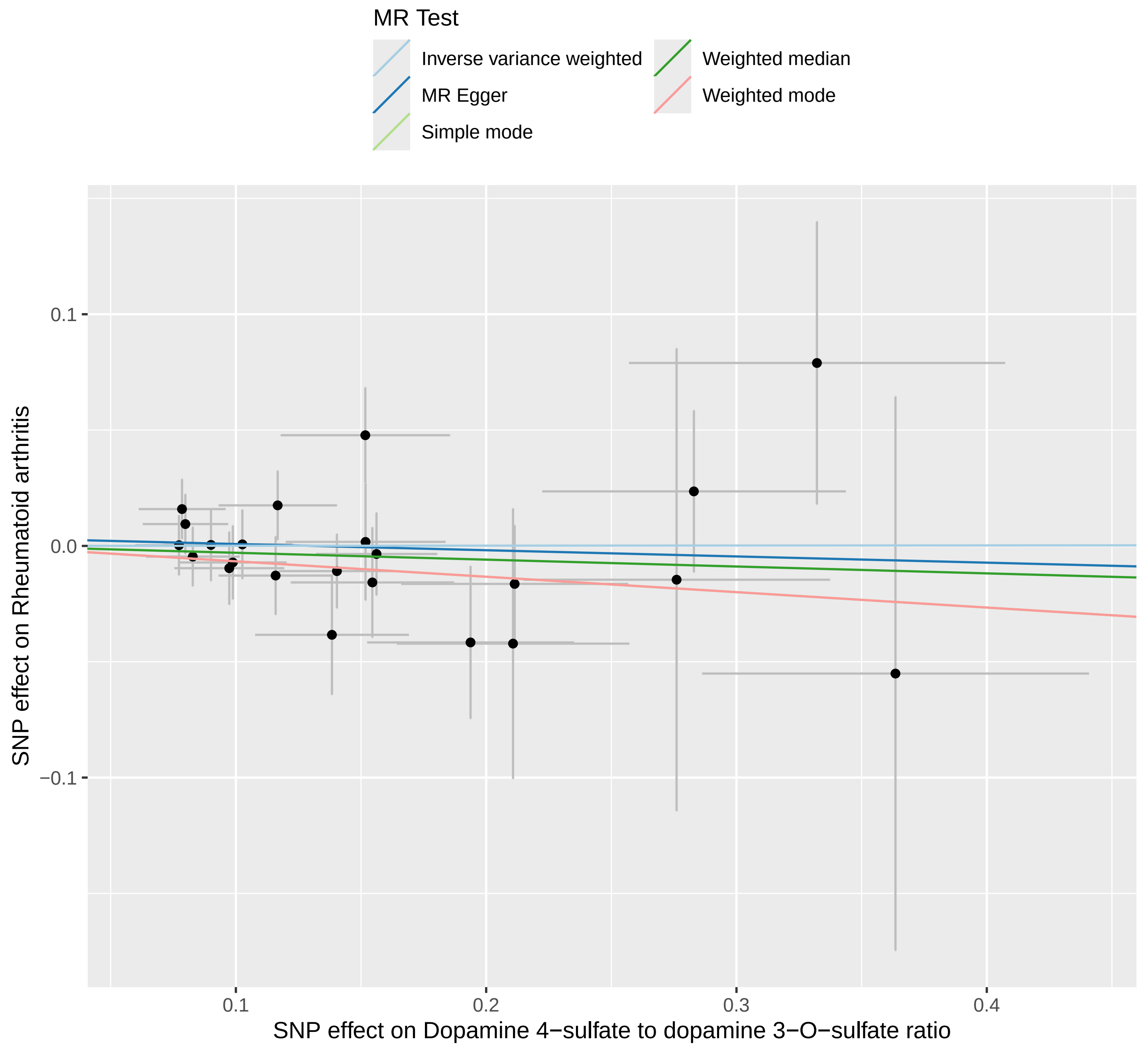
MR Test
Inverse variance weighted
MR Egger
Simple mode
Weighted median
Weighted mode
0.1
0.0
−0.1
0.1
0.2
0.3
0.4
SNP effect on Rheumatoid arthritis
SNP effect on Dopamine 4−sulfate to dopamine 3−O−sulfate ratio

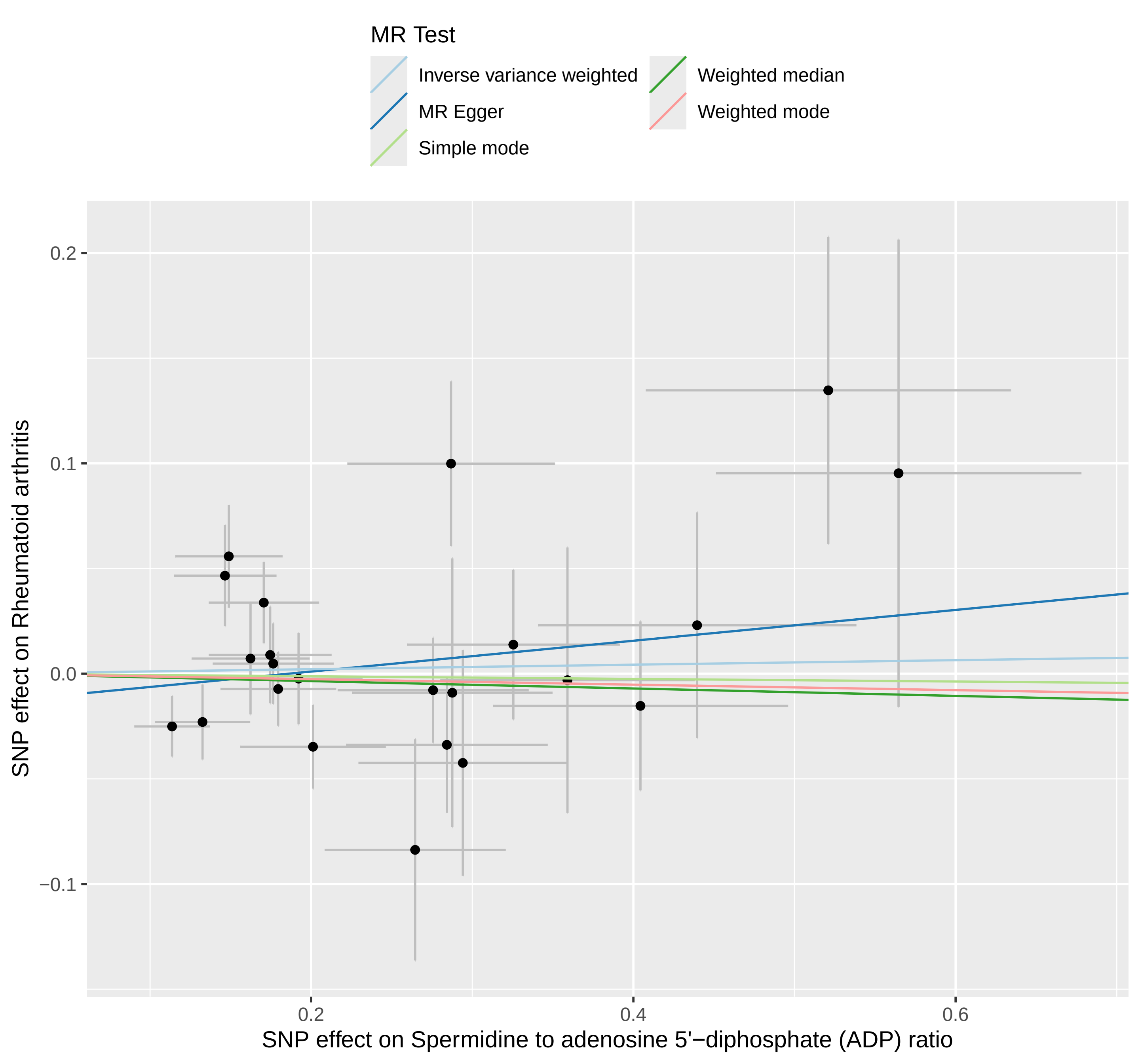

MR Test
Inverse variance weighted
MR Egger
Simple mode
Weighted median
Weighted mode
0.2
0.1
0.0
−0.1
SNP effect on Rheumatoid arthritis
0.2
0.4
0.6
SNP effect on Spermidine to adenosine 5'−diphosphate (ADP) ratio

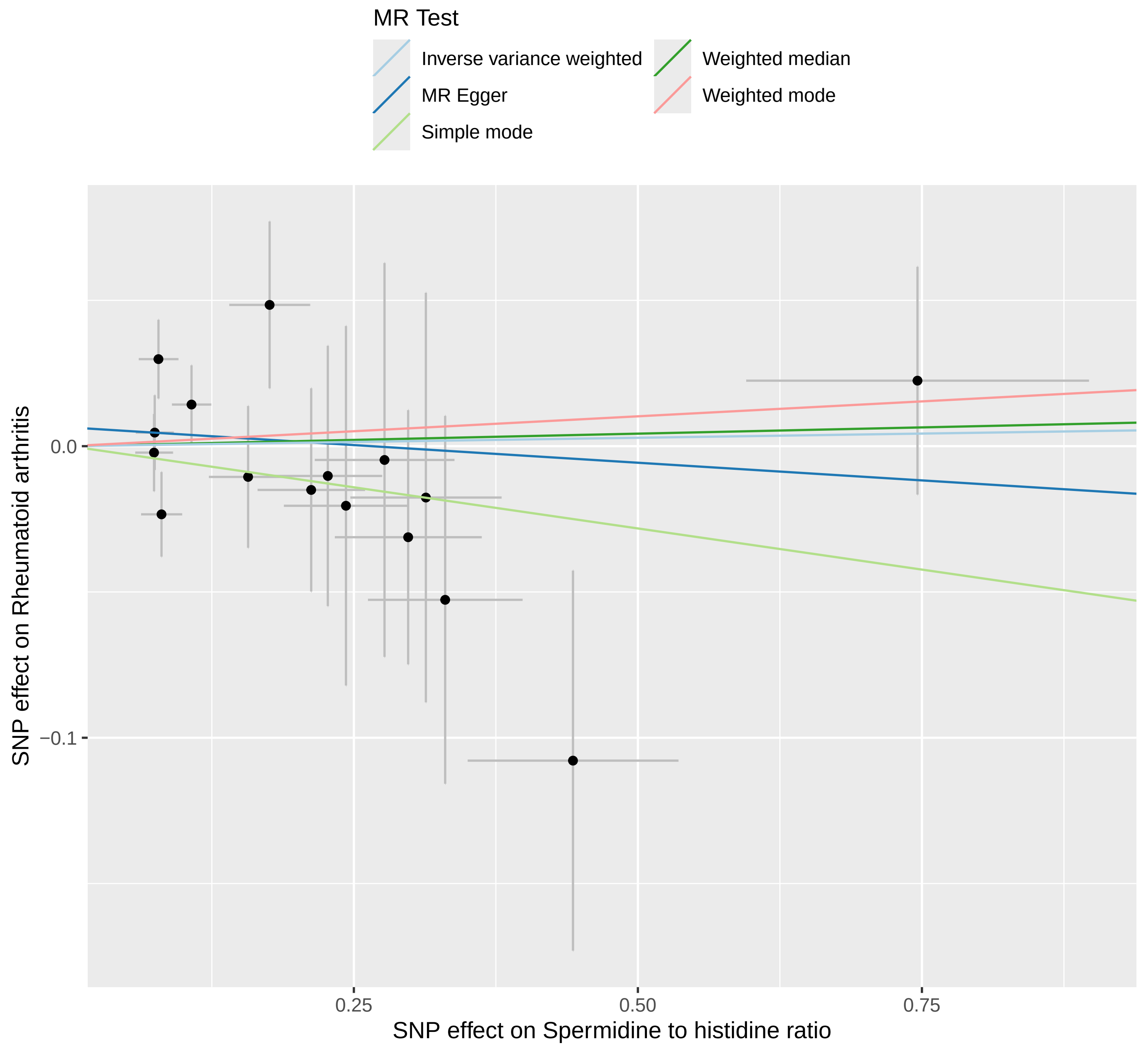
MR Test
Inverse variance weighted
MR Egger
Simple mode
Weighted median
Weighted mode
0.0
−0.1
0.25
0.50
0.75
SNP effect on Rheumatoid arthritis
SNP effect on Spermidine to histidine ratio

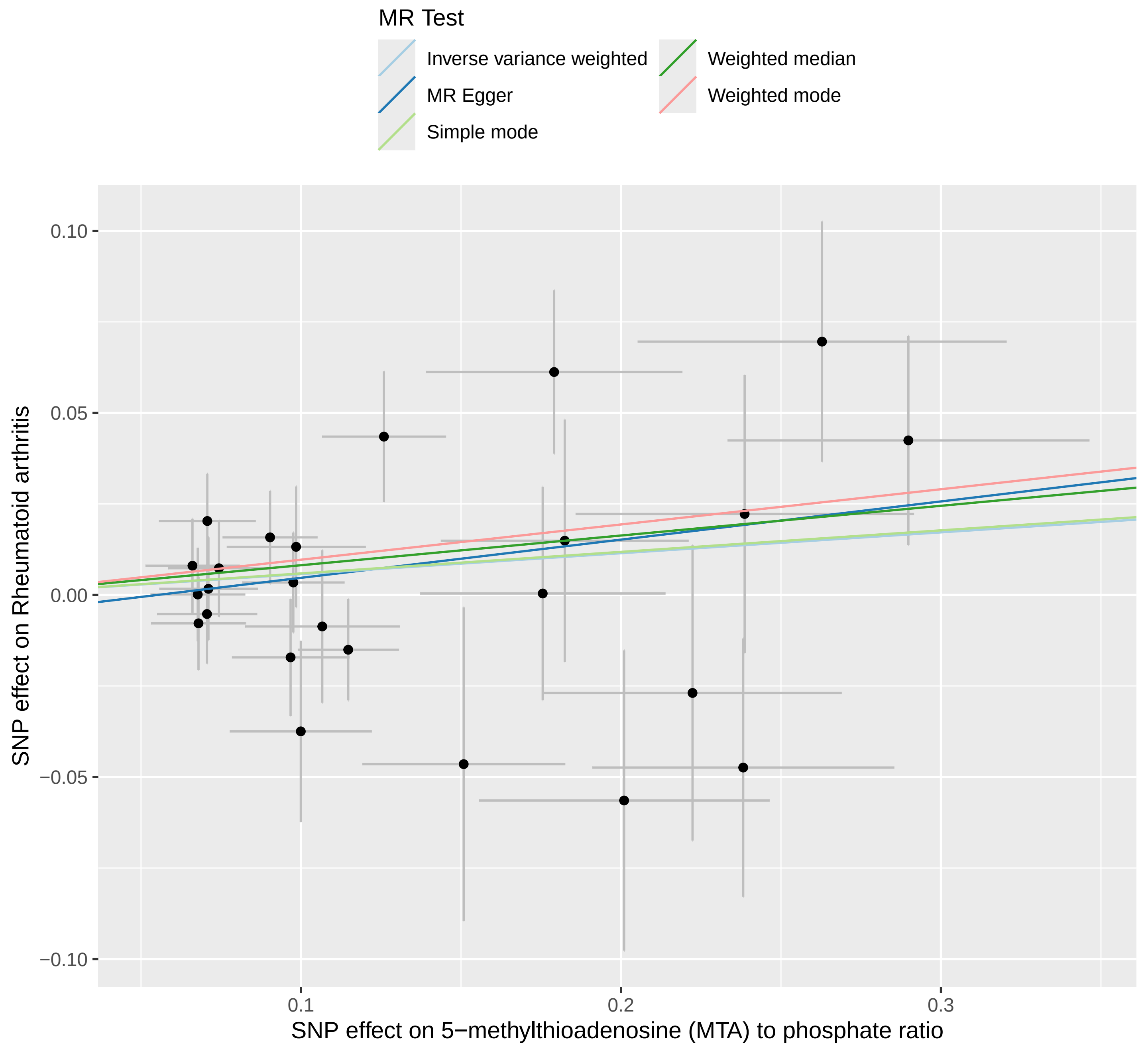
MR Test
Inverse variance weighted
MR Egger
Simple mode
Weighted median
Weighted mode
0.10
0.05
0.00
−0.05
−0.10
SNP effect on Rheumatoid arthritis
0.1
0.2
0.3
SNP effect on 5−methylthioadenosine (MTA) to phosphate ratio

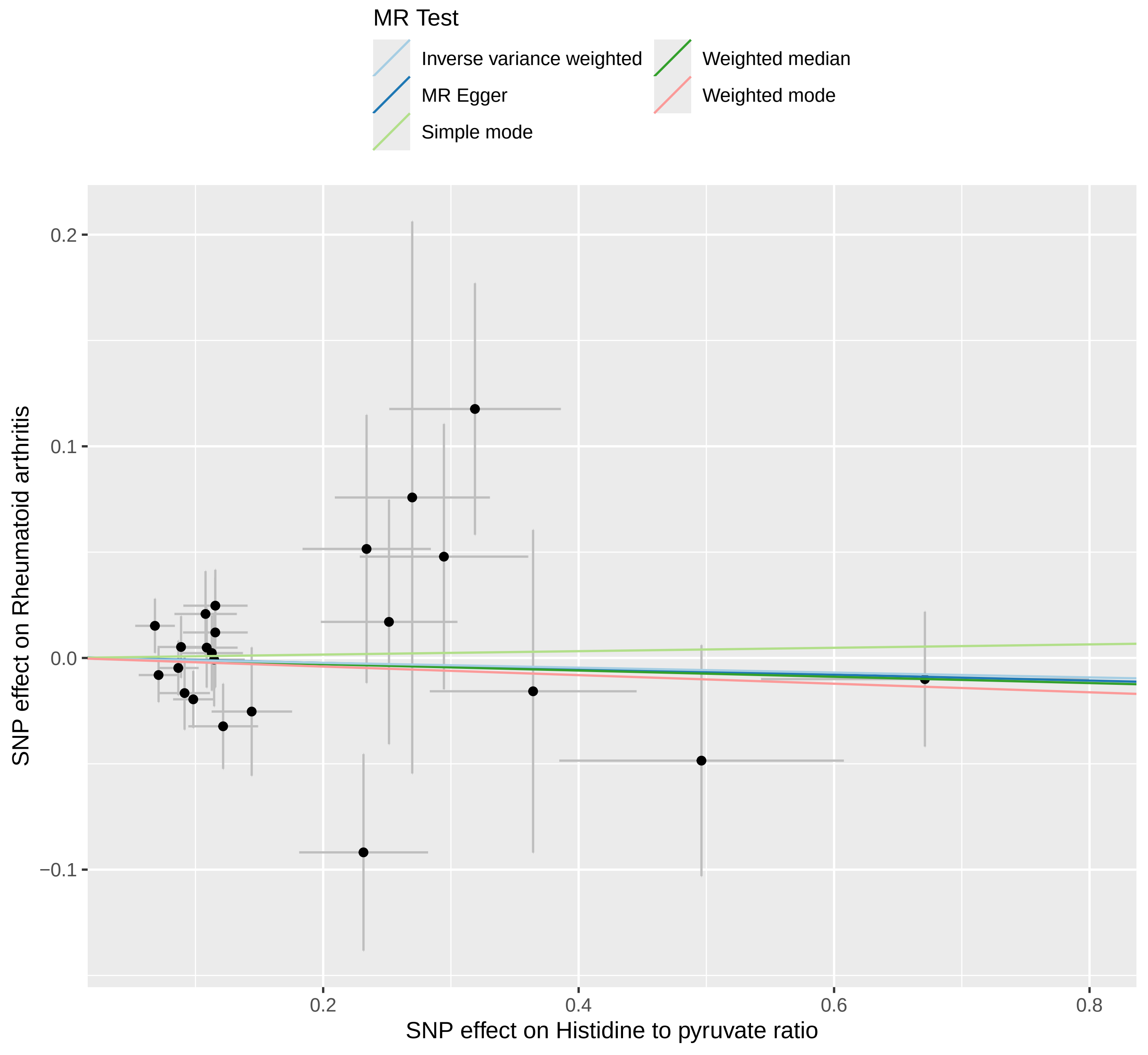
MR Test
Inverse variance weighted
MR Egger
Simple mode
Weighted median
Weighted mode
0.2
0.1
0.0
−0.1
0.2
0.4
0.6
0.8
SNP effect on Rheumatoid arthritis
SNP effect on Histidine to pyruvate ratio

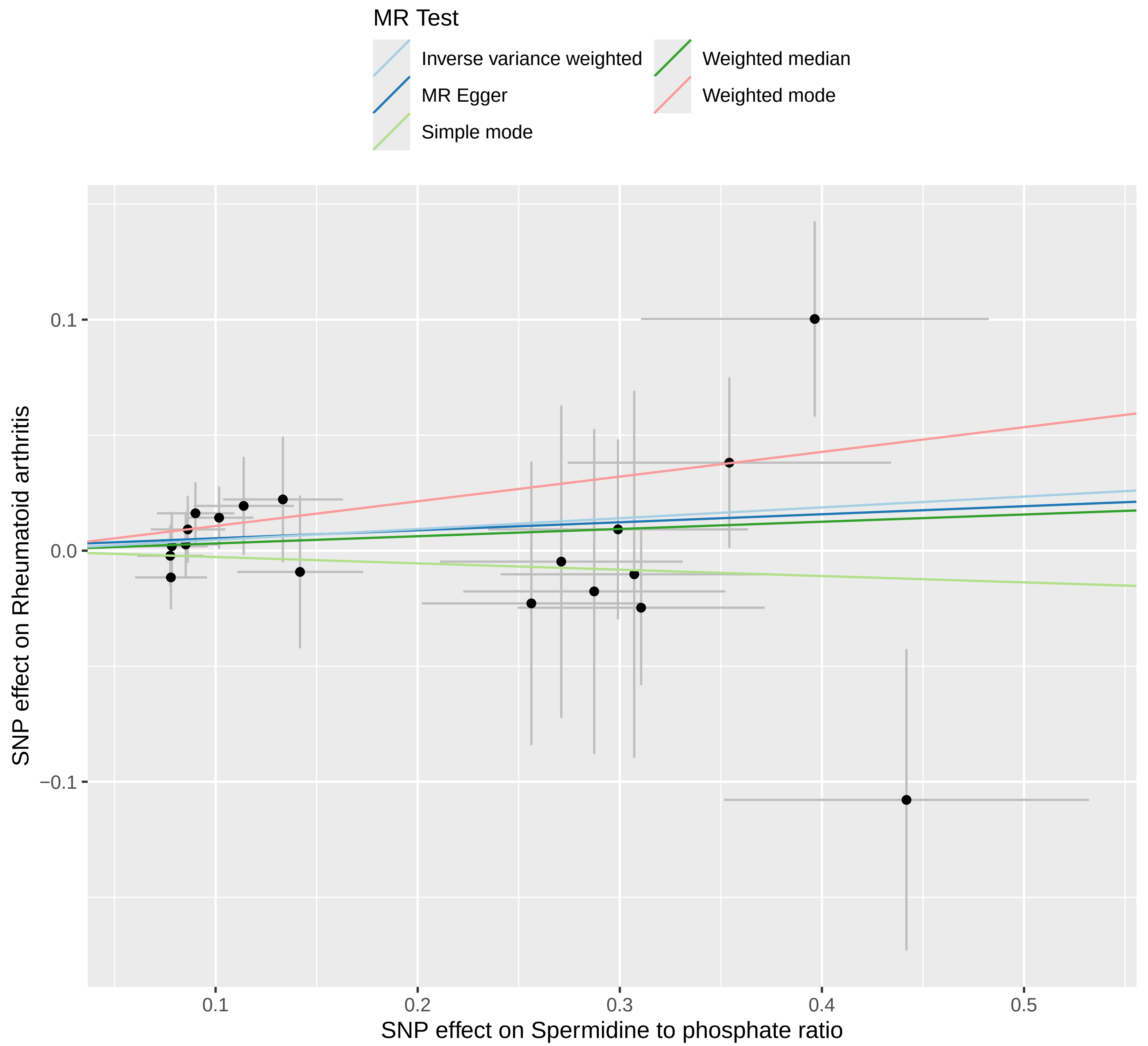

MR Test
Inverse variance weighted
MR Egger
Simple mode
Weighted median
Weighted mode
0.1
0.0
−0.1
SNP effect on Rheumatoid arthritis
0.1
0.2
0.3
0.4
0.5
SNP effect on Spermidine to phosphate ratio

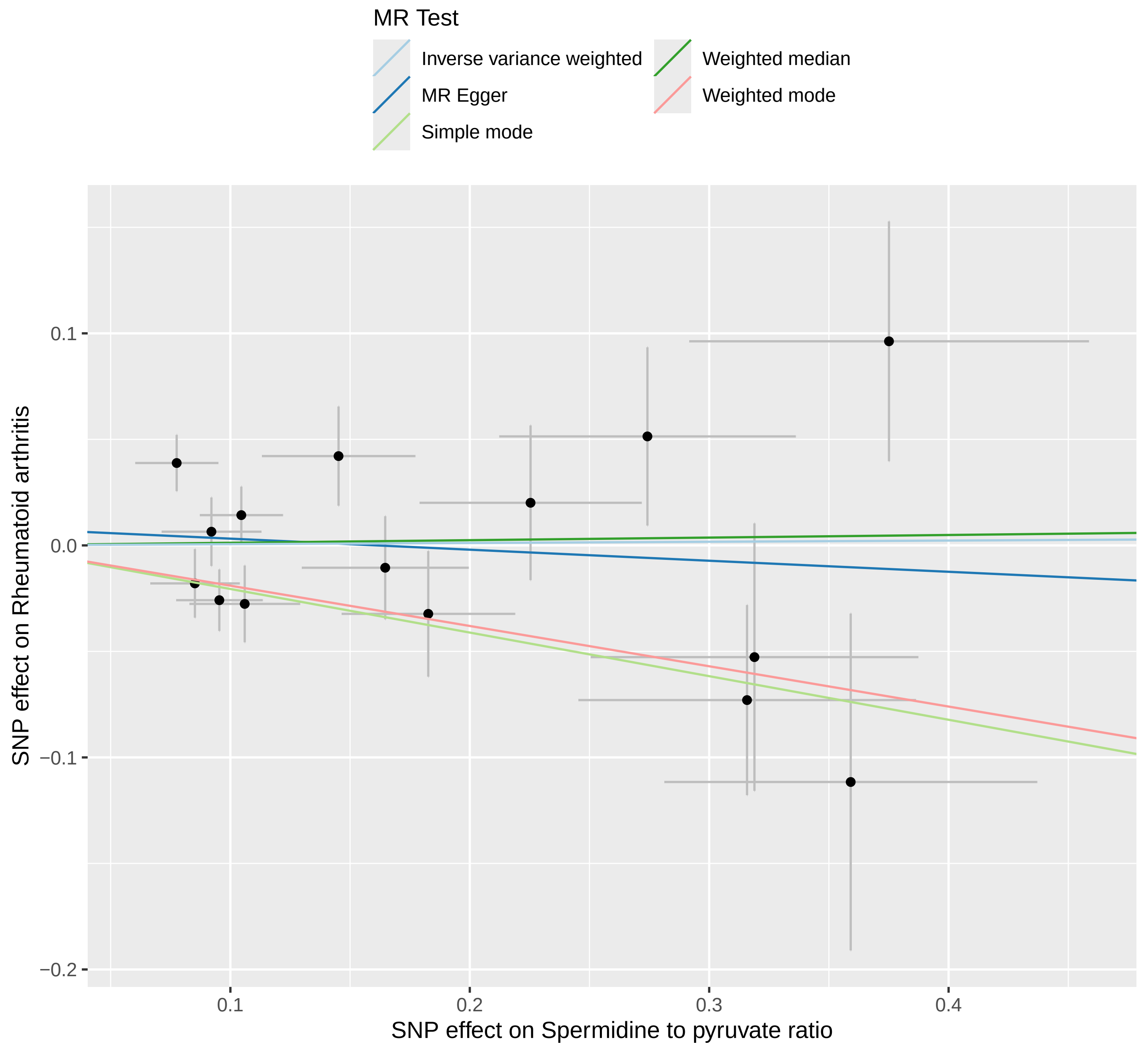

MR Test
Inverse variance weighted
MR Egger
Simple mode
Weighted median
Weighted mode
SNP effect on Rheumatoid arthritis
0.1
0.0
−0.1
−0.2
0.1
0.2
0.3
0.4
SNP effect on Spermidine to pyruvate ratio

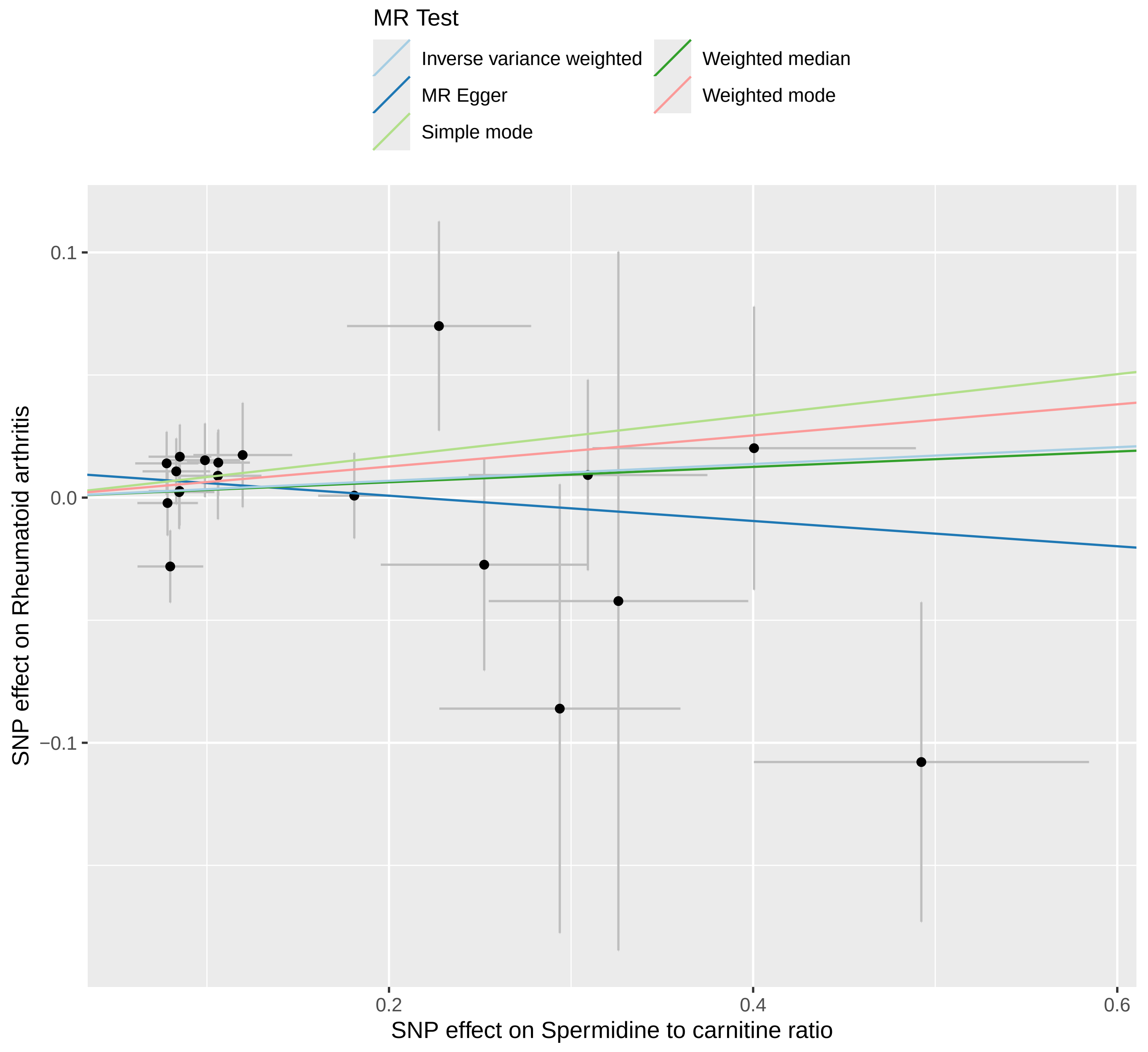
MR Test
Inverse variance weighted
MR Egger
Simple mode
Weighted median
Weighted mode
0.1
0.0
−0.1
0.2
0.4
0.6
SNP effect on Rheumatoid arthritis
SNP effect on Spermidine to carnitine ratio

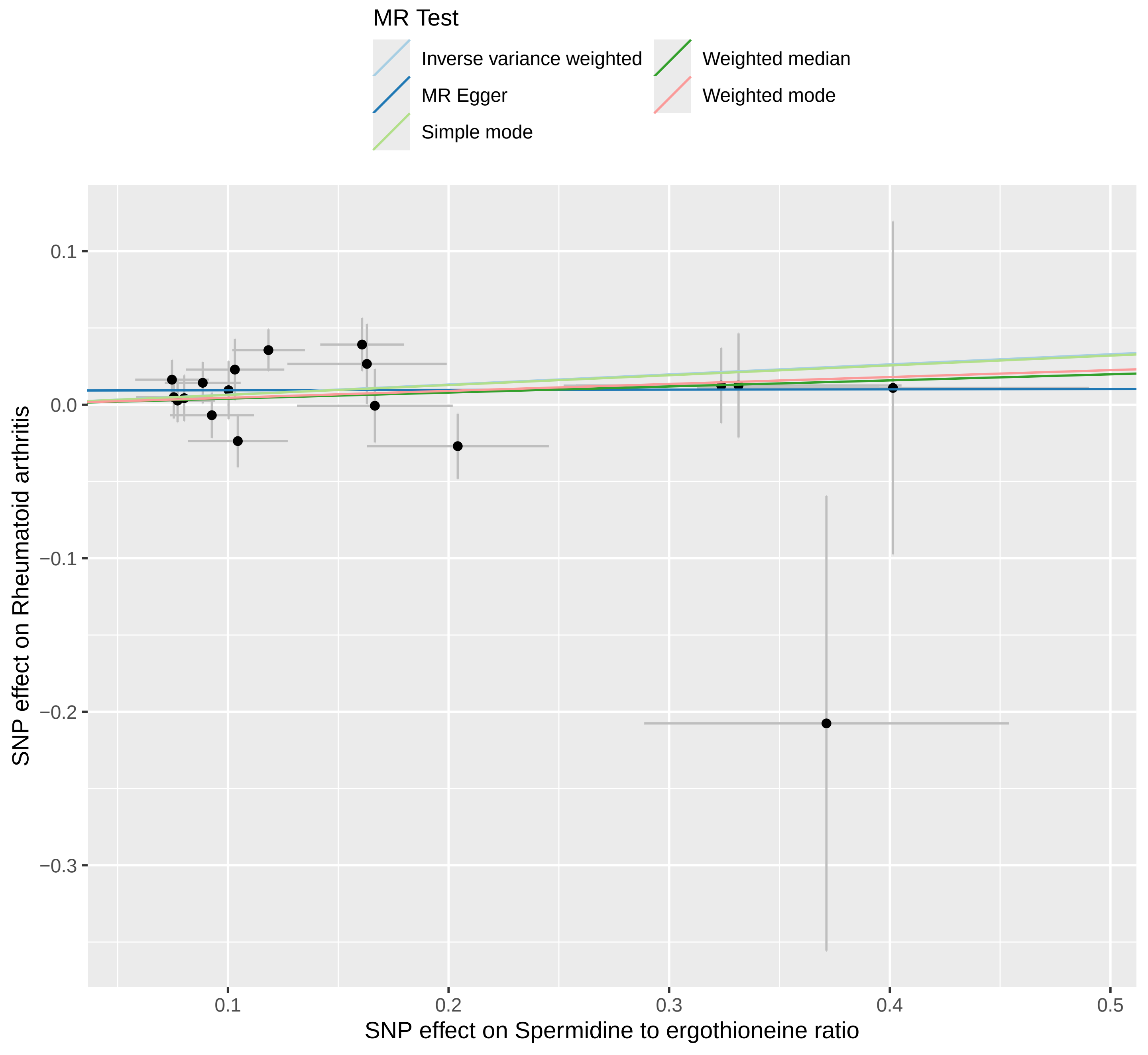
MR Test
Inverse variance weighted
MR Egger
Simple mode
Weighted median
Weighted mode
0.1
0.0
−0.1
−0.2
−0.3
SNP effect on Rheumatoid arthritis
0.1
0.2
0.3
0.4
0.5
SNP effect on Spermidine to ergothioneine ratio

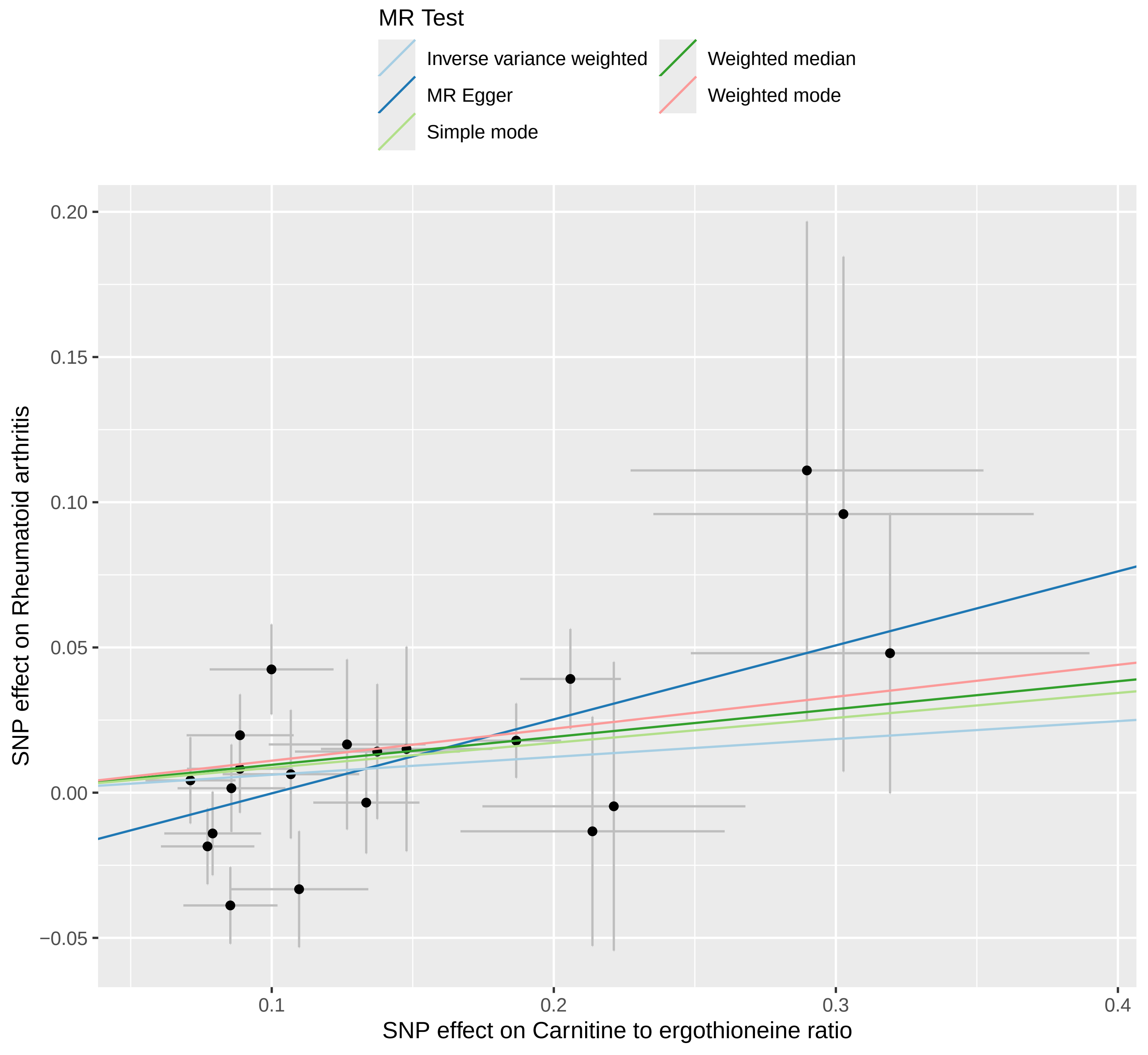

MR Test
Inverse variance weighted
MR Egger
Simple mode
Weighted median
Weighted mode
0.20
0.15
0.10
0.05
0.00
−0.05
0.1
0.2
0.3
0.4
SNP effect on Rheumatoid arthritis
SNP effect on Carnitine to ergothioneine ratio

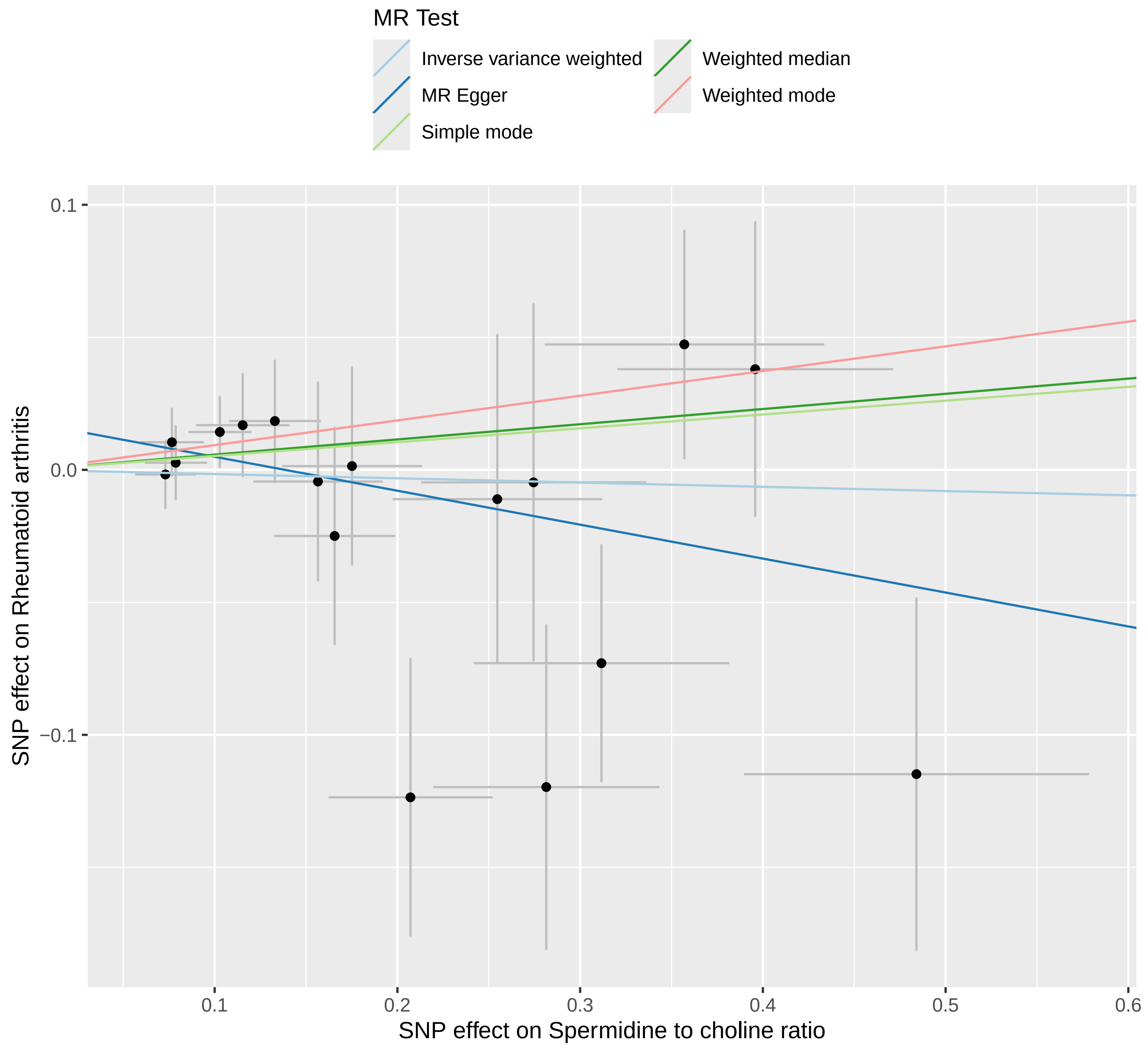

MR Test
Inverse variance weighted
MR Egger
Simple mode
Weighted median
Weighted mode
0.1
0.0
−0.1
SNP effect on Rheumatoid arthritis
0.1
0.2
0.3
0.4
0.5
0.6
SNP effect on Spermidine to choline ratio

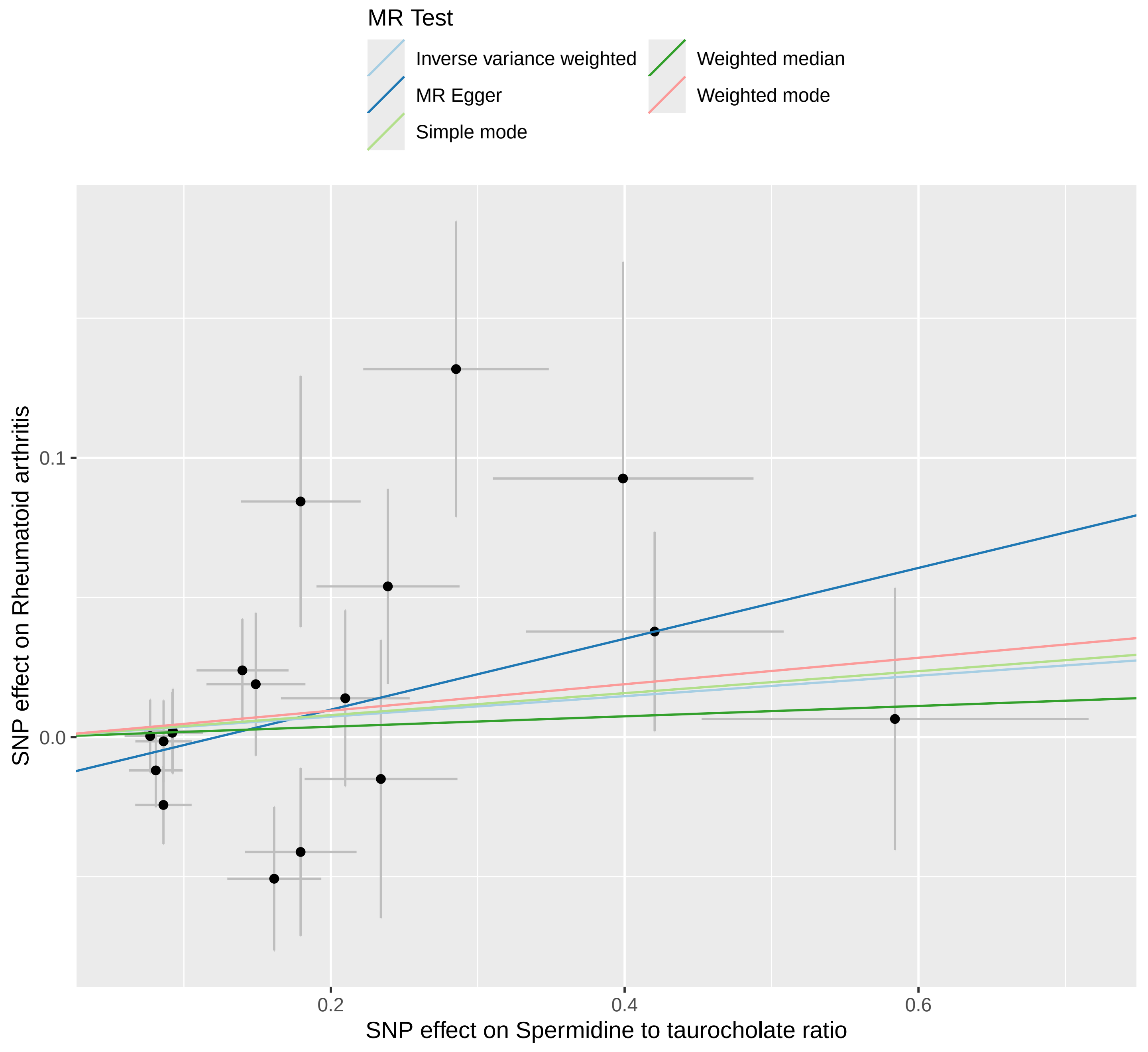
MR Test
Inverse variance weighted
MR Egger
Simple mode
Weighted median
Weighted mode
0.1
0.0
0.2
0.4
0.6
SNP effect on Rheumatoid arthritis
SNP effect on Spermidine to taurocholate ratio

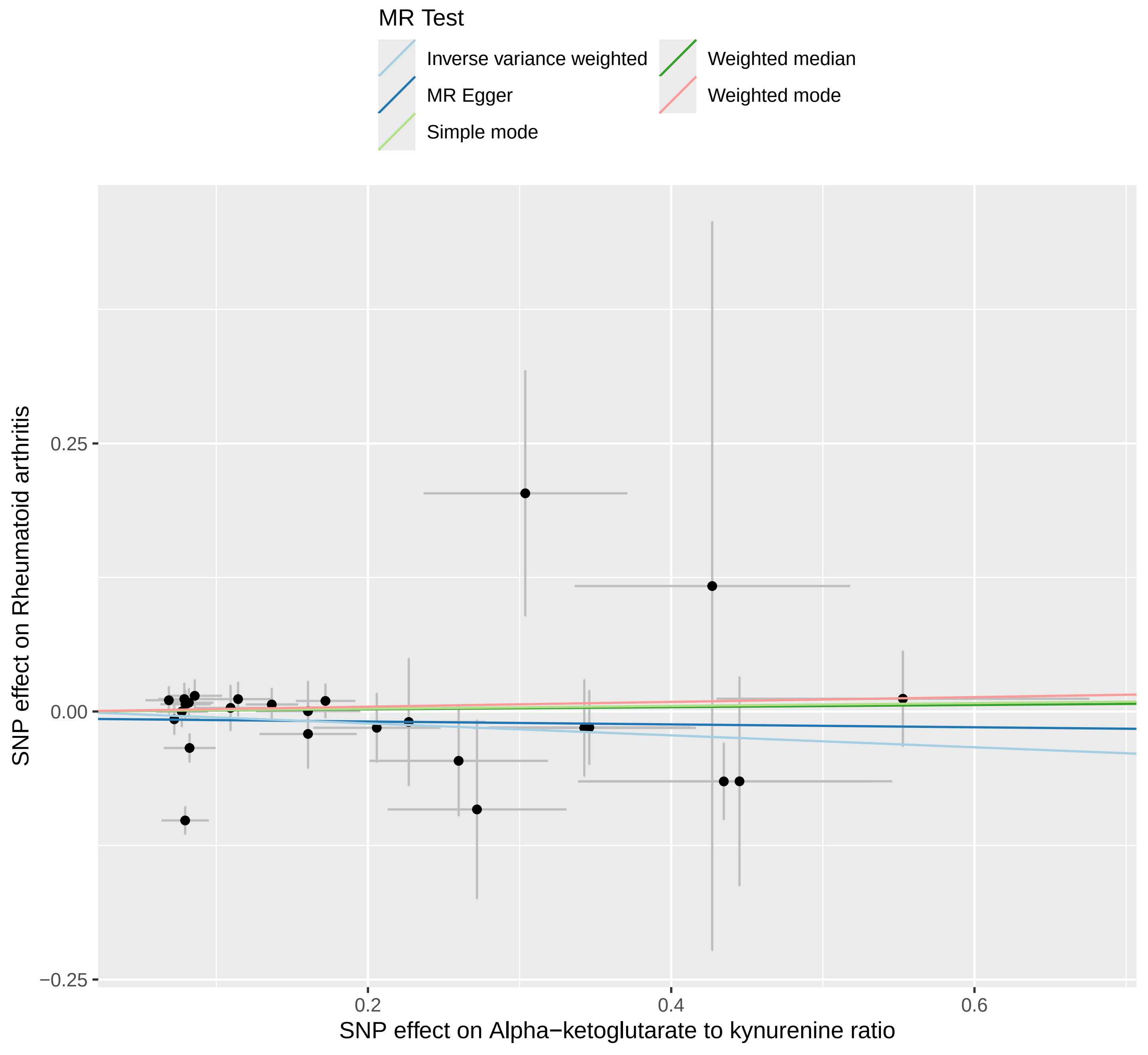
MR Test
Inverse variance weighted
MR Egger
Simple mode
Weighted median
Weighted mode
SNP effect on Rheumatoid arthritis
0.25
0.00
−0.25
0.2
0.4
0.6
SNP effect on Alpha−ketoglutarate to kynurenine ratio

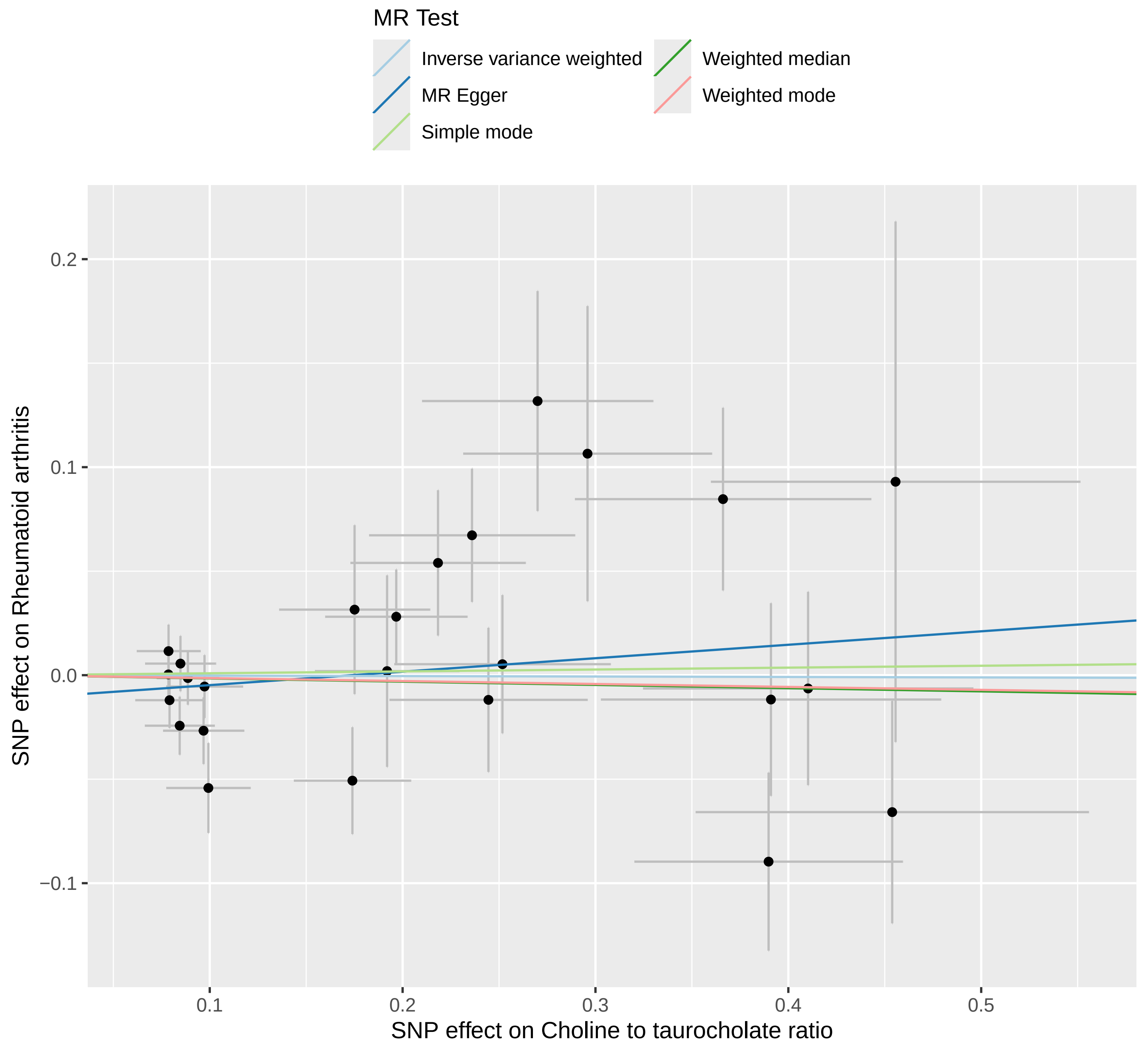
MR Test
Inverse variance weighted
MR Egger
Simple mode
Weighted median
Weighted mode
SNP effect on Rheumatoid arthritis
SNP effect on Choline to taurocholate ratio
0.2
0.1
0.0
−0.1
0.1
0.2
0.3
0.4
0.5

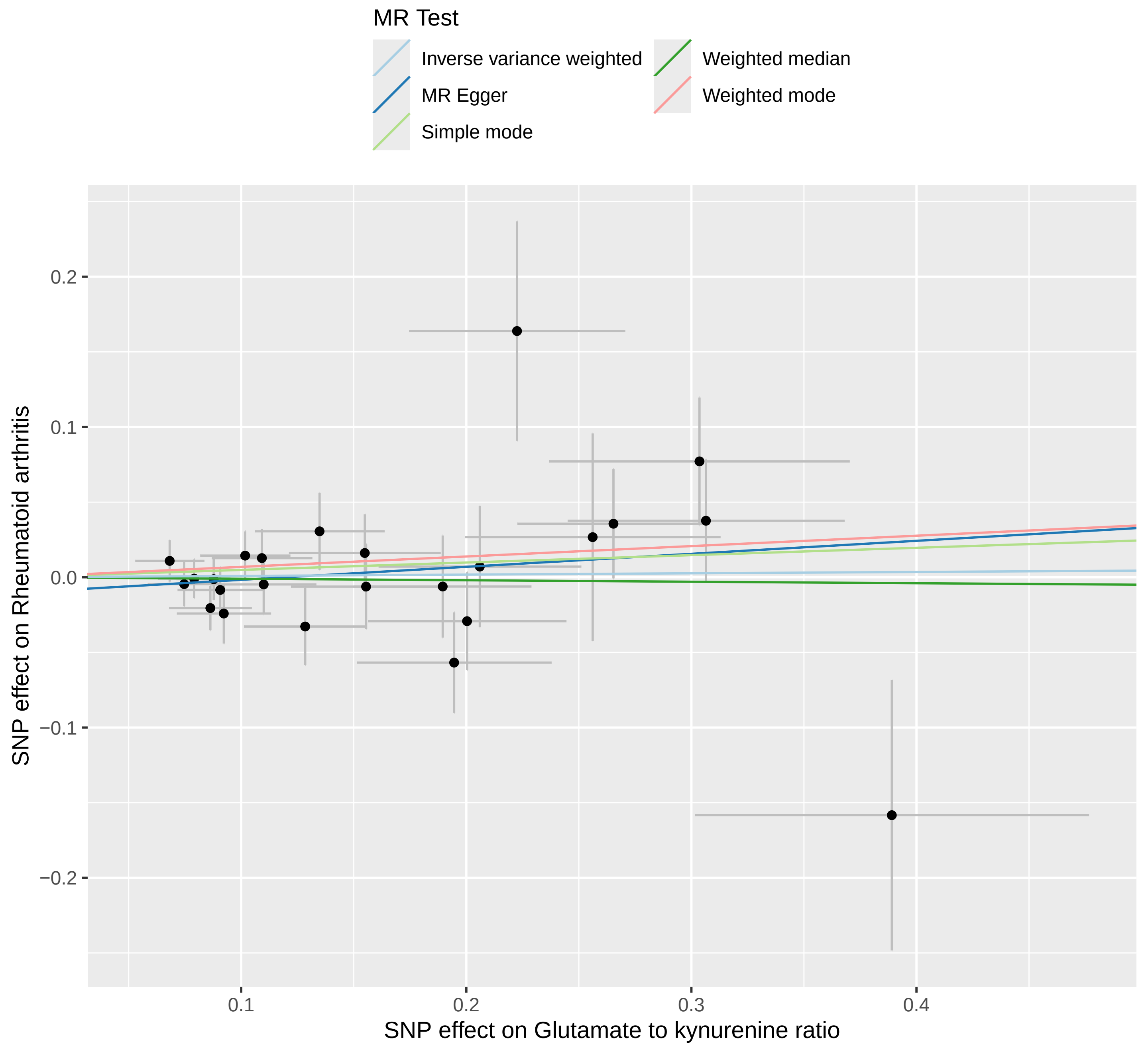

MR Test
Inverse variance weighted
MR Egger
Simple mode
Weighted median
Weighted mode
SNP effect on Rheumatoid arthritis
0.2
0.1
0.0
−0.1
−0.2
0.1
0.2
0.3
0.4
SNP effect on Glutamate to kynurenine ratio

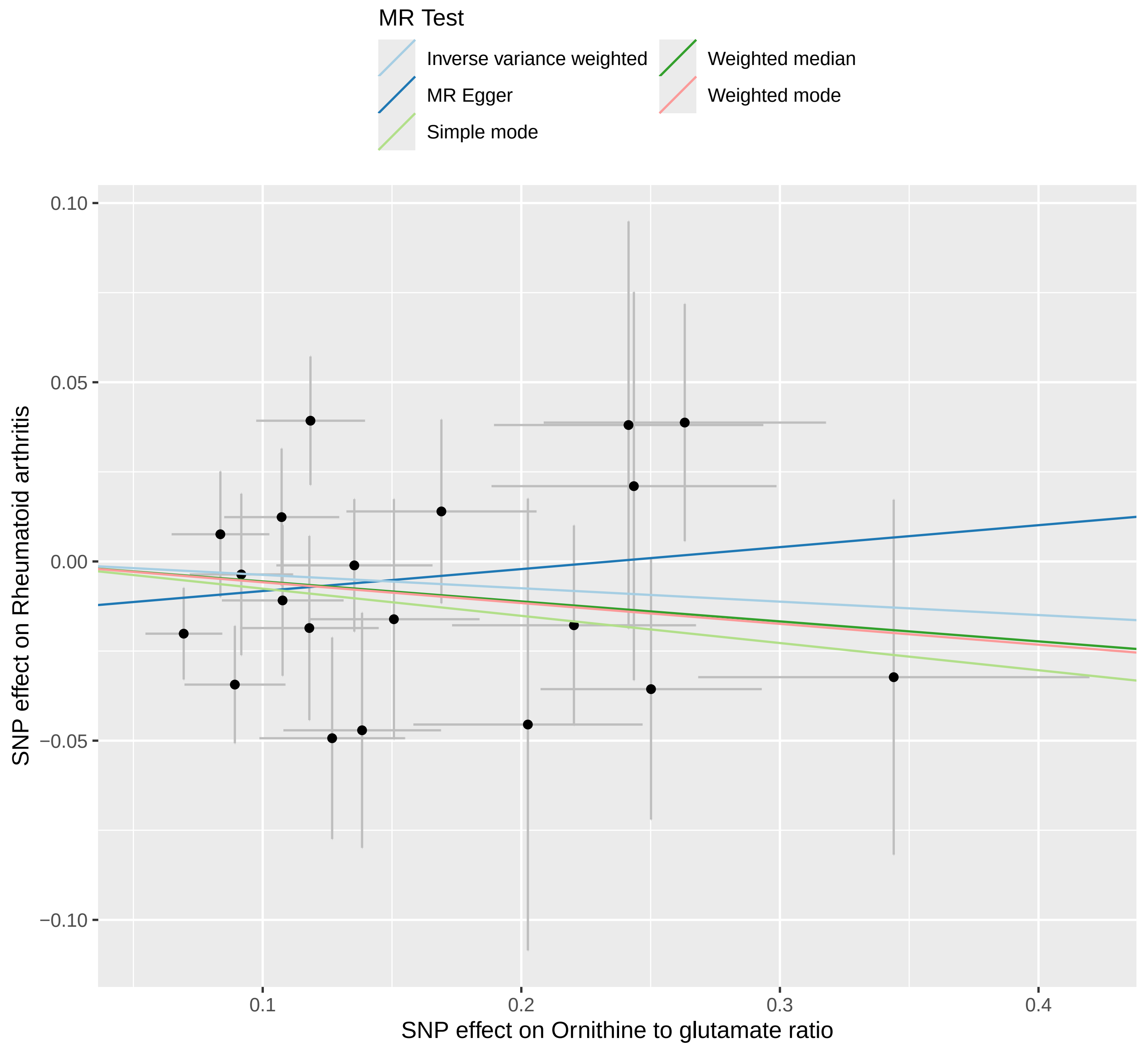

MR Test
Inverse variance weighted
MR Egger
Simple mode
Weighted median
Weighted mode
0.10
0.05
0.00
−0.05
−0.10
SNP effect on Rheumatoid arthritis
0.1
0.2
0.3
0.4
SNP effect on Ornithine to glutamate ratio

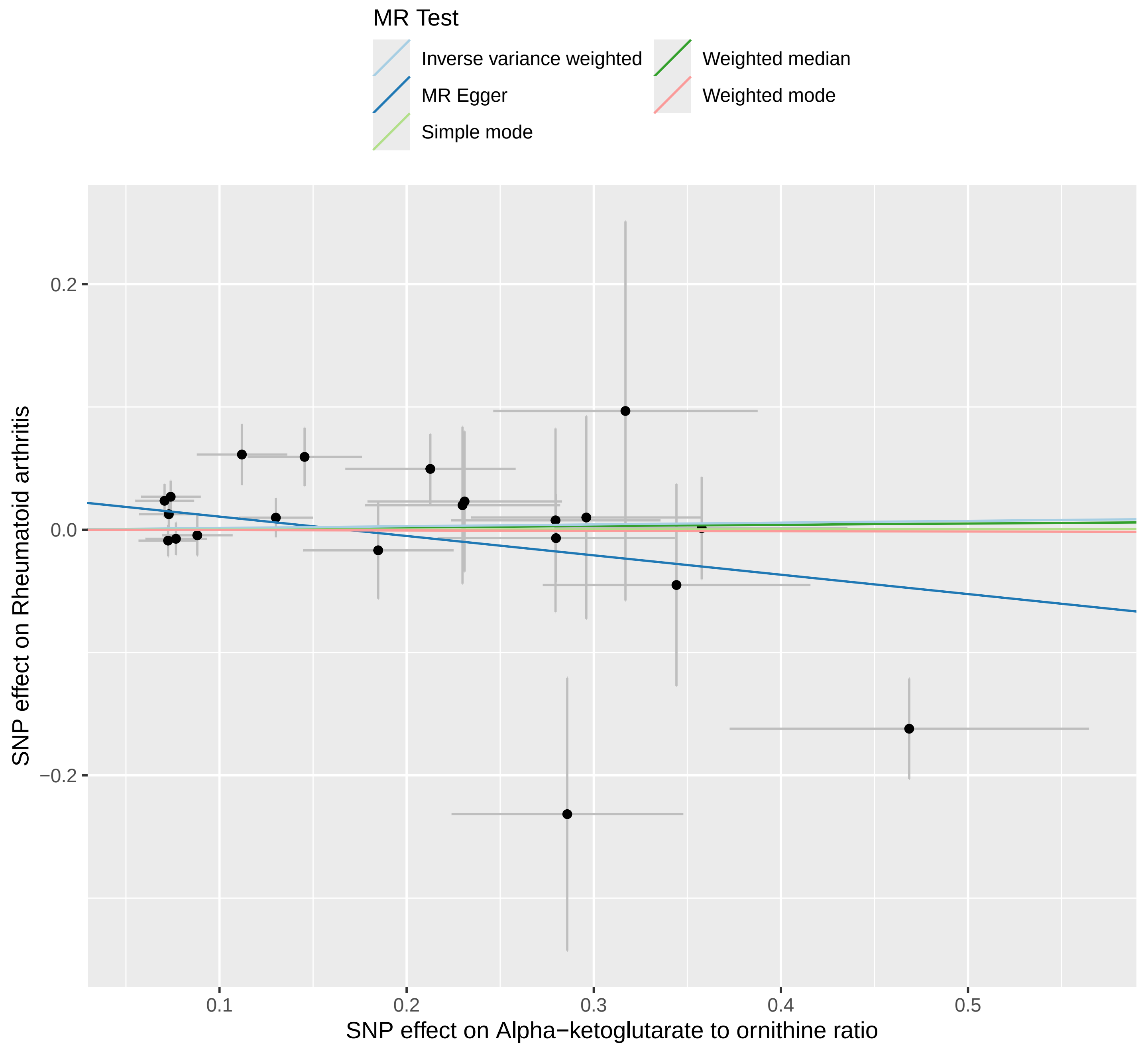
MR Test
Inverse variance weighted
MR Egger
Simple mode
Weighted median
Weighted mode
0.2
0.0
−0.2
0.1
0.2
0.3
0.4
0.5
SNP effect on Rheumatoid arthritis
SNP effect on Alpha−ketoglutarate to ornithine ratio

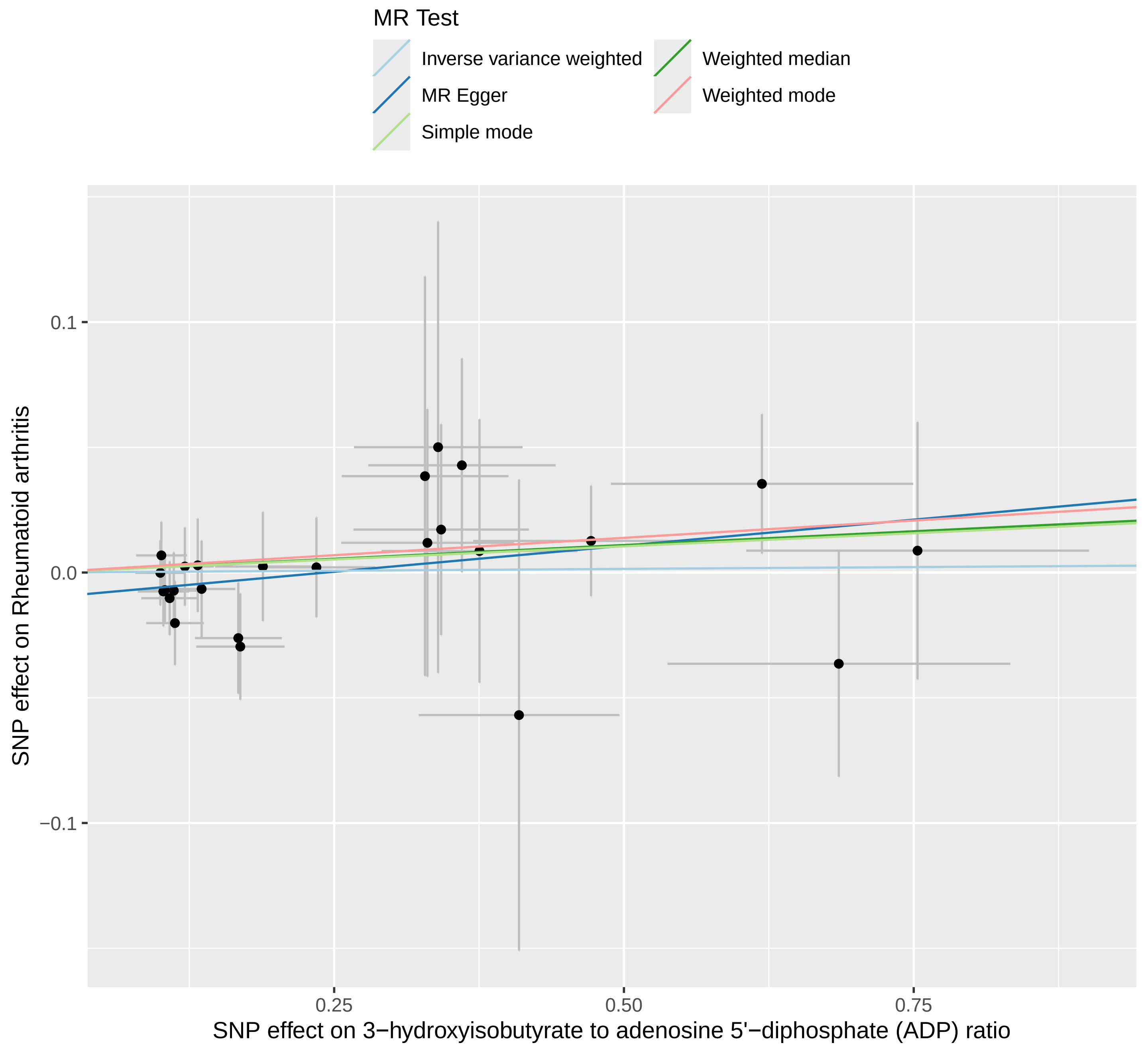
MR Test
Inverse variance weighted
MR Egger
Simple mode
Weighted median
Weighted mode
0.1
0.0
−0.1
0.25
0.50
0.75
SNP effect on Rheumatoid arthritis
SNP effect on 3−hydroxyisobutyrate to adenosine 5'−diphosphate (ADP) ratio

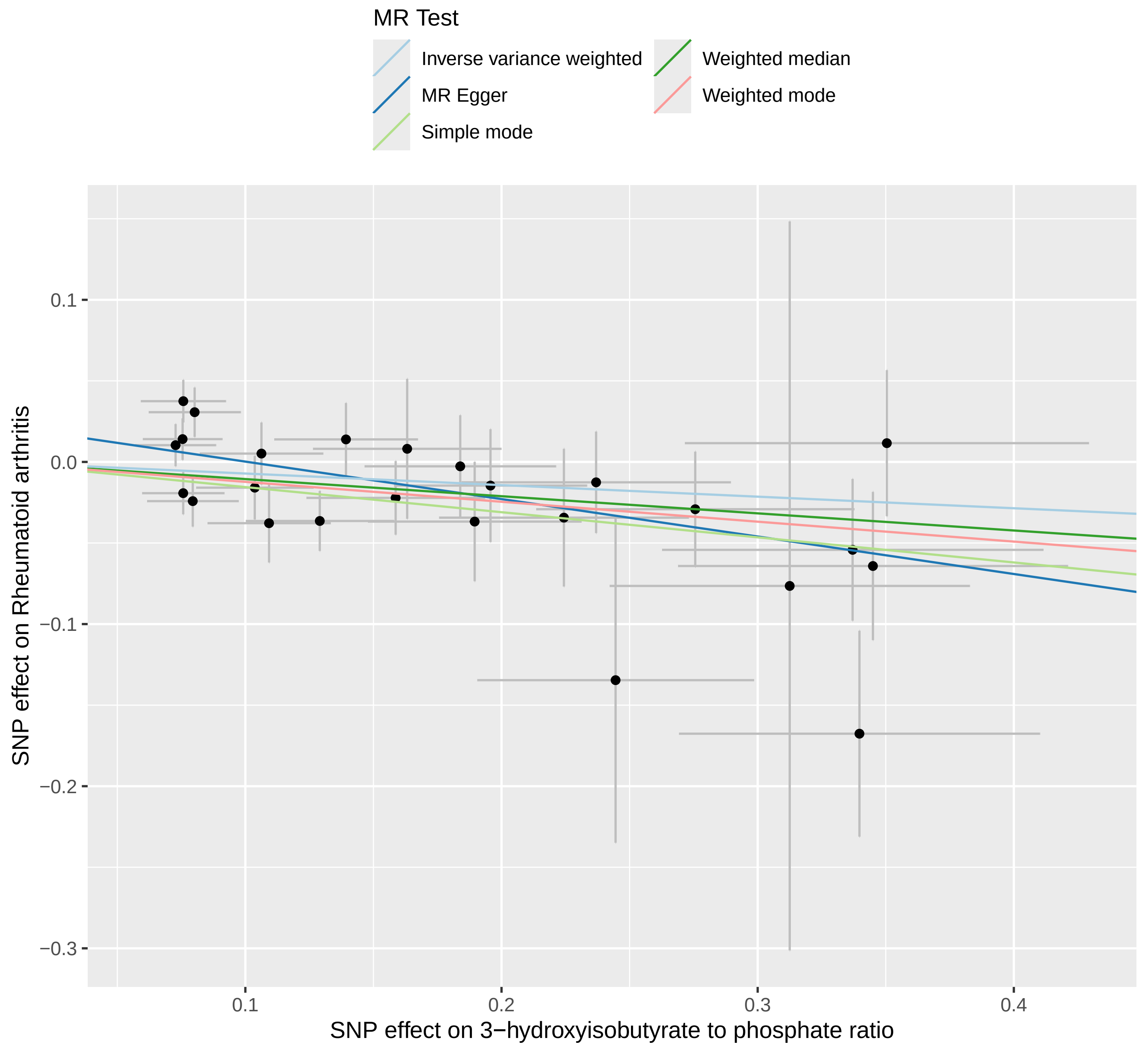

MR Test
Inverse variance weighted
MR Egger
Simple mode
Weighted median
Weighted mode
0.1
0.0
−0.1
−0.2
−0.3
SNP effect on Rheumatoid arthritis
0.1
0.2
0.3
0.4
SNP effect on 3−hydroxyisobutyrate to phosphate ratio

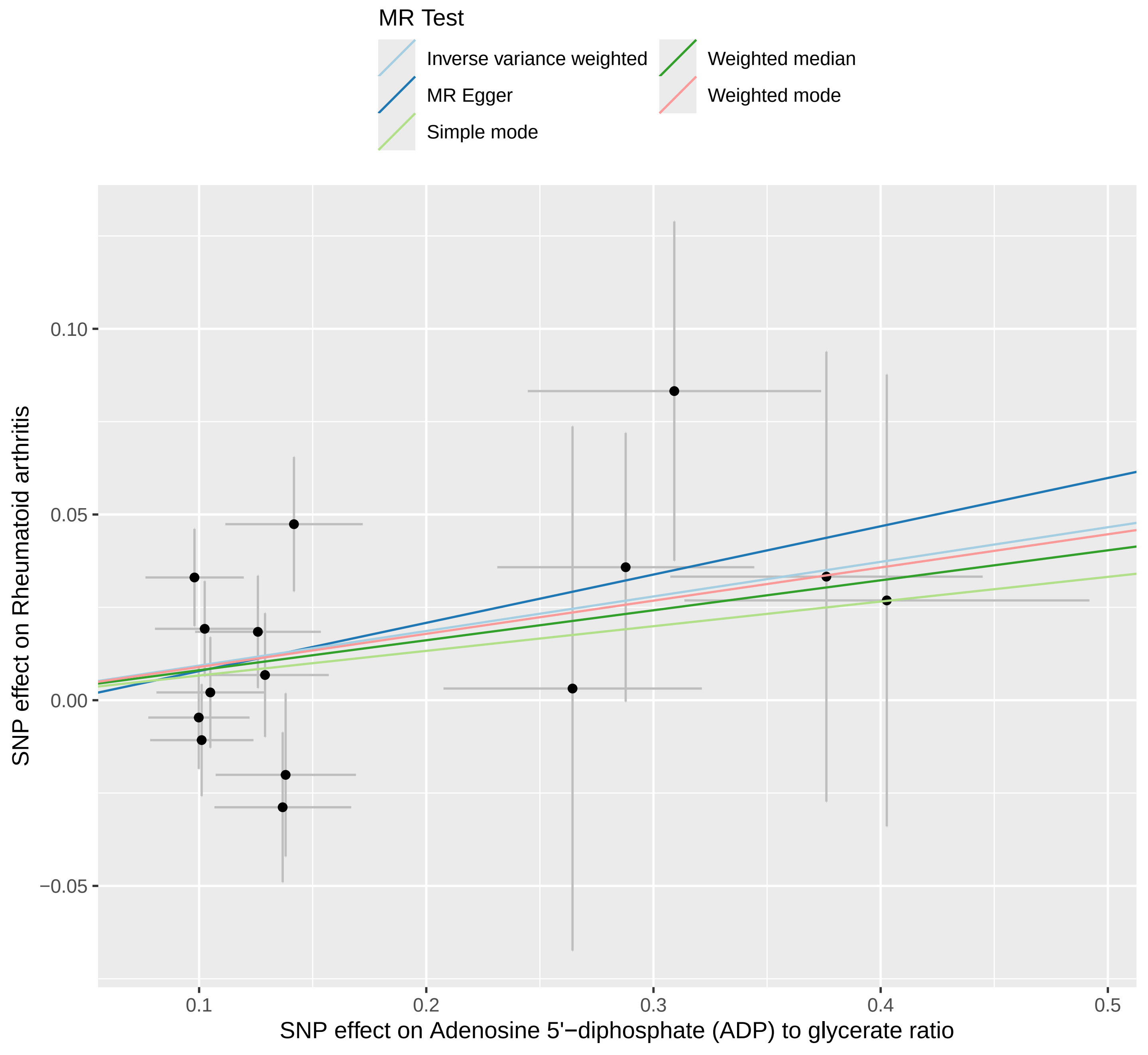

MR Test
Inverse variance weighted
MR Egger
Simple mode
Weighted median
Weighted mode
0.10
0.05
0.00
−0.05
0.1
0.2
0.3
0.4
0.5
SNP effect on Rheumatoid arthritis
SNP effect on Adenosine 5'−diphosphate (ADP) to glycerate ratio

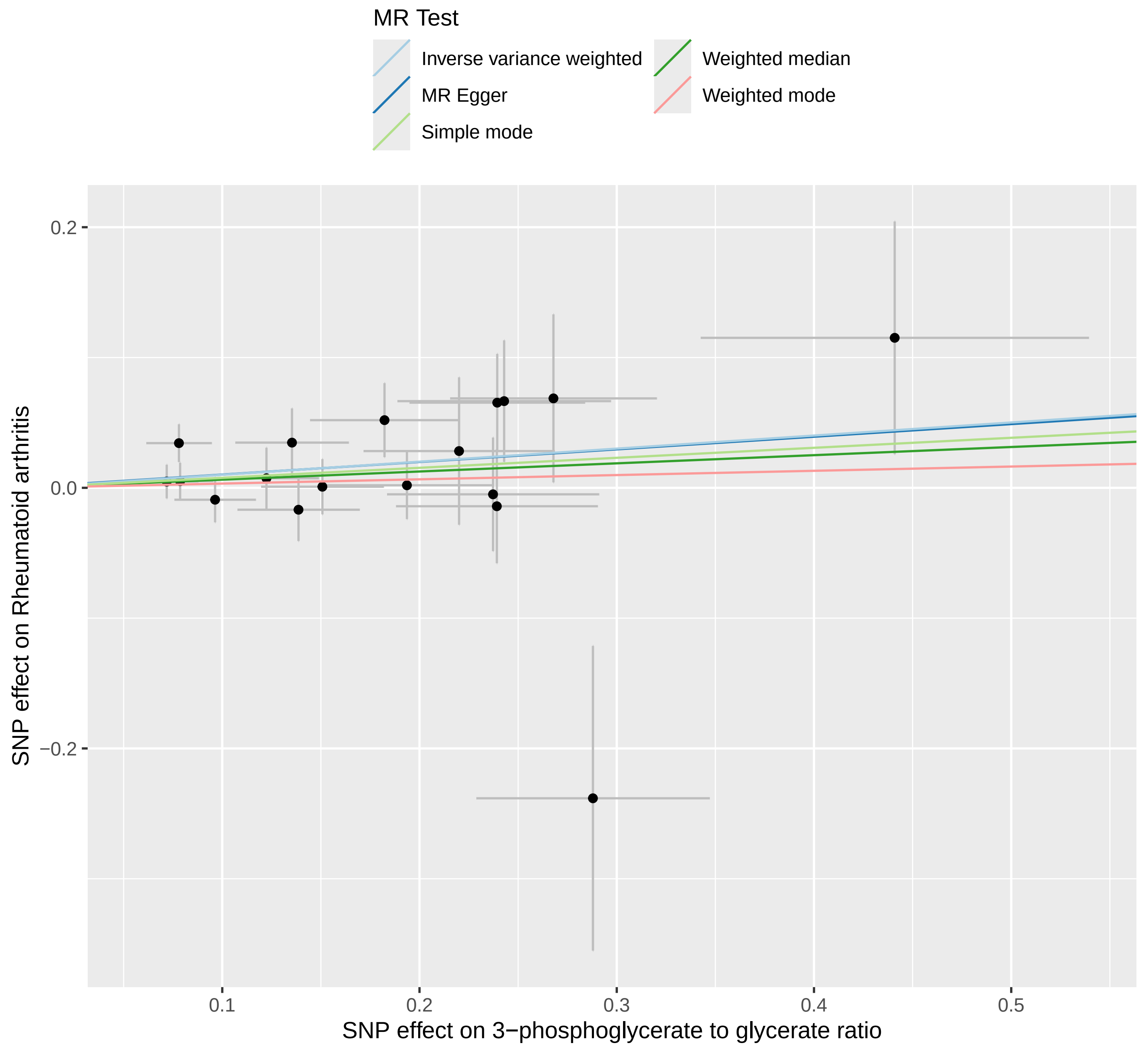
MR Test
Inverse variance weighted
MR Egger
Simple mode
Weighted median
Weighted mode
0.2
0.0
−0.2
SNP effect on Rheumatoid arthritis
0.1
0.2
0.3
0.4
0.5
SNP effect on 3−phosphoglycerate to glycerate ratio

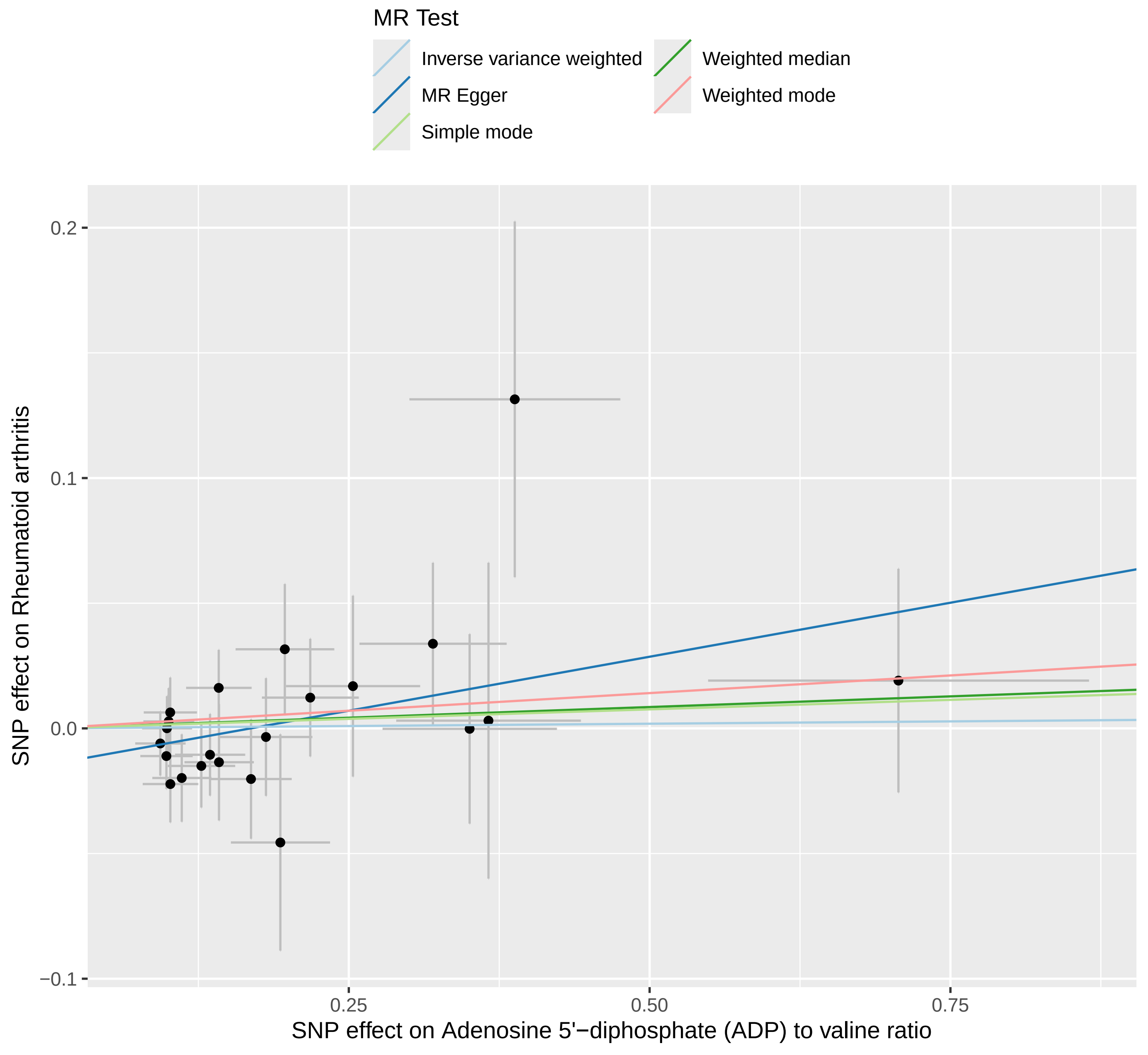
MR Test
Inverse variance weighted
MR Egger
Simple mode
Weighted median
Weighted mode
0.2
0.1
0.0
−0.1
0.25
0.50
0.75
SNP effect on Rheumatoid arthritis
SNP effect on Adenosine 5'−diphosphate (ADP) to valine ratio

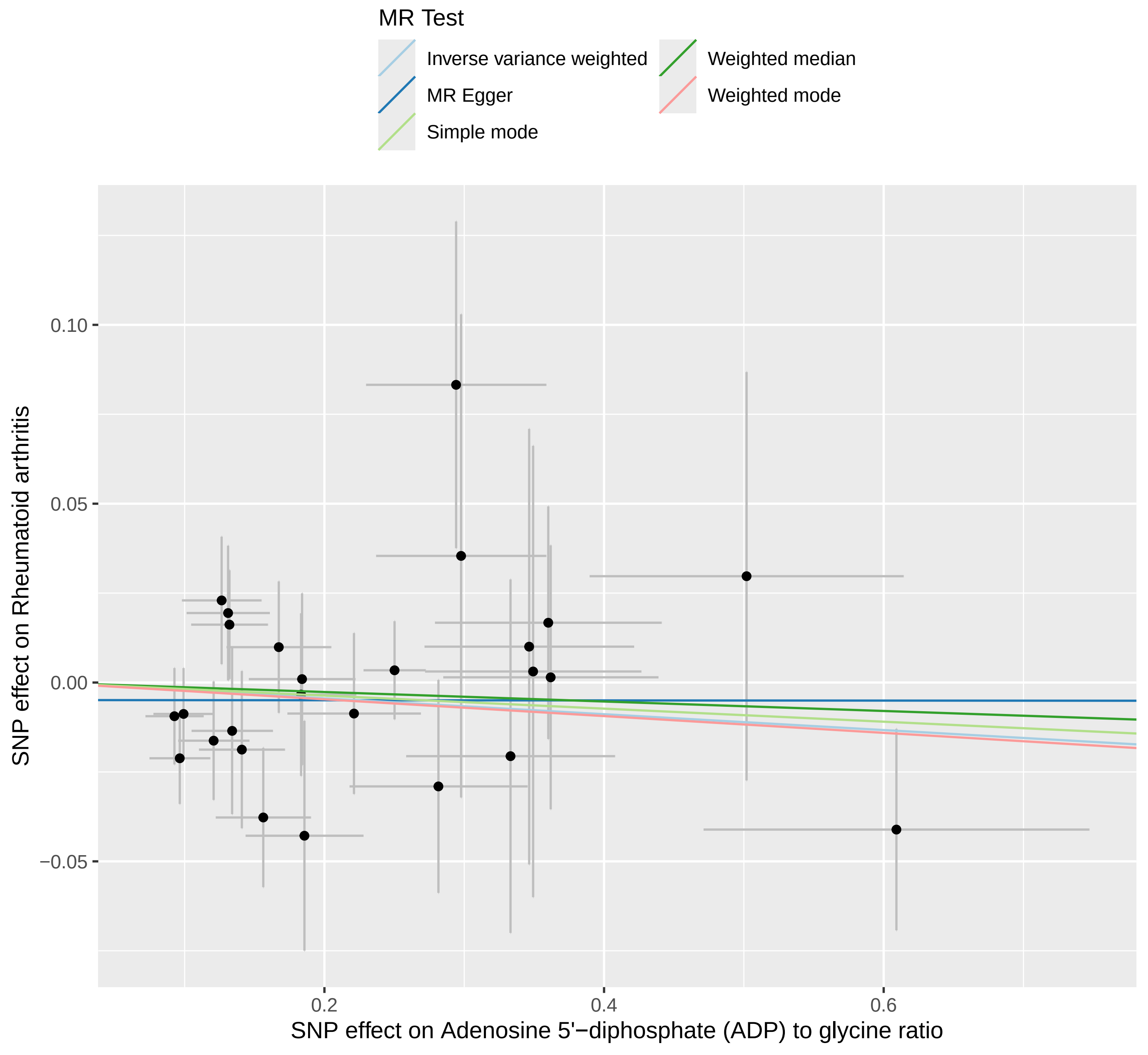
MR Test
Inverse variance weighted
MR Egger
Simple mode
Weighted median
Weighted mode
SNP effect on Rheumatoid arthritis
0.10
0.05
0.00
−0.05
0.2
0.4
0.6
SNP effect on Adenosine 5'−diphosphate (ADP) to glycine ratio

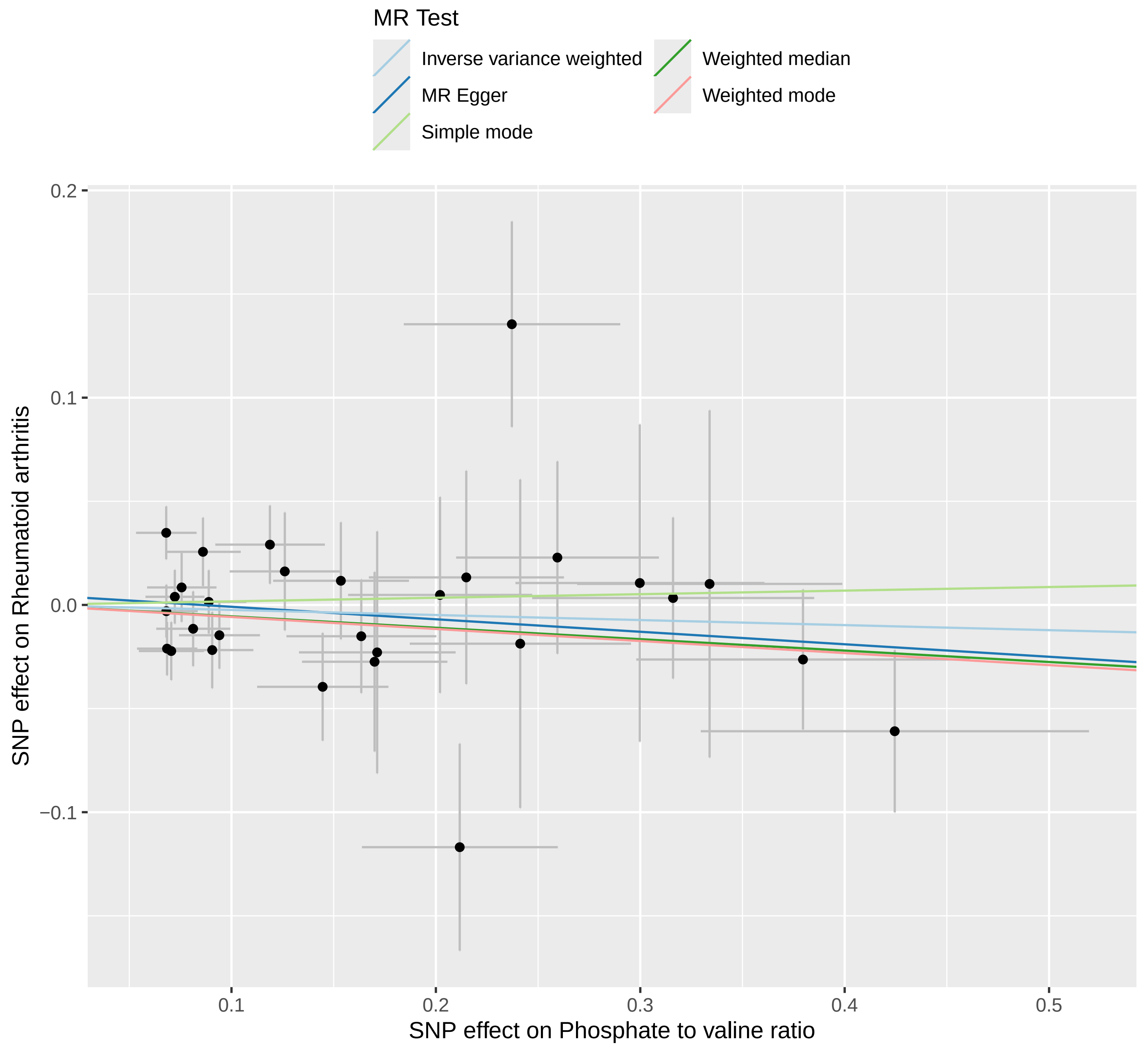
MR Test
Inverse variance weighted
MR Egger
Simple mode
Weighted median
Weighted mode
0.2
0.1
0.0
−0.1
0.1
0.2
0.3
0.4
0.5
SNP effect on Rheumatoid arthritis
SNP effect on Phosphate to valine ratio

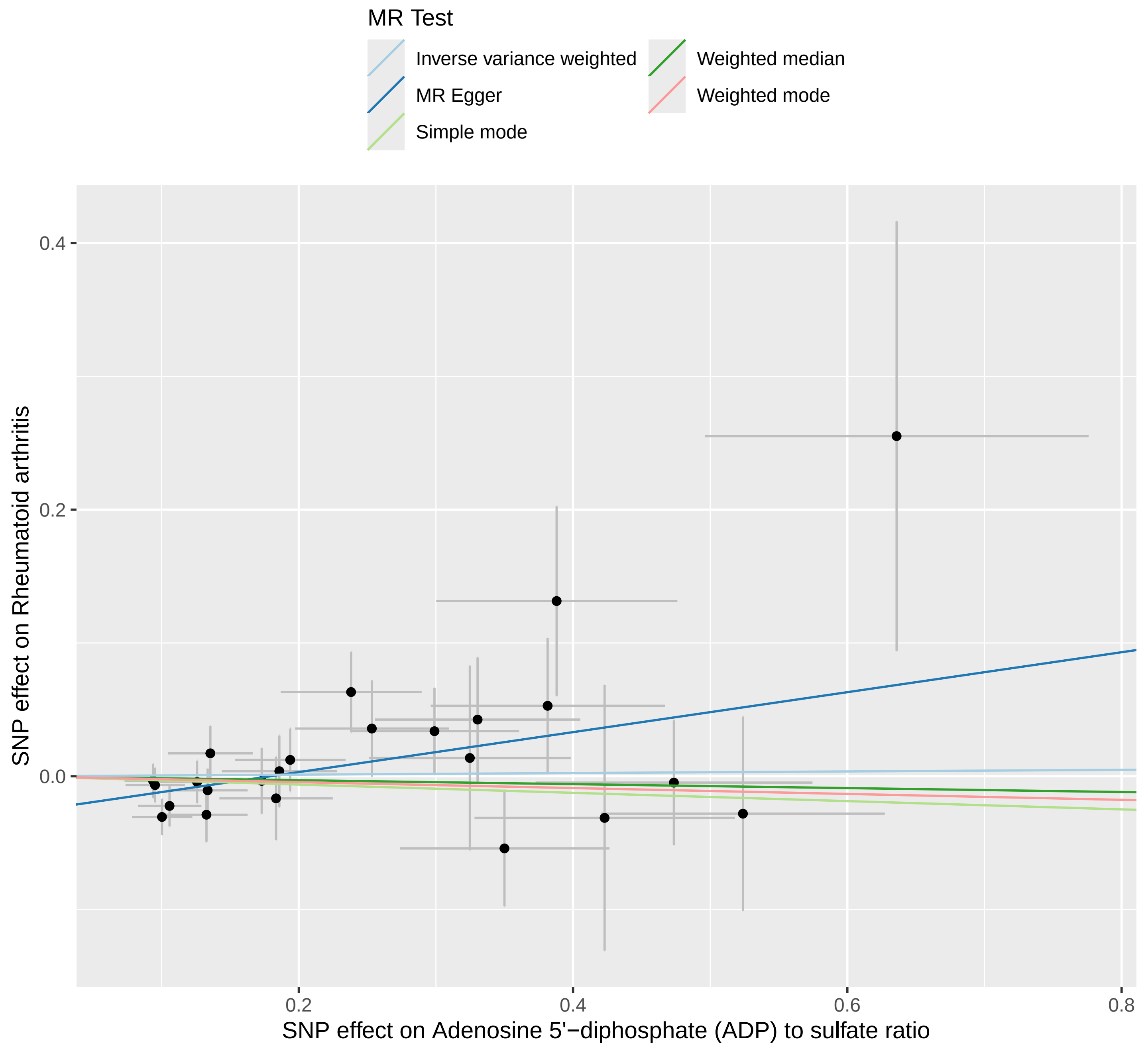
MR Test
Inverse variance weighted
MR Egger
Simple mode
Weighted median
Weighted mode
0.4
0.2
0.0
SNP effect on Rheumatoid arthritis
0.2
0.4
0.6
0.8
SNP effect on Adenosine 5'−diphosphate (ADP) to sulfate ratio

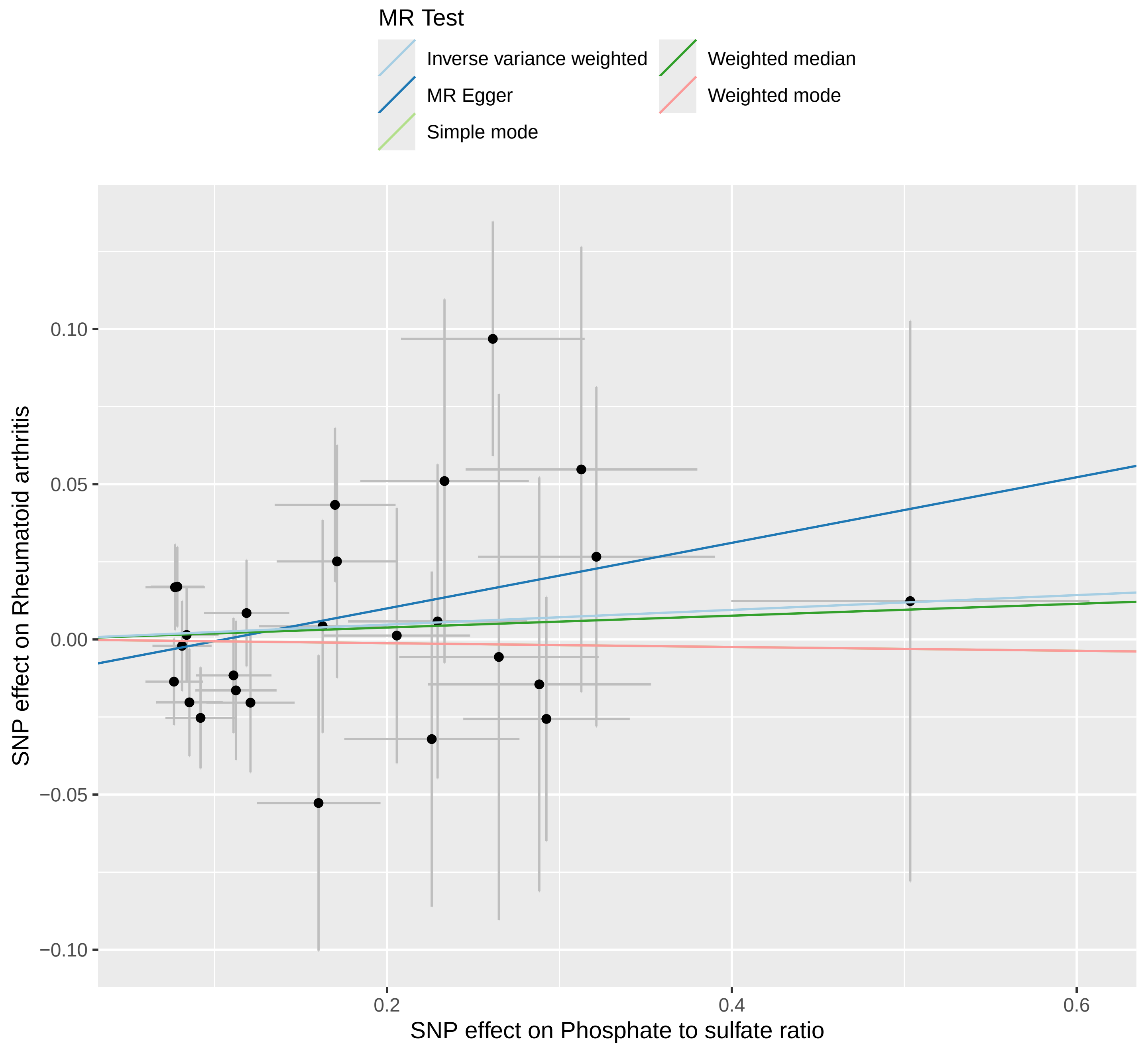
MR Test
Inverse variance weighted
MR Egger
Simple mode
Weighted median
Weighted mode
SNP effect on Rheumatoid arthritis
0.10
0.05
0.00
−0.05
−0.10
0.2
0.4
0.6
SNP effect on Phosphate to sulfate ratio

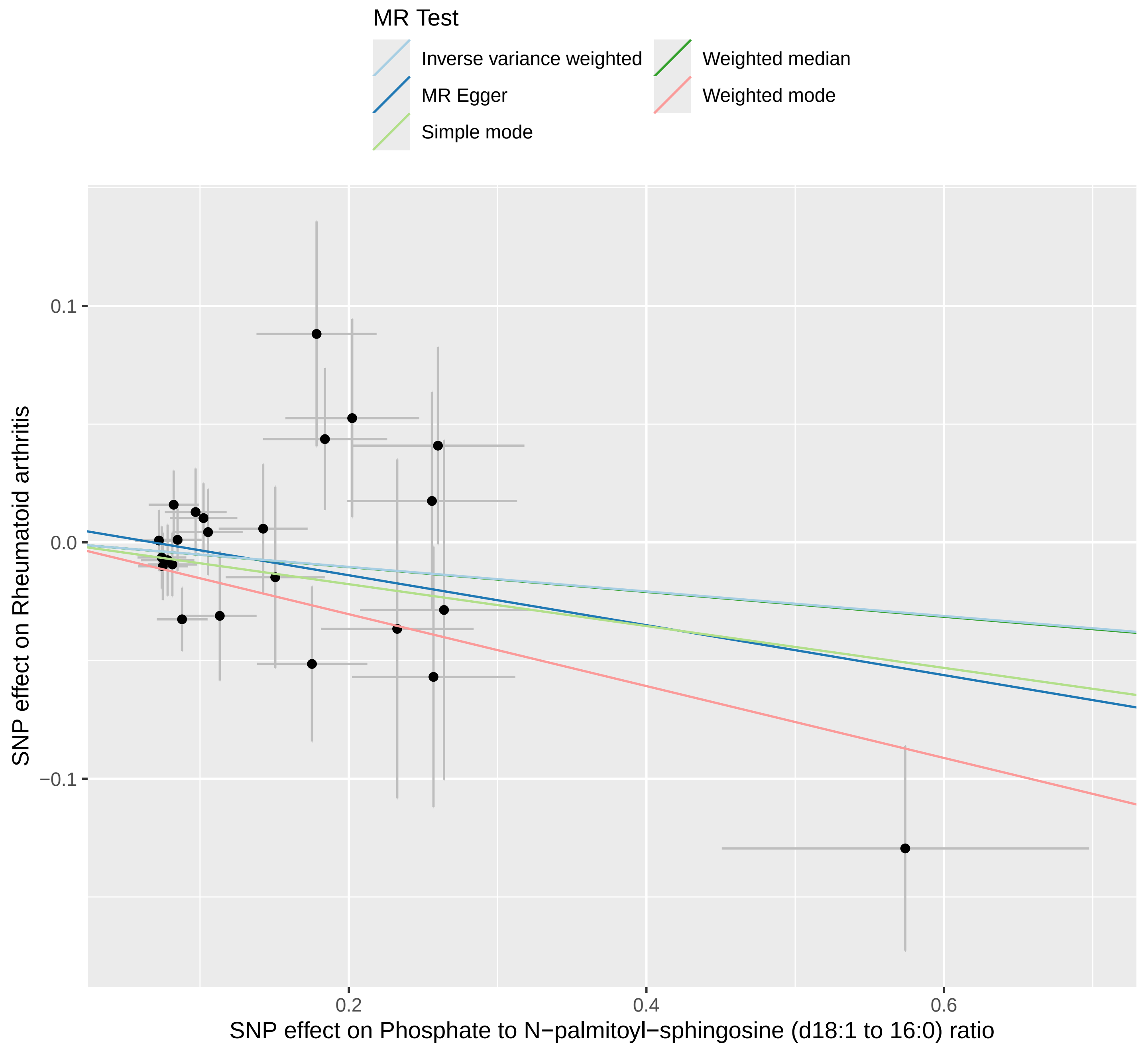
MR Test
Inverse variance weighted
MR Egger
Simple mode
Weighted median
Weighted mode
0.1
0.0
−0.1
0.2
0.4
0.6
SNP effect on Rheumatoid arthritis
SNP effect on Phosphate to N−palmitoyl−sphingosine (d18:1 to 16:0) ratio

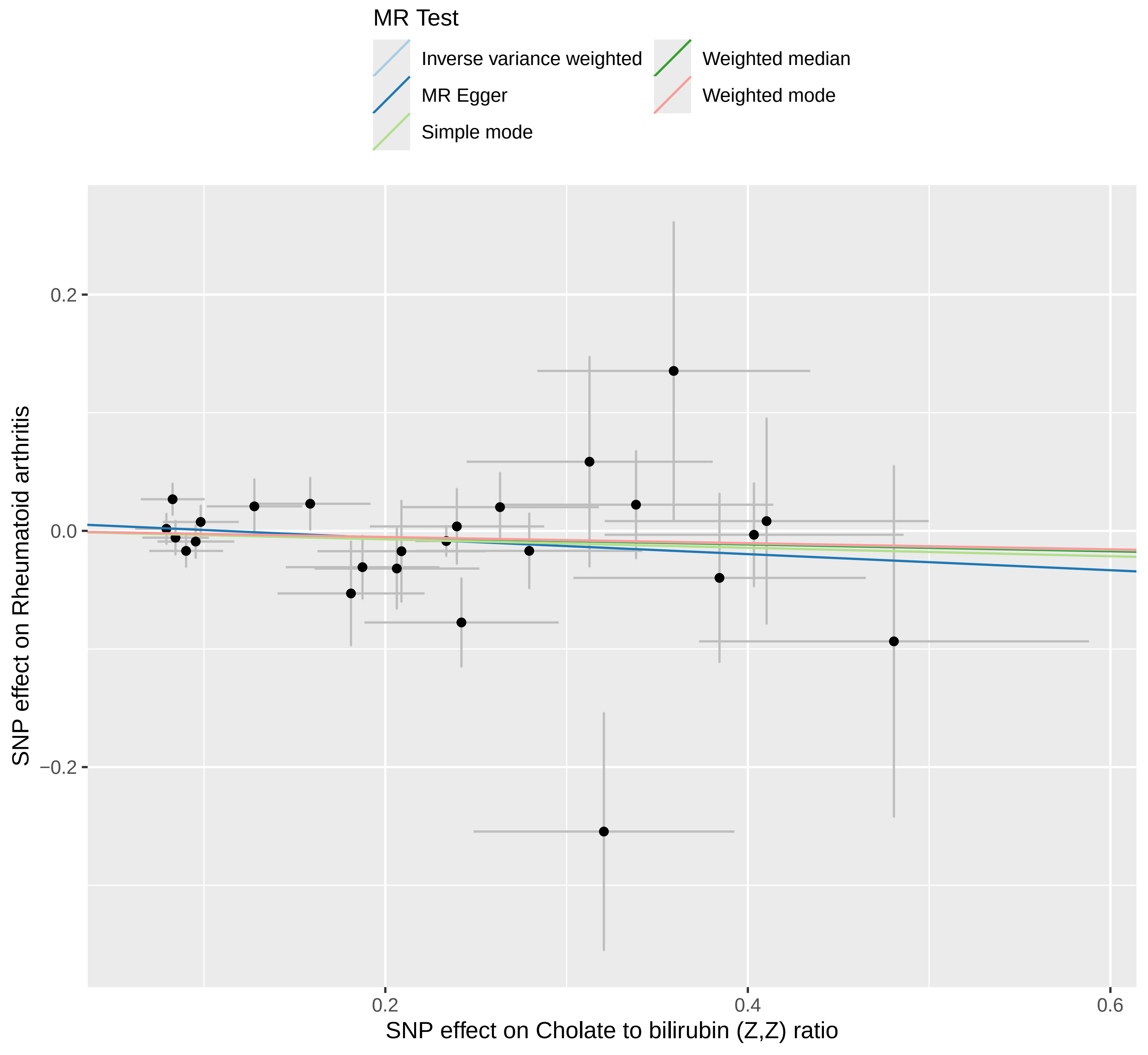
MR Test
Inverse variance weighted
MR Egger
Simple mode
Weighted median
Weighted mode
SNP effect on Rheumatoid arthritis
0.2
0.0
−0.2
0.2
0.4
0.6
SNP effect on Cholate to bilirubin (Z,Z) ratio

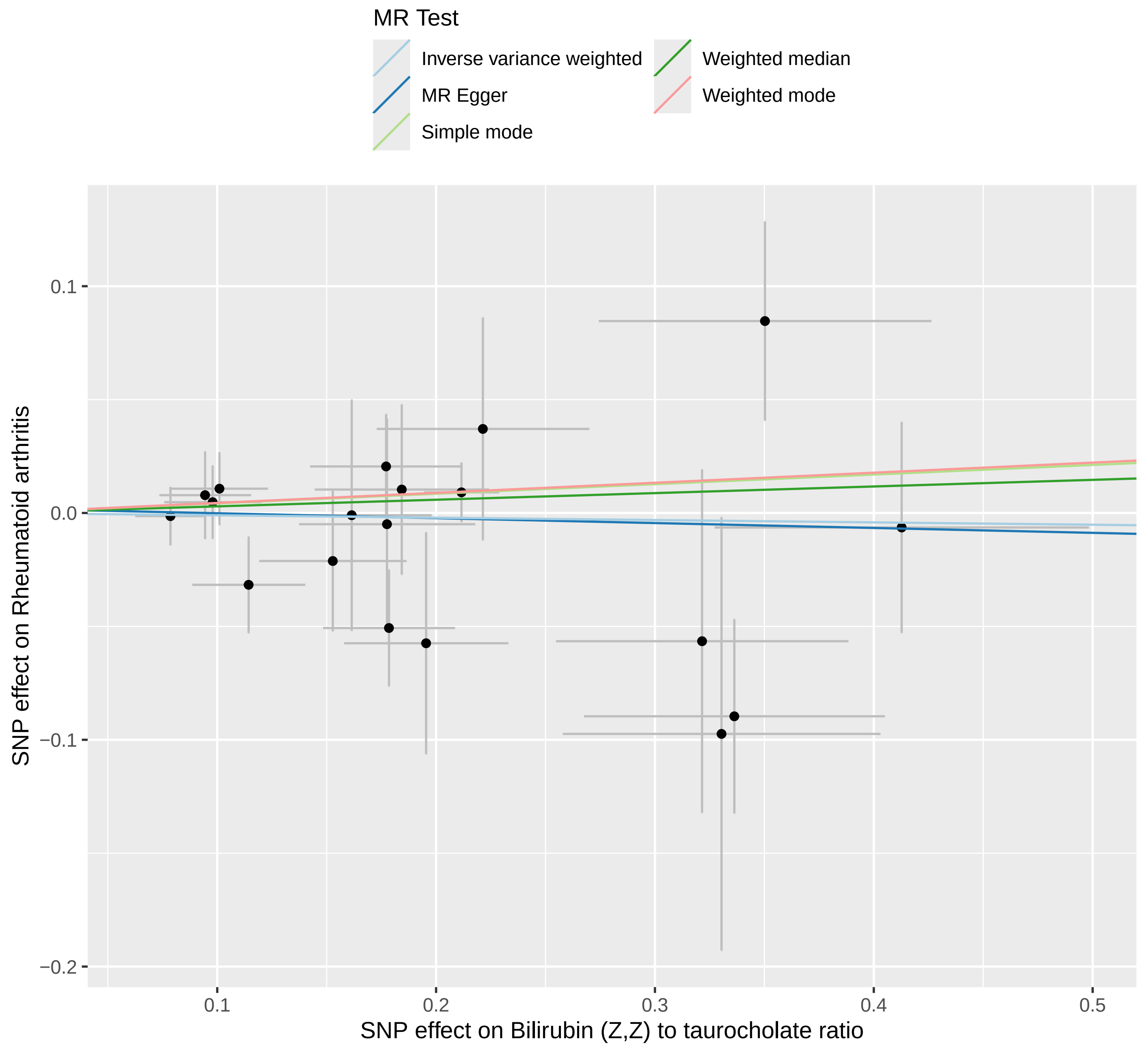

MR Test
Inverse variance weighted
MR Egger
Simple mode
Weighted median
Weighted mode
0.1
0.0
−0.1
−0.2
SNP effect on Rheumatoid arthritis
0.1
0.2
0.3
0.4
0.5
SNP effect on Bilirubin (Z,Z) to taurocholate ratio

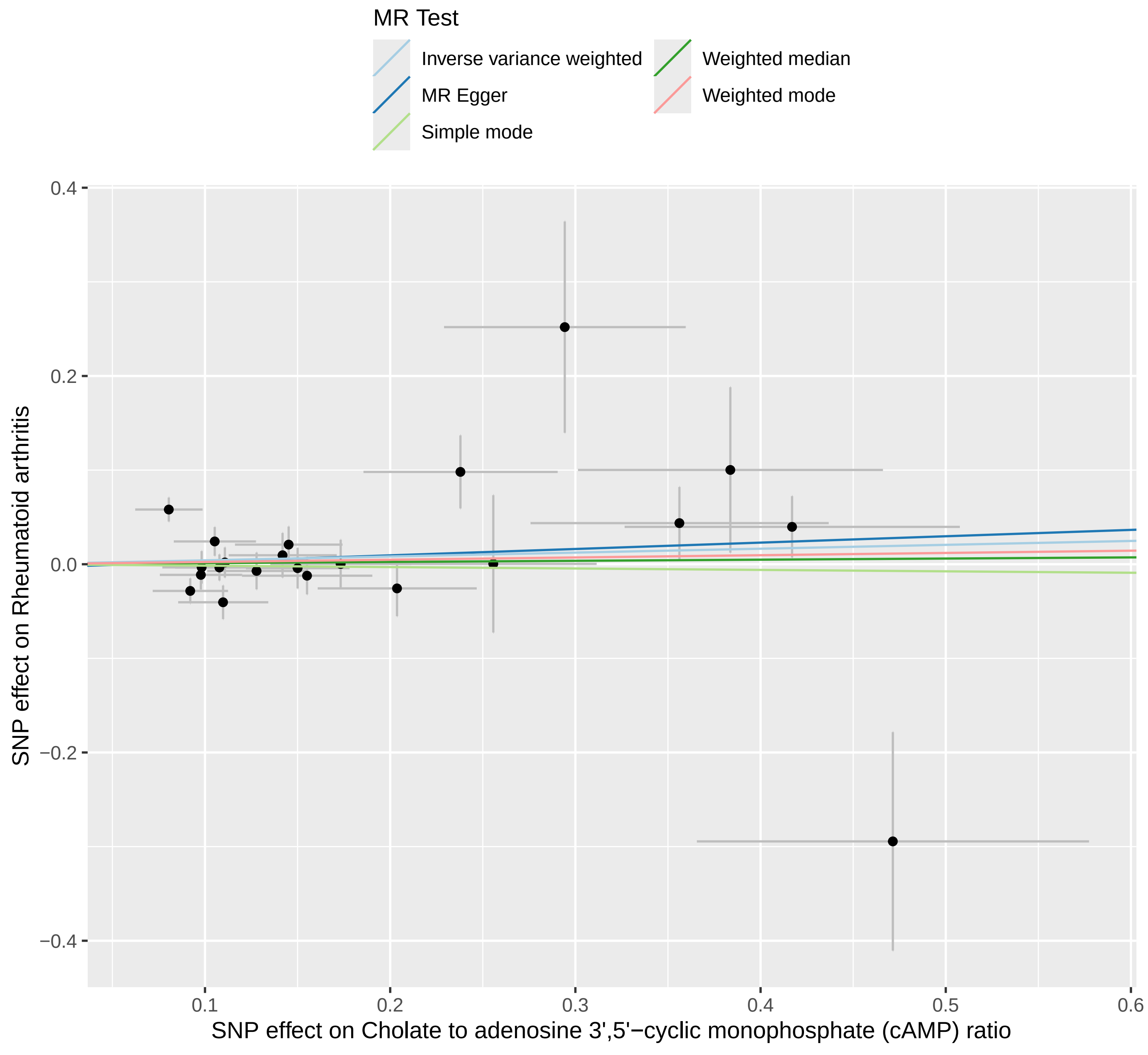
MR Test
Inverse variance weighted
MR Egger
Simple mode
Weighted median
Weighted mode
SNP effect on Rheumatoid arthritis
0.4
0.2
0.0
−0.2
−0.4
0.1
0.2
0.3
0.4
0.5
0.6
SNP effect on Cholate to adenosine 3',5'−cyclic monophosphate (cAMP) ratio

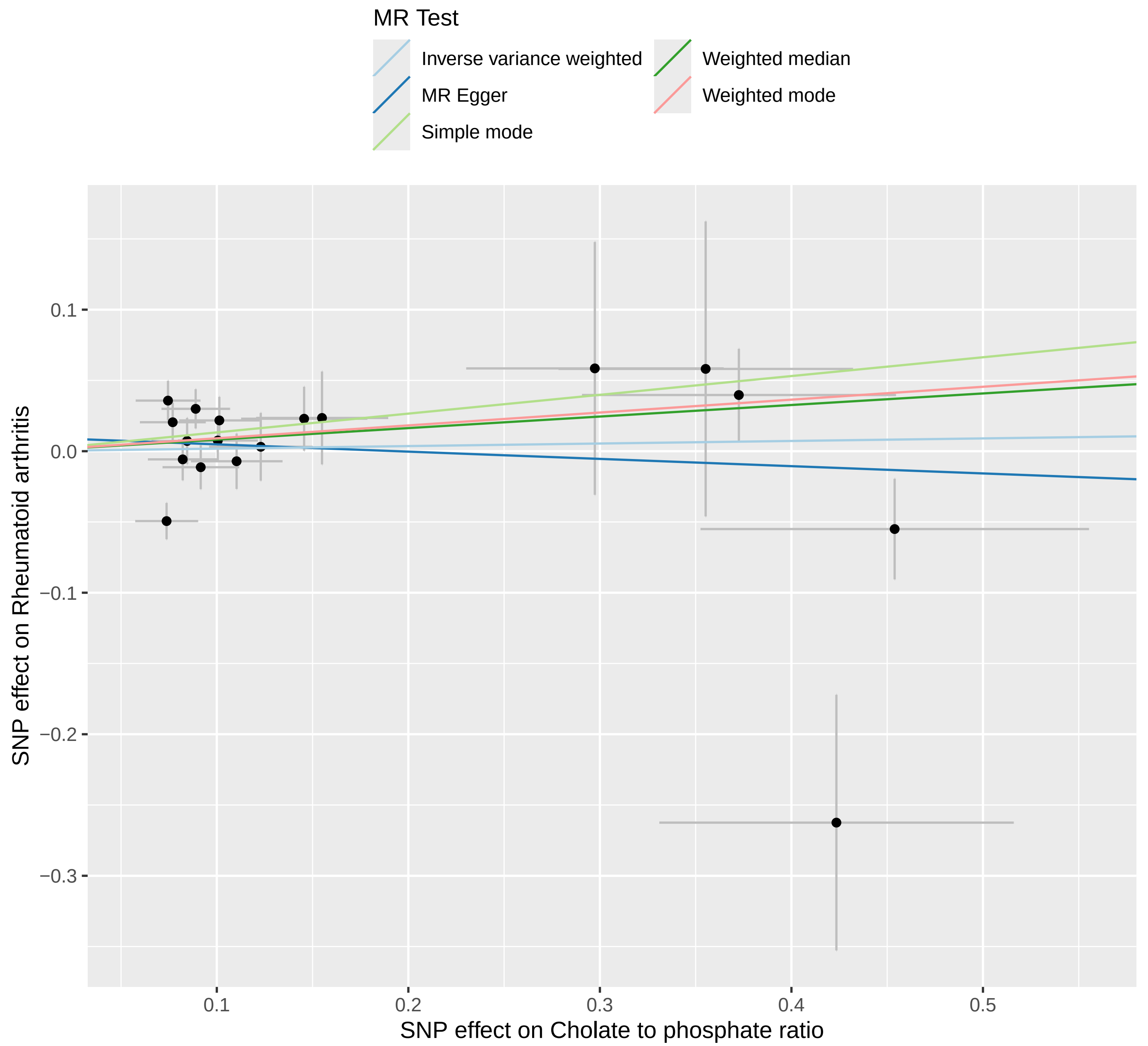

MR Test
Inverse variance weighted
MR Egger
Simple mode
Weighted median
Weighted mode
SNP effect on Rheumatoid arthritis
SNP effect on Cholate to phosphate ratio
0.1
0.0
−0.1
−0.2
−0.3
0.1
0.2
0.3
0.4
0.5

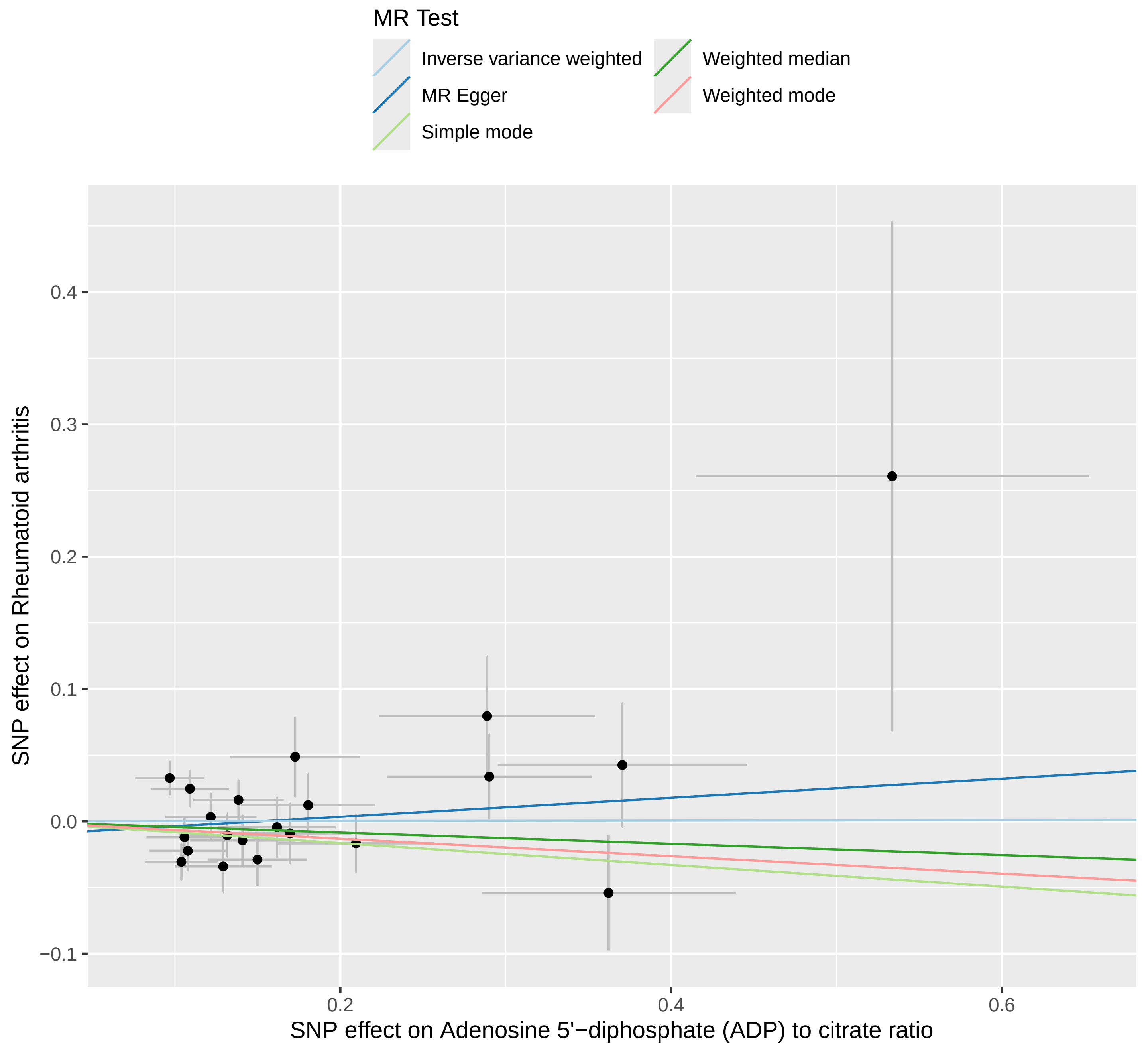

MR Test
Inverse variance weighted
MR Egger
Simple mode
Weighted median
Weighted mode
0.4
0.3
0.2
0.1
0.0
−0.1
SNP effect on Rheumatoid arthritis
0.2
0.4
0.6
SNP effect on Adenosine 5'−diphosphate (ADP) to citrate ratio

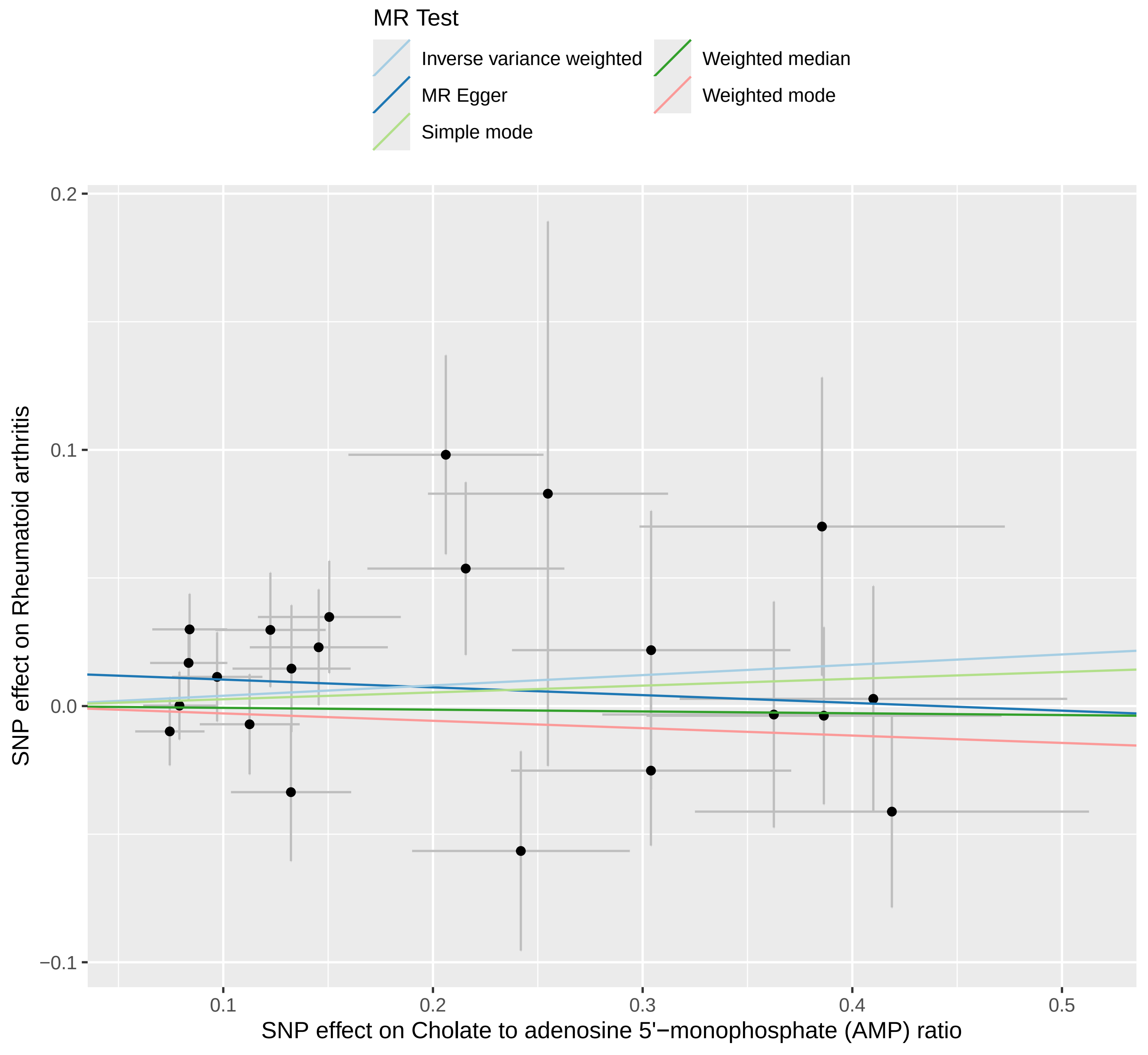
MR Test
Inverse variance weighted
MR Egger
Simple mode
Weighted median
Weighted mode
0.2
0.1
0.0
−0.1
0.1
0.2
0.3
0.4
0.5
SNP effect on Rheumatoid arthritis
SNP effect on Cholate to adenosine 5'−monophosphate (AMP) ratio

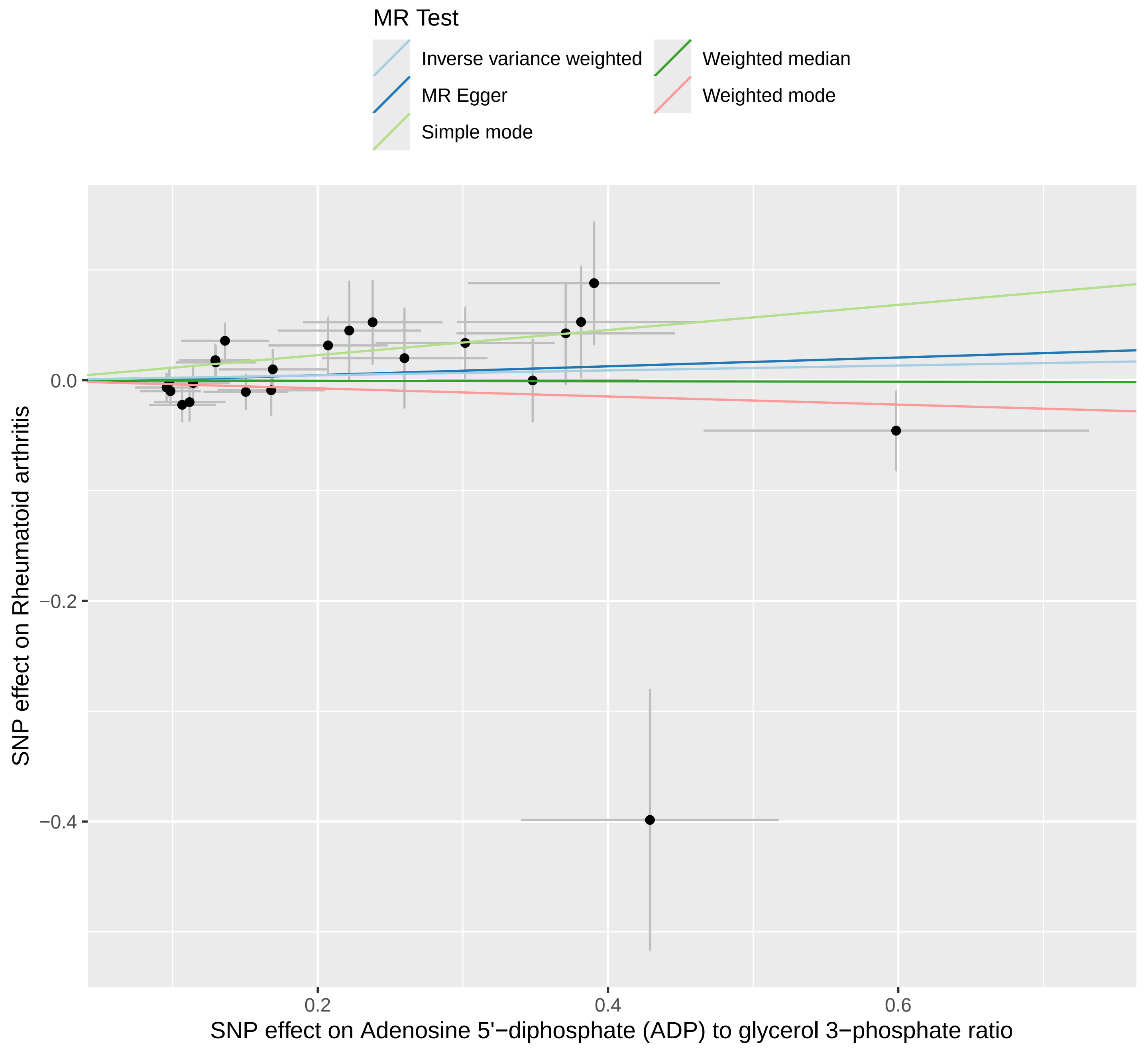
MR Test
Inverse variance weighted
MR Egger
Simple mode
Weighted median
Weighted mode
SNP effect on Rheumatoid arthritis
0.0
−0.2
−0.4
0.2
0.4
0.6
SNP effect on Adenosine 5'−diphosphate (ADP) to glycerol 3−phosphate ratio

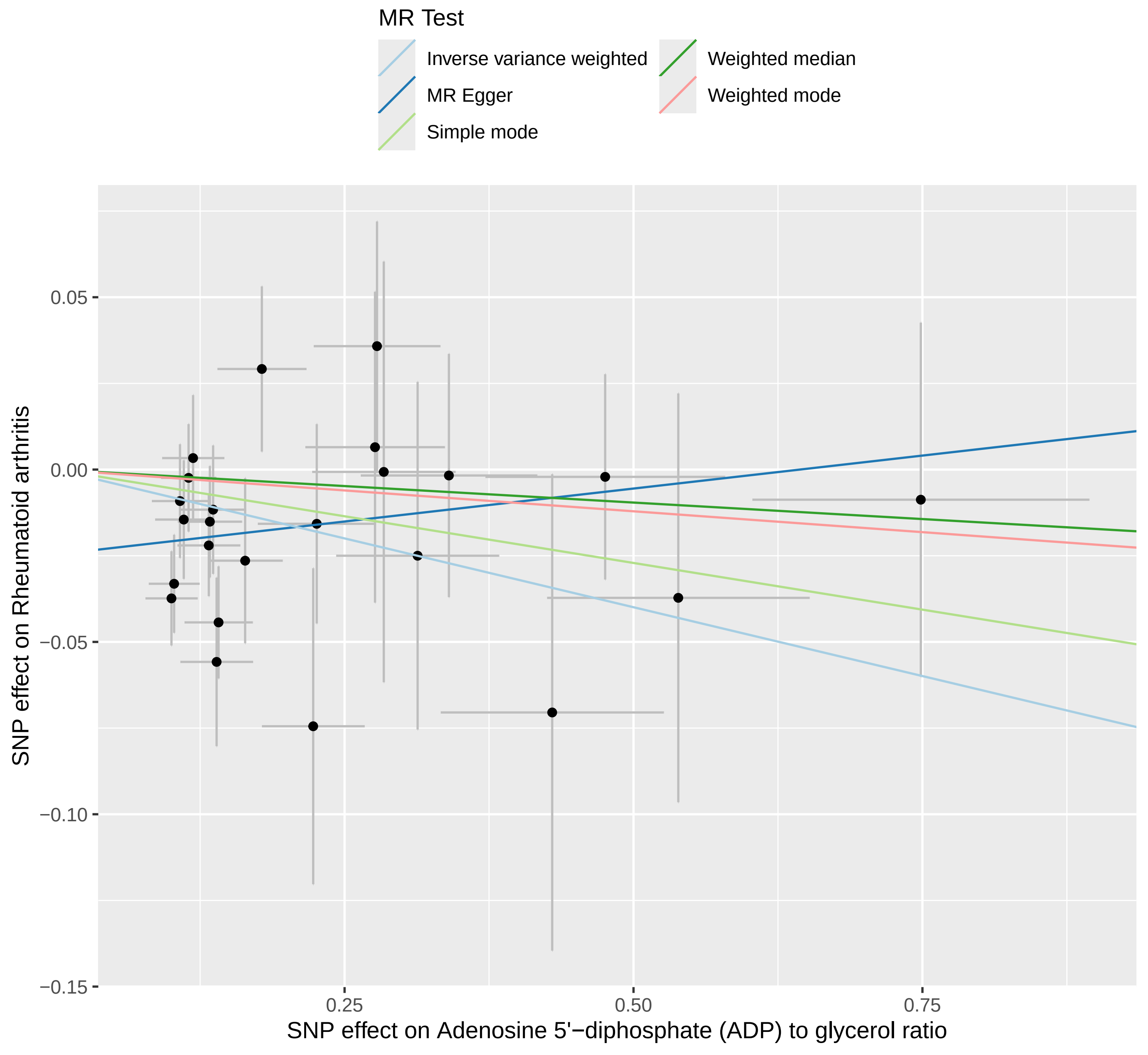
MR Test
Inverse variance weighted
MR Egger
Simple mode
Weighted median
Weighted mode
0.05
0.00
−0.05
−0.10
−0.15
0.25
0.50
0.75
SNP effect on Rheumatoid arthritis
SNP effect on Adenosine 5'−diphosphate (ADP) to glycerol ratio

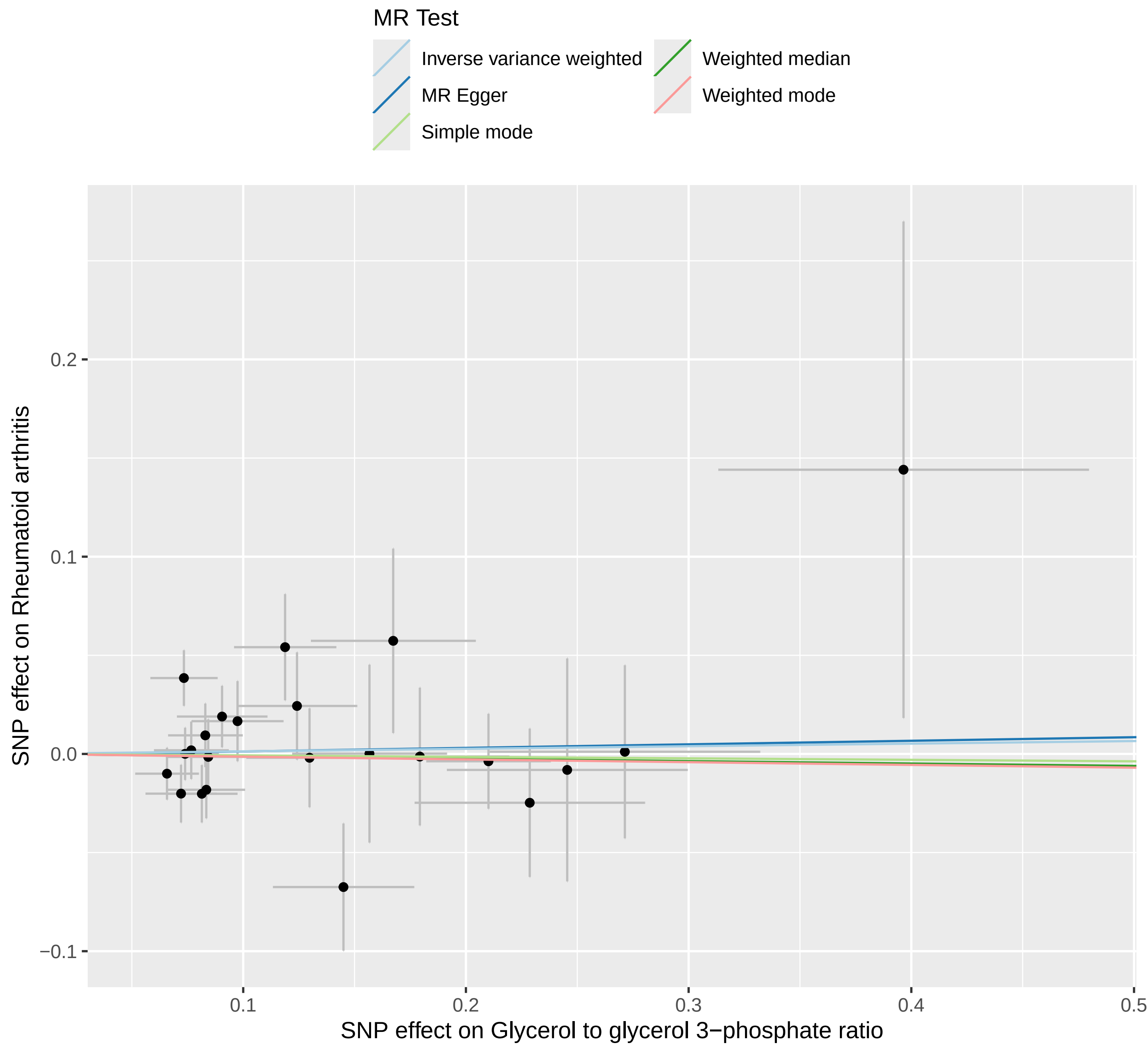
MR Test
Inverse variance weighted
MR Egger
Simple mode
Weighted median
Weighted mode
SNP effect on Rheumatoid arthritis
0.2
0.1
0.0
−0.1
0.1
0.2
0.3
0.4
0.5
SNP effect on Glycerol to glycerol 3−phosphate ratio

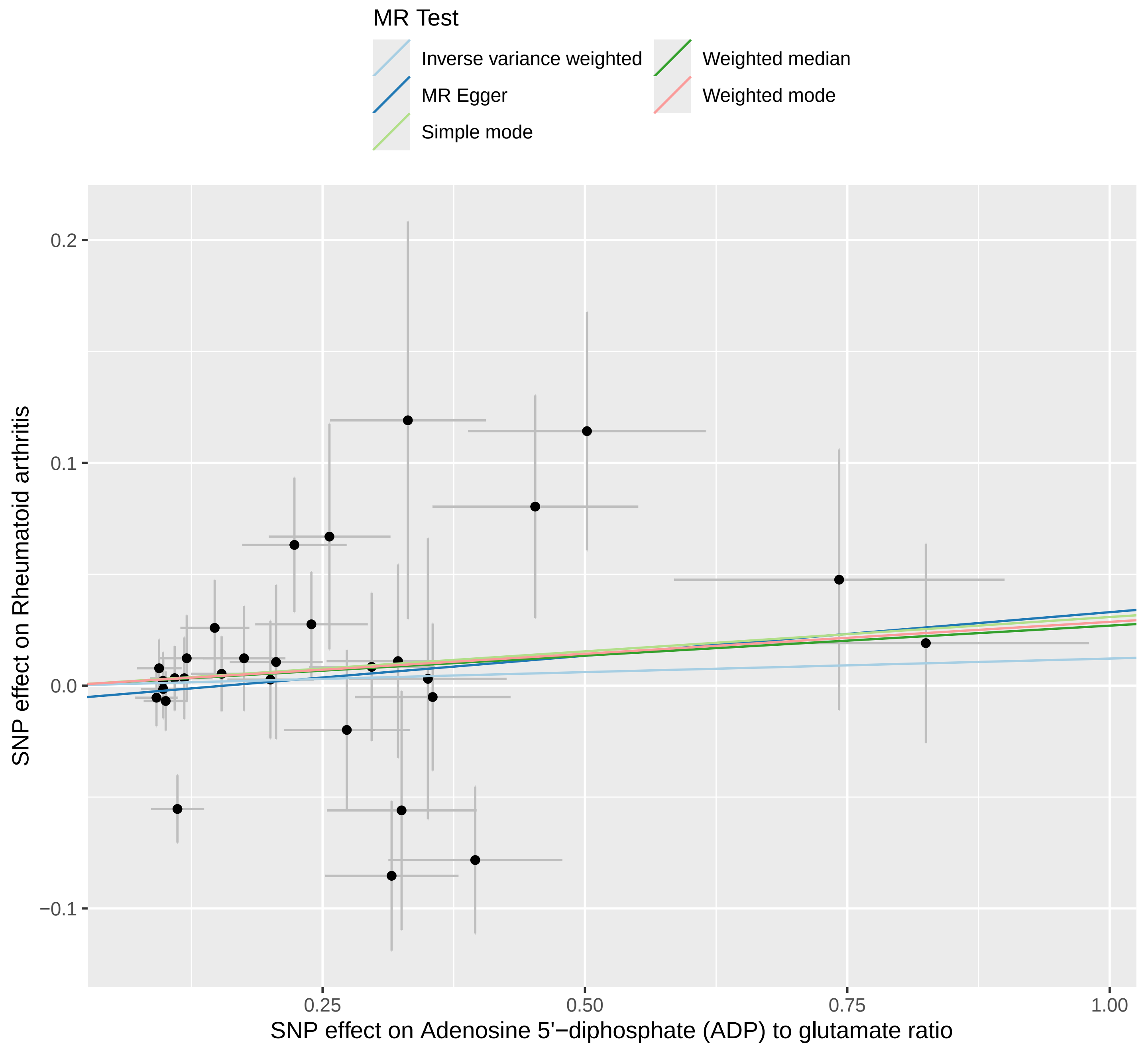
MR Test
Inverse variance weighted
MR Egger
Simple mode
Weighted median
Weighted mode
0.2
0.1
0.0
−0.1
0.25
0.50
0.75
1.00
SNP effect on Rheumatoid arthritis
SNP effect on Adenosine 5'−diphosphate (ADP) to glutamate ratio

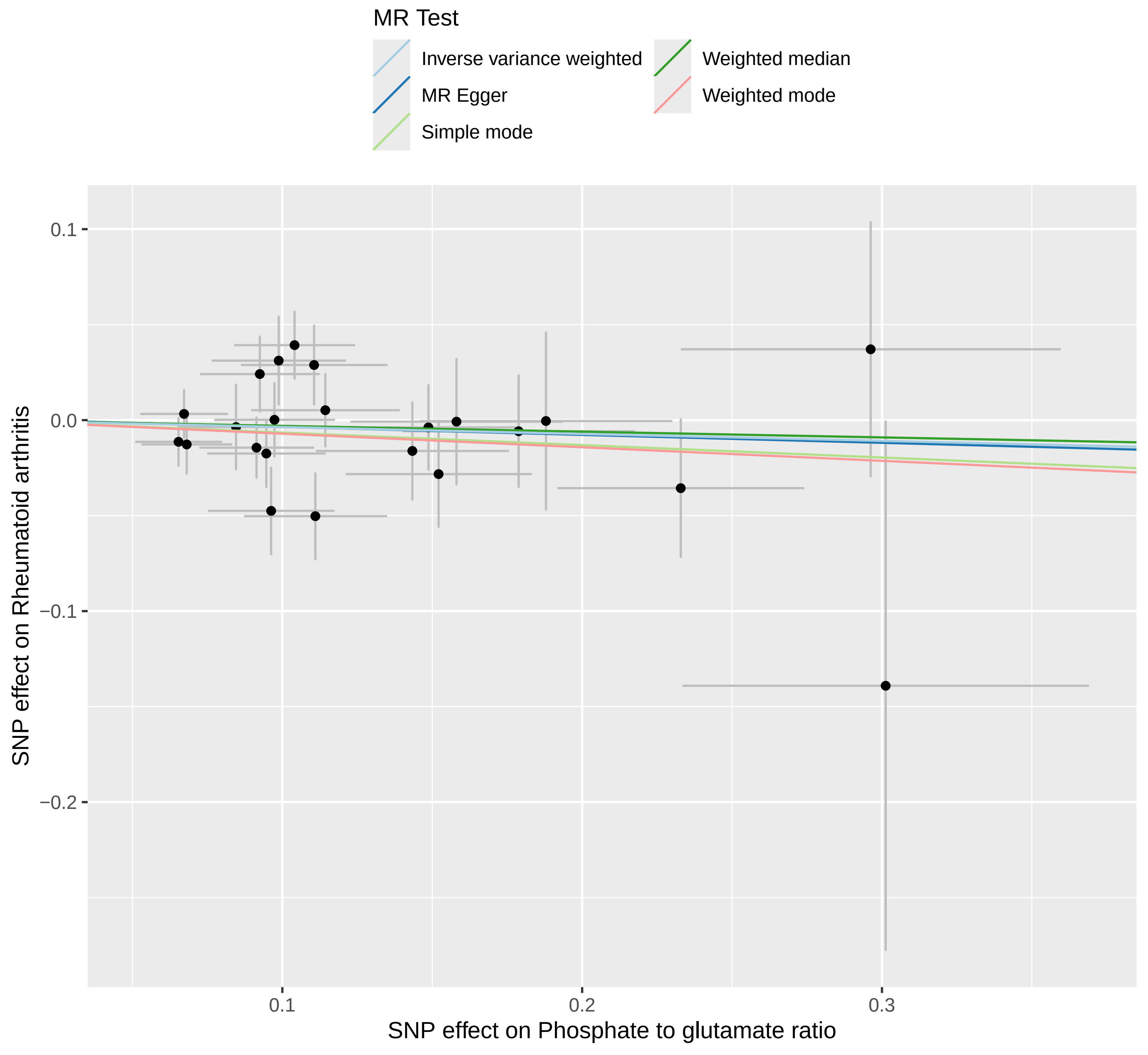

MR Test
Inverse variance weighted
MR Egger
Simple mode
Weighted median
Weighted mode
0.1
0.0
−0.1
−0.2
SNP effect on Rheumatoid arthritis
0.1
0.2
0.3
SNP effect on Phosphate to glutamate ratio

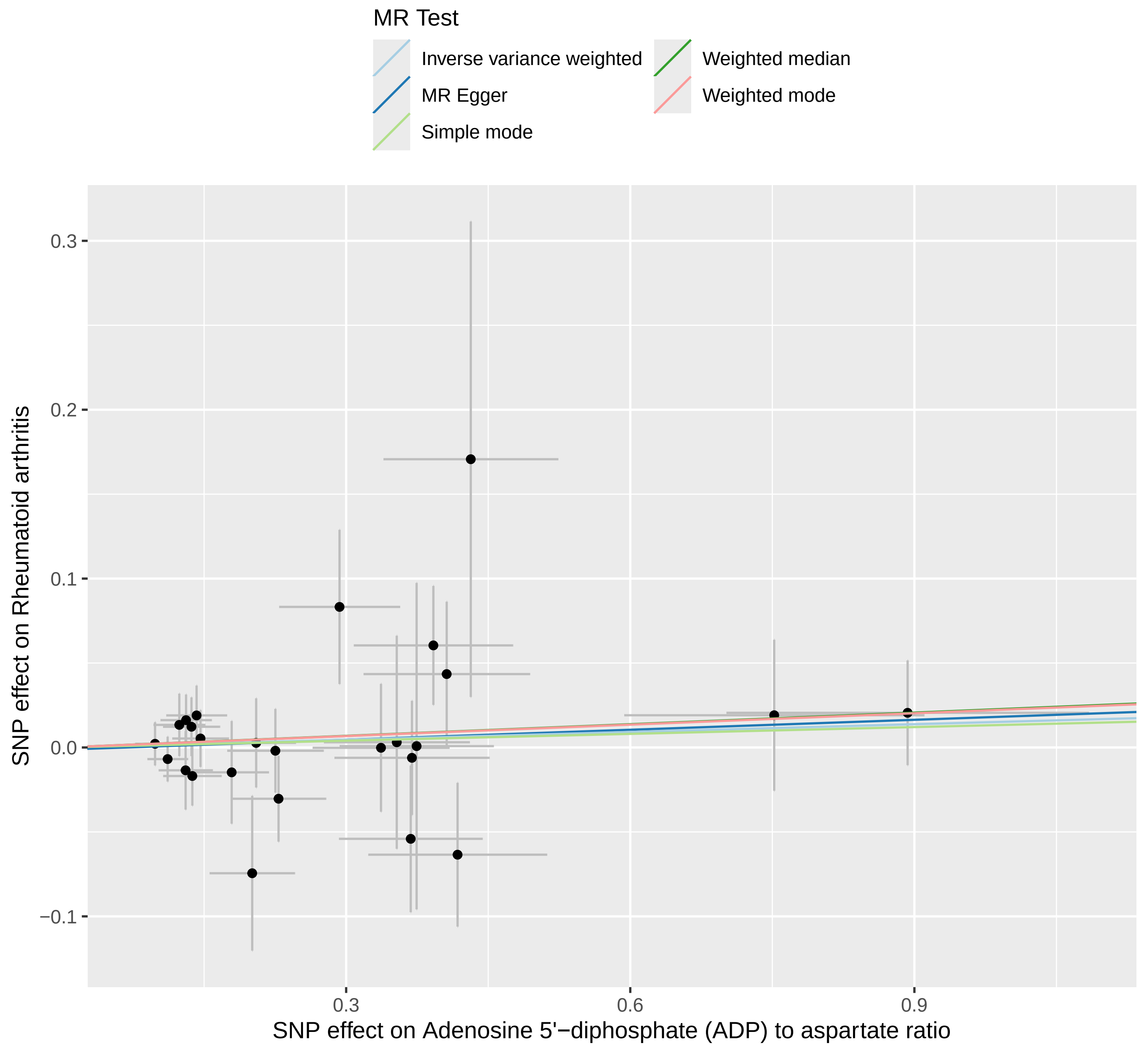
MR Test
Inverse variance weighted
MR Egger
Simple mode
Weighted median
Weighted mode
0.3
0.2
0.1
0.0
−0.1
SNP effect on Rheumatoid arthritis
0.3
0.6
0.9
SNP effect on Adenosine 5'−diphosphate (ADP) to aspartate ratio

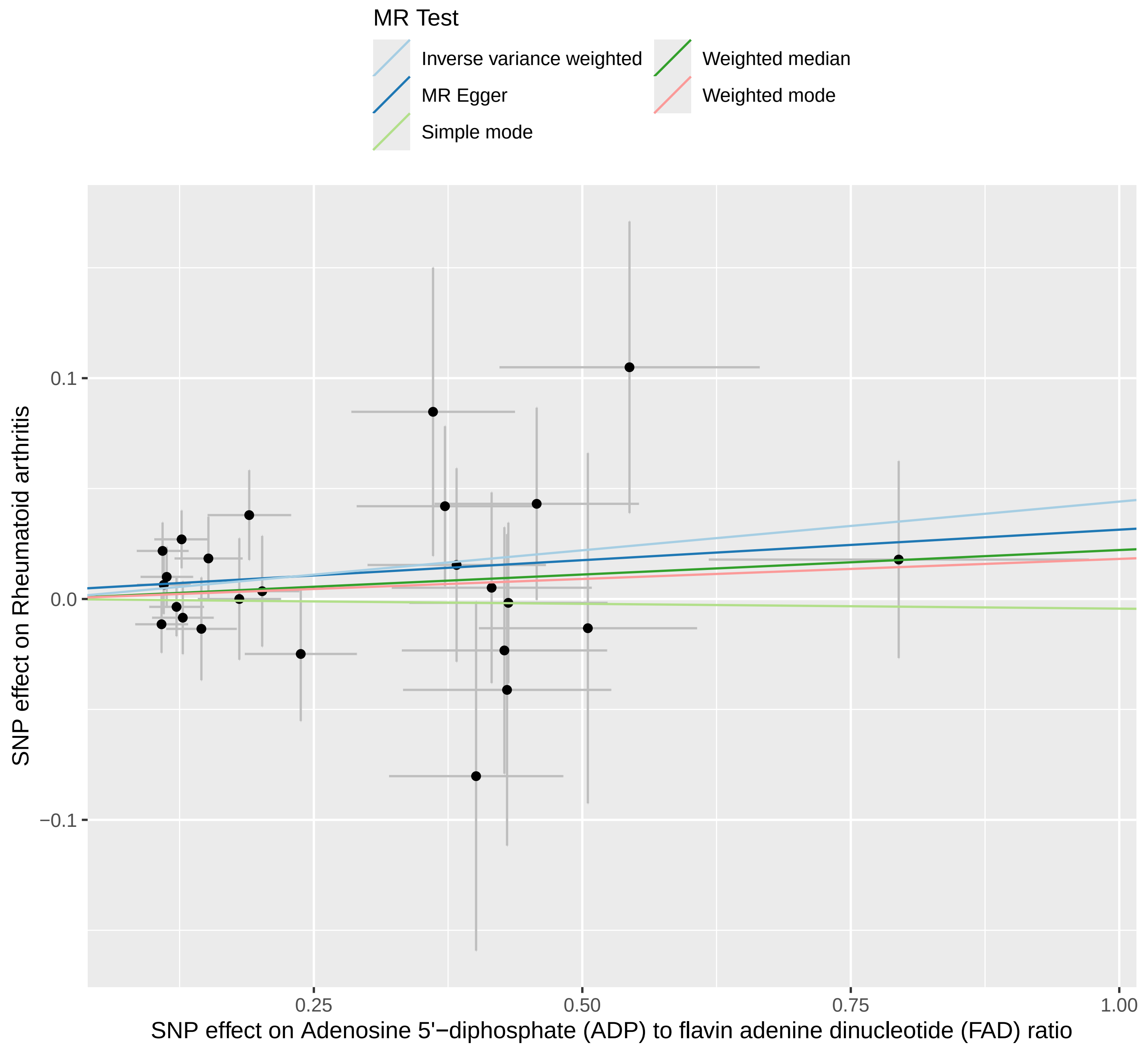
MR Test
Inverse variance weighted
MR Egger
Simple mode
Weighted median
Weighted mode
0.1
0.0
−0.1
SNP effect on Rheumatoid arthritis
0.25
0.50
0.75
1.00
SNP effect on Adenosine 5'−diphosphate (ADP) to flavin adenine dinucleotide (FAD) ratio

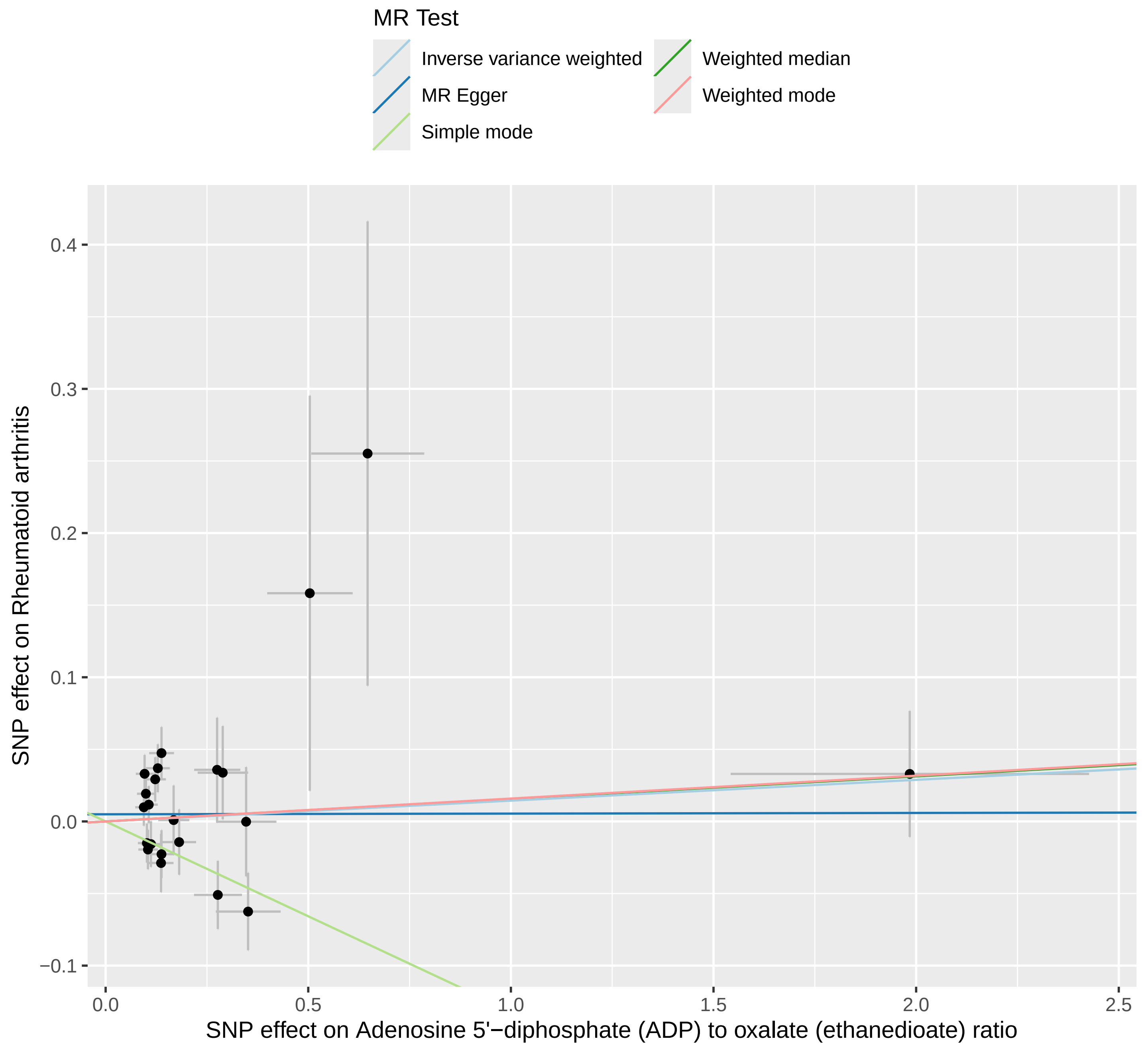

MR Test
Inverse variance weighted
MR Egger
Simple mode
Weighted median
Weighted mode
0.4
0.3
0.2
0.1
0.0
−0.1
0.0
0.5
1.0
1.5
2.0
2.5
SNP effect on Rheumatoid arthritis
SNP effect on Adenosine 5'−diphosphate (ADP) to oxalate (ethanedioate) ratio

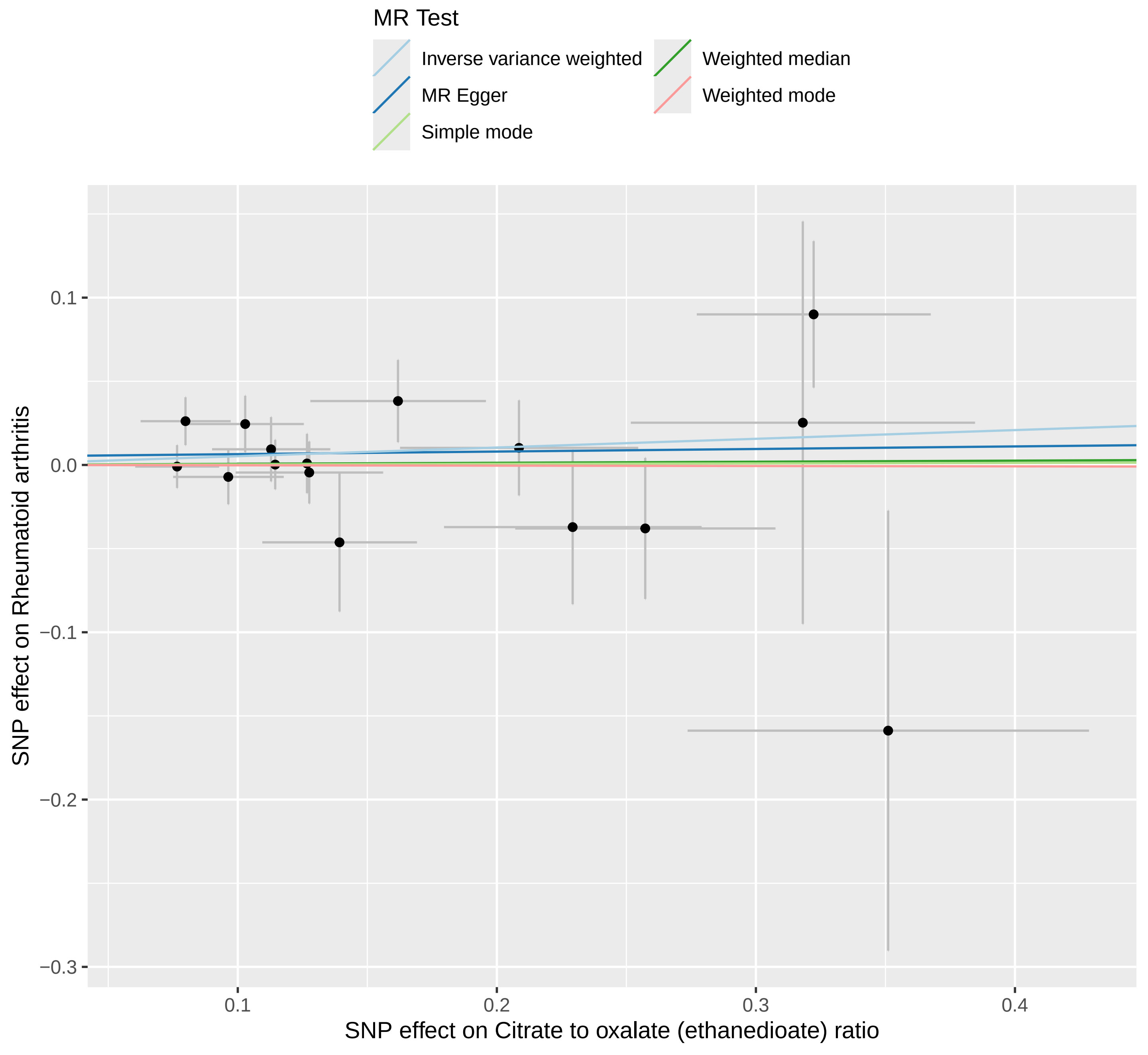
MR Test
Inverse variance weighted
MR Egger
Simple mode
Weighted median
Weighted mode
0.1
0.0
−0.1
−0.2
−0.3
SNP effect on Rheumatoid arthritis
0.1
0.2
0.3
0.4
SNP effect on Citrate to oxalate (ethanedioate) ratio

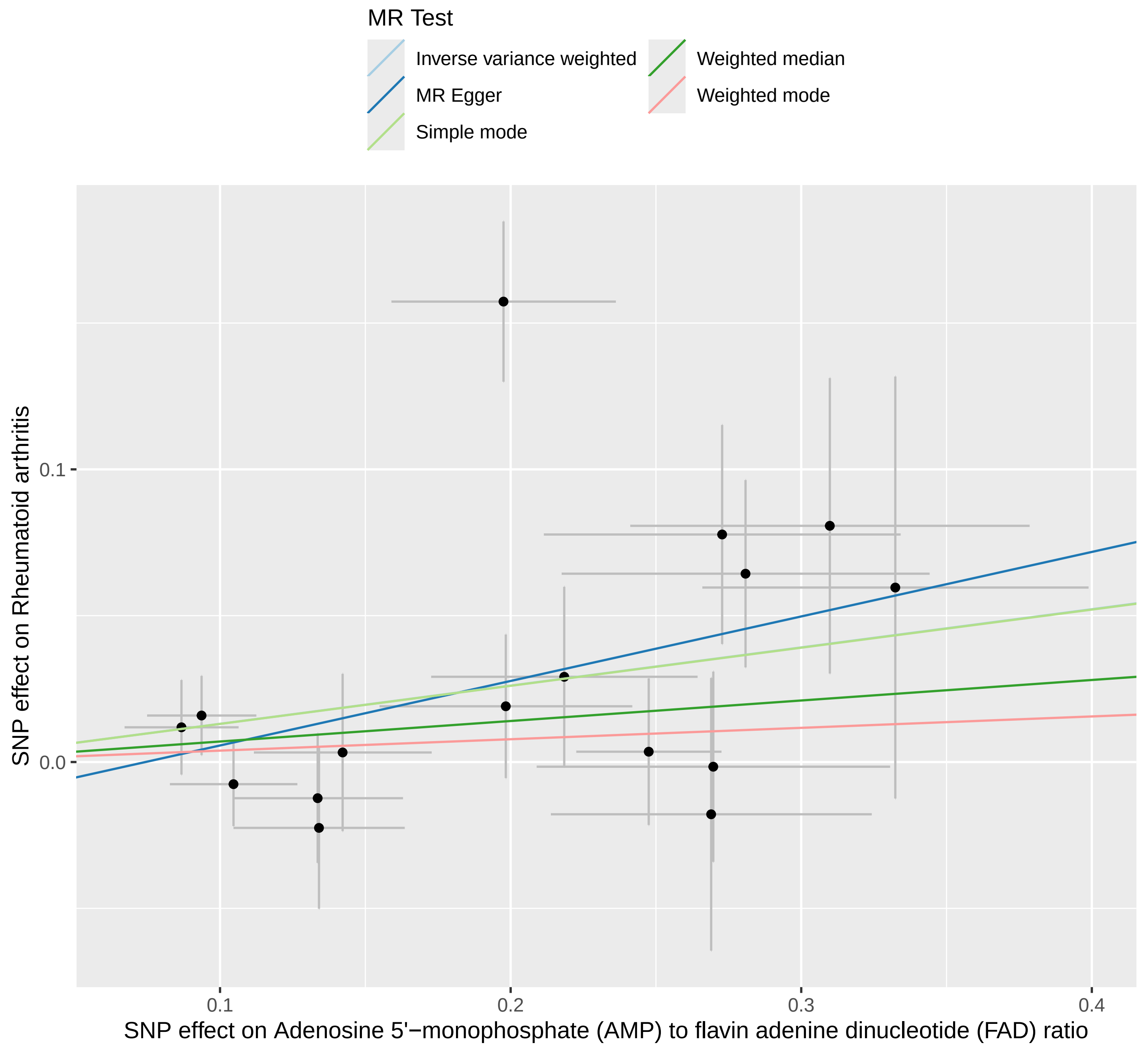

MR Test
Inverse variance weighted
MR Egger
Simple mode
Weighted median
Weighted mode
SNP effect on Rheumatoid arthritis
0.1
0.0
0.1
0.2
0.3
0.4
SNP effect on Adenosine 5'−monophosphate (AMP) to flavin adenine dinucleotide (FAD) ratio

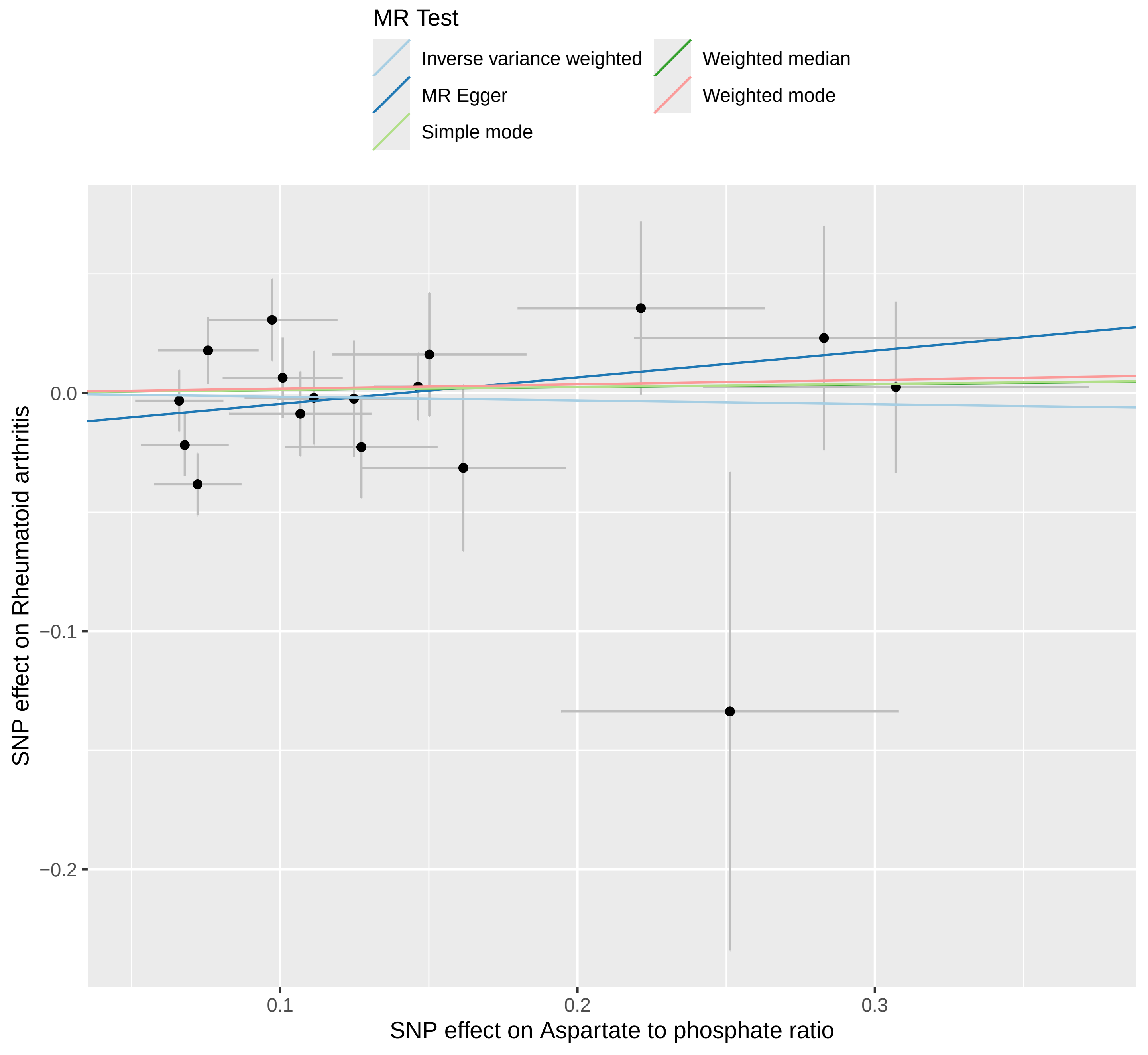

MR Test
Inverse variance weighted
MR Egger
Simple mode
Weighted median
Weighted mode
SNP effect on Rheumatoid arthritis
0.0
−0.1
−0.2
0.1
0.2
0.3
SNP effect on Aspartate to phosphate ratio

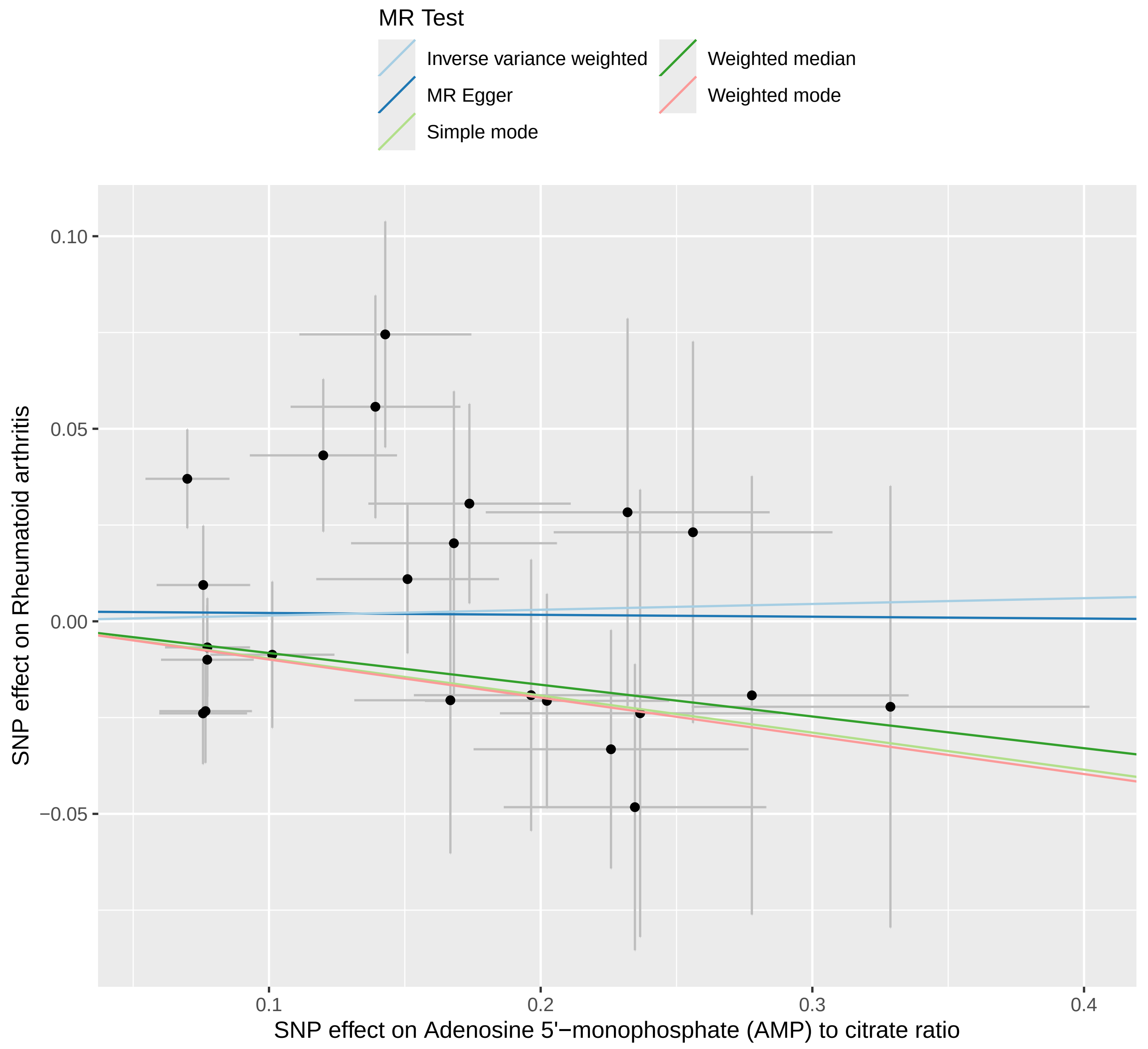

MR Test
Inverse variance weighted
MR Egger
Simple mode
Weighted median
Weighted mode
0.10
0.05
0.00
−0.05
0.1
0.2
0.3
0.4
SNP effect on Rheumatoid arthritis
SNP effect on Adenosine 5'−monophosphate (AMP) to citrate ratio

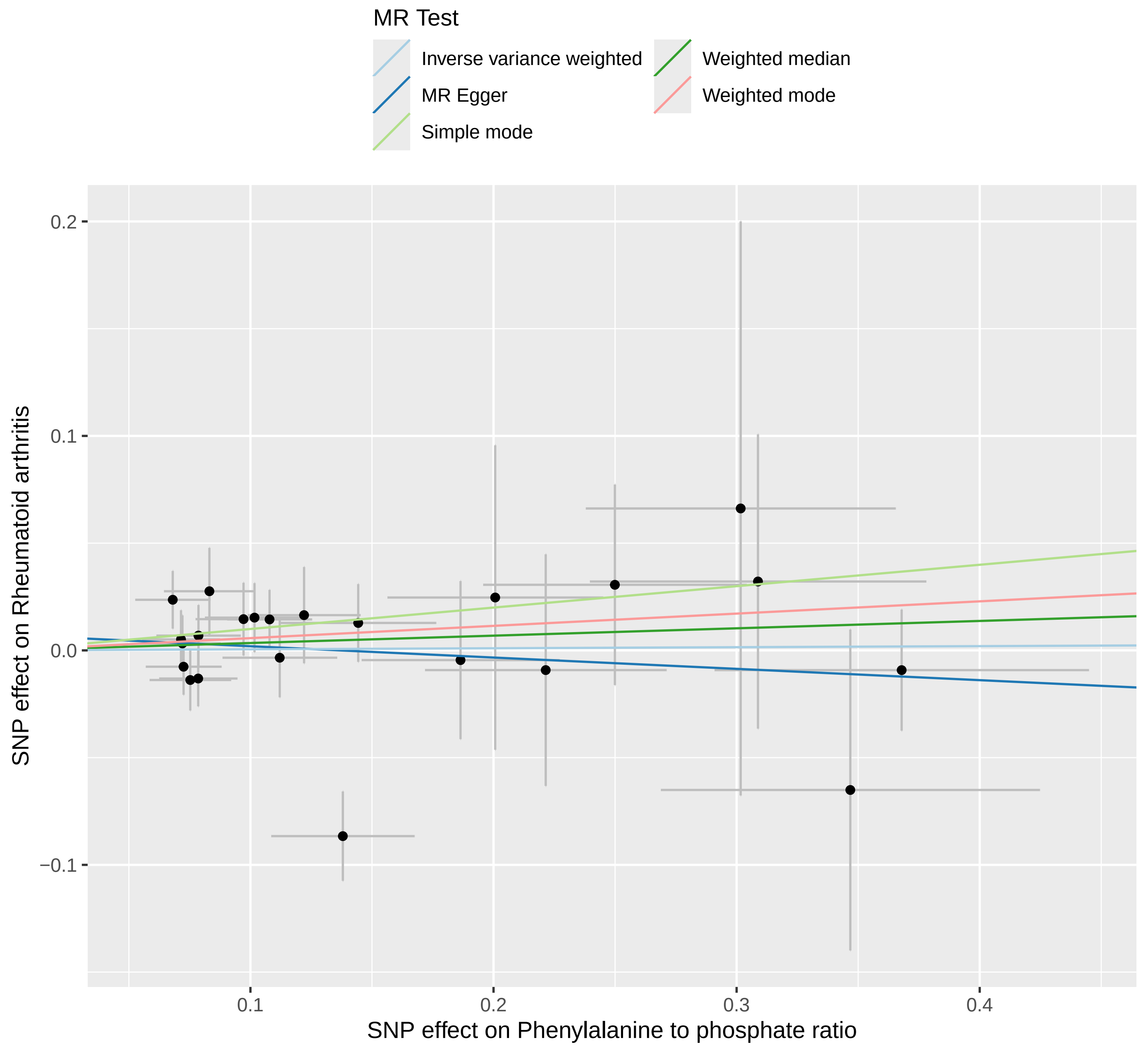
MR Test
Inverse variance weighted
MR Egger
Simple mode
Weighted median
Weighted mode
SNP effect on Rheumatoid arthritis
0.2
0.1
0.0
−0.1
0.1
0.2
0.3
0.4
SNP effect on Phenylalanine to phosphate ratio

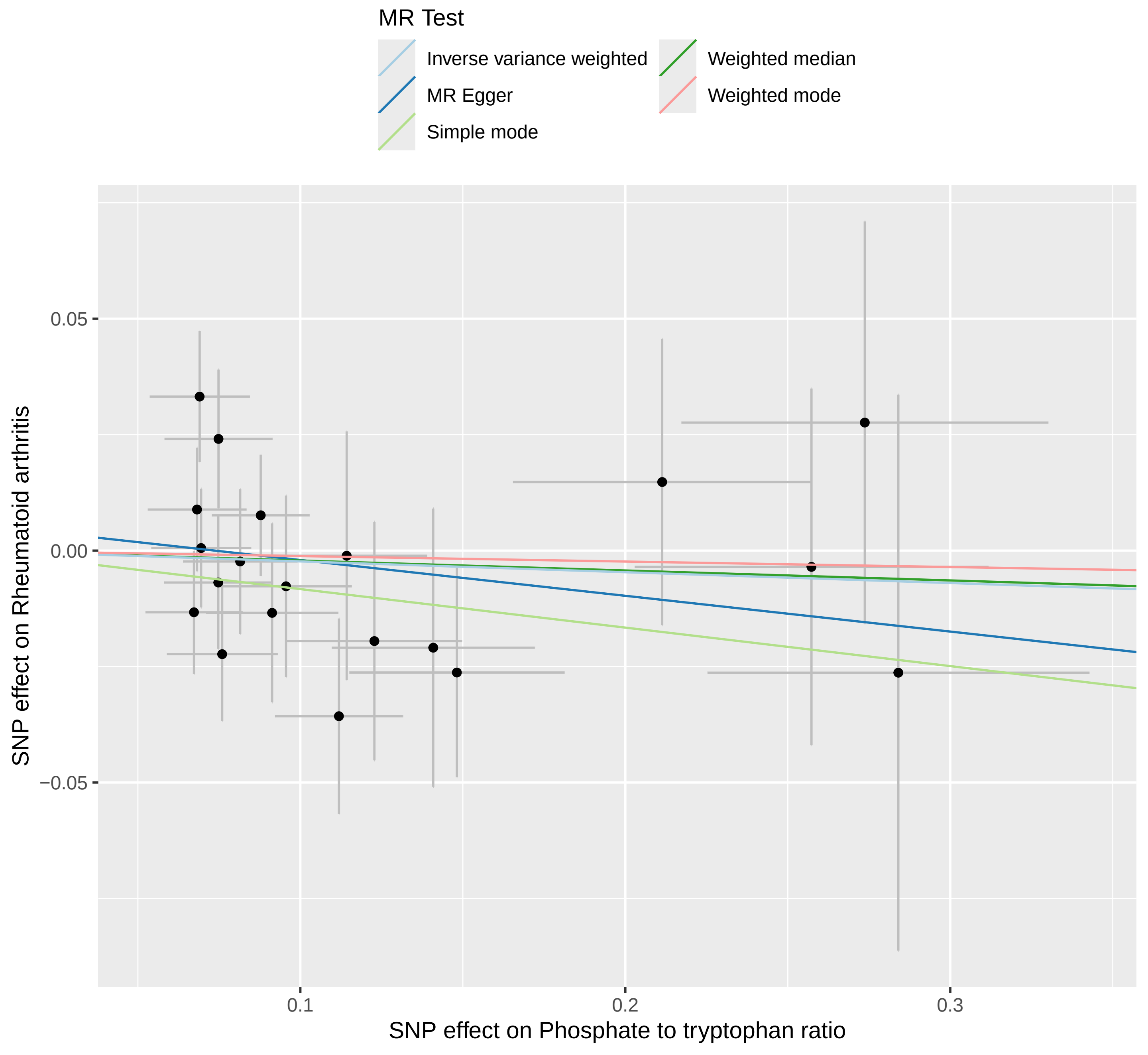
MR Test
Inverse variance weighted
MR Egger
Simple mode
Weighted median
Weighted mode
0.05
0.00
−0.05
0.1
0.2
0.3
SNP effect on Rheumatoid arthritis
SNP effect on Phosphate to tryptophan ratio

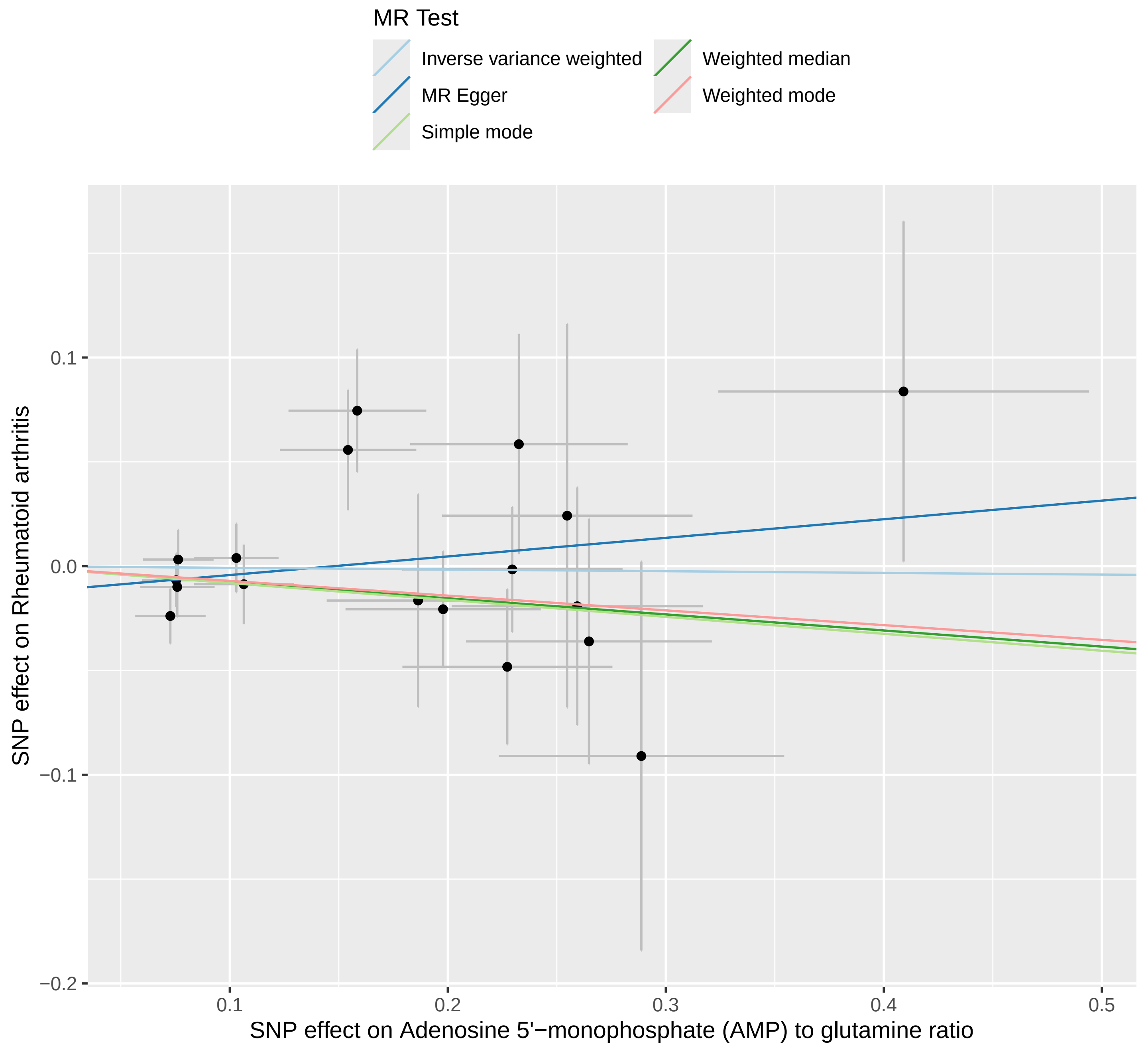

MR Test
Inverse variance weighted
MR Egger
Simple mode
Weighted median
Weighted mode
SNP effect on Rheumatoid arthritis
0.1
0.0
−0.1
−0.2
0.1
0.2
0.3
0.4
0.5
SNP effect on Adenosine 5'−monophosphate (AMP) to glutamine ratio

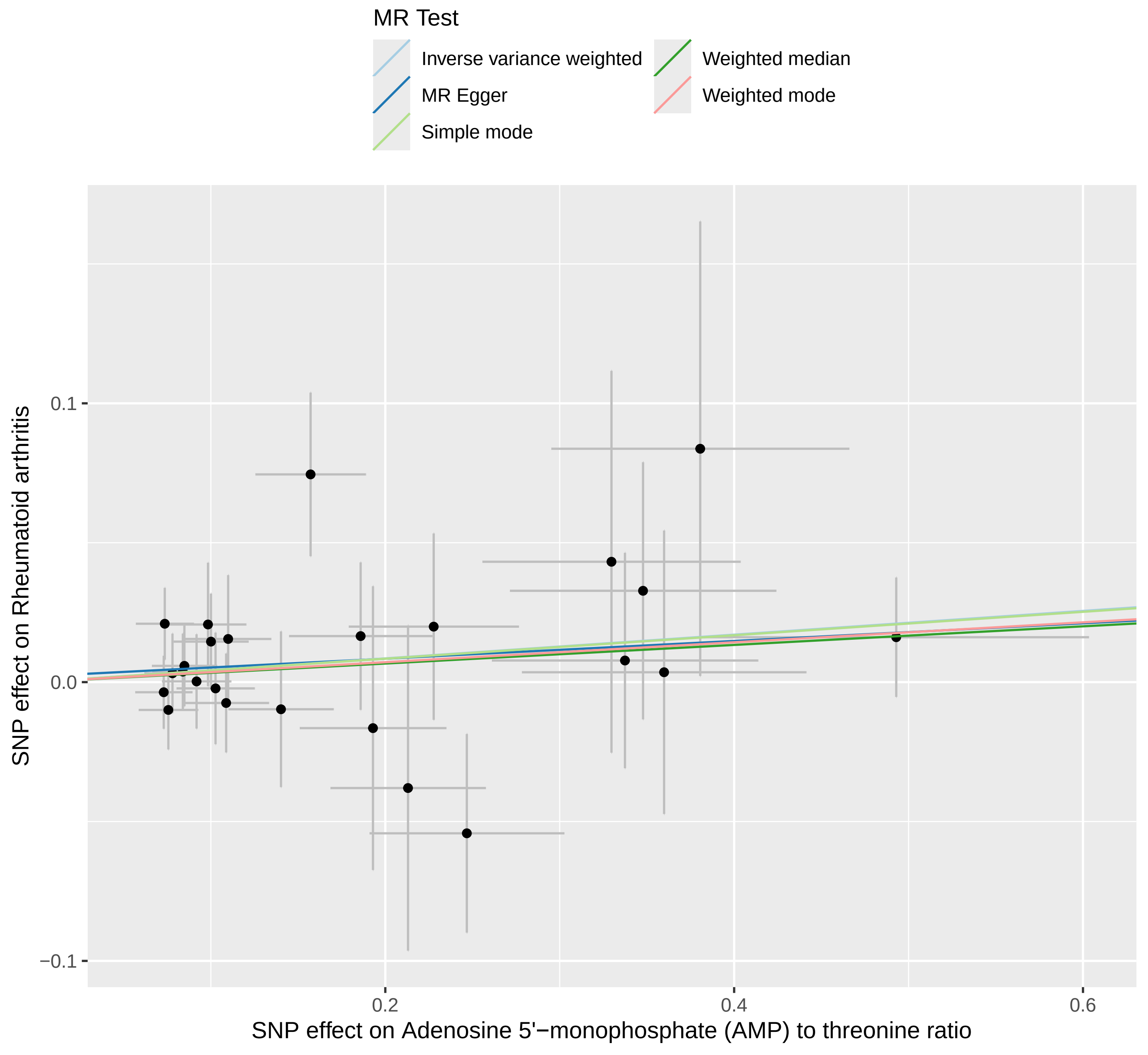
MR Test
Inverse variance weighted
MR Egger
Simple mode
Weighted median
Weighted mode
0.1
0.0
−0.1
0.2
0.4
0.6
SNP effect on Rheumatoid arthritis
SNP effect on Adenosine 5'−monophosphate (AMP) to threonine ratio

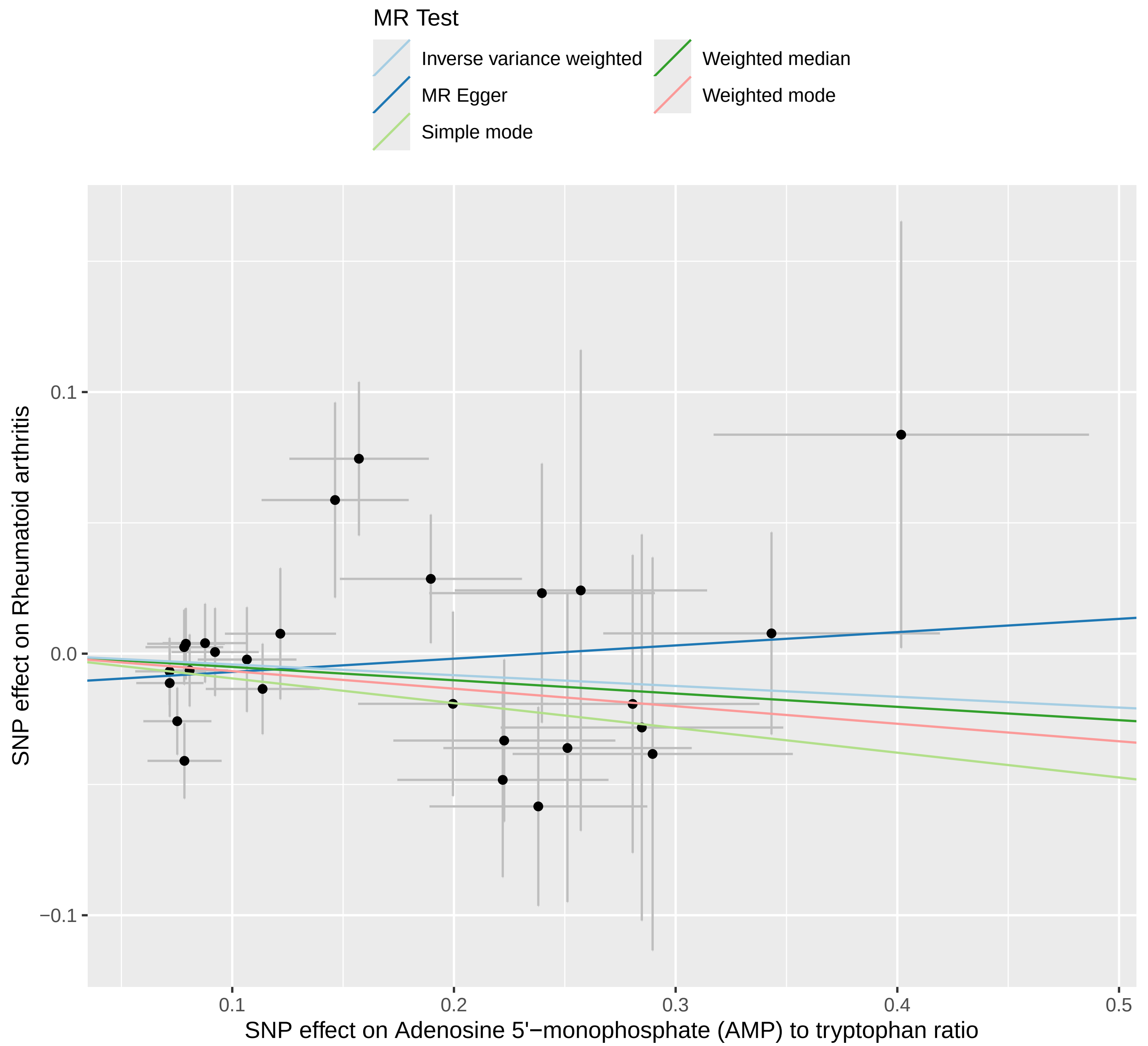
MR Test
Inverse variance weighted
MR Egger
Simple mode
Weighted median
Weighted mode
0.1
0.0
−0.1
0.1
0.2
0.3
0.4
0.5
SNP effect on Rheumatoid arthritis
SNP effect on Adenosine 5'−monophosphate (AMP) to tryptophan ratio

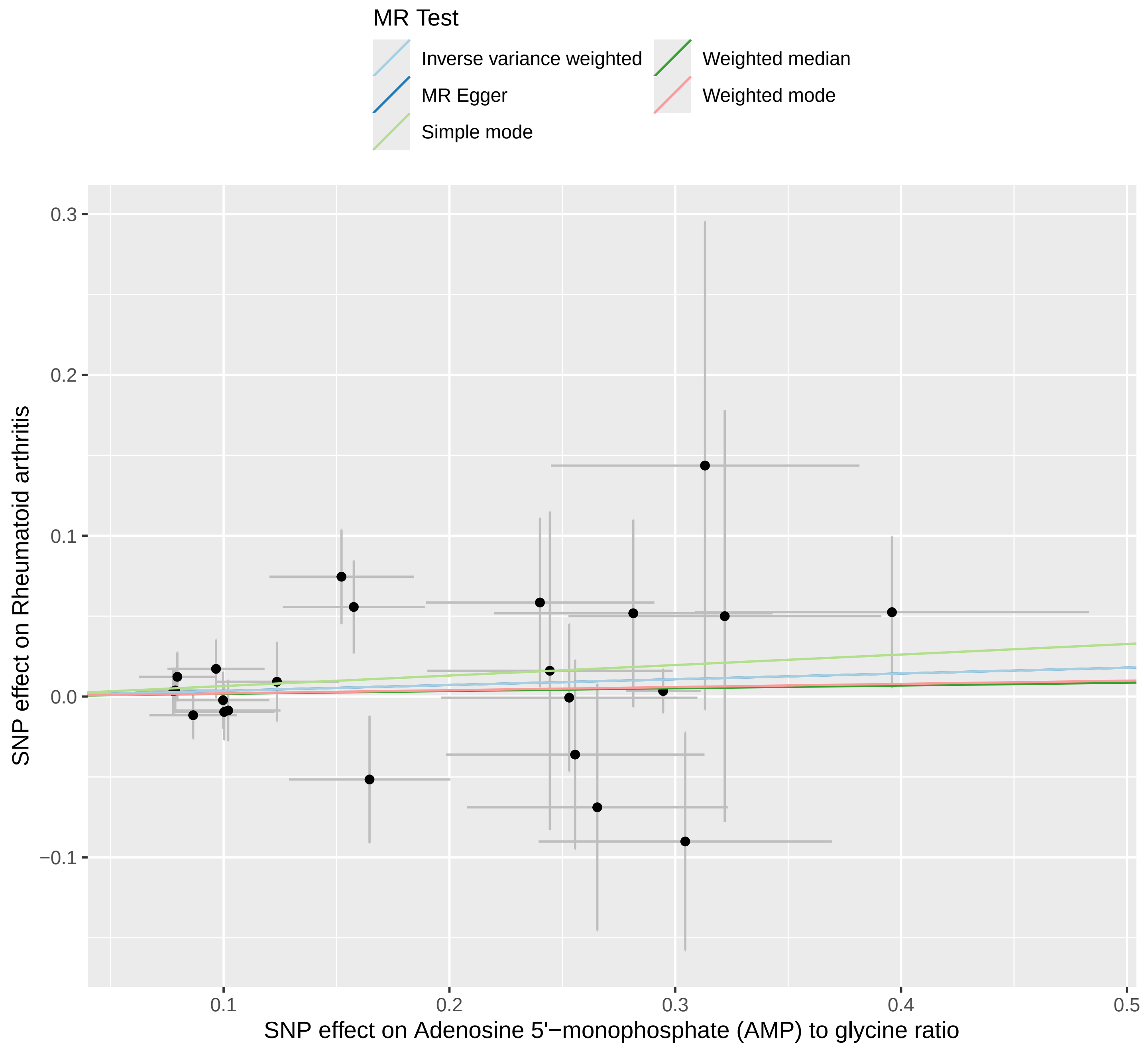

MR Test
Inverse variance weighted
MR Egger
Simple mode
Weighted median
Weighted mode
0.3
0.2
0.1
0.0
−0.1
SNP effect on Rheumatoid arthritis
0.1
0.2
0.3
0.4
0.5
SNP effect on Adenosine 5'−monophosphate (AMP) to glycine ratio

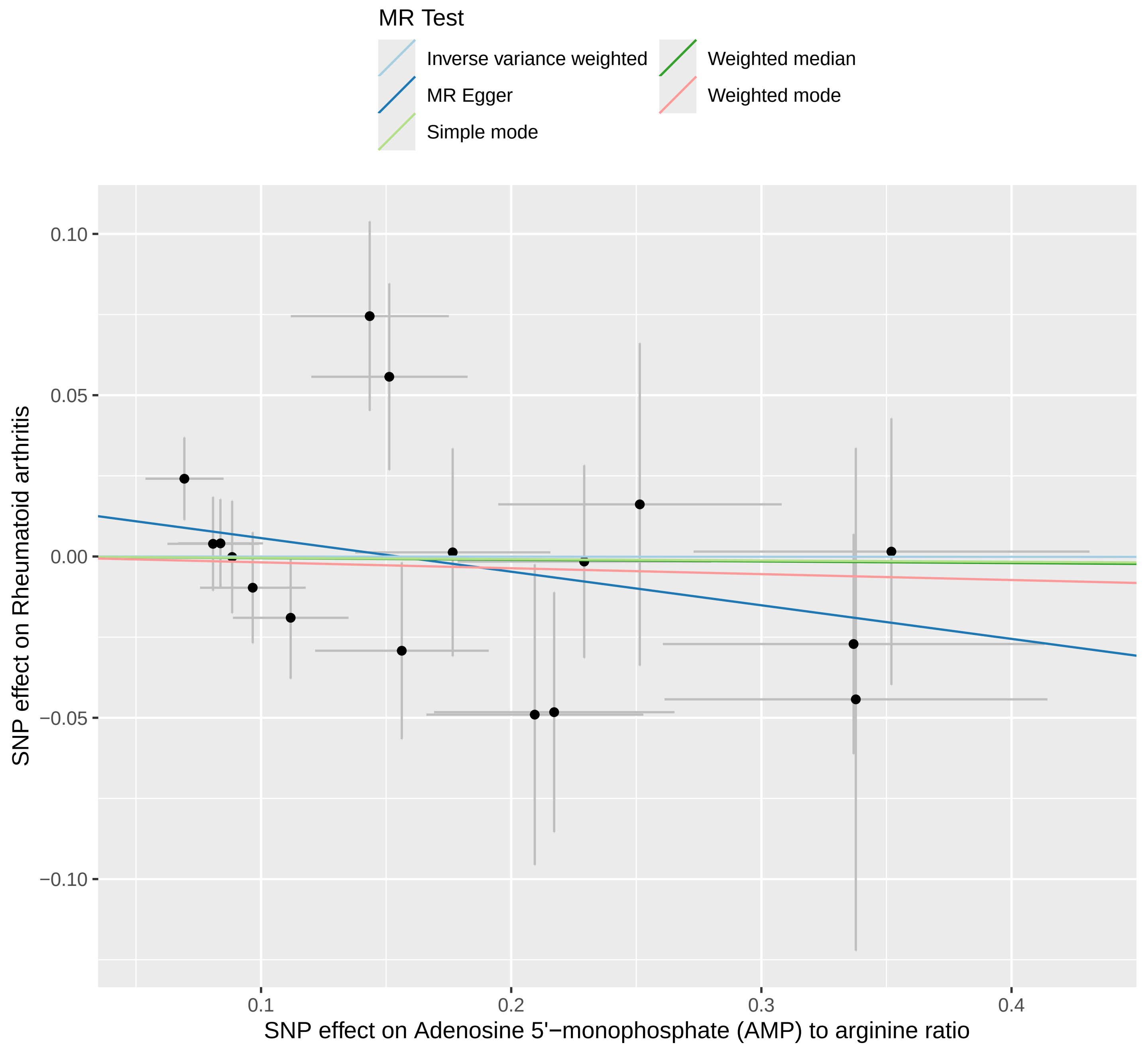

MR Test
Inverse variance weighted
MR Egger
Simple mode
Weighted median
Weighted mode
SNP effect on Rheumatoid arthritis
0.10
0.05
0.00
−0.05
−0.10
0.1
0.2
0.3
0.4
SNP effect on Adenosine 5'−monophosphate (AMP) to arginine ratio

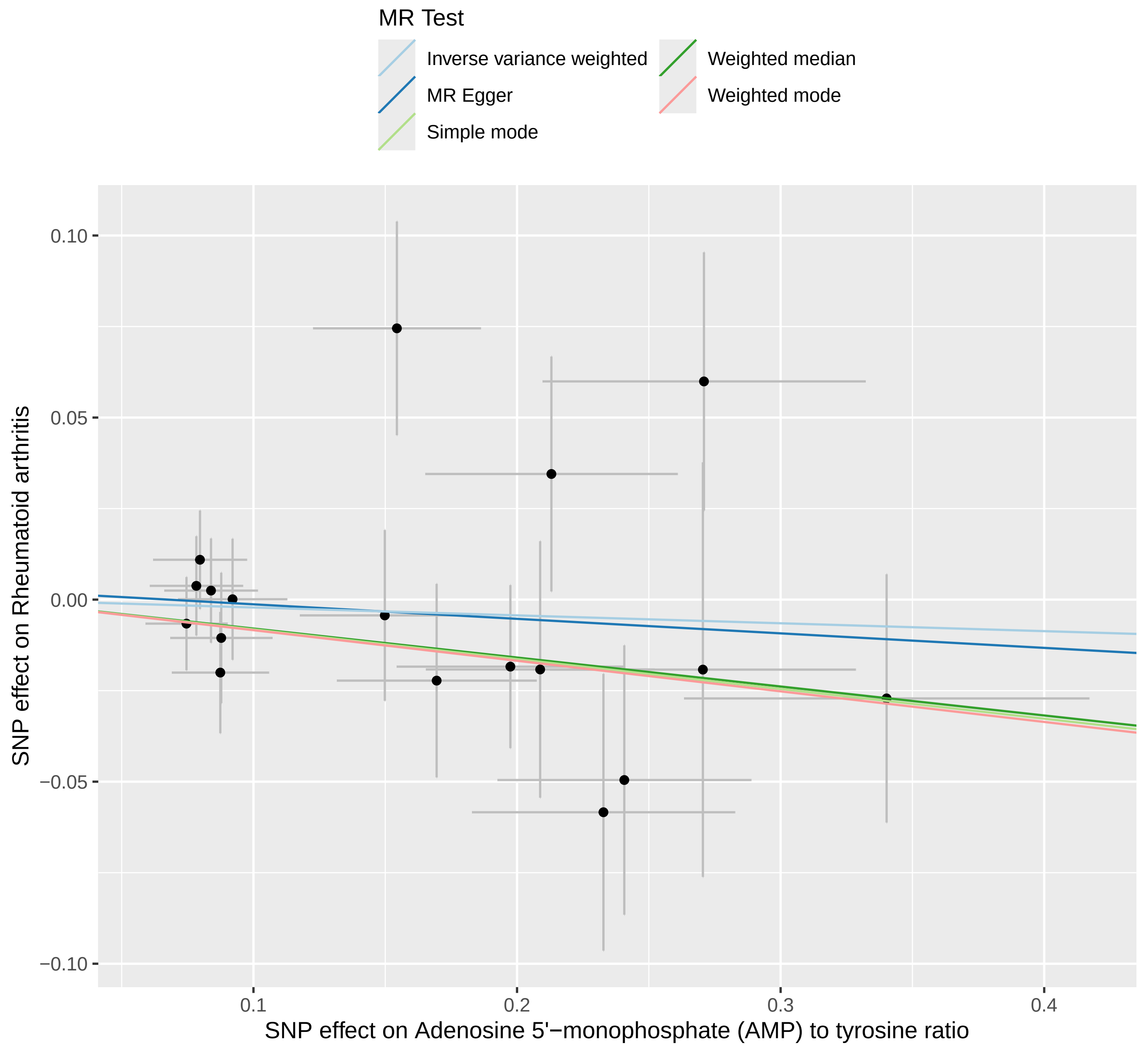
MR Test
Inverse variance weighted
MR Egger
Simple mode
Weighted median
Weighted mode
0.10
0.05
0.00
−0.05
−0.10
0.1
0.2
0.3
0.4
SNP effect on Rheumatoid arthritis
SNP effect on Adenosine 5'−monophosphate (AMP) to tyrosine ratio

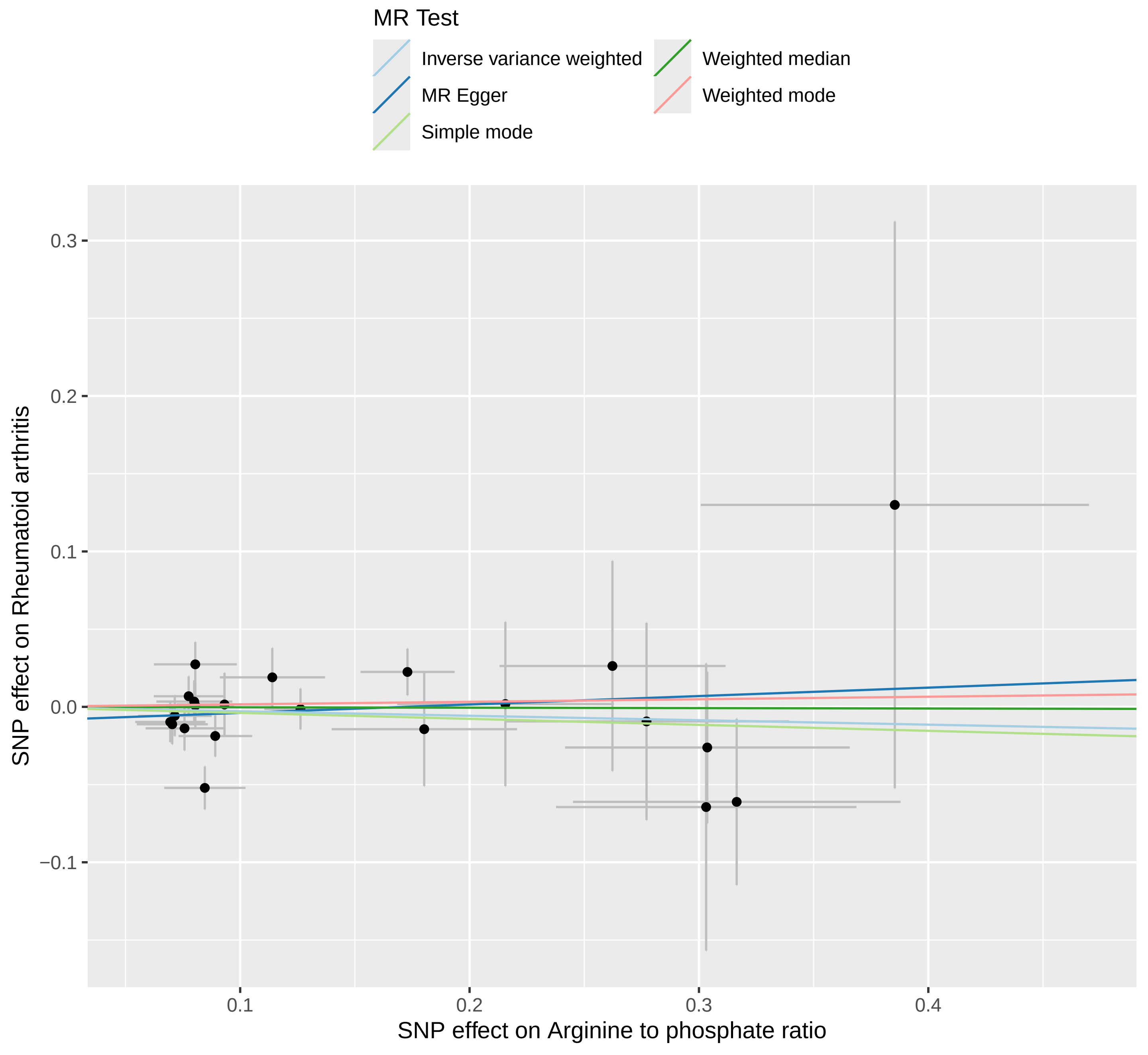
MR Test
Inverse variance weighted
MR Egger
Simple mode
Weighted median
Weighted mode
SNP effect on Rheumatoid arthritis
SNP effect on Arginine to phosphate ratio
0.3
0.2
0.1
0.0
−0.1
0.1
0.2
0.3
0.4

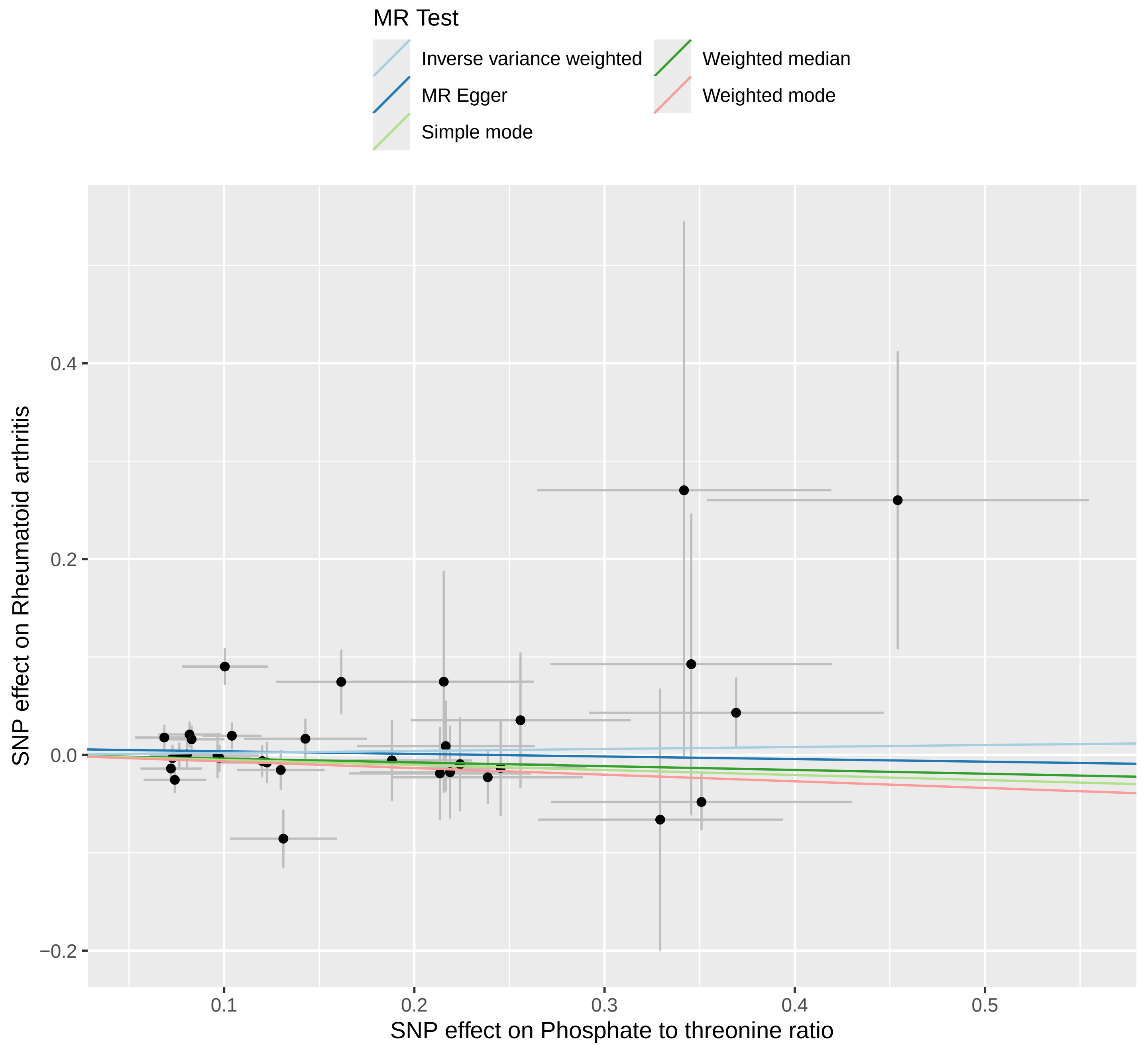

MR Test
Inverse variance weighted
MR Egger
Simple mode
Weighted median
Weighted mode
0.4
0.2
0.0
−0.2
0.1
0.2
0.3
0.4
0.5
SNP effect on Rheumatoid arthritis
SNP effect on Phosphate to threonine ratio

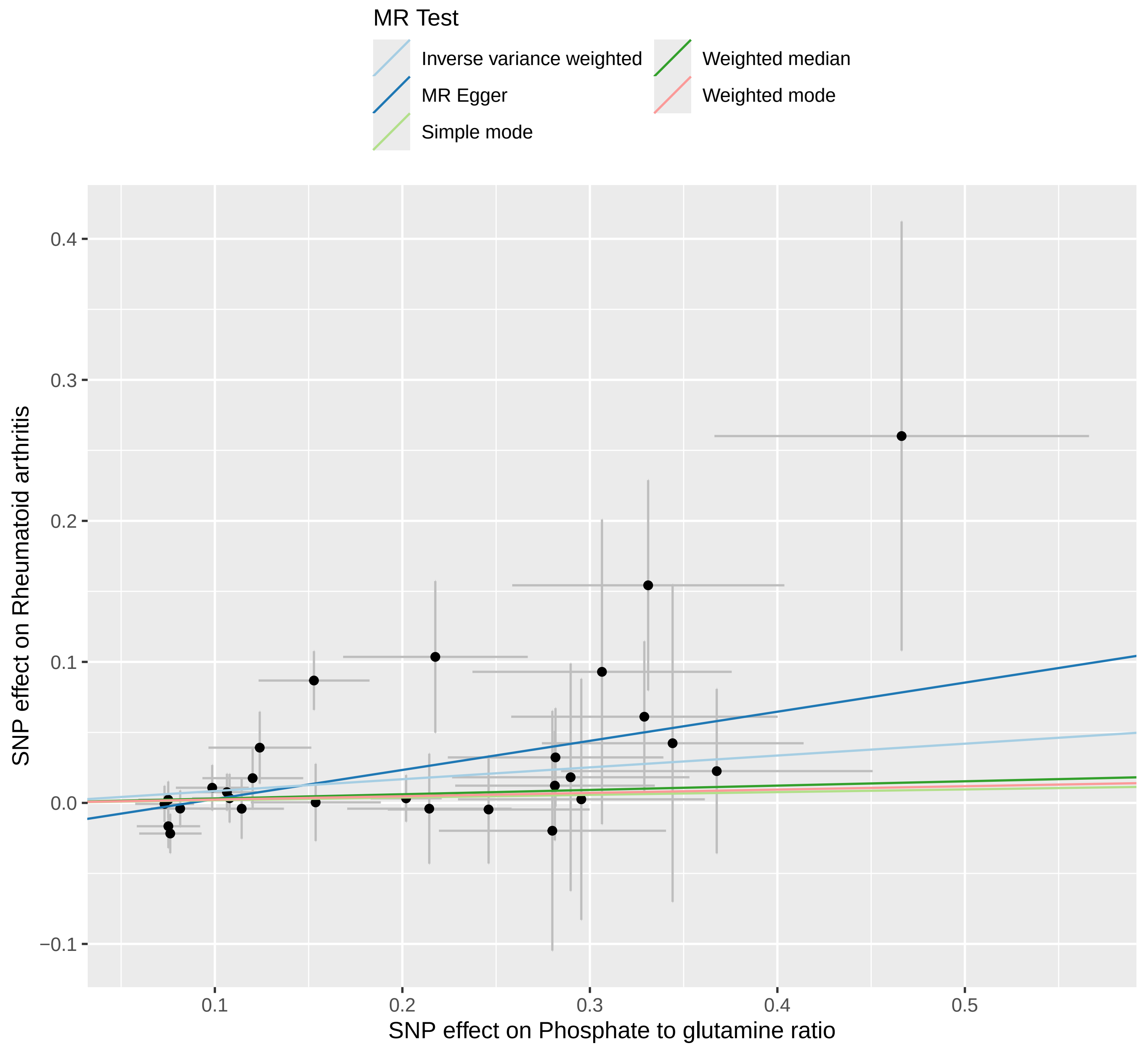

MR Test
Inverse variance weighted
MR Egger
Simple mode
Weighted median
Weighted mode
0.4
0.3
0.2
0.1
0.0
−0.1
SNP effect on Rheumatoid arthritis
0.1
0.2
0.3
0.4
0.5
SNP effect on Phosphate to glutamine ratio

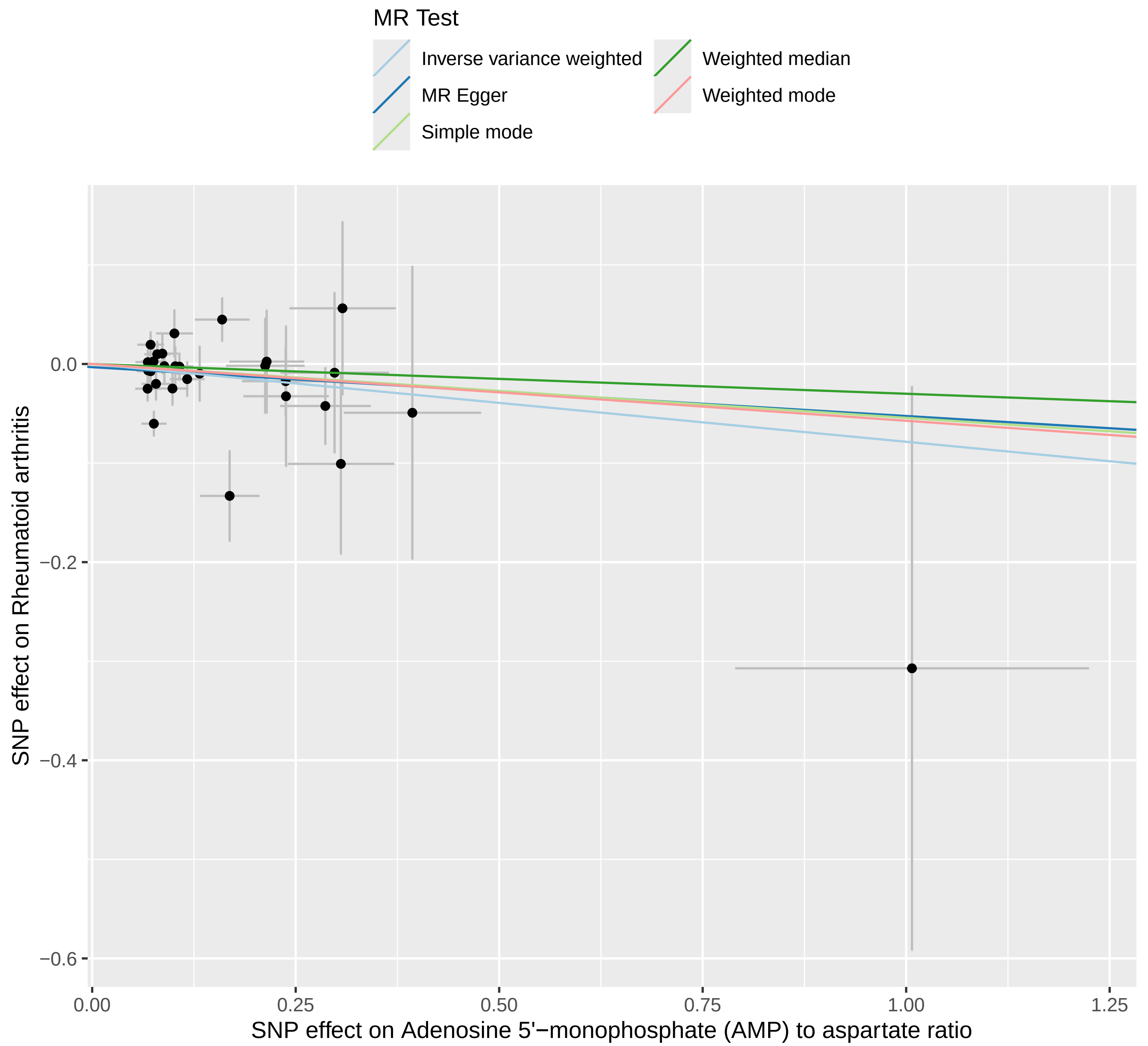

MR Test
Inverse variance weighted
MR Egger
Simple mode
Weighted median
Weighted mode
0.0
−0.2
−0.4
−0.6
0.00
0.25
0.50
0.75
1.00
1.25
SNP effect on Rheumatoid arthritis
SNP effect on Adenosine 5'−monophosphate (AMP) to aspartate ratio

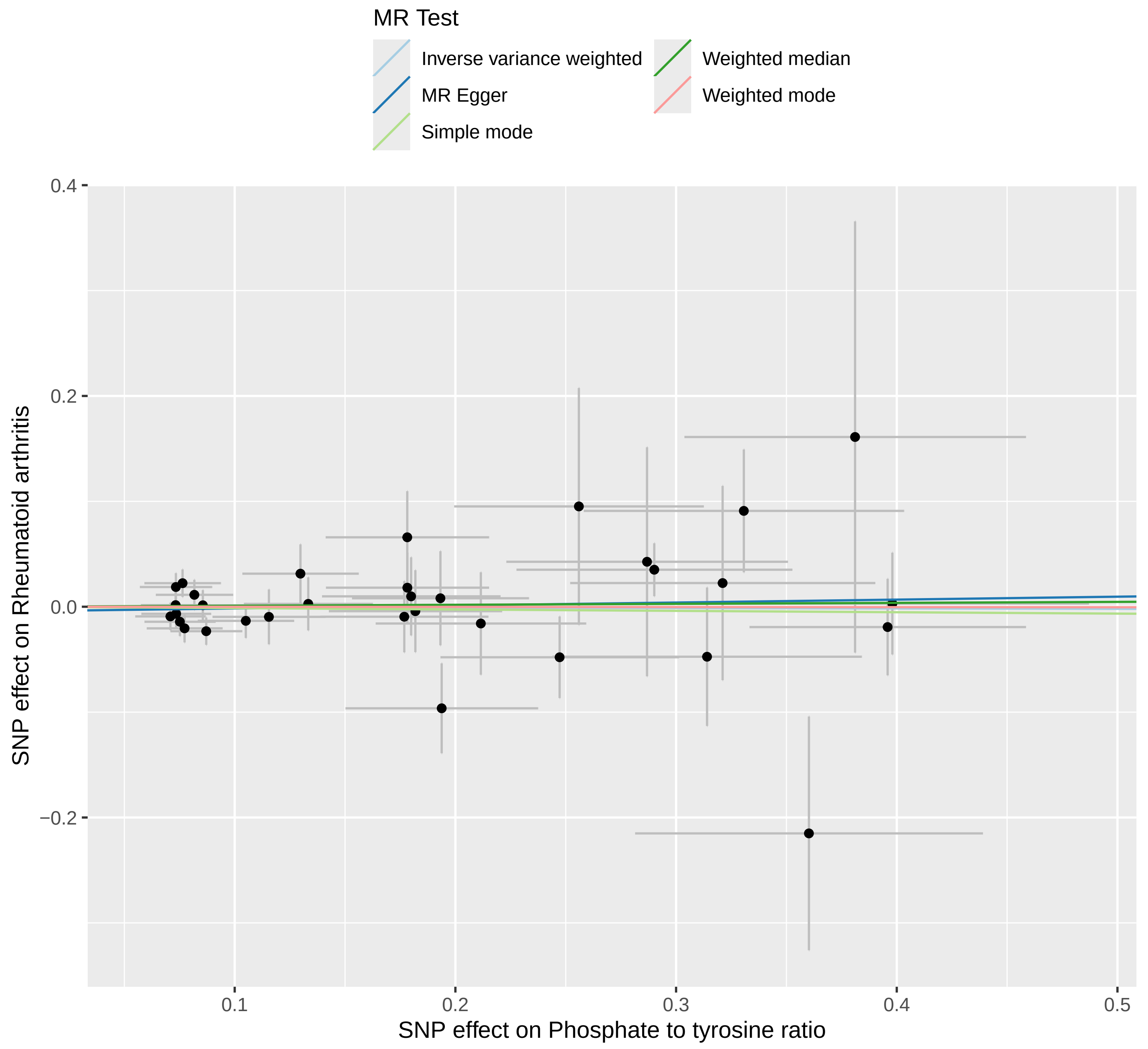

MR Test
Inverse variance weighted
MR Egger
Simple mode
Weighted median
Weighted mode
0.4
0.2
0.0
−0.2
0.1
0.2
0.3
0.4
0.5
SNP effect on Rheumatoid arthritis
SNP effect on Phosphate to tyrosine ratio

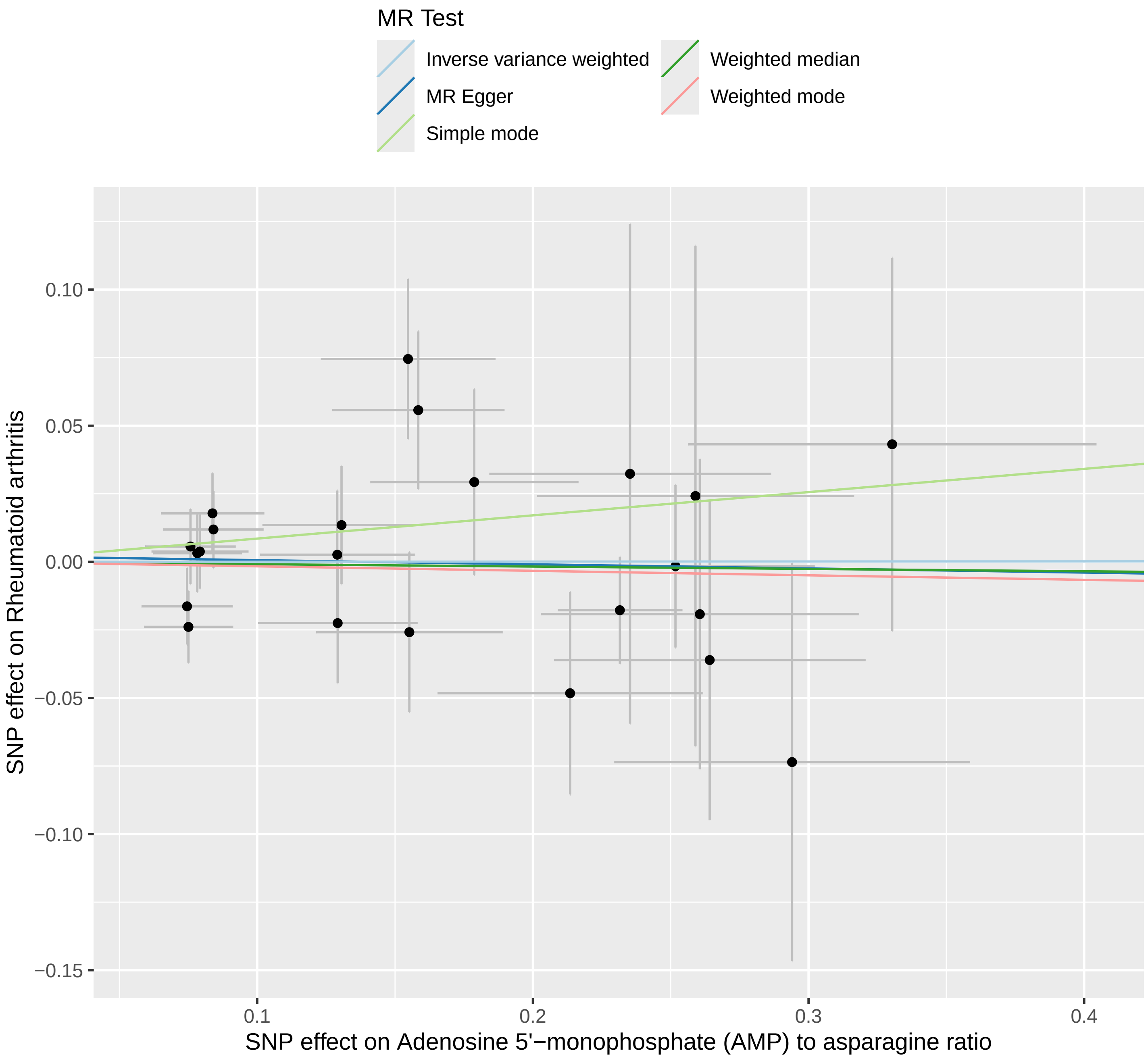
MR Test
Inverse variance weighted
MR Egger
Simple mode
Weighted median
Weighted mode
SNP effect on Rheumatoid arthritis
0.10
0.05
0.00
−0.05
−0.10
−0.15
0.1
0.2
0.3
0.4
SNP effect on Adenosine 5'−monophosphate (AMP) to asparagine ratio

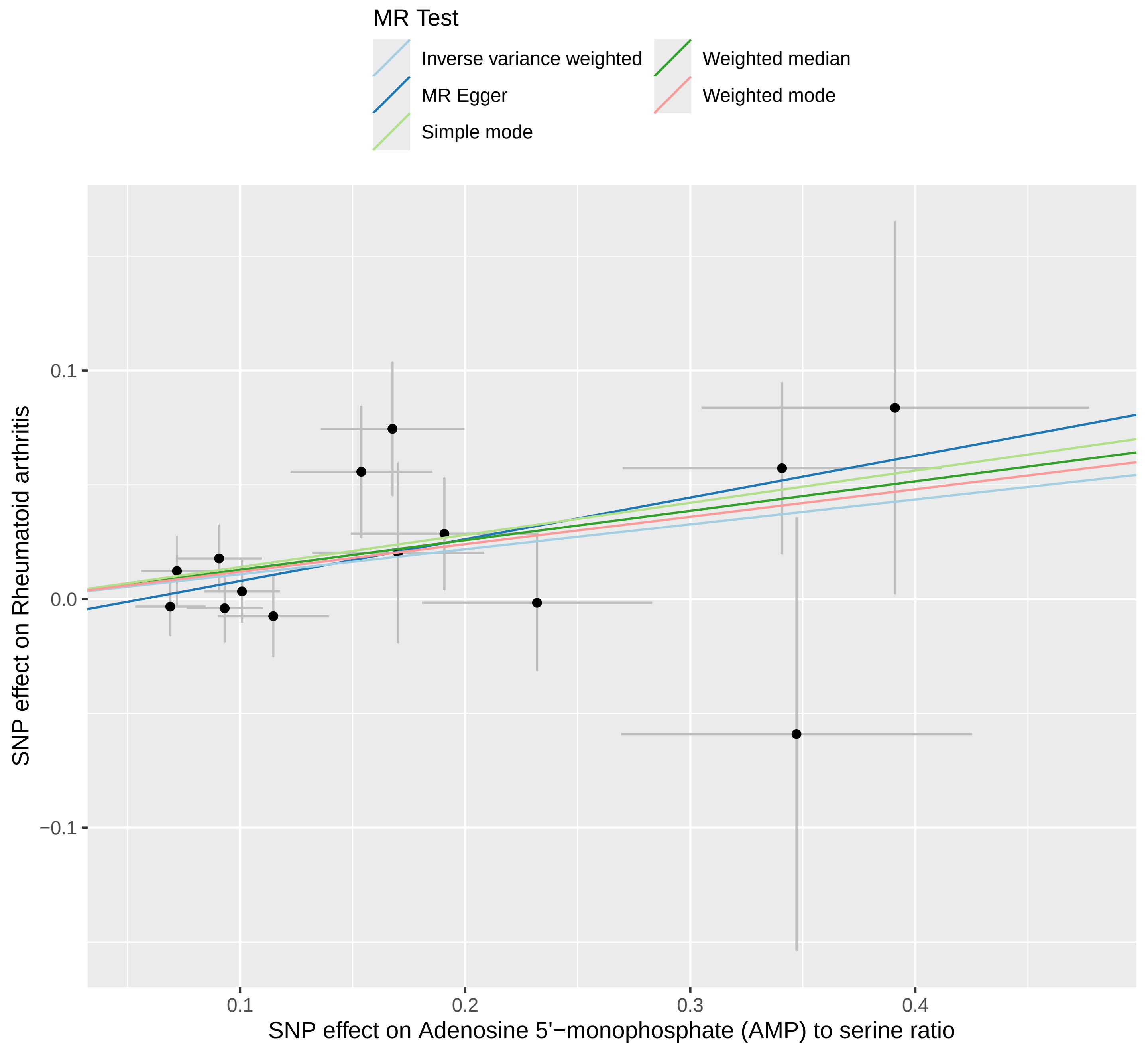
MR Test
Inverse variance weighted
MR Egger
Simple mode
Weighted median
Weighted mode
0.1
0.0
−0.1
0.1
0.2
0.3
0.4
SNP effect on Rheumatoid arthritis
SNP effect on Adenosine 5'−monophosphate (AMP) to serine ratio

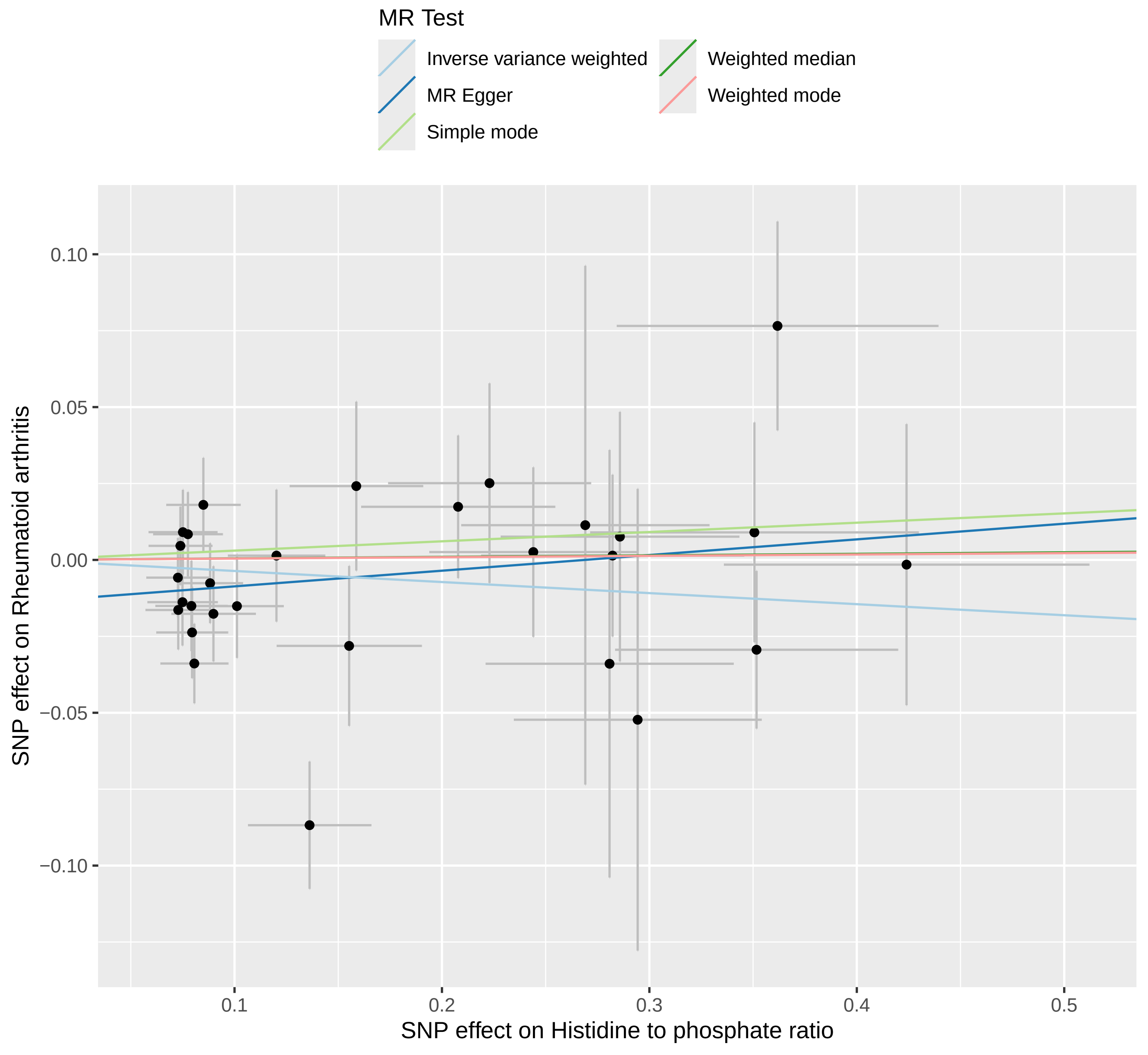

MR Test
Inverse variance weighted
MR Egger
Simple mode
Weighted median
Weighted mode
0.10
0.05
0.00
−0.05
−0.10
SNP effect on Rheumatoid arthritis
0.1
0.2
0.3
0.4
0.5
SNP effect on Histidine to phosphate ratio

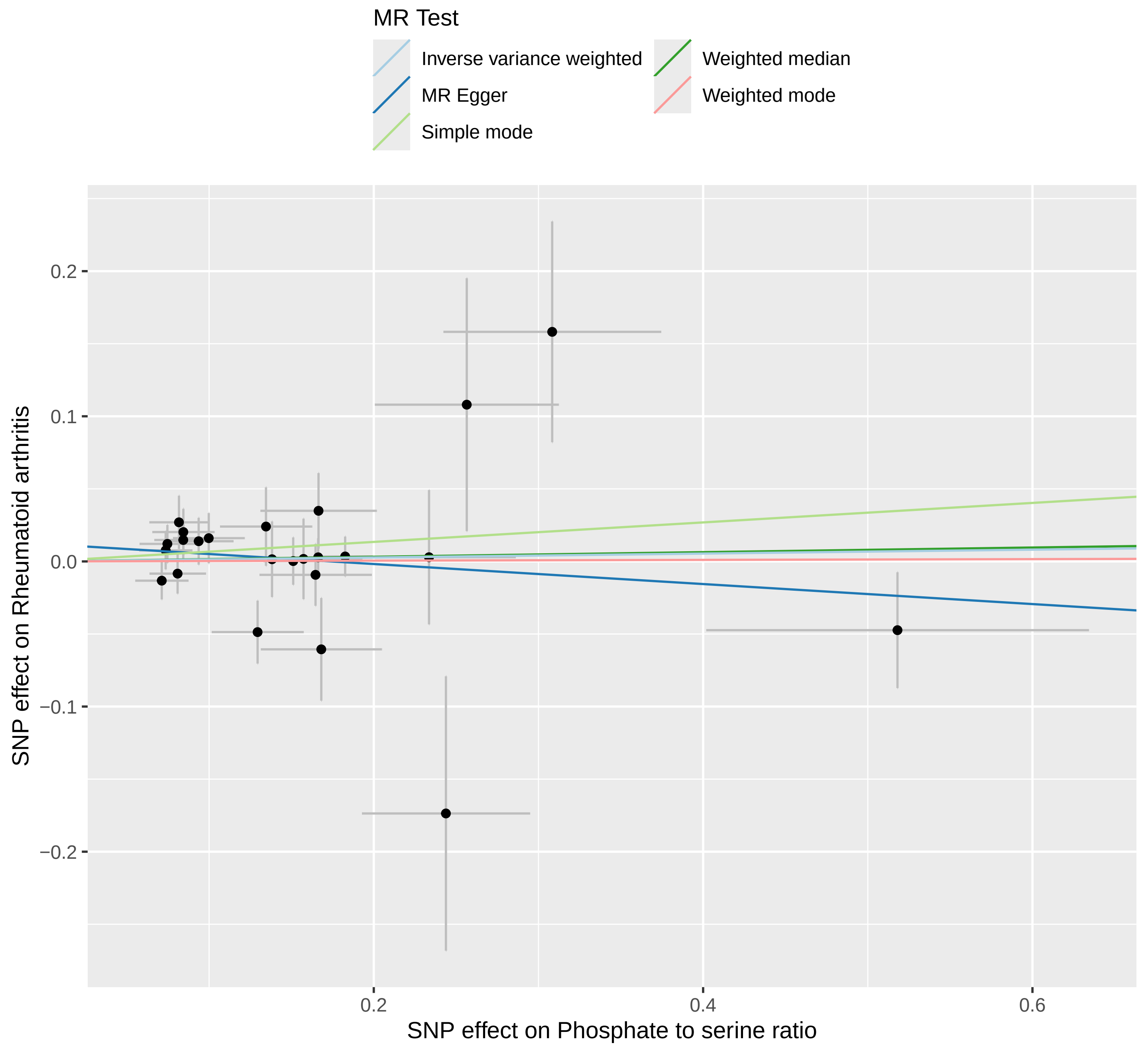
MR Test
Inverse variance weighted
MR Egger
Simple mode
Weighted median
Weighted mode
0.2
0.1
0.0
−0.1
−0.2
SNP effect on Rheumatoid arthritis
0.2
0.4
0.6
SNP effect on Phosphate to serine ratio

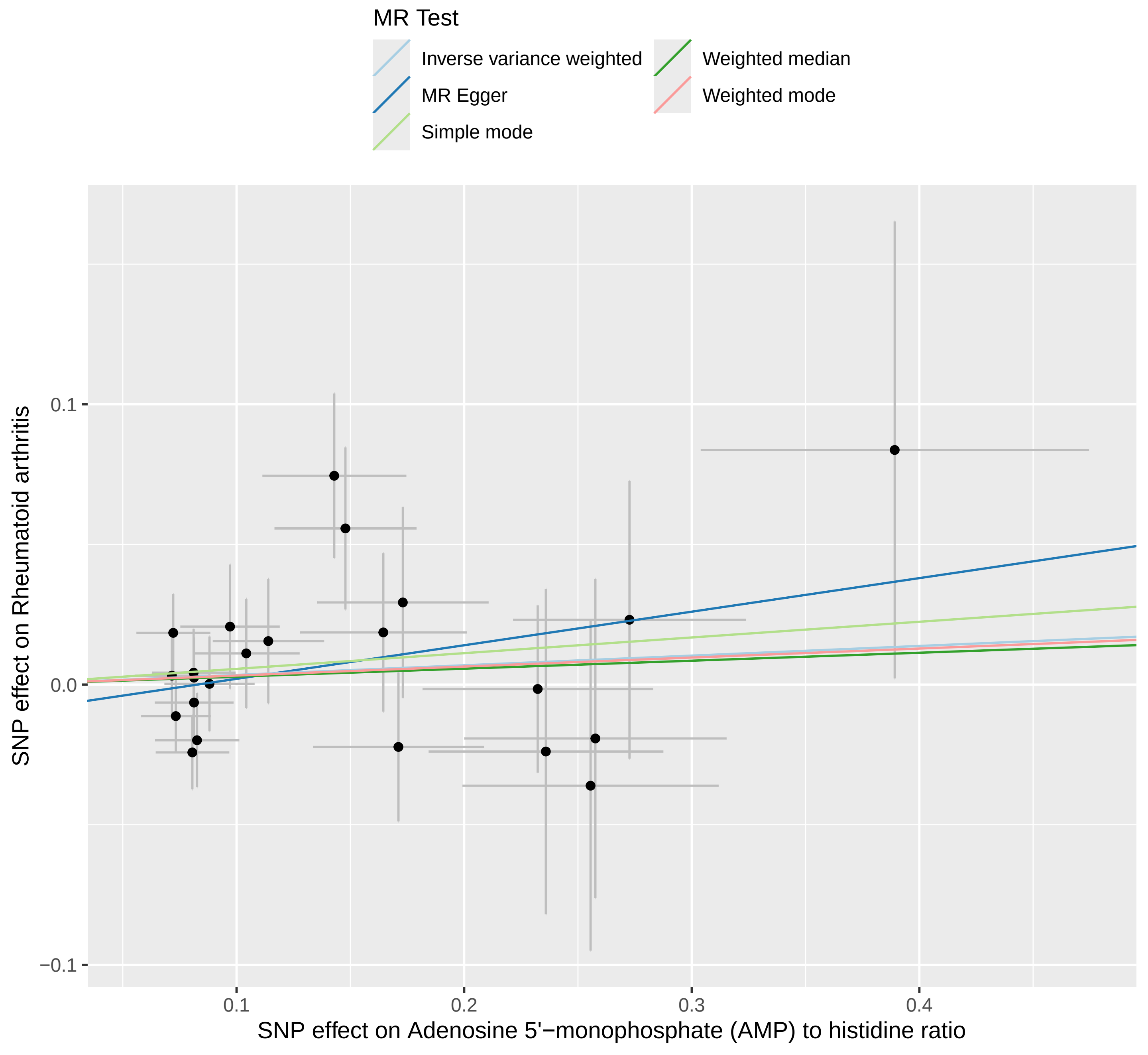

MR Test
Inverse variance weighted
MR Egger
Simple mode
Weighted median
Weighted mode
0.1
0.0
−0.1
SNP effect on Rheumatoid arthritis
0.1
0.2
0.3
0.4
SNP effect on Adenosine 5'−monophosphate (AMP) to histidine ratio

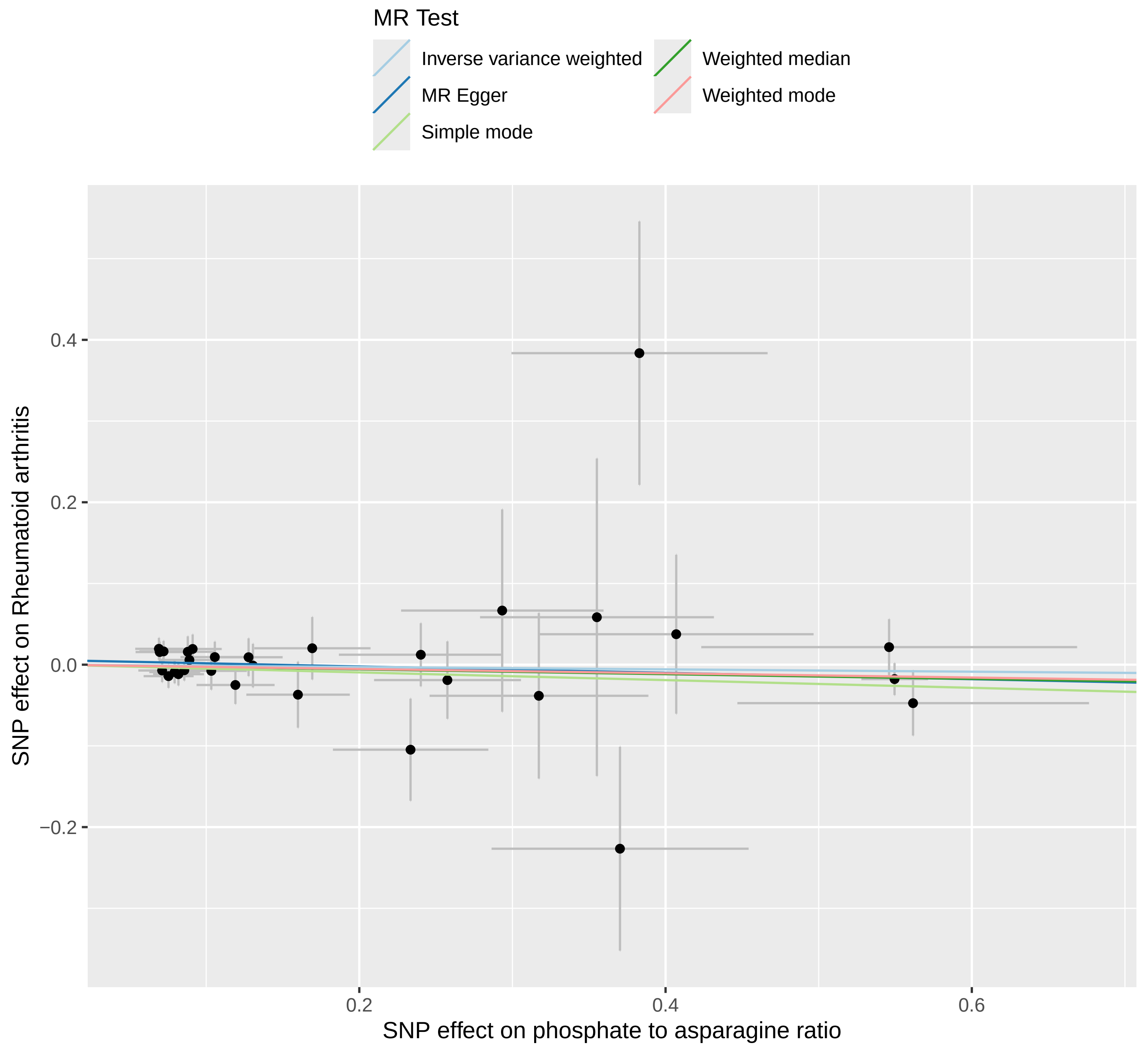

MR Test
Inverse variance weighted
MR Egger
Simple mode
Weighted median
Weighted mode
SNP effect on Rheumatoid arthritis
0.4
0.2
0.0
−0.2
0.2
0.4
0.6
SNP effect on phosphate to asparagine ratio

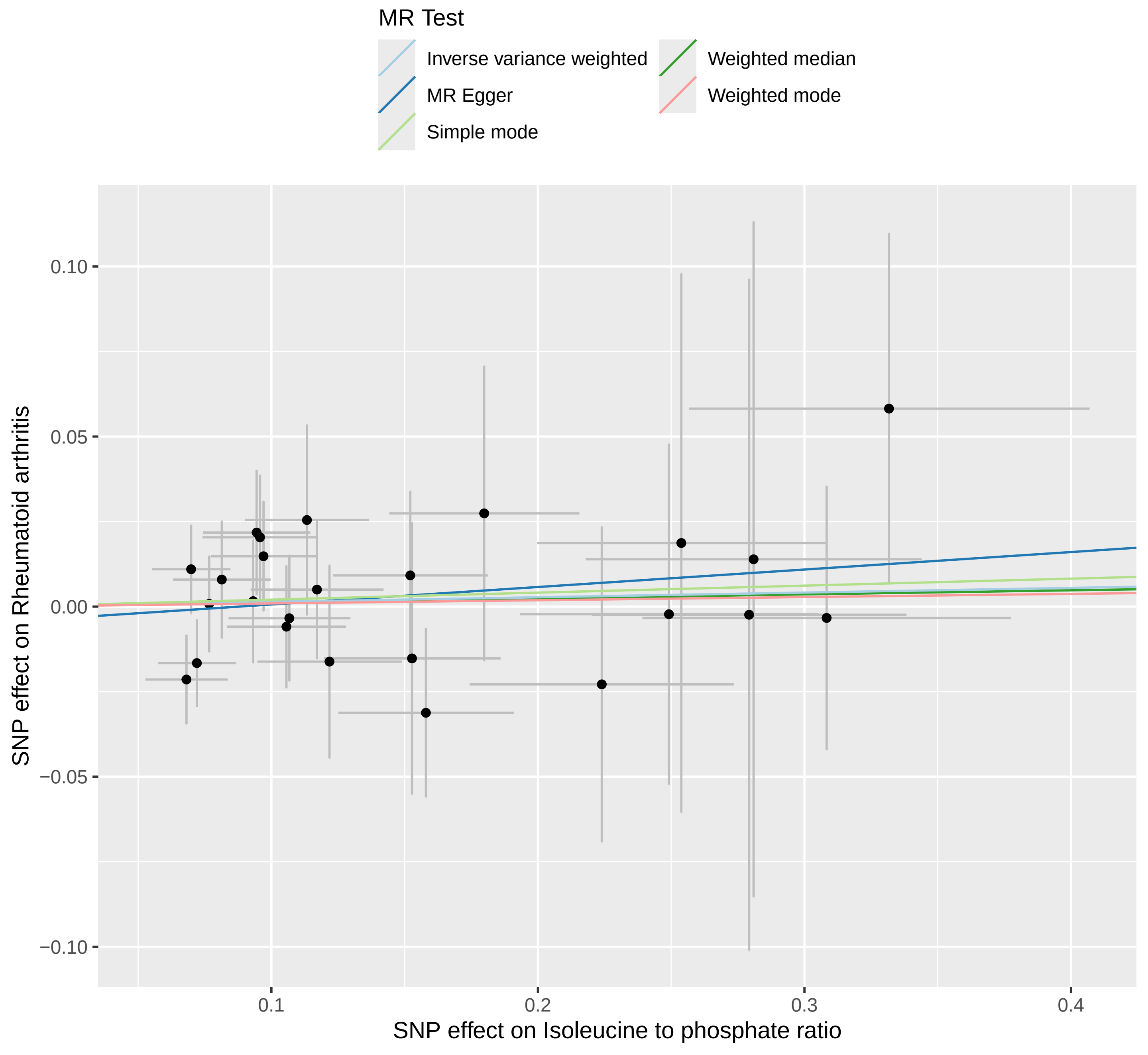

MR Test
Inverse variance weighted
MR Egger
Simple mode
Weighted median
Weighted mode
0.10
0.05
0.00
−0.05
−0.10
SNP effect on Rheumatoid arthritis
0.1
0.2
0.3
0.4
SNP effect on Isoleucine to phosphate ratio

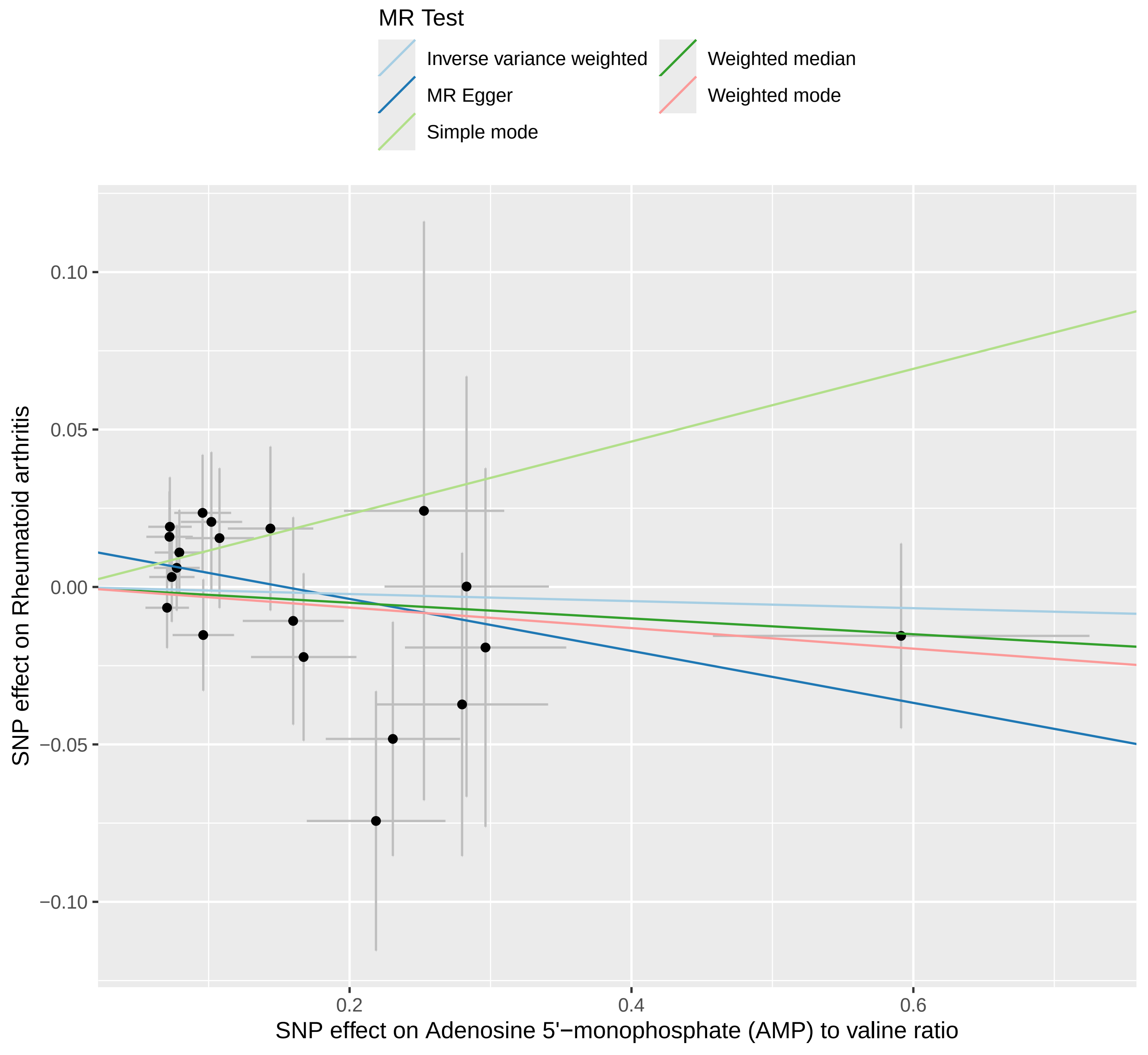
MR Test
Inverse variance weighted
MR Egger
Simple mode
Weighted median
Weighted mode
0.10
0.05
0.00
−0.05
−0.10
0.2
0.4
0.6
SNP effect on Rheumatoid arthritis
SNP effect on Adenosine 5'−monophosphate (AMP) to valine ratio

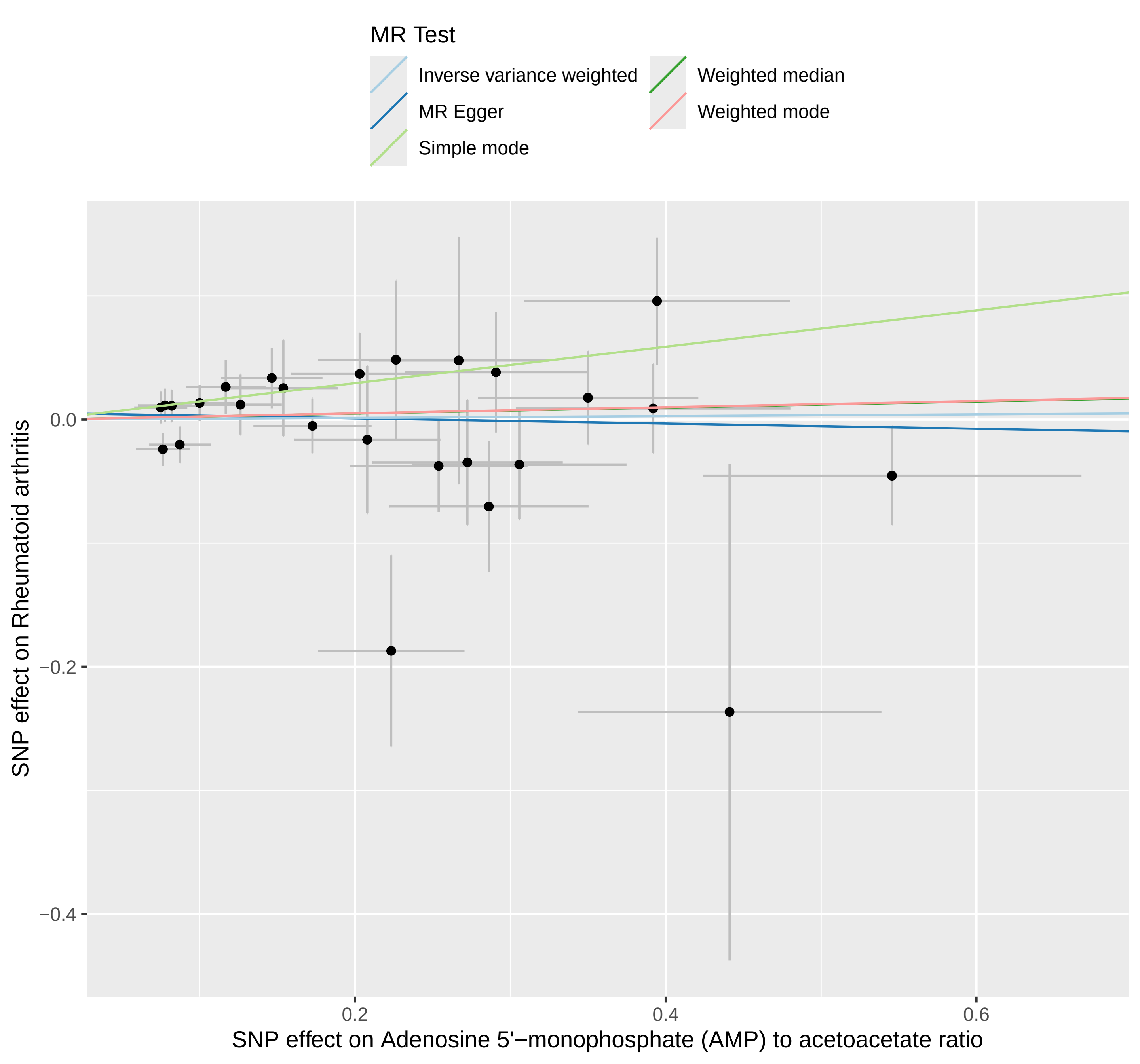
MR Test
Inverse variance weighted
MR Egger
Simple mode
Weighted median
Weighted mode
SNP effect on Rheumatoid arthritis
0.0
−0.2
−0.4
0.2
0.4
0.6
SNP effect on Adenosine 5'−monophosphate (AMP) to acetoacetate ratio

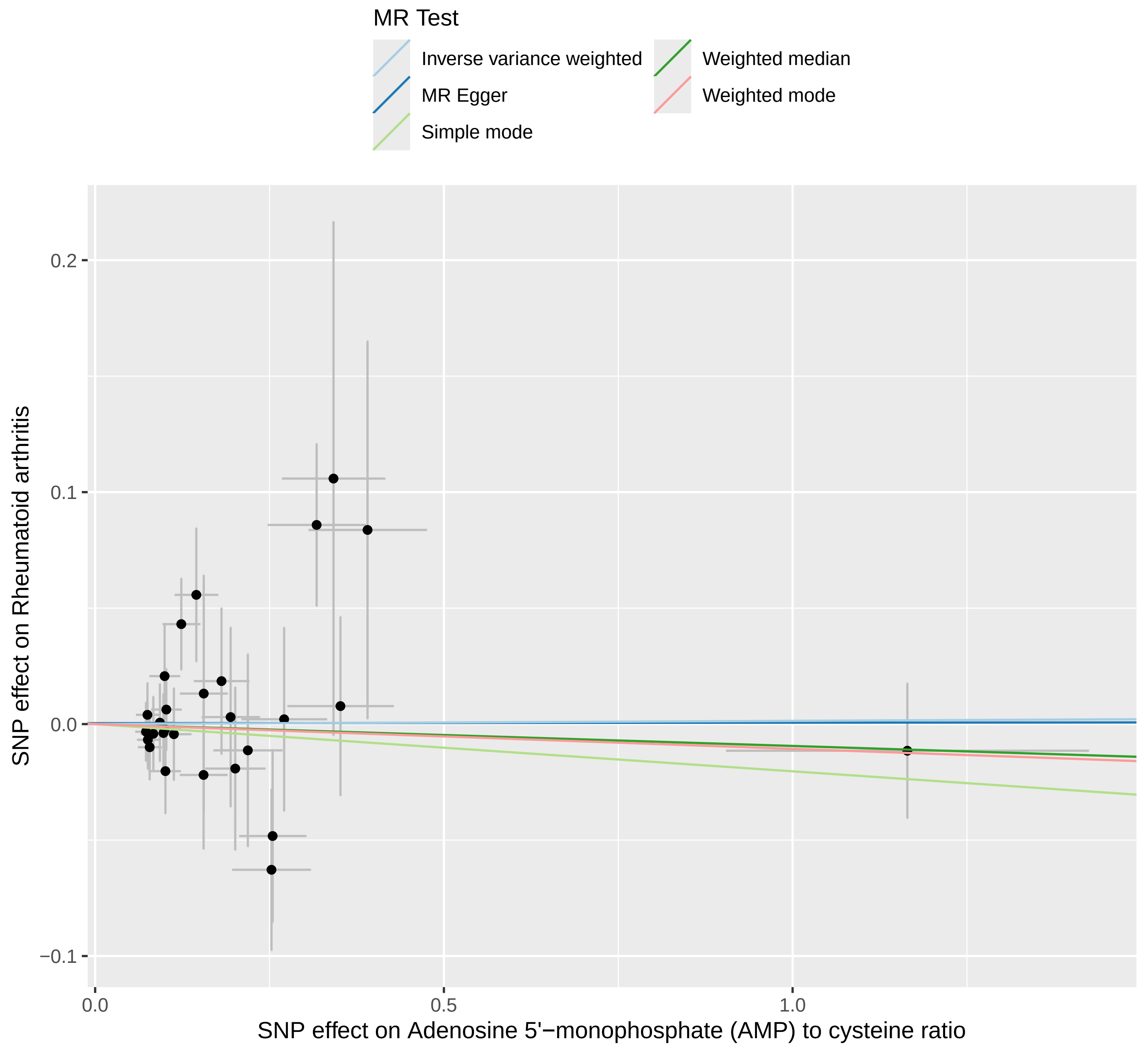
MR Test
Inverse variance weighted
MR Egger
Simple mode
Weighted median
Weighted mode
0.2
0.1
0.0
−0.1
0.0
0.5
1.0
SNP effect on Rheumatoid arthritis
SNP effect on Adenosine 5'−monophosphate (AMP) to cysteine ratio

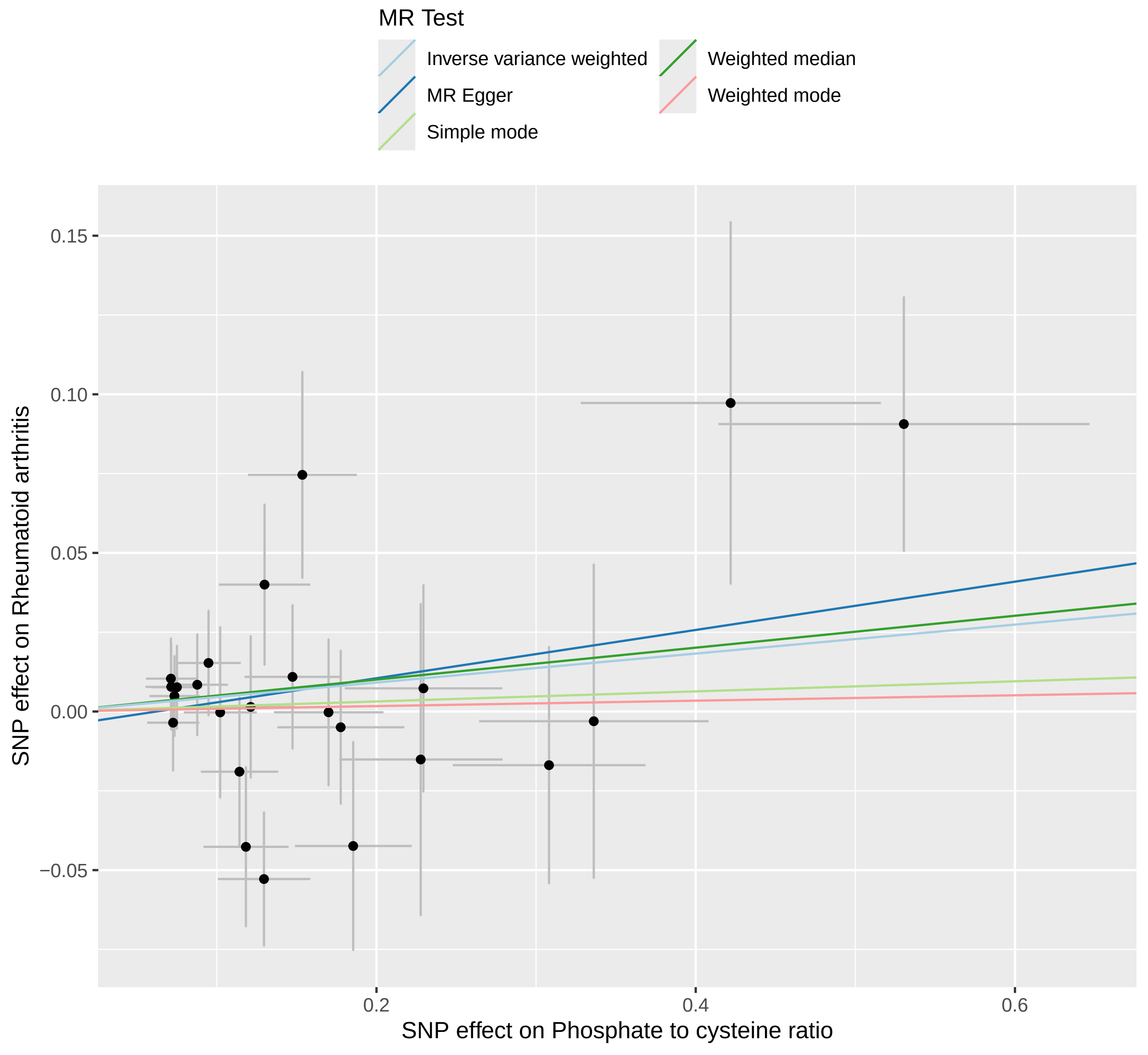

MR Test
Inverse variance weighted
MR Egger
Simple mode
Weighted median
Weighted mode
0.15
0.10
0.05
0.00
−0.05
0.2
0.4
0.6
SNP effect on Rheumatoid arthritis
SNP effect on Phosphate to cysteine ratio

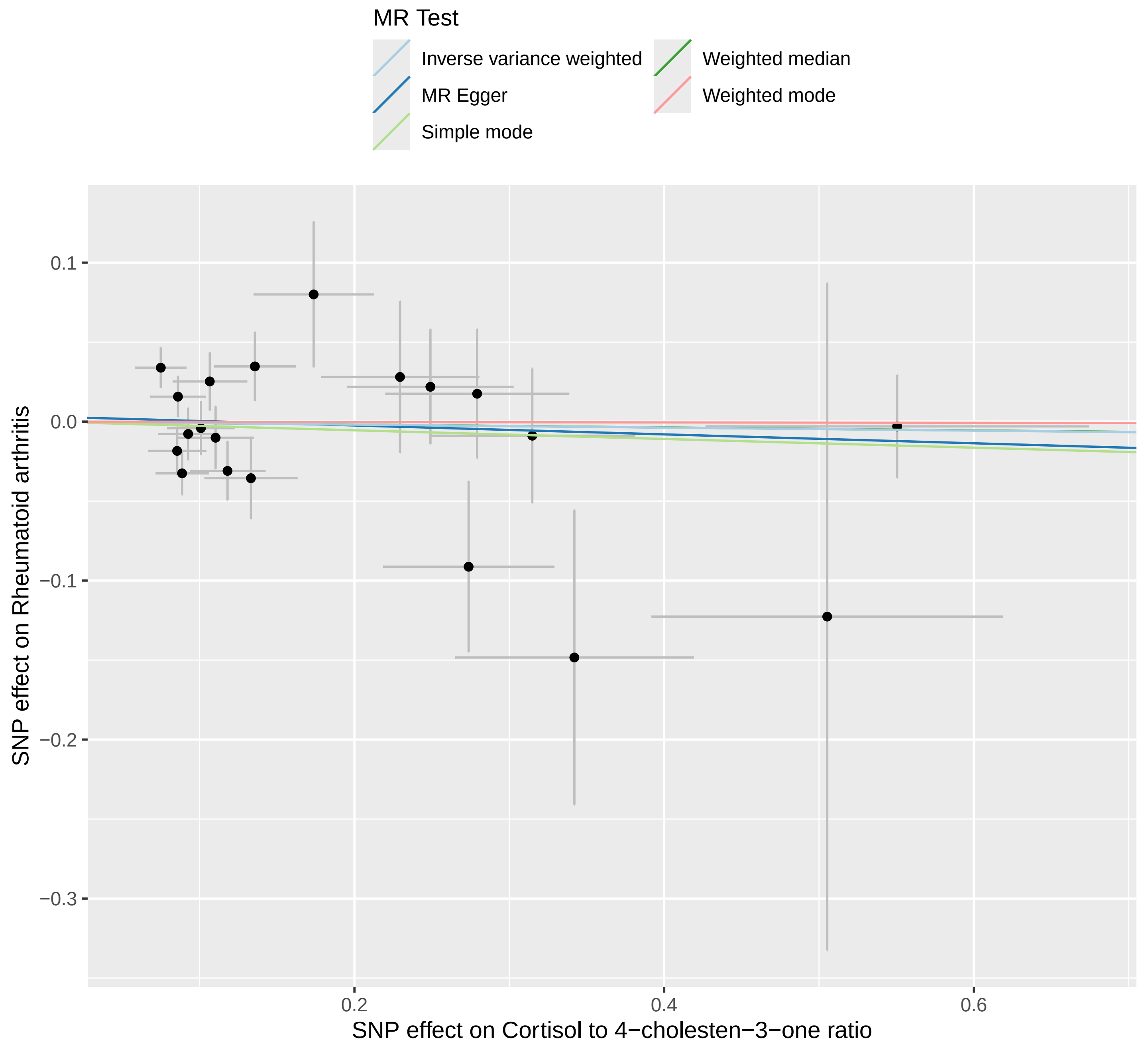
MR Test
Inverse variance weighted
MR Egger
Simple mode
Weighted median
Weighted mode
0.1
0.0
−0.1
−0.2
−0.3
SNP effect on Rheumatoid arthritis
0.2
0.4
0.6
SNP effect on Cortisol to 4−cholesten−3−one ratio

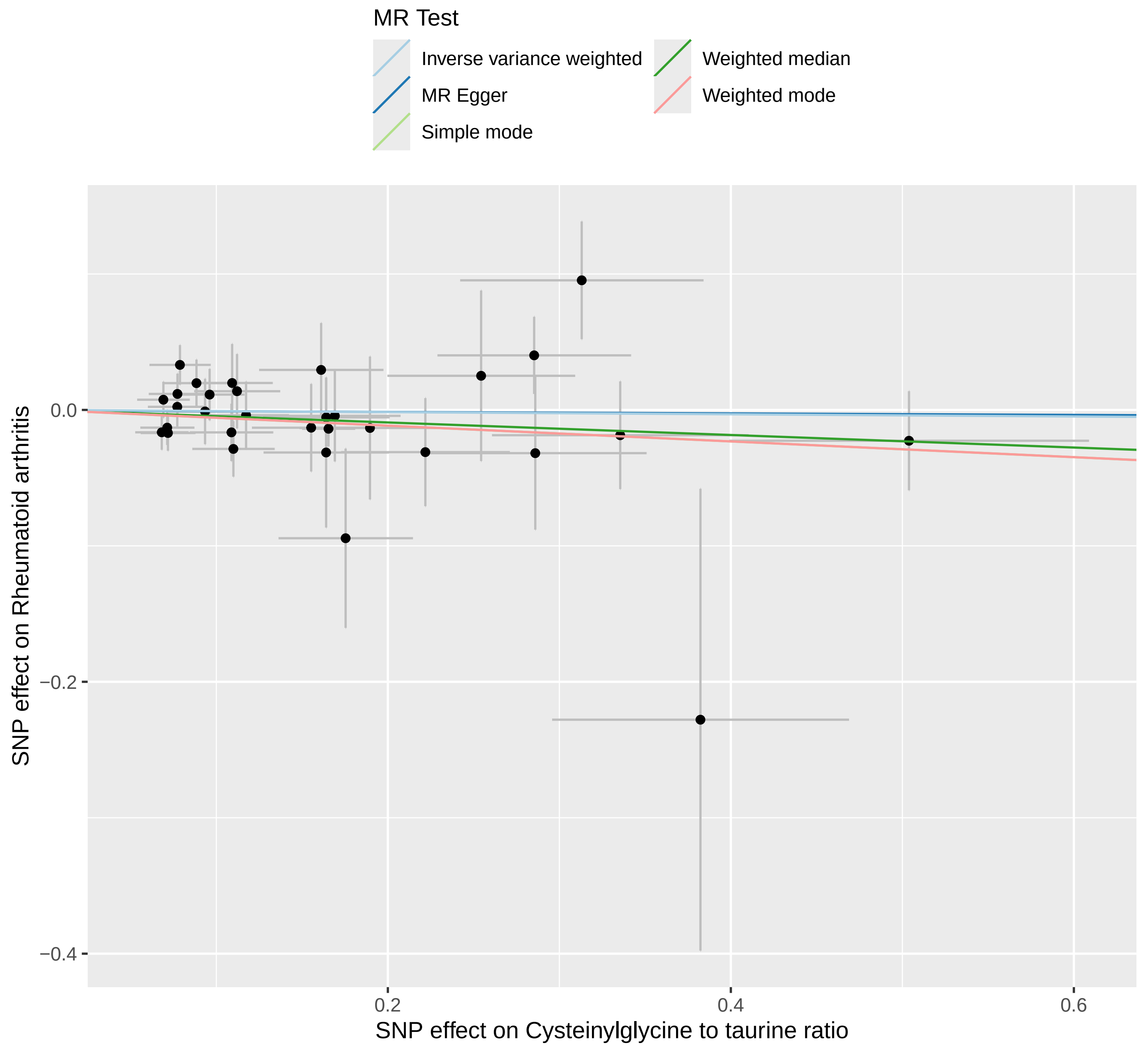
MR Test
Inverse variance weighted
MR Egger
Simple mode
Weighted median
Weighted mode
0.0
−0.2
−0.4
0.2
0.4
0.6
SNP effect on Rheumatoid arthritis
SNP effect on Cysteinylglycine to taurine ratio

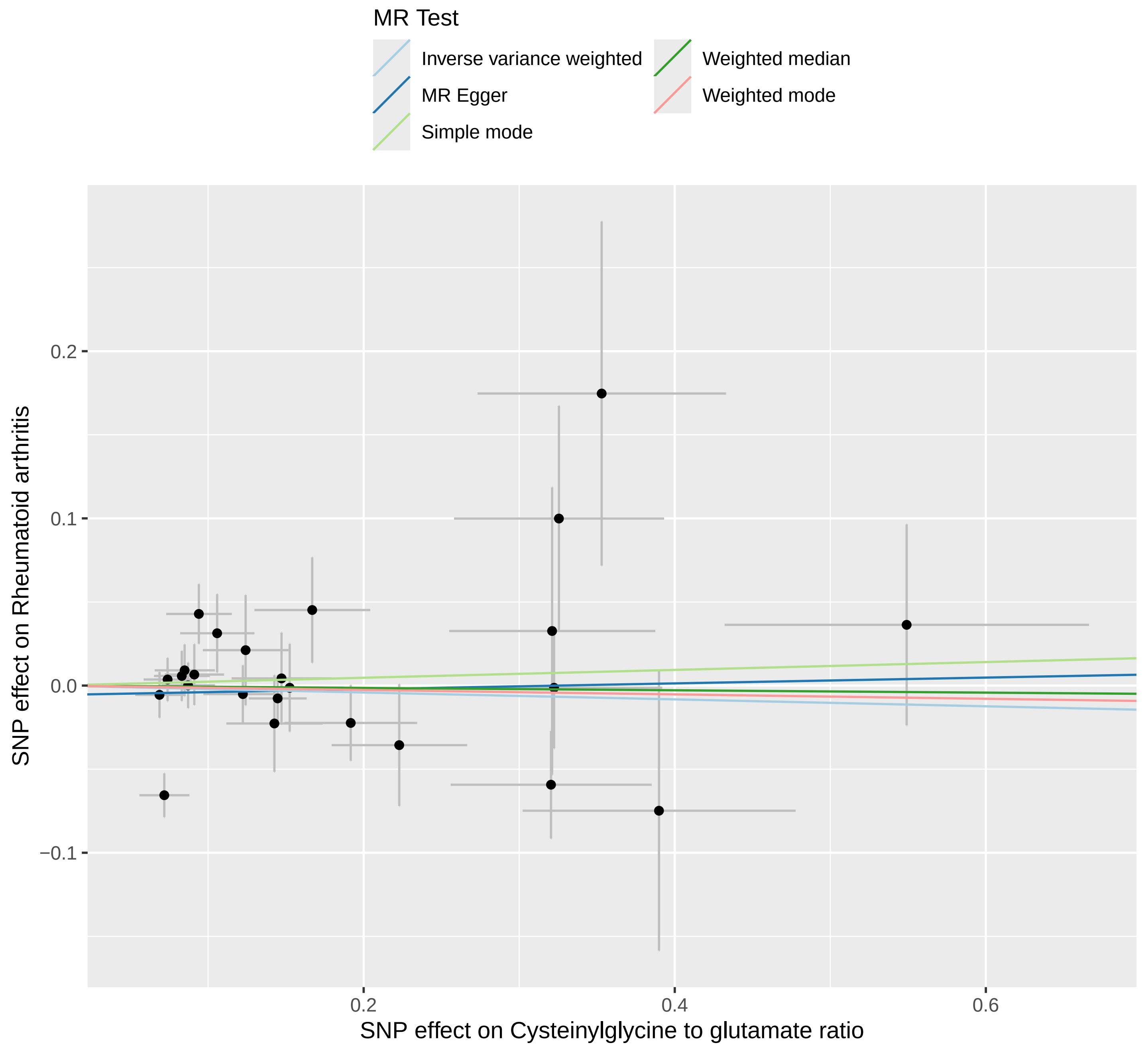

MR Test
Inverse variance weighted
MR Egger
Simple mode
Weighted median
Weighted mode
0.2
0.1
0.0
−0.1
SNP effect on Rheumatoid arthritis
0.2
0.4
0.6
SNP effect on Cysteinylglycine to glutamate ratio

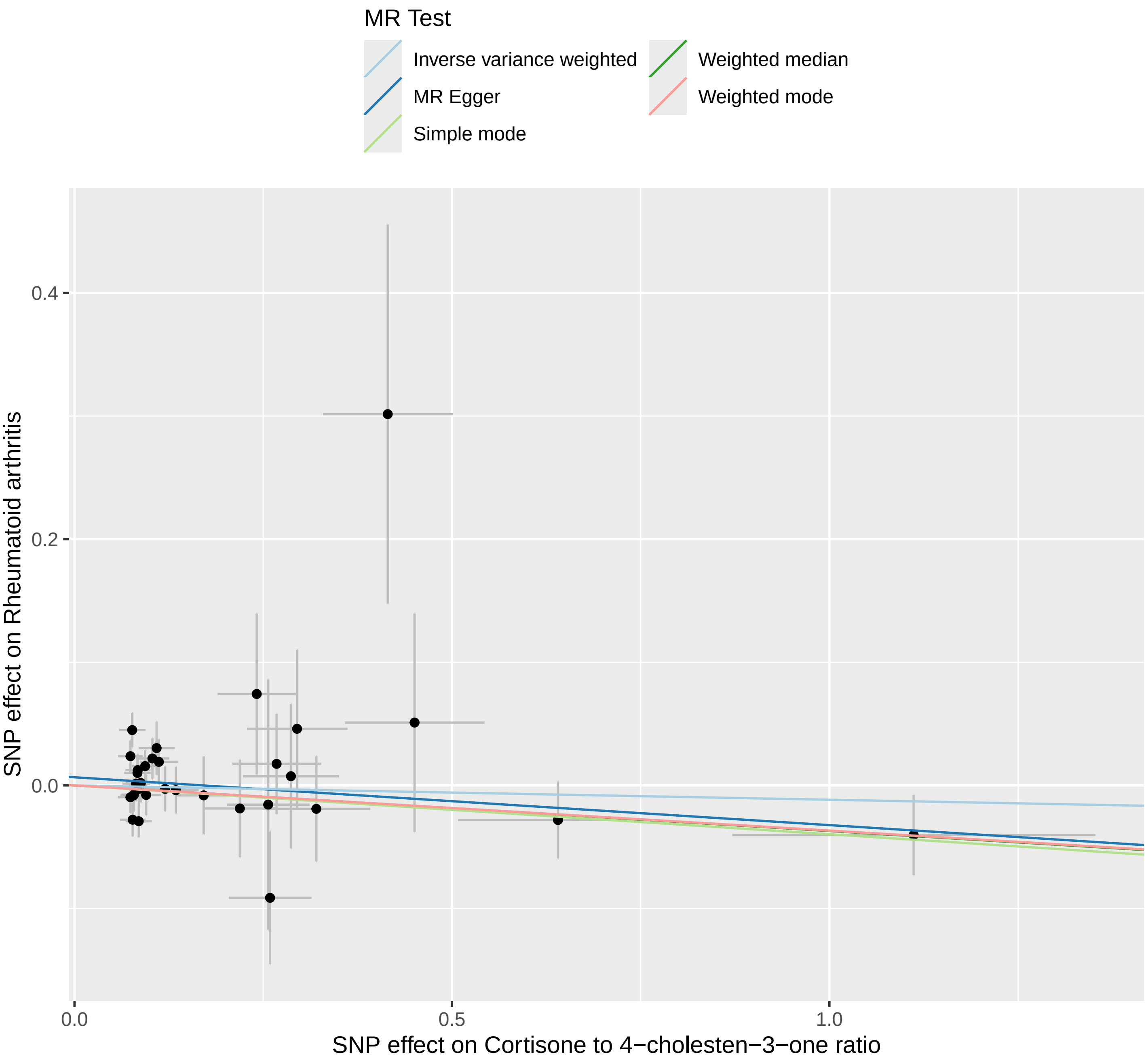

MR Test
Inverse variance weighted
MR Egger
Simple mode
Weighted median
Weighted mode
0.4
0.2
0.0
0.0
0.5
1.0
SNP effect on Rheumatoid arthritis
SNP effect on Cortisone to 4−cholesten−3−one ratio

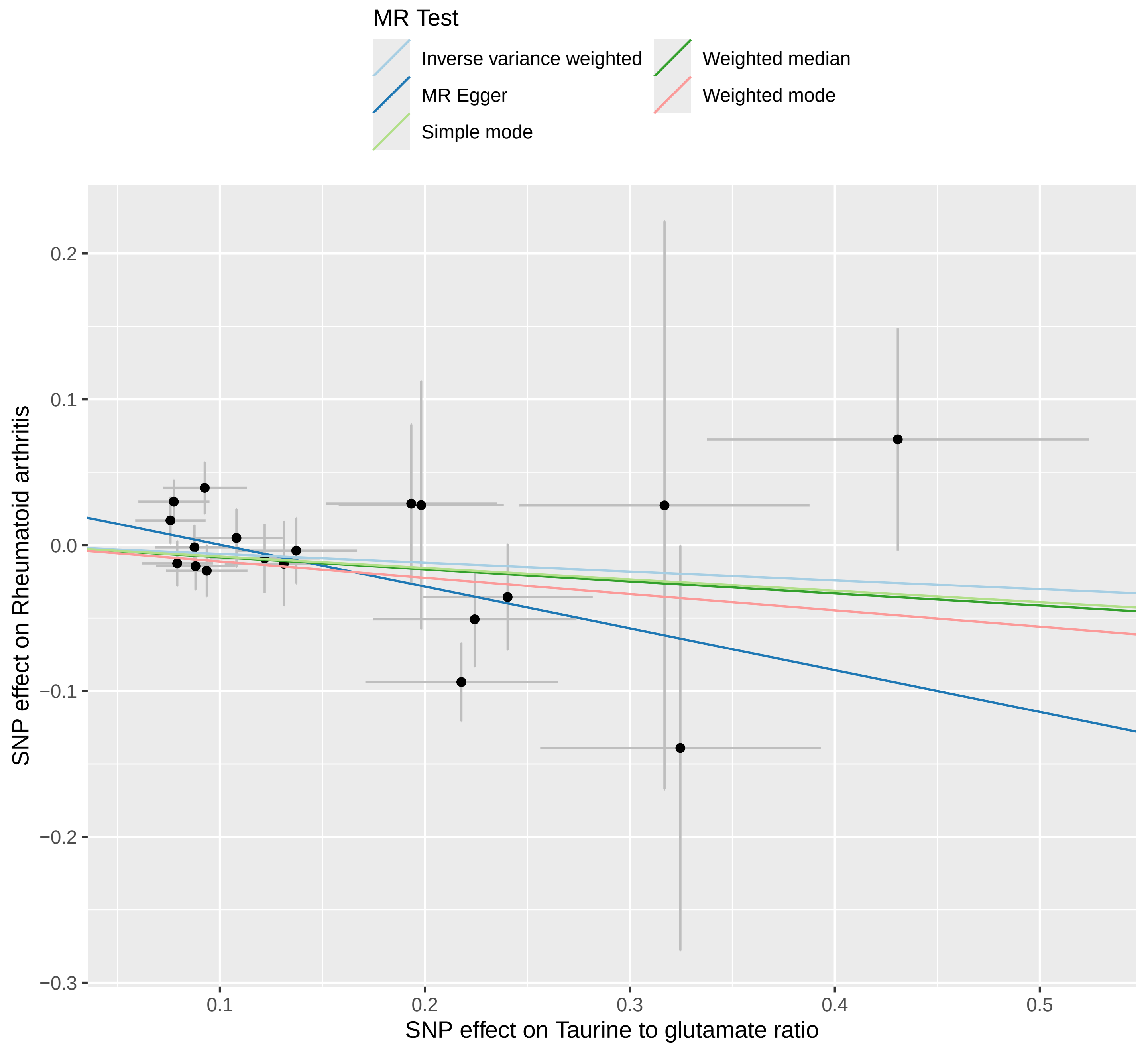
MR Test
Inverse variance weighted
MR Egger
Simple mode
Weighted median
Weighted mode
SNP effect on Rheumatoid arthritis
0.2
0.1
0.0
−0.1
−0.2
−0.3
0.1
0.2
0.3
0.4
0.5
SNP effect on Taurine to glutamate ratio

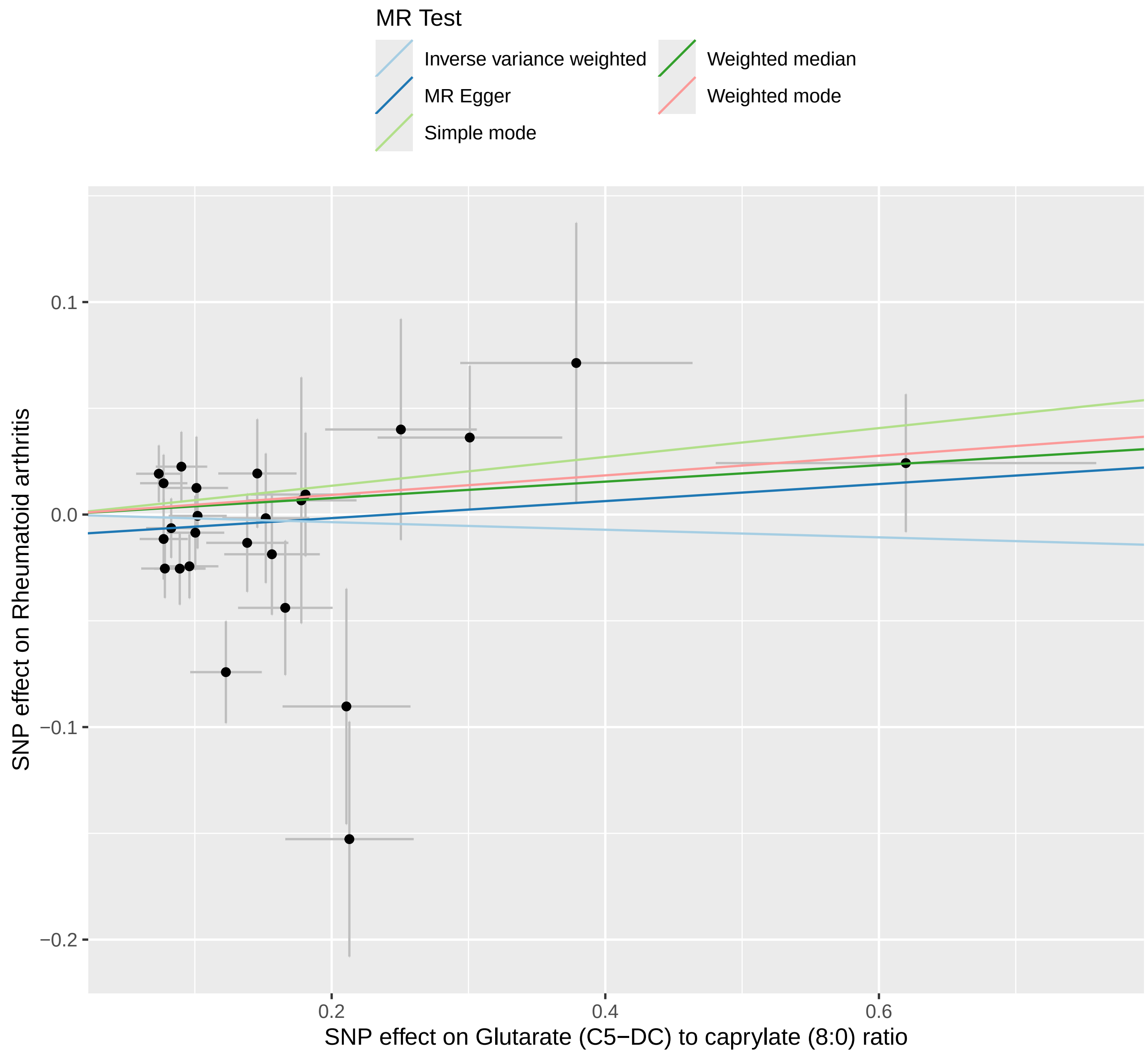
MR Test
Inverse variance weighted
MR Egger
Simple mode
Weighted median
Weighted mode
0.1
0.0
−0.1
−0.2
0.2
0.4
0.6
SNP effect on Rheumatoid arthritis
SNP effect on Glutarate (C5−DC) to caprylate (8:0) ratio

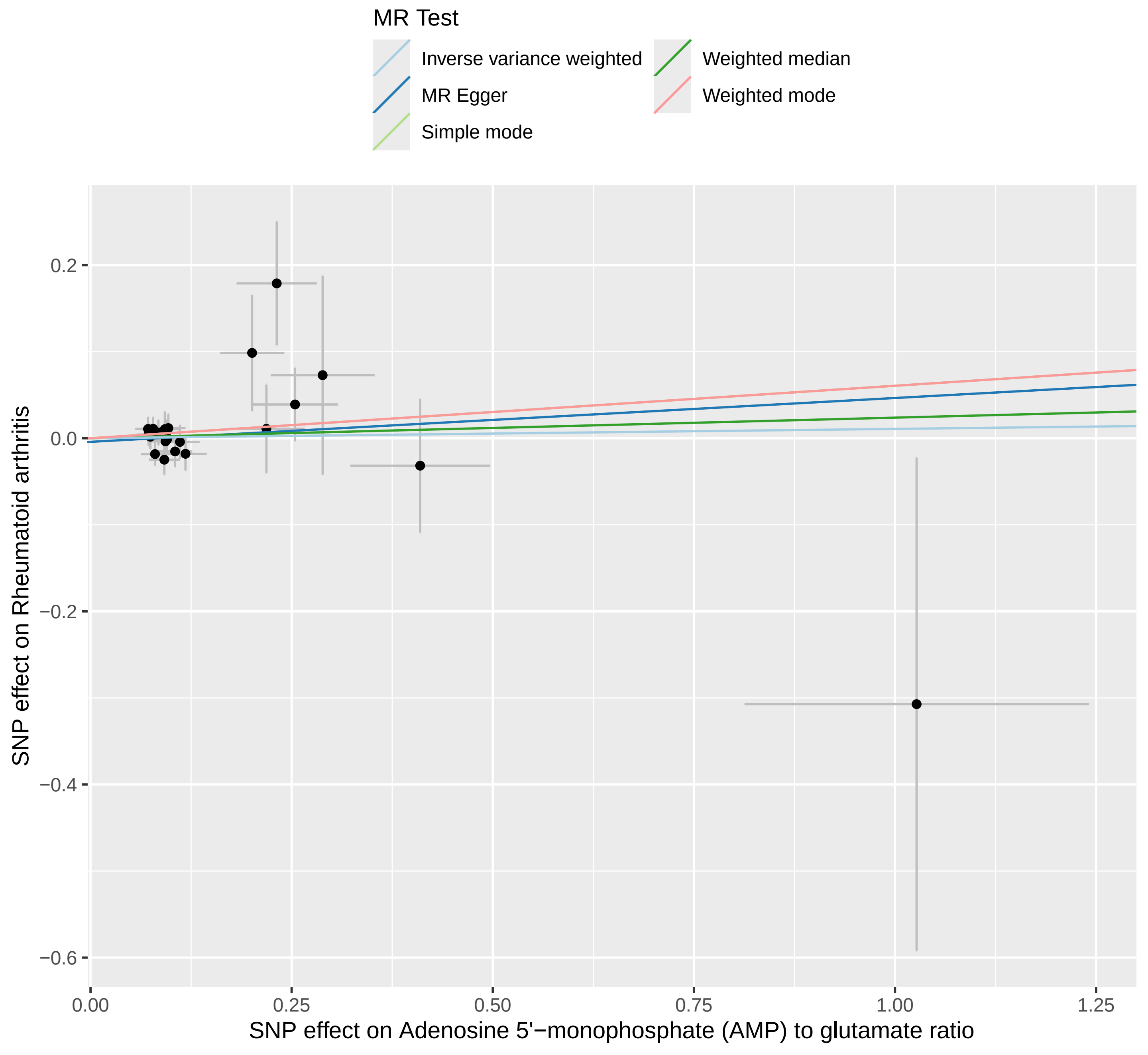
MR Test
Inverse variance weighted
MR Egger
Simple mode
Weighted median
Weighted mode
0.2
0.0
−0.2
−0.4
−0.6
SNP effect on Rheumatoid arthritis
0.00
0.25
0.50
0.75
1.00
1.25
SNP effect on Adenosine 5'−monophosphate (AMP) to glutamate ratio

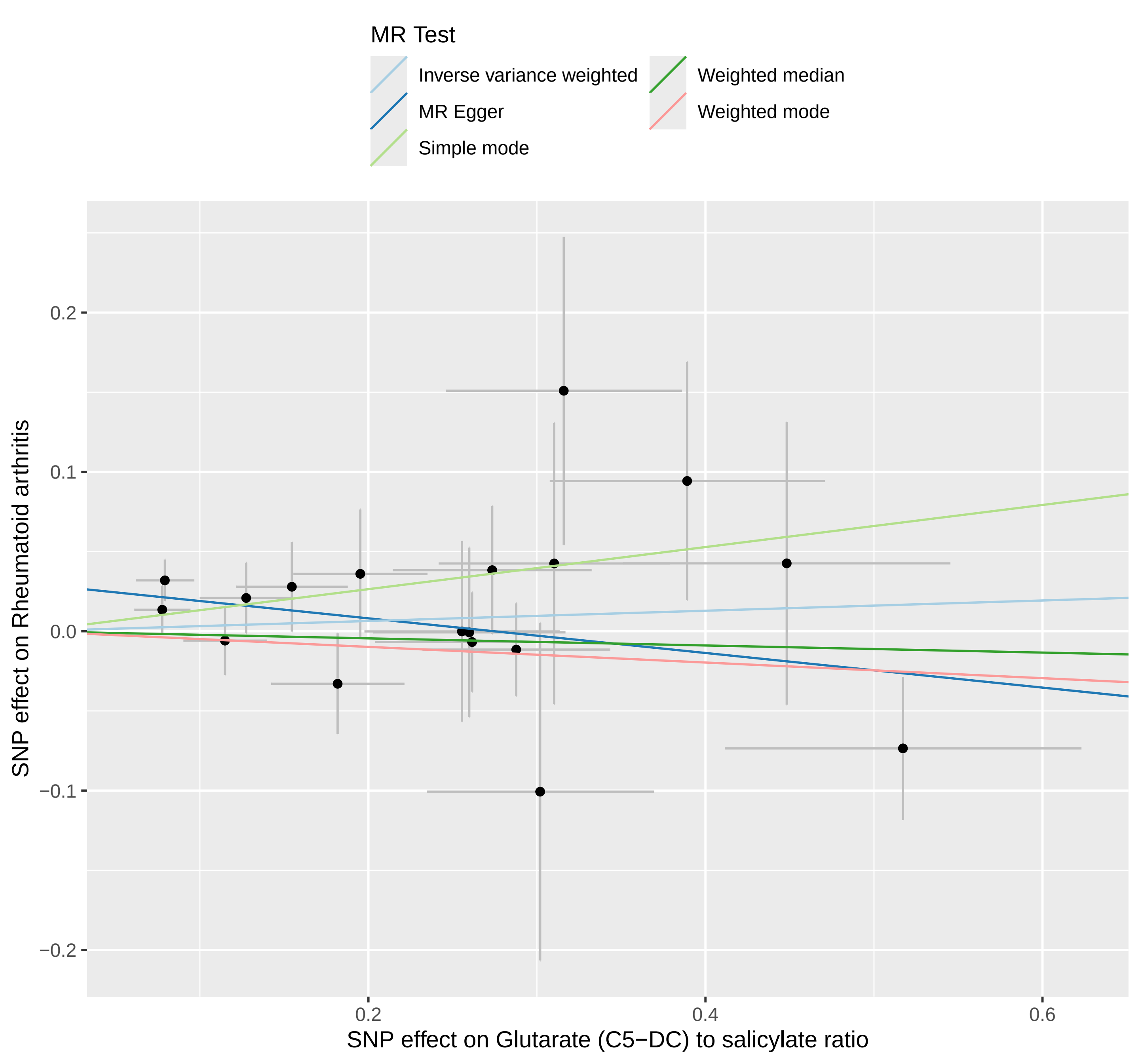
MR Test
Inverse variance weighted
MR Egger
Simple mode
Weighted median
Weighted mode
0.2
0.1
0.0
−0.1
−0.2
SNP effect on Rheumatoid arthritis
0.2
0.4
0.6
SNP effect on Glutarate (C5−DC) to salicylate ratio

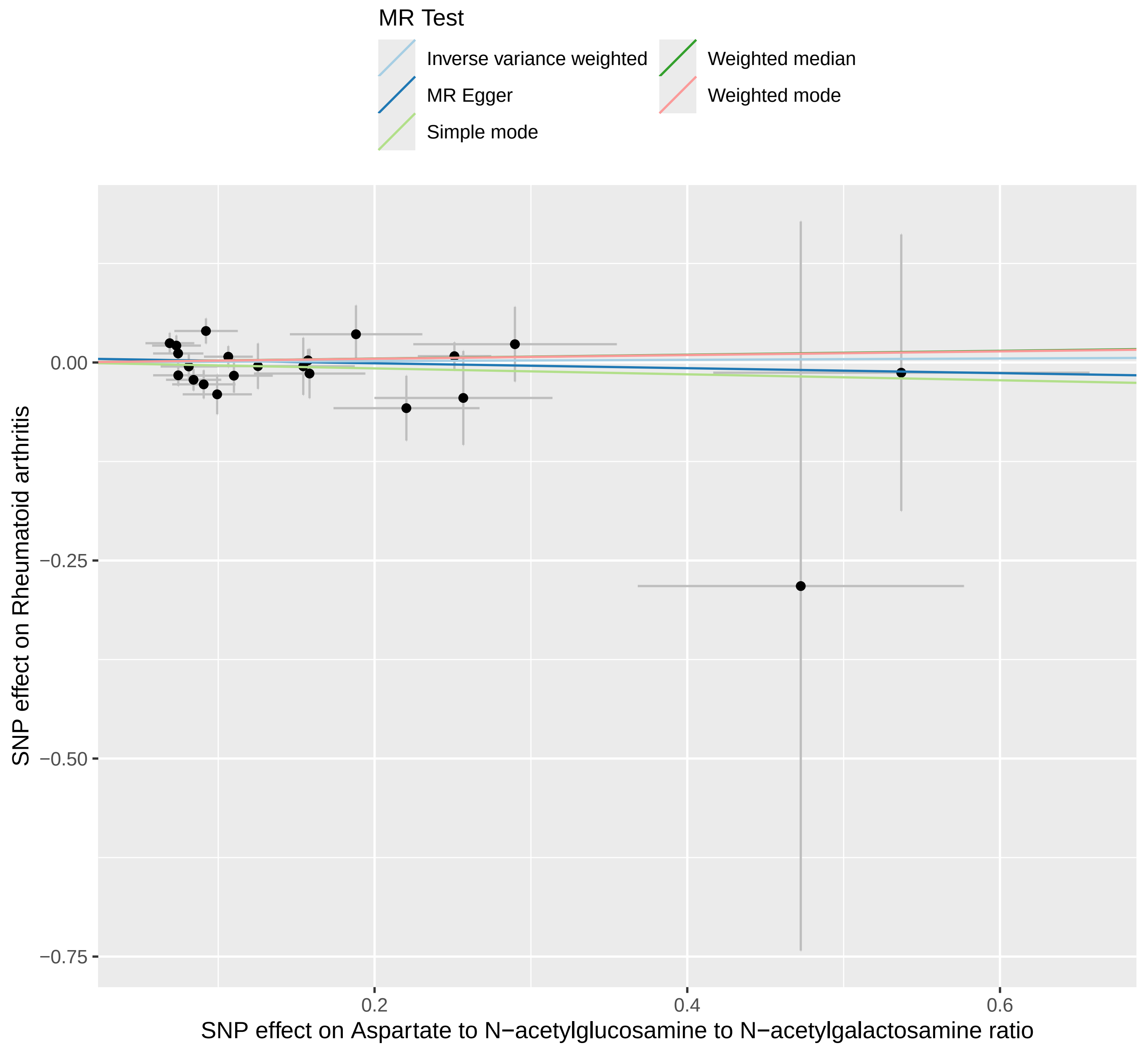

MR Test
Inverse variance weighted
MR Egger
Simple mode
Weighted median
Weighted mode
0.00
−0.25
−0.50
−0.75
0.2
0.4
0.6
SNP effect on Rheumatoid arthritis
SNP effect on Aspartate to N−acetylglucosamine to N−acetylgalactosamine ratio

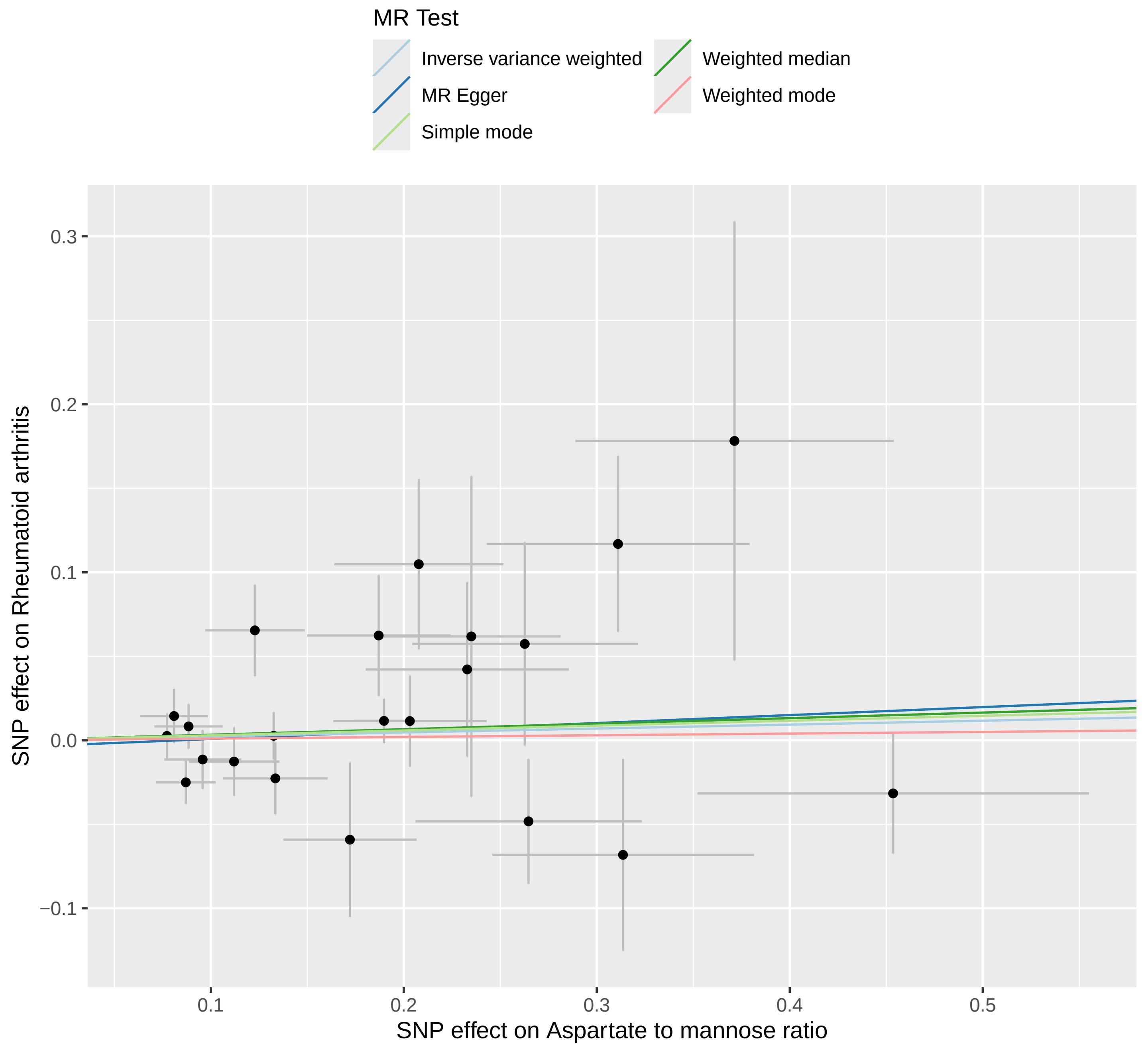
MR Test
Inverse variance weighted
MR Egger
Simple mode
Weighted median
Weighted mode
0.3
0.2
0.1
0.0
−0.1
0.1
0.2
0.3
0.4
0.5
SNP effect on Rheumatoid arthritis
SNP effect on Aspartate to mannose ratio

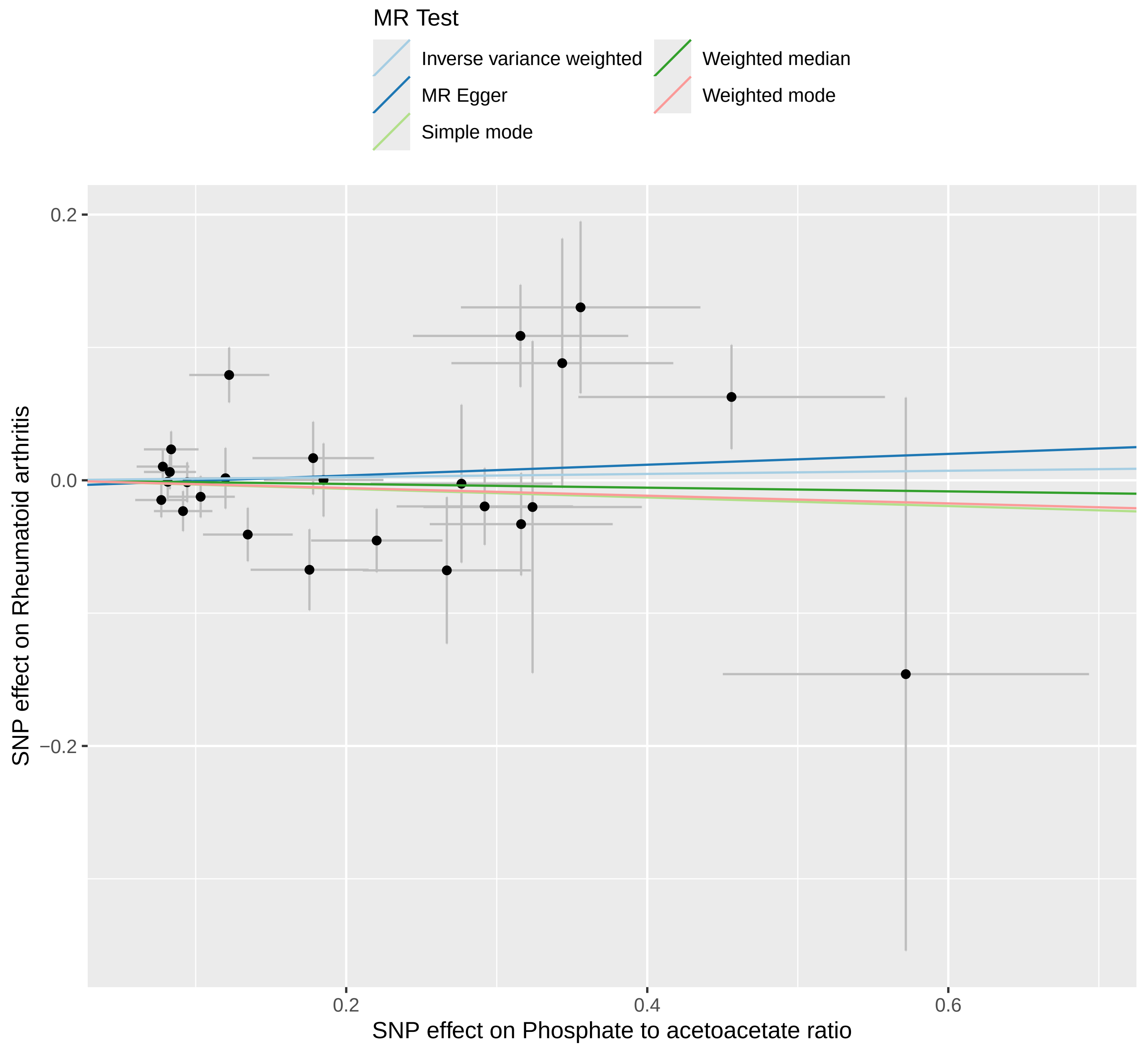
MR Test
Inverse variance weighted
MR Egger
Simple mode
Weighted median
Weighted mode
0.2
0.0
−0.2
SNP effect on Rheumatoid arthritis
0.2
0.4
0.6
SNP effect on Phosphate to acetoacetate ratio

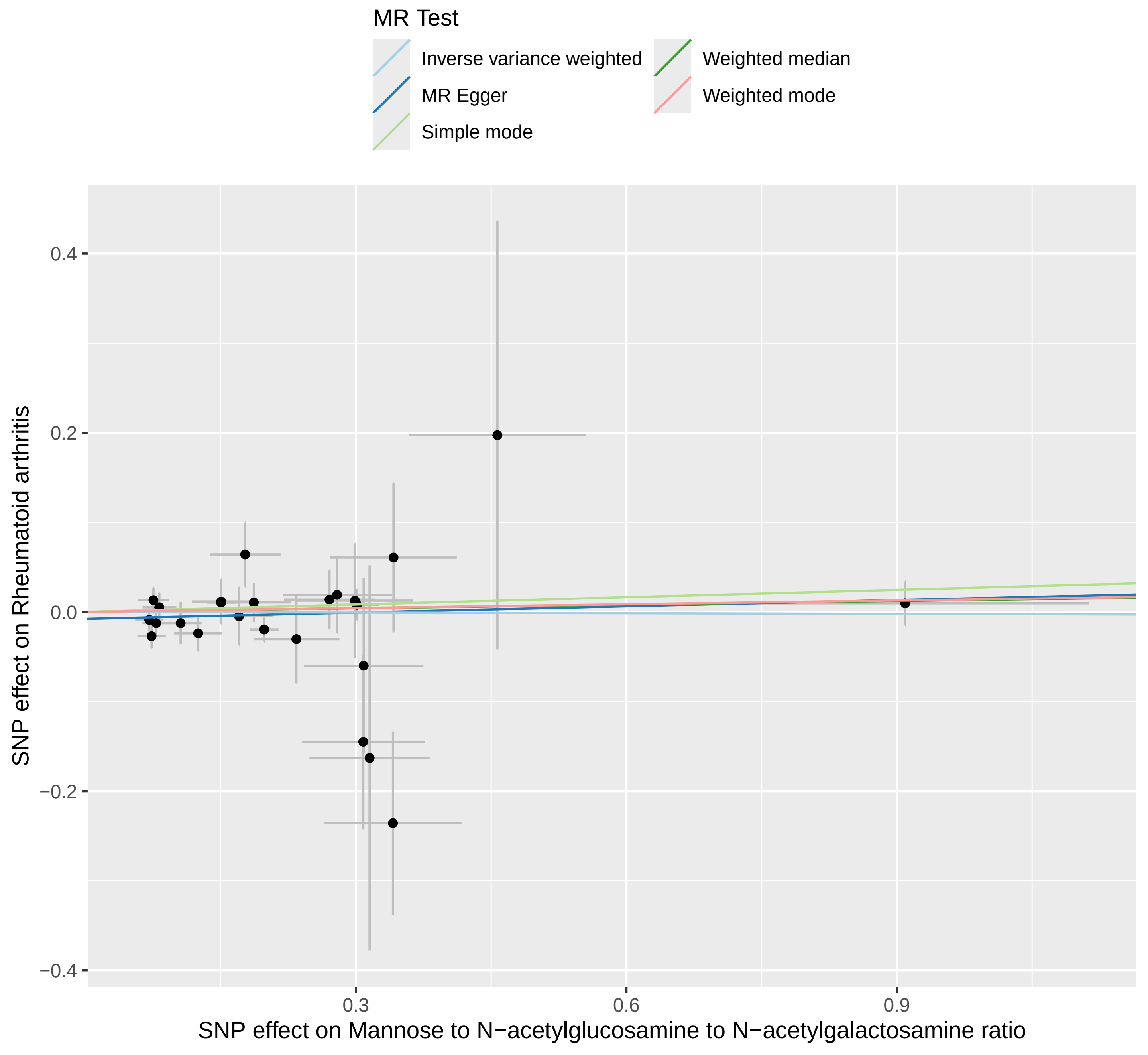
MR Test
Inverse variance weighted
MR Egger
Simple mode
Weighted median
Weighted mode
0.4
0.2
0.0
−0.2
−0.4
0.3
0.6
0.9
SNP effect on Rheumatoid arthritis
SNP effect on Mannose to N−acetylglucosamine to N−acetylgalactosamine ratio

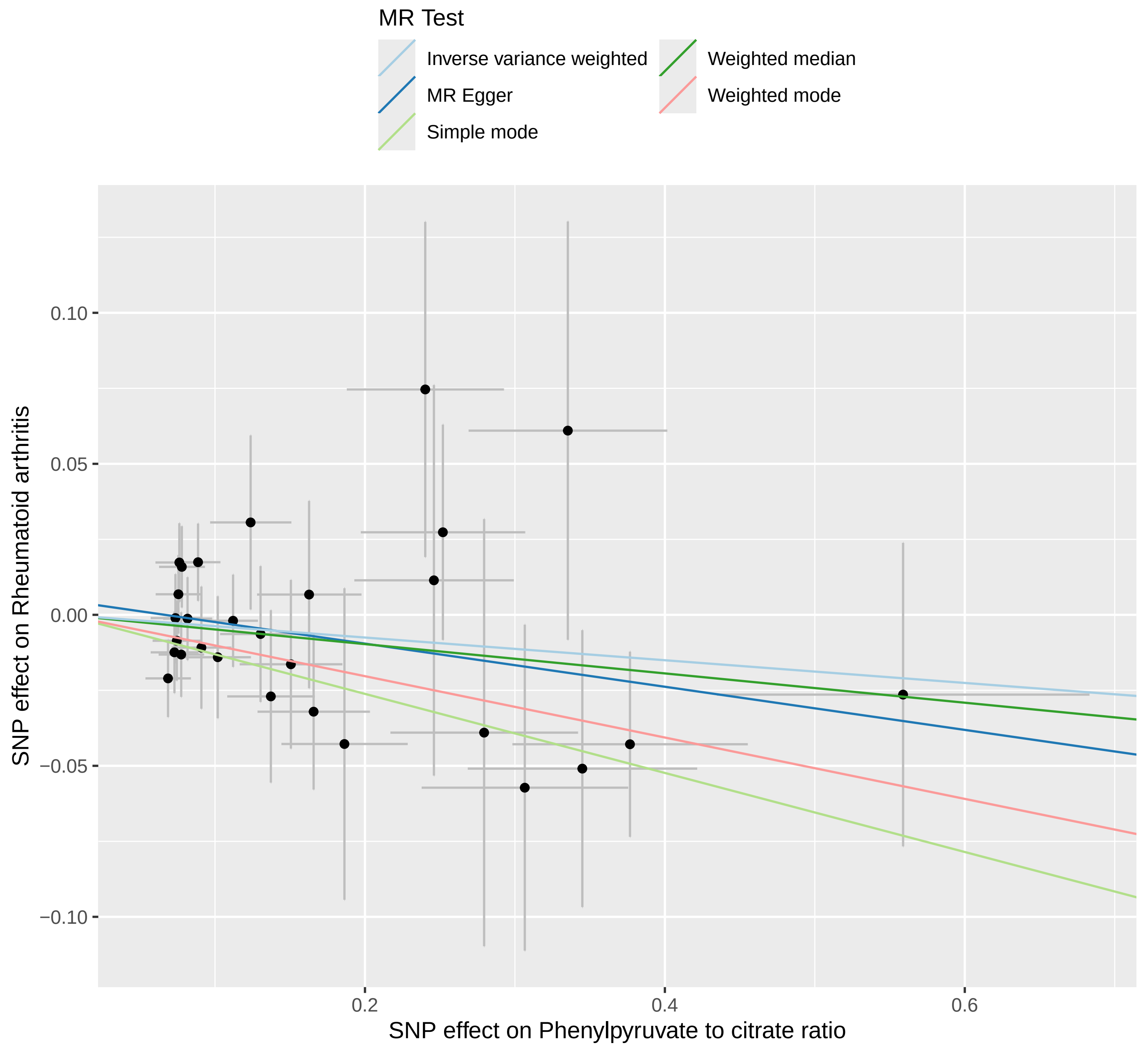

MR Test
Inverse variance weighted
MR Egger
Simple mode
Weighted median
Weighted mode
0.10
0.05
0.00
−0.05
−0.10
SNP effect on Rheumatoid arthritis
0.2
0.4
0.6
SNP effect on Phenylpyruvate to citrate ratio

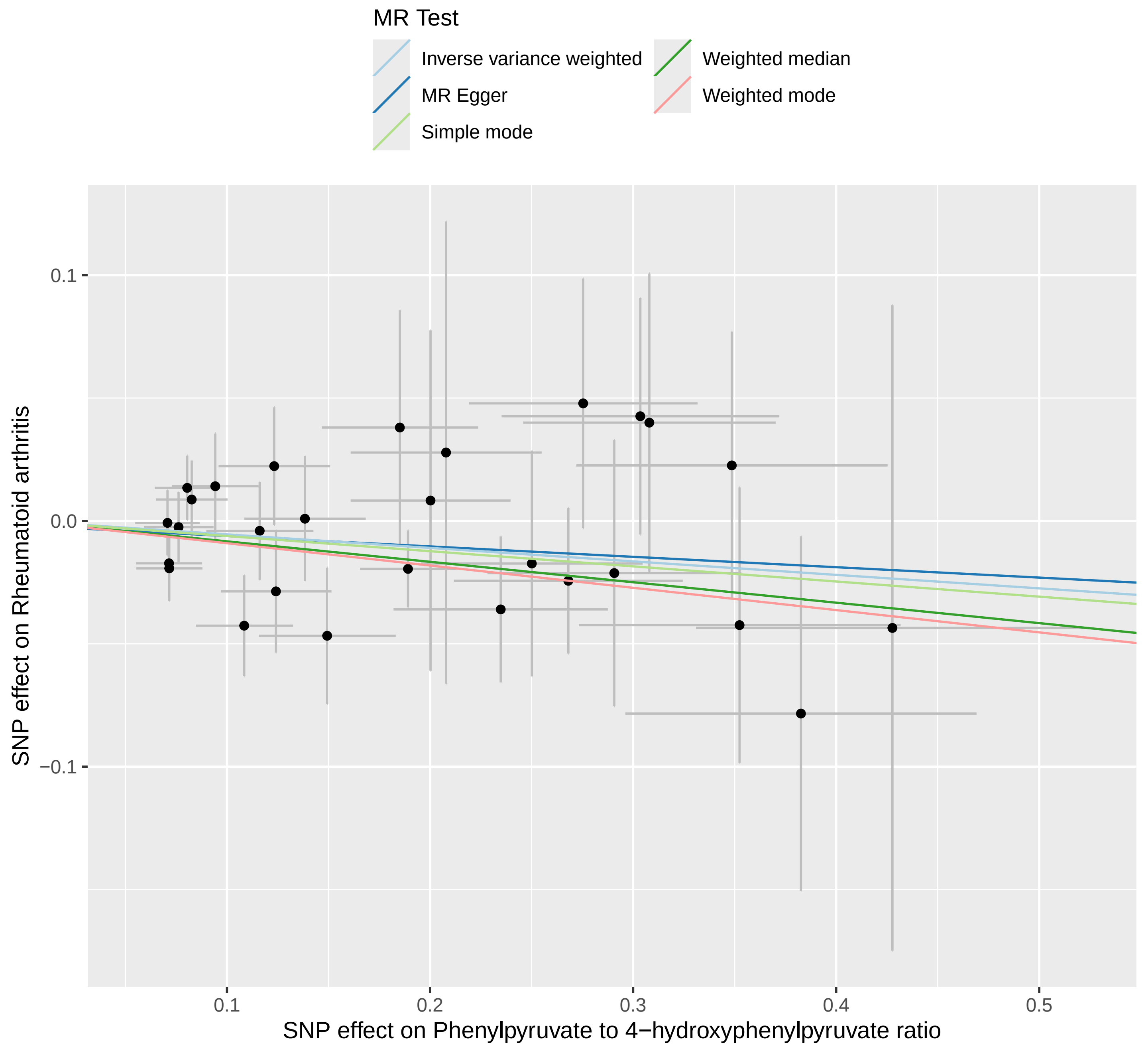
MR Test
Inverse variance weighted
MR Egger
Simple mode
Weighted median
Weighted mode
0.1
0.0
−0.1
0.1
0.2
0.3
0.4
0.5
SNP effect on Rheumatoid arthritis
SNP effect on Phenylpyruvate to 4−hydroxyphenylpyruvate ratio

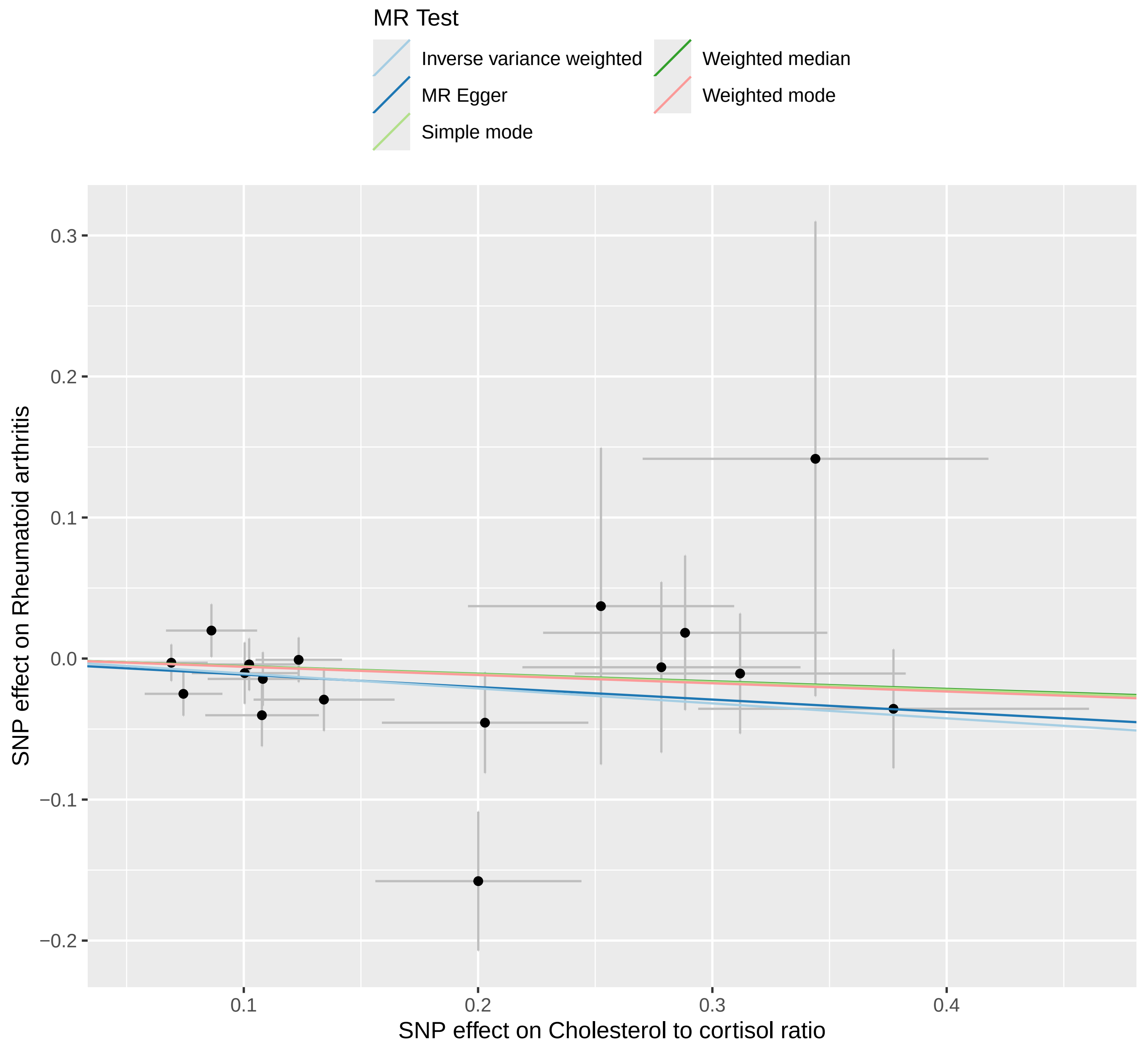
MR Test
Inverse variance weighted
MR Egger
Simple mode
Weighted median
Weighted mode
SNP effect on Rheumatoid arthritis
SNP effect on Cholesterol to cortisol ratio
0.3
0.2
0.1
0.0
−0.1
−0.2
0.1
0.2
0.3
0.4

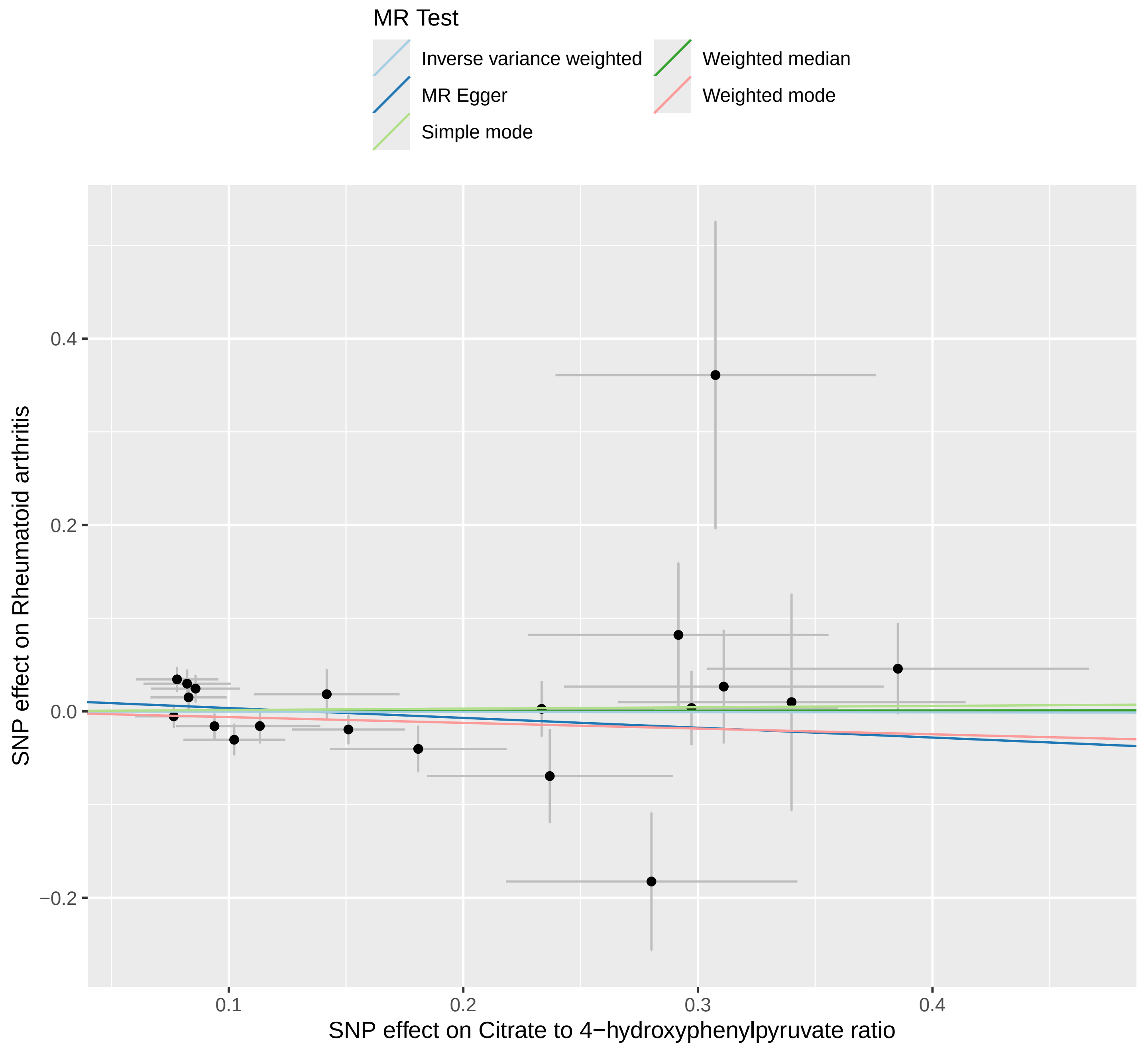

MR Test
Inverse variance weighted
Weighted median
MR Egger
Weighted mode
Simple mode
0.4
0.2
0.0
−0.2
SNP effect on Rheumatoid arthritis
0.1
0.2
0.3
0.4
SNP effect on Citrate to 4−hydroxyphenylpyruvate ratio

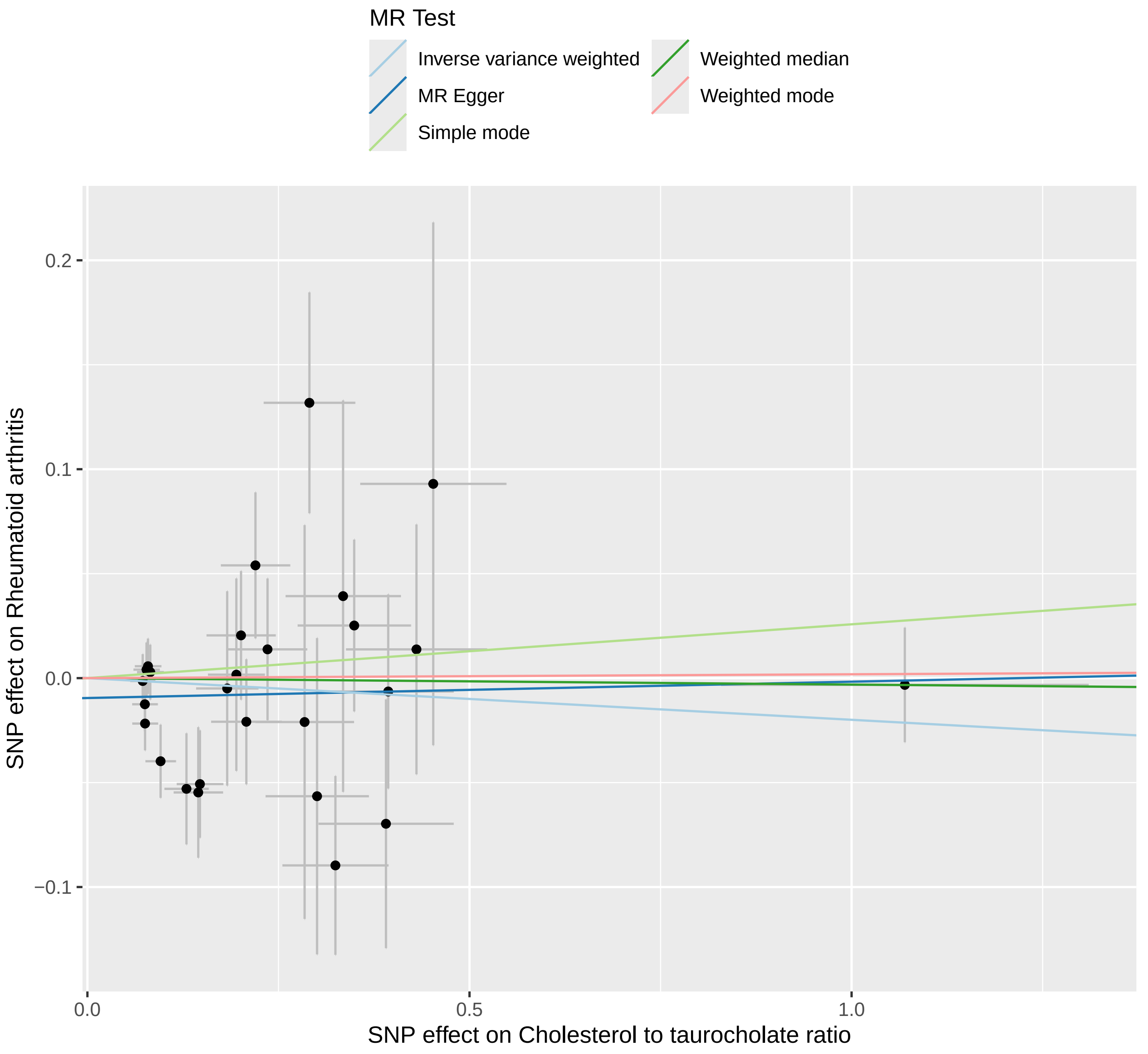
MR Test
Inverse variance weighted
MR Egger
Simple mode
Weighted median
Weighted mode
0.2
0.1
0.0
−0.1
SNP effect on Rheumatoid arthritis
0.0
0.5
1.0
SNP effect on Cholesterol to taurocholate ratio

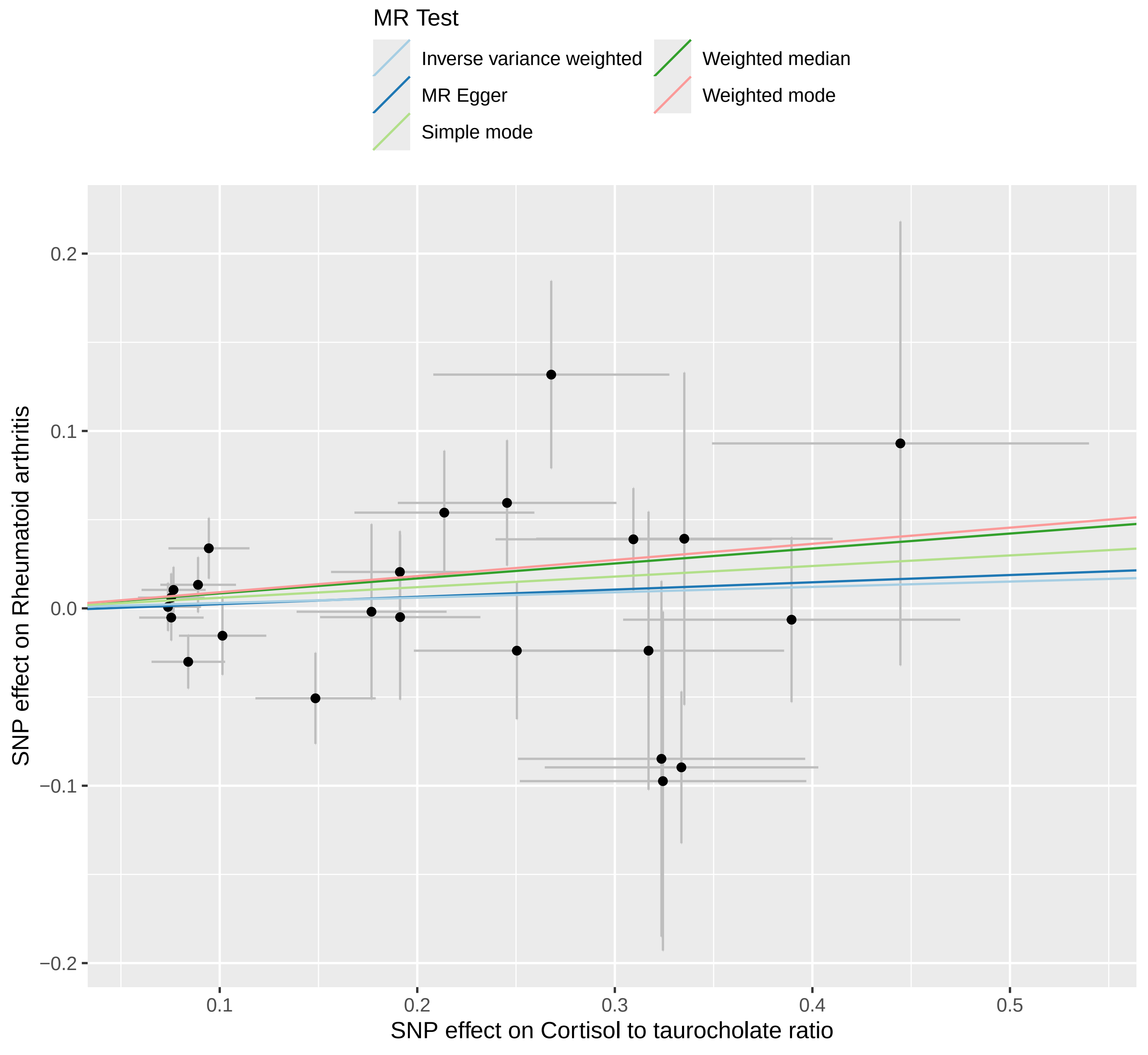
MR Test
Inverse variance weighted
MR Egger
Simple mode
Weighted median
Weighted mode
0.2
0.1
0.0
−0.1
−0.2
0.1
0.2
0.3
0.4
0.5
SNP effect on Rheumatoid arthritis
SNP effect on Cortisol to taurocholate ratio

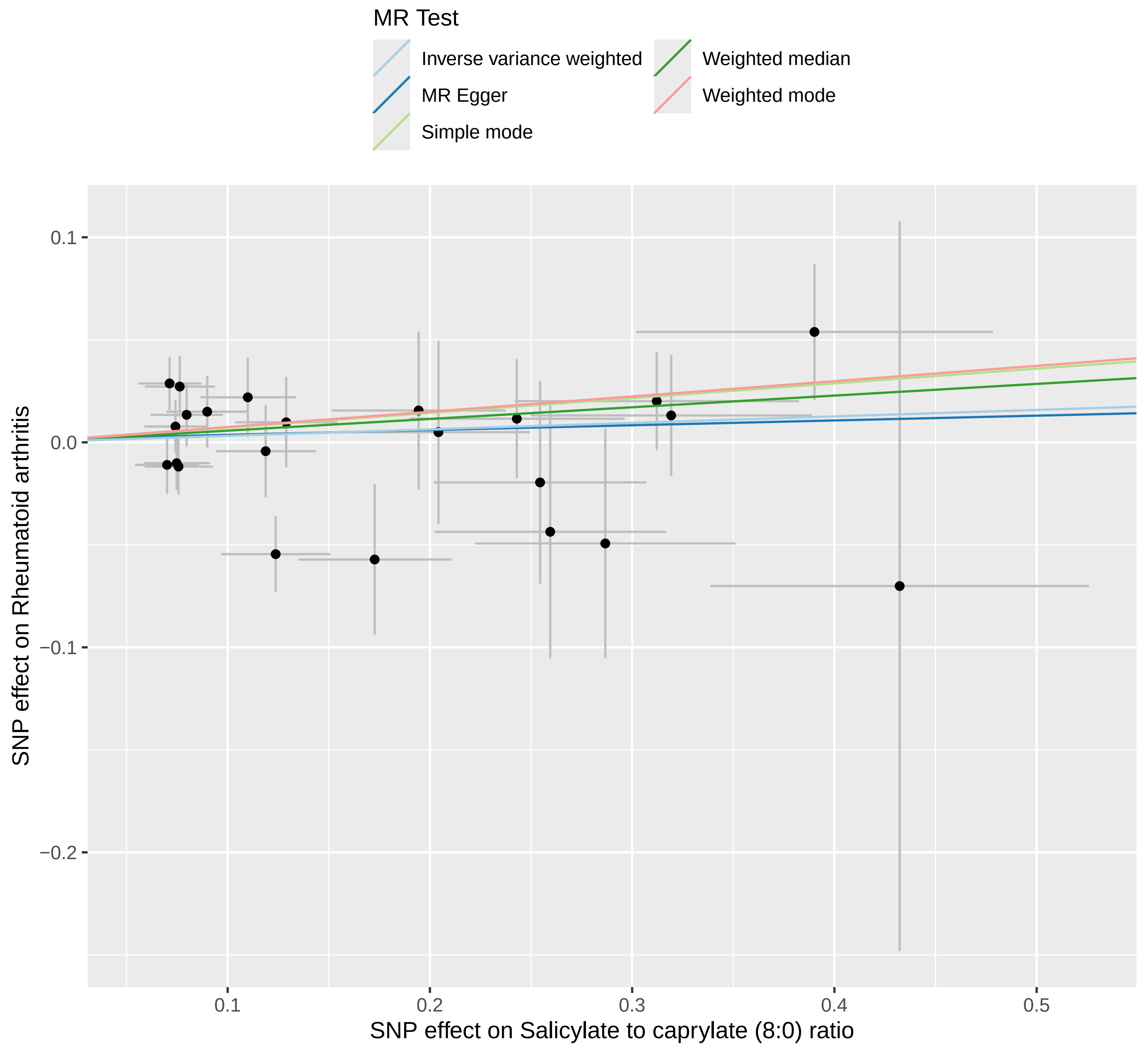
MR Test
Inverse variance weighted
MR Egger
Simple mode
Weighted median
Weighted mode
0.1
0.0
−0.1
−0.2
0.1
0.2
0.3
0.4
0.5
SNP effect on Rheumatoid arthritis
SNP effect on Salicylate to caprylate (8:0) ratio

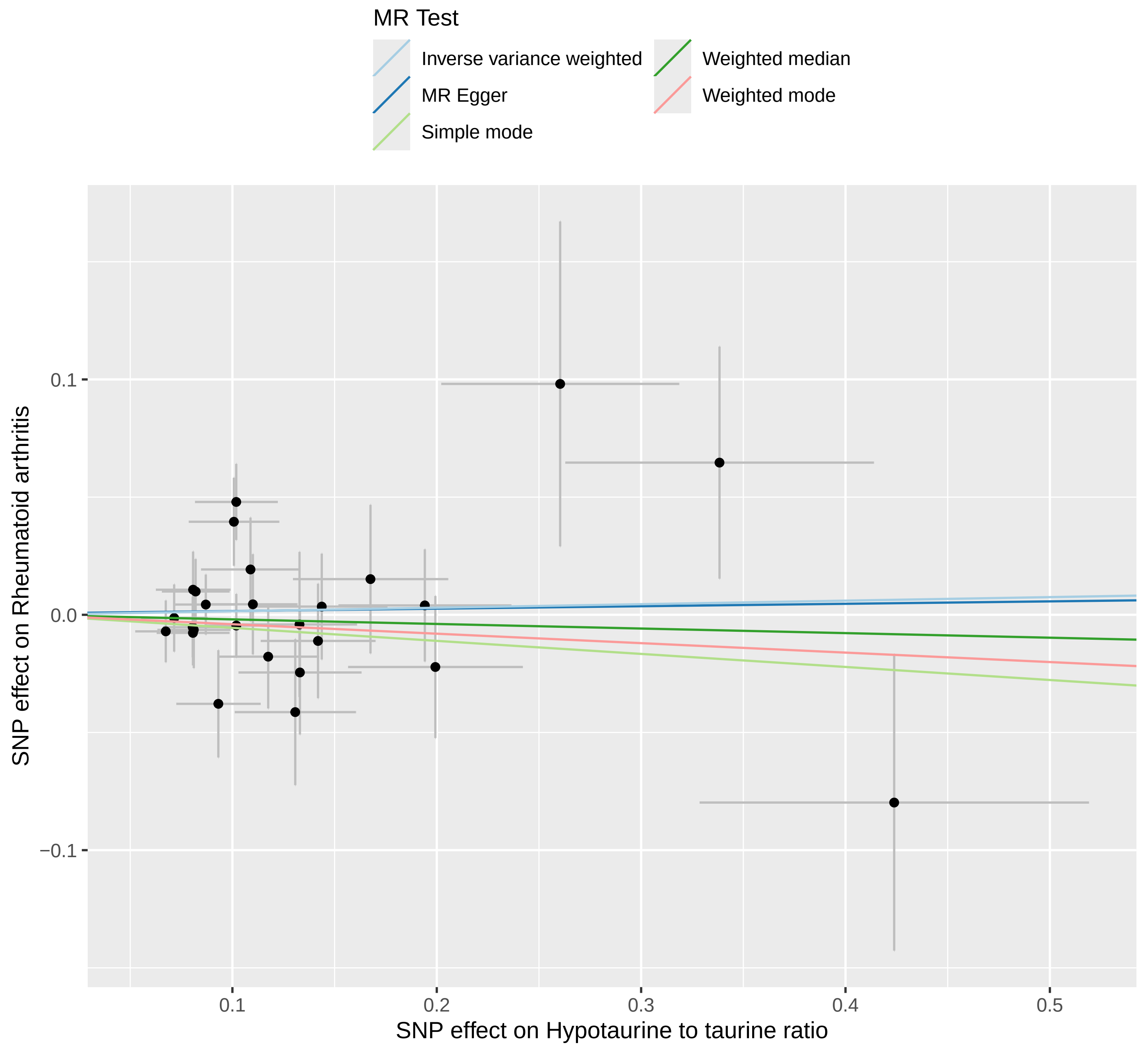
MR Test
Inverse variance weighted
MR Egger
Simple mode
Weighted median
Weighted mode
0.1
0.0
−0.1
SNP effect on Rheumatoid arthritis
0.1
0.2
0.3
0.4
0.5
SNP effect on Hypotaurine to taurine ratio

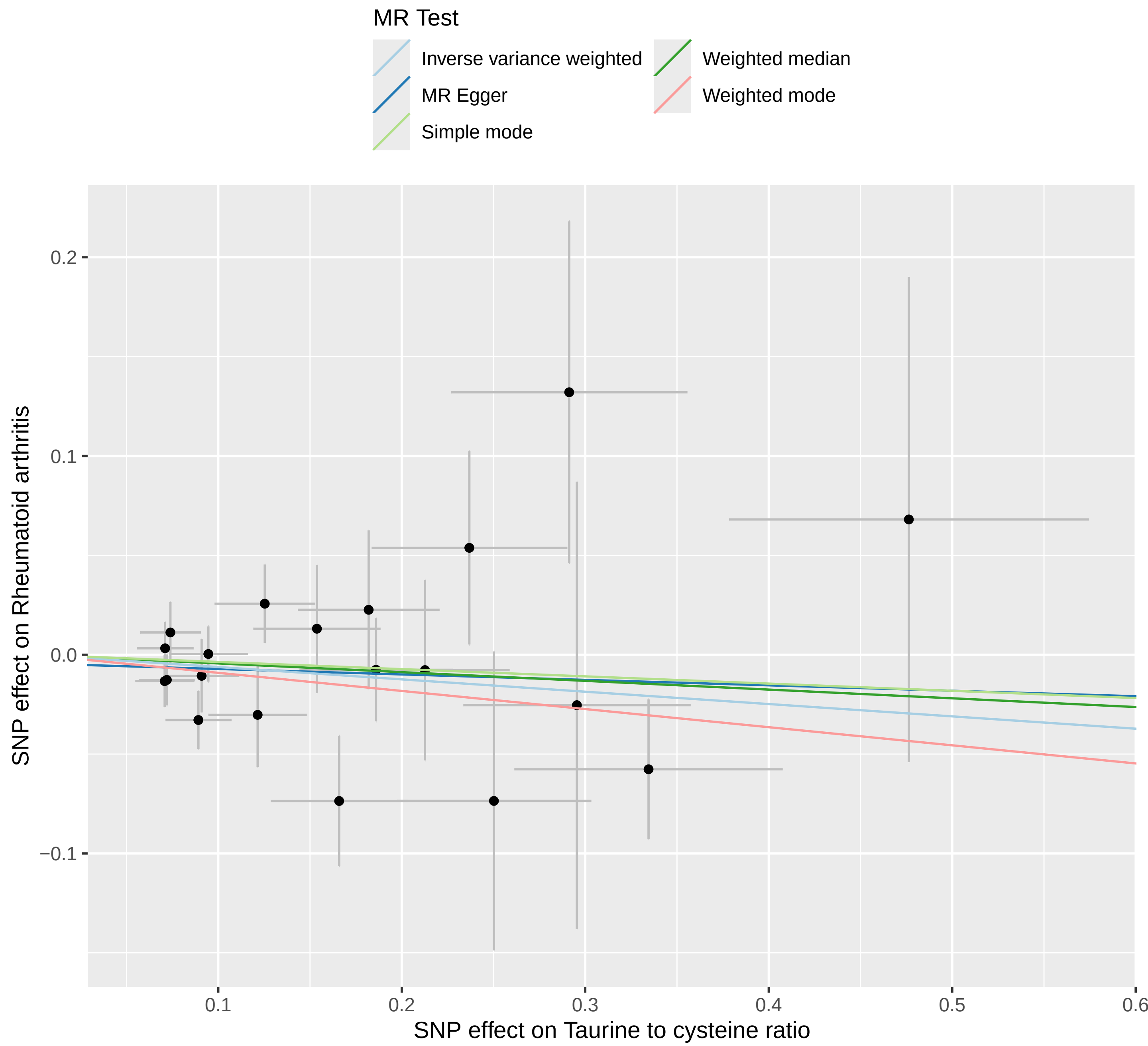
MR Test
Inverse variance weighted
MR Egger
Simple mode
Weighted median
Weighted mode
0.2
0.1
0.0
−0.1
SNP effect on Rheumatoid arthritis
0.1
0.2
0.3
0.4
0.5
0.6
SNP effect on Taurine to cysteine ratio

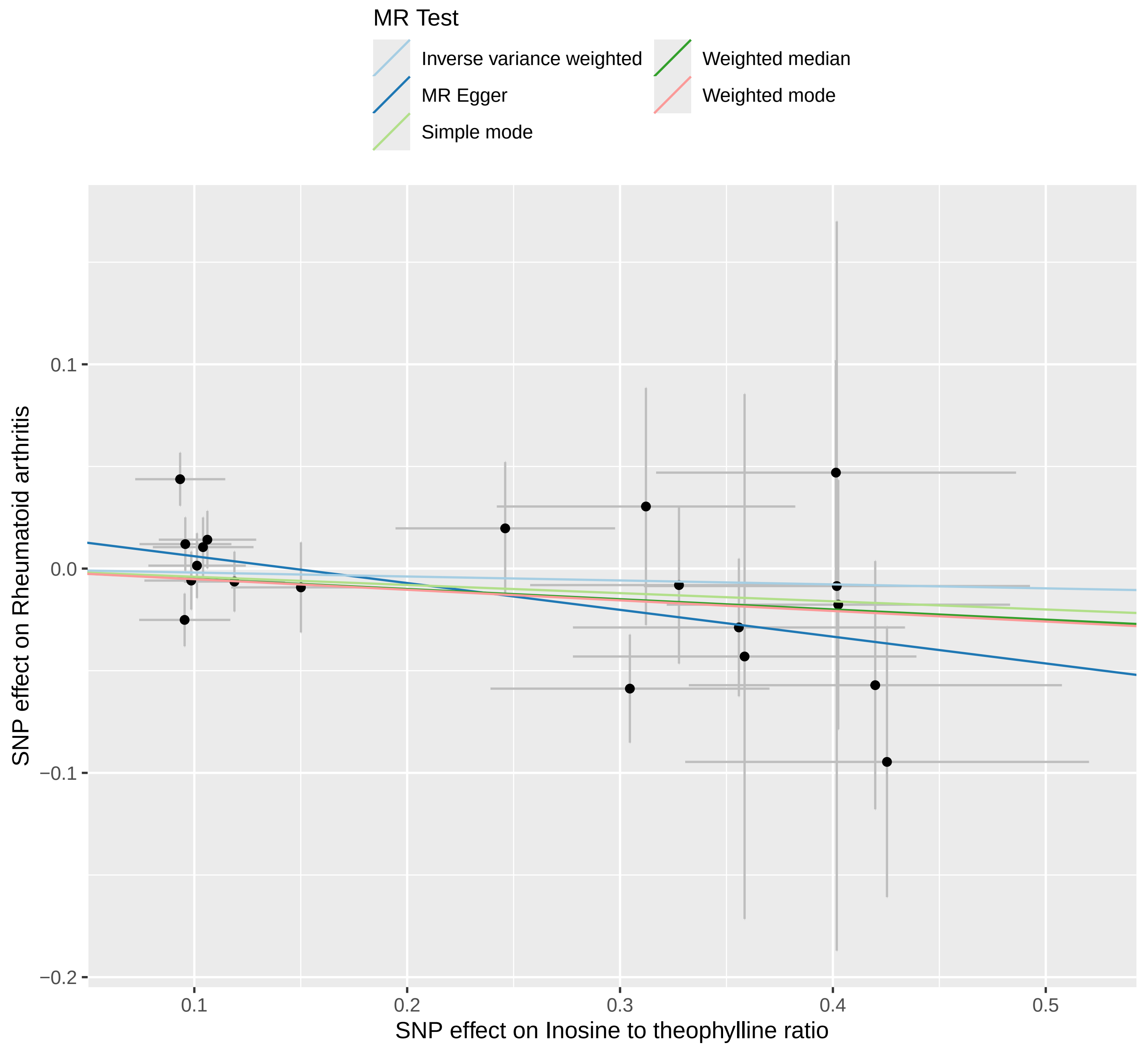

MR Test
Inverse variance weighted
MR Egger
Simple mode
Weighted median
Weighted mode
0.1
0.0
−0.1
−0.2
SNP effect on Rheumatoid arthritis
0.1
0.2
0.3
0.4
0.5
SNP effect on Inosine to theophylline ratio

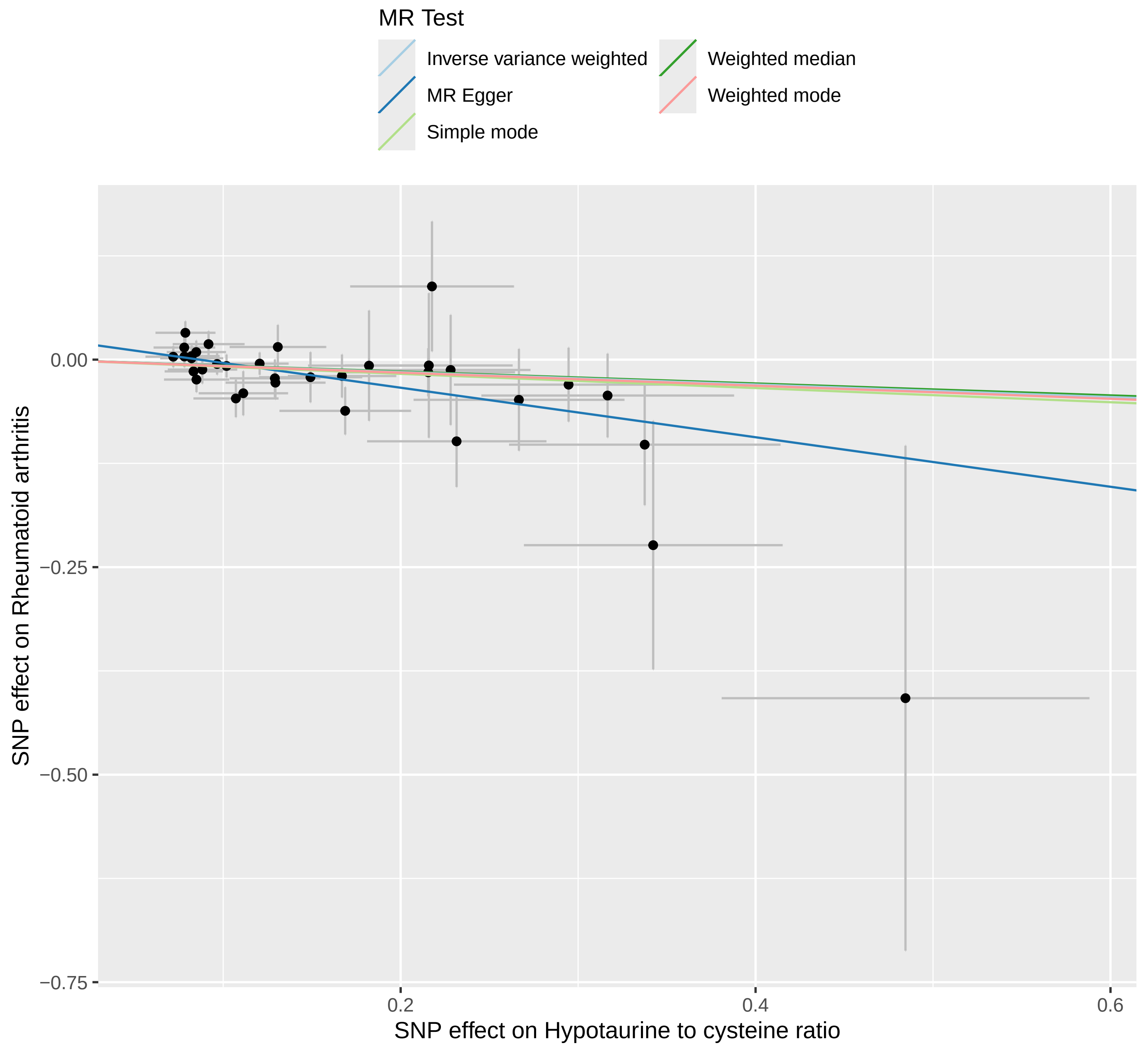
MR Test
Inverse variance weighted
MR Egger
Simple mode
Weighted median
Weighted mode
0.00
−0.25
−0.50
−0.75
0.2
0.4
0.6
SNP effect on Rheumatoid arthritis
SNP effect on Hypotaurine to cysteine ratio

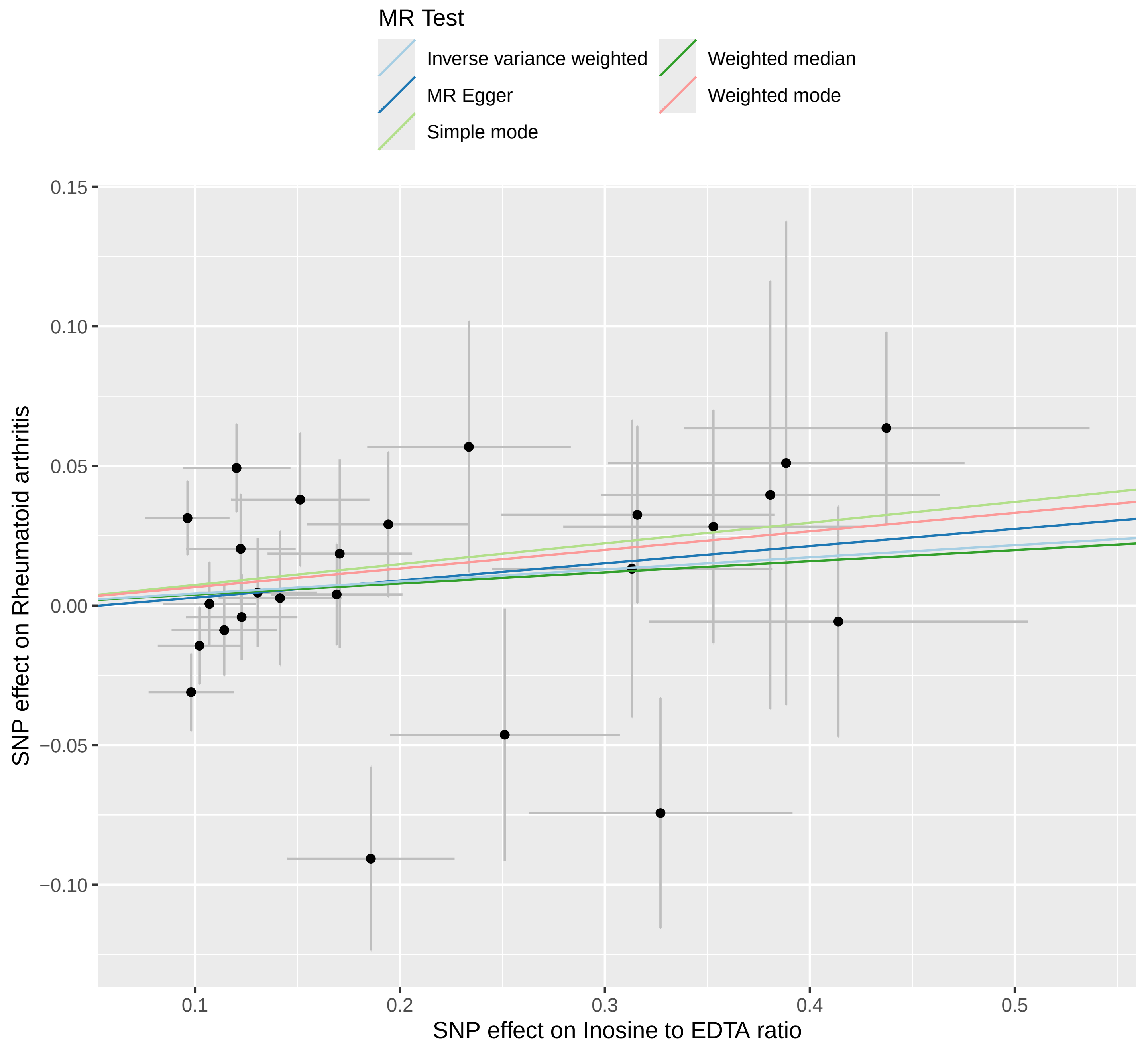
MR Test
Inverse variance weighted
MR Egger
Simple mode
Weighted median
Weighted mode
0.15
0.10
0.05
0.00
−0.05
−0.10
SNP effect on Rheumatoid arthritis
0.1
0.2
0.3
0.4
0.5
SNP effect on Inosine to EDTA ratio

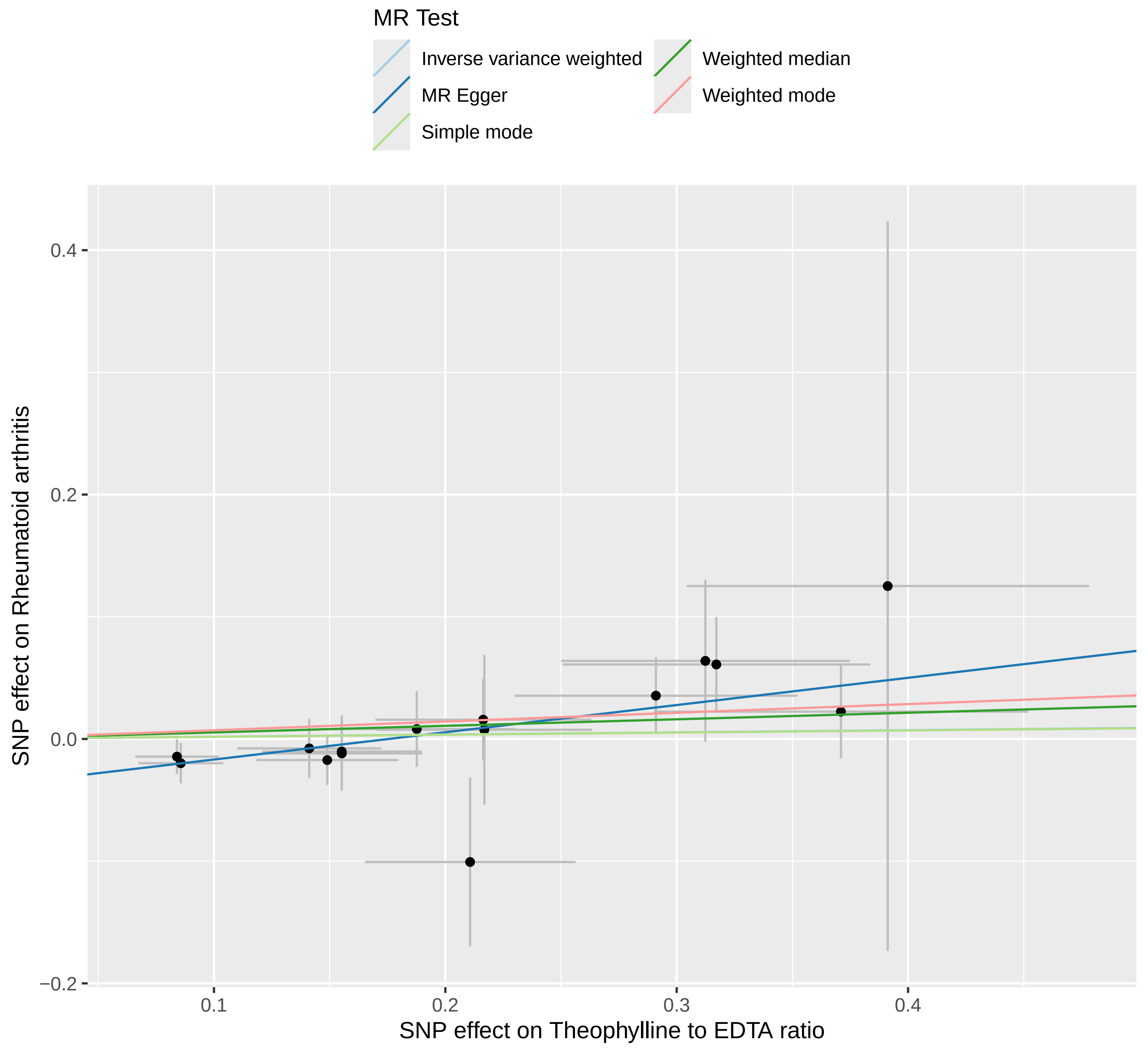

MR Test
Inverse variance weighted
MR Egger
Simple mode
Weighted median
Weighted mode
0.4
0.2
0.0
−0.2
0.1
0.2
0.3
0.4
SNP effect on Rheumatoid arthritis
SNP effect on Theophylline to EDTA ratio

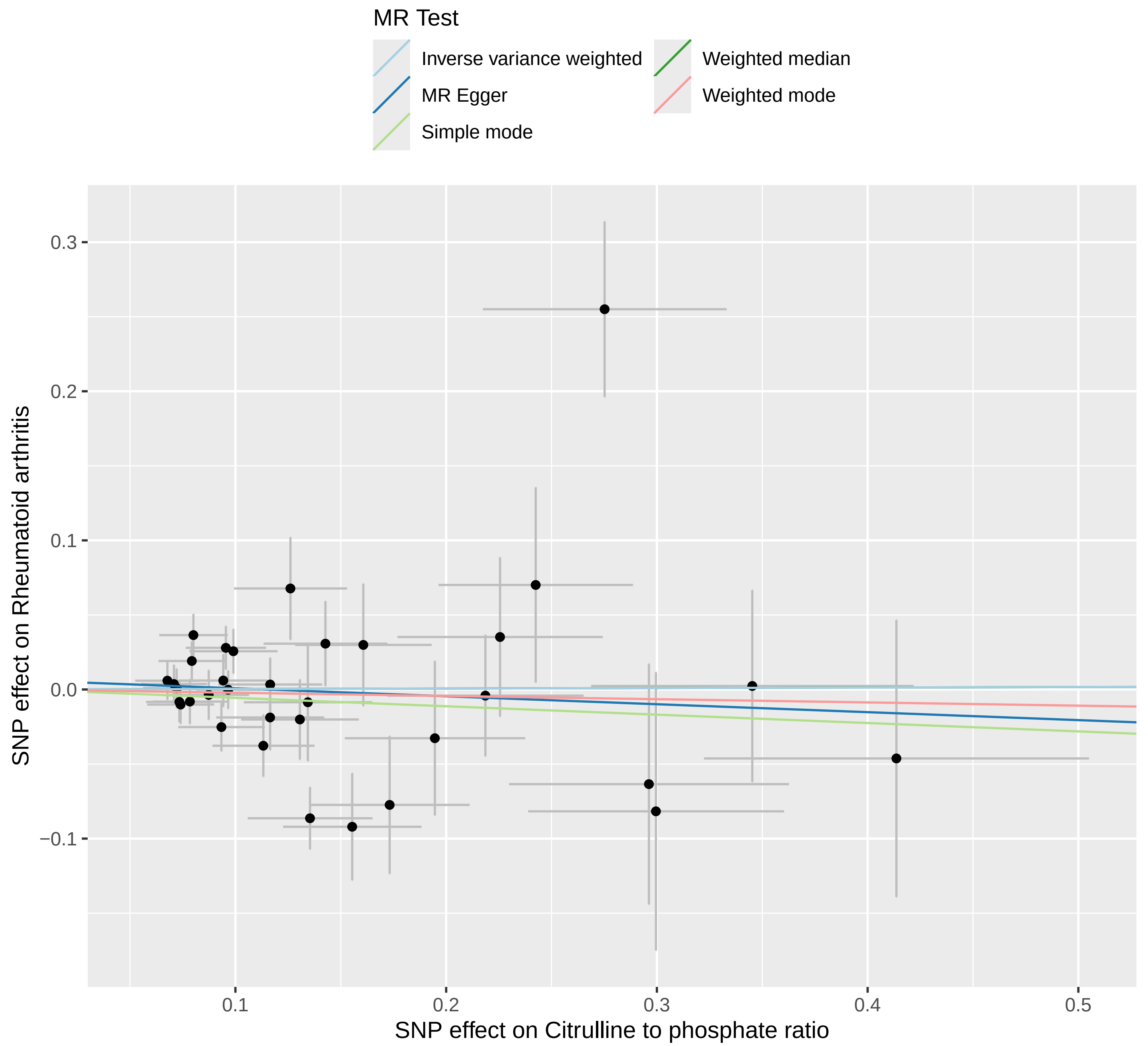

MR Test
Inverse variance weighted
MR Egger
Simple mode
Weighted median
Weighted mode
0.3
0.2
0.1
0.0
−0.1
SNP effect on Rheumatoid arthritis
0.1
0.2
0.3
0.4
0.5
SNP effect on Citrulline to phosphate ratio

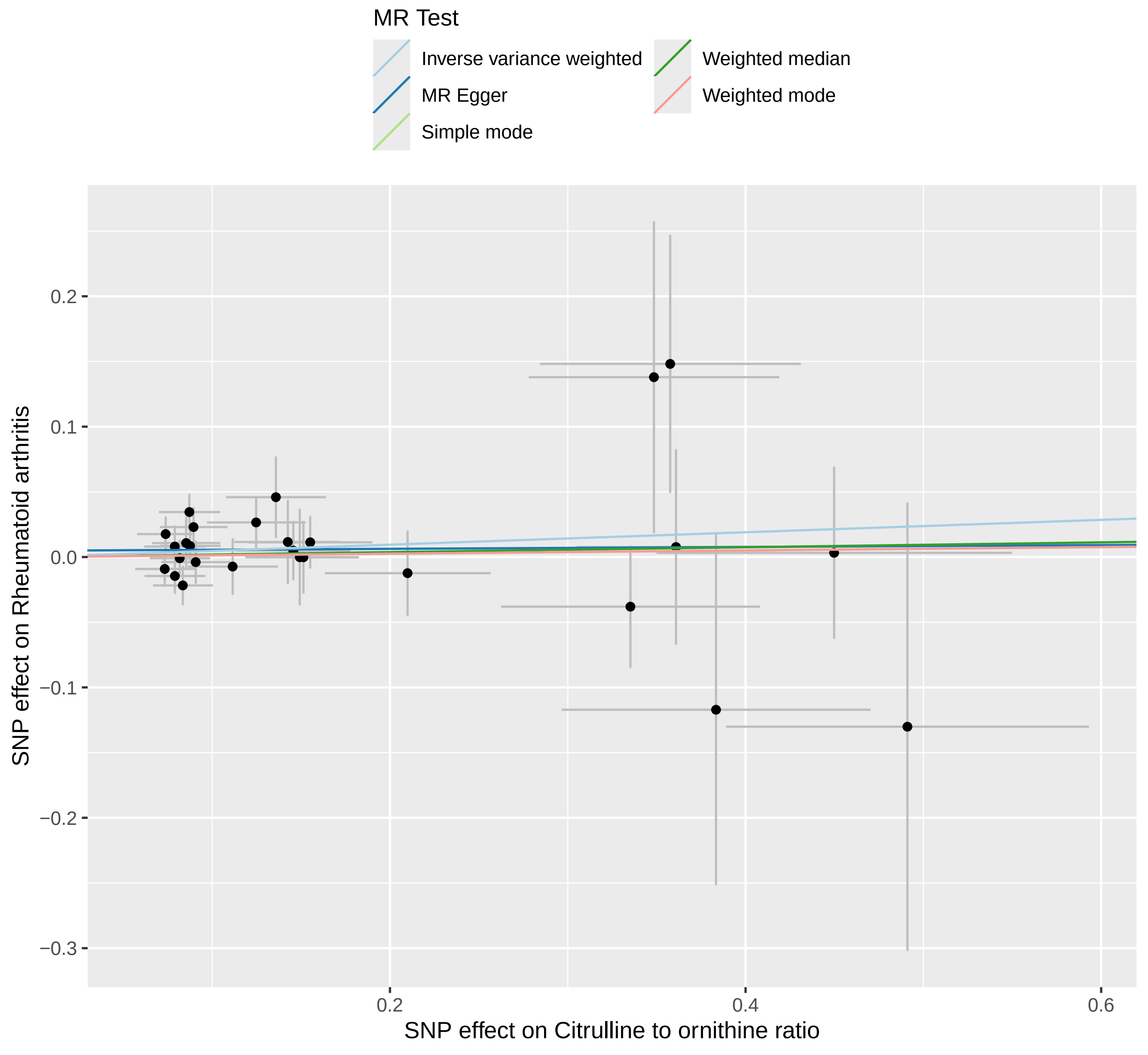
MR Test
Inverse variance weighted
MR Egger
Simple mode
Weighted median
Weighted mode
SNP effect on Rheumatoid arthritis
0.2
0.1
0.0
−0.1
−0.2
−0.3
0.2
0.4
0.6
SNP effect on Citrulline to ornithine ratio

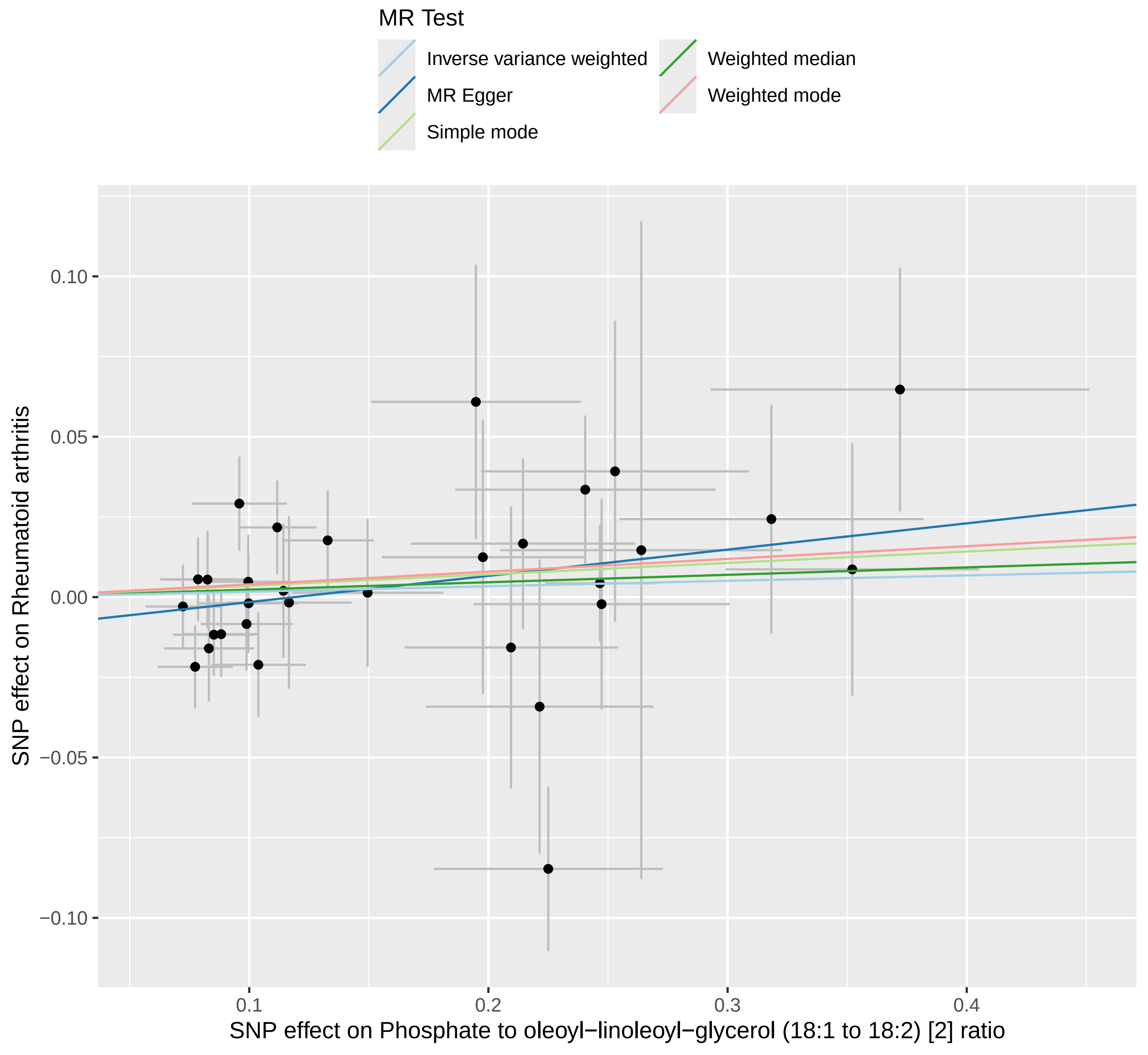
MR Test
Inverse variance weighted
MR Egger
Simple mode
Weighted median
Weighted mode
0.10
0.05
0.00
−0.05
−0.10
SNP effect on Rheumatoid arthritis
0.1
0.2
0.3
0.4
SNP effect on Phosphate to oleoyl−linoleoyl−glycerol (18:1 to 18:2) [2] ratio

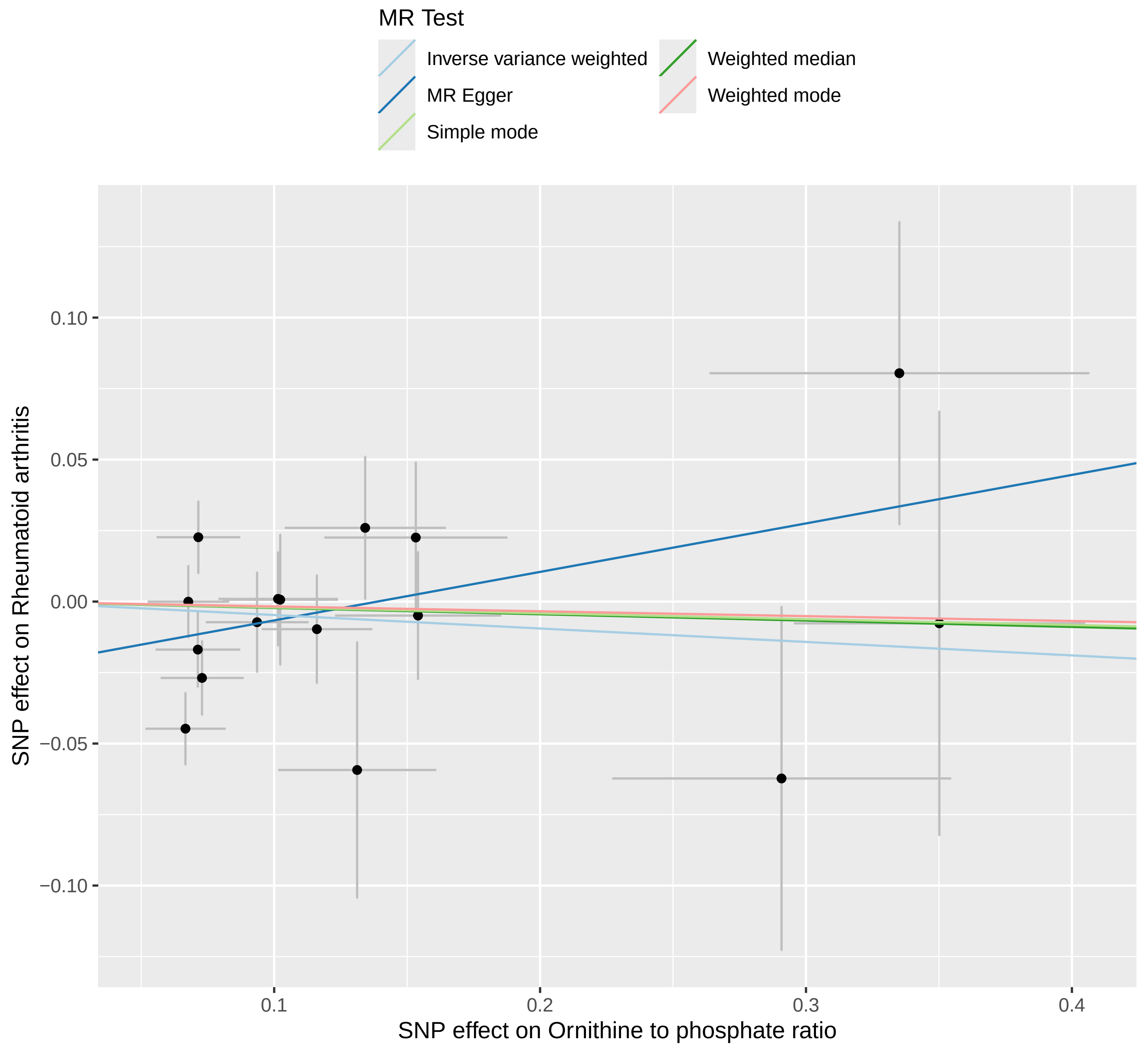

MR Test
Inverse variance weighted
MR Egger
Simple mode
Weighted median
Weighted mode
0.10
0.05
0.00
−0.05
−0.10
SNP effect on Rheumatoid arthritis
0.1
0.2
0.3
0.4
SNP effect on Ornithine to phosphate ratio

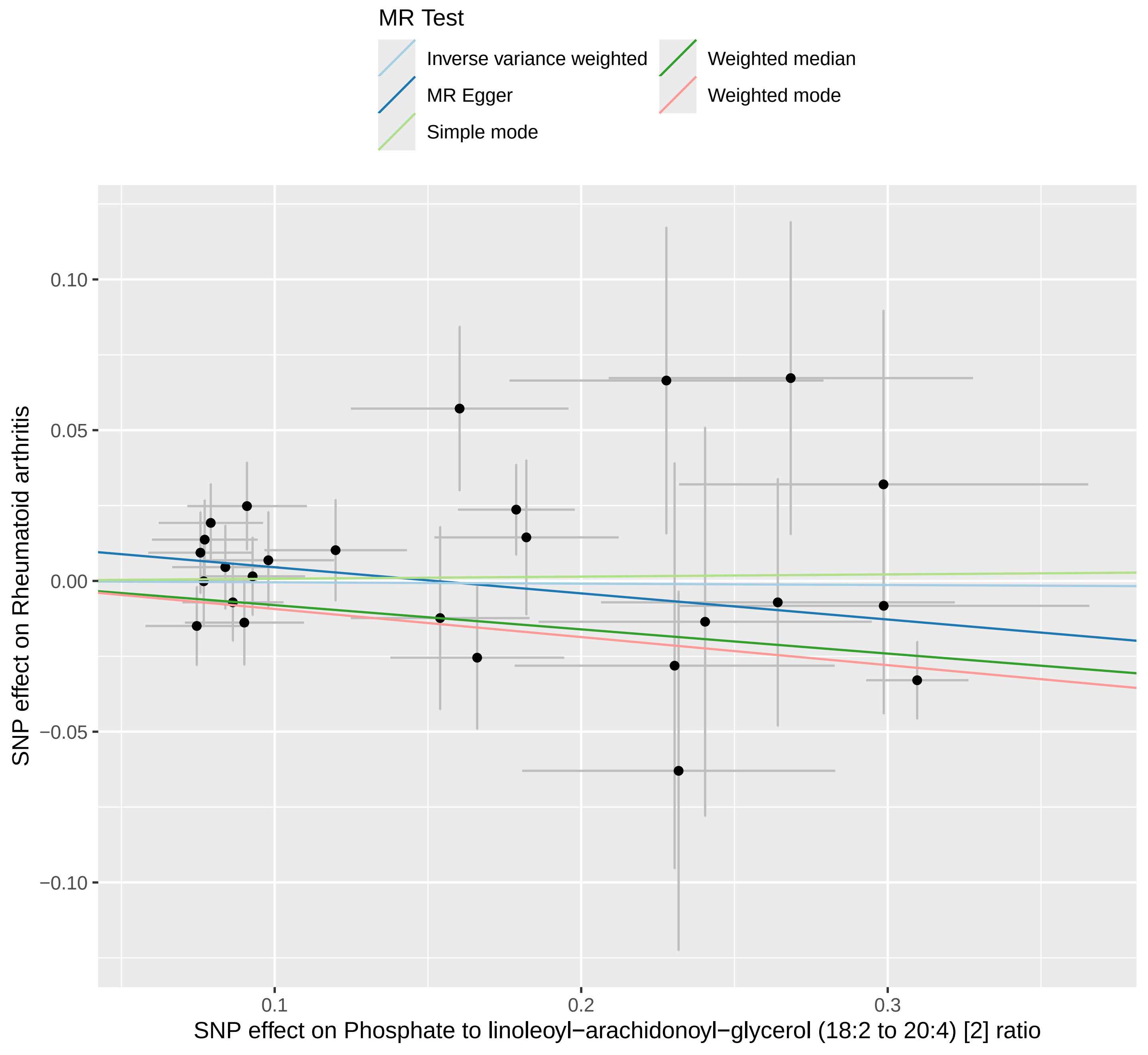
MR Test
Inverse variance weighted
MR Egger
Simple mode
Weighted median
Weighted mode
0.10
0.05
0.00
−0.05
−0.10
SNP effect on Rheumatoid arthritis
0.1
0.2
0.3
SNP effect on Phosphate to linoleoyl−arachidonoyl−glycerol (18:2 to 20:4) [2] ratio

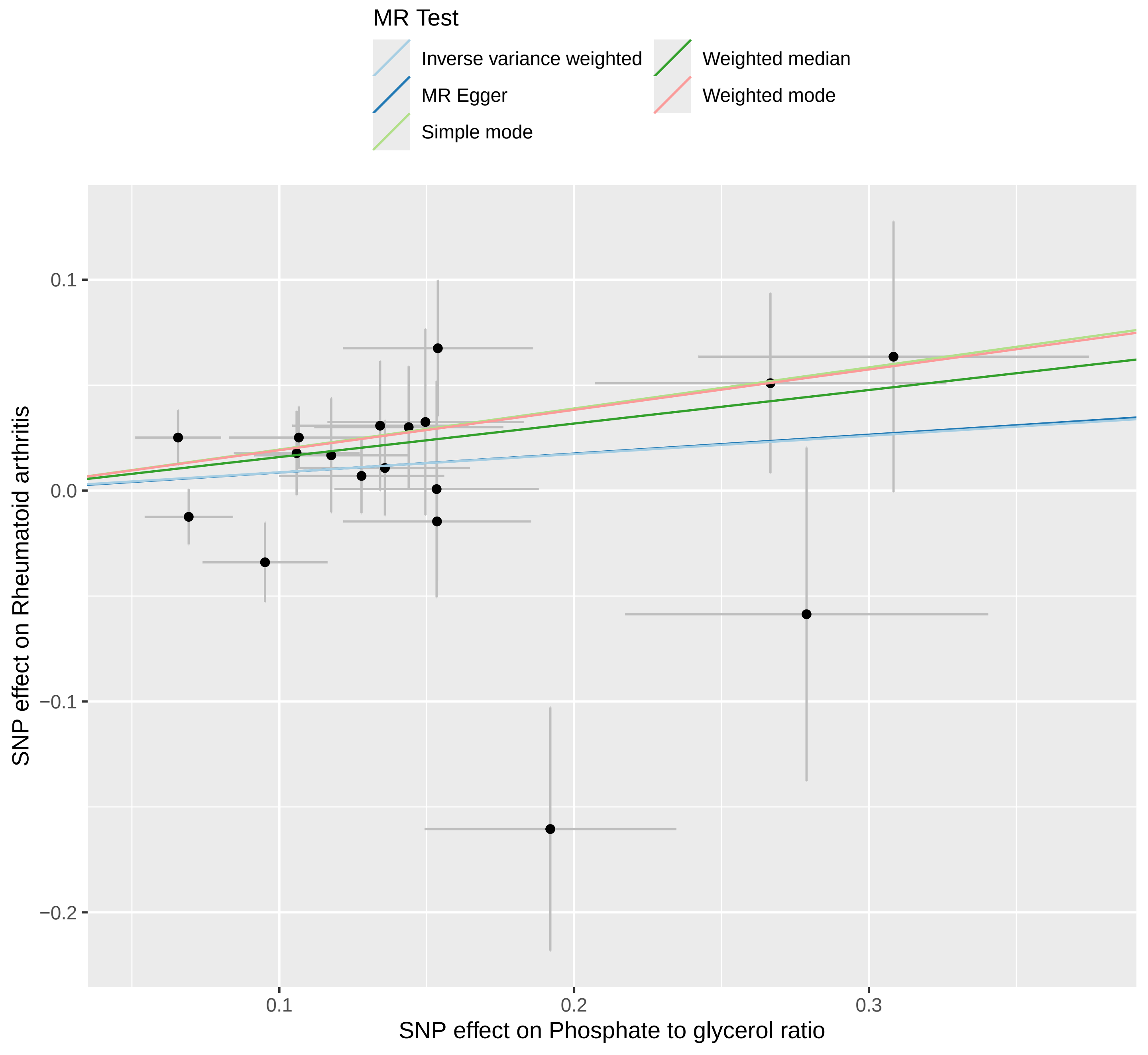

MR Test
Inverse variance weighted
MR Egger
Simple mode
Weighted median
Weighted mode
0.1
0.0
−0.1
−0.2
0.1
0.2
0.3
SNP effect on Rheumatoid arthritis
SNP effect on Phosphate to glycerol ratio

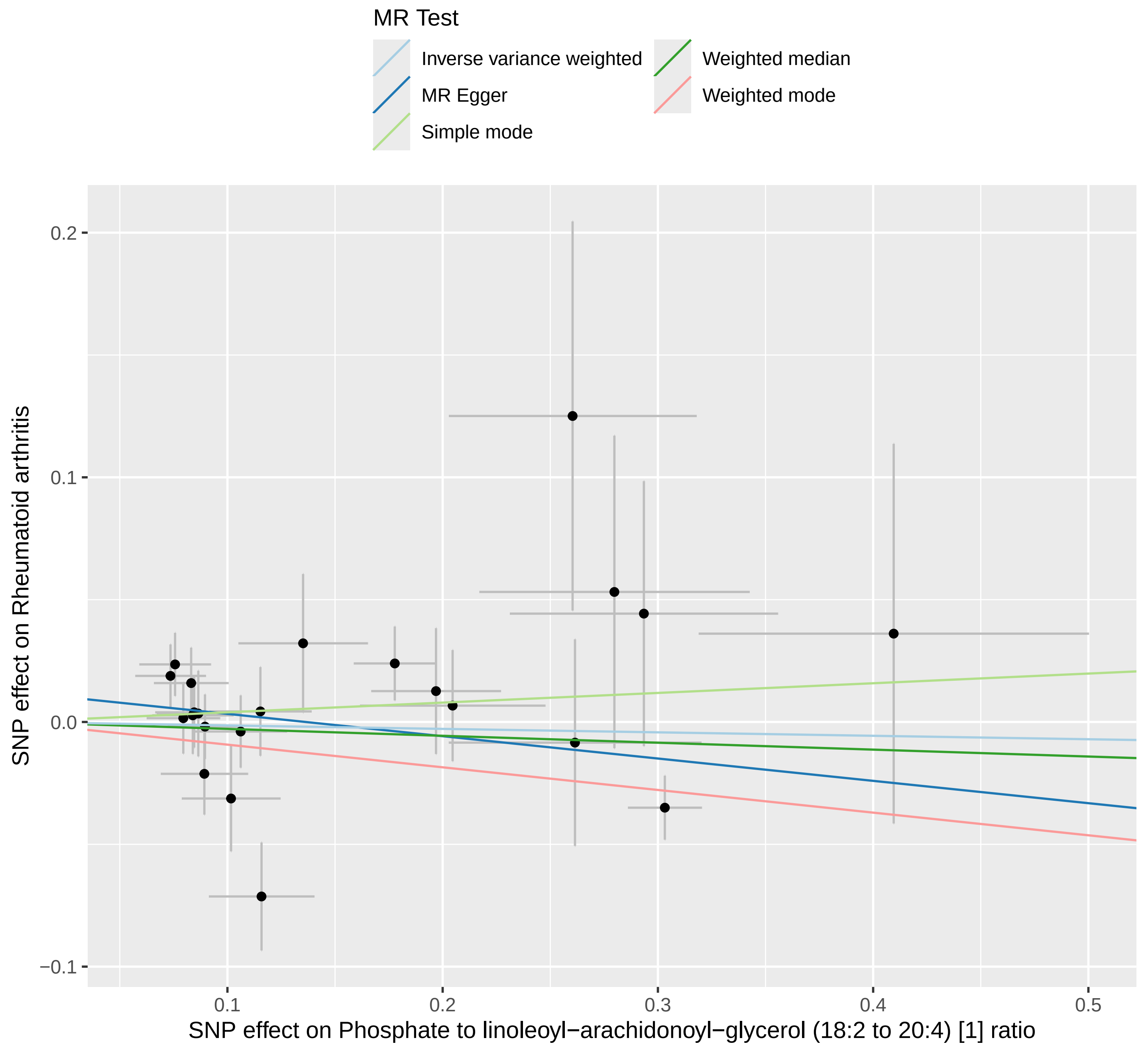
MR Test
Inverse variance weighted
MR Egger
Simple mode
Weighted median
Weighted mode
SNP effect on Rheumatoid arthritis
0.2
0.1
0.0
−0.1
0.1
0.2
0.3
0.4
0.5
SNP effect on Phosphate to linoleoyl−arachidonoyl−glycerol (18:2 to 20:4) [1] ratio

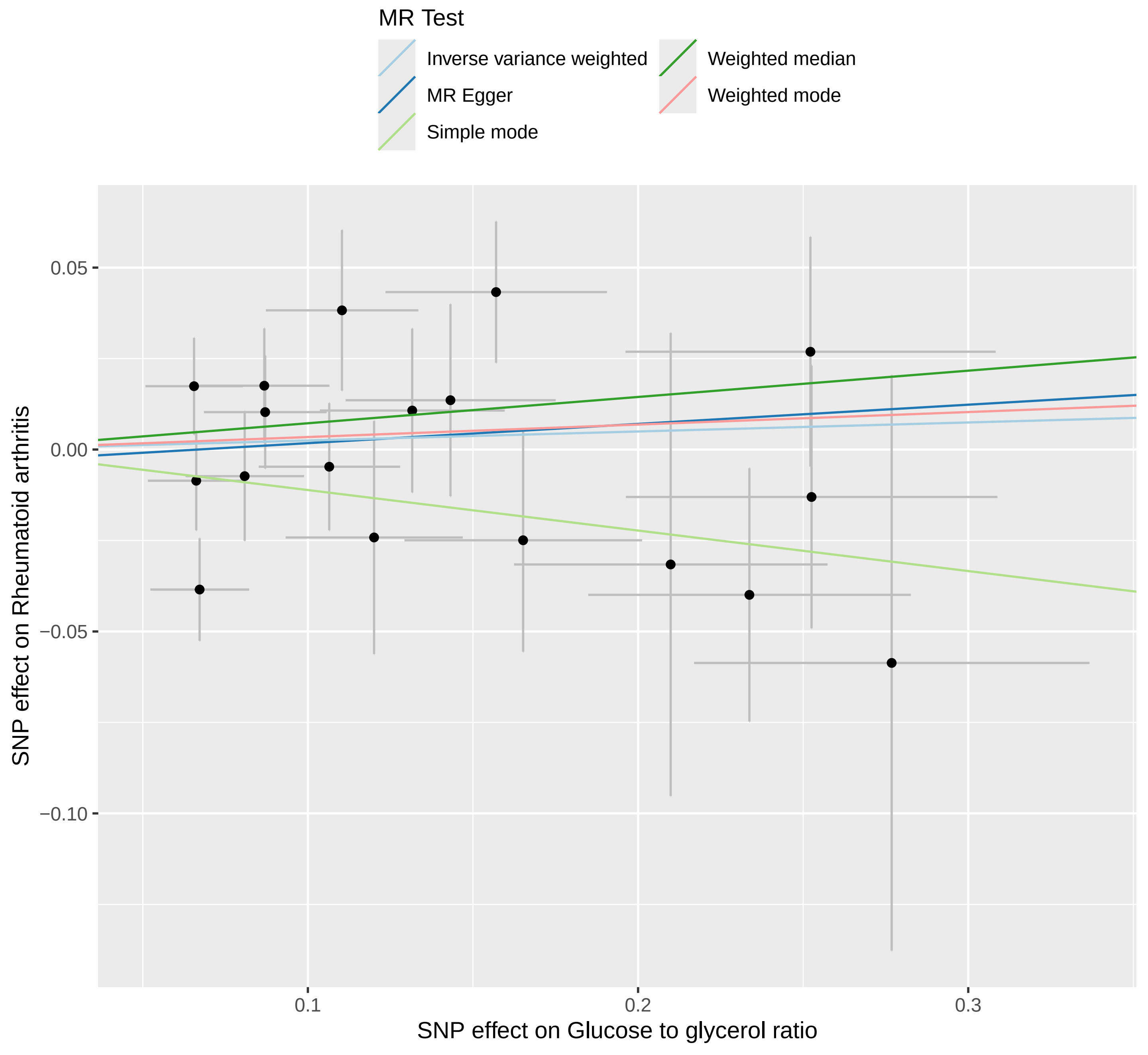
MR Test
Inverse variance weighted
MR Egger
Simple mode
Weighted median
Weighted mode
0.05
0.00
−0.05
−0.10
0.1
0.2
0.3
SNP effect on Rheumatoid arthritis
SNP effect on Glucose to glycerol ratio

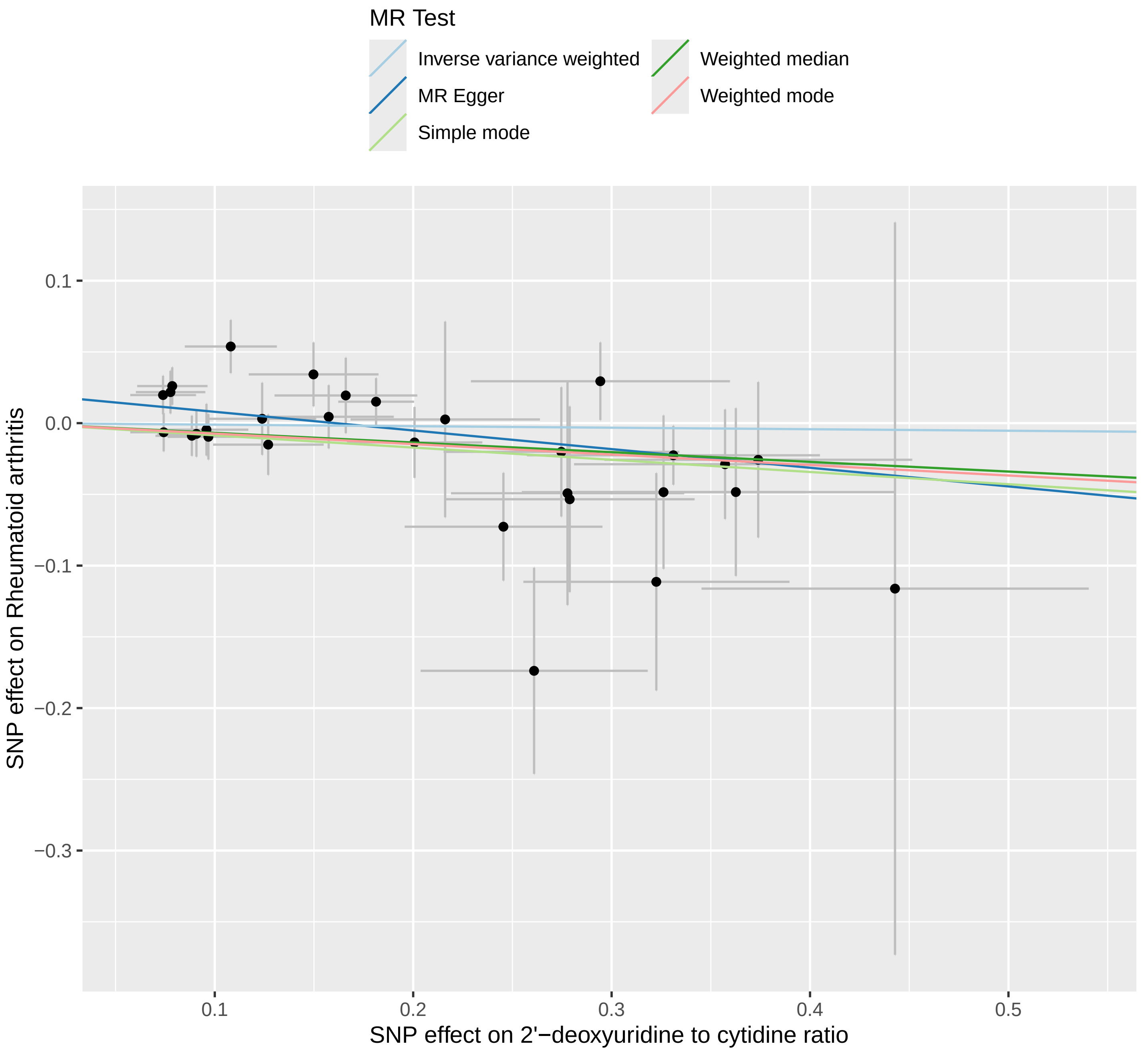

MR Test
Inverse variance weighted
MR Egger
Simple mode
Weighted median
Weighted mode
0.1
0.0
−0.1
−0.2
−0.3
SNP effect on Rheumatoid arthritis
0.1
0.2
0.3
0.4
0.5
SNP effect on 2'−deoxyuridine to cytidine ratio

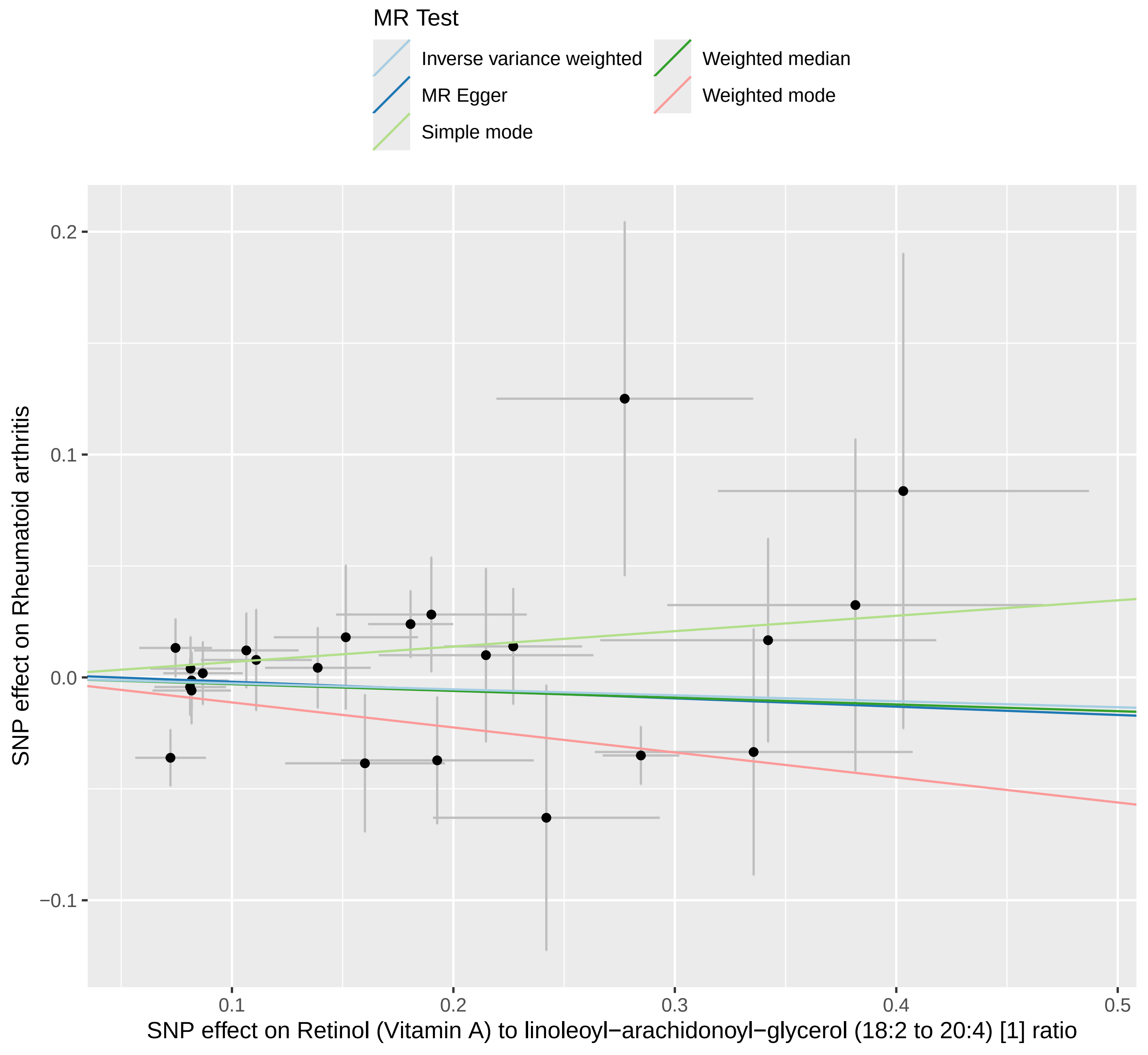
MR Test
Inverse variance weighted
MR Egger
Simple mode
Weighted median
Weighted mode
0.2
0.1
0.0
−0.1
0.1
0.2
0.3
0.4
0.5
SNP effect on Rheumatoid arthritis
SNP effect on Retinol (Vitamin A) to linoleoyl−arachidonoyl−glycerol (18:2 to 20:4) [1] ratio

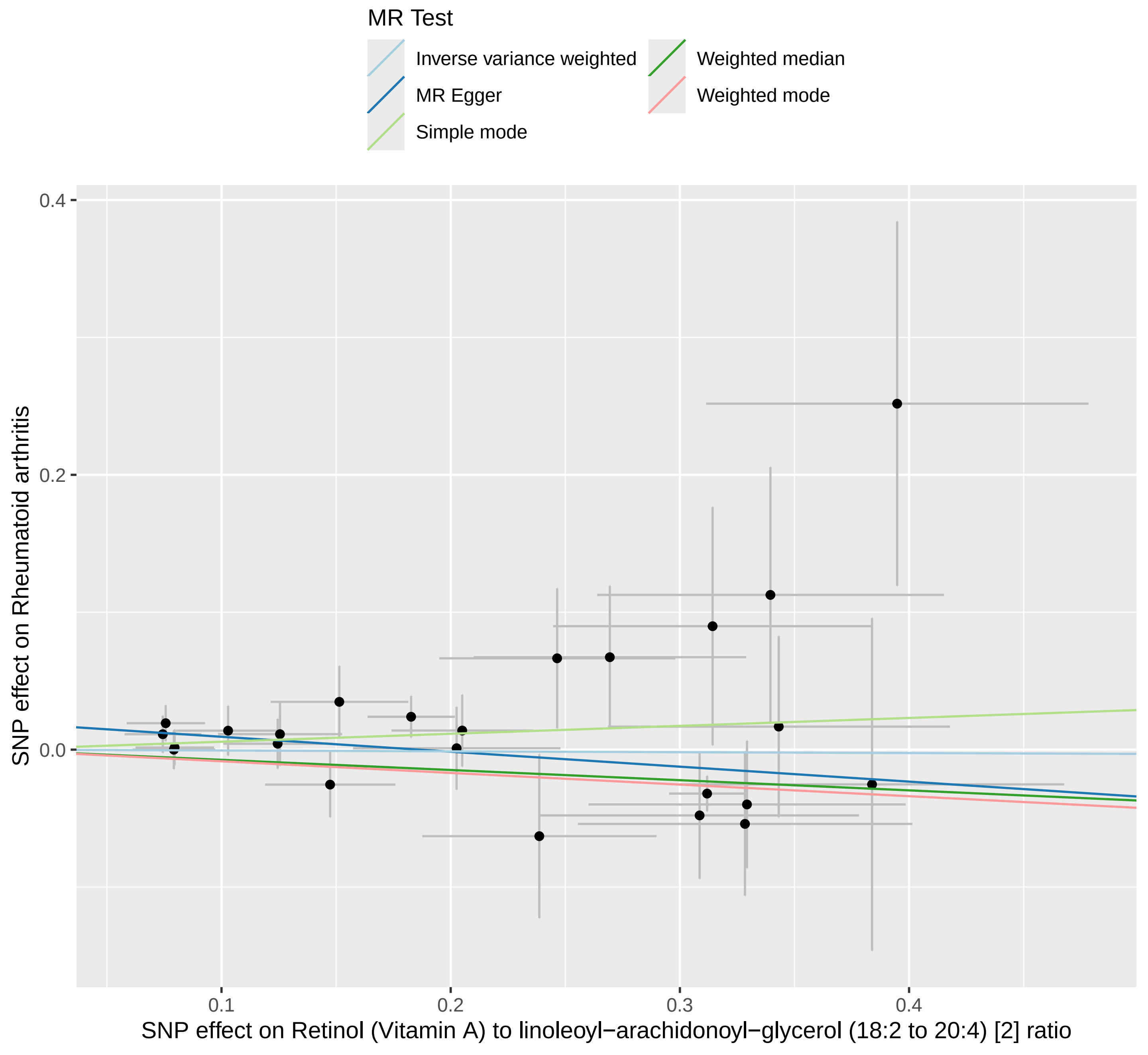

MR Test
Inverse variance weighted
MR Egger
Simple mode
Weighted median
Weighted mode
0.4
0.2
0.0
0.1
0.2
0.3
0.4
SNP effect on Rheumatoid arthritis
SNP effect on Retinol (Vitamin A) to linoleoyl−arachidonoyl−glycerol (18:2 to 20:4) [2] ratio

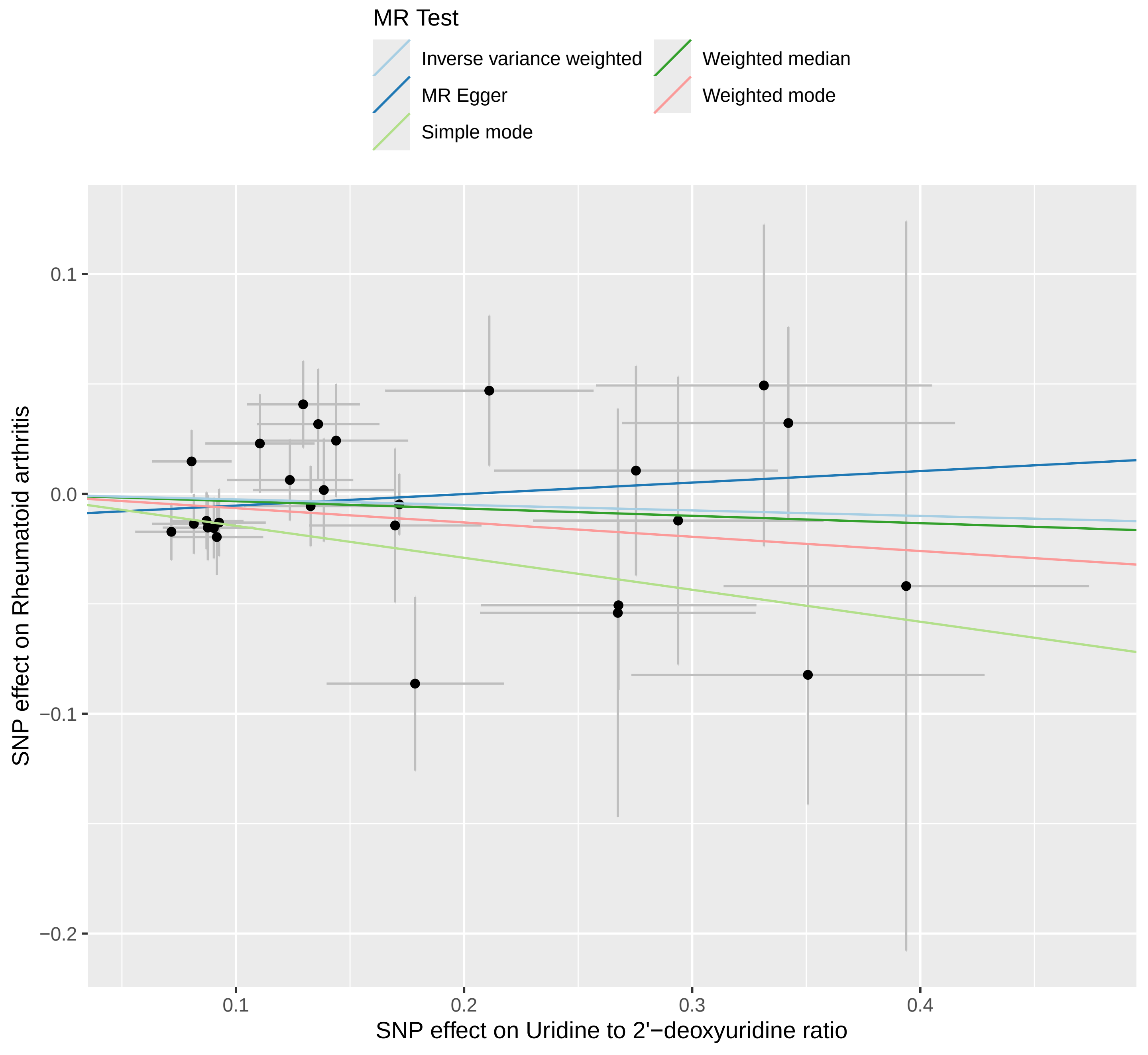

MR Test
Inverse variance weighted
MR Egger
Simple mode
Weighted median
Weighted mode
0.1
0.0
−0.1
−0.2
SNP effect on Rheumatoid arthritis
0.1
0.2
0.3
0.4
SNP effect on Uridine to 2'−deoxyuridine ratio

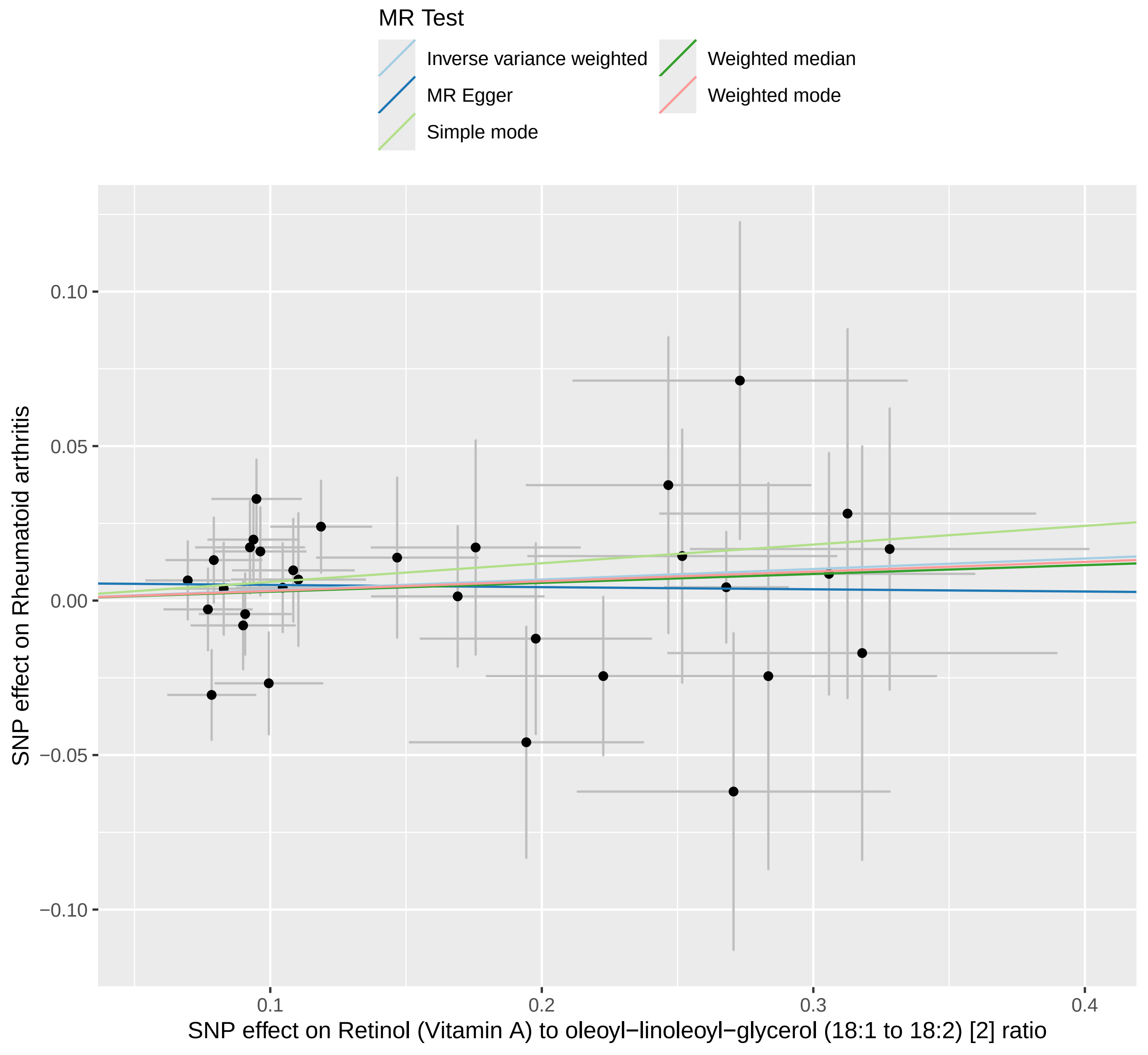

MR Test
Inverse variance weighted
MR Egger
Simple mode
Weighted median
Weighted mode
0.10
0.05
0.00
−0.05
−0.10
SNP effect on Rheumatoid arthritis
0.1
0.2
0.3
0.4
SNP effect on Retinol (Vitamin A) to oleoyl−linoleoyl−glycerol (18:1 to 18:2) [2] ratio

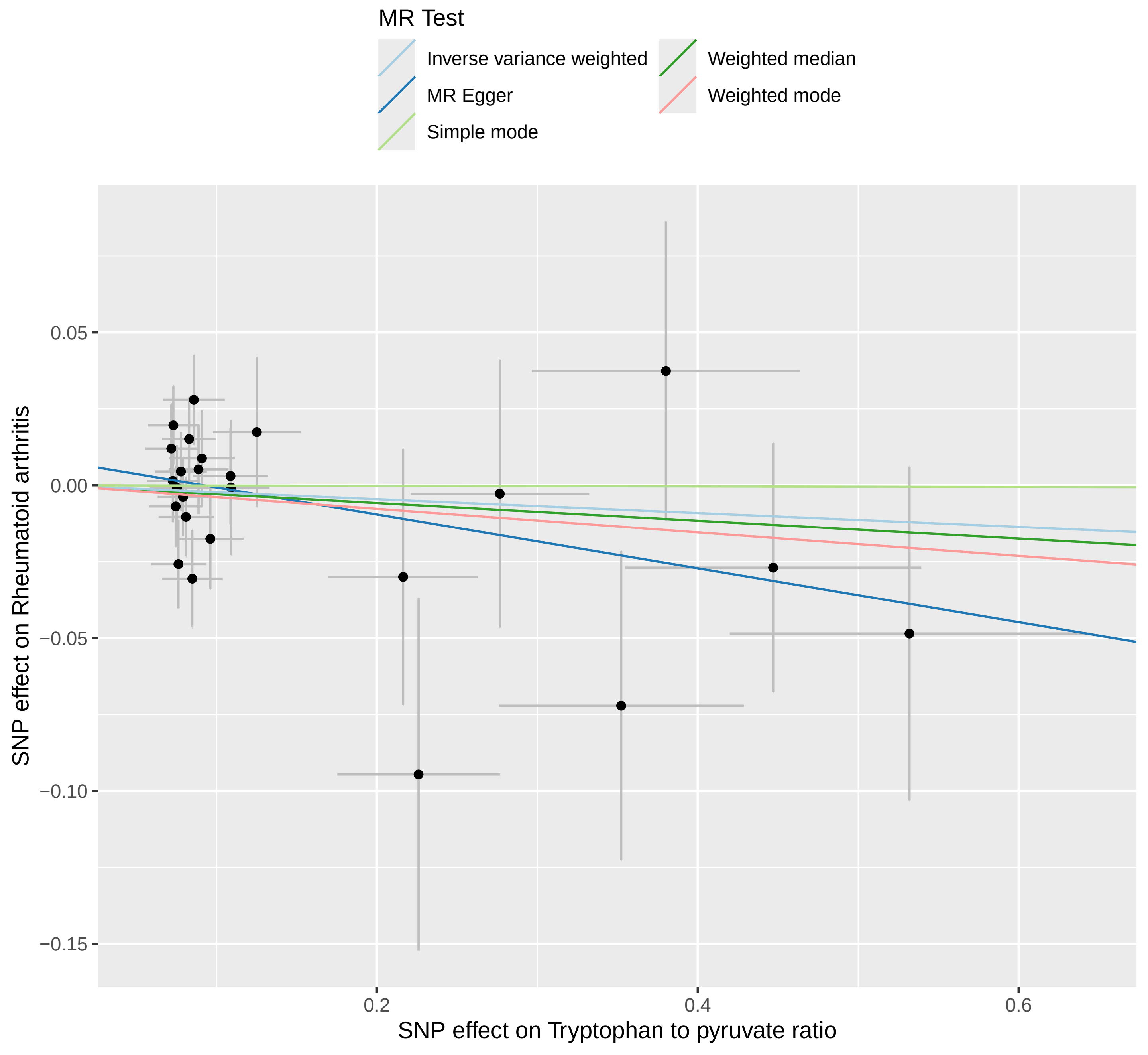

MR Test
Inverse variance weighted
MR Egger
Simple mode
Weighted median
Weighted mode
0.05
0.00
−0.05
−0.10
−0.15
0.2
0.4
0.6
SNP effect on Rheumatoid arthritis
SNP effect on Tryptophan to pyruvate ratio

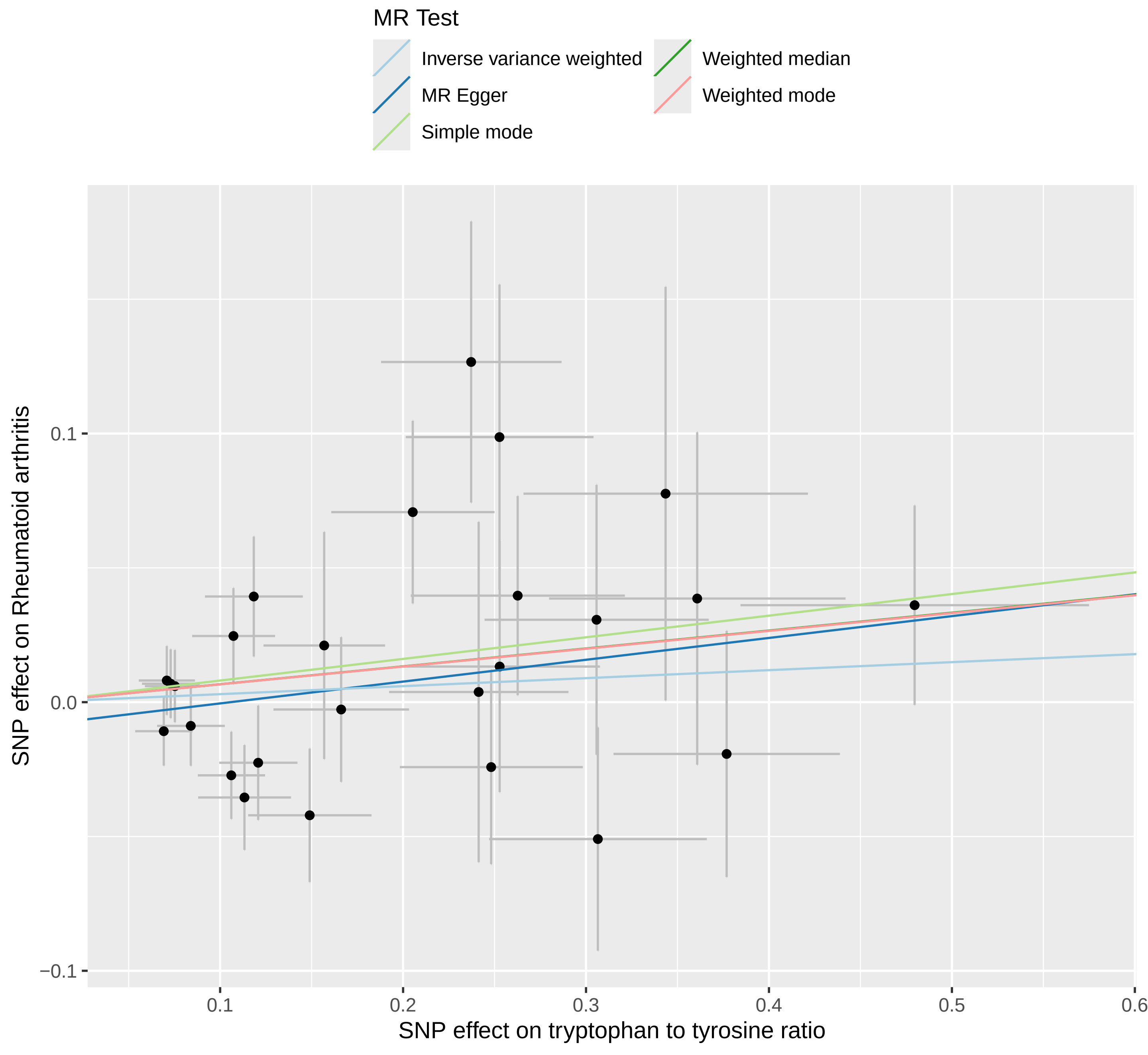

MR Test
Inverse variance weighted
MR Egger
Simple mode
Weighted median
Weighted mode
SNP effect on Rheumatoid arthritis
0.1
0.0
−0.1
0.1
0.2
0.3
0.4
0.5
0.6
SNP effect on tryptophan to tyrosine ratio

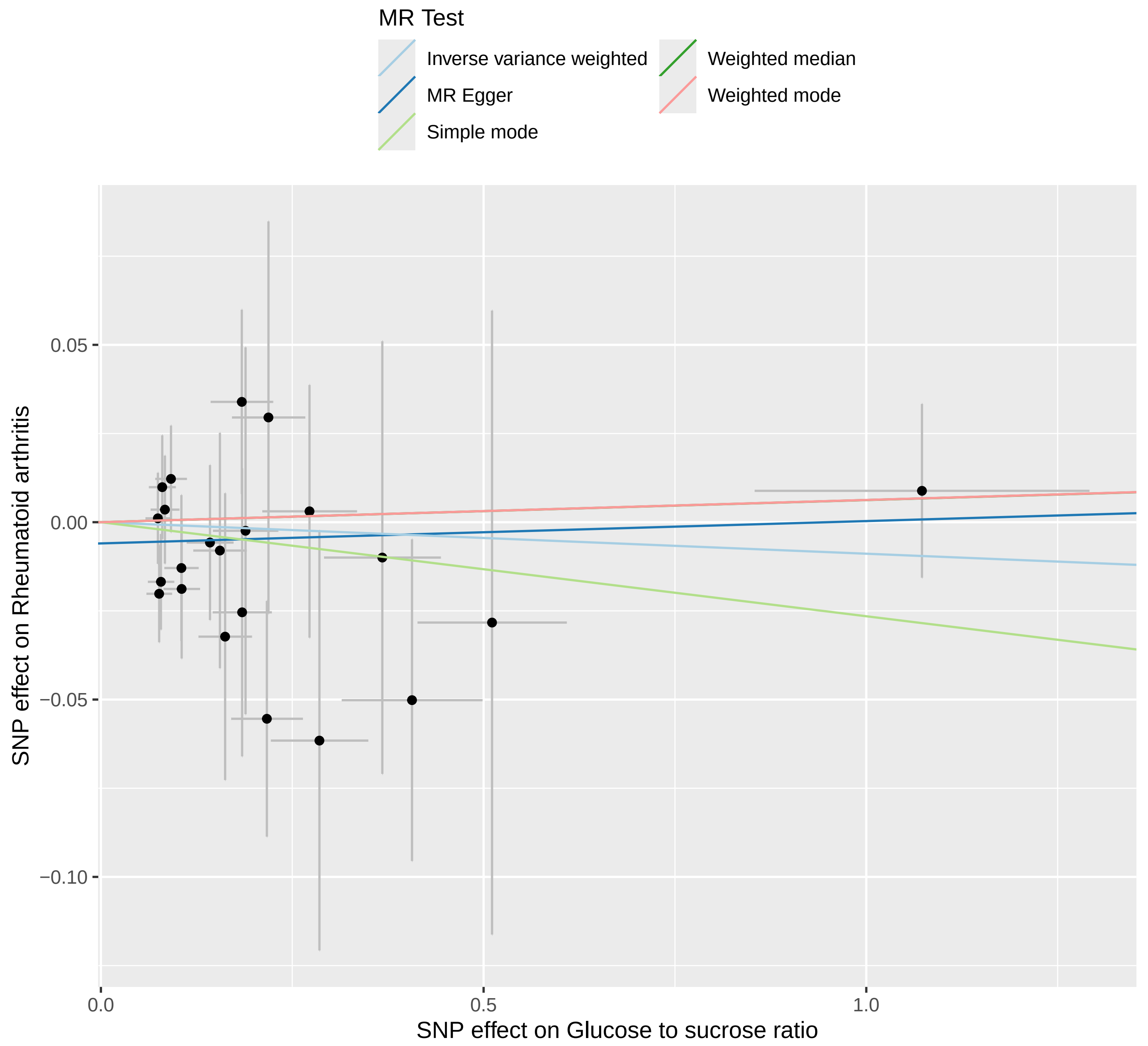

MR Test
Inverse variance weighted
MR Egger
Simple mode
Weighted median
Weighted mode
0.05
0.00
−0.05
−0.10
SNP effect on Rheumatoid arthritis
0.0
0.5
1.0
SNP effect on Glucose to sucrose ratio

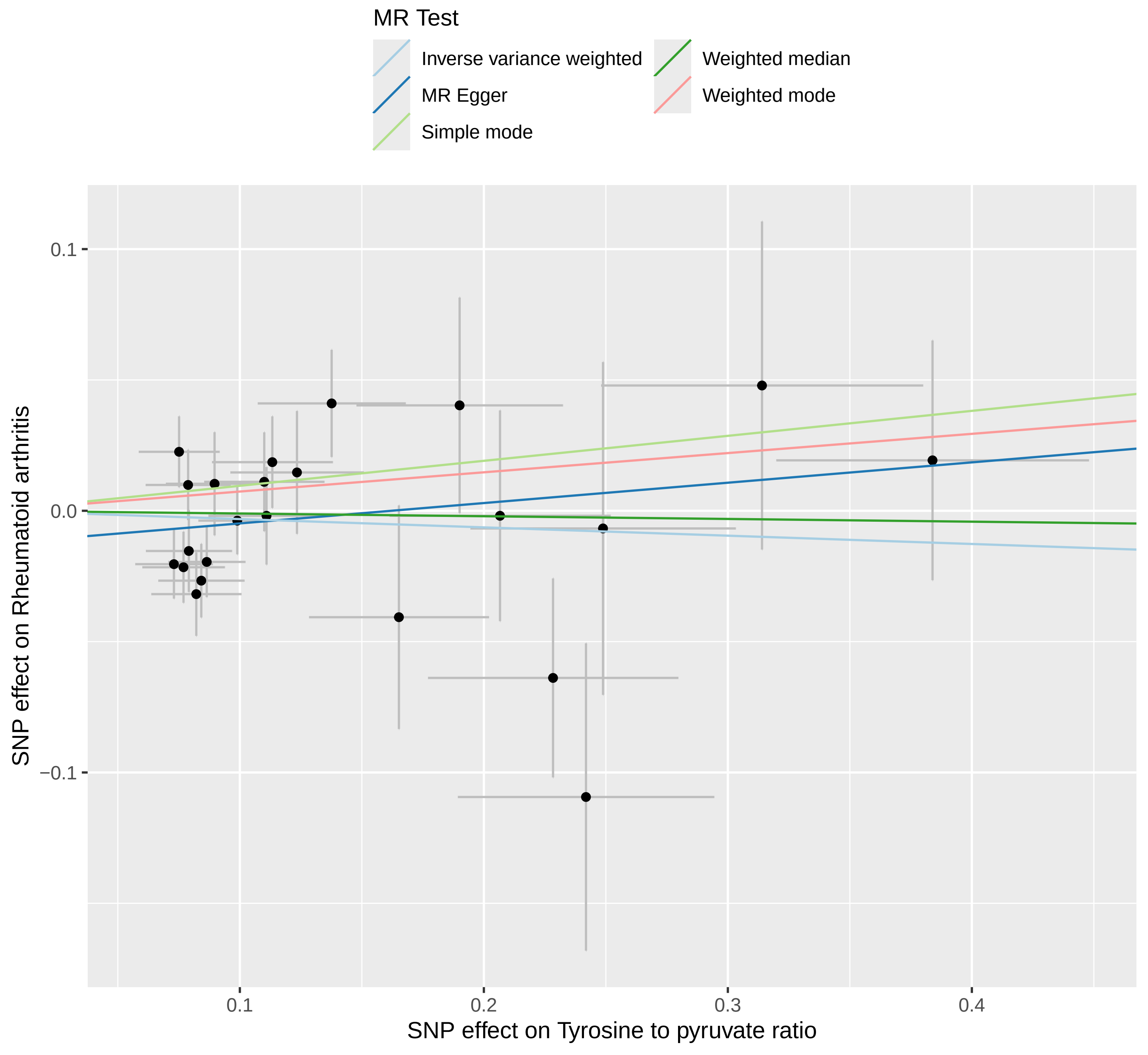

MR Test
Inverse variance weighted
MR Egger
Simple mode
Weighted median
Weighted mode
0.1
0.0
−0.1
SNP effect on Rheumatoid arthritis
0.1
0.2
0.3
0.4
SNP effect on Tyrosine to pyruvate ratio

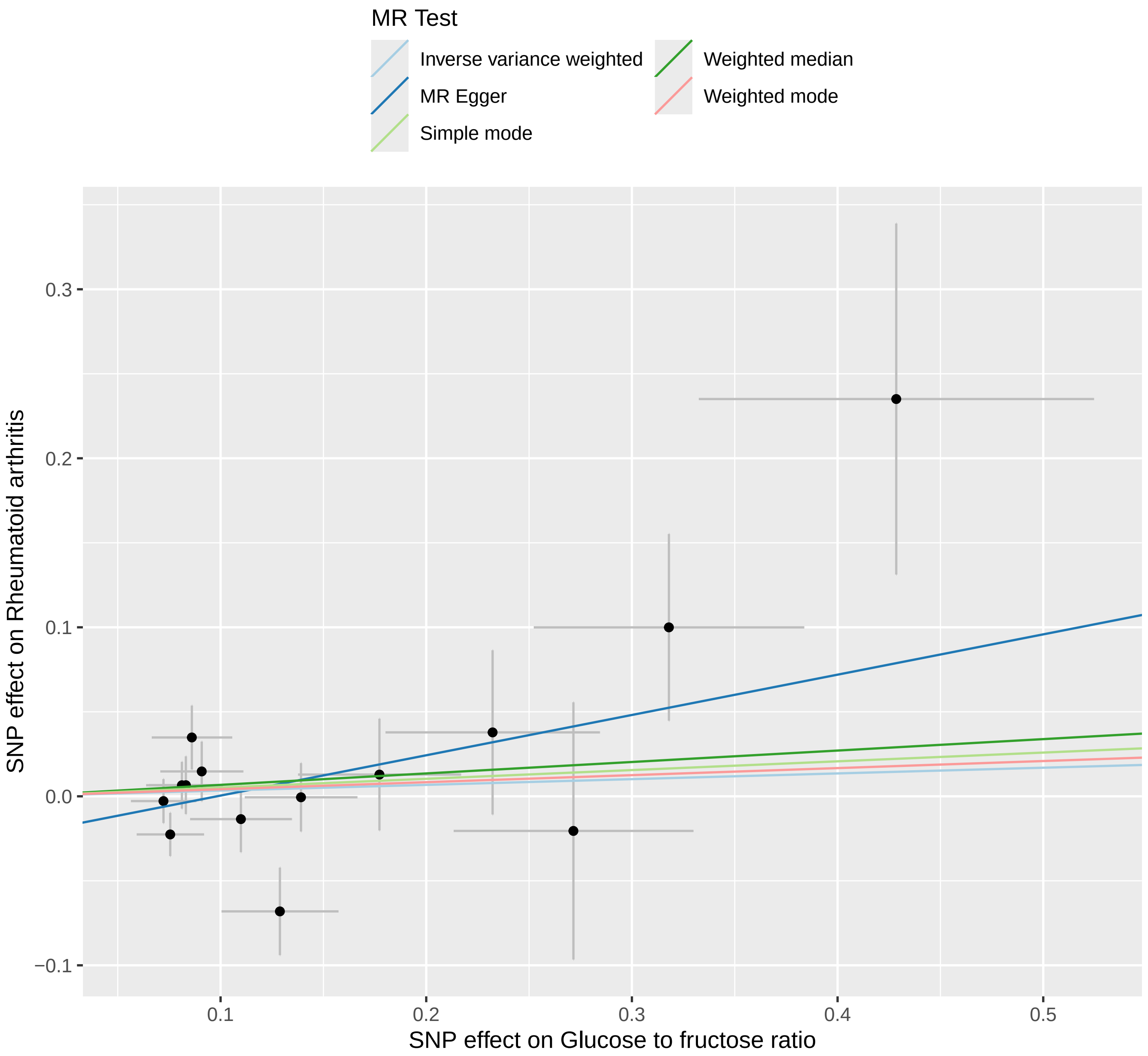
MR Test
Inverse variance weighted
MR Egger
Simple mode
Weighted median
Weighted mode
SNP effect on Rheumatoid arthritis
0.3
0.2
0.1
0.0
−0.1
0.1
0.2
0.3
0.4
0.5
SNP effect on Glucose to fructose ratio

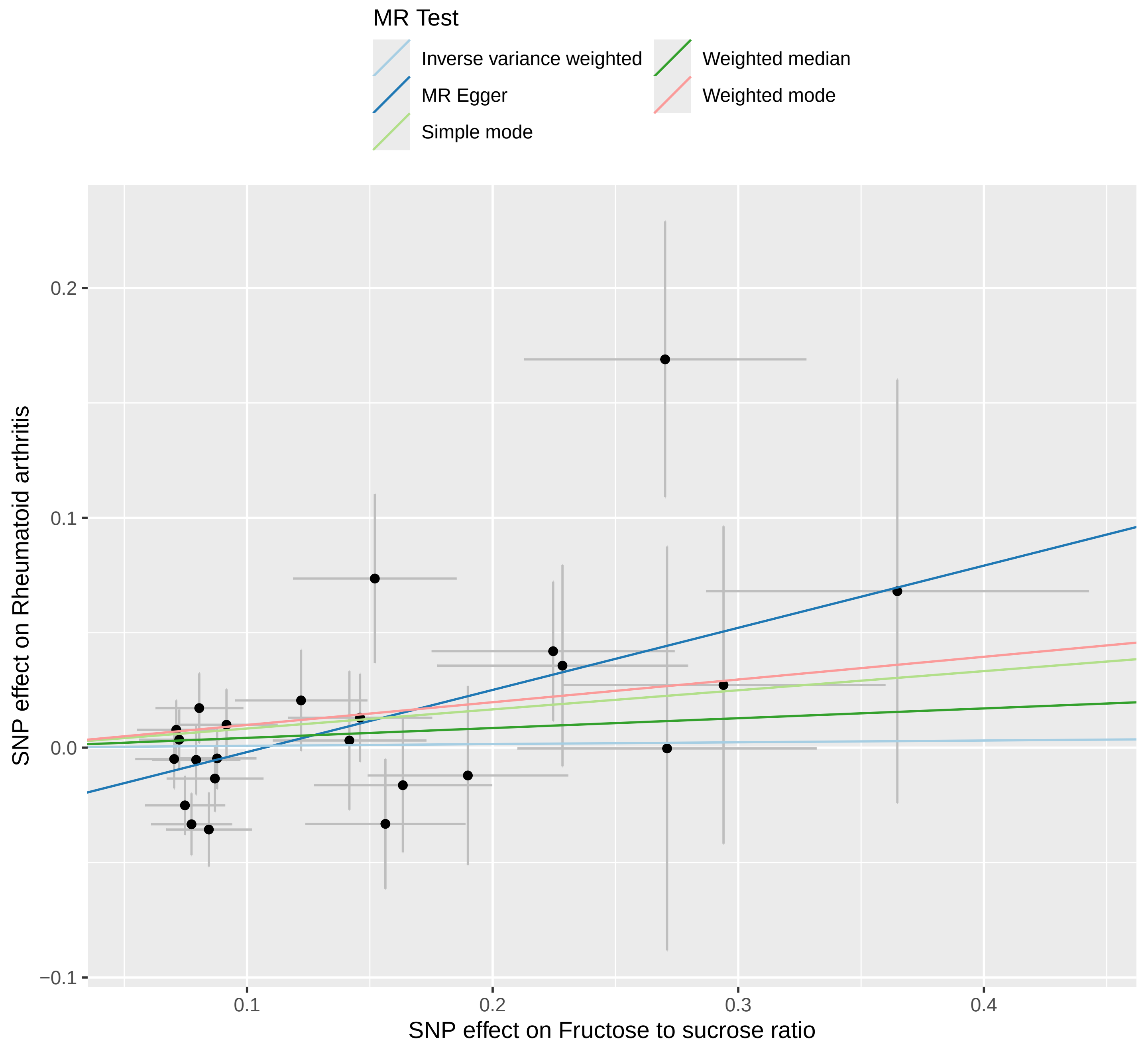

MR Test
Inverse variance weighted
MR Egger
Simple mode
Weighted median
Weighted mode
0.2
0.1
0.0
−0.1
0.1
0.2
0.3
0.4
SNP effect on Rheumatoid arthritis
SNP effect on Fructose to sucrose ratio

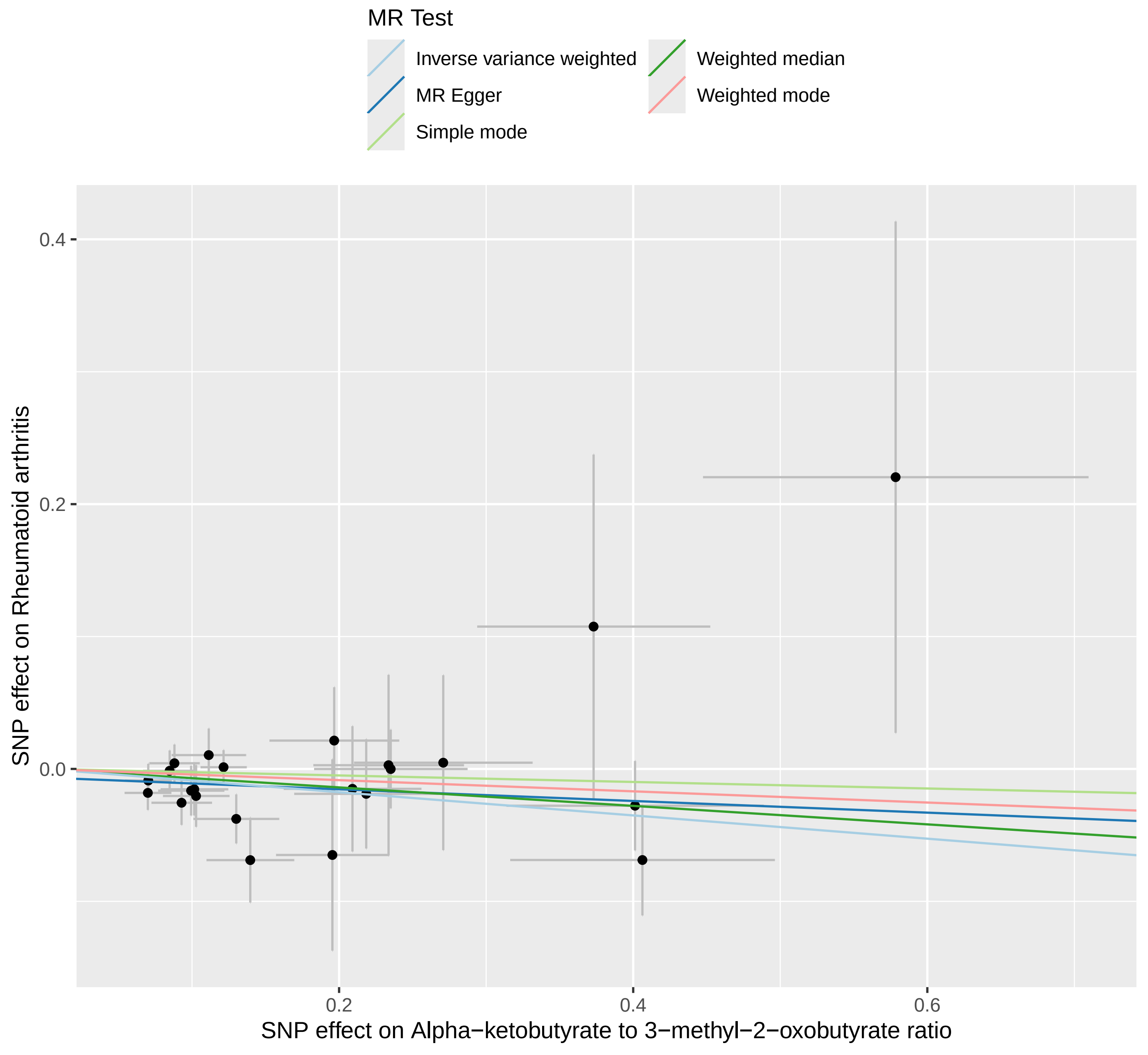

MR Test
Inverse variance weighted
MR Egger
Simple mode
Weighted median
Weighted mode
0.4
0.2
0.0
0.2
0.4
0.6
SNP effect on Rheumatoid arthritis
SNP effect on Alpha−ketobutyrate to 3−methyl−2−oxobutyrate ratio

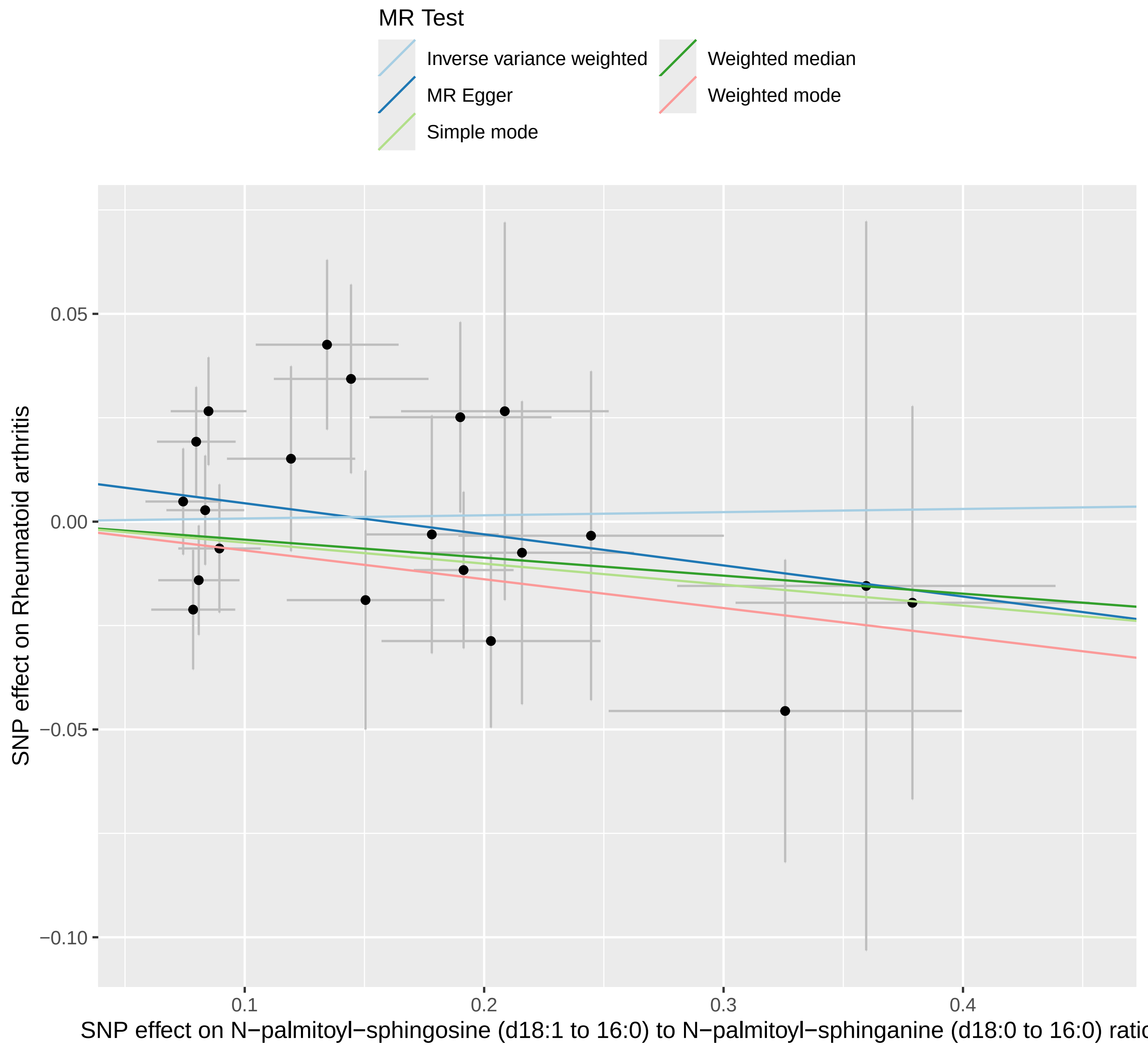
MR Test
Inverse variance weighted
MR Egger
Simple mode
Weighted median
Weighted mode
0.05
0.00
−0.05
−0.10
0.1
0.2
0.3
0.4
SNP effect on Rheumatoid arthritis
SNP effect on N−palmitoyl−sphingosine (d18:1 to 16:0) to N−palmitoyl−sphinganine (d18:0 to 16:0) ratio

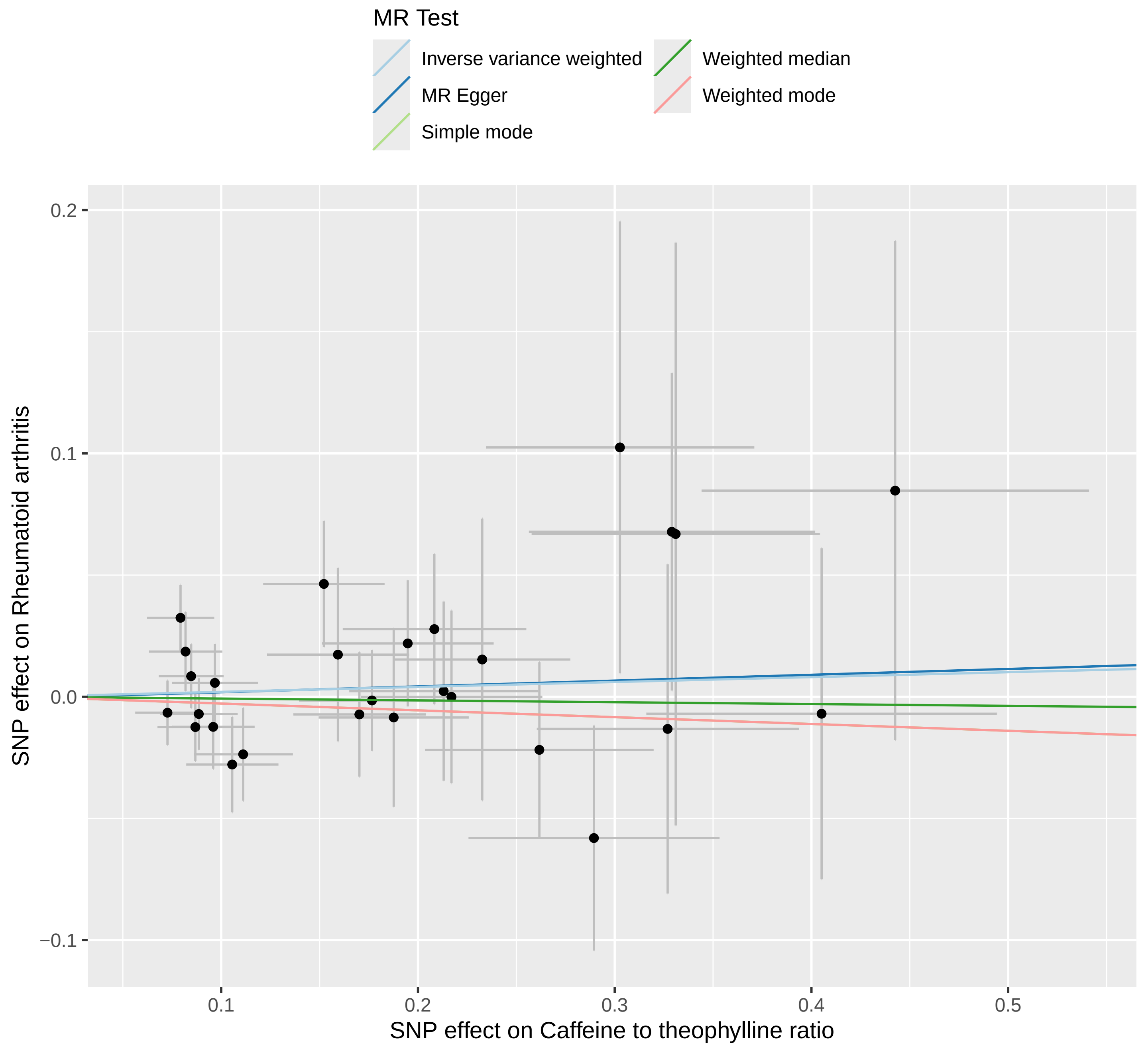

MR Test
Inverse variance weighted
MR Egger
Simple mode
Weighted median
Weighted mode
0.2
0.1
0.0
−0.1
0.1
0.2
0.3
0.4
0.5
SNP effect on Rheumatoid arthritis
SNP effect on Caffeine to theophylline ratio

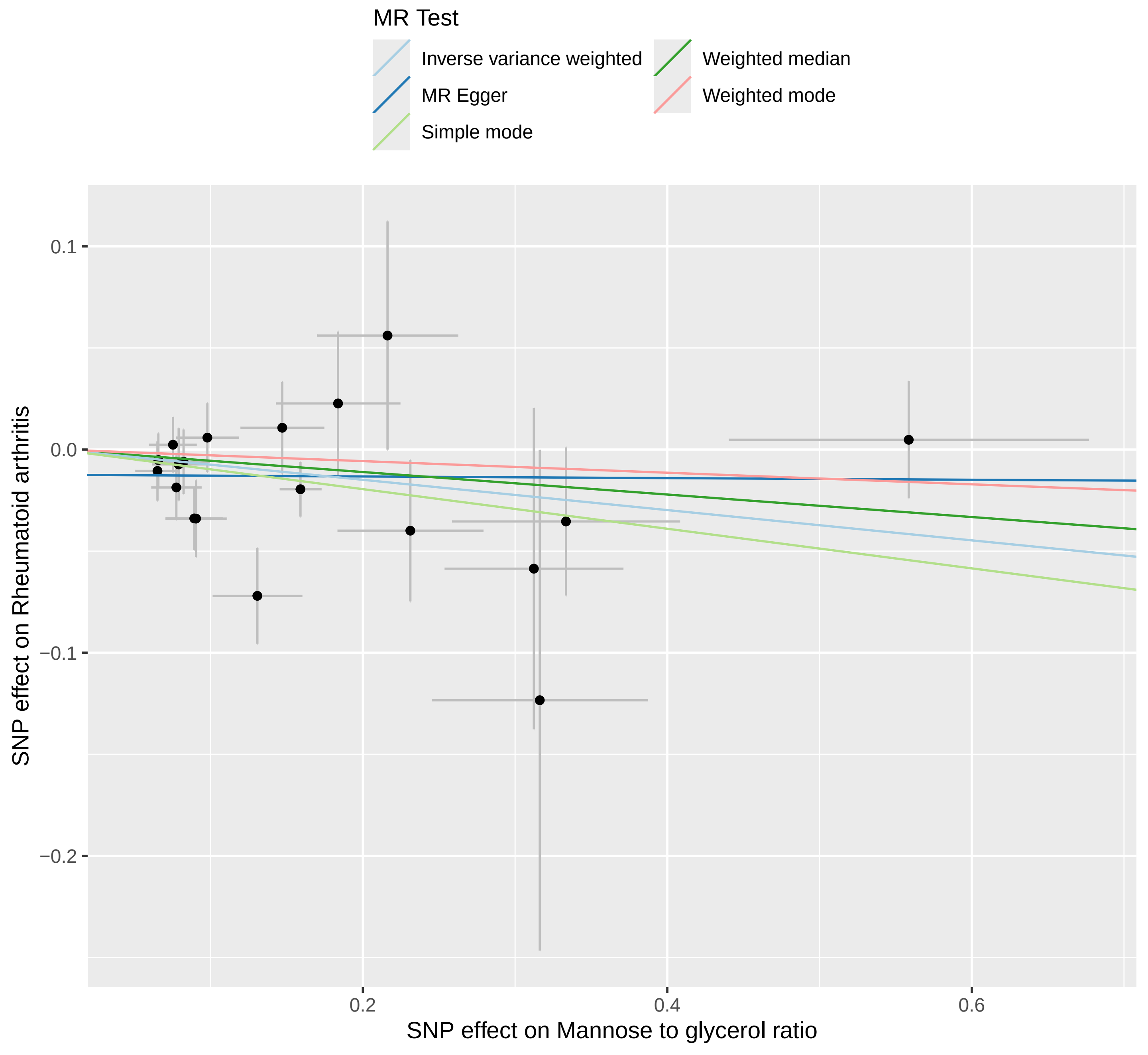
MR Test
Inverse variance weighted
MR Egger
Simple mode
Weighted median
Weighted mode
0.1
0.0
−0.1
−0.2
SNP effect on Rheumatoid arthritis
0.2
0.4
0.6
SNP effect on Mannose to glycerol ratio

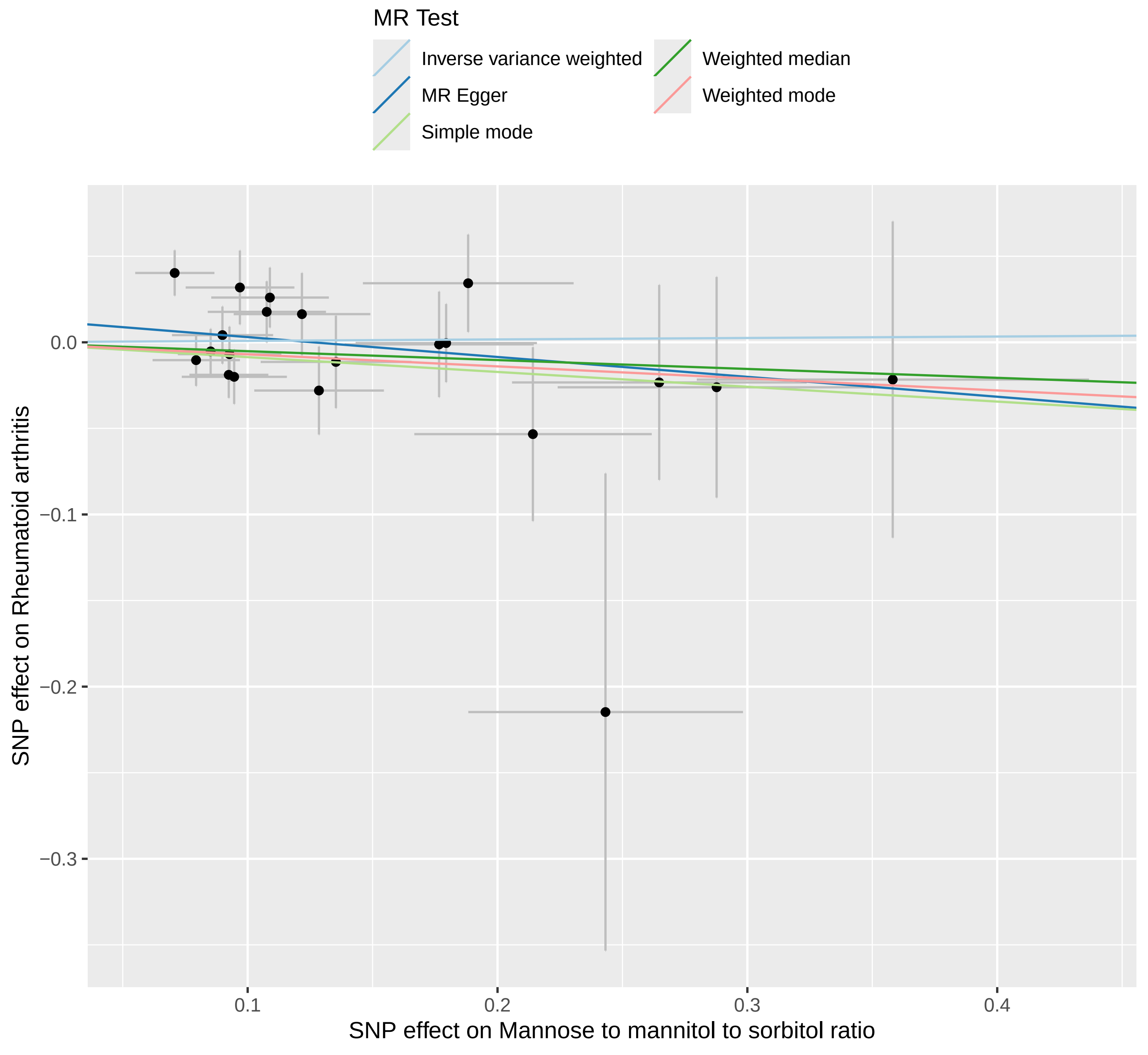

MR Test
Inverse variance weighted
MR Egger
Simple mode
Weighted median
Weighted mode
0.0
−0.1
−0.2
−0.3
0.1
0.2
0.3
0.4
SNP effect on Rheumatoid arthritis
SNP effect on Mannose to mannitol to sorbitol ratio

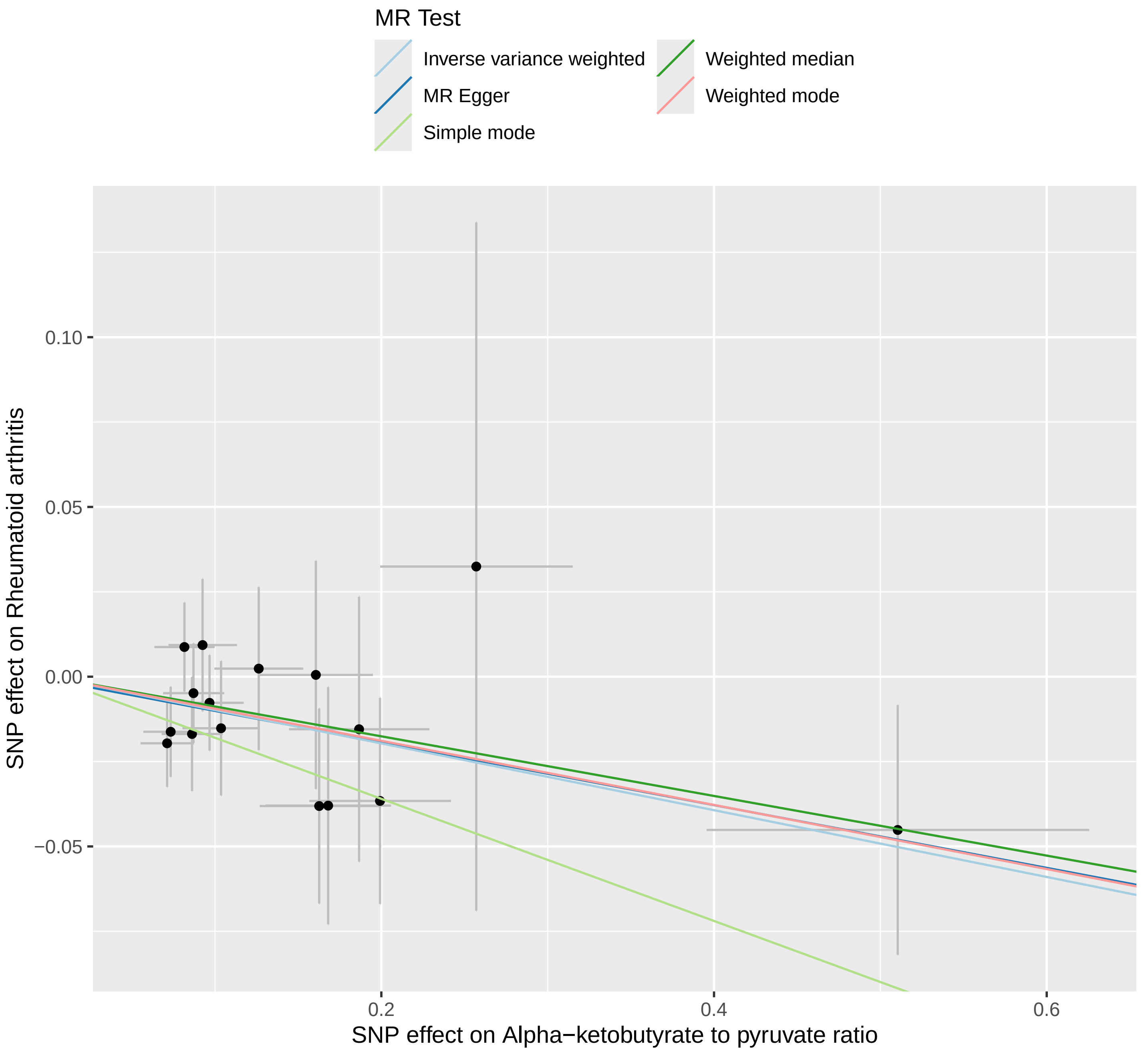
MR Test
Inverse variance weighted
MR Egger
Simple mode
Weighted median
Weighted mode
SNP effect on Rheumatoid arthritis
0.10
0.05
0.00
−0.05
0.2
0.4
0.6
SNP effect on Alpha−ketobutyrate to pyruvate ratio

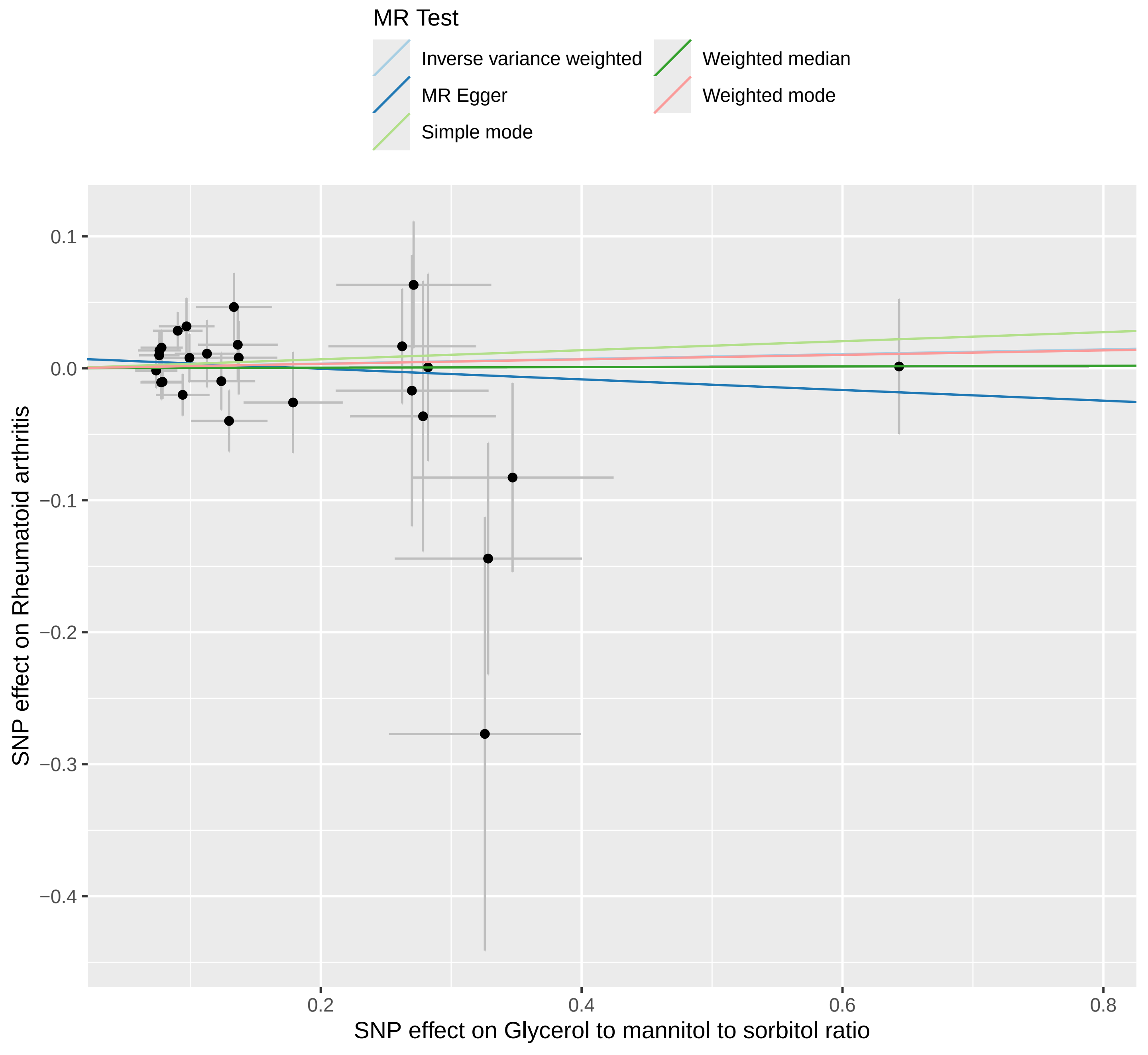
MR Test
Inverse variance weighted
MR Egger
Simple mode
Weighted median
Weighted mode
0.1
0.0
−0.1
−0.2
−0.3
−0.4
SNP effect on Rheumatoid arthritis
0.2
0.4
0.6
0.8
SNP effect on Glycerol to mannitol to sorbitol ratio

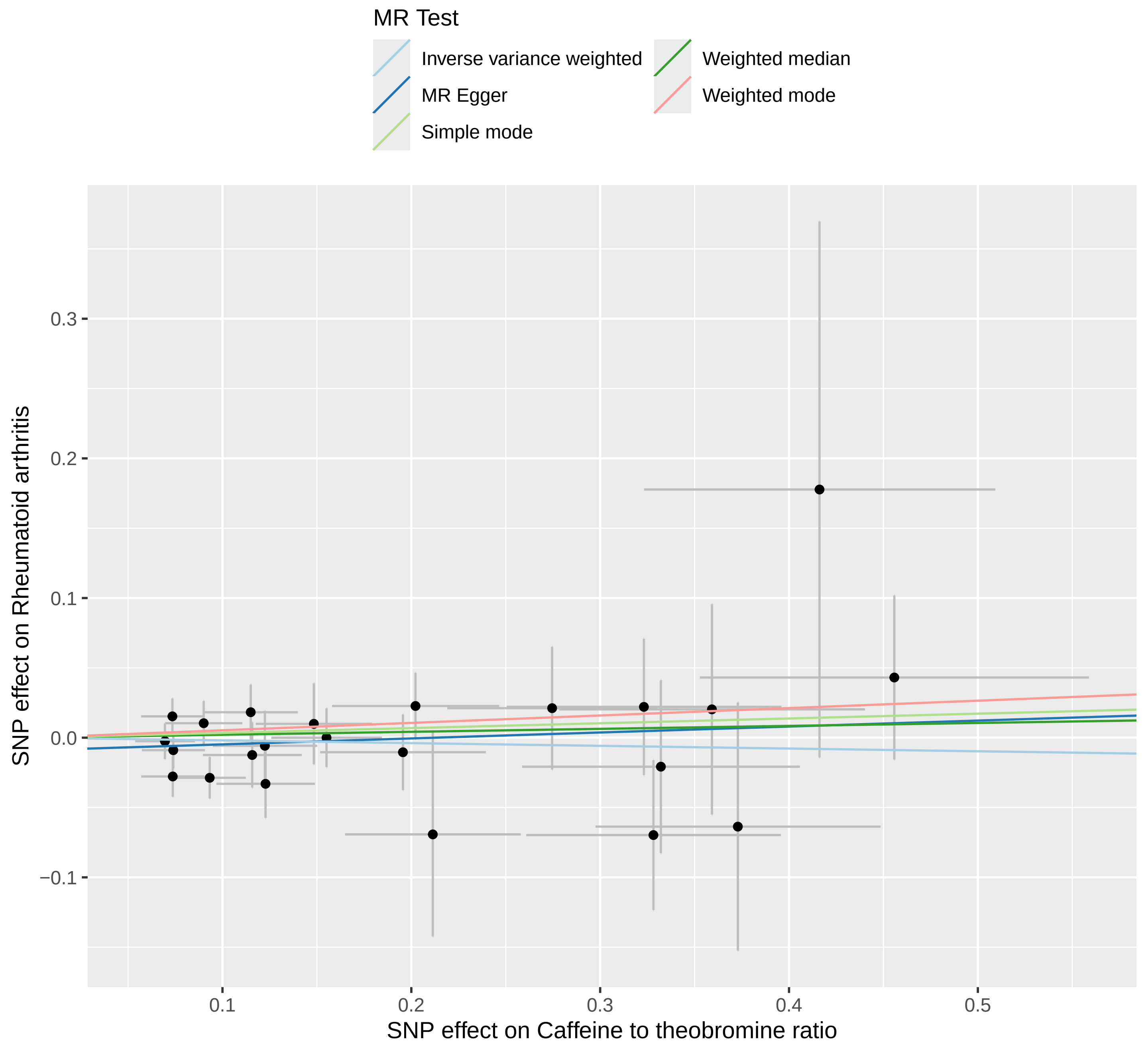
MR Test
Inverse variance weighted
MR Egger
Simple mode
Weighted median
Weighted mode
SNP effect on Rheumatoid arthritis
0.3
0.2
0.1
0.0
−0.1
0.1
0.2
0.3
0.4
0.5
SNP effect on Caffeine to theobromine ratio

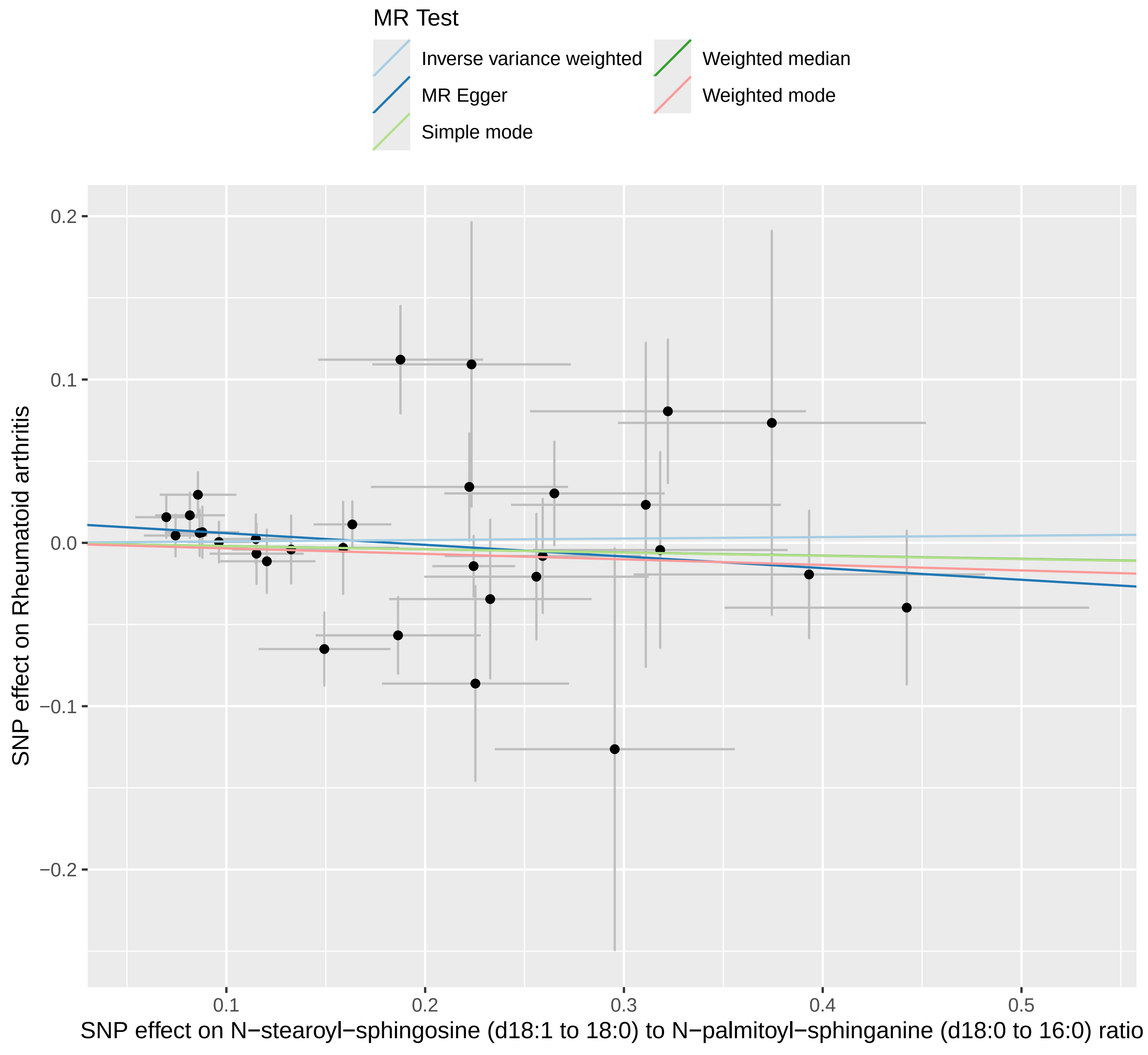
MR Test
Inverse variance weighted
MR Egger
Simple mode
Weighted median
Weighted mode
0.2
0.1
0.0
−0.1
−0.2
SNP effect on Rheumatoid arthritis
0.1
0.2
0.3
0.4
0.5
SNP effect on N−stearoyl−sphingosine (d18:1 to 18:0) to N−palmitoyl−sphinganine (d18:0 to 16:0) ratio

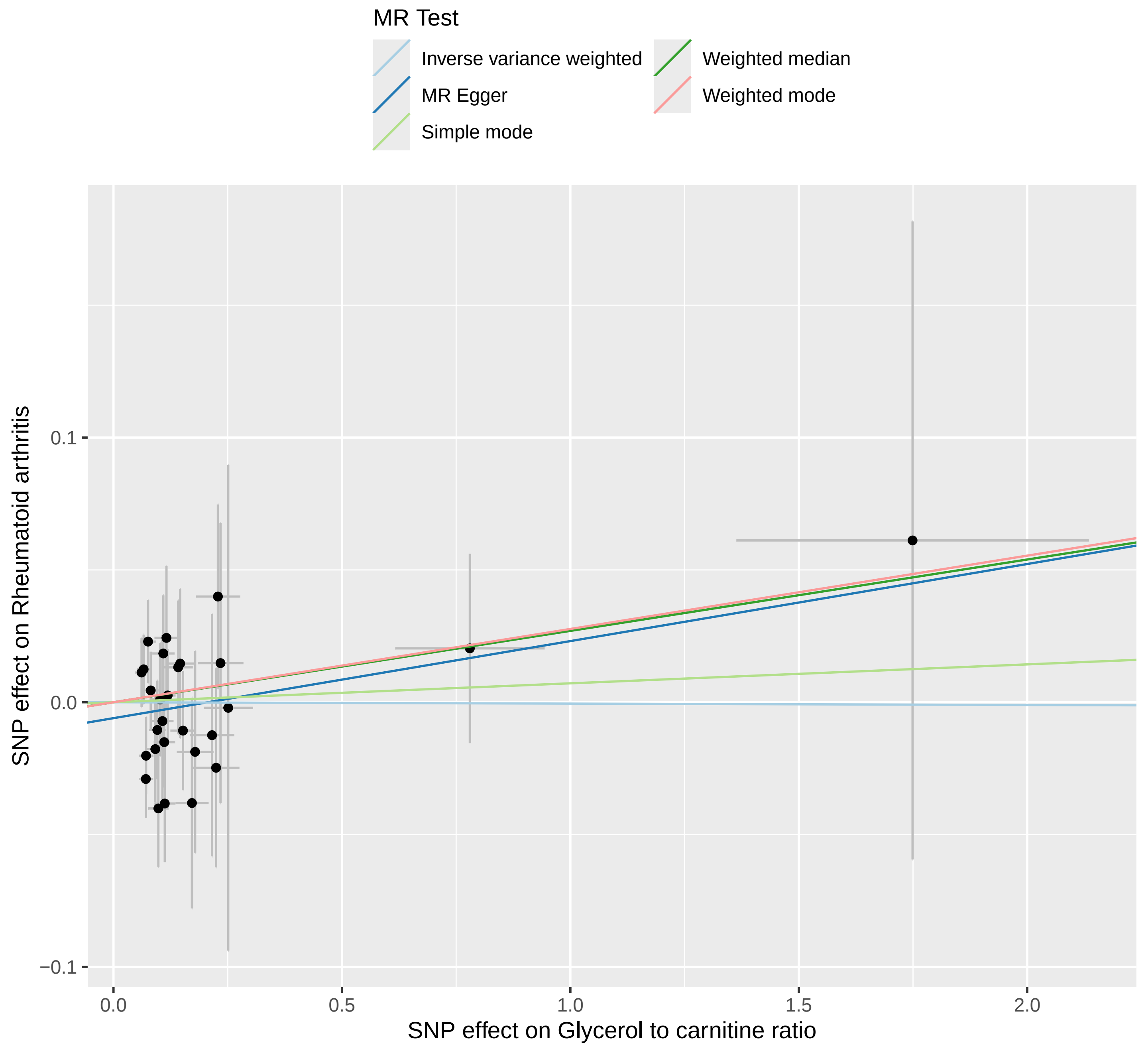
MR Test
Inverse variance weighted
MR Egger
Simple mode
Weighted median
Weighted mode
0.1
0.0
−0.1
0.0
0.5
1.0
1.5
2.0
SNP effect on Glycerol to carnitine ratio
SNP effect on Rheumatoid arthritis

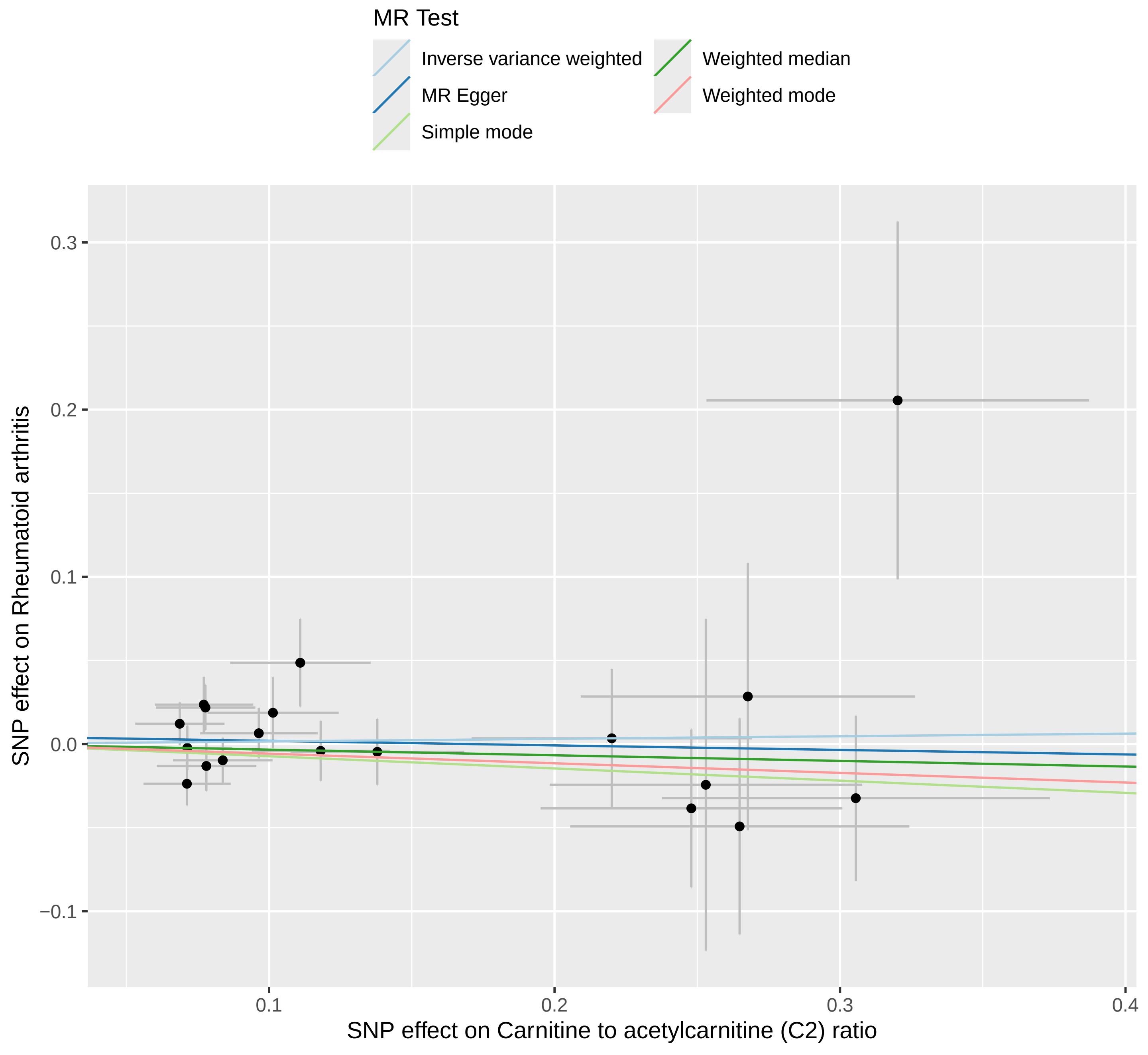

MR Test
Inverse variance weighted
MR Egger
Simple mode
Weighted median
Weighted mode
0.3
0.2
0.1
0.0
−0.1
0.1
0.2
0.3
0.4
SNP effect on Rheumatoid arthritis
SNP effect on Carnitine to acetylcarnitine (C2) ratio

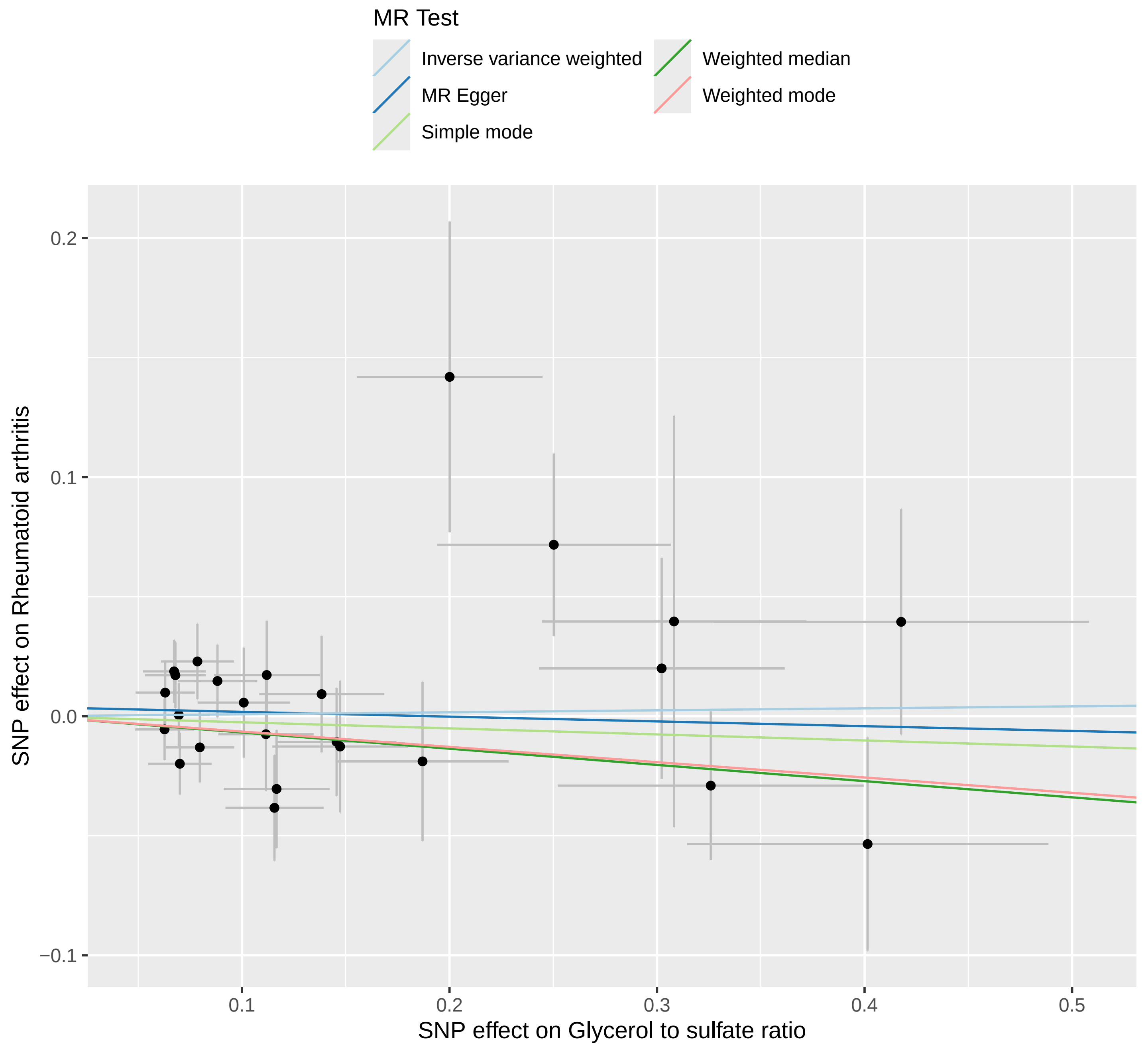

MR Test
Inverse variance weighted
MR Egger
Simple mode
Weighted median
Weighted mode
0.2
0.1
0.0
−0.1
0.1
0.2
0.3
0.4
0.5
SNP effect on Rheumatoid arthritis
SNP effect on Glycerol to sulfate ratio

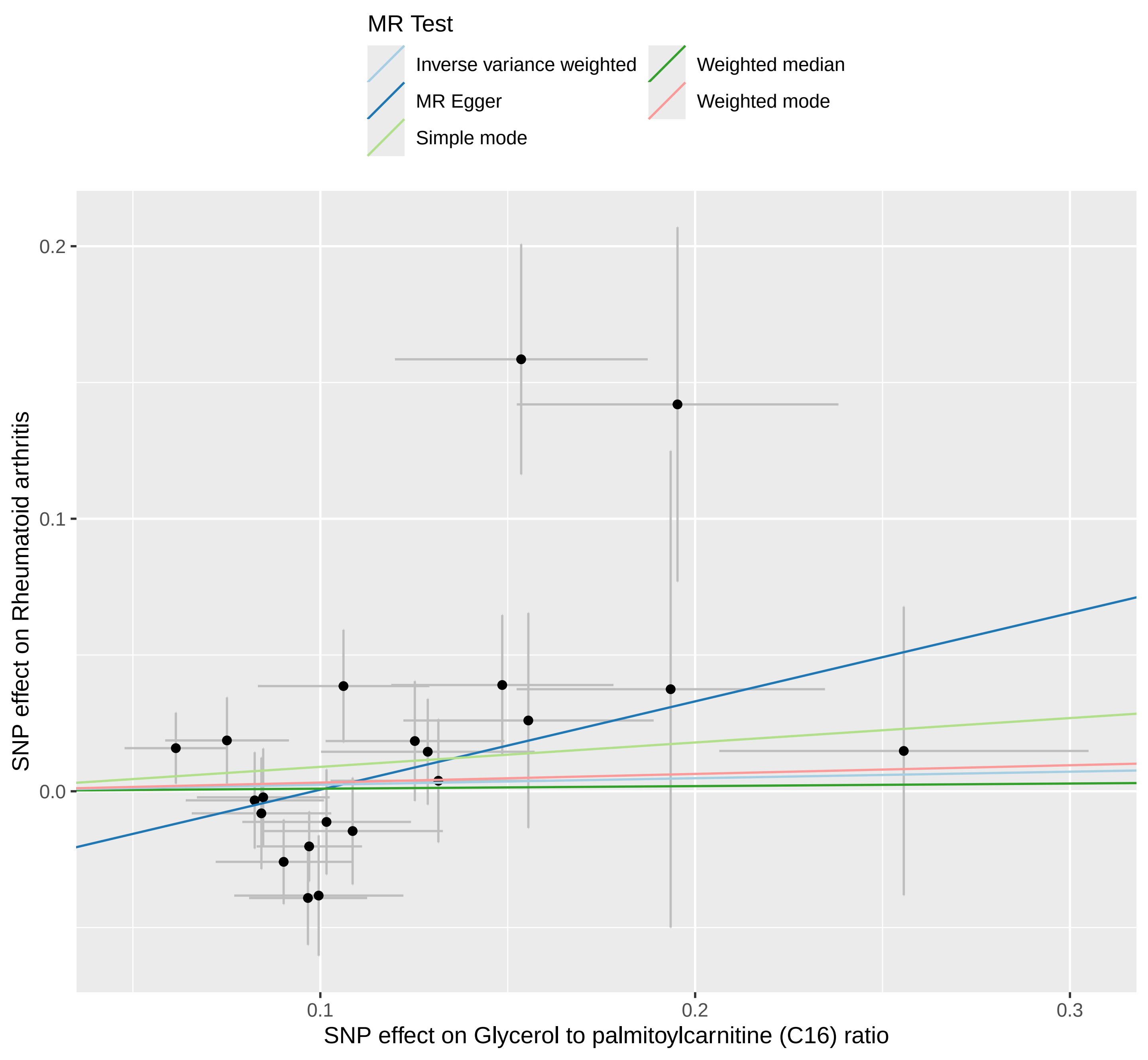
MR Test
Inverse variance weighted
MR Egger
Simple mode
Weighted median
Weighted mode
0.2
0.1
0.0
0.1
0.2
0.3
SNP effect on Rheumatoid arthritis
SNP effect on Glycerol to palmitoylcarnitine (C16) ratio

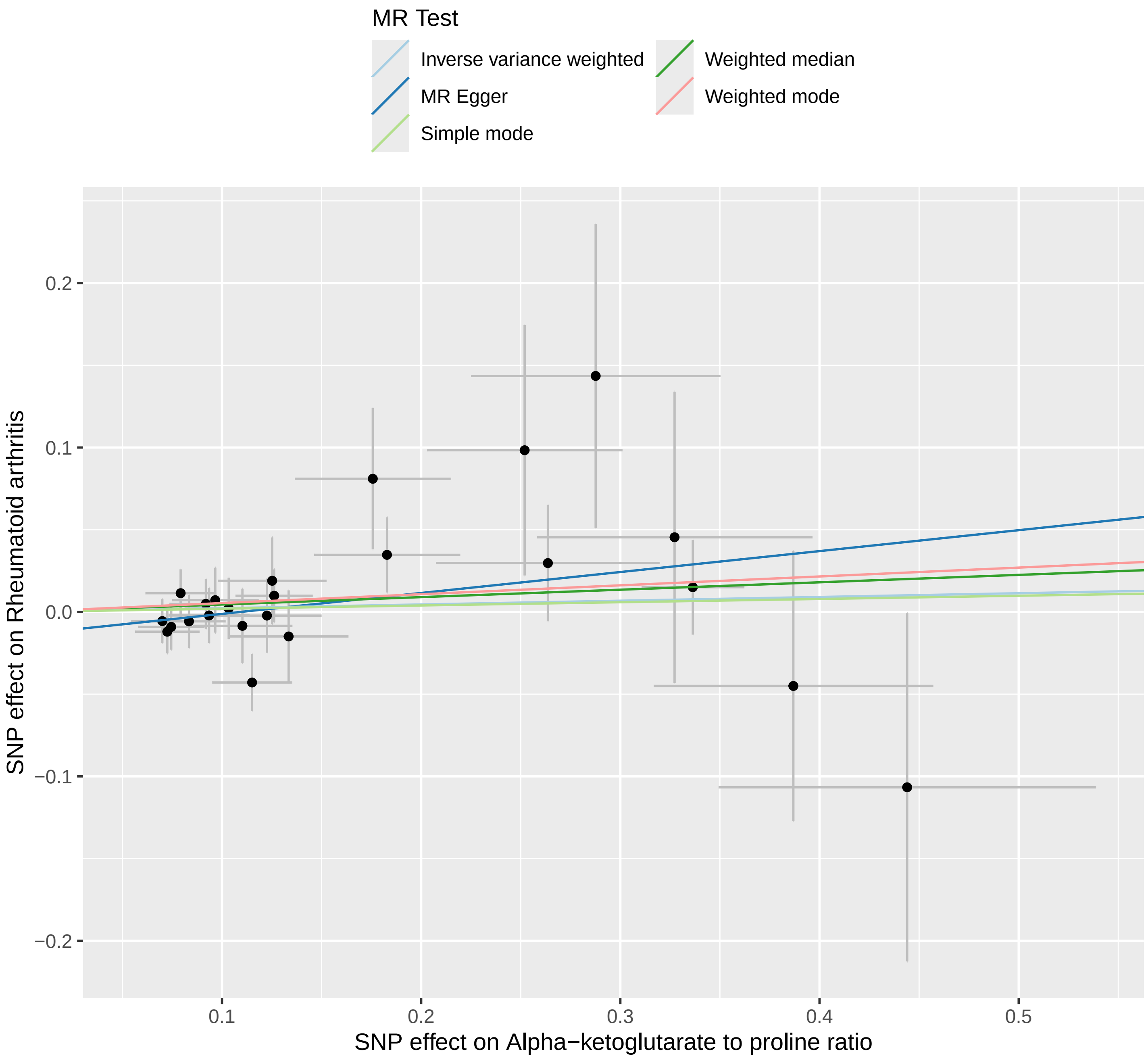
MR Test
Inverse variance weighted
MR Egger
Simple mode
Weighted median
Weighted mode
0.2
0.1
0.0
−0.1
−0.2
SNP effect on Rheumatoid arthritis
0.1
0.2
0.3
0.4
0.5
SNP effect on Alpha−ketoglutarate to proline ratio

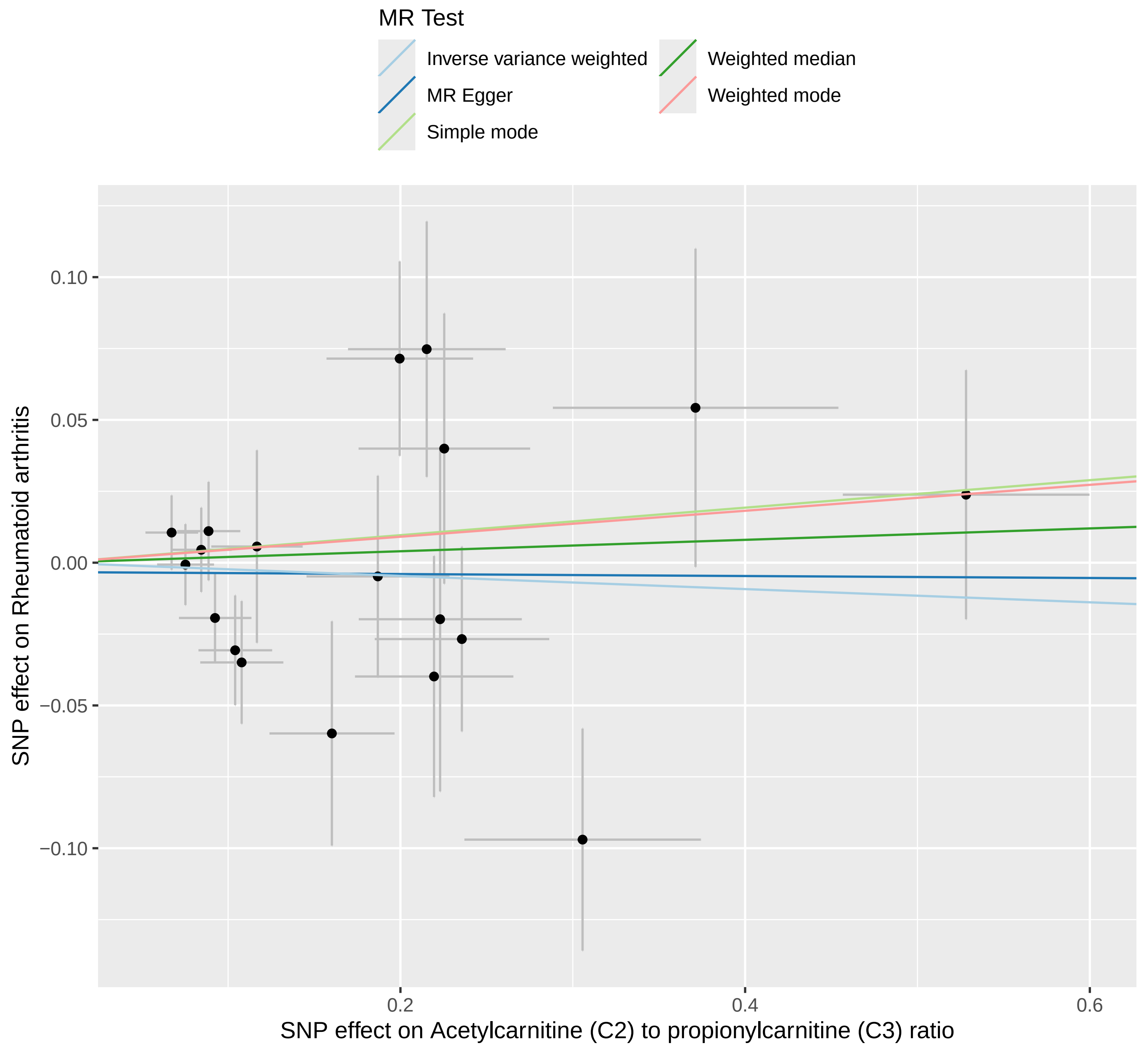
MR Test
Inverse variance weighted
MR Egger
Simple mode
Weighted median
Weighted mode
SNP effect on Rheumatoid arthritis
0.10
0.05
0.00
−0.05
−0.10
0.2
0.4
0.6
SNP effect on Acetylcarnitine (C2) to propionylcarnitine (C3) ratio

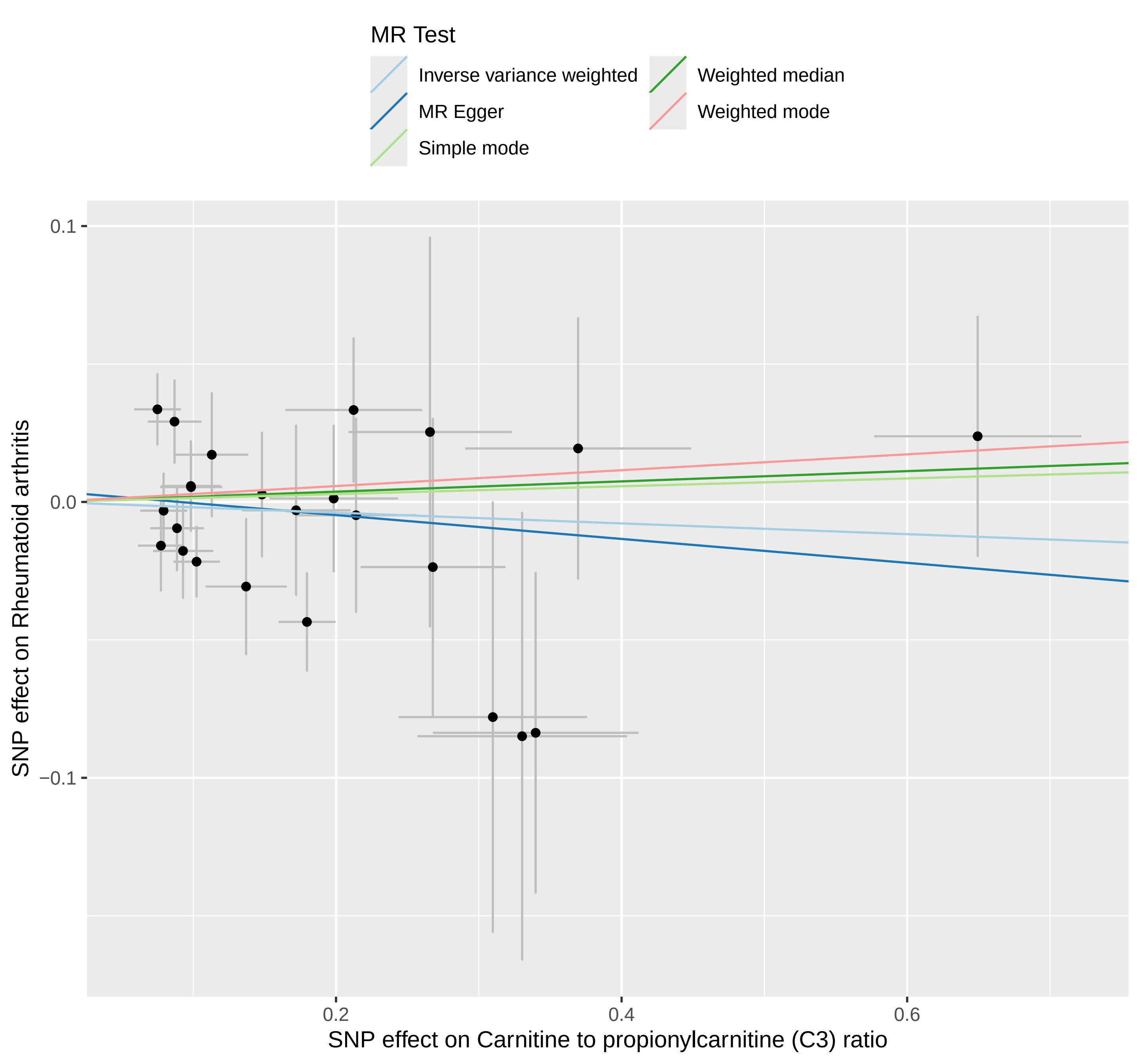

MR Test
Inverse variance weighted
MR Egger
Simple mode
Weighted median
Weighted mode
0.1
0.0
−0.1
0.2
0.4
0.6
SNP effect on Rheumatoid arthritis
SNP effect on Carnitine to propionylcarnitine (C3) ratio

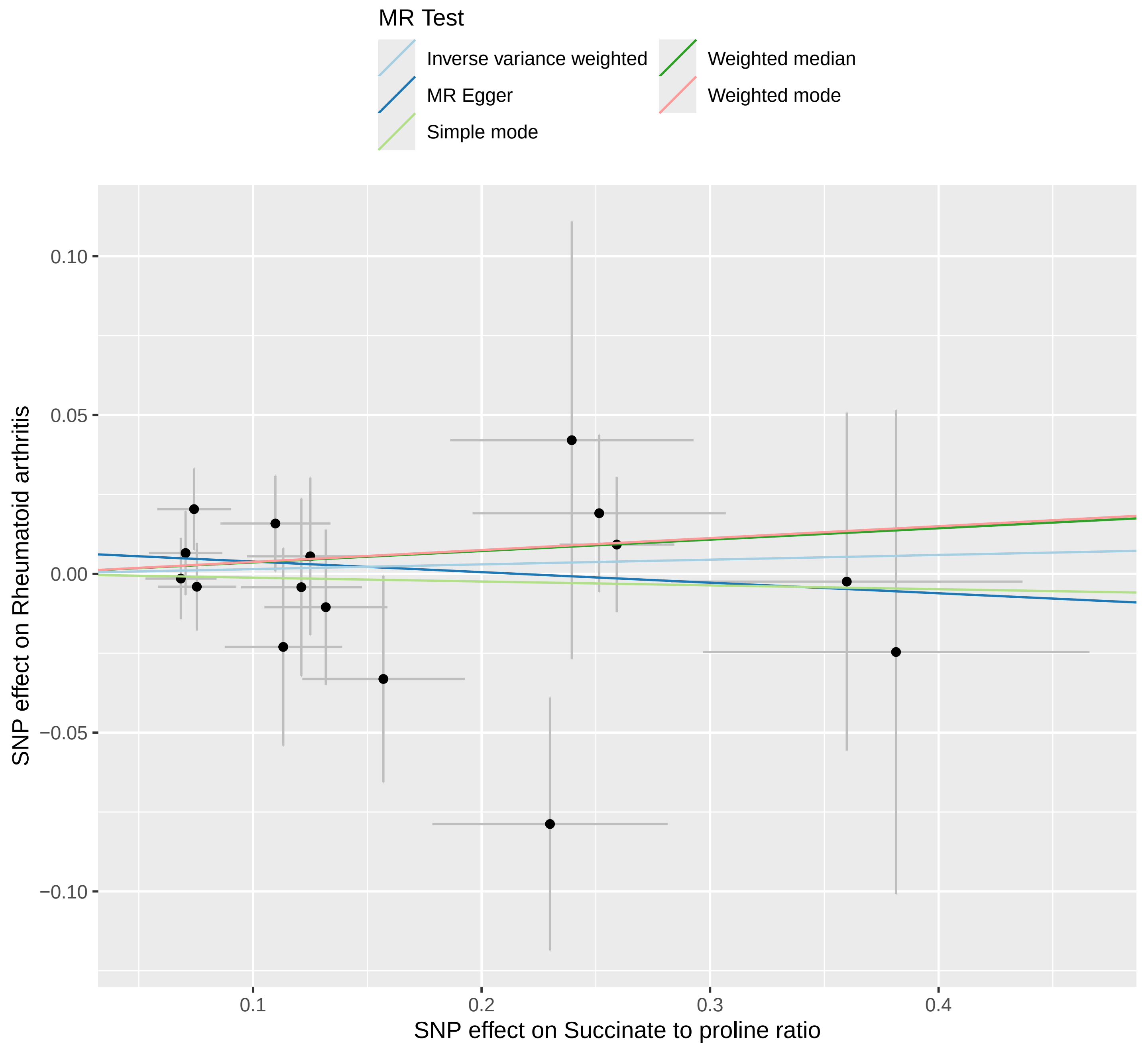
MR Test
Inverse variance weighted
MR Egger
Simple mode
Weighted median
Weighted mode
0.10
0.05
0.00
−0.05
−0.10
0.1
0.2
0.3
0.4
SNP effect on Rheumatoid arthritis
SNP effect on Succinate to proline ratio

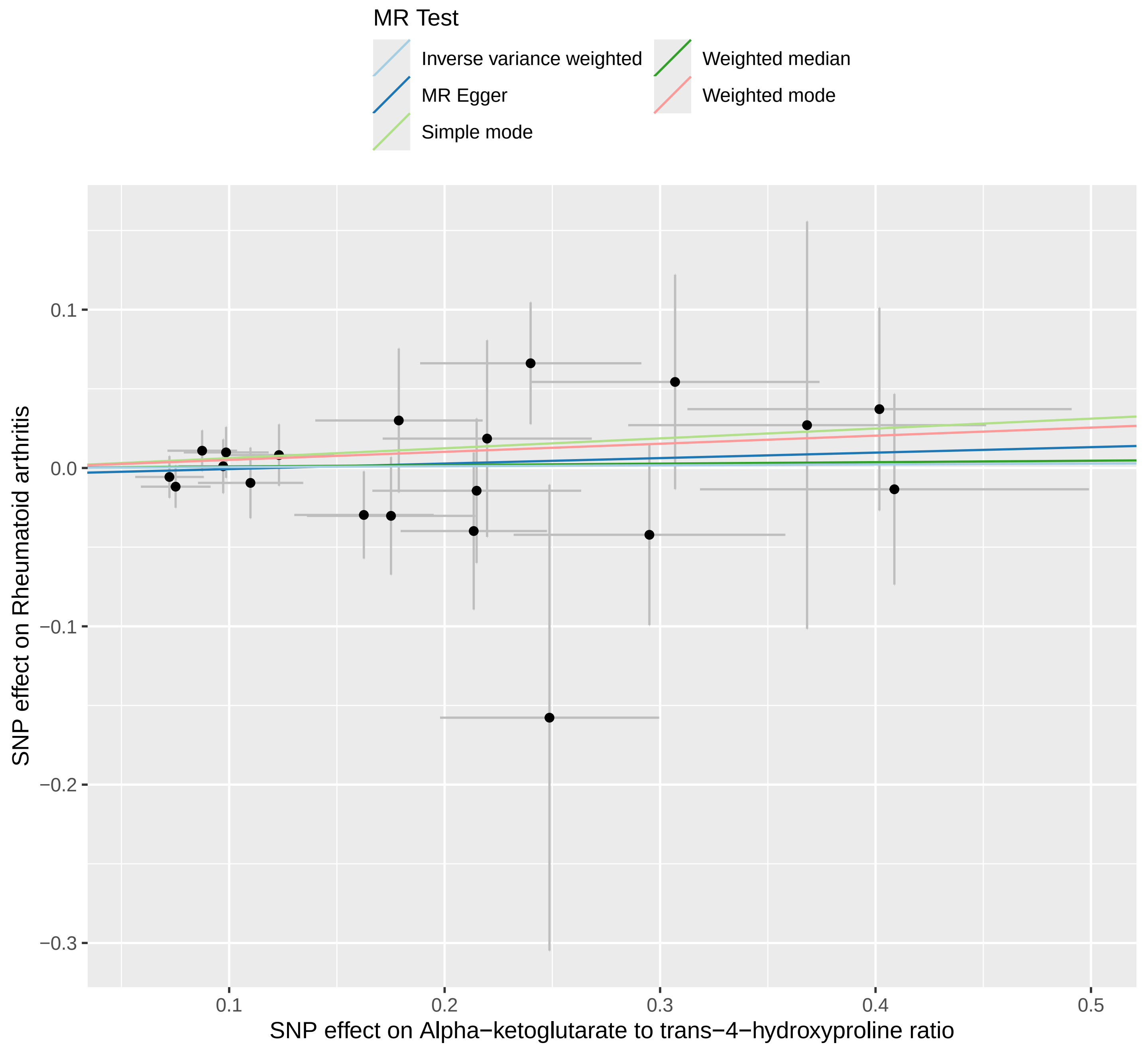
MR Test
Inverse variance weighted
MR Egger
Simple mode
Weighted median
Weighted mode
SNP effect on Rheumatoid arthritis
SNP effect on Alpha−ketoglutarate to trans−4−hydroxyproline ratio
0.1
0.0
−0.1
−0.2
−0.3
0.1
0.2
0.3
0.4
0.5

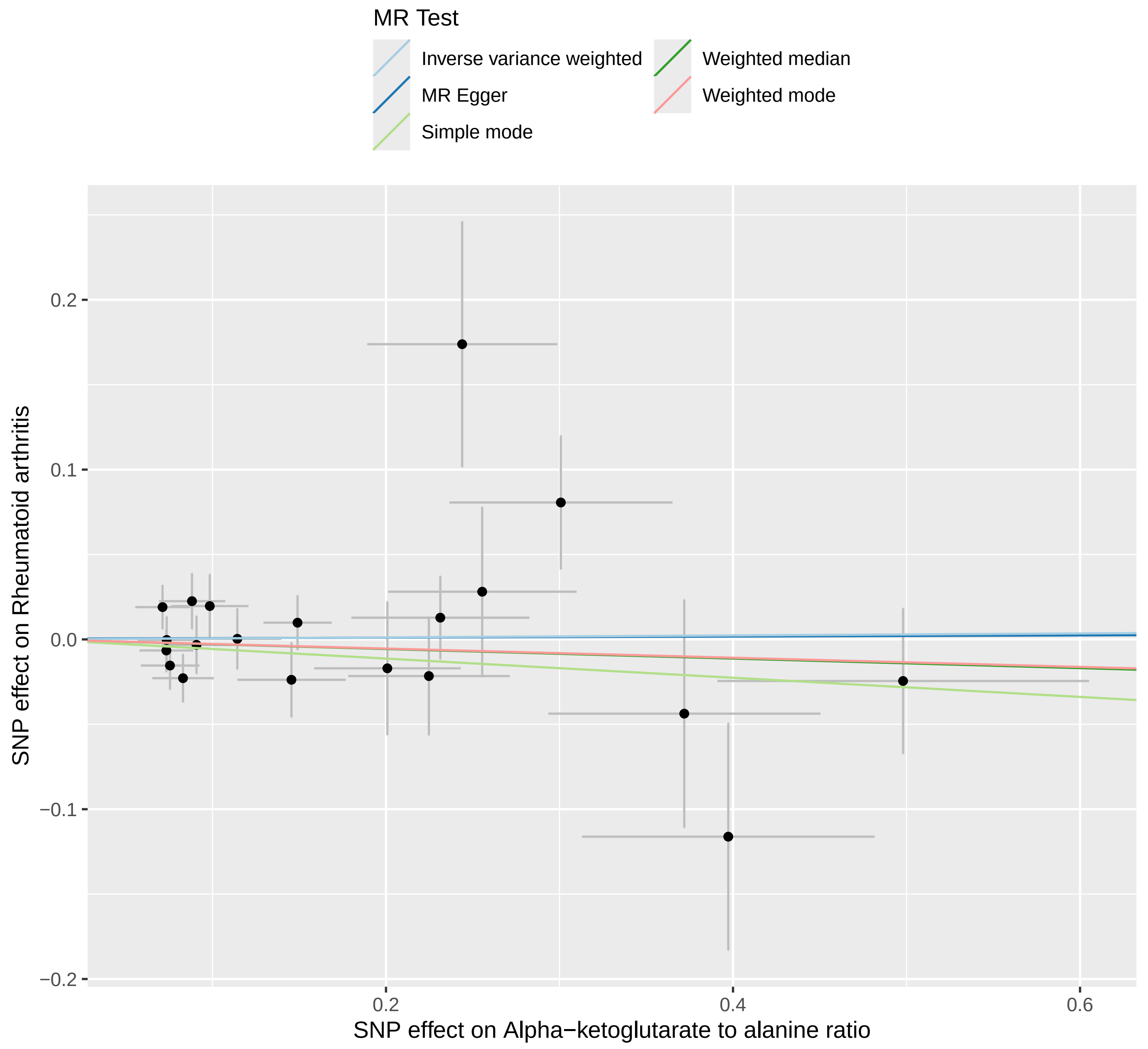
MR Test
Inverse variance weighted
MR Egger
Simple mode
Weighted median
Weighted mode
0.2
0.1
0.0
−0.1
−0.2
SNP effect on Rheumatoid arthritis
0.2
0.4
0.6
SNP effect on Alpha−ketoglutarate to alanine ratio

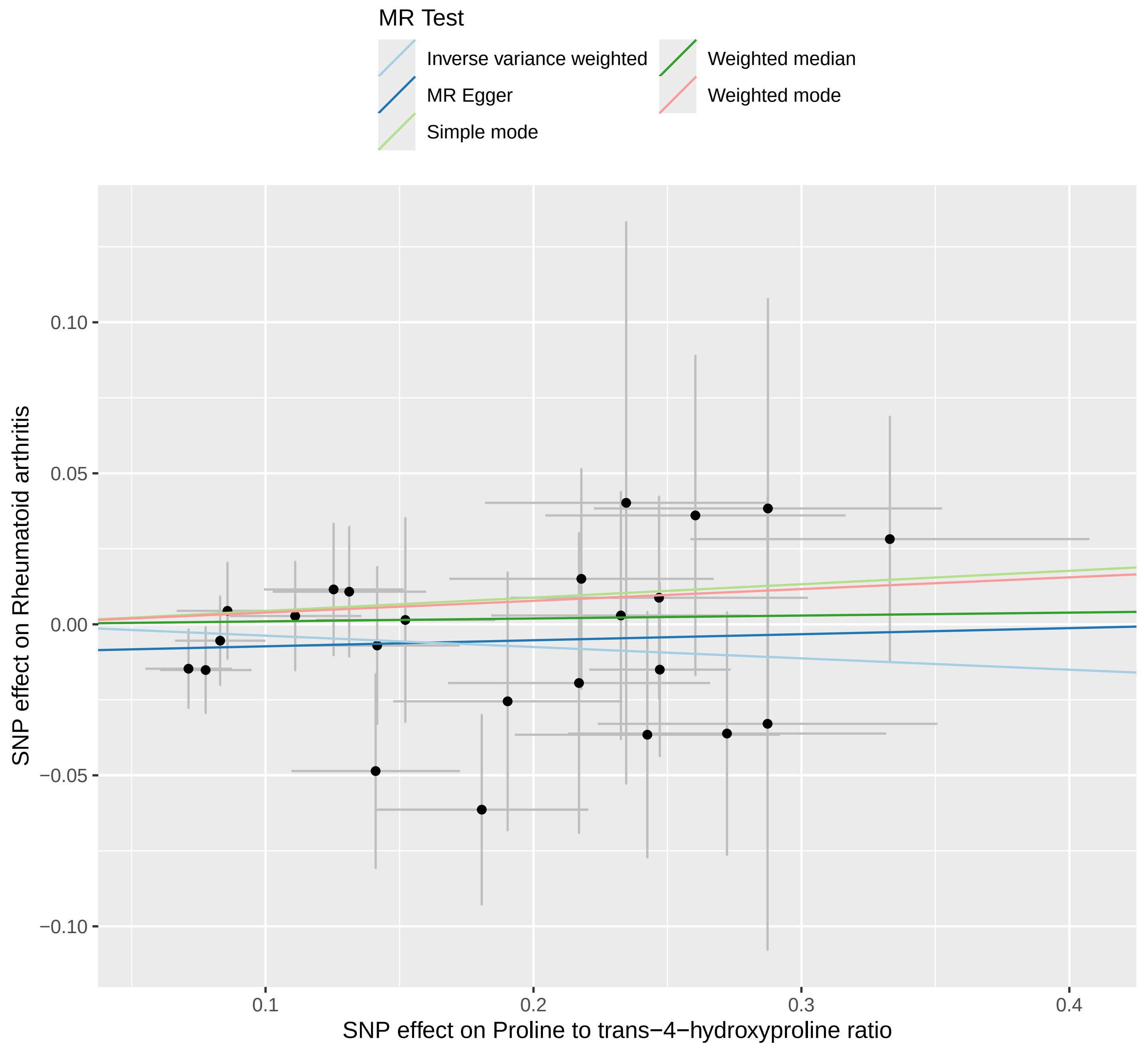
MR Test
Inverse variance weighted
MR Egger
Simple mode
Weighted median
Weighted mode
SNP effect on Rheumatoid arthritis
0.10
0.05
0.00
−0.05
−0.10
0.1
0.2
0.3
0.4
SNP effect on Proline to trans−4−hydroxyproline ratio

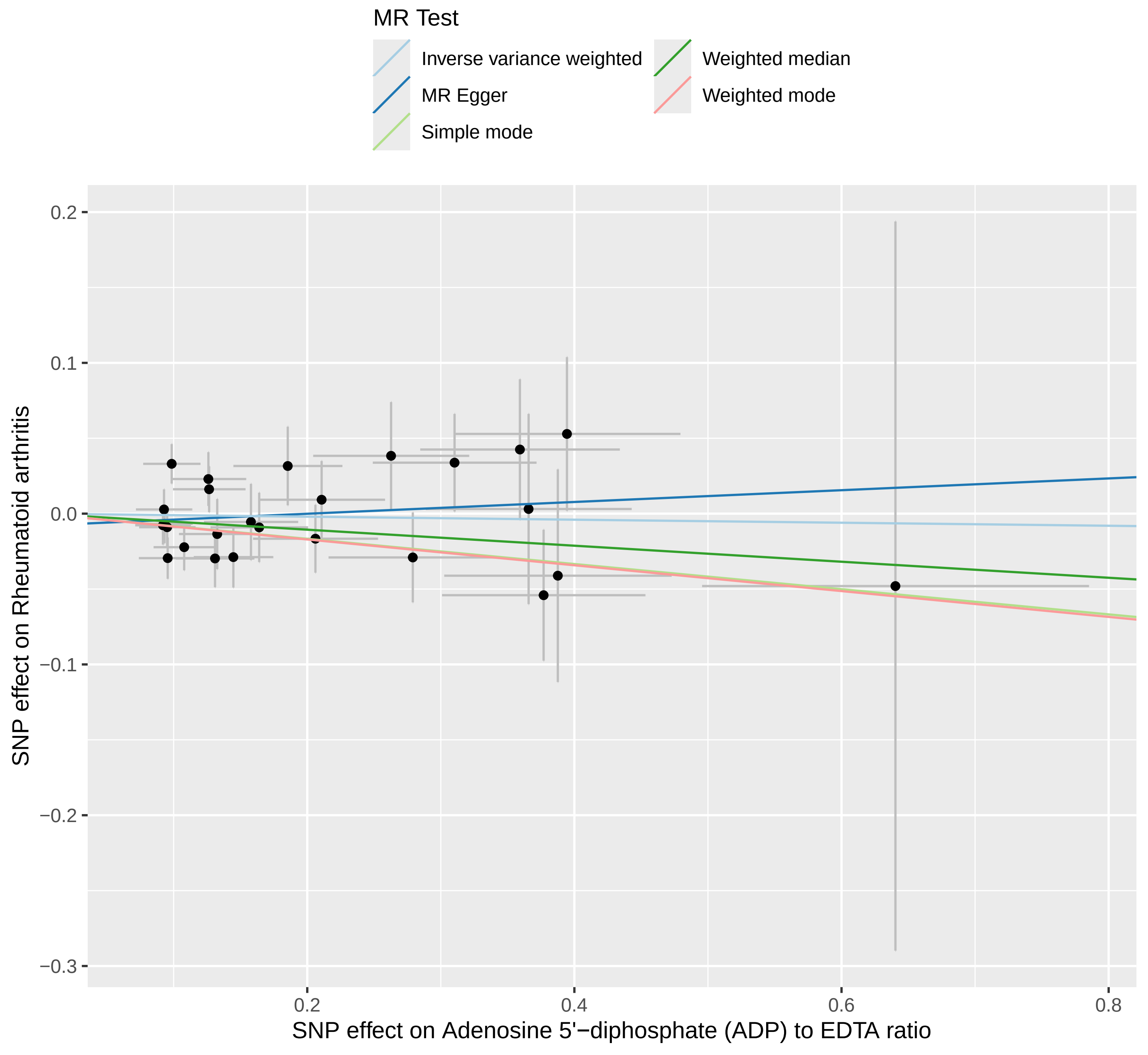

MR Test
Inverse variance weighted
MR Egger
Simple mode
Weighted median
Weighted mode
0.2
0.1
0.0
−0.1
−0.2
−0.3
SNP effect on Rheumatoid arthritis
0.2
0.4
0.6
0.8
SNP effect on Adenosine 5'−diphosphate (ADP) to EDTA ratio

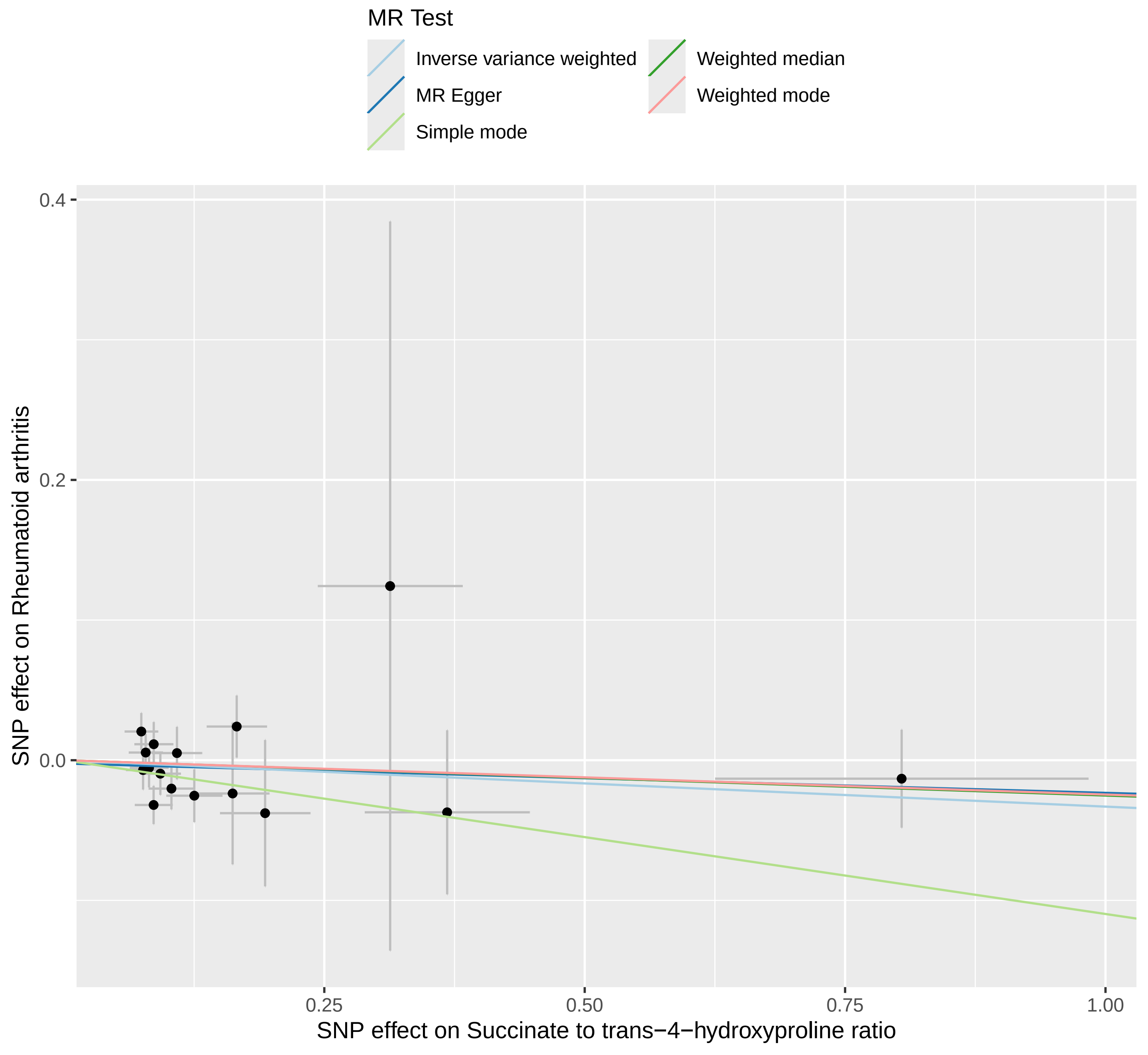
MR Test
Inverse variance weighted
MR Egger
Simple mode
Weighted median
Weighted mode
0.4
0.2
0.0
0.25
0.50
0.75
1.00
SNP effect on Rheumatoid arthritis
SNP effect on Succinate to trans−4−hydroxyproline ratio

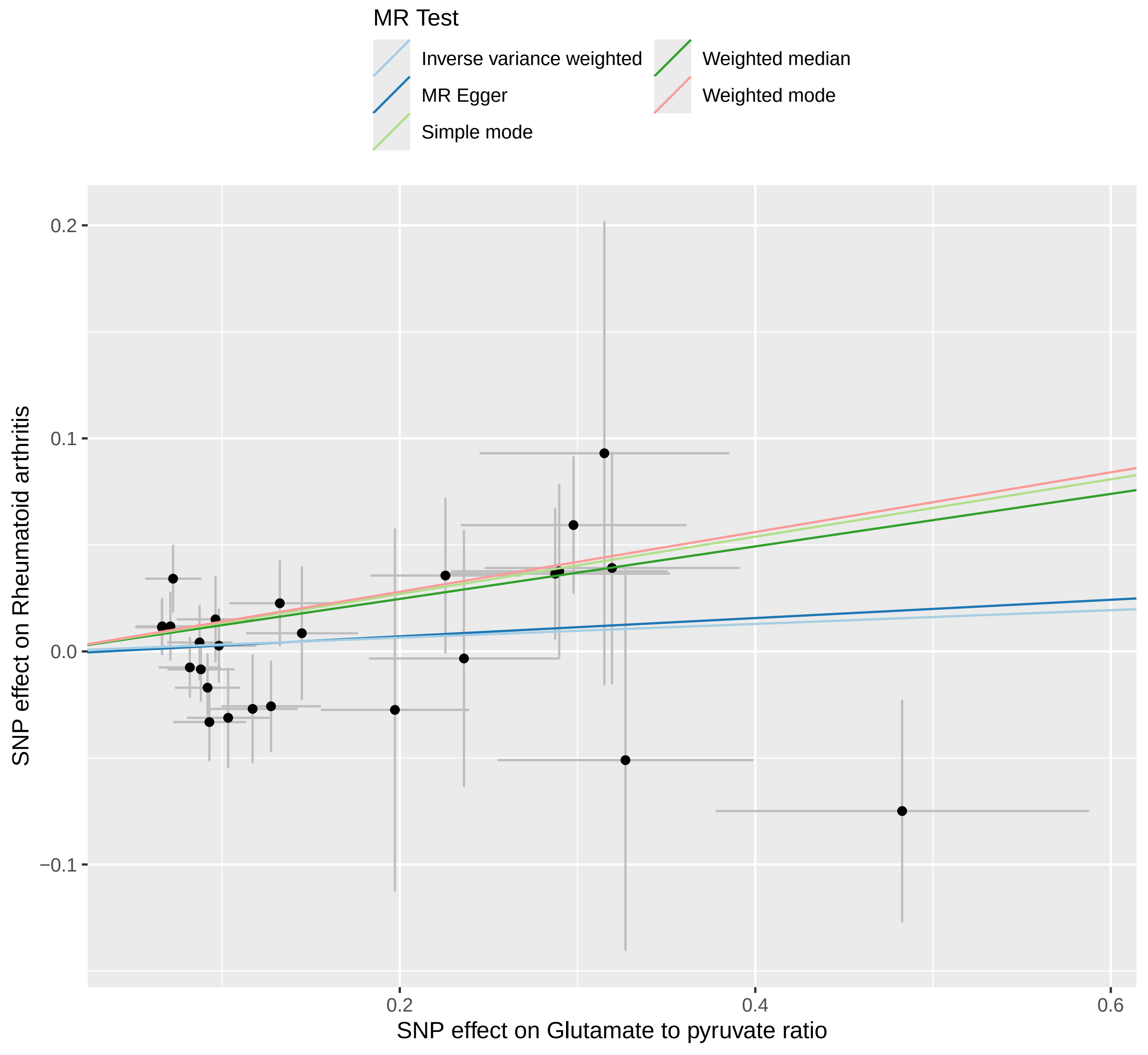

MR Test
Inverse variance weighted
MR Egger
Simple mode
Weighted median
Weighted mode
0.2
0.1
0.0
−0.1
0.2
0.4
0.6
SNP effect on Rheumatoid arthritis
SNP effect on Glutamate to pyruvate ratio

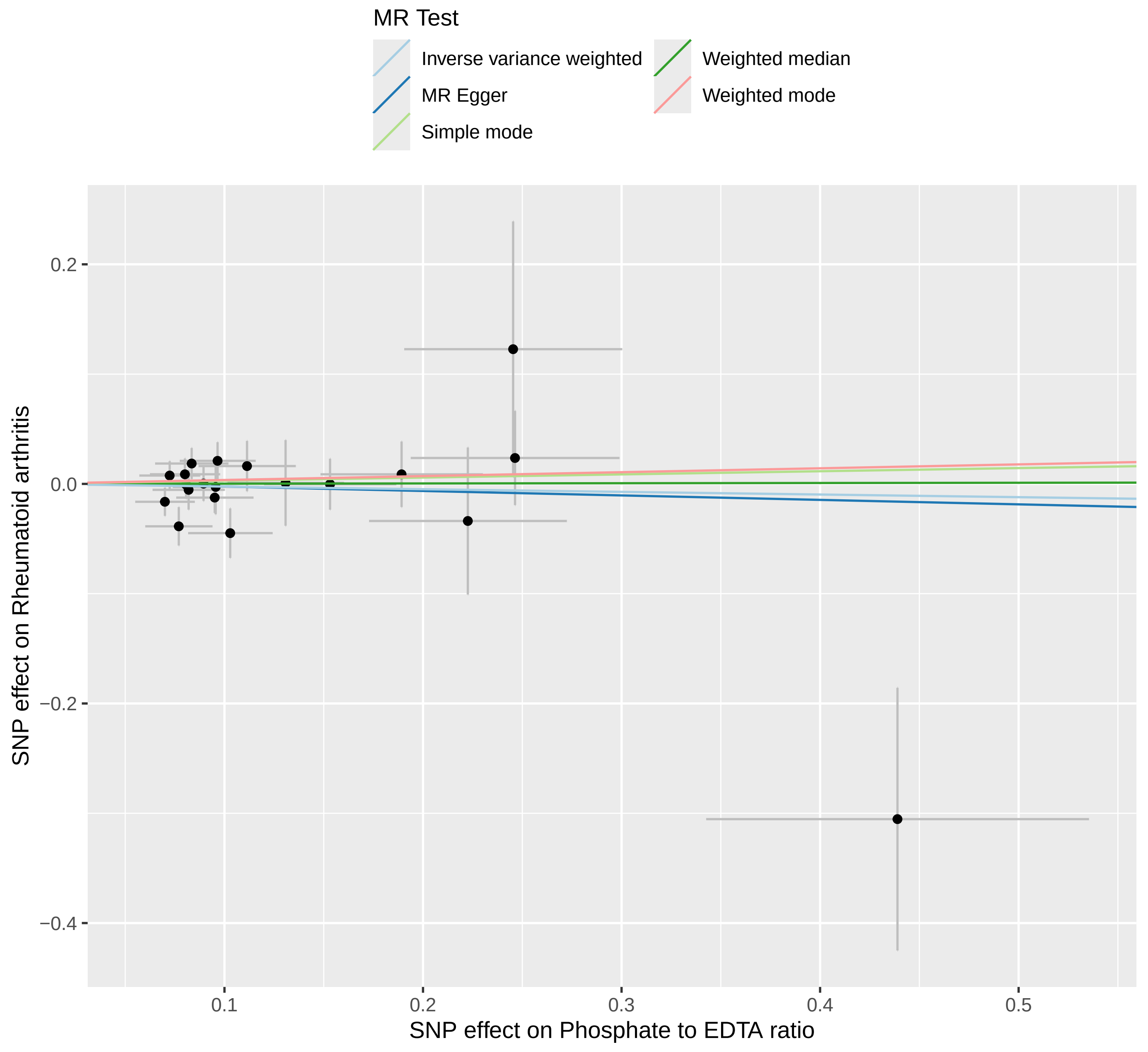
MR Test
Inverse variance weighted
MR Egger
Simple mode
Weighted median
Weighted mode
0.2
0.0
−0.2
−0.4
0.1
0.2
0.3
0.4
0.5
SNP effect on Rheumatoid arthritis
SNP effect on Phosphate to EDTA ratio

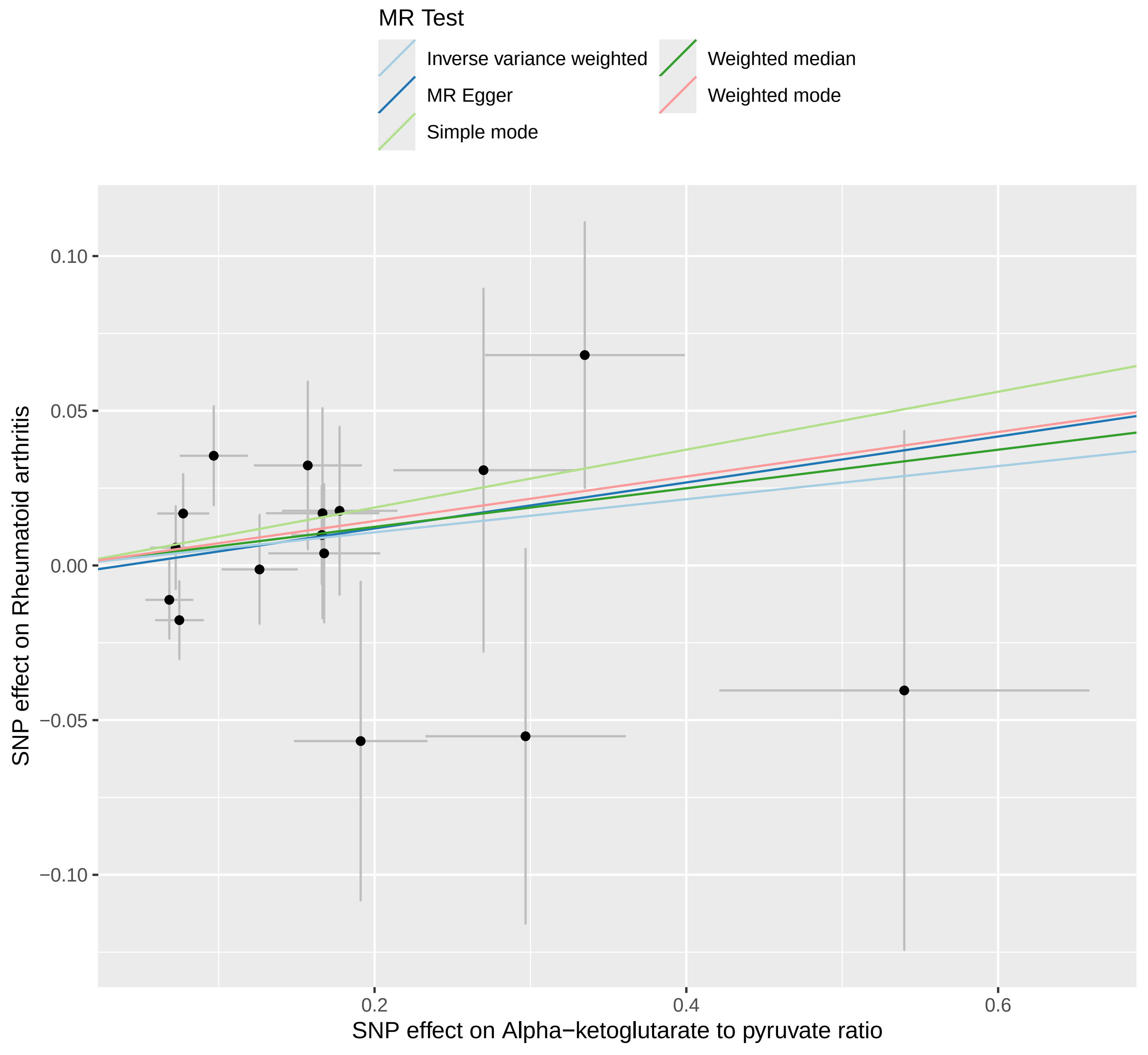
MR Test
Inverse variance weighted
MR Egger
Simple mode
Weighted median
Weighted mode
0.10
0.05
0.00
−0.05
−0.10
SNP effect on Rheumatoid arthritis
0.2
0.4
0.6
SNP effect on Alpha−ketoglutarate to pyruvate ratio

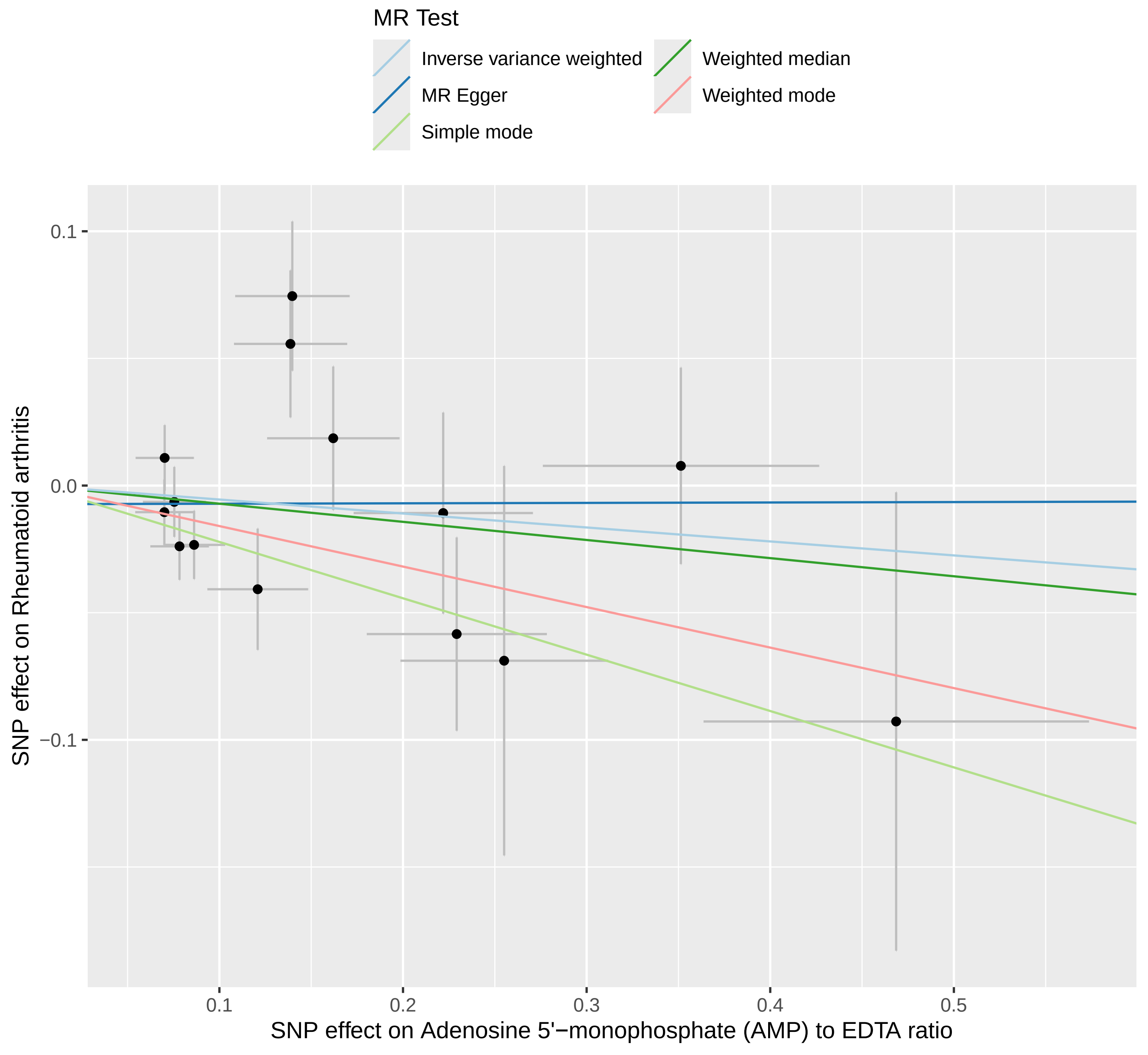

MR Test
Inverse variance weighted
MR Egger
Simple mode
Weighted median
Weighted mode
0.1
0.0
−0.1
SNP effect on Rheumatoid arthritis
0.1
0.2
0.3
0.4
0.5
SNP effect on Adenosine 5'−monophosphate (AMP) to EDTA ratio

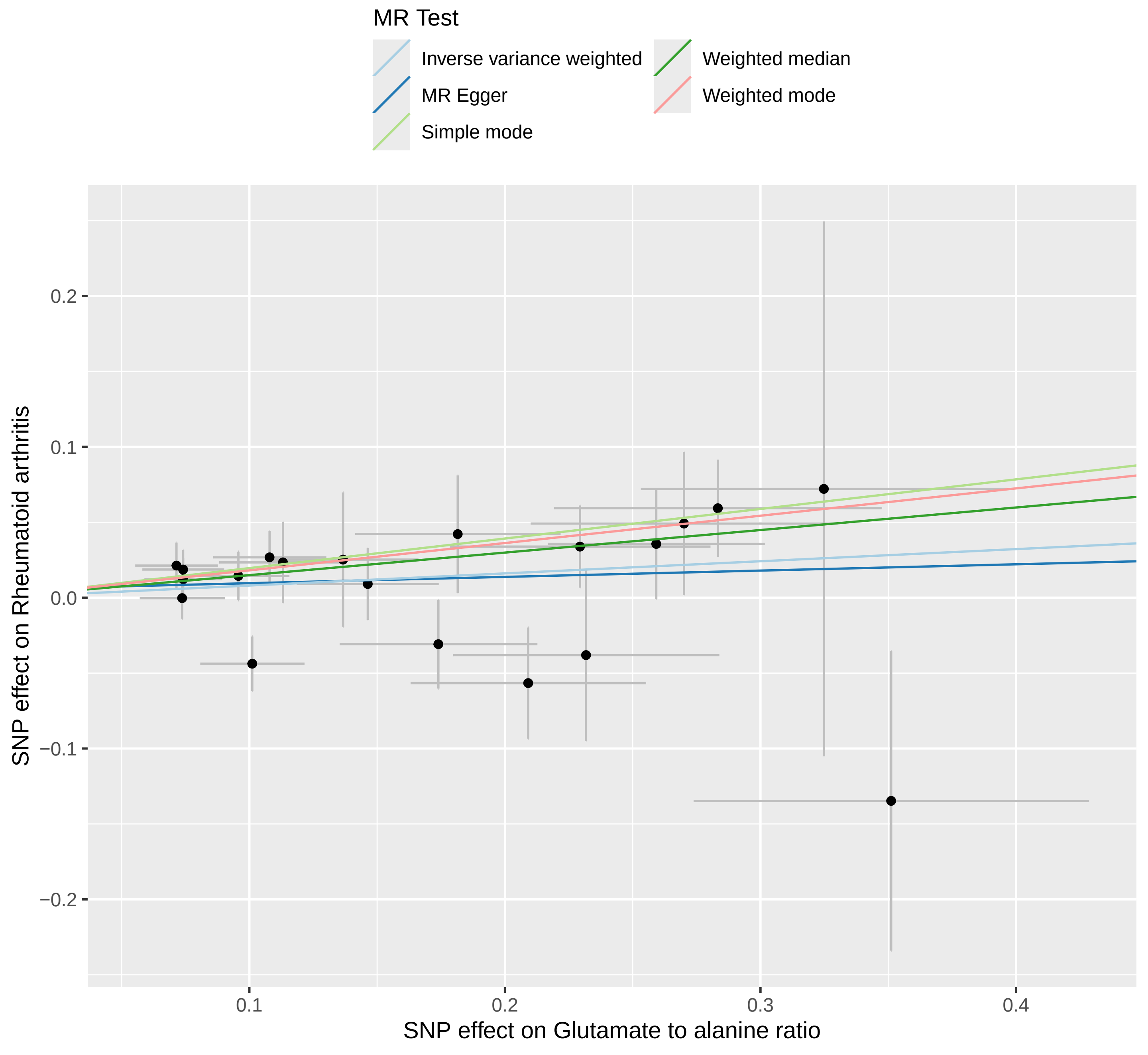
MR Test
Inverse variance weighted
MR Egger
Simple mode
Weighted median
Weighted mode
SNP effect on Rheumatoid arthritis
0.2
0.1
0.0
−0.1
−0.2
0.1
0.2
0.3
0.4
SNP effect on Glutamate to alanine ratio

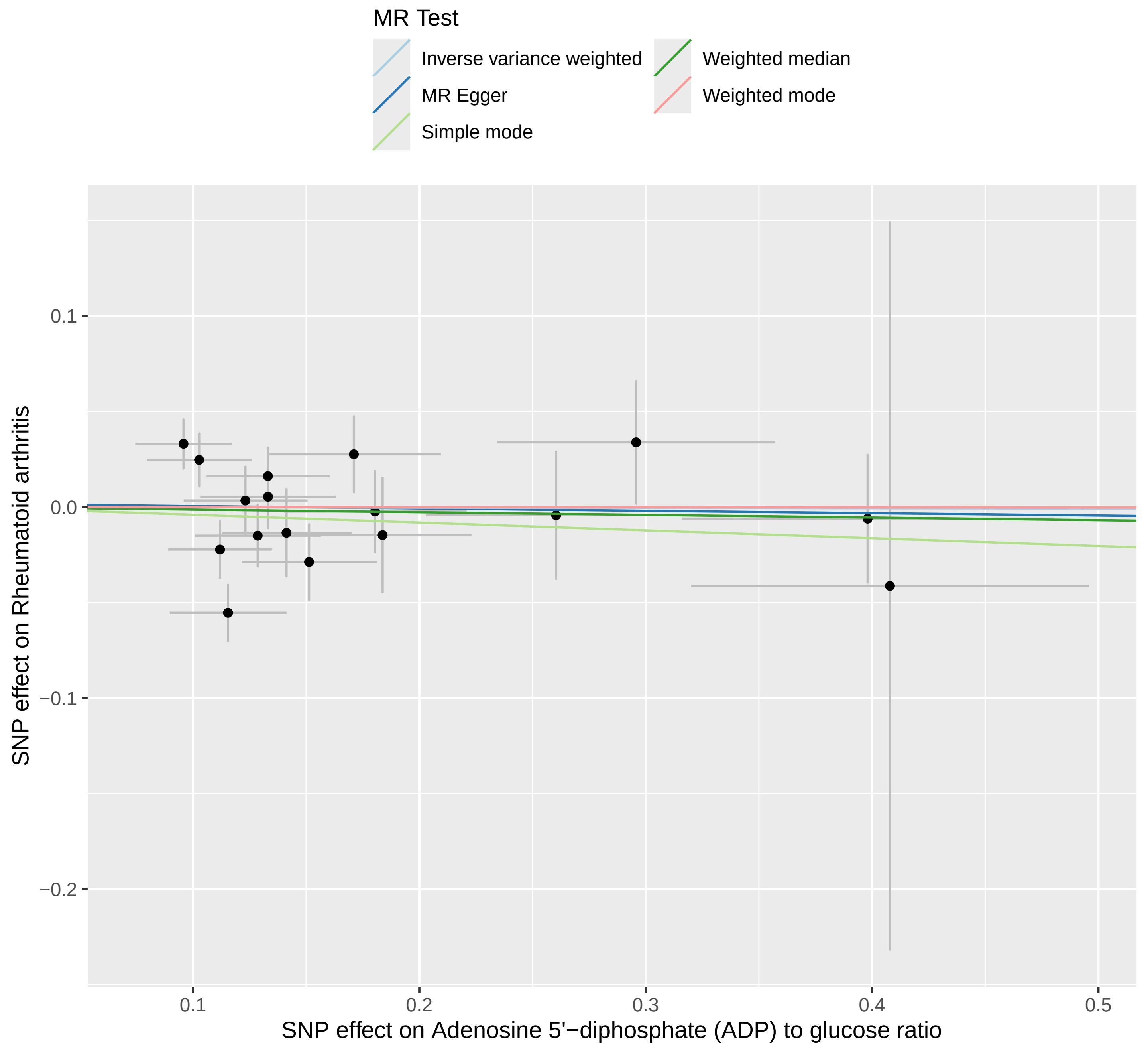
MR Test
Inverse variance weighted
MR Egger
Simple mode
Weighted median
Weighted mode
0.1
0.0
−0.1
−0.2
0.1
0.2
0.3
0.4
0.5
SNP effect on Rheumatoid arthritis
SNP effect on Adenosine 5'−diphosphate (ADP) to glucose ratio

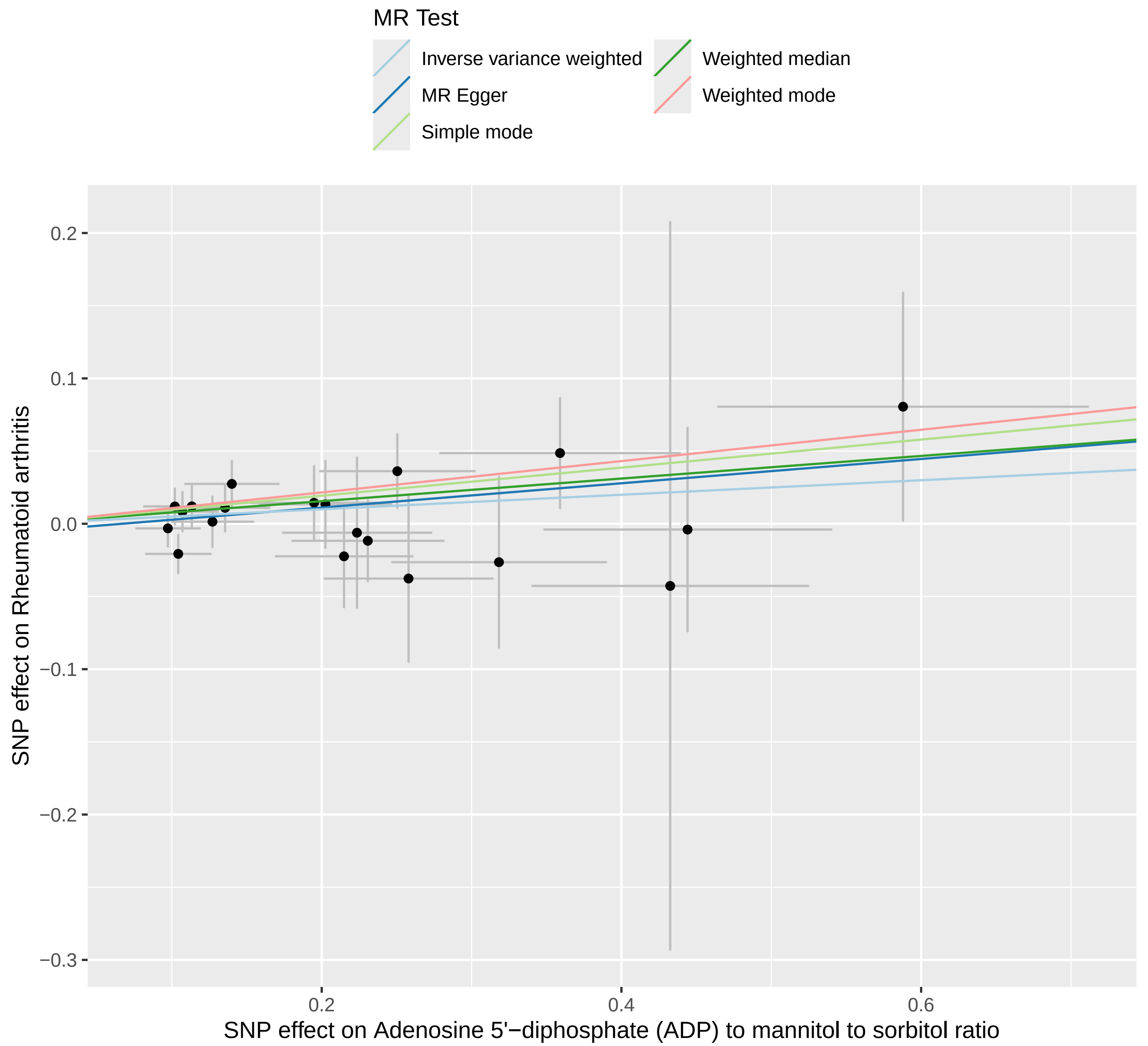
MR Test
Inverse variance weighted
MR Egger
Simple mode
Weighted median
Weighted mode
0.2
0.1
0.0
−0.1
−0.2
−0.3
SNP effect on Rheumatoid arthritis
0.2
0.4
0.6
SNP effect on Adenosine 5'−diphosphate (ADP) to mannitol to sorbitol ratio

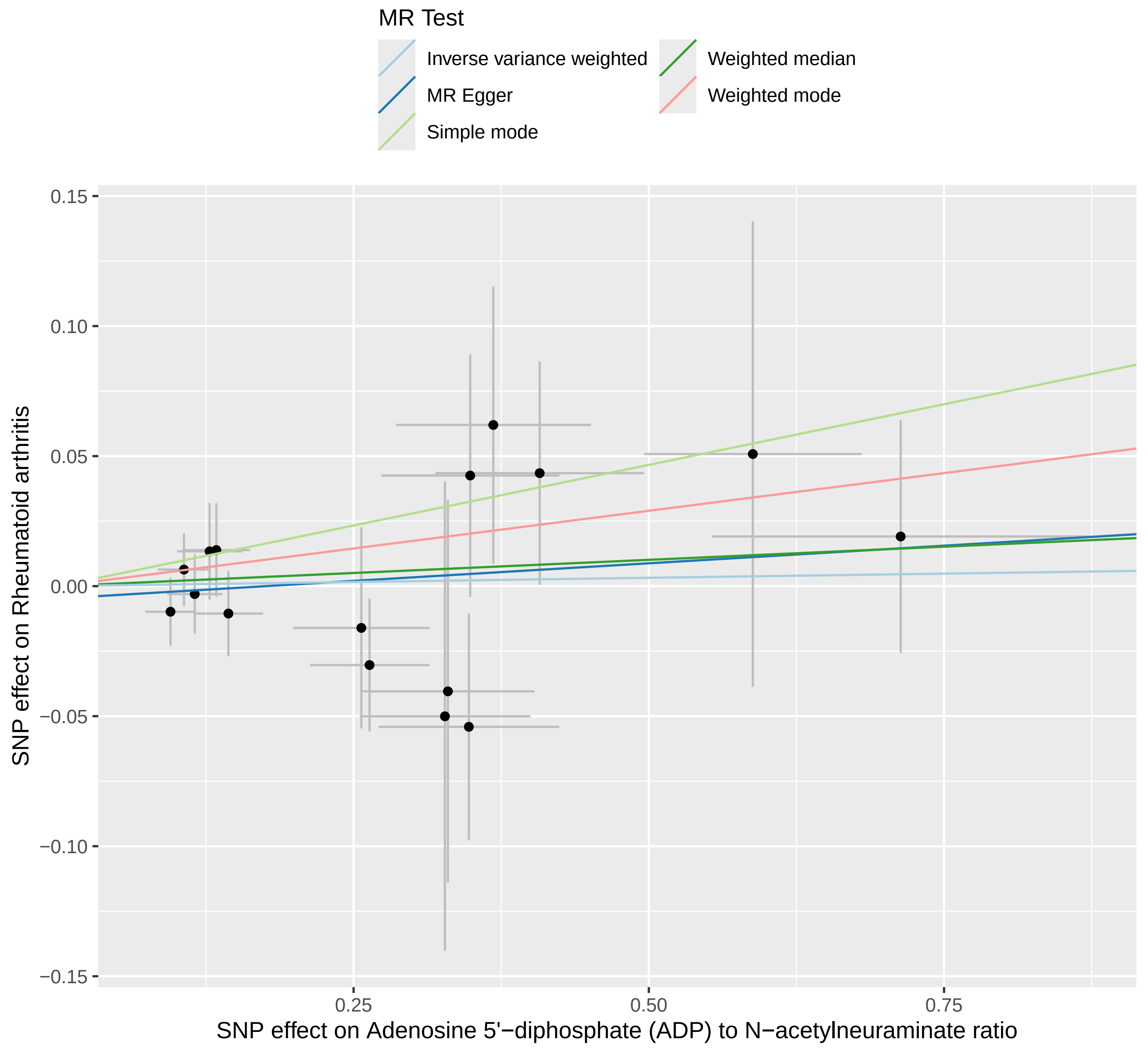
MR Test
Inverse variance weighted
MR Egger
Simple mode
Weighted median
Weighted mode
0.15
0.10
0.05
0.00
−0.05
−0.10
−0.15
SNP effect on Rheumatoid arthritis
0.25
0.50
0.75
SNP effect on Adenosine 5'−diphosphate (ADP) to N−acetylneuraminate ratio

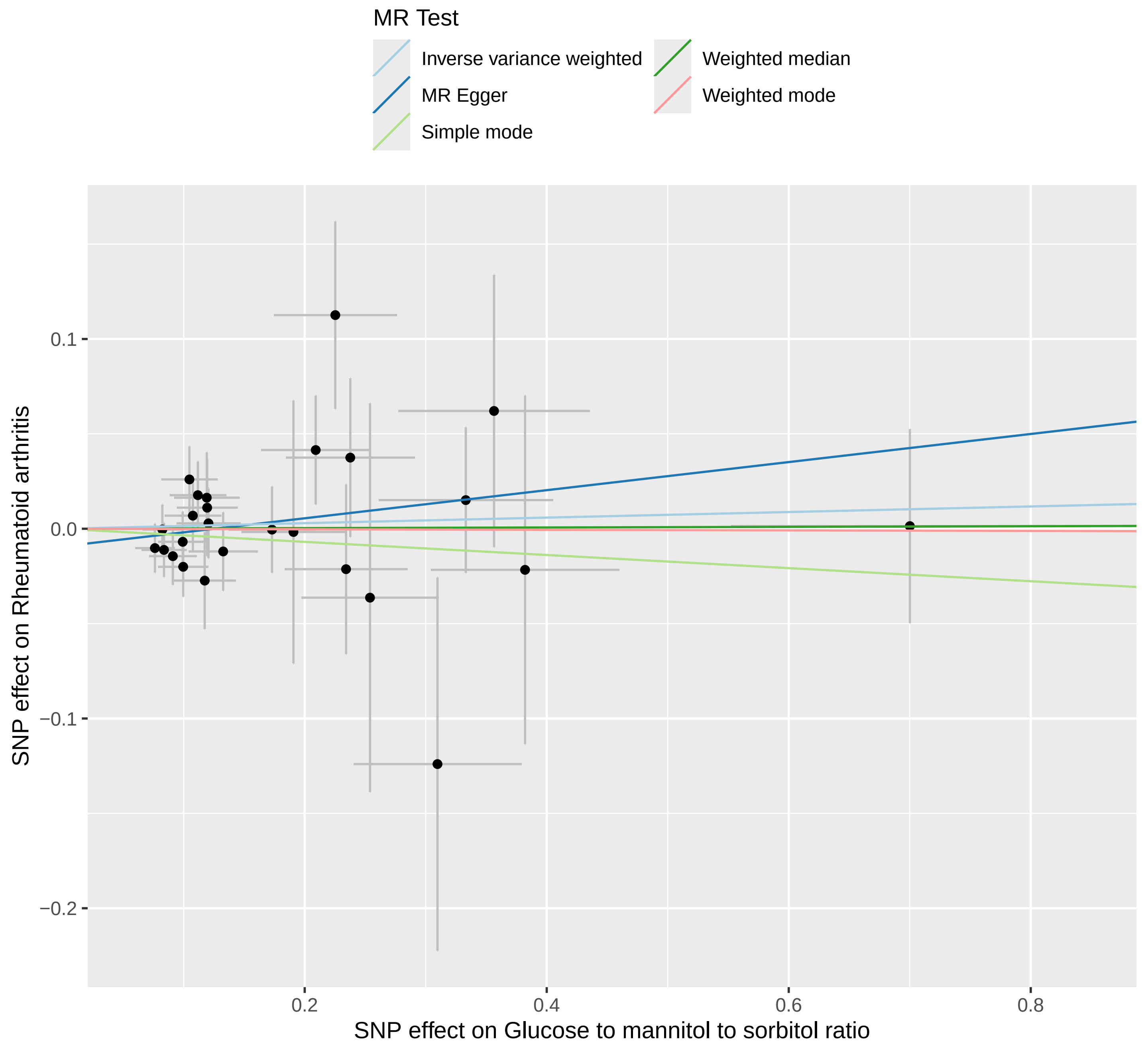
MR Test
Inverse variance weighted
MR Egger
Simple mode
Weighted median
Weighted mode
0.1
0.0
−0.1
−0.2
SNP effect on Rheumatoid arthritis
0.2
0.4
0.6
0.8
SNP effect on Glucose to mannitol to sorbitol ratio

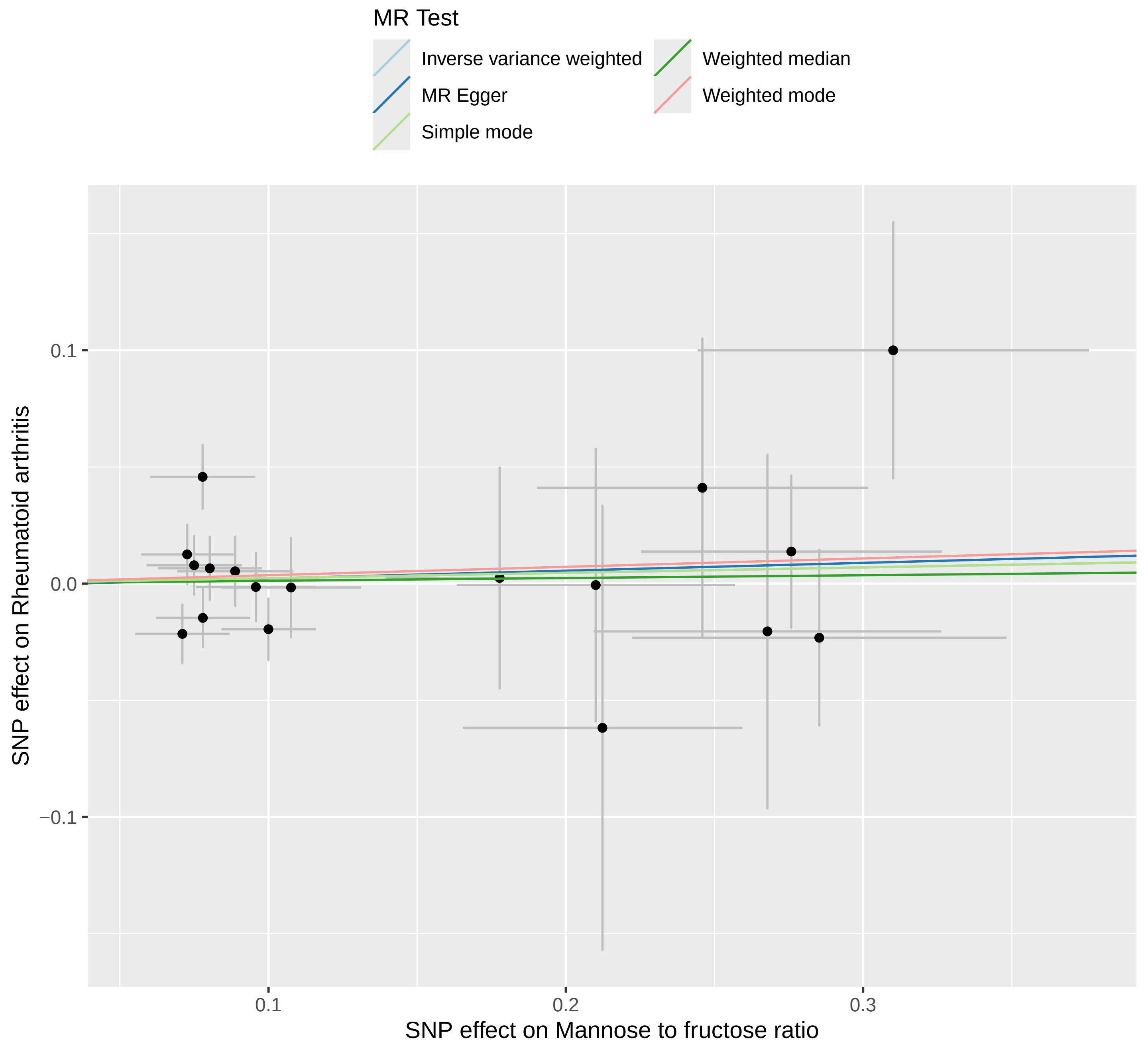
MR Test
Inverse variance weighted
MR Egger
Simple mode
Weighted median
Weighted mode
SNP effect on Rheumatoid arthritis
0.1
0.0
−0.1
0.1
0.2
0.3
SNP effect on Mannose to fructose ratio

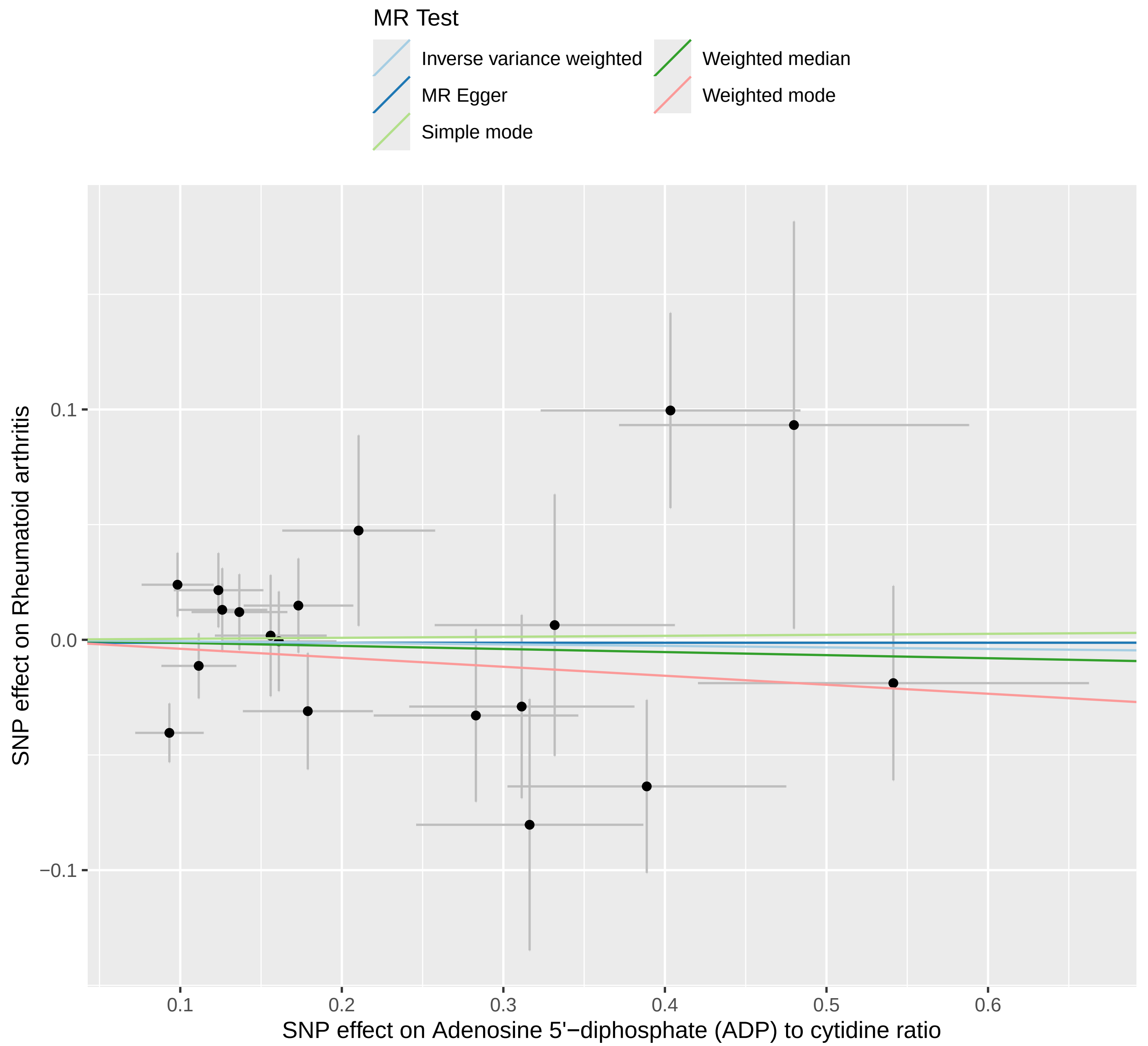

MR Test
Inverse variance weighted
MR Egger
Simple mode
Weighted median
Weighted mode
0.1
0.0
−0.1
SNP effect on Rheumatoid arthritis
0.1
0.2
0.3
0.4
0.5
0.6
SNP effect on Adenosine 5'−diphosphate (ADP) to cytidine ratio

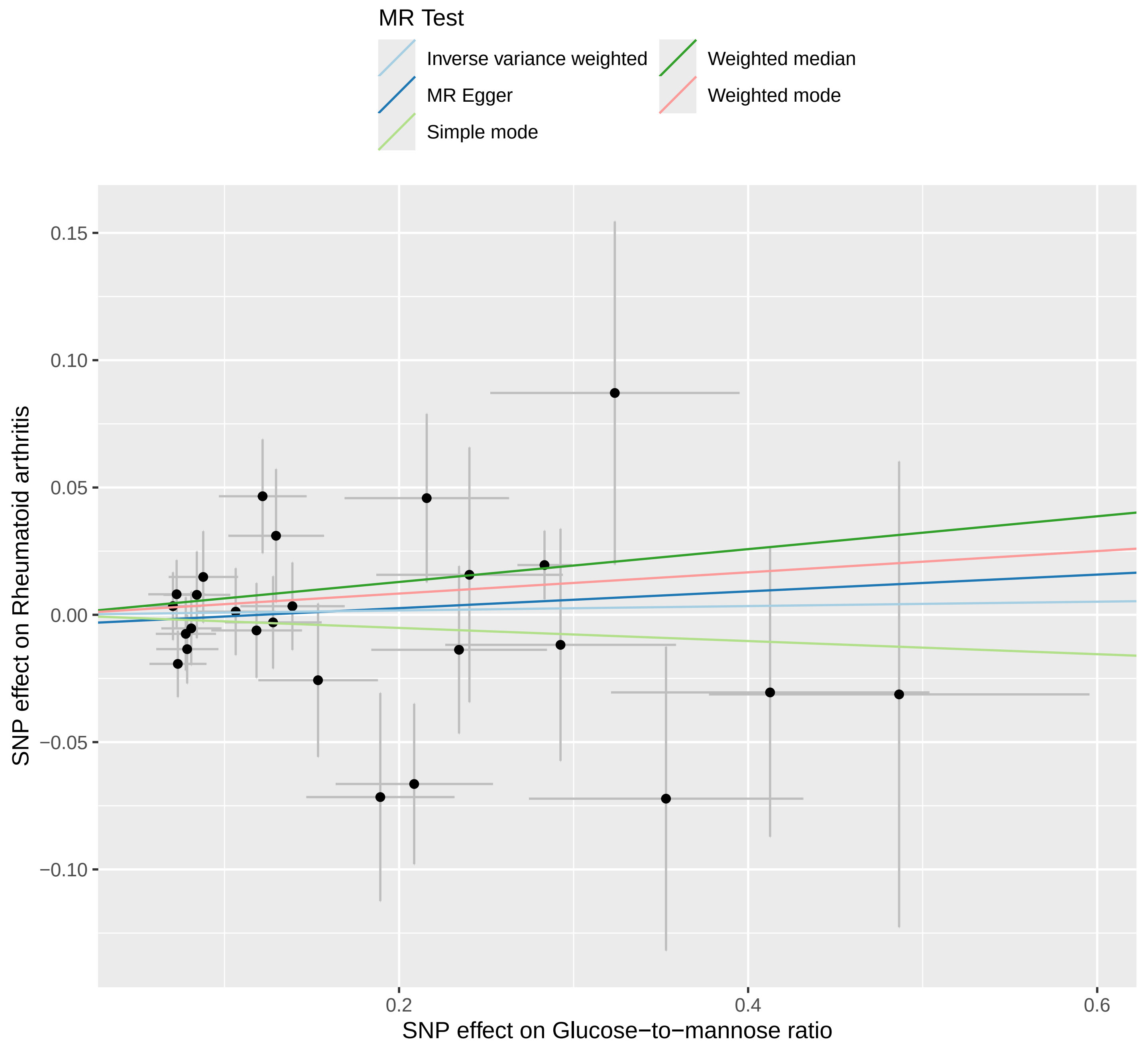
MR Test
Inverse variance weighted
MR Egger
Simple mode
Weighted median
Weighted mode
0.15
0.10
0.05
0.00
−0.05
−0.10
SNP effect on Rheumatoid arthritis
0.2
0.4
0.6
SNP effect on Glucose−to−mannose ratio

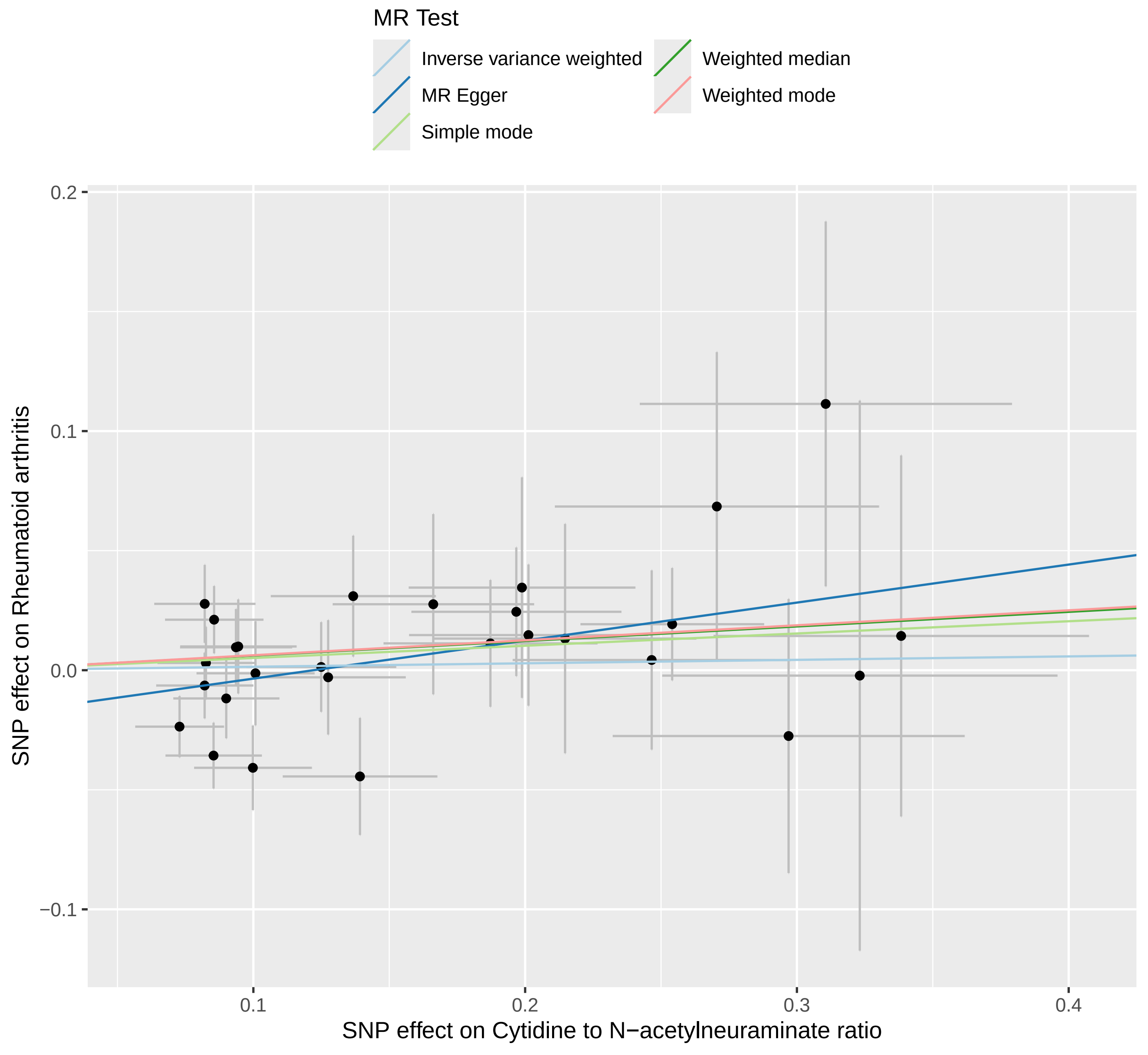
MR Test
Inverse variance weighted
MR Egger
Simple mode
Weighted median
Weighted mode
0.2
0.1
0.0
−0.1
SNP effect on Rheumatoid arthritis
0.1
0.2
0.3
0.4
SNP effect on Cytidine to N−acetylneuraminate ratio

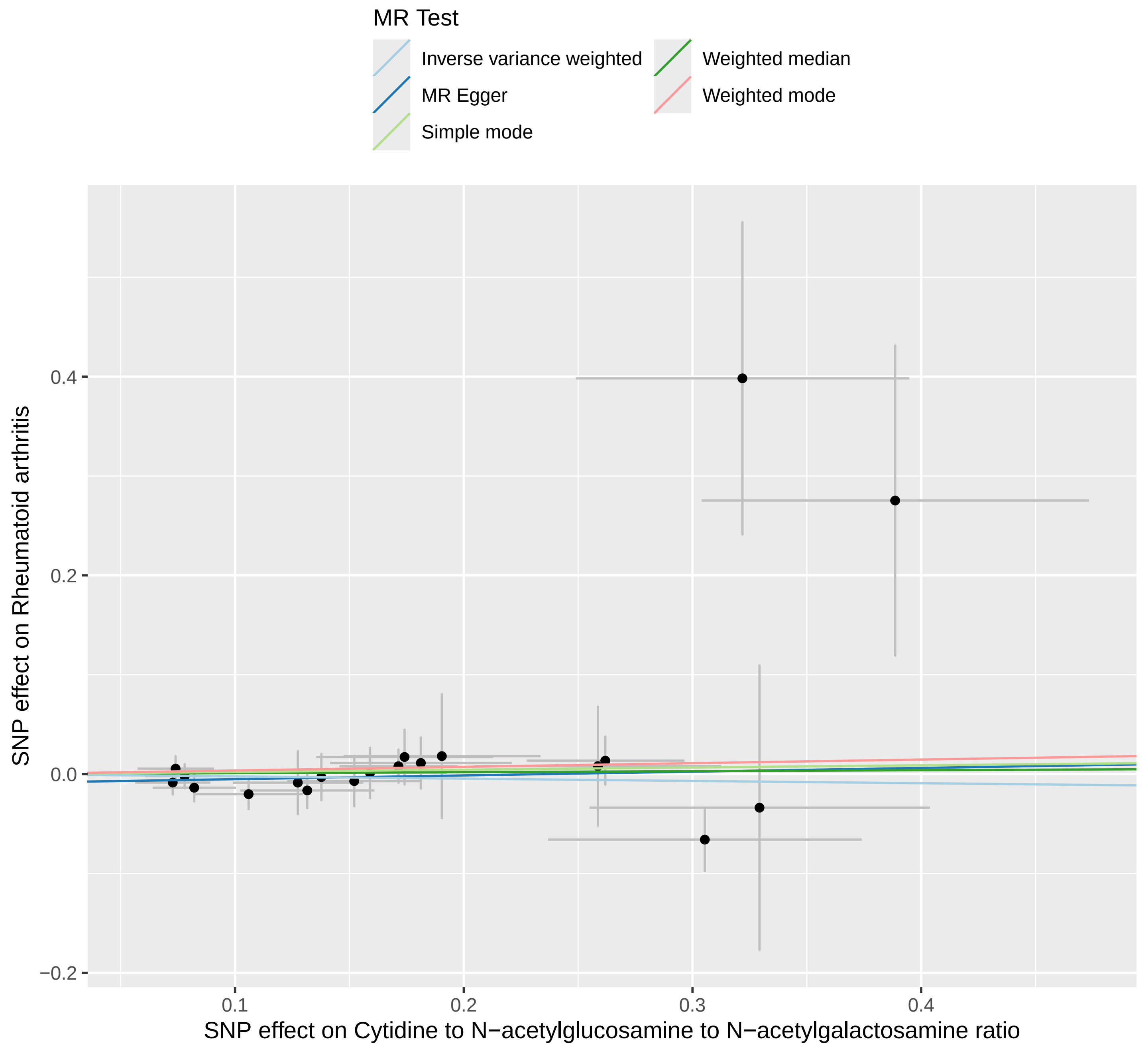
MR Test
Inverse variance weighted
MR Egger
Simple mode
Weighted median
Weighted mode
SNP effect on Rheumatoid arthritis
0.4
0.2
0.0
−0.2
0.1
0.2
0.3
0.4
SNP effect on Cytidine to N−acetylglucosamine to N−acetylgalactosamine ratio

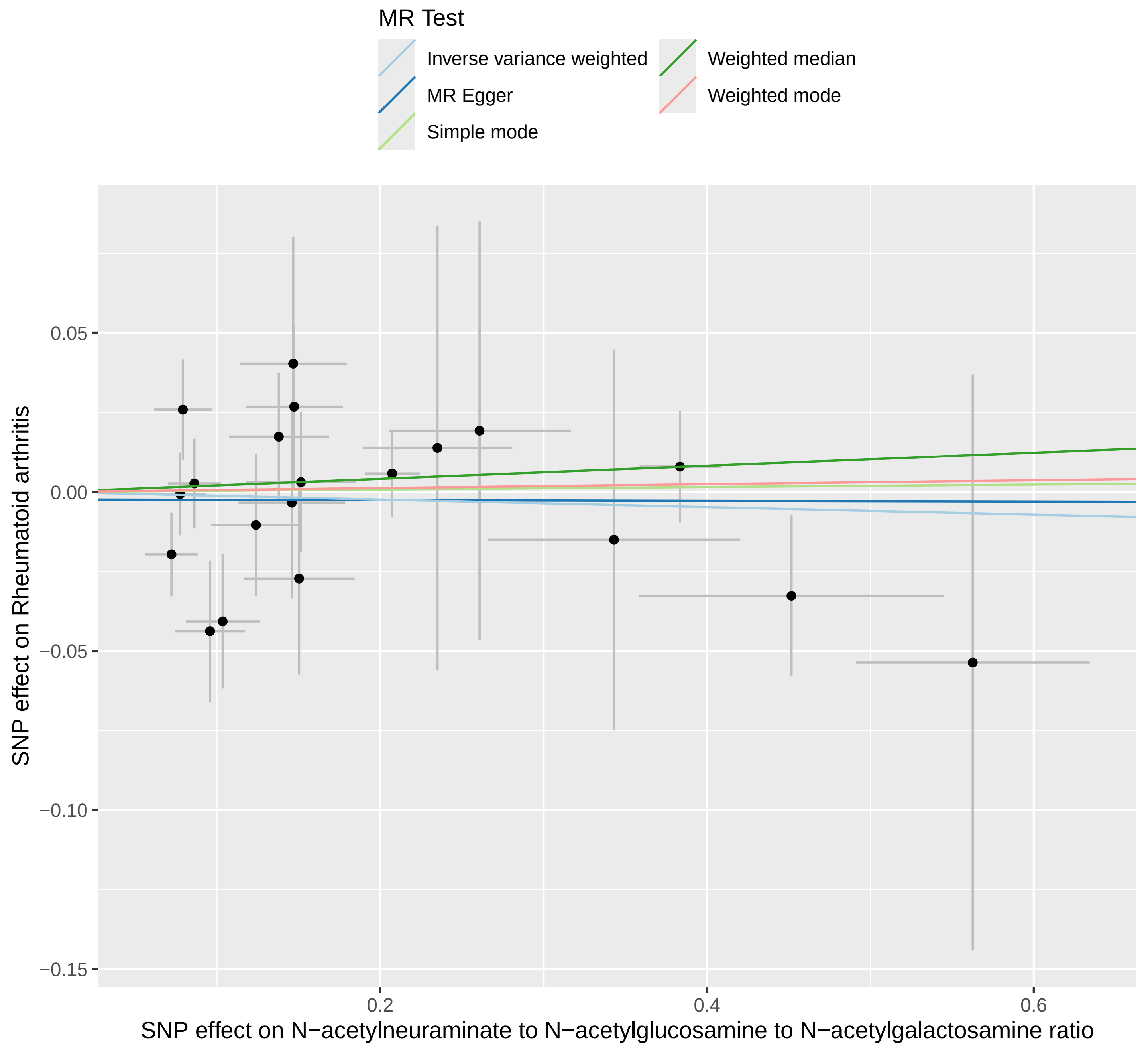

MR Test
Inverse variance weighted
MR Egger
Simple mode
Weighted median
Weighted mode
0.05
0.00
−0.05
−0.10
−0.15
0.2
0.4
0.6
SNP effect on Rheumatoid arthritis
SNP effect on N−acetylneuraminate to N−acetylglucosamine to N−acetylgalactosamine ratio

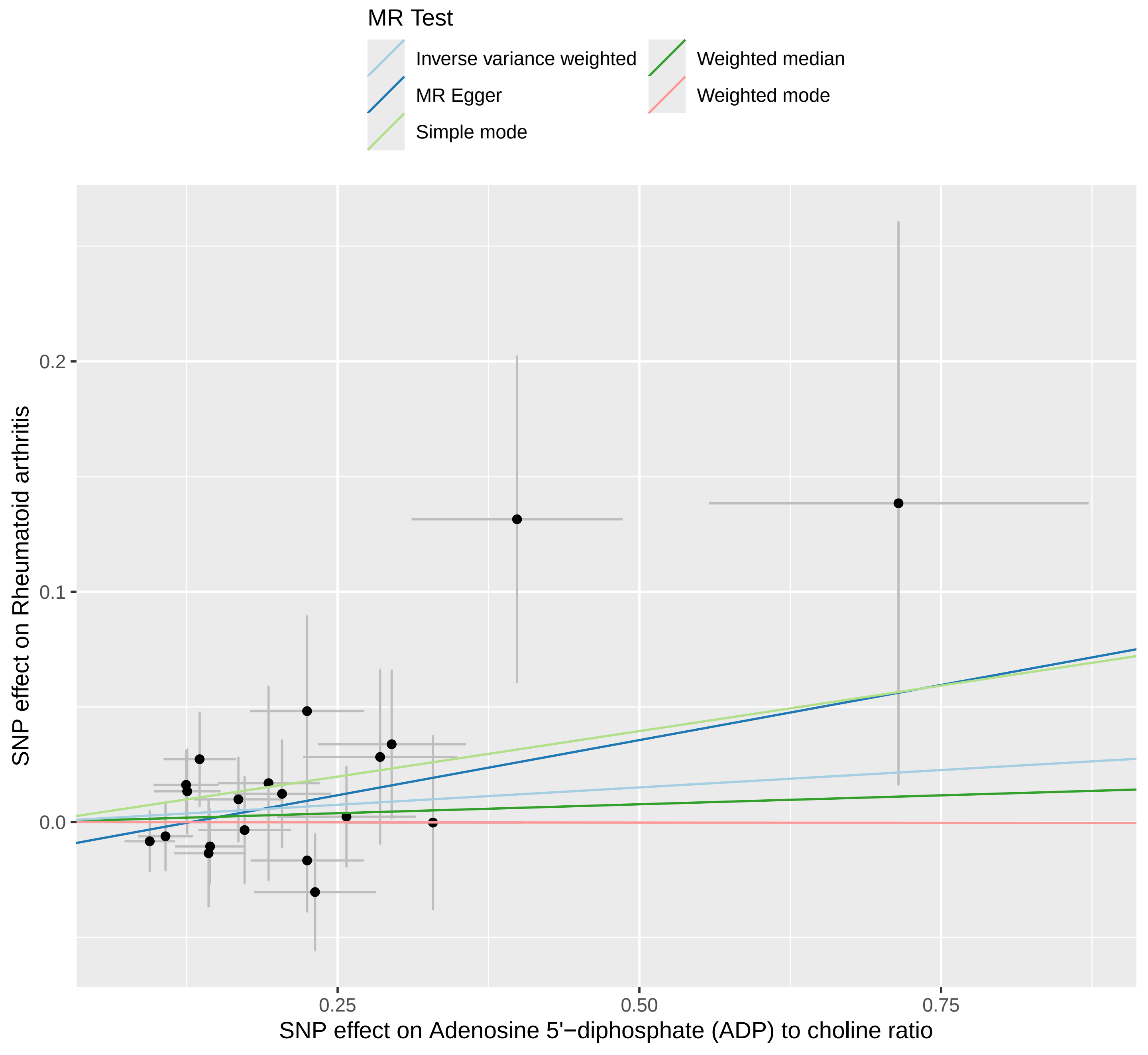
MR Test
Inverse variance weighted
MR Egger
Simple mode
Weighted median
Weighted mode
SNP effect on Rheumatoid arthritis
0.2
0.1
0.0
0.25
0.50
0.75
SNP effect on Adenosine 5'−diphosphate (ADP) to choline ratio

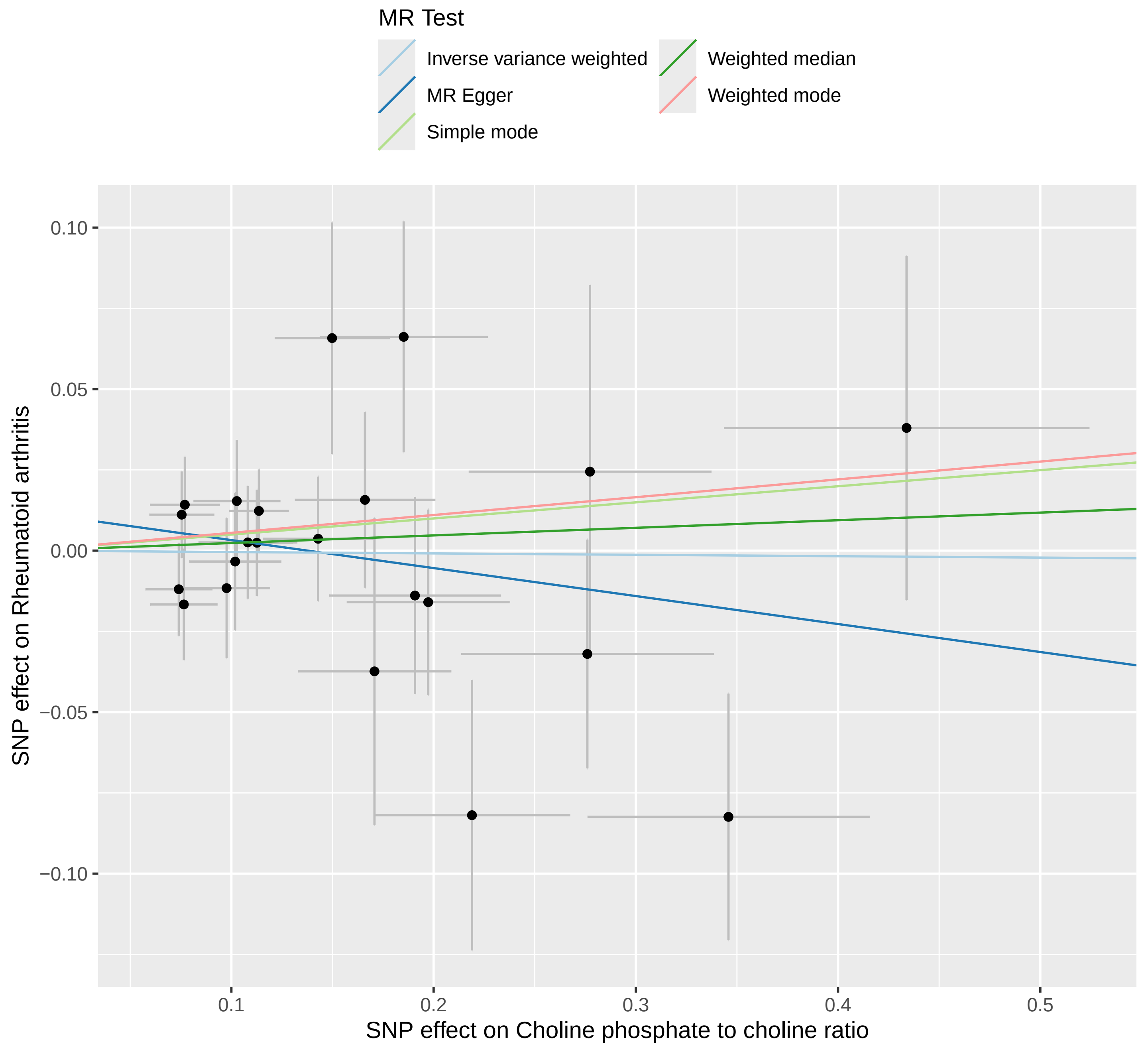

MR Test
Inverse variance weighted
MR Egger
Simple mode
Weighted median
Weighted mode
0.10
0.05
0.00
−0.05
−0.10
SNP effect on Rheumatoid arthritis
0.1
0.2
0.3
0.4
0.5
SNP effect on Choline phosphate to choline ratio

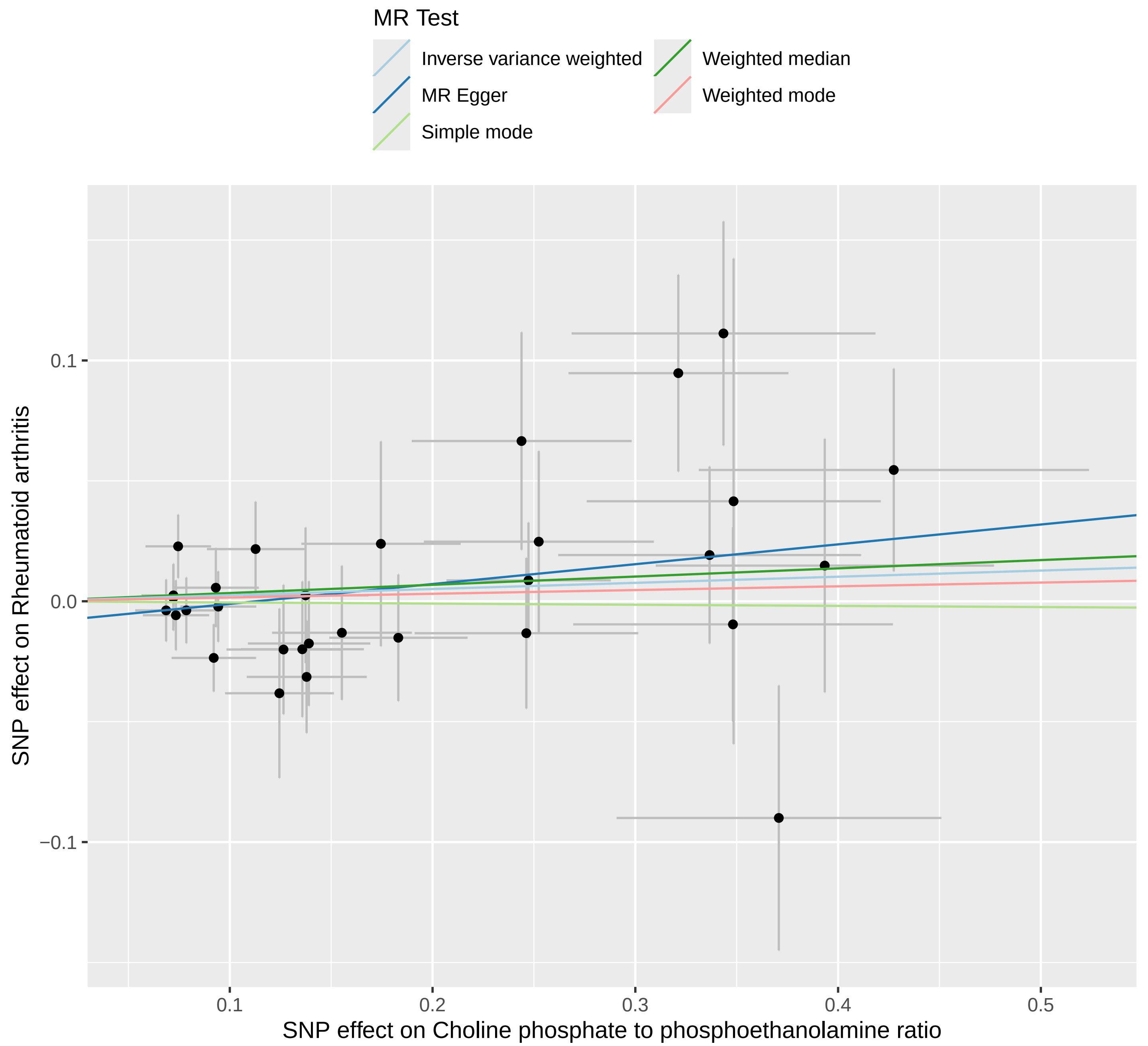
MR Test
Inverse variance weighted
MR Egger
Simple mode
Weighted median
Weighted mode
0.1
0.0
−0.1
SNP effect on Rheumatoid arthritis
0.1
0.2
0.3
0.4
0.5
SNP effect on Choline phosphate to phosphoethanolamine ratio

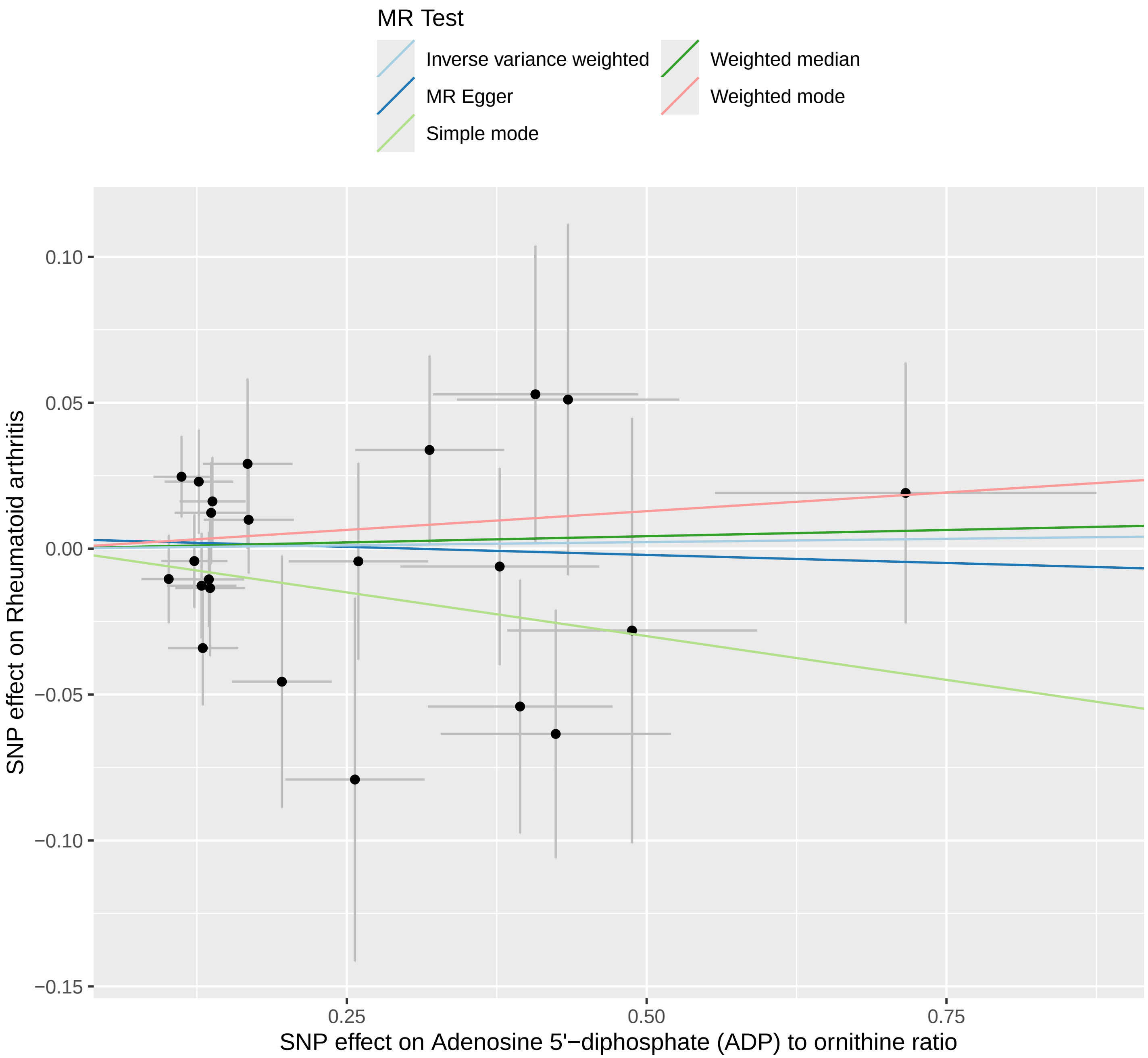

MR Test
Inverse variance weighted
MR Egger
Simple mode
Weighted median
Weighted mode
0.10
0.05
0.00
−0.05
−0.10
−0.15
SNP effect on Rheumatoid arthritis
0.25
0.50
0.75
SNP effect on Adenosine 5'−diphosphate (ADP) to ornithine ratio

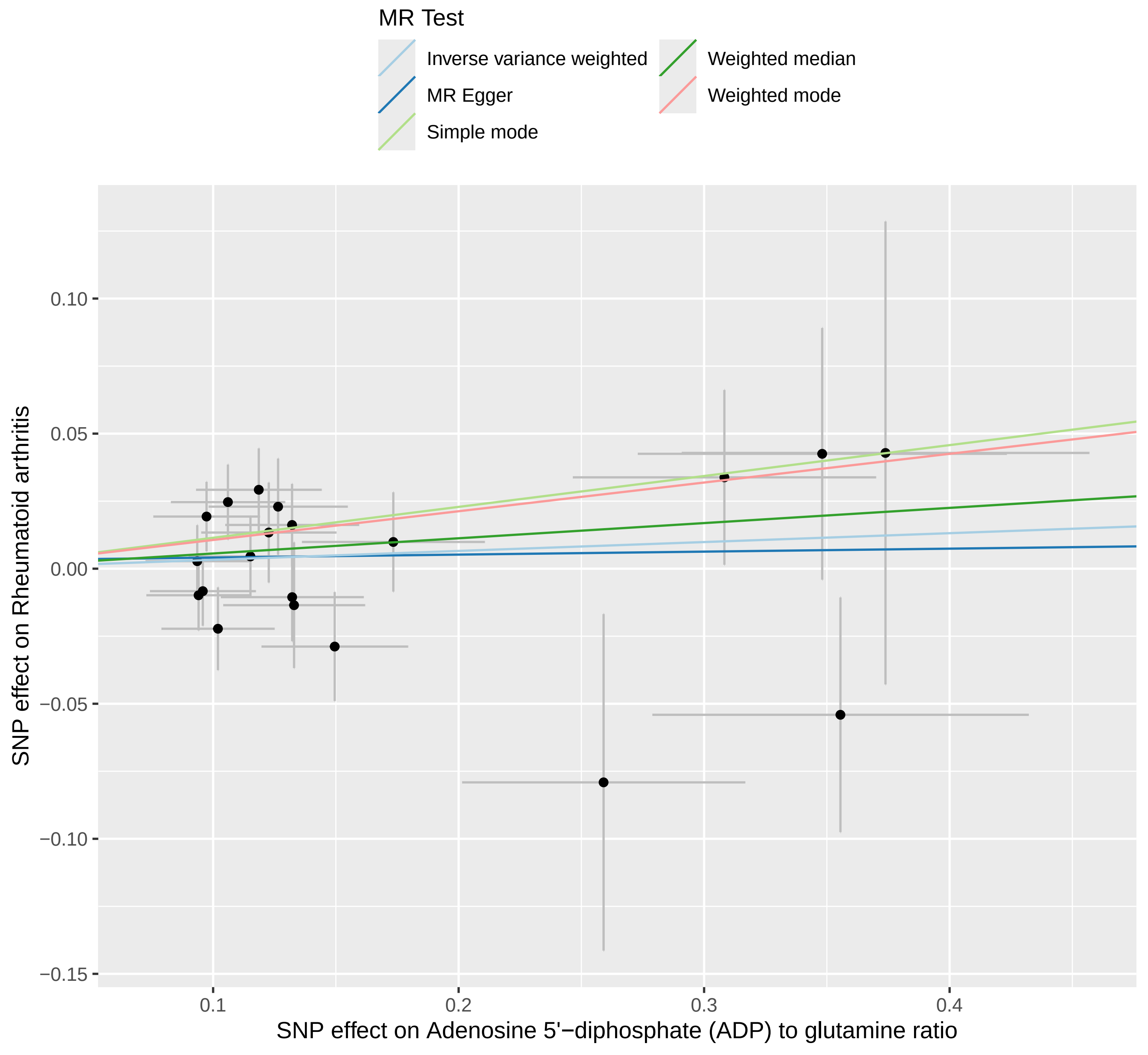

MR Test
Inverse variance weighted
MR Egger
Simple mode
Weighted median
Weighted mode
0.10
0.05
0.00
−0.05
−0.10
−0.15
SNP effect on Rheumatoid arthritis
0.1
0.2
0.3
0.4
SNP effect on Adenosine 5'−diphosphate (ADP) to glutamine ratio

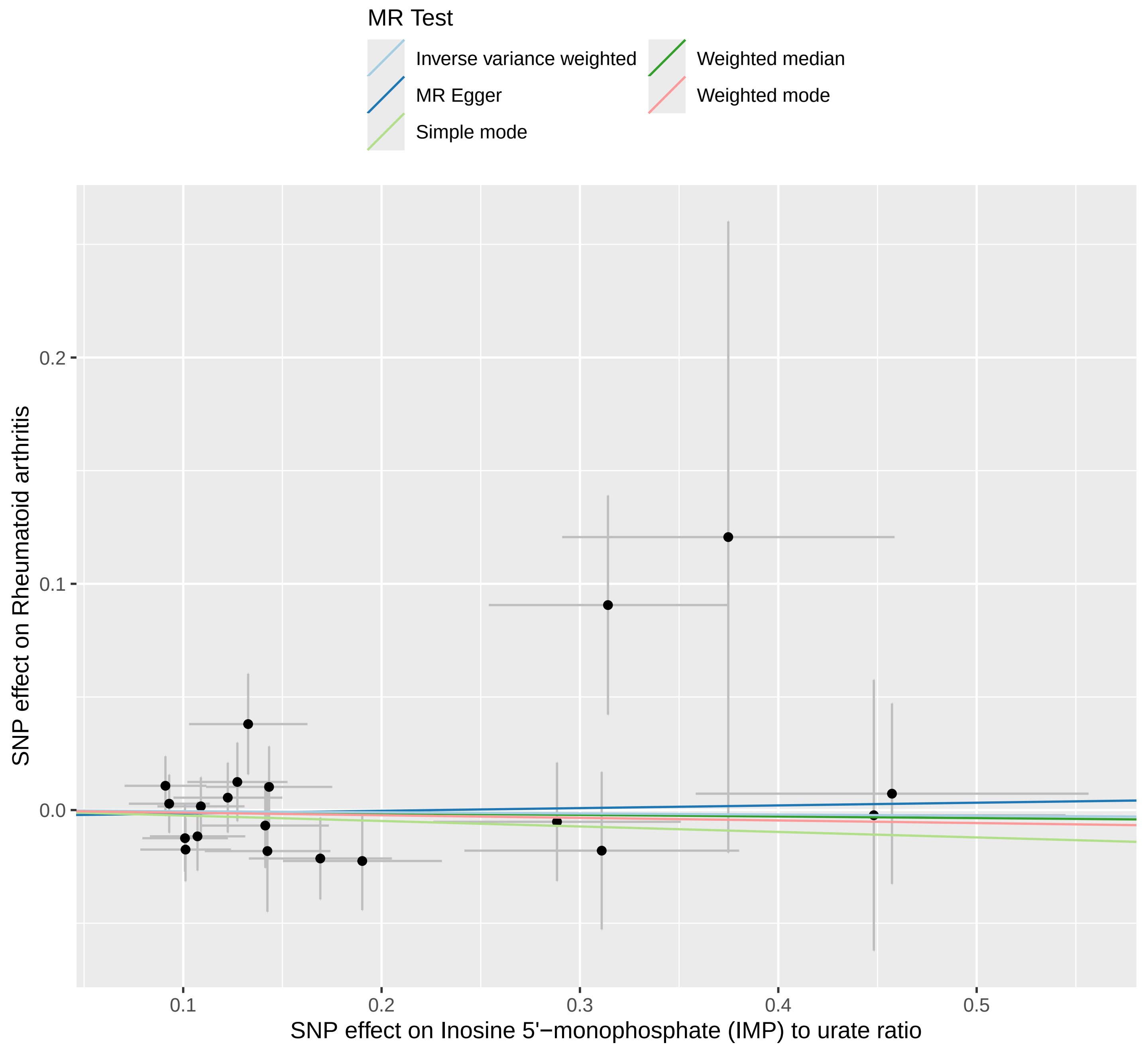
MR Test
Inverse variance weighted
MR Egger
Simple mode
Weighted median
Weighted mode
0.2
0.1
0.0
0.1
0.2
0.3
0.4
0.5
SNP effect on Rheumatoid arthritis
SNP effect on Inosine 5'−monophosphate (IMP) to urate ratio

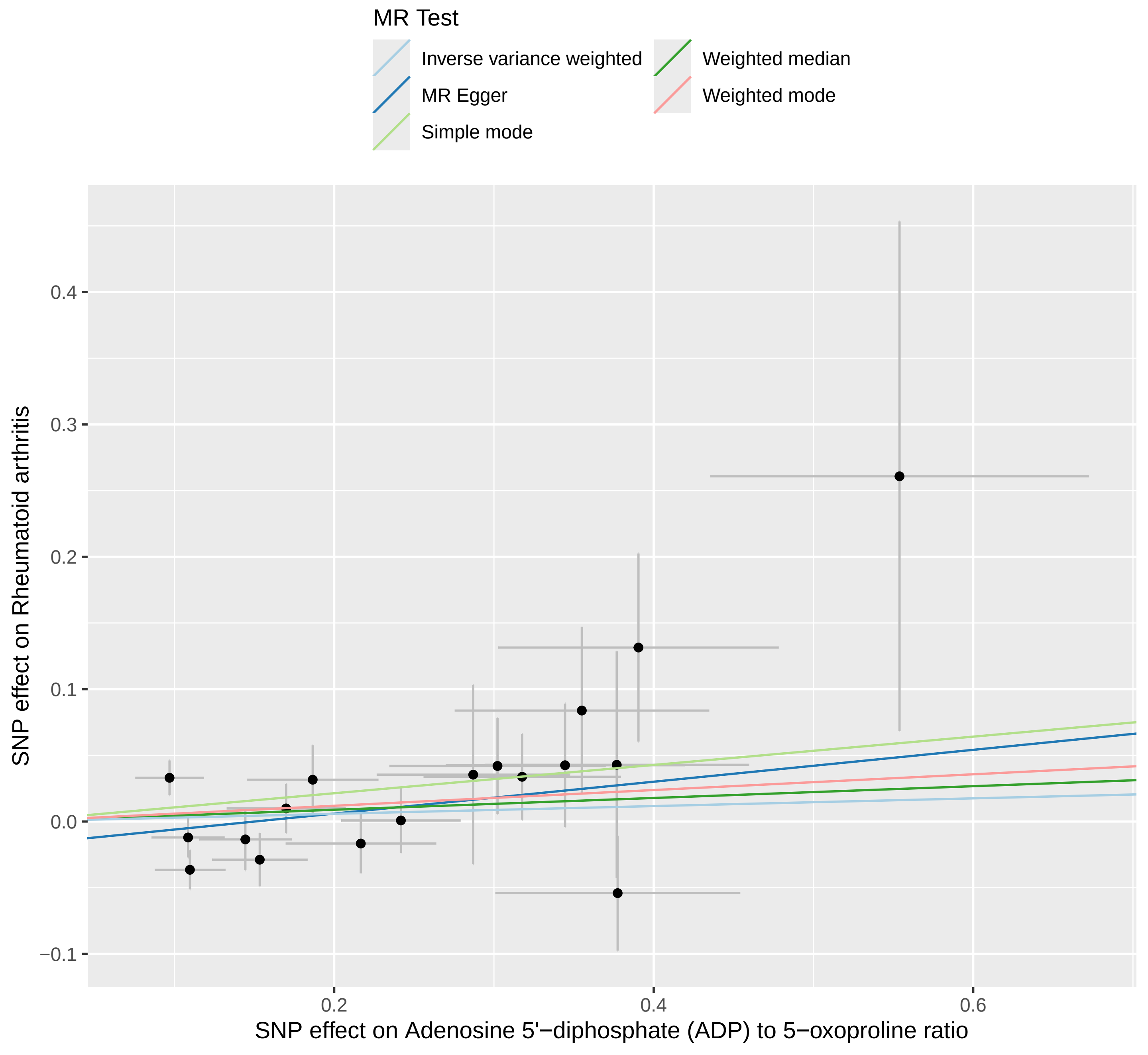

MR Test
Inverse variance weighted
MR Egger
Simple mode
Weighted median
Weighted mode
SNP effect on Rheumatoid arthritis
0.4
0.3
0.2
0.1
0.0
−0.1
0.2
0.4
0.6
SNP effect on Adenosine 5'−diphosphate (ADP) to 5−oxoproline ratio

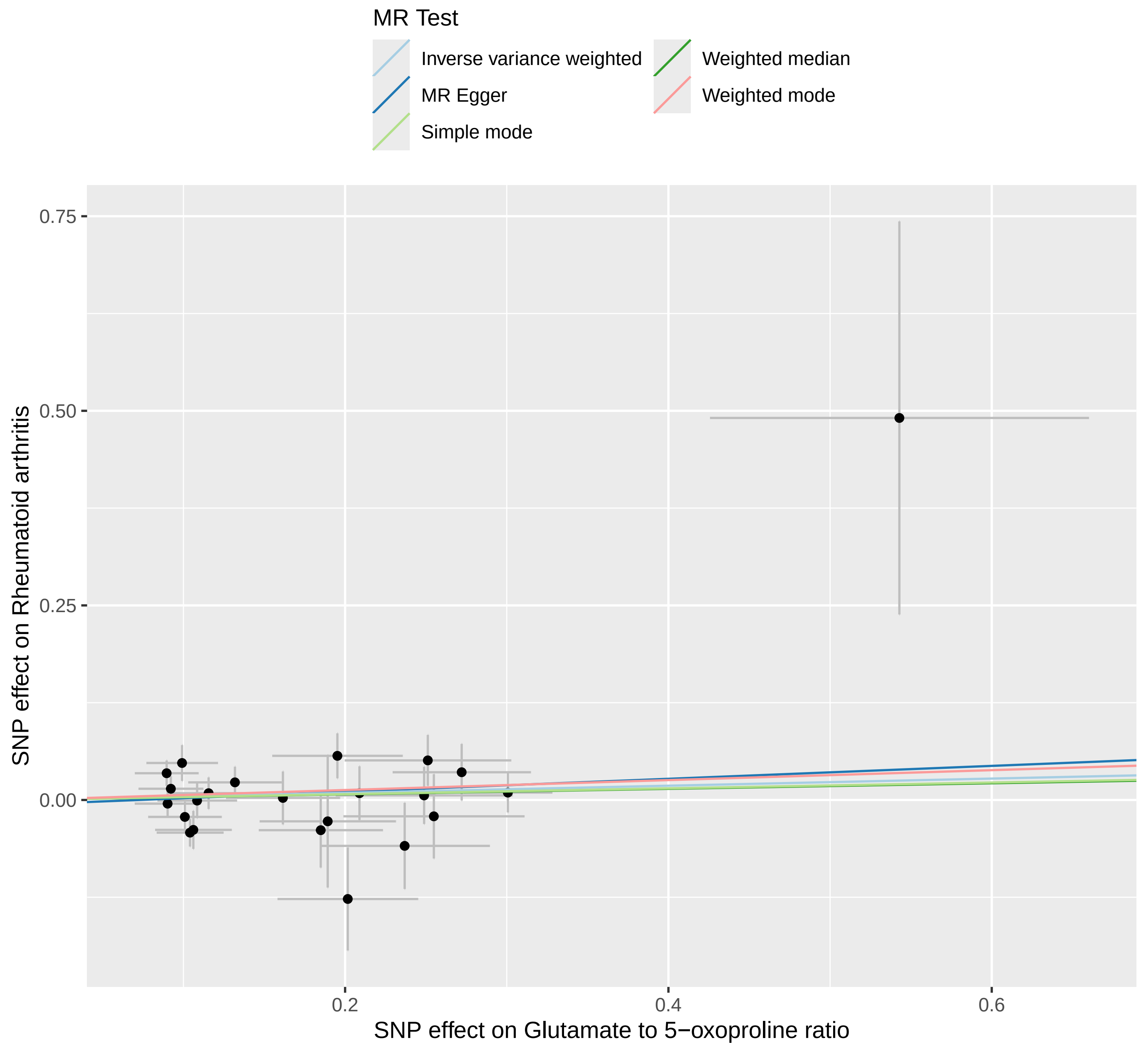

MR Test
Inverse variance weighted
MR Egger
Simple mode
Weighted median
Weighted mode
0.75
0.50
0.25
0.00
0.2
0.4
0.6
SNP effect on Rheumatoid arthritis
SNP effect on Glutamate to 5−oxoproline ratio

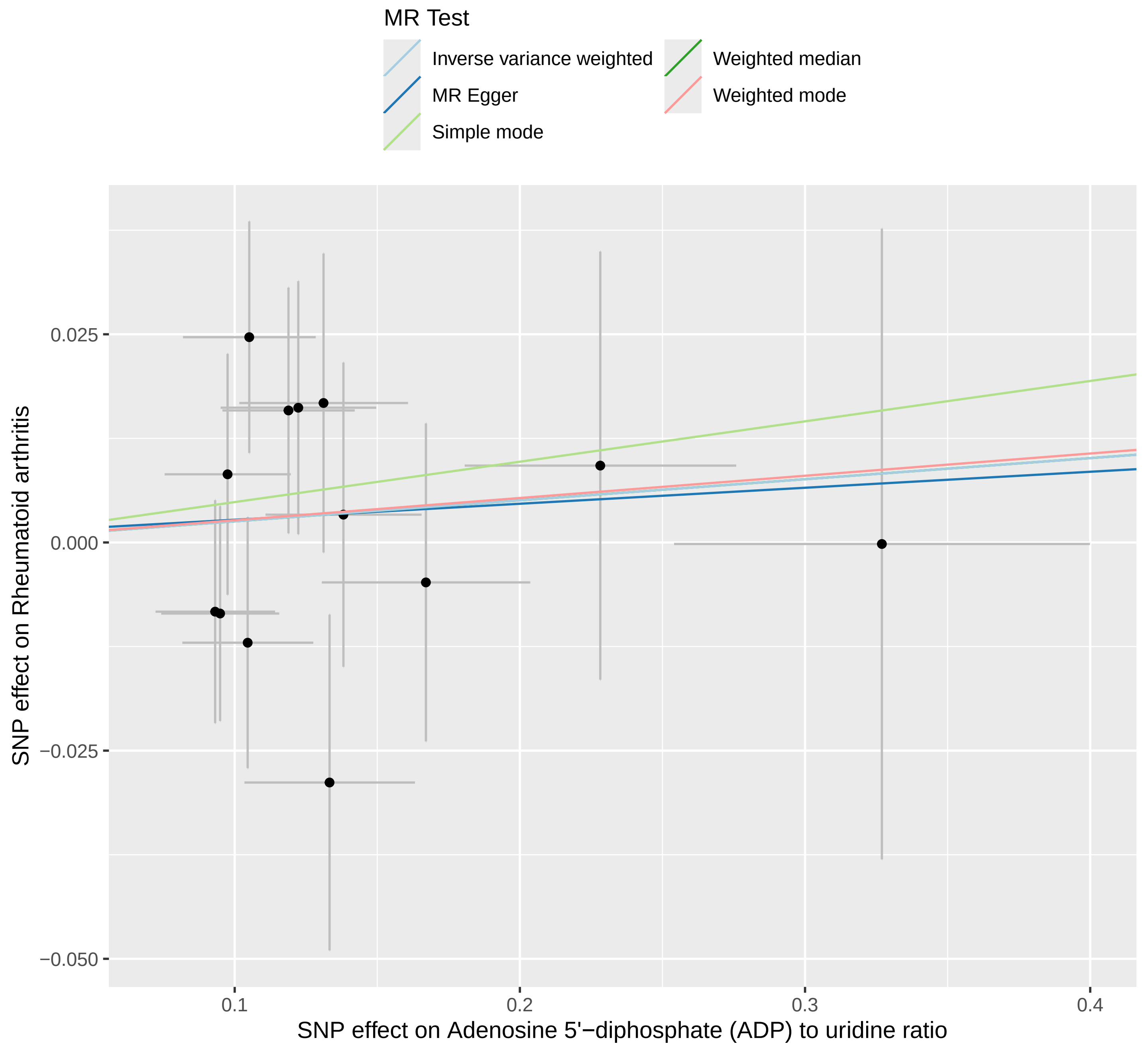

MR Test
Inverse variance weighted
MR Egger
Simple mode
Weighted median
Weighted mode
SNP effect on Rheumatoid arthritis
0.025
0.000
−0.025
−0.050
0.1
0.2
0.3
0.4
SNP effect on Adenosine 5'−diphosphate (ADP) to uridine ratio

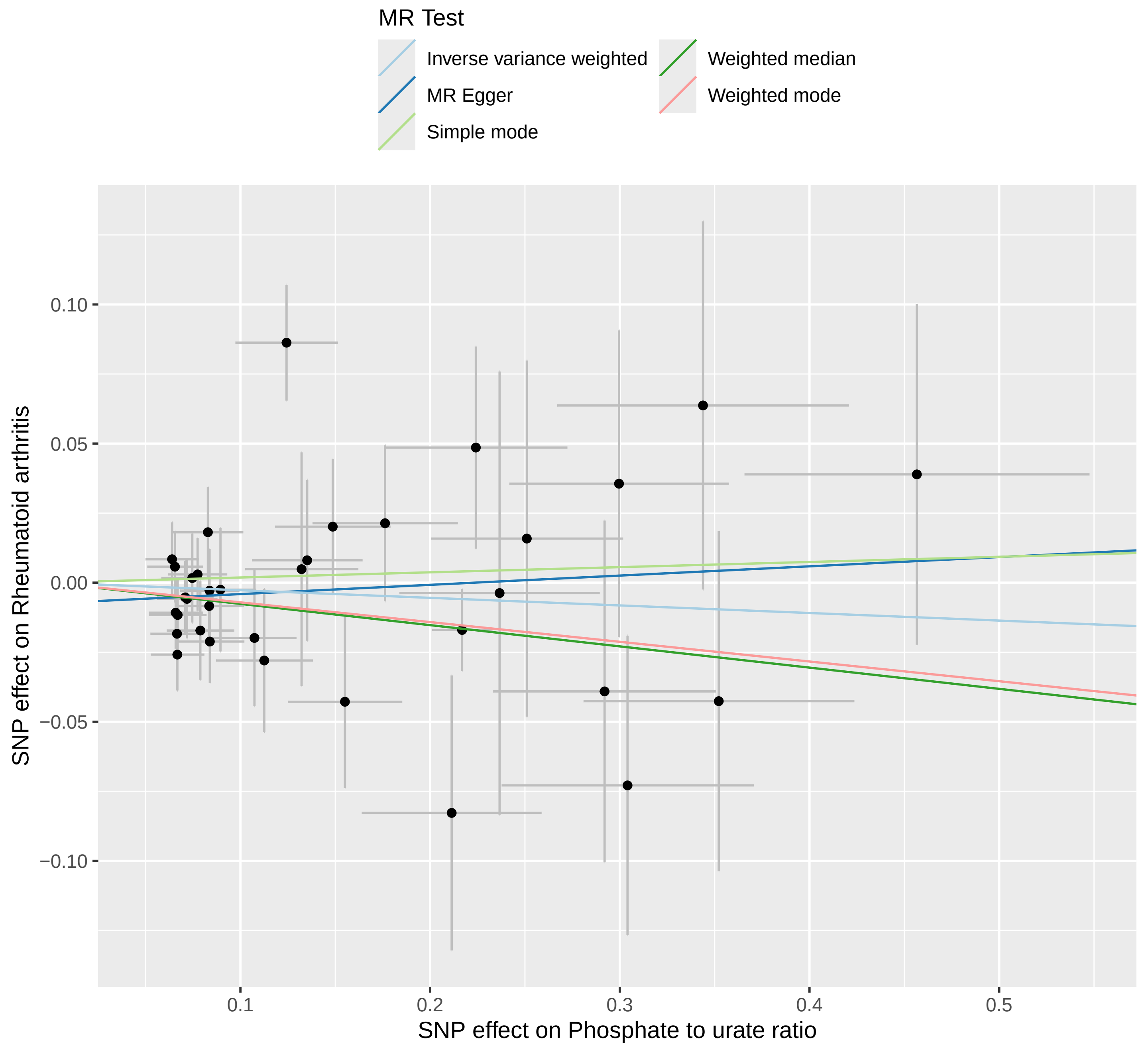

MR Test
Inverse variance weighted
MR Egger
Simple mode
Weighted median
Weighted mode
0.10
0.05
0.00
−0.05
−0.10
SNP effect on Rheumatoid arthritis
0.1
0.2
0.3
0.4
0.5
SNP effect on Phosphate to urate ratio

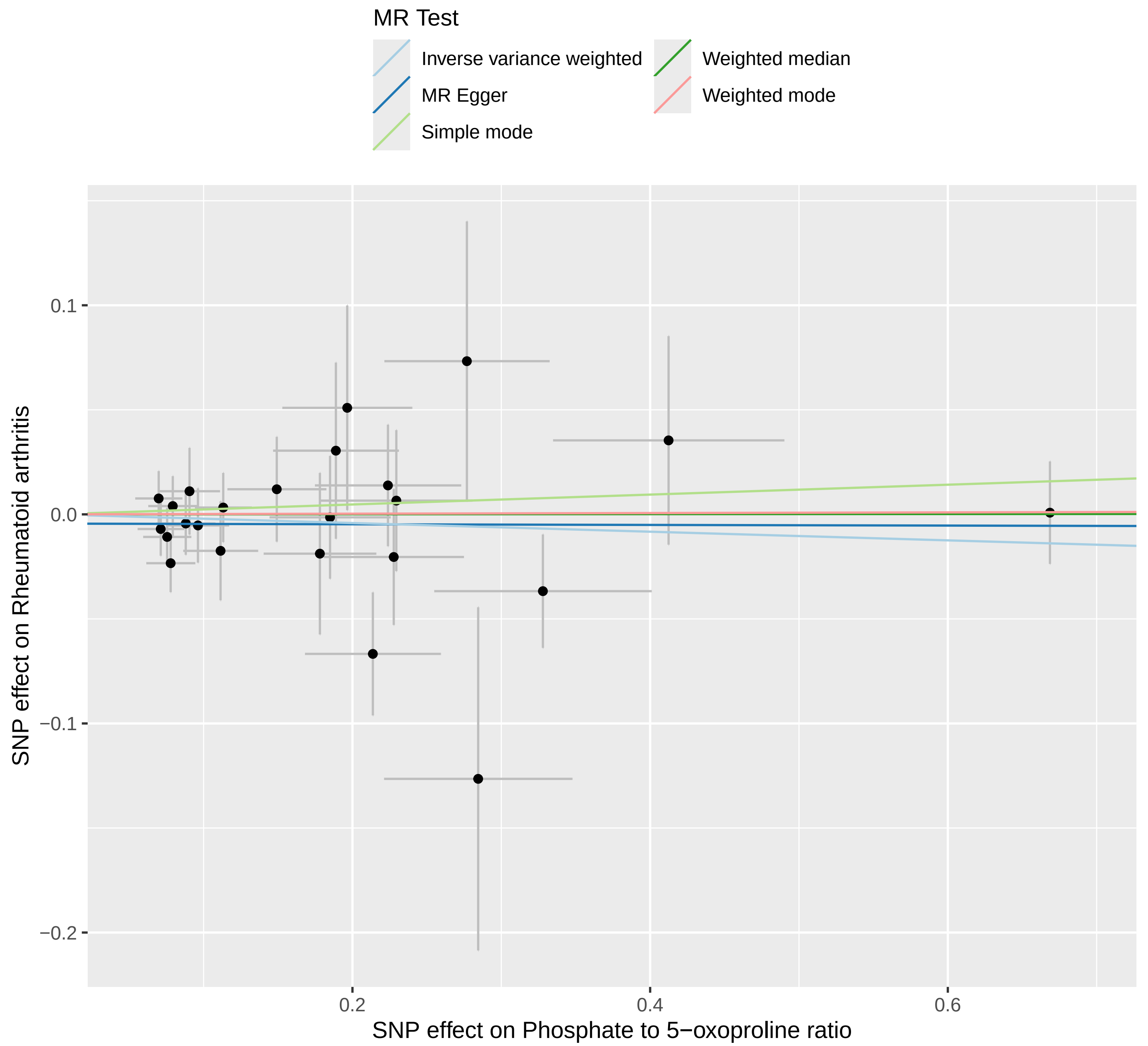
MR Test
Inverse variance weighted
MR Egger
Simple mode
Weighted median
Weighted mode
0.1
0.0
−0.1
−0.2
0.2
0.4
0.6
SNP effect on Rheumatoid arthritis
SNP effect on Phosphate to 5−oxoproline ratio

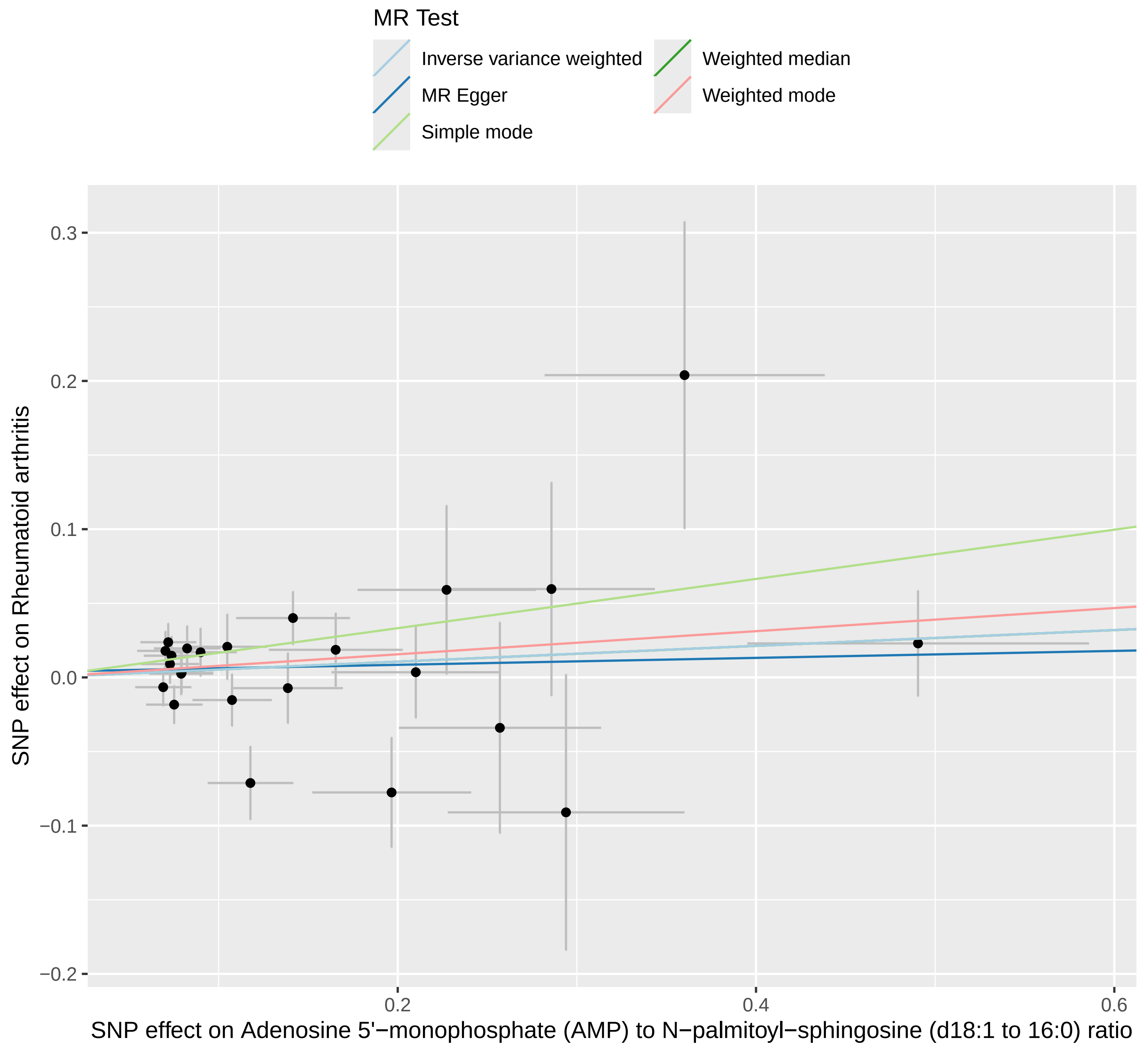

MR Test
Inverse variance weighted
MR Egger
Simple mode
Weighted median
Weighted mode
0.3
0.2
0.1
0.0
−0.1
−0.2
0.2
0.4
0.6
SNP effect on Rheumatoid arthritis
SNP effect on Adenosine 5'−monophosphate (AMP) to N−palmitoyl−sphingosine (d18:1 to 16:0) ratio

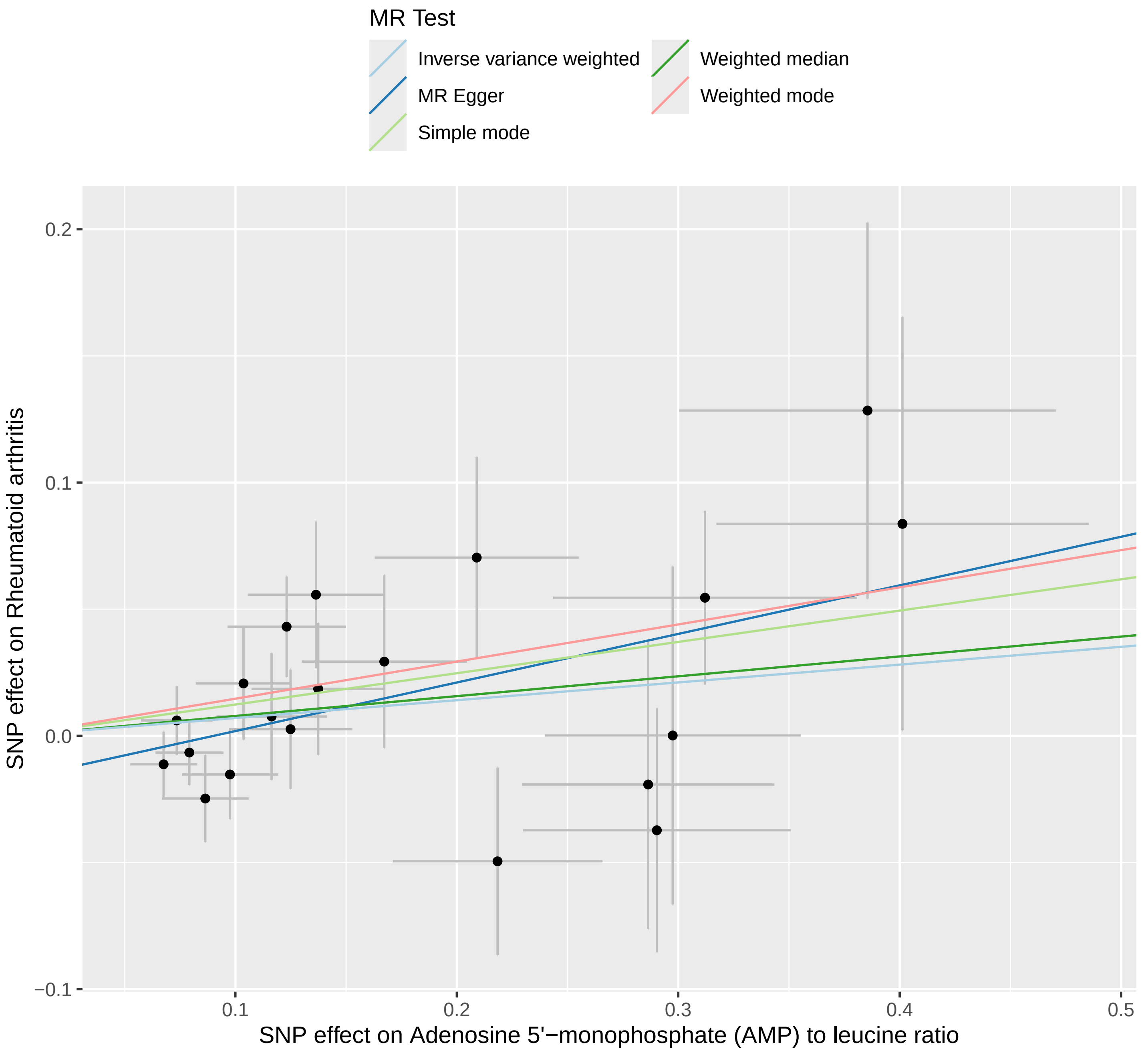

MR Test
Inverse variance weighted
MR Egger
Simple mode
Weighted median
Weighted mode
SNP effect on Rheumatoid arthritis
0.2
0.1
0.0
−0.1
0.1
0.2
0.3
0.4
0.5
SNP effect on Adenosine 5'−monophosphate (AMP) to leucine ratio

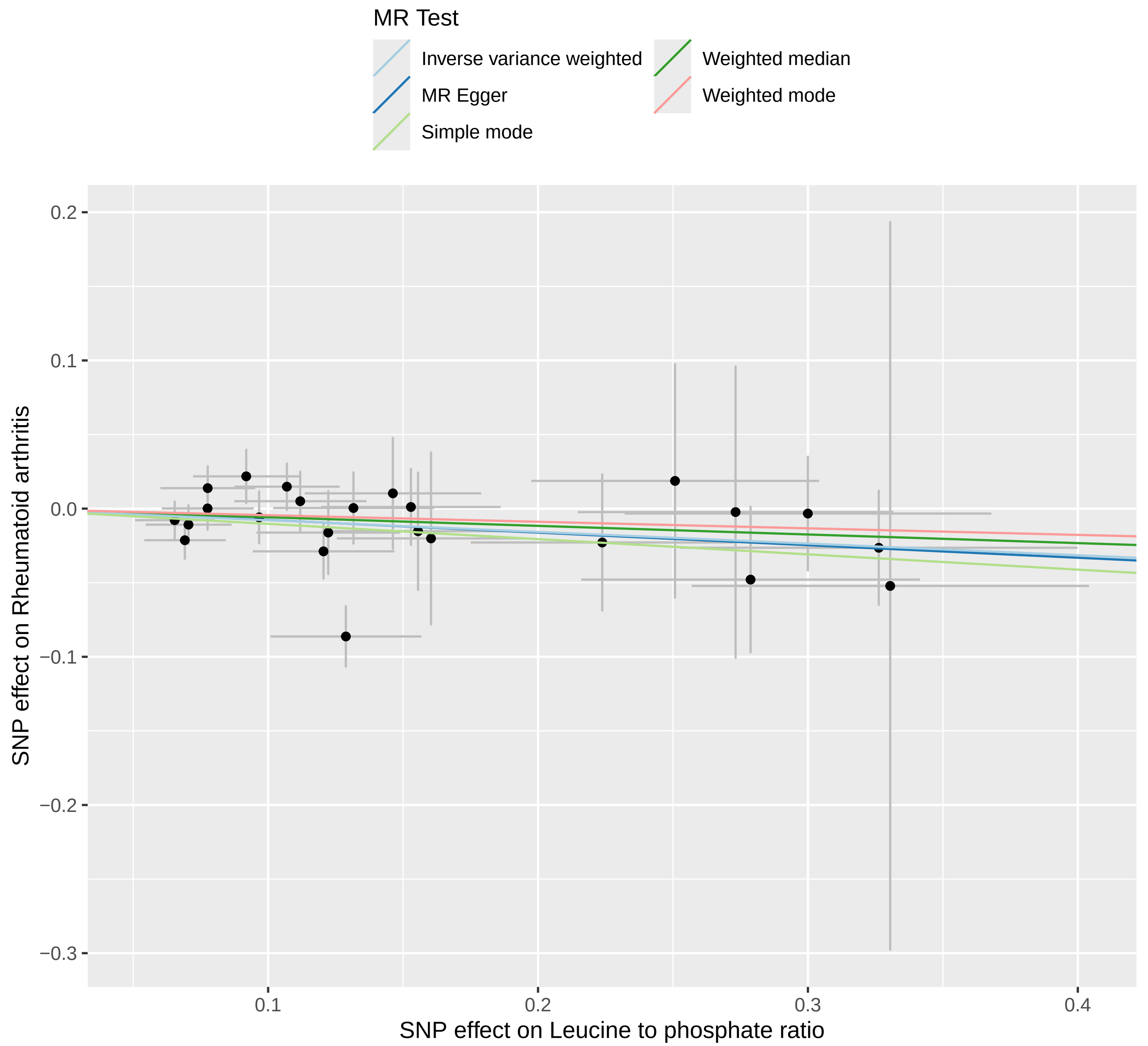

MR Test
Inverse variance weighted
MR Egger
Simple mode
Weighted median
Weighted mode
0.2
0.1
0.0
−0.1
−0.2
−0.3
SNP effect on Rheumatoid arthritis
0.1
0.2
0.3
0.4
SNP effect on Leucine to phosphate ratio

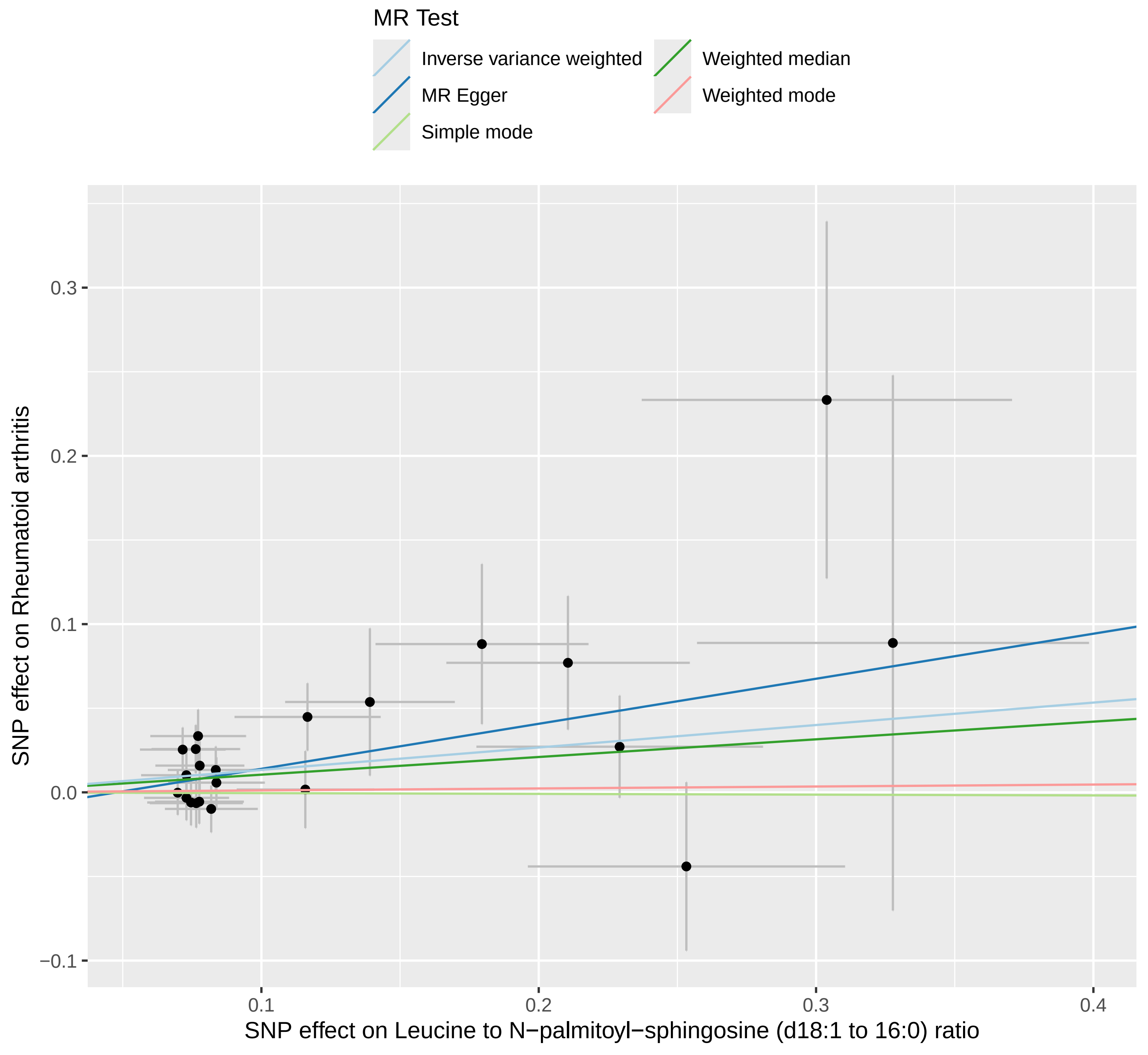
MR Test
Inverse variance weighted
MR Egger
Simple mode
Weighted median
Weighted mode
0.3
0.2
0.1
0.0
−0.1
0.1
0.2
0.3
0.4
SNP effect on Rheumatoid arthritis
SNP effect on Leucine to N−palmitoyl−sphingosine (d18:1 to 16:0) ratio

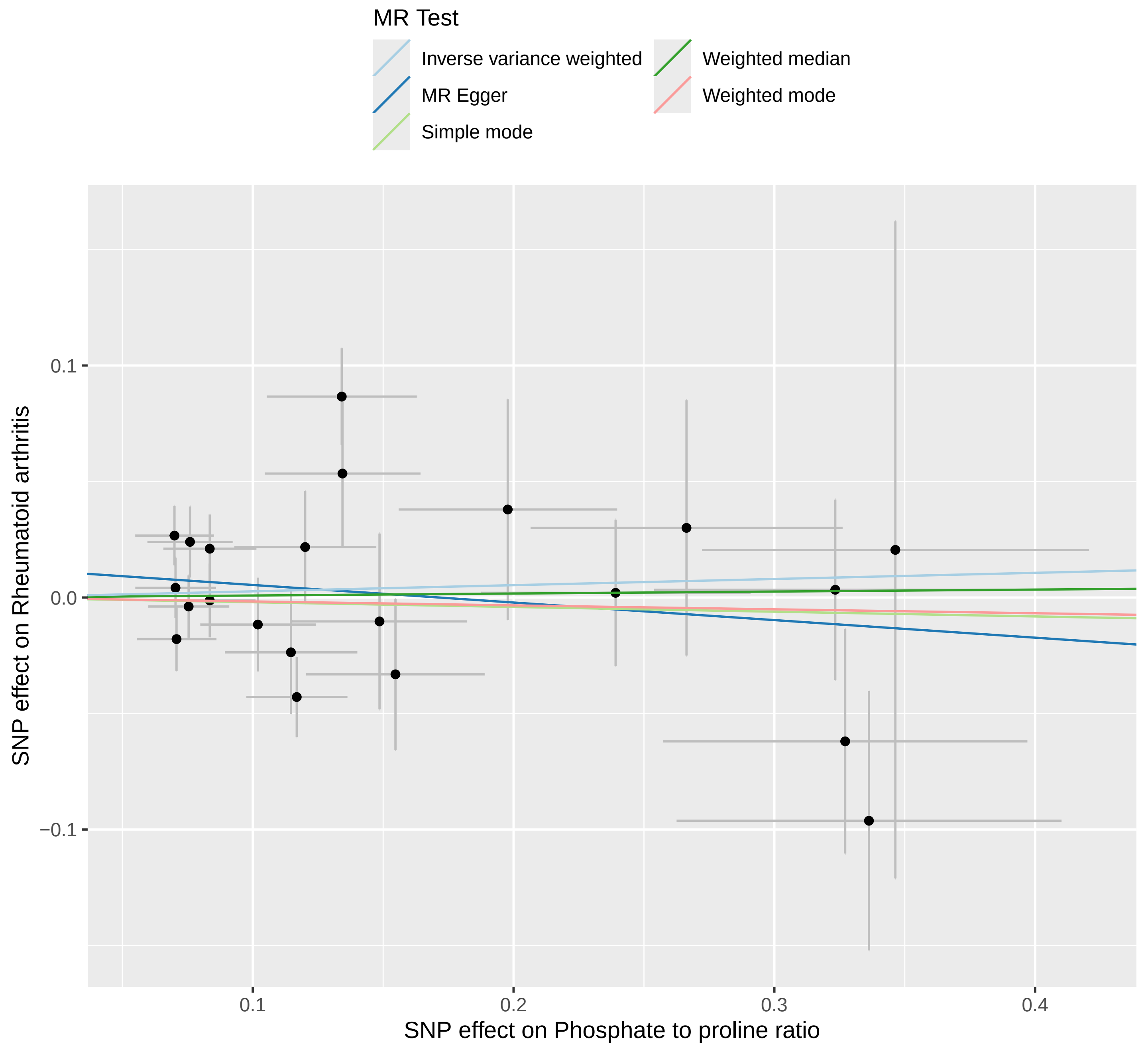

MR Test
Inverse variance weighted
MR Egger
Simple mode
Weighted median
Weighted mode
0.1
0.0
−0.1
0.1
0.2
0.3
0.4
SNP effect on Rheumatoid arthritis
SNP effect on Phosphate to proline ratio

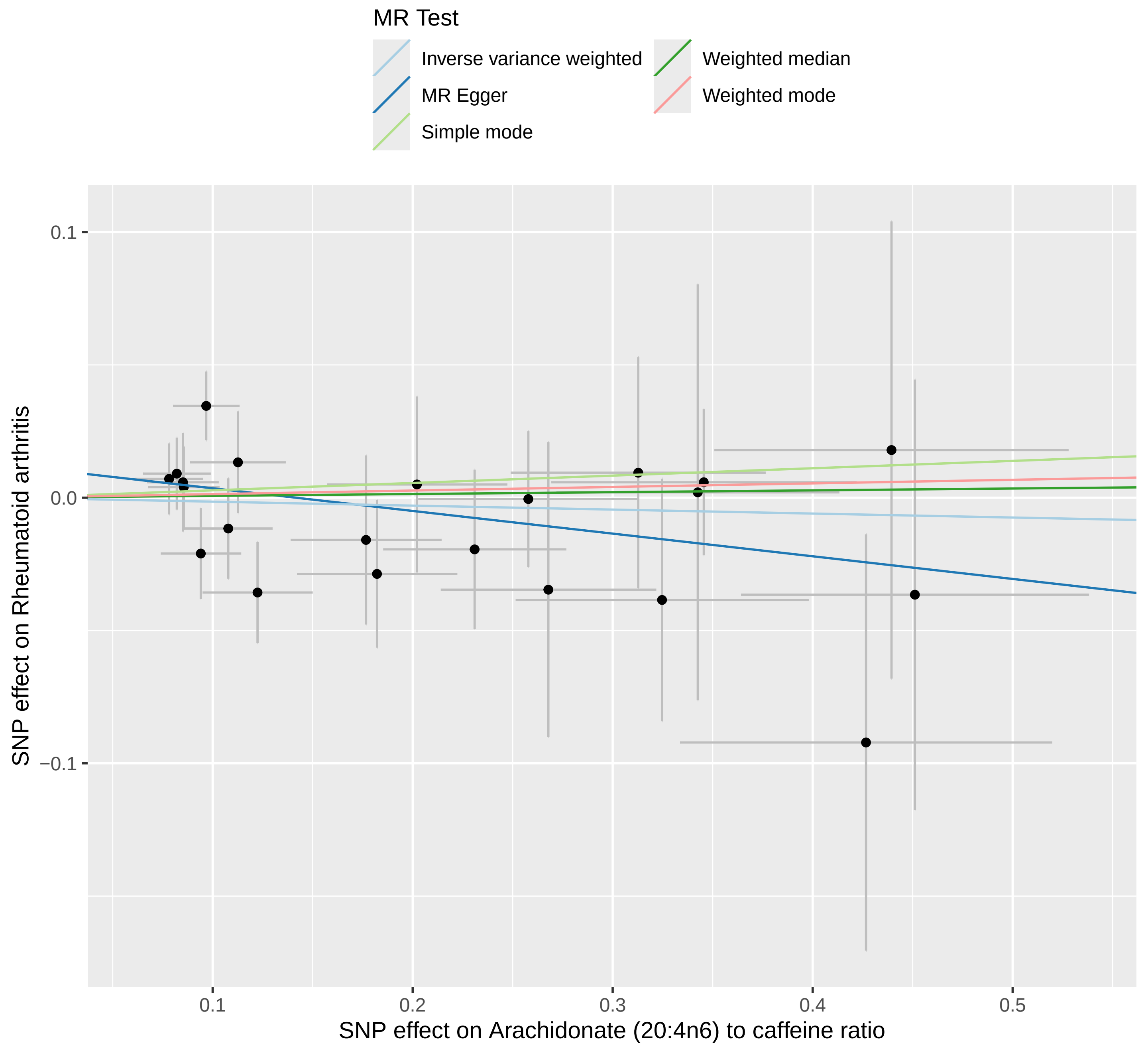

MR Test
Inverse variance weighted
MR Egger
Simple mode
Weighted median
Weighted mode
0.1
0.0
−0.1
0.1
0.2
0.3
0.4
0.5
SNP effect on Rheumatoid arthritis
SNP effect on Arachidonate (20:4n6) to caffeine ratio

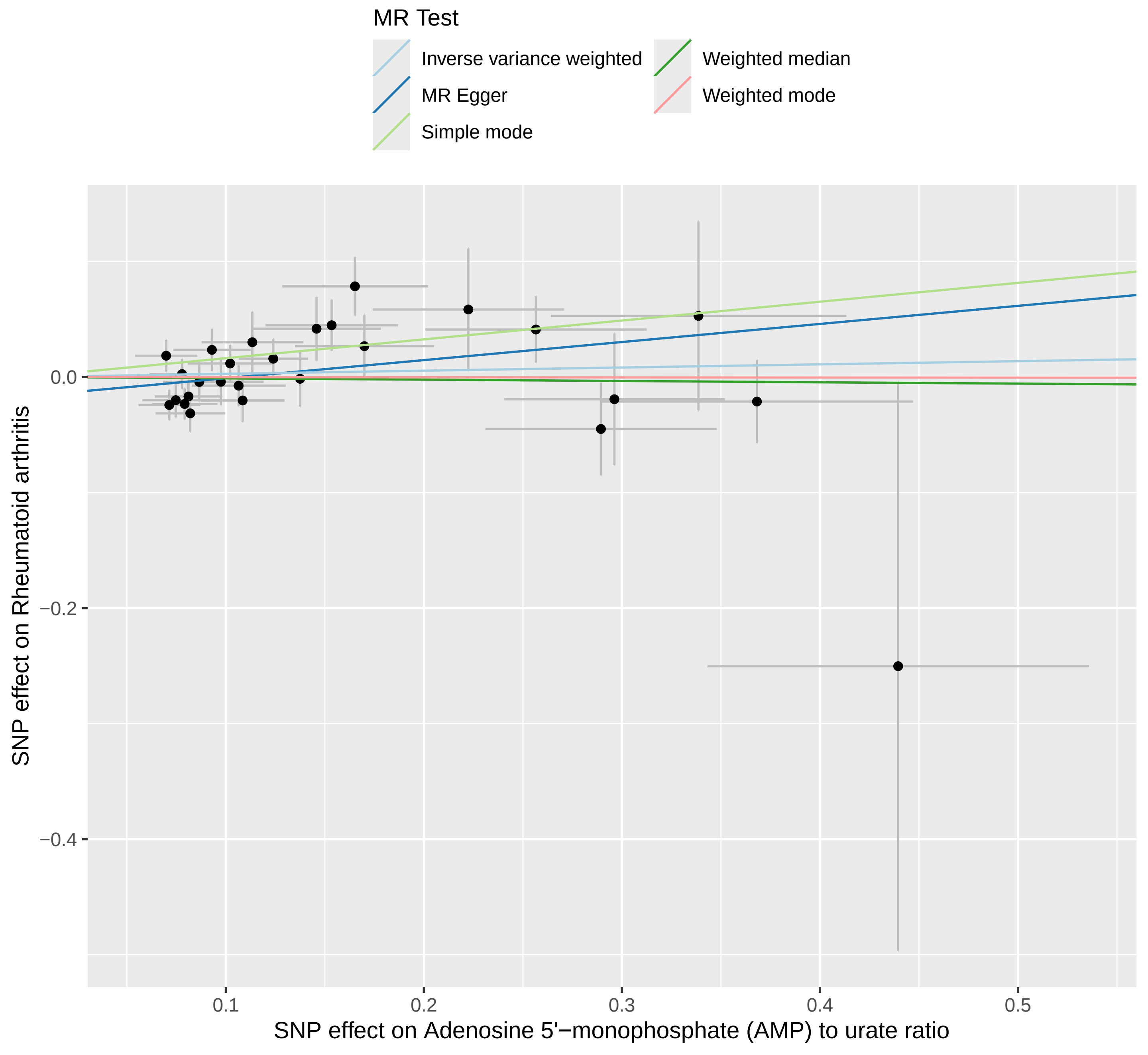
MR Test
Inverse variance weighted
MR Egger
Simple mode
Weighted median
Weighted mode
0.0
−0.2
−0.4
0.1
0.2
0.3
0.4
0.5
SNP effect on Rheumatoid arthritis
SNP effect on Adenosine 5'−monophosphate (AMP) to urate ratio

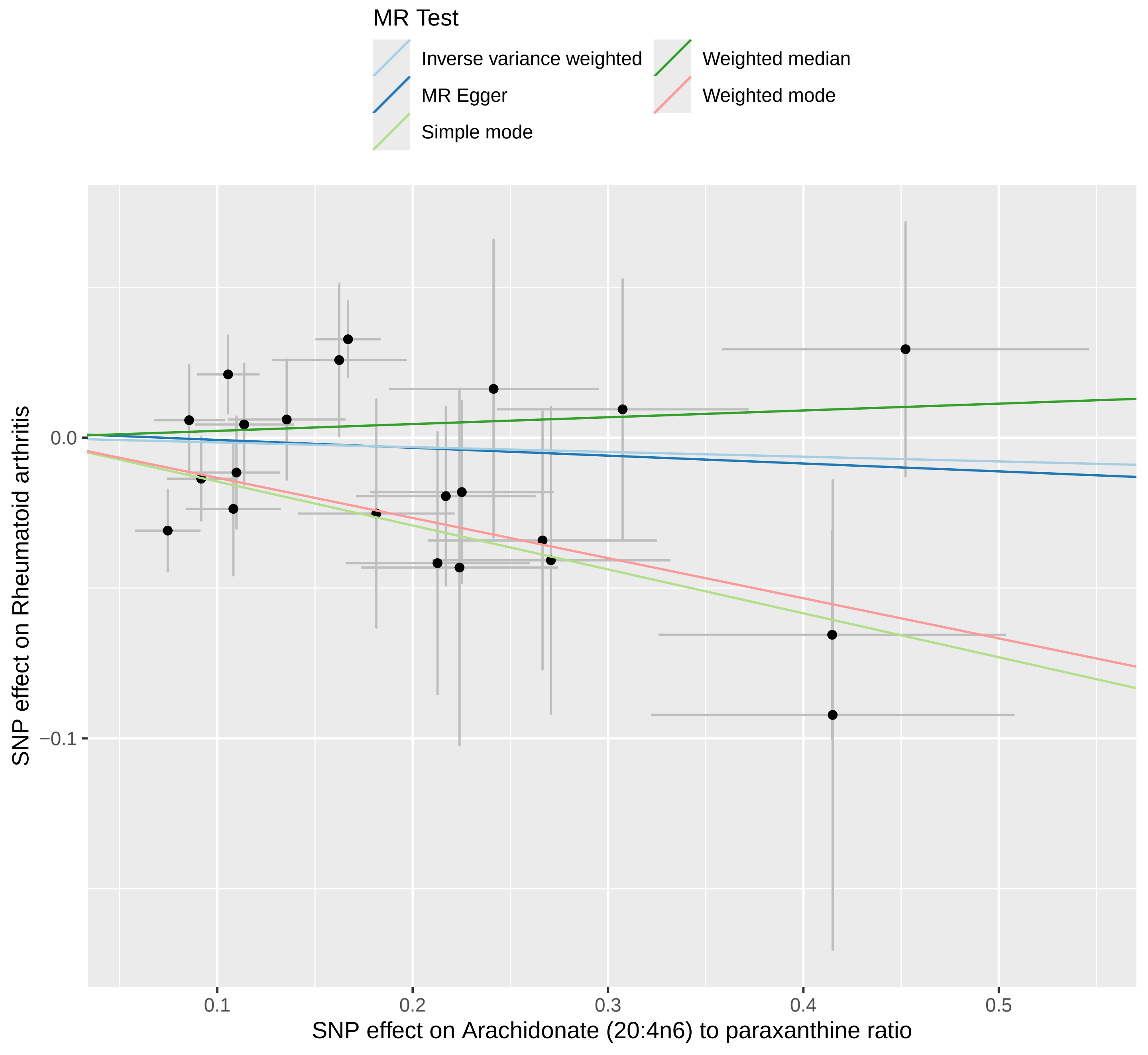
MR Test
Inverse variance weighted
MR Egger
Simple mode
Weighted median
Weighted mode
0.0
−0.1
0.1
0.2
0.3
0.4
0.5
SNP effect on Rheumatoid arthritis
SNP effect on Arachidonate (20:4n6) to paraxanthine ratio

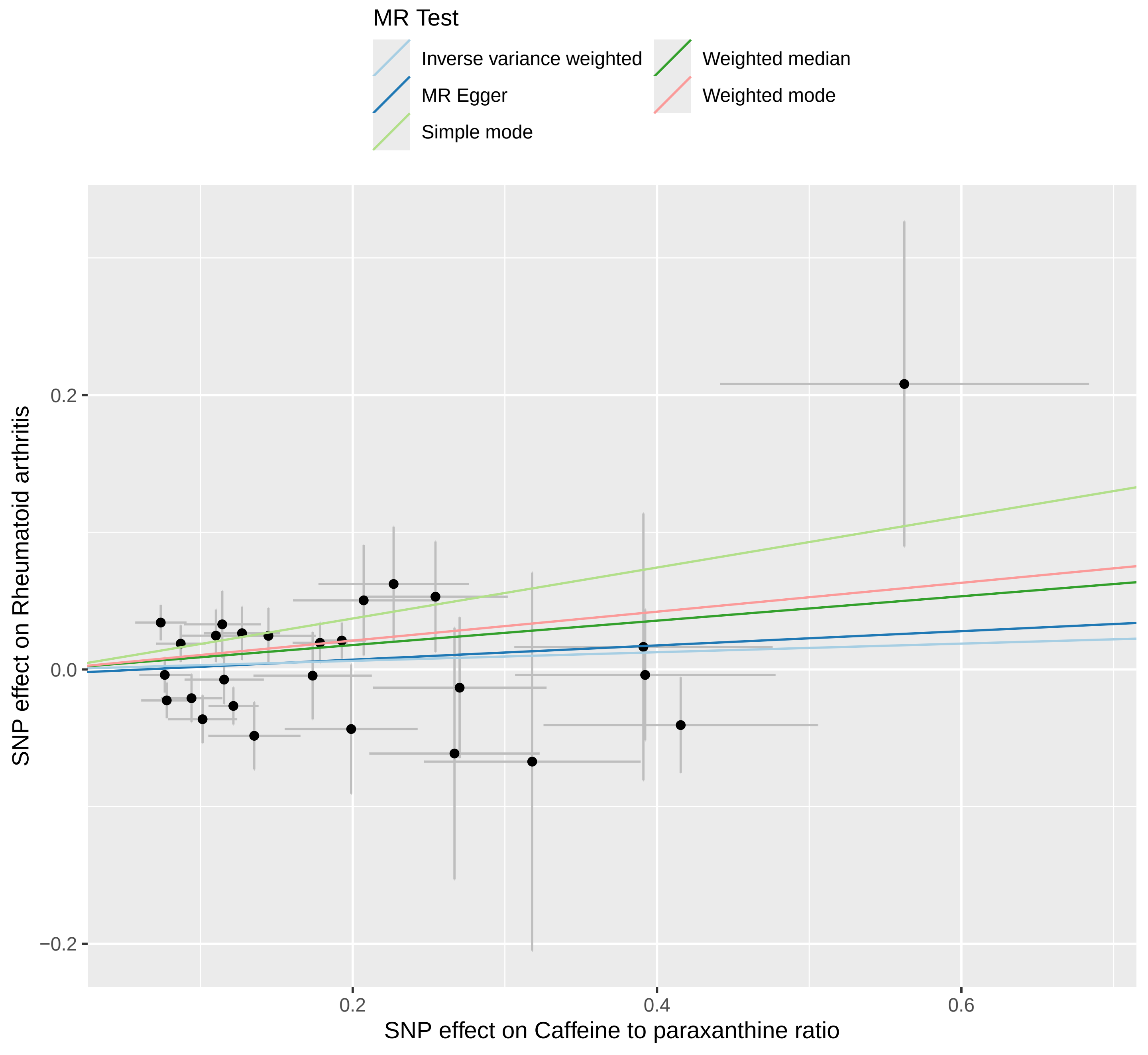
MR Test
Inverse variance weighted
MR Egger
Simple mode
Weighted median
Weighted mode
SNP effect on Rheumatoid arthritis
0.2
0.0
−0.2
0.2
0.4
0.6
SNP effect on Caffeine to paraxanthine ratio

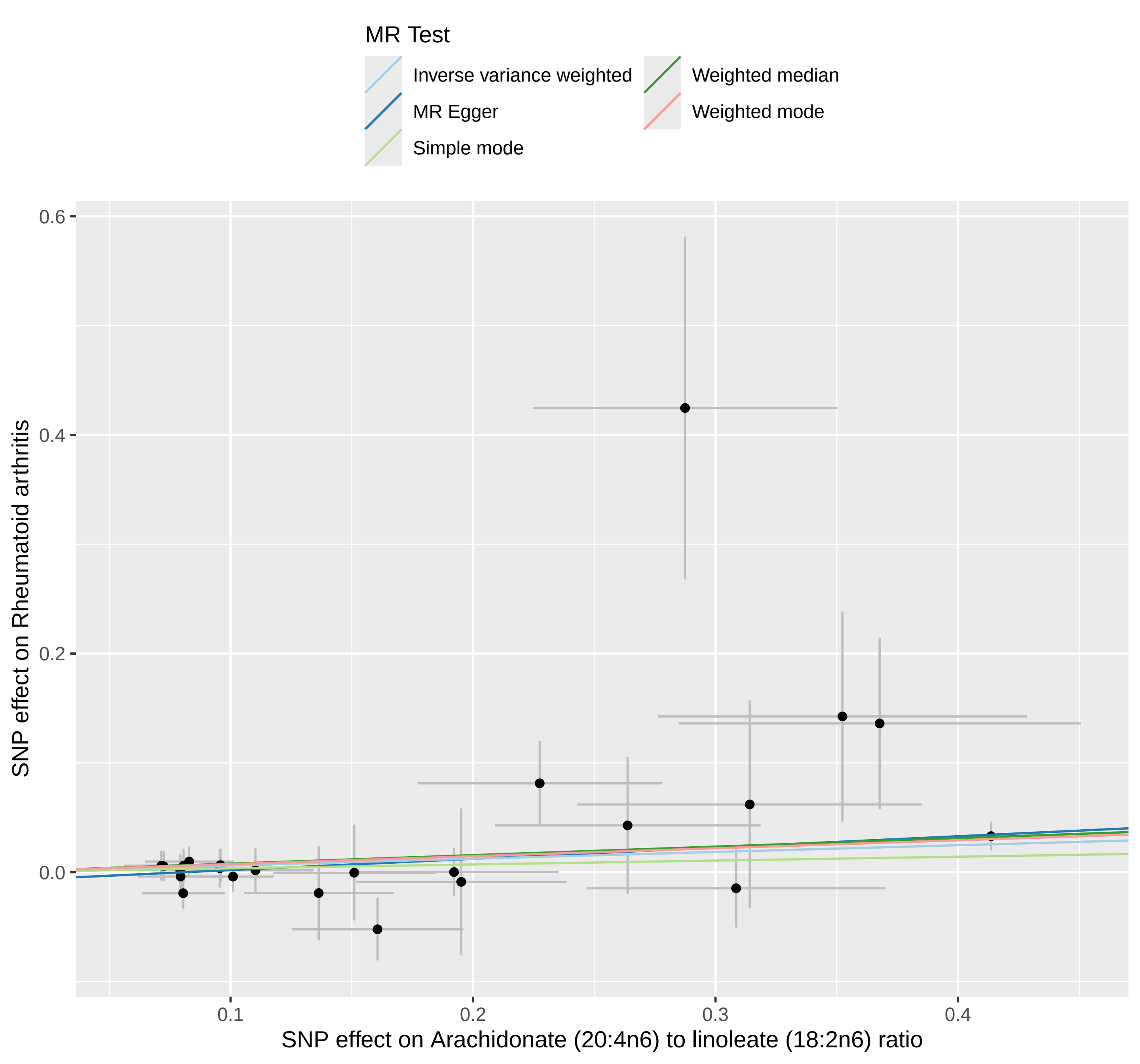
MR Test
Inverse variance weighted
MR Egger
Simple mode
Weighted median
Weighted mode
0.6
0.4
0.2
0.0
0.1
0.2
0.3
0.4
SNP effect on Rheumatoid arthritis
SNP effect on Arachidonate (20:4n6) to linoleate (18:2n6) ratio

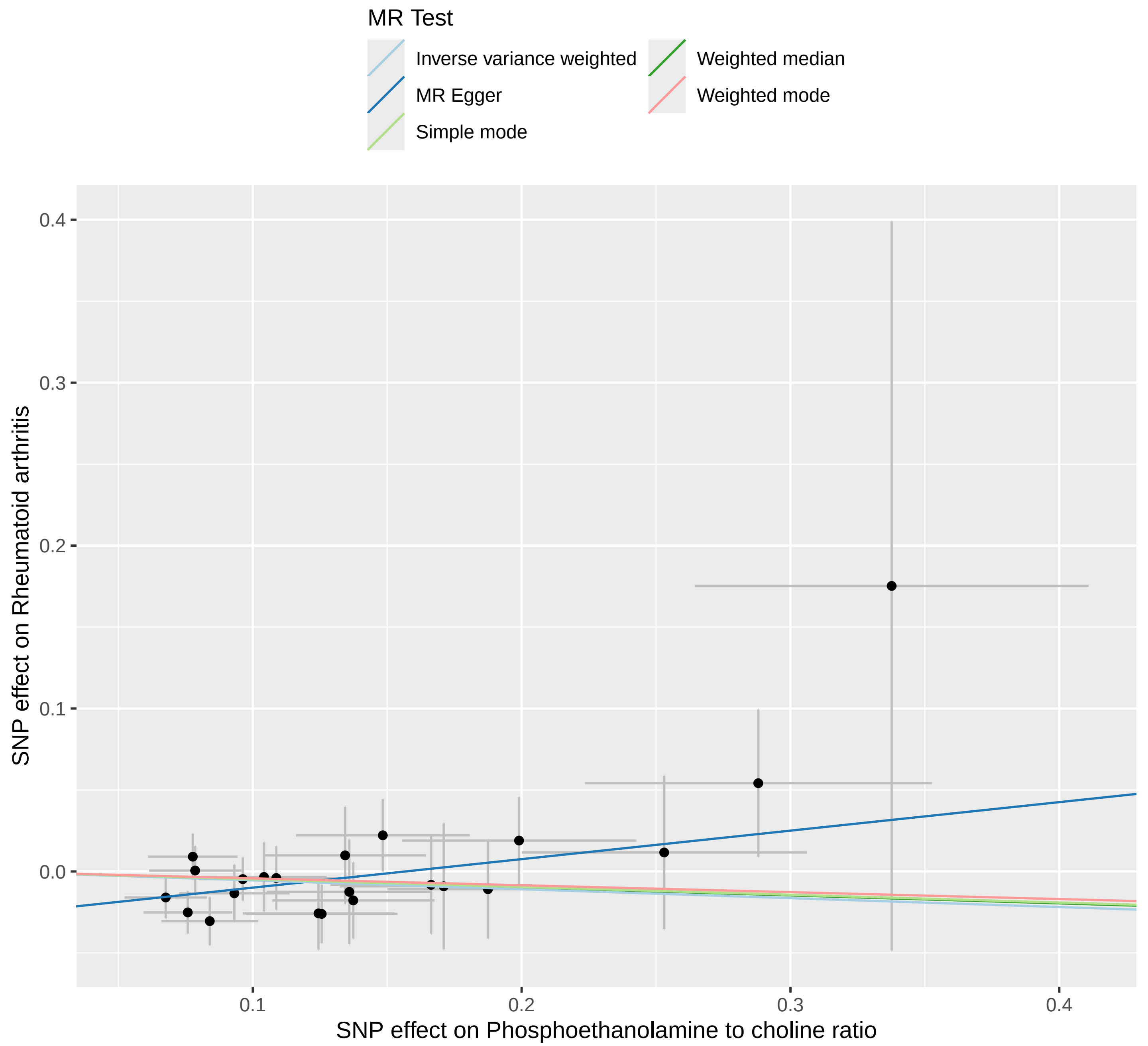

MR Test
Inverse variance weighted
MR Egger
Simple mode
Weighted median
Weighted mode
SNP effect on Rheumatoid arthritis
0.4
0.3
0.2
0.1
0.0
0.1
0.2
0.3
0.4
SNP effect on Phosphoethanolamine to choline ratio

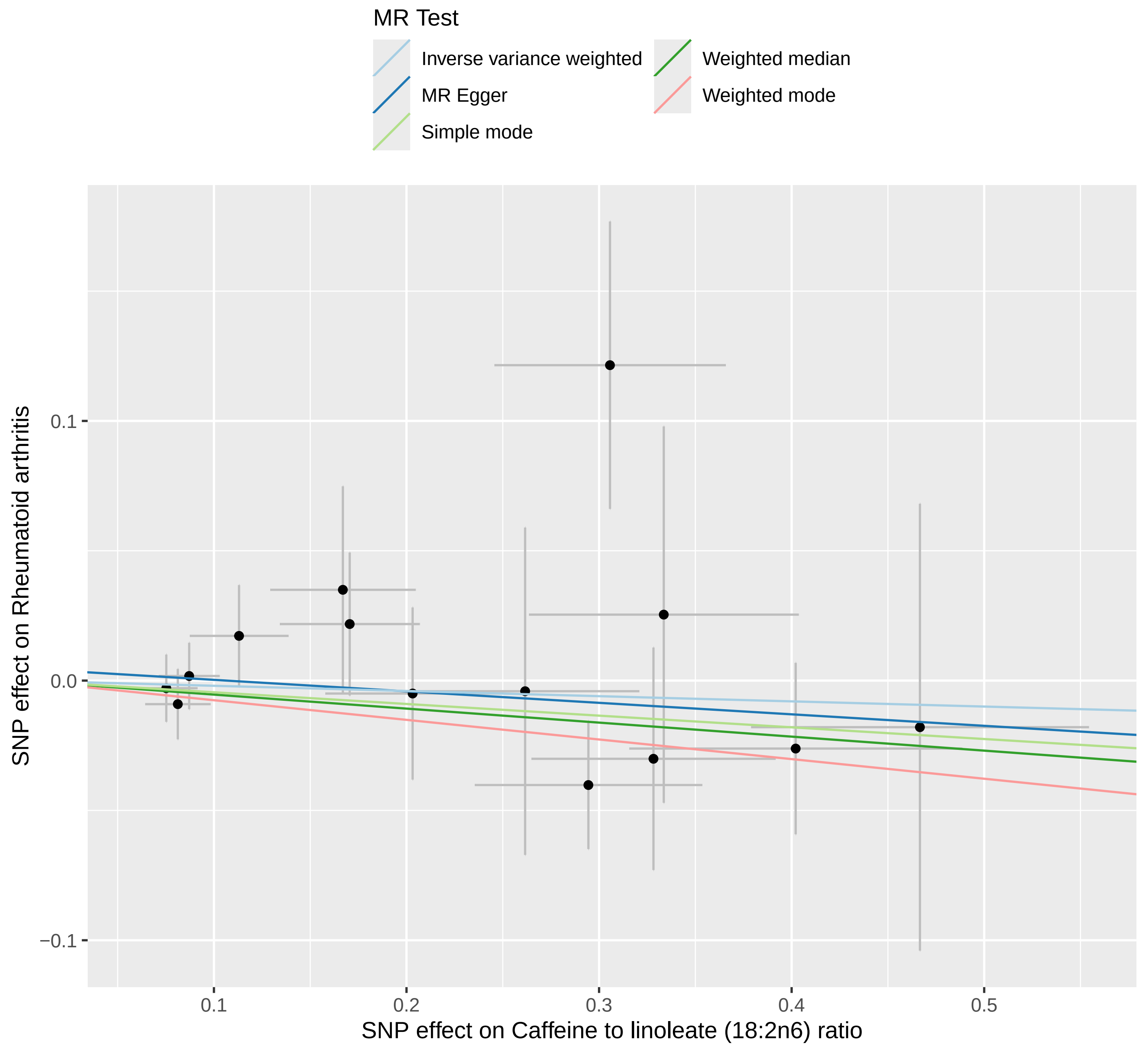

MR Test
Inverse variance weighted
MR Egger
Simple mode
Weighted median
Weighted mode
SNP effect on Rheumatoid arthritis
0.1
0.0
−0.1
0.1
0.2
0.3
0.4
0.5
SNP effect on Caffeine to linoleate (18:2n6) ratio

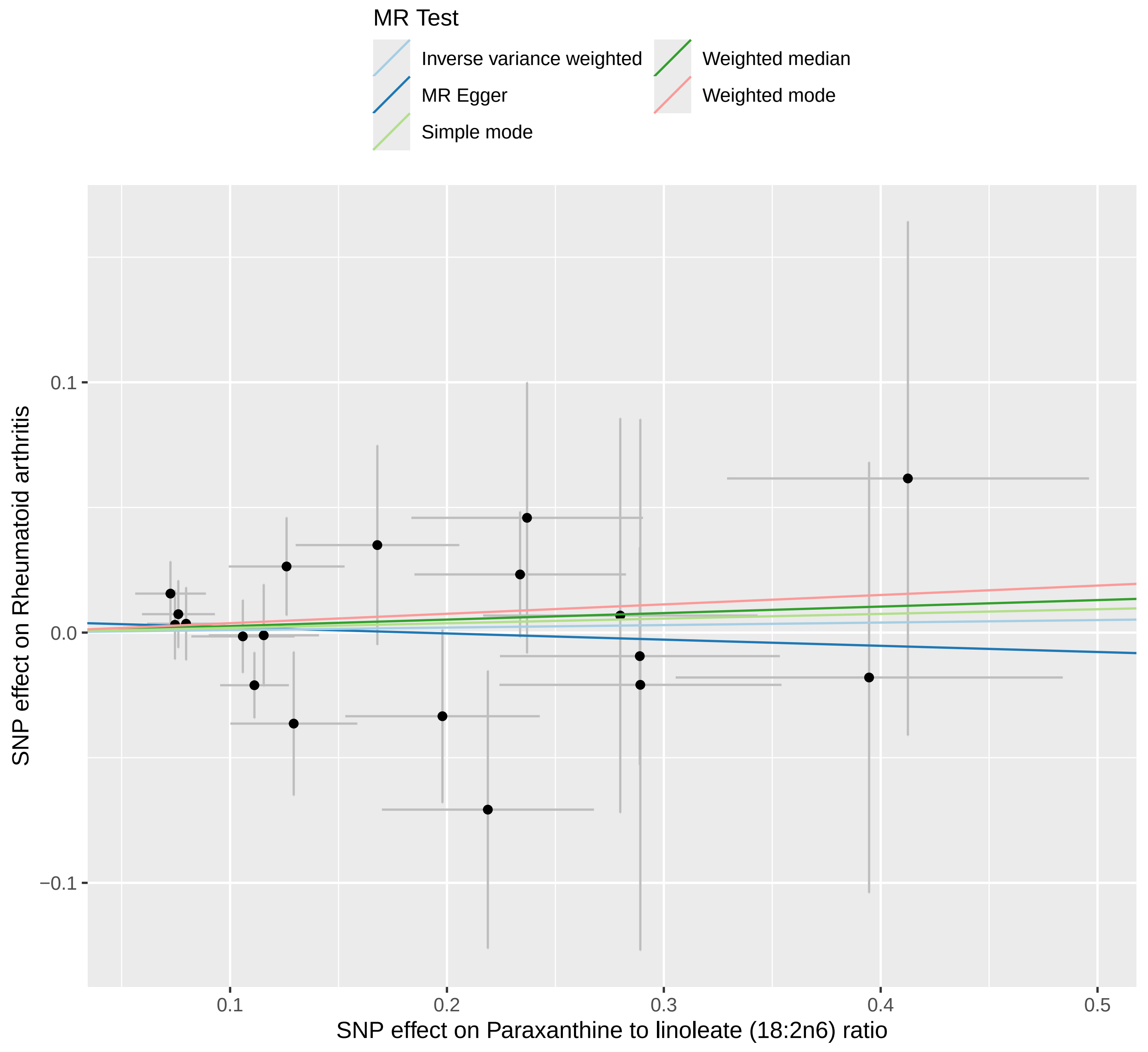
MR Test
Inverse variance weighted
MR Egger
Simple mode
Weighted median
Weighted mode
0.1
0.0
−0.1
0.1
0.2
0.3
0.4
0.5
SNP effect on Rheumatoid arthritis
SNP effect on Paraxanthine to linoleate (18:2n6) ratio

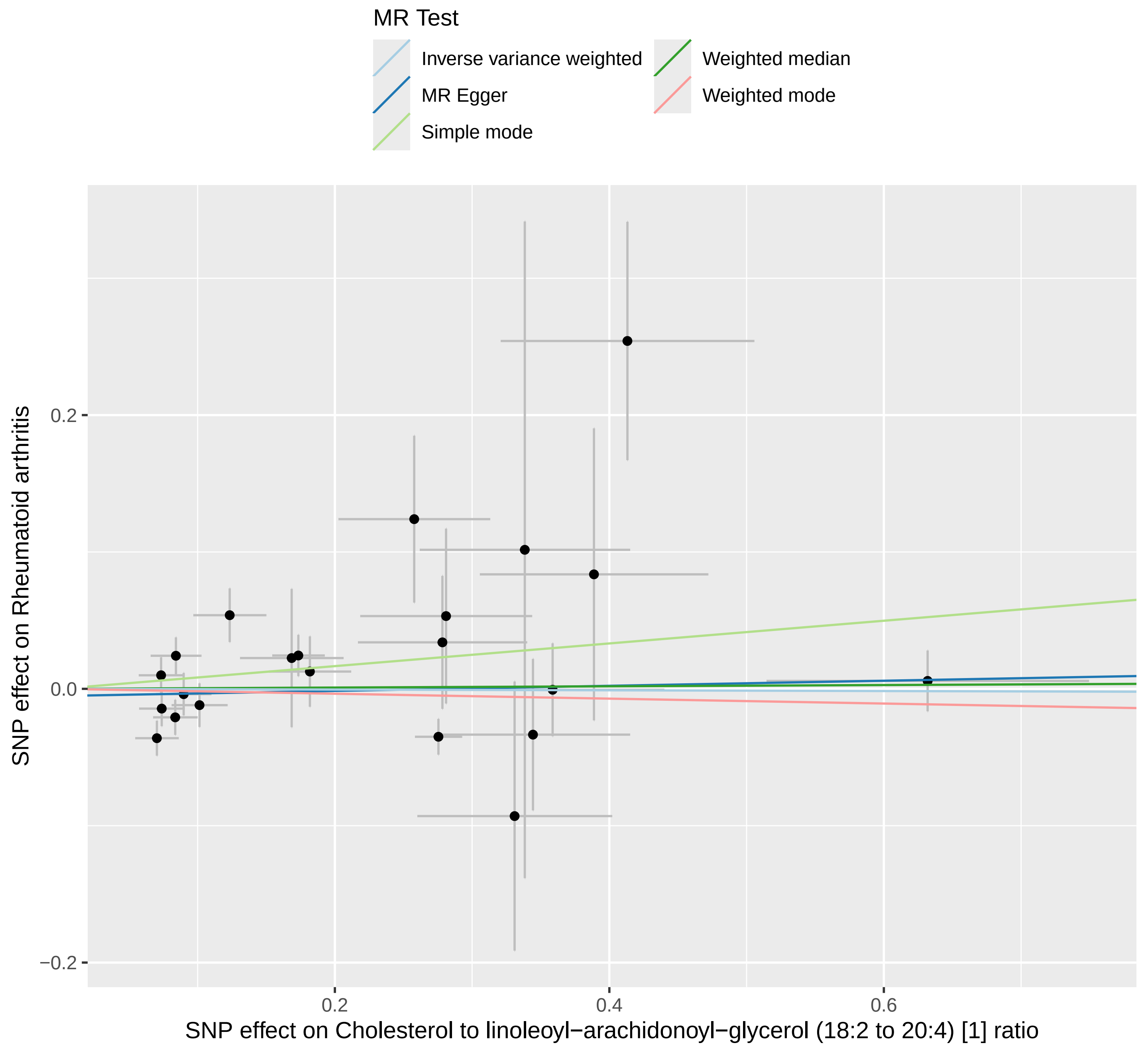

MR Test
Inverse variance weighted
MR Egger
Simple mode
Weighted median
Weighted mode
SNP effect on Rheumatoid arthritis
0.2
0.0
−0.2
0.2
0.4
0.6
SNP effect on Cholesterol to linoleoyl−arachidonoyl−glycerol (18:2 to 20:4) [1] ratio

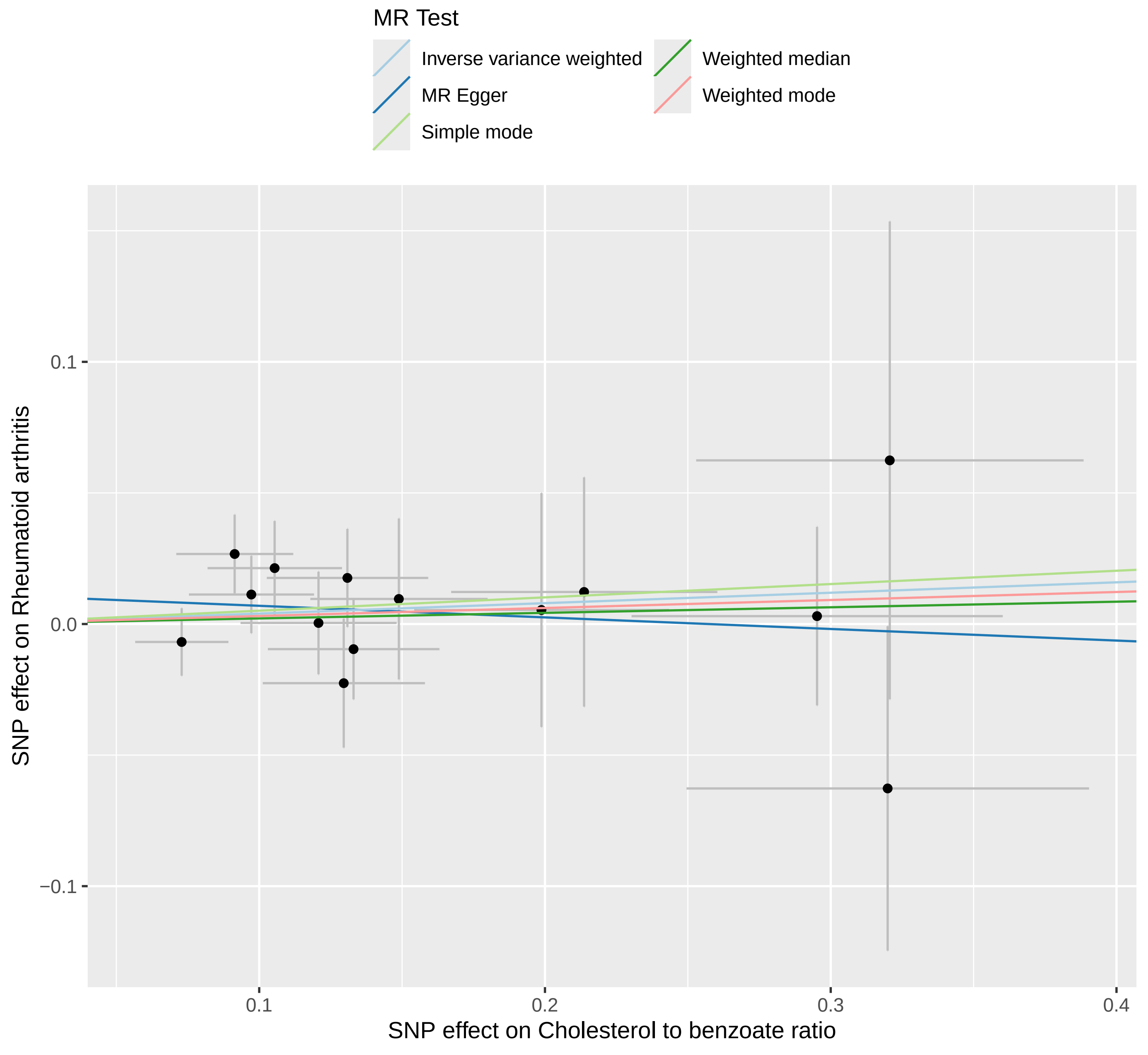

MR Test
Inverse variance weighted
MR Egger
Simple mode
Weighted median
Weighted mode
SNP effect on Rheumatoid arthritis
0.1
0.0
−0.1
0.1
0.2
0.3
0.4
SNP effect on Cholesterol to benzoate ratio

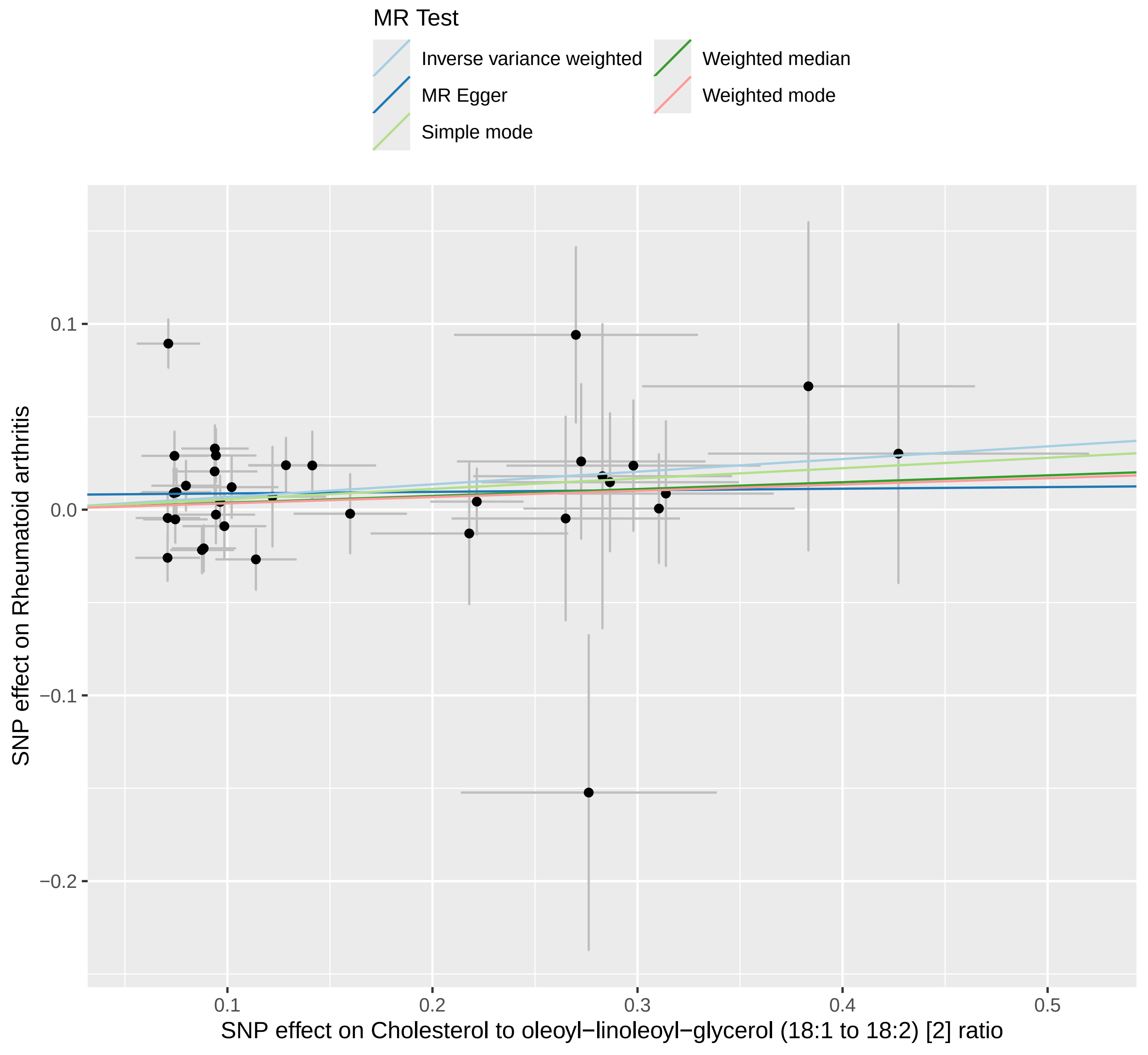
MR Test
Inverse variance weighted
MR Egger
Simple mode
Weighted median
Weighted mode
SNP effect on Rheumatoid arthritis
0.1
0.0
−0.1
−0.2
0.1
0.2
0.3
0.4
0.5
SNP effect on Cholesterol to oleoyl−linoleoyl−glycerol (18:1 to 18:2) [2] ratio

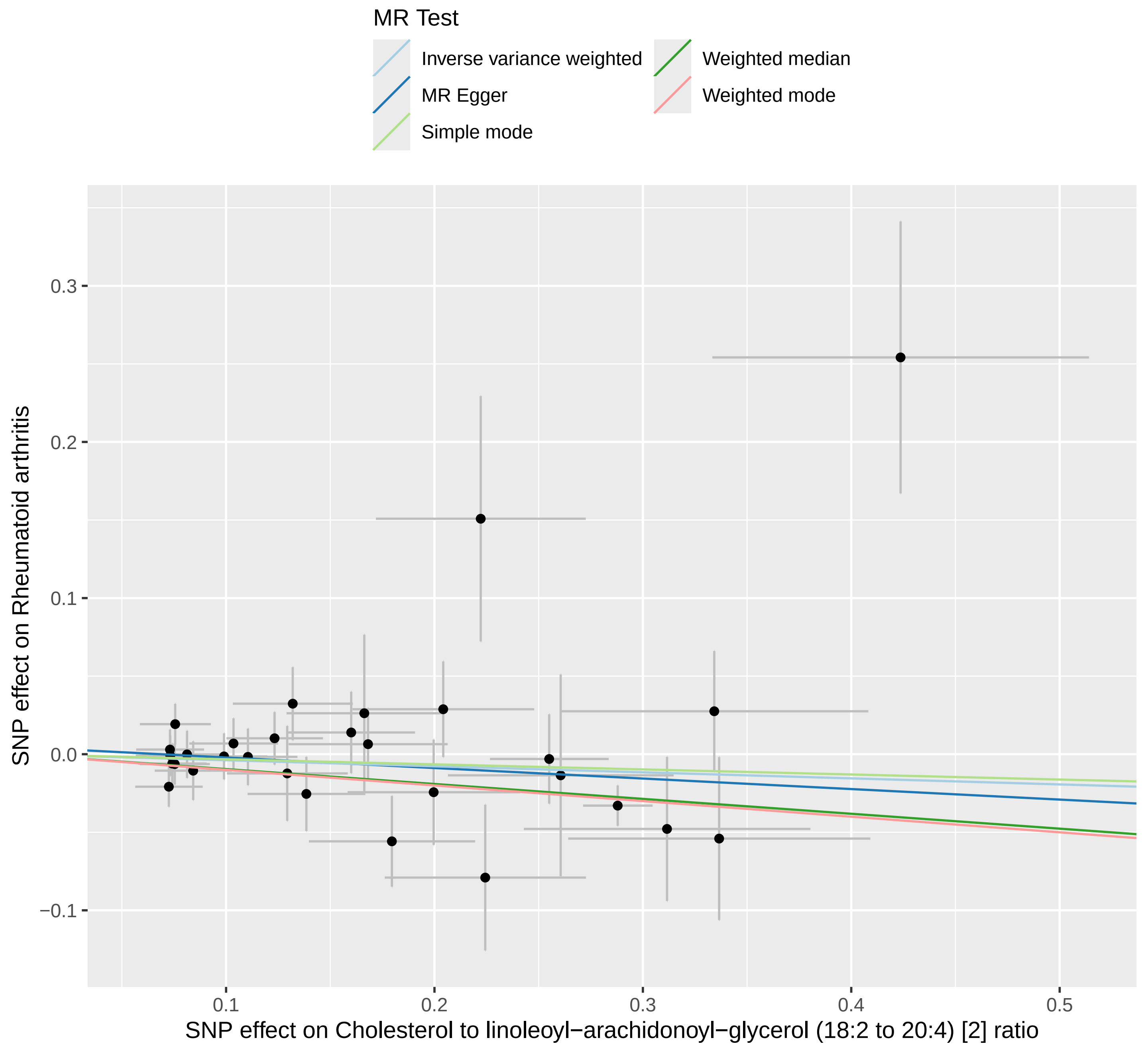

MR Test
Inverse variance weighted
MR Egger
Simple mode
Weighted median
Weighted mode
SNP effect on Rheumatoid arthritis
0.3
0.2
0.1
0.0
−0.1
0.1
0.2
0.3
0.4
0.5
SNP effect on Cholesterol to linoleoyl−arachidonoyl−glycerol (18:2 to 20:4) [2] ratio

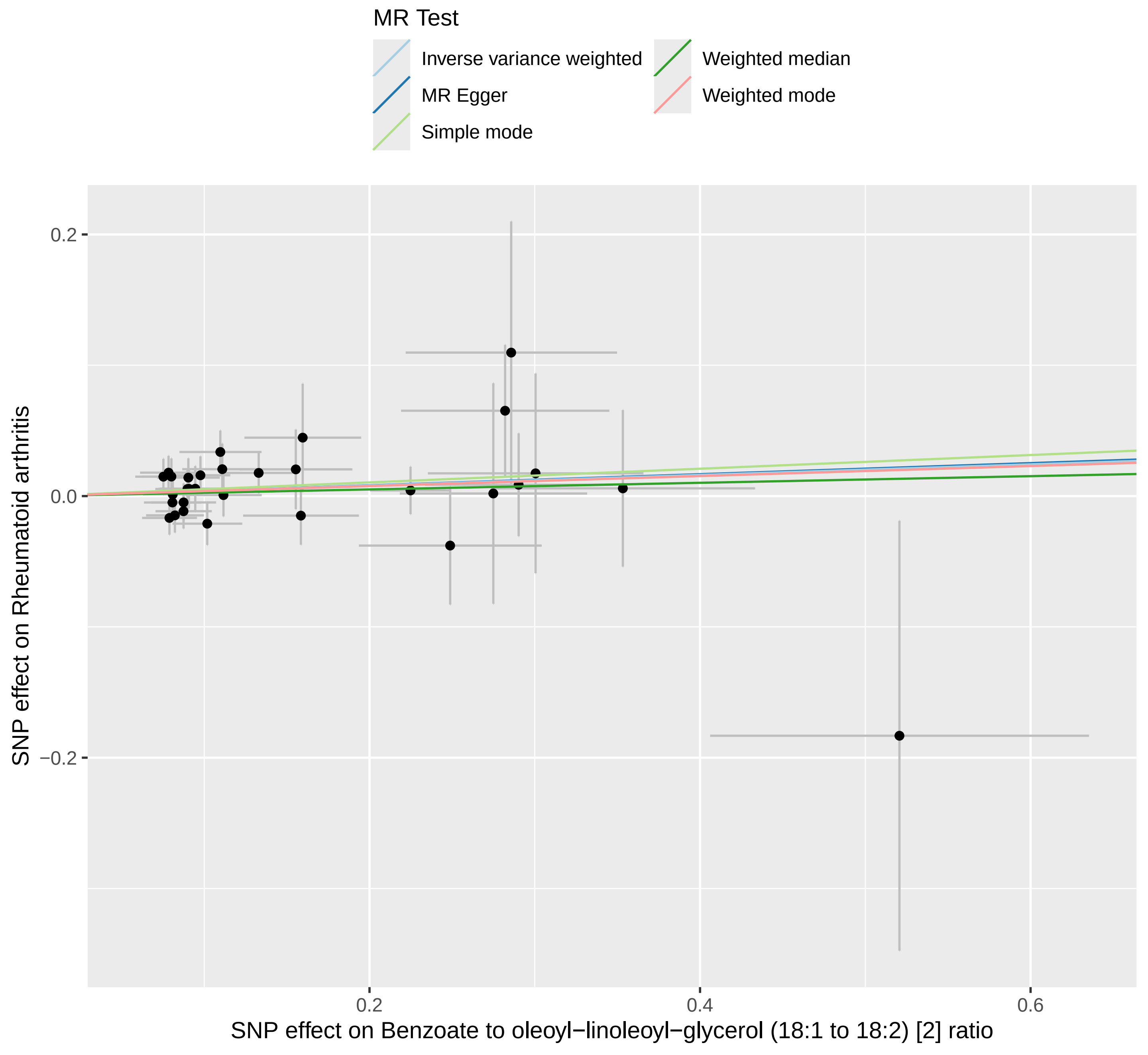
MR Test
Inverse variance weighted
MR Egger
Simple mode
Weighted median
Weighted mode
SNP effect on Rheumatoid arthritis
0.2
0.0
−0.2
0.2
0.4
0.6
SNP effect on Benzoate to oleoyl−linoleoyl−glycerol (18:1 to 18:2) [2] ratio

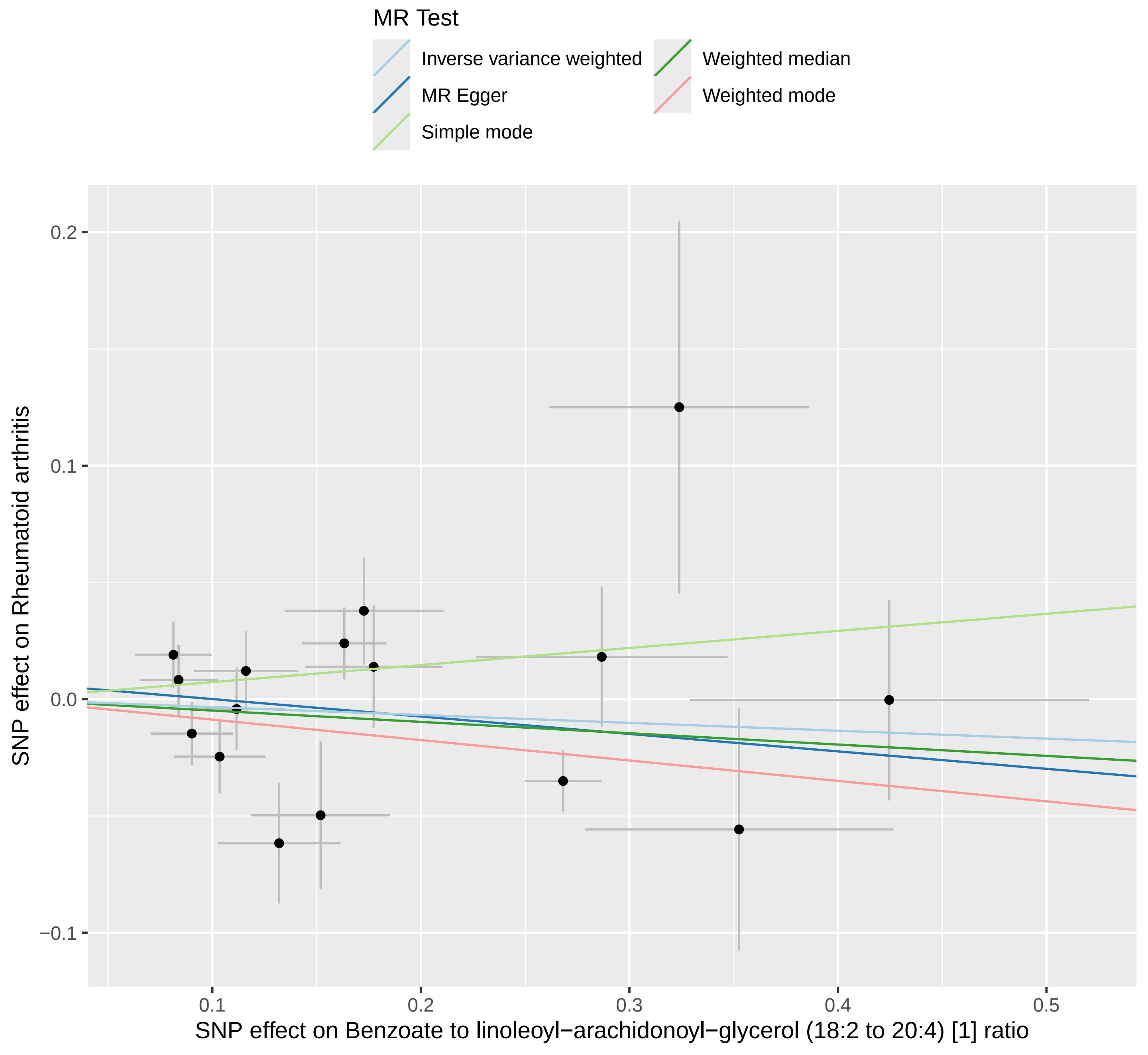

MR Test
Inverse variance weighted
MR Egger
Simple mode
Weighted median
Weighted mode
0.2
0.1
0.0
−0.1
0.1
0.2
0.3
0.4
0.5
SNP effect on Rheumatoid arthritis
SNP effect on Benzoate to linoleoyl−arachidonoyl−glycerol (18:2 to 20:4) [1] ratio

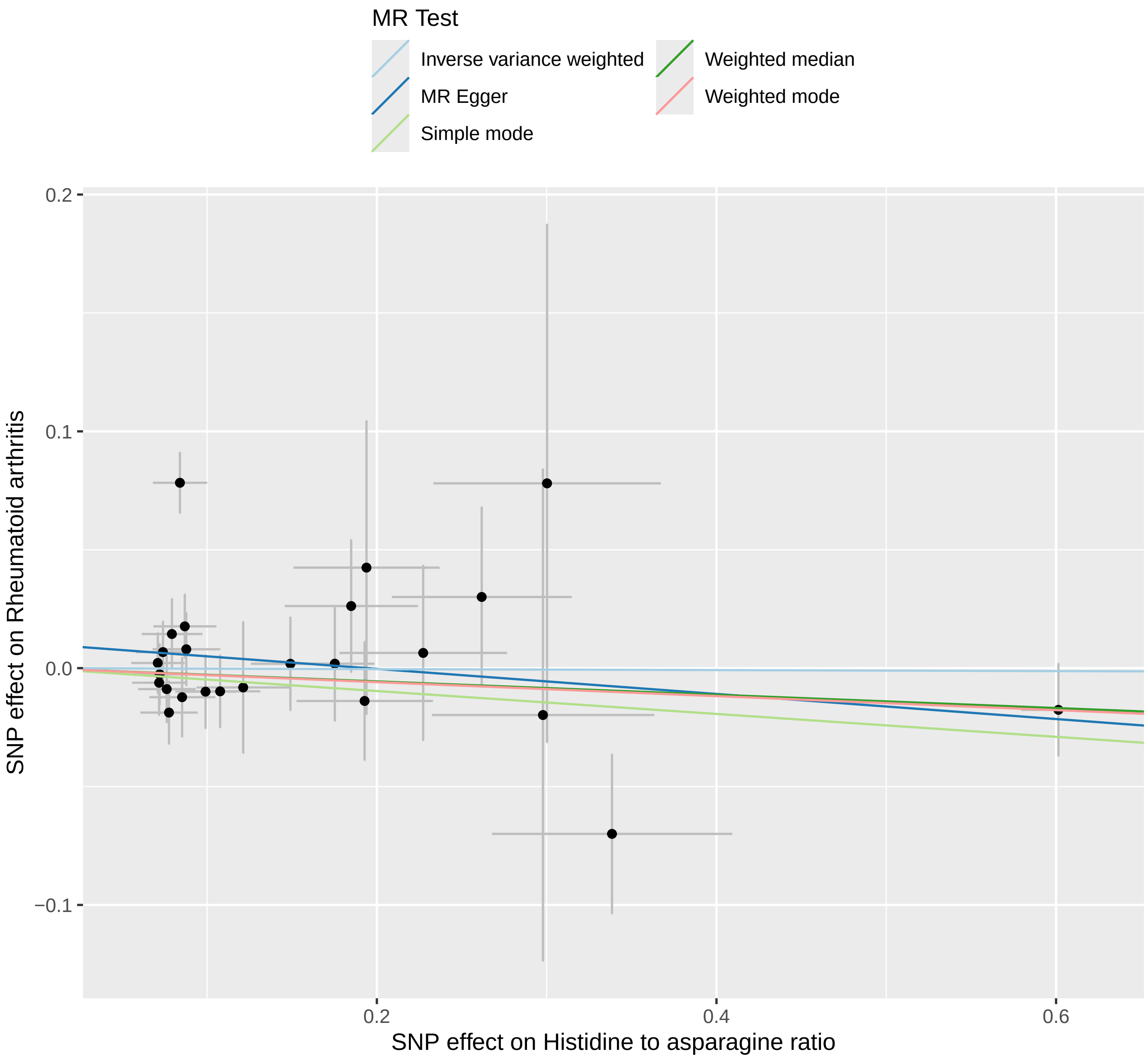
MR Test
Inverse variance weighted
MR Egger
Simple mode
Weighted median
Weighted mode
0.2
0.1
0.0
−0.1
0.2
0.4
0.6
SNP effect on Rheumatoid arthritis
SNP effect on Histidine to asparagine ratio

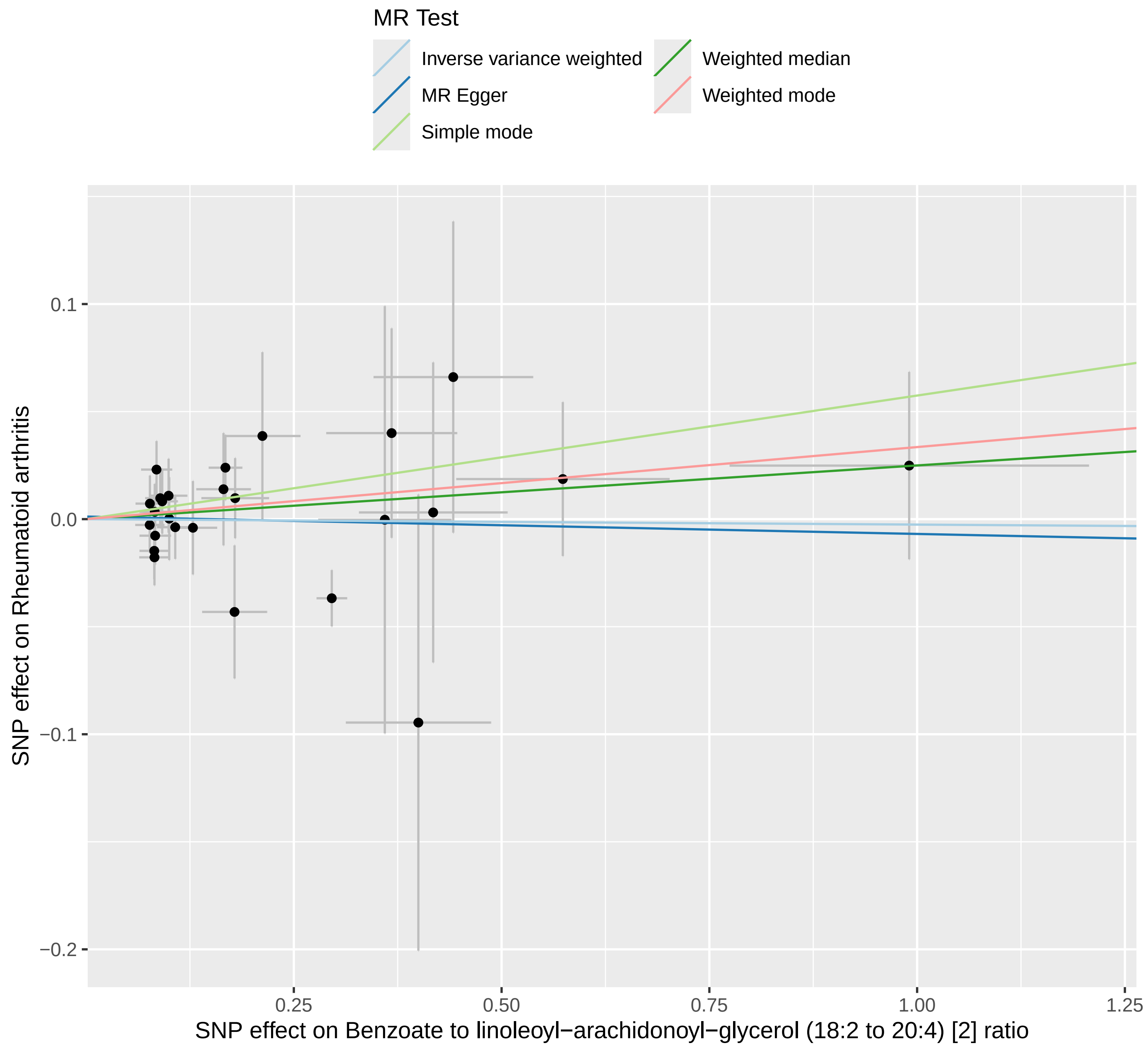
MR Test
Inverse variance weighted
MR Egger
Simple mode
Weighted median
Weighted mode
0.1
0.0
−0.1
−0.2
SNP effect on Rheumatoid arthritis
0.25
0.50
0.75
1.00
1.25
SNP effect on Benzoate to linoleoyl−arachidonoyl−glycerol (18:2 to 20:4) [2] ratio

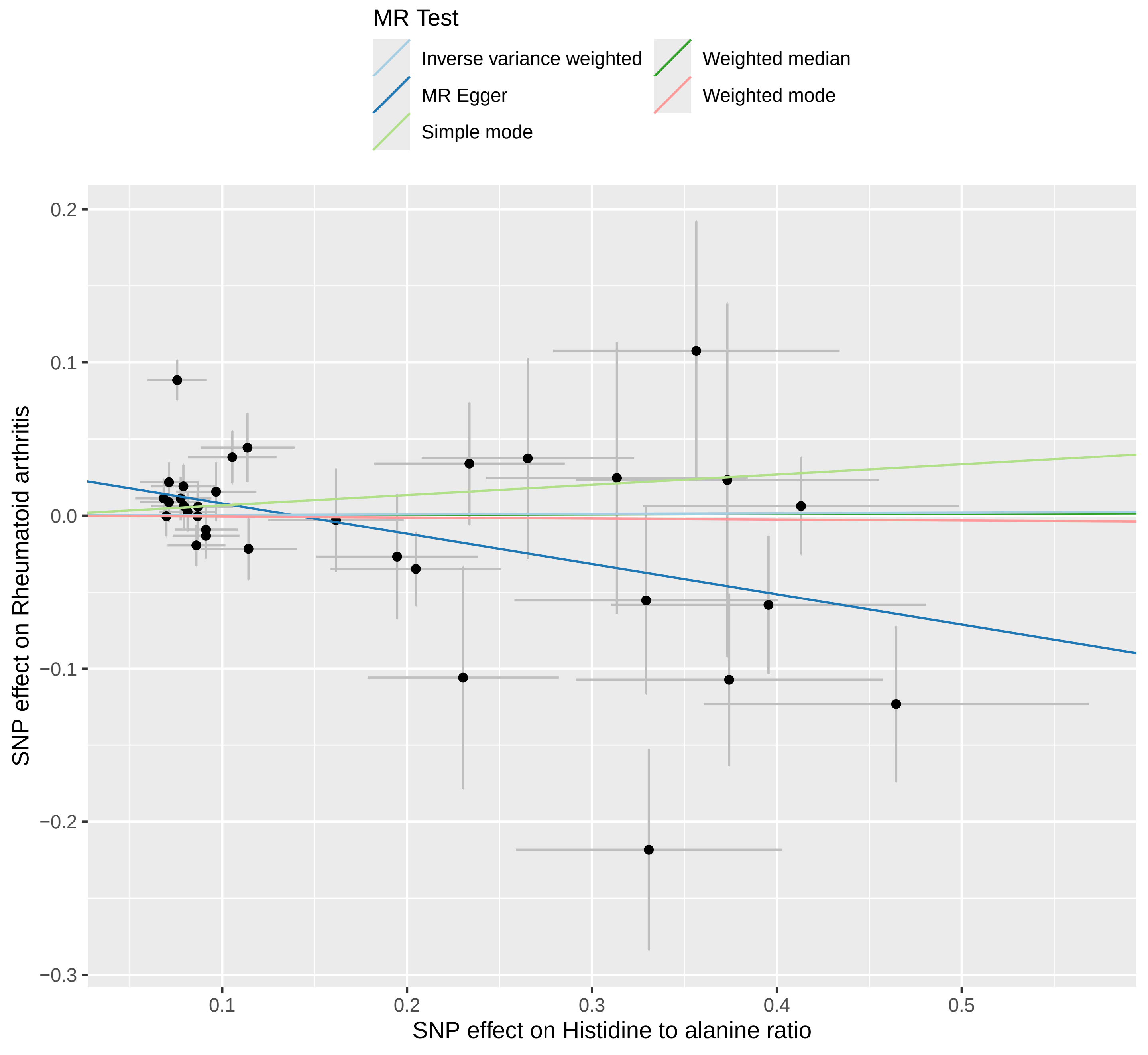
MR Test
Inverse variance weighted
MR Egger
Simple mode
Weighted median
Weighted mode
SNP effect on Rheumatoid arthritis
SNP effect on Histidine to alanine ratio
0.2
0.1
0.0
−0.1
−0.2
−0.3
0.1
0.2
0.3
0.4
0.5

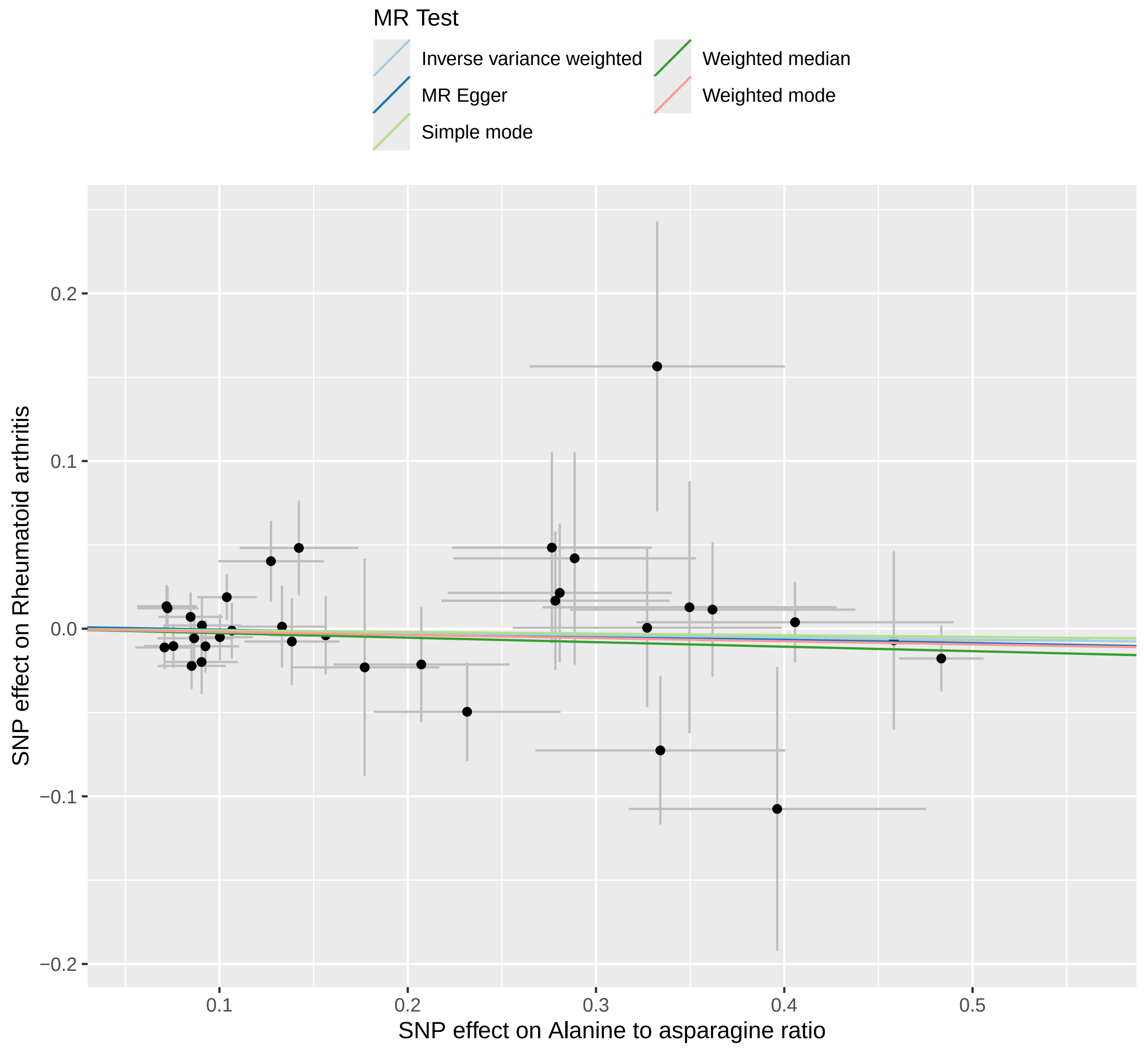

MR Test
Inverse variance weighted
MR Egger
Simple mode
Weighted median
Weighted mode
SNP effect on Rheumatoid arthritis
0.2
0.1
0.0
−0.1
−0.2
0.1
0.2
0.3
0.4
0.5
SNP effect on Alanine to asparagine ratio

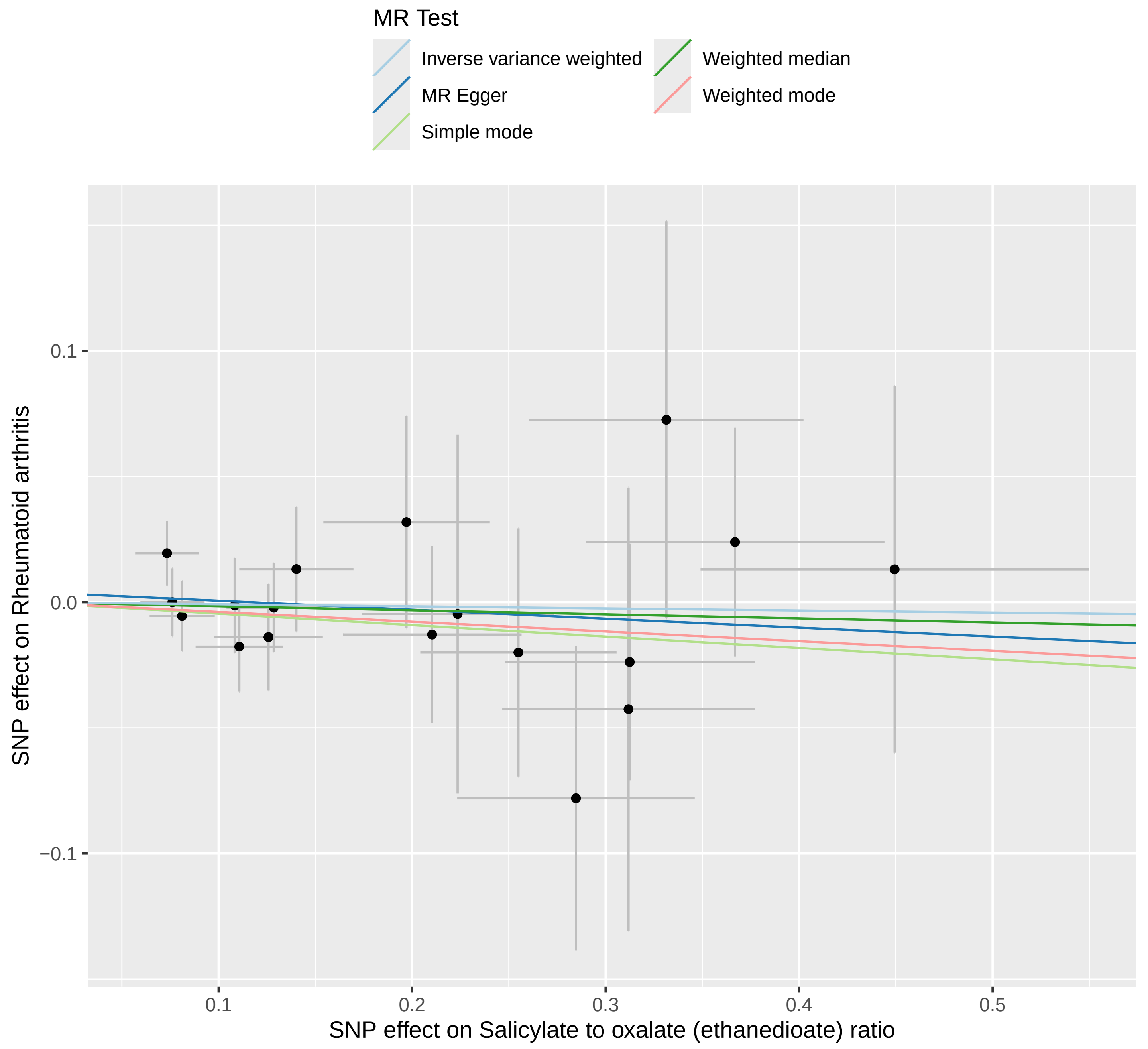

MR Test
Inverse variance weighted
MR Egger
Simple mode
Weighted median
Weighted mode
SNP effect on Rheumatoid arthritis
0.1
0.0
−0.1
0.1
0.2
0.3
0.4
0.5
SNP effect on Salicylate to oxalate (ethanedioate) ratio

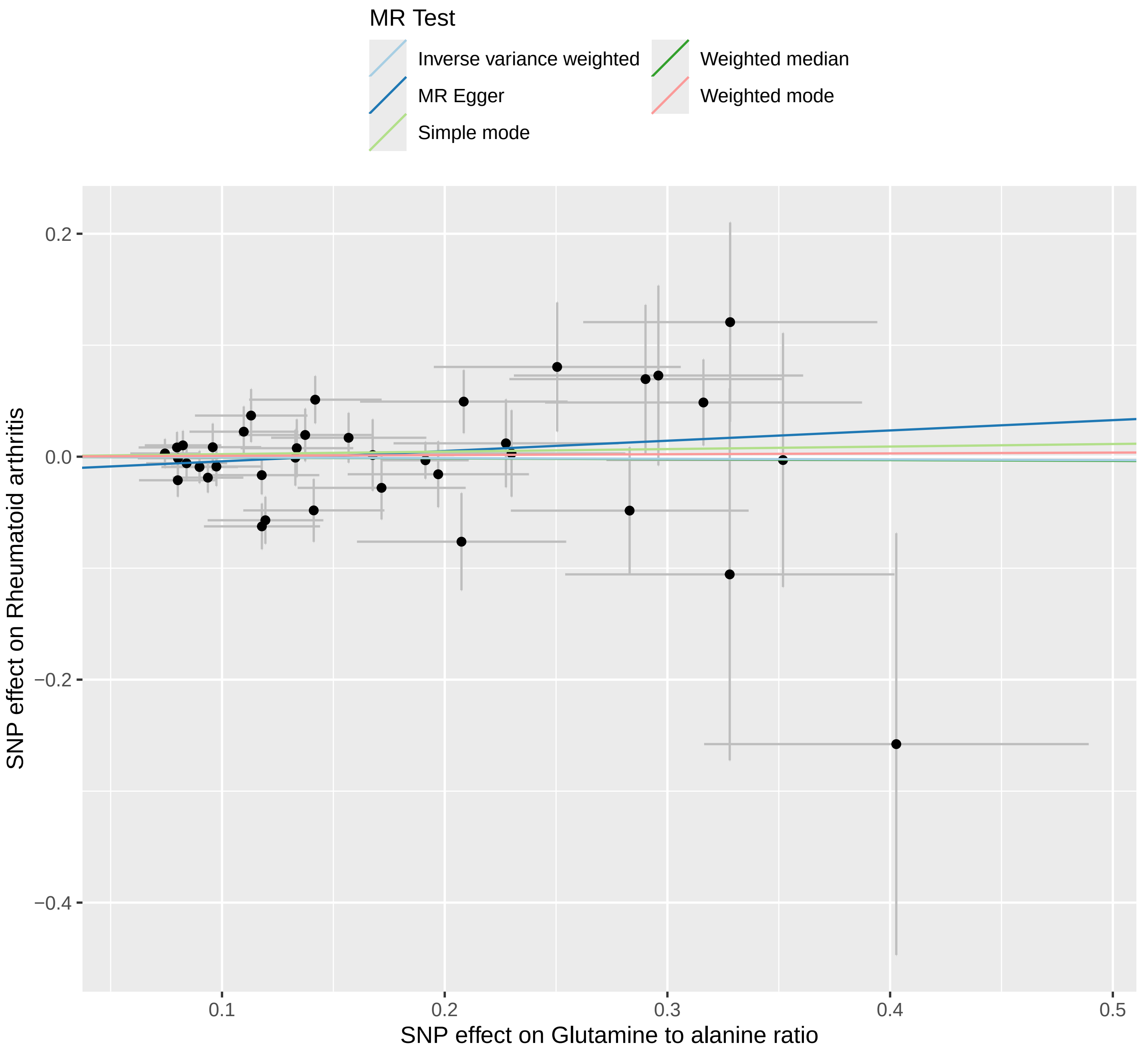
MR Test
Inverse variance weighted
MR Egger
Simple mode
Weighted median
Weighted mode
0.2
0.0
−0.2
−0.4
0.1
0.2
0.3
0.4
0.5
SNP effect on Rheumatoid arthritis
SNP effect on Glutamine to alanine ratio

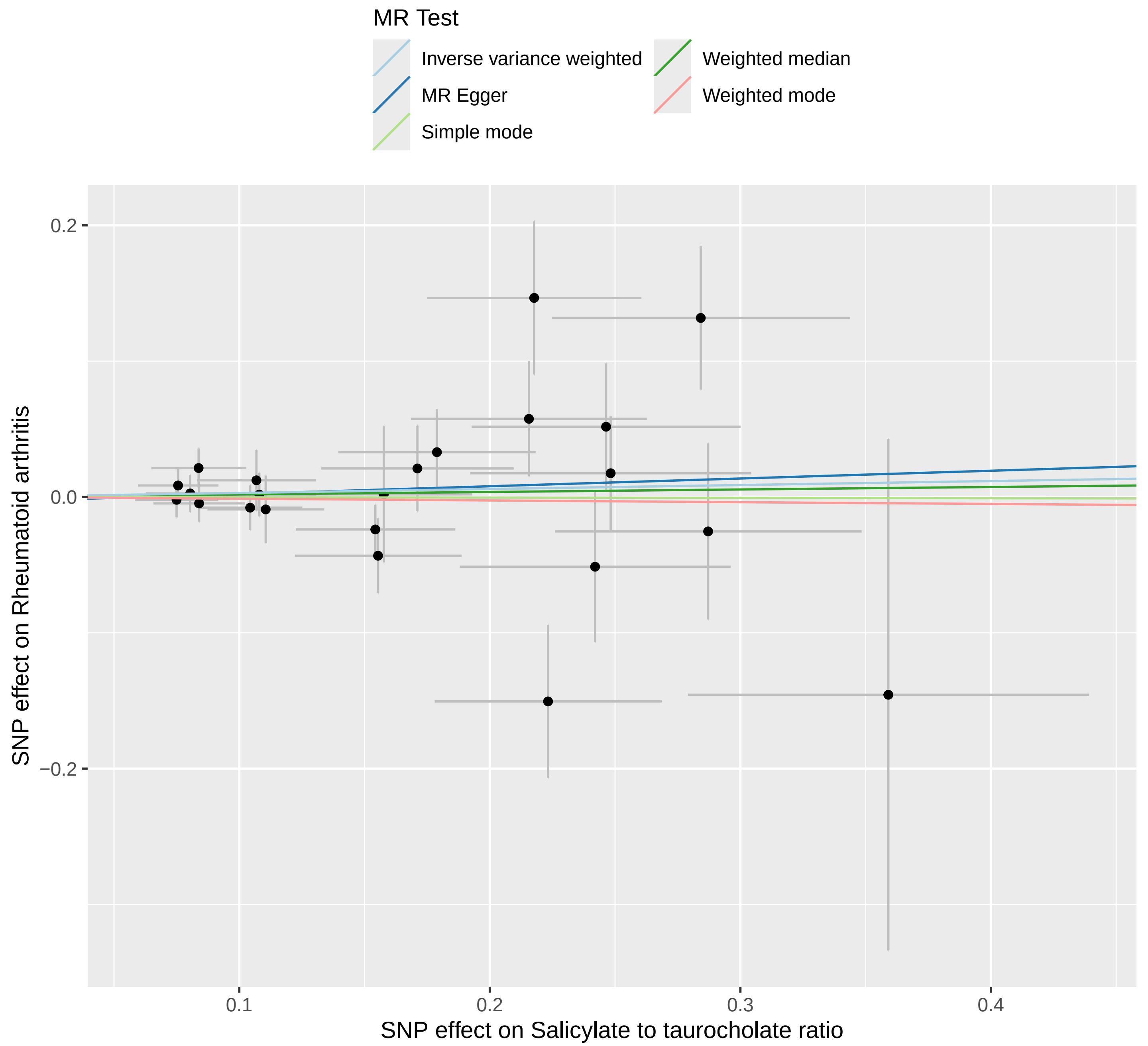
MR Test
Inverse variance weighted
MR Egger
Simple mode
Weighted median
Weighted mode
0.2
0.0
−0.2
0.1
0.2
0.3
0.4
SNP effect on Rheumatoid arthritis
SNP effect on Salicylate to taurocholate ratio

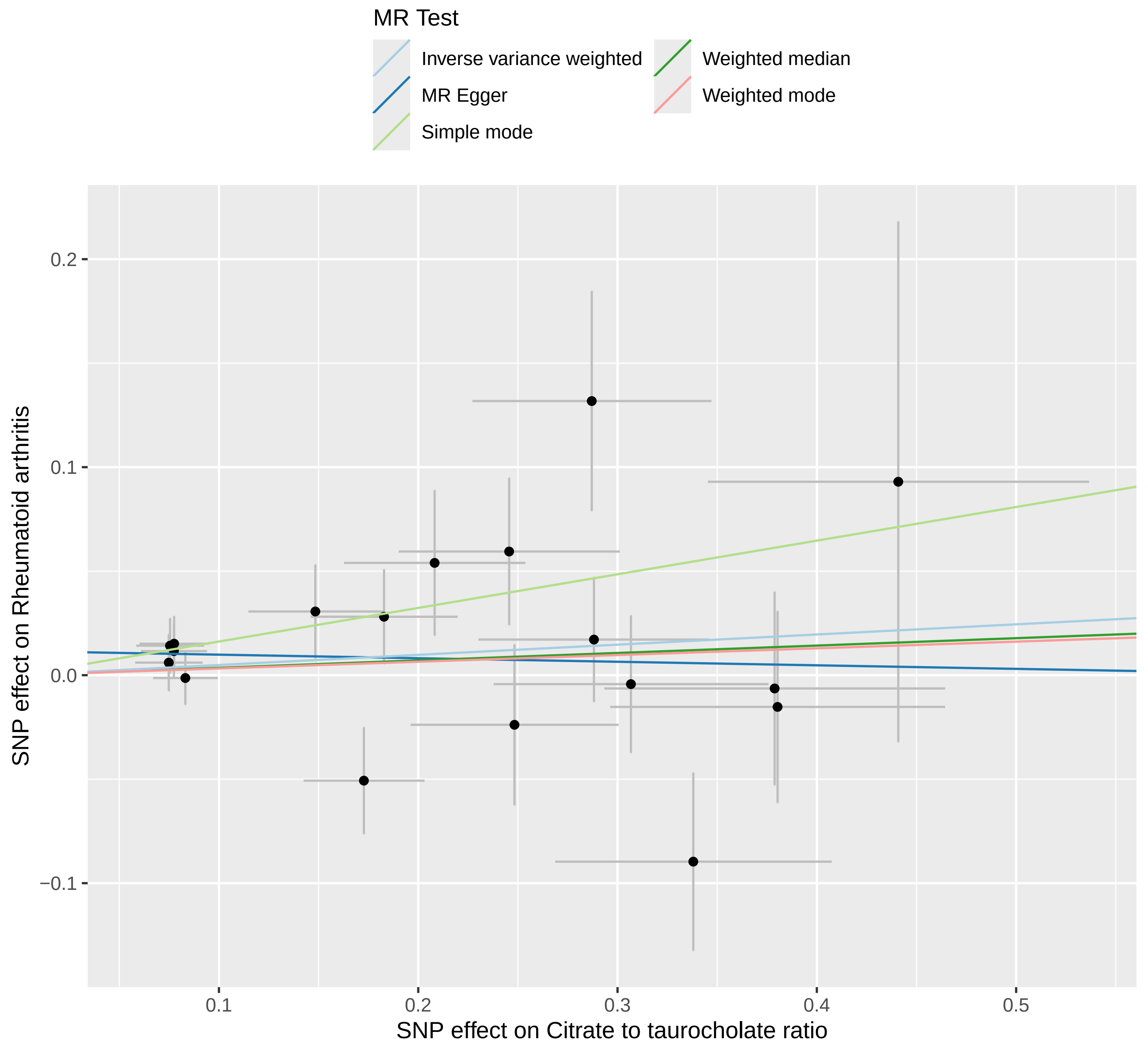

MR Test
Inverse variance weighted
MR Egger
Simple mode
Weighted median
Weighted mode
SNP effect on Rheumatoid arthritis
0.2
0.1
0.0
−0.1
0.1
0.2
0.3
0.4
0.5
SNP effect on Citrate to taurocholate ratio

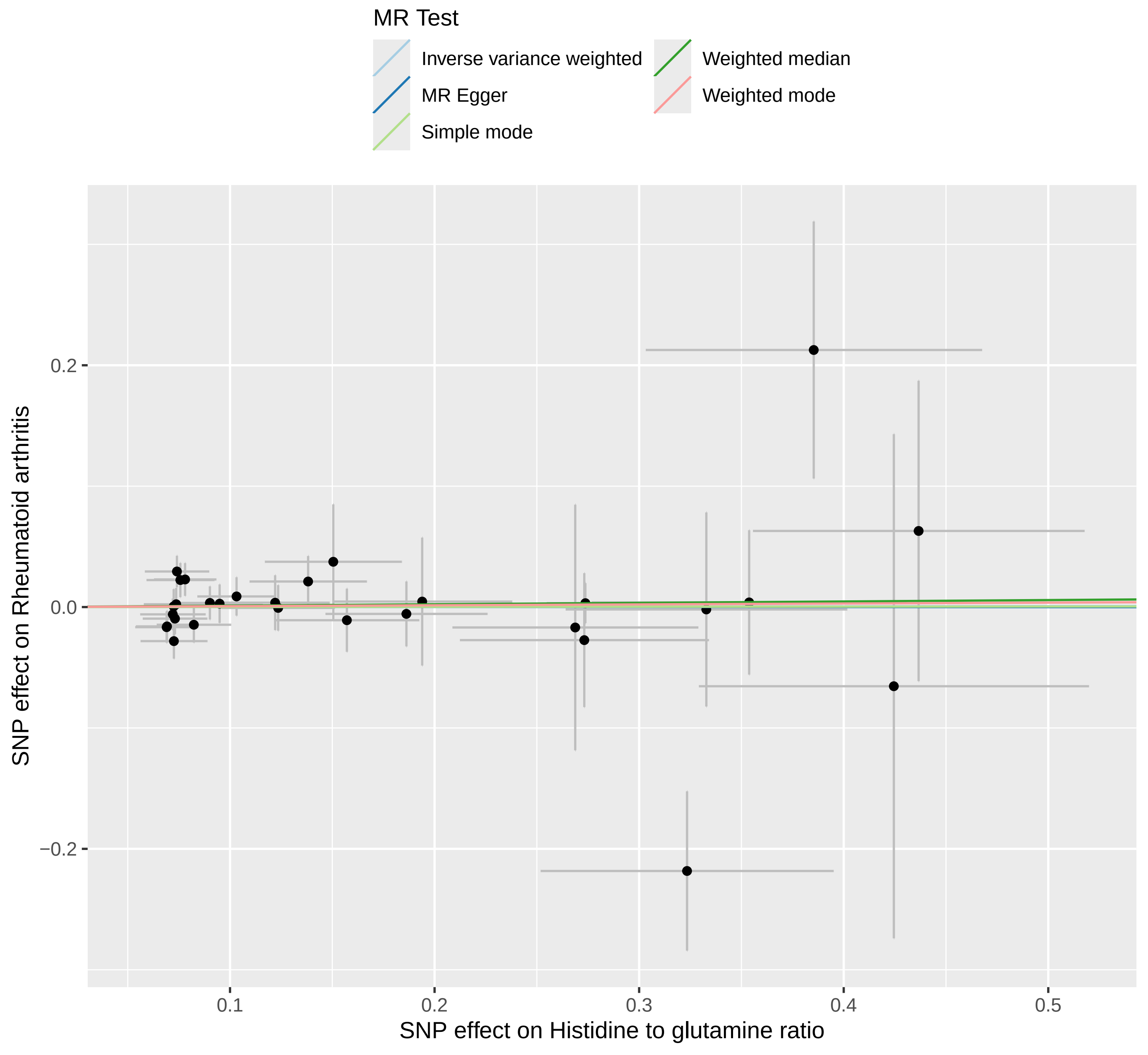
MR Test
Inverse variance weighted
MR Egger
Simple mode
Weighted median
Weighted mode
0.2
0.0
−0.2
0.1
0.2
0.3
0.4
0.5
SNP effect on Rheumatoid arthritis
SNP effect on Histidine to glutamine ratio

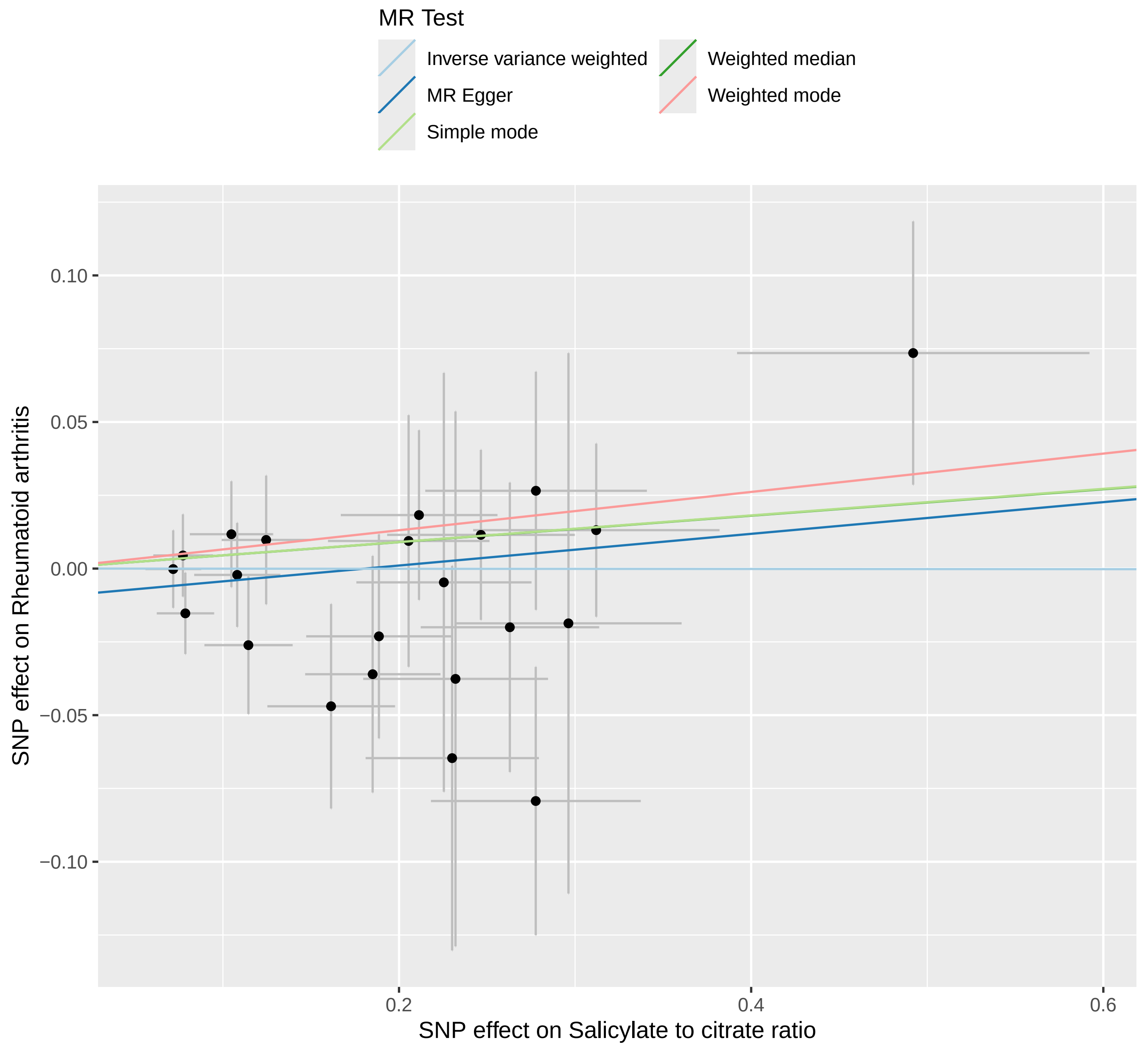
MR Test
Inverse variance weighted
MR Egger
Simple mode
Weighted median
Weighted mode
0.10
0.05
0.00
−0.05
−0.10
0.2
0.4
0.6
SNP effect on Rheumatoid arthritis
SNP effect on Salicylate to citrate ratio

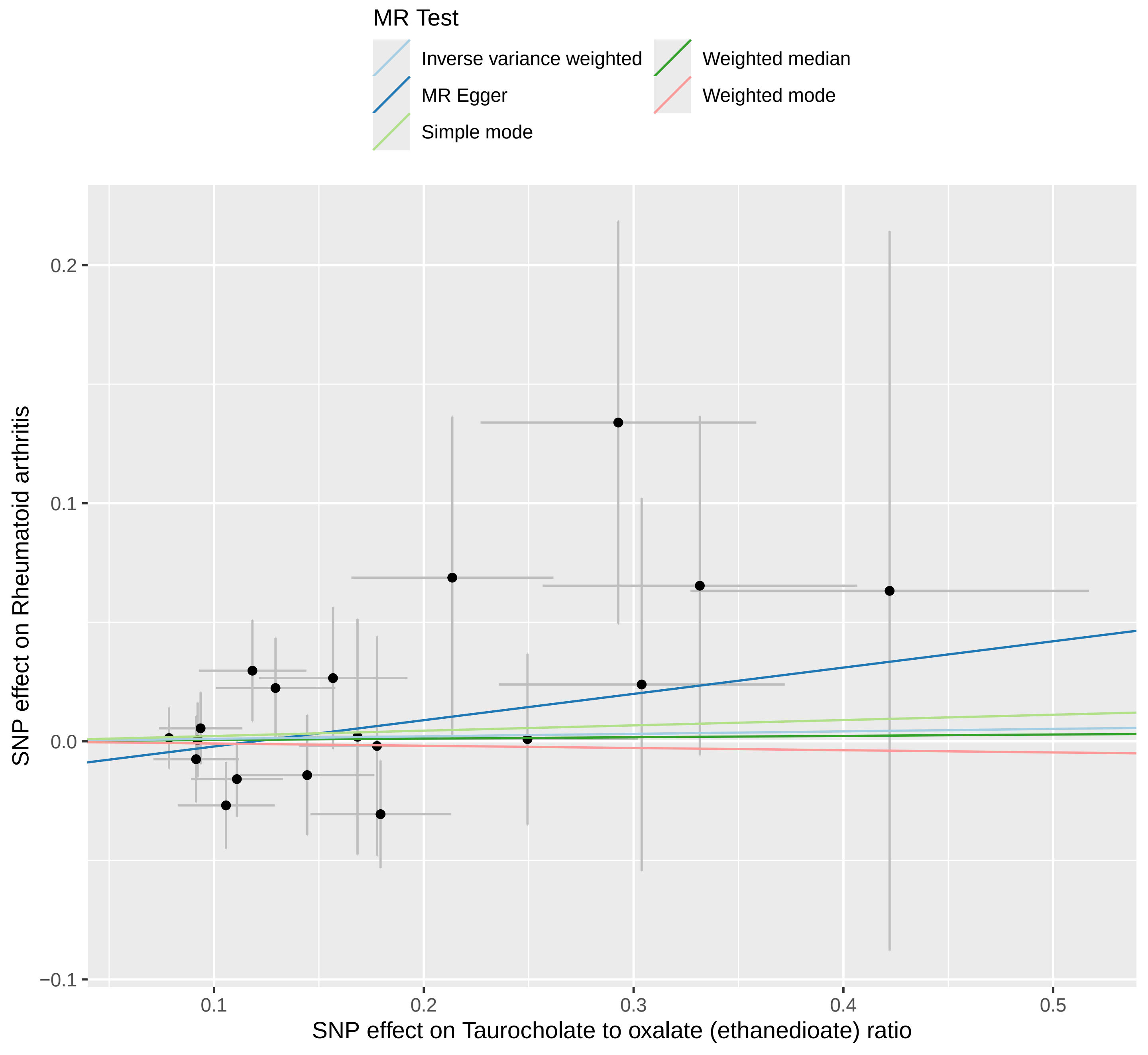

MR Test
Inverse variance weighted
MR Egger
Simple mode
Weighted median
Weighted mode
SNP effect on Rheumatoid arthritis
0.2
0.1
0.0
−0.1
0.1
0.2
0.3
0.4
0.5
SNP effect on Taurocholate to oxalate (ethanedioate) ratio

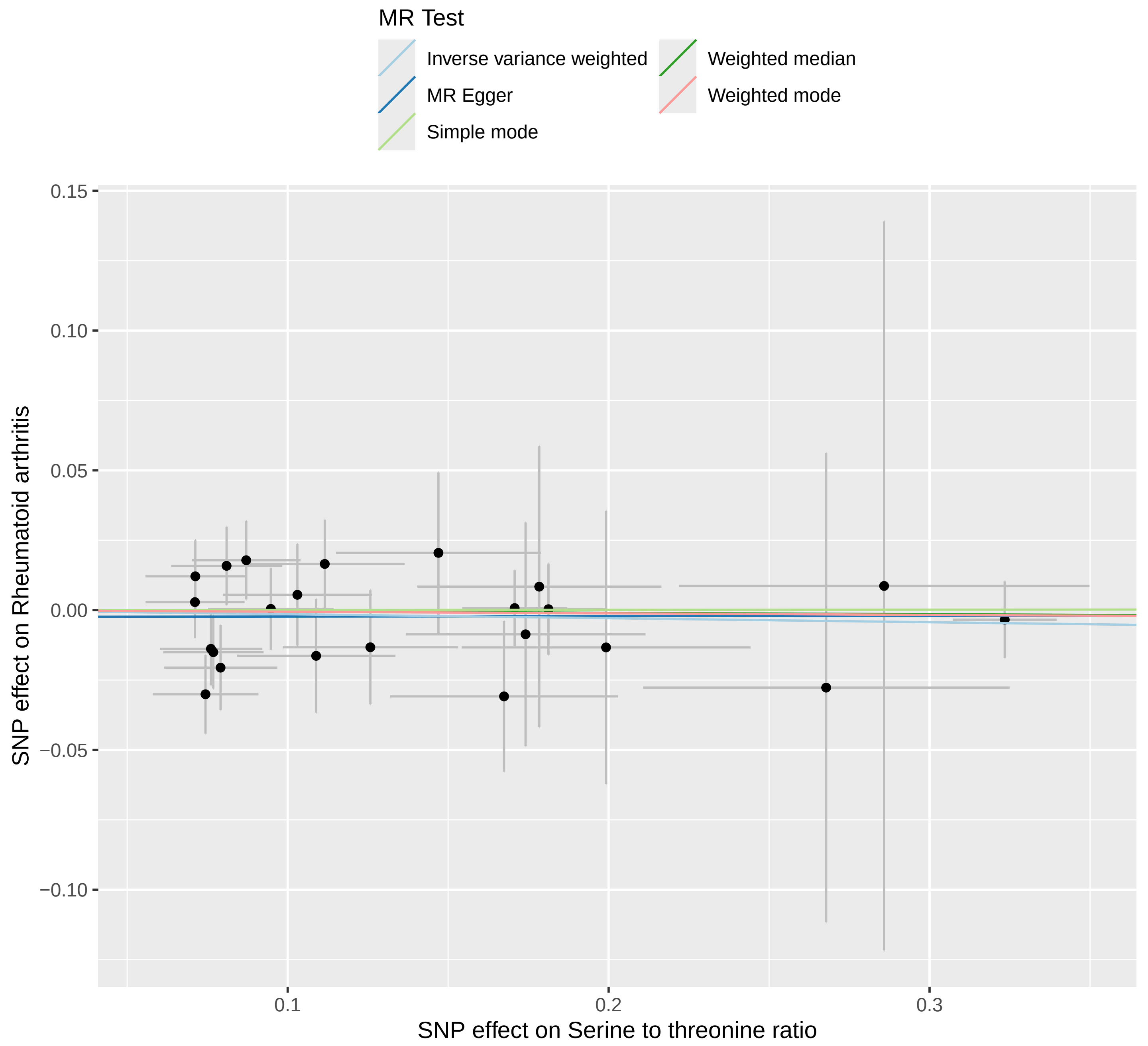

MR Test
Inverse variance weighted
MR Egger
Simple mode
Weighted median
Weighted mode
0.15
0.10
0.05
0.00
−0.05
−0.10
SNP effect on Rheumatoid arthritis
0.1
0.2
0.3
SNP effect on Serine to threonine ratio

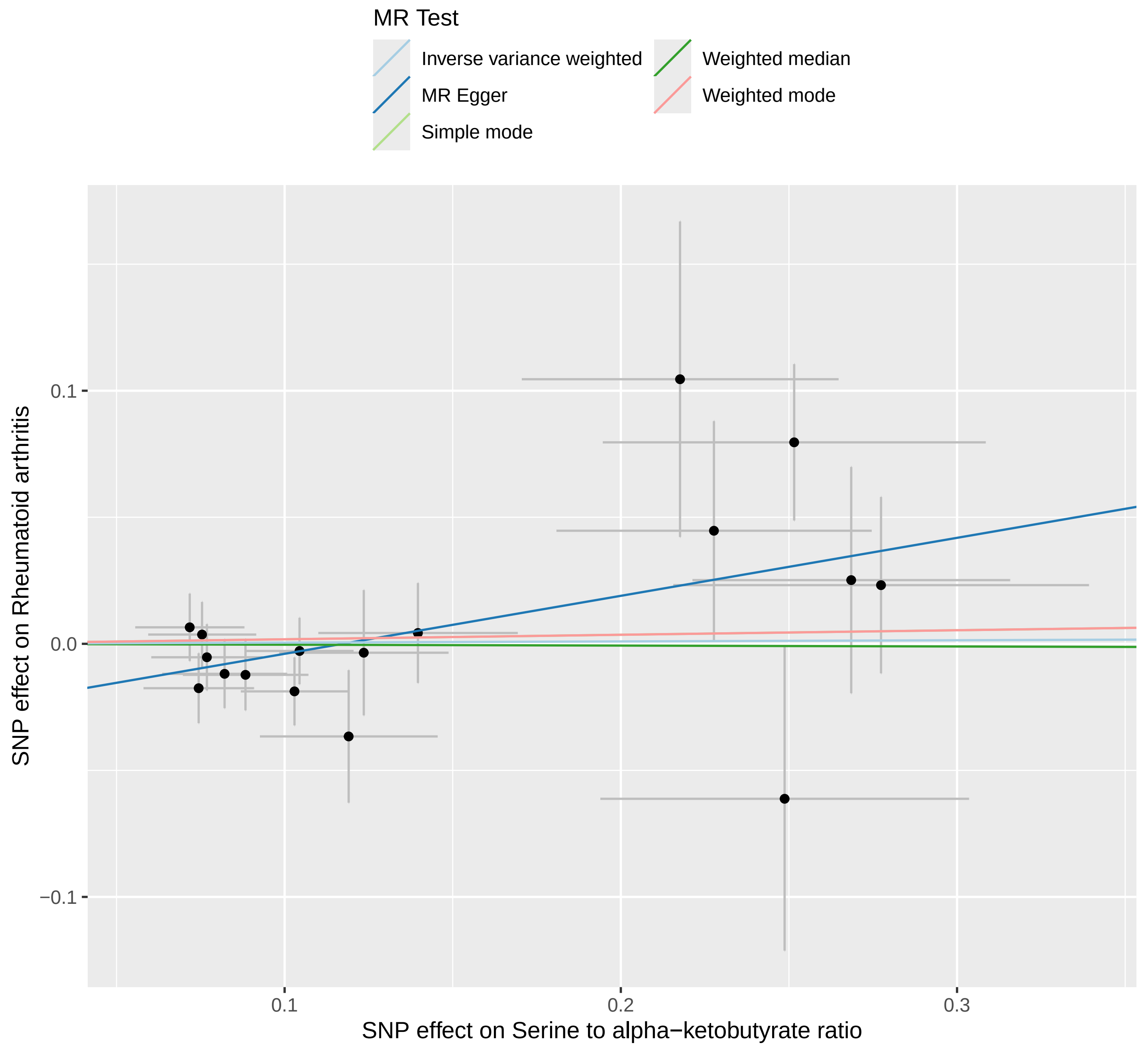

MR Test
Inverse variance weighted
MR Egger
Simple mode
Weighted median
Weighted mode
0.1
0.0
−0.1
0.1
0.2
0.3
SNP effect on Rheumatoid arthritis
SNP effect on Serine to alpha−ketobutyrate ratio

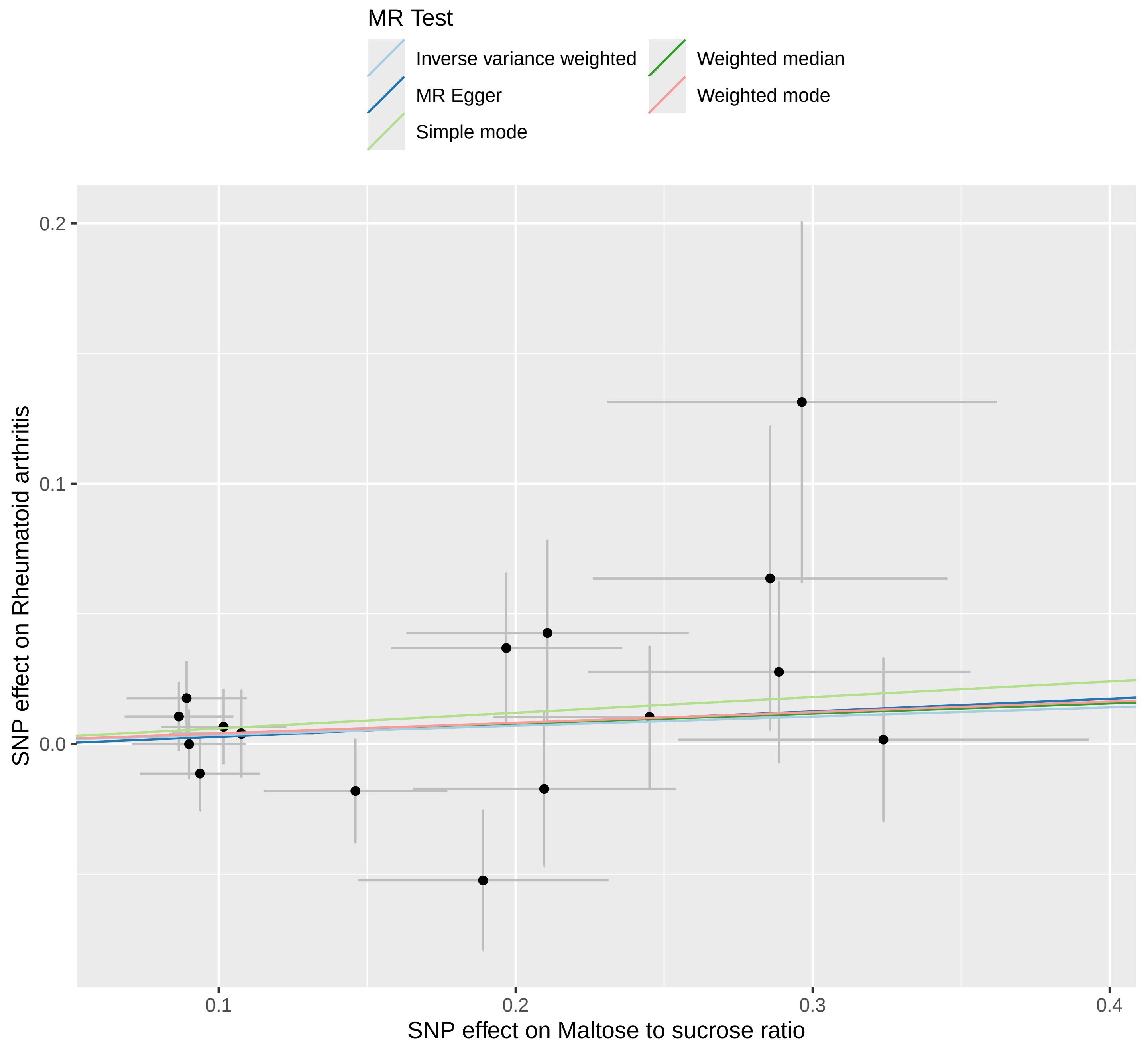

MR Test
Inverse variance weighted
MR Egger
Simple mode
Weighted median
Weighted mode
0.2
0.1
0.0
0.1
0.2
0.3
0.4
SNP effect on Rheumatoid arthritis
SNP effect on Maltose to sucrose ratio

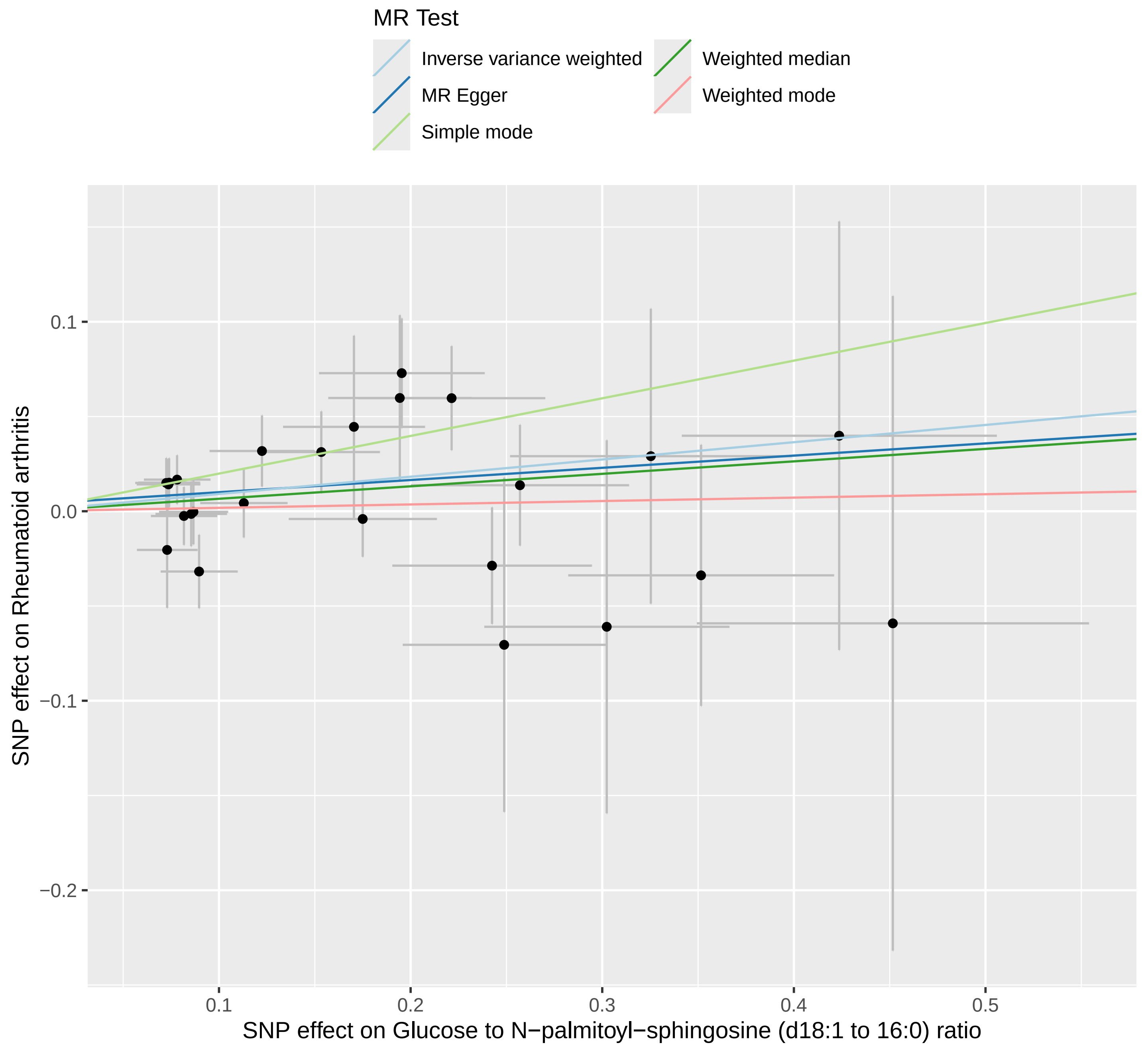

MR Test
Inverse variance weighted
MR Egger
Simple mode
Weighted median
Weighted mode
0.1
0.0
−0.1
−0.2
SNP effect on Rheumatoid arthritis
0.1
0.2
0.3
0.4
0.5
SNP effect on Glucose to N−palmitoyl−sphingosine (d18:1 to 16:0) ratio

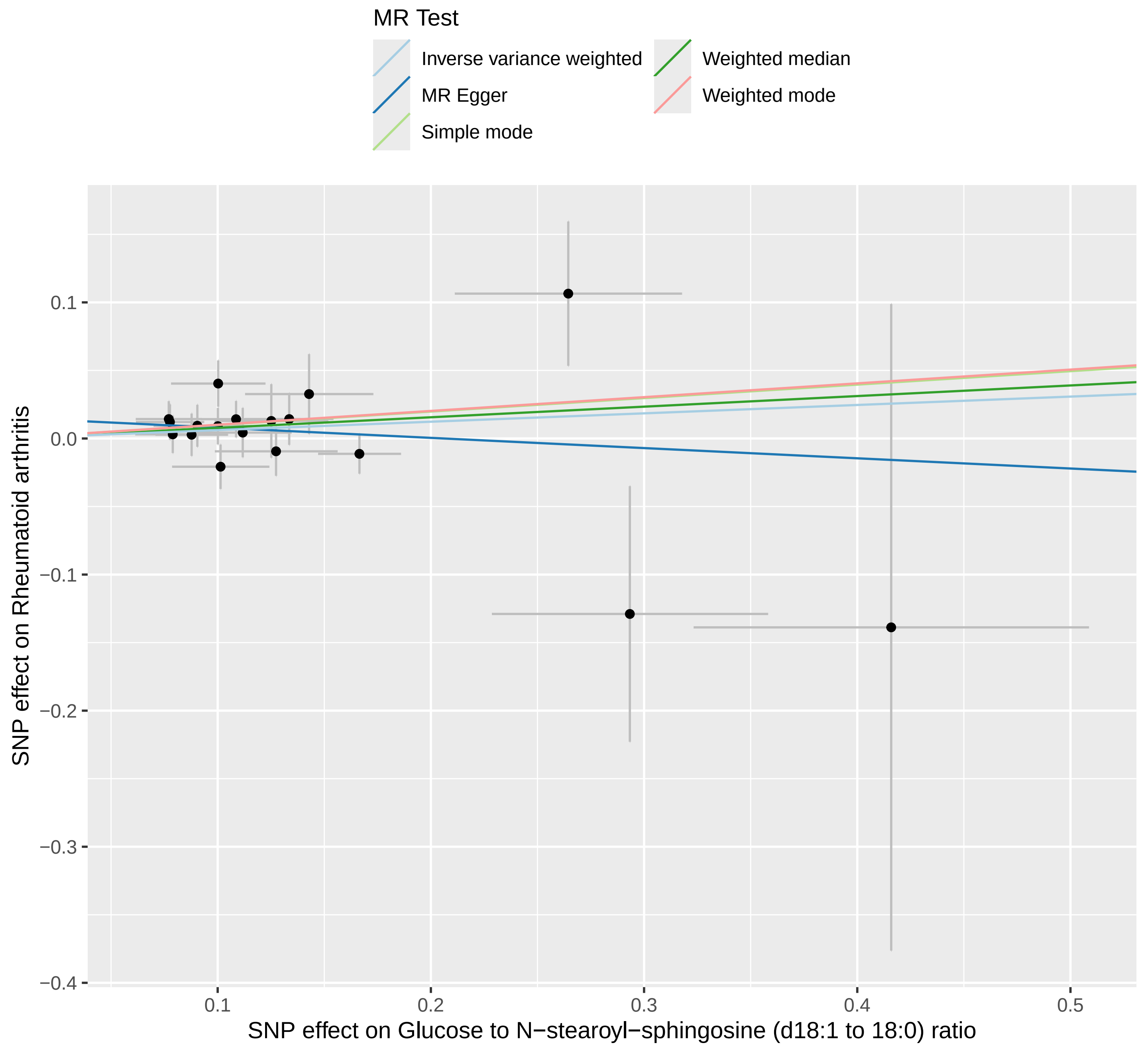
MR Test
Inverse variance weighted
MR Egger
Simple mode
Weighted median
Weighted mode
SNP effect on Rheumatoid arthritis
SNP effect on Glucose to N−stearoyl−sphingosine (d18:1 to 18:0) ratio
0.1
0.0
−0.1
−0.2
−0.3
−0.4
0.1
0.2
0.3
0.4
0.5

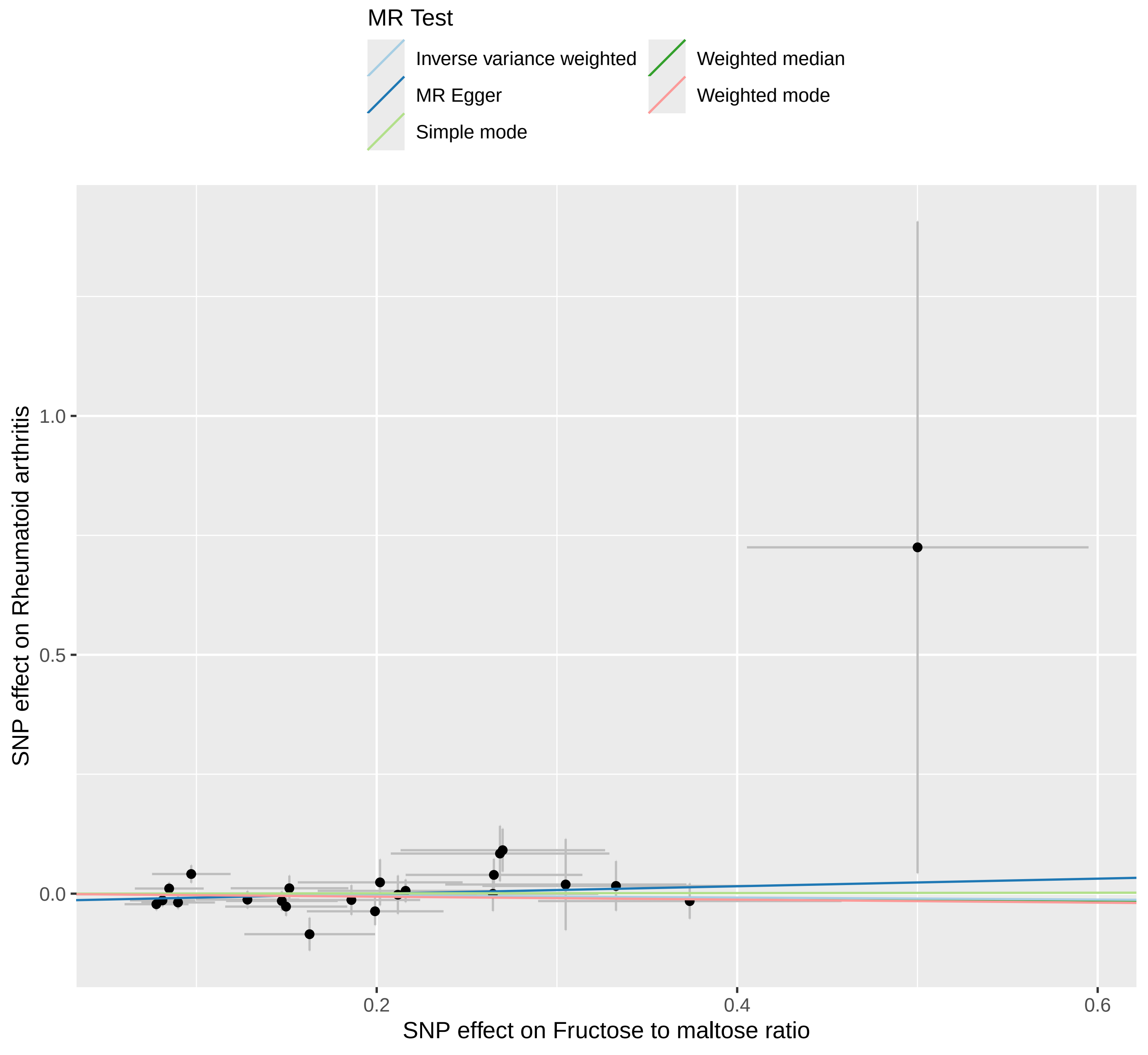
MR Test
Inverse variance weighted
MR Egger
Simple mode
Weighted median
Weighted mode
1.0
0.5
0.0
0.2
0.4
0.6
SNP effect on Rheumatoid arthritis
SNP effect on Fructose to maltose ratio

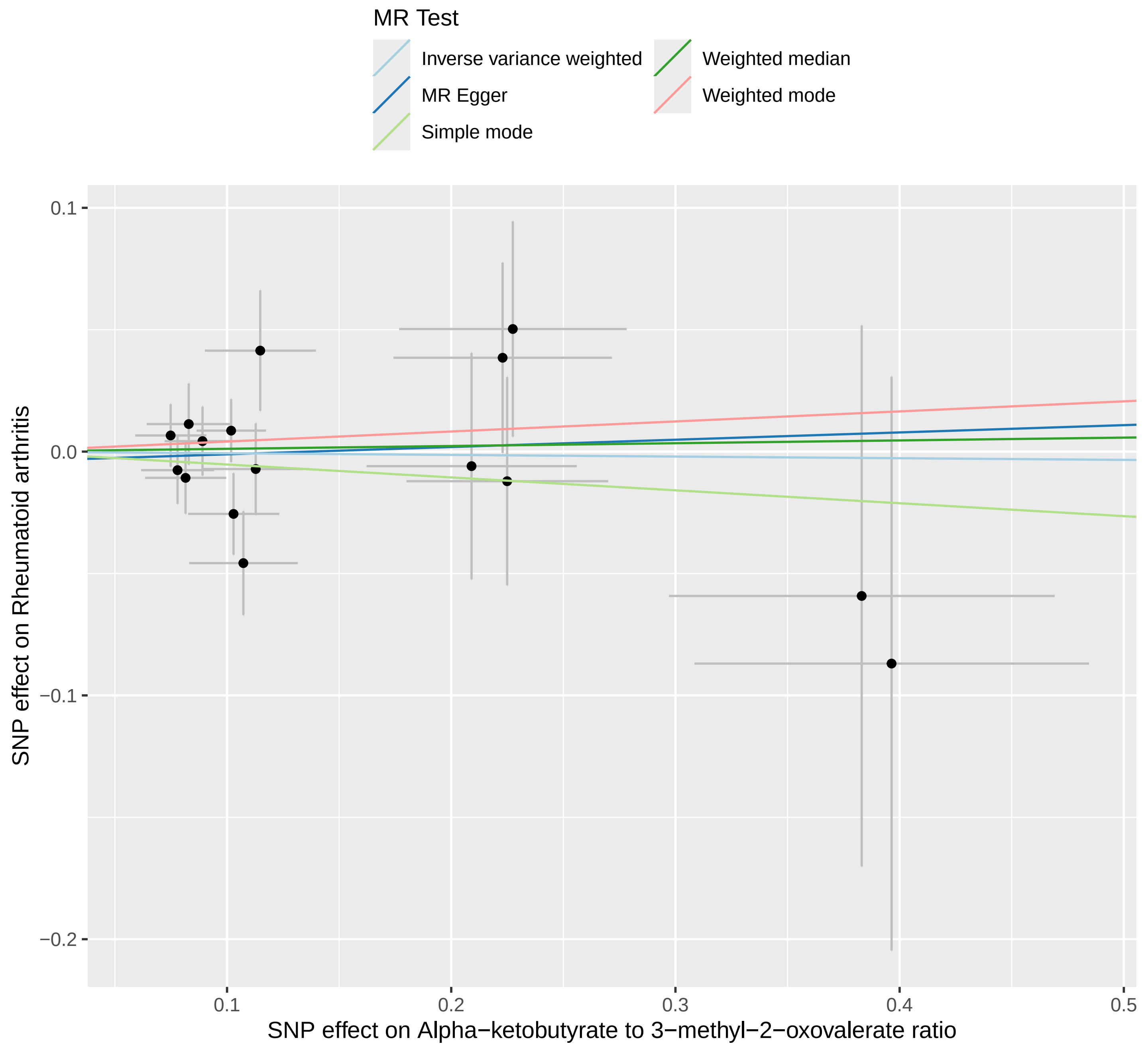
MR Test
Inverse variance weighted
MR Egger
Simple mode
Weighted median
Weighted mode
0.1
0.0
−0.1
−0.2
0.1
0.2
0.3
0.4
0.5
SNP effect on Rheumatoid arthritis
SNP effect on Alpha−ketobutyrate to 3−methyl−2−oxovalerate ratio

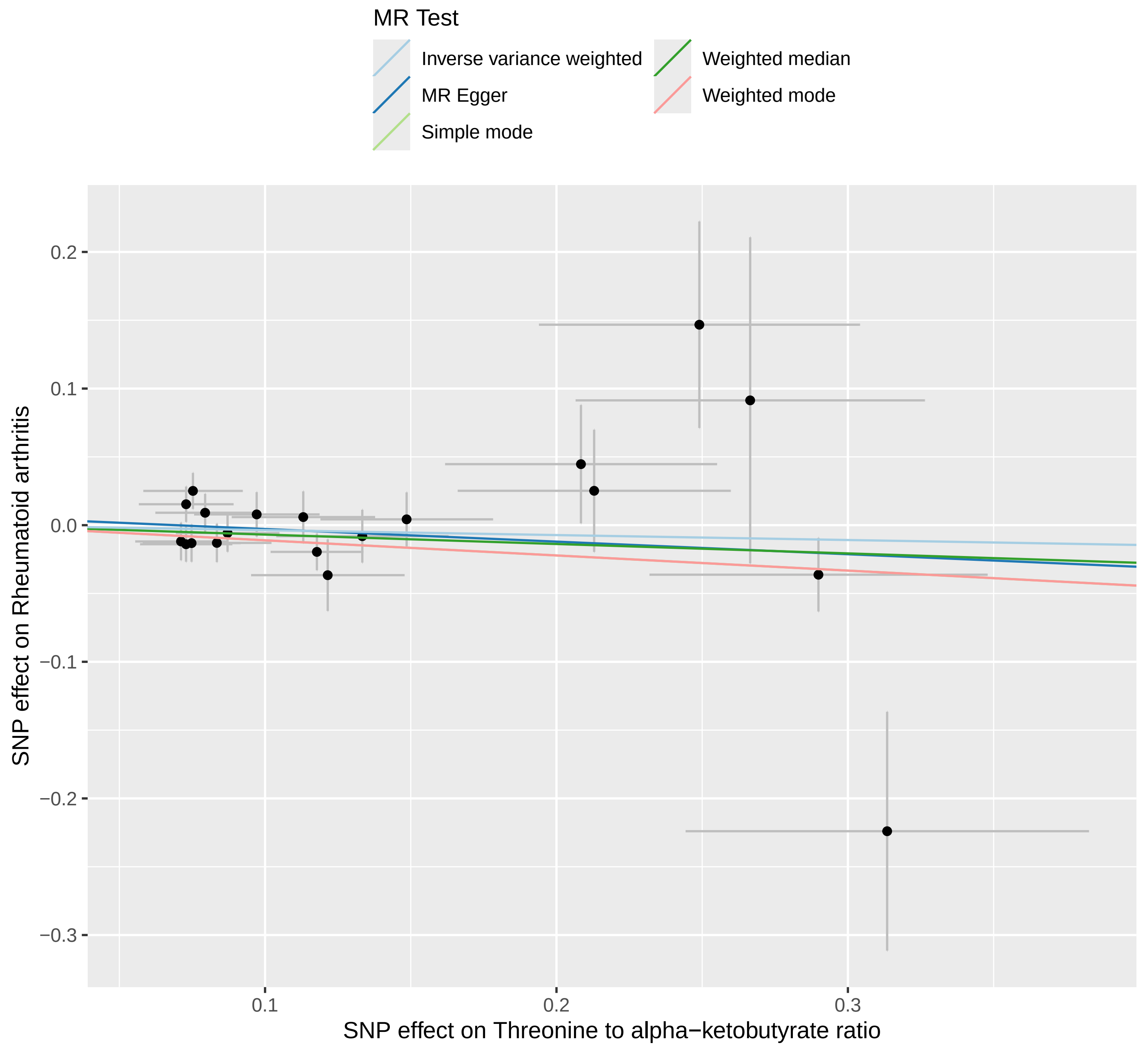
MR Test
Inverse variance weighted
MR Egger
Simple mode
Weighted median
Weighted mode
0.2
0.1
0.0
−0.1
−0.2
−0.3
SNP effect on Rheumatoid arthritis
0.1
0.2
0.3
SNP effect on Threonine to alpha−ketobutyrate ratio

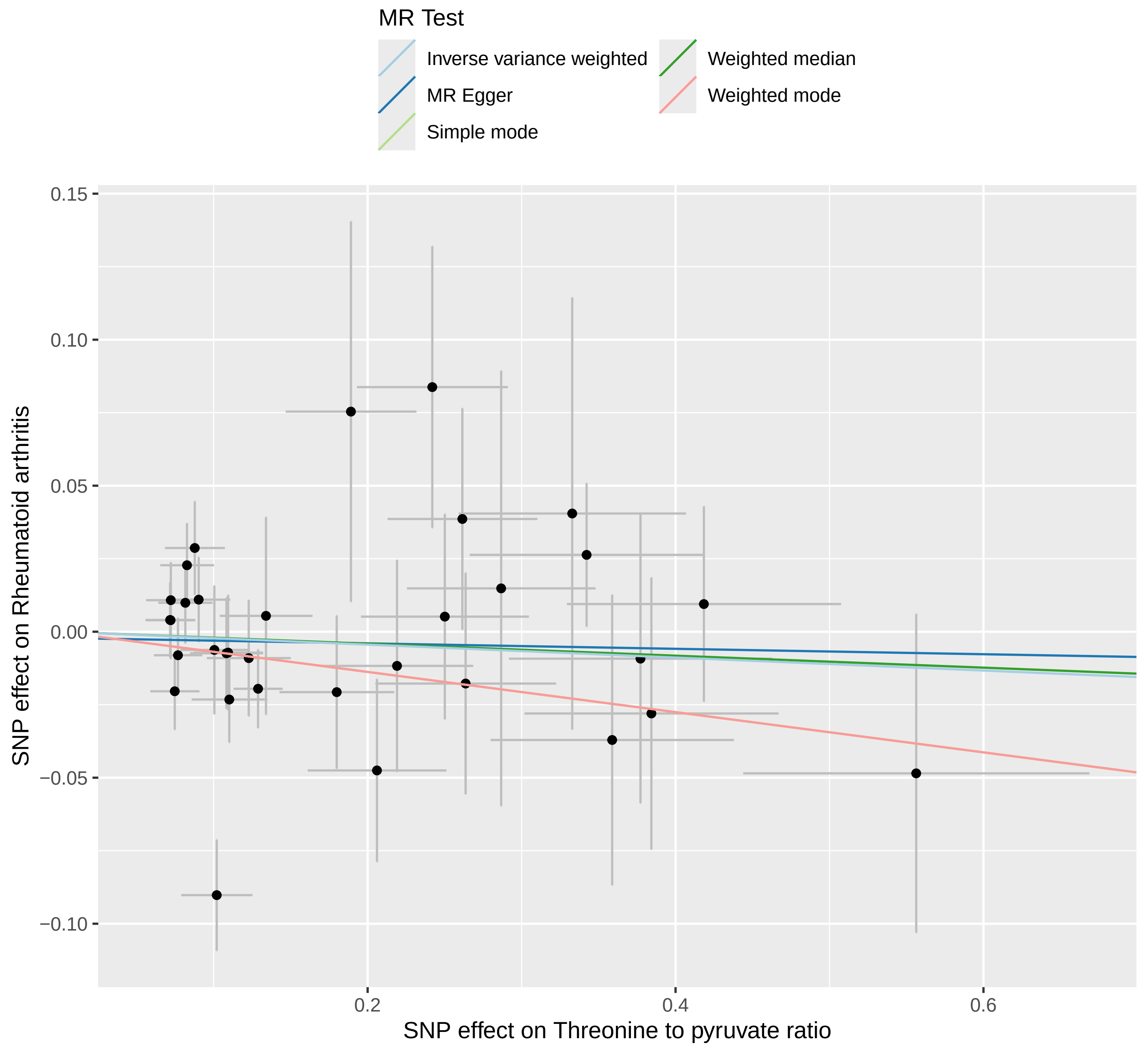

MR Test
Inverse variance weighted
MR Egger
Simple mode
Weighted median
Weighted mode
0.15
0.10
0.05
0.00
−0.05
−0.10
0.2
0.4
0.6
SNP effect on Rheumatoid arthritis
SNP effect on Threonine to pyruvate ratio

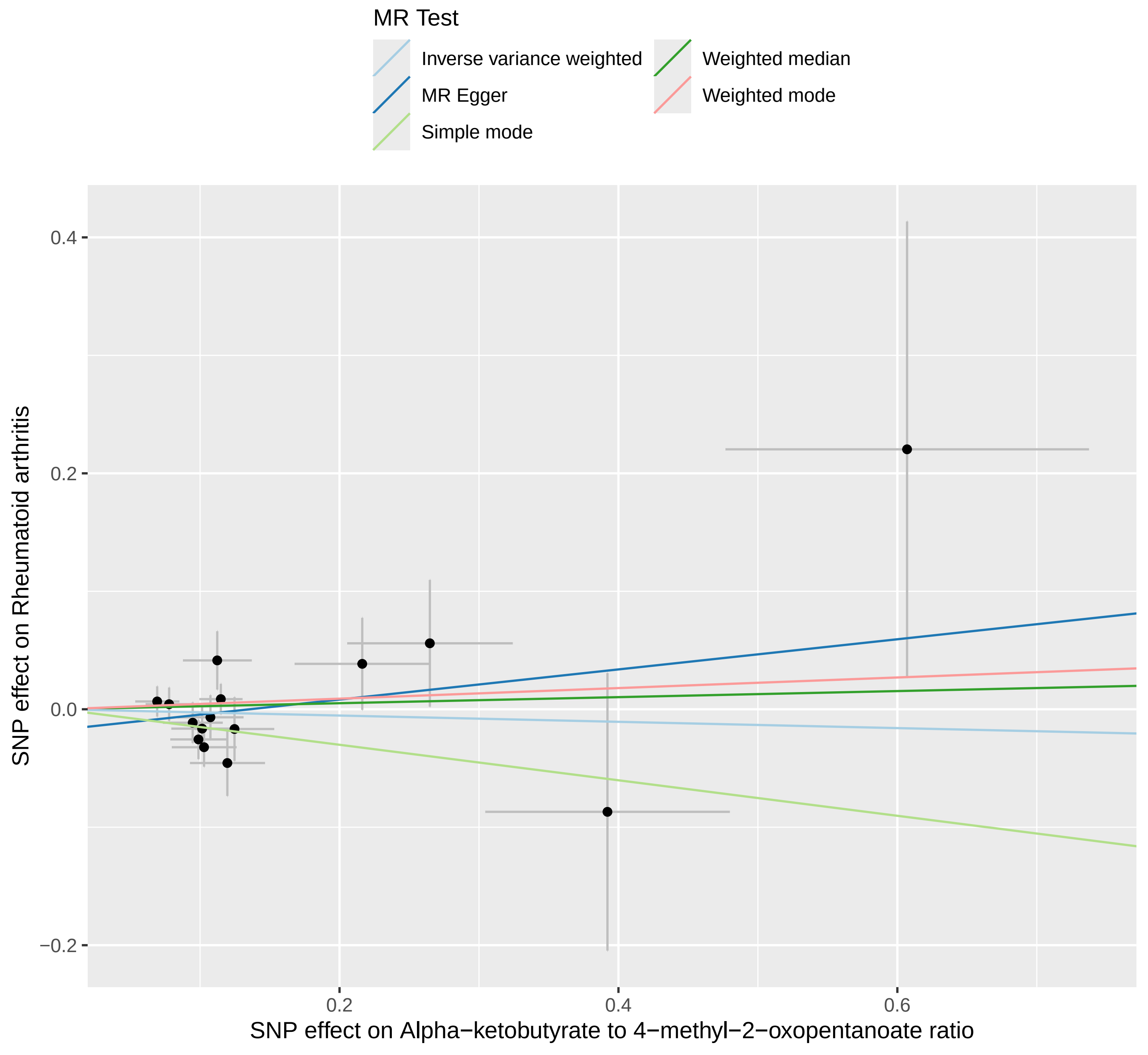
MR Test
Inverse variance weighted
MR Egger
Simple mode
Weighted median
Weighted mode
0.4
0.2
0.0
−0.2
0.2
0.4
0.6
SNP effect on Rheumatoid arthritis
SNP effect on Alpha−ketobutyrate to 4−methyl−2−oxopentanoate ratio

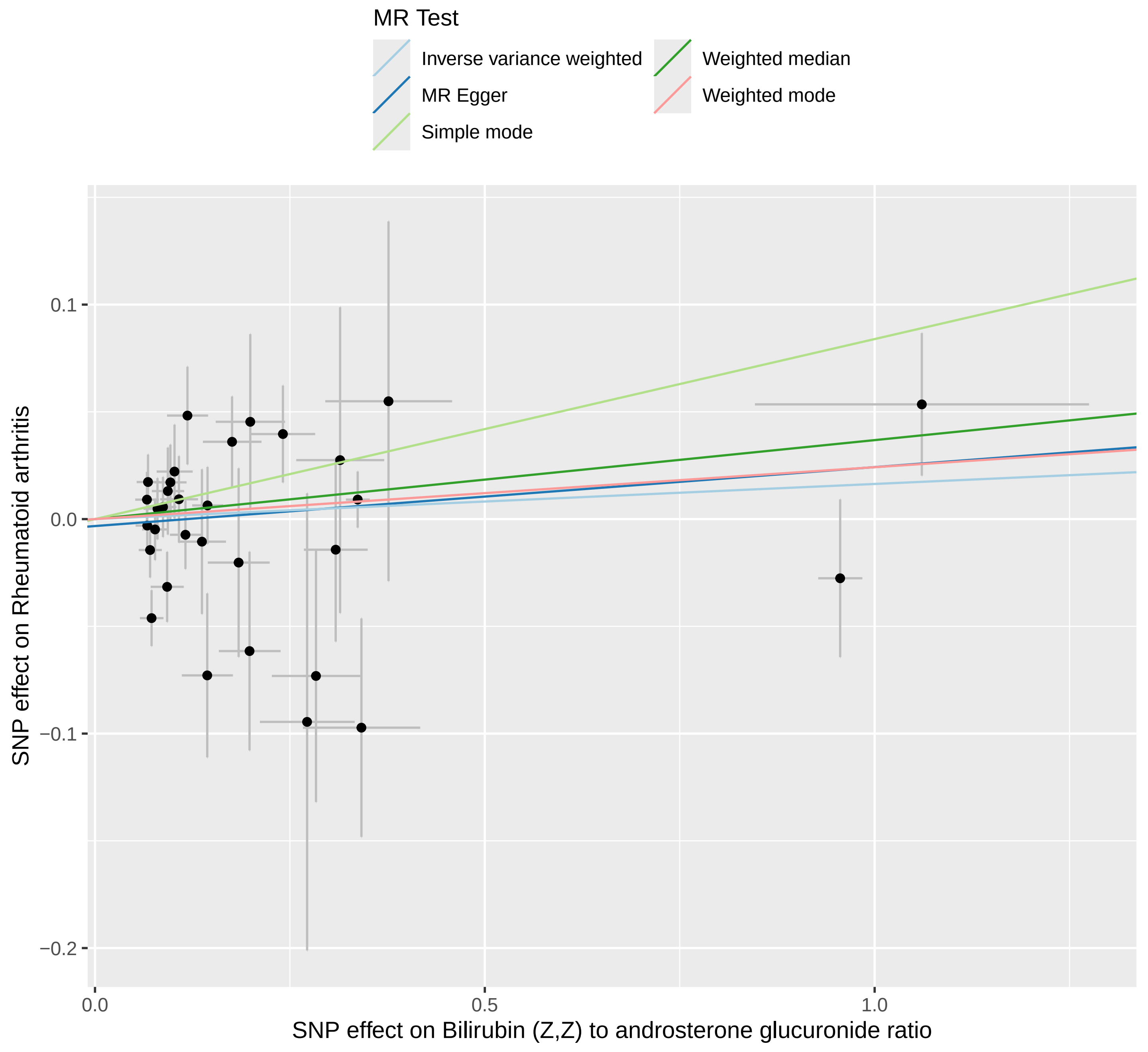
MR Test
Inverse variance weighted
MR Egger
Simple mode
Weighted median
Weighted mode
0.1
0.0
−0.1
−0.2
0.0
0.5
1.0
SNP effect on Rheumatoid arthritis
SNP effect on Bilirubin (Z,Z) to androsterone glucuronide ratio

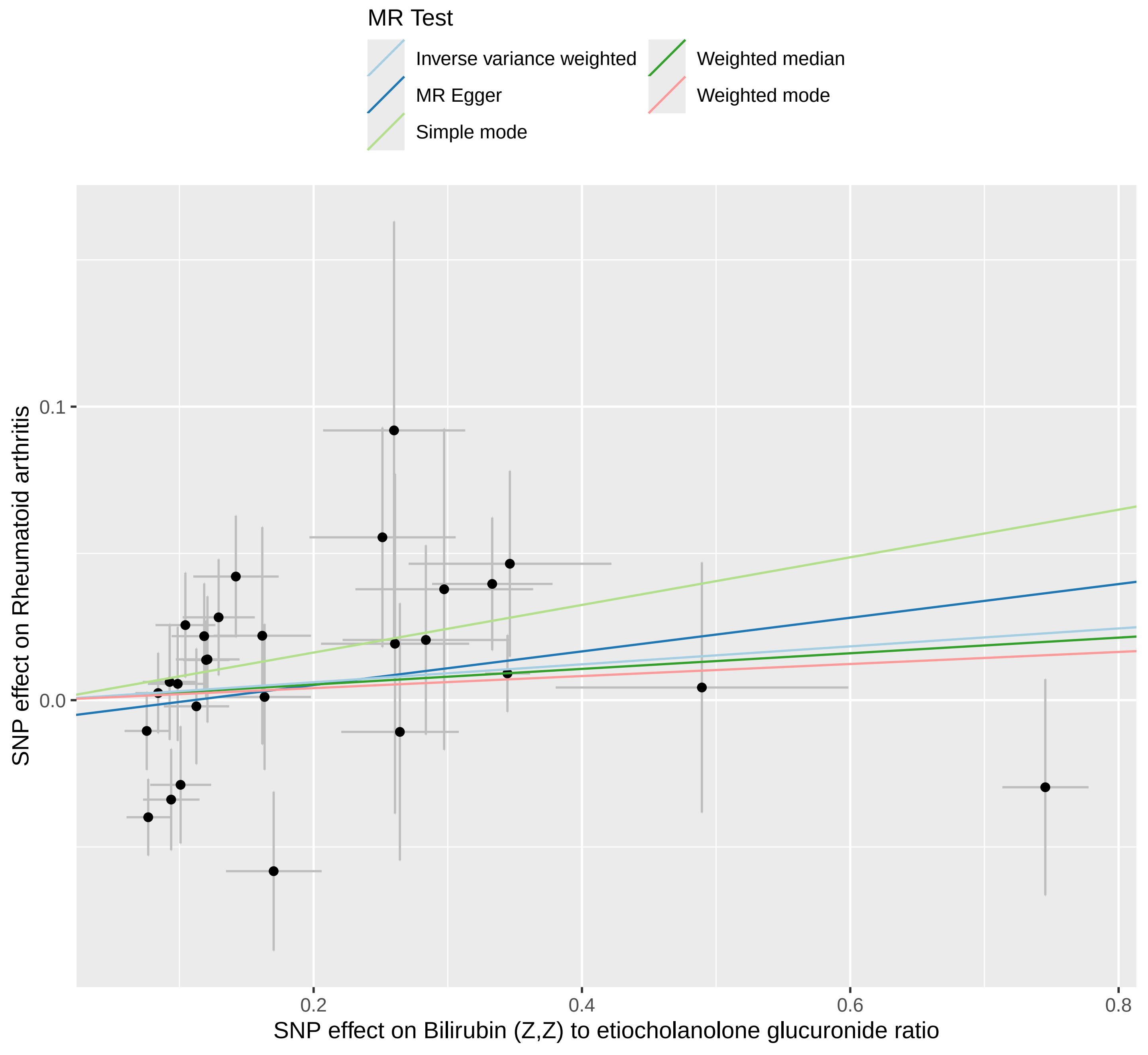
MR Test
Inverse variance weighted
MR Egger
Simple mode
Weighted median
Weighted mode
0.1
0.0
0.2
0.4
0.6
0.8
SNP effect on Rheumatoid arthritis
SNP effect on Bilirubin (Z,Z) to etiocholanolone glucuronide ratio

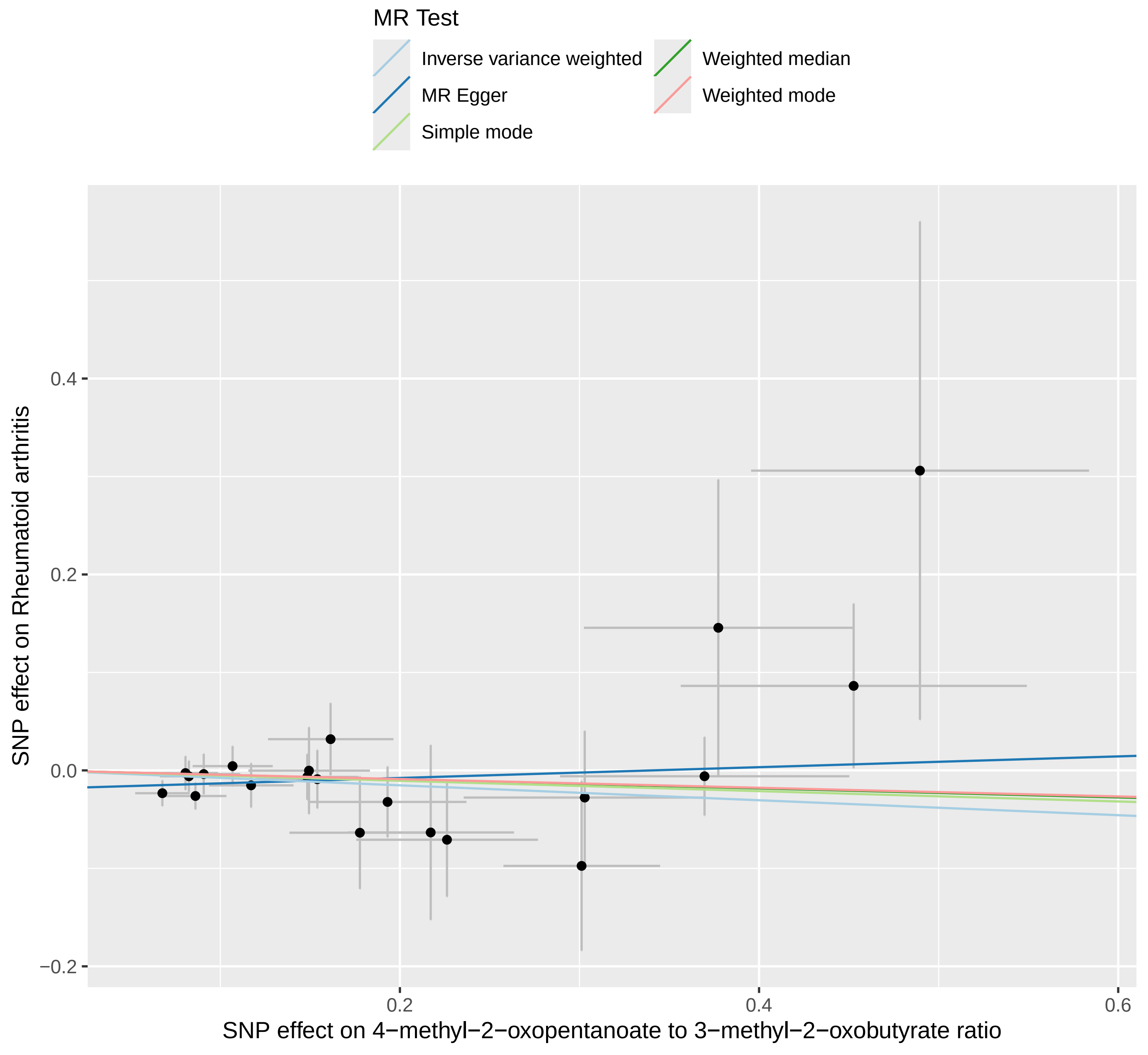

MR Test
Inverse variance weighted
MR Egger
Simple mode
Weighted median
Weighted mode
0.4
0.2
0.0
−0.2
SNP effect on Rheumatoid arthritis
0.2
0.4
0.6
SNP effect on 4−methyl−2−oxopentanoate to 3−methyl−2−oxobutyrate ratio

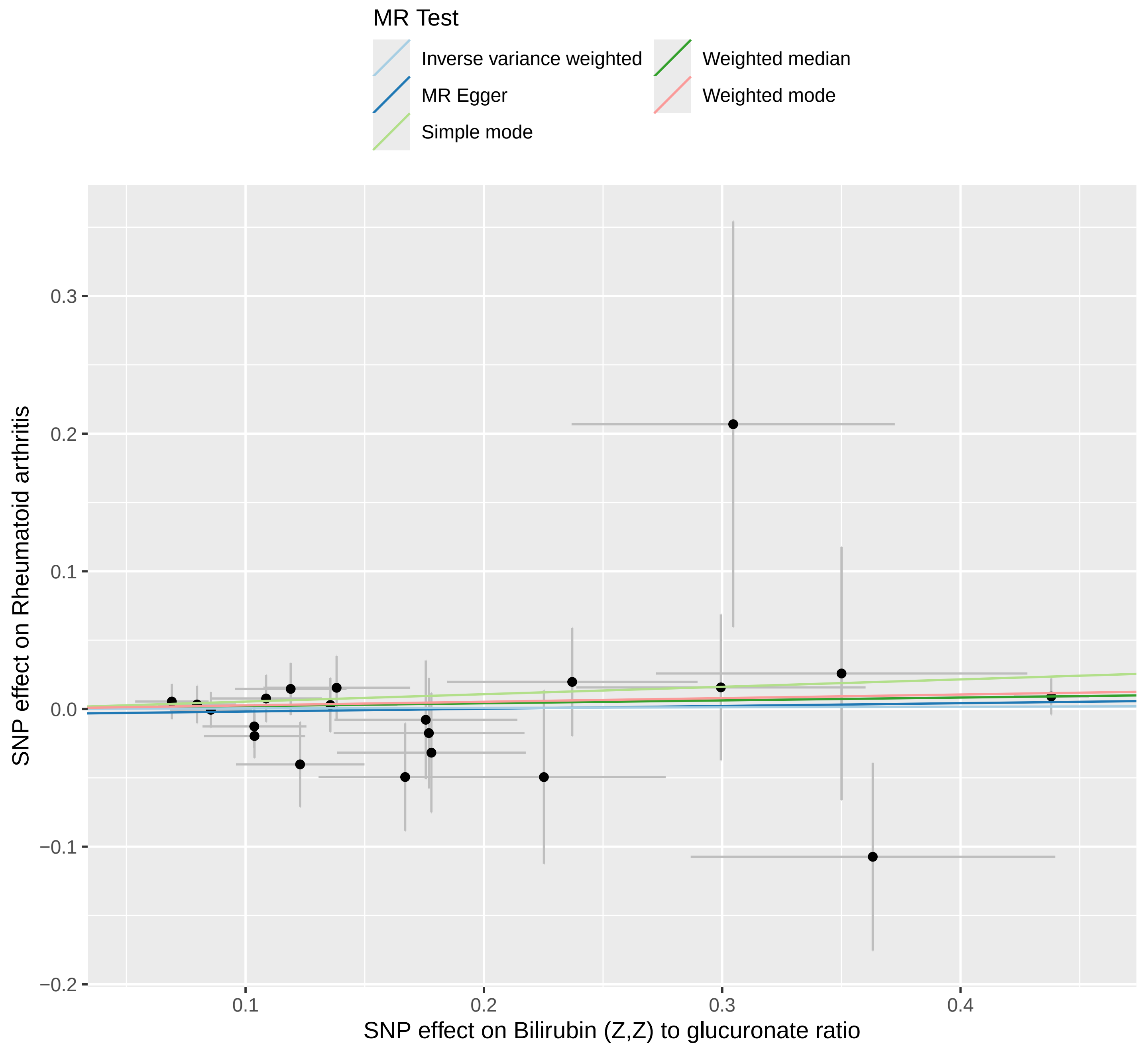
MR Test
Inverse variance weighted
MR Egger
Simple mode
Weighted median
Weighted mode
SNP effect on Rheumatoid arthritis
0.3
0.2
0.1
0.0
−0.1
−0.2
0.1
0.2
0.3
0.4
SNP effect on Bilirubin (Z,Z) to glucuronate ratio

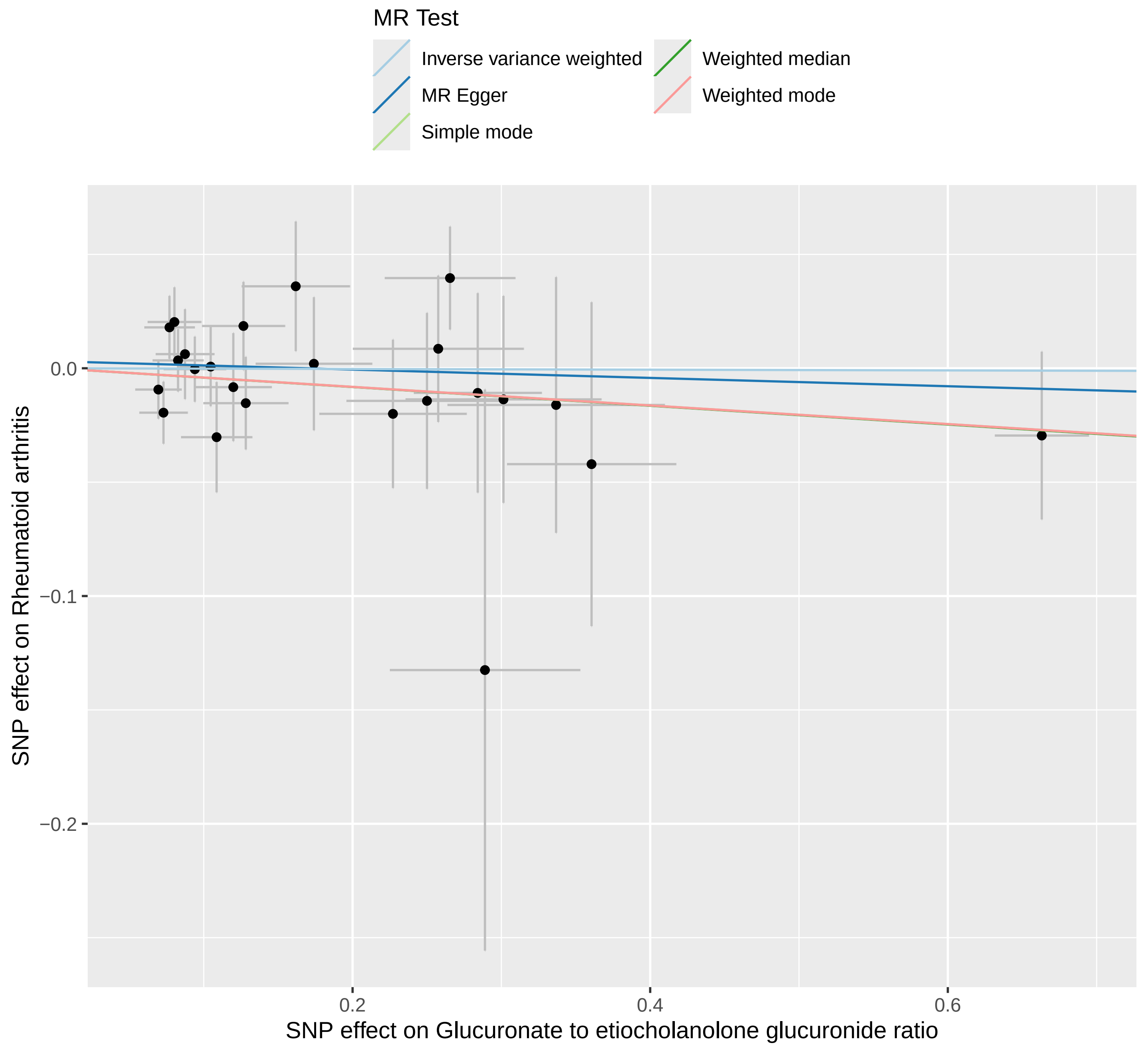
MR Test
Inverse variance weighted
MR Egger
Simple mode
Weighted median
Weighted mode
0.0
−0.1
−0.2
0.2
0.4
0.6
SNP effect on Rheumatoid arthritis
SNP effect on Glucuronate to etiocholanolone glucuronide ratio

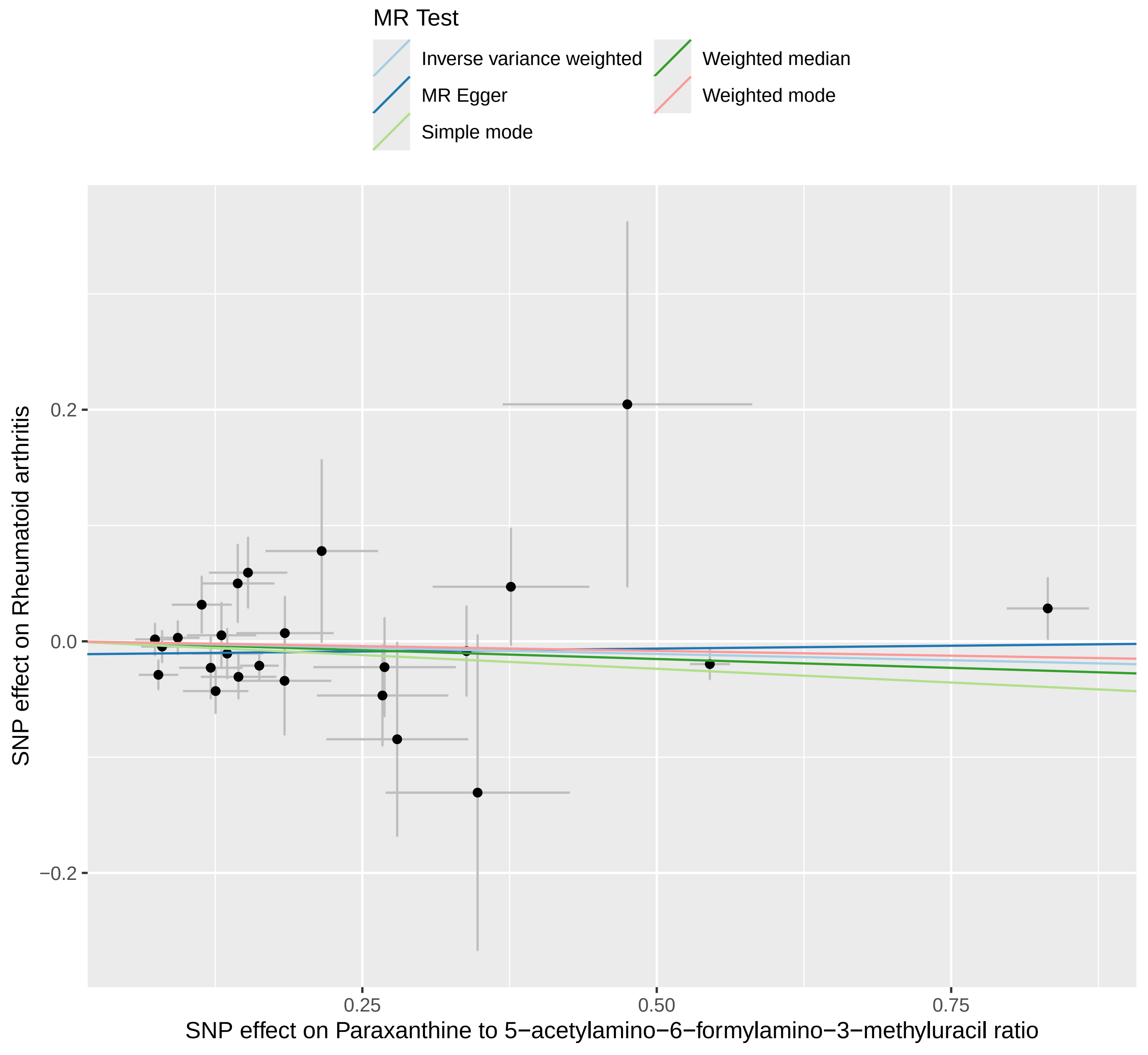
MR Test
Inverse variance weighted
MR Egger
Simple mode
Weighted median
Weighted mode
SNP effect on Rheumatoid arthritis
0.2
0.0
−0.2
0.25
0.50
0.75
SNP effect on Paraxanthine to 5−acetylamino−6−formylamino−3−methyluracil ratio
